# Assembly of highly repetitive genomes using short reads: the genome of discrete typing unit III *Trypanosoma cruzi* strain 231

**DOI:** 10.1099/mgen.0.000156

**Published:** 2018-02-14

**Authors:** Rodrigo P. Baptista, Joao Luis Reis-Cunha, Jeremy D. DeBarry, Egler Chiari, Jessica C. Kissinger, Daniella C. Bartholomeu, Andrea M. Macedo

**Affiliations:** ^1^​ Center for Tropical and Emerging Global Diseases, University of Georgia, Athens, GA, USA; ^2^​ Institute of Bioinformatics, University of Georgia, Athens, USA; ^3^​ Departamento de Parasitologia, Instituto de Ciências Biológicas, Universidade Federal de Minas Gerais, Belo Horizonte, Brazil; ^4^​ Department of Genetics, University of Georgia, Athens, USA; ^5^​ Departamento de Bioquímica e Imunologia, Instituto de Ciências Biológicas, Universidade Federal de Minas Gerais, Belo Horizonte, Minas Gerais, Brazil

**Keywords:** *Trypanosoma cruzi*, genome assembly, evolution, DTUs

## Abstract

Next-generation sequencing (NGS) methods are low-cost high-throughput technologies that produce thousands to millions of sequence reads. Despite the high number of raw sequence reads, their short length, relative to Sanger, PacBio or Nanopore reads, complicates the assembly of genomic repeats. Many genome tools are available, but the assembly of highly repetitive genome sequences using only NGS short reads remains challenging. Genome assembly of organisms responsible for important neglected diseases such as *Trypanosoma cruzi*, the aetiological agent of Chagas disease, is known to be challenging because of their repetitive nature. Only three of six recognized discrete typing units (DTUs) of the parasite have their draft genomes published and therefore genome evolution analyses in the taxon are limited. In this study, we developed a computational workflow to assemble highly repetitive genomes via a combination of *de novo* and reference-based assembly strategies to better overcome the intrinsic limitations of each, based on Illumina reads. The highly repetitive genome of the human-infecting parasite *T. cruzi* 231 strain was used as a test subject. The combined-assembly approach shown in this study benefits from the reference-based assembly ability to resolve highly repetitive sequences and from the *de novo* capacity to assemble genome-specific regions, improving the quality of the assembly. The acceptable confidence obtained by analyzing our results showed that our combined approach is an attractive option to assemble highly repetitive genomes with NGS short reads. Phylogenomic analysis including the 231 strain, the first representative of DTU III whose genome was sequenced, was also performed and provides new insights into *T. cruzi* genome evolution.

## Data Summary

The *T. cruzi* 231 strain whole genome shotgun project has been deposited at DDBJ/EMBL/GenBank [1] under accession number PRJEB9129 (url - http://www.ncbi. nlm.nih.gov/bioproject/PRJEB9129). The data described in this paper represent the first version of the raw reads, assembly and annotation. The data will also be made available at TriTrypDB [2]. The annotation obtained by RATT is available at https://figshare.com/s/0734907aea9 e93083727.

**Impact Statement**

The generation of short, accurate, genome sequence reads has become standard practice for the investigation of new genome sequences and the analysis of SNPs when existing genome sequences are available. However, assembly of the sequence reads into an inferred genome sequence and the generation of a corresponding annotation is much more informative. Existing tools vary greatly in their performance (speed, scalability, hardware requirements, acceptance of newer read technologies) and in their final output (composition of assembled sequence). Genomes that contain many repetitive sequences have traditionally been difficult to assemble when only short sequence reads are available. We designed a low-cost (relative to single-molecule long-read technologies, such as PacBio) approach to quickly assemble and annotate draft genomes that contain repeats and used it for assembly of *Trypanosoma cruzi* 231 (Tc231), the first representative of *T. cruzi* discrete typing unit III (DTU III) to be sequenced. Phylogenomic analysis including Tc231 genes was also performed to test the ability of current models to explain *T. cruzi* evolution.

## Introduction

Several DNA sequencing methods were developed in the mid- to late 1990s. These techniques comprise the first of the low-cost high-throughput next-generation sequencing (NGS) methods, producing thousands to millions of reads [3]. Despite the accurate generation of high-throughput raw sequences, NGS produces smaller reads when compared to Sanger sequencing [4]. The length of the sequences generated by low error rate NGS techniques typically ranges from 75 to 300 bp. Thus, the assembly of a complete eukaryotic genome sequence (millions to billions of base pairs) is very challenging. The basic assumption behind any assembly approach is that highly similar sequences are derived from the same genomic location. This allows the joining of overlapping individual reads into larger contiguous sequences. Genomic repeats complicate the assembly process, because repetitive regions that are scattered throughout the genome sequence are algorithmically collapsed during assembly into only a few sequences due to their similarity. The resulting genome assemblies are incorrect in terms of the number and location of repetitive sequences and, at times, artificial fusions of genomic regions that are not biologically contiguous are created. Importantly, it remains unclear how to best assess the quality of genome assemblies [5].

Trypanosomatids are protozoan parasites, many of which cause tropical human diseases. The genome sequences of several trypanosomatids have been published, including *Trypanosoma cruzi*, *Trypanosoma brucei* and *Leishmania major*, known as the ‘TriTryps’. The genome organization of these eukaryotic parasites is unique; they have long, strand-specific gene clusters that are polycistronically transcribed [6]; only a few promoters have been functionally characterized [7] and experimental evidence suggests that transcription initiates bi-directionally between two divergent gene clusters [8, 9].

*T. cruzi* is the aetiological agent of Chagas disease, which affects approximately 6–7 million people in a region that extends from southern Argentina to the southern United States, where at least 25 million people are at risk of infection [10, 11]. *T. cruzi* displays a high degree of intraspecific polymorphism and currently six different discrete typing units (DTUs), named TcI–TcVI, are recognized [12]. There is much speculation regarding whether this variability is associated with their geographical distribution, transmission cycle, drug resistance and/or disease prognoses [13–15]. A better understanding of the relationships between the distinct lineages is crucial for the establishment of effective control strategies.

In 2005, the first genome sequence of *T. cruzi* was published. CL Brener, a hybrid clone strain representative of DTU TcVI, was chosen as the reference strain for the *T. cruzi* genome project because it was well characterized biologically [16]. DTU TcVI is derived from a relatively recent hybridization event between strains from DTUs TcII and TcIII [17, 18]. Thus, the CL Brener hybrid genome sequence is composed of two distinct haplotypes named Esmo-like and non-Esmo-like, which are derived from TcII and TcIII, respectively [19]. The diploid CL Brener genome was sequenced using Sanger technology and has an estimated size of 110 MB, with approximately 22 000 genes. Among the TriTryps, the *T. cruzi* genome has the highest repeat content. Over 50 % of the genome is composed of repetitive sequences such as retrotransposons, tandem repeats and multi-copy gene families that encode thousands of surface proteins. The hybrid nature of CL Brener and its highly repetitive content imposed serious challenges for genome assembly [19].

In 2011, the genome sequence of a representative TcI DTU, clone Sylvio X10/1, was generated [20]. The core gene repertoire between the CL Brener and Sylvio clones is highly similar, but copy number variation in the genes encoding surface proteins, with potential functional and epidemiological implications, was observed [19–22]. In the Franzen *et al.* study [20], sequences derived from both long Roche 454 (~700 bp read length) and short Illumina (~100 bp read length) reads were used for assembly of the Sylvio X10/1 genome. There are drawbacks to using combined sequencing technologies or generating libraries of different insert size from the same sequencing platform. They include both higher costs and time-consuming procedures for preparing libraries specific to each sequencing platform and sometimes the need for hybrid sequence assembly algorithms. In 2013, Hoffman LaRoche announced discontinuation of the low-error longer-read 454 platform (http://www.roche.com/). Although there are new long-read platforms such as Pacbio [23] and Nanopore [24], they are still costly and the quantity of DNA needed to perform the sequencing, depending on the organism, can be a challenge. Since trypanosomatids are known to be endemic in tropical developing countries, and are diverse species, much can be gained from cheaper genome sequencing, such as provided by Illumina short reads. However, due to the highly repetitive nature of the *T. cruzi* genome, current short-read *de novo* assembly strategies generate a highly fragmented genome. For that reason, new methodologies combining different approaches are needed. Also, the availability of several reference genomes from different *T. cruzi* DTUs will allow genome-wide SNP discovery and population structure analysis in the taxon.

Although having some genomes available, the evolutionary history of *T. cruzi* is still ambiguous. For that reason, several groups had proposed four major models to explain the number of hybridization events and genetic exchange throughout this parasite's evolution [18, 25–27]. Overall, they disagree about which are the ancestral DTUs and the number of hybridization events during the species evolution, but they all agree that both known hybrid DTUs, TcV and TcVI, originated from parental TcII and TcIII strains. Most of the proposed models were based on just a few genes, and the differences between each model could be the result of differences in the evolution rate of each gene chosen for their analysis. From all the *T. cruzi* whole genome sequences available in public databases, only three of the six DTUs described (I, II and VI), which are mostly related to human infections, are available [12, 14, 19, 20]. Having the whole genome sequences as representatives from the remaining DTUs is needed to generate an overview of the parasite's population diversity. In the present study, we designed a low-cost short-read only approach to quickly assemble and annotate the repetitive genome sequence of *T. cruzi* 231 (Tc231), a representative of DTU III, believed to be a hybrid parental strain, isolated from a human patient. Phylogenomic analysis including Tc231 was also used to improve our understanding of the evolutionary history of this parasite.

## Methods

### Sequence generation, assembly and annotation

Tc231 was isolated by haemoculture from the blood of a Chagas disease patient, in the endemic region of Bambuí in Minas Gerais State, Brazil (20.0171° S 45.9795° W) [28]. Parasites were first cloned according to the Gomes *et al.* protocol [29] to minimize the presence of more than one genetic isolate in our sample. Briefly, 10^3^ *T. cruzi* epimastigotes were plated into medium comprising 48.4 % semi-solid liver infusion tryptose (LIT), 48.4 % brain heart infusion (BHI), 0.75 % low-melting-point agarose and 2.5 % defibrinated and complement inactivated blood. These plates were incubated for 30 days at 28 °C until the appearance of colonies. Each colony was inoculated into LIT liquid medium and upon reaching the exponential growth phase, DNA was extracted using phenol/chloroform/isoamyl alcohol (25 : 24 : 1) methodology [30]. The DNA sample was examined for *Mycoplasma* contamination via PCR.

**Fig. 1.**
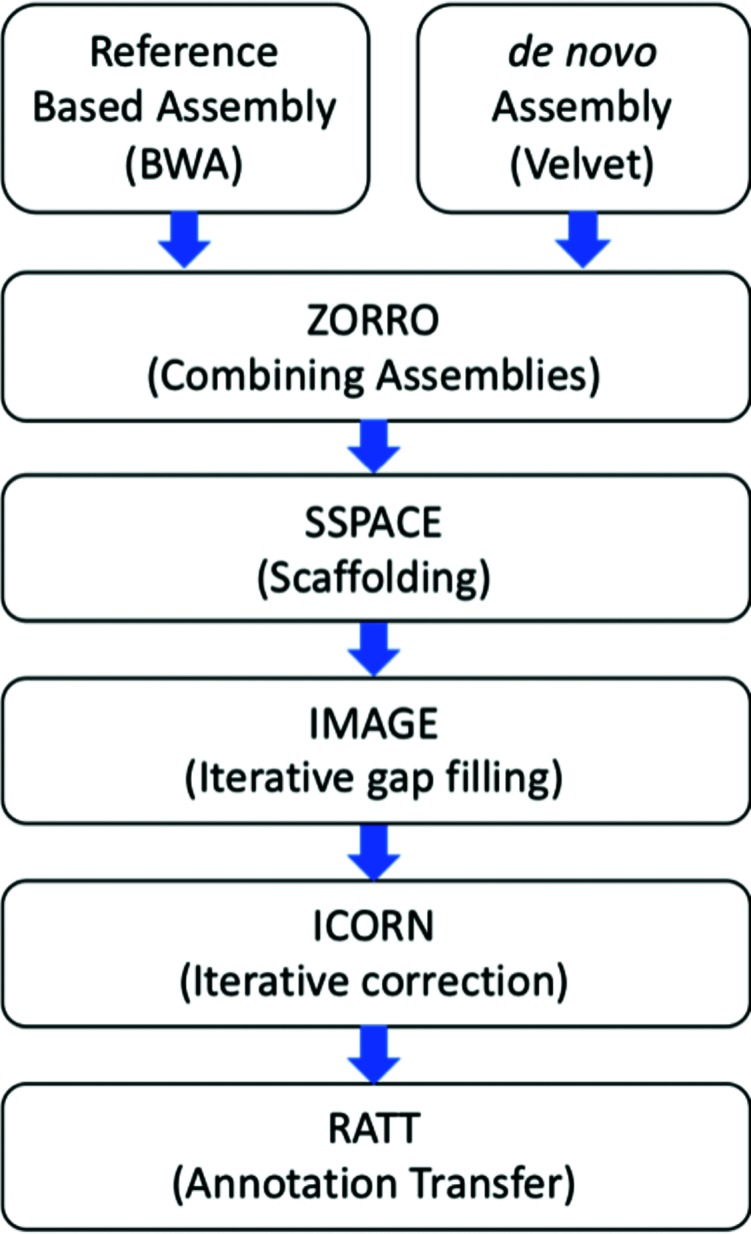
Outline of the combined-assembly pipeline.

**Fig. 2.**
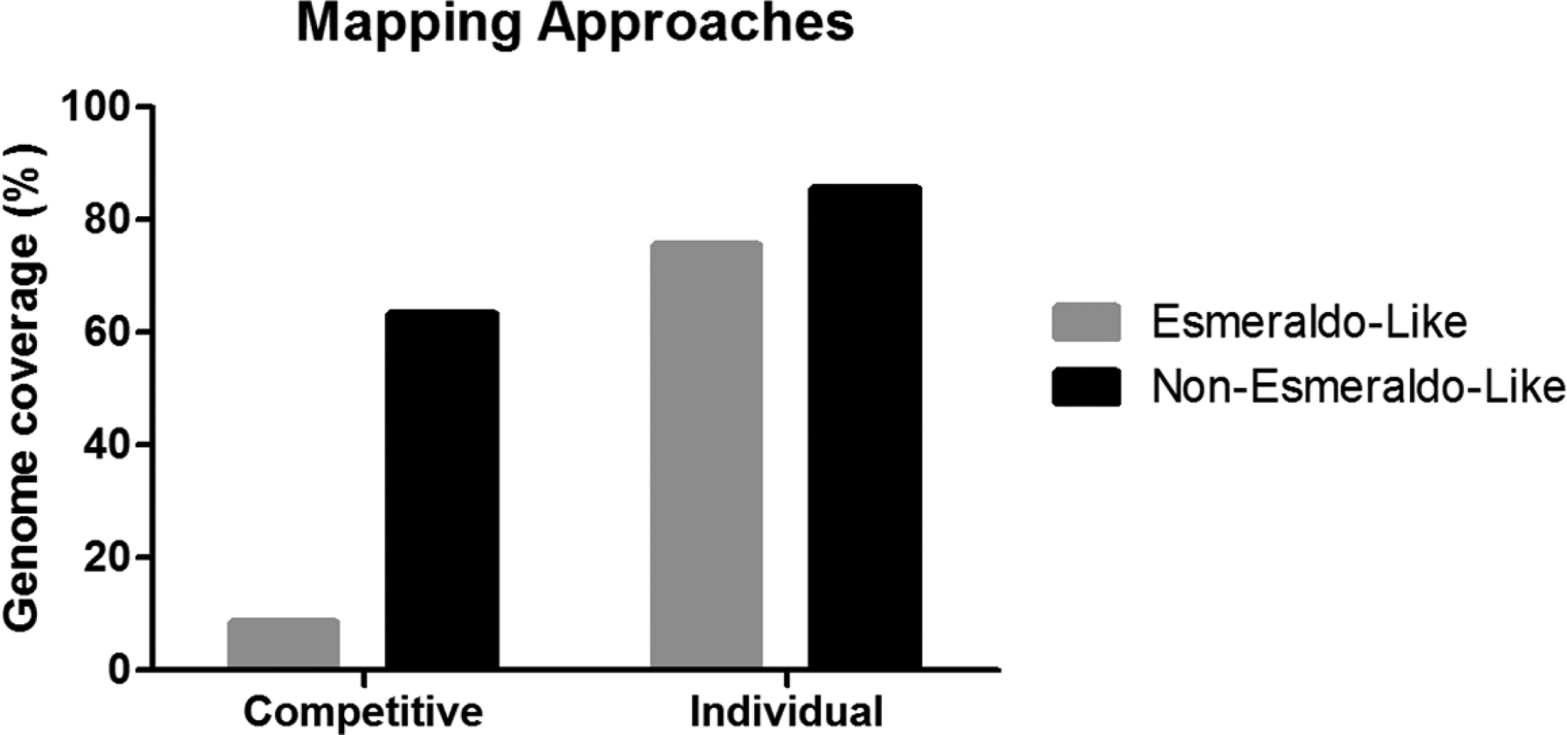
Competitive genome mapping results. Genome coverage results obtained by the competitive and individual mapping approaches of Tc231 reads against reference genome sequences of Esmeraldo-like and non-Esmeraldo-like CL Brener haplotypes.

The 100 bp paired-end sequence libraries were prepared using the TruSeq DNA PCR-free kit with a 350 bp insert size and sequenced using the NGS Illumina HiSeq 2000 platform. However, for repetitive genomes such as *T. cruzi*, disambiguation of repeats is essential. To facilitate repeat resolution, paired-end reads were trimmed to contain a minimum quality of 32 using Trimommatic [31]. Trimmed reads were used in a combined-approach assembly pipeline (Fig. 1). In the first approach, a reference-guided assembly was performed using the program BWA mem 0.7.12 [32] and SAMtools v1.4 [33] with the main purpose of mapping some of the highly repetitive regions present in the CL Brener reference assembly, which are assumed to be already resolved via the use of Sanger sequencing. In this genome-guided assembly, after comparison with different available reference genomes, the *T. cruzi* CL Brener Non-Esmeraldo-like haplotype, found to be more closely related to the Tc231 than the CL Brener Esmeraldo-like haplotype, was used as a reference. This conclusion was determined with two different mapping approaches: competitive mapping and single mapping. In the former, Tc231 reads were aligned to both CL Brener reference sequence haplotypes simultaneously, creating competition between them, where only the best matches were recovered, resulting in a genome coverage of 63.20 and 8.51 % for Non-Esmeraldo and Esmeraldo-like, respectively. In the individual mapping, the Tc231 read collections were individually mapped to each CL Brener reference haplotype, resulting in 85.56 and 75.60 % genome coverage, respectively (Fig. 2). As a second approach, the reads were subjected to *de novo* assembly using Velvet v.1.2.08 [34], with a K-mer size of 51 and the Velvetoptmizer.pl script [35]. The *de novo* approach assembled single-copy regions of the Tc231 genome sequence, but generated many gaps, primarily in repetitive regions. To incorporate reads into some of these gap regions, the Iterative Mapping and Assembly for Gap Elimination software v.2.4.1 (IMAGE) [36] and Iterative Correction of Reference Nucleotide software v.0.95 (ICORN2) [37] were utilized. The two assemblies were then combined using the assembly merger tool ZORRO (http://www.lge.ibi.unicamp.br/zorro/), which was chosen because of its ability to better detect/avoid repetitive regions, its higher genome coverage and its ability to handle large eukaryotic genomes [38]. The combined contigs were scaffolded using SSPACE 2.0 [39], and again subjected to analysis with IMAGE and ICORN. This final assembly was compared to existing *T. cruzi* assemblies available in public databases.

### Annotation transfer

Draft annotation was performed using the Rapid Annotation Transfer Tool, RATT [40]. This method first checks for synteny between the reference genome sequence and the query sequence using Nucmer [41]. The minimum nucleotide sequence identity between syntenic blocks must be at least 40 % to be included. Next, possible errors such as start and stop codons or frameshifts are corrected, and finally annotation files describing the corrections and statistics are generated. Following annotation transfer, the file containing non-transferred annotations was inspected to identify genes that are not syntenic or are not included in our assembly.

Assembly quality was evaluated by checking the percentage of CL Brener ORFs mapped in entirety to the Tc231 assembled sequences, with no partial matches. If a short sequence from the beginning, middle or the end of a feature can be placed within a syntenic region, mapping is attempted. In addition, if the feature maps to different contigs, the model is split and identified in the output file. A comparison between the assembled sequences generated by *de novo* and combined assembly methods was also performed.

### Repeat content analysis

To estimate the repetitive content of the assembled Tc231 genome, all scaffolds were analysed by both Repeat-Scout v1.0.5 [42] and Repeat-Masker v4.0.5 [43] software packages. Both software packages were used with default parameters and provided an estimation of the per cent of the assembly constituted by simple repeats, transposable elements or multi-copy gene families. The Tc231 repetitive content was compared to other reference assemblies.

### Evolutionary analysis of nuclear genes

To select the best data set to be used in our phylogenetic analysis we first determined which reads were mapping uniquely to each *T. cruzi* reference DTU and those that were shared, the latter representing *T. cruzi* conserved regions. All shared reads were aligned against the CL Brener Non-Esmeraldo genome reference, the most similar genome reference available to DTU III, using BWA MEM [29], and the consensus assembly FASTA sequence was generated by the Samtools package [33]. All assembled sequences obtained by this step were compared to the Tc231 assembly to ensure correct assembly. The assembled sequences obtained by reads conserved between all reference DTUs were submitted to a blastx search against the UniprotDB *T. cruzi* proteome (no. 5693) for protein identification. All identified protein sequences were grouped into orthologous clusters and recovered from OrthoMCLDB [44]. All paralogous genes were removed and pseudogenes were filtered to obtain a set of orthologous genes in each set. Finally, for phylogenetic analysis we identified clusters that represent proteins present in all DTU strains analysed (Fig. S1, available in the online version of this article).

### Phylogenetic analysis and divergence time estimation

Using the DTU conserved nuclear gene dataset identified above, a phylogenetic analysis was performed on the amino acid sequences. Sequences from each Tc231 gene group were aligned with other available *T. cruzi* DTU representatives (strains: Sylvio – DTU I; Esmeraldo – DTU II; CL Brener Esmeraldo-like – DTU II-like; CL Brener Non-Esmeraldo-like – DTU III-like), two *T. brucei* strains (strains: TREU927 and gambiense DAL972) and one *T. congolense* strain IL3000, using three different programs: muscle v3.8 [45], mafft v7.0 [46] and clustal w v2.0 [47]. The three resulting alignments were combined into a consensus alignment using M-Coffee v9.03 [48], which was subsequently trimmed using trimAl v1.4 [49], with a consistency score cut-off of 0.1667 and a gap score cut-off of 0.1, to remove poorly aligned regions [50].

Maximum-likelihood (ML) phylogenetic reconstruction used two different input datasets. First, alignments were evaluated using a gene by gene approach and, second, amino acid sequences for all genes were concatenated and analysed as a single sequence. The ability to reconstruct the reference phylogeny was used to rank individual reconstructions by comparing them to the reference species phylogeny. All ML trees were reconstructed using PhyML v3.0 [51]. The best substitution model was set to JTT+G [52] determined by using ProtTest 3 [53] according to the agreement between the Akaike information criterion (AIC) and Bayesian information criterion (BIC).

We performed a Likelihood Ratio Test (LRT) on the final ML trees to evaluate the null hypothesis that each locus of the concatenated dataset evolved under a molecular clock [54]. All loci in which the molecular clock was not rejected, and that had a homologue in *T. brucei*, were concatenated for these analyses. Divergence dates were estimated using beast v.2 [55]. Both the strict and the relaxed lognormal clock models were used to estimate divergence times for the concatenated nuclear loci datasets. All analyses were conducted without any topological constraints using the best-fit substitution model selected by ProtTest3, with four gamma categories, as well as partitioning of codons into three positions. All priors were set to default values, except for the Yule speciation process as a tree prior and the divergence estimate between *T. cruzi* and *T. brucei*, which was set to 100 million years ago (mya) under a normal distribution with 10 mya as the standard deviation [56].

## Results and discussion

### Sequence generation and comparative analyses

The genome sequence of the Tc231 cloned strain was generated using Illumina HiSeq 2000 NGS technology, totalling 55 031 792 paired-end reads. In-house PERL scripts were used to estimate coverage and size of the Tc231 genome sequence. Briefly, this approach calculates the coverage of each nucleotide derived from 1594 *T. cruzi* single-copy genes to estimate the depth of genome coverage [57]. Using this approach, coverage was estimated as 41.7×. To estimate the expected size of the Tc231 genome we divided the total number of nucleotides used in the assembly (2 823 893 082) by the estimated genome depth coverage, resulting in a diploid genome size of 67.7 Mb, comparable to that of the non-hybrid *T. cruzi* strain Sylvio [20].

First, two different approaches to assemble the Tc231 genome were used: *de novo* and reference-based. In the *de novo* assembly, the trimmed paired-end reads were submitted to the VelvetOptimizer.pl script resulting in an assembly with a best K-mer size of 51. After filling gaps using IMAGE and performing corrections using ICORN, the final size of the haploid genome sequence was estimated at 28.4 Mb, represented by 13 482 scaffolds. The haploid genome size obtained was smaller than previously estimated by our calculations (67.7/2=33.9 Mb), suggesting that repetitive regions may not have been resolved by the *de novo* assembly. As a second approach, a reference-based assembly was performed. Tc231 reads were initially mapped to all available *T. cruzi* genome sequences (both CL Brener Esmeraldo-like and non-Esmeraldo-like haplotypes and Sylvio) to determine the best reference genome sequence. Since the CL Brener strain is a hybrid of TcII and TcIII, the CL Brener Non-Emeraldo-like haplotype resulted in the best coverage and highest similarity to the Tc231 genome sequence, and therefore was selected as the reference genome sequence for the analysis.

Using the programs BWA-MEM, SAMtools and BCFtools, a haploid genome of ~32.3 Mb was obtained. This genome size is close to the expected size of 33.9 Mb, and covers about 90 % of the reference sequence. The reference-based assembly, however, generated a highly fragmented genome sequence with approximately 21 464 contigs and scaffolds containing large regions of ‘N's’, mainly due to differences between the Tc231 genome sequence and the reference genome.

To overcome the intrinsic limitations of each strategy, an alternative assembly approach combining reference-based and *de novo* assembly was used. This combined approach resulted in 13 576 contigs with the shortest sequence length at 50 % of the genome (N50) of 5300 bp, 8471 scaffolds (N50=14 202) (Table S1), and an estimated haploid genome size of 35.36 Mb, close to but larger than the calculated genome size of 33.9 Mb. This approach combines the best elements of the *de novo* assembly approach (assembles sequences specific to the Tc231 genome) and of the reference-based assembly approach (repetitive content better resolved), bypassing the inherent limitations in each assembly strategy.

To evaluate the combined assembly strategy, we compared the metrics of the final combined assembly with those obtained from the *de novo* and reference-based assemblies, as well as with the assembly metrics of other *T. cruzi* genomes available in public databases (GenBank and TriTrypDB) [1, 2]. As shown in Table 1, the combined assembly strategy resulted in an improvement of all metrics compared to the separate *de novo* and reference-based genome assembly, and similar metrics when compared to other *T. cruzi* strain assemblies using longer reads (Roche 454). Some of these genome sequences, such as CL Brener, exhibit larger genome sizes when compared to the others, probably due to the hybrid nature of the two strains.

**Table 1.** Comparison of *T. cruzi* assemblies available on public databases, and our new assembly obtained by using different assembly methodologies All data are available at: http://www.ncbi.nlm.nih.gov/genome/genomes/25. The Sylvio X10 genome does not have data for scaffolds, only contigs. na, Not applicable.

*T. cruzi* strain	Size* (MB)	G+C content (%)	No. of scaffolds	Scaffold N50	No. of contigs	Contig N50	Platform
CL Brener	89.94	51.7	29 495	88 624	32 746	14 669	Sanger
JR cl.4	41.48	51.3	15 312	83 591	18 103	7407	Roche 454
Tula cl.2	83.51	51.4	45 711	7772	53 083	2193	Roche 454
Esmeraldo	38.08	50.9	15 803	66 229	20 187	5353	Roche 454
Sylvio X10	38.59	51.1	–	–	27 019	2307	Roche 454+Illumina
231							
*de novo* assembly	28.41	50.0	13 482	3745	16 684	2242	Illumina
Reference based assembly	24.98	50.7	na	na	21 464	3239	
*de novo*+reference-based assembly methods	35.35	48.7	8471	14 202	13 576	5300	

*Genome sizes were obtained by counting nucleotides in the genome excluding ‘N's’.

**Fig. 3.**
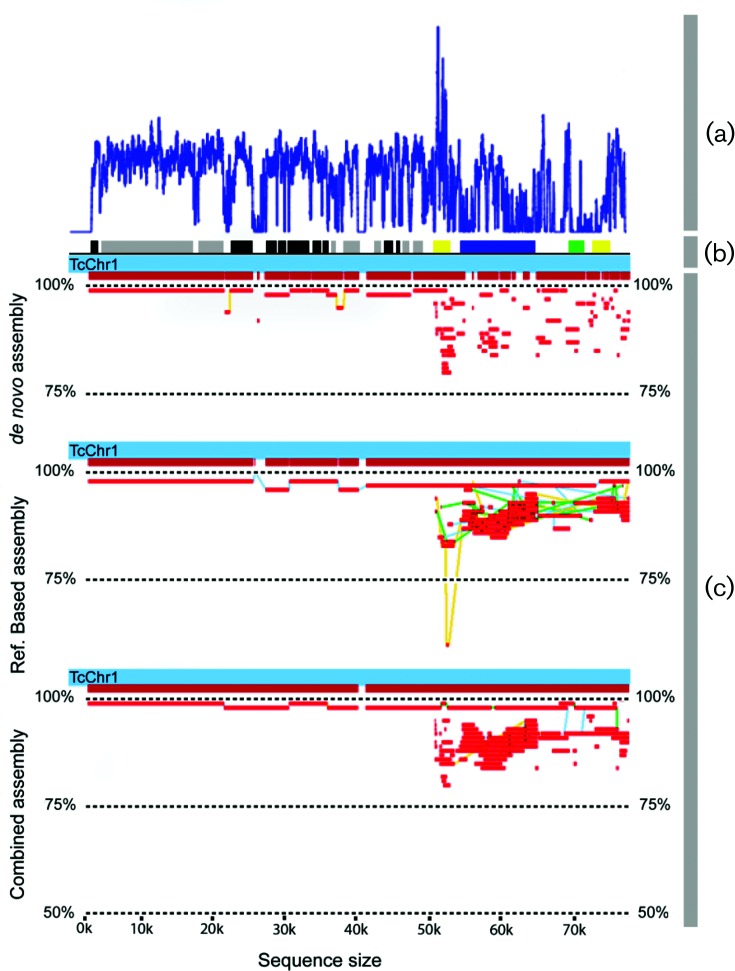
Comparison of results produced by each assembly approach. MapView of the three strategies used in this study: *de novo*, reference-based and combined assembly. (a) Read alignment frequency along chromosome 1 in the reference-based assembly; (b) schematic drawing of chromosome 1, where black=hypothetical proteins, grey=housekeeping genes and yellow, dark blue and green=multigene families; (c) scaffold alignments along the *T. cruzi* Non-Esmeraldo chromosome 1 (TcChr1-P) sequence reference and their degree of identity. The lines connecting the sequences in the alignment represent different gapped alignments.

The *de novo*, reference-based and combined draft assemblies were then mapped to CL Brener chromosome 1 to estimate overall assembly coverage for this chromosome. As shown in Fig. 3, using only the *de novo* approach, unique regions commonly lost in the reference-based approach were covered correctly, whereas the assembly of repetitive regions was more fragmented, as expected, due to the technical difficulties associated with the accurate assembly of repetitive regions. More importantly, the combined assembly strategy resulted in a better coverage of highly repetitive regions when compared to the *de novo* assembly (Fig. 3).

### Annotation transfer

The combined genome assembly consensus sequence was annotated using RATT. This software transfers annotation based on identity and synteny between the accepted reference genome sequence annotation and an unannotated genome. Note that our measure of correctness assumes that the CL Brener reference annotation and assembly are correct. Using this approach, a total of 31 182 out of 34 803 annotated features were transferred to the Tc231 genome sequence. Of the transferred features, 29 916 were transferred in entirety and 1266 were split between different contigs. A total of 10 592 of the 10 833 reference gene models were correctly transferred to the Tc231 DTU III draft genome sequence (Table 2).

**Table 2.** Overview of transferred annotation elements

**Transferred annotation elements**	
34 803	Elements were found on the reference
31 182	Elements were completely transferred
0	Elements were partially transferred
1266	Elements were split
3621*	Elements were not transferred
**Coding sequences**	
10 833	Gene models were transferred from the reference
10 592	Gene models were transferred correctly
0	Gene models were partially transferred
241*	Gene models were not transferred

*Multi-copy gene families, pseudogenes and some hypothetical proteins.

During the annotation process, no partial transfers were found, but 3621 elements and 241 gene models could not be transferred. To evaluate the completeness of the assembled dataset using these three different assembly approaches, we compared the datasets corresponding to the non-transferred content. The combined assembly had a mean of 19 % with no synteny to the query while the *de novo* and reference-based assemblies had respectively 33.41 and 18.22 %. The majority of the non-transferred annotations consisted of repetitive elements, such as transposons, multicopy gene families and tandem repeats, as well as hypothetical proteins. These results show that the combined assembly approach was able to generate sequence scaffolds with almost all housekeeping genes and a large proportion of the current described repeats. A file containing the Tc231 transferred and predicted protein sequences is available in the supplementary files.

### Estimation of repetitive content in the *T. cruzi* DTU III 231 genome sequence

To estimate the repetitive content of the Tc231 draft genome sequence, the consensus sequence from the combined assembly was analysed using Repeat Scout and Repeat masker software. All identified repetitive elements were filtered to remove low-complexity elements. Based on this analysis, we determined that approximately 32 % of the Tc231 genome sequence was composed of genomic repeats. Using the CL Brener genome assembly annotated repeat elements as a reference, only 19 % were not transferred to the Tc231 combined assembly, in contrast to the 32 % not transferred for the *de novo* only approach. The decrease in the number of non-transferrable annotations using the combined assembly approach, from 32 to 19 %, shows that the repetitive content information is partially improved in our final, combined, assembly.

### Gene selection

To select the best genes to use for an evolutionary analysis of the relationship of Tc231 to other *T. cruzi* strains, we evaluated gene similarity to different DTUs by mapping and comparing Tc231 read sequences to other *T. cruzi* reference genome sequences. Comparison of Tc231 reads mapped to Sylvio (TcI), Esmeraldo (TcII), and CL Brener Non-Esmeraldo-like (TcIII-like) genome sequences revealed that our sample was highly similar to the TcIII-like reference genome sequence at greater than 90 % coverage by Tc231 reads.

To select the most conserved regions between our genome assembly and all available DTU genome sequences, we classified Tc231 read sequences as TcIII-specific or shared between DTUs. After this read classification, we assembled the reads into scaffolds using the best reference genome sequence for each group of sorted reads. Using scaffolds that mapped to the regions shared between all *T. cruzi* reference DTUs, we identified protein sequences using blastx and searched for their orthologous groups in OrthoMCLDB.

**Fig. 4.**
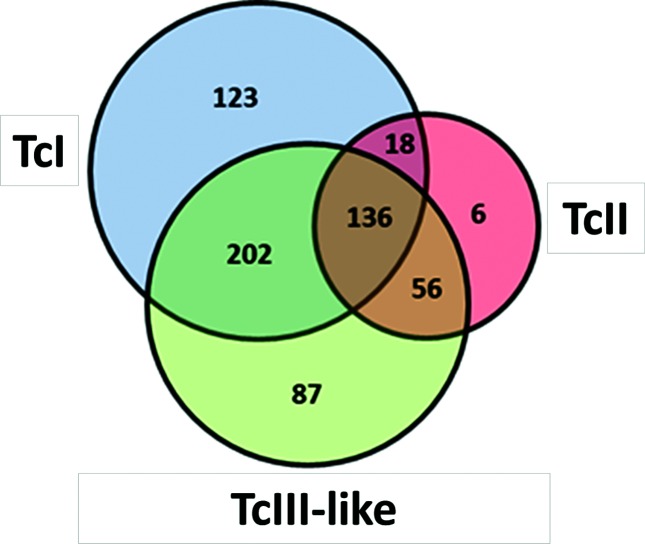
Venn diagram of Tc231 sequences indicating the number of single-copy gene orthologous gene clusters that are specific or shared between TcI, TcII and Non-Esmeraldo (TcIII-like).

A total of 6082 high-quality orthologous gene clusters were identified. After removing pseudogenes and multi-copy gene families that are phylogenetically unreliable due to increased variability, a total of 136 shared orthologous clusters were identified and subsequently used for evolutionary analysis (Fig. 4). From these 136 orthologous clusters, we identified 43 genes that were: (1) present in all genomes as single-exon genes with blast alignments covering >95 % of the *T. cruzi* reference sequences with an E-value <1e-30; and (2) also present in other trypanosomatids including *T. brucei* and *L. major.* These highly conserved gene sequences were used for subsequent phylogenetic analyses. All predicted orthologous groups, including paralogues and pseudo-genes, are available in the supplementary files.

### Phylogenetic analysis and divergence time estimation

The predominantly clonal mode of *T. cruzi* propagation and the lack of evidence for intragenic recombination in the data, due its high degree of conservation among different isolates, permitted the use of nuclear gene sequences for reconstruction of the intraspecific phylogeny. The 43 nuclear loci analysed are randomly distributed in the genome. They are located in 18 of the 41 *T. cruzi* CL Brener-predicted *T. cruzi* chromosomes [58] (Fig. S2). We aligned the sequences for all 43 protein-encoding loci, and submitted them to an ML phylogenetic analysis. For each tree, we performed an approximate LRT to evaluate if they evolved under a molecular clock. Thirty loci had a chi-squared-based parametric branch value close to 1, supporting the hypothesis that observed outcome was likely to occur under a molecular clock model. Subsequently, the 30 loci that were evolving at a similar rate were concatenated and used to reconstruct a reference ML phylogenetic tree [50].

**Fig. 5.**
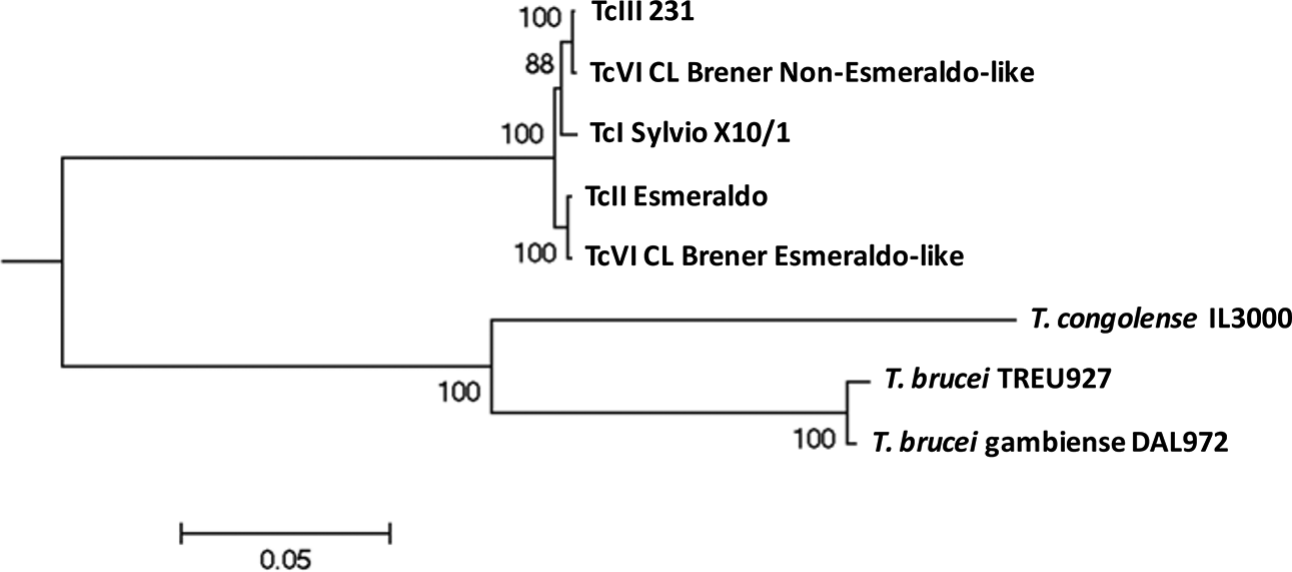
ML nuclear tree obtained from 30 concatenated nuclear gene sequences. For the reconstruction, the best amino acid substitution model was JTT+G obtained by ProtTest, with 1000 bootstrap resampling used for statistical support. The scale bar shows length of branch that represents an amount genetic change of 0.05.

Analyses of the individual loci produced phylogenetic trees, the majority of which had the same topology as our reference tree (Fig. 5). This topology is consistent with a history of divergence in which *T. cruzi* II strains are in a separate clade to the other DTUs analysed [56].

Interestingly, 38 of the 43 loci are characterized by OrthoMCLDB as hypothetical proteins that are restricted to the phylum *Euglenozoa* (Table S2). Thus, these genes may be used to describe this phylum, like a barcode. As is shown in the ML nuclear tree, Fig. 5, TcIII is more closely related to TcI than to TcII. The same observation can be made looking at the Venn diagram (Figs 4 and 5).

**Fig. 6.**
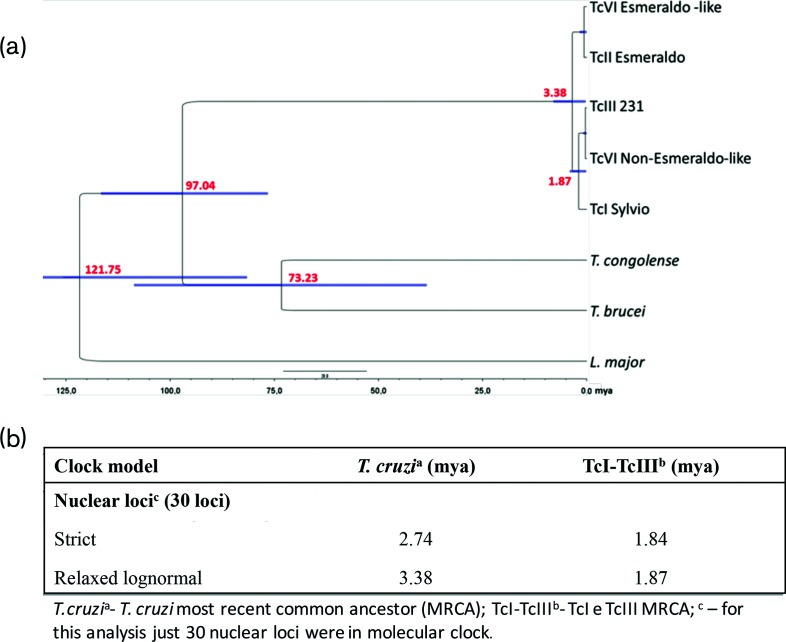
Average divergence times based on Bayesian analysis for the major *T. cruzi* lineages. (a) Divergence times estimated from 30 nuclear loci with the relaxed lognormal clock model. (b) Data for alternative models. Scale bar in million years ago (mya).

To estimate times of divergence, the 30 loci that passed the LTR molecular clock test were used for Bayesian divergence time analyses. The dates estimated with the concatenated data from 30 nuclear loci point towards a Pleistocene/Pliocene origin of *T. cruzi* [time to most recent common ancestor (tMRCA)=2.74 mya (strict); tMRCA=3.38 mya (relaxed)] (Fig. 6). These dates are similar to previously estimated divergence times using a single locus (Machado: tMRCA=3.91 mya and Flores-López: tMRCA=2.18 mya) [56, 59]. We also obtained very similar divergence time estimates from the concatenated data set of all nuclear loci that had a homologue in *T. brucei*, including genes that rejected the molecular clock hypothesis (tMRCA=3.38 mya).

Our results show that TcII strains have a distinct evolutionary history from the other DTUs, that they diverged from the most recent common ancestor of *T. cruzi* (TcMRCA) ~3 mya, and that samples from TcIII and TcI share more than one TcMRCA, and consequently share a more similar evolutionary history. Results also confirm that the hybrid DTUs originated from hybridization events between TcII and TcIII, agreeing with previously proposed models [18, 25, 27].

## Conclusion

The combined assembly pipeline described here benefits from the ability of the reference-based assembly approach to resolve highly repetitive sequences represented in the reference genome, and the *de novo* capacity to assemble genome-specific regions, improving the quality of the genome assembly using only short Illumina reads using an affordable and fast methodology. The only requirement for use of this combined assembly strategy is the availability of an acceptable quality genome assembly of a closely related species for reference-based mapping.

This pipeline was tested with other trypanosomatids including *Leishmania peruviana* using *Leishmania braziliensis* as the reference. These are distinct, yet similar species with approximately 5 % sequence divergence [60]. The results of the *L. peruviana* assembly using only Illumina reads are comparable to our conclusions [61], demonstrating that the use of a close, but not identical, reference genome sequence with this approach is a good choice for genome assembly. Given the myriad of other genomes currently being sequenced using NGS platforms, the assembly strategy proposed in this study represents an attractive option for genome assembly from related species, especially those with highly repetitive regions.

Using the genome data, we have also reconstructed the evolutionary history of the major lineages of the parasite *T. cruzi* using nucleotide sequences from 30 to 43 unlinked nuclear loci. Our results show that the DTU TcIII is not a hybrid of the TcI and TcII DTUs as proposed by others. Rather, *T. cruzi* diversification into the current extant lineages was recent. We also confirm that TcIII is more closely related to TcI than to TcII, refuting the original classification of *T. cruzi* into two major groups, *T. cruzi* I (TcI) and *T. cruzi* II (TcII–VI), reflecting a different evolutionary history for this *T. cruzi* lineage.

Conclusions drawn from *T. cruzi* studies that report results of analyses from one or a few strains do not encompass all existing genetic variability [18, 25, 26]. Thus, future multi-locus phylogenetic studies with additional DTUs and additional samples should be conducted to better reflect the differences among these DTUs and better resolve the evolutionary history of *T. cruzi*.

## Data bibliography

1. Baptista RP. NCBI BioProject PRJEB9129 (2015).

## Supplementary Data

159 Supplementary File 1

## Supplementary Data

869 Supplementary File 2

## Supplementary Data

25551 Supplementary File 3

## Supplementary Data

5074 Supplementary File 4
