# Supplementary material for: Assembly of highly repetitive genomes using short reads: the genome of discrete typing unit III Trypanosoma cruzi strain 231
Source: Microb Genom. 2018 Feb 14;4(4):e000156. doi: 10.1099/mgen.0.000156 (PMC5989580; doi:10.1099/mgen.0.000156)
Supplement: Supplementary File 1 [file mgen-4-156-s001.pdf]

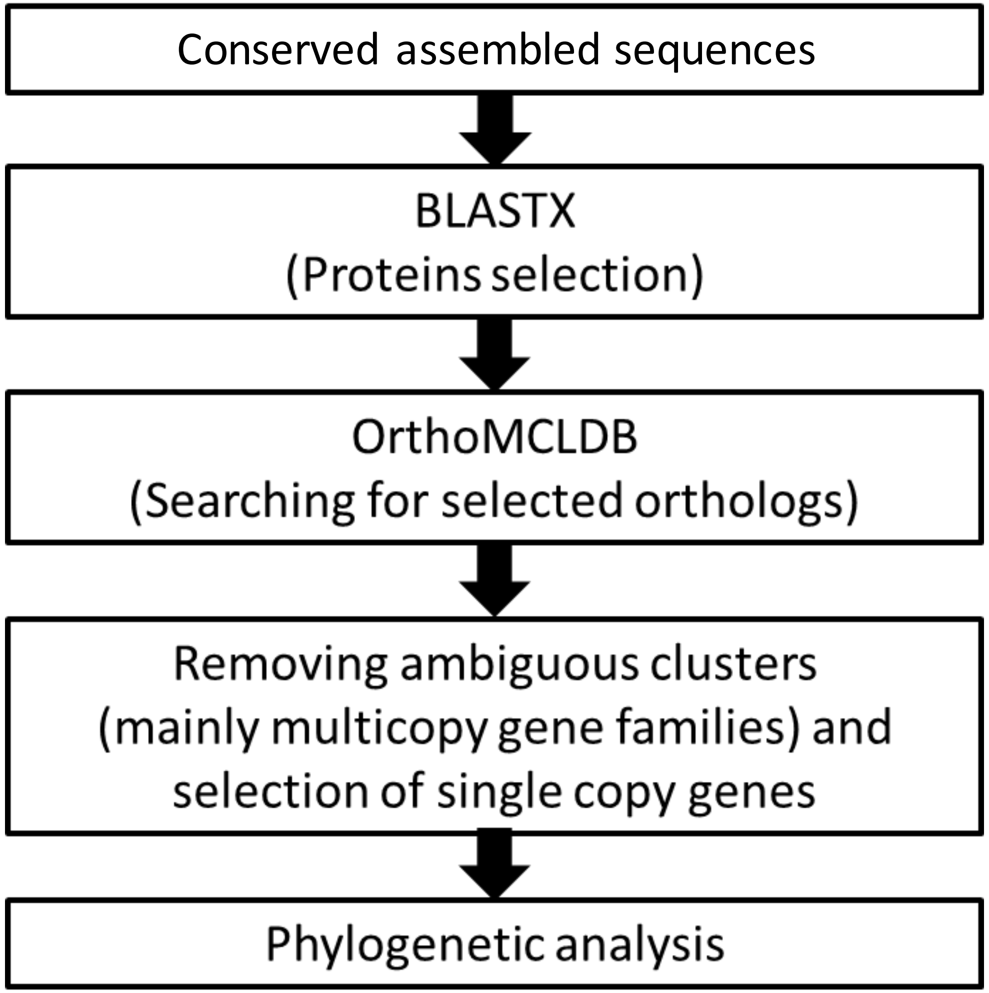

Supplementary Figure 1. Workflow illustrating how we performed the gene selection for the evolutionary analysis.

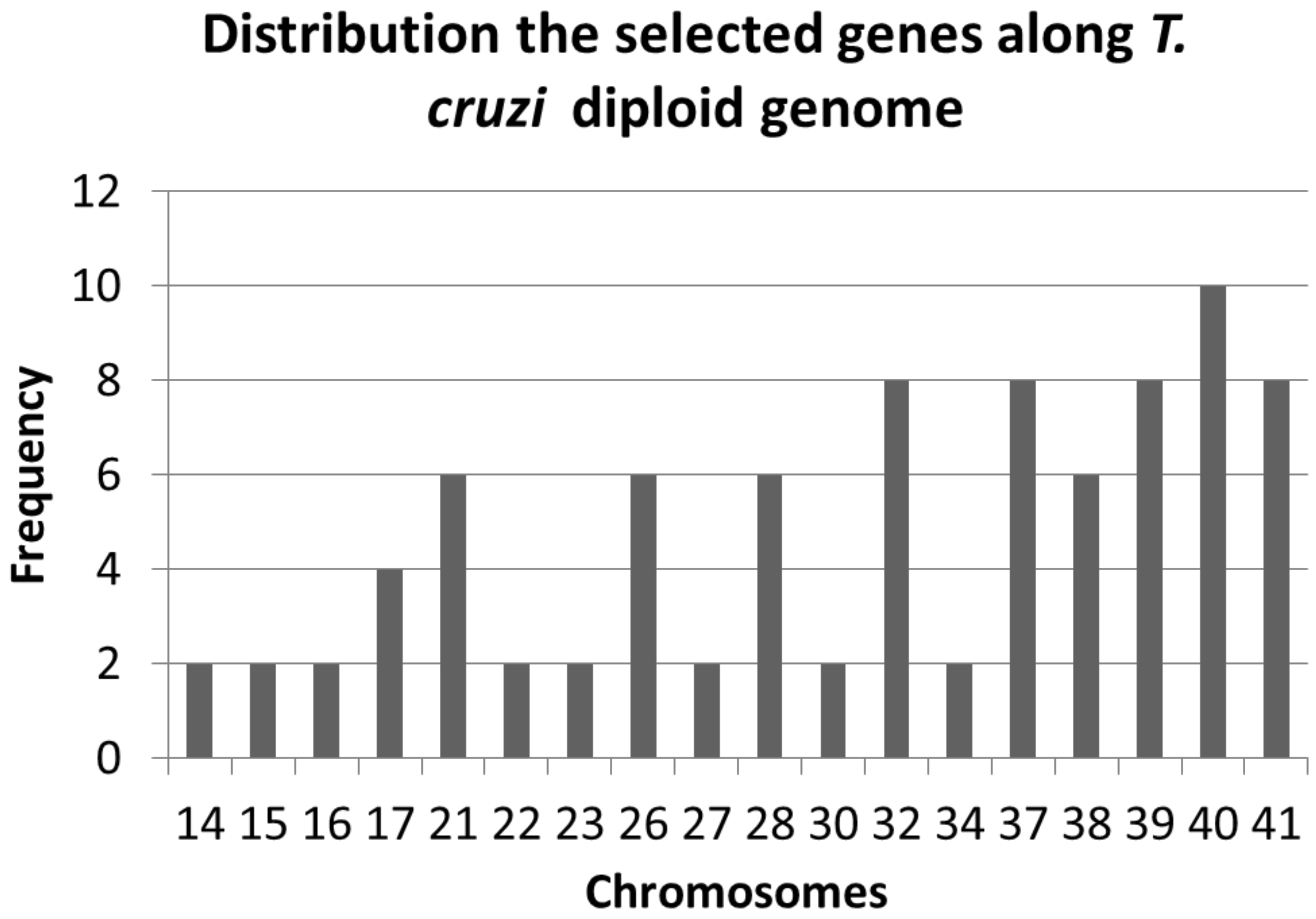

Supplementary Figure 2. The distribution of the identified 43 nuclear loci along the TcIII 231 diploid genome. They are located in 18 of the 41 predicted *T. cruzi* chromosomes.

Supplementary Table 1 – N statistics of *T. cruzi* strain 231 assemblies

	N50	N90
**Final assembly**		
**contigs**	5,300	963
**scaffolds**	14,202	1,354
**velvet assembly**		
**contigs**	13,482	658

Supplementary Table 2 – List of the 38 Orthologous groups characterized by OrthoMCLDB as *T. cruzi* hypothetical proteins

Ortholog Group ID	# of Sequences in the group	Connectivity (%)
OG5_142230	14	86.8
OG5_142346	13	85.9
OG5_143853	13	92.3
OG5_143918	13	87.2
OG5_144295	12	87.9
OG5_144869	12	80.3
OG5_145736	12	100
OG5_146021	12	86.4
OG5_146035	12	100
OG5_146079	12	84.8
OG5_146486	11	100
OG5_146646	11	96.4
OG5_147543	11	100
OG5_148085	11	100
OG5_148167	11	100
OG5_148255	11	87.3
OG5_148263	11	100
OG5_148303	11	100
OG5_148309	11	100
OG5_148314	11	100
OG5_148321	11	100
OG5_148474	11	100
OG5_148658	11	100
OG5_148684	11	100
OG5_148705	11	100
OG5_148709	11	100
OG5_148792	11	100
OG5_148810	11	85.5
OG5_148871	11	100
OG5_148902	11	100
OG5_148953	11	100
OG5_148969	11	83.6
OG5_148970	11	87.3

OG5_148982	11	90.9
OG5_149037	11	100
OG5_149105	11	100
OG5_149107	11	100
OG5_151361	10	100
