# Supplementary material for: Assembly of highly repetitive genomes using short reads: the genome of discrete typing unit III Trypanosoma cruzi strain 231
Source: Microb Genom. 2018 Feb 14;4(4):e000156. doi: 10.1099/mgen.0.000156 (PMC5989580; doi:10.1099/mgen.0.000156)
Supplement: Supplementary File 2 [file mgen-4-156-s002.pdf]

OG0000000: TCRU_10573 TCRU_10626 TCRU_2086 TCRU_213 TCRU_2614 TCRU_308 TCRU_3266 TCRU_3322 TCRU_3527 TCRU_374 TCRU_3953 TCRU_4430 TCRU_4496 TCRU_4524 TCRU_4732 TCRU_5257 TCRU_5295 TCRU_5302 TCRU_5328 TCRU_6417 TCRU_6435 TCRU_6513 TCRU_6552 TCRU_6606 TCRU_6905 TCRU_6941 TCRU_7312 TCRU_7320 TCRU_7332 TCRU_7412 TCRU_7692 TCRU_7711 TCRU_7810 TCRU_7878 TCRU_792 TCRU_7920 TCRU_8063 TCRU_8073 TCRU_8235 TCRU_8275 TCRU_8298 TCRU_8299 TCRU_8390 TCRU_8501 TCRU_8557 TCRU_9200 TCRU_9488 TcCLB-EL.503659.20_pseudogenic_transcript-p1 TcCLB-EL.503717.31_mRNA-p1 TcCLB-EL.503717.5_pseudogenic_transcript-p1 TcCLB-EL.503761.30_mRNA-p1 TcCLB-EL.503783.17_mRNA-p1 TcCLB-EL.503977.40_mRNA-p1 TcCLB-EL.504039.120_mRNA-p1 TcCLB-EL.504039.99_pseudogenic_transcript-p1 TcCLB-EL.504167.20_mRNA-p1 TcCLB-EL.504239.171_mRNA-p1 TcCLB-EL.504239.20_mRNA-p1 TcCLB-EL.504239.340_mRNA-p1 TcCLB-EL.505025.100_pseudogenic_transcript-p1 TcCLB-EL.505025.137_mRNA-p1 TcCLB-EL.506267.60_mRNA-p1 TcCLB-EL.506269.90_mRNA-p1 TcCLB-EL.506285.120_mRNA-p1 TcCLB-EL.506285.90_mRNA-p1 TcCLB-EL.506409.200_mRNA-p1 TcCLB-EL.506409.50_mRNA-p1 TcCLB-EL.506499.110_pseudogenic_transcript-p1 TcCLB-EL.506499.149_mRNA-p1 TcCLB-EL.506501.170_mRNA-p1 TcCLB-EL.506501.190_mRNA-p1 TcCLB-EL.506501.230_mRNA-p1 TcCLB-EL.506501.280_mRNA-p1 TcCLB-EL.506501.390_mRNA-p1 TcCLB-EL.506501.70_mRNA-p1 TcCLB-EL.506545.5_mRNA-p1 TcCLB-EL.506597.30_mRNA-p1 TcCLB-EL.506599.130_pseudogenic_transcript-p1 TcCLB-EL.506599.189_mRNA-p1 TcCLB-EL.506599.224_mRNA-p1 TcCLB-EL.506599.270_mRNA-p1 TcCLB-EL.506599.350_mRNA-p1 TcCLB-EL.506599.430_mRNA-p1 TcCLB-EL.506599.70_mRNA-p1 TcCLB-EL.506609.80_mRNA-p1 TcCLB-EL.506759.160_mRNA-p1 TcCLB-EL.506759.210_mRNA-p1 TcCLB-EL.506759.70_mRNA-p1 TcCLB-EL.506761.19_mRNA-p1 TcCLB-EL.506761.47_mRNA-p1 TcCLB-EL.506763.190_mRNA-p1 TcCLB-EL.506765.80_mRNA-p1 TcCLB-EL.506767.130_mRNA-p1 TcCLB-EL.506767.220_mRNA-p1 TcCLB-EL.506767.360_mRNA-p1 TcCLB-EL.506767.50_mRNA-p1 TcCLB-EL.506769.70_mRNA-p1 TcCLB-EL.506837.10_mRNA-p1 TcCLB-EL.506893.10_mRNA-p1 TcCLB-EL.506951.50_pseudogenic_transcript-p1 TcCLB-EL.506967.110_mRNA-p1 TcCLB-EL.506967.40_mRNA-p1 TcCLB-EL.506967.65_pseudogenic_transcript-p1 TcCLB-EL.506969.21_mRNA-p1 TcCLB-EL.506971.70_mRNA-p1 TcCLB-EL.506973.14_pseudogenic_transcript-p1 TcCLB-EL.507041.71_mRNA-p1 TcCLB-EL.507043.10_mRNA-p1 TcCLB-EL.507145.79_mRNA-p1 TcCLB-EL.507145.88_mRNA-p1 TcCLB-EL.507237.160_mRNA-p1 TcCLB-EL.507237.210_mRNA-p1 TcCLB-EL.507237.260_mRNA-p1 TcCLB-EL.507237.280_mRNA-p1 TcCLB-EL.507237.350_mRNA-p1 TcCLB-EL.507237.89_mRNA-p1 TcCLB-EL.507429.30_mRNA-p1 TcCLB-EL.507429.49_mRNA-p1 TcCLB-EL.507429.70_mRNA-p1 TcCLB-EL.507547.9_mRNA-p1 TcCLB-EL.507699.10_mRNA-p1 TcCLB-EL.507699.120_mRNA-p1 TcCLB-EL.507699.149_mRNA-p1 TcCLB-EL.507699.200_mRNA-p1 TcCLB-EL.507699.64_mRNA-p1 TcCLB-EL.507699.90_mRNA-p1 TcCLB-EL.507701.10_mRNA-p1 TcCLB-EL.507747.80_mRNA-p1 TcCLB-EL.507847.10_pseudogenic_transcript-p1 TcCLB-EL.507957.229_mRNA-p1 TcCLB-EL.507957.270_mRNA-p1 TcCLB-EL.507957.50_mRNA-p1 TcCLB-EL.507959.200_mRNA-p1 TcCLB-EL.507959.250_mRNA-p1 TcCLB-EL.507959.270_mRNA-p1 TcCLB-EL.507959.310_mRNA-p1 TcCLB-EL.508021.20_pseudogenic_transcript-p1 TcCLB-EL.508109.14_mRNA-p1

TcCLB-EL.508109.20_mRNA-p1 TcCLB-EL.508121.120_mRNA-p1 TcCLB-EL.508121.155_pseudogenic_transcript-p1 TcCLB-EL.508121.80_mRNA-p1 TcCLB-EL.508125.22_pseudogenic_transcript-p1 TcCLB-EL.508125.30_mRNA-p1 TcCLB-EL.508147.140_mRNA-p1 TcCLB-EL.508147.79_mRNA-p1 TcCLB-EL.508159.60_mRNA-p1 TcCLB-EL.508163.20_mRNA-p1 TcCLB-EL.508163.310_mRNA-p1 TcCLB-EL.508163.40_pseudogenic_transcript-p1 TcCLB-EL.508165.100_mRNA-p1 TcCLB-EL.508165.115_pseudogenic_transcript-p1 TcCLB-EL.508165.270_mRNA-p1 TcCLB-EL.508165.290_mRNA-p1 TcCLB-EL.508165.34_pseudogenic_transcript-p1 TcCLB-EL.508165.425_pseudogenic_transcript-p1 TcCLB-EL.508165.60_mRNA-p1 TcCLB-EL.508227.30_mRNA-p1 TcCLB-EL.508389.114_mRNA-p1 TcCLB-EL.508389.138_mRNA-p1 TcCLB-EL.509097.60_mRNA-p1 TcCLB-EL.509631.30_pseudogenic_transcript-p1 TcCLB-EL.509631.90_mRNA-p1 TcCLB-EL.509699.150_mRNA-p1 TcCLB-EL.509699.200_mRNA-p1 TcCLB-EL.509699.30_pseudogenic_transcript-p1 TcCLB-EL.509847.70_mRNA-p1 TcCLB-EL.509925.20_mRNA-p1 TcCLB-EL.509993.60_mRNA-p1 TcCLB-EL.510013.240_mRNA-p1 TcCLB-EL.510013.300_mRNA-p1 TcCLB-EL.510015.20_mRNA-p1 TcCLB-EL.510017.40_mRNA-p1 TcCLB-EL.510019.10_mRNA-p1 TcCLB-EL.510021.100_mRNA-p1 TcCLB-EL.510021.10_mRNA-p1 TcCLB-EL.510021.200_mRNA-p1 TcCLB-EL.510021.30_mRNA-p1 TcCLB-EL.510025.210_mRNA-p1 TcCLB-EL.510191.20_mRNA-p1 TcCLB-EL.510191.80_mRNA-p1 TcCLB-EL.510275.219_mRNA-p1 TcCLB-EL.510275.283_pseudogenic_transcript-p1 TcCLB-EL.510275.330_mRNA-p1 TcCLB-EL.510275.360_pseudogenic_transcript-p1 TcCLB-EL.510275.50_mRNA-p1 TcCLB-EL.510279.130_mRNA-p1 TcCLB-EL.510279.220_pseudogenic_transcript-p1 TcCLB-EL.510279.340_mRNA-p1 TcCLB-EL.510371.110_mRNA-p1 TcCLB-EL.510371.130_mRNA-p1 TcCLB-EL.510373.70_mRNA-p1 TcCLB-EL.510377.150_mRNA-p1 TcCLB-EL.510377.190_mRNA-p1 TcCLB-EL.510377.250_pseudogenic_transcript-p1 TcCLB-EL.510377.370_mRNA-p1 TcCLB-EL.510377.400_mRNA-p1 TcCLB-EL.510377.410_mRNA-p1 TcCLB-EL.510377.70_mRNA-p1 TcCLB-EL.510555.30_mRNA-p1 TcCLB-EL.510557.50_mRNA-p1 TcCLB-EL.510557.70_mRNA-p1 TcCLB-EL.510557.80_mRNA-p1 TcCLB-EL.510561.20_mRNA-p1 TcCLB-EL.510561.40_mRNA-p1 TcCLB-EL.510621.70_mRNA-p1 TcCLB-EL.510939.25_mRNA-p1 TcCLB-EL.510939.30_mRNA-p1 TcCLB-EL.510961.40_mRNA-p1 TcCLB-EL.510961.5_mRNA-p1 TcCLB-EL.511107.5_mRNA-p1 TcCLB-EL.511173.241_pseudogenic_transcript-p1 TcCLB-EL.511173.340_pseudogenic_transcript-p1 TcCLB-EL.511173.380_pseudogenic_transcript-p1 TcCLB-EL.511173.483_pseudogenic_transcript-p1 TcCLB-EL.511173.89_pseudogenic_transcript-p1 TcCLB-EL.511553.130_mRNA-p1 TcCLB-EL.511603.200_mRNA-p1 TcCLB-EL.511603.260_mRNA-p1 TcCLB-EL.511603.304_mRNA-p1 TcCLB-EL.511603.330_mRNA-p1 TcCLB-EL.511603.390_mRNA-p1 TcCLB-EL.511603.60_mRNA-p1 TcCLB-EL.511607.100_mRNA-p1 TcCLB-EL.511607.30_mRNA-p1 TcCLB-EL.511611.5_pseudogenic_transcript-p1 TcCLB-EL.511613.189_mRNA-p1 TcCLB-EL.511625.100_mRNA-p1 TcCLB-EL.511625.149_pseudogenic_transcript-p1 TcCLB-EL.511875.70_pseudogenic_transcript-p1 TcCLB-NE.430603.20_mRNA-p1 TcCLB-NE.442801.10_mRNA-p1 TcCLB-NE.503417.30_mRNA-p1 TcCLB-NE.503519.20_mRNA-p1 TcCLB-NE.503519.40_mRNA-p1 TcCLB-NE.

503771.74_pseudogenic_transcript-p1 TcCLB-NE.503787.20_mRNA-p1 TcCLB-NE.503787.50_mRNA-p1 TcCLB-NE.503889.10_mRNA-p1 TcCLB-NE.503973.250_pseudogenic_transcript-p1 TcCLB-NE.503973.290_mRNA-p1 TcCLB-NE.504081.110_pseudogenic_transcript-p1 TcCLB-NE.504081.270_mRNA-p1 TcCLB-NE.504081.330_mRNA-p1 TcCLB-NE.504081.409_pseudogenic_transcript-p1 TcCLB-NE.504081.520_mRNA-p1 TcCLB-NE.504155.170_mRNA-p1 TcCLB-NE.504233.70_mRNA-p1 TcCLB-NE.505297.70_mRNA-p1 TcCLB-NE.506137.40_mRNA-p1 TcCLB-NE.506139.4_mRNA-p1 TcCLB-NE.506139.80_mRNA-p1 TcCLB-NE.506279.200_mRNA-p1 TcCLB-NE.506279.30_mRNA-p1 TcCLB-NE.506279.90_mRNA-p1 TcCLB-NE.506281.117_pseudogenic_transcript-p1 TcCLB-NE.506281.20_mRNA-p1 TcCLB-NE.506281.70_mRNA-p1 TcCLB-NE.506281.90_mRNA-p1 TcCLB-NE.506321.10_mRNA-p1 TcCLB-NE.506321.130_mRNA-p1 TcCLB-NE.506321.40_mRNA-p1 TcCLB-NE.506329.20_mRNA-p1 TcCLB-NE.506329.50_mRNA-p1 TcCLB-NE.506329.70_mRNA-p1 TcCLB-NE.506459.160_pseudogenic_transcript-p1 TcCLB-NE.506459.210_mRNA-p1 TcCLB-NE.506459.219_mRNA-p1 TcCLB-NE.506611.20_mRNA-p1 TcCLB-NE.506613.100_pseudogenic_transcript-p1 TcCLB-NE.506613.24_mRNA-p1 TcCLB-NE.506821.60_mRNA-p1 TcCLB-NE.506917.60_mRNA-p1 TcCLB-NE.506923.21_mRNA-p1 TcCLB-NE.506993.110_mRNA-p1 TcCLB-NE.506993.20_mRNA-p1 TcCLB-NE.506993.40_mRNA-p1 TcCLB-NE.506993.63_mRNA-p1 TcCLB-NE.506995.40_mRNA-p1 TcCLB-NE.506995.80_mRNA-p1 TcCLB-NE.507065.70_mRNA-p1 TcCLB-NE.507071.164_pseudogenic_transcript-p1 TcCLB-NE.507071.250_mRNA-p1 TcCLB-NE.507071.34_mRNA-p1 TcCLB-NE.507071.60_mRNA-p1 TcCLB-NE.507071.89_pseudogenic_transcript-p1 TcCLB-NE.507085.10_mRNA-p1 TcCLB-NE.507085.140_mRNA-p1 TcCLB-NE.507085.170_mRNA-p1 TcCLB-NE.507085.60_mRNA-p1 TcCLB-NE.507633.40_mRNA-p1 TcCLB-NE.507637.100_mRNA-p1 TcCLB-NE.507637.130_mRNA-p1 TcCLB-NE.507637.30_mRNA-p1 TcCLB-NE.507935.10_pseudogenic_transcript-p1 TcCLB-NE.507937.10_mRNA-p1 TcCLB-NE.507937.14_pseudogenic_transcript-p1 TcCLB-NE.507973.10_mRNA-p1 TcCLB-NE.508119.30_mRNA-p1 TcCLB-NE.508119.70_mRNA-p1 TcCLB-NE.508221.338_mRNA-p1 TcCLB-NE.508221.518_mRNA-p1 TcCLB-NE.508221.560_mRNA-p1 TcCLB-NE.508221.585_pseudogenic_transcript-p1 TcCLB-NE.508221.910_mRNA-p1 TcCLB-NE.508233.30_mRNA-p1 TcCLB-NE.508235.30_mRNA-p1 TcCLB-NE.508243.23_mRNA-p1 TcCLB-NE.508243.60_mRNA-p1 TcCLB-NE.508245.60_mRNA-p1 TcCLB-NE.508247.40_mRNA-p1 TcCLB-NE.508251.40_mRNA-p1 TcCLB-NE.508261.29_pseudogenic_transcript-p1 TcCLB-NE.508273.100_mRNA-p1 TcCLB-NE.508273.120_mRNA-p1 TcCLB-NE.508361.30_mRNA-p1 TcCLB-NE.508365.130_mRNA-p1 TcCLB-NE.508365.210_mRNA-p1 TcCLB-NE.508365.240_mRNA-p1 TcCLB-NE.508365.280_mRNA-p1 TcCLB-NE.508365.290_mRNA-p1 TcCLB-NE.508365.30_mRNA-p1 TcCLB-NE.508365.50_mRNA-p1 TcCLB-NE.508433.170_mRNA-p1 TcCLB-NE.508433.20_pseudogenic_transcript-p1 TcCLB-NE.508433.210_pseudogenic_transcript-p1 TcCLB-NE.508441.110_mRNA-p1 TcCLB-NE.508441.94_mRNA-p1 TcCLB-NE.508493.100_mRNA-p1 TcCLB-NE.508493.10_mRNA-p1 TcCLB-NE.508493.130_mRNA-p1 TcCLB-NE.508493.40_mRNA-p1 TcCLB-NE.508495.140_mRNA-p1 TcCLB-NE.508495.170_mRNA-p1 TcCLB-NE.508495.200_mRNA-p1 TcCLB-NE.508495.50_mRNA-p1 TcCLB-NE.508495.70_mRNA-p1 TcCLB-NE.508499.10_mRNA-p1 TcCLB-NE.508501.110_mRNA-p1 TcCLB-NE.508501.140_mRNA-p1 TcCLB-NE.508501.40_mRNA-p1 TcCLB-NE.

508501.70_mRNA-p1 TcCLB-NE.508871.140_mRNA-p1 TcCLB-NE.
508873.116_pseudogenic_transcript-p1 TcCLB-NE.508873.150_mRNA-p1
TcCLB-NE.508873.240_mRNA-p1 TcCLB-NE.
508873.439_pseudogenic_transcript-p1 TcCLB-NE.
508873.454_pseudogenic_transcript-p1 TcCLB-NE.
508873.514_pseudogenic_transcript-p1 TcCLB-NE.508883.30_mRNA-p1
TcCLB-NE.508885.20_mRNA-p1 TcCLB-NE.508887.20_mRNA-p1 TcCLB-NE.
509493.110_mRNA-p1 TcCLB-NE.509493.20_mRNA-p1 TcCLB-NE.
509493.39_mRNA-p1 TcCLB-NE.509493.80_mRNA-p1 TcCLB-NE.
509495.49_mRNA-p1 TcCLB-NE.509525.120_mRNA-p1 TcCLB-NE.
509525.150_mRNA-p1 TcCLB-NE.509525.180_mRNA-p1 TcCLB-NE.
509525.220_mRNA-p1 TcCLB-NE.509525.234_mRNA-p1 TcCLB-NE.
509525.250_mRNA-p1 TcCLB-NE.509525.270_mRNA-p1 TcCLB-NE.
509525.320_mRNA-p1 TcCLB-NE.509525.350_mRNA-p1 TcCLB-NE.
509525.380_mRNA-p1 TcCLB-NE.509525.400_mRNA-p1 TcCLB-NE.
509525.420_mRNA-p1 TcCLB-NE.509525.60_mRNA-p1 TcCLB-NE.
509525.90_mRNA-p1 TcCLB-NE.509527.10_mRNA-p1 TcCLB-NE.
509657.40_mRNA-p1 TcCLB-NE.509753.150_mRNA-p1 TcCLB-NE.
509753.240_mRNA-p1 TcCLB-NE.509753.300_mRNA-p1 TcCLB-NE.
509869.10_mRNA-p1 TcCLB-NE.510047.109_mRNA-p1 TcCLB-NE.
510047.129_mRNA-p1 TcCLB-NE.510197.47_pseudogenic_transcript-p1
TcCLB-NE.510201.15_pseudogenic_transcript-p1 TcCLB-NE.
510239.60_mRNA-p1 TcCLB-NE.510359.580_mRNA-p1 TcCLB-NE.
510359.604_pseudogenic_transcript-p1 TcCLB-NE.510359.660_mRNA-p1
TcCLB-NE.510361.100_mRNA-p1 TcCLB-NE.510361.200_mRNA-p1 TcCLB-NE.
510363.170_mRNA-p1 TcCLB-NE.510363.270_mRNA-p1 TcCLB-NE.
510483.130_mRNA-p1 TcCLB-NE.510483.224_pseudogenic_transcript-p1
TcCLB-NE.510483.234_pseudogenic_transcript-p1 TcCLB-NE.
510483.300_mRNA-p1 TcCLB-NE.510489.44_pseudogenic_transcript-p1
TcCLB-NE.510627.119_pseudogenic_transcript-p1 TcCLB-NE.
510627.169_pseudogenic_transcript-p1 TcCLB-NE.
510629.329_pseudogenic_transcript-p1 TcCLB-NE.
510693.176_pseudogenic_transcript-p1 TcCLB-NE.
510693.274_pseudogenic_transcript-p1 TcCLB-NE.
510709.50_pseudogenic_transcript-p1 TcCLB-NE.510715.120_mRNA-p1
TcCLB-NE.510789.10_mRNA-p1 TcCLB-NE.510791.20_mRNA-p1 TcCLB-NE.
510791.50_mRNA-p1 TcCLB-NE.510979.30_pseudogenic_transcript-p1
TcCLB-NE.510979.60_mRNA-p1 TcCLB-NE.511255.210_mRNA-p1 TcCLB-NE.
511255.604_mRNA-p1 TcCLB-NE.511255.640_mRNA-p1 TcCLB-NE.
511259.189_mRNA-p1 TcCLB-NE.511259.210_mRNA-p1 TcCLB-NE.
511259.80_mRNA-p1 TcCLB-NE.511259.9_mRNA-p1 TcCLB-NE.511403.30_mRNA-
p1 TcCLB-NE.511405.10_mRNA-p1 TcCLB-NE.511645.21_mRNA-p1 TcCLB-NE.
511839.10_mRNA-p1 TcCLB-NE.511843.5_mRNA-p1 TcSYL_0017510.t1-p1
TcSYL_0018290.t1-p1 TcSYL_0065110.t1-p1 TcSYL_0065200.t1-p1
TcSYL_0065640.t1-p1 TcSYL_0066840.t1-p1 TcSYL_0068290.t1-p1
TcSYL_0068400.t1-p1 TcSYL_0069600.t1-p1 TcSYL_0074120.t1-p1
TcSYL_0092880.t1-p1 TcSYL_0105970.t1-p1 TcSYL_0108580.t1-p1
TcSYL_0109830.t1-p1 TcSYL_0110050.t1-p1 TcSYL_0110210.t1-p1
TcSYL_0125350.t1-p1 TcSYL_0127250.t1-p1 TcSYL_0128170.t1-p1
TcSYL_0133420.t1-p1 TcSYL_0133710.t1-p1 TcSYL_0134060.t1-p1
TcSYL_0135780.t1-p1 TcSYL_0149020.t1-p1 TcSYL_0152280.t1-p1
TcSYL_0154680.t1-p1 TcSYL_0155010.t1-p1 TcSYL_0160580.t1-p1
TcSYL_0191170.t1-p1
OG0000001: TCRU_10025 TCRU_1521 TCRU_2205 TCRU_261 TCRU_2745

TCRU_2746 TCRU_3172 TCRU_3741 TCRU_3828 TCRU_3989 TCRU_4091
TCRU_4180 TCRU_4181 TCRU_4508 TCRU_4536 TCRU_4571 TCRU_4664
TCRU_4764 TCRU_4803 TCRU_4945 TCRU_5081 TCRU_5181 TCRU_5252
TCRU_5353 TCRU_5392 TCRU_7488 TCRU_8170 TCRU_84 TCRU_8480 TCRU_9071
TCRU_9117 TCRU_98 TcCLB-EL.425435.10_mRNA-p1 TcCLB-EL.455171.9_mRNA-
p1 TcCLB-EL.503717.10_mRNA-p1 TcCLB-EL.503717.120_mRNA-p1 TcCLB-EL.
504101.50_mRNA-p1 TcCLB-EL.504239.260_mRNA-p1 TcCLB-EL.
504239.434_mRNA-p1 TcCLB-EL.506409.170_mRNA-p1 TcCLB-EL.
506499.170_mRNA-p1 TcCLB-EL.506597.40_mRNA-p1 TcCLB-EL.
506603.40_mRNA-p1 TcCLB-EL.506609.30_mRNA-p1 TcCLB-EL.
506667.160_mRNA-p1 TcCLB-EL.506667.50_pseudogenic_transcript-p1
TcCLB-EL.506667.90_mRNA-p1 TcCLB-EL.506737.90_mRNA-p1 TcCLB-EL.
506757.120_mRNA-p1 TcCLB-EL.506757.60_mRNA-p1 TcCLB-EL.
506759.90_pseudogenic_transcript-p1 TcCLB-EL.
506767.310_pseudogenic_transcript-p1 TcCLB-EL.506885.480_mRNA-p1
TcCLB-EL.506951.80_mRNA-p1 TcCLB-EL.506965.170_mRNA-p1 TcCLB-EL.
506967.10_mRNA-p1 TcCLB-EL.506967.60_mRNA-p1 TcCLB-EL.
506973.10_mRNA-p1 TcCLB-EL.506973.120_mRNA-p1 TcCLB-EL.
507091.60_mRNA-p1 TcCLB-EL.507145.60_mRNA-p1 TcCLB-EL.
507163.70_mRNA-p1 TcCLB-EL.507237.10_mRNA-p1 TcCLB-EL.
507283.10_mRNA-p1 TcCLB-EL.507747.180_mRNA-p1 TcCLB-EL.
507953.100_mRNA-p1 TcCLB-EL.507953.140_mRNA-p1 TcCLB-EL.
507997.30_mRNA-p1 TcCLB-EL.508047.60_mRNA-p1 TcCLB-EL.508101.9_mRNA-
p1 TcCLB-EL.508103.30_mRNA-p1 TcCLB-EL.508107.30_mRNA-p1 TcCLB-EL.
508109.60_mRNA-p1 TcCLB-EL.508121.150_mRNA-p1 TcCLB-EL.
508125.149_mRNA-p1 TcCLB-EL.508125.80_pseudogenic_transcript-p1
TcCLB-EL.508143.90_pseudogenic_transcript-p1 TcCLB-EL.
508161.20_mRNA-p1 TcCLB-EL.508161.50_mRNA-p1 TcCLB-EL.
508163.250_mRNA-p1 TcCLB-EL.508165.14_mRNA-p1 TcCLB-EL.
508165.200_pseudogenic_transcript-p1 TcCLB-EL.
508165.40_pseudogenic_transcript-p1 TcCLB-EL.508229.40_mRNA-p1
TcCLB-EL.508375.20_mRNA-p1 TcCLB-EL.508977.10_mRNA-p1 TcCLB-EL.
508977.120_mRNA-p1 TcCLB-EL.508977.40_mRNA-p1 TcCLB-EL.
508977.80_mRNA-p1 TcCLB-EL.509097.20_mRNA-p1 TcCLB-EL.
509699.40_mRNA-p1 TcCLB-EL.509979.320_mRNA-p1 TcCLB-EL.
510021.120_mRNA-p1 TcCLB-EL.510021.180_mRNA-p1 TcCLB-EL.
510021.220_mRNA-p1 TcCLB-EL.510025.50_mRNA-p1 TcCLB-EL.
510163.60_mRNA-p1 TcCLB-EL.510191.40_pseudogenic_transcript-p1
TcCLB-EL.510205.40_mRNA-p1 TcCLB-EL.510207.30_mRNA-p1 TcCLB-EL.
510269.60_pseudogenic_transcript-p1 TcCLB-EL.510275.350_mRNA-p1
TcCLB-EL.510279.10_pseudogenic_transcript-p1 TcCLB-EL.
510279.110_mRNA-p1 TcCLB-EL.510279.160_pseudogenic_transcript-p1
TcCLB-EL.510279.230_mRNA-p1 TcCLB-EL.510279.320_mRNA-p1 TcCLB-EL.
510371.90_mRNA-p1 TcCLB-EL.510377.10_mRNA-p1 TcCLB-EL.
510377.330_mRNA-p1 TcCLB-EL.510377.500_pseudogenic_transcript-p1
TcCLB-EL.510377.50_mRNA-p1 TcCLB-EL.510553.40_mRNA-p1 TcCLB-EL.
510557.10_pseudogenic_transcript-p1 TcCLB-EL.511123.11_mRNA-p1
TcCLB-EL.511173.130_mRNA-p1 TcCLB-EL.
511173.190_pseudogenic_transcript-p1 TcCLB-EL.
511173.230_pseudogenic_transcript-p1 TcCLB-EL.511173.280_mRNA-p1
TcCLB-EL.511173.370_mRNA-p1 TcCLB-EL.511173.440_mRNA-p1 TcCLB-EL.
511173.470_mRNA-p1 TcCLB-EL.511173.80_mRNA-p1 TcCLB-EL.
511399.24_mRNA-p1 TcCLB-EL.511401.120_mRNA-p1 TcCLB-EL.
511401.30_mRNA-p1 TcCLB-EL.511401.90_mRNA-p1 TcCLB-EL.

511599.80_mRNA-p1 TcCLB-EL.511603.450_mRNA-p1 TcCLB-EL.
511603.90_mRNA-p1 TcCLB-EL.511607.60_pseudogenic_transcript-p1
TcCLB-EL.511615.10_mRNA-p1 TcCLB-EL.511625.130_mRNA-p1 TcCLB-EL.
511797.110_mRNA-p1 TcCLB-EL.511797.240_pseudogenic_transcript-p1
TcCLB-EL.511873.10_mRNA-p1 TcCLB-NE.398915.5.1-p1 TcCLB-NE.
439991.10_pseudogenic_transcript-p1 TcCLB-NE.
504081.390_pseudogenic_transcript-p1 TcCLB-NE.504081.540_mRNA-p1
TcCLB-NE.504081.80_mRNA-p1 TcCLB-NE.504115.60_mRNA-p1 TcCLB-NE.
504155.20_mRNA-p1 TcCLB-NE.504155.280_pseudogenic_transcript-p1
TcCLB-NE.504261.20_mRNA-p1 TcCLB-NE.506001.40_mRNA-p1 TcCLB-NE.
506139.120_mRNA-p1 TcCLB-NE.506139.170_pseudogenic_transcript-p1
TcCLB-NE.506279.10_mRNA-p1 TcCLB-NE.506279.140_mRNA-p1 TcCLB-NE.
506279.250_pseudogenic_transcript-p1 TcCLB-NE.506279.60_mRNA-p1
TcCLB-NE.506335.130_mRNA-p1 TcCLB-NE.506339.10_mRNA-p1 TcCLB-NE.
506459.180_mRNA-p1 TcCLB-NE.506459.230_mRNA-p1 TcCLB-NE.
506613.150_pseudogenic_transcript-p1 TcCLB-NE.506615.150_mRNA-p1
TcCLB-NE.506751.90_pseudogenic_transcript-p1 TcCLB-NE.
506789.20_mRNA-p1 TcCLB-NE.506955.240_mRNA-p1 TcCLB-NE.
506955.270_mRNA-p1 TcCLB-NE.506993.160_pseudogenic_transcript-p1
TcCLB-NE.507059.20_mRNA-p1 TcCLB-NE.507059.50_mRNA-p1 TcCLB-NE.
507069.120_pseudogenic_transcript-p1 TcCLB-NE.507069.160_mRNA-p1
TcCLB-NE.507069.40_mRNA-p1 TcCLB-NE.507071.10_mRNA-p1 TcCLB-NE.
507071.190_mRNA-p1 TcCLB-NE.507071.230_mRNA-p1 TcCLB-NE.
507071.390_mRNA-p1 TcCLB-NE.507071.80_mRNA-p1 TcCLB-NE.
507633.80_mRNA-p1 TcCLB-NE.508219.170_pseudogenic_transcript-p1
TcCLB-NE.508219.20_mRNA-p1 TcCLB-NE.508221.180_mRNA-p1 TcCLB-NE.
508221.400_mRNA-p1 TcCLB-NE.508221.530_pseudogenic_transcript-p1
TcCLB-NE.508221.590_mRNA-p1 TcCLB-NE.508221.650_mRNA-p1 TcCLB-NE.
508221.750_mRNA-p1 TcCLB-NE.508221.790_mRNA-p1 TcCLB-NE.
508221.870_mRNA-p1 TcCLB-NE.508305.40_mRNA-p1 TcCLB-NE.
508365.190_mRNA-p1 TcCLB-NE.508433.200_pseudogenic_transcript-p1
TcCLB-NE.508433.40_mRNA-p1 TcCLB-NE.
508437.20_pseudogenic_transcript-p1 TcCLB-NE.508455.20_mRNA-p1
TcCLB-NE.508871.10_mRNA-p1 TcCLB-NE.508871.110_mRNA-p1 TcCLB-NE.
508871.170_mRNA-p1 TcCLB-NE.508873.330_mRNA-p1 TcCLB-NE.
508873.390_mRNA-p1 TcCLB-NE.508873.60_mRNA-p1 TcCLB-NE.
508883.48_mRNA-p1 TcCLB-NE.508979.50_mRNA-p1 TcCLB-NE.
508999.120_pseudogenic_transcript-p1 TcCLB-NE.509031.20_mRNA-p1
TcCLB-NE.509031.3_mRNA-p1 TcCLB-NE.509115.20_mRNA-p1 TcCLB-NE.
509217.40_mRNA-p1 TcCLB-NE.509251.20_mRNA-p1 TcCLB-NE.
509251.80_mRNA-p1 TcCLB-NE.509525.100_pseudogenic_transcript-p1
TcCLB-NE.509525.20_pseudogenic_transcript-p1 TcCLB-NE.
509527.120_mRNA-p1 TcCLB-NE.509545.40_pseudogenic_transcript-p1
TcCLB-NE.509655.10_pseudogenic_transcript-p1 TcCLB-NE.
509753.90_pseudogenic_transcript-p1 TcCLB-NE.509755.10_mRNA-p1
TcCLB-NE.509755.50_mRNA-p1 TcCLB-NE.509867.70_mRNA-p1 TcCLB-NE.
509897.110_mRNA-p1 TcCLB-NE.509897.140_mRNA-p1 TcCLB-NE.
509897.200_mRNA-p1 TcCLB-NE.509897.20_mRNA-p1 TcCLB-NE.
509897.270_mRNA-p1 TcCLB-NE.509897.70_mRNA-p1 TcCLB-NE.
509899.100_mRNA-p1 TcCLB-NE.509899.40_pseudogenic_transcript-p1
TcCLB-NE.509971.90_mRNA-p1 TcCLB-NE.510033.30_mRNA-p1 TcCLB-NE.
510197.130_mRNA-p1 TcCLB-NE.510197.180_mRNA-p1 TcCLB-NE.
510197.229_mRNA-p1 TcCLB-NE.510197.30_mRNA-p1 TcCLB-NE.
510197.80_mRNA-p1 TcCLB-NE.510201.10_mRNA-p1 TcCLB-NE.

510237.80_mRNA-p1 TcCLB-NE.510239.80_mRNA-p1 TcCLB-NE.
510359.530_mRNA-p1 TcCLB-NE.510361.180_pseudogenic_transcript-p1
TcCLB-NE.510361.80_mRNA-p1 TcCLB-NE.510363.250_mRNA-p1 TcCLB-NE.
510363.310_pseudogenic_transcript-p1 TcCLB-NE.
510363.60_pseudogenic_transcript-p1 TcCLB-NE.510419.20_mRNA-p1
TcCLB-NE.510483.210_pseudogenic_transcript-p1 TcCLB-NE.
510483.250_pseudogenic_transcript-p1 TcCLB-NE.510483.60_mRNA-p1
TcCLB-NE.510625.40_mRNA-p1 TcCLB-NE.
510693.120_pseudogenic_transcript-p1 TcCLB-NE.510693.240_mRNA-p1
TcCLB-NE.510693.270_mRNA-p1 TcCLB-NE.510697.50_mRNA-p1 TcCLB-NE.
510709.70_mRNA-p1 TcCLB-NE.510713.160_mRNA-p1 TcCLB-NE.
510713.90_mRNA-p1 TcCLB-NE.510715.150_mRNA-p1 TcCLB-NE.
510715.20_mRNA-p1 TcCLB-NE.510715.90_mRNA-p1 TcCLB-NE.
511255.10_mRNA-p1 TcCLB-NE.511255.190_mRNA-p1 TcCLB-NE.
511255.350_mRNA-p1 TcCLB-NE.511255.700_mRNA-p1 TcCLB-NE.
511259.170_pseudogenic_transcript-p1 TcCLB-NE.511643.110_mRNA-p1
TcCLB-NE.511643.40_mRNA-p1 TcCLB-NE.511643.70_mRNA-p1 TcCLB-NE.
511839.40_mRNA-p1 TcCLB-NE.511843.70_mRNA-p1 TcCLB-NE.
511917.10_mRNA-p1 TcCLB-NE.511919.20_mRNA-p1 TcCLB-NE.
511921.150_mRNA-p1 TcCLB-NE.511921.50_mRNA-p1 TcCLB-NE.
511923.100_pseudogenic_transcript-p1 TcCLB-NE.511923.30_mRNA-p1
TcCLB-NE.511923.60_mRNA-p1 TcSYL_0017800.t1-p1 TcSYL_0019140.t1-p1
TcSYL_0019150.t1-p1 TcSYL_0022150.t1-p1 TcSYL_0028490.t1-p1
TcSYL_0029610.t1-p1 TcSYL_0066720.t1-p1 TcSYL_0066910.t1-p1
TcSYL_0074170.t1-p1 TcSYL_0079880.t1-p1 TcSYL_0083760.t1-p1
TcSYL_0092570.t1-p1 TcSYL_0093150.t1-p1 TcSYL_0100220.t1-p1
TcSYL_0106860.t1-p1 TcSYL_0125690.t1-p1 TcSYL_0128000.t1-p1
TcSYL_0129550.t1-p1 TcSYL_0137390.t1-p1 TcSYL_0148750.t1-p1
TcSYL_0148900.t1-p1 TcSYL_0148910.t1-p1 TcSYL_0157200.t1-p1
TcSYL_0161320.t1-p1 TcSYL_0163970.t1-p1 TcSYL_0190730.t1-p1
OG0000002: TCRU_10238 TCRU_10420 TCRU_264 TCRU_2712 TCRU_3805
TCRU_4506 TCRU_4730 TCRU_6903 TCRU_7009 TCRU_7146 TCRU_850 TCRU_8902
TcCLB-EL.460061.20_mRNA-p1 TcCLB-EL.483813.10_mRNA-p1 TcCLB-EL.
492827.10_mRNA-p1 TcCLB-EL.503441.5_mRNA-p1 TcCLB-EL.503713.50_mRNA-
p1 TcCLB-EL.504193.15_mRNA-p1 TcCLB-EL.
505025.150_pseudogenic_transcript-p1 TcCLB-EL.505155.4_mRNA-p1
TcCLB-EL.506597.9_mRNA-p1 TcCLB-EL.507035.80_pseudogenic_transcript-
p1 TcCLB-EL.507045.5.1-p1 TcCLB-EL.507445.30_pseudogenic_transcript-
p1 TcCLB-EL.507867.5_mRNA-p1 TcCLB-EL.508047.11_mRNA-p1 TcCLB-EL.
508559.20_pseudogenic_transcript-p1 TcCLB-EL.
508687.11_pseudogenic_transcript-p1 TcCLB-EL.509081.166_mRNA-p1
TcCLB-EL.509123.5_pseudogenic_transcript-p1 TcCLB-EL.
509349.10_pseudogenic_transcript-p1 TcCLB-EL.509815.5_mRNA-p1 TcCLB-
EL.509821.20_pseudogenic_transcript-p1 TcCLB-EL.510107.15_mRNA-p1
TcCLB-EL.510157.5_mRNA-p1 TcCLB-EL.510207.50_pseudogenic_transcript-
p1 TcCLB-EL.510211.10_pseudogenic_transcript-p1 TcCLB-EL.
510971.30_mRNA-p1 TcCLB-EL.511567.11_mRNA-p1 TcCLB-EL.
511593.91_mRNA-p1 TcCLB-EL.511595.59_mRNA-p1 TcCLB-EL.
511597.50_mRNA-p1 TcCLB-NE.409407.10_pseudogenic_transcript-p1
TcCLB-NE.410199.30_mRNA-p1 TcCLB-NE.
410337.10_pseudogenic_transcript-p1 TcCLB-NE.423707.10_mRNA-p1
TcCLB-NE.433559.10_mRNA-p1 TcCLB-NE.
503973.340_pseudogenic_transcript-p1 TcCLB-NE.
504081.9_pseudogenic_transcript-p1 TcCLB-NE.506159.5_mRNA-p1 TcCLB-

NE.506281.7_mRNA-p1 TcCLB-NE.506615.10_pseudogenic_transcript-p1 TcCLB-NE.506809.10_pseudogenic_transcript-p1 TcCLB-NE.
507095.30_mRNA-p1 TcCLB-NE.507777.41_pseudogenic_transcript-p1 TcCLB-NE.508005.20_pseudogenic_transcript-p1 TcCLB-NE.
508433.70_mRNA-p1 TcCLB-NE.508849.11_mRNA-p1 TcCLB-NE.
508857.15_mRNA-p1 TcCLB-NE.509179.231_pseudogenic_transcript-p1 TcCLB-NE.509575.4_mRNA-p1 TcCLB-NE.509663.69_mRNA-p1 TcCLB-NE.
509915.10_pseudogenic_transcript-p1 TcCLB-NE.510363.19.1-p1 TcCLB-NE.510567.10_mRNA-p1 TcCLB-NE.510979.5_mRNA-p1 TcCLB-NE.
511485.30_pseudogenic_transcript-p1 TcCLB-NE.
511765.55_pseudogenic_transcript-p1 TcCLB-NE.
511827.125_pseudogenic_transcript-p1 TcSYL_0017810.t1-p1 TcSYL_0017890.t1-p1 TcSYL_0018150.t1-p1 TcSYL_0018330.t1-p1 TcSYL_0018930.t1-p1 TcSYL_0021260.t1-p1 TcSYL_0028090.t1-p1 TcSYL_0028480.t1-p1 TcSYL_0039680.t1-p1 TcSYL_0051260.t1-p1 TcSYL_0058180.t1-p1 TcSYL_0058470.t1-p1 TcSYL_0059060.t1-p1 TcSYL_0060000.t1-p1 TcSYL_0066240.t1-p1 TcSYL_0066920.t1-p1 TcSYL_0067230.t1-p1 TcSYL_0067800.t1-p1 TcSYL_0068000.t1-p1 TcSYL_0069360.t1-p1 TcSYL_0072000.t1-p1 TcSYL_0079840.t1-p1 TcSYL_0083770.t1-p1 TcSYL_0091320.t1-p1 TcSYL_0092760.t1-p1 TcSYL_0093000.t1-p1 TcSYL_0093140.t1-p1 TcSYL_0093420.t1-p1 TcSYL_0093820.t1-p1 TcSYL_0106650.t1-p1 TcSYL_0118890.t1-p1 TcSYL_0118950.t1-p1 TcSYL_0119010.t1-p1 TcSYL_0119370.t1-p1 TcSYL_0119680.t1-p1 TcSYL_0120020.t1-p1 TcSYL_0120270.t1-p1 TcSYL_0120660.t1-p1 TcSYL_0120860.t1-p1 TcSYL_0120980.t1-p1 TcSYL_0124060.t1-p1 TcSYL_0124710.t1-p1 TcSYL_0125700.t1-p1 TcSYL_0126130.t1-p1 TcSYL_0127320.t1-p1 TcSYL_0128970.t1-p1 TcSYL_0129960.t1-p1 TcSYL_0133990.t1-p1 TcSYL_0134730.t1-p1 TcSYL_0135980.t1-p1 TcSYL_0143120.t1-p1 TcSYL_0147790.t1-p1 TcSYL_0147900.t1-p1 TcSYL_0150340.t1-p1 TcSYL_0151110.t1-p1 TcSYL_0160610.t1-p1 TcSYL_0187070.t1-p1 TcSYL_0191120.t1-p1 TcSYL_0203820.t1-p1

OG0000003: TcCLB-EL.503547.20_pseudogenic_transcript-p1 TcCLB-EL.
504241.34_mRNA-p1 TcCLB-EL.504769.160_mRNA-p1 TcCLB-EL.
506183.20_mRNA-p1 TcCLB-EL.506243.50_pseudogenic_transcript-p1 TcCLB-EL.506375.50_mRNA-p1 TcCLB-EL.506671.70_mRNA-p1 TcCLB-EL.
506683.180_mRNA-p1 TcCLB-EL.506885.489_mRNA-p1 TcCLB-EL.
506961.50_mRNA-p1 TcCLB-EL.506973.70_mRNA-p1 TcCLB-EL.
507035.30_mRNA-p1 TcCLB-EL.507179.20_mRNA-p1 TcCLB-EL.
507477.10_mRNA-p1 TcCLB-EL.507521.150_mRNA-p1 TcCLB-EL.
507611.199_mRNA-p1 TcCLB-EL.507719.9_mRNA-p1 TcCLB-EL.
507827.10_mRNA-p1 TcCLB-EL.507835.70_mRNA-p1 TcCLB-EL.507921.9_mRNA-p1 TcCLB-EL.507975.40_mRNA-p1 TcCLB-EL.507999.10_mRNA-p1 TcCLB-EL.
508159.40_mRNA-p1 TcCLB-EL.508163.190_mRNA-p1 TcCLB-EL.
508163.360_mRNA-p1 TcCLB-EL.508163.70_mRNA-p1 TcCLB-EL.
508283.10_mRNA-p1 TcCLB-EL.508283.69_mRNA-p1 TcCLB-EL.
508325.30_mRNA-p1 TcCLB-EL.508391.10_mRNA-p1 TcCLB-EL.
508559.29_mRNA-p1 TcCLB-EL.508607.30_mRNA-p1 TcCLB-EL.
508775.30_mRNA-p1 TcCLB-EL.508831.250_mRNA-p1 TcCLB-EL.
508837.34_mRNA-p1 TcCLB-EL.509631.60_mRNA-p1 TcCLB-EL.
509735.84_mRNA-p1 TcCLB-EL.509815.120_pseudogenic_transcript-p1 TcCLB-EL.509871.80_mRNA-p1 TcCLB-EL.509925.70_mRNA-p1 TcCLB-EL.
510175.60_mRNA-p1 TcCLB-EL.510271.20_pseudogenic_transcript-p1 TcCLB-EL.510275.130_mRNA-p1 TcCLB-EL.510307.330_mRNA-p1 TcCLB-EL.

510367.10_mRNA-p1 TcCLB-EL.510441.20_mRNA-p1 TcCLB-EL.
510449.10_mRNA-p1 TcCLB-EL.510607.29_mRNA-p1 TcCLB-EL.
510607.80_mRNA-p1 TcCLB-EL.510847.70_mRNA-p1 TcCLB-EL.
510971.10_mRNA-p1 TcCLB-EL.511415.40_mRNA-p1 TcCLB-EL.
511469.110_mRNA-p1 TcCLB-EL.511599.40_mRNA-p1 TcCLB-EL.
511677.10_mRNA-p1 TcCLB-EL.511797.220_mRNA-p1 TcCLB-EL.
511797.54_mRNA-p1 TcCLB-NE.433717.10_pseudogenic_transcript-p1
TcCLB-NE.503655.20_mRNA-p1 TcCLB-NE.503861.80_mRNA-p1 TcCLB-NE.
503973.40_mRNA-p1 TcCLB-NE.504115.100_mRNA-p1 TcCLB-NE.
504689.60_mRNA-p1 TcCLB-NE.505037.100_mRNA-p1 TcCLB-NE.
505997.174_mRNA-p1 TcCLB-NE.506159.30_mRNA-p1 TcCLB-NE.
506191.39_mRNA-p1 TcCLB-NE.506241.19_mRNA-p1 TcCLB-NE.
506339.100_mRNA-p1 TcCLB-NE.506393.90_mRNA-p1 TcCLB-NE.
506549.9_mRNA-p1 TcCLB-NE.506613.130_mRNA-p1 TcCLB-NE.
506781.50_mRNA-p1 TcCLB-NE.507167.169_mRNA-p1 TcCLB-NE.
507643.50_mRNA-p1 TcCLB-NE.507687.20_mRNA-p1 TcCLB-NE.
507779.30_mRNA-p1 TcCLB-NE.507789.9_mRNA-p1 TcCLB-NE.507911.30_mRNA-p1 TcCLB-NE.508061.50_mRNA-p1 TcCLB-NE.508139.140_mRNA-p1 TcCLB-NE.
508139.20_mRNA-p1 TcCLB-NE.508139.210_mRNA-p1 TcCLB-NE.
508523.40_mRNA-p1 TcCLB-NE.508573.39_mRNA-p1 TcCLB-NE.
508577.40_mRNA-p1 TcCLB-NE.509085.60_mRNA-p1 TcCLB-NE.
509287.140_mRNA-p1 TcCLB-NE.509287.240_mRNA-p1 TcCLB-NE.
509295.10_mRNA-p1 TcCLB-NE.509657.20_mRNA-p1 TcCLB-NE.
509875.160_mRNA-p1 TcCLB-NE.509875.20_mRNA-p1 TcCLB-NE.
509915.20_mRNA-p1 TcCLB-NE.510197.110_mRNA-p1 TcCLB-NE.
510201.50_mRNA-p1 TcCLB-NE.510237.129_mRNA-p1 TcCLB-NE.
510355.10_mRNA-p1 TcCLB-NE.510355.90_mRNA-p1 TcCLB-NE.
510385.10_mRNA-p1 TcCLB-NE.510391.10_mRNA-p1 TcCLB-NE.
510399.39_mRNA-p1 TcCLB-NE.510415.9_mRNA-p1 TcCLB-NE.
510481.20_pseudogenic_transcript-p1 TcCLB-NE.510625.10_mRNA-p1
TcCLB-NE.510629.210_mRNA-p1 TcCLB-NE.510643.10_mRNA-p1 TcCLB-NE.
510677.9_mRNA-p1 TcCLB-NE.510713.20_mRNA-p1 TcCLB-NE.511475.24_mRNA-p1 TcCLB-NE.511475.50_mRNA-p1 TcCLB-NE.
511477.40_pseudogenic_transcript-p1 TcCLB-NE.511487.30_mRNA-p1
TcCLB-NE.511665.50_mRNA-p1 TcCLB-NE.511765.20_mRNA-p1 TcCLB-NE.
511767.20_mRNA-p1 TcCLB-NE.511769.20_mRNA-p1 TcCLB-NE.
511771.210_mRNA-p1 TcCLB-NE.511771.70_mRNA-p1 TcCLB-NE.
511827.130_mRNA-p1 TcCLB-NE.511829.10_mRNA-p1 TcCLB-NE.
511843.40_mRNA-p1 TcCLB-NE.511851.10_mRNA-p1 TcCLB-NE.
511889.40_mRNA-p1
OG0000004: TCRU_10071 TCRU_10325 TCRU_1206 TCRU_1436 TCRU_1485
TCRU_1677 TCRU_1738 TCRU_1821 TCRU_1891 TCRU_1967 TCRU_2138
TCRU_2388 TCRU_2390 TCRU_2524 TCRU_2546 TCRU_2557 TCRU_2648
TCRU_2708 TCRU_2775 TCRU_3366 TCRU_4060 TCRU_4345 TCRU_4361
TCRU_4465 TCRU_4760 TCRU_4872 TCRU_4879 TCRU_510 TCRU_5229 TCRU_6611
TCRU_7001 TCRU_709 TCRU_7404 TCRU_7723 TCRU_782 TCRU_7953 TCRU_8579
TCRU_8899 TCRU_9065 TCRU_9066 TCRU_9116 TCRU_9322 TCRU_9484
TCRU_9759 TCRU_983 TcCLB-EL.503601.20_mRNA-p1 TcCLB-EL.
504239.230_mRNA-p1 TcCLB-EL.504239.270_mRNA-p1 TcCLB-EL.
506269.25_mRNA-p1 TcCLB-EL.506499.100_mRNA-p1 TcCLB-EL.
506499.180_mRNA-p1 TcCLB-EL.506499.230_mRNA-p1 TcCLB-EL.
506667.180_mRNA-p1 TcCLB-EL.506667.20_mRNA-p1 TcCLB-EL.
506759.130_pseudogenic_transcript-p1 TcCLB-EL.506759.230_mRNA-p1
TcCLB-EL.506769.20_mRNA-p1 TcCLB-EL.506965.120_mRNA-p1 TcCLB-EL.

507237.180_mRNA-p1 TcCLB-EL.507699.210_mRNA-p1 TcCLB-EL.
507953.210_mRNA-p1 TcCLB-EL.507953.30_mRNA-p1 TcCLB-EL.
507953.80_mRNA-p1 TcCLB-EL.507981.20_mRNA-p1 TcCLB-EL.
507987.20_mRNA-p1 TcCLB-EL.508081.40_mRNA-p1 TcCLB-EL.
508081.60_mRNA-p1 TcCLB-EL.508081.80_mRNA-p1 TcCLB-EL.
508099.30_mRNA-p1 TcCLB-EL.508163.240_mRNA-p1 TcCLB-EL.
508227.10_mRNA-p1 TcCLB-EL.508229.20_mRNA-p1 TcCLB-EL.
509097.10_mRNA-p1 TcCLB-EL.510013.190_mRNA-p1 TcCLB-EL.
510013.330_mRNA-p1 TcCLB-EL.510021.60_mRNA-p1 TcCLB-EL.
510025.60_mRNA-p1 TcCLB-EL.510213.20_mRNA-p1 TcCLB-EL.
510275.410_mRNA-p1 TcCLB-EL.510279.100_mRNA-p1 TcCLB-EL.
510373.20_mRNA-p1 TcCLB-EL.510373.40_mRNA-p1 TcCLB-EL.
510377.110_mRNA-p1 TcCLB-EL.510557.30_mRNA-p1 TcCLB-EL.
511173.200_mRNA-p1 TcCLB-EL.511553.180_mRNA-p1 TcCLB-EL.
511611.40_mRNA-p1 TcCLB-EL.511613.70_mRNA-p1 TcCLB-NE.
433237.10_mRNA-p1 TcCLB-NE.503417.40_mRNA-p1 TcCLB-NE.
504081.342_pseudogenic_transcript-p1 TcCLB-NE.504689.10_mRNA-p1
TcCLB-NE.506789.40_mRNA-p1 TcCLB-NE.506993.140_mRNA-p1 TcCLB-NE.
507067.70_mRNA-p1 TcCLB-NE.507085.110_mRNA-p1 TcCLB-NE.
508221.1020_mRNA-p1 TcCLB-NE.508221.410_mRNA-p1 TcCLB-NE.
508221.880_mRNA-p1 TcCLB-NE.508261.120_mRNA-p1 TcCLB-NE.
508305.30_mRNA-p1 TcCLB-NE.508493.70_mRNA-p1 TcCLB-NE.
508495.30_mRNA-p1 TcCLB-NE.508501.10_mRNA-p1 TcCLB-NE.
508873.300_mRNA-p1 TcCLB-NE.508999.100_mRNA-p1 TcCLB-NE.
509755.30_mRNA-p1 TcCLB-NE.510239.100_mRNA-p1 TcCLB-NE.
510361.20_mRNA-p1 TcCLB-NE.510361.250_mRNA-p1 TcCLB-NE.
510623.30_mRNA-p1 TcCLB-NE.510625.130_mRNA-p1 TcCLB-NE.
510627.140_mRNA-p1 TcCLB-NE.510627.90_mRNA-p1 TcCLB-NE.
510629.300_mRNA-p1 TcCLB-NE.511233.150_mRNA-p1 TcCLB-NE.
511255.20_mRNA-p1 TcCLB-NE.511255.550_mRNA-p1 TcCLB-NE.
511255.90_mRNA-p1 TcSYL_0066150.t1-p1 TcSYL_0100430.t1-p1
TcSYL_0161780.t1-p1
OG0000005: TCRU_1125 TCRU_6935 TCRU_8463 TCRU_96 TcCLB-EL.
508241.140_mRNA-p1 TcCLB-EL.509149.9_mRNA-p1 TcCLB-EL.
510381.10_mRNA-p1 TcCLB-EL.511691.10_mRNA-p1 TcCLB-EL.
511691.20_mRNA-p1 TcCLB-NE.506905.50_mRNA-p1 TcCLB-NE.
508175.360_mRNA-p1 TcCLB-NE.508177.110_mRNA-p1 TcCLB-NE.
508177.120_mRNA-p1 TcCLB-NE.508177.129_mRNA-p1 TcSYL_0075620.t1-p1
TcSYL_0075630.t1-p1 TcSYL_0075640.t1-p1 TcSYL_0075650.t1-p1
TcSYL_0075660.t1-p1 TcSYL_0075670.t1-p1 TcSYL_0075680.t1-p1
TcSYL_0075690.t1-p1 TcSYL_0075700.t1-p1 TcSYL_0075710.t1-p1
TcSYL_0075720.t1-p1 TcSYL_0075730.t1-p1 TcSYL_0075760.t1-p1
TcSYL_0075800.t1-p1 TcSYL_0075820.t1-p1 TcSYL_0075890.t1-p1
TcSYL_0075930.t1-p1 TcSYL_0075970.t1-p1 TcSYL_0076150.t1-p1
TcSYL_0076180.t1-p1 TcSYL_0076260.t1-p1 TcSYL_0076290.t1-p1
TcSYL_0076320.t1-p1 TcSYL_0076340.t1-p1 TcSYL_0076360.t1-p1
TcSYL_0077770.t1-p1 TcSYL_0077800.t1-p1 TcSYL_0077820.t1-p1
TcSYL_0077850.t1-p1 TcSYL_0078110.t1-p1 TcSYL_0078190.t1-p1
TcSYL_0078220.t1-p1 TcSYL_0078250.t1-p1 TcSYL_0078270.t1-p1
TcSYL_0078290.t1-p1 TcSYL_0078310.t1-p1 TcSYL_0078360.t1-p1
TcSYL_0078380.t1-p1 TcSYL_0078420.t1-p1 TcSYL_0078440.t1-p1
TcSYL_0078470.t1-p1 TcSYL_0171490.t1-p1 TcSYL_0183670.t1-p1
TcSYL_0183680.t1-p1 TcSYL_0183690.t1-p1 TcSYL_0183700.t1-p1
TcSYL_0183710.t1-p1 TcSYL_0183720.t1-p1 TcSYL_0183730.t1-p1

TcSYL_0183740.t1-p1 TcSYL_0183750.t1-p1 TcSYL_0183760.t1-p1
TcSYL_0183790.t1-p1 TcSYL_0183850.t1-p1 TcSYL_0183860.t1-p1
TcSYL_0183880.t1-p1 TcSYL_0183910.t1-p1 TcSYL_0183930.t1-p1
TcSYL_0183980.t1-p1 TcSYL_0184080.t1-p1 TcSYL_0184100.t1-p1
TcSYL_0184120.t1-p1 TcSYL_0184170.t1-p1 TcSYL_0184200.t1-p1
TcSYL_0184220.t1-p1 TcSYL_0184250.t1-p1 TcSYL_0184270.t1-p1
TcSYL_0184310.t1-p1 TcSYL_0184340.t1-p1 TcSYL_0184580.t1-p1
TcSYL_0184640.t1-p1 TcSYL_0184670.t1-p1 TcSYL_0184930.t1-p1
TcSYL_0184940.t1-p1 TcSYL_0184950.t1-p1 TcSYL_0184960.t1-p1
TcSYL_0184970.t1-p1 TcSYL_0184980.t1-p1 TcSYL_0184990.t1-p1
TcSYL_0185000.t1-p1 TcSYL_0185010.t1-p1 TcSYL_0185030.t1-p1
TcSYL_0185060.t1-p1 TcSYL_0185120.t1-p1 TcSYL_0185180.t1-p1
TcSYL_0185210.t1-p1 TcSYL_0185240.t1-p1 TcSYL_0185280.t1-p1
TcSYL_0185310.t1-p1 TcSYL_0185360.t1-p1 TcSYL_0185390.t1-p1
TcSYL_0185430.t1-p1 TcSYL_0185550.t1-p1 TcSYL_0185610.t1-p1
TcSYL_0186050.t1-p1 TcSYL_0186130.t1-p1 TcSYL_0186150.t1-p1
TcSYL_0186190.t1-p1 TcSYL_0186260.t1-p1 TcSYL_0186350.t1-p1
OG0000006: TCRU_1796 TCRU_2455 TCRU_3165 TCRU_3260 TCRU_3369
TCRU_3370 TCRU_3481 TCRU_3531 TCRU_3549 TCRU_3713 TCRU_4228
TCRU_4525 TCRU_5073 TCRU_5223 TCRU_5240 TCRU_530 TCRU_58 TCRU_6187
TCRU_6241 TCRU_6882 TCRU_6952 TCRU_7010 TcCLB-EL.
416503.10_pseudogenic_transcript-p1 TcCLB-EL.420293.20_mRNA-p1
TcCLB-EL.424383.9_pseudogenic_transcript-p1 TcCLB-EL.433673.10.1-p1
TcCLB-EL.435601.10.1-p1 TcCLB-EL.503501.50_pseudogenic_transcript-p1
TcCLB-EL.503767.10_mRNA-p1 TcCLB-EL.
503957.60_pseudogenic_transcript-p1 TcCLB-EL.
504055.130_pseudogenic_transcript-p1 TcCLB-EL.504099.50_mRNA-p1
TcCLB-EL.504229.100_pseudogenic_transcript-p1 TcCLB-EL.
504343.10_mRNA-p1 TcCLB-EL.505363.19_mRNA-p1 TcCLB-EL.
506021.20_mRNA-p1 TcCLB-EL.506129.30_mRNA-p1 TcCLB-EL.
506345.40_mRNA-p1 TcCLB-EL.506345.90_mRNA-p1 TcCLB-EL.
506397.10_mRNA-p1 TcCLB-EL.506455.30_mRNA-p1 TcCLB-EL.
506683.190_mRNA-p1 TcCLB-EL.506757.90_mRNA-p1 TcCLB-EL.
506765.40_mRNA-p1 TcCLB-EL.507213.40_mRNA-p1 TcCLB-EL.
507479.20_mRNA-p1 TcCLB-EL.507611.170_mRNA-p1 TcCLB-EL.
507875.220_mRNA-p1 TcCLB-EL.507879.10_mRNA-p1 TcCLB-EL.
507907.20_mRNA-p1 TcCLB-EL.508225.4_mRNA-p1 TcCLB-EL.
508283.40_pseudogenic_transcript-p1 TcCLB-EL.508285.60_mRNA-p1
TcCLB-EL.508325.250_pseudogenic_transcript-p1 TcCLB-EL.
508325.290_pseudogenic_transcript-p1 TcCLB-EL.508563.20_mRNA-p1
TcCLB-EL.508775.40_pseudogenic_transcript-p1 TcCLB-EL.
508837.70_mRNA-p1 TcCLB-EL.509265.90_mRNA-p1 TcCLB-EL.
509735.100_pseudogenic_transcript-p1 TcCLB-EL.509739.10_mRNA-p1
TcCLB-EL.509921.20_pseudogenic_transcript-p1 TcCLB-EL.
510005.20_mRNA-p1 TcCLB-EL.510049.10_mRNA-p1 TcCLB-EL.
510307.210_mRNA-p1 TcCLB-EL.510307.230_mRNA-p1 TcCLB-EL.
510403.30_mRNA-p1 TcCLB-EL.510607.70_pseudogenic_transcript-p1
TcCLB-EL.511311.20_mRNA-p1 TcCLB-EL.511349.100_mRNA-p1 TcCLB-EL.
511413.19_mRNA-p1 TcCLB-EL.511471.20_pseudogenic_transcript-p1
TcCLB-EL.511569.30_pseudogenic_transcript-p1 TcCLB-EL.
511585.230_mRNA-p1 TcCLB-EL.511911.10_mRNA-p1 TcCLB-NE.477471.10.1-
p1 TcCLB-NE.504183.20_mRNA-p1 TcCLB-NE.
505267.20_pseudogenic_transcript-p1 TcCLB-NE.506157.40_mRNA-p1
TcCLB-NE.506241.30_mRNA-p1 TcCLB-NE.506331.90_mRNA-p1 TcCLB-NE.

506471.100_mRNA-p1 TcCLB-NE.506537.80_mRNA-p1 TcCLB-NE.
506751.50_mRNA-p1 TcCLB-NE.507167.80_mRNA-p1 TcCLB-NE.
508001.50_pseudogenic_transcript-p1 TcCLB-NE.508007.10_mRNA-p1
TcCLB-NE.508061.20_mRNA-p1 TcCLB-NE.508523.30_mRNA-p1 TcCLB-NE.
509257.10_mRNA-p1 TcCLB-NE.509417.9_mRNA-p1 TcCLB-NE.509771.10_mRNA-
p1 TcCLB-NE.510359.610_mRNA-p1 TcCLB-NE.510569.10_mRNA-p1 TcCLB-NE.
510817.50_mRNA-p1 TcCLB-NE.511301.160_mRNA-p1 TcCLB-NE.
511391.60_pseudogenic_transcript-p1 TcCLB-NE.511757.70_mRNA-p1
TcCLB-NE.511885.20_mRNA-p1 TcCLB-NE.511891.10_mRNA-p1
TcSYL_0006170.t1-p1 TcSYL_0084130.t1-p1 TcSYL_0099950.t1-p1
TcSYL_0100020.t1-p1 TcSYL_0100060.t1-p1 TcSYL_0191530.t1-p1
TcSYL_0200640.t1-p1 TcSYL_0203900.t1-p1
OG0000007: TCRU_10004 TCRU_10517 TCRU_10676 TCRU_1122 TCRU_1228
TCRU_1229 TCRU_1230 TCRU_1235 TCRU_1270 TCRU_1289 TCRU_1376
TCRU_1377 TCRU_142 TCRU_1460 TCRU_1814 TCRU_1836 TCRU_1865 TCRU_1960
TCRU_2336 TCRU_2589 TCRU_2644 TCRU_2759 TCRU_2782 TCRU_2927
TCRU_2929 TCRU_2996 TCRU_2997 TCRU_3000 TCRU_3187 TCRU_3477
TCRU_3643 TCRU_3743 TCRU_3746 TCRU_3899 TCRU_3969 TCRU_4093
TCRU_4199 TCRU_457 TCRU_4615 TCRU_4665 TCRU_4748 TCRU_4749 TCRU_4801
TCRU_4841 TCRU_4870 TCRU_4871 TCRU_4903 TCRU_4921 TCRU_5084
TCRU_5101 TCRU_5102 TCRU_5190 TCRU_5198 TCRU_5209 TCRU_5217
TCRU_5238 TCRU_5241 TCRU_5374 TCRU_5436 TCRU_574 TCRU_6163 TCRU_621
TCRU_6284 TCRU_6294 TCRU_6308 TCRU_6363 TCRU_6364 TCRU_6482
TCRU_6538 TCRU_6563 TCRU_6661 TCRU_6701 TCRU_6702 TCRU_6784
TCRU_6839 TCRU_6883 TCRU_6904 TCRU_6907 TCRU_7108 TCRU_7171
TCRU_7366 TCRU_747 TCRU_7513 TCRU_76 TCRU_8022 TCRU_8025 TCRU_8136
TCRU_8613 TCRU_886 TCRU_9159 TCRU_9170 TCRU_9187 TCRU_927 TCRU_9283
TCRU_9309 TCRU_9310 TCRU_9377 TCRU_9378 TCRU_9948 TcSYL_0061310.t1-
p1 TcSYL_0090400.t1-p1 TcSYL_0090720.t1-p1 TcSYL_0161550.t1-p1
TcSYL_0167450.t1-p1 TcSYL_0168750.t1-p1
OG0000008: TCRU_10137 TCRU_10188 TCRU_10190 TCRU_10196 TCRU_10197
TCRU_10199 TCRU_10201 TCRU_10202 TCRU_10466 TCRU_1052 TCRU_10590
TCRU_10603 TCRU_10604 TCRU_10619 TCRU_10620 TCRU_10658 TCRU_1248
TCRU_1286 TCRU_1416 TCRU_1424 TCRU_1425 TCRU_144 TCRU_1481 TCRU_1593
TCRU_1698 TCRU_1749 TCRU_1750 TCRU_214 TCRU_2161 TCRU_2177 TCRU_2179
TCRU_2297 TCRU_2298 TCRU_2656 TCRU_2692 TCRU_274 TCRU_2780 TCRU_2781
TCRU_2797 TCRU_2916 TCRU_300 TCRU_301 TCRU_3237 TCRU_350 TCRU_3594
TCRU_3665 TCRU_3992 TCRU_3993 TCRU_401 TCRU_4295 TCRU_4299 TCRU_4497
TCRU_5326 TCRU_5327 TCRU_543 TCRU_544 TCRU_6388 TCRU_6571 TCRU_6625
TCRU_6628 TCRU_663 TCRU_7023 TCRU_7024 TCRU_713 TCRU_7363 TCRU_7367
TCRU_7407 TCRU_7609 TCRU_7760 TCRU_7804 TCRU_7955 TCRU_8469 TCRU_891
TCRU_892 TCRU_893 TCRU_906 TCRU_9273 TCRU_9277 TCRU_929 TCRU_9337
TCRU_9379 TCRU_9441 TCRU_9474 TCRU_9495 TCRU_9653 TCRU_9654
TCRU_9727 TCRU_9762 TCRU_984 TCRU_985 TCRU_993 TCRU_9933 TCRU_9966
TcCLB-NE.507787.150.1-p1 TcSYL_0024560.t1-p1 TcSYL_0024800.t1-p1
TcSYL_0025010.t1-p1 TcSYL_0025320.t1-p1 TcSYL_0049460.t1-p1
TcSYL_0049940.t1-p1 TcSYL_0110150.t1-p1 TcSYL_0110470.t1-p1
TcSYL_0115000.t1-p1 TcSYL_0190370.t1-p1
OG0000009: TCRU_7265 TCRU_of TCRU_of TCRU_of TCRU_of TCRU_of TCRU_of
TCRU_of TCRU_of TCRU_of TCRU_of TCRU_of TCRU_of TCRU_of TCRU_of
TCRU_of TCRU_of TCRU_of TCRU_of TcCLB-EL.417939.9_mRNA-p1 TcCLB-EL.
503623.10_mRNA-p1 TcCLB-EL.504241.90_mRNA-p1 TcCLB-EL.
504887.10_mRNA-p1 TcCLB-EL.506623.60_mRNA-p1 TcCLB-EL.
506717.30_pseudogenic_transcript-p1 TcCLB-EL.

506753.15_pseudogenic_transcript-p1 TcCLB-EL.507839.30_mRNA-p1
TcCLB-EL.507859.120_mRNA-p1 TcCLB-EL.507863.20_mRNA-p1 TcCLB-EL.
508147.20_mRNA-p1 TcCLB-EL.508149.10_mRNA-p1 TcCLB-EL.508281.9_mRNA-
p1 TcCLB-EL.508681.40_mRNA-p1 TcCLB-EL.508773.9_mRNA-p1 TcCLB-EL.
508775.70_mRNA-p1 TcCLB-EL.509087.20_mRNA-p1 TcCLB-EL.509089.9_mRNA-
p1 TcCLB-EL.509121.20_pseudogenic_transcript-p1 TcCLB-EL.
509511.10_mRNA-p1 TcCLB-EL.509667.10_mRNA-p1 TcCLB-EL.509845.9_mRNA-
p1 TcCLB-EL.509919.9_mRNA-p1 TcCLB-EL.509921.60_mRNA-p1 TcCLB-EL.
510005.50_mRNA-p1 TcCLB-EL.510059.10_mRNA-p1 TcCLB-EL.
510447.10_mRNA-p1 TcCLB-EL.511171.10_mRNA-p1 TcCLB-EL.
511789.10_pseudogenic_transcript-p1 TcCLB-NE.450019.9_mRNA-p1 TcCLB-
NE.452699.30_mRNA-p1 TcCLB-NE.503845.20_pseudogenic_transcript-p1
TcCLB-NE.504067.10_mRNA-p1 TcCLB-NE.504169.10_mRNA-p1 TcCLB-NE.
504533.10_mRNA-p1 TcCLB-NE.505207.40_mRNA-p1 TcCLB-NE.
505267.110_mRNA-p1 TcCLB-NE.505773.20_mRNA-p1 TcCLB-NE.
505967.19_mRNA-p1 TcCLB-NE.506111.20_mRNA-p1 TcCLB-NE.
506233.10_mRNA-p1 TcCLB-NE.506595.10_mRNA-p1 TcCLB-NE.
507167.120_mRNA-p1 TcCLB-NE.507563.9_mRNA-p1 TcCLB-NE.
507801.200_mRNA-p1 TcCLB-NE.507901.10_mRNA-p1 TcCLB-NE.
508001.10_mRNA-p1 TcCLB-NE.508249.10_mRNA-p1 TcCLB-NE.
508259.20_pseudogenic_transcript-p1 TcCLB-NE.
508303.80_pseudogenic_transcript-p1 TcCLB-NE.
508419.20_pseudogenic_transcript-p1 TcCLB-NE.508573.120_mRNA-p1
TcCLB-NE.508629.10_mRNA-p1 TcCLB-NE.508641.10_mRNA-p1 TcCLB-NE.
508967.20_mRNA-p1 TcCLB-NE.508969.40_mRNA-p1 TcCLB-NE.509181.9_mRNA-
p1 TcCLB-NE.509223.10_pseudogenic_transcript-p1 TcCLB-NE.
509929.10_mRNA-p1 TcCLB-NE.510195.9_mRNA-p1 TcCLB-NE.510239.10_mRNA-
p1 TcCLB-NE.510355.180_mRNA-p1 TcCLB-NE.
510487.20_pseudogenic_transcript-p1 TcCLB-NE.
510625.240_pseudogenic_transcript-p1 TcCLB-NE.510627.230_mRNA-p1
TcCLB-NE.510639.10_pseudogenic_transcript-p1 TcCLB-NE.
510671.10_mRNA-p1 TcCLB-NE.511265.10_pseudogenic_transcript-p1
TcCLB-NE.511271.10_pseudogenic_transcript-p1 TcCLB-NE.
511561.20_mRNA-p1 TcSYL_0004680.t1-p1 TcSYL_0041080.t1-p1
TcSYL_0057130.t1-p1 TcSYL_0074540.t1-p1 TcSYL_0083910.t1-p1
TcSYL_0157650.t1-p1 TcSYL_0166040.t1-p1 TcSYL_0200100.t1-p1
TcSYL_0200510.t1-p1 TcSYL_0203670.t1-p1
OG0000010: TCRU_8152 TcCLB-EL.506779.150_mRNA-p1 TcCLB-NE.
511153.64_mRNA-p1 TcCLB-NE.511635.10_mRNA-p1 TcCLB-NE.
511635.20_mRNA-p1 TcSYL_0006270.t1-p1 TcSYL_0006280.t1-p1
TcSYL_0006290.t1-p1 TcSYL_0006300.t1-p1 TcSYL_0006310.t1-p1
TcSYL_0006320.t1-p1 TcSYL_0006330.t1-p1 TcSYL_0006340.t1-p1
TcSYL_0006360.t1-p1 TcSYL_0006380.t1-p1 TcSYL_0006420.t1-p1
TcSYL_0006440.t1-p1 TcSYL_0006480.t1-p1 TcSYL_0006500.t1-p1
TcSYL_0006520.t1-p1 TcSYL_0006560.t1-p1 TcSYL_0006580.t1-p1
TcSYL_0006620.t1-p1 TcSYL_0006630.t1-p1 TcSYL_0006660.t1-p1
TcSYL_0006670.t1-p1 TcSYL_0006800.t1-p1 TcSYL_0006850.t1-p1
TcSYL_0006870.t1-p1 TcSYL_0006880.t1-p1 TcSYL_0006900.t1-p1
TcSYL_0006920.t1-p1 TcSYL_0006940.t1-p1 TcSYL_0006950.t1-p1
TcSYL_0006970.t1-p1 TcSYL_0007000.t1-p1 TcSYL_0007020.t1-p1
TcSYL_0007040.t1-p1 TcSYL_0007060.t1-p1 TcSYL_0007080.t1-p1
TcSYL_0007090.t1-p1 TcSYL_0007110.t1-p1 TcSYL_0007120.t1-p1
TcSYL_0007140.t1-p1 TcSYL_0007160.t1-p1 TcSYL_0007180.t1-p1
TcSYL_0007200.t1-p1 TcSYL_0007220.t1-p1 TcSYL_0007240.t1-p1

TcSYL_0007260.t1-p1 TcSYL_0007280.t1-p1 TcSYL_0007290.t1-p1
TcSYL_0007300.t1-p1 TcSYL_0007320.t1-p1 TcSYL_0007340.t1-p1
TcSYL_0007420.t1-p1 TcSYL_0007440.t1-p1 TcSYL_0007470.t1-p1
TcSYL_0007490.t1-p1 TcSYL_0007510.t1-p1 TcSYL_0007530.t1-p1
TcSYL_0007550.t1-p1 TcSYL_0007570.t1-p1 TcSYL_0007590.t1-p1
TcSYL_0007610.t1-p1 TcSYL_0007630.t1-p1 TcSYL_0007650.t1-p1
TcSYL_0007660.t1-p1 TcSYL_0007680.t1-p1 TcSYL_0007700.t1-p1
TcSYL_0007710.t1-p1 TcSYL_0007720.t1-p1 TcSYL_0007730.t1-p1
TcSYL_0007740.t1-p1 TcSYL_0007750.t1-p1 TcSYL_0007760.t1-p1
TcSYL_0007770.t1-p1 TcSYL_0007780.t1-p1 TcSYL_0007790.t1-p1
TcSYL_0007800.t1-p1 TcSYL_0007810.t1-p1 TcSYL_0007820.t1-p1
TcSYL_0007830.t1-p1 TcSYL_0007840.t1-p1 TcSYL_0007850.t1-p1
TcSYL_0007860.t1-p1 TcSYL_0007870.t1-p1 TcSYL_0007880.t1-p1
TcSYL_0007890.t1-p1 TcSYL_0007900.t1-p1 TcSYL_0007910.t1-p1
TcSYL_0007920.t1-p1 TcSYL_0007930.t1-p1 TcSYL_0007940.t1-p1
TcSYL_0007950.t1-p1 TcSYL_0007960.t1-p1
OG0000011: TCRU_10520 TCRU_2018 TCRU_4638 TCRU_4829 TCRU_5050
TCRU_5116 TCRU_5136 TCRU_6425 TCRU_6821 TCRU_7021 TCRU_7087
TCRU_7095 TCRU_7308 TCRU_7361 TCRU_7564 TCRU_7570 TCRU_7657
TCRU_7708 TCRU_8048 TCRU_9755 TcCLB-EL.508539.110_mRNA-p1 TcCLB-EL.
508539.160_mRNA-p1 TcCLB-EL.508539.60_mRNA-p1 TcCLB-EL.
508541.110_mRNA-p1 TcCLB-EL.508541.20_mRNA-p1 TcCLB-EL.
510205.50_mRNA-p1 TcCLB-EL.510833.20_mRNA-p1 TcCLB-EL.
511171.90_mRNA-p1 TcCLB-EL.511173.270_mRNA-p1 TcCLB-EL.
511173.360_mRNA-p1 TcCLB-EL.511173.400_mRNA-p1 TcCLB-NE.
503491.39_mRNA-p1 TcCLB-NE.503645.40_mRNA-p1 TcCLB-NE.
503947.20_mRNA-p1 TcCLB-NE.504197.5_mRNA-p1 TcCLB-NE.
504249.111_mRNA-p1 TcCLB-NE.504261.100_pseudogenic_transcript-p1
TcCLB-NE.504493.10_mRNA-p1 TcCLB-NE.506139.1_mRNA-p1 TcCLB-NE.
506281.134_mRNA-p1 TcCLB-NE.506615.29_pseudogenic_transcript-p1
TcCLB-NE.506955.184_pseudogenic_transcript-p1 TcCLB-NE.
506955.244_mRNA-p1 TcCLB-NE.506955.99_mRNA-p1 TcCLB-NE.
507069.10_mRNA-p1 TcCLB-NE.507069.150_mRNA-p1 TcCLB-NE.
507069.180_mRNA-p1 TcCLB-NE.507069.220_mRNA-p1 TcCLB-NE.
507071.120_mRNA-p1 TcCLB-NE.507071.140_mRNA-p1 TcCLB-NE.
507937.50_mRNA-p1 TcCLB-NE.508117.20_pseudogenic_transcript-p1
TcCLB-NE.508221.170_mRNA-p1 TcCLB-NE.508221.490_mRNA-p1 TcCLB-NE.
508261.15_mRNA-p1 TcCLB-NE.508305.50_mRNA-p1 TcCLB-NE.508365.4_mRNA-
p1 TcCLB-NE.508427.20_mRNA-p1 TcCLB-NE.508427.40_mRNA-p1 TcCLB-NE.
508433.90_mRNA-p1 TcCLB-NE.509753.194_mRNA-p1 TcCLB-NE.
510391.50_mRNA-p1 TcCLB-NE.510417.40_mRNA-p1 TcCLB-NE.
510483.230_mRNA-p1 TcCLB-NE.510489.20_mRNA-p1 TcCLB-NE.
510489.5_mRNA-p1 TcCLB-NE.510627.190_mRNA-p1 TcCLB-NE.
510693.130_mRNA-p1 TcCLB-NE.510697.130_mRNA-p1 TcCLB-NE.
510697.40_mRNA-p1 TcCLB-NE.510701.10_mRNA-p1 TcCLB-NE.
510713.110_mRNA-p1 TcCLB-NE.510713.130_mRNA-p1 TcCLB-NE.
510713.170_mRNA-p1 TcCLB-NE.510715.40_mRNA-p1 TcCLB-NE.
511255.540_mRNA-p1 TcSYL_0020720.t1-p1 TcSYL_0020820.t1-p1
TcSYL_0069850.t1-p1 TcSYL_0070060.t1-p1 TcSYL_0092940.t1-p1
TcSYL_0124230.t1-p1 TcSYL_0130150.t1-p1 TcSYL_0137460.t1-p1
OG0000012: TCRU_10429 TCRU_165 TCRU_167 TCRU_3834 TCRU_6740
TCRU_8393 TCRU_8528 TcCLB-EL.405453.9_mRNA-p1 TcCLB-EL.
457547.9_mRNA-p1 TcCLB-EL.503667.39_mRNA-p1 TcCLB-EL.503909.19_mRNA-
p1 TcCLB-EL.504557.50_mRNA-p1 TcCLB-EL.505607.9_mRNA-p1 TcCLB-EL.

506117.9_mRNA-p1 TcCLB-EL.506487.24_mRNA-p1 TcCLB-EL.506523.9_mRNA-p1 TcCLB-EL.506625.19_mRNA-p1 TcCLB-EL.506651.9_mRNA-p1 TcCLB-EL.507229.29_mRNA-p1 TcCLB-EL.507243.9_mRNA-p1 TcCLB-EL.507445.9_mRNA-p1 TcCLB-EL.507551.9_mRNA-p1 TcCLB-EL.507725.9_pseudogenic_transcript-p1 TcCLB-EL.507861.14_mRNA-p1 TcCLB-EL.507983.10_mRNA-p1 TcCLB-EL.508147.159_mRNA-p1 TcCLB-EL.508393.9_mRNA-p1 TcCLB-EL.508685.9_mRNA-p1 TcCLB-EL.508755.20_pseudogenic_transcript-p1 TcCLB-EL.508803.19_mRNA-p1 TcCLB-EL.509503.9_mRNA-p1 TcCLB-EL.509883.9_mRNA-p1 TcCLB-EL.509887.19_mRNA-p1 TcCLB-EL.509921.9_mRNA-p1 TcCLB-EL.510815.9_mRNA-p1 TcCLB-EL.510913.10_mRNA-p1 TcCLB-EL.511119.10_mRNA-p1 TcCLB-EL.511125.9_mRNA-p1 TcCLB-EL.511213.89_mRNA-p1 TcCLB-EL.511569.14_mRNA-p1 TcCLB-NE.415789.9_mRNA-p1 TcCLB-NE.504639.19_mRNA-p1 TcCLB-NE.505297.10_pseudogenic_transcript-p1 TcCLB-NE.505401.19_mRNA-p1 TcCLB-NE.506095.9_mRNA-p1 TcCLB-NE.506471.90_pseudogenic_transcript-p1 TcCLB-NE.506787.9_mRNA-p1 TcCLB-NE.507155.9_pseudogenic_transcript-p1 TcCLB-NE.507201.9_mRNA-p1 TcCLB-NE.507203.9_mRNA-p1 TcCLB-NE.507379.9_mRNA-p1 TcCLB-NE.508063.9_mRNA-p1 TcCLB-NE.508247.149_mRNA-p1 TcCLB-NE.508399.49_mRNA-p1 TcCLB-NE.508417.29_mRNA-p1 TcCLB-NE.508455.9_mRNA-p1 TcCLB-NE.508919.189_mRNA-p1 TcCLB-NE.509593.59_mRNA-p1 TcCLB-NE.510079.49_mRNA-p1 TcCLB-NE.510627.14_mRNA-p1 TcCLB-NE.510633.9_mRNA-p1 TcCLB-NE.510637.9_mRNA-p1 TcCLB-NE.510675.9_mRNA-p1 TcCLB-NE.510867.9_mRNA-p1 TcCLB-NE.511563.9_mRNA-p1 TcCLB-NE.511663.19_mRNA-p1 TcCLB-NE.511665.15_mRNA-p1 TcSYL_0018080.t1-p1 TcSYL_0057110.t1-p1 TcSYL_0083950.t1-p1 TcSYL_0084050.t1-p1 TcSYL_0086800.t1-p1 TcSYL_0092450.t1-p1 TcSYL_0092530.t1-p1 TcSYL_0093720.t1-p1 TcSYL_0137960.t1-p1 TcSYL_0141440.t1-p1 TcSYL_0186840.t1-p1 TcSYL_0187660.t1-p1 TcSYL_0200620.t1-p1 TcSYL_0201880.t1-p1
OG0000013: TCRU_1301 TCRU_2705 TCRU_4032 TCRU_5222 TCRU_5339 TCRU_6378 TCRU_6624 TCRU_6634 TCRU_7514 TCRU_8236 TCRU_9213 TCRU_9828 TcCLB-EL.503761.40_mRNA-p1 TcCLB-EL.504039.230_mRNA-p1 TcCLB-EL.504239.161_pseudogenic_transcript-p1 TcCLB-EL.504239.290_mRNA-p1 TcCLB-EL.506269.79_pseudogenic_transcript-p1 TcCLB-EL.506409.30_mRNA-p1 TcCLB-EL.506597.20_pseudogenic_transcript-p1 TcCLB-EL.506667.40_mRNA-p1 TcCLB-EL.506667.80_mRNA-p1 TcCLB-EL.506757.129_pseudogenic_transcript-p1 TcCLB-EL.506757.150_mRNA-p1 TcCLB-EL.506761.54_pseudogenic_transcript-p1 TcCLB-EL.506767.110_mRNA-p1 TcCLB-EL.506767.183_pseudogenic_transcript-p1 TcCLB-EL.506769.80_mRNA-p1 TcCLB-EL.507145.70_pseudogenic_transcript-p1 TcCLB-EL.507163.40_mRNA-p1 TcCLB-EL.507237.20_mRNA-p1 TcCLB-EL.507237.270_mRNA-p1 TcCLB-EL.507237.80_mRNA-p1 TcCLB-EL.507747.170_mRNA-p1 TcCLB-EL.507867.10_mRNA-p1 TcCLB-EL.507957.160_mRNA-p1 TcCLB-EL.508165.50_mRNA-p1 TcCLB-EL.508227.20_mRNA-p1 TcCLB-EL.508229.30_mRNA-p1 TcCLB-EL.509699.110_mRNA-p1 TcCLB-EL.510013.230_mRNA-p1 TcCLB-EL.510021.20_mRNA-p1 TcCLB-EL.510021.210_pseudogenic_transcript-p1 TcCLB-EL.510021.50_mRNA-p1 TcCLB-EL.510371.50_mRNA-p1 TcCLB-EL.510375.10_mRNA-p1 TcCLB-EL.510375.40_mRNA-p1 TcCLB-EL.510377.200_mRNA-p1 TcCLB-EL.510377.30_pseudogenic_transcript-p1 TcCLB-EL.510377.450_mRNA-p1 TcCLB-EL.511603.190_mRNA-p1 TcCLB-EL.511603.320_mRNA-p1 TcCLB-EL.511625.140_pseudogenic_transcript-p1 TcCLB-NE.504081.170_mRNA-p1

TcCLB-NE.504081.40_mRNA-p1 TcCLB-NE.504081.490_mRNA-p1 TcCLB-NE.
504155.293_mRNA-p1 TcCLB-NE.504261.40_mRNA-p1 TcCLB-NE.
506459.10_mRNA-p1 TcCLB-NE.506751.100_mRNA-p1 TcCLB-NE.
506751.20_pseudogenic_transcript-p1 TcCLB-NE.508221.210_mRNA-p1
TcCLB-NE.508221.329_pseudogenic_transcript-p1 TcCLB-NE.
508221.810_pseudogenic_transcript-p1 TcCLB-NE.508221.894_mRNA-p1
TcCLB-NE.508245.40_mRNA-p1 TcCLB-NE.508365.160_mRNA-p1 TcCLB-NE.
508873.103_pseudogenic_transcript-p1 TcCLB-NE.
508873.520_pseudogenic_transcript-p1 TcCLB-NE.509525.240_mRNA-p1
TcCLB-NE.509545.50_mRNA-p1 TcCLB-NE.509755.40_mRNA-p1 TcCLB-NE.
510237.70_mRNA-p1 TcCLB-NE.510361.240_pseudogenic_transcript-p1
TcCLB-NE.510483.220_mRNA-p1 TcCLB-NE.
511259.70_pseudogenic_transcript-p1 TcCLB-NE.511839.30_mRNA-p1
TcSYL_0074260.t1-p1 TcSYL_0133890.t1-p1 TcSYL_0134130.t1-p1
TcSYL_0136960.t1-p1 TcSYL_0152290.t1-p1
OG0000014: TCRU_10005 TCRU_10100 TCRU_10189 TCRU_10621 TCRU_1121
TCRU_1140 TCRU_1164 TCRU_1201 TCRU_1317 TCRU_1336 TCRU_1368 TCRU_141
TCRU_1420 TCRU_1624 TCRU_1625 TCRU_173 TCRU_2091 TCRU_2615 TCRU_2660
TCRU_2758 TCRU_2945 TCRU_2969 TCRU_3019 TCRU_3135 TCRU_3173
TCRU_3258 TCRU_3478 TCRU_3587 TCRU_3701 TCRU_3815 TCRU_3841
TCRU_3845 TCRU_3850 TCRU_4046 TCRU_4112 TCRU_4177 TCRU_4200
TCRU_4280 TCRU_4919 TCRU_4933 TCRU_4988 TCRU_500 TCRU_5146 TCRU_535
TCRU_5419 TCRU_585 TCRU_6307 TCRU_6419 TCRU_6539 TCRU_6783 TCRU_6840
TCRU_7109 TCRU_7300 TCRU_7402 TCRU_748 TCRU_7637 TCRU_7909 TCRU_8053
TCRU_8135 TCRU_8243 TCRU_8264 TCRU_9311 TCRU_9530 TCRU_9550
TCRU_9602 TCRU_9721 TCRU_9869 TCRU_9949 TCRU_9950 TCRU_9960
TcSYL_0000210.t1-p1 TcSYL_0037990.t1-p1 TcSYL_0044540.t1-p1
TcSYL_0052200.t1-p1 TcSYL_0065190.t1-p1 TcSYL_0121130.t1-p1
TcSYL_0174390.t1-p1 TcSYL_0179990.t1-p1
OG0000015: TCRU_3474 TCRU_4590 TCRU_8568 TCRU_9137 TcCLB-EL.
503503.15_pseudogenic_transcript-p1 TcCLB-EL.504039.110_mRNA-p1
TcCLB-EL.505025.141_mRNA-p1 TcCLB-EL.506267.19_mRNA-p1 TcCLB-EL.
506267.48_mRNA-p1 TcCLB-EL.506269.59_pseudogenic_transcript-p1
TcCLB-EL.506409.10_mRNA-p1 TcCLB-EL.506409.70_mRNA-p1 TcCLB-EL.
506501.59_mRNA-p1 TcCLB-EL.506597.33_mRNA-p1 TcCLB-EL.
506767.80_mRNA-p1 TcCLB-EL.506769.55_mRNA-p1 TcCLB-EL.
507237.140_mRNA-p1 TcCLB-EL.507237.200_mRNA-p1 TcCLB-EL.
507429.24_mRNA-p1 TcCLB-EL.507429.65_mRNA-p1 TcCLB-EL.507429.6_mRNA-
p1 TcCLB-EL.508097.110_mRNA-p1 TcCLB-EL.508125.120_mRNA-p1 TcCLB-EL.
508147.75_mRNA-p1 TcCLB-EL.508163.300_mRNA-p1 TcCLB-EL.
508389.146_mRNA-p1 TcCLB-EL.509291.10_mRNA-p1 TcCLB-EL.
509973.40_mRNA-p1 TcCLB-EL.510015.40_mRNA-p1 TcCLB-EL.
510373.80_mRNA-p1 TcCLB-EL.510555.21_mRNA-p1 TcCLB-EL.
510557.65_mRNA-p1 TcCLB-EL.510557.76_mRNA-p1 TcCLB-EL.
510561.14_mRNA-p1 TcCLB-EL.510561.35_mRNA-p1 TcCLB-EL.
510561.60_mRNA-p1 TcCLB-EL.510621.40_mRNA-p1 TcCLB-EL.
510621.80_mRNA-p1 TcCLB-EL.511593.65_mRNA-p1 TcCLB-EL.
511793.20_mRNA-p1 TcCLB-EL.511797.150_mRNA-p1 TcCLB-NE.
503417.36_mRNA-p1 TcCLB-NE.503889.5_mRNA-p1 TcCLB-NE.506613.30_mRNA-
p1 TcCLB-NE.506923.30_pseudogenic_transcript-p1 TcCLB-NE.
506995.95_mRNA-p1 TcCLB-NE.508119.40_mRNA-p1 TcCLB-NE.
508363.10_mRNA-p1 TcCLB-NE.508365.140_mRNA-p1 TcCLB-NE.
508435.15_mRNA-p1 TcCLB-NE.508501.90_mRNA-p1 TcCLB-NE.
508873.224_mRNA-p1 TcCLB-NE.508999.140_mRNA-p1 TcCLB-NE.

509493.60_mRNA-p1 TcCLB-NE.509525.232_mRNA-p1 TcCLB-NE.
509525.244_mRNA-p1 TcCLB-NE.510359.590_mRNA-p1 TcCLB-NE.
510587.10_mRNA-p1 TcCLB-NE.510625.70_mRNA-p1 TcCLB-NE.
510629.60_mRNA-p1 TcCLB-NE.510791.11_mRNA-p1 TcCLB-NE.
510979.80_mRNA-p1 TcCLB-NE.511255.650_mRNA-p1 TcCLB-NE.
511645.30_mRNA-p1 TcSYL_0068770.t1-p1 TcSYL_0105940.t1-p1
TcSYL_0109800.t1-p1 TcSYL_0134650.t1-p1 TcSYL_0154640.t1-p1
TcSYL_0155610.t1-p1
OG0000016: TCRU_10116 TCRU_10361 TCRU_10481 TCRU_1181 TCRU_2561
TCRU_2706 TCRU_276 TCRU_299 TCRU_3137 TCRU_3566 TCRU_3677 TCRU_4332
TCRU_4348 TCRU_4503 TCRU_5053 TCRU_5099 TCRU_5298 TCRU_5410
TCRU_6333 TCRU_6487 TCRU_7958 TCRU_8500 TCRU_8632 TCRU_9211
TCRU_9964 TcCLB-EL.430281.10_pseudogenic_transcript-p1 TcCLB-EL.
504099.40_pseudogenic_transcript-p1 TcCLB-EL.
506495.30_pseudogenic_transcript-p1 TcCLB-EL.
507881.10_pseudogenic_transcript-p1 TcCLB-EL.
509765.20_pseudogenic_transcript-p1 TcCLB-NE.
504115.90_pseudogenic_transcript-p1 TcCLB-NE.506537.50_mRNA-p1
TcCLB-NE.506561.20_pseudogenic_transcript-p1 TcCLB-NE.
507661.10_pseudogenic_transcript-p1 TcCLB-NE.
508139.135_pseudogenic_transcript-p1 TcCLB-NE.
508193.20_pseudogenic_transcript-p1 TcCLB-NE.509875.11_mRNA-p1
TcCLB-NE.510397.30_pseudogenic_transcript-p1 TcCLB-NE.
511843.60_pseudogenic_transcript-p1 TcSYL_0004860.t1-p1
TcSYL_0027060.t1-p1 TcSYL_0028390.t1-p1 TcSYL_0029330.t1-p1
TcSYL_0049030.t1-p1 TcSYL_0084080.t1-p1 TcSYL_0084120.t1-p1
TcSYL_0092580.t1-p1 TcSYL_0103910.t1-p1 TcSYL_0109520.t1-p1
TcSYL_0112590.t1-p1 TcSYL_0112600.t1-p1 TcSYL_0118260.t1-p1
TcSYL_0120830.t1-p1 TcSYL_0121300.t1-p1 TcSYL_0121460.t1-p1
TcSYL_0121480.t1-p1 TcSYL_0121490.t1-p1 TcSYL_0137950.t1-p1
TcSYL_0155200.t1-p1 TcSYL_0163790.t1-p1 TcSYL_0164000.t1-p1
TcSYL_0172920.t1-p1 TcSYL_0190930.t1-p1 TcSYL_0200250.t1-p1
TcSYL_0203440.t1-p1 TcSYL_0203500.t1-p1 TcSYL_0203970.t1-p1
TcSYL_0204420.t1-p1
OG0000017: TCRU_1754 TCRU_1869 TCRU_4302 TCRU_4434 TCRU_887
TCRU_8903 TCRU_9336 TcCLB-EL.418405.10_mRNA-p1 TcCLB-EL.
503659.10_mRNA-p1 TcCLB-EL.506799.130_mRNA-p1 TcCLB-EL.
506801.40_mRNA-p1 TcCLB-EL.506965.130_mRNA-p1 TcCLB-EL.
506971.60_mRNA-p1 TcCLB-EL.506973.30_mRNA-p1 TcCLB-EL.
507237.190_mRNA-p1 TcCLB-EL.507701.4_mRNA-p1 TcCLB-EL.
507859.60_mRNA-p1 TcCLB-EL.508125.14_mRNA-p1 TcCLB-EL.
508389.154_mRNA-p1 TcCLB-EL.508389.50_mRNA-p1 TcCLB-EL.
509095.10_mRNA-p1 TcCLB-EL.509195.30_mRNA-p1 TcCLB-EL.
510621.60_mRNA-p1 TcCLB-EL.511607.110_mRNA-p1 TcCLB-EL.
511611.50_mRNA-p1 TcCLB-EL.511613.60_mRNA-p1 TcCLB-EL.
511787.10_mRNA-p1 TcCLB-EL.511793.30_mRNA-p1 TcCLB-EL.
511797.167_mRNA-p1 TcCLB-NE.506001.14_mRNA-p1 TcCLB-NE.
506781.19_mRNA-p1 TcCLB-NE.506781.39_mRNA-p1 TcCLB-NE.
506783.39_mRNA-p1 TcCLB-NE.506785.14_mRNA-p1 TcCLB-NE.
506789.11_mRNA-p1 TcCLB-NE.506789.50_mRNA-p1 TcCLB-NE.
508221.104_mRNA-p1 TcCLB-NE.508221.150_mRNA-p1 TcCLB-NE.
508261.130_mRNA-p1 TcCLB-NE.508261.50_mRNA-p1 TcCLB-NE.
508261.70_pseudogenic_transcript-p1 TcCLB-NE.510387.40_mRNA-p1
TcCLB-NE.510625.109_mRNA-p1 TcCLB-NE.510625.150_mRNA-p1 TcCLB-NE.

510625.190_mRNA-p1 TcCLB-NE.510625.88_mRNA-p1 TcCLB-NE.
510627.110_mRNA-p1 TcCLB-NE.510627.128_mRNA-p1 TcCLB-NE.
510627.80_mRNA-p1 TcCLB-NE.510629.150_mRNA-p1 TcCLB-NE.
510629.30_mRNA-p1 TcCLB-NE.510629.310_mRNA-p1 TcCLB-NE.
510629.350_mRNA-p1 TcCLB-NE.511259.180_mRNA-p1 TcSYL_0029460.t1-p1
TcSYL_0093310.t1-p1 TcSYL_0126840.t1-p1 TcSYL_0143410.t1-p1
TcSYL_0150460.t1-p1 TcSYL_0151220.t1-p1 TcSYL_0151480.t1-p1
TcSYL_0153750.t1-p1 TcSYL_0161770.t1-p1 TcSYL_0162240.t1-p1
OG0000018: TCRU_10138 TCRU_10421 TCRU_10436 TCRU_10486 TCRU_1291
TCRU_1294 TCRU_1548 TCRU_2502 TCRU_3038 TCRU_3163 TCRU_3681
TCRU_3698 TCRU_3717 TCRU_403 TCRU_4192 TCRU_4526 TCRU_4596 TCRU_4878
TCRU_4989 TCRU_5113 TCRU_5409 TCRU_5438 TCRU_596 TCRU_6383 TCRU_6542
TCRU_6969 TCRU_7121 TCRU_7136 TCRU_7323 TCRU_791 TCRU_8173 TCRU_8259
TCRU_9683 TCRU_9958 TcCLB-EL.506243.77_pseudogenic_transcript-p1
TcCLB-EL.506683.250_pseudogenic_transcript-p1 TcCLB-EL.
508563.30_pseudogenic_transcript-p1 TcCLB-EL.
508605.10_pseudogenic_transcript-p1 TcCLB-EL.508607.10_mRNA-p1
TcCLB-NE.503763.10_mRNA-p1 TcCLB-NE.
508139.40_pseudogenic_transcript-p1 TcCLB-NE.508529.10_mRNA-p1
TcCLB-NE.508969.30_pseudogenic_transcript-p1 TcCLB-NE.510571.5_mRNA-
p1 TcCLB-NE.511887.10_pseudogenic_transcript-p1 TcSYL_0014640.t1-p1
TcSYL_0017310.t1-p1 TcSYL_0022580.t1-p1 TcSYL_0058380.t1-p1
TcSYL_0059790.t1-p1 TcSYL_0070780.t1-p1 TcSYL_0079720.t1-p1
TcSYL_0080000.t1-p1 TcSYL_0106330.t1-p1 TcSYL_0106670.t1-p1
TcSYL_0155220.t1-p1 TcSYL_0190920.t1-p1 TcSYL_0204450.t1-p1
OG0000019: TCRU_10107 TCRU_10478 TCRU_1204 TCRU_1539 TCRU_1813
TCRU_2133 TCRU_2324 TCRU_2735 TCRU_4106 TCRU_4194 TCRU_8257
TCRU_9542 TcCLB-EL.504039.210_mRNA-p1 TcCLB-EL.506499.50_mRNA-p1
TcCLB-EL.506501.105_pseudogenic_transcript-p1 TcCLB-EL.
506501.310_mRNA-p1 TcCLB-EL.506501.370_mRNA-p1 TcCLB-EL.
506599.300_mRNA-p1 TcCLB-EL.506599.380_mRNA-p1 TcCLB-EL.
506759.10_mRNA-p1 TcCLB-EL.506763.160_mRNA-p1 TcCLB-EL.
506767.90_mRNA-p1 TcCLB-EL.506769.35_pseudogenic_transcript-p1
TcCLB-EL.507953.150_mRNA-p1 TcCLB-EL.507953.50_mRNA-p1 TcCLB-EL.
507957.290_mRNA-p1 TcCLB-EL.507959.290_mRNA-p1 TcCLB-EL.
510013.100_mRNA-p1 TcCLB-EL.510013.320_mRNA-p1 TcCLB-EL.
510371.80_mRNA-p1 TcCLB-EL.510373.50_mRNA-p1 TcCLB-NE.
503645.55_mRNA-p1 TcCLB-NE.503973.110_mRNA-p1 TcCLB-NE.
503973.350_mRNA-p1 TcCLB-NE.504081.360_mRNA-p1 TcCLB-NE.
506137.20_mRNA-p1 TcCLB-NE.506459.140_mRNA-p1 TcCLB-NE.
506459.200_mRNA-p1 TcCLB-NE.507069.20_mRNA-p1 TcCLB-NE.
507069.210_mRNA-p1 TcCLB-NE.507071.130_mRNA-p1 TcCLB-NE.
507937.60_mRNA-p1 TcCLB-NE.508219.100_mRNA-p1 TcCLB-NE.
508219.80_mRNA-p1 TcCLB-NE.508221.480_mRNA-p1 TcCLB-NE.
508871.100_mRNA-p1 TcCLB-NE.508871.160_mRNA-p1 TcCLB-NE.
508873.20_mRNA-p1 TcCLB-NE.508873.260_mRNA-p1 TcCLB-NE.
508873.470_mRNA-p1 TcCLB-NE.509971.60_mRNA-p1 TcCLB-NE.
510361.120_mRNA-p1 TcCLB-NE.510361.220_mRNA-p1 TcCLB-NE.
510363.290_mRNA-p1 TcCLB-NE.510363.90_mRNA-p1 TcCLB-NE.
511259.30_mRNA-p1 TcSYL_0133460.t1-p1 TcSYL_0136440.t1-p1
OG0000020: TCRU_10008 TCRU_10635 TCRU_1461 TCRU_335 TCRU_3883
TCRU_6814 TCRU_77 TCRU_8510 TCRU_8585 TcCLB-EL.
420293.40_pseudogenic_transcript-p1 TcCLB-EL.503447.60_mRNA-p1
TcCLB-EL.505365.50_pseudogenic_transcript-p1 TcCLB-EL.

506129.60_mRNA-p1 TcCLB-EL.506183.70_mRNA-p1 TcCLB-EL.
506341.30_mRNA-p1 TcCLB-EL.506345.100_pseudogenic_transcript-p1
TcCLB-EL.506683.140_pseudogenic_transcript-p1 TcCLB-EL.
507121.40_mRNA-p1 TcCLB-EL.507479.51_mRNA-p1 TcCLB-EL.
507875.60_mRNA-p1 TcCLB-EL.508325.220_pseudogenic_transcript-p1
TcCLB-EL.508607.60_mRNA-p1 TcCLB-EL.509581.20_mRNA-p1 TcCLB-EL.
509777.40_mRNA-p1 TcCLB-EL.509815.50_mRNA-p1 TcCLB-EL.
509959.40_mRNA-p1 TcCLB-EL.510847.20_mRNA-p1 TcCLB-EL.
510853.50_mRNA-p1 TcCLB-NE.503861.50_mRNA-p1 TcCLB-NE.
504115.30_mRNA-p1 TcCLB-NE.505207.20_mRNA-p1 TcCLB-NE.
505267.30_mRNA-p1 TcCLB-NE.506393.30_mRNA-p1 TcCLB-NE.
506471.40_mRNA-p1 TcCLB-NE.506507.30_mRNA-p1 TcCLB-NE.
506595.60_pseudogenic_transcript-p1 TcCLB-NE.507167.200_mRNA-p1
TcCLB-NE.507591.20_pseudogenic_transcript-p1 TcCLB-NE.
508521.112_pseudogenic_transcript-p1 TcCLB-NE.
508521.30_pseudogenic_transcript-p1 TcCLB-NE.508677.50_mRNA-p1
TcCLB-NE.509223.60_mRNA-p1 TcCLB-NE.509295.50_mRNA-p1 TcCLB-NE.
509875.50_mRNA-p1 TcCLB-NE.509931.10_mRNA-p1 TcCLB-NE.
510643.110_mRNA-p1 TcCLB-NE.511671.30_mRNA-p1 TcCLB-NE.
511771.30_pseudogenic_transcript-p1 TcCLB-NE.
511861.60_pseudogenic_transcript-p1 TcSYL_0000430.t1-p1
TcSYL_0004840.t1-p1 TcSYL_0022610.t1-p1 TcSYL_0112920.t1-p1
TcSYL_0166660.t1-p1 TcSYL_0190900.t1-p1 TcSYL_0200430.t1-p1
TcSYL_0201920.t1-p1
OG0000021: TCRU_6834 TcCLB-EL.506563.40_mRNA-p1 TcCLB-NE.
509003.70_pseudogenic_transcript-p1 TcSYL_0022180.t1-p1
TcSYL_0024300.t1-p1 TcSYL_0024540.t1-p1 TcSYL_0024680.t1-p1
TcSYL_0024810.t1-p1 TcSYL_0024840.t1-p1 TcSYL_0024850.t1-p1
TcSYL_0024890.t1-p1 TcSYL_0024930.t1-p1 TcSYL_0025070.t1-p1
TcSYL_0025230.t1-p1 TcSYL_0025280.t1-p1 TcSYL_0025350.t1-p1
TcSYL_0049190.t1-p1 TcSYL_0049260.t1-p1 TcSYL_0049270.t1-p1
TcSYL_0049340.t1-p1 TcSYL_0049360.t1-p1 TcSYL_0049410.t1-p1
TcSYL_0049570.t1-p1 TcSYL_0049580.t1-p1 TcSYL_0049640.t1-p1
TcSYL_0049650.t1-p1 TcSYL_0049700.t1-p1 TcSYL_0049880.t1-p1
TcSYL_0063460.t1-p1 TcSYL_0063470.t1-p1 TcSYL_0063560.t1-p1
TcSYL_0063580.t1-p1 TcSYL_0063610.t1-p1 TcSYL_0063620.t1-p1
TcSYL_0063650.t1-p1 TcSYL_0063690.t1-p1 TcSYL_0063700.t1-p1
TcSYL_0063710.t1-p1 TcSYL_0063740.t1-p1 TcSYL_0063760.t1-p1
TcSYL_0063770.t1-p1 TcSYL_0063780.t1-p1 TcSYL_0063810.t1-p1
TcSYL_0063850.t1-p1 TcSYL_0063870.t1-p1 TcSYL_0063880.t1-p1
TcSYL_0063890.t1-p1 TcSYL_0063900.t1-p1 TcSYL_0071010.t1-p1
TcSYL_0071270.t1-p1 TcSYL_0137590.t1-p1 TcSYL_0137600.t1-p1
TcSYL_0137620.t1-p1 TcSYL_0137640.t1-p1 TcSYL_0137660.t1-p1
TcSYL_0166610.t1-p1 TcSYL_0194460.t1-p1
OG0000022: TCRU_1367 TCRU_2433 TCRU_2662 TCRU_275 TCRU_2982
TCRU_3311 TCRU_3735 TcCLB-EL.421173.4_mRNA-p1 TcCLB-EL.
505365.60_mRNA-p1 TcCLB-EL.506341.50_mRNA-p1 TcCLB-EL.
506683.110_mRNA-p1 TcCLB-EL.506717.80_mRNA-p1 TcCLB-EL.
506961.150_mRNA-p1 TcCLB-EL.507121.20_mRNA-p1 TcCLB-EL.
507555.50_mRNA-p1 TcCLB-EL.507759.10_mRNA-p1 TcCLB-EL.
507869.20_pseudogenic_transcript-p1 TcCLB-EL.508515.150_mRNA-p1
TcCLB-EL.508903.10_pseudogenic_transcript-p1 TcCLB-EL.
508903.110_mRNA-p1 TcCLB-EL.509581.10_mRNA-p1 TcCLB-EL.
509765.50_mRNA-p1 TcCLB-EL.509843.20_mRNA-p1 TcCLB-EL.

509959.60_pseudogenic_transcript-p1 TcCLB-EL.510125.20_mRNA-p1
TcCLB-EL.510307.240_mRNA-p1 TcCLB-EL.510847.10_mRNA-p1 TcCLB-EL.
511587.90_mRNA-p1 TcCLB-NE.503861.40_mRNA-p1 TcCLB-NE.
504115.10_mRNA-p1 TcCLB-NE.505207.10_mRNA-p1 TcCLB-NE.
505997.80_mRNA-p1 TcCLB-NE.506083.20_pseudogenic_transcript-p1
TcCLB-NE.506537.200_mRNA-p1 TcCLB-NE.506723.20_mRNA-p1 TcCLB-NE.
506975.90_mRNA-p1 TcCLB-NE.508061.154_mRNA-p1 TcCLB-NE.
508171.10_mRNA-p1 TcCLB-NE.508521.100_mRNA-p1 TcCLB-NE.
508521.20_mRNA-p1 TcCLB-NE.508677.40_mRNA-p1 TcCLB-NE.
508963.30_mRNA-p1 TcCLB-NE.509217.160_mRNA-p1 TcCLB-NE.
509387.10_mRNA-p1 TcCLB-NE.509663.50_mRNA-p1 TcCLB-NE.
509931.20_mRNA-p1 TcCLB-NE.510643.40_mRNA-p1 TcCLB-NE.
511669.10_mRNA-p1 TcCLB-NE.511831.10_mRNA-p1 TcCLB-NE.
511833.10_mRNA-p1 TcCLB-NE.511861.50_mRNA-p1 TcSYL_0014610.t1-p1
TcSYL_0014620.t1-p1 TcSYL_0021230.t1-p1 TcSYL_0093650.t1-p1
TcSYL_0093810.t1-p1
OG0000023: TCRU_680 TcCLB-EL.504219.70_mRNA-p1 TcCLB-EL.
506409.110_mRNA-p1 TcCLB-EL.508125.110_mRNA-p1 TcCLB-EL.
509091.10_mRNA-p1 TcCLB-EL.509293.10_mRNA-p1 TcCLB-EL.
510015.10_mRNA-p1 TcCLB-EL.510015.50_pseudogenic_transcript-p1
TcCLB-NE.413859.10_mRNA-p1 TcCLB-NE.509753.220_mRNA-p1 TcCLB-NE.
510693.210_mRNA-p1 TcSYL_0017590.t1-p1 TcSYL_0022020.t1-p1
TcSYL_0023800.t1-p1 TcSYL_0024150.t1-p1 TcSYL_0028180.t1-p1
TcSYL_0039510.t1-p1 TcSYL_0067340.t1-p1 TcSYL_0068730.t1-p1
TcSYL_0068800.t1-p1 TcSYL_0069080.t1-p1 TcSYL_0069580.t1-p1
TcSYL_0070230.t1-p1 TcSYL_0070290.t1-p1 TcSYL_0070410.t1-p1
TcSYL_0071890.t1-p1 TcSYL_0093090.t1-p1 TcSYL_0100310.t1-p1
TcSYL_0100900.t1-p1 TcSYL_0101250.t1-p1 TcSYL_0123960.t1-p1
TcSYL_0128150.t1-p1 TcSYL_0128920.t1-p1 TcSYL_0129490.t1-p1
TcSYL_0129790.t1-p1 TcSYL_0133920.t1-p1 TcSYL_0134320.t1-p1
TcSYL_0134690.t1-p1 TcSYL_0135340.t1-p1 TcSYL_0137010.t1-p1
TcSYL_0149030.t1-p1 TcSYL_0149990.t1-p1 TcSYL_0150080.t1-p1
TcSYL_0150290.t1-p1 TcSYL_0150580.t1-p1 TcSYL_0151460.t1-p1
TcSYL_0152270.t1-p1 TcSYL_0155550.t1-p1 TcSYL_0155720.t1-p1
TcSYL_0160940.t1-p1 TcSYL_0161090.t1-p1 TcSYL_0162020.t1-p1
TcSYL_0190340.t1-p1 TcSYL_0190500.t1-p1 TcSYL_0190550.t1-p1
OG0000024: TCRU_10209 TCRU_4874 TCRU_6198 TCRU_6245 TCRU_6301
TCRU_9293 TCRU_of TCRU_of TCRU_of TCRU_of TCRU_of TCRU_of TCRU_of
TCRU_of TCRU_of TCRU_of TCRU_of TCRU_of TCRU_of TCRU_of TCRU_of
TCRU_of TCRU_of TCRU_of TCRU_of TCRU_of TCRU_of TCRU_of TCRU_of
TCRU_of TCRU_of TCRU_of TCRU_of TCRU_of TCRU_of TCRU_of TCRU_of
TCRU_of TCRU_of TCRU_of TCRU_of TCRU_of TCRU_of TCRU_of TCRU_of
TCRU_of TCRU_of TCRU_of TCRU_of TCRU_of TCRU_of TCRU_of TCRU_of
TcCLB-NE.506903.50_pseudogenic_transcript-p1
OG0000025: TCRU_1670 TCRU_3200 TCRU_4061 TCRU_4156 TCRU_4771
TCRU_511 TCRU_6306 TCRU_7050 TCRU_7062 TCRU_994 TcCLB-EL.
454787.10_mRNA-p1 TcCLB-EL.504239.280_mRNA-p1 TcCLB-EL.
505025.60_mRNA-p1 TcCLB-EL.506499.190_mRNA-p1 TcCLB-EL.
506499.220_mRNA-p1 TcCLB-EL.506499.90_mRNA-p1 TcCLB-EL.
506667.30_mRNA-p1 TcCLB-EL.506759.140_mRNA-p1 TcCLB-EL.
507163.30_mRNA-p1 TcCLB-EL.507953.200_mRNA-p1 TcCLB-EL.
507953.70_mRNA-p1 TcCLB-EL.507981.30_mRNA-p1 TcCLB-EL.
508099.20_mRNA-p1 TcCLB-EL.508157.10_pseudogenic_transcript-p1
TcCLB-EL.508163.234_mRNA-p1 TcCLB-EL.510013.200_mRNA-p1 TcCLB-EL.

510021.160_mRNA-p1 TcCLB-EL.510021.80_mRNA-p1 TcCLB-EL.
510025.70_mRNA-p1 TcCLB-EL.510261.41_pseudogenic_transcript-p1
TcCLB-EL.510279.40_mRNA-p1 TcCLB-EL.510373.30_mRNA-p1 TcCLB-EL.
511553.160_mRNA-p1 TcCLB-EL.511599.30_mRNA-p1 TcCLB-EL.
511603.470_mRNA-p1 TcCLB-EL.511605.55_mRNA-p1 TcCLB-NE.
504081.250_mRNA-p1 TcCLB-NE.504081.440_mRNA-p1 TcCLB-NE.
508219.90_mRNA-p1 TcCLB-NE.508221.260_mRNA-p1 TcCLB-NE.
508221.730_mRNA-p1 TcCLB-NE.508247.110_mRNA-p1 TcCLB-NE.
508853.30_mRNA-p1 TcCLB-NE.508873.289_mRNA-p1 TcCLB-NE.
510483.280_mRNA-p1 TcCLB-NE.510693.40_mRNA-p1 TcCLB-NE.
511233.160_mRNA-p1 TcCLB-NE.511487.150_mRNA-p1 TcSYL_0135650.t1-p1
TcSYL_0137570.t1-p1 TcSYL_0153720.t1-p1 TcSYL_0154240.t1-p1
OG0000026: TCRU_10447 TCRU_2968 TCRU_5768 TCRU_5813 TCRU_5920
TCRU_6073 TCRU_8707 TCRU_924 TCRU_9705 TcCLB-EL.424889.10_mRNA-p1
TcCLB-EL.503615.10_mRNA-p1 TcCLB-EL.503949.40_mRNA-p1 TcCLB-EL.
504033.21_mRNA-p1 TcCLB-EL.504557.10_mRNA-p1 TcCLB-EL.
504557.20_mRNA-p1 TcCLB-EL.507757.21_mRNA-p1 TcCLB-EL.
508367.20_mRNA-p1 TcCLB-EL.508605.20_mRNA-p1 TcCLB-EL.
508673.20_mRNA-p1 TcCLB-EL.508805.120_mRNA-p1 TcCLB-EL.
508805.136_mRNA-p1 TcCLB-EL.508975.30_mRNA-p1 TcCLB-EL.
510071.30_mRNA-p1 TcCLB-EL.510553.50_mRNA-p1 TcCLB-EL.
511129.80_mRNA-p1 TcCLB-EL.511395.120_mRNA-p1 TcCLB-NE.
503481.20_mRNA-p1 TcCLB-NE.507895.60_mRNA-p1 TcCLB-NE.
508065.10_mRNA-p1 TcCLB-NE.508193.60_mRNA-p1 TcCLB-NE.
508195.10_mRNA-p1 TcCLB-NE.508297.20_mRNA-p1 TcCLB-NE.508547.9_mRNA-
p1 TcCLB-NE.508595.20_mRNA-p1 TcCLB-NE.508929.10_mRNA-p1 TcCLB-NE.
508929.20_mRNA-p1 TcCLB-NE.508931.30_mRNA-p1 TcCLB-NE.
508971.10_mRNA-p1 TcCLB-NE.509263.10_mRNA-p1 TcCLB-NE.
509863.10_mRNA-p1 TcCLB-NE.510339.100_mRNA-p1 TcCLB-NE.
511861.80_mRNA-p1 TcSYL_0039850.t1-p1 TcSYL_0083970.t1-p1
TcSYL_0146150.t1-p1 TcSYL_0146550.t1-p1 TcSYL_0178080.t1-p1
TcSYL_0184920.t1-p1 TcSYL_0203430.t1-p1
OG0000027: TCRU_10241 TCRU_1123 TCRU_3868 TCRU_4063 TCRU_4111
TCRU_4588 TCRU_4592 TCRU_4780 TCRU_4916 TCRU_5286 TCRU_7316
TCRU_8992 TcCLB-EL.503665.40_pseudogenic_transcript-p1 TcCLB-EL.
504239.40_pseudogenic_transcript-p1 TcCLB-EL.
506501.150_pseudogenic_transcript-p1 TcCLB-EL.506501.20_mRNA-p1
TcCLB-EL.506759.31_pseudogenic_transcript-p1 TcCLB-EL.
506767.389_pseudogenic_transcript-p1 TcCLB-EL.
507237.359_pseudogenic_transcript-p1 TcCLB-EL.
507959.224_pseudogenic_transcript-p1 TcCLB-EL.
508165.360_pseudogenic_transcript-p1 TcCLB-EL.
508201.20_pseudogenic_transcript-p1 TcCLB-EL.
509975.15_pseudogenic_transcript-p1 TcCLB-EL.
509979.162_pseudogenic_transcript-p1 TcCLB-EL.
510013.26_pseudogenic_transcript-p1 TcCLB-EL.
510025.191_pseudogenic_transcript-p1 TcCLB-EL.
510375.5_pseudogenic_transcript-p1 TcCLB-EL.511603.151_mRNA-p1
TcCLB-EL.511603.434_mRNA-p1 TcCLB-EL.
511613.166_pseudogenic_transcript-p1 TcCLB-NE.
503771.19_pseudogenic_transcript-p1 TcCLB-NE.
504081.156_pseudogenic_transcript-p1 TcCLB-NE.
504155.221_pseudogenic_transcript-p1 TcCLB-NE.
506321.150_pseudogenic_transcript-p1 TcCLB-NE.

506321.60_pseudogenic_transcript-p1 TcCLB-NE.
507071.294_pseudogenic_transcript-p1 TcCLB-NE.
508117.10_pseudogenic_transcript-p1 TcCLB-NE.
508119.11_pseudogenic_transcript-p1 TcCLB-NE.
508221.990_pseudogenic_transcript-p1 TcCLB-NE.508245.81_mRNA-p1
TcCLB-NE.508521.50_pseudogenic_transcript-p1 TcCLB-NE.
510037.20_pseudogenic_transcript-p1 TcCLB-NE.511255.320_mRNA-p1
TcSYL_0020740.t1-p1 TcSYL_0094540.t1-p1 TcSYL_0133310.t1-p1
TcSYL_0148670.t1-p1 TcSYL_0156330.t1-p1
OG0000028: TCRU_10389 TCRU_10674 TCRU_1606 TCRU_1610 TCRU_1627
TCRU_377 TCRU_5251 TCRU_5982 TCRU_6527 TCRU_7133 TCRU_8042 TCRU_8483
TCRU_8533 TCRU_8615 TCRU_8784 TCRU_9054 TCRU_9560 TCRU_9691
TCRU_9830 TcCLB-EL.507907.50_mRNA-p1 TcCLB-EL.
509871.39_pseudogenic_transcript-p1 TcCLB-NE.
505997.40_pseudogenic_transcript-p1 TcCLB-NE.
506001.120_pseudogenic_transcript-p1 TcCLB-NE.
506017.20_pseudogenic_transcript-p1 TcCLB-NE.
506325.30_pseudogenic_transcript-p1 TcCLB-NE.
507499.50_pseudogenic_transcript-p1 TcCLB-NE.
508003.30_pseudogenic_transcript-p1 TcCLB-NE.
508479.90_pseudogenic_transcript-p1 TcCLB-NE.
508629.58_pseudogenic_transcript-p1 TcCLB-NE.
508631.40_pseudogenic_transcript-p1 TcCLB-NE.
509163.100_pseudogenic_transcript-p1 TcCLB-NE.
509217.140_pseudogenic_transcript-p1 TcCLB-NE.
509259.80_pseudogenic_transcript-p1 TcCLB-NE.
509579.40_pseudogenic_transcript-p1 TcCLB-NE.
509717.140_pseudogenic_transcript-p1 TcCLB-NE.
509725.100_pseudogenic_transcript-p1 TcCLB-NE.
509915.80_pseudogenic_transcript-p1 TcCLB-NE.
510081.11_pseudogenic_transcript-p1 TcCLB-NE.
511019.60_pseudogenic_transcript-p1 TcCLB-NE.
511843.90_pseudogenic_transcript-p1 TcSYL_0004730.t1-p1
TcSYL_0022490.t1-p1 TcSYL_0022560.t1-p1 TcSYL_0079610.t1-p1
TcSYL_0141640.t1-p1 TcSYL_0143230.t1-p1 TcSYL_0148070.t1-p1
OG0000029: TCRU_1315 TCRU_3040 TCRU_3843 TCRU_3951 TCRU_3990
TCRU_5699 TCRU_6047 TCRU_6654 TCRU_712 TCRU_8668 TCRU_8883 TCRU_9308
TCRU_9440 TCRU_990 TcCLB-EL.416761.10_mRNA-p1 TcCLB-EL.
479517.50_mRNA-p1 TcCLB-EL.505989.70_mRNA-p1 TcCLB-EL.
506401.380_mRNA-p1 TcCLB-EL.507993.350_mRNA-p1 TcCLB-EL.
508609.10_mRNA-p1 TcCLB-EL.508611.30_mRNA-p1 TcCLB-EL.
510263.30_mRNA-p1 TcCLB-EL.510747.40_mRNA-p1 TcCLB-EL.
510761.80_mRNA-p1 TcCLB-EL.511035.10_mRNA-p1 TcCLB-NE.
410797.10_mRNA-p1 TcCLB-NE.506321.240_mRNA-p1 TcCLB-NE.
506435.370_mRNA-p1 TcCLB-NE.507917.10_mRNA-p1 TcCLB-NE.
507919.10_mRNA-p1 TcCLB-NE.508187.30_mRNA-p1 TcCLB-NE.
508545.40_mRNA-p1 TcCLB-NE.508693.100_mRNA-p1 TcCLB-NE.
508999.170_mRNA-p1 TcCLB-NE.510327.20_pseudogenic_transcript-p1
TcCLB-NE.510657.200_mRNA-p1 TcCLB-NE.511257.60_mRNA-p1 TcCLB-NE.
511277.610_mRNA-p1 TcCLB-NE.511281.50_mRNA-p1 TcCLB-NE.
511723.10_mRNA-p1 TcSYL_0014690.t1-p1 TcSYL_0142410.t1-p1
TcSYL_0160120.t1-p1 TcSYL_0203070.t1-p1
OG0000030: TCRU_2259 TcCLB-EL.507941.150_mRNA-p1 TcCLB-EL.
507941.160_mRNA-p1 TcCLB-EL.507941.170_mRNA-p1 TcCLB-EL.

508203.20_mRNA-p1 TcCLB-EL.508203.29_mRNA-p1 TcCLB-EL.
508203.38_mRNA-p1 TcCLB-EL.508203.47_mRNA-p1 TcCLB-EL.
508203.56_mRNA-p1 TcCLB-EL.510351.11_mRNA-p1 TcCLB-EL.
510351.20_mRNA-p1 TcCLB-EL.510351.31_mRNA-p1 TcCLB-EL.
511681.20_mRNA-p1 TcCLB-NE.507601.150_mRNA-p1 TcCLB-NE.
507601.160_mRNA-p1 TcCLB-NE.507601.170_mRNA-p1 TcCLB-NE.
507943.10_mRNA-p1 TcCLB-NE.507943.20_mRNA-p1 TcCLB-NE.
507943.30_mRNA-p1 TcCLB-NE.507943.40_mRNA-p1 TcCLB-NE.507943.5_mRNA-p1 TcCLB-NE.508739.60_mRNA-p1 TcSYL_0062750.t1-p1 TcSYL_0062760.t1-p1 TcSYL_0062820.t1-p1 TcSYL_0062830.t1-p1 TcSYL_0062840.t1-p1 TcSYL_0062850.t1-p1 TcSYL_0062870.t1-p1 TcSYL_0062900.t1-p1 TcSYL_0062970.t1-p1 TcSYL_0062980.t1-p1 TcSYL_0063000.t1-p1 TcSYL_0063010.t1-p1 TcSYL_0063040.t1-p1 TcSYL_0063050.t1-p1 TcSYL_0063070.t1-p1 TcSYL_0063080.t1-p1 TcSYL_0063090.t1-p1 TcSYL_0063100.t1-p1 TcSYL_0063130.t1-p1 TcSYL_0063150.t1-p1 TcSYL_0063160.t1-p1 TcSYL_0192200.t1-p1
OG0000031: TCRU_10007 TCRU_10322 TCRU_10623 TCRU_1269 TCRU_140 TCRU_1421 TCRU_1675 TCRU_1753 TCRU_1815 TCRU_2092 TCRU_2338 TCRU_2659 TCRU_268 TCRU_2713 TCRU_2957 TCRU_3159 TCRU_3176 TCRU_3186 TCRU_3882 TCRU_3994 TCRU_4495 TCRU_4522 TCRU_4641 TCRU_59 TCRU_6618 TCRU_670 TCRU_6725 TCRU_6741 TCRU_7422 TCRU_75 TCRU_7987 TCRU_8134 TCRU_8199 TCRU_8471 TCRU_9169 TCRU_926 TCRU_9282 TCRU_9312 TCRU_9959 TcSYL_0052070.t1-p1 TcSYL_0061320.t1-p1 TcSYL_0165070.t1-p1 TcSYL_0174180.t1-p1
OG0000032: TCRU_255 TcCLB-EL.442427.10_pseudogenic_transcript-p1 TcCLB-EL.503717.130_pseudogenic_transcript-p1 TcCLB-EL.
504637.21_pseudogenic_transcript-p1 TcCLB-EL.
506671.50_pseudogenic_transcript-p1 TcCLB-EL.
506971.30_pseudogenic_transcript-p1 TcCLB-EL.
507553.30_pseudogenic_transcript-p1 TcCLB-EL.508103.14_mRNA-p1 TcCLB-EL.508107.20_pseudogenic_transcript-p1 TcCLB-EL.
508143.50_pseudogenic_transcript-p1 TcCLB-EL.
508163.170_pseudogenic_transcript-p1 TcCLB-EL.
508163.90_pseudogenic_transcript-p1 TcCLB-EL.
510191.31_pseudogenic_transcript-p1 TcCLB-EL.
510275.160_pseudogenic_transcript-p1 TcCLB-EL.
510279.263_pseudogenic_transcript-p1 TcCLB-EL.
510553.20_pseudogenic_transcript-p1 TcCLB-EL.
511871.170_pseudogenic_transcript-p1 TcCLB-NE.
504261.30_pseudogenic_transcript-p1 TcCLB-NE.
506339.60_pseudogenic_transcript-p1 TcCLB-NE.
507233.70_pseudogenic_transcript-p1 TcCLB-NE.
508113.7_pseudogenic_transcript-p1 TcCLB-NE.
509217.60_pseudogenic_transcript-p1 TcCLB-NE.
509225.30_pseudogenic_transcript-p1 TcCLB-NE.
509897.230_pseudogenic_transcript-p1 TcCLB-NE.
510483.90_pseudogenic_transcript-p1 TcCLB-NE.
511915.20_pseudogenic_transcript-p1 TcSYL_0017880.t1-p1 TcSYL_0067630.t1-p1 TcSYL_0068070.t1-p1 TcSYL_0070860.t1-p1 TcSYL_0101670.t1-p1 TcSYL_0124620.t1-p1 TcSYL_0124930.t1-p1 TcSYL_0126660.t1-p1 TcSYL_0127130.t1-p1 TcSYL_0134190.t1-p1 TcSYL_0148800.t1-p1 TcSYL_0150890.t1-p1 TcSYL_0151180.t1-p1 TcSYL_0161420.t1-p1 TcSYL_0187130.t1-p1
OG0000033: TCRU_1209 TCRU_1739 TCRU_1787 TCRU_2275 TCRU_2984

TCRU_3601 TCRU_4411 TCRU_4572 TCRU_4901 TCRU_4941 TCRU_5237
TCRU_5456 TCRU_5458 TCRU_6360 TCRU_6370 TCRU_666 TCRU_6727 TCRU_6804
TCRU_6897 TCRU_7120 TCRU_7191 TCRU_7202 TCRU_7217 TCRU_8114
TCRU_8268 TCRU_8465 TCRU_8573 TCRU_9198 TCRU_9206 TCRU_9605
TCRU_9829 TCRU_9969 TcCLB-EL.507861.24_mRNA-p1 TcCLB-EL.509883.18.1-
p1 TcCLB-NE.435665.10_mRNA-p1 TcCLB-NE.508887.5_mRNA-p1 TcCLB-NE.
510199.10_mRNA-p1 TcSYL_0023940.t1-p1 TcSYL_0066890.t1-p1
TcSYL_0067970.t1-p1 TcSYL_0137410.t1-p1
OG0000034: TCRU_1431 TCRU_296 TCRU_9990 TcCLB-EL.
506129.20_pseudogenic_transcript-p1 TcCLB-EL.
506183.109_pseudogenic_transcript-p1 TcCLB-EL.
507229.20_pseudogenic_transcript-p1 TcCLB-EL.
508563.11_pseudogenic_transcript-p1 TcCLB-EL.
509191.9_pseudogenic_transcript-p1 TcCLB-EL.
509501.10_pseudogenic_transcript-p1 TcCLB-EL.
509803.70_pseudogenic_transcript-p1 TcCLB-EL.509817.21_mRNA-p1
TcCLB-NE.398099.10_pseudogenic_transcript-p1 TcCLB-NE.506335.9_mRNA-
p1 TcCLB-NE.507901.51_mRNA-p1 TcCLB-NE.
508193.30_pseudogenic_transcript-p1 TcCLB-NE.
508303.130_pseudogenic_transcript-p1 TcCLB-NE.
508651.10_pseudogenic_transcript-p1 TcSYL_0015210.t1-p1
TcSYL_0015310.t1-p1 TcSYL_0019120.t1-p1 TcSYL_0020910.t1-p1
TcSYL_0022400.t1-p1 TcSYL_0022420.t1-p1 TcSYL_0041190.t1-p1
TcSYL_0060700.t1-p1 TcSYL_0061980.t1-p1 TcSYL_0092540.t1-p1
TcSYL_0093740.t1-p1 TcSYL_0106470.t1-p1 TcSYL_0111340.t1-p1
TcSYL_0140870.t1-p1 TcSYL_0148010.t1-p1 TcSYL_0163930.t1-p1
TcSYL_0165680.t1-p1 TcSYL_0166280.t1-p1 TcSYL_0187670.t1-p1
TcSYL_0187730.t1-p1 TcSYL_0191470.t1-p1 TcSYL_0194540.t1-p1
TcSYL_0200210.t1-p1
OG0000035: TCRU_gamma_ TCRU_gamma_ TCRU_gamma_ TCRU_gamma_
TCRU_gamma_ TCRU_gamma_ TCRU_gamma_ TCRU_gamma_ TCRU_gamma_
TCRU_gamma_ TCRU_gamma_ TCRU_gamma_ TCRU_gamma_ TCRU_gamma_ TcCLB-
EL.506603.29_pseudogenic_transcript-p1 TcCLB-EL.
506737.81_pseudogenic_transcript-p1 TcCLB-EL.
508163.105_pseudogenic_transcript-p1 TcCLB-EL.508163.150_mRNA-p1
TcCLB-EL.509979.281_pseudogenic_transcript-p1 TcCLB-EL.
510275.180_mRNA-p1 TcCLB-EL.511875.25_pseudogenic_transcript-p1
TcCLB-EL.511875.5_pseudogenic_transcript-p1 TcCLB-NE.
504081.344_pseudogenic_transcript-p1 TcCLB-NE.
506001.19_pseudogenic_transcript-p1 TcCLB-NE.
506389.39_pseudogenic_transcript-p1 TcCLB-NE.
506751.116_pseudogenic_transcript-p1 TcCLB-NE.
506751.30_pseudogenic_transcript-p1 TcCLB-NE.
507633.101_pseudogenic_transcript-p1 TcCLB-NE.507937.40_mRNA-p1
TcCLB-NE.508221.199_mRNA-p1 TcCLB-NE.508221.414_mRNA-p1 TcCLB-NE.
508221.610_mRNA-p1 TcCLB-NE.508261.145_pseudogenic_transcript-p1
TcCLB-NE.508873.425_pseudogenic_transcript-p1 TcCLB-NE.
510627.195_pseudogenic_transcript-p1 TcCLB-NE.511861.111_mRNA-p1
TcCLB-NE.511861.7_mRNA-p1 TcSYL_0070980.t1-p1 TcSYL_0099810.t1-p1
TcSYL_0156640.t1-p1
OG0000036: TCRU_10677 TCRU_2617 TCRU_3196 TCRU_5777 TCRU_5804
TCRU_5876 TCRU_7772 TCRU_846 TcCLB-EL.505931.10_mRNA-p1 TcCLB-EL.
505931.20_mRNA-p1 TcCLB-EL.505941.10_mRNA-p1 TcCLB-EL.
505965.10_mRNA-p1 TcCLB-EL.506163.10_mRNA-p1 TcCLB-EL.

506163.20_mRNA-p1 TcCLB-EL.506351.90_mRNA-p1 TcCLB-EL.
506867.40_mRNA-p1 TcCLB-EL.506929.10_mRNA-p1 TcCLB-EL.
507077.60_mRNA-p1 TcCLB-EL.508813.40_mRNA-p1 TcCLB-EL.
509205.100_mRNA-p1 TcCLB-EL.509205.90_pseudogenic_transcript-p1
TcCLB-EL.511211.90_mRNA-p1 TcCLB-NE.467465.10_mRNA-p1 TcCLB-NE.
503993.30_mRNA-p1 TcCLB-NE.506257.50_mRNA-p1 TcCLB-NE.
506587.100_mRNA-p1 TcCLB-NE.506587.90_mRNA-p1 TcCLB-NE.
507197.10_mRNA-p1 TcCLB-NE.507623.110_mRNA-p1 TcCLB-NE.
508699.100_mRNA-p1 TcCLB-NE.508699.90_mRNA-p1 TcCLB-NE.
508825.10_mRNA-p1 TcCLB-NE.510873.20_mRNA-p1 TcCLB-NE.
510899.10_mRNA-p1 TcCLB-NE.511203.10_mRNA-p1 TcCLB-NE.
511203.20_mRNA-p1 TcCLB-NE.511907.350_pseudogenic_transcript-p1
TcSYL_0044270.t1-p1 TcSYL_0044410.t1-p1 TcSYL_0095910.t1-p1
OG0000037: TCRU_10082 TCRU_10692 TCRU_10736 TCRU_3683 TCRU_4658
TCRU_5577 TCRU_5796 TCRU_8487 TCRU_8693 TCRU_8719 TCRU_8826
TCRU_8864 TCRU_9480 TcCLB-EL.504229.30_mRNA-p1 TcCLB-EL.
507611.40_mRNA-p1 TcCLB-EL.507881.60_mRNA-p1 TcCLB-EL.
508207.20_mRNA-p1 TcCLB-EL.508325.130_mRNA-p1 TcCLB-EL.
508325.320_mRNA-p1 TcCLB-EL.510175.160_mRNA-p1 TcCLB-EL.
510307.191_pseudogenic_transcript-p1 TcCLB-EL.
510911.45_pseudogenic_transcript-p1 TcCLB-EL.511055.50_mRNA-p1
TcCLB-EL.511471.60_mRNA-p1 TcCLB-NE.503423.20_mRNA-p1 TcCLB-NE.
503607.10_mRNA-p1 TcCLB-NE.506537.130_mRNA-p1 TcCLB-NE.
506537.260_mRNA-p1 TcCLB-NE.507503.11_pseudogenic_transcript-p1
TcCLB-NE.507519.190_pseudogenic_transcript-p1 TcCLB-NE.
508479.10_mRNA-p1 TcCLB-NE.509163.70_mRNA-p1 TcCLB-NE.
509259.130_mRNA-p1 TcCLB-NE.509723.10_mRNA-p1 TcCLB-NE.
509725.50_mRNA-p1 TcCLB-NE.510817.10_mRNA-p1 TcCLB-NE.
511665.130_mRNA-p1 TcCLB-NE.511773.50_mRNA-p1 TcSYL_0058600.t1-p1
OG0000038: TCRU_10397 TCRU_1478 TCRU_1974 TCRU_2141 TCRU_2171
TCRU_2278 TCRU_2486 TCRU_254 TCRU_3336 TCRU_3383 TCRU_3802 TCRU_3837
TCRU_4044 TCRU_4667 TCRU_4777 TCRU_4943 TCRU_5122 TCRU_5420
TCRU_6371 TCRU_7630 TCRU_7993 TCRU_8229 TCRU_9177 TCRU_9209 TCRU_946
TcCLB-EL.508121.30_pseudogenic_transcript-p1 TcCLB-EL.
508163.340_pseudogenic_transcript-p1 TcCLB-EL.
511875.110_pseudogenic_transcript-p1 TcCLB-NE.
508221.440_pseudogenic_transcript-p1 TcCLB-NE.
508873.90_pseudogenic_transcript-p1 TcCLB-NE.
509249.20_pseudogenic_transcript-p1 TcCLB-NE.
510711.10_pseudogenic_transcript-p1 TcSYL_0068060.t1-p1
TcSYL_0070890.t1-p1 TcSYL_0091420.t1-p1 TcSYL_0091540.t1-p1
TcSYL_0126640.t1-p1 TcSYL_0127100.t1-p1
OG0000039: TCRU_7831 TCRU_7832 TcCLB-EL.510565.11_mRNA-p1 TcCLB-NE.
510187.30_mRNA-p1 TcCLB-NE.510187.40_mRNA-p1 TcCLB-NE.
510187.50_mRNA-p1 TcCLB-NE.510795.10_mRNA-p1 TcCLB-NE.
511461.20_mRNA-p1 TcSYL_0011710.t1-p1 TcSYL_0011790.t1-p1
TcSYL_0011820.t1-p1 TcSYL_0012070.t1-p1 TcSYL_0012280.t1-p1
TcSYL_0012340.t1-p1 TcSYL_0012370.t1-p1 TcSYL_0012500.t1-p1
TcSYL_0012530.t1-p1 TcSYL_0012570.t1-p1 TcSYL_0012600.t1-p1
TcSYL_0130970.t1-p1 TcSYL_0167350.t1-p1 TcSYL_0167700.t1-p1
TcSYL_0167740.t1-p1 TcSYL_0167870.t1-p1 TcSYL_0167910.t1-p1
TcSYL_0168000.t1-p1 TcSYL_0168100.t1-p1 TcSYL_0168130.t1-p1
TcSYL_0168160.t1-p1 TcSYL_0168190.t1-p1 TcSYL_0168310.t1-p1
TcSYL_0168480.t1-p1 TcSYL_0168520.t1-p1 TcSYL_0168550.t1-p1

TcSYL_0168590.t1-p1 TcSYL_0168630.t1-p1 TcSYL_0168690.t1-p1
TcSYL_0168790.t1-p1
OG0000040: TCRU_10038 TCRU_1411 TCRU_2249 TCRU_4179 TCRU_4532
TCRU_4629 TCRU_4984 TCRU_5162 TCRU_6858 TCRU_6951 TCRU_7537
TCRU_8344 TCRU_8392 TCRU_9205 TCRU_9280 TCRU_9281 TCRU_9544 TcCLB-EL.503957.10_mRNA-p1 TcCLB-EL.507133.10_mRNA-p1 TcCLB-EL.507835.80_mRNA-p1 TcCLB-EL.509271.10_mRNA-p1 TcCLB-NE.504069.140_pseudogenic_transcript-p1 TcCLB-NE.506191.58_mRNA-p1
TcCLB-NE.507501.5.1-p1 TcCLB-NE.511667.140_pseudogenic_transcript-p1
TcCLB-NE.511767.10_mRNA-p1 TcCLB-NE.511767.60_pseudogenic_transcript-p1 TcSYL_0027050.t1-p1
TcSYL_0049980.t1-p1 TcSYL_0083640.t1-p1 TcSYL_0106520.t1-p1
TcSYL_0106630.t1-p1 TcSYL_0146450.t1-p1 TcSYL_0148250.t1-p1
TcSYL_0160010.t1-p1 TcSYL_0201930.t1-p1
OG0000041: TCRU_10017 TCRU_3593 TCRU_7127 TcCLB-EL.417203.9_mRNA-p1
TcCLB-EL.417993.10_pseudogenic_transcript-p1 TcCLB-EL.473619.9_mRNA-p1 TcCLB-EL.510995.9_mRNA-p1 TcCLB-NE.511883.30_pseudogenic_transcript-p1 TcSYL_0015360.t1-p1
TcSYL_0020940.t1-p1 TcSYL_0039890.t1-p1 TcSYL_0048710.t1-p1
TcSYL_0060160.t1-p1 TcSYL_0060450.t1-p1 TcSYL_0061930.t1-p1
TcSYL_0063990.t1-p1 TcSYL_0074580.t1-p1 TcSYL_0083920.t1-p1
TcSYL_0084030.t1-p1 TcSYL_0094070.t1-p1 TcSYL_0106210.t1-p1
TcSYL_0106780.t1-p1 TcSYL_0111570.t1-p1 TcSYL_0137970.t1-p1
TcSYL_0140900.t1-p1 TcSYL_0141470.t1-p1 TcSYL_0151680.t1-p1
TcSYL_0152750.t1-p1 TcSYL_0154060.t1-p1 TcSYL_0157710.t1-p1
TcSYL_0163750.t1-p1 TcSYL_0163820.t1-p1 TcSYL_0166550.t1-p1
TcSYL_0187710.t1-p1 TcSYL_0194500.t1-p1 TcSYL_0200160.t1-p1
OG0000042: TCRU_10491 TCRU_1536 TCRU_1657 TCRU_3034 TCRU_3084
TCRU_3608 TCRU_3806 TCRU_4224 TCRU_4882 TCRU_695 TCRU_812 TcCLB-EL.506689.20_pseudogenic_transcript-p1 TcCLB-EL.508163.371_pseudogenic_transcript-p1 TcCLB-EL.508163.81_pseudogenic_transcript-p1 TcCLB-EL.508367.50_pseudogenic_transcript-p1 TcCLB-EL.508835.10_pseudogenic_transcript-p1 TcCLB-EL.509631.45_pseudogenic_transcript-p1 TcCLB-EL.510279.276_pseudogenic_transcript-p1 TcCLB-EL.511471.10_pseudogenic_transcript-p1 TcCLB-NE.507459.10_pseudogenic_transcript-p1 TcCLB-NE.510197.5_pseudogenic_transcript-p1 TcCLB-NE.510237.21_pseudogenic_transcript-p1 TcCLB-NE.511667.90_pseudogenic_transcript-p1 TcSYL_0017940.t1-p1
TcSYL_0018600.t1-p1 TcSYL_0018980.t1-p1 TcSYL_0038700.t1-p1
TcSYL_0098500.t1-p1 TcSYL_0099440.t1-p1 TcSYL_0099690.t1-p1
TcSYL_0099700.t1-p1 TcSYL_0128650.t1-p1 TcSYL_0187030.t1-p1
TcSYL_0187460.t1-p1 TcSYL_0203760.t1-p1
OG0000043: TcCLB-EL.504099.26_pseudogenic_transcript-p1 TcCLB-EL.504149.201_pseudogenic_transcript-p1 TcCLB-EL.504193.51_pseudogenic_transcript-p1 TcCLB-EL.506047.13_pseudogenic_transcript-p1 TcCLB-EL.506113.50_pseudogenic_transcript-p1 TcCLB-EL.506113.83_pseudogenic_transcript-p1 TcCLB-EL.506961.104_pseudogenic_transcript-p1 TcCLB-EL.507105.132_pseudogenic_transcript-p1 TcCLB-EL.507907.55_pseudogenic_transcript-p1 TcCLB-EL.

508285.30_pseudogenic_transcript-p1 TcCLB-EL.
509265.36_pseudogenic_transcript-p1 TcCLB-EL.
509871.31_pseudogenic_transcript-p1 TcCLB-EL.
509959.198_pseudogenic_transcript-p1 TcCLB-EL.
510307.94_pseudogenic_transcript-p1 TcCLB-EL.
511587.39_pseudogenic_transcript-p1 TcCLB-NE.504109.189_mRNA-p1
TcCLB-NE.506001.111_pseudogenic_transcript-p1 TcCLB-NE.
506271.10_pseudogenic_transcript-p1 TcCLB-NE.
506325.33_pseudogenic_transcript-p1 TcCLB-NE.
507167.41_pseudogenic_transcript-p1 TcCLB-NE.
507911.36_pseudogenic_transcript-p1 TcCLB-NE.
508401.70_pseudogenic_transcript-p1 TcCLB-NE.
508629.63_pseudogenic_transcript-p1 TcCLB-NE.
508631.60_pseudogenic_transcript-p1 TcCLB-NE.
509163.105_pseudogenic_transcript-p1 TcCLB-NE.
509259.100_pseudogenic_transcript-p1 TcCLB-NE.
509579.34_pseudogenic_transcript-p1 TcCLB-NE.
509717.152_pseudogenic_transcript-p1 TcCLB-NE.
509917.6_pseudogenic_transcript-p1 TcCLB-NE.
511807.6_pseudogenic_transcript-p1 TcSYL_0017400.t1-p1
TcSYL_0020030.t1-p1 TcSYL_0020530.t1-p1 TcSYL_0120150.t1-p1
TcSYL_0166160.t1-p1
OG0000044: TCRU_10374 TCRU_164 TCRU_302 TCRU_453 TCRU_501 TCRU_560
TCRU_7061 TCRU_9478 TCRU_9825 TcCLB-EL.
503985.10_pseudogenic_transcript-p1 TcCLB-EL.
504049.30_pseudogenic_transcript-p1 TcCLB-EL.
505943.95_pseudogenic_transcript-p1 TcCLB-EL.
508559.80_pseudogenic_transcript-p1 TcCLB-EL.
509735.140_pseudogenic_transcript-p1 TcCLB-EL.
510769.202_pseudogenic_transcript-p1 TcCLB-EL.
510853.96_pseudogenic_transcript-p1 TcCLB-EL.
511371.5_pseudogenic_transcript-p1 TcCLB-EL.
511415.21_pseudogenic_transcript-p1 TcCLB-EL.
511585.310_pseudogenic_transcript-p1 TcCLB-NE.
452699.5_pseudogenic_transcript-p1 TcCLB-NE.
505037.157_pseudogenic_transcript-p1 TcCLB-NE.
506507.40_pseudogenic_transcript-p1 TcCLB-NE.
506595.70_pseudogenic_transcript-p1 TcCLB-NE.
507167.259_pseudogenic_transcript-p1 TcCLB-NE.
507621.5_pseudogenic_transcript-p1 TcCLB-NE.
509085.110_pseudogenic_transcript-p1 TcCLB-NE.
509429.10_pseudogenic_transcript-p1 TcCLB-NE.
509559.40_pseudogenic_transcript-p1 TcCLB-NE.
510643.121_pseudogenic_transcript-p1 TcCLB-NE.
511019.26_pseudogenic_transcript-p1 TcCLB-NE.511667.30_mRNA-p1
TcSYL_0079490.t1-p1 TcSYL_0154530.t1-p1 TcSYL_0155120.t1-p1
OG0000045: TCRU_1293 TCRU_3755 TCRU_5147 TCRU_6418 TCRU_6522
TCRU_6555 TCRU_7162 TCRU_7266 TCRU_9335 TCRU_9826 TcCLB-EL.
503501.70_mRNA-p1 TcCLB-EL.506269.10_mRNA-p1 TcCLB-EL.
506599.150_mRNA-p1 TcCLB-EL.506763.50_mRNA-p1 TcCLB-EL.
506767.140_mRNA-p1 TcCLB-EL.507959.300_mRNA-p1 TcCLB-EL.
508541.90_mRNA-p1 TcCLB-EL.510013.290_mRNA-p1 TcCLB-EL.
510013.70_mRNA-p1 TcCLB-EL.510021.130_mRNA-p1 TcCLB-EL.
510373.119_mRNA-p1 TcCLB-EL.510373.90_mRNA-p1 TcCLB-NE.

506279.74_pseudogenic_transcript-p1 TcCLB-NE.
506993.86_pseudogenic_transcript-p1 TcCLB-NE.506995.70_mRNA-p1
TcCLB-NE.508219.10_pseudogenic_transcript-p1 TcCLB-NE.
508221.860_pseudogenic_transcript-p1 TcCLB-NE.
508873.280_pseudogenic_transcript-p1 TcSYL_0068340.t1-p1
TcSYL_0069790.t1-p1 TcSYL_0101200.t1-p1 TcSYL_0123830.t1-p1
TcSYL_0129810.t1-p1 TcSYL_0154850.t1-p1
OG0000046: TCRU_1863 TCRU_3020 TCRU_5538 TCRU_5646 TCRU_6046
TCRU_6540 TCRU_6806 TCRU_8197 TCRU_8800 TcCLB-EL.
504049.40_pseudogenic_transcript-p1 TcCLB-EL.
504769.170_pseudogenic_transcript-p1 TcCLB-EL.
505943.110_pseudogenic_transcript-p1 TcCLB-EL.
506343.30_pseudogenic_transcript-p1 TcCLB-EL.506961.10_mRNA-p1
TcCLB-EL.506961.40_pseudogenic_transcript-p1 TcCLB-EL.
507875.110_pseudogenic_transcript-p1 TcCLB-EL.
507899.10_pseudogenic_transcript-p1 TcCLB-NE.
503845.30_pseudogenic_transcript-p1 TcCLB-NE.
504081.220_pseudogenic_transcript-p1 TcCLB-NE.
506331.104_pseudogenic_transcript-p1 TcCLB-NE.
506459.60_pseudogenic_transcript-p1 TcCLB-NE.
507167.110_pseudogenic_transcript-p1 TcCLB-NE.
508529.30_pseudogenic_transcript-p1 TcCLB-NE.
509085.40_pseudogenic_transcript-p1 TcCLB-NE.
509163.90_pseudogenic_transcript-p1 TcCLB-NE.
509437.100_pseudogenic_transcript-p1 TcCLB-NE.
509559.20_pseudogenic_transcript-p1 TcCLB-NE.
509941.30_pseudogenic_transcript-p1 TcCLB-NE.
510361.160_pseudogenic_transcript-p1 TcCLB-NE.510479.11_mRNA-p1
TcSYL_0058150.t1-p1 TcSYL_0093660.t1-p1 TcSYL_0093910.t1-p1
TcSYL_0094380.t1-p1
OG0000047: TcCLB-EL.508123.10_mRNA-p1 TcCLB-EL.509233.10_mRNA-p1
TcCLB-NE.473703.10_mRNA-p1 TcCLB-NE.509287.220_mRNA-p1
TcSYL_0021920.t1-p1 TcSYL_0039650.t1-p1 TcSYL_0039820.t1-p1
TcSYL_0065610.t1-p1 TcSYL_0067030.t1-p1 TcSYL_0067150.t1-p1
TcSYL_0067750.t1-p1 TcSYL_0068020.t1-p1 TcSYL_0069630.t1-p1
TcSYL_0070210.t1-p1 TcSYL_0093210.t1-p1 TcSYL_0100320.t1-p1
TcSYL_0100750.t1-p1 TcSYL_0124080.t1-p1 TcSYL_0125450.t1-p1
TcSYL_0127010.t1-p1 TcSYL_0127720.t1-p1 TcSYL_0128960.t1-p1
TcSYL_0133660.t1-p1 TcSYL_0133880.t1-p1 TcSYL_0134270.t1-p1
TcSYL_0134470.t1-p1 TcSYL_0135610.t1-p1 TcSYL_0149520.t1-p1
TcSYL_0149940.t1-p1 TcSYL_0150120.t1-p1 TcSYL_0155580.t1-p1
TcSYL_0161240.t1-p1 TcSYL_0161300.t1-p1 TcSYL_0187170.t1-p1
OG0000048: TCRU_3878 TCRU_454 TCRU_4868 TCRU_5406 TCRU_7053 TcCLB-
EL.503503.40_mRNA-p1 TcCLB-EL.504239.110_mRNA-p1 TcCLB-EL.
504239.420_mRNA-p1 TcCLB-EL.505025.10_mRNA-p1 TcCLB-EL.
506499.80_mRNA-p1 TcCLB-EL.506763.330_mRNA-p1 TcCLB-EL.
506767.230_mRNA-p1 TcCLB-EL.506767.400_mRNA-p1 TcCLB-EL.
506965.180_mRNA-p1 TcCLB-EL.507953.40_mRNA-p1 TcCLB-EL.
507957.280_mRNA-p1 TcCLB-EL.509079.20_mRNA-p1 TcCLB-EL.
509081.5_mRNA-p1 TcCLB-EL.510377.100_mRNA-p1 TcCLB-EL.
511553.10_mRNA-p1 TcCLB-EL.511599.120_mRNA-p1 TcCLB-EL.
511603.30_mRNA-p1 TcCLB-EL.511603.80_mRNA-p1 TcCLB-NE.
503973.80_mRNA-p1 TcCLB-NE.506459.130_mRNA-p1 TcCLB-NE.
507071.310_mRNA-p1 TcCLB-NE.510361.110_mRNA-p1 TcCLB-NE.

510483.70_mRNA-p1 TcCLB-NE.510487.50_mRNA-p1 TcCLB-NE.
510693.230_mRNA-p1 TcCLB-NE.511255.30_mRNA-p1 TcSYL_0129070.t1-p1
TcSYL_0133080.t1-p1
OG0000049: TCRU_212 TCRU_3606 TCRU_5901 TCRU_6097 TCRU_7372 TCRU_848
TCRU_8751 TCRU_8854 TcCLB-EL.503859.60_pseudogenic_transcript-p1
TcCLB-EL.504239.90_mRNA-p1 TcCLB-EL.
506601.11_pseudogenic_transcript-p1 TcCLB-EL.
506767.371_pseudogenic_transcript-p1 TcCLB-EL.
510025.139_pseudogenic_transcript-p1 TcCLB-EL.
510025.205_pseudogenic_transcript-p1 TcCLB-EL.511553.80_mRNA-p1
TcCLB-EL.511603.130_mRNA-p1 TcCLB-NE.506321.180_mRNA-p1 TcCLB-NE.
507605.39_pseudogenic_transcript-p1 TcCLB-NE.508221.52_mRNA-p1
TcCLB-NE.508243.71_pseudogenic_transcript-p1 TcCLB-NE.
508247.30_mRNA-p1 TcCLB-NE.508273.91_mRNA-p1 TcCLB-NE.
510359.671_pseudogenic_transcript-p1 TcCLB-NE.510629.262_mRNA-p1
TcCLB-NE.510709.41_mRNA-p1 TcCLB-NE.
511255.503_pseudogenic_transcript-p1 TcCLB-NE.
511255.611_pseudogenic_transcript-p1 TcSYL_0021990.t1-p1
TcSYL_0133390.t1-p1 TcSYL_0149040.t1-p1 TcSYL_0155530.t1-p1
TcSYL_0156290.t1-p1
OG0000050: TCRU_10020 TCRU_10203 TCRU_1145 TCRU_2085 TCRU_2243
TCRU_479 TCRU_536 TCRU_6434 TCRU_6551 TCRU_6627 TCRU_746 TCRU_7881
TCRU_7957 TCRU_8055 TCRU_9393 TCRU_944 TcCLB-EL.503717.60_mRNA-p1
TcCLB-NE.504233.60_mRNA-p1 TcCLB-NE.506917.10_mRNA-p1 TcCLB-NE.
506993.190_mRNA-p1 TcCLB-NE.507973.20_mRNA-p1 TcCLB-NE.
508365.70_mRNA-p1 TcCLB-NE.508883.20_mRNA-p1 TcCLB-NE.
509525.210_mRNA-p1 TcCLB-NE.509525.340_mRNA-p1 TcCLB-NE.
509525.50_mRNA-p1 TcCLB-NE.509545.60_mRNA-p1 TcSYL_0100800.t1-p1
TcSYL_0101100.t1-p1 TcSYL_0128910.t1-p1 TcSYL_0129780.t1-p1
TcSYL_0154270.t1-p1
OG0000051: TCRU_10384 TCRU_10675 TCRU_2525 TCRU_789 TCRU_923
TCRU_9291 TCRU_9390 TcCLB-EL.504149.220_pseudogenic_transcript-p1
TcCLB-EL.506345.70_pseudogenic_transcript-p1 TcCLB-EL.
506683.210_pseudogenic_transcript-p1 TcCLB-EL.
506717.140_pseudogenic_transcript-p1 TcCLB-EL.
506757.70_pseudogenic_transcript-p1 TcCLB-EL.
506767.280_pseudogenic_transcript-p1 TcCLB-EL.
507835.60_pseudogenic_transcript-p1 TcCLB-EL.
507953.110_pseudogenic_transcript-p1 TcCLB-EL.
508225.20_pseudogenic_transcript-p1 TcCLB-EL.
508991.10_pseudogenic_transcript-p1 TcCLB-EL.
509765.60_pseudogenic_transcript-p1 TcCLB-EL.
510175.10_pseudogenic_transcript-p1 TcCLB-EL.510845.10_mRNA-p1
TcCLB-EL.511349.110_pseudogenic_transcript-p1 TcCLB-EL.
511349.61_pseudogenic_transcript-p1 TcCLB-EL.
511567.20_pseudogenic_transcript-p1 TcCLB-EL.
511609.10_pseudogenic_transcript-p1 TcCLB-NE.506139.200_mRNA-p1
TcCLB-NE.506191.20_pseudogenic_transcript-p1 TcCLB-NE.
506433.10_pseudogenic_transcript-p1 TcCLB-NE.
508061.30_pseudogenic_transcript-p1 TcCLB-NE.
509899.20_pseudogenic_transcript-p1 TcCLB-NE.
510483.320_pseudogenic_transcript-p1 TcCLB-NE.
510693.60_pseudogenic_transcript-p1 TcSYL_0017290.t1-p1
OG0000052: TCRU_10018 TCRU_10476 TCRU_1346 TCRU_135 TCRU_137

TCRU_2022 TCRU_247 TCRU_2569 TCRU_3734 TCRU_4296 TCRU_4715 TCRU_4717
TCRU_4718 TCRU_4830 TCRU_6626 TCRU_7882 TCRU_8209 TCRU_8486
TCRU_9395 TCRU_9608 TcCLB-EL.498829.21_mRNA-p1 TcCLB-EL.
508325.90_mRNA-p1 TcCLB-NE.508243.36_mRNA-p1 TcCLB-NE.
509259.123_pseudogenic_transcript-p1 TcCLB-NE.511773.30_mRNA-p1
TcSYL_0017640.t1-p1 TcSYL_0018760.t1-p1 TcSYL_0133570.t1-p1
TcSYL_0149500.t1-p1 TcSYL_0149510.t1-p1 TcSYL_0154840.t1-p1
OG0000053: TCRU_10200 TCRU_10204 TCRU_10591 TCRU_10605 TCRU_1141
TCRU_1423 TCRU_1435 TCRU_1748 TCRU_1784 TCRU_2159 TCRU_273 TCRU_2744
TCRU_2779 TCRU_2915 TCRU_3596 TCRU_4051 TCRU_616 TCRU_714 TCRU_7406
TCRU_7888 TCRU_8381 TCRU_9276 TCRU_9338 TCRU_9475 TCRU_9491
TCRU_9496 TCRU_9590 TCRU_9763 TcCLB-NE.508623.10.1-p1
TcSYL_0040550.t1-p1 TcSYL_0049710.t1-p1
OG0000054: TCRU_10145 TCRU_10487 TCRU_10519 TCRU_1344 TCRU_1437
TCRU_2350 TCRU_3085 TCRU_390 TCRU_4840 TCRU_5284 TCRU_624 TCRU_6943
TCRU_7995 TCRU_9757 TCRU_9991 TcCLB-EL.
511173.261_pseudogenic_transcript-p1 TcCLB-NE.
503491.29_pseudogenic_transcript-p1 TcCLB-NE.
506955.115_pseudogenic_transcript-p1 TcCLB-NE.
506955.32_pseudogenic_transcript-p1 TcCLB-NE.
506955.79_pseudogenic_transcript-p1 TcCLB-NE.
506987.21_pseudogenic_transcript-p1 TcCLB-NE.
508223.10_pseudogenic_transcript-p1 TcCLB-NE.
508877.50_pseudogenic_transcript-p1 TcCLB-NE.
510489.28_pseudogenic_transcript-p1 TcCLB-NE.
510693.141_pseudogenic_transcript-p1 TcCLB-NE.
510693.295_pseudogenic_transcript-p1 TcCLB-NE.
511255.145_pseudogenic_transcript-p1 TcSYL_0069660.t1-p1
TcSYL_0123810.t1-p1 TcSYL_0149110.t1-p1
OG0000055: TCRU_10023 TCRU_1111 TCRU_1516 TCRU_2158 TCRU_4569
TCRU_555 TCRU_6376 TCRU_7490 TCRU_8479 TCRU_9323 TCRU_9676 TcCLB-EL.
503977.70_pseudogenic_transcript-p1 TcCLB-EL.507091.120_mRNA-p1
TcCLB-EL.508977.130_mRNA-p1 TcCLB-EL.508977.20_mRNA-p1 TcCLB-EL.
508977.99_pseudogenic_transcript-p1 TcCLB-EL.
511401.130_pseudogenic_transcript-p1 TcCLB-EL.
511401.40_pseudogenic_transcript-p1 TcCLB-NE.
506993.130_pseudogenic_transcript-p1 TcCLB-NE.508219.110_mRNA-p1
TcCLB-NE.508435.10_mRNA-p1 TcCLB-NE.
509897.170_pseudogenic_transcript-p1 TcCLB-NE.509897.279_mRNA-p1
TcCLB-NE.509897.30_pseudogenic_transcript-p1 TcCLB-NE.
509897.80_pseudogenic_transcript-p1 TcCLB-NE.
509899.50_pseudogenic_transcript-p1 TcCLB-NE.511921.140_mRNA-p1
TcCLB-NE.511923.80_pseudogenic_transcript-p1 TcSYL_0129190.t1-p1
TcSYL_0148990.t1-p1
OG0000056: TCRU_10666 TCRU_10667 TCRU_1924 TCRU_1925 TCRU_1926
TCRU_1927 TCRU_1963 TCRU_1964 TCRU_1965 TCRU_3125 TcCLB-EL.
506513.200_mRNA-p1 TcCLB-EL.507979.4_mRNA-p1 TcCLB-EL.
508321.11_mRNA-p1 TcCLB-EL.508321.21_mRNA-p1 TcCLB-EL.
510525.100_mRNA-p1 TcCLB-EL.510525.110_mRNA-p1 TcCLB-EL.
510525.80_mRNA-p1 TcCLB-EL.510525.90_mRNA-p1 TcCLB-NE.
509207.11_mRNA-p1 TcCLB-NE.509207.20_mRNA-p1 TcCLB-NE.
511323.40_mRNA-p1 TcCLB-NE.511809.135_mRNA-p1 TcCLB-NE.
511809.140_mRNA-p1 TcCLB-NE.511817.140_mRNA-p1 TcCLB-NE.
511817.151_mRNA-p1 TcCLB-NE.511817.161_mRNA-p1 TcCLB-NE.

511817.171_mRNA-p1 TcCLB-NE.511817.180_mRNA-p1 TcSYL_0158700.t1-p1 TcSYL_0201610.t1-p1
OG0000057: TCRU_10141 TCRU_10142 TCRU_10816 TCRU_4656 TCRU_7761 TCRU_8107 TCRU_9015 TCRU_9482 TCRU_9483 TcCLB-EL.
463155.40_pseudogenic_transcript-p1 TcCLB-EL.
504229.20_pseudogenic_transcript-p1 TcCLB-EL.
507611.30_pseudogenic_transcript-p1 TcCLB-EL.
508207.10_pseudogenic_transcript-p1 TcCLB-EL.
508325.340_pseudogenic_transcript-p1 TcCLB-EL.
510175.170_pseudogenic_transcript-p1 TcCLB-EL.
511471.90_pseudogenic_transcript-p1 TcCLB-NE.
503423.10_pseudogenic_transcript-p1 TcCLB-NE.
503439.80_pseudogenic_transcript-p1 TcCLB-NE.
506537.160_pseudogenic_transcript-p1 TcCLB-NE.
506537.280_pseudogenic_transcript-p1 TcCLB-NE.
507503.20_pseudogenic_transcript-p1 TcCLB-NE.
507519.210_pseudogenic_transcript-p1 TcCLB-NE.
508479.5_pseudogenic_transcript-p1 TcCLB-NE.
508531.10_pseudogenic_transcript-p1 TcCLB-NE.
509303.20_pseudogenic_transcript-p1 TcCLB-NE.
509723.20_pseudogenic_transcript-p1 TcCLB-NE.
509725.60_pseudogenic_transcript-p1 TcCLB-NE.
511773.60_pseudogenic_transcript-p1 TcSYL_0058750.t1-p1 TcSYL_0058950.t1-p1
OG0000058: TCRU_139 TCRU_4655 TCRU_5972 TCRU_5992 TCRU_8868 TcCLB-EL.463155.20_mRNA-p1 TcCLB-EL.503441.19_pseudogenic_transcript-p1 TcCLB-EL.503957.120_mRNA-p1 TcCLB-EL.506113.60_mRNA-p1 TcCLB-EL.
507881.20_mRNA-p1 TcCLB-EL.508325.160_mRNA-p1 TcCLB-EL.
510005.41_mRNA-p1 TcCLB-EL.510971.5_pseudogenic_transcript-p1 TcCLB-EL.511055.20_mRNA-p1 TcCLB-NE.503607.4_mRNA-p1 TcCLB-NE.
506313.10_mRNA-p1 TcCLB-NE.506537.180_pseudogenic_transcript-p1 TcCLB-NE.506537.299_mRNA-p1 TcCLB-NE.508401.20_mRNA-p1 TcCLB-NE.
509223.20_mRNA-p1 TcCLB-NE.509259.160_mRNA-p1 TcCLB-NE.
509259.50_pseudogenic_transcript-p1 TcCLB-NE.509295.80_mRNA-p1 TcCLB-NE.510629.200_pseudogenic_transcript-p1 TcCLB-NE.
511019.74_mRNA-p1 TcCLB-NE.511773.10_pseudogenic_transcript-p1 TcCLB-NE.511773.90_pseudogenic_transcript-p1 TcSYL_0166070.t1-p1 TcSYL_0204070.t1-p1
OG0000059: TCRU_987 TcCLB-EL.503859.10_pseudogenic_transcript-p1 TcCLB-EL.504039.25_pseudogenic_transcript-p1 TcCLB-EL.
506501.141_pseudogenic_transcript-p1 TcCLB-EL.
506501.27_pseudogenic_transcript-p1 TcCLB-EL.
506763.361_pseudogenic_transcript-p1 TcCLB-EL.
506767.123_pseudogenic_transcript-p1 TcCLB-EL.
506767.209_pseudogenic_transcript-p1 TcCLB-EL.
506767.21_pseudogenic_transcript-p1 TcCLB-EL.
506767.381_pseudogenic_transcript-p1 TcCLB-EL.
506967.27_pseudogenic_transcript-p1 TcCLB-EL.
507957.36_pseudogenic_transcript-p1 TcCLB-EL.
507959.323_pseudogenic_transcript-p1 TcCLB-EL.
509979.249_pseudogenic_transcript-p1 TcCLB-EL.
510013.13_pseudogenic_transcript-p1 TcCLB-EL.
510025.7_pseudogenic_transcript-p1 TcCLB-EL.
511553.71_pseudogenic_transcript-p1 TcCLB-EL.

511613.151_pseudogenic_transcript-p1 TcCLB-NE.
503775.30_pseudogenic_transcript-p1 TcCLB-NE.
507071.277_pseudogenic_transcript-p1 TcCLB-NE.
507971.20_pseudogenic_transcript-p1 TcCLB-NE.
510363.196_pseudogenic_transcript-p1 TcCLB-NE.
510387.15_pseudogenic_transcript-p1 TcCLB-NE.
510627.179_pseudogenic_transcript-p1 TcCLB-NE.
510709.31_pseudogenic_transcript-p1 TcCLB-NE.
511255.313_pseudogenic_transcript-p1 TcCLB-NE.
511255.618_pseudogenic_transcript-p1 TcSYL_0066060.t1-p1
TcSYL_0135420.t1-p1
OG0000060: TCRU_1357 TCRU_207 TCRU_2487 TCRU_2725 TCRU_3231
TCRU_3611 TCRU_4095 TCRU_4096 TCRU_4104 TCRU_4640 TCRU_5380
TCRU_6514 TCRU_6610 TCRU_6655 TCRU_7601 TCRU_8137 TCRU_9002
TCRU_9153 TCRU_9383 TCRU_9827 TcCLB-EL.504239.210_mRNA-p1 TcCLB-EL.
506759.50_mRNA-p1 TcCLB-EL.506767.410_mRNA-p1 TcSYL_0024160.t1-p1
TcSYL_0100290.t1-p1 TcSYL_0101260.t1-p1 TcSYL_0133690.t1-p1
TcSYL_0187360.t1-p1
OG0000061: TCRU_10437 TCRU_10673 TCRU_1152 TCRU_1626 TCRU_5250
TCRU_6526 TCRU_7305 TCRU_7857 TCRU_8043 TCRU_827 TCRU_8482 TCRU_8512
TCRU_8614 TCRU_9038 TCRU_9149 TCRU_9559 TCRU_9660 TCRU_9831 TcCLB-
EL.504149.190_pseudogenic_transcript-p1 TcCLB-EL.
511471.150_pseudogenic_transcript-p1 TcCLB-NE.
507911.40_pseudogenic_transcript-p1 TcCLB-NE.
508401.50_pseudogenic_transcript-p1 TcCLB-NE.
511807.10_pseudogenic_transcript-p1 TcSYL_0004720.t1-p1
TcSYL_0017340.t1-p1 TcSYL_0022570.t1-p1 TcSYL_0079600.t1-p1
TcSYL_0204020.t1-p1
OG0000062: TCRU_1233 TCRU_4617 TcCLB-EL.503447.50_mRNA-p1 TcCLB-EL.
504341.10_mRNA-p1 TcCLB-EL.506183.90_pseudogenic_transcript-p1
TcCLB-EL.506951.90_mRNA-p1 TcCLB-EL.507121.11_mRNA-p1 TcCLB-EL.
507479.70_mRNA-p1 TcCLB-EL.507997.14_mRNA-p1 TcCLB-EL.
509265.120_mRNA-p1 TcCLB-EL.510307.284_mRNA-p1 TcCLB-EL.
510491.60_mRNA-p1 TcCLB-EL.510853.40_mRNA-p1 TcCLB-NE.
504205.20_pseudogenic_transcript-p1 TcCLB-NE.504533.40_mRNA-p1
TcCLB-NE.506053.50_mRNA-p1 TcCLB-NE.506443.100_mRNA-p1 TcCLB-NE.
507723.50_mRNA-p1 TcCLB-NE.507773.10_mRNA-p1 TcCLB-NE.
508593.10_mRNA-p1 TcCLB-NE.508593.20_mRNA-p1 TcCLB-NE.
508677.30_pseudogenic_transcript-p1 TcCLB-NE.509387.19_mRNA-p1
TcSYL_0061100.t1-p1 TcSYL_0148140.t1-p1 TcSYL_0148160.t1-p1
TcSYL_0171890.t1-p1 TcSYL_0191540.t1-p1
OG0000063: TCRU_4059 TCRU_4157 TCRU_4507 TCRU_6300 TCRU_6401
TCRU_6847 TCRU_7123 TCRU_9006 TcSYL_0023370.t1-p1 TcSYL_0029480.t1-
p1 TcSYL_0051770.t1-p1 TcSYL_0070100.t1-p1 TcSYL_0071780.t1-p1
TcSYL_0072180.t1-p1 TcSYL_0100190.t1-p1 TcSYL_0100230.t1-p1
TcSYL_0100330.t1-p1 TcSYL_0101750.t1-p1 TcSYL_0125010.t1-p1
TcSYL_0125020.t1-p1 TcSYL_0126970.t1-p1 TcSYL_0127730.t1-p1
TcSYL_0127830.t1-p1 TcSYL_0129100.t1-p1 TcSYL_0129350.t1-p1
TcSYL_0134520.t1-p1 TcSYL_0191550.t1-p1 TcSYL_0203160.t1-p1
OG0000064: TCRU_6026 TcCLB-EL.507641.280_mRNA-p1 TcCLB-EL.
507641.290_mRNA-p1 TcCLB-EL.507641.300_mRNA-p1 TcCLB-NE.
510187.420_pseudogenic_transcript-p1 TcCLB-NE.510187.551_mRNA-p1
TcSYL_0011240.t1-p1 TcSYL_0011250.t1-p1 TcSYL_0011260.t1-p1
TcSYL_0011270.t1-p1 TcSYL_0011280.t1-p1 TcSYL_0011290.t1-p1

TcSYL_0011300.t1-p1 TcSYL_0011310.t1-p1 TcSYL_0011320.t1-p1
TcSYL_0011330.t1-p1 TcSYL_0011340.t1-p1 TcSYL_0011350.t1-p1
TcSYL_0011360.t1-p1 TcSYL_0011370.t1-p1 TcSYL_0011380.t1-p1
TcSYL_0011390.t1-p1 TcSYL_0011400.t1-p1 TcSYL_0011410.t1-p1
TcSYL_0011420.t1-p1 TcSYL_0011430.t1-p1 TcSYL_0011440.t1-p1
TcSYL_0011450.t1-p1
OG0000065: TcCLB-EL.504239.401_pseudogenic_transcript-p1 TcCLB-EL.
506501.131_pseudogenic_transcript-p1 TcCLB-EL.
506501.275_pseudogenic_transcript-p1 TcCLB-EL.
506501.34_pseudogenic_transcript-p1 TcCLB-EL.
506501.401_pseudogenic_transcript-p1 TcCLB-EL.
506599.445_pseudogenic_transcript-p1 TcCLB-EL.
506737.135_pseudogenic_transcript-p1 TcCLB-EL.
506763.350_pseudogenic_transcript-p1 TcCLB-EL.
506767.10_pseudogenic_transcript-p1 TcCLB-EL.
506767.127_pseudogenic_transcript-p1 TcCLB-EL.
506767.215_pseudogenic_transcript-p1 TcCLB-EL.
507237.353_pseudogenic_transcript-p1 TcCLB-EL.
507957.43_pseudogenic_transcript-p1 TcCLB-EL.
509081.80_pseudogenic_transcript-p1 TcCLB-EL.
509097.52_pseudogenic_transcript-p1 TcCLB-EL.
509993.5_pseudogenic_transcript-p1 TcCLB-EL.
510025.14_pseudogenic_transcript-p1 TcCLB-NE.
503771.60_pseudogenic_transcript-p1 TcCLB-NE.
503973.255_pseudogenic_transcript-p1 TcCLB-NE.
504081.125_pseudogenic_transcript-p1 TcCLB-NE.
504081.321_pseudogenic_transcript-p1 TcCLB-NE.
504155.125_pseudogenic_transcript-p1 TcCLB-NE.
504155.185_pseudogenic_transcript-p1 TcCLB-NE.
506321.101_pseudogenic_transcript-p1 TcCLB-NE.
507071.260_pseudogenic_transcript-p1 TcCLB-NE.
509753.118_pseudogenic_transcript-p1 TcCLB-NE.
511255.306_pseudogenic_transcript-p1 TcSYL_0066030.t1-p1
OG0000066: TCRU_4311 TCRU_710 TcCLB-EL.
503783.34_pseudogenic_transcript-p1 TcCLB-EL.505025.70_mRNA-p1
TcCLB-EL.506499.160_mRNA-p1 TcCLB-EL.506965.140_mRNA-p1 TcCLB-EL.
508121.110_mRNA-p1 TcCLB-EL.508163.230_mRNA-p1 TcCLB-EL.
510207.40_mRNA-p1 TcCLB-EL.510209.27_mRNA-p1 TcCLB-EL.
510261.10_mRNA-p1 TcCLB-EL.510373.10_mRNA-p1 TcCLB-EL.
511553.150_mRNA-p1 TcCLB-EL.511603.300_mRNA-p1 TcCLB-NE.
503973.120_mRNA-p1 TcCLB-NE.504197.20_mRNA-p1 TcCLB-NE.
506329.59_pseudogenic_transcript-p1 TcCLB-NE.
506993.31_pseudogenic_transcript-p1 TcCLB-NE.507067.60_mRNA-p1
TcCLB-NE.508221.30_mRNA-p1 TcCLB-NE.
508365.114_pseudogenic_transcript-p1 TcCLB-NE.508873.30_mRNA-p1
TcCLB-NE.509525.160_pseudogenic_transcript-p1 TcCLB-NE.
510623.20_mRNA-p1 TcCLB-NE.511259.200_pseudogenic_transcript-p1
TcSYL_0068830.t1-p1 TcSYL_0068840.t1-p1
OG0000067: TCRU_10834 TCRU_6008 TcCLB-EL.507689.30_mRNA-p1 TcCLB-EL.
507689.40_mRNA-p1 TcCLB-EL.507689.50_mRNA-p1 TcCLB-NE.
507657.10_mRNA-p1 TcCLB-NE.507657.20_mRNA-p1 TcSYL_0032900.t1-p1
TcSYL_0033090.t1-p1 TcSYL_0033110.t1-p1 TcSYL_0033220.t1-p1
TcSYL_0033460.t1-p1 TcSYL_0035000.t1-p1 TcSYL_0035830.t1-p1
TcSYL_0036190.t1-p1 TcSYL_0036390.t1-p1 TcSYL_0036590.t1-p1

TcSYL_0036810.t1-p1 TcSYL_0052830.t1-p1 TcSYL_0052870.t1-p1
TcSYL_0052910.t1-p1 TcSYL_0053140.t1-p1 TcSYL_0053490.t1-p1
TcSYL_0053500.t1-p1 TcSYL_0053540.t1-p1 TcSYL_0053550.t1-p1
TcSYL_0053960.t1-p1
OG0000068: TCRU_10135 TCRU_2241 TCRU_2749 TCRU_2778 TCRU_4932
TCRU_7020 TCRU_9344 TCRU_9714 TcCLB-EL.
506473.4_pseudogenic_transcript-p1 TcCLB-EL.507237.320_mRNA-p1
TcCLB-EL.508097.100_mRNA-p1 TcCLB-EL.
508109.70_pseudogenic_transcript-p1 TcCLB-EL.508165.250_mRNA-p1
TcCLB-EL.508165.80_mRNA-p1 TcCLB-EL.511349.40_mRNA-p1 TcCLB-NE.
506139.90_mRNA-p1 TcSYL_0028160.t1-p1 TcSYL_0028560.t1-p1
TcSYL_0068750.t1-p1 TcSYL_0069560.t1-p1 TcSYL_0070250.t1-p1
TcSYL_0093500.t1-p1 TcSYL_0100940.t1-p1 TcSYL_0134620.t1-p1
TcSYL_0160260.t1-p1 TcSYL_0160910.t1-p1
OG0000069: TCRU_3736 TCRU_5748 TCRU_5775 TCRU_5871 TCRU_6031
TCRU_6954 TcCLB-EL.507611.260_pseudogenic_transcript-p1 TcCLB-EL.
507979.30_mRNA-p1 TcCLB-EL.508089.10_mRNA-p1 TcCLB-EL.
509817.50_mRNA-p1 TcCLB-EL.510055.10_mRNA-p1 TcCLB-NE.
506923.10_pseudogenic_transcript-p1 TcCLB-NE.506975.80_mRNA-p1
TcCLB-NE.507085.30_mRNA-p1 TcCLB-NE.508857.30_mRNA-p1 TcCLB-NE.
508859.118_mRNA-p1 TcCLB-NE.508913.10_pseudogenic_transcript-p1
TcCLB-NE.509495.30_mRNA-p1 TcCLB-NE.511323.10_mRNA-p1
TcSYL_0025400.t1-p1 TcSYL_0027040.t1-p1 TcSYL_0065780.t1-p1
TcSYL_0106140.t1-p1 TcSYL_0110270.t1-p1 TcSYL_0158780.t1-p1
TcSYL_0159220.t1-p1
OG0000070: TcCLB-EL.504099.21_pseudogenic_transcript-p1 TcCLB-EL.
506113.86_pseudogenic_transcript-p1 TcCLB-EL.
506961.97_pseudogenic_transcript-p1 TcCLB-EL.
509959.236_pseudogenic_transcript-p1 TcCLB-EL.
510307.77_pseudogenic_transcript-p1 TcCLB-EL.
511587.111_pseudogenic_transcript-p1 TcCLB-EL.
511587.36_pseudogenic_transcript-p1 TcCLB-NE.
503437.21_pseudogenic_transcript-p1 TcCLB-NE.
504109.195_pseudogenic_transcript-p1 TcCLB-NE.
505997.21_pseudogenic_transcript-p1 TcCLB-NE.
506001.101_pseudogenic_transcript-p1 TcCLB-NE.
506017.41_pseudogenic_transcript-p1 TcCLB-NE.
506325.36_pseudogenic_transcript-p1 TcCLB-NE.
506393.166_pseudogenic_transcript-p1 TcCLB-NE.
507167.31_pseudogenic_transcript-p1 TcCLB-NE.
507499.56_pseudogenic_transcript-p1 TcCLB-NE.
508479.83_pseudogenic_transcript-p1 TcCLB-NE.
508629.68_pseudogenic_transcript-p1 TcCLB-NE.
508851.31_pseudogenic_transcript-p1 TcCLB-NE.
509217.146_pseudogenic_transcript-p1 TcCLB-NE.
509259.111_pseudogenic_transcript-p1 TcCLB-NE.
509717.164_pseudogenic_transcript-p1 TcCLB-NE.
509725.126_pseudogenic_transcript-p1 TcCLB-NE.
509917.12_pseudogenic_transcript-p1 TcCLB-NE.
511807.3_pseudogenic_transcript-p1 TcSYL_0079680.t1-p1
OG0000071: TcCLB-EL.503977.15_pseudogenic_transcript-p1 TcCLB-EL.
506267.101_pseudogenic_transcript-p1 TcCLB-EL.
506501.255_pseudogenic_transcript-p1 TcCLB-EL.
506501.341_pseudogenic_transcript-p1 TcCLB-EL.

506601.31_pseudogenic_transcript-p1 TcCLB-EL.
506737.151_pseudogenic_transcript-p1 TcCLB-EL.
506767.119_pseudogenic_transcript-p1 TcCLB-EL.
506767.31_pseudogenic_transcript-p1 TcCLB-EL.
506967.16_pseudogenic_transcript-p1 TcCLB-EL.
507957.29_pseudogenic_transcript-p1 TcCLB-EL.
507957.304_pseudogenic_transcript-p1 TcCLB-EL.
509081.40_pseudogenic_transcript-p1 TcCLB-EL.
509097.36_pseudogenic_transcript-p1 TcCLB-EL.
509699.85_pseudogenic_transcript-p1 TcCLB-EL.
510025.113_pseudogenic_transcript-p1 TcCLB-EL.
511553.45_pseudogenic_transcript-p1 TcCLB-EL.
511603.369_pseudogenic_transcript-p1 TcCLB-NE.
503973.181_pseudogenic_transcript-p1 TcCLB-NE.
503973.271_pseudogenic_transcript-p1 TcCLB-NE.
504155.91_pseudogenic_transcript-p1 TcCLB-NE.
507607.10_pseudogenic_transcript-p1 TcCLB-NE.
508221.941_pseudogenic_transcript-p1 TcCLB-NE.
509031.10_pseudogenic_transcript-p1 TcCLB-NE.
509969.20_pseudogenic_transcript-p1 TcCLB-NE.
510363.209_pseudogenic_transcript-p1 TcCLB-NE.
511255.65_pseudogenic_transcript-p1
OG0000072: TCRU_10191 TCRU_1042 TCRU_143 TCRU_4298 TCRU_6550
TCRU_7807 TCRU_7883 TCRU_7954 TCRU_9494 TCRU_9665 TcCLB-EL.
506285.80_mRNA-p1 TcCLB-EL.506769.40_mRNA-p1 TcCLB-EL.
507953.170_mRNA-p1 TcCLB-EL.509699.170_mRNA-p1 TcCLB-NE.
503417.20_mRNA-p1 TcCLB-NE.504081.340_pseudogenic_transcript-p1
TcCLB-NE.504233.80_mRNA-p1 TcCLB-NE.506279.179_mRNA-p1 TcCLB-NE.
506281.35_mRNA-p1 TcCLB-NE.506995.20_pseudogenic_transcript-p1
TcCLB-NE.508365.270_mRNA-p1 TcCLB-NE.509525.130_mRNA-p1 TcCLB-NE.
509525.190_mRNA-p1 TcCLB-NE.509525.280_mRNA-p1 TcCLB-NE.
509525.330_mRNA-p1
OG0000073: TCRU_10105 TCRU_10376 TCRU_1041 TCRU_1660 TCRU_172
TCRU_2134 TCRU_2201 TCRU_2691 TCRU_5110 TCRU_5212 TCRU_6179
TCRU_6228 TCRU_7350 TCRU_7802 TCRU_7892 TCRU_8021 TCRU_9381 TcCLB-
EL.506501.300_mRNA-p1 TcCLB-EL.506501.90_mRNA-p1 TcCLB-EL.
509699.240_mRNA-p1 TcCLB-EL.511175.20_mRNA-p1 TcCLB-EL.
511603.480_mRNA-p1 TcCLB-EL.511605.10_mRNA-p1 TcCLB-NE.
510701.20_mRNA-p1 TcSYL_0163850.t1-p1
OG0000074: TCRU_6940 TCRU_7051 TCRU_8373 TCRU_family_ TCRU_family_
TCRU_family_ TCRU_family_ TCRU_family_ TCRU_family_ TCRU_family_
TCRU_family_ TCRU_family_ TcCLB-EL.
511603.356_pseudogenic_transcript-p1 TcCLB-EL.
511603.420_pseudogenic_transcript-p1 TcCLB-EL.
511875.50_pseudogenic_transcript-p1 TcCLB-NE.
504081.141_pseudogenic_transcript-p1 TcCLB-NE.
504155.110_pseudogenic_transcript-p1 TcCLB-NE.
506321.160_pseudogenic_transcript-p1 TcCLB-NE.
508243.81_pseudogenic_transcript-p1 TcCLB-NE.508273.80_mRNA-p1
TcCLB-NE.509753.126_pseudogenic_transcript-p1 TcSYL_0091880.t1-p1
TcSYL_0133350.t1-p1 TcSYL_0149070.t1-p1 TcSYL_0156260.t1-p1
OG0000075: TCRU_10278 TCRU_10284 TCRU_1370 TCRU_1668 TCRU_2281
TCRU_2928 TCRU_3213 TCRU_3744 TCRU_3833 TCRU_5188 TCRU_5210 TCRU_534
TCRU_6281 TCRU_6387 TCRU_6619 TCRU_7693 TCRU_7856 TCRU_8052

TCRU_8612 TCRU_8951 TCRU_9135 TCRU_9476 TcCLB-NE.507951.215_mRNA-p1
TcSYL_0004820.t1-p1 TcSYL_0156310.t1-p1
OG0000076: TCRU_10398 TCRU_10773 TCRU_4501 TCRU_840 TcCLB-EL.
503441.10_pseudogenic_transcript-p1 TcCLB-EL.503957.30_mRNA-p1
TcCLB-EL.506961.160_mRNA-p1 TcCLB-EL.506961.90_mRNA-p1 TcCLB-EL.
507475.30_pseudogenic_transcript-p1 TcCLB-EL.507843.10_mRNA-p1
TcCLB-EL.508109.50_pseudogenic_transcript-p1 TcCLB-EL.
511587.100_mRNA-p1 TcCLB-NE.507233.60_pseudogenic_transcript-p1
TcCLB-NE.507499.60_pseudogenic_transcript-p1 TcCLB-NE.
507591.9_pseudogenic_transcript-p1 TcCLB-NE.
508641.40_pseudogenic_transcript-p1 TcCLB-NE.
509217.150_pseudogenic_transcript-p1 TcCLB-NE.
509217.30_pseudogenic_transcript-p1 TcCLB-NE.509575.10_mRNA-p1
TcCLB-NE.509875.90_pseudogenic_transcript-p1 TcCLB-NE.
510081.30_pseudogenic_transcript-p1 TcCLB-NE.
511665.30_pseudogenic_transcript-p1 TcSYL_0118240.t1-p1
TcSYL_0120640.t1-p1 TcSYL_0121600.t1-p1
OG0000077: TCRU_1015 TCRU_10435 TCRU_1288 TCRU_1601 TCRU_2107
TCRU_3354 TCRU_3851 TCRU_9391 TCRU_9392 TcCLB-EL.
504239.120_pseudogenic_transcript-p1 TcCLB-EL.
506499.30_pseudogenic_transcript-p1 TcCLB-EL.
506767.170_pseudogenic_transcript-p1 TcCLB-EL.
506767.250_pseudogenic_transcript-p1 TcCLB-EL.
506967.150_pseudogenic_transcript-p1 TcCLB-EL.
507957.140_pseudogenic_transcript-p1 TcCLB-EL.
510013.270_pseudogenic_transcript-p1 TcCLB-EL.510013.50_mRNA-p1
TcCLB-EL.510025.250_pseudogenic_transcript-p1 TcCLB-NE.
504081.30_pseudogenic_transcript-p1 TcCLB-NE.
504081.450_pseudogenic_transcript-p1 TcCLB-NE.
508221.310_pseudogenic_transcript-p1 TcCLB-NE.
510693.50_pseudogenic_transcript-p1 TcSYL_0029400.t1-p1
TcSYL_0080050.t1-p1 TcSYL_0137440.t1-p1
OG0000078: TCRU_2139 TcCLB-EL.506609.15_pseudogenic_transcript-p1
TcCLB-NE.506805.20_mRNA-p1 TcCLB-NE.508115.10_mRNA-p1
TcSYL_0029440.t1-p1 TcSYL_0029560.t1-p1 TcSYL_0066160.t1-p1
TcSYL_0067190.t1-p1 TcSYL_0068300.t1-p1 TcSYL_0068600.t1-p1
TcSYL_0079800.t1-p1 TcSYL_0091210.t1-p1 TcSYL_0093240.t1-p1
TcSYL_0093250.t1-p1 TcSYL_0110130.t1-p1 TcSYL_0125070.t1-p1
TcSYL_0126500.t1-p1 TcSYL_0127790.t1-p1 TcSYL_0129160.t1-p1
TcSYL_0130320.t1-p1 TcSYL_0136490.t1-p1 TcSYL_0136900.t1-p1
TcSYL_0148210.t1-p1 TcSYL_0152420.t1-p1 TcSYL_0161670.t1-p1
OG0000079: TCRU_10083 TCRU_3684 TCRU_6818 TCRU_6857 TCRU_7083
TCRU_8105 TCRU_9013 TCRU_9435 TcCLB-EL.507611.50_mRNA-p1 TcCLB-EL.
508207.40_mRNA-p1 TcCLB-EL.508325.110_mRNA-p1 TcCLB-EL.
508325.300_mRNA-p1 TcCLB-EL.510307.200_mRNA-p1 TcCLB-EL.
511055.60_mRNA-p1 TcCLB-NE.506537.120_mRNA-p1 TcCLB-NE.
507501.30_mRNA-p1 TcCLB-NE.507519.180_mRNA-p1 TcCLB-NE.
507781.10_pseudogenic_transcript-p1 TcCLB-NE.
508001.90_pseudogenic_transcript-p1 TcCLB-NE.509163.54_mRNA-p1
TcCLB-NE.509725.30_pseudogenic_transcript-p1 TcCLB-NE.
510817.30_pseudogenic_transcript-p1 TcCLB-NE.511667.10_mRNA-p1
TcCLB-NE.511887.50_mRNA-p1 TcSYL_0058670.t1-p1
OG0000080: TCRU_9448 TCRU_9449 TCRU_9450 TcCLB-EL.505931.40_mRNA-p1
TcCLB-EL.505931.50_mRNA-p1 TcCLB-EL.507817.18_mRNA-p1 TcCLB-EL.

507817.9_mRNA-p1 TcCLB-NE.509471.59_mRNA-p1 TcCLB-NE.509471.68_mRNA-p1 TcCLB-NE.509471.77_mRNA-p1 TcCLB-NE.509471.86_mRNA-p1 TcCLB-NE.509471.95_mRNA-p1 TcSYL_0041370.t1-p1 TcSYL_0041380.t1-p1 TcSYL_0041390.t1-p1 TcSYL_0041400.t1-p1 TcSYL_0041580.t1-p1 TcSYL_0041680.t1-p1 TcSYL_0041810.t1-p1 TcSYL_0041850.t1-p1 TcSYL_0041950.t1-p1 TcSYL_0041990.t1-p1 TcSYL_0042190.t1-p1 TcSYL_0042240.t1-p1 TcSYL_0042300.t1-p1
OG0000081: TCRU_10594 TCRU_10660 TCRU_1343 TCRU_234 TCRU_3749 TCRU_4338 TCRU_4663 TCRU_6704 TCRU_6961 TCRU_7380 TCRU_7889 TCRU_8071 TCRU_8110 TCRU_8261 TCRU_8581 TCRU_865 TCRU_898 TCRU_9069 TCRU_9600 TCRU_9764 TCRU_9880 TCRU_9954 TcSYL_0066570.t1-p1 TcSYL_0186910.t1-p1
OG0000082: TCRU_10547 TCRU_1973 TCRU_2044 TCRU_2485 TCRU_392 TCRU_4045 TCRU_4301 TCRU_4587 TCRU_4942 TCRU_8521 TCRU_9208 TcCLB-NE.503763.50_pseudogenic_transcript-p1 TcSYL_0017860.t1-p1 TcSYL_0091240.t1-p1 TcSYL_0091520.t1-p1 TcSYL_0099840.t1-p1 TcSYL_0109170.t1-p1 TcSYL_0109190.t1-p1 TcSYL_0140800.t1-p1 TcSYL_0151010.t1-p1 TcSYL_0152070.t1-p1 TcSYL_0152450.t1-p1 TcSYL_0156200.t1-p1 TcSYL_0190810.t1-p1
OG0000083: TCRU_10096 TCRU_1197 TCRU_1422 TCRU_1479 TCRU_3533 TCRU_4446 TCRU_8608 TCRU_9178 TCRU_9712 TcSYL_0018890.t1-p1 TcSYL_0067640.t1-p1 TcSYL_0100010.t1-p1 TcSYL_0100070.t1-p1 TcSYL_0100210.t1-p1 TcSYL_0121390.t1-p1 TcSYL_0124640.t1-p1 TcSYL_0127110.t1-p1 TcSYL_0127490.t1-p1 TcSYL_0140840.t1-p1 TcSYL_0147910.t1-p1 TcSYL_0150910.t1-p1 TcSYL_0153660.t1-p1 TcSYL_0154170.t1-p1 TcSYL_0186800.t1-p1
OG0000084: TCRU_10108 TCRU_1486 TCRU_5987 TCRU_6114 TCRU_8039 TCRU_8774 TCRU_9382 TcCLB-EL.505025.144_mRNA-p1 TcCLB-EL.506597.36_pseudogenic_transcript-p1 TcCLB-EL.506967.13_mRNA-p1 TcCLB-EL.509097.28_mRNA-p1 TcCLB-EL.510021.185_mRNA-p1 TcCLB-EL.510377.475_pseudogenic_transcript-p1 TcCLB-EL.510377.61_mRNA-p1 TcCLB-NE.504081.23_mRNA-p1 TcCLB-NE.506459.195_mRNA-p1 TcCLB-NE.506459.235_mRNA-p1 TcCLB-NE.508869.75_mRNA-p1 TcCLB-NE.508871.105_mRNA-p1 TcCLB-NE.509031.6_mRNA-p1 TcCLB-NE.509971.80_mRNA-p1 TcCLB-NE.511487.71_mRNA-p1 TcSYL_0154590.t1-p1 TcSYL_0155090.t1-p1
OG0000085: TCRU_1545 TCRU_6424 TCRU_7309 TcCLB-EL.505025.50_mRNA-p1 TcCLB-EL.506597.51_mRNA-p1 TcCLB-EL.507957.210_mRNA-p1 TcCLB-EL.511173.140_mRNA-p1 TcCLB-EL.511597.25_mRNA-p1 TcCLB-NE.506001.24_pseudogenic_transcript-p1 TcCLB-NE.508221.190_mRNA-p1 TcCLB-NE.508495.110_mRNA-p1 TcCLB-NE.509217.180_pseudogenic_transcript-p1 TcCLB-NE.509665.10_mRNA-p1 TcCLB-NE.509665.40_mRNA-p1 TcCLB-NE.510047.70_pseudogenic_transcript-p1 TcCLB-NE.510483.270_mRNA-p1 TcCLB-NE.510625.100_mRNA-p1 TcCLB-NE.510625.170_mRNA-p1 TcSYL_0022030.t1-p1 TcSYL_0070850.t1-p1 TcSYL_0091390.t1-p1 TcSYL_0109960.t1-p1 TcSYL_0124610.t1-p1 TcSYL_0134200.t1-p1
OG0000086: TCRU_2661 TCRU_2689 TCRU_2690 TCRU_5126 TCRU_6497 TCRU_8141 TCRU_8609 TCRU_9001 TcCLB-EL.509631.120_pseudogenic_transcript-p1 TcCLB-NE.404431.10_mRNA-p1 TcCLB-NE.459061.10_mRNA-p1 TcCLB-NE.508401.10_mRNA-p1 TcSYL_0067850.t1-p1 TcSYL_0118270.t1-p1 TcSYL_0118280.t1-p1 TcSYL_0118290.t1-p1 TcSYL_0118980.t1-p1 TcSYL_0119220.t1-p1 TcSYL_0119860.t1-p1 TcSYL_0120880.t1-p1 TcSYL_0121310.t1-p1

TcSYL_0121320.t1-p1 TcSYL_0121330.t1-p1 TcSYL_0160730.t1-p1
OG0000087: TCRU_4264 TCRU_7511 TcCLB-EL.457109.9_mRNA-p1 TcCLB-EL.462545.9_mRNA-p1 TcCLB-EL.466007.9_mRNA-p1 TcCLB-EL.468405.9_mRNA-p1 TcCLB-EL.485845.9_mRNA-p1 TcCLB-EL.507871.9_mRNA-p1 TcCLB-EL.508683.9_mRNA-p1 TcCLB-NE.483581.9_mRNA-p1 TcCLB-NE.506981.10_mRNA-p1 TcSYL_0018510.t1-p1 TcSYL_0019100.t1-p1 TcSYL_0020970.t1-p1 TcSYL_0060430.t1-p1 TcSYL_0084010.t1-p1 TcSYL_0106180.t1-p1 TcSYL_0121350.t1-p1 TcSYL_0140980.t1-p1 TcSYL_0153500.t1-p1 TcSYL_0157050.t1-p1 TcSYL_0187590.t1-p1 TcSYL_0190850.t1-p1 TcSYL_0201860.t1-p1
OG0000088: TCRU_10240 TCRU_10363 TCRU_10592 TCRU_266 TCRU_4476 TCRU_4889 TCRU_5141 TCRU_6585 TCRU_7267 TCRU_79 TCRU_8397 TCRU_8621 TCRU_9558 TcCLB-EL.421173.14_mRNA-p1 TcCLB-EL.506377.10_mRNA-p1 TcCLB-NE.507167.278_pseudogenic_transcript-p1 TcCLB-NE.507549.20_pseudogenic_transcript-p1 TcCLB-NE.508399.40_pseudogenic_transcript-p1 TcCLB-NE.509221.50_pseudogenic_transcript-p1 TcCLB-NE.510631.20_pseudogenic_transcript-p1 TcSYL_0020640.t1-p1 TcSYL_0059660.t1-p1 TcSYL_0093790.t1-p1
OG0000089: TCRU_1114 TCRU_2357 TCRU_6203 TCRU_7701 TCRU_8051 TCRU_8481 TCRU_9652 TCRU_9767 TcCLB-EL.506599.35_pseudogenic_transcript-p1 TcCLB-EL.506763.270_pseudogenic_transcript-p1 TcCLB-EL.507091.150_pseudogenic_transcript-p1 TcCLB-EL.510371.35_pseudogenic_transcript-p1 TcCLB-EL.510373.100_pseudogenic_transcript-p1 TcCLB-EL.511401.24_pseudogenic_transcript-p1 TcCLB-EL.511401.80_pseudogenic_transcript-p1 TcCLB-NE.508221.230_mRNA-p1 TcCLB-NE.508221.840_mRNA-p1 TcCLB-NE.508873.530_pseudogenic_transcript-p1 TcCLB-NE.509897.190_pseudogenic_transcript-p1 TcCLB-NE.509897.51_pseudogenic_transcript-p1 TcCLB-NE.511921.10_pseudogenic_transcript-p1 TcCLB-NE.511921.167_pseudogenic_transcript-p1 TcSYL_0125770.t1-p1
OG0000090: TCRU_1211 TCRU_1551 TCRU_2155 TCRU_2545 TCRU_3245 TCRU_4292 TCRU_4876 TCRU_9073 TCRU_9138 TCRU_9896 TCRU_9989 TcCLB-NE.503423.5_mRNA-p1 TcCLB-NE.505997.160_pseudogenic_transcript-p1 TcCLB-NE.508629.26_pseudogenic_transcript-p1 TcCLB-NE.508631.20_pseudogenic_transcript-p1 TcCLB-NE.508873.360_pseudogenic_transcript-p1 TcCLB-NE.509385.11_pseudogenic_transcript-p1 TcCLB-NE.510569.20_mRNA-p1 TcCLB-NE.510643.50_pseudogenic_transcript-p1 TcSYL_0048790.t1-p1 TcSYL_0061530.t1-p1 TcSYL_0079950.t1-p1 TcSYL_0153130.t1-p1
OG0000091: TCRU_3479 TcCLB-NE.506263.10_mRNA-p1 TcCLB-NE.506263.20_mRNA-p1 TcCLB-NE.510099.120_mRNA-p1 TcSYL_0044930.t1-p1 TcSYL_0197220.t1-p1 TcSYL_0197270.t1-p1 TcSYL_0197340.t1-p1 TcSYL_0197470.t1-p1 TcSYL_0197900.t1-p1 TcSYL_0198430.t1-p1 TcSYL_0198480.t1-p1 TcSYL_0198740.t1-p1 TcSYL_0198810.t1-p1 TcSYL_0198950.t1-p1 TcSYL_0199230.t1-p1 TcSYL_0199550.t1-p1 TcSYL_0199610.t1-p1 TcSYL_0199780.t1-p1 TcSYL_0199840.t1-p1 TcSYL_0199900.t1-p1 TcSYL_0199960.t1-p1 TcSYL_0200010.t1-p1
OG0000092: TCRU_5083 TcCLB-EL.506599.395_pseudogenic_transcript-p1 TcCLB-EL.506653.50_pseudogenic_transcript-p1 TcCLB-EL.506671.41_pseudogenic_transcript-p1 TcCLB-EL.

506965.60_pseudogenic_transcript-p1 TcCLB-EL.
507859.70_pseudogenic_transcript-p1 TcCLB-EL.
508047.25_pseudogenic_transcript-p1 TcCLB-EL.
508375.5_pseudogenic_transcript-p1 TcCLB-EL.
510105.322_pseudogenic_transcript-p1 TcCLB-EL.
510189.51_pseudogenic_transcript-p1 TcCLB-EL.
511877.15_pseudogenic_transcript-p1 TcCLB-NE.
506139.151_pseudogenic_transcript-p1 TcCLB-NE.
506279.235_pseudogenic_transcript-p1 TcCLB-NE.
506335.105_pseudogenic_transcript-p1 TcCLB-NE.
506611.55_pseudogenic_transcript-p1 TcCLB-NE.
506615.111_pseudogenic_transcript-p1 TcCLB-NE.
508117.30_pseudogenic_transcript-p1 TcCLB-NE.
508221.451_pseudogenic_transcript-p1 TcCLB-NE.
509665.16_pseudogenic_transcript-p1 TcCLB-NE.510037.5_mRNA-p1 TcCLB-NE.510361.274_pseudogenic_transcript-p1 TcSYL_0018720.t1-p1 TcSYL_0018860.t1-p1
OG0000093: TCRU_10009 TCRU_7356 TCRU_8113 TCRU_8201 TcCLB-EL.
504127.20_mRNA-p1 TcCLB-EL.506799.100_mRNA-p1 TcCLB-EL.
506799.110_mRNA-p1 TcCLB-EL.506799.20_mRNA-p1 TcCLB-EL.
506799.30_mRNA-p1 TcCLB-EL.506801.20_mRNA-p1 TcCLB-EL.
506801.60_mRNA-p1 TcCLB-EL.511047.20_mRNA-p1 TcCLB-NE.
503655.30_mRNA-p1 TcCLB-NE.507059.10_mRNA-p1 TcCLB-NE.
508429.20_mRNA-p1 TcCLB-NE.509115.50_mRNA-p1 TcCLB-NE.
509115.60_mRNA-p1 TcCLB-NE.511859.120_mRNA-p1 TcCLB-NE.
511859.130_mRNA-p1 TcCLB-NE.511859.150_mRNA-p1 TcCLB-NE.
511907.120_mRNA-p1 TcCLB-NE.511907.130_mRNA-p1 TcSYL_0090650.t1-p1
OG0000094: TCRU_1311 TCRU_1340 TCRU_1488 TCRU_2089 TCRU_3340 TCRU_344 TCRU_3936 TCRU_5128 TCRU_5258 TCRU_6664 TCRU_7319 TCRU_7566 TCRU_7952 TCRU_8374 TCRU_847 TcCLB-EL.
509081.60_pseudogenic_transcript-p1 TcCLB-NE.
504081.310_pseudogenic_transcript-p1 TcCLB-NE.
506785.62_pseudogenic_transcript-p1 TcCLB-NE.
507605.50_pseudogenic_transcript-p1 TcCLB-NE.
508221.64_pseudogenic_transcript-p1 TcSYL_0066050.t1-p1 TcSYL_0149050.t1-p1
OG0000095: TCRU_2539 TCRU_3081 TCRU_4041 TCRU_4593 TCRU_4888 TCRU_4915 TCRU_4918 TCRU_5356 TCRU_7763 TCRU_8379 TCRU_899 TCRU_8994 TcCLB-EL.504239.380_mRNA-p1 TcCLB-EL.506967.20_mRNA-p1 TcCLB-EL.
510089.30_mRNA-p1 TcCLB-NE.503973.170_mRNA-p1 TcCLB-NE.
504155.100_mRNA-p1 TcCLB-NE.504155.210_mRNA-p1 TcCLB-NE.
508247.10_mRNA-p1 TcSYL_0021940.t1-p1 TcSYL_0066090.t1-p1 TcSYL_0148690.t1-p1
OG0000096: TCRU_1113 TCRU_2328 TCRU_2718 TCRU_2930 TCRU_3829 TCRU_3955 TCRU_5233 TCRU_5516 TCRU_8610 TCRU_914 TcCLB-EL.
504219.50_pseudogenic_transcript-p1 TcCLB-EL.506599.40_mRNA-p1 TcCLB-EL.507163.20_mRNA-p1 TcCLB-EL.507163.60_mRNA-p1 TcCLB-EL.
510025.80_pseudogenic_transcript-p1 TcCLB-EL.510373.110_mRNA-p1 TcCLB-EL.511625.120_mRNA-p1 TcCLB-NE.
503973.140_pseudogenic_transcript-p1 TcCLB-NE.
506459.215_pseudogenic_transcript-p1 TcCLB-NE.
508221.830_pseudogenic_transcript-p1 TcCLB-NE.510483.200_mRNA-p1 TcSYL_0023920.t1-p1
OG0000097: TCRU_3866 TCRU_7128 TcCLB-EL.406999.10_mRNA-p1 TcCLB-EL.

419637.9_mRNA-p1 TcCLB-NE.507895.50_pseudogenic_transcript-p1 TcCLB-NE.510485.9_mRNA-p1 TcCLB-NE.511273.10_pseudogenic_transcript-p1 TcSYL_0015340.t1-p1 TcSYL_0038950.t1-p1 TcSYL_0039900.t1-p1 TcSYL_0041160.t1-p1 TcSYL_0057070.t1-p1 TcSYL_0066630.t1-p1 TcSYL_0084040.t1-p1 TcSYL_0092460.t1-p1 TcSYL_0094090.t1-p1 TcSYL_0111350.t1-p1 TcSYL_0111540.t1-p1 TcSYL_0148020.t1-p1 TcSYL_0165710.t1-p1 TcSYL_0194510.t1-p1 TcSYL_0200180.t1-p1
OG0000098: TCRU_7307 TcCLB-EL.511211.160_mRNA-p1 TcCLB-EL.511211.170_mRNA-p1 TcCLB-NE.510439.61_mRNA-p1 TcSYL_0182570.t1-p1 TcSYL_0182580.t1-p1 TcSYL_0182610.t1-p1 TcSYL_0182710.t1-p1 TcSYL_0182720.t1-p1 TcSYL_0182750.t1-p1 TcSYL_0182770.t1-p1 TcSYL_0182800.t1-p1 TcSYL_0182830.t1-p1 TcSYL_0183030.t1-p1 TcSYL_0183160.t1-p1 TcSYL_0183190.t1-p1 TcSYL_0183220.t1-p1 TcSYL_0183230.t1-p1 TcSYL_0183310.t1-p1 TcSYL_0183440.t1-p1 TcSYL_0183480.t1-p1 TcSYL_0183500.t1-p1
OG0000099: TCRU_2087 TCRU_754 TcCLB-EL.504039.160_mRNA-p1 TcCLB-EL.504039.57_pseudogenic_transcript-p1 TcCLB-EL.506267.90_mRNA-p1 TcCLB-EL.506501.380_mRNA-p1 TcCLB-EL.506599.100_mRNA-p1 TcCLB-EL.506599.210_mRNA-p1 TcCLB-EL.506759.20_mRNA-p1 TcCLB-EL.506759.80_mRNA-p1 TcCLB-EL.506763.30_mRNA-p1 TcCLB-EL.506769.120_mRNA-p1 TcCLB-EL.507957.320_mRNA-p1 TcCLB-EL.507957.334_mRNA-p1 TcCLB-EL.510369.10_mRNA-p1 TcCLB-EL.510371.120_mRNA-p1 TcCLB-EL.510371.70_mRNA-p1 TcCLB-EL.510377.390_mRNA-p1 TcCLB-NE.508221.960_mRNA-p1 TcCLB-NE.510699.80_mRNA-p1 TcCLB-NE.511255.330_mRNA-p1
OG0000100: TCRU_1142 TCRU_1203 TCRU_1659 TCRU_2325 TCRU_2510 TCRU_3199 TCRU_4531 TCRU_4900 TCRU_7222 TCRU_7491 TCRU_8458 TcCLB-EL.508143.110_mRNA-p1 TcCLB-NE.510363.130_mRNA-p1 TcSYL_0074090.t1-p1 TcSYL_0083720.t1-p1 TcSYL_0126370.t1-p1 TcSYL_0132970.t1-p1 TcSYL_0137160.t1-p1 TcSYL_0148940.t1-p1 TcSYL_0160230.t1-p1 TcSYL_0160520.t1-p1
OG0000101: TCRU_1430 TCRU_2558 TCRU_5325 TCRU_6812 TCRU_8024 TCRU_8274 TcCLB-EL.503909.40_mRNA-p1 TcCLB-EL.504769.140_mRNA-p1 TcCLB-EL.506961.70_pseudogenic_transcript-p1 TcCLB-EL.508831.220_pseudogenic_transcript-p1 TcCLB-NE.412393.10.1-p1 TcCLB-NE.503861.100_pseudogenic_transcript-p1 TcCLB-NE.504115.110_pseudogenic_transcript-p1 TcCLB-NE.506331.200_pseudogenic_transcript-p1 TcCLB-NE.506393.80_pseudogenic_transcript-p1 TcCLB-NE.506471.50_pseudogenic_transcript-p1 TcCLB-NE.507643.60_pseudogenic_transcript-p1 TcCLB-NE.508303.120_pseudogenic_transcript-p1 TcCLB-NE.508577.50_mRNA-p1 TcCLB-NE.511843.30_pseudogenic_transcript-p1 TcSYL_0121340.t1-p1
OG0000102: TCRU_10231 TCRU_1550 TCRU_1678 TCRU_4293 TCRU_4528 TCRU_4651 TCRU_4661 TCRU_6332 TCRU_6703 TCRU_6912 TCRU_7489 TCRU_8100 TCRU_9120 TcCLB-EL.511121.20_mRNA-p1 TcCLB-NE.509295.90_mRNA-p1 TcSYL_0028000.t1-p1 TcSYL_0029310.t1-p1 TcSYL_0079960.t1-p1 TcSYL_0106700.t1-p1 TcSYL_0166060.t1-p1 TcSYL_0203490.t1-p1
OG0000103: TCRU_1654 TCRU_4040 TCRU_5193 TcCLB-EL.508047.20_mRNA-p1 TcCLB-EL.508159.50_pseudogenic_transcript-p1 TcCLB-EL.508161.30_mRNA-p1 TcCLB-EL.508163.50_mRNA-p1 TcCLB-EL.508165.300_mRNA-p1 TcCLB-EL.508165.420_mRNA-p1 TcCLB-EL.510107.30_mRNA-p1 TcCLB-EL.510275.306_pseudogenic_transcript-p1

TcCLB-EL.510275.370_mRNA-p1 TcCLB-EL.510279.140_mRNA-p1 TcCLB-NE.
506335.100_mRNA-p1 TcCLB-NE.506613.110_pseudogenic_transcript-p1
TcCLB-NE.506613.50_mRNA-p1 TcCLB-NE.508873.10_mRNA-p1 TcCLB-NE.
508873.460_mRNA-p1 TcCLB-NE.509657.30_mRNA-p1 TcSYL_0017490.t1-p1
TcSYL_0018320.t1-p1
OG0000104: TCRU_5657 TCRU_5718 TCRU_5805 TCRU_6213 TCRU_8448 TcCLB-
EL.503783.50_pseudogenic_transcript-p1 TcCLB-EL.504239.180_mRNA-p1
TcCLB-EL.504239.330_pseudogenic_transcript-p1 TcCLB-EL.
506501.210_pseudogenic_transcript-p1 TcCLB-EL.
507237.310_pseudogenic_transcript-p1 TcCLB-EL.
507237.60_pseudogenic_transcript-p1 TcCLB-EL.
507957.260_pseudogenic_transcript-p1 TcCLB-EL.
508539.150_pseudogenic_transcript-p1 TcCLB-EL.
509081.130_pseudogenic_transcript-p1 TcCLB-EL.510013.120_mRNA-p1
TcCLB-EL.510377.420_mRNA-p1 TcCLB-EL.511553.120_mRNA-p1 TcCLB-NE.
504081.510_mRNA-p1 TcCLB-NE.506995.60_mRNA-p1 TcSYL_0021850.t1-p1
TcSYL_0092560.t1-p1
OG0000105: TCRU_6556 TCRU_9376 TcCLB-EL.
508771.55_pseudogenic_transcript-p1 TcCLB-NE.
508595.30_pseudogenic_transcript-p1 TcSYL_0015470.t1-p1
TcSYL_0024760.t1-p1 TcSYL_0031740.t1-p1 TcSYL_0038840.t1-p1
TcSYL_0038850.t1-p1 TcSYL_0039350.t1-p1 TcSYL_0039470.t1-p1
TcSYL_0048760.t1-p1 TcSYL_0056980.t1-p1 TcSYL_0071430.t1-p1
TcSYL_0083820.t1-p1 TcSYL_0087200.t1-p1 TcSYL_0128500.t1-p1
TcSYL_0128600.t1-p1 TcSYL_0128740.t1-p1 TcSYL_0177940.t1-p1
TcSYL_0203710.t1-p1
OG0000106: TCRU_8485 TcCLB-EL.511473.6_pseudogenic_transcript-p1
TcCLB-NE.503421.14_pseudogenic_transcript-p1 TcCLB-NE.
503437.31_pseudogenic_transcript-p1 TcCLB-NE.
505997.31_pseudogenic_transcript-p1 TcCLB-NE.
506017.31_pseudogenic_transcript-p1 TcCLB-NE.
506393.143_pseudogenic_transcript-p1 TcCLB-NE.
507499.53_pseudogenic_transcript-p1 TcCLB-NE.
508269.73_pseudogenic_transcript-p1 TcCLB-NE.
508479.465_pseudogenic_transcript-p1 TcCLB-NE.
508479.86_pseudogenic_transcript-p1 TcCLB-NE.
509217.143_pseudogenic_transcript-p1 TcCLB-NE.
509725.113_pseudogenic_transcript-p1 TcCLB-NE.
510081.21_pseudogenic_transcript-p1 TcCLB-NE.
510355.104_pseudogenic_transcript-p1 TcCLB-NE.
511019.54_pseudogenic_transcript-p1 TcCLB-NE.
511771.132_pseudogenic_transcript-p1 TcSYL_0021400.t1-p1
TcSYL_0079660.t1-p1 TcSYL_0120140.t1-p1 TcSYL_0204340.t1-p1
OG0000107: TcCLB-EL.504035.21_mRNA-p1 TcCLB-EL.507163.75_mRNA-p1
TcCLB-EL.507859.15_mRNA-p1 TcCLB-EL.507859.31_mRNA-p1 TcCLB-EL.
507859.46_mRNA-p1 TcCLB-EL.508049.40_mRNA-p1 TcCLB-EL.
508687.39_pseudogenic_transcript-p1 TcCLB-EL.508687.48_mRNA-p1
TcCLB-EL.508687.57_mRNA-p1 TcCLB-EL.508687.66_mRNA-p1 TcCLB-EL.
508777.210_mRNA-p1 TcCLB-EL.508777.220_mRNA-p1 TcCLB-EL.
508777.230_mRNA-p1 TcCLB-EL.509697.29_mRNA-p1 TcCLB-EL.
509697.39_mRNA-p1 TcCLB-EL.510275.255_mRNA-p1 TcCLB-EL.
511605.5_pseudogenic_transcript-p1 TcCLB-NE.
509751.28_pseudogenic_transcript-p1 TcSYL_0124580.t1-p1
TcSYL_0154690.t1-p1 TcSYL_0204990.t1-p1

OG0000108: TCRU_10390 TCRU_10439 TCRU_1605 TCRU_1609 TCRU_1628 TCRU_376 TCRU_3886 TCRU_4530 TCRU_6910 TCRU_7134 TCRU_8041 TCRU_826 TCRU_8484 TCRU_8514 TCRU_9053 TCRU_9148 TCRU_9903 TcCLB-EL.506047.10_pseudogenic_transcript-p1 TcSYL_0079620.t1-p1 TcSYL_0204300.t1-p1
OG0000109: TCRU_1337 TCRU_586 TCRU_6679 TCRU_7597 TCRU_9961 TcCLB-EL.504429.70_pseudogenic_transcript-p1 TcCLB-EL.506365.20_pseudogenic_transcript-p1 TcCLB-EL.506431.20_pseudogenic_transcript-p1 TcCLB-EL.506847.30_pseudogenic_transcript-p1 TcCLB-EL.509829.9_mRNA-p1 TcCLB-EL.509843.10_mRNA-p1 TcCLB-NE.402375.10_pseudogenic_transcript-p1 TcCLB-NE.506577.90_pseudogenic_transcript-p1 TcCLB-NE.509327.10_pseudogenic_transcript-p1 TcCLB-NE.509329.20_pseudogenic_transcript-p1 TcCLB-NE.509329.9_mRNA-p1 TcCLB-NE.509463.41_mRNA-p1 TcSYL_0082390.t1-p1 TcSYL_0171930.t1-p1 TcSYL_0171940.t1-p1
OG0000110: TCRU_1013 TCRU_10622 TCRU_1722 TCRU_3588 TCRU_4047 TCRU_4281 TCRU_4736 TCRU_5079 TCRU_5114 TCRU_6218 TCRU_6724 TCRU_6726 TCRU_694 TCRU_7596 TCRU_790 TCRU_7908 TCRU_8020 TCRU_9603 TcSYL_0000260.t1-p1 TcSYL_0114370.t1-p1
OG0000111: TCRU_1318 TcCLB-NE.509623.19_mRNA-p1 TcCLB-NE.510003.41_mRNA-p1 TcSYL_0002050.t1-p1 TcSYL_0002080.t1-p1 TcSYL_0002120.t1-p1 TcSYL_0002160.t1-p1 TcSYL_0002190.t1-p1 TcSYL_0002220.t1-p1 TcSYL_0002250.t1-p1 TcSYL_0002290.t1-p1 TcSYL_0002320.t1-p1 TcSYL_0002350.t1-p1 TcSYL_0002380.t1-p1 TcSYL_0002410.t1-p1 TcSYL_0002580.t1-p1 TcSYL_0002640.t1-p1 TcSYL_0002700.t1-p1 TcSYL_0002730.t1-p1 TcSYL_0002760.t1-p1
OG0000112: TCRU_1993 TCRU_2274 TCRU_5765 TcCLB-EL.511411.30_mRNA-p1 TcCLB-NE.506053.10_mRNA-p1 TcCLB-NE.506153.10_mRNA-p1 TcCLB-NE.506153.20_mRNA-p1 TcSYL_0098370.t1-p1 TcSYL_0098390.t1-p1 TcSYL_0098690.t1-p1 TcSYL_0098830.t1-p1 TcSYL_0098870.t1-p1 TcSYL_0098910.t1-p1 TcSYL_0098950.t1-p1 TcSYL_0098990.t1-p1 TcSYL_0099060.t1-p1 TcSYL_0099250.t1-p1 TcSYL_0099330.t1-p1 TcSYL_0099540.t1-p1 TcSYL_0099570.t1-p1
OG0000113: TCRU_10050 TCRU_2452 TCRU_3869 TCRU_6249 TcCLB-EL.504769.20_mRNA-p1 TcCLB-EL.508201.30_mRNA-p1 TcCLB-EL.509973.10_mRNA-p1 TcCLB-EL.509973.70_mRNA-p1 TcCLB-EL.509975.30_mRNA-p1 TcCLB-EL.509977.30_mRNA-p1 TcCLB-EL.509977.80_mRNA-p1 TcCLB-EL.509979.174_mRNA-p1 TcCLB-EL.509979.200_mRNA-p1 TcCLB-EL.509979.270_mRNA-p1 TcCLB-NE.506615.100_mRNA-p1 TcCLB-NE.508613.74_mRNA-p1 TcCLB-NE.510037.10_mRNA-p1 TcCLB-NE.510039.30_mRNA-p1 TcCLB-NE.510203.34_pseudogenic_transcript-p1 TcSYL_0156320.t1-p1
OG0000114: TCRU_10638 TCRU_4154 TCRU_8228 TCRU_9824 TcCLB-EL.503447.40_mRNA-p1 TcCLB-EL.503957.20_mRNA-p1 TcCLB-EL.504229.70_mRNA-p1 TcCLB-EL.508325.60_mRNA-p1 TcCLB-EL.510175.90_mRNA-p1 TcCLB-EL.510307.220_mRNA-p1 TcCLB-EL.511121.11_mRNA-p1 TcCLB-EL.511577.180_mRNA-p1 TcCLB-NE.453995.10_pseudogenic_transcript-p1 TcCLB-NE.507225.10_mRNA-p1 TcCLB-NE.511301.170_mRNA-p1 TcCLB-NE.511667.40_mRNA-p1 TcSYL_0103790.t1-p1 TcSYL_0143220.t1-p1 TcSYL_0143240.t1-p1 TcSYL_0190970.t1-p1
OG0000115: TCRU_5401 TCRU_5402 TCRU_5403 TcCLB-EL.507713.30_mRNA-p1 TcCLB-NE.509105.140_mRNA-p1 TcCLB-NE.509105.150_mRNA-p1 TcCLB-NE.

509643.130_mRNA-p1 TcSYL_0008070.t1-p1 TcSYL_0008080.t1-p1
TcSYL_0008090.t1-p1 TcSYL_0008170.t1-p1 TcSYL_0008200.t1-p1
TcSYL_0008250.t1-p1 TcSYL_0008340.t1-p1 TcSYL_0008390.t1-p1
TcSYL_0008420.t1-p1 TcSYL_0008470.t1-p1 TcSYL_0008490.t1-p1
TcSYL_0008510.t1-p1 TcSYL_0008670.t1-p1
OG0000116: TCRU_8565 TcCLB-EL.503717.90_mRNA-p1 TcCLB-EL.
503859.25_pseudogenic_transcript-p1 TcCLB-EL.
504239.365_pseudogenic_transcript-p1 TcCLB-EL.
506767.196_pseudogenic_transcript-p1 TcCLB-EL.
506951.70_pseudogenic_transcript-p1 TcCLB-EL.
507959.336_pseudogenic_transcript-p1 TcCLB-EL.
509973.55_pseudogenic_transcript-p1 TcCLB-EL.
509977.61_pseudogenic_transcript-p1 TcCLB-EL.
509979.187_pseudogenic_transcript-p1 TcCLB-EL.
509979.330_pseudogenic_transcript-p1 TcCLB-EL.
510017.15_pseudogenic_transcript-p1 TcCLB-EL.510089.20_mRNA-p1
TcCLB-EL.510371.61_pseudogenic_transcript-p1 TcCLB-NE.
508245.21_pseudogenic_transcript-p1 TcCLB-NE.
508613.100_pseudogenic_transcript-p1 TcCLB-NE.
510039.33_pseudogenic_transcript-p1 TcCLB-NE.
511255.529_pseudogenic_transcript-p1 TcCLB-NE.
511255.625_pseudogenic_transcript-p1 TcCLB-NE.
511849.50_pseudogenic_transcript-p1
OG0000117: TcCLB-EL.504193.30_pseudogenic_transcript-p1 TcCLB-EL.
506961.130_pseudogenic_transcript-p1 TcCLB-EL.
508803.5_pseudogenic_transcript-p1 TcCLB-EL.
510307.352_pseudogenic_transcript-p1 TcCLB-EL.
510607.51_pseudogenic_transcript-p1 TcCLB-EL.
511587.60_pseudogenic_transcript-p1 TcCLB-NE.
503439.61_pseudogenic_transcript-p1 TcCLB-NE.
503537.9_pseudogenic_transcript-p1 TcCLB-NE.
504115.50_pseudogenic_transcript-p1 TcCLB-NE.
506471.110_pseudogenic_transcript-p1 TcCLB-NE.
507643.30_pseudogenic_transcript-p1 TcCLB-NE.
507777.30_pseudogenic_transcript-p1 TcCLB-NE.
508519.20_pseudogenic_transcript-p1 TcCLB-NE.
508629.42_pseudogenic_transcript-p1 TcCLB-NE.
508629.74_pseudogenic_transcript-p1 TcCLB-NE.
509295.5_pseudogenic_transcript-p1 TcCLB-NE.
509437.140_pseudogenic_transcript-p1 TcCLB-NE.
510355.97_pseudogenic_transcript-p1 TcCLB-NE.
510699.110_pseudogenic_transcript-p1 TcSYL_0141080.t1-p1
OG0000118: TCRU_138 TCRU_2329 TCRU_7650 TcCLB-EL.
506671.30_pseudogenic_transcript-p1 TcCLB-EL.506971.80_mRNA-p1
TcCLB-EL.508167.10_pseudogenic_transcript-p1 TcCLB-EL.
508389.100_mRNA-p1 TcCLB-EL.510269.20_mRNA-p1 TcCLB-EL.
511875.9_mRNA-p1 TcCLB-NE.506137.60_mRNA-p1 TcCLB-NE.
507065.108_pseudogenic_transcript-p1 TcCLB-NE.507605.13_mRNA-p1
TcCLB-NE.508243.50_mRNA-p1 TcCLB-NE.508433.233_mRNA-p1 TcCLB-NE.
509755.80_pseudogenic_transcript-p1 TcCLB-NE.
510699.20_pseudogenic_transcript-p1 TcSYL_0125430.t1-p1
TcSYL_0129180.t1-p1 TcSYL_0162120.t1-p1
OG0000119: TCRU_538 TCRU_541 TCRU_9029 TcCLB-EL.507485.10_mRNA-p1
TcCLB-EL.507485.20_mRNA-p1 TcCLB-EL.507485.30_mRNA-p1 TcCLB-EL.

507485.40_mRNA-p1 TcCLB-EL.507673.50_mRNA-p1 TcCLB-EL.
507673.60_mRNA-p1 TcCLB-EL.507673.70_mRNA-p1 TcCLB-NE.
506199.10_mRNA-p1 TcCLB-NE.506199.20_mRNA-p1 TcCLB-NE.
507159.50_mRNA-p1 TcCLB-NE.507159.60_mRNA-p1 TcCLB-NE.
507159.70_mRNA-p1 TcCLB-NE.507159.80_mRNA-p1 TcSYL_0194800.t1-p1
TcSYL_0194930.t1-p1 TcSYL_0195090.t1-p1
OG0000120: TCRU_10319 TCRU_10549 TCRU_2182 TCRU_2185 TCRU_2366
TCRU_2367 TCRU_3943 TCRU_4880 TCRU_4891 TCRU_4894 TCRU_662 TCRU_7774
TCRU_7873 TCRU_7874 TCRU_8502 TCRU_8560 TCRU_9580 TCRU_9932
TcSYL_0065900.t1-p1
OG0000121: TCRU_1180 TCRU_2707 TCRU_2798 TCRU_4975 TCRU_7124 TcCLB-
EL.504343.30_mRNA-p1 TcCLB-EL.505943.79_pseudogenic_transcript-p1
TcCLB-EL.507907.30_mRNA-p1 TcCLB-EL.507907.60_mRNA-p1 TcCLB-EL.
508559.90_mRNA-p1 TcCLB-EL.509871.10_pseudogenic_transcript-p1
TcCLB-EL.511371.10_mRNA-p1 TcCLB-EL.511415.11_mRNA-p1 TcCLB-EL.
511585.320_mRNA-p1 TcCLB-NE.504109.200_mRNA-p1 TcCLB-NE.
505207.30_mRNA-p1 TcCLB-NE.506595.30_pseudogenic_transcript-p1
TcCLB-NE.508577.30_pseudogenic_transcript-p1 TcSYL_0020440.t1-p1
OG0000122: TCRU_1345 TCRU_3685 TCRU_4527 TCRU_9434 TCRU_9487 TcCLB-
EL.510175.125_mRNA-p1 TcCLB-NE.506329.35_pseudogenic_transcript-p1
TcCLB-NE.506537.110_mRNA-p1 TcCLB-NE.506537.230_mRNA-p1 TcCLB-NE.
506917.27_pseudogenic_transcript-p1 TcCLB-NE.507501.20_mRNA-p1
TcCLB-NE.507723.20_mRNA-p1 TcCLB-NE.
508365.220_pseudogenic_transcript-p1 TcCLB-NE.510817.40_mRNA-p1
TcCLB-NE.511887.40_mRNA-p1 TcSYL_0136390.t1-p1 TcSYL_0149470.t1-p1
TcSYL_0149580.t1-p1 TcSYL_0154910.t1-p1
OG0000123: TCRU_1369 TcCLB-EL.504011.80_mRNA-p1 TcCLB-EL.
507631.20_mRNA-p1 TcCLB-EL.509581.35_pseudogenic_transcript-p1
TcCLB-EL.510249.30_mRNA-p1 TcCLB-EL.511047.70_mRNA-p1 TcCLB-EL.
511053.10_mRNA-p1 TcCLB-EL.511129.60_mRNA-p1 TcCLB-EL.
511911.90_mRNA-p1 TcCLB-NE.504533.50_mRNA-p1 TcCLB-NE.
506011.10_mRNA-p1 TcCLB-NE.508637.40_mRNA-p1 TcCLB-NE.
508735.100_mRNA-p1 TcCLB-NE.508945.10_mRNA-p1 TcCLB-NE.
511859.200_mRNA-p1 TcSYL_0090480.t1-p1 TcSYL_0090710.t1-p1
TcSYL_0148280.t1-p1 TcSYL_0171510.t1-p1
OG0000124: TCRU_2143 TCRU_2965 TCRU_3041 TCRU_3970 TCRU_4105
TCRU_4731 TCRU_5226 TCRU_6430 TCRU_6965 TCRU_8447 TcCLB-EL.
507981.10_pseudogenic_transcript-p1 TcCLB-EL.507987.10_mRNA-p1
TcCLB-EL.508229.10_mRNA-p1 TcCLB-EL.510061.30_mRNA-p1 TcCLB-NE.
506389.10_mRNA-p1 TcCLB-NE.507687.10_mRNA-p1 TcCLB-NE.
511855.10_mRNA-p1 TcSYL_0112900.t1-p1 TcSYL_0112910.t1-p1
OG0000125: TCRU_3830 TCRU_6881 TCRU_7602 TCRU_8026 TCRU_8057
TCRU_8281 TcCLB-EL.506285.110_pseudogenic_transcript-p1 TcCLB-EL.
506409.190_pseudogenic_transcript-p1 TcCLB-EL.506501.110_mRNA-p1
TcCLB-EL.506501.240_mRNA-p1 TcCLB-EL.
506761.40_pseudogenic_transcript-p1 TcCLB-EL.506765.74_mRNA-p1
TcCLB-EL.506767.40_mRNA-p1 TcCLB-EL.
506967.140_pseudogenic_transcript-p1 TcCLB-EL.509195.50_mRNA-p1
TcCLB-EL.509699.210_mRNA-p1 TcCLB-EL.510621.49_mRNA-p1 TcCLB-EL.
511597.40_mRNA-p1 TcSYL_0100440.t1-p1
OG0000126: TCRU_4471 TCRU_4472 TCRU_8928 TcCLB-EL.506533.106_mRNA-p1
TcCLB-EL.506533.142_mRNA-p1 TcCLB-EL.509147.50_mRNA-p1 TcCLB-EL.
511679.10_mRNA-p1 TcCLB-EL.511685.10_mRNA-p1 TcCLB-EL.
511685.20_mRNA-p1 TcCLB-EL.511685.30_mRNA-p1 TcCLB-NE.

508739.21_pseudogenic_transcript-p1 TcCLB-NE.508741.40_mRNA-p1
TcCLB-NE.508741.50_mRNA-p1 TcCLB-NE.508741.60_mRNA-p1 TcCLB-NE.
508741.70_mRNA-p1 TcCLB-NE.508741.80_mRNA-p1 TcSYL_0193430.t1-p1
TcSYL_0193610.t1-p1 TcSYL_0193780.t1-p1
OG0000127: TCRU_10785 TCRU_5228 TCRU_5248 TCRU_5863 TCRU_8839
TCRU_8869 TCRU_9347 TcCLB-EL.508039.30_mRNA-p1 TcCLB-EL.
509265.10_mRNA-p1 TcCLB-EL.510853.73_pseudogenic_transcript-p1
TcCLB-NE.503421.50_pseudogenic_transcript-p1 TcCLB-NE.
507167.240_mRNA-p1 TcCLB-NE.507723.30_pseudogenic_transcript-p1
TcCLB-NE.508001.70_mRNA-p1 TcCLB-NE.508003.10_mRNA-p1 TcCLB-NE.
509259.120_mRNA-p1 TcCLB-NE.511667.20_mRNA-p1 TcCLB-NE.
511843.80_mRNA-p1 TcSYL_0138040.t1-p1
OG0000128: TCRU_of TCRU_of TCRU_of TcCLB-EL.420091.10_mRNA-p1 TcCLB-
EL.504099.80_pseudogenic_transcript-p1 TcCLB-EL.510061.60_mRNA-p1
TcCLB-EL.511123.40_mRNA-p1 TcCLB-NE.505401.40_mRNA-p1 TcCLB-NE.
506903.10_mRNA-p1 TcCLB-NE.509019.30_mRNA-p1 TcCLB-NE.
509295.110_pseudogenic_transcript-p1 TcCLB-NE.510399.10_mRNA-p1
TcCLB-NE.510401.10_mRNA-p1 TcCLB-NE.511663.50_mRNA-p1
TcSYL_0093670.t1-p1 TcSYL_0106150.t1-p1 TcSYL_0151760.t1-p1
TcSYL_0163780.t1-p1 TcSYL_0186940.t1-p1
OG0000129: TCRU_6547 TcCLB-EL.422319.10_mRNA-p1 TcCLB-EL.
506289.130_mRNA-p1 TcCLB-EL.506289.160_mRNA-p1 TcCLB-EL.
506289.200_mRNA-p1 TcCLB-EL.506289.240_mRNA-p1 TcCLB-EL.
506289.94_mRNA-p1 TcCLB-EL.506291.20_mRNA-p1 TcCLB-EL.
506799.120_mRNA-p1 TcCLB-EL.506799.40_mRNA-p1 TcCLB-EL.
506801.30_mRNA-p1 TcCLB-EL.506965.160_mRNA-p1 TcCLB-EL.
508047.40_pseudogenic_transcript-p1 TcCLB-EL.508081.20_mRNA-p1
TcCLB-EL.510279.30_mRNA-p1 TcCLB-NE.507633.90_mRNA-p1 TcCLB-NE.
508429.10_mRNA-p1 TcCLB-NE.511257.110_mRNA-p1 TcCLB-NE.
511257.80_mRNA-p1
OG0000130: TCRU_6666 TCRU_9152 TcCLB-EL.
447847.19_pseudogenic_transcript-p1 TcCLB-EL.
506713.70_pseudogenic_transcript-p1 TcCLB-EL.507611.110_mRNA-p1
TcCLB-EL.507841.14_mRNA-p1 TcCLB-EL.509815.10_mRNA-p1 TcCLB-EL.
509827.4_mRNA-p1 TcCLB-NE.506535.10_mRNA-p1 TcCLB-NE.
506595.149_mRNA-p1 TcCLB-NE.507015.10_mRNA-p1 TcCLB-NE.
508173.20_pseudogenic_transcript-p1 TcCLB-NE.508851.19_mRNA-p1
TcCLB-NE.510083.30_pseudogenic_transcript-p1 TcCLB-NE.
510355.150_pseudogenic_transcript-p1 TcCLB-NE.
510643.20_pseudogenic_transcript-p1 TcCLB-NE.511019.13_mRNA-p1
TcCLB-NE.511889.20_pseudogenic_transcript-p1 TcSYL_0125190.t1-p1
OG0000131: TcCLB-EL.503985.20_pseudogenic_transcript-p1 TcCLB-EL.
506021.10_pseudogenic_transcript-p1 TcCLB-EL.
506623.40_pseudogenic_transcript-p1 TcCLB-EL.
508687.20_pseudogenic_transcript-p1 TcCLB-EL.
508775.20_pseudogenic_transcript-p1 TcCLB-EL.
509921.40_pseudogenic_transcript-p1 TcCLB-EL.
509925.50_pseudogenic_transcript-p1 TcCLB-EL.
510061.40_pseudogenic_transcript-p1 TcCLB-EL.
510367.20_pseudogenic_transcript-p1 TcCLB-EL.
510591.30_pseudogenic_transcript-p1 TcCLB-EL.
510769.190_pseudogenic_transcript-p1 TcCLB-EL.
511415.50_pseudogenic_transcript-p1 TcCLB-NE.
504533.20_pseudogenic_transcript-p1 TcCLB-NE.

507687.30_pseudogenic_transcript-p1 TcCLB-NE.
508061.60_pseudogenic_transcript-p1 TcCLB-NE.
509429.30_pseudogenic_transcript-p1 TcCLB-NE.
511771.10_pseudogenic_transcript-p1 TcCLB-NE.
511807.22_pseudogenic_transcript-p1 TcSYL_0027940.t1-p1
OG0000132: TCRU_1184 TCRU_1501 TCRU_3170 TCRU_4227 TCRU_4337
TCRU_4628 TCRU_4691 TCRU_55 TCRU_7264 TCRU_7632 TCRU_7678 TCRU_7721
TCRU_8092 TCRU_9599 TCRU_9765 TcCLB-NE.507587.24_mRNA-p1 TcCLB-NE.
508395.9_mRNA-p1 TcCLB-NE.510397.39_mRNA-p1
OG0000133: TCRU_1463 TCRU_1881 TCRU_2187 TCRU_2363 TCRU_2375
TCRU_278 TCRU_8605 TcCLB-EL.418613.20_mRNA-p1 TcCLB-EL.
507429.40_pseudogenic_transcript-p1 TcCLB-NE.
507085.70_pseudogenic_transcript-p1 TcCLB-NE.
508435.30_pseudogenic_transcript-p1 TcCLB-NE.
508493.20_pseudogenic_transcript-p1 TcCLB-NE.
508493.50_pseudogenic_transcript-p1 TcCLB-NE.
508495.210_pseudogenic_transcript-p1 TcCLB-NE.
508499.30_pseudogenic_transcript-p1 TcCLB-NE.
509493.100_pseudogenic_transcript-p1 TcCLB-NE.
511643.130_pseudogenic_transcript-p1 TcSYL_0109850.t1-p1
OG0000134: TCRU_298 TCRU_5052 TCRU_562 TCRU_6607 TcCLB-EL.
511311.14_pseudogenic_transcript-p1 TcCLB-NE.
485335.10_pseudogenic_transcript-p1 TcCLB-NE.
508705.10_pseudogenic_transcript-p1 TcCLB-NE.
511771.156_pseudogenic_transcript-p1 TcSYL_0022590.t1-p1
TcSYL_0027930.t1-p1 TcSYL_0059950.t1-p1 TcSYL_0083780.t1-p1
TcSYL_0106350.t1-p1 TcSYL_0106690.t1-p1 TcSYL_0163860.t1-p1
TcSYL_0165860.t1-p1 TcSYL_0166220.t1-p1 TcSYL_0204620.t1-p1
OG0000135: TCRU_10320 TCRU_2181 TCRU_2186 TCRU_2365 TCRU_3666
TCRU_4498 TCRU_4895 TCRU_6461 TCRU_664 TCRU_7775 TCRU_7875 TCRU_8558
TCRU_9274 TCRU_9581 TCRU_9589 TcCLB-EL.507429.60_mRNA-p1
TcSYL_0134870.t1-p1 TcSYL_0190270.t1-p1
OG0000136: TCRU_2944 TCRU_6054 TCRU_8781 TCRU_8782 TcCLB-EL.
506551.10_mRNA-p1 TcCLB-EL.510811.10_mRNA-p1 TcCLB-EL.
510811.20_mRNA-p1 TcCLB-NE.508799.270_mRNA-p1 TcCLB-NE.
508799.280_mRNA-p1 TcCLB-NE.509713.10_mRNA-p1 TcCLB-NE.
509713.20_mRNA-p1 TcCLB-NE.510069.20_mRNA-p1 TcSYL_0047320.t1-p1
TcSYL_0047330.t1-p1 TcSYL_0047350.t1-p1 TcSYL_0047360.t1-p1
TcSYL_0075080.t1-p1 TcSYL_0075220.t1-p1
OG0000137: TCRU_5705 TCRU_5879 TcCLB-EL.
506289.110_pseudogenic_transcript-p1 TcCLB-EL.506289.140_mRNA-p1
TcCLB-EL.506289.170_mRNA-p1 TcCLB-EL.506289.210_mRNA-p1 TcCLB-EL.
506289.250_pseudogenic_transcript-p1 TcCLB-EL.
506291.31_pseudogenic_transcript-p1 TcCLB-EL.
506799.90_pseudogenic_transcript-p1 TcCLB-NE.468803.10_mRNA-p1
TcCLB-NE.507637.170_mRNA-p1 TcCLB-NE.508365.100_mRNA-p1 TcCLB-NE.
511257.100_mRNA-p1 TcCLB-NE.511257.70_mRNA-p1 TcCLB-NE.
511907.180_pseudogenic_transcript-p1 TcSYL_0101350.t1-p1
TcSYL_0101770.t1-p1 TcSYL_0121590.t1-p1
OG0000138: TCRU_7959 TcCLB-EL.508169.20_mRNA-p1 TcCLB-EL.
508169.9_mRNA-p1 TcCLB-EL.510963.90_mRNA-p1 TcCLB-NE.510965.5_mRNA-
p1 TcSYL_0112970.t1-p1 TcSYL_0112980.t1-p1 TcSYL_0112990.t1-p1
TcSYL_0113000.t1-p1 TcSYL_0113010.t1-p1 TcSYL_0113030.t1-p1
TcSYL_0113070.t1-p1 TcSYL_0113090.t1-p1 TcSYL_0113110.t1-p1

TcSYL_0113120.t1-p1 TcSYL_0113130.t1-p1 TcSYL_0113140.t1-p1
TcSYL_0113220.t1-p1
OG0000139: TcCLB-EL.506259.30_pseudogenic_transcript-p1 TcCLB-EL.
506717.60_pseudogenic_transcript-p1 TcCLB-EL.
506951.10_pseudogenic_transcript-p1 TcCLB-EL.
507035.20_pseudogenic_transcript-p1 TcCLB-EL.
508559.40_pseudogenic_transcript-p1 TcCLB-EL.
509735.70_pseudogenic_transcript-p1 TcCLB-EL.
509737.19_pseudogenic_transcript-p1 TcCLB-EL.
509957.30_pseudogenic_transcript-p1 TcCLB-EL.
511415.30_pseudogenic_transcript-p1 TcCLB-NE.
503605.10_pseudogenic_transcript-p1 TcCLB-NE.
505997.100_pseudogenic_transcript-p1 TcCLB-NE.
507895.31_pseudogenic_transcript-p1 TcCLB-NE.
508527.20_pseudogenic_transcript-p1 TcCLB-NE.
510079.10_pseudogenic_transcript-p1 TcCLB-NE.
511767.30_pseudogenic_transcript-p1 TcCLB-NE.
511851.20_pseudogenic_transcript-p1 TcSYL_0059580.t1-p1
TcSYL_0059960.t1-p1
OG0000140: TCRU_10482 TCRU_3136 TCRU_692 TCRU_8366 TCRU_9427
TCRU_9902 TcCLB-EL.506343.51_pseudogenic_transcript-p1 TcCLB-EL.
507611.114_pseudogenic_transcript-p1 TcCLB-EL.
508285.40_pseudogenic_transcript-p1 TcCLB-NE.
503537.18_pseudogenic_transcript-p1 TcCLB-NE.506271.30_mRNA-p1
TcCLB-NE.511771.143_pseudogenic_transcript-p1 TcSYL_0051500.t1-p1
TcSYL_0120210.t1-p1 TcSYL_0120630.t1-p1 TcSYL_0120810.t1-p1
TcSYL_0203480.t1-p1
OG0000141: TCRU_1112 TCRU_1581 TCRU_3134 TCRU_4158 TCRU_4201
TCRU_5097 TcCLB-EL.505025.40_mRNA-p1 TcCLB-EL.506759.220_mRNA-p1
TcCLB-EL.507953.10_mRNA-p1 TcCLB-EL.510019.30_mRNA-p1 TcCLB-EL.
511553.200_mRNA-p1 TcCLB-NE.503973.240_mRNA-p1 TcCLB-NE.
503973.50_mRNA-p1 TcCLB-NE.508221.820_mRNA-p1 TcCLB-NE.
510359.570_mRNA-p1 TcSYL_0136520.t1-p1 TcSYL_0136530.t1-p1
OG0000142: TCRU_10399 TCRU_1198 TCRU_2042 TCRU_6838 TCRU_7315 TcCLB-
EL.506965.40_pseudogenic_transcript-p1 TcCLB-EL.
508047.30_pseudogenic_transcript-p1 TcCLB-EL.
510275.80_pseudogenic_transcript-p1 TcCLB-EL.
511873.30_pseudogenic_transcript-p1 TcCLB-NE.
504155.250_pseudogenic_transcript-p1 TcCLB-NE.
506139.140_pseudogenic_transcript-p1 TcCLB-NE.
506615.120_pseudogenic_transcript-p1 TcCLB-NE.
508431.80_pseudogenic_transcript-p1 TcCLB-NE.
509899.10_pseudogenic_transcript-p1 TcCLB-NE.
510487.37_pseudogenic_transcript-p1 TcSYL_0067920.t1-p1
TcSYL_0124940.t1-p1
OG0000143: TCRU_2114 TCRU_2170 TCRU_3337 TCRU_8174 TcSYL_0017870.t1-
p1 TcSYL_0051590.t1-p1 TcSYL_0060010.t1-p1 TcSYL_0091410.t1-p1
TcSYL_0118830.t1-p1 TcSYL_0118960.t1-p1 TcSYL_0119690.t1-p1
TcSYL_0119760.t1-p1 TcSYL_0121040.t1-p1 TcSYL_0124630.t1-p1
TcSYL_0150900.t1-p1 TcSYL_0154180.t1-p1 TcSYL_0155340.t1-p1
OG0000144: TCRU_10117 TCRU_2449 TCRU_3384 TCRU_3565 TcCLB-EL.
508539.71_pseudogenic_transcript-p1 TcCLB-EL.
508541.10_pseudogenic_transcript-p1 TcCLB-EL.
508541.125_pseudogenic_transcript-p1 TcCLB-NE.

504261.35_pseudogenic_transcript-p1 TcCLB-NE.
506955.226_pseudogenic_transcript-p1 TcCLB-NE.
507063.315_pseudogenic_transcript-p1 TcCLB-NE.
508365.171_pseudogenic_transcript-p1 TcCLB-NE.
510487.44_pseudogenic_transcript-p1 TcCLB-NE.
510699.27_pseudogenic_transcript-p1 TcCLB-NE.511255.180_mRNA-p1
TcCLB-NE.511255.681_pseudogenic_transcript-p1 TcSYL_0067390.t1-p1
TcSYL_0101650.t1-p1
OG0000145: TCRU_10574 TCRU_2556 TCRU_6554 TcCLB-EL.
504239.140_pseudogenic_transcript-p1 TcCLB-EL.506499.40_mRNA-p1
TcCLB-EL.506767.340_mRNA-p1 TcCLB-EL.507957.150_mRNA-p1 TcCLB-EL.
510013.40_mRNA-p1 TcCLB-EL.510025.100_mRNA-p1 TcCLB-EL.
510025.260_mRNA-p1 TcCLB-EL.510377.220_pseudogenic_transcript-p1
TcCLB-NE.504081.90_mRNA-p1 TcCLB-NE.508873.250_mRNA-p1 TcCLB-NE.
508979.60_mRNA-p1 TcCLB-NE.511255.200_mRNA-p1 TcCLB-NE.
511255.230_pseudogenic_transcript-p1 TcSYL_0134030.t1-p1
OG0000146: TCRU_3827 TCRU_5465 TCRU_6369 TCRU_6622 TCRU_6685
TCRU_8617 TcCLB-EL.506501.290_mRNA-p1 TcCLB-EL.506967.100_mRNA-p1
TcCLB-EL.507237.340_mRNA-p1 TcCLB-EL.507959.280_mRNA-p1 TcCLB-EL.
511613.80_mRNA-p1 TcCLB-NE.506787.19_pseudogenic_transcript-p1
TcCLB-NE.508221.360_pseudogenic_transcript-p1 TcCLB-NE.
508221.580_mRNA-p1 TcCLB-NE.510693.19_pseudogenic_transcript-p1
TcSYL_0135630.t1-p1 TcSYL_0137220.t1-p1
OG0000147: TCRU_4375 TCRU_5195 TCRU_6623 TcCLB-EL.504039.180_mRNA-p1
TcCLB-EL.504239.220_mRNA-p1 TcCLB-EL.506269.40_mRNA-p1 TcCLB-EL.
506599.330_mRNA-p1 TcCLB-EL.506599.50_mRNA-p1 TcCLB-EL.
506763.260_mRNA-p1 TcCLB-EL.507955.20_mRNA-p1 TcCLB-EL.
507957.179_mRNA-p1 TcCLB-EL.507957.200_mRNA-p1 TcCLB-EL.
509699.140_pseudogenic_transcript-p1 TcCLB-EL.510011.9_mRNA-p1
TcCLB-EL.510013.170_mRNA-p1 TcCLB-EL.510377.134_mRNA-p1
TcSYL_0126020.t1-p1
OG0000148: TCRU_6212 TcCLB-NE.504261.80_mRNA-p1 TcSYL_0066970.t1-p1
TcSYL_0070390.t1-p1 TcSYL_0093080.t1-p1 TcSYL_0101110.t1-p1
TcSYL_0133040.t1-p1 TcSYL_0135000.t1-p1 TcSYL_0136350.t1-p1
TcSYL_0137090.t1-p1 TcSYL_0149440.t1-p1 TcSYL_0149610.t1-p1
TcSYL_0154930.t1-p1 TcSYL_0160270.t1-p1 TcSYL_0160920.t1-p1
TcSYL_0161070.t1-p1 TcSYL_0161180.t1-p1
OG0000149: TCRU_8383 TcCLB-EL.506667.170_mRNA-p1 TcCLB-EL.
506769.10_mRNA-p1 TcCLB-EL.510275.405_mRNA-p1 TcCLB-EL.
510377.120_mRNA-p1 TcCLB-NE.506001.30_mRNA-p1 TcCLB-NE.
508247.100_pseudogenic_transcript-p1 TcCLB-NE.509217.170_mRNA-p1
TcCLB-NE.509755.20_mRNA-p1 TcCLB-NE.510625.120_mRNA-p1 TcCLB-NE.
510715.11_mRNA-p1 TcSYL_0017760.t1-p1 TcSYL_0029640.t1-p1
TcSYL_0067610.t1-p1 TcSYL_0070180.t1-p1 TcSYL_0074200.t1-p1
TcSYL_0161130.t1-p1
OG0000150: TCRU_1504 TCRU_2356 TCRU_4568 TCRU_6569 TCRU_7138
TCRU_793 TCRU_8049 TCRU_83 TCRU_9513 TcCLB-EL.
504239.10_pseudogenic_transcript-p1 TcCLB-NE.
506279.131_pseudogenic_transcript-p1 TcCLB-NE.508979.40_mRNA-p1
TcCLB-NE.509867.40_mRNA-p1 TcCLB-NE.510483.290_mRNA-p1 TcCLB-NE.
510697.120_mRNA-p1 TcSYL_0071910.t1-p1
OG0000151: TCRU_10143 TCRU_10144 TCRU_3682 TCRU_4657 TCRU_545
TCRU_6819 TCRU_6856 TCRU_8106 TCRU_8445 TCRU_8488 TCRU_8489
TCRU_9014 TCRU_9481 TcCLB-NE.411915.10_pseudogenic_transcript-p1

TcCLB-NE.503607.7_pseudogenic_transcript-p1 TcSYL_0058930.t1-p1
OG0000152: TCRU_1998 TCRU_2547 TCRU_808 TcCLB-EL.409139.9_mRNA-p1
TcCLB-EL.503665.10_pseudogenic_transcript-p1 TcCLB-EL.
506685.5_pseudogenic_transcript-p1 TcCLB-EL.509927.10_mRNA-p1 TcCLB-
EL.511593.40_pseudogenic_transcript-p1 TcCLB-EL.
511595.10_pseudogenic_transcript-p1 TcCLB-NE.
508521.90_pseudogenic_transcript-p1 TcCLB-NE.
509259.10_pseudogenic_transcript-p1 TcCLB-NE.509287.80_mRNA-p1
TcCLB-NE.509663.47_pseudogenic_transcript-p1 TcCLB-NE.
511851.30_pseudogenic_transcript-p1 TcSYL_0099740.t1-p1
TcSYL_0191480.t1-p1
OG0000153: TCRU_10570 TCRU_10659 TCRU_1527 TCRU_1532 TCRU_2173
TCRU_2752 TCRU_4873 TCRU_6380 TCRU_8260 TCRU_8277 TCRU_8529
TCRU_8960 TCRU_918 TCRU_9662 TCRU_9957 TcSYL_0200200.t1-p1
OG0000154: TCRU_10464 TCRU_1049 TCRU_1124 TCRU_1500 TCRU_2450
TCRU_4589 TCRU_4693 TCRU_4739 TCRU_5337 TCRU_6298 TCRU_8993 TcCLB-
NE.509753.134_pseudogenic_transcript-p1 TcSYL_0065050.t1-p1
TcSYL_0065070.t1-p1 TcSYL_0091620.t1-p1 TcSYL_0157110.t1-p1
OG0000155: TCRU_1791 TCRU_3358 TCRU_4129 TCRU_4716 TCRU_5281 TcCLB-
NE.508427.10_mRNA-p1 TcSYL_0070370.t1-p1 TcSYL_0100840.t1-p1
TcSYL_0101130.t1-p1 TcSYL_0133030.t1-p1 TcSYL_0133610.t1-p1
TcSYL_0137110.t1-p1 TcSYL_0137120.t1-p1 TcSYL_0161060.t1-p1
TcSYL_0161170.t1-p1 TcSYL_0187330.t1-p1
OG0000156: TCRU_4142 TCRU_6520 TCRU_7322 TcCLB-EL.504039.200_mRNA-p1
TcCLB-EL.506269.50_pseudogenic_transcript-p1 TcCLB-EL.
506501.220_mRNA-p1 TcCLB-EL.506969.10_pseudogenic_transcript-p1
TcCLB-EL.506969.3_mRNA-p1 TcCLB-EL.507959.110_mRNA-p1 TcCLB-NE.
507069.130_pseudogenic_transcript-p1 TcCLB-NE.
510361.209_pseudogenic_transcript-p1 TcCLB-NE.510361.30_mRNA-p1
TcCLB-NE.510363.260_mRNA-p1 TcCLB-NE.
510625.30_pseudogenic_transcript-p1 TcCLB-NE.
510715.100_pseudogenic_transcript-p1 TcCLB-NE.
510715.140_pseudogenic_transcript-p1
OG0000157: TcCLB-EL.418405.20_pseudogenic_transcript-p1 TcCLB-EL.
503649.20_mRNA-p1 TcCLB-EL.506601.51_pseudogenic_transcript-p1
TcCLB-EL.507429.10_pseudogenic_transcript-p1 TcCLB-EL.
508165.185_pseudogenic_transcript-p1 TcCLB-EL.
510089.5_pseudogenic_transcript-p1 TcCLB-NE.
506613.60_pseudogenic_transcript-p1 TcCLB-NE.
506751.148_pseudogenic_transcript-p1 TcCLB-NE.
511405.40_pseudogenic_transcript-p1 TcSYL_0017740.t1-p1
TcSYL_0023890.t1-p1 TcSYL_0051660.t1-p1 TcSYL_0070010.t1-p1
TcSYL_0070430.t1-p1 TcSYL_0133910.t1-p1 TcSYL_0134700.t1-p1
OG0000158: TcCLB-EL.506759.150_pseudogenic_transcript-p1 TcCLB-EL.
510021.150_pseudogenic_transcript-p1 TcCLB-EL.
510023.10_pseudogenic_transcript-p1 TcCLB-EL.
510025.165_pseudogenic_transcript-p1 TcCLB-EL.
510371.141_pseudogenic_transcript-p1 TcCLB-EL.
510371.30_pseudogenic_transcript-p1 TcCLB-EL.
510377.405_pseudogenic_transcript-p1 TcCLB-NE.
503973.64_pseudogenic_transcript-p1 TcCLB-NE.
506339.25_pseudogenic_transcript-p1 TcCLB-NE.507069.70_mRNA-p1
TcCLB-NE.508247.60_pseudogenic_transcript-p1 TcCLB-NE.
508887.10_pseudogenic_transcript-p1 TcCLB-NE.

510627.70_pseudogenic_transcript-p1 TcSYL_0127410.t1-p1
TcSYL_0150530.t1-p1 TcSYL_0151410.t1-p1
OG0000159: TCRU_10283 TCRU_10485 TCRU_1549 TCRU_2710 TCRU_4990
TCRU_57 TCRU_599 TCRU_6177 TCRU_828 TCRU_9140 TCRU_9500 TCRU_9898
TcSYL_0048820.t1-p1 TcSYL_0150730.t1-p1 TcSYL_0153100.t1-p1
OG0000160: TCRU_10046 TCRU_10657 TCRU_2347 TCRU_258 TCRU_4980
TCRU_507 TCRU_7379 TCRU_8093 TCRU_8394 TCRU_8620 TCRU_8958 TCRU_919
TCRU_9848 TcSYL_0071520.t1-p1 TcSYL_0152610.t1-p1
OG0000161: TCRU_1533 TCRU_267 TCRU_2734 TCRU_3847 TCRU_3848
TCRU_4341 TCRU_456 TCRU_5282 TCRU_6810 TCRU_7509 TCRU_8441 TCRU_8582
TCRU_8619 TCRU_9956 TcSYL_0071540.t1-p1
OG0000162: TCRU_2140 TCRU_9207 TCRU_947 TcCLB-EL.
506609.93_pseudogenic_transcript-p1 TcCLB-EL.
506667.110_pseudogenic_transcript-p1 TcCLB-NE.
511841.10_pseudogenic_transcript-p1 TcSYL_0018910.t1-p1
TcSYL_0074220.t1-p1 TcSYL_0091230.t1-p1 TcSYL_0091510.t1-p1
TcSYL_0127500.t1-p1 TcSYL_0130300.t1-p1 TcSYL_0149380.t1-p1
TcSYL_0154190.t1-p1 TcSYL_0187410.t1-p1
OG0000163: TCRU_1084 TCRU_1085 TCRU_5638 TCRU_5639 TcCLB-EL.
511369.10_mRNA-p1 TcCLB-EL.511369.20_mRNA-p1 TcCLB-EL.
511369.30_mRNA-p1 TcCLB-NE.510119.20_mRNA-p1 TcCLB-NE.510119.9_mRNA-
p1 TcCLB-NE.511367.360_mRNA-p1 TcCLB-NE.511367.370_mRNA-p1
TcSYL_0169430.t1-p1 TcSYL_0169800.t1-p1 TcSYL_0169860.t1-p1
TcSYL_0169880.t1-p1
OG0000164: TCRU_1205 TCRU_3750 TcCLB-EL.504239.30_mRNA-p1 TcCLB-EL.
506501.10_mRNA-p1 TcCLB-EL.506599.240_mRNA-p1 TcCLB-EL.
506759.40_mRNA-p1 TcCLB-EL.507957.10_mRNA-p1 TcCLB-EL.
510013.150_pseudogenic_transcript-p1 TcCLB-EL.510377.350_mRNA-p1
TcCLB-NE.504081.460_mRNA-p1 TcCLB-NE.508219.50_mRNA-p1 TcCLB-NE.
508999.127_pseudogenic_transcript-p1 TcCLB-NE.508999.70_mRNA-p1
TcCLB-NE.510363.280_mRNA-p1 TcCLB-NE.510363.80_mRNA-p1
OG0000165: TCRU_10473 TCRU_1864 TCRU_4020 TcCLB-EL.
503501.60_pseudogenic_transcript-p1 TcCLB-EL.
504219.10_pseudogenic_transcript-p1 TcCLB-EL.
504239.115_pseudogenic_transcript-p1 TcCLB-EL.
506499.10_pseudogenic_transcript-p1 TcCLB-EL.
506765.66_pseudogenic_transcript-p1 TcCLB-EL.
506767.155_pseudogenic_transcript-p1 TcCLB-EL.508229.50_mRNA-p1
TcCLB-EL.510025.230_mRNA-p1 TcCLB-EL.
510375.30_pseudogenic_transcript-p1 TcCLB-NE.504081.20_mRNA-p1
TcCLB-NE.508221.539_mRNA-p1 TcSYL_0072170.t1-p1
OG0000166: TCRU_10400 TCRU_3790 TCRU_3900 TcCLB-EL.
508121.160_pseudogenic_transcript-p1 TcCLB-EL.
508121.90_pseudogenic_transcript-p1 TcCLB-EL.508539.80_mRNA-p1
TcCLB-EL.508541.130_mRNA-p1 TcCLB-EL.510213.70_mRNA-p1 TcCLB-NE.
503849.40_mRNA-p1 TcCLB-NE.506955.150_mRNA-p1 TcCLB-NE.
508433.220_mRNA-p1 TcCLB-NE.510629.290_pseudogenic_transcript-p1
TcCLB-NE.510693.280_mRNA-p1 TcCLB-NE.511255.690_mRNA-p1
TcSYL_0067020.t1-p1
OG0000167: TCRU_7720 TcCLB-EL.510157.20_mRNA-p1 TcSYL_0018180.t1-p1
TcSYL_0067350.t1-p1 TcSYL_0069610.t1-p1 TcSYL_0072040.t1-p1
TcSYL_0123950.t1-p1 TcSYL_0126030.t1-p1 TcSYL_0129270.t1-p1
TcSYL_0135350.t1-p1 TcSYL_0143430.t1-p1 TcSYL_0150470.t1-p1
TcSYL_0155560.t1-p1 TcSYL_0155730.t1-p1 TcSYL_0160320.t1-p1

OG0000168: TCRU_8267 TcCLB-EL.503665.21_pseudogenic_transcript-p1 TcCLB-EL.506499.215_pseudogenic_transcript-p1 TcCLB-EL.508125.65_pseudogenic_transcript-p1 TcCLB-EL.508143.65_pseudogenic_transcript-p1 TcCLB-EL.511197.50_pseudogenic_transcript-p1 TcCLB-EL.511593.51_pseudogenic_transcript-p1 TcCLB-EL.511599.85_pseudogenic_transcript-p1 TcCLB-NE.504015.21_pseudogenic_transcript-p1 TcCLB-NE.509259.21_pseudogenic_transcript-p1 TcCLB-NE.509657.61_pseudogenic_transcript-p1 TcCLB-NE.510199.20_pseudogenic_transcript-p1 TcCLB-NE.511839.25_pseudogenic_transcript-p1 TcSYL_0072270.t1-p1 TcSYL_0136220.t1-p1
OG0000169: TCRU_10423 TCRU_10852 TCRU_8637 TcCLB-EL.439653.10_mRNA-p1 TcCLB-EL.503467.9_mRNA-p1 TcCLB-EL.508535.10_mRNA-p1 TcCLB-EL.510213.110_mRNA-p1 TcCLB-EL.510215.10_mRNA-p1 TcCLB-NE.503849.60_mRNA-p1 TcCLB-NE.503849.80_mRNA-p1 TcCLB-NE.511461.60_pseudogenic_transcript-p1 TcSYL_0131100.t1-p1 TcSYL_0131110.t1-p1 TcSYL_0131120.t1-p1 TcSYL_0131150.t1-p1
OG0000170: TCRU_1862 TCRU_3064 TCRU_783 TcCLB-EL.506599.10_mRNA-p1 TcCLB-EL.506603.50_mRNA-p1 TcCLB-EL.510163.20_mRNA-p1 TcCLB-NE.506459.240_mRNA-p1 TcCLB-NE.506459.290_pseudogenic_transcript-p1 TcCLB-NE.508261.140_mRNA-p1 TcCLB-NE.510625.140_mRNA-p1 TcCLB-NE.510697.70_pseudogenic_transcript-p1 TcSYL_0072210.t1-p1 TcSYL_0072510.t1-p1 TcSYL_0115050.t1-p1
OG0000171: TCRU_3080 TCRU_4774 TCRU_916 TcCLB-EL.507555.70_pseudogenic_transcript-p1 TcCLB-EL.507875.40_pseudogenic_transcript-p1 TcCLB-EL.509165.10_pseudogenic_transcript-p1 TcCLB-EL.510591.10_pseudogenic_transcript-p1 TcCLB-NE.410923.40_pseudogenic_transcript-p1 TcCLB-NE.507895.10_pseudogenic_transcript-p1 TcCLB-NE.508111.10_pseudogenic_transcript-p1 TcSYL_0084140.t1-p1 TcSYL_0100100.t1-p1 TcSYL_0190910.t1-p1 TcSYL_0190960.t1-p1
OG0000172: TCRU_1440 TcCLB-EL.506409.90_mRNA-p1 TcCLB-EL.507747.114_pseudogenic_transcript-p1 TcCLB-EL.508125.50_pseudogenic_transcript-p1 TcCLB-EL.508143.80_mRNA-p1 TcCLB-NE.508365.200_mRNA-p1 TcCLB-NE.508365.40_mRNA-p1 TcCLB-NE.509525.230_mRNA-p1 TcCLB-NE.509525.390_mRNA-p1 TcCLB-NE.509527.80_mRNA-p1 TcCLB-NE.509545.10_mRNA-p1 TcCLB-NE.511259.18_pseudogenic_transcript-p1 TcCLB-NE.511259.220_mRNA-p1 TcSYL_0149410.t1-p1
OG0000173: TCRU_1475 TCRU_2349 TCRU_3420 TcCLB-EL.507711.100_mRNA-p1 TcCLB-EL.507711.110_mRNA-p1 TcCLB-EL.507711.90_mRNA-p1 TcCLB-NE.508173.120_mRNA-p1 TcCLB-NE.509637.36_mRNA-p1 TcCLB-NE.509639.10_mRNA-p1 TcCLB-NE.510129.20_mRNA-p1 TcCLB-NE.510129.30_mRNA-p1 TcSYL_0008870.t1-p1 TcSYL_0008990.t1-p1 TcSYL_0079370.t1-p1
OG0000174: TCRU_10445 TCRU_1633 TCRU_2646 TCRU_3687 TCRU_6511 TCRU_8807 TCRU_9086 TcCLB-EL.506529.546_mRNA-p1 TcCLB-EL.511695.10_mRNA-p1 TcSYL_0031810.t1-p1 TcSYL_0038520.t1-p1 TcSYL_0038780.t1-p1 TcSYL_0039410.t1-p1 TcSYL_0039420.t1-p1
OG0000175: TCRU_1655 TCRU_5098 TcCLB-EL.504769.150_mRNA-p1 TcCLB-EL.506487.37_mRNA-p1 TcCLB-EL.507179.14_mRNA-p1 TcCLB-EL.

507981.61_mRNA-p1 TcCLB-NE.508247.140_mRNA-p1 TcCLB-NE.
508431.5_mRNA-p1 TcCLB-NE.510201.70_mRNA-p1 TcCLB-NE.511843.35_mRNA-p1 TcSYL_0018390.t1-p1 TcSYL_0066670.t1-p1 TcSYL_0154130.t1-p1 TcSYL_0165630.t1-p1
OG0000176: TCRU_2682 TCRU_5810 TcCLB-EL.
504049.20_pseudogenic_transcript-p1 TcCLB-EL.506047.20_mRNA-p1 TcCLB-EL.507611.10_mRNA-p1 TcCLB-EL.
507875.210_pseudogenic_transcript-p1 TcCLB-EL.509581.5_mRNA-p1 TcCLB-NE.506017.51_mRNA-p1 TcCLB-NE.
506507.60_pseudogenic_transcript-p1 TcCLB-NE.507167.20_mRNA-p1 TcCLB-NE.507503.70_pseudogenic_transcript-p1 TcCLB-NE.
508527.39_mRNA-p1 TcCLB-NE.511391.10_pseudogenic_transcript-p1 TcSYL_0095840.t1-p1
OG0000177: TCRU_2747 TCRU_8499 TCRU_8587 TcCLB-EL.503665.30_mRNA-p1 TcCLB-EL.510269.50_mRNA-p1 TcCLB-EL.511593.60_mRNA-p1 TcCLB-EL.
511595.30_mRNA-p1 TcCLB-NE.508433.10_mRNA-p1 TcCLB-NE.
508521.70_pseudogenic_transcript-p1 TcCLB-NE.
509259.30_pseudogenic_transcript-p1 TcCLB-NE.509753.250_mRNA-p1 TcCLB-NE.510709.10_mRNA-p1 TcCLB-NE.510715.30_mRNA-p1 TcSYL_0018170.t1-p1
OG0000178: TCRU_10640 TCRU_2824 TCRU_3053 TCRU_8989 TcCLB-EL.
503881.30_mRNA-p1 TcCLB-EL.507039.30_pseudogenic_transcript-p1 TcCLB-NE.508119.140_mRNA-p1 TcCLB-NE.511019.80_mRNA-p1 TcCLB-NE.
511907.330_mRNA-p1 TcSYL_0103860.t1-p1 TcSYL_0103870.t1-p1 TcSYL_0103890.t1-p1 TcSYL_0103920.t1-p1 TcSYL_0178450.t1-p1
OG0000179: TCRU_6385 TCRU_8873 TCRU_9428 TcCLB-EL.506243.110_mRNA-p1 TcCLB-EL.507611.120_mRNA-p1 TcCLB-EL.509957.20_mRNA-p1 TcCLB-EL.
511437.20_mRNA-p1 TcCLB-EL.511439.10_mRNA-p1 TcCLB-NE.
508139.160_mRNA-p1 TcCLB-NE.510679.90_pseudogenic_transcript-p1 TcCLB-NE.511767.130_mRNA-p1 TcSYL_0031040.t1-p1 TcSYL_0031120.t1-p1 TcSYL_0031170.t1-p1
OG0000180: TCRU_8038 TCRU_9586 TcCLB-EL.
504035.41_pseudogenic_transcript-p1 TcCLB-EL.505025.130_mRNA-p1 TcCLB-EL.506501.350_mRNA-p1 TcCLB-EL.506757.50_mRNA-p1 TcCLB-EL.
506765.50_mRNA-p1 TcCLB-EL.506767.240_mRNA-p1 TcCLB-EL.
507957.300_mRNA-p1 TcCLB-EL.508687.30_pseudogenic_transcript-p1 TcCLB-NE.507687.70_mRNA-p1 TcCLB-NE.508871.60_mRNA-p1 TcCLB-NE.
510359.380_mRNA-p1 TcSYL_0133140.t1-p1
OG0000181: TCRU_10538 TCRU_8046 TcCLB-EL.506967.70_mRNA-p1 TcCLB-EL.
511173.180_mRNA-p1 TcCLB-NE.503771.10_mRNA-p1 TcCLB-NE.
504155.80_mRNA-p1 TcCLB-NE.506321.120_mRNA-p1 TcCLB-NE.
508251.50_mRNA-p1 TcCLB-NE.508433.180_mRNA-p1 TcCLB-NE.
508435.40_pseudogenic_transcript-p1 TcCLB-NE.509653.10_mRNA-p1 TcCLB-NE.509753.210_mRNA-p1 TcCLB-NE.
509969.31_pseudogenic_transcript-p1 TcSYL_0152190.t1-p1
OG0000182: TcCLB-EL.506529.550_mRNA-p1 TcCLB-EL.
510073.10_pseudogenic_transcript-p1 TcCLB-NE.509429.329_mRNA-p1 TcSYL_0175480.t1-p1 TcSYL_0175790.t1-p1 TcSYL_0176090.t1-p1 TcSYL_0176120.t1-p1 TcSYL_0176170.t1-p1 TcSYL_0176430.t1-p1 TcSYL_0176520.t1-p1 TcSYL_0176550.t1-p1 TcSYL_0176840.t1-p1 TcSYL_0176860.t1-p1 TcSYL_0178060.t1-p1
OG0000183: TcCLB-EL.506409.130_pseudogenic_transcript-p1 TcCLB-EL.
510557.73_pseudogenic_transcript-p1 TcCLB-NE.
442801.5_pseudogenic_transcript-p1 TcCLB-NE.

503889.20_pseudogenic_transcript-p1 TcCLB-NE.
508979.15_pseudogenic_transcript-p1 TcCLB-NE.
510789.20_pseudogenic_transcript-p1 TcCLB-NE.
510791.30_pseudogenic_transcript-p1 TcCLB-NE.
510791.60_pseudogenic_transcript-p1 TcCLB-NE.
511403.15_pseudogenic_transcript-p1 TcCLB-NE.
511405.20_pseudogenic_transcript-p1 TcSYL_0065180.t1-p1
TcSYL_0106000.t1-p1 TcSYL_0106010.t1-p1 TcSYL_0110230.t1-p1
OG0000184: TcCLB-EL.508535.50_mRNA-p1 TcCLB-NE.509041.10_mRNA-p1
TcCLB-NE.509043.10_pseudogenic_transcript-p1 TcSYL_0011970.t1-p1
TcSYL_0012110.t1-p1 TcSYL_0012150.t1-p1 TcSYL_0167570.t1-p1
TcSYL_0167660.t1-p1 TcSYL_0167780.t1-p1 TcSYL_0167880.t1-p1
TcSYL_0168010.t1-p1 TcSYL_0168060.t1-p1 TcSYL_0168390.t1-p1
TcSYL_0168450.t1-p1
OG0000185: TcCLB-EL.510213.39_mRNA-p1 TcCLB-EL.511173.24_mRNA-p1
TcCLB-EL.511173.309_pseudogenic_transcript-p1 TcCLB-NE.
503491.19_mRNA-p1 TcCLB-NE.506339.40_mRNA-p1 TcCLB-NE.
507065.29_pseudogenic_transcript-p1 TcCLB-NE.507069.94_mRNA-p1
TcCLB-NE.508429.40_pseudogenic_transcript-p1 TcCLB-NE.
509867.20_mRNA-p1 TcCLB-NE.510483.154_mRNA-p1 TcCLB-NE.
510487.79_mRNA-p1 TcCLB-NE.510699.89_mRNA-p1 TcSYL_0092850.t1-p1
TcSYL_0160210.t1-p1
OG0000186: TcSYL_0204920.t1-p1 TcSYL_0205010.t1-p1 TcSYL_0205060.t1-
p1 TcSYL_0205160.t1-p1 TcSYL_0205180.t1-p1 TcSYL_0205200.t1-p1
TcSYL_0205280.t1-p1 TcSYL_0205320.t1-p1 TcSYL_0205340.t1-p1
TcSYL_0205410.t1-p1 TcSYL_0205450.t1-p1 TcSYL_0205510.t1-p1
TcSYL_0205570.t1-p1 TcSYL_0205630.t1-p1
OG0000187: TCRU_552 TCRU_5708 TcCLB-EL.
508087.20_pseudogenic_transcript-p1 TcCLB-EL.
509233.20_pseudogenic_transcript-p1 TcCLB-EL.509349.20_mRNA-p1
TcCLB-EL.511871.130_mRNA-p1 TcCLB-NE.508479.80_mRNA-p1 TcCLB-NE.
509085.120_mRNA-p1 TcCLB-NE.509163.110_mRNA-p1 TcCLB-NE.
509437.110_mRNA-p1 TcCLB-NE.511861.90_mRNA-p1 TcCLB-NE.
511863.4_mRNA-p1 TcSYL_0069320.t1-p1
OG0000188: TCRU_1629 TCRU_1882 TCRU_1985 TCRU_3703 TCRU_3816
TCRU_4616 TCRU_667 TCRU_7599 TcCLB-EL.
507835.10_pseudogenic_transcript-p1 TcCLB-EL.509763.10_mRNA-p1
TcCLB-EL.509815.20_pseudogenic_transcript-p1 TcCLB-NE.
504639.60_mRNA-p1 TcSYL_0200340.t1-p1
OG0000189: TCRU_758 TCRU_9870 TcCLB-EL.503907.10_mRNA-p1 TcCLB-EL.
504427.230_mRNA-p1 TcCLB-EL.509157.170_mRNA-p1 TcCLB-NE.
432997.10_mRNA-p1 TcCLB-NE.503769.20_pseudogenic_transcript-p1
TcCLB-NE.506427.10_mRNA-p1 TcCLB-NE.506577.80_mRNA-p1 TcCLB-NE.
507219.9_mRNA-p1 TcCLB-NE.508355.80_mRNA-p1 TcSYL_0009090.t1-p1
TcSYL_0141650.t1-p1
OG0000190: TCRU_1234 TCRU_6498 TcCLB-EL.504011.70_mRNA-p1 TcCLB-EL.
506633.74_pseudogenic_transcript-p1 TcCLB-EL.
506953.10_pseudogenic_transcript-p1 TcCLB-EL.511049.30_mRNA-p1
TcCLB-EL.511129.54_pseudogenic_transcript-p1 TcCLB-EL.
511911.81_pseudogenic_transcript-p1 TcCLB-NE.506255.30_mRNA-p1
TcCLB-NE.506257.80_mRNA-p1 TcCLB-NE.507753.3_pseudogenic_transcript-
p1 TcCLB-NE.508637.30_pseudogenic_transcript-p1 TcSYL_0090270.t1-p1
OG0000191: TCRU_1578 TCRU_7408 TcCLB-EL.506767.70_mRNA-p1 TcCLB-EL.
508165.350_mRNA-p1 TcCLB-EL.508165.410_mRNA-p1 TcCLB-EL.

510275.190_mRNA-p1 TcCLB-EL.510279.120_mRNA-p1 TcCLB-EL.
510279.60_mRNA-p1 TcCLB-NE.506139.110_mRNA-p1 TcCLB-NE.
510201.5_mRNA-p1 TcCLB-NE.510831.10_pseudogenic_transcript-p1
TcSYL_0149530.t1-p1 TcSYL_0154860.t1-p1
OG0000192: TCRU_1741 TCRU_1745 TCRU_2985 TCRU_3312 TCRU_3529
TCRU_5076 TCRU_5077 TCRU_7496 TCRU_7629 TcCLB-EL.509699.250_mRNA-p1
TcCLB-NE.508873.50_mRNA-p1 TcCLB-NE.509527.40_mRNA-p1
TcSYL_0179980.t1-p1
OG0000193: TCRU_2523 TCRU_9486 TcCLB-EL.511599.90_mRNA-p1 TcCLB-NE.
408825.5_mRNA-p1 TcCLB-NE.506643.10_mRNA-p1 TcCLB-NE.
508247.130_mRNA-p1 TcCLB-NE.508853.10_mRNA-p1 TcCLB-NE.
509753.60_mRNA-p1 TcCLB-NE.509753.70_mRNA-p1 TcSYL_0150690.t1-p1
TcSYL_0150940.t1-p1 TcSYL_0152100.t1-p1 TcSYL_0154340.t1-p1
OG0000194: TCRU_5197 TCRU_7764 TcCLB-EL.473633.9_mRNA-p1 TcCLB-EL.
509881.9_mRNA-p1 TcCLB-NE.507903.19_pseudogenic_transcript-p1
TcSYL_0015350.t1-p1 TcSYL_0039980.t1-p1 TcSYL_0057060.t1-p1
TcSYL_0074570.t1-p1 TcSYL_0083830.t1-p1 TcSYL_0148030.t1-p1
TcSYL_0157020.t1-p1 TcSYL_0187650.t1-p1
OG0000195: TCRU_5460 TCRU_9965 TcCLB-EL.508125.140_mRNA-p1 TcCLB-EL.
510205.80_mRNA-p1 TcCLB-EL.511173.100_mRNA-p1 TcCLB-EL.
511173.496_mRNA-p1 TcCLB-EL.511175.10_mRNA-p1 TcCLB-NE.
503645.10_mRNA-p1 TcCLB-NE.507069.50_pseudogenic_transcript-p1
TcCLB-NE.510483.20_mRNA-p1 TcCLB-NE.
510483.240_pseudogenic_transcript-p1 TcCLB-NE.510697.60_mRNA-p1
TcCLB-NE.510701.40_pseudogenic_transcript-p1
OG0000196: TCRU_10705 TCRU_5600 TcCLB-EL.508551.30_mRNA-p1 TcCLB-EL.
508551.39_mRNA-p1 TcCLB-EL.511041.40_mRNA-p1 TcCLB-NE.
506355.10_mRNA-p1 TcCLB-NE.508231.9_mRNA-p1 TcSYL_0107860.t1-p1
TcSYL_0107950.t1-p1 TcSYL_0107960.t1-p1 TcSYL_0107970.t1-p1
TcSYL_0107980.t1-p1 TcSYL_0107990.t1-p1
OG0000197: TCRU_6093 TCRU_8492 TCRU_8872 TcCLB-EL.509011.90_mRNA-p1
TcCLB-EL.511437.30_mRNA-p1 TcCLB-EL.511439.20_mRNA-p1 TcCLB-EL.
511439.30_mRNA-p1 TcCLB-NE.510679.100_mRNA-p1 TcCLB-NE.
510679.60_mRNA-p1 TcCLB-NE.510679.70_mRNA-p1 TcCLB-NE.
510681.30_mRNA-p1 TcSYL_0030910.t1-p1 TcSYL_0030920.t1-p1
OG0000198: TCRU_6330 TCRU_6995 TcCLB-EL.508125.130_mRNA-p1 TcCLB-EL.
510207.10_mRNA-p1 TcCLB-EL.510213.80_mRNA-p1 TcCLB-EL.
511173.110_mRNA-p1 TcCLB-EL.511173.420_mRNA-p1 TcCLB-NE.
503849.51_pseudogenic_transcript-p1 TcCLB-NE.507065.150_mRNA-p1
TcCLB-NE.507065.89_pseudogenic_transcript-p1 TcCLB-NE.
507069.139_pseudogenic_transcript-p1 TcCLB-NE.507071.360_mRNA-p1
TcSYL_0067010.t1-p1
OG0000199: TCRU_8074 TCRU_9156 TcCLB-EL.504425.10_mRNA-p1 TcCLB-EL.
504429.50_pseudogenic_transcript-p1 TcCLB-EL.511129.40_mRNA-p1
TcCLB-EL.511911.60_mRNA-p1 TcCLB-NE.506253.39_mRNA-p1 TcCLB-NE.
507233.10_mRNA-p1 TcCLB-NE.507753.10_mRNA-p1 TcCLB-NE.
508045.120_mRNA-p1 TcCLB-NE.508637.10_mRNA-p1 TcCLB-NE.
509333.10_mRNA-p1 TcSYL_0171620.t1-p1
OG0000200: TcCLB-EL.479721.10_pseudogenic_transcript-p1 TcCLB-EL.
508121.15_pseudogenic_transcript-p1 TcCLB-EL.
508147.45_pseudogenic_transcript-p1 TcCLB-EL.
510553.11_pseudogenic_transcript-p1 TcCLB-EL.
511413.11_pseudogenic_transcript-p1 TcCLB-EL.
511599.61_pseudogenic_transcript-p1 TcCLB-EL.

511795.10_pseudogenic_transcript-p1 TcCLB-EL.
511797.215_pseudogenic_transcript-p1 TcCLB-NE.
506053.35_pseudogenic_transcript-p1 TcCLB-NE.
506613.141_pseudogenic_transcript-p1 TcCLB-NE.
508251.21_pseudogenic_transcript-p1 TcCLB-NE.
509657.11_pseudogenic_transcript-p1 TcCLB-NE.
510239.30_pseudogenic_transcript-p1
OG0000201: TcCLB-EL.511587.75_mRNA-p1 TcCLB-NE.508219.160_mRNA-p1
TcCLB-NE.510359.450_mRNA-p1 TcCLB-NE.511223.10_mRNA-p1
TcSYL_0020670.t1-p1 TcSYL_0059880.t1-p1 TcSYL_0068920.t1-p1
TcSYL_0071060.t1-p1 TcSYL_0118870.t1-p1 TcSYL_0119500.t1-p1
TcSYL_0126590.t1-p1 TcSYL_0128030.t1-p1 TcSYL_0147960.t1-p1
OG0000202: TCRU_1019 TCRU_1320 TCRU_343 TCRU_3876 TCRU_3884
TCRU_8922 TcCLB-NE.504689.30_pseudogenic_transcript-p1 TcCLB-NE.
507167.190_pseudogenic_transcript-p1 TcCLB-NE.
507549.40_pseudogenic_transcript-p1 TcCLB-NE.
508573.80_pseudogenic_transcript-p1 TcCLB-NE.
509223.80_pseudogenic_transcript-p1 TcSYL_0049060.t1-p1
OG0000203: TCRU_10192 TCRU_10518 TCRU_10521 TCRU_1505 TCRU_389
TcCLB-EL.508539.130_pseudogenic_transcript-p1 TcCLB-EL.
508539.169_pseudogenic_transcript-p1 TcCLB-EL.
508541.120_pseudogenic_transcript-p1 TcCLB-EL.
510833.40_pseudogenic_transcript-p1 TcCLB-NE.
504249.100_pseudogenic_transcript-p1 TcCLB-NE.
506955.130_pseudogenic_transcript-p1 TcCLB-NE.
506955.90_pseudogenic_transcript-p1
OG0000204: TCRU_gamma_ TCRU_gamma_ TCRU_gamma_ TCRU_gamma_ TcCLB-EL.
511613.100_mRNA-p1 TcCLB-NE.408825.10_pseudogenic_transcript-p1
TcCLB-NE.508221.670_mRNA-p1 TcCLB-NE.508247.120_mRNA-p1 TcCLB-NE.
509753.65_pseudogenic_transcript-p1 TcCLB-NE.
509753.81_pseudogenic_transcript-p1 TcCLB-NE.
511233.170_pseudogenic_transcript-p1 TcSYL_0109160.t1-p1
OG0000205: TCRU_537 TCRU_539 TCRU_540 TCRU_9030 TcCLB-EL.
507485.15_mRNA-p1 TcCLB-EL.507485.34_mRNA-p1 TcCLB-EL.
507673.65_mRNA-p1 TcCLB-NE.506199.5_mRNA-p1 TcCLB-NE.507159.44_mRNA-
p1 TcCLB-NE.507159.65_mRNA-p1 TcCLB-NE.507159.90_mRNA-p1
TcSYL_0195240.t1-p1
OG0000206: TCRU_10205 TCRU_2737 TCRU_4623 TCRU_4674 TCRU_4978
TCRU_4979 TCRU_693 TCRU_7608 TCRU_8350 TcSYL_0089000.t1-p1
TcSYL_0120220.t1-p1 TcSYL_0147850.t1-p1
OG0000207: TCRU_10427 TCRU_2043 TCRU_2361 TCRU_2369 TCRU_3826 TcCLB-
NE.508261.150_pseudogenic_transcript-p1 TcSYL_0018880.t1-p1
TcSYL_0099820.t1-p1 TcSYL_0099940.t1-p1 TcSYL_0100050.t1-p1
TcSYL_0136650.t1-p1 TcSYL_0174900.t1-p1
OG0000208: TCRU_2540 TCRU_7710 TCRU_8047 TCRU_9290 TcCLB-EL.
506667.130_pseudogenic_transcript-p1 TcCLB-NE.503947.11_mRNA-p1
TcCLB-NE.508221.496_pseudogenic_transcript-p1 TcCLB-NE.
509753.202_mRNA-p1 TcCLB-NE.510627.45_pseudogenic_transcript-p1
TcCLB-NE.510629.281_pseudogenic_transcript-p1 TcCLB-NE.
510713.101_pseudogenic_transcript-p1 TcSYL_0150130.t1-p1
OG0000209: TCRU_2635 TCRU_2637 TCRU_2639 TCRU_5819 TcCLB-EL.
506543.30_mRNA-p1 TcCLB-EL.506543.50_mRNA-p1 TcCLB-NE.
506041.10_mRNA-p1 TcCLB-NE.506041.30_mRNA-p1 TcCLB-NE.
508267.20_mRNA-p1 TcCLB-NE.508267.40_mRNA-p1 TcCLB-NE.

508267.60_mRNA-p1 TcSYL_0019430.t1-p1
OG0000210: TCRU_3603 TCRU_4963 TCRU_7088 TcCLB-EL.506505.30_mRNA-p1 TcCLB-EL.507529.30_mRNA-p1 TcCLB-EL.507677.160_mRNA-p1 TcCLB-EL.507707.20_mRNA-p1 TcCLB-EL.509669.170_mRNA-p1 TcCLB-NE.503837.10_mRNA-p1 TcCLB-NE.506175.110_mRNA-p1 TcSYL_0009260.t1-p1 TcSYL_0009430.t1-p1
OG0000211: TCRU_10270 TCRU_10271 TCRU_4229 TcCLB-EL.506749.20_mRNA-p1 TcCLB-EL.509391.10_mRNA-p1 TcCLB-EL.509391.20_mRNA-p1 TcCLB-EL.509391.30_mRNA-p1 TcCLB-NE.507491.151_mRNA-p1 TcCLB-NE.507491.162_mRNA-p1 TcCLB-NE.507891.29_mRNA-p1 TcCLB-NE.507891.38_mRNA-p1 TcCLB-NE.507891.47_mRNA-p1
OG0000212: TCRU_4699 TcCLB-EL.504703.20_mRNA-p1 TcCLB-EL.506673.69_mRNA-p1 TcCLB-NE.446067.9_mRNA-p1 TcCLB-NE.504827.21_mRNA-p1 TcCLB-NE.509577.9_mRNA-p1 TcSYL_0117760.t1-p1 TcSYL_0117770.t1-p1 TcSYL_0117820.t1-p1 TcSYL_0117850.t1-p1 TcSYL_0117870.t1-p1 TcSYL_0118090.t1-p1
OG0000213: TCRU_5412 TcCLB-EL.503977.79_mRNA-p1 TcCLB-EL.508121.43_pseudogenic_transcript-p1 TcCLB-EL.508165.341_pseudogenic_transcript-p1 TcCLB-EL.510107.41_pseudogenic_transcript-p1 TcCLB-EL.510269.55_pseudogenic_transcript-p1 TcCLB-EL.510833.15_pseudogenic_transcript-p1 TcCLB-NE.506339.81_pseudogenic_transcript-p1 TcCLB-NE.509545.25_pseudogenic_transcript-p1 TcCLB-NE.511259.161_pseudogenic_transcript-p1 TcCLB-NE.511643.61_pseudogenic_transcript-p1 TcSYL_0149540.t1-p1
OG0000214: TCRU_5489 TcCLB-EL.507603.260_mRNA-p1 TcCLB-EL.507603.270_mRNA-p1 TcCLB-NE.509401.30_mRNA-p1 TcCLB-NE.509429.320_mRNA-p1 TcSYL_0175340.t1-p1 TcSYL_0175840.t1-p1 TcSYL_0176410.t1-p1 TcSYL_0176470.t1-p1 TcSYL_0176810.t1-p1 TcSYL_0176830.t1-p1 TcSYL_0178010.t1-p1
OG0000215: TCRU_10514 TCRU_10799 TCRU_10850 TCRU_5632 TCRU_5919 TCRU_family_ TcCLB-EL.506501.270_pseudogenic_transcript-p1 TcCLB-EL.506737.140_mRNA-p1 TcCLB-NE.510629.270_pseudogenic_transcript-p1 TcSYL_0091630.t1-p1 TcSYL_0091660.t1-p1 TcSYL_0091890.t1-p1
OG0000216: TCRU_7003 TCRU_7005 TCRU_7007 TcCLB-EL.508209.100_mRNA-p1 TcCLB-EL.508209.120_mRNA-p1 TcCLB-EL.508209.90_mRNA-p1 TcCLB-NE.509505.30_mRNA-p1 TcCLB-NE.509505.50_mRNA-p1 TcCLB-NE.509505.70_mRNA-p1 TcSYL_0022910.t1-p1 TcSYL_0022960.t1-p1 TcSYL_0023000.t1-p1
OG0000217: TCRU_7778 TcCLB-EL.444331.10_mRNA-p1 TcCLB-EL.507429.20_mRNA-p1 TcCLB-EL.507429.3_mRNA-p1 TcCLB-EL.507699.37_pseudogenic_transcript-p1 TcCLB-EL.510561.50_mRNA-p1 TcCLB-NE.445783.10_pseudogenic_transcript-p1 TcCLB-NE.508495.80_pseudogenic_transcript-p1 TcCLB-NE.508495.9_pseudogenic_transcript-p1 TcCLB-NE.510483.310_mRNA-p1 TcSYL_0109860.t1-p1 TcSYL_0110060.t1-p1
OG0000218: TCRU_7837 TCRU_8551 TcCLB-EL.506289.120_mRNA-p1 TcCLB-EL.506289.150_mRNA-p1 TcCLB-EL.506289.180_pseudogenic_transcript-p1 TcCLB-EL.506289.230_pseudogenic_transcript-p1 TcCLB-EL.506291.10_mRNA-p1 TcCLB-EL.506799.50_mRNA-p1 TcCLB-NE.457979.10_mRNA-p1 TcCLB-NE.511257.90_mRNA-p1 TcCLB-NE.511907.160_pseudogenic_transcript-p1 TcSYL_0127810.t1-p1
OG0000219: TCRU_9339 TcCLB-EL.510555.41_pseudogenic_transcript-p1

TcCLB-EL.511175.50_pseudogenic_transcript-p1 TcCLB-NE.
503787.65_pseudogenic_transcript-p1 TcCLB-NE.
506917.44_pseudogenic_transcript-p1 TcCLB-NE.
507633.21_pseudogenic_transcript-p1 TcCLB-NE.
508361.21_pseudogenic_transcript-p1 TcCLB-NE.
508885.25_pseudogenic_transcript-p1 TcCLB-NE.511259.120_mRNA-p1
TcSYL_0065720.t1-p1 TcSYL_0065910.t1-p1 TcSYL_0125320.t1-p1
OG0000220: TcCLB-EL.503759.30.1-p1 TcCLB-EL.506431.5.1-p1 TcCLB-EL.
507839.35.1-p1 TcCLB-EL.507843.20.1-p1 TcCLB-EL.508837.39.1-p1
TcCLB-NE.507381.41.1-p1 TcCLB-NE.508521.18.1-p1 TcCLB-NE.
511665.21.1-p1 TcSYL_0069350.t1-p1 TcSYL_0118610.t1-p1
TcSYL_0120260.t1-p1 TcSYL_0174400.t1-p1
OG0000221: TcCLB-EL.504239.390_pseudogenic_transcript-p1 TcCLB-EL.
504239.80_pseudogenic_transcript-p1 TcCLB-EL.
506601.21_pseudogenic_transcript-p1 TcCLB-EL.
507237.356_pseudogenic_transcript-p1 TcCLB-EL.
507959.240_pseudogenic_transcript-p1 TcCLB-EL.
509097.44_pseudogenic_transcript-p1 TcCLB-EL.
510025.200_pseudogenic_transcript-p1 TcCLB-EL.
511603.141_pseudogenic_transcript-p1 TcCLB-NE.
503771.40_pseudogenic_transcript-p1 TcCLB-NE.
503973.260_pseudogenic_transcript-p1 TcCLB-NE.
508247.19_pseudogenic_transcript-p1 TcCLB-NE.
510693.310_pseudogenic_transcript-p1
OG0000222: TcCLB-EL.506653.40_pseudogenic_transcript-p1 TcCLB-EL.
508163.380_pseudogenic_transcript-p1 TcCLB-EL.
508165.330_pseudogenic_transcript-p1 TcCLB-EL.
509979.290_pseudogenic_transcript-p1 TcCLB-EL.
511171.40_pseudogenic_transcript-p1 TcCLB-EL.
511197.40_pseudogenic_transcript-p1 TcCLB-NE.
506335.110_pseudogenic_transcript-p1 TcCLB-NE.
509897.155_pseudogenic_transcript-p1 TcCLB-NE.
510035.10_pseudogenic_transcript-p1 TcCLB-NE.
510483.30_pseudogenic_transcript-p1 TcCLB-NE.
510693.260_pseudogenic_transcript-p1 TcCLB-NE.
510699.34_pseudogenic_transcript-p1
OG0000223: TcCLB-EL.508539.95_pseudogenic_transcript-p1 TcCLB-EL.
511171.60_pseudogenic_transcript-p1 TcCLB-NE.
503645.20_pseudogenic_transcript-p1 TcCLB-NE.
503775.10_pseudogenic_transcript-p1 TcCLB-NE.
504197.15_pseudogenic_transcript-p1 TcCLB-NE.
504249.15_pseudogenic_transcript-p1 TcCLB-NE.
508221.160_pseudogenic_transcript-p1 TcCLB-NE.
510699.11_pseudogenic_transcript-p1 TcCLB-NE.
510699.85_pseudogenic_transcript-p1 TcCLB-NE.
510709.21_pseudogenic_transcript-p1 TcCLB-NE.
510715.52_pseudogenic_transcript-p1 TcSYL_0066110.t1-p1
OG0000224: TCRU_1261 TCRU_1997 TCRU_2017 TCRU_70 TCRU_8941 TcCLB-EL.
511871.150_pseudogenic_transcript-p1 TcCLB-NE.
508493.60_pseudogenic_transcript-p1 TcCLB-NE.508495.100_mRNA-p1
TcCLB-NE.508495.20_pseudogenic_transcript-p1 TcCLB-NE.
508499.39_pseudogenic_transcript-p1 TcSYL_0024040.t1-p1
OG0000225: TCRU_3600 TCRU_375 TCRU_5074 TcCLB-EL.
510555.10_pseudogenic_transcript-p1 TcCLB-EL.511603.100_mRNA-p1

TcCLB-NE.506321.200_mRNA-p1 TcCLB-NE.508493.110_mRNA-p1 TcCLB-NE.508501.80_mRNA-p1 TcCLB-NE.510585.10_pseudogenic_transcript-p1 TcCLB-NE.511645.10_mRNA-p1 TcSYL_0072020.t1-p1
OG0000226: TCRU_458 TCRU_4928 TcCLB-EL.504039.90_pseudogenic_transcript-p1 TcCLB-EL.508097.60_mRNA-p1 TcCLB-EL.511173.150_mRNA-p1 TcCLB-EL.511173.210_mRNA-p1 TcCLB-EL.511173.460_mRNA-p1 TcCLB-NE.504155.230_mRNA-p1 TcCLB-NE.509547.40_mRNA-p1 TcSYL_0150800.t1-p1 TcSYL_0151100.t1-p1
OG0000227: TCRU_478 TcCLB-EL.503717.40_pseudogenic_transcript-p1 TcCLB-EL.510105.310_mRNA-p1 TcCLB-EL.510279.349_mRNA-p1 TcCLB-NE.503771.90_mRNA-p1 TcCLB-NE.507071.160_mRNA-p1 TcCLB-NE.507071.210_mRNA-p1 TcCLB-NE.508435.70_mRNA-p1 TcCLB-NE.510693.150_mRNA-p1 TcSYL_0021820.t1-p1 TcSYL_0130230.t1-p1
OG0000228: TCRU_10194 TCRU_755 TCRU_9756 TcCLB-EL.506609.50_mRNA-p1 TcCLB-EL.506667.140_mRNA-p1 TcCLB-EL.508165.380_mRNA-p1 TcCLB-NE.507071.110_mRNA-p1 TcCLB-NE.508433.80_mRNA-p1 TcSYL_0069680.t1-p1 TcSYL_0069830.t1-p1 TcSYL_0150650.t1-p1
OG0000229: TCRU_2955 TCRU_4092 TCRU_4223 TCRU_6714 TCRU_807 TcCLB-EL.504637.10_mRNA-p1 TcCLB-NE.509655.20_pseudogenic_transcript-p1 TcCLB-NE.509753.10_pseudogenic_transcript-p1 TcSYL_0018620.t1-p1 TcSYL_0018960.t1-p1 TcSYL_0128780.t1-p1
OG0000230: TCRU_821 TCRU_8618 TCRU_8907 TCRU_9345 TcCLB-EL.510269.40_mRNA-p1 TcCLB-NE.508365.80_mRNA-p1 TcCLB-NE.510385.20_mRNA-p1 TcSYL_0127910.t1-p1 TcSYL_0164110.t1-p1 TcSYL_0166460.t1-p1 TcSYL_0166470.t1-p1
OG0000231: TCRU_922 TcCLB-EL.506375.70_mRNA-p1 TcCLB-EL.509667.31_pseudogenic_transcript-p1 TcCLB-NE.508417.10_mRNA-p1 TcCLB-NE.508423.20_pseudogenic_transcript-p1 TcCLB-NE.508425.31_pseudogenic_transcript-p1 TcCLB-NE.510671.31_mRNA-p1 TcSYL_0039920.t1-p1 TcSYL_0040020.t1-p1 TcSYL_0040980.t1-p1 TcSYL_0157550.t1-p1
OG0000232: TCRU_925 TcCLB-NE.509005.90_mRNA-p1 TcSYL_0063310.t1-p1 TcSYL_0063320.t1-p1 TcSYL_0063350.t1-p1 TcSYL_0063390.t1-p1 TcSYL_0138860.t1-p1 TcSYL_0138870.t1-p1 TcSYL_0138910.t1-p1 TcSYL_0138930.t1-p1 TcSYL_0138950.t1-p1
OG0000233: TCRU_1116 TCRU_5208 TCRU_5303 TCRU_7129 TCRU_7130 TCRU_9041 TcCLB-EL.507237.30_mRNA-p1 TcSYL_0067000.t1-p1 TcSYL_0127950.t1-p1 TcSYL_0129480.t1-p1 TcSYL_0154260.t1-p1
OG0000234: TCRU_1264 TCRU_1265 TcCLB-EL.506565.4_mRNA-p1 TcCLB-EL.506565.8_mRNA-p1 TcCLB-EL.508145.10_mRNA-p1 TcCLB-EL.508145.20_mRNA-p1 TcCLB-EL.508145.30_mRNA-p1 TcCLB-NE.504243.10_mRNA-p1 TcCLB-NE.511837.138_mRNA-p1 TcSYL_0114820.t1-p1 TcSYL_0114860.t1-p1
OG0000235: TCRU_1341 TCRU_1969 TcCLB-EL.439245.10_mRNA-p1 TcCLB-EL.509195.40_mRNA-p1 TcCLB-EL.510025.170_mRNA-p1 TcCLB-EL.510621.30_mRNA-p1 TcCLB-EL.511793.10_mRNA-p1 TcCLB-NE.508261.60_mRNA-p1 TcCLB-NE.510391.30_mRNA-p1 TcCLB-NE.510391.40_mRNA-p1 TcSYL_0155000.t1-p1
OG0000236: TCRU_1736 TCRU_9292 TCRU_9493 TCRU_9690 TcCLB-EL.503767.20_mRNA-p1 TcCLB-EL.507611.154_pseudogenic_transcript-p1 TcCLB-NE.508873.170_pseudogenic_transcript-p1 TcCLB-NE.510641.10_pseudogenic_transcript-p1 TcCLB-NE.511225.20_pseudogenic_transcript-p1 TcSYL_0017280.t1-p1 TcSYL_0129670.t1-p1
OG0000237: TCRU_10522 TCRU_2541 TCRU_3496 TCRU_4203 TCRU_6195

TCRU_6304 TCRU_6809 TCRU_7702 TcCLB-EL.511173.10_mRNA-p1
TcSYL_0136870.t1-p1 TcSYL_0151380.t1-p1
OG0000238: TCRU_2865 TCRU_7111 TcCLB-EL.
504153.90_pseudogenic_transcript-p1 TcCLB-NE.507053.209_mRNA-p1
TcSYL_0047020.t1-p1 TcSYL_0047030.t1-p1 TcSYL_0047040.t1-p1
TcSYL_0047050.t1-p1 TcSYL_0047060.t1-p1 TcSYL_0047070.t1-p1
TcSYL_0138760.t1-p1
OG0000239: TCRU_3057 TcCLB-EL.503667.20_mRNA-p1 TcCLB-EL.
507875.50_mRNA-p1 TcCLB-EL.509349.30_mRNA-p1 TcCLB-NE.
506595.140_mRNA-p1 TcCLB-NE.508677.60_mRNA-p1 TcCLB-NE.
511827.115_mRNA-p1 TcSYL_0061080.t1-p1 TcSYL_0061190.t1-p1
TcSYL_0147820.t1-p1 TcSYL_0171790.t1-p1
OG0000240: TCRU_3599 TCRU_6188 TcCLB-EL.
506251.70_pseudogenic_transcript-p1 TcCLB-EL.509377.20_mRNA-p1
TcCLB-NE.509629.10_mRNA-p1 TcCLB-NE.510331.10_mRNA-p1
TcSYL_0000290.t1-p1 TcSYL_0001380.t1-p1 TcSYL_0001580.t1-p1
TcSYL_0001590.t1-p1 TcSYL_0001600.t1-p1
OG0000241: TCRU_3680 TcCLB-EL.511253.20_mRNA-p1 TcCLB-EL.
511253.31_mRNA-p1 TcCLB-NE.508867.9_mRNA-p1 TcCLB-NE.
511249.110_pseudogenic_transcript-p1 TcSYL_0027330.t1-p1
TcSYL_0027340.t1-p1 TcSYL_0027360.t1-p1 TcSYL_0027410.t1-p1
TcSYL_0027420.t1-p1 TcSYL_0027430.t1-p1
OG0000242: TCRU_3825 TCRU_6795 TcCLB-EL.506025.4_mRNA-p1 TcCLB-NE.
508827.10_mRNA-p1 TcCLB-NE.511209.69_mRNA-p1 TcSYL_0181460.t1-p1
TcSYL_0181470.t1-p1 TcSYL_0181480.t1-p1 TcSYL_0181490.t1-p1
TcSYL_0181530.t1-p1 TcSYL_0181560.t1-p1
OG0000243: TCRU_3831 TCRU_6846 TcCLB-EL.506649.20_mRNA-p1 TcCLB-EL.
507627.20_mRNA-p1 TcCLB-EL.509809.39_mRNA-p1 TcCLB-NE.
506333.10_mRNA-p1 TcCLB-NE.510575.5_mRNA-p1 TcCLB-NE.511261.4_mRNA-
p1 TcSYL_0016350.t1-p1 TcSYL_0016570.t1-p1 TcSYL_0016720.t1-p1
OG0000244: TCRU_4432 TCRU_8380 TcCLB-EL.511603.250_mRNA-p1 TcCLB-NE.
503973.190_mRNA-p1 TcCLB-NE.504081.400_mRNA-p1 TcCLB-NE.
508873.580_mRNA-p1 TcCLB-NE.509753.100_mRNA-p1 TcCLB-NE.
510237.120_mRNA-p1 TcCLB-NE.510483.180_mRNA-p1 TcSYL_0150450.t1-p1
TcSYL_0151490.t1-p1
OG0000245: TCRU_4611 TCRU_5598 TcCLB-EL.506717.20_mRNA-p1 TcCLB-EL.
511043.60_mRNA-p1 TcCLB-EL.511705.10_mRNA-p1 TcCLB-NE.
506355.20_mRNA-p1 TcCLB-NE.507965.10_mRNA-p1 TcCLB-NE.
509539.20_mRNA-p1 TcSYL_0083140.t1-p1 TcSYL_0107840.t1-p1
TcSYL_0107930.t1-p1
OG0000246: TCRU_10492 TCRU_5589 TcCLB-EL.
508143.30_pseudogenic_transcript-p1 TcCLB-EL.510491.50_mRNA-p1
TcCLB-EL.510493.4_mRNA-p1 TcCLB-NE.508259.11_pseudogenic_transcript-
p1 TcCLB-NE.510385.5_pseudogenic_transcript-p1 TcCLB-NE.
510389.10_pseudogenic_transcript-p1 TcCLB-NE.511631.9_mRNA-p1 TcCLB-
NE.511633.10_mRNA-p1 TcSYL_0152910.t1-p1
OG0000247: TCRU_10548 TCRU_6372 TCRU_9210 TcCLB-EL.503875.10_mRNA-p1
TcCLB-EL.505025.90_mRNA-p1 TcCLB-EL.507987.30_mRNA-p1 TcCLB-EL.
511553.140_mRNA-p1 TcCLB-NE.509251.70_pseudogenic_transcript-p1
TcCLB-NE.509897.150_mRNA-p1 TcCLB-NE.
509897.210_pseudogenic_transcript-p1 TcCLB-NE.511919.10_mRNA-p1
OG0000248: TCRU_6375 TcCLB-EL.507041.55_pseudogenic_transcript-p1
TcCLB-NE.504689.20_pseudogenic_transcript-p1 TcCLB-NE.
506281.100_pseudogenic_transcript-p1 TcCLB-NE.

509251.9_pseudogenic_transcript-p1 TcCLB-NE.
509897.124_pseudogenic_transcript-p1 TcCLB-NE.
511921.29_pseudogenic_transcript-p1 TcCLB-NE.
511923.10_pseudogenic_transcript-p1 TcCLB-NE.
511923.49_pseudogenic_transcript-p1 TcSYL_0126450.t1-p1
TcSYL_0149000.t1-p1
OG0000249: TCRU_7359 TCRU_7360 TCRU_8759 TcCLB-EL.510613.40_mRNA-p1
TcCLB-EL.510615.4_mRNA-p1 TcCLB-NE.506789.334_mRNA-p1 TcCLB-NE.
506789.340_mRNA-p1 TcCLB-NE.506789.352_mRNA-p1 TcCLB-NE.
508263.5_mRNA-p1 TcSYL_0163260.t1-p1 TcSYL_0163330.t1-p1
OG0000250: TCRU_8119 TcCLB-EL.505363.10_mRNA-p1 TcCLB-EL.
506413.89_mRNA-p1 TcCLB-EL.510307.250_pseudogenic_transcript-p1
TcCLB-EL.510431.300_mRNA-p1 TcCLB-EL.510535.109_mRNA-p1 TcCLB-NE.
507771.120_pseudogenic_transcript-p1 TcCLB-NE.
509527.90_pseudogenic_transcript-p1 TcCLB-NE.511237.130_mRNA-p1
TcCLB-NE.511827.110_mRNA-p1 TcSYL_0100120.t1-p1
OG0000251: TCRU_8845 TcCLB-EL.506971.20_mRNA-p1 TcCLB-EL.
508163.330_mRNA-p1 TcCLB-EL.508165.130_mRNA-p1 TcCLB-EL.
508165.310_mRNA-p1 TcCLB-EL.508389.90_mRNA-p1 TcCLB-EL.
510281.20_mRNA-p1 TcCLB-NE.504015.30_mRNA-p1 TcCLB-NE.
504155.150_mRNA-p1 TcCLB-NE.506613.9_mRNA-p1 TcSYL_0186830.t1-p1
OG0000252: TCRU_9272 TcCLB-EL.503501.30_pseudogenic_transcript-p1
TcCLB-EL.506599.250_pseudogenic_transcript-p1 TcCLB-EL.
506759.190_pseudogenic_transcript-p1 TcCLB-EL.
507699.170_pseudogenic_transcript-p1 TcCLB-EL.
511213.30_pseudogenic_transcript-p1 TcCLB-NE.
506459.100_pseudogenic_transcript-p1 TcCLB-NE.
508885.10_pseudogenic_transcript-p1 TcCLB-NE.
511255.410_pseudogenic_transcript-p1 TcSYL_0074310.t1-p1
TcSYL_0134370.t1-p1
OG0000253: TCRU_9847 TcCLB-EL.506345.120_mRNA-p1 TcCLB-EL.
507799.10_mRNA-p1 TcCLB-EL.508317.120_mRNA-p1 TcCLB-EL.
508317.80_mRNA-p1 TcCLB-EL.510513.10_mRNA-p1 TcCLB-NE.
505267.50_mRNA-p1 TcSYL_0022540.t1-p1 TcSYL_0140830.t1-p1
TcSYL_0174440.t1-p1 TcSYL_0174460.t1-p1
OG0000254: TCRU_10401 TcCLB-EL.507585.10_mRNA-p1 TcCLB-NE.
507659.20_mRNA-p1 TcCLB-NE.507659.30_mRNA-p1 TcSYL_0037300.t1-p1
TcSYL_0052620.t1-p1 TcSYL_0052630.t1-p1 TcSYL_0052660.t1-p1
TcSYL_0205850.t1-p1 TcSYL_0206000.t1-p1 TcSYL_0206030.t1-p1
OG0000255: TcCLB-EL.504229.37_mRNA-p1 TcCLB-EL.508207.31_mRNA-p1
TcCLB-EL.508325.310_mRNA-p1 TcCLB-EL.510175.150_mRNA-p1 TcCLB-EL.
510911.40_mRNA-p1 TcCLB-NE.503423.30_mRNA-p1 TcCLB-NE.
506537.250_mRNA-p1 TcCLB-NE.509723.5_mRNA-p1 TcCLB-NE.
510817.21_mRNA-p1 TcCLB-NE.511773.40_mRNA-p1 TcSYL_0058690.t1-p1
OG0000256: TcCLB-EL.504039.175_pseudogenic_transcript-p1 TcCLB-EL.
506267.75_pseudogenic_transcript-p1 TcCLB-EL.
506763.35_pseudogenic_transcript-p1 TcCLB-NE.
508221.502_pseudogenic_transcript-p1 TcCLB-NE.
509251.98_pseudogenic_transcript-p1 TcCLB-NE.
509897.95_pseudogenic_transcript-p1 TcCLB-NE.
510697.105_pseudogenic_transcript-p1 TcCLB-NE.
511921.130_pseudogenic_transcript-p1 TcCLB-NE.
511923.71_pseudogenic_transcript-p1 TcSYL_0125910.t1-p1
TcSYL_0125920.t1-p1

OG0000257: TcCLB-EL.506965.100_mRNA-p1 TcCLB-EL.506965.20_mRNA-p1
TcCLB-EL.507237.100_mRNA-p1 TcCLB-EL.507237.170_mRNA-p1 TcCLB-EL.
507957.240_mRNA-p1 TcCLB-EL.510377.260_mRNA-p1 TcCLB-EL.
511609.30_mRNA-p1 TcCLB-EL.511611.20_mRNA-p1 TcCLB-NE.
508247.50_pseudogenic_transcript-p1 TcSYL_0150930.t1-p1
TcSYL_0152090.t1-p1
OG0000258: TcCLB-EL.506671.61_pseudogenic_transcript-p1 TcCLB-EL.
508159.26_pseudogenic_transcript-p1 TcCLB-EL.
508163.181_pseudogenic_transcript-p1 TcCLB-EL.
510269.73_pseudogenic_transcript-p1 TcCLB-EL.
510275.150_pseudogenic_transcript-p1 TcCLB-EL.
510849.30_pseudogenic_transcript-p1 TcCLB-EL.
510851.50_pseudogenic_transcript-p1 TcCLB-EL.
511171.25_pseudogenic_transcript-p1 TcCLB-NE.
508001.30_pseudogenic_transcript-p1 TcCLB-NE.
508523.35_pseudogenic_transcript-p1 TcCLB-NE.
511477.25_pseudogenic_transcript-p1
OG0000259: TcCLB-EL.504277.11_mRNA-p1 TcCLB-EL.504277.20_mRNA-p1
TcCLB-EL.508767.10_mRNA-p1 TcCLB-EL.508767.20_mRNA-p1 TcCLB-NE.
504157.130_mRNA-p1 TcCLB-NE.506617.10_mRNA-p1 TcCLB-NE.
506617.20_mRNA-p1 TcCLB-NE.506617.5_mRNA-p1 TcCLB-NE.510433.20_mRNA-
p1 TcSYL_0087980.t1-p1 TcSYL_0087990.t1-p1
OG0000260: TcCLB-EL.430281.20_mRNA-p1 TcCLB-EL.508803.10_mRNA-p1
TcCLB-EL.510275.120_mRNA-p1 TcCLB-EL.511469.105_mRNA-p1 TcCLB-NE.
410199.50_mRNA-p1 TcCLB-NE.506335.15_mRNA-p1 TcSYL_0018090.t1-p1
TcSYL_0018410.t1-p1 TcSYL_0083560.t1-p1 TcSYL_0151600.t1-p1
TcSYL_0154120.t1-p1
OG0000261: TCRU_1540 TCRU_178 TCRU_1890 TCRU_9115 TcCLB-NE.
508219.70_mRNA-p1 TcCLB-NE.508873.210_mRNA-p1 TcCLB-NE.
510359.460_mRNA-p1 TcCLB-NE.510359.520_mRNA-p1 TcCLB-NE.
510359.550_mRNA-p1 TcCLB-NE.510359.600_mRNA-p1
OG0000262: TCRU_2093 TCRU_2793 TCRU_294 TcCLB-EL.
504101.40_pseudogenic_transcript-p1 TcCLB-EL.
509847.50_pseudogenic_transcript-p1 TcCLB-EL.
509925.40_pseudogenic_transcript-p1 TcCLB-NE.
505297.80_pseudogenic_transcript-p1 TcCLB-NE.
507801.230_pseudogenic_transcript-p1 TcCLB-NE.
511859.100_pseudogenic_transcript-p1 TcSYL_0191190.t1-p1
OG0000263: TCRU_502 TCRU_7600 TCRU_9666 TcCLB-EL.507107.30_mRNA-p1
TcCLB-NE.431357.20_mRNA-p1 TcCLB-NE.509261.40_mRNA-p1
TcSYL_0039490.t1-p1 TcSYL_0039710.t1-p1 TcSYL_0039860.t1-p1
TcSYL_0202680.t1-p1
OG0000264: TCRU_583 TCRU_7803 TCRU_900 TcCLB-NE.
508221.381_pseudogenic_transcript-p1 TcCLB-NE.
508221.509_pseudogenic_transcript-p1 TcCLB-NE.508221.970_mRNA-p1
TcCLB-NE.508873.344_pseudogenic_transcript-p1 TcCLB-NE.
510363.220_mRNA-p1 TcCLB-NE.511255.50_mRNA-p1 TcSYL_0135750.t1-p1
OG0000265: TCRU_1715 TCRU_1789 TCRU_4775 TCRU_6652 TCRU_825 TcCLB-
EL.510261.21_mRNA-p1 TcCLB-NE.506335.80_mRNA-p1 TcSYL_0018750.t1-p1
TcSYL_0154830.t1-p1 TcSYL_0187290.t1-p1
OG0000266: TCRU_1764 TCRU_5524 TCRU_921 TcCLB-EL.506375.10_mRNA-p1
TcCLB-EL.506375.60_mRNA-p1 TcCLB-NE.507589.14_mRNA-p1 TcCLB-NE.
508425.20_pseudogenic_transcript-p1 TcCLB-NE.510671.20_mRNA-p1
TcSYL_0022480.t1-p1 TcSYL_0039930.t1-p1

OG0000267: TCRU_986 TcCLB-EL.511315.56_mRNA-p1 TcCLB-NE.
508905.10_pseudogenic_transcript-p1 TcCLB-NE.508907.10_mRNA-p1
TcCLB-NE.511317.10_mRNA-p1 TcCLB-NE.511755.10_mRNA-p1 TcCLB-NE.
511755.19_mRNA-p1 TcSYL_0159570.t1-p1 TcSYL_0159580.t1-p1
TcSYL_0159590.t1-p1
OG0000268: TCRU_1144 TCRU_2790 TCRU_3714 TCRU_3880 TCRU_7219
TCRU_7365 TcCLB-NE.508999.49_mRNA-p1 TcSYL_0018240.t1-p1
TcSYL_0051450.t1-p1 TcSYL_0154290.t1-p1
OG0000269: TCRU_1196 TcCLB-EL.504769.190_pseudogenic_transcript-p1
TcCLB-EL.506343.41_pseudogenic_transcript-p1 TcCLB-EL.
507875.130_pseudogenic_transcript-p1 TcCLB-EL.
509735.60_pseudogenic_transcript-p1 TcCLB-EL.
510451.21_pseudogenic_transcript-p1 TcCLB-NE.
506273.10_pseudogenic_transcript-p1 TcCLB-NE.
507591.15_pseudogenic_transcript-p1 TcCLB-NE.
507723.41_pseudogenic_transcript-p1 TcCLB-NE.
509085.136_pseudogenic_transcript-p1
OG0000270: TCRU_1674 TCRU_2563 TCRU_8343 TcCLB-EL.487507.10_mRNA-p1
TcCLB-EL.505983.9_mRNA-p1 TcCLB-EL.509445.10_mRNA-p1 TcCLB-NE.
504839.28_mRNA-p1 TcCLB-NE.504839.44_mRNA-p1 TcCLB-NE.
507259.10_mRNA-p1 TcSYL_0064420.t1-p1
OG0000271: TCRU_1752 TCRU_2337 TCRU_2588 TCRU_3139 TCRU_6484
TCRU_9531 TcCLB-NE.510829.20_mRNA-p1 TcCLB-NE.511825.130_mRNA-p1
TcSYL_0089480.t1-p1 TcSYL_0090920.t1-p1
OG0000272: TCRU_2683 TCRU_3800 TCRU_9037 TcCLB-EL.507485.130_mRNA-p1
TcCLB-EL.507485.150_mRNA-p1 TcCLB-EL.507739.120_mRNA-p1 TcCLB-NE.
506437.10_mRNA-p1 TcCLB-NE.506437.30_mRNA-p1 TcCLB-NE.
509289.10_mRNA-p1 TcSYL_0195740.t1-p1
OG0000273: TCRU_4342 TCRU_4839 TCRU_6167 TcCLB-EL.506609.70_mRNA-p1
TcCLB-EL.507961.10_pseudogenic_transcript-p1 TcCLB-EL.
508163.120_pseudogenic_transcript-p1 TcCLB-EL.508165.30_mRNA-p1
TcCLB-EL.510191.10_mRNA-p1 TcCLB-EL.
511171.50_pseudogenic_transcript-p1 TcSYL_0069220.t1-p1
OG0000274: TCRU_10627 TCRU_4692 TcCLB-EL.503859.40_mRNA-p1 TcCLB-EL.
506501.184_pseudogenic_transcript-p1 TcCLB-EL.
508147.90_pseudogenic_transcript-p1 TcCLB-EL.510025.160_mRNA-p1
TcCLB-EL.510377.430_mRNA-p1 TcCLB-NE.504155.10_mRNA-p1 TcCLB-NE.
508245.70_mRNA-p1 TcCLB-NE.509545.79_mRNA-p1
OG0000275: TCRU_10263 TCRU_4763 TcCLB-EL.503659.30_mRNA-p1 TcCLB-EL.
510209.9_mRNA-p1 TcCLB-NE.506139.60_pseudogenic_transcript-p1 TcCLB-
NE.511487.90_mRNA-p1 TcSYL_0132950.t1-p1 TcSYL_0134930.t1-p1
TcSYL_0148770.t1-p1 TcSYL_0157210.t1-p1
OG0000276: TCRU_5082 TCRU_6286 TcCLB-EL.507527.40_mRNA-p1 TcCLB-EL.
507527.50_mRNA-p1 TcCLB-EL.509197.10_mRNA-p1 TcCLB-EL.
509197.20_mRNA-p1 TcCLB-NE.508701.20_mRNA-p1 TcSYL_0180970.t1-p1
TcSYL_0180980.t1-p1 TcSYL_0180990.t1-p1
OG0000277: TCRU_10490 TCRU_5118 TCRU_7691 TcCLB-EL.
504241.85_pseudogenic_transcript-p1 TcCLB-EL.
510607.75_pseudogenic_transcript-p1 TcCLB-EL.
511787.30_pseudogenic_transcript-p1 TcCLB-NE.447801.10_mRNA-p1
TcCLB-NE.508261.166_pseudogenic_transcript-p1 TcCLB-NE.
510627.215_pseudogenic_transcript-p1 TcSYL_0203770.t1-p1
OG0000278: TCRU_5121 TcCLB-EL.507761.10_pseudogenic_transcript-p1
TcCLB-EL.509101.9_mRNA-p1 TcCLB-EL.509889.9_mRNA-p1 TcCLB-EL.

509923.9_mRNA-p1 TcCLB-NE.506641.9_mRNA-p1 TcSYL_0092470.t1-p1
TcSYL_0111580.t1-p1 TcSYL_0157030.t1-p1 TcSYL_0165750.t1-p1
OG0000279: TCRU_5313 TcCLB-EL.503501.14_mRNA-p1 TcCLB-EL.
503859.45_mRNA-p1 TcCLB-EL.504239.100_mRNA-p1 TcCLB-EL.
504239.410_mRNA-p1 TcCLB-EL.506763.80_mRNA-p1 TcCLB-EL.
510025.150_mRNA-p1 TcCLB-EL.510377.439_mRNA-p1 TcCLB-NE.
506955.250_mRNA-p1 TcSYL_0135480.t1-p1
OG0000280: TCRU_10537 TCRU_6392 TCRU_7317 TCRU_9458 TcCLB-EL.
511553.60_mRNA-p1 TcCLB-NE.508221.76_mRNA-p1 TcSYL_0135710.t1-p1
TcSYL_0149090.t1-p1 TcSYL_0155460.t1-p1 TcSYL_0156240.t1-p1
OG0000281: TCRU_7884 TCRU_8023 TcCLB-EL.
400945.10_pseudogenic_transcript-p1 TcCLB-EL.
507835.35_pseudogenic_transcript-p1 TcCLB-NE.504189.5_mRNA-p1 TcCLB-
NE.506393.40_pseudogenic_transcript-p1 TcCLB-NE.
509033.10_pseudogenic_transcript-p1 TcSYL_0020130.t1-p1
TcSYL_0020590.t1-p1 TcSYL_0165580.t1-p1
OG0000282: TCRU_8244 TcCLB-EL.408573.5_mRNA-p1 TcCLB-EL.
509135.9_mRNA-p1 TcCLB-EL.511681.10_mRNA-p1 TcCLB-NE.508739.30_mRNA-
p1 TcCLB-NE.508739.50_mRNA-p1 TcSYL_0191720.t1-p1 TcSYL_0191970.t1-
p1 TcSYL_0191980.t1-p1 TcSYL_0192190.t1-p1
OG0000283: TCRU_8995 TcCLB-EL.504421.10_mRNA-p1 TcCLB-EL.
506467.40_mRNA-p1 TcCLB-EL.506467.50_mRNA-p1 TcCLB-NE.
505193.110_mRNA-p1 TcSYL_0104510.t1-p1 TcSYL_0104540.t1-p1
TcSYL_0104580.t1-p1 TcSYL_0104610.t1-p1 TcSYL_0104630.t1-p1
OG0000284: TcCLB-EL.507757.26_mRNA-p1 TcCLB-EL.510071.20_mRNA-p1
TcCLB-NE.508595.11_mRNA-p1 TcSYL_0054500.t1-p1 TcSYL_0054770.t1-p1
TcSYL_0055640.t1-p1 TcSYL_0202290.t1-p1 TcSYL_0202700.t1-p1
TcSYL_0202820.t1-p1 TcSYL_0202970.t1-p1
OG0000285: TcCLB-EL.503397.5_pseudogenic_transcript-p1 TcCLB-EL.
504193.41_pseudogenic_transcript-p1 TcCLB-EL.
509265.23_pseudogenic_transcript-p1 TcCLB-EL.
509871.21_pseudogenic_transcript-p1 TcCLB-NE.
503421.7_pseudogenic_transcript-p1 TcCLB-NE.
506271.20_pseudogenic_transcript-p1 TcCLB-NE.
508269.76_pseudogenic_transcript-p1 TcCLB-NE.
508401.90_pseudogenic_transcript-p1 TcCLB-NE.
509579.32_pseudogenic_transcript-p1 TcCLB-NE.
511019.47_pseudogenic_transcript-p1
OG0000286: TcCLB-EL.507611.180_mRNA-p1 TcCLB-EL.508087.10_mRNA-p1
TcCLB-EL.511873.5_mRNA-p1 TcCLB-NE.410199.20_mRNA-p1 TcCLB-NE.
506665.20_mRNA-p1 TcSYL_0021210.t1-p1 TcSYL_0028070.t1-p1
TcSYL_0057810.t1-p1 TcSYL_0119660.t1-p1 TcSYL_0141370.t1-p1
OG0000287: TcCLB-EL.506599.400_pseudogenic_transcript-p1 TcCLB-EL.
506973.50_pseudogenic_transcript-p1 TcCLB-EL.
507859.80_pseudogenic_transcript-p1 TcCLB-EL.
508389.160_pseudogenic_transcript-p1 TcCLB-EL.
509195.80_pseudogenic_transcript-p1 TcCLB-EL.
511787.20_pseudogenic_transcript-p1 TcCLB-EL.
511797.210_pseudogenic_transcript-p1 TcCLB-NE.
507687.60_pseudogenic_transcript-p1 TcCLB-NE.
510217.70_pseudogenic_transcript-p1 TcCLB-NE.
510627.200_pseudogenic_transcript-p1
OG0000288: TcCLB-EL.507105.120_pseudogenic_transcript-p1 TcCLB-EL.
508381.20_pseudogenic_transcript-p1 TcCLB-NE.

503421.20_pseudogenic_transcript-p1 TcCLB-NE.
506537.30_pseudogenic_transcript-p1 TcCLB-NE.
507167.50_pseudogenic_transcript-p1 TcCLB-NE.
507787.190_pseudogenic_transcript-p1 TcCLB-NE.
510355.110_pseudogenic_transcript-p1 TcSYL_0061450.t1-p1
TcSYL_0166110.t1-p1 TcSYL_0204110.t1-p1
OG0000289: TCRU_1110 TCRU_51 TCRU_6325 TCRU_6329 TCRU_7377 TCRU_8583
TCRU_895 TCRU_9176 TcSYL_0029170.t1-p1
OG0000290: TCRU_3567 TCRU_620 TCRU_72 TCRU_7598 TcCLB-NE.
483183.10_mRNA-p1 TcCLB-NE.509329.31_mRNA-p1 TcCLB-NE.
510097.10_mRNA-p1 TcSYL_0114340.t1-p1 TcSYL_0114610.t1-p1
OG0000291: TCRU_5096 TCRU_604 TCRU_6269 TcCLB-EL.511871.120_mRNA-p1
TcCLB-EL.511875.80_mRNA-p1 TcCLB-NE.506321.50_mRNA-p1 TcCLB-NE.
507063.340_mRNA-p1 TcCLB-NE.508985.10_pseudogenic_transcript-p1
TcCLB-NE.511863.10_mRNA-p1
OG0000292: TCRU_10489 TCRU_4300 TCRU_696 TCRU_811 TcCLB-NE.
506335.69_pseudogenic_transcript-p1 TcCLB-NE.
508251.31_pseudogenic_transcript-p1 TcCLB-NE.
510237.40_pseudogenic_transcript-p1 TcCLB-NE.
510239.40_pseudogenic_transcript-p1 TcSYL_0069210.t1-p1
OG0000293: TCRU_5631 TCRU_5723 TCRU_6068 TCRU_753 TCRU_8827 TcCLB-
NE.511809.130_mRNA-p1 TcCLB-NE.511809.99_mRNA-p1 TcCLB-NE.
511811.10_mRNA-p1 TcSYL_0201640.t1-p1
OG0000294: TCRU_1426 TCRU_1427 TCRU_879 TcCLB-EL.507211.10_mRNA-p1
TcCLB-EL.511353.4_mRNA-p1 TcCLB-NE.508621.30_pseudogenic_transcript-
p1 TcCLB-NE.511301.80_pseudogenic_transcript-p1 TcSYL_0121680.t1-p1
TcSYL_0147480.t1-p1
OG0000295: TCRU_902 TcCLB-EL.509011.20_mRNA-p1 TcCLB-NE.
510685.30_mRNA-p1 TcSYL_0030620.t1-p1 TcSYL_0030630.t1-p1
TcSYL_0030650.t1-p1 TcSYL_0038430.t1-p1 TcSYL_0038460.t1-p1
TcSYL_0038500.t1-p1
OG0000296: TCRU_1310 TCRU_3575 TCRU_964 TcCLB-EL.503447.20_mRNA-p1
TcCLB-NE.398477.10_mRNA-p1 TcCLB-NE.506331.130_mRNA-p1 TcCLB-NE.
507949.210_mRNA-p1 TcCLB-NE.508139.240_mRNA-p1 TcCLB-NE.
508139.250_mRNA-p1
OG0000297: TCRU_995 TcCLB-EL.504239.240_pseudogenic_transcript-p1
TcCLB-EL.506499.140_mRNA-p1 TcCLB-EL.506763.150_mRNA-p1 TcCLB-EL.
506769.30_mRNA-p1 TcCLB-EL.507957.220_mRNA-p1 TcCLB-NE.
508221.920_pseudogenic_transcript-p1 TcCLB-NE.510361.190_mRNA-p1
TcSYL_0136470.t1-p1
OG0000298: TCRU_1109 TCRU_5244 TCRU_9479 TcCLB-EL.
401041.10_pseudogenic_transcript-p1 TcCLB-NE.406933.10_mRNA-p1
TcCLB-NE.508139.10_pseudogenic_transcript-p1 TcCLB-NE.
508849.20_pseudogenic_transcript-p1 TcCLB-NE.
509287.250_pseudogenic_transcript-p1 TcSYL_0147870.t1-p1
OG0000299: TCRU_1287 TCRU_1300 TCRU_3351 TCRU_6389 TCRU_6390 TcCLB-
EL.506501.5_pseudogenic_transcript-p1 TcCLB-EL.511553.5.1-p1 TcCLB-
NE.509971.27_pseudogenic_transcript-p1 TcSYL_0133260.t1-p1
OG0000300: TCRU_10098 TCRU_1326 TCRU_6914 TCRU_7411 TCRU_8075
TCRU_9133 TCRU_9134 TCRU_9287 TcSYL_0172900.t1-p1
OG0000301: TCRU_1737 TCRU_3881 TCRU_4309 TCRU_5254 TCRU_7687 TcCLB-
NE.503605.20_mRNA-p1 TcCLB-NE.506325.10_mRNA-p1 TcCLB-NE.
510083.10_pseudogenic_transcript-p1 TcSYL_0204240.t1-p1
OG0000302: TCRU_2360 TCRU_8750 TcCLB-EL.

445635.10_pseudogenic_transcript-p1 TcCLB-EL.509449.10_mRNA-p1
TcCLB-NE.510581.20_mRNA-p1 TcCLB-NE.511895.10_mRNA-p1
TcSYL_0083170.t1-p1 TcSYL_0083260.t1-p1 TcSYL_0088720.t1-p1
OG0000303: TCRU_10321 TCRU_2368 TCRU_4892 TCRU_6459 TCRU_7777
TCRU_7877 TCRU_8504 TCRU_9275 TcSYL_0065260.t1-p1
OG0000304: TCRU_2435 TCRU_8566 TCRU_8567 TcCLB-EL.506767.324_mRNA-p1
TcCLB-EL.507747.160_mRNA-p1 TcCLB-NE.506781.10_mRNA-p1 TcCLB-NE.
506785.30_mRNA-p1 TcCLB-NE.510623.10_mRNA-p1 TcSYL_0136750.t1-p1
OG0000305: TCRU_2436 TcCLB-EL.504769.100_mRNA-p1 TcCLB-EL.
506713.80_mRNA-p1 TcCLB-EL.507035.11_mRNA-p1 TcCLB-EL.
508607.50_mRNA-p1 TcCLB-EL.509765.129_mRNA-p1 TcCLB-NE.
506471.120_mRNA-p1 TcCLB-NE.506471.30_mRNA-p1 TcCLB-NE.
506595.40_mRNA-p1
OG0000306: TCRU_2503 TCRU_3504 TCRU_8258 TcCLB-NE.
511827.120_pseudogenic_transcript-p1 TcSYL_0060350.t1-p1
TcSYL_0074030.t1-p1 TcSYL_0093640.t1-p1 TcSYL_0163810.t1-p1
TcSYL_0201830.t1-p1
OG0000307: TCRU_2554 TCRU_3502 TCRU_5961 TCRU_7858 TcCLB-EL.
508285.10_mRNA-p1 TcCLB-NE.506421.10_mRNA-p1 TcCLB-NE.
509873.10_mRNA-p1 TcCLB-NE.509915.60_mRNA-p1 TcSYL_0060540.t1-p1
OG0000308: TCRU_2663 TCRU_3534 TcCLB-EL.506667.10_mRNA-p1 TcCLB-EL.
508109.30_mRNA-p1 TcCLB-EL.508165.110_mRNA-p1 TcCLB-NE.
507071.20_mRNA-p1 TcCLB-NE.510483.120_mRNA-p1 TcCLB-NE.
510693.190_mRNA-p1 TcSYL_0069440.t1-p1
OG0000309: TCRU_2986 TCRU_4065 TcCLB-EL.510165.60_mRNA-p1 TcCLB-NE.
503471.19_mRNA-p1 TcCLB-NE.506975.97_mRNA-p1 TcCLB-NE.
507015.59_pseudogenic_transcript-p1 TcCLB-NE.511807.33_mRNA-p1
TcSYL_0165100.t1-p1 TcSYL_0165110.t1-p1
OG0000310: TCRU_10426 TCRU_3161 TCRU_3934 TCRU_5111 TcCLB-EL.
506667.120_mRNA-p1 TcCLB-EL.510189.40_mRNA-p1 TcCLB-NE.
510623.50_pseudogenic_transcript-p1 TcCLB-NE.510627.60_mRNA-p1
TcCLB-NE.510629.70_mRNA-p1
OG0000311: TCRU_3503 TCRU_4155 TCRU_5243 TCRU_7607 TCRU_7886
TCRU_8132 TCRU_8439 TcCLB-EL.506743.135_pseudogenic_transcript-p1
TcCLB-NE.503765.10_pseudogenic_transcript-p1
OG0000312: TCRU_3574 TCRU_5467 TCRU_6874 TcCLB-EL.503579.70_mRNA-p1
TcCLB-EL.504125.40_mRNA-p1 TcCLB-NE.504131.180_mRNA-p1 TcCLB-NE.
506977.70_mRNA-p1 TcCLB-NE.507517.60_mRNA-p1 TcSYL_0170410.t1-p1
OG0000313: TCRU_3725 TcCLB-EL.507639.110_mRNA-p1 TcCLB-EL.
507641.14_mRNA-p1 TcCLB-NE.503781.10_mRNA-p1 TcCLB-NE.
509729.29_mRNA-p1 TcSYL_0011000.t1-p1 TcSYL_0011030.t1-p1
TcSYL_0011040.t1-p1 TcSYL_0011050.t1-p1
OG0000314: TCRU_3853 TCRU_9340 TcCLB-EL.
507041.100_pseudogenic_transcript-p1 TcCLB-NE.
508233.70_pseudogenic_transcript-p1 TcSYL_0087940.t1-p1
TcSYL_0088570.t1-p1 TcSYL_0088580.t1-p1 TcSYL_0108320.t1-p1
TcSYL_0108480.t1-p1
OG0000315: TCRU_4006 TcCLB-NE.508931.10_mRNA-p1 TcSYL_0030080.t1-p1
TcSYL_0030370.t1-p1 TcSYL_0032010.t1-p1 TcSYL_0032590.t1-p1
TcSYL_0038410.t1-p1 TcSYL_0038450.t1-p1 TcSYL_0057030.t1-p1
OG0000316: TCRU_4050 TCRU_8607 TcCLB-EL.422207.10_mRNA-p1 TcCLB-EL.
506483.40_pseudogenic_transcript-p1 TcCLB-NE.
509215.45_pseudogenic_transcript-p1 TcCLB-NE.
511299.35_pseudogenic_transcript-p1 TcSYL_0202320.t1-p1

TcSYL_0205740.t1-p1 TcSYL_0205870.t1-p1
OG0000317: TCRU_4103 TCRU_4666 TCRU_5379 TCRU_6248 TCRU_7762 TCRU_7848 TCRU_7849 TcCLB-EL.508163.30_mRNA-p1 TcSYL_0100920.t1-p1
OG0000318: TCRU_4481 TCRU_4580 TCRU_4581 TcCLB-EL.506743.210_mRNA-p1 TcCLB-EL.510601.40_pseudogenic_transcript-p1 TcCLB-NE.509109.170_pseudogenic_transcript-p1 TcCLB-NE.509253.20_mRNA-p1 TcSYL_0014260.t1-p1 TcSYL_0164740.t1-p1
OG0000319: TCRU_10229 TCRU_4712 TCRU_5245 TCRU_6197 TcCLB-EL.507875.170_mRNA-p1 TcCLB-EL.509165.34_mRNA-p1 TcCLB-NE.506595.104_mRNA-p1 TcCLB-NE.507783.10_mRNA-p1 TcCLB-NE.510643.150_pseudogenic_transcript-p1
OG0000320: TCRU_4958 TCRU_4959 TCRU_4961 TcCLB-EL.508719.40_mRNA-p1 TcCLB-EL.508719.60_mRNA-p1 TcCLB-NE.509793.10_mRNA-p1 TcCLB-NE.509793.30_mRNA-p1 TcCLB-NE.509793.40_mRNA-p1 TcSYL_0107060.t1-p1
OG0000321: TCRU_5338 TcCLB-NE.503973.70_mRNA-p1 TcCLB-NE.508221.680_pseudogenic_transcript-p1 TcCLB-NE.508871.51_pseudogenic_transcript-p1 TcCLB-NE.510361.130_mRNA-p1 TcCLB-NE.510915.5_mRNA-p1 TcSYL_0065750.t1-p1 TcSYL_0148570.t1-p1 TcSYL_0153060.t1-p1
OG0000322: TCRU_6092 TCRU_6116 TCRU_8777 TCRU_9675 TcCLB-EL.507105.40_mRNA-p1 TcCLB-EL.509583.4_mRNA-p1 TcCLB-NE.503709.20_mRNA-p1 TcCLB-NE.506815.60_mRNA-p1 TcSYL_0088040.t1-p1
OG0000323: TCRU_6227 TcCLB-EL.506599.30_pseudogenic_transcript-p1 TcCLB-EL.506603.20_pseudogenic_transcript-p1 TcCLB-EL.510163.30_mRNA-p1 TcCLB-EL.511555.10_mRNA-p1 TcCLB-NE.506459.299_pseudogenic_transcript-p1 TcCLB-NE.508119.20_mRNA-p1 TcCLB-NE.508873.160_mRNA-p1 TcCLB-NE.511255.670_mRNA-p1
OG0000324: TCRU_6303 TcCLB-EL.506973.61_pseudogenic_transcript-p1 TcCLB-EL.507859.101_pseudogenic_transcript-p1 TcCLB-EL.507863.6_pseudogenic_transcript-p1 TcCLB-EL.508775.60_pseudogenic_transcript-p1 TcCLB-NE.510197.95_pseudogenic_transcript-p1 TcCLB-NE.510201.45_pseudogenic_transcript-p1 TcCLB-NE.510387.70_pseudogenic_transcript-p1 TcCLB-NE.510413.10_pseudogenic_transcript-p1
OG0000325: TCRU_6592 TCRU_6604 TcSYL_0069670.t1-p1 TcSYL_0069820.t1-p1 TcSYL_0091460.t1-p1 TcSYL_0091570.t1-p1 TcSYL_0124200.t1-p1 TcSYL_0130600.t1-p1 TcSYL_0148700.t1-p1
OG0000326: TCRU_6715 TcCLB-EL.504929.10_mRNA-p1 TcCLB-EL.508541.220_mRNA-p1 TcCLB-EL.508541.230_mRNA-p1 TcCLB-EL.508541.240_mRNA-p1 TcCLB-NE.507305.20_mRNA-p1 TcCLB-NE.507305.30_mRNA-p1 TcCLB-NE.507541.30_mRNA-p1 TcCLB-NE.507541.40_mRNA-p1
OG0000327: TCRU_6970 TcCLB-EL.508805.30_pseudogenic_transcript-p1 TcCLB-EL.511181.160_pseudogenic_transcript-p1 TcCLB-NE.487739.20_pseudogenic_transcript-p1 TcCLB-NE.503911.10_mRNA-p1 TcCLB-NE.508297.61_mRNA-p1 TcSYL_0203360.t1-p1 TcSYL_0203390.t1-p1 TcSYL_0203400.t1-p1
OG0000328: TCRU_7492 TCRU_8816 TcCLB-EL.511337.20_mRNA-p1 TcCLB-EL.511337.30_mRNA-p1 TcCLB-EL.511339.30_mRNA-p1 TcCLB-NE.441923.10_mRNA-p1 TcCLB-NE.509235.10_mRNA-p1 TcCLB-NE.509237.60_mRNA-p1 TcCLB-NE.509237.70_mRNA-p1
OG0000329: TCRU_7567 TcCLB-EL.448567.9_mRNA-p1 TcSYL_0043970.t1-p1 TcSYL_0084220.t1-p1 TcSYL_0089110.t1-p1 TcSYL_0113960.t1-p1

TcSYL_0174720.t1-p1 TcSYL_0174730.t1-p1 TcSYL_0174750.t1-p1
OG0000330: TCRU_8120 TcCLB-EL.507683.10_mRNA-p1 TcCLB-EL.
510029.80_mRNA-p1 TcCLB-EL.510029.90_mRNA-p1 TcCLB-NE.
511291.80_mRNA-p1 TcSYL_0122570.t1-p1 TcSYL_0122780.t1-p1
TcSYL_0122800.t1-p1 TcSYL_0122920.t1-p1
OG0000331: TCRU_10280 TCRU_8234 TcCLB-EL.503575.34_mRNA-p1 TcCLB-EL.
503881.39_mRNA-p1 TcCLB-EL.509351.4_mRNA-p1 TcCLB-NE.
508119.144_mRNA-p1 TcCLB-NE.510943.26_mRNA-p1 TcSYL_0001390.t1-p1
TcSYL_0103930.t1-p1
OG0000332: TCRU_8242 TcCLB-EL.504225.20_mRNA-p1 TcCLB-EL.
509129.10_mRNA-p1 TcCLB-EL.509141.40_mRNA-p1 TcCLB-EL.
511687.10_mRNA-p1 TcCLB-EL.511687.19_mRNA-p1 TcCLB-NE.
508737.210_mRNA-p1 TcCLB-NE.508741.229_mRNA-p1 TcSYL_0191690.t1-p1
OG0000333: TCRU_9498 TcCLB-EL.508241.120_mRNA-p1 TcSYL_0075750.t1-p1
TcSYL_0077590.t1-p1 TcSYL_0077600.t1-p1 TcSYL_0077610.t1-p1
TcSYL_0185970.t1-p1 TcSYL_0185980.t1-p1 TcSYL_0185990.t1-p1
OG0000334: TCRU_10680 TcCLB-EL.504117.50_pseudogenic_transcript-p1
TcCLB-NE.506619.30_mRNA-p1 TcSYL_0087270.t1-p1 TcSYL_0087280.t1-p1
TcSYL_0087290.t1-p1 TcSYL_0087300.t1-p1 TcSYL_0087310.t1-p1
TcSYL_0087320.t1-p1
OG0000335: TcCLB-EL.508813.30_pseudogenic_transcript-p1 TcCLB-EL.
510249.40_mRNA-p1 TcCLB-NE.509241.10_mRNA-p1 TcSYL_0044220.t1-p1
TcSYL_0044300.t1-p1 TcSYL_0044350.t1-p1 TcSYL_0044400.t1-p1
TcSYL_0089460.t1-p1 TcSYL_0179440.t1-p1
OG0000336: TcCLB-EL.420293.50_pseudogenic_transcript-p1 TcCLB-EL.
508681.11_pseudogenic_transcript-p1 TcCLB-NE.507051.20_mRNA-p1
TcSYL_0020580.t1-p1 TcSYL_0029370.t1-p1 TcSYL_0058860.t1-p1
TcSYL_0060990.t1-p1 TcSYL_0091610.t1-p1 TcSYL_0135510.t1-p1
OG0000337: TcCLB-EL.504229.44_pseudogenic_transcript-p1 TcCLB-EL.
508325.101_mRNA-p1 TcCLB-EL.510175.140_pseudogenic_transcript-p1
TcCLB-NE.506537.240_mRNA-p1 TcCLB-NE.
507501.25_pseudogenic_transcript-p1 TcCLB-NE.509163.50_mRNA-p1
TcCLB-NE.509259.129_pseudogenic_transcript-p1 TcCLB-NE.
511887.45_pseudogenic_transcript-p1 TcSYL_0058540.t1-p1
OG0000338: TcCLB-EL.505945.90_mRNA-p1 TcCLB-NE.506149.10_mRNA-p1
TcSYL_0045080.t1-p1 TcSYL_0094810.t1-p1 TcSYL_0094930.t1-p1
TcSYL_0095100.t1-p1 TcSYL_0095230.t1-p1 TcSYL_0095670.t1-p1
TcSYL_0095730.t1-p1
OG0000339: TcCLB-EL.506499.120_mRNA-p1 TcCLB-EL.506763.210_mRNA-p1
TcCLB-EL.511611.11_mRNA-p1 TcCLB-NE.506613.70_mRNA-p1 TcCLB-NE.
508221.350_mRNA-p1 TcCLB-NE.508873.130_mRNA-p1 TcCLB-NE.
511565.15_mRNA-p1 TcSYL_0066940.t1-p1 TcSYL_0110020.t1-p1
OG0000340: TcCLB-EL.507799.5_mRNA-p1 TcCLB-EL.508317.129_mRNA-p1
TcCLB-NE.506383.10_mRNA-p1 TcCLB-NE.509601.5_mRNA-p1 TcCLB-NE.
509849.40_mRNA-p1 TcCLB-NE.509851.10_mRNA-p1 TcCLB-NE.
509853.10_mRNA-p1 TcSYL_0061350.t1-p1 TcSYL_0174450.t1-p1
OG0000341: TcCLB-EL.506197.10_mRNA-p1 TcCLB-EL.506973.140_mRNA-p1
TcCLB-EL.508097.10_mRNA-p1 TcCLB-EL.509631.11_mRNA-p1 TcCLB-NE.
507609.150_pseudogenic_transcript-p1 TcCLB-NE.509527.200_mRNA-p1
TcCLB-NE.509547.10_mRNA-p1 TcCLB-NE.509755.89_mRNA-p1 TcCLB-NE.
510217.10_mRNA-p1
OG0000342: TcCLB-EL.507747.94_mRNA-p1 TcCLB-NE.
433237.20_pseudogenic_transcript-p1 TcCLB-NE.
511403.40_pseudogenic_transcript-p1 TcSYL_0129020.t1-p1

TcSYL_0129510.t1-p1 TcSYL_0190420.t1-p1 TcSYL_0190480.t1-p1
TcSYL_0190530.t1-p1 TcSYL_0190630.t1-p1
OG0000343: TcCLB-EL.504769.41_pseudogenic_transcript-p1 TcCLB-EL.
509979.261_pseudogenic_transcript-p1 TcCLB-EL.
509979.365_pseudogenic_transcript-p1 TcCLB-NE.
506615.21_pseudogenic_transcript-p1 TcCLB-NE.
506615.75_pseudogenic_transcript-p1 TcCLB-NE.
508119.90_pseudogenic_transcript-p1 TcCLB-NE.
510033.22_pseudogenic_transcript-p1 TcCLB-NE.
510203.19_pseudogenic_transcript-p1 TcCLB-NE.
510915.21_pseudogenic_transcript-p1
OG0000344: TcCLB-NE.508193.15.1-p1 TcCLB-NE.508251.10_mRNA-p1
TcSYL_0018550.t1-p1 TcSYL_0038740.t1-p1 TcSYL_0148460.t1-p1
TcSYL_0151810.t1-p1 TcSYL_0153820.t1-p1 TcSYL_0186980.t1-p1
TcSYL_0187510.t1-p1
OG0000345: TCRU_148 TCRU_3382 TcCLB-EL.507831.10_mRNA-p1 TcCLB-NE.
511277.614_mRNA-p1 TcCLB-NE.511281.53_mRNA-p1 TcSYL_0142170.t1-p1
TcSYL_0142480.t1-p1 TcSYL_0196210.t1-p1
OG0000346: TCRU_233 TCRU_245 TCRU_257 TCRU_4431 TCRU_506 TCRU_7302
TCRU_743 TcSYL_0152810.t1-p1
OG0000347: TCRU_271 TcCLB-EL.510603.169_mRNA-p1 TcCLB-EL.
510605.19_mRNA-p1 TcCLB-NE.509109.14_mRNA-p1 TcCLB-NE.511639.9_mRNA-
p1 TcSYL_0014400.t1-p1 TcSYL_0014410.t1-p1 TcSYL_0014470.t1-p1
OG0000348: TCRU_284 TcCLB-EL.511557.50_mRNA-p1 TcCLB-EL.
511559.10_mRNA-p1 TcCLB-NE.503995.10_mRNA-p1 TcCLB-NE.
505171.109_mRNA-p1 TcSYL_0073280.t1-p1 TcSYL_0073290.t1-p1
TcSYL_0073300.t1-p1
OG0000349: TCRU_3203 TCRU_346 TCRU_4792 TcCLB-EL.506763.370_mRNA-p1
TcSYL_0021950.t1-p1 TcSYL_0133330.t1-p1 TcSYL_0135410.t1-p1
TcSYL_0156460.t1-p1
OG0000350: TCRU_391 TCRU_5246 TCRU_917 TCRU_9349 TcCLB-EL.
507875.200_pseudogenic_transcript-p1 TcCLB-EL.
510769.170_pseudogenic_transcript-p1 TcCLB-NE.478071.10.1-p1 TcCLB-
NE.507499.90_pseudogenic_transcript-p1
OG0000351: TCRU_473 TCRU_8155 TcCLB-EL.507559.10_mRNA-p1 TcCLB-EL.
511701.10_mRNA-p1 TcCLB-EL.511703.10_mRNA-p1 TcCLB-NE.
508173.270_mRNA-p1 TcCLB-NE.511151.110_mRNA-p1 TcSYL_0079170.t1-p1
OG0000352: TCRU_1168 TCRU_2387 TCRU_481 TcCLB-NE.508221.600_mRNA-p1
TcCLB-NE.508495.150_pseudogenic_transcript-p1 TcCLB-NE.
510359.480_pseudogenic_transcript-p1 TcCLB-NE.
510359.680_pseudogenic_transcript-p1 TcCLB-NE.510359.690_mRNA-p1
OG0000353: TCRU_584 TcCLB-EL.506267.70_mRNA-p1 TcCLB-EL.
506599.170_mRNA-p1 TcCLB-EL.506599.390_mRNA-p1 TcCLB-EL.
506599.80_mRNA-p1 TcCLB-NE.504081.50_mRNA-p1 TcCLB-NE.
508221.1010_mRNA-p1 TcCLB-NE.511255.80_mRNA-p1
OG0000354: TCRU_4297 TCRU_4695 TCRU_593 TCRU_7809 TcCLB-NE.
508885.30_mRNA-p1 TcCLB-NE.509259.126_mRNA-p1 TcSYL_0100860.t1-p1
TcSYL_0136400.t1-p1
OG0000355: TCRU_688 TcCLB-EL.507547.90_mRNA-p1 TcCLB-NE.
508441.20_mRNA-p1 TcSYL_0086110.t1-p1 TcSYL_0086190.t1-p1
TcSYL_0086280.t1-p1 TcSYL_0086300.t1-p1 TcSYL_0086380.t1-p1
OG0000356: TCRU_6942 TCRU_915 TcCLB-EL.506269.70_mRNA-p1 TcCLB-EL.
506965.190_mRNA-p1 TcCLB-NE.508247.70_mRNA-p1 TcSYL_0151870.t1-p1
TcSYL_0153030.t1-p1 TcSYL_0153770.t1-p1

OG0000357: TCRU_1258 TcCLB-EL.508371.10_pseudogenic_transcript-p1 TcCLB-EL.508373.10_mRNA-p1 TcCLB-NE.507059.80_mRNA-p1 TcCLB-NE.511647.4_mRNA-p1 TcSYL_0014090.t1-p1 TcSYL_0014100.t1-p1 TcSYL_0014130.t1-p1
OG0000358: TCRU_1314 TCRU_3167 TCRU_5080 TcCLB-EL.508681.31_mRNA-p1 TcCLB-NE.510481.30_mRNA-p1 TcSYL_0017990.t1-p1 TcSYL_0148440.t1-p1 TcSYL_0204830.t1-p1
OG0000359: TCRU_1355 TCRU_3197 TCRU_4637 TCRU_5218 TCRU_7891 TcCLB-EL.506599.320_mRNA-p1 TcCLB-NE.508221.220_mRNA-p1 TcSYL_0134100.t1-p1
OG0000360: TCRU_1363 TCRU_1844 TCRU_4343 TCRU_4750 TCRU_5283 TCRU_5285 TCRU_7759 TcSYL_0133970.t1-p1
OG0000361: TCRU_1538 TCRU_4190 TcCLB-EL.456097.10_mRNA-p1 TcSYL_0068740.t1-p1 TcSYL_0069070.t1-p1 TcSYL_0069570.t1-p1 TcSYL_0093490.t1-p1 TcSYL_0124320.t1-p1
OG0000362: TCRU_1792 TcCLB-EL.509769.30_mRNA-p1 TcCLB-NE.429229.10_mRNA-p1 TcSYL_0174850.t1-p1 TcSYL_0174860.t1-p1 TcSYL_0174870.t1-p1 TcSYL_0174880.t1-p1 TcSYL_0174890.t1-p1
OG0000363: TCRU_1839 TCRU_6243 TcCLB-EL.510441.10_mRNA-p1 TcSYL_0018530.t1-p1 TcSYL_0019050.t1-p1 TcSYL_0038540.t1-p1 TcSYL_0071460.t1-p1 TcSYL_0128480.t1-p1
OG0000364: TCRU_2047 TCRU_8466 TcCLB-EL.509703.10_mRNA-p1 TcCLB-EL.509705.10_mRNA-p1 TcCLB-NE.503461.20_mRNA-p1 TcSYL_0038210.t1-p1 TcSYL_0056550.t1-p1 TcSYL_0056640.t1-p1
OG0000365: TCRU_2080 TCRU_5753 TcCLB-EL.510103.30_pseudogenic_transcript-p1 TcCLB-NE.508801.10_mRNA-p1 TcSYL_0047250.t1-p1 TcSYL_0047380.t1-p1 TcSYL_0075210.t1-p1 TcSYL_0075230.t1-p1
OG0000366: TCRU_2490 TcCLB-EL.510073.17_pseudogenic_transcript-p1 TcCLB-NE.460197.10_pseudogenic_transcript-p1 TcCLB-NE.508595.50_mRNA-p1 TcSYL_0175430.t1-p1 TcSYL_0176750.t1-p1 TcSYL_0176790.t1-p1 TcSYL_0176850.t1-p1
OG0000367: TCRU_2504 TCRU_2738 TcCLB-EL.509121.10_pseudogenic_transcript-p1 TcCLB-EL.511677.5_pseudogenic_transcript-p1 TcCLB-NE.511827.139_pseudogenic_transcript-p1 TcCLB-NE.511829.20_pseudogenic_transcript-p1 TcSYL_0027900.t1-p1 TcSYL_0057600.t1-p1
OG0000368: TCRU_2681 TCRU_4128 TCRU_4694 TCRU_4890 TCRU_7487 TcSYL_0070350.t1-p1 TcSYL_0126330.t1-p1 TcSYL_0133010.t1-p1
OG0000369: TCRU_2722 TCRU_5826 TcCLB-EL.509149.40_mRNA-p1 TcCLB-EL.509149.60_mRNA-p1 TcCLB-NE.508175.309_mRNA-p1 TcCLB-NE.508175.329_mRNA-p1 TcSYL_0078610.t1-p1 TcSYL_0078630.t1-p1
OG0000370: TCRU_2821 TCRU_3752 TCRU_3789 TCRU_7652 TCRU_8172 TCRU_9072 TcSYL_0079980.t1-p1 TcSYL_0128110.t1-p1
OG0000371: TCRU_10516 TCRU_2956 TCRU_3160 TCRU_4234 TCRU_8076 TcCLB-NE.511825.120_mRNA-p1 TcSYL_0052080.t1-p1 TcSYL_0173580.t1-p1
OG0000372: TCRU_2988 TCRU_6271 TCRU_6549 TcCLB-NE.506279.120_mRNA-p1 TcCLB-NE.507633.120_mRNA-p1 TcCLB-NE.507637.60_mRNA-p1 TcCLB-NE.508979.30_mRNA-p1 TcSYL_0127970.t1-p1
OG0000373: TCRU_2990 TCRU_8586 TCRU_8867 TcCLB-EL.510307.10_mRNA-p1 TcCLB-NE.509259.40_mRNA-p1 TcCLB-NE.509287.130_pseudogenic_transcript-p1 TcSYL_0022520.t1-p1 TcSYL_0094530.t1-p1

OG0000374: TCRU_3003 TcCLB-NE.509617.20_mRNA-p1 TcSYL_0000040.t1-p1 TcSYL_0000100.t1-p1 TcSYL_0000110.t1-p1 TcSYL_0001860.t1-p1 TcSYL_0001870.t1-p1 TcSYL_0001880.t1-p1
OG0000375: TCRU_3021 TCRU_3022 TcCLB-EL.510755.89_mRNA-p1 TcCLB-EL.510755.98_mRNA-p1 TcCLB-NE.508413.68_mRNA-p1 TcCLB-NE.508413.76_mRNA-p1 TcCLB-NE.508413.84_mRNA-p1 TcSYL_0115580.t1-p1
OG0000376: TCRU_3054 TCRU_3745 TcSYL_0058850.t1-p1 TcSYL_0059320.t1-p1 TcSYL_0059980.t1-p1 TcSYL_0155310.t1-p1 TcSYL_0165900.t1-p1 TcSYL_0203530.t1-p1
OG0000377: TCRU_3171 TCRU_4714 TCRU_8040 TcCLB-EL.506945.359_mRNA-p1 TcCLB-EL.509165.49_mRNA-p1 TcCLB-NE.506595.90_pseudogenic_transcript-p1 TcCLB-NE.509725.139_mRNA-p1 TcCLB-NE.510643.130_mRNA-p1
OG0000378: TCRU_3293 TcCLB-EL.509967.30_mRNA-p1 TcCLB-EL.511911.21_mRNA-p1 TcCLB-NE.507233.94_mRNA-p1 TcCLB-NE.509693.209_mRNA-p1 TcCLB-NE.509695.10_mRNA-p1 TcSYL_0171000.t1-p1 TcSYL_0171060.t1-p1
OG0000379: TCRU_3443 TCRU_9553 TcCLB-EL.509123.10_mRNA-p1 TcCLB-EL.509147.40_mRNA-p1 TcCLB-EL.511675.3_mRNA-p1 TcCLB-NE.508737.10_mRNA-p1 TcCLB-NE.508741.440_mRNA-p1 TcCLB-NE.508743.10_mRNA-p1
OG0000380: TCRU_3444 TCRU_9552 TcCLB-EL.508163.390_mRNA-p1 TcCLB-EL.509123.20_mRNA-p1 TcCLB-EL.509147.21_pseudogenic_transcript-p1 TcCLB-NE.508737.20_mRNA-p1 TcCLB-NE.508741.415_pseudogenic_transcript-p1 TcSYL_0191560.t1-p1
OG0000381: TCRU_3679 TCRU_6354 TcCLB-EL.506163.40_mRNA-p1 TcCLB-EL.506867.20_mRNA-p1 TcCLB-NE.507623.129_mRNA-p1 TcCLB-NE.508821.50_mRNA-p1 TcSYL_0043070.t1-p1 TcSYL_0095930.t1-p1
OG0000382: TCRU_3786 TCRU_5906 TCRU_8133 TcCLB-EL.507555.80_mRNA-p1 TcCLB-NE.503437.10_pseudogenic_transcript-p1 TcCLB-NE.506443.150_mRNA-p1 TcCLB-NE.511885.50_pseudogenic_transcript-p1 TcSYL_0154430.t1-p1
OG0000383: TCRU_3901 TCRU_5964 TcCLB-EL.510999.39_mRNA-p1 TcCLB-EL.511001.18_mRNA-p1 TcCLB-EL.511001.9_mRNA-p1 TcCLB-NE.503635.59_mRNA-p1 TcCLB-NE.503635.68_mRNA-p1 TcSYL_0132400.t1-p1
OG0000384: TCRU_10140 TCRU_4009 TCRU_6541 TcCLB-EL.504229.4_mRNA-p1 TcCLB-EL.511585.269_pseudogenic_transcript-p1 TcCLB-NE.403985.10_mRNA-p1 TcCLB-NE.509303.10_pseudogenic_transcript-p1 TcSYL_0093920.t1-p1
OG0000385: TCRU_4109 TcSYL_0044870.t1-p1 TcSYL_0044940.t1-p1 TcSYL_0198530.t1-p1 TcSYL_0198870.t1-p1 TcSYL_0199480.t1-p1 TcSYL_0199710.t1-p1 TcSYL_0200060.t1-p1
OG0000386: TCRU_4220 TCRU_6899 TcCLB-EL.429185.20_pseudogenic_transcript-p1 TcCLB-EL.439803.9_pseudogenic_transcript-p1 TcCLB-EL.509133.10_pseudogenic_transcript-p1 TcCLB-EL.511627.20_pseudogenic_transcript-p1 TcCLB-NE.508735.10_pseudogenic_transcript-p1 TcSYL_0191700.t1-p1
OG0000387: TCRU_4303 TCRU_4304 TcCLB-EL.507483.30_mRNA-p1 TcCLB-EL.507483.39_mRNA-p1 TcCLB-EL.507483.50_mRNA-p1 TcCLB-NE.506389.79_mRNA-p1 TcCLB-NE.506391.10_mRNA-p1 TcCLB-NE.506391.20_mRNA-p1
OG0000388: TCRU_4373 TCRU_5688 TcCLB-EL.510345.9_mRNA-p1 TcCLB-NE.506469.30_mRNA-p1 TcCLB-NE.506469.80_mRNA-p1 TcSYL_0156760.t1-p1 TcSYL_0156900.t1-p1 TcSYL_0156910.t1-p1

OG0000389: TCRU_4483 TCRU_4578 TcCLB-EL.506745.10_mRNA-p1 TcCLB-EL.
510601.20_mRNA-p1 TcCLB-NE.509109.190_mRNA-p1 TcCLB-NE.
511925.60_mRNA-p1 TcSYL_0014220.t1-p1 TcSYL_0164660.t1-p1
OG0000390: TCRU_4488 TCRU_4573 TcCLB-EL.507091.30_mRNA-p1 TcCLB-EL.
508375.60_mRNA-p1 TcCLB-NE.511643.10_mRNA-p1 TcCLB-NE.
511923.130_mRNA-p1 TcSYL_0110630.t1-p1 TcSYL_0164420.t1-p1
OG0000391: TCRU_4804 TcCLB-EL.511603.380_mRNA-p1 TcCLB-EL.
511605.80_mRNA-p1 TcCLB-EL.511613.110_mRNA-p1 TcCLB-NE.
508243.10_mRNA-p1 TcCLB-NE.508247.80_mRNA-p1 TcSYL_0150310.t1-p1
TcSYL_0155750.t1-p1
OG0000392: TCRU_5182 TCRU_5902 TcCLB-EL.
506747.10_pseudogenic_transcript-p1 TcCLB-EL.508373.29_mRNA-p1
TcCLB-NE.507059.60_mRNA-p1 TcCLB-NE.509899.110_mRNA-p1
TcSYL_0014150.t1-p1 TcSYL_0164150.t1-p1
OG0000393: TCRU_5219 TcCLB-EL.506269.99_mRNA-p1 TcCLB-EL.
506971.10_mRNA-p1 TcCLB-EL.506973.5_mRNA-p1 TcCLB-EL.
508147.150_mRNA-p1 TcCLB-EL.509631.80_mRNA-p1 TcCLB-NE.
508873.70_pseudogenic_transcript-p1 TcSYL_0137370.t1-p1
OG0000394: TCRU_5445 TcCLB-NE.508193.45_pseudogenic_transcript-p1
TcCLB-NE.509085.31_pseudogenic_transcript-p1 TcCLB-NE.
509429.41_pseudogenic_transcript-p1 TcCLB-NE.
509437.109_pseudogenic_transcript-p1 TcSYL_0058400.t1-p1
TcSYL_0204470.t1-p1 TcSYL_0204480.t1-p1
OG0000395: TCRU_5563 TCRU_9970 TcCLB-EL.503899.110_mRNA-p1 TcCLB-EL.
503899.119_mRNA-p1 TcCLB-EL.503899.130_mRNA-p1 TcCLB-NE.
507515.10_mRNA-p1 TcCLB-NE.507515.4_mRNA-p1 TcSYL_0023090.t1-p1
OG0000396: TCRU_5623 TCRU_5624 TcCLB-EL.510089.219_mRNA-p1 TcCLB-EL.
510091.7_mRNA-p1 TcCLB-NE.510421.260_mRNA-p1 TcSYL_0019290.t1-p1
TcSYL_0019310.t1-p1 TcSYL_0019330.t1-p1
OG0000397: TCRU_5719 TcCLB-EL.510531.109_mRNA-p1 TcCLB-EL.
510533.10_mRNA-p1 TcCLB-NE.511819.59_mRNA-p1 TcCLB-NE.
511821.10_mRNA-p1 TcSYL_0201390.t1-p1 TcSYL_0201410.t1-p1
TcSYL_0201430.t1-p1
OG0000398: TCRU_5739 TcCLB-EL.508943.4_mRNA-p1 TcCLB-EL.
511355.50_mRNA-p1 TcCLB-EL.511357.5_mRNA-p1 TcCLB-NE.
503809.60_pseudogenic_transcript-p1 TcCLB-NE.503809.90_mRNA-p1
TcSYL_0121760.t1-p1 TcSYL_0121770.t1-p1
OG0000399: TCRU_5782 TCRU_6053 TcCLB-EL.511621.220_mRNA-p1 TcCLB-EL.
511621.90_mRNA-p1 TcCLB-NE.504741.140_mRNA-p1 TcCLB-NE.
509317.20_mRNA-p1 TcSYL_0084350.t1-p1 TcSYL_0084800.t1-p1
OG0000400: TCRU_5834 TcCLB-EL.508369.50_mRNA-p1 TcCLB-EL.
510599.14_mRNA-p1 TcCLB-NE.474937.9_mRNA-p1 TcCLB-NE.507897.40_mRNA-
p1 TcSYL_0057320.t1-p1 TcSYL_0057340.t1-p1 TcSYL_0057360.t1-p1
OG0000401: TCRU_6151 TCRU_7815 TcCLB-EL.507257.60_mRNA-p1 TcCLB-EL.
507669.10_mRNA-p1 TcCLB-NE.509879.40_mRNA-p1 TcCLB-NE.
510329.270_mRNA-p1 TcSYL_0001340.t1-p1 TcSYL_0048440.t1-p1
OG0000402: TCRU_6305 TcCLB-EL.503717.71_pseudogenic_transcript-p1
TcCLB-EL.506269.45_pseudogenic_transcript-p1 TcCLB-EL.
506969.6_pseudogenic_transcript-p1 TcCLB-NE.
506139.2_pseudogenic_transcript-p1 TcCLB-NE.
507633.51_pseudogenic_transcript-p1 TcCLB-NE.
507937.6_pseudogenic_transcript-p1 TcCLB-NE.
508883.15_pseudogenic_transcript-p1
OG0000403: TCRU_10443 TCRU_6567 TCRU_8564 TCRU_9094

TcSYL_0031720.t1-p1 TcSYL_0031860.t1-p1 TcSYL_0038550.t1-p1
TcSYL_0128590.t1-p1
OG0000404: TCRU_6650 TcCLB-EL.506949.20_mRNA-p1 TcCLB-NE.
508959.20_mRNA-p1 TcCLB-NE.511379.9_mRNA-p1 TcCLB-NE.511381.19_mRNA-
p1 TcSYL_0188480.t1-p1 TcSYL_0188530.t1-p1 TcSYL_0188560.t1-p1
OG0000405: TCRU_6822 TcCLB-EL.498829.10_mRNA-p1 TcCLB-NE.
507071.170_mRNA-p1 TcCLB-NE.510699.40_mRNA-p1 TcSYL_0066850.t1-p1
TcSYL_0070020.t1-p1 TcSYL_0092810.t1-p1 TcSYL_0125330.t1-p1
OG0000406: TCRU_6887 TCRU_6888 TcCLB-EL.505945.20_mRNA-p1 TcCLB-EL.
505945.30_mRNA-p1 TcCLB-NE.509553.30_mRNA-p1 TcCLB-NE.
509553.40_mRNA-p1 TcSYL_0095780.t1-p1 TcSYL_0095790.t1-p1
OG0000407: TCRU_6901 TcCLB-EL.468217.14_mRNA-p1 TcCLB-EL.
509925.10_mRNA-p1 TcCLB-EL.511625.90_mRNA-p1 TcCLB-NE.
505297.60_mRNA-p1 TcCLB-NE.508735.30_mRNA-p1 TcSYL_0083700.t1-p1
TcSYL_0191160.t1-p1
OG0000408: TCRU_7515 TCRU_7516 TcCLB-EL.504163.40_mRNA-p1 TcCLB-EL.
504163.50_mRNA-p1 TcCLB-NE.510301.10_mRNA-p1 TcCLB-NE.
510301.20_mRNA-p1 TcSYL_0114290.t1-p1 TcSYL_0114300.t1-p1
OG0000409: TCRU_7927 TCRU_7928 TcCLB-EL.508671.20_mRNA-p1 TcCLB-NE.
509695.210_mRNA-p1 TcCLB-NE.509695.220_mRNA-p1 TcCLB-NE.
509695.230_mRNA-p1 TcSYL_0171220.t1-p1 TcSYL_0171400.t1-p1
OG0000410: TCRU_8211 TCRU_8572 TcCLB-EL.
510609.40_pseudogenic_transcript-p1 TcCLB-NE.503617.40_mRNA-p1
TcSYL_0163670.t1-p1 TcSYL_0163690.t1-p1 TcSYL_0163700.t1-p1
TcSYL_0163710.t1-p1
OG0000411: TCRU_8286 TCRU_8988 TcCLB-EL.507039.10_mRNA-p1 TcCLB-EL.
511715.10_mRNA-p1 TcCLB-NE.508445.20_mRNA-p1 TcCLB-NE.
511019.90_mRNA-p1 TcSYL_0112580.t1-p1 TcSYL_0141690.t1-p1
OG0000412: TCRU_8333 TCRU_8848 TcCLB-EL.511529.160_mRNA-p1 TcCLB-EL.
511529.170_mRNA-p1 TcCLB-NE.506727.100_mRNA-p1 TcCLB-NE.
506727.90_mRNA-p1 TcSYL_0112100.t1-p1 TcSYL_0112110.t1-p1
OG0000413: TCRU_8461 TCRU_9504 TcCLB-EL.506163.50_mRNA-p1 TcCLB-EL.
511691.39_mRNA-p1 TcCLB-NE.508177.90_mRNA-p1 TcCLB-NE.
508821.40_mRNA-p1 TcSYL_0043080.t1-p1 TcSYL_0183660.t1-p1
OG0000414: TCRU_8633 TcCLB-EL.507669.50_mRNA-p1 TcCLB-NE.
402647.9_mRNA-p1 TcCLB-NE.507857.80_pseudogenic_transcript-p1 TcCLB-
NE.509879.10_mRNA-p1 TcSYL_0048500.t1-p1 TcSYL_0048520.t1-p1
TcSYL_0048530.t1-p1
OG0000415: TCRU_8635 TCRU_8636 TcCLB-EL.503833.40_mRNA-p1 TcCLB-EL.
506213.60_mRNA-p1 TcCLB-NE.511287.120_mRNA-p1 TcCLB-NE.
511287.40_mRNA-p1 TcSYL_0123380.t1-p1 TcSYL_0123440.t1-p1
OG0000416: TCRU_10382 TcCLB-EL.510971.69_mRNA-p1 TcCLB-NE.
509913.30_mRNA-p1 TcSYL_0057670.t1-p1 TcSYL_0057700.t1-p1
TcSYL_0057710.t1-p1 TcSYL_0057720.t1-p1 TcSYL_0057750.t1-p1
OG0000417: TcCLB-EL.504341.20_mRNA-p1 TcCLB-EL.507907.10_mRNA-p1
TcCLB-EL.507995.20_mRNA-p1 TcCLB-NE.510929.10_mRNA-p1
TcSYL_0060950.t1-p1 TcSYL_0061090.t1-p1 TcSYL_0148150.t1-p1
TcSYL_0191510.t1-p1
OG0000418: TcCLB-EL.508277.100_mRNA-p1 TcCLB-EL.508277.110_mRNA-p1
TcCLB-NE.503541.5_mRNA-p1 TcCLB-NE.509943.20_mRNA-p1
TcSYL_0064050.t1-p1 TcSYL_0064060.t1-p1 TcSYL_0064070.t1-p1
TcSYL_0064110.t1-p1
OG0000419: TcCLB-EL.503759.10_mRNA-p1 TcCLB-EL.507047.40_mRNA-p1
TcCLB-EL.507839.40_mRNA-p1 TcCLB-EL.509513.10_mRNA-p1 TcCLB-EL.

511311.30_mRNA-p1 TcCLB-EL.511311.7_mRNA-p1 TcCLB-NE.509875.80_mRNA-p1 TcCLB-NE.511757.100_mRNA-p1
OG0000420: TcCLB-EL.506765.43_mRNA-p1 TcCLB-EL.509763.20_mRNA-p1 TcCLB-EL.510379.20_mRNA-p1 TcCLB-NE.506053.60_mRNA-p1 TcCLB-NE.508221.780_mRNA-p1 TcCLB-NE.510699.100_mRNA-p1 TcSYL_0029660.t1-p1 TcSYL_0147990.t1-p1
OG0000421: TcCLB-EL.507091.91_pseudogenic_transcript-p1 TcCLB-EL.507237.110_mRNA-p1 TcCLB-EL.508977.111_pseudogenic_transcript-p1 TcCLB-EL.508977.31_pseudogenic_transcript-p1 TcCLB-EL.508977.65_pseudogenic_transcript-p1 TcCLB-EL.511399.76_pseudogenic_transcript-p1 TcCLB-EL.511401.111_pseudogenic_transcript-p1 TcCLB-NE.509115.41_pseudogenic_transcript-p1
OG0000422: TcCLB-EL.503957.25_pseudogenic_transcript-p1 TcCLB-NE.479847.11_pseudogenic_transcript-p1 TcCLB-NE.503861.30_pseudogenic_transcript-p1 TcCLB-NE.508001.60_pseudogenic_transcript-p1 TcCLB-NE.508521.11_pseudogenic_transcript-p1 TcCLB-NE.508527.30_pseudogenic_transcript-p1 TcCLB-NE.511885.35_pseudogenic_transcript-p1 TcSYL_0079500.t1-p1
OG0000423: TcCLB-EL.504769.10_mRNA-p1 TcCLB-EL.508201.40_mRNA-p1 TcCLB-EL.509973.25_pseudogenic_transcript-p1 TcCLB-EL.509973.80_mRNA-p1 TcCLB-EL.509975.41_mRNA-p1 TcCLB-EL.509977.40_mRNA-p1 TcCLB-EL.509977.90_mRNA-p1 TcCLB-NE.506615.50_mRNA-p1
OG0000424: TcCLB-EL.507699.110_pseudogenic_transcript-p1 TcCLB-EL.507699.130_mRNA-p1 TcCLB-EL.507699.180_pseudogenic_transcript-p1 TcCLB-EL.510557.60_mRNA-p1 TcCLB-EL.510559.20_pseudogenic_transcript-p1 TcCLB-EL.510561.30_mRNA-p1 TcCLB-NE.506805.10_pseudogenic_transcript-p1 TcSYL_0066300.t1-p1
OG0000425: TcCLB-EL.507711.200_mRNA-p1 TcCLB-NE.507675.10_mRNA-p1 TcCLB-NE.509641.79_mRNA-p1 TcCLB-NE.509647.13_mRNA-p1 TcSYL_0008770.t1-p1 TcSYL_0010650.t1-p1 TcSYL_0010670.t1-p1 TcSYL_0010680.t1-p1
OG0000426: TcCLB-EL.504107.10_mRNA-p1 TcCLB-EL.506925.550_pseudogenic_transcript-p1 TcCLB-EL.509013.19_mRNA-p1 TcSYL_0080690.t1-p1 TcSYL_0080700.t1-p1 TcSYL_0080740.t1-p1 TcSYL_0080800.t1-p1 TcSYL_0080810.t1-p1
OG0000427: TcCLB-NE.506053.5_mRNA-p1 TcSYL_0098530.t1-p1 TcSYL_0098880.t1-p1 TcSYL_0098920.t1-p1 TcSYL_0098960.t1-p1 TcSYL_0099000.t1-p1 TcSYL_0099030.t1-p1 TcSYL_0099300.t1-p1
OG0000428: TCRU_3602 TCRU_71 TCRU_8102 TCRU_9271 TCRU_9901 TcCLB-EL.510279.70_mRNA-p1 TcSYL_0069030.t1-p1
OG0000429: TCRU_133 TCRU_7030 TCRU_7094 TCRU_824 TcCLB-NE.504233.50_mRNA-p1 TcSYL_0018810.t1-p1 TcSYL_0154920.t1-p1
OG0000430: TCRU_149 TCRU_4043 TcCLB-EL.507831.20_mRNA-p1 TcCLB-EL.509805.240_pseudogenic_transcript-p1 TcCLB-NE.511263.50_mRNA-p1 TcCLB-NE.511267.20_mRNA-p1 TcSYL_0015500.t1-p1
OG0000431: TCRU_187 TCRU_188 TcCLB-EL.503453.30_mRNA-p1 TcCLB-EL.503453.40_mRNA-p1 TcCLB-NE.506959.120_mRNA-p1 TcCLB-NE.506959.130_mRNA-p1 TcSYL_0132840.t1-p1
OG0000432: TCRU_226 TCRU_6415 TcCLB-EL.503449.10_mRNA-p1 TcCLB-EL.506213.80_mRNA-p1 TcCLB-NE.509875.210_mRNA-p1 TcCLB-NE.511287.30_mRNA-p1 TcSYL_0048680.t1-p1

OG0000433: TCRU_256 TCRU_3083 TcCLB-EL.
510449.20_pseudogenic_transcript-p1 TcCLB-NE.
503973.31_pseudogenic_transcript-p1 TcCLB-NE.
509907.50_pseudogenic_transcript-p1 TcSYL_0066470.t1-p1
TcSYL_0148500.t1-p1
OG0000434: TCRU_340 TcCLB-EL.461165.4_mRNA-p1 TcCLB-EL.
508539.10_mRNA-p1 TcCLB-NE.511459.70_mRNA-p1 TcCLB-NE.511461.4_mRNA-
p1 TcSYL_0130910.t1-p1 TcSYL_0130920.t1-p1
OG0000435: TCRU_2618 TCRU_451 TCRU_8912 TcCLB-EL.506399.10_mRNA-p1
TcCLB-NE.507989.20_mRNA-p1 TcCLB-NE.509243.50_mRNA-p1 TcCLB-NE.
510063.10_mRNA-p1
OG0000436: TCRU_protein_ TcCLB-NE.508441.10_mRNA-p1
TcSYL_0085640.t1-p1 TcSYL_0085650.t1-p1 TcSYL_0086070.t1-p1
TcSYL_0086080.t1-p1 TcSYL_0086270.t1-p1
OG0000437: TCRU_1669 TCRU_3937 TCRU_708 TcSYL_0056630.t1-p1
TcSYL_0136320.t1-p1 TcSYL_0154600.t1-p1 TcSYL_0155070.t1-p1
OG0000438: TCRU_875 TcCLB-EL.507211.40_mRNA-p1 TcCLB-EL.
511351.9_mRNA-p1 TcCLB-NE.511301.110_mRNA-p1 TcSYL_0147560.t1-p1
TcSYL_0147570.t1-p1 TcSYL_0147680.t1-p1
OG0000439: TCRU_876 TcCLB-EL.507211.30_mRNA-p1 TcCLB-EL.
511351.20_mRNA-p1 TcCLB-NE.511301.100_mRNA-p1 TcSYL_0121650.t1-p1
TcSYL_0147540.t1-p1 TcSYL_0147660.t1-p1
OG0000440: TCRU_1429 TCRU_877 TcCLB-EL.507211.20_mRNA-p1 TcCLB-EL.
511351.30_mRNA-p1 TcCLB-NE.508621.10_mRNA-p1 TcCLB-NE.
511301.90_mRNA-p1 TcSYL_0147520.t1-p1
OG0000441: TCRU_897 TcCLB-EL.507041.90_mRNA-p1 TcCLB-EL.
511107.30_mRNA-p1 TcCLB-NE.508665.30_mRNA-p1 TcSYL_0087910.t1-p1
TcSYL_0087920.t1-p1 TcSYL_0088560.t1-p1
OG0000442: TCRU_6994 TCRU_7994 TCRU_945 TcCLB-EL.508163.400_mRNA-p1
TcCLB-EL.511877.10_mRNA-p1 TcCLB-NE.511861.120_mRNA-p1
TcSYL_0070950.t1-p1
OG0000443: TCRU_3952 TCRU_4675 TCRU_991 TCRU_9910 TcCLB-EL.
508609.20_mRNA-p1 TcCLB-EL.509543.10_mRNA-p1 TcSYL_0142220.t1-p1
OG0000444: TCRU_10160 TCRU_7447 TCRU_8320 TCRU_992 TcCLB-EL.
479517.30_mRNA-p1 TcCLB-EL.506519.40_mRNA-p1 TcSYL_0142370.t1-p1
OG0000445: TCRU_1020 TcCLB-EL.424795.10_mRNA-p1 TcCLB-EL.
430895.16_mRNA-p1 TcCLB-EL.510309.10_mRNA-p1 TcCLB-NE.
508175.264_pseudogenic_transcript-p1 TcSYL_0000450.t1-p1
TcSYL_0000460.t1-p1
OG0000446: TCRU_10002 TCRU_1115 TcCLB-EL.509195.61_mRNA-p1 TcCLB-EL.
511797.184_pseudogenic_transcript-p1 TcCLB-NE.506781.30_mRNA-p1
TcCLB-NE.510629.160_mRNA-p1 TcCLB-NE.
510629.360_pseudogenic_transcript-p1
OG0000447: TCRU_1224 TCRU_6789 TcCLB-EL.503757.10_mRNA-p1 TcCLB-EL.
509825.14_mRNA-p1 TcCLB-NE.503719.20_mRNA-p1 TcCLB-NE.
510425.19_mRNA-p1 TcSYL_0121230.t1-p1
OG0000448: TCRU_1250 TCRU_4219 TcCLB-EL.506743.110_mRNA-p1 TcCLB-EL.
510603.80_mRNA-p1 TcCLB-NE.503471.10_mRNA-p1 TcCLB-NE.
509109.120_mRNA-p1 TcSYL_0014280.t1-p1
OG0000449: TCRU_1252 TCRU_9019 TcCLB-EL.506743.90_mRNA-p1 TcCLB-EL.
510603.100_mRNA-p1 TcCLB-NE.509109.110_mRNA-p1 TcCLB-NE.
511927.20_mRNA-p1 TcSYL_0165180.t1-p1
OG0000450: TCRU_1253 TCRU_9020 TcCLB-EL.506743.80_mRNA-p1 TcCLB-EL.
510603.110_mRNA-p1 TcCLB-NE.509109.100_mRNA-p1 TcCLB-NE.

511927.30_mRNA-p1 TcSYL_0165200.t1-p1
OG0000451: TCRU_1305 TCRU_1306 TcCLB-EL.506529.420_mRNA-p1 TcCLB-EL.
506529.430_mRNA-p1 TcCLB-NE.510889.120_mRNA-p1 TcCLB-NE.
510889.140_mRNA-p1 TcSYL_0178360.t1-p1
OG0000452: TCRU_1364 TCRU_2106 TCRU_3257 TcCLB-EL.510013.260_mRNA-p1
TcCLB-NE.510697.10_mRNA-p1 TcCLB-NE.510697.80_mRNA-p1 TcCLB-NE.
511255.420_pseudogenic_transcript-p1
OG0000453: TCRU_1402 TcCLB-EL.509599.130_mRNA-p1 TcCLB-EL.
509599.150_mRNA-p1 TcCLB-NE.463451.10_mRNA-p1 TcCLB-NE.
506327.90_mRNA-p1 TcCLB-NE.506327.99_mRNA-p1 TcSYL_0010880.t1-p1
OG0000454: TCRU_1614 TCRU_7833 TcCLB-EL.508537.10_mRNA-p1 TcCLB-EL.
510105.230_mRNA-p1 TcCLB-NE.509717.80_mRNA-p1 TcCLB-NE.
510187.60_mRNA-p1 TcSYL_0011690.t1-p1
OG0000455: TCRU_10324 TCRU_1676 TcCLB-EL.504239.350_mRNA-p1 TcCLB-
EL.506965.70_mRNA-p1 TcCLB-NE.506139.160_mRNA-p1 TcCLB-NE.
507637.150_pseudogenic_transcript-p1 TcCLB-NE.
507637.180_pseudogenic_transcript-p1
OG0000456: TCRU_10488 TCRU_1692 TcCLB-NE.508875.20_mRNA-p1 TcCLB-NE.
510363.320_mRNA-p1 TcCLB-NE.510693.91_pseudogenic_transcript-p1
TcSYL_0070200.t1-p1 TcSYL_0124770.t1-p1
OG0000457: TCRU_1701 TCRU_1702 TcCLB-EL.504769.5_mRNA-p1 TcCLB-EL.
506715.10_mRNA-p1 TcCLB-EL.510349.80_mRNA-p1 TcCLB-NE.
503925.119_mRNA-p1 TcSYL_0156660.t1-p1
OG0000458: TCRU_1717 TCRU_3974 TcCLB-EL.434945.4_mRNA-p1 TcCLB-EL.
509297.30_mRNA-p1 TcCLB-NE.508197.20_mRNA-p1 TcCLB-NE.
508199.10_mRNA-p1 TcSYL_0146680.t1-p1
OG0000459: TCRU_1816 TCRU_5486 TcCLB-EL.506531.50_mRNA-p1 TcCLB-EL.
507537.40_mRNA-p1 TcCLB-NE.507297.30_mRNA-p1 TcCLB-NE.
509399.20_mRNA-p1 TcSYL_0174920.t1-p1
OG0000460: TCRU_1867 TCRU_6593 TcCLB-EL.510565.150_mRNA-p1 TcCLB-NE.
506145.80_mRNA-p1 TcCLB-NE.506921.10_mRNA-p1 TcSYL_0105640.t1-p1
TcSYL_0105650.t1-p1
OG0000461: TCRU_1918 TCRU_1919 TcCLB-EL.508323.60_mRNA-p1 TcCLB-EL.
508323.70_mRNA-p1 TcCLB-NE.511825.40_mRNA-p1 TcCLB-NE.
511825.50_mRNA-p1 TcSYL_0200860.t1-p1
OG0000462: TCRU_1943 TCRU_1944 TcCLB-EL.510323.30_mRNA-p1 TcCLB-NE.
508153.280_mRNA-p1 TcCLB-NE.508153.290_mRNA-p1 TcSYL_0001000.t1-p1
TcSYL_0001010.t1-p1
OG0000463: TCRU_1966 TcCLB-EL.504039.170_pseudogenic_transcript-p1
TcCLB-EL.506267.80_mRNA-p1 TcCLB-EL.506599.310_mRNA-p1 TcCLB-EL.
508151.20_mRNA-p1 TcCLB-NE.509867.60_mRNA-p1 TcCLB-NE.
510629.10_mRNA-p1
OG0000464: TCRU_10422 TCRU_1991 TCRU_5112 TcCLB-EL.
511585.280_pseudogenic_transcript-p1 TcCLB-EL.
511675.30_pseudogenic_transcript-p1 TcCLB-NE.
509287.150_pseudogenic_transcript-p1 TcSYL_0148120.t1-p1
OG0000465: TCRU_2045 TcCLB-NE.508933.50_mRNA-p1 TcSYL_0038150.t1-p1
TcSYL_0038250.t1-p1 TcSYL_0056530.t1-p1 TcSYL_0056610.t1-p1
TcSYL_0056700.t1-p1
OG0000466: TCRU_2192 TCRU_5043 TcCLB-EL.510241.50_mRNA-p1 TcCLB-EL.
510243.40_mRNA-p1 TcCLB-NE.510575.190_mRNA-p1 TcCLB-NE.
510579.80_mRNA-p1 TcSYL_0147240.t1-p1
OG0000467: TCRU_2276 TCRU_7206 TcCLB-EL.509433.30_mRNA-p1 TcCLB-NE.
508015.10_mRNA-p1 TcCLB-NE.508015.20_mRNA-p1 TcCLB-NE.

508015.30_mRNA-p1 TcCLB-NE.510087.40_mRNA-p1
OG0000468: TCRU_2291 TcCLB-EL.509777.70_mRNA-p1 TcCLB-NE.
503563.10_mRNA-p1 TcCLB-NE.504141.9_mRNA-p1 TcSYL_0013870.t1-p1
TcSYL_0013880.t1-p1 TcSYL_0013890.t1-p1
OG0000469: TCRU_2484 TCRU_8027 TcCLB-EL.511873.20_mRNA-p1 TcCLB-NE.
464415.10_mRNA-p1 TcCLB-NE.506139.130_mRNA-p1 TcCLB-NE.
511907.140_mRNA-p1 TcSYL_0127140.t1-p1
OG0000470: TCRU_2636 TCRU_2638 TCRU_2640 TcCLB-EL.506543.40_mRNA-p1
TcCLB-NE.506041.20_mRNA-p1 TcCLB-NE.508267.30_mRNA-p1 TcCLB-NE.
508267.50_mRNA-p1
OG0000471: TCRU_2672 TCRU_2673 TcCLB-EL.508347.159_mRNA-p1 TcCLB-NE.
507615.19_mRNA-p1 TcSYL_0104690.t1-p1 TcSYL_0104750.t1-p1
TcSYL_0104770.t1-p1
OG0000472: TCRU_2731 TCRU_7680 TcCLB-EL.401661.10.1-p1 TcCLB-NE.
508919.180_pseudogenic_transcript-p1 TcCLB-NE.
511665.60_pseudogenic_transcript-p1 TcSYL_0171520.t1-p1
TcSYL_0171980.t1-p1
OG0000473: TCRU_3198 TcCLB-EL.506285.10_mRNA-p1 TcCLB-EL.
506499.200_mRNA-p1 TcCLB-EL.506501.100_mRNA-p1 TcCLB-EL.
506973.25_mRNA-p1 TcCLB-EL.510377.340_mRNA-p1 TcCLB-NE.
509527.140_mRNA-p1
OG0000474: TCRU_10841 TCRU_3215 TcCLB-NE.504081.260_mRNA-p1 TcCLB-
NE.507067.20_mRNA-p1 TcCLB-NE.508219.30_pseudogenic_transcript-p1
TcSYL_0070510.t1-p1 TcSYL_0071040.t1-p1
OG0000475: TCRU_3263 TCRU_3991 TCRU_6285 TCRU_9097 TcCLB-NE.
506321.230_mRNA-p1 TcSYL_0110580.t1-p1 TcSYL_0142200.t1-p1
OG0000476: TCRU_3273 TCRU_5894 TcCLB-EL.510737.70_mRNA-p1 TcCLB-EL.
510737.79_mRNA-p1 TcCLB-NE.510663.19_mRNA-p1 TcCLB-NE.
510663.30_mRNA-p1 TcSYL_0116390.t1-p1
OG0000477: TCRU_3302 TcCLB-EL.506267.39_pseudogenic_transcript-p1
TcCLB-EL.506763.130_mRNA-p1 TcCLB-EL.510015.30_mRNA-p1 TcCLB-EL.
510017.59_mRNA-p1 TcCLB-EL.510371.10_mRNA-p1 TcSYL_0065970.t1-p1
OG0000478: TCRU_family_ TcCLB-EL.508647.10_mRNA-p1 TcCLB-NE.
508645.50_mRNA-p1 TcSYL_0047710.t1-p1 TcSYL_0047740.t1-p1
TcSYL_0047770.t1-p1 TcSYL_0047810.t1-p1
OG0000479: TCRU_3512 TCRU_4255 TcCLB-EL.507837.50_mRNA-p1 TcCLB-EL.
511139.20_mRNA-p1 TcCLB-NE.508319.70_mRNA-p1 TcCLB-NE.
508405.40_mRNA-p1 TcSYL_0118400.t1-p1
OG0000480: TCRU_3535 TcCLB-EL.506671.90_mRNA-p1 TcCLB-EL.
508163.320_mRNA-p1 TcCLB-EL.510279.210_mRNA-p1 TcCLB-EL.
511613.40_mRNA-p1 TcCLB-NE.506335.50_mRNA-p1 TcSYL_0148870.t1-p1
OG0000481: TCRU_3537 TCRU_9871 TcCLB-EL.438059.10_mRNA-p1 TcCLB-EL.
509633.50_mRNA-p1 TcCLB-EL.509633.60_mRNA-p1 TcCLB-NE.
506201.70_mRNA-p1 TcCLB-NE.506201.80_mRNA-p1
OG0000482: TCRU_3572 TCRU_3973 TCRU_6854 TcCLB-EL.510767.20_mRNA-p1
TcCLB-NE.506977.49_mRNA-p1 TcCLB-NE.509671.64_mRNA-p1
TcSYL_0074510.t1-p1
OG0000483: TCRU_3715 TcSYL_0022290.t1-p1 TcSYL_0022350.t1-p1
TcSYL_0022380.t1-p1 TcSYL_0040050.t1-p1 TcSYL_0040070.t1-p1
TcSYL_0157380.t1-p1
OG0000484: TCRU_10634 TCRU_3718 TcCLB-EL.507639.20_mRNA-p1 TcCLB-EL.
507639.30_mRNA-p1 TcCLB-NE.503781.80_mRNA-p1 TcCLB-NE.
503781.90_mRNA-p1 TcSYL_0010950.t1-p1
OG0000485: TCRU_3838 TcCLB-EL.506285.40_pseudogenic_transcript-p1

TcCLB-EL.506501.80_pseudogenic_transcript-p1 TcCLB-EL.
510013.310_mRNA-p1 TcCLB-EL.510373.60_mRNA-p1 TcSYL_0039550.t1-p1
TcSYL_0039640.t1-p1
OG0000486: TCRU_3902 TCRU_4728 TCRU_6515 TcCLB-EL.507699.230_mRNA-p1
TcCLB-NE.508221.1050_mRNA-p1 TcCLB-NE.
511255.110_pseudogenic_transcript-p1 TcSYL_0115020.t1-p1
OG0000487: TCRU_3959 TCRU_5727 TcCLB-EL.505843.20_mRNA-p1 TcCLB-EL.
507709.50_mRNA-p1 TcCLB-NE.504949.14_mRNA-p1 TcCLB-NE.
506577.54_mRNA-p1 TcSYL_0009100.t1-p1
OG0000488: TCRU_3985 TCRU_9216 TcCLB-EL.503855.20_mRNA-p1 TcCLB-EL.
504033.130_mRNA-p1 TcCLB-NE.510337.40_mRNA-p1 TcCLB-NE.
510339.50_mRNA-p1 TcSYL_0146630.t1-p1
OG0000489: TCRU_4235 TcCLB-EL.504151.80_mRNA-p1 TcCLB-EL.
510511.9_mRNA-p1 TcCLB-NE.509459.60_mRNA-p1 TcSYL_0174310.t1-p1
TcSYL_0174320.t1-p1 TcSYL_0174330.t1-p1
OG0000490: TCRU_4266 TcCLB-EL.511593.30_mRNA-p1 TcCLB-EL.
511593.80_mRNA-p1 TcCLB-EL.511595.50_pseudogenic_transcript-p1
TcCLB-NE.504155.160_mRNA-p1 TcCLB-NE.506139.70_mRNA-p1 TcCLB-NE.
509753.310_mRNA-p1
OG0000491: TCRU_4312 TCRU_4751 TCRU_7921 TcCLB-EL.423205.10_mRNA-p1
TcCLB-NE.505297.50_pseudogenic_transcript-p1 TcSYL_0171970.t1-p1
TcSYL_0191840.t1-p1
OG0000492: TCRU_of TCRU_of TcCLB-EL.506961.20_mRNA-p1 TcCLB-EL.
508143.15_mRNA-p1 TcCLB-NE.510479.20_pseudogenic_transcript-p1
TcSYL_0066550.t1-p1 TcSYL_0204580.t1-p1
OG0000493: TCRU_4450 TCRU_9876 TcCLB-EL.506739.150_mRNA-p1 TcCLB-EL.
507673.20_mRNA-p1 TcCLB-NE.506201.20_mRNA-p1 TcCLB-NE.
506315.50_mRNA-p1 TcSYL_0050090.t1-p1
OG0000494: TCRU_4482 TCRU_4579 TcCLB-EL.506743.220_mRNA-p1 TcCLB-EL.
510601.30_mRNA-p1 TcCLB-NE.509109.180_mRNA-p1 TcCLB-NE.
509253.10_mRNA-p1 TcSYL_0164720.t1-p1
OG0000495: TCRU_4485 TCRU_4576 TcCLB-EL.506745.30_mRNA-p1 TcCLB-EL.
508379.20_mRNA-p1 TcCLB-NE.509111.19_mRNA-p1 TcCLB-NE.
511925.40_mRNA-p1 TcSYL_0164620.t1-p1
OG0000496: TCRU_4487 TCRU_4575 TcCLB-EL.507091.10_mRNA-p1 TcCLB-EL.
508375.80_mRNA-p1 TcCLB-NE.511641.10_mRNA-p1 TcCLB-NE.
511925.20_mRNA-p1 TcSYL_0014200.t1-p1
OG0000497: TCRU_4490 TCRU_6041 TcCLB-EL.507091.50_mRNA-p1 TcCLB-EL.
508375.30_mRNA-p1 TcCLB-NE.511643.30_mRNA-p1 TcCLB-NE.
511923.110_mRNA-p1 TcSYL_0164380.t1-p1
OG0000498: TCRU_4671 TCRU_5781 TcCLB-EL.511621.200_mRNA-p1 TcCLB-EL.
511621.70_mRNA-p1 TcCLB-NE.504741.160_mRNA-p1 TcCLB-NE.
509317.40_mRNA-p1 TcSYL_0084820.t1-p1
OG0000499: TCRU_4929 TCRU_7310 TcCLB-EL.508157.50_mRNA-p1 TcCLB-EL.
508165.430_mRNA-p1 TcCLB-NE.510047.80_mRNA-p1 TcSYL_0163980.t1-p1
TcSYL_0163990.t1-p1
OG0000500: TCRU_4935 TCRU_5140 TcCLB-EL.510205.10_mRNA-p1 TcCLB-EL.
511597.10_mRNA-p1 TcCLB-EL.511599.10_mRNA-p1 TcCLB-NE.
511233.140_mRNA-p1 TcSYL_0150370.t1-p1
OG0000501: TCRU_5056 TcCLB-EL.507739.110_mRNA-p1 TcCLB-NE.
506435.470_mRNA-p1 TcSYL_0195860.t1-p1 TcSYL_0195880.t1-p1
TcSYL_0195890.t1-p1 TcSYL_0195900.t1-p1
OG0000502: TCRU_5124 TcCLB-NE.508361.9_mRNA-p1 TcCLB-NE.
509525.40_mRNA-p1 TcCLB-NE.509527.135_mRNA-p1 TcCLB-NE.

509527.30_mRNA-p1 TcSYL_0069060.t1-p1 TcSYL_0127930.t1-p1
OG0000503: TCRU_5174 TCRU_6234 TcCLB-EL.510769.90_mRNA-p1 TcCLB-EL.
511145.20_mRNA-p1 TcCLB-NE.504069.30_mRNA-p1 TcCLB-NE.
506241.100_mRNA-p1 TcSYL_0115140.t1-p1
OG0000504: TCRU_5426 TCRU_8348 TcCLB-EL.510293.40_mRNA-p1 TcCLB-EL.
510409.39_mRNA-p1 TcCLB-NE.508661.20_mRNA-p1 TcCLB-NE.
509937.100_mRNA-p1 TcSYL_0113470.t1-p1
OG0000505: TCRU_5609 TcCLB-EL.462761.10_mRNA-p1 TcCLB-EL.
503755.60_pseudogenic_transcript-p1 TcCLB-NE.508073.10_mRNA-p1
TcCLB-NE.511203.29_mRNA-p1 TcCLB-NE.
511205.10_pseudogenic_transcript-p1 TcSYL_0043050.t1-p1
OG0000506: TCRU_5614 TCRU_6644 TcCLB-EL.506861.30_mRNA-p1 TcCLB-EL.
506937.30_mRNA-p1 TcCLB-NE.510437.20_mRNA-p1 TcCLB-NE.
511825.200_mRNA-p1 TcSYL_0200830.t1-p1
OG0000507: TCRU_5642 TCRU_9657 TcCLB-EL.510761.14_mRNA-p1 TcCLB-EL.
511067.20_mRNA-p1 TcCLB-NE.511903.130_mRNA-p1 TcSYL_0103320.t1-p1
TcSYL_0115410.t1-p1
OG0000508: TCRU_5678 TCRU_6168 TCRU_6957 TCRU_7031 TcCLB-EL.
508037.10_mRNA-p1 TcCLB-NE.399997.10_mRNA-p1 TcCLB-NE.
505419.20_pseudogenic_transcript-p1
OG0000509: TCRU_5800 TcCLB-EL.509013.10_mRNA-p1 TcCLB-EL.
511445.10_mRNA-p1 TcCLB-NE.506721.30_mRNA-p1 TcSYL_0080750.t1-p1
TcSYL_0080840.t1-p1 TcSYL_0080860.t1-p1
OG0000510: TCRU_5833 TCRU_9355 TcCLB-EL.506963.20_mRNA-p1 TcCLB-EL.
507831.90_mRNA-p1 TcCLB-NE.511233.20_mRNA-p1 TcCLB-NE.
511261.140_mRNA-p1 TcSYL_0015800.t1-p1
OG0000511: TCRU_5981 TCRU_7287 TcCLB-EL.511127.320_mRNA-p1 TcCLB-EL.
511127.330_mRNA-p1 TcCLB-NE.509027.60_mRNA-p1 TcCLB-NE.
509027.70_mRNA-p1 TcSYL_0156020.t1-p1
OG0000512: TCRU_6288 TCRU_6290 TcCLB-EL.503611.20_mRNA-p1 TcCLB-EL.
503611.40_mRNA-p1 TcCLB-NE.506679.230_mRNA-p1 TcCLB-NE.
506679.250_mRNA-p1 TcSYL_0114230.t1-p1
OG0000513: TCRU_6423 TCRU_9664 TcCLB-EL.
510271.30_pseudogenic_transcript-p1 TcCLB-EL.
510275.110_pseudogenic_transcript-p1 TcCLB-NE.
506335.20_pseudogenic_transcript-p1 TcCLB-NE.
508873.430_pseudogenic_transcript-p1 TcSYL_0074610.t1-p1
OG0000514: TCRU_6485 TcCLB-EL.510249.14_mRNA-p1 TcCLB-EL.
511129.47_pseudogenic_transcript-p1 TcCLB-EL.511911.71_mRNA-p1
TcCLB-NE.506253.21_mRNA-p1 TcCLB-NE.508637.19_mRNA-p1 TcCLB-NE.
508947.9_mRNA-p1
OG0000515: TCRU_10362 TCRU_6584 TCRU_7270 TcCLB-NE.
507167.140_pseudogenic_transcript-p1 TcCLB-NE.
510155.220_pseudogenic_transcript-p1 TcSYL_0127620.t1-p1
TcSYL_0201910.t1-p1
OG0000516: TCRU_6649 TcCLB-EL.401473.9_mRNA-p1 TcCLB-EL.
443683.9_mRNA-p1 TcCLB-EL.508839.89_mRNA-p1 TcCLB-NE.511381.40_mRNA-
p1 TcSYL_0188390.t1-p1 TcSYL_0188400.t1-p1
OG0000517: TCRU_7022 TcCLB-EL.508081.10_mRNA-p1 TcCLB-EL.
508081.30_mRNA-p1 TcCLB-EL.508081.50_mRNA-p1 TcCLB-EL.
508081.70_mRNA-p1 TcCLB-EL.508081.90_mRNA-p1 TcCLB-NE.
508433.120_pseudogenic_transcript-p1
OG0000518: TCRU_7190 TcCLB-EL.506743.60_mRNA-p1 TcCLB-EL.
510603.130_mRNA-p1 TcCLB-NE.509109.80_mRNA-p1 TcCLB-NE.

509255.10_mRNA-p1 TcSYL_0165320.t1-p1 TcSYL_0165360.t1-p1
OG0000519: TCRU_7560 TcCLB-EL.507559.110_mRNA-p1 TcCLB-NE.
510303.10_mRNA-p1 TcCLB-NE.510303.20_mRNA-p1 TcSYL_0079300.t1-p1
TcSYL_0079310.t1-p1 TcSYL_0079320.t1-p1
OG0000520: TCRU_7825 TCRU_7826 TcCLB-EL.511545.20_mRNA-p1 TcCLB-EL.
511545.40_mRNA-p1 TcCLB-NE.506817.30_mRNA-p1 TcCLB-NE.
506817.50_mRNA-p1 TcSYL_0088130.t1-p1
OG0000521: TCRU_7896 TCRU_7897 TcCLB-EL.507275.70_mRNA-p1 TcCLB-EL.
511217.209_mRNA-p1 TcCLB-NE.506573.10_mRNA-p1 TcCLB-NE.
507491.10_mRNA-p1 TcSYL_0189850.t1-p1
OG0000522: TCRU_7918 TCRU_7919 TcCLB-EL.511417.80_mRNA-p1 TcCLB-EL.
511417.90_mRNA-p1 TcCLB-NE.505999.10_mRNA-p1 TcCLB-NE.
505999.20_mRNA-p1 TcSYL_0111290.t1-p1
OG0000523: TCRU_7923 TCRU_7924 TcCLB-EL.504181.20_mRNA-p1 TcCLB-EL.
504181.30_mRNA-p1 TcCLB-NE.509695.180_mRNA-p1 TcCLB-NE.
509695.184_mRNA-p1 TcSYL_0171160.t1-p1
OG0000524: TCRU_7946 TCRU_8282 TcCLB-EL.504181.10_mRNA-p1 TcCLB-EL.
511729.40_mRNA-p1 TcCLB-NE.506835.30_mRNA-p1 TcCLB-NE.
509695.170_mRNA-p1 TcSYL_0140530.t1-p1
OG0000525: TCRU_10415 TCRU_8227 TcCLB-EL.
503531.30_pseudogenic_transcript-p1 TcCLB-EL.507083.60_mRNA-p1
TcCLB-NE.509901.140_mRNA-p1 TcCLB-NE.511649.60_mRNA-p1
TcSYL_0014030.t1-p1
OG0000526: TCRU_8297 TcCLB-EL.507875.70_mRNA-p1 TcCLB-EL.
511875.20_mRNA-p1 TcCLB-NE.510483.360_mRNA-p1 TcCLB-NE.
510635.10_mRNA-p1 TcCLB-NE.510713.30_mRNA-p1 TcCLB-NE.
511771.40_pseudogenic_transcript-p1
OG0000527: TCRU_8408 TCRU_8409 TcCLB-EL.506943.50_mRNA-p1 TcCLB-EL.
506943.60_mRNA-p1 TcCLB-NE.509065.60_mRNA-p1 TcCLB-NE.
509065.70_mRNA-p1 TcSYL_0140080.t1-p1
OG0000528: TCRU_8446 TcCLB-EL.422507.10_mRNA-p1 TcCLB-EL.
507881.30_pseudogenic_transcript-p1 TcCLB-EL.
508325.150_pseudogenic_transcript-p1 TcCLB-EL.
510307.180_pseudogenic_transcript-p1 TcCLB-NE.
506295.190_pseudogenic_transcript-p1 TcSYL_0058960.t1-p1
OG0000529: TCRU_8523 TCRU_8524 TcCLB-EL.510309.40_mRNA-p1 TcCLB-EL.
510309.50_mRNA-p1 TcCLB-NE.503505.10_mRNA-p1 TcCLB-NE.
510267.20_mRNA-p1 TcSYL_0000480.t1-p1
OG0000530: TCRU_10154 TCRU_8544 TcCLB-EL.430605.29_mRNA-p1 TcCLB-EL.
507677.39_mRNA-p1 TcCLB-NE.504021.109_mRNA-p1 TcCLB-NE.
509353.30_mRNA-p1 TcSYL_0010030.t1-p1
OG0000531: TCRU_8703 TcCLB-EL.508239.30_mRNA-p1 TcCLB-NE.
511157.9_mRNA-p1 TcCLB-NE.511159.7_mRNA-p1 TcSYL_0076620.t1-p1
TcSYL_0076630.t1-p1 TcSYL_0076690.t1-p1
OG0000532: TCRU_8956 TcCLB-EL.509803.40_mRNA-p1 TcCLB-NE.
508889.4_mRNA-p1 TcCLB-NE.511273.59_mRNA-p1 TcSYL_0015140.t1-p1
TcSYL_0015170.t1-p1 TcSYL_0060380.t1-p1
OG0000533: TCRU_9036 TcCLB-EL.507485.120_mRNA-p1 TcCLB-EL.
507485.140_mRNA-p1 TcCLB-NE.506437.20_mRNA-p1 TcCLB-NE.
509289.20_mRNA-p1 TcCLB-NE.509289.5_mRNA-p1 TcSYL_0195770.t1-p1
OG0000534: TCRU_9770 TCRU_9771 TcCLB-EL.510127.79_mRNA-p1 TcCLB-NE.
510571.30_mRNA-p1 TcCLB-NE.510573.10_mRNA-p1 TcSYL_0147460.t1-p1
TcSYL_0147470.t1-p1
OG0000535: TCRU_9978 TCRU_9979 TcCLB-EL.503899.20_mRNA-p1 TcCLB-EL.

503899.30_mRNA-p1 TcCLB-NE.507513.60_mRNA-p1 TcCLB-NE.
507513.70_mRNA-p1 TcSYL_0023130.t1-p1
OG0000536: TCRU_10086 TCRU_10087 TcCLB-EL.
506563.70_pseudogenic_transcript-p1 TcCLB-EL.
506563.79_pseudogenic_transcript-p1 TcCLB-NE.509003.30_mRNA-p1
TcCLB-NE.509003.40_mRNA-p1 TcSYL_0063490.t1-p1
OG0000537: TCRU_10434 TcCLB-EL.504019.9_mRNA-p1 TcCLB-EL.
511529.9_mRNA-p1 TcCLB-NE.511031.49_mRNA-p1 TcCLB-NE.511033.10_mRNA-
p1 TcSYL_0112240.t1-p1 TcSYL_0112250.t1-p1
OG0000538: TCRU_of TcCLB-EL.507595.10_pseudogenic_transcript-p1
TcCLB-EL.507865.9_mRNA-p1 TcCLB-NE.509021.9_mRNA-p1
TcSYL_0015390.t1-p1 TcSYL_0018520.t1-p1 TcSYL_0187570.t1-p1
OG0000539: TCRU_10808 TcCLB-EL.511107.20_pseudogenic_transcript-p1
TcCLB-EL.511189.10_pseudogenic_transcript-p1 TcCLB-NE.
508665.40_mRNA-p1 TcCLB-NE.510973.9_mRNA-p1 TcSYL_0086450.t1-p1
TcSYL_0087880.t1-p1
OG0000540: TcCLB-EL.504229.50_pseudogenic_transcript-p1 TcCLB-EL.
510853.30_pseudogenic_transcript-p1 TcCLB-NE.
506459.30_pseudogenic_transcript-p1 TcSYL_0143250.t1-p1
TcSYL_0146420.t1-p1 TcSYL_0146460.t1-p1 TcSYL_0146530.t1-p1
OG0000541: TcCLB-EL.506683.100_pseudogenic_transcript-p1 TcCLB-EL.
509959.90_pseudogenic_transcript-p1 TcCLB-NE.
503545.20_pseudogenic_transcript-p1 TcCLB-NE.
506157.30_pseudogenic_transcript-p1 TcCLB-NE.
506443.110_pseudogenic_transcript-p1 TcCLB-NE.
507519.260_pseudogenic_transcript-p1 TcCLB-NE.
508221.284_pseudogenic_transcript-p1
OG0000542: TcCLB-EL.508903.100_mRNA-p1 TcCLB-EL.511121.30_mRNA-p1
TcCLB-NE.508005.10_mRNA-p1 TcCLB-NE.508999.110_mRNA-p1
TcSYL_0127280.t1-p1 TcSYL_0152030.t1-p1 TcSYL_0161250.t1-p1
OG0000543: TcCLB-EL.510557.40_mRNA-p1 TcCLB-EL.511603.40_mRNA-p1
TcCLB-NE.503417.33_mRNA-p1 TcCLB-NE.504261.10_mRNA-p1 TcCLB-NE.
510979.70_mRNA-p1 TcSYL_0110040.t1-p1 TcSYL_0110180.t1-p1
OG0000544: TcCLB-EL.509769.148_mRNA-p1 TcCLB-NE.508817.5_mRNA-p1
TcSYL_0023210.t1-p1 TcSYL_0043910.t1-p1 TcSYL_0046850.t1-p1
TcSYL_0084150.t1-p1 TcSYL_0174760.t1-p1
OG0000545: TcCLB-EL.511617.9_pseudogenic_transcript-p1
TcSYL_0085570.t1-p1 TcSYL_0086120.t1-p1 TcSYL_0086130.t1-p1
TcSYL_0086140.t1-p1 TcSYL_0086290.t1-p1 TcSYL_0086360.t1-p1
OG0000546: TcCLB-EL.507559.120_mRNA-p1 TcCLB-EL.509155.39_mRNA-p1
TcCLB-EL.511701.19_mRNA-p1 TcCLB-EL.511703.19_mRNA-p1 TcCLB-NE.
511153.19_mRNA-p1 TcSYL_0077080.t1-p1 TcSYL_0079180.t1-p1
OG0000547: TcCLB-EL.506603.35_pseudogenic_transcript-p1 TcCLB-EL.
507283.39_pseudogenic_transcript-p1 TcCLB-EL.
508081.110_pseudogenic_transcript-p1 TcCLB-EL.
508389.70_pseudogenic_transcript-p1 TcCLB-NE.
509251.30_pseudogenic_transcript-p1 TcCLB-NE.
511255.341_pseudogenic_transcript-p1 TcSYL_0136910.t1-p1
OG0000548: TcCLB-EL.506501.177_pseudogenic_transcript-p1 TcCLB-EL.
506767.60_pseudogenic_transcript-p1 TcCLB-EL.
507953.185_pseudogenic_transcript-p1 TcCLB-EL.
509699.35_pseudogenic_transcript-p1 TcCLB-EL.
510015.5_pseudogenic_transcript-p1 TcCLB-EL.
510021.90_pseudogenic_transcript-p1 TcCLB-NE.

510693.11_pseudogenic_transcript-p1
OG0000549: TcCLB-EL.508833.10_mRNA-p1 TcCLB-NE.510437.45_mRNA-p1 TcSYL_0039460.t1-p1 TcSYL_0148450.t1-p1 TcSYL_0151800.t1-p1 TcSYL_0152970.t1-p1 TcSYL_0153400.t1-p1
OG0000550: TcCLB-EL.506545.10_mRNA-p1 TcSYL_0141970.t1-p1 TcSYL_0142080.t1-p1 TcSYL_0142140.t1-p1 TcSYL_0142260.t1-p1 TcSYL_0142300.t1-p1 TcSYL_0142340.t1-p1
OG0000551: TcCLB-EL.504039.240_mRNA-p1 TcCLB-EL.505025.30_mRNA-p1 TcCLB-EL.506501.120_mRNA-p1 TcCLB-EL.508541.80_mRNA-p1 TcCLB-EL.511553.209_pseudogenic_transcript-p1 TcCLB-NE.504081.470_mRNA-p1 TcSYL_0136550.t1-p1
OG0000552: TcCLB-EL.504039.140_pseudogenic_transcript-p1 TcCLB-EL.504039.80_pseudogenic_transcript-p1 TcCLB-EL.506599.91_pseudogenic_transcript-p1 TcCLB-EL.506769.90_mRNA-p1 TcCLB-EL.510163.40_pseudogenic_transcript-p1 TcSYL_0021930.t1-p1 TcSYL_0157190.t1-p1
OG0000553: TcCLB-EL.506653.10_mRNA-p1 TcCLB-EL.510379.10_mRNA-p1 TcCLB-NE.507937.3_mRNA-p1 TcCLB-NE.508115.20_mRNA-p1 TcCLB-NE.509225.11_mRNA-p1 TcSYL_0091350.t1-p1 TcSYL_0127170.t1-p1
OG0000554: TcCLB-EL.507953.60_mRNA-p1 TcCLB-NE.503417.50_mRNA-p1 TcCLB-NE.507095.10_mRNA-p1 TcCLB-NE.508869.10_mRNA-p1 TcSYL_0066180.t1-p1 TcSYL_0126380.t1-p1 TcSYL_0132960.t1-p1
OG0000555: TcCLB-EL.509081.110_mRNA-p1 TcCLB-EL.511553.100_mRNA-p1 TcCLB-NE.508365.284_pseudogenic_transcript-p1 TcCLB-NE.508887.24_pseudogenic_transcript-p1 TcCLB-NE.509525.10_pseudogenic_transcript-p1 TcCLB-NE.509525.410_pseudogenic_transcript-p1 TcCLB-NE.509525.94_pseudogenic_transcript-p1
OG0000556: TcCLB-NE.505939.10_mRNA-p1 TcSYL_0042780.t1-p1 TcSYL_0044260.t1-p1 TcSYL_0044290.t1-p1 TcSYL_0044330.t1-p1 TcSYL_0179450.t1-p1 TcSYL_0182440.t1-p1
OG0000557: TcSYL_0141980.t1-p1 TcSYL_0142030.t1-p1 TcSYL_0142110.t1-p1 TcSYL_0142150.t1-p1 TcSYL_0142270.t1-p1 TcSYL_0142350.t1-p1 TcSYL_0142450.t1-p1
OG0000558: TCRU_10242 TCRU_4176 TCRU_4586 TCRU_8578 TCRU_99 TcSYL_0091220.t1-p1
OG0000559: TCRU_102 TcCLB-EL.506773.10_mRNA-p1 TcCLB-NE.508799.200_mRNA-p1 TcCLB-NE.508799.220_pseudogenic_transcript-p1 TcCLB-NE.508799.230_mRNA-p1 TcSYL_0075260.t1-p1
OG0000560: TCRU_147 TCRU_3568 TCRU_7988 TcCLB-EL.508099.50_mRNA-p1 TcCLB-EL.510833.10_mRNA-p1 TcSYL_0152020.t1-p1
OG0000561: TCRU_224 TcCLB-EL.507625.180_mRNA-p1 TcCLB-NE.507787.10_pseudogenic_transcript-p1 TcCLB-NE.509741.50_pseudogenic_transcript-p1 TcSYL_0016990.t1-p1 TcSYL_0017000.t1-p1
OG0000562: TCRU_231 TcCLB-EL.506405.140_mRNA-p1 TcCLB-NE.507251.30_mRNA-p1 TcSYL_0046480.t1-p1 TcSYL_0046550.t1-p1 TcSYL_0046640.t1-p1
OG0000563: TCRU_252 TcCLB-EL.511545.100_mRNA-p1 TcCLB-NE.506819.10_mRNA-p1 TcSYL_0088200.t1-p1 TcSYL_0088220.t1-p1 TcSYL_0088230.t1-p1
OG0000564: TCRU_260 TCRU_5477 TcCLB-EL.508163.270_mRNA-p1 TcCLB-EL.508165.280_mRNA-p1 TcCLB-NE.510197.190_mRNA-p1 TcSYL_0187150.t1-p1
OG0000565: TCRU_272 TCRU_745 TcSYL_0031820.t1-p1 TcSYL_0038640.t1-p1

TcSYL_0071350.t1-p1 TcSYL_0087150.t1-p1
OG0000566: TCRU_336 TcCLB-EL.506947.10_mRNA-p1 TcCLB-EL.510605.10_mRNA-p1 TcCLB-NE.509107.9_mRNA-p1 TcCLB-NE.511639.20_mRNA-p1 TcSYL_0014460.t1-p1
OG0000567: TCRU_10106 TCRU_10581 TCRU_351 TcCLB-NE.510489.10_mRNA-p1 TcSYL_0125480.t1-p1 TcSYL_0136340.t1-p1
OG0000568: TCRU_2509 TCRU_3597 TCRU_480 TcSYL_0101280.t1-p1 TcSYL_0164120.t1-p1 TcSYL_0179970.t1-p1
OG0000569: TCRU_508 TcCLB-EL.505789.10_mRNA-p1 TcCLB-EL.505789.20_mRNA-p1 TcCLB-EL.509441.10_mRNA-p1 TcCLB-NE.510063.30_mRNA-p1 TcCLB-NE.510065.10_mRNA-p1
OG0000570: TCRU_10332 TCRU_4100 TCRU_622 TcCLB-EL.507257.180_mRNA-p1 TcCLB-NE.507103.20_mRNA-p1 TcSYL_0000200.t1-p1
OG0000571: TCRU_2728 TCRU_6247 TCRU_6820 TCRU_698 TcCLB-EL.433033.10_pseudogenic_transcript-p1 TcSYL_0157830.t1-p1
OG0000572: TCRU_724 TcCLB-EL.510747.170_mRNA-p1 TcCLB-EL.510749.4_mRNA-p1 TcCLB-NE.510657.70_pseudogenic_transcript-p1 TcSYL_0115800.t1-p1 TcSYL_0115810.t1-p1
OG0000573: TCRU_734 TcCLB-EL.508469.4_mRNA-p1 TcCLB-EL.510751.40_mRNA-p1 TcCLB-NE.510655.90_mRNA-p1 TcSYL_0115730.t1-p1 TcSYL_0115740.t1-p1
OG0000574: TCRU_741 TCRU_742 TcCLB-EL.508675.29_mRNA-p1 TcCLB-NE.506983.39_mRNA-p1 TcCLB-NE.506983.48_mRNA-p1 TcSYL_0027230.t1-p1
OG0000575: TCRU_779 TcCLB-EL.478671.9_mRNA-p1 TcCLB-EL.510613.10_mRNA-p1 TcCLB-NE.504175.9_mRNA-p1 TcSYL_0163520.t1-p1 TcSYL_0163540.t1-p1
OG0000576: TCRU_832 TcCLB-EL.415331.10.1-p1 TcCLB-EL.506183.15_pseudogenic_transcript-p1 TcCLB-NE.505401.31_pseudogenic_transcript-p1 TcCLB-NE.509875.165_pseudogenic_transcript-p1 TcCLB-NE.511663.40_pseudogenic_transcript-p1
OG0000577: TCRU_3621 TCRU_867 TcCLB-EL.508807.10_mRNA-p1 TcCLB-NE.506617.90_pseudogenic_transcript-p1 TcCLB-NE.506821.20_pseudogenic_transcript-p1 TcSYL_0086470.t1-p1
OG0000578: TCRU_1025 TcCLB-EL.503577.9_pseudogenic_transcript-p1 TcCLB-EL.509351.10_mRNA-p1 TcCLB-NE.510943.50_mRNA-p1 TcSYL_0001420.t1-p1 TcSYL_0001430.t1-p1
OG0000579: TCRU_1081 TcCLB-EL.508951.10_mRNA-p1 TcCLB-NE.416891.19_mRNA-p1 TcCLB-NE.510121.9_mRNA-p1 TcSYL_0169210.t1-p1 TcSYL_0169220.t1-p1
OG0000580: TCRU_1155 TcCLB-EL.510247.10_mRNA-p1 TcCLB-NE.506491.20_mRNA-p1 TcSYL_0148290.t1-p1 TcSYL_0148340.t1-p1 TcSYL_0170340.t1-p1
OG0000581: TCRU_1193 TCRU_8161 TcCLB-EL.506925.300_mRNA-p1 TcCLB-EL.507521.70_mRNA-p1 TcCLB-NE.508577.140_mRNA-p1 TcSYL_0041250.t1-p1
OG0000582: TCRU_1249 TcCLB-EL.506743.130_mRNA-p1 TcCLB-EL.510603.60_mRNA-p1 TcCLB-NE.503851.24_mRNA-p1 TcCLB-NE.509109.130_mRNA-p1 TcSYL_0014270.t1-p1
OG0000583: TCRU_1330 TcCLB-EL.504427.140_mRNA-p1 TcCLB-NE.507221.70_mRNA-p1 TcSYL_0172690.t1-p1 TcSYL_0172700.t1-p1 TcSYL_0172720.t1-p1
OG0000584: TCRU_1359 TcCLB-EL.506553.40_pseudogenic_transcript-p1 TcCLB-EL.510105.9_mRNA-p1 TcCLB-NE.507769.50_pseudogenic_transcript-p1 TcSYL_0073610.t1-p1 TcSYL_0073620.t1-p1

OG0000585: TCRU_E3 TcCLB-EL.507711.60_mRNA-p1 TcCLB-NE.
425785.9_mRNA-p1 TcCLB-NE.509637.9_mRNA-p1 TcSYL_0008800.t1-p1
TcSYL_0008830.t1-p1
OG0000586: TCRU_1462 TCRU_6115 TcCLB-NE.506559.524_mRNA-p1 TcCLB-NE.
506559.530_mRNA-p1 TcSYL_0045380.t1-p1 TcSYL_0045390.t1-p1
OG0000587: TCRU_1476 TCRU_1785 TCRU_3595 TCRU_8468 TCRU_9492
TcSYL_0173600.t1-p1
OG0000588: TCRU_1484 TCRU_6656 TcCLB-EL.510755.129_mRNA-p1 TcCLB-EL.
511071.171_mRNA-p1 TcCLB-NE.504037.30_mRNA-p1 TcCLB-NE.
508413.44_mRNA-p1
OG0000589: TCRU_1598 TcCLB-EL.507959.180_pseudogenic_transcript-p1
TcCLB-EL.508541.40_pseudogenic_transcript-p1 TcCLB-NE.
503973.300_pseudogenic_transcript-p1 TcCLB-NE.
508221.570_pseudogenic_transcript-p1 TcSYL_0149300.t1-p1
OG0000590: TCRU_1632 TcCLB-EL.503643.3_mRNA-p1 TcCLB-NE.
504121.30_mRNA-p1 TcCLB-NE.504121.39_mRNA-p1 TcSYL_0003330.t1-p1
TcSYL_0003360.t1-p1
OG0000591: TCRU_1691 TcCLB-EL.510297.110_mRNA-p1 TcCLB-NE.
506009.109_mRNA-p1 TcSYL_0113600.t1-p1 TcSYL_0113610.t1-p1
TcSYL_0113640.t1-p1
OG0000592: TCRU_1695 TcCLB-EL.509167.160_mRNA-p1 TcCLB-NE.
503597.5_mRNA-p1 TcCLB-NE.504027.20_mRNA-p1 TcSYL_0140230.t1-p1
TcSYL_0140240.t1-p1
OG0000593: TCRU_1697 TcCLB-EL.509167.180_mRNA-p1 TcCLB-NE.
425839.10_mRNA-p1 TcCLB-NE.504027.4_mRNA-p1 TcSYL_0140180.t1-p1
TcSYL_0140200.t1-p1
OG0000594: TCRU_1700 TcCLB-EL.506463.10_mRNA-p1 TcCLB-EL.
506715.20_mRNA-p1 TcCLB-EL.510349.90_mRNA-p1 TcCLB-NE.
416143.10_mRNA-p1 TcSYL_0156650.t1-p1
OG0000595: TCRU_1718 TcCLB-EL.434945.10_mRNA-p1 TcCLB-EL.
503855.10_mRNA-p1 TcCLB-NE.508197.30_mRNA-p1 TcCLB-NE.
510337.50_pseudogenic_transcript-p1 TcSYL_0146650.t1-p1
OG0000596: TCRU_1723 TCRU_9136 TcCLB-EL.504425.60_mRNA-p1 TcCLB-NE.
508677.80_mRNA-p1 TcCLB-NE.509331.210_mRNA-p1 TcSYL_0061230.t1-p1
OG0000597: TCRU_1755 TCRU_9490 TcCLB-NE.506137.10_mRNA-p1 TcCLB-NE.
506611.70_mRNA-p1 TcSYL_0068640.t1-p1 TcSYL_0137180.t1-p1
OG0000598: TCRU_1819 TcCLB-EL.506601.40_mRNA-p1 TcCLB-EL.
510961.10_mRNA-p1 TcCLB-EL.510961.30_mRNA-p1 TcCLB-NE.
508999.150_mRNA-p1 TcCLB-NE.508999.80_mRNA-p1
OG0000599: TCRU_1840 TcCLB-EL.511181.50_mRNA-p1 TcCLB-NE.
503465.49_mRNA-p1 TcCLB-NE.508301.20_mRNA-p1 TcSYL_0203230.t1-p1
TcSYL_0203240.t1-p1
OG0000600: TCRU_1856 TcCLB-EL.504835.29_mRNA-p1 TcCLB-EL.
507389.10_mRNA-p1 TcCLB-NE.509911.130_mRNA-p1 TcSYL_0057490.t1-p1
TcSYL_0057500.t1-p1
OG0000601: TCRU_1940 TcCLB-EL.506699.40_mRNA-p1 TcCLB-EL.
510323.4_mRNA-p1 TcCLB-NE.508153.330_mRNA-p1 TcSYL_0000970.t1-p1
TcSYL_0000980.t1-p1
OG0000602: TCRU_1968 TCRU_2465 TcCLB-EL.507629.30_mRNA-p1 TcCLB-NE.
503931.30_mRNA-p1 TcSYL_0089390.t1-p1 TcSYL_0089400.t1-p1
OG0000603: TCRU_1976 TcCLB-EL.504143.30_pseudogenic_transcript-p1
TcCLB-NE.506485.40_pseudogenic_transcript-p1 TcSYL_0003450.t1-p1
TcSYL_0003460.t1-p1 TcSYL_0003470.t1-p1
OG0000604: TCRU_2003 TcCLB-EL.506607.10_mRNA-p1 TcCLB-NE.

506321.300_mRNA-p1 TcSYL_0137760.t1-p1 TcSYL_0137830.t1-p1
TcSYL_0137840.t1-p1
OG0000605: TCRU_2006 TcCLB-EL.503777.4_mRNA-p1 TcCLB-EL.
510173.10_mRNA-p1 TcCLB-NE.506321.330_mRNA-p1 TcSYL_0137850.t1-p1
TcSYL_0137860.t1-p1
OG0000606: TCRU_2023 TCRU_2024 TcCLB-NE.508355.250_mRNA-p1 TcCLB-NE.
508355.260_mRNA-p1 TcSYL_0083010.t1-p1 TcSYL_0083020.t1-p1
OG0000607: TCRU_2041 TcCLB-EL.507091.80_mRNA-p1 TcCLB-EL.
508977.50_mRNA-p1 TcCLB-EL.511399.50_mRNA-p1 TcCLB-EL.
511401.100_mRNA-p1 TcCLB-NE.509115.30_mRNA-p1
OG0000608: TCRU_2046 TcCLB-NE.508933.40_mRNA-p1 TcSYL_0038170.t1-p1
TcSYL_0038290.t1-p1 TcSYL_0056590.t1-p1 TcSYL_0056670.t1-p1
OG0000609: TCRU_2121 TcCLB-EL.511217.90_mRNA-p1 TcCLB-NE.
506575.9_mRNA-p1 TcCLB-NE.508957.40_pseudogenic_transcript-p1
TcSYL_0189030.t1-p1 TcSYL_0189040.t1-p1
OG0000610: TCRU_2172 TcCLB-EL.506755.10_mRNA-p1 TcCLB-EL.
506755.5_mRNA-p1 TcCLB-NE.510357.150_mRNA-p1 TcCLB-NE.
510359.10_mRNA-p1 TcSYL_0112740.t1-p1
OG0000611: TCRU_2223 TcCLB-EL.507667.11_mRNA-p1 TcCLB-NE.
503979.10_mRNA-p1 TcSYL_0048330.t1-p1 TcSYL_0048340.t1-p1
TcSYL_0048350.t1-p1
OG0000612: TCRU_10022 TCRU_2280 TcCLB-EL.506409.230_mRNA-p1 TcCLB-
NE.508979.80_mRNA-p1 TcCLB-NE.511235.10_pseudogenic_transcript-p1
TcSYL_0101030.t1-p1
OG0000613: TCRU_2389 TcCLB-EL.509233.239_mRNA-p1 TcCLB-EL.
509451.70_mRNA-p1 TcCLB-NE.511879.9_mRNA-p1 TcSYL_0145920.t1-p1
TcSYL_0145940.t1-p1
OG0000614: TCRU_2396 TcCLB-EL.506579.10_mRNA-p1 TcCLB-NE.
507241.39_mRNA-p1 TcSYL_0064560.t1-p1 TcSYL_0064680.t1-p1
TcSYL_0064810.t1-p1
OG0000615: TCRU_2406 TCRU_4672 TcCLB-EL.511621.210_mRNA-p1 TcCLB-EL.
511621.80_mRNA-p1 TcCLB-NE.504741.149_mRNA-p1 TcCLB-NE.
509317.29_mRNA-p1
OG0000616: TCRU_2424 TcCLB-EL.503749.5_mRNA-p1 TcCLB-EL.
509067.70_mRNA-p1 TcCLB-NE.511023.10_mRNA-p1 TcSYL_0111740.t1-p1
TcSYL_0111750.t1-p1
OG0000617: TCRU_2431 TcCLB-EL.511537.40_mRNA-p1 TcCLB-NE.
508707.340_mRNA-p1 TcCLB-NE.511021.10_mRNA-p1 TcSYL_0111660.t1-p1
TcSYL_0111680.t1-p1
OG0000618: TCRU_2448 TcCLB-EL.507237.220_mRNA-p1 TcCLB-EL.
509093.10_mRNA-p1 TcCLB-EL.511607.90_pseudogenic_transcript-p1
TcCLB-EL.511613.10_mRNA-p1 TcCLB-NE.511255.169_mRNA-p1
OG0000619: TCRU_2464 TCRU_7651 TcCLB-EL.506963.14_mRNA-p1 TcCLB-NE.
511233.14_mRNA-p1 TcCLB-NE.511277.175_mRNA-p1 TcSYL_0014810.t1-p1
OG0000620: TCRU_2627 TcCLB-EL.468941.9_mRNA-p1 TcCLB-EL.
506691.14_mRNA-p1 TcCLB-NE.508153.1050_mRNA-p1 TcSYL_0000620.t1-p1
TcSYL_0000640.t1-p1
OG0000621: TCRU_2730 TCRU_2772 TCRU_3870 TCRU_9180 TCRU_9334
TcSYL_0171610.t1-p1
OG0000622: TCRU_2791 TcCLB-EL.506409.240_mRNA-p1 TcCLB-NE.
503723.90_mRNA-p1 TcCLB-NE.508979.71_pseudogenic_transcript-p1
TcCLB-NE.511235.20_mRNA-p1 TcSYL_0101070.t1-p1
OG0000623: TCRU_2816 TCRU_2817 TcCLB-EL.510593.49_mRNA-p1 TcCLB-EL.
510595.4_mRNA-p1 TcCLB-NE.507895.124_mRNA-p1 TcSYL_0057180.t1-p1

OG0000624: TCRU_2949 TcCLB-EL.503667.10_mRNA-p1 TcCLB-NE.
508139.130_mRNA-p1 TcCLB-NE.509287.230_mRNA-p1 TcSYL_0148240.t1-p1
TcSYL_0191110.t1-p1
OG0000625: TCRU_2994 TcCLB-EL.506953.49_mRNA-p1 TcCLB-EL.
507883.10_pseudogenic_transcript-p1 TcCLB-NE.
506193.70_pseudogenic_transcript-p1 TcCLB-NE.509287.50_mRNA-p1
TcSYL_0191490.t1-p1
OG0000626: TCRU_2999 TcCLB-EL.507009.10_mRNA-p1 TcCLB-NE.
508461.570_mRNA-p1 TcCLB-NE.510669.20_mRNA-p1 TcCLB-NE.
510669.49_mRNA-p1 TcSYL_0117590.t1-p1
OG0000627: TCRU_3010 TcCLB-EL.509669.50_mRNA-p1 TcCLB-NE.
506175.5_mRNA-p1 TcCLB-NE.509619.20_pseudogenic_transcript-p1 TcCLB-
NE.509621.10_mRNA-p1 TcSYL_0001950.t1-p1
OG0000628: TCRU_3039 TCRU_4193 TCRU_6420 TCRU_6737 TCRU_7122 TcCLB-
NE.506111.10_pseudogenic_transcript-p1
OG0000629: TCRU_3059 TCRU_7277 TcCLB-EL.507093.220_mRNA-p1 TcCLB-EL.
507093.229_mRNA-p1 TcSYL_0080400.t1-p1 TcSYL_0080410.t1-p1
OG0000630: TCRU_3073 TcCLB-EL.510055.150_mRNA-p1 TcCLB-NE.
508909.339_mRNA-p1 TcCLB-NE.508911.10_mRNA-p1 TcSYL_0159380.t1-p1
TcSYL_0159390.t1-p1
OG0000631: TCRU_3094 TCRU_3966 TcCLB-EL.510101.30_mRNA-p1 TcCLB-EL.
510101.40_mRNA-p1 TcCLB-NE.506297.270_mRNA-p1 TcSYL_0074750.t1-p1
OG0000632: TCRU_3105 TcCLB-EL.510101.170_mRNA-p1 TcCLB-EL.
510101.180_mRNA-p1 TcCLB-NE.506297.150_mRNA-p1 TcCLB-NE.
506297.160_mRNA-p1 TcSYL_0074870.t1-p1
OG0000633: TCRU_3123 TcCLB-EL.510101.420_mRNA-p1 TcCLB-EL.
510101.430_mRNA-p1 TcCLB-NE.507765.60_mRNA-p1 TcCLB-NE.
507765.70_mRNA-p1 TcSYL_0075000.t1-p1
OG0000634: TCRU_3153 TcCLB-EL.405179.10_mRNA-p1 TcCLB-EL.
433273.10_mRNA-p1 TcCLB-NE.508815.179_mRNA-p1 TcSYL_0043920.t1-p1
TcSYL_0043990.t1-p1
OG0000635: TCRU_3202 TcCLB-EL.507079.30_mRNA-p1 TcSYL_0022240.t1-p1
TcSYL_0022270.t1-p1 TcSYL_0022330.t1-p1 TcSYL_0040080.t1-p1
OG0000636: TCRU_3253 TCRU_3254 TcCLB-EL.506413.20_mRNA-p1 TcCLB-EL.
506413.30_mRNA-p1 TcCLB-NE.511237.50_mRNA-p1 TcCLB-NE.
511237.60_mRNA-p1
OG0000637: TCRU_3259 TCRU_4058 TCRU_6403 TCRU_6436 TcSYL_0142180.t1-
p1 TcSYL_0196200.t1-p1
OG0000638: TCRU_3269 TcCLB-EL.507011.210_mRNA-p1 TcCLB-NE.
503983.29_mRNA-p1 TcCLB-NE.507003.70_mRNA-p1 TcSYL_0116700.t1-p1
TcSYL_0116720.t1-p1
OG0000639: TCRU_3277 TcCLB-EL.510737.119_mRNA-p1 TcCLB-EL.
510739.10_mRNA-p1 TcCLB-NE.510661.260_mRNA-p1 TcSYL_0116330.t1-p1
TcSYL_0116360.t1-p1
OG0000640: TCRU_3304 TcCLB-EL.506945.290_mRNA-p1 TcCLB-EL.
510767.10_mRNA-p1 TcCLB-NE.509671.70_mRNA-p1 TcSYL_0074500.t1-p1
TcSYL_0140770.t1-p1
OG0000641: TCRU_3353 TcCLB-EL.507631.10_mRNA-p1 TcCLB-EL.
511049.20_mRNA-p1 TcCLB-NE.506011.30_mRNA-p1 TcCLB-NE.
506255.20_mRNA-p1 TcSYL_0090280.t1-p1
OG0000642: TCRU_3421 TcCLB-EL.508317.10_mRNA-p1 TcCLB-NE.
507849.50_mRNA-p1 TcCLB-NE.508173.110_mRNA-p1 TcSYL_0079380.t1-p1
TcSYL_0079390.t1-p1
OG0000643: TCRU_3429 TcCLB-EL.506203.10_mRNA-p1 TcCLB-NE.

508645.40_mRNA-p1 TcCLB-NE.508645.70_mRNA-p1 TcSYL_0047730.t1-p1 TcSYL_0047800.t1-p1
OG0000644: TCRU_3482 TcCLB-EL.511127.369_mRNA-p1 TcCLB-EL.511127.380_mRNA-p1 TcCLB-NE.503657.9_mRNA-p1 TcCLB-NE.503931.46_pseudogenic_transcript-p1 TcSYL_0156040.t1-p1
OG0000645: TCRU_3497 TCRU_4689 TCRU_5119 TCRU_9204 TCRU_9297 TcSYL_0186970.t1-p1
OG0000646: TCRU_3622 TCRU_6049 TcCLB-EL.506579.40_mRNA-p1 TcCLB-EL.506579.50_mRNA-p1 TcCLB-NE.506509.10_mRNA-p1 TcSYL_0064840.t1-p1
OG0000647: TCRU_3640 TcCLB-EL.511745.10_mRNA-p1 TcCLB-NE.507029.30_mRNA-p1 TcSYL_0139650.t1-p1 TcSYL_0139670.t1-p1 TcSYL_0139680.t1-p1
OG0000648: TCRU_3647 TCRU_7522 TcCLB-NE.507071.50_mRNA-p1 TcSYL_0018820.t1-p1 TcSYL_0070380.t1-p1 TcSYL_0154760.t1-p1
OG0000649: TCRU_3742 TcCLB-EL.508647.20_mRNA-p1 TcCLB-NE.508645.59_mRNA-p1 TcSYL_0047720.t1-p1 TcSYL_0047750.t1-p1 TcSYL_0047790.t1-p1
OG0000650: TCRU_10418 TCRU_3803 TCRU_6902 TcCLB-EL.468217.30_mRNA-p1 TcCLB-NE.508735.65_pseudogenic_transcript-p1 TcSYL_0065060.t1-p1
OG0000651: TCRU_3896 TcCLB-EL.505555.30_mRNA-p1 TcCLB-NE.510823.109_mRNA-p1 TcCLB-NE.510825.10_mRNA-p1 TcSYL_0050210.t1-p1 TcSYL_0050220.t1-p1
OG0000652: TCRU_3929 TcCLB-EL.507509.89_mRNA-p1 TcCLB-EL.507511.10_mRNA-p1 TcCLB-NE.506871.140_mRNA-p1 TcSYL_0142530.t1-p1 TcSYL_0142540.t1-p1
OG0000653: TCRU_3981 TcCLB-EL.504033.180_mRNA-p1 TcCLB-NE.508199.90_mRNA-p1 TcCLB-NE.510339.7_mRNA-p1 TcSYL_0147030.t1-p1 TcSYL_0147040.t1-p1
OG0000654: TCRU_4114 TcCLB-EL.509453.90_mRNA-p1 TcCLB-EL.509455.9_mRNA-p1 TcCLB-NE.503879.100_mRNA-p1 TcSYL_0145380.t1-p1 TcSYL_0145420.t1-p1
OG0000655: TCRU_4123 TcCLB-EL.503813.30_mRNA-p1 TcCLB-EL.509453.4_mRNA-p1 TcCLB-NE.506709.30_mRNA-p1 TcSYL_0145710.t1-p1 TcSYL_0145720.t1-p1
OG0000656: TCRU_4140 TcCLB-EL.510755.50_mRNA-p1 TcCLB-EL.510755.60_mRNA-p1 TcCLB-NE.508415.40_mRNA-p1 TcCLB-NE.508415.50_mRNA-p1 TcSYL_0115600.t1-p1
OG0000657: TCRU_4143 TcCLB-EL.510535.10_mRNA-p1 TcCLB-NE.509211.39_mRNA-p1 TcCLB-NE.511827.10_mRNA-p1 TcSYL_0200760.t1-p1 TcSYL_0200770.t1-p1
OG0000658: TCRU_4166 TcCLB-EL.511445.160_mRNA-p1 TcCLB-NE.503703.50_mRNA-p1 TcSYL_0080990.t1-p1 TcSYL_0082240.t1-p1 TcSYL_0082260.t1-p1
OG0000659: TCRU_4198 TcCLB-EL.506531.9_mRNA-p1 TcCLB-EL.510075.60_mRNA-p1 TcCLB-NE.509399.90_mRNA-p1 TcSYL_0177040.t1-p1 TcSYL_0177070.t1-p1
OG0000660: TCRU_4236 TcCLB-EL.504151.60_mRNA-p1 TcCLB-NE.509459.79_mRNA-p1 TcCLB-NE.509461.10_mRNA-p1 TcSYL_0174260.t1-p1 TcSYL_0174270.t1-p1
OG0000661: TCRU_4265 TcCLB-EL.507145.84_pseudogenic_transcript-p1 TcCLB-EL.507145.98_mRNA-p1 TcCLB-EL.507847.20_pseudogenic_transcript-p1 TcCLB-EL.511625.160_mRNA-p1 TcCLB-NE.508869.20_pseudogenic_transcript-p1
OG0000662: TCRU_4380 TcCLB-EL.506941.9_mRNA-p1 TcCLB-NE.

511511.10_mRNA-p1 TcSYL_0139520.t1-p1 TcSYL_0139530.t1-p1
TcSYL_0139540.t1-p1
OG0000663: TCRU_4554 TCRU_4555 TcCLB-EL.510719.30_mRNA-p1 TcCLB-EL.
510719.35_mRNA-p1 TcCLB-NE.509749.33_mRNA-p1 TcCLB-NE.
509749.36_mRNA-p1
OG0000664: TCRU_4574 TcCLB-EL.507091.20_mRNA-p1 TcCLB-EL.
508375.70_mRNA-p1 TcCLB-NE.511643.4_mRNA-p1 TcCLB-NE.511925.10_mRNA-
p1 TcSYL_0164430.t1-p1
OG0000665: TCRU_10268 TCRU_4597 TcCLB-EL.509965.390_mRNA-p1 TcCLB-
NE.509051.20_mRNA-p1 TcSYL_0042830.t1-p1 TcSYL_0042860.t1-p1
OG0000666: TCRU_4599 TcCLB-EL.449247.20_mRNA-p1 TcCLB-EL.
508205.4_mRNA-p1 TcCLB-NE.506123.60_mRNA-p1 TcSYL_0062110.t1-p1
TcSYL_0062120.t1-p1
OG0000667: TCRU_4711 TcCLB-EL.507875.180_mRNA-p1 TcCLB-EL.
509165.30_mRNA-p1 TcCLB-NE.506595.110_mRNA-p1 TcCLB-NE.
507783.20_mRNA-p1 TcCLB-NE.510643.160_mRNA-p1
OG0000668: TCRU_4713 TcCLB-EL.505977.26_mRNA-p1 TcCLB-EL.
509165.40_mRNA-p1 TcCLB-NE.506595.100_mRNA-p1 TcCLB-NE.
510643.140_mRNA-p1 TcSYL_0064350.t1-p1
OG0000669: TCRU_4838 TcCLB-EL.511867.209_mRNA-p1 TcSYL_0146240.t1-p1
TcSYL_0146290.t1-p1 TcSYL_0146300.t1-p1 TcSYL_0146310.t1-p1
OG0000670: TCRU_4843 TCRU_6214 TCRU_6499 TcCLB-NE.506679.140_mRNA-p1
TcCLB-NE.506679.150_mRNA-p1 TcSYL_0114020.t1-p1
OG0000671: TCRU_5199 TCRU_6108 TCRU_6109 TcCLB-NE.508823.120_mRNA-p1
TcCLB-NE.508823.140_mRNA-p1 TcSYL_0042980.t1-p1
OG0000672: TCRU_5205 TcCLB-EL.409117.20_mRNA-p1 TcCLB-EL.
506945.230_mRNA-p1 TcCLB-NE.508823.50_mRNA-p1 TcSYL_0043020.t1-p1
TcSYL_0140720.t1-p1
OG0000673: TCRU_5260 TcCLB-EL.507941.10_mRNA-p1 TcCLB-NE.
509985.78_mRNA-p1 TcCLB-NE.509987.10_mRNA-p1 TcSYL_0063270.t1-p1
TcSYL_0063280.t1-p1
OG0000674: TCRU_5308 TcCLB-EL.504215.30_mRNA-p1 TcCLB-NE.
508891.4_mRNA-p1 TcCLB-NE.511275.40_mRNA-p1 TcSYL_0015030.t1-p1
TcSYL_0015040.t1-p1
OG0000675: TCRU_5548 TCRU_5975 TcCLB-NE.506705.70_mRNA-p1 TcCLB-NE.
507251.10_mRNA-p1 TcSYL_0046510.t1-p1 TcSYL_0046590.t1-p1
OG0000676: TCRU_5560 TcCLB-EL.505965.86_pseudogenic_transcript-p1
TcCLB-NE.510901.260_pseudogenic_transcript-p1 TcCLB-NE.
510903.10_mRNA-p1 TcSYL_0178660.t1-p1 TcSYL_0178710.t1-p1
OG0000677: TCRU_5604 TcCLB-EL.508765.30_mRNA-p1 TcCLB-EL.
508807.5_mRNA-p1 TcCLB-NE.506617.70_mRNA-p1 TcCLB-NE.506821.30_mRNA-
p1 TcSYL_0086480.t1-p1
OG0000678: TCRU_5671 TcCLB-EL.509247.30_mRNA-p1 TcCLB-NE.
506977.139_mRNA-p1 TcSYL_0170430.t1-p1 TcSYL_0170440.t1-p1
TcSYL_0170450.t1-p1
OG0000679: TCRU_5673 TCRU_5675 TcCLB-NE.
506989.300_pseudogenic_transcript-p1 TcCLB-NE.
507951.30_pseudogenic_transcript-p1 TcSYL_0003630.t1-p1
TcSYL_0003690.t1-p1
OG0000680: TCRU_5747 TcCLB-EL.511443.10_pseudogenic_transcript-p1
TcCLB-NE.510801.10_mRNA-p1 TcSYL_0080720.t1-p1 TcSYL_0080770.t1-p1
TcSYL_0080830.t1-p1
OG0000681: TCRU_5774 TcCLB-EL.508997.20_pseudogenic_transcript-p1
TcCLB-NE.506475.14_mRNA-p1 TcSYL_0110690.t1-p1 TcSYL_0110700.t1-p1

TcSYL_0110710.t1-p1
OG0000682: TCRU_5884 TCRU_6136 TcCLB-EL.484311.10_mRNA-p1 TcCLB-NE.505985.9_mRNA-p1 TcSYL_0080710.t1-p1 TcSYL_0080820.t1-p1
OG0000683: TCRU_10515 TCRU_5997 TcCLB-EL.509979.230_mRNA-p1 TcCLB-NE.507971.29_mRNA-p1 TcCLB-NE.510203.49_pseudogenic_transcript-p1 TcSYL_0156490.t1-p1
OG0000684: TCRU_6010 TcCLB-EL.507465.10_mRNA-p1 TcCLB-EL.507467.10_mRNA-p1 TcCLB-EL.509449.5_mRNA-p1 TcCLB-NE.508355.5_mRNA-p1 TcCLB-NE.510581.9_mRNA-p1
OG0000685: TCRU_6078 TcCLB-EL.504171.10_mRNA-p1 TcCLB-EL.508719.9_mRNA-p1 TcCLB-NE.509793.69_mRNA-p1 TcCLB-NE.509795.10_mRNA-p1 TcSYL_0107050.t1-p1
OG0000686: TCRU_6211 TcCLB-EL.434931.10_mRNA-p1 TcCLB-EL.511215.119_mRNA-p1 TcCLB-NE.508961.79_mRNA-p1 TcSYL_0188020.t1-p1 TcSYL_0188080.t1-p1
OG0000687: TCRU_6291 TcCLB-EL.503611.10_mRNA-p1 TcCLB-EL.504097.4_mRNA-p1 TcCLB-NE.506679.220_mRNA-p1 TcSYL_0114110.t1-p1 TcSYL_0114120.t1-p1
OG0000688: TCRU_6339 TcCLB-EL.508987.9_mRNA-p1 TcCLB-NE.439301.9_mRNA-p1 TcCLB-NE.441967.9_mRNA-p1 TcSYL_0096610.t1-p1 TcSYL_0096640.t1-p1
OG0000689: TCRU_10528 TCRU_6600 TcCLB-EL.511001.120_mRNA-p1 TcCLB-NE.506957.90_mRNA-p1 TcCLB-NE.511727.129_mRNA-p1 TcSYL_0142760.t1-p1
OG0000690: TCRU_6698 TCRU_6699 TcCLB-EL.506605.70_mRNA-p1 TcCLB-NE.511239.60_mRNA-p1 TcSYL_0026070.t1-p1 TcSYL_0026090.t1-p1
OG0000691: TCRU_6778 TcCLB-EL.508465.60_mRNA-p1 TcCLB-NE.507001.140_mRNA-p1 TcCLB-NE.510659.4_mRNA-p1 TcSYL_0116020.t1-p1 TcSYL_0116030.t1-p1
OG0000692: TCRU_6824 TcCLB-EL.510953.49_mRNA-p1 TcCLB-EL.510955.4_mRNA-p1 TcCLB-NE.509005.50_mRNA-p1 TcSYL_0063410.t1-p1 TcSYL_0138840.t1-p1
OG0000693: TCRU_6924 TcCLB-EL.506779.10_mRNA-p1 TcCLB-EL.508239.49_mRNA-p1 TcCLB-NE.508793.14_mRNA-p1 TcSYL_0076730.t1-p1 TcSYL_0076740.t1-p1
OG0000694: TCRU_6964 TCRU_8870 TcCLB-NE.506393.189_mRNA-p1 TcCLB-NE.509259.180_mRNA-p1 TcCLB-NE.509429.4_mRNA-p1 TcSYL_0074010.t1-p1
OG0000695: TCRU_7090 TcCLB-EL.507529.50_mRNA-p1 TcCLB-NE.510001.59_mRNA-p1 TcCLB-NE.510003.9_mRNA-p1 TcSYL_0003020.t1-p1 TcSYL_0003030.t1-p1
OG0000696: TCRU_7092 TcCLB-EL.507531.19_mRNA-p1 TcCLB-EL.507533.10_mRNA-p1 TcCLB-NE.510003.30_mRNA-p1 TcSYL_0002820.t1-p1 TcSYL_0002850.t1-p1
OG0000697: TCRU_7297 TcCLB-EL.428999.20_mRNA-p1 TcCLB-NE.460757.10_mRNA-p1 TcCLB-NE.511895.30_mRNA-p1 TcSYL_0083160.t1-p1 TcSYL_0083250.t1-p1
OG0000698: TCRU_7364 TcCLB-EL.511555.110_mRNA-p1 TcCLB-NE.503691.9_mRNA-p1 TcCLB-NE.506621.70_mRNA-p1 TcSYL_0073340.t1-p1 TcSYL_0073350.t1-p1
OG0000699: TCRU_7531 TcCLB-EL.422041.9_mRNA-p1 TcCLB-EL.509537.10_mRNA-p1 TcCLB-NE.507681.220_mRNA-p1 TcSYL_0073850.t1-p1 TcSYL_0073870.t1-p1
OG0000700: TCRU_7565 TCRU_7568 TcCLB-EL.511473.10_pseudogenic_transcript-p1 TcCLB-NE.479847.20_mRNA-p1 TcCLB-NE.508139.230_pseudogenic_transcript-p1 TcSYL_0060360.t1-p1

OG0000701: TCRU_7616 TcCLB-EL.511001.220_mRNA-p1 TcCLB-NE.
509017.9_mRNA-p1 TcCLB-NE.511465.30_mRNA-p1 TcSYL_0131410.t1-p1
TcSYL_0131420.t1-p1
OG0000702: TCRU_7634 TCRU_9104 TcCLB-NE.511487.51_mRNA-p1
TcSYL_0128490.t1-p1 TcSYL_0152950.t1-p1 TcSYL_0153420.t1-p1
OG0000703: TCRU_component_ TcCLB-EL.408345.20_mRNA-p1 TcCLB-EL.
503793.10_mRNA-p1 TcCLB-NE.510797.10_mRNA-p1 TcSYL_0082360.t1-p1
TcSYL_0082370.t1-p1
OG0000704: TCRU_7758 TcCLB-EL.506163.30_mRNA-p1 TcCLB-EL.
506867.30_mRNA-p1 TcCLB-NE.507623.120_mRNA-p1 TcCLB-NE.
511203.5_mRNA-p1 TcSYL_0043060.t1-p1
OG0000705: TCRU_7801 TcCLB-EL.427247.4_mRNA-p1 TcCLB-EL.
510989.69_mRNA-p1 TcCLB-NE.511245.230_mRNA-p1 TcSYL_0027180.t1-p1
TcSYL_0027190.t1-p1
OG0000706: TCRU_7814 TcCLB-EL.507257.50_mRNA-p1 TcCLB-NE.
510329.260_mRNA-p1 TcSYL_0001290.t1-p1 TcSYL_0001300.t1-p1
TcSYL_0001310.t1-p1
OG0000707: TCRU_8036 TcCLB-EL.507507.10_mRNA-p1 TcCLB-EL.
509491.20_pseudogenic_transcript-p1 TcCLB-NE.506871.20_mRNA-p1
TcSYL_0142580.t1-p1 TcSYL_0142590.t1-p1
OG0000708: TCRU_8097 TcCLB-EL.509595.50_mRNA-p1 TcCLB-NE.
508817.180_pseudogenic_transcript-p1 TcSYL_0043350.t1-p1
TcSYL_0043360.t1-p1 TcSYL_0043370.t1-p1
OG0000709: TCRU_8180 TCRU_9588 TcCLB-NE.
510627.30_pseudogenic_transcript-p1 TcCLB-NE.
511259.230_pseudogenic_transcript-p1 TcSYL_0129290.t1-p1
TcSYL_0134890.t1-p1
OG0000710: TCRU_8210 TCRU_9055 TcCLB-EL.
504099.30_pseudogenic_transcript-p1 TcCLB-EL.
510307.110_pseudogenic_transcript-p1 TcCLB-NE.
508479.450_pseudogenic_transcript-p1 TcSYL_0022620.t1-p1
OG0000711: TCRU_8245 TcCLB-EL.503629.10_mRNA-p1 TcCLB-NE.
508739.40_mRNA-p1 TcSYL_0191770.t1-p1 TcSYL_0192050.t1-p1
TcSYL_0192140.t1-p1
OG0000712: TCRU_8273 TcCLB-EL.506895.80_mRNA-p1 TcCLB-NE.
508501.320_mRNA-p1 TcSYL_0025410.t1-p1 TcSYL_0110260.t1-p1
TcSYL_0110420.t1-p1
OG0000713: TCRU_8340 TcCLB-EL.504449.14_mRNA-p1 TcCLB-EL.
505983.40_mRNA-p1 TcCLB-NE.459389.10_mRNA-p1 TcCLB-NE.
504839.70_mRNA-p1 TcSYL_0064390.t1-p1
OG0000714: TCRU_8377 TcCLB-EL.503989.50_mRNA-p1 TcCLB-EL.
506221.20_mRNA-p1 TcCLB-NE.504207.29_mRNA-p1 TcCLB-NE.510727.9_mRNA-
p1 TcSYL_0141740.t1-p1
OG0000715: TCRU_8389 TcCLB-EL.414517.9_mRNA-p1 TcCLB-EL.
503529.10_mRNA-p1 TcCLB-NE.511647.50_mRNA-p1 TcSYL_0014050.t1-p1
TcSYL_0014060.t1-p1
OG0000716: TCRU_8597 TcCLB-EL.450155.9_mRNA-p1 TcCLB-EL.
503885.10_mRNA-p1 TcCLB-NE.511903.200_mRNA-p1 TcSYL_0103010.t1-p1
TcSYL_0103020.t1-p1
OG0000717: TCRU_8698 TcCLB-EL.503981.9_pseudogenic_transcript-p1
TcCLB-NE.504131.70_mRNA-p1 TcSYL_0048130.t1-p1 TcSYL_0048140.t1-p1
TcSYL_0048150.t1-p1
OG0000718: TCRU_8708 TCRU_9874 TcCLB-EL.507671.30_mRNA-p1 TcCLB-NE.
506201.39_mRNA-p1 TcCLB-NE.509733.100_mRNA-p1 TcSYL_0010760.t1-p1

OG0000719: TCRU_8815 TcCLB-EL.504203.20_mRNA-p1 TcCLB-NE.
503791.49_mRNA-p1 TcCLB-NE.508657.20_pseudogenic_transcript-p1
TcSYL_0113400.t1-p1 TcSYL_0113410.t1-p1
OG0000720: TCRU_8877 TcCLB-EL.508387.10_mRNA-p1 TcCLB-EL.
510617.19_mRNA-p1 TcCLB-NE.506789.290_mRNA-p1 TcSYL_0163090.t1-p1
TcSYL_0163140.t1-p1
OG0000721: TCRU_8897 TcCLB-EL.509589.10_mRNA-p1 TcCLB-NE.
509587.10_mRNA-p1 TcSYL_0089150.t1-p1 TcSYL_0089160.t1-p1
TcSYL_0089170.t1-p1
OG0000722: TCRU_8906 TcCLB-EL.509701.10_mRNA-p1 TcCLB-NE.
504233.4_mRNA-p1 TcCLB-NE.508981.39_mRNA-p1 TcSYL_0024260.t1-p1
TcSYL_0024270.t1-p1
OG0000723: TCRU_10824 TCRU_8940 TcCLB-EL.509979.90_mRNA-p1 TcCLB-EL.
509979.95_mRNA-p1 TcCLB-NE.510039.90_mRNA-p1 TcSYL_0156520.t1-p1
OG0000724: TCRU_9017 TcCLB-EL.506683.240_mRNA-p1 TcCLB-EL.
511309.80_pseudogenic_transcript-p1 TcCLB-NE.416041.14_mRNA-p1
TcCLB-NE.507949.190_pseudogenic_transcript-p1 TcCLB-NE.
511759.20_pseudogenic_transcript-p1
OG0000725: TCRU_9057 TCRU_9058 TCRU_9420 TcCLB-EL.507809.70_mRNA-p1
TcCLB-NE.511661.50_mRNA-p1 TcSYL_0013690.t1-p1
OG0000726: TCRU_9192 TcCLB-EL.506211.114_mRNA-p1 TcCLB-NE.
508895.10_mRNA-p1 TcCLB-NE.511289.119_mRNA-p1 TcSYL_0123010.t1-p1
TcSYL_0123020.t1-p1
OG0000727: TCRU_9221 TcCLB-EL.510029.30_mRNA-p1 TcCLB-NE.
507031.10_mRNA-p1 TcCLB-NE.511291.129_mRNA-p1 TcSYL_0122500.t1-p1
TcSYL_0122720.t1-p1
OG0000728: TCRU_9222 TcCLB-EL.507963.90_mRNA-p1 TcCLB-EL.
510029.10_pseudogenic_transcript-p1 TcCLB-NE.507031.20_mRNA-p1
TcSYL_0122490.t1-p1 TcSYL_0122680.t1-p1
OG0000729: TCRU_9360 TcCLB-EL.503455.30_mRNA-p1 TcCLB-NE.
511261.80_mRNA-p1 TcSYL_0016070.t1-p1 TcSYL_0016080.t1-p1
TcSYL_0016090.t1-p1
OG0000730: TCRU_9597 TcCLB-EL.504167.30_mRNA-p1 TcCLB-NE.
398235.10_mRNA-p1 TcCLB-NE.508235.40_mRNA-p1 TcSYL_0108330.t1-p1
TcSYL_0108610.t1-p1
OG0000731: TCRU_9651 TcCLB-NE.509753.290_pseudogenic_transcript-p1
TcCLB-NE.509899.70_pseudogenic_transcript-p1 TcCLB-NE.
511643.80_pseudogenic_transcript-p1 TcSYL_0150510.t1-p1
TcSYL_0151430.t1-p1
OG0000732: TCRU_9655 TCRU_9656 TcCLB-EL.504147.120_mRNA-p1 TcCLB-EL.
510719.160_mRNA-p1 TcCLB-NE.509747.20_mRNA-p1 TcSYL_0005370.t1-p1
OG0000733: TCRU_9736 TcCLB-EL.511211.120_mRNA-p1 TcCLB-EL.
511211.130_mRNA-p1 TcSYL_0182520.t1-p1 TcSYL_0182530.t1-p1
TcSYL_0182550.t1-p1
OG0000734: TCRU_9772 TcCLB-EL.508067.10_mRNA-p1 TcCLB-NE.
452039.9_mRNA-p1 TcCLB-NE.510573.19_mRNA-p1 TcSYL_0147440.t1-p1
TcSYL_0147450.t1-p1
OG0000735: TCRU_9814 TcCLB-EL.506695.4_mRNA-p1 TcCLB-EL.
510315.40_mRNA-p1 TcCLB-NE.508153.610_mRNA-p1 TcSYL_0000750.t1-p1
TcSYL_0000770.t1-p1
OG0000736: TCRU_10015 TcCLB-EL.510029.70_mRNA-p1 TcCLB-EL.
511189.30_mRNA-p1 TcCLB-NE.510973.30_mRNA-p1 TcCLB-NE.
511291.90_mRNA-p1 TcSYL_0087830.t1-p1
OG0000737: TCRU_10451 TcCLB-EL.511677.20_mRNA-p1 TcCLB-NE.

504081.195_pseudogenic_transcript-p1 TcCLB-NE.
506329.65_pseudogenic_transcript-p1 TcCLB-NE.
508245.45_pseudogenic_transcript-p1 TcSYL_0173110.t1-p1
OG0000738: TCRU_10534 TcCLB-EL.508989.90_mRNA-p1 TcCLB-NE.
504093.4_mRNA-p1 TcCLB-NE.509569.180_mRNA-p1 TcSYL_0097580.t1-p1
TcSYL_0097590.t1-p1
OG0000739: TCRU_10618 TcCLB-EL.509103.39_mRNA-p1 TcCLB-NE.
506347.10_pseudogenic_transcript-p1 TcCLB-NE.507801.19_mRNA-p1
TcSYL_0084180.t1-p1 TcSYL_0084240.t1-p1
OG0000740: TCRU_10707 TcCLB-EL.506929.60_pseudogenic_transcript-p1
TcCLB-EL.511199.9_mRNA-p1 TcCLB-NE.506587.40_mRNA-p1
TcSYL_0044810.t1-p1 TcSYL_0044820.t1-p1
OG0000741: TCRU_10738 TcCLB-EL.419043.10_mRNA-p1 TcCLB-NE.
506053.20_mRNA-p1 TcSYL_0098430.t1-p1 TcSYL_0099370.t1-p1
TcSYL_0099620.t1-p1
OG0000742: TCRU_10779 TcCLB-EL.504009.4_mRNA-p1 TcCLB-NE.
507049.130_pseudogenic_transcript-p1 TcSYL_0114640.t1-p1
TcSYL_0114670.t1-p1 TcSYL_0114680.t1-p1
OG0000743: TCRU_10794 TcCLB-EL.510073.30_mRNA-p1 TcCLB-NE.
509399.190_mRNA-p1 TcSYL_0177500.t1-p1 TcSYL_0177510.t1-p1
TcSYL_0177520.t1-p1
OG0000744: TCRU_10847 TcCLB-EL.508719.80_mRNA-p1 TcCLB-EL.
508721.4_mRNA-p1 TcCLB-NE.509791.180_mRNA-p1 TcSYL_0107080.t1-p1
TcSYL_0107090.t1-p1
OG0000745: TCRU_10863 TcCLB-EL.511001.160_mRNA-p1 TcCLB-NE.
506957.23_mRNA-p1 TcSYL_0131630.t1-p1 TcSYL_0131660.t1-p1
TcSYL_0131700.t1-p1
OG0000746: TcCLB-EL.504099.10_mRNA-p1 TcCLB-EL.
506113.90_pseudogenic_transcript-p1 TcCLB-EL.509735.120_mRNA-p1
TcCLB-EL.509735.30_pseudogenic_transcript-p1 TcCLB-EL.
510165.110_mRNA-p1 TcCLB-NE.509917.19_mRNA-p1
OG0000747: TcCLB-EL.505365.20_pseudogenic_transcript-p1 TcCLB-EL.
508325.180_pseudogenic_transcript-p1 TcCLB-EL.
509959.10_pseudogenic_transcript-p1 TcCLB-EL.
510847.40_pseudogenic_transcript-p1 TcCLB-NE.
416041.24_pseudogenic_transcript-p1 TcSYL_0165880.t1-p1
OG0000748: TcCLB-EL.479379.9_mRNA-p1 TcSYL_0046840.t1-p1
TcSYL_0084160.t1-p1 TcSYL_0089130.t1-p1 TcSYL_0146220.t1-p1
TcSYL_0146230.t1-p1
OG0000749: TcCLB-EL.507553.41_mRNA-p1 TcCLB-EL.510275.29_mRNA-p1
TcCLB-EL.510561.7_mRNA-p1 TcCLB-NE.510219.5_mRNA-p1
TcSYL_0126620.t1-p1 TcSYL_0127450.t1-p1
OG0000750: TcCLB-EL.508439.40_mRNA-p1 TcCLB-EL.510283.10_mRNA-p1
TcCLB-EL.510283.20_mRNA-p1 TcCLB-EL.510283.30_mRNA-p1 TcCLB-NE.
506275.110_mRNA-p1 TcCLB-NE.506275.121_mRNA-p1
OG0000751: TcCLB-EL.506967.34_pseudogenic_transcript-p1 TcCLB-EL.
507041.65_pseudogenic_transcript-p1 TcCLB-EL.
511603.343_pseudogenic_transcript-p1 TcCLB-EL.
511603.405_pseudogenic_transcript-p1 TcCLB-EL.
511613.135_pseudogenic_transcript-p1 TcCLB-NE.
510363.183_pseudogenic_transcript-p1
OG0000752: TcCLB-EL.511605.90_mRNA-p1 TcCLB-EL.511613.120_mRNA-p1
TcCLB-NE.506321.110_mRNA-p1 TcCLB-NE.510237.110_mRNA-p1
TcSYL_0150480.t1-p1 TcSYL_0151470.t1-p1

OG0000753: TcCLB-EL.506341.40_mRNA-p1 TcCLB-NE.508575.10_mRNA-p1 TcSYL_0020100.t1-p1 TcSYL_0020560.t1-p1 TcSYL_0061130.t1-p1 TcSYL_0171860.t1-p1
OG0000754: TcCLB-EL.507091.4_mRNA-p1 TcCLB-EL.508375.89_mRNA-p1 TcCLB-NE.511641.5_mRNA-p1 TcCLB-NE.511925.30_pseudogenic_transcript-p1 TcSYL_0164540.t1-p1 TcSYL_0164550.t1-p1
OG0000755: TcCLB-EL.504425.50_mRNA-p1 TcCLB-NE.508677.90_mRNA-p1 TcCLB-NE.509331.221_mRNA-p1 TcSYL_0061250.t1-p1 TcSYL_0171720.t1-p1 TcSYL_0172030.t1-p1
OG0000756: TcCLB-EL.507275.61_mRNA-p1 TcCLB-EL.508841.10_mRNA-p1 TcCLB-NE.506573.5_mRNA-p1 TcSYL_0189560.t1-p1 TcSYL_0189760.t1-p1 TcSYL_0190080.t1-p1
OG0000757: TcCLB-EL.506025.14_mRNA-p1 TcCLB-EL.509201.15_mRNA-p1 TcCLB-EL.511805.15_mRNA-p1 TcCLB-NE.511015.20_mRNA-p1 TcCLB-NE.511209.54_mRNA-p1 TcSYL_0180240.t1-p1
OG0000758: TcCLB-EL.412093.10_pseudogenic_transcript-p1 TcCLB-EL.510877.10_mRNA-p1 TcCLB-NE.506559.559_mRNA-p1 TcSYL_0045410.t1-p1 TcSYL_0045420.t1-p1 TcSYL_0045680.t1-p1
OG0000759: TcCLB-EL.438147.9_mRNA-p1 TcCLB-NE.509161.140_mRNA-p1 TcSYL_0142920.t1-p1 TcSYL_0142960.t1-p1 TcSYL_0143010.t1-p1 TcSYL_0143040.t1-p1
OG0000760: TcCLB-EL.507511.71_pseudogenic_transcript-p1 TcCLB-EL.507993.340_mRNA-p1 TcCLB-NE.510327.10_mRNA-p1 TcCLB-NE.510657.210_mRNA-p1 TcSYL_0087360.t1-p1 TcSYL_0142380.t1-p1
OG0000761: TcCLB-EL.430895.4_mRNA-p1 TcCLB-EL.479253.9_mRNA-p1 TcCLB-EL.508179.70_mRNA-p1 TcCLB-NE.504269.20_mRNA-p1 TcSYL_0000380.t1-p1 TcSYL_0000420.t1-p1
OG0000762: TcCLB-EL.505683.10_mRNA-p1 TcCLB-NE.504191.10_mRNA-p1 TcCLB-NE.504191.19_mRNA-p1 TcCLB-NE.510185.10_mRNA-p1 TcSYL_0012790.t1-p1 TcSYL_0013000.t1-p1
OG0000763: TcCLB-EL.508537.5_mRNA-p1 TcSYL_0011780.t1-p1 TcSYL_0012490.t1-p1 TcSYL_0167420.t1-p1 TcSYL_0168370.t1-p1 TcSYL_0168430.t1-p1
OG0000764: TcCLB-EL.511173.40_pseudogenic_transcript-p1 TcCLB-NE.508437.5_pseudogenic_transcript-p1 TcCLB-NE.510693.251_pseudogenic_transcript-p1 TcCLB-NE.510697.55_pseudogenic_transcript-p1 TcCLB-NE.510701.50_pseudogenic_transcript-p1 TcSYL_0160770.t1-p1
OG0000765: TcCLB-EL.506671.10_mRNA-p1 TcCLB-EL.508167.29_mRNA-p1 TcCLB-EL.510275.60_mRNA-p1 TcCLB-NE.506613.20_pseudogenic_transcript-p1 TcSYL_0187100.t1-p1 TcSYL_0187270.t1-p1
OG0000766: TcCLB-EL.507959.350_mRNA-p1 TcCLB-EL.507959.80_mRNA-p1 TcCLB-EL.510023.30_mRNA-p1 TcCLB-EL.511347.20_pseudogenic_transcript-p1 TcCLB-NE.504081.530_mRNA-p1 TcSYL_0134290.t1-p1
OG0000767: TcCLB-EL.506499.130_mRNA-p1 TcSYL_0022080.t1-p1 TcSYL_0093510.t1-p1 TcSYL_0124180.t1-p1 TcSYL_0134630.t1-p1 TcSYL_0155060.t1-p1
OG0000768: TcCLB-EL.506499.60_mRNA-p1 TcSYL_0101120.t1-p1 TcSYL_0149450.t1-p1 TcSYL_0149600.t1-p1 TcSYL_0149700.t1-p1 TcSYL_0154770.t1-p1
OG0000769: TcCLB-EL.506763.40_mRNA-p1 TcCLB-EL.510371.40_mRNA-p1 TcCLB-NE.508221.250_mRNA-p1 TcCLB-NE.508221.850_mRNA-p1

TcSYL_0125900.t1-p1 TcSYL_0164280.t1-p1
OG0000770: TcCLB-EL.506769.65_pseudogenic_transcript-p1 TcCLB-NE.506751.132_pseudogenic_transcript-p1 TcCLB-NE.508261.25.1-p1 TcCLB-NE.509545.55_pseudogenic_transcript-p1 TcSYL_0038560.t1-p1 TcSYL_0069190.t1-p1
OG0000771: TcCLB-EL.507959.170_mRNA-p1 TcCLB-EL.509081.20_mRNA-p1 TcCLB-EL.511553.30_mRNA-p1 TcCLB-NE.503973.280_mRNA-p1 TcCLB-NE.508221.550_mRNA-p1 TcSYL_0125440.t1-p1
OG0000772: TcCLB-EL.510377.310_pseudogenic_transcript-p1 TcCLB-NE.506279.19_pseudogenic_transcript-p1 TcCLB-NE.507071.70_pseudogenic_transcript-p1 TcSYL_0024110.t1-p1 TcSYL_0129820.t1-p1 TcSYL_0133290.t1-p1
OG0000773: TcCLB-NE.507095.20_mRNA-p1 TcSYL_0066170.t1-p1 TcSYL_0068610.t1-p1 TcSYL_0134810.t1-p1 TcSYL_0135150.t1-p1 TcSYL_0135920.t1-p1
OG0000774: TCRU_20 TcCLB-EL.447255.29_mRNA-p1 TcCLB-EL.510231.29_mRNA-p1 TcCLB-NE.506559.100_mRNA-p1 TcSYL_0045150.t1-p1
OG0000775: TCRU_130 TcCLB-EL.510879.110_mRNA-p1 TcCLB-EL.510879.120_mRNA-p1 TcCLB-NE.504013.11_mRNA-p1 TcSYL_0045950.t1-p1
OG0000776: TCRU_10477 TCRU_134 TCRU_5317 TcSYL_0133000.t1-p1 TcSYL_0137140.t1-p1
OG0000777: TCRU_186 TcCLB-EL.503453.50_mRNA-p1 TcCLB-NE.506959.110_mRNA-p1 TcSYL_0132820.t1-p1 TcSYL_0132830.t1-p1
OG0000778: TCRU_189 TcCLB-EL.503453.20_mRNA-p1 TcCLB-EL.511467.4_mRNA-p1 TcCLB-NE.506959.140_mRNA-p1 TcSYL_0132860.t1-p1
OG0000779: TCRU_235 TCRU_9329 TcCLB-EL.507467.50_mRNA-p1 TcCLB-NE.508355.50_mRNA-p1 TcSYL_0083130.t1-p1
OG0000780: TCRU_237 TcCLB-EL.511803.70_mRNA-p1 TcCLB-NE.510895.20_mRNA-p1 TcSYL_0180130.t1-p1 TcSYL_0180140.t1-p1
OG0000781: TCRU_1600 TCRU_244 TCRU_7303 TCRU_8196 TcSYL_0048730.t1-p1
OG0000782: TCRU_277 TCRU_4094 TcCLB-NE.509661.10_pseudogenic_transcript-p1 TcSYL_0200630.t1-p1 TcSYL_0200660.t1-p1
OG0000783: TCRU_326 TcCLB-EL.503811.10_mRNA-p1 TcCLB-EL.506591.4_mRNA-p1 TcCLB-NE.506989.190_mRNA-p1 TcSYL_0003830.t1-p1
OG0000784: TCRU_386 TcCLB-EL.511051.39_mRNA-p1 TcCLB-EL.511051.50_mRNA-p1 TcCLB-NE.509683.117_mRNA-p1 TcSYL_0089620.t1-p1
OG0000785: TCRU_388 TcCLB-EL.511051.70_mRNA-p1 TcCLB-NE.509683.130_mRNA-p1 TcSYL_0089540.t1-p1 TcSYL_0090990.t1-p1
OG0000786: TCRU_393 TCRU_6735 TCRU_7885 TcCLB-EL.447847.10_pseudogenic_transcript-p1 TcSYL_0190830.t1-p1
OG0000787: TCRU_2711 TCRU_402 TCRU_8276 TcCLB-NE.510401.31_pseudogenic_transcript-p1 TcSYL_0060570.t1-p1
OG0000788: TCRU_3385 TCRU_404 TCRU_5907 TcCLB-EL.511365.80_mRNA-p1 TcSYL_0121860.t1-p1
OG0000789: TCRU_408 TcCLB-EL.511363.10_mRNA-p1 TcCLB-EL.511365.4_mRNA-p1 TcCLB-NE.508899.40_mRNA-p1 TcSYL_0121830.t1-p1
OG0000790: TCRU_430 TcCLB-EL.507011.130_mRNA-p1 TcCLB-NE.510665.60_pseudogenic_transcript-p1 TcSYL_0116880.t1-p1 TcSYL_0116900.t1-p1
OG0000791: TCRU_438 TcCLB-EL.509379.29_mRNA-p1 TcCLB-NE.506701.29_mRNA-p1 TcSYL_0001730.t1-p1 TcSYL_0001740.t1-p1
OG0000792: TCRU_3747 TCRU_503 TCRU_6706 TcSYL_0106730.t1-p1

TcSYL_0187550.t1-p1
OG0000793: TCRU_509 TCRU_8910 TcCLB-EL.509441.5_mRNA-p1 TcCLB-NE.
510063.39_mRNA-p1 TcCLB-NE.510065.20_mRNA-p1
OG0000794: TCRU_572 TcCLB-EL.511545.140_mRNA-p1 TcCLB-NE.
506819.49_mRNA-p1 TcSYL_0088330.t1-p1 TcSYL_0088350.t1-p1
OG0000795: TCRU_578 TcCLB-EL.506811.50_mRNA-p1 TcCLB-NE.
506303.171_mRNA-p1 TcCLB-NE.506305.5_mRNA-p1 TcSYL_0138460.t1-p1
OG0000796: TCRU_589 TcCLB-EL.504025.10_mRNA-p1 TcCLB-EL.
506771.9_mRNA-p1 TcCLB-NE.506247.370_mRNA-p1 TcSYL_0108690.t1-p1
OG0000797: TCRU_597 TCRU_6807 TCRU_8256 TcSYL_0028040.t1-p1
TcSYL_0093870.t1-p1
OG0000798: TCRU_607 TcCLB-EL.509173.9_mRNA-p1 TcCLB-EL.
511749.9_mRNA-p1 TcCLB-NE.507023.220_mRNA-p1 TcSYL_0139360.t1-p1
OG0000799: TCRU_4099 TCRU_623 TcCLB-EL.508179.10_mRNA-p1 TcCLB-NE.
507103.10_mRNA-p1 TcCLB-NE.509629.20_mRNA-p1
OG0000800: TCRU_3591 TCRU_659 TcCLB-EL.510855.4_mRNA-p1 TcCLB-NE.
509611.189_mRNA-p1 TcCLB-NE.511883.49_mRNA-p1
OG0000801: TCRU_2685 TCRU_660 TCRU_8378 TcCLB-NE.
509613.20_pseudogenic_transcript-p1 TcSYL_0120930.t1-p1
OG0000802: TCRU_676 TcCLB-EL.509167.100_mRNA-p1 TcCLB-NE.
510443.9_mRNA-p1 TcSYL_0140390.t1-p1 TcSYL_0140400.t1-p1
OG0000803: TCRU_2931 TCRU_679 TcCLB-EL.506979.20_mRNA-p1 TcCLB-NE.
503499.30_pseudogenic_transcript-p1 TcSYL_0084860.t1-p1
OG0000804: TCRU_6546 TCRU_6836 TCRU_690 TCRU_7830 TcSYL_0028760.t1-
p1
OG0000805: TCRU_706 TcCLB-EL.509233.180_mRNA-p1 TcCLB-NE.
467287.30_mRNA-p1 TcSYL_0145270.t1-p1 TcSYL_0146090.t1-p1
OG0000806: TCRU_732 TcCLB-EL.510749.99_mRNA-p1 TcCLB-EL.
510751.4_mRNA-p1 TcCLB-NE.510655.120_mRNA-p1 TcSYL_0115760.t1-p1
OG0000807: TCRU_759 TcCLB-EL.503955.40_mRNA-p1 TcCLB-EL.
509777.30_mRNA-p1 TcCLB-NE.507063.70_mRNA-p1 TcSYL_0013620.t1-p1
OG0000808: TCRU_761 TcCLB-EL.503953.59_mRNA-p1 TcCLB-EL.
503955.10_mRNA-p1 TcCLB-NE.507063.100_mRNA-p1 TcSYL_0013600.t1-p1
OG0000809: TCRU_780 TcCLB-EL.504147.190_mRNA-p1 TcSYL_0005130.t1-p1
TcSYL_0005140.t1-p1 TcSYL_0005150.t1-p1
OG0000810: TCRU_795 TcCLB-EL.503821.19_mRNA-p1 TcCLB-NE.
509683.20_mRNA-p1 TcSYL_0089950.t1-p1 TcSYL_0089960.t1-p1
OG0000811: TCRU_7291 TCRU_806 TCRU_8351 TcCLB-NE.
505267.10_pseudogenic_transcript-p1 TcSYL_0136640.t1-p1
OG0000812: TCRU_815 TcCLB-EL.507757.50_mRNA-p1 TcCLB-NE.
508933.5_mRNA-p1 TcSYL_0038370.t1-p1 TcSYL_0055730.t1-p1
OG0000813: TCRU_816 TcCLB-EL.508727.44_mRNA-p1 TcCLB-NE.
503797.4_mRNA-p1 TcSYL_0107300.t1-p1 TcSYL_0107310.t1-p1
OG0000814: TCRU_819 TcCLB-EL.508725.10_mRNA-p1 TcCLB-EL.
508727.9_mRNA-p1 TcSYL_0107240.t1-p1 TcSYL_0107250.t1-p1
OG0000815: TCRU_836 TcCLB-EL.505123.20_mRNA-p1 TcCLB-EL.
508921.20_mRNA-p1 TcCLB-EL.510841.20_mRNA-p1 TcCLB-NE.
508141.60_mRNA-p1
OG0000816: TCRU_4008 TCRU_4965 TCRU_7773 TCRU_845 TcSYL_0182400.t1-
p1
OG0000817: TCRU_874 TcCLB-EL.507213.10_mRNA-p1 TcCLB-NE.
511301.120_mRNA-p1 TcSYL_0121640.t1-p1 TcSYL_0147710.t1-p1
OG0000818: TCRU_1428 TCRU_878 TcCLB-EL.511351.41_mRNA-p1
TcSYL_0147490.t1-p1 TcSYL_0147600.t1-p1

OG0000819: TCRU_890 TcCLB-EL.511417.40_mRNA-p1 TcCLB-NE.
505997.210_mRNA-p1 TcSYL_0111320.t1-p1 TcSYL_0111330.t1-p1
OG0000820: TCRU_965 TcCLB-EL.398345.10_mRNA-p1 TcCLB-EL.
509649.59_mRNA-p1 TcCLB-NE.511285.50_mRNA-p1 TcSYL_0123500.t1-p1
OG0000821: TCRU_968 TcCLB-EL.503435.14_mRNA-p1 TcCLB-NE.
511285.10_mRNA-p1 TcSYL_0123550.t1-p1 TcSYL_0123560.t1-p1
OG0000822: TCRU_988 TcCLB-EL.503717.20_mRNA-p1 TcCLB-EL.
510279.330_mRNA-p1 TcCLB-NE.508221.460_pseudogenic_transcript-p1
TcSYL_0161380.t1-p1
OG0000823: TCRU_996 TcCLB-EL.509205.120_mRNA-p1 TcCLB-NE.
508601.141_mRNA-p1 TcCLB-NE.508699.120_mRNA-p1 TcSYL_0180910.t1-p1
OG0000824: TCRU_997 TcCLB-EL.509197.39_mRNA-p1 TcCLB-NE.
450161.10_mRNA-p1 TcCLB-NE.508699.130_mRNA-p1 TcSYL_0180940.t1-p1
OG0000825: TCRU_1044 TcCLB-EL.504041.4_mRNA-p1 TcCLB-EL.
504075.10_mRNA-p1 TcCLB-EL.506933.107_mRNA-p1 TcCLB-NE.
508815.4_mRNA-p1
OG0000826: TCRU_1182 TCRU_7125 TCRU_8396 TcSYL_0141030.t1-p1
TcSYL_0141180.t1-p1
OG0000827: TCRU_1220 TcCLB-EL.506633.109_mRNA-p1 TcCLB-EL.
506635.10_pseudogenic_transcript-p1 TcCLB-NE.508027.60_mRNA-p1
TcSYL_0045070.t1-p1
OG0000828: TCRU_1257 TcCLB-EL.506747.20_mRNA-p1 TcCLB-EL.
508373.20_mRNA-p1 TcCLB-NE.507059.70_mRNA-p1 TcSYL_0014140.t1-p1
OG0000829: TCRU_1259 TcCLB-EL.507757.40_mRNA-p1 TcCLB-NE.
508931.50_mRNA-p1 TcSYL_0055700.t1-p1 TcSYL_0056330.t1-p1
OG0000830: TCRU_1298 TcCLB-EL.504153.310_mRNA-p1 TcCLB-NE.
506301.11_mRNA-p1 TcSYL_0138570.t1-p1 TcSYL_0138580.t1-p1
OG0000831: TCRU_1325 TcCLB-EL.504149.175_mRNA-p1 TcCLB-EL.
506129.89_mRNA-p1 TcCLB-EL.508039.70_mRNA-p1 TcCLB-NE.
509429.59_mRNA-p1
OG0000832: TCRU_1338 TcCLB-EL.506633.90_mRNA-p1 TcCLB-EL.
511575.130_mRNA-p1 TcCLB-NE.508027.40_mRNA-p1 TcSYL_0095520.t1-p1
OG0000833: TCRU_1366 TcCLB-EL.507957.310_mRNA-p1 TcCLB-NE.
508875.10_mRNA-p1 TcSYL_0068230.t1-p1 TcSYL_0124810.t1-p1
OG0000834: TCRU_1452 TcCLB-EL.404001.20_mRNA-p1 TcCLB-NE.
508533.10_mRNA-p1 TcSYL_0050330.t1-p1 TcSYL_0050740.t1-p1
OG0000835: TCRU_1453 TCRU_9052 TcCLB-NE.506001.90_mRNA-p1 TcCLB-NE.
507167.70_mRNA-p1 TcCLB-NE.508479.500_mRNA-p1
OG0000836: TCRU_1510 TcCLB-EL.508357.9_mRNA-p1 TcCLB-NE.
508355.330_mRNA-p1 TcSYL_0082930.t1-p1 TcSYL_0082940.t1-p1
OG0000837: TCRU_1537 TcCLB-NE.511667.80_pseudogenic_transcript-p1
TcSYL_0083870.t1-p1 TcSYL_0148060.t1-p1 TcSYL_0203830.t1-p1
OG0000838: TCRU_1561 TcCLB-EL.504147.20_mRNA-p1 TcCLB-EL.
506181.50_mRNA-p1 TcCLB-NE.503395.40_mRNA-p1 TcSYL_0005650.t1-p1
OG0000839: TCRU_1564 TcCLB-EL.506181.20_mRNA-p1 TcCLB-NE.
503395.10_mRNA-p1 TcCLB-NE.507663.9_mRNA-p1 TcSYL_0092310.t1-p1
OG0000840: TCRU_1608 TCRU_1612 TCRU_5249 TCRU_6908 TcCLB-NE.
509219.50_pseudogenic_transcript-p1
OG0000841: TCRU_10276 TCRU_1634 TCRU_6512 TcSYL_0038530.t1-p1
TcSYL_0038770.t1-p1
OG0000842: TCRU_1653 TcCLB-NE.506613.80_mRNA-p1 TcCLB-NE.
511565.20_mRNA-p1 TcSYL_0129460.t1-p1 TcSYL_0129860.t1-p1
OG0000843: TCRU_1714 TcCLB-EL.508777.180_mRNA-p1 TcCLB-NE.
511483.40_mRNA-p1 TcSYL_0127630.t1-p1 TcSYL_0156130.t1-p1

OG0000844: TCRU_1720 TcCLB-EL.506925.120_mRNA-p1 TcCLB-EL.
506925.130_mRNA-p1 TcSYL_0080540.t1-p1 TcSYL_0080550.t1-p1
OG0000845: TCRU_1756 TcCLB-EL.511607.40_mRNA-p1 TcCLB-NE.
504015.11_mRNA-p1 TcCLB-NE.506611.40_mRNA-p1 TcCLB-NE.
510239.70_mRNA-p1
OG0000846: TCRU_1770 TcCLB-EL.510407.60_pseudogenic_transcript-p1
TcCLB-NE.509937.180_mRNA-p1 TcSYL_0027620.t1-p1 TcSYL_0027630.t1-p1
OG0000847: TCRU_1834 TcCLB-EL.438147.18_mRNA-p1 TcCLB-NE.
509161.149_mRNA-p1 TcSYL_0142900.t1-p1 TcSYL_0142940.t1-p1
OG0000848: TCRU_1855 TcCLB-EL.507389.30_mRNA-p1 TcCLB-NE.
509911.120_mRNA-p1 TcSYL_0057460.t1-p1 TcSYL_0057470.t1-p1
OG0000849: TCRU_1929 TcCLB-EL.508321.40_mRNA-p1 TcCLB-NE.
509207.40_mRNA-p1 TcSYL_0201760.t1-p1 TcSYL_0201770.t1-p1
OG0000850: TCRU_1935 TcCLB-EL.434259.19_mRNA-p1 TcCLB-EL.
438897.10_mRNA-p1 TcCLB-NE.508153.390_mRNA-p1 TcSYL_0000900.t1-p1
OG0000851: TCRU_1937 TcCLB-EL.506699.3_mRNA-p1 TcCLB-EL.
510321.20_mRNA-p1 TcCLB-NE.508153.370_mRNA-p1 TcSYL_0000940.t1-p1
OG0000852: TCRU_2009 TcCLB-EL.510173.40_mRNA-p1 TcCLB-NE.
506321.359_mRNA-p1 TcCLB-NE.509721.10_mRNA-p1 TcSYL_0137890.t1-p1
OG0000853: TCRU_2064 TcCLB-EL.503909.120_mRNA-p1 TcCLB-EL.
511747.7_mRNA-p1 TcCLB-NE.511507.30_mRNA-p1 TcSYL_0139430.t1-p1
OG0000854: TCRU_2071 TcCLB-EL.503909.44_mRNA-p1 TcCLB-NE.
510901.70_mRNA-p1 TcCLB-NE.511507.120_mRNA-p1 TcSYL_0179170.t1-p1
OG0000855: TCRU_2090 TcCLB-EL.509099.20_mRNA-p1 TcCLB-NE.
509321.10_mRNA-p1 TcSYL_0085490.t1-p1 TcSYL_0086000.t1-p1
OG0000856: TCRU_2097 TcCLB-EL.506519.140_pseudogenic_transcript-p1
TcCLB-NE.508909.10_mRNA-p1 TcSYL_0159510.t1-p1 TcSYL_0159520.t1-p1
OG0000857: TCRU_2108 TCRU_5117 TcCLB-EL.506967.50_mRNA-p1 TcCLB-NE.
508221.660_mRNA-p1 TcSYL_0151320.t1-p1
OG0000858: TCRU_2129 TCRU_9172 TcCLB-EL.506925.104_mRNA-p1 TcCLB-NE.
510897.6_mRNA-p1 TcSYL_0080530.t1-p1
OG0000859: TCRU_2160 TcCLB-EL.508109.10_mRNA-p1 TcCLB-EL.
508161.10_mRNA-p1 TcCLB-EL.510191.70_mRNA-p1 TcCLB-NE.
511843.10_mRNA-p1
OG0000860: TCRU_2202 TcCLB-EL.511049.40_mRNA-p1 TcCLB-NE.
509679.9_mRNA-p1 TcSYL_0090210.t1-p1 TcSYL_0090250.t1-p1
OG0000861: TCRU_2235 TcCLB-EL.510147.9_mRNA-p1 TcCLB-EL.
510149.9_mRNA-p1 TcCLB-NE.508411.50_mRNA-p1 TcSYL_0004200.t1-p1
OG0000862: TCRU_2273 TcCLB-EL.449247.4_mRNA-p1 TcCLB-NE.
507601.10_mRNA-p1 TcSYL_0062130.t1-p1 TcSYL_0062140.t1-p1
OG0000863: TCRU_2277 TcCLB-EL.511421.230_mRNA-p1 TcCLB-NE.
507949.260_mRNA-p1 TcSYL_0111020.t1-p1 TcSYL_0111040.t1-p1
OG0000864: TCRU_2279 TCRU_4729 TcCLB-EL.506409.220_mRNA-p1 TcCLB-NE.
508979.90_mRNA-p1 TcSYL_0101010.t1-p1
OG0000865: TCRU_2300 TcCLB-EL.508163.280_mRNA-p1 TcCLB-EL.
511605.30_mRNA-p1 TcCLB-NE.511565.10_mRNA-p1 TcCLB-NE.
511565.30_mRNA-p1
OG0000866: TCRU_2302 TCRU_3877 TcCLB-EL.504769.80_mRNA-p1 TcCLB-NE.
506471.10_mRNA-p1 TcSYL_0156980.t1-p1
OG0000867: TCRU_2322 TCRU_6118 TcCLB-NE.506679.100_mRNA-p1 TcCLB-NE.
506679.94_mRNA-p1 TcSYL_0113930.t1-p1
OG0000868: TCRU_2326 TCRU_7131 TcCLB-EL.509269.4_mRNA-p1 TcCLB-NE.
507969.10_mRNA-p1 TcSYL_0088930.t1-p1
OG0000869: TCRU_2354 TcCLB-EL.511433.20_mRNA-p1 TcCLB-EL.

511435.10_mRNA-p1 TcCLB-NE.506475.80_mRNA-p1 TcSYL_0110730.t1-p1
OG0000870: TCRU_2394 TcCLB-EL.511735.50_mRNA-p1 TcCLB-NE.
511521.40_mRNA-p1 TcCLB-NE.511521.9_mRNA-p1 TcSYL_0139880.t1-p1
OG0000871: TCRU_2416 TcCLB-EL.511531.59_mRNA-p1 TcCLB-EL.
511533.5_pseudogenic_transcript-p1 TcCLB-NE.511025.30_mRNA-p1
TcSYL_0111830.t1-p1
OG0000872: TCRU_2418 TcCLB-EL.503927.9_mRNA-p1 TcCLB-EL.
511533.19_mRNA-p1 TcCLB-NE.511025.10_mRNA-p1 TcSYL_0111820.t1-p1
OG0000873: TCRU_2421 TcCLB-EL.511535.30_mRNA-p1 TcCLB-NE.
508713.10_mRNA-p1 TcSYL_0111790.t1-p1 TcSYL_0111800.t1-p1
OG0000874: TCRU_2453 TcSYL_0060790.t1-p1 TcSYL_0091270.t1-p1
TcSYL_0091550.t1-p1 TcSYL_0156430.t1-p1
OG0000875: TCRU_2456 TCRU_8171 TCRU_9389 TcCLB-NE.
509385.19_pseudogenic_transcript-p1 TcSYL_0153110.t1-p1
OG0000876: TCRU_2467 TcCLB-EL.506963.10_mRNA-p1 TcCLB-NE.
511233.7_mRNA-p1 TcCLB-NE.511277.160_mRNA-p1 TcSYL_0014830.t1-p1
OG0000877: TCRU_2553 TCRU_8212 TcCLB-EL.506599.420_mRNA-p1 TcCLB-NE.
508221.420_mRNA-p1 TcCLB-NE.510359.650_mRNA-p1
OG0000878: TCRU_2562 TCRU_5803 TcCLB-EL.509445.30_mRNA-p1 TcCLB-NE.
422031.10_mRNA-p1 TcCLB-NE.427091.10_mRNA-p1
OG0000879: TCRU_2584 TcCLB-EL.506605.229_mRNA-p1 TcCLB-NE.
508859.74_mRNA-p1 TcSYL_0025550.t1-p1 TcSYL_0025560.t1-p1
OG0000880: TCRU_2732 TCRU_3872 TCRU_7679 TcCLB-NE.
503439.40_pseudogenic_transcript-p1 TcCLB-NE.
508529.40_pseudogenic_transcript-p1
OG0000881: TCRU_2741 TcCLB-EL.507923.10_mRNA-p1 TcCLB-EL.
511753.120_mRNA-p1 TcSYL_0139030.t1-p1 TcSYL_0139090.t1-p1
OG0000882: TCRU_2792 TcCLB-NE.504155.30_pseudogenic_transcript-p1
TcCLB-NE.506231.40_pseudogenic_transcript-p1 TcCLB-NE.
508873.590_pseudogenic_transcript-p1 TcCLB-NE.
511255.470_pseudogenic_transcript-p1
OG0000883: TCRU_2850 TcCLB-EL.511751.260_mRNA-p1 TcCLB-EL.
511753.9_mRNA-p1 TcCLB-NE.507021.70_mRNA-p1 TcSYL_0139170.t1-p1
OG0000884: TCRU_2856 TcCLB-NE.510687.100_mRNA-p1 TcCLB-NE.
510687.94_mRNA-p1 TcSYL_0047140.t1-p1 TcSYL_0047150.t1-p1
OG0000885: TCRU_2891 TcCLB-EL.506097.9_mRNA-p1 TcCLB-NE.
508257.170_mRNA-p1 TcSYL_0046740.t1-p1 TcSYL_0046810.t1-p1
OG0000886: TCRU_2939 TcCLB-EL.510763.100_mRNA-p1 TcCLB-NE.
509671.130_pseudogenic_transcript-p1 TcSYL_0074440.t1-p1
TcSYL_0115370.t1-p1
OG0000887: TCRU_2943 TcCLB-EL.510765.60_mRNA-p1 TcCLB-EL.
510765.70_mRNA-p1 TcCLB-NE.509671.80_mRNA-p1 TcSYL_0115310.t1-p1
OG0000888: TCRU_2960 TcCLB-EL.503967.4_mRNA-p1 TcCLB-EL.
510057.4_mRNA-p1 TcCLB-NE.508909.170_mRNA-p1 TcSYL_0159460.t1-p1
OG0000889: TCRU_2983 TcCLB-EL.503425.29_mRNA-p1 TcCLB-EL.
504507.20_pseudogenic_transcript-p1 TcCLB-NE.503555.10_mRNA-p1
TcSYL_0006260.t1-p1
OG0000890: TCRU_2998 TcCLB-NE.508461.560_mRNA-p1 TcCLB-NE.
508461.589_mRNA-p1 TcCLB-NE.510669.30_mRNA-p1 TcSYL_0117600.t1-p1
OG0000891: TCRU_3009 TcCLB-EL.509669.40_mRNA-p1 TcCLB-NE.
509617.89_mRNA-p1 TcCLB-NE.509619.10_mRNA-p1 TcSYL_0001940.t1-p1
OG0000892: TCRU_3062 TcCLB-EL.510159.19_mRNA-p1 TcCLB-EL.
510161.10_mRNA-p1 TcCLB-NE.506459.260_mRNA-p1 TcSYL_0136130.t1-p1
OG0000893: TCRU_3068 TcCLB-EL.504427.64_mRNA-p1 TcCLB-EL.

504427.75_pseudogenic_transcript-p1 TcCLB-NE.509331.74_mRNA-p1
TcSYL_0172450.t1-p1
OG0000894: TCRU_3088 TcCLB-EL.508041.30_mRNA-p1 TcCLB-EL.
509381.20_mRNA-p1 TcCLB-NE.506297.330_mRNA-p1 TcSYL_0001610.t1-p1
OG0000895: TCRU_3092 TcCLB-EL.508041.80_pseudogenic_transcript-p1
TcCLB-EL.510101.10_pseudogenic_transcript-p1 TcCLB-NE.
506297.290_mRNA-p1 TcSYL_0074740.t1-p1
OG0000896: TCRU_3101 TcCLB-EL.510101.140_mRNA-p1 TcCLB-NE.
506297.190_mRNA-p1 TcSYL_0074830.t1-p1 TcSYL_0074860.t1-p1
OG0000897: TCRU_3225 TcCLB-EL.506249.20_mRNA-p1 TcCLB-NE.
508231.230_mRNA-p1 TcCLB-NE.508233.4_mRNA-p1 TcSYL_0108290.t1-p1
OG0000898: TCRU_3228 TcCLB-EL.507041.132_mRNA-p1 TcCLB-NE.
506821.50_mRNA-p1 TcCLB-NE.508233.40_mRNA-p1 TcSYL_0108410.t1-p1
OG0000899: TCRU_3255 TcCLB-EL.506411.39_mRNA-p1 TcCLB-EL.
506413.10_mRNA-p1 TcCLB-NE.511237.40_mRNA-p1 TcSYL_0100140.t1-p1
OG0000900: TCRU_3278 TcCLB-EL.510739.19_mRNA-p1 TcCLB-EL.
510741.10_mRNA-p1 TcCLB-NE.510661.250_mRNA-p1 TcSYL_0116320.t1-p1
OG0000901: TCRU_3333 TcCLB-EL.510105.160_mRNA-p1 TcCLB-NE.
509715.150_mRNA-p1 TcCLB-NE.509717.9_pseudogenic_transcript-p1
TcSYL_0073510.t1-p1
OG0000902: TCRU_3335 TcCLB-EL.510105.140_pseudogenic_transcript-p1
TcCLB-NE.509715.130_mRNA-p1 TcSYL_0073530.t1-p1 TcSYL_0073540.t1-p1
OG0000903: TCRU_3359 TcCLB-EL.507809.170_mRNA-p1 TcCLB-NE.
511657.10_mRNA-p1 TcSYL_0013790.t1-p1 TcSYL_0013810.t1-p1
OG0000904: TCRU_3389 TcCLB-EL.504057.30_mRNA-p1 TcCLB-NE.
481729.10_mRNA-p1 TcSYL_0046180.t1-p1 TcSYL_0046190.t1-p1
OG0000905: TCRU_3391 TcCLB-EL.508047.70_mRNA-p1 TcCLB-EL.
510307.19_pseudogenic_transcript-p1 TcCLB-NE.
506331.139_pseudogenic_transcript-p1 TcCLB-NE.
510643.90_pseudogenic_transcript-p1
OG0000906: TCRU_3435 TcCLB-EL.511181.110_mRNA-p1 TcCLB-NE.
508299.50_mRNA-p1 TcSYL_0203310.t1-p1 TcSYL_0203320.t1-p1
OG0000907: TCRU_3437 TcCLB-EL.511181.100_mRNA-p1 TcCLB-EL.
511527.34_mRNA-p1 TcCLB-NE.508299.60_mRNA-p1 TcSYL_0203300.t1-p1
OG0000908: TCRU_3492 TCRU_9000 TcCLB-NE.424771.10_mRNA-p1 TcCLB-NE.
461879.10_pseudogenic_transcript-p1 TcSYL_0120840.t1-p1
OG0000909: TCRU_3506 TcCLB-EL.503425.20_mRNA-p1 TcCLB-EL.
506893.80_mRNA-p1 TcCLB-NE.508501.230_mRNA-p1 TcSYL_0110330.t1-p1
OG0000910: TCRU_3526 TCRU_7374 TcCLB-NE.510707.10_mRNA-p1 TcCLB-NE.
511223.20_mRNA-p1 TcSYL_0028950.t1-p1
OG0000911: TCRU_3553 TcCLB-EL.507101.10_mRNA-p1 TcCLB-EL.
508923.10_mRNA-p1 TcCLB-EL.511325.40_mRNA-p1 TcCLB-NE.
510245.10_mRNA-p1
OG0000912: TCRU_3648 TcCLB-EL.504153.10_mRNA-p1 TcCLB-EL.
506127.20_mRNA-p1 TcCLB-EL.508271.4_mRNA-p1 TcSYL_0083380.t1-p1
OG0000913: TCRU_3663 TCRU_4334 TCRU_6816 TcSYL_0084070.t1-p1
TcSYL_0141400.t1-p1
OG0000914: TCRU_3697 TcCLB-EL.504103.109_mRNA-p1 TcCLB-EL.
504147.10_mRNA-p1 TcCLB-NE.503847.99_pseudogenic_transcript-p1
TcSYL_0005690.t1-p1
OG0000915: TCRU_3740 TcCLB-EL.506363.70_mRNA-p1 TcCLB-NE.
506975.40_mRNA-p1 TcSYL_0118490.t1-p1 TcSYL_0118550.t1-p1
OG0000916: TCRU_3766 TcCLB-EL.507933.10_mRNA-p1 TcCLB-NE.
509693.20_pseudogenic_transcript-p1 TcSYL_0170580.t1-p1

TcSYL_0170590.t1-p1
OG0000917: TCRU_3777 TcCLB-EL.506457.140_mRNA-p1 TcCLB-NE.
509693.180_mRNA-p1 TcSYL_0170920.t1-p1 TcSYL_0170930.t1-p1
OG0000918: TCRU_3813 TcCLB-EL.504883.30_mRNA-p1 TcCLB-NE.
437999.9_mRNA-p1 TcCLB-NE.506511.10_mRNA-p1 TcSYL_0064990.t1-p1
OG0000919: TCRU_3817 TcCLB-EL.507143.10_mRNA-p1 TcCLB-NE.
503459.20_mRNA-p1 TcCLB-NE.506319.5_mRNA-p1 TcSYL_0050810.t1-p1
OG0000920: TCRU_3821 TcCLB-NE.508827.50_mRNA-p1 TcSYL_0181800.t1-p1
TcSYL_0181810.t1-p1 TcSYL_0181840.t1-p1
OG0000921: TCRU_3832 TcSYL_0016360.t1-p1 TcSYL_0016580.t1-p1
TcSYL_0016680.t1-p1 TcSYL_0016740.t1-p1
OG0000922: TCRU_3898 TcCLB-EL.505555.10_pseudogenic_transcript-p1
TcCLB-NE.510825.30_mRNA-p1 TcSYL_0050230.t1-p1 TcSYL_0050240.t1-p1
OG0000923: TCRU_3903 TcCLB-EL.509381.29_mRNA-p1 TcCLB-NE.
467795.19_mRNA-p1 TcSYL_0001810.t1-p1 TcSYL_0001820.t1-p1
OG0000924: TCRU_3924 TcCLB-EL.506265.60_mRNA-p1 TcCLB-NE.
506435.70_pseudogenic_transcript-p1 TcSYL_0196930.t1-p1
TcSYL_0196960.t1-p1
OG0000925: TCRU_3938 TcCLB-EL.506351.80_mRNA-p1 TcCLB-NE.
505939.20_mRNA-p1 TcSYL_0042740.t1-p1 TcSYL_0042750.t1-p1
OG0000926: TCRU_3976 TcCLB-EL.509297.10_mRNA-p1 TcCLB-NE.
508199.30_mRNA-p1 TcSYL_0146840.t1-p1 TcSYL_0146890.t1-p1
OG0000927: TCRU_3984 TcCLB-EL.504033.140_pseudogenic_transcript-p1
TcCLB-NE.510339.40_mRNA-p1 TcSYL_0147130.t1-p1 TcSYL_0147140.t1-p1
OG0000928: TCRU_4000 TcCLB-EL.503865.80_pseudogenic_transcript-p1
TcCLB-EL.505987.70_mRNA-p1 TcCLB-NE.508695.20_mRNA-p1
TcSYL_0046430.t1-p1
OG0000929: TCRU_4019 TCRU_6394 TcCLB-NE.
510401.50_pseudogenic_transcript-p1 TcSYL_0083880.t1-p1
TcSYL_0166650.t1-p1
OG0000930: TCRU_4028 TcCLB-EL.508121.130_pseudogenic_transcript-p1
TcCLB-EL.511173.120_pseudogenic_transcript-p1 TcCLB-EL.
511173.430_mRNA-p1 TcSYL_0130060.t1-p1
OG0000931: TCRU_4055 TcCLB-EL.509707.20_mRNA-p1 TcCLB-EL.
509707.25_mRNA-p1 TcCLB-NE.508065.60_mRNA-p1 TcSYL_0056870.t1-p1
OG0000932: TCRU_4056 TcCLB-EL.504765.20_mRNA-p1 TcCLB-EL.
509707.10_mRNA-p1 TcCLB-NE.508065.70_mRNA-p1 TcSYL_0056840.t1-p1
OG0000933: TCRU_4062 TcCLB-EL.511753.110_mRNA-p1 TcCLB-EL.
511753.150_mRNA-p1 TcCLB-NE.511501.10_mRNA-p1 TcSYL_0139050.t1-p1
OG0000934: TCRU_4070 TcCLB-EL.506717.240_mRNA-p1 TcCLB-EL.
508085.20_mRNA-p1 TcCLB-EL.508095.10_mRNA-p1 TcCLB-NE.
511807.100_mRNA-p1
OG0000935: TCRU_4073 TcCLB-EL.506683.30_mRNA-p1 TcCLB-EL.
506717.200_mRNA-p1 TcCLB-NE.505101.30_mRNA-p1 TcCLB-NE.
511807.120_mRNA-p1
OG0000936: TCRU_4116 TcCLB-EL.509453.70_mRNA-p1 TcCLB-NE.
503879.119_mRNA-p1 TcCLB-NE.511881.40_mRNA-p1 TcSYL_0145540.t1-p1
OG0000937: TCRU_4117 TcCLB-EL.509453.60_mRNA-p1 TcCLB-NE.
511881.30_mRNA-p1 TcSYL_0145570.t1-p1 TcSYL_0145580.t1-p1
OG0000938: TCRU_4197 TcCLB-EL.506531.20_mRNA-p1 TcCLB-NE.
509399.80_mRNA-p1 TcSYL_0176960.t1-p1 TcSYL_0176970.t1-p1
OG0000939: TCRU_4243 TcCLB-EL.510507.60_mRNA-p1 TcCLB-NE.
509461.90_mRNA-p1 TcSYL_0173980.t1-p1 TcSYL_0173990.t1-p1
OG0000940: TCRU_4263 TcCLB-EL.507839.9_mRNA-p1 TcCLB-EL.

509819.49_mRNA-p1 TcCLB-NE.510635.50_mRNA-p1 TcSYL_0118310.t1-p1
OG0000941: TCRU_4286 TcCLB-EL.506211.30_mRNA-p1 TcCLB-NE.
508895.89_mRNA-p1 TcCLB-NE.511291.10_mRNA-p1 TcSYL_0122950.t1-p1
OG0000942: TCRU_4344 TcCLB-EL.504053.10_pseudogenic_transcript-p1
TcCLB-EL.507939.70_mRNA-p1 TcCLB-NE.508119.200_mRNA-p1
TcSYL_0103970.t1-p1
OG0000943: TCRU_4346 TcCLB-EL.442629.9_mRNA-p1 TcCLB-NE.
474339.9_mRNA-p1 TcCLB-NE.507945.30_mRNA-p1 TcSYL_0110780.t1-p1
OG0000944: TCRU_4352 TcCLB-EL.509167.140_mRNA-p1 TcCLB-NE.
485683.10_mRNA-p1 TcCLB-NE.504257.9_mRNA-p1 TcSYL_0140300.t1-p1
OG0000945: TCRU_4371 TcCLB-EL.510347.20_mRNA-p1 TcCLB-NE.
503925.3_mRNA-p1 TcCLB-NE.506469.10_mRNA-p1 TcSYL_0156750.t1-p1
OG0000946: TCRU_4396 TcCLB-EL.506941.194_mRNA-p1 TcCLB-NE.
507027.30_pseudogenic_transcript-p1 TcCLB-NE.511513.30_mRNA-p1
TcSYL_0139620.t1-p1
OG0000947: TCRU_4401 TcCLB-EL.507527.30_mRNA-p1 TcCLB-NE.
435519.10_mRNA-p1 TcSYL_0181350.t1-p1 TcSYL_0181360.t1-p1
OG0000948: TCRU_4403 TcCLB-EL.507527.10_mRNA-p1 TcCLB-NE.
503935.4_pseudogenic_transcript-p1 TcCLB-NE.506935.19_mRNA-p1
TcSYL_0181390.t1-p1
OG0000949: TCRU_4406 TcCLB-EL.506025.50_mRNA-p1 TcCLB-NE.
511209.10_mRNA-p1 TcSYL_0181420.t1-p1 TcSYL_0181430.t1-p1
OG0000950: TCRU_4537 TcCLB-EL.403153.10_pseudogenic_transcript-p1
TcCLB-EL.506591.120_pseudogenic_transcript-p1 TcSYL_0003700.t1-p1
TcSYL_0061990.t1-p1
OG0000951: TCRU_4538 TcSYL_0107750.t1-p1 TcSYL_0107810.t1-p1
TcSYL_0107880.t1-p1 TcSYL_0107890.t1-p1
OG0000952: TCRU_10024 TCRU_4570 TCRU_8375 TCRU_9677
TcSYL_0126420.t1-p1
OG0000953: TCRU_4577 TcCLB-EL.506745.20_mRNA-p1 TcCLB-NE.
509111.10_mRNA-p1 TcCLB-NE.511925.50_mRNA-p1 TcSYL_0164650.t1-p1
OG0000954: TCRU_4585 TcCLB-EL.506743.160_mRNA-p1 TcCLB-NE.
503473.10_mRNA-p1 TcSYL_0164920.t1-p1 TcSYL_0164930.t1-p1
OG0000955: TCRU_4612 TCRU_6894 TCRU_8045 TcCLB-EL.509267.3_mRNA-p1
TcSYL_0107830.t1-p1
OG0000956: TCRU_4668 TcCLB-EL.506979.5_pseudogenic_transcript-p1
TcCLB-EL.511621.20_mRNA-p1 TcCLB-NE.509317.100_mRNA-p1
TcSYL_0084840.t1-p1
OG0000957: TCRU_4884 TcCLB-EL.510073.90_mRNA-p1 TcCLB-NE.
509399.130_mRNA-p1 TcSYL_0177310.t1-p1 TcSYL_0177320.t1-p1
OG0000958: TCRU_4913 TcCLB-EL.398751.10_mRNA-p1 TcCLB-EL.
511527.9_mRNA-p1 TcCLB-NE.508525.60_mRNA-p1 TcSYL_0112330.t1-p1
OG0000959: TCRU_4969 TcCLB-EL.503671.10_mRNA-p1 TcCLB-EL.
510325.69_mRNA-p1 TcCLB-NE.508153.60_mRNA-p1 TcSYL_0001120.t1-p1
OG0000960: TCRU_4981 TCRU_8103 TcCLB-EL.
508039.10_pseudogenic_transcript-p1 TcCLB-NE.511773.110_mRNA-p1
TcSYL_0163800.t1-p1
OG0000961: TCRU_5025 TCRU_6107 TcCLB-EL.511467.30_mRNA-p1 TcCLB-NE.
506247.190_mRNA-p1 TcSYL_0108830.t1-p1
OG0000962: TCRU_5105 TcCLB-EL.503557.10_mRNA-p1 TcCLB-EL.
508465.149_mRNA-p1 TcCLB-NE.507001.40_mRNA-p1 TcSYL_0115960.t1-p1
OG0000963: TCRU_10559 TCRU_5169 TcCLB-EL.511145.50_mRNA-p1 TcCLB-NE.
504069.70_mRNA-p1 TcSYL_0040160.t1-p1
OG0000964: TCRU_5179 TcCLB-NE.506783.10_pseudogenic_transcript-p1

TcSYL_0017910.t1-p1 TcSYL_0018630.t1-p1 TcSYL_0099720.t1-p1
OG0000965: TCRU_5221 TcCLB-EL.504039.250_pseudogenic_transcript-p1 TcCLB-EL.505025.110_pseudogenic_transcript-p1 TcCLB-EL.505025.20_pseudogenic_transcript-p1 TcCLB-NE.508871.30_pseudogenic_transcript-p1
OG0000966: TCRU_5225 TCRU_9298 TCRU_9442 TcSYL_0153790.t1-p1 TcSYL_0187470.t1-p1
OG0000967: TCRU_5242 TcCLB-EL.511075.9_mRNA-p1 TcCLB-NE.440101.10_mRNA-p1 TcCLB-NE.509239.10_mRNA-p1 TcSYL_0103620.t1-p1
OG0000968: TCRU_5255 TcCLB-EL.509583.19_mRNA-p1 TcCLB-EL.509585.10_mRNA-p1 TcSYL_0089090.t1-p1 TcSYL_0089120.t1-p1
OG0000969: TCRU_5322 TcCLB-EL.503971.59_mRNA-p1 TcCLB-EL.507841.40_mRNA-p1 TcCLB-NE.508269.50_mRNA-p1 TcSYL_0121290.t1-p1
OG0000970: TCRU_5447 TCRU_6331 TcCLB-NE.507085.150_mRNA-p1 TcCLB-NE.508499.20_mRNA-p1 TcSYL_0109980.t1-p1
OG0000971: TCRU_5469 TcCLB-EL.503579.50_mRNA-p1 TcCLB-NE.507517.40_mRNA-p1 TcSYL_0022860.t1-p1 TcSYL_0022870.t1-p1
OG0000972: TCRU_5476 TcCLB-EL.508209.70_mRNA-p1 TcCLB-NE.503919.4_mRNA-p1 TcCLB-NE.509505.89_mRNA-p1 TcSYL_0022900.t1-p1
OG0000973: TCRU_5566 TcCLB-EL.507811.30_mRNA-p1 TcCLB-NE.511649.169_mRNA-p1 TcCLB-NE.511651.10_mRNA-p1 TcSYL_0013980.t1-p1
OG0000974: TCRU_5573 TCRU_8788 TcCLB-EL.509895.60_mRNA-p1 TcCLB-NE.460747.30_mRNA-p1 TcCLB-NE.510009.19_mRNA-p1
OG0000975: TCRU_5593 TCRU_6069 TcCLB-NE.511809.120_mRNA-p1 TcCLB-NE.511811.30_mRNA-p1 TcSYL_0201730.t1-p1
OG0000976: TCRU_5619 TcCLB-EL.506221.60_pseudogenic_transcript-p1 TcCLB-NE.427789.10_mRNA-p1 TcCLB-NE.504217.4_mRNA-p1 TcSYL_0141770.t1-p1
OG0000977: TCRU_5643 TcCLB-EL.511439.80_mRNA-p1 TcCLB-NE.504259.19_mRNA-p1 TcSYL_0031470.t1-p1 TcSYL_0031480.t1-p1
OG0000978: TCRU_5661 TcCLB-EL.508771.10_mRNA-p1 TcCLB-EL.508771.20_mRNA-p1 TcCLB-NE.403875.10_mRNA-p1 TcSYL_0086730.t1-p1
OG0000979: TCRU_5674 TcCLB-EL.506591.130_mRNA-p1 TcCLB-NE.506989.309_mRNA-p1 TcSYL_0003610.t1-p1 TcSYL_0003670.t1-p1
OG0000980: TCRU_5703 TcCLB-EL.509967.20_mRNA-p1 TcCLB-EL.511911.30_pseudogenic_transcript-p1 TcCLB-NE.507233.110_mRNA-p1 TcSYL_0171470.t1-p1
OG0000981: TCRU_5709 TCRU_6104 TcCLB-EL.507641.233_mRNA-p1 TcCLB-EL.511145.46_mRNA-p1 TcCLB-NE.504069.56_mRNA-p1
OG0000982: TCRU_5720 TcCLB-EL.510533.20_mRNA-p1 TcCLB-NE.511821.20_mRNA-p1 TcSYL_0201370.t1-p1 TcSYL_0201380.t1-p1
OG0000983: TCRU_5731 TcCLB-EL.503643.20_mRNA-p1 TcCLB-NE.442495.10_mRNA-p1 TcCLB-NE.506485.109_mRNA-p1 TcSYL_0003390.t1-p1
OG0000984: TCRU_5769 TcCLB-EL.504133.45_mRNA-p1 TcCLB-EL.509247.60_pseudogenic_transcript-p1 TcCLB-NE.509691.10_mRNA-p1 TcSYL_0170480.t1-p1
OG0000985: TCRU_5784 TcCLB-EL.503561.20_mRNA-p1 TcCLB-EL.511625.4_mRNA-p1 TcCLB-NE.504741.70_mRNA-p1 TcSYL_0084310.t1-p1
OG0000986: TCRU_5788 TcCLB-EL.509067.60_mRNA-p1 TcCLB-NE.508711.10_mRNA-p1 TcCLB-NE.511023.19_mRNA-p1 TcSYL_0111760.t1-p1
OG0000987: TCRU_5792 TcCLB-EL.510027.10_mRNA-p1 TcCLB-NE.508897.10_mRNA-p1 TcSYL_0122220.t1-p1 TcSYL_0122240.t1-p1
OG0000988: TCRU_5809 TcCLB-EL.510167.20_mRNA-p1 TcCLB-NE.508859.100_mRNA-p1 TcSYL_0025470.t1-p1 TcSYL_0025480.t1-p1

OG0000989: TCRU_5822 TcCLB-EL.504423.20_mRNA-p1 TcCLB-NE.
507569.4_mRNA-p1 TcCLB-NE.509535.9_mRNA-p1 TcSYL_0104640.t1-p1
OG0000990: TCRU_5851 TcCLB-EL.510765.30_mRNA-p1 TcCLB-NE.
509671.90_mRNA-p1 TcSYL_0074470.t1-p1 TcSYL_0115340.t1-p1
OG0000991: TCRU_5869 TcCLB-EL.506513.170_pseudogenic_transcript-p1
TcCLB-NE.508915.9_mRNA-p1 TcCLB-NE.508917.4_mRNA-p1
TcSYL_0158560.t1-p1
OG0000992: TCRU_5872 TCRU_8706 TcCLB-EL.510053.60_mRNA-p1 TcCLB-NE.
511321.23_mRNA-p1 TcSYL_0159050.t1-p1
OG0000993: TCRU_5881 TcCLB-EL.482097.10_mRNA-p1 TcCLB-EL.
504047.70_mRNA-p1 TcCLB-NE.507081.139_mRNA-p1 TcSYL_0019620.t1-p1
OG0000994: TCRU_5892 TcCLB-EL.510737.30_mRNA-p1 TcCLB-NE.
510663.64_mRNA-p1 TcSYL_0116510.t1-p1 TcSYL_0116520.t1-p1
OG0000995: TCRU_5914 TcCLB-EL.509203.10_mRNA-p1 TcCLB-EL.
511805.29_mRNA-p1 TcCLB-NE.511017.20_mRNA-p1 TcSYL_0180350.t1-p1
OG0000996: TCRU_6003 TcCLB-EL.504151.39_mRNA-p1 TcCLB-NE.
509461.30_mRNA-p1 TcSYL_0174210.t1-p1 TcSYL_0174220.t1-p1
OG0000997: TCRU_6032 TcCLB-EL.504137.10_mRNA-p1 TcCLB-NE.
509797.40_mRNA-p1 TcSYL_0106950.t1-p1 TcSYL_0106960.t1-p1
OG0000998: TCRU_6050 TcCLB-EL.509455.100_mRNA-p1 TcCLB-NE.
503539.10_mRNA-p1 TcCLB-NE.503877.19_mRNA-p1 TcSYL_0145200.t1-p1
OG0000999: TCRU_6071 TcCLB-EL.504247.70_pseudogenic_transcript-p1
TcCLB-NE.509991.70_pseudogenic_transcript-p1 TcSYL_0110760.t1-p1
TcSYL_0110770.t1-p1
OG0001000: TCRU_6074 TcCLB-EL.507035.110_mRNA-p1 TcCLB-NE.
508523.80_mRNA-p1 TcCLB-NE.508525.5_pseudogenic_transcript-p1
TcSYL_0112360.t1-p1
OG0001001: TCRU_6111 TcCLB-EL.508387.120_mRNA-p1 TcCLB-NE.
506789.180_mRNA-p1 TcSYL_0162580.t1-p1 TcSYL_0162600.t1-p1
OG0001002: TCRU_6141 TcCLB-EL.509455.120_mRNA-p1 TcCLB-NE.
439125.10_mRNA-p1 TcCLB-NE.506711.4_mRNA-p1 TcSYL_0144280.t1-p1
OG0001003: TCRU_6149 TcCLB-EL.507089.280_pseudogenic_transcript-p1
TcCLB-EL.511531.9_mRNA-p1 TcCLB-NE.511025.100_mRNA-p1
TcSYL_0111880.t1-p1
OG0001004: TCRU_6266 TcCLB-EL.503799.4_mRNA-p1 TcCLB-EL.
506165.16_mRNA-p1 TcCLB-NE.508817.80_mRNA-p1 TcSYL_0043650.t1-p1
OG0001005: TCRU_6267 TcCLB-EL.503801.9_mRNA-p1 TcCLB-NE.
508817.70_mRNA-p1 TcSYL_0043690.t1-p1 TcSYL_0043710.t1-p1
OG0001006: TCRU_6289 TcCLB-EL.503611.30_mRNA-p1 TcCLB-NE.
506679.240_mRNA-p1 TcSYL_0114150.t1-p1 TcSYL_0114210.t1-p1
OG0001007: TCRU_6323 TcCLB-EL.503875.40_mRNA-p1 TcSYL_0058640.t1-p1
TcSYL_0125460.t1-p1 TcSYL_0133870.t1-p1
OG0001008: TCRU_6386 TCRU_9429 TcCLB-EL.509955.50_mRNA-p1 TcCLB-NE.
508139.150_mRNA-p1 TcCLB-NE.511767.100_mRNA-p1
OG0001009: TCRU_6402 TCRU_6446 TcCLB-EL.509233.190_mRNA-p1 TcCLB-NE.
504105.94_mRNA-p1 TcSYL_0145250.t1-p1
OG0001010: TCRU_6471 TCRU_6480 TcCLB-EL.511577.70_mRNA-p1 TcCLB-NE.
504797.60_mRNA-p1 TcSYL_0109560.t1-p1
OG0001011: TCRU_6472 TCRU_6479 TcCLB-EL.511577.80_mRNA-p1 TcCLB-NE.
504797.50_mRNA-p1 TcSYL_0109550.t1-p1
OG0001012: TCRU_6473 TCRU_6478 TcCLB-EL.511577.83_mRNA-p1 TcCLB-NE.
504797.40_mRNA-p1 TcSYL_0109540.t1-p1
OG0001013: TCRU_6525 TCRU_7293 TCRU_9039 TCRU_9056 TcSYL_0017330.t1-
p1

OG0001014: TCRU_6534 TcCLB-EL.511619.5_mRNA-p1 TcCLB-NE.
507207.19_mRNA-p1 TcSYL_0084900.t1-p1 TcSYL_0084910.t1-p1
OG0001015: TCRU_6535 TcCLB-EL.506979.59_mRNA-p1 TcCLB-EL.
511619.10_mRNA-p1 TcCLB-NE.507207.10_mRNA-p1 TcSYL_0084890.t1-p1
OG0001016: TCRU_6537 TcCLB-EL.507237.250_pseudogenic_transcript-p1
TcCLB-NE.507205.10_pseudogenic_transcript-p1 TcCLB-NE.
509527.154_pseudogenic_transcript-p1 TcSYL_0153240.t1-p1
OG0001017: TCRU_6557 TcSYL_0071450.t1-p1 TcSYL_0151790.t1-p1
TcSYL_0152870.t1-p1 TcSYL_0153410.t1-p1
OG0001018: TCRU_6608 TcCLB-EL.507875.140_pseudogenic_transcript-p1
TcCLB-NE.503591.10_pseudogenic_transcript-p1 TcCLB-NE.
509717.176_mRNA-p1 TcCLB-NE.511845.10_mRNA-p1
OG0001019: TCRU_6645 TcCLB-EL.506937.40_mRNA-p1 TcCLB-EL.
508829.9_mRNA-p1 TcCLB-NE.510437.10_mRNA-p1 TcSYL_0174740.t1-p1
OG0001020: TCRU_6659 TcCLB-EL.510757.30_mRNA-p1 TcCLB-EL.
510759.4_mRNA-p1 TcCLB-NE.508413.10_mRNA-p1 TcSYL_0115540.t1-p1
OG0001021: TCRU_6660 TcCLB-EL.510759.10_mRNA-p1 TcCLB-EL.
510763.30_mRNA-p1 TcCLB-NE.506999.191_mRNA-p1 TcCLB-NE.
508413.5_mRNA-p1
OG0001022: TCRU_6690 TcCLB-EL.504213.110_mRNA-p1 TcCLB-NE.
506985.40_mRNA-p1 TcSYL_0026440.t1-p1 TcSYL_0026450.t1-p1
OG0001023: TCRU_6717 TcCLB-EL.458153.10_mRNA-p1 TcCLB-EL.
506465.50_mRNA-p1 TcCLB-NE.505193.10_mRNA-p1 TcSYL_0104360.t1-p1
OG0001024: TCRU_6752 TcCLB-EL.510253.29_mRNA-p1 TcCLB-EL.
510255.4_mRNA-p1 TcCLB-NE.509213.10_mRNA-p1 TcSYL_0114890.t1-p1
OG0001025: TCRU_6785 TcCLB-EL.503759.59_mRNA-p1 TcCLB-NE.
510423.40_mRNA-p1 TcSYL_0121140.t1-p1 TcSYL_0121150.t1-p1
OG0001026: TCRU_6788 TcCLB-EL.509825.20_mRNA-p1 TcCLB-NE.
510423.70_mRNA-p1 TcSYL_0118250.t1-p1 TcSYL_0121210.t1-p1
OG0001027: TCRU_6828 TcCLB-EL.510955.40_mRNA-p1 TcCLB-NE.
503869.9_mRNA-p1 TcCLB-NE.509005.10_mRNA-p1 TcSYL_0063420.t1-p1
OG0001028: TCRU_6920 TCRU_6921 TcCLB-EL.511127.260_mRNA-p1 TcCLB-NE.
509025.29_mRNA-p1 TcCLB-NE.509027.10_mRNA-p1
OG0001029: TCRU_6932 TcCLB-EL.510381.40_mRNA-p1 TcCLB-NE.
506905.20_mRNA-p1 TcSYL_0076440.t1-p1 TcSYL_0076450.t1-p1
OG0001030: TCRU_6979 TcCLB-EL.509599.20_mRNA-p1 TcCLB-NE.
509733.90_mRNA-p1 TcSYL_0010770.t1-p1 TcSYL_0010790.t1-p1
OG0001031: TCRU_7045 TcCLB-EL.506513.110_mRNA-p1 TcCLB-NE.
508917.49_mRNA-p1 TcCLB-NE.508919.10_pseudogenic_transcript-p1
TcSYL_0158330.t1-p1
OG0001032: TCRU_7070 TcCLB-EL.508207.194_mRNA-p1 TcCLB-NE.
507519.10_pseudogenic_transcript-p1 TcCLB-NE.509509.69_mRNA-p1
TcSYL_0022720.t1-p1
OG0001033: TCRU_7078 TcCLB-EL.508207.100_mRNA-p1 TcCLB-EL.
508207.110_mRNA-p1 TcCLB-NE.507519.110_mRNA-p1 TcSYL_0022670.t1-p1
OG0001034: TCRU_7098 TcCLB-EL.511217.10_mRNA-p1 TcCLB-NE.
511727.240_mRNA-p1 TcSYL_0188660.t1-p1 TcSYL_0188670.t1-p1
OG0001035: TCRU_7106 TCRU_9529 TcCLB-NE.511825.110_mRNA-p1
TcSYL_0156230.t1-p1 TcSYL_0171640.t1-p1
OG0001036: TCRU_7117 TcCLB-EL.503971.5_mRNA-p1 TcCLB-EL.
509823.4_mRNA-p1 TcCLB-NE.503997.10_mRNA-p1 TcCLB-NE.506807.39_mRNA-
p1
OG0001037: TCRU_7118 TcCLB-EL.466823.10_mRNA-p1 TcCLB-NE.
507589.30_mRNA-p1 TcSYL_0040400.t1-p1 TcSYL_0157370.t1-p1

OG0001038: TCRU_7157 TcCLB-EL.508973.9_mRNA-p1 TcCLB-NE.
508547.150_mRNA-p1 TcSYL_0202470.t1-p1 TcSYL_0202490.t1-p1
OG0001039: TCRU_7170 TcCLB-EL.511303.40_mRNA-p1 TcCLB-NE.
509179.10_pseudogenic_transcript-p1 TcCLB-NE.511763.19_mRNA-p1
TcSYL_0159900.t1-p1
OG0001040: TCRU_7173 TCRU_7186 TcCLB-EL.510275.250_mRNA-p1 TcCLB-EL.
510275.340_pseudogenic_transcript-p1 TcCLB-NE.
510197.160_pseudogenic_transcript-p1
OG0001041: TCRU_7181 TcCLB-NE.510687.149_mRNA-p1 TcCLB-NE.
510689.10_pseudogenic_transcript-p1 TcSYL_0047170.t1-p1
TcSYL_0047190.t1-p1
OG0001042: TCRU_7203 TCRU_7204 TCRU_7205 TcCLB-NE.508461.500_mRNA-p1
TcCLB-NE.508461.510_mRNA-p1
OG0001043: TCRU_7256 TCRU_7257 TcCLB-EL.503669.10_mRNA-p1 TcCLB-NE.
508459.9_mRNA-p1 TcCLB-NE.510731.130_mRNA-p1
OG0001044: TCRU_7311 TcCLB-NE.504249.30_mRNA-p1 TcCLB-NE.
510047.100_pseudogenic_transcript-p1 TcSYL_0051670.t1-p1
TcSYL_0066270.t1-p1
OG0001045: TCRU_7369 TCRU_7370 TcSYL_0074620.t1-p1 TcSYL_0129950.t1-
p1 TcSYL_0156140.t1-p1
OG0001046: TCRU_7396 TcCLB-EL.510517.50_mRNA-p1 TcCLB-NE.
504003.14_mRNA-p1 TcCLB-NE.507851.10_mRNA-p1 TcSYL_0174640.t1-p1
OG0001047: TCRU_7464 TcCLB-EL.505807.270_mRNA-p1 TcCLB-NE.
507017.120_mRNA-p1 TcSYL_0166960.t1-p1 TcSYL_0166970.t1-p1
OG0001048: TCRU_7477 TcCLB-EL.505807.130_mRNA-p1 TcCLB-NE.
503815.29_mRNA-p1 TcCLB-NE.507017.4_mRNA-p1 TcSYL_0166890.t1-p1
OG0001049: TCRU_7524 TCRU_7525 TcCLB-NE.507681.150_mRNA-p1 TcCLB-NE.
507681.160_mRNA-p1 TcSYL_0073760.t1-p1
OG0001050: TCRU_7543 TcCLB-EL.511303.110_mRNA-p1 TcCLB-NE.
509175.18_mRNA-p1 TcCLB-NE.509177.10_mRNA-p1 TcSYL_0159820.t1-p1
OG0001051: TCRU_7563 TCRU_8701 TcCLB-EL.507559.80_mRNA-p1 TcCLB-NE.
510303.50_mRNA-p1 TcSYL_0079230.t1-p1
OG0001052: TCRU_7590 TcCLB-EL.445849.10_mRNA-p1 TcCLB-EL.
506221.129_mRNA-p1 TcCLB-NE.511717.50_mRNA-p1 TcSYL_0141800.t1-p1
OG0001053: TCRU_7618 TcCLB-EL.503841.20_mRNA-p1 TcCLB-NE.
511463.20_mRNA-p1 TcSYL_0131350.t1-p1 TcSYL_0131360.t1-p1
OG0001054: TCRU_7656 TcCLB-EL.426397.9_mRNA-p1 TcCLB-NE.
509695.150_mRNA-p1 TcSYL_0171140.t1-p1 TcSYL_0171150.t1-p1
OG0001055: TCRU_7664 TcCLB-EL.508075.7_mRNA-p1 TcCLB-EL.
510143.129_mRNA-p1 TcCLB-NE.508409.320_mRNA-p1 TcSYL_0004270.t1-p1
OG0001056: TCRU_7732 TcCLB-EL.508217.9_mRNA-p1 TcCLB-NE.
508879.30_mRNA-p1 TcSYL_0023180.t1-p1 TcSYL_0023200.t1-p1
OG0001057: TCRU_7781 TcCLB-EL.511071.160_mRNA-p1 TcCLB-NE.
504037.40_mRNA-p1 TcCLB-NE.511899.20_mRNA-p1 TcSYL_0103600.t1-p1
OG0001058: TCRU_7791 TcCLB-EL.511069.10_mRNA-p1 TcCLB-EL.
511069.20_mRNA-p1 TcCLB-NE.511903.110_mRNA-p1 TcSYL_0103380.t1-p1
OG0001059: TCRU_7838 TcCLB-EL.420293.31_mRNA-p1 TcCLB-EL.
510591.20_mRNA-p1 TcCLB-NE.410923.30_mRNA-p1 TcSYL_0039130.t1-p1
OG0001060: TCRU_7895 TcCLB-EL.510275.10_mRNA-p1 TcCLB-EL.
510275.90_mRNA-p1 TcCLB-EL.510279.240_mRNA-p1 TcCLB-NE.
503973.10_mRNA-p1
OG0001061: TCRU_7933 TcCLB-EL.505965.50_mRNA-p1 TcCLB-NE.
510903.50_mRNA-p1 TcSYL_0178560.t1-p1 TcSYL_0178570.t1-p1
OG0001062: TCRU_7948 TcCLB-EL.506945.10_mRNA-p1 TcCLB-EL.

511729.9_mRNA-p1 TcCLB-NE.506835.60_mRNA-p1 TcSYL_0140540.t1-p1
OG0001063: TCRU_8032 TcCLB-EL.507507.49_mRNA-p1 TcCLB-EL.
507509.10_pseudogenic_transcript-p1 TcCLB-NE.
506871.60_pseudogenic_transcript-p1 TcSYL_0142560.t1-p1
OG0001064: TCRU_8082 TcCLB-EL.504427.270_mRNA-p1 TcCLB-NE.
508045.4_mRNA-p1 TcSYL_0173280.t1-p1 TcSYL_0173300.t1-p1
OG0001065: TCRU_8115 TcCLB-EL.503887.10_mRNA-p1 TcCLB-EL.
510431.340_mRNA-p1 TcCLB-NE.507771.90_mRNA-p1 TcSYL_0138370.t1-p1
OG0001066: TCRU_8185 TcCLB-EL.507795.20_mRNA-p1 TcCLB-NE.
508407.4_mRNA-p1 TcCLB-NE.510647.69_mRNA-p1 TcSYL_0004590.t1-p1
OG0001067: TCRU_8203 TcCLB-EL.511047.50_mRNA-p1 TcCLB-NE.
511859.180_mRNA-p1 TcSYL_0090780.t1-p1 TcSYL_0091070.t1-p1
OG0001068: TCRU_8285 TcCLB-EL.509595.44_mRNA-p1 TcCLB-NE.
508819.10_mRNA-p1 TcSYL_0043330.t1-p1 TcSYL_0043340.t1-p1
OG0001069: TCRU_8295 TcCLB-NE.506331.80_pseudogenic_transcript-p1
TcCLB-NE.507643.15_mRNA-p1 TcCLB-NE.
509929.21_pseudogenic_transcript-p1 TcSYL_0190820.t1-p1
OG0001070: TCRU_10597 TCRU_8349 TcCLB-NE.
479961.20_pseudogenic_transcript-p1 TcSYL_0082380.t1-p1
TcSYL_0172930.t1-p1
OG0001071: TCRU_8370 TcCLB-EL.510285.70_mRNA-p1 TcCLB-NE.
503823.169_mRNA-p1 TcCLB-NE.504827.170_mRNA-p1 TcSYL_0117690.t1-p1
OG0001072: TCRU_8430 TcCLB-EL.506829.39_mRNA-p1 TcCLB-NE.
506593.19_mRNA-p1 TcCLB-NE.506593.30_mRNA-p1 TcSYL_0109440.t1-p1
OG0001073: TCRU_8493 TcCLB-EL.511439.40_mRNA-p1 TcCLB-NE.
510679.40_mRNA-p1 TcSYL_0031250.t1-p1 TcSYL_0031350.t1-p1
OG0001074: TCRU_8595 TcCLB-EL.503885.30_pseudogenic_transcript-p1
TcCLB-NE.511903.230_mRNA-p1 TcSYL_0102940.t1-p1 TcSYL_0102950.t1-p1
OG0001075: TCRU_WW TcCLB-EL.511065.36_mRNA-p1 TcCLB-NE.
511903.170_mRNA-p1 TcSYL_0103130.t1-p1 TcSYL_0103140.t1-p1
OG0001076: TCRU_8601 TcCLB-EL.511065.50_mRNA-p1 TcCLB-EL.
511067.4_mRNA-p1 TcCLB-NE.511903.160_mRNA-p1 TcSYL_0103220.t1-p1
OG0001077: TCRU_8658 TcCLB-EL.509099.30_mRNA-p1 TcCLB-NE.
509319.109_mRNA-p1 TcSYL_0085460.t1-p1 TcSYL_0085480.t1-p1
OG0001078: TCRU_8682 TcCLB-EL.508463.20_mRNA-p1 TcCLB-NE.
510659.140_pseudogenic_transcript-p1 TcSYL_0116140.t1-p1
TcSYL_0116150.t1-p1
OG0001079: TCRU_8692 TcCLB-EL.508471.19_mRNA-p1 TcCLB-EL.
510769.10_mRNA-p1 TcCLB-NE.509671.10_mRNA-p1 TcSYL_0115210.t1-p1
OG0001080: TCRU_8761 TcCLB-EL.509473.10_pseudogenic_transcript-p1
TcCLB-NE.509555.10_mRNA-p1 TcSYL_0095810.t1-p1 TcSYL_0095820.t1-p1
OG0001081: TCRU_8814 TcCLB-EL.504203.4_mRNA-p1 TcCLB-EL.
510289.99_mRNA-p1 TcCLB-NE.510969.40_mRNA-p1 TcSYL_0113390.t1-p1
OG0001082: TCRU_8835 TcCLB-EL.507795.10_mRNA-p1 TcCLB-NE.
508407.10_mRNA-p1 TcCLB-NE.510649.24_mRNA-p1 TcSYL_0004580.t1-p1
OG0001083: TCRU_8842 TcCLB-EL.503881.50_mRNA-p1 TcCLB-EL.
507939.14_mRNA-p1 TcCLB-NE.508119.150_mRNA-p1 TcSYL_0103940.t1-p1
OG0001084: TCRU_8861 TcCLB-EL.506829.100_mRNA-p1 TcCLB-NE.
508079.20_pseudogenic_transcript-p1 TcCLB-NE.510151.10_mRNA-p1
TcSYL_0109410.t1-p1
OG0001085: TCRU_8878 TcCLB-EL.510309.20_mRNA-p1 TcCLB-NE.
503505.20_mRNA-p1 TcCLB-NE.506669.4_mRNA-p1 TcSYL_0000470.t1-p1
OG0001086: TCRU_8982 TcCLB-EL.507037.89_mRNA-p1 TcCLB-EL.
511543.10_mRNA-p1 TcCLB-NE.508707.30_mRNA-p1 TcSYL_0112540.t1-p1

OG0001087: TCRU_8991 TcCLB-EL.509979.379_mRNA-p1 TcCLB-NE.
508119.100_mRNA-p1 TcSYL_0103810.t1-p1 TcSYL_0156180.t1-p1
OG0001088: TCRU_9076 TcCLB-EL.508717.50_mRNA-p1 TcCLB-NE.
504079.20_mRNA-p1 TcCLB-NE.506841.4_mRNA-p1 TcSYL_0106850.t1-p1
OG0001089: TCRU_9113 TcCLB-EL.511043.20_mRNA-p1 TcCLB-NE.
506355.60_mRNA-p1 TcSYL_0107730.t1-p1 TcSYL_0107780.t1-p1
OG0001090: TCRU_9143 TcCLB-EL.508347.40_mRNA-p1 TcCLB-NE.
506145.10_mRNA-p1 TcSYL_0105290.t1-p1 TcSYL_0105320.t1-p1
OG0001091: TCRU_9155 TcCLB-EL.503601.10_mRNA-p1 TcCLB-EL.
511197.10_mRNA-p1 TcCLB-NE.510979.40_mRNA-p1 TcSYL_0086850.t1-p1
OG0001092: TCRU_9168 TcCLB-NE.464879.9_mRNA-p1 TcCLB-NE.
508637.150_mRNA-p1 TcCLB-NE.511913.9_pseudogenic_transcript-p1
TcSYL_0170380.t1-p1
OG0001093: TCRU_9171 TcCLB-EL.511803.10_mRNA-p1 TcCLB-NE.
510897.10_mRNA-p1 TcSYL_0180000.t1-p1 TcSYL_0180020.t1-p1
OG0001094: TCRU_9197 TcCLB-EL.508383.10_mRNA-p1 TcCLB-EL.
510611.79_mRNA-p1 TcCLB-NE.506791.50_mRNA-p1 TcSYL_0163560.t1-p1
OG0001095: TCRU_9220 TcCLB-EL.510029.40_mRNA-p1 TcCLB-NE.
511291.120_mRNA-p1 TcSYL_0122510.t1-p1 TcSYL_0122730.t1-p1
OG0001096: TCRU_9239 TcCLB-EL.506503.140_mRNA-p1 TcCLB-NE.
507033.29_mRNA-p1 TcCLB-NE.511293.10_mRNA-p1 TcSYL_0122340.t1-p1
OG0001097: TCRU_9269 TcCLB-EL.511581.10_mRNA-p1 TcCLB-NE.
504177.10_mRNA-p1 TcSYL_0109360.t1-p1 TcSYL_0109390.t1-p1
OG0001098: TCRU_9314 TcCLB-EL.506743.69_mRNA-p1 TcCLB-EL.
510603.119_mRNA-p1 TcCLB-NE.509109.90_mRNA-p1 TcSYL_0014310.t1-p1
OG0001099: TCRU_9319 TcCLB-EL.506579.90_mRNA-p1 TcCLB-NE.
506509.50_mRNA-p1 TcSYL_0064850.t1-p1 TcSYL_0064860.t1-p1
OG0001100: TCRU_9452 TcCLB-EL.503753.29_mRNA-p1 TcCLB-NE.
506585.70_mRNA-p1 TcSYL_0044620.t1-p1 TcSYL_0044630.t1-p1
OG0001101: TCRU_9471 TcCLB-EL.503639.10_mRNA-p1 TcCLB-EL.
510181.9_mRNA-p1 TcCLB-NE.507609.60_mRNA-p1 TcSYL_0013480.t1-p1
OG0001102: TCRU_9522 TcCLB-EL.506563.210_mRNA-p1 TcCLB-NE.
508999.200_mRNA-p1 TcSYL_0063540.t1-p1 TcSYL_0110540.t1-p1
OG0001103: TCRU_9524 TcCLB-EL.511393.30_mRNA-p1 TcCLB-NE.
503565.10_mRNA-p1 TcSYL_0202050.t1-p1 TcSYL_0202060.t1-p1
OG0001104: TCRU_9564 TcCLB-EL.506715.59_mRNA-p1 TcCLB-EL.
510341.4_mRNA-p1 TcCLB-NE.506469.120_mRNA-p1 TcSYL_0156920.t1-p1
OG0001105: TCRU_9610 TcCLB-EL.507929.20_mRNA-p1 TcCLB-NE.
509045.20_mRNA-p1 TcSYL_0167460.t1-p1 TcSYL_0168720.t1-p1
OG0001106: TCRU_9707 TcCLB-NE.508595.40_mRNA-p1 TcCLB-NE.
510889.261_mRNA-p1 TcSYL_0175920.t1-p1 TcSYL_0176200.t1-p1
OG0001107: TCRU_9731 TcCLB-EL.504047.5_mRNA-p1 TcCLB-NE.
504045.110_mRNA-p1 TcSYL_0019530.t1-p1 TcSYL_0019540.t1-p1
OG0001108: TCRU_9851 TcCLB-EL.508675.60_mRNA-p1 TcCLB-NE.
506983.10_mRNA-p1 TcCLB-NE.508865.2_mRNA-p1 TcSYL_0027250.t1-p1
OG0001109: TCRU_9916 TcCLB-EL.506369.9_mRNA-p1 TcCLB-EL.
509833.10_mRNA-p1 TcCLB-NE.444777.20_mRNA-p1 TcSYL_0082510.t1-p1
OG0001110: TCRU_9917 TcCLB-EL.509831.50_mRNA-p1 TcCLB-NE.
444777.29_mRNA-p1 TcCLB-NE.506629.10_mRNA-p1 TcSYL_0082500.t1-p1
OG0001111: TCRU_9938 TcCLB-EL.510777.10_mRNA-p1 TcCLB-NE.
509039.10_mRNA-p1 TcSYL_0167170.t1-p1 TcSYL_0167180.t1-p1
OG0001112: TCRU_10014 TcCLB-EL.511189.50_pseudogenic_transcript-p1
TcCLB-NE.510973.50_mRNA-p1 TcSYL_0087810.t1-p1 TcSYL_0087820.t1-p1
OG0001113: TCRU_10016 TcCLB-EL.511107.10_pseudogenic_transcript-p1

TcCLB-EL.511189.20_mRNA-p1 TcCLB-NE.510973.20_mRNA-p1 TcSYL_0086440.t1-p1
OG0001114: TCRU_10184 TcCLB-EL.511309.20_mRNA-p1 TcCLB-NE.503399.29_mRNA-p1 TcCLB-NE.507087.10_pseudogenic_transcript-p1 TcSYL_0159730.t1-p1
OG0001115: TCRU_10213 TcCLB-EL.504163.30_mRNA-p1 TcCLB-EL.506401.120_mRNA-p1 TcCLB-NE.508543.30_mRNA-p1 TcSYL_0114280.t1-p1
OG0001116: TCRU_10306 TcCLB-EL.506527.4_mRNA-p1 TcCLB-NE.511283.160_mRNA-p1 TcSYL_0123700.t1-p1 TcSYL_0123710.t1-p1
OG0001117: TCRU_10345 TcCLB-EL.447705.19_mRNA-p1 TcCLB-EL.508839.30_mRNA-p1 TcCLB-NE.508961.30_mRNA-p1 TcSYL_0188170.t1-p1
OG0001118: TCRU_methyltransferase_ TcCLB-EL.447705.9_mRNA-p1 TcCLB-EL.508839.40_mRNA-p1 TcCLB-NE.508961.20_mRNA-p1 TcSYL_0188190.t1-p1
OG0001119: TCRU_10353 TcCLB-EL.506697.80_mRNA-p1 TcCLB-EL.510317.7_mRNA-p1 TcCLB-NE.508153.460_mRNA-p1 TcSYL_0000860.t1-p1
OG0001120: TCRU_10364 TcCLB-EL.509011.50_mRNA-p1 TcCLB-NE.504575.10_mRNA-p1 TcSYL_0030720.t1-p1 TcSYL_0030730.t1-p1
OG0001121: TCRU_10367 TcCLB-EL.509011.80_mRNA-p1 TcCLB-NE.510681.41_mRNA-p1 TcSYL_0030880.t1-p1 TcSYL_0030970.t1-p1
OG0001122: TCRU_10383 TcCLB-EL.510971.60_mRNA-p1 TcCLB-NE.410199.4_mRNA-p1 TcSYL_0057510.t1-p1 TcSYL_0057760.t1-p1
OG0001123: TCRU_10395 TcCLB-EL.503535.9_mRNA-p1 TcCLB-EL.508721.20_mRNA-p1 TcCLB-NE.509791.160_mRNA-p1 TcSYL_0107120.t1-p1
OG0001124: TCRU_10403 TcCLB-EL.506107.20_mRNA-p1 TcCLB-EL.507689.10_pseudogenic_transcript-p1 TcCLB-NE.504173.50_mRNA-p1 TcSYL_0034450.t1-p1
OG0001125: TCRU_10462 TcCLB-EL.510311.90_mRNA-p1 TcCLB-NE.510265.10_mRNA-p1 TcSYL_0000540.t1-p1 TcSYL_0000550.t1-p1
OG0001126: TCRU_10561 TcCLB-EL.504149.20_mRNA-p1 TcCLB-NE.503573.9_mRNA-p1 TcSYL_0074660.t1-p1 TcSYL_0074670.t1-p1
OG0001127: TCRU_10589 TcCLB-EL.509777.100_mRNA-p1 TcCLB-NE.509117.10_mRNA-p1 TcCLB-NE.511655.69_mRNA-p1 TcSYL_0013920.t1-p1
OG0001128: TCRU_10693 TcCLB-EL.507485.100_mRNA-p1 TcCLB-NE.509289.39_pseudogenic_transcript-p1 TcSYL_0195620.t1-p1 TcSYL_0195630.t1-p1
OG0001129: TCRU_10723 TcCLB-EL.504881.10_mRNA-p1 TcCLB-EL.506579.110_mRNA-p1 TcCLB-NE.510043.20_pseudogenic_transcript-p1 TcSYL_0064880.t1-p1
OG0001130: TCRU_10729 TcCLB-EL.509827.20_mRNA-p1 TcCLB-NE.509575.30_mRNA-p1 TcSYL_0118230.t1-p1 TcSYL_0120560.t1-p1
OG0001131: TCRU_10749 TcCLB-EL.504167.40_mRNA-p1 TcCLB-NE.398235.20_mRNA-p1 TcSYL_0108350.t1-p1 TcSYL_0108620.t1-p1
OG0001132: TCRU_10756 TcCLB-EL.507105.10_mRNA-p1 TcCLB-NE.506815.30_mRNA-p1 TcSYL_0088000.t1-p1 TcSYL_0088010.t1-p1
OG0001133: TCRU_10787 TcCLB-EL.420989.10_mRNA-p1 TcCLB-EL.506659.39_mRNA-p1 TcCLB-NE.508865.4_mRNA-p1 TcSYL_0027260.t1-p1
OG0001134: TCRU_family TcCLB-EL.509979.60_mRNA-p1 TcCLB-NE.510039.109_mRNA-p1 TcCLB-NE.510041.10_mRNA-p1 TcSYL_0156540.t1-p1
OG0001135: TCRU_10848 TcCLB-EL.506107.10_mRNA-p1 TcCLB-NE.504173.60_mRNA-p1 TcSYL_0034460.t1-p1 TcSYL_0034470.t1-p1
OG0001136: TcCLB-EL.479517.40_pseudogenic_transcript-p1 TcCLB-EL.506529.370_mRNA-p1 TcCLB-NE.508187.20_pseudogenic_transcript-p1 TcSYL_0087370.t1-p1 TcSYL_0196310.t1-p1
OG0001137: TcCLB-EL.504149.210_pseudogenic_transcript-p1 TcCLB-EL.

506047.16_pseudogenic_transcript-p1 TcCLB-EL.
506113.55_pseudogenic_transcript-p1 TcCLB-EL.
511473.9_pseudogenic_transcript-p1 TcCLB-NE.
508479.480_pseudogenic_transcript-p1
OG0001138: TcCLB-EL.504213.10_mRNA-p1 TcCLB-EL.508089.71_mRNA-p1
TcCLB-NE.508857.90_mRNA-p1 TcSYL_0026820.t1-p1 TcSYL_0026840.t1-p1
OG0001139: TcCLB-EL.511407.30_mRNA-p1 TcCLB-NE.506151.4_mRNA-p1
TcCLB-NE.509567.60_mRNA-p1 TcSYL_0096870.t1-p1 TcSYL_0096890.t1-p1
OG0001140: TcCLB-EL.509993.15_pseudogenic_transcript-p1 TcCLB-NE.
509227.20_pseudogenic_transcript-p1 TcCLB-NE.
511851.44_pseudogenic_transcript-p1 TcCLB-NE.
511851.61_pseudogenic_transcript-p1 TcCLB-NE.
511853.21_pseudogenic_transcript-p1
OG0001141: TcCLB-EL.505063.30_pseudogenic_transcript-p1 TcCLB-EL.
509765.95_pseudogenic_transcript-p1 TcCLB-NE.
510355.41_pseudogenic_transcript-p1 TcCLB-NE.
510489.36_pseudogenic_transcript-p1 TcSYL_0067740.t1-p1
OG0001142: TcCLB-EL.506763.140_mRNA-p1 TcCLB-EL.506965.110_mRNA-p1
TcCLB-NE.510239.90_mRNA-p1 TcCLB-NE.510419.30_mRNA-p1
TcSYL_0102030.t1-p1
OG0001143: TcCLB-EL.504099.15_mRNA-p1 TcCLB-EL.511587.33_mRNA-p1
TcCLB-NE.508479.490_mRNA-p1 TcSYL_0154470.t1-p1 TcSYL_0155180.t1-p1
OG0001144: TcCLB-EL.504241.57_pseudogenic_transcript-p1 TcCLB-EL.
508283.60_pseudogenic_transcript-p1 TcCLB-EL.
508775.35_pseudogenic_transcript-p1 TcCLB-NE.
507703.10_pseudogenic_transcript-p1 TcCLB-NE.
507911.33_pseudogenic_transcript-p1
OG0001145: TcCLB-EL.507953.20_mRNA-p1 TcCLB-EL.508099.40_mRNA-p1
TcCLB-EL.510013.180_mRNA-p1 TcCLB-NE.509225.21_mRNA-p1
TcSYL_0130330.t1-p1
OG0001146: TcCLB-EL.506375.35_mRNA-p1 TcCLB-NE.507587.50_mRNA-p1
TcCLB-NE.508417.20_mRNA-p1 TcCLB-NE.508423.30_mRNA-p1
TcSYL_0157510.t1-p1
OG0001147: TcCLB-EL.404975.30_pseudogenic_transcript-p1 TcCLB-EL.
506847.50_pseudogenic_transcript-p1 TcCLB-NE.
507849.10_pseudogenic_transcript-p1 TcSYL_0114440.t1-p1
TcSYL_0114520.t1-p1
OG0001148: TcCLB-EL.503713.40_mRNA-p1 TcCLB-EL.510431.290_mRNA-p1
TcCLB-EL.510491.70_mRNA-p1 TcCLB-NE.504205.10_mRNA-p1 TcCLB-NE.
507723.60_mRNA-p1
OG0001149: TcCLB-EL.511217.60_mRNA-p1 TcCLB-NE.506575.40_mRNA-p1
TcSYL_0188890.t1-p1 TcSYL_0188900.t1-p1 TcSYL_0188910.t1-p1
OG0001150: TcCLB-EL.503717.15_mRNA-p1 TcCLB-EL.
511401.95_pseudogenic_transcript-p1 TcSYL_0068550.t1-p1
TcSYL_0069120.t1-p1 TcSYL_0151250.t1-p1
OG0001151: TcCLB-EL.511071.130_mRNA-p1 TcCLB-NE.511899.50_mRNA-p1
TcCLB-NE.511901.10_mRNA-p1 TcSYL_0103550.t1-p1 TcSYL_0103560.t1-p1
OG0001152: TcCLB-EL.511785.5_mRNA-p1 TcSYL_0069170.t1-p1
TcSYL_0124750.t1-p1 TcSYL_0128420.t1-p1 TcSYL_0150380.t1-p1
OG0001153: TcCLB-EL.466879.10_mRNA-p1 TcCLB-EL.511799.9_mRNA-p1
TcCLB-NE.510901.10_mRNA-p1 TcSYL_0179240.t1-p1 TcSYL_0179250.t1-p1
OG0001154: TcCLB-EL.509667.20_pseudogenic_transcript-p1 TcCLB-NE.
508419.30_pseudogenic_transcript-p1 TcCLB-NE.
508421.30_pseudogenic_transcript-p1 TcSYL_0040030.t1-p1

TcSYL_0157360.t1-p1
OG0001155: TcCLB-EL.403153.20_pseudogenic_transcript-p1 TcCLB-EL.
506591.81_pseudogenic_transcript-p1 TcCLB-NE.
506989.260_pseudogenic_transcript-p1 TcSYL_0003570.t1-p1
TcSYL_0088670.t1-p1
OG0001156: TcCLB-EL.507533.21_pseudogenic_transcript-p1 TcCLB-EL.
507721.20_pseudogenic_transcript-p1 TcSYL_0002180.t1-p1
TcSYL_0002430.t1-p1 TcSYL_0002720.t1-p1
OG0001157: TcCLB-EL.511173.250_pseudogenic_transcript-p1 TcCLB-EL.
511173.50_mRNA-p1 TcCLB-NE.507637.45_pseudogenic_transcript-p1
TcCLB-NE.511255.130_mRNA-p1 TcSYL_0080090.t1-p1
OG0001158: TcCLB-EL.508157.40_mRNA-p1 TcCLB-EL.510281.10_mRNA-p1
TcSYL_0066870.t1-p1 TcSYL_0070160.t1-p1 TcSYL_0129140.t1-p1
OG0001159: TcCLB-EL.508161.23_mRNA-p1 TcSYL_0092820.t1-p1
TcSYL_0093260.t1-p1 TcSYL_0160860.t1-p1 TcSYL_0161010.t1-p1
OG0001160: TcCLB-EL.508165.150_mRNA-p1 TcCLB-EL.510279.190_mRNA-p1
TcCLB-EL.510279.310_mRNA-p1 TcCLB-NE.511839.20_mRNA-p1
TcSYL_0074110.t1-p1
OG0001161: TcCLB-EL.510275.272_mRNA-p1 TcCLB-EL.510275.355_mRNA-p1
TcCLB-NE.505997.70_mRNA-p1 TcSYL_0029120.t1-p1 TcSYL_0074190.t1-p1
OG0001162: TcCLB-EL.503761.50_mRNA-p1 TcCLB-NE.508887.30_mRNA-p1
TcSYL_0074270.t1-p1 TcSYL_0101390.t1-p1 TcSYL_0137210.t1-p1
OG0001163: TcCLB-EL.504239.150_mRNA-p1 TcCLB-EL.506765.49_mRNA-p1
TcCLB-NE.510701.31_mRNA-p1 TcSYL_0133640.t1-p1 TcSYL_0137070.t1-p1
OG0001164: TcCLB-EL.505025.147_mRNA-p1 TcCLB-EL.506603.43_mRNA-p1
TcCLB-NE.504081.26_mRNA-p1 TcCLB-NE.506459.190_mRNA-p1
TcSYL_0154580.t1-p1
OG0001165: TcCLB-EL.508681.20_mRNA-p1 TcCLB-NE.506723.30_mRNA-p1
TcSYL_0061120.t1-p1 TcSYL_0171870.t1-p1 TcSYL_0200380.t1-p1
OG0001166: TcCLB-NE.511829.5_pseudogenic_transcript-p1
TcSYL_0070610.t1-p1 TcSYL_0106310.t1-p1 TcSYL_0106500.t1-p1
TcSYL_0106610.t1-p1
OG0001167: TcCLB-NE.510237.95_pseudogenic_transcript-p1 TcCLB-NE.
511919.30_pseudogenic_transcript-p1 TcCLB-NE.
511921.71_pseudogenic_transcript-p1 TcCLB-NE.
511923.40_pseudogenic_transcript-p1 TcSYL_0164230.t1-p1
OG0001168: TcCLB-NE.509527.50_pseudogenic_transcript-p1
TcSYL_0100470.t1-p1 TcSYL_0129500.t1-p1 TcSYL_0129800.t1-p1
TcSYL_0190290.t1-p1
OG0001169: TcCLB-NE.506425.5_mRNA-p1 TcSYL_0062000.t1-p1
TcSYL_0107760.t1-p1 TcSYL_0107820.t1-p1 TcSYL_0107940.t1-p1
OG0001170: TCRU_12 TcCLB-EL.508131.19_mRNA-p1 TcCLB-NE.
506559.240_mRNA-p1 TcSYL_0045200.t1-p1
OG0001171: TCRU_15 TcCLB-EL.510227.10_mRNA-p1 TcCLB-NE.
506559.210_mRNA-p1 TcSYL_0045190.t1-p1
OG0001172: TCRU_18 TcCLB-EL.510231.10_mRNA-p1 TcCLB-NE.
506559.129_mRNA-p1 TcSYL_0045160.t1-p1
OG0001173: TCRU_24 TcCLB-EL.506631.20_mRNA-p1 TcCLB-NE.
506559.40_mRNA-p1 TcSYL_0045140.t1-p1
OG0001174: TCRU_26 TcCLB-EL.506631.40_mRNA-p1 TcCLB-NE.
506559.20_mRNA-p1 TcSYL_0045120.t1-p1
OG0001175: TCRU_27 TcCLB-EL.508133.10_mRNA-p1 TcCLB-NE.
506559.10_mRNA-p1 TcSYL_0045110.t1-p1
OG0001176: TCRU_33 TcCLB-EL.503903.60_mRNA-p1 TcCLB-NE.

508153.760_mRNA-p1 TcSYL_0000720.t1-p1
OG0001177: TCRU_34 TcCLB-EL.503903.30_mRNA-p1 TcCLB-NE.
508153.780_mRNA-p1 TcSYL_0000710.t1-p1
OG0001178: TCRU_38 TcCLB-EL.508181.130_mRNA-p1 TcCLB-NE.
508153.830_mRNA-p1 TcSYL_0000690.t1-p1
OG0001179: TCRU_45 TcCLB-EL.508181.60_mRNA-p1 TcCLB-NE.
508153.900_mRNA-p1 TcSYL_0000680.t1-p1
OG0001180: TCRU_48 TcCLB-EL.508181.30_mRNA-p1 TcCLB-NE.
508153.940_pseudogenic_transcript-p1 TcSYL_0000670.t1-p1
OG0001181: TCRU_49 TcCLB-EL.508181.20_mRNA-p1 TcCLB-NE.
508153.950_mRNA-p1 TcSYL_0000660.t1-p1
OG0001182: TCRU_73 TcCLB-EL.504213.20_mRNA-p1 TcCLB-NE.
508857.100_mRNA-p1 TcSYL_0026790.t1-p1
OG0001183: TCRU_74 TcCLB-EL.504213.29_mRNA-p1 TcCLB-NE.
508857.110_mRNA-p1 TcSYL_0026770.t1-p1
OG0001184: TCRU_86 TcCLB-EL.504153.140_mRNA-p1 TcSYL_0138710.t1-p1
TcSYL_0138720.t1-p1
OG0001185: TCRU_105 TcCLB-EL.511425.30_mRNA-p1 TcCLB-NE.
506479.20_mRNA-p1 TcSYL_0110820.t1-p1
OG0001186: TCRU_106 TcCLB-EL.503803.4_mRNA-p1 TcCLB-NE.
506479.39_mRNA-p1 TcSYL_0110840.t1-p1
OG0001187: TCRU_107 TcCLB-EL.503803.10_mRNA-p1 TcCLB-NE.
506479.50_mRNA-p1 TcSYL_0110860.t1-p1
OG0001188: TCRU_110 TcCLB-EL.503805.10_mRNA-p1 TcCLB-NE.
506479.80_mRNA-p1 TcSYL_0110890.t1-p1
OG0001189: TCRU_111 TcCLB-EL.504427.80_mRNA-p1 TcCLB-NE.
509331.60_mRNA-p1 TcSYL_0172510.t1-p1
OG0001190: TCRU_112 TcCLB-EL.504427.90_mRNA-p1 TcCLB-NE.
509331.50_mRNA-p1 TcSYL_0172550.t1-p1
OG0001191: TCRU_113 TcCLB-EL.504427.100_mRNA-p1 TcCLB-NE.
509331.40_mRNA-p1 TcSYL_0172570.t1-p1
OG0001192: TCRU_114 TcCLB-EL.504427.110_mRNA-p1 TcCLB-NE.
509331.30_mRNA-p1 TcSYL_0172590.t1-p1
OG0001193: TCRU_123 TcCLB-EL.504089.60_mRNA-p1 TcCLB-NE.
504001.10_mRNA-p1 TcSYL_0092100.t1-p1
OG0001194: TCRU__Ubiquitin TcCLB-EL.510879.60_mRNA-p1 TcCLB-NE.
504013.55_mRNA-p1 TcSYL_0045920.t1-p1
OG0001195: TCRU_126 TcCLB-EL.510879.70_mRNA-p1 TcCLB-NE.
504013.50_mRNA-p1 TcSYL_0045930.t1-p1
OG0001196: TCRU_128 TcCLB-EL.510879.90_mRNA-p1 TcCLB-NE.
504013.30_mRNA-p1 TcSYL_0045940.t1-p1
OG0001197: TCRU_151 TcCLB-EL.509805.210_mRNA-p1 TcCLB-NE.
511267.40_mRNA-p1 TcSYL_0015290.t1-p1
OG0001198: TCRU_155 TcCLB-EL.509805.170_mRNA-p1 TcCLB-NE.
503939.40_mRNA-p1 TcSYL_0015280.t1-p1
OG0001199: TCRU_159 TcCLB-EL.509805.130_mRNA-p1 TcCLB-NE.
503939.80_mRNA-p1 TcSYL_0015260.t1-p1
OG0001200: TCRU_161 TcCLB-EL.509805.110_mRNA-p1 TcCLB-NE.
503939.90_mRNA-p1 TcSYL_0015250.t1-p1
OG0001201: TCRU_166 TcCLB-EL.506239.20_mRNA-p1 TcCLB-NE.
506177.10_mRNA-p1 TcSYL_0092440.t1-p1
OG0001202: TCRU_169 TcCLB-EL.511001.40_mRNA-p1 TcCLB-NE.
503635.40_mRNA-p1 TcSYL_0132340.t1-p1
OG0001203: TCRU_170 TcCLB-EL.511001.30_mRNA-p1 TcCLB-NE.

503635.50_mRNA-p1 TcSYL_0132370.t1-p1
OG0001204: TCRU_175 TcCLB-EL.509233.40_mRNA-p1 TcCLB-NE.
509437.80_mRNA-p1 TcSYL_0146140.t1-p1
OG0001205: TCRU_176 TcCLB-EL.509233.50_mRNA-p1 TcCLB-NE.
509437.70_mRNA-p1 TcSYL_0146130.t1-p1
OG0001206: TCRU_179 TcCLB-EL.510997.50_pseudogenic_transcript-p1
TcCLB-NE.506959.30_mRNA-p1 TcSYL_0132600.t1-p1
OG0001207: TCRU_180 TcCLB-EL.510997.40_mRNA-p1 TcCLB-NE.
506959.40_mRNA-p1 TcSYL_0132630.t1-p1
OG0001208: TCRU_181 TcCLB-EL.510997.30_mRNA-p1 TcCLB-NE.
506959.50_mRNA-p1 TcSYL_0132650.t1-p1
OG0001209: TCRU_182 TcCLB-EL.510997.20_mRNA-p1 TcCLB-NE.
506959.60_mRNA-p1 TcSYL_0132690.t1-p1
OG0001210: TCRU_183 TcCLB-EL.510997.10_mRNA-p1 TcCLB-NE.
506959.64_mRNA-p1 TcSYL_0132720.t1-p1
OG0001211: TCRU_184 TcCLB-EL.503453.100_mRNA-p1 TcCLB-NE.
506959.70_mRNA-p1 TcSYL_0132770.t1-p1
OG0001212: TCRU_185 TcCLB-EL.503453.80_mRNA-p1 TcCLB-NE.
506959.80_mRNA-p1 TcSYL_0132810.t1-p1
OG0001213: TCRU_190 TcCLB-EL.503453.10_mRNA-p1 TcCLB-EL.
511467.10_mRNA-p1 TcSYL_0132880.t1-p1
OG0001214: TCRU_193 TcCLB-EL.507625.190_mRNA-p1 TcCLB-NE.
509741.30_mRNA-p1 TcSYL_0016930.t1-p1
OG0001215: TCRU_194 TcCLB-EL.507627.9_mRNA-p1 TcCLB-NE.
506333.19_mRNA-p1 TcSYL_0016850.t1-p1
OG0001216: TCRU_209 TcCLB-EL.510409.50_mRNA-p1 TcCLB-NE.
509937.90_mRNA-p1 TcSYL_0027540.t1-p1
OG0001217: TCRU_210 TCRU_7638 TCRU_8372 TcSYL_0020850.t1-p1
OG0001218: TCRU_215 TcCLB-EL.507625.84_mRNA-p1 TcCLB-NE.
507787.120_mRNA-p1 TcSYL_0017180.t1-p1
OG0001219: TCRU_216 TcCLB-EL.507625.99_mRNA-p1 TcCLB-NE.
507787.100_mRNA-p1 TcSYL_0017140.t1-p1
OG0001220: TCRU_217 TcCLB-EL.507625.110_mRNA-p1 TcCLB-NE.
507787.90_mRNA-p1 TcSYL_0017120.t1-p1
OG0001221: TCRU_218 TcCLB-EL.507625.120_mRNA-p1 TcCLB-NE.
507787.80_mRNA-p1 TcSYL_0017110.t1-p1
OG0001222: TCRU_219 TcCLB-EL.507625.130_mRNA-p1 TcCLB-NE.
507787.70_mRNA-p1 TcSYL_0017090.t1-p1
OG0001223: TCRU_220 TcCLB-EL.507625.140_mRNA-p1 TcCLB-NE.
507787.50_pseudogenic_transcript-p1 TcSYL_0017060.t1-p1
OG0001224: TCRU_221 TcCLB-EL.507625.150_mRNA-p1 TcCLB-NE.
507787.40_mRNA-p1 TcSYL_0017050.t1-p1
OG0001225: TCRU_homologue TcCLB-EL.507625.160_mRNA-p1 TcCLB-NE.
507787.30_mRNA-p1 TcSYL_0017040.t1-p1
OG0001226: TCRU_223 TcCLB-EL.507625.170_mRNA-p1 TcCLB-NE.
507787.20_mRNA-p1 TcSYL_0017010.t1-p1
OG0001227: TCRU_232 TcCLB-EL.506405.149_mRNA-p1 TcCLB-NE.
507251.20_mRNA-p1 TcSYL_0046630.t1-p1
OG0001228: TCRU_236 TcCLB-EL.511803.60_mRNA-p1 TcCLB-NE.
510895.29_mRNA-p1 TcSYL_0180120.t1-p1
OG0001229: TCRU_240 TcCLB-EL.510431.110_mRNA-p1 TcSYL_0138060.t1-p1
TcSYL_0138210.t1-p1
OG0001230: TCRU_243 TCRU_628 TCRU_7381 TcSYL_0083400.t1-p1
OG0001231: TCRU_249 TcCLB-EL.511545.130_mRNA-p1 TcCLB-NE.

506819.40_mRNA-p1 TcSYL_0088300.t1-p1
OG0001232: TCRU_250 TcCLB-EL.511545.120_mRNA-p1 TcCLB-NE.
506819.30_mRNA-p1 TcSYL_0088270.t1-p1
OG0001233: TCRU_251 TcCLB-EL.511545.110_mRNA-p1 TcCLB-NE.
506819.20_mRNA-p1 TcSYL_0088240.t1-p1
OG0001234: TCRU_281 TcCLB-EL.511557.10_mRNA-p1 TcCLB-NE.
503995.50_mRNA-p1 TcSYL_0073330.t1-p1
OG0001235: TCRU_282 TcCLB-EL.511557.20_mRNA-p1 TcCLB-NE.
503995.40_mRNA-p1 TcSYL_0073320.t1-p1
OG0001236: TCRU_285 TcCLB-EL.511557.60_mRNA-p1 TcCLB-NE.
505171.100_mRNA-p1 TcSYL_0073270.t1-p1
OG0001237: TCRU_287 TcCLB-EL.509083.9_mRNA-p1 TcCLB-NE.
505171.70_mRNA-p1 TcSYL_0073260.t1-p1
OG0001238: TCRU_288 TcCLB-EL.511559.20_mRNA-p1 TcCLB-NE.
505171.60_mRNA-p1 TcSYL_0073250.t1-p1
OG0001239: TCRU_289 TcCLB-EL.511559.30_mRNA-p1 TcCLB-NE.
505171.50_mRNA-p1 TcSYL_0073240.t1-p1
OG0001240: TCRU_291 TcCLB-EL.511559.40_mRNA-p1 TcCLB-NE.
505171.40_mRNA-p1 TcSYL_0073230.t1-p1
OG0001241: TCRU_312 TcCLB-EL.510149.80_mRNA-p1 TcCLB-NE.
506989.30_mRNA-p1 TcSYL_0004150.t1-p1
OG0001242: TCRU_314 TcCLB-EL.510149.100_mRNA-p1 TcCLB-NE.
506989.50_mRNA-p1 TcSYL_0004110.t1-p1
OG0001243: TCRU_315 TcCLB-EL.510149.120_mRNA-p1 TcCLB-NE.
506989.70_mRNA-p1 TcSYL_0004090.t1-p1
OG0001244: TCRU_316 TcCLB-EL.510149.130_mRNA-p1 TcCLB-NE.
506989.80_mRNA-p1 TcSYL_0004080.t1-p1
OG0001245: TCRU_317 TcCLB-EL.510149.140_mRNA-p1 TcCLB-NE.
506989.100_pseudogenic_transcript-p1 TcSYL_0004070.t1-p1
OG0001246: TCRU_318 TcCLB-EL.510149.150_mRNA-p1 TcCLB-NE.
506989.110_mRNA-p1 TcSYL_0004060.t1-p1
OG0001247: TCRU_319 TcCLB-EL.503811.80_mRNA-p1 TcCLB-NE.
506989.120_mRNA-p1 TcSYL_0004040.t1-p1
OG0001248: TCRU_320 TcCLB-EL.503811.70_mRNA-p1 TcCLB-NE.
506989.130_mRNA-p1 TcSYL_0004010.t1-p1
OG0001249: TCRU_321 TcCLB-EL.503811.60_mRNA-p1 TcCLB-NE.
506989.140_mRNA-p1 TcSYL_0003980.t1-p1
OG0001250: TCRU_322 TcCLB-EL.503811.45_mRNA-p1 TcCLB-NE.
506989.155_mRNA-p1 TcSYL_0003940.t1-p1
OG0001251: TCRU_323 TcCLB-EL.503811.40_mRNA-p1 TcCLB-NE.
506989.160_mRNA-p1 TcSYL_0003920.t1-p1
OG0001252: TCRU_325 TcCLB-EL.503811.20_mRNA-p1 TcCLB-NE.
506989.180_mRNA-p1 TcSYL_0003880.t1-p1
OG0001253: TCRU_328 TcCLB-EL.506591.20_mRNA-p1 TcCLB-NE.
506989.210_mRNA-p1 TcSYL_0003760.t1-p1
OG0001254: TCRU_329 TcCLB-EL.506591.30_mRNA-p1 TcCLB-NE.
506989.220_mRNA-p1 TcSYL_0003740.t1-p1
OG0001255: TCRU_331 TcCLB-EL.506591.69_mRNA-p1 TcCLB-NE.
506989.250_mRNA-p1 TcSYL_0003730.t1-p1
OG0001256: TCRU_332 TcCLB-EL.506591.90_mRNA-p1 TcCLB-NE.
506989.270_mRNA-p1 TcSYL_0003720.t1-p1
OG0001257: TCRU_337 TcCLB-EL.506947.20_mRNA-p1 TcCLB-NE.
504251.30_mRNA-p1 TcSYL_0014510.t1-p1
OG0001258: TCRU_338 TcCLB-EL.506947.40_mRNA-p1 TcCLB-NE.

504251.9_mRNA-p1 TcSYL_0014540.t1-p1
OG0001259: TCRU_341 TcCLB-EL.508539.20_mRNA-p1 TcCLB-NE.
511459.60_mRNA-p1 TcSYL_0130900.t1-p1
OG0001260: TCRU_342 TcCLB-EL.508539.30_mRNA-p1 TcCLB-NE.
511459.50_mRNA-p1 TcSYL_0130880.t1-p1
OG0001261: TCRU_352 TcCLB-EL.507077.40_mRNA-p1 TcCLB-NE.
510899.30_mRNA-p1 TcSYL_0179270.t1-p1
OG0001262: TCRU_355 TcCLB-EL.507077.9_mRNA-p1 TcCLB-NE.
510899.59_mRNA-p1 TcSYL_0179260.t1-p1
OG0001263: TCRU_379 TcCLB-EL.508277.160_mRNA-p1 TcCLB-NE.
507913.39_mRNA-p1 TcSYL_0064150.t1-p1
OG0001264: TCRU_380 TcCLB-EL.508277.150_mRNA-p1 TcCLB-NE.
507913.30_mRNA-p1 TcSYL_0064140.t1-p1
OG0001265: TCRU_383 TcCLB-EL.508277.120_mRNA-p1 TcCLB-NE.
509943.30_mRNA-p1 TcSYL_0064130.t1-p1
OG0001266: TCRU_384 TcCLB-EL.511051.20_mRNA-p1 TcCLB-NE.
509683.100_mRNA-p1 TcSYL_0089690.t1-p1
OG0001267: TCRU_385 TcCLB-EL.511051.30_mRNA-p1 TcCLB-NE.
509683.110_mRNA-p1 TcSYL_0089650.t1-p1
OG0001268: TCRU_binding TcCLB-EL.511051.59_mRNA-p1 TcCLB-NE.
509683.124_mRNA-p1 TcSYL_0089560.t1-p1
OG0001269: TCRU_395 TcCLB-EL.511439.100_mRNA-p1 TcCLB-NE.
509261.10_mRNA-p1 TcSYL_0031570.t1-p1
OG0001270: TCRU_396 TcCLB-EL.511439.120_mRNA-p1 TcCLB-NE.
509261.20_mRNA-p1 TcSYL_0031600.t1-p1
OG0001271: TCRU_397 TcCLB-EL.507107.40_mRNA-p1 TcCLB-NE.
509261.30_mRNA-p1 TcSYL_0031620.t1-p1
OG0001272: TCRU_405 TcCLB-EL.511365.70_mRNA-p1 TcCLB-NE.
508899.10_mRNA-p1 TcSYL_0121850.t1-p1
OG0001273: TCRU_406 TcCLB-EL.511365.50_pseudogenic_transcript-p1
TcCLB-NE.508899.20_mRNA-p1 TcSYL_0121840.t1-p1
OG0001274: TCRU_410 TcCLB-EL.511361.70_mRNA-p1 TcCLB-NE.
508899.80_mRNA-p1 TcSYL_0121810.t1-p1
OG0001275: TCRU_411 TcCLB-EL.511361.50_mRNA-p1 TcCLB-NE.
508899.100_mRNA-p1 TcSYL_0121790.t1-p1
OG0001276: TCRU_412 TcCLB-EL.511361.40_mRNA-p1 TcCLB-NE.
508899.110_mRNA-p1 TcSYL_0121780.t1-p1
OG0001277: TCRU_424 TcCLB-EL.507011.60_mRNA-p1 TcCLB-NE.
507005.60_mRNA-p1 TcSYL_0117100.t1-p1
OG0001278: TCRU_426 TcCLB-EL.507011.70_mRNA-p1 TcCLB-NE.
507005.50_mRNA-p1 TcSYL_0117080.t1-p1
OG0001279: TCRU_427 TcCLB-EL.507011.90_mRNA-p1 TcCLB-NE.
507005.30_mRNA-p1 TcSYL_0117010.t1-p1
OG0001280: TCRU_429 TcCLB-EL.507011.120_mRNA-p1 TcCLB-NE.
507005.4_mRNA-p1 TcSYL_0116920.t1-p1
OG0001281: TCRU_431 TcCLB-EL.507011.140_mRNA-p1 TcCLB-NE.
510665.40_mRNA-p1 TcSYL_0116870.t1-p1
OG0001282: TCRU_433 TcCLB-EL.507011.160_mRNA-p1 TcCLB-NE.
510665.20_mRNA-p1 TcSYL_0116840.t1-p1
OG0001283: TCRU_434 TcCLB-EL.507011.180_mRNA-p1 TcCLB-NE.
510665.4_mRNA-p1 TcSYL_0116800.t1-p1
OG0001284: TCRU_439 TcCLB-EL.509379.20_mRNA-p1 TcCLB-NE.
506701.20_mRNA-p1 TcSYL_0001710.t1-p1
OG0001285: TCRU_450 TCRU_8911 TcCLB-EL.506399.20_mRNA-p1 TcCLB-NE.

507989.10_mRNA-p1
OG0001286: TCRU_452 TcCLB-EL.503391.10_mRNA-p1 TcCLB-NE.
509243.60_mRNA-p1 TcCLB-NE.510063.20_mRNA-p1
OG0001287: TCRU_461 TcCLB-EL.509779.40_mRNA-p1 TcCLB-NE.
511653.40_mRNA-p1 TcSYL_0013950.t1-p1
OG0001288: TCRU_464 TcCLB-EL.507811.20_mRNA-p1 TcCLB-NE.
511653.10_mRNA-p1 TcSYL_0013960.t1-p1
OG0001289: TCRU_468 TcCLB-EL.507811.60_mRNA-p1 TcCLB-NE.
511649.130_mRNA-p1 TcSYL_0013990.t1-p1
OG0001290: TCRU_472 TcCLB-EL.503595.10_mRNA-p1 TcCLB-NE.
503631.9_mRNA-p1 TcSYL_0094580.t1-p1
OG0001291: TCRU_474 TcCLB-EL.510999.10_mRNA-p1 TcCLB-NE.
506959.20_mRNA-p1 TcSYL_0132520.t1-p1
OG0001292: TCRU_486 TcCLB-EL.507993.40_mRNA-p1 TcCLB-NE.
511277.490_mRNA-p1 TcSYL_0014760.t1-p1
OG0001293: TCRU_513 TcCLB-EL.504077.50_mRNA-p1 TcCLB-NE.
507081.40_mRNA-p1 TcSYL_0019680.t1-p1
OG0001294: TCRU_514 TcCLB-EL.504077.40_mRNA-p1 TcCLB-NE.
507081.30_mRNA-p1 TcSYL_0019690.t1-p1
OG0001295: TCRU_515 TcCLB-EL.504077.30_mRNA-p1 TcCLB-NE.
507081.20_mRNA-p1 TcSYL_0019700.t1-p1
OG0001296: TCRU_517 TcCLB-EL.411427.30_mRNA-p1 TcCLB-NE.
509395.50_mRNA-p1 TcSYL_0194380.t1-p1
OG0001297: TCRU_518 TcCLB-EL.411427.10_mRNA-p1 TcCLB-EL.
463297.10_mRNA-p1 TcCLB-NE.509395.40_mRNA-p1
OG0001298: TCRU_529 TcCLB-EL.506825.40_mRNA-p1 TcCLB-NE.
506681.40_mRNA-p1 TcSYL_0078910.t1-p1
OG0001299: TCRU_532 TcCLB-EL.510609.20_mRNA-p1 TcCLB-NE.
510397.10_mRNA-p1 TcSYL_0163720.t1-p1
OG0001300: TCRU_542 TcCLB-EL.507747.240_mRNA-p1 TcCLB-NE.
503723.50_mRNA-p1 TcSYL_0023600.t1-p1
OG0001301: TCRU_550 TcCLB-EL.508571.10_mRNA-p1 TcCLB-EL.
510863.9_mRNA-p1 TcCLB-NE.503731.9_mRNA-p1
OG0001302: TCRU_563 TcCLB-EL.510609.80_mRNA-p1 TcCLB-NE.
510393.10_mRNA-p1 TcSYL_0163650.t1-p1
OG0001303: TCRU_566 TcCLB-EL.508277.320_mRNA-p1 TcCLB-NE.
431849.20_mRNA-p1 TcSYL_0064250.t1-p1
OG0001304: TCRU_567 TcCLB-EL.508277.330_mRNA-p1 TcCLB-NE.
506445.10_mRNA-p1 TcSYL_0064280.t1-p1
OG0001305: TCRU_568 TcCLB-EL.508277.340_mRNA-p1 TcCLB-NE.
506445.20_mRNA-p1 TcSYL_0064290.t1-p1
OG0001306: TCRU_569 TcCLB-EL.511545.180_mRNA-p1 TcCLB-NE.
510435.30_mRNA-p1 TcSYL_0088450.t1-p1
OG0001307: TCRU_570 TcCLB-EL.511545.170_mRNA-p1 TcCLB-NE.
510435.20_mRNA-p1 TcSYL_0088430.t1-p1
OG0001308: TCRU_571 TcCLB-EL.511545.150_mRNA-p1 TcCLB-NE.
510435.5_pseudogenic_transcript-p1 TcSYL_0088380.t1-p1
OG0001309: TCRU_575 TcCLB-EL.506811.100_mRNA-p1 TcCLB-NE.
506303.130_mRNA-p1 TcSYL_0138470.t1-p1
OG0001310: TCRU_580 TcCLB-EL.506811.30_mRNA-p1 TcCLB-NE.
506305.20_mRNA-p1 TcSYL_0138450.t1-p1
OG0001311: TCRU_581 TcCLB-EL.506811.20_mRNA-p1 TcCLB-NE.
506305.29_mRNA-p1 TcSYL_0138440.t1-p1
OG0001312: TCRU_582 TcCLB-EL.506811.10_mRNA-p1 TcCLB-NE.

506305.40_mRNA-p1 TcSYL_0138430.t1-p1
OG0001313: TCRU_590 TcCLB-EL.504025.19_mRNA-p1 TcCLB-NE.
506247.380_mRNA-p1 TcSYL_0108680.t1-p1
OG0001314: TCRU_591 TcCLB-EL.503779.20_mRNA-p1 TcCLB-NE.
506247.390_mRNA-p1 TcSYL_0108670.t1-p1
OG0001315: TCRU_594 TCRU_7808 TCRU_9126 TcSYL_0070320.t1-p1
OG0001316: TCRU_608 TcCLB-EL.509171.90_mRNA-p1 TcCLB-NE.
507023.230_mRNA-p1 TcSYL_0139390.t1-p1
OG0001317: TCRU_612 TcCLB-EL.509171.47_mRNA-p1 TcCLB-NE.
507023.280_mRNA-p1 TcSYL_0139400.t1-p1
OG0001318: TCRU_614 TcCLB-EL.509171.20_mRNA-p1 TcCLB-NE.
511505.20_mRNA-p1 TcSYL_0139410.t1-p1
OG0001319: TCRU_618 TcCLB-NE.509113.10_pseudogenic_transcript-p1
TcCLB-NE.511643.73_pseudogenic_transcript-p1 TcSYL_0164300.t1-p1
OG0001320: TCRU_631 TCRU_7724 TcCLB-EL.506207.50_mRNA-p1 TcCLB-NE.
510065.30_mRNA-p1
OG0001321: TCRU_669 TcCLB-EL.511871.100_mRNA-p1 TcCLB-NE.
511863.30_mRNA-p1 TcSYL_0146160.t1-p1
OG0001322: TCRU_671 TcCLB-EL.508137.20_mRNA-p1 TcCLB-NE.
453445.10_mRNA-p1 TcSYL_0147210.t1-p1
OG0001323: TCRU_673 TcCLB-EL.509167.130_mRNA-p1 TcCLB-NE.
504257.20_mRNA-p1 TcSYL_0140320.t1-p1
OG0001324: TCRU_674 TcCLB-EL.509167.120_mRNA-p1 TcCLB-NE.
504257.30_mRNA-p1 TcSYL_0140350.t1-p1
OG0001325: TCRU_675 TcCLB-EL.509167.110_mRNA-p1 TcCLB-NE.
504257.60_mRNA-p1 TcSYL_0140360.t1-p1
OG0001326: TCRU_677 TcCLB-EL.506979.50_mRNA-p1 TcCLB-NE.
503499.10_mRNA-p1 TcSYL_0084880.t1-p1
OG0001327: TCRU_678 TcCLB-EL.506979.40_mRNA-p1 TcCLB-NE.
503499.20_mRNA-p1 TcSYL_0084870.t1-p1
OG0001328: TCRU_683 TcCLB-EL.507547.40_mRNA-p1 TcCLB-NE.
508441.70_mRNA-p1 TcSYL_0086430.t1-p1
OG0001329: TCRU_684 TcCLB-EL.507547.50_mRNA-p1 TcCLB-NE.
508441.60_mRNA-p1 TcSYL_0086420.t1-p1
OG0001330: TCRU_687 TcCLB-EL.507547.80_mRNA-p1 TcCLB-NE.
508441.30_mRNA-p1 TcSYL_0086400.t1-p1
OG0001331: TCRU_699 TcCLB-EL.511867.20_mRNA-p1 TcCLB-NE.
507775.20_mRNA-p1 TcSYL_0146380.t1-p1
OG0001332: TCRU_702 TcCLB-EL.511867.50_mRNA-p1 TcCLB-NE.
507775.50_mRNA-p1 TcSYL_0146370.t1-p1
OG0001333: TCRU_705 TcCLB-EL.509233.160_mRNA-p1 TcCLB-NE.
467287.10_mRNA-p1 TcSYL_0145330.t1-p1
OG0001334: TCRU_716 TcCLB-EL.510747.80_mRNA-p1 TcCLB-NE.
510657.160_mRNA-p1 TcSYL_0115920.t1-p1
OG0001335: TCRU_720 TcCLB-EL.510747.120_mRNA-p1 TcCLB-NE.
510657.110_mRNA-p1 TcSYL_0115910.t1-p1
OG0001336: TCRU_721 TcCLB-EL.510747.130_mRNA-p1 TcCLB-NE.
510657.100_mRNA-p1 TcSYL_0115900.t1-p1
OG0001337: TCRU_722 TcCLB-EL.510747.140_mRNA-p1 TcCLB-NE.
510657.90_mRNA-p1 TcSYL_0115850.t1-p1
OG0001338: TCRU_723 TcCLB-EL.510747.150_mRNA-p1 TcCLB-NE.
510657.80_mRNA-p1 TcSYL_0115830.t1-p1
OG0001339: TCRU_727 TcCLB-EL.510749.30_mRNA-p1 TcCLB-NE.
510657.30_mRNA-p1 TcSYL_0115790.t1-p1

OG0001340: TCRU_729 TcCLB-EL.510749.50_mRNA-p1 TcCLB-NE.
510657.10_mRNA-p1 TcSYL_0115780.t1-p1
OG0001341: TCRU_736 TcCLB-EL.508469.20_mRNA-p1 TcCLB-NE.
510655.70_mRNA-p1 TcSYL_0115720.t1-p1
OG0001342: TCRU_739 TcCLB-EL.508469.50_pseudogenic_transcript-p1
TcCLB-NE.510655.40_mRNA-p1 TcSYL_0115700.t1-p1
OG0001343: TCRU_740 TcCLB-EL.508675.20_mRNA-p1 TcCLB-NE.
506983.60_mRNA-p1 TcSYL_0027210.t1-p1
OG0001344: TCRU_752 TcCLB-NE.511809.110_mRNA-p1 TcCLB-NE.
511811.20_mRNA-p1 TcSYL_0201680.t1-p1
OG0001345: TCRU_760 TcCLB-EL.503955.20_mRNA-p1 TcCLB-NE.
507063.90_mRNA-p1 TcSYL_0013610.t1-p1
OG0001346: TCRU_762 TcCLB-EL.503953.50_mRNA-p1 TcCLB-NE.
507063.110_mRNA-p1 TcSYL_0013590.t1-p1
OG0001347: TCRU_763 TcCLB-EL.503953.30_mRNA-p1 TcCLB-NE.
507063.130_mRNA-p1 TcSYL_0013580.t1-p1
OG0001348: TCRU_766 TcCLB-EL.503953.10_mRNA-p1 TcCLB-NE.
507063.150_mRNA-p1 TcSYL_0013560.t1-p1
OG0001349: TCRU_767 TcCLB-EL.507807.40_mRNA-p1 TcCLB-NE.
507063.160_mRNA-p1 TcSYL_0013550.t1-p1
OG0001350: TCRU_770 TcCLB-EL.507807.10_mRNA-p1 TcCLB-NE.
507063.240_pseudogenic_transcript-p1 TcSYL_0013530.t1-p1
OG0001351: TCRU_like_ TcCLB-EL.508385.60_mRNA-p1 TcCLB-NE.
504175.20_mRNA-p1 TcSYL_0163550.t1-p1
OG0001352: TCRU_794 TcCLB-EL.504087.20_mRNA-p1 TcCLB-NE.
509683.10_mRNA-p1 TcSYL_0090040.t1-p1
OG0001353: TCRU_796 TcCLB-EL.503821.10_mRNA-p1 TcCLB-NE.
509683.24_mRNA-p1 TcSYL_0089920.t1-p1
OG0001354: TCRU_797 TcCLB-EL.508731.9_mRNA-p1 TcCLB-NE.
509683.30_mRNA-p1 TcSYL_0089890.t1-p1
OG0001355: TCRU_798 TcCLB-EL.508731.20_mRNA-p1 TcCLB-NE.
509683.40_mRNA-p1 TcSYL_0089870.t1-p1
OG0001356: TCRU_800 TcCLB-EL.508731.40_mRNA-p1 TcCLB-NE.
509683.60_mRNA-p1 TcSYL_0089840.t1-p1
OG0001357: TCRU_801 TcCLB-EL.508731.60_mRNA-p1 TcCLB-NE.
509683.80_mRNA-p1 TcSYL_0089750.t1-p1
OG0001358: TCRU_804 TcCLB-EL.507875.20_mRNA-p1 TcCLB-NE.
508111.30_mRNA-p1 TcSYL_0074000.t1-p1
OG0001359: TCRU_813 TcCLB-EL.507757.70_mRNA-p1 TcCLB-NE.
508933.20_mRNA-p1 TcSYL_0038340.t1-p1
OG0001360: TCRU_814 TcCLB-EL.507757.60_mRNA-p1 TcCLB-NE.
508933.10_mRNA-p1 TcSYL_0056160.t1-p1
OG0001361: TCRU_817 TcCLB-EL.508727.30_mRNA-p1 TcCLB-NE.
503797.10_mRNA-p1 TcSYL_0107280.t1-p1
OG0001362: TCRU_818 TcCLB-EL.508727.18_mRNA-p1 TcCLB-NE.
503797.20_pseudogenic_transcript-p1 TcSYL_0107270.t1-p1
OG0001363: TCRU_833 TcCLB-EL.511559.70_pseudogenic_transcript-p1
TcCLB-NE.505171.10_mRNA-p1 TcSYL_0072910.t1-p1
OG0001364: TCRU_835 TcCLB-EL.505123.10_mRNA-p1 TcCLB-EL.
508921.10_mRNA-p1 TcCLB-NE.508141.50_mRNA-p1
OG0001365: TCRU_837 TcCLB-EL.508921.30_mRNA-p1 TcCLB-EL.
510841.10_mRNA-p1 TcCLB-NE.508141.70_mRNA-p1
OG0001366: TCRU_5754 TCRU_838 TcCLB-EL.508921.40_mRNA-p1 TcCLB-NE.
508141.80_mRNA-p1

OG0001367: TCRU_WW TcCLB-EL.511313.20_mRNA-p1 TcCLB-NE.
511755.90_mRNA-p1 TcSYL_0159660.t1-p1
OG0001368: TCRU_844 TcCLB-EL.511037.20_mRNA-p1 TcCLB-NE.
509799.140_mRNA-p1 TcSYL_0106870.t1-p1
OG0001369: TCRU_854 TcCLB-EL.508551.20_mRNA-p1 TcCLB-NE.
508231.20_mRNA-p1 TcSYL_0108040.t1-p1
OG0001370: TCRU_855 TcCLB-EL.508549.30_pseudogenic_transcript-p1
TcCLB-EL.508551.10_pseudogenic_transcript-p1 TcCLB-NE.
508231.30_mRNA-p1
OG0001371: TCRU_861 TcCLB-EL.509205.20_mRNA-p1 TcCLB-NE.
508699.30_mRNA-p1 TcSYL_0180680.t1-p1
OG0001372: TCRU_862 TcCLB-EL.509203.70_mRNA-p1 TcCLB-NE.
508699.10_mRNA-p1 TcSYL_0180600.t1-p1
OG0001373: TCRU_869 TcCLB-EL.504765.15_pseudogenic_transcript-p1
TcCLB-NE.503461.40_pseudogenic_transcript-p1 TcSYL_0056790.t1-p1
OG0001374: TCRU_870 TcCLB-EL.504765.9_mRNA-p1 TcCLB-NE.
503461.30_mRNA-p1 TcSYL_0056750.t1-p1
OG0001375: TCRU_872 TcCLB-EL.507213.30_mRNA-p1 TcCLB-NE.
511301.140_mRNA-p1 TcSYL_0121610.t1-p1
OG0001376: TCRU_873 TcCLB-EL.507213.20_mRNA-p1 TcCLB-NE.
511301.130_mRNA-p1 TcSYL_0147750.t1-p1
OG0001377: TCRU_880 TcCLB-EL.511353.20_mRNA-p1 TcCLB-NE.
511301.60_mRNA-p1 TcSYL_0121710.t1-p1
OG0001378: TCRU_883 TcCLB-EL.511353.50_mRNA-p1 TcCLB-NE.
511301.30_mRNA-p1 TcSYL_0121720.t1-p1
OG0001379: TCRU_884 TcCLB-EL.511353.69_mRNA-p1 TcCLB-NE.
511299.79_mRNA-p1 TcCLB-NE.511301.10_mRNA-p1
OG0001380: TCRU_901 TcCLB-EL.507641.80_mRNA-p1 TcSYL_0011100.t1-p1
TcSYL_0011110.t1-p1
OG0001381: TCRU_903 TcCLB-EL.509011.30_mRNA-p1 TcCLB-NE.
510685.20_mRNA-p1 TcSYL_0030680.t1-p1
OG0001382: TCRU_904 TcCLB-EL.509011.40_mRNA-p1 TcCLB-NE.
510685.10_mRNA-p1 TcSYL_0030690.t1-p1
OG0001383: TCRU_908 TcCLB-EL.506863.10_mRNA-p1 TcCLB-NE.
511825.220_mRNA-p1 TcSYL_0200820.t1-p1
OG0001384: TCRU_910 TcCLB-EL.506863.40_mRNA-p1 TcCLB-NE.
511825.250_mRNA-p1 TcSYL_0200790.t1-p1
OG0001385: TCRU_912 TcCLB-EL.506863.60_mRNA-p1 TcCLB-NE.
509211.10_mRNA-p1 TcSYL_0200780.t1-p1
OG0001386: TCRU_932 TcCLB-EL.504431.30_mRNA-p1 TcCLB-NE.
507951.80_mRNA-p1 TcSYL_0003560.t1-p1
OG0001387: TCRU_933 TcCLB-EL.504431.64_mRNA-p1 TcCLB-NE.
507951.114_mRNA-p1 TcSYL_0003550.t1-p1
OG0001388: TCRU_935 TcCLB-EL.504431.90_mRNA-p1 TcCLB-NE.
507951.140_mRNA-p1 TcSYL_0003490.t1-p1
OG0001389: TCRU_936 TcCLB-EL.504431.100_mRNA-p1 TcCLB-NE.
507951.150_mRNA-p1 TcSYL_0003510.t1-p1
OG0001390: TCRU_948 TcCLB-EL.508089.30_mRNA-p1 TcCLB-NE.
508857.50_mRNA-p1 TcSYL_0026950.t1-p1
OG0001391: TCRU_949 TcCLB-EL.508089.40_mRNA-p1 TcCLB-NE.
508857.60_mRNA-p1 TcSYL_0026910.t1-p1
OG0001392: TCRU_950 TcCLB-EL.508089.50_mRNA-p1 TcCLB-NE.
508857.70_mRNA-p1 TcSYL_0026890.t1-p1
OG0001393: TCRU_951 TcCLB-EL.508089.60_mRNA-p1 TcCLB-NE.

508857.80_mRNA-p1 TcSYL_0026850.t1-p1
OG0001394: TCRU_957 TcCLB-EL.506221.50_mRNA-p1 TcCLB-NE.
504217.10_mRNA-p1 TcSYL_0141760.t1-p1
OG0001395: TCRU_967 TcCLB-EL.503435.30_mRNA-p1 TcCLB-NE.
511285.20_mRNA-p1 TcSYL_0123520.t1-p1
OG0001396: TCRU_969 TcCLB-EL.506215.19_mRNA-p1 TcCLB-NE.
511285.4_mRNA-p1 TcSYL_0123600.t1-p1
OG0001397: TCRU_989 TcCLB-NE.510421.250_mRNA-p1 TcSYL_0019300.t1-p1
TcSYL_0019320.t1-p1
OG0001398: TCRU_998 TcCLB-EL.509197.30_mRNA-p1 TcCLB-NE.
508701.10_mRNA-p1 TcSYL_0180950.t1-p1
OG0001399: TCRU_1006 TcCLB-EL.509965.20_mRNA-p1 TcCLB-NE.
509059.10_mRNA-p1 TcCLB-NE.511499.69_mRNA-p1
OG0001400: TCRU_1023 TcCLB-EL.503575.60_mRNA-p1 TcCLB-NE.
510943.40_mRNA-p1 TcSYL_0001410.t1-p1
OG0001401: TCRU_1026 TcCLB-EL.503577.20_mRNA-p1 TcCLB-NE.
510943.60_mRNA-p1 TcSYL_0001440.t1-p1
OG0001402: TCRU_1028 TcCLB-EL.504867.20_mRNA-p1 TcCLB-NE.
510943.80_mRNA-p1 TcSYL_0001460.t1-p1
OG0001403: TCRU_1029 TcCLB-EL.504867.30_mRNA-p1 TcCLB-NE.
510943.90_mRNA-p1 TcSYL_0001470.t1-p1
OG0001404: TCRU_1030 TcCLB-EL.504867.40_mRNA-p1 TcCLB-NE.
510943.100_mRNA-p1 TcSYL_0001480.t1-p1
OG0001405: TCRU_1031 TcCLB-EL.504867.50_mRNA-p1 TcCLB-NE.
510943.110_mRNA-p1 TcSYL_0001490.t1-p1
OG0001406: TCRU_1032 TcCLB-EL.504867.60_mRNA-p1 TcCLB-NE.
510943.120_mRNA-p1 TcSYL_0001500.t1-p1
OG0001407: TCRU_1033 TcCLB-EL.504867.70_mRNA-p1 TcCLB-NE.
510943.130_mRNA-p1 TcSYL_0001510.t1-p1
OG0001408: TCRU_1034 TcCLB-EL.504867.80_mRNA-p1 TcCLB-NE.
510943.140_mRNA-p1 TcSYL_0001520.t1-p1
OG0001409: TCRU_1035 TcCLB-EL.504867.90_mRNA-p1 TcCLB-NE.
510943.150_mRNA-p1 TcSYL_0001540.t1-p1
OG0001410: TCRU_1036 TcCLB-EL.504867.99_mRNA-p1 TcCLB-NE.
510943.170_mRNA-p1 TcSYL_0001550.t1-p1
OG0001411: TCRU_1037 TcCLB-EL.504867.110_mRNA-p1 TcCLB-NE.
510943.180_mRNA-p1 TcSYL_0001560.t1-p1
OG0001412: TCRU_1039 TcCLB-EL.504867.120_mRNA-p1 TcCLB-NE.
510943.190_mRNA-p1 TcSYL_0001570.t1-p1
OG0001413: TCRU_1040 TCRU_1818 TcCLB-NE.
509657.70_pseudogenic_transcript-p1 TcCLB-NE.
510201.40_pseudogenic_transcript-p1
OG0001414: TCRU_1046 TcCLB-EL.504075.3_mRNA-p1 TcCLB-EL.
506933.89_mRNA-p1 TcSYL_0044080.t1-p1
OG0001415: TCRU_1051 TcCLB-EL.509537.90_mRNA-p1 TcCLB-NE.
402857.10_mRNA-p1 TcSYL_0073960.t1-p1
OG0001416: TCRU_1061 TcCLB-EL.508955.10_mRNA-p1 TcCLB-NE.
508059.40_mRNA-p1 TcSYL_0168950.t1-p1
OG0001417: TCRU_1065 TcCLB-EL.506493.90_mRNA-p1 TcCLB-NE.
510121.170_mRNA-p1 TcSYL_0168980.t1-p1
OG0001418: TCRU_1066 TcCLB-EL.506493.80_mRNA-p1 TcCLB-NE.
510121.160_mRNA-p1 TcSYL_0169000.t1-p1
OG0001419: TCRU_1067 TcCLB-EL.506493.70_mRNA-p1 TcCLB-NE.
510121.150_mRNA-p1 TcSYL_0169020.t1-p1

OG0001420: TCRU_1068 TcCLB-EL.506493.60_mRNA-p1 TcCLB-NE.
510121.140_mRNA-p1 TcSYL_0169030.t1-p1
OG0001421: TCRU_1069 TcCLB-EL.506493.50_mRNA-p1 TcCLB-NE.
510121.130_mRNA-p1 TcSYL_0169050.t1-p1
OG0001422: TCRU_1070 TcCLB-EL.506493.40_mRNA-p1 TcCLB-NE.
510121.120_mRNA-p1 TcSYL_0169060.t1-p1
OG0001423: TCRU_1071 TcCLB-EL.506493.30_mRNA-p1 TcCLB-NE.
510121.110_mRNA-p1 TcSYL_0169080.t1-p1
OG0001424: TCRU_1072 TcCLB-EL.506493.20_mRNA-p1 TcCLB-NE.
510121.100_mRNA-p1 TcSYL_0169090.t1-p1
OG0001425: TCRU_1073 TcCLB-EL.506493.9_mRNA-p1 TcCLB-NE.
510121.90_mRNA-p1 TcSYL_0169110.t1-p1
OG0001426: TCRU_1074 TcCLB-EL.508951.80_mRNA-p1 TcCLB-NE.
510121.80_mRNA-p1 TcSYL_0169130.t1-p1
OG0001427: TCRU_1075 TcCLB-EL.508951.70_mRNA-p1 TcCLB-NE.
510121.70_mRNA-p1 TcSYL_0169140.t1-p1
OG0001428: TCRU_1076 TcCLB-EL.508951.60_mRNA-p1 TcCLB-NE.
510121.60_mRNA-p1 TcSYL_0169150.t1-p1
OG0001429: TCRU_1077 TcCLB-EL.508951.50_mRNA-p1 TcCLB-NE.
510121.50_mRNA-p1 TcSYL_0169160.t1-p1
OG0001430: TCRU_1078 TcCLB-EL.508951.40_mRNA-p1 TcCLB-NE.
510121.40_mRNA-p1 TcSYL_0169170.t1-p1
OG0001431: TCRU_1079 TcCLB-EL.508951.29_mRNA-p1 TcCLB-NE.
510121.30_mRNA-p1 TcSYL_0169180.t1-p1
OG0001432: TCRU_1080 TcCLB-EL.508951.20_mRNA-p1 TcCLB-NE.
510121.20_mRNA-p1 TcSYL_0169200.t1-p1
OG0001433: TCRU_1083 TcCLB-EL.508949.10_mRNA-p1 TcCLB-NE.
510119.30_mRNA-p1 TcSYL_0169260.t1-p1
OG0001434: TCRU_1092 TcCLB-EL.510759.100_mRNA-p1 TcCLB-NE.
506999.120_mRNA-p1 TcSYL_0115490.t1-p1
OG0001435: TCRU_1095 TcCLB-EL.510759.120_mRNA-p1 TcCLB-NE.
506999.90_mRNA-p1 TcSYL_0115480.t1-p1
OG0001436: TCRU_1099 TcCLB-EL.510759.150_mRNA-p1 TcCLB-NE.
506999.50_mRNA-p1 TcSYL_0115470.t1-p1
OG0001437: TCRU_1102 TcCLB-EL.510759.180_mRNA-p1 TcCLB-NE.
506999.20_mRNA-p1 TcSYL_0115460.t1-p1
OG0001438: TCRU_1103 TcCLB-EL.503651.4_mRNA-p1 TcCLB-EL.
510759.200_mRNA-p1 TcSYL_0115450.t1-p1
OG0001439: TCRU_1127 TcCLB-EL.413605.10_mRNA-p1 TcCLB-NE.
511817.220_mRNA-p1 TcSYL_0201540.t1-p1
OG0001440: TCRU_1128 TcCLB-EL.503853.10_mRNA-p1 TcCLB-NE.
511817.230_mRNA-p1 TcSYL_0201530.t1-p1
OG0001441: TCRU_1130 TcCLB-EL.510529.20_mRNA-p1 TcCLB-NE.
511817.250_mRNA-p1 TcSYL_0201510.t1-p1
OG0001442: TCRU_1131 TcCLB-EL.510529.30_mRNA-p1 TcCLB-NE.
511817.260_mRNA-p1 TcSYL_0201500.t1-p1
OG0001443: TCRU_1133 TcCLB-EL.510531.10_mRNA-p1 TcCLB-NE.
511817.280_mRNA-p1 TcSYL_0201480.t1-p1
OG0001444: TCRU_1136 TcCLB-EL.510531.40_mRNA-p1 TcCLB-NE.
511817.319_mRNA-p1 TcSYL_0201470.t1-p1
OG0001445: TCRU_1137 TcCLB-EL.510531.54_mRNA-p1 TcCLB-NE.
511819.14_mRNA-p1 TcSYL_0201450.t1-p1
OG0001446: TCRU_1146 TcCLB-EL.507711.40_mRNA-p1 TcCLB-NE.
424123.40_mRNA-p1 TcSYL_0008850.t1-p1

OG0001447: TCRU_1154 TcCLB-EL.510247.20_mRNA-p1 TcCLB-NE.
506491.10_mRNA-p1 TcSYL_0148330.t1-p1
OG0001448: TCRU_1185 TcCLB-EL.507521.140_mRNA-p1 TcCLB-NE.
508577.60_mRNA-p1 TcSYL_0041200.t1-p1
OG0001449: TCRU_1186 TcCLB-EL.507521.130_mRNA-p1 TcCLB-NE.
508577.70_mRNA-p1 TcSYL_0041210.t1-p1
OG0001450: TCRU_1187 TcCLB-EL.507521.120_mRNA-p1 TcCLB-NE.
508577.80_mRNA-p1 TcSYL_0041220.t1-p1
OG0001451: TCRU_1190 TcCLB-EL.507521.100_mRNA-p1 TcCLB-NE.
508577.110_mRNA-p1 TcSYL_0041230.t1-p1
OG0001452: TCRU_1191 TcCLB-EL.507521.90_mRNA-p1 TcCLB-NE.
508577.120_mRNA-p1 TcSYL_0041240.t1-p1
OG0001453: TCRU_1194 TcCLB-EL.507521.60_mRNA-p1 TcCLB-NE.
508577.150_mRNA-p1 TcSYL_0041260.t1-p1
OG0001454: TCRU_1195 TcCLB-EL.507521.50_mRNA-p1 TcCLB-NE.
508577.160_mRNA-p1 TcSYL_0041270.t1-p1
OG0001455: TCRU_1210 TcCLB-EL.510431.80_mRNA-p1 TcCLB-NE.
508337.10_mRNA-p1 TcSYL_0138140.t1-p1
OG0001456: TCRU_1212 TcCLB-EL.506635.110_mRNA-p1 TcCLB-NE.
508029.50_mRNA-p1 TcSYL_0045020.t1-p1
OG0001457: TCRU_1213 TcCLB-EL.506635.100_mRNA-p1 TcCLB-NE.
508029.30_mRNA-p1 TcSYL_0045030.t1-p1
OG0001458: TCRU_1216 TcCLB-EL.506635.50_mRNA-p1 TcCLB-NE.
508027.100_mRNA-p1 TcSYL_0045040.t1-p1
OG0001459: TCRU_1219 TcCLB-EL.506635.20_mRNA-p1 TcCLB-NE.
508027.70_mRNA-p1 TcSYL_0045060.t1-p1
OG0001460: TCRU_1225 TcCLB-EL.503757.20_mRNA-p1 TcCLB-NE.
503719.30_mRNA-p1 TcSYL_0118200.t1-p1
OG0001461: TCRU_1236 TcCLB-EL.509199.10_mRNA-p1 TcCLB-NE.
508601.130_mRNA-p1 TcSYL_0179550.t1-p1
OG0001462: TCRU_1238 TcCLB-EL.511801.10_mRNA-p1 TcCLB-NE.
508601.100_mRNA-p1 TcSYL_0179670.t1-p1
OG0001463: TCRU_1239 TcCLB-EL.511801.14_mRNA-p1 TcCLB-NE.
508601.90_mRNA-p1 TcSYL_0179700.t1-p1
OG0001464: TCRU_1240 TcCLB-EL.511801.20_mRNA-p1 TcCLB-NE.
508601.70_mRNA-p1 TcSYL_0179730.t1-p1
OG0001465: TCRU_1241 TcCLB-EL.511801.30_mRNA-p1 TcCLB-NE.
508601.60_mRNA-p1 TcSYL_0179750.t1-p1
OG0001466: TCRU_1242 TcCLB-EL.511801.34_mRNA-p1 TcCLB-NE.
508601.50_mRNA-p1 TcSYL_0179770.t1-p1
OG0001467: TCRU_1244 TcCLB-EL.511801.44_mRNA-p1 TcCLB-NE.
508601.30_mRNA-p1 TcSYL_0179800.t1-p1
OG0001468: TCRU_1245 TcCLB-EL.511801.50_mRNA-p1 TcCLB-NE.
508601.20_mRNA-p1 TcSYL_0179830.t1-p1
OG0001469: TCRU_1246 TcCLB-EL.511801.70_mRNA-p1 TcCLB-NE.
510897.99_mRNA-p1 TcSYL_0179870.t1-p1
OG0001470: TCRU_1247 TcCLB-EL.456097.30_mRNA-p1 TcCLB-NE.
510897.70_pseudogenic_transcript-p1 TcSYL_0179900.t1-p1
OG0001471: TCRU_1251 TcCLB-EL.510603.90_mRNA-p1 TcCLB-NE.
509109.114_mRNA-p1 TcSYL_0014290.t1-p1
OG0001472: TCRU_C-terminal TcCLB-EL.503905.50_mRNA-p1 TcCLB-NE.
507705.10_mRNA-p1 TcSYL_0094570.t1-p1
OG0001473: TCRU_1260 TcCLB-EL.507757.30_mRNA-p1 TcCLB-NE.
508931.40_mRNA-p1 TcSYL_0056070.t1-p1

OG0001474: TCRU_1262 TcCLB-EL.506565.20_mRNA-p1 TcCLB-NE.
511837.120_mRNA-p1 TcSYL_0114790.t1-p1
OG0001475: TCRU_1266 TcCLB-EL.504507.5_mRNA-p1 TcCLB-NE.
503555.30_mRNA-p1 TcSYL_0006250.t1-p1
OG0001476: TCRU_1271 TcCLB-EL.511583.30_mRNA-p1 TcCLB-NE.
510153.10_mRNA-p1 TcSYL_0109350.t1-p1
OG0001477: TCRU_1272 TcCLB-EL.511583.20_mRNA-p1 TcCLB-NE.
431889.10_mRNA-p1 TcCLB-NE.510153.5_mRNA-p1
OG0001478: TCRU_1275 TcCLB-EL.510939.9_mRNA-p1 TcCLB-NE.
506821.90_mRNA-p1 TcSYL_0088590.t1-p1
OG0001479: TCRU_subunit TcCLB-EL.432787.10_mRNA-p1 TcCLB-NE.
506821.100_mRNA-p1 TcSYL_0088600.t1-p1
OG0001480: TCRU_1277 TcCLB-EL.504159.109_mRNA-p1 TcCLB-NE.
506821.110_mRNA-p1 TcSYL_0088610.t1-p1
OG0001481: TCRU_1281 TcCLB-EL.504159.70_mRNA-p1 TcCLB-NE.
506821.150_mRNA-p1 TcSYL_0088620.t1-p1
OG0001482: TCRU_1283 TcCLB-EL.504159.50_mRNA-p1 TcCLB-NE.
506821.170_mRNA-p1 TcSYL_0088630.t1-p1
OG0001483: TCRU_1296 TcCLB-EL.504153.330_mRNA-p1 TcCLB-NE.
506301.30_mRNA-p1 TcSYL_0138560.t1-p1
OG0001484: TCRU_1307 TcCLB-EL.506529.440_mRNA-p1 TcCLB-NE.
510889.150_mRNA-p1 TcSYL_0178340.t1-p1
OG0001485: TCRU_1308 TcCLB-EL.506529.460_mRNA-p1 TcCLB-NE.
510889.160_pseudogenic_transcript-p1 TcSYL_0178150.t1-p1
OG0001486: TCRU_1312 TcCLB-EL.503393.10_mRNA-p1 TcCLB-EL.
511325.25_mRNA-p1 TcCLB-NE.504223.10_mRNA-p1
OG0001487: TCRU_1319 TcCLB-EL.508805.20_mRNA-p1 TcCLB-NE.
503911.30_mRNA-p1 TcSYL_0203380.t1-p1
OG0001488: TCRU_1328 TcCLB-EL.504427.120_mRNA-p1 TcCLB-NE.
509331.20_mRNA-p1 TcSYL_0172640.t1-p1
OG0001489: TCRU_1329 TcCLB-EL.504427.130_mRNA-p1 TcCLB-NE.
507221.80_mRNA-p1 TcSYL_0172680.t1-p1
OG0001490: TCRU_1331 TcCLB-EL.504427.150_mRNA-p1 TcCLB-NE.
507221.60_pseudogenic_transcript-p1 TcSYL_0172730.t1-p1
OG0001491: TCRU_1332 TcCLB-EL.504427.170_mRNA-p1 TcCLB-NE.
507221.50_mRNA-p1 TcSYL_0172770.t1-p1
OG0001492: TCRU_1333 TcCLB-EL.504427.180_mRNA-p1 TcCLB-NE.
507221.40_mRNA-p1 TcSYL_0172810.t1-p1
OG0001493: TCRU_1334 TcCLB-EL.504427.190_mRNA-p1 TcCLB-NE.
507221.30_mRNA-p1 TcSYL_0172830.t1-p1
OG0001494: TCRU_1335 TcCLB-EL.504427.200_mRNA-p1 TcCLB-NE.
507221.20_mRNA-p1 TcSYL_0172890.t1-p1
OG0001495: TCRU_1348 TcCLB-EL.511113.9_mRNA-p1 TcCLB-NE.
507715.70_mRNA-p1 TcSYL_0086660.t1-p1
OG0001496: TCRU_1349 TcCLB-EL.511115.20_mRNA-p1 TcCLB-NE.
507715.40_mRNA-p1 TcSYL_0086680.t1-p1
OG0001497: TCRU_1351 TcCLB-EL.511115.40_mRNA-p1 TcCLB-NE.
507715.30_mRNA-p1 TcSYL_0086700.t1-p1
OG0001498: TCRU_1352 TcCLB-EL.480653.10_mRNA-p1 TcCLB-NE.
507715.20_mRNA-p1 TcSYL_0086710.t1-p1
OG0001499: TCRU_1353 TcCLB-EL.486465.9_mRNA-p1 TcCLB-NE.
507715.10_mRNA-p1 TcSYL_0086720.t1-p1
OG0001500: TCRU_1354 TCRU_8050 TcCLB-NE.
509753.180_pseudogenic_transcript-p1 TcCLB-NE.

509755.60_pseudogenic_transcript-p1
OG0001501: TCRU_1358 TcCLB-EL.506553.20_mRNA-p1 TcCLB-NE.
507769.30_mRNA-p1 TcSYL_0073640.t1-p1
OG0001502: TCRU_1360 TcCLB-EL.510105.20_mRNA-p1 TcCLB-NE.
509715.9_mRNA-p1 TcSYL_0073590.t1-p1
OG0001503: TCRU_1361 TcCLB-EL.510105.30_mRNA-p1 TcCLB-NE.
509715.20_mRNA-p1 TcSYL_0073580.t1-p1
OG0001504: TCRU_1405 TcCLB-EL.509599.170_mRNA-p1 TcCLB-NE.
506327.60_mRNA-p1 TcSYL_0010890.t1-p1
OG0001505: TCRU_1407 TcCLB-EL.506167.20_mRNA-p1 TcCLB-NE.
506327.30_mRNA-p1 TcSYL_0010920.t1-p1
OG0001506: TCRU_1408 TcCLB-EL.506167.30_mRNA-p1 TcCLB-NE.
506327.20_mRNA-p1 TcSYL_0010930.t1-p1
OG0001507: TCRU_1413 TcCLB-EL.507711.70_mRNA-p1 TcCLB-NE.
509637.18_mRNA-p1 TcSYL_0009050.t1-p1
OG0001508: TCRU_1414 TcCLB-EL.507711.80_mRNA-p1 TcCLB-NE.
509637.27_mRNA-p1 TcSYL_0009010.t1-p1
OG0001509: TCRU_1417 TcCLB-NE.507951.15_mRNA-p1 TcSYL_0003580.t1-p1
TcSYL_0003650.t1-p1
OG0001510: TCRU_1418 TcCLB-EL.508811.10_mRNA-p1 TcCLB-NE.
503413.19_mRNA-p1 TcCLB-NE.503415.10_pseudogenic_transcript-p1
OG0001511: TCRU_1470 TcCLB-EL.507711.150_mRNA-p1 TcCLB-NE.
509641.30_mRNA-p1 TcSYL_0008790.t1-p1
OG0001512: TCRU_1482 TcCLB-EL.511071.190_mRNA-p1 TcCLB-NE.
504037.10_mRNA-p1 TcSYL_0103610.t1-p1
OG0001513: TCRU_1487 TCRU_4102 TcCLB-NE.503973.320_mRNA-p1 TcCLB-NE.
504081.500_mRNA-p1
OG0001514: TCRU_1508 TcCLB-EL.508357.23_mRNA-p1 TcCLB-NE.
508355.350_mRNA-p1 TcSYL_0082910.t1-p1
OG0001515: TCRU_1522 TcCLB-EL.508539.50_mRNA-p1 TcCLB-NE.
511459.20_mRNA-p1 TcSYL_0130740.t1-p1
OG0001516: TCRU_1528 TcCLB-EL.510753.20_mRNA-p1 TcCLB-NE.
510655.20_mRNA-p1 TcSYL_0115680.t1-p1
OG0001517: TCRU_1529 TcCLB-EL.510753.30_mRNA-p1 TcCLB-NE.
510655.10_mRNA-p1 TcSYL_0115670.t1-p1
OG0001518: TCRU_1543 TcCLB-EL.510345.20_mRNA-p1 TcCLB-NE.
506469.70_mRNA-p1 TcSYL_0156880.t1-p1
OG0001519: TCRU_1552 TcCLB-EL.506181.150_mRNA-p1 TcCLB-NE.
506177.50_mRNA-p1 TcSYL_0092420.t1-p1
OG0001520: TCRU_1555 TcCLB-EL.506181.120_mRNA-p1 TcCLB-NE.
506177.80_mRNA-p1 TcSYL_0092410.t1-p1
OG0001521: TCRU_1556 TcCLB-EL.506181.110_mRNA-p1 TcCLB-NE.
506177.90_mRNA-p1 TcSYL_0092400.t1-p1
OG0001522: TCRU_1557 TcCLB-EL.506181.104_mRNA-p1 TcCLB-NE.
506177.100_mRNA-p1 TcSYL_0092390.t1-p1
OG0001523: TCRU_1558 TcCLB-EL.506181.97_mRNA-p1 TcCLB-NE.
506177.110_mRNA-p1 TcSYL_0092380.t1-p1
OG0001524: TCRU_1559 TcCLB-EL.506181.70_mRNA-p1 TcCLB-NE.
506177.150_mRNA-p1 TcSYL_0092350.t1-p1
OG0001525: TCRU_1562 TcCLB-EL.506181.40_mRNA-p1 TcCLB-NE.
503395.30_mRNA-p1 TcSYL_0092330.t1-p1
OG0001526: TCRU_1563 TcCLB-EL.506181.30_mRNA-p1 TcCLB-NE.
503395.20_mRNA-p1 TcSYL_0092320.t1-p1
OG0001527: TCRU_1565 TcCLB-EL.506181.10_mRNA-p1 TcCLB-NE.

507663.20_mRNA-p1 TcSYL_0092300.t1-p1
OG0001528: TCRU_1566 TcCLB-EL.509625.30_mRNA-p1 TcCLB-NE.
507663.30_mRNA-p1 TcSYL_0092290.t1-p1
OG0001529: TCRU_1568 TcCLB-EL.509625.10_mRNA-p1 TcCLB-NE.
507663.50_mRNA-p1 TcSYL_0092280.t1-p1
OG0001530: TCRU_1570 TcCLB-EL.506179.40_mRNA-p1 TcCLB-NE.
507663.70_mRNA-p1 TcSYL_0092250.t1-p1
OG0001531: TCRU_1571 TcCLB-EL.506179.20_mRNA-p1 TcCLB-NE.
507665.10_mRNA-p1 TcSYL_0092230.t1-p1
OG0001532: TCRU_1575 TcCLB-EL.508989.100_mRNA-p1 TcCLB-NE.
504093.10_pseudogenic_transcript-p1 TcSYL_0097640.t1-p1
OG0001533: TCRU_1576 TcCLB-EL.508989.110_mRNA-p1 TcCLB-NE.
466593.19_mRNA-p1 TcSYL_0097650.t1-p1
OG0001534: TCRU_1582 TcCLB-EL.506961.35_pseudogenic_transcript-p1
TcCLB-EL.508605.31_pseudogenic_transcript-p1 TcCLB-NE.
508297.14_pseudogenic_transcript-p1
OG0001535: TCRU_1596 TcCLB-EL.506239.40_mRNA-p1 TcCLB-NE.
506177.30_mRNA-p1 TcSYL_0092430.t1-p1
OG0001536: TCRU_1602 TcCLB-EL.510999.19_mRNA-p1 TcCLB-NE.
503635.89_mRNA-p1 TcSYL_0132470.t1-p1
OG0001537: TCRU_1603 TcCLB-EL.510999.30_mRNA-p1 TcCLB-NE.
503635.80_mRNA-p1 TcSYL_0132430.t1-p1
OG0001538: TCRU_1613 TcCLB-EL.510105.250_mRNA-p1 TcCLB-NE.
509717.100_mRNA-p1 TcSYL_0073410.t1-p1
OG0001539: TCRU_1640 TcCLB-EL.506287.90_mRNA-p1 TcCLB-NE.
506991.10_mRNA-p1 TcSYL_0023500.t1-p1
OG0001540: TCRU_1644 TcCLB-EL.506287.140_mRNA-p1 TcCLB-NE.
508881.120_mRNA-p1 TcSYL_0023490.t1-p1
OG0001541: TCRU_1645 TcCLB-EL.506287.150_mRNA-p1 TcCLB-NE.
508881.110_mRNA-p1 TcSYL_0023480.t1-p1
OG0001542: TCRU_1646 TcCLB-EL.506287.200_mRNA-p1 TcCLB-NE.
508881.80_mRNA-p1 TcSYL_0023440.t1-p1
OG0001543: TCRU_1648 TcCLB-EL.506289.30_mRNA-p1 TcCLB-NE.
508881.54_mRNA-p1 TcSYL_0023430.t1-p1
OG0001544: TCRU_1649 TcCLB-EL.506289.40_mRNA-p1 TcCLB-NE.
508881.50_mRNA-p1 TcSYL_0023420.t1-p1
OG0001545: TCRU_1650 TcCLB-EL.506289.50_mRNA-p1 TcCLB-NE.
508881.40_mRNA-p1 TcSYL_0023410.t1-p1
OG0001546: TCRU_1652 TcCLB-EL.506289.70_mRNA-p1 TcCLB-NE.
508881.20_mRNA-p1 TcSYL_0023400.t1-p1
OG0001547: TCRU_1658 TCRU_7930 TcSYL_0023360.t1-p1 TcSYL_0137510.t1-
p1
OG0001548: TCRU_1662 TcCLB-EL.511537.50_mRNA-p1 TcCLB-NE.
508707.330_mRNA-p1 TcSYL_0111650.t1-p1
OG0001549: TCRU_1680 TcCLB-EL.510295.40_mRNA-p1 TcCLB-NE.
506007.30_mRNA-p1 TcSYL_0113520.t1-p1
OG0001550: TCRU_1681 TcCLB-EL.510295.50_mRNA-p1 TcCLB-NE.
506007.40_mRNA-p1 TcSYL_0113530.t1-p1
OG0001551: TCRU_1683 TcCLB-EL.510297.20_mRNA-p1 TcCLB-NE.
506009.20_mRNA-p1 TcSYL_0113540.t1-p1
OG0001552: TCRU_1688 TcCLB-EL.510297.80_mRNA-p1 TcCLB-NE.
506009.80_mRNA-p1 TcSYL_0113550.t1-p1
OG0001553: TCRU_1689 TcCLB-EL.510297.90_mRNA-p1 TcCLB-NE.
506009.90_mRNA-p1 TcSYL_0113560.t1-p1

OG0001554: TCRU_1690 TcCLB-EL.510297.100_mRNA-p1 TcCLB-NE.
506009.100_mRNA-p1 TcSYL_0113580.t1-p1
OG0001555: TCRU_1693 TcCLB-EL.509341.10_mRNA-p1 TcCLB-NE.
510945.50_mRNA-p1 TcSYL_0048200.t1-p1
OG0001556: TCRU_1694 TcCLB-EL.509341.20_mRNA-p1 TcCLB-NE.
510945.60_mRNA-p1 TcSYL_0048240.t1-p1
OG0001557: TCRU_1696 TcCLB-EL.509167.170_mRNA-p1 TcCLB-NE.
504027.10_mRNA-p1 TcSYL_0140220.t1-p1
OG0001558: TCRU_1703 TcCLB-EL.510349.70_mRNA-p1 TcCLB-NE.
503925.110_mRNA-p1 TcSYL_0156670.t1-p1
OG0001559: TCRU_1704 TcCLB-EL.510349.64_mRNA-p1 TcCLB-NE.
503925.100_mRNA-p1 TcSYL_0156680.t1-p1
OG0001560: TCRU_1708 TcCLB-EL.508777.110_mRNA-p1 TcCLB-NE.
509029.140_mRNA-p1 TcSYL_0156100.t1-p1
OG0001561: TCRU_1711 TcCLB-EL.508777.150_mRNA-p1 TcCLB-NE.
511483.10_mRNA-p1 TcSYL_0127640.t1-p1
OG0001562: TCRU_1716 TcCLB-NE.508197.10_mRNA-p1 TcSYL_0146670.t1-p1
TcSYL_0146740.t1-p1
OG0001563: TCRU_1724 TcCLB-EL.504425.70_mRNA-p1 TcCLB-NE.
509331.200_mRNA-p1 TcSYL_0172090.t1-p1
OG0001564: TCRU_1725 TcCLB-EL.504425.80_mRNA-p1 TcCLB-NE.
509331.189_mRNA-p1 TcSYL_0172120.t1-p1
OG0001565: TCRU_1727 TcCLB-EL.504425.90_mRNA-p1 TcCLB-NE.
509331.180_mRNA-p1 TcSYL_0172170.t1-p1
OG0001566: TCRU_1728 TcCLB-EL.504425.100_mRNA-p1 TcCLB-NE.
509331.160_mRNA-p1 TcSYL_0172190.t1-p1
OG0001567: TCRU_1729 TcCLB-EL.504425.110_mRNA-p1 TcCLB-NE.
509331.150_mRNA-p1 TcSYL_0172220.t1-p1
OG0001568: TCRU_1734 TcCLB-EL.505977.13_mRNA-p1 TcCLB-NE.
470521.10_mRNA-p1 TcSYL_0064360.t1-p1
OG0001569: TCRU_1735 TcCLB-EL.504449.30_mRNA-p1 TcCLB-NE.
506447.19_mRNA-p1 TcSYL_0064370.t1-p1
OG0001570: TCRU_1740 TcCLB-EL.511311.80_mRNA-p1 TcCLB-NE.
511755.119_mRNA-p1 TcSYL_0159670.t1-p1
OG0001571: TCRU_1765 TcCLB-EL.503409.10_mRNA-p1 TcCLB-NE.
505193.60_mRNA-p1 TcSYL_0104480.t1-p1
OG0001572: TCRU_1766 TcCLB-EL.510407.100_mRNA-p1 TcCLB-NE.
509937.140_mRNA-p1 TcSYL_0027580.t1-p1
OG0001573: TCRU_1767 TcCLB-EL.510407.90_mRNA-p1 TcCLB-NE.
509937.150_mRNA-p1 TcSYL_0027590.t1-p1
OG0001574: TCRU_1768 TcCLB-EL.510407.80_mRNA-p1 TcCLB-NE.
509937.160_mRNA-p1 TcSYL_0027600.t1-p1
OG0001575: TCRU_1771 TcCLB-EL.510407.30_mRNA-p1 TcCLB-NE.
509937.200_mRNA-p1 TcSYL_0027640.t1-p1
OG0001576: TCRU_1773 TcCLB-EL.510407.9_mRNA-p1 TcCLB-NE.
509937.220_mRNA-p1 TcSYL_0027650.t1-p1
OG0001577: TCRU_1775 TcCLB-EL.508265.110_mRNA-p1 TcCLB-NE.
506441.10_mRNA-p1 TcSYL_0027670.t1-p1
OG0001578: TCRU_1776 TcCLB-EL.508265.100_mRNA-p1 TcCLB-NE.
506441.20_mRNA-p1 TcSYL_0027680.t1-p1
OG0001579: TCRU_1777 TcCLB-EL.508265.70_mRNA-p1 TcCLB-NE.
506443.20_mRNA-p1 TcSYL_0027690.t1-p1
OG0001580: TCRU_1779 TcCLB-EL.508265.44_mRNA-p1 TcCLB-NE.
506443.40_mRNA-p1 TcSYL_0027700.t1-p1

OG0001581: TCRU_1780 TcCLB-EL.508265.40_mRNA-p1 TcCLB-NE.
506443.50_mRNA-p1 TcSYL_0027710.t1-p1
OG0001582: TCRU_1781 TcCLB-EL.508265.30_mRNA-p1 TcCLB-NE.
506443.60_mRNA-p1 TcSYL_0027730.t1-p1
OG0001583: TCRU_1782 TcCLB-EL.508265.10_mRNA-p1 TcCLB-NE.
506443.70_mRNA-p1 TcSYL_0027750.t1-p1
OG0001584: TCRU_1803 TcCLB-EL.504029.80_mRNA-p1 TcCLB-NE.
509997.86_mRNA-p1 TcSYL_0003280.t1-p1
OG0001585: TCRU_1805 TcCLB-EL.504029.60_mRNA-p1 TcCLB-NE.
509999.20_mRNA-p1 TcSYL_0003270.t1-p1
OG0001586: TCRU_1806 TcCLB-EL.504029.50_mRNA-p1 TcCLB-NE.
509999.30_mRNA-p1 TcSYL_0003260.t1-p1
OG0001587: TCRU_1807 TcCLB-EL.504029.21_mRNA-p1 TcCLB-NE.
509999.50_mRNA-p1 TcSYL_0003250.t1-p1
OG0001588: TCRU_1811 TcCLB-EL.503685.30_mRNA-p1 TcCLB-NE.
509999.90_mRNA-p1 TcSYL_0003210.t1-p1
OG0001589: TCRU_1820 TCRU_5180 TcCLB-NE.508855.10_mRNA-p1
TcSYL_0100110.t1-p1
OG0001590: TCRU_1823 TcCLB-EL.504643.30_mRNA-p1 TcCLB-NE.
509161.10_mRNA-p1 TcSYL_0142840.t1-p1
OG0001591: TCRU_1824 TcCLB-EL.504643.20_mRNA-p1 TcCLB-NE.
509161.20_mRNA-p1 TcSYL_0142850.t1-p1
OG0001592: TCRU_1847 TcCLB-EL.510755.80_mRNA-p1 TcCLB-NE.
508415.10_pseudogenic_transcript-p1 TcSYL_0115590.t1-p1
OG0001593: TCRU_1849 TcCLB-EL.510599.50_mRNA-p1 TcCLB-NE.
509911.30_mRNA-p1 TcSYL_0057420.t1-p1
OG0001594: TCRU_1850 TcCLB-EL.505073.30_mRNA-p1 TcCLB-NE.
509911.70_mRNA-p1 TcSYL_0057430.t1-p1
OG0001595: TCRU_1852 TcCLB-EL.507389.79_mRNA-p1 TcCLB-NE.
509911.80_mRNA-p1 TcSYL_0057440.t1-p1
OG0001596: TCRU_1853 TcCLB-EL.507389.70_mRNA-p1 TcCLB-NE.
509911.90_mRNA-p1 TcSYL_0057450.t1-p1
OG0001597: TCRU_1861 TcCLB-EL.508837.80_mRNA-p1 TcCLB-NE.
511383.89_mRNA-p1 TcSYL_0187800.t1-p1
OG0001598: TCRU_1896 TcCLB-EL.510533.40_mRNA-p1 TcCLB-NE.
511821.30_mRNA-p1 TcSYL_0201360.t1-p1
OG0001599: TCRU_1898 TcCLB-EL.510533.60_mRNA-p1 TcCLB-NE.
511821.50_mRNA-p1 TcSYL_0201350.t1-p1
OG0001600: TCRU_1900 TcCLB-EL.510533.80_mRNA-p1 TcCLB-NE.
511821.70_mRNA-p1 TcSYL_0201340.t1-p1
OG0001601: TCRU_1902 TcCLB-EL.510533.100_mRNA-p1 TcCLB-NE.
511821.90_mRNA-p1 TcSYL_0201330.t1-p1
OG0001602: TCRU_1903 TcCLB-EL.510533.120_mRNA-p1 TcCLB-NE.
511821.110_mRNA-p1 TcSYL_0201320.t1-p1
OG0001603: TCRU_1904 TcCLB-EL.510533.130_mRNA-p1 TcCLB-NE.
511821.120_mRNA-p1 TcSYL_0201300.t1-p1
OG0001604: TCRU_1905 TcCLB-EL.510533.150_mRNA-p1 TcCLB-NE.
511821.130_mRNA-p1 TcSYL_0201290.t1-p1
OG0001605: TCRU_1906 TcCLB-EL.510533.160_mRNA-p1 TcCLB-NE.
511821.140_mRNA-p1 TcSYL_0201260.t1-p1
OG0001606: TCRU_1907 TcCLB-EL.510533.170_mRNA-p1 TcCLB-NE.
511821.150_mRNA-p1 TcSYL_0201250.t1-p1
OG0001607: TCRU_1908 TcCLB-EL.510533.180_mRNA-p1 TcCLB-NE.
511821.160_mRNA-p1 TcSYL_0201210.t1-p1

OG0001608: TCRU_1909 TcCLB-EL.510533.190_mRNA-p1 TcCLB-NE.
511821.170_mRNA-p1 TcSYL_0201060.t1-p1
OG0001609: TCRU_1910 TcCLB-NE.511821.179_mRNA-p1 TcSYL_0201000.t1-p1
TcSYL_0201010.t1-p1
OG0001610: TCRU_1911 TcCLB-EL.510533.210_pseudogenic_transcript-p1
TcCLB-NE.511823.14_mRNA-p1 TcSYL_0200950.t1-p1
OG0001611: TCRU_1912 TcCLB-EL.510533.220_mRNA-p1 TcCLB-NE.
511823.30_mRNA-p1 TcSYL_0200920.t1-p1
OG0001612: TCRU_1914 TcCLB-EL.508323.9_mRNA-p1 TcCLB-NE.
511823.70_mRNA-p1 TcSYL_0200910.t1-p1
OG0001613: TCRU_1915 TcCLB-EL.508323.20_mRNA-p1 TcCLB-NE.
511825.4_mRNA-p1 TcSYL_0200900.t1-p1
OG0001614: TCRU_1916 TcCLB-EL.508323.30_mRNA-p1 TcCLB-NE.
511825.10_mRNA-p1 TcSYL_0200890.t1-p1
OG0001615: TCRU_1920 TcCLB-EL.508323.80_mRNA-p1 TcCLB-NE.
511825.60_mRNA-p1 TcSYL_0200850.t1-p1
OG0001616: TCRU_1930 TcCLB-EL.508321.60_mRNA-p1 TcCLB-NE.
509207.70_mRNA-p1 TcSYL_0201790.t1-p1
OG0001617: TCRU_1931 TcCLB-EL.506857.10_mRNA-p1 TcCLB-NE.
509207.80_mRNA-p1 TcSYL_0201800.t1-p1
OG0001618: TCRU_1932 TcCLB-EL.506857.20_mRNA-p1 TcCLB-NE.
509207.90_mRNA-p1 TcSYL_0201810.t1-p1
OG0001619: TCRU_1933 TcCLB-EL.506857.30_mRNA-p1 TcCLB-NE.
509207.100_mRNA-p1 TcSYL_0201820.t1-p1
OG0001620: TCRU_1936 TcCLB-EL.434259.10_mRNA-p1 TcCLB-NE.
508153.380_mRNA-p1 TcSYL_0000910.t1-p1
OG0001621: TCRU_1939 TcCLB-EL.506699.34_pseudogenic_transcript-p1
TcCLB-NE.508153.340_mRNA-p1 TcSYL_0000960.t1-p1
OG0001622: TCRU_1941 TcCLB-EL.510323.10_mRNA-p1 TcCLB-NE.
508153.310_mRNA-p1 TcSYL_0000990.t1-p1
OG0001623: TCRU_1945 TcCLB-EL.510323.40_mRNA-p1 TcCLB-NE.
508153.270_mRNA-p1 TcSYL_0001020.t1-p1
OG0001624: TCRU_1946 TcCLB-EL.510323.50_mRNA-p1 TcCLB-NE.
508153.260_mRNA-p1 TcSYL_0001030.t1-p1
OG0001625: TCRU_1952 TcCLB-EL.505843.40_mRNA-p1 TcCLB-NE.
509353.10_mRNA-p1 TcSYL_0064470.t1-p1
OG0001626: TCRU_1961 TcCLB-EL.509069.20_mRNA-p1 TcCLB-NE.
508707.227_mRNA-p1 TcSYL_0112370.t1-p1
OG0001627: TCRU_1970 TcCLB-EL.506973.90_pseudogenic_transcript-p1
TcCLB-NE.510391.20_mRNA-p1 TcSYL_0166430.t1-p1
OG0001628: TCRU_1981 TcCLB-EL.503643.39_mRNA-p1 TcCLB-NE.
506485.90_mRNA-p1 TcSYL_0003420.t1-p1
OG0001629: TCRU_1982 TcCLB-EL.503643.30_mRNA-p1 TcCLB-NE.
506485.100_mRNA-p1 TcSYL_0003410.t1-p1
OG0001630: TCRU_1983 TcCLB-EL.503643.10_mRNA-p1 TcCLB-NE.
504121.10_mRNA-p1 TcSYL_0003370.t1-p1
OG0001631: TCRU_1987 TcCLB-EL.511423.149_mRNA-p1 TcCLB-NE.
507949.29_mRNA-p1 TcSYL_0110930.t1-p1
OG0001632: TCRU_1994 TcCLB-EL.507395.20_mRNA-p1 TcCLB-NE.
510087.10_mRNA-p1 TcSYL_0051870.t1-p1
OG0001633: TCRU_1996 TCRU_2188 TcSYL_0109890.t1-p1 TcSYL_0110080.t1-
p1
OG0001634: TCRU_1999 TCRU_5537 TcCLB-NE.410923.20_mRNA-p1 TcCLB-NE.
503861.10_mRNA-p1

OG0001635: TCRU_2001 TcCLB-NE.506321.280_mRNA-p1 TcSYL_0137700.t1-p1
TcSYL_0157170.t1-p1
OG0001636: TCRU_2004 TcCLB-EL.506607.19_mRNA-p1 TcCLB-NE.
506321.310_mRNA-p1 TcSYL_0157150.t1-p1
OG0001637: TCRU_2007 TcCLB-EL.510173.20_mRNA-p1 TcCLB-NE.
506321.340_mRNA-p1 TcSYL_0137870.t1-p1
OG0001638: TCRU_2008 TcCLB-EL.510173.30_mRNA-p1 TcCLB-NE.
506321.350_mRNA-p1 TcSYL_0137880.t1-p1
OG0001639: TCRU_2011 TcCLB-EL.510173.60_mRNA-p1 TcCLB-NE.
509721.30_mRNA-p1 TcSYL_0137910.t1-p1
OG0001640: TCRU_2012 TcCLB-EL.510173.70_mRNA-p1 TcCLB-NE.
509721.40_mRNA-p1 TcSYL_0137920.t1-p1
OG0001641: TCRU_2014 TcCLB-EL.510173.90_mRNA-p1 TcCLB-NE.
509721.60_mRNA-p1 TcSYL_0137930.t1-p1
OG0001642: TCRU_2016 TcCLB-EL.510173.110_mRNA-p1 TcCLB-NE.
509721.79_mRNA-p1 TcSYL_0137940.t1-p1
OG0001643: TCRU_2036 TcCLB-EL.511211.20_mRNA-p1 TcCLB-NE.
508827.139_mRNA-p1 TcSYL_0182170.t1-p1
OG0001644: TCRU_2049 TcCLB-EL.504147.240_mRNA-p1 TcSYL_0004990.t1-p1
TcSYL_0005000.t1-p1
OG0001645: TCRU_2053 TcCLB-EL.508515.40_mRNA-p1 TcCLB-NE.
508641.170_mRNA-p1 TcSYL_0047460.t1-p1
OG0001646: TCRU_2054 TcCLB-EL.508515.80_mRNA-p1 TcCLB-NE.
508641.120_mRNA-p1 TcSYL_0047440.t1-p1
OG0001647: TCRU_2056 TcCLB-EL.508515.110_mRNA-p1 TcCLB-NE.
508641.90_mRNA-p1 TcSYL_0047420.t1-p1
OG0001648: TCRU_2062 TcCLB-EL.511747.30_mRNA-p1 TcCLB-NE.
511507.10_mRNA-p1 TcSYL_0139420.t1-p1
OG0001649: TCRU_2065 TcCLB-EL.503909.110_mRNA-p1 TcCLB-NE.
511507.39_mRNA-p1 TcSYL_0139440.t1-p1
OG0001650: TCRU_2066 TcCLB-EL.503909.100_mRNA-p1 TcCLB-NE.
511507.50_mRNA-p1 TcSYL_0139450.t1-p1
OG0001651: TCRU_2068 TcCLB-EL.503909.76_mRNA-p1 TcCLB-NE.
511507.80_mRNA-p1 TcSYL_0139460.t1-p1
OG0001652: TCRU_2070 TcCLB-EL.503909.60_mRNA-p1 TcCLB-NE.
511507.100_mRNA-p1 TcSYL_0139470.t1-p1
OG0001653: TCRU_2098 TcCLB-EL.506519.130_mRNA-p1 TcCLB-NE.
508909.20_mRNA-p1 TcSYL_0159500.t1-p1
OG0001654: TCRU_2099 TcCLB-EL.506519.110_mRNA-p1 TcCLB-NE.
508909.40_mRNA-p1 TcSYL_0159490.t1-p1
OG0001655: TCRU_2100 TcCLB-EL.506519.100_mRNA-p1 TcCLB-NE.
508909.50_mRNA-p1 TcSYL_0159480.t1-p1
OG0001656: TCRU_2102 TcCLB-EL.506519.80_mRNA-p1 TcCLB-NE.
508909.70_mRNA-p1 TcSYL_0159470.t1-p1
OG0001657: TCRU_2110 TcCLB-EL.506743.4_mRNA-p1 TcCLB-NE.
503945.10_mRNA-p1 TcSYL_0165460.t1-p1
OG0001658: TCRU_like TcCLB-EL.503713.10_mRNA-p1 TcCLB-NE.
503945.20_mRNA-p1 TcSYL_0165490.t1-p1
OG0001659: TCRU_2112 TcCLB-EL.503713.20_mRNA-p1 TcCLB-NE.
503945.30_mRNA-p1 TcSYL_0165510.t1-p1
OG0001660: TCRU_2113 TcCLB-EL.503713.30_mRNA-p1 TcCLB-NE.
503945.40_mRNA-p1 TcSYL_0165520.t1-p1
OG0001661: TCRU_2117 TcCLB-EL.511217.20_mRNA-p1 TcCLB-NE.
506575.70_mRNA-p1 TcSYL_0188710.t1-p1

OG0001662: TCRU_2118 TcCLB-EL.511217.30_mRNA-p1 TcCLB-NE.
506575.60_mRNA-p1 TcSYL_0188780.t1-p1
OG0001663: TCRU_2119 TcCLB-EL.511217.50_mRNA-p1 TcCLB-NE.
506575.50_mRNA-p1 TcSYL_0188870.t1-p1
OG0001664: TCRU_2120 TcCLB-EL.511217.80_mRNA-p1 TcCLB-NE.
506575.20_mRNA-p1 TcSYL_0189010.t1-p1
OG0001665: TCRU_2122 TcCLB-EL.511217.100_mRNA-p1 TcCLB-NE.
508957.30_mRNA-p1 TcSYL_0189080.t1-p1
OG0001666: TCRU_2123 TcCLB-EL.511217.120_mRNA-p1 TcCLB-NE.
503581.20_mRNA-p1 TcSYL_0189180.t1-p1
OG0001667: TCRU_2124 TcCLB-EL.511217.130_mRNA-p1 TcCLB-NE.
503581.30_mRNA-p1 TcSYL_0189230.t1-p1
OG0001668: TCRU_2125 TcCLB-EL.511217.140_mRNA-p1 TcCLB-NE.
503581.39_mRNA-p1 TcSYL_0189270.t1-p1
OG0001669: TCRU_2126 TcCLB-EL.511217.150_mRNA-p1 TcCLB-NE.
506573.70_mRNA-p1 TcSYL_0189340.t1-p1
OG0001670: TCRU_2127 TcCLB-EL.511217.170_mRNA-p1 TcCLB-NE.
506573.50_mRNA-p1 TcSYL_0189420.t1-p1
OG0001671: TCRU_2152 TcCLB-EL.508535.40_mRNA-p1 TcCLB-NE.
511461.30_mRNA-p1 TcSYL_0131010.t1-p1
OG0001672: TCRU_2156 TcCLB-EL.509007.10_mRNA-p1 TcCLB-NE.
508965.100_mRNA-p1 TcSYL_0171430.t1-p1
OG0001673: TCRU_2157 TcCLB-EL.506753.120_mRNA-p1 TcCLB-NE.
510357.10_mRNA-p1 TcSYL_0112650.t1-p1
OG0001674: TCRU_2167 TcCLB-EL.511075.30_mRNA-p1 TcCLB-NE.
511897.10_mRNA-p1 TcSYL_0103630.t1-p1
OG0001675: TCRU_2168 TcCLB-EL.511075.20_mRNA-p1 TcCLB-NE.
440101.20_pseudogenic_transcript-p1 TcCLB-NE.511897.28_mRNA-p1
OG0001676: TCRU_2184 TcCLB-EL.401569.10_mRNA-p1 TcCLB-EL.
508343.10_mRNA-p1 TcSYL_0109680.t1-p1
OG0001677: TCRU_2189 TcCLB-EL.510241.10_mRNA-p1 TcCLB-NE.
510579.119_mRNA-p1 TcSYL_0147220.t1-p1
OG0001678: TCRU_2193 TcCLB-EL.510241.60_mRNA-p1 TcCLB-NE.
510579.70_mRNA-p1 TcSYL_0147250.t1-p1
OG0001679: TCRU_C TcCLB-EL.510241.110_mRNA-p1 TcCLB-NE.
510579.30_mRNA-p1 TcSYL_0147260.t1-p1
OG0001680: TCRU_2200 TcCLB-EL.510241.130_pseudogenic_transcript-p1
TcCLB-NE.510579.10_mRNA-p1 TcSYL_0147270.t1-p1
OG0001681: TCRU_2209 TcCLB-EL.511127.110_mRNA-p1 TcCLB-NE.
509023.110_mRNA-p1 TcSYL_0155920.t1-p1
OG0001682: TCRU_2210 TcCLB-EL.511127.100_mRNA-p1 TcCLB-NE.
509023.100_mRNA-p1 TcSYL_0155900.t1-p1
OG0001683: TCRU_2214 TcCLB-EL.511127.60_mRNA-p1 TcCLB-NE.
509023.50_mRNA-p1 TcSYL_0155890.t1-p1
OG0001684: TCRU_2215 TcCLB-EL.511127.50_mRNA-p1 TcCLB-NE.
509023.40_mRNA-p1 TcSYL_0155860.t1-p1
OG0001685: TCRU_2217 TcCLB-EL.511127.30_mRNA-p1 TcCLB-NE.
509023.20_mRNA-p1 TcSYL_0155850.t1-p1
OG0001686: TCRU_2218 TcCLB-EL.511127.20_mRNA-p1 TcCLB-NE.
509023.10_mRNA-p1 TcSYL_0155840.t1-p1
OG0001687: TCRU_2222 TcCLB-EL.503819.30_mRNA-p1 TcCLB-NE.
510359.310_mRNA-p1 TcSYL_0112870.t1-p1
OG0001688: TCRU_2232 TcCLB-EL.510149.50_mRNA-p1 TcCLB-NE.
508411.90_mRNA-p1 TcSYL_0004170.t1-p1

OG0001689: TCRU_2234 TcCLB-EL.510149.30_mRNA-p1 TcCLB-NE.
508411.70_mRNA-p1 TcSYL_0004180.t1-p1
OG0001690: TCRU_2236 TcCLB-EL.510145.59_mRNA-p1 TcCLB-NE.
508411.40_mRNA-p1 TcSYL_0004210.t1-p1
OG0001691: TCRU_2239 TcCLB-EL.510145.20_mRNA-p1 TcCLB-NE.
508411.10_mRNA-p1 TcSYL_0004220.t1-p1
OG0001692: TCRU_2240 TcCLB-EL.508075.60_mRNA-p1 TcCLB-NE.
456021.10_mRNA-p1 TcSYL_0004240.t1-p1
OG0001693: TCRU_2250 TcCLB-EL.507941.14_mRNA-p1 TcCLB-NE.
509985.69_mRNA-p1 TcSYL_0063260.t1-p1
OG0001694: TCRU_2251 TcCLB-EL.507941.30_mRNA-p1 TcCLB-NE.
509985.50_mRNA-p1 TcSYL_0063250.t1-p1
OG0001695: TCRU_2252 TcCLB-EL.507941.40_mRNA-p1 TcCLB-NE.
509985.40_mRNA-p1 TcSYL_0063240.t1-p1
OG0001696: TCRU_2253 TcCLB-EL.507941.50_mRNA-p1 TcCLB-NE.
509985.30_mRNA-p1 TcSYL_0063230.t1-p1
OG0001697: TCRU_2254 TcCLB-EL.507941.60_mRNA-p1 TcCLB-NE.
509985.20_mRNA-p1 TcSYL_0063220.t1-p1
OG0001698: TCRU_2255 TcCLB-EL.507941.70_mRNA-p1 TcCLB-NE.
509985.10_pseudogenic_transcript-p1 TcSYL_0063210.t1-p1
OG0001699: TCRU_2257 TcCLB-EL.507941.120_mRNA-p1 TcCLB-NE.
507943.60_mRNA-p1 TcSYL_0063180.t1-p1
OG0001700: TCRU_2260 TcCLB-EL.510351.50_mRNA-p1 TcCLB-NE.
507601.130_mRNA-p1 TcSYL_0062240.t1-p1
OG0001701: TCRU_2265 TcCLB-EL.510351.70_mRNA-p1 TcCLB-NE.
507601.90_mRNA-p1 TcSYL_0062210.t1-p1
OG0001702: TCRU_2267 TcCLB-EL.510351.90_mRNA-p1 TcCLB-NE.
507601.70_mRNA-p1 TcSYL_0062200.t1-p1
OG0001703: TCRU_2268 TcCLB-EL.510351.100_mRNA-p1 TcCLB-NE.
507601.60_mRNA-p1 TcSYL_0062180.t1-p1
OG0001704: TCRU_2269 TcCLB-EL.510351.110_mRNA-p1 TcCLB-NE.
507601.50_mRNA-p1 TcSYL_0062170.t1-p1
OG0001705: TCRU_2271 TcCLB-EL.510351.130_mRNA-p1 TcCLB-NE.
507601.30_mRNA-p1 TcSYL_0062160.t1-p1
OG0001706: TCRU_2287 TcCLB-EL.509775.40_mRNA-p1 TcCLB-NE.
507061.30_mRNA-p1 TcSYL_0013850.t1-p1
OG0001707: TCRU_2288 TcCLB-EL.509777.4_mRNA-p1 TcCLB-NE.
507061.20_mRNA-p1 TcSYL_0013860.t1-p1
OG0001708: TCRU_2292 TcCLB-EL.509777.80_mRNA-p1 TcCLB-NE.
504141.20_mRNA-p1 TcSYL_0013900.t1-p1
OG0001709: TCRU_2301 TcCLB-EL.506795.50_mRNA-p1 TcCLB-NE.
509937.10_mRNA-p1 TcSYL_0027510.t1-p1
OG0001710: TCRU_2303 TcCLB-EL.504769.70_mRNA-p1 TcCLB-NE.
503647.9_mRNA-p1 TcSYL_0156950.t1-p1
OG0001711: TCRU_2308 TcCLB-EL.508813.50_mRNA-p1 TcCLB-NE.
507197.20_mRNA-p1 TcSYL_0044210.t1-p1
OG0001712: TCRU_2312 TcCLB-EL.458015.4_mRNA-p1 TcCLB-NE.
506155.10_mRNA-p1 TcSYL_0118180.t1-p1
OG0001713: TCRU_2313 TcCLB-EL.458015.10_mRNA-p1 TcCLB-NE.
506155.20_mRNA-p1 TcSYL_0118170.t1-p1
OG0001714: TCRU_2351 TcCLB-EL.511435.40_mRNA-p1 TcCLB-NE.
506475.30_mRNA-p1 TcSYL_0110720.t1-p1
OG0001715: TCRU_2370 TcCLB-EL.503529.20_mRNA-p1 TcCLB-NE.
511647.60_mRNA-p1 TcSYL_0014040.t1-p1

OG0001716: TCRU_2372 TcCLB-EL.506127.40_mRNA-p1 TcCLB-EL.
508271.20_mRNA-p1 TcSYL_0083360.t1-p1
OG0001717: TCRU_2373 TcCLB-EL.506127.30_mRNA-p1 TcCLB-EL.
508271.10_mRNA-p1 TcSYL_0083370.t1-p1
OG0001718: TCRU_2377 TcCLB-EL.510323.120_mRNA-p1 TcCLB-NE.
508153.200_mRNA-p1 TcSYL_0001040.t1-p1
OG0001719: TCRU_2379 TcCLB-EL.508183.10_mRNA-p1 TcCLB-NE.
508153.170_mRNA-p1 TcSYL_0001070.t1-p1
OG0001720: TCRU_2384 TcCLB-EL.508185.10_mRNA-p1 TcCLB-NE.
508153.130_mRNA-p1 TcSYL_0001080.t1-p1
OG0001721: TCRU_2392 TCRU_3067 TcCLB-NE.
508983.10_pseudogenic_transcript-p1 TcSYL_0129990.t1-p1
OG0001722: TCRU_2397 TcCLB-EL.482369.29_mRNA-p1 TcCLB-NE.
507241.30_mRNA-p1 TcSYL_0064780.t1-p1
OG0001723: TCRU_2399 TcCLB-EL.511621.110_mRNA-p1 TcCLB-NE.
504741.250_mRNA-p1 TcSYL_0084700.t1-p1
OG0001724: TCRU_2400 TcCLB-EL.511621.130_mRNA-p1 TcCLB-NE.
504741.230_mRNA-p1 TcSYL_0084620.t1-p1
OG0001725: TCRU_2401 TcCLB-EL.511621.140_mRNA-p1 TcCLB-NE.
504741.220_mRNA-p1 TcSYL_0084600.t1-p1
OG0001726: TCRU_2402 TcCLB-EL.511621.150_mRNA-p1 TcCLB-NE.
504741.210_mRNA-p1 TcSYL_0084550.t1-p1
OG0001727: TCRU_2403 TcCLB-EL.511621.160_mRNA-p1 TcCLB-NE.
504741.200_mRNA-p1 TcSYL_0084490.t1-p1
OG0001728: TCRU_2404 TcCLB-EL.511621.180_mRNA-p1 TcCLB-NE.
504741.180_mRNA-p1 TcSYL_0084440.t1-p1
OG0001729: TCRU_2405 TcCLB-EL.511621.190_mRNA-p1 TcCLB-NE.
504741.170_mRNA-p1 TcSYL_0084410.t1-p1
OG0001730: TCRU_methyltransferase_ TcCLB-EL.511621.259_mRNA-p1
TcCLB-NE.504741.110_mRNA-p1 TcSYL_0084340.t1-p1
OG0001731: TCRU_2412 TcCLB-EL.503561.10_mRNA-p1 TcCLB-NE.
504741.80_mRNA-p1 TcSYL_0084320.t1-p1
OG0001732: TCRU_2413 TcCLB-EL.511625.10_mRNA-p1 TcCLB-NE.
504741.60_mRNA-p1 TcSYL_0084300.t1-p1
OG0001733: TCRU_2415 TcCLB-EL.511625.50_mRNA-p1 TcCLB-NE.
504741.20_mRNA-p1 TcSYL_0084290.t1-p1
OG0001734: TCRU_2419 TcCLB-EL.511535.10_mRNA-p1 TcCLB-NE.
508713.30_mRNA-p1 TcSYL_0111810.t1-p1
OG0001735: TCRU_2422 TcCLB-EL.509067.14_mRNA-p1 TcCLB-NE.
508711.49_mRNA-p1 TcSYL_0111780.t1-p1
OG0001736: TCRU_2423 TcCLB-EL.509067.40_mRNA-p1 TcCLB-NE.
508711.30_mRNA-p1 TcSYL_0111770.t1-p1
OG0001737: TCRU_2425 TcCLB-EL.503749.10_mRNA-p1 TcCLB-NE.
508709.10_mRNA-p1 TcSYL_0111730.t1-p1
OG0001738: TCRU_2426 TcCLB-EL.503749.20_mRNA-p1 TcCLB-NE.
511021.90_mRNA-p1 TcSYL_0111720.t1-p1
OG0001739: TCRU_2427 TcCLB-EL.503749.30_mRNA-p1 TcCLB-NE.
511021.80_mRNA-p1 TcSYL_0111710.t1-p1
OG0001740: TCRU_2429 TcCLB-EL.511537.30_mRNA-p1 TcCLB-NE.
511021.30_mRNA-p1 TcSYL_0111690.t1-p1
OG0001741: TCRU_2437 TcCLB-EL.506289.74_mRNA-p1 TcCLB-NE.
508881.10_mRNA-p1 TcSYL_0023390.t1-p1
OG0001742: TCRU_2447 TcCLB-EL.510027.20_mRNA-p1 TcCLB-NE.
437575.18_mRNA-p1 TcSYL_0122250.t1-p1

OG0001743: TCRU_2454 TcCLB-EL.511309.7_mRNA-p1 TcCLB-NE.
507087.20_mRNA-p1 TcSYL_0159740.t1-p1
OG0001744: TCRU_2468 TcCLB-EL.464807.10_mRNA-p1 TcCLB-NE.
511277.140_mRNA-p1 TcSYL_0014840.t1-p1
OG0001745: TCRU_2470 TcCLB-EL.506357.29_mRNA-p1 TcCLB-NE.
511277.84_mRNA-p1 TcSYL_0014880.t1-p1
OG0001746: TCRU_2471 TcCLB-EL.506357.40_mRNA-p1 TcCLB-NE.
511277.70_mRNA-p1 TcSYL_0014900.t1-p1
OG0001747: TCRU_2472 TcCLB-EL.506357.50_mRNA-p1 TcCLB-NE.
511277.60_mRNA-p1 TcSYL_0014910.t1-p1
OG0001748: TCRU_2476 TcCLB-EL.506357.90_mRNA-p1 TcCLB-NE.
511277.20_mRNA-p1 TcSYL_0014920.t1-p1
OG0001749: TCRU_2477 TcCLB-EL.506357.100_mRNA-p1 TcCLB-NE.
511277.10_mRNA-p1 TcSYL_0014930.t1-p1
OG0001750: TCRU_2478 TcCLB-EL.506357.110_mRNA-p1 TcCLB-NE.
511277.4_mRNA-p1 TcSYL_0014940.t1-p1
OG0001751: TCRU_2479 TcCLB-EL.506357.120_mRNA-p1 TcCLB-NE.
503681.20_mRNA-p1 TcSYL_0014950.t1-p1
OG0001752: TCRU_2480 TcCLB-EL.506357.130_mRNA-p1 TcCLB-NE.
503681.9_mRNA-p1 TcSYL_0014960.t1-p1
OG0001753: TCRU_2481 TcCLB-EL.506357.140_mRNA-p1 TcCLB-NE.
508891.120_pseudogenic_transcript-p1 TcSYL_0014990.t1-p1
OG0001754: TCRU_2491 TcCLB-EL.511439.60_mRNA-p1 TcCLB-NE.
510679.14_mRNA-p1 TcSYL_0031420.t1-p1
OG0001755: TCRU_2492 TcCLB-EL.511439.50_mRNA-p1 TcCLB-NE.
510679.30_mRNA-p1 TcSYL_0031400.t1-p1
OG0001756: TCRU_2507 TcCLB-EL.506473.14_mRNA-p1 TcCLB-NE.
508999.20_mRNA-p1 TcSYL_0110660.t1-p1
OG0001757: TCRU_2508 TcCLB-EL.506473.20_mRNA-p1 TcCLB-NE.
508999.10_mRNA-p1 TcSYL_0110670.t1-p1
OG0001758: TCRU_2521 TcCLB-EL.511067.30_mRNA-p1 TcCLB-NE.
511903.120_mRNA-p1 TcSYL_0103350.t1-p1
OG0001759: TCRU_2526 TcCLB-EL.511409.10_mRNA-p1 TcCLB-NE.
509571.4_mRNA-p1 TcSYL_0097750.t1-p1
OG0001760: TCRU_2527 TcCLB-EL.511409.20_mRNA-p1 TcCLB-NE.
509571.10_mRNA-p1 TcSYL_0097760.t1-p1
OG0001761: TCRU_2528 TcCLB-EL.511409.30_mRNA-p1 TcCLB-NE.
509571.30_mRNA-p1 TcSYL_0097800.t1-p1
OG0001762: TCRU_2530 TcCLB-EL.511409.60_mRNA-p1 TcCLB-NE.
509571.60_mRNA-p1 TcSYL_0097890.t1-p1
OG0001763: TCRU_2531 TcCLB-EL.503689.4_mRNA-p1 TcCLB-NE.
509571.79_mRNA-p1 TcSYL_0097930.t1-p1
OG0001764: TCRU_2532 TcCLB-EL.503689.10_mRNA-p1 TcCLB-NE.
509573.10_mRNA-p1 TcSYL_0097960.t1-p1
OG0001765: TCRU_2534 TcCLB-EL.503689.30_mRNA-p1 TcCLB-NE.
509573.30_mRNA-p1 TcSYL_0098010.t1-p1
OG0001766: TCRU_2535 TcCLB-EL.503689.34_mRNA-p1 TcCLB-NE.
509573.40_mRNA-p1 TcSYL_0098020.t1-p1
OG0001767: TCRU_2536 TcCLB-EL.503689.40_mRNA-p1 TcCLB-NE.
509573.50_mRNA-p1 TcSYL_0098030.t1-p1
OG0001768: TCRU_2537 TcCLB-EL.511411.8_mRNA-p1 TcCLB-NE.
473755.9_mRNA-p1 TcSYL_0098110.t1-p1
OG0001769: TCRU_2565 TCRU_3352 TcCLB-NE.
506011.20_pseudogenic_transcript-p1 TcCLB-NE.

506255.10_pseudogenic_transcript-p1
OG0001770: TCRU_2570 TcCLB-EL.506605.90_mRNA-p1 TcCLB-NE.
511239.80_mRNA-p1 TcSYL_0025990.t1-p1
OG0001771: TCRU_2571 TcCLB-EL.506605.100_mRNA-p1 TcCLB-NE.
511239.90_mRNA-p1 TcSYL_0025960.t1-p1
OG0001772: TCRU_2574 TcCLB-EL.506605.139_mRNA-p1 TcCLB-NE.
511239.130_mRNA-p1 TcSYL_0025870.t1-p1
OG0001773: TCRU_2575 TcCLB-EL.506605.150_mRNA-p1 TcCLB-NE.
511239.140_mRNA-p1 TcSYL_0025860.t1-p1
OG0001774: TCRU_2576 TcCLB-EL.506605.160_mRNA-p1 TcCLB-NE.
511239.150_mRNA-p1 TcSYL_0025840.t1-p1
OG0001775: TCRU_2578 TcCLB-EL.506605.174_mRNA-p1 TcCLB-NE.
414243.20_mRNA-p1 TcSYL_0025780.t1-p1
OG0001776: TCRU_2579 TcCLB-EL.506605.190_mRNA-p1 TcCLB-NE.
508859.10_mRNA-p1 TcSYL_0025750.t1-p1
OG0001777: TCRU_2580 TcCLB-EL.506605.194_mRNA-p1 TcCLB-NE.
508859.20_mRNA-p1 TcSYL_0025690.t1-p1
OG0001778: TCRU_2581 TcCLB-EL.506605.200_mRNA-p1 TcCLB-NE.
508859.30_mRNA-p1 TcSYL_0025670.t1-p1
OG0001779: TCRU_2582 TcCLB-EL.506605.210_mRNA-p1 TcCLB-NE.
508859.40_mRNA-p1 TcSYL_0025650.t1-p1
OG0001780: TCRU_2583 TcCLB-EL.506605.214_mRNA-p1 TcCLB-NE.
508859.50_mRNA-p1 TcSYL_0025630.t1-p1
OG0001781: TCRU_2585 TcCLB-EL.510167.4_mRNA-p1 TcCLB-NE.
508859.80_mRNA-p1 TcSYL_0025530.t1-p1
OG0001782: TCRU_2586 TcCLB-EL.510167.10_mRNA-p1 TcCLB-NE.
508859.90_mRNA-p1 TcSYL_0025490.t1-p1
OG0001783: TCRU_2587 TcCLB-EL.510167.29_mRNA-p1 TcCLB-NE.
508859.109_mRNA-p1 TcSYL_0025460.t1-p1
OG0001784: TCRU_2621 TcCLB-EL.510311.130_mRNA-p1 TcCLB-NE.
508153.1120_mRNA-p1 TcSYL_0000560.t1-p1
OG0001785: TCRU_2622 TcCLB-EL.510311.140_mRNA-p1 TcCLB-NE.
508153.1110_mRNA-p1 TcSYL_0000570.t1-p1
OG0001786: TCRU_2623 TcCLB-EL.510311.150_mRNA-p1 TcCLB-NE.
508153.1100_mRNA-p1 TcSYL_0000580.t1-p1
OG0001787: TCRU_2634 TcCLB-EL.506543.20_mRNA-p1 TcCLB-NE.
508267.10_mRNA-p1 TcSYL_0019410.t1-p1
OG0001788: TCRU_2642 TcCLB-EL.506543.90_mRNA-p1 TcCLB-NE.
506041.50_mRNA-p1 TcSYL_0019450.t1-p1
OG0001789: TCRU_2649 TcCLB-EL.511867.80_mRNA-p1 TcCLB-NE.
440363.19_mRNA-p1 TcSYL_0146350.t1-p1
OG0001790: TCRU_2666 TcCLB-EL.506755.20_mRNA-p1 TcCLB-NE.
510359.20_mRNA-p1 TcSYL_0112750.t1-p1
OG0001791: TCRU_2671 TcCLB-EL.504423.30_mRNA-p1 TcCLB-NE.
507569.10_mRNA-p1 TcSYL_0104650.t1-p1
OG0001792: TCRU_2675 TcCLB-EL.508347.140_mRNA-p1 TcCLB-NE.
507615.60_mRNA-p1 TcSYL_0104790.t1-p1
OG0001793: TCRU_2676 TcCLB-EL.508347.130_mRNA-p1 TcCLB-NE.
507615.70_mRNA-p1 TcSYL_0104810.t1-p1
OG0001794: TCRU_2677 TcCLB-EL.508347.120_mRNA-p1 TcCLB-NE.
507615.73_mRNA-p1 TcSYL_0104840.t1-p1
OG0001795: TCRU_2678 TcCLB-EL.508347.110_mRNA-p1 TcCLB-NE.
507615.76_mRNA-p1 TcSYL_0104920.t1-p1
OG0001796: TCRU_2679 TcCLB-EL.508347.100_mRNA-p1 TcCLB-NE.

507615.80_mRNA-p1 TcSYL_0104950.t1-p1
OG0001797: TCRU_2680 TcCLB-EL.508347.90_mRNA-p1 TcCLB-NE.
507615.90_mRNA-p1 TcSYL_0105030.t1-p1
OG0001798: TCRU_2684 TcCLB-EL.507739.130_mRNA-p1 TcCLB-NE.
506437.40_mRNA-p1 TcSYL_0195730.t1-p1
OG0001799: TCRU_2688 TcCLB-NE.511491.170_mRNA-p1 TcCLB-NE.
511493.10_mRNA-p1 TcSYL_0167120.t1-p1
OG0001800: TCRU_2693 TcCLB-EL.505989.74_mRNA-p1 TcCLB-NE.
508693.90_mRNA-p1 TcSYL_0046400.t1-p1
OG0001801: TCRU_2695 TcCLB-EL.505989.82_mRNA-p1 TcCLB-NE.
508693.80_mRNA-p1 TcSYL_0046390.t1-p1
OG0001802: TCRU_2696 TcCLB-EL.505989.90_mRNA-p1 TcCLB-NE.
508693.70_mRNA-p1 TcSYL_0046380.t1-p1
OG0001803: TCRU_2698 TcCLB-EL.505989.110_mRNA-p1 TcCLB-NE.
508693.50_mRNA-p1 TcSYL_0046370.t1-p1
OG0001804: TCRU_2704 TcCLB-EL.509167.150_mRNA-p1 TcCLB-NE.
503597.10_mRNA-p1 TcSYL_0140250.t1-p1
OG0001805: TCRU_2723 TcCLB-EL.509149.50_mRNA-p1 TcCLB-NE.
508175.320_mRNA-p1 TcSYL_0078620.t1-p1
OG0001806: TCRU_2726 TcCLB-EL.506513.14_mRNA-p1 TcCLB-NE.
508919.110_mRNA-p1 TcSYL_0158010.t1-p1
OG0001807: TCRU_2727 TcCLB-EL.506513.7_mRNA-p1 TcCLB-NE.
508919.130_mRNA-p1 TcSYL_0157980.t1-p1
OG0001808: TCRU_2740 TcCLB-EL.507923.20_mRNA-p1 TcCLB-EL.
511753.130_mRNA-p1 TcSYL_0139020.t1-p1
OG0001809: TCRU_2742 TcCLB-EL.507831.30_mRNA-p1 TcCLB-NE.
511263.40_mRNA-p1 TcSYL_0015550.t1-p1
OG0001810: TCRU_2754 TCRU_3867 TCRU_7805 TcCLB-NE.
508877.10_pseudogenic_transcript-p1
OG0001811: TCRU_2762 TcCLB-EL.511423.60_mRNA-p1 TcCLB-NE.
507949.140_mRNA-p1 TcSYL_0110980.t1-p1
OG0001812: TCRU_2764 TcCLB-EL.511423.80_mRNA-p1 TcCLB-NE.
507949.120_mRNA-p1 TcSYL_0110970.t1-p1
OG0001813: TCRU_2765 TcCLB-EL.511423.90_mRNA-p1 TcCLB-NE.
507949.110_mRNA-p1 TcSYL_0110960.t1-p1
OG0001814: TCRU_2767 TcCLB-EL.511423.110_mRNA-p1 TcCLB-NE.
507949.60_mRNA-p1 TcSYL_0110950.t1-p1
OG0001815: TCRU_2768 TcCLB-EL.511423.130_pseudogenic_transcript-p1
TcCLB-NE.507949.50_mRNA-p1 TcSYL_0110940.t1-p1
OG0001816: TCRU_2773 TcCLB-EL.504153.20_mRNA-p1 TcCLB-EL.
506127.9_mRNA-p1 TcSYL_0083390.t1-p1
OG0001817: TCRU_2774 TcCLB-EL.504153.30_mRNA-p1 TcCLB-EL.
506125.20_mRNA-p1 TcSYL_0138820.t1-p1
OG0001818: TCRU_2777 TcCLB-EL.511411.24_mRNA-p1 TcCLB-NE.
506153.6_mRNA-p1 TcSYL_0098170.t1-p1
OG0001819: TCRU_2802 TcCLB-EL.508369.40_pseudogenic_transcript-p1
TcCLB-NE.507897.30_mRNA-p1 TcSYL_0057310.t1-p1
OG0001820: TCRU_2803 TcCLB-EL.508369.20_mRNA-p1 TcCLB-NE.
507897.20_mRNA-p1 TcSYL_0057300.t1-p1
OG0001821: TCRU_2804 TcCLB-EL.508369.10_mRNA-p1 TcCLB-NE.
507897.10_mRNA-p1 TcSYL_0057290.t1-p1
OG0001822: TCRU_2807 TcCLB-EL.510597.40_mRNA-p1 TcCLB-NE.
506419.60_mRNA-p1 TcSYL_0057260.t1-p1
OG0001823: TCRU_2808 TcCLB-EL.510597.30_mRNA-p1 TcCLB-NE.

506419.40_mRNA-p1 TcSYL_0057250.t1-p1
OG0001824: TCRU_2809 TcCLB-EL.510597.20_mRNA-p1 TcCLB-NE.
506419.30_mRNA-p1 TcSYL_0057240.t1-p1
OG0001825: TCRU_2811 TcCLB-EL.510595.58_mRNA-p1 TcCLB-NE.
507895.170_mRNA-p1 TcSYL_0057220.t1-p1
OG0001826: TCRU_regulator_ TcCLB-EL.510595.40_mRNA-p1 TcCLB-NE.
507895.160_mRNA-p1 TcSYL_0057200.t1-p1
OG0001827: TCRU_2813 TcCLB-EL.510595.20_mRNA-p1 TcCLB-NE.
507895.150_mRNA-p1 TcSYL_0057190.t1-p1
OG0001828: TCRU_2818 TcCLB-EL.510593.40_mRNA-p1 TcCLB-NE.
507895.110_mRNA-p1 TcSYL_0057170.t1-p1
OG0001829: TCRU_2819 TcCLB-EL.510593.30_mRNA-p1 TcCLB-NE.
507895.100_pseudogenic_transcript-p1 TcSYL_0057160.t1-p1
OG0001830: TCRU_2827 TcCLB-EL.510157.10_mRNA-p1 TcCLB-EL.
510205.20_mRNA-p1 TcCLB-NE.510693.320_mRNA-p1
OG0001831: TCRU_2830 TcCLB-EL.511751.50_mRNA-p1 TcCLB-NE.
507023.180_mRNA-p1 TcSYL_0139310.t1-p1
OG0001832: TCRU_2831 TcCLB-EL.511751.60_mRNA-p1 TcCLB-NE.
507023.170_mRNA-p1 TcSYL_0139300.t1-p1
OG0001833: TCRU_2832 TcCLB-EL.511751.70_mRNA-p1 TcCLB-NE.
507023.160_mRNA-p1 TcSYL_0139290.t1-p1
OG0001834: TCRU_2835 TcCLB-EL.511751.110_mRNA-p1 TcCLB-NE.
507023.130_mRNA-p1 TcSYL_0139270.t1-p1
OG0001835: TCRU_2837 TcCLB-EL.511751.130_mRNA-p1 TcCLB-NE.
507023.110_mRNA-p1 TcSYL_0139260.t1-p1
OG0001836: TCRU_2838 TcCLB-EL.511751.140_mRNA-p1 TcCLB-NE.
507023.100_mRNA-p1 TcSYL_0139250.t1-p1
OG0001837: TCRU_2839 TcCLB-EL.511751.144_mRNA-p1 TcCLB-NE.
507023.90_mRNA-p1 TcSYL_0139240.t1-p1
OG0001838: TCRU_2840 TcCLB-EL.511751.150_mRNA-p1 TcCLB-NE.
507023.80_mRNA-p1 TcSYL_0139230.t1-p1
OG0001839: TCRU_2841 TcCLB-EL.511751.160_mRNA-p1 TcCLB-NE.
507023.70_mRNA-p1 TcSYL_0139220.t1-p1
OG0001840: TCRU_2844 TcCLB-EL.511751.180_mRNA-p1 TcCLB-NE.
507023.30_mRNA-p1 TcSYL_0139210.t1-p1
OG0001841: TCRU_2846 TcCLB-EL.511751.200_mRNA-p1 TcCLB-NE.
507023.10_mRNA-p1 TcSYL_0139200.t1-p1
OG0001842: TCRU_2847 TcCLB-EL.511751.220_mRNA-p1 TcCLB-NE.
507021.100_mRNA-p1 TcSYL_0139180.t1-p1
OG0001843: TCRU_2851 TcCLB-EL.511753.30_mRNA-p1 TcCLB-NE.
507021.50_mRNA-p1 TcSYL_0139150.t1-p1
OG0001844: TCRU_2852 TcCLB-EL.511753.40_mRNA-p1 TcCLB-NE.
507021.40_mRNA-p1 TcSYL_0139140.t1-p1
OG0001845: TCRU_2853 TcCLB-EL.511753.50_mRNA-p1 TcCLB-NE.
507021.30_mRNA-p1 TcSYL_0139130.t1-p1
OG0001846: TCRU_2854 TcCLB-EL.511753.60_mRNA-p1 TcCLB-NE.
507021.20_mRNA-p1 TcSYL_0139120.t1-p1
OG0001847: TCRU_family_ TcCLB-EL.511753.70_mRNA-p1 TcCLB-NE.
507021.10_mRNA-p1 TcSYL_0139110.t1-p1
OG0001848: TCRU_2875 TcCLB-NE.507053.100_mRNA-p1 TcSYL_0046960.t1-p1
TcSYL_0046970.t1-p1
OG0001849: TCRU_2898 TcCLB-EL.469435.4_mRNA-p1 TcCLB-NE.
508257.104_mRNA-p1 TcSYL_0046720.t1-p1
OG0001850: TCRU_2901 TcCLB-EL.506405.10_mRNA-p1 TcCLB-NE.

508257.70_mRNA-p1 TcSYL_0046700.t1-p1
OG0001851: TCRU_2905 TcCLB-EL.506405.50_mRNA-p1 TcCLB-NE.
508257.10_mRNA-p1 TcSYL_0046690.t1-p1
OG0001852: TCRU_2907 TcCLB-EL.506405.70_mRNA-p1 TcCLB-NE.
503929.60_mRNA-p1 TcSYL_0046680.t1-p1
OG0001853: TCRU_2908 TcCLB-EL.506405.80_mRNA-p1 TcCLB-NE.
503929.50_mRNA-p1 TcSYL_0046670.t1-p1
OG0001854: TCRU_2912 TcCLB-EL.506405.120_mRNA-p1 TcCLB-NE.
503929.10_mRNA-p1 TcSYL_0046650.t1-p1
OG0001855: TCRU_2913 TcCLB-EL.504159.10_mRNA-p1 TcCLB-NE.
506821.200_mRNA-p1 TcSYL_0088640.t1-p1
OG0001856: TCRU_2914 TcCLB-EL.503873.10_mRNA-p1 TcCLB-NE.
506821.210_mRNA-p1 TcSYL_0088650.t1-p1
OG0001857: TCRU_2917 TcCLB-EL.507129.30_mRNA-p1 TcCLB-NE.
507969.60_mRNA-p1 TcSYL_0088800.t1-p1
OG0001858: TCRU_2918 TcCLB-EL.507129.19_mRNA-p1 TcCLB-NE.
507969.54_mRNA-p1 TcSYL_0088820.t1-p1
OG0001859: TCRU_2919 TcCLB-EL.507129.10_mRNA-p1 TcCLB-NE.
507969.50_mRNA-p1 TcSYL_0088840.t1-p1
OG0001860: TCRU_2920 TcCLB-EL.507129.5_mRNA-p1 TcCLB-NE.
507969.40_mRNA-p1 TcSYL_0088860.t1-p1
OG0001861: TCRU_2921 TcCLB-EL.509269.10_mRNA-p1 TcCLB-NE.
507969.20_mRNA-p1 TcSYL_0088910.t1-p1
OG0001862: TCRU_2922 TcCLB-EL.504073.30_mRNA-p1 TcCLB-EL.
506933.20_mRNA-p1 TcSYL_0044150.t1-p1
OG0001863: TCRU_2924 TCRU_2925 TcCLB-EL.506933.30_mRNA-p1
TcSYL_0044130.t1-p1
OG0001864: TCRU_2933 TcCLB-EL.511407.60_mRNA-p1 TcCLB-NE.
506151.30_mRNA-p1 TcSYL_0097050.t1-p1
OG0001865: TCRU_2935 TcCLB-EL.510763.34_mRNA-p1 TcCLB-NE.
509671.189_mRNA-p1 TcSYL_0115380.t1-p1
OG0001866: TCRU_2940 TcCLB-EL.510763.109_mRNA-p1 TcCLB-NE.
509671.120_mRNA-p1 TcSYL_0115360.t1-p1
OG0001867: TCRU_2941 TcCLB-EL.510765.10_mRNA-p1 TcCLB-NE.
509671.110_mRNA-p1 TcSYL_0115350.t1-p1
OG0001868: TCRU_2946 TcCLB-EL.506753.150_mRNA-p1 TcCLB-NE.
510357.40_mRNA-p1 TcSYL_0112670.t1-p1
OG0001869: TCRU_2951 TCRU_7163 TcCLB-EL.510911.10_mRNA-p1 TcCLB-NE.
458759.10_mRNA-p1
OG0001870: TCRU_2952 TcCLB-EL.510911.20_mRNA-p1 TcCLB-NE.
458759.20_mRNA-p1 TcSYL_0143070.t1-p1
OG0001871: TCRU_2953 TcCLB-EL.510911.30_mRNA-p1 TcCLB-NE.
509163.3_mRNA-p1 TcSYL_0143090.t1-p1
OG0001872: TCRU_2961 TcCLB-EL.503967.10_mRNA-p1 TcCLB-NE.
508909.190_mRNA-p1 TcSYL_0159450.t1-p1
OG0001873: TCRU_2964 TcCLB-EL.504057.11_mRNA-p1 TcCLB-NE.
511003.10_mRNA-p1 TcSYL_0046200.t1-p1
OG0001874: TCRU_2978 TcCLB-EL.509153.40_mRNA-p1 TcCLB-NE.
510305.50_mRNA-p1 TcSYL_0078920.t1-p1
OG0001875: TCRU_2980 TcCLB-EL.509153.60_mRNA-p1 TcCLB-NE.
510305.30_mRNA-p1 TcSYL_0078930.t1-p1
OG0001876: TCRU_2991 TcCLB-EL.448197.10_mRNA-p1 TcCLB-EL.
506951.60_mRNA-p1 TcCLB-NE.509287.110_mRNA-p1
OG0001877: TCRU_2995 TcCLB-EL.506953.30_mRNA-p1 TcCLB-NE.

509287.20_mRNA-p1 TcSYL_0191500.t1-p1
OG0001878: TCRU_3004 TcCLB-EL.506237.10_mRNA-p1 TcCLB-NE.
509617.30_mRNA-p1 TcSYL_0001910.t1-p1
OG0001879: TCRU_3008 TcCLB-EL.509669.30_mRNA-p1 TcCLB-NE.
509617.80_mRNA-p1 TcSYL_0001930.t1-p1
OG0001880: TCRU_3014 TcCLB-EL.509669.90_mRNA-p1 TcCLB-NE.
506175.40_mRNA-p1 TcSYL_0001980.t1-p1
OG0001881: TCRU_3016 TcCLB-EL.509669.110_mRNA-p1 TcCLB-NE.
506175.60_mRNA-p1 TcSYL_0001990.t1-p1
OG0001882: TCRU_3018 TcCLB-EL.509669.140_mRNA-p1 TcCLB-NE.
506175.80_mRNA-p1 TcSYL_0002000.t1-p1
OG0001883: TCRU_3023 TcCLB-EL.510755.110_mRNA-p1 TcCLB-NE.
508413.60_mRNA-p1 TcSYL_0115570.t1-p1
OG0001884: TCRU_3025 TcCLB-EL.507011.20_mRNA-p1 TcCLB-NE.
510667.14_mRNA-p1 TcSYL_0117190.t1-p1
OG0001885: TCRU_3026 TcCLB-EL.507011.10_mRNA-p1 TcCLB-NE.
510667.20_mRNA-p1 TcSYL_0117220.t1-p1
OG0001886: TCRU_3027 TcCLB-EL.507009.130_mRNA-p1 TcCLB-NE.
510667.30_mRNA-p1 TcSYL_0117280.t1-p1
OG0001887: TCRU_3028 TcCLB-EL.507009.120_mRNA-p1 TcCLB-NE.
510667.40_mRNA-p1 TcSYL_0117300.t1-p1
OG0001888: TCRU_3029 TcCLB-EL.507009.110_mRNA-p1 TcCLB-NE.
510667.50_mRNA-p1 TcSYL_0117330.t1-p1
OG0001889: TCRU_3030 TcCLB-EL.507009.100_mRNA-p1 TcCLB-NE.
510667.60_mRNA-p1 TcSYL_0117350.t1-p1
OG0001890: TCRU_3031 TcCLB-EL.507009.90_mRNA-p1 TcCLB-NE.
510667.70_mRNA-p1 TcSYL_0117380.t1-p1
OG0001891: TCRU_3032 TcCLB-EL.507009.80_mRNA-p1 TcCLB-NE.
510667.80_mRNA-p1 TcSYL_0117400.t1-p1
OG0001892: TCRU_3033 TcCLB-EL.506637.120_mRNA-p1 TcCLB-NE.
510099.110_mRNA-p1 TcSYL_0197240.t1-p1
OG0001893: TCRU_3042 TcCLB-EL.511419.80_mRNA-p1 TcCLB-NE.
505999.130_mRNA-p1 TcSYL_0111170.t1-p1
OG0001894: TCRU_3044 TcCLB-EL.506891.20_mRNA-p1 TcCLB-NE.
505999.150_mRNA-p1 TcSYL_0111160.t1-p1
OG0001895: TCRU_3045 TcCLB-EL.506891.30_mRNA-p1 TcCLB-NE.
505999.160_mRNA-p1 TcSYL_0111150.t1-p1
OG0001896: TCRU_3046 TcCLB-EL.511421.10_mRNA-p1 TcCLB-NE.
505999.170_mRNA-p1 TcSYL_0111140.t1-p1
OG0001897: TCRU_3048 TcCLB-EL.511421.20_mRNA-p1 TcCLB-NE.
505999.180_mRNA-p1 TcSYL_0111130.t1-p1
OG0001898: TCRU_3063 TcCLB-EL.510159.10_mRNA-p1 TcCLB-NE.
506459.250_mRNA-p1 TcSYL_0136080.t1-p1
OG0001899: TCRU_3069 TcCLB-EL.504427.70_mRNA-p1 TcCLB-NE.
509331.70_mRNA-p1 TcSYL_0172470.t1-p1
OG0001900: TCRU_3074 TcCLB-EL.510055.140_mRNA-p1 TcCLB-NE.
508911.20_mRNA-p1 TcSYL_0159360.t1-p1
OG0001901: TCRU_3077 TcCLB-EL.510055.100_mRNA-p1 TcCLB-NE.
508911.60_mRNA-p1 TcSYL_0159350.t1-p1
OG0001902: TCRU_3078 TcCLB-EL.510055.80_mRNA-p1 TcCLB-NE.
508911.70_mRNA-p1 TcSYL_0159340.t1-p1
OG0001903: TCRU_3079 TcCLB-EL.510055.70_mRNA-p1 TcCLB-NE.
508911.80_mRNA-p1 TcSYL_0159280.t1-p1
OG0001904: TCRU_3086 TcCLB-EL.508041.10_mRNA-p1 TcCLB-NE.

506297.350_mRNA-p1 TcSYL_0074680.t1-p1
OG0001905: TCRU_3087 TcCLB-EL.508041.20_mRNA-p1 TcCLB-NE.
506297.340_mRNA-p1 TcSYL_0074690.t1-p1
OG0001906: TCRU_3090 TcCLB-EL.508041.50_mRNA-p1 TcCLB-NE.
506297.310_mRNA-p1 TcSYL_0074710.t1-p1
OG0001907: TCRU_3091 TcCLB-EL.508041.60_mRNA-p1 TcCLB-NE.
506297.300_mRNA-p1 TcSYL_0074730.t1-p1
OG0001908: TCRU_3095 TcCLB-EL.510101.50_mRNA-p1 TcCLB-NE.
506297.260_mRNA-p1 TcSYL_0074770.t1-p1
OG0001909: TCRU_subunit TcCLB-EL.510101.70_mRNA-p1 TcCLB-NE.
506297.240_mRNA-p1 TcSYL_0074790.t1-p1
OG0001910: TCRU_3098 TcCLB-EL.510101.90_mRNA-p1 TcCLB-NE.
506297.220_mRNA-p1 TcSYL_0074800.t1-p1
OG0001911: TCRU_3100 TcCLB-EL.510101.130_mRNA-p1 TcCLB-NE.
506297.200_mRNA-p1 TcSYL_0074810.t1-p1
OG0001912: TCRU_3110 TcCLB-EL.510101.250_mRNA-p1 TcCLB-NE.
506297.80_mRNA-p1 TcSYL_0074890.t1-p1
OG0001913: TCRU_3112 TcCLB-EL.510101.270_mRNA-p1 TcCLB-NE.
506297.60_mRNA-p1 TcSYL_0074920.t1-p1
OG0001914: TCRU_3114 TcCLB-EL.510101.300_mRNA-p1 TcCLB-NE.
506297.30_mRNA-p1 TcSYL_0074940.t1-p1
OG0001915: TCRU_3116 TcCLB-EL.510101.320_mRNA-p1 TcCLB-NE.
506297.10_mRNA-p1 TcSYL_0074950.t1-p1
OG0001916: TCRU_3117 TcCLB-EL.510101.340_mRNA-p1 TcCLB-NE.
507765.140_mRNA-p1 TcSYL_0074960.t1-p1
OG0001917: TCRU_3119 TcCLB-EL.510101.380_mRNA-p1 TcCLB-NE.
507765.110_mRNA-p1 TcSYL_0074990.t1-p1
OG0001918: TCRU_3124 TcCLB-EL.506513.180_mRNA-p1 TcCLB-NE.
511323.60_mRNA-p1 TcSYL_0158620.t1-p1
OG0001919: TCRU_3126 TcCLB-EL.507979.10_mRNA-p1 TcCLB-NE.
511323.30_mRNA-p1 TcSYL_0158710.t1-p1
OG0001920: TCRU_3127 TcCLB-EL.507979.20_mRNA-p1 TcCLB-NE.
511323.20_mRNA-p1 TcSYL_0158740.t1-p1
OG0001921: TCRU_3128 TcCLB-EL.507979.50_mRNA-p1 TcCLB-NE.
511321.70_mRNA-p1 TcSYL_0158840.t1-p1
OG0001922: TCRU_3129 TcCLB-EL.510053.20_mRNA-p1 TcCLB-NE.
511321.50_mRNA-p1 TcSYL_0158920.t1-p1
OG0001923: TCRU_3130 TcCLB-EL.510053.30_mRNA-p1 TcCLB-NE.
511321.40_mRNA-p1 TcSYL_0158970.t1-p1
OG0001924: TCRU_3131 TcCLB-EL.510053.40_mRNA-p1 TcCLB-NE.
511321.30_mRNA-p1 TcSYL_0158990.t1-p1
OG0001925: TCRU_3133 TcCLB-EL.510053.50_mRNA-p1 TcCLB-NE.
511321.26_mRNA-p1 TcSYL_0159030.t1-p1
OG0001926: TCRU_3154 TcCLB-EL.503801.79_mRNA-p1 TcCLB-NE.
508817.10_mRNA-p1 TcSYL_0043900.t1-p1
OG0001927: TCRU_3155 TcCLB-EL.503801.70_mRNA-p1 TcCLB-NE.
508817.20_mRNA-p1 TcSYL_0043860.t1-p1
OG0001928: TCRU_3156 TcCLB-EL.503801.60_mRNA-p1 TcCLB-NE.
508817.30_mRNA-p1 TcSYL_0043830.t1-p1
OG0001929: TCRU_3157 TcCLB-EL.503801.50_mRNA-p1 TcCLB-NE.
508817.40_mRNA-p1 TcSYL_0043810.t1-p1
OG0001930: TCRU_3158 TcCLB-EL.509247.50_mRNA-p1 TcSYL_0170460.t1-p1
TcSYL_0170470.t1-p1
OG0001931: TCRU_3177 TcCLB-EL.509233.150_mRNA-p1 TcCLB-NE.

503769.40_mRNA-p1 TcSYL_0145350.t1-p1
OG0001932: TCRU_3189 TcCLB-EL.511419.40_mRNA-p1 TcCLB-NE.
505999.90_mRNA-p1 TcSYL_0111210.t1-p1
OG0001933: TCRU_3191 TcCLB-EL.506797.40_mRNA-p1 TcCLB-NE.
511907.10_mRNA-p1 TcSYL_0102460.t1-p1
OG0001934: TCRU_3192 TcCLB-EL.506797.70_mRNA-p1 TcCLB-NE.
511907.50_mRNA-p1 TcSYL_0102340.t1-p1
OG0001935: TCRU_3193 TcCLB-EL.506797.100_mRNA-p1 TcCLB-NE.
511907.80_mRNA-p1 TcSYL_0102290.t1-p1
OG0001936: TCRU_3195 TcCLB-EL.506797.120_mRNA-p1 TcCLB-NE.
511907.100_mRNA-p1 TcSYL_0102260.t1-p1
OG0001937: TCRU_3206 TcCLB-EL.510283.100_mRNA-p1 TcCLB-NE.
503823.70_mRNA-p1 TcSYL_0117620.t1-p1
OG0001938: TCRU_3207 TcCLB-EL.510283.110_mRNA-p1 TcCLB-NE.
503823.80_mRNA-p1 TcSYL_0117630.t1-p1
OG0001939: TCRU_3208 TcCLB-EL.510283.120_mRNA-p1 TcCLB-NE.
503823.90_mRNA-p1 TcSYL_0117640.t1-p1
OG0001940: TCRU_3210 TcCLB-EL.510283.140_mRNA-p1 TcCLB-NE.
503823.104_mRNA-p1 TcSYL_0117650.t1-p1
OG0001941: TCRU_3211 TcCLB-EL.510283.150_mRNA-p1 TcCLB-NE.
503823.110_mRNA-p1 TcSYL_0117660.t1-p1
OG0001942: TCRU_specific TcCLB-EL.510285.20_mRNA-p1 TcCLB-NE.
503823.130_mRNA-p1 TcSYL_0117670.t1-p1
OG0001943: TCRU_3216 TcCLB-EL.507395.10_mRNA-p1 TcCLB-NE.
510087.30_mRNA-p1 TcSYL_0052040.t1-p1
OG0001944: TCRU_3218 TcCLB-EL.506251.40_mRNA-p1 TcCLB-NE.
508231.150_mRNA-p1 TcSYL_0108090.t1-p1
OG0001945: TCRU_3219 TcCLB-EL.506251.30_mRNA-p1 TcCLB-NE.
508231.170_mRNA-p1 TcSYL_0108160.t1-p1
OG0001946: TCRU_3220 TcCLB-EL.506251.10_mRNA-p1 TcCLB-NE.
508231.180_mRNA-p1 TcSYL_0108210.t1-p1
OG0001947: TCRU_3221 TcCLB-EL.506249.70_mRNA-p1 TcCLB-NE.
508231.190_mRNA-p1 TcSYL_0108250.t1-p1
OG0001948: TCRU_3222 TcCLB-EL.506249.60_mRNA-p1 TcCLB-NE.
508231.200_mRNA-p1 TcSYL_0108260.t1-p1
OG0001949: TCRU_3223 TcCLB-EL.506249.50_mRNA-p1 TcCLB-NE.
508231.204_mRNA-p1 TcSYL_0108270.t1-p1
OG0001950: TCRU_3224 TcCLB-EL.506249.40_mRNA-p1 TcCLB-NE.
508231.210_mRNA-p1 TcSYL_0108280.t1-p1
OG0001951: TCRU_3226 TcCLB-EL.506249.10_mRNA-p1 TcCLB-NE.
508233.10_mRNA-p1 TcSYL_0108300.t1-p1
OG0001952: TCRU_3227 TcCLB-EL.504167.7_mRNA-p1 TcCLB-NE.
508233.19_mRNA-p1 TcSYL_0108310.t1-p1
OG0001953: TCRU_3232 TcCLB-EL.511715.100_mRNA-p1 TcCLB-NE.
401469.20_mRNA-p1 TcCLB-NE.508445.99_mRNA-p1
OG0001954: TCRU_3239 TcCLB-EL.505943.40_mRNA-p1 TcCLB-NE.
507623.30_mRNA-p1 TcSYL_0095850.t1-p1
OG0001955: TCRU_3240 TcCLB-EL.505943.30_mRNA-p1 TcCLB-NE.
507623.40_mRNA-p1 TcSYL_0095860.t1-p1
OG0001956: TCRU_3241 TcCLB-EL.505943.20_mRNA-p1 TcCLB-NE.
507623.50_mRNA-p1 TcSYL_0095870.t1-p1
OG0001957: TCRU_subunit TcCLB-EL.505941.49_mRNA-p1 TcCLB-NE.
507623.60_mRNA-p1 TcSYL_0095880.t1-p1
OG0001958: TCRU_3243 TcCLB-EL.505941.40_mRNA-p1 TcCLB-NE.

507623.80_mRNA-p1 TcSYL_0095890.t1-p1
OG0001959: TCRU_family_ TcCLB-EL.505941.30_mRNA-p1 TcCLB-NE.
507623.90_mRNA-p1 TcSYL_0095900.t1-p1
OG0001960: TCRU_3248 TcCLB-EL.506413.80_mRNA-p1 TcCLB-NE.
511237.110_mRNA-p1 TcSYL_0100130.t1-p1
OG0001961: TCRU_3267 TcCLB-EL.507011.190_mRNA-p1 TcCLB-NE.
503983.10_mRNA-p1 TcSYL_0116770.t1-p1
OG0001962: TCRU_domain/DAK2 TcCLB-EL.507011.200_mRNA-p1 TcCLB-NE.
503983.20_mRNA-p1 TcSYL_0116750.t1-p1
OG0001963: TCRU_3270 TcCLB-EL.507011.240_mRNA-p1 TcCLB-NE.
507003.50_mRNA-p1 TcSYL_0116660.t1-p1
OG0001964: TCRU_3271 TcCLB-EL.510737.40_mRNA-p1 TcCLB-NE.
510663.57_mRNA-p1 TcSYL_0116480.t1-p1
OG0001965: TCRU_3272 TcCLB-EL.510737.60_mRNA-p1 TcCLB-NE.
510663.40_mRNA-p1 TcSYL_0116420.t1-p1
OG0001966: TCRU_3275 TcCLB-EL.510737.100_mRNA-p1 TcCLB-NE.
510661.279_mRNA-p1 TcSYL_0116370.t1-p1
OG0001967: TCRU_3276 TcCLB-EL.510737.110_mRNA-p1 TcCLB-NE.
510661.270_mRNA-p1 TcSYL_0116350.t1-p1
OG0001968: TCRU_3279 TcCLB-EL.510741.30_mRNA-p1 TcCLB-NE.
510661.240_mRNA-p1 TcSYL_0116310.t1-p1
OG0001969: TCRU_3280 TcCLB-EL.510741.40_mRNA-p1 TcCLB-NE.
510661.230_mRNA-p1 TcSYL_0116300.t1-p1
OG0001970: TCRU_3283 TcCLB-EL.510741.70_mRNA-p1 TcCLB-NE.
510661.190_mRNA-p1 TcSYL_0116290.t1-p1
OG0001971: TCRU_3284 TcCLB-EL.510741.80_mRNA-p1 TcCLB-NE.
510661.174_mRNA-p1 TcSYL_0116280.t1-p1
OG0001972: TCRU_3285 TcCLB-EL.510741.90_mRNA-p1 TcCLB-NE.
510661.160_mRNA-p1 TcSYL_0116270.t1-p1
OG0001973: TCRU_3289 TcCLB-EL.510741.124_mRNA-p1 TcCLB-NE.
510661.129_mRNA-p1 TcSYL_0116260.t1-p1
OG0001974: TCRU_3292 TcCLB-EL.510741.180_mRNA-p1 TcCLB-NE.
510661.90_mRNA-p1 TcSYL_0116250.t1-p1
OG0001975: TCRU_3294 TcCLB-EL.509967.40_mRNA-p1 TcCLB-NE.
509695.20_mRNA-p1 TcSYL_0171070.t1-p1
OG0001976: TCRU_3299 TcCLB-EL.509967.100_pseudogenic_transcript-p1
TcCLB-NE.509695.80_mRNA-p1 TcSYL_0171080.t1-p1
OG0001977: TCRU_3300 TcCLB-EL.509967.120_pseudogenic_transcript-p1
TcCLB-NE.509695.90_mRNA-p1 TcSYL_0171100.t1-p1
OG0001978: TCRU_3309 TcCLB-EL.503405.10_mRNA-p1 TcCLB-NE.
508175.370_mRNA-p1 TcSYL_0078590.t1-p1
OG0001979: TCRU_3323 TcCLB-EL.507667.20_mRNA-p1 TcCLB-NE.
506403.9_mRNA-p1 TcSYL_0048390.t1-p1
OG0001980: TCRU_3327 TcCLB-EL.510105.210_mRNA-p1 TcCLB-NE.
509717.60_mRNA-p1 TcSYL_0073440.t1-p1
OG0001981: TCRU_3328 TcCLB-EL.510105.200_mRNA-p1 TcCLB-NE.
509717.50_mRNA-p1 TcSYL_0073450.t1-p1
OG0001982: TCRU_3331 TcCLB-EL.510105.180_mRNA-p1 TcCLB-NE.
509717.30_mRNA-p1 TcSYL_0073480.t1-p1
OG0001983: TCRU_3332 TcCLB-EL.510105.170_mRNA-p1 TcCLB-NE.
509717.20_mRNA-p1 TcSYL_0073500.t1-p1
OG0001984: TCRU_3334 TcCLB-EL.510105.150_mRNA-p1 TcCLB-NE.
509715.140_mRNA-p1 TcSYL_0073520.t1-p1
OG0001985: TCRU_3338 TcCLB-EL.511603.110_mRNA-p1 TcCLB-NE.

504155.56_mRNA-p1 TcCLB-NE.506321.190_mRNA-p1
OG0001986: TCRU_3343 TcCLB-EL.509979.30_mRNA-p1 TcCLB-NE.
508613.10_mRNA-p1 TcSYL_0156570.t1-p1
OG0001987: TCRU_3346 TcCLB-EL.509979.10_mRNA-p1 TcCLB-NE.
508613.50_mRNA-p1 TcSYL_0156620.t1-p1
OG0001988: TCRU_3347 TcCLB-EL.509977.100_mRNA-p1 TcCLB-NE.
508613.60_mRNA-p1 TcSYL_0156630.t1-p1
OG0001989: TCRU_3348 TcCLB-NE.421231.10_mRNA-p1 TcSYL_0065590.t1-p1
TcSYL_0065920.t1-p1
OG0001990: TCRU_3360 TcCLB-EL.509773.9_mRNA-p1 TcCLB-NE.
511657.20_mRNA-p1 TcSYL_0013800.t1-p1
OG0001991: TCRU_3362 TcCLB-EL.507809.150_mRNA-p1 TcCLB-NE.
511657.40_mRNA-p1 TcSYL_0013780.t1-p1
OG0001992: TCRU_3364 TcCLB-EL.507809.130_mRNA-p1 TcCLB-NE.
511657.60_mRNA-p1 TcSYL_0013760.t1-p1
OG0001993: TCRU_3379 TcCLB-EL.509489.29_mRNA-p1 TcCLB-NE.
511725.10_mRNA-p1 TcSYL_0142610.t1-p1
OG0001994: TCRU_3386 TcCLB-EL.506533.34_mRNA-p1 TcCLB-NE.
509395.20_mRNA-p1 TcSYL_0194350.t1-p1
OG0001995: TCRU_3387 TcCLB-EL.506533.70_mRNA-p1 TcCLB-NE.
509395.10_mRNA-p1 TcSYL_0194330.t1-p1
OG0001996: TCRU_3392 TcCLB-EL.504017.70_mRNA-p1 TcCLB-NE.
511229.120_mRNA-p1 TcCLB-NE.511231.14_mRNA-p1
OG0001997: TCRU_3432 TcCLB-EL.510759.80_mRNA-p1 TcCLB-NE.
506999.130_mRNA-p1 TcSYL_0115500.t1-p1
OG0001998: TCRU_3434 TcCLB-EL.511181.140_mRNA-p1 TcCLB-NE.
508299.30_mRNA-p1 TcSYL_0203330.t1-p1
OG0001999: TCRU_receptor TcCLB-EL.511181.90_mRNA-p1 TcCLB-NE.
508299.70_mRNA-p1 TcSYL_0203280.t1-p1
OG0002000: TCRU_3439 TcCLB-EL.511181.80_mRNA-p1 TcCLB-NE.
508299.80_mRNA-p1 TcSYL_0203270.t1-p1
OG0002001: TCRU_3440 TcCLB-EL.511181.70_mRNA-p1 TcCLB-NE.
508299.89_mRNA-p1 TcSYL_0203260.t1-p1
OG0002002: TCRU_3445 TcCLB-EL.508323.120_mRNA-p1 TcCLB-NE.
511825.100_mRNA-p1 TcSYL_0200840.t1-p1
OG0002003: TCRU_3457 TcCLB-EL.507093.10_mRNA-p1 TcCLB-NE.
504109.60_mRNA-p1 TcSYL_0080300.t1-p1
OG0002004: TCRU_3469 TcCLB-EL.509201.20_mRNA-p1 TcCLB-EL.
511805.10_mRNA-p1 TcCLB-NE.511015.10_mRNA-p1
OG0002005: TCRU_3470 TcCLB-EL.509203.14_mRNA-p1 TcCLB-NE.
511017.25_mRNA-p1 TcSYL_0180380.t1-p1
OG0002006: TCRU_3471 TcCLB-EL.509203.40_mRNA-p1 TcCLB-NE.
511017.50_mRNA-p1 TcSYL_0180520.t1-p1
OG0002007: TCRU_3472 TcCLB-EL.509203.50_mRNA-p1 TcCLB-NE.
511017.60_mRNA-p1 TcSYL_0180550.t1-p1
OG0002008: TCRU_3473 TcCLB-EL.509203.60_mRNA-p1 TcCLB-NE.
511017.69_mRNA-p1 TcSYL_0180570.t1-p1
OG0002009: TCRU_3485 TcCLB-EL.511127.410_mRNA-p1 TcCLB-NE.
509029.20_mRNA-p1 TcSYL_0156050.t1-p1
OG0002010: TCRU_3486 TcCLB-EL.511127.420_mRNA-p1 TcCLB-NE.
509029.30_mRNA-p1 TcSYL_0156060.t1-p1
OG0002011: TCRU_3495 TcCLB-EL.511737.10_mRNA-p1 TcCLB-NE.
511519.9_mRNA-p1 TcSYL_0139870.t1-p1
OG0002012: TCRU_3500 TcCLB-EL.510507.40_mRNA-p1 TcCLB-NE.

509463.10_mRNA-p1 TcSYL_0173820.t1-p1
OG0002013: TCRU_3511 TcCLB-EL.508783.10_pseudogenic_transcript-p1
TcCLB-EL.511137.20_mRNA-p1 TcCLB-NE.507073.10_mRNA-p1
OG0002014: TCRU_3518 TcCLB-EL.511141.30_mRNA-p1 TcCLB-EL.
511143.6_mRNA-p1 TcCLB-NE.506851.10_mRNA-p1
OG0002015: TCRU_3550 TcCLB-EL.510345.40_mRNA-p1 TcCLB-NE.
506469.50_mRNA-p1 TcSYL_0156830.t1-p1
OG0002016: TCRU_3555 TcCLB-NE.508869.50_mRNA-p1 TcCLB-NE.
508871.20_mRNA-p1 TcCLB-NE.509971.10_mRNA-p1
OG0002017: TCRU_3576 TcCLB-EL.507041.50_mRNA-p1 TcCLB-NE.
508273.70_mRNA-p1 TcSYL_0088550.t1-p1
OG0002018: TCRU_3577 TcCLB-EL.507041.40_mRNA-p1 TcCLB-NE.
508273.60_mRNA-p1 TcSYL_0088540.t1-p1
OG0002019: TCRU_3578 TcCLB-EL.507041.30_mRNA-p1 TcCLB-NE.
508273.50_mRNA-p1 TcSYL_0088530.t1-p1
OG0002020: TCRU_3581 TcCLB-EL.410589.10_mRNA-p1 TcCLB-NE.
508273.10_mRNA-p1 TcSYL_0088520.t1-p1
OG0002021: TCRU_3582 TcCLB-EL.511545.190_mRNA-p1 TcCLB-NE.
510435.40_mRNA-p1 TcSYL_0088480.t1-p1
OG0002022: TCRU_10277 TCRU_3598 TcCLB-NE.
508231.120_pseudogenic_transcript-p1 TcCLB-NE.508639.10_mRNA-p1
OG0002023: TCRU_3604 TcCLB-EL.509233.70_mRNA-p1 TcCLB-NE.
509437.50_mRNA-p1 TcSYL_0146100.t1-p1
OG0002024: TCRU_3609 TcCLB-EL.504073.20_mRNA-p1 TcCLB-EL.
506933.10_mRNA-p1 TcSYL_0044170.t1-p1
OG0002025: TCRU_3612 TcCLB-EL.503685.20_mRNA-p1 TcCLB-NE.
509999.100_mRNA-p1 TcSYL_0003200.t1-p1
OG0002026: TCRU_3613 TcCLB-EL.503685.10_mRNA-p1 TcCLB-NE.
509999.110_mRNA-p1 TcSYL_0003190.t1-p1
OG0002027: TCRU_3616 TcCLB-EL.507529.10_mRNA-p1 TcCLB-NE.
510001.10_mRNA-p1 TcSYL_0003180.t1-p1
OG0002028: TCRU_3617 TcCLB-EL.507529.20_mRNA-p1 TcCLB-NE.
510001.20_mRNA-p1 TcSYL_0003170.t1-p1
OG0002029: TCRU_3618 TcSYL_0009330.t1-p1 TcSYL_0009410.t1-p1
TcSYL_0009510.t1-p1
OG0002030: TCRU_3623 TcCLB-EL.511737.30_mRNA-p1 TcCLB-NE.
511517.164_mRNA-p1 TcSYL_0139860.t1-p1
OG0002031: TCRU_3624 TcCLB-EL.511737.40_mRNA-p1 TcCLB-NE.
511517.160_mRNA-p1 TcSYL_0139850.t1-p1
OG0002032: TCRU_3627 TcCLB-EL.511737.80_mRNA-p1 TcCLB-NE.
511517.140_mRNA-p1 TcSYL_0139840.t1-p1
OG0002033: TCRU_3628 TcCLB-EL.511737.90_mRNA-p1 TcCLB-NE.
511517.130_mRNA-p1 TcSYL_0139830.t1-p1
OG0002034: TCRU_3629 TcCLB-EL.511739.20_mRNA-p1 TcCLB-NE.
511517.110_mRNA-p1 TcSYL_0139790.t1-p1
OG0002035: TCRU_3630 TcCLB-EL.511739.30_mRNA-p1 TcCLB-NE.
511517.99_mRNA-p1 TcSYL_0139780.t1-p1
OG0002036: TCRU_3632 TcCLB-EL.511741.30_mRNA-p1 TcCLB-NE.
511517.80_mRNA-p1 TcSYL_0139750.t1-p1
OG0002037: TCRU_3636 TcCLB-EL.506941.280_mRNA-p1 TcCLB-NE.
432677.10_pseudogenic_transcript-p1 TcCLB-NE.511515.40_mRNA-p1
OG0002038: TCRU_3637 TcCLB-EL.511745.20_mRNA-p1 TcCLB-NE.
511515.20_pseudogenic_transcript-p1 TcSYL_0139660.t1-p1
OG0002039: TCRU_3652 TcCLB-EL.510609.10_pseudogenic_transcript-p1

TcCLB-NE.511477.10_pseudogenic_transcript-p1 TcCLB-NE.
511479.10_pseudogenic_transcript-p1
OG0002040: TCRU_3667 TcCLB-EL.510565.20_mRNA-p1 TcCLB-NE.
510795.30_pseudogenic_transcript-p1 TcSYL_0105880.t1-p1
OG0002041: TCRU_3668 TcCLB-EL.510565.30_mRNA-p1 TcCLB-NE.
508503.20_mRNA-p1 TcSYL_0110430.t1-p1
OG0002042: TCRU_3669 TcCLB-EL.510565.70_mRNA-p1 TcCLB-NE.
508503.60_mRNA-p1 TcSYL_0105800.t1-p1
OG0002043: TCRU_3670 TcCLB-EL.510565.100_mRNA-p1 TcCLB-NE.
508503.70_mRNA-p1 TcSYL_0105750.t1-p1
OG0002044: TCRU_subunit TcCLB-EL.510565.110_mRNA-p1 TcCLB-NE.
508503.80_mRNA-p1 TcSYL_0105740.t1-p1
OG0002045: TCRU_3672 TcCLB-EL.510565.120_mRNA-p1 TcCLB-NE.
508503.90_mRNA-p1 TcSYL_0105730.t1-p1
OG0002046: TCRU_3691 TcCLB-EL.504103.30_mRNA-p1 TcCLB-NE.
503847.20_mRNA-p1 TcSYL_0005930.t1-p1
OG0002047: TCRU_3692 TcCLB-EL.504103.50_mRNA-p1 TcCLB-NE.
503847.40_mRNA-p1 TcSYL_0005880.t1-p1
OG0002048: TCRU_3693 TcCLB-EL.504103.70_mRNA-p1 TcCLB-NE.
503847.60_mRNA-p1 TcSYL_0005850.t1-p1
OG0002049: TCRU_3694 TcCLB-EL.504103.80_mRNA-p1 TcCLB-NE.
503847.70_mRNA-p1 TcSYL_0005800.t1-p1
OG0002050: TCRU_C-terminal TcCLB-EL.504103.90_mRNA-p1 TcCLB-NE.
503847.80_mRNA-p1 TcSYL_0005750.t1-p1
OG0002051: TCRU_protein_ TcCLB-EL.504103.100_mRNA-p1 TcCLB-NE.
503847.90_mRNA-p1 TcSYL_0005710.t1-p1
OG0002052: TCRU_3700 TcCLB-NE.460111.10_mRNA-p1 TcCLB-NE.
509665.30_mRNA-p1 TcSYL_0086840.t1-p1
OG0002053: TCRU_3719 TcCLB-EL.507639.40_mRNA-p1 TcCLB-NE.
503781.70_mRNA-p1 TcSYL_0010960.t1-p1
OG0002054: TCRU_3720 TcCLB-EL.507639.50_mRNA-p1 TcCLB-NE.
503781.60_mRNA-p1 TcSYL_0010970.t1-p1
OG0002055: TCRU_3722 TcCLB-EL.507639.70_mRNA-p1 TcCLB-NE.
503781.40_mRNA-p1 TcSYL_0010980.t1-p1
OG0002056: TCRU_3723 TcCLB-EL.507639.90_mRNA-p1 TcCLB-NE.
503781.30_mRNA-p1 TcSYL_0010990.t1-p1
OG0002057: TCRU_3726 TcCLB-EL.507641.30_mRNA-p1 TcCLB-NE.
509729.20_mRNA-p1 TcSYL_0011060.t1-p1
OG0002058: TCRU_3727 TcCLB-EL.507641.40_mRNA-p1 TcCLB-NE.
509729.10_mRNA-p1 TcSYL_0011080.t1-p1
OG0002059: TCRU_3729 TcCLB-EL.507817.80_mRNA-p1 TcCLB-NE.
427303.10_mRNA-p1 TcSYL_0042460.t1-p1
OG0002060: TCRU_3738 TcCLB-EL.506363.50_mRNA-p1 TcCLB-NE.
506975.60_mRNA-p1 TcSYL_0118560.t1-p1
OG0002061: TCRU_3757 TcCLB-EL.503493.30_mRNA-p1 TcCLB-NE.
511761.30_mRNA-p1 TcSYL_0159790.t1-p1
OG0002062: TCRU_3759 TcCLB-EL.511305.10_mRNA-p1 TcCLB-NE.
507087.80_mRNA-p1 TcSYL_0159780.t1-p1
OG0002063: TCRU_3764 TcCLB-EL.503801.30_mRNA-p1 TcCLB-NE.
508817.50_mRNA-p1 TcSYL_0043750.t1-p1
OG0002064: TCRU_3765 TcCLB-EL.504133.15_mRNA-p1 TcSYL_0170520.t1-p1
TcSYL_0170560.t1-p1
OG0002065: TCRU_3767 TcCLB-EL.507933.20_mRNA-p1 TcCLB-NE.
509693.30_mRNA-p1 TcSYL_0170600.t1-p1

OG0002066: TCRU_3768 TcCLB-EL.507933.29_mRNA-p1 TcCLB-NE.509693.40_mRNA-p1 TcSYL_0170610.t1-p1
OG0002067: TCRU_3769 TcCLB-EL.506457.10_mRNA-p1 TcCLB-NE.509693.50_mRNA-p1 TcSYL_0170620.t1-p1
OG0002068: TCRU_3770 TcCLB-EL.506457.30_mRNA-p1 TcCLB-NE.509693.70_mRNA-p1 TcSYL_0170650.t1-p1
OG0002069: TCRU_3771 TcCLB-EL.506457.40_mRNA-p1 TcCLB-NE.509693.80_mRNA-p1 TcSYL_0170670.t1-p1
OG0002070: TCRU_3772 TcCLB-EL.506457.50_mRNA-p1 TcCLB-NE.509693.90_mRNA-p1 TcSYL_0170690.t1-p1
OG0002071: TCRU_3773 TcCLB-EL.506457.70_mRNA-p1 TcCLB-NE.509693.110_mRNA-p1 TcSYL_0170710.t1-p1
OG0002072: TCRU_3774 TcCLB-EL.506457.80_mRNA-p1 TcCLB-NE.509693.120_mRNA-p1 TcSYL_0170720.t1-p1
OG0002073: TCRU_3775 TcCLB-EL.506457.90_mRNA-p1 TcCLB-NE.509693.130_mRNA-p1 TcSYL_0170780.t1-p1
OG0002074: TCRU_3776 TcCLB-EL.506457.110_mRNA-p1 TcCLB-NE.509693.150_mRNA-p1 TcSYL_0170820.t1-p1
OG0002075: TCRU_3778 TcCLB-EL.506457.149_mRNA-p1 TcCLB-NE.509693.190_mRNA-p1 TcSYL_0170950.t1-p1
OG0002076: TCRU_3779 TcCLB-EL.504077.60_pseudogenic_transcript-p1 TcCLB-NE.507081.50_mRNA-p1 TcSYL_0019670.t1-p1
OG0002077: TCRU_3783 TcCLB-EL.503991.10_mRNA-p1 TcCLB-NE.507081.100_mRNA-p1 TcSYL_0019660.t1-p1
OG0002078: TCRU_3784 TcCLB-EL.503991.30_mRNA-p1 TcCLB-NE.507081.120_mRNA-p1 TcSYL_0019640.t1-p1
OG0002079: TCRU_3785 TcCLB-EL.503991.39_mRNA-p1 TcCLB-NE.507081.130_mRNA-p1 TcSYL_0019630.t1-p1
OG0002080: TCRU_3791 TcCLB-EL.506779.200_pseudogenic_transcript-p1 TcCLB-NE.511151.90_mRNA-p1 TcSYL_0077240.t1-p1
OG0002081: TCRU_kDa TcCLB-EL.508241.20_mRNA-p1 TcCLB-NE.511151.80_mRNA-p1 TcSYL_0077260.t1-p1
OG0002082: TCRU_3793 TcCLB-EL.508241.30_mRNA-p1 TcCLB-NE.511151.70_mRNA-p1 TcSYL_0077280.t1-p1
OG0002083: TCRU_3794 TcCLB-EL.508241.40_mRNA-p1 TcCLB-NE.511151.60_mRNA-p1 TcSYL_0077320.t1-p1
OG0002084: TCRU_3795 TcCLB-EL.508241.60_mRNA-p1 TcCLB-NE.511151.50_mRNA-p1 TcSYL_0077380.t1-p1
OG0002085: TCRU_family_ TcCLB-EL.508241.70_mRNA-p1 TcCLB-NE.511151.39_mRNA-p1 TcSYL_0077400.t1-p1
OG0002086: TCRU_3797 TcCLB-EL.508241.80_mRNA-p1 TcCLB-NE.511151.30_mRNA-p1 TcSYL_0077420.t1-p1
OG0002087: TCRU_3798 TcCLB-EL.508241.90_mRNA-p1 TcCLB-NE.511151.20_mRNA-p1 TcSYL_0077440.t1-p1
OG0002088: TCRU_3799 TcCLB-EL.508241.100_mRNA-p1 TcCLB-NE.511151.11_mRNA-p1 TcSYL_0077480.t1-p1
OG0002089: TCRU_3811 TcCLB-EL.504883.50_mRNA-p1 TcCLB-NE.506511.30_mRNA-p1 TcSYL_0065010.t1-p1
OG0002090: TCRU_3812 TcCLB-EL.504883.40_mRNA-p1 TcCLB-NE.506511.20_mRNA-p1 TcSYL_0065000.t1-p1
OG0002091: TCRU_10409 TCRU_3819 TcCLB-NE.508827.70_mRNA-p1 TcCLB-NE.508827.79_mRNA-p1
OG0002092: TCRU_3839 TcCLB-EL.511529.59_mRNA-p1 TcCLB-NE.425969.5_mRNA-p1 TcSYL_0112200.t1-p1

OG0002093: TCRU_3840 TcCLB-EL.511529.68_mRNA-p1 TcCLB-NE.425969.9_mRNA-p1 TcSYL_0112190.t1-p1
OG0002094: TCRU_3889 TcCLB-EL.505229.10_mRNA-p1 TcCLB-NE.510823.30_mRNA-p1 TcSYL_0050160.t1-p1
OG0002095: TCRU_3893 TcCLB-EL.505555.61_mRNA-p1 TcCLB-NE.510823.80_mRNA-p1 TcSYL_0050190.t1-p1
OG0002096: TCRU_3895 TcCLB-EL.505555.40_mRNA-p1 TcCLB-NE.510823.100_mRNA-p1 TcSYL_0050200.t1-p1
OG0002097: TCRU_3904 TcCLB-NE.467795.9_mRNA-p1 TcSYL_0001620.t1-p1 TcSYL_0001840.t1-p1
OG0002098: TCRU_3906 TcCLB-EL.504055.60_mRNA-p1 TcCLB-NE.506435.290_mRNA-p1 TcSYL_0196390.t1-p1
OG0002099: TCRU_3907 TcCLB-EL.504055.40_mRNA-p1 TcCLB-NE.506435.270_mRNA-p1 TcSYL_0196470.t1-p1
OG0002100: TCRU_3908 TcCLB-EL.504055.30_mRNA-p1 TcCLB-NE.506435.260_mRNA-p1 TcSYL_0196480.t1-p1
OG0002101: TCRU_3909 TcCLB-EL.504055.20_mRNA-p1 TcCLB-NE.506435.250_mRNA-p1 TcSYL_0196490.t1-p1
OG0002102: TCRU_3910 TcCLB-EL.504055.10_mRNA-p1 TcCLB-NE.506435.230_pseudogenic_transcript-p1 TcSYL_0196510.t1-p1
OG0002103: TCRU_3912 TcCLB-EL.506265.180_mRNA-p1 TcCLB-NE.506435.200_mRNA-p1 TcSYL_0196570.t1-p1
OG0002104: TCRU_3913 TcCLB-EL.506265.170_mRNA-p1 TcCLB-NE.506435.190_mRNA-p1 TcSYL_0196590.t1-p1
OG0002105: TCRU_3914 TcCLB-EL.506265.150_mRNA-p1 TcCLB-NE.506435.170_mRNA-p1 TcSYL_0196630.t1-p1
OG0002106: TCRU_3915 TcCLB-EL.506265.140_mRNA-p1 TcCLB-NE.506435.160_mRNA-p1 TcSYL_0196640.t1-p1
OG0002107: TCRU_3916 TcCLB-EL.506265.130_mRNA-p1 TcCLB-NE.506435.150_mRNA-p1 TcSYL_0196680.t1-p1
OG0002108: TCRU_3917 TcCLB-EL.506265.120_mRNA-p1 TcCLB-NE.506435.140_mRNA-p1 TcSYL_0196730.t1-p1
OG0002109: TCRU_3918 TcCLB-EL.506265.114_mRNA-p1 TcCLB-NE.506435.134_mRNA-p1 TcSYL_0196760.t1-p1
OG0002110: TCRU_3919 TcCLB-EL.506265.110_mRNA-p1 TcCLB-NE.506435.130_mRNA-p1 TcSYL_0196780.t1-p1
OG0002111: TCRU_3920 TcCLB-EL.506265.100_mRNA-p1 TcCLB-NE.506435.120_mRNA-p1 TcSYL_0196820.t1-p1
OG0002112: TCRU_3921 TcCLB-EL.506265.90_mRNA-p1 TcCLB-NE.506435.110_mRNA-p1 TcSYL_0196830.t1-p1
OG0002113: TCRU_3922 TcCLB-EL.506265.80_mRNA-p1 TcCLB-NE.506435.100_mRNA-p1 TcSYL_0196850.t1-p1
OG0002114: TCRU_3923 TcCLB-EL.506265.70_mRNA-p1 TcCLB-NE.506435.90_mRNA-p1 TcSYL_0196870.t1-p1
OG0002115: TCRU_3925 TcCLB-EL.506265.50_mRNA-p1 TcCLB-NE.506435.60_mRNA-p1 TcSYL_0196980.t1-p1
OG0002116: TCRU_3927 TcCLB-EL.507511.40_mRNA-p1 TcCLB-NE.506871.180_mRNA-p1 TcSYL_0142510.t1-p1
OG0002117: TCRU_3928 TcCLB-EL.507511.20_mRNA-p1 TcCLB-NE.506871.150_mRNA-p1 TcSYL_0142520.t1-p1
OG0002118: TCRU_3935 TcCLB-EL.511303.60_mRNA-p1 TcCLB-NE.509177.68_mRNA-p1 TcSYL_0159880.t1-p1
OG0002119: TCRU_3939 TcCLB-EL.506351.70_mRNA-p1 TcCLB-NE.505939.30_mRNA-p1 TcSYL_0042690.t1-p1

OG0002120: TCRU_3940 TcCLB-EL.506351.60_mRNA-p1 TcCLB-NE.
505939.40_mRNA-p1 TcSYL_0042630.t1-p1
OG0002121: TCRU_3942 TcCLB-EL.506351.20_mRNA-p1 TcCLB-NE.
505939.80_mRNA-p1 TcSYL_0042510.t1-p1
OG0002122: TCRU_3947 TcCLB-EL.509801.10_mRNA-p1 TcCLB-NE.
511277.120_mRNA-p1 TcSYL_0014860.t1-p1
OG0002123: TCRU_3949 TcCLB-EL.509801.30_mRNA-p1 TcCLB-NE.
511277.110_mRNA-p1 TcSYL_0014870.t1-p1
OG0002124: TCRU_3956 TcCLB-EL.506759.170_mRNA-p1 TcCLB-EL.
510371.100_mRNA-p1 TcCLB-NE.511255.630_mRNA-p1
OG0002125: TCRU_3963 TcCLB-EL.507709.10_mRNA-p1 TcCLB-NE.
506577.10_mRNA-p1 TcSYL_0009150.t1-p1
OG0002126: TCRU_3965 TcCLB-EL.507677.150_mRNA-p1 TcCLB-NE.
504021.10_mRNA-p1 TcSYL_0009610.t1-p1
OG0002127: TCRU_3975 TcCLB-EL.509297.20_mRNA-p1 TcCLB-NE.
508199.20_mRNA-p1 TcSYL_0146760.t1-p1
OG0002128: TCRU_3977 TcCLB-EL.504033.220_mRNA-p1 TcCLB-NE.
508199.50_mRNA-p1 TcSYL_0146930.t1-p1
OG0002129: TCRU_3978 TcCLB-EL.504033.210_mRNA-p1 TcCLB-NE.
508199.60_mRNA-p1 TcSYL_0146960.t1-p1
OG0002130: TCRU_3979 TcCLB-EL.504033.200_mRNA-p1 TcCLB-NE.
508199.70_mRNA-p1 TcSYL_0146980.t1-p1
OG0002131: TCRU_3980 TcCLB-EL.504033.190_mRNA-p1 TcCLB-NE.
508199.80_mRNA-p1 TcSYL_0147010.t1-p1
OG0002132: TCRU_3982 TcCLB-EL.504033.170_mRNA-p1 TcCLB-NE.
510339.14_mRNA-p1 TcSYL_0147080.t1-p1
OG0002133: TCRU_3983 TcCLB-EL.504033.160_mRNA-p1 TcCLB-NE.
510339.20_mRNA-p1 TcSYL_0147100.t1-p1
OG0002134: TCRU_3997 TcCLB-EL.503865.50_mRNA-p1 TcCLB-NE.
508693.180_mRNA-p1 TcSYL_0046410.t1-p1
OG0002135: TCRU_3999 TcCLB-EL.503865.70_mRNA-p1 TcCLB-NE.
508693.199_mRNA-p1 TcSYL_0046420.t1-p1
OG0002136: TCRU_4004 TcCLB-EL.505987.20_mRNA-p1 TcCLB-NE.
506705.50_mRNA-p1 TcSYL_0046440.t1-p1
OG0002137: TCRU_4013 TcCLB-EL.506753.90_mRNA-p1 TcCLB-NE.
510355.250_mRNA-p1 TcSYL_0112640.t1-p1
OG0002138: TCRU_4023 TcCLB-EL.509591.60_mRNA-p1 TcCLB-NE.
503999.100_mRNA-p1 TcSYL_0089260.t1-p1
OG0002139: TCRU_4026 TcCLB-EL.509591.10_mRNA-p1 TcCLB-NE.
503999.50_mRNA-p1 TcSYL_0089240.t1-p1
OG0002140: TCRU_4027 TcCLB-EL.509589.40_mRNA-p1 TcCLB-NE.
503999.30_mRNA-p1 TcSYL_0089220.t1-p1
OG0002141: TCRU_4034 TcCLB-EL.506775.160_mRNA-p1 TcCLB-NE.
511167.20_mRNA-p1 TcSYL_0075480.t1-p1
OG0002142: TCRU_4035 TcCLB-EL.506775.140_mRNA-p1 TcCLB-NE.
511167.40_mRNA-p1 TcSYL_0075470.t1-p1
OG0002143: TCRU_4036 TcCLB-EL.506775.130_mRNA-p1 TcCLB-NE.
511167.50_mRNA-p1 TcSYL_0075450.t1-p1
OG0002144: TCRU_4037 TcCLB-EL.506775.110_mRNA-p1 TcCLB-NE.
511167.60_mRNA-p1 TcSYL_0075430.t1-p1
OG0002145: TCRU_4038 TcCLB-EL.506775.100_mRNA-p1 TcCLB-NE.
511167.70_mRNA-p1 TcSYL_0075420.t1-p1
OG0002146: TCRU_4039 TcCLB-EL.506775.80_mRNA-p1 TcCLB-NE.
511167.90_mRNA-p1 TcSYL_0075380.t1-p1

OG0002147: TCRU_4054 TcCLB-EL.509707.29_mRNA-p1 TcCLB-NE.
508065.50_mRNA-p1 TcSYL_0056920.t1-p1
OG0002148: TCRU_4057 TcCLB-EL.506625.50_mRNA-p1 TcSYL_0039360.t1-p1
TcSYL_0142360.t1-p1
OG0002149: TCRU_4080 TcCLB-EL.506733.80_mRNA-p1 TcCLB-NE.
511857.20_mRNA-p1 TcSYL_0091760.t1-p1
OG0002150: TCRU_4082 TcCLB-EL.506733.60_mRNA-p1 TcCLB-NE.
511857.40_mRNA-p1 TcSYL_0091750.t1-p1
OG0002151: TCRU_4083 TcCLB-EL.506733.50_mRNA-p1 TcCLB-NE.
511857.50_mRNA-p1 TcSYL_0091740.t1-p1
OG0002152: TCRU_4084 TcCLB-EL.506733.40_mRNA-p1 TcCLB-NE.
511857.59_mRNA-p1 TcSYL_0091730.t1-p1
OG0002153: TCRU_4085 TcCLB-EL.506733.30_mRNA-p1 TcCLB-NE.
511857.70_mRNA-p1 TcSYL_0091720.t1-p1
OG0002154: TCRU_4086 TcCLB-EL.506733.20_mRNA-p1 TcCLB-NE.
511857.80_mRNA-p1 TcSYL_0091710.t1-p1
OG0002155: TCRU_4090 TcCLB-EL.511045.50_mRNA-p1 TcCLB-NE.
511859.70_mRNA-p1 TcSYL_0091680.t1-p1
OG0002156: TCRU_4108 TcCLB-EL.511417.70_mRNA-p1 TcCLB-NE.
505999.4_mRNA-p1 TcSYL_0111300.t1-p1
OG0002157: TCRU_4115 TcCLB-EL.509453.79_mRNA-p1 TcCLB-NE.
503879.110_mRNA-p1 TcSYL_0145500.t1-p1
OG0002158: TCRU__GCN5L1__ TcCLB-EL.509453.30_mRNA-p1 TcCLB-NE.
511881.10_mRNA-p1 TcSYL_0145640.t1-p1
OG0002159: TCRU_4121 TcCLB-EL.509453.20_mRNA-p1 TcCLB-NE.
506709.50_mRNA-p1 TcSYL_0145660.t1-p1
OG0002160: TCRU_4122 TcCLB-EL.509453.10_mRNA-p1 TcCLB-NE.
506709.40_mRNA-p1 TcSYL_0145680.t1-p1
OG0002161: TCRU_4124 TcCLB-EL.503813.20_mRNA-p1 TcCLB-NE.
506709.20_mRNA-p1 TcSYL_0145760.t1-p1
OG0002162: TCRU_4125 TcCLB-EL.503813.10_mRNA-p1 TcCLB-NE.
506709.10_mRNA-p1 TcSYL_0145810.t1-p1
OG0002163: TCRU_4134 TcCLB-EL.511469.30_mRNA-p1 TcCLB-NE.
506295.90_mRNA-p1 TcSYL_0132910.t1-p1
OG0002164: TCRU_4137 TcCLB-EL.511469.70_mRNA-p1 TcCLB-NE.
506295.130_mRNA-p1 TcSYL_0132920.t1-p1
OG0002165: TCRU_4139 TcCLB-EL.511469.100_mRNA-p1 TcCLB-NE.
506295.160_mRNA-p1 TcSYL_0132940.t1-p1
OG0002166: TCRU_4144 TcCLB-EL.510535.20_mRNA-p1 TcCLB-NE.
511827.20_mRNA-p1 TcSYL_0200740.t1-p1
OG0002167: TCRU_4145 TcCLB-EL.510535.30_mRNA-p1 TcCLB-NE.
511827.30_mRNA-p1 TcSYL_0200720.t1-p1
OG0002168: TCRU_4146 TcCLB-EL.510535.40_mRNA-p1 TcCLB-NE.
511827.40_mRNA-p1 TcSYL_0200710.t1-p1
OG0002169: TCRU_4148 TcCLB-EL.510535.60_mRNA-p1 TcCLB-NE.
511827.60_mRNA-p1 TcSYL_0200700.t1-p1
OG0002170: TCRU_4151 TcCLB-EL.510535.80_mRNA-p1 TcCLB-NE.
511827.80_mRNA-p1 TcSYL_0200690.t1-p1
OG0002171: TCRU_4152 TcCLB-EL.510535.90_mRNA-p1 TcCLB-NE.
511827.90_mRNA-p1 TcSYL_0200680.t1-p1
OG0002172: TCRU_4159 TcCLB-EL.503893.150_mRNA-p1 TcCLB-NE.
508505.40_mRNA-p1 TcSYL_0082310.t1-p1
OG0002173: TCRU_4161 TcCLB-EL.503893.90_mRNA-p1 TcCLB-NE.
508507.50_mRNA-p1 TcSYL_0082290.t1-p1

OG0002174: TCRU_4163 TcCLB-EL.503893.60_mRNA-p1 TcCLB-NE.
503703.10_mRNA-p1 TcSYL_0082270.t1-p1
OG0002175: TCRU_4167 TcCLB-EL.511445.130_mRNA-p1 TcCLB-NE.
503703.70_mRNA-p1 TcSYL_0080910.t1-p1
OG0002176: TCRU_4171 TcCLB-EL.511445.90_mRNA-p1 TcCLB-NE.
510799.9_mRNA-p1 TcSYL_0080900.t1-p1
OG0002177: TCRU_4175 TcCLB-EL.508179.60_mRNA-p1 TcCLB-NE.
504269.10_pseudogenic_transcript-p1 TcCLB-NE.510941.3_mRNA-p1
OG0002178: TCRU_4182 TcCLB-EL.511127.140_mRNA-p1 TcCLB-NE.
509023.130_mRNA-p1 TcSYL_0155940.t1-p1
OG0002179: TCRU_4184 TcCLB-EL.511127.160_mRNA-p1 TcCLB-NE.
509023.150_mRNA-p1 TcSYL_0155950.t1-p1
OG0002180: TCRU_4187 TcCLB-EL.511127.220_mRNA-p1 TcCLB-NE.
509023.200_mRNA-p1 TcSYL_0155970.t1-p1
OG0002181: TCRU_4196 TcCLB-EL.506531.30_mRNA-p1 TcCLB-NE.
509399.70_mRNA-p1 TcSYL_0176930.t1-p1
OG0002182: TCRU_4204 TcCLB-EL.510879.50_mRNA-p1 TcCLB-NE.
504013.60_mRNA-p1 TcSYL_0045910.t1-p1
OG0002183: TCRU_4206 TcCLB-EL.510879.30_mRNA-p1 TcCLB-NE.
504013.90_mRNA-p1 TcSYL_0045900.t1-p1
OG0002184: TCRU_4207 TcCLB-EL.510879.20_mRNA-p1 TcCLB-NE.
504013.100_mRNA-p1 TcSYL_0045890.t1-p1
OG0002185: TCRU_4214 TcCLB-EL.510877.150_mRNA-p1 TcCLB-NE.
508689.20_mRNA-p1 TcSYL_0045880.t1-p1
OG0002186: TCRU_4216 TcCLB-EL.510877.130_mRNA-p1 TcCLB-NE.
508689.5_pseudogenic_transcript-p1 TcSYL_0045870.t1-p1
OG0002187: TCRU_4222 TcCLB-NE.504081.365_mRNA-p1 TcSYL_0136420.t1-p1
TcSYL_0150420.t1-p1
OG0002188: TCRU_4237 TcCLB-EL.504151.50_mRNA-p1 TcCLB-NE.
509461.20_mRNA-p1 TcSYL_0174240.t1-p1
OG0002189: TCRU_4238 TcCLB-EL.504151.30_mRNA-p1 TcCLB-NE.
509461.40_mRNA-p1 TcSYL_0174190.t1-p1
OG0002190: TCRU_4239 TcCLB-EL.504151.20_mRNA-p1 TcCLB-NE.
509461.50_mRNA-p1 TcSYL_0174170.t1-p1
OG0002191: TCRU_4241 TcCLB-EL.510509.50_mRNA-p1 TcCLB-NE.
509461.60_mRNA-p1 TcSYL_0174140.t1-p1
OG0002192: TCRU_4242 TcCLB-EL.510509.30_mRNA-p1 TcCLB-NE.
509461.70_mRNA-p1 TcSYL_0174100.t1-p1
OG0002193: TCRU_4244 TcCLB-EL.510507.50_mRNA-p1 TcCLB-NE.
509461.100_mRNA-p1 TcSYL_0173940.t1-p1
OG0002194: TCRU_4245 TcCLB-EL.506363.100_mRNA-p1 TcCLB-NE.
506975.20_mRNA-p1 TcSYL_0118440.t1-p1
OG0002195: TCRU_4248 TcCLB-EL.506363.130_mRNA-p1 TcCLB-NE.
508405.110_mRNA-p1 TcSYL_0118430.t1-p1
OG0002196: TCRU_4250 TcCLB-EL.506363.150_mRNA-p1 TcCLB-NE.
508405.90_mRNA-p1 TcSYL_0118420.t1-p1
OG0002197: TCRU_4253 TcCLB-EL.507837.30_mRNA-p1 TcCLB-NE.
508405.60_mRNA-p1 TcSYL_0118410.t1-p1
OG0002198: TCRU_domain TcCLB-EL.507837.80_mRNA-p1 TcCLB-NE.
508405.20_mRNA-p1 TcSYL_0118380.t1-p1
OG0002199: TCRU_4257 TcCLB-EL.507837.90_mRNA-p1 TcCLB-NE.
508405.10_mRNA-p1 TcSYL_0118370.t1-p1
OG0002200: TCRU_4258 TcCLB-EL.507837.100_mRNA-p1 TcCLB-NE.
508403.20_pseudogenic_transcript-p1 TcSYL_0118350.t1-p1

OG0002201: TCRU_4259 TcCLB-EL.509819.10_mRNA-p1 TcCLB-NE.
508403.10_mRNA-p1 TcSYL_0118340.t1-p1
OG0002202: TCRU_4260 TcCLB-EL.509819.20_mRNA-p1 TcCLB-NE.
503663.20_mRNA-p1 TcSYL_0118330.t1-p1
OG0002203: TCRU_4261 TcCLB-EL.509819.30_mRNA-p1 TcCLB-NE.
503663.10_mRNA-p1 TcSYL_0118320.t1-p1
OG0002204: TCRU_4271 TcCLB-EL.503613.60_mRNA-p1 TcCLB-NE.
511165.40_mRNA-p1 TcSYL_0075520.t1-p1
OG0002205: TCRU_4273 TcCLB-EL.503613.30_mRNA-p1 TcCLB-NE.
511165.60_mRNA-p1 TcSYL_0075510.t1-p1
OG0002206: TCRU_4275 TcCLB-EL.506775.190_mRNA-p1 TcCLB-NE.
511165.100_mRNA-p1 TcSYL_0075490.t1-p1
OG0002207: TCRU_4282 TcCLB-EL.506211.100_mRNA-p1 TcCLB-NE.
508895.30_mRNA-p1 TcSYL_0123000.t1-p1
OG0002208: TCRU_4284 TcCLB-EL.506211.60_mRNA-p1 TcCLB-NE.
508895.60_mRNA-p1 TcSYL_0122980.t1-p1
OG0002209: TCRU_4285 TcCLB-EL.506211.40_mRNA-p1 TcCLB-NE.
508895.80_mRNA-p1 TcSYL_0122970.t1-p1
OG0002210: TCRU_4287 TcCLB-EL.506211.20_mRNA-p1 TcCLB-NE.
511291.20_mRNA-p1 TcSYL_0122940.t1-p1
OG0002211: TCRU_4288 TcCLB-EL.506211.10_mRNA-p1 TcCLB-NE.
511291.30_mRNA-p1 TcSYL_0122930.t1-p1
OG0002212: TCRU_4290 TcCLB-EL.506973.20_mRNA-p1 TcCLB-NE.
507071.180_mRNA-p1 TcSYL_0124680.t1-p1
OG0002213: TCRU_4305 TcCLB-EL.510105.130_mRNA-p1 TcCLB-NE.
509715.120_mRNA-p1 TcSYL_0073550.t1-p1
OG0002214: TCRU_4306 TcCLB-EL.510105.100_mRNA-p1 TcCLB-NE.
509715.90_mRNA-p1 TcSYL_0073560.t1-p1
OG0002215: TCRU_4308 TcCLB-EL.510105.70_mRNA-p1 TcCLB-NE.
509715.60_mRNA-p1 TcSYL_0073570.t1-p1
OG0002216: TCRU_4315 TcCLB-EL.483623.10_mRNA-p1 TcCLB-EL.
510431.20_mRNA-p1 TcCLB-NE.406377.10_pseudogenic_transcript-p1
OG0002217: TCRU_4316 TcCLB-EL.408799.19_mRNA-p1 TcCLB-EL.
510431.10_mRNA-p1 TcCLB-NE.506883.9_mRNA-p1
OG0002218: TCRU_4326 TcCLB-EL.510427.5_mRNA-p1 TcCLB-NE.
504123.50_pseudogenic_transcript-p1 TcCLB-NE.
506883.130_pseudogenic_transcript-p1
OG0002219: TCRU_4353 TcCLB-EL.507735.50_pseudogenic_transcript-p1
TcCLB-NE.504253.10_mRNA-p1 TcSYL_0197150.t1-p1
OG0002220: TCRU_4364 TcCLB-EL.510349.40_mRNA-p1 TcCLB-NE.
503925.70_mRNA-p1 TcSYL_0156690.t1-p1
OG0002221: TCRU_4365 TcCLB-EL.510349.30_mRNA-p1 TcCLB-NE.
503925.60_mRNA-p1 TcSYL_0156710.t1-p1
OG0002222: TCRU_4368 TcCLB-EL.510347.50_mRNA-p1 TcCLB-NE.
503925.20_mRNA-p1 TcSYL_0156720.t1-p1
OG0002223: TCRU_4369 TcCLB-EL.510347.40_mRNA-p1 TcCLB-NE.
503925.10_mRNA-p1 TcSYL_0156730.t1-p1
OG0002224: TCRU_4370 TcCLB-EL.510347.29_mRNA-p1 TcCLB-NE.
503925.6_mRNA-p1 TcSYL_0156740.t1-p1
OG0002225: TCRU_4374 TcCLB-EL.510345.50_mRNA-p1 TcCLB-NE.
506469.40_mRNA-p1 TcSYL_0156780.t1-p1
OG0002226: TCRU_4381 TcCLB-EL.506941.20_mRNA-p1 TcCLB-NE.
511511.20_mRNA-p1 TcSYL_0139550.t1-p1
OG0002227: TCRU_4385 TcCLB-EL.506941.70_mRNA-p1 TcCLB-NE.

511511.70_mRNA-p1 TcSYL_0139560.t1-p1
OG0002228: TCRU_4387 TcCLB-EL.506941.90_mRNA-p1 TcCLB-NE.
511511.90_mRNA-p1 TcSYL_0139570.t1-p1
OG0002229: TCRU_4389 TcCLB-EL.506941.120_mRNA-p1 TcCLB-NE.
511511.120_mRNA-p1 TcSYL_0139580.t1-p1
OG0002230: TCRU_4392 TcCLB-EL.506941.150_mRNA-p1 TcCLB-NE.
511511.150_mRNA-p1 TcSYL_0139590.t1-p1
OG0002231: TCRU_4394 TcCLB-EL.506941.170_mRNA-p1 TcCLB-NE.
511511.170_mRNA-p1 TcSYL_0139600.t1-p1
OG0002232: TCRU_4395 TcCLB-EL.506941.180_mRNA-p1 TcCLB-NE.
511513.14_mRNA-p1 TcSYL_0139610.t1-p1
OG0002233: TCRU_4397 TcCLB-EL.506941.210_mRNA-p1 TcCLB-NE.
507027.40_mRNA-p1 TcSYL_0139630.t1-p1
OG0002234: TCRU_4402 TcCLB-EL.507527.20_mRNA-p1 TcCLB-NE.
506935.10_mRNA-p1 TcSYL_0181380.t1-p1
OG0002235: TCRU_4405 TcCLB-EL.506025.60_mRNA-p1 TcCLB-NE.
503935.20_mRNA-p1 TcSYL_0181410.t1-p1
OG0002236: TCRU_4410 TcCLB-EL.506025.10_mRNA-p1 TcCLB-NE.
511209.60_mRNA-p1 TcSYL_0181440.t1-p1
OG0002237: TCRU_4416 TcCLB-EL.506227.20_mRNA-p1 TcCLB-NE.
503837.30_mRNA-p1 TcSYL_0091940.t1-p1
OG0002238: TCRU_4426 TcCLB-EL.510611.40_mRNA-p1 TcCLB-NE.
506793.20_mRNA-p1 TcSYL_0163580.t1-p1
OG0002239: TCRU_4428 TcCLB-EL.510609.110_mRNA-p1 TcCLB-NE.
506793.80_mRNA-p1 TcSYL_0163630.t1-p1
OG0002240: TCRU_4429 TcCLB-EL.510609.90_mRNA-p1 TcCLB-NE.
506793.100_mRNA-p1 TcSYL_0163640.t1-p1
OG0002241: TCRU_4440 TcCLB-EL.509805.40_mRNA-p1 TcCLB-NE.
511269.20_mRNA-p1 TcSYL_0015220.t1-p1
OG0002242: TCRU_4442 TcCLB-EL.509805.60_mRNA-p1 TcCLB-NE.
506425.169_mRNA-p1 TcCLB-NE.511269.4_mRNA-p1
OG0002243: TCRU_4444 TcCLB-EL.511531.50_mRNA-p1 TcCLB-NE.
511025.50_mRNA-p1 TcSYL_0111840.t1-p1
OG0002244: TCRU_4448 TcCLB-EL.506739.180_mRNA-p1 TcCLB-NE.
506315.80_mRNA-p1 TcSYL_0050100.t1-p1
OG0002245: TCRU_4453 TcCLB-EL.506739.90_mRNA-p1 TcCLB-NE.
510819.110_mRNA-p1 TcSYL_0050060.t1-p1
OG0002246: TCRU_4457 TcCLB-EL.506739.50_mRNA-p1 TcCLB-NE.
510819.60_mRNA-p1 TcSYL_0050050.t1-p1
OG0002247: TCRU_4458 TcCLB-EL.506739.40_mRNA-p1 TcCLB-NE.
510819.40_mRNA-p1 TcSYL_0050040.t1-p1
OG0002248: TCRU_4459 TcCLB-EL.506739.30_mRNA-p1 TcCLB-NE.
510819.30_mRNA-p1 TcSYL_0050030.t1-p1
OG0002249: TCRU_4463 TcCLB-EL.511339.20_mRNA-p1 TcCLB-NE.
509235.20_mRNA-p1 TcCLB-NE.511895.69_mRNA-p1
OG0002250: TCRU_4464 TcCLB-NE.506611.10_mRNA-p1 TcCLB-NE.
509657.50_mRNA-p1 TcCLB-NE.510201.20_mRNA-p1
OG0002251: TCRU_4467 TcCLB-EL.503627.20_mRNA-p1 TcCLB-NE.
508739.70_mRNA-p1 TcSYL_0192210.t1-p1
OG0002252: TCRU_4469 TcCLB-EL.511683.20_mRNA-p1 TcCLB-NE.
508741.20_mRNA-p1 TcSYL_0194270.t1-p1
OG0002253: TCRU_4470 TcCLB-EL.511683.30_mRNA-p1 TcCLB-NE.
508741.30_mRNA-p1 TcSYL_0192230.t1-p1
OG0002254: TCRU_4486 TcCLB-EL.508379.10_mRNA-p1 TcCLB-NE.

509111.28_mRNA-p1 TcSYL_0164610.t1-p1
OG0002255: TCRU_4489 TcCLB-EL.507091.40_mRNA-p1 TcCLB-EL.
508375.50_mRNA-p1 TcCLB-NE.511643.20_mRNA-p1
OG0002256: TCRU_4512 TcCLB-EL.504137.60_mRNA-p1 TcCLB-NE.
509799.10_mRNA-p1 TcSYL_0106940.t1-p1
OG0002257: TCRU_4513 TcCLB-EL.504137.70_mRNA-p1 TcCLB-NE.
509799.20_mRNA-p1 TcSYL_0106920.t1-p1
OG0002258: TCRU_4514 TcCLB-EL.504137.80_mRNA-p1 TcCLB-NE.
509799.30_mRNA-p1 TcSYL_0106910.t1-p1
OG0002259: TCRU_4516 TcCLB-EL.504137.110_mRNA-p1 TcCLB-NE.
509799.60_mRNA-p1 TcSYL_0106880.t1-p1
OG0002260: TCRU_4539 TcSYL_0051730.t1-p1 TcSYL_0161720.t1-p1
TcSYL_0190710.t1-p1
OG0002261: TCRU_4582 TcCLB-EL.506743.200_mRNA-p1 TcCLB-NE.
509253.30_mRNA-p1 TcSYL_0164780.t1-p1
OG0002262: TCRU_4583 TcCLB-EL.506743.190_mRNA-p1 TcCLB-NE.
509253.40_mRNA-p1 TcSYL_0164800.t1-p1
OG0002263: TCRU_4584 TcCLB-EL.506743.180_mRNA-p1 TcCLB-NE.
509253.50_mRNA-p1 TcSYL_0164810.t1-p1
OG0002264: TCRU_4600 TcCLB-EL.508205.10_mRNA-p1 TcCLB-NE.
506123.50_mRNA-p1 TcSYL_0062100.t1-p1
OG0002265: TCRU_4601 TcCLB-EL.508205.20_mRNA-p1 TcCLB-NE.
506123.40_mRNA-p1 TcSYL_0062090.t1-p1
OG0002266: TCRU_4602 TcCLB-EL.508205.30_mRNA-p1 TcCLB-NE.
506123.30_mRNA-p1 TcSYL_0062080.t1-p1
OG0002267: TCRU_4603 TcCLB-EL.508205.39_mRNA-p1 TcCLB-NE.
506123.24_mRNA-p1 TcSYL_0062070.t1-p1
OG0002268: TCRU_4605 TcCLB-EL.510353.10_mRNA-p1 TcCLB-NE.
509541.19_mRNA-p1 TcSYL_0062050.t1-p1
OG0002269: TCRU_4606 TcCLB-EL.510353.20_mRNA-p1 TcCLB-NE.
509541.10_mRNA-p1 TcSYL_0062040.t1-p1
OG0002270: TCRU_4607 TcCLB-EL.510353.30_mRNA-p1 TcCLB-NE.
509541.4_mRNA-p1 TcSYL_0062020.t1-p1
OG0002271: TCRU_4633 TCRU_7132 TCRU_7779 TcSYL_0066370.t1-p1
OG0002272: TCRU_4634 TcCLB-EL.511685.60_mRNA-p1 TcCLB-NE.
508741.110_mRNA-p1 TcSYL_0192980.t1-p1
OG0002273: TCRU_4644 TcCLB-EL.509455.50_mRNA-p1 TcCLB-NE.
503879.30_mRNA-p1 TcSYL_0145240.t1-p1
OG0002274: TCRU_4645 TcCLB-EL.509455.60_mRNA-p1 TcCLB-NE.
503879.20_mRNA-p1 TcSYL_0145230.t1-p1
OG0002275: TCRU_4646 TcCLB-EL.509455.70_mRNA-p1 TcCLB-NE.
503879.10_mRNA-p1 TcSYL_0145220.t1-p1
OG0002276: TCRU_4647 TcCLB-EL.509455.80_mRNA-p1 TcCLB-NE.
503539.30_mRNA-p1 TcSYL_0145210.t1-p1
OG0002277: TCRU_4660 TCRU_5408 TcCLB-NE.445777.10_mRNA-p1
TcSYL_0203980.t1-p1
OG0002278: TCRU_4670 TcCLB-EL.511621.60_mRNA-p1 TcCLB-NE.
509317.50_mRNA-p1 TcSYL_0084390.t1-p1
OG0002279: TCRU_4673 TcCLB-EL.511621.100_mRNA-p1 TcCLB-NE.
509317.10_mRNA-p1 TcSYL_0084780.t1-p1
OG0002280: TCRU_4678 TcCLB-EL.506737.70_mRNA-p1 TcCLB-NE.
511855.20_mRNA-p1 TcSYL_0091850.t1-p1
OG0002281: TCRU_4681 TcCLB-EL.506737.40_mRNA-p1 TcCLB-NE.
511855.50_mRNA-p1 TcSYL_0091840.t1-p1

OG0002282: TCRU_4683 TcCLB-EL.506737.20_mRNA-p1 TcCLB-NE.
511855.70_mRNA-p1 TcSYL_0091830.t1-p1
OG0002283: TCRU_4685 TcCLB-EL.503501.7_mRNA-p1 TcCLB-EL.
506761.11_mRNA-p1 TcSYL_0133280.t1-p1
OG0002284: TCRU_4700 TcCLB-EL.506673.60_mRNA-p1 TcCLB-NE.
504827.40_mRNA-p1 TcSYL_0117740.t1-p1
OG0002285: TCRU_4706 TcCLB-EL.510285.120_mRNA-p1 TcCLB-NE.
504827.110_mRNA-p1 TcSYL_0117730.t1-p1
OG0002286: TCRU_4707 TcCLB-EL.510285.110_mRNA-p1 TcCLB-NE.
504827.120_mRNA-p1 TcSYL_0117710.t1-p1
OG0002287: TCRU_4710 TcCLB-EL.510285.80_mRNA-p1 TcCLB-NE.
504827.150_mRNA-p1 TcSYL_0117700.t1-p1
OG0002288: TCRU_4719 TcCLB-EL.510091.90_mRNA-p1 TcCLB-NE.
510421.330_mRNA-p1 TcSYL_0019390.t1-p1
OG0002289: TCRU_beta TcCLB-EL.510091.80_mRNA-p1 TcCLB-NE.
510421.320_mRNA-p1 TcSYL_0019380.t1-p1
OG0002290: TCRU_4723 TcCLB-EL.510091.50_mRNA-p1 TcCLB-NE.
510421.300_mRNA-p1 TcSYL_0019370.t1-p1
OG0002291: TCRU_4724 TcCLB-EL.510091.40_mRNA-p1 TcCLB-NE.
510421.290_mRNA-p1 TcSYL_0019360.t1-p1
OG0002292: TCRU_4725 TcCLB-EL.510091.30_mRNA-p1 TcCLB-NE.
510421.280_mRNA-p1 TcSYL_0019350.t1-p1
OG0002293: TCRU_4726 TcCLB-EL.510091.20_mRNA-p1 TcCLB-NE.
510421.270_mRNA-p1 TcSYL_0019340.t1-p1
OG0002294: TCRU_4742 TcCLB-EL.511109.30_mRNA-p1 TcCLB-NE.
504157.110_mRNA-p1 TcSYL_0086490.t1-p1
OG0002295: TCRU_4746 TcCLB-EL.511109.70_mRNA-p1 TcCLB-NE.
504157.70_mRNA-p1 TcSYL_0086510.t1-p1
OG0002296: TCRU_4753 TcCLB-EL.507809.60_mRNA-p1 TcCLB-NE.
511661.60_mRNA-p1 TcSYL_0013680.t1-p1
OG0002297: TCRU_4756 TcCLB-EL.507809.30_mRNA-p1 TcCLB-NE.
511661.90_mRNA-p1 TcSYL_0013670.t1-p1
OG0002298: TCRU_4757 TcCLB-EL.503955.100_mRNA-p1 TcCLB-NE.
511661.120_mRNA-p1 TcSYL_0013660.t1-p1
OG0002299: TCRU_4762 TcCLB-EL.504103.3_mRNA-p1 TcCLB-EL.
508307.180_mRNA-p1 TcSYL_0006000.t1-p1
OG0002300: TCRU_4782 TcCLB-EL.510963.70_mRNA-p1 TcCLB-NE.
508625.30_mRNA-p1 TcSYL_0112960.t1-p1
OG0002301: TCRU_4784 TcCLB-EL.510963.39_mRNA-p1 TcCLB-NE.
508625.60_mRNA-p1 TcSYL_0112950.t1-p1
OG0002302: TCRU_4786 TcCLB-EL.509757.10_mRNA-p1 TcCLB-EL.
510963.5_mRNA-p1 TcCLB-NE.508625.100_pseudogenic_transcript-p1
OG0002303: TCRU_4787 TcCLB-EL.509757.30_mRNA-p1 TcCLB-NE.
508625.130_mRNA-p1 TcSYL_0112930.t1-p1
OG0002304: TCRU_4794 TcCLB-EL.508995.60_mRNA-p1 TcCLB-NE.
506477.10_mRNA-p1 TcSYL_0110790.t1-p1
OG0002305: TCRU_4796 TcCLB-EL.508995.40_mRNA-p1 TcCLB-NE.
506477.20_mRNA-p1 TcSYL_0110800.t1-p1
OG0002306: TCRU_4802 TCRU_8604 TcCLB-EL.511175.40_mRNA-p1 TcCLB-NE.
508435.20_mRNA-p1
OG0002307: TCRU_4818 TcCLB-EL.507993.150_mRNA-p1 TcCLB-NE.
511277.590_mRNA-p1 TcSYL_0014700.t1-p1
OG0002308: TCRU_4820 TcCLB-EL.507993.130_mRNA-p1 TcCLB-NE.
511277.570_mRNA-p1 TcSYL_0014710.t1-p1

OG0002309: TCRU_4821 TcCLB-EL.507993.120_mRNA-p1 TcCLB-NE.
511277.560_mRNA-p1 TcSYL_0014720.t1-p1
OG0002310: TCRU_4823 TcCLB-EL.507993.100_mRNA-p1 TcCLB-NE.
511277.550_mRNA-p1 TcSYL_0014730.t1-p1
OG0002311: TCRU_4825 TcCLB-EL.507993.80_mRNA-p1 TcCLB-NE.
511277.520_pseudogenic_transcript-p1 TcSYL_0014740.t1-p1
OG0002312: TCRU_4826 TcCLB-EL.507993.70_mRNA-p1 TcCLB-NE.
511277.510_mRNA-p1 TcSYL_0014750.t1-p1
OG0002313: TCRU_4833 TcCLB-EL.511871.60_pseudogenic_transcript-p1
TcCLB-NE.511865.40_mRNA-p1 TcSYL_0146170.t1-p1
OG0002314: TCRU_4834 TcCLB-EL.511871.40_mRNA-p1 TcCLB-NE.
511865.50_mRNA-p1 TcSYL_0146180.t1-p1
OG0002315: TCRU_protein TcCLB-EL.511871.10_mRNA-p1 TcCLB-NE.
511865.80_mRNA-p1 TcSYL_0146200.t1-p1
OG0002316: TCRU_4848 TcCLB-EL.511429.10_mRNA-p1 TcCLB-NE.
509991.40_pseudogenic_transcript-p1 TcSYL_0110750.t1-p1
OG0002317: TCRU_4857 TcCLB-EL.508277.350_mRNA-p1 TcCLB-NE.
506445.30_mRNA-p1 TcSYL_0064310.t1-p1
OG0002318: TCRU_4866 TcCLB-EL.505977.40_pseudogenic_transcript-p1
TcCLB-NE.430727.9_mRNA-p1 TcSYL_0064340.t1-p1
OG0002319: TCRU_4883 TcCLB-EL.510073.80_mRNA-p1 TcCLB-NE.
509399.140_mRNA-p1 TcSYL_0177350.t1-p1
OG0002320: TCRU_4885 TcCLB-EL.510075.30_mRNA-p1 TcCLB-NE.
509399.120_mRNA-p1 TcSYL_0177240.t1-p1
OG0002321: TCRU_4886 TcCLB-EL.510075.40_mRNA-p1 TcCLB-NE.
509399.110_mRNA-p1 TcSYL_0177170.t1-p1
OG0002322: TCRU_4893 TcSYL_0065440.t1-p1 TcSYL_0065470.t1-p1
TcSYL_0109990.t1-p1
OG0002323: TCRU_4897 TcCLB-EL.506893.40_mRNA-p1 TcCLB-NE.
508501.190_mRNA-p1 TcSYL_0110280.t1-p1
OG0002324: TCRU_4898 TcCLB-EL.506893.50_mRNA-p1 TcCLB-NE.
508501.200_mRNA-p1 TcSYL_0110290.t1-p1
OG0002325: TCRU_4899 TcCLB-EL.506893.60_mRNA-p1 TcCLB-NE.
508501.210_mRNA-p1 TcSYL_0110300.t1-p1
OG0002326: TCRU_4910 TcCLB-EL.507035.120_mRNA-p1 TcCLB-NE.
508525.10_mRNA-p1 TcSYL_0112350.t1-p1
OG0002327: TCRU_4911 TcCLB-EL.507035.130_mRNA-p1 TcCLB-NE.
508525.20_mRNA-p1 TcSYL_0112340.t1-p1
OG0002328: TCRU_of TcCLB-NE.508421.20_pseudogenic_transcript-p1
TcCLB-NE.508425.10_mRNA-p1 TcSYL_0157230.t1-p1
OG0002329: TCRU_4923 TcCLB-EL.506637.20_mRNA-p1 TcCLB-EL.
506637.40_mRNA-p1 TcCLB-NE.510099.20_mRNA-p1
OG0002330: TCRU_4946 TcCLB-EL.504179.30_mRNA-p1 TcCLB-NE.
507823.39_mRNA-p1 TcSYL_0107020.t1-p1
OG0002331: TCRU_4949 TcCLB-EL.504179.60_mRNA-p1 TcCLB-NE.
507823.10_mRNA-p1 TcSYL_0107030.t1-p1
OG0002332: TCRU_4952 TcCLB-EL.504171.59_mRNA-p1 TcCLB-NE.
509795.60_mRNA-p1 TcSYL_0107040.t1-p1
OG0002333: TCRU_4960 TcCLB-EL.508719.50_mRNA-p1 TcCLB-NE.
509793.20_mRNA-p1 TcSYL_0107070.t1-p1
OG0002334: TCRU_4966 TcCLB-EL.503671.39_mRNA-p1 TcCLB-NE.
508153.20_mRNA-p1 TcSYL_0001150.t1-p1
OG0002335: TCRU_4967 TcCLB-EL.503671.30_mRNA-p1 TcCLB-NE.
508153.30_mRNA-p1 TcSYL_0001140.t1-p1

OG0002336: TCRU_4968 TcCLB-EL.503671.20_mRNA-p1 TcCLB-NE.
508153.40_mRNA-p1 TcSYL_0001130.t1-p1
OG0002337: TCRU_4971 TcCLB-EL.510325.50_mRNA-p1 TcCLB-NE.
508153.80_mRNA-p1 TcSYL_0001110.t1-p1
OG0002338: TCRU_4991 TcCLB-EL.506755.210_mRNA-p1 TcCLB-NE.
510359.220_mRNA-p1 TcSYL_0112820.t1-p1
OG0002339: TCRU_4995 TcCLB-EL.506755.160_mRNA-p1 TcCLB-NE.
510359.170_mRNA-p1 TcSYL_0112800.t1-p1
OG0002340: TCRU_4997 TcCLB-EL.506755.140_mRNA-p1 TcCLB-NE.
510359.150_mRNA-p1 TcSYL_0112790.t1-p1
OG0002341: TCRU_4998 TcCLB-EL.506755.120_mRNA-p1 TcCLB-NE.
510359.130_mRNA-p1 TcSYL_0112780.t1-p1
OG0002342: TCRU_5002 TcCLB-EL.506755.80_mRNA-p1 TcCLB-NE.
510359.80_mRNA-p1 TcSYL_0112770.t1-p1
OG0002343: TCRU_5005 TcCLB-EL.506755.50_mRNA-p1 TcCLB-NE.
510359.40_mRNA-p1 TcSYL_0112760.t1-p1
OG0002344: TCRU_5011 TcCLB-EL.507611.398_mRNA-p1 TcCLB-NE.
506247.30_mRNA-p1 TcSYL_0108910.t1-p1
OG0002345: TCRU_5042 TcCLB-EL.510243.30_mRNA-p1 TcCLB-NE.
510575.200_mRNA-p1 TcSYL_0147300.t1-p1
OG0002346: TCRU_5057 TcCLB-EL.507739.100_mRNA-p1 TcCLB-NE.
506435.460_mRNA-p1 TcSYL_0195910.t1-p1
OG0002347: TCRU_5058 TcCLB-EL.507739.90_mRNA-p1 TcCLB-NE.
506435.450_mRNA-p1 TcSYL_0195940.t1-p1
OG0002348: TCRU_5059 TcCLB-EL.507739.80_mRNA-p1 TcCLB-NE.
506435.440_mRNA-p1 TcSYL_0195970.t1-p1
OG0002349: TCRU_5060 TcCLB-EL.507739.70_mRNA-p1 TcCLB-NE.
506435.430_mRNA-p1 TcSYL_0196000.t1-p1
OG0002350: TCRU_5061 TcCLB-EL.507739.50_mRNA-p1 TcCLB-NE.
506435.420_mRNA-p1 TcSYL_0196060.t1-p1
OG0002351: TCRU_5062 TcCLB-EL.507739.40_mRNA-p1 TcCLB-NE.
506435.410_mRNA-p1 TcSYL_0196090.t1-p1
OG0002352: TCRU_5063 TcCLB-EL.507739.30_mRNA-p1 TcCLB-NE.
506435.400_mRNA-p1 TcSYL_0196120.t1-p1
OG0002353: TCRU_5064 TcCLB-EL.507739.20_mRNA-p1 TcCLB-NE.
506435.390_mRNA-p1 TcSYL_0196150.t1-p1
OG0002354: TCRU_5072 TcCLB-EL.503795.10_mRNA-p1 TcCLB-NE.
506679.10_mRNA-p1 TcSYL_0113690.t1-p1
OG0002355: TCRU_5085 TcCLB-EL.506127.50_mRNA-p1 TcCLB-EL.
508271.30_mRNA-p1 TcSYL_0083350.t1-p1
OG0002356: TCRU_5090 TcCLB-EL.506127.110_mRNA-p1 TcCLB-NE.
503593.70_mRNA-p1 TcSYL_0083290.t1-p1
OG0002357: TCRU_5094 TcCLB-EL.506127.160_mRNA-p1 TcCLB-NE.
503593.20_mRNA-p1 TcSYL_0083280.t1-p1
OG0002358: TCRU_5100 TcCLB-EL.503989.60_mRNA-p1 TcCLB-EL.
506221.30_mRNA-p1 TcSYL_0141750.t1-p1
OG0002359: TCRU_5103 TcCLB-EL.508465.120_mRNA-p1 TcCLB-NE.
507001.70_mRNA-p1 TcSYL_0115970.t1-p1
OG0002360: TCRU_5123 TcCLB-EL.428999.10_mRNA-p1 TcCLB-NE.
460757.20_mRNA-p1 TcCLB-NE.511895.40_mRNA-p1
OG0002361: TCRU_5148 TcCLB-EL.509155.10_mRNA-p1 TcCLB-NE.
510303.170_mRNA-p1 TcSYL_0079050.t1-p1
OG0002362: TCRU_5157 TcCLB-EL.509153.139_mRNA-p1 TcCLB-NE.
510303.270_mRNA-p1 TcSYL_0078980.t1-p1

OG0002363: TCRU_5159 TcCLB-EL.509153.120_mRNA-p1 TcCLB-NE.
510303.290_mRNA-p1 TcSYL_0078970.t1-p1
OG0002364: TCRU_5183 TcCLB-EL.506747.30_mRNA-p1 TcCLB-NE.
507887.20_mRNA-p1 TcSYL_0164130.t1-p1
OG0002365: TCRU_5187 TcCLB-EL.507083.10_mRNA-p1 TcCLB-NE.
470687.9_mRNA-p1 TcCLB-NE.506415.30_mRNA-p1
OG0002366: TCRU_5206 TcCLB-EL.409117.10_mRNA-p1 TcCLB-NE.
508823.40_mRNA-p1 TcSYL_0043030.t1-p1
OG0002367: TCRU_5213 TcCLB-EL.508387.130_mRNA-p1 TcCLB-NE.
506789.170_mRNA-p1 TcSYL_0162550.t1-p1
OG0002368: TCRU_5214 TcCLB-EL.508387.150_mRNA-p1 TcCLB-NE.
506789.150_mRNA-p1 TcSYL_0162440.t1-p1
OG0002369: TCRU_5216 TcCLB-EL.508389.10_mRNA-p1 TcCLB-NE.
506789.120_mRNA-p1 TcSYL_0161470.t1-p1
OG0002370: TCRU_10230 TCRU_5247 TCRU_8131 TcCLB-EL.478711.10.1-p1
OG0002371: TCRU_C TcCLB-EL.509767.200_mRNA-p1 TcCLB-NE.
506219.10_mRNA-p1 TcCLB-NE.507645.11_mRNA-p1
OG0002372: TCRU_5279 TcCLB-NE.506679.110_mRNA-p1 TcSYL_0113950.t1-p1
TcSYL_0113970.t1-p1
OG0002373: TCRU_5301 TcCLB-EL.504033.53_pseudogenic_transcript-p1
TcSYL_0038510.t1-p1 TcSYL_0147190.t1-p1
OG0002374: TCRU_5304 TcCLB-EL.506357.170_mRNA-p1 TcCLB-NE.
508891.60_mRNA-p1 TcSYL_0015000.t1-p1
OG0002375: TCRU_5305 TcCLB-EL.506357.199_mRNA-p1 TcCLB-NE.
508891.30_mRNA-p1 TcSYL_0015010.t1-p1
OG0002376: TCRU_5307 TcCLB-EL.504215.20_mRNA-p1 TcCLB-NE.
508891.10_mRNA-p1 TcSYL_0015020.t1-p1
OG0002377: TCRU_5310 TcCLB-EL.504215.50_mRNA-p1 TcCLB-NE.
511275.20_mRNA-p1 TcSYL_0015050.t1-p1
OG0002378: TCRU_5318 TcCLB-EL.503971.20_mRNA-p1 TcCLB-NE.
508269.10_mRNA-p1 TcSYL_0121270.t1-p1
OG0002379: TCRU_5321 TcCLB-EL.503971.50_mRNA-p1 TcCLB-NE.
508269.40_mRNA-p1 TcSYL_0121280.t1-p1
OG0002380: TCRU_5329 TcCLB-EL.507143.20_mRNA-p1 TcCLB-NE.
503459.10_mRNA-p1 TcSYL_0050820.t1-p1
OG0002381: TCRU_5330 TcCLB-EL.507143.30_mRNA-p1 TcCLB-NE.
506319.10_mRNA-p1 TcSYL_0050830.t1-p1
OG0002382: TCRU_5331 TcCLB-EL.507143.40_mRNA-p1 TcCLB-NE.
506319.20_mRNA-p1 TcSYL_0050880.t1-p1
OG0002383: TCRU_5332 TcCLB-EL.507143.50_mRNA-p1 TcCLB-NE.
506319.30_mRNA-p1 TcSYL_0050940.t1-p1
OG0002384: TCRU_5333 TcCLB-EL.507143.60_mRNA-p1 TcCLB-NE.
506319.40_mRNA-p1 TcSYL_0050990.t1-p1
OG0002385: TCRU_5334 TcCLB-EL.507143.70_mRNA-p1 TcCLB-NE.
506319.50_mRNA-p1 TcSYL_0051060.t1-p1
OG0002386: TCRU_5335 TcCLB-EL.507143.90_mRNA-p1 TcCLB-NE.
506319.70_mRNA-p1 TcSYL_0051100.t1-p1
OG0002387: TCRU_5345 TcCLB-EL.510491.40_mRNA-p1 TcCLB-NE.
511633.20_mRNA-p1 TcSYL_0006180.t1-p1
OG0002388: TCRU_5346 TcCLB-EL.510491.30_pseudogenic_transcript-p1
TcCLB-NE.511633.30_mRNA-p1 TcSYL_0006190.t1-p1
OG0002389: TCRU_5348 TcCLB-EL.510491.10_mRNA-p1 TcCLB-NE.
511633.50_mRNA-p1 TcSYL_0006200.t1-p1
OG0002390: TCRU_subunit TcCLB-EL.510089.110_mRNA-p1 TcCLB-NE.

510421.130_mRNA-p1 TcSYL_0019210.t1-p1
OG0002391: TCRU_5366 TcCLB-EL.510089.160_pseudogenic_transcript-p1 TcCLB-NE.510421.170_mRNA-p1 TcSYL_0019230.t1-p1
OG0002392: TCRU_5368 TcCLB-EL.510089.190_mRNA-p1 TcCLB-NE.510421.200_mRNA-p1 TcSYL_0019260.t1-p1
OG0002393: TCRU_ubiquitin TcCLB-EL.510089.200_mRNA-p1 TcCLB-NE.510421.210_mRNA-p1 TcSYL_0019270.t1-p1
OG0002394: TCRU_5370 TcCLB-EL.507809.100_mRNA-p1 TcCLB-NE.511661.10_mRNA-p1 TcSYL_0013750.t1-p1
OG0002395: TCRU_5371 TcCLB-EL.507809.90_mRNA-p1 TcCLB-NE.511661.20_mRNA-p1 TcSYL_0013730.t1-p1
OG0002396: TCRU_5372 TcCLB-EL.507809.80_mRNA-p1 TcCLB-NE.511661.30_mRNA-p1 TcSYL_0013720.t1-p1
OG0002397: TCRU_5387 TcCLB-EL.511329.10_mRNA-p1 TcCLB-NE.504933.10_mRNA-p1 TcCLB-NE.509237.151_mRNA-p1
OG0002398: TCRU_5391 TcCLB-EL.510877.30_mRNA-p1 TcCLB-EL.510877.40_mRNA-p1 TcSYL_0045790.t1-p1
OG0002399: TCRU_5396 TcCLB-EL.507711.320_mRNA-p1 TcCLB-NE.509643.80_mRNA-p1 TcSYL_0008730.t1-p1
OG0002400: TCRU_5398 TcCLB-EL.507711.340_mRNA-p1 TcCLB-NE.509643.100_mRNA-p1 TcSYL_0008720.t1-p1
OG0002401: TCRU_5411 TcCLB-NE.506139.10_mRNA-p1 TcSYL_0149140.t1-p1 TcSYL_0150110.t1-p1
OG0002402: TCRU_5413 TcCLB-EL.508387.50_mRNA-p1 TcCLB-NE.506789.249_mRNA-p1 TcSYL_0162950.t1-p1
OG0002403: TCRU_5414 TcCLB-EL.508387.70_mRNA-p1 TcCLB-NE.506789.230_mRNA-p1 TcSYL_0162870.t1-p1
OG0002404: TCRU_5415 TcCLB-EL.508387.90_mRNA-p1 TcCLB-NE.506789.210_mRNA-p1 TcSYL_0162710.t1-p1
OG0002405: TCRU_5416 TcCLB-EL.508387.100_mRNA-p1 TcCLB-NE.506789.200_mRNA-p1 TcSYL_0162690.t1-p1
OG0002406: TCRU_5417 TcCLB-EL.508387.110_mRNA-p1 TcCLB-NE.506789.190_mRNA-p1 TcSYL_0162640.t1-p1
OG0002407: TCRU_5422 TcCLB-EL.510293.79_mRNA-p1 TcCLB-NE.508661.60_mRNA-p1 TcSYL_0113510.t1-p1
OG0002408: TCRU_5424 TcCLB-EL.510293.60_mRNA-p1 TcCLB-NE.508661.40_mRNA-p1 TcSYL_0113500.t1-p1
OG0002409: TCRU_5425 TcCLB-EL.510293.50_mRNA-p1 TcCLB-NE.508661.30_mRNA-p1 TcSYL_0113480.t1-p1
OG0002410: TCRU_5427 TcCLB-EL.510293.30_mRNA-p1 TcCLB-NE.508661.10_mRNA-p1 TcSYL_0113460.t1-p1
OG0002411: TCRU_5428 TcCLB-EL.506677.39_mRNA-p1 TcCLB-NE.506583.60_mRNA-p1 TcSYL_0113450.t1-p1
OG0002412: TCRU_5434 TcCLB-EL.510291.7_mRNA-p1 TcCLB-NE.508659.9_mRNA-p1 TcSYL_0113440.t1-p1
OG0002413: TCRU_5440 TcCLB-EL.507683.30_mRNA-p1 TcCLB-NE.511291.70_mRNA-p1 TcSYL_0122660.t1-p1
OG0002414: TCRU_5446 TcCLB-EL.507511.60_mRNA-p1 TcSYL_0087350.t1-p1 TcSYL_0196290.t1-p1
OG0002415: TCRU_5463 TcCLB-EL.509455.140_mRNA-p1 TcCLB-NE.506711.30_mRNA-p1 TcSYL_0145170.t1-p1
OG0002416: TCRU_5464 TcCLB-EL.509455.130_mRNA-p1 TcCLB-NE.506711.10_mRNA-p1 TcSYL_0145190.t1-p1
OG0002417: TCRU_5471 TcCLB-EL.503579.20_mRNA-p1 TcCLB-NE.

507517.10_mRNA-p1 TcSYL_0022890.t1-p1
OG0002418: TCRU_5487 TcCLB-EL.507537.30_mRNA-p1 TcCLB-NE.
507297.20_mRNA-p1 TcSYL_0176870.t1-p1
OG0002419: TCRU_5492 TcCLB-EL.507603.230_mRNA-p1 TcCLB-NE.
509429.290_mRNA-p1 TcSYL_0175010.t1-p1
OG0002420: TCRU_5494 TcCLB-EL.507603.200_mRNA-p1 TcCLB-NE.
509429.270_mRNA-p1 TcSYL_0175000.t1-p1
OG0002421: TCRU_5495 TcCLB-EL.507603.170_mRNA-p1 TcCLB-NE.
509429.240_mRNA-p1 TcSYL_0174990.t1-p1
OG0002422: TCRU_5499 TcCLB-EL.507603.120_mRNA-p1 TcCLB-NE.
509429.190_mRNA-p1 TcSYL_0174980.t1-p1
OG0002423: TCRU_5501 TcCLB-EL.507603.100_mRNA-p1 TcCLB-NE.
509429.170_mRNA-p1 TcSYL_0174970.t1-p1
OG0002424: TCRU_5502 TcCLB-EL.507603.90_mRNA-p1 TcCLB-NE.
509429.160_mRNA-p1 TcSYL_0174960.t1-p1
OG0002425: TCRU_5503 TcCLB-EL.507603.80_mRNA-p1 TcCLB-NE.
509429.150_mRNA-p1 TcSYL_0174950.t1-p1
OG0002426: TCRU_5505 TcCLB-EL.507603.50_mRNA-p1 TcCLB-NE.
509429.120_mRNA-p1 TcSYL_0174930.t1-p1
OG0002427: TCRU_5509 TcCLB-EL.507089.260_mRNA-p1 TcCLB-NE.
511025.120_mRNA-p1 TcSYL_0111900.t1-p1
OG0002428: TCRU_5510 TcCLB-EL.507089.270_mRNA-p1 TcCLB-NE.
511025.110_mRNA-p1 TcSYL_0111890.t1-p1
OG0002429: TCRU_5511 TcCLB-EL.511531.20_mRNA-p1 TcCLB-NE.
511025.90_mRNA-p1 TcSYL_0111870.t1-p1
OG0002430: TCRU_5513 TcCLB-EL.511531.40_mRNA-p1 TcCLB-NE.
511025.70_mRNA-p1 TcSYL_0111850.t1-p1
OG0002431: TCRU_5515 TcCLB-EL.511735.40_mRNA-p1 TcCLB-EL.
511735.88_mRNA-p1 TcCLB-NE.511521.20_mRNA-p1
OG0002432: TCRU_5518 TcCLB-EL.510227.29_mRNA-p1 TcCLB-NE.
506559.200_mRNA-p1 TcSYL_0045180.t1-p1
OG0002433: TCRU_5522 TcCLB-EL.508181.140_mRNA-p1 TcCLB-NE.
508153.820_mRNA-p1 TcSYL_0000700.t1-p1
OG0002434: TCRU_5527 TcCLB-EL.504213.40_mRNA-p1 TcCLB-NE.
508857.120_mRNA-p1 TcSYL_0026740.t1-p1
OG0002435: TCRU_5530 TcCLB-EL.507711.240_mRNA-p1 TcCLB-NE.
469735.9_mRNA-p1 TcSYL_0008740.t1-p1
OG0002436: TCRU_5533 TcCLB-EL.511425.19_pseudogenic_transcript-p1
TcCLB-NE.506479.30_mRNA-p1 TcSYL_0110830.t1-p1
OG0002437: TCRU_5534 TcCLB-EL.503805.3_mRNA-p1 TcCLB-NE.
506479.60_mRNA-p1 TcSYL_0110880.t1-p1
OG0002438: TCRU_5536 TcCLB-EL.509805.220_mRNA-p1 TcCLB-NE.
511267.30_mRNA-p1 TcSYL_0015300.t1-p1
OG0002439: TCRU_5539 TcCLB-EL.503453.90_mRNA-p1 TcCLB-NE.
506959.74_mRNA-p1 TcSYL_0132800.t1-p1
OG0002440: TCRU_5543 TcCLB-EL.507625.200_mRNA-p1 TcCLB-NE.
509741.20_mRNA-p1 TcSYL_0016880.t1-p1
OG0002441: TCRU_5546 TcCLB-EL.507625.80_mRNA-p1 TcCLB-NE.
507787.130_mRNA-p1 TcSYL_0017210.t1-p1
OG0002442: TCRU_5547 TcCLB-EL.507625.90_mRNA-p1 TcCLB-NE.
507787.110_mRNA-p1 TcSYL_0017160.t1-p1
OG0002443: TCRU_10725 TCRU_5549 TcCLB-EL.507467.70_mRNA-p1 TcCLB-NE.
508355.70_mRNA-p1
OG0002444: TCRU_5553 TcCLB-EL.461165.10_mRNA-p1 TcCLB-NE.

511461.10_mRNA-p1 TcSYL_0130930.t1-p1
OG0002445: TCRU_5561 TcCLB-EL.507907.40_mRNA-p1 TcCLB-NE.
507911.50_mRNA-p1 TcSYL_0004700.t1-p1
OG0002446: TCRU_5562 TcCLB-EL.508277.180_mRNA-p1 TcCLB-NE.
416511.9_mRNA-p1 TcSYL_0064170.t1-p1
OG0002447: TCRU_5564 TcCLB-EL.511363.4_mRNA-p1 TcCLB-NE.
508899.54_mRNA-p1 TcSYL_0121820.t1-p1
OG0002448: TCRU_5565 TcCLB-EL.507011.170_mRNA-p1 TcCLB-NE.
510665.10_mRNA-p1 TcSYL_0116820.t1-p1
OG0002449: TCRU_5567 TcCLB-EL.507811.70_mRNA-p1 TcCLB-NE.
511649.120_mRNA-p1 TcSYL_0014000.t1-p1
OG0002450: TCRU_5568 TcCLB-EL.507811.90_mRNA-p1 TcCLB-NE.
511649.110_mRNA-p1 TcSYL_0014010.t1-p1
OG0002451: TCRU_5570 TcCLB-EL.507993.33_mRNA-p1 TcCLB-NE.
511277.483_mRNA-p1 TcSYL_0014780.t1-p1
OG0002452: TCRU_5579 TcCLB-EL.511315.44_pseudogenic_transcript-p1
TcCLB-NE.508905.20_pseudogenic_transcript-p1 TcCLB-NE.
511755.24_pseudogenic_transcript-p1
OG0002453: TCRU_5590 TcCLB-EL.509233.170_mRNA-p1 TcCLB-NE.
467287.20_mRNA-p1 TcSYL_0145300.t1-p1
OG0002454: TCRU_5592 TcCLB-EL.510749.74_mRNA-p1 TcCLB-NE.
510655.140_mRNA-p1 TcSYL_0115770.t1-p1
OG0002455: TCRU_5596 TcCLB-EL.508731.50_mRNA-p1 TcCLB-NE.
509683.70_mRNA-p1 TcSYL_0089810.t1-p1
OG0002456: TCRU_5603 TcCLB-EL.509203.79_mRNA-p1 TcCLB-NE.
508699.20_mRNA-p1 TcSYL_0180640.t1-p1
OG0002457: TCRU_5605 TcCLB-EL.511353.10_mRNA-p1 TcCLB-NE.
511301.70_mRNA-p1 TcSYL_0121690.t1-p1
OG0002458: TCRU_5620 TcCLB-EL.509651.10_mRNA-p1 TcCLB-NE.
511285.30_mRNA-p1 TcSYL_0123510.t1-p1
OG0002459: TCRU_5627 TcCLB-EL.503575.50_mRNA-p1 TcCLB-NE.
510943.33_mRNA-p1 TcSYL_0001400.t1-p1
OG0002460: TCRU_5634 TcCLB-EL.506493.110_mRNA-p1 TcCLB-NE.
510121.190_mRNA-p1 TcSYL_0168960.t1-p1
OG0002461: TCRU_5635 TcCLB-EL.506493.100_mRNA-p1 TcCLB-NE.
510121.180_mRNA-p1 TcSYL_0168970.t1-p1
OG0002462: TCRU_5636 TcCLB-EL.508949.50_mRNA-p1 TcCLB-NE.
510119.70_mRNA-p1 TcSYL_0169230.t1-p1
OG0002463: TCRU_5637 TcCLB-EL.508949.30_mRNA-p1 TcCLB-NE.
510119.50_mRNA-p1 TcSYL_0169240.t1-p1
OG0002464: TCRU_5645 TcCLB-EL.510531.50_mRNA-p1 TcCLB-NE.
511819.10_mRNA-p1 TcSYL_0201460.t1-p1
OG0002465: TCRU_5651 TcCLB-EL.511467.20_mRNA-p1 TcCLB-NE.
503451.9_mRNA-p1 TcSYL_0132900.t1-p1
OG0002466: TCRU_5654 TcCLB-EL.463673.9_mRNA-p1 TcCLB-NE.
508601.110_mRNA-p1 TcSYL_0179620.t1-p1
OG0002467: TCRU_5655 TcCLB-EL.511801.60_mRNA-p1 TcCLB-NE.
508601.10_mRNA-p1 TcSYL_0179850.t1-p1
OG0002468: TCRU_5656 TcCLB-EL.511583.40_mRNA-p1 TcCLB-NE.
510153.19_mRNA-p1 TcSYL_0109340.t1-p1
OG0002469: TCRU_5658 TcCLB-EL.504153.340_mRNA-p1 TcCLB-NE.
506301.40_mRNA-p1 TcSYL_0138550.t1-p1
OG0002470: TCRU_5660 TcCLB-EL.508805.10_pseudogenic_transcript-p1
TcCLB-NE.503911.20_mRNA-p1 TcSYL_0203370.t1-p1

OG0002471: TCRU_5662 TcCLB-EL.506553.30_mRNA-p1 TcCLB-NE.
507769.40_mRNA-p1 TcSYL_0073630.t1-p1
OG0002472: TCRU_5668 TcCLB-EL.509599.180_mRNA-p1 TcCLB-NE.
506327.50_mRNA-p1 TcSYL_0010900.t1-p1
OG0002473: TCRU_5669 TcCLB-EL.506167.50_mRNA-p1 TcCLB-NE.
481435.9_mRNA-p1 TcSYL_0010940.t1-p1
OG0002474: TCRU_5670 TcCLB-EL.507711.50_mRNA-p1 TcCLB-NE.
425785.18_mRNA-p1 TcSYL_0008840.t1-p1
OG0002475: TCRU_5690 TcCLB-EL.506181.80_mRNA-p1 TcCLB-NE.
506177.130_mRNA-p1 TcSYL_0092360.t1-p1
OG0002476: TCRU_5691 TcCLB-EL.508989.120_mRNA-p1 TcCLB-NE.
466593.10_mRNA-p1 TcSYL_0097670.t1-p1
OG0002477: TCRU_5695 TcCLB-EL.506287.170_mRNA-p1 TcCLB-NE.
508881.100_mRNA-p1 TcSYL_0023450.t1-p1
OG0002478: TCRU_5697 TcCLB-EL.506287.209_mRNA-p1 TcCLB-EL.
506289.9_mRNA-p1 TcCLB-NE.508881.70_mRNA-p1
OG0002479: TCRU_5701 TcCLB-EL.508777.140_mRNA-p1 TcCLB-NE.
509029.170_mRNA-p1 TcSYL_0156110.t1-p1
OG0002480: TCRU_5712 TcCLB-EL.504643.10_pseudogenic_transcript-p1
TcCLB-NE.509161.30_mRNA-p1 TcSYL_0142860.t1-p1
OG0002481: TCRU_5717 TcCLB-NE.510187.240_mRNA-p1 TcSYL_0011570.t1-p1
TcSYL_0011580.t1-p1
OG0002482: TCRU_5724 TcCLB-EL.508321.50_mRNA-p1 TcCLB-NE.
509207.50_mRNA-p1 TcSYL_0201780.t1-p1
OG0002483: TCRU_5725 TcCLB-NE.508153.400_mRNA-p1 TcSYL_0000880.t1-p1
TcSYL_0000890.t1-p1
OG0002484: TCRU_5728 TcCLB-EL.505843.24_mRNA-p1 TcCLB-NE.
504949.30_mRNA-p1 TcSYL_0064460.t1-p1
OG0002485: TCRU_5734 TcCLB-EL.507721.10_mRNA-p1 TcCLB-NE.
509623.10_mRNA-p1 TcSYL_0002040.t1-p1
OG0002486: TCRU_5738 TcCLB-EL.508515.120_mRNA-p1 TcCLB-NE.
508641.80_mRNA-p1 TcSYL_0047410.t1-p1
OG0002487: TCRU_5741 TcCLB-EL.509233.230_mRNA-p1 TcCLB-EL.
509451.50_mRNA-p1 TcSYL_0145960.t1-p1
OG0002488: TCRU_5744 TcCLB-EL.509099.10_mRNA-p1 TcCLB-NE.
509321.19_mRNA-p1 TcSYL_0085520.t1-p1
OG0002489: TCRU_5749 TcCLB-EL.511217.70_mRNA-p1 TcCLB-NE.
506575.30_pseudogenic_transcript-p1 TcSYL_0188960.t1-p1
OG0002490: TCRU_5750 TcCLB-EL.511217.110_mRNA-p1 TcCLB-NE.
503581.10_mRNA-p1 TcSYL_0189140.t1-p1
OG0002491: TCRU_5751 TcCLB-EL.511217.160_mRNA-p1 TcCLB-NE.
506573.60_mRNA-p1 TcSYL_0189380.t1-p1
OG0002492: TCRU_5755 TcCLB-EL.510241.30_mRNA-p1 TcCLB-NE.
510579.100_mRNA-p1 TcSYL_0147230.t1-p1
OG0002493: TCRU_5759 TcCLB-EL.510149.20_mRNA-p1 TcCLB-NE.
508411.60_mRNA-p1 TcSYL_0004190.t1-p1
OG0002494: TCRU_5760 TcCLB-EL.510145.10_mRNA-p1 TcCLB-NE.
415803.10_mRNA-p1 TcSYL_0004230.t1-p1
OG0002495: TCRU_5762 TcCLB-EL.507941.80_mRNA-p1 TcCLB-NE.
507943.99_mRNA-p1 TcSYL_0063200.t1-p1
OG0002496: TCRU_5763 TcCLB-EL.507941.100_mRNA-p1 TcCLB-NE.
507943.80_pseudogenic_transcript-p1 TcSYL_0063190.t1-p1
OG0002497: TCRU_5764 TcCLB-EL.510351.40_mRNA-p1 TcCLB-NE.
507601.140_mRNA-p1 TcSYL_0062260.t1-p1

OG0002498: TCRU_5766 TcCLB-EL.509775.30_mRNA-p1 TcCLB-NE.
507061.40_pseudogenic_transcript-p1 TcSYL_0013840.t1-p1
OG0002499: TCRU_5779 TcCLB-EL.510325.10_mRNA-p1 TcCLB-NE.
508153.120_mRNA-p1 TcSYL_0001090.t1-p1
OG0002500: TCRU_5783 TcCLB-EL.511623.10_mRNA-p1 TcCLB-NE.
504741.100_mRNA-p1 TcSYL_0084330.t1-p1
OG0002501: TCRU_5789 TcCLB-EL.511537.8_mRNA-p1 TcCLB-NE.
511021.70_mRNA-p1 TcSYL_0111700.t1-p1
OG0002502: TCRU_5797 TcCLB-EL.511409.50_mRNA-p1 TcCLB-NE.
509571.50_mRNA-p1 TcSYL_0097880.t1-p1
OG0002503: TCRU_5798 TcCLB-EL.511409.70_mRNA-p1 TcCLB-NE.
509571.70_mRNA-p1 TcSYL_0097910.t1-p1
OG0002504: TCRU_5807 TcCLB-EL.506605.180_mRNA-p1 TcCLB-NE.
414243.10_mRNA-p1 TcSYL_0025770.t1-p1
OG0002505: TCRU_5808 TcCLB-EL.506605.220_mRNA-p1 TcCLB-NE.
508859.70_mRNA-p1 TcSYL_0025590.t1-p1
OG0002506: TCRU_5816 TcCLB-EL.510311.170_mRNA-p1 TcCLB-NE.
508153.1080_mRNA-p1 TcSYL_0000600.t1-p1
OG0002507: TCRU_5818 TcCLB-EL.506691.80_mRNA-p1 TcCLB-NE.
508153.980_mRNA-p1 TcSYL_0000650.t1-p1
OG0002508: TCRU_5821 TcCLB-EL.506637.30_mRNA-p1 TcCLB-NE.
510099.10_mRNA-p1 TcSYL_0044970.t1-p1
OG0002509: TCRU_5824 TcCLB-EL.505843.10_mRNA-p1 TcCLB-EL.
509445.39_mRNA-p1 TcSYL_0064450.t1-p1
OG0002510: TCRU_5827 TcCLB-EL.507975.80_mRNA-p1 TcCLB-NE.
508919.140_mRNA-p1 TcSYL_0157890.t1-p1
OG0002511: TCRU_5828 TcCLB-EL.511581.20_mRNA-p1 TcCLB-NE.
504177.20_mRNA-p1 TcSYL_0109370.t1-p1
OG0002512: TCRU_5829 TcCLB-NE.455721.9_mRNA-p1 TcCLB-NE.
508651.20_mRNA-p1 TcSYL_0157470.t1-p1
OG0002513: TCRU_5830 TcCLB-EL.511423.50_mRNA-p1 TcCLB-NE.
507949.150_mRNA-p1 TcSYL_0110990.t1-p1
OG0002514: TCRU_5831 TcCLB-EL.510125.10_mRNA-p1 TcCLB-NE.
507049.199_mRNA-p1 TcSYL_0114620.t1-p1
OG0002515: TCRU_5835 TcCLB-EL.510597.9_mRNA-p1 TcCLB-NE.
506419.20_mRNA-p1 TcSYL_0057230.t1-p1
OG0002516: TCRU_5836 TcCLB-EL.510595.49_mRNA-p1 TcCLB-NE.
507895.164_mRNA-p1 TcSYL_0057210.t1-p1
OG0002517: TCRU_5837 TcCLB-EL.510593.10_mRNA-p1 TcCLB-NE.
507895.69_mRNA-p1 TcSYL_0057150.t1-p1
OG0002518: TCRU_5842 TcCLB-EL.511751.210_mRNA-p1 TcCLB-NE.
507021.110_mRNA-p1 TcSYL_0139190.t1-p1
OG0002519: TCRU_5843 TcCLB-EL.511753.20_mRNA-p1 TcCLB-NE.
507021.60_mRNA-p1 TcSYL_0139160.t1-p1
OG0002520: TCRU_5847 TcCLB-EL.469435.10_mRNA-p1 TcCLB-NE.
508257.100_mRNA-p1 TcSYL_0046710.t1-p1
OG0002521: TCRU_5853 TcCLB-EL.510911.14_mRNA-p1 TcCLB-NE.
458759.14_mRNA-p1 TcSYL_0143060.t1-p1
OG0002522: TCRU_5858 TcCLB-EL.509669.70_mRNA-p1 TcCLB-NE.
506175.20_mRNA-p1 TcSYL_0001970.t1-p1
OG0002523: TCRU_5866 TcCLB-EL.510101.240_mRNA-p1 TcCLB-NE.
506297.94_mRNA-p1 TcSYL_0074880.t1-p1
OG0002524: TCRU_5868 TcCLB-EL.510101.360_mRNA-p1 TcCLB-NE.
507765.120_mRNA-p1 TcSYL_0074970.t1-p1

OG0002525: TCRU_5874 TcCLB-EL.506797.80_mRNA-p1 TcCLB-NE.
511907.60_mRNA-p1 TcSYL_0102330.t1-p1
OG0002526: TCRU_5875 TcCLB-EL.506797.90_mRNA-p1 TcCLB-NE.
511907.70_mRNA-p1 TcSYL_0102320.t1-p1
OG0002527: TCRU_5880 TcCLB-EL.506251.50_mRNA-p1 TcCLB-NE.
508231.130_mRNA-p1 TcSYL_0108050.t1-p1
OG0002528: TCRU_5888 TcCLB-EL.507011.230_pseudogenic_transcript-p1
TcCLB-NE.507003.60_mRNA-p1 TcSYL_0116680.t1-p1
OG0002529: TCRU_5889 TcCLB-EL.507011.250_mRNA-p1 TcCLB-NE.
507003.24_mRNA-p1 TcSYL_0116600.t1-p1
OG0002530: TCRU_5890 TcCLB-EL.507011.260_mRNA-p1 TcCLB-NE.
510663.80_mRNA-p1 TcSYL_0116570.t1-p1
OG0002531: TCRU_5891 TcCLB-EL.510737.10_mRNA-p1 TcCLB-NE.
510663.70_mRNA-p1 TcSYL_0116560.t1-p1
OG0002532: TCRU_5893 TcCLB-EL.510737.50_mRNA-p1 TcCLB-NE.
510663.50_mRNA-p1 TcSYL_0116450.t1-p1
OG0002533: TCRU_5896 TcCLB-EL.510741.190_pseudogenic_transcript-p1
TcCLB-NE.510661.70_mRNA-p1 TcSYL_0116230.t1-p1
OG0002534: TCRU_5908 TcCLB-EL.421321.9_mRNA-p1 TcCLB-NE.
509395.30_mRNA-p1 TcSYL_0194370.t1-p1
OG0002535: TCRU_5912 TcCLB-EL.507093.44_mRNA-p1 TcCLB-NE.
504109.24_mRNA-p1 TcSYL_0080310.t1-p1
OG0002536: TCRU_5915 TcCLB-EL.509203.30_mRNA-p1 TcCLB-NE.
511017.40_mRNA-p1 TcSYL_0180500.t1-p1
OG0002537: TCRU_5917 TcCLB-EL.508777.30_mRNA-p1 TcCLB-NE.
509029.60_mRNA-p1 TcSYL_0156080.t1-p1
OG0002538: TCRU_5922 TcCLB-EL.509233.60_mRNA-p1 TcCLB-NE.
509437.60_mRNA-p1 TcSYL_0146110.t1-p1
OG0002539: TCRU_5924 TcCLB-EL.510877.20_mRNA-p1 TcSYL_0045450.t1-p1
TcSYL_0045710.t1-p1
OG0002540: TCRU_5926 TcCLB-EL.511739.10_mRNA-p1 TcCLB-NE.
511517.120_mRNA-p1 TcSYL_0139810.t1-p1
OG0002541: TCRU_5929 TcCLB-EL.511743.10_mRNA-p1 TcCLB-NE.
511517.30_mRNA-p1 TcSYL_0139710.t1-p1
OG0002542: TCRU_5930 TcCLB-EL.511743.20_mRNA-p1 TcCLB-NE.
511517.20_mRNA-p1 TcSYL_0139700.t1-p1
OG0002543: TCRU_5934 TcCLB-EL.510565.50_mRNA-p1 TcCLB-NE.
508503.40_mRNA-p1 TcSYL_0105830.t1-p1
OG0002544: TCRU_5936 TcCLB-EL.504103.20_mRNA-p1 TcCLB-NE.
503847.10_pseudogenic_transcript-p1 TcSYL_0005950.t1-p1
OG0002545: TCRU_5937 TcCLB-EL.504103.40_mRNA-p1 TcCLB-NE.
503847.30_mRNA-p1 TcSYL_0005900.t1-p1
OG0002546: TCRU_5944 TcCLB-EL.506457.20_mRNA-p1 TcCLB-NE.
509693.60_mRNA-p1 TcSYL_0170640.t1-p1
OG0002547: TCRU_5945 TcCLB-EL.506457.99_mRNA-p1 TcCLB-NE.
509693.140_mRNA-p1 TcSYL_0170800.t1-p1
OG0002548: TCRU_5946 TcCLB-EL.506457.130_mRNA-p1 TcCLB-NE.
509693.170_mRNA-p1 TcSYL_0170910.t1-p1
OG0002549: TCRU_5948 TcCLB-EL.503991.20_mRNA-p1 TcCLB-NE.
507081.110_mRNA-p1 TcSYL_0019650.t1-p1
OG0002550: TCRU_5950 TcCLB-EL.508241.50_mRNA-p1 TcCLB-NE.
511151.54_mRNA-p1 TcSYL_0077350.t1-p1
OG0002551: TCRU_5956 TcCLB-EL.509645.10_mRNA-p1 TcCLB-EL.
509645.19_mRNA-p1 TcCLB-NE.509733.190_mRNA-p1

OG0002552: TCRU_5965 TcCLB-EL.504055.50_mRNA-p1 TcCLB-NE.
506435.280_mRNA-p1 TcSYL_0196430.t1-p1
OG0002553: TCRU_5966 TcCLB-EL.506265.160_mRNA-p1 TcCLB-NE.
506435.180_mRNA-p1 TcSYL_0196620.t1-p1
OG0002554: TCRU_5976 TcCLB-EL.506753.70_mRNA-p1 TcCLB-NE.
510355.230_mRNA-p1 TcSYL_0112620.t1-p1
OG0002555: TCRU_5978 TcCLB-EL.509591.40_mRNA-p1 TcCLB-NE.
503999.80_mRNA-p1 TcSYL_0089250.t1-p1
OG0002556: TCRU_5980 TcCLB-EL.506775.90_mRNA-p1 TcCLB-NE.
511167.80_mRNA-p1 TcSYL_0075390.t1-p1
OG0002557: TCRU_5985 TcCLB-EL.511045.10_mRNA-p1 TcCLB-NE.
511859.30_mRNA-p1 TcSYL_0091690.t1-p1
OG0002558: TCRU_5991 TcCLB-EL.511469.80_mRNA-p1 TcCLB-NE.
506295.140_mRNA-p1 TcSYL_0132930.t1-p1
OG0002559: TCRU_5994 TcCLB-EL.503893.80_mRNA-p1 TcCLB-NE.
508507.59_mRNA-p1 TcSYL_0082280.t1-p1
OG0002560: TCRU_6000 TcCLB-EL.506847.4_mRNA-p1 TcCLB-NE.
509459.40_mRNA-p1 TcSYL_0174370.t1-p1
OG0002561: TCRU_6001 TcCLB-EL.420495.10_pseudogenic_transcript-p1
TcCLB-NE.509459.50_mRNA-p1 TcSYL_0174360.t1-p1
OG0002562: TCRU_6004 TcCLB-EL.510509.20_mRNA-p1 TcCLB-NE.
509461.80_mRNA-p1 TcSYL_0174050.t1-p1
OG0002563: TCRU_6005 TcCLB-EL.507837.60_mRNA-p1 TcCLB-NE.
508405.30_mRNA-p1 TcSYL_0118390.t1-p1
OG0002564: TCRU_6006 TcCLB-EL.509835.30_mRNA-p1 TcCLB-NE.
504235.9_mRNA-p1 TcSYL_0082550.t1-p1
OG0002565: TCRU_6009 TcCLB-EL.506211.70_mRNA-p1 TcCLB-NE.
508895.50_mRNA-p1 TcSYL_0122990.t1-p1
OG0002566: TCRU_6018 TcCLB-EL.506941.220_mRNA-p1 TcCLB-NE.
507027.50_mRNA-p1 TcSYL_0139640.t1-p1
OG0002567: TCRU_6020 TcCLB-EL.506227.80_mRNA-p1 TcCLB-NE.
511847.60_mRNA-p1 TcSYL_0091960.t1-p1
OG0002568: TCRU_6023 TcCLB-EL.510609.130_mRNA-p1 TcCLB-NE.
506793.60_mRNA-p1 TcSYL_0163590.t1-p1
OG0002569: TCRU_6024 TcCLB-EL.510609.120_mRNA-p1 TcCLB-NE.
506793.70_mRNA-p1 TcSYL_0163600.t1-p1
OG0002570: TCRU_6028 TcCLB-EL.506739.140_mRNA-p1 TcCLB-NE.
506315.40_mRNA-p1 TcSYL_0050080.t1-p1
OG0002571: TCRU_6034 TcCLB-EL.504137.100_mRNA-p1 TcCLB-NE.
509799.50_mRNA-p1 TcSYL_0106900.t1-p1
OG0002572: TCRU_6036 TcCLB-EL.506743.30_mRNA-p1 TcCLB-NE.
422921.20_mRNA-p1 TcSYL_0165410.t1-p1
OG0002573: TCRU_6037 TCRU_8720 TcCLB-EL.510911.50_mRNA-p1 TcCLB-NE.
509163.80_mRNA-p1
OG0002574: TCRU_6043 TcCLB-EL.506743.170_mRNA-p1 TcCLB-NE.
503473.20_mRNA-p1 TcSYL_0164910.t1-p1
OG0002575: TCRU_6044 TcCLB-EL.506743.150_mRNA-p1 TcCLB-NE.
503473.4_mRNA-p1 TcSYL_0164950.t1-p1
OG0002576: TCRU_6052 TcCLB-EL.511621.50_mRNA-p1 TcCLB-NE.
509317.60_mRNA-p1 TcSYL_0084830.t1-p1
OG0002577: TCRU_6056 TcCLB-EL.504703.10_pseudogenic_transcript-p1
TcCLB-NE.506155.120_mRNA-p1 TcSYL_0118120.t1-p1
OG0002578: TCRU_6063 TcCLB-EL.510963.20_mRNA-p1 TcCLB-NE.
508625.80_mRNA-p1 TcSYL_0112940.t1-p1

OG0002579: TCRU_6065 TcCLB-EL.397937.10_mRNA-p1 TcCLB-EL.
509759.19_mRNA-p1 TcCLB-NE.508625.160_mRNA-p1
OG0002580: TCRU_6072 TcCLB-EL.511429.30_mRNA-p1 TcCLB-NE.
509991.20_mRNA-p1 TcSYL_0110740.t1-p1
OG0002581: TCRU_6080 TcCLB-EL.510325.40_mRNA-p1 TcCLB-NE.
508153.100_mRNA-p1 TcSYL_0001100.t1-p1
OG0002582: TCRU_6081 TcCLB-EL.506755.200_mRNA-p1 TcCLB-NE.
510359.210_mRNA-p1 TcSYL_0112810.t1-p1
OG0002583: TCRU_6089 TcCLB-EL.510243.100_mRNA-p1 TcCLB-NE.
510575.130_mRNA-p1 TcSYL_0147320.t1-p1
OG0002584: TCRU_6090 TcCLB-EL.507739.59_mRNA-p1 TcCLB-NE.
506435.424_mRNA-p1 TcSYL_0196040.t1-p1
OG0002585: TCRU_6094 TcCLB-EL.506127.170_mRNA-p1 TcCLB-NE.
503593.10_mRNA-p1 TcSYL_0083270.t1-p1
OG0002586: TCRU_6100 TcCLB-EL.509155.30_mRNA-p1 TcCLB-NE.
510303.160_mRNA-p1 TcSYL_0079150.t1-p1
OG0002587: TCRU_6102 TcCLB-EL.509153.90_mRNA-p1 TcCLB-NE.
510303.320_mRNA-p1 TcSYL_0078960.t1-p1
OG0002588: TCRU_6112 TcCLB-EL.508387.140_mRNA-p1 TcCLB-NE.
506789.160_mRNA-p1 TcSYL_0162500.t1-p1
OG0002589: TCRU_6113 TcCLB-EL.508387.160_mRNA-p1 TcCLB-NE.
506789.140_mRNA-p1 TcSYL_0162390.t1-p1
OG0002590: TCRU_6122 TcCLB-EL.507841.34_mRNA-p1 TcCLB-NE.
508269.60_mRNA-p1 TcSYL_0120090.t1-p1
OG0002591: TCRU_6123 TcCLB-EL.507841.30_mRNA-p1 TcCLB-NE.
508269.70_mRNA-p1 TcSYL_0120120.t1-p1
OG0002592: TCRU_6128 TcCLB-EL.510089.130_mRNA-p1 TcCLB-NE.
510421.150_mRNA-p1 TcSYL_0019220.t1-p1
OG0002593: TCRU_6129 TcCLB-EL.510089.170_mRNA-p1 TcCLB-NE.
510421.180_mRNA-p1 TcSYL_0019240.t1-p1
OG0002594: TCRU_6130 TcCLB-EL.510089.210_mRNA-p1 TcCLB-NE.
510421.220_mRNA-p1 TcSYL_0019280.t1-p1
OG0002595: TCRU_6132 TcCLB-EL.508555.60_mRNA-p1 TcCLB-EL.
511333.4_mRNA-p1 TcCLB-NE.509237.130_mRNA-p1
OG0002596: TCRU_6147 TcCLB-EL.507603.70_mRNA-p1 TcCLB-NE.
509429.140_mRNA-p1 TcSYL_0174940.t1-p1
OG0002597: TCRU_6152 TcCLB-EL.507669.14_mRNA-p1 TcCLB-NE.
509879.30_mRNA-p1 TcSYL_0048470.t1-p1
OG0002598: TCRU_6153 TcCLB-EL.507669.20_mRNA-p1 TcCLB-NE.
509879.20_mRNA-p1 TcSYL_0048490.t1-p1
OG0002599: TCRU_6154 TcCLB-EL.507669.60_mRNA-p1 TcCLB-NE.
507857.70_mRNA-p1 TcSYL_0048570.t1-p1
OG0002600: TCRU_6155 TcCLB-EL.507669.70_mRNA-p1 TcCLB-NE.
507857.60_mRNA-p1 TcSYL_0048600.t1-p1
OG0002601: TCRU_6157 TcCLB-EL.507669.84_mRNA-p1 TcCLB-NE.
507857.40_mRNA-p1 TcSYL_0048620.t1-p1
OG0002602: TCRU_6170 TcCLB-EL.506213.50_pseudogenic_transcript-p1
TcCLB-NE.511287.49_mRNA-p1 TcSYL_0123350.t1-p1
OG0002603: TCRU_6171 TcCLB-EL.506213.40_mRNA-p1 TcCLB-NE.
511287.60_mRNA-p1 TcSYL_0123270.t1-p1
OG0002604: TCRU_6172 TcCLB-EL.506213.30_mRNA-p1 TcCLB-NE.
511287.64_mRNA-p1 TcSYL_0123220.t1-p1
OG0002605: TCRU_6173 TcCLB-EL.506213.10_mRNA-p1 TcCLB-NE.
511287.80_mRNA-p1 TcSYL_0123200.t1-p1

OG0002606: TCRU_6174 TcCLB-EL.503833.69_mRNA-p1 TcCLB-NE.
511287.90_mRNA-p1 TcSYL_0123170.t1-p1
OG0002607: TCRU_6175 TcCLB-EL.503833.60_mRNA-p1 TcCLB-NE.
511287.100_mRNA-p1 TcSYL_0123150.t1-p1
OG0002608: TCRU_6176 TcCLB-EL.503833.50_mRNA-p1 TcCLB-NE.
511287.110_mRNA-p1 TcSYL_0123130.t1-p1
OG0002609: TCRU_6181 TcCLB-EL.508535.30_mRNA-p1 TcCLB-NE.
511461.40_mRNA-p1 TcSYL_0131020.t1-p1
OG0002610: TCRU_6182 TcCLB-EL.508535.20_mRNA-p1 TcCLB-NE.
511461.50_mRNA-p1 TcSYL_0131050.t1-p1
OG0002611: TCRU_6183 TcCLB-EL.503841.70_mRNA-p1 TcCLB-NE.
503679.10_mRNA-p1 TcSYL_0131200.t1-p1
OG0002612: TCRU_6184 TcCLB-EL.503841.60_mRNA-p1 TcCLB-NE.
503679.20_mRNA-p1 TcSYL_0131240.t1-p1
OG0002613: TCRU_6185 TcCLB-EL.503841.50_mRNA-p1 TcCLB-NE.
503679.30_mRNA-p1 TcSYL_0131270.t1-p1
OG0002614: TCRU_6201 TcCLB-EL.462761.16_mRNA-p1 TcCLB-NE.
508823.10_mRNA-p1 TcCLB-NE.511207.10_mRNA-p1
OG0002615: TCRU_6202 TcCLB-EL.511181.150_mRNA-p1 TcCLB-NE.
508299.20_mRNA-p1 TcSYL_0203350.t1-p1
OG0002616: TCRU_6204 TcCLB-EL.506773.130_mRNA-p1 TcCLB-NE.
508799.70_mRNA-p1 TcSYL_0075310.t1-p1
OG0002617: TCRU_6205 TcCLB-EL.506773.140_mRNA-p1 TcCLB-NE.
508799.60_mRNA-p1 TcSYL_0075320.t1-p1
OG0002618: TCRU_6208 TcCLB-EL.506773.170_mRNA-p1 TcCLB-NE.
508799.30_mRNA-p1 TcSYL_0075330.t1-p1
OG0002619: TCRU_6216 TcCLB-EL.503915.9_mRNA-p1 TcCLB-NE.
506679.170_mRNA-p1 TcSYL_0114050.t1-p1
OG0002620: TCRU_6217 TcCLB-EL.504097.30_mRNA-p1 TcCLB-NE.
506679.190_mRNA-p1 TcSYL_0114070.t1-p1
OG0002621: TCRU_6221 TcCLB-EL.506211.160_mRNA-p1 TcCLB-NE.
511289.70_mRNA-p1 TcSYL_0123030.t1-p1
OG0002622: TCRU_6222 TcCLB-EL.506211.170_mRNA-p1 TcCLB-NE.
511289.59_mRNA-p1 TcSYL_0123040.t1-p1
OG0002623: TCRU_6223 TcCLB-EL.506211.180_mRNA-p1 TcCLB-NE.
511289.50_mRNA-p1 TcSYL_0123050.t1-p1
OG0002624: TCRU_6225 TcCLB-EL.506211.200_mRNA-p1 TcCLB-NE.
511289.30_mRNA-p1 TcSYL_0123060.t1-p1
OG0002625: TCRU_6226 TcCLB-EL.506211.210_mRNA-p1 TcCLB-NE.
511289.14_mRNA-p1 TcSYL_0123070.t1-p1
OG0002626: TCRU_6229 TcCLB-EL.510769.30_mRNA-p1 TcCLB-NE.
506241.200_mRNA-p1 TcSYL_0115200.t1-p1
OG0002627: TCRU_6230 TcCLB-EL.510769.49_mRNA-p1 TcCLB-NE.
506241.170_mRNA-p1 TcSYL_0115180.t1-p1
OG0002628: TCRU_6231 TcCLB-EL.510769.60_pseudogenic_transcript-p1
TcCLB-NE.506241.160_mRNA-p1 TcSYL_0115170.t1-p1
OG0002629: TCRU_6233 TcCLB-EL.510769.80_mRNA-p1 TcCLB-NE.
506241.124_mRNA-p1 TcSYL_0115150.t1-p1
OG0002630: TCRU_6235 TcCLB-EL.510769.100_mRNA-p1 TcCLB-NE.
506241.90_mRNA-p1 TcSYL_0115130.t1-p1
OG0002631: TCRU_6237 TcCLB-EL.510769.120_mRNA-p1 TcCLB-NE.
506241.70_mRNA-p1 TcSYL_0115100.t1-p1
OG0002632: TCRU_6238 TcCLB-EL.510769.130_mRNA-p1 TcCLB-NE.
506241.60_mRNA-p1 TcSYL_0115090.t1-p1

OG0002633: TCRU_6239 TcCLB-EL.510769.140_mRNA-p1 TcCLB-NE.
506241.50_mRNA-p1 TcSYL_0115080.t1-p1
OG0002634: TCRU_6261 TcCLB-EL.508715.20_pseudogenic_transcript-p1
TcSYL_0071830.t1-p1 TcSYL_0135290.t1-p1
OG0002635: TCRU_6264 TcCLB-EL.506165.4_mRNA-p1 TcCLB-NE.
508817.110_mRNA-p1 TcSYL_0043620.t1-p1
OG0002636: TCRU_6265 TcCLB-EL.506165.10_mRNA-p1 TcCLB-NE.
508817.90_pseudogenic_transcript-p1 TcSYL_0043640.t1-p1
OG0002637: TCRU_6268 TcCLB-EL.503801.20_mRNA-p1 TcCLB-NE.
508817.60_mRNA-p1 TcSYL_0043730.t1-p1
OG0002638: TCRU_6272 TcCLB-EL.399033.19_mRNA-p1 TcCLB-NE.
508737.30_mRNA-p1 TcSYL_0191580.t1-p1
OG0002639: TCRU_6276 TcCLB-EL.429257.20_mRNA-p1 TcCLB-NE.
508737.70_mRNA-p1 TcSYL_0191590.t1-p1
OG0002640: TCRU_6277 TcCLB-EL.509125.10_mRNA-p1 TcCLB-NE.
508737.80_mRNA-p1 TcSYL_0191600.t1-p1
OG0002641: TCRU_6278 TcCLB-EL.509125.20_mRNA-p1 TcCLB-NE.
508737.100_mRNA-p1 TcSYL_0191620.t1-p1
OG0002642: TCRU_6279 TcCLB-EL.509127.20_mRNA-p1 TcCLB-NE.
508737.110_mRNA-p1 TcSYL_0191630.t1-p1
OG0002643: TCRU_6280 TcCLB-EL.509127.30_mRNA-p1 TcCLB-NE.
508737.130_mRNA-p1 TcSYL_0191650.t1-p1
OG0002644: TCRU_6292 TcCLB-EL.504097.10_mRNA-p1 TcCLB-NE.
506679.210_mRNA-p1 TcSYL_0114090.t1-p1
OG0002645: TCRU_6293 TcCLB-EL.504097.20_mRNA-p1 TcCLB-NE.
506679.200_mRNA-p1 TcSYL_0114080.t1-p1
OG0002646: TCRU_6314 TcCLB-EL.509157.279_mRNA-p1 TcCLB-NE.
510725.99_mRNA-p1 TcSYL_0141670.t1-p1
OG0002647: TCRU_6319 TcCLB-EL.506753.190_mRNA-p1 TcCLB-NE.
510357.90_mRNA-p1 TcSYL_0112710.t1-p1
OG0002648: TCRU_6320 TcCLB-EL.506753.180_mRNA-p1 TcCLB-NE.
510357.80_mRNA-p1 TcSYL_0112700.t1-p1
OG0002649: TCRU_6321 TcCLB-EL.506753.170_mRNA-p1 TcCLB-NE.
510357.70_mRNA-p1 TcSYL_0112690.t1-p1
OG0002650: TCRU_6322 TcCLB-EL.506753.164_mRNA-p1 TcCLB-NE.
510357.60_mRNA-p1 TcSYL_0112680.t1-p1
OG0002651: TCRU_6334 TcCLB-EL.511407.50_mRNA-p1 TcCLB-NE.
506151.20_mRNA-p1 TcSYL_0096970.t1-p1
OG0002652: TCRU_6335 TcCLB-EL.511407.40_mRNA-p1 TcCLB-NE.
506151.10_mRNA-p1 TcSYL_0096940.t1-p1
OG0002653: TCRU_6337 TcCLB-EL.511407.10_mRNA-p1 TcCLB-NE.
509567.40_mRNA-p1 TcSYL_0096820.t1-p1
OG0002654: TCRU_6338 TcCLB-EL.508987.39_mRNA-p1 TcCLB-NE.
509567.34_mRNA-p1 TcSYL_0096790.t1-p1
OG0002655: TCRU_6340 TcCLB-EL.503897.20_mRNA-p1 TcCLB-NE.
509563.50_mRNA-p1 TcSYL_0096370.t1-p1
OG0002656: TCRU_6342 TcCLB-EL.503897.40_mRNA-p1 TcCLB-NE.
509563.30_mRNA-p1 TcSYL_0096300.t1-p1
OG0002657: TCRU_6343 TcCLB-EL.503897.50_mRNA-p1 TcCLB-NE.
509563.20_mRNA-p1 TcSYL_0096270.t1-p1
OG0002658: TCRU_6344 TcCLB-EL.503897.60_mRNA-p1 TcCLB-NE.
509563.10_mRNA-p1 TcSYL_0096260.t1-p1
OG0002659: TCRU_6345 TcCLB-EL.503897.70_mRNA-p1 TcCLB-NE.
509561.130_mRNA-p1 TcSYL_0096210.t1-p1

OG0002660: TCRU_6346 TcCLB-EL.503897.80_mRNA-p1 TcCLB-NE.
509561.120_mRNA-p1 TcSYL_0096170.t1-p1
OG0002661: TCRU_6347 TcCLB-EL.503897.90_mRNA-p1 TcCLB-NE.
509561.110_mRNA-p1 TcSYL_0096130.t1-p1
OG0002662: TCRU_6348 TcCLB-EL.503897.100_mRNA-p1 TcCLB-NE.
509561.100_mRNA-p1 TcSYL_0096060.t1-p1
OG0002663: TCRU_6349 TcCLB-EL.503897.110_mRNA-p1 TcCLB-NE.
509561.80_mRNA-p1 TcSYL_0096030.t1-p1
OG0002664: TCRU_6353 TcCLB-EL.503571.10_mRNA-p1 TcCLB-NE.
509561.20_mRNA-p1 TcSYL_0095970.t1-p1
OG0002665: TCRU_6358 TcCLB-EL.511179.150_mRNA-p1 TcCLB-NE.
508303.10_mRNA-p1 TcSYL_0203190.t1-p1
OG0002666: TCRU_6366 TcCLB-EL.511311.60_mRNA-p1 TcCLB-NE.
511757.20_mRNA-p1 TcSYL_0159700.t1-p1
OG0002667: TCRU_6367 TcCLB-EL.511311.70_mRNA-p1 TcCLB-NE.
511757.10_mRNA-p1 TcSYL_0159690.t1-p1
OG0002668: TCRU_6410 TcCLB-EL.509649.30_mRNA-p1 TcCLB-NE.
511285.90_mRNA-p1 TcSYL_0123490.t1-p1
OG0002669: TCRU_6411 TcCLB-EL.506213.120_mRNA-p1 TcCLB-NE.
511285.120_mRNA-p1 TcSYL_0123480.t1-p1
OG0002670: TCRU_6413 TcCLB-EL.506213.100_mRNA-p1 TcCLB-NE.
511287.10_mRNA-p1 TcSYL_0123470.t1-p1
OG0002671: TCRU_6414 TcCLB-EL.506213.90_mRNA-p1 TcCLB-NE.
511287.20_mRNA-p1 TcSYL_0123460.t1-p1
OG0002672: TCRU_6426 TcCLB-EL.508083.20_mRNA-p1 TcCLB-NE.
508873.410_mRNA-p1 TcSYL_0136160.t1-p1
OG0002673: TCRU_6427 TcCLB-EL.508083.10_mRNA-p1 TcCLB-NE.
508873.420_mRNA-p1 TcSYL_0136180.t1-p1
OG0002674: TCRU_6463 TcCLB-EL.511577.4_mRNA-p1 TcCLB-NE.
504797.140_mRNA-p1 TcSYL_0109650.t1-p1
OG0002675: TCRU_small TcCLB-EL.511577.10_mRNA-p1 TcCLB-NE.
504797.130_mRNA-p1 TcSYL_0109640.t1-p1
OG0002676: TCRU_6465 TcCLB-EL.511577.19_mRNA-p1 TcCLB-NE.
504797.120_mRNA-p1 TcSYL_0109630.t1-p1
OG0002677: TCRU_6466 TcCLB-EL.511577.30_mRNA-p1 TcCLB-NE.
504797.110_mRNA-p1 TcSYL_0109620.t1-p1
OG0002678: TCRU_6467 TcCLB-EL.511577.40_mRNA-p1 TcCLB-NE.
504797.100_mRNA-p1 TcSYL_0109600.t1-p1
OG0002679: TCRU_6468 TcCLB-EL.511577.44_mRNA-p1 TcCLB-NE.
504797.90_mRNA-p1 TcSYL_0109590.t1-p1
OG0002680: TCRU_6469 TcCLB-EL.511577.50_mRNA-p1 TcCLB-NE.
504797.80_mRNA-p1 TcSYL_0109580.t1-p1
OG0002681: TCRU_6488 TcCLB-EL.509099.120_mRNA-p1 TcCLB-NE.
509319.10_mRNA-p1 TcSYL_0085140.t1-p1
OG0002682: TCRU_6490 TcCLB-EL.509099.100_mRNA-p1 TcCLB-NE.
509319.30_mRNA-p1 TcSYL_0085180.t1-p1
OG0002683: TCRU_6492 TcCLB-EL.509099.80_mRNA-p1 TcCLB-NE.
509319.50_mRNA-p1 TcSYL_0085290.t1-p1
OG0002684: TCRU_6493 TcCLB-EL.509099.70_mRNA-p1 TcCLB-NE.
509319.60_mRNA-p1 TcSYL_0085330.t1-p1
OG0002685: TCRU_6494 TcCLB-EL.509099.59_mRNA-p1 TcCLB-NE.
509319.70_mRNA-p1 TcSYL_0085370.t1-p1
OG0002686: TCRU_6495 TcCLB-EL.509099.50_mRNA-p1 TcCLB-NE.
509319.90_mRNA-p1 TcSYL_0085390.t1-p1

OG0002687: TCRU_6496 TcCLB-EL.509099.40_mRNA-p1 TcCLB-NE.
509319.100_mRNA-p1 TcSYL_0085450.t1-p1
OG0002688: TCRU_6501 TcCLB-EL.510257.90_mRNA-p1 TcCLB-NE.
509213.130_mRNA-p1 TcSYL_0114940.t1-p1
OG0002689: TCRU_6503 TcCLB-EL.510257.120_mRNA-p1 TcCLB-NE.
509213.150_mRNA-p1 TcSYL_0114950.t1-p1
OG0002690: TCRU_6504 TcCLB-EL.510257.130_mRNA-p1 TcCLB-NE.
509213.160_mRNA-p1 TcSYL_0114960.t1-p1
OG0002691: TCRU_6505 TcCLB-EL.510257.139_mRNA-p1 TcCLB-NE.
509213.170_mRNA-p1 TcSYL_0114980.t1-p1
OG0002692: TCRU_6506 TcCLB-EL.510259.6_mRNA-p1 TcCLB-NE.
509215.10_mRNA-p1 TcSYL_0114990.t1-p1
OG0002693: TCRU_6528 TcCLB-EL.509099.130_mRNA-p1 TcCLB-NE.
507209.60_mRNA-p1 TcSYL_0085100.t1-p1
OG0002694: TCRU_6530 TcCLB-EL.509099.150_mRNA-p1 TcCLB-NE.
507209.40_mRNA-p1 TcSYL_0085010.t1-p1
OG0002695: TCRU_6531 TcCLB-EL.509099.160_mRNA-p1 TcCLB-NE.
507209.30_mRNA-p1 TcSYL_0084960.t1-p1
OG0002696: TCRU_6532 TcCLB-EL.509099.170_mRNA-p1 TcCLB-NE.
507209.20_mRNA-p1 TcSYL_0084930.t1-p1
OG0002697: TCRU_6559 TcCLB-EL.511355.7_mRNA-p1 TcCLB-NE.
503809.149_mRNA-p1 TcSYL_0121740.t1-p1
OG0002698: TCRU_6562 TcCLB-EL.511355.30_mRNA-p1 TcCLB-NE.
503809.110_mRNA-p1 TcSYL_0121750.t1-p1
OG0002699: TCRU_6587 TcCLB-EL.506265.20_mRNA-p1 TcCLB-NE.
506435.20_mRNA-p1 TcSYL_0197080.t1-p1
OG0002700: TCRU_6588 TcCLB-EL.506265.40_mRNA-p1 TcCLB-NE.
506435.40_mRNA-p1 TcSYL_0197010.t1-p1
OG0002701: TCRU_6589 TcCLB-EL.506265.45_mRNA-p1 TcCLB-NE.
506435.50_mRNA-p1 TcSYL_0197000.t1-p1
OG0002702: TCRU_6594 TcCLB-EL.510565.170_mRNA-p1 TcCLB-NE.
506145.50_mRNA-p1 TcSYL_0105540.t1-p1
OG0002703: TCRU_6595 TcCLB-EL.510565.179_mRNA-p1 TcCLB-NE.
506145.40_mRNA-p1 TcSYL_0105510.t1-p1
OG0002704: TCRU_6612 TcCLB-EL.506517.149_mRNA-p1 TcCLB-NE.
508909.214_mRNA-p1 TcSYL_0159440.t1-p1
OG0002705: TCRU_6614 TcCLB-EL.506517.140_mRNA-p1 TcCLB-NE.
508909.240_mRNA-p1 TcSYL_0159420.t1-p1
OG0002706: TCRU_6615 TcCLB-EL.506517.130_mRNA-p1 TcCLB-NE.
508909.250_mRNA-p1 TcSYL_0159410.t1-p1
OG0002707: TCRU_6620 TcCLB-EL.506517.50_mRNA-p1 TcCLB-NE.
508909.300_mRNA-p1 TcSYL_0159400.t1-p1
OG0002708: TCRU_6633 TcCLB-EL.504043.10_mRNA-p1 TcCLB-NE.
504045.5_mRNA-p1 TcSYL_0019460.t1-p1
OG0002709: TCRU_6646 TCRU_7887 TcCLB-EL.503467.30_mRNA-p1 TcCLB-NE.
505163.80_mRNA-p1
OG0002710: TCRU_6651 TcCLB-EL.506949.30_mRNA-p1 TcCLB-NE.
508959.10_mRNA-p1 TcSYL_0188580.t1-p1
OG0002711: TCRU_6658 TcCLB-EL.510757.9_mRNA-p1 TcCLB-NE.
508413.30_mRNA-p1 TcSYL_0115550.t1-p1
OG0002712: TCRU_6662 TcCLB-EL.510759.60_mRNA-p1 TcCLB-NE.
506999.150_mRNA-p1 TcSYL_0115510.t1-p1
OG0002713: TCRU_6667 TcCLB-EL.507089.60_mRNA-p1 TcCLB-NE.
504085.40_mRNA-p1 TcSYL_0112030.t1-p1

OG0002714: TCRU_6668 TcCLB-EL.507089.80_mRNA-p1 TcCLB-NE.
504085.20_mRNA-p1 TcSYL_0112010.t1-p1
OG0002715: TCRU_6673 TcCLB-EL.507089.130_mRNA-p1 TcCLB-NE.
506725.50_mRNA-p1 TcSYL_0112000.t1-p1
OG0002716: TCRU_6675 TcCLB-EL.507089.190_mRNA-p1 TcCLB-NE.
511027.20_mRNA-p1 TcSYL_0111970.t1-p1
OG0002717: TCRU_6676 TcCLB-EL.507089.200_mRNA-p1 TcCLB-NE.
511027.10_mRNA-p1 TcSYL_0111950.t1-p1
OG0002718: TCRU_6678 TcCLB-EL.507089.230_mRNA-p1 TcCLB-NE.
503839.30_mRNA-p1 TcSYL_0111940.t1-p1
OG0002719: TCRU_6680 TcCLB-EL.509829.20_mRNA-p1 TcCLB-NE.
506629.220_mRNA-p1 TcSYL_0082400.t1-p1
OG0002720: TCRU_6682 TcCLB-EL.506367.30_mRNA-p1 TcCLB-NE.
506629.200_mRNA-p1 TcSYL_0082410.t1-p1
OG0002721: TCRU_6688 TcCLB-EL.504213.90_mRNA-p1 TcCLB-NE.
506985.60_mRNA-p1 TcSYL_0026510.t1-p1
OG0002722: TCRU_6689 TcCLB-EL.504213.100_mRNA-p1 TcCLB-NE.
506985.50_mRNA-p1 TcSYL_0026480.t1-p1
OG0002723: TCRU_6691 TcCLB-EL.504213.120_mRNA-p1 TcCLB-NE.
506985.30_mRNA-p1 TcSYL_0026390.t1-p1
OG0002724: TCRU_6692 TcCLB-EL.504213.130_mRNA-p1 TcCLB-NE.
506985.20_mRNA-p1 TcSYL_0026370.t1-p1
OG0002725: TCRU_6694 TcCLB-EL.506605.10_mRNA-p1 TcCLB-NE.
511239.10_mRNA-p1 TcSYL_0026270.t1-p1
OG0002726: TCRU_6695 TcCLB-EL.506605.40_mRNA-p1 TcCLB-NE.
511239.30_mRNA-p1 TcSYL_0026160.t1-p1
OG0002727: TCRU_6697 TcCLB-EL.506605.59_mRNA-p1 TcCLB-NE.
511239.49_mRNA-p1 TcSYL_0026110.t1-p1
OG0002728: TCRU_6700 TcCLB-EL.506605.80_mRNA-p1 TcCLB-NE.
511239.70_mRNA-p1 TcSYL_0026010.t1-p1
OG0002729: TCRU_6716 TcCLB-EL.503407.20_mRNA-p1 TcCLB-NE.
505193.40_mRNA-p1 TcSYL_0104410.t1-p1
OG0002730: TCRU_6718 TcCLB-EL.506465.40_mRNA-p1 TcCLB-NE.
507317.60_mRNA-p1 TcSYL_0104240.t1-p1
OG0002731: TCRU_6728 TcCLB-EL.413977.10_mRNA-p1 TcCLB-NE.
506235.20_mRNA-p1 TcSYL_0086600.t1-p1
OG0002732: TCRU_6729 TcCLB-EL.511109.130_mRNA-p1 TcCLB-NE.
504157.10_mRNA-p1 TcSYL_0086580.t1-p1
OG0002733: TCRU_6730 TcCLB-EL.511109.120_mRNA-p1 TcCLB-NE.
504157.20_mRNA-p1 TcSYL_0086570.t1-p1
OG0002734: TCRU_6732 TcCLB-EL.511109.100_mRNA-p1 TcCLB-NE.
504157.40_mRNA-p1 TcSYL_0086550.t1-p1
OG0002735: TCRU_6733 TcCLB-EL.511109.90_mRNA-p1 TcCLB-NE.
504157.50_mRNA-p1 TcSYL_0086530.t1-p1
OG0002736: TCRU_6742 TcCLB-EL.510257.80_mRNA-p1 TcCLB-NE.
509213.120_mRNA-p1 TcSYL_0114930.t1-p1
OG0002737: TCRU_6744 TcCLB-EL.510257.60_mRNA-p1 TcCLB-NE.
509213.100_mRNA-p1 TcSYL_0114920.t1-p1
OG0002738: TCRU_6747 TcCLB-EL.510257.30_mRNA-p1 TcCLB-NE.
509213.70_mRNA-p1 TcSYL_0114910.t1-p1
OG0002739: TCRU_6750 TcCLB-EL.510257.10_mRNA-p1 TcCLB-NE.
509213.40_mRNA-p1 TcSYL_0114900.t1-p1
OG0002740: TCRU_6754 TcCLB-EL.510253.10_mRNA-p1 TcCLB-NE.
504243.40_mRNA-p1 TcSYL_0114880.t1-p1

OG0002741: TCRU_6755 TcCLB-EL.508145.49_mRNA-p1 TcCLB-NE.
504243.30_mRNA-p1 TcSYL_0114870.t1-p1
OG0002742: TCRU_6761 TcCLB-EL.510743.70_mRNA-p1 TcCLB-NE.
510659.240_mRNA-p1 TcSYL_0116190.t1-p1
OG0002743: TCRU_6763 TcCLB-EL.510743.90_mRNA-p1 TcCLB-NE.
510659.219_mRNA-p1 TcSYL_0116180.t1-p1
OG0002744: TCRU_6764 TcCLB-EL.510743.100_mRNA-p1 TcCLB-NE.
510659.210_mRNA-p1 TcSYL_0116170.t1-p1
OG0002745: TCRU_6765 TcCLB-EL.510743.110_mRNA-p1 TcCLB-NE.
510659.194_mRNA-p1 TcSYL_0116160.t1-p1
OG0002746: TCRU_6767 TcCLB-EL.508463.10_mRNA-p1 TcCLB-EL.
510743.130_pseudogenic_transcript-p1 TcCLB-NE.510659.170_mRNA-p1
OG0002747: TCRU_6768 TcCLB-EL.507013.20_mRNA-p1 TcCLB-NE.
510659.110_mRNA-p1 TcSYL_0116110.t1-p1
OG0002748: TCRU_6772 TcCLB-EL.510745.20_mRNA-p1 TcCLB-NE.
510659.80_mRNA-p1 TcSYL_0116100.t1-p1
OG0002749: TCRU_6774 TcCLB-EL.510745.40_mRNA-p1 TcCLB-NE.
510659.70_mRNA-p1 TcSYL_0116090.t1-p1
OG0002750: TCRU_6780 TcCLB-EL.508465.90_mRNA-p1 TcCLB-NE.
507001.110_mRNA-p1 TcSYL_0116000.t1-p1
OG0002751: TCRU_6782 TcCLB-EL.508465.110_mRNA-p1 TcCLB-NE.
507001.80_mRNA-p1 TcSYL_0115980.t1-p1
OG0002752: TCRU_6786 TcCLB-EL.509825.40_mRNA-p1 TcCLB-NE.
510423.50_mRNA-p1 TcSYL_0121180.t1-p1
OG0002753: TCRU_6787 TcCLB-EL.509825.30_mRNA-p1 TcCLB-NE.
510423.60_mRNA-p1 TcSYL_0121200.t1-p1
OG0002754: TCRU_6791 TCRU_8478 TcSYL_0160880.t1-p1 TcSYL_0161030.t1-
p1
OG0002755: TCRU_6793 TcCLB-EL.511315.20_mRNA-p1 TcCLB-NE.
511755.50_mRNA-p1 TcSYL_0159610.t1-p1
OG0002756: TCRU_6794 TcCLB-EL.511315.30_mRNA-p1 TcCLB-NE.
511755.40_mRNA-p1 TcSYL_0159600.t1-p1
OG0002757: TCRU_6831 TcCLB-EL.506563.10_mRNA-p1 TcCLB-NE.
503869.40_mRNA-p1 TcSYL_0063430.t1-p1
OG0002758: TCRU_6833 TcCLB-EL.506563.30_mRNA-p1 TcCLB-NE.
503869.60_pseudogenic_transcript-p1 TcSYL_0063440.t1-p1
OG0002759: TCRU_6841 TcCLB-EL.510243.128_mRNA-p1 TcCLB-NE.
510575.50_mRNA-p1 TcSYL_0147330.t1-p1
OG0002760: TCRU_6843 TcCLB-EL.506647.20_mRNA-p1 TcCLB-NE.
510575.30_mRNA-p1 TcSYL_0147340.t1-p1
OG0002761: TCRU_6849 TcCLB-EL.510767.69_mRNA-p1 TcCLB-NE.
509671.20_mRNA-p1 TcSYL_0115230.t1-p1
OG0002762: TCRU_6850 TcCLB-EL.510767.60_mRNA-p1 TcCLB-NE.
509671.30_mRNA-p1 TcSYL_0115240.t1-p1
OG0002763: TCRU_protein_ TcCLB-EL.510767.50_mRNA-p1 TcCLB-NE.
509671.40_mRNA-p1 TcSYL_0115250.t1-p1
OG0002764: TCRU_6852 TcCLB-EL.510767.40_mRNA-p1 TcCLB-NE.
509671.50_mRNA-p1 TcSYL_0115260.t1-p1
OG0002765: TCRU_6861 TcCLB-EL.507227.9_mRNA-p1 TcCLB-NE.
510945.40_mRNA-p1 TcSYL_0048190.t1-p1
OG0002766: TCRU_6864 TcCLB-EL.419703.20_mRNA-p1 TcCLB-NE.
510945.10_mRNA-p1 TcSYL_0048160.t1-p1
OG0002767: TCRU_6867 TcCLB-EL.503981.30_mRNA-p1 TcCLB-NE.
504131.90_mRNA-p1 TcSYL_0048100.t1-p1

OG0002768: TCRU_6868 TcCLB-EL.503981.50_mRNA-p1 TcCLB-NE.
504131.110_mRNA-p1 TcSYL_0048080.t1-p1
OG0002769: TCRU_6869 TcCLB-EL.503981.60_mRNA-p1 TcCLB-NE.
504131.120_mRNA-p1 TcSYL_0048070.t1-p1
OG0002770: TCRU_6870 TcCLB-EL.503981.70_mRNA-p1 TcCLB-NE.
504131.130_mRNA-p1 TcSYL_0048060.t1-p1
OG0002771: TCRU_6872 TcCLB-EL.504125.10_mRNA-p1 TcCLB-NE.
504131.150_mRNA-p1 TcSYL_0048050.t1-p1
OG0002772: TCRU_6875 TcCLB-EL.504125.50_mRNA-p1 TcCLB-NE.
504131.190_mRNA-p1 TcSYL_0048040.t1-p1
OG0002773: TCRU_6879 TcCLB-EL.507559.60_mRNA-p1 TcCLB-NE.
510303.70_mRNA-p1 TcSYL_0079210.t1-p1
OG0002774: TCRU_6880 TcCLB-EL.507559.70_mRNA-p1 TcCLB-NE.
510303.60_mRNA-p1 TcSYL_0079220.t1-p1
OG0002775: TCRU_6885 TcCLB-EL.505945.60_mRNA-p1 TcCLB-NE.
509553.10_mRNA-p1 TcSYL_0095750.t1-p1
OG0002776: TCRU_6886 TcCLB-EL.505945.50_mRNA-p1 TcCLB-NE.
509553.20_mRNA-p1 TcSYL_0095760.t1-p1
OG0002777: TCRU_6889 TcCLB-EL.505945.10_mRNA-p1 TcCLB-NE.
509553.50_mRNA-p1 TcSYL_0095800.t1-p1
OG0002778: TCRU_10438 TCRU_6909 TCRU_7306 TcSYL_0141630.t1-p1
OG0002779: TCRU_6913 TCRU_7135 TcCLB-NE.509219.20_mRNA-p1 TcCLB-NE.
511019.40_pseudogenic_transcript-p1
OG0002780: TCRU_6916 TcCLB-EL.511127.280_mRNA-p1 TcCLB-NE.
509027.30_mRNA-p1 TcSYL_0156000.t1-p1
OG0002781: TCRU_6919 TcCLB-EL.511127.270_mRNA-p1 TcCLB-NE.
509027.20_mRNA-p1 TcSYL_0155980.t1-p1
OG0002782: TCRU_6925 TcCLB-EL.508239.40_mRNA-p1 TcCLB-NE.
508793.30_mRNA-p1 TcSYL_0076710.t1-p1
OG0002783: TCRU_subunit_ TcCLB-EL.508239.20_mRNA-p1 TcCLB-NE.
511159.14_mRNA-p1 TcSYL_0076590.t1-p1
OG0002784: TCRU__ORC1__ TcCLB-EL.508239.10_mRNA-p1 TcCLB-NE.
511159.20_mRNA-p1 TcSYL_0076530.t1-p1
OG0002785: TCRU_6929 TcCLB-EL.510381.70_mRNA-p1 TcCLB-NE.
511161.10_pseudogenic_transcript-p1 TcSYL_0076500.t1-p1
OG0002786: TCRU_methyltransferase TcCLB-EL.510381.60_mRNA-p1 TcCLB-
NE.506905.4_mRNA-p1 TcSYL_0076490.t1-p1
OG0002787: TCRU_6931 TcCLB-EL.510381.50_mRNA-p1 TcCLB-NE.
506905.10_mRNA-p1 TcSYL_0076460.t1-p1
OG0002788: TCRU_6933 TcCLB-EL.510381.30_mRNA-p1 TcCLB-NE.
506905.30_mRNA-p1 TcSYL_0076430.t1-p1
OG0002789: TCRU_6934 TcCLB-EL.510381.20_mRNA-p1 TcCLB-NE.
506905.40_mRNA-p1 TcSYL_0076410.t1-p1
OG0002790: TCRU_6937 TcCLB-EL.506777.110_mRNA-p1 TcCLB-NE.
504113.20_mRNA-p1 TcSYL_0075590.t1-p1
OG0002791: TCRU_6938 TcCLB-EL.506777.100_mRNA-p1 TcCLB-NE.
504113.30_mRNA-p1 TcSYL_0075580.t1-p1
OG0002792: TCRU_6955 TcCLB-EL.506515.20_mRNA-p1 TcCLB-NE.
508913.39_mRNA-p1 TcSYL_0159180.t1-p1
OG0002793: TCRU_6956 TcCLB-EL.510053.70_mRNA-p1 TcCLB-NE.
511321.20_mRNA-p1 TcSYL_0159110.t1-p1
OG0002794: TCRU_6963 TcCLB-EL.504625.70_mRNA-p1 TcCLB-NE.
503583.100_mRNA-p1 TcCLB-NE.511635.80_mRNA-p1
OG0002795: TCRU_6971 TcCLB-EL.509599.110_mRNA-p1 TcCLB-NE.

509733.10_mRNA-p1 TcSYL_0010850.t1-p1
OG0002796: TCRU_6973 TcCLB-EL.509599.90_mRNA-p1 TcCLB-NE.
509733.30_mRNA-p1 TcSYL_0010840.t1-p1
OG0002797: TCRU_6974 TcCLB-EL.509599.80_mRNA-p1 TcCLB-NE.
509733.40_mRNA-p1 TcSYL_0010830.t1-p1
OG0002798: TCRU_6978 TcCLB-EL.509599.40_mRNA-p1 TcCLB-NE.
509733.80_mRNA-p1 TcSYL_0010820.t1-p1
OG0002799: TCRU_6989 TcCLB-EL.511539.9_mRNA-p1 TcCLB-NE.
508707.310_mRNA-p1 TcSYL_0111640.t1-p1
OG0002800: TCRU_6996 TcCLB-EL.506529.669_mRNA-p1 TcCLB-NE.
510889.340_mRNA-p1 TcSYL_0178090.t1-p1
OG0002801: TCRU_6999 TcCLB-EL.506529.640_mRNA-p1 TcCLB-NE.
510889.310_mRNA-p1 TcSYL_0178100.t1-p1
OG0002802: TCRU_7004 TcCLB-EL.508209.94_mRNA-p1 TcCLB-NE.
509505.60_mRNA-p1 TcSYL_0022940.t1-p1
OG0002803: TCRU_7006 TcCLB-EL.508209.110_mRNA-p1 TcCLB-NE.
509505.40_mRNA-p1 TcSYL_0022980.t1-p1
OG0002804: TCRU_7008 TcCLB-EL.508209.140_mRNA-p1 TcCLB-NE.
509505.10_mRNA-p1 TcSYL_0023020.t1-p1
OG0002805: TCRU_7026 TcCLB-EL.506481.10_pseudogenic_transcript-p1
TcCLB-NE.511295.40_mRNA-p1 TcSYL_0121890.t1-p1
OG0002806: TCRU_7027 TcCLB-EL.506481.20_mRNA-p1 TcCLB-NE.
511295.50_mRNA-p1 TcSYL_0121880.t1-p1
OG0002807: TCRU_7035 TcCLB-EL.506513.20_mRNA-p1 TcCLB-NE.
508919.100_mRNA-p1 TcSYL_0158070.t1-p1
OG0002808: TCRU_7036 TcCLB-EL.506513.24_mRNA-p1 TcCLB-NE.
508919.94_mRNA-p1 TcSYL_0158100.t1-p1
OG0002809: TCRU_7037 TcCLB-EL.506513.30_mRNA-p1 TcCLB-NE.
508919.90_mRNA-p1 TcSYL_0158120.t1-p1
OG0002810: TCRU_7039 TcCLB-EL.506513.50_mRNA-p1 TcCLB-NE.
508919.70_mRNA-p1 TcSYL_0158180.t1-p1
OG0002811: TCRU_7041 TcCLB-EL.506513.70_mRNA-p1 TcCLB-NE.
508919.50_mRNA-p1 TcSYL_0158230.t1-p1
OG0002812: TCRU_7042 TcCLB-EL.506513.80_mRNA-p1 TcCLB-NE.
508919.40_mRNA-p1 TcSYL_0158270.t1-p1
OG0002813: TCRU_7043 TcCLB-EL.506513.90_mRNA-p1 TcCLB-NE.
508919.30_mRNA-p1 TcSYL_0158280.t1-p1
OG0002814: TCRU_7046 TcCLB-EL.506513.120_mRNA-p1 TcCLB-NE.
508917.40_mRNA-p1 TcSYL_0158370.t1-p1
OG0002815: TCRU_7047 TcCLB-EL.506513.130_pseudogenic_transcript-p1
TcCLB-NE.508917.30_mRNA-p1 TcSYL_0158400.t1-p1
OG0002816: TCRU_7048 TcCLB-EL.506513.150_mRNA-p1 TcCLB-NE.
508917.20_mRNA-p1 TcSYL_0158460.t1-p1
OG0002817: TCRU_7049 TcCLB-EL.506513.160_mRNA-p1 TcCLB-NE.
508917.10_mRNA-p1 TcSYL_0158520.t1-p1
OG0002818: TCRU_7052 TcCLB-EL.511411.16_mRNA-p1 TcCLB-NE.
506153.3_mRNA-p1 TcSYL_0098140.t1-p1
OG0002819: TCRU_7056 TcCLB-EL.503887.70_mRNA-p1 TcCLB-NE.
507771.40_mRNA-p1 TcSYL_0138410.t1-p1
OG0002820: TCRU_7057 TcCLB-EL.503887.60_mRNA-p1 TcCLB-NE.
507771.50_mRNA-p1 TcSYL_0138400.t1-p1
OG0002821: TCRU_7059 TcCLB-EL.503887.40_mRNA-p1 TcCLB-NE.
507771.70_mRNA-p1 TcSYL_0138390.t1-p1
OG0002822: TCRU_7060 TcCLB-EL.503887.30_mRNA-p1 TcCLB-NE.

507771.80_mRNA-p1 TcSYL_0138380.t1-p1
OG0002823: TCRU_7065 TcCLB-EL.508207.240_mRNA-p1 TcCLB-NE.
509509.20_mRNA-p1 TcSYL_0022760.t1-p1
OG0002824: TCRU_7066 TcCLB-EL.508207.230_mRNA-p1 TcCLB-NE.
509509.30_mRNA-p1 TcSYL_0022750.t1-p1
OG0002825: TCRU_7069 TcCLB-EL.508207.200_mRNA-p1 TcCLB-NE.
509509.60_mRNA-p1 TcSYL_0022740.t1-p1
OG0002826: TCRU_7072 TcCLB-EL.508207.180_mRNA-p1 TcCLB-NE.
507519.30_mRNA-p1 TcSYL_0022710.t1-p1
OG0002827: TCRU_7077 TcCLB-EL.508207.130_mRNA-p1 TcCLB-NE.
507519.90_mRNA-p1 TcSYL_0022690.t1-p1
OG0002828: TCRU_7080 TcCLB-EL.508207.70_mRNA-p1 TcCLB-NE.
507519.150_mRNA-p1 TcSYL_0022640.t1-p1
OG0002829: TCRU_7089 TcCLB-EL.507529.40_mRNA-p1 TcCLB-NE.
510001.40_mRNA-p1 TcSYL_0003070.t1-p1
OG0002830: TCRU_7091 TcCLB-EL.507531.10_pseudogenic_transcript-p1
TcCLB-NE.510003.20_mRNA-p1 TcSYL_0002960.t1-p1
OG0002831: TCRU_7101 TcCLB-NE.511727.270_mRNA-p1 TcCLB-NE.
511727.290_mRNA-p1 TcSYL_0142820.t1-p1
OG0002832: TCRU_7113 TCRU_8265 TcCLB-EL.418405.30_mRNA-p1
TcSYL_0129380.t1-p1
OG0002833: TCRU_7115 TcCLB-EL.503969.20_mRNA-p1 TcCLB-NE.
506807.20_mRNA-p1 TcSYL_0121240.t1-p1
OG0002834: TCRU_7116 TcCLB-EL.509823.10_mRNA-p1 TcCLB-NE.
506807.30_mRNA-p1 TcSYL_0121250.t1-p1
OG0002835: TCRU_homolog TcCLB-EL.508975.20_mRNA-p1 TcCLB-NE.
508547.20_mRNA-p1 TcSYL_0202570.t1-p1
OG0002836: TCRU_7148 TcCLB-EL.508975.4_mRNA-p1 TcCLB-NE.
508547.40_mRNA-p1 TcSYL_0202540.t1-p1
OG0002837: TCRU_7149 TcCLB-EL.508973.110_mRNA-p1 TcCLB-NE.
508547.60_mRNA-p1 TcSYL_0202590.t1-p1
OG0002838: TCRU_7151 TcCLB-EL.508973.69_mRNA-p1 TcCLB-NE.
508547.90_mRNA-p1 TcSYL_0202510.t1-p1
OG0002839: TCRU_7153 TcCLB-EL.508973.50_mRNA-p1 TcCLB-NE.
508547.110_mRNA-p1 TcSYL_0202500.t1-p1
OG0002840: TCRU_7159 TcCLB-EL.508207.250_mRNA-p1 TcCLB-NE.
509507.49_mRNA-p1 TcSYL_0022780.t1-p1
OG0002841: TCRU_7160 TcCLB-EL.508207.270_mRNA-p1 TcCLB-NE.
509507.30_mRNA-p1 TcSYL_0022800.t1-p1
OG0002842: TCRU_7167 TcCLB-EL.511303.24_mRNA-p1 TcCLB-NE.
509179.40_mRNA-p1 TcSYL_0159930.t1-p1
OG0002843: TCRU_7168 TcCLB-EL.511303.30_mRNA-p1 TcCLB-NE.
509179.30_mRNA-p1 TcSYL_0159920.t1-p1
OG0002844: TCRU_7169 TcCLB-EL.511303.34_mRNA-p1 TcCLB-NE.
509179.20_mRNA-p1 TcSYL_0159910.t1-p1
OG0002845: TCRU_7178 TcCLB-NE.510689.40_mRNA-p1 TcSYL_0047220.t1-p1
TcSYL_0194360.t1-p1
OG0002846: TCRU_7188 TcCLB-EL.510603.160_mRNA-p1 TcCLB-NE.
509109.30_mRNA-p1 TcSYL_0014390.t1-p1
OG0002847: TCRU_7193 TcCLB-EL.508837.90_mRNA-p1 TcCLB-NE.
511383.80_mRNA-p1 TcSYL_0187830.t1-p1
OG0002848: TCRU_7194 TcCLB-EL.508837.100_mRNA-p1 TcCLB-NE.
511383.70_mRNA-p1 TcSYL_0187850.t1-p1
OG0002849: TCRU_7195 TcCLB-EL.508837.110_mRNA-p1 TcCLB-NE.

511383.60_mRNA-p1 TcSYL_0187860.t1-p1
OG0002850: TCRU_7196 TcCLB-EL.508837.120_mRNA-p1 TcCLB-NE.
511383.50_mRNA-p1 TcSYL_0187870.t1-p1
OG0002851: TCRU_7197 TcCLB-EL.508837.130_mRNA-p1 TcCLB-NE.
511383.40_mRNA-p1 TcSYL_0187880.t1-p1
OG0002852: TCRU_7213 TcCLB-EL.507709.120_mRNA-p1 TcCLB-NE.
506577.150_mRNA-p1 TcSYL_0009060.t1-p1
OG0002853: TCRU_7214 TcCLB-EL.507709.110_mRNA-p1 TcCLB-NE.
506577.140_mRNA-p1 TcSYL_0009070.t1-p1
OG0002854: TCRU_7220 TcCLB-EL.506529.480_mRNA-p1 TcCLB-NE.
510889.190_mRNA-p1 TcSYL_0178130.t1-p1
OG0002855: TCRU_7223 TcCLB-EL.511393.88_mRNA-p1 TcCLB-NE.
505071.110_mRNA-p1 TcSYL_0202080.t1-p1
OG0002856: TCRU_7225 TcCLB-EL.511395.20_mRNA-p1 TcCLB-NE.
505071.100_mRNA-p1 TcSYL_0202100.t1-p1
OG0002857: TCRU_7226 TcCLB-EL.511395.40_mRNA-p1 TcCLB-NE.
505071.80_mRNA-p1 TcSYL_0202120.t1-p1
OG0002858: TCRU_7228 TcCLB-EL.511395.70_mRNA-p1 TcCLB-NE.
505071.50_mRNA-p1 TcSYL_0202140.t1-p1
OG0002859: TCRU_7229 TcCLB-EL.511395.80_mRNA-p1 TcCLB-NE.
505071.40_mRNA-p1 TcSYL_0202150.t1-p1
OG0002860: TCRU_7230 TcCLB-EL.511395.90_mRNA-p1 TcCLB-NE.
505071.30_mRNA-p1 TcSYL_0202160.t1-p1
OG0002861: TCRU_7271 TcCLB-EL.511585.220_mRNA-p1 TcCLB-NE.
510155.210_mRNA-p1 TcSYL_0109200.t1-p1
OG0002862: TCRU_7273 TcCLB-EL.511585.190_mRNA-p1 TcCLB-NE.
510155.180_mRNA-p1 TcSYL_0109220.t1-p1
OG0002863: TCRU_7281 TcCLB-EL.508811.30_mRNA-p1 TcCLB-NE.
503415.30_mRNA-p1 TcSYL_0086860.t1-p1
OG0002864: TCRU_7301 TcCLB-EL.482971.9_mRNA-p1 TcSYL_0157140.t1-p1
TcSYL_0203650.t1-p1
OG0002865: TCRU_7325 TcCLB-EL.509267.90_mRNA-p1 TcCLB-NE.
510031.50_mRNA-p1 TcSYL_0088960.t1-p1
OG0002866: TCRU_7351 TcCLB-EL.506649.80_mRNA-p1 TcCLB-NE.
508349.39_mRNA-p1 TcSYL_0147420.t1-p1
OG0002867: TCRU_7353 TcCLB-EL.511445.20_mRNA-p1 TcCLB-NE.
506721.20_mRNA-p1 TcSYL_0080870.t1-p1
OG0002868: TCRU_7354 TcCLB-EL.511445.30_mRNA-p1 TcCLB-NE.
510799.80_mRNA-p1 TcSYL_0080880.t1-p1
OG0002869: TCRU_7357 TcCLB-EL.510613.20_mRNA-p1 TcCLB-NE.
508263.30_mRNA-p1 TcSYL_0163460.t1-p1
OG0002870: TCRU_7358 TcCLB-EL.510613.30_mRNA-p1 TcCLB-NE.
508263.10_mRNA-p1 TcSYL_0163400.t1-p1
OG0002871: TCRU_7368 TcCLB-EL.445553.10_mRNA-p1 TcCLB-NE.
507619.10_mRNA-p1 TcCLB-NE.509557.10_mRNA-p1
OG0002872: TCRU_7384 TcCLB-EL.510515.30_mRNA-p1 TcCLB-NE.
509859.10_mRNA-p1 TcSYL_0174530.t1-p1
OG0002873: TCRU_7385 TcCLB-EL.510515.40_mRNA-p1 TcCLB-NE.
509859.20_mRNA-p1 TcSYL_0174540.t1-p1
OG0002874: TCRU_7389 TcCLB-EL.510515.100_mRNA-p1 TcCLB-NE.
509859.80_mRNA-p1 TcSYL_0174560.t1-p1
OG0002875: TCRU_7390 TcCLB-EL.510515.110_mRNA-p1 TcCLB-NE.
504003.90_mRNA-p1 TcSYL_0174570.t1-p1
OG0002876: TCRU_7391 TcCLB-EL.510515.120_mRNA-p1 TcCLB-NE.

504003.80_mRNA-p1 TcSYL_0174580.t1-p1
OG0002877: TCRU_7392 TcCLB-EL.510515.130_mRNA-p1 TcCLB-NE.
504003.70_mRNA-p1 TcSYL_0174590.t1-p1
OG0002878: TCRU_7393 TcCLB-EL.510517.10_mRNA-p1 TcCLB-NE.
504003.60_mRNA-p1 TcSYL_0174610.t1-p1
OG0002879: TCRU_7394 TcCLB-EL.510517.30_mRNA-p1 TcCLB-NE.
504003.40_mRNA-p1 TcSYL_0174620.t1-p1
OG0002880: TCRU_7395 TcCLB-EL.510517.40_mRNA-p1 TcCLB-NE.
504003.30_mRNA-p1 TcSYL_0174630.t1-p1
OG0002881: TCRU_7398 TcCLB-EL.510517.70_mRNA-p1 TcCLB-NE.
507851.30_mRNA-p1 TcSYL_0174650.t1-p1
OG0002882: TCRU_7400 TcCLB-EL.510517.90_mRNA-p1 TcCLB-NE.
507851.50_mRNA-p1 TcSYL_0174660.t1-p1
OG0002883: TCRU_7401 TcCLB-EL.510517.100_mRNA-p1 TcCLB-NE.
507851.60_mRNA-p1 TcSYL_0174680.t1-p1
OG0002884: TCRU_7403 TcSYL_0188040.t1-p1 TcSYL_0188060.t1-p1
TcSYL_0188100.t1-p1
OG0002885: TCRU_7413 TcCLB-EL.509167.90_mRNA-p1 TcCLB-NE.
510443.20_mRNA-p1 TcSYL_0140410.t1-p1
OG0002886: TCRU_7414 TcCLB-EL.509167.60_mRNA-p1 TcCLB-NE.
506831.5_mRNA-p1 TcSYL_0140440.t1-p1
OG0002887: TCRU_7415 TcCLB-EL.509167.50_mRNA-p1 TcCLB-NE.
506831.10_mRNA-p1 TcSYL_0140450.t1-p1
OG0002888: TCRU_7417 TcCLB-EL.509167.30_mRNA-p1 TcCLB-NE.
506831.30_mRNA-p1 TcSYL_0140460.t1-p1
OG0002889: TCRU_7462 TcCLB-EL.509443.20_mRNA-p1 TcCLB-NE.
507017.140_mRNA-p1 TcSYL_0166980.t1-p1
OG0002890: TCRU_7465 TcCLB-EL.505807.260_mRNA-p1 TcCLB-NE.
507017.110_mRNA-p1 TcSYL_0166950.t1-p1
OG0002891: TCRU_7469 TcCLB-EL.505807.220_mRNA-p1 TcCLB-NE.
507017.70_mRNA-p1 TcSYL_0166940.t1-p1
OG0002892: TCRU_7472 TcCLB-EL.505807.190_mRNA-p1 TcCLB-NE.
507017.50_mRNA-p1 TcSYL_0166930.t1-p1
OG0002893: TCRU_7473 TcCLB-EL.505807.180_mRNA-p1 TcCLB-NE.
507017.40_mRNA-p1 TcSYL_0166920.t1-p1
OG0002894: TCRU_7474 TcCLB-EL.505807.160_mRNA-p1 TcCLB-NE.
507017.30_mRNA-p1 TcSYL_0166910.t1-p1
OG0002895: TCRU_7475 TcCLB-EL.505807.150_mRNA-p1 TcCLB-NE.
507017.20_mRNA-p1 TcSYL_0166900.t1-p1
OG0002896: TCRU_7478 TcCLB-EL.505807.120_mRNA-p1 TcCLB-NE.
503815.20_mRNA-p1 TcSYL_0166880.t1-p1
OG0002897: TCRU_7479 TcCLB-EL.505807.110_mRNA-p1 TcCLB-NE.
503815.10_mRNA-p1 TcSYL_0166870.t1-p1
OG0002898: TCRU_7480 TcCLB-EL.505807.90_mRNA-p1 TcCLB-NE.
509033.100_mRNA-p1 TcSYL_0166860.t1-p1
OG0002899: TCRU_7481 TcCLB-EL.505807.60_mRNA-p1 TcCLB-NE.
509033.80_mRNA-p1 TcSYL_0166840.t1-p1
OG0002900: TCRU_7483 TcCLB-EL.505807.40_mRNA-p1 TcCLB-NE.
509033.50_mRNA-p1 TcSYL_0166780.t1-p1
OG0002901: TCRU_7484 TcCLB-EL.505807.29_mRNA-p1 TcCLB-NE.
509033.44_mRNA-p1 TcSYL_0166750.t1-p1
OG0002902: TCRU_7485 TcCLB-EL.505807.20_mRNA-p1 TcCLB-NE.
509033.40_mRNA-p1 TcSYL_0166740.t1-p1
OG0002903: TCRU_7486 TcCLB-EL.505807.10_mRNA-p1 TcCLB-NE.

509033.30_mRNA-p1 TcSYL_0166720.t1-p1
OG0002904: TCRU_7499 TcCLB-EL.506945.160_mRNA-p1 TcCLB-NE.
510445.50_mRNA-p1 TcSYL_0140680.t1-p1
OG0002905: TCRU_7501 TcCLB-EL.506945.120_mRNA-p1 TcCLB-NE.
510445.20_mRNA-p1 TcSYL_0140660.t1-p1
OG0002906: TCRU_7506 TcCLB-EL.506945.80_mRNA-p1 TcCLB-NE.
506835.120_mRNA-p1 TcSYL_0140640.t1-p1
OG0002907: TCRU_7507 TcCLB-EL.506945.70_mRNA-p1 TcCLB-NE.
506835.110_mRNA-p1 TcSYL_0140590.t1-p1
OG0002908: TCRU_7532 TcCLB-EL.509537.20_mRNA-p1 TcCLB-NE.
507681.230_mRNA-p1 TcSYL_0073880.t1-p1
OG0002909: TCRU_7534 TcCLB-EL.509537.40_mRNA-p1 TcCLB-NE.
507681.250_mRNA-p1 TcSYL_0073890.t1-p1
OG0002910: TCRU_7535 TcCLB-EL.509537.50_mRNA-p1 TcCLB-NE.
507681.259_mRNA-p1 TcSYL_0073900.t1-p1
OG0002911: TCRU_7538 TcCLB-EL.511303.160_mRNA-p1 TcCLB-NE.
511761.50_mRNA-p1 TcSYL_0159800.t1-p1
OG0002912: TCRU_7542 TcCLB-EL.511303.120_mRNA-p1 TcCLB-NE.
511761.89_mRNA-p1 TcSYL_0159810.t1-p1
OG0002913: TCRU_7545 TcCLB-EL.511303.90_mRNA-p1 TcCLB-NE.
509177.30_mRNA-p1 TcSYL_0159830.t1-p1
OG0002914: TCRU_7546 TcCLB-EL.511303.80_mRNA-p1 TcCLB-NE.
509177.40_mRNA-p1 TcSYL_0159850.t1-p1
OG0002915: TCRU_7547 TcCLB-EL.511303.70_mRNA-p1 TcCLB-NE.
509177.50_mRNA-p1 TcSYL_0159860.t1-p1
OG0002916: TCRU_7561 TcCLB-EL.507559.100_mRNA-p1 TcCLB-NE.
510303.30_mRNA-p1 TcSYL_0079290.t1-p1
OG0002917: TCRU_7562 TcCLB-EL.507559.90_mRNA-p1 TcCLB-NE.
510303.40_mRNA-p1 TcSYL_0079270.t1-p1
OG0002918: TCRU_7571 TcCLB-EL.504051.20_mRNA-p1 TcCLB-NE.
509159.10_mRNA-p1 TcSYL_0141930.t1-p1
OG0002919: TCRU_7574 TcCLB-EL.504051.49_mRNA-p1 TcCLB-NE.
511719.30_mRNA-p1 TcSYL_0141920.t1-p1
OG0002920: TCRU_7576 TcCLB-EL.506223.100_mRNA-p1 TcCLB-NE.
511717.190_mRNA-p1 TcSYL_0141880.t1-p1
OG0002921: TCRU_7577 TcCLB-EL.506223.90_mRNA-p1 TcCLB-NE.
511717.180_mRNA-p1 TcSYL_0141870.t1-p1
OG0002922: TCRU_7579 TcCLB-EL.506223.80_mRNA-p1 TcCLB-NE.
511717.170_mRNA-p1 TcSYL_0141860.t1-p1
OG0002923: TCRU_7581 TcCLB-EL.506223.60_mRNA-p1 TcCLB-NE.
511717.150_mRNA-p1 TcSYL_0141850.t1-p1
OG0002924: TCRU_C-terminal TcCLB-EL.506223.40_mRNA-p1 TcCLB-NE.
511717.130_mRNA-p1 TcSYL_0141840.t1-p1
OG0002925: TCRU_7586 TcCLB-EL.503677.10_mRNA-p1 TcCLB-NE.
511717.90_mRNA-p1 TcSYL_0141820.t1-p1
OG0002926: TCRU_7587 TcCLB-EL.503677.20_mRNA-p1 TcCLB-NE.
511717.80_mRNA-p1 TcSYL_0141810.t1-p1
OG0002927: TCRU_7592 TcCLB-EL.506221.110_mRNA-p1 TcCLB-NE.
511717.30_mRNA-p1 TcSYL_0141790.t1-p1
OG0002928: TCRU_7593 TcCLB-EL.506221.100_mRNA-p1 TcCLB-NE.
511717.20_mRNA-p1 TcSYL_0141780.t1-p1
OG0002929: TCRU_7594 TcCLB-EL.506221.90_mRNA-p1 TcCLB-NE.
427789.39_mRNA-p1 TcCLB-NE.511717.10_pseudogenic_transcript-p1
OG0002930: TCRU_7595 TcCLB-EL.507709.80_mRNA-p1 TcCLB-NE.

506577.110_mRNA-p1 TcSYL_0009080.t1-p1
OG0002931: TCRU_7606 TcCLB-EL.510101.490_mRNA-p1 TcCLB-NE.
507763.30_mRNA-p1 TcSYL_0075040.t1-p1
OG0002932: TCRU_7612 TcCLB-EL.511001.170_mRNA-p1 TcCLB-NE.
509017.50_mRNA-p1 TcSYL_0131580.t1-p1
OG0002933: TCRU_7613 TcCLB-EL.511001.180_mRNA-p1 TcCLB-NE.
509017.40_mRNA-p1 TcSYL_0131490.t1-p1
OG0002934: TCRU_7614 TcCLB-EL.511001.190_mRNA-p1 TcCLB-NE.
509017.30_mRNA-p1 TcSYL_0131480.t1-p1
OG0002935: TCRU_7615 TcCLB-EL.511001.210_mRNA-p1 TcCLB-NE.
509017.20_mRNA-p1 TcSYL_0131440.t1-p1
OG0002936: TCRU_7617 TcCLB-EL.503841.10_mRNA-p1 TcCLB-NE.
511465.10_mRNA-p1 TcSYL_0131380.t1-p1
OG0002937: TCRU_7619 TcCLB-EL.503841.30_mRNA-p1 TcCLB-NE.
511463.10_mRNA-p1 TcSYL_0131320.t1-p1
OG0002938: TCRU_7622 TcCLB-EL.507669.180_mRNA-p1 TcCLB-NE.
509877.20_mRNA-p1 TcSYL_0048660.t1-p1
OG0002939: TCRU_7624 TcCLB-EL.507669.160_mRNA-p1 TcCLB-NE.
509877.40_mRNA-p1 TcSYL_0048650.t1-p1
OG0002940: TCRU_7626 TcCLB-EL.507669.130_mRNA-p1 TcCLB-NE.
509877.70_mRNA-p1 TcSYL_0048640.t1-p1
OG0002941: TCRU_7628 TcCLB-EL.507669.110_mRNA-p1 TcCLB-NE.
509877.89_mRNA-p1 TcSYL_0048630.t1-p1
OG0002942: TCRU_7635 TcCLB-NE.509943.11_mRNA-p1 TcSYL_0064080.t1-p1
TcSYL_0064120.t1-p1
OG0002943: TCRU_7644 TcCLB-EL.508723.30_mRNA-p1 TcCLB-NE.
509791.50_mRNA-p1 TcSYL_0107170.t1-p1
OG0002944: TCRU_7645 TcCLB-EL.508723.50_mRNA-p1 TcCLB-NE.
509791.30_mRNA-p1 TcSYL_0107180.t1-p1
OG0002945: TCRU_7647 TcCLB-EL.508723.70_mRNA-p1 TcCLB-NE.
509789.59_mRNA-p1 TcSYL_0107190.t1-p1
OG0002946: TCRU_7648 TcCLB-EL.508723.80_mRNA-p1 TcCLB-NE.
509789.50_mRNA-p1 TcSYL_0107220.t1-p1
OG0002947: TCRU_7649 TcCLB-EL.508723.90_mRNA-p1 TcCLB-NE.
509789.40_mRNA-p1 TcSYL_0107230.t1-p1
OG0002948: TCRU_7654 TcCLB-EL.509967.160_mRNA-p1 TcCLB-NE.
509695.120_mRNA-p1 TcSYL_0171120.t1-p1
OG0002949: TCRU_7659 TcCLB-EL.508075.50_mRNA-p1 TcCLB-NE.
510651.20_mRNA-p1 TcSYL_0004250.t1-p1
OG0002950: TCRU_7663 TcCLB-EL.508075.14_mRNA-p1 TcCLB-NE.
508409.329_mRNA-p1 TcSYL_0004260.t1-p1
OG0002951: TCRU_7666 TcCLB-EL.510143.110_mRNA-p1 TcCLB-NE.
508409.300_mRNA-p1 TcSYL_0004280.t1-p1
OG0002952: TCRU_7667 TcCLB-EL.510143.90_mRNA-p1 TcCLB-NE.
508409.280_mRNA-p1 TcSYL_0004290.t1-p1
OG0002953: TCRU_7668 TcCLB-EL.510143.80_mRNA-p1 TcCLB-NE.
508409.270_mRNA-p1 TcSYL_0004300.t1-p1
OG0002954: TCRU_7669 TcCLB-EL.510143.70_mRNA-p1 TcCLB-NE.
508409.260_mRNA-p1 TcSYL_0004320.t1-p1
OG0002955: TCRU_7670 TcCLB-EL.510143.60_mRNA-p1 TcCLB-NE.
508409.250_mRNA-p1 TcSYL_0004330.t1-p1
OG0002956: TCRU_7671 TcCLB-EL.510143.50_mRNA-p1 TcCLB-NE.
508409.240_mRNA-p1 TcSYL_0004360.t1-p1
OG0002957: TCRU_7672 TcCLB-EL.510143.40_mRNA-p1 TcCLB-NE.

508409.230_mRNA-p1 TcSYL_0004370.t1-p1
OG0002958: TCRU_7673 TcCLB-EL.510143.30_mRNA-p1 TcCLB-NE.
508409.220_mRNA-p1 TcSYL_0004380.t1-p1
OG0002959: TCRU_7674 TcCLB-EL.510143.24_mRNA-p1 TcCLB-NE.
508409.210_mRNA-p1 TcSYL_0004390.t1-p1
OG0002960: TCRU_7677 TcCLB-EL.510141.10_mRNA-p1 TcCLB-NE.
508409.160_mRNA-p1 TcSYL_0004410.t1-p1
OG0002961: TCRU_7698 TcCLB-EL.504255.30_mRNA-p1 TcCLB-NE.
510577.10_mRNA-p1 TcSYL_0147290.t1-p1
OG0002962: TCRU_7704 TcCLB-EL.506927.30_mRNA-p1 TcCLB-NE.
510797.30_mRNA-p1 TcSYL_0082330.t1-p1
OG0002963: TCRU_7707 TcCLB-EL.503893.170_mRNA-p1 TcCLB-NE.
508505.20_mRNA-p1 TcSYL_0082320.t1-p1
OG0002964: TCRU_subunit TcCLB-EL.506135.110_mRNA-p1 TcCLB-NE.
508879.170_mRNA-p1 TcSYL_0023280.t1-p1
OG0002965: TCRU_7743 TcCLB-EL.506135.100_mRNA-p1 TcCLB-NE.
508879.180_mRNA-p1 TcSYL_0023290.t1-p1
OG0002966: TCRU_7749 TcCLB-EL.506135.30_mRNA-p1 TcCLB-NE.
503721.20_mRNA-p1 TcSYL_0023300.t1-p1
OG0002967: TCRU_7755 TcCLB-EL.509127.70_mRNA-p1 TcCLB-NE.
508737.160_mRNA-p1 TcSYL_0191680.t1-p1
OG0002968: TCRU_7756 TcCLB-EL.509127.59_mRNA-p1 TcCLB-NE.
508737.154_mRNA-p1 TcSYL_0191670.t1-p1
OG0002969: TCRU_7757 TcCLB-EL.509127.50_mRNA-p1 TcCLB-NE.
508737.150_mRNA-p1 TcSYL_0191660.t1-p1
OG0002970: TCRU_7767 TcCLB-EL.505007.10_mRNA-p1 TcCLB-EL.
507885.10_mRNA-p1 TcCLB-NE.510007.30_mRNA-p1
OG0002971: TCRU_7769 TcCLB-EL.506929.40_mRNA-p1 TcCLB-NE.
506587.60_mRNA-p1 TcSYL_0044850.t1-p1
OG0002972: TCRU_7770 TcCLB-EL.506929.30_mRNA-p1 TcCLB-NE.
506587.70_mRNA-p1 TcSYL_0044860.t1-p1
OG0002973: TCRU_7783 TcCLB-EL.511071.140_mRNA-p1 TcCLB-NE.
511899.40_mRNA-p1 TcSYL_0103590.t1-p1
OG0002974: TCRU_7786 TcCLB-EL.511071.70_mRNA-p1 TcCLB-NE.
511903.30_mRNA-p1 TcSYL_0103510.t1-p1
OG0002975: TCRU_7787 TcCLB-EL.511071.50_pseudogenic_transcript-p1
TcCLB-NE.511903.40_mRNA-p1 TcSYL_0103500.t1-p1
OG0002976: TCRU_7788 TcCLB-EL.511071.30_mRNA-p1 TcCLB-NE.
511903.60_mRNA-p1 TcSYL_0103470.t1-p1
OG0002977: TCRU_7790 TcCLB-EL.511071.10_mRNA-p1 TcCLB-NE.
511903.90_mRNA-p1 TcSYL_0103440.t1-p1
OG0002978: TCRU_7794 TcCLB-EL.506661.50_mRNA-p1 TcCLB-NE.
511245.110_mRNA-p1 TcSYL_0027110.t1-p1
OG0002979: TCRU_subclass_ TcCLB-EL.506661.40_mRNA-p1 TcCLB-NE.
511245.120_mRNA-p1 TcSYL_0027120.t1-p1
OG0002980: TCRU_7796 TcCLB-EL.506661.20_mRNA-p1 TcCLB-NE.
511245.140_mRNA-p1 TcSYL_0027140.t1-p1
OG0002981: TCRU_7800 TcCLB-EL.510989.50_mRNA-p1 TcCLB-NE.
511245.200_mRNA-p1 TcSYL_0027170.t1-p1
OG0002982: TCRU_7812 TcCLB-EL.507257.10_mRNA-p1 TcCLB-NE.
510329.220_mRNA-p1 TcSYL_0001280.t1-p1
OG0002983: TCRU_7819 TcCLB-EL.507257.110_mRNA-p1 TcCLB-NE.
510329.320_mRNA-p1 TcSYL_0001360.t1-p1
OG0002984: TCRU_7823 TcCLB-EL.507257.160_mRNA-p1 TcCLB-NE.

510329.370_mRNA-p1 TcSYL_0001370.t1-p1
OG0002985: TCRU_7827 TcCLB-EL.511545.50_mRNA-p1 TcCLB-NE.
506817.60_mRNA-p1 TcSYL_0088140.t1-p1
OG0002986: TCRU_subunit TcCLB-EL.511545.70_mRNA-p1 TcCLB-NE.
506817.79_mRNA-p1 TcSYL_0088150.t1-p1
OG0002987: TCRU_7844 TcCLB-EL.469785.10_mRNA-p1 TcCLB-NE.
507025.20_mRNA-p1 TcSYL_0139490.t1-p1
OG0002988: TCRU_7847 TcCLB-EL.469785.40_mRNA-p1 TcCLB-NE.
507025.50_mRNA-p1 TcSYL_0139500.t1-p1
OG0002989: TCRU_7851 TcCLB-EL.508213.20_mRNA-p1 TcCLB-NE.
507515.60_mRNA-p1 TcSYL_0023070.t1-p1
OG0002990: TCRU_7853 TcCLB-EL.508211.70_mRNA-p1 TcCLB-NE.
507515.80_mRNA-p1 TcSYL_0023060.t1-p1
OG0002991: TCRU_7854 TcCLB-EL.508211.50_mRNA-p1 TcCLB-NE.
507515.100_mRNA-p1 TcSYL_0023050.t1-p1
OG0002992: TCRU_7863 TcCLB-EL.510289.50_mRNA-p1 TcCLB-NE.
510967.16_mRNA-p1 TcSYL_0113370.t1-p1
OG0002993: TCRU_7866 TcCLB-EL.510289.70_mRNA-p1 TcCLB-NE.
510969.10_mRNA-p1 TcSYL_0113380.t1-p1
OG0002994: TCRU_7870 TcCLB-EL.504203.29_mRNA-p1 TcCLB-NE.
503791.40_mRNA-p1 TcSYL_0113420.t1-p1
OG0002995: TCRU_7871 TcCLB-EL.504203.40_mRNA-p1 TcCLB-NE.
503791.30_mRNA-p1 TcSYL_0113430.t1-p1
OG0002996: TCRU_7893 TcCLB-EL.511217.190_mRNA-p1 TcCLB-NE.
506573.34_mRNA-p1 TcSYL_0189490.t1-p1
OG0002997: TCRU_7894 TcCLB-EL.511217.200_mRNA-p1 TcCLB-NE.
506573.30_mRNA-p1 TcSYL_0189510.t1-p1
OG0002998: TCRU_7898 TcCLB-NE.507491.20_mRNA-p1 TcSYL_0189810.t1-p1
TcSYL_0189940.t1-p1
OG0002999: TCRU_7899 TcCLB-EL.507275.30_mRNA-p1 TcCLB-NE.
507491.50_mRNA-p1 TcSYL_0190210.t1-p1
OG0003000: TCRU_7902 TcCLB-EL.507275.10_mRNA-p1 TcCLB-NE.
507491.90_mRNA-p1 TcSYL_0190220.t1-p1
OG0003001: TCRU_7903 TcCLB-EL.509393.50_mRNA-p1 TcCLB-NE.
507491.100_mRNA-p1 TcSYL_0190240.t1-p1
OG0003002: TCRU_7907 TcCLB-EL.509393.10_mRNA-p1 TcCLB-NE.
507491.140_mRNA-p1 TcSYL_0190250.t1-p1
OG0003003: TCRU_7912 TcCLB-EL.511419.20_mRNA-p1 TcCLB-NE.
505999.70_mRNA-p1 TcSYL_0111220.t1-p1
OG0003004: TCRU_7913 TcCLB-EL.511419.10_mRNA-p1 TcCLB-NE.
505999.60_mRNA-p1 TcSYL_0111240.t1-p1
OG0003005: TCRU_7925 TcCLB-EL.504181.40_mRNA-p1 TcCLB-NE.
509695.190_mRNA-p1 TcSYL_0171170.t1-p1
OG0003006: TCRU_7926 TcCLB-EL.504181.50_mRNA-p1 TcCLB-NE.
509695.200_mRNA-p1 TcSYL_0171190.t1-p1
OG0003007: TCRU_7931 TcCLB-EL.505965.70_pseudogenic_transcript-p1
TcCLB-NE.510903.30_mRNA-p1 TcSYL_0178620.t1-p1
OG0003008: TCRU_7932 TcCLB-EL.505965.60_mRNA-p1 TcCLB-NE.
510903.40_mRNA-p1 TcSYL_0178590.t1-p1
OG0003009: TCRU_7934 TcCLB-EL.505965.30_mRNA-p1 TcCLB-NE.
510903.60_mRNA-p1 TcSYL_0178540.t1-p1
OG0003010: TCRU_7935 TcCLB-EL.505965.20_mRNA-p1 TcCLB-NE.
510903.70_mRNA-p1 TcSYL_0178510.t1-p1
OG0003011: TCRU_7937 TcCLB-EL.511731.40_mRNA-p1 TcCLB-NE.

506833.9_mRNA-p1 TcSYL_0140480.t1-p1
OG0003012: TCRU_7941 TcCLB-EL.511731.10_mRNA-p1 TcCLB-NE.
506833.60_mRNA-p1 TcSYL_0140500.t1-p1
OG0003013: TCRU_7943 TcCLB-EL.511729.60_mRNA-p1 TcCLB-NE.
506833.79_mRNA-p1 TcSYL_0140510.t1-p1
OG0003014: TCRU_7944 TcCLB-EL.511729.50_mRNA-p1 TcCLB-NE.
506835.10_mRNA-p1 TcSYL_0140520.t1-p1
OG0003015: TCRU_7949 TcCLB-EL.506945.20_mRNA-p1 TcCLB-NE.
506835.70_mRNA-p1 TcSYL_0140550.t1-p1
OG0003016: TCRU_7950 TcCLB-EL.506945.30_mRNA-p1 TcCLB-NE.
506835.80_mRNA-p1 TcSYL_0140560.t1-p1
OG0003017: TCRU_7951 TcCLB-EL.506945.40_mRNA-p1 TcCLB-NE.
506835.90_mRNA-p1 TcSYL_0140570.t1-p1
OG0003018: TCRU_7962 TcCLB-EL.508169.60_mRNA-p1 TcCLB-NE.
506581.10_mRNA-p1 TcSYL_0113240.t1-p1
OG0003019: TCRU_7963 TcCLB-EL.508169.69_mRNA-p1 TcCLB-NE.
506581.14_mRNA-p1 TcSYL_0113250.t1-p1
OG0003020: TCRU_7965 TcCLB-EL.508169.90_mRNA-p1 TcCLB-NE.
506581.30_mRNA-p1 TcSYL_0113260.t1-p1
OG0003021: TCRU_7966 TcCLB-EL.508169.100_mRNA-p1 TcCLB-NE.
506581.40_mRNA-p1 TcSYL_0113270.t1-p1
OG0003022: TCRU_7967 TcCLB-EL.508169.110_mRNA-p1 TcCLB-NE.
506581.50_mRNA-p1 TcSYL_0113280.t1-p1
OG0003023: TCRU_7969 TcCLB-EL.510287.10_mRNA-p1 TcCLB-NE.
503891.120_mRNA-p1 TcSYL_0113300.t1-p1
OG0003024: TCRU_7971 TcCLB-EL.510287.30_mRNA-p1 TcCLB-NE.
503891.100_mRNA-p1 TcSYL_0113310.t1-p1
OG0003025: TCRU_7972 TcCLB-EL.510287.40_mRNA-p1 TcCLB-NE.
503891.90_mRNA-p1 TcSYL_0113320.t1-p1
OG0003026: TCRU_7973 TcCLB-EL.510287.60_mRNA-p1 TcCLB-NE.
503891.70_mRNA-p1 TcSYL_0113330.t1-p1
OG0003027: TCRU_7975 TcCLB-EL.506661.70_mRNA-p1 TcCLB-NE.
511245.90_mRNA-p1 TcSYL_0027090.t1-p1
OG0003028: TCRU_7979 TcCLB-EL.506661.110_mRNA-p1 TcCLB-NE.
511245.50_mRNA-p1 TcSYL_0027080.t1-p1
OG0003029: TCRU_7983 TcCLB-EL.506661.150_mRNA-p1 TcCLB-NE.
511245.20_mRNA-p1 TcSYL_0027070.t1-p1
OG0003030: TCRU_homolog_ TcCLB-EL.508515.20_mRNA-p1 TcCLB-NE.
508641.184_mRNA-p1 TcSYL_0047470.t1-p1
OG0003031: TCRU_nuclear TcCLB-EL.508515.10_mRNA-p1 TcCLB-NE.
508641.190_mRNA-p1 TcSYL_0047480.t1-p1
OG0003032: TCRU_7999 TcCLB-EL.503487.70_mRNA-p1 TcCLB-NE.
508641.200_mRNA-p1 TcSYL_0047490.t1-p1
OG0003033: TCRU_8001 TcCLB-EL.503487.40_mRNA-p1 TcCLB-NE.
508641.230_mRNA-p1 TcSYL_0047510.t1-p1
OG0003034: TCRU_8003 TcCLB-EL.503487.20_mRNA-p1 TcCLB-NE.
508641.250_mRNA-p1 TcSYL_0047520.t1-p1
OG0003035: TCRU_8004 TcCLB-EL.506203.120_mRNA-p1 TcCLB-NE.
508641.280_mRNA-p1 TcSYL_0047560.t1-p1
OG0003036: TCRU_8005 TcCLB-EL.506203.110_mRNA-p1 TcCLB-NE.
508641.290_mRNA-p1 TcSYL_0047570.t1-p1
OG0003037: TCRU_8006 TcCLB-EL.506203.100_mRNA-p1 TcCLB-NE.
508641.300_mRNA-p1 TcSYL_0047580.t1-p1
OG0003038: TCRU_8007 TcCLB-EL.506203.90_mRNA-p1 TcCLB-NE.

508641.310_mRNA-p1 TcSYL_0047590.t1-p1
OG0003039: TCRU_8008 TcCLB-EL.506203.80_mRNA-p1 TcCLB-NE.508641.320_mRNA-p1 TcSYL_0047600.t1-p1
OG0003040: TCRU_8009 TcCLB-EL.506203.70_mRNA-p1 TcCLB-NE.508643.9_mRNA-p1 TcSYL_0047610.t1-p1
OG0003041: TCRU_8010 TcCLB-EL.506203.60_mRNA-p1 TcCLB-NE.508643.20_mRNA-p1 TcSYL_0047630.t1-p1
OG0003042: TCRU_8011 TcCLB-EL.506203.50_mRNA-p1 TcCLB-NE.508643.30_mRNA-p1 TcSYL_0047640.t1-p1
OG0003043: TCRU_8012 TcCLB-EL.506203.30_mRNA-p1 TcCLB-NE.508645.10_mRNA-p1 TcSYL_0047670.t1-p1
OG0003044: TCRU_8014 TcCLB-EL.506203.20_mRNA-p1 TcCLB-NE.508645.30_mRNA-p1 TcSYL_0047690.t1-p1
OG0003045: TCRU_8016 TcCLB-EL.506359.50_mRNA-p1 TcCLB-NE.503521.60_mRNA-p1 TcSYL_0015060.t1-p1
OG0003046: TCRU_8017 TcCLB-EL.506359.60_mRNA-p1 TcCLB-NE.503521.50_mRNA-p1 TcSYL_0015070.t1-p1
OG0003047: TCRU_8019 TcCLB-EL.506359.80_mRNA-p1 TcCLB-NE.503521.30_mRNA-p1 TcSYL_0015080.t1-p1
OG0003048: TCRU_N-terminus/PTH.1 TcCLB-EL.507507.30_mRNA-p1 TcCLB-NE.506871.40_mRNA-p1 TcSYL_0142570.t1-p1
OG0003049: TCRU_8037 TcCLB-EL.509491.10_mRNA-p1 TcCLB-NE.506871.10_mRNA-p1 TcSYL_0142600.t1-p1
OG0003050: TCRU_8067 TcCLB-EL.509979.130_mRNA-p1 TcCLB-NE.510039.60_mRNA-p1 TcSYL_0156500.t1-p1
OG0003051: TCRU_8069 TcCLB-EL.509979.110_mRNA-p1 TcCLB-NE.510039.70_mRNA-p1 TcSYL_0156510.t1-p1
OG0003052: TCRU_8077 TcCLB-EL.503831.40_pseudogenic_transcript-p1 TcCLB-NE.508045.100_mRNA-p1 TcSYL_0173560.t1-p1
OG0003053: TCRU_8078 TcCLB-EL.503831.20_mRNA-p1 TcCLB-NE.508045.90_mRNA-p1 TcSYL_0173540.t1-p1
OG0003054: TCRU_8079 TcCLB-EL.503829.89_mRNA-p1 TcCLB-NE.508045.79_mRNA-p1 TcSYL_0173530.t1-p1
OG0003055: TCRU_8081 TcCLB-EL.503829.40_mRNA-p1 TcCLB-NE.508045.30_mRNA-p1 TcSYL_0173420.t1-p1
OG0003056: TCRU_8083 TcCLB-EL.504427.260_mRNA-p1 TcCLB-NE.508043.40_mRNA-p1 TcSYL_0173270.t1-p1
OG0003057: TCRU_8084 TcCLB-EL.504427.250_mRNA-p1 TcCLB-NE.508043.30_mRNA-p1 TcSYL_0173220.t1-p1
OG0003058: TCRU_8085 TcCLB-EL.504427.240_mRNA-p1 TcCLB-NE.508043.20_mRNA-p1 TcSYL_0173200.t1-p1
OG0003059: TCRU_8098 TcCLB-EL.509595.70_mRNA-p1 TcCLB-NE.508817.160_mRNA-p1 TcSYL_0043390.t1-p1
OG0003060: TCRU_8099 TcCLB-EL.509597.10_mRNA-p1 TcCLB-NE.508817.140_mRNA-p1 TcSYL_0043480.t1-p1
OG0003061: TCRU_8117 TcCLB-EL.510431.320_mRNA-p1 TcCLB-NE.507771.110_mRNA-p1 TcSYL_0138360.t1-p1
OG0003062: TCRU_8127 TcCLB-NE.507019.83_mRNA-p1 TcCLB-NE.507019.86_mRNA-p1 TcSYL_0168900.t1-p1
OG0003063: TCRU_8142 TcCLB-EL.506779.20_mRNA-p1 TcCLB-NE.511155.10_pseudogenic_transcript-p1 TcSYL_0076760.t1-p1
OG0003064: TCRU_8143 TcCLB-EL.506779.30_pseudogenic_transcript-p1 TcCLB-NE.511155.4_mRNA-p1 TcSYL_0076810.t1-p1
OG0003065: TCRU_8144 TcCLB-EL.506779.50_mRNA-p1 TcCLB-NE.

511153.140_mRNA-p1 TcSYL_0076860.t1-p1
OG0003066: TCRU_8146 TcCLB-EL.506779.80_mRNA-p1 TcCLB-NE.
511153.120_mRNA-p1 TcSYL_0076950.t1-p1
OG0003067: TCRU_8147 TcCLB-EL.506779.90_mRNA-p1 TcCLB-NE.
511153.110_mRNA-p1 TcSYL_0076960.t1-p1
OG0003068: TCRU_8149 TcCLB-EL.506779.110_mRNA-p1 TcCLB-NE.
511153.90_mRNA-p1 TcSYL_0076970.t1-p1
OG0003069: TCRU_8153 TcCLB-EL.506779.170_mRNA-p1 TcCLB-NE.
511153.50_mRNA-p1 TcSYL_0077030.t1-p1
OG0003070: TCRU_8175 TcCLB-NE.507067.40_mRNA-p1 TcCLB-NE.
508435.60_mRNA-p1 TcCLB-NE.510693.200_mRNA-p1
OG0003071: TCRU_8176 TcCLB-EL.506465.30_mRNA-p1 TcCLB-NE.
507317.50_mRNA-p1 TcSYL_0104180.t1-p1
OG0003072: TCRU_8177 TcCLB-EL.506465.20_mRNA-p1 TcCLB-NE.
507317.40_mRNA-p1 TcSYL_0104130.t1-p1
OG0003073: TCRU_8178 TcCLB-EL.506465.14_mRNA-p1 TcCLB-NE.
507317.34_mRNA-p1 TcSYL_0104100.t1-p1
OG0003074: TCRU_8179 TcCLB-EL.506465.10_mRNA-p1 TcCLB-NE.
507317.30_mRNA-p1 TcSYL_0104080.t1-p1
OG0003075: TCRU_8184 TcCLB-NE.504221.20_mRNA-p1 TcCLB-NE.
508409.9_mRNA-p1 TcSYL_0004570.t1-p1
OG0003076: TCRU_8186 TcCLB-EL.507795.30_mRNA-p1 TcCLB-NE.
510647.60_mRNA-p1 TcSYL_0004600.t1-p1
OG0003077: TCRU_8187 TcCLB-EL.507795.40_mRNA-p1 TcCLB-NE.
510647.50_mRNA-p1 TcSYL_0004610.t1-p1
OG0003078: TCRU_8188 TcCLB-EL.507795.50_mRNA-p1 TcCLB-NE.
510647.40_mRNA-p1 TcSYL_0004620.t1-p1
OG0003079: TCRU_8189 TcCLB-EL.507795.60_pseudogenic_transcript-p1
TcCLB-NE.510647.30_mRNA-p1 TcSYL_0004640.t1-p1
OG0003080: TCRU_8191 TcCLB-EL.507795.90_mRNA-p1 TcCLB-NE.
510647.10_mRNA-p1 TcSYL_0004650.t1-p1
OG0003081: TCRU_8192 TcCLB-EL.507795.100_mRNA-p1 TcCLB-NE.
510645.49_mRNA-p1 TcSYL_0004660.t1-p1
OG0003082: TCRU_8193 TcCLB-EL.509761.10_mRNA-p1 TcCLB-NE.
510645.40_pseudogenic_transcript-p1 TcSYL_0004670.t1-p1
OG0003083: TCRU_8202 TcCLB-EL.511047.40_mRNA-p1 TcCLB-NE.
511859.170_mRNA-p1 TcSYL_0091100.t1-p1
OG0003084: TCRU_8206 TcCLB-EL.503579.100_mRNA-p1 TcCLB-NE.
507517.90_mRNA-p1 TcSYL_0022850.t1-p1
OG0003085: TCRU_8208 TcCLB-EL.503579.130_mRNA-p1 TcCLB-NE.
507517.110_mRNA-p1 TcSYL_0022840.t1-p1
OG0003086: TCRU_8217 TcCLB-EL.508647.260_mRNA-p1 TcCLB-NE.
505183.10_mRNA-p1 TcSYL_0047930.t1-p1
OG0003087: TCRU_8218 TcCLB-EL.508647.264_mRNA-p1 TcCLB-NE.
505183.14_mRNA-p1 TcSYL_0047940.t1-p1
OG0003088: TCRU_8220 TcCLB-EL.508647.280_mRNA-p1 TcCLB-NE.
505183.30_mRNA-p1 TcSYL_0047970.t1-p1
OG0003089: TCRU_8221 TcCLB-EL.508647.290_mRNA-p1 TcCLB-NE.
505183.40_mRNA-p1 TcSYL_0047980.t1-p1
OG0003090: TCRU_8252 TcCLB-EL.506755.250_mRNA-p1 TcCLB-NE.
510359.260_mRNA-p1 TcSYL_0112840.t1-p1
OG0003091: TCRU_8254 TcCLB-EL.506755.230_mRNA-p1 TcCLB-NE.
510359.240_mRNA-p1 TcSYL_0112830.t1-p1
OG0003092: TCRU_8262 TcCLB-EL.503975.20_mRNA-p1 TcCLB-NE.

508669.30_mRNA-p1 TcSYL_0086950.t1-p1
OG0003093: TCRU_8263 TcCLB-EL.508809.40_mRNA-p1 TcCLB-NE.
508669.10_mRNA-p1 TcSYL_0087050.t1-p1
OG0003094: TCRU_8269 TcCLB-EL.506895.20_mRNA-p1 TcCLB-NE.
508501.270_mRNA-p1 TcSYL_0110350.t1-p1
OG0003095: TCRU_8270 TcCLB-EL.506895.30_mRNA-p1 TcCLB-NE.
508501.280_mRNA-p1 TcSYL_0110360.t1-p1
OG0003096: TCRU_8271 TcCLB-EL.506895.40_mRNA-p1 TcCLB-NE.
508501.290_mRNA-p1 TcSYL_0110370.t1-p1
OG0003097: TCRU_8272 TcCLB-EL.506895.50_mRNA-p1 TcCLB-NE.
508501.300_mRNA-p1 TcSYL_0110390.t1-p1
OG0003098: TCRU_8283 TcCLB-EL.509595.20_mRNA-p1 TcCLB-NE.
508819.40_mRNA-p1 TcSYL_0043230.t1-p1
OG0003099: TCRU_8284 TcCLB-EL.509595.40_mRNA-p1 TcCLB-NE.
508819.20_mRNA-p1 TcSYL_0043290.t1-p1
OG0003100: TCRU_8290 TcCLB-EL.511715.50_pseudogenic_transcript-p1
TcCLB-NE.508445.60_mRNA-p1 TcSYL_0141700.t1-p1
OG0003101: TCRU_8291 TcCLB-EL.511715.70_mRNA-p1 TcCLB-NE.
508445.70_mRNA-p1 TcSYL_0141710.t1-p1
OG0003102: TCRU_8292 TcCLB-EL.511715.80_mRNA-p1 TcCLB-NE.
508445.80_mRNA-p1 TcSYL_0141720.t1-p1
OG0003103: TCRU_8293 TcCLB-EL.511715.90_mRNA-p1 TcCLB-NE.
508445.90_mRNA-p1 TcSYL_0141730.t1-p1
OG0003104: TCRU_8319 TcCLB-EL.437121.9_mRNA-p1 TcCLB-NE.
510329.10_mRNA-p1 TcSYL_0001160.t1-p1
OG0003105: TCRU_8322 TcCLB-EL.507089.40_mRNA-p1 TcCLB-NE.
504085.60_mRNA-p1 TcSYL_0112040.t1-p1
OG0003106: TCRU_8324 TcCLB-EL.511529.259_mRNA-p1 TcCLB-NE.
506727.4_mRNA-p1 TcSYL_0112050.t1-p1
OG0003107: TCRU_8326 TcCLB-EL.511529.240_mRNA-p1 TcCLB-NE.
506727.20_mRNA-p1 TcSYL_0112060.t1-p1
OG0003108: TCRU_8328 TcCLB-EL.511529.220_mRNA-p1 TcCLB-NE.
506727.40_mRNA-p1 TcSYL_0112070.t1-p1
OG0003109: TCRU_8329 TcCLB-EL.511529.210_mRNA-p1 TcCLB-NE.
506727.50_mRNA-p1 TcSYL_0112080.t1-p1
OG0003110: TCRU_8331 TcCLB-EL.511529.190_mRNA-p1 TcCLB-NE.
506727.70_mRNA-p1 TcSYL_0112090.t1-p1
OG0003111: TCRU_8335 TcCLB-EL.511529.140_mRNA-p1 TcCLB-NE.
506727.120_mRNA-p1 TcSYL_0112120.t1-p1
OG0003112: TCRU_8337 TcCLB-EL.511529.110_mRNA-p1 TcCLB-NE.
506727.150_mRNA-p1 TcSYL_0112150.t1-p1
OG0003113: TCRU_8339 TcCLB-EL.511529.90_mRNA-p1 TcCLB-NE.
511029.10_mRNA-p1 TcSYL_0112170.t1-p1
OG0003114: TCRU_8342 TcCLB-EL.505983.20_mRNA-p1 TcCLB-NE.
504839.50_mRNA-p1 TcSYL_0064410.t1-p1
OG0003115: TCRU_8359 TcCLB-EL.507881.110_pseudogenic_transcript-p1
TcCLB-NE.506193.5_pseudogenic_transcript-p1 TcCLB-NE.
509941.85_pseudogenic_transcript-p1
OG0003116: TCRU_8363 TcCLB-EL.508277.70_mRNA-p1 TcCLB-NE.
503541.30_mRNA-p1 TcSYL_0064030.t1-p1
OG0003117: TCRU_8369 TcCLB-EL.510285.60_mRNA-p1 TcCLB-NE.
503823.160_mRNA-p1 TcSYL_0117680.t1-p1
OG0003118: TCRU_8386 TcCLB-EL.503619.10_mRNA-p1 TcCLB-NE.
511647.30_mRNA-p1 TcSYL_0014080.t1-p1

OG0003119: TCRU_8387 TcCLB-EL.503619.20_mRNA-p1 TcCLB-NE.
511647.40_mRNA-p1 TcSYL_0014070.t1-p1
OG0003120: TCRU_8400 TcCLB-EL.506943.160_mRNA-p1 TcCLB-NE.
509065.160_mRNA-p1 TcSYL_0140150.t1-p1
OG0003121: TCRU_8403 TcCLB-EL.506943.110_mRNA-p1 TcCLB-NE.
509065.120_mRNA-p1 TcSYL_0140130.t1-p1
OG0003122: TCRU_8404 TcCLB-EL.506943.100_mRNA-p1 TcCLB-NE.
509065.110_mRNA-p1 TcSYL_0140120.t1-p1
OG0003123: TCRU_8415 TcCLB-EL.511733.20_mRNA-p1 TcCLB-NE.
511523.90_mRNA-p1 TcSYL_0140050.t1-p1
OG0003124: TCRU_8417 TcCLB-EL.511733.50_mRNA-p1 TcCLB-NE.
511523.60_mRNA-p1 TcSYL_0140040.t1-p1
OG0003125: TCRU_8419 TcCLB-EL.511733.70_mRNA-p1 TcCLB-NE.
511523.40_mRNA-p1 TcSYL_0140030.t1-p1
OG0003126: TCRU_8420 TcCLB-EL.511733.80_mRNA-p1 TcCLB-NE.
511523.30_mRNA-p1 TcSYL_0140020.t1-p1
OG0003127: TCRU_8421 TcCLB-EL.511733.90_mRNA-p1 TcCLB-NE.
511523.20_mRNA-p1 TcSYL_0139990.t1-p1
OG0003128: TCRU_8422 TcCLB-EL.511733.100_mRNA-p1 TcCLB-NE.
511523.14_mRNA-p1 TcSYL_0139960.t1-p1
OG0003129: TCRU_8423 TcCLB-EL.511733.110_mRNA-p1 TcCLB-NE.
511523.10_mRNA-p1 TcSYL_0139930.t1-p1
OG0003130: TCRU_8424 TcCLB-EL.511733.120_mRNA-p1 TcCLB-NE.
511523.4_mRNA-p1 TcSYL_0139920.t1-p1
OG0003131: TCRU_8425 TcCLB-EL.511735.10_mRNA-p1 TcCLB-NE.
509063.30_mRNA-p1 TcSYL_0139910.t1-p1
OG0003132: TCRU_8426 TcCLB-EL.511735.20_mRNA-p1 TcCLB-NE.
509063.20_mRNA-p1 TcSYL_0139900.t1-p1
OG0003133: TCRU_8427 TcCLB-EL.506829.90_mRNA-p1 TcCLB-NE.
508077.9_mRNA-p1 TcCLB-NE.508079.10_mRNA-p1
OG0003134: TCRU_8433 TcCLB-EL.506829.20_mRNA-p1 TcCLB-NE.
504231.30_mRNA-p1 TcSYL_0109450.t1-p1
OG0003135: TCRU_8435 TcCLB-EL.509805.70_mRNA-p1 TcCLB-NE.
503939.130_mRNA-p1 TcSYL_0015230.t1-p1
OG0003136: TCRU_8437 TcCLB-EL.509805.84_mRNA-p1 TcCLB-NE.
503939.114_mRNA-p1 TcSYL_0015240.t1-p1
OG0003137: TCRU_8452 TcCLB-EL.506735.50_mRNA-p1 TcCLB-NE.
509229.40_mRNA-p1 TcSYL_0091800.t1-p1
OG0003138: TCRU_8459 TcCLB-EL.511693.20_mRNA-p1 TcCLB-NE.
508177.64_mRNA-p1 TcSYL_0183640.t1-p1
OG0003139: TCRU_8460 TcCLB-EL.511693.10_mRNA-p1 TcCLB-NE.
508177.70_mRNA-p1 TcSYL_0183650.t1-p1
OG0003140: TCRU_8462 TcCLB-EL.511691.30_mRNA-p1 TcCLB-NE.
508177.100_mRNA-p1 TcSYL_0186390.t1-p1
OG0003141: TCRU_8470 TcCLB-EL.509891.20_pseudogenic_transcript-p1
TcCLB-NE.510691.30_mRNA-p1 TcSYL_0047240.t1-p1
OG0003142: TCRU_8473 TcCLB-EL.509205.30_mRNA-p1 TcCLB-NE.
508699.40_mRNA-p1 TcSYL_0180710.t1-p1
OG0003143: TCRU_8474 TcCLB-EL.509205.40_mRNA-p1 TcCLB-NE.
508699.50_mRNA-p1 TcSYL_0180740.t1-p1
OG0003144: TCRU_8475 TcCLB-EL.509205.50_mRNA-p1 TcCLB-NE.
508699.60_mRNA-p1 TcSYL_0180790.t1-p1
OG0003145: TCRU_8476 TcCLB-EL.509205.60_mRNA-p1 TcCLB-NE.
508699.70_mRNA-p1 TcSYL_0180800.t1-p1

OG0003146: TCRU_8477 TcCLB-EL.509205.70_mRNA-p1 TcCLB-NE.
508699.80_mRNA-p1 TcSYL_0180830.t1-p1
OG0003147: TCRU_8494 TcCLB-EL.506947.110_pseudogenic_transcript-p1
TcCLB-EL.507445.50_mRNA-p1 TcCLB-NE.507057.4_mRNA-p1
OG0003148: TCRU_8495 TcCLB-EL.506947.100_mRNA-p1 TcCLB-NE.
507057.10_mRNA-p1 TcSYL_0014600.t1-p1
OG0003149: TCRU_8496 TcCLB-EL.506947.90_mRNA-p1 TcCLB-NE.
507057.20_mRNA-p1 TcSYL_0014590.t1-p1
OG0003150: TCRU_8497 TcCLB-EL.506947.70_mRNA-p1 TcCLB-NE.
507057.40_mRNA-p1 TcSYL_0014560.t1-p1
OG0003151: TCRU_8498 TcCLB-EL.506947.60_mRNA-p1 TcCLB-NE.
507057.50_mRNA-p1 TcSYL_0014550.t1-p1
OG0003152: TCRU_8515 TcCLB-EL.508387.40_mRNA-p1 TcCLB-NE.
506789.260_mRNA-p1 TcSYL_0162990.t1-p1
OG0003153: TCRU_8516 TcCLB-EL.508387.30_mRNA-p1 TcCLB-NE.
506789.270_mRNA-p1 TcSYL_0163020.t1-p1
OG0003154: TCRU_8517 TcCLB-EL.508387.20_mRNA-p1 TcCLB-NE.
506789.280_mRNA-p1 TcSYL_0163070.t1-p1
OG0003155: TCRU_8518 TcCLB-EL.510617.10_mRNA-p1 TcCLB-NE.
506789.300_mRNA-p1 TcSYL_0163150.t1-p1
OG0003156: TCRU_8519 TcCLB-EL.445869.10_mRNA-p1 TcCLB-NE.
506789.310_mRNA-p1 TcSYL_0163170.t1-p1
OG0003157: TCRU_family_ TcCLB-EL.504103.6_mRNA-p1 TcCLB-EL.
508307.184_mRNA-p1 TcSYL_0005980.t1-p1
OG0003158: TCRU_8530 TcCLB-EL.504213.50_mRNA-p1 TcCLB-NE.
508857.130_mRNA-p1 TcSYL_0026700.t1-p1
OG0003159: TCRU_8531 TcCLB-EL.504213.60_mRNA-p1 TcCLB-NE.
508857.140_mRNA-p1 TcSYL_0026620.t1-p1
OG0003160: TCRU_8532 TcCLB-EL.504213.70_mRNA-p1 TcCLB-NE.
508857.150_mRNA-p1 TcSYL_0026590.t1-p1
OG0003161: TCRU_8535 TcCLB-EL.507677.140_mRNA-p1 TcCLB-NE.
504021.20_mRNA-p1 TcSYL_0009690.t1-p1
OG0003162: TCRU_8536 TcCLB-EL.507677.129_mRNA-p1 TcCLB-NE.
504021.30_mRNA-p1 TcSYL_0009780.t1-p1
OG0003163: TCRU_8537 TcCLB-EL.507677.120_mRNA-p1 TcCLB-NE.
504021.40_mRNA-p1 TcSYL_0009810.t1-p1
OG0003164: TCRU_8538 TcCLB-EL.507677.110_mRNA-p1 TcCLB-NE.
504021.50_mRNA-p1 TcSYL_0009860.t1-p1
OG0003165: TCRU_8539 TcCLB-EL.507677.90_mRNA-p1 TcCLB-NE.
504021.70_mRNA-p1 TcSYL_0009890.t1-p1
OG0003166: TCRU_8540 TcCLB-EL.507677.80_mRNA-p1 TcCLB-NE.
504021.74_mRNA-p1 TcSYL_0009910.t1-p1
OG0003167: TCRU_8541 TcCLB-EL.507677.70_mRNA-p1 TcCLB-NE.
504021.80_mRNA-p1 TcSYL_0009950.t1-p1
OG0003168: TCRU_8542 TcCLB-EL.507677.60_mRNA-p1 TcCLB-NE.
504021.90_mRNA-p1 TcSYL_0009970.t1-p1
OG0003169: TCRU_8545 TcCLB-EL.507677.30_mRNA-p1 TcCLB-NE.
504021.120_mRNA-p1 TcSYL_0010060.t1-p1
OG0003170: TCRU_8546 TcCLB-EL.507677.20_mRNA-p1 TcCLB-NE.
504021.130_mRNA-p1 TcSYL_0010090.t1-p1
OG0003171: TCRU_8547 TcCLB-EL.507677.10_mRNA-p1 TcCLB-NE.
504021.140_mRNA-p1 TcSYL_0010100.t1-p1
OG0003172: TCRU_8550 TcCLB-EL.504883.10_mRNA-p1 TcCLB-NE.
510045.30_mRNA-p1 TcSYL_0064980.t1-p1

OG0003173: TCRU_8555 TcCLB-EL.506775.50_mRNA-p1 TcCLB-NE.
511167.119_mRNA-p1 TcSYL_0075370.t1-p1
OG0003174: TCRU_8561 TcCLB-EL.507993.390_mRNA-p1 TcCLB-NE.
511281.60_mRNA-p1 TcSYL_0123770.t1-p1
OG0003175: TCRU_8562 TcCLB-EL.507993.380_mRNA-p1 TcCLB-NE.
511281.59_mRNA-p1 TcSYL_0123780.t1-p1
OG0003176: TCRU_8570 TcCLB-EL.506739.209_mRNA-p1 TcCLB-NE.
510821.10_mRNA-p1 TcSYL_0050120.t1-p1
OG0003177: TCRU_8571 TcCLB-EL.506739.200_mRNA-p1 TcCLB-NE.
506315.100_mRNA-p1 TcSYL_0050110.t1-p1
OG0003178: TCRU_8588 TcCLB-EL.503885.100_mRNA-p1 TcCLB-NE.
511903.300_pseudogenic_transcript-p1 TcSYL_0102740.t1-p1
OG0003179: TCRU_8589 TcCLB-EL.503885.90_mRNA-p1 TcCLB-NE.
511903.290_mRNA-p1 TcSYL_0102790.t1-p1
OG0003180: TCRU_8590 TcCLB-EL.503885.80_mRNA-p1 TcCLB-NE.
511903.280_mRNA-p1 TcSYL_0102810.t1-p1
OG0003181: TCRU_8591 TcCLB-EL.503885.70_mRNA-p1 TcCLB-NE.
511903.270_mRNA-p1 TcSYL_0102850.t1-p1
OG0003182: TCRU_8592 TcCLB-EL.503885.60_mRNA-p1 TcCLB-NE.
511903.260_mRNA-p1 TcSYL_0102880.t1-p1
OG0003183: TCRU_8593 TcCLB-EL.503885.50_mRNA-p1 TcCLB-NE.
511903.250_mRNA-p1 TcSYL_0102900.t1-p1
OG0003184: TCRU_8594 TcCLB-EL.503885.40_mRNA-p1 TcCLB-NE.
511903.240_mRNA-p1 TcSYL_0102920.t1-p1
OG0003185: TCRU_8596 TcCLB-EL.503885.20_mRNA-p1 TcCLB-NE.
511903.220_mRNA-p1 TcSYL_0102980.t1-p1
OG0003186: TCRU_8598 TcCLB-EL.511065.10_mRNA-p1 TcCLB-NE.
511903.190_mRNA-p1 TcSYL_0103070.t1-p1
OG0003187: TCRU_8599 TcCLB-EL.511065.23_mRNA-p1 TcCLB-NE.
511903.180_mRNA-p1 TcSYL_0103100.t1-p1
OG0003188: TCRU_8602 TcCLB-EL.511067.10_mRNA-p1 TcCLB-NE.
511903.140_pseudogenic_transcript-p1 TcSYL_0103260.t1-p1
OG0003189: TCRU_8622 TcCLB-EL.510431.270_mRNA-p1 TcCLB-NE.
507773.40_mRNA-p1 TcSYL_0138340.t1-p1
OG0003190: TCRU_8623 TcCLB-EL.510431.260_mRNA-p1 TcCLB-NE.
507773.50_mRNA-p1 TcSYL_0138330.t1-p1
OG0003191: TCRU_8624 TcCLB-EL.510431.250_mRNA-p1 TcCLB-NE.
507773.60_mRNA-p1 TcSYL_0138310.t1-p1
OG0003192: TCRU_8643 TcCLB-EL.509677.10_mRNA-p1 TcCLB-NE.
506679.270_mRNA-p1 TcSYL_0114260.t1-p1
OG0003193: TCRU_8644 TcCLB-EL.503611.50_mRNA-p1 TcCLB-NE.
506679.260_mRNA-p1 TcSYL_0114240.t1-p1
OG0003194: TCRU_8645 TcCLB-EL.506753.230_mRNA-p1 TcCLB-NE.
510357.130_mRNA-p1 TcSYL_0112720.t1-p1
OG0003195: TCRU_8647 TcCLB-EL.508987.20_mRNA-p1 TcCLB-NE.
509567.10_mRNA-p1 TcSYL_0096660.t1-p1
OG0003196: TCRU_8648 TcCLB-EL.503897.120_mRNA-p1 TcCLB-NE.
509561.70_mRNA-p1 TcSYL_0096000.t1-p1
OG0003197: TCRU_8649 TcCLB-EL.503571.19_mRNA-p1 TcCLB-NE.
509561.9_mRNA-p1 TcSYL_0095960.t1-p1
OG0003198: TCRU_8650 TcCLB-EL.506867.10_mRNA-p1 TcCLB-NE.
507623.140_mRNA-p1 TcSYL_0095940.t1-p1
OG0003199: TCRU_8662 TcCLB-EL.506483.10_mRNA-p1 TcCLB-NE.
503809.158_mRNA-p1 TcCLB-NE.511299.10_mRNA-p1

OG0003200: TCRU_8665 TcCLB-EL.506265.30_mRNA-p1 TcCLB-NE.
506435.30_mRNA-p1 TcSYL_0197020.t1-p1
OG0003201: TCRU_8670 TcCLB-EL.508839.80_mRNA-p1 TcCLB-NE.
511381.50_mRNA-p1 TcSYL_0188330.t1-p1
OG0003202: TCRU_8671 TcCLB-EL.510755.120_mRNA-p1 TcCLB-NE.
508413.50_mRNA-p1 TcSYL_0115560.t1-p1
OG0003203: TCRU_8673 TcCLB-EL.507089.70_mRNA-p1 TcCLB-NE.
504085.30_mRNA-p1 TcSYL_0112020.t1-p1
OG0003204: TCRU_8675 TcCLB-EL.507089.154_mRNA-p1 TcCLB-NE.
506725.30_mRNA-p1 TcSYL_0111990.t1-p1
OG0003205: TCRU_8683 TcCLB-EL.508463.29_mRNA-p1 TcCLB-NE.
510659.130_mRNA-p1 TcSYL_0116130.t1-p1
OG0003206: TCRU_8686 TcCLB-EL.508465.40_mRNA-p1 TcCLB-NE.
510659.20_mRNA-p1 TcSYL_0116040.t1-p1
OG0003207: TCRU_8688 TcCLB-EL.508465.70_mRNA-p1 TcCLB-NE.
507001.130_mRNA-p1 TcSYL_0116010.t1-p1
OG0003208: TCRU_8689 TcCLB-EL.511313.40_mRNA-p1 TcCLB-NE.
511755.60_mRNA-p1 TcSYL_0159640.t1-p1
OG0003209: TCRU_8691 TcCLB-EL.510953.30_mRNA-p1 TcCLB-NE.
509005.70_mRNA-p1 TcSYL_0138850.t1-p1
OG0003210: TCRU_8699 TcCLB-EL.503981.40_mRNA-p1 TcCLB-NE.
504131.100_mRNA-p1 TcSYL_0048090.t1-p1
OG0003211: TCRU_8704 TcCLB-EL.508237.18_mRNA-p1 TcCLB-NE.
506905.59_mRNA-p1 TcCLB-NE.508795.10_pseudogenic_transcript-p1
OG0003212: TCRU_8705 TcCLB-EL.506515.10_mRNA-p1 TcCLB-NE.
511321.10_mRNA-p1 TcSYL_0159160.t1-p1
OG0003213: TCRU_8710 TcCLB-EL.508209.130_mRNA-p1 TcCLB-NE.
509505.20_mRNA-p1 TcSYL_0023010.t1-p1
OG0003214: TCRU_8715 TcCLB-EL.508207.140_mRNA-p1 TcCLB-NE.
507519.70_mRNA-p1 TcSYL_0022700.t1-p1
OG0003215: TCRU_8716 TcCLB-EL.508207.90_mRNA-p1 TcCLB-NE.
507519.120_mRNA-p1 TcSYL_0022660.t1-p1
OG0003216: TCRU_8717 TcCLB-EL.508207.74_mRNA-p1 TcCLB-NE.
507519.140_mRNA-p1 TcSYL_0022650.t1-p1
OG0003217: TCRU_8723 TcCLB-EL.503971.10_mRNA-p1 TcCLB-NE.
503997.19_mRNA-p1 TcSYL_0121260.t1-p1
OG0003218: TCRU_8725 TcCLB-EL.511421.80_pseudogenic_transcript-p1
TcSYL_0111080.t1-p1 TcSYL_0111090.t1-p1
OG0003219: TCRU_8726 TcCLB-EL.508975.10_mRNA-p1 TcCLB-NE.
508547.30_mRNA-p1 TcSYL_0202550.t1-p1
OG0003220: TCRU_8727 TcCLB-NE.508547.50_mRNA-p1 TcSYL_0202530.t1-p1
TcSYL_0206280.t1-p1
OG0003221: TCRU_C TcCLB-EL.508973.80_mRNA-p1 TcCLB-NE.
508547.80_mRNA-p1 TcSYL_0202520.t1-p1
OG0003222: TCRU_8729 TcCLB-EL.503901.9_pseudogenic_transcript-p1
TcCLB-NE.509507.10_mRNA-p1 TcSYL_0022810.t1-p1
OG0003223: TCRU_8737 TcCLB-EL.511395.30_mRNA-p1 TcCLB-NE.
505071.90_mRNA-p1 TcSYL_0202110.t1-p1
OG0003224: TCRU_8738 TcCLB-EL.511395.60_mRNA-p1 TcCLB-NE.
505071.60_mRNA-p1 TcSYL_0202130.t1-p1
OG0003225: TCRU_8742 TcCLB-EL.509551.20_mRNA-p1 TcCLB-NE.
507007.108_mRNA-p1 TcCLB-NE.510733.5_mRNA-p1
OG0003226: TCRU_8746 TcCLB-EL.506773.80_mRNA-p1 TcCLB-NE.
508799.130_mRNA-p1 TcSYL_0075300.t1-p1

OG0003227: TCRU_8749 TcCLB-EL.511127.360_mRNA-p1 TcCLB-NE.
503657.20_mRNA-p1 TcSYL_0156030.t1-p1
OG0003228: TCRU_8758 TcCLB-EL.511867.10_mRNA-p1 TcCLB-NE.
507775.10_mRNA-p1 TcSYL_0146400.t1-p1
OG0003229: TCRU_8760 TcCLB-EL.511555.90_mRNA-p1 TcCLB-NE.
506621.60_mRNA-p1 TcSYL_0073360.t1-p1
OG0003230: TCRU_8762 TcCLB-EL.510515.60_mRNA-p1 TcCLB-NE.
509859.40_mRNA-p1 TcSYL_0174550.t1-p1
OG0003231: TCRU_8764 TcCLB-EL.509167.80_mRNA-p1 TcCLB-NE.
510443.30_mRNA-p1 TcSYL_0140420.t1-p1
OG0003232: TCRU_8765 TcCLB-EL.509167.70_mRNA-p1 TcCLB-NE.
510443.40_mRNA-p1 TcSYL_0140430.t1-p1
OG0003233: TCRU_8766 TcCLB-EL.509167.20_mRNA-p1 TcCLB-NE.
506831.40_mRNA-p1 TcSYL_0140470.t1-p1
OG0003234: TCRU_8767 TcCLB-EL.509167.10_mRNA-p1 TcCLB-EL.
511731.89_mRNA-p1 TcCLB-NE.506831.50_mRNA-p1
OG0003235: TCRU_8773 TcCLB-EL.505807.50_mRNA-p1 TcCLB-NE.
509033.60_mRNA-p1 TcSYL_0166820.t1-p1
OG0003236: TCRU_8778 TcCLB-EL.504163.100_mRNA-p1 TcCLB-NE.
510301.70_mRNA-p1 TcSYL_0114320.t1-p1
OG0003237: TCRU_8779 TcCLB-EL.506223.120_mRNA-p1 TcCLB-NE.
511719.10_mRNA-p1 TcSYL_0141910.t1-p1
OG0003238: TCRU_8780 TcCLB-EL.506223.110_mRNA-p1 TcCLB-NE.
511717.200_mRNA-p1 TcSYL_0141890.t1-p1
OG0003239: TCRU_8785 TcCLB-EL.511001.200_mRNA-p1 TcCLB-NE.
509017.24_mRNA-p1 TcSYL_0131450.t1-p1
OG0003240: TCRU_8786 TcCLB-EL.511001.230_mRNA-p1 TcCLB-NE.
511465.20_mRNA-p1 TcSYL_0131400.t1-p1
OG0003241: TCRU_8790 TcCLB-EL.510141.20_mRNA-p1 TcCLB-NE.
508409.170_mRNA-p1 TcSYL_0004400.t1-p1
OG0003242: TCRU_family_ TcCLB-EL.510141.4_mRNA-p1 TcCLB-NE.
508409.150_mRNA-p1 TcSYL_0004420.t1-p1
OG0003243: TCRU_8793 TcCLB-EL.503793.20_mRNA-p1 TcCLB-NE.
506719.10_mRNA-p1 TcCLB-NE.510797.4_mRNA-p1
OG0003244: TCRU_8799 TcCLB-EL.511071.100_mRNA-p1 TcCLB-NE.
511901.30_mRNA-p1 TcSYL_0103520.t1-p1
OG0003245: TCRU_8801 TcCLB-EL.506661.30_mRNA-p1 TcCLB-NE.
511245.130_mRNA-p1 TcSYL_0027130.t1-p1
OG0003246: TCRU_8802 TcCLB-EL.506661.10_mRNA-p1 TcCLB-NE.
511245.150_mRNA-p1 TcSYL_0027150.t1-p1
OG0003247: TCRU_8803 TcCLB-EL.510989.10_mRNA-p1 TcCLB-NE.
511245.160_mRNA-p1 TcSYL_0027160.t1-p1
OG0003248: TCRU_8804 TcCLB-EL.427247.10_mRNA-p1 TcCLB-NE.
511245.240_mRNA-p1 TcSYL_0027200.t1-p1
OG0003249: TCRU_8808 TcCLB-EL.511545.30_mRNA-p1 TcCLB-NE.
506817.40_mRNA-p1 TcSYL_0088100.t1-p1
OG0003250: TCRU_8820 TcCLB-EL.506203.129_mRNA-p1 TcCLB-NE.
508641.260_mRNA-p1 TcSYL_0047550.t1-p1
OG0003251: TCRU_8821 TcCLB-EL.506203.40_mRNA-p1 TcCLB-NE.
508643.40_mRNA-p1 TcSYL_0047660.t1-p1
OG0003252: TCRU_8823 TcCLB-EL.507509.40_mRNA-p1 TcCLB-NE.
506871.90_mRNA-p1 TcSYL_0142550.t1-p1
OG0003253: TCRU_8824 TcCLB-EL.503829.70_mRNA-p1 TcCLB-NE.
508045.60_mRNA-p1 TcSYL_0173510.t1-p1

OG0003254: TCRU_8828 TcCLB-EL.509007.20_mRNA-p1 TcCLB-NE.
508965.90_mRNA-p1 TcSYL_0171440.t1-p1
OG0003255: TCRU_8830 TcCLB-EL.506779.140_mRNA-p1 TcCLB-NE.
511153.70_mRNA-p1 TcSYL_0076980.t1-p1
OG0003256: TCRU_8838 TcCLB-EL.503579.150_mRNA-p1 TcCLB-NE.
507517.130_mRNA-p1 TcSYL_0022830.t1-p1
OG0003257: TCRU_8843 TcCLB-EL.506755.279_mRNA-p1 TcCLB-NE.
510359.300_mRNA-p1 TcSYL_0112850.t1-p1
OG0003258: TCRU_8844 TcCLB-EL.508213.40_mRNA-p1 TcCLB-NE.
507515.40_mRNA-p1 TcSYL_0023080.t1-p1
OG0003259: TCRU_8846 TcCLB-EL.509595.30_mRNA-p1 TcCLB-NE.
508819.30_mRNA-p1 TcSYL_0043260.t1-p1
OG0003260: TCRU_8853 TcCLB-EL.508277.90_mRNA-p1 TcCLB-NE.
503541.10_mRNA-p1 TcSYL_0064040.t1-p1
OG0003261: TCRU_8855 TcCLB-EL.509167.200_mRNA-p1 TcCLB-NE.
509065.170_mRNA-p1 TcSYL_0140160.t1-p1
OG0003262: TCRU_8856 TcCLB-EL.506943.120_mRNA-p1 TcCLB-NE.
509065.130_mRNA-p1 TcSYL_0140140.t1-p1
OG0003263: TCRU_8858 TcCLB-EL.506943.40_mRNA-p1 TcCLB-NE.
509065.50_mRNA-p1 TcSYL_0140070.t1-p1
OG0003264: TCRU_8859 TcCLB-EL.511735.30_mRNA-p1 TcCLB-NE.
509063.10_mRNA-p1 TcSYL_0139890.t1-p1
OG0003265: TCRU_8860 TcCLB-EL.506829.110_mRNA-p1 TcCLB-NE.
510151.19_mRNA-p1 TcSYL_0109400.t1-p1
OG0003266: TCRU_8862 TcCLB-EL.506829.50_mRNA-p1 TcCLB-NE.
506593.40_mRNA-p1 TcSYL_0109420.t1-p1
OG0003267: TCRU_8871 TcCLB-EL.509011.100_mRNA-p1 TcCLB-NE.
510681.20_mRNA-p1 TcSYL_0030940.t1-p1
OG0003268: TCRU_8874 TcCLB-EL.506947.80_mRNA-p1 TcCLB-NE.
507057.30_mRNA-p1 TcSYL_0014580.t1-p1
OG0003269: TCRU_8879 TcCLB-EL.504213.80_mRNA-p1 TcCLB-NE.
508857.160_mRNA-p1 TcSYL_0026570.t1-p1
OG0003270: TCRU_8880 TcCLB-EL.507677.100_mRNA-p1 TcCLB-NE.
504021.60_mRNA-p1 TcSYL_0009880.t1-p1
OG0003271: TCRU_8881 TcCLB-EL.504881.50_mRNA-p1 TcCLB-NE.
510045.20_mRNA-p1 TcSYL_0064950.t1-p1
OG0003272: TCRU_8890 TcCLB-EL.507735.10_mRNA-p1 TcCLB-NE.
506263.40_mRNA-p1 TcSYL_0197180.t1-p1
OG0003273: TCRU_8891 TcCLB-EL.507735.20_mRNA-p1 TcCLB-NE.
506263.49_mRNA-p1 TcSYL_0197160.t1-p1
OG0003274: TCRU_8895 TcCLB-EL.509589.30_mRNA-p1 TcCLB-NE.
503999.20_mRNA-p1 TcSYL_0089190.t1-p1
OG0003275: TCRU_8896 TcCLB-EL.509589.20_mRNA-p1 TcCLB-NE.
503999.10_mRNA-p1 TcSYL_0089180.t1-p1
OG0003276: TCRU_8904 TcCLB-EL.504165.20_mRNA-p1 TcCLB-NE.
508981.20_mRNA-p1 TcSYL_0127670.t1-p1
OG0003277: TCRU_8914 TcCLB-EL.510513.20_mRNA-p1 TcCLB-NE.
509855.10_mRNA-p1 TcSYL_0174470.t1-p1
OG0003278: TCRU_8916 TcCLB-EL.510513.70_mRNA-p1 TcCLB-NE.
509857.40_mRNA-p1 TcSYL_0174500.t1-p1
OG0003279: TCRU_8924 TcCLB-EL.506795.29_mRNA-p1 TcCLB-NE.
509937.39_mRNA-p1 TcSYL_0027520.t1-p1
OG0003280: TCRU_8925 TcCLB-EL.506795.20_mRNA-p1 TcCLB-NE.
509937.50_mRNA-p1 TcSYL_0027530.t1-p1

OG0003281: TCRU_8937 TcCLB-EL.503755.10_mRNA-p1 TcCLB-NE.
510131.50_mRNA-p1 TcSYL_0044750.t1-p1
OG0003282: TCRU_8939 TcCLB-EL.503755.30_mRNA-p1 TcCLB-NE.
510131.70_mRNA-p1 TcSYL_0044760.t1-p1
OG0003283: TCRU_8942 TcCLB-EL.506729.90_mRNA-p1 TcCLB-NE.
503843.10_mRNA-p1 TcSYL_0023160.t1-p1
OG0003284: TCRU_8944 TcCLB-EL.506773.20_mRNA-p1 TcCLB-NE.
508799.190_mRNA-p1 TcSYL_0075270.t1-p1
OG0003285: TCRU_8946 TcCLB-EL.506773.44_mRNA-p1 TcCLB-NE.
508799.160_mRNA-p1 TcSYL_0075290.t1-p1
OG0003286: TCRU_8963 TcCLB-EL.509069.30_mRNA-p1 TcCLB-NE.
508707.224_mRNA-p1 TcSYL_0112380.t1-p1
OG0003287: TCRU_8967 TcCLB-EL.509073.20_mRNA-p1 TcCLB-NE.
508707.190_mRNA-p1 TcSYL_0112400.t1-p1
OG0003288: TCRU_8969 TcCLB-EL.509073.40_mRNA-p1 TcCLB-NE.
508707.170_mRNA-p1 TcSYL_0112410.t1-p1
OG0003289: TCRU_8970 TcCLB-EL.509073.50_mRNA-p1 TcCLB-NE.
508707.160_mRNA-p1 TcSYL_0112420.t1-p1
OG0003290: TCRU_8971 TcCLB-EL.509073.60_mRNA-p1 TcCLB-NE.
508707.149_mRNA-p1 TcSYL_0112430.t1-p1
OG0003291: TCRU_8974 TcCLB-EL.509073.90_mRNA-p1 TcCLB-NE.
508707.120_mRNA-p1 TcSYL_0112440.t1-p1
OG0003292: TCRU_8975 TcCLB-EL.509073.100_mRNA-p1 TcCLB-NE.
508707.110_mRNA-p1 TcSYL_0112460.t1-p1
OG0003293: TCRU_8976 TcCLB-EL.507037.10_pseudogenic_transcript-p1
TcCLB-NE.508707.90_mRNA-p1 TcSYL_0112470.t1-p1
OG0003294: TCRU_8977 TcCLB-EL.507037.20_mRNA-p1 TcCLB-NE.
508707.80_mRNA-p1 TcSYL_0112480.t1-p1
OG0003295: TCRU_8978 TcCLB-EL.507037.40_pseudogenic_transcript-p1
TcCLB-NE.508707.70_mRNA-p1 TcSYL_0112490.t1-p1
OG0003296: TCRU_8979 TcCLB-EL.507037.60_mRNA-p1 TcCLB-NE.
508707.60_mRNA-p1 TcSYL_0112510.t1-p1
OG0003297: TCRU_8980 TcCLB-EL.507037.70_mRNA-p1 TcCLB-NE.
508707.50_mRNA-p1 TcSYL_0112520.t1-p1
OG0003298: TCRU_8984 TcCLB-EL.511543.30_mRNA-p1 TcCLB-NE.
508707.10_mRNA-p1 TcSYL_0112550.t1-p1
OG0003299: TCRU_8986 TcCLB-EL.511543.50_mRNA-p1 TcCLB-NE.
511019.108_mRNA-p1 TcSYL_0112560.t1-p1
OG0003300: TCRU_8987 TcCLB-EL.511543.60_mRNA-p1 TcCLB-NE.
511019.99_mRNA-p1 TcSYL_0112570.t1-p1
OG0003301: TCRU_8990 TcCLB-EL.503881.10_mRNA-p1 TcCLB-NE.
508119.110_mRNA-p1 TcSYL_0103830.t1-p1
OG0003302: TCRU_8999 TCRU_9367 TcCLB-NE.506809.5_mRNA-p1
TcSYL_0120250.t1-p1
OG0003303: TCRU_9004 TcCLB-EL.504881.40_mRNA-p1 TcCLB-NE.
510043.59_mRNA-p1 TcSYL_0064930.t1-p1
OG0003304: TCRU_9005 TcCLB-EL.504881.20_mRNA-p1 TcCLB-NE.
510043.40_mRNA-p1 TcSYL_0064900.t1-p1
OG0003305: TCRU_9008 TcCLB-EL.511181.20_mRNA-p1 TcCLB-NE.
503465.20_pseudogenic_transcript-p1 TcSYL_0203210.t1-p1
OG0003306: TCRU_9009 TcCLB-EL.511181.30_mRNA-p1 TcCLB-NE.
503465.30_mRNA-p1 TcSYL_0203220.t1-p1
OG0003307: TCRU_9016 TcCLB-EL.511309.70_mRNA-p1 TcCLB-NE.
511759.30_mRNA-p1 TcSYL_0159710.t1-p1

OG0003308: TCRU_9018 TcCLB-EL.506743.100_mRNA-p1 TcCLB-NE.
511927.10_mRNA-p1 TcSYL_0165160.t1-p1
OG0003309: TCRU_9022 TcCLB-EL.503833.20_mRNA-p1 TcCLB-NE.
511287.130_mRNA-p1 TcSYL_0123100.t1-p1
OG0003310: TCRU_9024 TcCLB-EL.506211.250_mRNA-p1 TcCLB-NE.
511287.150_mRNA-p1 TcSYL_0123090.t1-p1
OG0003311: TCRU_9027 TcCLB-EL.506211.220_mRNA-p1 TcCLB-NE.
420719.9_mRNA-p1 TcSYL_0123080.t1-p1
OG0003312: TCRU_9031 TcCLB-EL.507485.60_mRNA-p1 TcCLB-NE.
507159.30_mRNA-p1 TcSYL_0195520.t1-p1
OG0003313: TCRU_9032 TcCLB-EL.507485.70_mRNA-p1 TcCLB-NE.
507159.20_mRNA-p1 TcSYL_0195550.t1-p1
OG0003314: TCRU_9033 TcCLB-EL.507485.80_mRNA-p1 TcCLB-NE.
507159.14_mRNA-p1 TcSYL_0195570.t1-p1
OG0003315: TCRU_9034 TcCLB-EL.507485.90_mRNA-p1 TcCLB-NE.
507159.10_pseudogenic_transcript-p1 TcSYL_0195590.t1-p1
OG0003316: TCRU_9035 TcCLB-EL.507485.110_mRNA-p1 TcCLB-NE.
509289.30_mRNA-p1 TcSYL_0195680.t1-p1
OG0003317: TCRU_9040 TcCLB-NE.505997.60_mRNA-p1 TcCLB-NE.
510643.190_mRNA-p1 TcSYL_0191520.t1-p1
OG0003318: TCRU_9042 TcCLB-EL.504153.370_mRNA-p1 TcCLB-NE.
506301.60_mRNA-p1 TcSYL_0138530.t1-p1
OG0003319: TCRU_9045 TcCLB-EL.506811.190_mRNA-p1 TcCLB-NE.
506303.50_mRNA-p1 TcSYL_0138510.t1-p1
OG0003320: TCRU_9046 TcCLB-EL.506811.180_mRNA-p1 TcCLB-NE.
506303.60_mRNA-p1 TcSYL_0138500.t1-p1
OG0003321: TCRU_9048 TcCLB-EL.506811.160_mRNA-p1 TcCLB-NE.
506303.80_mRNA-p1 TcSYL_0138490.t1-p1
OG0003322: TCRU_9077 TcCLB-EL.508717.40_mRNA-p1 TcCLB-NE.
504079.10_mRNA-p1 TcSYL_0106840.t1-p1
OG0003323: TCRU_9079 TcCLB-EL.508717.30_mRNA-p1 TcCLB-NE.
506839.80_mRNA-p1 TcSYL_0106830.t1-p1
OG0003324: TCRU_9080 TcCLB-EL.508717.20_mRNA-p1 TcCLB-NE.
506839.70_mRNA-p1 TcSYL_0106820.t1-p1
OG0003325: TCRU_9082 TcCLB-EL.506797.30_mRNA-p1 TcCLB-NE.
506915.30_mRNA-p1 TcSYL_0102490.t1-p1
OG0003326: TCRU_9083 TcCLB-EL.506797.20_mRNA-p1 TcCLB-NE.
506915.20_mRNA-p1 TcSYL_0102530.t1-p1
OG0003327: TCRU_9084 TcCLB-EL.506797.10_mRNA-p1 TcCLB-NE.
506915.10_mRNA-p1 TcSYL_0102590.t1-p1
OG0003328: TCRU_9085 TcCLB-EL.503551.20_mRNA-p1 TcCLB-NE.
511905.9_mRNA-p1 TcSYL_0102660.t1-p1
OG0003329: TCRU_9089 TcCLB-EL.511693.90_mRNA-p1 TcCLB-NE.
503559.109_mRNA-p1 TcSYL_0184900.t1-p1
OG0003330: TCRU_9090 TcCLB-EL.511693.70_mRNA-p1 TcCLB-NE.
508177.10_mRNA-p1 TcSYL_0184890.t1-p1
OG0003331: TCRU_9091 TcCLB-EL.511693.60_mRNA-p1 TcCLB-NE.
508177.20_mRNA-p1 TcSYL_0184880.t1-p1
OG0003332: TCRU_9092 TcCLB-EL.511693.50_mRNA-p1 TcCLB-NE.
508177.30_mRNA-p1 TcSYL_0186750.t1-p1
OG0003333: TCRU_9093 TcCLB-EL.511693.40_mRNA-p1 TcCLB-NE.
508177.40_mRNA-p1 TcSYL_0186690.t1-p1
OG0003334: TCRU_C-terminal TcCLB-EL.508727.54_mRNA-p1 TcCLB-NE.
506355.170_mRNA-p1 TcSYL_0107330.t1-p1

OG0003335: TCRU_domain TcCLB-EL.508727.60_mRNA-p1 TcCLB-NE.
506355.160_mRNA-p1 TcSYL_0107340.t1-p1
OG0003336: TCRU_9107 TcCLB-EL.508729.9_mRNA-p1 TcCLB-NE.
506355.150_mRNA-p1 TcSYL_0107370.t1-p1
OG0003337: TCRU_9108 TcCLB-EL.508729.18_mRNA-p1 TcCLB-NE.
506355.140_mRNA-p1 TcSYL_0107390.t1-p1
OG0003338: TCRU_9109 TcCLB-EL.511041.10_mRNA-p1 TcCLB-NE.
506355.130_mRNA-p1 TcSYL_0107410.t1-p1
OG0003339: TCRU_9110 TcCLB-EL.511041.20_mRNA-p1 TcCLB-NE.
506355.120_mRNA-p1 TcSYL_0107450.t1-p1
OG0003340: TCRU_9111 TcCLB-EL.511041.50_mRNA-p1 TcCLB-NE.
506355.80_mRNA-p1 TcSYL_0107570.t1-p1
OG0003341: TCRU_9112 TcCLB-EL.511043.10_mRNA-p1 TcCLB-NE.
506355.70_mRNA-p1 TcSYL_0107770.t1-p1
OG0003342: TCRU_9114 TcCLB-EL.511043.30_mRNA-p1 TcCLB-NE.
506355.50_mRNA-p1 TcSYL_0107740.t1-p1
OG0003343: TCRU_9123 TcCLB-EL.511199.20_mRNA-p1 TcCLB-NE.
506587.30_mRNA-p1 TcSYL_0044800.t1-p1
OG0003344: TCRU_9125 TcCLB-EL.503855.60_mRNA-p1 TcCLB-NE.
510335.10_mRNA-p1 TcSYL_0146590.t1-p1
OG0003345: TCRU_9144 TcCLB-EL.508347.30_mRNA-p1 TcCLB-NE.
506145.20_mRNA-p1 TcSYL_0105390.t1-p1
OG0003346: TCRU_9145 TcCLB-EL.508347.24_mRNA-p1 TcCLB-NE.
506145.24_mRNA-p1 TcSYL_0105410.t1-p1
OG0003347: TCRU_9146 TcCLB-EL.508347.20_mRNA-p1 TcCLB-NE.
506145.30_mRNA-p1 TcSYL_0105430.t1-p1
OG0003348: TCRU_9163 TcCLB-EL.511911.102_mRNA-p1 TcCLB-NE.
508637.69_mRNA-p1 TcSYL_0170350.t1-p1
OG0003349: TCRU_9164 TcCLB-EL.511911.110_mRNA-p1 TcCLB-NE.
508637.70_mRNA-p1 TcSYL_0170360.t1-p1
OG0003350: TCRU_9173 TcCLB-EL.511803.20_mRNA-p1 TcCLB-NE.
510897.3_pseudogenic_transcript-p1 TcSYL_0180040.t1-p1
OG0003351: TCRU_9184 TcCLB-EL.511191.10_mRNA-p1 TcCLB-NE.
506619.40_mRNA-p1 TcSYL_0087250.t1-p1
OG0003352: TCRU_9195 TcCLB-EL.510611.50_mRNA-p1 TcCLB-NE.
506791.79_mRNA-p1 TcCLB-NE.506793.10_pseudogenic_transcript-p1
OG0003353: TCRU_9196 TcCLB-EL.510611.60_mRNA-p1 TcCLB-NE.
506791.70_mRNA-p1 TcSYL_0163570.t1-p1
OG0003354: TCRU_9212 TcCLB-EL.511437.10_mRNA-p1 TcCLB-NE.
504575.60_mRNA-p1 TcCLB-NE.510681.9_mRNA-p1
OG0003355: TCRU_9214 TcCLB-EL.503855.40_mRNA-p1 TcCLB-NE.
510337.20_mRNA-p1 TcSYL_0146610.t1-p1
OG0003356: TCRU_9218 TcCLB-EL.510029.60_mRNA-p1 TcCLB-NE.
511291.100_mRNA-p1 TcSYL_0122760.t1-p1
OG0003357: TCRU_9219 TcCLB-EL.510029.50_mRNA-p1 TcCLB-NE.
511291.110_mRNA-p1 TcSYL_0122520.t1-p1
OG0003358: TCRU_9223 TcCLB-EL.507963.79_mRNA-p1 TcCLB-NE.
507031.29_mRNA-p1 TcSYL_0122430.t1-p1
OG0003359: TCRU_9234 TcCLB-EL.506505.40_mRNA-p1 TcCLB-NE.
507031.170_pseudogenic_transcript-p1 TcSYL_0122360.t1-p1
OG0003360: TCRU_9238 TcCLB-EL.506503.150_mRNA-p1 TcCLB-NE.
507033.20_mRNA-p1 TcSYL_0122350.t1-p1
OG0003361: TCRU_9240 TcCLB-EL.506503.120_mRNA-p1 TcCLB-NE.
511293.20_mRNA-p1 TcSYL_0122330.t1-p1

OG0003362: TCRU_9241 TcCLB-EL.506503.100_mRNA-p1 TcCLB-NE.511293.40_mRNA-p1 TcSYL_0122320.t1-p1
OG0003363: TCRU_9242 TcCLB-EL.506503.90_mRNA-p1 TcCLB-NE.511293.50_mRNA-p1 TcSYL_0122310.t1-p1
OG0003364: TCRU_9243 TcCLB-EL.506503.80_mRNA-p1 TcCLB-NE.511293.60_mRNA-p1 TcSYL_0122300.t1-p1
OG0003365: TCRU_9244 TcCLB-EL.506503.69_mRNA-p1 TcCLB-NE.511293.69_mRNA-p1 TcSYL_0122290.t1-p1
OG0003366: TCRU_9247 TcCLB-EL.506503.50_mRNA-p1 TcCLB-NE.504161.20_mRNA-p1 TcSYL_0122260.t1-p1
OG0003367: TCRU_9251 TcCLB-EL.506825.230_mRNA-p1 TcCLB-NE.508175.133_pseudogenic_transcript-p1 TcSYL_0078790.t1-p1
OG0003368: TCRU_9252 TcCLB-EL.509151.140_mRNA-p1 TcCLB-NE.508175.146_mRNA-p1 TcSYL_0078780.t1-p1
OG0003369: TCRU_9253 TcCLB-EL.509151.130_mRNA-p1 TcCLB-NE.508175.160_mRNA-p1 TcSYL_0078770.t1-p1
OG0003370: TCRU_9254 TcCLB-EL.509151.120_mRNA-p1 TcCLB-NE.508175.170_mRNA-p1 TcSYL_0078760.t1-p1
OG0003371: TCRU_9258 TcCLB-EL.509151.70_mRNA-p1 TcCLB-NE.508175.220_mRNA-p1 TcSYL_0078720.t1-p1
OG0003372: TCRU_9259 TcCLB-EL.509151.60_mRNA-p1 TcCLB-NE.508175.230_mRNA-p1 TcSYL_0078710.t1-p1
OG0003373: TCRU_9260 TcCLB-EL.509151.50_mRNA-p1 TcCLB-NE.508175.250_mRNA-p1 TcSYL_0078680.t1-p1
OG0003374: TCRU_9261 TcCLB-EL.509151.40_mRNA-p1 TcCLB-NE.508175.260_mRNA-p1 TcSYL_0078670.t1-p1
OG0003375: TCRU_9263 TcCLB-EL.509151.10_mRNA-p1 TcCLB-NE.508175.280_mRNA-p1 TcSYL_0078650.t1-p1
OG0003376: TCRU_9264 TcCLB-EL.509149.80_mRNA-p1 TcCLB-NE.508175.290_mRNA-p1 TcSYL_0078640.t1-p1
OG0003377: TCRU_9267 TcCLB-EL.507483.80_mRNA-p1 TcCLB-NE.506389.60_mRNA-p1 TcSYL_0072530.t1-p1
OG0003378: TCRU_9268 TcCLB-EL.507483.60_mRNA-p1 TcCLB-NE.506389.70_mRNA-p1 TcSYL_0072540.t1-p1
OG0003379: TCRU_9279 TcCLB-EL.508805.100_mRNA-p1 TcCLB-NE.508297.30_mRNA-p1 TcSYL_0203420.t1-p1
OG0003380: TCRU_9284 TcCLB-EL.510289.30_mRNA-p1 TcCLB-NE.503891.30_mRNA-p1 TcSYL_0113360.t1-p1
OG0003381: TCRU_9285 TcCLB-EL.510289.20_mRNA-p1 TcCLB-NE.503891.40_mRNA-p1 TcSYL_0113350.t1-p1
OG0003382: TCRU_9288 TcCLB-EL.510507.20_mRNA-p1 TcCLB-NE.509463.30_mRNA-p1 TcSYL_0173730.t1-p1
OG0003383: TCRU_9303 TcCLB-EL.506825.120_mRNA-p1 TcCLB-NE.508175.20_mRNA-p1 TcSYL_0078840.t1-p1
OG0003384: TCRU_9304 TcCLB-EL.506825.100_mRNA-p1 TcCLB-NE.508175.9_mRNA-p1 TcSYL_0078870.t1-p1
OG0003385: TCRU_9305 TcCLB-EL.506825.90_mRNA-p1 TcCLB-NE.476733.10_mRNA-p1 TcSYL_0078880.t1-p1
OG0003386: TCRU_9306 TcCLB-EL.506825.80_mRNA-p1 TcCLB-NE.476733.20_mRNA-p1 TcSYL_0078890.t1-p1
OG0003387: TCRU_9327 TcCLB-EL.503527.50_mRNA-p1 TcCLB-NE.510299.60_mRNA-p1 TcSYL_0114270.t1-p1
OG0003388: TCRU_9350 TcCLB-EL.507831.40_mRNA-p1 TcCLB-NE.511263.30_mRNA-p1 TcSYL_0015610.t1-p1

OG0003389: TCRU_9351 TcCLB-EL.507831.50_mRNA-p1 TcCLB-NE.
511263.20_mRNA-p1 TcSYL_0015680.t1-p1
OG0003390: TCRU_9352 TcCLB-EL.507831.60_mRNA-p1 TcCLB-NE.
511263.9_mRNA-p1 TcSYL_0015710.t1-p1
OG0003391: TCRU_alpha TcCLB-EL.507831.70_mRNA-p1 TcCLB-NE.
511261.160_mRNA-p1 TcSYL_0015750.t1-p1
OG0003392: TCRU_9354 TcCLB-EL.507831.80_mRNA-p1 TcCLB-NE.
511261.150_mRNA-p1 TcSYL_0015790.t1-p1
OG0003393: TCRU_9356 TcCLB-EL.507831.100_mRNA-p1 TcCLB-NE.
511261.130_mRNA-p1 TcSYL_0015830.t1-p1
OG0003394: TCRU_9357 TcCLB-EL.507831.110_mRNA-p1 TcCLB-NE.
511261.120_mRNA-p1 TcSYL_0015880.t1-p1
OG0003395: TCRU_9358 TcCLB-EL.503455.10_mRNA-p1 TcCLB-NE.
511261.100_mRNA-p1 TcSYL_0016000.t1-p1
OG0003396: TCRU_9359 TcCLB-EL.503455.20_mRNA-p1 TcCLB-NE.
511261.90_mRNA-p1 TcSYL_0016040.t1-p1
OG0003397: TCRU_9361 TcCLB-EL.509807.20_mRNA-p1 TcCLB-NE.
511261.60_mRNA-p1 TcSYL_0016170.t1-p1
OG0003398: TCRU_9362 TcCLB-EL.503457.10_mRNA-p1 TcCLB-NE.
511261.40_mRNA-p1 TcSYL_0016210.t1-p1
OG0003399: TCRU_9363 TcCLB-EL.509809.20_mRNA-p1 TcCLB-NE.
511261.20_mRNA-p1 TcSYL_0016290.t1-p1
OG0003400: TCRU_9364 TcCLB-EL.503757.40_mRNA-p1 TcCLB-NE.
509575.50_mRNA-p1 TcSYL_0118220.t1-p1
OG0003401: TCRU_9365 TcCLB-EL.509827.10_mRNA-p1 TcCLB-NE.
509575.20_mRNA-p1 TcSYL_0120580.t1-p1
OG0003402: TCRU_9372 TcCLB-EL.510101.470_mRNA-p1 TcCLB-NE.
507765.20_mRNA-p1 TcSYL_0075020.t1-p1
OG0003403: TCRU_9375 TcCLB-EL.510101.440_mRNA-p1 TcCLB-NE.
507765.50_mRNA-p1 TcSYL_0075010.t1-p1
OG0003404: TCRU_9387 TcCLB-EL.506735.70_mRNA-p1 TcCLB-NE.
509229.20_mRNA-p1 TcSYL_0091820.t1-p1
OG0003405: TCRU_9396 TcCLB-EL.504427.30_mRNA-p1 TcCLB-NE.
509331.110_mRNA-p1 TcSYL_0172340.t1-p1
OG0003406: TCRU_9397 TcCLB-EL.504427.40_mRNA-p1 TcCLB-NE.
509331.100_mRNA-p1 TcSYL_0172370.t1-p1
OG0003407: TCRU_9398 TcCLB-EL.504427.50_mRNA-p1 TcCLB-NE.
509331.90_mRNA-p1 TcSYL_0172400.t1-p1
OG0003408: TCRU_9414 TcCLB-EL.507641.236_mRNA-p1 TcCLB-NE.
508479.190_mRNA-p1 TcSYL_0158730.t1-p1
OG0003409: TCRU_9418 TcCLB-NE.509525.260_mRNA-p1 TcCLB-NE.
509525.70_mRNA-p1 TcSYL_0129520.t1-p1
OG0003410: TCRU_9421 TcCLB-EL.448387.9_mRNA-p1 TcCLB-NE.
509395.70_mRNA-p1 TcSYL_0194400.t1-p1
OG0003411: TCRU_9425 TcCLB-EL.503653.50_mRNA-p1 TcCLB-NE.
509395.120_mRNA-p1 TcSYL_0194410.t1-p1
OG0003412: TCRU_9443 TcCLB-EL.507817.70_mRNA-p1 TcCLB-NE.
509471.10_mRNA-p1 TcSYL_0042410.t1-p1
OG0003413: TCRU_9444 TcCLB-EL.507817.60_mRNA-p1 TcCLB-NE.
509471.20_mRNA-p1 TcSYL_0042390.t1-p1
OG0003414: TCRU_9445 TcCLB-EL.507817.50_mRNA-p1 TcCLB-NE.
509471.30_mRNA-p1 TcSYL_0042340.t1-p1
OG0003415: TCRU_9446 TcCLB-EL.507817.40_mRNA-p1 TcCLB-NE.
509471.40_mRNA-p1 TcSYL_0042330.t1-p1

OG0003416: TCRU_9447 TcCLB-EL.507817.30_mRNA-p1 TcCLB-NE.
509471.50_mRNA-p1 TcSYL_0042320.t1-p1
OG0003417: TCRU__mRPN1_ TcCLB-EL.506931.64_mRNA-p1 TcCLB-NE.
506585.90_mRNA-p1 TcSYL_0044650.t1-p1
OG0003418: TCRU_9455 TcCLB-EL.506931.60_mRNA-p1 TcCLB-NE.
506585.100_mRNA-p1 TcSYL_0044660.t1-p1
OG0003419: TCRU_9460 TcCLB-EL.510181.130_mRNA-p1 TcCLB-NE.
503431.30_mRNA-p1 TcSYL_0013380.t1-p1
OG0003420: TCRU_9461 TcCLB-EL.510181.120_mRNA-p1 TcCLB-NE.
503431.40_mRNA-p1 TcSYL_0013400.t1-p1
OG0003421: TCRU_9462 TcCLB-EL.510181.110_mRNA-p1 TcCLB-NE.
503431.50_mRNA-p1 TcSYL_0013430.t1-p1
OG0003422: TCRU_9467 TcCLB-EL.510181.50_mRNA-p1 TcCLB-NE.
507609.10_mRNA-p1 TcSYL_0013450.t1-p1
OG0003423: TCRU_9469 TcCLB-EL.510181.30_mRNA-p1 TcCLB-NE.
507609.30_mRNA-p1 TcSYL_0013460.t1-p1
OG0003424: TCRU_9470 TcCLB-EL.510181.20_mRNA-p1 TcCLB-NE.
507609.40_mRNA-p1 TcSYL_0013470.t1-p1
OG0003425: TCRU_9472 TcCLB-EL.503639.20_mRNA-p1 TcCLB-NE.
507609.70_mRNA-p1 TcSYL_0013490.t1-p1
OG0003426: TCRU_9501 TcCLB-EL.506163.80_mRNA-p1 TcCLB-NE.
508819.50_mRNA-p1 TcSYL_0043160.t1-p1
OG0003427: TCRU_9502 TcCLB-EL.506163.70_mRNA-p1 TcCLB-NE.
508821.20_mRNA-p1 TcSYL_0043150.t1-p1
OG0003428: TCRU_9503 TcCLB-EL.506163.60_mRNA-p1 TcCLB-NE.
508821.30_mRNA-p1 TcSYL_0043100.t1-p1
OG0003429: TCRU_9508 TcCLB-EL.503747.10_mRNA-p1 TcCLB-NE.
510187.20_mRNA-p1 TcSYL_0012630.t1-p1
OG0003430: TCRU_9512 TcCLB-EL.503747.40_mRNA-p1 TcCLB-NE.
510187.10_mRNA-p1 TcSYL_0012900.t1-p1
OG0003431: TCRU_9515 TcCLB-EL.506563.110_pseudogenic_transcript-p1
TcCLB-NE.509001.40_mRNA-p1 TcSYL_0063510.t1-p1
OG0003432: TCRU_9518 TcCLB-EL.506563.170_pseudogenic_transcript-p1
TcCLB-NE.508999.260_pseudogenic_transcript-p1 TcSYL_0063520.t1-p1
OG0003433: TCRU_9521 TcCLB-EL.506563.200_mRNA-p1 TcCLB-NE.
508999.220_mRNA-p1 TcSYL_0063530.t1-p1
OG0003434: TCRU_9523 TcCLB-EL.510957.9_mRNA-p1 TcCLB-NE.
508999.190_mRNA-p1 TcSYL_0063550.t1-p1
OG0003435: TCRU_9534 TcCLB-EL.506777.80_mRNA-p1 TcCLB-NE.
511163.10_mRNA-p1 TcSYL_0075560.t1-p1
OG0003436: TCRU_9535 TcCLB-EL.506777.70_mRNA-p1 TcCLB-NE.
511163.20_mRNA-p1 TcSYL_0075550.t1-p1
OG0003437: TCRU_interacting TcCLB-EL.506777.60_mRNA-p1 TcCLB-NE.
511163.30_mRNA-p1 TcSYL_0075540.t1-p1
OG0003438: TCRU_9566 TcCLB-EL.506715.40_mRNA-p1 TcCLB-NE.
506469.148_mRNA-p1 TcSYL_0156940.t1-p1
OG0003439: TCRU_9570 TcCLB-NE.508965.14_mRNA-p1 TcSYL_0022260.t1-p1
TcSYL_0040090.t1-p1
OG0003440: TCRU_9574 TcCLB-EL.506907.14_mRNA-p1 TcCLB-EL.
510773.100_mRNA-p1 TcCLB-NE.511385.90_mRNA-p1
OG0003441: TCRU_9594 TcCLB-EL.503779.60_mRNA-p1 TcCLB-NE.
506247.430_mRNA-p1 TcSYL_0108660.t1-p1
OG0003442: TCRU_9595 TcCLB-EL.503779.80_pseudogenic_transcript-p1
TcCLB-NE.506247.450_mRNA-p1 TcSYL_0108650.t1-p1

OG0003443: TCRU_9596 TcCLB-EL.503779.100_mRNA-p1 TcCLB-NE.
506247.460_mRNA-p1 TcSYL_0108640.t1-p1
OG0003444: TCRU_9609 TcCLB-EL.507929.30_mRNA-p1 TcCLB-NE.
509045.10_mRNA-p1 TcSYL_0168740.t1-p1
OG0003445: TCRU_9611 TcCLB-EL.507929.10_mRNA-p1 TcCLB-NE.
509045.29_mRNA-p1 TcSYL_0167480.t1-p1
OG0003446: TCRU_9612 TcCLB-EL.507927.100_mRNA-p1 TcCLB-NE.
509047.10_mRNA-p1 TcSYL_0167520.t1-p1
OG0003447: TCRU_9658 TCRU_9659 TcSYL_0129730.t1-p1 TcSYL_0134950.t1-
p1
OG0003448: TCRU_subunit TcCLB-EL.507105.20_mRNA-p1 TcCLB-NE.
506815.40_mRNA-p1 TcSYL_0088020.t1-p1
OG0003449: TCRU_9679 TcCLB-EL.510747.10_mRNA-p1 TcCLB-NE.
504185.30_mRNA-p1 TcSYL_0115940.t1-p1
OG0003450: TCRU_9694 TcCLB-EL.511585.130_mRNA-p1 TcCLB-NE.
510155.120_mRNA-p1 TcSYL_0109250.t1-p1
OG0003451: TCRU_9699 TcCLB-EL.511585.90_mRNA-p1 TcCLB-NE.
510155.90_mRNA-p1 TcSYL_0109260.t1-p1
OG0003452: TCRU_9700 TcCLB-EL.511585.80_mRNA-p1 TcCLB-NE.
510155.80_mRNA-p1 TcSYL_0109270.t1-p1
OG0003453: TCRU_9701 TcCLB-EL.511585.50_mRNA-p1 TcCLB-NE.
510155.50_mRNA-p1 TcSYL_0109290.t1-p1
OG0003454: TCRU_9702 TcCLB-EL.511585.40_mRNA-p1 TcCLB-NE.
510155.40_mRNA-p1 TcSYL_0109300.t1-p1
OG0003455: TCRU_9716 TcCLB-EL.507611.280_mRNA-p1 TcCLB-NE.
507723.100_mRNA-p1 TcSYL_0108930.t1-p1
OG0003456: TCRU_9717 TcCLB-EL.507611.310_mRNA-p1 TcCLB-NE.
507723.130_mRNA-p1 TcSYL_0108920.t1-p1
OG0003457: TCRU_9722 TcCLB-EL.507009.60_mRNA-p1 TcCLB-NE.
510667.100_mRNA-p1 TcSYL_0117470.t1-p1
OG0003458: TCRU_9723 TcCLB-EL.507009.50_mRNA-p1 TcCLB-NE.
510667.110_mRNA-p1 TcSYL_0117530.t1-p1
OG0003459: TCRU_9724 TcCLB-EL.507009.40_mRNA-p1 TcCLB-NE.
510667.120_mRNA-p1 TcSYL_0117560.t1-p1
OG0003460: TCRU_9728 TcCLB-EL.504047.30_mRNA-p1 TcCLB-NE.
504045.130_mRNA-p1 TcSYL_0019570.t1-p1
OG0003461: TCRU_9729 TcCLB-EL.504047.20_mRNA-p1 TcCLB-NE.
504045.120_mRNA-p1 TcSYL_0019560.t1-p1
OG0003462: TCRU_9730 TcCLB-EL.504047.10_mRNA-p1 TcCLB-NE.
504045.114_mRNA-p1 TcSYL_0019550.t1-p1
OG0003463: TCRU_9733 TcCLB-EL.506743.120_mRNA-p1 TcCLB-NE.
503851.10_mRNA-p1 TcSYL_0165050.t1-p1
OG0003464: TCRU_9734 TcCLB-EL.511211.150_mRNA-p1 TcSYL_0182540.t1-p1
TcSYL_0182560.t1-p1
OG0003465: TCRU_9741 TcCLB-EL.508989.60_mRNA-p1 TcCLB-NE.
509569.150_mRNA-p1 TcSYL_0097430.t1-p1
OG0003466: TCRU_9742 TcCLB-EL.508989.50_mRNA-p1 TcCLB-NE.
509569.140_mRNA-p1 TcSYL_0097380.t1-p1
OG0003467: TCRU_9743 TcCLB-EL.508989.30_mRNA-p1 TcCLB-NE.
509569.120_mRNA-p1 TcSYL_0097340.t1-p1
OG0003468: TCRU_9744 TcCLB-EL.508989.20_mRNA-p1 TcCLB-NE.
509569.110_mRNA-p1 TcSYL_0097330.t1-p1
OG0003469: TCRU_9745 TcCLB-EL.506865.10_mRNA-p1 TcCLB-NE.
509569.90_mRNA-p1 TcSYL_0097270.t1-p1

OG0003470: TCRU_9746 TcCLB-EL.506865.20_mRNA-p1 TcCLB-NE.
509569.80_mRNA-p1 TcSYL_0097250.t1-p1
OG0003471: TCRU_9747 TcCLB-EL.506865.40_mRNA-p1 TcCLB-NE.
509569.50_mRNA-p1 TcSYL_0097200.t1-p1
OG0003472: TCRU_9748 TcCLB-EL.506865.60_mRNA-p1 TcCLB-NE.
509569.40_mRNA-p1 TcSYL_0097150.t1-p1
OG0003473: TCRU_9773 TcCLB-EL.506635.130_mRNA-p1 TcCLB-NE.
504199.20_mRNA-p1 TcSYL_0044980.t1-p1
OG0003474: TCRU_9788 TcCLB-EL.504009.20_mRNA-p1 TcCLB-NE.
507049.150_mRNA-p1 TcSYL_0114630.t1-p1
OG0003475: TCRU_9793 TcCLB-EL.506567.100_mRNA-p1 TcCLB-NE.
507049.80_mRNA-p1 TcSYL_0114690.t1-p1
OG0003476: TCRU_9794 TcCLB-EL.506567.90_mRNA-p1 TcCLB-NE.
507049.70_mRNA-p1 TcSYL_0114700.t1-p1
OG0003477: TCRU_9797 TcCLB-EL.506567.70_mRNA-p1 TcCLB-NE.
507049.60_mRNA-p1 TcSYL_0114710.t1-p1
OG0003478: TCRU_9801 TcCLB-EL.510123.70_mRNA-p1 TcCLB-NE.
511837.10_mRNA-p1 TcSYL_0114740.t1-p1
OG0003479: TCRU_9802 TcCLB-EL.510123.60_mRNA-p1 TcCLB-NE.
511837.20_mRNA-p1 TcSYL_0114750.t1-p1
OG0003480: TCRU_9804 TcCLB-EL.510123.20_mRNA-p1 TcCLB-NE.
511837.70_mRNA-p1 TcSYL_0114770.t1-p1
OG0003481: TCRU_9805 TcCLB-EL.510123.10_mRNA-p1 TcCLB-NE.
511837.80_mRNA-p1 TcSYL_0114780.t1-p1
OG0003482: TCRU_9810 TcCLB-EL.506695.30_mRNA-p1 TcCLB-NE.
508153.570_mRNA-p1 TcSYL_0000790.t1-p1
OG0003483: TCRU_9811 TcCLB-EL.506695.20_mRNA-p1 TcCLB-NE.
508153.590_mRNA-p1 TcSYL_0000780.t1-p1
OG0003484: TCRU_9819 TcCLB-EL.506693.50_mRNA-p1 TcCLB-NE.
508153.650_pseudogenic_transcript-p1 TcSYL_0000740.t1-p1
OG0003485: TCRU_9822 TcCLB-EL.506693.4_mRNA-p1 TcCLB-NE.
508153.730_mRNA-p1 TcSYL_0000730.t1-p1
OG0003486: TCRU_9832 TcCLB-EL.511201.49_mRNA-p1 TcCLB-NE.
508823.20_mRNA-p1 TcSYL_0043040.t1-p1
OG0003487: TCRU_9834 TcCLB-EL.506649.40_mRNA-p1 TcCLB-NE.
508351.50_mRNA-p1 TcSYL_0147380.t1-p1
OG0003488: TCRU_9835 TcCLB-EL.506649.50_mRNA-p1 TcCLB-NE.
508351.40_mRNA-p1 TcSYL_0147410.t1-p1
OG0003489: TCRU_9843 TcCLB-EL.509893.10_mRNA-p1 TcCLB-NE.
507583.70_mRNA-p1 TcCLB-NE.511893.10_mRNA-p1
OG0003490: TCRU_9849 TcCLB-EL.506659.20_mRNA-p1 TcCLB-NE.
508865.20_mRNA-p1 TcSYL_0027280.t1-p1
OG0003491: TCRU_9850 TcCLB-EL.506659.30_mRNA-p1 TcCLB-NE.
508865.10_mRNA-p1 TcSYL_0027270.t1-p1
OG0003492: TCRU_9857 TcCLB-EL.511585.150_mRNA-p1 TcCLB-NE.
510155.140_mRNA-p1 TcSYL_0109240.t1-p1
OG0003493: TCRU_9866 TcCLB-EL.507467.104_mRNA-p1 TcCLB-NE.
508355.114_mRNA-p1 TcSYL_0083100.t1-p1
OG0003494: TCRU_9867 TcCLB-EL.507467.94_mRNA-p1 TcCLB-NE.
508355.104_mRNA-p1 TcSYL_0083110.t1-p1
OG0003495: TCRU_9868 TcCLB-EL.507467.90_mRNA-p1 TcCLB-NE.
508355.100_mRNA-p1 TcSYL_0083120.t1-p1
OG0003496: TCRU_9882 TcCLB-EL.508347.50_mRNA-p1 TcCLB-NE.
507615.130_mRNA-p1 TcSYL_0105230.t1-p1

OG0003497: TCRU_9883 TcCLB-EL.508347.60_mRNA-p1 TcCLB-NE.
507615.120_mRNA-p1 TcSYL_0105180.t1-p1
OG0003498: TCRU_9884 TcCLB-EL.508347.70_mRNA-p1 TcCLB-NE.
507615.110_mRNA-p1 TcSYL_0105160.t1-p1
OG0003499: TCRU_9885 TcCLB-EL.508347.80_mRNA-p1 TcCLB-NE.
507615.100_mRNA-p1 TcSYL_0105130.t1-p1
OG0003500: TCRU_9893 TcCLB-EL.509497.20_mRNA-p1 TcCLB-NE.
510947.30_mRNA-p1 TcSYL_0022310.t1-p1
OG0003501: TCRU_9895 TcCLB-EL.503589.10_mRNA-p1 TcCLB-NE.
510947.10_mRNA-p1 TcSYL_0022320.t1-p1
OG0003502: TCRU_9905 TcCLB-EL.503637.10_mRNA-p1 TcCLB-NE.
510183.40_mRNA-p1 TcSYL_0013100.t1-p1
OG0003503: TCRU_9906 TcCLB-EL.503637.20_mRNA-p1 TcCLB-NE.
510183.30_mRNA-p1 TcSYL_0013120.t1-p1
OG0003504: TCRU_9907 TcCLB-EL.503637.30_mRNA-p1 TcCLB-NE.
510183.20_mRNA-p1 TcSYL_0013180.t1-p1
OG0003505: TCRU_9908 TcCLB-EL.510073.40_mRNA-p1 TcCLB-NE.
509399.180_mRNA-p1 TcSYL_0177470.t1-p1
OG0003506: TCRU_9909 TcCLB-EL.510073.50_mRNA-p1 TcCLB-NE.
509399.170_mRNA-p1 TcSYL_0177440.t1-p1
OG0003507: TCRU_9913 TcCLB-EL.506369.40_mRNA-p1 TcCLB-NE.
510225.40_mRNA-p1 TcSYL_0082520.t1-p1
OG0003508: TCRU_9919 TcCLB-EL.509831.36_mRNA-p1 TcCLB-NE.
506629.30_mRNA-p1 TcSYL_0082490.t1-p1
OG0003509: TCRU_9921 TcCLB-EL.509831.20_mRNA-p1 TcCLB-NE.
506629.60_mRNA-p1 TcSYL_0082480.t1-p1
OG0003510: TCRU_9923 TcCLB-EL.506367.180_mRNA-p1 TcCLB-NE.
506629.80_mRNA-p1 TcSYL_0082470.t1-p1
OG0003511: TCRU_9925 TcCLB-EL.506367.140_mRNA-p1 TcCLB-NE.
506629.120_mRNA-p1 TcSYL_0082440.t1-p1
OG0003512: TCRU_9926 TcCLB-EL.506367.130_mRNA-p1 TcCLB-NE.
506629.130_mRNA-p1 TcSYL_0082430.t1-p1
OG0003513: TCRU_9927 TcCLB-EL.506367.120_mRNA-p1 TcCLB-NE.
506629.140_mRNA-p1 TcSYL_0082420.t1-p1
OG0003514: TCRU_9935 TcCLB-EL.504935.20_mRNA-p1 TcCLB-NE.
509037.30_mRNA-p1 TcSYL_0167140.t1-p1
OG0003515: TCRU_9936 TcCLB-EL.421959.10_mRNA-p1 TcCLB-EL.
504935.10_mRNA-p1 TcCLB-NE.509037.40_mRNA-p1
OG0003516: TCRU_9937 TcCLB-EL.510777.20_mRNA-p1 TcCLB-NE.
509037.50_mRNA-p1 TcSYL_0167150.t1-p1
OG0003517: TCRU_9940 TcCLB-EL.508489.40_mRNA-p1 TcCLB-NE.
509039.30_mRNA-p1 TcSYL_0167200.t1-p1
OG0003518: TCRU_9943 TcCLB-EL.508489.10_mRNA-p1 TcCLB-NE.
511495.30_mRNA-p1 TcSYL_0167210.t1-p1
OG0003519: TCRU_9946 TcCLB-NE.507019.130_mRNA-p1 TcSYL_0167340.t1-p1
TcSYL_0168840.t1-p1
OG0003520: TCRU_9953 TcCLB-EL.509543.50_mRNA-p1 TcCLB-NE.
511257.10_mRNA-p1 TcSYL_0023320.t1-p1
OG0003521: TCRU_9967 TcCLB-EL.511197.60_mRNA-p1 TcCLB-NE.
506001.50_mRNA-p1 TcCLB-NE.509665.20_mRNA-p1
OG0003522: TCRU_9972 TcCLB-EL.503899.90_mRNA-p1 TcCLB-NE.
503625.10_mRNA-p1 TcSYL_0023100.t1-p1
OG0003523: TCRU_9974 TcCLB-EL.503899.70_mRNA-p1 TcCLB-NE.
507513.110_mRNA-p1 TcSYL_0023110.t1-p1

OG0003524: TCRU_9976 TcCLB-EL.503899.50_mRNA-p1 TcCLB-NE.
507513.90_mRNA-p1 TcSYL_0023120.t1-p1
OG0003525: TCRU_9982 TcCLB-EL.506729.50_mRNA-p1 TcCLB-NE.
503843.40_mRNA-p1 TcCLB-NE.507513.10_mRNA-p1
OG0003526: TCRU_9983 TcCLB-EL.511423.160_mRNA-p1 TcCLB-NE.
507949.20_mRNA-p1 TcSYL_0110920.t1-p1
OG0003527: TCRU_9994 TcCLB-EL.506657.70_mRNA-p1 TcCLB-NE.
511247.27_mRNA-p1 TcSYL_0027300.t1-p1
OG0003528: TCRU_9996 TcCLB-EL.506657.40_mRNA-p1 TcCLB-NE.
511249.20_mRNA-p1 TcSYL_0027310.t1-p1
OG0003529: TCRU_10010 TcCLB-EL.511189.80_mRNA-p1 TcCLB-NE.
508667.49_mRNA-p1 TcSYL_0087560.t1-p1
OG0003530: TCRU_10011 TcCLB-EL.511189.70_mRNA-p1 TcCLB-NE.
508667.40_mRNA-p1 TcSYL_0087600.t1-p1
OG0003531: TCRU_10012 TcCLB-EL.511189.60_mRNA-p1 TcCLB-NE.
508667.30_mRNA-p1 TcSYL_0087770.t1-p1
OG0003532: TCRU_10013 TcCLB-EL.511189.54_mRNA-p1 TcCLB-NE.
508667.20_mRNA-p1 TcSYL_0087800.t1-p1
OG0003533: TCRU_10026 TcCLB-EL.507047.180_mRNA-p1 TcCLB-NE.
509179.70_mRNA-p1 TcSYL_0159940.t1-p1
OG0003534: TCRU_10029 TcCLB-EL.507047.140_mRNA-p1 TcCLB-NE.
509179.110_mRNA-p1 TcSYL_0159960.t1-p1
OG0003535: TCRU_10031 TcCLB-EL.507047.110_mRNA-p1 TcCLB-NE.
509179.150_mRNA-p1 TcSYL_0159970.t1-p1
OG0003536: TCRU_10033 TcCLB-EL.507047.80_mRNA-p1 TcCLB-NE.
509179.180_mRNA-p1 TcSYL_0159980.t1-p1
OG0003537: TCRU_10035 TcCLB-EL.507047.70_mRNA-p1 TcCLB-NE.
509179.200_mRNA-p1 TcSYL_0159990.t1-p1
OG0003538: TCRU_10036 TcCLB-EL.507047.60_mRNA-p1 TcCLB-NE.
509179.210_mRNA-p1 TcSYL_0160000.t1-p1
OG0003539: TCRU_10039 TcCLB-EL.511573.58_mRNA-p1 TcCLB-NE.
503773.50_mRNA-p1 TcSYL_0094680.t1-p1
OG0003540: TCRU_10041 TcCLB-EL.511575.10_mRNA-p1 TcCLB-NE.
503773.30_mRNA-p1 TcSYL_0094700.t1-p1
OG0003541: TCRU_10042 TcCLB-EL.511575.40_mRNA-p1 TcCLB-NE.
503773.9_mRNA-p1 TcSYL_0094710.t1-p1
OG0003542: TCRU_10048 TcCLB-EL.511307.10_mRNA-p1 TcCLB-NE.
507087.40_mRNA-p1 TcSYL_0159760.t1-p1
OG0003543: TCRU_10052 TcCLB-EL.511571.40_mRNA-p1 TcCLB-NE.
506229.60_mRNA-p1 TcSYL_0094600.t1-p1
OG0003544: TCRU_10053 TcCLB-EL.511573.10_mRNA-p1 TcCLB-NE.
506229.40_mRNA-p1 TcSYL_0094640.t1-p1
OG0003545: TCRU_10054 TcCLB-EL.511573.20_mRNA-p1 TcCLB-NE.
506229.30_mRNA-p1 TcSYL_0094650.t1-p1
OG0003546: TCRU_10055 TcCLB-EL.511573.30_mRNA-p1 TcCLB-NE.
506229.20_mRNA-p1 TcSYL_0094660.t1-p1
OG0003547: TCRU_10056 TcCLB-EL.511573.40_mRNA-p1 TcCLB-NE.
506229.10_mRNA-p1 TcSYL_0094670.t1-p1
OG0003548: TCRU_10057 TcCLB-EL.506931.40_mRNA-p1 TcCLB-NE.
510131.10_mRNA-p1 TcSYL_0044700.t1-p1
OG0003549: TCRU_10058 TcCLB-EL.506931.30_mRNA-p1 TcCLB-NE.
510131.20_mRNA-p1 TcSYL_0044720.t1-p1
OG0003550: TCRU_10060 TcCLB-EL.506931.10_mRNA-p1 TcCLB-NE.
510131.40_mRNA-p1 TcSYL_0044730.t1-p1

OG0003551: TCRU_10065 TcCLB-EL.504179.20_mRNA-p1 TcCLB-NE.
507825.10_mRNA-p1 TcSYL_0107010.t1-p1
OG0003552: TCRU_10084 TcCLB-EL.506563.100_mRNA-p1 TcCLB-NE.
509003.10_mRNA-p1 TcSYL_0063500.t1-p1
OG0003553: TCRU_10088 TcCLB-EL.506563.60_mRNA-p1 TcCLB-NE.
509003.50_mRNA-p1 TcSYL_0063480.t1-p1
OG0003554: TCRU_10093 TcCLB-EL.508777.90_mRNA-p1 TcCLB-NE.
509029.120_mRNA-p1 TcSYL_0156090.t1-p1
OG0003555: TCRU_10101 TcCLB-EL.506529.516_mRNA-p1 TcCLB-NE.
510889.231_mRNA-p1 TcSYL_0178120.t1-p1
OG0003556: TCRU_10109 TcCLB-EL.510077.30_mRNA-p1 TcCLB-NE.
505037.60_mRNA-p1 TcSYL_0194450.t1-p1
OG0003557: TCRU_10111 TcCLB-EL.503653.9_mRNA-p1 TcCLB-NE.
505037.40_mRNA-p1 TcSYL_0194440.t1-p1
OG0003558: TCRU_10112 TcCLB-EL.503653.20_mRNA-p1 TcCLB-NE.
505037.30_mRNA-p1 TcSYL_0194430.t1-p1
OG0003559: TCRU_10113 TcCLB-EL.503653.30_mRNA-p1 TcCLB-NE.
505037.20_mRNA-p1 TcSYL_0194420.t1-p1
OG0003560: TCRU_10115 TcCLB-EL.506739.10_mRNA-p1 TcCLB-NE.
510819.10_mRNA-p1 TcSYL_0050010.t1-p1
OG0003561: TCRU_10119 TcCLB-EL.503789.70_mRNA-p1 TcCLB-NE.
505183.50_mRNA-p1 TcSYL_0047990.t1-p1
OG0003562: TCRU_10121 TcCLB-EL.503789.40_pseudogenic_transcript-p1
TcCLB-NE.505183.80_mRNA-p1 TcSYL_0048000.t1-p1
OG0003563: TCRU_10125 TcCLB-EL.504125.100_pseudogenic_transcript-p1
TcCLB-NE.505183.130_mRNA-p1 TcSYL_0048010.t1-p1
OG0003564: TCRU_10149 TcCLB-EL.506649.100_mRNA-p1 TcCLB-NE.
508349.20_mRNA-p1 TcSYL_0147430.t1-p1
OG0003565: TCRU_10151 TcCLB-EL.506481.39_mRNA-p1 TcCLB-EL.
511365.100_mRNA-p1 TcCLB-NE.511297.10_mRNA-p1
OG0003566: TCRU_10155 TcCLB-EL.430605.20_mRNA-p1 TcCLB-NE.
509353.40_mRNA-p1 TcSYL_0064480.t1-p1
OG0003567: TCRU_10156 TcCLB-EL.430605.10_mRNA-p1 TcCLB-NE.
509353.50_mRNA-p1 TcSYL_0064490.t1-p1
OG0003568: TCRU_10161 TcCLB-EL.509669.160_mRNA-p1 TcCLB-NE.
506175.100_mRNA-p1 TcSYL_0002010.t1-p1
OG0003569: TCRU_10163 TcCLB-EL.504625.99_mRNA-p1 TcCLB-NE.
511635.40_mRNA-p1 TcSYL_0007970.t1-p1
OG0003570: TCRU_10164 TcCLB-EL.504625.90_mRNA-p1 TcCLB-NE.
511635.50_mRNA-p1 TcSYL_0007980.t1-p1
OG0003571: TCRU_10167 TcCLB-EL.504625.60_mRNA-p1 TcCLB-NE.
503583.90_mRNA-p1 TcSYL_0007990.t1-p1
OG0003572: TCRU_10171 TcCLB-EL.504625.10_mRNA-p1 TcCLB-NE.
503583.40_mRNA-p1 TcSYL_0008010.t1-p1
OG0003573: TCRU_10172 TcCLB-EL.503489.30_mRNA-p1 TcCLB-NE.
503583.30_mRNA-p1 TcSYL_0008020.t1-p1
OG0003574: TCRU_10175 TcCLB-EL.503489.10_mRNA-p1 TcCLB-NE.
503583.10_mRNA-p1 TcCLB-NE.511637.9_mRNA-p1
OG0003575: TCRU_10176 TcCLB-EL.506311.30_mRNA-p1 TcCLB-NE.
509105.10_mRNA-p1 TcSYL_0008030.t1-p1
OG0003576: TCRU_10178 TcCLB-EL.503419.30_mRNA-p1 TcCLB-NE.
509105.70_mRNA-p1 TcSYL_0008040.t1-p1
OG0003577: TCRU_10226 TcCLB-EL.509979.40_mRNA-p1 TcCLB-NE.
510041.30_mRNA-p1 TcSYL_0156550.t1-p1

OG0003578: TCRU_10227 TcCLB-EL.509979.70_mRNA-p1 TcCLB-NE.
510039.100_mRNA-p1 TcSYL_0156530.t1-p1
OG0003579: TCRU_10233 TcCLB-EL.510721.10_mRNA-p1 TcCLB-NE.
511909.10_mRNA-p1 TcSYL_0121430.t1-p1
OG0003580: TCRU_10235 TcCLB-EL.506399.89_mRNA-p1 TcCLB-NE.
511909.40_mRNA-p1 TcSYL_0121410.t1-p1
OG0003581: TCRU_10265 TcCLB-EL.507521.30_mRNA-p1 TcCLB-NE.
508579.20_mRNA-p1 TcSYL_0041280.t1-p1
OG0003582: TCRU_10273 TcCLB-EL.511753.80_mRNA-p1 TcCLB-NE.
511501.50_mRNA-p1 TcSYL_0139100.t1-p1
OG0003583: TCRU_10305 TcCLB-EL.506527.10_mRNA-p1 TcCLB-NE.
511283.170_mRNA-p1 TcSYL_0123690.t1-p1
OG0003584: TCRU_10309 TcCLB-EL.506525.104_mRNA-p1 TcCLB-NE.
511283.109_mRNA-p1 TcSYL_0123730.t1-p1
OG0003585: TCRU_10311 TcCLB-EL.506525.80_mRNA-p1 TcCLB-NE.
511283.90_mRNA-p1 TcSYL_0123740.t1-p1
OG0003586: TCRU_10314 TcCLB-EL.506525.45_pseudogenic_transcript-p1
TcCLB-NE.511283.50_mRNA-p1 TcSYL_0123750.t1-p1
OG0003587: TCRU_10317 TcCLB-EL.506525.20_mRNA-p1 TcCLB-NE.
511283.20_mRNA-p1 TcSYL_0014650.t1-p1
OG0003588: TCRU_10318 TcCLB-EL.506525.10_mRNA-p1 TcCLB-NE.
511283.10_mRNA-p1 TcSYL_0123760.t1-p1
OG0003589: TCRU_10323 TcCLB-EL.507689.25_mRNA-p1 TcCLB-NE.
504173.30_mRNA-p1 TcSYL_0033660.t1-p1
OG0003590: TCRU_10328 TcCLB-EL.507669.220_mRNA-p1 TcCLB-NE.
509875.240_mRNA-p1 TcSYL_0048670.t1-p1
OG0003591: TCRU_10330 TcCLB-EL.508179.50_mRNA-p1 TcCLB-NE.
510941.6_mRNA-p1 TcSYL_0000370.t1-p1
OG0003592: TCRU_10331 TcCLB-EL.508179.30_mRNA-p1 TcCLB-NE.
510941.20_mRNA-p1 TcSYL_0000360.t1-p1
OG0003593: TCRU_10343 TcCLB-EL.434931.20_mRNA-p1 TcCLB-NE.
508961.70_mRNA-p1 TcSYL_0188120.t1-p1
OG0003594: TCRU_10344 TcCLB-EL.508839.20_mRNA-p1 TcCLB-NE.
508961.40_mRNA-p1 TcSYL_0188160.t1-p1
OG0003595: TCRU_10347 TcCLB-EL.508839.50_mRNA-p1 TcCLB-NE.
508961.10_mRNA-p1 TcSYL_0188240.t1-p1
OG0003596: TCRU_10348 TcCLB-EL.508839.60_mRNA-p1 TcCLB-NE.
508961.4_mRNA-p1 TcSYL_0188280.t1-p1
OG0003597: TCRU_10352 TcCLB-EL.510317.20_mRNA-p1 TcCLB-NE.
508153.450_mRNA-p1 TcSYL_0000870.t1-p1
OG0003598: TCRU_10354 TcCLB-EL.506697.70_mRNA-p1 TcCLB-NE.
508153.470_mRNA-p1 TcSYL_0000850.t1-p1
OG0003599: TCRU_10355 TcCLB-EL.506697.60_mRNA-p1 TcCLB-NE.
508153.480_mRNA-p1 TcSYL_0000840.t1-p1
OG0003600: TCRU_10356 TcCLB-EL.506697.50_mRNA-p1 TcCLB-NE.
508153.490_mRNA-p1 TcSYL_0000820.t1-p1
OG0003601: TCRU_10357 TcCLB-EL.506697.40_mRNA-p1 TcCLB-NE.
508153.500_mRNA-p1 TcSYL_0000810.t1-p1
OG0003602: TCRU_10358 TcCLB-EL.506697.30_mRNA-p1 TcCLB-NE.
508153.510_mRNA-p1 TcSYL_0000800.t1-p1
OG0003603: TCRU_10365 TcCLB-EL.509011.60_mRNA-p1 TcCLB-NE.
504575.20_mRNA-p1 TcSYL_0030790.t1-p1
OG0003604: TCRU_10366 TcCLB-EL.509011.70_mRNA-p1 TcCLB-NE.
504575.40_mRNA-p1 TcSYL_0030860.t1-p1

OG0003605: TCRU_10368 TcCLB-EL.508277.190_mRNA-p1 TcCLB-NE.
509945.10_mRNA-p1 TcSYL_0064180.t1-p1
OG0003606: TCRU_10370 TcCLB-EL.508277.230_mRNA-p1 TcCLB-NE.
503913.4_pseudogenic_transcript-p1 TcSYL_0064220.t1-p1
OG0003607: TCRU_10371 TcCLB-EL.508277.250_mRNA-p1 TcCLB-NE.
503913.10_mRNA-p1 TcSYL_0064230.t1-p1
OG0003608: TCRU_10372 TcCLB-EL.508277.260_mRNA-p1 TcCLB-NE.
503913.20_mRNA-p1 TcSYL_0064240.t1-p1
OG0003609: TCRU_10377 TcCLB-NE.507067.30_pseudogenic_transcript-p1
TcSYL_0068350.t1-p1 TcSYL_0069780.t1-p1
OG0003610: TCRU_10379 TcCLB-EL.504835.20_mRNA-p1 TcCLB-NE.
509913.10_mRNA-p1 TcSYL_0057610.t1-p1
OG0003611: TCRU_10380 TcCLB-EL.504835.10_mRNA-p1 TcCLB-NE.
509913.20_mRNA-p1 TcSYL_0057630.t1-p1
OG0003612: TCRU_10385 TcCLB-EL.511111.50_mRNA-p1 TcCLB-NE.
507715.80_mRNA-p1 TcSYL_0086650.t1-p1
OG0003613: TCRU_10388 TcCLB-EL.511111.10_mRNA-p1 TcCLB-NE.
506235.10_mRNA-p1 TcSYL_0086630.t1-p1
OG0003614: TCRU_10391 TcCLB-EL.508723.10_mRNA-p1 TcCLB-NE.
509791.80_mRNA-p1 TcSYL_0107150.t1-p1
OG0003615: TCRU_10392 TcCLB-EL.511039.20_mRNA-p1 TcCLB-NE.
509791.110_mRNA-p1 TcSYL_0107130.t1-p1
OG0003616: TCRU_10402 TcCLB-EL.507585.20_mRNA-p1 TcCLB-NE.
507659.10_mRNA-p1 TcSYL_0037050.t1-p1
OG0003617: TCRU_10404 TcCLB-EL.507689.20_mRNA-p1 TcCLB-NE.
504173.40_mRNA-p1 TcSYL_0034020.t1-p1
OG0003618: TCRU_10431 TcCLB-EL.511529.50_mRNA-p1 TcCLB-NE.
511031.10_mRNA-p1 TcSYL_0112210.t1-p1
OG0003619: TCRU_protein_ TcCLB-EL.511529.40_mRNA-p1 TcCLB-NE.
511031.20_mRNA-p1 TcSYL_0112220.t1-p1
OG0003620: TCRU_10433 TcCLB-EL.511529.20_mRNA-p1 TcCLB-NE.
511031.40_mRNA-p1 TcSYL_0112230.t1-p1
OG0003621: TCRU_10440 TcCLB-EL.511555.80_mRNA-p1 TcCLB-NE.
506621.30_mRNA-p1 TcSYL_0073370.t1-p1
OG0003622: TCRU_10441 TcCLB-EL.511555.60_mRNA-p1 TcCLB-NE.
506621.14_mRNA-p1 TcSYL_0073380.t1-p1
OG0003623: TCRU_10448 TcCLB-EL.511577.160_mRNA-p1 TcCLB-NE.
509337.10_mRNA-p1 TcSYL_0109530.t1-p1
OG0003624: TCRU_10455 TcCLB-EL.510311.10_mRNA-p1 TcCLB-NE.
510265.100_mRNA-p1 TcSYL_0000490.t1-p1
OG0003625: TCRU_10459 TcCLB-EL.510311.50_mRNA-p1 TcCLB-NE.
510265.40_mRNA-p1 TcSYL_0000500.t1-p1
OG0003626: TCRU_10460 TcCLB-EL.510311.60_mRNA-p1 TcCLB-NE.
510265.30_mRNA-p1 TcSYL_0000520.t1-p1
OG0003627: TCRU_10461 TcCLB-EL.510311.70_mRNA-p1 TcCLB-NE.
510265.20_mRNA-p1 TcSYL_0000530.t1-p1
OG0003628: TCRU_10505 TcCLB-EL.503955.70_mRNA-p1 TcCLB-NE.
507063.20_mRNA-p1 TcSYL_0013630.t1-p1
OG0003629: TCRU_10507 TcCLB-EL.508555.20_mRNA-p1 TcCLB-EL.
511337.4_mRNA-p1 TcCLB-NE.509237.90_mRNA-p1
OG0003630: TCRU_10525 TcCLB-NE.509647.150_mRNA-p1 TcSYL_0010260.t1-
p1 TcSYL_0010270.t1-p1
OG0003631: TCRU_10526 TcCLB-EL.511001.140_mRNA-p1 TcCLB-NE.
506957.70_mRNA-p1 TcSYL_0131770.t1-p1

OG0003632: TCRU_10527 TcCLB-EL.511001.130_mRNA-p1 TcCLB-NE.506957.80_mRNA-p1 TcSYL_0131910.t1-p1
OG0003633: TCRU_10529 TcCLB-EL.511001.100_mRNA-p1 TcCLB-NE.506957.110_mRNA-p1 TcSYL_0132010.t1-p1
OG0003634: TCRU_10530 TcCLB-EL.511001.90_mRNA-p1 TcCLB-NE.506957.120_mRNA-p1 TcSYL_0132060.t1-p1
OG0003635: TCRU_10531 TcCLB-EL.511001.80_mRNA-p1 TcCLB-NE.506957.130_mRNA-p1 TcSYL_0132140.t1-p1
OG0003636: TCRU_10532 TcCLB-EL.511001.70_mRNA-p1 TcCLB-NE.506957.140_mRNA-p1 TcSYL_0132180.t1-p1
OG0003637: TCRU_10533 TcCLB-EL.511001.60_mRNA-p1 TcCLB-NE.503635.10_mRNA-p1 TcSYL_0132270.t1-p1
OG0003638: TCRU_10535 TcCLB-EL.508989.80_mRNA-p1 TcCLB-NE.509569.170_mRNA-p1 TcSYL_0097480.t1-p1
OG0003639: TCRU_10545 TcCLB-EL.506499.210_mRNA-p1 TcCLB-EL.506763.280_mRNA-p1 TcCLB-NE.510197.20_mRNA-p1
OG0003640: TCRU_10576 TcCLB-EL.506733.100_mRNA-p1 TcCLB-NE.509229.140_mRNA-p1 TcSYL_0091770.t1-p1
OG0003641: TCRU_10578 TcCLB-EL.506733.130_mRNA-p1 TcCLB-NE.509229.110_mRNA-p1 TcSYL_0091780.t1-p1
OG0003642: TCRU_10580 TcCLB-EL.506735.10_mRNA-p1 TcCLB-NE.509229.80_mRNA-p1 TcSYL_0091790.t1-p1
OG0003643: TCRU_10586 TcCLB-EL.509777.140_mRNA-p1 TcCLB-NE.511655.40_mRNA-p1 TcSYL_0013940.t1-p1
OG0003644: TCRU_of TcCLB-EL.506129.10_pseudogenic_transcript-p1 TcCLB-EL.506343.20_pseudogenic_transcript-p1 TcSYL_0083990.t1-p1
OG0003645: TCRU_10611 TcCLB-EL.511627.130_mRNA-p1 TcCLB-NE.507801.110_mRNA-p1 TcSYL_0084280.t1-p1
OG0003646: TCRU_10616 TcCLB-EL.509103.20_mRNA-p1 TcCLB-NE.507801.50_mRNA-p1 TcSYL_0084260.t1-p1
OG0003647: TCRU_10617 TcCLB-EL.509103.30_mRNA-p1 TcCLB-NE.507801.40_mRNA-p1 TcSYL_0084250.t1-p1
OG0003648: TCRU_10624 TcCLB-EL.506893.100_mRNA-p1 TcCLB-NE.508501.250_mRNA-p1 TcSYL_0105250.t1-p1
OG0003649: TCRU_10625 TcCLB-EL.506893.90_mRNA-p1 TcCLB-NE.508501.240_mRNA-p1 TcSYL_0110340.t1-p1
OG0003650: TCRU_10631 TcCLB-EL.507747.220_mRNA-p1 TcCLB-NE.503723.70_mRNA-p1 TcSYL_0023690.t1-p1
OG0003651: TCRU_10632 TcCLB-EL.507747.210_mRNA-p1 TcCLB-NE.503723.80_mRNA-p1 TcSYL_0023700.t1-p1
OG0003652: TCRU_10649 TcCLB-EL.506801.120_mRNA-p1 TcCLB-NE.511907.240_mRNA-p1 TcSYL_0121540.t1-p1
OG0003653: TCRU_10650 TcCLB-EL.506801.90_mRNA-p1 TcCLB-NE.511907.210_mRNA-p1 TcSYL_0121560.t1-p1
OG0003654: TCRU_10651 TcCLB-EL.506801.80_mRNA-p1 TcCLB-NE.511907.200_mRNA-p1 TcSYL_0121570.t1-p1
OG0003655: TCRU_10652 TcCLB-EL.506801.70_mRNA-p1 TcCLB-NE.511907.190_mRNA-p1 TcSYL_0101800.t1-p1
OG0003656: TCRU_10655 TcCLB-EL.510741.229_mRNA-p1 TcCLB-NE.510661.19_mRNA-p1 TcSYL_0116220.t1-p1
OG0003657: TCRU_10662 TcCLB-EL.417091.10_mRNA-p1 TcCLB-EL.506625.249_mRNA-p1 TcCLB-NE.510221.10_mRNA-p1
OG0003658: TCRU_10664 TcCLB-EL.461927.9_mRNA-p1 TcCLB-EL.508129.9_mRNA-p1 TcCLB-NE.510221.39_mRNA-p1

OG0003659: TCRU_10671 TcCLB-EL.510055.60_mRNA-p1 TcCLB-NE.
511319.10_mRNA-p1 TcSYL_0159240.t1-p1
OG0003660: TCRU_10679 TcCLB-EL.504117.20_mRNA-p1 TcCLB-NE.
506619.10_mRNA-p1 TcSYL_0087410.t1-p1
OG0003661: TCRU_10684 TcCLB-EL.510513.50_mRNA-p1 TcCLB-NE.
509857.20_mRNA-p1 TcSYL_0174480.t1-p1
OG0003662: TCRU_10685 TcCLB-EL.510513.60_mRNA-p1 TcCLB-NE.
509857.30_mRNA-p1 TcSYL_0174490.t1-p1
OG0003663: TCRU_10686 TcCLB-EL.462761.4_mRNA-p1 TcCLB-EL.
503755.54_mRNA-p1 TcCLB-NE.510131.99_mRNA-p1
OG0003664: TCRU_10688 TcCLB-EL.506773.40_mRNA-p1 TcCLB-NE.
508799.170_mRNA-p1 TcSYL_0075280.t1-p1
OG0003665: TCRU_10689 TcCLB-EL.507037.50_mRNA-p1 TcCLB-NE.
508707.64_mRNA-p1 TcSYL_0112500.t1-p1
OG0003666: TCRU_10690 TcCLB-EL.506467.10_mRNA-p1 TcCLB-NE.
505193.80_mRNA-p1 TcSYL_0104500.t1-p1
OG0003667: TCRU_10691 TcCLB-EL.504881.30_mRNA-p1 TcCLB-NE.
510043.50_mRNA-p1 TcSYL_0064910.t1-p1
OG0003668: TCRU_10694 TcCLB-EL.504153.360_mRNA-p1 TcCLB-NE.
506301.50_mRNA-p1 TcSYL_0138540.t1-p1
OG0003669: TCRU_10695 TcCLB-EL.506811.200_mRNA-p1 TcCLB-NE.
506303.30_mRNA-p1 TcSYL_0138520.t1-p1
OG0003670: TCRU_10699 TcCLB-EL.508211.40_mRNA-p1 TcCLB-NE.
507515.110_mRNA-p1 TcSYL_0023040.t1-p1
OG0003671: TCRU_10704 TcCLB-EL.511041.30_mRNA-p1 TcCLB-NE.
506355.110_mRNA-p1 TcSYL_0107490.t1-p1
OG0003672: TCRU_10711 TcCLB-EL.511911.120_mRNA-p1 TcCLB-NE.
508637.79_mRNA-p1 TcSYL_0170370.t1-p1
OG0003673: TCRU_10715 TcCLB-EL.507963.44_mRNA-p1 TcCLB-NE.
507031.64_mRNA-p1 TcSYL_0122380.t1-p1
OG0003674: TCRU_10718 TcCLB-EL.506503.60_mRNA-p1 TcCLB-NE.
511293.80_mRNA-p1 TcSYL_0122280.t1-p1
OG0003675: TCRU_10719 TcCLB-EL.506825.210_mRNA-p1 TcCLB-NE.
508175.110_mRNA-p1 TcSYL_0078800.t1-p1
OG0003676: TCRU_10726 TcCLB-EL.509807.29_mRNA-p1 TcCLB-NE.
511261.50_mRNA-p1 TcSYL_0016180.t1-p1
OG0003677: TCRU_10728 TcCLB-EL.509809.30_mRNA-p1 TcCLB-NE.
511261.10_mRNA-p1 TcSYL_0016330.t1-p1
OG0003678: TCRU_10739 TcCLB-EL.510181.150_mRNA-p1 TcCLB-NE.
503431.10_mRNA-p1 TcSYL_0013340.t1-p1
OG0003679: TCRU_10745 TcCLB-EL.506777.30_mRNA-p1 TcCLB-NE.
511163.59_mRNA-p1 TcSYL_0075530.t1-p1
OG0003680: TCRU_10747 TcCLB-EL.509233.220_mRNA-p1 TcCLB-EL.
509451.24_mRNA-p1 TcSYL_0145980.t1-p1
OG0003681: TCRU_10757 TcCLB-EL.507105.30_mRNA-p1 TcCLB-NE.
506815.50_mRNA-p1 TcSYL_0088030.t1-p1
OG0003682: TCRU_10758 TcCLB-EL.511585.70_mRNA-p1 TcCLB-NE.
510155.70_mRNA-p1 TcSYL_0109280.t1-p1
OG0003683: TCRU_10760 TcCLB-EL.511585.30_mRNA-p1 TcCLB-NE.
510155.30_mRNA-p1 TcSYL_0109310.t1-p1
OG0003684: TCRU_10761 TcCLB-EL.511585.20_mRNA-p1 TcCLB-NE.
510155.20_mRNA-p1 TcSYL_0109330.t1-p1
OG0003685: TCRU_10768 TcCLB-EL.506743.140_mRNA-p1 TcCLB-NE.
503851.40_mRNA-p1 TcSYL_0164960.t1-p1

OG0003686: TCRU_10769 TcCLB-EL.508989.9_mRNA-p1 TcCLB-NE.
509569.100_mRNA-p1 TcSYL_0097300.t1-p1
OG0003687: TCRU_10770 TcCLB-EL.506865.30_mRNA-p1 TcCLB-NE.
509569.60_mRNA-p1 TcSYL_0097220.t1-p1
OG0003688: TCRU_10771 TcCLB-EL.506865.70_mRNA-p1 TcCLB-NE.
509569.30_mRNA-p1 TcSYL_0097100.t1-p1
OG0003689: TCRU_10780 TcCLB-EL.506567.134_mRNA-p1 TcCLB-NE.
507049.119_mRNA-p1 TcSYL_0114650.t1-p1
OG0003690: TCRU_protein_ TcCLB-EL.506567.60_mRNA-p1 TcCLB-NE.
507049.50_mRNA-p1 TcSYL_0114720.t1-p1
OG0003691: TCRU_10788 TcCLB-EL.508675.40_mRNA-p1 TcCLB-NE.
506983.30_mRNA-p1 TcSYL_0027240.t1-p1
OG0003692: TCRU_10795 TcCLB-EL.510073.70_mRNA-p1 TcCLB-NE.
509399.160_mRNA-p1 TcSYL_0177410.t1-p1
OG0003693: TCRU_10801 TcCLB-EL.503899.10_mRNA-p1 TcCLB-NE.
507513.50_mRNA-p1 TcSYL_0023150.t1-p1
OG0003694: TCRU_10803 TcCLB-EL.508993.10_mRNA-p1 TcCLB-NE.
506479.130_mRNA-p1 TcSYL_0110910.t1-p1
OG0003695: TCRU_10804 TcCLB-EL.503805.20_mRNA-p1 TcCLB-NE.
506479.90_mRNA-p1 TcSYL_0110900.t1-p1
OG0003696: TCRU_10807 TcCLB-EL.511189.84_mRNA-p1 TcCLB-NE.
510975.10_pseudogenic_transcript-p1 TcSYL_0087530.t1-p1
OG0003697: TCRU_10809 TcCLB-EL.507047.150_mRNA-p1 TcCLB-NE.
509179.100_mRNA-p1 TcSYL_0159950.t1-p1
OG0003698: TCRU_10812 TcCLB-EL.507047.50_mRNA-p1 TcCLB-NE.
509179.220_mRNA-p1 TcSYL_0106540.t1-p1
OG0003699: TCRU_10813 TcCLB-EL.503525.50_mRNA-p1 TcCLB-NE.
507825.20_mRNA-p1 TcSYL_0106990.t1-p1
OG0003700: TCRU_10814 TcCLB-EL.503525.30_mRNA-p1 TcCLB-NE.
507825.40_mRNA-p1 TcSYL_0106980.t1-p1
OG0003701: TCRU_10820 TcCLB-EL.504625.50_mRNA-p1 TcCLB-NE.
503583.80_mRNA-p1 TcSYL_0008000.t1-p1
OG0003702: TCRU_10831 TcCLB-EL.508809.30_mRNA-p1 TcCLB-NE.
506619.90_mRNA-p1 TcSYL_0087140.t1-p1
OG0003703: TCRU_10845 TcCLB-EL.511039.30_mRNA-p1 TcCLB-NE.
509791.100_mRNA-p1 TcSYL_0107140.t1-p1
OG0003704: TCRU_10846 TcCLB-EL.508721.10_mRNA-p1 TcCLB-NE.
509791.170_mRNA-p1 TcSYL_0107110.t1-p1
OG0003705: TCRU_10862 TcCLB-EL.507105.70_mRNA-p1 TcCLB-NE.
506817.20_mRNA-p1 TcSYL_0088060.t1-p1
OG0003706: TCRU_family_ TcCLB-EL.509777.90_mRNA-p1 TcCLB-NE.
509117.19_mRNA-p1 TcSYL_0013910.t1-p1
OG0003707: TCRU_10873 TcCLB-EL.511627.159_mRNA-p1 TcCLB-NE.
507801.80_mRNA-p1 TcSYL_0084270.t1-p1
OG0003708: TCRU_10875 TcCLB-EL.506801.110_mRNA-p1 TcCLB-NE.
511907.230_mRNA-p1 TcSYL_0121550.t1-p1
OG0003709: TCRU_10877 TcCLB-EL.510525.120_mRNA-p1 TcCLB-NE.
511817.190_mRNA-p1 TcSYL_0201560.t1-p1
OG0003710: TcCLB-EL.506717.130_pseudogenic_transcript-p1 TcCLB-EL.
507981.51_pseudogenic_transcript-p1 TcCLB-EL.
507985.30_pseudogenic_transcript-p1 TcSYL_0114400.t1-p1
OG0003711: TcCLB-EL.509279.10_mRNA-p1 TcCLB-NE.510829.10_mRNA-p1
TcSYL_0051170.t1-p1 TcSYL_0051180.t1-p1
OG0003712: TcCLB-EL.508949.4_mRNA-p1 TcCLB-EL.511369.5_mRNA-p1

TcSYL_0169270.t1-p1 TcSYL_0169350.t1-p1
OG0003713: TcCLB-EL.503855.50_mRNA-p1 TcCLB-NE.510335.30_pseudogenic_transcript-p1 TcCLB-NE.510337.11_mRNA-p1 TcSYL_0146600.t1-p1
OG0003714: TcCLB-EL.506623.30_mRNA-p1 TcCLB-EL.509513.5_pseudogenic_transcript-p1 TcCLB-NE.508455.30_mRNA-p1 TcSYL_0028460.t1-p1
OG0003715: TcCLB-EL.506625.35_pseudogenic_transcript-p1 TcCLB-EL.506961.30_mRNA-p1 TcCLB-NE.509437.106_pseudogenic_transcript-p1 TcSYL_0204490.t1-p1
OG0003716: TcCLB-EL.506409.60_mRNA-p1 TcCLB-EL.508165.70_mRNA-p1 TcCLB-EL.510961.50_mRNA-p1 TcSYL_0155030.t1-p1
OG0003717: TcCLB-EL.503905.10_mRNA-p1 TcCLB-EL.506685.50_mRNA-p1 TcCLB-NE.509663.10_mRNA-p1 TcSYL_0094550.t1-p1
OG0003718: TcCLB-EL.511571.49_mRNA-p1 TcCLB-NE.506229.50_mRNA-p1 TcSYL_0094610.t1-p1 TcSYL_0094620.t1-p1
OG0003719: TcCLB-EL.506633.80_mRNA-p1 TcCLB-EL.511575.120_mRNA-p1 TcCLB-NE.508027.30_mRNA-p1 TcSYL_0094790.t1-p1
OG0003720: TcCLB-EL.504127.10_pseudogenic_transcript-p1 TcCLB-NE.503655.40_pseudogenic_transcript-p1 TcCLB-NE.511859.161_mRNA-p1 TcSYL_0091150.t1-p1
OG0003721: TcCLB-EL.506515.29_mRNA-p1 TcCLB-EL.510055.20_mRNA-p1 TcCLB-NE.508913.25_mRNA-p1 TcSYL_0159200.t1-p1
OG0003722: TcCLB-EL.507979.40_mRNA-p1 TcCLB-EL.510055.30_mRNA-p1 TcCLB-NE.511319.40_mRNA-p1 TcSYL_0158810.t1-p1
OG0003723: TcCLB-EL.506961.25_mRNA-p1 TcCLB-NE.508297.7_mRNA-p1 TcSYL_0204520.t1-p1 TcSYL_0204540.t1-p1
OG0003724: TcCLB-EL.503975.50_mRNA-p1 TcCLB-NE.510977.20_pseudogenic_transcript-p1 TcSYL_0086920.t1-p1 TcSYL_0086930.t1-p1
OG0003725: TcCLB-EL.511107.41_mRNA-p1 TcCLB-NE.508665.14_mRNA-p1 TcSYL_0086460.t1-p1 TcSYL_0087930.t1-p1
OG0003726: TcCLB-EL.511189.100_mRNA-p1 TcCLB-NE.510975.40_mRNA-p1 TcSYL_0087450.t1-p1 TcSYL_0087460.t1-p1
OG0003727: TcCLB-EL.398343.9_mRNA-p1 TcCLB-EL.507747.10_mRNA-p1 TcCLB-NE.504233.10_mRNA-p1 TcSYL_0100160.t1-p1
OG0003728: TcCLB-EL.408437.10_mRNA-p1 TcCLB-EL.503627.9_mRNA-p1 TcCLB-NE.508739.80_mRNA-p1 TcSYL_0192220.t1-p1
OG0003729: TcCLB-EL.511675.10_pseudogenic_transcript-p1 TcCLB-NE.508139.80_pseudogenic_transcript-p1 TcCLB-NE.509287.170_pseudogenic_transcript-p1 TcCLB-NE.510359.430_pseudogenic_transcript-p1
OG0003730: TcCLB-EL.506745.4_mRNA-p1 TcCLB-NE.511925.70_mRNA-p1 TcSYL_0014230.t1-p1 TcSYL_0164700.t1-p1
OG0003731: TcCLB-EL.506183.80_mRNA-p1 TcCLB-NE.508575.20_mRNA-p1 TcCLB-NE.510483.350_mRNA-p1 TcCLB-NE.511771.35_mRNA-p1
OG0003732: TcCLB-EL.510721.31_mRNA-p1 TcCLB-NE.509005.81_pseudogenic_transcript-p1 TcSYL_0044420.t1-p1 TcSYL_0179390.t1-p1
OG0003733: TcCLB-EL.508389.20_mRNA-p1 TcCLB-NE.506789.110_mRNA-p1 TcSYL_0161530.t1-p1 TcSYL_0162260.t1-p1
OG0003734: TcCLB-EL.505025.80_mRNA-p1 TcCLB-EL.509195.20_mRNA-p1 TcCLB-EL.510021.190_mRNA-p1 TcSYL_0150040.t1-p1
OG0003735: TcCLB-EL.418069.10_mRNA-p1 TcSYL_0044430.t1-p1

TcSYL_0044500.t1-p1 TcSYL_0044590.t1-p1
OG0003736: TcCLB-EL.505965.92_pseudogenic_transcript-p1 TcCLB-NE.510901.250_mRNA-p1 TcSYL_0178720.t1-p1 TcSYL_0178730.t1-p1
OG0003737: TcCLB-EL.508835.30_pseudogenic_transcript-p1 TcCLB-NE.473709.10_mRNA-p1 TcCLB-NE.507051.11_mRNA-p1 TcCLB-NE.511835.10_mRNA-p1
OG0003738: TcCLB-EL.509205.110_mRNA-p1 TcCLB-NE.508699.110_mRNA-p1 TcSYL_0179540.t1-p1 TcSYL_0180900.t1-p1
OG0003739: TcCLB-EL.506945.270_mRNA-p1 TcCLB-NE.511727.199_mRNA-p1 TcSYL_0140750.t1-p1 TcSYL_0142780.t1-p1
OG0003740: TcCLB-EL.511735.79_mRNA-p1 TcCLB-EL.511737.9_mRNA-p1 TcCLB-NE.511519.18_mRNA-p1 TcCLB-NE.511521.24_mRNA-p1
OG0003741: TcCLB-EL.506637.110_mRNA-p1 TcCLB-NE.510099.100_mRNA-p1 TcCLB-NE.510099.131_mRNA-p1 TcSYL_0198560.t1-p1
OG0003742: TcCLB-EL.503677.4_mRNA-p1 TcCLB-EL.506223.10_mRNA-p1 TcCLB-NE.511717.100_mRNA-p1 TcSYL_0141830.t1-p1
OG0003743: TcCLB-EL.507041.76_pseudogenic_transcript-p1 TcCLB-NE.508235.10_pseudogenic_transcript-p1 TcCLB-NE.508273.130_mRNA-p1 TcSYL_0108550.t1-p1
OG0003744: TcCLB-EL.503779.109_mRNA-p1 TcCLB-NE.506247.470_mRNA-p1 TcSYL_0108360.t1-p1 TcSYL_0108630.t1-p1
OG0003745: TcCLB-EL.510491.80_mRNA-p1 TcCLB-NE.504245.9_mRNA-p1 TcCLB-NE.507723.70_pseudogenic_transcript-p1 TcSYL_0006160.t1-p1
OG0003746: TcCLB-EL.508121.70_mRNA-p1 TcCLB-EL.508125.40_mRNA-p1 TcCLB-EL.511173.410_mRNA-p1 TcSYL_0137200.t1-p1
OG0003747: TcCLB-EL.508541.140_pseudogenic_transcript-p1 TcCLB-NE.508427.30_pseudogenic_transcript-p1 TcCLB-NE.510695.10_pseudogenic_transcript-p1 TcCLB-NE.510697.25_pseudogenic_transcript-p1
OG0003748: TcCLB-EL.504039.70_mRNA-p1 TcCLB-EL.510205.60_mRNA-p1 TcCLB-NE.504197.10_mRNA-p1 TcSYL_0150170.t1-p1
OG0003749: TcCLB-EL.503893.10_mRNA-p1 TcCLB-EL.509015.10_mRNA-p1 TcSYL_0081000.t1-p1 TcSYL_0081370.t1-p1
OG0003750: TcCLB-EL.506885.14_mRNA-p1 TcCLB-EL.510431.274_mRNA-p1 TcCLB-NE.507773.34_mRNA-p1 TcSYL_0138350.t1-p1
OG0003751: TcCLB-EL.510755.19_mRNA-p1 TcCLB-NE.510653.19_mRNA-p1 TcSYL_0115650.t1-p1 TcSYL_0115660.t1-p1
OG0003752: TcCLB-EL.509807.11_pseudogenic_transcript-p1 TcCLB-NE.511261.71_pseudogenic_transcript-p1 TcSYL_0016130.t1-p1 TcSYL_0016140.t1-p1
OG0003753: TcCLB-EL.503875.30_mRNA-p1 TcCLB-EL.510061.11_mRNA-p1 TcSYL_0127070.t1-p1 TcSYL_0127080.t1-p1
OG0003754: TcCLB-EL.506603.46_pseudogenic_transcript-p1 TcCLB-EL.506767.317_pseudogenic_transcript-p1 TcCLB-NE.503973.345_pseudogenic_transcript-p1 TcCLB-NE.508873.395_pseudogenic_transcript-p1
OG0003755: TcCLB-EL.506763.90_pseudogenic_transcript-p1 TcCLB-EL.506769.60_pseudogenic_transcript-p1 TcCLB-EL.507957.100_pseudogenic_transcript-p1 TcCLB-NE.508221.320_pseudogenic_transcript-p1
OG0003756: TcCLB-EL.506765.58_pseudogenic_transcript-p1 TcCLB-EL.510377.300_pseudogenic_transcript-p1 TcCLB-NE.508221.697_pseudogenic_transcript-p1 TcSYL_0137420.t1-p1
OG0003757: TcCLB-EL.507953.130_mRNA-p1 TcCLB-NE.509897.260_mRNA-p1

TcSYL_0058980.t1-p1 TcSYL_0059720.t1-p1
OG0003758: TcCLB-EL.507959.140_mRNA-p1 TcCLB-EL.507959.90_mRNA-p1 TcCLB-EL.510013.110_mRNA-p1 TcCLB-NE.506459.120_mRNA-p1
OG0003759: TcCLB-EL.510375.20_pseudogenic_transcript-p1 TcCLB-EL.510377.210_pseudogenic_transcript-p1 TcCLB-NE.511255.430_pseudogenic_transcript-p1 TcCLB-NE.511487.130_pseudogenic_transcript-p1
OG0003760: TcCLB-NE.469113.10_pseudogenic_transcript-p1 TcCLB-NE.503973.20_pseudogenic_transcript-p1 TcCLB-NE.511861.130_pseudogenic_transcript-p1 TcCLB-NE.511861.14_pseudogenic_transcript-p1
OG0003761: TcCLB-NE.511771.185_mRNA-p1 TcSYL_0020390.t1-p1 TcSYL_0178480.t1-p1 TcSYL_0200500.t1-p1
OG0003762: TcCLB-NE.508139.155_pseudogenic_transcript-p1 TcSYL_0031060.t1-p1 TcSYL_0031140.t1-p1 TcSYL_0048990.t1-p1
OG0003763: TcCLB-NE.504081.370_mRNA-p1 TcCLB-NE.508873.25_mRNA-p1 TcSYL_0134940.t1-p1 TcSYL_0136970.t1-p1
OG0003764: TcCLB-NE.506751.70_pseudogenic_transcript-p1 TcCLB-NE.508219.140_pseudogenic_transcript-p1 TcCLB-NE.508221.87_pseudogenic_transcript-p1 TcCLB-NE.510359.630_pseudogenic_transcript-p1
OG0003765: TcSYL_0021420.t1-p1 TcSYL_0154490.t1-p1 TcSYL_0155170.t1-p1 TcSYL_0203940.t1-p1
OG0003766: TcSYL_0022230.t1-p1 TcSYL_0022250.t1-p1 TcSYL_0040100.t1-p1 TcSYL_0040760.t1-p1
OG0003767: TcSYL_0155520.t1-p1 TcSYL_0156210.t1-p1 TcSYL_0156280.t1-p1 TcSYL_0156390.t1-p1
OG0003768: TCRU_0 TcCLB-NE.506559.370_mRNA-p1 TcSYL_0045280.t1-p1
OG0003769: TCRU_2 TcCLB-NE.506559.350_mRNA-p1 TcSYL_0045260.t1-p1
OG0003770: TCRU_3 TcCLB-NE.506559.330_mRNA-p1 TcSYL_0045250.t1-p1
OG0003771: TCRU_4 TcCLB-NE.506559.320_mRNA-p1 TcSYL_0045240.t1-p1
OG0003772: TCRU_6 TcCLB-NE.506559.300_mRNA-p1 TcSYL_0045230.t1-p1
OG0003773: TCRU_7 TcCLB-NE.506559.290_mRNA-p1 TcSYL_0045220.t1-p1
OG0003774: TCRU_9 TcCLB-NE.506559.270_mRNA-p1 TcSYL_0045210.t1-p1
OG0003775: TCRU_11 TcCLB-EL.508131.10_pseudogenic_transcript-p1 TcCLB-NE.506559.250_mRNA-p1
OG0003776: TCRU_16 TcCLB-EL.510229.10_mRNA-p1 TcCLB-NE.506559.180_mRNA-p1
OG0003777: TCRU_19 TcCLB-EL.510231.20_mRNA-p1 TcCLB-NE.506559.110_mRNA-p1
OG0003778: TCRU_21 TcCLB-EL.447255.20_mRNA-p1 TcCLB-NE.506559.80_mRNA-p1
OG0003779: TCRU_22 TcCLB-EL.447255.4_mRNA-p1 TcCLB-NE.506559.59_mRNA-p1
OG0003780: TCRU_23 TcCLB-EL.506631.10_mRNA-p1 TcCLB-NE.506559.50_mRNA-p1
OG0003781: TCRU_25 TcCLB-EL.506631.30_mRNA-p1 TcCLB-NE.506559.30_mRNA-p1
OG0003782: TCRU_28 TcCLB-EL.508133.19_mRNA-p1 TcCLB-NE.506559.4_mRNA-p1
OG0003783: TCRU_29 TcCLB-EL.506633.10_mRNA-p1 TcCLB-NE.508051.30_mRNA-p1
OG0003784: TCRU_30 TcCLB-EL.506633.20_mRNA-p1 TcCLB-NE.508051.20_mRNA-p1

OG0003785: TCRU_31 TcCLB-EL.506633.30_mRNA-p1 TcCLB-NE.
508051.10_mRNA-p1
OG0003786: TCRU_32 TcCLB-EL.503903.70_mRNA-p1 TcCLB-NE.
508153.750_mRNA-p1
OG0003787: TCRU_35 TcCLB-EL.503903.20_mRNA-p1 TcCLB-NE.
508153.790_mRNA-p1
OG0003788: TCRU_36 TcCLB-EL.503903.10_mRNA-p1 TcCLB-NE.
508153.800_mRNA-p1
OG0003789: TCRU_37 TcCLB-EL.508181.149_mRNA-p1 TcCLB-NE.
508153.810_mRNA-p1
OG0003790: TCRU_39 TcCLB-EL.508181.124_mRNA-p1 TcCLB-NE.
508153.840_mRNA-p1
OG0003791: TCRU_40 TcCLB-EL.508181.120_mRNA-p1 TcCLB-NE.
508153.850_mRNA-p1
OG0003792: TCRU_41 TcCLB-EL.508181.110_mRNA-p1 TcCLB-NE.
508153.860_mRNA-p1
OG0003793: TCRU_42 TcCLB-EL.508181.90_pseudogenic_transcript-p1
TcCLB-NE.508153.870_mRNA-p1
OG0003794: TCRU_43 TcCLB-EL.508181.80_mRNA-p1 TcCLB-NE.
508153.880_mRNA-p1
OG0003795: TCRU_44 TcCLB-EL.508181.70_mRNA-p1 TcCLB-NE.
508153.890_mRNA-p1
OG0003796: TCRU_46 TcCLB-EL.508181.50_mRNA-p1 TcCLB-NE.
508153.910_mRNA-p1
OG0003797: TCRU_47 TcCLB-EL.508181.40_mRNA-p1 TcCLB-NE.
508153.920_mRNA-p1
OG0003798: TCRU_50 TcCLB-EL.484949.9_mRNA-p1 TcCLB-NE.
508153.970_mRNA-p1
OG0003799: TCRU_61 TcCLB-NE.508479.370_mRNA-p1 TcSYL_0019710.t1-p1
OG0003800: TCRU_62 TcCLB-EL.505009.30_mRNA-p1 TcCLB-NE.
506489.50_mRNA-p1
OG0003801: TCRU_63 TcCLB-EL.505009.40_mRNA-p1 TcCLB-NE.
506489.40_mRNA-p1
OG0003802: TCRU_64 TcCLB-EL.505009.49_mRNA-p1 TcCLB-NE.
506489.30_mRNA-p1
OG0003803: TCRU_85 TcCLB-EL.504153.129_mRNA-p1 TcSYL_0138730.t1-p1
OG0003804: TCRU_87 TcCLB-EL.504153.150_mRNA-p1 TcSYL_0138690.t1-p1
OG0003805: TCRU_88 TcCLB-EL.507711.230_mRNA-p1 TcCLB-NE.
507675.39_mRNA-p1
OG0003806: TCRU_89 TcCLB-EL.507711.220_mRNA-p1 TcCLB-NE.
507675.30_mRNA-p1
OG0003807: TCRU_90 TcCLB-EL.507711.210_mRNA-p1 TcCLB-NE.
507675.20_mRNA-p1
OG0003808: TCRU_97 TcCLB-EL.509149.20_mRNA-p1 TcCLB-NE.
508175.350_mRNA-p1
OG0003809: TCRU_101 TcCLB-NE.508799.240_mRNA-p1 TcSYL_0075250.t1-p1
OG0003810: TCRU_103 TcCLB-EL.511427.10_mRNA-p1 TcCLB-NE.
507947.10_mRNA-p1
OG0003811: TCRU_104 TcCLB-EL.511425.40_mRNA-p1 TcCLB-NE.
506479.10_mRNA-p1
OG0003812: TCRU_108 TcCLB-EL.503805.6_mRNA-p1 TcCLB-NE.
506479.67_mRNA-p1
OG0003813: TCRU_109 TcCLB-EL.503805.9_mRNA-p1 TcCLB-NE.
506479.74_mRNA-p1

OG0003814: TCRU_117 TcCLB-EL.506227.230_mRNA-p1 TcSYL_0092050.t1-p1
OG0003815: TCRU_118 TcCLB-EL.506227.240_mRNA-p1 TcSYL_0092060.t1-p1
OG0003816: TCRU_120 TcCLB-EL.504089.40_mRNA-p1 TcSYL_0092090.t1-p1
OG0003817: TCRU_121 TcCLB-EL.504089.50_mRNA-p1 TcCLB-NE.
504001.3_mRNA-p1
OG0003818: TCRU_122 TcCLB-EL.504089.54_mRNA-p1 TcCLB-NE.
504001.6_mRNA-p1
OG0003819: TCRU_124 TcCLB-EL.504089.70_mRNA-p1 TcCLB-NE.
504001.20_mRNA-p1
OG0003820: TCRU_127 TcCLB-EL.510879.80_mRNA-p1 TcCLB-NE.
504013.40_mRNA-p1
OG0003821: TCRU_129 TcCLB-EL.510879.100_mRNA-p1 TcCLB-NE.
504013.20_mRNA-p1
OG0003822: TCRU_136 TcCLB-EL.509087.10_pseudogenic_transcript-p1
TcCLB-EL.511349.80_pseudogenic_transcript-p1
OG0003823: TCRU_150 TcCLB-EL.509805.230_mRNA-p1 TcCLB-NE.
511267.24_mRNA-p1
OG0003824: TCRU_152 TcCLB-EL.509805.200_mRNA-p1 TcCLB-NE.
503939.10_mRNA-p1
OG0003825: TCRU_153 TcCLB-EL.509805.190_mRNA-p1 TcCLB-NE.
503939.20_mRNA-p1
OG0003826: TCRU_154 TcCLB-EL.509805.180_mRNA-p1 TcCLB-NE.
503939.30_mRNA-p1
OG0003827: TCRU_156 TcCLB-EL.509805.160_mRNA-p1 TcCLB-NE.
503939.50_mRNA-p1
OG0003828: TCRU_157 TcCLB-EL.509805.150_mRNA-p1 TcCLB-NE.
503939.60_mRNA-p1
OG0003829: TCRU_158 TcCLB-EL.509805.140_mRNA-p1 TcCLB-NE.
503939.70_mRNA-p1
OG0003830: TCRU_protein TcCLB-EL.509805.120_mRNA-p1 TcCLB-NE.
503939.84_mRNA-p1
OG0003831: TCRU_162 TcCLB-EL.509805.100_mRNA-p1 TcCLB-NE.
503939.100_mRNA-p1
OG0003832: TCRU_171 TcCLB-EL.510609.50_mRNA-p1 TcCLB-NE.
503617.31_mRNA-p1
OG0003833: TCRU_191 TcCLB-EL.511867.190_mRNA-p1 TcSYL_0146330.t1-p1
OG0003834: TCRU_195 TcCLB-EL.506401.220_mRNA-p1 TcCLB-NE.
508543.140_mRNA-p1
OG0003835: TCRU_repeat_ TcCLB-EL.506401.230_mRNA-p1 TcCLB-NE.
508543.150_mRNA-p1
OG0003836: TCRU_197 TcCLB-EL.506401.240_mRNA-p1 TcCLB-NE.
508543.160_mRNA-p1
OG0003837: TCRU_198 TcCLB-EL.511555.40_mRNA-p1 TcCLB-NE.
510219.30_mRNA-p1
OG0003838: TCRU_199 TcCLB-EL.511555.30_mRNA-p1 TcCLB-NE.
510219.20_mRNA-p1
OG0003839: TCRU_subunit_ TcCLB-EL.506713.40_mRNA-p1 TcCLB-NE.
509719.9_mRNA-p1
OG0003840: TCRU_201 TcCLB-EL.506713.30_mRNA-p1 TcCLB-NE.
509719.20_mRNA-p1
OG0003841: TCRU_204 TcCLB-EL.506529.50_mRNA-p1 TcCLB-NE.
508593.110_mRNA-p1
OG0003842: TCRU_205 TcCLB-EL.506529.60_mRNA-p1 TcCLB-NE.
508593.120_mRNA-p1

OG0003843: TCRU_206 TcCLB-EL.506529.70_mRNA-p1 TcCLB-NE.
508593.130_mRNA-p1
OG0003844: TCRU_208 TcCLB-EL.510409.60_mRNA-p1 TcCLB-NE.
509937.80_mRNA-p1
OG0003845: TCRU_225 TcCLB-EL.507625.183_mRNA-p1 TcCLB-NE.
509741.40_mRNA-p1
OG0003846: TCRU_227 TcCLB-EL.503449.14_mRNA-p1 TcCLB-NE.
509875.204_mRNA-p1
OG0003847: TCRU_228 TcCLB-EL.503449.20_mRNA-p1 TcCLB-NE.
509875.200_mRNA-p1
OG0003848: TCRU_229 TcCLB-EL.503449.30_mRNA-p1 TcCLB-NE.
509875.190_mRNA-p1
OG0003849: TCRU_230 TcCLB-EL.503449.40_mRNA-p1 TcCLB-NE.
509875.180_mRNA-p1
OG0003850: TCRU_238 TcCLB-NE.511295.20_mRNA-p1 TcSYL_0121910.t1-p1
OG0003851: TCRU_239 TcCLB-EL.510431.100_mRNA-p1 TcSYL_0138190.t1-p1
OG0003852: TCRU_241 TcCLB-EL.510431.130_mRNA-p1 TcSYL_0138110.t1-p1
OG0003853: TCRU_246 TcCLB-NE.506279.160_pseudogenic_transcript-p1
TcCLB-NE.506993.180_mRNA-p1
OG0003854: TCRU_253 TcCLB-EL.511545.90_mRNA-p1 TcCLB-NE.
426897.10_mRNA-p1
OG0003855: TCRU_259 TcCLB-NE.510635.30_pseudogenic_transcript-p1
TcSYL_0028290.t1-p1
OG0003856: TCRU_262 TcCLB-EL.504227.10_mRNA-p1 TcCLB-NE.
506439.18_mRNA-p1
OG0003857: TCRU_263 TcCLB-EL.506795.90_mRNA-p1 TcCLB-NE.
509935.20_mRNA-p1
OG0003858: TCRU_283 TcCLB-EL.511557.40_mRNA-p1 TcCLB-NE.
503995.20_mRNA-p1
OG0003859: TCRU_286 TcCLB-EL.511557.64_mRNA-p1 TcCLB-NE.
505171.90_mRNA-p1
OG0003860: TCRU_290 TcCLB-EL.511559.34_mRNA-p1 TcCLB-NE.
505171.44_mRNA-p1
OG0003861: TCRU_292 TcCLB-EL.511559.50_mRNA-p1 TcCLB-NE.
505171.30_mRNA-p1
OG0003862: TCRU_293 TcCLB-EL.504101.10_mRNA-p1 TcCLB-NE.
511859.90_mRNA-p1
OG0003863: TCRU_303 TcCLB-EL.509769.60_mRNA-p1 TcSYL_0174840.t1-p1
OG0003864: TCRU_305 TcCLB-EL.509769.70_mRNA-p1 TcSYL_0174830.t1-p1
OG0003865: TCRU_306 TcCLB-NE.509647.190_mRNA-p1 TcSYL_0010130.t1-p1
OG0003866: TCRU_307 TcCLB-NE.509647.180_mRNA-p1 TcSYL_0010190.t1-p1
OG0003867: TCRU_311 TcCLB-EL.510149.70_mRNA-p1 TcCLB-NE.
506989.20_mRNA-p1
OG0003868: TCRU_313 TcCLB-EL.510149.90_mRNA-p1 TcCLB-NE.
506989.40_mRNA-p1
OG0003869: TCRU_327 TcCLB-EL.506591.10_mRNA-p1 TcCLB-NE.
506989.199_mRNA-p1
OG0003870: TCRU_330 TcCLB-EL.506591.50_pseudogenic_transcript-p1
TcCLB-NE.506989.240_pseudogenic_transcript-p1
OG0003871: TCRU_334 TcCLB-EL.506341.20_mRNA-p1 TcCLB-NE.
506507.20_mRNA-p1
OG0003872: TCRU_347 TcCLB-NE.503787.30_mRNA-p1 TcSYL_0100550.t1-p1
OG0003873: TCRU_353 TcCLB-EL.507077.30_mRNA-p1 TcCLB-NE.
510899.40_mRNA-p1

OG0003874: TCRU_354 TcCLB-EL.507077.20_mRNA-p1 TcCLB-NE.
510899.50_mRNA-p1
OG0003875: TCRU_357 TcCLB-NE.510901.30_mRNA-p1 TcSYL_0179220.t1-p1
OG0003876: TCRU_358 TcCLB-NE.510901.40_mRNA-p1 TcSYL_0179210.t1-p1
OG0003877: TCRU_359 TcCLB-NE.510901.50_mRNA-p1 TcSYL_0179200.t1-p1
OG0003878: TCRU_360 TcCLB-NE.510901.60_mRNA-p1 TcSYL_0179190.t1-p1
OG0003879: TCRU_361 TcCLB-NE.510901.90_mRNA-p1 TcSYL_0179150.t1-p1
OG0003880: TCRU_362 TcCLB-NE.510901.100_mRNA-p1 TcSYL_0179140.t1-p1
OG0003881: TCRU_365 TcCLB-NE.510901.150_mRNA-p1 TcSYL_0179100.t1-p1
OG0003882: TCRU_366 TcCLB-NE.510901.160_mRNA-p1 TcSYL_0179050.t1-p1
OG0003883: TCRU_367 TcCLB-NE.510901.170_mRNA-p1 TcSYL_0179020.t1-p1
OG0003884: TCRU_368 TcCLB-NE.510901.180_mRNA-p1 TcSYL_0178960.t1-p1
OG0003885: TCRU_369 TcCLB-NE.510901.190_mRNA-p1 TcSYL_0178920.t1-p1
OG0003886: TCRU_370 TcCLB-NE.510901.200_mRNA-p1 TcSYL_0178900.t1-p1
OG0003887: TCRU_371 TcCLB-NE.510901.220_mRNA-p1 TcSYL_0178810.t1-p1
OG0003888: TCRU_372 TcCLB-NE.510901.230_mRNA-p1 TcSYL_0178780.t1-p1
OG0003889: TCRU_378 TcCLB-EL.508277.170_mRNA-p1 TcCLB-NE.
507915.10_mRNA-p1
OG0003890: TCRU_381 TcCLB-EL.508277.140_mRNA-p1 TcCLB-NE.
507913.20_mRNA-p1
OG0003891: TCRU_382 TcCLB-EL.508277.130_mRNA-p1 TcCLB-NE.
507913.10_mRNA-p1
OG0003892: TCRU_398 TcCLB-EL.503899.139_mRNA-p1 TcCLB-NE.
507515.20_mRNA-p1
OG0003893: TCRU_399 TcCLB-EL.508213.49_mRNA-p1 TcCLB-NE.
507515.30_mRNA-p1
OG0003894: TCRU_407 TcCLB-EL.511365.10_mRNA-p1 TcCLB-NE.
508899.30_mRNA-p1
OG0003895: TCRU_409 TcCLB-EL.511361.80_mRNA-p1 TcCLB-NE.
508899.70_mRNA-p1
OG0003896: TCRU_413 TcCLB-EL.511361.30_mRNA-p1 TcCLB-NE.
508899.120_mRNA-p1
OG0003897: TCRU_414 TcCLB-EL.511361.20_mRNA-p1 TcCLB-NE.
508899.130_mRNA-p1
OG0003898: TCRU_415 TcCLB-EL.511361.10_pseudogenic_transcript-p1
TcCLB-NE.508899.140_mRNA-p1
OG0003899: TCRU_416 TcCLB-EL.511361.4_mRNA-p1 TcCLB-NE.
508901.10_mRNA-p1
OG0003900: TCRU_417 TcCLB-EL.424195.9_mRNA-p1 TcCLB-NE.
508901.20_mRNA-p1
OG0003901: TCRU_418 TcCLB-EL.503641.10_mRNA-p1 TcCLB-NE.
503809.10_mRNA-p1
OG0003902: TCRU_419 TcCLB-EL.503641.19_mRNA-p1 TcCLB-NE.
503809.20_mRNA-p1
OG0003903: TCRU_420 TcCLB-EL.508943.40_mRNA-p1 TcCLB-NE.
503809.24_mRNA-p1
OG0003904: TCRU_421 TcCLB-EL.508943.30_mRNA-p1 TcCLB-NE.
503809.30_mRNA-p1
OG0003905: TCRU_422 TcCLB-EL.508943.20_mRNA-p1 TcCLB-NE.
503809.40_mRNA-p1
OG0003906: TCRU_423 TcCLB-EL.507011.50_mRNA-p1 TcCLB-NE.
507005.70_mRNA-p1
OG0003907: TCRU_432 TcCLB-EL.507011.150_mRNA-p1 TcCLB-NE.
510665.30_mRNA-p1

OG0003908: TCRU_435 TcCLB-EL.509837.10_mRNA-p1 TcSYL_0082560.t1-p1
OG0003909: TCRU_436 TcCLB-EL.509837.20_mRNA-p1 TcSYL_0082570.t1-p1
OG0003910: TCRU_437 TcCLB-EL.509381.10_mRNA-p1 TcSYL_0001770.t1-p1
OG0003911: TCRU_440 TcCLB-EL.509379.10_mRNA-p1 TcCLB-NE.
506701.10_mRNA-p1
OG0003912: TCRU_442 TcCLB-EL.507641.120_mRNA-p1 TcSYL_0011130.t1-p1
OG0003913: TCRU_449 TcCLB-EL.506399.30_mRNA-p1 TcCLB-NE.
509243.30_mRNA-p1
OG0003914: TCRU_459 TcCLB-EL.509779.20_mRNA-p1 TcCLB-NE.
511653.60_mRNA-p1
OG0003915: TCRU_460 TcCLB-EL.509779.30_mRNA-p1 TcCLB-NE.
511653.50_mRNA-p1
OG0003916: TCRU_462 TcCLB-EL.509779.50_mRNA-p1 TcCLB-NE.
511653.30_mRNA-p1
OG0003917: TCRU_463 TcCLB-EL.509779.60_mRNA-p1 TcCLB-NE.
511653.20_mRNA-p1
OG0003918: TCRU_465 TcCLB-EL.507811.40_mRNA-p1 TcCLB-NE.
511649.160_mRNA-p1
OG0003919: TCRU_466 TcCLB-EL.507811.44_mRNA-p1 TcCLB-NE.
511649.150_mRNA-p1
OG0003920: TCRU_467 TcCLB-EL.507811.50_mRNA-p1 TcCLB-NE.
511649.140_mRNA-p1
OG0003921: TCRU_469 TcCLB-EL.507811.100_mRNA-p1 TcCLB-NE.
511649.100_mRNA-p1
OG0003922: TCRU_470 TcCLB-EL.507811.114_mRNA-p1 TcCLB-NE.
511649.90_mRNA-p1
OG0003923: TCRU_471 TcCLB-EL.507813.10_mRNA-p1 TcCLB-NE.
511649.70_mRNA-p1
OG0003924: TCRU_484 TcCLB-NE.506321.140_mRNA-p1 TcCLB-NE.
506321.20_mRNA-p1
OG0003925: TCRU_485 TcCLB-EL.507993.60_mRNA-p1 TcCLB-NE.
511277.500_mRNA-p1
OG0003926: TCRU_487 TcCLB-EL.507993.36_mRNA-p1 TcCLB-NE.
511277.486_mRNA-p1
OG0003927: TCRU_488 TcCLB-EL.507993.30_mRNA-p1 TcCLB-NE.
511277.480_mRNA-p1
OG0003928: TCRU_489 TcCLB-EL.507993.20_mRNA-p1 TcCLB-NE.
511277.470_mRNA-p1
OG0003929: TCRU_490 TcCLB-EL.507993.10_mRNA-p1 TcCLB-NE.
511277.460_mRNA-p1
OG0003930: TCRU_491 TcCLB-EL.507993.5_pseudogenic_transcript-p1
TcCLB-NE.511277.450_mRNA-p1
OG0003931: TCRU_498 TcCLB-EL.509895.50_mRNA-p1 TcCLB-NE.
460747.20_mRNA-p1
OG0003932: TCRU_499 TcCLB-EL.509895.40_mRNA-p1 TcCLB-NE.
460747.9_mRNA-p1
OG0003933: TCRU_505 TCRU_6739 TcSYL_0141590.t1-p1
OG0003934: TCRU_525 TcCLB-NE.510187.448_mRNA-p1 TcSYL_0011460.t1-p1
OG0003935: TCRU_527 TcCLB-NE.510187.430_mRNA-p1 TcSYL_0011470.t1-p1
OG0003936: TCRU_528 TcCLB-EL.506825.50_mRNA-p1 TcCLB-NE.
506681.50_mRNA-p1
OG0003937: TCRU_533 TcCLB-EL.510609.30_mRNA-p1 TcCLB-NE.
510395.30_mRNA-p1
OG0003938: TCRU_546 TcCLB-EL.508569.80_mRNA-p1 TcCLB-NE.

503733.70_mRNA-p1
OG0003939: TCRU_547 TcCLB-EL.508569.90_mRNA-p1 TcCLB-NE.
503733.50_pseudogenic_transcript-p1
OG0003940: TCRU_548 TcCLB-EL.508569.100_mRNA-p1 TcCLB-NE.
503733.40_mRNA-p1
OG0003941: TCRU_549 TcCLB-EL.508569.130_mRNA-p1 TcCLB-NE.
503733.20_mRNA-p1
OG0003942: TCRU_551 TcCLB-EL.510863.20_mRNA-p1 TcCLB-NE.
503729.19_mRNA-p1
OG0003943: TCRU_553 TcCLB-EL.511877.20_mRNA-p1 TcCLB-NE.
511861.100_pseudogenic_transcript-p1
OG0003944: TCRU_556 TcCLB-EL.508439.30_mRNA-p1 TcCLB-NE.
506275.90_mRNA-p1
OG0003945: TCRU_557 TcCLB-EL.510283.40_mRNA-p1 TcCLB-NE.
503823.10_mRNA-p1
OG0003946: TCRU_558 TcCLB-EL.510283.50_mRNA-p1 TcCLB-NE.
503823.20_mRNA-p1
OG0003947: TCRU_559 TcCLB-EL.510283.60_mRNA-p1 TcCLB-NE.
503823.30_mRNA-p1
OG0003948: TCRU_564 TcCLB-EL.510609.70_mRNA-p1 TcCLB-NE.
503617.10_mRNA-p1
OG0003949: TCRU_565 TcCLB-EL.510609.60_mRNA-p1 TcCLB-NE.
503617.20_mRNA-p1
OG0003950: TCRU_576 TcCLB-EL.506811.90_mRNA-p1 TcCLB-NE.
506303.140_mRNA-p1
OG0003951: TCRU_577 TcCLB-EL.506811.80_mRNA-p1 TcCLB-NE.
506303.150_mRNA-p1
OG0003952: TCRU_579 TcCLB-EL.506811.40_mRNA-p1 TcCLB-NE.
506305.10_mRNA-p1
OG0003953: TCRU_592 TcCLB-EL.503779.40_mRNA-p1 TcCLB-NE.
506247.410_mRNA-p1
OG0003954: TCRU_601 TcCLB-EL.510771.9_mRNA-p1 TcCLB-NE.
511385.14_mRNA-p1
OG0003955: TCRU_1599 TCRU_603 TcSYL_0136600.t1-p1
OG0003956: TCRU_605 TCRU_7792 TcCLB-NE.511861.20_mRNA-p1
OG0003957: TCRU_606 TcCLB-EL.511751.30_mRNA-p1 TcCLB-NE.
507023.200_mRNA-p1
OG0003958: TCRU_609 TcCLB-EL.509171.80_mRNA-p1 TcCLB-NE.
507023.240_mRNA-p1
OG0003959: TCRU_610 TcCLB-EL.509171.70_mRNA-p1 TcCLB-NE.
507023.250_mRNA-p1
OG0003960: TCRU_611 TcCLB-EL.509171.54_mRNA-p1 TcCLB-NE.
507023.270_mRNA-p1
OG0003961: TCRU_613 TcCLB-EL.509171.29_mRNA-p1 TcCLB-NE.
511505.10_mRNA-p1
OG0003962: TCRU_615 TcCLB-EL.509171.10_mRNA-p1 TcCLB-NE.
511505.30_mRNA-p1
OG0003963: TCRU_3619 TCRU_617 TcSYL_0164310.t1-p1
OG0003964: TCRU_625 TcCLB-NE.508827.120_mRNA-p1 TcSYL_0182060.t1-p1
OG0003965: TCRU_630 TcCLB-EL.506367.70_mRNA-p1 TcCLB-NE.
508355.210_mRNA-p1
OG0003966: TCRU_632 TcCLB-EL.506207.30_mRNA-p1 TcCLB-NE.
510065.50_mRNA-p1
OG0003967: TCRU_633 TcCLB-EL.506207.20_mRNA-p1 TcCLB-NE.

510067.9_mRNA-p1
OG0003968: TCRU_634 TcCLB-EL.506207.10_mRNA-p1 TcCLB-NE.
507991.10_mRNA-p1
OG0003969: TCRU_635 TcCLB-EL.510861.30_mRNA-p1 TcCLB-NE.
507649.10_mRNA-p1
OG0003970: TCRU_636 TcCLB-EL.510861.14_mRNA-p1 TcCLB-NE.
507649.20_mRNA-p1
OG0003971: TCRU_637 TcCLB-EL.510859.40_mRNA-p1 TcCLB-NE.
507649.40_mRNA-p1
OG0003972: TCRU_638 TcCLB-EL.510859.30_mRNA-p1 TcCLB-NE.
507649.50_mRNA-p1
OG0003973: TCRU_641 TcCLB-EL.508567.100_mRNA-p1 TcCLB-NE.
507649.100_mRNA-p1
OG0003974: TCRU_642 TcCLB-EL.508567.89_mRNA-p1 TcCLB-NE.
509609.10_mRNA-p1
OG0003975: TCRU_643 TcCLB-EL.508567.80_mRNA-p1 TcCLB-NE.
509611.10_mRNA-p1
OG0003976: TCRU_644 TcCLB-EL.508567.70_mRNA-p1 TcCLB-NE.
509611.20_mRNA-p1
OG0003977: TCRU_645 TcCLB-EL.508567.60_mRNA-p1 TcCLB-NE.
509611.30_mRNA-p1
OG0003978: TCRU_646 TcCLB-EL.508567.49_mRNA-p1 TcCLB-NE.
509611.40_mRNA-p1
OG0003979: TCRU_repeat_ TcCLB-EL.508567.20_mRNA-p1 TcCLB-NE.
509611.70_mRNA-p1
OG0003980: TCRU_648 TcCLB-EL.510857.60_mRNA-p1 TcCLB-NE.
509611.80_mRNA-p1
OG0003981: TCRU_649 TcCLB-EL.510857.54_mRNA-p1 TcCLB-NE.
509611.89_mRNA-p1
OG0003982: TCRU_650 TcCLB-EL.510857.50_mRNA-p1 TcCLB-NE.
509611.100_mRNA-p1
OG0003983: TCRU_651 TcCLB-EL.510857.40_mRNA-p1 TcCLB-NE.
509611.110_mRNA-p1
OG0003984: TCRU_652 TcCLB-EL.510857.30_mRNA-p1 TcCLB-NE.
509611.120_mRNA-p1
OG0003985: TCRU_653 TcCLB-EL.510857.20_mRNA-p1 TcCLB-NE.
509611.130_mRNA-p1
OG0003986: TCRU_655 TcCLB-EL.510855.40_mRNA-p1 TcCLB-NE.
509611.160_mRNA-p1
OG0003987: TCRU_656 TcCLB-EL.510855.20_mRNA-p1 TcCLB-NE.
509611.170_mRNA-p1
OG0003988: TCRU_657 TcCLB-EL.510855.10_mRNA-p1 TcCLB-NE.
509611.180_mRNA-p1
OG0003989: TCRU_668 TcCLB-EL.511871.110_mRNA-p1 TcCLB-NE.
511863.20_mRNA-p1
OG0003990: TCRU_672 TcCLB-EL.508137.30_mRNA-p1 TcCLB-NE.
453445.20_mRNA-p1
OG0003991: TCRU_682 TcCLB-EL.507547.30_mRNA-p1 TcCLB-NE.
508441.80_mRNA-p1
OG0003992: TCRU_685 TcCLB-EL.507547.59_mRNA-p1 TcCLB-NE.
508441.50_mRNA-p1
OG0003993: TCRU_686 TcCLB-EL.507547.70_mRNA-p1 TcCLB-NE.
508441.40_mRNA-p1
OG0003994: TCRU_4349 TCRU_4626 TCRU_697

OG0003995: TCRU_700 TcCLB-EL.511867.30_mRNA-p1 TcCLB-NE.
507775.30_mRNA-p1
OG0003996: TCRU_701 TcCLB-EL.511867.40_mRNA-p1 TcCLB-NE.
507775.40_mRNA-p1
OG0003997: TCRU_703 TcCLB-EL.511867.60_mRNA-p1 TcSYL_0146360.t1-p1
OG0003998: TCRU_707 TcCLB-EL.506885.210_mRNA-p1 TcCLB-NE.
510729.280_mRNA-p1
OG0003999: TCRU_715 TcCLB-EL.510747.60_mRNA-p1 TcCLB-NE.
510657.170_mRNA-p1
OG0004000: TCRU_717 TcCLB-EL.510747.90_mRNA-p1 TcCLB-NE.
510657.150_mRNA-p1
OG0004001: TCRU_718 TcCLB-EL.510747.100_mRNA-p1 TcCLB-NE.
510657.140_mRNA-p1
OG0004002: TCRU_719 TcCLB-EL.510747.114_mRNA-p1 TcCLB-NE.
510657.120_mRNA-p1
OG0004003: TCRU_725 TcCLB-EL.510749.10_mRNA-p1 TcCLB-NE.
510657.50_mRNA-p1
OG0004004: TCRU_726 TcCLB-EL.510749.20_mRNA-p1 TcCLB-NE.
510657.40_mRNA-p1
OG0004005: TCRU_728 TcCLB-EL.510749.40_mRNA-p1 TcCLB-NE.
510657.20_mRNA-p1
OG0004006: TCRU_730 TcCLB-EL.510749.60_mRNA-p1 TcCLB-NE.
510655.150_mRNA-p1
OG0004007: TCRU_731 TcCLB-EL.510749.90_mRNA-p1 TcCLB-NE.
510655.130_mRNA-p1
OG0004008: TCRU_733 TcCLB-EL.510751.10_mRNA-p1 TcCLB-NE.
510655.110_mRNA-p1
OG0004009: TCRU_735 TcCLB-EL.508469.10_mRNA-p1 TcCLB-NE.
510655.80_mRNA-p1
OG0004010: TCRU_737 TcCLB-EL.508469.30_mRNA-p1 TcCLB-NE.
510655.60_mRNA-p1
OG0004011: TCRU_757 TcCLB-EL.506529.590_mRNA-p1 TcCLB-NE.
510889.270_mRNA-p1
OG0004012: TCRU_764 TcCLB-EL.503953.20_mRNA-p1 TcCLB-NE.
507063.140_mRNA-p1
OG0004013: TCRU_765 TcCLB-EL.503953.14_mRNA-p1 TcCLB-NE.
507063.144_mRNA-p1
OG0004014: TCRU_768 TcCLB-EL.507807.30_mRNA-p1 TcCLB-NE.
507063.170_mRNA-p1
OG0004015: TCRU_769 TcCLB-EL.507807.20_mRNA-p1 TcCLB-NE.
507063.180_mRNA-p1
OG0004016: TCRU_771 TcCLB-EL.507805.20_mRNA-p1 TcCLB-NE.
507063.250_mRNA-p1
OG0004017: TCRU_772 TcCLB-EL.507805.9_mRNA-p1 TcCLB-NE.
507063.270_mRNA-p1
OG0004018: TCRU_773 TcCLB-EL.507803.30_mRNA-p1 TcCLB-NE.
507063.290_mRNA-p1
OG0004019: TCRU_774 TcCLB-EL.508385.10_mRNA-p1 TcCLB-NE.
506791.30_mRNA-p1
OG0004020: TCRU_775 TcCLB-EL.508385.30_mRNA-p1 TcCLB-NE.
506791.10_mRNA-p1
OG0004021: TCRU_776 TcCLB-EL.508385.40_mRNA-p1 TcCLB-NE.
504175.40_mRNA-p1
OG0004022: TCRU_777 TcCLB-EL.508385.50_mRNA-p1 TcCLB-NE.

504175.30_mRNA-p1
OG0004023: TCRU_781 TcCLB-EL.504147.200_mRNA-p1 TcSYL_0005090.t1-p1
OG0004024: TCRU_786 TcSYL_0018450.t1-p1 TcSYL_0141530.t1-p1
OG0004025: TCRU_799 TcCLB-EL.508731.30_mRNA-p1 TcCLB-NE.
509683.50_mRNA-p1
OG0004026: TCRU_805 TcCLB-EL.507875.30_mRNA-p1 TcCLB-NE.
508111.20_mRNA-p1
OG0004027: TCRU_809 TcCLB-NE.508971.40_mRNA-p1 TcSYL_0202370.t1-p1
OG0004028: TCRU_810 TcCLB-NE.508971.30_mRNA-p1 TcSYL_0202360.t1-p1
OG0004029: TCRU_842 TcCLB-EL.511313.10_mRNA-p1 TcCLB-NE.
511755.100_mRNA-p1
OG0004030: TCRU_843 TcCLB-EL.511037.30_mRNA-p1 TcCLB-NE.
509799.130_mRNA-p1
OG0004031: TCRU_851 TcCLB-EL.511575.90_mRNA-p1 TcSYL_0094760.t1-p1
OG0004032: TCRU_856 TcCLB-EL.508549.20_mRNA-p1 TcCLB-NE.
508231.40_mRNA-p1
OG0004033: TCRU_857 TcCLB-EL.508549.10_mRNA-p1 TcCLB-NE.
508231.50_mRNA-p1
OG0004034: TCRU_858 TcCLB-EL.506251.130_pseudogenic_transcript-p1
TcCLB-NE.508231.60_mRNA-p1
OG0004035: TCRU_859 TcCLB-EL.506251.120_mRNA-p1 TcCLB-NE.
508231.64_mRNA-p1
OG0004036: TCRU_860 TcCLB-EL.506251.110_mRNA-p1 TcCLB-NE.
508231.70_mRNA-p1
OG0004037: TCRU_881 TcCLB-EL.511353.30_mRNA-p1 TcCLB-NE.
511301.50_mRNA-p1
OG0004038: TCRU_882 TcCLB-EL.511353.40_mRNA-p1 TcCLB-NE.
511301.40_mRNA-p1
OG0004039: TCRU_885 TcCLB-EL.506483.69_mRNA-p1 TcCLB-NE.
511299.70_mRNA-p1
OG0004040: TCRU_894 TcCLB-EL.511201.58_mRNA-p1 TcCLB-NE.
508073.20_mRNA-p1
OG0004041: TCRU_907 TcCLB-EL.506863.4_mRNA-p1 TcCLB-NE.
511825.210_mRNA-p1
OG0004042: TCRU_909 TcCLB-EL.506863.20_mRNA-p1 TcCLB-NE.
511825.230_mRNA-p1
OG0004043: TCRU_911 TcCLB-EL.506863.50_mRNA-p1 TcCLB-NE.
511825.260_mRNA-p1
OG0004044: TCRU_913 TcCLB-EL.506863.80_mRNA-p1 TcCLB-NE.
509211.30_mRNA-p1
OG0004045: TCRU_7861 TCRU_920 TcSYL_0191390.t1-p1
OG0004046: TCRU_930 TcCLB-EL.504431.10_mRNA-p1 TcCLB-NE.
507951.60_mRNA-p1
OG0004047: TCRU_931 TcCLB-EL.504431.20_mRNA-p1 TcCLB-NE.
507951.70_mRNA-p1
OG0004048: TCRU_934 TcCLB-EL.504431.80_mRNA-p1 TcCLB-NE.
507951.130_mRNA-p1
OG0004049: TCRU_937 TcCLB-EL.504431.109_mRNA-p1 TcCLB-NE.
507951.154_mRNA-p1
OG0004050: TCRU_938 TcCLB-EL.504431.118_mRNA-p1 TcCLB-NE.
507951.160_mRNA-p1
OG0004051: TCRU_939 TcCLB-EL.504433.10_mRNA-p1 TcCLB-NE.
507951.170_mRNA-p1
OG0004052: TCRU_940 TcCLB-EL.509893.29_mRNA-p1 TcCLB-NE.

432867.10_mRNA-p1
OG0004053: TCRU_subunit TcCLB-EL.509895.10_mRNA-p1 TcCLB-NE.
432867.20_mRNA-p1
OG0004054: TCRU_952 TcCLB-EL.508569.10_mRNA-p1 TcCLB-NE.
509607.20_mRNA-p1
OG0004055: TCRU_953 TcCLB-EL.508569.20_mRNA-p1 TcCLB-NE.
509607.10_mRNA-p1
OG0004056: TCRU_954 TcCLB-EL.510861.60_mRNA-p1 TcCLB-NE.
507647.20_pseudogenic_transcript-p1
OG0004057: TCRU_955 TcCLB-EL.506221.80_mRNA-p1 TcCLB-NE.
427789.30_mRNA-p1
OG0004058: TCRU_956 TcCLB-EL.506221.70_mRNA-p1 TcCLB-NE.
427789.20_mRNA-p1
OG0004059: TCRU_958 TcCLB-EL.506221.40_mRNA-p1 TcCLB-NE.
504217.19_mRNA-p1
OG0004060: TCRU_959 TcCLB-EL.509767.240_mRNA-p1 TcCLB-NE.
507645.59_mRNA-p1
OG0004061: TCRU_960 TcCLB-EL.509767.230_mRNA-p1 TcCLB-NE.
507645.50_mRNA-p1
OG0004062: TCRU_961 TcCLB-EL.509767.220_mRNA-p1 TcCLB-NE.
507645.40_mRNA-p1
OG0004063: TCRU_962 TcCLB-EL.509767.215_mRNA-p1 TcCLB-NE.
507645.30_mRNA-p1
OG0004064: TCRU_963 TcCLB-EL.509767.210_mRNA-p1 TcCLB-NE.
507645.20_mRNA-p1
OG0004065: TCRU_966 TcCLB-EL.453917.9_mRNA-p1 TcCLB-NE.
511285.40_mRNA-p1
OG0004066: TCRU_971 TcCLB-NE.511283.290_mRNA-p1 TcSYL_0123650.t1-p1
OG0004067: TCRU_980 TcCLB-NE.511283.190_mRNA-p1 TcSYL_0123660.t1-p1
OG0004068: TCRU_family_ TcCLB-EL.508723.120_mRNA-p1 TcCLB-NE.
509789.10_mRNA-p1
OG0004069: TCRU_982 TcCLB-EL.508723.110_mRNA-p1 TcCLB-NE.
509789.20_mRNA-p1
OG0004070: TCRU_1000 TcCLB-EL.507923.40_mRNA-p1 TcCLB-NE.
509059.80_mRNA-p1
OG0004071: TCRU_1001 TcCLB-EL.507925.20_mRNA-p1 TcCLB-NE.
509059.70_mRNA-p1
OG0004072: TCRU_1002 TcCLB-EL.507925.30_mRNA-p1 TcCLB-NE.
509059.60_mRNA-p1
OG0004073: TCRU_1003 TcCLB-EL.507925.40_mRNA-p1 TcCLB-NE.
509059.50_mRNA-p1
OG0004074: TCRU_1004 TcCLB-EL.507925.60_mRNA-p1 TcCLB-NE.
509059.30_mRNA-p1
OG0004075: TCRU_1005 TcCLB-EL.509965.10_pseudogenic_transcript-p1
TcCLB-NE.509059.20_mRNA-p1
OG0004076: TCRU_1007 TcCLB-EL.509965.30_mRNA-p1 TcCLB-NE.
511499.59_mRNA-p1
OG0004077: TCRU_1008 TcCLB-EL.509965.40_mRNA-p1 TcCLB-NE.
511499.50_mRNA-p1
OG0004078: TCRU_1009 TcCLB-EL.509965.60_mRNA-p1 TcCLB-NE.
511499.30_mRNA-p1
OG0004079: TCRU_1010 TcCLB-EL.509965.70_mRNA-p1 TcCLB-NE.
511499.20_mRNA-p1
OG0004080: TCRU_1011 TcCLB-EL.509965.90_mRNA-p1 TcCLB-NE.

511499.10_mRNA-p1
OG0004081: TCRU_subunit TcCLB-EL.510091.110_mRNA-p1 TcCLB-NE.
503603.10_mRNA-p1
OG0004082: TCRU_1018 TcCLB-EL.510091.100_mRNA-p1 TcCLB-NE.
503603.20_mRNA-p1
OG0004083: TCRU_1021 TcCLB-EL.430895.10_mRNA-p1 TcCLB-NE.
506669.10_mRNA-p1
OG0004084: TCRU_1022 TcCLB-EL.503575.54_mRNA-p1 TcCLB-NE.
510943.36_mRNA-p1
OG0004085: TCRU_1024 TcCLB-EL.509351.6_mRNA-p1 TcCLB-NE.
510943.44_mRNA-p1
OG0004086: TCRU_1027 TcCLB-EL.504867.10_mRNA-p1 TcCLB-NE.
510943.70_mRNA-p1
OG0004087: TCRU_1038 TcCLB-EL.504867.114_mRNA-p1 TcCLB-NE.
510943.184_mRNA-p1
OG0004088: TCRU_1043 TcCLB-EL.504041.10_mRNA-p1 TcCLB-NE.
508815.10_mRNA-p1
OG0004089: TCRU_1045 TcCLB-EL.504075.6_mRNA-p1 TcCLB-EL.
506933.98_mRNA-p1
OG0004090: TCRU_1047 TcCLB-EL.506933.80_mRNA-p1 TcSYL_0044090.t1-p1
OG0004091: TCRU_1053 TcCLB-EL.511215.40_mRNA-p1 TcSYL_0187960.t1-p1
OG0004092: TCRU_1054 TcCLB-EL.511215.50_mRNA-p1 TcSYL_0187970.t1-p1
OG0004093: TCRU_1056 TcCLB-EL.506495.24_mRNA-p1 TcCLB-NE.
506561.14_mRNA-p1
OG0004094: TCRU_1057 TcCLB-EL.506495.20_mRNA-p1 TcCLB-NE.
506561.10_mRNA-p1
OG0004095: TCRU_1058 TcCLB-EL.506495.10_mRNA-p1 TcCLB-NE.
503965.10_mRNA-p1
OG0004096: TCRU_1059 TcCLB-EL.506495.9_mRNA-p1 TcCLB-NE.
503965.20_mRNA-p1
OG0004097: TCRU_1060 TcCLB-EL.506495.3_mRNA-p1 TcCLB-NE.
503965.39_mRNA-p1
OG0004098: TCRU_1062 TcCLB-EL.508953.40_mRNA-p1 TcCLB-NE.
508059.30_mRNA-p1
OG0004099: TCRU_1063 TcCLB-EL.508953.28_mRNA-p1 TcCLB-NE.
508059.24_mRNA-p1
OG0004100: TCRU_1064 TcCLB-EL.508953.19_mRNA-p1 TcCLB-NE.
508059.20_mRNA-p1
OG0004101: TCRU_1082 TcCLB-EL.508949.40_mRNA-p1 TcCLB-NE.
510119.60_mRNA-p1
OG0004102: TCRU_small TcCLB-NE.511367.354_mRNA-p1 TcSYL_0169900.t1-
p1
OG0004103: TCRU_1087 TcCLB-NE.511367.350_mRNA-p1 TcSYL_0169910.t1-p1
OG0004104: TCRU_1088 TcCLB-NE.511367.330_mRNA-p1 TcSYL_0169930.t1-p1
OG0004105: TCRU_1091 TcCLB-NE.511367.290_mRNA-p1 TcSYL_0169940.t1-p1
OG0004106: TCRU_1093 TcCLB-EL.510759.110_mRNA-p1 TcCLB-NE.
506999.110_mRNA-p1
OG0004107: TCRU_1094 TcCLB-EL.510759.114_mRNA-p1 TcCLB-NE.
506999.100_mRNA-p1
OG0004108: TCRU_1096 TcCLB-EL.510759.130_mRNA-p1 TcCLB-NE.
506999.80_mRNA-p1
OG0004109: TCRU_1097 TcCLB-EL.510759.134_mRNA-p1 TcCLB-NE.
506999.70_mRNA-p1
OG0004110: TCRU_1098 TcCLB-EL.510759.140_mRNA-p1 TcCLB-NE.

506999.60_mRNA-p1
OG0004111: TCRU_1100 TcCLB-EL.510759.160_mRNA-p1 TcCLB-NE.
506999.40_mRNA-p1
OG0004112: TCRU_1101 TcCLB-EL.510759.170_mRNA-p1 TcCLB-NE.
506999.30_mRNA-p1
OG0004113: TCRU_1105 TcCLB-EL.503651.20_mRNA-p1 TcSYL_0115440.t1-p1
OG0004114: TCRU_1106 TcCLB-EL.503651.30_mRNA-p1 TcSYL_0115430.t1-p1
OG0004115: TCRU_1107 TcCLB-EL.510761.4_mRNA-p1 TcSYL_0115420.t1-p1
OG0004116: TCRU_1118 TcCLB-EL.507483.4_pseudogenic_transcript-p1
TcCLB-NE.506391.50_pseudogenic_transcript-p1
OG0004117: TCRU_1120 TcCLB-EL.507483.20_mRNA-p1 TcCLB-NE.
506391.30_mRNA-p1
OG0004118: TCRU_1126 TcCLB-EL.508541.170_mRNA-p1 TcCLB-NE.
507539.20_mRNA-p1
OG0004119: TCRU_1129 TcCLB-EL.510529.10_mRNA-p1 TcCLB-NE.
511817.240_mRNA-p1
OG0004120: TCRU_1132 TcCLB-EL.432027.9_mRNA-p1 TcCLB-NE.
511817.270_mRNA-p1
OG0004121: TCRU_1134 TcCLB-EL.510531.20_mRNA-p1 TcCLB-NE.
511817.300_mRNA-p1
OG0004122: TCRU_1135 TcCLB-EL.510531.30_mRNA-p1 TcCLB-NE.
511817.310_mRNA-p1
OG0004123: TCRU_1138 TcCLB-EL.504047.50_mRNA-p1 TcSYL_0019600.t1-p1
OG0004124: TCRU_1147 TcCLB-EL.507711.30_mRNA-p1 TcCLB-NE.
424123.30_mRNA-p1
OG0004125: TCRU_1148 TcCLB-EL.507711.20_mRNA-p1 TcCLB-NE.
424123.20_mRNA-p1
OG0004126: TCRU_1149 TcCLB-EL.507711.10_mRNA-p1 TcCLB-NE.
424123.10_pseudogenic_transcript-p1
OG0004127: TCRU_1150 TcCLB-EL.504051.9_mRNA-p1 TcCLB-NE.
511721.10_mRNA-p1
OG0004128: TCRU_1151 TcCLB-NE.511721.20_mRNA-p1 TcSYL_0141940.t1-p1
OG0004129: TCRU_1160 TcCLB-EL.508541.200_mRNA-p1 TcCLB-NE.
507541.10_mRNA-p1
OG0004130: TCRU_1163 TcCLB-NE.443035.10_pseudogenic_transcript-p1
TcSYL_0020920.t1-p1
OG0004131: TCRU_1165 TcCLB-NE.510821.60_mRNA-p1 TcSYL_0050140.t1-p1
OG0004132: TCRU_1189 TcCLB-EL.507521.110_mRNA-p1 TcCLB-NE.
508577.100_mRNA-p1
OG0004133: TCRU_1192 TcCLB-EL.507521.80_mRNA-p1 TcCLB-NE.
508577.130_mRNA-p1
OG0004134: TCRU_1214 TcCLB-EL.506635.90_mRNA-p1 TcCLB-NE.
508029.20_mRNA-p1
OG0004135: TCRU_1215 TcCLB-EL.506635.80_mRNA-p1 TcCLB-NE.
508029.10_mRNA-p1
OG0004136: TCRU_1217 TcCLB-EL.506635.40_mRNA-p1 TcCLB-NE.
508027.90_mRNA-p1
OG0004137: TCRU_1218 TcCLB-EL.506635.30_mRNA-p1 TcCLB-NE.
508027.80_mRNA-p1
OG0004138: TCRU_1221 TcCLB-EL.506633.100_mRNA-p1 TcCLB-NE.
508027.50_mRNA-p1
OG0004139: TCRU_1223 TCRU_4944 TcSYL_0067070.t1-p1
OG0004140: TCRU_1226 TcCLB-EL.511421.200_mRNA-p1 TcSYL_0111070.t1-p1
OG0004141: TCRU_1227 TCRU_7383 TcSYL_0160280.t1-p1

OG0004142: TCRU_1232 TcCLB-EL.508831.20_mRNA-p1 TcCLB-NE.
509119.10_mRNA-p1
OG0004143: TCRU_1237 TcCLB-EL.509199.24_mRNA-p1 TcCLB-NE.
508601.119_mRNA-p1
OG0004144: TCRU_1243 TcCLB-EL.511801.40_mRNA-p1 TcCLB-NE.
508601.40_mRNA-p1
OG0004145: TCRU_1254 TcCLB-EL.503905.30_mRNA-p1 TcCLB-NE.
507705.30_mRNA-p1
OG0004146: TCRU_1255 TcCLB-EL.503905.40_mRNA-p1 TcCLB-NE.
507705.20_mRNA-p1
OG0004147: TCRU_1263 TcCLB-EL.506565.12_mRNA-p1 TcCLB-NE.
511837.129_mRNA-p1
OG0004148: TCRU_1267 TcCLB-EL.484299.4_mRNA-p1 TcCLB-NE.
503555.40_mRNA-p1
OG0004149: TCRU_1268 TcCLB-EL.507447.10_mRNA-p1 TcCLB-NE.
503555.50_mRNA-p1
OG0004150: TCRU_1273 TcCLB-EL.511583.10_mRNA-p1 TcCLB-NE.
504177.30_mRNA-p1
OG0004151: TCRU_1274 TcCLB-EL.510939.20_mRNA-p1 TcCLB-NE.
506821.80_mRNA-p1
OG0004152: TCRU_1278 TcCLB-EL.504159.100_mRNA-p1 TcCLB-NE.
506821.120_mRNA-p1
OG0004153: TCRU_1279 TcCLB-EL.504159.90_mRNA-p1 TcCLB-NE.
506821.130_mRNA-p1
OG0004154: TCRU_1280 TcCLB-EL.504159.80_mRNA-p1 TcCLB-NE.
506821.140_mRNA-p1
OG0004155: TCRU_1282 TcCLB-EL.504159.60_mRNA-p1 TcCLB-NE.
506821.160_mRNA-p1
OG0004156: TCRU_1284 TcCLB-EL.504159.40_mRNA-p1 TcCLB-NE.
506821.180_mRNA-p1
OG0004157: TCRU_1285 TcCLB-EL.504159.30_mRNA-p1 TcCLB-NE.
506821.190_mRNA-p1
OG0004158: TCRU_1290 TcCLB-EL.506529.250_mRNA-p1 TcCLB-NE.
510885.160_mRNA-p1
OG0004159: TCRU_1297 TcCLB-EL.504153.320_mRNA-p1 TcCLB-NE.
506301.20_mRNA-p1
OG0004160: TCRU_1302 TcCLB-EL.506529.380_mRNA-p1 TcCLB-NE.
510889.80_mRNA-p1
OG0004161: TCRU_1303 TcCLB-EL.506529.400_mRNA-p1 TcCLB-NE.
510889.100_mRNA-p1
OG0004162: TCRU_1304 TcCLB-EL.506529.410_mRNA-p1 TcCLB-NE.
510889.110_mRNA-p1
OG0004163: TCRU_1309 TcCLB-EL.506529.470_mRNA-p1 TcCLB-NE.
510889.170_mRNA-p1
OG0004164: TCRU_1313 TcCLB-EL.508557.20_mRNA-p1 TcCLB-NE.
504223.20_mRNA-p1
OG0004165: TCRU_1316 TcCLB-EL.507993.160_mRNA-p1 TcCLB-NE.
511277.600_mRNA-p1
OG0004166: TCRU_1324 TcCLB-EL.504149.160_mRNA-p1 TcCLB-EL.
508039.90_pseudogenic_transcript-p1
OG0004167: TCRU_1327 TcCLB-NE.510627.150_mRNA-p1 TcCLB-NE.
510629.240_pseudogenic_transcript-p1
OG0004168: TCRU_1347 TcCLB-NE.506139.40_mRNA-p1 TcSYL_0154990.t1-p1
OG0004169: TCRU_1350 TcCLB-EL.511115.30_mRNA-p1 TcCLB-NE.

507715.34_mRNA-p1
OG0004170: TCRU_1356 TcCLB-NE.508247.90_mRNA-p1 TcCLB-NE.
508853.20_mRNA-p1
OG0004171: TCRU_1362 TcCLB-EL.510105.33_mRNA-p1 TcCLB-NE.
509715.23_mRNA-p1
OG0004172: TCRU_1372 TcCLB-EL.506885.170_mRNA-p1 TcCLB-NE.
510729.240_mRNA-p1
OG0004173: TCRU_1373 TcCLB-EL.506885.180_mRNA-p1 TcCLB-NE.
510729.230_mRNA-p1
OG0004174: TCRU_1374 TcCLB-EL.506885.200_mRNA-p1 TcCLB-NE.
510729.220_mRNA-p1
OG0004175: TCRU_1375 TcCLB-EL.506885.204_mRNA-p1 TcCLB-NE.
510729.210_mRNA-p1
OG0004176: TCRU_1379 TcCLB-EL.506885.250_mRNA-p1 TcCLB-NE.
510729.180_mRNA-p1
OG0004177: TCRU_1380 TcCLB-EL.506885.260_mRNA-p1 TcCLB-NE.
510729.160_mRNA-p1
OG0004178: TCRU_1381 TcCLB-EL.506885.270_mRNA-p1 TcCLB-NE.
510729.150_mRNA-p1
OG0004179: TCRU_1382 TcCLB-EL.506885.280_mRNA-p1 TcCLB-NE.
510729.140_mRNA-p1
OG0004180: TCRU_1383 TcCLB-EL.506885.290_mRNA-p1 TcCLB-NE.
510729.130_mRNA-p1
OG0004181: TCRU_1384 TcCLB-EL.506885.300_mRNA-p1 TcCLB-NE.
510729.119_mRNA-p1
OG0004182: TCRU_1386 TcCLB-EL.506885.310_mRNA-p1 TcCLB-NE.
510729.110_mRNA-p1
OG0004183: TCRU_1387 TcCLB-EL.506885.320_mRNA-p1 TcCLB-NE.
510729.100_mRNA-p1
OG0004184: TCRU_1388 TcCLB-EL.506885.330_mRNA-p1 TcCLB-NE.
510729.90_mRNA-p1
OG0004185: TCRU_1389 TcCLB-EL.506885.340_mRNA-p1 TcCLB-NE.
510729.80_mRNA-p1
OG0004186: TCRU_subunit_ TcCLB-EL.506885.350_mRNA-p1 TcCLB-NE.
510729.70_mRNA-p1
OG0004187: TCRU_1391 TcCLB-EL.506885.354_mRNA-p1 TcCLB-NE.
510729.60_mRNA-p1
OG0004188: TCRU_1392 TcCLB-EL.506885.360_mRNA-p1 TcCLB-NE.
510729.50_mRNA-p1
OG0004189: TCRU_1393 TcCLB-EL.506885.364_mRNA-p1 TcCLB-NE.
510729.40_mRNA-p1
OG0004190: TCRU_1394 TcCLB-EL.506885.380_mRNA-p1 TcCLB-NE.
510729.20_mRNA-p1
OG0004191: TCRU_1395 TcCLB-EL.506885.390_mRNA-p1 TcCLB-NE.
510729.10_mRNA-p1
OG0004192: TCRU_1396 TcCLB-EL.506885.400_mRNA-p1 TcCLB-NE.
503687.40_mRNA-p1
OG0004193: TCRU_1397 TcCLB-EL.506885.410_mRNA-p1 TcCLB-NE.
503687.30_mRNA-p1
OG0004194: TCRU_1398 TcCLB-EL.506885.416_mRNA-p1 TcCLB-NE.
503687.10_mRNA-p1
OG0004195: TCRU_1399 TcCLB-EL.506885.420_mRNA-p1 TcCLB-NE.
508457.40_mRNA-p1
OG0004196: TCRU_1400 TcCLB-EL.506885.430_mRNA-p1 TcCLB-NE.

508457.30_mRNA-p1
OG0004197: TCRU_1401 TcCLB-EL.506885.450_mRNA-p1 TcCLB-NE.
508457.10_mRNA-p1
OG0004198: TCRU_1403 TcCLB-EL.509599.157_mRNA-p1 TcCLB-NE.
506327.80_mRNA-p1
OG0004199: TCRU_1404 TcCLB-EL.509599.164_mRNA-p1 TcCLB-NE.
506327.70_mRNA-p1
OG0004200: TCRU_1406 TcCLB-EL.506167.9_mRNA-p1 TcCLB-NE.
506327.40_mRNA-p1
OG0004201: TCRU_1409 TcCLB-EL.506167.40_mRNA-p1 TcCLB-NE.
506327.10_mRNA-p1
OG0004202: TCRU_1415 TcCLB-EL.509247.10_mRNA-p1 TcCLB-NE.
506977.120_mRNA-p1
OG0004203: TCRU_1419 TcCLB-EL.508837.170_mRNA-p1 TcSYL_0187900.t1-p1
OG0004204: TCRU_1432 TcCLB-EL.510127.60_mRNA-p1 TcCLB-NE.
510571.10_mRNA-p1
OG0004205: TCRU_1433 TcCLB-EL.510127.70_mRNA-p1 TcCLB-NE.
510571.20_mRNA-p1
OG0004206: TCRU_1434 TcCLB-NE.509017.60_mRNA-p1 TcSYL_0131600.t1-p1
OG0004207: TCRU_1442 TcCLB-EL.507163.80_mRNA-p1 TcCLB-NE.
509751.20_mRNA-p1
OG0004208: TCRU_1443 TcCLB-EL.507165.10_mRNA-p1 TcCLB-NE.
507793.60_mRNA-p1
OG0004209: TCRU_1444 TcCLB-EL.507165.30_mRNA-p1 TcCLB-NE.
507793.40_mRNA-p1
OG0004210: TCRU_1448 TcCLB-NE.506317.10_mRNA-p1 TcSYL_0050780.t1-p1
OG0004211: TCRU_1450 TcCLB-NE.508533.40_mRNA-p1 TcSYL_0050760.t1-p1
OG0004212: TCRU_1451 TcCLB-EL.404001.4_mRNA-p1 TcCLB-NE.
508533.30_mRNA-p1
OG0004213: TCRU_1455 TcCLB-EL.504153.290_mRNA-p1 TcSYL_0138610.t1-p1
OG0004214: TCRU_1456 TcCLB-NE.471901.10_mRNA-p1 TcSYL_0122230.t1-p1
OG0004215: TCRU_1466 TcCLB-EL.507711.190_mRNA-p1 TcCLB-NE.
509641.70_mRNA-p1
OG0004216: TCRU_1467 TcCLB-EL.507711.180_mRNA-p1 TcCLB-NE.
509641.60_mRNA-p1
OG0004217: TCRU_1468 TcCLB-EL.507711.170_mRNA-p1 TcCLB-NE.
509641.50_mRNA-p1
OG0004218: TCRU_1469 TcCLB-EL.507711.160_mRNA-p1 TcCLB-NE.
509641.40_mRNA-p1
OG0004219: TCRU_1471 TcCLB-EL.507711.140_mRNA-p1 TcCLB-NE.
509641.20_mRNA-p1
OG0004220: TCRU_1472 TcCLB-EL.507711.130_mRNA-p1 TcCLB-NE.
509641.10_mRNA-p1
OG0004221: TCRU_1474 TcCLB-EL.507711.120_mRNA-p1 TcCLB-NE.
509639.20_mRNA-p1
OG0004222: TCRU_1483 TcCLB-EL.511071.181_mRNA-p1 TcCLB-NE.
504037.20_mRNA-p1
OG0004223: TCRU_1491 TcCLB-NE.511277.350_mRNA-p1 TcSYL_0014790.t1-p1
OG0004224: TCRU_1495 TcCLB-NE.511277.290_mRNA-p1 TcSYL_0014800.t1-p1
OG0004225: TCRU_1502 TcCLB-EL.506183.9_mRNA-p1 TcCLB-NE.
509875.170_mRNA-p1
OG0004226: TCRU_1506 TcCLB-EL.508357.40_mRNA-p1 TcCLB-NE.
508355.380_mRNA-p1
OG0004227: TCRU_1507 TcCLB-EL.508357.26_mRNA-p1 TcCLB-NE.

508355.360_mRNA-p1
OG0004228: TCRU_1509 TcCLB-EL.508357.20_mRNA-p1 TcCLB-NE.
508355.340_mRNA-p1
OG0004229: TCRU_1511 TcCLB-NE.508355.320_mRNA-p1 TcSYL_0082950.t1-p1
OG0004230: TCRU_1512 TcCLB-NE.508355.310_mRNA-p1 TcSYL_0082970.t1-p1
OG0004231: TCRU_1514 TcCLB-NE.508355.290_mRNA-p1 TcSYL_0082980.t1-p1
OG0004232: TCRU_1524 TcSYL_0156380.t1-p1 TcSYL_0156480.t1-p1
OG0004233: TCRU_1525 TcCLB-EL.506885.124_mRNA-p1 TcCLB-NE.
510729.290_mRNA-p1
OG0004234: TCRU_1526 TcCLB-EL.506885.120_mRNA-p1 TcCLB-NE.
510729.299_mRNA-p1
OG0004235: TCRU_1553 TcCLB-EL.506181.140_mRNA-p1 TcCLB-NE.
506177.60_mRNA-p1
OG0004236: TCRU_1554 TcCLB-EL.506181.130_mRNA-p1 TcCLB-NE.
506177.70_mRNA-p1
OG0004237: TCRU_1560 TcCLB-EL.506181.59_mRNA-p1 TcCLB-NE.
503395.50_mRNA-p1
OG0004238: TCRU_1567 TcCLB-EL.509625.20_mRNA-p1 TcCLB-NE.
507663.40_mRNA-p1
OG0004239: TCRU_1569 TcCLB-EL.506179.50_mRNA-p1 TcCLB-NE.
507663.60_mRNA-p1
OG0004240: TCRU_1572 TcCLB-EL.506179.5_mRNA-p1 TcCLB-NE.
507665.50_mRNA-p1
OG0004241: TCRU_1573 TcCLB-EL.511127.240_mRNA-p1 TcCLB-NE.
509025.10_mRNA-p1
OG0004242: TCRU_1579 TcCLB-EL.509433.10_mRNA-p1 TcCLB-NE.
508015.40_mRNA-p1
OG0004243: TCRU_1584 TcCLB-EL.503513.10_mRNA-p1 TcCLB-NE.
508741.320_mRNA-p1
OG0004244: TCRU_1585 TcCLB-EL.503513.20_mRNA-p1 TcCLB-NE.
508741.310_mRNA-p1
OG0004245: TCRU_1586 TcCLB-EL.503513.40_mRNA-p1 TcCLB-NE.
508741.300_mRNA-p1
OG0004246: TCRU_1587 TcCLB-EL.503515.7_mRNA-p1 TcCLB-NE.
508741.290_mRNA-p1
OG0004247: TCRU_1588 TcCLB-EL.503515.14_mRNA-p1 TcCLB-NE.
508741.280_mRNA-p1
OG0004248: TCRU_1589 TcCLB-EL.503515.20_mRNA-p1 TcCLB-NE.
508741.270_mRNA-p1
OG0004249: TCRU_1590 TcCLB-EL.511689.19_mRNA-p1 TcCLB-NE.
508741.260_mRNA-p1
OG0004250: TCRU_1591 TcCLB-EL.511689.10_mRNA-p1 TcCLB-NE.
508741.250_mRNA-p1
OG0004251: TCRU_1592 TcCLB-EL.504225.10_mRNA-p1 TcCLB-NE.
508741.240_mRNA-p1
OG0004252: TCRU_1594 TcCLB-NE.509647.140_mRNA-p1 TcSYL_0010330.t1-p1
OG0004253: TCRU_1595 TcCLB-NE.509647.130_mRNA-p1 TcSYL_0010340.t1-p1
OG0004254: TCRU_1621 TcCLB-EL.504153.240_mRNA-p1 TcSYL_0138620.t1-p1
OG0004255: TCRU_1623 TcCLB-EL.510105.50_mRNA-p1 TcCLB-NE.
509715.40_mRNA-p1
OG0004256: TCRU_1630 TcCLB-NE.509995.29_mRNA-p1 TcSYL_0003300.t1-p1
OG0004257: TCRU_1631 TcCLB-NE.509995.20_mRNA-p1 TcSYL_0003310.t1-p1
OG0004258: TCRU_1636 TcCLB-EL.506287.9_mRNA-p1 TcCLB-NE.
503723.20_mRNA-p1

OG0004259: TCRU_1637 TcCLB-EL.506287.20_mRNA-p1 TcCLB-NE.
503723.10_mRNA-p1
OG0004260: TCRU_1638 TcCLB-EL.506287.30_mRNA-p1 TcCLB-NE.
410049.20_mRNA-p1
OG0004261: TCRU_1639 TcCLB-EL.506287.80_mRNA-p1 TcCLB-NE.
506991.19_mRNA-p1
OG0004262: TCRU_1641 TcCLB-EL.506287.100_mRNA-p1 TcCLB-NE.
508881.150_mRNA-p1
OG0004263: TCRU_1642 TcCLB-EL.506287.120_mRNA-p1 TcCLB-NE.
508881.140_mRNA-p1
OG0004264: TCRU_1643 TcCLB-EL.506287.130_mRNA-p1 TcCLB-NE.
508881.130_mRNA-p1
OG0004265: TCRU_1647 TcCLB-EL.506289.20_mRNA-p1 TcCLB-NE.
508881.60_mRNA-p1
OG0004266: TCRU_1651 TcCLB-EL.506289.60_mRNA-p1 TcCLB-NE.
508881.30_mRNA-p1
OG0004267: TCRU_1663 TcCLB-EL.511537.60_mRNA-p1 TcCLB-NE.
508707.320_mRNA-p1
OG0004268: TCRU_1664 TcCLB-NE.511391.140_mRNA-p1 TcSYL_0201950.t1-p1
OG0004269: TCRU_1672 TcCLB-EL.510761.30_mRNA-p1 TcSYL_0115400.t1-p1
OG0004270: TCRU_1682 TcCLB-EL.510295.59_mRNA-p1 TcCLB-NE.
506009.10_mRNA-p1
OG0004271: TCRU_1684 TcCLB-EL.510297.30_mRNA-p1 TcCLB-NE.
506009.30_mRNA-p1
OG0004272: TCRU_1685 TcCLB-EL.510297.40_mRNA-p1 TcCLB-NE.
506009.40_mRNA-p1
OG0004273: TCRU_1686 TcCLB-EL.510297.50_mRNA-p1 TcCLB-NE.
506009.50_mRNA-p1
OG0004274: TCRU_1687 TcCLB-EL.510297.70_mRNA-p1 TcCLB-NE.
506009.70_mRNA-p1
OG0004275: TCRU_1705 TcCLB-EL.510349.50_mRNA-p1 TcCLB-NE.
503925.80_mRNA-p1
OG0004276: TCRU_1709 TcCLB-EL.508777.120_mRNA-p1 TcCLB-NE.
509029.150_mRNA-p1
OG0004277: TCRU_1710 TcCLB-EL.508777.130_mRNA-p1 TcCLB-NE.
509029.160_mRNA-p1
OG0004278: TCRU_1712 TcCLB-EL.508777.160_mRNA-p1 TcCLB-NE.
511483.20_mRNA-p1
OG0004279: TCRU_1713 TcCLB-EL.508777.170_mRNA-p1 TcCLB-NE.
511483.30_mRNA-p1
OG0004280: TCRU_1719 TcCLB-EL.506925.150_mRNA-p1 TcSYL_0080560.t1-p1
OG0004281: TCRU_1721 TcCLB-EL.507165.59_mRNA-p1 TcCLB-NE.
507793.10_mRNA-p1
OG0004282: TCRU_1730 TcCLB-EL.504427.10_mRNA-p1 TcCLB-NE.
509331.134_mRNA-p1
OG0004283: TCRU_1731 TcCLB-EL.510173.120_mRNA-p1 TcCLB-NE.
507777.10_mRNA-p1
OG0004284: TCRU_1732 TcCLB-EL.510173.130_mRNA-p1 TcCLB-NE.
507777.20_mRNA-p1
OG0004285: TCRU_1757 TcCLB-EL.504057.145_mRNA-p1 TcSYL_0046040.t1-p1
OG0004286: TCRU_1758 TcCLB-EL.504057.150_mRNA-p1 TcSYL_0046030.t1-p1
OG0004287: TCRU_1761 TcCLB-EL.504057.178_mRNA-p1 TcCLB-EL.
510879.220_mRNA-p1
OG0004288: TCRU_1769 TcCLB-EL.510407.70_mRNA-p1 TcCLB-NE.

509937.170_mRNA-p1
OG0004289: TCRU_1772 TcCLB-EL.510407.20_mRNA-p1 TcCLB-NE.
509937.210_mRNA-p1
OG0004290: TCRU_1774 TcCLB-EL.405165.10_mRNA-p1 TcCLB-NE.
509939.10_mRNA-p1
OG0004291: TCRU_1778 TcCLB-EL.508265.60_pseudogenic_transcript-p1
TcCLB-NE.506443.30_mRNA-p1
OG0004292: TCRU_1783 TcCLB-EL.510403.89_mRNA-p1 TcCLB-NE.
506443.80_mRNA-p1
OG0004293: TCRU_1788 TCRU_2355 TcCLB-NE.459199.10_mRNA-p1
OG0004294: TCRU_1793 TcCLB-EL.507641.230_mRNA-p1 TcSYL_0011220.t1-p1
OG0004295: TCRU_1795 TcCLB-EL.507641.210_mRNA-p1 TcSYL_0011210.t1-p1
OG0004296: TCRU_1802 TcCLB-EL.504029.90_mRNA-p1 TcCLB-NE.
509997.73_mRNA-p1
OG0004297: TCRU_1804 TcCLB-EL.504029.70_mRNA-p1 TcCLB-NE.
509999.10_mRNA-p1
OG0004298: TCRU_1809 TcCLB-NE.509999.70_mRNA-p1 TcSYL_0003240.t1-p1
OG0004299: TCRU_1810 TcCLB-NE.509999.80_mRNA-p1 TcSYL_0003230.t1-p1
OG0004300: TCRU_1817 TcCLB-EL.504057.110_mRNA-p1 TcSYL_0046050.t1-p1
OG0004301: TCRU_1822 TcCLB-NE.507937.20_pseudogenic_transcript-p1
TcCLB-NE.511255.280_mRNA-p1
OG0004302: TCRU_1826 TcCLB-NE.509161.40_mRNA-p1 TcSYL_0142880.t1-p1
OG0004303: TCRU_1838 TcCLB-NE.509061.20_mRNA-p1 TcCLB-NE.
511509.5_mRNA-p1
OG0004304: TCRU_1842 TcCLB-EL.504035.80_mRNA-p1 TcCLB-NE.
507991.126_mRNA-p1
OG0004305: TCRU_1843 TcCLB-EL.504035.60_mRNA-p1 TcCLB-NE.
507991.130_mRNA-p1
OG0004306: TCRU_1846 TcCLB-EL.510755.70_mRNA-p1 TcCLB-NE.
508415.20_mRNA-p1
OG0004307: TCRU_1848 TcCLB-EL.506859.180_mRNA-p1 TcCLB-NE.
511815.180_mRNA-p1
OG0004308: TCRU_1854 TcCLB-EL.507389.50_mRNA-p1 TcCLB-NE.
509911.110_mRNA-p1
OG0004309: TCRU_1857 TCRU_1858 TcCLB-EL.508647.110_mRNA-p1
OG0004310: TCRU_1866 TcCLB-EL.506409.100_mRNA-p1 TcCLB-NE.
506281.50_mRNA-p1
OG0004311: TCRU_1868 TcCLB-EL.508541.190_mRNA-p1 TcCLB-NE.
507539.40_mRNA-p1
OG0004312: TCRU_1871 TcCLB-NE.510187.250_mRNA-p1 TcSYL_0011560.t1-p1
OG0004313: TCRU_1877 TcCLB-NE.510187.190_mRNA-p1 TcSYL_0011600.t1-p1
OG0004314: TCRU_1879 TcCLB-NE.510187.160_mRNA-p1 TcSYL_0011610.t1-p1
OG0004315: TCRU_1880 TcCLB-EL.508717.10_mRNA-p1 TcCLB-NE.
506839.60_mRNA-p1
OG0004316: TCRU_1885 TcCLB-EL.504147.90_mRNA-p1 TcSYL_0005450.t1-p1
OG0004317: TCRU_1887 TcCLB-EL.504147.70_mRNA-p1 TcSYL_0005540.t1-p1
OG0004318: TCRU_1888 TcCLB-EL.504147.60_mRNA-p1 TcSYL_0005560.t1-p1
OG0004319: TCRU_1889 TcCLB-EL.504147.50_pseudogenic_transcript-p1
TcSYL_0005590.t1-p1
OG0004320: TCRU_1892 TcCLB-EL.510531.60_mRNA-p1 TcCLB-NE.
511819.20_mRNA-p1
OG0004321: TCRU_1893 TcCLB-EL.510531.70_mRNA-p1 TcCLB-NE.
511819.30_mRNA-p1
OG0004322: TCRU_1894 TcCLB-EL.510531.90_pseudogenic_transcript-p1

TcCLB-NE.511819.40_mRNA-p1
OG0004323: TCRU_1895 TcCLB-EL.510531.100_mRNA-p1 TcCLB-NE.511819.50_mRNA-p1
OG0004324: TCRU_1897 TcCLB-EL.510533.50_mRNA-p1 TcCLB-NE.511821.40_mRNA-p1
OG0004325: TCRU_1899 TcCLB-EL.510533.70_mRNA-p1 TcCLB-NE.511821.60_mRNA-p1
OG0004326: TCRU_1901 TcCLB-EL.510533.90_mRNA-p1 TcCLB-NE.511821.80_mRNA-p1
OG0004327: TCRU_1913 TcCLB-EL.510533.230_mRNA-p1 TcCLB-NE.511823.50_mRNA-p1
OG0004328: TCRU_1917 TcCLB-EL.508323.40_mRNA-p1 TcCLB-NE.511825.20_mRNA-p1
OG0004329: TCRU_1921 TcCLB-EL.508323.84_mRNA-p1 TcCLB-NE.511825.63_mRNA-p1
OG0004330: TCRU_1922 TcCLB-EL.508323.94_mRNA-p1 TcCLB-NE.511825.69_mRNA-p1
OG0004331: TCRU_1923 TcCLB-EL.508323.100_mRNA-p1 TcCLB-NE.511825.80_mRNA-p1
OG0004332: TCRU_1928 TcCLB-EL.508321.30_mRNA-p1 TcCLB-NE.509207.30_mRNA-p1
OG0004333: TCRU_1934 TcCLB-EL.506857.40_mRNA-p1 TcCLB-NE.509207.110_mRNA-p1
OG0004334: TCRU_1938 TcCLB-EL.506699.10_mRNA-p1 TcCLB-NE.508153.350_mRNA-p1
OG0004335: TCRU_1942 TcCLB-EL.510323.20_mRNA-p1 TcCLB-NE.508153.300_mRNA-p1
OG0004336: TCRU_1947 TcCLB-EL.510323.60_mRNA-p1 TcCLB-NE.508153.250_mRNA-p1
OG0004337: TCRU_1948 TcCLB-EL.510323.70_mRNA-p1 TcCLB-NE.508153.240_mRNA-p1
OG0004338: TCRU_1949 TcCLB-EL.510323.80_mRNA-p1 TcCLB-NE.508153.230_mRNA-p1
OG0004339: TCRU_1950 TcCLB-EL.510323.90_mRNA-p1 TcCLB-NE.508153.220_mRNA-p1
OG0004340: TCRU_1951 TcCLB-EL.505843.30_mRNA-p1 TcCLB-NE.504949.39_mRNA-p1
OG0004341: TCRU_1953 TcCLB-EL.430605.40_mRNA-p1 TcCLB-NE.509353.20_mRNA-p1
OG0004342: TCRU_1955 TcCLB-EL.506925.190_mRNA-p1 TcSYL_0080570.t1-p1
OG0004343: TCRU_1958 TcCLB-EL.504057.97_mRNA-p1 TcSYL_0046090.t1-p1
OG0004344: TCRU_1962 TcCLB-EL.510525.70_mRNA-p1 TcCLB-NE.511817.134_mRNA-p1
OG0004345: TCRU_1975 TcCLB-EL.504143.10_mRNA-p1 TcCLB-NE.506485.10_mRNA-p1
OG0004346: TCRU_1979 TcCLB-NE.506485.70_mRNA-p1 TcSYL_0003440.t1-p1
OG0004347: TCRU_1984 TcCLB-EL.503643.6_mRNA-p1 TcCLB-NE.504121.20_mRNA-p1
OG0004348: TCRU_1986 TcCLB-EL.511423.140_mRNA-p1 TcCLB-NE.507949.40_mRNA-p1
OG0004349: TCRU_1988 TcCLB-EL.504147.160_mRNA-p1 TcSYL_0005260.t1-p1
OG0004350: TCRU_1989 TcCLB-EL.504147.170_mRNA-p1 TcSYL_0005230.t1-p1
OG0004351: TCRU_1990 TcCLB-EL.504147.180_mRNA-p1 TcSYL_0005200.t1-p1
OG0004352: TCRU_1992 TcCLB-NE.508547.180_mRNA-p1 TcSYL_0202430.t1-p1

OG0004353: TCRU_1995 TcCLB-EL.507395.14_mRNA-p1 TcCLB-NE.
510087.20_mRNA-p1
OG0004354: TCRU_2002 TcCLB-NE.506321.290_mRNA-p1 TcSYL_0157160.t1-p1
OG0004355: TCRU_2005 TcCLB-EL.503777.10_mRNA-p1 TcCLB-NE.
506321.320_mRNA-p1
OG0004356: TCRU_2010 TcCLB-EL.510173.50_mRNA-p1 TcCLB-NE.
509721.20_mRNA-p1
OG0004357: TCRU_2013 TcCLB-EL.510173.80_mRNA-p1 TcCLB-NE.
509721.50_mRNA-p1
OG0004358: TCRU_2015 TcCLB-EL.510173.100_mRNA-p1 TcCLB-NE.
509721.70_mRNA-p1
OG0004359: TCRU_2019 TcCLB-NE.508173.40_mRNA-p1 TcSYL_0079450.t1-p1
OG0004360: TCRU_2021 TcCLB-EL.508307.100_mRNA-p1 TcSYL_0006100.t1-p1
OG0004361: TCRU_2025 TcCLB-NE.508355.240_mRNA-p1 TcSYL_0083030.t1-p1
OG0004362: TCRU_2028 TcCLB-NE.506559.400_mRNA-p1 TcSYL_0045300.t1-p1
OG0004363: TCRU_2030 TcCLB-NE.506559.420_mRNA-p1 TcSYL_0045310.t1-p1
OG0004364: TCRU_2033 TcCLB-NE.506559.450_mRNA-p1 TcSYL_0045330.t1-p1
OG0004365: TCRU_2034 TcCLB-NE.506559.454_mRNA-p1 TcSYL_0045340.t1-p1
OG0004366: TCRU_2038 TcCLB-EL.511211.40_mRNA-p1 TcSYL_0182270.t1-p1
OG0004367: TCRU_2039 TcCLB-EL.511211.49_mRNA-p1 TcSYL_0182310.t1-p1
OG0004368: TCRU_2040 TcCLB-EL.511211.60_mRNA-p1 TcSYL_0182320.t1-p1
OG0004369: TCRU_2052 TcCLB-EL.508515.30_mRNA-p1 TcCLB-NE.
508641.180_mRNA-p1
OG0004370: TCRU_2055 TcCLB-EL.508515.100_mRNA-p1 TcCLB-NE.
508641.100_mRNA-p1
OG0004371: TCRU_2057 TcCLB-EL.511355.40_mRNA-p1 TcCLB-NE.
503809.100_mRNA-p1
OG0004372: TCRU_2059 TcCLB-EL.508307.170_mRNA-p1 TcSYL_0006030.t1-p1
OG0004373: TCRU_2061 TcCLB-EL.506859.204_mRNA-p1 TcCLB-NE.
503567.9_mRNA-p1
OG0004374: TCRU_2063 TcCLB-EL.511747.20_mRNA-p1 TcCLB-NE.
511507.20_mRNA-p1
OG0004375: TCRU_2067 TcCLB-EL.503909.84_mRNA-p1 TcCLB-NE.
511507.70_mRNA-p1
OG0004376: TCRU_2069 TcCLB-EL.503909.68_mRNA-p1 TcCLB-NE.
511507.90_mRNA-p1
OG0004377: TCRU_2072 TcCLB-EL.506925.460_mRNA-p1 TcSYL_0080650.t1-p1
OG0004378: TCRU_2073 TcCLB-EL.506925.480_mRNA-p1 TcSYL_0080670.t1-p1
OG0004379: TCRU_2076 TcCLB-EL.506925.510_mRNA-p1 TcSYL_0080680.t1-p1
OG0004380: TCRU_2081 TcCLB-NE.510187.130_mRNA-p1 TcSYL_0011640.t1-p1
OG0004381: TCRU_2083 TcCLB-NE.510187.110_mRNA-p1 TcSYL_0011650.t1-p1
OG0004382: TCRU_2084 TcCLB-NE.510187.100_mRNA-p1 TcSYL_0011660.t1-p1
OG0004383: TCRU_2095 TcCLB-NE.511317.50_mRNA-p1 TcSYL_0159550.t1-p1
OG0004384: TCRU_2101 TcCLB-EL.506519.90_mRNA-p1 TcCLB-NE.
508909.60_mRNA-p1
OG0004385: TCRU_2103 TcCLB-EL.506519.70_mRNA-p1 TcCLB-NE.
508909.80_mRNA-p1
OG0004386: TCRU_2104 TcCLB-EL.506519.60_mRNA-p1 TcCLB-NE.
508909.90_mRNA-p1
OG0004387: TCRU_2105 TcCLB-EL.506519.51_mRNA-p1 TcCLB-NE.
508909.100_mRNA-p1
OG0004388: TCRU_2109 TcCLB-EL.506743.10_mRNA-p1 TcSYL_0165440.t1-p1
OG0004389: TCRU_2115 TcCLB-NE.511367.220_mRNA-p1 TcSYL_0169990.t1-p1
OG0004390: TCRU_2116 TcCLB-EL.506949.50_mRNA-p1 TcCLB-NE.

508959.4_mRNA-p1
OG0004391: TCRU_2135 TcCLB-EL.508943.10_mRNA-p1 TcCLB-NE.
503809.50_mRNA-p1
OG0004392: TCRU_2136 TcCLB-EL.504047.40_mRNA-p1 TcSYL_0019580.t1-p1
OG0004393: TCRU_2142 TCRU_3493 TcSYL_0135870.t1-p1
OG0004394: TCRU_2144 TcCLB-EL.504035.90_mRNA-p1 TcCLB-NE.
507991.120_mRNA-p1
OG0004395: TCRU_2145 TcCLB-EL.504035.100_mRNA-p1 TcCLB-NE.
507991.110_mRNA-p1
OG0004396: TCRU_2146 TcCLB-EL.504035.120_mRNA-p1 TcCLB-NE.
507991.80_mRNA-p1
OG0004397: TCRU_2147 TcCLB-EL.504035.130_mRNA-p1 TcCLB-NE.
507991.70_mRNA-p1
OG0004398: TCRU_2148 TcCLB-EL.504035.140_mRNA-p1 TcCLB-NE.
507991.60_mRNA-p1
OG0004399: TCRU_2149 TcCLB-EL.506205.10_mRNA-p1 TcCLB-NE.
507991.50_mRNA-p1
OG0004400: TCRU_2150 TcCLB-EL.506205.20_mRNA-p1 TcCLB-NE.
507991.40_mRNA-p1
OG0004401: TCRU_2154 TcCLB-EL.504133.30_mRNA-p1 TcCLB-NE.
507743.9_mRNA-p1
OG0004402: TCRU_2163 TcCLB-EL.511075.60_mRNA-p1 TcCLB-NE.
440099.28_mRNA-p1
OG0004403: TCRU_2164 TcCLB-EL.511075.54_mRNA-p1 TcCLB-NE.
440099.19_mRNA-p1
OG0004404: TCRU_2165 TcCLB-EL.511075.50_mRNA-p1 TcCLB-NE.
440099.10_mRNA-p1
OG0004405: TCRU_2166 TcCLB-EL.511075.40_mRNA-p1 TcSYL_0103640.t1-p1
OG0004406: TCRU_2169 TcCLB-EL.510309.70_mRNA-p1 TcCLB-NE.
510265.110_mRNA-p1
OG0004407: TCRU_2174 TcCLB-EL.508557.29_mRNA-p1 TcCLB-NE.
508141.90_mRNA-p1
OG0004408: TCRU_2190 TcCLB-EL.510241.20_mRNA-p1 TcCLB-NE.
510579.110_mRNA-p1
OG0004409: TCRU_2191 TcCLB-EL.510241.40_mRNA-p1 TcCLB-NE.
510579.90_mRNA-p1
OG0004410: TCRU_2194 TcCLB-EL.510241.70_mRNA-p1 TcCLB-NE.
510579.60_mRNA-p1
OG0004411: TCRU_2195 TcCLB-EL.510241.90_mRNA-p1 TcCLB-NE.
510579.50_mRNA-p1
OG0004412: TCRU_2196 TcCLB-EL.510241.100_mRNA-p1 TcCLB-NE.
510579.40_mRNA-p1
OG0004413: TCRU_2198 TcCLB-EL.510241.120_mRNA-p1 TcCLB-NE.
510579.20_mRNA-p1
OG0004414: TCRU_2199 TcCLB-EL.510241.124_mRNA-p1 TcCLB-NE.
510579.14_mRNA-p1
OG0004415: TCRU_2208 TcCLB-EL.511127.120_mRNA-p1 TcCLB-NE.
509023.120_mRNA-p1
OG0004416: TCRU_2211 TcCLB-EL.511127.90_mRNA-p1 TcCLB-NE.
509023.90_mRNA-p1
OG0004417: TCRU_2212 TcCLB-EL.511127.80_mRNA-p1 TcCLB-NE.
509023.80_mRNA-p1
OG0004418: TCRU_2213 TcCLB-EL.511127.70_mRNA-p1 TcCLB-NE.
509023.60_mRNA-p1

OG0004419: TCRU_2216 TcCLB-EL.511127.40_mRNA-p1 TcCLB-NE.
509023.30_mRNA-p1
OG0004420: TCRU_2219 TcCLB-EL.511527.70_mRNA-p1 TcSYL_0112300.t1-p1
OG0004421: TCRU_2221 TcCLB-EL.503819.20_mRNA-p1 TcCLB-NE.
510359.320_mRNA-p1
OG0004422: TCRU_2224 TcCLB-NE.510945.70_mRNA-p1 TcSYL_0048280.t1-p1
OG0004423: TCRU_2226 TcCLB-EL.507093.150_pseudogenic_transcript-p1
TcSYL_0080350.t1-p1
OG0004424: TCRU_2228 TcCLB-EL.507093.140_mRNA-p1 TcSYL_0080340.t1-p1
OG0004425: TCRU_2230 TcCLB-EL.507093.120_mRNA-p1 TcSYL_0080330.t1-p1
OG0004426: TCRU_2233 TcCLB-EL.510149.40_mRNA-p1 TcCLB-NE.
508411.80_mRNA-p1
OG0004427: TCRU_2237 TcCLB-EL.510145.50_mRNA-p1 TcCLB-NE.
508411.30_mRNA-p1
OG0004428: TCRU_2238 TcCLB-EL.510145.30_mRNA-p1 TcCLB-NE.
508411.20_mRNA-p1
OG0004429: TCRU_2245 TcCLB-EL.510879.150_mRNA-p1 TcSYL_0045980.t1-p1
OG0004430: TCRU_2246 TcCLB-EL.510879.160_mRNA-p1 TcSYL_0045990.t1-p1
OG0004431: TCRU_2248 TcCLB-EL.510879.190_mRNA-p1 TcSYL_0046010.t1-p1
OG0004432: TCRU_2256 TcCLB-EL.507941.110_mRNA-p1 TcCLB-NE.
507943.70_mRNA-p1
OG0004433: TCRU_2258 TcCLB-EL.507941.130_mRNA-p1 TcCLB-NE.
507943.50_mRNA-p1
OG0004434: TCRU_2261 TcCLB-EL.510351.60_mRNA-p1 TcCLB-NE.
507601.120_mRNA-p1
OG0004435: TCRU_2266 TcCLB-EL.510351.80_mRNA-p1 TcCLB-NE.
507601.80_mRNA-p1
OG0004436: TCRU_2270 TcCLB-EL.510351.120_mRNA-p1 TcCLB-NE.
507601.40_mRNA-p1
OG0004437: TCRU_2272 TcCLB-EL.510351.140_mRNA-p1 TcCLB-NE.
507601.20_mRNA-p1
OG0004438: TCRU_2285 TcCLB-EL.509775.20_mRNA-p1 TcCLB-NE.
507061.49_mRNA-p1
OG0004439: TCRU_2286 TcCLB-EL.509775.34_mRNA-p1 TcCLB-NE.
507061.34_mRNA-p1
OG0004440: TCRU_2289 TcCLB-EL.509777.60_mRNA-p1 TcCLB-NE.
503563.20_mRNA-p1
OG0004441: TCRU_2290 TcCLB-EL.509777.64_mRNA-p1 TcCLB-NE.
503563.14_mRNA-p1
OG0004442: TCRU_2294 TcCLB-EL.503527.4_pseudogenic_transcript-p1
TcCLB-NE.510299.10_mRNA-p1
OG0004443: TCRU_2295 TcCLB-EL.503527.10_mRNA-p1 TcCLB-NE.
510299.20_mRNA-p1
OG0004444: TCRU_2296 TcCLB-NE.506585.40_mRNA-p1 TcSYL_0044440.t1-p1
OG0004445: TCRU_2299 TcCLB-NE.511565.40_mRNA-p1 TcSYL_0070550.t1-p1
OG0004446: TCRU_2304 TcCLB-EL.504769.60_mRNA-p1 TcCLB-NE.
503647.20_mRNA-p1
OG0004447: TCRU_2306 TcCLB-EL.508813.70_mRNA-p1 TcSYL_0044190.t1-p1
OG0004448: TCRU_2307 TcCLB-EL.508813.60_mRNA-p1 TcSYL_0044200.t1-p1
OG0004449: TCRU_2309 TcCLB-EL.511731.60_mRNA-p1 TcCLB-NE.
506831.70_mRNA-p1
OG0004450: TCRU_2310 TcCLB-EL.511731.50_mRNA-p1 TcCLB-NE.
432067.10_mRNA-p1
OG0004451: TCRU_2311 TcCLB-EL.442383.19_mRNA-p1 TcCLB-NE.

503719.10_mRNA-p1
OG0004452: TCRU_2315 TcCLB-NE.506155.40_mRNA-p1 TcSYL_0118160.t1-p1
OG0004453: TCRU_2316 TcCLB-NE.506155.50_mRNA-p1 TcSYL_0118150.t1-p1
OG0004454: TCRU_2317 TcCLB-NE.506155.70_mRNA-p1 TcSYL_0118130.t1-p1
OG0004455: TCRU_2327 TcCLB-EL.509267.110_mRNA-p1 TcCLB-NE.
510031.70_mRNA-p1
OG0004456: TCRU_2330 TcCLB-EL.508779.40_mRNA-p1 TcCLB-NE.
506855.130_mRNA-p1
OG0004457: TCRU_domain TcCLB-EL.508779.50_mRNA-p1 TcCLB-NE.
506855.120_mRNA-p1
OG0004458: TCRU_2332 TcCLB-EL.508779.70_mRNA-p1 TcCLB-NE.
506855.110_mRNA-p1
OG0004459: TCRU_2333 TcCLB-EL.508779.90_mRNA-p1 TcCLB-NE.
506855.90_mRNA-p1
OG0004460: TCRU_domain TcCLB-EL.508781.30_mRNA-p1 TcCLB-NE.
506855.70_mRNA-p1
OG0004461: TCRU_2335 TcCLB-EL.508781.40_mRNA-p1 TcCLB-NE.
506855.60_mRNA-p1
OG0004462: TCRU_2339 TcCLB-EL.507093.300_mRNA-p1 TcSYL_0080430.t1-p1
OG0004463: TCRU_2340 TcCLB-EL.507093.310_mRNA-p1 TcSYL_0080440.t1-p1
OG0004464: TCRU_2343 TcCLB-EL.504057.80_mRNA-p1 TcSYL_0046140.t1-p1
OG0004465: TCRU_2345 TcCLB-EL.504057.60_mRNA-p1 TcSYL_0046160.t1-p1
OG0004466: TCRU_2352 TcCLB-EL.511435.30_mRNA-p1 TcCLB-NE.
506475.40_mRNA-p1
OG0004467: TCRU_2353 TcCLB-EL.511435.20_mRNA-p1 TcCLB-NE.
506475.50_mRNA-p1
OG0004468: TCRU_2358 TCRU_4339 TcSYL_0157570.t1-p1
OG0004469: TCRU_2376 TcCLB-EL.510323.110_mRNA-p1 TcCLB-NE.
508153.210_mRNA-p1
OG0004470: TCRU_2380 TcCLB-EL.508183.20_mRNA-p1 TcCLB-NE.
508153.164_mRNA-p1
OG0004471: TCRU_2381 TcCLB-EL.508183.29_mRNA-p1 TcCLB-NE.
508153.160_mRNA-p1
OG0004472: TCRU_2385 TcCLB-EL.510325.20_mRNA-p1 TcCLB-NE.
508153.110_mRNA-p1
OG0004473: TCRU_2393 TcCLB-EL.511735.60_mRNA-p1 TcCLB-NE.
511521.30_mRNA-p1
OG0004474: TCRU_2395 TcCLB-EL.506579.20_mRNA-p1 TcSYL_0064710.t1-p1
OG0004475: TCRU_2398 TcSYL_0064610.t1-p1 TcSYL_0064740.t1-p1
OG0004476: TCRU_2407 TcCLB-EL.511621.240_mRNA-p1 TcCLB-NE.
504741.130_mRNA-p1
OG0004477: TCRU_2408 TcCLB-EL.511621.244_mRNA-p1 TcCLB-NE.
504741.124_mRNA-p1
OG0004478: TCRU_2409 TcCLB-EL.511621.250_mRNA-p1 TcCLB-NE.
504741.120_mRNA-p1
OG0004479: TCRU_2411 TcCLB-EL.511623.20_mRNA-p1 TcCLB-NE.
504741.90_mRNA-p1
OG0004480: TCRU_2414 TcCLB-EL.511625.20_mRNA-p1 TcCLB-NE.
504741.50_mRNA-p1
OG0004481: TCRU_2417 TcCLB-EL.511533.10_mRNA-p1 TcCLB-NE.
511025.20_mRNA-p1
OG0004482: TCRU_2420 TcCLB-EL.511535.20_mRNA-p1 TcCLB-NE.
508713.20_mRNA-p1
OG0004483: TCRU_2428 TcCLB-EL.511537.24_mRNA-p1 TcCLB-NE.

511021.40_mRNA-p1
OG0004484: TCRU_2430 TcCLB-NE.511021.20_mRNA-p1 TcSYL_0196320.t1-p1
OG0004485: TCRU_2438 TcCLB-NE.508897.110_mRNA-p1 TcSYL_0121970.t1-p1
OG0004486: TCRU_2439 TcCLB-NE.508897.100_mRNA-p1 TcSYL_0121990.t1-p1
OG0004487: TCRU_2440 TcCLB-NE.508897.90_mRNA-p1 TcSYL_0122010.t1-p1
OG0004488: TCRU_2441 TcCLB-NE.508897.80_mRNA-p1 TcSYL_0122020.t1-p1
OG0004489: TCRU_2443 TcCLB-NE.508897.50_mRNA-p1 TcSYL_0122080.t1-p1
OG0004490: TCRU_2444 TcCLB-NE.508897.40_mRNA-p1 TcSYL_0122130.t1-p1
OG0004491: TCRU_2445 TcCLB-NE.508897.30_mRNA-p1 TcSYL_0122170.t1-p1
OG0004492: TCRU_2446 TcCLB-NE.508897.20_mRNA-p1 TcSYL_0122200.t1-p1
OG0004493: TCRU_2457 TcCLB-EL.509537.80_mRNA-p1 TcSYL_0073950.t1-p1
OG0004494: TCRU_2458 TcCLB-EL.509537.70_mRNA-p1 TcSYL_0073940.t1-p1
OG0004495: TCRU_2469 TcCLB-EL.506357.20_mRNA-p1 TcCLB-NE.
511277.92_mRNA-p1
OG0004496: TCRU_2473 TcCLB-EL.506357.60_mRNA-p1 TcCLB-NE.
511277.50_mRNA-p1
OG0004497: TCRU_2474 TcCLB-EL.506357.69_mRNA-p1 TcCLB-NE.
511277.39_mRNA-p1
OG0004498: TCRU_2475 TcCLB-EL.506357.80_mRNA-p1 TcCLB-NE.
511277.30_pseudogenic_transcript-p1
OG0004499: TCRU_2482 TcCLB-EL.506357.150_mRNA-p1 TcCLB-NE.
508891.90_mRNA-p1
OG0004500: TCRU_2483 TcCLB-EL.506357.160_mRNA-p1 TcCLB-NE.
508891.80_pseudogenic_transcript-p1
OG0004501: TCRU_2506 TcCLB-EL.506473.6_mRNA-p1 TcCLB-NE.
508999.40_mRNA-p1
OG0004502: TCRU_2511 TcCLB-NE.509647.26_mRNA-p1 TcSYL_0010610.t1-p1
OG0004503: TCRU_2512 TcCLB-NE.509647.40_mRNA-p1 TcSYL_0010600.t1-p1
OG0004504: TCRU_2513 TcCLB-NE.509647.50_mRNA-p1 TcSYL_0010590.t1-p1
OG0004505: TCRU_2514 TcCLB-NE.509647.60_mRNA-p1 TcSYL_0010550.t1-p1
OG0004506: TCRU_2516 TcCLB-NE.509647.80_mRNA-p1 TcSYL_0010460.t1-p1
OG0004507: TCRU_2517 TcCLB-NE.509647.90_mRNA-p1 TcSYL_0010410.t1-p1
OG0004508: TCRU_2518 TcCLB-NE.509647.100_mRNA-p1 TcSYL_0010390.t1-p1
OG0004509: TCRU_2519 TcCLB-NE.509647.110_mRNA-p1 TcSYL_0010380.t1-p1
OG0004510: TCRU_2520 TcCLB-NE.509647.120_mRNA-p1 TcSYL_0010370.t1-p1
OG0004511: TCRU_2529 TcCLB-EL.511409.40_mRNA-p1 TcCLB-NE.
509571.40_mRNA-p1
OG0004512: TCRU_2533 TcCLB-EL.503689.20_mRNA-p1 TcCLB-NE.
509573.20_mRNA-p1
OG0004513: TCRU_2548 TcCLB-EL.506685.10_mRNA-p1 TcCLB-NE.
509663.44_mRNA-p1
OG0004514: TCRU_2549 TcCLB-EL.506685.20_mRNA-p1 TcCLB-NE.
509663.37_mRNA-p1
OG0004515: TCRU_2550 TcCLB-EL.506685.30_mRNA-p1 TcCLB-NE.
509663.30_mRNA-p1
OG0004516: TCRU_2552 TcCLB-EL.506933.40_mRNA-p1 TcSYL_0044110.t1-p1
OG0004517: TCRU_2555 TcCLB-EL.503975.40_mRNA-p1 TcCLB-NE.
510977.9_mRNA-p1
OG0004518: TCRU_2566 TCRU_7700 TcSYL_0023350.t1-p1
OG0004519: TCRU_2573 TcCLB-EL.506605.120_mRNA-p1 TcCLB-NE.
511239.110_mRNA-p1
OG0004520: TCRU_2577 TcCLB-EL.506605.170_mRNA-p1 TcCLB-NE.
511239.159_pseudogenic_transcript-p1
OG0004521: TCRU_2591 TcCLB-EL.511421.40_mRNA-p1 TcSYL_0111120.t1-p1

OG0004522: TCRU_2593 TcCLB-EL.506227.150_mRNA-p1 TcSYL_0092010.t1-p1
OG0004523: TCRU_2594 TcCLB-EL.503783.80_mRNA-p1 TcCLB-NE.510979.50_mRNA-p1
OG0004524: TCRU_2595 TcCLB-NE.511367.138_mRNA-p1 TcSYL_0170030.t1-p1
OG0004525: TCRU_2597 TcCLB-NE.508461.480_mRNA-p1 TcCLB-NE.508461.490_mRNA-p1
OG0004526: TCRU_2619 TcCLB-EL.510311.110_mRNA-p1 TcCLB-NE.508153.1140_mRNA-p1
OG0004527: TCRU_2620 TcCLB-EL.510311.120_mRNA-p1 TcCLB-NE.508153.1130_mRNA-p1
OG0004528: TCRU_2624 TcCLB-EL.510311.180_mRNA-p1 TcCLB-NE.508153.1074_mRNA-p1
OG0004529: TCRU_2625 TcCLB-EL.510311.190_mRNA-p1 TcCLB-NE.508153.1070_mRNA-p1
OG0004530: TCRU_2626 TcCLB-EL.510313.10_mRNA-p1 TcCLB-NE.508153.1060_mRNA-p1
OG0004531: TCRU_2628 TcCLB-EL.506691.22_mRNA-p1 TcCLB-NE.508153.1040_mRNA-p1
OG0004532: TCRU_2629 TcCLB-EL.506691.30_mRNA-p1 TcCLB-NE.508153.1030_mRNA-p1
OG0004533: TCRU_2630 TcCLB-EL.506691.40_mRNA-p1 TcCLB-NE.508153.1020_mRNA-p1
OG0004534: TCRU_2631 TcCLB-EL.506691.50_mRNA-p1 TcCLB-NE.508153.1010_mRNA-p1
OG0004535: TCRU_2632 TcCLB-EL.506691.70_mRNA-p1 TcCLB-NE.508153.990_mRNA-p1
OG0004536: TCRU_2633 TcCLB-EL.506543.10_mRNA-p1 TcCLB-NE.425855.9_mRNA-p1
OG0004537: TCRU_2641 TcCLB-EL.506543.70_mRNA-p1 TcCLB-NE.506041.40_mRNA-p1
OG0004538: TCRU_2643 TcCLB-EL.506543.100_mRNA-p1 TcCLB-NE.506041.60_mRNA-p1
OG0004539: TCRU_2650 TcCLB-EL.511867.90_mRNA-p1 TcCLB-NE.440363.10_mRNA-p1
OG0004540: TCRU__FEN-1__ TcCLB-EL.511867.110_mRNA-p1 TcSYL_0146340.t1-p1
OG0004541: TCRU_2664 TcCLB-EL.508647.140_mRNA-p1 TcSYL_0047860.t1-p1
OG0004542: TCRU_2667 TcCLB-NE.508963.10_pseudogenic_transcript-p1 TcSYL_0187750.t1-p1
OG0004543: TCRU_2674 TcCLB-EL.508347.150_mRNA-p1 TcCLB-NE.507615.40_mRNA-p1
OG0004544: TCRU_2694 TcCLB-EL.505989.78_mRNA-p1 TcCLB-NE.508693.84_mRNA-p1
OG0004545: TCRU_2697 TcCLB-EL.505989.100_mRNA-p1 TcCLB-NE.508693.60_mRNA-p1
OG0004546: TCRU_2699 TcCLB-NE.508693.40_mRNA-p1 TcSYL_0046360.t1-p1
OG0004547: TCRU_2700 TcCLB-NE.508693.30_mRNA-p1 TcSYL_0046350.t1-p1
OG0004548: TCRU_2701 TcCLB-NE.508693.20_mRNA-p1 TcSYL_0046330.t1-p1
OG0004549: TCRU_2702 TcCLB-EL.506945.200_mRNA-p1 TcSYL_0140700.t1-p1
OG0004550: TCRU_2703 TcCLB-EL.506945.220_mRNA-p1 TcSYL_0140710.t1-p1
OG0004551: TCRU_2743 TcCLB-EL.511579.9_mRNA-p1 TcSYL_0109380.t1-p1
OG0004552: TCRU_2751 TCRU_8959 TcSYL_0106450.t1-p1
OG0004553: TCRU_2760 TcCLB-EL.511423.40_mRNA-p1 TcCLB-NE.507949.154_mRNA-p1

OG0004554: TCRU_2763 TcCLB-EL.511423.70_mRNA-p1 TcCLB-NE.
507949.130_mRNA-p1
OG0004555: TCRU_2766 TcCLB-EL.511423.100_mRNA-p1 TcCLB-NE.
507949.80_mRNA-p1
OG0004556: TCRU_2771 TcSYL_0173610.t1-p1 TcSYL_0174430.t1-p1
OG0004557: TCRU_2783 TcCLB-EL.506963.100_mRNA-p1 TcCLB-NE.
511233.90_mRNA-p1
OG0004558: TCRU_2784 TcCLB-EL.506963.90_mRNA-p1 TcCLB-NE.
511233.80_mRNA-p1
OG0004559: TCRU_2785 TcCLB-EL.506963.80_mRNA-p1 TcCLB-NE.
511233.70_mRNA-p1
OG0004560: TCRU_2786 TcCLB-EL.506963.70_mRNA-p1 TcCLB-NE.
511233.59_mRNA-p1
OG0004561: TCRU_2787 TcCLB-EL.506963.60_pseudogenic_transcript-p1
TcCLB-NE.511233.50_mRNA-p1
OG0004562: TCRU_2788 TcCLB-EL.506963.40_mRNA-p1 TcCLB-NE.
511233.40_mRNA-p1
OG0004563: TCRU_2789 TcCLB-EL.506963.30_mRNA-p1 TcCLB-NE.
511233.30_mRNA-p1
OG0004564: TCRU_2796 TcCLB-EL.508831.180_pseudogenic_transcript-p1
TcSYL_0190890.t1-p1
OG0004565: TCRU_2801 TcCLB-EL.510431.70_mRNA-p1 TcCLB-NE.
460125.10_mRNA-p1
OG0004566: TCRU_2805 TcCLB-NE.507897.4_mRNA-p1 TcSYL_0057280.t1-p1
OG0004567: TCRU_2806 TcCLB-NE.507897.2_mRNA-p1 TcSYL_0057270.t1-p1
OG0004568: TCRU_2815 TcCLB-EL.510595.10_mRNA-p1 TcCLB-NE.
507895.140_mRNA-p1
OG0004569: TCRU_2820 TcCLB-EL.510593.20_mRNA-p1 TcCLB-NE.
507895.80_mRNA-p1
OG0004570: TCRU_2825 TcCLB-EL.508357.90_mRNA-p1 TcCLB-NE.
508355.431_mRNA-p1
OG0004571: TCRU_2829 TcCLB-EL.511751.40_mRNA-p1 TcCLB-NE.
507023.190_mRNA-p1
OG0004572: TCRU_2833 TcCLB-EL.511751.80_mRNA-p1 TcCLB-NE.
507023.150_mRNA-p1
OG0004573: TCRU_2834 TcCLB-EL.511751.90_mRNA-p1 TcCLB-NE.
507023.140_mRNA-p1
OG0004574: TCRU_2836 TcCLB-EL.511751.114_mRNA-p1 TcCLB-NE.
507023.124_mRNA-p1
OG0004575: TCRU_2842 TcCLB-EL.511751.163_mRNA-p1 TcCLB-NE.
507023.60_mRNA-p1
OG0004576: TCRU_2843 TcCLB-EL.511751.166_mRNA-p1 TcCLB-NE.
507023.50_mRNA-p1
OG0004577: TCRU_2845 TcCLB-EL.511751.191_pseudogenic_transcript-p1
TcCLB-NE.507023.20_mRNA-p1
OG0004578: TCRU_2848 TcCLB-EL.511751.240_pseudogenic_transcript-p1
TcCLB-NE.507021.90_mRNA-p1
OG0004579: TCRU_2849 TcCLB-EL.511751.250_mRNA-p1 TcCLB-NE.
507021.80_mRNA-p1
OG0004580: TCRU_2861 TcCLB-NE.510687.50_mRNA-p1 TcSYL_0047130.t1-p1
OG0004581: TCRU_2862 TcCLB-NE.510687.40_mRNA-p1 TcSYL_0047120.t1-p1
OG0004582: TCRU_2863 TcCLB-NE.510687.30_mRNA-p1 TcSYL_0047110.t1-p1
OG0004583: TCRU_2864 TcCLB-NE.510687.20_mRNA-p1 TcSYL_0047100.t1-p1
OG0004584: TCRU_2872 TcCLB-NE.507053.140_mRNA-p1 TcSYL_0047010.t1-p1

OG0004585: TCRU_2873 TcCLB-NE.507053.130_mRNA-p1 TcSYL_0047000.t1-p1
OG0004586: TCRU_2876 TcCLB-NE.507053.90_mRNA-p1 TcSYL_0046950.t1-p1
OG0004587: TCRU_2880 TcCLB-NE.507053.30_mRNA-p1 TcSYL_0046930.t1-p1
OG0004588: TCRU_2883 TcCLB-NE.508257.260_mRNA-p1 TcSYL_0046920.t1-p1
OG0004589: TCRU_2885 TcCLB-NE.508257.230_mRNA-p1 TcSYL_0046890.t1-p1
OG0004590: TCRU_2887 TcCLB-NE.508257.204_mRNA-p1 TcSYL_0046870.t1-p1
OG0004591: TCRU_2890 TcCLB-NE.508257.180_mRNA-p1 TcSYL_0046860.t1-p1
OG0004592: TCRU_2893 TcCLB-NE.508257.150_mRNA-p1 TcSYL_0046730.t1-p1
OG0004593: TCRU_2900 TcCLB-EL.506405.4_mRNA-p1 TcCLB-NE.508257.80_mRNA-p1
OG0004594: TCRU_2902 TcCLB-EL.506405.20_mRNA-p1 TcCLB-NE.508257.60_mRNA-p1
OG0004595: TCRU_2903 TcCLB-EL.506405.30_mRNA-p1 TcCLB-NE.508257.50_mRNA-p1
OG0004596: TCRU_2904 TcCLB-EL.506405.43_mRNA-p1 TcCLB-NE.508257.30_mRNA-p1
OG0004597: TCRU_2906 TcCLB-EL.506405.60_mRNA-p1 TcCLB-NE.503929.70_mRNA-p1
OG0004598: TCRU_2909 TcCLB-EL.506405.90_mRNA-p1 TcCLB-NE.503929.40_mRNA-p1
OG0004599: TCRU_2910 TcCLB-EL.506405.100_mRNA-p1 TcCLB-NE.503929.30_mRNA-p1
OG0004600: TCRU_2911 TcCLB-EL.506405.110_mRNA-p1 TcCLB-NE.503929.20_mRNA-p1
OG0004601: TCRU_2932 TcCLB-EL.506979.10_mRNA-p1 TcCLB-NE.490507.10_mRNA-p1
OG0004602: TCRU_2934 TcCLB-EL.507099.80_mRNA-p1 TcSYL_0089330.t1-p1
OG0004603: TCRU_2936 TcCLB-EL.510763.40_mRNA-p1 TcCLB-NE.509671.180_mRNA-p1
OG0004604: TCRU_2937 TcCLB-EL.510763.80_mRNA-p1 TcCLB-NE.509671.160_mRNA-p1
OG0004605: TCRU_2938 TcCLB-EL.510763.90_mRNA-p1 TcCLB-NE.509671.150_mRNA-p1
OG0004606: TCRU_2942 TcCLB-EL.510765.50_mRNA-p1 TcCLB-NE.509671.84_mRNA-p1
OG0004607: TCRU_2947 TcCLB-EL.506753.140_mRNA-p1 TcCLB-NE.510357.30_mRNA-p1
OG0004608: TCRU_2948 TcCLB-EL.506753.130_mRNA-p1 TcCLB-NE.510357.20_mRNA-p1
OG0004609: TCRU_2950 TcCLB-EL.510091.130_mRNA-p1 TcCLB-NE.457251.19_mRNA-p1
OG0004610: TCRU_2954 TcCLB-EL.504147.30_mRNA-p1 TcSYL_0005620.t1-p1
OG0004611: TCRU_2958 TcCLB-EL.510057.30_mRNA-p1 TcCLB-NE.508909.140_mRNA-p1
OG0004612: TCRU_2959 TcCLB-EL.510057.10_mRNA-p1 TcCLB-NE.508909.160_mRNA-p1
OG0004613: TCRU_2962 TcCLB-EL.503751.10_mRNA-p1 TcCLB-NE.508909.200_mRNA-p1
OG0004614: TCRU_2963 TcCLB-EL.503751.30_pseudogenic_transcript-p1 TcCLB-NE.508909.210_mRNA-p1
OG0004615: TCRU_2966 TcCLB-EL.504147.110_mRNA-p1 TcSYL_0005390.t1-p1
OG0004616: TCRU_2967 TcCLB-EL.504147.99_mRNA-p1 TcSYL_0005430.t1-p1
OG0004617: TCRU_2971 TcCLB-EL.506825.30_mRNA-p1 TcCLB-NE.506681.30_mRNA-p1

OG0004618: TCRU_2972 TcCLB-EL.506825.24_mRNA-p1 TcCLB-NE.
506681.24_mRNA-p1
OG0004619: TCRU_2973 TcCLB-EL.506825.20_mRNA-p1 TcCLB-NE.
506681.20_mRNA-p1
OG0004620: TCRU_2974 TcCLB-EL.506825.10_mRNA-p1 TcCLB-NE.
506681.10_mRNA-p1
OG0004621: TCRU_2975 TcCLB-EL.509153.10_mRNA-p1 TcCLB-NE.
510305.79_mRNA-p1
OG0004622: TCRU_2976 TcCLB-EL.509153.20_mRNA-p1 TcCLB-NE.
510305.70_mRNA-p1
OG0004623: TCRU_2977 TcCLB-EL.509153.30_mRNA-p1 TcCLB-NE.
510305.60_mRNA-p1
OG0004624: TCRU_2979 TcCLB-EL.509153.50_mRNA-p1 TcCLB-NE.
510305.40_mRNA-p1
OG0004625: TCRU_2981 TcCLB-EL.509153.70_mRNA-p1 TcCLB-NE.
510305.20_mRNA-p1
OG0004626: TCRU_2993 TcCLB-EL.436535.10_mRNA-p1 TcCLB-NE.
509287.60_mRNA-p1
OG0004627: TCRU_3001 TcCLB-EL.508647.170_mRNA-p1 TcSYL_0047870.t1-p1
OG0004628: TCRU_3005 TcCLB-EL.480997.10_mRNA-p1 TcCLB-NE.
509617.40_mRNA-p1
OG0004629: TCRU_3006 TcCLB-EL.509669.14_mRNA-p1 TcCLB-NE.
509617.60_mRNA-p1
OG0004630: TCRU_3007 TcCLB-EL.509669.20_mRNA-p1 TcCLB-NE.
509617.70_mRNA-p1
OG0004631: TCRU_3011 TcCLB-EL.509669.60_mRNA-p1 TcCLB-NE.
506175.10_mRNA-p1
OG0004632: TCRU_3012 TcCLB-EL.509669.75_mRNA-p1 TcCLB-NE.
506175.25_mRNA-p1
OG0004633: TCRU_3013 TcCLB-EL.509669.80_mRNA-p1 TcCLB-NE.
506175.30_mRNA-p1
OG0004634: TCRU_3015 TcCLB-EL.509669.100_mRNA-p1 TcCLB-NE.
506175.50_mRNA-p1
OG0004635: TCRU_3017 TcCLB-EL.509669.120_pseudogenic_transcript-p1
TcCLB-NE.506175.70_mRNA-p1
OG0004636: TCRU_3024 TcCLB-EL.507011.30_mRNA-p1 TcCLB-NE.
510667.10_mRNA-p1
OG0004637: TCRU_3036 TcCLB-EL.507905.30_pseudogenic_transcript-p1
TcCLB-NE.508061.130_pseudogenic_transcript-p1
OG0004638: TCRU_3037 TcCLB-EL.507905.39_mRNA-p1 TcCLB-NE.
508629.120_mRNA-p1
OG0004639: TCRU_3043 TcCLB-EL.506891.10_mRNA-p1 TcCLB-NE.
505999.140_mRNA-p1
OG0004640: TCRU_3052 TcCLB-EL.509779.10_mRNA-p1 TcCLB-NE.
511653.69_mRNA-p1
OG0004641: TCRU_3061 TcCLB-EL.510163.10_mRNA-p1 TcCLB-NE.
506459.280_mRNA-p1
OG0004642: TCRU_3070 TcCLB-EL.508277.300_mRNA-p1 TcCLB-NE.
504091.5_mRNA-p1
OG0004643: TCRU_3071 TcCLB-EL.506517.30_mRNA-p1 TcCLB-NE.
508909.320_mRNA-p1
OG0004644: TCRU_3072 TcCLB-EL.510055.160_mRNA-p1 TcCLB-NE.
508909.330_mRNA-p1
OG0004645: TCRU_3075 TcCLB-EL.510055.130_pseudogenic_transcript-p1

TcCLB-NE.508911.30_pseudogenic_transcript-p1
OG0004646: TCRU_3076 TcCLB-EL.510055.110_mRNA-p1 TcCLB-NE.508911.50_mRNA-p1
OG0004647: TCRU_3082 TCRU_5459 TcSYL_0079770.t1-p1
OG0004648: TCRU_3089 TcCLB-EL.508041.40_mRNA-p1 TcCLB-NE.506297.320_mRNA-p1
OG0004649: TCRU_3093 TcCLB-EL.510101.20_mRNA-p1 TcCLB-NE.506297.280_mRNA-p1
OG0004650: TCRU_3096 TcCLB-EL.510101.59_mRNA-p1 TcCLB-NE.506297.250_mRNA-p1
OG0004651: TCRU_3099 TcCLB-EL.510101.110_mRNA-p1 TcCLB-NE.506297.210_mRNA-p1
OG0004652: TCRU_3102 TcCLB-EL.510101.150_mRNA-p1 TcCLB-NE.506297.180_mRNA-p1
OG0004653: TCRU_3103 TcCLB-EL.510101.160_mRNA-p1 TcCLB-NE.506297.170_mRNA-p1
OG0004654: TCRU_3106 TcCLB-EL.510101.190_mRNA-p1 TcCLB-NE.506297.140_mRNA-p1
OG0004655: TCRU_3107 TcCLB-EL.510101.200_mRNA-p1 TcCLB-NE.506297.130_mRNA-p1
OG0004656: TCRU_3108 TcCLB-EL.510101.210_mRNA-p1 TcCLB-NE.506297.120_mRNA-p1
OG0004657: TCRU_3109 TcCLB-EL.510101.230_pseudogenic_transcript-p1 TcCLB-NE.506297.110_mRNA-p1
OG0004658: TCRU_3111 TcCLB-EL.510101.260_mRNA-p1 TcCLB-NE.506297.70_mRNA-p1
OG0004659: TCRU_3113 TcCLB-EL.510101.290_mRNA-p1 TcCLB-NE.506297.40_mRNA-p1
OG0004660: TCRU_3115 TcCLB-EL.510101.310_mRNA-p1 TcCLB-NE.506297.20_mRNA-p1
OG0004661: TCRU_3118 TcCLB-EL.510101.350_mRNA-p1 TcCLB-NE.507765.130_mRNA-p1
OG0004662: TCRU_3120 TcCLB-EL.510101.390_mRNA-p1 TcCLB-NE.507765.100_mRNA-p1
OG0004663: TCRU_3121 TcCLB-EL.510101.400_mRNA-p1 TcCLB-NE.507765.90_mRNA-p1
OG0004664: TCRU_3122 TcCLB-EL.510101.410_mRNA-p1 TcCLB-NE.507765.80_mRNA-p1
OG0004665: TCRU_3140 TcCLB-EL.504041.29_mRNA-p1 TcCLB-NE.508815.30_mRNA-p1
OG0004666: TCRU_3143 TcCLB-NE.508815.70_mRNA-p1 TcSYL_0044070.t1-p1
OG0004667: TCRU_3147 TcCLB-NE.508815.110_mRNA-p1 TcSYL_0044050.t1-p1
OG0004668: TCRU_3152 TcCLB-NE.508815.170_mRNA-p1 TcSYL_0044030.t1-p1
OG0004669: TCRU_3162 TcCLB-EL.511603.160_mRNA-p1 TcCLB-NE.509753.140_mRNA-p1
OG0004670: TCRU_3174 TcCLB-EL.509967.140_mRNA-p1 TcCLB-NE.509695.100_mRNA-p1
OG0004671: TCRU_3178 TcCLB-NE.507681.100_mRNA-p1 TcSYL_0073730.t1-p1
OG0004672: TCRU_3181 TcCLB-NE.507681.70_mRNA-p1 TcSYL_0073710.t1-p1
OG0004673: TCRU_3182 TcCLB-NE.507681.60_mRNA-p1 TcSYL_0073700.t1-p1
OG0004674: TCRU_3183 TcCLB-NE.507681.40_mRNA-p1 TcSYL_0073690.t1-p1
OG0004675: TCRU_3184 TcCLB-NE.507681.30_mRNA-p1 TcSYL_0073680.t1-p1
OG0004676: TCRU_3194 TcCLB-EL.506797.110_mRNA-p1 TcCLB-NE.511907.89_mRNA-p1

OG0004677: TCRU_3204 TcCLB-EL.510283.70_mRNA-p1 TcCLB-NE.
503823.40_mRNA-p1
OG0004678: TCRU_3205 TcCLB-EL.510283.80_mRNA-p1 TcCLB-NE.
503823.50_mRNA-p1
OG0004679: TCRU_3209 TcCLB-EL.510283.130_mRNA-p1 TcCLB-NE.
503823.100_mRNA-p1
OG0004680: TCRU_3217 TcCLB-NE.508017.20_pseudogenic_transcript-p1
TcSYL_0055540.t1-p1
OG0004681: TCRU_3230 TcCLB-EL.504769.89_mRNA-p1 TcCLB-NE.
506471.19_mRNA-p1
OG0004682: TCRU_3233 TcCLB-EL.503989.10_mRNA-p1 TcCLB-NE.
401469.10_mRNA-p1
OG0004683: TCRU_3238 TcCLB-EL.505943.50_mRNA-p1 TcCLB-NE.
507623.20_mRNA-p1
OG0004684: TCRU_3249 TcCLB-EL.506413.70_mRNA-p1 TcCLB-NE.
511237.100_mRNA-p1
OG0004685: TCRU_3250 TcCLB-EL.506413.60_mRNA-p1 TcCLB-NE.
511237.90_mRNA-p1
OG0004686: TCRU_3251 TcCLB-EL.506413.50_mRNA-p1 TcCLB-NE.
511237.80_mRNA-p1
OG0004687: TCRU_3252 TcCLB-EL.506413.40_mRNA-p1 TcCLB-NE.
511237.70_mRNA-p1
OG0004688: TCRU_3256 TcCLB-EL.506411.10_mRNA-p1 TcCLB-NE.
511237.10_mRNA-p1
OG0004689: TCRU_3261 TcCLB-EL.507083.100_mRNA-p1 TcCLB-NE.
509901.100_mRNA-p1
OG0004690: TCRU_3262 TcCLB-EL.507083.109_mRNA-p1 TcCLB-NE.
509901.90_pseudogenic_transcript-p1
OG0004691: TCRU_3264 TcCLB-EL.506649.110_mRNA-p1 TcCLB-NE.
508349.10_mRNA-p1
OG0004692: TCRU_3265 TcCLB-EL.508227.40_mRNA-p1 TcCLB-NE.
508871.150_mRNA-p1
OG0004693: TCRU_3274 TcCLB-EL.510737.90_mRNA-p1 TcCLB-NE.
510663.10_mRNA-p1
OG0004694: TCRU_3281 TcCLB-EL.510741.50_mRNA-p1 TcCLB-NE.
510661.220_mRNA-p1
OG0004695: TCRU_3282 TcCLB-EL.510741.60_mRNA-p1 TcCLB-NE.
510661.200_mRNA-p1
OG0004696: TCRU_3286 TcCLB-EL.510741.100_mRNA-p1 TcCLB-NE.
510661.150_mRNA-p1
OG0004697: TCRU_3287 TcCLB-EL.510741.110_mRNA-p1 TcCLB-NE.
510661.140_mRNA-p1
OG0004698: TCRU_3290 TcCLB-EL.510741.140_mRNA-p1 TcCLB-NE.
510661.120_mRNA-p1
OG0004699: TCRU_3291 TcCLB-EL.510741.170_mRNA-p1 TcCLB-NE.
510661.100_mRNA-p1
OG0004700: TCRU_3295 TcCLB-EL.509967.50_mRNA-p1 TcCLB-NE.
509695.30_mRNA-p1
OG0004701: TCRU_3296 TcCLB-EL.509967.70_mRNA-p1 TcCLB-NE.
509695.50_mRNA-p1
OG0004702: TCRU_3297 TcCLB-EL.509967.80_mRNA-p1 TcCLB-NE.
509695.60_mRNA-p1
OG0004703: TCRU_3298 TcCLB-EL.509967.90_mRNA-p1 TcCLB-NE.
509695.70_mRNA-p1

OG0004704: TCRU_3301 TcCLB-NE.508479.110_mRNA-p1 TcSYL_0019870.t1-p1
OG0004705: TCRU_3303 TcCLB-NE.510047.60_pseudogenic_transcript-p1 TcSYL_0065980.t1-p1
OG0004706: TCRU_3305 TcCLB-EL.506945.280_mRNA-p1 TcSYL_0140760.t1-p1
OG0004707: TCRU_3307 TcCLB-EL.506945.250_mRNA-p1 TcSYL_0140740.t1-p1
OG0004708: TCRU_3308 TcCLB-EL.506945.240_mRNA-p1 TcSYL_0140730.t1-p1
OG0004709: TCRU_3314 TcCLB-NE.511725.280_mRNA-p1 TcSYL_0142700.t1-p1
OG0004710: TCRU_3316 TcCLB-NE.511725.250_mRNA-p1 TcSYL_0142680.t1-p1
OG0004711: TCRU_3318 TcCLB-NE.511725.210_mRNA-p1 TcSYL_0142670.t1-p1
OG0004712: TCRU_3319 TcCLB-NE.511725.200_mRNA-p1 TcSYL_0142660.t1-p1
OG0004713: TCRU_3321 TcCLB-NE.511725.180_mRNA-p1 TcSYL_0142650.t1-p1
OG0004714: TCRU_3326 TcCLB-EL.510105.220_mRNA-p1 TcCLB-NE.509717.70_mRNA-p1
OG0004715: TCRU_3329 TcCLB-EL.510105.190_mRNA-p1 TcCLB-NE.509717.40_mRNA-p1
OG0004716: TCRU_3330 TcCLB-EL.510105.184_mRNA-p1 TcCLB-NE.509717.34_mRNA-p1
OG0004717: TCRU_3342 TcCLB-EL.504125.60_mRNA-p1 TcSYL_0048020.t1-p1
OG0004718: TCRU_3344 TcCLB-EL.509979.20_mRNA-p1 TcCLB-NE.508613.20_mRNA-p1
OG0004719: TCRU_3345 TcCLB-EL.509979.14_mRNA-p1 TcCLB-NE.508613.30_mRNA-p1
OG0004720: TCRU_3361 TcCLB-EL.507809.160_mRNA-p1 TcCLB-NE.511657.30_mRNA-p1
OG0004721: TCRU_3363 TcCLB-EL.507809.140_mRNA-p1 TcCLB-NE.511657.50_mRNA-p1
OG0004722: TCRU_3365 TcCLB-EL.507809.110_mRNA-p1 TcCLB-NE.511659.9_mRNA-p1
OG0004723: TCRU_3372 TcCLB-NE.511725.160_mRNA-p1 TcSYL_0142630.t1-p1
OG0004724: TCRU_3373 TcCLB-NE.511725.150_mRNA-p1 TcSYL_0142620.t1-p1
OG0004725: TCRU_3377 TcCLB-EL.509489.10_mRNA-p1 TcCLB-NE.511725.40_mRNA-p1
OG0004726: TCRU_3378 TcCLB-EL.509489.20_mRNA-p1 TcCLB-NE.511725.30_mRNA-p1
OG0004727: TCRU_3390 TcCLB-EL.504057.21_mRNA-p1 TcCLB-NE.481729.20_mRNA-p1
OG0004728: TCRU_3393 TcCLB-EL.504017.79_mRNA-p1 TcCLB-NE.511229.110_mRNA-p1
OG0004729: TCRU_3394 TcCLB-EL.511589.250_mRNA-p1 TcCLB-NE.511229.100_mRNA-p1
OG0004730: TCRU_3395 TcCLB-EL.511589.240_mRNA-p1 TcCLB-NE.511229.90_mRNA-p1
OG0004731: TCRU_3396 TcCLB-EL.511589.230_mRNA-p1 TcCLB-NE.511229.80_mRNA-p1
OG0004732: TCRU_3397 TcCLB-EL.511589.209_mRNA-p1 TcCLB-NE.511229.60_mRNA-p1
OG0004733: TCRU_3398 TcCLB-EL.511589.200_mRNA-p1 TcCLB-NE.511229.50_mRNA-p1
OG0004734: TCRU_3399 TcCLB-EL.511589.190_mRNA-p1 TcCLB-NE.511229.39_mRNA-p1
OG0004735: TCRU_3400 TcCLB-EL.511589.180_mRNA-p1 TcCLB-NE.511229.30_mRNA-p1
OG0004736: TCRU_3401 TcCLB-EL.511589.170_mRNA-p1 TcCLB-NE.511229.20_mRNA-p1

OG0004737: TCRU_3402 TcCLB-EL.511589.160_mRNA-p1 TcCLB-NE.
508851.210_mRNA-p1
OG0004738: TCRU_3403 TcCLB-EL.511589.150_mRNA-p1 TcCLB-NE.
508851.200_mRNA-p1
OG0004739: TCRU_3404 TcCLB-EL.511589.130_mRNA-p1 TcCLB-NE.
508851.180_mRNA-p1
OG0004740: TCRU_3405 TcCLB-EL.511589.120_mRNA-p1 TcCLB-NE.
508851.170_mRNA-p1
OG0004741: TCRU_3406 TcCLB-EL.511589.100_mRNA-p1 TcCLB-NE.
508851.154_mRNA-p1
OG0004742: TCRU_3407 TcCLB-EL.511589.90_mRNA-p1 TcCLB-NE.
508851.150_mRNA-p1
OG0004743: TCRU_3408 TcCLB-EL.511589.80_mRNA-p1 TcCLB-NE.
508851.140_mRNA-p1
OG0004744: TCRU_3409 TcCLB-EL.511589.70_mRNA-p1 TcCLB-NE.
508851.120_mRNA-p1
OG0004745: TCRU_3410 TcCLB-EL.511589.60_mRNA-p1 TcCLB-NE.
508851.110_mRNA-p1
OG0004746: TCRU_3416 TcCLB-EL.504255.4_mRNA-p1 TcCLB-NE.
510577.50_mRNA-p1
OG0004747: TCRU_3422 TcCLB-NE.508173.100_mRNA-p1 TcSYL_0079400.t1-p1
OG0004748: TCRU_3423 TcCLB-NE.508173.90_mRNA-p1 TcSYL_0079410.t1-p1
OG0004749: TCRU_3426 TcCLB-NE.508173.70_mRNA-p1 TcSYL_0079420.t1-p1
OG0004750: TCRU_3446 TcCLB-EL.507093.90_mRNA-p1 TcCLB-NE.
503917.50_mRNA-p1
OG0004751: TCRU_3447 TcCLB-EL.507093.80_mRNA-p1 TcCLB-NE.
503917.40_mRNA-p1
OG0004752: TCRU_3448 TcCLB-EL.507093.75_mRNA-p1 TcCLB-NE.
503917.35_mRNA-p1
OG0004753: TCRU_3449 TcCLB-EL.507093.72_mRNA-p1 TcCLB-NE.
503917.28_mRNA-p1
OG0004754: TCRU_3450 TcCLB-EL.507093.66_mRNA-p1 TcCLB-NE.
503917.14_mRNA-p1
OG0004755: TCRU_3451 TcCLB-EL.507093.63_mRNA-p1 TcCLB-NE.
503917.7_mRNA-p1
OG0004756: TCRU_3452 TcCLB-EL.507093.60_mRNA-p1 TcCLB-NE.
504109.10_mRNA-p1
OG0004757: TCRU_3453 TcCLB-EL.507093.50_mRNA-p1 TcCLB-NE.
504109.20_mRNA-p1
OG0004758: TCRU_3454 TcCLB-EL.507093.40_mRNA-p1 TcCLB-NE.
504109.30_mRNA-p1
OG0004759: TCRU_3455 TcCLB-EL.507093.30_mRNA-p1 TcCLB-NE.
504109.40_mRNA-p1
OG0004760: TCRU_3456 TcCLB-EL.507093.20_mRNA-p1 TcCLB-NE.
504109.50_mRNA-p1
OG0004761: TCRU_3461 TcCLB-NE.504109.110_mRNA-p1 TcSYL_0080290.t1-p1
OG0004762: TCRU_3466 TcCLB-NE.504109.160_mRNA-p1 TcSYL_0080280.t1-p1
OG0004763: TCRU_3483 TcCLB-EL.511127.390_mRNA-p1 TcCLB-NE.
503931.14_mRNA-p1
OG0004764: TCRU_3484 TcCLB-EL.511127.400_mRNA-p1 TcCLB-NE.
509029.10_mRNA-p1
OG0004765: TCRU_3487 TcCLB-EL.508777.10_mRNA-p1 TcCLB-NE.
509029.40_mRNA-p1
OG0004766: TCRU_3488 TcCLB-EL.508777.20_mRNA-p1 TcCLB-NE.

509029.50_mRNA-p1
OG0004767: TCRU_3489 TcCLB-EL.508777.40_mRNA-p1 TcCLB-NE.
509029.70_mRNA-p1
OG0004768: TCRU_3490 TcCLB-EL.508777.50_mRNA-p1 TcCLB-NE.
509029.80_mRNA-p1
OG0004769: TCRU_3494 TcCLB-NE.511893.40_mRNA-p1 TcSYL_0088700.t1-p1
OG0004770: TCRU_3507 TcCLB-EL.511313.30_mRNA-p1 TcCLB-NE.
511755.80_mRNA-p1
OG0004771: TCRU_3508 TcCLB-EL.511135.40_mRNA-p1 TcCLB-NE.
507073.40_mRNA-p1
OG0004772: TCRU_3510 TcCLB-EL.511137.10_mRNA-p1 TcCLB-NE.
507073.20_mRNA-p1
OG0004773: TCRU_3513 TcCLB-EL.482471.19_mRNA-p1 TcCLB-NE.
508319.40_mRNA-p1
OG0004774: TCRU_3514 TcCLB-EL.508785.4_mRNA-p1 TcCLB-NE.
508319.30_mRNA-p1
OG0004775: TCRU_3515 TcCLB-EL.508785.10_mRNA-p1 TcCLB-NE.
508319.20_mRNA-p1
OG0004776: TCRU_3516 TcCLB-EL.511141.10_mRNA-p1 TcCLB-NE.
506851.30_mRNA-p1
OG0004777: TCRU_3517 TcCLB-EL.511141.20_mRNA-p1 TcCLB-NE.
506851.20_mRNA-p1
OG0004778: TCRU_3519 TcCLB-EL.511143.12_mRNA-p1 TcCLB-NE.
510519.149_mRNA-p1
OG0004779: TCRU_3520 TcCLB-EL.511143.19_mRNA-p1 TcCLB-NE.
510519.140_mRNA-p1
OG0004780: TCRU_3521 TcCLB-EL.511143.30_mRNA-p1 TcCLB-NE.
510519.130_mRNA-p1
OG0004781: TCRU_3522 TcCLB-EL.506869.4_mRNA-p1 TcCLB-NE.
510519.120_mRNA-p1
OG0004782: TCRU_3523 TcCLB-EL.506869.10_mRNA-p1 TcCLB-NE.
510519.110_mRNA-p1
OG0004783: TCRU_3524 TcCLB-EL.506869.20_mRNA-p1 TcCLB-NE.
510519.100_mRNA-p1
OG0004784: TCRU_3525 TcCLB-EL.506869.30_mRNA-p1 TcCLB-NE.
510519.90_mRNA-p1
OG0004785: TCRU_3528 TcCLB-EL.504153.300_mRNA-p1 TcSYL_0138600.t1-p1
OG0004786: TCRU_3530 TcCLB-EL.508307.90_mRNA-p1 TcSYL_0006110.t1-p1
OG0004787: TCRU_3538 TcCLB-EL.509633.40_mRNA-p1 TcCLB-NE.
506201.84_mRNA-p1
OG0004788: TCRU_3539 TcCLB-EL.509633.30_mRNA-p1 TcCLB-NE.
506201.90_mRNA-p1
OG0004789: TCRU_3540 TcCLB-EL.509633.20_mRNA-p1 TcCLB-NE.
506201.100_mRNA-p1
OG0004790: TCRU_family_ TcCLB-EL.509633.10_mRNA-p1 TcCLB-NE.
506201.110_mRNA-p1
OG0004791: TCRU_3542 TcCLB-EL.504129.40_mRNA-p1 TcCLB-NE.
506201.120_mRNA-p1
OG0004792: TCRU_3543 TcCLB-EL.504129.30_mRNA-p1 TcCLB-NE.
506201.130_mRNA-p1
OG0004793: TCRU_3544 TcCLB-EL.504129.20_mRNA-p1 TcCLB-NE.
506201.140_mRNA-p1
OG0004794: TCRU_3545 TcCLB-EL.504129.10_mRNA-p1 TcCLB-NE.
506201.150_mRNA-p1

OG0004795: TCRU_3546 TcCLB-EL.509631.160_mRNA-p1 TcCLB-NE.
506201.154_mRNA-p1
OG0004796: TCRU_3547 TcCLB-EL.509631.150_mRNA-p1 TcCLB-NE.
506201.160_mRNA-p1
OG0004797: TCRU_3548 TcCLB-EL.509631.140_mRNA-p1 TcCLB-NE.
506201.170_mRNA-p1
OG0004798: TCRU_3556 TcCLB-EL.407477.20_mRNA-p1 TcCLB-NE.
511389.140_mRNA-p1
OG0004799: TCRU_3557 TcCLB-EL.407477.30_mRNA-p1 TcCLB-NE.
511389.130_mRNA-p1
OG0004800: TCRU_3558 TcCLB-EL.407477.40_mRNA-p1 TcCLB-NE.
511389.120_mRNA-p1
OG0004801: TCRU_3559 TcCLB-EL.407477.44_mRNA-p1 TcCLB-NE.
511389.114_mRNA-p1
OG0004802: TCRU_3560 TcCLB-EL.407477.50_mRNA-p1 TcCLB-NE.
511389.110_mRNA-p1
OG0004803: TCRU_3561 TcCLB-EL.407477.60_mRNA-p1 TcCLB-NE.
511389.100_mRNA-p1
OG0004804: TCRU_/ TcCLB-EL.407477.69_mRNA-p1 TcCLB-NE.
511389.90_mRNA-p1
OG0004805: TCRU_3570 TcCLB-NE.506977.30_mRNA-p1 TcSYL_0170390.t1-p1
OG0004806: TCRU_3571 TCRU_3972 TcCLB-NE.506977.40_mRNA-p1
OG0004807: TCRU_3579 TcCLB-EL.507041.20_mRNA-p1 TcCLB-NE.
508273.40_mRNA-p1
OG0004808: TCRU_3580 TcCLB-EL.507041.10_mRNA-p1 TcCLB-NE.
508273.30_mRNA-p1
OG0004809: TCRU_3589 TcCLB-EL.509465.10_mRNA-p1 TcCLB-NE.
509613.10_mRNA-p1
OG0004810: TCRU_3605 TcCLB-EL.509233.80_mRNA-p1 TcCLB-NE.
509437.40_mRNA-p1
OG0004811: TCRU_3614 TcCLB-EL.503683.20_mRNA-p1 TcCLB-NE.
509999.130_mRNA-p1
OG0004812: TCRU_3615 TcCLB-EL.503683.11_mRNA-p1 TcCLB-NE.
509999.140_mRNA-p1
OG0004813: TCRU_3625 TcCLB-EL.511737.50_mRNA-p1 TcCLB-NE.
511517.156_mRNA-p1
OG0004814: TCRU_3626 TcCLB-EL.511737.60_mRNA-p1 TcCLB-NE.
511517.153_mRNA-p1
OG0004815: TCRU_3631 TcCLB-EL.511741.10_mRNA-p1 TcCLB-NE.
511517.95_mRNA-p1
OG0004816: TCRU_3633 TcCLB-EL.511741.40_mRNA-p1 TcCLB-NE.
511517.70_mRNA-p1
OG0004817: TCRU_3634 TcCLB-EL.511741.60_mRNA-p1 TcCLB-NE.
511517.50_mRNA-p1
OG0004818: TCRU_3638 TcCLB-EL.511745.30_mRNA-p1 TcCLB-NE.
511515.9_mRNA-p1
OG0004819: TCRU_3639 TcCLB-EL.511745.50_mRNA-p1 TcCLB-NE.
507029.50_mRNA-p1
OG0004820: TCRU_3641 TcCLB-EL.506941.270_mRNA-p1 TcCLB-NE.
507029.20_mRNA-p1
OG0004821: TCRU_3642 TcCLB-EL.506941.260_mRNA-p1 TcCLB-NE.
507029.10_mRNA-p1
OG0004822: TCRU_3649 TCRU_7164 TcSYL_0123900.t1-p1
OG0004823: TCRU_3650 TcCLB-NE.507071.240_mRNA-p1 TcCLB-NE.

510709.60_mRNA-p1
OG0004824: TCRU_3653 TcCLB-EL.503867.10_mRNA-p1 TcCLB-NE.
511479.20_mRNA-p1
OG0004825: TCRU_3654 TcCLB-EL.504005.60_mRNA-p1 TcCLB-NE.
511479.30_mRNA-p1
OG0004826: TCRU_3655 TcCLB-EL.504005.54_mRNA-p1 TcCLB-NE.
511479.39_mRNA-p1
OG0004827: TCRU__PEX11__ TcCLB-EL.504005.40_mRNA-p1 TcCLB-NE.
511481.20_mRNA-p1
OG0004828: TCRU_3657 TcCLB-EL.504005.29_mRNA-p1 TcCLB-NE.
511481.30_mRNA-p1
OG0004829: TCRU_3658 TcCLB-EL.504005.20_mRNA-p1 TcCLB-NE.
511481.40_mRNA-p1
OG0004830: TCRU_3659 TcCLB-EL.504005.10_mRNA-p1 TcCLB-NE.
511481.50_mRNA-p1
OG0004831: TCRU_3660 TcCLB-EL.504005.3_mRNA-p1 TcCLB-NE.
511481.60_mRNA-p1
OG0004832: TCRU_3661 TcCLB-EL.508375.10_mRNA-p1 TcCLB-NE.
511643.50_pseudogenic_transcript-p1
OG0004833: TCRU_3674 TcCLB-EL.510565.141_mRNA-p1 TcCLB-NE.
506921.20_mRNA-p1
OG0004834: TCRU_3675 TcCLB-EL.511393.79_mRNA-p1 TcCLB-NE.
505071.119_mRNA-p1
OG0004835: TCRU_3699 TcCLB-EL.420369.40_mRNA-p1 TcCLB-NE.
506913.10_mRNA-p1
OG0004836: TCRU_3702 TCRU_4632 TcCLB-EL.509955.10_mRNA-p1
OG0004837: TCRU_3721 TcCLB-EL.507639.60_mRNA-p1 TcCLB-NE.
503781.50_mRNA-p1
OG0004838: TCRU_3724 TcCLB-EL.507639.100_mRNA-p1 TcCLB-NE.
503781.20_mRNA-p1
OG0004839: TCRU_3728 TcCLB-EL.507641.60_mRNA-p1 TcSYL_0011090.t1-p1
OG0004840: TCRU_3731 TcCLB-EL.508165.170_mRNA-p1 TcSYL_0074140.t1-p1
OG0004841: TCRU_3737 TcCLB-EL.506363.10_mRNA-p1 TcCLB-NE.
506975.70_mRNA-p1
OG0004842: TCRU_3739 TcCLB-EL.506363.60_mRNA-p1 TcCLB-NE.
506975.49_mRNA-p1
OG0004843: TCRU_3753 TcCLB-EL.510615.10_mRNA-p1 TcCLB-NE.
506789.330_mRNA-p1
OG0004844: TCRU_3756 TcCLB-EL.503493.40_mRNA-p1 TcCLB-NE.
511761.40_mRNA-p1
OG0004845: TCRU_3758 TcCLB-EL.503493.10_mRNA-p1 TcCLB-NE.
511761.10_mRNA-p1
OG0004846: TCRU_3760 TcCLB-EL.511305.20_mRNA-p1 TcCLB-NE.
507087.70_mRNA-p1
OG0004847: TCRU_3761 TcCLB-EL.511305.40_mRNA-p1 TcCLB-NE.
507087.60_mRNA-p1
OG0004848: TCRU_3782 TcCLB-EL.503485.20_pseudogenic_transcript-p1
TcCLB-NE.507081.90_mRNA-p1
OG0004849: TCRU_3787 TcCLB-EL.504047.60_mRNA-p1 TcSYL_0019610.t1-p1
OG0004850: TCRU_10419 TCRU_3804 TcSYL_0148130.t1-p1
OG0004851: TCRU_3809 TcCLB-NE.506511.60_mRNA-p1 TcSYL_0065020.t1-p1
OG0004852: TCRU_3810 TcCLB-EL.504883.70_pseudogenic_transcript-p1
TcCLB-NE.506511.50_mRNA-p1
OG0004853: TCRU_3818 TcCLB-NE.503459.29_mRNA-p1 TcSYL_0050800.t1-p1

OG0004854: TCRU_3820 TcCLB-NE.508827.60_mRNA-p1 TcSYL_0181900.t1-p1
OG0004855: TCRU_3822 TcCLB-NE.508827.44_mRNA-p1 TcSYL_0181760.t1-p1
OG0004856: TCRU_3823 TcCLB-NE.508827.30_mRNA-p1 TcSYL_0181650.t1-p1
OG0004857: TCRU_3824 TcCLB-NE.508827.20_mRNA-p1 TcSYL_0181600.t1-p1
OG0004858: TCRU_3854 TcCLB-EL.506859.240_mRNA-p1 TcCLB-NE.511817.20_mRNA-p1
OG0004859: TCRU_3855 TcCLB-EL.506859.249_mRNA-p1 TcCLB-NE.511817.30_mRNA-p1
OG0004860: TCRU_3856 TcCLB-EL.510521.10_pseudogenic_transcript-p1 TcCLB-NE.511817.40_mRNA-p1
OG0004861: TCRU_3857 TcCLB-EL.510523.20_mRNA-p1 TcCLB-NE.511817.60_mRNA-p1
OG0004862: TCRU_3858 TcCLB-EL.510523.29_mRNA-p1 TcCLB-NE.511817.70_mRNA-p1
OG0004863: TCRU_3859 TcCLB-EL.510525.10_mRNA-p1 TcCLB-NE.511817.80_mRNA-p1
OG0004864: TCRU_3860 TcCLB-EL.510525.30_mRNA-p1 TcCLB-NE.511817.100_mRNA-p1
OG0004865: TCRU_3861 TcCLB-EL.510525.40_mRNA-p1 TcCLB-NE.511817.110_mRNA-p1
OG0004866: TCRU_3862 TcCLB-EL.510525.50_mRNA-p1 TcCLB-NE.511817.120_mRNA-p1
OG0004867: TCRU_3863 TcCLB-EL.510525.60_mRNA-p1 TcCLB-NE.511817.130_mRNA-p1
OG0004868: TCRU_3871 TcSYL_0172970.t1-p1 TcSYL_0174420.t1-p1
OG0004869: TCRU_3885 TCRU_4029 TcSYL_0069270.t1-p1
OG0004870: TCRU_3888 TcCLB-EL.505229.20_mRNA-p1 TcCLB-NE.510823.20_mRNA-p1
OG0004871: TCRU_3890 TcCLB-EL.505229.5_mRNA-p1 TcCLB-NE.510823.40_mRNA-p1
OG0004872: TCRU_3891 TcCLB-NE.510823.60_mRNA-p1 TcSYL_0050170.t1-p1
OG0004873: TCRU_3892 TcCLB-NE.510823.70_mRNA-p1 TcSYL_0050180.t1-p1
OG0004874: TCRU_3894 TcCLB-EL.505555.50_mRNA-p1 TcCLB-NE.510823.90_mRNA-p1
OG0004875: TCRU_3897 TcCLB-EL.505555.20_mRNA-p1 TcCLB-NE.510825.20_mRNA-p1
OG0004876: TCRU_3905 TcCLB-EL.504055.81_mRNA-p1 TcCLB-NE.506435.300_mRNA-p1
OG0004877: TCRU_3911 TcCLB-EL.506265.200_mRNA-p1 TcCLB-NE.506435.220_mRNA-p1
OG0004878: TCRU_3926 TcCLB-EL.507511.50_mRNA-p1 TcCLB-NE.506871.190_mRNA-p1
OG0004879: TCRU_3930 TcCLB-EL.507509.80_mRNA-p1 TcCLB-NE.506871.130_mRNA-p1
OG0004880: TCRU_3931 TcCLB-EL.507509.70_mRNA-p1 TcCLB-NE.506871.120_mRNA-p1
OG0004881: TCRU_3932 TcCLB-EL.507509.60_mRNA-p1 TcCLB-NE.506871.110_mRNA-p1
OG0004882: TCRU_3933 TCRU_5968 TcSYL_0040480.t1-p1
OG0004883: TCRU_3941 TcCLB-EL.506351.40_mRNA-p1 TcCLB-NE.505939.60_mRNA-p1
OG0004884: TCRU_3948 TcCLB-EL.509801.20_mRNA-p1 TcCLB-NE.511277.115_mRNA-p1
OG0004885: TCRU_3950 TcCLB-EL.506357.10_mRNA-p1 TcCLB-NE.

511277.100_mRNA-p1
OG0004886: TCRU_3957 TcCLB-EL.507709.70_mRNA-p1 TcCLB-NE.
506577.70_mRNA-p1
OG0004887: TCRU_3958 TcCLB-EL.507709.60_mRNA-p1 TcCLB-NE.
506577.60_mRNA-p1
OG0004888: TCRU_3960 TcCLB-EL.507709.44_mRNA-p1 TcCLB-NE.
506577.47_mRNA-p1
OG0004889: TCRU_3961 TcCLB-EL.507709.40_mRNA-p1 TcCLB-NE.
506577.40_mRNA-p1
OG0004890: TCRU_3962 TcCLB-EL.507709.30_mRNA-p1 TcCLB-NE.
506577.30_mRNA-p1
OG0004891: TCRU_3964 TcCLB-NE.506577.5_mRNA-p1 TcSYL_0009180.t1-p1
OG0004892: TCRU_3971 TcSYL_0091560.t1-p1 TcSYL_0091900.t1-p1
OG0004893: TCRU_3986 TcCLB-EL.504033.120_mRNA-p1 TcCLB-NE.
510339.54_mRNA-p1
OG0004894: TCRU_3987 TcCLB-EL.504033.90_mRNA-p1 TcCLB-NE.
510339.74_mRNA-p1
OG0004895: TCRU_3988 TcCLB-EL.504033.80_mRNA-p1 TcCLB-NE.
510339.80_mRNA-p1
OG0004896: TCRU_3995 TcCLB-EL.503865.30_mRNA-p1 TcCLB-NE.
508693.160_mRNA-p1
OG0004897: TCRU_3996 TcCLB-EL.503865.40_mRNA-p1 TcCLB-NE.
508693.170_mRNA-p1
OG0004898: TCRU_3998 TcCLB-EL.503865.60_mRNA-p1 TcCLB-NE.
508693.190_mRNA-p1
OG0004899: TCRU_4001 TcCLB-EL.505987.60_mRNA-p1 TcCLB-NE.
508695.30_mRNA-p1
OG0004900: TCRU_4002 TcCLB-EL.505987.50_mRNA-p1 TcCLB-NE.
506705.20_mRNA-p1
OG0004901: TCRU_4003 TcCLB-EL.505987.30_mRNA-p1 TcCLB-NE.
506705.40_mRNA-p1
OG0004902: TCRU_4005 TcCLB-EL.508813.80_mRNA-p1 TcSYL_0044180.t1-p1
OG0004903: TCRU_4011 TcCLB-EL.506753.40_mRNA-p1 TcCLB-NE.
510355.200_mRNA-p1
OG0004904: TCRU_4012 TcCLB-EL.506753.50_mRNA-p1 TcCLB-NE.
510355.210_mRNA-p1
OG0004905: TCRU_4014 TcCLB-EL.506753.100_mRNA-p1 TcCLB-NE.
510355.260_mRNA-p1
OG0004906: TCRU_4015 TcCLB-EL.506753.110_mRNA-p1 TcCLB-NE.
510355.269_mRNA-p1
OG0004907: TCRU_4024 TcCLB-EL.509591.50_mRNA-p1 TcCLB-NE.
503999.90_mRNA-p1
OG0004908: TCRU_4025 TcCLB-EL.509591.20_mRNA-p1 TcCLB-NE.
503999.60_mRNA-p1
OG0004909: TCRU_4033 TcCLB-EL.506775.170_mRNA-p1 TcCLB-NE.
511167.10_mRNA-p1
OG0004910: TCRU_subunit TcCLB-EL.508307.80_mRNA-p1 TcSYL_0006120.t1-
p1
OG0004911: TCRU_4053 TcCLB-NE.508065.40_mRNA-p1 TcSYL_0056950.t1-p1
OG0004912: TCRU_4066 TcCLB-EL.510165.50_mRNA-p1 TcCLB-NE.
511807.40_mRNA-p1
OG0004913: TCRU_4067 TcCLB-EL.510165.40_mRNA-p1 TcCLB-NE.
511807.50_mRNA-p1
OG0004914: TCRU_4068 TcCLB-EL.510165.10_mRNA-p1 TcCLB-NE.

511807.70_mRNA-p1
OG0004915: TCRU_4069 TcCLB-EL.508095.20_mRNA-p1 TcCLB-NE.
511807.80_mRNA-p1
OG0004916: TCRU_4071 TcCLB-EL.506683.19_pseudogenic_transcript-p1
TcCLB-NE.511807.110_mRNA-p1
OG0004917: TCRU_4079 TcCLB-EL.506733.90_mRNA-p1 TcCLB-NE.
511857.10_mRNA-p1
OG0004918: TCRU_4081 TcCLB-EL.506733.70_mRNA-p1 TcCLB-NE.
511857.30_mRNA-p1
OG0004919: TCRU_4087 TcCLB-EL.506733.10_mRNA-p1 TcCLB-NE.
511859.20_mRNA-p1
OG0004920: TCRU_4088 TcCLB-EL.511045.20_mRNA-p1 TcCLB-NE.
511859.40_mRNA-p1
OG0004921: TCRU_4089 TcCLB-EL.511045.29_mRNA-p1 TcCLB-NE.
511859.50_mRNA-p1
OG0004922: TCRU_4098 TcCLB-EL.507099.30_mRNA-p1 TcSYL_0089290.t1-p1
OG0004923: TCRU_4113 TcCLB-EL.509455.20_mRNA-p1 TcCLB-NE.
503879.90_mRNA-p1
OG0004924: TCRU_4118 TcCLB-EL.509453.50_mRNA-p1 TcCLB-NE.
511881.20_mRNA-p1
OG0004925: TCRU_4119 TcCLB-EL.509453.40_mRNA-p1 TcCLB-NE.
511881.14_mRNA-p1
OG0004926: TCRU_4126 TcCLB-EL.506529.300_mRNA-p1 TcCLB-NE.
510887.50_mRNA-p1
OG0004927: TCRU_4127 TcCLB-EL.506227.180_mRNA-p1 TcSYL_0092030.t1-p1
OG0004928: TCRU_4130 TcCLB-EL.511467.50_mRNA-p1 TcCLB-NE.
506295.30_mRNA-p1
OG0004929: TCRU_4131 TcCLB-EL.511467.60_mRNA-p1 TcCLB-NE.
506295.40_mRNA-p1
OG0004930: TCRU_4132 TcCLB-EL.511469.10_mRNA-p1 TcCLB-NE.
506295.60_mRNA-p1
OG0004931: TCRU_4133 TcCLB-EL.511469.20_mRNA-p1 TcCLB-NE.
506295.70_mRNA-p1
OG0004932: TCRU_4135 TcCLB-EL.511469.40_mRNA-p1 TcCLB-NE.
506295.100_mRNA-p1
OG0004933: TCRU_4136 TcCLB-EL.511469.50_mRNA-p1 TcCLB-NE.
506295.110_mRNA-p1
OG0004934: TCRU_4138 TcCLB-EL.511469.90_mRNA-p1 TcCLB-NE.
506295.150_mRNA-p1
OG0004935: TCRU_family_ TCRU_family_ TcSYL_0156470.t1-p1
OG0004936: TCRU_4147 TcCLB-EL.510535.50_mRNA-p1 TcCLB-NE.
511827.50_mRNA-p1
OG0004937: TCRU_4149 TcCLB-EL.510535.64_mRNA-p1 TcCLB-NE.
511827.64_mRNA-p1
OG0004938: TCRU_4150 TcCLB-EL.510535.70_mRNA-p1 TcCLB-NE.
511827.70_mRNA-p1
OG0004939: TCRU_4153 TcCLB-EL.510535.100_mRNA-p1 TcCLB-NE.
511827.100_mRNA-p1
OG0004940: TCRU_4160 TcCLB-EL.503893.140_mRNA-p1 TcCLB-NE.
508507.20_mRNA-p1
OG0004941: TCRU_4162 TcCLB-EL.503893.70_mRNA-p1 TcCLB-NE.
443397.9_mRNA-p1
OG0004942: TCRU_4164 TcCLB-EL.503893.40_mRNA-p1 TcCLB-NE.
503703.30_mRNA-p1

OG0004943: TCRU_4165 TcCLB-EL.503893.30_mRNA-p1 TcCLB-NE.
503703.40_mRNA-p1
OG0004944: TCRU_4168 TcCLB-EL.511445.120_mRNA-p1 TcCLB-NE.
503703.80_mRNA-p1
OG0004945: TCRU_4169 TcCLB-EL.511445.110_mRNA-p1 TcCLB-NE.
503703.90_mRNA-p1
OG0004946: TCRU_4170 TcCLB-EL.511445.100_mRNA-p1 TcCLB-NE.
503703.100_mRNA-p1
OG0004947: TCRU_4172 TcCLB-EL.511445.80_mRNA-p1 TcCLB-NE.
510799.18_mRNA-p1
OG0004948: TCRU_4173 TcCLB-EL.511445.70_mRNA-p1 TcCLB-NE.
510799.30_mRNA-p1
OG0004949: TCRU_4183 TcCLB-EL.511127.150_mRNA-p1 TcCLB-NE.
509023.140_mRNA-p1
OG0004950: TCRU_4185 TcCLB-EL.511127.180_mRNA-p1 TcCLB-NE.
509023.160_mRNA-p1
OG0004951: TCRU_4186 TcCLB-EL.511127.190_mRNA-p1 TcCLB-NE.
509023.170_mRNA-p1
OG0004952: TCRU_4188 TcCLB-EL.511127.230_mRNA-p1 TcCLB-NE.
509023.209_mRNA-p1
OG0004953: TCRU_4191 TcCLB-NE.511391.120_mRNA-p1 TcSYL_0201940.t1-p1
OG0004954: TCRU_4205 TcCLB-EL.510879.40_mRNA-p1 TcCLB-NE.
504013.70_mRNA-p1
OG0004955: TCRU_4208 TcCLB-EL.508585.29_mRNA-p1 TcCLB-NE.
504013.110_mRNA-p1
OG0004956: TCRU_4209 TcCLB-EL.508585.20_mRNA-p1 TcCLB-NE.
503923.10_mRNA-p1
OG0004957: TCRU_4210 TcCLB-EL.508585.10_mRNA-p1 TcCLB-NE.
503923.20_mRNA-p1
OG0004958: TCRU_4211 TcCLB-EL.508583.10_mRNA-p1 TcCLB-NE.
503923.30_mRNA-p1
OG0004959: TCRU_4212 TcCLB-EL.510877.180_mRNA-p1 TcCLB-NE.
503923.50_mRNA-p1
OG0004960: TCRU_4213 TcCLB-EL.510877.160_mRNA-p1 TcCLB-NE.
508689.30_mRNA-p1
OG0004961: TCRU_4215 TcCLB-EL.510877.140_mRNA-p1 TcCLB-NE.
508689.10_mRNA-p1
OG0004962: TCRU_4217 TcCLB-EL.510877.120_mRNA-p1 TcSYL_0045860.t1-p1
OG0004963: TCRU_4225 TCRU_7690 TcSYL_0087170.t1-p1
OG0004964: TCRU_4246 TcCLB-EL.506363.110_mRNA-p1 TcCLB-NE.
506975.9_mRNA-p1
OG0004965: TCRU_4247 TcCLB-EL.506363.120_mRNA-p1 TcCLB-NE.
508405.119_mRNA-p1
OG0004966: TCRU_4249 TcCLB-EL.506363.140_mRNA-p1 TcCLB-NE.
508405.100_mRNA-p1
OG0004967: TCRU_4251 TcCLB-EL.507837.10_mRNA-p1 TcCLB-NE.
508405.80_mRNA-p1
OG0004968: TCRU_4252 TcCLB-EL.507837.20_mRNA-p1 TcCLB-NE.
508405.70_mRNA-p1
OG0004969: TCRU_4254 TcCLB-EL.507837.40_mRNA-p1 TcCLB-NE.
508405.50_mRNA-p1
OG0004970: TCRU_4262 TcCLB-EL.509819.40_mRNA-p1 TcCLB-NE.
510635.60_mRNA-p1
OG0004971: TCRU_4269 TcCLB-EL.506777.10_mRNA-p1 TcCLB-NE.

511165.10_mRNA-p1
OG0004972: TCRU_4270 TcCLB-EL.503613.79_mRNA-p1 TcCLB-NE.
511165.20_mRNA-p1
OG0004973: TCRU_4272 TcCLB-EL.503613.50_mRNA-p1 TcCLB-NE.
511165.50_mRNA-p1
OG0004974: TCRU_4274 TcCLB-EL.503613.20_mRNA-p1 TcCLB-NE.
511165.70_mRNA-p1
OG0004975: TCRU_4276 TcCLB-EL.510877.55_mRNA-p1 TcSYL_0045810.t1-p1
OG0004976: TCRU_4277 TcCLB-EL.510877.50_mRNA-p1 TcSYL_0045800.t1-p1
OG0004977: TCRU_4283 TcCLB-EL.506211.80_mRNA-p1 TcCLB-NE.
508895.40_mRNA-p1
OG0004978: TCRU_4307 TcCLB-EL.510105.90_mRNA-p1 TcCLB-NE.
509715.80_mRNA-p1
OG0004979: TCRU_4310 TCRU_7686 TcSYL_0049160.t1-p1
OG0004980: TCRU_4313 TcCLB-EL.510431.40_mRNA-p1 TcCLB-NE.
460127.20_mRNA-p1
OG0004981: TCRU_4314 TcCLB-EL.510431.30_mRNA-p1 TcCLB-NE.
460127.30_mRNA-p1
OG0004982: TCRU_4318 TcCLB-EL.510429.59_mRNA-p1 TcCLB-NE.
506883.34_mRNA-p1
OG0004983: TCRU_4319 TcCLB-EL.510429.50_mRNA-p1 TcCLB-NE.
506883.40_mRNA-p1
OG0004984: TCRU_4320 TcCLB-EL.510429.40_mRNA-p1 TcCLB-NE.
506883.50_mRNA-p1
OG0004985: TCRU_4321 TcCLB-EL.510429.24_mRNA-p1 TcCLB-NE.
506883.69_mRNA-p1
OG0004986: TCRU_4322 TcCLB-EL.510429.20_mRNA-p1 TcCLB-NE.
506883.80_mRNA-p1
OG0004987: TCRU_4323 TcCLB-EL.510429.10_mRNA-p1 TcCLB-NE.
506883.90_mRNA-p1
OG0004988: TCRU_4325 TcCLB-EL.510427.10_mRNA-p1 TcCLB-NE.
506883.120_mRNA-p1
OG0004989: TCRU_4330 TcCLB-EL.506885.4_mRNA-p1 TcCLB-NE.
504123.10_mRNA-p1
OG0004990: TCRU_4347 TcCLB-EL.508771.40_mRNA-p1 TcCLB-NE.
503785.20_mRNA-p1
OG0004991: TCRU_4351 TcSYL_0109460.t1-p1 TcSYL_0109470.t1-p1
OG0004992: TCRU_4354 TcCLB-EL.507735.60_mRNA-p1 TcCLB-NE.
504253.20_mRNA-p1
OG0004993: TCRU_4355 TcCLB-EL.507737.10_mRNA-p1 TcCLB-NE.
504253.40_mRNA-p1
OG0004994: TCRU_4356 TcCLB-EL.507737.20_mRNA-p1 TcCLB-NE.
504253.50_mRNA-p1
OG0004995: TCRU_4357 TcCLB-EL.507737.30_mRNA-p1 TcCLB-NE.
509933.10_mRNA-p1
OG0004996: TCRU_4358 TcCLB-EL.456139.10_mRNA-p1 TcCLB-NE.
509933.20_mRNA-p1
OG0004997: TCRU_4359 TCRU_5457 TcSYL_0074050.t1-p1
OG0004998: TCRU_4360 TcCLB-NE.511893.30_pseudogenic_transcript-p1
TcSYL_0088680.t1-p1
OG0004999: TCRU_4362 TcCLB-EL.506625.100_mRNA-p1 TcCLB-EL.
506625.120_mRNA-p1
OG0005000: TCRU_4366 TcCLB-EL.510349.20_mRNA-p1 TcCLB-NE.
503925.50_mRNA-p1

OG0005001: TCRU_4367 TcCLB-EL.510349.10_mRNA-p1 TcCLB-NE.
503925.40_mRNA-p1
OG0005002: TCRU_4372 TcCLB-EL.510347.10_mRNA-p1 TcCLB-NE.
506469.20_mRNA-p1
OG0005003: TCRU_4378 TcCLB-NE.506537.100_pseudogenic_transcript-p1
TcCLB-NE.506537.210_pseudogenic_transcript-p1
OG0005004: TCRU_4382 TcCLB-EL.506941.30_mRNA-p1 TcCLB-NE.
511511.30_mRNA-p1
OG0005005: TCRU_4383 TcCLB-EL.506941.40_mRNA-p1 TcCLB-NE.
511511.40_mRNA-p1
OG0005006: TCRU_4384 TcCLB-EL.506941.50_mRNA-p1 TcCLB-NE.
511511.50_mRNA-p1
OG0005007: TCRU_4386 TcCLB-EL.506941.80_mRNA-p1 TcCLB-NE.
511511.80_mRNA-p1
OG0005008: TCRU_4388 TcCLB-EL.506941.100_mRNA-p1 TcCLB-NE.
511511.100_mRNA-p1
OG0005009: TCRU_4390 TcCLB-EL.506941.130_mRNA-p1 TcCLB-NE.
511511.130_mRNA-p1
OG0005010: TCRU_4391 TcCLB-EL.506941.139_mRNA-p1 TcCLB-NE.
511511.139_mRNA-p1
OG0005011: TCRU_4393 TcCLB-EL.506941.159_mRNA-p1 TcCLB-NE.
511511.159_mRNA-p1
OG0005012: TCRU_4398 TcCLB-EL.506941.229_mRNA-p1 TcCLB-NE.
507027.59_mRNA-p1
OG0005013: TCRU_4399 TcCLB-EL.506941.238_mRNA-p1 TcCLB-NE.
507027.70_mRNA-p1
OG0005014: TCRU_4400 TcCLB-EL.506941.250_mRNA-p1 TcCLB-NE.
507027.80_mRNA-p1
OG0005015: TCRU_4404 TcCLB-EL.506025.70_mRNA-p1 TcCLB-NE.
503935.10_mRNA-p1
OG0005016: TCRU_4407 TcCLB-EL.506025.40_mRNA-p1 TcCLB-NE.
511209.30_pseudogenic_transcript-p1
OG0005017: TCRU_4408 TcCLB-EL.506025.30_mRNA-p1 TcCLB-NE.
511209.40_mRNA-p1
OG0005018: TCRU_4409 TcCLB-EL.506025.20_mRNA-p1 TcCLB-NE.
511209.50_mRNA-p1
OG0005019: TCRU_4414 TcCLB-EL.511211.80_mRNA-p1 TcSYL_0182370.t1-p1
OG0005020: TCRU_4415 TcCLB-EL.511211.70_mRNA-p1 TcSYL_0182330.t1-p1
OG0005021: TCRU_4417 TcCLB-EL.506227.30_mRNA-p1 TcCLB-NE.
511847.89_mRNA-p1
OG0005022: TCRU_4418 TcCLB-EL.506227.40_mRNA-p1 TcCLB-NE.
511847.80_mRNA-p1
OG0005023: TCRU_4419 TcCLB-EL.506227.100_mRNA-p1 TcCLB-NE.
511847.40_mRNA-p1
OG0005024: TCRU_4420 TcCLB-EL.506227.130_mRNA-p1 TcCLB-NE.
511847.10_mRNA-p1
OG0005025: TCRU_4421 TcCLB-EL.506227.140_mRNA-p1 TcSYL_0091990.t1-p1
OG0005026: TCRU_4422 TcCLB-EL.504643.49_mRNA-p1 TcCLB-NE.
457339.19_mRNA-p1
OG0005027: TCRU_4423 TcCLB-EL.504643.40_mRNA-p1 TcCLB-NE.
457339.10_mRNA-p1
OG0005028: TCRU_4427 TcCLB-EL.510611.30_mRNA-p1 TcCLB-NE.
506793.30_mRNA-p1
OG0005029: TCRU_4433 TcCLB-EL.506775.180_mRNA-p1 TcCLB-NE.

511165.109_mRNA-p1
OG0005030: TCRU_4435 TcCLB-EL.507829.10_mRNA-p1 TcCLB-NE.
511269.70_mRNA-p1
OG0005031: TCRU_4436 TcCLB-EL.507829.19_mRNA-p1 TcCLB-NE.
511269.60_mRNA-p1
OG0005032: TCRU_4437 TcCLB-EL.509805.10_mRNA-p1 TcCLB-NE.
511269.50_mRNA-p1
OG0005033: TCRU_4438 TcCLB-EL.509805.20_mRNA-p1 TcCLB-NE.
511269.40_mRNA-p1
OG0005034: TCRU_4439 TcCLB-EL.509805.30_mRNA-p1 TcCLB-NE.
511269.30_mRNA-p1
OG0005035: TCRU_4441 TcCLB-EL.509805.50_mRNA-p1 TcCLB-NE.
511269.10_mRNA-p1
OG0005036: TCRU_4447 TcCLB-EL.506739.190_mRNA-p1 TcCLB-NE.
506315.90_mRNA-p1
OG0005037: TCRU_4449 TcCLB-EL.506739.160_mRNA-p1 TcCLB-NE.
506315.60_mRNA-p1
OG0005038: TCRU_4451 TcCLB-EL.506739.120_mRNA-p1 TcCLB-NE.
506315.20_mRNA-p1
OG0005039: TCRU_4452 TcCLB-EL.506739.99_mRNA-p1 TcCLB-NE.
510819.119_mRNA-p1
OG0005040: TCRU_4454 TcCLB-EL.506739.80_mRNA-p1 TcCLB-NE.
510819.100_mRNA-p1
OG0005041: TCRU_4455 TcCLB-EL.506739.70_mRNA-p1 TcCLB-NE.
510819.90_mRNA-p1
OG0005042: TCRU_4456 TcCLB-EL.506739.60_mRNA-p1 TcCLB-NE.
510819.70_mRNA-p1
OG0005043: TCRU_4460 TcCLB-EL.506945.320_mRNA-p1 TcSYL_0140790.t1-p1
OG0005044: TCRU_4461 TcCLB-EL.506945.310_mRNA-p1 TcSYL_0140780.t1-p1
OG0005045: TCRU_4468 TcCLB-EL.408437.20_mRNA-p1 TcCLB-NE.
508739.90_mRNA-p1
OG0005046: TCRU_4473 TcCLB-EL.511685.50_mRNA-p1 TcCLB-NE.
508741.100_mRNA-p1
OG0005047: TCRU_4477 TcCLB-EL.506579.30_mRNA-p1 TcSYL_0064820.t1-p1
OG0005048: TCRU_4478 TcCLB-EL.510603.40_mRNA-p1 TcCLB-NE.
509109.140_mRNA-p1
OG0005049: TCRU_4479 TcCLB-EL.510603.20_mRNA-p1 TcCLB-NE.
509109.150_mRNA-p1
OG0005050: TCRU_4480 TcCLB-EL.510603.10_mRNA-p1 TcCLB-NE.
509109.160_mRNA-p1
OG0005051: TCRU_4491 TCRU_9003 TCRU_9070
OG0005052: TCRU_4505 TcCLB-EL.504147.340_mRNA-p1 TcSYL_0004900.t1-p1
OG0005053: TCRU_4509 TcCLB-EL.504137.30_mRNA-p1 TcCLB-NE.
509797.60_mRNA-p1
OG0005054: TCRU_4510 TcCLB-EL.504137.40_mRNA-p1 TcCLB-NE.
509797.69_mRNA-p1
OG0005055: TCRU_4511 TcCLB-EL.504137.50_mRNA-p1 TcCLB-NE.
509799.5_mRNA-p1
OG0005056: TCRU_4515 TcCLB-EL.504137.90_mRNA-p1 TcCLB-NE.
509799.40_mRNA-p1
OG0005057: TCRU_4517 TcCLB-EL.504137.120_mRNA-p1 TcCLB-NE.
509799.70_mRNA-p1
OG0005058: TCRU_WW TcCLB-EL.504137.130_mRNA-p1 TcCLB-NE.
509799.80_mRNA-p1

OG0005059: TCRU_4519 TcCLB-EL.504137.150_mRNA-p1 TcCLB-NE.
509799.100_mRNA-p1
OG0005060: TCRU_4520 TcCLB-EL.504137.160_mRNA-p1 TcCLB-NE.
509799.110_mRNA-p1
OG0005061: TCRU_C-terminal TcCLB-EL.511037.40_mRNA-p1 TcCLB-NE.
509799.120_mRNA-p1
OG0005062: TCRU_4535 TCRU_4973 TcSYL_0115010.t1-p1
OG0005063: TCRU_component_ TcCLB-EL.510717.30_mRNA-p1 TcCLB-NE.
506337.70_mRNA-p1
OG0005064: TCRU_4549 TcCLB-EL.503675.10_mRNA-p1 TcCLB-NE.
506337.40_mRNA-p1
OG0005065: TCRU_4550 TcCLB-EL.503675.20_mRNA-p1 TcCLB-NE.
506337.30_mRNA-p1
OG0005066: TCRU_4551 TcCLB-EL.503675.40_mRNA-p1 TcCLB-NE.
506337.20_mRNA-p1
OG0005067: TCRU_4552 TcCLB-EL.510719.10_mRNA-p1 TcCLB-NE.
506337.10_mRNA-p1
OG0005068: TCRU_4553 TcCLB-EL.510719.20_mRNA-p1 TcCLB-NE.
506337.4_mRNA-p1
OG0005069: TCRU_4556 TcCLB-EL.510719.40_mRNA-p1 TcCLB-NE.
509749.30_mRNA-p1
OG0005070: TCRU_4557 TcCLB-EL.510719.50_mRNA-p1 TcCLB-NE.
509749.20_mRNA-p1
OG0005071: TCRU_4558 TcCLB-EL.510719.70_mRNA-p1 TcCLB-NE.
509747.110_mRNA-p1
OG0005072: TCRU_4560 TcCLB-EL.510719.74_mRNA-p1 TcCLB-NE.
509747.104_mRNA-p1
OG0005073: TCRU_4561 TcCLB-EL.510719.80_mRNA-p1 TcCLB-NE.
509747.100_mRNA-p1
OG0005074: TCRU_4562 TcCLB-EL.510719.90_mRNA-p1 TcCLB-NE.
509747.90_mRNA-p1
OG0005075: TCRU_4563 TcCLB-EL.510719.110_mRNA-p1 TcCLB-NE.
509747.70_mRNA-p1
OG0005076: TCRU_4564 TcCLB-EL.510719.120_mRNA-p1 TcCLB-NE.
509747.60_mRNA-p1
OG0005077: TCRU_4565 TcCLB-EL.510719.130_mRNA-p1 TcCLB-NE.
509747.50_mRNA-p1
OG0005078: TCRU_4566 TcCLB-EL.510719.140_mRNA-p1 TcCLB-NE.
509747.40_mRNA-p1
OG0005079: TCRU_4567 TcCLB-EL.510719.150_mRNA-p1 TcCLB-NE.
509747.30_mRNA-p1
OG0005080: TCRU_4591 TcCLB-EL.509979.350_mRNA-p1 TcCLB-NE.
510033.14_pseudogenic_transcript-p1
OG0005081: TCRU_4598 TcCLB-EL.449247.10_mRNA-p1 TcCLB-NE.
506123.70_mRNA-p1
OG0005082: TCRU_4604 TcCLB-EL.508205.48_mRNA-p1 TcCLB-NE.
506123.20_mRNA-p1
OG0005083: TCRU_4608 TcCLB-EL.419417.4_mRNA-p1 TcCLB-NE.
509539.50_mRNA-p1
OG0005084: TCRU_4609 TcCLB-EL.419417.10_mRNA-p1 TcCLB-NE.
509539.44_mRNA-p1
OG0005085: TCRU_4610 TcCLB-EL.419417.19_mRNA-p1 TcCLB-NE.
509539.40_mRNA-p1
OG0005086: TCRU_4613 TcCLB-EL.507641.140_mRNA-p1 TcSYL_0011140.t1-p1

OG0005087: TCRU_4618 TcCLB-EL.509145.10_mRNA-p1 TcCLB-NE.
508741.380_mRNA-p1
OG0005088: TCRU_4619 TcCLB-EL.509143.10_mRNA-p1 TcCLB-NE.
508741.370_mRNA-p1
OG0005089: TCRU_4620 TcCLB-EL.503509.10_mRNA-p1 TcCLB-NE.
508741.360_mRNA-p1
OG0005090: TCRU_4622 TcCLB-EL.503511.10_mRNA-p1 TcCLB-NE.
508741.340_mRNA-p1
OG0005091: TCRU_4625 TcCLB-EL.508157.30_mRNA-p1 TcSYL_0133490.t1-p1
OG0005092: TCRU_4630 TcCLB-EL.503867.20_pseudogenic_transcript-p1
TcCLB-NE.505037.70_pseudogenic_transcript-p1
OG0005093: TCRU_4635 TcCLB-EL.509137.30_mRNA-p1 TcCLB-NE.
508741.140_mRNA-p1
OG0005094: TCRU_4642 TcCLB-EL.509455.30_mRNA-p1 TcCLB-NE.
503879.60_mRNA-p1
OG0005095: TCRU_4643 TcCLB-EL.509455.40_mRNA-p1 TcCLB-NE.
503879.50_mRNA-p1
OG0005096: TCRU_4648 TcCLB-EL.509455.90_mRNA-p1 TcCLB-NE.
503539.20_mRNA-p1
OG0005097: TCRU_4649 TcCLB-EL.509455.110_mRNA-p1 TcCLB-NE.
503877.10_mRNA-p1
OG0005098: TCRU_4654 TcCLB-EL.507093.100_mRNA-p1 TcSYL_0080320.t1-p1
OG0005099: TCRU_4669 TcCLB-EL.511621.30_mRNA-p1 TcCLB-NE.
509317.90_mRNA-p1
OG0005100: TCRU_4679 TcCLB-EL.506737.60_mRNA-p1 TcCLB-NE.
511855.30_mRNA-p1
OG0005101: TCRU_4680 TcCLB-EL.506737.50_mRNA-p1 TcCLB-NE.
511855.40_mRNA-p1
OG0005102: TCRU_4682 TcCLB-EL.506737.30_mRNA-p1 TcCLB-NE.
511855.60_mRNA-p1
OG0005103: TCRU_4684 TcCLB-EL.506737.10_mRNA-p1 TcCLB-NE.
511855.80_mRNA-p1
OG0005104: TCRU_4687 TcCLB-EL.511393.20_mRNA-p1 TcSYL_0202040.t1-p1
OG0005105: TCRU_4698 TcCLB-EL.414145.9_mRNA-p1 TcCLB-NE.
506155.110_mRNA-p1
OG0005106: TCRU_4701 TcCLB-EL.506673.40_mRNA-p1 TcCLB-NE.
504827.60_mRNA-p1
OG0005107: TCRU_4702 TcCLB-EL.506673.30_mRNA-p1 TcCLB-NE.
504827.70_mRNA-p1
OG0005108: TCRU_4703 TcCLB-EL.506673.20_mRNA-p1 TcCLB-NE.
504827.80_mRNA-p1
OG0005109: TCRU_4704 TcCLB-EL.506673.10_mRNA-p1 TcCLB-NE.
504827.90_mRNA-p1
OG0005110: TCRU_4705 TcCLB-EL.510285.130_mRNA-p1 TcCLB-NE.
504827.100_mRNA-p1
OG0005111: TCRU_4708 TcCLB-EL.510285.100_mRNA-p1 TcCLB-NE.
504827.130_mRNA-p1
OG0005112: TCRU_4709 TcCLB-EL.510285.90_mRNA-p1 TcCLB-NE.
504827.140_mRNA-p1
OG0005113: TCRU_4721 TcCLB-EL.510091.70_mRNA-p1 TcCLB-NE.
510421.314_mRNA-p1
OG0005114: TCRU_4722 TcCLB-EL.510091.60_mRNA-p1 TcCLB-NE.
510421.310_mRNA-p1
OG0005115: TCRU_4741 TcCLB-EL.511109.20_mRNA-p1 TcCLB-NE.

504157.120_mRNA-p1
OG0005116: TCRU_4743 TcCLB-EL.511109.40_mRNA-p1 TcCLB-NE.
504157.100_mRNA-p1
OG0005117: TCRU_4744 TcCLB-EL.511109.50_pseudogenic_transcript-p1
TcCLB-NE.504157.90_mRNA-p1
OG0005118: TCRU_4745 TcCLB-EL.511109.60_mRNA-p1 TcCLB-NE.
504157.80_mRNA-p1
OG0005119: TCRU_4754 TcCLB-EL.507809.50_mRNA-p1 TcCLB-NE.
511661.70_mRNA-p1
OG0005120: TCRU_4755 TcCLB-EL.507809.39_mRNA-p1 TcCLB-NE.
511661.80_mRNA-p1
OG0005121: TCRU_4758 TcCLB-NE.508445.10_mRNA-p1 TcSYL_0141680.t1-p1
OG0005122: TCRU_4765 TcCLB-NE.511391.220_mRNA-p1 TcSYL_0202020.t1-p1
OG0005123: TCRU_4767 TcCLB-NE.511391.200_mRNA-p1 TcSYL_0202010.t1-p1
OG0005124: TCRU_4768 TcCLB-NE.511391.180_mRNA-p1 TcSYL_0201980.t1-p1
OG0005125: TCRU_4769 TcCLB-NE.511391.170_mRNA-p1 TcSYL_0201970.t1-p1
OG0005126: TCRU_4770 TcCLB-NE.511391.160_mRNA-p1 TcSYL_0201960.t1-p1
OG0005127: TCRU_4772 TcCLB-EL.508307.50_mRNA-p1 TcSYL_0006140.t1-p1
OG0005128: TCRU_4781 TcCLB-EL.510963.80_mRNA-p1 TcCLB-NE.
508625.20_mRNA-p1
OG0005129: TCRU_4783 TcCLB-EL.510963.60_mRNA-p1 TcCLB-NE.
508625.40_mRNA-p1
OG0005130: TCRU_4785 TcCLB-EL.510963.10_mRNA-p1 TcCLB-NE.
508625.90_mRNA-p1
OG0005131: TCRU_4788 TcCLB-EL.509757.40_mRNA-p1 TcCLB-NE.
508625.140_mRNA-p1
OG0005132: TCRU_4789 TcCLB-EL.509759.10_mRNA-p1 TcCLB-NE.
508625.150_mRNA-p1
OG0005133: TCRU_4791 TcCLB-EL.508163.140_mRNA-p1 TcSYL_0017610.t1-p1
OG0005134: TCRU_4795 TcCLB-EL.508995.50_mRNA-p1 TcCLB-NE.
506477.14_mRNA-p1
OG0005135: TCRU_4797 TcCLB-EL.508995.30_mRNA-p1 TcCLB-NE.
506477.30_mRNA-p1
OG0005136: TCRU_4798 TcCLB-EL.508995.20_mRNA-p1 TcCLB-NE.
506477.40_mRNA-p1
OG0005137: TCRU_4799 TcCLB-EL.511427.19_mRNA-p1 TcCLB-NE.
504209.10_mRNA-p1
OG0005138: TCRU_4806 TcCLB-EL.508831.150_mRNA-p1 TcCLB-NE.
511671.50_mRNA-p1
OG0005139: TCRU_4807 TcCLB-EL.508831.140_mRNA-p1 TcCLB-NE.
511671.60_mRNA-p1
OG0005140: TCRU_4808 TcCLB-EL.508831.120_mRNA-p1 TcCLB-NE.
511671.70_mRNA-p1
OG0005141: TCRU_4809 TcCLB-EL.508831.110_mRNA-p1 TcCLB-NE.
511671.80_mRNA-p1
OG0005142: TCRU_4810 TcCLB-EL.508831.100_mRNA-p1 TcCLB-NE.
511671.90_mRNA-p1
OG0005143: TCRU_4811 TcCLB-EL.508831.90_mRNA-p1 TcCLB-NE.
511671.100_mRNA-p1
OG0005144: TCRU_4812 TcCLB-EL.508831.80_mRNA-p1 TcCLB-NE.
511671.110_mRNA-p1
OG0005145: TCRU_4813 TcCLB-EL.508831.70_mRNA-p1 TcCLB-NE.
511671.120_mRNA-p1
OG0005146: TCRU_4814 TcCLB-EL.508831.66_mRNA-p1 TcCLB-NE.

511671.130_mRNA-p1
OG0005147: TCRU_4815 TcCLB-EL.508831.63_mRNA-p1 TcCLB-NE.
511671.140_mRNA-p1
OG0005148: TCRU_4816 TcCLB-EL.508831.60_mRNA-p1 TcCLB-NE.
511671.149_mRNA-p1
OG0005149: TCRU_4819 TcCLB-EL.507993.140_mRNA-p1 TcCLB-NE.
511277.580_mRNA-p1
OG0005150: TCRU_4822 TcCLB-EL.507993.110_mRNA-p1 TcCLB-NE.
511277.554_mRNA-p1
OG0005151: TCRU_4824 TcCLB-EL.507993.89_mRNA-p1 TcCLB-NE.
511277.539_mRNA-p1
OG0005152: TCRU_4831 TcCLB-EL.511871.90_mRNA-p1 TcCLB-NE.
511865.10_mRNA-p1
OG0005153: TCRU_4832 TcCLB-EL.511871.70_mRNA-p1 TcCLB-NE.
511865.30_mRNA-p1
OG0005154: TCRU_4835 TcCLB-EL.511871.30_mRNA-p1 TcCLB-NE.
511865.60_mRNA-p1
OG0005155: TCRU_4836 TcCLB-EL.511871.20_mRNA-p1 TcCLB-NE.
511865.70_mRNA-p1
OG0005156: TCRU_4844 TcCLB-EL.504247.20_mRNA-p1 TcCLB-NE.
507945.10_mRNA-p1
OG0005157: TCRU_homolog TcCLB-EL.504247.30_mRNA-p1 TcCLB-NE.
509991.110_mRNA-p1
OG0005158: TCRU_4846 TcCLB-EL.504247.40_mRNA-p1 TcCLB-NE.
509991.90_mRNA-p1
OG0005159: TCRU_4847 TcCLB-EL.504247.50_mRNA-p1 TcCLB-NE.
509991.80_mRNA-p1
OG0005160: TCRU_4849 TcCLB-EL.511429.20_mRNA-p1 TcCLB-NE.
509991.29_mRNA-p1
OG0005161: TCRU_4850 TcCLB-EL.511431.10_mRNA-p1 TcCLB-NE.
509991.9_mRNA-p1
OG0005162: TCRU_4851 TcCLB-EL.511431.20_mRNA-p1 TcCLB-NE.
506475.130_mRNA-p1
OG0005163: TCRU_4852 TcCLB-EL.511431.30_mRNA-p1 TcCLB-NE.
506475.120_mRNA-p1
OG0005164: TCRU_4853 TcCLB-EL.511431.40_mRNA-p1 TcCLB-NE.
506475.116_mRNA-p1
OG0005165: TCRU_4854 TcCLB-EL.511431.54_mRNA-p1 TcCLB-NE.
506475.110_mRNA-p1
OG0005166: TCRU_4855 TcCLB-EL.511431.60_mRNA-p1 TcCLB-NE.
506475.105_mRNA-p1
OG0005167: TCRU_4856 TcCLB-EL.511431.90_mRNA-p1 TcCLB-NE.
506475.100_mRNA-p1
OG0005168: TCRU_4858 TcCLB-EL.508277.360_mRNA-p1 TcCLB-NE.
506445.40_pseudogenic_transcript-p1
OG0005169: TCRU_4859 TcCLB-EL.508277.370_mRNA-p1 TcCLB-NE.
506445.60_mRNA-p1
OG0005170: TCRU_4860 TcCLB-EL.508277.380_mRNA-p1 TcCLB-NE.
506445.70_mRNA-p1
OG0005171: TCRU_4861 TcCLB-EL.508277.390_mRNA-p1 TcCLB-NE.
506445.80_mRNA-p1
OG0005172: TCRU_4862 TcCLB-EL.508277.401_mRNA-p1 TcCLB-NE.
506445.90_mRNA-p1
OG0005173: TCRU_4864 TcCLB-NE.506445.110_mRNA-p1 TcSYL_0064320.t1-p1

OG0005174: TCRU_4869 TcCLB-EL.507089.240_mRNA-p1 TcCLB-NE.
503839.39_mRNA-p1
OG0005175: TCRU_4896 TcCLB-EL.506893.25_mRNA-p1 TcCLB-NE.
508501.165_mRNA-p1
OG0005176: TCRU_4912 TcCLB-EL.511525.29_mRNA-p1 TcCLB-NE.
508525.40_mRNA-p1
OG0005177: TCRU_4922 TCRU_8072 TcSYL_0161630.t1-p1
OG0005178: TCRU_4924 TcCLB-EL.506637.50_mRNA-p1 TcCLB-NE.
510099.30_mRNA-p1
OG0005179: TCRU_4925 TcCLB-EL.506637.60_mRNA-p1 TcCLB-NE.
510099.40_mRNA-p1
OG0005180: TCRU_4926 TcCLB-EL.506637.90_mRNA-p1 TcCLB-NE.
510099.70_mRNA-p1
OG0005181: TCRU_4927 TcCLB-EL.506637.100_mRNA-p1 TcCLB-NE.
510099.90_mRNA-p1
OG0005182: TCRU_4936 TcCLB-NE.508409.80_mRNA-p1 TcSYL_0004500.t1-p1
OG0005183: TCRU_4937 TcCLB-NE.508409.90_mRNA-p1 TcSYL_0004480.t1-p1
OG0005184: TCRU_4938 TcCLB-NE.508409.110_mRNA-p1 TcSYL_0004450.t1-p1
OG0005185: TCRU_4939 TcCLB-NE.508409.130_mRNA-p1 TcSYL_0004440.t1-p1
OG0005186: TCRU_4940 TcCLB-NE.508409.140_mRNA-p1 TcSYL_0004430.t1-p1
OG0005187: TCRU_4947 TcCLB-EL.504179.40_mRNA-p1 TcCLB-NE.
507823.30_mRNA-p1
OG0005188: TCRU_4948 TcCLB-EL.504179.50_mRNA-p1 TcCLB-NE.
507823.20_mRNA-p1
OG0005189: TCRU_4950 TcCLB-EL.504179.80_mRNA-p1 TcCLB-NE.
509795.80_mRNA-p1
OG0005190: TCRU_4951 TcCLB-EL.504179.90_mRNA-p1 TcCLB-NE.
509795.70_mRNA-p1
OG0005191: TCRU_4953 TcCLB-EL.504171.50_mRNA-p1 TcCLB-NE.
509795.50_mRNA-p1
OG0005192: TCRU_4954 TcCLB-EL.504171.40_mRNA-p1 TcCLB-NE.
509795.40_mRNA-p1
OG0005193: TCRU_4955 TcCLB-EL.504171.30_mRNA-p1 TcCLB-NE.
509795.30_mRNA-p1
OG0005194: TCRU_4956 TcCLB-EL.504171.20_mRNA-p1 TcCLB-NE.
509795.20_mRNA-p1
OG0005195: TCRU_4957 TcCLB-EL.508719.30_mRNA-p1 TcCLB-NE.
509793.50_mRNA-p1
OG0005196: TCRU_4970 TcCLB-EL.510325.60_mRNA-p1 TcCLB-NE.
508153.70_mRNA-p1
OG0005197: TCRU_4972 TcCLB-EL.510325.45_mRNA-p1 TcCLB-NE.
508153.90_mRNA-p1
OG0005198: TCRU_family_ TcCLB-EL.506715.30_mRNA-p1 TcCLB-NE.
503647.40_mRNA-p1
OG0005199: TCRU_10037 TCRU_4985 TcSYL_0070570.t1-p1
OG0005200: TCRU_4992 TcCLB-EL.506755.190_mRNA-p1 TcCLB-NE.
510359.200_mRNA-p1
OG0005201: TCRU_4993 TcCLB-EL.506755.180_mRNA-p1 TcCLB-NE.
510359.190_mRNA-p1
OG0005202: TCRU_4994 TcCLB-EL.506755.170_mRNA-p1 TcCLB-NE.
510359.180_mRNA-p1
OG0005203: TCRU_4996 TcCLB-EL.506755.150_mRNA-p1 TcCLB-NE.
510359.160_mRNA-p1
OG0005204: TCRU_4999 TcCLB-EL.506755.110_mRNA-p1 TcCLB-NE.

510359.120_pseudogenic_transcript-p1
OG0005205: TCRU_5000 TcCLB-EL.506755.100_mRNA-p1 TcCLB-NE.
510359.100_mRNA-p1
OG0005206: TCRU_5001 TcCLB-EL.506755.90_mRNA-p1 TcCLB-NE.
510359.90_mRNA-p1
OG0005207: TCRU_5003 TcCLB-EL.506755.74_mRNA-p1 TcCLB-NE.
510359.69_mRNA-p1
OG0005208: TCRU_5004 TcCLB-EL.506755.70_mRNA-p1 TcCLB-NE.
510359.60_mRNA-p1
OG0005209: TCRU_5006 TcCLB-EL.506755.40_mRNA-p1 TcCLB-NE.
510359.34_mRNA-p1
OG0005210: TCRU_5007 TcCLB-EL.506755.30_mRNA-p1 TcCLB-NE.
510359.30_mRNA-p1
OG0005211: TCRU_5008 TcCLB-EL.507611.370_mRNA-p1 TcCLB-NE.
506247.10_mRNA-p1
OG0005212: TCRU_5009 TcCLB-EL.507611.380_mRNA-p1 TcCLB-NE.
506247.20_mRNA-p1
OG0005213: TCRU_5010 TcCLB-EL.507611.389_mRNA-p1 TcCLB-NE.
506247.24_mRNA-p1
OG0005214: TCRU_5014 TcCLB-NE.506247.80_mRNA-p1 TcSYL_0108880.t1-p1
OG0005215: TCRU_5015 TcCLB-NE.506247.90_mRNA-p1 TcSYL_0108870.t1-p1
OG0005216: TCRU_5021 TcCLB-NE.506247.140_mRNA-p1 TcSYL_0108860.t1-p1
OG0005217: TCRU_5022 TcCLB-NE.506247.150_mRNA-p1 TcSYL_0108850.t1-p1
OG0005218: TCRU_5023 TcCLB-NE.506247.160_mRNA-p1 TcSYL_0108840.t1-p1
OG0005219: TCRU_subunit_ TcCLB-NE.506247.200_mRNA-p1
TcSYL_0108820.t1-p1
OG0005220: TCRU_5027 TcCLB-NE.506247.210_mRNA-p1 TcSYL_0108810.t1-p1
OG0005221: TCRU_5028 TcCLB-NE.506247.220_mRNA-p1 TcSYL_0108800.t1-p1
OG0005222: TCRU_5029 TcCLB-NE.506247.230_mRNA-p1 TcSYL_0108790.t1-p1
OG0005223: TCRU_5031 TcCLB-NE.506247.250_mRNA-p1 TcSYL_0108780.t1-p1
OG0005224: TCRU_5033 TcCLB-NE.506247.270_mRNA-p1 TcSYL_0108760.t1-p1
OG0005225: TCRU_5035 TcCLB-NE.506247.300_mRNA-p1 TcSYL_0108740.t1-p1
OG0005226: TCRU_5038 TcCLB-NE.506247.330_mRNA-p1 TcSYL_0108730.t1-p1
OG0005227: TCRU_5039 TcCLB-NE.506247.340_mRNA-p1 TcSYL_0108710.t1-p1
OG0005228: TCRU_5040 TcCLB-NE.506247.350_mRNA-p1 TcSYL_0108700.t1-p1
OG0005229: TCRU_5041 TcCLB-EL.506771.18_mRNA-p1 TcCLB-NE.
506247.360_mRNA-p1
OG0005230: TCRU_5044 TcCLB-EL.510243.50_mRNA-p1 TcCLB-NE.
510575.180_mRNA-p1
OG0005231: TCRU_5045 TcCLB-EL.510243.54_mRNA-p1 TcCLB-NE.
510575.170_mRNA-p1
OG0005232: TCRU_5046 TcCLB-EL.510243.60_mRNA-p1 TcCLB-NE.
510575.160_mRNA-p1
OG0005233: TCRU_5047 TcCLB-EL.510243.110_mRNA-p1 TcCLB-NE.
510575.120_mRNA-p1
OG0005234: TCRU_5048 TcCLB-EL.510243.119_mRNA-p1 TcCLB-NE.
510575.100_mRNA-p1
OG0005235: TCRU_5066 TcCLB-NE.506679.80_mRNA-p1 TcSYL_0113920.t1-p1
OG0005236: TCRU_5067 TcCLB-NE.506679.70_mRNA-p1 TcSYL_0113900.t1-p1
OG0005237: TCRU_5068 TcCLB-NE.506679.60_mRNA-p1 TcSYL_0113880.t1-p1
OG0005238: TCRU_5069 TcCLB-NE.506679.50_mRNA-p1 TcSYL_0113860.t1-p1
OG0005239: TCRU_5070 TcCLB-NE.506679.40_mRNA-p1 TcSYL_0113830.t1-p1
OG0005240: TCRU_5071 TcCLB-NE.506679.30_mRNA-p1 TcSYL_0113800.t1-p1
OG0005241: TCRU_5078 TcCLB-EL.511179.60_mRNA-p1 TcCLB-NE.

510481.90_mRNA-p1
OG0005242: TCRU_5086 TcCLB-EL.506127.70_mRNA-p1 TcCLB-EL.
508271.50_mRNA-p1
OG0005243: TCRU_5088 TcCLB-EL.506127.90_mRNA-p1 TcSYL_0083310.t1-p1
OG0005244: TCRU_5091 TcCLB-EL.506127.120_mRNA-p1 TcCLB-NE.
503593.60_mRNA-p1
OG0005245: TCRU_5092 TcCLB-EL.506127.130_mRNA-p1 TcCLB-NE.
503593.50_mRNA-p1
OG0005246: TCRU_5093 TcCLB-EL.506127.150_mRNA-p1 TcCLB-NE.
503593.30_mRNA-p1
OG0005247: TCRU_5104 TcCLB-EL.508465.140_mRNA-p1 TcCLB-NE.
507001.50_mRNA-p1
OG0005248: TCRU_5106 TcCLB-EL.503557.14_mRNA-p1 TcCLB-NE.
507001.34_mRNA-p1
OG0005249: TCRU_5107 TcCLB-EL.503557.20_mRNA-p1 TcCLB-NE.
507001.30_mRNA-p1
OG0005250: TCRU_5108 TcCLB-EL.503557.30_mRNA-p1 TcCLB-NE.
507001.20_mRNA-p1
OG0005251: TCRU_5109 TcCLB-EL.508467.10_mRNA-p1 TcCLB-NE.
507001.10_mRNA-p1
OG0005252: TCRU_5129 TcCLB-EL.508787.20_mRNA-p1 TcCLB-NE.
510519.10_mRNA-p1
OG0005253: TCRU_5130 TcCLB-EL.508787.10_mRNA-p1 TcCLB-NE.
510519.20_mRNA-p1
OG0005254: TCRU_5131 TcCLB-EL.506869.70_mRNA-p1 TcCLB-NE.
510519.30_mRNA-p1
OG0005255: TCRU_5132 TcCLB-EL.506869.60_mRNA-p1 TcCLB-NE.
510519.40_mRNA-p1
OG0005256: TCRU_5133 TcCLB-EL.506869.50_mRNA-p1 TcCLB-NE.
510519.60_mRNA-p1
OG0005257: TCRU_subunit TcCLB-EL.506869.40_mRNA-p1 TcCLB-NE.
510519.70_mRNA-p1
OG0005258: TCRU_5135 TcCLB-EL.506869.34_mRNA-p1 TcCLB-NE.
510519.80_mRNA-p1
OG0005259: TCRU_5142 TcCLB-EL.508715.10_mRNA-p1 TcCLB-NE.
506839.34_mRNA-p1
OG0005260: TCRU_5145 TcCLB-EL.480785.10_mRNA-p1 TcCLB-NE.
506839.10_mRNA-p1
OG0005261: TCRU_5150 TcCLB-NE.510303.190_mRNA-p1 TcSYL_0079030.t1-p1
OG0005262: TCRU_5151 TcCLB-NE.510303.200_mRNA-p1 TcSYL_0079020.t1-p1
OG0005263: TCRU_5153 TcCLB-NE.510303.220_mRNA-p1 TcSYL_0079000.t1-p1
OG0005264: TCRU_5154 TcCLB-NE.510303.230_mRNA-p1 TcSYL_0078990.t1-p1
OG0005265: TCRU_5158 TcCLB-EL.509153.130_mRNA-p1 TcCLB-NE.
510303.280_mRNA-p1
OG0005266: TCRU_5160 TcCLB-EL.509153.110_mRNA-p1 TcCLB-NE.
510303.299_mRNA-p1
OG0005267: TCRU_5161 TcCLB-EL.509153.100_mRNA-p1 TcCLB-NE.
510303.310_mRNA-p1
OG0005268: TCRU_5165 TcCLB-EL.504229.110_mRNA-p1 TcCLB-NE.
504069.120_mRNA-p1
OG0005269: TCRU_5166 TcCLB-EL.511145.89_mRNA-p1 TcCLB-NE.
504069.110_mRNA-p1
OG0005270: TCRU_5167 TcCLB-EL.511145.80_mRNA-p1 TcCLB-NE.
504069.100_mRNA-p1

OG0005271: TCRU_5168 TcCLB-EL.511145.70_mRNA-p1 TcCLB-NE.
504069.90_mRNA-p1
OG0005272: TCRU_5170 TcCLB-EL.511145.49_mRNA-p1 TcCLB-NE.
504069.60_mRNA-p1
OG0005273: TCRU_5171 TcCLB-EL.511145.43_mRNA-p1 TcCLB-NE.
504069.53_mRNA-p1
OG0005274: TCRU_5172 TcCLB-EL.511145.30_mRNA-p1 TcCLB-NE.
504069.40_mRNA-p1
OG0005275: TCRU_5173 TcCLB-EL.511145.24_mRNA-p1 TcCLB-NE.
504069.34_mRNA-p1
OG0005276: TCRU_5175 TcCLB-EL.511145.10_mRNA-p1 TcCLB-NE.
504069.20_mRNA-p1
OG0005277: TCRU_5176 TcCLB-EL.416883.9_mRNA-p1 TcCLB-NE.
504069.4_mRNA-p1
OG0005278: TCRU_5177 TcCLB-EL.508787.30_mRNA-p1 TcCLB-NE.
468005.9_mRNA-p1
OG0005279: TCRU_5184 TcCLB-EL.503477.30_mRNA-p1 TcCLB-NE.
507887.30_mRNA-p1
OG0005280: TCRU_5185 TcCLB-EL.503477.20_mRNA-p1 TcCLB-NE.
507889.10_mRNA-p1
OG0005281: TCRU_5186 TcCLB-EL.503477.10_mRNA-p1 TcCLB-NE.
507889.20_mRNA-p1
OG0005282: TCRU_5202 TcCLB-NE.508823.90_mRNA-p1 TcSYL_0043000.t1-p1
OG0005283: TCRU_5203 TcCLB-NE.508823.70_mRNA-p1 TcSYL_0043010.t1-p1
OG0005284: TCRU_homolog_ TcCLB-EL.508387.170_mRNA-p1 TcCLB-NE.
506789.130_mRNA-p1
OG0005285: TCRU_5231 TcCLB-EL.504147.140_mRNA-p1 TcSYL_0005330.t1-p1
OG0005286: TCRU_5239 TcCLB-EL.511421.60_mRNA-p1 TcSYL_0111100.t1-p1
OG0005287: TCRU_5256 TcCLB-EL.509583.10_mRNA-p1 TcSYL_0089080.t1-p1
OG0005288: TCRU_5261 TcCLB-EL.509767.10_mRNA-p1 TcCLB-NE.
509601.10_mRNA-p1
OG0005289: TCRU_5262 TcCLB-EL.509767.20_mRNA-p1 TcCLB-NE.
509601.20_mRNA-p1
OG0005290: TCRU_5263 TcCLB-EL.509767.30_mRNA-p1 TcCLB-NE.
509601.30_mRNA-p1
OG0005291: TCRU_5264 TcCLB-EL.509767.40_mRNA-p1 TcCLB-NE.
509601.40_mRNA-p1
OG0005292: TCRU_5265 TcCLB-EL.509767.50_mRNA-p1 TcCLB-NE.
509601.50_mRNA-p1
OG0005293: TCRU_5266 TcCLB-EL.509767.59_mRNA-p1 TcCLB-NE.
509601.59_mRNA-p1
OG0005294: TCRU_5267 TcCLB-EL.509767.70_mRNA-p1 TcCLB-NE.
509601.70_mRNA-p1
OG0005295: TCRU_5268 TcCLB-EL.509767.80_mRNA-p1 TcCLB-NE.
509601.80_mRNA-p1
OG0005296: TCRU_5269 TcCLB-EL.509767.90_mRNA-p1 TcCLB-NE.
509601.90_mRNA-p1
OG0005297: TCRU_5270 TcCLB-EL.509767.100_mRNA-p1 TcCLB-NE.
509601.100_mRNA-p1
OG0005298: TCRU_5271 TcCLB-EL.509767.110_mRNA-p1 TcCLB-NE.
509601.110_mRNA-p1
OG0005299: TCRU_5272 TcCLB-EL.509767.120_mRNA-p1 TcCLB-NE.
509601.120_mRNA-p1
OG0005300: TCRU_5273 TcCLB-EL.509767.130_mRNA-p1 TcCLB-NE.

509601.130_mRNA-p1
OG0005301: TCRU_5274 TcCLB-EL.509767.150_mRNA-p1 TcCLB-NE.
509601.150_mRNA-p1
OG0005302: TCRU_5275 TcCLB-EL.509767.160_mRNA-p1 TcCLB-NE.
509601.160_mRNA-p1
OG0005303: TCRU_5276 TcCLB-EL.509767.170_mRNA-p1 TcCLB-NE.
506219.40_mRNA-p1
OG0005304: TCRU_5277 TcCLB-EL.509767.180_mRNA-p1 TcCLB-NE.
506219.30_mRNA-p1
OG0005305: TCRU_5287 TcCLB-NE.508461.142_mRNA-p1 TcCLB-NE.
508461.146_mRNA-p1
OG0005306: TCRU_5306 TcCLB-EL.504215.10_mRNA-p1 TcCLB-NE.
508891.20_mRNA-p1
OG0005307: TCRU_5309 TcCLB-EL.504215.40_mRNA-p1 TcCLB-NE.
511275.30_mRNA-p1
OG0005308: TCRU_5311 TcCLB-EL.504215.59_mRNA-p1 TcCLB-NE.
511275.10_pseudogenic_transcript-p1
OG0005309: TCRU_5312 TcCLB-EL.506359.10_mRNA-p1 TcCLB-NE.
482319.19_mRNA-p1
OG0005310: TCRU_5314 TcCLB-EL.507099.40_mRNA-p1 TcSYL_0089310.t1-p1
OG0005311: TCRU_5315 TcCLB-EL.507099.50_mRNA-p1 TcSYL_0089320.t1-p1
OG0005312: TCRU_5319 TcCLB-EL.503971.30_mRNA-p1 TcCLB-NE.
508269.20_mRNA-p1
OG0005313: TCRU_5320 TcCLB-EL.503971.40_mRNA-p1 TcCLB-NE.
508269.30_mRNA-p1
OG0005314: TCRU_5336 TcCLB-NE.510387.30_pseudogenic_transcript-p1
TcCLB-NE.510627.184_mRNA-p1
OG0005315: TCRU_5340 TcCLB-EL.510719.250_mRNA-p1 TcCLB-NE.
506275.9_mRNA-p1
OG0005316: TCRU_5341 TcCLB-EL.510719.240_mRNA-p1 TcCLB-NE.
405737.14_mRNA-p1
OG0005317: TCRU_5342 TcCLB-EL.510719.230_mRNA-p1 TcCLB-NE.
509745.10_mRNA-p1
OG0005318: TCRU_5343 TcCLB-EL.510719.200_mRNA-p1 TcCLB-NE.
509745.60_mRNA-p1
OG0005319: TCRU_5347 TcCLB-EL.510491.20_mRNA-p1 TcCLB-NE.
511633.40_mRNA-p1
OG0005320: TCRU_5349 TcCLB-NE.511633.60_mRNA-p1 TcSYL_0006210.t1-p1
OG0005321: TCRU_5350 TcCLB-NE.511633.70_mRNA-p1 TcSYL_0006240.t1-p1
OG0005322: TCRU_5352 TcCLB-EL.511417.20_pseudogenic_transcript-p1
TcSYL_0111460.t1-p1
OG0005323: TCRU_5357 TcCLB-EL.510089.50_mRNA-p1 TcCLB-NE.
510421.50_mRNA-p1
OG0005324: TCRU_5358 TcCLB-EL.510089.60_mRNA-p1 TcCLB-NE.
510421.60_mRNA-p1
OG0005325: TCRU_5359 TcCLB-EL.510089.64_mRNA-p1 TcCLB-NE.
510421.70_mRNA-p1
OG0005326: TCRU_5360 TcCLB-EL.510089.70_mRNA-p1 TcCLB-NE.
510421.80_mRNA-p1
OG0005327: TCRU_5361 TcCLB-EL.510089.80_mRNA-p1 TcCLB-NE.
510421.90_mRNA-p1
OG0005328: TCRU_5362 TcCLB-EL.510089.90_mRNA-p1 TcCLB-NE.
510421.100_mRNA-p1
OG0005329: TCRU_5363 TcCLB-EL.510089.100_mRNA-p1 TcCLB-NE.

510421.110_mRNA-p1
OG0005330: TCRU_5365 TcCLB-EL.510089.120_mRNA-p1 TcCLB-NE.
510421.140_mRNA-p1
OG0005331: TCRU_5367 TcCLB-EL.510089.180_mRNA-p1 TcCLB-NE.
510421.190_mRNA-p1
OG0005332: TCRU_10072 TCRU_5375 TcCLB-EL.
508941.10_pseudogenic_transcript-p1
OG0005333: TCRU_5381 TcCLB-EL.509951.20_mRNA-p1 TcCLB-NE.
507583.10_mRNA-p1
OG0005334: TCRU_5382 TcCLB-EL.509951.10_mRNA-p1 TcCLB-NE.
507583.20_mRNA-p1
OG0005335: TCRU_5385 TcCLB-EL.510839.20_mRNA-p1 TcCLB-NE.
504933.30_mRNA-p1
OG0005336: TCRU_5386 TcCLB-EL.510839.11_mRNA-p1 TcCLB-NE.
504933.20_mRNA-p1
OG0005337: TCRU_5388 TcCLB-EL.508555.70_mRNA-p1 TcCLB-NE.
509237.140_mRNA-p1
OG0005338: TCRU_5389 TcCLB-EL.508555.50_mRNA-p1 TcCLB-NE.
509237.120_mRNA-p1
OG0005339: TCRU_5393 TcCLB-NE.510187.140_mRNA-p1 TcSYL_0011630.t1-p1
OG0005340: TCRU_5394 TcCLB-EL.507711.270_mRNA-p1 TcCLB-NE.
509643.40_mRNA-p1
OG0005341: TCRU_5395 TcCLB-EL.507711.290_mRNA-p1 TcCLB-NE.
509643.60_mRNA-p1
OG0005342: TCRU_5397 TcCLB-EL.507711.330_mRNA-p1 TcCLB-NE.
509643.90_mRNA-p1
OG0005343: TCRU_5399 TcCLB-EL.507711.349_mRNA-p1 TcCLB-NE.
509643.110_mRNA-p1
OG0005344: TCRU_5400 TcCLB-EL.507713.20_mRNA-p1 TcCLB-NE.
509643.120_mRNA-p1
OG0005345: TCRU_5404 TcCLB-NE.509105.130_mRNA-p1 TcSYL_0008060.t1-p1
OG0005346: TCRU_5405 TcCLB-NE.509105.120_mRNA-p1 TcSYL_0008050.t1-p1
OG0005347: TCRU_5418 TcCLB-EL.511173.64_mRNA-p1 TcCLB-NE.
508433.30_mRNA-p1
OG0005348: TCRU_5423 TcCLB-EL.510293.70_mRNA-p1 TcCLB-NE.
508661.50_mRNA-p1
OG0005349: TCRU_5429 TcCLB-EL.506677.20_mRNA-p1 TcCLB-NE.
506583.40_mRNA-p1
OG0005350: TCRU_5430 TcCLB-EL.506677.10_mRNA-p1 TcCLB-NE.
506583.30_mRNA-p1
OG0005351: TCRU_5432 TcCLB-EL.510291.20_mRNA-p1 TcCLB-NE.
508659.30_mRNA-p1
OG0005352: TCRU_5433 TcCLB-EL.510291.14_mRNA-p1 TcCLB-NE.
508659.18_mRNA-p1
OG0005353: TCRU_5435 TcCLB-EL.504203.50_mRNA-p1 TcCLB-NE.
503791.20_mRNA-p1
OG0005354: TCRU_5441 TcCLB-EL.507683.34_mRNA-p1 TcCLB-NE.
511291.50_mRNA-p1
OG0005355: TCRU_5442 TcCLB-EL.507683.40_mRNA-p1 TcCLB-NE.
511291.40_mRNA-p1
OG0005356: TCRU_5448 TcCLB-NE.511003.190_mRNA-p1 TcSYL_0046290.t1-p1
OG0005357: TCRU_5449 TcCLB-NE.511003.180_mRNA-p1 TcSYL_0046280.t1-p1
OG0005358: TCRU_5450 TcCLB-NE.511003.170_mRNA-p1 TcSYL_0046270.t1-p1
OG0005359: TCRU_5454 TcCLB-NE.511003.80_mRNA-p1 TcSYL_0046240.t1-p1

OG0005360: TCRU_5455 TcCLB-NE.511003.70_mRNA-p1 TcSYL_0046230.t1-p1
OG0005361: TCRU_5462 TcSYL_0074550.t1-p1 TcSYL_0143310.t1-p1
OG0005362: TCRU_5468 TcCLB-EL.503579.60_mRNA-p1 TcCLB-NE.
507517.50_mRNA-p1
OG0005363: TCRU_5470 TcCLB-EL.503579.40_mRNA-p1 TcCLB-NE.
507517.30_mRNA-p1
OG0005364: TCRU_5472 TcCLB-EL.503579.10_mRNA-p1 TcCLB-NE.
503919.60_mRNA-p1
OG0005365: TCRU_5473 TcCLB-EL.508209.20_mRNA-p1 TcCLB-NE.
503919.50_mRNA-p1
OG0005366: TCRU_5474 TcCLB-EL.508209.30_mRNA-p1 TcCLB-NE.
503919.40_mRNA-p1
OG0005367: TCRU_5475 TcCLB-EL.508209.60_mRNA-p1 TcCLB-NE.
503919.10_mRNA-p1
OG0005368: TCRU_5479 TcCLB-NE.511367.50_mRNA-p1 TcSYL_0170250.t1-p1
OG0005369: TCRU_5480 TcCLB-NE.511367.60_mRNA-p1 TcSYL_0170230.t1-p1
OG0005370: TCRU_5481 TcCLB-NE.511367.74_mRNA-p1 TcSYL_0170060.t1-p1
OG0005371: TCRU_5485 TcCLB-NE.511367.110_mRNA-p1 TcSYL_0170050.t1-p1
OG0005372: TCRU_5488 TcCLB-NE.507297.10_mRNA-p1 TcSYL_0175930.t1-p1
OG0005373: TCRU_5490 TcCLB-EL.507603.250_mRNA-p1 TcCLB-NE.
509429.310_mRNA-p1
OG0005374: TCRU_5491 TcCLB-EL.507603.240_mRNA-p1 TcCLB-NE.
509429.300_mRNA-p1
OG0005375: TCRU_5493 TcCLB-EL.507603.220_mRNA-p1 TcCLB-NE.
509429.280_mRNA-p1
OG0005376: TCRU_5496 TcCLB-EL.507603.160_mRNA-p1 TcCLB-NE.
509429.230_mRNA-p1
OG0005377: TCRU_5497 TcCLB-EL.507603.150_mRNA-p1 TcCLB-NE.
509429.220_mRNA-p1
OG0005378: TCRU_5498 TcCLB-EL.507603.140_mRNA-p1 TcCLB-NE.
509429.210_mRNA-p1
OG0005379: TCRU_5500 TcCLB-EL.507603.110_mRNA-p1 TcCLB-NE.
509429.180_mRNA-p1
OG0005380: TCRU_5504 TcCLB-EL.507603.60_mRNA-p1 TcCLB-NE.
509429.130_mRNA-p1
OG0005381: TCRU_5506 TcCLB-EL.507603.30_mRNA-p1 TcCLB-NE.
509429.100_mRNA-p1
OG0005382: TCRU_5507 TcCLB-EL.507603.20_mRNA-p1 TcCLB-NE.
509429.90_mRNA-p1
OG0005383: TCRU_5508 TcCLB-EL.507603.10_pseudogenic_transcript-p1
TcCLB-NE.509429.80_mRNA-p1
OG0005384: TCRU_5512 TcCLB-EL.511531.30_mRNA-p1 TcCLB-NE.
511025.80_mRNA-p1
OG0005385: TCRU_5514 TcCLB-EL.511531.44_mRNA-p1 TcCLB-NE.
511025.60_mRNA-p1
OG0005386: TCRU_5517 TcCLB-NE.506559.364_mRNA-p1 TcSYL_0045270.t1-p1
OG0005387: TCRU_5519 TcCLB-NE.506559.190_mRNA-p1 TcSYL_0045170.t1-p1
OG0005388: TCRU_5520 TcCLB-EL.510229.20_mRNA-p1 TcCLB-NE.
506559.170_mRNA-p1
OG0005389: TCRU_5521 TcCLB-EL.503903.50_mRNA-p1 TcCLB-NE.
508153.770_mRNA-p1
OG0005390: TCRU_5523 TcCLB-EL.508181.10_mRNA-p1 TcCLB-NE.
508153.960_mRNA-p1
OG0005391: TCRU_binding TcCLB-NE.508479.360_mRNA-p1

TcSYL_0019720.t1-p1
OG0005392: TCRU_5528 TcCLB-EL.504153.160_mRNA-p1 TcSYL_0138670.t1-p1
OG0005393: TCRU_5529 TcCLB-EL.504153.170_mRNA-p1 TcSYL_0138630.t1-p1
OG0005394: TCRU_5531 TcCLB-EL.509149.30_mRNA-p1 TcCLB-NE.
508175.340_mRNA-p1
OG0005395: TCRU_5535 TcCLB-EL.504089.14_mRNA-p1 TcSYL_0092080.t1-p1
OG0005396: TCRU_5540 TcCLB-EL.503453.70_mRNA-p1 TcCLB-NE.
506959.90_mRNA-p1
OG0005397: TCRU_5544 TcCLB-EL.509231.10_mRNA-p1 TcCLB-NE.
509719.40_mRNA-p1
OG0005398: TCRU_5545 TcCLB-EL.510877.60_mRNA-p1 TcSYL_0045820.t1-p1
OG0005399: TCRU_5550 TcCLB-EL.511803.50_mRNA-p1 TcSYL_0180110.t1-p1
OG0005400: TCRU_5551 TcCLB-EL.510431.120_mRNA-p1 TcSYL_0138080.t1-p1
OG0005401: TCRU_5552 TcCLB-EL.511557.70_mRNA-p1 TcCLB-NE.
505171.80_mRNA-p1
OG0005402: TCRU_5556 TcCLB-NE.510901.80_mRNA-p1 TcSYL_0179160.t1-p1
OG0005403: TCRU_5557 TcCLB-NE.510901.110_mRNA-p1 TcSYL_0179130.t1-p1
OG0005404: TCRU_5558 TcCLB-NE.510901.140_mRNA-p1 TcSYL_0179120.t1-p1
OG0005405: TCRU_5559 TcCLB-NE.510901.210_mRNA-p1 TcSYL_0178850.t1-p1
OG0005406: TCRU_5569 TcCLB-EL.507811.107_mRNA-p1 TcCLB-NE.
511649.94_mRNA-p1
OG0005407: TCRU_5576 TcCLB-EL.510609.34_mRNA-p1 TcCLB-NE.
510395.20_mRNA-p1
OG0005408: TCRU_5578 TcCLB-EL.508569.70_mRNA-p1 TcCLB-NE.
503733.80_mRNA-p1
OG0005409: TCRU_5580 TcCLB-EL.506811.110_mRNA-p1 TcCLB-NE.
506303.120_mRNA-p1
OG0005410: TCRU_5581 TcCLB-EL.509171.40_mRNA-p1 TcCLB-NE.
511503.9_pseudogenic_transcript-p1
OG0005411: TCRU_5582 TcCLB-NE.495369.10_mRNA-p1 TcCLB-NE.
511003.210_mRNA-p1
OG0005412: TCRU_5583 TcCLB-EL.506207.40_mRNA-p1 TcCLB-NE.
510065.40_mRNA-p1
OG0005413: TCRU_5584 TcCLB-EL.506205.40_mRNA-p1 TcCLB-NE.
507991.20_mRNA-p1
OG0005414: TCRU_5585 TcCLB-EL.510859.24_mRNA-p1 TcCLB-NE.
507649.60_mRNA-p1
OG0005415: TCRU_5586 TcCLB-EL.508567.40_mRNA-p1 TcCLB-NE.
509611.50_mRNA-p1
OG0005416: TCRU_5587 TcCLB-EL.510857.10_mRNA-p1 TcCLB-NE.
509611.140_mRNA-p1
OG0005417: TCRU_5588 TcCLB-EL.510857.4_mRNA-p1 TcCLB-NE.
509611.150_mRNA-p1
OG0005418: TCRU_5591 TcCLB-EL.510747.110_mRNA-p1 TcCLB-NE.
510657.130_mRNA-p1
OG0005419: TCRU_5594 TcCLB-EL.503955.30_mRNA-p1 TcCLB-NE.
507063.80_mRNA-p1
OG0005420: TCRU_5595 TcCLB-EL.503953.40_mRNA-p1 TcCLB-NE.
507063.120_mRNA-p1
OG0005421: TCRU_5599 TcCLB-EL.511575.100_mRNA-p1 TcSYL_0094770.t1-p1
OG0005422: TCRU__CLPTM1__ TcCLB-EL.503745.20_mRNA-p1 TcCLB-NE.
506193.20_mRNA-p1
OG0005423: TCRU_5602 TcCLB-EL.503745.10_mRNA-p1 TcCLB-NE.
506193.10_mRNA-p1

OG0005424: TCRU_5606 TcCLB-EL.511353.60_mRNA-p1 TcCLB-NE.
511301.20_mRNA-p1
OG0005425: TCRU_5607 TcCLB-EL.506483.60_mRNA-p1 TcCLB-NE.
511299.60_mRNA-p1
OG0005426: TCRU_5608 TcCLB-EL.511417.60_mRNA-p1 TcCLB-NE.
509487.20_mRNA-p1
OG0005427: TCRU_5611 TcCLB-EL.508323.190_mRNA-p1 TcCLB-NE.
511825.170_mRNA-p1
OG0005428: TCRU_5612 TcCLB-EL.506861.10_mRNA-p1 TcCLB-NE.
511825.180_mRNA-p1
OG0005429: TCRU_5613 TcCLB-EL.506861.20_mRNA-p1 TcCLB-NE.
511825.190_mRNA-p1
OG0005430: TCRU_5615 TcCLB-EL.506863.70_mRNA-p1 TcCLB-NE.
509211.20_mRNA-p1
OG0005431: TCRU_5617 TcCLB-EL.504431.50_mRNA-p1 TcCLB-NE.
507951.100_mRNA-p1
OG0005432: TCRU_5618 TcCLB-EL.504433.14_mRNA-p1 TcCLB-NE.
507951.179_mRNA-p1
OG0005433: TCRU_5622 TcCLB-EL.508723.100_mRNA-p1 TcCLB-NE.
509789.30_mRNA-p1
OG0005434: TCRU_5626 TcCLB-EL.507925.50_mRNA-p1 TcCLB-NE.
509059.40_mRNA-p1
OG0005435: TCRU_5628 TcCLB-EL.504041.20_mRNA-p1 TcCLB-NE.
508815.20_mRNA-p1
OG0005436: TCRU_5633 TcCLB-EL.506495.6_mRNA-p1 TcCLB-NE.
503965.30_mRNA-p1
OG0005437: TCRU_5640 TcCLB-NE.511367.340_mRNA-p1 TcSYL_0169920.t1-p1
OG0005438: TCRU_5641 TcCLB-EL.510759.190_mRNA-p1 TcCLB-NE.
506999.10_mRNA-p1
OG0005439: TCRU_5648 TcCLB-EL.508541.210_mRNA-p1 TcCLB-NE.
507541.20_mRNA-p1
OG0005440: TCRU_5649 TcCLB-EL.506635.70_mRNA-p1 TcCLB-NE.
508027.120_mRNA-p1
OG0005441: TCRU_5650 TcCLB-EL.506635.60_mRNA-p1 TcCLB-NE.
508027.110_mRNA-p1
OG0005442: TCRU_5653 TcCLB-EL.508831.10_mRNA-p1 TcCLB-NE.
509119.30_mRNA-p1
OG0005443: TCRU_5659 TcCLB-EL.430539.10_mRNA-p1 TcCLB-NE.
504223.30_mRNA-p1
OG0005444: TCRU_5663 TcCLB-EL.510105.36_mRNA-p1 TcCLB-NE.
509715.26_mRNA-p1
OG0005445: TCRU_5664 TcCLB-EL.506885.370_mRNA-p1 TcCLB-NE.
510729.30_mRNA-p1
OG0005446: TCRU_5665 TcCLB-EL.506885.394_mRNA-p1 TcCLB-NE.
503687.49_mRNA-p1
OG0005447: TCRU_5666 TcCLB-EL.506885.413_mRNA-p1 TcCLB-NE.
503687.20_mRNA-p1
OG0005448: TCRU_5667 TcCLB-EL.506885.440_mRNA-p1 TcCLB-NE.
508457.20_mRNA-p1
OG0005449: TCRU_5672 TcCLB-EL.509247.20_mRNA-p1 TcCLB-NE.
506977.130_mRNA-p1
OG0005450: TCRU_5679 TcCLB-EL.507163.90_mRNA-p1 TcCLB-NE.
509751.10_mRNA-p1
OG0005451: TCRU_5680 TcCLB-EL.507165.40_mRNA-p1 TcCLB-NE.

507793.30_mRNA-p1
OG0005452: TCRU_5686 TcCLB-EL.506251.100_mRNA-p1 TcCLB-NE.
508231.74_mRNA-p1
OG0005453: TCRU_5687 TcCLB-EL.510755.40_mRNA-p1 TcCLB-NE.
510653.10_mRNA-p1
OG0005454: TCRU_5693 TcCLB-EL.510105.40_mRNA-p1 TcCLB-NE.
509715.30_mRNA-p1
OG0005455: TCRU_5696 TcCLB-EL.506287.180_mRNA-p1 TcCLB-NE.
508881.90_mRNA-p1
OG0005456: TCRU_5698 TcCLB-EL.510295.30_mRNA-p1 TcCLB-NE.
506007.20_mRNA-p1
OG0005457: TCRU_5700 TcCLB-EL.510349.60_mRNA-p1 TcCLB-NE.
503925.90_mRNA-p1
OG0005458: TCRU_5702 TcCLB-EL.508777.200_mRNA-p1 TcCLB-NE.
511483.60_mRNA-p1
OG0005459: TCRU_5706 TcCLB-EL.511307.6_mRNA-p1 TcCLB-NE.
507087.50_mRNA-p1
OG0005460: TCRU_5707 TcCLB-EL.430061.9_mRNA-p1 TcCLB-NE.
504023.10_mRNA-p1
OG0005461: TCRU_5710 TcCLB-NE.509997.20_mRNA-p1 TcSYL_0003290.t1-p1
OG0005462: TCRU_5711 TcCLB-EL.504029.101_mRNA-p1 TcCLB-NE.
509997.60_mRNA-p1
OG0005463: TCRU_5715 TcCLB-EL.504035.70_mRNA-p1 TcCLB-NE.
507991.129_mRNA-p1
OG0005464: TCRU_5716 TcCLB-EL.507389.60_mRNA-p1 TcCLB-NE.
509911.100_mRNA-p1
OG0005465: TCRU_5721 TcCLB-EL.510533.110_mRNA-p1 TcCLB-NE.
511821.100_mRNA-p1
OG0005466: TCRU_5722 TcCLB-EL.508323.110_mRNA-p1 TcCLB-NE.
511825.90_mRNA-p1
OG0005467: TCRU_5726 TcCLB-EL.506699.9_mRNA-p1 TcCLB-NE.
508153.360_mRNA-p1
OG0005468: TCRU_5729 TcCLB-EL.504143.20_mRNA-p1 TcCLB-NE.
506485.20_mRNA-p1
OG0005469: TCRU_5730 TcCLB-NE.506485.80_mRNA-p1 TcSYL_0003430.t1-p1
OG0005470: TCRU_5732 TcCLB-NE.508547.170_mRNA-p1 TcSYL_0202440.t1-p1
OG0005471: TCRU_5735 TcCLB-NE.506559.430_mRNA-p1 TcSYL_0045320.t1-p1
OG0005472: TCRU_5736 TcCLB-EL.508515.60_mRNA-p1 TcCLB-NE.
508641.140_mRNA-p1
OG0005473: TCRU_5737 TcCLB-EL.508515.90_mRNA-p1 TcCLB-NE.
508641.110_mRNA-p1
OG0005474: TCRU_5740 TcCLB-EL.508307.160_mRNA-p1 TcSYL_0006060.t1-p1
OG0005475: TCRU_5742 TcCLB-EL.506925.470_mRNA-p1 TcSYL_0080660.t1-p1
OG0005476: TCRU_5745 TcCLB-NE.511317.60_mRNA-p1 TcSYL_0159540.t1-p1
OG0005477: TCRU_5746 TcCLB-EL.506519.120_mRNA-p1 TcCLB-NE.
508909.30_mRNA-p1
OG0005478: TCRU_5752 TcCLB-EL.506205.30_mRNA-p1 TcCLB-NE.
507991.30_mRNA-p1
OG0005479: TCRU_5757 TcCLB-NE.503979.20_mRNA-p1 TcSYL_0048320.t1-p1
OG0005480: TCRU_5761 TcCLB-EL.507941.20_mRNA-p1 TcCLB-NE.
509985.60_mRNA-p1
OG0005481: TCRU_5767 TcCLB-EL.506795.44_mRNA-p1 TcCLB-NE.
509937.20_mRNA-p1
OG0005482: TCRU_5770 TcCLB-EL.508781.20_mRNA-p1 TcCLB-NE.

506855.80_mRNA-p1
OG0005483: TCRU_5773 TcCLB-EL.508307.70_mRNA-p1 TcSYL_0006130.t1-p1
OG0005484: TCRU_5778 TcCLB-EL.508183.4_mRNA-p1 TcCLB-NE.
508153.180_mRNA-p1
OG0005485: TCRU_5780 TcCLB-EL.511621.120_mRNA-p1 TcCLB-NE.
504741.240_mRNA-p1
OG0005486: TCRU_5785 TcCLB-EL.511625.40_mRNA-p1 TcCLB-NE.
504741.30_mRNA-p1
OG0005487: TCRU_5786 TcCLB-EL.509067.30_mRNA-p1 TcCLB-NE.
508711.40_mRNA-p1
OG0005488: TCRU_5787 TcCLB-EL.509067.50_mRNA-p1 TcCLB-NE.
508711.20_mRNA-p1
OG0005489: TCRU_5790 TcCLB-EL.511537.16_mRNA-p1 TcCLB-NE.
511021.60_mRNA-p1
OG0005490: TCRU_5793 TcCLB-EL.504147.150_mRNA-p1 TcSYL_0005300.t1-p1
OG0005491: TCRU_5794 TcCLB-EL.506473.10_mRNA-p1 TcCLB-NE.
508999.30_mRNA-p1
OG0005492: TCRU_5799 TcCLB-EL.511393.10_mRNA-p1 TcSYL_0202030.t1-p1
OG0005493: TCRU_5802 TcCLB-EL.503599.60_mRNA-p1 TcCLB-NE.
505163.70_mRNA-p1
OG0005494: TCRU_5814 TcCLB-EL.508837.74_mRNA-p1 TcCLB-NE.
508963.20_mRNA-p1
OG0005495: TCRU_5815 TcCLB-EL.510311.160_mRNA-p1 TcCLB-NE.
508153.1090_mRNA-p1
OG0005496: TCRU_5817 TcCLB-EL.506691.60_mRNA-p1 TcCLB-NE.
508153.1000_mRNA-p1
OG0005497: TCRU_5832 TcCLB-EL.506963.110_mRNA-p1 TcCLB-NE.
511233.100_mRNA-p1
OG0005498: TCRU_5838 TcCLB-EL.508357.80_mRNA-p1 TcCLB-NE.
508355.420_mRNA-p1
OG0005499: TCRU_5840 TcCLB-EL.511751.120_mRNA-p1 TcCLB-NE.
507023.120_mRNA-p1
OG0005500: TCRU_5841 TcCLB-EL.511751.170_mRNA-p1 TcCLB-NE.
507023.40_mRNA-p1
OG0005501: TCRU_5844 TcCLB-NE.507053.110_mRNA-p1 TcSYL_0046990.t1-p1
OG0005502: TCRU_5845 TcCLB-NE.507053.70_mRNA-p1 TcSYL_0046940.t1-p1
OG0005503: TCRU_5848 TcCLB-EL.506405.40_mRNA-p1 TcCLB-NE.
508257.40_mRNA-p1
OG0005504: TCRU_5849 TcCLB-EL.510763.50_mRNA-p1 TcCLB-NE.
509671.172_mRNA-p1
OG0005505: TCRU_5850 TcCLB-EL.510763.70_mRNA-p1 TcCLB-NE.
509671.164_mRNA-p1
OG0005506: TCRU_5852 TcCLB-EL.437545.10_pseudogenic_transcript-p1
TcCLB-NE.457251.10_mRNA-p1
OG0005507: TCRU_5854 TcCLB-EL.504147.40_mRNA-p1 TcSYL_0005600.t1-p1
OG0005508: TCRU_5855 TcCLB-EL.509153.80_mRNA-p1 TcCLB-NE.
510305.9_mRNA-p1
OG0005509: TCRU_5856 TcCLB-EL.504507.10_mRNA-p1 TcCLB-NE.
503555.20_mRNA-p1
OG0005510: TCRU_5857 TcCLB-EL.506953.40_mRNA-p1 TcCLB-NE.
509287.30_mRNA-p1
OG0005511: TCRU_5859 TcCLB-EL.510431.160_mRNA-p1 TcSYL_0138260.t1-p1
OG0005512: TCRU_5861 TcCLB-EL.511419.60_mRNA-p1 TcCLB-NE.
505999.110_mRNA-p1

OG0005513: TCRU_5862 TcCLB-EL.511421.24_mRNA-p1 TcCLB-NE.
505999.189_pseudogenic_transcript-p1
OG0005514: TCRU_5864 TcCLB-EL.508277.290_mRNA-p1 TcCLB-NE.
504091.10_mRNA-p1
OG0005515: TCRU_5865 TcCLB-EL.510101.80_mRNA-p1 TcCLB-NE.
506297.230_mRNA-p1
OG0005516: TCRU_5867 TcCLB-EL.510101.330_mRNA-p1 TcCLB-NE.
507765.149_mRNA-p1
OG0005517: TCRU_5870 TcCLB-EL.506513.190_mRNA-p1 TcCLB-NE.
511323.50_mRNA-p1
OG0005518: TCRU_5873 TcCLB-EL.504057.40_mRNA-p1 TcSYL_0046170.t1-p1
OG0005519: TCRU_5877 TcCLB-EL.510283.90_mRNA-p1 TcCLB-NE.
503823.60_mRNA-p1
OG0005520: TCRU_5878 TcCLB-EL.510283.160_mRNA-p1 TcCLB-NE.
503823.120_mRNA-p1
OG0005521: TCRU_5882 TcCLB-EL.506411.30_mRNA-p1 TcCLB-NE.
511237.30_pseudogenic_transcript-p1
OG0005522: TCRU_5883 TcCLB-EL.506411.20_mRNA-p1 TcCLB-NE.
511237.20_mRNA-p1
OG0005523: TCRU_5885 TcCLB-EL.507083.90_mRNA-p1 TcCLB-NE.
509901.110_mRNA-p1
OG0005524: TCRU_5886 TcCLB-EL.508647.180_mRNA-p1 TcSYL_0047880.t1-p1
OG0005525: TCRU_5887 TcCLB-EL.508647.190_mRNA-p1 TcSYL_0047890.t1-p1
OG0005526: TCRU_5895 TcCLB-EL.510741.150_mRNA-p1 TcCLB-NE.
510661.110_mRNA-p1
OG0005527: TCRU_5897 TcCLB-EL.509967.60_mRNA-p1 TcCLB-NE.
509695.40_mRNA-p1
OG0005528: TCRU_5899 TcCLB-NE.510873.10_mRNA-p1 TcSYL_0041290.t1-p1
OG0005529: TCRU_5903 TcCLB-EL.507809.119_mRNA-p1 TcCLB-NE.
511657.70_mRNA-p1
OG0005530: TCRU_5909 TcCLB-EL.511589.220_mRNA-p1 TcCLB-NE.
511229.70_mRNA-p1
OG0005531: TCRU_5910 TcCLB-EL.511589.110_mRNA-p1 TcCLB-NE.
508851.160_mRNA-p1
OG0005532: TCRU_5916 TcCLB-EL.508307.20_mRNA-p1 TcCLB-NE.
511629.10_mRNA-p1
OG0005533: TCRU_5921 TcCLB-EL.507093.260_mRNA-p1 TcSYL_0080420.t1-p1
OG0005534: TCRU_5923 TcCLB-EL.509233.120_mRNA-p1 TcCLB-NE.
509437.30_mRNA-p1
OG0005535: TCRU_5925 TcCLB-EL.511737.70_mRNA-p1 TcCLB-NE.
511517.150_mRNA-p1
OG0005536: TCRU_5927 TcCLB-NE.511517.44_mRNA-p1 TcSYL_0139730.t1-p1
OG0005537: TCRU_5928 TcCLB-NE.511517.37_mRNA-p1 TcSYL_0139720.t1-p1
OG0005538: TCRU_5931 TcCLB-EL.511745.40_mRNA-p1 TcCLB-NE.
507029.60_mRNA-p1
OG0005539: TCRU_5932 TcCLB-EL.504005.50_mRNA-p1 TcCLB-NE.
511481.10_mRNA-p1
OG0005540: TCRU_5933 TcCLB-EL.504005.6_mRNA-p1 TcCLB-NE.
511481.55_mRNA-p1
OG0005541: TCRU_5939 TcCLB-EL.504557.60_mRNA-p1 TcCLB-NE.
506627.110_mRNA-p1
OG0005542: TCRU_5941 TcCLB-EL.506363.20_mRNA-p1 TcCLB-NE.
506975.69_mRNA-p1
OG0005543: TCRU_5942 TcCLB-EL.506363.30_mRNA-p1 TcCLB-NE.

506975.66_mRNA-p1
OG0005544: TCRU_5943 TcCLB-EL.511421.50_mRNA-p1 TcSYL_0111110.t1-p1
OG0005545: TCRU_5952 TcCLB-EL.504883.60_mRNA-p1 TcCLB-NE.
506511.40_mRNA-p1
OG0005546: TCRU_5953 TcCLB-NE.508827.40_mRNA-p1 TcSYL_0181710.t1-p1
OG0005547: TCRU_5954 TcCLB-EL.510877.90_mRNA-p1 TcSYL_0045830.t1-p1
OG0005548: TCRU_5957 TcCLB-NE.506977.10_mRNA-p1 TcCLB-NE.
508637.160_mRNA-p1
OG0005549: TCRU_5958 TcCLB-EL.507041.120_mRNA-p1 TcCLB-NE.
508233.50_mRNA-p1
OG0005550: TCRU_5959 TcCLB-EL.510523.10_pseudogenic_transcript-p1
TcCLB-NE.511817.50_mRNA-p1
OG0005551: TCRU_5960 TcCLB-EL.510525.20_mRNA-p1 TcCLB-NE.
511817.90_mRNA-p1
OG0005552: TCRU_5962 TcCLB-EL.509769.10_mRNA-p1 TcCLB-NE.
447483.10_mRNA-p1
OG0005553: TCRU_5963 TcCLB-NE.510823.10_mRNA-p1 TcSYL_0050150.t1-p1
OG0005554: TCRU_5967 TcCLB-EL.507511.30_mRNA-p1 TcCLB-NE.
506871.160_mRNA-p1
OG0005555: TCRU_5970 TcCLB-EL.464807.20_mRNA-p1 TcCLB-NE.
511277.130_mRNA-p1
OG0005556: TCRU_5971 TcCLB-EL.473111.10_mRNA-p1 TcCLB-NE.
507853.10_mRNA-p1
OG0005557: TCRU_protein TcCLB-EL.505987.41_mRNA-p1 TcCLB-NE.
506705.30_mRNA-p1
OG0005558: TCRU_5974 TcCLB-EL.505987.10_pseudogenic_transcript-p1
TcCLB-NE.506705.60_mRNA-p1
OG0005559: TCRU_5977 TcCLB-EL.506753.80_mRNA-p1 TcCLB-NE.
510355.240_mRNA-p1
OG0005560: TCRU_5979 TcCLB-EL.509591.30_mRNA-p1 TcCLB-NE.
503999.70_mRNA-p1
OG0005561: TCRU_5983 TcCLB-EL.510165.30_mRNA-p1 TcCLB-NE.
511807.60_mRNA-p1
OG0005562: TCRU_5984 TcCLB-EL.510165.20_mRNA-p1 TcCLB-NE.
511807.64_mRNA-p1
OG0005563: TCRU_5986 TcCLB-EL.511045.40_mRNA-p1 TcCLB-NE.
511859.60_mRNA-p1
OG0005564: TCRU_5988 TcCLB-EL.511467.70_mRNA-p1 TcCLB-NE.
506295.50_mRNA-p1
OG0005565: TCRU_5989 TcCLB-EL.511469.24_mRNA-p1 TcCLB-NE.
506295.80_mRNA-p1
OG0005566: TCRU_5990 TcCLB-EL.511469.60_mRNA-p1 TcCLB-NE.
506295.120_mRNA-p1
OG0005567: TCRU_5993 TcCLB-EL.503893.120_mRNA-p1 TcCLB-NE.
508507.30_mRNA-p1
OG0005568: TCRU_5995 TcCLB-EL.503893.50_mRNA-p1 TcCLB-NE.
503703.20_mRNA-p1
OG0005569: TCRU_5999 TcCLB-EL.506531.40_mRNA-p1 TcCLB-NE.
509399.60_mRNA-p1
OG0005570: TCRU_6002 TcCLB-EL.504151.69_mRNA-p1 TcCLB-NE.
509459.70_mRNA-p1
OG0005571: TCRU_6007 TcCLB-EL.506777.20_mRNA-p1 TcCLB-NE.
511165.4_mRNA-p1
OG0005572: TCRU_6011 TcCLB-EL.510105.80_mRNA-p1 TcCLB-NE.

509715.70_mRNA-p1
OG0005573: TCRU_6012 TcCLB-EL.511575.60_mRNA-p1 TcSYL_0094740.t1-p1
OG0005574: TCRU_6013 TcCLB-EL.408799.10_mRNA-p1 TcCLB-NE.
506883.20_mRNA-p1
OG0005575: TCRU_6014 TcCLB-EL.510429.30_mRNA-p1 TcCLB-NE.
506883.60_mRNA-p1
OG0005576: TCRU_6015 TcCLB-EL.507735.69_mRNA-p1 TcCLB-NE.
504253.30_mRNA-p1
OG0005577: TCRU_6016 TcCLB-EL.510347.60_mRNA-p1 TcCLB-NE.
503925.30_mRNA-p1
OG0005578: TCRU_6017 TcCLB-EL.506941.60_mRNA-p1 TcCLB-NE.
511511.60_mRNA-p1
OG0005579: TCRU_6019 TcCLB-EL.506227.10_mRNA-p1 TcCLB-NE.
503837.20_mRNA-p1
OG0005580: TCRU_family_ TcCLB-EL.506227.90_mRNA-p1 TcCLB-NE.
511847.50_mRNA-p1
OG0005581: TCRU_6022 TcCLB-EL.506227.120_mRNA-p1 TcCLB-NE.
511847.20_mRNA-p1
OG0005582: TCRU_6025 TcCLB-EL.510609.100_mRNA-p1 TcCLB-NE.
506793.90_mRNA-p1
OG0005583: TCRU_6027 TcCLB-EL.506739.170_mRNA-p1 TcCLB-NE.
506315.70_mRNA-p1
OG0005584: TCRU_6029 TcCLB-EL.506739.130_mRNA-p1 TcCLB-NE.
506315.30_mRNA-p1
OG0005585: TCRU_6030 TcCLB-EL.508307.130_mRNA-p1 TcSYL_0006090.t1-p1
OG0005586: TCRU_6033 TcCLB-EL.504137.20_mRNA-p1 TcCLB-NE.
509797.50_mRNA-p1
OG0005587: TCRU_6035 TcCLB-EL.504137.140_mRNA-p1 TcCLB-NE.
509799.90_mRNA-p1
OG0005588: TCRU_6039 TcCLB-EL.510719.60_mRNA-p1 TcCLB-NE.
509749.10_mRNA-p1
OG0005589: TCRU_6040 TcCLB-EL.510719.100_mRNA-p1 TcCLB-NE.
509747.80_mRNA-p1
OG0005590: TCRU_6045 TcCLB-EL.506717.10_mRNA-p1 TcCLB-NE.
509539.30_mRNA-p1
OG0005591: TCRU_6048 TcCLB-EL.450061.10_mRNA-p1 TcCLB-NE.
508741.390_mRNA-p1
OG0005592: TCRU_6051 TcCLB-EL.511621.40_mRNA-p1 TcCLB-NE.
509317.80_mRNA-p1
OG0005593: TCRU_6055 TcCLB-EL.509835.20_mRNA-p1 TcCLB-NE.
427743.9_mRNA-p1
OG0005594: TCRU_6057 TcCLB-EL.506673.50_mRNA-p1 TcCLB-NE.
504827.50_mRNA-p1
OG0005595: TCRU_6059 TcCLB-EL.507809.14_mRNA-p1 TcCLB-NE.
511661.100_mRNA-p1
OG0005596: TCRU_6060 TcCLB-EL.508307.44_mRNA-p1 TcSYL_0006150.t1-p1
OG0005597: TCRU_6061 TcCLB-EL.510963.50_mRNA-p1 TcCLB-NE.
508625.50_mRNA-p1
OG0005598: TCRU_6062 TcCLB-EL.510963.30_mRNA-p1 TcCLB-NE.
508625.70_mRNA-p1
OG0005599: TCRU_6064 TcCLB-EL.509757.20_mRNA-p1 TcCLB-NE.
508625.120_mRNA-p1
OG0005600: TCRU_6066 TcCLB-EL.508995.69_mRNA-p1 TcCLB-NE.
474887.9_mRNA-p1

OG0005601: TCRU_6067 TcCLB-EL.508995.10_mRNA-p1 TcCLB-NE.
506477.60_mRNA-p1
OG0005602: TCRU_6070 TcCLB-EL.504247.10_mRNA-p1 TcCLB-NE.
507945.20_mRNA-p1
OG0005603: TCRU_6075 TcCLB-EL.506637.70_mRNA-p1 TcCLB-NE.
510099.50_mRNA-p1
OG0005604: TCRU_6076 TcCLB-NE.508409.60_mRNA-p1 TcSYL_0004510.t1-p1
OG0005605: TCRU_6077 TcCLB-NE.508409.100_mRNA-p1 TcSYL_0004470.t1-p1
OG0005606: TCRU_6079 TcCLB-EL.508719.20_mRNA-p1 TcCLB-NE.
509793.60_mRNA-p1
OG0005607: TCRU_6082 TcCLB-EL.506755.130_mRNA-p1 TcCLB-NE.
510359.140_mRNA-p1
OG0005608: TCRU_6083 TcCLB-EL.506755.60_mRNA-p1 TcCLB-NE.
510359.50_mRNA-p1
OG0005609: TCRU_6084 TcCLB-NE.506247.50_mRNA-p1 TcSYL_0108900.t1-p1
OG0005610: TCRU_6086 TcCLB-NE.506247.290_mRNA-p1 TcSYL_0108750.t1-p1
OG0005611: TCRU_6087 TcCLB-EL.510243.70_mRNA-p1 TcCLB-NE.
510575.150_mRNA-p1
OG0005612: TCRU_6088 TcCLB-EL.510243.80_mRNA-p1 TcCLB-NE.
510575.140_mRNA-p1
OG0005613: TCRU_6091 TcCLB-NE.510187.260_mRNA-p1 TcSYL_0011550.t1-p1
OG0005614: TCRU_6095 TcCLB-EL.508465.130_mRNA-p1 TcCLB-NE.
507001.60_mRNA-p1
OG0005615: TCRU_6096 TcCLB-EL.503557.40_mRNA-p1 TcCLB-NE.
507001.14_mRNA-p1
OG0005616: TCRU_6098 TcCLB-EL.506869.54_mRNA-p1 TcCLB-NE.
510519.50_mRNA-p1
OG0005617: TCRU_6101 TcCLB-NE.510303.210_mRNA-p1 TcSYL_0079010.t1-p1
OG0005618: TCRU_subunit TcCLB-EL.511145.60_mRNA-p1 TcCLB-NE.
504069.80_mRNA-p1
OG0005619: TCRU_6105 TcCLB-EL.511145.40_mRNA-p1 TcCLB-NE.
504069.50_mRNA-p1
OG0005620: TCRU_6106 TcCLB-EL.416883.18_mRNA-p1 TcCLB-NE.
504069.10_mRNA-p1
OG0005621: TCRU_6117 TcCLB-EL.509767.44_mRNA-p1 TcCLB-NE.
509601.44_mRNA-p1
OG0005622: TCRU_6121 TcCLB-EL.506357.190_mRNA-p1 TcCLB-NE.
508891.40_mRNA-p1
OG0005623: TCRU_6124 TcCLB-EL.510719.260_mRNA-p1 TcCLB-NE.
506275.20_mRNA-p1
OG0005624: TCRU_6125 TcCLB-EL.510719.210_mRNA-p1 TcCLB-NE.
509745.40_mRNA-p1
OG0005625: TCRU_6126 TcCLB-EL.510719.180_mRNA-p1 TcCLB-NE.
509745.70_mRNA-p1
OG0005626: TCRU_6127 TcCLB-EL.510089.39_mRNA-p1 TcCLB-NE.
510421.29_mRNA-p1
OG0005627: TCRU_6131 TcCLB-EL.509951.30_mRNA-p1 TcCLB-NE.
507583.4_mRNA-p1
OG0005628: TCRU_6133 TcCLB-EL.507711.280_mRNA-p1 TcCLB-NE.
509643.50_mRNA-p1
OG0005629: TCRU_6134 TcCLB-EL.506677.4_mRNA-p1 TcCLB-NE.
506583.20_mRNA-p1
OG0005630: TCRU_6135 TcCLB-EL.510291.40_mRNA-p1 TcCLB-NE.
508659.50_mRNA-p1

OG0005631: TCRU_6139 TcCLB-NE.511003.120_mRNA-p1 TcSYL_0046250.t1-p1
OG0005632: TCRU_6143 TcCLB-EL.508209.50_mRNA-p1 TcCLB-NE.
503919.20_mRNA-p1
OG0005633: TCRU_6144 TcCLB-NE.511367.70_mRNA-p1 TcSYL_0170070.t1-p1
OG0005634: TCRU_6145 TcCLB-NE.511367.129_mRNA-p1 TcSYL_0170040.t1-p1
OG0005635: TCRU_protein_ TcCLB-EL.507603.130_mRNA-p1 TcCLB-NE.
509429.200_mRNA-p1
OG0005636: TCRU_subunit_ TcCLB-EL.507603.40_mRNA-p1 TcCLB-NE.
509429.110_mRNA-p1
OG0005637: TCRU_6156 TcCLB-EL.507669.80_mRNA-p1 TcCLB-NE.
507857.50_mRNA-p1
OG0005638: TCRU_6158 TcCLB-EL.507669.90_mRNA-p1 TcCLB-NE.
507857.30_mRNA-p1
OG0005639: TCRU_6160 TcCLB-EL.507669.100_mRNA-p1 TcCLB-NE.
507857.10_mRNA-p1
OG0005640: TCRU_6161 TcCLB-EL.507669.104_mRNA-p1 TcCLB-NE.
507857.4_mRNA-p1
OG0005641: TCRU_6162 TcCLB-NE.508305.9_mRNA-p1 TcSYL_0068160.t1-p1
OG0005642: TCRU_6164 TcCLB-NE.508547.190_mRNA-p1 TcSYL_0202420.t1-p1
OG0005643: TCRU_6165 TcCLB-EL.508831.30_mRNA-p1 TcCLB-NE.
452895.10_mRNA-p1
OG0005644: TCRU_6166 TcCLB-EL.508831.40_mRNA-p1 TcCLB-NE.
511673.9_mRNA-p1
OG0005645: TCRU_6169 TcSYL_0123370.t1-p1 TcSYL_0123420.t1-p1
OG0005646: TCRU_6186 TcCLB-NE.503679.40_mRNA-p1 TcSYL_0131280.t1-p1
OG0005647: TCRU_6193 TcCLB-NE.511727.40_mRNA-p1 TcSYL_0142720.t1-p1
OG0005648: TCRU_6194 TcCLB-NE.511727.30_mRNA-p1 TcSYL_0142710.t1-p1
OG0005649: TCRU_6196 TcCLB-EL.511073.10_mRNA-p1 TcCLB-NE.
426435.9_mRNA-p1
OG0005650: TCRU_6206 TcCLB-EL.506773.150_mRNA-p1 TcCLB-NE.
508799.50_mRNA-p1
OG0005651: TCRU_6207 TcCLB-EL.506773.160_mRNA-p1 TcCLB-NE.
508799.40_mRNA-p1
OG0005652: TCRU_6209 TcCLB-EL.506773.180_mRNA-p1 TcCLB-NE.
508799.20_mRNA-p1
OG0005653: TCRU_6210 TcCLB-NE.508799.10_mRNA-p1 TcSYL_0075340.t1-p1
OG0005654: TCRU_6215 TcCLB-NE.506679.160_mRNA-p1 TcSYL_0114040.t1-p1
OG0005655: TCRU_6219 TcSYL_0203700.t1-p1 TcSYL_0204840.t1-p1
OG0005656: TCRU_6220 TcCLB-EL.506211.150_mRNA-p1 TcCLB-NE.
511289.80_mRNA-p1
OG0005657: TCRU_6224 TcCLB-EL.506211.190_mRNA-p1 TcCLB-NE.
511289.40_mRNA-p1
OG0005658: TCRU_6232 TcCLB-EL.510769.70_mRNA-p1 TcCLB-NE.
506241.150_mRNA-p1
OG0005659: TCRU_6236 TcCLB-EL.510769.110_mRNA-p1 TcCLB-NE.
506241.80_mRNA-p1
OG0005660: TCRU_6240 TcCLB-EL.510769.150_mRNA-p1 TcCLB-NE.
506241.40_mRNA-p1
OG0005661: TCRU_6251 TcCLB-NE.510187.320_mRNA-p1 TcSYL_0011530.t1-p1
OG0005662: TCRU_6253 TcCLB-NE.510187.340_mRNA-p1 TcSYL_0011520.t1-p1
OG0005663: TCRU_6255 TcCLB-NE.510187.370_mRNA-p1 TcSYL_0011500.t1-p1
OG0005664: TCRU_6256 TcCLB-NE.510187.390_mRNA-p1 TcSYL_0011490.t1-p1
OG0005665: TCRU_6273 TcCLB-EL.399033.10_mRNA-p1 TcCLB-NE.
508737.40_mRNA-p1

OG0005666: TCRU_RNA TcCLB-EL.429257.10_mRNA-p1 TcCLB-NE.
508737.50_mRNA-p1
OG0005667: TCRU_6282 TcCLB-EL.506363.80_mRNA-p1 TcCLB-NE.
506975.34_mRNA-p1
OG0005668: TCRU_6287 TcCLB-EL.486901.10_mRNA-p1 TcCLB-NE.
506679.280_mRNA-p1
OG0005669: TCRU_6309 TcCLB-EL.509157.210_mRNA-p1 TcCLB-NE.
510725.30_mRNA-p1
OG0005670: TCRU_6310 TcCLB-EL.509157.230_mRNA-p1 TcCLB-NE.
510725.50_mRNA-p1
OG0005671: TCRU_6311 TcCLB-EL.509157.250_mRNA-p1 TcCLB-NE.
510725.70_mRNA-p1
OG0005672: TCRU_6312 TcCLB-EL.509157.260_mRNA-p1 TcCLB-NE.
510725.80_mRNA-p1
OG0005673: TCRU_6313 TcCLB-EL.509157.270_mRNA-p1 TcCLB-NE.
510725.90_mRNA-p1
OG0005674: TCRU_6316 TcCLB-EL.506753.220_mRNA-p1 TcCLB-NE.
510357.120_mRNA-p1
OG0005675: TCRU_6317 TcCLB-EL.506753.210_mRNA-p1 TcCLB-NE.
510357.110_mRNA-p1
OG0005676: TCRU_6318 TcCLB-EL.506753.200_mRNA-p1 TcCLB-NE.
510357.100_mRNA-p1
OG0005677: TCRU_6336 TcCLB-EL.511407.20_mRNA-p1 TcCLB-NE.
509567.50_mRNA-p1
OG0005678: TCRU_6341 TcCLB-EL.503897.30_mRNA-p1 TcCLB-NE.
509563.40_mRNA-p1
OG0005679: TCRU_6350 TcCLB-EL.503897.130_mRNA-p1 TcCLB-NE.
509561.60_mRNA-p1
OG0005680: TCRU_6351 TcCLB-EL.503897.150_mRNA-p1 TcCLB-NE.
509561.39_mRNA-p1
OG0005681: TCRU_6352 TcCLB-EL.503897.170_mRNA-p1 TcCLB-NE.
509561.30_mRNA-p1
OG0005682: TCRU_6355 TcCLB-EL.511179.100_mRNA-p1 TcCLB-NE.
508303.65_mRNA-p1
OG0005683: TCRU_6356 TcCLB-EL.511179.114_mRNA-p1 TcCLB-NE.
508303.44_mRNA-p1
OG0005684: TCRU_6357 TcCLB-EL.511179.120_mRNA-p1 TcCLB-NE.
508303.40_mRNA-p1
OG0005685: TCRU_6359 TcCLB-EL.511179.160_mRNA-p1 TcCLB-NE.
508303.4_mRNA-p1
OG0005686: TCRU_6396 TcCLB-EL.509769.110_mRNA-p1 TcSYL_0174810.t1-p1
OG0005687: TCRU_6397 TcCLB-EL.509769.120_mRNA-p1 TcSYL_0174800.t1-p1
OG0005688: TCRU_6398 TcCLB-EL.509769.130_mRNA-p1 TcSYL_0174780.t1-p1
OG0005689: TCRU_6404 TcCLB-EL.507993.330_mRNA-p1 TcCLB-NE.
511281.40_mRNA-p1
OG0005690: TCRU_6405 TcCLB-EL.507993.320_mRNA-p1 TcCLB-NE.
511281.30_mRNA-p1
OG0005691: TCRU_6406 TcCLB-EL.507993.310_mRNA-p1 TcCLB-NE.
511281.20_mRNA-p1
OG0005692: TCRU_6407 TcCLB-EL.507993.300_mRNA-p1 TcCLB-NE.
511281.10_mRNA-p1
OG0005693: TCRU_6408 TcCLB-EL.507993.280_mRNA-p1 TcCLB-NE.
508893.20_mRNA-p1
OG0005694: TCRU_6409 TcCLB-EL.507993.270_mRNA-p1 TcCLB-NE.

508893.10_mRNA-p1
OG0005695: TCRU_6412 TcCLB-EL.506213.110_mRNA-p1 TcCLB-NE.
511287.4_mRNA-p1
OG0005696: TCRU_6416 TcCLB-EL.506213.70_mRNA-p1 TcCLB-NE.
511287.34_mRNA-p1
OG0005697: TCRU_6437 TcCLB-EL.503959.78_mRNA-p1 TcCLB-NE.
506195.290_mRNA-p1
OG0005698: TCRU_6438 TcCLB-EL.504119.29_mRNA-p1 TcCLB-NE.
506195.300_mRNA-p1
OG0005699: TCRU_6439 TcCLB-EL.504119.20_mRNA-p1 TcCLB-NE.
506195.309_mRNA-p1
OG0005700: TCRU_6440 TcCLB-EL.504119.10_mRNA-p1 TcCLB-NE.
504105.20_mRNA-p1
OG0005701: TCRU_6441 TcCLB-EL.504119.4_mRNA-p1 TcCLB-NE.
504105.30_mRNA-p1
OG0005702: TCRU_6454 TcCLB-EL.503697.120_mRNA-p1 TcCLB-NE.
504105.180_mRNA-p1
OG0005703: TCRU_6455 TcCLB-EL.503697.110_mRNA-p1 TcCLB-NE.
504105.190_mRNA-p1
OG0005704: TCRU_6456 TcCLB-EL.503697.100_mRNA-p1 TcCLB-NE.
504105.200_mRNA-p1
OG0005705: TCRU_6457 TcCLB-EL.503697.90_mRNA-p1 TcCLB-NE.
504105.210_mRNA-p1
OG0005706: TCRU_6458 TcCLB-EL.503697.70_mRNA-p1 TcCLB-NE.
504105.220_mRNA-p1
OG0005707: TCRU_6470 TcCLB-EL.511577.60_mRNA-p1 TcCLB-NE.
504797.70_mRNA-p1
OG0005708: TCRU_6474 TcCLB-EL.511577.86_mRNA-p1 TcCLB-NE.
504797.34_mRNA-p1
OG0005709: TCRU_6475 TcCLB-EL.511577.90_mRNA-p1 TcCLB-NE.
504797.30_mRNA-p1
OG0005710: TCRU_6476 TcCLB-EL.511577.100_mRNA-p1 TcCLB-NE.
504797.20_mRNA-p1
OG0005711: TCRU_6477 TcCLB-EL.511577.110_mRNA-p1 TcCLB-NE.
504797.10_mRNA-p1
OG0005712: TCRU_6486 TcCLB-NE.510187.150_mRNA-p1 TcSYL_0011620.t1-p1
OG0005713: TCRU_6489 TcCLB-EL.509099.110_mRNA-p1 TcCLB-NE.
509319.20_mRNA-p1
OG0005714: TCRU_6491 TcCLB-EL.509099.89_mRNA-p1 TcCLB-NE.
509319.40_mRNA-p1
OG0005715: TCRU_subunit TcCLB-EL.510257.100_mRNA-p1 TcCLB-NE.
509213.140_mRNA-p1
OG0005716: TCRU_6507 TcCLB-EL.510259.12_mRNA-p1 TcCLB-NE.
509215.13_mRNA-p1
OG0005717: TCRU_6508 TcCLB-EL.510259.18_mRNA-p1 TcCLB-NE.
509215.16_mRNA-p1
OG0005718: TCRU_6509 TcCLB-EL.510259.24_mRNA-p1 TcCLB-NE.
509215.19_mRNA-p1
OG0005719: TCRU_6510 TcCLB-EL.510259.40_mRNA-p1 TcCLB-NE.
509215.30_mRNA-p1
OG0005720: TCRU_6517 TcCLB-EL.503715.40_mRNA-p1 TcCLB-NE.
509607.50_mRNA-p1
OG0005721: TCRU_6518 TcCLB-EL.510861.140_mRNA-p1 TcCLB-NE.
509607.70_mRNA-p1

OG0005722: TCRU_6529 TcCLB-EL.509099.140_mRNA-p1 TcCLB-NE.
507209.50_mRNA-p1
OG0005723: TCRU_6533 TcCLB-NE.507209.10_mRNA-p1 TcSYL_0084920.t1-p1
OG0005724: TCRU_10425 TCRU_6544 TcSYL_0161150.t1-p1
OG0005725: TCRU_6548 TcCLB-NE.430603.10_pseudogenic_transcript-p1
TcCLB-NE.509525.432_pseudogenic_transcript-p1
OG0005726: TCRU_6553 TcCLB-NE.507069.60_mRNA-p1 TcCLB-NE.
508433.145_pseudogenic_transcript-p1
OG0005727: TCRU_6560 TcCLB-EL.511355.14_mRNA-p1 TcCLB-NE.
503809.140_mRNA-p1
OG0005728: TCRU_6561 TcCLB-EL.511355.20_mRNA-p1 TcCLB-NE.
503809.130_mRNA-p1
OG0005729: TCRU_6572 TcCLB-EL.507867.60_mRNA-p1 TcCLB-NE.
510629.420_mRNA-p1
OG0005730: TCRU_6573 TcCLB-EL.507867.80_mRNA-p1 TcCLB-NE.
510629.440_mRNA-p1
OG0005731: TCRU_6574 TcCLB-EL.507867.100_mRNA-p1 TcCLB-NE.
510629.460_mRNA-p1
OG0005732: TCRU_6575 TcCLB-EL.509885.10_mRNA-p1 TcCLB-NE.
508397.10_mRNA-p1
OG0005733: TCRU_6576 TcCLB-EL.509885.20_mRNA-p1 TcCLB-NE.
508397.20_mRNA-p1
OG0005734: TCRU_6577 TcCLB-EL.509885.30_mRNA-p1 TcCLB-NE.
508397.30_mRNA-p1
OG0005735: TCRU_6578 TcCLB-EL.509885.40_mRNA-p1 TcCLB-NE.
508397.40_mRNA-p1
OG0005736: TCRU_6579 TcCLB-EL.509885.50_mRNA-p1 TcCLB-NE.
508397.50_mRNA-p1
OG0005737: TCRU_6580 TcCLB-EL.509885.60_mRNA-p1 TcCLB-NE.
508397.60_mRNA-p1
OG0005738: TCRU_6581 TcCLB-EL.509885.70_mRNA-p1 TcCLB-NE.
508397.70_mRNA-p1
OG0005739: TCRU_6582 TcCLB-EL.509885.80_mRNA-p1 TcCLB-NE.
508397.79_mRNA-p1
OG0005740: TCRU_6583 TcCLB-EL.507869.10_mRNA-p1 TcCLB-NE.
508399.10_mRNA-p1
OG0005741: TCRU_6586 TcCLB-EL.506265.10_mRNA-p1 TcCLB-NE.
506435.10_mRNA-p1
OG0005742: TCRU_6591 TcCLB-EL.507237.120_mRNA-p1 TcCLB-EL.
511613.90_mRNA-p1
OG0005743: TCRU_6596 TcCLB-EL.510621.20_mRNA-p1 TcCLB-EL.
511785.10_mRNA-p1
OG0005744: TCRU_6599 TcCLB-NE.511727.140_mRNA-p1 TcSYL_0142770.t1-p1
OG0005745: TCRU_6601 TcCLB-NE.511727.120_mRNA-p1 TcSYL_0142750.t1-p1
OG0005746: TCRU_6602 TcCLB-NE.511727.100_mRNA-p1 TcSYL_0142740.t1-p1
OG0005747: TCRU_6609 TCRU_7872 TcCLB-NE.
508523.10_pseudogenic_transcript-p1
OG0005748: TCRU_6613 TcCLB-NE.508909.230_pseudogenic_transcript-p1
TcSYL_0159430.t1-p1
OG0005749: TCRU_6616 TcCLB-EL.506517.110_mRNA-p1 TcCLB-NE.
508909.260_mRNA-p1
OG0005750: TCRU_6617 TcCLB-EL.506517.100_mRNA-p1 TcCLB-NE.
508909.270_mRNA-p1
OG0005751: TCRU_6621 TcCLB-EL.506517.40_mRNA-p1 TcCLB-NE.

508909.310_mRNA-p1
OG0005752: TCRU_6629 TcCLB-NE.504045.80_mRNA-p1 TcSYL_0019500.t1-p1
OG0005753: TCRU_6632 TcCLB-NE.504045.10_mRNA-p1 TcSYL_0019470.t1-p1
OG0005754: TCRU_6636 TcCLB-EL.511211.180_mRNA-p1 TcCLB-NE.
510439.50_mRNA-p1
OG0005755: TCRU_6637 TcCLB-EL.511211.190_mRNA-p1 TcCLB-NE.
510439.40_mRNA-p1
OG0005756: TCRU_6638 TcCLB-EL.511211.200_mRNA-p1 TcCLB-NE.
510439.30_mRNA-p1
OG0005757: TCRU_6639 TcCLB-EL.511211.220_mRNA-p1 TcCLB-NE.
510439.10_mRNA-p1
OG0005758: TCRU_6640 TcCLB-EL.511211.235_mRNA-p1 TcCLB-NE.
510437.60_mRNA-p1
OG0005759: TCRU_6641 TcCLB-EL.511211.240_mRNA-p1 TcCLB-NE.
510437.50_mRNA-p1
OG0005760: TCRU_6642 TcCLB-EL.506937.10_mRNA-p1 TcCLB-NE.
510437.40_mRNA-p1
OG0005761: TCRU_6643 TcCLB-EL.506937.20_mRNA-p1 TcCLB-NE.
510437.30_mRNA-p1
OG0005762: TCRU_6648 TcCLB-EL.508839.70_mRNA-p1 TcCLB-NE.
511381.60_mRNA-p1
OG0005763: TCRU_6657 TcCLB-EL.510755.138_mRNA-p1 TcCLB-NE.
508413.40_mRNA-p1
OG0005764: TCRU_6663 TcCLB-EL.510759.70_mRNA-p1 TcCLB-NE.
506999.140_mRNA-p1
OG0005765: TCRU_6670 TcCLB-EL.507089.100_mRNA-p1 TcCLB-NE.
506725.80_mRNA-p1
OG0005766: TCRU_6671 TcCLB-EL.507089.110_mRNA-p1 TcCLB-NE.
506725.70_mRNA-p1
OG0005767: TCRU_6672 TcCLB-EL.507089.120_mRNA-p1 TcCLB-NE.
506725.60_mRNA-p1
OG0005768: TCRU_6674 TcCLB-EL.507089.140_mRNA-p1 TcCLB-NE.
506725.40_mRNA-p1
OG0005769: TCRU_6677 TcCLB-EL.507089.210_mRNA-p1 TcCLB-NE.
503839.10_mRNA-p1
OG0005770: TCRU_6681 TcCLB-EL.506367.20_mRNA-p1 TcCLB-NE.
506629.210_mRNA-p1
OG0005771: TCRU_6683 TcCLB-EL.506367.40_mRNA-p1 TcCLB-NE.
506629.190_mRNA-p1
OG0005772: TCRU_6684 TcCLB-EL.506367.50_mRNA-p1 TcCLB-NE.
506629.180_mRNA-p1
OG0005773: TCRU_6686 TCRU_6687 TcSYL_0134380.t1-p1
OG0005774: TCRU_6693 TcCLB-EL.504213.140_mRNA-p1 TcCLB-NE.
506985.10_mRNA-p1
OG0005775: TCRU_6696 TcCLB-EL.506605.50_mRNA-p1 TcCLB-NE.
511239.40_mRNA-p1
OG0005776: TCRU_6708 TcCLB-EL.506847.70_mRNA-p1 TcCLB-NE.
507849.40_mRNA-p1
OG0005777: TCRU_6709 TcCLB-EL.508317.20_mRNA-p1 TcCLB-NE.
507849.60_mRNA-p1
OG0005778: TCRU_6710 TcCLB-EL.508317.30_mRNA-p1 TcCLB-NE.
507849.70_mRNA-p1
OG0005779: TCRU_6711 TcCLB-EL.508317.50_mRNA-p1 TcCLB-NE.
509849.10_mRNA-p1

OG0005780: TCRU_6712 TcSYL_0079750.t1-p1 TcSYL_0164030.t1-p1
OG0005781: TCRU_6719 TcCLB-EL.503955.89_mRNA-p1 TcSYL_0013650.t1-p1
OG0005782: TCRU_6721 TcCLB-EL.506401.24_mRNA-p1 TcCLB-NE.509605.20_mRNA-p1
OG0005783: TCRU_6722 TcCLB-EL.506401.70_mRNA-p1 TcCLB-NE.503727.29_mRNA-p1
OG0005784: TCRU_protein TcCLB-EL.511109.110_mRNA-p1 TcCLB-NE.504157.30_mRNA-p1
OG0005785: TCRU_6743 TcCLB-EL.510257.70_mRNA-p1 TcCLB-NE.509213.110_mRNA-p1
OG0005786: TCRU_6745 TcCLB-EL.510257.50_mRNA-p1 TcCLB-NE.509213.90_mRNA-p1
OG0005787: TCRU_6746 TcCLB-EL.510257.40_mRNA-p1 TcCLB-NE.509213.80_mRNA-p1
OG0005788: TCRU_6748 TcCLB-EL.510257.24_mRNA-p1 TcCLB-NE.509213.60_mRNA-p1
OG0005789: TCRU_6749 TcCLB-EL.510257.20_mRNA-p1 TcCLB-NE.509213.50_mRNA-p1
OG0005790: TCRU_6751 TcCLB-EL.510255.20_mRNA-p1 TcCLB-NE.509213.30_mRNA-p1
OG0005791: TCRU_6753 TcCLB-EL.510253.20_mRNA-p1 TcCLB-NE.504243.49_mRNA-p1
OG0005792: TCRU_6756 TcCLB-EL.508145.40_mRNA-p1 TcCLB-NE.504243.20_mRNA-p1
OG0005793: TCRU_6758 TcCLB-EL.510743.20_mRNA-p1 TcCLB-NE.510659.279_mRNA-p1
OG0005794: TCRU_6759 TcCLB-EL.510743.30_mRNA-p1 TcCLB-NE.510659.270_mRNA-p1
OG0005795: TCRU_6760 TcCLB-EL.510743.40_mRNA-p1 TcCLB-NE.510659.264_mRNA-p1
OG0005796: TCRU_6762 TcCLB-EL.510743.80_mRNA-p1 TcCLB-NE.510659.230_mRNA-p1
OG0005797: TCRU_6766 TcCLB-EL.510743.120_mRNA-p1 TcCLB-NE.510659.180_mRNA-p1
OG0005798: TCRU_6769 TcCLB-EL.507013.24_mRNA-p1 TcCLB-NE.510659.104_mRNA-p1
OG0005799: TCRU_6770 TcCLB-EL.507013.30_mRNA-p1 TcCLB-NE.510659.100_mRNA-p1
OG0005800: TCRU_6771 TcCLB-EL.510745.10_mRNA-p1 TcCLB-NE.510659.90_mRNA-p1
OG0005801: TCRU_6773 TcCLB-EL.510745.30_mRNA-p1 TcCLB-NE.510659.74_mRNA-p1
OG0005802: TCRU_6775 TcCLB-NE.510659.50_mRNA-p1 TcSYL_0116050.t1-p1
OG0005803: TCRU_6776 TcCLB-EL.508465.10_mRNA-p1 TcCLB-NE.510659.44_mRNA-p1
OG0005804: TCRU_6777 TcCLB-EL.508465.20_mRNA-p1 TcCLB-NE.510659.36_mRNA-p1
OG0005805: TCRU_6779 TcCLB-EL.508465.80_mRNA-p1 TcCLB-NE.507001.120_mRNA-p1
OG0005806: TCRU_6781 TcCLB-EL.508465.100_mRNA-p1 TcCLB-NE.507001.100_mRNA-p1
OG0005807: TCRU_6790 TcCLB-EL.509825.7_mRNA-p1 TcCLB-NE.510425.28_mRNA-p1
OG0005808: TCRU_6798 TcCLB-EL.508607.70_pseudogenic_transcript-p1

TcCLB-NE.508963.40_pseudogenic_transcript-p1
OG0005809: TCRU_6823 TcCLB-EL.510953.40_mRNA-p1 TcCLB-NE.509005.60_mRNA-p1
OG0005810: TCRU_6825 TcCLB-EL.510955.10_mRNA-p1 TcCLB-NE.509005.40_mRNA-p1
OG0005811: TCRU_6826 TcCLB-EL.510955.20_mRNA-p1 TcCLB-NE.509005.30_mRNA-p1
OG0005812: TCRU_6827 TcCLB-EL.510955.30_mRNA-p1 TcCLB-NE.509005.20_mRNA-p1
OG0005813: TCRU_6829 TcCLB-EL.510955.50_mRNA-p1 TcCLB-NE.503869.20_mRNA-p1
OG0005814: TCRU_6830 TcCLB-EL.510955.60_mRNA-p1 TcCLB-NE.503869.30_mRNA-p1
OG0005815: TCRU_6832 TcCLB-EL.506563.20_mRNA-p1 TcCLB-NE.503869.50_mRNA-p1
OG0005816: TCRU_6842 TcCLB-EL.506647.10_mRNA-p1 TcCLB-NE.510575.40_mRNA-p1
OG0005817: TCRU_6844 TcCLB-EL.506649.4_mRNA-p1 TcCLB-NE.510575.20_mRNA-p1
OG0005818: TCRU_6845 TcCLB-EL.506649.10_mRNA-p1 TcCLB-NE.510575.10_mRNA-p1
OG0005819: TCRU_6853 TcCLB-EL.510767.30_mRNA-p1 TcCLB-NE.509671.60_mRNA-p1
OG0005820: TCRU_6862 TcCLB-NE.510945.30_mRNA-p1 TcSYL_0048180.t1-p1
OG0005821: TCRU_6865 TcCLB-EL.419703.10_mRNA-p1 TcCLB-NE.504131.10_pseudogenic_transcript-p1
OG0005822: TCRU_6871 TcCLB-EL.503981.80_mRNA-p1 TcCLB-NE.504131.140_mRNA-p1
OG0005823: TCRU_6873 TcCLB-EL.504125.30_mRNA-p1 TcCLB-NE.504131.170_mRNA-p1
OG0005824: TCRU_6876 TcCLB-EL.507559.20_mRNA-p1 TcCLB-NE.510303.119_mRNA-p1
OG0005825: TCRU_6877 TcCLB-EL.507559.30_mRNA-p1 TcCLB-NE.510303.100_mRNA-p1
OG0005826: TCRU_6878 TcCLB-EL.507559.50_mRNA-p1 TcCLB-NE.510303.80_mRNA-p1
OG0005827: TCRU_6884 TcCLB-EL.505945.80_mRNA-p1 TcCLB-NE.506149.20_mRNA-p1
OG0005828: TCRU_6890 TcCLB-EL.509475.10_mRNA-p1 TcCLB-NE.509553.60_mRNA-p1
OG0005829: TCRU_6900 TcCLB-EL.429185.31_pseudogenic_transcript-p1 TcSYL_0191710.t1-p1
OG0005830: TCRU_6915 TcCLB-EL.511127.290_mRNA-p1 TcCLB-NE.509027.40_mRNA-p1
OG0005831: TCRU_6917 TcCLB-EL.511127.274_mRNA-p1 TcCLB-NE.509027.24_mRNA-p1
OG0005832: TCRU_6928 TcCLB-NE.511159.30_mRNA-p1 TcSYL_0076520.t1-p1
OG0005833: TCRU_6936 TcCLB-EL.506777.120_mRNA-p1 TcCLB-NE.504113.10_mRNA-p1
OG0005834: TCRU_6939 TcCLB-EL.506777.90_mRNA-p1 TcCLB-NE.504113.41_mRNA-p1
OG0005835: TCRU_6944 TcCLB-EL.508439.10_mRNA-p1 TcCLB-NE.506275.80_mRNA-p1
OG0005836: TCRU_6945 TcCLB-EL.510719.310_mRNA-p1 TcCLB-NE.

506275.70_mRNA-p1
OG0005837: TCRU_6946 TcCLB-EL.510719.300_mRNA-p1 TcCLB-NE.
506275.60_mRNA-p1
OG0005838: TCRU_6947 TcCLB-EL.510719.290_mRNA-p1 TcCLB-NE.
506275.50_mRNA-p1
OG0005839: TCRU_6948 TcCLB-EL.510719.280_mRNA-p1 TcCLB-NE.
506275.40_mRNA-p1
OG0005840: TCRU_6949 TcCLB-EL.510719.270_mRNA-p1 TcCLB-NE.
506275.30_mRNA-p1
OG0005841: TCRU_6975 TcCLB-EL.509599.70_mRNA-p1 TcCLB-NE.
509733.50_mRNA-p1
OG0005842: TCRU_6976 TcCLB-EL.509599.60_mRNA-p1 TcCLB-NE.
509733.60_mRNA-p1
OG0005843: TCRU_6977 TcCLB-EL.509599.50_mRNA-p1 TcCLB-NE.
509733.70_mRNA-p1
OG0005844: TCRU_6982 TcCLB-NE.509733.130_mRNA-p1 TcSYL_0010750.t1-p1
OG0005845: TCRU_6985 TcCLB-NE.509733.150_mRNA-p1 TcSYL_0010740.t1-p1
OG0005846: TCRU_6986 TcCLB-NE.509733.160_mRNA-p1 TcSYL_0010730.t1-p1
OG0005847: TCRU_6987 TcCLB-NE.509733.170_mRNA-p1 TcSYL_0010700.t1-p1
OG0005848: TCRU_6988 TcCLB-NE.509733.180_mRNA-p1 TcSYL_0010690.t1-p1
OG0005849: TCRU_6990 TcCLB-EL.511541.9_mRNA-p1 TcCLB-NE.
508707.300_mRNA-p1
OG0005850: TCRU_6991 TcCLB-EL.511541.30_mRNA-p1 TcCLB-NE.
508707.280_mRNA-p1
OG0005851: TCRU_6992 TcCLB-EL.511541.40_mRNA-p1 TcCLB-NE.
508707.270_mRNA-p1
OG0005852: TCRU_6993 TcCLB-NE.432629.30_mRNA-p1 TcSYL_0164090.t1-p1
OG0005853: TCRU_6997 TcCLB-EL.506529.660_mRNA-p1 TcCLB-NE.
510889.330_mRNA-p1
OG0005854: TCRU_6998 TcCLB-EL.506529.650_mRNA-p1 TcCLB-NE.
510889.320_mRNA-p1
OG0005855: TCRU_7000 TcCLB-EL.506529.610_mRNA-p1 TcCLB-NE.
510889.290_mRNA-p1
OG0005856: TCRU_7002 TcCLB-EL.508209.80_mRNA-p1 TcCLB-NE.
509505.80_mRNA-p1
OG0005857: TCRU_7013 TcCLB-EL.507993.260_mRNA-p1 TcCLB-NE.
511279.60_mRNA-p1
OG0005858: TCRU_7014 TcCLB-EL.507993.230_pseudogenic_transcript-p1
TcCLB-NE.511279.40_mRNA-p1
OG0005859: TCRU_7015 TcCLB-EL.507993.220_mRNA-p1 TcCLB-NE.
511279.30_mRNA-p1
OG0005860: TCRU_7016 TcCLB-EL.507993.210_mRNA-p1 TcCLB-NE.
511279.20_mRNA-p1
OG0005861: TCRU_7017 TcCLB-EL.507993.190_mRNA-p1 TcCLB-NE.
511277.630_mRNA-p1
OG0005862: TCRU_7018 TcCLB-EL.507993.180_mRNA-p1 TcCLB-NE.
511277.620_mRNA-p1
OG0005863: TCRU_7019 TcCLB-EL.510719.170_mRNA-p1 TcCLB-NE.
509747.10_mRNA-p1
OG0005864: TCRU_7025 TcCLB-NE.511295.30_mRNA-p1 TcSYL_0121900.t1-p1
OG0005865: TCRU_7028 TcCLB-EL.506481.30_mRNA-p1 TcCLB-NE.
511295.60_pseudogenic_transcript-p1
OG0005866: TCRU_7032 TcCLB-EL.507277.30_mRNA-p1 TcCLB-NE.
510007.40_mRNA-p1

OG0005867: TCRU_7033 TcCLB-EL.507277.20_mRNA-p1 TcCLB-NE.
510007.50_mRNA-p1
OG0005868: TCRU_7034 TcCLB-EL.509895.70_mRNA-p1 TcCLB-NE.
510009.10_mRNA-p1
OG0005869: TCRU_7038 TcCLB-EL.506513.40_mRNA-p1 TcCLB-NE.
508919.80_mRNA-p1
OG0005870: TCRU_7040 TcCLB-EL.506513.60_mRNA-p1 TcCLB-NE.
508919.60_mRNA-p1
OG0005871: TCRU_7044 TcCLB-EL.506513.100_mRNA-p1 TcCLB-NE.
508919.20_mRNA-p1
OG0005872: TCRU_7054 TcCLB-EL.419469.30_mRNA-p1 TcCLB-NE.
507771.20_mRNA-p1
OG0005873: TCRU_7055 TcCLB-EL.503887.79_mRNA-p1 TcCLB-NE.
507771.30_mRNA-p1
OG0005874: TCRU_7058 TcCLB-EL.503887.50_mRNA-p1 TcCLB-NE.
507771.60_mRNA-p1
OG0005875: TCRU_7063 TcCLB-EL.507875.10_mRNA-p1 TcCLB-NE.
508111.40_mRNA-p1
OG0005876: TCRU_7067 TcCLB-EL.508207.220_mRNA-p1 TcCLB-NE.
509509.40_mRNA-p1
OG0005877: TCRU_7068 TcCLB-EL.508207.210_mRNA-p1 TcCLB-NE.
509509.50_mRNA-p1
OG0005878: TCRU_7071 TcCLB-EL.508207.190_mRNA-p1 TcCLB-NE.
507519.20_mRNA-p1
OG0005879: TCRU_7073 TcCLB-EL.508207.170_mRNA-p1 TcCLB-NE.
507519.40_mRNA-p1
OG0005880: TCRU_7074 TcCLB-EL.508207.160_mRNA-p1 TcCLB-NE.
507519.50_mRNA-p1
OG0005881: TCRU_7075 TcCLB-EL.508207.150_mRNA-p1 TcCLB-NE.
507519.60_mRNA-p1
OG0005882: TCRU_7076 TcCLB-EL.508207.135_mRNA-p1 TcCLB-NE.
507519.80_mRNA-p1
OG0005883: TCRU_7079 TcCLB-EL.508207.80_mRNA-p1 TcCLB-NE.
507519.130_mRNA-p1
OG0005884: TCRU_7081 TcCLB-EL.508207.54_mRNA-p1 TcCLB-NE.
507519.164_mRNA-p1
OG0005885: TCRU_7082 TcCLB-EL.508207.45_mRNA-p1 TcCLB-NE.
507519.170_mRNA-p1
OG0005886: TCRU_7084 TcCLB-NE.511389.10_mRNA-p1 TcSYL_0171420.t1-p1
OG0005887: TCRU_7097 TcCLB-NE.511727.234_mRNA-p1 TcSYL_0089300.t1-p1
OG0005888: TCRU_7099 TcCLB-NE.511727.250_mRNA-p1 TcSYL_0142790.t1-p1
OG0005889: TCRU_7100 TcCLB-NE.511727.260_mRNA-p1 TcSYL_0142800.t1-p1
OG0005890: TCRU_7104 TcCLB-NE.511727.300_mRNA-p1 TcSYL_0142830.t1-p1
OG0005891: TCRU_7110 TcCLB-EL.504153.70_mRNA-p1 TcSYL_0138810.t1-p1
OG0005892: TCRU_7114 TcCLB-EL.503969.10_mRNA-p1 TcCLB-NE.
506807.10_mRNA-p1
OG0005893: TCRU_7141 TcCLB-NE.511003.30_mRNA-p1 TcSYL_0046210.t1-p1
OG0005894: TCRU_7143 TcCLB-EL.510879.200_mRNA-p1 TcSYL_0046020.t1-p1
OG0005895: TCRU_7150 TcCLB-EL.508973.90_mRNA-p1 TcCLB-NE.
508547.70_mRNA-p1
OG0005896: TCRU_7152 TcCLB-EL.508973.60_mRNA-p1 TcCLB-NE.
508547.100_mRNA-p1
OG0005897: TCRU_7155 TcCLB-EL.508973.30_mRNA-p1 TcCLB-NE.
508547.130_mRNA-p1

OG0005898: TCRU_7156 TcCLB-EL.508973.20_mRNA-p1 TcCLB-NE.
508547.140_mRNA-p1
OG0005899: TCRU_7158 TcCLB-NE.508547.160_mRNA-p1 TcSYL_0202460.t1-p1
OG0005900: TCRU_7161 TcCLB-EL.503901.18_mRNA-p1 TcCLB-NE.
509507.20_mRNA-p1
OG0005901: TCRU_7165 TcCLB-EL.503779.30_mRNA-p1 TcCLB-NE.
506247.400_mRNA-p1
OG0005902: TCRU_7166 TcCLB-EL.511303.20_mRNA-p1 TcCLB-NE.
509179.50_mRNA-p1
OG0005903: TCRU_7174 TcCLB-EL.507873.60_mRNA-p1 TcCLB-NE.
510691.10_mRNA-p1
OG0005904: TCRU_7175 TcCLB-EL.507873.50_mRNA-p1 TcCLB-NE.
510691.4_mRNA-p1
OG0005905: TCRU_7176 TcCLB-EL.507873.40_mRNA-p1 TcCLB-NE.
510689.80_mRNA-p1
OG0005906: TCRU_7177 TcCLB-EL.507873.30_mRNA-p1 TcCLB-NE.
510689.60_mRNA-p1
OG0005907: TCRU_7179 TcCLB-NE.510689.30_mRNA-p1 TcSYL_0047200.t1-p1
OG0005908: TCRU_7183 TcCLB-NE.510687.120_mRNA-p1 TcSYL_0047160.t1-p1
OG0005909: TCRU_7189 TcCLB-EL.510603.140_mRNA-p1 TcCLB-NE.
509109.50_mRNA-p1
OG0005910: TCRU_small TcCLB-EL.508837.140_mRNA-p1 TcCLB-NE.
511383.30_mRNA-p1
OG0005911: TCRU_7199 TcCLB-EL.508837.147_mRNA-p1 TcCLB-NE.
511383.20_mRNA-p1
OG0005912: TCRU_7200 TcCLB-EL.508837.154_mRNA-p1 TcCLB-NE.
511383.10_mRNA-p1
OG0005913: TCRU_7201 TcCLB-EL.508837.160_mRNA-p1 TcSYL_0187890.t1-p1
OG0005914: TCRU_7209 TcCLB-NE.510087.90_mRNA-p1 TcSYL_0051800.t1-p1
OG0005915: TCRU_7212 TcCLB-EL.507709.140_mRNA-p1 TcCLB-NE.
506577.170_mRNA-p1
OG0005916: TCRU_7215 TcCLB-EL.507709.100_mRNA-p1 TcCLB-NE.
506577.130_mRNA-p1
OG0005917: TCRU_7216 TcCLB-EL.507709.90_mRNA-p1 TcCLB-NE.
506577.120_mRNA-p1
OG0005918: TCRU_7221 TcCLB-EL.506529.490_mRNA-p1 TcCLB-NE.
510889.200_mRNA-p1
OG0005919: TCRU_7224 TcCLB-EL.511395.14_mRNA-p1 TcCLB-NE.
505071.104_mRNA-p1
OG0005920: TCRU_7227 TcCLB-EL.511395.50_mRNA-p1 TcCLB-NE.
505071.70_mRNA-p1
OG0005921: TCRU_7231 TcCLB-EL.506147.100_mRNA-p1 TcCLB-NE.
508461.4_mRNA-p1
OG0005922: TCRU_7232 TcCLB-EL.506147.110_mRNA-p1 TcCLB-NE.
510735.90_mRNA-p1
OG0005923: TCRU_7233 TcCLB-EL.506147.120_mRNA-p1 TcCLB-NE.
510735.70_mRNA-p1
OG0005924: TCRU_7234 TcCLB-EL.506147.130_mRNA-p1 TcCLB-NE.
510735.60_mRNA-p1
OG0005925: TCRU_7235 TcCLB-EL.506147.140_mRNA-p1 TcCLB-NE.
510735.50_mRNA-p1
OG0005926: TCRU_7236 TcCLB-EL.506147.150_mRNA-p1 TcCLB-NE.
510735.40_mRNA-p1
OG0005927: TCRU_7237 TcCLB-EL.506147.160_mRNA-p1 TcCLB-NE.

510735.20_mRNA-p1
OG0005928: TCRU_7238 TcCLB-EL.506147.184_mRNA-p1 TcCLB-NE.
510733.54_mRNA-p1
OG0005929: TCRU_7240 TcCLB-EL.506147.200_mRNA-p1 TcCLB-NE.
510733.40_mRNA-p1
OG0005930: TCRU_7241 TcCLB-EL.506147.220_mRNA-p1 TcCLB-NE.
510733.20_mRNA-p1
OG0005931: TCRU_7242 TcCLB-EL.509551.10_mRNA-p1 TcCLB-NE.
510733.10_mRNA-p1
OG0005932: TCRU_7243 TcCLB-EL.509551.30_mRNA-p1 TcCLB-NE.
507007.99_pseudogenic_transcript-p1
OG0005933: TCRU_7244 TcCLB-EL.509551.40_mRNA-p1 TcCLB-NE.
507007.90_mRNA-p1
OG0005934: TCRU_7245 TcCLB-EL.509551.50_mRNA-p1 TcCLB-NE.
507007.80_mRNA-p1
OG0005935: TCRU_7247 TcCLB-EL.509551.70_mRNA-p1 TcCLB-NE.
507007.70_mRNA-p1
OG0005936: TCRU_7248 TcCLB-EL.509551.80_mRNA-p1 TcCLB-NE.
507007.60_mRNA-p1
OG0005937: TCRU_7249 TcCLB-EL.509551.90_mRNA-p1 TcCLB-NE.
507007.50_mRNA-p1
OG0005938: TCRU_7250 TcCLB-EL.509551.95_mRNA-p1 TcCLB-NE.
507007.45_mRNA-p1
OG0005939: TCRU_7251 TcCLB-EL.509551.100_mRNA-p1 TcCLB-NE.
507007.40_mRNA-p1
OG0005940: TCRU_7252 TcCLB-EL.509551.110_mRNA-p1 TcCLB-NE.
507007.34_mRNA-p1
OG0005941: TCRU_7253 TcCLB-EL.509551.120_mRNA-p1 TcCLB-NE.
507007.30_mRNA-p1
OG0005942: TCRU_7254 TcCLB-EL.509551.130_mRNA-p1 TcCLB-NE.
507007.20_mRNA-p1
OG0005943: TCRU_7255 TcCLB-EL.509551.140_mRNA-p1 TcCLB-NE.
507007.9_pseudogenic_transcript-p1
OG0005944: TCRU_7258 TcCLB-EL.503669.20_mRNA-p1 TcCLB-NE.
510731.120_mRNA-p1
OG0005945: TCRU_7259 TcCLB-EL.503669.30_mRNA-p1 TcCLB-NE.
510731.110_mRNA-p1
OG0005946: TCRU_7260 TcCLB-EL.503669.39_mRNA-p1 TcCLB-NE.
510731.104_mRNA-p1
OG0005947: TCRU_7261 TcCLB-EL.506887.110_pseudogenic_transcript-p1
TcCLB-NE.510731.100_mRNA-p1
OG0005948: TCRU_7262 TcCLB-EL.506887.100_mRNA-p1 TcCLB-NE.
510731.90_mRNA-p1
OG0005949: TCRU_7263 TcCLB-EL.506887.90_mRNA-p1 TcCLB-NE.
510731.80_mRNA-p1
OG0005950: TCRU_7272 TcCLB-EL.511585.200_mRNA-p1 TcCLB-NE.
510155.190_mRNA-p1
OG0005951: TCRU_7274 TcCLB-EL.511585.180_mRNA-p1 TcCLB-NE.
510155.170_mRNA-p1
OG0005952: TCRU_7278 TcCLB-EL.506773.104_mRNA-p1 TcCLB-NE.
508799.104_mRNA-p1
OG0005953: TCRU_7279 TcCLB-EL.506773.100_mRNA-p1 TcCLB-NE.
508799.110_mRNA-p1
OG0005954: TCRU_7280 TcCLB-EL.506773.90_mRNA-p1 TcCLB-NE.

508799.120_mRNA-p1
OG0005955: TCRU_7285 TcSYL_0039020.t1-p1 TcSYL_0071620.t1-p1
OG0005956: TCRU_7288 TcCLB-EL.511127.340_mRNA-p1 TcCLB-NE.
503657.70_mRNA-p1
OG0005957: TCRU_7289 TcCLB-EL.511127.350_mRNA-p1 TcCLB-NE.
503657.50_mRNA-p1
OG0005958: TCRU_7298 TcCLB-EL.428999.30_mRNA-p1 TcCLB-NE.
511895.20_mRNA-p1
OG0005959: TCRU_7324 TcCLB-EL.509267.100_mRNA-p1 TcCLB-NE.
510031.60_mRNA-p1
OG0005960: TCRU_7326 TcCLB-EL.509267.70_mRNA-p1 TcCLB-NE.
510031.30_mRNA-p1
OG0005961: TCRU_7327 TcCLB-EL.509267.60_mRNA-p1 TcCLB-NE.
510031.20_mRNA-p1
OG0005962: TCRU_7328 TcCLB-EL.509267.50_mRNA-p1 TcCLB-NE.
510031.9_mRNA-p1
OG0005963: TCRU_7329 TcCLB-EL.509267.40_mRNA-p1 TcCLB-NE.
507967.30_mRNA-p1
OG0005964: TCRU_7330 TcCLB-EL.509267.30_mRNA-p1 TcCLB-NE.
507967.20_mRNA-p1
OG0005965: TCRU_7331 TcCLB-EL.509267.20_mRNA-p1 TcCLB-NE.
507967.10_mRNA-p1
OG0005966: TCRU_7333 TcCLB-EL.511705.20_mRNA-p1 TcCLB-NE.
506425.10_mRNA-p1
OG0005967: TCRU_7334 TcCLB-EL.511707.20_mRNA-p1 TcCLB-NE.
506425.20_mRNA-p1
OG0005968: TCRU_7335 TcCLB-EL.511707.30_mRNA-p1 TcCLB-NE.
506425.30_mRNA-p1
OG0005969: TCRU_7336 TcCLB-EL.511707.39_mRNA-p1 TcCLB-NE.
506425.40_mRNA-p1
OG0005970: TCRU_7338 TcCLB-EL.511709.10_mRNA-p1 TcCLB-NE.
506425.70_mRNA-p1
OG0005971: TCRU_7339 TcCLB-EL.511711.10_mRNA-p1 TcCLB-NE.
506425.100_mRNA-p1
OG0005972: TCRU_7340 TcCLB-EL.511711.20_mRNA-p1 TcCLB-NE.
506425.110_mRNA-p1
OG0005973: TCRU_methyltransferase TcCLB-EL.511711.30_mRNA-p1 TcCLB-
NE.506425.120_mRNA-p1
OG0005974: TCRU_7342 TcCLB-EL.511711.40_mRNA-p1 TcCLB-NE.
506425.130_mRNA-p1
OG0005975: TCRU_7343 TcCLB-EL.511711.60_mRNA-p1 TcCLB-NE.
506425.150_mRNA-p1
OG0005976: TCRU_7344 TcCLB-EL.511711.70_mRNA-p1 TcCLB-NE.
506425.160_mRNA-p1
OG0005977: TCRU_7345 TcCLB-EL.511711.80_mRNA-p1 TcCLB-NE.
503987.4_mRNA-p1
OG0005978: TCRU_7346 TcCLB-EL.509231.20_mRNA-p1 TcCLB-NE.
509719.50_mRNA-p1
OG0005979: TCRU_7347 TcCLB-EL.509231.26_mRNA-p1 TcCLB-NE.
509719.56_mRNA-p1
OG0005980: TCRU_7348 TcCLB-EL.509231.30_mRNA-p1 TcCLB-NE.
509719.60_mRNA-p1
OG0005981: TCRU_7349 TcCLB-EL.509231.50_mRNA-p1 TcCLB-NE.
509719.80_mRNA-p1

OG0005982: TCRU_7355 TcCLB-EL.511445.40_mRNA-p1 TcCLB-NE.
510799.70_mRNA-p1
OG0005983: TCRU_7386 TcCLB-EL.510515.50_mRNA-p1 TcCLB-NE.
509859.30_mRNA-p1
OG0005984: TCRU_7387 TcCLB-EL.510515.70_mRNA-p1 TcCLB-NE.
509859.50_mRNA-p1
OG0005985: TCRU_7388 TcCLB-EL.510515.80_mRNA-p1 TcCLB-NE.
509859.60_mRNA-p1
OG0005986: TCRU_7397 TcCLB-EL.510517.60_mRNA-p1 TcCLB-NE.
507851.20_mRNA-p1
OG0005987: TCRU_7399 TcCLB-EL.510517.80_mRNA-p1 TcCLB-NE.
507851.40_mRNA-p1
OG0005988: TCRU_7416 TcCLB-EL.509167.40_mRNA-p1 TcCLB-NE.
506831.20_mRNA-p1
OG0005989: TCRU_7418 TcCLB-EL.511731.74_mRNA-p1 TcCLB-NE.
506831.63_mRNA-p1
OG0005990: TCRU_7419 TcCLB-EL.511731.70_mRNA-p1 TcCLB-NE.
506831.66_mRNA-p1
OG0005991: TCRU_7423 TcCLB-EL.503745.30_mRNA-p1 TcCLB-NE.
506193.60_mRNA-p1
OG0005992: TCRU_7425 TcCLB-EL.436535.19_mRNA-p1 TcCLB-NE.
506195.10_pseudogenic_transcript-p1
OG0005993: TCRU_7426 TcCLB-EL.507883.50_mRNA-p1 TcCLB-NE.
506195.50_mRNA-p1
OG0005994: TCRU_7427 TcCLB-EL.507883.60_mRNA-p1 TcCLB-NE.
506195.60_mRNA-p1
OG0005995: TCRU_7428 TcCLB-EL.507883.70_mRNA-p1 TcCLB-NE.
506195.70_mRNA-p1
OG0005996: TCRU_7429 TcCLB-EL.507883.90_mRNA-p1 TcCLB-NE.
506195.90_mRNA-p1
OG0005997: TCRU_7430 TcCLB-EL.506407.10_mRNA-p1 TcCLB-NE.
506195.120_mRNA-p1
OG0005998: TCRU_7431 TcCLB-EL.506407.20_mRNA-p1 TcCLB-NE.
506195.130_mRNA-p1
OG0005999: TCRU_7432 TcCLB-EL.506407.30_mRNA-p1 TcCLB-NE.
506195.140_mRNA-p1
OG0006000: TCRU_7433 TcCLB-EL.506407.40_mRNA-p1 TcCLB-NE.
506195.150_mRNA-p1
OG0006001: TCRU_7434 TcCLB-EL.506407.50_mRNA-p1 TcCLB-NE.
506195.160_mRNA-p1
OG0006002: TCRU_7435 TcCLB-EL.506407.60_mRNA-p1 TcCLB-NE.
506195.170_mRNA-p1
OG0006003: TCRU_7436 TcCLB-EL.506407.70_mRNA-p1 TcCLB-NE.
506195.180_mRNA-p1
OG0006004: TCRU_7437 TcCLB-EL.506407.80_mRNA-p1 TcCLB-NE.
506195.190_mRNA-p1
OG0006005: TCRU_7438 TcCLB-EL.506407.90_mRNA-p1 TcCLB-NE.
506195.200_mRNA-p1
OG0006006: TCRU_7439 TcCLB-EL.506407.100_mRNA-p1 TcCLB-NE.
506195.210_mRNA-p1
OG0006007: TCRU_7440 TcCLB-EL.506407.110_mRNA-p1 TcCLB-NE.
506195.220_mRNA-p1
OG0006008: TCRU_7441 TcCLB-EL.503959.10_mRNA-p1 TcCLB-NE.
506195.230_mRNA-p1

OG0006009: TCRU_7442 TcCLB-EL.503959.20_mRNA-p1 TcCLB-NE.
506195.240_mRNA-p1
OG0006010: TCRU_7443 TcCLB-EL.503959.30_mRNA-p1 TcCLB-NE.
506195.250_mRNA-p1
OG0006011: TCRU_7444 TcCLB-EL.503959.40_mRNA-p1 TcCLB-NE.
506195.260_mRNA-p1
OG0006012: TCRU_7445 TcCLB-EL.503959.50_mRNA-p1 TcCLB-NE.
506195.270_mRNA-p1
OG0006013: TCRU_7446 TcCLB-EL.503959.60_mRNA-p1 TcCLB-NE.
506195.280_mRNA-p1
OG0006014: TCRU_7449 TcCLB-NE.511491.140_mRNA-p1 TcSYL_0167110.t1-p1
OG0006015: TCRU_7450 TcCLB-NE.511491.120_mRNA-p1 TcSYL_0167100.t1-p1
OG0006016: TCRU_7451 TcCLB-NE.511491.100_mRNA-p1 TcSYL_0167080.t1-p1
OG0006017: TCRU_7453 TcCLB-NE.511491.80_mRNA-p1 TcSYL_0167070.t1-p1
OG0006018: TCRU_7454 TcCLB-NE.511491.60_mRNA-p1 TcSYL_0167050.t1-p1
OG0006019: TCRU_7455 TcCLB-NE.511491.50_mRNA-p1 TcSYL_0167040.t1-p1
OG0006020: TCRU_7456 TcCLB-NE.511491.40_mRNA-p1 TcSYL_0167020.t1-p1
OG0006021: TCRU_/ TcCLB-NE.511491.30_mRNA-p1 TcSYL_0167010.t1-p1
OG0006022: TCRU_7458 TcCLB-NE.511491.20_mRNA-p1 TcSYL_0167000.t1-p1
OG0006023: TCRU_7459 TcCLB-EL.509443.60_mRNA-p1 TcCLB-NE.
511491.9_mRNA-p1
OG0006024: TCRU_7460 TcCLB-EL.509443.39_mRNA-p1 TcCLB-NE.
507017.159_mRNA-p1
OG0006025: TCRU_7461 TcCLB-EL.509443.30_mRNA-p1 TcCLB-NE.
507017.150_mRNA-p1
OG0006026: TCRU_7463 TcCLB-EL.509443.10_mRNA-p1 TcCLB-NE.
507017.130_mRNA-p1
OG0006027: TCRU_7466 TcCLB-EL.505807.254_mRNA-p1 TcCLB-NE.
507017.104_mRNA-p1
OG0006028: TCRU_7467 TcCLB-EL.505807.250_mRNA-p1 TcCLB-NE.
507017.100_mRNA-p1
OG0006029: TCRU_7468 TcCLB-EL.505807.240_mRNA-p1 TcCLB-NE.
507017.90_mRNA-p1
OG0006030: TCRU_7470 TcCLB-EL.505807.209_mRNA-p1 TcCLB-NE.
507017.64_mRNA-p1
OG0006031: TCRU_7471 TcCLB-EL.505807.200_mRNA-p1 TcCLB-NE.
507017.60_mRNA-p1
OG0006032: TCRU_domain TcCLB-EL.505807.140_mRNA-p1 TcCLB-NE.
507017.10_mRNA-p1
OG0006033: TCRU_7482 TcCLB-EL.505807.54_mRNA-p1 TcCLB-NE.
509033.70_mRNA-p1
OG0006034: TCRU_7493 TcCLB-EL.511337.39_mRNA-p1 TcCLB-NE.
509237.50_mRNA-p1
OG0006035: TCRU_7494 TcCLB-EL.508927.30_mRNA-p1 TcCLB-NE.
509237.20_mRNA-p1
OG0006036: TCRU_7495 TcCLB-EL.436521.9_mRNA-p1 TcCLB-NE.
509237.10_mRNA-p1
OG0006037: TCRU_7497 TcCLB-EL.506945.190_mRNA-p1 TcSYL_0140690.t1-p1
OG0006038: TCRU_7498 TcCLB-EL.506945.170_mRNA-p1 TcCLB-NE.
510445.61_mRNA-p1
OG0006039: TCRU_7500 TcCLB-EL.506945.140_pseudogenic_transcript-p1
TcCLB-NE.510445.30_mRNA-p1
OG0006040: TCRU_7502 TcCLB-EL.506945.110_mRNA-p1 TcCLB-NE.
510445.10_mRNA-p1

OG0006041: TCRU_7503 TcCLB-EL.506945.105_mRNA-p1 TcCLB-NE.
506835.150_mRNA-p1
OG0006042: TCRU_7504 TcCLB-EL.506945.100_mRNA-p1 TcCLB-NE.
506835.140_mRNA-p1
OG0006043: TCRU_7505 TcCLB-EL.506945.90_mRNA-p1 TcCLB-NE.
506835.131_mRNA-p1
OG0006044: TCRU_7508 TcCLB-EL.506945.50_mRNA-p1 TcCLB-NE.
506835.99_mRNA-p1
OG0006045: TCRU_7517 TcCLB-EL.504163.60_mRNA-p1 TcCLB-NE.
510301.30_mRNA-p1
OG0006046: TCRU_7518 TcCLB-EL.504163.70_mRNA-p1 TcCLB-NE.
510301.40_mRNA-p1
OG0006047: TCRU_7519 TcCLB-EL.504163.80_mRNA-p1 TcCLB-NE.
510301.50_mRNA-p1
OG0006048: TCRU_7520 TcCLB-EL.504163.90_mRNA-p1 TcCLB-NE.
510301.60_mRNA-p1
OG0006049: TCRU_7521 TcCLB-EL.504163.110_mRNA-p1 TcCLB-NE.
510301.80_mRNA-p1
OG0006050: TCRU_7523 TcCLB-NE.507681.130_mRNA-p1 TcSYL_0073740.t1-p1
OG0006051: TCRU_7526 TcCLB-NE.507681.170_mRNA-p1 TcSYL_0073770.t1-p1
OG0006052: TCRU_7527 TcCLB-NE.507681.180_mRNA-p1 TcSYL_0073780.t1-p1
OG0006053: TCRU_7529 TcCLB-NE.507681.200_mRNA-p1 TcSYL_0073800.t1-p1
OG0006054: TCRU_7530 TcCLB-NE.507681.210_mRNA-p1 TcSYL_0073810.t1-p1
OG0006055: TCRU_7533 TcCLB-EL.509537.30_mRNA-p1 TcCLB-NE.
507681.240_mRNA-p1
OG0006056: TCRU_7539 TcCLB-EL.511303.150_mRNA-p1 TcCLB-NE.
511761.60_mRNA-p1
OG0006057: TCRU_7540 TcCLB-EL.511303.140_mRNA-p1 TcCLB-NE.
511761.70_mRNA-p1
OG0006058: TCRU_7541 TcCLB-EL.511303.130_mRNA-p1 TcCLB-NE.
511761.80_mRNA-p1
OG0006059: TCRU_7544 TcCLB-EL.511303.100_mRNA-p1 TcCLB-NE.
509177.20_mRNA-p1
OG0006060: TCRU_7552 TcCLB-EL.506529.40_mRNA-p1 TcCLB-NE.
508593.100_mRNA-p1
OG0006061: TCRU_7553 TcCLB-EL.506529.14_mRNA-p1 TcCLB-NE.
508593.80_mRNA-p1
OG0006062: TCRU_7554 TcCLB-EL.507997.80_mRNA-p1 TcCLB-NE.
508593.69_mRNA-p1
OG0006063: TCRU_N-terminus/FAM.1 TcCLB-EL.507997.70_mRNA-p1 TcCLB-
NE.508593.60_mRNA-p1
OG0006064: TCRU_7556 TcCLB-EL.507997.60_mRNA-p1 TcCLB-NE.
508593.50_mRNA-p1
OG0006065: TCRU_7557 TcCLB-EL.507997.54_mRNA-p1 TcCLB-NE.
508593.44_mRNA-p1
OG0006066: TCRU_7558 TcCLB-EL.507997.50_mRNA-p1 TcCLB-NE.
508593.40_mRNA-p1
OG0006067: TCRU_7559 TcCLB-EL.507997.40_mRNA-p1 TcCLB-NE.
508593.30_mRNA-p1
OG0006068: TCRU_7572 TcCLB-EL.504051.30_mRNA-p1 TcCLB-NE.
506873.10_mRNA-p1
OG0006069: TCRU_7573 TcCLB-EL.504051.40_mRNA-p1 TcCLB-NE.
511719.41_mRNA-p1
OG0006070: TCRU_7575 TcCLB-EL.506223.130_mRNA-p1 TcCLB-NE.

511719.20_mRNA-p1
OG0006071: TCRU_7578 TcCLB-EL.506223.84_mRNA-p1 TcCLB-NE.
511717.174_mRNA-p1
OG0006072: TCRU_7580 TcCLB-EL.506223.70_mRNA-p1 TcCLB-NE.
511717.160_mRNA-p1
OG0006073: TCRU_7582 TcCLB-EL.506223.50_mRNA-p1 TcCLB-NE.
511717.140_mRNA-p1
OG0006074: TCRU_7584 TcCLB-EL.506223.30_mRNA-p1 TcCLB-NE.
511717.120_mRNA-p1
OG0006075: TCRU_7585 TcCLB-EL.506223.20_mRNA-p1 TcCLB-NE.
511717.110_mRNA-p1
OG0006076: TCRU_7588 TcCLB-EL.503677.29_mRNA-p1 TcCLB-NE.
511717.70_mRNA-p1
OG0006077: TCRU_7591 TcCLB-EL.506221.120_mRNA-p1 TcCLB-NE.
511717.40_mRNA-p1
OG0006078: TCRU_7603 TcCLB-EL.510103.24_mRNA-p1 TcCLB-NE.
509713.30_mRNA-p1
OG0006079: TCRU_7604 TcCLB-EL.510103.17_mRNA-p1 TcCLB-NE.
507763.10_mRNA-p1
OG0006080: TCRU_7605 TcCLB-EL.510103.10_mRNA-p1 TcCLB-NE.
507763.20_mRNA-p1
OG0006081: TCRU_7611 TcCLB-NE.506977.90_mRNA-p1 TcSYL_0170420.t1-p1
OG0006082: TCRU_7621 TcCLB-EL.507669.190_mRNA-p1 TcCLB-NE.
509877.10_mRNA-p1
OG0006083: TCRU_7623 TcCLB-EL.507669.170_mRNA-p1 TcCLB-NE.
509877.30_mRNA-p1
OG0006084: TCRU_7625 TcCLB-EL.507669.139_mRNA-p1 TcCLB-NE.
509877.60_mRNA-p1
OG0006085: TCRU_7627 TcCLB-EL.507669.120_mRNA-p1 TcCLB-NE.
509877.80_mRNA-p1
OG0006086: TCRU_7643 TcCLB-EL.508723.24_mRNA-p1 TcCLB-NE.
509791.60_mRNA-p1
OG0006087: TCRU_7646 TcCLB-EL.508723.59_mRNA-p1 TcCLB-NE.
509791.20_mRNA-p1
OG0006088: TCRU_7653 TcCLB-EL.509967.150_mRNA-p1 TcCLB-NE.
509695.110_mRNA-p1
OG0006089: TCRU_7655 TcCLB-EL.509967.180_mRNA-p1 TcCLB-NE.
509695.140_mRNA-p1
OG0006090: TCRU_7661 TcCLB-EL.508075.30_pseudogenic_transcript-p1
TcCLB-NE.490165.19_mRNA-p1
OG0006091: TCRU_7662 TcCLB-EL.508075.20_mRNA-p1 TcCLB-NE.
508409.338_mRNA-p1
OG0006092: TCRU_7665 TcCLB-EL.510143.120_mRNA-p1 TcCLB-NE.
508409.310_mRNA-p1
OG0006093: TCRU_7675 TcCLB-EL.510143.10_mRNA-p1 TcCLB-NE.
508409.190_mRNA-p1
OG0006094: TCRU_7676 TcCLB-EL.510143.5_mRNA-p1 TcCLB-NE.
508409.180_mRNA-p1
OG0006095: TCRU_7681 TcCLB-EL.506887.10_mRNA-p1 TcCLB-NE.
510731.10_mRNA-p1
OG0006096: TCRU_7682 TcCLB-EL.506887.20_mRNA-p1 TcCLB-NE.
510731.20_mRNA-p1
OG0006097: TCRU_7683 TcCLB-EL.506887.40_mRNA-p1 TcCLB-NE.
510731.30_mRNA-p1

OG0006098: TCRU_7684 TcCLB-EL.506887.60_mRNA-p1 TcCLB-NE.
510731.50_mRNA-p1
OG0006099: TCRU_7685 TcCLB-EL.506887.80_mRNA-p1 TcCLB-NE.
510731.68_mRNA-p1
OG0006100: TCRU_7695 TcCLB-EL.510243.20_mRNA-p1 TcCLB-NE.
510575.210_mRNA-p1
OG0006101: TCRU_7696 TcCLB-EL.510243.10_mRNA-p1 TcCLB-NE.
510575.220_mRNA-p1
OG0006102: TCRU_family_ TcCLB-EL.504255.50_mRNA-p1 TcCLB-NE.
510575.230_mRNA-p1
OG0006103: TCRU_7699 TcCLB-EL.504255.20_mRNA-p1 TcCLB-NE.
510577.30_mRNA-p1
OG0006104: TCRU_7705 TcCLB-EL.506927.20_mRNA-p1 TcCLB-NE.
510797.39_mRNA-p1
OG0006105: TCRU_7706 TcCLB-EL.506927.10_pseudogenic_transcript-p1
TcCLB-NE.508505.10_mRNA-p1
OG0006106: TCRU_7712 TcCLB-EL.509157.100_mRNA-p1 TcCLB-NE.
508443.40_mRNA-p1
OG0006107: TCRU_7713 TcCLB-EL.509157.90_mRNA-p1 TcCLB-NE.
508443.30_mRNA-p1
OG0006108: TCRU_7714 TcCLB-EL.509157.80_mRNA-p1 TcCLB-NE.
508443.20_mRNA-p1
OG0006109: TCRU_7715 TcCLB-EL.509157.70_mRNA-p1 TcCLB-NE.
508443.10_mRNA-p1
OG0006110: TCRU_7716 TcCLB-EL.509157.50_mRNA-p1 TcCLB-NE.
510723.20_mRNA-p1
OG0006111: TCRU_7717 TcCLB-EL.509157.40_mRNA-p1 TcCLB-NE.
510723.10_mRNA-p1
OG0006112: TCRU_7726 TcCLB-EL.506529.90_mRNA-p1 TcCLB-NE.
508593.150_mRNA-p1
OG0006113: TCRU_7727 TcCLB-EL.506529.100_mRNA-p1 TcCLB-NE.
510885.10_mRNA-p1
OG0006114: TCRU_7728 TcCLB-EL.506529.120_mRNA-p1 TcCLB-NE.
510885.30_mRNA-p1
OG0006115: TCRU_7730 TcCLB-EL.508215.10_mRNA-p1 TcCLB-NE.
508879.10_mRNA-p1
OG0006116: TCRU_7734 TcCLB-NE.508879.60_mRNA-p1 TcSYL_0023230.t1-p1
OG0006117: TCRU_7736 TcCLB-NE.508879.80_mRNA-p1 TcSYL_0023240.t1-p1
OG0006118: TCRU_7739 TcCLB-NE.508879.120_mRNA-p1 TcSYL_0023250.t1-p1
OG0006119: TCRU_7740 TcCLB-NE.508879.130_pseudogenic_transcript-p1
TcSYL_0023260.t1-p1
OG0006120: TCRU_7741 TcCLB-NE.508879.150_mRNA-p1 TcSYL_0023270.t1-p1
OG0006121: TCRU_7744 TcCLB-EL.506135.90_mRNA-p1 TcCLB-NE.
508879.190_mRNA-p1
OG0006122: TCRU_7745 TcCLB-EL.506135.80_mRNA-p1 TcCLB-NE.
508879.200_mRNA-p1
OG0006123: TCRU_7746 TcCLB-EL.506135.70_mRNA-p1 TcCLB-NE.
508879.210_mRNA-p1
OG0006124: TCRU_7747 TcCLB-EL.506135.50_mRNA-p1 TcCLB-NE.
508879.220_mRNA-p1
OG0006125: TCRU_7748 TcCLB-EL.506135.40_mRNA-p1 TcCLB-NE.
503721.10_mRNA-p1
OG0006126: TCRU_7750 TcCLB-EL.506135.20_mRNA-p1 TcCLB-NE.
503721.30_mRNA-p1

OG0006127: TCRU_7751 TcCLB-EL.509127.110_mRNA-p1 TcCLB-NE.
508737.200_mRNA-p1
OG0006128: TCRU_7752 TcCLB-EL.509127.104_mRNA-p1 TcCLB-NE.
508737.194_mRNA-p1
OG0006129: TCRU_7753 TcCLB-EL.509127.97_mRNA-p1 TcCLB-NE.
508737.187_mRNA-p1
OG0006130: TCRU_7754 TcCLB-EL.509127.80_mRNA-p1 TcCLB-NE.
508737.170_mRNA-p1
OG0006131: TCRU_7766 TcCLB-EL.505007.39_mRNA-p1 TcCLB-NE.
510007.10_mRNA-p1
OG0006132: TCRU_7768 TcCLB-EL.506929.50_mRNA-p1 TcCLB-NE.
506587.50_mRNA-p1
OG0006133: TCRU_7771 TcCLB-EL.506929.20_mRNA-p1 TcCLB-NE.
506587.80_mRNA-p1
OG0006134: TCRU_7780 TcCLB-NE.511899.10_mRNA-p1 TcSYL_0103580.t1-p1
OG0006135: TCRU_7782 TcCLB-EL.511071.150_mRNA-p1 TcCLB-NE.
511899.30_mRNA-p1
OG0006136: TCRU_7784 TcCLB-EL.511071.90_mRNA-p1 TcCLB-NE.
511903.10_mRNA-p1
OG0006137: TCRU_7785 TcCLB-EL.511071.80_mRNA-p1 TcCLB-NE.
511903.20_mRNA-p1
OG0006138: TCRU_7789 TcCLB-EL.511071.20_mRNA-p1 TcCLB-NE.
511903.70_mRNA-p1
OG0006139: TCRU_7797 TcCLB-EL.510989.20_mRNA-p1 TcCLB-NE.
511245.170_mRNA-p1
OG0006140: TCRU_7798 TcCLB-EL.510989.30_mRNA-p1 TcCLB-NE.
511245.180_mRNA-p1
OG0006141: TCRU_7799 TcCLB-EL.510989.40_mRNA-p1 TcCLB-NE.
511245.190_mRNA-p1
OG0006142: TCRU_7813 TcCLB-EL.507257.20_mRNA-p1 TcCLB-NE.
510329.230_mRNA-p1
OG0006143: TCRU_7816 TcCLB-EL.507257.70_mRNA-p1 TcCLB-NE.
510329.280_mRNA-p1
OG0006144: TCRU_7817 TcCLB-EL.507257.90_mRNA-p1 TcCLB-NE.
510329.300_mRNA-p1
OG0006145: TCRU_7818 TcCLB-EL.507257.100_mRNA-p1 TcCLB-NE.
510329.310_mRNA-p1
OG0006146: TCRU_7820 TcCLB-EL.507257.120_mRNA-p1 TcCLB-NE.
510329.330_mRNA-p1
OG0006147: TCRU_7821 TcCLB-EL.507257.130_mRNA-p1 TcCLB-NE.
510329.340_mRNA-p1
OG0006148: TCRU_7822 TcCLB-EL.507257.140_mRNA-p1 TcCLB-NE.
510329.350_mRNA-p1
OG0006149: TCRU_7824 TcCLB-EL.507257.170_mRNA-p1 TcCLB-NE.
510329.380_mRNA-p1
OG0006150: TCRU_7828 TcCLB-EL.511545.60_mRNA-p1 TcCLB-NE.
506817.70_mRNA-p1
OG0006151: TCRU_7834 TcCLB-NE.510187.70_mRNA-p1 TcSYL_0011680.t1-p1
OG0006152: TCRU_7835 TcCLB-NE.510187.80_mRNA-p1 TcSYL_0011670.t1-p1
OG0006153: TCRU_7841 TcCLB-EL.509169.39_mRNA-p1 TcCLB-NE.
511509.30_mRNA-p1
OG0006154: TCRU_7842 TcCLB-EL.509169.30_mRNA-p1 TcCLB-NE.
511509.40_mRNA-p1
OG0006155: TCRU_7843 TcCLB-EL.509169.20_mRNA-p1 TcCLB-NE.

511509.50_mRNA-p1
OG0006156: TCRU_7845 TcCLB-EL.469785.20_mRNA-p1 TcCLB-NE.
507025.30_mRNA-p1
OG0006157: TCRU_7846 TcCLB-EL.469785.30_mRNA-p1 TcCLB-NE.
507025.40_mRNA-p1
OG0006158: TCRU_7850 TcCLB-EL.508213.30_mRNA-p1 TcCLB-NE.
507515.50_mRNA-p1
OG0006159: TCRU_7852 TcCLB-EL.508213.9_mRNA-p1 TcCLB-NE.
507515.70_mRNA-p1
OG0006160: TCRU_7862 TcCLB-EL.510289.44_mRNA-p1 TcCLB-NE.
510967.13_mRNA-p1
OG0006161: TCRU_7864 TcCLB-EL.510289.54_mRNA-p1 TcCLB-NE.
510967.20_mRNA-p1
OG0006162: TCRU_7865 TcCLB-EL.510289.60_mRNA-p1 TcCLB-NE.
510967.30_mRNA-p1
OG0006163: TCRU_7867 TcCLB-EL.510289.80_mRNA-p1 TcCLB-NE.
510969.20_mRNA-p1
OG0006164: TCRU_7868 TcCLB-EL.510289.90_mRNA-p1 TcCLB-NE.
510969.30_mRNA-p1
OG0006165: TCRU_7869 TcCLB-EL.504203.10_mRNA-p1 TcCLB-NE.
508657.10_mRNA-p1
OG0006166: TCRU_7900 TcCLB-EL.507275.24_mRNA-p1 TcCLB-NE.
507491.59_mRNA-p1
OG0006167: TCRU_7901 TcCLB-EL.507275.20_mRNA-p1 TcCLB-NE.
507491.70_pseudogenic_transcript-p1
OG0006168: TCRU_7904 TcCLB-EL.509393.40_mRNA-p1 TcCLB-NE.
507491.110_mRNA-p1
OG0006169: TCRU_7905 TcCLB-EL.509393.30_mRNA-p1 TcCLB-NE.
507491.119_mRNA-p1
OG0006170: TCRU_7906 TcCLB-EL.509393.20_mRNA-p1 TcCLB-NE.
507491.130_mRNA-p1
OG0006171: TCRU_7911 TcCLB-EL.511419.30_mRNA-p1 TcCLB-NE.
505999.80_mRNA-p1
OG0006172: TCRU_7914 TcCLB-EL.511419.4_mRNA-p1 TcCLB-NE.
505999.50_mRNA-p1
OG0006173: TCRU_7915 TcCLB-EL.511417.120_pseudogenic_transcript-p1
TcCLB-NE.505999.40_mRNA-p1
OG0006174: TCRU_like TcCLB-EL.511417.100_mRNA-p1 TcCLB-NE.
505999.30_mRNA-p1
OG0006175: TCRU_7917 TcCLB-EL.511417.94_mRNA-p1 TcCLB-NE.
505999.24_mRNA-p1
OG0006176: TCRU_7929 TcCLB-NE.510819.4_mRNA-p1 TcSYL_0050000.t1-p1
OG0006177: TCRU_7938 TcCLB-EL.511731.30_mRNA-p1 TcCLB-NE.
506833.30_mRNA-p1
OG0006178: TCRU_7939 TcCLB-EL.511731.24_mRNA-p1 TcCLB-NE.
506833.40_mRNA-p1
OG0006179: TCRU_7940 TcCLB-EL.511731.20_mRNA-p1 TcCLB-NE.
506833.50_mRNA-p1
OG0006180: TCRU_7942 TcCLB-EL.511729.70_mRNA-p1 TcCLB-NE.
506833.70_mRNA-p1
OG0006181: TCRU_7947 TcCLB-EL.511729.20_mRNA-p1 TcCLB-NE.
506835.50_mRNA-p1
OG0006182: TCRU_7960 TcCLB-EL.508169.30_mRNA-p1 TcCLB-NE.
510965.10_mRNA-p1

OG0006183: TCRU_7961 TcCLB-EL.508169.50_mRNA-p1 TcCLB-NE.
510965.29_mRNA-p1
OG0006184: TCRU_7964 TcCLB-EL.508169.80_mRNA-p1 TcCLB-NE.
506581.20_mRNA-p1
OG0006185: TCRU_7968 TcCLB-EL.508169.129_mRNA-p1 TcCLB-NE.
506581.69_pseudogenic_transcript-p1
OG0006186: TCRU_7970 TcCLB-EL.510287.20_mRNA-p1 TcCLB-NE.
503891.110_mRNA-p1
OG0006187: TCRU_7976 TcCLB-EL.506661.80_mRNA-p1 TcCLB-NE.
511245.79_mRNA-p1
OG0006188: TCRU_7977 TcCLB-EL.506661.90_mRNA-p1 TcCLB-NE.
511245.70_mRNA-p1
OG0006189: TCRU_7978 TcCLB-EL.506661.100_mRNA-p1 TcCLB-NE.
511245.60_mRNA-p1
OG0006190: TCRU_7980 TcCLB-EL.506661.120_mRNA-p1 TcCLB-NE.
511245.40_mRNA-p1
OG0006191: TCRU_7981 TcCLB-EL.506661.130_mRNA-p1 TcCLB-NE.
511245.30_mRNA-p1
OG0006192: TCRU_7982 TcCLB-EL.506661.140_mRNA-p1 TcCLB-NE.
511245.24_mRNA-p1
OG0006193: TCRU_7984 TcCLB-EL.506661.160_mRNA-p1 TcCLB-NE.
511245.10_mRNA-p1
OG0006194: TCRU_7985 TcCLB-EL.506661.170_mRNA-p1 TcCLB-NE.
508863.20_mRNA-p1
OG0006195: TCRU_8000 TcCLB-EL.503487.60_mRNA-p1 TcCLB-NE.
508641.210_mRNA-p1
OG0006196: TCRU_8002 TcCLB-EL.503487.30_mRNA-p1 TcCLB-NE.
508641.240_mRNA-p1
OG0006197: TCRU_8013 TcCLB-EL.506203.25_mRNA-p1 TcCLB-NE.
508645.20_mRNA-p1
OG0006198: TCRU_8015 TcCLB-EL.506359.40_mRNA-p1 TcCLB-NE.
503521.80_mRNA-p1
OG0006199: TCRU_8018 TcCLB-EL.506359.70_mRNA-p1 TcCLB-NE.
503521.39_mRNA-p1
OG0006200: TCRU_8028 TcCLB-EL.507509.50_mRNA-p1 TcCLB-NE.
506871.100_mRNA-p1
OG0006201: TCRU_8030 TcCLB-EL.507509.30_mRNA-p1 TcCLB-NE.
506871.80_mRNA-p1
OG0006202: TCRU_8031 TcCLB-EL.507509.20_mRNA-p1 TcCLB-NE.
506871.70_mRNA-p1
OG0006203: TCRU_8033 TcCLB-EL.507507.40_mRNA-p1 TcCLB-NE.
506871.50_mRNA-p1
OG0006204: TCRU_8035 TcCLB-EL.507507.20_mRNA-p1 TcCLB-NE.
506871.30_mRNA-p1
OG0006205: TCRU_8060 TcCLB-EL.503697.9_mRNA-p1 TcCLB-NE.
509627.30_mRNA-p1
OG0006206: TCRU_8061 TcCLB-EL.503697.20_mRNA-p1 TcCLB-NE.
509627.20_mRNA-p1
OG0006207: TCRU_8062 TcCLB-EL.503697.30_mRNA-p1 TcCLB-NE.
509627.10_mRNA-p1
OG0006208: TCRU_8065 TcCLB-EL.509979.150_mRNA-p1 TcCLB-NE.
510039.40_mRNA-p1
OG0006209: TCRU_8066 TcCLB-EL.509979.140_mRNA-p1 TcCLB-NE.
510039.50_mRNA-p1

OG0006210: TCRU_8068 TcCLB-EL.509979.119_mRNA-p1 TcCLB-NE.
510039.64_mRNA-p1
OG0006211: TCRU_8070 TcCLB-EL.509979.100_mRNA-p1 TcCLB-NE.
510039.80_mRNA-p1
OG0006212: TCRU_8080 TcCLB-EL.503829.80_mRNA-p1 TcCLB-NE.
508045.70_mRNA-p1
OG0006213: TCRU_8086 TcCLB-EL.506401.310_mRNA-p1 TcCLB-NE.
510835.30_mRNA-p1
OG0006214: TCRU_8087 TcCLB-EL.506401.320_mRNA-p1 TcCLB-NE.
510835.40_mRNA-p1
OG0006215: TCRU_8088 TcCLB-EL.506401.330_mRNA-p1 TcCLB-NE.
510835.50_mRNA-p1
OG0006216: TCRU_8089 TcCLB-EL.506401.350_mRNA-p1 TcCLB-NE.
508545.9_mRNA-p1
OG0006217: TCRU_8090 TcCLB-EL.506401.360_mRNA-p1 TcCLB-NE.
508545.20_mRNA-p1
OG0006218: TCRU_8091 TcCLB-EL.506401.370_mRNA-p1 TcCLB-NE.
508545.30_mRNA-p1
OG0006219: TCRU_8116 TcCLB-EL.510431.330_mRNA-p1 TcCLB-NE.
507771.99_mRNA-p1
OG0006220: TCRU_8118 TcCLB-EL.510431.310_mRNA-p1 TcCLB-NE.
507771.114_mRNA-p1
OG0006221: TCRU_8121 TcCLB-NE.507019.20_mRNA-p1 TcSYL_0167320.t1-p1
OG0006222: TCRU_8123 TcCLB-NE.507019.40_mRNA-p1 TcSYL_0167330.t1-p1
OG0006223: TCRU_8129 TcCLB-EL.509007.40_mRNA-p1 TcCLB-NE.
508965.70_mRNA-p1
OG0006224: TCRU_8130 TcCLB-EL.509007.30_mRNA-p1 TcCLB-NE.
508965.80_mRNA-p1
OG0006225: TCRU_8140 TcCLB-NE.511367.190_mRNA-p1 TcSYL_0170000.t1-p1
OG0006226: TCRU_8145 TcCLB-EL.506779.60_mRNA-p1 TcCLB-NE.
511153.130_mRNA-p1
OG0006227: TCRU_8148 TcCLB-EL.506779.100_mRNA-p1 TcCLB-NE.
511153.100_mRNA-p1
OG0006228: TCRU_8150 TcCLB-EL.506779.120_mRNA-p1 TcCLB-NE.
511153.84_mRNA-p1
OG0006229: TCRU_8151 TcCLB-EL.506779.130_mRNA-p1 TcCLB-NE.
511153.80_mRNA-p1
OG0006230: TCRU_8157 TcCLB-EL.506925.250_pseudogenic_transcript-p1
TcSYL_0080580.t1-p1
OG0006231: TCRU_8158 TcCLB-EL.506925.260_mRNA-p1 TcSYL_0080590.t1-p1
OG0006232: TCRU_8159 TcCLB-EL.506925.270_mRNA-p1 TcSYL_0080610.t1-p1
OG0006233: TCRU_8163 TcCLB-EL.506925.319_mRNA-p1 TcSYL_0080620.t1-p1
OG0006234: TCRU_8181 TcCLB-NE.508409.40_mRNA-p1 TcSYL_0004520.t1-p1
OG0006235: TCRU_8182 TcCLB-NE.508409.30_mRNA-p1 TcSYL_0004540.t1-p1
OG0006236: TCRU_8183 TcCLB-NE.508409.20_mRNA-p1 TcSYL_0004560.t1-p1
OG0006237: TCRU_8190 TcCLB-EL.507795.80_mRNA-p1 TcCLB-NE.
510647.20_mRNA-p1
OG0006238: TCRU_8194 TcCLB-EL.509761.20_pseudogenic_transcript-p1
TcCLB-NE.510645.30_mRNA-p1
OG0006239: TCRU_8195 TcCLB-EL.507797.18_mRNA-p1 TcCLB-NE.
510645.9_mRNA-p1
OG0006240: TCRU_8200 TcCLB-EL.507803.10_mRNA-p1 TcCLB-EL.
510959.30_mRNA-p1
OG0006241: TCRU_8204 TcCLB-EL.503579.80_mRNA-p1 TcCLB-NE.

507517.70_mRNA-p1
OG0006242: TCRU_8205 TcCLB-EL.503579.90_mRNA-p1 TcCLB-NE.
507517.80_mRNA-p1
OG0006243: TCRU_8207 TcCLB-EL.503579.110_mRNA-p1 TcCLB-NE.
507517.100_mRNA-p1
OG0006244: TCRU_8213 TcCLB-EL.508647.200_mRNA-p1 TcSYL_0047900.t1-p1
OG0006245: TCRU_8215 TcCLB-EL.508647.220_mRNA-p1 TcSYL_0047910.t1-p1
OG0006246: TCRU_8216 TcCLB-EL.508647.230_pseudogenic_transcript-p1
TcSYL_0047920.t1-p1
OG0006247: TCRU_8219 TcCLB-EL.508647.270_mRNA-p1 TcCLB-NE.
505183.20_mRNA-p1
OG0006248: TCRU_8223 TcCLB-EL.503529.30_mRNA-p1 TcCLB-NE.
511647.70_mRNA-p1
OG0006249: TCRU_8225 TcCLB-EL.509781.10_mRNA-p1 TcCLB-NE.
511649.30_mRNA-p1
OG0006250: TCRU_8226 TcCLB-EL.503531.20_mRNA-p1 TcCLB-NE.
511649.50_mRNA-p1
OG0006251: TCRU_8230 TcCLB-EL.507939.50_mRNA-p1 TcCLB-NE.
508119.190_mRNA-p1
OG0006252: TCRU_8231 TcCLB-EL.507939.40_mRNA-p1 TcCLB-NE.
508119.180_mRNA-p1
OG0006253: TCRU_8233 TcCLB-EL.507939.30_mRNA-p1 TcCLB-NE.
508119.160_mRNA-p1
OG0006254: TCRU_8237 TcCLB-EL.509139.20_mRNA-p1 TcCLB-NE.
508741.180_mRNA-p1
OG0006255: TCRU_8238 TcCLB-EL.485889.4_mRNA-p1 TcCLB-NE.
508741.190_mRNA-p1
OG0006256: TCRU_8239 TcCLB-EL.485889.10_mRNA-p1 TcCLB-NE.
508741.200_mRNA-p1
OG0006257: TCRU_8240 TcCLB-EL.509141.10_mRNA-p1 TcCLB-NE.
508741.210_mRNA-p1
OG0006258: TCRU_8241 TcCLB-EL.509141.20_mRNA-p1 TcCLB-NE.
508741.220_mRNA-p1
OG0006259: TCRU_8246 TcCLB-EL.510861.125_mRNA-p1 TcCLB-NE.
503735.10_mRNA-p1
OG0006260: TCRU_8247 TcCLB-EL.510861.110_mRNA-p1 TcCLB-NE.
503735.20_mRNA-p1
OG0006261: TCRU_8248 TcCLB-EL.510861.94_mRNA-p1 TcCLB-NE.
503735.30_mRNA-p1
OG0006262: TCRU_8249 TcCLB-EL.510861.80_mRNA-p1 TcCLB-NE.
503735.40_mRNA-p1
OG0006263: TCRU_8250 TcCLB-EL.510861.70_mRNA-p1 TcCLB-NE.
503735.49_mRNA-p1
OG0006264: TCRU_8251 TcCLB-EL.506755.260_mRNA-p1 TcCLB-NE.
510359.270_mRNA-p1
OG0006265: TCRU_8253 TcCLB-EL.506755.240_mRNA-p1 TcCLB-NE.
510359.250_mRNA-p1
OG0006266: TCRU_8255 TcCLB-EL.506755.220_mRNA-p1 TcCLB-NE.
510359.230_mRNA-p1
OG0006267: TCRU_8280 TcCLB-EL.511577.120_mRNA-p1 TcCLB-NE.
509339.10_mRNA-p1
OG0006268: TCRU_8287 TcCLB-EL.511715.20_mRNA-p1 TcCLB-NE.
508445.30_mRNA-p1
OG0006269: TCRU_8288 TcCLB-EL.511715.30_mRNA-p1 TcCLB-NE.

508445.40_mRNA-p1
OG0006270: TCRU_8289 TcCLB-EL.511715.40_mRNA-p1 TcCLB-NE.
508445.50_mRNA-p1
OG0006271: TCRU_8302 TcCLB-NE.510329.190_mRNA-p1 TcSYL_0001270.t1-p1
OG0006272: TCRU_8304 TcCLB-NE.510329.170_mRNA-p1 TcSYL_0001250.t1-p1
OG0006273: TCRU_8305 TcCLB-NE.510329.160_mRNA-p1 TcSYL_0001240.t1-p1
OG0006274: TCRU_8307 TcCLB-NE.510329.140_mRNA-p1 TcSYL_0001210.t1-p1
OG0006275: TCRU_8308 TcCLB-NE.510329.130_mRNA-p1 TcSYL_0001200.t1-p1
OG0006276: TCRU_8312 TcCLB-NE.510329.90_mRNA-p1 TcSYL_0001180.t1-p1
OG0006277: TCRU_8315 TcCLB-NE.510329.60_mRNA-p1 TcSYL_0001170.t1-p1
OG0006278: TCRU_8321 TcCLB-EL.507089.50_mRNA-p1 TcCLB-NE.
504085.50_mRNA-p1
OG0006279: TCRU_type-like TcCLB-EL.507089.30_mRNA-p1 TcCLB-NE.
504085.70_mRNA-p1
OG0006280: TCRU_8325 TcCLB-EL.511529.250_mRNA-p1 TcCLB-NE.
506727.10_mRNA-p1
OG0006281: TCRU_8327 TcCLB-EL.511529.230_mRNA-p1 TcCLB-NE.
506727.30_mRNA-p1
OG0006282: TCRU_8330 TcCLB-EL.511529.200_mRNA-p1 TcCLB-NE.
506727.60_mRNA-p1
OG0006283: TCRU_8332 TcCLB-EL.511529.180_mRNA-p1 TcCLB-NE.
506727.80_mRNA-p1
OG0006284: TCRU_8334 TcCLB-EL.511529.150_mRNA-p1 TcCLB-NE.
506727.110_mRNA-p1
OG0006285: TCRU_8336 TcCLB-EL.511529.130_mRNA-p1 TcCLB-NE.
506727.130_mRNA-p1
OG0006286: TCRU_8338 TcCLB-EL.511529.100_mRNA-p1 TcSYL_0112160.t1-p1
OG0006287: TCRU_8341 TcCLB-EL.505983.30_mRNA-p1 TcCLB-NE.
504839.60_mRNA-p1
OG0006288: TCRU_8345 TcCLB-EL.510409.10_mRNA-p1 TcCLB-NE.
509937.130_mRNA-p1
OG0006289: TCRU_8346 TcCLB-EL.510409.20_pseudogenic_transcript-p1
TcCLB-NE.509937.120_mRNA-p1
OG0006290: TCRU_8347 TcCLB-EL.510409.30_mRNA-p1 TcCLB-NE.
509937.110_mRNA-p1
OG0006291: TCRU_8360 TcCLB-EL.508277.20_mRNA-p1 TcCLB-NE.
509941.120_mRNA-p1
OG0006292: TCRU_8361 TcCLB-EL.508277.40_mRNA-p1 TcCLB-NE.
503543.20_mRNA-p1
OG0006293: TCRU_8362 TcCLB-EL.508277.50_mRNA-p1 TcCLB-NE.
503543.10_mRNA-p1
OG0006294: TCRU_8364 TcCLB-EL.508277.80_mRNA-p1 TcCLB-NE.
503541.20_mRNA-p1
OG0006295: TCRU_8367 TcCLB-EL.510285.30_mRNA-p1 TcCLB-NE.
503823.140_mRNA-p1
OG0006296: TCRU_8368 TcCLB-EL.510285.40_mRNA-p1 TcCLB-NE.
503823.150_mRNA-p1
OG0006297: TCRU_8376 TcCLB-EL.503989.20_mRNA-p1 TcCLB-NE.
504207.10_mRNA-p1
OG0006298: TCRU_8384 TcCLB-EL.404843.20_mRNA-p1 TcCLB-NE.
511647.10_mRNA-p1
OG0006299: TCRU_8385 TcCLB-EL.404843.10_mRNA-p1 TcCLB-NE.
511647.20_mRNA-p1
OG0006300: TCRU_8388 TcCLB-EL.503619.29_mRNA-p1 TcCLB-NE.

511647.44_mRNA-p1
OG0006301: TCRU_8399 TcCLB-EL.509167.190_mRNA-p1 TcCLB-NE.
509065.180_mRNA-p1
OG0006302: TCRU_8401 TcCLB-EL.506943.150_pseudogenic_transcript-p1
TcCLB-NE.509065.150_mRNA-p1
OG0006303: TCRU_8402 TcCLB-EL.506943.114_mRNA-p1 TcCLB-NE.
509065.124_mRNA-p1
OG0006304: TCRU_8405 TcCLB-EL.506943.90_mRNA-p1 TcCLB-NE.
509065.100_mRNA-p1
OG0006305: TCRU_8406 TcCLB-EL.506943.80_mRNA-p1 TcCLB-NE.
509065.90_mRNA-p1
OG0006306: TCRU_8410 TcCLB-EL.506943.30_mRNA-p1 TcCLB-NE.
509065.40_mRNA-p1
OG0006307: TCRU_8411 TcCLB-EL.506943.23_mRNA-p1 TcCLB-NE.
509065.33_mRNA-p1
OG0006308: TCRU_8412 TcCLB-EL.506943.20_mRNA-p1 TcCLB-NE.
509065.30_mRNA-p1
OG0006309: TCRU_8413 TcCLB-EL.506943.10_mRNA-p1 TcCLB-NE.
509065.20_mRNA-p1
OG0006310: TCRU_8414 TcCLB-EL.506943.4_mRNA-p1 TcCLB-NE.
509065.9_mRNA-p1
OG0006311: TCRU_8416 TcCLB-EL.511733.30_mRNA-p1 TcCLB-NE.
511523.80_mRNA-p1
OG0006312: TCRU_8418 TcCLB-EL.511733.60_mRNA-p1 TcCLB-NE.
511523.50_mRNA-p1
OG0006313: TCRU_8428 TcCLB-EL.506829.80_mRNA-p1 TcCLB-NE.
506593.60_mRNA-p1
OG0006314: TCRU_8429 TcCLB-EL.506829.60_mRNA-p1 TcCLB-NE.
506593.50_mRNA-p1
OG0006315: TCRU_8432 TcCLB-EL.506829.30_mRNA-p1 TcCLB-NE.
506593.10_mRNA-p1
OG0006316: TCRU_8434 TcCLB-EL.506829.10_mRNA-p1 TcCLB-NE.
504231.20_mRNA-p1
OG0006317: TCRU_8436 TcCLB-EL.509805.80_mRNA-p1 TcCLB-NE.
503939.120_mRNA-p1
OG0006318: TCRU_8438 TcCLB-EL.509805.90_mRNA-p1 TcCLB-NE.
503939.110_mRNA-p1
OG0006319: TCRU_8453 TcCLB-EL.506735.40_mRNA-p1 TcCLB-NE.
509229.50_mRNA-p1
OG0006320: TCRU_8454 TcCLB-EL.506735.30_mRNA-p1 TcCLB-NE.
509229.60_mRNA-p1
OG0006321: TCRU_8455 TcCLB-EL.506735.20_mRNA-p1 TcCLB-NE.
509229.70_mRNA-p1
OG0006322: TCRU_8457 TcCLB-EL.510879.140_mRNA-p1 TcSYL_0045970.t1-p1
OG0006323: TCRU_8467 TcCLB-EL.509891.50_mRNA-p1 TcCLB-NE.
510691.80_mRNA-p1
OG0006324: TCRU_8491 TcCLB-EL.463155.10_pseudogenic_transcript-p1
TcCLB-NE.503763.30_pseudogenic_transcript-p1
OG0006325: TCRU_8522 TcCLB-EL.510309.30_mRNA-p1 TcCLB-NE.
503505.14_mRNA-p1
OG0006326: TCRU_8525 TcCLB-EL.510309.60_mRNA-p1 TcCLB-NE.
510267.10_mRNA-p1
OG0006327: TCRU_8543 TcCLB-EL.507677.50_mRNA-p1 TcCLB-NE.
504021.100_mRNA-p1

OG0006328: TCRU_8553 TcCLB-EL.506775.22_mRNA-p1 TcCLB-NE.
508797.20_mRNA-p1
OG0006329: TCRU_8554 TcCLB-EL.506775.40_mRNA-p1 TcCLB-NE.
511169.20_mRNA-p1
OG0006330: TCRU_8556 TcCLB-EL.506775.60_mRNA-p1 TcCLB-NE.
511167.110_mRNA-p1
OG0006331: TCRU_8563 TcCLB-EL.507993.369_mRNA-p1 TcCLB-NE.
511281.56_mRNA-p1
OG0006332: TCRU_8569 TcCLB-NE.510821.20_mRNA-p1 TcSYL_0050130.t1-p1
OG0006333: TCRU_8574 TcCLB-EL.511713.40_mRNA-p1 TcCLB-NE.
503987.70_mRNA-p1
OG0006334: TCRU_8575 TcCLB-EL.511713.30_mRNA-p1 TcCLB-NE.
503987.60_mRNA-p1
OG0006335: TCRU_8576 TcCLB-EL.511713.20_mRNA-p1 TcCLB-NE.
503987.50_mRNA-p1
OG0006336: TCRU_8577 TcCLB-EL.511711.90_mRNA-p1 TcCLB-NE.
503987.10_mRNA-p1
OG0006337: TCRU_8603 TcCLB-EL.511067.14_mRNA-p1 TcCLB-NE.
511903.134_mRNA-p1
OG0006338: TCRU_10430 TCRU_8606 TcSYL_0164100.t1-p1
OG0006339: TCRU_8629 TcCLB-EL.510431.210_mRNA-p1 TcSYL_0138300.t1-p1
OG0006340: TCRU_8630 TcCLB-EL.510431.190_mRNA-p1 TcSYL_0138280.t1-p1
OG0006341: TCRU_8631 TcCLB-EL.510431.180_mRNA-p1 TcSYL_0138270.t1-p1
OG0006342: TCRU_8634 TcCLB-NE.508971.50_mRNA-p1 TcSYL_0202380.t1-p1
OG0006343: TCRU_8638 TcCLB-NE.511727.60_mRNA-p1 TcSYL_0142730.t1-p1
OG0006344: TCRU_8639 TcCLB-EL.504097.40_mRNA-p1 TcCLB-NE.
506679.180_mRNA-p1
OG0006345: TCRU_8640 TcCLB-EL.510769.40_mRNA-p1 TcCLB-NE.
506241.190_mRNA-p1
OG0006346: TCRU_8642 TcCLB-EL.509127.40_mRNA-p1 TcCLB-NE.
508737.140_mRNA-p1
OG0006347: TCRU_8646 TcCLB-EL.506753.160_mRNA-p1 TcCLB-NE.
510357.50_mRNA-p1
OG0006348: TCRU_8651 TcCLB-EL.511179.110_mRNA-p1 TcCLB-NE.
508303.50_mRNA-p1
OG0006349: TCRU_8652 TcCLB-EL.511179.130_mRNA-p1 TcCLB-NE.
508303.30_mRNA-p1
OG0006350: TCRU_8654 TcCLB-EL.509769.100_mRNA-p1 TcSYL_0174820.t1-p1
OG0006351: TCRU_8655 TcCLB-EL.507993.290_mRNA-p1 TcCLB-NE.
508893.30_mRNA-p1
OG0006352: TCRU_8659 TcCLB-EL.510259.30_mRNA-p1 TcCLB-NE.
509215.20_mRNA-p1
OG0006353: TCRU_8660 TcCLB-EL.510259.50_mRNA-p1 TcCLB-NE.
509215.40_mRNA-p1
OG0006354: TCRU_8661 TcCLB-EL.507809.74_mRNA-p1 TcCLB-NE.
511661.40_mRNA-p1
OG0006355: TCRU_8663 TcCLB-EL.507867.50_mRNA-p1 TcCLB-NE.
510629.410_mRNA-p1
OG0006356: TCRU_8664 TcCLB-EL.507867.90_mRNA-p1 TcCLB-NE.
510629.450_mRNA-p1
OG0006357: TCRU_8666 TcCLB-NE.504045.60_mRNA-p1 TcSYL_0019490.t1-p1
OG0006358: TCRU_founding TcCLB-NE.504045.20_mRNA-p1
TcSYL_0019480.t1-p1
OG0006359: TCRU_8669 TcCLB-EL.511211.230_mRNA-p1 TcCLB-NE.

510437.70_mRNA-p1
OG0006360: TCRU_8672 TcCLB-EL.510757.20_mRNA-p1 TcCLB-NE.
508413.20_mRNA-p1
OG0006361: TCRU_8674 TcCLB-EL.507089.90_mRNA-p1 TcCLB-NE.
504085.10_mRNA-p1
OG0006362: TCRU_8676 TcCLB-EL.507089.180_mRNA-p1 TcCLB-NE.
511027.29_pseudogenic_transcript-p1
OG0006363: TCRU_8677 TcCLB-EL.508317.40_mRNA-p1 TcCLB-NE.
507849.80_mRNA-p1
OG0006364: TCRU_8678 TcCLB-EL.506401.50_mRNA-p1 TcCLB-NE.
509605.10_mRNA-p1
OG0006365: TCRU_8679 TcCLB-EL.506401.90_mRNA-p1 TcCLB-NE.
503727.10_mRNA-p1
OG0006366: TCRU_8680 TcCLB-EL.510255.10_mRNA-p1 TcCLB-NE.
509213.20_mRNA-p1
OG0006367: TCRU_8681 TcCLB-EL.510743.60_mRNA-p1 TcCLB-NE.
510659.250_mRNA-p1
OG0006368: TCRU_8684 TcCLB-EL.507013.10_mRNA-p1 TcCLB-NE.
510659.120_mRNA-p1
OG0006369: TCRU_8685 TcCLB-EL.508465.30_mRNA-p1 TcCLB-NE.
510659.28_mRNA-p1
OG0006370: TCRU_8687 TcCLB-EL.508465.50_mRNA-p1 TcCLB-NE.
510659.10_mRNA-p1
OG0006371: TCRU_8694 TcCLB-NE.510817.60_mRNA-p1 TcSYL_0049990.t1-p1
OG0006372: TCRU_8700 TcCLB-EL.507559.40_mRNA-p1 TcCLB-NE.
510303.90_mRNA-p1
OG0006373: TCRU_8702 TcCLB-EL.511127.300_mRNA-p1 TcCLB-NE.
509027.50_mRNA-p1
OG0006374: TCRU_8711 TcCLB-EL.507993.250_mRNA-p1 TcCLB-NE.
511279.50_mRNA-p1
OG0006375: TCRU_8712 TcCLB-EL.507993.200_mRNA-p1 TcCLB-NE.
511279.10_mRNA-p1
OG0006376: TCRU_8713 TcCLB-EL.509895.80_pseudogenic_transcript-p1
TcCLB-NE.510007.70_mRNA-p1
OG0006377: TCRU_8718 TcCLB-EL.508207.64_mRNA-p1 TcCLB-NE.
507519.154_mRNA-p1
OG0006378: TCRU_8730 TcCLB-EL.511303.9_mRNA-p1 TcCLB-NE.
509179.60_mRNA-p1
OG0006379: TCRU_8731 TcCLB-EL.509891.10_mRNA-p1 TcCLB-NE.
510691.20_mRNA-p1
OG0006380: TCRU_8732 TcCLB-EL.507873.20_mRNA-p1 TcCLB-NE.
510689.50_mRNA-p1
OG0006381: TCRU_8734 TcCLB-EL.510603.150_mRNA-p1 TcCLB-NE.
509109.40_mRNA-p1
OG0006382: TCRU_8735 TcCLB-NE.510087.110_mRNA-p1 TcSYL_0051790.t1-p1
OG0006383: TCRU_8736 TcCLB-EL.507709.130_mRNA-p1 TcCLB-NE.
506577.160_mRNA-p1
OG0006384: TCRU_8739 TcCLB-EL.506147.80_mRNA-p1 TcCLB-NE.
508461.10_mRNA-p1
OG0006385: TCRU_8740 TcCLB-EL.506147.170_mRNA-p1 TcCLB-NE.
510735.9_mRNA-p1
OG0006386: TCRU_8741 TcCLB-EL.506147.180_mRNA-p1 TcCLB-NE.
510733.60_mRNA-p1
OG0006387: TCRU_8743 TcCLB-EL.509551.149_mRNA-p1 TcCLB-NE.

407335.9_mRNA-p1
OG0006388: TCRU_8744 TcCLB-EL.506773.120_mRNA-p1 TcCLB-NE.
508799.80_mRNA-p1
OG0006389: TCRU_8745 TcCLB-EL.506773.110_mRNA-p1 TcCLB-NE.
508799.100_mRNA-p1
OG0006390: TCRU_8747 TcCLB-EL.506773.70_mRNA-p1 TcCLB-NE.
508799.140_mRNA-p1
OG0006391: TCRU_8748 TcCLB-EL.506773.50_mRNA-p1 TcCLB-NE.
508799.150_mRNA-p1
OG0006392: TCRU_8752 TcCLB-EL.509267.10_mRNA-p1 TcCLB-NE.
507967.4_mRNA-p1
OG0006393: TCRU_8754 TcCLB-EL.511709.19_mRNA-p1 TcCLB-NE.
506425.80_mRNA-p1
OG0006394: TCRU_8756 TcCLB-EL.511711.50_mRNA-p1 TcCLB-NE.
506425.140_mRNA-p1
OG0006395: TCRU_8757 TcCLB-EL.509231.39_mRNA-p1 TcCLB-NE.
509719.69_mRNA-p1
OG0006396: TCRU_8763 TcCLB-EL.510515.90_mRNA-p1 TcCLB-NE.
509859.70_mRNA-p1
OG0006397: TCRU_8768 TcCLB-EL.511731.80_mRNA-p1 TcCLB-NE.
506831.60_mRNA-p1
OG0006398: TCRU_8769 TcCLB-EL.507883.80_mRNA-p1 TcCLB-NE.
506195.80_mRNA-p1
OG0006399: TCRU_8770 TcCLB-EL.507883.100_mRNA-p1 TcCLB-NE.
506195.100_pseudogenic_transcript-p1
OG0006400: TCRU_8771 TcCLB-EL.507883.109_mRNA-p1 TcCLB-NE.
506195.110_mRNA-p1
OG0006401: TCRU_8772 TcCLB-NE.511491.70_mRNA-p1 TcSYL_0167060.t1-p1
OG0006402: TCRU_8775 TcCLB-EL.508927.20_mRNA-p1 TcCLB-NE.
509237.30_mRNA-p1
OG0006403: TCRU_8787 TcCLB-EL.508723.40_mRNA-p1 TcCLB-NE.
509791.40_mRNA-p1
OG0006404: TCRU_8789 TcCLB-EL.509967.170_mRNA-p1 TcCLB-NE.
509695.130_mRNA-p1
OG0006405: TCRU_8792 TcCLB-EL.506887.50_mRNA-p1 TcCLB-NE.
510731.40_mRNA-p1
OG0006406: TCRU_8794 TcCLB-EL.503893.160_mRNA-p1 TcCLB-NE.
508505.30_mRNA-p1
OG0006407: TCRU_8795 TcCLB-EL.509157.60_mRNA-p1 TcCLB-NE.
510723.29_mRNA-p1
OG0006408: TCRU_8796 TcCLB-NE.508879.40_mRNA-p1 TcSYL_0023220.t1-p1
OG0006409: TCRU_8798 TcCLB-EL.509127.90_mRNA-p1 TcCLB-NE.
508737.180_mRNA-p1
OG0006410: TCRU_8805 TcCLB-EL.507257.30_mRNA-p1 TcCLB-NE.
510329.240_mRNA-p1
OG0006411: TCRU_8806 TcCLB-EL.507257.150_mRNA-p1 TcCLB-NE.
510329.360_mRNA-p1
OG0006412: TCRU_8809 TcCLB-EL.511545.80_mRNA-p1 TcSYL_0088160.t1-p1
OG0006413: TCRU_8811 TcCLB-EL.509169.10_mRNA-p1 TcCLB-NE.
507025.10_mRNA-p1
OG0006414: TCRU_8812 TcCLB-EL.508211.60_mRNA-p1 TcCLB-NE.
507515.90_mRNA-p1
OG0006415: TCRU_8813 TcCLB-EL.510289.40_mRNA-p1 TcCLB-NE.
510967.10_mRNA-p1

OG0006416: TCRU_8817 TcCLB-EL.511729.30_mRNA-p1 TcCLB-NE.
506835.40_mRNA-p1
OG0006417: TCRU_8818 TcCLB-EL.508169.40_mRNA-p1 TcCLB-NE.
510965.20_mRNA-p1
OG0006418: TCRU_8822 TcCLB-EL.506359.30_pseudogenic_transcript-p1
TcCLB-NE.503521.89_mRNA-p1
OG0006419: TCRU_8825 TcCLB-EL.506401.304_mRNA-p1 TcCLB-NE.
510835.24_mRNA-p1
OG0006420: TCRU_8829 TcCLB-NE.511367.170_mRNA-p1 TcSYL_0170020.t1-p1
OG0006421: TCRU_8831 TcCLB-EL.506779.160_mRNA-p1 TcCLB-NE.
511153.60_mRNA-p1
OG0006422: TCRU_8833 TcCLB-EL.506925.330_mRNA-p1 TcSYL_0080640.t1-p1
OG0006423: TCRU_8836 TcCLB-EL.507797.9_mRNA-p1 TcCLB-NE.
510645.20_mRNA-p1
OG0006424: TCRU_8837 TcCLB-EL.503579.119_mRNA-p1 TcCLB-NE.
507517.104_mRNA-p1
OG0006425: TCRU_8840 TcCLB-EL.508647.250_mRNA-p1 TcCLB-NE.
505183.5_mRNA-p1
OG0006426: TCRU_8841 TcCLB-EL.503531.10_mRNA-p1 TcCLB-NE.
511649.39_mRNA-p1
OG0006427: TCRU_8850 TcCLB-EL.508277.10_mRNA-p1 TcCLB-NE.
509941.110_mRNA-p1
OG0006428: TCRU_8851 TcCLB-EL.508277.30_mRNA-p1 TcCLB-NE.
509941.140_mRNA-p1
OG0006429: TCRU_8852 TcCLB-EL.508277.60_mRNA-p1 TcCLB-NE.
503543.4_mRNA-p1
OG0006430: TCRU_8857 TcCLB-EL.506943.69_mRNA-p1 TcCLB-NE.
509065.79_mRNA-p1
OG0006431: TCRU_8863 TcCLB-EL.506829.5_mRNA-p1 TcCLB-NE.
504231.10_mRNA-p1
OG0006432: TCRU_8865 TcCLB-EL.509891.60_mRNA-p1 TcCLB-NE.
510691.95_mRNA-p1
OG0006433: TCRU_8866 TcCLB-EL.509891.40_mRNA-p1 TcCLB-NE.
510691.70_mRNA-p1
OG0006434: TCRU_8875 TcCLB-EL.506947.50_mRNA-p1 TcCLB-NE.
507057.60_mRNA-p1
OG0006435: TCRU_8876 TcCLB-NE.508461.280_mRNA-p1 TcCLB-NE.
508461.310_mRNA-p1
OG0006436: TCRU_8884 TcCLB-EL.511711.110_mRNA-p1 TcCLB-NE.
503987.30_mRNA-p1
OG0006437: TCRU_8885 TcCLB-EL.511711.100_mRNA-p1 TcCLB-NE.
503987.20_mRNA-p1
OG0006438: TCRU_8886 TcCLB-EL.510431.280_mRNA-p1 TcCLB-NE.
507773.30_mRNA-p1
OG0006439: TCRU_8887 TcCLB-EL.510431.200_mRNA-p1 TcSYL_0138290.t1-p1
OG0006440: TCRU_8905 TcCLB-EL.504165.10_mRNA-p1 TcCLB-NE.
508981.30_mRNA-p1
OG0006441: TCRU_8915 TcCLB-EL.510513.30_mRNA-p1 TcCLB-NE.
509855.20_mRNA-p1
OG0006442: TCRU_8917 TcCLB-EL.510515.20_pseudogenic_transcript-p1
TcCLB-NE.509857.60_mRNA-p1
OG0006443: TCRU_8923 TcCLB-EL.506795.40_mRNA-p1 TcCLB-NE.
509937.30_mRNA-p1
OG0006444: TCRU_8926 TcCLB-EL.506795.10_mRNA-p1 TcCLB-NE.

509937.60_mRNA-p1
OG0006445: TCRU_8929 TcCLB-EL.504017.60_mRNA-p1 TcCLB-NE.
511231.30_mRNA-p1
OG0006446: TCRU_8930 TcCLB-EL.504017.54_mRNA-p1 TcCLB-NE.
511231.34_mRNA-p1
OG0006447: TCRU_8931 TcCLB-EL.504017.50_mRNA-p1 TcCLB-NE.
511231.40_mRNA-p1
OG0006448: TCRU_8932 TcCLB-EL.504017.40_mRNA-p1 TcCLB-NE.
511231.50_mRNA-p1
OG0006449: TCRU_8933 TcCLB-EL.504017.30_mRNA-p1 TcCLB-NE.
511231.60_mRNA-p1
OG0006450: TCRU_8934 TcCLB-EL.504017.20_mRNA-p1 TcCLB-NE.
511231.69_mRNA-p1
OG0006451: TCRU_8935 TcCLB-EL.504017.10_mRNA-p1 TcCLB-NE.
511231.80_mRNA-p1
OG0006452: TCRU_8938 TcCLB-EL.503755.20_mRNA-p1 TcCLB-NE.
510131.60_mRNA-p1
OG0006453: TCRU_8943 TcCLB-EL.506729.70_mRNA-p1 TcCLB-NE.
503843.30_mRNA-p1
OG0006454: TCRU_8945 TcCLB-EL.506773.30_mRNA-p1 TcCLB-NE.
508799.180_mRNA-p1
OG0006455: TCRU_8952 TcCLB-EL.506359.90_mRNA-p1 TcCLB-NE.
503521.20_mRNA-p1
OG0006456: TCRU_8953 TcCLB-EL.506359.99_mRNA-p1 TcCLB-NE.
503521.10_mRNA-p1
OG0006457: TCRU_8954 TcCLB-EL.509803.10_mRNA-p1 TcCLB-NE.
508889.19_mRNA-p1
OG0006458: TCRU_8955 TcCLB-EL.509803.30_mRNA-p1 TcCLB-NE.
508889.10_mRNA-p1
OG0006459: TCRU_8957 TcCLB-EL.509803.50_mRNA-p1 TcCLB-NE.
511273.50_mRNA-p1
OG0006460: TCRU_8964 TcCLB-EL.509069.49_mRNA-p1 TcCLB-NE.
508707.210_mRNA-p1
OG0006461: TCRU_8965 TcCLB-NE.508707.200_mRNA-p1 TcSYL_0112390.t1-p1
OG0006462: TCRU_8966 TcCLB-EL.509073.10_mRNA-p1 TcCLB-NE.
508707.194_mRNA-p1
OG0006463: TCRU_8968 TcCLB-EL.509073.30_mRNA-p1 TcCLB-NE.
508707.180_mRNA-p1
OG0006464: TCRU_8972 TcCLB-EL.509073.70_mRNA-p1 TcCLB-NE.
508707.140_mRNA-p1
OG0006465: TCRU_8973 TcCLB-EL.509073.80_mRNA-p1 TcCLB-NE.
508707.130_mRNA-p1
OG0006466: TCRU_8981 TcCLB-EL.507037.80_mRNA-p1 TcCLB-NE.
508707.40_mRNA-p1
OG0006467: TCRU_8983 TcCLB-EL.511543.20_mRNA-p1 TcCLB-NE.
508707.20_mRNA-p1
OG0006468: TCRU_8985 TcCLB-EL.511543.40_mRNA-p1 TcCLB-NE.
511019.120_mRNA-p1
OG0006469: TCRU_8996 TcCLB-EL.506467.29_mRNA-p1 TcCLB-NE.
505193.100_mRNA-p1
OG0006470: TCRU_8997 TcCLB-EL.506467.20_mRNA-p1 TcCLB-NE.
505193.90_mRNA-p1
OG0006471: TCRU_9007 TcCLB-EL.511181.10_mRNA-p1 TcCLB-NE.
503465.10_mRNA-p1

OG0006472: TCRU_9010 TcCLB-EL.511181.40_mRNA-p1 TcCLB-NE.
503465.40_mRNA-p1
OG0006473: TCRU_9021 TcCLB-EL.503833.30_mRNA-p1 TcCLB-NE.
511287.124_mRNA-p1
OG0006474: TCRU_9023 TcCLB-EL.503833.10_mRNA-p1 TcCLB-NE.
511287.140_mRNA-p1
OG0006475: TCRU_9025 TcCLB-EL.506211.240_mRNA-p1 TcCLB-NE.
511287.160_mRNA-p1
OG0006476: TCRU_9026 TcCLB-EL.506211.230_mRNA-p1 TcCLB-NE.
511287.170_mRNA-p1
OG0006477: TCRU_9043 TcCLB-EL.506811.220_mRNA-p1 TcCLB-NE.
506303.10_mRNA-p1
OG0006478: TCRU_9044 TcCLB-EL.506811.210_mRNA-p1 TcCLB-NE.
506303.20_mRNA-p1
OG0006479: TCRU_9047 TcCLB-EL.506811.170_mRNA-p1 TcCLB-NE.
506303.70_mRNA-p1
OG0006480: TCRU_9049 TcCLB-EL.506811.150_mRNA-p1 TcCLB-NE.
506303.90_mRNA-p1
OG0006481: TCRU_9050 TcCLB-EL.506811.140_mRNA-p1 TcCLB-NE.
506303.100_mRNA-p1
OG0006482: TCRU_9051 TcCLB-EL.506811.120_mRNA-p1 TcCLB-NE.
506303.111_mRNA-p1
OG0006483: TCRU_9061 TcCLB-NE.506559.500_mRNA-p1 TcSYL_0045370.t1-p1
OG0006484: TCRU_9063 TcCLB-NE.506559.480_mRNA-p1 TcSYL_0045360.t1-p1
OG0006485: TCRU_9064 TcCLB-NE.506559.470_mRNA-p1 TcSYL_0045350.t1-p1
OG0006486: TCRU_9074 TcCLB-EL.508717.60_mRNA-p1 TcCLB-NE.
506841.20_mRNA-p1
OG0006487: TCRU_9075 TcCLB-EL.508717.53_mRNA-p1 TcCLB-NE.
506841.8_mRNA-p1
OG0006488: TCRU_9078 TcCLB-EL.508717.33_mRNA-p1 TcCLB-NE.
504079.3_mRNA-p1
OG0006489: TCRU_9087 TcCLB-EL.511693.120_mRNA-p1 TcCLB-NE.
503559.80_mRNA-p1
OG0006490: TCRU_9088 TcCLB-EL.511693.100_mRNA-p1 TcCLB-NE.
503559.100_mRNA-p1
OG0006491: TCRU_9096 TcCLB-EL.508211.30_mRNA-p1 TcCLB-NE.
507515.120_mRNA-p1
OG0006492: TCRU_9100 TcCLB-EL.511589.10_mRNA-p1 TcCLB-NE.
508851.59_mRNA-p1
OG0006493: TCRU_9101 TcCLB-EL.511589.20_mRNA-p1 TcCLB-NE.
508851.70_mRNA-p1
OG0006494: TCRU_9102 TcCLB-EL.511589.29_mRNA-p1 TcCLB-NE.
508851.79_mRNA-p1
OG0006495: TCRU_9121 TcCLB-EL.511201.40_mRNA-p1 TcCLB-NE.
506587.10_mRNA-p1
OG0006496: TCRU_9122 TcCLB-EL.511201.19_mRNA-p1 TcCLB-NE.
506587.20_mRNA-p1
OG0006497: TCRU_9124 TcCLB-EL.506929.54_mRNA-p1 TcCLB-NE.
506587.44_mRNA-p1
OG0006498: TCRU_9128 TcCLB-EL.506375.100_mRNA-p1 TcCLB-NE.
510993.19_mRNA-p1
OG0006499: TCRU_9129 TcCLB-EL.506375.110_mRNA-p1 TcCLB-NE.
510993.10_mRNA-p1
OG0006500: TCRU_9130 TcCLB-EL.503401.40_mRNA-p1 TcCLB-NE.

508677.150_mRNA-p1
OG0006501: TCRU_9131 TcCLB-EL.503401.29_mRNA-p1 TcCLB-NE.
508677.134_mRNA-p1
OG0006502: TCRU_9132 TcCLB-EL.503401.20_mRNA-p1 TcCLB-NE.
508677.120_mRNA-p1
OG0006503: TCRU_9147 TcCLB-NE.503445.9_mRNA-p1 TcSYL_0014630.t1-p1
OG0006504: TCRU_9161 TcCLB-EL.511911.94_mRNA-p1 TcCLB-NE.
508637.63_mRNA-p1
OG0006505: TCRU_9162 TcCLB-EL.511911.98_mRNA-p1 TcCLB-NE.
508637.66_mRNA-p1
OG0006506: TCRU_9165 TcCLB-EL.511911.140_mRNA-p1 TcCLB-NE.
508637.100_mRNA-p1
OG0006507: TCRU_9166 TcCLB-EL.511911.150_mRNA-p1 TcCLB-NE.
508637.110_mRNA-p1
OG0006508: TCRU_9167 TcCLB-EL.509245.10_mRNA-p1 TcCLB-NE.
508637.120_mRNA-p1
OG0006509: TCRU_9174 TcCLB-EL.511803.30_mRNA-p1 TcSYL_0180050.t1-p1
OG0006510: TCRU_9175 TcCLB-EL.511803.40_mRNA-p1 TcSYL_0180080.t1-p1
OG0006511: TCRU_9181 TcCLB-EL.507445.20_pseudogenic_transcript-p1
TcSYL_0194550.t1-p1
OG0006512: TCRU_9189 TcCLB-NE.510187.290_mRNA-p1 TcSYL_0011540.t1-p1
OG0006513: TCRU_9191 TcCLB-EL.506211.107_mRNA-p1 TcCLB-NE.
508895.20_mRNA-p1
OG0006514: TCRU_9193 TcCLB-EL.506211.120_mRNA-p1 TcCLB-NE.
511289.110_mRNA-p1
OG0006515: TCRU_protein TcCLB-EL.506211.140_mRNA-p1 TcCLB-NE.
511289.90_mRNA-p1
OG0006516: TCRU_9215 TcCLB-EL.503855.30_mRNA-p1 TcCLB-NE.
510337.30_mRNA-p1
OG0006517: TCRU_9224 TcCLB-EL.507963.70_mRNA-p1 TcCLB-NE.
507031.40_mRNA-p1
OG0006518: TCRU_9225 TcCLB-EL.507963.60_mRNA-p1 TcCLB-NE.
507031.50_mRNA-p1
OG0006519: TCRU_9226 TcCLB-EL.507963.50_mRNA-p1 TcCLB-NE.
507031.60_mRNA-p1
OG0006520: TCRU_9227 TcCLB-EL.507963.40_mRNA-p1 TcCLB-NE.
507031.70_mRNA-p1
OG0006521: TCRU_9228 TcCLB-EL.507963.30_mRNA-p1 TcCLB-NE.
507031.80_mRNA-p1
OG0006522: TCRU_9229 TcCLB-EL.507963.20_mRNA-p1 TcCLB-NE.
507031.90_mRNA-p1
OG0006523: TCRU_9230 TcCLB-EL.507963.10_mRNA-p1 TcCLB-NE.
507031.100_mRNA-p1
OG0006524: TCRU_9231 TcCLB-EL.504111.30_mRNA-p1 TcCLB-NE.
507031.130_mRNA-p1
OG0006525: TCRU_9233 TcCLB-EL.504111.49_mRNA-p1 TcCLB-NE.
507031.150_mRNA-p1
OG0006526: TCRU_9235 TcCLB-EL.506505.10_mRNA-p1 TcCLB-NE.
507031.179_mRNA-p1
OG0006527: TCRU_9236 TcCLB-EL.506503.170_mRNA-p1 TcCLB-NE.
507033.4_mRNA-p1
OG0006528: TCRU_9237 TcCLB-EL.506503.160_mRNA-p1 TcCLB-NE.
507033.10_mRNA-p1
OG0006529: TCRU_9245 TcCLB-EL.506503.56_mRNA-p1 TcCLB-NE.

511293.90_mRNA-p1
OG0006530: TCRU_9246 TcCLB-EL.506503.53_mRNA-p1 TcCLB-NE.
504161.10_mRNA-p1
OG0006531: TCRU_9248 TcCLB-EL.506503.30_mRNA-p1 TcCLB-NE.
504161.40_mRNA-p1
OG0006532: TCRU_9249 TcCLB-EL.506503.10_mRNA-p1 TcCLB-NE.
504161.50_mRNA-p1
OG0006533: TCRU_9250 TcCLB-EL.506825.219_mRNA-p1 TcCLB-NE.
508175.114_mRNA-p1
OG0006534: TCRU_9255 TcCLB-EL.509151.110_mRNA-p1 TcCLB-NE.
508175.180_mRNA-p1
OG0006535: TCRU_9256 TcCLB-EL.509151.100_mRNA-p1 TcCLB-NE.
508175.189_mRNA-p1
OG0006536: TCRU_9257 TcCLB-EL.509151.80_mRNA-p1 TcCLB-NE.
508175.210_mRNA-p1
OG0006537: TCRU_9262 TcCLB-EL.509151.20_mRNA-p1 TcCLB-NE.
508175.270_mRNA-p1
OG0006538: TCRU_9265 TcCLB-EL.509149.70_mRNA-p1 TcCLB-NE.
508175.300_mRNA-p1
OG0006539: TCRU_9278 TcCLB-EL.508805.90_mRNA-p1 TcCLB-NE.
508297.41_mRNA-p1
OG0006540: TCRU_9289 TcCLB-EL.510507.29_mRNA-p1 TcCLB-NE.
509463.20_mRNA-p1
OG0006541: TCRU_9299 TcCLB-EL.506825.160_mRNA-p1 TcCLB-NE.
508175.60_mRNA-p1
OG0006542: TCRU_9300 TcCLB-EL.506825.150_mRNA-p1 TcCLB-NE.
508175.50_mRNA-p1
OG0006543: TCRU_like_ TcCLB-EL.506825.140_mRNA-p1 TcCLB-NE.
508175.39_mRNA-p1
OG0006544: TCRU_9302 TcCLB-EL.506825.130_mRNA-p1 TcCLB-NE.
508175.30_mRNA-p1
OG0006545: TCRU_9307 TcCLB-EL.506825.60_mRNA-p1 TcCLB-NE.
506681.60_mRNA-p1
OG0006546: TCRU_9316 TcCLB-EL.506579.60_mRNA-p1 TcCLB-NE.
506509.20_mRNA-p1
OG0006547: TCRU_9317 TcCLB-EL.506579.70_mRNA-p1 TcCLB-NE.
506509.30_mRNA-p1
OG0006548: TCRU_9318 TcCLB-EL.506579.80_mRNA-p1 TcCLB-NE.
506509.40_mRNA-p1
OG0006549: TCRU_9320 TcCLB-EL.506579.100_mRNA-p1 TcCLB-NE.
506509.60_mRNA-p1
OG0006550: TCRU_9324 TcCLB-EL.503527.20_mRNA-p1 TcCLB-NE.
510299.30_mRNA-p1
OG0006551: TCRU_9325 TcCLB-EL.503527.29_mRNA-p1 TcCLB-NE.
510299.40_mRNA-p1
OG0006552: TCRU_9326 TcCLB-EL.503527.40_mRNA-p1 TcCLB-NE.
510299.50_mRNA-p1
OG0006553: TCRU_9331 TcCLB-EL.507467.30_mRNA-p1 TcCLB-NE.
508355.20_mRNA-p1
OG0006554: TCRU_9332 TcCLB-EL.507467.20_mRNA-p1 TcCLB-NE.
508355.10_mRNA-p1
OG0006555: TCRU_9368 TcCLB-NE.504071.100_mRNA-p1 TcSYL_0167220.t1-p1
OG0006556: TCRU_9371 TcCLB-EL.510101.480_mRNA-p1 TcCLB-NE.
507765.10_mRNA-p1

OG0006557: TCRU_9373 TcCLB-EL.510101.460_mRNA-p1 TcCLB-NE.
507765.30_mRNA-p1
OG0006558: TCRU_9374 TcCLB-EL.510101.450_mRNA-p1 TcCLB-NE.
507765.40_mRNA-p1
OG0006559: TCRU_9384 TcCLB-NE.508173.180_mRNA-p1 TcSYL_0079340.t1-p1
OG0006560: TCRU_9386 TcCLB-EL.506735.60_mRNA-p1 TcCLB-NE.
509229.30_mRNA-p1
OG0006561: TCRU_9388 TcCLB-EL.505965.80_mRNA-p1 TcCLB-NE.
510903.20_mRNA-p1
OG0006562: TCRU_9402 TcCLB-NE.508479.280_mRNA-p1 TcSYL_0019740.t1-p1
OG0006563: TCRU_9404 TcCLB-NE.508479.270_mRNA-p1 TcSYL_0019760.t1-p1
OG0006564: TCRU_9405 TcCLB-NE.508479.260_mRNA-p1 TcSYL_0019770.t1-p1
OG0006565: TCRU_9407 TcCLB-NE.508479.240_mRNA-p1 TcSYL_0019790.t1-p1
OG0006566: TCRU_9413 TcCLB-NE.508479.200_mRNA-p1 TcSYL_0019800.t1-p1
OG0006567: TCRU_9415 TcCLB-NE.508479.180_mRNA-p1 TcSYL_0019810.t1-p1
OG0006568: TCRU_9416 TcCLB-NE.508479.174_mRNA-p1 TcSYL_0019830.t1-p1
OG0006569: TCRU_9417 TcCLB-NE.508479.160_mRNA-p1 TcSYL_0019850.t1-p1
OG0006570: TCRU_9422 TcCLB-EL.503653.90_mRNA-p1 TcCLB-NE.
509395.80_mRNA-p1
OG0006571: TCRU_9423 TcCLB-EL.503653.70_mRNA-p1 TcCLB-NE.
509395.90_mRNA-p1
OG0006572: TCRU_9424 TcCLB-EL.503653.60_mRNA-p1 TcCLB-NE.
509395.100_mRNA-p1
OG0006573: TCRU_9431 TcCLB-EL.506529.290_mRNA-p1 TcCLB-NE.
510887.40_mRNA-p1
OG0006574: TCRU_9432 TcCLB-EL.506529.280_mRNA-p1 TcCLB-NE.
510887.30_mRNA-p1
OG0006575: TCRU_9433 TcCLB-EL.506529.260_mRNA-p1 TcCLB-NE.
510887.14_mRNA-p1
OG0006576: TCRU_9436 TcCLB-EL.508539.155_mRNA-p1 TcCLB-NE.
511255.370_mRNA-p1
OG0006577: TCRU_9437 TcCLB-EL.503697.40_mRNA-p1 TcCLB-NE.
504105.250_mRNA-p1
OG0006578: TCRU_9438 TcCLB-EL.503697.50_mRNA-p1 TcCLB-NE.
504105.240_mRNA-p1
OG0006579: TCRU_9439 TcCLB-EL.503697.60_mRNA-p1 TcCLB-NE.
504105.230_mRNA-p1
OG0006580: TCRU_9451 TcCLB-EL.503753.20_mRNA-p1 TcCLB-NE.
506585.60_mRNA-p1
OG0006581: TCRU_9453 TcCLB-EL.506931.70_mRNA-p1 TcCLB-NE.
506585.80_mRNA-p1
OG0006582: TCRU_9457 TcSYL_0023720.t1-p1 TcSYL_0101760.t1-p1
OG0006583: TCRU_9459 TcCLB-EL.510181.140_mRNA-p1 TcCLB-NE.
503431.20_mRNA-p1
OG0006584: TCRU_9463 TcCLB-EL.510181.100_mRNA-p1 TcCLB-NE.
503431.60_mRNA-p1
OG0006585: TCRU_9464 TcCLB-EL.510181.90_mRNA-p1 TcCLB-NE.
503431.70_mRNA-p1
OG0006586: TCRU_9465 TcCLB-EL.510181.69_mRNA-p1 TcCLB-NE.
503431.90_mRNA-p1
OG0006587: TCRU_subunit TcCLB-EL.510181.60_mRNA-p1 TcCLB-NE.
503431.100_mRNA-p1
OG0006588: TCRU_9468 TcCLB-EL.510181.40_mRNA-p1 TcCLB-NE.
507609.20_mRNA-p1

OG0006589: TCRU_9497 TcCLB-EL.508241.130_mRNA-p1 TcSYL_0186000.t1-p1
OG0006590: TCRU_9499 TcCLB-EL.508241.104_mRNA-p1 TcSYL_0077540.t1-p1
OG0006591: TCRU_9516 TcCLB-EL.506563.130_pseudogenic_transcript-p1 TcCLB-NE.509001.20_mRNA-p1
OG0006592: TCRU_9517 TcCLB-EL.506563.160_mRNA-p1 TcCLB-NE.509001.10_mRNA-p1
OG0006593: TCRU_9519 TcCLB-EL.506563.180_pseudogenic_transcript-p1 TcCLB-NE.508999.250_pseudogenic_transcript-p1
OG0006594: TCRU_9520 TcCLB-EL.506563.190_mRNA-p1 TcCLB-NE.508999.230_pseudogenic_transcript-p1
OG0006595: TCRU_9525 TcCLB-EL.511393.40_mRNA-p1 TcCLB-NE.503565.20_mRNA-p1
OG0006596: TCRU_9526 TcCLB-EL.511393.60_mRNA-p1 TcCLB-NE.505071.140_mRNA-p1
OG0006597: TCRU_9527 TcCLB-EL.509157.120_mRNA-p1 TcCLB-NE.508443.60_mRNA-p1
OG0006598: TCRU_9533 TcCLB-EL.480975.10_mRNA-p1 TcCLB-NE.511249.100_mRNA-p1
OG0006599: TCRU_9536 TcCLB-EL.506777.64_mRNA-p1 TcCLB-NE.511163.24_mRNA-p1
OG0006600: TCRU_9538 TcCLB-EL.506777.50_mRNA-p1 TcCLB-NE.511163.40_mRNA-p1
OG0006601: TCRU_9539 TcCLB-EL.506777.40_mRNA-p1 TcCLB-NE.511163.50_mRNA-p1
OG0006602: TCRU_9546 TcCLB-NE.511367.280_mRNA-p1 TcSYL_0169950.t1-p1
OG0006603: TCRU_9548 TcCLB-NE.511367.240_mRNA-p1 TcSYL_0169970.t1-p1
OG0006604: TCRU_9556 TcCLB-EL.506953.20_mRNA-p1 TcCLB-NE.509287.10_mRNA-p1
OG0006605: TCRU_9561 TcCLB-EL.510343.10_mRNA-p1 TcCLB-NE.506469.90_mRNA-p1
OG0006606: TCRU_9562 TcCLB-EL.510343.5_mRNA-p1 TcCLB-NE.506469.100_mRNA-p1
OG0006607: TCRU_9563 TcCLB-EL.510341.10_mRNA-p1 TcCLB-NE.506469.110_mRNA-p1
OG0006608: TCRU_9565 TcCLB-EL.506715.50_mRNA-p1 TcCLB-NE.506469.130_mRNA-p1
OG0006609: TCRU_9567 TcCLB-EL.509007.60_mRNA-p1 TcCLB-NE.508965.60_mRNA-p1
OG0006610: TCRU_9568 TcCLB-EL.509007.70_mRNA-p1 TcCLB-NE.508965.50_mRNA-p1
OG0006611: TCRU_9569 TcCLB-EL.509007.80_mRNA-p1 TcCLB-NE.508965.39_mRNA-p1
OG0006612: TCRU_9571 TcCLB-EL.507079.20_mRNA-p1 TcCLB-EL.509009.10_mRNA-p1
OG0006613: TCRU_9572 TcCLB-EL.507079.10_mRNA-p1 TcCLB-NE.511385.120_mRNA-p1
OG0006614: TCRU_9573 TcCLB-EL.510773.120_mRNA-p1 TcCLB-NE.511385.110_mRNA-p1
OG0006615: TCRU_9575 TcCLB-EL.510773.70_mRNA-p1 TcCLB-NE.511385.80_mRNA-p1
OG0006616: TCRU_9576 TcCLB-EL.510773.50_mRNA-p1 TcCLB-NE.511385.60_mRNA-p1
OG0006617: TCRU_9577 TcCLB-EL.510773.40_mRNA-p1 TcCLB-NE.511385.50_mRNA-p1

OG0006618: TCRU_9578 TcCLB-EL.510773.30_mRNA-p1 TcCLB-NE.
511385.40_mRNA-p1
OG0006619: TCRU_9579 TcCLB-EL.510773.20_mRNA-p1 TcCLB-NE.
511385.30_mRNA-p1
OG0006620: TCRU_9584 TcCLB-EL.503819.10_mRNA-p1 TcCLB-NE.
510359.330_mRNA-p1
OG0006621: TCRU_9585 TcCLB-EL.506757.20_mRNA-p1 TcCLB-NE.
510359.350_mRNA-p1
OG0006622: TCRU_9587 TcCLB-EL.504039.130_mRNA-p1 TcCLB-NE.
510359.420_pseudogenic_transcript-p1
OG0006623: TCRU_9591 TcCLB-EL.509233.200_mRNA-p1 TcSYL_0146050.t1-p1
OG0006624: TCRU_9592 TcCLB-EL.509233.210_mRNA-p1 TcSYL_0146030.t1-p1
OG0006625: TCRU_9593 TcCLB-EL.503779.50_mRNA-p1 TcCLB-NE.
506247.420_mRNA-p1
OG0006626: TCRU_9613 TcCLB-EL.507927.90_mRNA-p1 TcCLB-NE.
509047.14_mRNA-p1
OG0006627: TCRU_9614 TcCLB-EL.507927.80_mRNA-p1 TcCLB-NE.
509047.20_mRNA-p1
OG0006628: TCRU_family TcCLB-EL.507927.60_mRNA-p1 TcCLB-NE.
509047.40_mRNA-p1
OG0006629: TCRU_9616 TcCLB-EL.507927.50_mRNA-p1 TcCLB-NE.
509047.50_mRNA-p1
OG0006630: TCRU_9617 TcCLB-EL.507927.40_mRNA-p1 TcCLB-NE.
509047.60_mRNA-p1
OG0006631: TCRU_9618 TcCLB-EL.507927.30_mRNA-p1 TcCLB-NE.
509047.70_mRNA-p1
OG0006632: TCRU_9619 TcCLB-EL.507927.20_mRNA-p1 TcCLB-NE.
509047.80_pseudogenic_transcript-p1
OG0006633: TCRU_9620 TcCLB-EL.507927.10_mRNA-p1 TcCLB-NE.
509047.100_mRNA-p1
OG0006634: TCRU_9621 TcCLB-EL.507927.4_mRNA-p1 TcCLB-NE.
509047.110_mRNA-p1
OG0006635: TCRU_9622 TcCLB-EL.503835.10_mRNA-p1 TcCLB-NE.
509047.119_mRNA-p1
OG0006636: TCRU_9623 TcCLB-EL.503835.20_mRNA-p1 TcCLB-NE.
509049.10_mRNA-p1
OG0006637: TCRU_9624 TcCLB-EL.503835.30_mRNA-p1 TcCLB-NE.
509049.20_mRNA-p1
OG0006638: TCRU_9625 TcCLB-EL.503835.39_mRNA-p1 TcCLB-NE.
509049.30_mRNA-p1
OG0006639: TCRU_9626 TcCLB-EL.509965.400_mRNA-p1 TcCLB-NE.
509049.51_mRNA-p1
OG0006640: TCRU_9627 TcCLB-EL.509965.380_mRNA-p1 TcCLB-NE.
509051.30_mRNA-p1
OG0006641: TCRU_9628 TcCLB-EL.509965.370_mRNA-p1 TcCLB-NE.
509051.40_mRNA-p1
OG0006642: TCRU_9629 TcCLB-EL.509965.340_mRNA-p1 TcCLB-NE.
509053.20_mRNA-p1
OG0006643: TCRU_9630 TcCLB-EL.509965.330_mRNA-p1 TcCLB-NE.
509053.30_mRNA-p1
OG0006644: TCRU_9631 TcCLB-EL.509965.319_mRNA-p1 TcCLB-NE.
509053.40_mRNA-p1
OG0006645: TCRU_9633 TcCLB-EL.509965.300_mRNA-p1 TcCLB-NE.
509053.60_mRNA-p1

OG0006646: TCRU_9634 TcCLB-EL.509965.290_mRNA-p1 TcCLB-NE.
509053.70_mRNA-p1
OG0006647: TCRU_9635 TcCLB-EL.509965.280_mRNA-p1 TcCLB-NE.
509053.80_mRNA-p1
OG0006648: TCRU_9636 TcCLB-EL.509965.270_mRNA-p1 TcCLB-NE.
509053.90_mRNA-p1
OG0006649: TCRU_9637 TcCLB-EL.509965.260_mRNA-p1 TcCLB-NE.
509053.100_mRNA-p1
OG0006650: TCRU_9638 TcCLB-EL.509965.250_mRNA-p1 TcCLB-NE.
509053.110_mRNA-p1
OG0006651: TCRU_9639 TcCLB-EL.509965.229_pseudogenic_transcript-p1
TcCLB-NE.509053.130_mRNA-p1
OG0006652: TCRU_9640 TcCLB-EL.509965.220_mRNA-p1 TcCLB-NE.
509053.140_mRNA-p1
OG0006653: TCRU_9641 TcCLB-EL.509965.200_mRNA-p1 TcCLB-NE.
509053.160_mRNA-p1
OG0006654: TCRU_9642 TcCLB-EL.509965.180_mRNA-p1 TcCLB-NE.
509053.179_mRNA-p1
OG0006655: TCRU_9643 TcCLB-EL.509965.170_mRNA-p1 TcCLB-NE.
509055.20_mRNA-p1
OG0006656: TCRU_9644 TcCLB-EL.509965.160_mRNA-p1 TcCLB-NE.
509055.30_mRNA-p1
OG0006657: TCRU_9645 TcCLB-EL.509965.144_mRNA-p1 TcCLB-NE.
509055.44_mRNA-p1
OG0006658: TCRU_9646 TcCLB-EL.509965.140_mRNA-p1 TcCLB-NE.
509055.50_mRNA-p1
OG0006659: TCRU_9647 TcCLB-EL.509965.134_mRNA-p1 TcCLB-NE.
509055.54_mRNA-p1
OG0006660: TCRU_9648 TcCLB-EL.509965.130_mRNA-p1 TcCLB-NE.
509055.60_mRNA-p1
OG0006661: TCRU_9649 TcCLB-EL.509965.120_mRNA-p1 TcCLB-NE.
509057.10_mRNA-p1
OG0006662: TCRU_9650 TcCLB-EL.509965.110_mRNA-p1 TcCLB-NE.
509057.20_mRNA-p1
OG0006663: TCRU_9661 TCRU_9955 TcSYL_0186890.t1-p1
OG0006664: TCRU_9667 TcCLB-EL.507629.20_mRNA-p1 TcSYL_0089370.t1-p1
OG0006665: TCRU_9668 TcCLB-EL.507629.10_mRNA-p1 TcCLB-NE.
504059.11_mRNA-p1
OG0006666: TCRU_9669 TcCLB-EL.507099.140_mRNA-p1 TcCLB-NE.
504059.20_mRNA-p1
OG0006667: TCRU_9670 TcCLB-EL.507099.130_mRNA-p1 TcCLB-NE.
504059.24_mRNA-p1
OG0006668: TCRU_9671 TcCLB-EL.507099.120_mRNA-p1 TcCLB-NE.
504059.30_mRNA-p1
OG0006669: TCRU_9673 TcCLB-EL.504277.30_mRNA-p1 TcCLB-NE.
506815.20_pseudogenic_transcript-p1
OG0006670: TCRU_9680 TcCLB-EL.510747.18_mRNA-p1 TcCLB-NE.
504185.20_mRNA-p1
OG0006671: TCRU_9681 TcCLB-EL.510747.26_mRNA-p1 TcCLB-NE.
504185.9_mRNA-p1
OG0006672: TCRU_9682 TcCLB-EL.510747.34_mRNA-p1 TcCLB-NE.
510657.220_mRNA-p1
OG0006673: TCRU_9685 TcCLB-NE.504071.50_mRNA-p1 TcSYL_0167260.t1-p1
OG0006674: TCRU_9687 TcCLB-NE.504071.30_mRNA-p1 TcSYL_0167290.t1-p1

OG0006675: TCRU_9688 TcCLB-NE.504071.20_mRNA-p1 TcSYL_0167300.t1-p1
OG0006676: TCRU_9693 TcCLB-EL.511585.140_mRNA-p1 TcCLB-NE.
510155.130_mRNA-p1
OG0006677: TCRU_9695 TcCLB-EL.511585.120_mRNA-p1 TcCLB-NE.
510155.110_mRNA-p1
OG0006678: TCRU_9696 TcCLB-EL.511585.110_mRNA-p1 TcCLB-NE.
510155.100_mRNA-p1
OG0006679: TCRU_9698 TcCLB-EL.511585.100_mRNA-p1 TcCLB-NE.
510155.94_mRNA-p1
OG0006680: TCRU_9703 TcCLB-EL.511585.10_mRNA-p1 TcCLB-NE.
510155.10_mRNA-p1
OG0006681: TCRU_9708 TcCLB-EL.506529.320_mRNA-p1 TcCLB-NE.
510887.70_mRNA-p1
OG0006682: TCRU_9709 TcCLB-EL.506529.324_mRNA-p1 TcCLB-NE.
510889.10_mRNA-p1
OG0006683: TCRU_9710 TcCLB-EL.506529.330_mRNA-p1 TcCLB-NE.
510889.20_mRNA-p1
OG0006684: TCRU_9711 TcCLB-EL.506529.340_mRNA-p1 TcCLB-NE.
510889.30_mRNA-p1
OG0006685: TCRU_9718 TcCLB-EL.507611.340_mRNA-p1 TcCLB-NE.
507723.160_mRNA-p1
OG0006686: TCRU_9725 TcCLB-EL.507009.30_mRNA-p1 TcCLB-NE.
510667.140_mRNA-p1
OG0006687: TCRU_9726 TcCLB-EL.507009.20_mRNA-p1 TcCLB-NE.
510669.9_mRNA-p1
OG0006688: TCRU_9732 TcCLB-NE.504045.100_mRNA-p1 TcSYL_0019520.t1-p1
OG0006689: TCRU_9739 TcCLB-EL.511211.100_mRNA-p1 TcSYL_0182510.t1-p1
OG0006690: TCRU_9749 TcCLB-EL.510171.10_mRNA-p1 TcCLB-NE.
509875.120_mRNA-p1
OG0006691: TCRU_9752 TcCLB-EL.507641.190_mRNA-p1 TcSYL_0011200.t1-p1
OG0006692: TCRU_9753 TcCLB-EL.507641.160_mRNA-p1 TcSYL_0011170.t1-p1
OG0006693: TCRU_9754 TcCLB-EL.507641.150_mRNA-p1 TcSYL_0011150.t1-p1
OG0006694: TCRU_9774 TcCLB-EL.506637.10_mRNA-p1 TcCLB-NE.
504199.10_mRNA-p1
OG0006695: TCRU_9776 TcCLB-EL.508781.120_mRNA-p1 TcCLB-NE.
506853.40_mRNA-p1
OG0006696: TCRU_9777 TcCLB-EL.508781.110_mRNA-p1 TcCLB-NE.
506853.50_mRNA-p1
OG0006697: TCRU_9778 TcCLB-EL.508781.90_mRNA-p1 TcCLB-NE.
506853.60_mRNA-p1
OG0006698: TCRU_9779 TcCLB-EL.508781.80_mRNA-p1 TcCLB-NE.
506853.70_mRNA-p1
OG0006699: TCRU_9780 TcCLB-EL.508781.60_mRNA-p1 TcCLB-NE.
506855.20_mRNA-p1
OG0006700: TCRU_9783 TcCLB-NE.508173.260_mRNA-p1 TcSYL_0079330.t1-p1
OG0006701: TCRU_9785 TcCLB-EL.504009.70_mRNA-p1 TcCLB-NE.
507049.190_mRNA-p1
OG0006702: TCRU_9786 TcCLB-EL.504009.60_mRNA-p1 TcCLB-NE.
507049.179_mRNA-p1
OG0006703: TCRU_9787 TcCLB-EL.504009.40_mRNA-p1 TcCLB-NE.
507049.170_mRNA-p1
OG0006704: TCRU_9789 TcCLB-EL.504009.10_mRNA-p1 TcCLB-NE.
507049.140_mRNA-p1
OG0006705: TCRU_9790 TcCLB-EL.506567.130_mRNA-p1 TcCLB-NE.

507049.110_mRNA-p1
OG0006706: TCRU_9791 TcCLB-EL.506567.120_mRNA-p1 TcCLB-NE.
507049.100_mRNA-p1
OG0006707: TCRU_9792 TcCLB-EL.506567.110_mRNA-p1 TcCLB-NE.
507049.90_mRNA-p1
OG0006708: TCRU_9795 TcCLB-EL.506567.80_mRNA-p1 TcCLB-NE.
507049.66_mRNA-p1
OG0006709: TCRU_9796 TcCLB-EL.506567.74_mRNA-p1 TcCLB-NE.
507049.63_mRNA-p1
OG0006710: TCRU_9798 TcCLB-EL.506567.50_mRNA-p1 TcCLB-NE.
507049.41_mRNA-p1
OG0006711: TCRU_9799 TcCLB-EL.506567.34_mRNA-p1 TcCLB-NE.
507049.30_mRNA-p1
OG0006712: TCRU_9800 TcCLB-EL.506567.10_mRNA-p1 TcCLB-NE.
507049.10_mRNA-p1
OG0006713: TCRU_9803 TcCLB-EL.510123.30_mRNA-p1 TcCLB-NE.
511837.50_mRNA-p1
OG0006714: TCRU_9806 TcCLB-EL.476421.9_mRNA-p1 TcCLB-NE.
511837.90_mRNA-p1
OG0006715: TCRU_9807 TcCLB-EL.510251.10_mRNA-p1 TcCLB-NE.
511837.100_mRNA-p1
OG0006716: TCRU_9808 TcCLB-EL.510251.19_mRNA-p1 TcCLB-NE.
511837.110_mRNA-p1
OG0006717: TCRU_9809 TcCLB-EL.506695.40_mRNA-p1 TcCLB-NE.
508153.560_mRNA-p1
OG0006718: TCRU_9812 TcCLB-EL.506695.10_mRNA-p1 TcCLB-NE.
508153.600_mRNA-p1
OG0006719: TCRU_9813 TcCLB-EL.506695.7_mRNA-p1 TcCLB-NE.
508153.605_mRNA-p1
OG0006720: TCRU_9815 TcCLB-EL.510315.30_mRNA-p1 TcCLB-NE.
508153.620_mRNA-p1
OG0006721: TCRU_9816 TcCLB-EL.510315.20_mRNA-p1 TcCLB-NE.
508153.630_mRNA-p1
OG0006722: TCRU_9817 TcCLB-EL.510315.14_mRNA-p1 TcCLB-NE.
508153.634_mRNA-p1
OG0006723: TCRU_9818 TcCLB-EL.510315.10_mRNA-p1 TcCLB-NE.
508153.640_mRNA-p1
OG0006724: TCRU_9820 TcCLB-EL.506693.40_mRNA-p1 TcCLB-NE.
508153.670_mRNA-p1
OG0006725: TCRU_9821 TcCLB-EL.506693.7_mRNA-p1 TcCLB-NE.
508153.720_mRNA-p1
OG0006726: TCRU_9823 TcCLB-EL.511213.60_mRNA-p1 TcCLB-NE.
511667.50_mRNA-p1
OG0006727: TCRU_9833 TcCLB-EL.506649.30_mRNA-p1 TcCLB-NE.
508351.60_mRNA-p1
OG0006728: TCRU_9836 TcCLB-EL.506649.60_mRNA-p1 TcCLB-NE.
508351.30_mRNA-p1
OG0006729: TCRU_9837 TcCLB-EL.506649.64_mRNA-p1 TcCLB-NE.
508351.20_mRNA-p1
OG0006730: TCRU_9838 TcCLB-EL.506649.70_mRNA-p1 TcCLB-NE.
508351.10_mRNA-p1
OG0006731: TCRU_9839 TcCLB-EL.503599.40_mRNA-p1 TcCLB-NE.
505163.30_mRNA-p1
OG0006732: TCRU_9840 TcCLB-EL.503599.30_mRNA-p1 TcCLB-NE.

505163.20_mRNA-p1
OG0006733: TCRU_9841 TcCLB-EL.503599.20_mRNA-p1 TcCLB-NE.
505163.10_mRNA-p1
OG0006734: TCRU_9845 TcCLB-EL.505123.4_mRNA-p1 TcCLB-NE.
508141.40_mRNA-p1
OG0006735: TCRU_9852 TcCLB-EL.508675.50_mRNA-p1 TcCLB-NE.
506983.20_mRNA-p1
OG0006736: TCRU_9858 TcCLB-NE.508355.180_mRNA-p1 TcSYL_0083050.t1-p1
OG0006737: TCRU_9859 TcCLB-NE.508355.170_mRNA-p1 TcSYL_0083060.t1-p1
OG0006738: TCRU_family_ TcCLB-NE.508355.160_mRNA-p1
TcSYL_0083080.t1-p1
OG0006739: TCRU_9861 TcCLB-NE.508355.150_mRNA-p1 TcSYL_0083090.t1-p1
OG0006740: TCRU_9862 TcCLB-EL.503433.10_mRNA-p1 TcCLB-NE.
508355.140_mRNA-p1
OG0006741: TCRU_9863 TcCLB-EL.503433.20_mRNA-p1 TcCLB-NE.
508355.130_mRNA-p1
OG0006742: TCRU_9864 TcCLB-EL.503433.30_mRNA-p1 TcCLB-NE.
508355.127_mRNA-p1
OG0006743: TCRU_9865 TcCLB-EL.507467.110_mRNA-p1 TcCLB-NE.
508355.120_mRNA-p1
OG0006744: TCRU_9872 TcCLB-EL.507671.10_mRNA-p1 TcCLB-NE.
506201.60_mRNA-p1
OG0006745: TCRU_9873 TcCLB-EL.507671.20_mRNA-p1 TcCLB-NE.
506201.50_mRNA-p1
OG0006746: TCRU_9875 TcCLB-EL.507673.10_mRNA-p1 TcCLB-NE.
506201.30_mRNA-p1
OG0006747: TCRU_9877 TcCLB-EL.507673.30_mRNA-p1 TcCLB-NE.
506201.10_mRNA-p1
OG0006748: TCRU_9878 TcCLB-EL.507673.40_mRNA-p1 TcCLB-NE.
506199.30_mRNA-p1
OG0006749: TCRU_9888 TcCLB-EL.509499.38_mRNA-p1 TcCLB-NE.
508649.20_mRNA-p1
OG0006750: TCRU_9889 TcCLB-EL.509499.29_mRNA-p1 TcCLB-NE.
508649.14_mRNA-p1
OG0006751: TCRU_9890 TcCLB-EL.509499.20_mRNA-p1 TcCLB-NE.
508649.10_mRNA-p1
OG0006752: TCRU_9891 TcCLB-EL.509499.14_mRNA-p1 TcCLB-NE.
508649.5_mRNA-p1
OG0006753: TCRU_9892 TcCLB-EL.509497.30_mRNA-p1 TcCLB-NE.
510947.40_mRNA-p1
OG0006754: TCRU_9894 TcCLB-EL.509497.9_mRNA-p1 TcCLB-NE.
510947.20_mRNA-p1
OG0006755: TCRU_9911 TcCLB-EL.506369.60_mRNA-p1 TcCLB-NE.
510225.20_mRNA-p1
OG0006756: TCRU_9912 TcCLB-EL.506369.50_mRNA-p1 TcCLB-NE.
510225.30_mRNA-p1
OG0006757: TCRU_9914 TcCLB-EL.506369.30_mRNA-p1 TcCLB-NE.
510225.50_mRNA-p1
OG0006758: TCRU_9915 TcCLB-EL.506369.20_mRNA-p1 TcCLB-NE.
510225.59_mRNA-p1
OG0006759: TCRU_9918 TcCLB-EL.509831.40_mRNA-p1 TcCLB-NE.
506629.20_mRNA-p1
OG0006760: TCRU_9920 TcCLB-EL.509831.33_mRNA-p1 TcCLB-NE.
506629.40_mRNA-p1

OG0006761: TCRU_9922 TcCLB-EL.509831.10_mRNA-p1 TcCLB-NE.
506629.70_mRNA-p1
OG0006762: TCRU_9924 TcCLB-EL.506367.174_mRNA-p1 TcCLB-NE.
506629.90_mRNA-p1
OG0006763: TCRU_9928 TcCLB-EL.506367.89_mRNA-p1 TcCLB-NE.
506629.160_mRNA-p1
OG0006764: TCRU_9929 TcCLB-EL.506367.80_mRNA-p1 TcCLB-NE.
506629.170_mRNA-p1
OG0006765: TCRU_9939 TcCLB-NE.509039.20_mRNA-p1 TcSYL_0167190.t1-p1
OG0006766: TCRU_9941 TcCLB-EL.508489.30_mRNA-p1 TcCLB-NE.
511495.11_mRNA-p1
OG0006767: TCRU_9942 TcCLB-EL.508489.20_mRNA-p1 TcCLB-NE.
511495.20_mRNA-p1
OG0006768: TCRU_9945 TcCLB-NE.507019.120_mRNA-p1 TcSYL_0168860.t1-p1
OG0006769: TCRU_9947 TcCLB-NE.507019.140_mRNA-p1 TcSYL_0168810.t1-p1
OG0006770: TCRU_9951 TcCLB-EL.509543.30_mRNA-p1 TcCLB-NE.
511257.30_mRNA-p1
OG0006771: TCRU_9952 TcCLB-EL.509543.40_mRNA-p1 TcCLB-NE.
511257.20_mRNA-p1
OG0006772: TCRU_9971 TcCLB-EL.503899.100_mRNA-p1 TcCLB-NE.
503625.20_mRNA-p1
OG0006773: TCRU_9973 TcCLB-EL.503899.80_mRNA-p1 TcCLB-NE.
507513.130_pseudogenic_transcript-p1
OG0006774: TCRU_9975 TcCLB-EL.503899.60_mRNA-p1 TcCLB-NE.
507513.100_mRNA-p1
OG0006775: TCRU_9977 TcCLB-EL.503899.40_mRNA-p1 TcCLB-NE.
507513.80_mRNA-p1
OG0006776: TCRU_9980 TcCLB-EL.506729.20_mRNA-p1 TcCLB-NE.
507513.40_mRNA-p1
OG0006777: TCRU_9981 TcCLB-EL.506729.40_mRNA-p1 TcCLB-NE.
507513.20_mRNA-p1
OG0006778: TCRU_9984 TcCLB-EL.511423.170_mRNA-p1 TcCLB-NE.
507949.10_mRNA-p1
OG0006779: TCRU_9985 TcCLB-EL.508993.20_mRNA-p1 TcCLB-NE.
506479.120_mRNA-p1
OG0006780: TCRU_9986 TcCLB-EL.503805.40_mRNA-p1 TcCLB-NE.
506479.110_mRNA-p1
OG0006781: TCRU_9987 TcCLB-EL.503805.30_mRNA-p1 TcCLB-NE.
506479.100_mRNA-p1
OG0006782: TCRU_9992 TcCLB-EL.506657.88_mRNA-p1 TcCLB-NE.
511247.9_mRNA-p1
OG0006783: TCRU_9993 TcCLB-EL.506657.79_mRNA-p1 TcCLB-NE.
511247.18_mRNA-p1
OG0006784: TCRU_9995 TcCLB-EL.506657.50_pseudogenic_transcript-p1
TcCLB-NE.511249.10_mRNA-p1
OG0006785: TCRU_9997 TcCLB-EL.506657.20_mRNA-p1 TcCLB-NE.
511249.34_mRNA-p1
OG0006786: TCRU_9998 TcCLB-EL.506657.10_mRNA-p1 TcCLB-NE.
511249.40_mRNA-p1
OG0006787: TCRU_10027 TcCLB-EL.507047.170_mRNA-p1 TcCLB-NE.
509179.80_mRNA-p1
OG0006788: TCRU_10028 TcCLB-EL.507047.160_mRNA-p1 TcCLB-NE.
509179.90_mRNA-p1
OG0006789: TCRU_10030 TcCLB-EL.507047.120_mRNA-p1 TcCLB-NE.

509179.140_mRNA-p1
OG0006790: TCRU_10032 TcCLB-EL.507047.100_mRNA-p1 TcCLB-NE.
509179.160_mRNA-p1
OG0006791: TCRU_10040 TcCLB-NE.503773.40_mRNA-p1 TcSYL_0094690.t1-p1
OG0006792: TCRU_10043 TcCLB-EL.511575.50_mRNA-p1 TcSYL_0094720.t1-p1
OG0006793: TCRU_10049 TcCLB-EL.511307.20_mRNA-p1 TcCLB-NE.
507087.30_mRNA-p1
OG0006794: TCRU_10051 TcCLB-EL.511571.20_mRNA-p1 TcCLB-NE.
506229.64_mRNA-p1
OG0006795: TCRU_10059 TcCLB-EL.506931.20_mRNA-p1 TcCLB-NE.
510131.30_mRNA-p1
OG0006796: TCRU_10061 TcCLB-EL.506931.4_mRNA-p1 TcCLB-NE.
510131.44_mRNA-p1
OG0006797: TCRU_10063 TcCLB-EL.511215.20_mRNA-p1 TcSYL_0187920.t1-p1
OG0006798: TCRU_10064 TcCLB-EL.511215.30_mRNA-p1 TcSYL_0187950.t1-p1
OG0006799: TCRU_10066 TcCLB-EL.503525.40_mRNA-p1 TcCLB-NE.
507825.30_mRNA-p1
OG0006800: TCRU_10067 TcCLB-EL.503525.20_mRNA-p1 TcCLB-NE.
507825.49_mRNA-p1
OG0006801: TCRU_10068 TcCLB-EL.503525.10_mRNA-p1 TcCLB-NE.
509797.20_mRNA-p1
OG0006802: TCRU_family TcCLB-EL.503525.4_mRNA-p1 TcCLB-NE.
509797.30_mRNA-p1
OG0006803: TCRU_10073 TcCLB-EL.506659.10_mRNA-p1 TcCLB-NE.
508865.30_mRNA-p1
OG0006804: TCRU_10074 TcCLB-EL.506529.130_mRNA-p1 TcCLB-NE.
510885.40_mRNA-p1
OG0006805: TCRU_10075 TcCLB-EL.506529.140_mRNA-p1 TcCLB-NE.
510885.50_mRNA-p1
OG0006806: TCRU_10076 TcCLB-EL.506529.150_mRNA-p1 TcCLB-NE.
510885.60_mRNA-p1
OG0006807: TCRU_10077 TcCLB-EL.506529.170_mRNA-p1 TcCLB-NE.
510885.80_mRNA-p1
OG0006808: TCRU_10078 TcCLB-EL.506529.180_mRNA-p1 TcCLB-NE.
510885.90_mRNA-p1
OG0006809: TCRU_10079 TcCLB-EL.506529.190_mRNA-p1 TcCLB-NE.
510885.100_mRNA-p1
OG0006810: TCRU_10080 TcCLB-EL.506529.200_mRNA-p1 TcCLB-NE.
510885.110_mRNA-p1
OG0006811: TCRU_10081 TcCLB-EL.506529.230_mRNA-p1 TcCLB-NE.
510885.140_mRNA-p1
OG0006812: TCRU_10085 TcCLB-EL.506563.90_pseudogenic_transcript-p1
TcCLB-NE.509003.20_mRNA-p1
OG0006813: TCRU_10089 TcCLB-EL.506563.50_pseudogenic_transcript-p1
TcCLB-NE.509003.60_mRNA-p1
OG0006814: TCRU_10090 TcCLB-EL.508777.60_mRNA-p1 TcCLB-NE.
509029.90_mRNA-p1
OG0006815: TCRU_10091 TcCLB-EL.508777.70_mRNA-p1 TcCLB-NE.
509029.100_mRNA-p1
OG0006816: TCRU_10092 TcCLB-EL.508777.80_mRNA-p1 TcCLB-NE.
509029.110_mRNA-p1
OG0006817: TCRU_10094 TcCLB-EL.508777.100_mRNA-p1 TcCLB-NE.
509029.130_mRNA-p1
OG0006818: TCRU_10102 TcCLB-EL.506529.524_mRNA-p1 TcCLB-NE.

510889.240_mRNA-p1
OG0006819: TCRU_10103 TcCLB-EL.506529.530_mRNA-p1 TcCLB-NE.
510889.251_mRNA-p1
OG0006820: TCRU_10110 TcCLB-EL.510077.40_mRNA-p1 TcCLB-NE.
505037.50_mRNA-p1
OG0006821: TCRU_10114 TcCLB-EL.506739.20_mRNA-p1 TcCLB-NE.
510819.20_mRNA-p1
OG0006822: TCRU_10120 TcCLB-EL.503789.60_mRNA-p1 TcCLB-NE.
505183.60_mRNA-p1
OG0006823: TCRU_10122 TcCLB-EL.503789.30_mRNA-p1 TcCLB-NE.
505183.90_mRNA-p1
OG0006824: TCRU_10124 TcCLB-EL.504125.120_mRNA-p1 TcCLB-NE.
505183.124_mRNA-p1
OG0006825: TCRU_10126 TcCLB-EL.504125.90_mRNA-p1 TcCLB-NE.
505183.140_mRNA-p1
OG0006826: TCRU_10127 TcCLB-EL.504125.80_mRNA-p1 TcCLB-NE.
505183.160_mRNA-p1
OG0006827: TCRU_10128 TcCLB-EL.504125.70_mRNA-p1 TcCLB-NE.
503693.20_mRNA-p1
OG0006828: TCRU_10129 TcCLB-EL.504125.64_mRNA-p1 TcCLB-NE.
503693.29_mRNA-p1
OG0006829: TCRU_10132 TcCLB-EL.507093.184_mRNA-p1 TcSYL_0080390.t1-
p1
OG0006830: TCRU_10133 TcCLB-EL.507093.180_mRNA-p1 TcSYL_0080380.t1-
p1
OG0006831: TCRU_10134 TcCLB-EL.507093.160_mRNA-p1 TcSYL_0080360.t1-
p1
OG0006832: TCRU_10146 TcCLB-EL.506401.280_mRNA-p1 TcCLB-NE.
508543.180_mRNA-p1
OG0006833: TCRU_10147 TcCLB-EL.506401.250_mRNA-p1 TcCLB-NE.
508543.170_mRNA-p1
OG0006834: TCRU_10148 TcCLB-EL.506649.90_mRNA-p1 TcCLB-NE.
508349.30_mRNA-p1
OG0006835: TCRU_10152 TcCLB-EL.511365.94_mRNA-p1 TcCLB-NE.
511297.20_mRNA-p1
OG0006836: TCRU_10153 TcCLB-EL.511365.90_mRNA-p1 TcCLB-NE.
511297.30_mRNA-p1
OG0006837: TCRU_10158 TcCLB-EL.482369.10_mRNA-p1 TcCLB-NE.
507241.10_mRNA-p1
OG0006838: TCRU_10159 TcCLB-EL.482369.20_mRNA-p1 TcCLB-NE.
507241.20_mRNA-p1
OG0006839: TCRU_10162 TcCLB-EL.509669.150_mRNA-p1 TcCLB-NE.
506175.90_mRNA-p1
OG0006840: TCRU_10165 TcCLB-EL.504625.80_mRNA-p1 TcCLB-NE.
511635.60_mRNA-p1
OG0006841: TCRU_10166 TcCLB-EL.504625.74_mRNA-p1 TcCLB-NE.
511635.69_mRNA-p1
OG0006842: TCRU_10168 TcCLB-EL.504625.40_mRNA-p1 TcCLB-NE.
503583.70_mRNA-p1
OG0006843: TCRU_10169 TcCLB-EL.504625.30_mRNA-p1 TcCLB-NE.
503583.60_mRNA-p1
OG0006844: TCRU_10170 TcCLB-EL.504625.20_mRNA-p1 TcCLB-NE.
503583.50_mRNA-p1
OG0006845: TCRU_10173 TcCLB-EL.503489.24_mRNA-p1 TcCLB-NE.

503583.24_mRNA-p1
OG0006846: TCRU_10174 TcCLB-EL.503489.20_mRNA-p1 TcCLB-NE.
503583.20_mRNA-p1
OG0006847: TCRU_10177 TcCLB-EL.503419.20_mRNA-p1 TcCLB-NE.
509105.60_mRNA-p1
OG0006848: TCRU_10179 TcCLB-EL.503419.40_mRNA-p1 TcCLB-NE.
509105.80_mRNA-p1
OG0006849: TCRU_10180 TcCLB-EL.503419.50_mRNA-p1 TcCLB-NE.
509105.90_mRNA-p1
OG0006850: TCRU_10181 TcCLB-EL.503419.54_mRNA-p1 TcCLB-NE.
509105.94_mRNA-p1
OG0006851: TCRU_10182 TcCLB-EL.511309.40_mRNA-p1 TcCLB-NE.
503399.10_mRNA-p1
OG0006852: TCRU_10183 TcCLB-EL.511309.30_mRNA-p1 TcCLB-NE.
503399.20_mRNA-p1
OG0006853: TCRU_10185 TcCLB-EL.510761.60_mRNA-p1 TcSYL_0115390.t1-p1
OG0006854: TCRU_10198 TcCLB-EL.508765.40_mRNA-p1 TcCLB-NE.
506617.50_mRNA-p1
OG0006855: TCRU_10206 TcCLB-EL.508781.129_pseudogenic_transcript-p1
TcCLB-NE.506853.30_mRNA-p1
OG0006856: TCRU_10207 TcCLB-EL.417797.9_mRNA-p1 TcCLB-NE.
506853.20_mRNA-p1
OG0006857: TCRU_10208 TcCLB-EL.511135.20_mRNA-p1 TcCLB-NE.
506853.10_mRNA-p1
OG0006858: TCRU_10211 TcCLB-EL.506401.100_mRNA-p1 TcCLB-NE.
508543.10_mRNA-p1
OG0006859: TCRU_10212 TcCLB-EL.506401.110_mRNA-p1 TcCLB-NE.
508543.20_mRNA-p1
OG0006860: TCRU_10214 TcCLB-EL.506401.130_mRNA-p1 TcCLB-NE.
508543.40_mRNA-p1
OG0006861: TCRU_10215 TcCLB-EL.506401.140_mRNA-p1 TcCLB-NE.
508543.60_mRNA-p1
OG0006862: TCRU_10216 TcCLB-EL.506401.150_mRNA-p1 TcCLB-NE.
508543.70_mRNA-p1
OG0006863: TCRU_10217 TcCLB-EL.506401.160_mRNA-p1 TcCLB-NE.
508543.80_mRNA-p1
OG0006864: TCRU_10218 TcCLB-EL.506401.170_mRNA-p1 TcCLB-NE.
508543.90_mRNA-p1
OG0006865: TCRU_10219 TcCLB-EL.506401.180_mRNA-p1 TcCLB-NE.
508543.100_mRNA-p1
OG0006866: TCRU_10220 TcCLB-EL.506401.190_mRNA-p1 TcCLB-NE.
508543.110_mRNA-p1
OG0006867: TCRU_10221 TcCLB-EL.506401.200_mRNA-p1 TcCLB-NE.
508543.120_mRNA-p1
OG0006868: TCRU_10222 TcCLB-EL.506401.210_mRNA-p1 TcCLB-NE.
508543.129_mRNA-p1
OG0006869: TCRU_10224 TcCLB-NE.509085.20_pseudogenic_transcript-p1
TcCLB-NE.511563.30_pseudogenic_transcript-p1
OG0006870: TCRU_10236 TcCLB-EL.506399.60_mRNA-p1 TcCLB-NE.
511909.70_mRNA-p1
OG0006871: TCRU_10243 TcCLB-EL.506857.50_mRNA-p1 TcCLB-NE.
509207.120_mRNA-p1
OG0006872: TCRU_10244 TcCLB-EL.506857.60_mRNA-p1 TcCLB-NE.
509207.130_mRNA-p1

OG0006873: TCRU_10245 TcCLB-EL.506857.80_mRNA-p1 TcCLB-NE.509207.150_mRNA-p1
OG0006874: TCRU_10246 TcCLB-EL.506857.90_mRNA-p1 TcCLB-NE.509207.160_mRNA-p1
OG0006875: TCRU_10247 TcCLB-EL.506857.100_mRNA-p1 TcCLB-NE.509209.10_mRNA-p1
OG0006876: TCRU_10248 TcCLB-EL.506857.110_mRNA-p1 TcCLB-NE.509209.20_mRNA-p1
OG0006877: TCRU_10249 TcCLB-EL.506859.4_mRNA-p1 TcCLB-NE.511813.10_mRNA-p1
OG0006878: TCRU_10250 TcCLB-EL.506859.10_mRNA-p1 TcCLB-NE.511813.20_mRNA-p1
OG0006879: TCRU_10251 TcCLB-EL.506859.20_mRNA-p1 TcCLB-NE.511815.4_mRNA-p1
OG0006880: TCRU_10252 TcCLB-EL.506859.30_mRNA-p1 TcCLB-NE.511815.10_mRNA-p1
OG0006881: TCRU_10253 TcCLB-EL.506859.70_mRNA-p1 TcCLB-NE.511815.40_mRNA-p1
OG0006882: TCRU_10254 TcCLB-EL.506859.80_mRNA-p1 TcCLB-NE.511815.50_mRNA-p1
OG0006883: TCRU_10255 TcCLB-EL.506859.90_mRNA-p1 TcCLB-NE.511815.60_mRNA-p1
OG0006884: TCRU_10256 TcCLB-EL.506859.100_mRNA-p1 TcCLB-NE.511815.70_mRNA-p1
OG0006885: TCRU_10257 TcCLB-EL.506859.110_mRNA-p1 TcCLB-NE.511815.80_mRNA-p1
OG0006886: TCRU_10258 TcCLB-EL.506859.120_mRNA-p1 TcCLB-NE.511815.90_mRNA-p1
OG0006887: TCRU_10259 TcCLB-EL.506859.130_mRNA-p1 TcCLB-NE.511815.100_mRNA-p1
OG0006888: TCRU_10260 TcCLB-EL.506859.150_mRNA-p1 TcCLB-NE.511815.150_mRNA-p1
OG0006889: TCRU_10261 TcCLB-EL.506859.160_mRNA-p1 TcCLB-NE.511815.160_mRNA-p1
OG0006890: TCRU_10264 TcCLB-EL.507521.40_mRNA-p1 TcCLB-NE.508579.10_mRNA-p1
OG0006891: TCRU_10266 TcCLB-EL.507521.20_mRNA-p1 TcCLB-NE.508579.30_mRNA-p1
OG0006892: TCRU_10267 TcCLB-EL.507521.10_mRNA-p1 TcCLB-NE.508579.40_mRNA-p1
OG0006893: TCRU_10272 TcCLB-EL.506749.30_mRNA-p1 TcCLB-NE.507891.56_mRNA-p1
OG0006894: TCRU_10274 TcCLB-EL.511753.90_mRNA-p1 TcCLB-NE.511501.40_mRNA-p1
OG0006895: TCRU_10275 TcCLB-EL.511753.100_mRNA-p1 TcCLB-NE.511501.30_mRNA-p1
OG0006896: TCRU_10279 TcCLB-EL.503575.27_mRNA-p1 TcCLB-NE.510943.23_mRNA-p1
OG0006897: TCRU_10281 TcCLB-EL.503575.40_mRNA-p1 TcCLB-NE.510943.30_mRNA-p1
OG0006898: TCRU_10285 TcCLB-EL.511131.20_mRNA-p1 TcCLB-NE.506855.370_mRNA-p1
OG0006899: TCRU_10286 TcCLB-EL.511131.30_mRNA-p1 TcCLB-NE.506855.360_mRNA-p1

OG0006900: TCRU_10287 TcCLB-EL.511131.50_mRNA-p1 TcCLB-NE.
506855.340_mRNA-p1
OG0006901: TCRU_10288 TcCLB-EL.511131.60_mRNA-p1 TcCLB-NE.
506855.330_mRNA-p1
OG0006902: TCRU_10289 TcCLB-EL.511131.70_mRNA-p1 TcCLB-NE.
506855.320_mRNA-p1
OG0006903: TCRU_10290 TcCLB-EL.511131.80_mRNA-p1 TcCLB-NE.
506855.310_mRNA-p1
OG0006904: TCRU_10291 TcCLB-EL.511131.89_mRNA-p1 TcCLB-NE.
506855.300_mRNA-p1
OG0006905: TCRU_10292 TcCLB-EL.511131.98_mRNA-p1 TcCLB-NE.
506855.290_mRNA-p1
OG0006906: TCRU_10293 TcCLB-EL.511133.4_mRNA-p1 TcCLB-NE.
506855.280_mRNA-p1
OG0006907: TCRU_10294 TcCLB-EL.511133.20_mRNA-p1 TcCLB-NE.
506855.260_mRNA-p1
OG0006908: TCRU_10295 TcCLB-EL.511133.30_mRNA-p1 TcCLB-NE.
506855.250_mRNA-p1
OG0006909: TCRU_10296 TcCLB-EL.503479.70_mRNA-p1 TcCLB-NE.
506855.240_pseudogenic_transcript-p1
OG0006910: TCRU_10297 TcCLB-EL.503479.60_mRNA-p1 TcCLB-NE.
506855.220_mRNA-p1
OG0006911: TCRU_10298 TcCLB-EL.503479.49_mRNA-p1 TcCLB-NE.
506855.209_mRNA-p1
OG0006912: TCRU_10299 TcCLB-EL.503479.40_mRNA-p1 TcCLB-NE.
506855.190_mRNA-p1
OG0006913: TCRU_10300 TcCLB-EL.503479.30_mRNA-p1 TcCLB-NE.
506855.180_mRNA-p1
OG0006914: TCRU_10301 TcCLB-EL.503479.20_pseudogenic_transcript-p1
TcCLB-NE.506855.170_mRNA-p1
OG0006915: TCRU_10302 TcCLB-EL.508779.9_mRNA-p1 TcCLB-NE.
506855.160_mRNA-p1
OG0006916: TCRU_10303 TcCLB-EL.508779.20_mRNA-p1 TcCLB-NE.
506855.150_mRNA-p1
OG0006917: TCRU_10307 TcCLB-EL.506525.150_mRNA-p1 TcCLB-NE.
511283.140_mRNA-p1
OG0006918: TCRU_10308 TcCLB-EL.506525.140_mRNA-p1 TcCLB-NE.
511283.130_mRNA-p1
OG0006919: TCRU_10310 TcCLB-EL.506525.90_mRNA-p1 TcCLB-NE.
511283.100_mRNA-p1
OG0006920: TCRU_10312 TcCLB-EL.506525.70_mRNA-p1 TcCLB-NE.
511283.80_mRNA-p1
OG0006921: TCRU_10313 TcCLB-EL.506525.50_mRNA-p1 TcCLB-NE.
511283.60_mRNA-p1
OG0006922: TCRU_10315 TcCLB-EL.506525.40_mRNA-p1 TcCLB-NE.
511283.40_mRNA-p1
OG0006923: TCRU_10316 TcCLB-EL.506525.30_mRNA-p1 TcCLB-NE.
511283.30_mRNA-p1
OG0006924: TCRU_10326 TcCLB-EL.507669.200_mRNA-p1 TcCLB-NE.
509875.260_mRNA-p1
OG0006925: TCRU_10327 TcCLB-EL.507669.210_mRNA-p1 TcCLB-NE.
509875.250_mRNA-p1
OG0006926: TCRU_10329 TcCLB-EL.507669.230_mRNA-p1 TcCLB-NE.
509875.230_mRNA-p1

OG0006927: TCRU_10335 TcCLB-EL.506925.60_mRNA-p1 TcSYL_0080520.t1-p1
OG0006928: TCRU_10336 TcCLB-EL.506925.50_mRNA-p1 TcSYL_0080510.t1-p1
OG0006929: TCRU_10337 TcCLB-EL.506925.40_mRNA-p1 TcSYL_0080500.t1-p1
OG0006930: TCRU_10340 TcCLB-EL.506925.14_mRNA-p1 TcSYL_0080460.t1-p1
OG0006931: TCRU_10341 TcCLB-EL.506925.10_mRNA-p1 TcSYL_0080450.t1-p1
OG0006932: TCRU_10342 TcCLB-NE.506323.30_mRNA-p1 TcCLB-NE.
511929.30_mRNA-p1
OG0006933: TCRU_10351 TcCLB-EL.510317.40_mRNA-p1 TcCLB-NE.
508153.420_mRNA-p1
OG0006934: TCRU_10359 TcCLB-EL.506697.10_mRNA-p1 TcCLB-NE.
508153.540_mRNA-p1
OG0006935: TCRU_10369 TcCLB-EL.508277.200_mRNA-p1 TcCLB-NE.
509945.20_mRNA-p1
OG0006936: TCRU_10373 TcCLB-EL.508277.280_mRNA-p1 TcCLB-NE.
503913.40_mRNA-p1
OG0006937: TCRU_10381 TcCLB-NE.509913.24_mRNA-p1 TcSYL_0057660.t1-p1
OG0006938: TCRU_10386 TcCLB-EL.511111.40_mRNA-p1 TcCLB-NE.
507715.90_mRNA-p1
OG0006939: TCRU_10387 TcCLB-EL.511111.20_mRNA-p1 TcCLB-NE.
507717.20_mRNA-p1
OG0006940: TCRU_10393 TcCLB-EL.511039.6_mRNA-p1 TcCLB-NE.
509791.140_mRNA-p1
OG0006941: TCRU_10394 TcCLB-EL.511039.3_mRNA-p1 TcCLB-NE.
509791.150_mRNA-p1
OG0006942: TCRU_10396 TcCLB-EL.508719.70_mRNA-p1 TcCLB-NE.
509791.189_mRNA-p1
OG0006943: TCRU_10411 TcCLB-NE.508827.100_mRNA-p1 TcSYL_0182000.t1-
p1
OG0006944: TCRU_10413 TcCLB-EL.507083.30_mRNA-p1 TcCLB-NE.
509901.170_mRNA-p1
OG0006945: TCRU_10414 TcCLB-EL.507083.40_mRNA-p1 TcCLB-NE.
509901.160_mRNA-p1
OG0006946: TCRU_10416 TcCLB-EL.507083.70_mRNA-p1 TcCLB-NE.
509901.130_mRNA-p1
OG0006947: TCRU_10417 TcCLB-EL.507083.80_mRNA-p1 TcCLB-NE.
509901.120_mRNA-p1
OG0006948: TCRU_10449 TcCLB-EL.511577.150_mRNA-p1 TcCLB-NE.
509337.15_mRNA-p1
OG0006949: TCRU_10450 TcCLB-EL.511577.140_mRNA-p1 TcCLB-NE.
509337.28_pseudogenic_transcript-p1
OG0006950: TCRU_10454 TcCLB-EL.503975.60_mRNA-p1 TcCLB-NE.
503411.10_mRNA-p1
OG0006951: TCRU_10456 TcCLB-EL.510311.20_mRNA-p1 TcCLB-NE.
510265.90_mRNA-p1
OG0006952: TCRU_10457 TcCLB-EL.510311.30_mRNA-p1 TcCLB-NE.
510265.80_mRNA-p1
OG0006953: TCRU_10458 TcCLB-EL.510311.40_mRNA-p1 TcCLB-NE.
510265.70_mRNA-p1
OG0006954: TCRU_10463 TcCLB-EL.510311.100_mRNA-p1 TcCLB-NE.
510265.4_mRNA-p1
OG0006955: TCRU_10467 TcCLB-EL.504147.250_mRNA-p1 TcSYL_0004960.t1-
p1
OG0006956: TCRU_10468 TcCLB-EL.504147.260_mRNA-p1 TcSYL_0004950.t1-
p1

OG0006957: TCRU_10470 TcCLB-EL.504147.320_mRNA-p1 TcSYL_0004920.t1-p1
OG0006958: TCRU_10471 TcCLB-EL.504147.330_mRNA-p1 TcSYL_0004910.t1-p1
OG0006959: TCRU_10474 TcCLB-EL.508215.9_mRNA-p1 TcCLB-NE.503463.10_mRNA-p1
OG0006960: TCRU_10475 TcCLB-EL.508215.3_mRNA-p1 TcCLB-NE.503463.30_mRNA-p1
OG0006961: TCRU_10494 TcCLB-EL.506147.50_mRNA-p1 TcCLB-NE.508461.40_mRNA-p1
OG0006962: TCRU_10495 TcCLB-EL.506147.30_mRNA-p1 TcCLB-NE.508461.60_mRNA-p1
OG0006963: TCRU_10496 TcCLB-EL.506147.20_mRNA-p1 TcCLB-NE.508461.70_mRNA-p1
OG0006964: TCRU_10497 TcCLB-EL.507617.9_mRNA-p1 TcCLB-NE.508461.80_mRNA-p1
OG0006965: TCRU_10502 TcCLB-EL.503955.50_mRNA-p1 TcCLB-NE.507063.60_mRNA-p1
OG0006966: TCRU_10503 TcCLB-EL.503955.55_mRNA-p1 TcCLB-NE.507063.40_mRNA-p1
OG0006967: TCRU_10504 TcCLB-EL.503955.60_mRNA-p1 TcCLB-NE.507063.30_mRNA-p1
OG0006968: TCRU_10506 TcCLB-EL.508555.34_mRNA-p1 TcCLB-NE.509237.100_mRNA-p1
OG0006969: TCRU_10508 TcCLB-EL.511337.10_mRNA-p1 TcCLB-NE.509237.80_mRNA-p1
OG0006970: TCRU_10512 TcCLB-EL.503715.10_mRNA-p1 TcCLB-NE.509607.30_mRNA-p1
OG0006971: TCRU_10513 TcCLB-EL.507105.60_mRNA-p1 TcCLB-NE.506817.10_mRNA-p1
OG0006972: TCRU_10523 TcCLB-NE.509647.170_mRNA-p1 TcSYL_0010210.t1-p1
OG0006973: TCRU_10524 TcCLB-NE.509647.160_mRNA-p1 TcSYL_0010240.t1-p1
OG0006974: TCRU_10539 TcCLB-EL.508647.30_mRNA-p1 TcSYL_0047820.t1-p1
OG0006975: TCRU_10540 TcCLB-EL.508647.40_mRNA-p1 TcSYL_0047840.t1-p1
OG0006976: TCRU_10544 TcCLB-EL.508647.100_mRNA-p1 TcSYL_0047850.t1-p1
OG0006977: TCRU_10546 TcCLB-EL.506763.110_mRNA-p1 TcCLB-EL.508229.60_mRNA-p1
OG0006978: TCRU_10551 TcCLB-EL.510493.20_mRNA-p1 TcCLB-NE.511629.30_mRNA-p1
OG0006979: TCRU_10552 TcCLB-EL.508307.10_mRNA-p1 TcCLB-NE.511629.20_mRNA-p1
OG0006980: TCRU_10565 TcCLB-EL.504149.70_mRNA-p1 TcSYL_0074640.t1-p1
OG0006981: TCRU_10567 TcSYL_0018540.t1-p1 TcSYL_0019040.t1-p1
OG0006982: TCRU_10577 TcCLB-EL.506733.110_mRNA-p1 TcCLB-NE.509229.130_mRNA-p1
OG0006983: TCRU_10579 TcCLB-EL.506733.140_mRNA-p1 TcCLB-NE.509229.90_mRNA-p1
OG0006984: TCRU_homologue_ TcCLB-EL.507611.270_mRNA-p1 TcCLB-NE.507723.90_mRNA-p1
OG0006985: TCRU_10583 TcCLB-EL.509779.4_mRNA-p1 TcCLB-NE.511655.10_mRNA-p1

OG0006986: TCRU_10587 TcCLB-EL.509777.110_mRNA-p1 TcCLB-NE.
511655.60_mRNA-p1
OG0006987: TCRU_10606 TcCLB-EL.511627.60_mRNA-p1 TcCLB-NE.
507801.179_mRNA-p1
OG0006988: TCRU_10607 TcCLB-EL.511627.70_mRNA-p1 TcCLB-NE.
507801.160_mRNA-p1
OG0006989: TCRU_10608 TcCLB-EL.511627.100_mRNA-p1 TcCLB-NE.
507801.140_mRNA-p1
OG0006990: TCRU_10609 TcCLB-EL.511627.110_mRNA-p1 TcCLB-NE.
507801.130_pseudogenic_transcript-p1
OG0006991: TCRU_10610 TcCLB-EL.511627.120_mRNA-p1 TcCLB-NE.
507801.114_pseudogenic_transcript-p1
OG0006992: TCRU_10612 TcCLB-EL.511627.140_mRNA-p1 TcCLB-NE.
507801.100_mRNA-p1
OG0006993: TCRU_10613 TcCLB-EL.511627.150_mRNA-p1 TcCLB-NE.
507801.90_mRNA-p1
OG0006994: TCRU_10614 TcCLB-EL.509103.4_mRNA-p1 TcCLB-NE.
507801.70_mRNA-p1
OG0006995: TCRU_10615 TcCLB-EL.509103.10_mRNA-p1 TcCLB-NE.
507801.60_mRNA-p1
OG0006996: TCRU_10628 TcCLB-EL.506529.600_mRNA-p1 TcCLB-NE.
510889.280_mRNA-p1
OG0006997: TCRU_10629 TcCLB-EL.506401.290_mRNA-p1 TcCLB-NE.
510835.10_mRNA-p1
OG0006998: TCRU_10630 TcCLB-EL.506401.300_mRNA-p1 TcCLB-NE.
510835.20_mRNA-p1
OG0006999: TCRU_10633 TcCLB-EL.507639.10_mRNA-p1 TcCLB-NE.
509731.10_mRNA-p1
OG0007000: TCRU_10636 TcCLB-NE.506665.30_mRNA-p1 TcCLB-NE.
509437.160_mRNA-p1
OG0007001: TCRU_10645 TcCLB-NE.511907.280_mRNA-p1 TcSYL_0121520.t1-
p1
OG0007002: TCRU_10646 TcCLB-NE.511907.270_mRNA-p1 TcSYL_0121530.t1-
p1
OG0007003: TCRU_10648 TcCLB-EL.506801.129_mRNA-p1 TcCLB-NE.
511907.250_mRNA-p1
OG0007004: TCRU_10653 TcCLB-EL.510741.210_pseudogenic_transcript-p1
TcCLB-NE.510661.60_mRNA-p1
OG0007005: TCRU_10654 TcCLB-EL.510741.220_mRNA-p1 TcCLB-NE.
510661.40_mRNA-p1
OG0007006: TCRU_10663 TcCLB-EL.508127.9_mRNA-p1 TcCLB-NE.
510221.30_mRNA-p1
OG0007007: TCRU_10668 TcCLB-EL.510525.130_mRNA-p1 TcCLB-NE.
511817.200_mRNA-p1
OG0007008: TCRU_10669 TcCLB-EL.510525.139_mRNA-p1 TcCLB-NE.
511817.210_mRNA-p1
OG0007009: TCRU_10670 TcCLB-EL.510055.50_mRNA-p1 TcCLB-NE.
511319.20_mRNA-p1
OG0007010: TCRU_10678 TcCLB-EL.504011.60_mRNA-p1 TcCLB-NE.
506257.70_mRNA-p1
OG0007011: TCRU_10681 TcCLB-EL.507433.9_mRNA-p1 TcCLB-NE.
507989.40_mRNA-p1
OG0007012: TCRU_10682 TcCLB-EL.508317.60_mRNA-p1 TcCLB-NE.
509849.20_mRNA-p1

OG0007013: TCRU_10683 TcCLB-EL.510513.40_mRNA-p1 TcCLB-NE.
509857.10_mRNA-p1
OG0007014: TCRU_10687 TcCLB-EL.506729.80_mRNA-p1 TcCLB-NE.
503843.20_mRNA-p1
OG0007015: TCRU_10696 TcCLB-EL.508717.56_mRNA-p1 TcCLB-NE.
506841.12_mRNA-p1
OG0007016: TCRU_10697 TcCLB-EL.508717.36_mRNA-p1 TcCLB-NE.
504079.6_mRNA-p1
OG0007017: TCRU_10698 TcCLB-EL.511693.110_mRNA-p1 TcCLB-NE.
503559.90_mRNA-p1
OG0007018: TCRU_10700 TcCLB-EL.511587.120_mRNA-p1 TcCLB-NE.
508851.40_mRNA-p1
OG0007019: TCRU_10702 TcCLB-EL.511589.40_mRNA-p1 TcCLB-NE.
508851.90_mRNA-p1
OG0007020: TCRU_10703 TcCLB-EL.511589.50_mRNA-p1 TcCLB-NE.
508851.100_mRNA-p1
OG0007021: TCRU_10709 TcCLB-EL.506375.120_mRNA-p1 TcCLB-NE.
508677.160_mRNA-p1
OG0007022: TCRU_10712 TcCLB-EL.511911.159_mRNA-p1 TcCLB-NE.
508637.114_mRNA-p1
OG0007023: TCRU_10714 TcCLB-EL.506211.130_mRNA-p1 TcCLB-NE.
511289.100_mRNA-p1
OG0007024: TCRU_10716 TcCLB-EL.504111.20_mRNA-p1 TcCLB-NE.
507031.120_mRNA-p1
OG0007025: TCRU_10717 TcCLB-EL.506503.110_mRNA-p1 TcCLB-NE.
511293.30_mRNA-p1
OG0007026: TCRU_10720 TcCLB-NE.506389.50_mRNA-p1 TcSYL_0072520.t1-p1
OG0007027: TCRU_10721 TcCLB-EL.506825.180_mRNA-p1 TcCLB-NE.
508175.80_mRNA-p1
OG0007028: TCRU_10722 TcCLB-EL.506825.170_mRNA-p1 TcCLB-NE.
508175.70_mRNA-p1
OG0007029: TCRU_10724 TcCLB-EL.504163.10_mRNA-p1 TcCLB-NE.
510299.79_mRNA-p1
OG0007030: TCRU_10727 TcCLB-EL.509809.10_mRNA-p1 TcCLB-NE.
511261.30_mRNA-p1
OG0007031: TCRU_10730 TcCLB-NE.504071.70_mRNA-p1 TcSYL_0167240.t1-p1
OG0007032: TCRU_10731 TcCLB-NE.508173.170_mRNA-p1 TcSYL_0079350.t1-
p1
OG0007033: TCRU_10734 TcCLB-NE.508479.150_mRNA-p1 TcSYL_0019860.t1-
p1
OG0007034: TCRU_10740 TcCLB-EL.503639.30_mRNA-p1 TcCLB-NE.
507609.80_mRNA-p1
OG0007035: TCRU_10741 TcCLB-EL.506737.100_mRNA-p1 TcSYL_0156150.t1-
p1
OG0007036: TCRU_10742 TcCLB-EL.508241.110_mRNA-p1 TcSYL_0185950.t1-
p1
OG0007037: TCRU_10743 TcCLB-EL.511393.49_mRNA-p1 TcCLB-NE.
503565.29_mRNA-p1
OG0007038: TCRU_10746 TcCLB-EL.506757.30_mRNA-p1 TcCLB-NE.
510359.360_mRNA-p1
OG0007039: TCRU_10748 TcCLB-EL.503779.70_mRNA-p1 TcCLB-NE.
506247.440_mRNA-p1
OG0007040: TCRU_10750 TcCLB-EL.507927.70_mRNA-p1 TcCLB-NE.
509047.30_mRNA-p1

OG0007041: TCRU_10751 TcCLB-EL.509965.420_mRNA-p1 TcCLB-NE.
509049.40_mRNA-p1
OG0007042: TCRU_10752 TcCLB-EL.509965.350_mRNA-p1 TcCLB-NE.
509053.10_mRNA-p1
OG0007043: TCRU_10753 TcCLB-EL.509965.240_mRNA-p1 TcCLB-NE.
509053.120_mRNA-p1
OG0007044: TCRU_10754 TcCLB-EL.509965.190_mRNA-p1 TcCLB-NE.
509053.170_mRNA-p1
OG0007045: TCRU_10755 TcCLB-EL.509965.150_mRNA-p1 TcCLB-NE.
509055.40_mRNA-p1
OG0007046: TCRU_10759 TcCLB-EL.511585.60_mRNA-p1 TcCLB-NE.
510155.60_mRNA-p1
OG0007047: TCRU_10762 TcCLB-EL.506529.310_mRNA-p1 TcCLB-NE.
510887.60_mRNA-p1
OG0007048: TCRU_10763 TcCLB-EL.506529.360_mRNA-p1 TcCLB-NE.
510889.50_mRNA-p1
OG0007049: TCRU_10764 TcCLB-EL.507611.290_mRNA-p1 TcCLB-NE.
507723.110_mRNA-p1
OG0007050: TCRU_10765 TcCLB-EL.507611.300_mRNA-p1 TcCLB-NE.
507723.120_mRNA-p1
OG0007051: TCRU_10766 TcCLB-EL.507611.320_mRNA-p1 TcCLB-NE.
507723.140_mRNA-p1
OG0007052: TCRU_10767 TcCLB-EL.507611.330_mRNA-p1 TcCLB-NE.
507723.150_mRNA-p1
OG0007053: TCRU_10772 TcCLB-EL.511407.80_mRNA-p1 TcCLB-NE.
509569.10_mRNA-p1
OG0007054: TCRU_10775 TcCLB-EL.506635.120_mRNA-p1 TcCLB-NE.
504199.30_mRNA-p1
OG0007055: TCRU_10776 TcCLB-EL.508781.70_mRNA-p1 TcCLB-NE.
506855.10_mRNA-p1
OG0007056: TCRU_10778 TcCLB-EL.504009.30_mRNA-p1 TcCLB-NE.
507049.160_mRNA-p1
OG0007057: TCRU_10782 TcCLB-EL.506695.49_mRNA-p1 TcCLB-NE.
508153.550_mRNA-p1
OG0007058: TCRU_10783 TcCLB-EL.506693.30_mRNA-p1 TcCLB-NE.
508153.680_mRNA-p1
OG0007059: TCRU_10784 TcCLB-EL.506693.20_mRNA-p1 TcCLB-NE.
508153.700_mRNA-p1
OG0007060: TCRU_10786 TcCLB-EL.509893.20_mRNA-p1 TcCLB-NE.
508141.20_mRNA-p1
OG0007061: TCRU_10789 TcCLB-EL.511585.160_mRNA-p1 TcCLB-NE.
510155.150_mRNA-p1
OG0007062: TCRU_10790 TcCLB-NE.508355.190_mRNA-p1 TcSYL_0083040.t1-
p1
OG0007063: TCRU_10791 TcCLB-EL.507467.119_mRNA-p1 TcCLB-NE.
508355.124_mRNA-p1
OG0007064: TCRU_10793 TcCLB-EL.509499.10_mRNA-p1 TcCLB-NE.
510947.50_mRNA-p1
OG0007065: TCRU_10796 TcCLB-EL.509831.30_mRNA-p1 TcCLB-NE.
506629.50_mRNA-p1
OG0007066: TCRU_10797 TcCLB-EL.506367.100_mRNA-p1 TcCLB-NE.
506629.150_mRNA-p1
OG0007067: TCRU_10802 TcCLB-EL.506729.30_mRNA-p1 TcCLB-NE.
507513.30_mRNA-p1

OG0007068: TCRU_10805 TcCLB-EL.506657.30_mRNA-p1 TcCLB-NE.
511249.30_mRNA-p1
OG0007069: TCRU_10810 TcCLB-EL.507047.134_mRNA-p1 TcCLB-NE.
509179.120_mRNA-p1
OG0007070: TCRU_10811 TcCLB-EL.507047.130_mRNA-p1 TcCLB-NE.
509179.130_mRNA-p1
OG0007071: TCRU_10815 TcCLB-EL.506529.160_mRNA-p1 TcCLB-NE.
510885.70_mRNA-p1
OG0007072: TCRU_10817 TcCLB-EL.506529.508_mRNA-p1 TcCLB-NE.
510889.221_mRNA-p1
OG0007073: TCRU_10818 TcCLB-EL.503789.20_mRNA-p1 TcCLB-NE.
505183.100_mRNA-p1
OG0007074: TCRU_10819 TcCLB-EL.507093.170_mRNA-p1 TcSYL_0080370.t1-
p1
OG0007075: TCRU_protein_ TcCLB-EL.410943.10_mRNA-p1 TcCLB-NE.
509105.4_mRNA-p1
OG0007076: TCRU_10822 TcCLB-EL.506311.20_mRNA-p1 TcCLB-NE.
509105.20_mRNA-p1
OG0007077: TCRU_10825 TcCLB-EL.506399.70_mRNA-p1 TcCLB-NE.
511909.60_mRNA-p1
OG0007078: TCRU_10826 TcCLB-EL.506857.70_mRNA-p1 TcCLB-NE.
509207.140_mRNA-p1
OG0007079: TCRU_10827 TcCLB-EL.506859.49_mRNA-p1 TcCLB-NE.
511815.24_mRNA-p1
OG0007080: TCRU_10829 TcCLB-EL.507521.44_mRNA-p1 TcCLB-NE.
508579.4_mRNA-p1
OG0007081: TCRU_10832 TcCLB-EL.508779.30_mRNA-p1 TcCLB-NE.
506855.140_mRNA-p1
OG0007082: TCRU_10833 TcCLB-EL.506525.130_mRNA-p1 TcCLB-NE.
511283.124_mRNA-p1
OG0007083: TCRU_10835 TcCLB-EL.507669.239_mRNA-p1 TcCLB-NE.
509875.220_mRNA-p1
OG0007084: TCRU_10836 TcCLB-EL.508179.40_mRNA-p1 TcCLB-NE.
510941.10_mRNA-p1
OG0007085: TCRU_10837 TcCLB-EL.506925.20_mRNA-p1 TcSYL_0080480.t1-p1
OG0007086: TCRU_10839 TcCLB-EL.506697.20_mRNA-p1 TcCLB-NE.
508153.520_pseudogenic_transcript-p1
OG0007087: TCRU_10840 TcCLB-EL.508277.269_pseudogenic_transcript-p1
TcCLB-NE.503913.29_mRNA-p1
OG0007088: TCRU_10842 TcCLB-EL.511111.30_mRNA-p1 TcCLB-NE.
507715.100_mRNA-p1
OG0007089: TCRU_10844 TcCLB-EL.511039.39_mRNA-p1 TcCLB-NE.
509791.90_mRNA-p1
OG0007090: TCRU_10849 TcCLB-NE.508479.340_mRNA-p1 TcSYL_0019730.t1-
p1
OG0007091: TCRU_10851 TcCLB-NE.508827.110_mRNA-p1 TcSYL_0182030.t1-
p1
OG0007092: TCRU_10853 TcCLB-EL.511529.30_mRNA-p1 TcCLB-NE.
511031.30_mRNA-p1
OG0007093: TCRU_10854 TcCLB-EL.511577.130_mRNA-p1 TcCLB-NE.
509339.4_mRNA-p1
OG0007094: TCRU_10855 TcCLB-EL.504147.310_mRNA-p1 TcSYL_0004930.t1-
p1
OG0007095: TCRU_10856 TcCLB-EL.508215.6_mRNA-p1 TcCLB-NE.

503463.20_mRNA-p1
OG0007096: TCRU_10858 TcCLB-EL.506147.70_mRNA-p1 TcCLB-NE.
508461.19_mRNA-p1
OG0007097: TCRU_10859 TcCLB-EL.506147.60_mRNA-p1 TcCLB-NE.
508461.30_mRNA-p1
OG0007098: TCRU_10864 TcCLB-EL.508989.70_mRNA-p1 TcCLB-NE.
509569.160_mRNA-p1
OG0007099: TCRU_10869 TcCLB-EL.506733.120_mRNA-p1 TcCLB-NE.
509229.120_mRNA-p1
OG0007100: TCRU_10870 TcCLB-EL.509777.130_mRNA-p1 TcCLB-NE.
511655.50_pseudogenic_transcript-p1
OG0007101: TCRU_10872 TcCLB-EL.511627.80_mRNA-p1 TcCLB-NE.
507801.150_mRNA-p1
OG0007102: TCRU_10874 TcCLB-EL.506895.10_mRNA-p1 TcCLB-NE.
508501.260_mRNA-p1
OG0007103: TCRU_10876 TcCLB-EL.506801.100_mRNA-p1 TcCLB-NE.
511907.220_mRNA-p1
OG0007104: TCRU_10878 TcCLB-EL.504011.40_mRNA-p1 TcCLB-NE.
506257.60_mRNA-p1
OG0007105: TcCLB-EL.510105.240_mRNA-p1 TcCLB-NE.509717.90_mRNA-p1
TcSYL_0073420.t1-p1
OG0007106: TcCLB-EL.508955.20_mRNA-p1 TcCLB-NE.508059.50_mRNA-p1
TcSYL_0168940.t1-p1
OG0007107: TcCLB-EL.510249.22_pseudogenic_transcript-p1 TcCLB-NE.
506253.11_pseudogenic_transcript-p1 TcCLB-NE.
509287.5_pseudogenic_transcript-p1
OG0007108: TcCLB-EL.506529.425_mRNA-p1 TcCLB-NE.510889.130_mRNA-p1
TcSYL_0178280.t1-p1
OG0007109: TcCLB-EL.510075.50_mRNA-p1 TcCLB-NE.509399.100_mRNA-p1
TcSYL_0177140.t1-p1
OG0007110: TcCLB-EL.504881.14_mRNA-p1 TcCLB-EL.506579.119_mRNA-p1
TcCLB-NE.510043.29_mRNA-p1
OG0007111: TcCLB-EL.508277.220_mRNA-p1 TcCLB-NE.509945.30_mRNA-p1
TcSYL_0064200.t1-p1
OG0007112: TcCLB-EL.509265.50_pseudogenic_transcript-p1 TcCLB-EL.
509959.160_pseudogenic_transcript-p1 TcCLB-NE.
506393.120_pseudogenic_transcript-p1
OG0007113: TcCLB-EL.504255.10_mRNA-p1 TcCLB-NE.510577.40_mRNA-p1
TcSYL_0147280.t1-p1
OG0007114: TcCLB-EL.506457.120_mRNA-p1 TcCLB-NE.509693.160_mRNA-p1
TcSYL_0170870.t1-p1
OG0007115: TcCLB-EL.506457.60_mRNA-p1 TcCLB-NE.509693.100_mRNA-p1
TcSYL_0170700.t1-p1
OG0007116: TcCLB-EL.509967.10_mRNA-p1 TcCLB-NE.509693.200_mRNA-p1
TcSYL_0170990.t1-p1
OG0007117: TcCLB-EL.504227.5_mRNA-p1 TcCLB-NE.506439.9_mRNA-p1
TcSYL_0027440.t1-p1
OG0007118: TcCLB-EL.506605.130_mRNA-p1 TcCLB-NE.511239.120_mRNA-p1
TcSYL_0025890.t1-p1
OG0007119: TcCLB-EL.506605.30_mRNA-p1 TcCLB-NE.511239.20_mRNA-p1
TcSYL_0026200.t1-p1
OG0007120: TcCLB-EL.506661.60_mRNA-p1 TcCLB-NE.511245.100_mRNA-p1
TcSYL_0027100.t1-p1
OG0007121: TcCLB-EL.508089.20_mRNA-p1 TcCLB-NE.508857.40_mRNA-p1

TcSYL_0026980.t1-p1
OG0007122: TcCLB-EL.510167.37_pseudogenic_transcript-p1
TcSYL_0027030.t1-p1 TcSYL_0065810.t1-p1
OG0007123: TcCLB-EL.511253.10_mRNA-p1 TcCLB-NE.511251.10_mRNA-p1
TcSYL_0027350.t1-p1
OG0007124: TcCLB-EL.503595.19_mRNA-p1 TcCLB-NE.506229.70_mRNA-p1
TcSYL_0094590.t1-p1
OG0007125: TcCLB-EL.503895.19_mRNA-p1 TcCLB-NE.509565.9_mRNA-p1
TcSYL_0096520.t1-p1
OG0007126: TcCLB-EL.505941.20_pseudogenic_transcript-p1 TcCLB-NE.
507623.101_pseudogenic_transcript-p1 TcSYL_0041300.t1-p1
OG0007127: TcCLB-EL.506865.4_mRNA-p1 TcCLB-NE.509569.94_mRNA-p1
TcSYL_0097280.t1-p1
OG0007128: TcCLB-EL.506865.50_mRNA-p1 TcCLB-NE.509569.44_mRNA-p1
TcSYL_0097190.t1-p1
OG0007129: TcCLB-EL.508987.30_mRNA-p1 TcCLB-NE.509567.30_mRNA-p1
TcSYL_0096720.t1-p1
OG0007130: TcCLB-EL.508991.30_pseudogenic_transcript-p1 TcCLB-EL.
510451.10_pseudogenic_transcript-p1 TcCLB-NE.
507381.20_pseudogenic_transcript-p1
OG0007131: TcCLB-EL.511407.70_mRNA-p1 TcCLB-NE.412435.10_mRNA-p1
TcSYL_0097060.t1-p1
OG0007132: TcCLB-EL.506863.30_mRNA-p1 TcCLB-NE.511825.240_mRNA-p1
TcSYL_0200800.t1-p1
OG0007133: TcCLB-EL.508323.50_mRNA-p1 TcCLB-NE.511825.30_mRNA-p1
TcSYL_0200880.t1-p1
OG0007134: TcCLB-EL.459793.10_mRNA-p1 TcCLB-NE.511851.50_mRNA-p1
TcCLB-NE.511853.10_mRNA-p1
OG0007135: TcCLB-EL.504087.10_mRNA-p1 TcCLB-NE.509681.10_mRNA-p1
TcSYL_0090140.t1-p1
OG0007136: TcCLB-EL.506179.10_mRNA-p1 TcCLB-NE.507665.30_mRNA-p1
TcSYL_0092160.t1-p1
OG0007137: TcCLB-EL.506179.30_mRNA-p1 TcCLB-NE.507663.80_mRNA-p1
TcSYL_0092240.t1-p1
OG0007138: TcCLB-EL.506181.90_mRNA-p1 TcCLB-NE.506177.120_mRNA-p1
TcSYL_0092370.t1-p1
OG0007139: TcCLB-EL.506227.110_mRNA-p1 TcCLB-NE.511847.30_mRNA-p1
TcSYL_0091980.t1-p1
OG0007140: TcCLB-EL.507099.10_mRNA-p1 TcCLB-NE.503999.110_mRNA-p1
TcSYL_0089280.t1-p1
OG0007141: TcCLB-EL.511051.80_mRNA-p1 TcCLB-NE.506257.10_mRNA-p1
TcSYL_0090950.t1-p1
OG0007142: TcCLB-EL.509231.23_mRNA-p1 TcCLB-NE.509719.53_mRNA-p1
TcSYL_0146410.t1-p1
OG0007143: TcCLB-EL.511871.34_mRNA-p1 TcCLB-NE.511865.54_mRNA-p1
TcSYL_0146190.t1-p1
OG0007144: TcCLB-EL.511871.4_mRNA-p1 TcCLB-NE.511865.90_mRNA-p1
TcSYL_0146210.t1-p1
OG0007145: TcCLB-EL.503757.30_mRNA-p1 TcCLB-NE.503719.39_mRNA-p1
TcSYL_0118210.t1-p1
OG0007146: TcCLB-EL.510717.10_mRNA-p1 TcCLB-EL.510717.20_mRNA-p1
TcCLB-NE.506337.90_mRNA-p1
OG0007147: TcCLB-EL.507975.70_pseudogenic_transcript-p1 TcCLB-NE.
505037.140_pseudogenic_transcript-p1 TcCLB-NE.

511665.100_pseudogenic_transcript-p1
OG0007148: TcCLB-EL.511303.64_mRNA-p1 TcCLB-NE.509177.59_mRNA-p1 TcSYL_0159870.t1-p1
OG0007149: TcCLB-EL.511309.60_mRNA-p1 TcCLB-NE.511759.40_mRNA-p1 TcSYL_0159720.t1-p1
OG0007150: TcCLB-EL.507237.24_pseudogenic_transcript-p1 TcCLB-EL.511613.50_mRNA-p1 TcCLB-NE.510237.60_mRNA-p1
OG0007151: TcCLB-EL.511587.10_mRNA-p1 TcSYL_0004710.t1-p1 TcSYL_0021330.t1-p1
OG0007152: TcCLB-EL.508149.65_mRNA-p1 TcCLB-EL.511603.310_mRNA-p1 TcSYL_0126900.t1-p1
OG0007153: TcCLB-EL.510859.10_mRNA-p1 TcCLB-EL.510859.17_mRNA-p1 TcCLB-NE.507649.70_mRNA-p1
OG0007154: TcCLB-EL.510861.50_mRNA-p1 TcCLB-NE.507647.30_mRNA-p1 TcCLB-NE.507649.5_mRNA-p1
OG0007155: TcCLB-EL.511393.70_mRNA-p1 TcCLB-NE.505071.130_mRNA-p1 TcSYL_0202070.t1-p1
OG0007156: TcCLB-EL.506667.70_mRNA-p1 TcCLB-EL.508149.70_mRNA-p1 TcSYL_0018280.t1-p1
OG0007157: TcCLB-EL.508169.120_mRNA-p1 TcCLB-NE.506581.60_mRNA-p1 TcSYL_0113290.t1-p1
OG0007158: TcCLB-EL.510123.50_mRNA-p1 TcCLB-NE.511837.29_mRNA-p1 TcSYL_0114760.t1-p1
OG0007159: TcCLB-EL.510289.3_mRNA-p1 TcCLB-NE.503891.60_mRNA-p1 TcSYL_0113340.t1-p1
OG0007160: TcCLB-EL.510293.54_mRNA-p1 TcCLB-NE.508661.34_mRNA-p1 TcSYL_0113490.t1-p1
OG0007161: TcCLB-EL.503531.40_mRNA-p1 TcCLB-EL.507083.50_mRNA-p1 TcCLB-NE.509901.150_mRNA-p1
OG0007162: TcCLB-EL.507803.40_mRNA-p1 TcCLB-NE.507063.280_mRNA-p1 TcSYL_0013500.t1-p1
OG0007163: TcCLB-EL.509775.9_mRNA-p1 TcCLB-NE.503825.19_mRNA-p1 TcSYL_0013830.t1-p1
OG0007164: TcCLB-EL.503829.10_mRNA-p1 TcCLB-NE.508045.14_mRNA-p1 TcSYL_0173380.t1-p1
OG0007165: TcCLB-EL.503829.30_mRNA-p1 TcCLB-NE.508045.20_mRNA-p1 TcSYL_0173400.t1-p1
OG0007166: TcCLB-EL.503829.50_mRNA-p1 TcCLB-NE.508045.40_mRNA-p1 TcSYL_0173500.t1-p1
OG0007167: TcCLB-EL.504427.20_mRNA-p1 TcCLB-NE.509331.120_mRNA-p1 TcSYL_0172280.t1-p1
OG0007168: TcCLB-EL.504427.280_mRNA-p1 TcCLB-NE.508045.10_mRNA-p1 TcSYL_0173350.t1-p1
OG0007169: TcCLB-EL.504427.60_mRNA-p1 TcCLB-NE.509331.80_mRNA-p1 TcSYL_0172430.t1-p1
OG0007170: TcCLB-EL.506849.20_pseudogenic_transcript-p1 TcCLB-NE.507853.20_pseudogenic_transcript-p1 TcSYL_0174690.t1-p1
OG0007171: TcCLB-EL.510513.80_mRNA-p1 TcCLB-NE.509857.50_mRNA-p1 TcSYL_0174520.t1-p1
OG0007172: TcCLB-EL.503975.100_mRNA-p1 TcCLB-NE.503413.4_mRNA-p1 TcSYL_0086880.t1-p1
OG0007173: TcCLB-EL.503975.70_mRNA-p1 TcCLB-NE.503411.20_mRNA-p1 TcSYL_0086910.t1-p1
OG0007174: TcCLB-EL.508771.34_mRNA-p1 TcCLB-NE.503785.30_mRNA-p1

TcSYL_0086740.t1-p1
OG0007175: TcCLB-EL.508809.50_mRNA-p1 TcCLB-NE.508669.21_mRNA-p1 TcSYL_0087000.t1-p1
OG0007176: TcCLB-EL.511189.90_mRNA-p1 TcCLB-NE.510975.30_mRNA-p1 TcSYL_0087510.t1-p1
OG0007177: TcCLB-EL.511191.19_mRNA-p1 TcCLB-NE.506619.49_mRNA-p1 TcSYL_0087240.t1-p1
OG0007178: TcCLB-EL.511621.170_mRNA-p1 TcCLB-NE.504741.190_mRNA-p1 TcSYL_0084460.t1-p1
OG0007179: TcCLB-EL.511621.9_mRNA-p1 TcCLB-NE.509317.109_mRNA-p1 TcSYL_0084850.t1-p1
OG0007180: TcCLB-EL.511675.6_pseudogenic_transcript-p1 TcSYL_0189590.t1-p1 TcSYL_0189970.t1-p1
OG0007181: TcCLB-EL.511685.40_mRNA-p1 TcCLB-NE.508741.90_mRNA-p1 TcSYL_0192920.t1-p1
OG0007182: TcCLB-EL.506743.20_mRNA-p1 TcCLB-NE.422921.10_mRNA-p1 TcSYL_0165420.t1-p1
OG0007183: TcCLB-EL.507275.50_mRNA-p1 TcCLB-NE.507491.30_mRNA-p1 TcSYL_0190200.t1-p1
OG0007184: TcCLB-EL.511217.145_mRNA-p1 TcCLB-NE.506573.81_mRNA-p1 TcCLB-NE.508153.184_mRNA-p1
OG0007185: TcCLB-EL.511217.180_mRNA-p1 TcCLB-NE.506573.40_mRNA-p1 TcSYL_0189460.t1-p1
OG0007186: TcCLB-EL.503487.50_mRNA-p1 TcCLB-NE.508641.220_mRNA-p1 TcSYL_0047500.t1-p1
OG0007187: TcCLB-EL.503981.20_mRNA-p1 TcCLB-NE.504131.80_mRNA-p1 TcSYL_0048120.t1-p1
OG0007188: TcCLB-EL.506797.50_mRNA-p1 TcCLB-NE.511907.30_mRNA-p1 TcSYL_0102400.t1-p1
OG0007189: TcCLB-EL.506797.60_mRNA-p1 TcCLB-NE.511907.40_mRNA-p1 TcSYL_0102370.t1-p1
OG0007190: TcCLB-EL.506799.10_mRNA-p1 TcCLB-NE.511907.110_mRNA-p1 TcSYL_0102190.t1-p1
OG0007191: TcCLB-EL.508515.170_pseudogenic_transcript-p1 TcCLB-NE.506629.230_pseudogenic_transcript-p1 TcCLB-NE.507219.20_pseudogenic_transcript-p1
OG0007192: TcCLB-EL.508515.50_mRNA-p1 TcCLB-NE.508641.160_mRNA-p1 TcSYL_0047450.t1-p1
OG0007193: TcCLB-EL.510721.21_mRNA-p1 TcCLB-NE.508825.20_mRNA-p1 TcSYL_0179360.t1-p1
OG0007194: TcCLB-EL.511071.120_mRNA-p1 TcCLB-NE.511901.20_mRNA-p1 TcSYL_0103530.t1-p1
OG0007195: TcCLB-EL.503613.10_mRNA-p1 TcCLB-NE.511165.80_mRNA-p1 TcSYL_0075500.t1-p1
OG0007196: TcCLB-EL.506775.14_mRNA-p1 TcCLB-NE.508797.29_mRNA-p1 TcSYL_0075350.t1-p1
OG0007197: TcCLB-EL.506779.190_mRNA-p1 TcCLB-NE.511151.100_mRNA-p1 TcSYL_0077170.t1-p1
OG0007198: TcCLB-EL.506779.44_mRNA-p1 TcCLB-NE.511153.144_mRNA-p1 TcSYL_0076850.t1-p1
OG0007199: TcCLB-EL.506825.200_mRNA-p1 TcCLB-NE.508175.100_mRNA-p1 TcSYL_0078820.t1-p1
OG0007200: TcCLB-EL.506825.70_mRNA-p1 TcCLB-NE.506681.70_mRNA-p1 TcSYL_0078900.t1-p1

OG0007201: TcCLB-EL.508237.9_mRNA-p1 TcCLB-NE.508795.19_mRNA-p1 TcSYL_0075610.t1-p1
OG0007202: TcCLB-EL.509151.90_mRNA-p1 TcCLB-NE.508175.200_mRNA-p1 TcSYL_0078740.t1-p1
OG0007203: TcCLB-EL.510101.280_mRNA-p1 TcCLB-NE.506297.50_mRNA-p1 TcSYL_0074930.t1-p1
OG0007204: TcCLB-EL.511693.130_mRNA-p1 TcCLB-NE.503559.70_mRNA-p1 TcSYL_0184910.t1-p1
OG0007205: TcCLB-EL.511693.30_mRNA-p1 TcCLB-NE.508177.60_mRNA-p1 TcSYL_0186630.t1-p1
OG0007206: TcCLB-EL.511697.40_pseudogenic_transcript-p1 TcSYL_0076010.t1-p1 TcSYL_0078520.t1-p1
OG0007207: TcCLB-EL.437805.9_pseudogenic_transcript-p1 TcCLB-EL.456627.10_pseudogenic_transcript-p1 TcSYL_0163960.t1-p1
OG0007208: TcCLB-EL.507863.10_mRNA-p1 TcSYL_0018560.t1-p1 TcSYL_0019020.t1-p1
OG0007209: TcCLB-EL.506767.5_mRNA-p1 TcCLB-EL.507867.31_pseudogenic_transcript-p1 TcSYL_0129260.t1-p1
OG0007210: TcCLB-EL.508387.60_mRNA-p1 TcCLB-NE.506789.240_mRNA-p1 TcSYL_0162910.t1-p1
OG0007211: TcCLB-EL.508387.80_mRNA-p1 TcCLB-NE.506789.220_mRNA-p1 TcSYL_0162780.t1-p1
OG0007212: TcCLB-EL.509195.10_mRNA-p1 TcCLB-NE.509493.120_mRNA-p1 TcSYL_0093520.t1-p1
OG0007213: TcCLB-EL.503579.30_mRNA-p1 TcCLB-NE.507517.20_mRNA-p1 TcSYL_0022880.t1-p1
OG0007214: TcCLB-EL.506135.9_mRNA-p1 TcCLB-NE.503721.39_mRNA-p1 TcSYL_0023310.t1-p1
OG0007215: TcCLB-EL.507747.230_mRNA-p1 TcCLB-NE.503723.60_mRNA-p1 TcSYL_0023670.t1-p1
OG0007216: TcCLB-EL.507747.260_mRNA-p1 TcCLB-NE.503723.40_mRNA-p1 TcSYL_0023510.t1-p1
OG0007217: TcCLB-EL.508207.260_mRNA-p1 TcCLB-NE.509507.40_mRNA-p1 TcSYL_0022790.t1-p1
OG0007218: TcCLB-EL.508207.60_mRNA-p1 TcCLB-NE.507519.160_mRNA-p1 TcSYL_0022630.t1-p1
OG0007219: TcCLB-EL.508209.159_mRNA-p1 TcCLB-NE.472777.9_mRNA-p1 TcSYL_0023030.t1-p1
OG0007220: TcCLB-EL.503755.50_mRNA-p1 TcCLB-NE.510131.90_mRNA-p1 TcSYL_0044770.t1-p1
OG0007221: TcCLB-EL.506351.30_mRNA-p1 TcCLB-NE.505939.70_mRNA-p1 TcSYL_0042550.t1-p1
OG0007222: TcCLB-EL.506351.35_mRNA-p1 TcCLB-NE.505939.65_mRNA-p1 TcSYL_0042560.t1-p1
OG0007223: TcCLB-EL.506351.50_mRNA-p1 TcCLB-NE.505939.50_mRNA-p1 TcSYL_0042610.t1-p1
OG0007224: TcCLB-EL.506351.9_mRNA-p1 TcCLB-NE.505939.89_mRNA-p1 TcSYL_0042470.t1-p1
OG0007225: TcCLB-EL.507077.50_mRNA-p1 TcCLB-NE.510899.19_mRNA-p1 TcSYL_0179280.t1-p1
OG0007226: TcCLB-EL.507527.35_pseudogenic_transcript-p1 TcSYL_0179500.t1-p1 TcSYL_0180930.t1-p1
OG0007227: TcCLB-EL.509595.60_mRNA-p1 TcCLB-NE.508817.170_mRNA-p1 TcSYL_0043380.t1-p1

OG0007228: TcCLB-EL.509597.20_mRNA-p1 TcCLB-NE.508817.130_mRNA-p1 TcSYL_0043530.t1-p1
OG0007229: TcCLB-EL.509597.29_mRNA-p1 TcCLB-NE.508817.119_mRNA-p1 TcSYL_0043550.t1-p1
OG0007230: TcCLB-EL.509597.4_mRNA-p1 TcCLB-NE.508817.150_mRNA-p1 TcSYL_0043450.t1-p1
OG0007231: TcCLB-EL.511211.10_mRNA-p1 TcCLB-NE.508827.130_mRNA-p1 TcSYL_0182130.t1-p1
OG0007232: TcCLB-EL.506907.30_mRNA-p1 TcCLB-EL.510773.110_mRNA-p1 TcCLB-NE.511385.100_mRNA-p1
OG0007233: TcCLB-EL.507277.10_mRNA-p1 TcCLB-EL.509895.100_mRNA-p1 TcCLB-NE.510007.60_mRNA-p1
OG0007234: TcCLB-EL.509011.54_mRNA-p1 TcCLB-NE.504575.15_mRNA-p1 TcSYL_0030760.t1-p1
OG0007235: TcCLB-EL.509957.10_mRNA-p1 TcSYL_0031160.t1-p1 TcSYL_0048880.t1-p1
OG0007236: TcCLB-EL.510599.30_mRNA-p1 TcCLB-NE.509911.4_mRNA-p1 TcSYL_0057390.t1-p1
OG0007237: TcCLB-EL.510599.40_mRNA-p1 TcCLB-NE.509911.10_mRNA-p1 TcSYL_0057400.t1-p1
OG0007238: TcCLB-EL.503909.50_mRNA-p1 TcCLB-NE.511507.110_mRNA-p1 TcSYL_0139480.t1-p1
OG0007239: TcCLB-EL.509443.48_mRNA-p1 TcCLB-NE.507017.170_mRNA-p1 TcSYL_0166990.t1-p1
OG0007240: TcCLB-EL.511733.10_mRNA-p1 TcCLB-NE.511523.100_mRNA-p1 TcSYL_0140060.t1-p1
OG0007241: TcCLB-EL.511735.70_mRNA-p1 TcCLB-EL.511737.6_mRNA-p1 TcCLB-NE.511521.27_mRNA-p1
OG0007242: TcCLB-EL.511741.20_mRNA-p1 TcCLB-NE.511517.90_mRNA-p1 TcSYL_0139770.t1-p1
OG0007243: TcCLB-EL.511741.50_mRNA-p1 TcCLB-NE.511517.60_mRNA-p1 TcSYL_0139740.t1-p1
OG0007244: TcCLB-EL.511743.30_mRNA-p1 TcCLB-NE.511517.10_mRNA-p1 TcSYL_0139690.t1-p1
OG0007245: TcCLB-EL.511751.22_mRNA-p1 TcCLB-NE.507023.210_mRNA-p1 TcSYL_0139320.t1-p1
OG0007246: TcCLB-EL.503407.10_mRNA-p1 TcCLB-NE.505193.20_mRNA-p1 TcSYL_0104400.t1-p1
OG0007247: TcCLB-EL.503881.20_mRNA-p1 TcCLB-NE.508119.120_mRNA-p1 TcSYL_0103850.t1-p1
OG0007248: TcCLB-EL.506465.5_mRNA-p1 TcCLB-NE.507317.20_mRNA-p1 TcSYL_0104050.t1-p1
OG0007249: TcCLB-EL.506715.44_mRNA-p1 TcCLB-NE.506469.139_mRNA-p1 TcSYL_0156930.t1-p1
OG0007250: TcCLB-EL.506893.70_mRNA-p1 TcCLB-NE.508501.220_mRNA-p1 TcSYL_0110320.t1-p1
OG0007251: TcCLB-EL.506895.60_pseudogenic_transcript-p1 TcCLB-NE.508501.310_mRNA-p1 TcSYL_0110400.t1-p1
OG0007252: TcCLB-EL.510345.30_mRNA-p1 TcCLB-NE.506469.60_mRNA-p1 TcSYL_0156870.t1-p1
OG0007253: TcCLB-EL.510565.160_mRNA-p1 TcCLB-NE.506145.70_mRNA-p1 TcSYL_0105590.t1-p1
OG0007254: TcCLB-EL.510565.40_mRNA-p1 TcCLB-NE.508503.30_mRNA-p1 TcSYL_0105860.t1-p1

OG0007255: TcCLB-EL.506265.190_mRNA-p1 TcCLB-NE.506435.210_mRNA-p1
TcSYL_0196560.t1-p1
OG0007256: TcCLB-EL.507485.45_mRNA-p1 TcCLB-NE.507159.41_mRNA-p1
TcSYL_0195460.t1-p1
OG0007257: TcCLB-EL.507485.50_mRNA-p1 TcCLB-NE.507159.37_mRNA-p1
TcSYL_0195490.t1-p1
OG0007258: TcCLB-EL.504019.3_mRNA-p1 TcCLB-NE.511033.29_mRNA-p1
TcSYL_0112260.t1-p1
OG0007259: TcCLB-EL.507089.170_mRNA-p1 TcCLB-NE.506725.20_mRNA-p1
TcSYL_0111980.t1-p1
OG0007260: TcCLB-EL.507129.40_mRNA-p1 TcCLB-NE.507969.70_mRNA-p1
TcSYL_0088780.t1-p1
OG0007261: TcCLB-EL.507511.81_mRNA-p1 TcCLB-EL.507511.91_mRNA-p1
TcCLB-NE.508235.20_mRNA-p1
OG0007262: TcCLB-EL.509157.220_mRNA-p1 TcCLB-NE.510725.40_mRNA-p1
TcSYL_0141660.t1-p1
OG0007263: TcCLB-EL.509269.19_mRNA-p1 TcCLB-NE.507969.30_mRNA-p1
TcSYL_0088880.t1-p1
OG0007264: TcCLB-EL.511529.120_mRNA-p1 TcCLB-NE.506727.140_mRNA-p1
TcSYL_0112140.t1-p1
OG0007265: TcCLB-EL.511529.80_mRNA-p1 TcCLB-NE.511029.20_mRNA-p1
TcSYL_0112180.t1-p1
OG0007266: TcCLB-EL.511545.199_mRNA-p1 TcCLB-NE.
510435.50_pseudogenic_transcript-p1 TcSYL_0088490.t1-p1
OG0007267: TcCLB-EL.511043.45_pseudogenic_transcript-p1 TcCLB-EL.
511705.5_pseudogenic_transcript-p1 TcCLB-NE.
506355.35_pseudogenic_transcript-p1
OG0007268: TcCLB-EL.503811.50_mRNA-p1 TcCLB-NE.506989.150_mRNA-p1
TcSYL_0003960.t1-p1
OG0007269: TcCLB-EL.506699.6_mRNA-p1 TcCLB-NE.508153.364_mRNA-p1
TcSYL_0000950.t1-p1
OG0007270: TcCLB-EL.507529.45_mRNA-p1 TcCLB-NE.510001.50_mRNA-p1
TcSYL_0003060.t1-p1
OG0007271: TcCLB-EL.509669.10_mRNA-p1 TcCLB-NE.509617.50_mRNA-p1
TcSYL_0001920.t1-p1
OG0007272: TcCLB-EL.510149.110_mRNA-p1 TcCLB-NE.506989.60_mRNA-p1
TcSYL_0004100.t1-p1
OG0007273: TcCLB-EL.510149.60_mRNA-p1 TcCLB-NE.506989.10_mRNA-p1
TcSYL_0004160.t1-p1
OG0007274: TcCLB-EL.504103.10_mRNA-p1 TcCLB-EL.508307.190_mRNA-p1
TcSYL_0005970.t1-p1
OG0007275: TcCLB-EL.504103.60_mRNA-p1 TcCLB-NE.503847.50_mRNA-p1
TcSYL_0005860.t1-p1
OG0007276: TcCLB-EL.506251.20_mRNA-p1 TcCLB-NE.508231.174_mRNA-p1
TcSYL_0108200.t1-p1
OG0007277: TcCLB-EL.507677.4_mRNA-p1 TcCLB-NE.504021.149_mRNA-p1
TcSYL_0010110.t1-p1
OG0007278: TcCLB-EL.507713.40_mRNA-p1 TcSYL_0008580.t1-p1
TcSYL_0008600.t1-p1
OG0007279: TcCLB-EL.508723.20_mRNA-p1 TcCLB-NE.509791.70_mRNA-p1
TcSYL_0107160.t1-p1
OG0007280: TcCLB-EL.510181.80_mRNA-p1 TcCLB-NE.503431.80_mRNA-p1
TcSYL_0013440.t1-p1
OG0007281: TcCLB-EL.503841.40_mRNA-p1 TcCLB-NE.511463.4_mRNA-p1

TcSYL_0131290.t1-p1
OG0007282: TcCLB-EL.508539.40_mRNA-p1 TcCLB-NE.511459.40_mRNA-p1 TcSYL_0130850.t1-p1
OG0007283: TcCLB-EL.508805.80_mRNA-p1 TcCLB-NE.508297.50_mRNA-p1 TcSYL_0203410.t1-p1
OG0007284: TcCLB-EL.510205.70_mRNA-p1 TcSYL_0124210.t1-p1 TcSYL_0155450.t1-p1
OG0007285: TcCLB-EL.511001.110_mRNA-p1 TcCLB-NE.506957.100_mRNA-p1 TcSYL_0131970.t1-p1
OG0007286: TcCLB-EL.511001.50_mRNA-p1 TcCLB-NE.503635.20_mRNA-p1 TcSYL_0132300.t1-p1
OG0007287: TcCLB-EL.511173.57_pseudogenic_transcript-p1 TcCLB-NE.507071.105_pseudogenic_transcript-p1 TcCLB-NE.507071.151_pseudogenic_transcript-p1
OG0007288: TcCLB-EL.511179.140_mRNA-p1 TcCLB-NE.508303.20_mRNA-p1 TcSYL_0203180.t1-p1
OG0007289: TcCLB-EL.511181.60_mRNA-p1 TcCLB-NE.508301.10_mRNA-p1 TcSYL_0203250.t1-p1
OG0007290: TcCLB-EL.511473.3_pseudogenic_transcript-p1 TcSYL_0021370.t1-p1 TcSYL_0204150.t1-p1
OG0007291: TcCLB-EL.408345.10_mRNA-p1 TcCLB-NE.510797.20_mRNA-p1 TcSYL_0082350.t1-p1
OG0007292: TcCLB-EL.419469.14_mRNA-p1 TcCLB-NE.507771.9_mRNA-p1 TcSYL_0138420.t1-p1
OG0007293: TcCLB-EL.506367.160_mRNA-p1 TcCLB-NE.506629.110_mRNA-p1 TcSYL_0082460.t1-p1
OG0007294: TcCLB-EL.507009.70_mRNA-p1 TcCLB-NE.510667.90_mRNA-p1 TcSYL_0117430.t1-p1
OG0007295: TcCLB-EL.507011.100_mRNA-p1 TcCLB-NE.507005.20_mRNA-p1 TcSYL_0116990.t1-p1
OG0007296: TcCLB-EL.507011.40_mRNA-p1 TcCLB-NE.510667.4_mRNA-p1 TcSYL_0117160.t1-p1
OG0007297: TcCLB-EL.507011.80_mRNA-p1 TcCLB-NE.507005.40_mRNA-p1 TcSYL_0117040.t1-p1
OG0007298: TcCLB-EL.508467.19_mRNA-p1 TcCLB-NE.507001.4_mRNA-p1 TcSYL_0115950.t1-p1
OG0007299: TcCLB-EL.508083.5_pseudogenic_transcript-p1 TcCLB-EL.510553.25_pseudogenic_transcript-p1 TcSYL_0072250.t1-p1
OG0007300: TcCLB-EL.510743.50_mRNA-p1 TcCLB-NE.510659.257_mRNA-p1 TcSYL_0116200.t1-p1
OG0007301: TcCLB-EL.510745.49_mRNA-p1 TcCLB-NE.510659.60_mRNA-p1 TcSYL_0116070.t1-p1
OG0007302: TcCLB-EL.510751.30_mRNA-p1 TcCLB-NE.510655.100_mRNA-p1 TcSYL_0115750.t1-p1
OG0007303: TcCLB-EL.510753.9_mRNA-p1 TcCLB-NE.510655.30_mRNA-p1 TcSYL_0115690.t1-p1
OG0007304: TcCLB-EL.510759.40_mRNA-p1 TcCLB-NE.506999.180_mRNA-p1 TcSYL_0115530.t1-p1
OG0007305: TcCLB-EL.510759.50_mRNA-p1 TcCLB-NE.506999.170_mRNA-p1 TcSYL_0115520.t1-p1
OG0007306: TcCLB-EL.503649.10_pseudogenic_transcript-p1 TcCLB-EL.510089.10_mRNA-p1 TcCLB-NE.507071.349_mRNA-p1
OG0007307: TcCLB-EL.506213.20_mRNA-p1 TcCLB-NE.511287.70_mRNA-p1 TcSYL_0123210.t1-p1

OG0007308: TcCLB-EL.506525.120_mRNA-p1 TcCLB-NE.511283.120_mRNA-p1 TcSYL_0123720.t1-p1
OG0007309: TcCLB-EL.506671.20_pseudogenic_transcript-p1 TcCLB-EL.508167.20_mRNA-p1 TcSYL_0025150.t1-p1
OG0007310: TcCLB-EL.507625.186_mRNA-p1 TcCLB-NE.509741.34_mRNA-p1 TcSYL_0016940.t1-p1
OG0007311: TcCLB-EL.507625.70_mRNA-p1 TcCLB-NE.507787.140_mRNA-p1 TcSYL_0017240.t1-p1
OG0007312: TcCLB-EL.439307.9_mRNA-p1 TcCLB-EL.506753.249_mRNA-p1 TcCLB-NE.510357.160_mRNA-p1
OG0007313: TcCLB-EL.504083.20_pseudogenic_transcript-p1 TcCLB-NE.509753.50_pseudogenic_transcript-p1 TcCLB-NE.509897.250_pseudogenic_transcript-p1
OG0007314: TcCLB-EL.506655.20_pseudogenic_transcript-p1 TcCLB-NE.505169.10_pseudogenic_transcript-p1 TcSYL_0072650.t1-p1
OG0007315: TcCLB-EL.506655.7_mRNA-p1 TcCLB-NE.505169.21_pseudogenic_transcript-p1 TcSYL_0073210.t1-p1
OG0007316: TcCLB-EL.506753.240_mRNA-p1 TcCLB-NE.510357.140_mRNA-p1 TcSYL_0112730.t1-p1
OG0007317: TcCLB-EL.506753.60_mRNA-p1 TcCLB-NE.510355.220_mRNA-p1 TcSYL_0112610.t1-p1
OG0007318: TcCLB-EL.511127.210_mRNA-p1 TcCLB-NE.509023.190_mRNA-p1 TcSYL_0155960.t1-p1
OG0007319: TcCLB-EL.511557.29_mRNA-p1 TcCLB-NE.503995.30_mRNA-p1 TcSYL_0073310.t1-p1
OG0007320: TcCLB-NE.507681.10_mRNA-p1 TcCLB-NE.507769.10_mRNA-p1 TcSYL_0073650.t1-p1
OG0007321: TcCLB-NE.507681.20_mRNA-p1 TcCLB-NE.507767.10_mRNA-p1 TcSYL_0073670.t1-p1
OG0007322: TcCLB-NE.508269.80_pseudogenic_transcript-p1 TcSYL_0051350.t1-p1 TcSYL_0051490.t1-p1
OG0007323: TcCLB-NE.511769.10_mRNA-p1 TcCLB-NE.511771.60_mRNA-p1 TcSYL_0165640.t1-p1
OG0007324: TcCLB-NE.511771.199_mRNA-p1 TcSYL_0165910.t1-p1 TcSYL_0166260.t1-p1
OG0007325: TcCLB-NE.504155.140_mRNA-p1 TcCLB-NE.507605.26_mRNA-p1 TcSYL_0068720.t1-p1
OG0007326: TcCLB-NE.509219.10_mRNA-p1 TcSYL_0049020.t1-p1 TcSYL_0069310.t1-p1
OG0007327: TcCLB-NE.504081.70_mRNA-p1 TcCLB-NE.506789.30_mRNA-p1 TcSYL_0109950.t1-p1
OG0007328: TcCLB-NE.506331.64_mRNA-p1 TcCLB-NE.506595.65_mRNA-p1 TcSYL_0200400.t1-p1
OG0007329: TcCLB-NE.508691.10_mRNA-p1 TcCLB-NE.511003.200_mRNA-p1 TcSYL_0046300.t1-p1
OG0007330: TcCLB-NE.510687.10_pseudogenic_transcript-p1 TcSYL_0047090.t1-p1 TcSYL_0138980.t1-p1
OG0007331: TcCLB-NE.509995.10_mRNA-p1 TcSYL_0003320.t1-p1 TcSYL_0003340.t1-p1
OG0007332: TcCLB-NE.510697.90_mRNA-p1 TcSYL_0125600.t1-p1 TcSYL_0125760.t1-p1
OG0007333: TcCLB-NE.508897.119_mRNA-p1 TcCLB-NE.511295.10_mRNA-p1 TcSYL_0121950.t1-p1
OG0007334: TcCLB-NE.508871.180_mRNA-p1 TcSYL_0028440.t1-p1

TcSYL_0141350.t1-p1
OG0007335: TcSYL_0066980.t1-p1 TcSYL_0070400.t1-p1 TcSYL_0161080.t1-p1
OG0007336: TcSYL_0072030.t1-p1 TcSYL_0123940.t1-p1 TcSYL_0161650.t1-p1
OG0007337: TCRU_1 TcCLB-NE.506559.360_mRNA-p1
OG0007338: TCRU_5 TcCLB-NE.506559.310_mRNA-p1
OG0007339: TCRU_8 TcCLB-NE.506559.280_mRNA-p1
OG0007340: TCRU_10 TcCLB-NE.506559.260_mRNA-p1
OG0007341: TCRU_13 TcCLB-NE.506559.230_mRNA-p1
OG0007342: TCRU_14 TcCLB-NE.506559.220_mRNA-p1
OG0007343: TCRU_17 TcCLB-NE.506559.148_mRNA-p1
OG0007344: TCRU_54 TcSYL_0119130.t1-p1
OG0007345: TCRU_60 TcCLB-NE.508479.390_mRNA-p1
OG0007346: TCRU_65 TcCLB-NE.506489.20_mRNA-p1
OG0007347: TCRU_78 TcSYL_0059070.t1-p1
OG0007348: TCRU_82 TcSYL_0130010.t1-p1
OG0007349: TCRU_91 TcCLB-NE.511809.10_mRNA-p1
OG0007350: TCRU_92 TcCLB-NE.511807.290_mRNA-p1
OG0007351: TCRU_93 TcCLB-NE.511807.284_mRNA-p1
OG0007352: TCRU_94 TcCLB-NE.511807.280_mRNA-p1
OG0007353: TCRU_100 TcCLB-NE.508799.250_mRNA-p1
OG0007354: TCRU_115 TcCLB-EL.506227.210_mRNA-p1
OG0007355: TCRU_116 TcCLB-EL.506227.220_mRNA-p1
OG0007356: TCRU_119 TcCLB-EL.504089.30_mRNA-p1
OG0007357: TCRU_131 TcCLB-EL.510879.130_mRNA-p1
OG0007358: TCRU_145 TcSYL_0130030.t1-p1
OG0007359: TCRU_192 TcCLB-EL.511867.160_mRNA-p1
OG0007360: TCRU_202 TcCLB-EL.510877.70_mRNA-p1
OG0007361: TCRU_203 TcCLB-EL.510877.80_mRNA-p1
OG0007362: TCRU_242 TcCLB-EL.510431.140_mRNA-p1
OG0007363: TCRU_265 TcSYL_0020140.t1-p1
OG0007364: TCRU_270 TcSYL_0014480.t1-p1
OG0007365: TCRU_280 TcCLB-NE.503519.30_mRNA-p1
OG0007366: TCRU_297 TcCLB-NE.508461.140_mRNA-p1
OG0007367: TCRU_304 TcCLB-EL.509769.63_mRNA-p1
OG0007368: TCRU_339 TcCLB-NE.511461.14_mRNA-p1
OG0007369: TCRU_356 TcCLB-NE.510901.20_mRNA-p1
OG0007370: TCRU_363 TcCLB-NE.510901.120_mRNA-p1
OG0007371: TCRU_364 TcCLB-NE.510901.130_mRNA-p1
OG0007372: TCRU_400 TcCLB-NE.510943.206_mRNA-p1
OG0007373: TCRU_425 TcSYL_0117090.t1-p1
OG0007374: TCRU_428 TcCLB-EL.507011.110_mRNA-p1
OG0007375: TCRU_441 TcCLB-EL.507641.130_mRNA-p1
OG0007376: TCRU_443 TcCLB-EL.507641.110_mRNA-p1
OG0007377: TCRU_444 TcCLB-EL.507641.104_mRNA-p1
OG0007378: TCRU_445 TcCLB-EL.507641.100_mRNA-p1
OG0007379: TCRU_446 TcCLB-NE.506279.220_pseudogenic_transcript-p1
OG0007380: TCRU_492 TcCLB-NE.511277.440_mRNA-p1
OG0007381: TCRU_493 TcCLB-NE.511277.420_mRNA-p1
OG0007382: TCRU_494 TcCLB-NE.511277.410_mRNA-p1
OG0007383: TCRU_495 TcCLB-NE.511277.400_mRNA-p1
OG0007384: TCRU_496 TcCLB-NE.511277.380_mRNA-p1
OG0007385: TCRU_516 TcSYL_0194390.t1-p1

OG0007386: TCRU_519 TcCLB-NE.510187.540_mRNA-p1
OG0007387: TCRU_520 TcCLB-NE.510187.530_mRNA-p1
OG0007388: TCRU_521 TcCLB-NE.510187.520_mRNA-p1
OG0007389: TCRU_522 TcCLB-NE.510187.510_mRNA-p1
OG0007390: TCRU_523 TcCLB-NE.510187.480_mRNA-p1
OG0007391: TCRU_526 TcCLB-NE.510187.439_mRNA-p1
OG0007392: TCRU_573 TcCLB-NE.511755.28_mRNA-p1
OG0007393: TCRU_595 TcSYL_0029340.t1-p1
OG0007394: TCRU_602 TcSYL_0072130.t1-p1
OG0007395: TCRU_639 TcCLB-NE.507649.80_mRNA-p1
OG0007396: TCRU_640 TcCLB-NE.507649.90_mRNA-p1
OG0007397: TCRU_2686 TCRU_661
OG0007398: TCRU_681 TcCLB-EL.508811.55_mRNA-p1
OG0007399: TCRU_704 TcCLB-EL.511867.70_mRNA-p1
OG0007400: TCRU_711 TcCLB-NE.504183.10_pseudogenic_transcript-p1
OG0007401: TCRU_750 TcCLB-NE.506231.10_mRNA-p1
OG0007402: TCRU_802 TcSYL_0089710.t1-p1
OG0007403: TCRU_820 TcSYL_0028820.t1-p1
OG0007404: TCRU_831 TcSYL_0028720.t1-p1
OG0007405: TCRU_839 TcCLB-EL.506885.110_mRNA-p1
OG0007406: TCRU_849 TcCLB-NE.509753.110_mRNA-p1
OG0007407: TCRU_864 TcCLB-NE.507019.10_mRNA-p1
OG0007408: TCRU_866 TcCLB-NE.510193.10_mRNA-p1
OG0007409: TCRU_942 TcCLB-EL.509895.20_mRNA-p1
OG0007410: TCRU_943 TcCLB-EL.509895.30_mRNA-p1
OG0007411: TCRU_970 TcCLB-NE.511283.300_mRNA-p1
OG0007412: TCRU_972 TcCLB-NE.511283.280_mRNA-p1
OG0007413: TCRU_973 TcCLB-NE.511283.260_mRNA-p1
OG0007414: TCRU_974 TcCLB-NE.511283.240_mRNA-p1
OG0007415: TCRU_975 TcCLB-NE.511283.230_mRNA-p1
OG0007416: TCRU_976 TcCLB-NE.511283.220_mRNA-p1
OG0007417: TCRU_977 TcCLB-NE.511283.210_mRNA-p1
OG0007418: TCRU_978 TcCLB-NE.511283.200_mRNA-p1
OG0007419: TCRU_979 TcCLB-NE.511283.194_mRNA-p1
OG0007420: TCRU_1048 TcCLB-EL.506933.70_mRNA-p1
OG0007421: TCRU_1050 TcCLB-NE.402857.20_mRNA-p1
OG0007422: TCRU_1055 TcCLB-EL.511215.60_mRNA-p1
OG0007423: TCRU_1089 TcCLB-NE.511367.320_mRNA-p1
OG0007424: TCRU_1090 TcCLB-NE.511367.310_mRNA-p1
OG0007425: TCRU_1104 TcCLB-EL.503651.10_mRNA-p1
OG0007426: TCRU_1108 TcCLB-EL.510761.10_mRNA-p1
OG0007427: TCRU_1139 TcSYL_0200090.t1-p1
OG0007428: TCRU_1143 TcSYL_0166450.t1-p1
OG0007429: TCRU_1156 TcCLB-NE.511367.10_mRNA-p1
OG0007430: TCRU_1157 TcCLB-NE.511367.40_mRNA-p1
OG0007431: TCRU_1158 TcCLB-EL.511215.100_mRNA-p1
OG0007432: TCRU_1162 TcSYL_0061830.t1-p1
OG0007433: TCRU_1166 TcCLB-NE.510821.50_mRNA-p1
OG0007434: TCRU_1167 TcCLB-NE.510821.40_mRNA-p1
OG0007435: TCRU_1169 TcCLB-NE.511727.220_mRNA-p1
OG0007436: TCRU_1170 TcCLB-NE.511727.210_mRNA-p1
OG0007437: TCRU_1171 TcCLB-EL.506625.220_mRNA-p1
OG0007438: TCRU_1172 TcCLB-EL.506625.210_mRNA-p1
OG0007439: TCRU_1173 TcCLB-EL.506625.200_mRNA-p1

OG0007440: TCRU_1174 TcCLB-EL.506625.190_mRNA-p1
OG0007441: TCRU_1175 TcCLB-EL.506625.180_mRNA-p1
OG0007442: TCRU_1177 TcCLB-EL.506625.160_mRNA-p1
OG0007443: TCRU_1178 TcCLB-EL.506625.140_mRNA-p1
OG0007444: TCRU_1179 TcCLB-EL.506625.130_mRNA-p1
OG0007445: TCRU_1188 TcCLB-EL.507521.115_mRNA-p1
OG0007446: TCRU_1222 TcCLB-NE.508873.510_mRNA-p1
OG0007447: TCRU_1231 TcSYL_0093190.t1-p1
OG0007448: TCRU_1292 TcSYL_0203510.t1-p1
OG0007449: TCRU_family_ TcCLB-EL.504149.130_mRNA-p1
OG0007450: TCRU_1322 TcCLB-EL.504149.140_mRNA-p1
OG0007451: TCRU_1323 TcCLB-EL.504149.150_mRNA-p1
OG0007452: TCRU_1342 TcSYL_0060020.t1-p1
OG0007453: TCRU_1385 TcCLB-NE.510729.115_mRNA-p1
OG0007454: TCRU_1439 TcSYL_0133360.t1-p1
OG0007455: TCRU_1447 TcCLB-NE.506317.20_mRNA-p1
OG0007456: TCRU_1449 TcCLB-NE.508533.50_mRNA-p1
OG0007457: TCRU_1454 TcCLB-EL.504153.280_mRNA-p1
OG0007458: TCRU_1457 TcCLB-NE.471901.20_mRNA-p1
OG0007459: TCRU_1458 TcCLB-EL.506945.340_mRNA-p1
OG0007460: TCRU_1459 TcCLB-EL.506945.350_mRNA-p1
OG0007461: TCRU_1492 TcCLB-NE.511277.330_mRNA-p1
OG0007462: TCRU_1493 TcCLB-NE.511277.310_mRNA-p1
OG0007463: TCRU_1494 TcCLB-NE.511277.300_mRNA-p1
OG0007464: TCRU_1496 TcCLB-NE.511277.280_mRNA-p1
OG0007465: TCRU_1497 TcCLB-NE.511277.270_mRNA-p1
OG0007466: TCRU_1498 TcCLB-NE.511277.260_mRNA-p1
OG0007467: TCRU_1499 TcCLB-NE.511277.250_mRNA-p1
OG0007468: TCRU_1513 TcCLB-NE.508355.300_mRNA-p1
OG0007469: TCRU_1515 TcCLB-NE.508355.270_mRNA-p1
OG0007470: TCRU_1517 TcSYL_0031910.t1-p1
OG0007471: TCRU_1531 TcSYL_0084060.t1-p1
OG0007472: TCRU_1534 TcSYL_0106220.t1-p1
OG0007473: TCRU_1535 TcSYL_0094030.t1-p1
OG0007474: TCRU_1541 TcCLB-NE.508219.60_pseudogenic_transcript-p1
OG0007475: TCRU_1542 TcSYL_0129740.t1-p1
OG0007476: TCRU_1546 TcSYL_0068080.t1-p1
OG0007477: TCRU_1547 TcCLB-EL.506885.60_mRNA-p1
OG0007478: TCRU_1574 TcCLB-NE.509869.30_mRNA-p1
OG0007479: TCRU_1580 TcCLB-NE.508015.50_mRNA-p1
OG0007480: TCRU_1604 TcCLB-EL.508307.140_mRNA-p1
OG0007481: TCRU_1615 TcCLB-EL.504153.180_mRNA-p1
OG0007482: TCRU_1616 TcCLB-EL.504153.190_mRNA-p1
OG0007483: TCRU_1617 TcCLB-EL.504153.200_mRNA-p1
OG0007484: TCRU_1618 TcCLB-EL.504153.210_mRNA-p1
OG0007485: TCRU_1619 TcCLB-EL.504153.220_mRNA-p1
OG0007486: TCRU_1620 TcCLB-EL.504153.230_mRNA-p1
OG0007487: TCRU_1622 TcCLB-EL.504153.250_mRNA-p1
OG0007488: TCRU_1661 TcSYL_0069040.t1-p1
OG0007489: TCRU_1667 TcCLB-NE.511391.130_mRNA-p1
OG0007490: TCRU_1671 TcCLB-EL.510761.44_mRNA-p1
OG0007491: TCRU_1673 TcCLB-EL.510761.22_mRNA-p1
OG0007492: TCRU_1679 TcCLB-EL.510295.15_mRNA-p1
OG0007493: TCRU_1699 TcCLB-NE.508173.160_mRNA-p1

OG0007494: TCRU_1706 TcCLB-NE.511391.100_mRNA-p1
OG0007495: TCRU_1707 TcCLB-NE.511391.110_mRNA-p1
OG0007496: TCRU_1726 TcSYL_0172150.t1-p1
OG0007497: TCRU_1759 TcCLB-EL.504057.160_mRNA-p1
OG0007498: TCRU_1760 TcCLB-EL.504057.170_mRNA-p1
OG0007499: TCRU_1762 TcSYL_0027970.t1-p1
OG0007500: TCRU_1786 TcSYL_0111600.t1-p1
OG0007501: TCRU_1794 TcCLB-EL.507641.220_mRNA-p1
OG0007502: TCRU_1798 TcSYL_0111490.t1-p1
OG0007503: TCRU_1800 TcCLB-NE.509997.30_mRNA-p1
OG0007504: TCRU_1801 TcCLB-NE.509997.40_mRNA-p1
OG0007505: TCRU_1808 TcCLB-NE.509999.60_mRNA-p1
OG0007506: TCRU_1812 TcCLB-EL.511575.110_mRNA-p1
OG0007507: TCRU_1825 TcCLB-NE.509161.34_mRNA-p1
OG0007508: TCRU_1827 TcCLB-NE.509161.50_mRNA-p1
OG0007509: TCRU_1828 TcCLB-NE.509161.60_mRNA-p1
OG0007510: TCRU_1829 TcCLB-NE.509161.80_mRNA-p1
OG0007511: TCRU_1830 TcCLB-NE.509161.110_mRNA-p1
OG0007512: TCRU_1831 TcCLB-NE.509161.120_mRNA-p1
OG0007513: TCRU_1832 TcCLB-NE.509161.130_mRNA-p1
OG0007514: TCRU_1833 TcCLB-NE.509161.135_mRNA-p1
OG0007515: TCRU_1841 TcCLB-EL.511215.10_mRNA-p1
OG0007516: TCRU_1845 TcSYL_0067840.t1-p1
OG0007517: TCRU_1851 TcCLB-NE.509911.74_mRNA-p1
OG0007518: TCRU_1859 TcCLB-EL.508647.120_mRNA-p1
OG0007519: TCRU_1860 TcCLB-EL.508647.130_mRNA-p1
OG0007520: TCRU_1872 TcCLB-NE.510187.234_mRNA-p1
OG0007521: TCRU_1873 TcCLB-NE.510187.230_mRNA-p1
OG0007522: TCRU_1874 TcCLB-NE.510187.220_mRNA-p1
OG0007523: TCRU_1875 TcCLB-NE.510187.210_mRNA-p1
OG0007524: TCRU_C TcCLB-NE.510187.199_mRNA-p1
OG0007525: TCRU_1878 TcCLB-NE.510187.180_mRNA-p1
OG0007526: TCRU_1883 TcSYL_0048810.t1-p1
OG0007527: TCRU_1886 TcCLB-EL.504147.80_mRNA-p1
OG0007528: TCRU_1954 TcCLB-EL.506925.180_mRNA-p1
OG0007529: TCRU_1956 TcCLB-EL.506925.200_mRNA-p1
OG0007530: TCRU_1957 TcCLB-EL.504057.104_mRNA-p1
OG0007531: TCRU_1959 TcCLB-EL.504057.90_mRNA-p1
OG0007532: TCRU_1972 TcCLB-EL.509951.49_mRNA-p1
OG0007533: TCRU_1977 TcCLB-NE.506485.50_mRNA-p1
OG0007534: TCRU_1978 TcCLB-NE.506485.60_mRNA-p1
OG0007535: TCRU_2000 TcCLB-NE.506321.260_mRNA-p1
OG0007536: TCRU_2020 TcCLB-EL.508307.110_mRNA-p1
OG0007537: TCRU_2026 TcCLB-NE.508355.230_mRNA-p1
OG0007538: TCRU_2027 TcCLB-NE.506559.380_mRNA-p1
OG0007539: TCRU_2029 TcCLB-NE.506559.410_mRNA-p1
OG0007540: TCRU_2031 TcCLB-NE.506559.434_mRNA-p1
OG0007541: TCRU_2032 TcCLB-NE.506559.440_mRNA-p1
OG0007542: TCRU_2035 TcCLB-NE.506559.460_mRNA-p1
OG0007543: TCRU_2037 TcCLB-EL.511211.30_mRNA-p1
OG0007544: TCRU_2050 TcSYL_0066770.t1-p1
OG0007545: TCRU_2051 TcCLB-NE.510713.120_mRNA-p1
OG0007546: TCRU_2058 TcCLB-EL.508307.150_mRNA-p1
OG0007547: TCRU_2074 TcCLB-EL.506925.490_mRNA-p1

OG0007548: TCRU_2075 TcCLB-EL.506925.500_mRNA-p1
OG0007549: TCRU_2077 TcCLB-EL.506925.520_mRNA-p1
OG0007550: TCRU_2078 TcCLB-EL.506925.530_mRNA-p1
OG0007551: TCRU_2079 TcCLB-EL.506925.540_mRNA-p1
OG0007552: TCRU_2082 TcCLB-NE.510187.120_mRNA-p1
OG0007553: TCRU_2096 TcCLB-NE.511317.70_mRNA-p1
OG0007554: TCRU_2128 TcCLB-EL.506925.110_mRNA-p1
OG0007555: TCRU_2130 TcCLB-EL.506925.100_mRNA-p1
OG0007556: TCRU_2131 TcCLB-EL.506925.90_mRNA-p1
OG0007557: TCRU_2132 TcCLB-EL.506925.80_mRNA-p1
OG0007558: TCRU_2137 TcCLB-EL.504047.34_mRNA-p1
OG0007559: TCRU_2151 TcCLB-NE.510625.19_mRNA-p1
OG0007560: TCRU_2153 TcCLB-NE.504249.65_mRNA-p1
OG0007561: TCRU_2207 TcCLB-NE.508061.10_mRNA-p1
OG0007562: TCRU_2225 TcCLB-EL.511867.130_mRNA-p1
OG0007563: TCRU_2229 TcCLB-EL.507093.130_mRNA-p1
OG0007564: TCRU_2242 TcCLB-EL.507099.70_mRNA-p1
OG0007565: TCRU_2244 TcCLB-EL.507093.280_mRNA-p1
OG0007566: TCRU_2247 TcCLB-EL.510879.170_mRNA-p1
OG0007567: TCRU_2262 TcSYL_0062230.t1-p1
OG0007568: TCRU_2263 TcCLB-NE.507601.110_pseudogenic_transcript-p1
OG0007569: TCRU_2264 TcSYL_0062220.t1-p1
OG0007570: TCRU_2284 TcSYL_0039340.t1-p1
OG0007571: TCRU_family_ TcCLB-NE.504141.31_pseudogenic_transcript-p1
OG0007572: TCRU_2314 TcCLB-NE.506155.30_mRNA-p1
OG0007573: TCRU_2318 TcCLB-NE.506155.80_mRNA-p1
OG0007574: TCRU_2319 TcCLB-NE.506155.90_pseudogenic_transcript-p1
OG0007575: TCRU_2321 TcCLB-NE.506679.90_mRNA-p1
OG0007576: TCRU_2323 TcSYL_0022010.t1-p1
OG0007577: TCRU_2341 TcCLB-EL.507093.320_mRNA-p1
OG0007578: TCRU_2342 TcCLB-EL.507093.339_mRNA-p1
OG0007579: TCRU_2344 TcCLB-EL.504057.65_mRNA-p1
OG0007580: TCRU_2346 TcCLB-EL.508307.60_mRNA-p1
OG0007581: TCRU_2359 TcSYL_0072070.t1-p1
OG0007582: TCRU_2362 TcCLB-NE.506783.30_mRNA-p1
OG0007583: TCRU_2374 TcCLB-EL.504153.270_mRNA-p1
OG0007584: TCRU_2378 TcCLB-NE.508153.190_mRNA-p1
OG0007585: TCRU_2382 TcCLB-NE.508153.150_mRNA-p1
OG0007586: TCRU_2383 TcCLB-NE.508153.140_mRNA-p1
OG0007587: TCRU_2432 TcSYL_0071050.t1-p1
OG0007588: TCRU_2434 TcCLB-NE.506785.46_mRNA-p1
OG0007589: TCRU_2442 TcCLB-NE.508897.70_mRNA-p1
OG0007590: TCRU_2459 TcCLB-EL.509537.60_mRNA-p1
OG0007591: TCRU_2460 TcCLB-NE.511277.220_mRNA-p1
OG0007592: TCRU_2461 TcCLB-NE.511277.200_mRNA-p1
OG0007593: TCRU_2462 TcCLB-NE.511277.190_mRNA-p1
OG0007594: TCRU_2463 TcCLB-NE.511277.180_mRNA-p1
OG0007595: TCRU_2466 TcCLB-NE.511277.170_mRNA-p1
OG0007596: TCRU_2488 TcCLB-EL.506933.43_mRNA-p1
OG0007597: TCRU_2489 TcCLB-EL.506933.46_mRNA-p1
OG0007598: TCRU_2493 TcCLB-NE.511807.190_mRNA-p1
OG0007599: TCRU_2494 TcCLB-NE.511807.200_mRNA-p1
OG0007600: TCRU_2495 TcCLB-NE.511807.210_pseudogenic_transcript-p1
OG0007601: TCRU_2496 TcCLB-NE.511807.220_mRNA-p1

OG0007602: TCRU_2498 TcCLB-NE.511807.250_mRNA-p1
OG0007603: TCRU_2499 TcCLB-NE.511807.253_mRNA-p1
OG0007604: TCRU_2500 TcCLB-NE.511807.260_mRNA-p1
OG0007605: TCRU_2501 TcCLB-EL.507629.39_mRNA-p1
OG0007606: TCRU_2515 TcCLB-NE.509647.70_mRNA-p1
OG0007607: TCRU_2551 TcSYL_0094560.t1-p1
OG0007608: TCRU_2564 TcCLB-EL.509445.20_mRNA-p1
OG0007609: TCRU_2568 TcCLB-EL.511527.60_mRNA-p1
OG0007610: TCRU_2590 TcCLB-EL.511421.30_mRNA-p1
OG0007611: TCRU_2592 TcCLB-EL.504153.260_mRNA-p1
OG0007612: TCRU_2596 TcCLB-NE.511367.150_mRNA-p1
OG0007613: TCRU_2598 TcCLB-NE.508461.470_mRNA-p1
OG0007614: TCRU_2599 TcCLB-NE.508461.460_mRNA-p1
OG0007615: TCRU_2600 TcCLB-NE.508461.450_mRNA-p1
OG0007616: TCRU_2601 TcCLB-NE.508461.440_mRNA-p1
OG0007617: TCRU_2602 TcCLB-NE.508461.430_mRNA-p1
OG0007618: TCRU_2603 TcCLB-NE.508461.410_mRNA-p1
OG0007619: TCRU_2604 TcCLB-NE.508461.400_mRNA-p1
OG0007620: TCRU_2605 TcCLB-NE.508461.390_mRNA-p1
OG0007621: TCRU_2606 TcCLB-NE.508461.380_mRNA-p1
OG0007622: TCRU_2607 TcCLB-NE.508461.370_mRNA-p1
OG0007623: TCRU_2608 TcCLB-NE.508461.360_mRNA-p1
OG0007624: TCRU_2609 TcCLB-NE.508461.350_mRNA-p1
OG0007625: TCRU_2610 TcCLB-NE.508461.340_mRNA-p1
OG0007626: TCRU_2611 TcCLB-NE.508461.330_mRNA-p1
OG0007627: TCRU_2616 TcCLB-NE.508173.10_pseudogenic_transcript-p1
OG0007628: TCRU_2651 TcCLB-EL.511867.100_mRNA-p1
OG0007629: TCRU_2653 TcCLB-EL.511867.120_mRNA-p1
OG0007630: TCRU_2654 TcCLB-EL.504057.130_mRNA-p1
OG0007631: TCRU_2655 TcCLB-EL.506795.60_mRNA-p1
OG0007632: TCRU_2665 TcCLB-EL.508647.160_mRNA-p1
OG0007633: TCRU_2668 TcCLB-EL.507099.100_mRNA-p1
OG0007634: TCRU_2669 TcCLB-EL.507099.90_mRNA-p1
OG0007635: TCRU_2670 TcCLB-EL.504423.15_mRNA-p1
OG0007636: TCRU_2709 TcSYL_0154790.t1-p1
OG0007637: TCRU_2714 TcCLB-EL.506885.40_mRNA-p1
OG0007638: TCRU_2717 TcCLB-EL.506885.50_mRNA-p1
OG0007639: TCRU_2720 TcCLB-EL.506227.200_mRNA-p1
OG0007640: TCRU_2729 TcSYL_0157820.t1-p1
OG0007641: TCRU_2733 TcSYL_0200600.t1-p1
OG0007642: TCRU_2736 TcSYL_0126280.t1-p1
OG0007643: TCRU_2739 TcCLB-EL.511753.140_mRNA-p1
OG0007644: TCRU_2750 TcSYL_0154310.t1-p1
OG0007645: TCRU_2755 TcCLB-EL.510877.100_mRNA-p1
OG0007646: TCRU_2810 TcCLB-NE.506419.10_mRNA-p1
OG0007647: TCRU_2826 TcCLB-EL.508357.100_mRNA-p1
OG0007648: TCRU_2857 TcCLB-NE.510687.90_mRNA-p1
OG0007649: TCRU_2858 TcCLB-NE.510687.80_mRNA-p1
OG0007650: TCRU_2859 TcCLB-NE.510687.70_mRNA-p1
OG0007651: TCRU_2860 TcCLB-NE.510687.60_mRNA-p1
OG0007652: TCRU_2866 TcCLB-NE.507053.200_mRNA-p1
OG0007653: TCRU_2867 TcCLB-NE.507053.190_mRNA-p1
OG0007654: TCRU_2868 TcCLB-NE.507053.180_mRNA-p1
OG0007655: TCRU_2869 TcCLB-NE.507053.170_mRNA-p1

OG0007656: TCRU_2870 TcCLB-NE.507053.160_mRNA-p1
OG0007657: TCRU_2871 TcCLB-NE.507053.150_mRNA-p1
OG0007658: TCRU_2874 TcCLB-NE.507053.120_mRNA-p1
OG0007659: TCRU_2877 TcCLB-NE.507053.80_mRNA-p1
OG0007660: TCRU_2878 TcCLB-NE.507053.60_mRNA-p1
OG0007661: TCRU_2879 TcCLB-NE.507053.50_mRNA-p1
OG0007662: TCRU_2881 TcCLB-NE.507053.20_mRNA-p1
OG0007663: TCRU_2882 TcCLB-NE.507053.10_mRNA-p1
OG0007664: TCRU_2884 TcCLB-NE.508257.250_mRNA-p1
OG0007665: TCRU_2886 TcCLB-NE.508257.210_mRNA-p1
OG0007666: TCRU_2888 TcCLB-NE.508257.200_mRNA-p1
OG0007667: TCRU_2889 TcCLB-NE.508257.190_mRNA-p1
OG0007668: TCRU_2892 TcCLB-NE.508257.160_mRNA-p1
OG0007669: TCRU_2894 TcCLB-NE.508257.140_mRNA-p1
OG0007670: TCRU_2895 TcCLB-NE.508257.130_mRNA-p1
OG0007671: TCRU_2896 TcCLB-NE.508257.120_mRNA-p1
OG0007672: TCRU_2897 TcCLB-NE.508257.110_mRNA-p1
OG0007673: TCRU_2899 TcCLB-NE.508257.90_mRNA-p1
OG0007674: TCRU_2923 TcCLB-EL.506933.24_mRNA-p1
OG0007675: TCRU_2926 TcCLB-EL.511441.10_mRNA-p1
OG0007676: TCRU_2970 TcCLB-EL.508307.134_mRNA-p1
OG0007677: TCRU_2992 TcCLB-NE.509287.100_mRNA-p1
OG0007678: TCRU_3002 TcCLB-NE.511671.20_pseudogenic_transcript-p1
OG0007679: TCRU_3049 TcCLB-EL.504149.124_mRNA-p1
OG0007680: TCRU_3050 TcCLB-EL.504149.117_mRNA-p1
OG0007681: TCRU_3051 TcCLB-EL.504149.110_mRNA-p1
OG0007682: TCRU_3055 TcCLB-EL.506625.110_mRNA-p1
OG0007683: TCRU_3058 TcCLB-EL.507093.240_mRNA-p1
OG0007684: TCRU_3132 TcSYL_0159010.t1-p1
OG0007685: TCRU_3141 TcCLB-NE.508815.50_mRNA-p1
OG0007686: TCRU_3142 TcCLB-NE.508815.60_mRNA-p1
OG0007687: TCRU_3144 TcCLB-NE.508815.80_mRNA-p1
OG0007688: TCRU_3145 TcCLB-NE.508815.90_mRNA-p1
OG0007689: TCRU_3146 TcCLB-NE.508815.100_mRNA-p1
OG0007690: TCRU_3148 TcCLB-NE.508815.120_mRNA-p1
OG0007691: TCRU_3149 TcCLB-NE.508815.130_mRNA-p1
OG0007692: TCRU_3150 TcCLB-NE.508815.140_mRNA-p1
OG0007693: TCRU_3151 TcCLB-NE.508815.160_mRNA-p1
OG0007694: TCRU_3166 TcSYL_0038690.t1-p1
OG0007695: TCRU_3179 TcCLB-NE.507681.90_mRNA-p1
OG0007696: TCRU_3188 TcCLB-EL.504057.45_mRNA-p1
OG0007697: TCRU_3190 TcSYL_0111200.t1-p1
OG0007698: TCRU_3234 TcCLB-EL.504057.140_mRNA-p1
OG0007699: TCRU_3235 TcSYL_0070340.t1-p1
OG0007700: TCRU_3247 TcSYL_0202270.t1-p1
OG0007701: TCRU_3306 TcCLB-EL.506945.260_mRNA-p1
OG0007702: TCRU_3310 TcSYL_0203680.t1-p1
OG0007703: TCRU_3315 TcCLB-NE.511725.270_mRNA-p1
OG0007704: TCRU_3317 TcCLB-NE.511725.230_mRNA-p1
OG0007705: TCRU_3320 TcCLB-NE.511725.190_mRNA-p1
OG0007706: TCRU_3324 TcSYL_0042810.t1-p1
OG0007707: TCRU_3341 TcCLB-NE.503845.60_pseudogenic_transcript-p1
OG0007708: TCRU_3350 TcCLB-NE.510339.90_pseudogenic_transcript-p1
OG0007709: TCRU_3355 TcCLB-NE.448329.10_mRNA-p1

OG0007710: TCRU_3367 TcCLB-EL.511421.170_mRNA-p1
OG0007711: TCRU_3368 TcCLB-EL.511421.180_mRNA-p1
OG0007712: TCRU_3371 TcCLB-NE.511725.170_mRNA-p1
OG0007713: TCRU_3374 TcCLB-NE.511725.140_mRNA-p1
OG0007714: TCRU_3375 TcCLB-NE.511725.120_mRNA-p1
OG0007715: TCRU_3376 TcCLB-NE.511725.110_mRNA-p1
OG0007716: TCRU_3380 TcSYL_0051300.t1-p1
OG0007717: TCRU_3388 TcCLB-EL.506925.170_mRNA-p1
OG0007718: TCRU_3412 TcCLB-EL.511421.120_mRNA-p1
OG0007719: TCRU_3413 TcCLB-EL.511421.110_mRNA-p1
OG0007720: TCRU_3414 TcCLB-EL.511421.100_mRNA-p1
OG0007721: TCRU_3417 TcCLB-EL.506945.330_mRNA-p1
OG0007722: TCRU_3419 TcCLB-NE.508173.129_mRNA-p1
OG0007723: TCRU_3424 TcCLB-NE.508173.84_mRNA-p1
OG0007724: TCRU_3425 TcCLB-NE.508173.80_mRNA-p1
OG0007725: TCRU_3427 TcCLB-NE.508173.60_mRNA-p1
OG0007726: TCRU_3428 TcCLB-NE.508173.54_mRNA-p1
OG0007727: TCRU_3441 TcCLB-NE.511809.20_mRNA-p1
OG0007728: TCRU_3458 TcCLB-NE.504109.70_mRNA-p1
OG0007729: TCRU_3459 TcCLB-NE.504109.80_mRNA-p1
OG0007730: TCRU_3460 TcCLB-NE.504109.90_mRNA-p1
OG0007731: TCRU_3462 TcCLB-NE.504109.120_mRNA-p1
OG0007732: TCRU_3463 TcCLB-NE.504109.130_mRNA-p1
OG0007733: TCRU_3464 TcCLB-NE.504109.140_mRNA-p1
OG0007734: TCRU_3465 TcCLB-NE.504109.150_mRNA-p1
OG0007735: TCRU_3467 TcCLB-NE.504109.170_mRNA-p1
OG0007736: TCRU_3480 TcSYL_0179920.t1-p1
OG0007737: TCRU_3491 TcCLB-NE.509429.50_pseudogenic_transcript-p1
OG0007738: TCRU_3505 TcCLB-EL.503425.10_mRNA-p1
OG0007739: TCRU_3509 TcCLB-NE.507073.30_mRNA-p1
OG0007740: TCRU_3536 TcCLB-EL.506625.80_mRNA-p1
OG0007741: TCRU_3552 TcCLB-NE.508969.20_pseudogenic_transcript-p1
OG0007742: TCRU_3563 TcCLB-NE.511389.80_mRNA-p1
OG0007743: TCRU_3564 TcCLB-NE.511389.70_mRNA-p1
OG0007744: TCRU_3573 TcCLB-NE.506977.60_mRNA-p1
OG0007745: TCRU_3583 TcCLB-EL.506885.20_mRNA-p1
OG0007746: TCRU_3584 TcCLB-EL.506885.30_mRNA-p1
OG0007747: TCRU_3585 TcSYL_0094240.t1-p1
OG0007748: TCRU_3590 TcCLB-EL.425311.9_mRNA-p1
OG0007749: TCRU_3610 TcCLB-EL.504073.10_mRNA-p1
OG0007750: TCRU_3620 TcCLB-EL.504153.100_mRNA-p1
OG0007751: TCRU_3645 TcSYL_0093710.t1-p1
OG0007752: TCRU_3664 TcCLB-NE.506331.20_pseudogenic_transcript-p1
OG0007753: TCRU_3673 TcCLB-EL.510565.130_mRNA-p1
OG0007754: TCRU_3686 TcCLB-NE.511887.30_pseudogenic_transcript-p1
OG0007755: TCRU_3690 TcCLB-NE.511367.210_mRNA-p1
OG0007756: TCRU_3704 TcCLB-NE.506627.40_mRNA-p1
OG0007757: TCRU_3705 TcCLB-NE.506627.50_mRNA-p1
OG0007758: TCRU_3706 TcCLB-NE.506627.70_mRNA-p1
OG0007759: TCRU_3707 TcCLB-NE.506627.80_mRNA-p1
OG0007760: TCRU_3708 TcCLB-NE.506627.100_mRNA-p1
OG0007761: TCRU_3709 TcSYL_0018490.t1-p1
OG0007762: TCRU_3710 TcSYL_0191460.t1-p1
OG0007763: TCRU_3733 TcSYL_0114330.t1-p1

OG0007764: TCRU_3762 TcCLB-EL.504147.210_mRNA-p1
OG0007765: TCRU_3780 TcCLB-NE.507081.60_mRNA-p1
OG0007766: TCRU_3781 TcCLB-NE.507081.70_mRNA-p1
OG0007767: TCRU_3801 TcSYL_0195810.t1-p1
OG0007768: TCRU_3807 TcCLB-NE.510047.10_mRNA-p1
OG0007769: TCRU_3808 TcCLB-NE.506511.80_mRNA-p1
OG0007770: TCRU_3835 TcSYL_0074590.t1-p1
OG0007771: TCRU_3852 TcCLB-NE.508233.60_mRNA-p1
OG0007772: TCRU_3887 TcCLB-EL.511421.150_mRNA-p1
OG0007773: TCRU_3954 TcCLB-EL.507093.270_mRNA-p1
OG0007774: TCRU_3967 TcCLB-EL.510101.34_mRNA-p1
OG0007775: TCRU_4017 TCRU_8128
OG0007776: TCRU_4030 TcCLB-EL.507099.110_mRNA-p1
OG0007777: TCRU_4049 TcSYL_0056030.t1-p1
OG0007778: TCRU_4052 TcSYL_0056960.t1-p1
OG0007779: TCRU_4074 TcCLB-NE.511807.130_mRNA-p1
OG0007780: TCRU_4075 TcCLB-NE.511807.140_mRNA-p1
OG0007781: TCRU_4076 TcCLB-NE.511807.150_mRNA-p1
OG0007782: TCRU_4077 TcCLB-NE.511807.160_mRNA-p1
OG0007783: TCRU_4078 TcCLB-NE.511807.180_mRNA-p1
OG0007784: TCRU_4097 TcCLB-EL.507099.20_mRNA-p1
OG0007785: TCRU_4101 TcCLB-NE.511727.150_mRNA-p1
OG0007786: TCRU_4174 TcCLB-NE.509753.20_pseudogenic_transcript-p1
OG0007787: TCRU_4202 TcCLB-NE.510361.10_mRNA-p1
OG0007788: TCRU_4218 TcCLB-NE.510831.21_mRNA-p1
OG0007789: TCRU_4221 TCRU_4278
OG0007790: TCRU_4230 TcCLB-NE.510699.50_mRNA-p1
OG0007791: TCRU_4231 TcSYL_0058480.t1-p1
OG0007792: TCRU_4232 TcSYL_0114590.t1-p1
OG0007793: TCRU_4240 TcSYL_0174150.t1-p1
OG0007794: TCRU_4268 TcCLB-EL.506777.24_mRNA-p1
OG0007795: TCRU_4289 TcSYL_0018780.t1-p1
OG0007796: TCRU_family_ TcCLB-EL.506925.160_mRNA-p1
OG0007797: TCRU_4317 TcCLB-NE.506883.30_mRNA-p1
OG0007798: TCRU_4324 TcCLB-NE.506883.100_mRNA-p1
OG0007799: TCRU_4327 TcCLB-NE.504123.40_mRNA-p1
OG0007800: TCRU_4328 TcCLB-NE.504123.30_mRNA-p1
OG0007801: TCRU_4329 TcCLB-NE.504123.20_mRNA-p1
OG0007802: TCRU_4331 TcCLB-EL.506885.10_mRNA-p1
OG0007803: TCRU_4336 TcSYL_0128220.t1-p1
OG0007804: TCRU_4376 TcSYL_0072150.t1-p1
OG0007805: TCRU_4377 TcSYL_0019130.t1-p1
OG0007806: TCRU_4379 TcCLB-NE.511511.6_mRNA-p1
OG0007807: TCRU_4425 TcSYL_0056970.t1-p1
OG0007808: TCRU_4462 TcCLB-EL.506945.300_mRNA-p1
OG0007809: TCRU_4466 TcCLB-NE.511319.30_mRNA-p1
OG0007810: TCRU_4484 TcCLB-EL.510601.10_mRNA-p1
OG0007811: TCRU_4500 TcSYL_0079690.t1-p1
OG0007812: TCRU_4529 TcSYL_0028400.t1-p1
OG0007813: TCRU_C-terminal TcCLB-EL.506239.10_mRNA-p1
OG0007814: TCRU_4540 TcCLB-NE.506337.210_mRNA-p1
OG0007815: TCRU_4542 TcCLB-NE.506337.180_mRNA-p1
OG0007816: TCRU_4543 TcCLB-NE.506337.160_mRNA-p1
OG0007817: TCRU_4544 TcCLB-NE.506337.150_mRNA-p1

OG0007818: TCRU_4545 TcCLB-NE.506337.140_mRNA-p1
OG0007819: TCRU_4546 TcCLB-NE.506337.130_mRNA-p1
OG0007820: TCRU_4547 TcCLB-NE.506337.120_mRNA-p1
OG0007821: TCRU_4594 TcSYL_0102130.t1-p1
OG0007822: TCRU_4621 TcCLB-NE.508741.350_mRNA-p1
OG0007823: TCRU_4624 TcCLB-EL.506625.240_mRNA-p1
OG0007824: TCRU_4636 TcCLB-NE.508741.160_mRNA-p1
OG0007825: TCRU_4659 TcCLB-NE.509163.60_mRNA-p1
OG0007826: TCRU_4697 TcCLB-NE.506155.99_mRNA-p1
OG0007827: TCRU_4733 TcCLB-EL.509951.40_mRNA-p1
OG0007828: TCRU_4766 TcCLB-NE.511391.210_mRNA-p1
OG0007829: TCRU_4773 TcCLB-EL.508307.40_mRNA-p1
OG0007830: TCRU_4779 TcCLB-EL.510879.210_mRNA-p1
OG0007831: TCRU_4817 TcCLB-EL.507093.250_mRNA-p1
OG0007832: TCRU_4842 TcCLB-NE.506679.130_mRNA-p1
OG0007833: TCRU_4863 TcCLB-NE.506445.100_mRNA-p1
OG0007834: TCRU_4865 TcCLB-NE.506445.121_mRNA-p1
OG0007835: TCRU_of TcSYL_0020980.t1-p1
OG0007836: TCRU_4908 TcCLB-NE.508523.50_mRNA-p1
OG0007837: TCRU_4914 TcCLB-EL.511527.20_mRNA-p1
OG0007838: TCRU_4974 TcSYL_0022130.t1-p1
OG0007839: TCRU_4976 TcSYL_0040750.t1-p1
OG0007840: TCRU_4987 TcSYL_0125150.t1-p1
OG0007841: TCRU_5012 TcCLB-NE.506247.40_mRNA-p1
OG0007842: TCRU_5013 TcCLB-NE.506247.70_mRNA-p1
OG0007843: TCRU_5016 TcCLB-NE.506247.100_mRNA-p1
OG0007844: TCRU_5017 TcCLB-NE.506247.110_mRNA-p1
OG0007845: TCRU_5018 TcCLB-NE.506247.120_mRNA-p1
OG0007846: TCRU_5020 TcCLB-NE.506247.130_mRNA-p1
OG0007847: TCRU_5024 TcCLB-NE.506247.170_mRNA-p1
OG0007848: TCRU_5030 TcCLB-NE.506247.240_mRNA-p1
OG0007849: TCRU_5032 TcCLB-NE.506247.260_mRNA-p1
OG0007850: TCRU_5034 TcCLB-NE.506247.280_mRNA-p1
OG0007851: TCRU_5036 TcCLB-NE.506247.310_mRNA-p1
OG0007852: TCRU_5037 TcCLB-NE.506247.320_mRNA-p1
OG0007853: TCRU_5087 TcCLB-EL.506127.80_mRNA-p1
OG0007854: TCRU_5089 TcCLB-EL.506127.100_mRNA-p1
OG0007855: TCRU_5095 TcCLB-EL.507641.239_mRNA-p1
OG0007856: TCRU_5137 TcCLB-EL.506625.84_mRNA-p1
OG0007857: TCRU_5138 TcCLB-EL.506625.90_mRNA-p1
OG0007858: TCRU_5143 TcCLB-NE.506839.20_mRNA-p1
OG0007859: TCRU_5144 TcCLB-NE.506839.14_mRNA-p1
OG0007860: TCRU_5149 TcCLB-NE.510303.180_mRNA-p1
OG0007861: TCRU_5152 TcCLB-NE.510303.204_mRNA-p1
OG0007862: TCRU_5155 TcCLB-NE.510303.250_mRNA-p1
OG0007863: TCRU_5156 TcCLB-NE.510303.260_mRNA-p1
OG0007864: TCRU_5194 TcCLB-NE.506613.40_mRNA-p1
OG0007865: TCRU_5200 TcCLB-NE.508823.130_mRNA-p1
OG0007866: TCRU_5201 TcCLB-NE.508823.100_mRNA-p1
OG0007867: TCRU_5204 TcCLB-NE.508823.60_mRNA-p1
OG0007868: TCRU_5230 TcCLB-NE.511487.170_mRNA-p1
OG0007869: TCRU_5232 TcCLB-EL.504147.130_mRNA-p1
OG0007870: TCRU_5235 TcCLB-NE.506559.550_mRNA-p1
OG0007871: TCRU_5236 TcCLB-NE.506559.540_mRNA-p1

OG0007872: TCRU_5253 TcSYL_0040240.t1-p1
OG0007873: TCRU_5259 TcSYL_0012950.t1-p1
OG0007874: TCRU_5280 TcCLB-NE.506679.120_mRNA-p1
OG0007875: TCRU_5288 TcCLB-NE.508461.149_mRNA-p1
OG0007876: TCRU_5289 TcCLB-NE.508461.150_mRNA-p1
OG0007877: TCRU_5290 TcCLB-NE.508461.174_mRNA-p1
OG0007878: TCRU_5291 TcCLB-NE.508461.190_mRNA-p1
OG0007879: TCRU_5292 TcCLB-NE.508461.200_mRNA-p1
OG0007880: TCRU_5293 TcCLB-NE.508461.210_mRNA-p1
OG0007881: TCRU_5294 TcCLB-NE.508461.230_mRNA-p1
OG0007882: TCRU_5296 TcSYL_0187990.t1-p1
OG0007883: TCRU_5297 TcSYL_0093750.t1-p1
OG0007884: TCRU_5299 TcSYL_0022340.t1-p1
OG0007885: TCRU_5316 TcCLB-EL.507099.60_mRNA-p1
OG0007886: TCRU_5376 TcSYL_0187770.t1-p1
OG0007887: TCRU_5377 TcSYL_0103650.t1-p1
OG0007888: TCRU_5383 TcCLB-NE.507583.30_mRNA-p1
OG0007889: TCRU_5384 TcCLB-NE.507583.40_mRNA-p1
OG0007890: TCRU_5431 TcCLB-EL.510291.30_mRNA-p1
OG0007891: TCRU_5443 TcSYL_0129900.t1-p1
OG0007892: TCRU_5451 TcCLB-NE.511003.140_mRNA-p1
OG0007893: TCRU_5452 TcCLB-NE.511003.110_mRNA-p1
OG0007894: TCRU_5453 TcCLB-NE.511003.100_mRNA-p1
OG0007895: TCRU_5483 TcCLB-NE.511367.90_mRNA-p1
OG0007896: TCRU_5484 TcCLB-NE.511367.100_mRNA-p1
OG0007897: TCRU_5525 TcCLB-NE.508479.380_mRNA-p1
OG0007898: TCRU_5532 TcCLB-NE.508799.260_mRNA-p1
OG0007899: TCRU_5541 TcCLB-EL.511867.180_mRNA-p1
OG0007900: TCRU_5542 TcCLB-EL.511867.150_mRNA-p1
OG0007901: TCRU_5554 TcCLB-NE.507025.60_mRNA-p1
OG0007902: TCRU_5571 TcCLB-NE.511277.390_mRNA-p1
OG0007903: TCRU_5572 TcCLB-NE.511277.359_mRNA-p1
OG0007904: TCRU_5574 TcCLB-NE.510187.500_mRNA-p1
OG0007905: TCRU_5575 TcCLB-NE.510187.490_mRNA-p1
OG0007906: TCRU_5597 TcCLB-EL.506885.90_mRNA-p1
OG0007907: TCRU_5610 TcCLB-EL.507641.70_mRNA-p1
OG0007908: TCRU_5621 TcCLB-NE.511283.310_mRNA-p1
OG0007909: TCRU_5629 TcCLB-EL.506933.60_mRNA-p1
OG0007910: TCRU_5630 TcCLB-EL.506933.50_mRNA-p1
OG0007911: TCRU_5647 TcCLB-EL.511215.110_mRNA-p1
OG0007912: TCRU_5652 TcCLB-EL.511421.190_mRNA-p1
OG0007913: TCRU_5676 TcCLB-EL.508837.180_mRNA-p1
OG0007914: TCRU_5677 TcCLB-EL.508837.189_mRNA-p1
OG0007915: TCRU_5681 TcCLB-NE.506559.517_mRNA-p1
OG0007916: TCRU_5683 TcCLB-NE.511277.340_mRNA-p1
OG0007917: TCRU_5684 TcCLB-NE.511277.320_mRNA-p1
OG0007918: TCRU_5685 TcCLB-NE.511277.240_mRNA-p1
OG0007919: TCRU__Poly_A_-binding TcCLB-EL.506885.70_mRNA-p1
OG0007920: TCRU_C-terminal TcCLB-NE.509997.10_mRNA-p1
OG0007921: TCRU_5704 TcCLB-EL.506925.140_mRNA-p1
OG0007922: TCRU_5713 TcCLB-NE.509161.90_mRNA-p1
OG0007923: TCRU_5714 TcCLB-NE.509161.100_mRNA-p1
OG0007924: TCRU_5733 TcCLB-NE.506321.270_mRNA-p1
OG0007925: TCRU_5758 TcCLB-NE.505997.10_mRNA-p1

OG0007926: TCRU_5771 TcCLB-EL.507093.330_mRNA-p1
OG0007927: TCRU_5772 TcCLB-EL.504057.70_mRNA-p1
OG0007928: TCRU_5791 TcCLB-EL.506925.450_mRNA-p1
OG0007929: TCRU_5795 TcCLB-EL.508307.120_mRNA-p1
OG0007930: TCRU_5801 TcSYL_0112270.t1-p1
OG0007931: TCRU_5806 TcCLB-EL.511527.50_mRNA-p1
OG0007932: TCRU_5811 TcCLB-NE.508461.420_mRNA-p1
OG0007933: TCRU_5812 TcCLB-NE.508461.344_mRNA-p1
OG0007934: TCRU_5823 TcCLB-EL.506945.210_mRNA-p1
OG0007935: TCRU_5825 TcCLB-NE.508479.120_mRNA-p1
OG0007936: TCRU_5846 TcCLB-NE.507053.40_mRNA-p1
OG0007937: TCRU_5860 TcCLB-EL.510431.150_mRNA-p1
OG0007938: TCRU_5898 TcCLB-NE.511725.240_mRNA-p1
OG0007939: TCRU_5904 TcCLB-EL.511421.160_mRNA-p1
OG0007940: TCRU_5905 TcCLB-NE.511725.134_mRNA-p1
OG0007941: TCRU_5911 TcCLB-EL.511421.217_mRNA-p1
OG0007942: TCRU_5913 TcCLB-NE.504109.100_mRNA-p1
OG0007943: TCRU_5918 TcSYL_0035450.t1-p1
OG0007944: TCRU_5938 TcCLB-NE.506627.60_mRNA-p1
OG0007945: TCRU_5940 TcCLB-EL.507641.50_mRNA-p1
OG0007946: TCRU_5947 TcCLB-NE.507081.80_mRNA-p1
OG0007947: TCRU_5951 TcCLB-NE.506511.70_mRNA-p1
OG0007948: TCRU_5955 TcCLB-NE.511807.270_mRNA-p1
OG0007949: TCRU_5996 TcCLB-EL.504057.120_mRNA-p1
OG0007950: TCRU_6038 TcCLB-NE.506337.200_mRNA-p1
OG0007951: TCRU_6058 TcCLB-EL.503467.20_mRNA-p1
OG0007952: TCRU_6085 TcCLB-NE.506247.180_mRNA-p1
OG0007953: TCRU_6099 TcCLB-NE.506839.30_mRNA-p1
OG0007954: TCRU_6110 TcCLB-NE.508823.110_mRNA-p1
OG0007955: TCRU_6119 TcCLB-NE.508461.160_mRNA-p1
OG0007956: TCRU_6137 TcCLB-NE.511003.160_mRNA-p1
OG0007957: TCRU_6138 TcCLB-NE.511003.130_mRNA-p1
OG0007958: TCRU_6140 TcCLB-NE.511003.90_mRNA-p1
OG0007959: TCRU_6142 TcCLB-EL.509455.114_mRNA-p1
OG0007960: TCRU_6150 TcCLB-EL.506227.205_mRNA-p1
OG0007961: TCRU_6180 TcSYL_0023380.t1-p1
OG0007962: TCRU_6191 TcCLB-NE.511727.70_mRNA-p1
OG0007963: TCRU_6192 TcCLB-NE.511727.50_mRNA-p1
OG0007964: TCRU_6250 TcCLB-NE.510363.160_pseudogenic_transcript-p1
OG0007965: TCRU_6252 TcCLB-NE.510187.330_mRNA-p1
OG0007966: TCRU_6254 TcCLB-NE.510187.360_mRNA-p1
OG0007967: TCRU_6257 TcCLB-NE.510187.394_mRNA-p1
OG0007968: TCRU_6258 TcCLB-NE.510187.400_mRNA-p1
OG0007969: TCRU_6259 TcCLB-NE.510187.404_mRNA-p1
OG0007970: TCRU_6260 TcCLB-NE.510187.410_mRNA-p1
OG0007971: TCRU_6270 TcCLB-EL.506925.210_mRNA-p1
OG0007972: TCRU_6315 TcCLB-EL.507735.30_mRNA-p1
OG0007973: TCRU_6393 TcSYL_0156190.t1-p1
OG0007974: TCRU_6395 TcCLB-EL.509769.80_mRNA-p1
OG0007975: TCRU_6399 TcCLB-EL.509769.134_mRNA-p1
OG0007976: TCRU_6400 TcCLB-EL.509769.140_mRNA-p1
OG0007977: TCRU_gamma_ TcCLB-EL.454787.20_pseudogenic_transcript-p1
OG0007978: TCRU_6442 TcCLB-NE.504105.40_mRNA-p1
OG0007979: TCRU_6443 TcCLB-NE.504105.60_mRNA-p1

OG0007980: TCRU_6444 TcCLB-NE.504105.70_mRNA-p1
OG0007981: TCRU_6445 TcCLB-NE.504105.80_mRNA-p1
OG0007982: TCRU_6447 TcCLB-NE.504105.100_mRNA-p1
OG0007983: TCRU_6448 TcCLB-NE.504105.110_mRNA-p1
OG0007984: TCRU_6449 TcCLB-NE.504105.120_mRNA-p1
OG0007985: TCRU_6450 TcCLB-NE.504105.130_mRNA-p1
OG0007986: TCRU_6451 TcCLB-NE.504105.140_mRNA-p1
OG0007987: TCRU_subunit_ TcCLB-NE.504105.150_mRNA-p1
OG0007988: TCRU_interacting TcCLB-NE.504105.160_mRNA-p1
OG0007989: TCRU_6462 TcCLB-NE.504797.144_mRNA-p1
OG0007990: TCRU_6500 TcSYL_0187320.t1-p1
OG0007991: TCRU_6516 TcCLB-EL.503715.30_mRNA-p1
OG0007992: TCRU_6543 TcCLB-EL.511867.140_mRNA-p1
OG0007993: TCRU_6545 TcCLB-NE.510197.10_pseudogenic_transcript-p1
OG0007994: TCRU_6558 TcSYL_0031840.t1-p1
OG0007995: TCRU_6597 TcCLB-NE.511809.30_mRNA-p1
OG0007996: TCRU_6598 TcCLB-NE.511809.40_mRNA-p1
OG0007997: TCRU_6603 TcCLB-NE.511727.90_mRNA-p1
OG0007998: TCRU_6630 TcCLB-NE.504045.70_mRNA-p1
OG0007999: TCRU_6631 TcCLB-NE.504045.50_mRNA-p1
OG0008000: TCRU_6736 TcSYL_0191270.t1-p1
OG0008001: TCRU_6757 TcSYL_0103800.t1-p1
OG0008002: TCRU_6800 TcCLB-NE.507951.280_mRNA-p1
OG0008003: TCRU_and TcCLB-NE.507951.270_mRNA-p1
OG0008004: TCRU_6802 TcCLB-NE.507951.250_mRNA-p1
OG0008005: TCRU_6813 TcSYL_0118300.t1-p1
OG0008006: TCRU_6815 TcSYL_0018850.t1-p1
OG0008007: TCRU_6835 TcSYL_0063930.t1-p1
OG0008008: TCRU_6848 TcSYL_0149060.t1-p1
OG0008009: TCRU_6863 TcCLB-NE.510945.20_mRNA-p1
OG0008010: TCRU_6866 TcCLB-NE.504131.40_mRNA-p1
OG0008011: TCRU_6893 TcSYL_0134710.t1-p1
OG0008012: TCRU_6898 TcCLB-NE.507779.20_pseudogenic_transcript-p1
OG0008013: TCRU_6906 TcCLB-EL.510279.90_pseudogenic_transcript-p1
OG0008014: TCRU_6918 TcSYL_0155990.t1-p1
OG0008015: TCRU_6922 TcCLB-NE.508187.10_mRNA-p1
OG0008016: TCRU_6953 TcSYL_0136850.t1-p1
OG0008017: TCRU_6966 TcSYL_0186870.t1-p1
OG0008018: TCRU_6980 TcCLB-NE.509733.95_mRNA-p1
OG0008019: TCRU_6981 TcCLB-NE.509733.110_mRNA-p1
OG0008020: TCRU_6983 TcCLB-NE.509733.134_mRNA-p1
OG0008021: TCRU_6984 TcCLB-NE.509733.140_mRNA-p1
OG0008022: TCRU_7012 TcSYL_0134610.t1-p1
OG0008023: TCRU_7029 TcSYL_0121870.t1-p1
OG0008024: TCRU_7085 TcCLB-NE.511389.40_mRNA-p1
OG0008025: TCRU_7086 TcCLB-NE.511389.50_mRNA-p1
OG0008026: TCRU_7102 TcCLB-NE.511727.280_mRNA-p1
OG0008027: TCRU_7107 TcCLB-EL.506795.70_mRNA-p1
OG0008028: TCRU_7112 TcCLB-EL.506289.80_mRNA-p1
OG0008029: TCRU_7140 TcCLB-NE.511003.40_mRNA-p1
OG0008030: TCRU_7144 TcCLB-EL.511421.90_mRNA-p1
OG0008031: TCRU_7172 TcSYL_0014500.t1-p1
OG0008032: TCRU_7180 TcCLB-NE.510689.20_mRNA-p1
OG0008033: TCRU_7182 TcCLB-NE.510687.140_mRNA-p1

OG0008034: TCRU_7184 TcCLB-NE.510687.110_mRNA-p1
OG0008035: TCRU_7208 TcCLB-NE.510087.80_mRNA-p1
OG0008036: TCRU_7210 TcCLB-NE.510087.100_mRNA-p1
OG0008037: TCRU_7246 TcCLB-NE.507007.76_mRNA-p1
OG0008038: TCRU_7268 TcSYL_0022600.t1-p1
OG0008039: TCRU_7295 TcCLB-NE.511895.60_mRNA-p1
OG0008040: TCRU_7296 TcCLB-NE.511895.50_mRNA-p1
OG0008041: TCRU_7313 TcCLB-NE.510047.120_mRNA-p1
OG0008042: TCRU_7314 TcSYL_0019920.t1-p1
OG0008043: TCRU_7318 TcCLB-NE.504155.200_pseudogenic_transcript-p1
OG0008044: TCRU_7337 TcCLB-NE.506425.60_mRNA-p1
OG0008045: TCRU_7371 TcCLB-EL.510961.21_mRNA-p1
OG0008046: TCRU_7378 TcSYL_0151650.t1-p1
OG0008047: TCRU_7405 TcCLB-NE.510489.50_mRNA-p1
OG0008048: TCRU_7410 TcSYL_0092980.t1-p1
OG0008049: TCRU_7420 TcSYL_0068120.t1-p1
OG0008050: TCRU_7448 TcCLB-NE.511491.150_mRNA-p1
OG0008051: TCRU_7452 TcCLB-NE.511491.90_mRNA-p1
OG0008052: TCRU_7528 TcCLB-NE.507681.189_mRNA-p1
OG0008053: TCRU_7550 TcCLB-NE.511893.20_mRNA-p1
OG0008054: TCRU_7589 TcCLB-NE.511717.60_mRNA-p1
OG0008055: TCRU_7610 TcCLB-NE.506977.80_mRNA-p1
OG0008056: TCRU_7631 TcSYL_0018420.t1-p1
OG0008057: TCRU_7639 TcSYL_0068030.t1-p1
OG0008058: TCRU_7658 TcCLB-NE.503973.150_mRNA-p1
OG0008059: TCRU_7660 TcCLB-NE.510651.10_mRNA-p1
OG0008060: TCRU_7709 TcSYL_0124040.t1-p1
OG0008061: TCRU_7725 TcSYL_0101270.t1-p1
OG0008062: TCRU_7731 TcCLB-NE.508879.20_mRNA-p1
OG0008063: TCRU_7733 TcCLB-NE.508879.50_mRNA-p1
OG0008064: TCRU_7735 TcCLB-NE.508879.70_mRNA-p1
OG0008065: TCRU_7737 TcCLB-NE.508879.100_mRNA-p1
OG0008066: TCRU_7738 TcCLB-NE.508879.110_mRNA-p1
OG0008067: TCRU_of TcCLB-NE.509437.103_pseudogenic_transcript-p1
OG0008068: TCRU_7806 TcCLB-NE.511259.130_mRNA-p1
OG0008069: TCRU_7811 TcCLB-NE.510329.210_mRNA-p1
OG0008070: TCRU_7839 TcCLB-NE.511509.10_mRNA-p1
OG0008071: TCRU_7840 TcCLB-NE.511509.21_mRNA-p1
OG0008072: TCRU_7855 TcSYL_0147180.t1-p1
OG0008073: TCRU_7890 TcCLB-NE.510483.190_mRNA-p1
OG0008074: TCRU_7910 TcSYL_0186860.t1-p1
OG0008075: TCRU_7936 TcSYL_0072080.t1-p1
OG0008076: TCRU_7945 TcCLB-NE.506835.20_mRNA-p1
OG0008077: TCRU_7956 TcSYL_0154800.t1-p1
OG0008078: TCRU_7986 TcCLB-NE.508863.10_mRNA-p1
OG0008079: TCRU_protease TcCLB-EL.506885.80_mRNA-p1
OG0008080: TCRU_8044 TcCLB-NE.509725.80_pseudogenic_transcript-p1
OG0008081: TCRU_8059 TcCLB-NE.509627.40_mRNA-p1
OG0008082: TCRU_8095 TcSYL_0129430.t1-p1
OG0008083: TCRU_8122 TcCLB-NE.507019.30_mRNA-p1
OG0008084: TCRU_8124 TcCLB-NE.507019.50_mRNA-p1
OG0008085: TCRU_8125 TcCLB-NE.507019.60_mRNA-p1
OG0008086: TCRU_8126 TcCLB-NE.507019.80_mRNA-p1
OG0008087: TCRU_8138 TcCLB-NE.511367.159_pseudogenic_transcript-p1

OG0008088: TCRU_8139 TcCLB-NE.511367.180_mRNA-p1
OG0008089: TCRU_8156 TcCLB-EL.506925.220_mRNA-p1
OG0008090: TCRU_8160 TcCLB-EL.506925.290_mRNA-p1
OG0008091: TCRU_8162 TcCLB-EL.506925.310_mRNA-p1
OG0008092: TCRU_8164 TcCLB-EL.506925.340_mRNA-p1
OG0008093: TCRU_8165 TcCLB-EL.506925.350_mRNA-p1
OG0008094: TCRU_8166 TcCLB-EL.506925.360_mRNA-p1
OG0008095: TCRU_8167 TcCLB-EL.506925.390_mRNA-p1
OG0008096: TCRU_8168 TcCLB-EL.506925.400_mRNA-p1
OG0008097: TCRU_8169 TcCLB-EL.506925.410_mRNA-p1
OG0008098: TCRU_8214 TcCLB-EL.508647.210_mRNA-p1
OG0008099: TCRU_8224 TcCLB-NE.511649.14_mRNA-p1
OG0008100: TCRU_8266 TcCLB-NE.510199.30_mRNA-p1
OG0008101: TCRU_8296 TcSYL_0171820.t1-p1
OG0008102: TCRU_8300 TcCLB-NE.510329.200_mRNA-p1
OG0008103: TCRU_8303 TcCLB-NE.510329.180_mRNA-p1
OG0008104: TCRU_8306 TcCLB-NE.510329.150_mRNA-p1
OG0008105: TCRU_8309 TcCLB-NE.510329.120_mRNA-p1
OG0008106: TCRU_8310 TcCLB-NE.510329.110_mRNA-p1
OG0008107: TCRU_8311 TcCLB-NE.510329.100_mRNA-p1
OG0008108: TCRU_8313 TcCLB-NE.510329.80_mRNA-p1
OG0008109: TCRU_8314 TcCLB-NE.510329.70_mRNA-p1
OG0008110: TCRU_8316 TcCLB-NE.510329.50_mRNA-p1
OG0008111: TCRU_8317 TcCLB-NE.510329.40_mRNA-p1
OG0008112: TCRU_8318 TcCLB-NE.510329.20_mRNA-p1
OG0008113: TCRU_8352 TcCLB-NE.508461.234_mRNA-p1
OG0008114: TCRU_8353 TcCLB-NE.508461.240_mRNA-p1
OG0008115: TCRU_8354 TcCLB-NE.508461.250_mRNA-p1
OG0008116: TCRU_8355 TcCLB-NE.508461.260_mRNA-p1
OG0008117: TCRU_8356 TcCLB-NE.508461.270_mRNA-p1
OG0008118: TCRU_8456 TcCLB-NE.507067.50_pseudogenic_transcript-p1
OG0008119: TCRU_8490 TcSYL_0058770.t1-p1
OG0008120: TCRU_8505 TcCLB-NE.508461.284_mRNA-p1
OG0008121: TCRU_8506 TcCLB-NE.508461.290_mRNA-p1
OG0008122: TCRU_8507 TcCLB-NE.508461.300_mRNA-p1
OG0008123: TCRU_8527 TcSYL_0123800.t1-p1
OG0008124: TCRU_8548 TcSYL_0018440.t1-p1
OG0008125: TCRU_8580 TcSYL_0187560.t1-p1
OG0008126: TCRU_8611 TcCLB-NE.509217.90_mRNA-p1
OG0008127: TCRU_8625 TcCLB-EL.510431.245_pseudogenic_transcript-p1
OG0008128: TCRU_8626 TcCLB-EL.510431.240_mRNA-p1
OG0008129: TCRU_8627 TcCLB-EL.510431.230_mRNA-p1
OG0008130: TCRU_8628 TcCLB-EL.510431.220_mRNA-p1
OG0008131: TCRU_8641 TcCLB-NE.510187.380_mRNA-p1
OG0008132: TCRU_8653 TcCLB-EL.509769.90_mRNA-p1
OG0008133: TCRU_8690 TcCLB-NE.507951.260_mRNA-p1
OG0008134: TCRU_8695 TcCLB-NE.504131.20_mRNA-p1
OG0008135: TCRU_8696 TcCLB-NE.504131.30_mRNA-p1
OG0008136: TCRU_8697 TcCLB-NE.504131.60_mRNA-p1
OG0008137: TCRU_8709 TcCLB-NE.509733.120_mRNA-p1
OG0008138: TCRU_8721 TcCLB-NE.511389.20_mRNA-p1
OG0008139: TCRU_8722 TcCLB-NE.511727.310_mRNA-p1
OG0008140: TCRU_8724 TcCLB-NE.511003.50_mRNA-p1
OG0008141: TCRU_8733 TcCLB-NE.510689.14_mRNA-p1

OG0008142: TCRU_8753 TcCLB-NE.506425.50_mRNA-p1
OG0008143: TCRU_8755 TcCLB-NE.506425.90_mRNA-p1
OG0008144: TCRU_domain TcCLB-EL.506945.180_mRNA-p1
OG0008145: TCRU_8797 TcCLB-NE.508879.90_mRNA-p1
OG0008146: TCRU_8810 TcCLB-NE.510187.74_mRNA-p1
OG0008147: TCRU_8832 TcCLB-EL.506925.280_mRNA-p1
OG0008148: TCRU_8834 TcCLB-NE.508409.50_mRNA-p1
OG0008149: TCRU_8847 TcCLB-NE.510329.30_mRNA-p1
OG0008150: TCRU_8849 TcCLB-NE.509941.100_mRNA-p1
OG0008151: TCRU_8888 TcCLB-EL.510431.170_mRNA-p1
OG0008152: TCRU_8889 TcCLB-NE.506263.30_mRNA-p1
OG0008153: TCRU_8898 TcSYL_0092920.t1-p1
OG0008154: TCRU_8900 TcCLB-NE.506955.212_mRNA-p1
OG0008155: TCRU_8901 TcCLB-NE.506955.198_mRNA-p1
OG0008156: TCRU_8908 TcSYL_0203090.t1-p1
OG0008157: TCRU_8913 TcSYL_0004790.t1-p1
OG0008158: TCRU_8918 TcCLB-NE.511809.80_mRNA-p1
OG0008159: TCRU_8919 TcCLB-NE.511809.70_mRNA-p1
OG0008160: TCRU_8920 TcCLB-NE.511809.60_mRNA-p1
OG0008161: TCRU_8921 TcCLB-NE.511809.50_mRNA-p1
OG0008162: TCRU_8936 TcCLB-EL.506625.230_mRNA-p1
OG0008163: TCRU_8947 TcCLB-NE.508461.520_mRNA-p1
OG0008164: TCRU_8948 TcCLB-NE.508461.530_mRNA-p1
OG0008165: TCRU_8949 TcCLB-NE.508461.540_mRNA-p1
OG0008166: TCRU_8950 TcCLB-NE.508461.550_mRNA-p1
OG0008167: TCRU_8961 TcCLB-NE.511809.90_mRNA-p1
OG0008168: TCRU_9060 TcCLB-NE.506559.510_mRNA-p1
OG0008169: TCRU_9062 TcCLB-NE.506559.490_mRNA-p1
OG0008170: TCRU_9098 TcCLB-NE.508851.50_mRNA-p1
OG0008171: TCRU_9103 TcSYL_0164350.t1-p1
OG0008172: TCRU_9127 TcCLB-EL.506375.80_mRNA-p1
OG0008173: TCRU_9154 TcCLB-NE.510979.10_mRNA-p1
OG0008174: TCRU_9179 TcSYL_0163940.t1-p1
OG0008175: TCRU_9182 TcSYL_0083940.t1-p1
OG0008176: TCRU_9183 TcSYL_0141460.t1-p1
OG0008177: TCRU_9188 TcCLB-NE.510187.300_mRNA-p1
OG0008178: TCRU_9190 TcCLB-NE.510187.280_mRNA-p1
OG0008179: TCRU_9202 TcSYL_0191340.t1-p1
OG0008180: TCRU_9217 TcSYL_0122770.t1-p1
OG0008181: TCRU_9232 TcCLB-EL.504111.40_mRNA-p1
OG0008182: TCRU_9313 TcSYL_0165350.t1-p1
OG0008183: TCRU_9328 TcCLB-EL.504163.20_mRNA-p1
OG0008184: TCRU_9330 TcCLB-NE.508355.40_mRNA-p1
OG0008185: TCRU_9366 TcSYL_0125180.t1-p1
OG0008186: TCRU_9369 TcCLB-NE.504071.90_mRNA-p1
OG0008187: TCRU_9370 TcCLB-NE.504071.80_mRNA-p1
OG0008188: TCRU_9385 TcCLB-NE.508173.219_mRNA-p1
OG0008189: TCRU_9400 TcCLB-NE.508479.300_mRNA-p1
OG0008190: TCRU_9401 TcCLB-NE.508479.290_mRNA-p1
OG0008191: TCRU_9403 TcCLB-NE.508479.274_mRNA-p1
OG0008192: TCRU_9406 TcCLB-NE.508479.250_mRNA-p1
OG0008193: TCRU_9408 TcCLB-NE.508479.230_mRNA-p1
OG0008194: TCRU_9409 TcCLB-NE.508479.224_mRNA-p1
OG0008195: TCRU_9410 TcCLB-NE.508479.220_mRNA-p1

OG0008196: TCRU_9411 TcCLB-NE.508479.216_mRNA-p1
OG0008197: TCRU_9412 TcCLB-NE.508479.210_mRNA-p1
OG0008198: TCRU_9430 TcSYL_0109020.t1-p1
OG0008199: TCRU_9505 TcCLB-NE.511317.20_mRNA-p1
OG0008200: TCRU_9506 TcCLB-NE.511317.30_mRNA-p1
OG0008201: TCRU_9510 TcCLB-EL.503747.20_mRNA-p1
OG0008202: TCRU_9511 TcCLB-EL.503747.30_mRNA-p1
OG0008203: TCRU_9514 TcSYL_0072780.t1-p1
OG0008204: TCRU_9532 TcCLB-NE.511249.90_mRNA-p1
OG0008205: TCRU_9541 TcSYL_0129360.t1-p1
OG0008206: TCRU_9543 TcCLB-NE.504081.350_mRNA-p1
OG0008207: TCRU_9545 TcSYL_0203630.t1-p1
OG0008208: TCRU_9547 TcCLB-NE.511367.260_mRNA-p1
OG0008209: TCRU_9554 TcCLB-NE.508947.5_mRNA-p1
OG0008210: TCRU_9557 TcSYL_0071330.t1-p1
OG0008211: TCRU_9632 TcCLB-NE.509053.50_mRNA-p1
OG0008212: TCRU_9672 TcCLB-NE.510433.10_mRNA-p1
OG0008213: TCRU_9686 TcCLB-NE.504071.40_mRNA-p1
OG0008214: TCRU_9704 TcSYL_0177990.t1-p1
OG0008215: TCRU_9735 TcCLB-EL.511211.140_mRNA-p1
OG0008216: TCRU_9737 TcCLB-EL.511211.110_mRNA-p1
OG0008217: TCRU_9738 TcCLB-EL.511211.104_mRNA-p1
OG0008218: TCRU_9751 TcCLB-EL.507641.200_mRNA-p1
OG0008219: TCRU_9758 TcCLB-NE.510715.64_mRNA-p1
OG0008220: TCRU_9766 TcSYL_0029490.t1-p1
OG0008221: TCRU_9775 TcSYL_0194560.t1-p1
OG0008222: TCRU_9782 TcCLB-NE.508173.264_mRNA-p1
OG0008223: TCRU_9784 TcCLB-NE.508173.230_mRNA-p1
OG0008224: TCRU_9844 TcCLB-NE.508141.30_mRNA-p1
OG0008225: TCRU_9846 TcSYL_0100080.t1-p1
OG0008226: TCRU_9886 TcSYL_0050510.t1-p1
OG0008227: TCRU_9887 TcCLB-NE.510827.10_mRNA-p1
OG0008228: TCRU_9899 TcCLB-EL.506625.60_mRNA-p1
OG0008229: TCRU_9900 TcCLB-EL.506625.70_mRNA-p1
OG0008230: TCRU_9930 TcCLB-NE.511727.190_mRNA-p1
OG0008231: TCRU_9931 TcCLB-NE.511727.160_mRNA-p1
OG0008232: TCRU_9962 TcCLB-NE.511849.30_mRNA-p1
OG0008233: TCRU_9968 TcSYL_0115030.t1-p1
OG0008234: TCRU_9999 TcCLB-NE.511249.44_mRNA-p1
OG0008235: TCRU_10000 TcCLB-NE.511249.60_mRNA-p1
OG0008236: TCRU_10047 TcSYL_0191430.t1-p1
OG0008237: TCRU_10062 TcCLB-EL.511215.14_mRNA-p1
OG0008238: TCRU_10070 TcSYL_0027850.t1-p1
OG0008239: TCRU_10123 TcCLB-NE.505183.120_mRNA-p1
OG0008240: TCRU_10130 TcCLB-EL.507093.210_mRNA-p1
OG0008241: TCRU_10131 TcCLB-EL.507093.200_mRNA-p1
OG0008242: TCRU_10139 TcSYL_0049150.t1-p1
OG0008243: TCRU_10150 TcSYL_0166370.t1-p1
OG0008244: TCRU_10157 TcCLB-NE.509353.60_mRNA-p1
OG0008245: TCRU_10186 TcCLB-EL.510761.70_mRNA-p1
OG0008246: TCRU_10187 TcCLB-EL.510761.73_mRNA-p1
OG0008247: TCRU_10193 TcSYL_0150630.t1-p1
OG0008248: TCRU_10195 TcCLB-NE.506955.70_mRNA-p1
OG0008249: TCRU_10234 TcCLB-NE.511909.30_mRNA-p1

OG0008250: TCRU_10239 TcSYL_0165590.t1-p1
OG0008251: TCRU_10269 TcCLB-NE.508461.320_mRNA-p1
OG0008252: TCRU_10304 TcCLB-NE.511283.180_mRNA-p1
OG0008253: TCRU_10333 TcCLB-EL.506925.70_mRNA-p1
OG0008254: TCRU_10334 TcCLB-EL.506925.64_mRNA-p1
OG0008255: TCRU_10338 TcCLB-EL.506925.30_mRNA-p1
OG0008256: TCRU_10349 TcCLB-NE.510627.174_mRNA-p1
OG0008257: TCRU_10350 TcSYL_0040250.t1-p1
OG0008258: TCRU_10360 TcSYL_0029320.t1-p1
OG0008259: TCRU_10405 TcCLB-NE.508479.350_mRNA-p1
OG0008260: TCRU_10406 TcCLB-NE.508479.330_mRNA-p1
OG0008261: TCRU_10407 TcCLB-NE.508479.320_mRNA-p1
OG0008262: TCRU_10408 TcCLB-NE.508479.310_mRNA-p1
OG0008263: TCRU_10410 TcCLB-NE.508827.90_mRNA-p1
OG0008264: TCRU_10424 TcSYL_0067310.t1-p1
OG0008265: TCRU_10446 TcSYL_0039400.t1-p1
OG0008266: TCRU_10452 TcSYL_0151630.t1-p1
OG0008267: TCRU_10469 TcCLB-EL.504147.280_mRNA-p1
OG0008268: TCRU_10472 TcCLB-NE.510359.490_pseudogenic_transcript-p1
OG0008269: TCRU_10480 TcSYL_0191290.t1-p1
OG0008270: TCRU_10483 TcSYL_0126250.t1-p1
OG0008271: TCRU_10498 TcCLB-NE.508461.90_mRNA-p1
OG0008272: TCRU_protein TcCLB-NE.508461.100_mRNA-p1
OG0008273: TCRU_10500 TcCLB-NE.508461.110_mRNA-p1
OG0008274: TCRU_10541 TcCLB-EL.508647.50_pseudogenic_transcript-p1
OG0008275: TCRU_10542 TcCLB-EL.508647.80_mRNA-p1
OG0008276: TCRU_10543 TcCLB-EL.508647.90_mRNA-p1
OG0008277: TCRU_10553 TcSYL_0040790.t1-p1
OG0008278: TCRU_10554 TcSYL_0040110.t1-p1
OG0008279: TCRU_10555 TcSYL_0040820.t1-p1
OG0008280: TCRU_10556 TcSYL_0040130.t1-p1
OG0008281: TCRU_10557 TcSYL_0040870.t1-p1
OG0008282: TCRU_10558 TcSYL_0040150.t1-p1
OG0008283: TCRU_10560 TcSYL_0040930.t1-p1
OG0008284: TCRU_10562 TcCLB-EL.504149.50_mRNA-p1
OG0008285: TCRU_10563 TcCLB-EL.504149.55_mRNA-p1
OG0008286: TCRU_10564 TcCLB-EL.504149.60_mRNA-p1
OG0008287: TCRU_10566 TcCLB-EL.504149.90_mRNA-p1
OG0008288: TCRU_10584 TcCLB-NE.511655.20_mRNA-p1
OG0008289: TCRU_10585 TcCLB-NE.511655.30_mRNA-p1
OG0008290: TCRU_10588 TcSYL_0013930.t1-p1
OG0008291: TCRU_10596 TcSYL_0173040.t1-p1
OG0008292: TCRU_10598 TcSYL_0099850.t1-p1
OG0008293: TCRU_10599 TcCLB-NE.507801.210_pseudogenic_transcript-p1
OG0008294: TCRU_10637 TcSYL_0060960.t1-p1
OG0008295: TCRU_10641 TcCLB-NE.511907.320_mRNA-p1
OG0008296: TCRU_10642 TcCLB-NE.511907.310_mRNA-p1
OG0008297: TCRU_10643 TcCLB-NE.511907.300_mRNA-p1
OG0008298: TCRU_10644 TcCLB-NE.511907.290_mRNA-p1
OG0008299: TCRU_10647 TcCLB-NE.511907.260_mRNA-p1
OG0008300: TCRU_10665 TcCLB-NE.506627.20_mRNA-p1
OG0008301: TCRU_10708 TcCLB-EL.506375.90_mRNA-p1
OG0008302: TCRU_10713 TcCLB-NE.510187.270_mRNA-p1
OG0008303: TCRU_10732 TcCLB-NE.508173.200_mRNA-p1

OG0008304: TCRU_10733 TcCLB-NE.508173.210_mRNA-p1
OG0008305: TCRU_10744 TcCLB-NE.511249.80_mRNA-p1
OG0008306: TCRU_family_ TcCLB-EL.507641.170_mRNA-p1
OG0008307: TCRU_10777 TcCLB-NE.508173.240_mRNA-p1
OG0008308: TCRU_10798 TcCLB-NE.507019.100_mRNA-p1
OG0008309: TCRU_10806 TcCLB-NE.511249.50_mRNA-p1
OG0008310: TCRU_10860 TcCLB-NE.508461.120_mRNA-p1
OG0008311: TCRU_10861 TcCLB-NE.508461.130_mRNA-p1
OG0008312: TCRU_10865 TcCLB-EL.504149.30_mRNA-p1
OG0008313: TCRU_10866 TcCLB-EL.504149.40_mRNA-p1
OG0008314: TCRU_10867 TcCLB-EL.504149.80_mRNA-p1
OG0008315: TCRU_10868 TcCLB-EL.504149.100_mRNA-p1
OG0008316: TcCLB-EL.411427.40_mRNA-p1 TcCLB-NE.509395.59_mRNA-p1
OG0008317: TcCLB-EL.506717.219_pseudogenic_transcript-p1 TcCLB-NE.505101.50_pseudogenic_transcript-p1
OG0008318: TcCLB-EL.510351.150_mRNA-p1 TcSYL_0062150.t1-p1
OG0008319: TcCLB-EL.404001.10_mRNA-p1 TcCLB-NE.508533.20_mRNA-p1
OG0008320: TcCLB-EL.506739.110_mRNA-p1 TcCLB-NE.506315.10_mRNA-p1
OG0008321: TcCLB-EL.507143.80_mRNA-p1 TcCLB-NE.506319.60_mRNA-p1
OG0008322: TcCLB-EL.511055.40_pseudogenic_transcript-p1 TcCLB-NE.509259.140_pseudogenic_transcript-p1
OG0008323: TcCLB-EL.506553.10_mRNA-p1 TcCLB-NE.507769.20_mRNA-p1
OG0008324: TcCLB-EL.510105.120_mRNA-p1 TcCLB-NE.509715.104_mRNA-p1
OG0008325: TcCLB-EL.470169.9_mRNA-p1 TcCLB-NE.508059.4_mRNA-p1
OG0008326: TcCLB-EL.508949.20_mRNA-p1 TcCLB-NE.510119.40_mRNA-p1
OG0008327: TcCLB-EL.508951.4_mRNA-p1 TcCLB-NE.416891.9_mRNA-p1
OG0008328: TcCLB-EL.508951.90_mRNA-p1 TcSYL_0169120.t1-p1
OG0008329: TcCLB-EL.508953.10_mRNA-p1 TcCLB-NE.508059.10_mRNA-p1
OG0008330: TcCLB-EL.506529.110_mRNA-p1 TcCLB-NE.510885.20_mRNA-p1
OG0008331: TcCLB-EL.506529.220_mRNA-p1 TcCLB-NE.510885.120_mRNA-p1
OG0008332: TcCLB-EL.506529.224_mRNA-p1 TcCLB-NE.510885.130_mRNA-p1
OG0008333: TcCLB-EL.506529.30_mRNA-p1 TcCLB-NE.508593.90_mRNA-p1
OG0008334: TcCLB-EL.506529.314_mRNA-p1 TcCLB-NE.510887.64_mRNA-p1
OG0008335: TcCLB-EL.506529.500_mRNA-p1 TcCLB-NE.510889.210_mRNA-p1
OG0008336: TcCLB-EL.506529.541_mRNA-p1 TcSYL_0177930.t1-p1
OG0008337: TcCLB-EL.506529.620_mRNA-p1 TcCLB-NE.510889.300_mRNA-p1
OG0008338: TcCLB-EL.506529.680_mRNA-p1 TcCLB-NE.510889.352_mRNA-p1
OG0008339: TcCLB-EL.506529.80_mRNA-p1 TcCLB-NE.508593.140_mRNA-p1
OG0008340: TcCLB-EL.506531.60_mRNA-p1 TcCLB-NE.509399.9_mRNA-p1
OG0008341: TcCLB-EL.507603.180_mRNA-p1 TcCLB-NE.509429.250_mRNA-p1
OG0008342: TcCLB-EL.507603.190_mRNA-p1 TcCLB-NE.509429.260_mRNA-p1
OG0008343: TcCLB-EL.507603.54_mRNA-p1 TcCLB-NE.509429.124_mRNA-p1
OG0008344: TcCLB-EL.507995.10_pseudogenic_transcript-p1 TcCLB-NE.508629.100_pseudogenic_transcript-p1
OG0008345: TcCLB-EL.510073.75_mRNA-p1 TcCLB-NE.509399.150_mRNA-p1
OG0008346: TcCLB-EL.504449.40_mRNA-p1 TcCLB-NE.506447.10_mRNA-p1
OG0008347: TcCLB-EL.506579.129_mRNA-p1 TcSYL_0064890.t1-p1
OG0008348: TcCLB-EL.508277.210_mRNA-p1 TcCLB-NE.509945.24_mRNA-p1
OG0008349: TcCLB-EL.508277.310_mRNA-p1 TcCLB-NE.431849.10_mRNA-p1
OG0008350: TcCLB-EL.508277.65_mRNA-p1 TcCLB-NE.503541.40_mRNA-p1
OG0008351: TcCLB-EL.510441.30_pseudogenic_transcript-p1 TcCLB-NE.508479.50_pseudogenic_transcript-p1
OG0008352: TcCLB-EL.503855.80_mRNA-p1 TcCLB-NE.510333.10_mRNA-p1
OG0008353: TcCLB-EL.504033.100_mRNA-p1 TcCLB-NE.510339.70_mRNA-p1

OG0008354: TcCLB-EL.504033.110_mRNA-p1 TcCLB-NE.510339.60_mRNA-p1
OG0008355: TcCLB-EL.504033.40_mRNA-p1 TcSYL_0055340.t1-p1
OG0008356: TcCLB-EL.506623.20_pseudogenic_transcript-p1 TcCLB-NE.506331.50_pseudogenic_transcript-p1
OG0008357: TcCLB-EL.508127.5_mRNA-p1 TcCLB-NE.510221.20_mRNA-p1
OG0008358: TcCLB-EL.508137.40_mRNA-p1 TcCLB-NE.458241.10_mRNA-p1
OG0008359: TcCLB-EL.510241.5_mRNA-p1 TcCLB-NE.509161.70_mRNA-p1
OG0008360: TcCLB-EL.503803.20_mRNA-p1 TcSYL_0110870.t1-p1
OG0008361: TcCLB-EL.504247.34_mRNA-p1 TcCLB-NE.509991.100_mRNA-p1
OG0008362: TcCLB-EL.506563.120_mRNA-p1 TcCLB-NE.509001.30_mRNA-p1
OG0008363: TcCLB-EL.511417.30_mRNA-p1 TcCLB-NE.505997.200_mRNA-p1
OG0008364: TcCLB-EL.511417.50_mRNA-p1 TcCLB-NE.509487.10_mRNA-p1
OG0008365: TcCLB-EL.511419.50_mRNA-p1 TcCLB-NE.505999.100_mRNA-p1
OG0008366: TcCLB-EL.511419.70_mRNA-p1 TcCLB-NE.505999.120_mRNA-p1
OG0008367: TcCLB-EL.511423.104_mRNA-p1 TcCLB-NE.507949.69_mRNA-p1
OG0008368: TcCLB-EL.511431.50_mRNA-p1 TcCLB-NE.506475.113_mRNA-p1
OG0008369: TcCLB-EL.511433.10_mRNA-p1 TcCLB-NE.506475.90_mRNA-p1
OG0008370: TcCLB-EL.508671.10_mRNA-p1 TcSYL_0171280.t1-p1
OG0008371: TcCLB-EL.508779.80_mRNA-p1 TcCLB-NE.506855.100_mRNA-p1
OG0008372: TcCLB-EL.508787.14_mRNA-p1 TcCLB-NE.510519.14_mRNA-p1
OG0008373: TcCLB-EL.509245.20_mRNA-p1 TcCLB-NE.508637.130_mRNA-p1
OG0008374: TcCLB-EL.509245.29_mRNA-p1 TcCLB-NE.508637.140_mRNA-p1
OG0008375: TcCLB-EL.509967.189_mRNA-p1 TcSYL_0171130.t1-p1
OG0008376: TcCLB-EL.509967.3_pseudogenic_transcript-p1 TcSYL_0170960.t1-p1
OG0008377: TcCLB-EL.509967.7_mRNA-p1 TcCLB-NE.509693.194_mRNA-p1
OG0008378: TcCLB-EL.511131.40_mRNA-p1 TcCLB-NE.506855.350_mRNA-p1
OG0008379: TcCLB-EL.511133.10_mRNA-p1 TcCLB-NE.506855.270_mRNA-p1
OG0008380: TcCLB-EL.511135.30_mRNA-p1 TcCLB-NE.507073.49_mRNA-p1
OG0008381: TcCLB-EL.511139.10_pseudogenic_transcript-p1 TcCLB-NE.508319.79_mRNA-p1
OG0008382: TcCLB-EL.511139.30_mRNA-p1 TcCLB-NE.508319.60_mRNA-p1
OG0008383: TcCLB-EL.511911.130_mRNA-p1 TcCLB-NE.508637.90_mRNA-p1
OG0008384: TcCLB-EL.405165.19_mRNA-p1 TcCLB-NE.509939.19_mRNA-p1
OG0008385: TcCLB-EL.466965.10_pseudogenic_transcript-p1 TcSYL_0128680.t1-p1
OG0008386: TcCLB-EL.506795.100_mRNA-p1 TcCLB-NE.509935.10_mRNA-p1
OG0008387: TcCLB-EL.506795.80_mRNA-p1 TcCLB-NE.509935.29_mRNA-p1
OG0008388: TcCLB-EL.508265.80_mRNA-p1 TcCLB-NE.506443.10_mRNA-p1
OG0008389: TcCLB-EL.510403.71_pseudogenic_transcript-p1 TcCLB-NE.508523.21_pseudogenic_transcript-p1
OG0008390: TcCLB-EL.510407.40_mRNA-p1 TcCLB-NE.509937.190_mRNA-p1
OG0008391: TcCLB-EL.510989.60_mRNA-p1 TcCLB-NE.511245.210_mRNA-p1
OG0008392: TcCLB-EL.503895.10_mRNA-p1 TcSYL_0096590.t1-p1
OG0008393: TcCLB-EL.503897.10_mRNA-p1 TcCLB-NE.509563.60_mRNA-p1
OG0008394: TcCLB-EL.503897.140_mRNA-p1 TcCLB-NE.509561.50_mRNA-p1
OG0008395: TcCLB-EL.505945.69_mRNA-p1 TcCLB-NE.506149.29_mRNA-p1
OG0008396: TcCLB-EL.506685.40_mRNA-p1 TcCLB-NE.509663.20_mRNA-p1
OG0008397: TcCLB-EL.511575.20_mRNA-p1 TcCLB-NE.503773.24_mRNA-p1
OG0008398: TcCLB-EL.511575.30_mRNA-p1 TcCLB-NE.503773.20_mRNA-p1
OG0008399: TcCLB-EL.511575.70_pseudogenic_transcript-p1 TcSYL_0094750.t1-p1
OG0008400: TcCLB-EL.506859.170_mRNA-p1 TcCLB-NE.511815.170_mRNA-p1
OG0008401: TcCLB-EL.506859.230_mRNA-p1 TcCLB-NE.511817.10_mRNA-p1

OG0008402: TcCLB-EL.508323.90_mRNA-p1 TcCLB-NE.511825.66_mRNA-p1
OG0008403: TcCLB-EL.510165.54_mRNA-p1 TcCLB-NE.511807.36_mRNA-p1
OG0008404: TcCLB-EL.510165.90_pseudogenic_transcript-p1 TcCLB-NE.507015.30_pseudogenic_transcript-p1
OG0008405: TcCLB-EL.510527.9_mRNA-p1 TcSYL_0201550.t1-p1
OG0008406: TcCLB-EL.421619.10_mRNA-p1 TcCLB-NE.511859.80_mRNA-p1
OG0008407: TcCLB-EL.506179.59_mRNA-p1 TcSYL_0092260.t1-p1
OG0008408: TcCLB-EL.506227.160_mRNA-p1 TcCLB-NE.450489.10_mRNA-p1
OG0008409: TcCLB-EL.506227.170_mRNA-p1 TcSYL_0092020.t1-p1
OG0008410: TcCLB-EL.506227.190_mRNA-p1 TcSYL_0092040.t1-p1
OG0008411: TcCLB-EL.506227.50_mRNA-p1 TcCLB-NE.511847.70_mRNA-p1
OG0008412: TcCLB-EL.506239.30_mRNA-p1 TcCLB-NE.506177.20_mRNA-p1
OG0008413: TcCLB-EL.506239.50_mRNA-p1 TcCLB-NE.506177.40_mRNA-p1
OG0008414: TcCLB-EL.506731.10_mRNA-p1 TcSYL_0089410.t1-p1
OG0008415: TcCLB-EL.506735.80_mRNA-p1 TcCLB-NE.509229.10_mRNA-p1
OG0008416: TcCLB-EL.507631.15_pseudogenic_transcript-p1 TcCLB-NE.507753.6_pseudogenic_transcript-p1
OG0008417: TcCLB-EL.509581.50_mRNA-p1 TcCLB-NE.503709.10_mRNA-p1
OG0008418: TcCLB-EL.509589.50_mRNA-p1 TcCLB-NE.503999.40_mRNA-p1
OG0008419: TcCLB-EL.511049.10_pseudogenic_transcript-p1 TcSYL_0090310.t1-p1
OG0008420: TcCLB-EL.511051.10_mRNA-p1 TcCLB-NE.509683.90_mRNA-p1
OG0008421: TcCLB-EL.474341.9_mRNA-p1 TcSYL_0146260.t1-p1
OG0008422: TcCLB-EL.506713.10_mRNA-p1 TcCLB-NE.509719.30_mRNA-p1
OG0008423: TcCLB-EL.506713.14_mRNA-p1 TcCLB-NE.509719.28_mRNA-p1
OG0008424: TcCLB-EL.506713.20_mRNA-p1 TcCLB-NE.509719.26_mRNA-p1
OG0008425: TcCLB-EL.506713.24_mRNA-p1 TcCLB-NE.509719.23_mRNA-p1
OG0008426: TcCLB-EL.511867.200_mRNA-p1 TcSYL_0146320.t1-p1
OG0008427: TcCLB-EL.511869.9_mRNA-p1 TcSYL_0146280.t1-p1
OG0008428: TcCLB-EL.511871.80_mRNA-p1 TcCLB-NE.511865.19_mRNA-p1
OG0008429: TcCLB-EL.432621.9_mRNA-p1 TcSYL_0154070.t1-p1
OG0008430: TcCLB-EL.442383.9_mRNA-p1 TcSYL_0118190.t1-p1
OG0008431: TcCLB-EL.506363.40_mRNA-p1 TcCLB-NE.506975.63_mRNA-p1
OG0008432: TcCLB-EL.506363.90_mRNA-p1 TcCLB-NE.506975.30_mRNA-p1
OG0008433: TcCLB-EL.507165.20_mRNA-p1 TcCLB-NE.507793.50_mRNA-p1
OG0008434: TcCLB-EL.507165.50_mRNA-p1 TcCLB-NE.507793.20_mRNA-p1
OG0008435: TcCLB-EL.507839.18_mRNA-p1 TcCLB-NE.510635.40_mRNA-p1
OG0008436: TcCLB-EL.510283.114_mRNA-p1 TcCLB-NE.503823.84_mRNA-p1
OG0008437: TcCLB-EL.510285.74_mRNA-p1 TcCLB-NE.504827.159_mRNA-p1
OG0008438: TcCLB-EL.510717.40_mRNA-p1 TcCLB-NE.506337.50_mRNA-p1
OG0008439: TcCLB-EL.510719.220_mRNA-p1 TcCLB-NE.509745.20_mRNA-p1
OG0008440: TcCLB-EL.503493.20_mRNA-p1 TcCLB-NE.511761.20_mRNA-p1
OG0008441: TcCLB-EL.507047.90_mRNA-p1 TcCLB-NE.509179.170_mRNA-p1
OG0008442: TcCLB-EL.507977.9_pseudogenic_transcript-p1 TcSYL_0157920.t1-p1
OG0008443: TcCLB-EL.510053.10_mRNA-p1 TcCLB-NE.511321.60_mRNA-p1
OG0008444: TcCLB-EL.510053.79_mRNA-p1 TcSYL_0159150.t1-p1
OG0008445: TcCLB-EL.510057.20_mRNA-p1 TcCLB-NE.508909.150_mRNA-p1
OG0008446: TcCLB-EL.511303.50_mRNA-p1 TcCLB-NE.511763.10_mRNA-p1
OG0008447: TcCLB-EL.511309.14_mRNA-p1 TcCLB-NE.507087.14_mRNA-p1
OG0008448: TcCLB-EL.511315.9_mRNA-p1 TcSYL_0159620.t1-p1
OG0008449: TcCLB-EL.506963.150_pseudogenic_transcript-p1 TcSYL_0106480.t1-p1
OG0008450: TcCLB-EL.507237.150_mRNA-p1 TcSYL_0070280.t1-p1

OG0008451: TcCLB-EL.511589.140_mRNA-p1 TcCLB-NE.508851.189_mRNA-p1
OG0008452: TcCLB-EL.511589.74_mRNA-p1 TcCLB-NE.508851.130_mRNA-p1
OG0008453: TcCLB-EL.511591.10_mRNA-p1 TcCLB-NE.511233.130_mRNA-p1
OG0008454: TcCLB-EL.511611.30_mRNA-p1 TcSYL_0079790.t1-p1
OG0008455: TcCLB-EL.503715.20_mRNA-p1 TcCLB-NE.509607.40_mRNA-p1
OG0008456: TcCLB-EL.506401.12_mRNA-p1 TcCLB-NE.509605.30_mRNA-p1
OG0008457: TcCLB-EL.506401.80_mRNA-p1 TcCLB-NE.503727.20_mRNA-p1
OG0008458: TcCLB-EL.508279.10_pseudogenic_transcript-p1 TcCLB-NE.
507203.5_pseudogenic_transcript-p1
OG0008459: TcCLB-EL.508283.25_pseudogenic_transcript-p1 TcCLB-NE.
509915.35_pseudogenic_transcript-p1
OG0008460: TcCLB-EL.508285.5_pseudogenic_transcript-p1 TcCLB-NE.
509915.55_pseudogenic_transcript-p1
OG0008461: TcCLB-EL.508567.30_mRNA-p1 TcCLB-NE.509611.60_mRNA-p1
OG0008462: TcCLB-EL.508569.120_mRNA-p1 TcCLB-NE.503733.30_mRNA-p1
OG0008463: TcCLB-EL.508569.140_mRNA-p1 TcCLB-NE.503733.10_mRNA-p1
OG0008464: TcCLB-EL.510005.11_pseudogenic_transcript-p1 TcCLB-NE.
511391.90_mRNA-p1
OG0008465: TcCLB-EL.510861.150_mRNA-p1 TcCLB-NE.509607.60_mRNA-p1
OG0008466: TcCLB-EL.510861.40_mRNA-p1 TcCLB-NE.507647.39_mRNA-p1
OG0008467: TcCLB-EL.510863.30_mRNA-p1 TcCLB-NE.503729.10_mRNA-p1
OG0008468: TcCLB-EL.511395.10_mRNA-p1 TcSYL_0202090.t1-p1
OG0008469: TcCLB-EL.445011.10_mRNA-p1 TcSYL_0114060.t1-p1
OG0008470: TcCLB-EL.503527.60_mRNA-p1 TcCLB-NE.510299.70_mRNA-p1
OG0008471: TcCLB-EL.504203.60_mRNA-p1 TcCLB-NE.503791.10_mRNA-p1
OG0008472: TcCLB-EL.506341.10_mRNA-p1 TcCLB-NE.506507.10_mRNA-p1
OG0008473: TcCLB-EL.506567.140_mRNA-p1 TcSYL_0114660.t1-p1
OG0008474: TcCLB-EL.506567.20_mRNA-p1 TcCLB-NE.507049.20_mRNA-p1
OG0008475: TcCLB-EL.506567.77_pseudogenic_transcript-p1 TcCLB-NE.
507049.65_pseudogenic_transcript-p1
OG0008476: TcCLB-EL.506677.30_mRNA-p1 TcCLB-NE.506583.50_mRNA-p1
OG0008477: TcCLB-EL.508151.10_mRNA-p1 TcCLB-NE.510417.30_mRNA-p1
OG0008478: TcCLB-EL.510123.24_mRNA-p1 TcCLB-NE.511837.60_mRNA-p1
OG0008479: TcCLB-EL.510123.40_mRNA-p1 TcCLB-NE.511837.40_mRNA-p1
OG0008480: TcCLB-EL.510287.50_mRNA-p1 TcCLB-NE.503891.80_mRNA-p1
OG0008481: TcCLB-EL.510289.10_mRNA-p1 TcCLB-NE.503891.50_mRNA-p1
OG0008482: TcCLB-EL.510289.6_mRNA-p1 TcCLB-NE.503891.54_mRNA-p1
OG0008483: TcCLB-EL.510293.20_mRNA-p1 TcCLB-NE.506583.70_mRNA-p1
OG0008484: TcCLB-EL.510295.10_mRNA-p1 TcCLB-NE.508661.70_mRNA-p1
OG0008485: TcCLB-EL.510295.20_mRNA-p1 TcCLB-NE.506007.10_mRNA-p1
OG0008486: TcCLB-EL.510297.60_mRNA-p1 TcCLB-NE.506009.60_mRNA-p1
OG0008487: TcCLB-EL.503619.25_mRNA-p1 TcCLB-NE.511647.42_mRNA-p1
OG0008488: TcCLB-EL.503955.80_mRNA-p1 TcCLB-NE.507063.10_mRNA-p1
OG0008489: TcCLB-EL.506947.30_mRNA-p1 TcCLB-NE.504251.20_mRNA-p1
OG0008490: TcCLB-EL.507805.30_pseudogenic_transcript-p1
TcSYL_0013510.t1-p1
OG0008491: TcCLB-EL.507811.120_mRNA-p1 TcCLB-NE.511649.80_mRNA-p1
OG0008492: TcCLB-EL.508377.9_mRNA-p1 TcSYL_0014210.t1-p1
OG0008493: TcCLB-EL.509777.10_mRNA-p1 TcCLB-NE.507061.10_mRNA-p1
OG0008494: TcCLB-EL.509777.21_mRNA-p1 TcSYL_0071650.t1-p1
OG0008495: TcCLB-EL.510603.30_mRNA-p1 TcCLB-NE.
509109.145_pseudogenic_transcript-p1
OG0008496: TcCLB-EL.441061.9_mRNA-p1 TcSYL_0174700.t1-p1
OG0008497: TcCLB-EL.503401.11_mRNA-p1 TcCLB-NE.506629.240_mRNA-p1

OG0008498: TcCLB-EL.508317.70_mRNA-p1 TcCLB-NE.509849.30_mRNA-p1
OG0008499: TcCLB-EL.509499.4_mRNA-p1 TcCLB-NE.510947.44_mRNA-p1
OG0008500: TcCLB-EL.509767.140_mRNA-p1 TcCLB-NE.509601.140_mRNA-p1
OG0008501: TcCLB-EL.509769.4_mRNA-p1 TcCLB-NE.507645.70_mRNA-p1
OG0008502: TcCLB-EL.510509.9_mRNA-p1 TcSYL_0174010.t1-p1
OG0008503: TcCLB-EL.510515.139_mRNA-p1 TcSYL_0174600.t1-p1
OG0008504: TcCLB-EL.510517.110_mRNA-p1 TcCLB-NE.507851.70_mRNA-p1
OG0008505: TcCLB-EL.510517.20_mRNA-p1 TcCLB-NE.504003.50_mRNA-p1
OG0008506: TcCLB-EL.504117.9_mRNA-p1 TcSYL_0087440.t1-p1
OG0008507: TcCLB-EL.506979.30_mRNA-p1 TcCLB-NE.503499.24_mRNA-p1
OG0008508: TcCLB-EL.508769.10_pseudogenic_transcript-p1
TcSYL_0086590.t1-p1
OG0008509: TcCLB-EL.508771.30_mRNA-p1 TcCLB-NE.403875.19_mRNA-p1
OG0008510: TcCLB-EL.508771.50_mRNA-p1 TcCLB-NE.
503785.10_pseudogenic_transcript-p1
OG0008511: TcCLB-EL.508811.40_mRNA-p1 TcCLB-NE.503415.40_mRNA-p1
OG0008512: TcCLB-EL.509737.9_mRNA-p1 TcCLB-NE.506347.5_mRNA-p1
OG0008513: TcCLB-EL.511109.80_mRNA-p1 TcCLB-NE.504157.60_mRNA-p1
OG0008514: TcCLB-EL.511115.10_mRNA-p1 TcSYL_0086670.t1-p1
OG0008515: TcCLB-EL.511625.30_mRNA-p1 TcCLB-NE.504741.40_mRNA-p1
OG0008516: TcCLB-EL.511625.60_mRNA-p1 TcCLB-NE.504741.10_mRNA-p1
OG0008517: TcCLB-EL.511625.74_mRNA-p1 TcCLB-NE.504741.4_mRNA-p1
OG0008518: TcCLB-EL.511627.90_mRNA-p1 TcCLB-NE.507801.144_mRNA-p1
OG0008519: TcCLB-EL.408437.29_mRNA-p1 TcCLB-NE.508741.10_mRNA-p1
OG0008520: TcCLB-EL.408573.9_mRNA-p1 TcSYL_0194260.t1-p1
OG0008521: TcCLB-EL.425851.9_mRNA-p1 TcSYL_0192070.t1-p1
OG0008522: TcCLB-EL.475705.9_mRNA-p1 TcSYL_0192100.t1-p1
OG0008523: TcCLB-EL.503667.31_pseudogenic_transcript-p1
TcSYL_0083580.t1-p1
OG0008524: TcCLB-EL.503863.9_mRNA-p1 TcCLB-NE.508741.330_mRNA-p1
OG0008525: TcCLB-EL.506409.255_mRNA-p1 TcCLB-NE.511235.31_mRNA-p1
OG0008526: TcCLB-EL.506951.103_mRNA-p1 TcCLB-NE.511859.140_mRNA-p1
OG0008527: TcCLB-EL.509123.30_mRNA-p1 TcSYL_0191570.t1-p1
OG0008528: TcCLB-EL.509137.20_mRNA-p1 TcCLB-NE.508741.130_mRNA-p1
OG0008529: TcCLB-EL.509139.10_mRNA-p1 TcCLB-NE.508741.170_mRNA-p1
OG0008530: TcCLB-EL.509925.5.1-p1 TcSYL_0127990.t1-p1
OG0008531: TcCLB-EL.403789.9_mRNA-p1 TcSYL_0188380.t1-p1
OG0008532: TcCLB-EL.506749.10_mRNA-p1 TcCLB-NE.507891.20_mRNA-p1
OG0008533: TcCLB-EL.507083.20_mRNA-p1 TcCLB-NE.506415.10_mRNA-p1
OG0008534: TcCLB-EL.507275.40_mRNA-p1 TcCLB-NE.507491.39_mRNA-p1
OG0008535: TcCLB-EL.511215.70_mRNA-p1 TcSYL_0187980.t1-p1
OG0008536: TcCLB-EL.511217.39_mRNA-p1 TcCLB-NE.506575.54_mRNA-p1
OG0008537: TcCLB-EL.503487.10_mRNA-p1 TcSYL_0047530.t1-p1
OG0008538: TcCLB-EL.503789.10_mRNA-p1 TcCLB-NE.505183.110_mRNA-p1
OG0008539: TcCLB-EL.503789.34_mRNA-p1 TcCLB-NE.505183.84_mRNA-p1
OG0008540: TcCLB-EL.504035.110_mRNA-p1 TcCLB-NE.507991.90_mRNA-p1
OG0008541: TcCLB-EL.504035.84_mRNA-p1 TcCLB-NE.507991.123_mRNA-p1
OG0008542: TcCLB-EL.504125.20_mRNA-p1 TcCLB-NE.504131.160_mRNA-p1
OG0008543: TcCLB-EL.506399.40_mRNA-p1 TcCLB-NE.509243.20_mRNA-p1
OG0008544: TcCLB-EL.506399.80_mRNA-p1 TcCLB-NE.511909.50_mRNA-p1
OG0008545: TcCLB-EL.506801.10_pseudogenic_transcript-p1
TcSYL_0024050.t1-p1
OG0008546: TcCLB-EL.507669.150_mRNA-p1 TcCLB-NE.509877.50_mRNA-p1
OG0008547: TcCLB-EL.511071.40_mRNA-p1 TcCLB-NE.511903.50_mRNA-p1

OG0008548: TcCLB-EL.511075.24_mRNA-p1 TcCLB-NE.511897.19_mRNA-p1
OG0008549: TcCLB-EL.503613.40_mRNA-p1 TcCLB-NE.511165.54_mRNA-p1
OG0008550: TcCLB-EL.503613.70_mRNA-p1 TcCLB-NE.511165.30_mRNA-p1
OG0008551: TcCLB-EL.506775.150_mRNA-p1 TcCLB-NE.511167.30_mRNA-p1
OG0008552: TcCLB-EL.506775.30_mRNA-p1 TcCLB-NE.511169.29_mRNA-p1
OG0008553: TcCLB-EL.506775.70_mRNA-p1 TcCLB-NE.511167.100_mRNA-p1
OG0008554: TcCLB-EL.506779.70_mRNA-p1 TcCLB-NE.511153.124_mRNA-p1
OG0008555: TcCLB-EL.506825.190_mRNA-p1 TcCLB-NE.508175.90_mRNA-p1
OG0008556: TcCLB-EL.508039.50_pseudogenic_transcript-p1 TcSYL_0194470.t1-p1
OG0008557: TcCLB-EL.508241.149_mRNA-p1 TcSYL_0076240.t1-p1
OG0008558: TcCLB-EL.507867.70_mRNA-p1 TcCLB-NE.510629.430_mRNA-p1
OG0008559: TcCLB-EL.508385.20_mRNA-p1 TcCLB-NE.506791.20_mRNA-p1
OG0008560: TcCLB-EL.509885.64_mRNA-p1 TcCLB-NE.508397.64_mRNA-p1
OG0008561: TcCLB-EL.509887.10_mRNA-p1 TcSYL_0020890.t1-p1
OG0008562: TcCLB-EL.510609.65_mRNA-p1 TcCLB-NE.503617.15_mRNA-p1
OG0008563: TcCLB-EL.510611.10_mRNA-p1 TcCLB-NE.506793.50_mRNA-p1
OG0008564: TcCLB-EL.510611.20_mRNA-p1 TcCLB-NE.506793.40_mRNA-p1
OG0008565: TcCLB-EL.510611.70_mRNA-p1 TcCLB-NE.506791.60_mRNA-p1
OG0008566: TcCLB-EL.503579.140_mRNA-p1 TcCLB-NE.507517.120_mRNA-p1
OG0008567: TcCLB-EL.507747.130_pseudogenic_transcript-p1 TcCLB-NE.511259.50_pseudogenic_transcript-p1
OG0008568: TcCLB-EL.508207.120_mRNA-p1 TcCLB-NE.507519.100_mRNA-p1
OG0008569: TcCLB-EL.508207.50_mRNA-p1 TcCLB-NE.507519.167_mRNA-p1
OG0008570: TcCLB-EL.508209.144_mRNA-p1 TcCLB-NE.472777.30_mRNA-p1
OG0008571: TcCLB-EL.508209.150_mRNA-p1 TcCLB-NE.472777.20_mRNA-p1
OG0008572: TcCLB-EL.508209.39_mRNA-p1 TcCLB-NE.503919.30_mRNA-p1
OG0008573: TcCLB-EL.508211.10_mRNA-p1 TcCLB-NE.507515.140_mRNA-p1
OG0008574: TcCLB-EL.508211.19_mRNA-p1 TcCLB-NE.507515.130_mRNA-p1
OG0008575: TcCLB-EL.509543.20_mRNA-p1 TcCLB-NE.511257.40_mRNA-p1
OG0008576: TcCLB-EL.462617.10_pseudogenic_transcript-p1 TcSYL_0043940.t1-p1
OG0008577: TcCLB-EL.503753.10_mRNA-p1 TcCLB-NE.506585.50_mRNA-p1
OG0008578: TcCLB-EL.503799.10_mRNA-p1 TcSYL_0043670.t1-p1
OG0008579: TcCLB-EL.505931.30_mRNA-p1 TcCLB-NE.503993.10_mRNA-p1
OG0008580: TcCLB-EL.506163.35_mRNA-p1 TcCLB-NE.508821.60_mRNA-p1
OG0008581: TcCLB-EL.506931.80_mRNA-p1 TcSYL_0044640.t1-p1
OG0008582: TcCLB-EL.508831.4_mRNA-p1 TcCLB-NE.508275.9_mRNA-p1
OG0008583: TcCLB-EL.509199.40_mRNA-p1 TcSYL_0179610.t1-p1
OG0008584: TcCLB-EL.509201.10_mRNA-p1 TcCLB-NE.510895.9_mRNA-p1
OG0008585: TcCLB-EL.509203.20_mRNA-p1 TcCLB-NE.511017.30_mRNA-p1
OG0008586: TcCLB-EL.509205.10_mRNA-p1 TcSYL_0180650.t1-p1
OG0008587: TcCLB-EL.511211.210_mRNA-p1 TcCLB-NE.510439.20_mRNA-p1
OG0008588: TcCLB-EL.511801.4_mRNA-p1 TcSYL_0179630.t1-p1
OG0008589: TcCLB-EL.511805.20_mRNA-p1 TcCLB-NE.511015.30_mRNA-p1
OG0008590: TcCLB-EL.407477.10_mRNA-p1 TcCLB-NE.511389.150_mRNA-p1
OG0008591: TcCLB-EL.505007.30_mRNA-p1 TcCLB-NE.510007.20_mRNA-p1
OG0008592: TcCLB-EL.505009.20_mRNA-p1 TcCLB-NE.506489.59_mRNA-p1
OG0008593: TcCLB-EL.505073.14_mRNA-p1 TcCLB-NE.509911.40_mRNA-p1
OG0008594: TcCLB-EL.505073.20_mRNA-p1 TcCLB-NE.509911.60_mRNA-p1
OG0008595: TcCLB-EL.506455.10_pseudogenic_transcript-p1 TcCLB-NE.506159.10_pseudogenic_transcript-p1
OG0008596: TcCLB-EL.507389.20_mRNA-p1 TcCLB-NE.509911.124_mRNA-p1
OG0008597: TcCLB-EL.507395.30_mRNA-p1 TcSYL_0051930.t1-p1

OG0008598: TcCLB-EL.507689.5_mRNA-p1 TcSYL_0052730.t1-p1
OG0008599: TcCLB-EL.507755.10_mRNA-p1 TcSYL_0032370.t1-p1
OG0008600: TcCLB-EL.507757.10_mRNA-p1 TcSYL_0055610.t1-p1
OG0008601: TcCLB-EL.508927.10_mRNA-p1 TcCLB-NE.509237.40_mRNA-p1
OG0008602: TcCLB-EL.509007.14_mRNA-p1 TcCLB-NE.508965.94_mRNA-p1
OG0008603: TcCLB-EL.509007.90_mRNA-p1 TcCLB-NE.508965.30_mRNA-p1
OG0008604: TcCLB-EL.510773.60_mRNA-p1 TcCLB-NE.511385.70_mRNA-p1
OG0008605: TcCLB-EL.511439.70_mRNA-p1 TcSYL_0031440.t1-p1
OG0008606: TcCLB-EL.511439.90_mRNA-p1 TcCLB-NE.504259.10_mRNA-p1
OG0008607: TcCLB-EL.503483.9_mRNA-p1 TcCLB-NE.507501.10_mRNA-p1
OG0008608: TcCLB-EL.503909.90_mRNA-p1 TcCLB-NE.511507.60_mRNA-p1
OG0008609: TcCLB-EL.505807.100_mRNA-p1 TcCLB-NE.509033.109_mRNA-p1
OG0008610: TcCLB-EL.505807.230_mRNA-p1 TcCLB-NE.507017.80_mRNA-p1
OG0008611: TcCLB-EL.506943.130_mRNA-p1 TcCLB-NE.509065.140_mRNA-p1
OG0008612: TcCLB-EL.506943.27_mRNA-p1 TcCLB-NE.509065.36_mRNA-p1
OG0008613: TcCLB-EL.506945.150_mRNA-p1 TcCLB-NE.510445.40_mRNA-p1
OG0008614: TcCLB-EL.507923.30_mRNA-p1 TcCLB-NE.463359.9_mRNA-p1
OG0008615: TcCLB-EL.509171.100_mRNA-p1 TcSYL_0139370.t1-p1
OG0008616: TcCLB-EL.509171.60_mRNA-p1 TcCLB-NE.507023.260_mRNA-p1
OG0008617: TcCLB-EL.509965.210_mRNA-p1 TcCLB-NE.509053.150_mRNA-p1
OG0008618: TcCLB-EL.509965.360_mRNA-p1 TcCLB-NE.509051.50_mRNA-p1
OG0008619: TcCLB-EL.509965.394_mRNA-p1 TcCLB-NE.511497.19_mRNA-p1
OG0008620: TcCLB-EL.509965.50_mRNA-p1 TcCLB-NE.511499.40_mRNA-p1
OG0008621: TcCLB-EL.511731.35_mRNA-p1 TcCLB-NE.506833.20_mRNA-p1
OG0008622: TcCLB-EL.511733.40_mRNA-p1 TcCLB-NE.511523.70_mRNA-p1
OG0008623: TcCLB-EL.511735.34_mRNA-p1 TcCLB-NE.509063.4_mRNA-p1
OG0008624: TcCLB-EL.511737.20_mRNA-p1 TcCLB-NE.511517.170_mRNA-p1
OG0008625: TcCLB-EL.511745.60_mRNA-p1 TcCLB-NE.507029.41_mRNA-p1
OG0008626: TcCLB-EL.511747.14_mRNA-p1 TcCLB-NE.511507.24_mRNA-p1
OG0008627: TcCLB-EL.511751.14_mRNA-p1 TcSYL_0139330.t1-p1
OG0008628: TcCLB-EL.441241.10_mRNA-p1 TcSYL_0103950.t1-p1
OG0008629: TcCLB-EL.503409.20_mRNA-p1 TcCLB-NE.505193.70_mRNA-p1
OG0008630: TcCLB-EL.503881.4_mRNA-p1 TcSYL_0103820.t1-p1
OG0008631: TcCLB-EL.504421.19_mRNA-p1 TcCLB-NE.503943.14_mRNA-p1
OG0008632: TcCLB-EL.504423.10_mRNA-p1 TcCLB-NE.503943.20_mRNA-p1
OG0008633: TcCLB-EL.506467.14_mRNA-p1 TcCLB-NE.505193.84_mRNA-p1
OG0008634: TcCLB-EL.506467.4_mRNA-p1 TcCLB-NE.505193.74_mRNA-p1
OG0008635: TcCLB-EL.508345.9_pseudogenic_transcript-p1 TcCLB-NE.
510795.4_pseudogenic_transcript-p1
OG0008636: TcCLB-EL.508347.10_mRNA-p1 TcSYL_0105450.t1-p1
OG0008637: TcCLB-EL.509979.51_mRNA-p1 TcCLB-NE.510041.20_mRNA-p1
OG0008638: TcCLB-EL.510565.125_mRNA-p1 TcCLB-NE.508503.100_mRNA-p1
OG0008639: TcCLB-EL.511577.104_mRNA-p1 TcCLB-NE.504797.14_mRNA-p1
OG0008640: TcCLB-EL.511577.144_mRNA-p1 TcCLB-NE.509337.19_mRNA-p1
OG0008641: TcCLB-EL.511577.170_pseudogenic_transcript-p1 TcCLB-NE.
509337.5_pseudogenic_transcript-p1
OG0008642: TcCLB-EL.511577.188_mRNA-p1 TcCLB-NE.
504231.5_pseudogenic_transcript-p1
OG0008643: TcCLB-EL.511585.125_mRNA-p1 TcCLB-NE.510155.115_mRNA-p1
OG0008644: TcCLB-EL.511585.170_mRNA-p1 TcCLB-NE.510155.160_mRNA-p1
OG0008645: TcCLB-EL.511585.210_mRNA-p1 TcCLB-NE.510155.200_mRNA-p1
OG0008646: TcCLB-EL.447255.10_mRNA-p1 TcCLB-NE.506559.70_mRNA-p1
OG0008647: TcCLB-EL.503865.20_mRNA-p1 TcCLB-NE.508693.150_mRNA-p1
OG0008648: TcCLB-EL.504055.100_pseudogenic_transcript-p1 TcCLB-NE.

509417.30_pseudogenic_transcript-p1
OG0008649: TcCLB-EL.506635.104_mRNA-p1 TcCLB-NE.508029.40_mRNA-p1
OG0008650: TcCLB-EL.506635.44_mRNA-p1 TcCLB-NE.508027.94_mRNA-p1
OG0008651: TcCLB-EL.506637.80_mRNA-p1 TcCLB-NE.510099.60_mRNA-p1
OG0008652: TcCLB-EL.507485.159_mRNA-p1 TcSYL_0195720.t1-p1
OG0008653: TcCLB-EL.507873.34_mRNA-p1 TcCLB-NE.510689.70_mRNA-p1
OG0008654: TcCLB-EL.507875.90_pseudogenic_transcript-p1 TcCLB-NE.
510079.40_pseudogenic_transcript-p1
OG0008655: TcCLB-EL.508135.10_mRNA-p1 TcCLB-NE.508051.40_mRNA-p1
OG0008656: TcCLB-EL.509891.70_pseudogenic_transcript-p1 TcCLB-NE.
507055.20_mRNA-p1
OG0008657: TcCLB-EL.510227.20_mRNA-p1 TcCLB-NE.506559.205_mRNA-p1
OG0008658: TcCLB-EL.510877.110_mRNA-p1 TcSYL_0045840.t1-p1
OG0008659: TcCLB-EL.510877.170_mRNA-p1 TcCLB-NE.508689.40_mRNA-p1
OG0008660: TcCLB-EL.510877.190_mRNA-p1 TcCLB-NE.503923.40_mRNA-p1
OG0008661: TcCLB-EL.510879.180_mRNA-p1 TcSYL_0046000.t1-p1
OG0008662: TcCLB-EL.503989.30_pseudogenic_transcript-p1 TcCLB-NE.
504207.20_mRNA-p1
OG0008663: TcCLB-EL.504019.6_mRNA-p1 TcCLB-NE.511033.20_mRNA-p1
OG0008664: TcCLB-EL.507089.220_mRNA-p1 TcCLB-NE.503839.19_mRNA-p1
OG0008665: TcCLB-EL.507105.50_mRNA-p1 TcSYL_0088050.t1-p1
OG0008666: TcCLB-EL.508607.40_pseudogenic_transcript-p1 TcCLB-NE.
506241.10_pseudogenic_transcript-p1
OG0008667: TcCLB-EL.509069.14_mRNA-p1 TcCLB-NE.508707.234_mRNA-p1
OG0008668: TcCLB-EL.509069.40_mRNA-p1 TcCLB-NE.508707.220_mRNA-p1
OG0008669: TcCLB-EL.509073.110_mRNA-p1 TcCLB-NE.508707.100_mRNA-p1
OG0008670: TcCLB-EL.509157.110_mRNA-p1 TcCLB-NE.508443.50_mRNA-p1
OG0008671: TcCLB-EL.509157.240_mRNA-p1 TcCLB-NE.510725.60_mRNA-p1
OG0008672: TcCLB-EL.509267.80_mRNA-p1 TcCLB-NE.510031.40_mRNA-p1
OG0008673: TcCLB-EL.511525.20_mRNA-p1 TcCLB-NE.508525.30_mRNA-p1
OG0008674: TcCLB-EL.511527.82_mRNA-p1 TcSYL_0112280.t1-p1
OG0008675: TcCLB-EL.511537.21_mRNA-p1 TcCLB-NE.511021.51_mRNA-p1
OG0008676: TcCLB-EL.511541.20_mRNA-p1 TcCLB-NE.508707.290_mRNA-p1
OG0008677: TcCLB-EL.511545.160_mRNA-p1 TcCLB-NE.510435.10_mRNA-p1
OG0008678: TcCLB-EL.511713.9_mRNA-p1 TcCLB-NE.503987.39_mRNA-p1
OG0008679: TcCLB-EL.503683.30_mRNA-p1 TcCLB-NE.509999.120_mRNA-p1
OG0008680: TcCLB-EL.503697.130_mRNA-p1 TcCLB-NE.504105.170_mRNA-p1
OG0008681: TcCLB-EL.503903.79_mRNA-p1 TcCLB-NE.508153.740_mRNA-p1
OG0008682: TcCLB-EL.504029.40_mRNA-p1 TcCLB-NE.509999.40_mRNA-p1
OG0008683: TcCLB-EL.504431.40_mRNA-p1 TcCLB-NE.507951.90_mRNA-p1
OG0008684: TcCLB-EL.504867.129_mRNA-p1 TcCLB-NE.510943.198_mRNA-p1
OG0008685: TcCLB-EL.506691.64_mRNA-p1 TcCLB-NE.508153.994_mRNA-p1
OG0008686: TcCLB-EL.506693.10_mRNA-p1 TcCLB-NE.508153.710_mRNA-p1
OG0008687: TcCLB-EL.507257.40_mRNA-p1 TcCLB-NE.510329.250_mRNA-p1
OG0008688: TcCLB-EL.507257.80_mRNA-p1 TcCLB-NE.510329.290_mRNA-p1
OG0008689: TcCLB-EL.507883.25_mRNA-p1 TcCLB-NE.506195.25_mRNA-p1
OG0008690: TcCLB-EL.507883.40_mRNA-p1 TcCLB-NE.506195.40_mRNA-p1
OG0008691: TcCLB-EL.510143.17_mRNA-p1 TcCLB-NE.508409.199_mRNA-p1
OG0008692: TcCLB-EL.510311.80_mRNA-p1 TcCLB-NE.510265.14_mRNA-p1
OG0008693: TcCLB-EL.510313.20_mRNA-p1 TcSYL_0000610.t1-p1
OG0008694: TcCLB-EL.510317.14_mRNA-p1 TcCLB-NE.508153.454_mRNA-p1
OG0008695: TcCLB-EL.510317.30_mRNA-p1 TcCLB-NE.508153.440_mRNA-p1
OG0008696: TcCLB-EL.510319.9_mRNA-p1 TcCLB-NE.508153.410_mRNA-p1
OG0008697: TcCLB-EL.510321.10_pseudogenic_transcript-p1

TcSYL_0000930.t1-p1
OG0008698: TcCLB-EL.442297.10_mRNA-p1 TcCLB-NE.511635.30_mRNA-p1
OG0008699: TcCLB-EL.466529.9_mRNA-p1 TcCLB-NE.509105.110_mRNA-p1
OG0008700: TcCLB-EL.503419.10_mRNA-p1 TcCLB-NE.509105.50_mRNA-p1
OG0008701: TcCLB-EL.503419.60_mRNA-p1 TcCLB-NE.509105.100_mRNA-p1
OG0008702: TcCLB-EL.504147.224_mRNA-p1 TcSYL_0005040.t1-p1
OG0008703: TcCLB-EL.504167.14_mRNA-p1 TcCLB-NE.508233.25_mRNA-p1
OG0008704: TcCLB-EL.504179.69_mRNA-p1 TcCLB-NE.509795.89_mRNA-p1
OG0008705: TcCLB-EL.504179.9_mRNA-p1 TcSYL_0107000.t1-p1
OG0008706: TcCLB-EL.505683.5_mRNA-p1 TcSYL_0013070.t1-p1
OG0008707: TcCLB-EL.506249.30_mRNA-p1 TcCLB-NE.508231.220_mRNA-p1
OG0008708: TcCLB-EL.507447.19_mRNA-p1 TcCLB-NE.511633.79_mRNA-p1
OG0008709: TcCLB-EL.507611.131_pseudogenic_transcript-p1
TcSYL_0048850.t1-p1
OG0008710: TcCLB-EL.507641.180_mRNA-p1 TcSYL_0011180.t1-p1
OG0008711: TcCLB-EL.507641.90_mRNA-p1 TcSYL_0011120.t1-p1
OG0008712: TcCLB-EL.507709.20_mRNA-p1 TcCLB-NE.506577.20_mRNA-p1
OG0008713: TcCLB-EL.507711.250_mRNA-p1 TcCLB-NE.509643.20_mRNA-p1
OG0008714: TcCLB-EL.507711.260_mRNA-p1 TcCLB-NE.509643.30_mRNA-p1
OG0008715: TcCLB-EL.507711.300_mRNA-p1 TcCLB-NE.509643.70_mRNA-p1
OG0008716: TcCLB-EL.508097.81_mRNA-p1 TcCLB-NE.509755.70_mRNA-p1
OG0008717: TcCLB-EL.508727.40_mRNA-p1 TcCLB-NE.
503797.7_pseudogenic_transcript-p1
OG0008718: TcCLB-EL.508727.50_mRNA-p1 TcSYL_0107320.t1-p1
OG0008719: TcCLB-EL.509599.100_mRNA-p1 TcCLB-NE.509733.20_mRNA-p1
OG0008720: TcCLB-EL.509599.120_mRNA-p1 TcCLB-NE.463451.4_mRNA-p1
OG0008721: TcCLB-EL.509645.30_mRNA-p1 TcSYL_0008760.t1-p1
OG0008722: TcCLB-EL.510493.10_mRNA-p1 TcCLB-NE.511629.40_mRNA-p1
OG0008723: TcCLB-EL.511039.10_mRNA-p1 TcCLB-NE.509791.120_mRNA-p1
OG0008724: TcCLB-EL.511041.4_mRNA-p1 TcSYL_0107400.t1-p1
OG0008725: TcCLB-EL.503453.60_mRNA-p1 TcCLB-NE.506959.100_mRNA-p1
OG0008726: TcCLB-EL.503453.64_mRNA-p1 TcCLB-NE.506959.94_mRNA-p1
OG0008727: TcCLB-EL.503599.10_mRNA-p1 TcCLB-NE.509409.10_mRNA-p1
OG0008728: TcCLB-EL.503599.50_mRNA-p1 TcCLB-NE.505163.40_mRNA-p1
OG0008729: TcCLB-EL.504929.5_mRNA-p1 TcCLB-NE.507305.40_mRNA-p1
OG0008730: TcCLB-EL.508541.160_mRNA-p1 TcCLB-NE.507539.10_mRNA-p1
OG0008731: TcCLB-EL.510205.30_mRNA-p1 TcSYL_0133650.t1-p1
OG0008732: TcCLB-EL.510207.21_pseudogenic_transcript-p1 TcCLB-NE.
510363.70_mRNA-p1
OG0008733: TcCLB-EL.510213.9_mRNA-p1 TcCLB-NE.511255.360_mRNA-p1
OG0008734: TcCLB-EL.511001.150_mRNA-p1 TcCLB-NE.506957.46_mRNA-p1
OG0008735: TcCLB-EL.511181.130_mRNA-p1 TcCLB-NE.508299.40_mRNA-p1
OG0008736: TcCLB-EL.511467.16_mRNA-p1 TcCLB-NE.449759.9_mRNA-p1
OG0008737: TcCLB-EL.445281.9_mRNA-p1 TcSYL_0080850.t1-p1
OG0008738: TcCLB-EL.503887.20_mRNA-p1 TcCLB-NE.507771.85_mRNA-p1
OG0008739: TcCLB-EL.503893.100_mRNA-p1 TcCLB-NE.508507.40_mRNA-p1
OG0008740: TcCLB-EL.503893.130_mRNA-p1 TcCLB-NE.508507.24_mRNA-p1
OG0008741: TcCLB-EL.504153.120_mRNA-p1 TcSYL_0138750.t1-p1
OG0008742: TcCLB-EL.506127.140_mRNA-p1 TcCLB-NE.503593.40_mRNA-p1
OG0008743: TcCLB-EL.506147.190_mRNA-p1 TcCLB-NE.510733.50_mRNA-p1
OG0008744: TcCLB-EL.506147.210_mRNA-p1 TcCLB-NE.510733.30_mRNA-p1
OG0008745: TcCLB-EL.506147.40_mRNA-p1 TcCLB-NE.508461.50_mRNA-p1
OG0008746: TcCLB-EL.506367.170_mRNA-p1 TcCLB-NE.506629.100_mRNA-p1
OG0008747: TcCLB-EL.506367.60_mRNA-p1 TcCLB-NE.508355.200_mRNA-p1

OG0008748: TcCLB-EL.506369.70_mRNA-p1 TcCLB-NE.510225.10_mRNA-p1
OG0008749: TcCLB-EL.506811.70_mRNA-p1 TcCLB-NE.506303.160_mRNA-p1
OG0008750: TcCLB-EL.506887.69_mRNA-p1 TcCLB-NE.510731.59_mRNA-p1
OG0008751: TcCLB-EL.507093.69_mRNA-p1 TcCLB-NE.503917.21_mRNA-p1
OG0008752: TcCLB-EL.507467.100_mRNA-p1 TcCLB-NE.508355.110_mRNA-p1
OG0008753: TcCLB-EL.508357.30_mRNA-p1 TcCLB-NE.508355.370_mRNA-p1
OG0008754: TcCLB-EL.508471.10_mRNA-p1 TcSYL_0115220.t1-p1
OG0008755: TcCLB-EL.509551.60_mRNA-p1 TcCLB-NE.507007.77_mRNA-p1
OG0008756: TcCLB-EL.509551.64_mRNA-p1 TcCLB-NE.507007.74_mRNA-p1
OG0008757: TcCLB-EL.509835.10_mRNA-p1 TcCLB-NE.472833.10_mRNA-p1
OG0008758: TcCLB-EL.510431.90_mRNA-p1 TcSYL_0138170.t1-p1
OG0008759: TcCLB-EL.510741.20_mRNA-p1 TcCLB-NE.510661.244_mRNA-p1
OG0008760: TcCLB-EL.510743.10_mRNA-p1 TcSYL_0116210.t1-p1
OG0008761: TcCLB-EL.510747.5_mRNA-p1 TcCLB-NE.504185.40_mRNA-p1
OG0008762: TcCLB-EL.510755.65_mRNA-p1 TcCLB-NE.508415.30_mRNA-p1
OG0008763: TcCLB-EL.510759.54_mRNA-p1 TcCLB-NE.506999.160_mRNA-p1
OG0008764: TcCLB-EL.510763.60_mRNA-p1 TcCLB-NE.509671.168_mRNA-p1
OG0008765: TcCLB-EL.510765.20_mRNA-p1 TcCLB-NE.509671.99_mRNA-p1
OG0008766: TcCLB-EL.511441.20_mRNA-p1 TcSYL_0080730.t1-p1
OG0008767: TcCLB-EL.511445.140_mRNA-p1 TcCLB-NE.503703.60_mRNA-p1
OG0008768: TcCLB-EL.460061.10_mRNA-p1 TcSYL_0072010.t1-p1
OG0008769: TcCLB-EL.504111.10_mRNA-p1 TcCLB-NE.507031.110_mRNA-p1
OG0008770: TcCLB-EL.506211.50_mRNA-p1 TcCLB-NE.508895.70_mRNA-p1
OG0008771: TcCLB-EL.506357.180_mRNA-p1 TcCLB-NE.508891.50_mRNA-p1
OG0008772: TcCLB-EL.506503.40_mRNA-p1 TcCLB-NE.504161.30_mRNA-p1
OG0008773: TcCLB-EL.506503.4_mRNA-p1 TcCLB-NE.504161.59_mRNA-p1
OG0008774: TcCLB-EL.508165.220_pseudogenic_transcript-p1 TcCLB-NE.
510197.210_mRNA-p1
OG0008775: TcCLB-EL.508165.400_mRNA-p1 TcSYL_0017600.t1-p1
OG0008776: TcCLB-EL.509649.20_mRNA-p1 TcCLB-NE.511285.100_mRNA-p1
OG0008777: TcCLB-EL.509649.40_mRNA-p1 TcCLB-NE.511285.80_mRNA-p1
OG0008778: TcCLB-EL.509649.50_mRNA-p1 TcCLB-NE.511285.70_mRNA-p1
OG0008779: TcCLB-EL.510091.14_mRNA-p1 TcCLB-NE.510421.264_mRNA-p1
OG0008780: TcCLB-EL.510279.290_pseudogenic_transcript-p1 TcCLB-NE.
506537.60_pseudogenic_transcript-p1
OG0008781: TcCLB-EL.511353.34_mRNA-p1 TcCLB-NE.511301.44_mRNA-p1
OG0008782: TcCLB-EL.511355.24_mRNA-p1 TcCLB-NE.503809.120_mRNA-p1
OG0008783: TcCLB-EL.511357.9_mRNA-p1 TcCLB-NE.503809.75_mRNA-p1
OG0008784: TcCLB-EL.511361.60_mRNA-p1 TcCLB-NE.508899.90_mRNA-p1
OG0008785: TcCLB-EL.503859.50_mRNA-p1 TcSYL_0135460.t1-p1
OG0008786: TcCLB-EL.506499.105_pseudogenic_transcript-p1 TcCLB-NE.
511255.580_pseudogenic_transcript-p1
OG0008787: TcCLB-EL.506599.315_pseudogenic_transcript-p1 TcCLB-NE.
509867.50_pseudogenic_transcript-p1
OG0008788: TcCLB-EL.506753.30_mRNA-p1 TcCLB-NE.510355.190_mRNA-p1
OG0008789: TcCLB-EL.506755.270_mRNA-p1 TcCLB-NE.510359.290_mRNA-p1
OG0008790: TcCLB-EL.506757.100_pseudogenic_transcript-p1 TcCLB-NE.
509875.140_pseudogenic_transcript-p1
OG0008791: TcCLB-EL.506757.10_mRNA-p1 TcCLB-NE.510359.340_mRNA-p1
OG0008792: TcCLB-EL.507981.40_mRNA-p1 TcSYL_0153210.t1-p1
OG0008793: TcCLB-EL.508687.25_mRNA-p1 TcSYL_0126230.t1-p1
OG0008794: TcCLB-EL.508777.190_mRNA-p1 TcCLB-NE.511483.50_mRNA-p1
OG0008795: TcCLB-EL.510025.126_pseudogenic_transcript-p1 TcCLB-NE.
511255.516_pseudogenic_transcript-p1

OG0008796: TcCLB-EL.510025.30_pseudogenic_transcript-p1 TcCLB-NE.
510361.50_pseudogenic_transcript-p1
OG0008797: TcCLB-EL.510061.20_mRNA-p1 TcSYL_0039790.t1-p1
OG0008798: TcCLB-EL.510161.20_mRNA-p1 TcCLB-NE.506459.270_mRNA-p1
OG0008799: TcCLB-EL.511127.10_mRNA-p1 TcCLB-NE.511481.70_mRNA-p1
OG0008800: TcCLB-EL.511127.200_mRNA-p1 TcCLB-NE.509023.180_mRNA-p1
OG0008801: TcCLB-EL.511127.250_mRNA-p1 TcCLB-NE.509025.20_mRNA-p1
OG0008802: TcCLB-EL.511127.74_mRNA-p1 TcCLB-NE.509023.70_mRNA-p1
OG0008803: TcCLB-EL.511555.20_mRNA-p1 TcCLB-NE.510219.10_mRNA-p1
OG0008804: TcCLB-EL.511555.50_mRNA-p1 TcCLB-NE.510219.40_mRNA-p1
OG0008805: TcCLB-NE.504639.40.1-p1 TcSYL_0060520.t1-p1
OG0008806: TcCLB-NE.506123.10_mRNA-p1 TcSYL_0062060.t1-p1
OG0008807: TcCLB-NE.509539.67_mRNA-p1 TcSYL_0062010.t1-p1
OG0008808: TcCLB-NE.506317.30_pseudogenic_transcript-p1
TcSYL_0050790.t1-p1
OG0008809: TcCLB-NE.507681.80_mRNA-p1 TcSYL_0073720.t1-p1
OG0008810: TcCLB-NE.443971.10_mRNA-p1 TcSYL_0170320.t1-p1
OG0008811: TcCLB-NE.511367.230_mRNA-p1 TcSYL_0169980.t1-p1
OG0008812: TcCLB-NE.509401.20_pseudogenic_transcript-p1
TcSYL_0175300.t1-p1
OG0008813: TcCLB-NE.404073.10_mRNA-p1 TcSYL_0064260.t1-p1
OG0008814: TcCLB-NE.404073.20_mRNA-p1 TcSYL_0064270.t1-p1
OG0008815: TcCLB-NE.507915.19_mRNA-p1 TcSYL_0064160.t1-p1
OG0008816: TcCLB-NE.507949.250_mRNA-p1 TcSYL_0111010.t1-p1
OG0008817: TcCLB-NE.509003.79_mRNA-p1 TcSYL_0063450.t1-p1
OG0008818: TcCLB-NE.509487.4_mRNA-p1 TcSYL_0111310.t1-p1
OG0008819: TcCLB-NE.509989.10_mRNA-p1 TcSYL_0110680.t1-p1
OG0008820: TcCLB-NE.478283.10_pseudogenic_transcript-p1
TcSYL_0095950.t1-p1
OG0008821: TcCLB-NE.509567.4_mRNA-p1 TcSYL_0096650.t1-p1
OG0008822: TcCLB-NE.509661.21_pseudogenic_transcript-p1
TcSYL_0092150.t1-p1
OG0008823: TcCLB-NE.511859.10_mRNA-p1 TcSYL_0091700.t1-p1
OG0008824: TcCLB-NE.506665.10_mRNA-p1 TcSYL_0057780.t1-p1
OG0008825: TcCLB-NE.506155.60_mRNA-p1 TcSYL_0118140.t1-p1
OG0008826: TcCLB-NE.510425.10_mRNA-p1 TcSYL_0121220.t1-p1
OG0008827: TcCLB-NE.511317.40_mRNA-p1 TcSYL_0159560.t1-p1
OG0008828: TcCLB-NE.511317.79_mRNA-p1 TcSYL_0159530.t1-p1
OG0008829: TcCLB-NE.511771.75_mRNA-p1 TcSYL_0060600.t1-p1
OG0008830: TcCLB-NE.508245.50_mRNA-p1 TcSYL_0150490.t1-p1
OG0008831: TcCLB-NE.508547.201_mRNA-p1 TcSYL_0202410.t1-p1
OG0008832: TcCLB-NE.508625.10_mRNA-p1 TcSYL_0113020.t1-p1
OG0008833: TcCLB-NE.511651.19_mRNA-p1 TcSYL_0013970.t1-p1
OG0008834: TcCLB-NE.508043.10_mRNA-p1 TcSYL_0172000.t1-p1
OG0008835: TcCLB-NE.509331.9_mRNA-p1 TcSYL_0172670.t1-p1
OG0008836: TcCLB-NE.509727.10_pseudogenic_transcript-p1
TcSYL_0201050.t1-p1
OG0008837: TcCLB-NE.503411.30_mRNA-p1 TcSYL_0086900.t1-p1
OG0008838: TcCLB-NE.463955.20_mRNA-p1 TcSYL_0128940.t1-p1
OG0008839: TcCLB-NE.508139.110_mRNA-p1 TcSYL_0157090.t1-p1
OG0008840: TcCLB-NE.508743.5_pseudogenic_transcript-p1
TcSYL_0190120.t1-p1
OG0008841: TcCLB-NE.511237.120_mRNA-p1 TcSYL_0028470.t1-p1
OG0008842: TcCLB-NE.506573.91_mRNA-p1 TcSYL_0189290.t1-p1

OG0008843: TcCLB-NE.508957.14_mRNA-p1 TcSYL_0189120.t1-p1
OG0008844: TcCLB-NE.509259.45_mRNA-p1 TcSYL_0049130.t1-p1
OG0008845: TcCLB-NE.451263.9_mRNA-p1 TcSYL_0102650.t1-p1
OG0008846: TcCLB-NE.509431.10_pseudogenic_transcript-p1 TcSYL_0047260.t1-p1
OG0008847: TcCLB-NE.511907.340_mRNA-p1 TcSYL_0178500.t1-p1
OG0008848: TcCLB-NE.420533.9_mRNA-p1 TcSYL_0078950.t1-p1
OG0008849: TcCLB-NE.508173.150_mRNA-p1 TcSYL_0079360.t1-p1
OG0008850: TcCLB-NE.508173.47_mRNA-p1 TcSYL_0079430.t1-p1
OG0008851: TcCLB-NE.508177.50_mRNA-p1 TcSYL_0186680.t1-p1
OG0008852: TcCLB-NE.508177.81_pseudogenic_transcript-p1 TcSYL_0184790.t1-p1
OG0008853: TcCLB-NE.508799.290_mRNA-p1 TcSYL_0075190.t1-p1
OG0008854: TcCLB-NE.511153.150_mRNA-p1 TcSYL_0076820.t1-p1
OG0008855: TcCLB-NE.510395.10_mRNA-p1 TcSYL_0163680.t1-p1
OG0008856: TcCLB-NE.506279.100_mRNA-p1 TcSYL_0190460.t1-p1
OG0008857: TcCLB-NE.506281.14_mRNA-p1 TcSYL_0069720.t1-p1
OG0008858: TcCLB-NE.435519.19_mRNA-p1 TcSYL_0181370.t1-p1
OG0008859: TcCLB-NE.505939.5_mRNA-p1 TcSYL_0180890.t1-p1
OG0008860: TcCLB-NE.508821.70_mRNA-p1 TcSYL_0095920.t1-p1
OG0008861: TcCLB-NE.510897.80_mRNA-p1 TcSYL_0179890.t1-p1
OG0008862: TcCLB-NE.511669.20_mRNA-p1 TcSYL_0059520.t1-p1
OG0008863: TcCLB-NE.431357.10_mRNA-p1 TcSYL_0029710.t1-p1
OG0008864: TcCLB-NE.508017.30_mRNA-p1 TcSYL_0051830.t1-p1
OG0008865: TcCLB-NE.510087.50_mRNA-p1 TcSYL_0051810.t1-p1
OG0008866: TcCLB-NE.422955.10_pseudogenic_transcript-p1 TcSYL_0167390.t1-p1
OG0008867: TcCLB-NE.504071.60_mRNA-p1 TcSYL_0167250.t1-p1
OG0008868: TcCLB-NE.504257.70_mRNA-p1 TcSYL_0140380.t1-p1
OG0008869: TcCLB-NE.507019.110_mRNA-p1 TcSYL_0168880.t1-p1
OG0008870: TcCLB-NE.509039.4_mRNA-p1 TcSYL_0167160.t1-p1
OG0008871: TcCLB-NE.511491.110_mRNA-p1 TcSYL_0167090.t1-p1
OG0008872: TcCLB-NE.436083.9_mRNA-p1 TcSYL_0156970.t1-p1
OG0008873: TcCLB-NE.503943.10_mRNA-p1 TcSYL_0104550.t1-p1
OG0008874: TcCLB-NE.506595.21_mRNA-p1 TcSYL_0021050.t1-p1
OG0008875: TcCLB-NE.507085.120_mRNA-p1 TcSYL_0000310.t1-p1
OG0008876: TcCLB-NE.508503.10_mRNA-p1 TcSYL_0065290.t1-p1
OG0008877: TcCLB-NE.509413.10_mRNA-p1 TcSYL_0103960.t1-p1
OG0008878: TcCLB-NE.510039.15_mRNA-p1 TcSYL_0156400.t1-p1
OG0008879: TcCLB-NE.510039.36_mRNA-p1 TcSYL_0149920.t1-p1
OG0008880: TcCLB-NE.426799.19_mRNA-p1 TcSYL_0195600.t1-p1
OG0008881: TcCLB-NE.508257.220_mRNA-p1 TcSYL_0046880.t1-p1
OG0008882: TcCLB-NE.511003.150_mRNA-p1 TcSYL_0046260.t1-p1
OG0008883: TcCLB-NE.511003.221_mRNA-p1 TcSYL_0046310.t1-p1
OG0008884: TcCLB-NE.511003.60_mRNA-p1 TcSYL_0046220.t1-p1
OG0008885: TcCLB-NE.426897.4_mRNA-p1 TcSYL_0088170.t1-p1
OG0008886: TcCLB-NE.506723.10_mRNA-p1 TcSYL_0021250.t1-p1
OG0008887: TcCLB-NE.509163.10_mRNA-p1 TcSYL_0143110.t1-p1
OG0008888: TcCLB-NE.507103.29_mRNA-p1 TcSYL_0000350.t1-p1
OG0008889: TcCLB-NE.509617.10_pseudogenic_transcript-p1 TcSYL_0000150.t1-p1
OG0008890: TcCLB-NE.510001.31_mRNA-p1 TcSYL_0003100.t1-p1
OG0008891: TcCLB-NE.506247.60_mRNA-p1 TcSYL_0108890.t1-p1
OG0008892: TcCLB-NE.509643.139_mRNA-p1 TcSYL_0008310.t1-p1

OG0008893: TcCLB-NE.510183.10_mRNA-p1 TcSYL_0013240.t1-p1
OG0008894: TcCLB-NE.413893.10_pseudogenic_transcript-p1
TcSYL_0039700.t1-p1
OG0008895: TcCLB-NE.436761.10_mRNA-p1 TcSYL_0132890.t1-p1
OG0008896: TcCLB-NE.507071.340_mRNA-p1 TcSYL_0067950.t1-p1
OG0008897: TcCLB-NE.507071.380_pseudogenic_transcript-p1
TcSYL_0093580.t1-p1
OG0008898: TcCLB-NE.508437.10_mRNA-p1 TcSYL_0110110.t1-p1
OG0008899: TcCLB-NE.510479.30_mRNA-p1 TcSYL_0204500.t1-p1
OG0008900: TcCLB-NE.507005.10_mRNA-p1 TcSYL_0116940.t1-p1
OG0008901: TcCLB-NE.507005.80_mRNA-p1 TcSYL_0117150.t1-p1
OG0008902: TcCLB-NE.507773.20_mRNA-p1 TcSYL_0165540.t1-p1
OG0008903: TcCLB-NE.508355.280_mRNA-p1 TcSYL_0082990.t1-p1
OG0008904: TcCLB-NE.508507.9_mRNA-p1 TcSYL_0082300.t1-p1
OG0008905: TcCLB-NE.508479.170_mRNA-p1 TcSYL_0019840.t1-p1
OG0008906: TcCLB-NE.508891.130_mRNA-p1 TcSYL_0014980.t1-p1
OG0008907: TcCLB-NE.511283.294_mRNA-p1 TcSYL_0123640.t1-p1
OG0008908: TcCLB-NE.511283.320_mRNA-p1 TcSYL_0123630.t1-p1
OG0008909: TcCLB-NE.508221.240_mRNA-p1 TcSYL_0127380.t1-p1
OG0008910: TcCLB-NE.509031.4.1-p1 TcSYL_0135890.t1-p1
OG0008911: TcCLB-NE.511487.19.1-p1 TcSYL_0196230.t1-p1
OG0008912: TCRU_52
OG0008913: TCRU_of
OG0008914: TCRU_56
OG0008915: TCRU_66
OG0008916: TCRU_67
OG0008917: TCRU_68
OG0008918: TCRU_69
OG0008919: TCRU_80
OG0008920: TCRU_95
OG0008921: TCRU_132
OG0008922: TCRU_146
OG0008923: TCRU_163
OG0008924: TCRU_168
OG0008925: TCRU_174
OG0008926: TCRU_177
OG0008927: TCRU_248
OG0008928: TCRU_269
OG0008929: TCRU_279
OG0008930: TCRU_295
OG0008931: TCRU_309
OG0008932: TCRU_310
OG0008933: TCRU_324
OG0008934: TCRU_333
OG0008935: TCRU_348
OG0008936: TCRU_349
OG0008937: TCRU_373
OG0008938: TCRU_394
OG0008939: TCRU_448
OG0008940: TCRU_455
OG0008941: TCRU_475
OG0008942: TCRU_476
OG0008943: TCRU_477
OG0008944: TCRU_482

OG0008945: TCRU_483
OG0008946: TCRU_497
OG0008947: TCRU_504
OG0008948: TCRU_524
OG0008949: TCRU_531
OG0008950: TCRU_561
OG0008951: TCRU_587
OG0008952: TCRU_588
OG0008953: TCRU_598
OG0008954: TCRU_600
OG0008955: TCRU_gamma_
OG0008956: TCRU_626
OG0008957: TCRU_627
OG0008958: TCRU_629
OG0008959: TCRU_654
OG0008960: TCRU_658
OG0008961: TCRU_665
OG0008962: TCRU_691
OG0008963: TCRU_738
OG0008964: TCRU_744
OG0008965: TCRU_749
OG0008966: TCRU_756
OG0008967: TCRU_784
OG0008968: TCRU_785
OG0008969: TCRU_787
OG0008970: TCRU_788
OG0008971: TCRU_803
OG0008972: TCRU_822
OG0008973: TCRU_823
OG0008974: TCRU_829
OG0008975: TCRU_834
OG0008976: TCRU_852
OG0008977: TCRU_853
OG0008978: TCRU_863
OG0008979: TCRU_871
OG0008980: TCRU_888
OG0008981: TCRU_889
OG0008982: TCRU_896
OG0008983: TCRU_905
OG0008984: TCRU_928
OG0008985: TCRU_999
OG0008986: TCRU_1012
OG0008987: TCRU_1014
OG0008988: TCRU_1016
OG0008989: TCRU_1117
OG0008990: TCRU_1119
OG0008991: TCRU_1153
OG0008992: TCRU_1159
OG0008993: TCRU_1161
OG0008994: TCRU_1176
OG0008995: TCRU_1199
OG0008996: TCRU_1202
OG0008997: TCRU_1207
OG0008998: TCRU_1208

OG0008999: TCRU_1295
OG0009000: TCRU_1299
OG0009001: TCRU_family_
OG0009002: TCRU_1365
OG0009003: TCRU_1371
OG0009004: TCRU_1378
OG0009005: TCRU_1410
OG0009006: TCRU_family_
OG0009007: TCRU_1441
OG0009008: TCRU_1445
OG0009009: TCRU_1446
OG0009010: TCRU_1464
OG0009011: TCRU_of
OG0009012: TCRU_1473
OG0009013: TCRU_1477
OG0009014: TCRU_1480
OG0009015: TCRU_1490
OG0009016: TCRU_1503
OG0009017: TCRU_1518
OG0009018: TCRU_1519
OG0009019: TCRU_1520
OG0009020: TCRU_family_
OG0009021: TCRU_1530
OG0009022: TCRU_1544
OG0009023: TCRU_1577
OG0009024: TCRU_1583
OG0009025: TCRU_1597
OG0009026: TCRU_1607
OG0009027: TCRU_1611
OG0009028: TCRU_1635
OG0009029: TCRU_1665
OG0009030: TCRU_1666
OG0009031: TCRU_1733
OG0009032: TCRU_1742
OG0009033: TCRU_1743
OG0009034: TCRU_1744
OG0009035: TCRU_1746
OG0009036: TCRU_1747
OG0009037: TCRU_1751
OG0009038: TCRU_1790
OG0009039: TCRU_1797
OG0009040: TCRU_1799
OG0009041: TCRU_1835
OG0009042: TCRU_1837
OG0009043: TCRU_1870
OG0009044: TCRU_1884
OG0009045: TCRU_1971
OG0009046: TCRU_1980
OG0009047: TCRU_2048
OG0009048: TCRU_2060
OG0009049: TCRU_family_
OG0009050: TCRU_2094
OG0009051: TCRU_2162
OG0009052: TCRU_2175

OG0009053: TCRU_2176
OG0009054: TCRU_2178
OG0009055: TCRU_2180
OG0009056: TCRU_2183
OG0009057: TCRU_2203
OG0009058: TCRU_2204
OG0009059: TCRU_2206
OG0009060: TCRU_2220
OG0009061: TCRU_2227
OG0009062: TCRU_2231
OG0009063: TCRU_2282
OG0009064: TCRU_2283
OG0009065: TCRU_2305
OG0009066: TCRU_2320
OG0009067: TCRU_2348
OG0009068: TCRU_2364
OG0009069: TCRU_2371
OG0009070: TCRU_2386
OG0009071: TCRU_2391
OG0009072: TCRU_2497
OG0009073: TCRU_2505
OG0009074: TCRU_2542
OG0009075: TCRU_2543
OG0009076: TCRU_2544
OG0009077: TCRU_2559
OG0009078: TCRU_2567
OG0009079: TCRU_2572
OG0009080: TCRU_2612
OG0009081: TCRU_2613
OG0009082: TCRU_2645
OG0009083: TCRU_2647
OG0009084: TCRU_2657
OG0009085: TCRU_2687
OG0009086: TCRU_2715
OG0009087: TCRU_2716
OG0009088: TCRU_2719
OG0009089: TCRU_2721
OG0009090: TCRU_2724
OG0009091: TCRU_2748
OG0009092: TCRU_2753
OG0009093: TCRU_2756
OG0009094: TCRU_2757
OG0009095: TCRU_2761
OG0009096: TCRU_2770
OG0009097: TCRU_2776
OG0009098: TCRU_2794
OG0009099: TCRU_2795
OG0009100: TCRU_2799
OG0009101: TCRU_2800
OG0009102: TCRU_2814
OG0009103: TCRU_2822
OG0009104: TCRU_2823
OG0009105: TCRU_2828
OG0009106: TCRU_2987

OG0009107: TCRU_3035
OG0009108: TCRU_3047
OG0009109: TCRU_3056
OG0009110: TCRU_3065
OG0009111: TCRU_3066
OG0009112: TCRU_3104
OG0009113: TCRU_3138
OG0009114: TCRU_3164
OG0009115: TCRU_3169
OG0009116: TCRU_3180
OG0009117: TCRU_3185
OG0009118: TCRU_3201
OG0009119: TCRU_3214
OG0009120: TCRU_3229
OG0009121: TCRU_3246
OG0009122: TCRU_3288
OG0009123: TCRU_3325
OG0009124: TCRU_3339
OG0009125: TCRU_3349
OG0009126: TCRU_3356
OG0009127: TCRU_3357
OG0009128: TCRU_3381
OG0009129: TCRU_3411
OG0009130: TCRU_3415
OG0009131: TCRU_3418
OG0009132: TCRU_3431
OG0009133: TCRU_3433
OG0009134: TCRU_3436
OG0009135: TCRU_3442
OG0009136: TCRU_3468
OG0009137: TCRU_3475
OG0009138: TCRU_3476
OG0009139: TCRU_3499
OG0009140: TCRU_3501
OG0009141: TCRU_3532
OG0009142: TCRU_3551
OG0009143: TCRU_3554
OG0009144: TCRU_3569
OG0009145: TCRU_3586
OG0009146: TCRU_3607
OG0009147: TCRU_3635
OG0009148: TCRU_3646
OG0009149: TCRU_3651
OG0009150: TCRU_3662
OG0009151: TCRU_3676
OG0009152: TCRU_3678
OG0009153: TCRU_3688
OG0009154: TCRU_3689
OG0009155: TCRU_3711
OG0009156: TCRU_3712
OG0009157: TCRU_3716
OG0009158: TCRU_3748
OG0009159: TCRU_3751
OG0009160: TCRU_3754

OG0009161: TCRU_3763
OG0009162: TCRU_3788
OG0009163: TCRU_3814
OG0009164: TCRU_3842
OG0009165: TCRU_3844
OG0009166: TCRU_3846
OG0009167: TCRU_3864
OG0009168: TCRU_3865
OG0009169: TCRU_3874
OG0009170: TCRU_3875
OG0009171: TCRU_3879
OG0009172: TCRU_3944
OG0009173: TCRU_3945
OG0009174: TCRU_3946
OG0009175: TCRU_3968
OG0009176: TCRU_4007
OG0009177: TCRU_4016
OG0009178: TCRU_4018
OG0009179: TCRU_4021
OG0009180: TCRU_4022
OG0009181: TCRU_4031
OG0009182: TCRU_family_
OG0009183: TCRU_4064
OG0009184: TCRU_4072
OG0009185: TCRU_4107
OG0009186: TCRU_4110
OG0009187: TCRU_4178
OG0009188: TCRU_4189
OG0009189: TCRU_4195
OG0009190: TCRU_4233
OG0009191: TCRU_4267
OG0009192: TCRU_4279
OG0009193: TCRU_4294
OG0009194: TCRU_4333
OG0009195: TCRU_4335
OG0009196: TCRU_4340
OG0009197: TCRU_of
OG0009198: TCRU_4363
OG0009199: TCRU_4413
OG0009200: TCRU_4424
OG0009201: TCRU_4443
OG0009202: TCRU_gamma_
OG0009203: TCRU_4474
OG0009204: TCRU_4475
OG0009205: TCRU_4492
OG0009206: TCRU_4493
OG0009207: TCRU_4499
OG0009208: TCRU_4502
OG0009209: TCRU_4504
OG0009210: TCRU_4523
OG0009211: TCRU_4533
OG0009212: TCRU_4541
OG0009213: TCRU_4559
OG0009214: TCRU_4595

OG0009215: TCRU_4614
OG0009216: TCRU_4631
OG0009217: TCRU_4639
OG0009218: TCRU_4650
OG0009219: TCRU_4652
OG0009220: TCRU_4653
OG0009221: TCRU_4676
OG0009222: TCRU_gamma_
OG0009223: TCRU_4686
OG0009224: TCRU_4688
OG0009225: TCRU_4690
OG0009226: TCRU_4696
OG0009227: TCRU_4727
OG0009228: TCRU_4734
OG0009229: TCRU_4735
OG0009230: TCRU_4737
OG0009231: TCRU_4738
OG0009232: TCRU_4740
OG0009233: TCRU_4747
OG0009234: TCRU_4752
OG0009235: TCRU_4759
OG0009236: TCRU_4761
OG0009237: TCRU_4776
OG0009238: TCRU_4778
OG0009239: TCRU_4790
OG0009240: TCRU_4800
OG0009241: TCRU_4805
OG0009242: TCRU_4827
OG0009243: TCRU_4828
OG0009244: TCRU_4867
OG0009245: TCRU_4877
OG0009246: TCRU_4881
OG0009247: TCRU_4887
OG0009248: TCRU_4902
OG0009249: TCRU_4904
OG0009250: TCRU_4905
OG0009251: TCRU_4906
OG0009252: TCRU_4907
OG0009253: TCRU_4909
OG0009254: TCRU_family_
OG0009255: TCRU_4930
OG0009256: TCRU_4931
OG0009257: TCRU_4934
OG0009258: TCRU_4962
OG0009259: TCRU_4964
OG0009260: TCRU_4982
OG0009261: TCRU_4986
OG0009262: TCRU_5019
OG0009263: TCRU_5049
OG0009264: TCRU_5051
OG0009265: TCRU_5054
OG0009266: TCRU_5065
OG0009267: TCRU_5075
OG0009268: TCRU_5115

OG0009269: TCRU_5125
OG0009270: TCRU_5127
OG0009271: TCRU_5139
OG0009272: TCRU_5163
OG0009273: TCRU_5164
OG0009274: TCRU_5178
OG0009275: TCRU_5189
OG0009276: TCRU_5191
OG0009277: TCRU_5192
OG0009278: TCRU_5196
OG0009279: TCRU_5207
OG0009280: TCRU_5211
OG0009281: TCRU_5220
OG0009282: TCRU_5227
OG0009283: TCRU_5300
OG0009284: TCRU_5323
OG0009285: TCRU_5324
OG0009286: TCRU_5344
OG0009287: TCRU_5351
OG0009288: TCRU_5354
OG0009289: TCRU_5355
OG0009290: TCRU_5373
OG0009291: TCRU_5378
OG0009292: TCRU_5390
OG0009293: TCRU_5407
OG0009294: TCRU_5421
OG0009295: TCRU_5437
OG0009296: TCRU_5439
OG0009297: TCRU_5444
OG0009298: TCRU_5461
OG0009299: TCRU_5466
OG0009300: TCRU_5478
OG0009301: TCRU_5482
OG0009302: TCRU_5555
OG0009303: TCRU_5616
OG0009304: TCRU_5625
OG0009305: TCRU_5644
OG0009306: TCRU_5682
OG0009307: TCRU_5692
OG0009308: TCRU_5743
OG0009309: TCRU_5756
OG0009310: TCRU_5776
OG0009311: TCRU_5820
OG0009312: TCRU_5839
OG0009313: TCRU_5900
OG0009314: TCRU_5935
OG0009315: TCRU_5949
OG0009316: TCRU_5969
OG0009317: TCRU_5998
OG0009318: TCRU_6042
OG0009319: TCRU_6120
OG0009320: TCRU_6159
OG0009321: TCRU_6178
OG0009322: TCRU_6189

OG0009323: TCRU_6190
OG0009324: TCRU_6200
OG0009325: TCRU_6242
OG0009326: TCRU_6246
OG0009327: TCRU_6262
OG0009328: TCRU_6263
OG0009329: TCRU_6275
OG0009330: TCRU_6283
OG0009331: TCRU_6295
OG0009332: TCRU_6297
OG0009333: TCRU_6299
OG0009334: TCRU_6324
OG0009335: TCRU_6327
OG0009336: TCRU_6328
OG0009337: TCRU_6361
OG0009338: TCRU_6362
OG0009339: TCRU_6365
OG0009340: TCRU_6368
OG0009341: TCRU_6373
OG0009342: TCRU_6374
OG0009343: TCRU_6379
OG0009344: TCRU_6382
OG0009345: TCRU_6384
OG0009346: TCRU_6391
OG0009347: TCRU_6422
OG0009348: TCRU_6431
OG0009349: TCRU_6432
OG0009350: TCRU_6433
OG0009351: TCRU_6460
OG0009352: TCRU_6481
OG0009353: TCRU_6483
OG0009354: TCRU_6519
OG0009355: TCRU_6521
OG0009356: TCRU_6523
OG0009357: TCRU_6524
OG0009358: TCRU_6536
OG0009359: TCRU_6564
OG0009360: TCRU_6565
OG0009361: TCRU_6566
OG0009362: TCRU_6570
OG0009363: TCRU_6590
OG0009364: TCRU_6605
OG0009365: TCRU_6635
OG0009366: TCRU_6647
OG0009367: TCRU_6653
OG0009368: TCRU_6665
OG0009369: TCRU_6669
OG0009370: TCRU_6705
OG0009371: TCRU_6707
OG0009372: TCRU_6720
OG0009373: TCRU_6723
OG0009374: TCRU_6734
OG0009375: TCRU_of
OG0009376: TCRU_6792

OG0009377: TCRU_6796
OG0009378: TCRU_6797
OG0009379: TCRU_6799
OG0009380: TCRU_6803
OG0009381: TCRU_6808
OG0009382: TCRU_6811
OG0009383: TCRU_6817
OG0009384: TCRU_6837
OG0009385: TCRU_6855
OG0009386: TCRU_6859
OG0009387: TCRU_6860
OG0009388: TCRU_6891
OG0009389: TCRU_6892
OG0009390: TCRU_6895
OG0009391: TCRU_6896
OG0009392: TCRU_6911
OG0009393: TCRU_6923
OG0009394: TCRU_6950
OG0009395: TCRU_6958
OG0009396: TCRU_6959
OG0009397: TCRU_6960
OG0009398: TCRU_of
OG0009399: TCRU_6968
OG0009400: TCRU_6972
OG0009401: TCRU_7011
OG0009402: TCRU_7064
OG0009403: TCRU_7093
OG0009404: TCRU_7096
OG0009405: TCRU_7103
OG0009406: TCRU_7105
OG0009407: TCRU_7119
OG0009408: TCRU_7137
OG0009409: TCRU_7139
OG0009410: TCRU_7142
OG0009411: TCRU_7145
OG0009412: TCRU_7154
OG0009413: TCRU_7187
OG0009414: TCRU_7192
OG0009415: TCRU_7207
OG0009416: TCRU_7211
OG0009417: TCRU_7218
OG0009418: TCRU_7239
OG0009419: TCRU_7269
OG0009420: TCRU_7275
OG0009421: TCRU_7276
OG0009422: TCRU_7282
OG0009423: TCRU_7283
OG0009424: TCRU_7284
OG0009425: TCRU_7286
OG0009426: TCRU_7290
OG0009427: TCRU_7294
OG0009428: TCRU_7299
OG0009429: TCRU_7304
OG0009430: TCRU_7321

OG0009431: TCRU_7352
OG0009432: TCRU_7373
OG0009433: TCRU_7375
OG0009434: TCRU_7376
OG0009435: TCRU_7382
OG0009436: TCRU_7409
OG0009437: TCRU_7421
OG0009438: TCRU_7424
OG0009439: TCRU_7510
OG0009440: TCRU_7536
OG0009441: TCRU_7548
OG0009442: TCRU_7549
OG0009443: TCRU_7551
OG0009444: TCRU_7569
OG0009445: TCRU_7620
OG0009446: TCRU_7636
OG0009447: TCRU_7641
OG0009448: TCRU_7642
OG0009449: TCRU_7688
OG0009450: TCRU_7694
OG0009451: TCRU_7718
OG0009452: TCRU_7729
OG0009453: TCRU_7765
OG0009454: TCRU_7776
OG0009455: TCRU_gamma_
OG0009456: TCRU_7859
OG0009457: TCRU_7860
OG0009458: TCRU_7876
OG0009459: TCRU_7879
OG0009460: TCRU_7880
OG0009461: TCRU_7922
OG0009462: TCRU_7974
OG0009463: TCRU_7990
OG0009464: TCRU_7991
OG0009465: TCRU_7992
OG0009466: TCRU_8029
OG0009467: TCRU_8054
OG0009468: TCRU_8056
OG0009469: TCRU_8058
OG0009470: TCRU_8064
OG0009471: TCRU_8096
OG0009472: TCRU_8101
OG0009473: TCRU_8104
OG0009474: TCRU_8108
OG0009475: TCRU_8109
OG0009476: TCRU_8112
OG0009477: TCRU_8154
OG0009478: TCRU_8222
OG0009479: TCRU_8232
OG0009480: TCRU_8278
OG0009481: TCRU_8294
OG0009482: TCRU_8301
OG0009483: TCRU_8357
OG0009484: TCRU_8358

OG0009485: TCRU_8365
OG0009486: TCRU_8371
OG0009487: TCRU_8382
OG0009488: TCRU_8391
OG0009489: TCRU_8398
OG0009490: TCRU_8407
OG0009491: TCRU_8431
OG0009492: TCRU_8440
OG0009493: TCRU_8443
OG0009494: TCRU_8444
OG0009495: TCRU_8449
OG0009496: TCRU_8450
OG0009497: TCRU_8451
OG0009498: TCRU_8464
OG0009499: TCRU_of
OG0009500: TCRU_8503
OG0009501: TCRU_8508
OG0009502: TCRU_8509
OG0009503: TCRU_8511
OG0009504: TCRU_8513
OG0009505: TCRU_8520
OG0009506: TCRU_8534
OG0009507: TCRU_8549
OG0009508: TCRU_8552
OG0009509: TCRU_8559
OG0009510: TCRU_8584
OG0009511: TCRU_8616
OG0009512: TCRU_8656
OG0009513: TCRU_8657
OG0009514: TCRU_8714
OG0009515: TCRU_8783
OG0009516: TCRU_8819
OG0009517: TCRU_8882
OG0009518: TCRU_8892
OG0009519: TCRU_8893
OG0009520: TCRU_8894
OG0009521: TCRU_8909
OG0009522: TCRU_8927
OG0009523: TCRU_8962
OG0009524: TCRU_8998
OG0009525: TCRU_9011
OG0009526: TCRU_9012
OG0009527: TCRU_9059
OG0009528: TCRU_9068
OG0009529: TCRU_9081
OG0009530: TCRU_9099
OG0009531: TCRU_9118
OG0009532: TCRU_9139
OG0009533: TCRU_9141
OG0009534: TCRU_9142
OG0009535: TCRU_9150
OG0009536: TCRU_9151
OG0009537: TCRU_9157
OG0009538: TCRU_9158

OG0009539: TCRU_9160
OG0009540: TCRU_9185
OG0009541: TCRU_9186
OG0009542: TCRU_9199
OG0009543: TCRU_9201
OG0009544: TCRU_of
OG0009545: TCRU_9270
OG0009546: TCRU_9286
OG0009547: TCRU_9315
OG0009548: TCRU_9321
OG0009549: TCRU_9333
OG0009550: TCRU_9341
OG0009551: TCRU_9342
OG0009552: TCRU_9343
OG0009553: TCRU_9346
OG0009554: TCRU_9348
OG0009555: TCRU_9380
OG0009556: TCRU_9394
OG0009557: TCRU_9399
OG0009558: TCRU_9419
OG0009559: TCRU_9426
OG0009560: TCRU_9456
OG0009561: TCRU_9473
OG0009562: TCRU_9477
OG0009563: TCRU_9489
OG0009564: TCRU_9507
OG0009565: TCRU_9509
OG0009566: TCRU_9528
OG0009567: TCRU_9540
OG0009568: TCRU_9549
OG0009569: TCRU_9551
OG0009570: TCRU_9555
OG0009571: TCRU_9582
OG0009572: TCRU_9583
OG0009573: TCRU_9598
OG0009574: TCRU_9601
OG0009575: TCRU_9606
OG0009576: TCRU_9607
OG0009577: TCRU_9663
OG0009578: TCRU_9678
OG0009579: TCRU_9684
OG0009580: TCRU_9689
OG0009581: TCRU_9692
OG0009582: TCRU_9697
OG0009583: TCRU_9706
OG0009584: TCRU_9713
OG0009585: TCRU_9715
OG0009586: TCRU_9719
OG0009587: TCRU_9720
OG0009588: TCRU_9740
OG0009589: TCRU_9750
OG0009590: TCRU_9760
OG0009591: TCRU_9761
OG0009592: TCRU_9768

OG0009593: TCRU_9769
OG0009594: TCRU_9781
OG0009595: TCRU_9842
OG0009596: TCRU_9853
OG0009597: TCRU_9854
OG0009598: TCRU_9855
OG0009599: TCRU_9856
OG0009600: TCRU_9879
OG0009601: TCRU_9881
OG0009602: TCRU_9897
OG0009603: TCRU_9904
OG0009604: TCRU_9934
OG0009605: TCRU_9944
OG0009606: TCRU_9963
OG0009607: TCRU_10001
OG0009608: TCRU_10003
OG0009609: TCRU_10006
OG0009610: TCRU_10019
OG0009611: TCRU_10021
OG0009612: TCRU_10034
OG0009613: TCRU_10044
OG0009614: TCRU_10095
OG0009615: TCRU_10097
OG0009616: TCRU_10099
OG0009617: TCRU_10104
OG0009618: TCRU_10118
OG0009619: TCRU_10136
OG0009620: TCRU_10223
OG0009621: TCRU_10225
OG0009622: TCRU_10228
OG0009623: TCRU_10237
OG0009624: TCRU_10262
OG0009625: TCRU_10339
OG0009626: TCRU_10375
OG0009627: TCRU_10378
OG0009628: TCRU_of
OG0009629: TCRU_10442
OG0009630: TCRU_10444
OG0009631: TCRU_10453
OG0009632: TCRU_family_
OG0009633: TCRU_10479
OG0009634: TCRU_10484
OG0009635: TCRU_10493
OG0009636: TCRU_10501
OG0009637: TCRU_10509
OG0009638: TCRU_10510
OG0009639: TCRU_10511
OG0009640: TCRU_10550
OG0009641: TCRU_10572
OG0009642: TCRU_10575
OG0009643: TCRU_10595
OG0009644: TCRU_10600
OG0009645: TCRU_10661
OG0009646: TCRU_10672

OG0009647: TCRU_10701
OG0009648: TCRU_10706
OG0009649: TCRU_10710
OG0009650: TCRU_10735
OG0009651: TCRU_10737
OG0009652: TCRU_10792
OG0009653: TCRU_10800
OG0009654: TCRU_10828
OG0009655: TCRU_10830
OG0009656: TCRU_10838
OG0009657: TCRU_10843
OG0009658: TCRU_10857
OG0009659: TcCLB-EL.410961.10_mRNA-p1
OG0009660: TcCLB-EL.411427.49_mRNA-p1
OG0009661: TcCLB-EL.503653.104_mRNA-p1
OG0009662: TcCLB-EL.510077.20_pseudogenic_transcript-p1
OG0009663: TcCLB-EL.506717.100_pseudogenic_transcript-p1
OG0009664: TcCLB-EL.506717.25_pseudogenic_transcript-p1
OG0009665: TcCLB-EL.506717.4_mRNA-p1
OG0009666: TcCLB-EL.507145.31_pseudogenic_transcript-p1
OG0009667: TcCLB-EL.511055.10_pseudogenic_transcript-p1
OG0009668: TcCLB-EL.508047.80_pseudogenic_transcript-p1
OG0009669: TcCLB-EL.438923.10_mRNA-p1
OG0009670: TcCLB-EL.506495.40_mRNA-p1
OG0009671: TcCLB-EL.506495.50_mRNA-p1
OG0009672: TcCLB-EL.511369.15_pseudogenic_transcript-p1
OG0009673: TcCLB-EL.422207.21_pseudogenic_transcript-p1
OG0009674: TcCLB-EL.506129.50_mRNA-p1
OG0009675: TcCLB-EL.506129.80_pseudogenic_transcript-p1
OG0009676: TcCLB-EL.506529.535_mRNA-p1
OG0009677: TcCLB-EL.506529.595_mRNA-p1
OG0009678: TcCLB-EL.507537.10_pseudogenic_transcript-p1
OG0009679: TcCLB-EL.507537.20_mRNA-p1
OG0009680: TcCLB-EL.507997.89_mRNA-p1
OG0009681: TcCLB-EL.510073.24_mRNA-p1
OG0009682: TcCLB-EL.505843.49_mRNA-p1
OG0009683: TcCLB-EL.506113.10_pseudogenic_transcript-p1
OG0009684: TcCLB-EL.506113.40_pseudogenic_transcript-p1
OG0009685: TcCLB-EL.506113.80_pseudogenic_transcript-p1
OG0009686: TcCLB-EL.509265.110_mRNA-p1
OG0009687: TcCLB-EL.509265.60_pseudogenic_transcript-p1
OG0009688: TcCLB-EL.510441.41_pseudogenic_transcript-p1
OG0009689: TcCLB-EL.504033.66_pseudogenic_transcript-p1
OG0009690: TcCLB-EL.504557.40_mRNA-p1
OG0009691: TcCLB-EL.505155.10_pseudogenic_transcript-p1
OG0009692: TcCLB-EL.506625.150_mRNA-p1
OG0009693: TcCLB-EL.507213.60_pseudogenic_transcript-p1
OG0009694: TcCLB-EL.504247.79_mRNA-p1
OG0009695: TcCLB-EL.506563.220_mRNA-p1
OG0009696: TcCLB-EL.506563.4_mRNA-p1
OG0009697: TcCLB-EL.510953.10_pseudogenic_transcript-p1
OG0009698: TcCLB-EL.510959.15_pseudogenic_transcript-p1
OG0009699: TcCLB-EL.511417.129_mRNA-p1
OG0009700: TcCLB-EL.511421.155_mRNA-p1

OG0009701: TcCLB-EL.511421.185_mRNA-p1
OG0009702: TcCLB-EL.511421.210_mRNA-p1
OG0009703: TcCLB-EL.511421.224_pseudogenic_transcript-p1
OG0009704: TcCLB-EL.511425.10_mRNA-p1
OG0009705: TcCLB-EL.511425.49_mRNA-p1
OG0009706: TcCLB-EL.511435.49_mRNA-p1
OG0009707: TcCLB-EL.482471.10_mRNA-p1
OG0009708: TcCLB-EL.503479.10_mRNA-p1
OG0009709: TcCLB-EL.506457.4_mRNA-p1
OG0009710: TcCLB-EL.508781.10_pseudogenic_transcript-p1
OG0009711: TcCLB-EL.508787.5_mRNA-p1
OG0009712: TcCLB-EL.511135.10_pseudogenic_transcript-p1
OG0009713: TcCLB-EL.511139.40_mRNA-p1
OG0009714: TcCLB-EL.511143.39_mRNA-p1
OG0009715: TcCLB-EL.422843.10_pseudogenic_transcript-p1
OG0009716: TcCLB-EL.430737.10_mRNA-p1
OG0009717: TcCLB-EL.430737.20_mRNA-p1
OG0009718: TcCLB-EL.506659.35_mRNA-p1
OG0009719: TcCLB-EL.506689.10_pseudogenic_transcript-p1
OG0009720: TcCLB-EL.508091.10_pseudogenic_transcript-p1
OG0009721: TcCLB-EL.508265.4_mRNA-p1
OG0009722: TcCLB-EL.508675.9_mRNA-p1
OG0009723: TcCLB-EL.510307.140_pseudogenic_transcript-p1
OG0009724: TcCLB-EL.510307.245_pseudogenic_transcript-p1
OG0009725: TcCLB-EL.510307.320_pseudogenic_transcript-p1
OG0009726: TcCLB-EL.510307.60_pseudogenic_transcript-p1
OG0009727: TcCLB-EL.510403.20_pseudogenic_transcript-p1
OG0009728: TcCLB-EL.510403.50_pseudogenic_transcript-p1
OG0009729: TcCLB-EL.510405.9_mRNA-p1
OG0009730: TcCLB-EL.505943.10_mRNA-p1
OG0009731: TcCLB-EL.505945.5_mRNA-p1
OG0009732: TcCLB-EL.506683.10_pseudogenic_transcript-p1
OG0009733: TcCLB-EL.506865.2_mRNA-p1
OG0009734: TcCLB-EL.506865.79_mRNA-p1
OG0009735: TcCLB-EL.511571.10_mRNA-p1
OG0009736: TcCLB-EL.511573.49_mRNA-p1
OG0009737: TcCLB-EL.506857.120_mRNA-p1
OG0009738: TcCLB-EL.506861.39_mRNA-p1
OG0009739: TcCLB-EL.508085.10_mRNA-p1
OG0009740: TcCLB-EL.508323.199_mRNA-p1
OG0009741: TcCLB-EL.508325.20_pseudogenic_transcript-p1
OG0009742: TcCLB-EL.508325.230_mRNA-p1
OG0009743: TcCLB-EL.508325.25_pseudogenic_transcript-p1
OG0009744: TcCLB-EL.508325.270_pseudogenic_transcript-p1
OG0009745: TcCLB-EL.508325.80_pseudogenic_transcript-p1
OG0009746: TcCLB-EL.510165.70_pseudogenic_transcript-p1
OG0009747: TcCLB-EL.510529.39_mRNA-p1
OG0009748: TcCLB-EL.459793.20_pseudogenic_transcript-p1
OG0009749: TcCLB-EL.503821.5_mRNA-p1
OG0009750: TcCLB-EL.504011.30_pseudogenic_transcript-p1
OG0009751: TcCLB-EL.504089.79_pseudogenic_transcript-p1
OG0009752: TcCLB-EL.504127.30.1-p1
OG0009753: TcCLB-EL.506227.250_mRNA-p1
OG0009754: TcCLB-EL.506239.60_mRNA-p1

OG0009755: TcCLB-EL.506737.107_pseudogenic_transcript-p1
OG0009756: TcCLB-EL.506737.114_mRNA-p1
OG0009757: TcCLB-EL.506737.121_pseudogenic_transcript-p1
OG0009758: TcCLB-EL.506737.128_mRNA-p1
OG0009759: TcCLB-EL.506737.161_mRNA-p1
OG0009760: TcCLB-EL.509993.10_mRNA-p1
OG0009761: TcCLB-EL.509993.20_mRNA-p1
OG0009762: TcCLB-EL.509993.31_pseudogenic_transcript-p1
OG0009763: TcCLB-EL.509993.40_mRNA-p1
OG0009764: TcCLB-EL.509993.51_pseudogenic_transcript-p1
OG0009765: TcCLB-EL.509231.5_mRNA-p1
OG0009766: TcCLB-EL.509233.140_pseudogenic_transcript-p1
OG0009767: TcCLB-EL.511867.170_mRNA-p1
OG0009768: TcCLB-EL.511873.41_pseudogenic_transcript-p1
OG0009769: TcCLB-EL.511875.123_pseudogenic_transcript-p1
OG0009770: TcCLB-EL.511875.29_pseudogenic_transcript-p1
OG0009771: TcCLB-EL.511875.40_pseudogenic_transcript-p1
OG0009772: TcCLB-EL.511875.64_pseudogenic_transcript-p1
OG0009773: TcCLB-EL.511877.30_pseudogenic_transcript-p1
OG0009774: TcCLB-EL.408547.10_mRNA-p1
OG0009775: TcCLB-EL.408547.5_mRNA-p1
OG0009776: TcCLB-EL.503757.49_mRNA-p1
OG0009777: TcCLB-EL.506267.10_mRNA-p1
OG0009778: TcCLB-EL.506267.30_mRNA-p1
OG0009779: TcCLB-EL.506363.159_mRNA-p1
OG0009780: TcCLB-EL.507163.11_mRNA-p1
OG0009781: TcCLB-EL.507163.50_mRNA-p1
OG0009782: TcCLB-EL.507179.7_pseudogenic_transcript-p1
OG0009783: TcCLB-EL.509291.20_mRNA-p1
OG0009784: TcCLB-EL.509815.70_pseudogenic_transcript-p1
OG0009785: TcCLB-EL.509815.90_pseudogenic_transcript-p1
OG0009786: TcCLB-EL.509817.40_pseudogenic_transcript-p1
OG0009787: TcCLB-EL.510285.140_mRNA-p1
OG0009788: TcCLB-EL.503751.20_mRNA-p1
OG0009789: TcCLB-EL.503967.19_mRNA-p1
OG0009790: TcCLB-EL.506517.14_mRNA-p1
OG0009791: TcCLB-EL.506517.158_mRNA-p1
OG0009792: TcCLB-EL.507045.10_pseudogenic_transcript-p1
OG0009793: TcCLB-EL.507047.20_pseudogenic_transcript-p1
OG0009794: TcCLB-EL.507975.10_pseudogenic_transcript-p1
OG0009795: TcCLB-EL.507975.50_pseudogenic_transcript-p1
OG0009796: TcCLB-EL.508903.30_pseudogenic_transcript-p1
OG0009797: TcCLB-EL.508903.90_pseudogenic_transcript-p1
OG0009798: TcCLB-EL.509711.10_mRNA-p1
OG0009799: TcCLB-EL.509711.20_pseudogenic_transcript-p1
OG0009800: TcCLB-EL.509765.30_pseudogenic_transcript-p1
OG0009801: TcCLB-EL.511307.3_mRNA-p1
OG0009802: TcCLB-EL.503937.40_pseudogenic_transcript-p1
OG0009803: TcCLB-EL.506961.110_pseudogenic_transcript-p1
OG0009804: TcCLB-EL.506965.10_mRNA-p1
OG0009805: TcCLB-EL.506965.90_mRNA-p1
OG0009806: TcCLB-EL.507237.40_mRNA-p1
OG0009807: TcCLB-EL.507237.70_mRNA-p1
OG0009808: TcCLB-EL.511587.30_pseudogenic_transcript-p1

OG0009809: TcCLB-EL.511587.40_pseudogenic_transcript-p1
OG0009810: TcCLB-EL.511589.260_mRNA-p1
OG0009811: TcCLB-EL.511593.20_mRNA-p1
OG0009812: TcCLB-EL.511593.70_mRNA-p1
OG0009813: TcCLB-EL.511595.39_mRNA-p1
OG0009814: TcCLB-EL.511599.110_mRNA-p1
OG0009815: TcCLB-EL.511603.170_mRNA-p1
OG0009816: TcCLB-EL.511603.20_mRNA-p1
OG0009817: TcCLB-EL.511603.500_mRNA-p1
OG0009818: TcCLB-EL.511603.70_mRNA-p1
OG0009819: TcCLB-EL.511605.20_mRNA-p1
OG0009820: TcCLB-EL.511611.16_pseudogenic_transcript-p1
OG0009821: TcCLB-EL.511613.180_mRNA-p1
OG0009822: TcCLB-EL.504139.10_pseudogenic_transcript-p1
OG0009823: TcCLB-EL.506487.50_pseudogenic_transcript-p1
OG0009824: TcCLB-EL.506487.60_mRNA-p1
OG0009825: TcCLB-EL.508285.20_pseudogenic_transcript-p1
OG0009826: TcCLB-EL.508285.50_pseudogenic_transcript-p1
OG0009827: TcCLB-EL.510005.30_pseudogenic_transcript-p1
OG0009828: TcCLB-EL.510849.11_pseudogenic_transcript-p1
OG0009829: TcCLB-EL.510849.20_pseudogenic_transcript-p1
OG0009830: TcCLB-EL.510851.21_pseudogenic_transcript-p1
OG0009831: TcCLB-EL.510851.40_pseudogenic_transcript-p1
OG0009832: TcCLB-EL.510853.10_pseudogenic_transcript-p1
OG0009833: TcCLB-EL.510853.120_mRNA-p1
OG0009834: TcCLB-EL.510859.50_mRNA-p1
OG0009835: TcCLB-EL.397937.5_mRNA-p1
OG0009836: TcCLB-EL.445011.19_mRNA-p1
OG0009837: TcCLB-EL.506565.29_mRNA-p1
OG0009838: TcCLB-EL.506609.60_pseudogenic_transcript-p1
OG0009839: TcCLB-EL.506667.5_pseudogenic_transcript-p1
OG0009840: TcCLB-EL.508099.9_pseudogenic_transcript-p1
OG0009841: TcCLB-EL.508147.70_pseudogenic_transcript-p1
OG0009842: TcCLB-EL.508149.40_pseudogenic_transcript-p1
OG0009843: TcCLB-EL.508149.60_pseudogenic_transcript-p1
OG0009844: TcCLB-EL.508151.5_pseudogenic_transcript-p1
OG0009845: TcCLB-EL.509757.49_mRNA-p1
OG0009846: TcCLB-EL.510189.30_pseudogenic_transcript-p1
OG0009847: TcCLB-EL.510189.60_pseudogenic_transcript-p1
OG0009848: TcCLB-EL.510255.29_mRNA-p1
OG0009849: TcCLB-EL.510261.31_pseudogenic_transcript-p1
OG0009850: TcCLB-EL.510287.4_mRNA-p1
OG0009851: TcCLB-EL.510297.10_mRNA-p1
OG0009852: TcCLB-EL.510297.119_mRNA-p1
OG0009853: TcCLB-EL.404711.10_pseudogenic_transcript-p1
OG0009854: TcCLB-EL.503531.49_mRNA-p1
OG0009855: TcCLB-EL.503619.39_mRNA-p1
OG0009856: TcCLB-EL.503777.19_mRNA-p1
OG0009857: TcCLB-EL.503955.110_mRNA-p1
OG0009858: TcCLB-EL.507477.20_pseudogenic_transcript-p1
OG0009859: TcCLB-EL.507477.5_mRNA-p1
OG0009860: TcCLB-EL.507479.29_pseudogenic_transcript-p1
OG0009861: TcCLB-EL.507803.49_mRNA-p1
OG0009862: TcCLB-EL.508093.29_pseudogenic_transcript-p1

OG0009863: TcCLB-EL.508093.39_pseudogenic_transcript-p1
OG0009864: TcCLB-EL.508379.29_mRNA-p1
OG0009865: TcCLB-EL.509773.20_mRNA-p1
OG0009866: TcCLB-EL.509775.49_mRNA-p1
OG0009867: TcCLB-EL.510175.110_pseudogenic_transcript-p1
OG0009868: TcCLB-EL.510175.200_pseudogenic_transcript-p1
OG0009869: TcCLB-EL.510175.30_pseudogenic_transcript-p1
OG0009870: TcCLB-EL.420495.19_pseudogenic_transcript-p1
OG0009871: TcCLB-EL.466823.20_mRNA-p1
OG0009872: TcCLB-EL.503589.19_mRNA-p1
OG0009873: TcCLB-EL.506345.30_pseudogenic_transcript-p1
OG0009874: TcCLB-EL.506375.20_pseudogenic_transcript-p1
OG0009875: TcCLB-EL.507133.20_mRNA-p1
OG0009876: TcCLB-EL.509769.20_mRNA-p1
OG0009877: TcCLB-EL.509769.33_mRNA-p1
OG0009878: TcCLB-EL.509769.36_mRNA-p1
OG0009879: TcCLB-EL.509769.40_mRNA-p1
OG0009880: TcCLB-EL.509769.66_mRNA-p1
OG0009881: TcCLB-EL.509871.70_pseudogenic_transcript-p1
OG0009882: TcCLB-EL.411337.10_pseudogenic_transcript-p1
OG0009883: TcCLB-EL.461515.10_mRNA-p1
OG0009884: TcCLB-EL.503783.70_mRNA-p1
OG0009885: TcCLB-EL.503951.10_pseudogenic_transcript-p1
OG0009886: TcCLB-EL.507547.18_mRNA-p1
OG0009887: TcCLB-EL.507847.30_pseudogenic_transcript-p1
OG0009888: TcCLB-EL.508765.50_pseudogenic_transcript-p1
OG0009889: TcCLB-EL.508771.60_pseudogenic_transcript-p1
OG0009890: TcCLB-EL.509099.5_mRNA-p1
OG0009891: TcCLB-EL.509735.150_pseudogenic_transcript-p1
OG0009892: TcCLB-EL.509847.10_pseudogenic_transcript-p1
OG0009893: TcCLB-EL.511109.10_pseudogenic_transcript-p1
OG0009894: TcCLB-EL.511115.49_mRNA-p1
OG0009895: TcCLB-EL.511193.9_pseudogenic_transcript-p1
OG0009896: TcCLB-EL.511195.10_pseudogenic_transcript-p1
OG0009897: TcCLB-EL.441401.10_mRNA-p1
OG0009898: TcCLB-EL.485889.16_mRNA-p1
OG0009899: TcCLB-EL.503665.15.1-p1
OG0009900: TcCLB-EL.503957.90_pseudogenic_transcript-p1
OG0009901: TcCLB-EL.505363.15_pseudogenic_transcript-p1
OG0009902: TcCLB-EL.505365.80_pseudogenic_transcript-p1
OG0009903: TcCLB-EL.506409.140_mRNA-p1
OG0009904: TcCLB-EL.506411.25_pseudogenic_transcript-p1
OG0009905: TcCLB-EL.506951.30_pseudogenic_transcript-p1
OG0009906: TcCLB-EL.506953.45_pseudogenic_transcript-p1
OG0009907: TcCLB-EL.507899.5_pseudogenic_transcript-p1
OG0009908: TcCLB-EL.509127.10_pseudogenic_transcript-p1
OG0009909: TcCLB-EL.509137.10_mRNA-p1
OG0009910: TcCLB-EL.509141.30_mRNA-p1
OG0009911: TcCLB-EL.509145.19_mRNA-p1
OG0009912: TcCLB-EL.509921.51_pseudogenic_transcript-p1
OG0009913: TcCLB-EL.511683.10_mRNA-p1
OG0009914: TcCLB-EL.506949.10_mRNA-p1
OG0009915: TcCLB-EL.507091.100_pseudogenic_transcript-p1
OG0009916: TcCLB-EL.507091.160_pseudogenic_transcript-p1

OG0009917: TcCLB-EL.507229.25_pseudogenic_transcript-p1
OG0009918: TcCLB-EL.511215.90_mRNA-p1
OG0009919: TcCLB-EL.511397.10_pseudogenic_transcript-p1
OG0009920: TcCLB-EL.511397.21_pseudogenic_transcript-p1
OG0009921: TcCLB-EL.420369.20_pseudogenic_transcript-p1
OG0009922: TcCLB-EL.503449.5_mRNA-p1
OG0009923: TcCLB-EL.503981.90_mRNA-p1
OG0009924: TcCLB-EL.504035.149_mRNA-p1
OG0009925: TcCLB-EL.504099.70_pseudogenic_transcript-p1
OG0009926: TcCLB-EL.504241.80_pseudogenic_transcript-p1
OG0009927: TcCLB-EL.506183.40_pseudogenic_transcript-p1
OG0009928: TcCLB-EL.506205.49_mRNA-p1
OG0009929: TcCLB-EL.506243.10_pseudogenic_transcript-p1
OG0009930: TcCLB-EL.506243.135_pseudogenic_transcript-p1
OG0009931: TcCLB-EL.506243.94_pseudogenic_transcript-p1
OG0009932: TcCLB-EL.506799.150_pseudogenic_transcript-p1
OG0009933: TcCLB-EL.506801.50_pseudogenic_transcript-p1
OG0009934: TcCLB-EL.508647.174_mRNA-p1
OG0009935: TcCLB-EL.508755.11_pseudogenic_transcript-p1
OG0009936: TcCLB-EL.511069.4_mRNA-p1
OG0009937: TcCLB-EL.511071.202_mRNA-p1
OG0009938: TcCLB-EL.511071.45_pseudogenic_transcript-p1
OG0009939: TcCLB-EL.447925.10_mRNA-p1
OG0009940: TcCLB-EL.506779.180_mRNA-p1
OG0009941: TcCLB-EL.508241.10_mRNA-p1
OG0009942: TcCLB-EL.509151.29_pseudogenic_transcript-p1
OG0009943: TcCLB-EL.510381.5_pseudogenic_transcript-p1
OG0009944: TcCLB-EL.511697.30_mRNA-p1
OG0009945: TcCLB-EL.506973.105_pseudogenic_transcript-p1
OG0009946: TcCLB-EL.507863.3_pseudogenic_transcript-p1
OG0009947: TcCLB-EL.508381.40_pseudogenic_transcript-p1
OG0009948: TcCLB-EL.508389.130_mRNA-p1
OG0009949: TcCLB-EL.508389.174_pseudogenic_transcript-p1
OG0009950: TcCLB-EL.509195.70_mRNA-p1
OG0009951: TcCLB-EL.510621.55_pseudogenic_transcript-p1
OG0009952: TcCLB-EL.511791.10_mRNA-p1
OG0009953: TcCLB-EL.511797.120_mRNA-p1
OG0009954: TcCLB-EL.511797.130_pseudogenic_transcript-p1
OG0009955: TcCLB-EL.511797.200_mRNA-p1
OG0009956: TcCLB-EL.511797.260_pseudogenic_transcript-p1
OG0009957: TcCLB-EL.477079.10_mRNA-p1
OG0009958: TcCLB-EL.506285.30_pseudogenic_transcript-p1
OG0009959: TcCLB-EL.506285.50_mRNA-p1
OG0009960: TcCLB-EL.506729.9_mRNA-p1
OG0009961: TcCLB-EL.507747.110_mRNA-p1
OG0009962: TcCLB-EL.507747.195_pseudogenic_transcript-p1
OG0009963: TcCLB-EL.508207.279_mRNA-p1
OG0009964: TcCLB-EL.508207.5_pseudogenic_transcript-p1
OG0009965: TcCLB-EL.508209.10_pseudogenic_transcript-p1
OG0009966: TcCLB-EL.509697.15_pseudogenic_transcript-p1
OG0009967: TcCLB-EL.509699.220_pseudogenic_transcript-p1
OG0009968: TcCLB-EL.509699.268_pseudogenic_transcript-p1
OG0009969: TcCLB-EL.509699.60_pseudogenic_transcript-p1
OG0009970: TcCLB-EL.507075.30_pseudogenic_transcript-p1

OG0009971: TcCLB-EL.507075.40_mRNA-p1
OG0009972: TcCLB-EL.507527.4_mRNA-p1
OG0009973: TcCLB-EL.508813.15_mRNA-p1
OG0009974: TcCLB-EL.508831.190_pseudogenic_transcript-p1
OG0009975: TcCLB-EL.509197.50_mRNA-p1
OG0009976: TcCLB-EL.511213.15_mRNA-p1
OG0009977: TcCLB-EL.511213.50_mRNA-p1
OG0009978: TcCLB-EL.511213.70_pseudogenic_transcript-p1
OG0009979: TcCLB-EL.511213.81_pseudogenic_transcript-p1
OG0009980: TcCLB-EL.511803.15_pseudogenic_transcript-p1
OG0009981: TcCLB-EL.511803.80_mRNA-p1
OG0009982: TcCLB-EL.430539.20_mRNA-p1
OG0009983: TcCLB-EL.477983.10_mRNA-p1
OG0009984: TcCLB-EL.503883.10_pseudogenic_transcript-p1
OG0009985: TcCLB-EL.505009.10_mRNA-p1
OG0009986: TcCLB-EL.505009.4_mRNA-p1
OG0009987: TcCLB-EL.505073.10_mRNA-p1
OG0009988: TcCLB-EL.505123.28_mRNA-p1
OG0009989: TcCLB-EL.506387.9_mRNA-p1
OG0009990: TcCLB-EL.506907.40_mRNA-p1
OG0009991: TcCLB-EL.507101.20_pseudogenic_transcript-p1
OG0009992: TcCLB-EL.507107.50_mRNA-p1
OG0009993: TcCLB-EL.507395.40_mRNA-p1
OG0009994: TcCLB-EL.508367.70_pseudogenic_transcript-p1
OG0009995: TcCLB-EL.508555.10_mRNA-p1
OG0009996: TcCLB-EL.509007.50_mRNA-p1
OG0009997: TcCLB-EL.509007.99_pseudogenic_transcript-p1
OG0009998: TcCLB-EL.509011.10_pseudogenic_transcript-p1
OG0009999: TcCLB-EL.509895.110_mRNA-p1
OG0010000: TcCLB-EL.509953.9_mRNA-p1
OG0010001: TcCLB-EL.510595.30_mRNA-p1
OG0010002: TcCLB-EL.510599.70_mRNA-p1
OG0010003: TcCLB-EL.510773.9_mRNA-p1
OG0010004: TcCLB-EL.510841.30_mRNA-p1
OG0010005: TcCLB-EL.511325.10_pseudogenic_transcript-p1
OG0010006: TcCLB-EL.511325.50_mRNA-p1
OG0010007: TcCLB-EL.511327.20_mRNA-p1
OG0010008: TcCLB-EL.511331.10_mRNA-p1
OG0010009: TcCLB-EL.511333.10_mRNA-p1
OG0010010: TcCLB-EL.511335.19_pseudogenic_transcript-p1
OG0010011: TcCLB-EL.511335.9_mRNA-p1
OG0010012: TcCLB-EL.511339.10_mRNA-p1
OG0010013: TcCLB-EL.511437.40_mRNA-p1
OG0010014: TcCLB-EL.469785.5_mRNA-p1
OG0010015: TcCLB-EL.503835.5_mRNA-p1
OG0010016: TcCLB-EL.507925.10_pseudogenic_transcript-p1
OG0010017: TcCLB-EL.507925.69_mRNA-p1
OG0010018: TcCLB-EL.509965.310_pseudogenic_transcript-p1
OG0010019: TcCLB-EL.509965.429_mRNA-p1
OG0010020: TcCLB-EL.510777.29_mRNA-p1
OG0010021: TcCLB-EL.511731.5_pseudogenic_transcript-p1
OG0010022: TcCLB-EL.511733.130_mRNA-p1
OG0010023: TcCLB-EL.511737.3_mRNA-p1
OG0010024: TcCLB-EL.511751.205_mRNA-p1

OG0010025: TcCLB-EL.506829.71_pseudogenic_transcript-p1
OG0010026: TcCLB-EL.506895.5_mRNA-p1
OG0010027: TcCLB-EL.508341.10_mRNA-p1
OG0010028: TcCLB-EL.509977.15_pseudogenic_transcript-p1
OG0010029: TcCLB-EL.510343.15_mRNA-p1
OG0010030: TcCLB-EL.510347.5_mRNA-p1
OG0010031: TcCLB-EL.511585.260_pseudogenic_transcript-p1
OG0010032: TcCLB-EL.409101.9_mRNA-p1
OG0010033: TcCLB-EL.410243.10_mRNA-p1
OG0010034: TcCLB-EL.504057.50_mRNA-p1
OG0010035: TcCLB-EL.504129.50_pseudogenic_transcript-p1
OG0010036: TcCLB-EL.506265.209_mRNA-p1
OG0010037: TcCLB-EL.506631.50_mRNA-p1
OG0010038: TcCLB-EL.506635.139_pseudogenic_transcript-p1
OG0010039: TcCLB-EL.507671.39_mRNA-p1
OG0010040: TcCLB-EL.507737.40_mRNA-p1
OG0010041: TcCLB-EL.507875.150_pseudogenic_transcript-p1
OG0010042: TcCLB-EL.507875.159_pseudogenic_transcript-p1
OG0010043: TcCLB-EL.508135.20_mRNA-p1
OG0010044: TcCLB-EL.509631.100_pseudogenic_transcript-p1
OG0010045: TcCLB-EL.509631.20_mRNA-p1
OG0010046: TcCLB-EL.509635.9_mRNA-p1
OG0010047: TcCLB-EL.510879.10_mRNA-p1
OG0010048: TcCLB-EL.510879.229_mRNA-p1
OG0010049: TcCLB-EL.410589.19_mRNA-p1
OG0010050: TcCLB-EL.436497.10_mRNA-p1
OG0010051: TcCLB-EL.503397.10_pseudogenic_transcript-p1
OG0010052: TcCLB-EL.507035.70_mRNA-p1
OG0010053: TcCLB-EL.507035.75_pseudogenic_transcript-p1
OG0010054: TcCLB-EL.507039.20_pseudogenic_transcript-p1
OG0010055: TcCLB-EL.507039.41_mRNA-p1
OG0010056: TcCLB-EL.507041.60_pseudogenic_transcript-p1
OG0010057: TcCLB-EL.507041.80_mRNA-p1
OG0010058: TcCLB-EL.507041.85_pseudogenic_transcript-p1
OG0010059: TcCLB-EL.507043.21_mRNA-p1
OG0010060: TcCLB-EL.507105.100_pseudogenic_transcript-p1
OG0010061: TcCLB-EL.507105.80_mRNA-p1
OG0010062: TcCLB-EL.508605.40_pseudogenic_transcript-p1
OG0010063: TcCLB-EL.509071.10_pseudogenic_transcript-p1
OG0010064: TcCLB-EL.509073.119_mRNA-p1
OG0010065: TcCLB-EL.509267.6_pseudogenic_transcript-p1
OG0010066: TcCLB-EL.510911.58_pseudogenic_transcript-p1
OG0010067: TcCLB-EL.511525.11_mRNA-p1
OG0010068: TcCLB-EL.511535.39_mRNA-p1
OG0010069: TcCLB-EL.511537.69_mRNA-p1
OG0010070: TcCLB-EL.511539.18_mRNA-p1
OG0010071: TcCLB-EL.511707.10_mRNA-p1
OG0010072: TcCLB-EL.412943.9_mRNA-p1
OG0010073: TcCLB-EL.503685.5_mRNA-p1
OG0010074: TcCLB-EL.503697.140_mRNA-p1
OG0010075: TcCLB-EL.503745.25_mRNA-p1
OG0010076: TcCLB-EL.504433.20_mRNA-p1
OG0010077: TcCLB-EL.506237.19_mRNA-p1
OG0010078: TcCLB-EL.506343.10_mRNA-p1

OG0010079: TcCLB-EL.506691.90_mRNA-p1
OG0010080: TcCLB-EL.507529.58_mRNA-p1
OG0010081: TcCLB-EL.507881.90_pseudogenic_transcript-p1
OG0010082: TcCLB-EL.508181.4_mRNA-p1
OG0010083: TcCLB-EL.508185.19_mRNA-p1
OG0010084: TcCLB-EL.510311.199_mRNA-p1
OG0010085: TcCLB-EL.510315.4_mRNA-p1
OG0010086: TcCLB-EL.484299.10_mRNA-p1
OG0010087: TcCLB-EL.503425.5_mRNA-p1
OG0010088: TcCLB-EL.503779.10_mRNA-p1
OG0010089: TcCLB-EL.504179.100_mRNA-p1
OG0010090: TcCLB-EL.507611.142_pseudogenic_transcript-p1
OG0010091: TcCLB-EL.507611.190_pseudogenic_transcript-p1
OG0010092: TcCLB-EL.507611.210_mRNA-p1
OG0010093: TcCLB-EL.507611.230_pseudogenic_transcript-p1
OG0010094: TcCLB-EL.507611.70_pseudogenic_transcript-p1
OG0010095: TcCLB-EL.507641.240_mRNA-p1
OG0010096: TcCLB-EL.507641.250_mRNA-p1
OG0010097: TcCLB-EL.507641.260_pseudogenic_transcript-p1
OG0010098: TcCLB-EL.507641.64_mRNA-p1
OG0010099: TcCLB-EL.507711.235_pseudogenic_transcript-p1
OG0010100: TcCLB-EL.507713.10_mRNA-p1
OG0010101: TcCLB-EL.508097.40_pseudogenic_transcript-p1
OG0010102: TcCLB-EL.508307.125_mRNA-p1
OG0010103: TcCLB-EL.508307.200_mRNA-p1
OG0010104: TcCLB-EL.508725.4_mRNA-p1
OG0010105: TcCLB-EL.508727.24_mRNA-p1
OG0010106: TcCLB-EL.509645.5_pseudogenic_transcript-p1
OG0010107: TcCLB-EL.427355.10_mRNA-p1
OG0010108: TcCLB-EL.461165.16_mRNA-p1
OG0010109: TcCLB-EL.503453.4_mRNA-p1
OG0010110: TcCLB-EL.503599.70_mRNA-p1
OG0010111: TcCLB-EL.503841.80_mRNA-p1
OG0010112: TcCLB-EL.504929.15_mRNA-p1
OG0010113: TcCLB-EL.508121.101_pseudogenic_transcript-p1
OG0010114: TcCLB-EL.508121.56_pseudogenic_transcript-p1
OG0010115: TcCLB-EL.508125.100_mRNA-p1
OG0010116: TcCLB-EL.508541.100_mRNA-p1
OG0010117: TcCLB-EL.508541.225_mRNA-p1
OG0010118: TcCLB-EL.508541.30_mRNA-p1
OG0010119: TcCLB-EL.508805.110_mRNA-p1
OG0010120: TcCLB-EL.510205.15_pseudogenic_transcript-p1
OG0010121: TcCLB-EL.510207.35_pseudogenic_transcript-p1
OG0010122: TcCLB-EL.510209.18_pseudogenic_transcript-p1
OG0010123: TcCLB-EL.511001.240_mRNA-p1
OG0010124: TcCLB-EL.511173.160_pseudogenic_transcript-p1
OG0010125: TcCLB-EL.511173.171_pseudogenic_transcript-p1
OG0010126: TcCLB-EL.511467.13_mRNA-p1
OG0010127: TcCLB-EL.511471.120_pseudogenic_transcript-p1
OG0010128: TcCLB-EL.503557.49_mRNA-p1
OG0010129: TcCLB-EL.503651.40_mRNA-p1
OG0010130: TcCLB-EL.503651.49_pseudogenic_transcript-p1
OG0010131: TcCLB-EL.503893.181_mRNA-p1
OG0010132: TcCLB-EL.504153.110_mRNA-p1

OG0010133: TcCLB-EL.506125.30_mRNA-p1
OG0010134: TcCLB-EL.506147.9_mRNA-p1
OG0010135: TcCLB-EL.506367.10_mRNA-p1
OG0010136: TcCLB-EL.506811.60_mRNA-p1
OG0010137: TcCLB-EL.506885.140_pseudogenic_transcript-p1
OG0010138: TcCLB-EL.506925.230_mRNA-p1
OG0010139: TcCLB-EL.506925.370_pseudogenic_transcript-p1
OG0010140: TcCLB-EL.506925.380_mRNA-p1
OG0010141: TcCLB-EL.506925.420_mRNA-p1
OG0010142: TcCLB-EL.506925.440_pseudogenic_transcript-p1
OG0010143: TcCLB-EL.507011.269_mRNA-p1
OG0010144: TcCLB-EL.507093.190_mRNA-p1
OG0010145: TcCLB-EL.507467.40_pseudogenic_transcript-p1
OG0010146: TcCLB-EL.508469.59_mRNA-p1
OG0010147: TcCLB-EL.508473.10_mRNA-p1
OG0010148: TcCLB-EL.509831.5_mRNA-p1
OG0010149: TcCLB-EL.509837.30_mRNA-p1
OG0010150: TcCLB-EL.509837.40_mRNA-p1
OG0010151: TcCLB-EL.510553.30_mRNA-p1
OG0010152: TcCLB-EL.510755.147_mRNA-p1
OG0010153: TcCLB-EL.510761.18_mRNA-p1
OG0010154: TcCLB-EL.510761.76_mRNA-p1
OG0010155: TcCLB-EL.424195.5_mRNA-p1
OG0010156: TcCLB-EL.472253.10_pseudogenic_transcript-p1
OG0010157: TcCLB-EL.482097.20_mRNA-p1
OG0010158: TcCLB-EL.503457.4_mRNA-p1
OG0010159: TcCLB-EL.503717.50_pseudogenic_transcript-p1
OG0010160: TcCLB-EL.503717.80_pseudogenic_transcript-p1
OG0010161: TcCLB-EL.503767.5_mRNA-p1
OG0010162: TcCLB-EL.506211.259_mRNA-p1
OG0010163: TcCLB-EL.506215.10_pseudogenic_transcript-p1
OG0010164: TcCLB-EL.506505.50_mRNA-p1
OG0010165: TcCLB-EL.506525.162_mRNA-p1
OG0010166: TcCLB-EL.506543.113_mRNA-p1
OG0010167: TcCLB-EL.507625.10_mRNA-p1
OG0010168: TcCLB-EL.507625.60_pseudogenic_transcript-p1
OG0010169: TcCLB-EL.508021.10_mRNA-p1
OG0010170: TcCLB-EL.508157.20_mRNA-p1
OG0010171: TcCLB-EL.508159.13_pseudogenic_transcript-p1
OG0010172: TcCLB-EL.508159.70_mRNA-p1
OG0010173: TcCLB-EL.508159.80_mRNA-p1
OG0010174: TcCLB-EL.508161.26_pseudogenic_transcript-p1
OG0010175: TcCLB-EL.508161.40_pseudogenic_transcript-p1
OG0010176: TcCLB-EL.508163.10_pseudogenic_transcript-p1
OG0010177: TcCLB-EL.508163.210_pseudogenic_transcript-p1
OG0010178: TcCLB-EL.508165.120_mRNA-p1
OG0010179: TcCLB-EL.508165.140_pseudogenic_transcript-p1
OG0010180: TcCLB-EL.508165.210_mRNA-p1
OG0010181: TcCLB-EL.509803.60_pseudogenic_transcript-p1
OG0010182: TcCLB-EL.509805.260_pseudogenic_transcript-p1
OG0010183: TcCLB-EL.510091.119_pseudogenic_transcript-p1
OG0010184: TcCLB-EL.510269.10_pseudogenic_transcript-p1
OG0010185: TcCLB-EL.510275.21_pseudogenic_transcript-p1
OG0010186: TcCLB-EL.510275.260_pseudogenic_transcript-p1

OG0010187: TcCLB-EL.510275.390_pseudogenic_transcript-p1
OG0010188: TcCLB-EL.510275.400_mRNA-p1
OG0010189: TcCLB-EL.510279.180_pseudogenic_transcript-p1
OG0010190: TcCLB-EL.510279.250_mRNA-p1
OG0010191: TcCLB-EL.510279.314_pseudogenic_transcript-p1
OG0010192: TcCLB-EL.510279.50_pseudogenic_transcript-p1
OG0010193: TcCLB-EL.510281.5_pseudogenic_transcript-p1
OG0010194: TcCLB-EL.511347.11_mRNA-p1
OG0010195: TcCLB-EL.511347.31_pseudogenic_transcript-p1
OG0010196: TcCLB-EL.511349.20_pseudogenic_transcript-p1
OG0010197: TcCLB-EL.439307.19_mRNA-p1
OG0010198: TcCLB-EL.503503.30_mRNA-p1
OG0010199: TcCLB-EL.503761.20_pseudogenic_transcript-p1
OG0010200: TcCLB-EL.503867.5_mRNA-p1
OG0010201: TcCLB-EL.503977.30_mRNA-p1
OG0010202: TcCLB-EL.503977.50_pseudogenic_transcript-p1
OG0010203: TcCLB-EL.504039.50_pseudogenic_transcript-p1
OG0010204: TcCLB-EL.504219.40_pseudogenic_transcript-p1
OG0010205: TcCLB-EL.504239.195_pseudogenic_transcript-p1
OG0010206: TcCLB-EL.506499.70_mRNA-p1
OG0010207: TcCLB-EL.506501.160_pseudogenic_transcript-p1
OG0010208: TcCLB-EL.506501.329_pseudogenic_transcript-p1
OG0010209: TcCLB-EL.506501.40_mRNA-p1
OG0010210: TcCLB-EL.506501.50_pseudogenic_transcript-p1
OG0010211: TcCLB-EL.506599.120_pseudogenic_transcript-p1
OG0010212: TcCLB-EL.506599.280_pseudogenic_transcript-p1
OG0010213: TcCLB-EL.506599.360_pseudogenic_transcript-p1
OG0010214: TcCLB-EL.506763.121_pseudogenic_transcript-p1
OG0010215: TcCLB-EL.506763.234_pseudogenic_transcript-p1
OG0010216: TcCLB-EL.506763.290_mRNA-p1
OG0010217: TcCLB-EL.506763.340_mRNA-p1
OG0010218: TcCLB-EL.506763.4_pseudogenic_transcript-p1
OG0010219: TcCLB-EL.506765.10_pseudogenic_transcript-p1
OG0010220: TcCLB-EL.506765.46_pseudogenic_transcript-p1
OG0010221: TcCLB-EL.506767.74_pseudogenic_transcript-p1
OG0010222: TcCLB-EL.506769.4_pseudogenic_transcript-p1
OG0010223: TcCLB-EL.506769.50_mRNA-p1
OG0010224: TcCLB-EL.507953.180_mRNA-p1
OG0010225: TcCLB-EL.507953.190_mRNA-p1
OG0010226: TcCLB-EL.507957.60_pseudogenic_transcript-p1
OG0010227: TcCLB-EL.507957.90_mRNA-p1
OG0010228: TcCLB-EL.507959.120_pseudogenic_transcript-p1
OG0010229: TcCLB-EL.507959.210_mRNA-p1
OG0010230: TcCLB-EL.507959.260_mRNA-p1
OG0010231: TcCLB-EL.507959.41_mRNA-p1
OG0010232: TcCLB-EL.507985.20_pseudogenic_transcript-p1
OG0010233: TcCLB-EL.508227.51_pseudogenic_transcript-p1
OG0010234: TcCLB-EL.509079.10_pseudogenic_transcript-p1
OG0010235: TcCLB-EL.509081.100_mRNA-p1
OG0010236: TcCLB-EL.509081.10_mRNA-p1
OG0010237: TcCLB-EL.509081.140_mRNA-p1
OG0010238: TcCLB-EL.509081.150_mRNA-p1
OG0010239: TcCLB-EL.510013.59.1-p1
OG0010240: TcCLB-EL.510017.30_pseudogenic_transcript-p1

OG0010241: TcCLB-EL.510017.50_pseudogenic_transcript-p1
OG0010242: TcCLB-EL.510021.109_pseudogenic_transcript-p1
OG0010243: TcCLB-EL.510021.140_pseudogenic_transcript-p1
OG0010244: TcCLB-EL.510023.20_mRNA-p1
OG0010245: TcCLB-EL.510025.180_mRNA-p1
OG0010246: TcCLB-EL.510025.20_mRNA-p1
OG0010247: TcCLB-EL.510025.220_mRNA-p1
OG0010248: TcCLB-EL.510025.90_mRNA-p1
OG0010249: TcCLB-EL.510365.10_pseudogenic_transcript-p1
OG0010250: TcCLB-EL.510371.5_pseudogenic_transcript-p1
OG0010251: TcCLB-EL.510377.270_pseudogenic_transcript-p1
OG0010252: TcCLB-EL.511123.20_pseudogenic_transcript-p1
OG0010253: TcCLB-EL.511127.429_mRNA-p1
OG0010254: TcCLB-EL.511127.5_mRNA-p1
OG0010255: TcCLB-EL.511553.90_mRNA-p1
OG0010256: TcCLB-NE.504639.50_pseudogenic_transcript-p1
OG0010257: TcCLB-NE.509539.10_pseudogenic_transcript-p1
OG0010258: TcCLB-NE.509539.59_mRNA-p1
OG0010259: TcCLB-NE.510817.5_mRNA-p1
OG0010260: TcCLB-NE.510823.50_mRNA-p1
OG0010261: TcCLB-NE.510827.40_mRNA-p1
OG0010262: TcCLB-NE.507681.120_pseudogenic_transcript-p1
OG0010263: TcCLB-NE.507681.50_mRNA-p1
OG0010264: TcCLB-NE.508945.20_pseudogenic_transcript-p1
OG0010265: TcCLB-NE.511367.120_mRNA-p1
OG0010266: TcCLB-NE.511367.199_pseudogenic_transcript-p1
OG0010267: TcCLB-NE.511367.20_mRNA-p1
OG0010268: TcCLB-NE.511367.30_mRNA-p1
OG0010269: TcCLB-NE.511367.80_mRNA-p1
OG0010270: TcCLB-NE.508595.35_pseudogenic_transcript-p1
OG0010271: TcCLB-NE.509429.70_mRNA-p1
OG0010272: TcCLB-NE.510889.205_mRNA-p1
OG0010273: TcCLB-NE.510889.4_mRNA-p1
OG0010274: TcCLB-NE.503545.10_pseudogenic_transcript-p1
OG0010275: TcCLB-NE.503913.49_mRNA-p1
OG0010276: TcCLB-NE.504839.36_mRNA-p1
OG0010277: TcCLB-NE.507259.16_mRNA-p1
OG0010278: TcCLB-NE.507259.4_mRNA-p1
OG0010279: TcCLB-NE.507911.15_pseudogenic_transcript-p1
OG0010280: TcCLB-NE.507911.60_pseudogenic_transcript-p1
OG0010281: TcCLB-NE.507915.5_mRNA-p1
OG0010282: TcCLB-NE.509941.70_pseudogenic_transcript-p1
OG0010283: TcCLB-NE.419207.9_mRNA-p1
OG0010284: TcCLB-NE.506627.10_mRNA-p1
OG0010285: TcCLB-NE.506627.130_pseudogenic_transcript-p1
OG0010286: TcCLB-NE.506627.90_mRNA-p1
OG0010287: TcCLB-NE.507901.30_pseudogenic_transcript-p1
OG0010288: TcCLB-NE.508197.39_mRNA-p1
OG0010289: TcCLB-NE.508621.51_pseudogenic_transcript-p1
OG0010290: TcCLB-NE.510571.39_mRNA-p1
OG0010291: TcCLB-NE.510575.239_mRNA-p1
OG0010292: TcCLB-NE.505997.190_pseudogenic_transcript-p1
OG0010293: TcCLB-NE.505997.65_pseudogenic_transcript-p1
OG0010294: TcCLB-NE.506477.4_mRNA-p1

OG0010295: TcCLB-NE.507949.219_pseudogenic_transcript-p1
OG0010296: TcCLB-NE.507949.4_mRNA-p1
OG0010297: TcCLB-NE.508999.134_mRNA-p1
OG0010298: TcCLB-NE.508999.269_mRNA-p1
OG0010299: TcCLB-NE.508999.60_mRNA-p1
OG0010300: TcCLB-NE.506253.30_mRNA-p1
OG0010301: TcCLB-NE.506855.200_mRNA-p1
OG0010302: TcCLB-NE.506977.110_pseudogenic_transcript-p1
OG0010303: TcCLB-NE.507499.30_pseudogenic_transcript-p1
OG0010304: TcCLB-NE.508319.10_mRNA-p1
OG0010305: TcCLB-NE.508635.10_mRNA-p1
OG0010306: TcCLB-NE.414243.29_mRNA-p1
OG0010307: TcCLB-NE.506443.130_pseudogenic_transcript-p1
OG0010308: TcCLB-NE.509937.70_mRNA-p1
OG0010309: TcCLB-NE.511249.4_mRNA-p1
OG0010310: TcCLB-NE.503861.110_pseudogenic_transcript-p1
OG0010311: TcCLB-NE.507621.10_pseudogenic_transcript-p1
OG0010312: TcCLB-NE.507623.125_mRNA-p1
OG0010313: TcCLB-NE.509573.60_mRNA-p1
OG0010314: TcCLB-NE.509663.25_pseudogenic_transcript-p1
OG0010315: TcCLB-NE.509663.5_pseudogenic_transcript-p1
OG0010316: TcCLB-NE.509663.60_pseudogenic_transcript-p1
OG0010317: TcCLB-NE.507015.50_pseudogenic_transcript-p1
OG0010318: TcCLB-NE.511807.125_mRNA-p1
OG0010319: TcCLB-NE.511807.230_mRNA-p1
OG0010320: TcCLB-NE.511807.256_mRNA-p1
OG0010321: TcCLB-NE.511813.30_mRNA-p1
OG0010322: TcCLB-NE.511823.79_mRNA-p1
OG0010323: TcCLB-NE.432629.10_pseudogenic_transcript-p1
OG0010324: TcCLB-NE.503395.59_mRNA-p1
OG0010325: TcCLB-NE.503837.40_mRNA-p1
OG0010326: TcCLB-NE.503837.5_mRNA-p1
OG0010327: TcCLB-NE.504911.20_pseudogenic_transcript-p1
OG0010328: TcCLB-NE.504911.31_pseudogenic_transcript-p1
OG0010329: TcCLB-NE.506257.5_mRNA-p1
OG0010330: TcCLB-NE.506257.98_pseudogenic_transcript-p1
OG0010331: TcCLB-NE.509227.10_mRNA-p1
OG0010332: TcCLB-NE.509587.20_mRNA-p1
OG0010333: TcCLB-NE.511849.10_mRNA-p1
OG0010334: TcCLB-NE.511849.15_pseudogenic_transcript-p1
OG0010335: TcCLB-NE.511849.20_mRNA-p1
OG0010336: TcCLB-NE.511851.37_mRNA-p1
OG0010337: TcCLB-NE.511851.5_mRNA-p1
OG0010338: TcCLB-NE.404845.10_pseudogenic_transcript-p1
OG0010339: TcCLB-NE.503775.41_pseudogenic_transcript-p1
OG0010340: TcCLB-NE.503775.50_pseudogenic_transcript-p1
OG0010341: TcCLB-NE.503775.60_mRNA-p1
OG0010342: TcCLB-NE.509085.50_pseudogenic_transcript-p1
OG0010343: TcCLB-NE.509085.90_pseudogenic_transcript-p1
OG0010344: TcCLB-NE.509437.125_pseudogenic_transcript-p1
OG0010345: TcCLB-NE.509437.170_mRNA-p1
OG0010346: TcCLB-NE.509437.183_pseudogenic_transcript-p1
OG0010347: TcCLB-NE.510197.220_pseudogenic_transcript-p1
OG0010348: TcCLB-NE.510197.64_pseudogenic_transcript-p1

OG0010349: TcCLB-NE.511561.11_pseudogenic_transcript-p1
OG0010350: TcCLB-NE.511563.20_pseudogenic_transcript-p1
OG0010351: TcCLB-NE.405737.24_mRNA-p1
OG0010352: TcCLB-NE.405737.4_mRNA-p1
OG0010353: TcCLB-NE.503663.4_mRNA-p1
OG0010354: TcCLB-NE.506155.130_mRNA-p1
OG0010355: TcCLB-NE.506337.100_mRNA-p1
OG0010356: TcCLB-NE.506337.171_pseudogenic_transcript-p1
OG0010357: TcCLB-NE.506337.190_mRNA-p1
OG0010358: TcCLB-NE.506337.220_mRNA-p1
OG0010359: TcCLB-NE.506337.80_mRNA-p1
OG0010360: TcCLB-NE.509749.39_mRNA-p1
OG0010361: TcCLB-NE.509751.4_mRNA-p1
OG0010362: TcCLB-NE.509753.270_pseudogenic_transcript-p1
OG0010363: TcCLB-NE.510643.100_pseudogenic_transcript-p1
OG0010364: TcCLB-NE.510643.180_pseudogenic_transcript-p1
OG0010365: TcCLB-NE.402449.10.1-p1
OG0010366: TcCLB-NE.508905.30_pseudogenic_transcript-p1
OG0010367: TcCLB-NE.508919.150_pseudogenic_transcript-p1
OG0010368: TcCLB-NE.509175.9_mRNA-p1
OG0010369: TcCLB-NE.509387.5_mRNA-p1
OG0010370: TcCLB-NE.511321.4_mRNA-p1
OG0010371: TcCLB-NE.511765.10_pseudogenic_transcript-p1
OG0010372: TcCLB-NE.511765.40_pseudogenic_transcript-p1
OG0010373: TcCLB-NE.511771.120_pseudogenic_transcript-p1
OG0010374: TcCLB-NE.511771.171_mRNA-p1
OG0010375: TcCLB-NE.511771.80_pseudogenic_transcript-p1
OG0010376: TcCLB-NE.511773.35_pseudogenic_transcript-p1
OG0010377: TcCLB-NE.504155.43_pseudogenic_transcript-p1
OG0010378: TcCLB-NE.504155.70_mRNA-p1
OG0010379: TcCLB-NE.506137.27_pseudogenic_transcript-p1
OG0010380: TcCLB-NE.506139.100_mRNA-p1
OG0010381: TcCLB-NE.509545.70_mRNA-p1
OG0010382: TcCLB-NE.511227.20_pseudogenic_transcript-p1
OG0010383: TcCLB-NE.511229.9_mRNA-p1
OG0010384: TcCLB-NE.419833.10_mRNA-p1
OG0010385: TcCLB-NE.503733.5_mRNA-p1
OG0010386: TcCLB-NE.509603.10_mRNA-p1
OG0010387: TcCLB-NE.509603.19_mRNA-p1
OG0010388: TcCLB-NE.509609.19_mRNA-p1
OG0010389: TcCLB-NE.510835.60_mRNA-p1
OG0010390: TcCLB-NE.511391.24_pseudogenic_transcript-p1
OG0010391: TcCLB-NE.511391.80_mRNA-p1
OG0010392: TcCLB-NE.506581.4_mRNA-p1
OG0010393: TcCLB-NE.506583.10_mRNA-p1
OG0010394: TcCLB-NE.506583.4_mRNA-p1
OG0010395: TcCLB-NE.506679.20_mRNA-p1
OG0010396: TcCLB-NE.507049.5_pseudogenic_transcript-p1
OG0010397: TcCLB-NE.508659.40_pseudogenic_transcript-p1
OG0010398: TcCLB-NE.509217.70_pseudogenic_transcript-p1
OG0010399: TcCLB-NE.510969.4_mRNA-p1
OG0010400: TcCLB-NE.437349.10_mRNA-p1
OG0010401: TcCLB-NE.503563.29_mRNA-p1
OG0010402: TcCLB-NE.503825.10_pseudogenic_transcript-p1

OG0010403: TcCLB-NE.506321.210_mRNA-p1
OG0010404: TcCLB-NE.506321.30_mRNA-p1
OG0010405: TcCLB-NE.506321.90_pseudogenic_transcript-p1
OG0010406: TcCLB-NE.507059.30_pseudogenic_transcript-p1
OG0010407: TcCLB-NE.507063.260_mRNA-p1
OG0010408: TcCLB-NE.511657.79_mRNA-p1
OG0010409: TcCLB-NE.441729.9_mRNA-p1
OG0010410: TcCLB-NE.447483.4_mRNA-p1
OG0010411: TcCLB-NE.503445.20_pseudogenic_transcript-p1
OG0010412: TcCLB-NE.506219.20_mRNA-p1
OG0010413: TcCLB-NE.506323.10_pseudogenic_transcript-p1
OG0010414: TcCLB-NE.506323.40_pseudogenic_transcript-p1
OG0010415: TcCLB-NE.506325.40_pseudogenic_transcript-p1
OG0010416: TcCLB-NE.507643.90_pseudogenic_transcript-p1
OG0010417: TcCLB-NE.508045.131_pseudogenic_transcript-p1
OG0010418: TcCLB-NE.508421.40_pseudogenic_transcript-p1
OG0010419: TcCLB-NE.509727.20_pseudogenic_transcript-p1
OG0010420: TcCLB-NE.509859.90_mRNA-p1
OG0010421: TcCLB-NE.510673.10_pseudogenic_transcript-p1
OG0010422: TcCLB-NE.503411.4_mRNA-p1
OG0010423: TcCLB-NE.506233.20_pseudogenic_transcript-p1
OG0010424: TcCLB-NE.506537.10_pseudogenic_transcript-p1
OG0010425: TcCLB-NE.506537.71_pseudogenic_transcript-p1
OG0010426: TcCLB-NE.506619.5_mRNA-p1
OG0010427: TcCLB-NE.507717.9_mRNA-p1
OG0010428: TcCLB-NE.507801.240_pseudogenic_transcript-p1
OG0010429: TcCLB-NE.508663.10_pseudogenic_transcript-p1
OG0010430: TcCLB-NE.508667.9_mRNA-p1
OG0010431: TcCLB-NE.509665.13_pseudogenic_transcript-p1
OG0010432: TcCLB-NE.510081.40_pseudogenic_transcript-p1
OG0010433: TcCLB-NE.463955.10_mRNA-p1
OG0010434: TcCLB-NE.508139.120_mRNA-p1
OG0010435: TcCLB-NE.508139.170_pseudogenic_transcript-p1
OG0010436: TcCLB-NE.508365.10_mRNA-p1
OG0010437: TcCLB-NE.508365.180_mRNA-p1
OG0010438: TcCLB-NE.508737.60_mRNA-p1
OG0010439: TcCLB-NE.508739.11_pseudogenic_transcript-p1
OG0010440: TcCLB-NE.509525.360_pseudogenic_transcript-p1
OG0010441: TcCLB-NE.509527.130_mRNA-p1
OG0010442: TcCLB-NE.509527.70_mRNA-p1
OG0010443: TcCLB-NE.510587.20_pseudogenic_transcript-p1
OG0010444: TcCLB-NE.463269.10_mRNA-p1
OG0010445: TcCLB-NE.503851.32_pseudogenic_transcript-p1
OG0010446: TcCLB-NE.503851.54_mRNA-p1
OG0010447: TcCLB-NE.504115.5_pseudogenic_transcript-p1
OG0010448: TcCLB-NE.507887.10_pseudogenic_transcript-p1
OG0010449: TcCLB-NE.508573.90_pseudogenic_transcript-p1
OG0010450: TcCLB-NE.508575.30_mRNA-p1
OG0010451: TcCLB-NE.509897.132_pseudogenic_transcript-p1
OG0010452: TcCLB-NE.509897.160_pseudogenic_transcript-p1
OG0010453: TcCLB-NE.510865.10_pseudogenic_transcript-p1
OG0010454: TcCLB-NE.510867.19_pseudogenic_transcript-p1
OG0010455: TcCLB-NE.511921.100_pseudogenic_transcript-p1
OG0010456: TcCLB-NE.511921.90_pseudogenic_transcript-p1

OG0010457: TcCLB-NE.511923.120_mRNA-p1
OG0010458: TcCLB-NE.511929.20_pseudogenic_transcript-p1
OG0010459: TcCLB-NE.511929.40_pseudogenic_transcript-p1
OG0010460: TcCLB-NE.428403.10_mRNA-p1
OG0010461: TcCLB-NE.428403.5_mRNA-p1
OG0010462: TcCLB-NE.479883.10_mRNA-p1
OG0010463: TcCLB-NE.503693.11_mRNA-p1
OG0010464: TcCLB-NE.505773.10_pseudogenic_transcript-p1
OG0010465: TcCLB-NE.506913.20_mRNA-p1
OG0010466: TcCLB-NE.507381.31_pseudogenic_transcript-p1
OG0010467: TcCLB-NE.507383.10_mRNA-p1
OG0010468: TcCLB-NE.508641.330_mRNA-p1
OG0010469: TcCLB-NE.508645.4_mRNA-p1
OG0010470: TcCLB-NE.509243.40_pseudogenic_transcript-p1
OG0010471: TcCLB-NE.509875.110_mRNA-p1
OG0010472: TcCLB-NE.509875.25.1-p1
OG0010473: TcCLB-NE.509875.30_pseudogenic_transcript-p1
OG0010474: TcCLB-NE.509875.60_mRNA-p1
OG0010475: TcCLB-NE.510063.5_mRNA-p1
OG0010476: TcCLB-NE.510065.5_mRNA-p1
OG0010477: TcCLB-NE.510065.60_mRNA-p1
OG0010478: TcCLB-NE.511903.45_pseudogenic_transcript-p1
OG0010479: TcCLB-NE.511909.20_mRNA-p1
OG0010480: TcCLB-NE.476733.29_mRNA-p1
OG0010481: TcCLB-NE.506297.4_pseudogenic_transcript-p1
OG0010482: TcCLB-NE.508173.140_mRNA-p1
OG0010483: TcCLB-NE.508173.30_pseudogenic_transcript-p1
OG0010484: TcCLB-NE.508797.10_mRNA-p1
OG0010485: TcCLB-NE.510303.140_mRNA-p1
OG0010486: TcCLB-NE.511153.40_mRNA-p1
OG0010487: TcCLB-NE.511169.10_mRNA-p1
OG0010488: TcCLB-NE.506783.20_pseudogenic_transcript-p1
OG0010489: TcCLB-NE.507167.150_pseudogenic_transcript-p1
OG0010490: TcCLB-NE.508261.80_pseudogenic_transcript-p1
OG0010491: TcCLB-NE.509221.10_pseudogenic_transcript-p1
OG0010492: TcCLB-NE.509295.30_pseudogenic_transcript-p1
OG0010493: TcCLB-NE.510387.60_pseudogenic_transcript-p1
OG0010494: TcCLB-NE.510397.20_pseudogenic_transcript-p1
OG0010495: TcCLB-NE.510397.5_mRNA-p1
OG0010496: TcCLB-NE.510623.39_pseudogenic_transcript-p1
OG0010497: TcCLB-NE.510623.45_pseudogenic_transcript-p1
OG0010498: TcCLB-NE.510623.80_pseudogenic_transcript-p1
OG0010499: TcCLB-NE.510625.210_pseudogenic_transcript-p1
OG0010500: TcCLB-NE.510625.225_pseudogenic_transcript-p1
OG0010501: TcCLB-NE.510625.54_mRNA-p1
OG0010502: TcCLB-NE.510625.79_pseudogenic_transcript-p1
OG0010503: TcCLB-NE.510629.101_pseudogenic_transcript-p1
OG0010504: TcCLB-NE.510629.120_pseudogenic_transcript-p1
OG0010505: TcCLB-NE.510629.254_pseudogenic_transcript-p1
OG0010506: TcCLB-NE.510629.44_pseudogenic_transcript-p1
OG0010507: TcCLB-NE.510629.80_pseudogenic_transcript-p1
OG0010508: TcCLB-NE.510631.11_pseudogenic_transcript-p1
OG0010509: TcCLB-NE.410049.10_mRNA-p1
OG0010510: TcCLB-NE.506329.79_pseudogenic_transcript-p1

OG0010511: TcCLB-NE.506329.9_pseudogenic_transcript-p1
OG0010512: TcCLB-NE.507633.110_pseudogenic_transcript-p1
OG0010513: TcCLB-NE.507633.59_pseudogenic_transcript-p1
OG0010514: TcCLB-NE.508879.160_mRNA-p1
OG0010515: TcCLB-NE.508879.229_mRNA-p1
OG0010516: TcCLB-NE.508879.4_mRNA-p1
OG0010517: TcCLB-NE.508883.10_pseudogenic_transcript-p1
OG0010518: TcCLB-NE.508883.39_pseudogenic_transcript-p1
OG0010519: TcCLB-NE.508885.5_mRNA-p1
OG0010520: TcCLB-NE.508983.20_pseudogenic_transcript-p1
OG0010521: TcCLB-NE.508985.21_mRNA-p1
OG0010522: TcCLB-NE.511259.149_pseudogenic_transcript-p1
OG0010523: TcCLB-NE.503993.20_mRNA-p1
OG0010524: TcCLB-NE.508577.10_pseudogenic_transcript-p1
OG0010525: TcCLB-NE.508815.150_mRNA-p1
OG0010526: TcCLB-NE.508815.40_mRNA-p1
OG0010527: TcCLB-NE.508821.10_mRNA-p1
OG0010528: TcCLB-NE.508823.152_mRNA-p1
OG0010529: TcCLB-NE.508825.30_pseudogenic_transcript-p1
OG0010530: TcCLB-NE.509119.39_mRNA-p1
OG0010531: TcCLB-NE.510437.79_mRNA-p1
OG0010532: TcCLB-NE.510903.80_pseudogenic_transcript-p1
OG0010533: TcCLB-NE.511665.110_pseudogenic_transcript-p1
OG0010534: TcCLB-NE.511667.120_pseudogenic_transcript-p1
OG0010535: TcCLB-NE.410199.10_mRNA-p1
OG0010536: TcCLB-NE.410199.40_mRNA-p1
OG0010537: TcCLB-NE.410199.7_mRNA-p1
OG0010538: TcCLB-NE.505267.100_pseudogenic_transcript-p1
OG0010539: TcCLB-NE.505267.60_pseudogenic_transcript-p1
OG0010540: TcCLB-NE.506419.69_mRNA-p1
OG0010541: TcCLB-NE.506489.10_mRNA-p1
OG0010542: TcCLB-NE.507583.60_mRNA-p1
OG0010543: TcCLB-NE.507591.40_pseudogenic_transcript-p1
OG0010544: TcCLB-NE.508017.10_mRNA-p1
OG0010545: TcCLB-NE.509579.30_pseudogenic_transcript-p1
OG0010546: TcCLB-NE.509593.30_pseudogenic_transcript-p1
OG0010547: TcCLB-NE.509593.50_pseudogenic_transcript-p1
OG0010548: TcCLB-NE.509907.60_pseudogenic_transcript-p1
OG0010549: TcCLB-NE.509911.20_mRNA-p1
OG0010550: TcCLB-NE.509915.50_pseudogenic_transcript-p1
OG0010551: TcCLB-NE.432067.20_pseudogenic_transcript-p1
OG0010552: TcCLB-NE.432677.20_mRNA-p1
OG0010553: TcCLB-NE.485683.5_mRNA-p1
OG0010554: TcCLB-NE.503815.4_mRNA-p1
OG0010555: TcCLB-NE.504071.110_mRNA-p1
OG0010556: TcCLB-NE.506831.79_mRNA-p1
OG0010557: TcCLB-NE.506835.159_mRNA-p1
OG0010558: TcCLB-NE.507019.45_mRNA-p1
OG0010559: TcCLB-NE.507019.90_mRNA-p1
OG0010560: TcCLB-NE.507023.289_mRNA-p1
OG0010561: TcCLB-NE.509033.20_mRNA-p1
OG0010562: TcCLB-NE.509035.20_mRNA-p1
OG0010563: TcCLB-NE.509037.20_mRNA-p1
OG0010564: TcCLB-NE.509039.40_mRNA-p1

OG0010565: TcCLB-NE.509039.49_mRNA-p1
OG0010566: TcCLB-NE.509049.5_mRNA-p1
OG0010567: TcCLB-NE.509051.60_mRNA-p1
OG0010568: TcCLB-NE.509055.10_mRNA-p1
OG0010569: TcCLB-NE.509055.69_mRNA-p1
OG0010570: TcCLB-NE.509059.89_mRNA-p1
OG0010571: TcCLB-NE.509061.10_pseudogenic_transcript-p1
OG0010572: TcCLB-NE.509063.2_mRNA-p1
OG0010573: TcCLB-NE.511491.130_mRNA-p1
OG0010574: TcCLB-NE.511493.5_mRNA-p1
OG0010575: TcCLB-NE.511497.10_mRNA-p1
OG0010576: TcCLB-NE.511511.3_mRNA-p1
OG0010577: TcCLB-NE.511511.5_mRNA-p1
OG0010578: TcCLB-NE.511517.5_mRNA-p1
OG0010579: TcCLB-NE.511885.10_pseudogenic_transcript-p1
OG0010580: TcCLB-NE.511889.11_pseudogenic_transcript-p1
OG0010581: TcCLB-NE.511889.50_pseudogenic_transcript-p1
OG0010582: TcCLB-NE.505193.50_mRNA-p1
OG0010583: TcCLB-NE.506595.120_pseudogenic_transcript-p1
OG0010584: TcCLB-NE.507085.130_mRNA-p1
OG0010585: TcCLB-NE.507085.80_pseudogenic_transcript-p1
OG0010586: TcCLB-NE.507225.19_mRNA-p1
OG0010587: TcCLB-NE.508113.14_pseudogenic_transcript-p1
OG0010588: TcCLB-NE.508113.20_pseudogenic_transcript-p1
OG0010589: TcCLB-NE.508495.130_mRNA-p1
OG0010590: TcCLB-NE.510155.270_pseudogenic_transcript-p1
OG0010591: TcCLB-NE.510787.10_mRNA-p1
OG0010592: TcCLB-NE.510795.21_pseudogenic_transcript-p1
OG0010593: TcCLB-NE.510915.10_mRNA-p1
OG0010594: TcCLB-NE.426799.9_mRNA-p1
OG0010595: TcCLB-NE.503929.5_mRNA-p1
OG0010596: TcCLB-NE.503929.79_mRNA-p1
OG0010597: TcCLB-NE.504199.4_mRNA-p1
OG0010598: TcCLB-NE.504253.60_mRNA-p1
OG0010599: TcCLB-NE.506201.4_mRNA-p1
OG0010600: TcCLB-NE.506559.314_mRNA-p1
OG0010601: TcCLB-NE.506559.484_mRNA-p1
OG0010602: TcCLB-NE.506705.10_mRNA-p1
OG0010603: TcCLB-NE.507055.10_mRNA-p1
OG0010604: TcCLB-NE.508027.129_mRNA-p1
OG0010605: TcCLB-NE.508029.59_mRNA-p1
OG0010606: TcCLB-NE.508257.240_mRNA-p1
OG0010607: TcCLB-NE.508257.244_mRNA-p1
OG0010608: TcCLB-NE.508689.50_mRNA-p1
OG0010609: TcCLB-NE.508691.20_mRNA-p1
OG0010610: TcCLB-NE.508693.10_mRNA-p1
OG0010611: TcCLB-NE.508695.10_mRNA-p1
OG0010612: TcCLB-NE.510687.130_mRNA-p1
OG0010613: TcCLB-NE.510689.89_mRNA-p1
OG0010614: TcCLB-NE.503737.20_mRNA-p1
OG0010615: TcCLB-NE.503839.5_mRNA-p1
OG0010616: TcCLB-NE.504207.5_mRNA-p1
OG0010617: TcCLB-NE.506723.41_pseudogenic_transcript-p1
OG0010618: TcCLB-NE.506725.10_mRNA-p1

OG0010619: TcCLB-NE.506821.10_mRNA-p1
OG0010620: TcCLB-NE.506873.20_mRNA-p1
OG0010621: TcCLB-NE.506873.4_mRNA-p1
OG0010622: TcCLB-NE.508521.40_mRNA-p1
OG0010623: TcCLB-NE.508521.81_mRNA-p1
OG0010624: TcCLB-NE.509163.30_pseudogenic_transcript-p1
OG0010625: TcCLB-NE.509163.6_mRNA-p1
OG0010626: TcCLB-NE.511019.84_mRNA-p1
OG0010627: TcCLB-NE.511021.99_mRNA-p1
OG0010628: TcCLB-NE.511717.209_mRNA-p1
OG0010629: TcCLB-NE.511725.100_mRNA-p1
OG0010630: TcCLB-NE.511725.130_mRNA-p1
OG0010631: TcCLB-NE.511725.174_mRNA-p1
OG0010632: TcCLB-NE.511725.300_mRNA-p1
OG0010633: TcCLB-NE.511725.80_mRNA-p1
OG0010634: TcCLB-NE.511725.90_mRNA-p1
OG0010635: TcCLB-NE.511727.110_mRNA-p1
OG0010636: TcCLB-NE.511727.145_mRNA-p1
OG0010637: TcCLB-NE.511727.14_mRNA-p1
OG0010638: TcCLB-NE.511727.230_mRNA-p1
OG0010639: TcCLB-NE.442287.10_pseudogenic_transcript-p1
OG0010640: TcCLB-NE.442495.19_mRNA-p1
OG0010641: TcCLB-NE.490165.9_mRNA-p1
OG0010642: TcCLB-NE.504105.10_mRNA-p1
OG0010643: TcCLB-NE.504105.260_mRNA-p1
OG0010644: TcCLB-NE.504105.87_mRNA-p1
OG0010645: TcCLB-NE.504221.9_mRNA-p1
OG0010646: TcCLB-NE.506175.120_mRNA-p1
OG0010647: TcCLB-NE.507951.290_mRNA-p1
OG0010648: TcCLB-NE.507951.299_mRNA-p1
OG0010649: TcCLB-NE.508153.690_mRNA-p1
OG0010650: TcCLB-NE.509629.30_mRNA-p1
OG0010651: TcCLB-NE.510327.5_pseudogenic_transcript-p1
OG0010652: TcCLB-NE.510331.5_mRNA-p1
OG0010653: TcCLB-NE.510943.20_mRNA-p1
OG0010654: TcCLB-NE.411059.18_pseudogenic_transcript-p1
OG0010655: TcCLB-NE.424937.10_mRNA-p1
OG0010656: TcCLB-NE.424937.20_mRNA-p1
OG0010657: TcCLB-NE.503431.109_mRNA-p1
OG0010658: TcCLB-NE.506247.355_mRNA-p1
OG0010659: TcCLB-NE.506247.74_mRNA-p1
OG0010660: TcCLB-NE.506355.100_pseudogenic_transcript-p1
OG0010661: TcCLB-NE.506355.5_mRNA-p1
OG0010662: TcCLB-NE.506839.89_mRNA-p1
OG0010663: TcCLB-NE.507723.189_mRNA-p1
OG0010664: TcCLB-NE.507785.10_mRNA-p1
OG0010665: TcCLB-NE.507785.20_mRNA-p1
OG0010666: TcCLB-NE.509643.10_mRNA-p1
OG0010667: TcCLB-NE.509733.185_pseudogenic_transcript-p1
OG0010668: TcCLB-NE.509733.199_pseudogenic_transcript-p1
OG0010669: TcCLB-NE.509791.10_mRNA-p1
OG0010670: TcCLB-NE.509797.9_pseudogenic_transcript-p1
OG0010671: TcCLB-NE.510129.11_mRNA-p1
OG0010672: TcCLB-NE.510187.310_mRNA-p1

OG0010673: TcCLB-NE.510187.460_mRNA-p1
OG0010674: TcCLB-NE.510187.470_mRNA-p1
OG0010675: TcCLB-NE.436761.20_mRNA-p1
OG0010676: TcCLB-NE.452699.10_pseudogenic_transcript-p1
OG0010677: TcCLB-NE.457979.5_pseudogenic_transcript-p1
OG0010678: TcCLB-NE.503439.14_pseudogenic_transcript-p1
OG0010679: TcCLB-NE.504189.10_pseudogenic_transcript-p1
OG0010680: TcCLB-NE.505163.50_mRNA-p1
OG0010681: TcCLB-NE.506295.200_pseudogenic_transcript-p1
OG0010682: TcCLB-NE.506955.14_pseudogenic_transcript-p1
OG0010683: TcCLB-NE.506955.161_pseudogenic_transcript-p1
OG0010684: TcCLB-NE.506955.170_pseudogenic_transcript-p1
OG0010685: TcCLB-NE.506955.261_pseudogenic_transcript-p1
OG0010686: TcCLB-NE.506955.50_pseudogenic_transcript-p1
OG0010687: TcCLB-NE.507065.127_pseudogenic_transcript-p1
OG0010688: TcCLB-NE.507065.60_mRNA-p1
OG0010689: TcCLB-NE.507071.100_mRNA-p1
OG0010690: TcCLB-NE.507071.220_pseudogenic_transcript-p1
OG0010691: TcCLB-NE.507305.11_pseudogenic_transcript-p1
OG0010692: TcCLB-NE.507539.49_mRNA-p1
OG0010693: TcCLB-NE.507541.50_mRNA-p1
OG0010694: TcCLB-NE.508001.106_pseudogenic_transcript-p1
OG0010695: TcCLB-NE.508305.20_mRNA-p1
OG0010696: TcCLB-NE.508431.10_pseudogenic_transcript-p1
OG0010697: TcCLB-NE.508431.60_pseudogenic_transcript-p1
OG0010698: TcCLB-NE.508433.50_pseudogenic_transcript-p1
OG0010699: TcCLB-NE.510479.40.1-p1
OG0010700: TcCLB-NE.510483.340_pseudogenic_transcript-p1
OG0010701: TcCLB-NE.510487.30_pseudogenic_transcript-p1
OG0010702: TcCLB-NE.510487.70_pseudogenic_transcript-p1
OG0010703: TcCLB-NE.510693.163_mRNA-p1
OG0010704: TcCLB-NE.510693.220_mRNA-p1
OG0010705: TcCLB-NE.510693.314_pseudogenic_transcript-p1
OG0010706: TcCLB-NE.510705.10_mRNA-p1
OG0010707: TcCLB-NE.510705.20_pseudogenic_transcript-p1
OG0010708: TcCLB-NE.510707.20_mRNA-p1
OG0010709: TcCLB-NE.510713.11_pseudogenic_transcript-p1
OG0010710: TcCLB-NE.510713.50_pseudogenic_transcript-p1
OG0010711: TcCLB-NE.510713.70_pseudogenic_transcript-p1
OG0010712: TcCLB-NE.407335.18_mRNA-p1
OG0010713: TcCLB-NE.444777.10_mRNA-p1
OG0010714: TcCLB-NE.452283.10_pseudogenic_transcript-p1
OG0010715: TcCLB-NE.452283.4_mRNA-p1
OG0010716: TcCLB-NE.503497.10_pseudogenic_transcript-p1
OG0010717: TcCLB-NE.503687.58_mRNA-p1
OG0010718: TcCLB-NE.504109.174_mRNA-p1
OG0010719: TcCLB-NE.504109.180_mRNA-p1
OG0010720: TcCLB-NE.506721.10_mRNA-p1
OG0010721: TcCLB-NE.506721.50_mRNA-p1
OG0010722: TcCLB-NE.507007.37_mRNA-p1
OG0010723: TcCLB-NE.508415.59_mRNA-p1
OG0010724: TcCLB-NE.508459.18_mRNA-p1
OG0010725: TcCLB-NE.508461.143_mRNA-p1
OG0010726: TcCLB-NE.508461.580_mRNA-p1

OG0010727: TcCLB-NE.508461.84_mRNA-p1
OG0010728: TcCLB-NE.508505.4_mRNA-p1
OG0010729: TcCLB-NE.510223.5_mRNA-p1
OG0010730: TcCLB-NE.510223.9_mRNA-p1
OG0010731: TcCLB-NE.510655.159_mRNA-p1
OG0010732: TcCLB-NE.510661.80_mRNA-p1
OG0010733: TcCLB-NE.510665.70_mRNA-p1
OG0010734: TcCLB-NE.510669.40_mRNA-p1
OG0010735: TcCLB-NE.510669.5_mRNA-p1
OG0010736: TcCLB-NE.510735.100_mRNA-p1
OG0010737: TcCLB-NE.510799.60_mRNA-p1
OG0010738: TcCLB-NE.437575.9_mRNA-p1
OG0010739: TcCLB-NE.471901.5_mRNA-p1
OG0010740: TcCLB-NE.477935.9_pseudogenic_transcript-p1
OG0010741: TcCLB-NE.482319.10_mRNA-p1
OG0010742: TcCLB-NE.503681.29_mRNA-p1
OG0010743: TcCLB-NE.503809.68_pseudogenic_transcript-p1
OG0010744: TcCLB-NE.503939.4_mRNA-p1
OG0010745: TcCLB-NE.504045.30_mRNA-p1
OG0010746: TcCLB-NE.504045.55_mRNA-p1
OG0010747: TcCLB-NE.504045.90_pseudogenic_transcript-p1
OG0010748: TcCLB-NE.506331.120_pseudogenic_transcript-p1
OG0010749: TcCLB-NE.506331.174_pseudogenic_transcript-p1
OG0010750: TcCLB-NE.506331.30_mRNA-p1
OG0010751: TcCLB-NE.506335.150_pseudogenic_transcript-p1
OG0010752: TcCLB-NE.506335.55_pseudogenic_transcript-p1
OG0010753: TcCLB-NE.506335.60_pseudogenic_transcript-p1
OG0010754: TcCLB-NE.507031.140_pseudogenic_transcript-p1
OG0010755: TcCLB-NE.507787.170_pseudogenic_transcript-p1
OG0010756: TcCLB-NE.508479.140_mRNA-p1
OG0010757: TcCLB-NE.508479.20_pseudogenic_transcript-p1
OG0010758: TcCLB-NE.508479.213_mRNA-p1
OG0010759: TcCLB-NE.508893.40_mRNA-p1
OG0010760: TcCLB-NE.508899.151_mRNA-p1
OG0010761: TcCLB-NE.510417.20_pseudogenic_transcript-p1
OG0010762: TcCLB-NE.510419.11_pseudogenic_transcript-p1
OG0010763: TcCLB-NE.510421.10_pseudogenic_transcript-p1
OG0010764: TcCLB-NE.510421.240_pseudogenic_transcript-p1
OG0010765: TcCLB-NE.511277.150_mRNA-p1
OG0010766: TcCLB-NE.511277.210_mRNA-p1
OG0010767: TcCLB-NE.511277.370_mRNA-p1
OG0010768: TcCLB-NE.511283.184_mRNA-p1
OG0010769: TcCLB-NE.511287.180_mRNA-p1
OG0010770: TcCLB-NE.511295.70_mRNA-p1
OG0010771: TcCLB-NE.511301.180_pseudogenic_transcript-p1
OG0010772: TcCLB-NE.503973.57_pseudogenic_transcript-p1
OG0010773: TcCLB-NE.504081.280_pseudogenic_transcript-p1
OG0010774: TcCLB-NE.504081.295_pseudogenic_transcript-p1
OG0010775: TcCLB-NE.504081.420_mRNA-p1
OG0010776: TcCLB-NE.504081.45_pseudogenic_transcript-p1
OG0010777: TcCLB-NE.505171.20_mRNA-p1
OG0010778: TcCLB-NE.506389.20_pseudogenic_transcript-p1
OG0010779: TcCLB-NE.506459.149_mRNA-p1
OG0010780: TcCLB-NE.506459.40_pseudogenic_transcript-p1

OG0010781: TcCLB-NE.506987.10_pseudogenic_transcript-p1
OG0010782: TcCLB-NE.507935.20_pseudogenic_transcript-p1
OG0010783: TcCLB-NE.508221.120_mRNA-p1
OG0010784: TcCLB-NE.508221.130_pseudogenic_transcript-p1
OG0010785: TcCLB-NE.508221.20_pseudogenic_transcript-p1
OG0010786: TcCLB-NE.508221.40_mRNA-p1
OG0010787: TcCLB-NE.508221.714_pseudogenic_transcript-p1
OG0010788: TcCLB-NE.508221.769_pseudogenic_transcript-p1
OG0010789: TcCLB-NE.508223.30_pseudogenic_transcript-p1
OG0010790: TcCLB-NE.508869.59_mRNA-p1
OG0010791: TcCLB-NE.508871.40_mRNA-p1
OG0010792: TcCLB-NE.508873.370_mRNA-p1
OG0010793: TcCLB-NE.508873.399_mRNA-p1
OG0010794: TcCLB-NE.508873.40_pseudogenic_transcript-p1
OG0010795: TcCLB-NE.508873.450_mRNA-p1
OG0010796: TcCLB-NE.508877.30_pseudogenic_transcript-p1
OG0010797: TcCLB-NE.509019.10_pseudogenic_transcript-p1
OG0010798: TcCLB-NE.509019.21_pseudogenic_transcript-p1
OG0010799: TcCLB-NE.509971.44_pseudogenic_transcript-p1
OG0010800: TcCLB-NE.509971.70_mRNA-p1
OG0010801: TcCLB-NE.510355.70_pseudogenic_transcript-p1
OG0010802: TcCLB-NE.510359.371_pseudogenic_transcript-p1
OG0010803: TcCLB-NE.510359.540_mRNA-p1
OG0010804: TcCLB-NE.510361.260_mRNA-p1
OG0010805: TcCLB-NE.510361.90_mRNA-p1
OG0010806: TcCLB-NE.510363.30_pseudogenic_transcript-p1
OG0010807: TcCLB-NE.511255.160_pseudogenic_transcript-p1
OG0010808: TcCLB-NE.511255.270_pseudogenic_transcript-p1
OG0010809: TcCLB-NE.511255.299_mRNA-p1
OG0010810: TcCLB-NE.511255.490_pseudogenic_transcript-p1
OG0010811: TcCLB-NE.511255.655_mRNA-p1
OG0010812: TcCLB-NE.511255.660_mRNA-p1
OG0010813: TcCLB-NE.511479.48_mRNA-p1
OG0010814: TcCLB-NE.511485.11_pseudogenic_transcript-p1
OG0010815: TcCLB-NE.511485.20_pseudogenic_transcript-p1
OG0010816: TcCLB-NE.511487.100_pseudogenic_transcript-p1
OG0010817: TcSYL_0000010.t1-p1
OG0010818: TcSYL_0000020.t1-p1
OG0010819: TcSYL_0000030.t1-p1
OG0010820: TcSYL_0000050.t1-p1
OG0010821: TcSYL_0000060.t1-p1
OG0010822: TcSYL_0000070.t1-p1
OG0010823: TcSYL_0000080.t1-p1
OG0010824: TcSYL_0000090.t1-p1
OG0010825: TcSYL_0000120.t1-p1
OG0010826: TcSYL_0000130.t1-p1
OG0010827: TcSYL_0000140.t1-p1
OG0010828: TcSYL_0000160.t1-p1
OG0010829: TcSYL_0000170.t1-p1
OG0010830: TcSYL_0000180.t1-p1
OG0010831: TcSYL_0000190.t1-p1
OG0010832: TcSYL_0000220.t1-p1
OG0010833: TcSYL_0000230.t1-p1
OG0010834: TcSYL_0000240.t1-p1

OG0010835: TcSYL_0000250.t1-p1
OG0010836: TcSYL_0000270.t1-p1
OG0010837: TcSYL_0000280.t1-p1
OG0010838: TcSYL_0000300.t1-p1
OG0010839: TcSYL_0000320.t1-p1
OG0010840: TcSYL_0000330.t1-p1
OG0010841: TcSYL_0000340.t1-p1
OG0010842: TcSYL_0000390.t1-p1
OG0010843: TcSYL_0000400.t1-p1
OG0010844: TcSYL_0000410.t1-p1
OG0010845: TcSYL_0000440.t1-p1
OG0010846: TcSYL_0000510.t1-p1
OG0010847: TcSYL_0000590.t1-p1
OG0010848: TcSYL_0000630.t1-p1
OG0010849: TcSYL_0000760.t1-p1
OG0010850: TcSYL_0000830.t1-p1
OG0010851: TcSYL_0000920.t1-p1
OG0010852: TcSYL_0001050.t1-p1
OG0010853: TcSYL_0001060.t1-p1
OG0010854: TcSYL_0001190.t1-p1
OG0010855: TcSYL_0001220.t1-p1
OG0010856: TcSYL_0001230.t1-p1
OG0010857: TcSYL_0001260.t1-p1
OG0010858: TcSYL_0001320.t1-p1
OG0010859: TcSYL_0001330.t1-p1
OG0010860: TcSYL_0001350.t1-p1
OG0010861: TcSYL_0001450.t1-p1
OG0010862: TcSYL_0001530.t1-p1
OG0010863: TcSYL_0001630.t1-p1
OG0010864: TcSYL_0001640.t1-p1
OG0010865: TcSYL_0001650.t1-p1
OG0010866: TcSYL_0001660.t1-p1
OG0010867: TcSYL_0001670.t1-p1
OG0010868: TcSYL_0001680.t1-p1
OG0010869: TcSYL_0001690.t1-p1
OG0010870: TcSYL_0001700.t1-p1
OG0010871: TcSYL_0001720.t1-p1
OG0010872: TcSYL_0001750.t1-p1
OG0010873: TcSYL_0001760.t1-p1
OG0010874: TcSYL_0001780.t1-p1
OG0010875: TcSYL_0001790.t1-p1
OG0010876: TcSYL_0001800.t1-p1
OG0010877: TcSYL_0001830.t1-p1
OG0010878: TcSYL_0001850.t1-p1
OG0010879: TcSYL_0001890.t1-p1
OG0010880: TcSYL_0001900.t1-p1
OG0010881: TcSYL_0001960.t1-p1
OG0010882: TcSYL_0002020.t1-p1
OG0010883: TcSYL_0002030.t1-p1
OG0010884: TcSYL_0002060.t1-p1
OG0010885: TcSYL_0002070.t1-p1
OG0010886: TcSYL_0002090.t1-p1
OG0010887: TcSYL_0002100.t1-p1
OG0010888: TcSYL_0002110.t1-p1

OG0010889: TcSYL_0002130.t1-p1
OG0010890: TcSYL_0002140.t1-p1
OG0010891: TcSYL_0002150.t1-p1
OG0010892: TcSYL_0002170.t1-p1
OG0010893: TcSYL_0002200.t1-p1
OG0010894: TcSYL_0002210.t1-p1
OG0010895: TcSYL_0002230.t1-p1
OG0010896: TcSYL_0002240.t1-p1
OG0010897: TcSYL_0002260.t1-p1
OG0010898: TcSYL_0002270.t1-p1
OG0010899: TcSYL_0002280.t1-p1
OG0010900: TcSYL_0002300.t1-p1
OG0010901: TcSYL_0002310.t1-p1
OG0010902: TcSYL_0002330.t1-p1
OG0010903: TcSYL_0002340.t1-p1
OG0010904: TcSYL_0002360.t1-p1
OG0010905: TcSYL_0002370.t1-p1
OG0010906: TcSYL_0002390.t1-p1
OG0010907: TcSYL_0002400.t1-p1
OG0010908: TcSYL_0002420.t1-p1
OG0010909: TcSYL_0002440.t1-p1
OG0010910: TcSYL_0002450.t1-p1
OG0010911: TcSYL_0002460.t1-p1
OG0010912: TcSYL_0002470.t1-p1
OG0010913: TcSYL_0002480.t1-p1
OG0010914: TcSYL_0002490.t1-p1
OG0010915: TcSYL_0002500.t1-p1
OG0010916: TcSYL_0002510.t1-p1
OG0010917: TcSYL_0002520.t1-p1
OG0010918: TcSYL_0002530.t1-p1
OG0010919: TcSYL_0002540.t1-p1
OG0010920: TcSYL_0002550.t1-p1
OG0010921: TcSYL_0002560.t1-p1
OG0010922: TcSYL_0002570.t1-p1
OG0010923: TcSYL_0002590.t1-p1
OG0010924: TcSYL_0002600.t1-p1
OG0010925: TcSYL_0002610.t1-p1
OG0010926: TcSYL_0002620.t1-p1
OG0010927: TcSYL_0002630.t1-p1
OG0010928: TcSYL_0002650.t1-p1
OG0010929: TcSYL_0002660.t1-p1
OG0010930: TcSYL_0002670.t1-p1
OG0010931: TcSYL_0002680.t1-p1
OG0010932: TcSYL_0002690.t1-p1
OG0010933: TcSYL_0002710.t1-p1
OG0010934: TcSYL_0002740.t1-p1
OG0010935: TcSYL_0002750.t1-p1
OG0010936: TcSYL_0002770.t1-p1
OG0010937: TcSYL_0002780.t1-p1
OG0010938: TcSYL_0002790.t1-p1
OG0010939: TcSYL_0002800.t1-p1
OG0010940: TcSYL_0002810.t1-p1
OG0010941: TcSYL_0002830.t1-p1
OG0010942: TcSYL_0002840.t1-p1

OG0010943: TcSYL_0002860.t1-p1
OG0010944: TcSYL_0002870.t1-p1
OG0010945: TcSYL_0002880.t1-p1
OG0010946: TcSYL_0002890.t1-p1
OG0010947: TcSYL_0002900.t1-p1
OG0010948: TcSYL_0002910.t1-p1
OG0010949: TcSYL_0002920.t1-p1
OG0010950: TcSYL_0002930.t1-p1
OG0010951: TcSYL_0002940.t1-p1
OG0010952: TcSYL_0002950.t1-p1
OG0010953: TcSYL_0002970.t1-p1
OG0010954: TcSYL_0002980.t1-p1
OG0010955: TcSYL_0002990.t1-p1
OG0010956: TcSYL_0003000.t1-p1
OG0010957: TcSYL_0003010.t1-p1
OG0010958: TcSYL_0003040.t1-p1
OG0010959: TcSYL_0003050.t1-p1
OG0010960: TcSYL_0003080.t1-p1
OG0010961: TcSYL_0003090.t1-p1
OG0010962: TcSYL_0003110.t1-p1
OG0010963: TcSYL_0003120.t1-p1
OG0010964: TcSYL_0003130.t1-p1
OG0010965: TcSYL_0003140.t1-p1
OG0010966: TcSYL_0003150.t1-p1
OG0010967: TcSYL_0003160.t1-p1
OG0010968: TcSYL_0003220.t1-p1
OG0010969: TcSYL_0003350.t1-p1
OG0010970: TcSYL_0003380.t1-p1
OG0010971: TcSYL_0003400.t1-p1
OG0010972: TcSYL_0003480.t1-p1
OG0010973: TcSYL_0003500.t1-p1
OG0010974: TcSYL_0003520.t1-p1
OG0010975: TcSYL_0003530.t1-p1
OG0010976: TcSYL_0003540.t1-p1
OG0010977: TcSYL_0003590.t1-p1
OG0010978: TcSYL_0003600.t1-p1
OG0010979: TcSYL_0003620.t1-p1
OG0010980: TcSYL_0003640.t1-p1
OG0010981: TcSYL_0003660.t1-p1
OG0010982: TcSYL_0003680.t1-p1
OG0010983: TcSYL_0003710.t1-p1
OG0010984: TcSYL_0003750.t1-p1
OG0010985: TcSYL_0003770.t1-p1
OG0010986: TcSYL_0003780.t1-p1
OG0010987: TcSYL_0003790.t1-p1
OG0010988: TcSYL_0003800.t1-p1
OG0010989: TcSYL_0003810.t1-p1
OG0010990: TcSYL_0003820.t1-p1
OG0010991: TcSYL_0003840.t1-p1
OG0010992: TcSYL_0003850.t1-p1
OG0010993: TcSYL_0003860.t1-p1
OG0010994: TcSYL_0003870.t1-p1
OG0010995: TcSYL_0003890.t1-p1
OG0010996: TcSYL_0003900.t1-p1

OG0010997: TcSYL_0003910.t1-p1
OG0010998: TcSYL_0003930.t1-p1
OG0010999: TcSYL_0003950.t1-p1
OG0011000: TcSYL_0003970.t1-p1
OG0011001: TcSYL_0003990.t1-p1
OG0011002: TcSYL_0004000.t1-p1
OG0011003: TcSYL_0004020.t1-p1
OG0011004: TcSYL_0004030.t1-p1
OG0011005: TcSYL_0004050.t1-p1
OG0011006: TcSYL_0004120.t1-p1
OG0011007: TcSYL_0004130.t1-p1
OG0011008: TcSYL_0004140.t1-p1
OG0011009: TcSYL_0004310.t1-p1
OG0011010: TcSYL_0004340.t1-p1
OG0011011: TcSYL_0004350.t1-p1
OG0011012: TcSYL_0004460.t1-p1
OG0011013: TcSYL_0004490.t1-p1
OG0011014: TcSYL_0004530.t1-p1
OG0011015: TcSYL_0004550.t1-p1
OG0011016: TcSYL_0004630.t1-p1
OG0011017: TcSYL_0004690.t1-p1
OG0011018: TcSYL_0004740.t1-p1
OG0011019: TcSYL_0004750.t1-p1
OG0011020: TcSYL_0004760.t1-p1
OG0011021: TcSYL_0004770.t1-p1
OG0011022: TcSYL_0004780.t1-p1
OG0011023: TcSYL_0004800.t1-p1
OG0011024: TcSYL_0004810.t1-p1
OG0011025: TcSYL_0004830.t1-p1
OG0011026: TcSYL_0004850.t1-p1
OG0011027: TcSYL_0004870.t1-p1
OG0011028: TcSYL_0004880.t1-p1
OG0011029: TcSYL_0004890.t1-p1
OG0011030: TcSYL_0004940.t1-p1
OG0011031: TcSYL_0004970.t1-p1
OG0011032: TcSYL_0004980.t1-p1
OG0011033: TcSYL_0005010.t1-p1
OG0011034: TcSYL_0005020.t1-p1
OG0011035: TcSYL_0005030.t1-p1
OG0011036: TcSYL_0005050.t1-p1
OG0011037: TcSYL_0005060.t1-p1
OG0011038: TcSYL_0005070.t1-p1
OG0011039: TcSYL_0005080.t1-p1
OG0011040: TcSYL_0005100.t1-p1
OG0011041: TcSYL_0005110.t1-p1
OG0011042: TcSYL_0005120.t1-p1
OG0011043: TcSYL_0005160.t1-p1
OG0011044: TcSYL_0005170.t1-p1
OG0011045: TcSYL_0005180.t1-p1
OG0011046: TcSYL_0005190.t1-p1
OG0011047: TcSYL_0005210.t1-p1
OG0011048: TcSYL_0005220.t1-p1
OG0011049: TcSYL_0005240.t1-p1
OG0011050: TcSYL_0005250.t1-p1

OG0011051: TcSYL_0005270.t1-p1
OG0011052: TcSYL_0005280.t1-p1
OG0011053: TcSYL_0005290.t1-p1
OG0011054: TcSYL_0005310.t1-p1
OG0011055: TcSYL_0005320.t1-p1
OG0011056: TcSYL_0005340.t1-p1
OG0011057: TcSYL_0005350.t1-p1
OG0011058: TcSYL_0005360.t1-p1
OG0011059: TcSYL_0005380.t1-p1
OG0011060: TcSYL_0005400.t1-p1
OG0011061: TcSYL_0005410.t1-p1
OG0011062: TcSYL_0005420.t1-p1
OG0011063: TcSYL_0005440.t1-p1
OG0011064: TcSYL_0005460.t1-p1
OG0011065: TcSYL_0005470.t1-p1
OG0011066: TcSYL_0005480.t1-p1
OG0011067: TcSYL_0005490.t1-p1
OG0011068: TcSYL_0005500.t1-p1
OG0011069: TcSYL_0005510.t1-p1
OG0011070: TcSYL_0005520.t1-p1
OG0011071: TcSYL_0005530.t1-p1
OG0011072: TcSYL_0005550.t1-p1
OG0011073: TcSYL_0005570.t1-p1
OG0011074: TcSYL_0005580.t1-p1
OG0011075: TcSYL_0005610.t1-p1
OG0011076: TcSYL_0005630.t1-p1
OG0011077: TcSYL_0005640.t1-p1
OG0011078: TcSYL_0005660.t1-p1
OG0011079: TcSYL_0005670.t1-p1
OG0011080: TcSYL_0005680.t1-p1
OG0011081: TcSYL_0005700.t1-p1
OG0011082: TcSYL_0005720.t1-p1
OG0011083: TcSYL_0005730.t1-p1
OG0011084: TcSYL_0005740.t1-p1
OG0011085: TcSYL_0005760.t1-p1
OG0011086: TcSYL_0005770.t1-p1
OG0011087: TcSYL_0005780.t1-p1
OG0011088: TcSYL_0005790.t1-p1
OG0011089: TcSYL_0005810.t1-p1
OG0011090: TcSYL_0005820.t1-p1
OG0011091: TcSYL_0005830.t1-p1
OG0011092: TcSYL_0005840.t1-p1
OG0011093: TcSYL_0005870.t1-p1
OG0011094: TcSYL_0005890.t1-p1
OG0011095: TcSYL_0005910.t1-p1
OG0011096: TcSYL_0005920.t1-p1
OG0011097: TcSYL_0005940.t1-p1
OG0011098: TcSYL_0005960.t1-p1
OG0011099: TcSYL_0005990.t1-p1
OG0011100: TcSYL_0006010.t1-p1
OG0011101: TcSYL_0006020.t1-p1
OG0011102: TcSYL_0006040.t1-p1
OG0011103: TcSYL_0006050.t1-p1
OG0011104: TcSYL_0006070.t1-p1

OG0011105: TcSYL_0006080.t1-p1
OG0011106: TcSYL_0006220.t1-p1
OG0011107: TcSYL_0006230.t1-p1
OG0011108: TcSYL_0006350.t1-p1
OG0011109: TcSYL_0006370.t1-p1
OG0011110: TcSYL_0006390.t1-p1
OG0011111: TcSYL_0006400.t1-p1
OG0011112: TcSYL_0006410.t1-p1
OG0011113: TcSYL_0006430.t1-p1
OG0011114: TcSYL_0006450.t1-p1
OG0011115: TcSYL_0006460.t1-p1
OG0011116: TcSYL_0006470.t1-p1
OG0011117: TcSYL_0006490.t1-p1
OG0011118: TcSYL_0006510.t1-p1
OG0011119: TcSYL_0006530.t1-p1
OG0011120: TcSYL_0006540.t1-p1
OG0011121: TcSYL_0006550.t1-p1
OG0011122: TcSYL_0006570.t1-p1
OG0011123: TcSYL_0006590.t1-p1
OG0011124: TcSYL_0006600.t1-p1
OG0011125: TcSYL_0006610.t1-p1
OG0011126: TcSYL_0006640.t1-p1
OG0011127: TcSYL_0006650.t1-p1
OG0011128: TcSYL_0006680.t1-p1
OG0011129: TcSYL_0006690.t1-p1
OG0011130: TcSYL_0006700.t1-p1
OG0011131: TcSYL_0006710.t1-p1
OG0011132: TcSYL_0006720.t1-p1
OG0011133: TcSYL_0006730.t1-p1
OG0011134: TcSYL_0006740.t1-p1
OG0011135: TcSYL_0006750.t1-p1
OG0011136: TcSYL_0006760.t1-p1
OG0011137: TcSYL_0006770.t1-p1
OG0011138: TcSYL_0006780.t1-p1
OG0011139: TcSYL_0006790.t1-p1
OG0011140: TcSYL_0006810.t1-p1
OG0011141: TcSYL_0006820.t1-p1
OG0011142: TcSYL_0006830.t1-p1
OG0011143: TcSYL_0006840.t1-p1
OG0011144: TcSYL_0006860.t1-p1
OG0011145: TcSYL_0006890.t1-p1
OG0011146: TcSYL_0006910.t1-p1
OG0011147: TcSYL_0006930.t1-p1
OG0011148: TcSYL_0006960.t1-p1
OG0011149: TcSYL_0006980.t1-p1
OG0011150: TcSYL_0006990.t1-p1
OG0011151: TcSYL_0007010.t1-p1
OG0011152: TcSYL_0007030.t1-p1
OG0011153: TcSYL_0007050.t1-p1
OG0011154: TcSYL_0007070.t1-p1
OG0011155: TcSYL_0007100.t1-p1
OG0011156: TcSYL_0007130.t1-p1
OG0011157: TcSYL_0007150.t1-p1
OG0011158: TcSYL_0007170.t1-p1

OG0011159: TcSYL_0007190.t1-p1
OG0011160: TcSYL_0007210.t1-p1
OG0011161: TcSYL_0007230.t1-p1
OG0011162: TcSYL_0007250.t1-p1
OG0011163: TcSYL_0007270.t1-p1
OG0011164: TcSYL_0007310.t1-p1
OG0011165: TcSYL_0007330.t1-p1
OG0011166: TcSYL_0007350.t1-p1
OG0011167: TcSYL_0007360.t1-p1
OG0011168: TcSYL_0007370.t1-p1
OG0011169: TcSYL_0007380.t1-p1
OG0011170: TcSYL_0007390.t1-p1
OG0011171: TcSYL_0007400.t1-p1
OG0011172: TcSYL_0007410.t1-p1
OG0011173: TcSYL_0007430.t1-p1
OG0011174: TcSYL_0007450.t1-p1
OG0011175: TcSYL_0007460.t1-p1
OG0011176: TcSYL_0007480.t1-p1
OG0011177: TcSYL_0007500.t1-p1
OG0011178: TcSYL_0007520.t1-p1
OG0011179: TcSYL_0007540.t1-p1
OG0011180: TcSYL_0007560.t1-p1
OG0011181: TcSYL_0007580.t1-p1
OG0011182: TcSYL_0007600.t1-p1
OG0011183: TcSYL_0007620.t1-p1
OG0011184: TcSYL_0007640.t1-p1
OG0011185: TcSYL_0007670.t1-p1
OG0011186: TcSYL_0007690.t1-p1
OG0011187: TcSYL_0008100.t1-p1
OG0011188: TcSYL_0008110.t1-p1
OG0011189: TcSYL_0008120.t1-p1
OG0011190: TcSYL_0008130.t1-p1
OG0011191: TcSYL_0008140.t1-p1
OG0011192: TcSYL_0008150.t1-p1
OG0011193: TcSYL_0008160.t1-p1
OG0011194: TcSYL_0008180.t1-p1
OG0011195: TcSYL_0008190.t1-p1
OG0011196: TcSYL_0008210.t1-p1
OG0011197: TcSYL_0008220.t1-p1
OG0011198: TcSYL_0008230.t1-p1
OG0011199: TcSYL_0008240.t1-p1
OG0011200: TcSYL_0008260.t1-p1
OG0011201: TcSYL_0008270.t1-p1
OG0011202: TcSYL_0008280.t1-p1
OG0011203: TcSYL_0008290.t1-p1
OG0011204: TcSYL_0008300.t1-p1
OG0011205: TcSYL_0008320.t1-p1
OG0011206: TcSYL_0008330.t1-p1
OG0011207: TcSYL_0008350.t1-p1
OG0011208: TcSYL_0008360.t1-p1
OG0011209: TcSYL_0008370.t1-p1
OG0011210: TcSYL_0008380.t1-p1
OG0011211: TcSYL_0008400.t1-p1
OG0011212: TcSYL_0008410.t1-p1

OG0011213: TcSYL_0008430.t1-p1
OG0011214: TcSYL_0008440.t1-p1
OG0011215: TcSYL_0008450.t1-p1
OG0011216: TcSYL_0008460.t1-p1
OG0011217: TcSYL_0008480.t1-p1
OG0011218: TcSYL_0008500.t1-p1
OG0011219: TcSYL_0008520.t1-p1
OG0011220: TcSYL_0008530.t1-p1
OG0011221: TcSYL_0008540.t1-p1
OG0011222: TcSYL_0008550.t1-p1
OG0011223: TcSYL_0008560.t1-p1
OG0011224: TcSYL_0008570.t1-p1
OG0011225: TcSYL_0008590.t1-p1
OG0011226: TcSYL_0008610.t1-p1
OG0011227: TcSYL_0008620.t1-p1
OG0011228: TcSYL_0008630.t1-p1
OG0011229: TcSYL_0008640.t1-p1
OG0011230: TcSYL_0008650.t1-p1
OG0011231: TcSYL_0008660.t1-p1
OG0011232: TcSYL_0008680.t1-p1
OG0011233: TcSYL_0008690.t1-p1
OG0011234: TcSYL_0008700.t1-p1
OG0011235: TcSYL_0008710.t1-p1
OG0011236: TcSYL_0008750.t1-p1
OG0011237: TcSYL_0008780.t1-p1
OG0011238: TcSYL_0008810.t1-p1
OG0011239: TcSYL_0008820.t1-p1
OG0011240: TcSYL_0008860.t1-p1
OG0011241: TcSYL_0008880.t1-p1
OG0011242: TcSYL_0008890.t1-p1
OG0011243: TcSYL_0008900.t1-p1
OG0011244: TcSYL_0008910.t1-p1
OG0011245: TcSYL_0008920.t1-p1
OG0011246: TcSYL_0008930.t1-p1
OG0011247: TcSYL_0008940.t1-p1
OG0011248: TcSYL_0008950.t1-p1
OG0011249: TcSYL_0008960.t1-p1
OG0011250: TcSYL_0008970.t1-p1
OG0011251: TcSYL_0008980.t1-p1
OG0011252: TcSYL_0009000.t1-p1
OG0011253: TcSYL_0009020.t1-p1
OG0011254: TcSYL_0009030.t1-p1
OG0011255: TcSYL_0009040.t1-p1
OG0011256: TcSYL_0009110.t1-p1
OG0011257: TcSYL_0009120.t1-p1
OG0011258: TcSYL_0009130.t1-p1
OG0011259: TcSYL_0009140.t1-p1
OG0011260: TcSYL_0009160.t1-p1
OG0011261: TcSYL_0009170.t1-p1
OG0011262: TcSYL_0009190.t1-p1
OG0011263: TcSYL_0009200.t1-p1
OG0011264: TcSYL_0009210.t1-p1
OG0011265: TcSYL_0009220.t1-p1
OG0011266: TcSYL_0009230.t1-p1

OG0011267: TcSYL_0009240.t1-p1
OG0011268: TcSYL_0009250.t1-p1
OG0011269: TcSYL_0009270.t1-p1
OG0011270: TcSYL_0009280.t1-p1
OG0011271: TcSYL_0009290.t1-p1
OG0011272: TcSYL_0009300.t1-p1
OG0011273: TcSYL_0009310.t1-p1
OG0011274: TcSYL_0009320.t1-p1
OG0011275: TcSYL_0009340.t1-p1
OG0011276: TcSYL_0009350.t1-p1
OG0011277: TcSYL_0009360.t1-p1
OG0011278: TcSYL_0009370.t1-p1
OG0011279: TcSYL_0009380.t1-p1
OG0011280: TcSYL_0009390.t1-p1
OG0011281: TcSYL_0009400.t1-p1
OG0011282: TcSYL_0009420.t1-p1
OG0011283: TcSYL_0009440.t1-p1
OG0011284: TcSYL_0009450.t1-p1
OG0011285: TcSYL_0009460.t1-p1
OG0011286: TcSYL_0009470.t1-p1
OG0011287: TcSYL_0009480.t1-p1
OG0011288: TcSYL_0009490.t1-p1
OG0011289: TcSYL_0009500.t1-p1
OG0011290: TcSYL_0009520.t1-p1
OG0011291: TcSYL_0009530.t1-p1
OG0011292: TcSYL_0009540.t1-p1
OG0011293: TcSYL_0009550.t1-p1
OG0011294: TcSYL_0009560.t1-p1
OG0011295: TcSYL_0009570.t1-p1
OG0011296: TcSYL_0009580.t1-p1
OG0011297: TcSYL_0009590.t1-p1
OG0011298: TcSYL_0009600.t1-p1
OG0011299: TcSYL_0009620.t1-p1
OG0011300: TcSYL_0009630.t1-p1
OG0011301: TcSYL_0009640.t1-p1
OG0011302: TcSYL_0009650.t1-p1
OG0011303: TcSYL_0009660.t1-p1
OG0011304: TcSYL_0009670.t1-p1
OG0011305: TcSYL_0009680.t1-p1
OG0011306: TcSYL_0009700.t1-p1
OG0011307: TcSYL_0009710.t1-p1
OG0011308: TcSYL_0009720.t1-p1
OG0011309: TcSYL_0009730.t1-p1
OG0011310: TcSYL_0009740.t1-p1
OG0011311: TcSYL_0009750.t1-p1
OG0011312: TcSYL_0009760.t1-p1
OG0011313: TcSYL_0009770.t1-p1
OG0011314: TcSYL_0009790.t1-p1
OG0011315: TcSYL_0009800.t1-p1
OG0011316: TcSYL_0009820.t1-p1
OG0011317: TcSYL_0009830.t1-p1
OG0011318: TcSYL_0009840.t1-p1
OG0011319: TcSYL_0009850.t1-p1
OG0011320: TcSYL_0009870.t1-p1

OG0011321: TcSYL_0009900.t1-p1
OG0011322: TcSYL_0009920.t1-p1
OG0011323: TcSYL_0009930.t1-p1
OG0011324: TcSYL_0009940.t1-p1
OG0011325: TcSYL_0009960.t1-p1
OG0011326: TcSYL_0009980.t1-p1
OG0011327: TcSYL_0009990.t1-p1
OG0011328: TcSYL_0010000.t1-p1
OG0011329: TcSYL_0010010.t1-p1
OG0011330: TcSYL_0010020.t1-p1
OG0011331: TcSYL_0010040.t1-p1
OG0011332: TcSYL_0010050.t1-p1
OG0011333: TcSYL_0010070.t1-p1
OG0011334: TcSYL_0010080.t1-p1
OG0011335: TcSYL_0010120.t1-p1
OG0011336: TcSYL_0010140.t1-p1
OG0011337: TcSYL_0010150.t1-p1
OG0011338: TcSYL_0010160.t1-p1
OG0011339: TcSYL_0010170.t1-p1
OG0011340: TcSYL_0010180.t1-p1
OG0011341: TcSYL_0010200.t1-p1
OG0011342: TcSYL_0010220.t1-p1
OG0011343: TcSYL_0010230.t1-p1
OG0011344: TcSYL_0010250.t1-p1
OG0011345: TcSYL_0010280.t1-p1
OG0011346: TcSYL_0010290.t1-p1
OG0011347: TcSYL_0010300.t1-p1
OG0011348: TcSYL_0010310.t1-p1
OG0011349: TcSYL_0010320.t1-p1
OG0011350: TcSYL_0010350.t1-p1
OG0011351: TcSYL_0010360.t1-p1
OG0011352: TcSYL_0010400.t1-p1
OG0011353: TcSYL_0010420.t1-p1
OG0011354: TcSYL_0010430.t1-p1
OG0011355: TcSYL_0010440.t1-p1
OG0011356: TcSYL_0010450.t1-p1
OG0011357: TcSYL_0010470.t1-p1
OG0011358: TcSYL_0010480.t1-p1
OG0011359: TcSYL_0010490.t1-p1
OG0011360: TcSYL_0010500.t1-p1
OG0011361: TcSYL_0010510.t1-p1
OG0011362: TcSYL_0010520.t1-p1
OG0011363: TcSYL_0010530.t1-p1
OG0011364: TcSYL_0010540.t1-p1
OG0011365: TcSYL_0010560.t1-p1
OG0011366: TcSYL_0010570.t1-p1
OG0011367: TcSYL_0010580.t1-p1
OG0011368: TcSYL_0010620.t1-p1
OG0011369: TcSYL_0010630.t1-p1
OG0011370: TcSYL_0010640.t1-p1
OG0011371: TcSYL_0010660.t1-p1
OG0011372: TcSYL_0010710.t1-p1
OG0011373: TcSYL_0010720.t1-p1
OG0011374: TcSYL_0010780.t1-p1

OG0011375: TcSYL_0010800.t1-p1
OG0011376: TcSYL_0010810.t1-p1
OG0011377: TcSYL_0010860.t1-p1
OG0011378: TcSYL_0010870.t1-p1
OG0011379: TcSYL_0010910.t1-p1
OG0011380: TcSYL_0011010.t1-p1
OG0011381: TcSYL_0011020.t1-p1
OG0011382: TcSYL_0011070.t1-p1
OG0011383: TcSYL_0011160.t1-p1
OG0011384: TcSYL_0011190.t1-p1
OG0011385: TcSYL_0011230.t1-p1
OG0011386: TcSYL_0011480.t1-p1
OG0011387: TcSYL_0011510.t1-p1
OG0011388: TcSYL_0011590.t1-p1
OG0011389: TcSYL_0011700.t1-p1
OG0011390: TcSYL_0011720.t1-p1
OG0011391: TcSYL_0011730.t1-p1
OG0011392: TcSYL_0011740.t1-p1
OG0011393: TcSYL_0011750.t1-p1
OG0011394: TcSYL_0011760.t1-p1
OG0011395: TcSYL_0011770.t1-p1
OG0011396: TcSYL_0011800.t1-p1
OG0011397: TcSYL_0011810.t1-p1
OG0011398: TcSYL_0011830.t1-p1
OG0011399: TcSYL_0011840.t1-p1
OG0011400: TcSYL_0011850.t1-p1
OG0011401: TcSYL_0011860.t1-p1
OG0011402: TcSYL_0011870.t1-p1
OG0011403: TcSYL_0011880.t1-p1
OG0011404: TcSYL_0011890.t1-p1
OG0011405: TcSYL_0011900.t1-p1
OG0011406: TcSYL_0011910.t1-p1
OG0011407: TcSYL_0011920.t1-p1
OG0011408: TcSYL_0011930.t1-p1
OG0011409: TcSYL_0011940.t1-p1
OG0011410: TcSYL_0011950.t1-p1
OG0011411: TcSYL_0011960.t1-p1
OG0011412: TcSYL_0011980.t1-p1
OG0011413: TcSYL_0011990.t1-p1
OG0011414: TcSYL_0012000.t1-p1
OG0011415: TcSYL_0012010.t1-p1
OG0011416: TcSYL_0012020.t1-p1
OG0011417: TcSYL_0012030.t1-p1
OG0011418: TcSYL_0012040.t1-p1
OG0011419: TcSYL_0012050.t1-p1
OG0011420: TcSYL_0012060.t1-p1
OG0011421: TcSYL_0012080.t1-p1
OG0011422: TcSYL_0012090.t1-p1
OG0011423: TcSYL_0012100.t1-p1
OG0011424: TcSYL_0012120.t1-p1
OG0011425: TcSYL_0012130.t1-p1
OG0011426: TcSYL_0012140.t1-p1
OG0011427: TcSYL_0012160.t1-p1
OG0011428: TcSYL_0012170.t1-p1

OG0011429: TcSYL_0012180.t1-p1
OG0011430: TcSYL_0012190.t1-p1
OG0011431: TcSYL_0012200.t1-p1
OG0011432: TcSYL_0012210.t1-p1
OG0011433: TcSYL_0012220.t1-p1
OG0011434: TcSYL_0012230.t1-p1
OG0011435: TcSYL_0012240.t1-p1
OG0011436: TcSYL_0012250.t1-p1
OG0011437: TcSYL_0012260.t1-p1
OG0011438: TcSYL_0012270.t1-p1
OG0011439: TcSYL_0012290.t1-p1
OG0011440: TcSYL_0012300.t1-p1
OG0011441: TcSYL_0012310.t1-p1
OG0011442: TcSYL_0012320.t1-p1
OG0011443: TcSYL_0012330.t1-p1
OG0011444: TcSYL_0012350.t1-p1
OG0011445: TcSYL_0012360.t1-p1
OG0011446: TcSYL_0012380.t1-p1
OG0011447: TcSYL_0012390.t1-p1
OG0011448: TcSYL_0012400.t1-p1
OG0011449: TcSYL_0012410.t1-p1
OG0011450: TcSYL_0012420.t1-p1
OG0011451: TcSYL_0012430.t1-p1
OG0011452: TcSYL_0012440.t1-p1
OG0011453: TcSYL_0012450.t1-p1
OG0011454: TcSYL_0012460.t1-p1
OG0011455: TcSYL_0012470.t1-p1
OG0011456: TcSYL_0012480.t1-p1
OG0011457: TcSYL_0012510.t1-p1
OG0011458: TcSYL_0012520.t1-p1
OG0011459: TcSYL_0012540.t1-p1
OG0011460: TcSYL_0012550.t1-p1
OG0011461: TcSYL_0012560.t1-p1
OG0011462: TcSYL_0012580.t1-p1
OG0011463: TcSYL_0012590.t1-p1
OG0011464: TcSYL_0012610.t1-p1
OG0011465: TcSYL_0012620.t1-p1
OG0011466: TcSYL_0012640.t1-p1
OG0011467: TcSYL_0012650.t1-p1
OG0011468: TcSYL_0012660.t1-p1
OG0011469: TcSYL_0012670.t1-p1
OG0011470: TcSYL_0012680.t1-p1
OG0011471: TcSYL_0012690.t1-p1
OG0011472: TcSYL_0012700.t1-p1
OG0011473: TcSYL_0012710.t1-p1
OG0011474: TcSYL_0012720.t1-p1
OG0011475: TcSYL_0012730.t1-p1
OG0011476: TcSYL_0012740.t1-p1
OG0011477: TcSYL_0012750.t1-p1
OG0011478: TcSYL_0012760.t1-p1
OG0011479: TcSYL_0012770.t1-p1
OG0011480: TcSYL_0012780.t1-p1
OG0011481: TcSYL_0012800.t1-p1
OG0011482: TcSYL_0012810.t1-p1

OG0011483: TcSYL_0012820.t1-p1
OG0011484: TcSYL_0012830.t1-p1
OG0011485: TcSYL_0012840.t1-p1
OG0011486: TcSYL_0012850.t1-p1
OG0011487: TcSYL_0012860.t1-p1
OG0011488: TcSYL_0012870.t1-p1
OG0011489: TcSYL_0012880.t1-p1
OG0011490: TcSYL_0012890.t1-p1
OG0011491: TcSYL_0012910.t1-p1
OG0011492: TcSYL_0012920.t1-p1
OG0011493: TcSYL_0012930.t1-p1
OG0011494: TcSYL_0012940.t1-p1
OG0011495: TcSYL_0012960.t1-p1
OG0011496: TcSYL_0012970.t1-p1
OG0011497: TcSYL_0012980.t1-p1
OG0011498: TcSYL_0012990.t1-p1
OG0011499: TcSYL_0013010.t1-p1
OG0011500: TcSYL_0013020.t1-p1
OG0011501: TcSYL_0013030.t1-p1
OG0011502: TcSYL_0013040.t1-p1
OG0011503: TcSYL_0013050.t1-p1
OG0011504: TcSYL_0013060.t1-p1
OG0011505: TcSYL_0013080.t1-p1
OG0011506: TcSYL_0013090.t1-p1
OG0011507: TcSYL_0013110.t1-p1
OG0011508: TcSYL_0013130.t1-p1
OG0011509: TcSYL_0013140.t1-p1
OG0011510: TcSYL_0013150.t1-p1
OG0011511: TcSYL_0013160.t1-p1
OG0011512: TcSYL_0013170.t1-p1
OG0011513: TcSYL_0013190.t1-p1
OG0011514: TcSYL_0013200.t1-p1
OG0011515: TcSYL_0013210.t1-p1
OG0011516: TcSYL_0013220.t1-p1
OG0011517: TcSYL_0013230.t1-p1
OG0011518: TcSYL_0013250.t1-p1
OG0011519: TcSYL_0013260.t1-p1
OG0011520: TcSYL_0013270.t1-p1
OG0011521: TcSYL_0013280.t1-p1
OG0011522: TcSYL_0013290.t1-p1
OG0011523: TcSYL_0013300.t1-p1
OG0011524: TcSYL_0013310.t1-p1
OG0011525: TcSYL_0013320.t1-p1
OG0011526: TcSYL_0013330.t1-p1
OG0011527: TcSYL_0013350.t1-p1
OG0011528: TcSYL_0013360.t1-p1
OG0011529: TcSYL_0013370.t1-p1
OG0011530: TcSYL_0013390.t1-p1
OG0011531: TcSYL_0013410.t1-p1
OG0011532: TcSYL_0013420.t1-p1
OG0011533: TcSYL_0013520.t1-p1
OG0011534: TcSYL_0013540.t1-p1
OG0011535: TcSYL_0013570.t1-p1
OG0011536: TcSYL_0013640.t1-p1

OG0011537: TcSYL_0013700.t1-p1
OG0011538: TcSYL_0013710.t1-p1
OG0011539: TcSYL_0013740.t1-p1
OG0011540: TcSYL_0013770.t1-p1
OG0011541: TcSYL_0013820.t1-p1
OG0011542: TcSYL_0014020.t1-p1
OG0011543: TcSYL_0014110.t1-p1
OG0011544: TcSYL_0014120.t1-p1
OG0011545: TcSYL_0014160.t1-p1
OG0011546: TcSYL_0014170.t1-p1
OG0011547: TcSYL_0014180.t1-p1
OG0011548: TcSYL_0014190.t1-p1
OG0011549: TcSYL_0014240.t1-p1
OG0011550: TcSYL_0014250.t1-p1
OG0011551: TcSYL_0014300.t1-p1
OG0011552: TcSYL_0014320.t1-p1
OG0011553: TcSYL_0014330.t1-p1
OG0011554: TcSYL_0014340.t1-p1
OG0011555: TcSYL_0014350.t1-p1
OG0011556: TcSYL_0014360.t1-p1
OG0011557: TcSYL_0014370.t1-p1
OG0011558: TcSYL_0014380.t1-p1
OG0011559: TcSYL_0014420.t1-p1
OG0011560: TcSYL_0014430.t1-p1
OG0011561: TcSYL_0014440.t1-p1
OG0011562: TcSYL_0014450.t1-p1
OG0011563: TcSYL_0014490.t1-p1
OG0011564: TcSYL_0014520.t1-p1
OG0011565: TcSYL_0014530.t1-p1
OG0011566: TcSYL_0014570.t1-p1
OG0011567: TcSYL_0014660.t1-p1
OG0011568: TcSYL_0014670.t1-p1
OG0011569: TcSYL_0014680.t1-p1
OG0011570: TcSYL_0014770.t1-p1
OG0011571: TcSYL_0014820.t1-p1
OG0011572: TcSYL_0014850.t1-p1
OG0011573: TcSYL_0014890.t1-p1
OG0011574: TcSYL_0014970.t1-p1
OG0011575: TcSYL_0015090.t1-p1
OG0011576: TcSYL_0015100.t1-p1
OG0011577: TcSYL_0015110.t1-p1
OG0011578: TcSYL_0015120.t1-p1
OG0011579: TcSYL_0015130.t1-p1
OG0011580: TcSYL_0015150.t1-p1
OG0011581: TcSYL_0015160.t1-p1
OG0011582: TcSYL_0015180.t1-p1
OG0011583: TcSYL_0015190.t1-p1
OG0011584: TcSYL_0015200.t1-p1
OG0011585: TcSYL_0015270.t1-p1
OG0011586: TcSYL_0015320.t1-p1
OG0011587: TcSYL_0015330.t1-p1
OG0011588: TcSYL_0015370.t1-p1
OG0011589: TcSYL_0015380.t1-p1
OG0011590: TcSYL_0015400.t1-p1

OG0011591: TcSYL_0015410.t1-p1
OG0011592: TcSYL_0015420.t1-p1
OG0011593: TcSYL_0015430.t1-p1
OG0011594: TcSYL_0015440.t1-p1
OG0011595: TcSYL_0015450.t1-p1
OG0011596: TcSYL_0015460.t1-p1
OG0011597: TcSYL_0015480.t1-p1
OG0011598: TcSYL_0015490.t1-p1
OG0011599: TcSYL_0015510.t1-p1
OG0011600: TcSYL_0015520.t1-p1
OG0011601: TcSYL_0015530.t1-p1
OG0011602: TcSYL_0015540.t1-p1
OG0011603: TcSYL_0015560.t1-p1
OG0011604: TcSYL_0015570.t1-p1
OG0011605: TcSYL_0015580.t1-p1
OG0011606: TcSYL_0015590.t1-p1
OG0011607: TcSYL_0015600.t1-p1
OG0011608: TcSYL_0015620.t1-p1
OG0011609: TcSYL_0015630.t1-p1
OG0011610: TcSYL_0015640.t1-p1
OG0011611: TcSYL_0015650.t1-p1
OG0011612: TcSYL_0015660.t1-p1
OG0011613: TcSYL_0015670.t1-p1
OG0011614: TcSYL_0015690.t1-p1
OG0011615: TcSYL_0015700.t1-p1
OG0011616: TcSYL_0015720.t1-p1
OG0011617: TcSYL_0015730.t1-p1
OG0011618: TcSYL_0015740.t1-p1
OG0011619: TcSYL_0015760.t1-p1
OG0011620: TcSYL_0015770.t1-p1
OG0011621: TcSYL_0015780.t1-p1
OG0011622: TcSYL_0015810.t1-p1
OG0011623: TcSYL_0015820.t1-p1
OG0011624: TcSYL_0015840.t1-p1
OG0011625: TcSYL_0015850.t1-p1
OG0011626: TcSYL_0015860.t1-p1
OG0011627: TcSYL_0015870.t1-p1
OG0011628: TcSYL_0015890.t1-p1
OG0011629: TcSYL_0015900.t1-p1
OG0011630: TcSYL_0015910.t1-p1
OG0011631: TcSYL_0015920.t1-p1
OG0011632: TcSYL_0015930.t1-p1
OG0011633: TcSYL_0015940.t1-p1
OG0011634: TcSYL_0015950.t1-p1
OG0011635: TcSYL_0015960.t1-p1
OG0011636: TcSYL_0015970.t1-p1
OG0011637: TcSYL_0015980.t1-p1
OG0011638: TcSYL_0015990.t1-p1
OG0011639: TcSYL_0016010.t1-p1
OG0011640: TcSYL_0016020.t1-p1
OG0011641: TcSYL_0016030.t1-p1
OG0011642: TcSYL_0016050.t1-p1
OG0011643: TcSYL_0016060.t1-p1
OG0011644: TcSYL_0016100.t1-p1

OG0011645: TcSYL_0016110.t1-p1
OG0011646: TcSYL_0016120.t1-p1
OG0011647: TcSYL_0016150.t1-p1
OG0011648: TcSYL_0016160.t1-p1
OG0011649: TcSYL_0016190.t1-p1
OG0011650: TcSYL_0016200.t1-p1
OG0011651: TcSYL_0016220.t1-p1
OG0011652: TcSYL_0016230.t1-p1
OG0011653: TcSYL_0016240.t1-p1
OG0011654: TcSYL_0016250.t1-p1
OG0011655: TcSYL_0016260.t1-p1
OG0011656: TcSYL_0016270.t1-p1
OG0011657: TcSYL_0016280.t1-p1
OG0011658: TcSYL_0016300.t1-p1
OG0011659: TcSYL_0016310.t1-p1
OG0011660: TcSYL_0016320.t1-p1
OG0011661: TcSYL_0016340.t1-p1
OG0011662: TcSYL_0016370.t1-p1
OG0011663: TcSYL_0016380.t1-p1
OG0011664: TcSYL_0016390.t1-p1
OG0011665: TcSYL_0016400.t1-p1
OG0011666: TcSYL_0016410.t1-p1
OG0011667: TcSYL_0016420.t1-p1
OG0011668: TcSYL_0016430.t1-p1
OG0011669: TcSYL_0016440.t1-p1
OG0011670: TcSYL_0016450.t1-p1
OG0011671: TcSYL_0016460.t1-p1
OG0011672: TcSYL_0016470.t1-p1
OG0011673: TcSYL_0016480.t1-p1
OG0011674: TcSYL_0016490.t1-p1
OG0011675: TcSYL_0016500.t1-p1
OG0011676: TcSYL_0016510.t1-p1
OG0011677: TcSYL_0016520.t1-p1
OG0011678: TcSYL_0016530.t1-p1
OG0011679: TcSYL_0016540.t1-p1
OG0011680: TcSYL_0016550.t1-p1
OG0011681: TcSYL_0016560.t1-p1
OG0011682: TcSYL_0016590.t1-p1
OG0011683: TcSYL_0016600.t1-p1
OG0011684: TcSYL_0016610.t1-p1
OG0011685: TcSYL_0016620.t1-p1
OG0011686: TcSYL_0016630.t1-p1
OG0011687: TcSYL_0016640.t1-p1
OG0011688: TcSYL_0016650.t1-p1
OG0011689: TcSYL_0016660.t1-p1
OG0011690: TcSYL_0016670.t1-p1
OG0011691: TcSYL_0016690.t1-p1
OG0011692: TcSYL_0016700.t1-p1
OG0011693: TcSYL_0016710.t1-p1
OG0011694: TcSYL_0016730.t1-p1
OG0011695: TcSYL_0016750.t1-p1
OG0011696: TcSYL_0016760.t1-p1
OG0011697: TcSYL_0016770.t1-p1
OG0011698: TcSYL_0016780.t1-p1

OG0011699: TcSYL_0016790.t1-p1
OG0011700: TcSYL_0016800.t1-p1
OG0011701: TcSYL_0016810.t1-p1
OG0011702: TcSYL_0016820.t1-p1
OG0011703: TcSYL_0016830.t1-p1
OG0011704: TcSYL_0016840.t1-p1
OG0011705: TcSYL_0016860.t1-p1
OG0011706: TcSYL_0016870.t1-p1
OG0011707: TcSYL_0016890.t1-p1
OG0011708: TcSYL_0016900.t1-p1
OG0011709: TcSYL_0016910.t1-p1
OG0011710: TcSYL_0016920.t1-p1
OG0011711: TcSYL_0016950.t1-p1
OG0011712: TcSYL_0016960.t1-p1
OG0011713: TcSYL_0016970.t1-p1
OG0011714: TcSYL_0016980.t1-p1
OG0011715: TcSYL_0017020.t1-p1
OG0011716: TcSYL_0017030.t1-p1
OG0011717: TcSYL_0017070.t1-p1
OG0011718: TcSYL_0017080.t1-p1
OG0011719: TcSYL_0017100.t1-p1
OG0011720: TcSYL_0017130.t1-p1
OG0011721: TcSYL_0017150.t1-p1
OG0011722: TcSYL_0017170.t1-p1
OG0011723: TcSYL_0017190.t1-p1
OG0011724: TcSYL_0017200.t1-p1
OG0011725: TcSYL_0017220.t1-p1
OG0011726: TcSYL_0017230.t1-p1
OG0011727: TcSYL_0017250.t1-p1
OG0011728: TcSYL_0017260.t1-p1
OG0011729: TcSYL_0017270.t1-p1
OG0011730: TcSYL_0017300.t1-p1
OG0011731: TcSYL_0017320.t1-p1
OG0011732: TcSYL_0017350.t1-p1
OG0011733: TcSYL_0017360.t1-p1
OG0011734: TcSYL_0017370.t1-p1
OG0011735: TcSYL_0017380.t1-p1
OG0011736: TcSYL_0017390.t1-p1
OG0011737: TcSYL_0017410.t1-p1
OG0011738: TcSYL_0017420.t1-p1
OG0011739: TcSYL_0017430.t1-p1
OG0011740: TcSYL_0017440.t1-p1
OG0011741: TcSYL_0017450.t1-p1
OG0011742: TcSYL_0017460.t1-p1
OG0011743: TcSYL_0017470.t1-p1
OG0011744: TcSYL_0017480.t1-p1
OG0011745: TcSYL_0017500.t1-p1
OG0011746: TcSYL_0017520.t1-p1
OG0011747: TcSYL_0017530.t1-p1
OG0011748: TcSYL_0017540.t1-p1
OG0011749: TcSYL_0017550.t1-p1
OG0011750: TcSYL_0017560.t1-p1
OG0011751: TcSYL_0017570.t1-p1
OG0011752: TcSYL_0017580.t1-p1

OG0011753: TcSYL_0017620.t1-p1
OG0011754: TcSYL_0017630.t1-p1
OG0011755: TcSYL_0017650.t1-p1
OG0011756: TcSYL_0017660.t1-p1
OG0011757: TcSYL_0017670.t1-p1
OG0011758: TcSYL_0017680.t1-p1
OG0011759: TcSYL_0017690.t1-p1
OG0011760: TcSYL_0017700.t1-p1
OG0011761: TcSYL_0017710.t1-p1
OG0011762: TcSYL_0017720.t1-p1
OG0011763: TcSYL_0017730.t1-p1
OG0011764: TcSYL_0017750.t1-p1
OG0011765: TcSYL_0017770.t1-p1
OG0011766: TcSYL_0017780.t1-p1
OG0011767: TcSYL_0017790.t1-p1
OG0011768: TcSYL_0017820.t1-p1
OG0011769: TcSYL_0017830.t1-p1
OG0011770: TcSYL_0017840.t1-p1
OG0011771: TcSYL_0017850.t1-p1
OG0011772: TcSYL_0017900.t1-p1
OG0011773: TcSYL_0017920.t1-p1
OG0011774: TcSYL_0017930.t1-p1
OG0011775: TcSYL_0017950.t1-p1
OG0011776: TcSYL_0017960.t1-p1
OG0011777: TcSYL_0017970.t1-p1
OG0011778: TcSYL_0017980.t1-p1
OG0011779: TcSYL_0018000.t1-p1
OG0011780: TcSYL_0018010.t1-p1
OG0011781: TcSYL_0018020.t1-p1
OG0011782: TcSYL_0018030.t1-p1
OG0011783: TcSYL_0018040.t1-p1
OG0011784: TcSYL_0018050.t1-p1
OG0011785: TcSYL_0018060.t1-p1
OG0011786: TcSYL_0018070.t1-p1
OG0011787: TcSYL_0018100.t1-p1
OG0011788: TcSYL_0018110.t1-p1
OG0011789: TcSYL_0018120.t1-p1
OG0011790: TcSYL_0018130.t1-p1
OG0011791: TcSYL_0018140.t1-p1
OG0011792: TcSYL_0018160.t1-p1
OG0011793: TcSYL_0018190.t1-p1
OG0011794: TcSYL_0018200.t1-p1
OG0011795: TcSYL_0018210.t1-p1
OG0011796: TcSYL_0018220.t1-p1
OG0011797: TcSYL_0018230.t1-p1
OG0011798: TcSYL_0018250.t1-p1
OG0011799: TcSYL_0018260.t1-p1
OG0011800: TcSYL_0018270.t1-p1
OG0011801: TcSYL_0018300.t1-p1
OG0011802: TcSYL_0018310.t1-p1
OG0011803: TcSYL_0018340.t1-p1
OG0011804: TcSYL_0018350.t1-p1
OG0011805: TcSYL_0018360.t1-p1
OG0011806: TcSYL_0018370.t1-p1

OG0011807: TcSYL_0018380.t1-p1
OG0011808: TcSYL_0018400.t1-p1
OG0011809: TcSYL_0018430.t1-p1
OG0011810: TcSYL_0018460.t1-p1
OG0011811: TcSYL_0018470.t1-p1
OG0011812: TcSYL_0018480.t1-p1
OG0011813: TcSYL_0018500.t1-p1
OG0011814: TcSYL_0018570.t1-p1
OG0011815: TcSYL_0018580.t1-p1
OG0011816: TcSYL_0018590.t1-p1
OG0011817: TcSYL_0018610.t1-p1
OG0011818: TcSYL_0018640.t1-p1
OG0011819: TcSYL_0018650.t1-p1
OG0011820: TcSYL_0018660.t1-p1
OG0011821: TcSYL_0018670.t1-p1
OG0011822: TcSYL_0018680.t1-p1
OG0011823: TcSYL_0018690.t1-p1
OG0011824: TcSYL_0018700.t1-p1
OG0011825: TcSYL_0018710.t1-p1
OG0011826: TcSYL_0018730.t1-p1
OG0011827: TcSYL_0018740.t1-p1
OG0011828: TcSYL_0018770.t1-p1
OG0011829: TcSYL_0018790.t1-p1
OG0011830: TcSYL_0018800.t1-p1
OG0011831: TcSYL_0018830.t1-p1
OG0011832: TcSYL_0018840.t1-p1
OG0011833: TcSYL_0018870.t1-p1
OG0011834: TcSYL_0018900.t1-p1
OG0011835: TcSYL_0018920.t1-p1
OG0011836: TcSYL_0018940.t1-p1
OG0011837: TcSYL_0018950.t1-p1
OG0011838: TcSYL_0018970.t1-p1
OG0011839: TcSYL_0018990.t1-p1
OG0011840: TcSYL_0019000.t1-p1
OG0011841: TcSYL_0019010.t1-p1
OG0011842: TcSYL_0019030.t1-p1
OG0011843: TcSYL_0019060.t1-p1
OG0011844: TcSYL_0019070.t1-p1
OG0011845: TcSYL_0019080.t1-p1
OG0011846: TcSYL_0019090.t1-p1
OG0011847: TcSYL_0019110.t1-p1
OG0011848: TcSYL_0019160.t1-p1
OG0011849: TcSYL_0019170.t1-p1
OG0011850: TcSYL_0019180.t1-p1
OG0011851: TcSYL_0019190.t1-p1
OG0011852: TcSYL_0019200.t1-p1
OG0011853: TcSYL_0019250.t1-p1
OG0011854: TcSYL_0019400.t1-p1
OG0011855: TcSYL_0019420.t1-p1
OG0011856: TcSYL_0019440.t1-p1
OG0011857: TcSYL_0019510.t1-p1
OG0011858: TcSYL_0019590.t1-p1
OG0011859: TcSYL_0019750.t1-p1
OG0011860: TcSYL_0019780.t1-p1

OG0011861: TcSYL_0019820.t1-p1
OG0011862: TcSYL_0019880.t1-p1
OG0011863: TcSYL_0019890.t1-p1
OG0011864: TcSYL_0019900.t1-p1
OG0011865: TcSYL_0019910.t1-p1
OG0011866: TcSYL_0019930.t1-p1
OG0011867: TcSYL_0019940.t1-p1
OG0011868: TcSYL_0019950.t1-p1
OG0011869: TcSYL_0019960.t1-p1
OG0011870: TcSYL_0019970.t1-p1
OG0011871: TcSYL_0019980.t1-p1
OG0011872: TcSYL_0019990.t1-p1
OG0011873: TcSYL_0020000.t1-p1
OG0011874: TcSYL_0020010.t1-p1
OG0011875: TcSYL_0020020.t1-p1
OG0011876: TcSYL_0020040.t1-p1
OG0011877: TcSYL_0020050.t1-p1
OG0011878: TcSYL_0020060.t1-p1
OG0011879: TcSYL_0020070.t1-p1
OG0011880: TcSYL_0020080.t1-p1
OG0011881: TcSYL_0020090.t1-p1
OG0011882: TcSYL_0020110.t1-p1
OG0011883: TcSYL_0020120.t1-p1
OG0011884: TcSYL_0020150.t1-p1
OG0011885: TcSYL_0020160.t1-p1
OG0011886: TcSYL_0020170.t1-p1
OG0011887: TcSYL_0020180.t1-p1
OG0011888: TcSYL_0020190.t1-p1
OG0011889: TcSYL_0020200.t1-p1
OG0011890: TcSYL_0020210.t1-p1
OG0011891: TcSYL_0020220.t1-p1
OG0011892: TcSYL_0020230.t1-p1
OG0011893: TcSYL_0020240.t1-p1
OG0011894: TcSYL_0020250.t1-p1
OG0011895: TcSYL_0020260.t1-p1
OG0011896: TcSYL_0020270.t1-p1
OG0011897: TcSYL_0020280.t1-p1
OG0011898: TcSYL_0020290.t1-p1
OG0011899: TcSYL_0020300.t1-p1
OG0011900: TcSYL_0020310.t1-p1
OG0011901: TcSYL_0020320.t1-p1
OG0011902: TcSYL_0020330.t1-p1
OG0011903: TcSYL_0020340.t1-p1
OG0011904: TcSYL_0020350.t1-p1
OG0011905: TcSYL_0020360.t1-p1
OG0011906: TcSYL_0020370.t1-p1
OG0011907: TcSYL_0020380.t1-p1
OG0011908: TcSYL_0020400.t1-p1
OG0011909: TcSYL_0020410.t1-p1
OG0011910: TcSYL_0020420.t1-p1
OG0011911: TcSYL_0020430.t1-p1
OG0011912: TcSYL_0020450.t1-p1
OG0011913: TcSYL_0020460.t1-p1
OG0011914: TcSYL_0020470.t1-p1

OG0011915: TcSYL_0020480.t1-p1
OG0011916: TcSYL_0020490.t1-p1
OG0011917: TcSYL_0020500.t1-p1
OG0011918: TcSYL_0020510.t1-p1
OG0011919: TcSYL_0020520.t1-p1
OG0011920: TcSYL_0020540.t1-p1
OG0011921: TcSYL_0020550.t1-p1
OG0011922: TcSYL_0020570.t1-p1
OG0011923: TcSYL_0020600.t1-p1
OG0011924: TcSYL_0020610.t1-p1
OG0011925: TcSYL_0020620.t1-p1
OG0011926: TcSYL_0020630.t1-p1
OG0011927: TcSYL_0020650.t1-p1
OG0011928: TcSYL_0020660.t1-p1
OG0011929: TcSYL_0020680.t1-p1
OG0011930: TcSYL_0020690.t1-p1
OG0011931: TcSYL_0020700.t1-p1
OG0011932: TcSYL_0020710.t1-p1
OG0011933: TcSYL_0020730.t1-p1
OG0011934: TcSYL_0020750.t1-p1
OG0011935: TcSYL_0020760.t1-p1
OG0011936: TcSYL_0020770.t1-p1
OG0011937: TcSYL_0020780.t1-p1
OG0011938: TcSYL_0020790.t1-p1
OG0011939: TcSYL_0020800.t1-p1
OG0011940: TcSYL_0020810.t1-p1
OG0011941: TcSYL_0020830.t1-p1
OG0011942: TcSYL_0020840.t1-p1
OG0011943: TcSYL_0020860.t1-p1
OG0011944: TcSYL_0020870.t1-p1
OG0011945: TcSYL_0020880.t1-p1
OG0011946: TcSYL_0020900.t1-p1
OG0011947: TcSYL_0020930.t1-p1
OG0011948: TcSYL_0020950.t1-p1
OG0011949: TcSYL_0020960.t1-p1
OG0011950: TcSYL_0020990.t1-p1
OG0011951: TcSYL_0021000.t1-p1
OG0011952: TcSYL_0021010.t1-p1
OG0011953: TcSYL_0021020.t1-p1
OG0011954: TcSYL_0021030.t1-p1
OG0011955: TcSYL_0021040.t1-p1
OG0011956: TcSYL_0021060.t1-p1
OG0011957: TcSYL_0021070.t1-p1
OG0011958: TcSYL_0021080.t1-p1
OG0011959: TcSYL_0021090.t1-p1
OG0011960: TcSYL_0021100.t1-p1
OG0011961: TcSYL_0021110.t1-p1
OG0011962: TcSYL_0021120.t1-p1
OG0011963: TcSYL_0021130.t1-p1
OG0011964: TcSYL_0021140.t1-p1
OG0011965: TcSYL_0021150.t1-p1
OG0011966: TcSYL_0021160.t1-p1
OG0011967: TcSYL_0021170.t1-p1
OG0011968: TcSYL_0021180.t1-p1

OG0011969: TcSYL_0021190.t1-p1
OG0011970: TcSYL_0021200.t1-p1
OG0011971: TcSYL_0021220.t1-p1
OG0011972: TcSYL_0021240.t1-p1
OG0011973: TcSYL_0021270.t1-p1
OG0011974: TcSYL_0021280.t1-p1
OG0011975: TcSYL_0021290.t1-p1
OG0011976: TcSYL_0021300.t1-p1
OG0011977: TcSYL_0021310.t1-p1
OG0011978: TcSYL_0021320.t1-p1
OG0011979: TcSYL_0021340.t1-p1
OG0011980: TcSYL_0021350.t1-p1
OG0011981: TcSYL_0021360.t1-p1
OG0011982: TcSYL_0021380.t1-p1
OG0011983: TcSYL_0021390.t1-p1
OG0011984: TcSYL_0021410.t1-p1
OG0011985: TcSYL_0021430.t1-p1
OG0011986: TcSYL_0021440.t1-p1
OG0011987: TcSYL_0021450.t1-p1
OG0011988: TcSYL_0021460.t1-p1
OG0011989: TcSYL_0021470.t1-p1
OG0011990: TcSYL_0021480.t1-p1
OG0011991: TcSYL_0021490.t1-p1
OG0011992: TcSYL_0021500.t1-p1
OG0011993: TcSYL_0021510.t1-p1
OG0011994: TcSYL_0021520.t1-p1
OG0011995: TcSYL_0021530.t1-p1
OG0011996: TcSYL_0021540.t1-p1
OG0011997: TcSYL_0021550.t1-p1
OG0011998: TcSYL_0021560.t1-p1
OG0011999: TcSYL_0021570.t1-p1
OG0012000: TcSYL_0021580.t1-p1
OG0012001: TcSYL_0021590.t1-p1
OG0012002: TcSYL_0021600.t1-p1
OG0012003: TcSYL_0021610.t1-p1
OG0012004: TcSYL_0021620.t1-p1
OG0012005: TcSYL_0021630.t1-p1
OG0012006: TcSYL_0021640.t1-p1
OG0012007: TcSYL_0021650.t1-p1
OG0012008: TcSYL_0021660.t1-p1
OG0012009: TcSYL_0021670.t1-p1
OG0012010: TcSYL_0021680.t1-p1
OG0012011: TcSYL_0021690.t1-p1
OG0012012: TcSYL_0021700.t1-p1
OG0012013: TcSYL_0021710.t1-p1
OG0012014: TcSYL_0021720.t1-p1
OG0012015: TcSYL_0021730.t1-p1
OG0012016: TcSYL_0021740.t1-p1
OG0012017: TcSYL_0021750.t1-p1
OG0012018: TcSYL_0021760.t1-p1
OG0012019: TcSYL_0021770.t1-p1
OG0012020: TcSYL_0021780.t1-p1
OG0012021: TcSYL_0021790.t1-p1
OG0012022: TcSYL_0021800.t1-p1

OG0012023: TcSYL_0021810.t1-p1
OG0012024: TcSYL_0021830.t1-p1
OG0012025: TcSYL_0021840.t1-p1
OG0012026: TcSYL_0021860.t1-p1
OG0012027: TcSYL_0021870.t1-p1
OG0012028: TcSYL_0021880.t1-p1
OG0012029: TcSYL_0021890.t1-p1
OG0012030: TcSYL_0021900.t1-p1
OG0012031: TcSYL_0021910.t1-p1
OG0012032: TcSYL_0021960.t1-p1
OG0012033: TcSYL_0021970.t1-p1
OG0012034: TcSYL_0021980.t1-p1
OG0012035: TcSYL_0022000.t1-p1
OG0012036: TcSYL_0022040.t1-p1
OG0012037: TcSYL_0022050.t1-p1
OG0012038: TcSYL_0022060.t1-p1
OG0012039: TcSYL_0022070.t1-p1
OG0012040: TcSYL_0022090.t1-p1
OG0012041: TcSYL_0022100.t1-p1
OG0012042: TcSYL_0022110.t1-p1
OG0012043: TcSYL_0022120.t1-p1
OG0012044: TcSYL_0022140.t1-p1
OG0012045: TcSYL_0022160.t1-p1
OG0012046: TcSYL_0022170.t1-p1
OG0012047: TcSYL_0022190.t1-p1
OG0012048: TcSYL_0022200.t1-p1
OG0012049: TcSYL_0022210.t1-p1
OG0012050: TcSYL_0022220.t1-p1
OG0012051: TcSYL_0022280.t1-p1
OG0012052: TcSYL_0022300.t1-p1
OG0012053: TcSYL_0022360.t1-p1
OG0012054: TcSYL_0022370.t1-p1
OG0012055: TcSYL_0022390.t1-p1
OG0012056: TcSYL_0022410.t1-p1
OG0012057: TcSYL_0022430.t1-p1
OG0012058: TcSYL_0022440.t1-p1
OG0012059: TcSYL_0022450.t1-p1
OG0012060: TcSYL_0022460.t1-p1
OG0012061: TcSYL_0022470.t1-p1
OG0012062: TcSYL_0022500.t1-p1
OG0012063: TcSYL_0022510.t1-p1
OG0012064: TcSYL_0022530.t1-p1
OG0012065: TcSYL_0022550.t1-p1
OG0012066: TcSYL_0022680.t1-p1
OG0012067: TcSYL_0022730.t1-p1
OG0012068: TcSYL_0022770.t1-p1
OG0012069: TcSYL_0022820.t1-p1
OG0012070: TcSYL_0022920.t1-p1
OG0012071: TcSYL_0022930.t1-p1
OG0012072: TcSYL_0022950.t1-p1
OG0012073: TcSYL_0022970.t1-p1
OG0012074: TcSYL_0022990.t1-p1
OG0012075: TcSYL_0023140.t1-p1
OG0012076: TcSYL_0023170.t1-p1

OG0012077: TcSYL_0023190.t1-p1
OG0012078: TcSYL_0023330.t1-p1
OG0012079: TcSYL_0023340.t1-p1
OG0012080: TcSYL_0023460.t1-p1
OG0012081: TcSYL_0023470.t1-p1
OG0012082: TcSYL_0023520.t1-p1
OG0012083: TcSYL_0023530.t1-p1
OG0012084: TcSYL_0023540.t1-p1
OG0012085: TcSYL_0023550.t1-p1
OG0012086: TcSYL_0023560.t1-p1
OG0012087: TcSYL_0023570.t1-p1
OG0012088: TcSYL_0023580.t1-p1
OG0012089: TcSYL_0023590.t1-p1
OG0012090: TcSYL_0023610.t1-p1
OG0012091: TcSYL_0023620.t1-p1
OG0012092: TcSYL_0023630.t1-p1
OG0012093: TcSYL_0023640.t1-p1
OG0012094: TcSYL_0023650.t1-p1
OG0012095: TcSYL_0023660.t1-p1
OG0012096: TcSYL_0023680.t1-p1
OG0012097: TcSYL_0023710.t1-p1
OG0012098: TcSYL_0023730.t1-p1
OG0012099: TcSYL_0023740.t1-p1
OG0012100: TcSYL_0023750.t1-p1
OG0012101: TcSYL_0023760.t1-p1
OG0012102: TcSYL_0023770.t1-p1
OG0012103: TcSYL_0023780.t1-p1
OG0012104: TcSYL_0023790.t1-p1
OG0012105: TcSYL_0023810.t1-p1
OG0012106: TcSYL_0023820.t1-p1
OG0012107: TcSYL_0023830.t1-p1
OG0012108: TcSYL_0023840.t1-p1
OG0012109: TcSYL_0023850.t1-p1
OG0012110: TcSYL_0023860.t1-p1
OG0012111: TcSYL_0023870.t1-p1
OG0012112: TcSYL_0023880.t1-p1
OG0012113: TcSYL_0023900.t1-p1
OG0012114: TcSYL_0023910.t1-p1
OG0012115: TcSYL_0023930.t1-p1
OG0012116: TcSYL_0023950.t1-p1
OG0012117: TcSYL_0023960.t1-p1
OG0012118: TcSYL_0023970.t1-p1
OG0012119: TcSYL_0023980.t1-p1
OG0012120: TcSYL_0023990.t1-p1
OG0012121: TcSYL_0024000.t1-p1
OG0012122: TcSYL_0024010.t1-p1
OG0012123: TcSYL_0024020.t1-p1
OG0012124: TcSYL_0024030.t1-p1
OG0012125: TcSYL_0024060.t1-p1
OG0012126: TcSYL_0024070.t1-p1
OG0012127: TcSYL_0024080.t1-p1
OG0012128: TcSYL_0024090.t1-p1
OG0012129: TcSYL_0024100.t1-p1
OG0012130: TcSYL_0024120.t1-p1

OG0012131: TcSYL_0024130.t1-p1
OG0012132: TcSYL_0024140.t1-p1
OG0012133: TcSYL_0024170.t1-p1
OG0012134: TcSYL_0024180.t1-p1
OG0012135: TcSYL_0024190.t1-p1
OG0012136: TcSYL_0024200.t1-p1
OG0012137: TcSYL_0024210.t1-p1
OG0012138: TcSYL_0024220.t1-p1
OG0012139: TcSYL_0024230.t1-p1
OG0012140: TcSYL_0024240.t1-p1
OG0012141: TcSYL_0024250.t1-p1
OG0012142: TcSYL_0024280.t1-p1
OG0012143: TcSYL_0024290.t1-p1
OG0012144: TcSYL_0024310.t1-p1
OG0012145: TcSYL_0024320.t1-p1
OG0012146: TcSYL_0024330.t1-p1
OG0012147: TcSYL_0024340.t1-p1
OG0012148: TcSYL_0024350.t1-p1
OG0012149: TcSYL_0024360.t1-p1
OG0012150: TcSYL_0024370.t1-p1
OG0012151: TcSYL_0024380.t1-p1
OG0012152: TcSYL_0024390.t1-p1
OG0012153: TcSYL_0024400.t1-p1
OG0012154: TcSYL_0024410.t1-p1
OG0012155: TcSYL_0024420.t1-p1
OG0012156: TcSYL_0024430.t1-p1
OG0012157: TcSYL_0024440.t1-p1
OG0012158: TcSYL_0024450.t1-p1
OG0012159: TcSYL_0024460.t1-p1
OG0012160: TcSYL_0024470.t1-p1
OG0012161: TcSYL_0024480.t1-p1
OG0012162: TcSYL_0024490.t1-p1
OG0012163: TcSYL_0024500.t1-p1
OG0012164: TcSYL_0024510.t1-p1
OG0012165: TcSYL_0024520.t1-p1
OG0012166: TcSYL_0024530.t1-p1
OG0012167: TcSYL_0024550.t1-p1
OG0012168: TcSYL_0024570.t1-p1
OG0012169: TcSYL_0024580.t1-p1
OG0012170: TcSYL_0024590.t1-p1
OG0012171: TcSYL_0024600.t1-p1
OG0012172: TcSYL_0024610.t1-p1
OG0012173: TcSYL_0024620.t1-p1
OG0012174: TcSYL_0024630.t1-p1
OG0012175: TcSYL_0024640.t1-p1
OG0012176: TcSYL_0024650.t1-p1
OG0012177: TcSYL_0024660.t1-p1
OG0012178: TcSYL_0024670.t1-p1
OG0012179: TcSYL_0024690.t1-p1
OG0012180: TcSYL_0024700.t1-p1
OG0012181: TcSYL_0024710.t1-p1
OG0012182: TcSYL_0024720.t1-p1
OG0012183: TcSYL_0024730.t1-p1
OG0012184: TcSYL_0024740.t1-p1

OG0012185: TcSYL_0024750.t1-p1
OG0012186: TcSYL_0024770.t1-p1
OG0012187: TcSYL_0024780.t1-p1
OG0012188: TcSYL_0024790.t1-p1
OG0012189: TcSYL_0024820.t1-p1
OG0012190: TcSYL_0024830.t1-p1
OG0012191: TcSYL_0024860.t1-p1
OG0012192: TcSYL_0024870.t1-p1
OG0012193: TcSYL_0024880.t1-p1
OG0012194: TcSYL_0024900.t1-p1
OG0012195: TcSYL_0024910.t1-p1
OG0012196: TcSYL_0024920.t1-p1
OG0012197: TcSYL_0024940.t1-p1
OG0012198: TcSYL_0024950.t1-p1
OG0012199: TcSYL_0024960.t1-p1
OG0012200: TcSYL_0024970.t1-p1
OG0012201: TcSYL_0024980.t1-p1
OG0012202: TcSYL_0024990.t1-p1
OG0012203: TcSYL_0025000.t1-p1
OG0012204: TcSYL_0025020.t1-p1
OG0012205: TcSYL_0025030.t1-p1
OG0012206: TcSYL_0025040.t1-p1
OG0012207: TcSYL_0025050.t1-p1
OG0012208: TcSYL_0025060.t1-p1
OG0012209: TcSYL_0025080.t1-p1
OG0012210: TcSYL_0025090.t1-p1
OG0012211: TcSYL_0025100.t1-p1
OG0012212: TcSYL_0025110.t1-p1
OG0012213: TcSYL_0025120.t1-p1
OG0012214: TcSYL_0025130.t1-p1
OG0012215: TcSYL_0025140.t1-p1
OG0012216: TcSYL_0025180.t1-p1
OG0012217: TcSYL_0025190.t1-p1
OG0012218: TcSYL_0025200.t1-p1
OG0012219: TcSYL_0025210.t1-p1
OG0012220: TcSYL_0025220.t1-p1
OG0012221: TcSYL_0025240.t1-p1
OG0012222: TcSYL_0025250.t1-p1
OG0012223: TcSYL_0025260.t1-p1
OG0012224: TcSYL_0025270.t1-p1
OG0012225: TcSYL_0025290.t1-p1
OG0012226: TcSYL_0025300.t1-p1
OG0012227: TcSYL_0025310.t1-p1
OG0012228: TcSYL_0025330.t1-p1
OG0012229: TcSYL_0025340.t1-p1
OG0012230: TcSYL_0025360.t1-p1
OG0012231: TcSYL_0025370.t1-p1
OG0012232: TcSYL_0025380.t1-p1
OG0012233: TcSYL_0025390.t1-p1
OG0012234: TcSYL_0025420.t1-p1
OG0012235: TcSYL_0025430.t1-p1
OG0012236: TcSYL_0025440.t1-p1
OG0012237: TcSYL_0025450.t1-p1
OG0012238: TcSYL_0025500.t1-p1

OG0012239: TcSYL_0025510.t1-p1
OG0012240: TcSYL_0025520.t1-p1
OG0012241: TcSYL_0025540.t1-p1
OG0012242: TcSYL_0025570.t1-p1
OG0012243: TcSYL_0025580.t1-p1
OG0012244: TcSYL_0025600.t1-p1
OG0012245: TcSYL_0025610.t1-p1
OG0012246: TcSYL_0025620.t1-p1
OG0012247: TcSYL_0025640.t1-p1
OG0012248: TcSYL_0025660.t1-p1
OG0012249: TcSYL_0025680.t1-p1
OG0012250: TcSYL_0025700.t1-p1
OG0012251: TcSYL_0025710.t1-p1
OG0012252: TcSYL_0025720.t1-p1
OG0012253: TcSYL_0025730.t1-p1
OG0012254: TcSYL_0025740.t1-p1
OG0012255: TcSYL_0025760.t1-p1
OG0012256: TcSYL_0025790.t1-p1
OG0012257: TcSYL_0025800.t1-p1
OG0012258: TcSYL_0025810.t1-p1
OG0012259: TcSYL_0025820.t1-p1
OG0012260: TcSYL_0025830.t1-p1
OG0012261: TcSYL_0025850.t1-p1
OG0012262: TcSYL_0025880.t1-p1
OG0012263: TcSYL_0025900.t1-p1
OG0012264: TcSYL_0025910.t1-p1
OG0012265: TcSYL_0025920.t1-p1
OG0012266: TcSYL_0025930.t1-p1
OG0012267: TcSYL_0025940.t1-p1
OG0012268: TcSYL_0025950.t1-p1
OG0012269: TcSYL_0025970.t1-p1
OG0012270: TcSYL_0025980.t1-p1
OG0012271: TcSYL_0026000.t1-p1
OG0012272: TcSYL_0026020.t1-p1
OG0012273: TcSYL_0026030.t1-p1
OG0012274: TcSYL_0026040.t1-p1
OG0012275: TcSYL_0026050.t1-p1
OG0012276: TcSYL_0026060.t1-p1
OG0012277: TcSYL_0026080.t1-p1
OG0012278: TcSYL_0026100.t1-p1
OG0012279: TcSYL_0026120.t1-p1
OG0012280: TcSYL_0026130.t1-p1
OG0012281: TcSYL_0026140.t1-p1
OG0012282: TcSYL_0026150.t1-p1
OG0012283: TcSYL_0026170.t1-p1
OG0012284: TcSYL_0026180.t1-p1
OG0012285: TcSYL_0026190.t1-p1
OG0012286: TcSYL_0026210.t1-p1
OG0012287: TcSYL_0026220.t1-p1
OG0012288: TcSYL_0026230.t1-p1
OG0012289: TcSYL_0026240.t1-p1
OG0012290: TcSYL_0026250.t1-p1
OG0012291: TcSYL_0026260.t1-p1
OG0012292: TcSYL_0026280.t1-p1

OG0012293: TcSYL_0026290.t1-p1
OG0012294: TcSYL_0026300.t1-p1
OG0012295: TcSYL_0026310.t1-p1
OG0012296: TcSYL_0026320.t1-p1
OG0012297: TcSYL_0026330.t1-p1
OG0012298: TcSYL_0026340.t1-p1
OG0012299: TcSYL_0026350.t1-p1
OG0012300: TcSYL_0026360.t1-p1
OG0012301: TcSYL_0026380.t1-p1
OG0012302: TcSYL_0026400.t1-p1
OG0012303: TcSYL_0026410.t1-p1
OG0012304: TcSYL_0026420.t1-p1
OG0012305: TcSYL_0026430.t1-p1
OG0012306: TcSYL_0026460.t1-p1
OG0012307: TcSYL_0026470.t1-p1
OG0012308: TcSYL_0026490.t1-p1
OG0012309: TcSYL_0026500.t1-p1
OG0012310: TcSYL_0026520.t1-p1
OG0012311: TcSYL_0026530.t1-p1
OG0012312: TcSYL_0026540.t1-p1
OG0012313: TcSYL_0026550.t1-p1
OG0012314: TcSYL_0026560.t1-p1
OG0012315: TcSYL_0026580.t1-p1
OG0012316: TcSYL_0026600.t1-p1
OG0012317: TcSYL_0026610.t1-p1
OG0012318: TcSYL_0026630.t1-p1
OG0012319: TcSYL_0026640.t1-p1
OG0012320: TcSYL_0026650.t1-p1
OG0012321: TcSYL_0026660.t1-p1
OG0012322: TcSYL_0026670.t1-p1
OG0012323: TcSYL_0026680.t1-p1
OG0012324: TcSYL_0026690.t1-p1
OG0012325: TcSYL_0026710.t1-p1
OG0012326: TcSYL_0026720.t1-p1
OG0012327: TcSYL_0026730.t1-p1
OG0012328: TcSYL_0026750.t1-p1
OG0012329: TcSYL_0026760.t1-p1
OG0012330: TcSYL_0026780.t1-p1
OG0012331: TcSYL_0026800.t1-p1
OG0012332: TcSYL_0026810.t1-p1
OG0012333: TcSYL_0026830.t1-p1
OG0012334: TcSYL_0026860.t1-p1
OG0012335: TcSYL_0026870.t1-p1
OG0012336: TcSYL_0026880.t1-p1
OG0012337: TcSYL_0026900.t1-p1
OG0012338: TcSYL_0026920.t1-p1
OG0012339: TcSYL_0026930.t1-p1
OG0012340: TcSYL_0026940.t1-p1
OG0012341: TcSYL_0026960.t1-p1
OG0012342: TcSYL_0026970.t1-p1
OG0012343: TcSYL_0026990.t1-p1
OG0012344: TcSYL_0027000.t1-p1
OG0012345: TcSYL_0027010.t1-p1
OG0012346: TcSYL_0027020.t1-p1

OG0012347: TcSYL_0027220.t1-p1
OG0012348: TcSYL_0027290.t1-p1
OG0012349: TcSYL_0027320.t1-p1
OG0012350: TcSYL_0027370.t1-p1
OG0012351: TcSYL_0027380.t1-p1
OG0012352: TcSYL_0027390.t1-p1
OG0012353: TcSYL_0027400.t1-p1
OG0012354: TcSYL_0027450.t1-p1
OG0012355: TcSYL_0027460.t1-p1
OG0012356: TcSYL_0027470.t1-p1
OG0012357: TcSYL_0027480.t1-p1
OG0012358: TcSYL_0027490.t1-p1
OG0012359: TcSYL_0027500.t1-p1
OG0012360: TcSYL_0027550.t1-p1
OG0012361: TcSYL_0027560.t1-p1
OG0012362: TcSYL_0027570.t1-p1
OG0012363: TcSYL_0027610.t1-p1
OG0012364: TcSYL_0027660.t1-p1
OG0012365: TcSYL_0027720.t1-p1
OG0012366: TcSYL_0027740.t1-p1
OG0012367: TcSYL_0027760.t1-p1
OG0012368: TcSYL_0027770.t1-p1
OG0012369: TcSYL_0027780.t1-p1
OG0012370: TcSYL_0027790.t1-p1
OG0012371: TcSYL_0027800.t1-p1
OG0012372: TcSYL_0027810.t1-p1
OG0012373: TcSYL_0027820.t1-p1
OG0012374: TcSYL_0027830.t1-p1
OG0012375: TcSYL_0027840.t1-p1
OG0012376: TcSYL_0027860.t1-p1
OG0012377: TcSYL_0027870.t1-p1
OG0012378: TcSYL_0027880.t1-p1
OG0012379: TcSYL_0027890.t1-p1
OG0012380: TcSYL_0027910.t1-p1
OG0012381: TcSYL_0027920.t1-p1
OG0012382: TcSYL_0027950.t1-p1
OG0012383: TcSYL_0027960.t1-p1
OG0012384: TcSYL_0027980.t1-p1
OG0012385: TcSYL_0027990.t1-p1
OG0012386: TcSYL_0028010.t1-p1
OG0012387: TcSYL_0028020.t1-p1
OG0012388: TcSYL_0028030.t1-p1
OG0012389: TcSYL_0028050.t1-p1
OG0012390: TcSYL_0028060.t1-p1
OG0012391: TcSYL_0028080.t1-p1
OG0012392: TcSYL_0028100.t1-p1
OG0012393: TcSYL_0028110.t1-p1
OG0012394: TcSYL_0028120.t1-p1
OG0012395: TcSYL_0028130.t1-p1
OG0012396: TcSYL_0028140.t1-p1
OG0012397: TcSYL_0028150.t1-p1
OG0012398: TcSYL_0028170.t1-p1
OG0012399: TcSYL_0028190.t1-p1
OG0012400: TcSYL_0028200.t1-p1

OG0012401: TcSYL_0028210.t1-p1
OG0012402: TcSYL_0028220.t1-p1
OG0012403: TcSYL_0028230.t1-p1
OG0012404: TcSYL_0028240.t1-p1
OG0012405: TcSYL_0028250.t1-p1
OG0012406: TcSYL_0028260.t1-p1
OG0012407: TcSYL_0028270.t1-p1
OG0012408: TcSYL_0028280.t1-p1
OG0012409: TcSYL_0028300.t1-p1
OG0012410: TcSYL_0028310.t1-p1
OG0012411: TcSYL_0028320.t1-p1
OG0012412: TcSYL_0028330.t1-p1
OG0012413: TcSYL_0028340.t1-p1
OG0012414: TcSYL_0028350.t1-p1
OG0012415: TcSYL_0028360.t1-p1
OG0012416: TcSYL_0028370.t1-p1
OG0012417: TcSYL_0028380.t1-p1
OG0012418: TcSYL_0028410.t1-p1
OG0012419: TcSYL_0028420.t1-p1
OG0012420: TcSYL_0028430.t1-p1
OG0012421: TcSYL_0028450.t1-p1
OG0012422: TcSYL_0028500.t1-p1
OG0012423: TcSYL_0028510.t1-p1
OG0012424: TcSYL_0028520.t1-p1
OG0012425: TcSYL_0028530.t1-p1
OG0012426: TcSYL_0028540.t1-p1
OG0012427: TcSYL_0028550.t1-p1
OG0012428: TcSYL_0028570.t1-p1
OG0012429: TcSYL_0028580.t1-p1
OG0012430: TcSYL_0028590.t1-p1
OG0012431: TcSYL_0028600.t1-p1
OG0012432: TcSYL_0028610.t1-p1
OG0012433: TcSYL_0028620.t1-p1
OG0012434: TcSYL_0028630.t1-p1
OG0012435: TcSYL_0028640.t1-p1
OG0012436: TcSYL_0028650.t1-p1
OG0012437: TcSYL_0028660.t1-p1
OG0012438: TcSYL_0028670.t1-p1
OG0012439: TcSYL_0028680.t1-p1
OG0012440: TcSYL_0028690.t1-p1
OG0012441: TcSYL_0028700.t1-p1
OG0012442: TcSYL_0028710.t1-p1
OG0012443: TcSYL_0028730.t1-p1
OG0012444: TcSYL_0028740.t1-p1
OG0012445: TcSYL_0028750.t1-p1
OG0012446: TcSYL_0028770.t1-p1
OG0012447: TcSYL_0028780.t1-p1
OG0012448: TcSYL_0028790.t1-p1
OG0012449: TcSYL_0028800.t1-p1
OG0012450: TcSYL_0028810.t1-p1
OG0012451: TcSYL_0028830.t1-p1
OG0012452: TcSYL_0028840.t1-p1
OG0012453: TcSYL_0028850.t1-p1
OG0012454: TcSYL_0028860.t1-p1

OG0012455: TcSYL_0028870.t1-p1
OG0012456: TcSYL_0028880.t1-p1
OG0012457: TcSYL_0028890.t1-p1
OG0012458: TcSYL_0028900.t1-p1
OG0012459: TcSYL_0028910.t1-p1
OG0012460: TcSYL_0028920.t1-p1
OG0012461: TcSYL_0028930.t1-p1
OG0012462: TcSYL_0028940.t1-p1
OG0012463: TcSYL_0028960.t1-p1
OG0012464: TcSYL_0028970.t1-p1
OG0012465: TcSYL_0028980.t1-p1
OG0012466: TcSYL_0028990.t1-p1
OG0012467: TcSYL_0029000.t1-p1
OG0012468: TcSYL_0029010.t1-p1
OG0012469: TcSYL_0029020.t1-p1
OG0012470: TcSYL_0029030.t1-p1
OG0012471: TcSYL_0029040.t1-p1
OG0012472: TcSYL_0029050.t1-p1
OG0012473: TcSYL_0029060.t1-p1
OG0012474: TcSYL_0029070.t1-p1
OG0012475: TcSYL_0029080.t1-p1
OG0012476: TcSYL_0029090.t1-p1
OG0012477: TcSYL_0029100.t1-p1
OG0012478: TcSYL_0029110.t1-p1
OG0012479: TcSYL_0029130.t1-p1
OG0012480: TcSYL_0029140.t1-p1
OG0012481: TcSYL_0029150.t1-p1
OG0012482: TcSYL_0029160.t1-p1
OG0012483: TcSYL_0029180.t1-p1
OG0012484: TcSYL_0029190.t1-p1
OG0012485: TcSYL_0029200.t1-p1
OG0012486: TcSYL_0029210.t1-p1
OG0012487: TcSYL_0029220.t1-p1
OG0012488: TcSYL_0029230.t1-p1
OG0012489: TcSYL_0029240.t1-p1
OG0012490: TcSYL_0029250.t1-p1
OG0012491: TcSYL_0029260.t1-p1
OG0012492: TcSYL_0029270.t1-p1
OG0012493: TcSYL_0029280.t1-p1
OG0012494: TcSYL_0029290.t1-p1
OG0012495: TcSYL_0029300.t1-p1
OG0012496: TcSYL_0029350.t1-p1
OG0012497: TcSYL_0029360.t1-p1
OG0012498: TcSYL_0029380.t1-p1
OG0012499: TcSYL_0029390.t1-p1
OG0012500: TcSYL_0029410.t1-p1
OG0012501: TcSYL_0029420.t1-p1
OG0012502: TcSYL_0029430.t1-p1
OG0012503: TcSYL_0029450.t1-p1
OG0012504: TcSYL_0029470.t1-p1
OG0012505: TcSYL_0029500.t1-p1
OG0012506: TcSYL_0029510.t1-p1
OG0012507: TcSYL_0029520.t1-p1
OG0012508: TcSYL_0029530.t1-p1

OG0012509: TcSYL_0029540.t1-p1
OG0012510: TcSYL_0029550.t1-p1
OG0012511: TcSYL_0029570.t1-p1
OG0012512: TcSYL_0029580.t1-p1
OG0012513: TcSYL_0029590.t1-p1
OG0012514: TcSYL_0029600.t1-p1
OG0012515: TcSYL_0029620.t1-p1
OG0012516: TcSYL_0029630.t1-p1
OG0012517: TcSYL_0029650.t1-p1
OG0012518: TcSYL_0029670.t1-p1
OG0012519: TcSYL_0029680.t1-p1
OG0012520: TcSYL_0029690.t1-p1
OG0012521: TcSYL_0029700.t1-p1
OG0012522: TcSYL_0029720.t1-p1
OG0012523: TcSYL_0029730.t1-p1
OG0012524: TcSYL_0029740.t1-p1
OG0012525: TcSYL_0029750.t1-p1
OG0012526: TcSYL_0029760.t1-p1
OG0012527: TcSYL_0029770.t1-p1
OG0012528: TcSYL_0029780.t1-p1
OG0012529: TcSYL_0029790.t1-p1
OG0012530: TcSYL_0029800.t1-p1
OG0012531: TcSYL_0029810.t1-p1
OG0012532: TcSYL_0029820.t1-p1
OG0012533: TcSYL_0029830.t1-p1
OG0012534: TcSYL_0029840.t1-p1
OG0012535: TcSYL_0029850.t1-p1
OG0012536: TcSYL_0029860.t1-p1
OG0012537: TcSYL_0029870.t1-p1
OG0012538: TcSYL_0029880.t1-p1
OG0012539: TcSYL_0029890.t1-p1
OG0012540: TcSYL_0029900.t1-p1
OG0012541: TcSYL_0029910.t1-p1
OG0012542: TcSYL_0029920.t1-p1
OG0012543: TcSYL_0029930.t1-p1
OG0012544: TcSYL_0029940.t1-p1
OG0012545: TcSYL_0029950.t1-p1
OG0012546: TcSYL_0029960.t1-p1
OG0012547: TcSYL_0029970.t1-p1
OG0012548: TcSYL_0029980.t1-p1
OG0012549: TcSYL_0029990.t1-p1
OG0012550: TcSYL_0030000.t1-p1
OG0012551: TcSYL_0030010.t1-p1
OG0012552: TcSYL_0030020.t1-p1
OG0012553: TcSYL_0030030.t1-p1
OG0012554: TcSYL_0030040.t1-p1
OG0012555: TcSYL_0030050.t1-p1
OG0012556: TcSYL_0030060.t1-p1
OG0012557: TcSYL_0030070.t1-p1
OG0012558: TcSYL_0030090.t1-p1
OG0012559: TcSYL_0030100.t1-p1
OG0012560: TcSYL_0030110.t1-p1
OG0012561: TcSYL_0030120.t1-p1
OG0012562: TcSYL_0030130.t1-p1

OG0012563: TcSYL_0030140.t1-p1
OG0012564: TcSYL_0030150.t1-p1
OG0012565: TcSYL_0030160.t1-p1
OG0012566: TcSYL_0030170.t1-p1
OG0012567: TcSYL_0030180.t1-p1
OG0012568: TcSYL_0030190.t1-p1
OG0012569: TcSYL_0030200.t1-p1
OG0012570: TcSYL_0030210.t1-p1
OG0012571: TcSYL_0030220.t1-p1
OG0012572: TcSYL_0030230.t1-p1
OG0012573: TcSYL_0030240.t1-p1
OG0012574: TcSYL_0030250.t1-p1
OG0012575: TcSYL_0030260.t1-p1
OG0012576: TcSYL_0030270.t1-p1
OG0012577: TcSYL_0030280.t1-p1
OG0012578: TcSYL_0030290.t1-p1
OG0012579: TcSYL_0030300.t1-p1
OG0012580: TcSYL_0030310.t1-p1
OG0012581: TcSYL_0030320.t1-p1
OG0012582: TcSYL_0030330.t1-p1
OG0012583: TcSYL_0030340.t1-p1
OG0012584: TcSYL_0030350.t1-p1
OG0012585: TcSYL_0030360.t1-p1
OG0012586: TcSYL_0030380.t1-p1
OG0012587: TcSYL_0030390.t1-p1
OG0012588: TcSYL_0030400.t1-p1
OG0012589: TcSYL_0030410.t1-p1
OG0012590: TcSYL_0030420.t1-p1
OG0012591: TcSYL_0030430.t1-p1
OG0012592: TcSYL_0030440.t1-p1
OG0012593: TcSYL_0030450.t1-p1
OG0012594: TcSYL_0030460.t1-p1
OG0012595: TcSYL_0030470.t1-p1
OG0012596: TcSYL_0030480.t1-p1
OG0012597: TcSYL_0030490.t1-p1
OG0012598: TcSYL_0030500.t1-p1
OG0012599: TcSYL_0030510.t1-p1
OG0012600: TcSYL_0030520.t1-p1
OG0012601: TcSYL_0030530.t1-p1
OG0012602: TcSYL_0030540.t1-p1
OG0012603: TcSYL_0030550.t1-p1
OG0012604: TcSYL_0030560.t1-p1
OG0012605: TcSYL_0030570.t1-p1
OG0012606: TcSYL_0030580.t1-p1
OG0012607: TcSYL_0030590.t1-p1
OG0012608: TcSYL_0030600.t1-p1
OG0012609: TcSYL_0030610.t1-p1
OG0012610: TcSYL_0030640.t1-p1
OG0012611: TcSYL_0030660.t1-p1
OG0012612: TcSYL_0030670.t1-p1
OG0012613: TcSYL_0030700.t1-p1
OG0012614: TcSYL_0030710.t1-p1
OG0012615: TcSYL_0030740.t1-p1
OG0012616: TcSYL_0030750.t1-p1

OG0012617: TcSYL_0030770.t1-p1
OG0012618: TcSYL_0030780.t1-p1
OG0012619: TcSYL_0030800.t1-p1
OG0012620: TcSYL_0030810.t1-p1
OG0012621: TcSYL_0030820.t1-p1
OG0012622: TcSYL_0030830.t1-p1
OG0012623: TcSYL_0030840.t1-p1
OG0012624: TcSYL_0030850.t1-p1
OG0012625: TcSYL_0030870.t1-p1
OG0012626: TcSYL_0030890.t1-p1
OG0012627: TcSYL_0030900.t1-p1
OG0012628: TcSYL_0030930.t1-p1
OG0012629: TcSYL_0030950.t1-p1
OG0012630: TcSYL_0030960.t1-p1
OG0012631: TcSYL_0030980.t1-p1
OG0012632: TcSYL_0030990.t1-p1
OG0012633: TcSYL_0031000.t1-p1
OG0012634: TcSYL_0031010.t1-p1
OG0012635: TcSYL_0031020.t1-p1
OG0012636: TcSYL_0031030.t1-p1
OG0012637: TcSYL_0031050.t1-p1
OG0012638: TcSYL_0031070.t1-p1
OG0012639: TcSYL_0031080.t1-p1
OG0012640: TcSYL_0031090.t1-p1
OG0012641: TcSYL_0031100.t1-p1
OG0012642: TcSYL_0031110.t1-p1
OG0012643: TcSYL_0031130.t1-p1
OG0012644: TcSYL_0031150.t1-p1
OG0012645: TcSYL_0031180.t1-p1
OG0012646: TcSYL_0031190.t1-p1
OG0012647: TcSYL_0031200.t1-p1
OG0012648: TcSYL_0031210.t1-p1
OG0012649: TcSYL_0031220.t1-p1
OG0012650: TcSYL_0031230.t1-p1
OG0012651: TcSYL_0031240.t1-p1
OG0012652: TcSYL_0031260.t1-p1
OG0012653: TcSYL_0031270.t1-p1
OG0012654: TcSYL_0031280.t1-p1
OG0012655: TcSYL_0031290.t1-p1
OG0012656: TcSYL_0031300.t1-p1
OG0012657: TcSYL_0031310.t1-p1
OG0012658: TcSYL_0031320.t1-p1
OG0012659: TcSYL_0031330.t1-p1
OG0012660: TcSYL_0031340.t1-p1
OG0012661: TcSYL_0031360.t1-p1
OG0012662: TcSYL_0031370.t1-p1
OG0012663: TcSYL_0031380.t1-p1
OG0012664: TcSYL_0031390.t1-p1
OG0012665: TcSYL_0031410.t1-p1
OG0012666: TcSYL_0031430.t1-p1
OG0012667: TcSYL_0031450.t1-p1
OG0012668: TcSYL_0031460.t1-p1
OG0012669: TcSYL_0031490.t1-p1
OG0012670: TcSYL_0031500.t1-p1

OG0012671: TcSYL_0031510.t1-p1
OG0012672: TcSYL_0031520.t1-p1
OG0012673: TcSYL_0031530.t1-p1
OG0012674: TcSYL_0031540.t1-p1
OG0012675: TcSYL_0031550.t1-p1
OG0012676: TcSYL_0031560.t1-p1
OG0012677: TcSYL_0031580.t1-p1
OG0012678: TcSYL_0031590.t1-p1
OG0012679: TcSYL_0031610.t1-p1
OG0012680: TcSYL_0031630.t1-p1
OG0012681: TcSYL_0031640.t1-p1
OG0012682: TcSYL_0031650.t1-p1
OG0012683: TcSYL_0031660.t1-p1
OG0012684: TcSYL_0031670.t1-p1
OG0012685: TcSYL_0031680.t1-p1
OG0012686: TcSYL_0031690.t1-p1
OG0012687: TcSYL_0031700.t1-p1
OG0012688: TcSYL_0031710.t1-p1
OG0012689: TcSYL_0031730.t1-p1
OG0012690: TcSYL_0031750.t1-p1
OG0012691: TcSYL_0031760.t1-p1
OG0012692: TcSYL_0031770.t1-p1
OG0012693: TcSYL_0031780.t1-p1
OG0012694: TcSYL_0031790.t1-p1
OG0012695: TcSYL_0031800.t1-p1
OG0012696: TcSYL_0031830.t1-p1
OG0012697: TcSYL_0031850.t1-p1
OG0012698: TcSYL_0031870.t1-p1
OG0012699: TcSYL_0031880.t1-p1
OG0012700: TcSYL_0031890.t1-p1
OG0012701: TcSYL_0031900.t1-p1
OG0012702: TcSYL_0031920.t1-p1
OG0012703: TcSYL_0031930.t1-p1
OG0012704: TcSYL_0031940.t1-p1
OG0012705: TcSYL_0031950.t1-p1
OG0012706: TcSYL_0031960.t1-p1
OG0012707: TcSYL_0031970.t1-p1
OG0012708: TcSYL_0031980.t1-p1
OG0012709: TcSYL_0031990.t1-p1
OG0012710: TcSYL_0032000.t1-p1
OG0012711: TcSYL_0032020.t1-p1
OG0012712: TcSYL_0032030.t1-p1
OG0012713: TcSYL_0032040.t1-p1
OG0012714: TcSYL_0032050.t1-p1
OG0012715: TcSYL_0032060.t1-p1
OG0012716: TcSYL_0032070.t1-p1
OG0012717: TcSYL_0032080.t1-p1
OG0012718: TcSYL_0032090.t1-p1
OG0012719: TcSYL_0032100.t1-p1
OG0012720: TcSYL_0032110.t1-p1
OG0012721: TcSYL_0032120.t1-p1
OG0012722: TcSYL_0032130.t1-p1
OG0012723: TcSYL_0032140.t1-p1
OG0012724: TcSYL_0032150.t1-p1

OG0012725: TcSYL_0032160.t1-p1
OG0012726: TcSYL_0032170.t1-p1
OG0012727: TcSYL_0032180.t1-p1
OG0012728: TcSYL_0032190.t1-p1
OG0012729: TcSYL_0032200.t1-p1
OG0012730: TcSYL_0032210.t1-p1
OG0012731: TcSYL_0032220.t1-p1
OG0012732: TcSYL_0032230.t1-p1
OG0012733: TcSYL_0032240.t1-p1
OG0012734: TcSYL_0032250.t1-p1
OG0012735: TcSYL_0032260.t1-p1
OG0012736: TcSYL_0032270.t1-p1
OG0012737: TcSYL_0032280.t1-p1
OG0012738: TcSYL_0032290.t1-p1
OG0012739: TcSYL_0032300.t1-p1
OG0012740: TcSYL_0032310.t1-p1
OG0012741: TcSYL_0032320.t1-p1
OG0012742: TcSYL_0032330.t1-p1
OG0012743: TcSYL_0032340.t1-p1
OG0012744: TcSYL_0032350.t1-p1
OG0012745: TcSYL_0032360.t1-p1
OG0012746: TcSYL_0032380.t1-p1
OG0012747: TcSYL_0032390.t1-p1
OG0012748: TcSYL_0032400.t1-p1
OG0012749: TcSYL_0032410.t1-p1
OG0012750: TcSYL_0032420.t1-p1
OG0012751: TcSYL_0032430.t1-p1
OG0012752: TcSYL_0032440.t1-p1
OG0012753: TcSYL_0032450.t1-p1
OG0012754: TcSYL_0032460.t1-p1
OG0012755: TcSYL_0032470.t1-p1
OG0012756: TcSYL_0032480.t1-p1
OG0012757: TcSYL_0032490.t1-p1
OG0012758: TcSYL_0032500.t1-p1
OG0012759: TcSYL_0032510.t1-p1
OG0012760: TcSYL_0032520.t1-p1
OG0012761: TcSYL_0032530.t1-p1
OG0012762: TcSYL_0032540.t1-p1
OG0012763: TcSYL_0032550.t1-p1
OG0012764: TcSYL_0032560.t1-p1
OG0012765: TcSYL_0032570.t1-p1
OG0012766: TcSYL_0032580.t1-p1
OG0012767: TcSYL_0032600.t1-p1
OG0012768: TcSYL_0032610.t1-p1
OG0012769: TcSYL_0032620.t1-p1
OG0012770: TcSYL_0032630.t1-p1
OG0012771: TcSYL_0032640.t1-p1
OG0012772: TcSYL_0032650.t1-p1
OG0012773: TcSYL_0032660.t1-p1
OG0012774: TcSYL_0032670.t1-p1
OG0012775: TcSYL_0032680.t1-p1
OG0012776: TcSYL_0032690.t1-p1
OG0012777: TcSYL_0032700.t1-p1
OG0012778: TcSYL_0032710.t1-p1

OG0012779: TcSYL_0032720.t1-p1
OG0012780: TcSYL_0032730.t1-p1
OG0012781: TcSYL_0032740.t1-p1
OG0012782: TcSYL_0032750.t1-p1
OG0012783: TcSYL_0032760.t1-p1
OG0012784: TcSYL_0032770.t1-p1
OG0012785: TcSYL_0032780.t1-p1
OG0012786: TcSYL_0032790.t1-p1
OG0012787: TcSYL_0032800.t1-p1
OG0012788: TcSYL_0032810.t1-p1
OG0012789: TcSYL_0032820.t1-p1
OG0012790: TcSYL_0032830.t1-p1
OG0012791: TcSYL_0032840.t1-p1
OG0012792: TcSYL_0032850.t1-p1
OG0012793: TcSYL_0032860.t1-p1
OG0012794: TcSYL_0032870.t1-p1
OG0012795: TcSYL_0032880.t1-p1
OG0012796: TcSYL_0032890.t1-p1
OG0012797: TcSYL_0032910.t1-p1
OG0012798: TcSYL_0032920.t1-p1
OG0012799: TcSYL_0032930.t1-p1
OG0012800: TcSYL_0032940.t1-p1
OG0012801: TcSYL_0032950.t1-p1
OG0012802: TcSYL_0032960.t1-p1
OG0012803: TcSYL_0032970.t1-p1
OG0012804: TcSYL_0032980.t1-p1
OG0012805: TcSYL_0032990.t1-p1
OG0012806: TcSYL_0033000.t1-p1
OG0012807: TcSYL_0033010.t1-p1
OG0012808: TcSYL_0033020.t1-p1
OG0012809: TcSYL_0033030.t1-p1
OG0012810: TcSYL_0033040.t1-p1
OG0012811: TcSYL_0033050.t1-p1
OG0012812: TcSYL_0033060.t1-p1
OG0012813: TcSYL_0033070.t1-p1
OG0012814: TcSYL_0033080.t1-p1
OG0012815: TcSYL_0033100.t1-p1
OG0012816: TcSYL_0033120.t1-p1
OG0012817: TcSYL_0033130.t1-p1
OG0012818: TcSYL_0033140.t1-p1
OG0012819: TcSYL_0033150.t1-p1
OG0012820: TcSYL_0033160.t1-p1
OG0012821: TcSYL_0033170.t1-p1
OG0012822: TcSYL_0033180.t1-p1
OG0012823: TcSYL_0033190.t1-p1
OG0012824: TcSYL_0033200.t1-p1
OG0012825: TcSYL_0033210.t1-p1
OG0012826: TcSYL_0033230.t1-p1
OG0012827: TcSYL_0033240.t1-p1
OG0012828: TcSYL_0033250.t1-p1
OG0012829: TcSYL_0033260.t1-p1
OG0012830: TcSYL_0033270.t1-p1
OG0012831: TcSYL_0033280.t1-p1
OG0012832: TcSYL_0033290.t1-p1

OG0012833: TcSYL_0033300.t1-p1
OG0012834: TcSYL_0033310.t1-p1
OG0012835: TcSYL_0033320.t1-p1
OG0012836: TcSYL_0033330.t1-p1
OG0012837: TcSYL_0033340.t1-p1
OG0012838: TcSYL_0033350.t1-p1
OG0012839: TcSYL_0033360.t1-p1
OG0012840: TcSYL_0033370.t1-p1
OG0012841: TcSYL_0033380.t1-p1
OG0012842: TcSYL_0033390.t1-p1
OG0012843: TcSYL_0033400.t1-p1
OG0012844: TcSYL_0033410.t1-p1
OG0012845: TcSYL_0033420.t1-p1
OG0012846: TcSYL_0033430.t1-p1
OG0012847: TcSYL_0033440.t1-p1
OG0012848: TcSYL_0033450.t1-p1
OG0012849: TcSYL_0033470.t1-p1
OG0012850: TcSYL_0033480.t1-p1
OG0012851: TcSYL_0033490.t1-p1
OG0012852: TcSYL_0033500.t1-p1
OG0012853: TcSYL_0033510.t1-p1
OG0012854: TcSYL_0033520.t1-p1
OG0012855: TcSYL_0033530.t1-p1
OG0012856: TcSYL_0033540.t1-p1
OG0012857: TcSYL_0033550.t1-p1
OG0012858: TcSYL_0033560.t1-p1
OG0012859: TcSYL_0033570.t1-p1
OG0012860: TcSYL_0033580.t1-p1
OG0012861: TcSYL_0033590.t1-p1
OG0012862: TcSYL_0033600.t1-p1
OG0012863: TcSYL_0033610.t1-p1
OG0012864: TcSYL_0033620.t1-p1
OG0012865: TcSYL_0033630.t1-p1
OG0012866: TcSYL_0033640.t1-p1
OG0012867: TcSYL_0033650.t1-p1
OG0012868: TcSYL_0033670.t1-p1
OG0012869: TcSYL_0033680.t1-p1
OG0012870: TcSYL_0033690.t1-p1
OG0012871: TcSYL_0033700.t1-p1
OG0012872: TcSYL_0033710.t1-p1
OG0012873: TcSYL_0033720.t1-p1
OG0012874: TcSYL_0033730.t1-p1
OG0012875: TcSYL_0033740.t1-p1
OG0012876: TcSYL_0033750.t1-p1
OG0012877: TcSYL_0033760.t1-p1
OG0012878: TcSYL_0033770.t1-p1
OG0012879: TcSYL_0033780.t1-p1
OG0012880: TcSYL_0033790.t1-p1
OG0012881: TcSYL_0033800.t1-p1
OG0012882: TcSYL_0033810.t1-p1
OG0012883: TcSYL_0033820.t1-p1
OG0012884: TcSYL_0033830.t1-p1
OG0012885: TcSYL_0033840.t1-p1
OG0012886: TcSYL_0033850.t1-p1

OG0012887: TcSYL_0033860.t1-p1
OG0012888: TcSYL_0033870.t1-p1
OG0012889: TcSYL_0033880.t1-p1
OG0012890: TcSYL_0033890.t1-p1
OG0012891: TcSYL_0033900.t1-p1
OG0012892: TcSYL_0033910.t1-p1
OG0012893: TcSYL_0033920.t1-p1
OG0012894: TcSYL_0033930.t1-p1
OG0012895: TcSYL_0033940.t1-p1
OG0012896: TcSYL_0033950.t1-p1
OG0012897: TcSYL_0033960.t1-p1
OG0012898: TcSYL_0033970.t1-p1
OG0012899: TcSYL_0033980.t1-p1
OG0012900: TcSYL_0033990.t1-p1
OG0012901: TcSYL_0034000.t1-p1
OG0012902: TcSYL_0034010.t1-p1
OG0012903: TcSYL_0034030.t1-p1
OG0012904: TcSYL_0034040.t1-p1
OG0012905: TcSYL_0034050.t1-p1
OG0012906: TcSYL_0034060.t1-p1
OG0012907: TcSYL_0034070.t1-p1
OG0012908: TcSYL_0034080.t1-p1
OG0012909: TcSYL_0034090.t1-p1
OG0012910: TcSYL_0034100.t1-p1
OG0012911: TcSYL_0034110.t1-p1
OG0012912: TcSYL_0034120.t1-p1
OG0012913: TcSYL_0034130.t1-p1
OG0012914: TcSYL_0034140.t1-p1
OG0012915: TcSYL_0034150.t1-p1
OG0012916: TcSYL_0034160.t1-p1
OG0012917: TcSYL_0034170.t1-p1
OG0012918: TcSYL_0034180.t1-p1
OG0012919: TcSYL_0034190.t1-p1
OG0012920: TcSYL_0034200.t1-p1
OG0012921: TcSYL_0034210.t1-p1
OG0012922: TcSYL_0034220.t1-p1
OG0012923: TcSYL_0034230.t1-p1
OG0012924: TcSYL_0034240.t1-p1
OG0012925: TcSYL_0034250.t1-p1
OG0012926: TcSYL_0034260.t1-p1
OG0012927: TcSYL_0034270.t1-p1
OG0012928: TcSYL_0034280.t1-p1
OG0012929: TcSYL_0034290.t1-p1
OG0012930: TcSYL_0034300.t1-p1
OG0012931: TcSYL_0034310.t1-p1
OG0012932: TcSYL_0034320.t1-p1
OG0012933: TcSYL_0034330.t1-p1
OG0012934: TcSYL_0034340.t1-p1
OG0012935: TcSYL_0034350.t1-p1
OG0012936: TcSYL_0034360.t1-p1
OG0012937: TcSYL_0034370.t1-p1
OG0012938: TcSYL_0034380.t1-p1
OG0012939: TcSYL_0034390.t1-p1
OG0012940: TcSYL_0034400.t1-p1

OG0012941: TcSYL_0034410.t1-p1
OG0012942: TcSYL_0034420.t1-p1
OG0012943: TcSYL_0034430.t1-p1
OG0012944: TcSYL_0034440.t1-p1
OG0012945: TcSYL_0034480.t1-p1
OG0012946: TcSYL_0034490.t1-p1
OG0012947: TcSYL_0034500.t1-p1
OG0012948: TcSYL_0034510.t1-p1
OG0012949: TcSYL_0034520.t1-p1
OG0012950: TcSYL_0034530.t1-p1
OG0012951: TcSYL_0034540.t1-p1
OG0012952: TcSYL_0034550.t1-p1
OG0012953: TcSYL_0034560.t1-p1
OG0012954: TcSYL_0034570.t1-p1
OG0012955: TcSYL_0034580.t1-p1
OG0012956: TcSYL_0034590.t1-p1
OG0012957: TcSYL_0034600.t1-p1
OG0012958: TcSYL_0034610.t1-p1
OG0012959: TcSYL_0034620.t1-p1
OG0012960: TcSYL_0034630.t1-p1
OG0012961: TcSYL_0034640.t1-p1
OG0012962: TcSYL_0034650.t1-p1
OG0012963: TcSYL_0034660.t1-p1
OG0012964: TcSYL_0034670.t1-p1
OG0012965: TcSYL_0034680.t1-p1
OG0012966: TcSYL_0034690.t1-p1
OG0012967: TcSYL_0034700.t1-p1
OG0012968: TcSYL_0034710.t1-p1
OG0012969: TcSYL_0034720.t1-p1
OG0012970: TcSYL_0034730.t1-p1
OG0012971: TcSYL_0034740.t1-p1
OG0012972: TcSYL_0034750.t1-p1
OG0012973: TcSYL_0034760.t1-p1
OG0012974: TcSYL_0034770.t1-p1
OG0012975: TcSYL_0034780.t1-p1
OG0012976: TcSYL_0034790.t1-p1
OG0012977: TcSYL_0034800.t1-p1
OG0012978: TcSYL_0034810.t1-p1
OG0012979: TcSYL_0034820.t1-p1
OG0012980: TcSYL_0034830.t1-p1
OG0012981: TcSYL_0034840.t1-p1
OG0012982: TcSYL_0034850.t1-p1
OG0012983: TcSYL_0034860.t1-p1
OG0012984: TcSYL_0034870.t1-p1
OG0012985: TcSYL_0034880.t1-p1
OG0012986: TcSYL_0034890.t1-p1
OG0012987: TcSYL_0034900.t1-p1
OG0012988: TcSYL_0034910.t1-p1
OG0012989: TcSYL_0034920.t1-p1
OG0012990: TcSYL_0034930.t1-p1
OG0012991: TcSYL_0034940.t1-p1
OG0012992: TcSYL_0034950.t1-p1
OG0012993: TcSYL_0034960.t1-p1
OG0012994: TcSYL_0034970.t1-p1

OG0012995: TcSYL_0034980.t1-p1
OG0012996: TcSYL_0034990.t1-p1
OG0012997: TcSYL_0035010.t1-p1
OG0012998: TcSYL_0035020.t1-p1
OG0012999: TcSYL_0035030.t1-p1
OG0013000: TcSYL_0035040.t1-p1
OG0013001: TcSYL_0035050.t1-p1
OG0013002: TcSYL_0035060.t1-p1
OG0013003: TcSYL_0035070.t1-p1
OG0013004: TcSYL_0035080.t1-p1
OG0013005: TcSYL_0035090.t1-p1
OG0013006: TcSYL_0035100.t1-p1
OG0013007: TcSYL_0035110.t1-p1
OG0013008: TcSYL_0035120.t1-p1
OG0013009: TcSYL_0035130.t1-p1
OG0013010: TcSYL_0035140.t1-p1
OG0013011: TcSYL_0035150.t1-p1
OG0013012: TcSYL_0035160.t1-p1
OG0013013: TcSYL_0035170.t1-p1
OG0013014: TcSYL_0035180.t1-p1
OG0013015: TcSYL_0035190.t1-p1
OG0013016: TcSYL_0035200.t1-p1
OG0013017: TcSYL_0035210.t1-p1
OG0013018: TcSYL_0035220.t1-p1
OG0013019: TcSYL_0035230.t1-p1
OG0013020: TcSYL_0035240.t1-p1
OG0013021: TcSYL_0035250.t1-p1
OG0013022: TcSYL_0035260.t1-p1
OG0013023: TcSYL_0035270.t1-p1
OG0013024: TcSYL_0035280.t1-p1
OG0013025: TcSYL_0035290.t1-p1
OG0013026: TcSYL_0035300.t1-p1
OG0013027: TcSYL_0035310.t1-p1
OG0013028: TcSYL_0035320.t1-p1
OG0013029: TcSYL_0035330.t1-p1
OG0013030: TcSYL_0035340.t1-p1
OG0013031: TcSYL_0035350.t1-p1
OG0013032: TcSYL_0035360.t1-p1
OG0013033: TcSYL_0035370.t1-p1
OG0013034: TcSYL_0035380.t1-p1
OG0013035: TcSYL_0035390.t1-p1
OG0013036: TcSYL_0035400.t1-p1
OG0013037: TcSYL_0035410.t1-p1
OG0013038: TcSYL_0035420.t1-p1
OG0013039: TcSYL_0035430.t1-p1
OG0013040: TcSYL_0035440.t1-p1
OG0013041: TcSYL_0035460.t1-p1
OG0013042: TcSYL_0035470.t1-p1
OG0013043: TcSYL_0035480.t1-p1
OG0013044: TcSYL_0035490.t1-p1
OG0013045: TcSYL_0035500.t1-p1
OG0013046: TcSYL_0035510.t1-p1
OG0013047: TcSYL_0035520.t1-p1
OG0013048: TcSYL_0035530.t1-p1

OG0013049: TcSYL_0035540.t1-p1
OG0013050: TcSYL_0035550.t1-p1
OG0013051: TcSYL_0035560.t1-p1
OG0013052: TcSYL_0035570.t1-p1
OG0013053: TcSYL_0035580.t1-p1
OG0013054: TcSYL_0035590.t1-p1
OG0013055: TcSYL_0035600.t1-p1
OG0013056: TcSYL_0035610.t1-p1
OG0013057: TcSYL_0035620.t1-p1
OG0013058: TcSYL_0035630.t1-p1
OG0013059: TcSYL_0035640.t1-p1
OG0013060: TcSYL_0035650.t1-p1
OG0013061: TcSYL_0035660.t1-p1
OG0013062: TcSYL_0035670.t1-p1
OG0013063: TcSYL_0035680.t1-p1
OG0013064: TcSYL_0035690.t1-p1
OG0013065: TcSYL_0035700.t1-p1
OG0013066: TcSYL_0035710.t1-p1
OG0013067: TcSYL_0035720.t1-p1
OG0013068: TcSYL_0035730.t1-p1
OG0013069: TcSYL_0035740.t1-p1
OG0013070: TcSYL_0035750.t1-p1
OG0013071: TcSYL_0035760.t1-p1
OG0013072: TcSYL_0035770.t1-p1
OG0013073: TcSYL_0035780.t1-p1
OG0013074: TcSYL_0035790.t1-p1
OG0013075: TcSYL_0035800.t1-p1
OG0013076: TcSYL_0035810.t1-p1
OG0013077: TcSYL_0035820.t1-p1
OG0013078: TcSYL_0035840.t1-p1
OG0013079: TcSYL_0035850.t1-p1
OG0013080: TcSYL_0035860.t1-p1
OG0013081: TcSYL_0035870.t1-p1
OG0013082: TcSYL_0035880.t1-p1
OG0013083: TcSYL_0035890.t1-p1
OG0013084: TcSYL_0035900.t1-p1
OG0013085: TcSYL_0035910.t1-p1
OG0013086: TcSYL_0035920.t1-p1
OG0013087: TcSYL_0035930.t1-p1
OG0013088: TcSYL_0035940.t1-p1
OG0013089: TcSYL_0035950.t1-p1
OG0013090: TcSYL_0035960.t1-p1
OG0013091: TcSYL_0035970.t1-p1
OG0013092: TcSYL_0035980.t1-p1
OG0013093: TcSYL_0035990.t1-p1
OG0013094: TcSYL_0036000.t1-p1
OG0013095: TcSYL_0036010.t1-p1
OG0013096: TcSYL_0036020.t1-p1
OG0013097: TcSYL_0036030.t1-p1
OG0013098: TcSYL_0036040.t1-p1
OG0013099: TcSYL_0036050.t1-p1
OG0013100: TcSYL_0036060.t1-p1
OG0013101: TcSYL_0036070.t1-p1
OG0013102: TcSYL_0036080.t1-p1

OG0013103: TcSYL_0036090.t1-p1
OG0013104: TcSYL_0036100.t1-p1
OG0013105: TcSYL_0036110.t1-p1
OG0013106: TcSYL_0036120.t1-p1
OG0013107: TcSYL_0036130.t1-p1
OG0013108: TcSYL_0036140.t1-p1
OG0013109: TcSYL_0036150.t1-p1
OG0013110: TcSYL_0036160.t1-p1
OG0013111: TcSYL_0036170.t1-p1
OG0013112: TcSYL_0036180.t1-p1
OG0013113: TcSYL_0036200.t1-p1
OG0013114: TcSYL_0036210.t1-p1
OG0013115: TcSYL_0036220.t1-p1
OG0013116: TcSYL_0036230.t1-p1
OG0013117: TcSYL_0036240.t1-p1
OG0013118: TcSYL_0036250.t1-p1
OG0013119: TcSYL_0036260.t1-p1
OG0013120: TcSYL_0036270.t1-p1
OG0013121: TcSYL_0036280.t1-p1
OG0013122: TcSYL_0036290.t1-p1
OG0013123: TcSYL_0036300.t1-p1
OG0013124: TcSYL_0036310.t1-p1
OG0013125: TcSYL_0036320.t1-p1
OG0013126: TcSYL_0036330.t1-p1
OG0013127: TcSYL_0036340.t1-p1
OG0013128: TcSYL_0036350.t1-p1
OG0013129: TcSYL_0036360.t1-p1
OG0013130: TcSYL_0036370.t1-p1
OG0013131: TcSYL_0036380.t1-p1
OG0013132: TcSYL_0036400.t1-p1
OG0013133: TcSYL_0036410.t1-p1
OG0013134: TcSYL_0036420.t1-p1
OG0013135: TcSYL_0036430.t1-p1
OG0013136: TcSYL_0036440.t1-p1
OG0013137: TcSYL_0036450.t1-p1
OG0013138: TcSYL_0036460.t1-p1
OG0013139: TcSYL_0036470.t1-p1
OG0013140: TcSYL_0036480.t1-p1
OG0013141: TcSYL_0036490.t1-p1
OG0013142: TcSYL_0036500.t1-p1
OG0013143: TcSYL_0036510.t1-p1
OG0013144: TcSYL_0036520.t1-p1
OG0013145: TcSYL_0036530.t1-p1
OG0013146: TcSYL_0036540.t1-p1
OG0013147: TcSYL_0036550.t1-p1
OG0013148: TcSYL_0036560.t1-p1
OG0013149: TcSYL_0036570.t1-p1
OG0013150: TcSYL_0036580.t1-p1
OG0013151: TcSYL_0036600.t1-p1
OG0013152: TcSYL_0036610.t1-p1
OG0013153: TcSYL_0036620.t1-p1
OG0013154: TcSYL_0036630.t1-p1
OG0013155: TcSYL_0036640.t1-p1
OG0013156: TcSYL_0036650.t1-p1

OG0013157: TcSYL_0036660.t1-p1
OG0013158: TcSYL_0036670.t1-p1
OG0013159: TcSYL_0036680.t1-p1
OG0013160: TcSYL_0036690.t1-p1
OG0013161: TcSYL_0036700.t1-p1
OG0013162: TcSYL_0036710.t1-p1
OG0013163: TcSYL_0036720.t1-p1
OG0013164: TcSYL_0036730.t1-p1
OG0013165: TcSYL_0036740.t1-p1
OG0013166: TcSYL_0036750.t1-p1
OG0013167: TcSYL_0036760.t1-p1
OG0013168: TcSYL_0036770.t1-p1
OG0013169: TcSYL_0036780.t1-p1
OG0013170: TcSYL_0036790.t1-p1
OG0013171: TcSYL_0036800.t1-p1
OG0013172: TcSYL_0036820.t1-p1
OG0013173: TcSYL_0036830.t1-p1
OG0013174: TcSYL_0036840.t1-p1
OG0013175: TcSYL_0036850.t1-p1
OG0013176: TcSYL_0036860.t1-p1
OG0013177: TcSYL_0036870.t1-p1
OG0013178: TcSYL_0036880.t1-p1
OG0013179: TcSYL_0036890.t1-p1
OG0013180: TcSYL_0036900.t1-p1
OG0013181: TcSYL_0036910.t1-p1
OG0013182: TcSYL_0036920.t1-p1
OG0013183: TcSYL_0036930.t1-p1
OG0013184: TcSYL_0036940.t1-p1
OG0013185: TcSYL_0036950.t1-p1
OG0013186: TcSYL_0036960.t1-p1
OG0013187: TcSYL_0036970.t1-p1
OG0013188: TcSYL_0036980.t1-p1
OG0013189: TcSYL_0036990.t1-p1
OG0013190: TcSYL_0037000.t1-p1
OG0013191: TcSYL_0037010.t1-p1
OG0013192: TcSYL_0037020.t1-p1
OG0013193: TcSYL_0037030.t1-p1
OG0013194: TcSYL_0037040.t1-p1
OG0013195: TcSYL_0037060.t1-p1
OG0013196: TcSYL_0037070.t1-p1
OG0013197: TcSYL_0037080.t1-p1
OG0013198: TcSYL_0037090.t1-p1
OG0013199: TcSYL_0037100.t1-p1
OG0013200: TcSYL_0037110.t1-p1
OG0013201: TcSYL_0037120.t1-p1
OG0013202: TcSYL_0037130.t1-p1
OG0013203: TcSYL_0037140.t1-p1
OG0013204: TcSYL_0037150.t1-p1
OG0013205: TcSYL_0037160.t1-p1
OG0013206: TcSYL_0037170.t1-p1
OG0013207: TcSYL_0037180.t1-p1
OG0013208: TcSYL_0037190.t1-p1
OG0013209: TcSYL_0037200.t1-p1
OG0013210: TcSYL_0037210.t1-p1

OG0013211: TcSYL_0037220.t1-p1
OG0013212: TcSYL_0037230.t1-p1
OG0013213: TcSYL_0037240.t1-p1
OG0013214: TcSYL_0037250.t1-p1
OG0013215: TcSYL_0037260.t1-p1
OG0013216: TcSYL_0037270.t1-p1
OG0013217: TcSYL_0037280.t1-p1
OG0013218: TcSYL_0037290.t1-p1
OG0013219: TcSYL_0037310.t1-p1
OG0013220: TcSYL_0037320.t1-p1
OG0013221: TcSYL_0037330.t1-p1
OG0013222: TcSYL_0037340.t1-p1
OG0013223: TcSYL_0037350.t1-p1
OG0013224: TcSYL_0037360.t1-p1
OG0013225: TcSYL_0037370.t1-p1
OG0013226: TcSYL_0037380.t1-p1
OG0013227: TcSYL_0037390.t1-p1
OG0013228: TcSYL_0037400.t1-p1
OG0013229: TcSYL_0037410.t1-p1
OG0013230: TcSYL_0037420.t1-p1
OG0013231: TcSYL_0037430.t1-p1
OG0013232: TcSYL_0037440.t1-p1
OG0013233: TcSYL_0037450.t1-p1
OG0013234: TcSYL_0037460.t1-p1
OG0013235: TcSYL_0037470.t1-p1
OG0013236: TcSYL_0037480.t1-p1
OG0013237: TcSYL_0037490.t1-p1
OG0013238: TcSYL_0037500.t1-p1
OG0013239: TcSYL_0037510.t1-p1
OG0013240: TcSYL_0037520.t1-p1
OG0013241: TcSYL_0037530.t1-p1
OG0013242: TcSYL_0037540.t1-p1
OG0013243: TcSYL_0037550.t1-p1
OG0013244: TcSYL_0037560.t1-p1
OG0013245: TcSYL_0037570.t1-p1
OG0013246: TcSYL_0037580.t1-p1
OG0013247: TcSYL_0037590.t1-p1
OG0013248: TcSYL_0037600.t1-p1
OG0013249: TcSYL_0037610.t1-p1
OG0013250: TcSYL_0037620.t1-p1
OG0013251: TcSYL_0037630.t1-p1
OG0013252: TcSYL_0037640.t1-p1
OG0013253: TcSYL_0037650.t1-p1
OG0013254: TcSYL_0037660.t1-p1
OG0013255: TcSYL_0037670.t1-p1
OG0013256: TcSYL_0037680.t1-p1
OG0013257: TcSYL_0037690.t1-p1
OG0013258: TcSYL_0037700.t1-p1
OG0013259: TcSYL_0037710.t1-p1
OG0013260: TcSYL_0037720.t1-p1
OG0013261: TcSYL_0037730.t1-p1
OG0013262: TcSYL_0037740.t1-p1
OG0013263: TcSYL_0037750.t1-p1
OG0013264: TcSYL_0037760.t1-p1

OG0013265: TcSYL_0037770.t1-p1
OG0013266: TcSYL_0037780.t1-p1
OG0013267: TcSYL_0037790.t1-p1
OG0013268: TcSYL_0037800.t1-p1
OG0013269: TcSYL_0037810.t1-p1
OG0013270: TcSYL_0037820.t1-p1
OG0013271: TcSYL_0037830.t1-p1
OG0013272: TcSYL_0037840.t1-p1
OG0013273: TcSYL_0037850.t1-p1
OG0013274: TcSYL_0037860.t1-p1
OG0013275: TcSYL_0037870.t1-p1
OG0013276: TcSYL_0037880.t1-p1
OG0013277: TcSYL_0037890.t1-p1
OG0013278: TcSYL_0037900.t1-p1
OG0013279: TcSYL_0037910.t1-p1
OG0013280: TcSYL_0037920.t1-p1
OG0013281: TcSYL_0037930.t1-p1
OG0013282: TcSYL_0037940.t1-p1
OG0013283: TcSYL_0037950.t1-p1
OG0013284: TcSYL_0037960.t1-p1
OG0013285: TcSYL_0037970.t1-p1
OG0013286: TcSYL_0037980.t1-p1
OG0013287: TcSYL_0038000.t1-p1
OG0013288: TcSYL_0038010.t1-p1
OG0013289: TcSYL_0038020.t1-p1
OG0013290: TcSYL_0038030.t1-p1
OG0013291: TcSYL_0038040.t1-p1
OG0013292: TcSYL_0038050.t1-p1
OG0013293: TcSYL_0038060.t1-p1
OG0013294: TcSYL_0038070.t1-p1
OG0013295: TcSYL_0038080.t1-p1
OG0013296: TcSYL_0038090.t1-p1
OG0013297: TcSYL_0038100.t1-p1
OG0013298: TcSYL_0038110.t1-p1
OG0013299: TcSYL_0038120.t1-p1
OG0013300: TcSYL_0038130.t1-p1
OG0013301: TcSYL_0038140.t1-p1
OG0013302: TcSYL_0038160.t1-p1
OG0013303: TcSYL_0038180.t1-p1
OG0013304: TcSYL_0038190.t1-p1
OG0013305: TcSYL_0038200.t1-p1
OG0013306: TcSYL_0038220.t1-p1
OG0013307: TcSYL_0038230.t1-p1
OG0013308: TcSYL_0038240.t1-p1
OG0013309: TcSYL_0038260.t1-p1
OG0013310: TcSYL_0038270.t1-p1
OG0013311: TcSYL_0038280.t1-p1
OG0013312: TcSYL_0038300.t1-p1
OG0013313: TcSYL_0038310.t1-p1
OG0013314: TcSYL_0038320.t1-p1
OG0013315: TcSYL_0038330.t1-p1
OG0013316: TcSYL_0038350.t1-p1
OG0013317: TcSYL_0038360.t1-p1
OG0013318: TcSYL_0038380.t1-p1

OG0013319: TcSYL_0038390.t1-p1
OG0013320: TcSYL_0038400.t1-p1
OG0013321: TcSYL_0038420.t1-p1
OG0013322: TcSYL_0038440.t1-p1
OG0013323: TcSYL_0038470.t1-p1
OG0013324: TcSYL_0038480.t1-p1
OG0013325: TcSYL_0038490.t1-p1
OG0013326: TcSYL_0038570.t1-p1
OG0013327: TcSYL_0038580.t1-p1
OG0013328: TcSYL_0038590.t1-p1
OG0013329: TcSYL_0038600.t1-p1
OG0013330: TcSYL_0038610.t1-p1
OG0013331: TcSYL_0038620.t1-p1
OG0013332: TcSYL_0038630.t1-p1
OG0013333: TcSYL_0038650.t1-p1
OG0013334: TcSYL_0038660.t1-p1
OG0013335: TcSYL_0038670.t1-p1
OG0013336: TcSYL_0038680.t1-p1
OG0013337: TcSYL_0038710.t1-p1
OG0013338: TcSYL_0038720.t1-p1
OG0013339: TcSYL_0038730.t1-p1
OG0013340: TcSYL_0038750.t1-p1
OG0013341: TcSYL_0038760.t1-p1
OG0013342: TcSYL_0038790.t1-p1
OG0013343: TcSYL_0038800.t1-p1
OG0013344: TcSYL_0038810.t1-p1
OG0013345: TcSYL_0038820.t1-p1
OG0013346: TcSYL_0038830.t1-p1
OG0013347: TcSYL_0038860.t1-p1
OG0013348: TcSYL_0038870.t1-p1
OG0013349: TcSYL_0038880.t1-p1
OG0013350: TcSYL_0038890.t1-p1
OG0013351: TcSYL_0038900.t1-p1
OG0013352: TcSYL_0038910.t1-p1
OG0013353: TcSYL_0038920.t1-p1
OG0013354: TcSYL_0038930.t1-p1
OG0013355: TcSYL_0038940.t1-p1
OG0013356: TcSYL_0038960.t1-p1
OG0013357: TcSYL_0038970.t1-p1
OG0013358: TcSYL_0038980.t1-p1
OG0013359: TcSYL_0038990.t1-p1
OG0013360: TcSYL_0039000.t1-p1
OG0013361: TcSYL_0039010.t1-p1
OG0013362: TcSYL_0039030.t1-p1
OG0013363: TcSYL_0039040.t1-p1
OG0013364: TcSYL_0039050.t1-p1
OG0013365: TcSYL_0039060.t1-p1
OG0013366: TcSYL_0039070.t1-p1
OG0013367: TcSYL_0039080.t1-p1
OG0013368: TcSYL_0039090.t1-p1
OG0013369: TcSYL_0039100.t1-p1
OG0013370: TcSYL_0039110.t1-p1
OG0013371: TcSYL_0039120.t1-p1
OG0013372: TcSYL_0039140.t1-p1

OG0013373: TcSYL_0039150.t1-p1
OG0013374: TcSYL_0039160.t1-p1
OG0013375: TcSYL_0039170.t1-p1
OG0013376: TcSYL_0039180.t1-p1
OG0013377: TcSYL_0039190.t1-p1
OG0013378: TcSYL_0039200.t1-p1
OG0013379: TcSYL_0039210.t1-p1
OG0013380: TcSYL_0039220.t1-p1
OG0013381: TcSYL_0039230.t1-p1
OG0013382: TcSYL_0039240.t1-p1
OG0013383: TcSYL_0039250.t1-p1
OG0013384: TcSYL_0039260.t1-p1
OG0013385: TcSYL_0039270.t1-p1
OG0013386: TcSYL_0039280.t1-p1
OG0013387: TcSYL_0039290.t1-p1
OG0013388: TcSYL_0039300.t1-p1
OG0013389: TcSYL_0039310.t1-p1
OG0013390: TcSYL_0039320.t1-p1
OG0013391: TcSYL_0039330.t1-p1
OG0013392: TcSYL_0039370.t1-p1
OG0013393: TcSYL_0039380.t1-p1
OG0013394: TcSYL_0039390.t1-p1
OG0013395: TcSYL_0039430.t1-p1
OG0013396: TcSYL_0039440.t1-p1
OG0013397: TcSYL_0039450.t1-p1
OG0013398: TcSYL_0039480.t1-p1
OG0013399: TcSYL_0039500.t1-p1
OG0013400: TcSYL_0039520.t1-p1
OG0013401: TcSYL_0039530.t1-p1
OG0013402: TcSYL_0039540.t1-p1
OG0013403: TcSYL_0039560.t1-p1
OG0013404: TcSYL_0039570.t1-p1
OG0013405: TcSYL_0039580.t1-p1
OG0013406: TcSYL_0039590.t1-p1
OG0013407: TcSYL_0039600.t1-p1
OG0013408: TcSYL_0039610.t1-p1
OG0013409: TcSYL_0039620.t1-p1
OG0013410: TcSYL_0039630.t1-p1
OG0013411: TcSYL_0039660.t1-p1
OG0013412: TcSYL_0039670.t1-p1
OG0013413: TcSYL_0039690.t1-p1
OG0013414: TcSYL_0039720.t1-p1
OG0013415: TcSYL_0039730.t1-p1
OG0013416: TcSYL_0039740.t1-p1
OG0013417: TcSYL_0039750.t1-p1
OG0013418: TcSYL_0039760.t1-p1
OG0013419: TcSYL_0039770.t1-p1
OG0013420: TcSYL_0039780.t1-p1
OG0013421: TcSYL_0039800.t1-p1
OG0013422: TcSYL_0039810.t1-p1
OG0013423: TcSYL_0039830.t1-p1
OG0013424: TcSYL_0039840.t1-p1
OG0013425: TcSYL_0039870.t1-p1
OG0013426: TcSYL_0039880.t1-p1

OG0013427: TcSYL_0039910.t1-p1
OG0013428: TcSYL_0039940.t1-p1
OG0013429: TcSYL_0039950.t1-p1
OG0013430: TcSYL_0039960.t1-p1
OG0013431: TcSYL_0039970.t1-p1
OG0013432: TcSYL_0039990.t1-p1
OG0013433: TcSYL_0040000.t1-p1
OG0013434: TcSYL_0040010.t1-p1
OG0013435: TcSYL_0040040.t1-p1
OG0013436: TcSYL_0040060.t1-p1
OG0013437: TcSYL_0040120.t1-p1
OG0013438: TcSYL_0040140.t1-p1
OG0013439: TcSYL_0040170.t1-p1
OG0013440: TcSYL_0040180.t1-p1
OG0013441: TcSYL_0040190.t1-p1
OG0013442: TcSYL_0040200.t1-p1
OG0013443: TcSYL_0040210.t1-p1
OG0013444: TcSYL_0040220.t1-p1
OG0013445: TcSYL_0040230.t1-p1
OG0013446: TcSYL_0040260.t1-p1
OG0013447: TcSYL_0040270.t1-p1
OG0013448: TcSYL_0040280.t1-p1
OG0013449: TcSYL_0040290.t1-p1
OG0013450: TcSYL_0040300.t1-p1
OG0013451: TcSYL_0040310.t1-p1
OG0013452: TcSYL_0040320.t1-p1
OG0013453: TcSYL_0040330.t1-p1
OG0013454: TcSYL_0040340.t1-p1
OG0013455: TcSYL_0040350.t1-p1
OG0013456: TcSYL_0040360.t1-p1
OG0013457: TcSYL_0040370.t1-p1
OG0013458: TcSYL_0040380.t1-p1
OG0013459: TcSYL_0040390.t1-p1
OG0013460: TcSYL_0040410.t1-p1
OG0013461: TcSYL_0040430.t1-p1
OG0013462: TcSYL_0040440.t1-p1
OG0013463: TcSYL_0040450.t1-p1
OG0013464: TcSYL_0040460.t1-p1
OG0013465: TcSYL_0040470.t1-p1
OG0013466: TcSYL_0040490.t1-p1
OG0013467: TcSYL_0040500.t1-p1
OG0013468: TcSYL_0040510.t1-p1
OG0013469: TcSYL_0040520.t1-p1
OG0013470: TcSYL_0040540.t1-p1
OG0013471: TcSYL_0040560.t1-p1
OG0013472: TcSYL_0040570.t1-p1
OG0013473: TcSYL_0040580.t1-p1
OG0013474: TcSYL_0040590.t1-p1
OG0013475: TcSYL_0040600.t1-p1
OG0013476: TcSYL_0040610.t1-p1
OG0013477: TcSYL_0040620.t1-p1
OG0013478: TcSYL_0040630.t1-p1
OG0013479: TcSYL_0040640.t1-p1
OG0013480: TcSYL_0040650.t1-p1

OG0013481: TcSYL_0040660.t1-p1
OG0013482: TcSYL_0040670.t1-p1
OG0013483: TcSYL_0040700.t1-p1
OG0013484: TcSYL_0040710.t1-p1
OG0013485: TcSYL_0040720.t1-p1
OG0013486: TcSYL_0040730.t1-p1
OG0013487: TcSYL_0040740.t1-p1
OG0013488: TcSYL_0040770.t1-p1
OG0013489: TcSYL_0040780.t1-p1
OG0013490: TcSYL_0040800.t1-p1
OG0013491: TcSYL_0040810.t1-p1
OG0013492: TcSYL_0040830.t1-p1
OG0013493: TcSYL_0040840.t1-p1
OG0013494: TcSYL_0040860.t1-p1
OG0013495: TcSYL_0040880.t1-p1
OG0013496: TcSYL_0040900.t1-p1
OG0013497: TcSYL_0040920.t1-p1
OG0013498: TcSYL_0040940.t1-p1
OG0013499: TcSYL_0040950.t1-p1
OG0013500: TcSYL_0040960.t1-p1
OG0013501: TcSYL_0040970.t1-p1
OG0013502: TcSYL_0040990.t1-p1
OG0013503: TcSYL_0041000.t1-p1
OG0013504: TcSYL_0041010.t1-p1
OG0013505: TcSYL_0041020.t1-p1
OG0013506: TcSYL_0041030.t1-p1
OG0013507: TcSYL_0041040.t1-p1
OG0013508: TcSYL_0041050.t1-p1
OG0013509: TcSYL_0041060.t1-p1
OG0013510: TcSYL_0041070.t1-p1
OG0013511: TcSYL_0041090.t1-p1
OG0013512: TcSYL_0041100.t1-p1
OG0013513: TcSYL_0041110.t1-p1
OG0013514: TcSYL_0041120.t1-p1
OG0013515: TcSYL_0041130.t1-p1
OG0013516: TcSYL_0041140.t1-p1
OG0013517: TcSYL_0041150.t1-p1
OG0013518: TcSYL_0041170.t1-p1
OG0013519: TcSYL_0041180.t1-p1
OG0013520: TcSYL_0041310.t1-p1
OG0013521: TcSYL_0041320.t1-p1
OG0013522: TcSYL_0041330.t1-p1
OG0013523: TcSYL_0041340.t1-p1
OG0013524: TcSYL_0041350.t1-p1
OG0013525: TcSYL_0041360.t1-p1
OG0013526: TcSYL_0041410.t1-p1
OG0013527: TcSYL_0041420.t1-p1
OG0013528: TcSYL_0041430.t1-p1
OG0013529: TcSYL_0041440.t1-p1
OG0013530: TcSYL_0041450.t1-p1
OG0013531: TcSYL_0041460.t1-p1
OG0013532: TcSYL_0041470.t1-p1
OG0013533: TcSYL_0041480.t1-p1
OG0013534: TcSYL_0041490.t1-p1

OG0013535: TcSYL_0041500.t1-p1
OG0013536: TcSYL_0041510.t1-p1
OG0013537: TcSYL_0041520.t1-p1
OG0013538: TcSYL_0041530.t1-p1
OG0013539: TcSYL_0041540.t1-p1
OG0013540: TcSYL_0041550.t1-p1
OG0013541: TcSYL_0041560.t1-p1
OG0013542: TcSYL_0041570.t1-p1
OG0013543: TcSYL_0041590.t1-p1
OG0013544: TcSYL_0041600.t1-p1
OG0013545: TcSYL_0041610.t1-p1
OG0013546: TcSYL_0041620.t1-p1
OG0013547: TcSYL_0041630.t1-p1
OG0013548: TcSYL_0041640.t1-p1
OG0013549: TcSYL_0041650.t1-p1
OG0013550: TcSYL_0041660.t1-p1
OG0013551: TcSYL_0041670.t1-p1
OG0013552: TcSYL_0041690.t1-p1
OG0013553: TcSYL_0041700.t1-p1
OG0013554: TcSYL_0041710.t1-p1
OG0013555: TcSYL_0041720.t1-p1
OG0013556: TcSYL_0041730.t1-p1
OG0013557: TcSYL_0041740.t1-p1
OG0013558: TcSYL_0041750.t1-p1
OG0013559: TcSYL_0041760.t1-p1
OG0013560: TcSYL_0041770.t1-p1
OG0013561: TcSYL_0041780.t1-p1
OG0013562: TcSYL_0041790.t1-p1
OG0013563: TcSYL_0041800.t1-p1
OG0013564: TcSYL_0041820.t1-p1
OG0013565: TcSYL_0041830.t1-p1
OG0013566: TcSYL_0041840.t1-p1
OG0013567: TcSYL_0041860.t1-p1
OG0013568: TcSYL_0041870.t1-p1
OG0013569: TcSYL_0041880.t1-p1
OG0013570: TcSYL_0041890.t1-p1
OG0013571: TcSYL_0041900.t1-p1
OG0013572: TcSYL_0041910.t1-p1
OG0013573: TcSYL_0041920.t1-p1
OG0013574: TcSYL_0041930.t1-p1
OG0013575: TcSYL_0041940.t1-p1
OG0013576: TcSYL_0041960.t1-p1
OG0013577: TcSYL_0041970.t1-p1
OG0013578: TcSYL_0041980.t1-p1
OG0013579: TcSYL_0042000.t1-p1
OG0013580: TcSYL_0042010.t1-p1
OG0013581: TcSYL_0042020.t1-p1
OG0013582: TcSYL_0042030.t1-p1
OG0013583: TcSYL_0042040.t1-p1
OG0013584: TcSYL_0042050.t1-p1
OG0013585: TcSYL_0042060.t1-p1
OG0013586: TcSYL_0042070.t1-p1
OG0013587: TcSYL_0042080.t1-p1
OG0013588: TcSYL_0042090.t1-p1

OG0013589: TcSYL_0042100.t1-p1
OG0013590: TcSYL_0042110.t1-p1
OG0013591: TcSYL_0042120.t1-p1
OG0013592: TcSYL_0042130.t1-p1
OG0013593: TcSYL_0042140.t1-p1
OG0013594: TcSYL_0042150.t1-p1
OG0013595: TcSYL_0042160.t1-p1
OG0013596: TcSYL_0042170.t1-p1
OG0013597: TcSYL_0042180.t1-p1
OG0013598: TcSYL_0042200.t1-p1
OG0013599: TcSYL_0042210.t1-p1
OG0013600: TcSYL_0042220.t1-p1
OG0013601: TcSYL_0042230.t1-p1
OG0013602: TcSYL_0042250.t1-p1
OG0013603: TcSYL_0042260.t1-p1
OG0013604: TcSYL_0042270.t1-p1
OG0013605: TcSYL_0042280.t1-p1
OG0013606: TcSYL_0042290.t1-p1
OG0013607: TcSYL_0042310.t1-p1
OG0013608: TcSYL_0042350.t1-p1
OG0013609: TcSYL_0042360.t1-p1
OG0013610: TcSYL_0042370.t1-p1
OG0013611: TcSYL_0042380.t1-p1
OG0013612: TcSYL_0042400.t1-p1
OG0013613: TcSYL_0042420.t1-p1
OG0013614: TcSYL_0042430.t1-p1
OG0013615: TcSYL_0042440.t1-p1
OG0013616: TcSYL_0042450.t1-p1
OG0013617: TcSYL_0042480.t1-p1
OG0013618: TcSYL_0042490.t1-p1
OG0013619: TcSYL_0042500.t1-p1
OG0013620: TcSYL_0042520.t1-p1
OG0013621: TcSYL_0042530.t1-p1
OG0013622: TcSYL_0042540.t1-p1
OG0013623: TcSYL_0042570.t1-p1
OG0013624: TcSYL_0042580.t1-p1
OG0013625: TcSYL_0042590.t1-p1
OG0013626: TcSYL_0042600.t1-p1
OG0013627: TcSYL_0042620.t1-p1
OG0013628: TcSYL_0042640.t1-p1
OG0013629: TcSYL_0042650.t1-p1
OG0013630: TcSYL_0042660.t1-p1
OG0013631: TcSYL_0042670.t1-p1
OG0013632: TcSYL_0042680.t1-p1
OG0013633: TcSYL_0042700.t1-p1
OG0013634: TcSYL_0042710.t1-p1
OG0013635: TcSYL_0042720.t1-p1
OG0013636: TcSYL_0042730.t1-p1
OG0013637: TcSYL_0042760.t1-p1
OG0013638: TcSYL_0042770.t1-p1
OG0013639: TcSYL_0042790.t1-p1
OG0013640: TcSYL_0042800.t1-p1
OG0013641: TcSYL_0042820.t1-p1
OG0013642: TcSYL_0042840.t1-p1

OG0013643: TcSYL_0042850.t1-p1
OG0013644: TcSYL_0042870.t1-p1
OG0013645: TcSYL_0042880.t1-p1
OG0013646: TcSYL_0042890.t1-p1
OG0013647: TcSYL_0042900.t1-p1
OG0013648: TcSYL_0042910.t1-p1
OG0013649: TcSYL_0042920.t1-p1
OG0013650: TcSYL_0042930.t1-p1
OG0013651: TcSYL_0042940.t1-p1
OG0013652: TcSYL_0042950.t1-p1
OG0013653: TcSYL_0042960.t1-p1
OG0013654: TcSYL_0042970.t1-p1
OG0013655: TcSYL_0042990.t1-p1
OG0013656: TcSYL_0043090.t1-p1
OG0013657: TcSYL_0043110.t1-p1
OG0013658: TcSYL_0043120.t1-p1
OG0013659: TcSYL_0043130.t1-p1
OG0013660: TcSYL_0043140.t1-p1
OG0013661: TcSYL_0043170.t1-p1
OG0013662: TcSYL_0043180.t1-p1
OG0013663: TcSYL_0043190.t1-p1
OG0013664: TcSYL_0043200.t1-p1
OG0013665: TcSYL_0043210.t1-p1
OG0013666: TcSYL_0043220.t1-p1
OG0013667: TcSYL_0043240.t1-p1
OG0013668: TcSYL_0043250.t1-p1
OG0013669: TcSYL_0043270.t1-p1
OG0013670: TcSYL_0043280.t1-p1
OG0013671: TcSYL_0043300.t1-p1
OG0013672: TcSYL_0043310.t1-p1
OG0013673: TcSYL_0043320.t1-p1
OG0013674: TcSYL_0043400.t1-p1
OG0013675: TcSYL_0043410.t1-p1
OG0013676: TcSYL_0043420.t1-p1
OG0013677: TcSYL_0043430.t1-p1
OG0013678: TcSYL_0043440.t1-p1
OG0013679: TcSYL_0043460.t1-p1
OG0013680: TcSYL_0043470.t1-p1
OG0013681: TcSYL_0043490.t1-p1
OG0013682: TcSYL_0043500.t1-p1
OG0013683: TcSYL_0043510.t1-p1
OG0013684: TcSYL_0043520.t1-p1
OG0013685: TcSYL_0043540.t1-p1
OG0013686: TcSYL_0043560.t1-p1
OG0013687: TcSYL_0043570.t1-p1
OG0013688: TcSYL_0043580.t1-p1
OG0013689: TcSYL_0043590.t1-p1
OG0013690: TcSYL_0043600.t1-p1
OG0013691: TcSYL_0043610.t1-p1
OG0013692: TcSYL_0043630.t1-p1
OG0013693: TcSYL_0043660.t1-p1
OG0013694: TcSYL_0043680.t1-p1
OG0013695: TcSYL_0043700.t1-p1
OG0013696: TcSYL_0043720.t1-p1

OG0013697: TcSYL_0043740.t1-p1
OG0013698: TcSYL_0043760.t1-p1
OG0013699: TcSYL_0043770.t1-p1
OG0013700: TcSYL_0043780.t1-p1
OG0013701: TcSYL_0043790.t1-p1
OG0013702: TcSYL_0043800.t1-p1
OG0013703: TcSYL_0043820.t1-p1
OG0013704: TcSYL_0043840.t1-p1
OG0013705: TcSYL_0043850.t1-p1
OG0013706: TcSYL_0043870.t1-p1
OG0013707: TcSYL_0043880.t1-p1
OG0013708: TcSYL_0043890.t1-p1
OG0013709: TcSYL_0043930.t1-p1
OG0013710: TcSYL_0043950.t1-p1
OG0013711: TcSYL_0043960.t1-p1
OG0013712: TcSYL_0043980.t1-p1
OG0013713: TcSYL_0044000.t1-p1
OG0013714: TcSYL_0044010.t1-p1
OG0013715: TcSYL_0044020.t1-p1
OG0013716: TcSYL_0044040.t1-p1
OG0013717: TcSYL_0044060.t1-p1
OG0013718: TcSYL_0044100.t1-p1
OG0013719: TcSYL_0044120.t1-p1
OG0013720: TcSYL_0044140.t1-p1
OG0013721: TcSYL_0044160.t1-p1
OG0013722: TcSYL_0044230.t1-p1
OG0013723: TcSYL_0044240.t1-p1
OG0013724: TcSYL_0044250.t1-p1
OG0013725: TcSYL_0044280.t1-p1
OG0013726: TcSYL_0044310.t1-p1
OG0013727: TcSYL_0044320.t1-p1
OG0013728: TcSYL_0044340.t1-p1
OG0013729: TcSYL_0044360.t1-p1
OG0013730: TcSYL_0044370.t1-p1
OG0013731: TcSYL_0044380.t1-p1
OG0013732: TcSYL_0044390.t1-p1
OG0013733: TcSYL_0044450.t1-p1
OG0013734: TcSYL_0044460.t1-p1
OG0013735: TcSYL_0044470.t1-p1
OG0013736: TcSYL_0044480.t1-p1
OG0013737: TcSYL_0044490.t1-p1
OG0013738: TcSYL_0044510.t1-p1
OG0013739: TcSYL_0044520.t1-p1
OG0013740: TcSYL_0044530.t1-p1
OG0013741: TcSYL_0044550.t1-p1
OG0013742: TcSYL_0044560.t1-p1
OG0013743: TcSYL_0044570.t1-p1
OG0013744: TcSYL_0044580.t1-p1
OG0013745: TcSYL_0044600.t1-p1
OG0013746: TcSYL_0044610.t1-p1
OG0013747: TcSYL_0044670.t1-p1
OG0013748: TcSYL_0044680.t1-p1
OG0013749: TcSYL_0044690.t1-p1
OG0013750: TcSYL_0044710.t1-p1

OG0013751: TcSYL_0044740.t1-p1
OG0013752: TcSYL_0044780.t1-p1
OG0013753: TcSYL_0044790.t1-p1
OG0013754: TcSYL_0044830.t1-p1
OG0013755: TcSYL_0044840.t1-p1
OG0013756: TcSYL_0044880.t1-p1
OG0013757: TcSYL_0044890.t1-p1
OG0013758: TcSYL_0044900.t1-p1
OG0013759: TcSYL_0044910.t1-p1
OG0013760: TcSYL_0044920.t1-p1
OG0013761: TcSYL_0044950.t1-p1
OG0013762: TcSYL_0044960.t1-p1
OG0013763: TcSYL_0044990.t1-p1
OG0013764: TcSYL_0045000.t1-p1
OG0013765: TcSYL_0045010.t1-p1
OG0013766: TcSYL_0045050.t1-p1
OG0013767: TcSYL_0045090.t1-p1
OG0013768: TcSYL_0045100.t1-p1
OG0013769: TcSYL_0045130.t1-p1
OG0013770: TcSYL_0045290.t1-p1
OG0013771: TcSYL_0045400.t1-p1
OG0013772: TcSYL_0045430.t1-p1
OG0013773: TcSYL_0045440.t1-p1
OG0013774: TcSYL_0045460.t1-p1
OG0013775: TcSYL_0045470.t1-p1
OG0013776: TcSYL_0045480.t1-p1
OG0013777: TcSYL_0045490.t1-p1
OG0013778: TcSYL_0045500.t1-p1
OG0013779: TcSYL_0045510.t1-p1
OG0013780: TcSYL_0045520.t1-p1
OG0013781: TcSYL_0045530.t1-p1
OG0013782: TcSYL_0045540.t1-p1
OG0013783: TcSYL_0045550.t1-p1
OG0013784: TcSYL_0045560.t1-p1
OG0013785: TcSYL_0045570.t1-p1
OG0013786: TcSYL_0045580.t1-p1
OG0013787: TcSYL_0045590.t1-p1
OG0013788: TcSYL_0045600.t1-p1
OG0013789: TcSYL_0045610.t1-p1
OG0013790: TcSYL_0045620.t1-p1
OG0013791: TcSYL_0045630.t1-p1
OG0013792: TcSYL_0045640.t1-p1
OG0013793: TcSYL_0045650.t1-p1
OG0013794: TcSYL_0045660.t1-p1
OG0013795: TcSYL_0045670.t1-p1
OG0013796: TcSYL_0045690.t1-p1
OG0013797: TcSYL_0045700.t1-p1
OG0013798: TcSYL_0045720.t1-p1
OG0013799: TcSYL_0045730.t1-p1
OG0013800: TcSYL_0045740.t1-p1
OG0013801: TcSYL_0045750.t1-p1
OG0013802: TcSYL_0045760.t1-p1
OG0013803: TcSYL_0045770.t1-p1
OG0013804: TcSYL_0045780.t1-p1

OG0013805: TcSYL_0045850.t1-p1
OG0013806: TcSYL_0045960.t1-p1
OG0013807: TcSYL_0046060.t1-p1
OG0013808: TcSYL_0046070.t1-p1
OG0013809: TcSYL_0046080.t1-p1
OG0013810: TcSYL_0046100.t1-p1
OG0013811: TcSYL_0046110.t1-p1
OG0013812: TcSYL_0046120.t1-p1
OG0013813: TcSYL_0046130.t1-p1
OG0013814: TcSYL_0046150.t1-p1
OG0013815: TcSYL_0046320.t1-p1
OG0013816: TcSYL_0046340.t1-p1
OG0013817: TcSYL_0046450.t1-p1
OG0013818: TcSYL_0046460.t1-p1
OG0013819: TcSYL_0046470.t1-p1
OG0013820: TcSYL_0046490.t1-p1
OG0013821: TcSYL_0046500.t1-p1
OG0013822: TcSYL_0046520.t1-p1
OG0013823: TcSYL_0046530.t1-p1
OG0013824: TcSYL_0046540.t1-p1
OG0013825: TcSYL_0046560.t1-p1
OG0013826: TcSYL_0046570.t1-p1
OG0013827: TcSYL_0046580.t1-p1
OG0013828: TcSYL_0046600.t1-p1
OG0013829: TcSYL_0046610.t1-p1
OG0013830: TcSYL_0046620.t1-p1
OG0013831: TcSYL_0046660.t1-p1
OG0013832: TcSYL_0046750.t1-p1
OG0013833: TcSYL_0046760.t1-p1
OG0013834: TcSYL_0046770.t1-p1
OG0013835: TcSYL_0046780.t1-p1
OG0013836: TcSYL_0046790.t1-p1
OG0013837: TcSYL_0046800.t1-p1
OG0013838: TcSYL_0046820.t1-p1
OG0013839: TcSYL_0046830.t1-p1
OG0013840: TcSYL_0046900.t1-p1
OG0013841: TcSYL_0046910.t1-p1
OG0013842: TcSYL_0046980.t1-p1
OG0013843: TcSYL_0047080.t1-p1
OG0013844: TcSYL_0047180.t1-p1
OG0013845: TcSYL_0047210.t1-p1
OG0013846: TcSYL_0047230.t1-p1
OG0013847: TcSYL_0047270.t1-p1
OG0013848: TcSYL_0047280.t1-p1
OG0013849: TcSYL_0047290.t1-p1
OG0013850: TcSYL_0047300.t1-p1
OG0013851: TcSYL_0047310.t1-p1
OG0013852: TcSYL_0047340.t1-p1
OG0013853: TcSYL_0047370.t1-p1
OG0013854: TcSYL_0047390.t1-p1
OG0013855: TcSYL_0047400.t1-p1
OG0013856: TcSYL_0047430.t1-p1
OG0013857: TcSYL_0047540.t1-p1
OG0013858: TcSYL_0047620.t1-p1

OG0013859: TcSYL_0047650.t1-p1
OG0013860: TcSYL_0047680.t1-p1
OG0013861: TcSYL_0047700.t1-p1
OG0013862: TcSYL_0047760.t1-p1
OG0013863: TcSYL_0047780.t1-p1
OG0013864: TcSYL_0047830.t1-p1
OG0013865: TcSYL_0047950.t1-p1
OG0013866: TcSYL_0047960.t1-p1
OG0013867: TcSYL_0048030.t1-p1
OG0013868: TcSYL_0048110.t1-p1
OG0013869: TcSYL_0048170.t1-p1
OG0013870: TcSYL_0048210.t1-p1
OG0013871: TcSYL_0048220.t1-p1
OG0013872: TcSYL_0048230.t1-p1
OG0013873: TcSYL_0048250.t1-p1
OG0013874: TcSYL_0048260.t1-p1
OG0013875: TcSYL_0048270.t1-p1
OG0013876: TcSYL_0048290.t1-p1
OG0013877: TcSYL_0048300.t1-p1
OG0013878: TcSYL_0048310.t1-p1
OG0013879: TcSYL_0048360.t1-p1
OG0013880: TcSYL_0048370.t1-p1
OG0013881: TcSYL_0048380.t1-p1
OG0013882: TcSYL_0048400.t1-p1
OG0013883: TcSYL_0048410.t1-p1
OG0013884: TcSYL_0048420.t1-p1
OG0013885: TcSYL_0048430.t1-p1
OG0013886: TcSYL_0048450.t1-p1
OG0013887: TcSYL_0048460.t1-p1
OG0013888: TcSYL_0048480.t1-p1
OG0013889: TcSYL_0048510.t1-p1
OG0013890: TcSYL_0048540.t1-p1
OG0013891: TcSYL_0048550.t1-p1
OG0013892: TcSYL_0048560.t1-p1
OG0013893: TcSYL_0048580.t1-p1
OG0013894: TcSYL_0048590.t1-p1
OG0013895: TcSYL_0048610.t1-p1
OG0013896: TcSYL_0048690.t1-p1
OG0013897: TcSYL_0048700.t1-p1
OG0013898: TcSYL_0048720.t1-p1
OG0013899: TcSYL_0048740.t1-p1
OG0013900: TcSYL_0048750.t1-p1
OG0013901: TcSYL_0048770.t1-p1
OG0013902: TcSYL_0048780.t1-p1
OG0013903: TcSYL_0048800.t1-p1
OG0013904: TcSYL_0048830.t1-p1
OG0013905: TcSYL_0048840.t1-p1
OG0013906: TcSYL_0048860.t1-p1
OG0013907: TcSYL_0048870.t1-p1
OG0013908: TcSYL_0048890.t1-p1
OG0013909: TcSYL_0048900.t1-p1
OG0013910: TcSYL_0048910.t1-p1
OG0013911: TcSYL_0048920.t1-p1
OG0013912: TcSYL_0048930.t1-p1

OG0013913: TcSYL_0048940.t1-p1
OG0013914: TcSYL_0048950.t1-p1
OG0013915: TcSYL_0048960.t1-p1
OG0013916: TcSYL_0048970.t1-p1
OG0013917: TcSYL_0048980.t1-p1
OG0013918: TcSYL_0049000.t1-p1
OG0013919: TcSYL_0049010.t1-p1
OG0013920: TcSYL_0049040.t1-p1
OG0013921: TcSYL_0049050.t1-p1
OG0013922: TcSYL_0049070.t1-p1
OG0013923: TcSYL_0049080.t1-p1
OG0013924: TcSYL_0049090.t1-p1
OG0013925: TcSYL_0049100.t1-p1
OG0013926: TcSYL_0049110.t1-p1
OG0013927: TcSYL_0049120.t1-p1
OG0013928: TcSYL_0049140.t1-p1
OG0013929: TcSYL_0049170.t1-p1
OG0013930: TcSYL_0049180.t1-p1
OG0013931: TcSYL_0049200.t1-p1
OG0013932: TcSYL_0049210.t1-p1
OG0013933: TcSYL_0049220.t1-p1
OG0013934: TcSYL_0049230.t1-p1
OG0013935: TcSYL_0049240.t1-p1
OG0013936: TcSYL_0049250.t1-p1
OG0013937: TcSYL_0049280.t1-p1
OG0013938: TcSYL_0049290.t1-p1
OG0013939: TcSYL_0049300.t1-p1
OG0013940: TcSYL_0049310.t1-p1
OG0013941: TcSYL_0049320.t1-p1
OG0013942: TcSYL_0049330.t1-p1
OG0013943: TcSYL_0049350.t1-p1
OG0013944: TcSYL_0049370.t1-p1
OG0013945: TcSYL_0049380.t1-p1
OG0013946: TcSYL_0049390.t1-p1
OG0013947: TcSYL_0049400.t1-p1
OG0013948: TcSYL_0049420.t1-p1
OG0013949: TcSYL_0049430.t1-p1
OG0013950: TcSYL_0049440.t1-p1
OG0013951: TcSYL_0049450.t1-p1
OG0013952: TcSYL_0049470.t1-p1
OG0013953: TcSYL_0049480.t1-p1
OG0013954: TcSYL_0049490.t1-p1
OG0013955: TcSYL_0049500.t1-p1
OG0013956: TcSYL_0049510.t1-p1
OG0013957: TcSYL_0049520.t1-p1
OG0013958: TcSYL_0049530.t1-p1
OG0013959: TcSYL_0049540.t1-p1
OG0013960: TcSYL_0049550.t1-p1
OG0013961: TcSYL_0049560.t1-p1
OG0013962: TcSYL_0049590.t1-p1
OG0013963: TcSYL_0049600.t1-p1
OG0013964: TcSYL_0049610.t1-p1
OG0013965: TcSYL_0049620.t1-p1
OG0013966: TcSYL_0049630.t1-p1

OG0013967: TcSYL_0049660.t1-p1
OG0013968: TcSYL_0049670.t1-p1
OG0013969: TcSYL_0049680.t1-p1
OG0013970: TcSYL_0049690.t1-p1
OG0013971: TcSYL_0049720.t1-p1
OG0013972: TcSYL_0049730.t1-p1
OG0013973: TcSYL_0049740.t1-p1
OG0013974: TcSYL_0049750.t1-p1
OG0013975: TcSYL_0049760.t1-p1
OG0013976: TcSYL_0049770.t1-p1
OG0013977: TcSYL_0049780.t1-p1
OG0013978: TcSYL_0049790.t1-p1
OG0013979: TcSYL_0049800.t1-p1
OG0013980: TcSYL_0049810.t1-p1
OG0013981: TcSYL_0049820.t1-p1
OG0013982: TcSYL_0049830.t1-p1
OG0013983: TcSYL_0049840.t1-p1
OG0013984: TcSYL_0049850.t1-p1
OG0013985: TcSYL_0049860.t1-p1
OG0013986: TcSYL_0049870.t1-p1
OG0013987: TcSYL_0049890.t1-p1
OG0013988: TcSYL_0049900.t1-p1
OG0013989: TcSYL_0049910.t1-p1
OG0013990: TcSYL_0049920.t1-p1
OG0013991: TcSYL_0049930.t1-p1
OG0013992: TcSYL_0049950.t1-p1
OG0013993: TcSYL_0049960.t1-p1
OG0013994: TcSYL_0049970.t1-p1
OG0013995: TcSYL_0050020.t1-p1
OG0013996: TcSYL_0050070.t1-p1
OG0013997: TcSYL_0050250.t1-p1
OG0013998: TcSYL_0050260.t1-p1
OG0013999: TcSYL_0050270.t1-p1
OG0014000: TcSYL_0050280.t1-p1
OG0014001: TcSYL_0050290.t1-p1
OG0014002: TcSYL_0050300.t1-p1
OG0014003: TcSYL_0050310.t1-p1
OG0014004: TcSYL_0050320.t1-p1
OG0014005: TcSYL_0050340.t1-p1
OG0014006: TcSYL_0050350.t1-p1
OG0014007: TcSYL_0050360.t1-p1
OG0014008: TcSYL_0050370.t1-p1
OG0014009: TcSYL_0050380.t1-p1
OG0014010: TcSYL_0050390.t1-p1
OG0014011: TcSYL_0050400.t1-p1
OG0014012: TcSYL_0050410.t1-p1
OG0014013: TcSYL_0050420.t1-p1
OG0014014: TcSYL_0050430.t1-p1
OG0014015: TcSYL_0050440.t1-p1
OG0014016: TcSYL_0050450.t1-p1
OG0014017: TcSYL_0050460.t1-p1
OG0014018: TcSYL_0050470.t1-p1
OG0014019: TcSYL_0050480.t1-p1
OG0014020: TcSYL_0050490.t1-p1

OG0014021: TcSYL_0050500.t1-p1
OG0014022: TcSYL_0050520.t1-p1
OG0014023: TcSYL_0050530.t1-p1
OG0014024: TcSYL_0050540.t1-p1
OG0014025: TcSYL_0050550.t1-p1
OG0014026: TcSYL_0050560.t1-p1
OG0014027: TcSYL_0050570.t1-p1
OG0014028: TcSYL_0050580.t1-p1
OG0014029: TcSYL_0050590.t1-p1
OG0014030: TcSYL_0050600.t1-p1
OG0014031: TcSYL_0050610.t1-p1
OG0014032: TcSYL_0050620.t1-p1
OG0014033: TcSYL_0050630.t1-p1
OG0014034: TcSYL_0050640.t1-p1
OG0014035: TcSYL_0050650.t1-p1
OG0014036: TcSYL_0050660.t1-p1
OG0014037: TcSYL_0050670.t1-p1
OG0014038: TcSYL_0050680.t1-p1
OG0014039: TcSYL_0050690.t1-p1
OG0014040: TcSYL_0050700.t1-p1
OG0014041: TcSYL_0050710.t1-p1
OG0014042: TcSYL_0050720.t1-p1
OG0014043: TcSYL_0050730.t1-p1
OG0014044: TcSYL_0050750.t1-p1
OG0014045: TcSYL_0050770.t1-p1
OG0014046: TcSYL_0050840.t1-p1
OG0014047: TcSYL_0050850.t1-p1
OG0014048: TcSYL_0050860.t1-p1
OG0014049: TcSYL_0050870.t1-p1
OG0014050: TcSYL_0050890.t1-p1
OG0014051: TcSYL_0050900.t1-p1
OG0014052: TcSYL_0050910.t1-p1
OG0014053: TcSYL_0050920.t1-p1
OG0014054: TcSYL_0050930.t1-p1
OG0014055: TcSYL_0050950.t1-p1
OG0014056: TcSYL_0050960.t1-p1
OG0014057: TcSYL_0050970.t1-p1
OG0014058: TcSYL_0050980.t1-p1
OG0014059: TcSYL_0051000.t1-p1
OG0014060: TcSYL_0051010.t1-p1
OG0014061: TcSYL_0051020.t1-p1
OG0014062: TcSYL_0051030.t1-p1
OG0014063: TcSYL_0051040.t1-p1
OG0014064: TcSYL_0051050.t1-p1
OG0014065: TcSYL_0051070.t1-p1
OG0014066: TcSYL_0051080.t1-p1
OG0014067: TcSYL_0051090.t1-p1
OG0014068: TcSYL_0051110.t1-p1
OG0014069: TcSYL_0051120.t1-p1
OG0014070: TcSYL_0051130.t1-p1
OG0014071: TcSYL_0051140.t1-p1
OG0014072: TcSYL_0051150.t1-p1
OG0014073: TcSYL_0051160.t1-p1
OG0014074: TcSYL_0051190.t1-p1

OG0014075: TcSYL_0051200.t1-p1
OG0014076: TcSYL_0051210.t1-p1
OG0014077: TcSYL_0051220.t1-p1
OG0014078: TcSYL_0051230.t1-p1
OG0014079: TcSYL_0051240.t1-p1
OG0014080: TcSYL_0051250.t1-p1
OG0014081: TcSYL_0051270.t1-p1
OG0014082: TcSYL_0051280.t1-p1
OG0014083: TcSYL_0051290.t1-p1
OG0014084: TcSYL_0051310.t1-p1
OG0014085: TcSYL_0051320.t1-p1
OG0014086: TcSYL_0051330.t1-p1
OG0014087: TcSYL_0051340.t1-p1
OG0014088: TcSYL_0051360.t1-p1
OG0014089: TcSYL_0051370.t1-p1
OG0014090: TcSYL_0051380.t1-p1
OG0014091: TcSYL_0051390.t1-p1
OG0014092: TcSYL_0051400.t1-p1
OG0014093: TcSYL_0051410.t1-p1
OG0014094: TcSYL_0051420.t1-p1
OG0014095: TcSYL_0051430.t1-p1
OG0014096: TcSYL_0051440.t1-p1
OG0014097: TcSYL_0051460.t1-p1
OG0014098: TcSYL_0051470.t1-p1
OG0014099: TcSYL_0051480.t1-p1
OG0014100: TcSYL_0051510.t1-p1
OG0014101: TcSYL_0051520.t1-p1
OG0014102: TcSYL_0051530.t1-p1
OG0014103: TcSYL_0051540.t1-p1
OG0014104: TcSYL_0051550.t1-p1
OG0014105: TcSYL_0051560.t1-p1
OG0014106: TcSYL_0051570.t1-p1
OG0014107: TcSYL_0051580.t1-p1
OG0014108: TcSYL_0051600.t1-p1
OG0014109: TcSYL_0051610.t1-p1
OG0014110: TcSYL_0051620.t1-p1
OG0014111: TcSYL_0051630.t1-p1
OG0014112: TcSYL_0051640.t1-p1
OG0014113: TcSYL_0051650.t1-p1
OG0014114: TcSYL_0051680.t1-p1
OG0014115: TcSYL_0051690.t1-p1
OG0014116: TcSYL_0051700.t1-p1
OG0014117: TcSYL_0051710.t1-p1
OG0014118: TcSYL_0051720.t1-p1
OG0014119: TcSYL_0051740.t1-p1
OG0014120: TcSYL_0051750.t1-p1
OG0014121: TcSYL_0051760.t1-p1
OG0014122: TcSYL_0051780.t1-p1
OG0014123: TcSYL_0051820.t1-p1
OG0014124: TcSYL_0051840.t1-p1
OG0014125: TcSYL_0051850.t1-p1
OG0014126: TcSYL_0051860.t1-p1
OG0014127: TcSYL_0051880.t1-p1
OG0014128: TcSYL_0051890.t1-p1

OG0014129: TcSYL_0051900.t1-p1
OG0014130: TcSYL_0051910.t1-p1
OG0014131: TcSYL_0051920.t1-p1
OG0014132: TcSYL_0051940.t1-p1
OG0014133: TcSYL_0051950.t1-p1
OG0014134: TcSYL_0051960.t1-p1
OG0014135: TcSYL_0051970.t1-p1
OG0014136: TcSYL_0051980.t1-p1
OG0014137: TcSYL_0051990.t1-p1
OG0014138: TcSYL_0052000.t1-p1
OG0014139: TcSYL_0052010.t1-p1
OG0014140: TcSYL_0052020.t1-p1
OG0014141: TcSYL_0052030.t1-p1
OG0014142: TcSYL_0052050.t1-p1
OG0014143: TcSYL_0052060.t1-p1
OG0014144: TcSYL_0052090.t1-p1
OG0014145: TcSYL_0052100.t1-p1
OG0014146: TcSYL_0052110.t1-p1
OG0014147: TcSYL_0052120.t1-p1
OG0014148: TcSYL_0052130.t1-p1
OG0014149: TcSYL_0052140.t1-p1
OG0014150: TcSYL_0052150.t1-p1
OG0014151: TcSYL_0052160.t1-p1
OG0014152: TcSYL_0052170.t1-p1
OG0014153: TcSYL_0052180.t1-p1
OG0014154: TcSYL_0052190.t1-p1
OG0014155: TcSYL_0052210.t1-p1
OG0014156: TcSYL_0052220.t1-p1
OG0014157: TcSYL_0052230.t1-p1
OG0014158: TcSYL_0052240.t1-p1
OG0014159: TcSYL_0052250.t1-p1
OG0014160: TcSYL_0052260.t1-p1
OG0014161: TcSYL_0052270.t1-p1
OG0014162: TcSYL_0052280.t1-p1
OG0014163: TcSYL_0052290.t1-p1
OG0014164: TcSYL_0052300.t1-p1
OG0014165: TcSYL_0052310.t1-p1
OG0014166: TcSYL_0052320.t1-p1
OG0014167: TcSYL_0052330.t1-p1
OG0014168: TcSYL_0052340.t1-p1
OG0014169: TcSYL_0052350.t1-p1
OG0014170: TcSYL_0052360.t1-p1
OG0014171: TcSYL_0052370.t1-p1
OG0014172: TcSYL_0052380.t1-p1
OG0014173: TcSYL_0052390.t1-p1
OG0014174: TcSYL_0052400.t1-p1
OG0014175: TcSYL_0052410.t1-p1
OG0014176: TcSYL_0052420.t1-p1
OG0014177: TcSYL_0052430.t1-p1
OG0014178: TcSYL_0052440.t1-p1
OG0014179: TcSYL_0052450.t1-p1
OG0014180: TcSYL_0052460.t1-p1
OG0014181: TcSYL_0052470.t1-p1
OG0014182: TcSYL_0052480.t1-p1

OG0014183: TcSYL_0052490.t1-p1
OG0014184: TcSYL_0052500.t1-p1
OG0014185: TcSYL_0052510.t1-p1
OG0014186: TcSYL_0052520.t1-p1
OG0014187: TcSYL_0052530.t1-p1
OG0014188: TcSYL_0052540.t1-p1
OG0014189: TcSYL_0052550.t1-p1
OG0014190: TcSYL_0052560.t1-p1
OG0014191: TcSYL_0052570.t1-p1
OG0014192: TcSYL_0052580.t1-p1
OG0014193: TcSYL_0052590.t1-p1
OG0014194: TcSYL_0052600.t1-p1
OG0014195: TcSYL_0052610.t1-p1
OG0014196: TcSYL_0052640.t1-p1
OG0014197: TcSYL_0052650.t1-p1
OG0014198: TcSYL_0052670.t1-p1
OG0014199: TcSYL_0052680.t1-p1
OG0014200: TcSYL_0052690.t1-p1
OG0014201: TcSYL_0052700.t1-p1
OG0014202: TcSYL_0052710.t1-p1
OG0014203: TcSYL_0052720.t1-p1
OG0014204: TcSYL_0052740.t1-p1
OG0014205: TcSYL_0052750.t1-p1
OG0014206: TcSYL_0052760.t1-p1
OG0014207: TcSYL_0052770.t1-p1
OG0014208: TcSYL_0052780.t1-p1
OG0014209: TcSYL_0052790.t1-p1
OG0014210: TcSYL_0052800.t1-p1
OG0014211: TcSYL_0052810.t1-p1
OG0014212: TcSYL_0052820.t1-p1
OG0014213: TcSYL_0052840.t1-p1
OG0014214: TcSYL_0052850.t1-p1
OG0014215: TcSYL_0052860.t1-p1
OG0014216: TcSYL_0052880.t1-p1
OG0014217: TcSYL_0052890.t1-p1
OG0014218: TcSYL_0052900.t1-p1
OG0014219: TcSYL_0052920.t1-p1
OG0014220: TcSYL_0052930.t1-p1
OG0014221: TcSYL_0052940.t1-p1
OG0014222: TcSYL_0052950.t1-p1
OG0014223: TcSYL_0052960.t1-p1
OG0014224: TcSYL_0052970.t1-p1
OG0014225: TcSYL_0052980.t1-p1
OG0014226: TcSYL_0052990.t1-p1
OG0014227: TcSYL_0053000.t1-p1
OG0014228: TcSYL_0053010.t1-p1
OG0014229: TcSYL_0053020.t1-p1
OG0014230: TcSYL_0053030.t1-p1
OG0014231: TcSYL_0053040.t1-p1
OG0014232: TcSYL_0053050.t1-p1
OG0014233: TcSYL_0053060.t1-p1
OG0014234: TcSYL_0053070.t1-p1
OG0014235: TcSYL_0053080.t1-p1
OG0014236: TcSYL_0053090.t1-p1

OG0014237: TcSYL_0053100.t1-p1
OG0014238: TcSYL_0053110.t1-p1
OG0014239: TcSYL_0053120.t1-p1
OG0014240: TcSYL_0053130.t1-p1
OG0014241: TcSYL_0053150.t1-p1
OG0014242: TcSYL_0053160.t1-p1
OG0014243: TcSYL_0053170.t1-p1
OG0014244: TcSYL_0053180.t1-p1
OG0014245: TcSYL_0053190.t1-p1
OG0014246: TcSYL_0053200.t1-p1
OG0014247: TcSYL_0053210.t1-p1
OG0014248: TcSYL_0053220.t1-p1
OG0014249: TcSYL_0053230.t1-p1
OG0014250: TcSYL_0053240.t1-p1
OG0014251: TcSYL_0053250.t1-p1
OG0014252: TcSYL_0053260.t1-p1
OG0014253: TcSYL_0053270.t1-p1
OG0014254: TcSYL_0053280.t1-p1
OG0014255: TcSYL_0053290.t1-p1
OG0014256: TcSYL_0053300.t1-p1
OG0014257: TcSYL_0053310.t1-p1
OG0014258: TcSYL_0053320.t1-p1
OG0014259: TcSYL_0053330.t1-p1
OG0014260: TcSYL_0053340.t1-p1
OG0014261: TcSYL_0053350.t1-p1
OG0014262: TcSYL_0053360.t1-p1
OG0014263: TcSYL_0053370.t1-p1
OG0014264: TcSYL_0053380.t1-p1
OG0014265: TcSYL_0053390.t1-p1
OG0014266: TcSYL_0053400.t1-p1
OG0014267: TcSYL_0053410.t1-p1
OG0014268: TcSYL_0053420.t1-p1
OG0014269: TcSYL_0053430.t1-p1
OG0014270: TcSYL_0053440.t1-p1
OG0014271: TcSYL_0053450.t1-p1
OG0014272: TcSYL_0053460.t1-p1
OG0014273: TcSYL_0053470.t1-p1
OG0014274: TcSYL_0053480.t1-p1
OG0014275: TcSYL_0053510.t1-p1
OG0014276: TcSYL_0053520.t1-p1
OG0014277: TcSYL_0053530.t1-p1
OG0014278: TcSYL_0053560.t1-p1
OG0014279: TcSYL_0053570.t1-p1
OG0014280: TcSYL_0053580.t1-p1
OG0014281: TcSYL_0053590.t1-p1
OG0014282: TcSYL_0053600.t1-p1
OG0014283: TcSYL_0053610.t1-p1
OG0014284: TcSYL_0053620.t1-p1
OG0014285: TcSYL_0053630.t1-p1
OG0014286: TcSYL_0053640.t1-p1
OG0014287: TcSYL_0053650.t1-p1
OG0014288: TcSYL_0053660.t1-p1
OG0014289: TcSYL_0053670.t1-p1
OG0014290: TcSYL_0053680.t1-p1

OG0014291: TcSYL_0053690.t1-p1
OG0014292: TcSYL_0053700.t1-p1
OG0014293: TcSYL_0053710.t1-p1
OG0014294: TcSYL_0053720.t1-p1
OG0014295: TcSYL_0053730.t1-p1
OG0014296: TcSYL_0053740.t1-p1
OG0014297: TcSYL_0053750.t1-p1
OG0014298: TcSYL_0053760.t1-p1
OG0014299: TcSYL_0053770.t1-p1
OG0014300: TcSYL_0053780.t1-p1
OG0014301: TcSYL_0053790.t1-p1
OG0014302: TcSYL_0053800.t1-p1
OG0014303: TcSYL_0053810.t1-p1
OG0014304: TcSYL_0053820.t1-p1
OG0014305: TcSYL_0053830.t1-p1
OG0014306: TcSYL_0053840.t1-p1
OG0014307: TcSYL_0053850.t1-p1
OG0014308: TcSYL_0053860.t1-p1
OG0014309: TcSYL_0053870.t1-p1
OG0014310: TcSYL_0053880.t1-p1
OG0014311: TcSYL_0053890.t1-p1
OG0014312: TcSYL_0053900.t1-p1
OG0014313: TcSYL_0053910.t1-p1
OG0014314: TcSYL_0053920.t1-p1
OG0014315: TcSYL_0053930.t1-p1
OG0014316: TcSYL_0053940.t1-p1
OG0014317: TcSYL_0053950.t1-p1
OG0014318: TcSYL_0053970.t1-p1
OG0014319: TcSYL_0053980.t1-p1
OG0014320: TcSYL_0053990.t1-p1
OG0014321: TcSYL_0054000.t1-p1
OG0014322: TcSYL_0054010.t1-p1
OG0014323: TcSYL_0054020.t1-p1
OG0014324: TcSYL_0054030.t1-p1
OG0014325: TcSYL_0054040.t1-p1
OG0014326: TcSYL_0054050.t1-p1
OG0014327: TcSYL_0054060.t1-p1
OG0014328: TcSYL_0054070.t1-p1
OG0014329: TcSYL_0054080.t1-p1
OG0014330: TcSYL_0054090.t1-p1
OG0014331: TcSYL_0054100.t1-p1
OG0014332: TcSYL_0054110.t1-p1
OG0014333: TcSYL_0054120.t1-p1
OG0014334: TcSYL_0054130.t1-p1
OG0014335: TcSYL_0054140.t1-p1
OG0014336: TcSYL_0054150.t1-p1
OG0014337: TcSYL_0054160.t1-p1
OG0014338: TcSYL_0054170.t1-p1
OG0014339: TcSYL_0054180.t1-p1
OG0014340: TcSYL_0054190.t1-p1
OG0014341: TcSYL_0054200.t1-p1
OG0014342: TcSYL_0054210.t1-p1
OG0014343: TcSYL_0054220.t1-p1
OG0014344: TcSYL_0054230.t1-p1

OG0014345: TcSYL_0054240.t1-p1
OG0014346: TcSYL_0054250.t1-p1
OG0014347: TcSYL_0054260.t1-p1
OG0014348: TcSYL_0054270.t1-p1
OG0014349: TcSYL_0054280.t1-p1
OG0014350: TcSYL_0054290.t1-p1
OG0014351: TcSYL_0054300.t1-p1
OG0014352: TcSYL_0054310.t1-p1
OG0014353: TcSYL_0054320.t1-p1
OG0014354: TcSYL_0054330.t1-p1
OG0014355: TcSYL_0054340.t1-p1
OG0014356: TcSYL_0054350.t1-p1
OG0014357: TcSYL_0054360.t1-p1
OG0014358: TcSYL_0054370.t1-p1
OG0014359: TcSYL_0054380.t1-p1
OG0014360: TcSYL_0054390.t1-p1
OG0014361: TcSYL_0054400.t1-p1
OG0014362: TcSYL_0054410.t1-p1
OG0014363: TcSYL_0054420.t1-p1
OG0014364: TcSYL_0054430.t1-p1
OG0014365: TcSYL_0054440.t1-p1
OG0014366: TcSYL_0054450.t1-p1
OG0014367: TcSYL_0054460.t1-p1
OG0014368: TcSYL_0054470.t1-p1
OG0014369: TcSYL_0054480.t1-p1
OG0014370: TcSYL_0054490.t1-p1
OG0014371: TcSYL_0054510.t1-p1
OG0014372: TcSYL_0054520.t1-p1
OG0014373: TcSYL_0054530.t1-p1
OG0014374: TcSYL_0054540.t1-p1
OG0014375: TcSYL_0054550.t1-p1
OG0014376: TcSYL_0054560.t1-p1
OG0014377: TcSYL_0054570.t1-p1
OG0014378: TcSYL_0054580.t1-p1
OG0014379: TcSYL_0054590.t1-p1
OG0014380: TcSYL_0054600.t1-p1
OG0014381: TcSYL_0054610.t1-p1
OG0014382: TcSYL_0054620.t1-p1
OG0014383: TcSYL_0054630.t1-p1
OG0014384: TcSYL_0054640.t1-p1
OG0014385: TcSYL_0054650.t1-p1
OG0014386: TcSYL_0054660.t1-p1
OG0014387: TcSYL_0054670.t1-p1
OG0014388: TcSYL_0054680.t1-p1
OG0014389: TcSYL_0054690.t1-p1
OG0014390: TcSYL_0054700.t1-p1
OG0014391: TcSYL_0054710.t1-p1
OG0014392: TcSYL_0054720.t1-p1
OG0014393: TcSYL_0054730.t1-p1
OG0014394: TcSYL_0054740.t1-p1
OG0014395: TcSYL_0054750.t1-p1
OG0014396: TcSYL_0054760.t1-p1
OG0014397: TcSYL_0054780.t1-p1
OG0014398: TcSYL_0054790.t1-p1

OG0014399: TcSYL_0054800.t1-p1
OG0014400: TcSYL_0054810.t1-p1
OG0014401: TcSYL_0054820.t1-p1
OG0014402: TcSYL_0054830.t1-p1
OG0014403: TcSYL_0054840.t1-p1
OG0014404: TcSYL_0054850.t1-p1
OG0014405: TcSYL_0054860.t1-p1
OG0014406: TcSYL_0054870.t1-p1
OG0014407: TcSYL_0054880.t1-p1
OG0014408: TcSYL_0054890.t1-p1
OG0014409: TcSYL_0054900.t1-p1
OG0014410: TcSYL_0054910.t1-p1
OG0014411: TcSYL_0054920.t1-p1
OG0014412: TcSYL_0054930.t1-p1
OG0014413: TcSYL_0054940.t1-p1
OG0014414: TcSYL_0054950.t1-p1
OG0014415: TcSYL_0054960.t1-p1
OG0014416: TcSYL_0054970.t1-p1
OG0014417: TcSYL_0054980.t1-p1
OG0014418: TcSYL_0054990.t1-p1
OG0014419: TcSYL_0055000.t1-p1
OG0014420: TcSYL_0055010.t1-p1
OG0014421: TcSYL_0055020.t1-p1
OG0014422: TcSYL_0055030.t1-p1
OG0014423: TcSYL_0055040.t1-p1
OG0014424: TcSYL_0055050.t1-p1
OG0014425: TcSYL_0055060.t1-p1
OG0014426: TcSYL_0055070.t1-p1
OG0014427: TcSYL_0055080.t1-p1
OG0014428: TcSYL_0055090.t1-p1
OG0014429: TcSYL_0055100.t1-p1
OG0014430: TcSYL_0055110.t1-p1
OG0014431: TcSYL_0055120.t1-p1
OG0014432: TcSYL_0055130.t1-p1
OG0014433: TcSYL_0055140.t1-p1
OG0014434: TcSYL_0055150.t1-p1
OG0014435: TcSYL_0055160.t1-p1
OG0014436: TcSYL_0055170.t1-p1
OG0014437: TcSYL_0055180.t1-p1
OG0014438: TcSYL_0055190.t1-p1
OG0014439: TcSYL_0055200.t1-p1
OG0014440: TcSYL_0055210.t1-p1
OG0014441: TcSYL_0055220.t1-p1
OG0014442: TcSYL_0055230.t1-p1
OG0014443: TcSYL_0055240.t1-p1
OG0014444: TcSYL_0055250.t1-p1
OG0014445: TcSYL_0055260.t1-p1
OG0014446: TcSYL_0055270.t1-p1
OG0014447: TcSYL_0055280.t1-p1
OG0014448: TcSYL_0055290.t1-p1
OG0014449: TcSYL_0055300.t1-p1
OG0014450: TcSYL_0055310.t1-p1
OG0014451: TcSYL_0055320.t1-p1
OG0014452: TcSYL_0055330.t1-p1

OG0014453: TcSYL_0055350.t1-p1
OG0014454: TcSYL_0055360.t1-p1
OG0014455: TcSYL_0055370.t1-p1
OG0014456: TcSYL_0055380.t1-p1
OG0014457: TcSYL_0055390.t1-p1
OG0014458: TcSYL_0055400.t1-p1
OG0014459: TcSYL_0055410.t1-p1
OG0014460: TcSYL_0055420.t1-p1
OG0014461: TcSYL_0055430.t1-p1
OG0014462: TcSYL_0055440.t1-p1
OG0014463: TcSYL_0055450.t1-p1
OG0014464: TcSYL_0055460.t1-p1
OG0014465: TcSYL_0055470.t1-p1
OG0014466: TcSYL_0055480.t1-p1
OG0014467: TcSYL_0055490.t1-p1
OG0014468: TcSYL_0055500.t1-p1
OG0014469: TcSYL_0055510.t1-p1
OG0014470: TcSYL_0055520.t1-p1
OG0014471: TcSYL_0055530.t1-p1
OG0014472: TcSYL_0055550.t1-p1
OG0014473: TcSYL_0055560.t1-p1
OG0014474: TcSYL_0055570.t1-p1
OG0014475: TcSYL_0055580.t1-p1
OG0014476: TcSYL_0055590.t1-p1
OG0014477: TcSYL_0055600.t1-p1
OG0014478: TcSYL_0055620.t1-p1
OG0014479: TcSYL_0055630.t1-p1
OG0014480: TcSYL_0055650.t1-p1
OG0014481: TcSYL_0055660.t1-p1
OG0014482: TcSYL_0055670.t1-p1
OG0014483: TcSYL_0055680.t1-p1
OG0014484: TcSYL_0055690.t1-p1
OG0014485: TcSYL_0055710.t1-p1
OG0014486: TcSYL_0055720.t1-p1
OG0014487: TcSYL_0055740.t1-p1
OG0014488: TcSYL_0055750.t1-p1
OG0014489: TcSYL_0055760.t1-p1
OG0014490: TcSYL_0055770.t1-p1
OG0014491: TcSYL_0055780.t1-p1
OG0014492: TcSYL_0055790.t1-p1
OG0014493: TcSYL_0055800.t1-p1
OG0014494: TcSYL_0055810.t1-p1
OG0014495: TcSYL_0055820.t1-p1
OG0014496: TcSYL_0055830.t1-p1
OG0014497: TcSYL_0055840.t1-p1
OG0014498: TcSYL_0055850.t1-p1
OG0014499: TcSYL_0055860.t1-p1
OG0014500: TcSYL_0055870.t1-p1
OG0014501: TcSYL_0055880.t1-p1
OG0014502: TcSYL_0055890.t1-p1
OG0014503: TcSYL_0055900.t1-p1
OG0014504: TcSYL_0055910.t1-p1
OG0014505: TcSYL_0055920.t1-p1
OG0014506: TcSYL_0055930.t1-p1

OG0014507: TcSYL_0055940.t1-p1
OG0014508: TcSYL_0055950.t1-p1
OG0014509: TcSYL_0055960.t1-p1
OG0014510: TcSYL_0055970.t1-p1
OG0014511: TcSYL_0055980.t1-p1
OG0014512: TcSYL_0055990.t1-p1
OG0014513: TcSYL_0056000.t1-p1
OG0014514: TcSYL_0056010.t1-p1
OG0014515: TcSYL_0056020.t1-p1
OG0014516: TcSYL_0056040.t1-p1
OG0014517: TcSYL_0056050.t1-p1
OG0014518: TcSYL_0056060.t1-p1
OG0014519: TcSYL_0056080.t1-p1
OG0014520: TcSYL_0056090.t1-p1
OG0014521: TcSYL_0056100.t1-p1
OG0014522: TcSYL_0056110.t1-p1
OG0014523: TcSYL_0056120.t1-p1
OG0014524: TcSYL_0056130.t1-p1
OG0014525: TcSYL_0056140.t1-p1
OG0014526: TcSYL_0056150.t1-p1
OG0014527: TcSYL_0056170.t1-p1
OG0014528: TcSYL_0056180.t1-p1
OG0014529: TcSYL_0056190.t1-p1
OG0014530: TcSYL_0056200.t1-p1
OG0014531: TcSYL_0056210.t1-p1
OG0014532: TcSYL_0056220.t1-p1
OG0014533: TcSYL_0056230.t1-p1
OG0014534: TcSYL_0056240.t1-p1
OG0014535: TcSYL_0056250.t1-p1
OG0014536: TcSYL_0056260.t1-p1
OG0014537: TcSYL_0056270.t1-p1
OG0014538: TcSYL_0056280.t1-p1
OG0014539: TcSYL_0056290.t1-p1
OG0014540: TcSYL_0056300.t1-p1
OG0014541: TcSYL_0056310.t1-p1
OG0014542: TcSYL_0056320.t1-p1
OG0014543: TcSYL_0056340.t1-p1
OG0014544: TcSYL_0056350.t1-p1
OG0014545: TcSYL_0056360.t1-p1
OG0014546: TcSYL_0056370.t1-p1
OG0014547: TcSYL_0056380.t1-p1
OG0014548: TcSYL_0056390.t1-p1
OG0014549: TcSYL_0056400.t1-p1
OG0014550: TcSYL_0056410.t1-p1
OG0014551: TcSYL_0056420.t1-p1
OG0014552: TcSYL_0056430.t1-p1
OG0014553: TcSYL_0056440.t1-p1
OG0014554: TcSYL_0056450.t1-p1
OG0014555: TcSYL_0056460.t1-p1
OG0014556: TcSYL_0056470.t1-p1
OG0014557: TcSYL_0056480.t1-p1
OG0014558: TcSYL_0056490.t1-p1
OG0014559: TcSYL_0056500.t1-p1
OG0014560: TcSYL_0056510.t1-p1

OG0014561: TcSYL_0056520.t1-p1
OG0014562: TcSYL_0056540.t1-p1
OG0014563: TcSYL_0056560.t1-p1
OG0014564: TcSYL_0056570.t1-p1
OG0014565: TcSYL_0056580.t1-p1
OG0014566: TcSYL_0056600.t1-p1
OG0014567: TcSYL_0056620.t1-p1
OG0014568: TcSYL_0056650.t1-p1
OG0014569: TcSYL_0056660.t1-p1
OG0014570: TcSYL_0056680.t1-p1
OG0014571: TcSYL_0056690.t1-p1
OG0014572: TcSYL_0056710.t1-p1
OG0014573: TcSYL_0056720.t1-p1
OG0014574: TcSYL_0056730.t1-p1
OG0014575: TcSYL_0056740.t1-p1
OG0014576: TcSYL_0056760.t1-p1
OG0014577: TcSYL_0056770.t1-p1
OG0014578: TcSYL_0056780.t1-p1
OG0014579: TcSYL_0056800.t1-p1
OG0014580: TcSYL_0056810.t1-p1
OG0014581: TcSYL_0056820.t1-p1
OG0014582: TcSYL_0056830.t1-p1
OG0014583: TcSYL_0056850.t1-p1
OG0014584: TcSYL_0056860.t1-p1
OG0014585: TcSYL_0056880.t1-p1
OG0014586: TcSYL_0056890.t1-p1
OG0014587: TcSYL_0056900.t1-p1
OG0014588: TcSYL_0056910.t1-p1
OG0014589: TcSYL_0056930.t1-p1
OG0014590: TcSYL_0056940.t1-p1
OG0014591: TcSYL_0056990.t1-p1
OG0014592: TcSYL_0057000.t1-p1
OG0014593: TcSYL_0057010.t1-p1
OG0014594: TcSYL_0057020.t1-p1
OG0014595: TcSYL_0057040.t1-p1
OG0014596: TcSYL_0057050.t1-p1
OG0014597: TcSYL_0057080.t1-p1
OG0014598: TcSYL_0057090.t1-p1
OG0014599: TcSYL_0057100.t1-p1
OG0014600: TcSYL_0057120.t1-p1
OG0014601: TcSYL_0057140.t1-p1
OG0014602: TcSYL_0057330.t1-p1
OG0014603: TcSYL_0057350.t1-p1
OG0014604: TcSYL_0057370.t1-p1
OG0014605: TcSYL_0057380.t1-p1
OG0014606: TcSYL_0057410.t1-p1
OG0014607: TcSYL_0057480.t1-p1
OG0014608: TcSYL_0057520.t1-p1
OG0014609: TcSYL_0057530.t1-p1
OG0014610: TcSYL_0057540.t1-p1
OG0014611: TcSYL_0057550.t1-p1
OG0014612: TcSYL_0057560.t1-p1
OG0014613: TcSYL_0057570.t1-p1
OG0014614: TcSYL_0057580.t1-p1

OG0014615: TcSYL_0057590.t1-p1
OG0014616: TcSYL_0057620.t1-p1
OG0014617: TcSYL_0057640.t1-p1
OG0014618: TcSYL_0057650.t1-p1
OG0014619: TcSYL_0057680.t1-p1
OG0014620: TcSYL_0057690.t1-p1
OG0014621: TcSYL_0057730.t1-p1
OG0014622: TcSYL_0057740.t1-p1
OG0014623: TcSYL_0057770.t1-p1
OG0014624: TcSYL_0057790.t1-p1
OG0014625: TcSYL_0057800.t1-p1
OG0014626: TcSYL_0057820.t1-p1
OG0014627: TcSYL_0057830.t1-p1
OG0014628: TcSYL_0057840.t1-p1
OG0014629: TcSYL_0057850.t1-p1
OG0014630: TcSYL_0057860.t1-p1
OG0014631: TcSYL_0057870.t1-p1
OG0014632: TcSYL_0057880.t1-p1
OG0014633: TcSYL_0057890.t1-p1
OG0014634: TcSYL_0057900.t1-p1
OG0014635: TcSYL_0057910.t1-p1
OG0014636: TcSYL_0057920.t1-p1
OG0014637: TcSYL_0057930.t1-p1
OG0014638: TcSYL_0057940.t1-p1
OG0014639: TcSYL_0057950.t1-p1
OG0014640: TcSYL_0057960.t1-p1
OG0014641: TcSYL_0057970.t1-p1
OG0014642: TcSYL_0057980.t1-p1
OG0014643: TcSYL_0057990.t1-p1
OG0014644: TcSYL_0058000.t1-p1
OG0014645: TcSYL_0058010.t1-p1
OG0014646: TcSYL_0058020.t1-p1
OG0014647: TcSYL_0058030.t1-p1
OG0014648: TcSYL_0058040.t1-p1
OG0014649: TcSYL_0058050.t1-p1
OG0014650: TcSYL_0058060.t1-p1
OG0014651: TcSYL_0058070.t1-p1
OG0014652: TcSYL_0058080.t1-p1
OG0014653: TcSYL_0058090.t1-p1
OG0014654: TcSYL_0058100.t1-p1
OG0014655: TcSYL_0058110.t1-p1
OG0014656: TcSYL_0058120.t1-p1
OG0014657: TcSYL_0058130.t1-p1
OG0014658: TcSYL_0058140.t1-p1
OG0014659: TcSYL_0058160.t1-p1
OG0014660: TcSYL_0058170.t1-p1
OG0014661: TcSYL_0058190.t1-p1
OG0014662: TcSYL_0058200.t1-p1
OG0014663: TcSYL_0058210.t1-p1
OG0014664: TcSYL_0058220.t1-p1
OG0014665: TcSYL_0058230.t1-p1
OG0014666: TcSYL_0058240.t1-p1
OG0014667: TcSYL_0058250.t1-p1
OG0014668: TcSYL_0058260.t1-p1

OG0014669: TcSYL_0058270.t1-p1
OG0014670: TcSYL_0058280.t1-p1
OG0014671: TcSYL_0058290.t1-p1
OG0014672: TcSYL_0058300.t1-p1
OG0014673: TcSYL_0058310.t1-p1
OG0014674: TcSYL_0058320.t1-p1
OG0014675: TcSYL_0058330.t1-p1
OG0014676: TcSYL_0058340.t1-p1
OG0014677: TcSYL_0058350.t1-p1
OG0014678: TcSYL_0058360.t1-p1
OG0014679: TcSYL_0058370.t1-p1
OG0014680: TcSYL_0058390.t1-p1
OG0014681: TcSYL_0058410.t1-p1
OG0014682: TcSYL_0058420.t1-p1
OG0014683: TcSYL_0058430.t1-p1
OG0014684: TcSYL_0058440.t1-p1
OG0014685: TcSYL_0058450.t1-p1
OG0014686: TcSYL_0058460.t1-p1
OG0014687: TcSYL_0058490.t1-p1
OG0014688: TcSYL_0058500.t1-p1
OG0014689: TcSYL_0058510.t1-p1
OG0014690: TcSYL_0058520.t1-p1
OG0014691: TcSYL_0058530.t1-p1
OG0014692: TcSYL_0058550.t1-p1
OG0014693: TcSYL_0058560.t1-p1
OG0014694: TcSYL_0058570.t1-p1
OG0014695: TcSYL_0058580.t1-p1
OG0014696: TcSYL_0058590.t1-p1
OG0014697: TcSYL_0058610.t1-p1
OG0014698: TcSYL_0058620.t1-p1
OG0014699: TcSYL_0058630.t1-p1
OG0014700: TcSYL_0058650.t1-p1
OG0014701: TcSYL_0058660.t1-p1
OG0014702: TcSYL_0058680.t1-p1
OG0014703: TcSYL_0058700.t1-p1
OG0014704: TcSYL_0058710.t1-p1
OG0014705: TcSYL_0058720.t1-p1
OG0014706: TcSYL_0058730.t1-p1
OG0014707: TcSYL_0058740.t1-p1
OG0014708: TcSYL_0058760.t1-p1
OG0014709: TcSYL_0058780.t1-p1
OG0014710: TcSYL_0058790.t1-p1
OG0014711: TcSYL_0058800.t1-p1
OG0014712: TcSYL_0058810.t1-p1
OG0014713: TcSYL_0058820.t1-p1
OG0014714: TcSYL_0058830.t1-p1
OG0014715: TcSYL_0058840.t1-p1
OG0014716: TcSYL_0058870.t1-p1
OG0014717: TcSYL_0058880.t1-p1
OG0014718: TcSYL_0058890.t1-p1
OG0014719: TcSYL_0058900.t1-p1
OG0014720: TcSYL_0058910.t1-p1
OG0014721: TcSYL_0058920.t1-p1
OG0014722: TcSYL_0058940.t1-p1

OG0014723: TcSYL_0058970.t1-p1
OG0014724: TcSYL_0058990.t1-p1
OG0014725: TcSYL_0059000.t1-p1
OG0014726: TcSYL_0059010.t1-p1
OG0014727: TcSYL_0059020.t1-p1
OG0014728: TcSYL_0059030.t1-p1
OG0014729: TcSYL_0059040.t1-p1
OG0014730: TcSYL_0059050.t1-p1
OG0014731: TcSYL_0059080.t1-p1
OG0014732: TcSYL_0059090.t1-p1
OG0014733: TcSYL_0059100.t1-p1
OG0014734: TcSYL_0059110.t1-p1
OG0014735: TcSYL_0059120.t1-p1
OG0014736: TcSYL_0059130.t1-p1
OG0014737: TcSYL_0059140.t1-p1
OG0014738: TcSYL_0059150.t1-p1
OG0014739: TcSYL_0059160.t1-p1
OG0014740: TcSYL_0059170.t1-p1
OG0014741: TcSYL_0059180.t1-p1
OG0014742: TcSYL_0059190.t1-p1
OG0014743: TcSYL_0059200.t1-p1
OG0014744: TcSYL_0059210.t1-p1
OG0014745: TcSYL_0059220.t1-p1
OG0014746: TcSYL_0059230.t1-p1
OG0014747: TcSYL_0059240.t1-p1
OG0014748: TcSYL_0059250.t1-p1
OG0014749: TcSYL_0059260.t1-p1
OG0014750: TcSYL_0059270.t1-p1
OG0014751: TcSYL_0059280.t1-p1
OG0014752: TcSYL_0059290.t1-p1
OG0014753: TcSYL_0059300.t1-p1
OG0014754: TcSYL_0059310.t1-p1
OG0014755: TcSYL_0059330.t1-p1
OG0014756: TcSYL_0059340.t1-p1
OG0014757: TcSYL_0059350.t1-p1
OG0014758: TcSYL_0059360.t1-p1
OG0014759: TcSYL_0059370.t1-p1
OG0014760: TcSYL_0059380.t1-p1
OG0014761: TcSYL_0059390.t1-p1
OG0014762: TcSYL_0059400.t1-p1
OG0014763: TcSYL_0059410.t1-p1
OG0014764: TcSYL_0059420.t1-p1
OG0014765: TcSYL_0059430.t1-p1
OG0014766: TcSYL_0059440.t1-p1
OG0014767: TcSYL_0059450.t1-p1
OG0014768: TcSYL_0059460.t1-p1
OG0014769: TcSYL_0059470.t1-p1
OG0014770: TcSYL_0059480.t1-p1
OG0014771: TcSYL_0059490.t1-p1
OG0014772: TcSYL_0059500.t1-p1
OG0014773: TcSYL_0059510.t1-p1
OG0014774: TcSYL_0059530.t1-p1
OG0014775: TcSYL_0059540.t1-p1
OG0014776: TcSYL_0059550.t1-p1

OG0014777: TcSYL_0059560.t1-p1
OG0014778: TcSYL_0059570.t1-p1
OG0014779: TcSYL_0059590.t1-p1
OG0014780: TcSYL_0059600.t1-p1
OG0014781: TcSYL_0059610.t1-p1
OG0014782: TcSYL_0059620.t1-p1
OG0014783: TcSYL_0059630.t1-p1
OG0014784: TcSYL_0059640.t1-p1
OG0014785: TcSYL_0059650.t1-p1
OG0014786: TcSYL_0059670.t1-p1
OG0014787: TcSYL_0059680.t1-p1
OG0014788: TcSYL_0059690.t1-p1
OG0014789: TcSYL_0059700.t1-p1
OG0014790: TcSYL_0059710.t1-p1
OG0014791: TcSYL_0059730.t1-p1
OG0014792: TcSYL_0059740.t1-p1
OG0014793: TcSYL_0059750.t1-p1
OG0014794: TcSYL_0059760.t1-p1
OG0014795: TcSYL_0059770.t1-p1
OG0014796: TcSYL_0059780.t1-p1
OG0014797: TcSYL_0059800.t1-p1
OG0014798: TcSYL_0059810.t1-p1
OG0014799: TcSYL_0059820.t1-p1
OG0014800: TcSYL_0059830.t1-p1
OG0014801: TcSYL_0059840.t1-p1
OG0014802: TcSYL_0059850.t1-p1
OG0014803: TcSYL_0059860.t1-p1
OG0014804: TcSYL_0059870.t1-p1
OG0014805: TcSYL_0059890.t1-p1
OG0014806: TcSYL_0059900.t1-p1
OG0014807: TcSYL_0059910.t1-p1
OG0014808: TcSYL_0059920.t1-p1
OG0014809: TcSYL_0059930.t1-p1
OG0014810: TcSYL_0059940.t1-p1
OG0014811: TcSYL_0059970.t1-p1
OG0014812: TcSYL_0059990.t1-p1
OG0014813: TcSYL_0060030.t1-p1
OG0014814: TcSYL_0060040.t1-p1
OG0014815: TcSYL_0060050.t1-p1
OG0014816: TcSYL_0060060.t1-p1
OG0014817: TcSYL_0060070.t1-p1
OG0014818: TcSYL_0060080.t1-p1
OG0014819: TcSYL_0060090.t1-p1
OG0014820: TcSYL_0060100.t1-p1
OG0014821: TcSYL_0060110.t1-p1
OG0014822: TcSYL_0060120.t1-p1
OG0014823: TcSYL_0060130.t1-p1
OG0014824: TcSYL_0060140.t1-p1
OG0014825: TcSYL_0060150.t1-p1
OG0014826: TcSYL_0060170.t1-p1
OG0014827: TcSYL_0060180.t1-p1
OG0014828: TcSYL_0060190.t1-p1
OG0014829: TcSYL_0060200.t1-p1
OG0014830: TcSYL_0060210.t1-p1

OG0014831: TcSYL_0060220.t1-p1
OG0014832: TcSYL_0060230.t1-p1
OG0014833: TcSYL_0060240.t1-p1
OG0014834: TcSYL_0060250.t1-p1
OG0014835: TcSYL_0060260.t1-p1
OG0014836: TcSYL_0060270.t1-p1
OG0014837: TcSYL_0060280.t1-p1
OG0014838: TcSYL_0060290.t1-p1
OG0014839: TcSYL_0060300.t1-p1
OG0014840: TcSYL_0060310.t1-p1
OG0014841: TcSYL_0060320.t1-p1
OG0014842: TcSYL_0060330.t1-p1
OG0014843: TcSYL_0060340.t1-p1
OG0014844: TcSYL_0060370.t1-p1
OG0014845: TcSYL_0060390.t1-p1
OG0014846: TcSYL_0060400.t1-p1
OG0014847: TcSYL_0060410.t1-p1
OG0014848: TcSYL_0060420.t1-p1
OG0014849: TcSYL_0060440.t1-p1
OG0014850: TcSYL_0060460.t1-p1
OG0014851: TcSYL_0060470.t1-p1
OG0014852: TcSYL_0060480.t1-p1
OG0014853: TcSYL_0060490.t1-p1
OG0014854: TcSYL_0060500.t1-p1
OG0014855: TcSYL_0060510.t1-p1
OG0014856: TcSYL_0060530.t1-p1
OG0014857: TcSYL_0060550.t1-p1
OG0014858: TcSYL_0060560.t1-p1
OG0014859: TcSYL_0060580.t1-p1
OG0014860: TcSYL_0060590.t1-p1
OG0014861: TcSYL_0060610.t1-p1
OG0014862: TcSYL_0060620.t1-p1
OG0014863: TcSYL_0060630.t1-p1
OG0014864: TcSYL_0060640.t1-p1
OG0014865: TcSYL_0060650.t1-p1
OG0014866: TcSYL_0060660.t1-p1
OG0014867: TcSYL_0060670.t1-p1
OG0014868: TcSYL_0060680.t1-p1
OG0014869: TcSYL_0060690.t1-p1
OG0014870: TcSYL_0060710.t1-p1
OG0014871: TcSYL_0060720.t1-p1
OG0014872: TcSYL_0060730.t1-p1
OG0014873: TcSYL_0060740.t1-p1
OG0014874: TcSYL_0060750.t1-p1
OG0014875: TcSYL_0060760.t1-p1
OG0014876: TcSYL_0060770.t1-p1
OG0014877: TcSYL_0060780.t1-p1
OG0014878: TcSYL_0060800.t1-p1
OG0014879: TcSYL_0060810.t1-p1
OG0014880: TcSYL_0060820.t1-p1
OG0014881: TcSYL_0060830.t1-p1
OG0014882: TcSYL_0060840.t1-p1
OG0014883: TcSYL_0060850.t1-p1
OG0014884: TcSYL_0060860.t1-p1

OG0014885: TcSYL_0060870.t1-p1
OG0014886: TcSYL_0060880.t1-p1
OG0014887: TcSYL_0060890.t1-p1
OG0014888: TcSYL_0060900.t1-p1
OG0014889: TcSYL_0060910.t1-p1
OG0014890: TcSYL_0060920.t1-p1
OG0014891: TcSYL_0060930.t1-p1
OG0014892: TcSYL_0060940.t1-p1
OG0014893: TcSYL_0060970.t1-p1
OG0014894: TcSYL_0060980.t1-p1
OG0014895: TcSYL_0061000.t1-p1
OG0014896: TcSYL_0061010.t1-p1
OG0014897: TcSYL_0061020.t1-p1
OG0014898: TcSYL_0061030.t1-p1
OG0014899: TcSYL_0061040.t1-p1
OG0014900: TcSYL_0061050.t1-p1
OG0014901: TcSYL_0061060.t1-p1
OG0014902: TcSYL_0061070.t1-p1
OG0014903: TcSYL_0061110.t1-p1
OG0014904: TcSYL_0061140.t1-p1
OG0014905: TcSYL_0061150.t1-p1
OG0014906: TcSYL_0061160.t1-p1
OG0014907: TcSYL_0061170.t1-p1
OG0014908: TcSYL_0061180.t1-p1
OG0014909: TcSYL_0061200.t1-p1
OG0014910: TcSYL_0061210.t1-p1
OG0014911: TcSYL_0061220.t1-p1
OG0014912: TcSYL_0061240.t1-p1
OG0014913: TcSYL_0061260.t1-p1
OG0014914: TcSYL_0061270.t1-p1
OG0014915: TcSYL_0061280.t1-p1
OG0014916: TcSYL_0061290.t1-p1
OG0014917: TcSYL_0061300.t1-p1
OG0014918: TcSYL_0061330.t1-p1
OG0014919: TcSYL_0061340.t1-p1
OG0014920: TcSYL_0061360.t1-p1
OG0014921: TcSYL_0061370.t1-p1
OG0014922: TcSYL_0061380.t1-p1
OG0014923: TcSYL_0061390.t1-p1
OG0014924: TcSYL_0061400.t1-p1
OG0014925: TcSYL_0061410.t1-p1
OG0014926: TcSYL_0061420.t1-p1
OG0014927: TcSYL_0061430.t1-p1
OG0014928: TcSYL_0061440.t1-p1
OG0014929: TcSYL_0061460.t1-p1
OG0014930: TcSYL_0061470.t1-p1
OG0014931: TcSYL_0061480.t1-p1
OG0014932: TcSYL_0061490.t1-p1
OG0014933: TcSYL_0061500.t1-p1
OG0014934: TcSYL_0061510.t1-p1
OG0014935: TcSYL_0061520.t1-p1
OG0014936: TcSYL_0061540.t1-p1
OG0014937: TcSYL_0061550.t1-p1
OG0014938: TcSYL_0061560.t1-p1

OG0014939: TcSYL_0061570.t1-p1
OG0014940: TcSYL_0061580.t1-p1
OG0014941: TcSYL_0061590.t1-p1
OG0014942: TcSYL_0061600.t1-p1
OG0014943: TcSYL_0061610.t1-p1
OG0014944: TcSYL_0061620.t1-p1
OG0014945: TcSYL_0061630.t1-p1
OG0014946: TcSYL_0061640.t1-p1
OG0014947: TcSYL_0061650.t1-p1
OG0014948: TcSYL_0061660.t1-p1
OG0014949: TcSYL_0061670.t1-p1
OG0014950: TcSYL_0061680.t1-p1
OG0014951: TcSYL_0061690.t1-p1
OG0014952: TcSYL_0061700.t1-p1
OG0014953: TcSYL_0061710.t1-p1
OG0014954: TcSYL_0061720.t1-p1
OG0014955: TcSYL_0061730.t1-p1
OG0014956: TcSYL_0061740.t1-p1
OG0014957: TcSYL_0061750.t1-p1
OG0014958: TcSYL_0061760.t1-p1
OG0014959: TcSYL_0061770.t1-p1
OG0014960: TcSYL_0061780.t1-p1
OG0014961: TcSYL_0061790.t1-p1
OG0014962: TcSYL_0061800.t1-p1
OG0014963: TcSYL_0061810.t1-p1
OG0014964: TcSYL_0061820.t1-p1
OG0014965: TcSYL_0061840.t1-p1
OG0014966: TcSYL_0061850.t1-p1
OG0014967: TcSYL_0061860.t1-p1
OG0014968: TcSYL_0061870.t1-p1
OG0014969: TcSYL_0061880.t1-p1
OG0014970: TcSYL_0061890.t1-p1
OG0014971: TcSYL_0061900.t1-p1
OG0014972: TcSYL_0061910.t1-p1
OG0014973: TcSYL_0061920.t1-p1
OG0014974: TcSYL_0061940.t1-p1
OG0014975: TcSYL_0061950.t1-p1
OG0014976: TcSYL_0061960.t1-p1
OG0014977: TcSYL_0061970.t1-p1
OG0014978: TcSYL_0062030.t1-p1
OG0014979: TcSYL_0062190.t1-p1
OG0014980: TcSYL_0062250.t1-p1
OG0014981: TcSYL_0062270.t1-p1
OG0014982: TcSYL_0062280.t1-p1
OG0014983: TcSYL_0062290.t1-p1
OG0014984: TcSYL_0062300.t1-p1
OG0014985: TcSYL_0062310.t1-p1
OG0014986: TcSYL_0062320.t1-p1
OG0014987: TcSYL_0062330.t1-p1
OG0014988: TcSYL_0062340.t1-p1
OG0014989: TcSYL_0062350.t1-p1
OG0014990: TcSYL_0062360.t1-p1
OG0014991: TcSYL_0062370.t1-p1
OG0014992: TcSYL_0062380.t1-p1

OG0014993: TcSYL_0062390.t1-p1
OG0014994: TcSYL_0062400.t1-p1
OG0014995: TcSYL_0062410.t1-p1
OG0014996: TcSYL_0062420.t1-p1
OG0014997: TcSYL_0062430.t1-p1
OG0014998: TcSYL_0062440.t1-p1
OG0014999: TcSYL_0062450.t1-p1
OG0015000: TcSYL_0062460.t1-p1
OG0015001: TcSYL_0062470.t1-p1
OG0015002: TcSYL_0062480.t1-p1
OG0015003: TcSYL_0062490.t1-p1
OG0015004: TcSYL_0062500.t1-p1
OG0015005: TcSYL_0062510.t1-p1
OG0015006: TcSYL_0062520.t1-p1
OG0015007: TcSYL_0062530.t1-p1
OG0015008: TcSYL_0062540.t1-p1
OG0015009: TcSYL_0062550.t1-p1
OG0015010: TcSYL_0062560.t1-p1
OG0015011: TcSYL_0062570.t1-p1
OG0015012: TcSYL_0062580.t1-p1
OG0015013: TcSYL_0062590.t1-p1
OG0015014: TcSYL_0062600.t1-p1
OG0015015: TcSYL_0062610.t1-p1
OG0015016: TcSYL_0062620.t1-p1
OG0015017: TcSYL_0062630.t1-p1
OG0015018: TcSYL_0062640.t1-p1
OG0015019: TcSYL_0062650.t1-p1
OG0015020: TcSYL_0062660.t1-p1
OG0015021: TcSYL_0062670.t1-p1
OG0015022: TcSYL_0062680.t1-p1
OG0015023: TcSYL_0062690.t1-p1
OG0015024: TcSYL_0062700.t1-p1
OG0015025: TcSYL_0062710.t1-p1
OG0015026: TcSYL_0062720.t1-p1
OG0015027: TcSYL_0062730.t1-p1
OG0015028: TcSYL_0062740.t1-p1
OG0015029: TcSYL_0062770.t1-p1
OG0015030: TcSYL_0062780.t1-p1
OG0015031: TcSYL_0062790.t1-p1
OG0015032: TcSYL_0062800.t1-p1
OG0015033: TcSYL_0062810.t1-p1
OG0015034: TcSYL_0062860.t1-p1
OG0015035: TcSYL_0062880.t1-p1
OG0015036: TcSYL_0062890.t1-p1
OG0015037: TcSYL_0062910.t1-p1
OG0015038: TcSYL_0062920.t1-p1
OG0015039: TcSYL_0062930.t1-p1
OG0015040: TcSYL_0062940.t1-p1
OG0015041: TcSYL_0062950.t1-p1
OG0015042: TcSYL_0062960.t1-p1
OG0015043: TcSYL_0062990.t1-p1
OG0015044: TcSYL_0063020.t1-p1
OG0015045: TcSYL_0063030.t1-p1
OG0015046: TcSYL_0063060.t1-p1

OG0015047: TcSYL_0063110.t1-p1
OG0015048: TcSYL_0063120.t1-p1
OG0015049: TcSYL_0063140.t1-p1
OG0015050: TcSYL_0063170.t1-p1
OG0015051: TcSYL_0063290.t1-p1
OG0015052: TcSYL_0063300.t1-p1
OG0015053: TcSYL_0063330.t1-p1
OG0015054: TcSYL_0063340.t1-p1
OG0015055: TcSYL_0063360.t1-p1
OG0015056: TcSYL_0063370.t1-p1
OG0015057: TcSYL_0063380.t1-p1
OG0015058: TcSYL_0063400.t1-p1
OG0015059: TcSYL_0063570.t1-p1
OG0015060: TcSYL_0063590.t1-p1
OG0015061: TcSYL_0063600.t1-p1
OG0015062: TcSYL_0063630.t1-p1
OG0015063: TcSYL_0063640.t1-p1
OG0015064: TcSYL_0063660.t1-p1
OG0015065: TcSYL_0063670.t1-p1
OG0015066: TcSYL_0063680.t1-p1
OG0015067: TcSYL_0063720.t1-p1
OG0015068: TcSYL_0063730.t1-p1
OG0015069: TcSYL_0063750.t1-p1
OG0015070: TcSYL_0063790.t1-p1
OG0015071: TcSYL_0063800.t1-p1
OG0015072: TcSYL_0063820.t1-p1
OG0015073: TcSYL_0063830.t1-p1
OG0015074: TcSYL_0063840.t1-p1
OG0015075: TcSYL_0063860.t1-p1
OG0015076: TcSYL_0063910.t1-p1
OG0015077: TcSYL_0063920.t1-p1
OG0015078: TcSYL_0063940.t1-p1
OG0015079: TcSYL_0063950.t1-p1
OG0015080: TcSYL_0063960.t1-p1
OG0015081: TcSYL_0063970.t1-p1
OG0015082: TcSYL_0063980.t1-p1
OG0015083: TcSYL_0064000.t1-p1
OG0015084: TcSYL_0064010.t1-p1
OG0015085: TcSYL_0064020.t1-p1
OG0015086: TcSYL_0064090.t1-p1
OG0015087: TcSYL_0064100.t1-p1
OG0015088: TcSYL_0064190.t1-p1
OG0015089: TcSYL_0064210.t1-p1
OG0015090: TcSYL_0064300.t1-p1
OG0015091: TcSYL_0064330.t1-p1
OG0015092: TcSYL_0064380.t1-p1
OG0015093: TcSYL_0064400.t1-p1
OG0015094: TcSYL_0064430.t1-p1
OG0015095: TcSYL_0064440.t1-p1
OG0015096: TcSYL_0064500.t1-p1
OG0015097: TcSYL_0064510.t1-p1
OG0015098: TcSYL_0064520.t1-p1
OG0015099: TcSYL_0064530.t1-p1
OG0015100: TcSYL_0064540.t1-p1

OG0015101: TcSYL_0064550.t1-p1
OG0015102: TcSYL_0064570.t1-p1
OG0015103: TcSYL_0064580.t1-p1
OG0015104: TcSYL_0064590.t1-p1
OG0015105: TcSYL_0064600.t1-p1
OG0015106: TcSYL_0064620.t1-p1
OG0015107: TcSYL_0064630.t1-p1
OG0015108: TcSYL_0064640.t1-p1
OG0015109: TcSYL_0064650.t1-p1
OG0015110: TcSYL_0064660.t1-p1
OG0015111: TcSYL_0064670.t1-p1
OG0015112: TcSYL_0064690.t1-p1
OG0015113: TcSYL_0064700.t1-p1
OG0015114: TcSYL_0064720.t1-p1
OG0015115: TcSYL_0064730.t1-p1
OG0015116: TcSYL_0064750.t1-p1
OG0015117: TcSYL_0064760.t1-p1
OG0015118: TcSYL_0064770.t1-p1
OG0015119: TcSYL_0064790.t1-p1
OG0015120: TcSYL_0064800.t1-p1
OG0015121: TcSYL_0064830.t1-p1
OG0015122: TcSYL_0064870.t1-p1
OG0015123: TcSYL_0064920.t1-p1
OG0015124: TcSYL_0064940.t1-p1
OG0015125: TcSYL_0064960.t1-p1
OG0015126: TcSYL_0064970.t1-p1
OG0015127: TcSYL_0065030.t1-p1
OG0015128: TcSYL_0065040.t1-p1
OG0015129: TcSYL_0065080.t1-p1
OG0015130: TcSYL_0065090.t1-p1
OG0015131: TcSYL_0065100.t1-p1
OG0015132: TcSYL_0065120.t1-p1
OG0015133: TcSYL_0065130.t1-p1
OG0015134: TcSYL_0065140.t1-p1
OG0015135: TcSYL_0065150.t1-p1
OG0015136: TcSYL_0065160.t1-p1
OG0015137: TcSYL_0065170.t1-p1
OG0015138: TcSYL_0065210.t1-p1
OG0015139: TcSYL_0065220.t1-p1
OG0015140: TcSYL_0065230.t1-p1
OG0015141: TcSYL_0065240.t1-p1
OG0015142: TcSYL_0065250.t1-p1
OG0015143: TcSYL_0065270.t1-p1
OG0015144: TcSYL_0065280.t1-p1
OG0015145: TcSYL_0065300.t1-p1
OG0015146: TcSYL_0065310.t1-p1
OG0015147: TcSYL_0065320.t1-p1
OG0015148: TcSYL_0065330.t1-p1
OG0015149: TcSYL_0065340.t1-p1
OG0015150: TcSYL_0065350.t1-p1
OG0015151: TcSYL_0065360.t1-p1
OG0015152: TcSYL_0065370.t1-p1
OG0015153: TcSYL_0065380.t1-p1
OG0015154: TcSYL_0065390.t1-p1

OG0015155: TcSYL_0065400.t1-p1
OG0015156: TcSYL_0065410.t1-p1
OG0015157: TcSYL_0065420.t1-p1
OG0015158: TcSYL_0065430.t1-p1
OG0015159: TcSYL_0065450.t1-p1
OG0015160: TcSYL_0065460.t1-p1
OG0015161: TcSYL_0065480.t1-p1
OG0015162: TcSYL_0065490.t1-p1
OG0015163: TcSYL_0065500.t1-p1
OG0015164: TcSYL_0065510.t1-p1
OG0015165: TcSYL_0065520.t1-p1
OG0015166: TcSYL_0065530.t1-p1
OG0015167: TcSYL_0065540.t1-p1
OG0015168: TcSYL_0065550.t1-p1
OG0015169: TcSYL_0065560.t1-p1
OG0015170: TcSYL_0065570.t1-p1
OG0015171: TcSYL_0065580.t1-p1
OG0015172: TcSYL_0065600.t1-p1
OG0015173: TcSYL_0065620.t1-p1
OG0015174: TcSYL_0065630.t1-p1
OG0015175: TcSYL_0065650.t1-p1
OG0015176: TcSYL_0065660.t1-p1
OG0015177: TcSYL_0065670.t1-p1
OG0015178: TcSYL_0065680.t1-p1
OG0015179: TcSYL_0065690.t1-p1
OG0015180: TcSYL_0065700.t1-p1
OG0015181: TcSYL_0065710.t1-p1
OG0015182: TcSYL_0065730.t1-p1
OG0015183: TcSYL_0065740.t1-p1
OG0015184: TcSYL_0065760.t1-p1
OG0015185: TcSYL_0065770.t1-p1
OG0015186: TcSYL_0065790.t1-p1
OG0015187: TcSYL_0065800.t1-p1
OG0015188: TcSYL_0065820.t1-p1
OG0015189: TcSYL_0065830.t1-p1
OG0015190: TcSYL_0065840.t1-p1
OG0015191: TcSYL_0065850.t1-p1
OG0015192: TcSYL_0065860.t1-p1
OG0015193: TcSYL_0065870.t1-p1
OG0015194: TcSYL_0065880.t1-p1
OG0015195: TcSYL_0065890.t1-p1
OG0015196: TcSYL_0065930.t1-p1
OG0015197: TcSYL_0065940.t1-p1
OG0015198: TcSYL_0065950.t1-p1
OG0015199: TcSYL_0065960.t1-p1
OG0015200: TcSYL_0065990.t1-p1
OG0015201: TcSYL_0066000.t1-p1
OG0015202: TcSYL_0066010.t1-p1
OG0015203: TcSYL_0066020.t1-p1
OG0015204: TcSYL_0066040.t1-p1
OG0015205: TcSYL_0066070.t1-p1
OG0015206: TcSYL_0066080.t1-p1
OG0015207: TcSYL_0066100.t1-p1
OG0015208: TcSYL_0066120.t1-p1

OG0015209: TcSYL_0066130.t1-p1
OG0015210: TcSYL_0066140.t1-p1
OG0015211: TcSYL_0066190.t1-p1
OG0015212: TcSYL_0066200.t1-p1
OG0015213: TcSYL_0066210.t1-p1
OG0015214: TcSYL_0066220.t1-p1
OG0015215: TcSYL_0066230.t1-p1
OG0015216: TcSYL_0066250.t1-p1
OG0015217: TcSYL_0066260.t1-p1
OG0015218: TcSYL_0066280.t1-p1
OG0015219: TcSYL_0066290.t1-p1
OG0015220: TcSYL_0066310.t1-p1
OG0015221: TcSYL_0066320.t1-p1
OG0015222: TcSYL_0066330.t1-p1
OG0015223: TcSYL_0066340.t1-p1
OG0015224: TcSYL_0066350.t1-p1
OG0015225: TcSYL_0066360.t1-p1
OG0015226: TcSYL_0066380.t1-p1
OG0015227: TcSYL_0066390.t1-p1
OG0015228: TcSYL_0066400.t1-p1
OG0015229: TcSYL_0066410.t1-p1
OG0015230: TcSYL_0066420.t1-p1
OG0015231: TcSYL_0066430.t1-p1
OG0015232: TcSYL_0066440.t1-p1
OG0015233: TcSYL_0066450.t1-p1
OG0015234: TcSYL_0066460.t1-p1
OG0015235: TcSYL_0066480.t1-p1
OG0015236: TcSYL_0066490.t1-p1
OG0015237: TcSYL_0066500.t1-p1
OG0015238: TcSYL_0066510.t1-p1
OG0015239: TcSYL_0066520.t1-p1
OG0015240: TcSYL_0066530.t1-p1
OG0015241: TcSYL_0066540.t1-p1
OG0015242: TcSYL_0066560.t1-p1
OG0015243: TcSYL_0066580.t1-p1
OG0015244: TcSYL_0066590.t1-p1
OG0015245: TcSYL_0066600.t1-p1
OG0015246: TcSYL_0066610.t1-p1
OG0015247: TcSYL_0066620.t1-p1
OG0015248: TcSYL_0066640.t1-p1
OG0015249: TcSYL_0066650.t1-p1
OG0015250: TcSYL_0066660.t1-p1
OG0015251: TcSYL_0066680.t1-p1
OG0015252: TcSYL_0066690.t1-p1
OG0015253: TcSYL_0066700.t1-p1
OG0015254: TcSYL_0066710.t1-p1
OG0015255: TcSYL_0066730.t1-p1
OG0015256: TcSYL_0066740.t1-p1
OG0015257: TcSYL_0066750.t1-p1
OG0015258: TcSYL_0066760.t1-p1
OG0015259: TcSYL_0066780.t1-p1
OG0015260: TcSYL_0066790.t1-p1
OG0015261: TcSYL_0066800.t1-p1
OG0015262: TcSYL_0066810.t1-p1

OG0015263: TcSYL_0066820.t1-p1
OG0015264: TcSYL_0066830.t1-p1
OG0015265: TcSYL_0066860.t1-p1
OG0015266: TcSYL_0066880.t1-p1
OG0015267: TcSYL_0066900.t1-p1
OG0015268: TcSYL_0066930.t1-p1
OG0015269: TcSYL_0066950.t1-p1
OG0015270: TcSYL_0066960.t1-p1
OG0015271: TcSYL_0066990.t1-p1
OG0015272: TcSYL_0067040.t1-p1
OG0015273: TcSYL_0067050.t1-p1
OG0015274: TcSYL_0067060.t1-p1
OG0015275: TcSYL_0067080.t1-p1
OG0015276: TcSYL_0067090.t1-p1
OG0015277: TcSYL_0067100.t1-p1
OG0015278: TcSYL_0067110.t1-p1
OG0015279: TcSYL_0067120.t1-p1
OG0015280: TcSYL_0067130.t1-p1
OG0015281: TcSYL_0067140.t1-p1
OG0015282: TcSYL_0067160.t1-p1
OG0015283: TcSYL_0067170.t1-p1
OG0015284: TcSYL_0067180.t1-p1
OG0015285: TcSYL_0067200.t1-p1
OG0015286: TcSYL_0067210.t1-p1
OG0015287: TcSYL_0067220.t1-p1
OG0015288: TcSYL_0067240.t1-p1
OG0015289: TcSYL_0067250.t1-p1
OG0015290: TcSYL_0067260.t1-p1
OG0015291: TcSYL_0067270.t1-p1
OG0015292: TcSYL_0067280.t1-p1
OG0015293: TcSYL_0067290.t1-p1
OG0015294: TcSYL_0067300.t1-p1
OG0015295: TcSYL_0067320.t1-p1
OG0015296: TcSYL_0067330.t1-p1
OG0015297: TcSYL_0067360.t1-p1
OG0015298: TcSYL_0067370.t1-p1
OG0015299: TcSYL_0067380.t1-p1
OG0015300: TcSYL_0067400.t1-p1
OG0015301: TcSYL_0067410.t1-p1
OG0015302: TcSYL_0067420.t1-p1
OG0015303: TcSYL_0067430.t1-p1
OG0015304: TcSYL_0067440.t1-p1
OG0015305: TcSYL_0067450.t1-p1
OG0015306: TcSYL_0067460.t1-p1
OG0015307: TcSYL_0067470.t1-p1
OG0015308: TcSYL_0067480.t1-p1
OG0015309: TcSYL_0067490.t1-p1
OG0015310: TcSYL_0067500.t1-p1
OG0015311: TcSYL_0067510.t1-p1
OG0015312: TcSYL_0067520.t1-p1
OG0015313: TcSYL_0067530.t1-p1
OG0015314: TcSYL_0067540.t1-p1
OG0015315: TcSYL_0067550.t1-p1
OG0015316: TcSYL_0067560.t1-p1

OG0015317: TcSYL_0067570.t1-p1
OG0015318: TcSYL_0067580.t1-p1
OG0015319: TcSYL_0067590.t1-p1
OG0015320: TcSYL_0067600.t1-p1
OG0015321: TcSYL_0067620.t1-p1
OG0015322: TcSYL_0067650.t1-p1
OG0015323: TcSYL_0067660.t1-p1
OG0015324: TcSYL_0067670.t1-p1
OG0015325: TcSYL_0067680.t1-p1
OG0015326: TcSYL_0067690.t1-p1
OG0015327: TcSYL_0067700.t1-p1
OG0015328: TcSYL_0067710.t1-p1
OG0015329: TcSYL_0067720.t1-p1
OG0015330: TcSYL_0067730.t1-p1
OG0015331: TcSYL_0067760.t1-p1
OG0015332: TcSYL_0067770.t1-p1
OG0015333: TcSYL_0067780.t1-p1
OG0015334: TcSYL_0067790.t1-p1
OG0015335: TcSYL_0067810.t1-p1
OG0015336: TcSYL_0067820.t1-p1
OG0015337: TcSYL_0067830.t1-p1
OG0015338: TcSYL_0067860.t1-p1
OG0015339: TcSYL_0067870.t1-p1
OG0015340: TcSYL_0067880.t1-p1
OG0015341: TcSYL_0067890.t1-p1
OG0015342: TcSYL_0067900.t1-p1
OG0015343: TcSYL_0067910.t1-p1
OG0015344: TcSYL_0067930.t1-p1
OG0015345: TcSYL_0067940.t1-p1
OG0015346: TcSYL_0067960.t1-p1
OG0015347: TcSYL_0067980.t1-p1
OG0015348: TcSYL_0067990.t1-p1
OG0015349: TcSYL_0068010.t1-p1
OG0015350: TcSYL_0068040.t1-p1
OG0015351: TcSYL_0068050.t1-p1
OG0015352: TcSYL_0068090.t1-p1
OG0015353: TcSYL_0068100.t1-p1
OG0015354: TcSYL_0068110.t1-p1
OG0015355: TcSYL_0068130.t1-p1
OG0015356: TcSYL_0068140.t1-p1
OG0015357: TcSYL_0068150.t1-p1
OG0015358: TcSYL_0068170.t1-p1
OG0015359: TcSYL_0068180.t1-p1
OG0015360: TcSYL_0068190.t1-p1
OG0015361: TcSYL_0068200.t1-p1
OG0015362: TcSYL_0068210.t1-p1
OG0015363: TcSYL_0068220.t1-p1
OG0015364: TcSYL_0068240.t1-p1
OG0015365: TcSYL_0068250.t1-p1
OG0015366: TcSYL_0068260.t1-p1
OG0015367: TcSYL_0068270.t1-p1
OG0015368: TcSYL_0068280.t1-p1
OG0015369: TcSYL_0068310.t1-p1
OG0015370: TcSYL_0068320.t1-p1

OG0015371: TcSYL_0068330.t1-p1
OG0015372: TcSYL_0068360.t1-p1
OG0015373: TcSYL_0068370.t1-p1
OG0015374: TcSYL_0068380.t1-p1
OG0015375: TcSYL_0068390.t1-p1
OG0015376: TcSYL_0068410.t1-p1
OG0015377: TcSYL_0068420.t1-p1
OG0015378: TcSYL_0068430.t1-p1
OG0015379: TcSYL_0068440.t1-p1
OG0015380: TcSYL_0068450.t1-p1
OG0015381: TcSYL_0068460.t1-p1
OG0015382: TcSYL_0068470.t1-p1
OG0015383: TcSYL_0068480.t1-p1
OG0015384: TcSYL_0068490.t1-p1
OG0015385: TcSYL_0068500.t1-p1
OG0015386: TcSYL_0068510.t1-p1
OG0015387: TcSYL_0068520.t1-p1
OG0015388: TcSYL_0068530.t1-p1
OG0015389: TcSYL_0068540.t1-p1
OG0015390: TcSYL_0068560.t1-p1
OG0015391: TcSYL_0068570.t1-p1
OG0015392: TcSYL_0068580.t1-p1
OG0015393: TcSYL_0068590.t1-p1
OG0015394: TcSYL_0068620.t1-p1
OG0015395: TcSYL_0068630.t1-p1
OG0015396: TcSYL_0068650.t1-p1
OG0015397: TcSYL_0068660.t1-p1
OG0015398: TcSYL_0068670.t1-p1
OG0015399: TcSYL_0068680.t1-p1
OG0015400: TcSYL_0068690.t1-p1
OG0015401: TcSYL_0068700.t1-p1
OG0015402: TcSYL_0068710.t1-p1
OG0015403: TcSYL_0068760.t1-p1
OG0015404: TcSYL_0068780.t1-p1
OG0015405: TcSYL_0068790.t1-p1
OG0015406: TcSYL_0068810.t1-p1
OG0015407: TcSYL_0068820.t1-p1
OG0015408: TcSYL_0068850.t1-p1
OG0015409: TcSYL_0068860.t1-p1
OG0015410: TcSYL_0068870.t1-p1
OG0015411: TcSYL_0068880.t1-p1
OG0015412: TcSYL_0068890.t1-p1
OG0015413: TcSYL_0068900.t1-p1
OG0015414: TcSYL_0068910.t1-p1
OG0015415: TcSYL_0068930.t1-p1
OG0015416: TcSYL_0068940.t1-p1
OG0015417: TcSYL_0068950.t1-p1
OG0015418: TcSYL_0068960.t1-p1
OG0015419: TcSYL_0068970.t1-p1
OG0015420: TcSYL_0068980.t1-p1
OG0015421: TcSYL_0068990.t1-p1
OG0015422: TcSYL_0069000.t1-p1
OG0015423: TcSYL_0069010.t1-p1
OG0015424: TcSYL_0069020.t1-p1

OG0015425: TcSYL_0069050.t1-p1
OG0015426: TcSYL_0069090.t1-p1
OG0015427: TcSYL_0069100.t1-p1
OG0015428: TcSYL_0069110.t1-p1
OG0015429: TcSYL_0069130.t1-p1
OG0015430: TcSYL_0069140.t1-p1
OG0015431: TcSYL_0069150.t1-p1
OG0015432: TcSYL_0069160.t1-p1
OG0015433: TcSYL_0069180.t1-p1
OG0015434: TcSYL_0069200.t1-p1
OG0015435: TcSYL_0069230.t1-p1
OG0015436: TcSYL_0069240.t1-p1
OG0015437: TcSYL_0069250.t1-p1
OG0015438: TcSYL_0069260.t1-p1
OG0015439: TcSYL_0069280.t1-p1
OG0015440: TcSYL_0069290.t1-p1
OG0015441: TcSYL_0069300.t1-p1
OG0015442: TcSYL_0069330.t1-p1
OG0015443: TcSYL_0069340.t1-p1
OG0015444: TcSYL_0069370.t1-p1
OG0015445: TcSYL_0069380.t1-p1
OG0015446: TcSYL_0069390.t1-p1
OG0015447: TcSYL_0069400.t1-p1
OG0015448: TcSYL_0069410.t1-p1
OG0015449: TcSYL_0069420.t1-p1
OG0015450: TcSYL_0069430.t1-p1
OG0015451: TcSYL_0069450.t1-p1
OG0015452: TcSYL_0069460.t1-p1
OG0015453: TcSYL_0069470.t1-p1
OG0015454: TcSYL_0069480.t1-p1
OG0015455: TcSYL_0069490.t1-p1
OG0015456: TcSYL_0069500.t1-p1
OG0015457: TcSYL_0069510.t1-p1
OG0015458: TcSYL_0069520.t1-p1
OG0015459: TcSYL_0069530.t1-p1
OG0015460: TcSYL_0069540.t1-p1
OG0015461: TcSYL_0069550.t1-p1
OG0015462: TcSYL_0069590.t1-p1
OG0015463: TcSYL_0069620.t1-p1
OG0015464: TcSYL_0069640.t1-p1
OG0015465: TcSYL_0069650.t1-p1
OG0015466: TcSYL_0069690.t1-p1
OG0015467: TcSYL_0069700.t1-p1
OG0015468: TcSYL_0069710.t1-p1
OG0015469: TcSYL_0069730.t1-p1
OG0015470: TcSYL_0069740.t1-p1
OG0015471: TcSYL_0069750.t1-p1
OG0015472: TcSYL_0069760.t1-p1
OG0015473: TcSYL_0069770.t1-p1
OG0015474: TcSYL_0069800.t1-p1
OG0015475: TcSYL_0069810.t1-p1
OG0015476: TcSYL_0069840.t1-p1
OG0015477: TcSYL_0069860.t1-p1
OG0015478: TcSYL_0069870.t1-p1

OG0015479: TcSYL_0069880.t1-p1
OG0015480: TcSYL_0069890.t1-p1
OG0015481: TcSYL_0069900.t1-p1
OG0015482: TcSYL_0069910.t1-p1
OG0015483: TcSYL_0069920.t1-p1
OG0015484: TcSYL_0069930.t1-p1
OG0015485: TcSYL_0069940.t1-p1
OG0015486: TcSYL_0069950.t1-p1
OG0015487: TcSYL_0069960.t1-p1
OG0015488: TcSYL_0069970.t1-p1
OG0015489: TcSYL_0069980.t1-p1
OG0015490: TcSYL_0069990.t1-p1
OG0015491: TcSYL_0070000.t1-p1
OG0015492: TcSYL_0070030.t1-p1
OG0015493: TcSYL_0070040.t1-p1
OG0015494: TcSYL_0070050.t1-p1
OG0015495: TcSYL_0070070.t1-p1
OG0015496: TcSYL_0070080.t1-p1
OG0015497: TcSYL_0070090.t1-p1
OG0015498: TcSYL_0070110.t1-p1
OG0015499: TcSYL_0070120.t1-p1
OG0015500: TcSYL_0070130.t1-p1
OG0015501: TcSYL_0070140.t1-p1
OG0015502: TcSYL_0070150.t1-p1
OG0015503: TcSYL_0070170.t1-p1
OG0015504: TcSYL_0070190.t1-p1
OG0015505: TcSYL_0070220.t1-p1
OG0015506: TcSYL_0070240.t1-p1
OG0015507: TcSYL_0070260.t1-p1
OG0015508: TcSYL_0070270.t1-p1
OG0015509: TcSYL_0070300.t1-p1
OG0015510: TcSYL_0070310.t1-p1
OG0015511: TcSYL_0070330.t1-p1
OG0015512: TcSYL_0070360.t1-p1
OG0015513: TcSYL_0070420.t1-p1
OG0015514: TcSYL_0070440.t1-p1
OG0015515: TcSYL_0070450.t1-p1
OG0015516: TcSYL_0070460.t1-p1
OG0015517: TcSYL_0070470.t1-p1
OG0015518: TcSYL_0070480.t1-p1
OG0015519: TcSYL_0070490.t1-p1
OG0015520: TcSYL_0070500.t1-p1
OG0015521: TcSYL_0070520.t1-p1
OG0015522: TcSYL_0070530.t1-p1
OG0015523: TcSYL_0070540.t1-p1
OG0015524: TcSYL_0070560.t1-p1
OG0015525: TcSYL_0070580.t1-p1
OG0015526: TcSYL_0070590.t1-p1
OG0015527: TcSYL_0070600.t1-p1
OG0015528: TcSYL_0070620.t1-p1
OG0015529: TcSYL_0070630.t1-p1
OG0015530: TcSYL_0070640.t1-p1
OG0015531: TcSYL_0070650.t1-p1
OG0015532: TcSYL_0070660.t1-p1

OG0015533: TcSYL_0070670.t1-p1
OG0015534: TcSYL_0070680.t1-p1
OG0015535: TcSYL_0070690.t1-p1
OG0015536: TcSYL_0070700.t1-p1
OG0015537: TcSYL_0070710.t1-p1
OG0015538: TcSYL_0070720.t1-p1
OG0015539: TcSYL_0070730.t1-p1
OG0015540: TcSYL_0070740.t1-p1
OG0015541: TcSYL_0070750.t1-p1
OG0015542: TcSYL_0070760.t1-p1
OG0015543: TcSYL_0070770.t1-p1
OG0015544: TcSYL_0070790.t1-p1
OG0015545: TcSYL_0070800.t1-p1
OG0015546: TcSYL_0070810.t1-p1
OG0015547: TcSYL_0070820.t1-p1
OG0015548: TcSYL_0070830.t1-p1
OG0015549: TcSYL_0070840.t1-p1
OG0015550: TcSYL_0070870.t1-p1
OG0015551: TcSYL_0070880.t1-p1
OG0015552: TcSYL_0070900.t1-p1
OG0015553: TcSYL_0070910.t1-p1
OG0015554: TcSYL_0070920.t1-p1
OG0015555: TcSYL_0070930.t1-p1
OG0015556: TcSYL_0070940.t1-p1
OG0015557: TcSYL_0070960.t1-p1
OG0015558: TcSYL_0070970.t1-p1
OG0015559: TcSYL_0070990.t1-p1
OG0015560: TcSYL_0071000.t1-p1
OG0015561: TcSYL_0071020.t1-p1
OG0015562: TcSYL_0071030.t1-p1
OG0015563: TcSYL_0071070.t1-p1
OG0015564: TcSYL_0071080.t1-p1
OG0015565: TcSYL_0071090.t1-p1
OG0015566: TcSYL_0071100.t1-p1
OG0015567: TcSYL_0071110.t1-p1
OG0015568: TcSYL_0071120.t1-p1
OG0015569: TcSYL_0071130.t1-p1
OG0015570: TcSYL_0071140.t1-p1
OG0015571: TcSYL_0071150.t1-p1
OG0015572: TcSYL_0071160.t1-p1
OG0015573: TcSYL_0071170.t1-p1
OG0015574: TcSYL_0071180.t1-p1
OG0015575: TcSYL_0071190.t1-p1
OG0015576: TcSYL_0071200.t1-p1
OG0015577: TcSYL_0071210.t1-p1
OG0015578: TcSYL_0071220.t1-p1
OG0015579: TcSYL_0071230.t1-p1
OG0015580: TcSYL_0071240.t1-p1
OG0015581: TcSYL_0071250.t1-p1
OG0015582: TcSYL_0071260.t1-p1
OG0015583: TcSYL_0071280.t1-p1
OG0015584: TcSYL_0071290.t1-p1
OG0015585: TcSYL_0071300.t1-p1
OG0015586: TcSYL_0071310.t1-p1

OG0015587: TcSYL_0071320.t1-p1
OG0015588: TcSYL_0071340.t1-p1
OG0015589: TcSYL_0071360.t1-p1
OG0015590: TcSYL_0071370.t1-p1
OG0015591: TcSYL_0071380.t1-p1
OG0015592: TcSYL_0071390.t1-p1
OG0015593: TcSYL_0071400.t1-p1
OG0015594: TcSYL_0071410.t1-p1
OG0015595: TcSYL_0071420.t1-p1
OG0015596: TcSYL_0071440.t1-p1
OG0015597: TcSYL_0071470.t1-p1
OG0015598: TcSYL_0071480.t1-p1
OG0015599: TcSYL_0071490.t1-p1
OG0015600: TcSYL_0071500.t1-p1
OG0015601: TcSYL_0071510.t1-p1
OG0015602: TcSYL_0071530.t1-p1
OG0015603: TcSYL_0071550.t1-p1
OG0015604: TcSYL_0071560.t1-p1
OG0015605: TcSYL_0071570.t1-p1
OG0015606: TcSYL_0071580.t1-p1
OG0015607: TcSYL_0071590.t1-p1
OG0015608: TcSYL_0071600.t1-p1
OG0015609: TcSYL_0071610.t1-p1
OG0015610: TcSYL_0071630.t1-p1
OG0015611: TcSYL_0071640.t1-p1
OG0015612: TcSYL_0071660.t1-p1
OG0015613: TcSYL_0071670.t1-p1
OG0015614: TcSYL_0071680.t1-p1
OG0015615: TcSYL_0071690.t1-p1
OG0015616: TcSYL_0071700.t1-p1
OG0015617: TcSYL_0071710.t1-p1
OG0015618: TcSYL_0071720.t1-p1
OG0015619: TcSYL_0071730.t1-p1
OG0015620: TcSYL_0071740.t1-p1
OG0015621: TcSYL_0071750.t1-p1
OG0015622: TcSYL_0071760.t1-p1
OG0015623: TcSYL_0071770.t1-p1
OG0015624: TcSYL_0071790.t1-p1
OG0015625: TcSYL_0071800.t1-p1
OG0015626: TcSYL_0071810.t1-p1
OG0015627: TcSYL_0071820.t1-p1
OG0015628: TcSYL_0071840.t1-p1
OG0015629: TcSYL_0071850.t1-p1
OG0015630: TcSYL_0071860.t1-p1
OG0015631: TcSYL_0071870.t1-p1
OG0015632: TcSYL_0071880.t1-p1
OG0015633: TcSYL_0071900.t1-p1
OG0015634: TcSYL_0071920.t1-p1
OG0015635: TcSYL_0071930.t1-p1
OG0015636: TcSYL_0071940.t1-p1
OG0015637: TcSYL_0071950.t1-p1
OG0015638: TcSYL_0071960.t1-p1
OG0015639: TcSYL_0071970.t1-p1
OG0015640: TcSYL_0071980.t1-p1

OG0015641: TcSYL_0071990.t1-p1
OG0015642: TcSYL_0072050.t1-p1
OG0015643: TcSYL_0072060.t1-p1
OG0015644: TcSYL_0072090.t1-p1
OG0015645: TcSYL_0072100.t1-p1
OG0015646: TcSYL_0072110.t1-p1
OG0015647: TcSYL_0072120.t1-p1
OG0015648: TcSYL_0072140.t1-p1
OG0015649: TcSYL_0072160.t1-p1
OG0015650: TcSYL_0072190.t1-p1
OG0015651: TcSYL_0072200.t1-p1
OG0015652: TcSYL_0072220.t1-p1
OG0015653: TcSYL_0072230.t1-p1
OG0015654: TcSYL_0072240.t1-p1
OG0015655: TcSYL_0072260.t1-p1
OG0015656: TcSYL_0072280.t1-p1
OG0015657: TcSYL_0072290.t1-p1
OG0015658: TcSYL_0072300.t1-p1
OG0015659: TcSYL_0072310.t1-p1
OG0015660: TcSYL_0072320.t1-p1
OG0015661: TcSYL_0072330.t1-p1
OG0015662: TcSYL_0072340.t1-p1
OG0015663: TcSYL_0072350.t1-p1
OG0015664: TcSYL_0072360.t1-p1
OG0015665: TcSYL_0072370.t1-p1
OG0015666: TcSYL_0072380.t1-p1
OG0015667: TcSYL_0072390.t1-p1
OG0015668: TcSYL_0072400.t1-p1
OG0015669: TcSYL_0072410.t1-p1
OG0015670: TcSYL_0072420.t1-p1
OG0015671: TcSYL_0072430.t1-p1
OG0015672: TcSYL_0072440.t1-p1
OG0015673: TcSYL_0072450.t1-p1
OG0015674: TcSYL_0072460.t1-p1
OG0015675: TcSYL_0072470.t1-p1
OG0015676: TcSYL_0072480.t1-p1
OG0015677: TcSYL_0072490.t1-p1
OG0015678: TcSYL_0072500.t1-p1
OG0015679: TcSYL_0072550.t1-p1
OG0015680: TcSYL_0072560.t1-p1
OG0015681: TcSYL_0072570.t1-p1
OG0015682: TcSYL_0072580.t1-p1
OG0015683: TcSYL_0072590.t1-p1
OG0015684: TcSYL_0072600.t1-p1
OG0015685: TcSYL_0072610.t1-p1
OG0015686: TcSYL_0072620.t1-p1
OG0015687: TcSYL_0072630.t1-p1
OG0015688: TcSYL_0072640.t1-p1
OG0015689: TcSYL_0072660.t1-p1
OG0015690: TcSYL_0072670.t1-p1
OG0015691: TcSYL_0072680.t1-p1
OG0015692: TcSYL_0072690.t1-p1
OG0015693: TcSYL_0072700.t1-p1
OG0015694: TcSYL_0072710.t1-p1

OG0015695: TcSYL_0072720.t1-p1
OG0015696: TcSYL_0072730.t1-p1
OG0015697: TcSYL_0072740.t1-p1
OG0015698: TcSYL_0072750.t1-p1
OG0015699: TcSYL_0072760.t1-p1
OG0015700: TcSYL_0072770.t1-p1
OG0015701: TcSYL_0072790.t1-p1
OG0015702: TcSYL_0072800.t1-p1
OG0015703: TcSYL_0072810.t1-p1
OG0015704: TcSYL_0072820.t1-p1
OG0015705: TcSYL_0072830.t1-p1
OG0015706: TcSYL_0072840.t1-p1
OG0015707: TcSYL_0072850.t1-p1
OG0015708: TcSYL_0072860.t1-p1
OG0015709: TcSYL_0072870.t1-p1
OG0015710: TcSYL_0072880.t1-p1
OG0015711: TcSYL_0072890.t1-p1
OG0015712: TcSYL_0072900.t1-p1
OG0015713: TcSYL_0072920.t1-p1
OG0015714: TcSYL_0072930.t1-p1
OG0015715: TcSYL_0072940.t1-p1
OG0015716: TcSYL_0072950.t1-p1
OG0015717: TcSYL_0072960.t1-p1
OG0015718: TcSYL_0072970.t1-p1
OG0015719: TcSYL_0072980.t1-p1
OG0015720: TcSYL_0072990.t1-p1
OG0015721: TcSYL_0073000.t1-p1
OG0015722: TcSYL_0073010.t1-p1
OG0015723: TcSYL_0073020.t1-p1
OG0015724: TcSYL_0073030.t1-p1
OG0015725: TcSYL_0073040.t1-p1
OG0015726: TcSYL_0073050.t1-p1
OG0015727: TcSYL_0073060.t1-p1
OG0015728: TcSYL_0073070.t1-p1
OG0015729: TcSYL_0073080.t1-p1
OG0015730: TcSYL_0073090.t1-p1
OG0015731: TcSYL_0073100.t1-p1
OG0015732: TcSYL_0073110.t1-p1
OG0015733: TcSYL_0073120.t1-p1
OG0015734: TcSYL_0073130.t1-p1
OG0015735: TcSYL_0073140.t1-p1
OG0015736: TcSYL_0073150.t1-p1
OG0015737: TcSYL_0073160.t1-p1
OG0015738: TcSYL_0073170.t1-p1
OG0015739: TcSYL_0073180.t1-p1
OG0015740: TcSYL_0073190.t1-p1
OG0015741: TcSYL_0073200.t1-p1
OG0015742: TcSYL_0073220.t1-p1
OG0015743: TcSYL_0073390.t1-p1
OG0015744: TcSYL_0073400.t1-p1
OG0015745: TcSYL_0073430.t1-p1
OG0015746: TcSYL_0073460.t1-p1
OG0015747: TcSYL_0073470.t1-p1
OG0015748: TcSYL_0073490.t1-p1

OG0015749: TcSYL_0073600.t1-p1
OG0015750: TcSYL_0073660.t1-p1
OG0015751: TcSYL_0073750.t1-p1
OG0015752: TcSYL_0073790.t1-p1
OG0015753: TcSYL_0073820.t1-p1
OG0015754: TcSYL_0073830.t1-p1
OG0015755: TcSYL_0073840.t1-p1
OG0015756: TcSYL_0073860.t1-p1
OG0015757: TcSYL_0073910.t1-p1
OG0015758: TcSYL_0073920.t1-p1
OG0015759: TcSYL_0073930.t1-p1
OG0015760: TcSYL_0073970.t1-p1
OG0015761: TcSYL_0073980.t1-p1
OG0015762: TcSYL_0073990.t1-p1
OG0015763: TcSYL_0074020.t1-p1
OG0015764: TcSYL_0074040.t1-p1
OG0015765: TcSYL_0074060.t1-p1
OG0015766: TcSYL_0074070.t1-p1
OG0015767: TcSYL_0074080.t1-p1
OG0015768: TcSYL_0074100.t1-p1
OG0015769: TcSYL_0074130.t1-p1
OG0015770: TcSYL_0074150.t1-p1
OG0015771: TcSYL_0074160.t1-p1
OG0015772: TcSYL_0074180.t1-p1
OG0015773: TcSYL_0074210.t1-p1
OG0015774: TcSYL_0074230.t1-p1
OG0015775: TcSYL_0074240.t1-p1
OG0015776: TcSYL_0074250.t1-p1
OG0015777: TcSYL_0074280.t1-p1
OG0015778: TcSYL_0074290.t1-p1
OG0015779: TcSYL_0074300.t1-p1
OG0015780: TcSYL_0074320.t1-p1
OG0015781: TcSYL_0074330.t1-p1
OG0015782: TcSYL_0074340.t1-p1
OG0015783: TcSYL_0074350.t1-p1
OG0015784: TcSYL_0074360.t1-p1
OG0015785: TcSYL_0074370.t1-p1
OG0015786: TcSYL_0074380.t1-p1
OG0015787: TcSYL_0074390.t1-p1
OG0015788: TcSYL_0074400.t1-p1
OG0015789: TcSYL_0074410.t1-p1
OG0015790: TcSYL_0074420.t1-p1
OG0015791: TcSYL_0074430.t1-p1
OG0015792: TcSYL_0074450.t1-p1
OG0015793: TcSYL_0074460.t1-p1
OG0015794: TcSYL_0074480.t1-p1
OG0015795: TcSYL_0074490.t1-p1
OG0015796: TcSYL_0074520.t1-p1
OG0015797: TcSYL_0074530.t1-p1
OG0015798: TcSYL_0074560.t1-p1
OG0015799: TcSYL_0074600.t1-p1
OG0015800: TcSYL_0074630.t1-p1
OG0015801: TcSYL_0074650.t1-p1
OG0015802: TcSYL_0074700.t1-p1

OG0015803: TcSYL_0074720.t1-p1
OG0015804: TcSYL_0074760.t1-p1
OG0015805: TcSYL_0074780.t1-p1
OG0015806: TcSYL_0074820.t1-p1
OG0015807: TcSYL_0074840.t1-p1
OG0015808: TcSYL_0074850.t1-p1
OG0015809: TcSYL_0074900.t1-p1
OG0015810: TcSYL_0074910.t1-p1
OG0015811: TcSYL_0074980.t1-p1
OG0015812: TcSYL_0075030.t1-p1
OG0015813: TcSYL_0075050.t1-p1
OG0015814: TcSYL_0075060.t1-p1
OG0015815: TcSYL_0075070.t1-p1
OG0015816: TcSYL_0075090.t1-p1
OG0015817: TcSYL_0075100.t1-p1
OG0015818: TcSYL_0075110.t1-p1
OG0015819: TcSYL_0075120.t1-p1
OG0015820: TcSYL_0075130.t1-p1
OG0015821: TcSYL_0075140.t1-p1
OG0015822: TcSYL_0075150.t1-p1
OG0015823: TcSYL_0075160.t1-p1
OG0015824: TcSYL_0075170.t1-p1
OG0015825: TcSYL_0075180.t1-p1
OG0015826: TcSYL_0075200.t1-p1
OG0015827: TcSYL_0075240.t1-p1
OG0015828: TcSYL_0075360.t1-p1
OG0015829: TcSYL_0075400.t1-p1
OG0015830: TcSYL_0075410.t1-p1
OG0015831: TcSYL_0075440.t1-p1
OG0015832: TcSYL_0075460.t1-p1
OG0015833: TcSYL_0075570.t1-p1
OG0015834: TcSYL_0075600.t1-p1
OG0015835: TcSYL_0075740.t1-p1
OG0015836: TcSYL_0075770.t1-p1
OG0015837: TcSYL_0075780.t1-p1
OG0015838: TcSYL_0075790.t1-p1
OG0015839: TcSYL_0075810.t1-p1
OG0015840: TcSYL_0075830.t1-p1
OG0015841: TcSYL_0075840.t1-p1
OG0015842: TcSYL_0075850.t1-p1
OG0015843: TcSYL_0075860.t1-p1
OG0015844: TcSYL_0075870.t1-p1
OG0015845: TcSYL_0075880.t1-p1
OG0015846: TcSYL_0075900.t1-p1
OG0015847: TcSYL_0075910.t1-p1
OG0015848: TcSYL_0075920.t1-p1
OG0015849: TcSYL_0075940.t1-p1
OG0015850: TcSYL_0075950.t1-p1
OG0015851: TcSYL_0075960.t1-p1
OG0015852: TcSYL_0075980.t1-p1
OG0015853: TcSYL_0075990.t1-p1
OG0015854: TcSYL_0076000.t1-p1
OG0015855: TcSYL_0076020.t1-p1
OG0015856: TcSYL_0076030.t1-p1

OG0015857: TcSYL_0076040.t1-p1
OG0015858: TcSYL_0076050.t1-p1
OG0015859: TcSYL_0076060.t1-p1
OG0015860: TcSYL_0076070.t1-p1
OG0015861: TcSYL_0076080.t1-p1
OG0015862: TcSYL_0076090.t1-p1
OG0015863: TcSYL_0076100.t1-p1
OG0015864: TcSYL_0076110.t1-p1
OG0015865: TcSYL_0076120.t1-p1
OG0015866: TcSYL_0076130.t1-p1
OG0015867: TcSYL_0076140.t1-p1
OG0015868: TcSYL_0076160.t1-p1
OG0015869: TcSYL_0076170.t1-p1
OG0015870: TcSYL_0076190.t1-p1
OG0015871: TcSYL_0076200.t1-p1
OG0015872: TcSYL_0076210.t1-p1
OG0015873: TcSYL_0076220.t1-p1
OG0015874: TcSYL_0076230.t1-p1
OG0015875: TcSYL_0076250.t1-p1
OG0015876: TcSYL_0076270.t1-p1
OG0015877: TcSYL_0076280.t1-p1
OG0015878: TcSYL_0076300.t1-p1
OG0015879: TcSYL_0076310.t1-p1
OG0015880: TcSYL_0076330.t1-p1
OG0015881: TcSYL_0076350.t1-p1
OG0015882: TcSYL_0076370.t1-p1
OG0015883: TcSYL_0076380.t1-p1
OG0015884: TcSYL_0076390.t1-p1
OG0015885: TcSYL_0076400.t1-p1
OG0015886: TcSYL_0076420.t1-p1
OG0015887: TcSYL_0076470.t1-p1
OG0015888: TcSYL_0076480.t1-p1
OG0015889: TcSYL_0076510.t1-p1
OG0015890: TcSYL_0076540.t1-p1
OG0015891: TcSYL_0076550.t1-p1
OG0015892: TcSYL_0076560.t1-p1
OG0015893: TcSYL_0076570.t1-p1
OG0015894: TcSYL_0076580.t1-p1
OG0015895: TcSYL_0076600.t1-p1
OG0015896: TcSYL_0076610.t1-p1
OG0015897: TcSYL_0076640.t1-p1
OG0015898: TcSYL_0076650.t1-p1
OG0015899: TcSYL_0076660.t1-p1
OG0015900: TcSYL_0076670.t1-p1
OG0015901: TcSYL_0076680.t1-p1
OG0015902: TcSYL_0076700.t1-p1
OG0015903: TcSYL_0076720.t1-p1
OG0015904: TcSYL_0076750.t1-p1
OG0015905: TcSYL_0076770.t1-p1
OG0015906: TcSYL_0076780.t1-p1
OG0015907: TcSYL_0076790.t1-p1
OG0015908: TcSYL_0076800.t1-p1
OG0015909: TcSYL_0076830.t1-p1
OG0015910: TcSYL_0076840.t1-p1

OG0015911: TcSYL_0076870.t1-p1
OG0015912: TcSYL_0076880.t1-p1
OG0015913: TcSYL_0076890.t1-p1
OG0015914: TcSYL_0076900.t1-p1
OG0015915: TcSYL_0076910.t1-p1
OG0015916: TcSYL_0076920.t1-p1
OG0015917: TcSYL_0076930.t1-p1
OG0015918: TcSYL_0076940.t1-p1
OG0015919: TcSYL_0076990.t1-p1
OG0015920: TcSYL_0077000.t1-p1
OG0015921: TcSYL_0077010.t1-p1
OG0015922: TcSYL_0077020.t1-p1
OG0015923: TcSYL_0077040.t1-p1
OG0015924: TcSYL_0077050.t1-p1
OG0015925: TcSYL_0077060.t1-p1
OG0015926: TcSYL_0077070.t1-p1
OG0015927: TcSYL_0077090.t1-p1
OG0015928: TcSYL_0077100.t1-p1
OG0015929: TcSYL_0077110.t1-p1
OG0015930: TcSYL_0077120.t1-p1
OG0015931: TcSYL_0077130.t1-p1
OG0015932: TcSYL_0077140.t1-p1
OG0015933: TcSYL_0077150.t1-p1
OG0015934: TcSYL_0077160.t1-p1
OG0015935: TcSYL_0077180.t1-p1
OG0015936: TcSYL_0077190.t1-p1
OG0015937: TcSYL_0077200.t1-p1
OG0015938: TcSYL_0077210.t1-p1
OG0015939: TcSYL_0077220.t1-p1
OG0015940: TcSYL_0077230.t1-p1
OG0015941: TcSYL_0077250.t1-p1
OG0015942: TcSYL_0077270.t1-p1
OG0015943: TcSYL_0077290.t1-p1
OG0015944: TcSYL_0077300.t1-p1
OG0015945: TcSYL_0077310.t1-p1
OG0015946: TcSYL_0077330.t1-p1
OG0015947: TcSYL_0077340.t1-p1
OG0015948: TcSYL_0077360.t1-p1
OG0015949: TcSYL_0077370.t1-p1
OG0015950: TcSYL_0077390.t1-p1
OG0015951: TcSYL_0077410.t1-p1
OG0015952: TcSYL_0077430.t1-p1
OG0015953: TcSYL_0077450.t1-p1
OG0015954: TcSYL_0077460.t1-p1
OG0015955: TcSYL_0077470.t1-p1
OG0015956: TcSYL_0077490.t1-p1
OG0015957: TcSYL_0077500.t1-p1
OG0015958: TcSYL_0077510.t1-p1
OG0015959: TcSYL_0077520.t1-p1
OG0015960: TcSYL_0077530.t1-p1
OG0015961: TcSYL_0077550.t1-p1
OG0015962: TcSYL_0077560.t1-p1
OG0015963: TcSYL_0077570.t1-p1
OG0015964: TcSYL_0077580.t1-p1

OG0015965: TcSYL_0077620.t1-p1
OG0015966: TcSYL_0077630.t1-p1
OG0015967: TcSYL_0077640.t1-p1
OG0015968: TcSYL_0077650.t1-p1
OG0015969: TcSYL_0077660.t1-p1
OG0015970: TcSYL_0077670.t1-p1
OG0015971: TcSYL_0077680.t1-p1
OG0015972: TcSYL_0077690.t1-p1
OG0015973: TcSYL_0077700.t1-p1
OG0015974: TcSYL_0077710.t1-p1
OG0015975: TcSYL_0077720.t1-p1
OG0015976: TcSYL_0077730.t1-p1
OG0015977: TcSYL_0077740.t1-p1
OG0015978: TcSYL_0077750.t1-p1
OG0015979: TcSYL_0077760.t1-p1
OG0015980: TcSYL_0077780.t1-p1
OG0015981: TcSYL_0077790.t1-p1
OG0015982: TcSYL_0077810.t1-p1
OG0015983: TcSYL_0077830.t1-p1
OG0015984: TcSYL_0077840.t1-p1
OG0015985: TcSYL_0077860.t1-p1
OG0015986: TcSYL_0077870.t1-p1
OG0015987: TcSYL_0077880.t1-p1
OG0015988: TcSYL_0077890.t1-p1
OG0015989: TcSYL_0077900.t1-p1
OG0015990: TcSYL_0077910.t1-p1
OG0015991: TcSYL_0077920.t1-p1
OG0015992: TcSYL_0077930.t1-p1
OG0015993: TcSYL_0077940.t1-p1
OG0015994: TcSYL_0077950.t1-p1
OG0015995: TcSYL_0077960.t1-p1
OG0015996: TcSYL_0077970.t1-p1
OG0015997: TcSYL_0077980.t1-p1
OG0015998: TcSYL_0077990.t1-p1
OG0015999: TcSYL_0078000.t1-p1
OG0016000: TcSYL_0078010.t1-p1
OG0016001: TcSYL_0078020.t1-p1
OG0016002: TcSYL_0078030.t1-p1
OG0016003: TcSYL_0078040.t1-p1
OG0016004: TcSYL_0078050.t1-p1
OG0016005: TcSYL_0078060.t1-p1
OG0016006: TcSYL_0078070.t1-p1
OG0016007: TcSYL_0078080.t1-p1
OG0016008: TcSYL_0078090.t1-p1
OG0016009: TcSYL_0078100.t1-p1
OG0016010: TcSYL_0078120.t1-p1
OG0016011: TcSYL_0078130.t1-p1
OG0016012: TcSYL_0078140.t1-p1
OG0016013: TcSYL_0078150.t1-p1
OG0016014: TcSYL_0078160.t1-p1
OG0016015: TcSYL_0078170.t1-p1
OG0016016: TcSYL_0078180.t1-p1
OG0016017: TcSYL_0078200.t1-p1
OG0016018: TcSYL_0078210.t1-p1

OG0016019: TcSYL_0078230.t1-p1
OG0016020: TcSYL_0078240.t1-p1
OG0016021: TcSYL_0078260.t1-p1
OG0016022: TcSYL_0078280.t1-p1
OG0016023: TcSYL_0078300.t1-p1
OG0016024: TcSYL_0078320.t1-p1
OG0016025: TcSYL_0078330.t1-p1
OG0016026: TcSYL_0078340.t1-p1
OG0016027: TcSYL_0078350.t1-p1
OG0016028: TcSYL_0078370.t1-p1
OG0016029: TcSYL_0078390.t1-p1
OG0016030: TcSYL_0078400.t1-p1
OG0016031: TcSYL_0078410.t1-p1
OG0016032: TcSYL_0078430.t1-p1
OG0016033: TcSYL_0078450.t1-p1
OG0016034: TcSYL_0078460.t1-p1
OG0016035: TcSYL_0078480.t1-p1
OG0016036: TcSYL_0078490.t1-p1
OG0016037: TcSYL_0078500.t1-p1
OG0016038: TcSYL_0078510.t1-p1
OG0016039: TcSYL_0078530.t1-p1
OG0016040: TcSYL_0078540.t1-p1
OG0016041: TcSYL_0078550.t1-p1
OG0016042: TcSYL_0078560.t1-p1
OG0016043: TcSYL_0078570.t1-p1
OG0016044: TcSYL_0078580.t1-p1
OG0016045: TcSYL_0078600.t1-p1
OG0016046: TcSYL_0078660.t1-p1
OG0016047: TcSYL_0078690.t1-p1
OG0016048: TcSYL_0078700.t1-p1
OG0016049: TcSYL_0078730.t1-p1
OG0016050: TcSYL_0078750.t1-p1
OG0016051: TcSYL_0078810.t1-p1
OG0016052: TcSYL_0078830.t1-p1
OG0016053: TcSYL_0078850.t1-p1
OG0016054: TcSYL_0078860.t1-p1
OG0016055: TcSYL_0078940.t1-p1
OG0016056: TcSYL_0079040.t1-p1
OG0016057: TcSYL_0079060.t1-p1
OG0016058: TcSYL_0079070.t1-p1
OG0016059: TcSYL_0079080.t1-p1
OG0016060: TcSYL_0079090.t1-p1
OG0016061: TcSYL_0079100.t1-p1
OG0016062: TcSYL_0079110.t1-p1
OG0016063: TcSYL_0079120.t1-p1
OG0016064: TcSYL_0079130.t1-p1
OG0016065: TcSYL_0079140.t1-p1
OG0016066: TcSYL_0079160.t1-p1
OG0016067: TcSYL_0079190.t1-p1
OG0016068: TcSYL_0079200.t1-p1
OG0016069: TcSYL_0079240.t1-p1
OG0016070: TcSYL_0079250.t1-p1
OG0016071: TcSYL_0079260.t1-p1
OG0016072: TcSYL_0079280.t1-p1

OG0016073: TcSYL_0079440.t1-p1
OG0016074: TcSYL_0079460.t1-p1
OG0016075: TcSYL_0079470.t1-p1
OG0016076: TcSYL_0079480.t1-p1
OG0016077: TcSYL_0079510.t1-p1
OG0016078: TcSYL_0079520.t1-p1
OG0016079: TcSYL_0079530.t1-p1
OG0016080: TcSYL_0079540.t1-p1
OG0016081: TcSYL_0079550.t1-p1
OG0016082: TcSYL_0079560.t1-p1
OG0016083: TcSYL_0079570.t1-p1
OG0016084: TcSYL_0079580.t1-p1
OG0016085: TcSYL_0079590.t1-p1
OG0016086: TcSYL_0079630.t1-p1
OG0016087: TcSYL_0079640.t1-p1
OG0016088: TcSYL_0079650.t1-p1
OG0016089: TcSYL_0079670.t1-p1
OG0016090: TcSYL_0079700.t1-p1
OG0016091: TcSYL_0079710.t1-p1
OG0016092: TcSYL_0079730.t1-p1
OG0016093: TcSYL_0079740.t1-p1
OG0016094: TcSYL_0079760.t1-p1
OG0016095: TcSYL_0079780.t1-p1
OG0016096: TcSYL_0079810.t1-p1
OG0016097: TcSYL_0079820.t1-p1
OG0016098: TcSYL_0079830.t1-p1
OG0016099: TcSYL_0079850.t1-p1
OG0016100: TcSYL_0079860.t1-p1
OG0016101: TcSYL_0079870.t1-p1
OG0016102: TcSYL_0079890.t1-p1
OG0016103: TcSYL_0079900.t1-p1
OG0016104: TcSYL_0079910.t1-p1
OG0016105: TcSYL_0079920.t1-p1
OG0016106: TcSYL_0079930.t1-p1
OG0016107: TcSYL_0079940.t1-p1
OG0016108: TcSYL_0079970.t1-p1
OG0016109: TcSYL_0079990.t1-p1
OG0016110: TcSYL_0080010.t1-p1
OG0016111: TcSYL_0080020.t1-p1
OG0016112: TcSYL_0080030.t1-p1
OG0016113: TcSYL_0080040.t1-p1
OG0016114: TcSYL_0080060.t1-p1
OG0016115: TcSYL_0080070.t1-p1
OG0016116: TcSYL_0080080.t1-p1
OG0016117: TcSYL_0080100.t1-p1
OG0016118: TcSYL_0080110.t1-p1
OG0016119: TcSYL_0080120.t1-p1
OG0016120: TcSYL_0080130.t1-p1
OG0016121: TcSYL_0080140.t1-p1
OG0016122: TcSYL_0080150.t1-p1
OG0016123: TcSYL_0080160.t1-p1
OG0016124: TcSYL_0080170.t1-p1
OG0016125: TcSYL_0080180.t1-p1
OG0016126: TcSYL_0080190.t1-p1

OG0016127: TcSYL_0080200.t1-p1
OG0016128: TcSYL_0080210.t1-p1
OG0016129: TcSYL_0080220.t1-p1
OG0016130: TcSYL_0080230.t1-p1
OG0016131: TcSYL_0080240.t1-p1
OG0016132: TcSYL_0080250.t1-p1
OG0016133: TcSYL_0080260.t1-p1
OG0016134: TcSYL_0080270.t1-p1
OG0016135: TcSYL_0080470.t1-p1
OG0016136: TcSYL_0080490.t1-p1
OG0016137: TcSYL_0080600.t1-p1
OG0016138: TcSYL_0080630.t1-p1
OG0016139: TcSYL_0080760.t1-p1
OG0016140: TcSYL_0080780.t1-p1
OG0016141: TcSYL_0080790.t1-p1
OG0016142: TcSYL_0080890.t1-p1
OG0016143: TcSYL_0080920.t1-p1
OG0016144: TcSYL_0080930.t1-p1
OG0016145: TcSYL_0080940.t1-p1
OG0016146: TcSYL_0080950.t1-p1
OG0016147: TcSYL_0080960.t1-p1
OG0016148: TcSYL_0080970.t1-p1
OG0016149: TcSYL_0080980.t1-p1
OG0016150: TcSYL_0081010.t1-p1
OG0016151: TcSYL_0081020.t1-p1
OG0016152: TcSYL_0081030.t1-p1
OG0016153: TcSYL_0081040.t1-p1
OG0016154: TcSYL_0081050.t1-p1
OG0016155: TcSYL_0081060.t1-p1
OG0016156: TcSYL_0081070.t1-p1
OG0016157: TcSYL_0081080.t1-p1
OG0016158: TcSYL_0081090.t1-p1
OG0016159: TcSYL_0081100.t1-p1
OG0016160: TcSYL_0081110.t1-p1
OG0016161: TcSYL_0081120.t1-p1
OG0016162: TcSYL_0081130.t1-p1
OG0016163: TcSYL_0081140.t1-p1
OG0016164: TcSYL_0081150.t1-p1
OG0016165: TcSYL_0081160.t1-p1
OG0016166: TcSYL_0081170.t1-p1
OG0016167: TcSYL_0081180.t1-p1
OG0016168: TcSYL_0081190.t1-p1
OG0016169: TcSYL_0081200.t1-p1
OG0016170: TcSYL_0081210.t1-p1
OG0016171: TcSYL_0081220.t1-p1
OG0016172: TcSYL_0081230.t1-p1
OG0016173: TcSYL_0081240.t1-p1
OG0016174: TcSYL_0081250.t1-p1
OG0016175: TcSYL_0081260.t1-p1
OG0016176: TcSYL_0081270.t1-p1
OG0016177: TcSYL_0081280.t1-p1
OG0016178: TcSYL_0081290.t1-p1
OG0016179: TcSYL_0081300.t1-p1
OG0016180: TcSYL_0081310.t1-p1

OG0016181: TcSYL_0081320.t1-p1
OG0016182: TcSYL_0081330.t1-p1
OG0016183: TcSYL_0081340.t1-p1
OG0016184: TcSYL_0081350.t1-p1
OG0016185: TcSYL_0081360.t1-p1
OG0016186: TcSYL_0081380.t1-p1
OG0016187: TcSYL_0081390.t1-p1
OG0016188: TcSYL_0081400.t1-p1
OG0016189: TcSYL_0081410.t1-p1
OG0016190: TcSYL_0081420.t1-p1
OG0016191: TcSYL_0081430.t1-p1
OG0016192: TcSYL_0081440.t1-p1
OG0016193: TcSYL_0081450.t1-p1
OG0016194: TcSYL_0081460.t1-p1
OG0016195: TcSYL_0081470.t1-p1
OG0016196: TcSYL_0081480.t1-p1
OG0016197: TcSYL_0081490.t1-p1
OG0016198: TcSYL_0081500.t1-p1
OG0016199: TcSYL_0081510.t1-p1
OG0016200: TcSYL_0081520.t1-p1
OG0016201: TcSYL_0081530.t1-p1
OG0016202: TcSYL_0081540.t1-p1
OG0016203: TcSYL_0081550.t1-p1
OG0016204: TcSYL_0081560.t1-p1
OG0016205: TcSYL_0081570.t1-p1
OG0016206: TcSYL_0081580.t1-p1
OG0016207: TcSYL_0081590.t1-p1
OG0016208: TcSYL_0081600.t1-p1
OG0016209: TcSYL_0081610.t1-p1
OG0016210: TcSYL_0081620.t1-p1
OG0016211: TcSYL_0081630.t1-p1
OG0016212: TcSYL_0081640.t1-p1
OG0016213: TcSYL_0081650.t1-p1
OG0016214: TcSYL_0081660.t1-p1
OG0016215: TcSYL_0081670.t1-p1
OG0016216: TcSYL_0081680.t1-p1
OG0016217: TcSYL_0081690.t1-p1
OG0016218: TcSYL_0081700.t1-p1
OG0016219: TcSYL_0081710.t1-p1
OG0016220: TcSYL_0081720.t1-p1
OG0016221: TcSYL_0081730.t1-p1
OG0016222: TcSYL_0081740.t1-p1
OG0016223: TcSYL_0081750.t1-p1
OG0016224: TcSYL_0081760.t1-p1
OG0016225: TcSYL_0081770.t1-p1
OG0016226: TcSYL_0081780.t1-p1
OG0016227: TcSYL_0081790.t1-p1
OG0016228: TcSYL_0081800.t1-p1
OG0016229: TcSYL_0081810.t1-p1
OG0016230: TcSYL_0081820.t1-p1
OG0016231: TcSYL_0081830.t1-p1
OG0016232: TcSYL_0081840.t1-p1
OG0016233: TcSYL_0081850.t1-p1
OG0016234: TcSYL_0081860.t1-p1

OG0016235: TcSYL_0081870.t1-p1
OG0016236: TcSYL_0081880.t1-p1
OG0016237: TcSYL_0081890.t1-p1
OG0016238: TcSYL_0081900.t1-p1
OG0016239: TcSYL_0081910.t1-p1
OG0016240: TcSYL_0081920.t1-p1
OG0016241: TcSYL_0081930.t1-p1
OG0016242: TcSYL_0081940.t1-p1
OG0016243: TcSYL_0081950.t1-p1
OG0016244: TcSYL_0081960.t1-p1
OG0016245: TcSYL_0081970.t1-p1
OG0016246: TcSYL_0081980.t1-p1
OG0016247: TcSYL_0081990.t1-p1
OG0016248: TcSYL_0082000.t1-p1
OG0016249: TcSYL_0082010.t1-p1
OG0016250: TcSYL_0082020.t1-p1
OG0016251: TcSYL_0082030.t1-p1
OG0016252: TcSYL_0082040.t1-p1
OG0016253: TcSYL_0082050.t1-p1
OG0016254: TcSYL_0082060.t1-p1
OG0016255: TcSYL_0082070.t1-p1
OG0016256: TcSYL_0082080.t1-p1
OG0016257: TcSYL_0082090.t1-p1
OG0016258: TcSYL_0082100.t1-p1
OG0016259: TcSYL_0082110.t1-p1
OG0016260: TcSYL_0082120.t1-p1
OG0016261: TcSYL_0082130.t1-p1
OG0016262: TcSYL_0082140.t1-p1
OG0016263: TcSYL_0082150.t1-p1
OG0016264: TcSYL_0082160.t1-p1
OG0016265: TcSYL_0082170.t1-p1
OG0016266: TcSYL_0082180.t1-p1
OG0016267: TcSYL_0082190.t1-p1
OG0016268: TcSYL_0082200.t1-p1
OG0016269: TcSYL_0082210.t1-p1
OG0016270: TcSYL_0082220.t1-p1
OG0016271: TcSYL_0082230.t1-p1
OG0016272: TcSYL_0082250.t1-p1
OG0016273: TcSYL_0082340.t1-p1
OG0016274: TcSYL_0082450.t1-p1
OG0016275: TcSYL_0082530.t1-p1
OG0016276: TcSYL_0082540.t1-p1
OG0016277: TcSYL_0082580.t1-p1
OG0016278: TcSYL_0082590.t1-p1
OG0016279: TcSYL_0082600.t1-p1
OG0016280: TcSYL_0082610.t1-p1
OG0016281: TcSYL_0082620.t1-p1
OG0016282: TcSYL_0082630.t1-p1
OG0016283: TcSYL_0082640.t1-p1
OG0016284: TcSYL_0082650.t1-p1
OG0016285: TcSYL_0082660.t1-p1
OG0016286: TcSYL_0082670.t1-p1
OG0016287: TcSYL_0082680.t1-p1
OG0016288: TcSYL_0082690.t1-p1

OG0016289: TcSYL_0082700.t1-p1
OG0016290: TcSYL_0082710.t1-p1
OG0016291: TcSYL_0082720.t1-p1
OG0016292: TcSYL_0082730.t1-p1
OG0016293: TcSYL_0082740.t1-p1
OG0016294: TcSYL_0082750.t1-p1
OG0016295: TcSYL_0082760.t1-p1
OG0016296: TcSYL_0082770.t1-p1
OG0016297: TcSYL_0082780.t1-p1
OG0016298: TcSYL_0082790.t1-p1
OG0016299: TcSYL_0082800.t1-p1
OG0016300: TcSYL_0082810.t1-p1
OG0016301: TcSYL_0082820.t1-p1
OG0016302: TcSYL_0082830.t1-p1
OG0016303: TcSYL_0082840.t1-p1
OG0016304: TcSYL_0082850.t1-p1
OG0016305: TcSYL_0082860.t1-p1
OG0016306: TcSYL_0082870.t1-p1
OG0016307: TcSYL_0082880.t1-p1
OG0016308: TcSYL_0082890.t1-p1
OG0016309: TcSYL_0082900.t1-p1
OG0016310: TcSYL_0082920.t1-p1
OG0016311: TcSYL_0082960.t1-p1
OG0016312: TcSYL_0083000.t1-p1
OG0016313: TcSYL_0083070.t1-p1
OG0016314: TcSYL_0083150.t1-p1
OG0016315: TcSYL_0083180.t1-p1
OG0016316: TcSYL_0083190.t1-p1
OG0016317: TcSYL_0083200.t1-p1
OG0016318: TcSYL_0083210.t1-p1
OG0016319: TcSYL_0083220.t1-p1
OG0016320: TcSYL_0083230.t1-p1
OG0016321: TcSYL_0083240.t1-p1
OG0016322: TcSYL_0083300.t1-p1
OG0016323: TcSYL_0083320.t1-p1
OG0016324: TcSYL_0083330.t1-p1
OG0016325: TcSYL_0083340.t1-p1
OG0016326: TcSYL_0083410.t1-p1
OG0016327: TcSYL_0083420.t1-p1
OG0016328: TcSYL_0083430.t1-p1
OG0016329: TcSYL_0083440.t1-p1
OG0016330: TcSYL_0083450.t1-p1
OG0016331: TcSYL_0083460.t1-p1
OG0016332: TcSYL_0083470.t1-p1
OG0016333: TcSYL_0083480.t1-p1
OG0016334: TcSYL_0083490.t1-p1
OG0016335: TcSYL_0083500.t1-p1
OG0016336: TcSYL_0083510.t1-p1
OG0016337: TcSYL_0083520.t1-p1
OG0016338: TcSYL_0083530.t1-p1
OG0016339: TcSYL_0083540.t1-p1
OG0016340: TcSYL_0083550.t1-p1
OG0016341: TcSYL_0083570.t1-p1
OG0016342: TcSYL_0083590.t1-p1

OG0016343: TcSYL_0083600.t1-p1
OG0016344: TcSYL_0083610.t1-p1
OG0016345: TcSYL_0083620.t1-p1
OG0016346: TcSYL_0083630.t1-p1
OG0016347: TcSYL_0083650.t1-p1
OG0016348: TcSYL_0083660.t1-p1
OG0016349: TcSYL_0083670.t1-p1
OG0016350: TcSYL_0083680.t1-p1
OG0016351: TcSYL_0083690.t1-p1
OG0016352: TcSYL_0083710.t1-p1
OG0016353: TcSYL_0083730.t1-p1
OG0016354: TcSYL_0083740.t1-p1
OG0016355: TcSYL_0083750.t1-p1
OG0016356: TcSYL_0083790.t1-p1
OG0016357: TcSYL_0083800.t1-p1
OG0016358: TcSYL_0083810.t1-p1
OG0016359: TcSYL_0083840.t1-p1
OG0016360: TcSYL_0083850.t1-p1
OG0016361: TcSYL_0083860.t1-p1
OG0016362: TcSYL_0083890.t1-p1
OG0016363: TcSYL_0083900.t1-p1
OG0016364: TcSYL_0083930.t1-p1
OG0016365: TcSYL_0083960.t1-p1
OG0016366: TcSYL_0083980.t1-p1
OG0016367: TcSYL_0084000.t1-p1
OG0016368: TcSYL_0084020.t1-p1
OG0016369: TcSYL_0084090.t1-p1
OG0016370: TcSYL_0084100.t1-p1
OG0016371: TcSYL_0084110.t1-p1
OG0016372: TcSYL_0084170.t1-p1
OG0016373: TcSYL_0084190.t1-p1
OG0016374: TcSYL_0084200.t1-p1
OG0016375: TcSYL_0084210.t1-p1
OG0016376: TcSYL_0084230.t1-p1
OG0016377: TcSYL_0084360.t1-p1
OG0016378: TcSYL_0084370.t1-p1
OG0016379: TcSYL_0084380.t1-p1
OG0016380: TcSYL_0084400.t1-p1
OG0016381: TcSYL_0084420.t1-p1
OG0016382: TcSYL_0084430.t1-p1
OG0016383: TcSYL_0084450.t1-p1
OG0016384: TcSYL_0084470.t1-p1
OG0016385: TcSYL_0084480.t1-p1
OG0016386: TcSYL_0084500.t1-p1
OG0016387: TcSYL_0084510.t1-p1
OG0016388: TcSYL_0084520.t1-p1
OG0016389: TcSYL_0084530.t1-p1
OG0016390: TcSYL_0084540.t1-p1
OG0016391: TcSYL_0084560.t1-p1
OG0016392: TcSYL_0084570.t1-p1
OG0016393: TcSYL_0084580.t1-p1
OG0016394: TcSYL_0084590.t1-p1
OG0016395: TcSYL_0084610.t1-p1
OG0016396: TcSYL_0084630.t1-p1

OG0016397: TcSYL_0084640.t1-p1
OG0016398: TcSYL_0084650.t1-p1
OG0016399: TcSYL_0084660.t1-p1
OG0016400: TcSYL_0084670.t1-p1
OG0016401: TcSYL_0084680.t1-p1
OG0016402: TcSYL_0084690.t1-p1
OG0016403: TcSYL_0084710.t1-p1
OG0016404: TcSYL_0084720.t1-p1
OG0016405: TcSYL_0084730.t1-p1
OG0016406: TcSYL_0084740.t1-p1
OG0016407: TcSYL_0084750.t1-p1
OG0016408: TcSYL_0084760.t1-p1
OG0016409: TcSYL_0084770.t1-p1
OG0016410: TcSYL_0084790.t1-p1
OG0016411: TcSYL_0084810.t1-p1
OG0016412: TcSYL_0084940.t1-p1
OG0016413: TcSYL_0084950.t1-p1
OG0016414: TcSYL_0084970.t1-p1
OG0016415: TcSYL_0084980.t1-p1
OG0016416: TcSYL_0084990.t1-p1
OG0016417: TcSYL_0085000.t1-p1
OG0016418: TcSYL_0085020.t1-p1
OG0016419: TcSYL_0085030.t1-p1
OG0016420: TcSYL_0085040.t1-p1
OG0016421: TcSYL_0085050.t1-p1
OG0016422: TcSYL_0085060.t1-p1
OG0016423: TcSYL_0085070.t1-p1
OG0016424: TcSYL_0085080.t1-p1
OG0016425: TcSYL_0085090.t1-p1
OG0016426: TcSYL_0085110.t1-p1
OG0016427: TcSYL_0085120.t1-p1
OG0016428: TcSYL_0085130.t1-p1
OG0016429: TcSYL_0085150.t1-p1
OG0016430: TcSYL_0085160.t1-p1
OG0016431: TcSYL_0085170.t1-p1
OG0016432: TcSYL_0085190.t1-p1
OG0016433: TcSYL_0085200.t1-p1
OG0016434: TcSYL_0085210.t1-p1
OG0016435: TcSYL_0085220.t1-p1
OG0016436: TcSYL_0085230.t1-p1
OG0016437: TcSYL_0085240.t1-p1
OG0016438: TcSYL_0085250.t1-p1
OG0016439: TcSYL_0085260.t1-p1
OG0016440: TcSYL_0085270.t1-p1
OG0016441: TcSYL_0085280.t1-p1
OG0016442: TcSYL_0085300.t1-p1
OG0016443: TcSYL_0085310.t1-p1
OG0016444: TcSYL_0085320.t1-p1
OG0016445: TcSYL_0085340.t1-p1
OG0016446: TcSYL_0085350.t1-p1
OG0016447: TcSYL_0085360.t1-p1
OG0016448: TcSYL_0085380.t1-p1
OG0016449: TcSYL_0085400.t1-p1
OG0016450: TcSYL_0085410.t1-p1

OG0016451: TcSYL_0085420.t1-p1
OG0016452: TcSYL_0085430.t1-p1
OG0016453: TcSYL_0085440.t1-p1
OG0016454: TcSYL_0085470.t1-p1
OG0016455: TcSYL_0085500.t1-p1
OG0016456: TcSYL_0085510.t1-p1
OG0016457: TcSYL_0085530.t1-p1
OG0016458: TcSYL_0085540.t1-p1
OG0016459: TcSYL_0085550.t1-p1
OG0016460: TcSYL_0085560.t1-p1
OG0016461: TcSYL_0085580.t1-p1
OG0016462: TcSYL_0085590.t1-p1
OG0016463: TcSYL_0085600.t1-p1
OG0016464: TcSYL_0085610.t1-p1
OG0016465: TcSYL_0085620.t1-p1
OG0016466: TcSYL_0085630.t1-p1
OG0016467: TcSYL_0085660.t1-p1
OG0016468: TcSYL_0085670.t1-p1
OG0016469: TcSYL_0085680.t1-p1
OG0016470: TcSYL_0085690.t1-p1
OG0016471: TcSYL_0085700.t1-p1
OG0016472: TcSYL_0085710.t1-p1
OG0016473: TcSYL_0085720.t1-p1
OG0016474: TcSYL_0085730.t1-p1
OG0016475: TcSYL_0085740.t1-p1
OG0016476: TcSYL_0085750.t1-p1
OG0016477: TcSYL_0085760.t1-p1
OG0016478: TcSYL_0085770.t1-p1
OG0016479: TcSYL_0085780.t1-p1
OG0016480: TcSYL_0085790.t1-p1
OG0016481: TcSYL_0085800.t1-p1
OG0016482: TcSYL_0085810.t1-p1
OG0016483: TcSYL_0085820.t1-p1
OG0016484: TcSYL_0085830.t1-p1
OG0016485: TcSYL_0085840.t1-p1
OG0016486: TcSYL_0085850.t1-p1
OG0016487: TcSYL_0085860.t1-p1
OG0016488: TcSYL_0085870.t1-p1
OG0016489: TcSYL_0085880.t1-p1
OG0016490: TcSYL_0085890.t1-p1
OG0016491: TcSYL_0085900.t1-p1
OG0016492: TcSYL_0085910.t1-p1
OG0016493: TcSYL_0085920.t1-p1
OG0016494: TcSYL_0085930.t1-p1
OG0016495: TcSYL_0085940.t1-p1
OG0016496: TcSYL_0085950.t1-p1
OG0016497: TcSYL_0085960.t1-p1
OG0016498: TcSYL_0085970.t1-p1
OG0016499: TcSYL_0085980.t1-p1
OG0016500: TcSYL_0085990.t1-p1
OG0016501: TcSYL_0086010.t1-p1
OG0016502: TcSYL_0086020.t1-p1
OG0016503: TcSYL_0086030.t1-p1
OG0016504: TcSYL_0086040.t1-p1

OG0016505: TcSYL_0086050.t1-p1
OG0016506: TcSYL_0086060.t1-p1
OG0016507: TcSYL_0086090.t1-p1
OG0016508: TcSYL_0086100.t1-p1
OG0016509: TcSYL_0086150.t1-p1
OG0016510: TcSYL_0086160.t1-p1
OG0016511: TcSYL_0086170.t1-p1
OG0016512: TcSYL_0086180.t1-p1
OG0016513: TcSYL_0086200.t1-p1
OG0016514: TcSYL_0086210.t1-p1
OG0016515: TcSYL_0086220.t1-p1
OG0016516: TcSYL_0086230.t1-p1
OG0016517: TcSYL_0086240.t1-p1
OG0016518: TcSYL_0086250.t1-p1
OG0016519: TcSYL_0086260.t1-p1
OG0016520: TcSYL_0086310.t1-p1
OG0016521: TcSYL_0086320.t1-p1
OG0016522: TcSYL_0086330.t1-p1
OG0016523: TcSYL_0086340.t1-p1
OG0016524: TcSYL_0086350.t1-p1
OG0016525: TcSYL_0086370.t1-p1
OG0016526: TcSYL_0086390.t1-p1
OG0016527: TcSYL_0086410.t1-p1
OG0016528: TcSYL_0086500.t1-p1
OG0016529: TcSYL_0086520.t1-p1
OG0016530: TcSYL_0086540.t1-p1
OG0016531: TcSYL_0086560.t1-p1
OG0016532: TcSYL_0086610.t1-p1
OG0016533: TcSYL_0086620.t1-p1
OG0016534: TcSYL_0086640.t1-p1
OG0016535: TcSYL_0086690.t1-p1
OG0016536: TcSYL_0086750.t1-p1
OG0016537: TcSYL_0086760.t1-p1
OG0016538: TcSYL_0086770.t1-p1
OG0016539: TcSYL_0086780.t1-p1
OG0016540: TcSYL_0086790.t1-p1
OG0016541: TcSYL_0086810.t1-p1
OG0016542: TcSYL_0086820.t1-p1
OG0016543: TcSYL_0086830.t1-p1
OG0016544: TcSYL_0086870.t1-p1
OG0016545: TcSYL_0086890.t1-p1
OG0016546: TcSYL_0086940.t1-p1
OG0016547: TcSYL_0086960.t1-p1
OG0016548: TcSYL_0086970.t1-p1
OG0016549: TcSYL_0086980.t1-p1
OG0016550: TcSYL_0086990.t1-p1
OG0016551: TcSYL_0087010.t1-p1
OG0016552: TcSYL_0087020.t1-p1
OG0016553: TcSYL_0087030.t1-p1
OG0016554: TcSYL_0087040.t1-p1
OG0016555: TcSYL_0087060.t1-p1
OG0016556: TcSYL_0087070.t1-p1
OG0016557: TcSYL_0087080.t1-p1
OG0016558: TcSYL_0087090.t1-p1

OG0016559: TcSYL_0087100.t1-p1
OG0016560: TcSYL_0087110.t1-p1
OG0016561: TcSYL_0087120.t1-p1
OG0016562: TcSYL_0087130.t1-p1
OG0016563: TcSYL_0087160.t1-p1
OG0016564: TcSYL_0087180.t1-p1
OG0016565: TcSYL_0087190.t1-p1
OG0016566: TcSYL_0087210.t1-p1
OG0016567: TcSYL_0087220.t1-p1
OG0016568: TcSYL_0087230.t1-p1
OG0016569: TcSYL_0087260.t1-p1
OG0016570: TcSYL_0087330.t1-p1
OG0016571: TcSYL_0087340.t1-p1
OG0016572: TcSYL_0087380.t1-p1
OG0016573: TcSYL_0087390.t1-p1
OG0016574: TcSYL_0087400.t1-p1
OG0016575: TcSYL_0087420.t1-p1
OG0016576: TcSYL_0087430.t1-p1
OG0016577: TcSYL_0087470.t1-p1
OG0016578: TcSYL_0087480.t1-p1
OG0016579: TcSYL_0087490.t1-p1
OG0016580: TcSYL_0087500.t1-p1
OG0016581: TcSYL_0087520.t1-p1
OG0016582: TcSYL_0087540.t1-p1
OG0016583: TcSYL_0087550.t1-p1
OG0016584: TcSYL_0087570.t1-p1
OG0016585: TcSYL_0087580.t1-p1
OG0016586: TcSYL_0087590.t1-p1
OG0016587: TcSYL_0087610.t1-p1
OG0016588: TcSYL_0087620.t1-p1
OG0016589: TcSYL_0087630.t1-p1
OG0016590: TcSYL_0087640.t1-p1
OG0016591: TcSYL_0087650.t1-p1
OG0016592: TcSYL_0087660.t1-p1
OG0016593: TcSYL_0087670.t1-p1
OG0016594: TcSYL_0087680.t1-p1
OG0016595: TcSYL_0087690.t1-p1
OG0016596: TcSYL_0087700.t1-p1
OG0016597: TcSYL_0087710.t1-p1
OG0016598: TcSYL_0087720.t1-p1
OG0016599: TcSYL_0087730.t1-p1
OG0016600: TcSYL_0087740.t1-p1
OG0016601: TcSYL_0087750.t1-p1
OG0016602: TcSYL_0087760.t1-p1
OG0016603: TcSYL_0087780.t1-p1
OG0016604: TcSYL_0087790.t1-p1
OG0016605: TcSYL_0087840.t1-p1
OG0016606: TcSYL_0087850.t1-p1
OG0016607: TcSYL_0087860.t1-p1
OG0016608: TcSYL_0087870.t1-p1
OG0016609: TcSYL_0087890.t1-p1
OG0016610: TcSYL_0087900.t1-p1
OG0016611: TcSYL_0087950.t1-p1
OG0016612: TcSYL_0087960.t1-p1

OG0016613: TcSYL_0087970.t1-p1
OG0016614: TcSYL_0088070.t1-p1
OG0016615: TcSYL_0088080.t1-p1
OG0016616: TcSYL_0088090.t1-p1
OG0016617: TcSYL_0088110.t1-p1
OG0016618: TcSYL_0088120.t1-p1
OG0016619: TcSYL_0088180.t1-p1
OG0016620: TcSYL_0088190.t1-p1
OG0016621: TcSYL_0088210.t1-p1
OG0016622: TcSYL_0088250.t1-p1
OG0016623: TcSYL_0088260.t1-p1
OG0016624: TcSYL_0088280.t1-p1
OG0016625: TcSYL_0088290.t1-p1
OG0016626: TcSYL_0088310.t1-p1
OG0016627: TcSYL_0088320.t1-p1
OG0016628: TcSYL_0088340.t1-p1
OG0016629: TcSYL_0088360.t1-p1
OG0016630: TcSYL_0088370.t1-p1
OG0016631: TcSYL_0088390.t1-p1
OG0016632: TcSYL_0088400.t1-p1
OG0016633: TcSYL_0088410.t1-p1
OG0016634: TcSYL_0088420.t1-p1
OG0016635: TcSYL_0088440.t1-p1
OG0016636: TcSYL_0088460.t1-p1
OG0016637: TcSYL_0088470.t1-p1
OG0016638: TcSYL_0088500.t1-p1
OG0016639: TcSYL_0088510.t1-p1
OG0016640: TcSYL_0088660.t1-p1
OG0016641: TcSYL_0088690.t1-p1
OG0016642: TcSYL_0088710.t1-p1
OG0016643: TcSYL_0088730.t1-p1
OG0016644: TcSYL_0088740.t1-p1
OG0016645: TcSYL_0088750.t1-p1
OG0016646: TcSYL_0088760.t1-p1
OG0016647: TcSYL_0088770.t1-p1
OG0016648: TcSYL_0088790.t1-p1
OG0016649: TcSYL_0088810.t1-p1
OG0016650: TcSYL_0088830.t1-p1
OG0016651: TcSYL_0088850.t1-p1
OG0016652: TcSYL_0088870.t1-p1
OG0016653: TcSYL_0088890.t1-p1
OG0016654: TcSYL_0088900.t1-p1
OG0016655: TcSYL_0088920.t1-p1
OG0016656: TcSYL_0088940.t1-p1
OG0016657: TcSYL_0088950.t1-p1
OG0016658: TcSYL_0088970.t1-p1
OG0016659: TcSYL_0088980.t1-p1
OG0016660: TcSYL_0088990.t1-p1
OG0016661: TcSYL_0089010.t1-p1
OG0016662: TcSYL_0089020.t1-p1
OG0016663: TcSYL_0089030.t1-p1
OG0016664: TcSYL_0089040.t1-p1
OG0016665: TcSYL_0089050.t1-p1
OG0016666: TcSYL_0089060.t1-p1

OG0016667: TcSYL_0089070.t1-p1
OG0016668: TcSYL_0089100.t1-p1
OG0016669: TcSYL_0089140.t1-p1
OG0016670: TcSYL_0089200.t1-p1
OG0016671: TcSYL_0089210.t1-p1
OG0016672: TcSYL_0089230.t1-p1
OG0016673: TcSYL_0089270.t1-p1
OG0016674: TcSYL_0089340.t1-p1
OG0016675: TcSYL_0089350.t1-p1
OG0016676: TcSYL_0089360.t1-p1
OG0016677: TcSYL_0089380.t1-p1
OG0016678: TcSYL_0089420.t1-p1
OG0016679: TcSYL_0089430.t1-p1
OG0016680: TcSYL_0089440.t1-p1
OG0016681: TcSYL_0089450.t1-p1
OG0016682: TcSYL_0089470.t1-p1
OG0016683: TcSYL_0089490.t1-p1
OG0016684: TcSYL_0089500.t1-p1
OG0016685: TcSYL_0089510.t1-p1
OG0016686: TcSYL_0089520.t1-p1
OG0016687: TcSYL_0089530.t1-p1
OG0016688: TcSYL_0089550.t1-p1
OG0016689: TcSYL_0089570.t1-p1
OG0016690: TcSYL_0089580.t1-p1
OG0016691: TcSYL_0089590.t1-p1
OG0016692: TcSYL_0089600.t1-p1
OG0016693: TcSYL_0089610.t1-p1
OG0016694: TcSYL_0089630.t1-p1
OG0016695: TcSYL_0089640.t1-p1
OG0016696: TcSYL_0089660.t1-p1
OG0016697: TcSYL_0089670.t1-p1
OG0016698: TcSYL_0089680.t1-p1
OG0016699: TcSYL_0089700.t1-p1
OG0016700: TcSYL_0089720.t1-p1
OG0016701: TcSYL_0089730.t1-p1
OG0016702: TcSYL_0089740.t1-p1
OG0016703: TcSYL_0089760.t1-p1
OG0016704: TcSYL_0089770.t1-p1
OG0016705: TcSYL_0089780.t1-p1
OG0016706: TcSYL_0089790.t1-p1
OG0016707: TcSYL_0089800.t1-p1
OG0016708: TcSYL_0089820.t1-p1
OG0016709: TcSYL_0089830.t1-p1
OG0016710: TcSYL_0089850.t1-p1
OG0016711: TcSYL_0089860.t1-p1
OG0016712: TcSYL_0089880.t1-p1
OG0016713: TcSYL_0089900.t1-p1
OG0016714: TcSYL_0089910.t1-p1
OG0016715: TcSYL_0089930.t1-p1
OG0016716: TcSYL_0089940.t1-p1
OG0016717: TcSYL_0089970.t1-p1
OG0016718: TcSYL_0089980.t1-p1
OG0016719: TcSYL_0089990.t1-p1
OG0016720: TcSYL_0090000.t1-p1

OG0016721: TcSYL_0090010.t1-p1
OG0016722: TcSYL_0090020.t1-p1
OG0016723: TcSYL_0090030.t1-p1
OG0016724: TcSYL_0090050.t1-p1
OG0016725: TcSYL_0090060.t1-p1
OG0016726: TcSYL_0090070.t1-p1
OG0016727: TcSYL_0090080.t1-p1
OG0016728: TcSYL_0090090.t1-p1
OG0016729: TcSYL_0090100.t1-p1
OG0016730: TcSYL_0090110.t1-p1
OG0016731: TcSYL_0090120.t1-p1
OG0016732: TcSYL_0090130.t1-p1
OG0016733: TcSYL_0090150.t1-p1
OG0016734: TcSYL_0090160.t1-p1
OG0016735: TcSYL_0090170.t1-p1
OG0016736: TcSYL_0090180.t1-p1
OG0016737: TcSYL_0090190.t1-p1
OG0016738: TcSYL_0090200.t1-p1
OG0016739: TcSYL_0090220.t1-p1
OG0016740: TcSYL_0090230.t1-p1
OG0016741: TcSYL_0090240.t1-p1
OG0016742: TcSYL_0090260.t1-p1
OG0016743: TcSYL_0090290.t1-p1
OG0016744: TcSYL_0090300.t1-p1
OG0016745: TcSYL_0090320.t1-p1
OG0016746: TcSYL_0090330.t1-p1
OG0016747: TcSYL_0090340.t1-p1
OG0016748: TcSYL_0090350.t1-p1
OG0016749: TcSYL_0090360.t1-p1
OG0016750: TcSYL_0090370.t1-p1
OG0016751: TcSYL_0090380.t1-p1
OG0016752: TcSYL_0090390.t1-p1
OG0016753: TcSYL_0090410.t1-p1
OG0016754: TcSYL_0090420.t1-p1
OG0016755: TcSYL_0090430.t1-p1
OG0016756: TcSYL_0090440.t1-p1
OG0016757: TcSYL_0090450.t1-p1
OG0016758: TcSYL_0090460.t1-p1
OG0016759: TcSYL_0090470.t1-p1
OG0016760: TcSYL_0090490.t1-p1
OG0016761: TcSYL_0090500.t1-p1
OG0016762: TcSYL_0090510.t1-p1
OG0016763: TcSYL_0090520.t1-p1
OG0016764: TcSYL_0090530.t1-p1
OG0016765: TcSYL_0090540.t1-p1
OG0016766: TcSYL_0090550.t1-p1
OG0016767: TcSYL_0090560.t1-p1
OG0016768: TcSYL_0090570.t1-p1
OG0016769: TcSYL_0090580.t1-p1
OG0016770: TcSYL_0090590.t1-p1
OG0016771: TcSYL_0090600.t1-p1
OG0016772: TcSYL_0090610.t1-p1
OG0016773: TcSYL_0090620.t1-p1
OG0016774: TcSYL_0090630.t1-p1

OG0016775: TcSYL_0090640.t1-p1
OG0016776: TcSYL_0090660.t1-p1
OG0016777: TcSYL_0090670.t1-p1
OG0016778: TcSYL_0090680.t1-p1
OG0016779: TcSYL_0090690.t1-p1
OG0016780: TcSYL_0090700.t1-p1
OG0016781: TcSYL_0090730.t1-p1
OG0016782: TcSYL_0090740.t1-p1
OG0016783: TcSYL_0090750.t1-p1
OG0016784: TcSYL_0090760.t1-p1
OG0016785: TcSYL_0090770.t1-p1
OG0016786: TcSYL_0090790.t1-p1
OG0016787: TcSYL_0090800.t1-p1
OG0016788: TcSYL_0090810.t1-p1
OG0016789: TcSYL_0090820.t1-p1
OG0016790: TcSYL_0090830.t1-p1
OG0016791: TcSYL_0090840.t1-p1
OG0016792: TcSYL_0090850.t1-p1
OG0016793: TcSYL_0090860.t1-p1
OG0016794: TcSYL_0090870.t1-p1
OG0016795: TcSYL_0090880.t1-p1
OG0016796: TcSYL_0090890.t1-p1
OG0016797: TcSYL_0090900.t1-p1
OG0016798: TcSYL_0090910.t1-p1
OG0016799: TcSYL_0090930.t1-p1
OG0016800: TcSYL_0090940.t1-p1
OG0016801: TcSYL_0090960.t1-p1
OG0016802: TcSYL_0090970.t1-p1
OG0016803: TcSYL_0090980.t1-p1
OG0016804: TcSYL_0091000.t1-p1
OG0016805: TcSYL_0091010.t1-p1
OG0016806: TcSYL_0091020.t1-p1
OG0016807: TcSYL_0091030.t1-p1
OG0016808: TcSYL_0091040.t1-p1
OG0016809: TcSYL_0091050.t1-p1
OG0016810: TcSYL_0091060.t1-p1
OG0016811: TcSYL_0091080.t1-p1
OG0016812: TcSYL_0091090.t1-p1
OG0016813: TcSYL_0091110.t1-p1
OG0016814: TcSYL_0091120.t1-p1
OG0016815: TcSYL_0091130.t1-p1
OG0016816: TcSYL_0091140.t1-p1
OG0016817: TcSYL_0091160.t1-p1
OG0016818: TcSYL_0091170.t1-p1
OG0016819: TcSYL_0091180.t1-p1
OG0016820: TcSYL_0091190.t1-p1
OG0016821: TcSYL_0091200.t1-p1
OG0016822: TcSYL_0091250.t1-p1
OG0016823: TcSYL_0091260.t1-p1
OG0016824: TcSYL_0091280.t1-p1
OG0016825: TcSYL_0091290.t1-p1
OG0016826: TcSYL_0091300.t1-p1
OG0016827: TcSYL_0091310.t1-p1
OG0016828: TcSYL_0091330.t1-p1

OG0016829: TcSYL_0091340.t1-p1
OG0016830: TcSYL_0091360.t1-p1
OG0016831: TcSYL_0091370.t1-p1
OG0016832: TcSYL_0091380.t1-p1
OG0016833: TcSYL_0091400.t1-p1
OG0016834: TcSYL_0091430.t1-p1
OG0016835: TcSYL_0091440.t1-p1
OG0016836: TcSYL_0091450.t1-p1
OG0016837: TcSYL_0091470.t1-p1
OG0016838: TcSYL_0091480.t1-p1
OG0016839: TcSYL_0091490.t1-p1
OG0016840: TcSYL_0091500.t1-p1
OG0016841: TcSYL_0091530.t1-p1
OG0016842: TcSYL_0091580.t1-p1
OG0016843: TcSYL_0091590.t1-p1
OG0016844: TcSYL_0091600.t1-p1
OG0016845: TcSYL_0091640.t1-p1
OG0016846: TcSYL_0091650.t1-p1
OG0016847: TcSYL_0091670.t1-p1
OG0016848: TcSYL_0091810.t1-p1
OG0016849: TcSYL_0091860.t1-p1
OG0016850: TcSYL_0091870.t1-p1
OG0016851: TcSYL_0091910.t1-p1
OG0016852: TcSYL_0091920.t1-p1
OG0016853: TcSYL_0091930.t1-p1
OG0016854: TcSYL_0091950.t1-p1
OG0016855: TcSYL_0091970.t1-p1
OG0016856: TcSYL_0092000.t1-p1
OG0016857: TcSYL_0092070.t1-p1
OG0016858: TcSYL_0092110.t1-p1
OG0016859: TcSYL_0092120.t1-p1
OG0016860: TcSYL_0092130.t1-p1
OG0016861: TcSYL_0092140.t1-p1
OG0016862: TcSYL_0092170.t1-p1
OG0016863: TcSYL_0092180.t1-p1
OG0016864: TcSYL_0092190.t1-p1
OG0016865: TcSYL_0092200.t1-p1
OG0016866: TcSYL_0092210.t1-p1
OG0016867: TcSYL_0092220.t1-p1
OG0016868: TcSYL_0092270.t1-p1
OG0016869: TcSYL_0092340.t1-p1
OG0016870: TcSYL_0092480.t1-p1
OG0016871: TcSYL_0092490.t1-p1
OG0016872: TcSYL_0092500.t1-p1
OG0016873: TcSYL_0092510.t1-p1
OG0016874: TcSYL_0092520.t1-p1
OG0016875: TcSYL_0092550.t1-p1
OG0016876: TcSYL_0092590.t1-p1
OG0016877: TcSYL_0092600.t1-p1
OG0016878: TcSYL_0092610.t1-p1
OG0016879: TcSYL_0092620.t1-p1
OG0016880: TcSYL_0092630.t1-p1
OG0016881: TcSYL_0092640.t1-p1
OG0016882: TcSYL_0092650.t1-p1

OG0016883: TcSYL_0092660.t1-p1
OG0016884: TcSYL_0092670.t1-p1
OG0016885: TcSYL_0092680.t1-p1
OG0016886: TcSYL_0092690.t1-p1
OG0016887: TcSYL_0092700.t1-p1
OG0016888: TcSYL_0092710.t1-p1
OG0016889: TcSYL_0092720.t1-p1
OG0016890: TcSYL_0092730.t1-p1
OG0016891: TcSYL_0092740.t1-p1
OG0016892: TcSYL_0092750.t1-p1
OG0016893: TcSYL_0092770.t1-p1
OG0016894: TcSYL_0092780.t1-p1
OG0016895: TcSYL_0092790.t1-p1
OG0016896: TcSYL_0092800.t1-p1
OG0016897: TcSYL_0092830.t1-p1
OG0016898: TcSYL_0092840.t1-p1
OG0016899: TcSYL_0092860.t1-p1
OG0016900: TcSYL_0092870.t1-p1
OG0016901: TcSYL_0092890.t1-p1
OG0016902: TcSYL_0092900.t1-p1
OG0016903: TcSYL_0092910.t1-p1
OG0016904: TcSYL_0092930.t1-p1
OG0016905: TcSYL_0092950.t1-p1
OG0016906: TcSYL_0092960.t1-p1
OG0016907: TcSYL_0092970.t1-p1
OG0016908: TcSYL_0092990.t1-p1
OG0016909: TcSYL_0093010.t1-p1
OG0016910: TcSYL_0093020.t1-p1
OG0016911: TcSYL_0093030.t1-p1
OG0016912: TcSYL_0093040.t1-p1
OG0016913: TcSYL_0093050.t1-p1
OG0016914: TcSYL_0093060.t1-p1
OG0016915: TcSYL_0093070.t1-p1
OG0016916: TcSYL_0093100.t1-p1
OG0016917: TcSYL_0093110.t1-p1
OG0016918: TcSYL_0093120.t1-p1
OG0016919: TcSYL_0093130.t1-p1
OG0016920: TcSYL_0093160.t1-p1
OG0016921: TcSYL_0093170.t1-p1
OG0016922: TcSYL_0093180.t1-p1
OG0016923: TcSYL_0093200.t1-p1
OG0016924: TcSYL_0093220.t1-p1
OG0016925: TcSYL_0093230.t1-p1
OG0016926: TcSYL_0093270.t1-p1
OG0016927: TcSYL_0093280.t1-p1
OG0016928: TcSYL_0093290.t1-p1
OG0016929: TcSYL_0093300.t1-p1
OG0016930: TcSYL_0093320.t1-p1
OG0016931: TcSYL_0093330.t1-p1
OG0016932: TcSYL_0093340.t1-p1
OG0016933: TcSYL_0093350.t1-p1
OG0016934: TcSYL_0093360.t1-p1
OG0016935: TcSYL_0093370.t1-p1
OG0016936: TcSYL_0093380.t1-p1

OG0016937: TcSYL_0093390.t1-p1
OG0016938: TcSYL_0093400.t1-p1
OG0016939: TcSYL_0093410.t1-p1
OG0016940: TcSYL_0093430.t1-p1
OG0016941: TcSYL_0093440.t1-p1
OG0016942: TcSYL_0093450.t1-p1
OG0016943: TcSYL_0093460.t1-p1
OG0016944: TcSYL_0093470.t1-p1
OG0016945: TcSYL_0093480.t1-p1
OG0016946: TcSYL_0093530.t1-p1
OG0016947: TcSYL_0093540.t1-p1
OG0016948: TcSYL_0093550.t1-p1
OG0016949: TcSYL_0093560.t1-p1
OG0016950: TcSYL_0093570.t1-p1
OG0016951: TcSYL_0093590.t1-p1
OG0016952: TcSYL_0093600.t1-p1
OG0016953: TcSYL_0093610.t1-p1
OG0016954: TcSYL_0093620.t1-p1
OG0016955: TcSYL_0093630.t1-p1
OG0016956: TcSYL_0093680.t1-p1
OG0016957: TcSYL_0093690.t1-p1
OG0016958: TcSYL_0093700.t1-p1
OG0016959: TcSYL_0093730.t1-p1
OG0016960: TcSYL_0093760.t1-p1
OG0016961: TcSYL_0093770.t1-p1
OG0016962: TcSYL_0093780.t1-p1
OG0016963: TcSYL_0093800.t1-p1
OG0016964: TcSYL_0093830.t1-p1
OG0016965: TcSYL_0093840.t1-p1
OG0016966: TcSYL_0093850.t1-p1
OG0016967: TcSYL_0093860.t1-p1
OG0016968: TcSYL_0093880.t1-p1
OG0016969: TcSYL_0093890.t1-p1
OG0016970: TcSYL_0093900.t1-p1
OG0016971: TcSYL_0093930.t1-p1
OG0016972: TcSYL_0093940.t1-p1
OG0016973: TcSYL_0093950.t1-p1
OG0016974: TcSYL_0093960.t1-p1
OG0016975: TcSYL_0093970.t1-p1
OG0016976: TcSYL_0093980.t1-p1
OG0016977: TcSYL_0093990.t1-p1
OG0016978: TcSYL_0094000.t1-p1
OG0016979: TcSYL_0094010.t1-p1
OG0016980: TcSYL_0094020.t1-p1
OG0016981: TcSYL_0094040.t1-p1
OG0016982: TcSYL_0094050.t1-p1
OG0016983: TcSYL_0094060.t1-p1
OG0016984: TcSYL_0094080.t1-p1
OG0016985: TcSYL_0094100.t1-p1
OG0016986: TcSYL_0094110.t1-p1
OG0016987: TcSYL_0094120.t1-p1
OG0016988: TcSYL_0094130.t1-p1
OG0016989: TcSYL_0094140.t1-p1
OG0016990: TcSYL_0094150.t1-p1

OG0016991: TcSYL_0094160.t1-p1
OG0016992: TcSYL_0094170.t1-p1
OG0016993: TcSYL_0094180.t1-p1
OG0016994: TcSYL_0094190.t1-p1
OG0016995: TcSYL_0094200.t1-p1
OG0016996: TcSYL_0094210.t1-p1
OG0016997: TcSYL_0094220.t1-p1
OG0016998: TcSYL_0094230.t1-p1
OG0016999: TcSYL_0094250.t1-p1
OG0017000: TcSYL_0094260.t1-p1
OG0017001: TcSYL_0094270.t1-p1
OG0017002: TcSYL_0094280.t1-p1
OG0017003: TcSYL_0094290.t1-p1
OG0017004: TcSYL_0094300.t1-p1
OG0017005: TcSYL_0094310.t1-p1
OG0017006: TcSYL_0094320.t1-p1
OG0017007: TcSYL_0094330.t1-p1
OG0017008: TcSYL_0094340.t1-p1
OG0017009: TcSYL_0094350.t1-p1
OG0017010: TcSYL_0094360.t1-p1
OG0017011: TcSYL_0094370.t1-p1
OG0017012: TcSYL_0094390.t1-p1
OG0017013: TcSYL_0094400.t1-p1
OG0017014: TcSYL_0094410.t1-p1
OG0017015: TcSYL_0094420.t1-p1
OG0017016: TcSYL_0094430.t1-p1
OG0017017: TcSYL_0094440.t1-p1
OG0017018: TcSYL_0094450.t1-p1
OG0017019: TcSYL_0094460.t1-p1
OG0017020: TcSYL_0094470.t1-p1
OG0017021: TcSYL_0094480.t1-p1
OG0017022: TcSYL_0094490.t1-p1
OG0017023: TcSYL_0094500.t1-p1
OG0017024: TcSYL_0094510.t1-p1
OG0017025: TcSYL_0094520.t1-p1
OG0017026: TcSYL_0094630.t1-p1
OG0017027: TcSYL_0094730.t1-p1
OG0017028: TcSYL_0094780.t1-p1
OG0017029: TcSYL_0094800.t1-p1
OG0017030: TcSYL_0094820.t1-p1
OG0017031: TcSYL_0094830.t1-p1
OG0017032: TcSYL_0094840.t1-p1
OG0017033: TcSYL_0094850.t1-p1
OG0017034: TcSYL_0094860.t1-p1
OG0017035: TcSYL_0094870.t1-p1
OG0017036: TcSYL_0094880.t1-p1
OG0017037: TcSYL_0094890.t1-p1
OG0017038: TcSYL_0094900.t1-p1
OG0017039: TcSYL_0094910.t1-p1
OG0017040: TcSYL_0094920.t1-p1
OG0017041: TcSYL_0094940.t1-p1
OG0017042: TcSYL_0094950.t1-p1
OG0017043: TcSYL_0094960.t1-p1
OG0017044: TcSYL_0094970.t1-p1

OG0017045: TcSYL_0094980.t1-p1
OG0017046: TcSYL_0094990.t1-p1
OG0017047: TcSYL_0095000.t1-p1
OG0017048: TcSYL_0095010.t1-p1
OG0017049: TcSYL_0095020.t1-p1
OG0017050: TcSYL_0095030.t1-p1
OG0017051: TcSYL_0095040.t1-p1
OG0017052: TcSYL_0095050.t1-p1
OG0017053: TcSYL_0095060.t1-p1
OG0017054: TcSYL_0095070.t1-p1
OG0017055: TcSYL_0095080.t1-p1
OG0017056: TcSYL_0095090.t1-p1
OG0017057: TcSYL_0095110.t1-p1
OG0017058: TcSYL_0095120.t1-p1
OG0017059: TcSYL_0095130.t1-p1
OG0017060: TcSYL_0095140.t1-p1
OG0017061: TcSYL_0095150.t1-p1
OG0017062: TcSYL_0095160.t1-p1
OG0017063: TcSYL_0095170.t1-p1
OG0017064: TcSYL_0095180.t1-p1
OG0017065: TcSYL_0095190.t1-p1
OG0017066: TcSYL_0095200.t1-p1
OG0017067: TcSYL_0095210.t1-p1
OG0017068: TcSYL_0095220.t1-p1
OG0017069: TcSYL_0095240.t1-p1
OG0017070: TcSYL_0095250.t1-p1
OG0017071: TcSYL_0095260.t1-p1
OG0017072: TcSYL_0095270.t1-p1
OG0017073: TcSYL_0095280.t1-p1
OG0017074: TcSYL_0095290.t1-p1
OG0017075: TcSYL_0095300.t1-p1
OG0017076: TcSYL_0095310.t1-p1
OG0017077: TcSYL_0095320.t1-p1
OG0017078: TcSYL_0095330.t1-p1
OG0017079: TcSYL_0095340.t1-p1
OG0017080: TcSYL_0095350.t1-p1
OG0017081: TcSYL_0095360.t1-p1
OG0017082: TcSYL_0095370.t1-p1
OG0017083: TcSYL_0095380.t1-p1
OG0017084: TcSYL_0095390.t1-p1
OG0017085: TcSYL_0095400.t1-p1
OG0017086: TcSYL_0095410.t1-p1
OG0017087: TcSYL_0095420.t1-p1
OG0017088: TcSYL_0095430.t1-p1
OG0017089: TcSYL_0095440.t1-p1
OG0017090: TcSYL_0095450.t1-p1
OG0017091: TcSYL_0095460.t1-p1
OG0017092: TcSYL_0095470.t1-p1
OG0017093: TcSYL_0095480.t1-p1
OG0017094: TcSYL_0095490.t1-p1
OG0017095: TcSYL_0095500.t1-p1
OG0017096: TcSYL_0095510.t1-p1
OG0017097: TcSYL_0095530.t1-p1
OG0017098: TcSYL_0095540.t1-p1

OG0017099: TcSYL_0095550.t1-p1
OG0017100: TcSYL_0095560.t1-p1
OG0017101: TcSYL_0095570.t1-p1
OG0017102: TcSYL_0095580.t1-p1
OG0017103: TcSYL_0095590.t1-p1
OG0017104: TcSYL_0095600.t1-p1
OG0017105: TcSYL_0095610.t1-p1
OG0017106: TcSYL_0095620.t1-p1
OG0017107: TcSYL_0095630.t1-p1
OG0017108: TcSYL_0095640.t1-p1
OG0017109: TcSYL_0095650.t1-p1
OG0017110: TcSYL_0095660.t1-p1
OG0017111: TcSYL_0095680.t1-p1
OG0017112: TcSYL_0095690.t1-p1
OG0017113: TcSYL_0095700.t1-p1
OG0017114: TcSYL_0095710.t1-p1
OG0017115: TcSYL_0095720.t1-p1
OG0017116: TcSYL_0095740.t1-p1
OG0017117: TcSYL_0095770.t1-p1
OG0017118: TcSYL_0095830.t1-p1
OG0017119: TcSYL_0095980.t1-p1
OG0017120: TcSYL_0095990.t1-p1
OG0017121: TcSYL_0096010.t1-p1
OG0017122: TcSYL_0096020.t1-p1
OG0017123: TcSYL_0096040.t1-p1
OG0017124: TcSYL_0096050.t1-p1
OG0017125: TcSYL_0096070.t1-p1
OG0017126: TcSYL_0096080.t1-p1
OG0017127: TcSYL_0096090.t1-p1
OG0017128: TcSYL_0096100.t1-p1
OG0017129: TcSYL_0096110.t1-p1
OG0017130: TcSYL_0096120.t1-p1
OG0017131: TcSYL_0096140.t1-p1
OG0017132: TcSYL_0096150.t1-p1
OG0017133: TcSYL_0096160.t1-p1
OG0017134: TcSYL_0096180.t1-p1
OG0017135: TcSYL_0096190.t1-p1
OG0017136: TcSYL_0096200.t1-p1
OG0017137: TcSYL_0096220.t1-p1
OG0017138: TcSYL_0096230.t1-p1
OG0017139: TcSYL_0096240.t1-p1
OG0017140: TcSYL_0096250.t1-p1
OG0017141: TcSYL_0096280.t1-p1
OG0017142: TcSYL_0096290.t1-p1
OG0017143: TcSYL_0096310.t1-p1
OG0017144: TcSYL_0096320.t1-p1
OG0017145: TcSYL_0096330.t1-p1
OG0017146: TcSYL_0096340.t1-p1
OG0017147: TcSYL_0096350.t1-p1
OG0017148: TcSYL_0096360.t1-p1
OG0017149: TcSYL_0096380.t1-p1
OG0017150: TcSYL_0096390.t1-p1
OG0017151: TcSYL_0096400.t1-p1
OG0017152: TcSYL_0096410.t1-p1

OG0017153: TcSYL_0096420.t1-p1
OG0017154: TcSYL_0096430.t1-p1
OG0017155: TcSYL_0096440.t1-p1
OG0017156: TcSYL_0096450.t1-p1
OG0017157: TcSYL_0096460.t1-p1
OG0017158: TcSYL_0096470.t1-p1
OG0017159: TcSYL_0096480.t1-p1
OG0017160: TcSYL_0096490.t1-p1
OG0017161: TcSYL_0096500.t1-p1
OG0017162: TcSYL_0096510.t1-p1
OG0017163: TcSYL_0096530.t1-p1
OG0017164: TcSYL_0096540.t1-p1
OG0017165: TcSYL_0096550.t1-p1
OG0017166: TcSYL_0096560.t1-p1
OG0017167: TcSYL_0096570.t1-p1
OG0017168: TcSYL_0096580.t1-p1
OG0017169: TcSYL_0096600.t1-p1
OG0017170: TcSYL_0096620.t1-p1
OG0017171: TcSYL_0096630.t1-p1
OG0017172: TcSYL_0096670.t1-p1
OG0017173: TcSYL_0096680.t1-p1
OG0017174: TcSYL_0096690.t1-p1
OG0017175: TcSYL_0096700.t1-p1
OG0017176: TcSYL_0096710.t1-p1
OG0017177: TcSYL_0096730.t1-p1
OG0017178: TcSYL_0096740.t1-p1
OG0017179: TcSYL_0096750.t1-p1
OG0017180: TcSYL_0096760.t1-p1
OG0017181: TcSYL_0096770.t1-p1
OG0017182: TcSYL_0096780.t1-p1
OG0017183: TcSYL_0096800.t1-p1
OG0017184: TcSYL_0096810.t1-p1
OG0017185: TcSYL_0096830.t1-p1
OG0017186: TcSYL_0096840.t1-p1
OG0017187: TcSYL_0096850.t1-p1
OG0017188: TcSYL_0096860.t1-p1
OG0017189: TcSYL_0096880.t1-p1
OG0017190: TcSYL_0096900.t1-p1
OG0017191: TcSYL_0096910.t1-p1
OG0017192: TcSYL_0096920.t1-p1
OG0017193: TcSYL_0096930.t1-p1
OG0017194: TcSYL_0096950.t1-p1
OG0017195: TcSYL_0096960.t1-p1
OG0017196: TcSYL_0096980.t1-p1
OG0017197: TcSYL_0096990.t1-p1
OG0017198: TcSYL_0097000.t1-p1
OG0017199: TcSYL_0097010.t1-p1
OG0017200: TcSYL_0097020.t1-p1
OG0017201: TcSYL_0097030.t1-p1
OG0017202: TcSYL_0097040.t1-p1
OG0017203: TcSYL_0097070.t1-p1
OG0017204: TcSYL_0097080.t1-p1
OG0017205: TcSYL_0097090.t1-p1
OG0017206: TcSYL_0097110.t1-p1

OG0017207: TcSYL_0097120.t1-p1
OG0017208: TcSYL_0097130.t1-p1
OG0017209: TcSYL_0097140.t1-p1
OG0017210: TcSYL_0097160.t1-p1
OG0017211: TcSYL_0097170.t1-p1
OG0017212: TcSYL_0097180.t1-p1
OG0017213: TcSYL_0097210.t1-p1
OG0017214: TcSYL_0097230.t1-p1
OG0017215: TcSYL_0097240.t1-p1
OG0017216: TcSYL_0097260.t1-p1
OG0017217: TcSYL_0097290.t1-p1
OG0017218: TcSYL_0097310.t1-p1
OG0017219: TcSYL_0097320.t1-p1
OG0017220: TcSYL_0097350.t1-p1
OG0017221: TcSYL_0097360.t1-p1
OG0017222: TcSYL_0097370.t1-p1
OG0017223: TcSYL_0097390.t1-p1
OG0017224: TcSYL_0097400.t1-p1
OG0017225: TcSYL_0097410.t1-p1
OG0017226: TcSYL_0097420.t1-p1
OG0017227: TcSYL_0097440.t1-p1
OG0017228: TcSYL_0097450.t1-p1
OG0017229: TcSYL_0097460.t1-p1
OG0017230: TcSYL_0097470.t1-p1
OG0017231: TcSYL_0097490.t1-p1
OG0017232: TcSYL_0097500.t1-p1
OG0017233: TcSYL_0097510.t1-p1
OG0017234: TcSYL_0097520.t1-p1
OG0017235: TcSYL_0097530.t1-p1
OG0017236: TcSYL_0097540.t1-p1
OG0017237: TcSYL_0097550.t1-p1
OG0017238: TcSYL_0097560.t1-p1
OG0017239: TcSYL_0097570.t1-p1
OG0017240: TcSYL_0097600.t1-p1
OG0017241: TcSYL_0097610.t1-p1
OG0017242: TcSYL_0097620.t1-p1
OG0017243: TcSYL_0097630.t1-p1
OG0017244: TcSYL_0097660.t1-p1
OG0017245: TcSYL_0097680.t1-p1
OG0017246: TcSYL_0097690.t1-p1
OG0017247: TcSYL_0097700.t1-p1
OG0017248: TcSYL_0097710.t1-p1
OG0017249: TcSYL_0097720.t1-p1
OG0017250: TcSYL_0097730.t1-p1
OG0017251: TcSYL_0097740.t1-p1
OG0017252: TcSYL_0097770.t1-p1
OG0017253: TcSYL_0097780.t1-p1
OG0017254: TcSYL_0097790.t1-p1
OG0017255: TcSYL_0097810.t1-p1
OG0017256: TcSYL_0097820.t1-p1
OG0017257: TcSYL_0097830.t1-p1
OG0017258: TcSYL_0097840.t1-p1
OG0017259: TcSYL_0097850.t1-p1
OG0017260: TcSYL_0097860.t1-p1

OG0017261: TcSYL_0097870.t1-p1
OG0017262: TcSYL_0097900.t1-p1
OG0017263: TcSYL_0097920.t1-p1
OG0017264: TcSYL_0097940.t1-p1
OG0017265: TcSYL_0097950.t1-p1
OG0017266: TcSYL_0097970.t1-p1
OG0017267: TcSYL_0097980.t1-p1
OG0017268: TcSYL_0097990.t1-p1
OG0017269: TcSYL_0098000.t1-p1
OG0017270: TcSYL_0098040.t1-p1
OG0017271: TcSYL_0098050.t1-p1
OG0017272: TcSYL_0098060.t1-p1
OG0017273: TcSYL_0098070.t1-p1
OG0017274: TcSYL_0098080.t1-p1
OG0017275: TcSYL_0098090.t1-p1
OG0017276: TcSYL_0098100.t1-p1
OG0017277: TcSYL_0098120.t1-p1
OG0017278: TcSYL_0098130.t1-p1
OG0017279: TcSYL_0098150.t1-p1
OG0017280: TcSYL_0098160.t1-p1
OG0017281: TcSYL_0098180.t1-p1
OG0017282: TcSYL_0098190.t1-p1
OG0017283: TcSYL_0098200.t1-p1
OG0017284: TcSYL_0098210.t1-p1
OG0017285: TcSYL_0098220.t1-p1
OG0017286: TcSYL_0098230.t1-p1
OG0017287: TcSYL_0098240.t1-p1
OG0017288: TcSYL_0098250.t1-p1
OG0017289: TcSYL_0098260.t1-p1
OG0017290: TcSYL_0098270.t1-p1
OG0017291: TcSYL_0098280.t1-p1
OG0017292: TcSYL_0098290.t1-p1
OG0017293: TcSYL_0098300.t1-p1
OG0017294: TcSYL_0098310.t1-p1
OG0017295: TcSYL_0098320.t1-p1
OG0017296: TcSYL_0098330.t1-p1
OG0017297: TcSYL_0098340.t1-p1
OG0017298: TcSYL_0098350.t1-p1
OG0017299: TcSYL_0098360.t1-p1
OG0017300: TcSYL_0098380.t1-p1
OG0017301: TcSYL_0098400.t1-p1
OG0017302: TcSYL_0098410.t1-p1
OG0017303: TcSYL_0098420.t1-p1
OG0017304: TcSYL_0098440.t1-p1
OG0017305: TcSYL_0098450.t1-p1
OG0017306: TcSYL_0098460.t1-p1
OG0017307: TcSYL_0098470.t1-p1
OG0017308: TcSYL_0098480.t1-p1
OG0017309: TcSYL_0098490.t1-p1
OG0017310: TcSYL_0098510.t1-p1
OG0017311: TcSYL_0098520.t1-p1
OG0017312: TcSYL_0098540.t1-p1
OG0017313: TcSYL_0098550.t1-p1
OG0017314: TcSYL_0098560.t1-p1

OG0017315: TcSYL_0098570.t1-p1
OG0017316: TcSYL_0098580.t1-p1
OG0017317: TcSYL_0098590.t1-p1
OG0017318: TcSYL_0098600.t1-p1
OG0017319: TcSYL_0098610.t1-p1
OG0017320: TcSYL_0098620.t1-p1
OG0017321: TcSYL_0098630.t1-p1
OG0017322: TcSYL_0098640.t1-p1
OG0017323: TcSYL_0098650.t1-p1
OG0017324: TcSYL_0098660.t1-p1
OG0017325: TcSYL_0098670.t1-p1
OG0017326: TcSYL_0098680.t1-p1
OG0017327: TcSYL_0098700.t1-p1
OG0017328: TcSYL_0098710.t1-p1
OG0017329: TcSYL_0098720.t1-p1
OG0017330: TcSYL_0098730.t1-p1
OG0017331: TcSYL_0098740.t1-p1
OG0017332: TcSYL_0098750.t1-p1
OG0017333: TcSYL_0098760.t1-p1
OG0017334: TcSYL_0098770.t1-p1
OG0017335: TcSYL_0098780.t1-p1
OG0017336: TcSYL_0098790.t1-p1
OG0017337: TcSYL_0098800.t1-p1
OG0017338: TcSYL_0098810.t1-p1
OG0017339: TcSYL_0098820.t1-p1
OG0017340: TcSYL_0098840.t1-p1
OG0017341: TcSYL_0098850.t1-p1
OG0017342: TcSYL_0098860.t1-p1
OG0017343: TcSYL_0098890.t1-p1
OG0017344: TcSYL_0098900.t1-p1
OG0017345: TcSYL_0098930.t1-p1
OG0017346: TcSYL_0098940.t1-p1
OG0017347: TcSYL_0098970.t1-p1
OG0017348: TcSYL_0098980.t1-p1
OG0017349: TcSYL_0099010.t1-p1
OG0017350: TcSYL_0099020.t1-p1
OG0017351: TcSYL_0099040.t1-p1
OG0017352: TcSYL_0099050.t1-p1
OG0017353: TcSYL_0099070.t1-p1
OG0017354: TcSYL_0099080.t1-p1
OG0017355: TcSYL_0099090.t1-p1
OG0017356: TcSYL_0099100.t1-p1
OG0017357: TcSYL_0099110.t1-p1
OG0017358: TcSYL_0099120.t1-p1
OG0017359: TcSYL_0099130.t1-p1
OG0017360: TcSYL_0099140.t1-p1
OG0017361: TcSYL_0099150.t1-p1
OG0017362: TcSYL_0099160.t1-p1
OG0017363: TcSYL_0099170.t1-p1
OG0017364: TcSYL_0099180.t1-p1
OG0017365: TcSYL_0099190.t1-p1
OG0017366: TcSYL_0099200.t1-p1
OG0017367: TcSYL_0099210.t1-p1
OG0017368: TcSYL_0099220.t1-p1

OG0017369: TcSYL_0099230.t1-p1
OG0017370: TcSYL_0099240.t1-p1
OG0017371: TcSYL_0099260.t1-p1
OG0017372: TcSYL_0099270.t1-p1
OG0017373: TcSYL_0099280.t1-p1
OG0017374: TcSYL_0099290.t1-p1
OG0017375: TcSYL_0099310.t1-p1
OG0017376: TcSYL_0099320.t1-p1
OG0017377: TcSYL_0099340.t1-p1
OG0017378: TcSYL_0099350.t1-p1
OG0017379: TcSYL_0099360.t1-p1
OG0017380: TcSYL_0099380.t1-p1
OG0017381: TcSYL_0099390.t1-p1
OG0017382: TcSYL_0099400.t1-p1
OG0017383: TcSYL_0099410.t1-p1
OG0017384: TcSYL_0099420.t1-p1
OG0017385: TcSYL_0099430.t1-p1
OG0017386: TcSYL_0099450.t1-p1
OG0017387: TcSYL_0099460.t1-p1
OG0017388: TcSYL_0099470.t1-p1
OG0017389: TcSYL_0099480.t1-p1
OG0017390: TcSYL_0099490.t1-p1
OG0017391: TcSYL_0099500.t1-p1
OG0017392: TcSYL_0099510.t1-p1
OG0017393: TcSYL_0099520.t1-p1
OG0017394: TcSYL_0099530.t1-p1
OG0017395: TcSYL_0099550.t1-p1
OG0017396: TcSYL_0099560.t1-p1
OG0017397: TcSYL_0099580.t1-p1
OG0017398: TcSYL_0099590.t1-p1
OG0017399: TcSYL_0099600.t1-p1
OG0017400: TcSYL_0099610.t1-p1
OG0017401: TcSYL_0099630.t1-p1
OG0017402: TcSYL_0099640.t1-p1
OG0017403: TcSYL_0099650.t1-p1
OG0017404: TcSYL_0099660.t1-p1
OG0017405: TcSYL_0099670.t1-p1
OG0017406: TcSYL_0099680.t1-p1
OG0017407: TcSYL_0099710.t1-p1
OG0017408: TcSYL_0099730.t1-p1
OG0017409: TcSYL_0099750.t1-p1
OG0017410: TcSYL_0099760.t1-p1
OG0017411: TcSYL_0099770.t1-p1
OG0017412: TcSYL_0099780.t1-p1
OG0017413: TcSYL_0099790.t1-p1
OG0017414: TcSYL_0099800.t1-p1
OG0017415: TcSYL_0099830.t1-p1
OG0017416: TcSYL_0099860.t1-p1
OG0017417: TcSYL_0099870.t1-p1
OG0017418: TcSYL_0099880.t1-p1
OG0017419: TcSYL_0099890.t1-p1
OG0017420: TcSYL_0099900.t1-p1
OG0017421: TcSYL_0099910.t1-p1
OG0017422: TcSYL_0099920.t1-p1

OG0017423: TcSYL_0099930.t1-p1
OG0017424: TcSYL_0099960.t1-p1
OG0017425: TcSYL_0099970.t1-p1
OG0017426: TcSYL_0099980.t1-p1
OG0017427: TcSYL_0099990.t1-p1
OG0017428: TcSYL_0100000.t1-p1
OG0017429: TcSYL_0100030.t1-p1
OG0017430: TcSYL_0100040.t1-p1
OG0017431: TcSYL_0100090.t1-p1
OG0017432: TcSYL_0100150.t1-p1
OG0017433: TcSYL_0100170.t1-p1
OG0017434: TcSYL_0100180.t1-p1
OG0017435: TcSYL_0100200.t1-p1
OG0017436: TcSYL_0100240.t1-p1
OG0017437: TcSYL_0100250.t1-p1
OG0017438: TcSYL_0100260.t1-p1
OG0017439: TcSYL_0100270.t1-p1
OG0017440: TcSYL_0100280.t1-p1
OG0017441: TcSYL_0100300.t1-p1
OG0017442: TcSYL_0100340.t1-p1
OG0017443: TcSYL_0100350.t1-p1
OG0017444: TcSYL_0100360.t1-p1
OG0017445: TcSYL_0100370.t1-p1
OG0017446: TcSYL_0100380.t1-p1
OG0017447: TcSYL_0100390.t1-p1
OG0017448: TcSYL_0100400.t1-p1
OG0017449: TcSYL_0100410.t1-p1
OG0017450: TcSYL_0100420.t1-p1
OG0017451: TcSYL_0100450.t1-p1
OG0017452: TcSYL_0100460.t1-p1
OG0017453: TcSYL_0100480.t1-p1
OG0017454: TcSYL_0100490.t1-p1
OG0017455: TcSYL_0100500.t1-p1
OG0017456: TcSYL_0100510.t1-p1
OG0017457: TcSYL_0100520.t1-p1
OG0017458: TcSYL_0100530.t1-p1
OG0017459: TcSYL_0100540.t1-p1
OG0017460: TcSYL_0100560.t1-p1
OG0017461: TcSYL_0100570.t1-p1
OG0017462: TcSYL_0100580.t1-p1
OG0017463: TcSYL_0100590.t1-p1
OG0017464: TcSYL_0100600.t1-p1
OG0017465: TcSYL_0100610.t1-p1
OG0017466: TcSYL_0100620.t1-p1
OG0017467: TcSYL_0100630.t1-p1
OG0017468: TcSYL_0100640.t1-p1
OG0017469: TcSYL_0100650.t1-p1
OG0017470: TcSYL_0100660.t1-p1
OG0017471: TcSYL_0100670.t1-p1
OG0017472: TcSYL_0100680.t1-p1
OG0017473: TcSYL_0100690.t1-p1
OG0017474: TcSYL_0100700.t1-p1
OG0017475: TcSYL_0100710.t1-p1
OG0017476: TcSYL_0100720.t1-p1

OG0017477: TcSYL_0100730.t1-p1
OG0017478: TcSYL_0100740.t1-p1
OG0017479: TcSYL_0100760.t1-p1
OG0017480: TcSYL_0100770.t1-p1
OG0017481: TcSYL_0100780.t1-p1
OG0017482: TcSYL_0100790.t1-p1
OG0017483: TcSYL_0100810.t1-p1
OG0017484: TcSYL_0100820.t1-p1
OG0017485: TcSYL_0100830.t1-p1
OG0017486: TcSYL_0100850.t1-p1
OG0017487: TcSYL_0100870.t1-p1
OG0017488: TcSYL_0100880.t1-p1
OG0017489: TcSYL_0100890.t1-p1
OG0017490: TcSYL_0100910.t1-p1
OG0017491: TcSYL_0100930.t1-p1
OG0017492: TcSYL_0100950.t1-p1
OG0017493: TcSYL_0100960.t1-p1
OG0017494: TcSYL_0100970.t1-p1
OG0017495: TcSYL_0100980.t1-p1
OG0017496: TcSYL_0100990.t1-p1
OG0017497: TcSYL_0101000.t1-p1
OG0017498: TcSYL_0101020.t1-p1
OG0017499: TcSYL_0101040.t1-p1
OG0017500: TcSYL_0101050.t1-p1
OG0017501: TcSYL_0101060.t1-p1
OG0017502: TcSYL_0101080.t1-p1
OG0017503: TcSYL_0101090.t1-p1
OG0017504: TcSYL_0101140.t1-p1
OG0017505: TcSYL_0101150.t1-p1
OG0017506: TcSYL_0101160.t1-p1
OG0017507: TcSYL_0101170.t1-p1
OG0017508: TcSYL_0101180.t1-p1
OG0017509: TcSYL_0101190.t1-p1
OG0017510: TcSYL_0101210.t1-p1
OG0017511: TcSYL_0101220.t1-p1
OG0017512: TcSYL_0101230.t1-p1
OG0017513: TcSYL_0101240.t1-p1
OG0017514: TcSYL_0101290.t1-p1
OG0017515: TcSYL_0101300.t1-p1
OG0017516: TcSYL_0101310.t1-p1
OG0017517: TcSYL_0101320.t1-p1
OG0017518: TcSYL_0101330.t1-p1
OG0017519: TcSYL_0101340.t1-p1
OG0017520: TcSYL_0101360.t1-p1
OG0017521: TcSYL_0101370.t1-p1
OG0017522: TcSYL_0101380.t1-p1
OG0017523: TcSYL_0101400.t1-p1
OG0017524: TcSYL_0101410.t1-p1
OG0017525: TcSYL_0101420.t1-p1
OG0017526: TcSYL_0101430.t1-p1
OG0017527: TcSYL_0101440.t1-p1
OG0017528: TcSYL_0101450.t1-p1
OG0017529: TcSYL_0101460.t1-p1
OG0017530: TcSYL_0101470.t1-p1

OG0017531: TcSYL_0101480.t1-p1
OG0017532: TcSYL_0101490.t1-p1
OG0017533: TcSYL_0101500.t1-p1
OG0017534: TcSYL_0101510.t1-p1
OG0017535: TcSYL_0101520.t1-p1
OG0017536: TcSYL_0101530.t1-p1
OG0017537: TcSYL_0101540.t1-p1
OG0017538: TcSYL_0101550.t1-p1
OG0017539: TcSYL_0101560.t1-p1
OG0017540: TcSYL_0101570.t1-p1
OG0017541: TcSYL_0101580.t1-p1
OG0017542: TcSYL_0101590.t1-p1
OG0017543: TcSYL_0101600.t1-p1
OG0017544: TcSYL_0101610.t1-p1
OG0017545: TcSYL_0101620.t1-p1
OG0017546: TcSYL_0101630.t1-p1
OG0017547: TcSYL_0101640.t1-p1
OG0017548: TcSYL_0101660.t1-p1
OG0017549: TcSYL_0101680.t1-p1
OG0017550: TcSYL_0101690.t1-p1
OG0017551: TcSYL_0101700.t1-p1
OG0017552: TcSYL_0101710.t1-p1
OG0017553: TcSYL_0101720.t1-p1
OG0017554: TcSYL_0101730.t1-p1
OG0017555: TcSYL_0101740.t1-p1
OG0017556: TcSYL_0101780.t1-p1
OG0017557: TcSYL_0101790.t1-p1
OG0017558: TcSYL_0101810.t1-p1
OG0017559: TcSYL_0101820.t1-p1
OG0017560: TcSYL_0101830.t1-p1
OG0017561: TcSYL_0101840.t1-p1
OG0017562: TcSYL_0101850.t1-p1
OG0017563: TcSYL_0101860.t1-p1
OG0017564: TcSYL_0101870.t1-p1
OG0017565: TcSYL_0101880.t1-p1
OG0017566: TcSYL_0101890.t1-p1
OG0017567: TcSYL_0101900.t1-p1
OG0017568: TcSYL_0101910.t1-p1
OG0017569: TcSYL_0101920.t1-p1
OG0017570: TcSYL_0101930.t1-p1
OG0017571: TcSYL_0101940.t1-p1
OG0017572: TcSYL_0101950.t1-p1
OG0017573: TcSYL_0101960.t1-p1
OG0017574: TcSYL_0101970.t1-p1
OG0017575: TcSYL_0101980.t1-p1
OG0017576: TcSYL_0101990.t1-p1
OG0017577: TcSYL_0102000.t1-p1
OG0017578: TcSYL_0102010.t1-p1
OG0017579: TcSYL_0102020.t1-p1
OG0017580: TcSYL_0102040.t1-p1
OG0017581: TcSYL_0102050.t1-p1
OG0017582: TcSYL_0102060.t1-p1
OG0017583: TcSYL_0102070.t1-p1
OG0017584: TcSYL_0102080.t1-p1

OG0017585: TcSYL_0102090.t1-p1
OG0017586: TcSYL_0102100.t1-p1
OG0017587: TcSYL_0102110.t1-p1
OG0017588: TcSYL_0102120.t1-p1
OG0017589: TcSYL_0102140.t1-p1
OG0017590: TcSYL_0102150.t1-p1
OG0017591: TcSYL_0102160.t1-p1
OG0017592: TcSYL_0102170.t1-p1
OG0017593: TcSYL_0102180.t1-p1
OG0017594: TcSYL_0102200.t1-p1
OG0017595: TcSYL_0102210.t1-p1
OG0017596: TcSYL_0102220.t1-p1
OG0017597: TcSYL_0102230.t1-p1
OG0017598: TcSYL_0102240.t1-p1
OG0017599: TcSYL_0102250.t1-p1
OG0017600: TcSYL_0102270.t1-p1
OG0017601: TcSYL_0102280.t1-p1
OG0017602: TcSYL_0102300.t1-p1
OG0017603: TcSYL_0102310.t1-p1
OG0017604: TcSYL_0102350.t1-p1
OG0017605: TcSYL_0102360.t1-p1
OG0017606: TcSYL_0102380.t1-p1
OG0017607: TcSYL_0102390.t1-p1
OG0017608: TcSYL_0102410.t1-p1
OG0017609: TcSYL_0102420.t1-p1
OG0017610: TcSYL_0102430.t1-p1
OG0017611: TcSYL_0102440.t1-p1
OG0017612: TcSYL_0102450.t1-p1
OG0017613: TcSYL_0102470.t1-p1
OG0017614: TcSYL_0102480.t1-p1
OG0017615: TcSYL_0102500.t1-p1
OG0017616: TcSYL_0102510.t1-p1
OG0017617: TcSYL_0102520.t1-p1
OG0017618: TcSYL_0102540.t1-p1
OG0017619: TcSYL_0102550.t1-p1
OG0017620: TcSYL_0102560.t1-p1
OG0017621: TcSYL_0102570.t1-p1
OG0017622: TcSYL_0102580.t1-p1
OG0017623: TcSYL_0102600.t1-p1
OG0017624: TcSYL_0102610.t1-p1
OG0017625: TcSYL_0102620.t1-p1
OG0017626: TcSYL_0102630.t1-p1
OG0017627: TcSYL_0102640.t1-p1
OG0017628: TcSYL_0102670.t1-p1
OG0017629: TcSYL_0102680.t1-p1
OG0017630: TcSYL_0102690.t1-p1
OG0017631: TcSYL_0102700.t1-p1
OG0017632: TcSYL_0102710.t1-p1
OG0017633: TcSYL_0102720.t1-p1
OG0017634: TcSYL_0102730.t1-p1
OG0017635: TcSYL_0102750.t1-p1
OG0017636: TcSYL_0102760.t1-p1
OG0017637: TcSYL_0102770.t1-p1
OG0017638: TcSYL_0102780.t1-p1

OG0017639: TcSYL_0102800.t1-p1
OG0017640: TcSYL_0102820.t1-p1
OG0017641: TcSYL_0102830.t1-p1
OG0017642: TcSYL_0102840.t1-p1
OG0017643: TcSYL_0102860.t1-p1
OG0017644: TcSYL_0102870.t1-p1
OG0017645: TcSYL_0102890.t1-p1
OG0017646: TcSYL_0102910.t1-p1
OG0017647: TcSYL_0102930.t1-p1
OG0017648: TcSYL_0102960.t1-p1
OG0017649: TcSYL_0102970.t1-p1
OG0017650: TcSYL_0102990.t1-p1
OG0017651: TcSYL_0103000.t1-p1
OG0017652: TcSYL_0103030.t1-p1
OG0017653: TcSYL_0103040.t1-p1
OG0017654: TcSYL_0103050.t1-p1
OG0017655: TcSYL_0103060.t1-p1
OG0017656: TcSYL_0103080.t1-p1
OG0017657: TcSYL_0103090.t1-p1
OG0017658: TcSYL_0103110.t1-p1
OG0017659: TcSYL_0103120.t1-p1
OG0017660: TcSYL_0103150.t1-p1
OG0017661: TcSYL_0103160.t1-p1
OG0017662: TcSYL_0103170.t1-p1
OG0017663: TcSYL_0103180.t1-p1
OG0017664: TcSYL_0103190.t1-p1
OG0017665: TcSYL_0103200.t1-p1
OG0017666: TcSYL_0103210.t1-p1
OG0017667: TcSYL_0103230.t1-p1
OG0017668: TcSYL_0103240.t1-p1
OG0017669: TcSYL_0103250.t1-p1
OG0017670: TcSYL_0103270.t1-p1
OG0017671: TcSYL_0103280.t1-p1
OG0017672: TcSYL_0103290.t1-p1
OG0017673: TcSYL_0103300.t1-p1
OG0017674: TcSYL_0103310.t1-p1
OG0017675: TcSYL_0103330.t1-p1
OG0017676: TcSYL_0103340.t1-p1
OG0017677: TcSYL_0103360.t1-p1
OG0017678: TcSYL_0103370.t1-p1
OG0017679: TcSYL_0103390.t1-p1
OG0017680: TcSYL_0103400.t1-p1
OG0017681: TcSYL_0103410.t1-p1
OG0017682: TcSYL_0103420.t1-p1
OG0017683: TcSYL_0103430.t1-p1
OG0017684: TcSYL_0103450.t1-p1
OG0017685: TcSYL_0103460.t1-p1
OG0017686: TcSYL_0103480.t1-p1
OG0017687: TcSYL_0103490.t1-p1
OG0017688: TcSYL_0103540.t1-p1
OG0017689: TcSYL_0103570.t1-p1
OG0017690: TcSYL_0103660.t1-p1
OG0017691: TcSYL_0103670.t1-p1
OG0017692: TcSYL_0103680.t1-p1

OG0017693: TcSYL_0103690.t1-p1
OG0017694: TcSYL_0103700.t1-p1
OG0017695: TcSYL_0103710.t1-p1
OG0017696: TcSYL_0103720.t1-p1
OG0017697: TcSYL_0103730.t1-p1
OG0017698: TcSYL_0103740.t1-p1
OG0017699: TcSYL_0103750.t1-p1
OG0017700: TcSYL_0103760.t1-p1
OG0017701: TcSYL_0103770.t1-p1
OG0017702: TcSYL_0103780.t1-p1
OG0017703: TcSYL_0103840.t1-p1
OG0017704: TcSYL_0103880.t1-p1
OG0017705: TcSYL_0103900.t1-p1
OG0017706: TcSYL_0103980.t1-p1
OG0017707: TcSYL_0103990.t1-p1
OG0017708: TcSYL_0104000.t1-p1
OG0017709: TcSYL_0104010.t1-p1
OG0017710: TcSYL_0104020.t1-p1
OG0017711: TcSYL_0104030.t1-p1
OG0017712: TcSYL_0104040.t1-p1
OG0017713: TcSYL_0104060.t1-p1
OG0017714: TcSYL_0104070.t1-p1
OG0017715: TcSYL_0104090.t1-p1
OG0017716: TcSYL_0104110.t1-p1
OG0017717: TcSYL_0104120.t1-p1
OG0017718: TcSYL_0104140.t1-p1
OG0017719: TcSYL_0104150.t1-p1
OG0017720: TcSYL_0104160.t1-p1
OG0017721: TcSYL_0104170.t1-p1
OG0017722: TcSYL_0104190.t1-p1
OG0017723: TcSYL_0104200.t1-p1
OG0017724: TcSYL_0104210.t1-p1
OG0017725: TcSYL_0104220.t1-p1
OG0017726: TcSYL_0104230.t1-p1
OG0017727: TcSYL_0104250.t1-p1
OG0017728: TcSYL_0104260.t1-p1
OG0017729: TcSYL_0104270.t1-p1
OG0017730: TcSYL_0104280.t1-p1
OG0017731: TcSYL_0104290.t1-p1
OG0017732: TcSYL_0104300.t1-p1
OG0017733: TcSYL_0104310.t1-p1
OG0017734: TcSYL_0104320.t1-p1
OG0017735: TcSYL_0104330.t1-p1
OG0017736: TcSYL_0104340.t1-p1
OG0017737: TcSYL_0104350.t1-p1
OG0017738: TcSYL_0104370.t1-p1
OG0017739: TcSYL_0104380.t1-p1
OG0017740: TcSYL_0104390.t1-p1
OG0017741: TcSYL_0104420.t1-p1
OG0017742: TcSYL_0104430.t1-p1
OG0017743: TcSYL_0104440.t1-p1
OG0017744: TcSYL_0104450.t1-p1
OG0017745: TcSYL_0104460.t1-p1
OG0017746: TcSYL_0104470.t1-p1

OG0017747: TcSYL_0104490.t1-p1
OG0017748: TcSYL_0104520.t1-p1
OG0017749: TcSYL_0104530.t1-p1
OG0017750: TcSYL_0104560.t1-p1
OG0017751: TcSYL_0104570.t1-p1
OG0017752: TcSYL_0104590.t1-p1
OG0017753: TcSYL_0104600.t1-p1
OG0017754: TcSYL_0104620.t1-p1
OG0017755: TcSYL_0104660.t1-p1
OG0017756: TcSYL_0104670.t1-p1
OG0017757: TcSYL_0104680.t1-p1
OG0017758: TcSYL_0104700.t1-p1
OG0017759: TcSYL_0104710.t1-p1
OG0017760: TcSYL_0104720.t1-p1
OG0017761: TcSYL_0104730.t1-p1
OG0017762: TcSYL_0104740.t1-p1
OG0017763: TcSYL_0104760.t1-p1
OG0017764: TcSYL_0104780.t1-p1
OG0017765: TcSYL_0104800.t1-p1
OG0017766: TcSYL_0104820.t1-p1
OG0017767: TcSYL_0104830.t1-p1
OG0017768: TcSYL_0104850.t1-p1
OG0017769: TcSYL_0104860.t1-p1
OG0017770: TcSYL_0104870.t1-p1
OG0017771: TcSYL_0104880.t1-p1
OG0017772: TcSYL_0104890.t1-p1
OG0017773: TcSYL_0104900.t1-p1
OG0017774: TcSYL_0104910.t1-p1
OG0017775: TcSYL_0104930.t1-p1
OG0017776: TcSYL_0104940.t1-p1
OG0017777: TcSYL_0104960.t1-p1
OG0017778: TcSYL_0104970.t1-p1
OG0017779: TcSYL_0104980.t1-p1
OG0017780: TcSYL_0104990.t1-p1
OG0017781: TcSYL_0105000.t1-p1
OG0017782: TcSYL_0105010.t1-p1
OG0017783: TcSYL_0105020.t1-p1
OG0017784: TcSYL_0105040.t1-p1
OG0017785: TcSYL_0105050.t1-p1
OG0017786: TcSYL_0105060.t1-p1
OG0017787: TcSYL_0105070.t1-p1
OG0017788: TcSYL_0105080.t1-p1
OG0017789: TcSYL_0105090.t1-p1
OG0017790: TcSYL_0105100.t1-p1
OG0017791: TcSYL_0105110.t1-p1
OG0017792: TcSYL_0105120.t1-p1
OG0017793: TcSYL_0105140.t1-p1
OG0017794: TcSYL_0105150.t1-p1
OG0017795: TcSYL_0105170.t1-p1
OG0017796: TcSYL_0105190.t1-p1
OG0017797: TcSYL_0105200.t1-p1
OG0017798: TcSYL_0105210.t1-p1
OG0017799: TcSYL_0105220.t1-p1
OG0017800: TcSYL_0105240.t1-p1

OG0017801: TcSYL_0105260.t1-p1
OG0017802: TcSYL_0105270.t1-p1
OG0017803: TcSYL_0105280.t1-p1
OG0017804: TcSYL_0105300.t1-p1
OG0017805: TcSYL_0105310.t1-p1
OG0017806: TcSYL_0105330.t1-p1
OG0017807: TcSYL_0105340.t1-p1
OG0017808: TcSYL_0105350.t1-p1
OG0017809: TcSYL_0105360.t1-p1
OG0017810: TcSYL_0105370.t1-p1
OG0017811: TcSYL_0105380.t1-p1
OG0017812: TcSYL_0105400.t1-p1
OG0017813: TcSYL_0105420.t1-p1
OG0017814: TcSYL_0105440.t1-p1
OG0017815: TcSYL_0105460.t1-p1
OG0017816: TcSYL_0105470.t1-p1
OG0017817: TcSYL_0105480.t1-p1
OG0017818: TcSYL_0105490.t1-p1
OG0017819: TcSYL_0105500.t1-p1
OG0017820: TcSYL_0105520.t1-p1
OG0017821: TcSYL_0105530.t1-p1
OG0017822: TcSYL_0105550.t1-p1
OG0017823: TcSYL_0105560.t1-p1
OG0017824: TcSYL_0105570.t1-p1
OG0017825: TcSYL_0105580.t1-p1
OG0017826: TcSYL_0105600.t1-p1
OG0017827: TcSYL_0105610.t1-p1
OG0017828: TcSYL_0105620.t1-p1
OG0017829: TcSYL_0105630.t1-p1
OG0017830: TcSYL_0105660.t1-p1
OG0017831: TcSYL_0105670.t1-p1
OG0017832: TcSYL_0105680.t1-p1
OG0017833: TcSYL_0105690.t1-p1
OG0017834: TcSYL_0105700.t1-p1
OG0017835: TcSYL_0105710.t1-p1
OG0017836: TcSYL_0105720.t1-p1
OG0017837: TcSYL_0105760.t1-p1
OG0017838: TcSYL_0105770.t1-p1
OG0017839: TcSYL_0105780.t1-p1
OG0017840: TcSYL_0105790.t1-p1
OG0017841: TcSYL_0105810.t1-p1
OG0017842: TcSYL_0105820.t1-p1
OG0017843: TcSYL_0105840.t1-p1
OG0017844: TcSYL_0105850.t1-p1
OG0017845: TcSYL_0105870.t1-p1
OG0017846: TcSYL_0105890.t1-p1
OG0017847: TcSYL_0105900.t1-p1
OG0017848: TcSYL_0105910.t1-p1
OG0017849: TcSYL_0105920.t1-p1
OG0017850: TcSYL_0105930.t1-p1
OG0017851: TcSYL_0105950.t1-p1
OG0017852: TcSYL_0105960.t1-p1
OG0017853: TcSYL_0105980.t1-p1
OG0017854: TcSYL_0105990.t1-p1

OG0017855: TcSYL_0106020.t1-p1
OG0017856: TcSYL_0106030.t1-p1
OG0017857: TcSYL_0106040.t1-p1
OG0017858: TcSYL_0106050.t1-p1
OG0017859: TcSYL_0106060.t1-p1
OG0017860: TcSYL_0106070.t1-p1
OG0017861: TcSYL_0106080.t1-p1
OG0017862: TcSYL_0106090.t1-p1
OG0017863: TcSYL_0106100.t1-p1
OG0017864: TcSYL_0106110.t1-p1
OG0017865: TcSYL_0106120.t1-p1
OG0017866: TcSYL_0106130.t1-p1
OG0017867: TcSYL_0106160.t1-p1
OG0017868: TcSYL_0106170.t1-p1
OG0017869: TcSYL_0106190.t1-p1
OG0017870: TcSYL_0106200.t1-p1
OG0017871: TcSYL_0106230.t1-p1
OG0017872: TcSYL_0106240.t1-p1
OG0017873: TcSYL_0106250.t1-p1
OG0017874: TcSYL_0106260.t1-p1
OG0017875: TcSYL_0106270.t1-p1
OG0017876: TcSYL_0106280.t1-p1
OG0017877: TcSYL_0106290.t1-p1
OG0017878: TcSYL_0106300.t1-p1
OG0017879: TcSYL_0106320.t1-p1
OG0017880: TcSYL_0106340.t1-p1
OG0017881: TcSYL_0106360.t1-p1
OG0017882: TcSYL_0106370.t1-p1
OG0017883: TcSYL_0106380.t1-p1
OG0017884: TcSYL_0106390.t1-p1
OG0017885: TcSYL_0106400.t1-p1
OG0017886: TcSYL_0106410.t1-p1
OG0017887: TcSYL_0106420.t1-p1
OG0017888: TcSYL_0106430.t1-p1
OG0017889: TcSYL_0106440.t1-p1
OG0017890: TcSYL_0106460.t1-p1
OG0017891: TcSYL_0106490.t1-p1
OG0017892: TcSYL_0106510.t1-p1
OG0017893: TcSYL_0106530.t1-p1
OG0017894: TcSYL_0106550.t1-p1
OG0017895: TcSYL_0106560.t1-p1
OG0017896: TcSYL_0106570.t1-p1
OG0017897: TcSYL_0106580.t1-p1
OG0017898: TcSYL_0106590.t1-p1
OG0017899: TcSYL_0106600.t1-p1
OG0017900: TcSYL_0106620.t1-p1
OG0017901: TcSYL_0106640.t1-p1
OG0017902: TcSYL_0106660.t1-p1
OG0017903: TcSYL_0106680.t1-p1
OG0017904: TcSYL_0106710.t1-p1
OG0017905: TcSYL_0106720.t1-p1
OG0017906: TcSYL_0106740.t1-p1
OG0017907: TcSYL_0106750.t1-p1
OG0017908: TcSYL_0106760.t1-p1

OG0017909: TcSYL_0106770.t1-p1
OG0017910: TcSYL_0106790.t1-p1
OG0017911: TcSYL_0106800.t1-p1
OG0017912: TcSYL_0106810.t1-p1
OG0017913: TcSYL_0106890.t1-p1
OG0017914: TcSYL_0106930.t1-p1
OG0017915: TcSYL_0106970.t1-p1
OG0017916: TcSYL_0107100.t1-p1
OG0017917: TcSYL_0107200.t1-p1
OG0017918: TcSYL_0107210.t1-p1
OG0017919: TcSYL_0107260.t1-p1
OG0017920: TcSYL_0107290.t1-p1
OG0017921: TcSYL_0107350.t1-p1
OG0017922: TcSYL_0107360.t1-p1
OG0017923: TcSYL_0107380.t1-p1
OG0017924: TcSYL_0107420.t1-p1
OG0017925: TcSYL_0107430.t1-p1
OG0017926: TcSYL_0107440.t1-p1
OG0017927: TcSYL_0107460.t1-p1
OG0017928: TcSYL_0107470.t1-p1
OG0017929: TcSYL_0107480.t1-p1
OG0017930: TcSYL_0107500.t1-p1
OG0017931: TcSYL_0107510.t1-p1
OG0017932: TcSYL_0107520.t1-p1
OG0017933: TcSYL_0107530.t1-p1
OG0017934: TcSYL_0107540.t1-p1
OG0017935: TcSYL_0107550.t1-p1
OG0017936: TcSYL_0107560.t1-p1
OG0017937: TcSYL_0107580.t1-p1
OG0017938: TcSYL_0107590.t1-p1
OG0017939: TcSYL_0107600.t1-p1
OG0017940: TcSYL_0107610.t1-p1
OG0017941: TcSYL_0107620.t1-p1
OG0017942: TcSYL_0107630.t1-p1
OG0017943: TcSYL_0107640.t1-p1
OG0017944: TcSYL_0107650.t1-p1
OG0017945: TcSYL_0107660.t1-p1
OG0017946: TcSYL_0107670.t1-p1
OG0017947: TcSYL_0107680.t1-p1
OG0017948: TcSYL_0107690.t1-p1
OG0017949: TcSYL_0107700.t1-p1
OG0017950: TcSYL_0107710.t1-p1
OG0017951: TcSYL_0107720.t1-p1
OG0017952: TcSYL_0107790.t1-p1
OG0017953: TcSYL_0107800.t1-p1
OG0017954: TcSYL_0107850.t1-p1
OG0017955: TcSYL_0107870.t1-p1
OG0017956: TcSYL_0107900.t1-p1
OG0017957: TcSYL_0107910.t1-p1
OG0017958: TcSYL_0107920.t1-p1
OG0017959: TcSYL_0108000.t1-p1
OG0017960: TcSYL_0108010.t1-p1
OG0017961: TcSYL_0108020.t1-p1
OG0017962: TcSYL_0108030.t1-p1

OG0017963: TcSYL_0108060.t1-p1
OG0017964: TcSYL_0108070.t1-p1
OG0017965: TcSYL_0108080.t1-p1
OG0017966: TcSYL_0108100.t1-p1
OG0017967: TcSYL_0108110.t1-p1
OG0017968: TcSYL_0108120.t1-p1
OG0017969: TcSYL_0108130.t1-p1
OG0017970: TcSYL_0108140.t1-p1
OG0017971: TcSYL_0108150.t1-p1
OG0017972: TcSYL_0108170.t1-p1
OG0017973: TcSYL_0108180.t1-p1
OG0017974: TcSYL_0108190.t1-p1
OG0017975: TcSYL_0108220.t1-p1
OG0017976: TcSYL_0108230.t1-p1
OG0017977: TcSYL_0108240.t1-p1
OG0017978: TcSYL_0108340.t1-p1
OG0017979: TcSYL_0108370.t1-p1
OG0017980: TcSYL_0108380.t1-p1
OG0017981: TcSYL_0108390.t1-p1
OG0017982: TcSYL_0108400.t1-p1
OG0017983: TcSYL_0108420.t1-p1
OG0017984: TcSYL_0108430.t1-p1
OG0017985: TcSYL_0108440.t1-p1
OG0017986: TcSYL_0108450.t1-p1
OG0017987: TcSYL_0108460.t1-p1
OG0017988: TcSYL_0108470.t1-p1
OG0017989: TcSYL_0108490.t1-p1
OG0017990: TcSYL_0108500.t1-p1
OG0017991: TcSYL_0108510.t1-p1
OG0017992: TcSYL_0108520.t1-p1
OG0017993: TcSYL_0108530.t1-p1
OG0017994: TcSYL_0108540.t1-p1
OG0017995: TcSYL_0108560.t1-p1
OG0017996: TcSYL_0108570.t1-p1
OG0017997: TcSYL_0108590.t1-p1
OG0017998: TcSYL_0108600.t1-p1
OG0017999: TcSYL_0108720.t1-p1
OG0018000: TcSYL_0108770.t1-p1
OG0018001: TcSYL_0108940.t1-p1
OG0018002: TcSYL_0108950.t1-p1
OG0018003: TcSYL_0108960.t1-p1
OG0018004: TcSYL_0108970.t1-p1
OG0018005: TcSYL_0108980.t1-p1
OG0018006: TcSYL_0108990.t1-p1
OG0018007: TcSYL_0109000.t1-p1
OG0018008: TcSYL_0109010.t1-p1
OG0018009: TcSYL_0109030.t1-p1
OG0018010: TcSYL_0109040.t1-p1
OG0018011: TcSYL_0109050.t1-p1
OG0018012: TcSYL_0109060.t1-p1
OG0018013: TcSYL_0109070.t1-p1
OG0018014: TcSYL_0109080.t1-p1
OG0018015: TcSYL_0109090.t1-p1
OG0018016: TcSYL_0109100.t1-p1

OG0018017: TcSYL_0109110.t1-p1
OG0018018: TcSYL_0109120.t1-p1
OG0018019: TcSYL_0109130.t1-p1
OG0018020: TcSYL_0109140.t1-p1
OG0018021: TcSYL_0109150.t1-p1
OG0018022: TcSYL_0109180.t1-p1
OG0018023: TcSYL_0109210.t1-p1
OG0018024: TcSYL_0109230.t1-p1
OG0018025: TcSYL_0109320.t1-p1
OG0018026: TcSYL_0109430.t1-p1
OG0018027: TcSYL_0109480.t1-p1
OG0018028: TcSYL_0109490.t1-p1
OG0018029: TcSYL_0109500.t1-p1
OG0018030: TcSYL_0109510.t1-p1
OG0018031: TcSYL_0109570.t1-p1
OG0018032: TcSYL_0109610.t1-p1
OG0018033: TcSYL_0109660.t1-p1
OG0018034: TcSYL_0109670.t1-p1
OG0018035: TcSYL_0109690.t1-p1
OG0018036: TcSYL_0109700.t1-p1
OG0018037: TcSYL_0109710.t1-p1
OG0018038: TcSYL_0109720.t1-p1
OG0018039: TcSYL_0109730.t1-p1
OG0018040: TcSYL_0109740.t1-p1
OG0018041: TcSYL_0109750.t1-p1
OG0018042: TcSYL_0109760.t1-p1
OG0018043: TcSYL_0109770.t1-p1
OG0018044: TcSYL_0109780.t1-p1
OG0018045: TcSYL_0109790.t1-p1
OG0018046: TcSYL_0109810.t1-p1
OG0018047: TcSYL_0109820.t1-p1
OG0018048: TcSYL_0109840.t1-p1
OG0018049: TcSYL_0109870.t1-p1
OG0018050: TcSYL_0109880.t1-p1
OG0018051: TcSYL_0109900.t1-p1
OG0018052: TcSYL_0109910.t1-p1
OG0018053: TcSYL_0109920.t1-p1
OG0018054: TcSYL_0109930.t1-p1
OG0018055: TcSYL_0109940.t1-p1
OG0018056: TcSYL_0109970.t1-p1
OG0018057: TcSYL_0110000.t1-p1
OG0018058: TcSYL_0110010.t1-p1
OG0018059: TcSYL_0110030.t1-p1
OG0018060: TcSYL_0110070.t1-p1
OG0018061: TcSYL_0110090.t1-p1
OG0018062: TcSYL_0110100.t1-p1
OG0018063: TcSYL_0110120.t1-p1
OG0018064: TcSYL_0110140.t1-p1
OG0018065: TcSYL_0110160.t1-p1
OG0018066: TcSYL_0110170.t1-p1
OG0018067: TcSYL_0110190.t1-p1
OG0018068: TcSYL_0110200.t1-p1
OG0018069: TcSYL_0110220.t1-p1
OG0018070: TcSYL_0110240.t1-p1

OG0018071: TcSYL_0110250.t1-p1
OG0018072: TcSYL_0110310.t1-p1
OG0018073: TcSYL_0110380.t1-p1
OG0018074: TcSYL_0110410.t1-p1
OG0018075: TcSYL_0110440.t1-p1
OG0018076: TcSYL_0110450.t1-p1
OG0018077: TcSYL_0110460.t1-p1
OG0018078: TcSYL_0110480.t1-p1
OG0018079: TcSYL_0110490.t1-p1
OG0018080: TcSYL_0110500.t1-p1
OG0018081: TcSYL_0110510.t1-p1
OG0018082: TcSYL_0110520.t1-p1
OG0018083: TcSYL_0110530.t1-p1
OG0018084: TcSYL_0110550.t1-p1
OG0018085: TcSYL_0110560.t1-p1
OG0018086: TcSYL_0110570.t1-p1
OG0018087: TcSYL_0110590.t1-p1
OG0018088: TcSYL_0110600.t1-p1
OG0018089: TcSYL_0110610.t1-p1
OG0018090: TcSYL_0110620.t1-p1
OG0018091: TcSYL_0110640.t1-p1
OG0018092: TcSYL_0110650.t1-p1
OG0018093: TcSYL_0110810.t1-p1
OG0018094: TcSYL_0110850.t1-p1
OG0018095: TcSYL_0111000.t1-p1
OG0018096: TcSYL_0111030.t1-p1
OG0018097: TcSYL_0111050.t1-p1
OG0018098: TcSYL_0111060.t1-p1
OG0018099: TcSYL_0111180.t1-p1
OG0018100: TcSYL_0111190.t1-p1
OG0018101: TcSYL_0111230.t1-p1
OG0018102: TcSYL_0111250.t1-p1
OG0018103: TcSYL_0111260.t1-p1
OG0018104: TcSYL_0111270.t1-p1
OG0018105: TcSYL_0111280.t1-p1
OG0018106: TcSYL_0111360.t1-p1
OG0018107: TcSYL_0111370.t1-p1
OG0018108: TcSYL_0111380.t1-p1
OG0018109: TcSYL_0111390.t1-p1
OG0018110: TcSYL_0111400.t1-p1
OG0018111: TcSYL_0111410.t1-p1
OG0018112: TcSYL_0111420.t1-p1
OG0018113: TcSYL_0111430.t1-p1
OG0018114: TcSYL_0111440.t1-p1
OG0018115: TcSYL_0111450.t1-p1
OG0018116: TcSYL_0111470.t1-p1
OG0018117: TcSYL_0111480.t1-p1
OG0018118: TcSYL_0111500.t1-p1
OG0018119: TcSYL_0111510.t1-p1
OG0018120: TcSYL_0111520.t1-p1
OG0018121: TcSYL_0111530.t1-p1
OG0018122: TcSYL_0111550.t1-p1
OG0018123: TcSYL_0111560.t1-p1
OG0018124: TcSYL_0111590.t1-p1

OG0018125: TcSYL_0111610.t1-p1
OG0018126: TcSYL_0111620.t1-p1
OG0018127: TcSYL_0111630.t1-p1
OG0018128: TcSYL_0111670.t1-p1
OG0018129: TcSYL_0111860.t1-p1
OG0018130: TcSYL_0111910.t1-p1
OG0018131: TcSYL_0111920.t1-p1
OG0018132: TcSYL_0111930.t1-p1
OG0018133: TcSYL_0111960.t1-p1
OG0018134: TcSYL_0112130.t1-p1
OG0018135: TcSYL_0112290.t1-p1
OG0018136: TcSYL_0112310.t1-p1
OG0018137: TcSYL_0112320.t1-p1
OG0018138: TcSYL_0112450.t1-p1
OG0018139: TcSYL_0112530.t1-p1
OG0018140: TcSYL_0112630.t1-p1
OG0018141: TcSYL_0112660.t1-p1
OG0018142: TcSYL_0112860.t1-p1
OG0018143: TcSYL_0112880.t1-p1
OG0018144: TcSYL_0112890.t1-p1
OG0018145: TcSYL_0113040.t1-p1
OG0018146: TcSYL_0113050.t1-p1
OG0018147: TcSYL_0113060.t1-p1
OG0018148: TcSYL_0113080.t1-p1
OG0018149: TcSYL_0113100.t1-p1
OG0018150: TcSYL_0113150.t1-p1
OG0018151: TcSYL_0113160.t1-p1
OG0018152: TcSYL_0113170.t1-p1
OG0018153: TcSYL_0113180.t1-p1
OG0018154: TcSYL_0113190.t1-p1
OG0018155: TcSYL_0113200.t1-p1
OG0018156: TcSYL_0113210.t1-p1
OG0018157: TcSYL_0113230.t1-p1
OG0018158: TcSYL_0113570.t1-p1
OG0018159: TcSYL_0113590.t1-p1
OG0018160: TcSYL_0113620.t1-p1
OG0018161: TcSYL_0113630.t1-p1
OG0018162: TcSYL_0113650.t1-p1
OG0018163: TcSYL_0113660.t1-p1
OG0018164: TcSYL_0113670.t1-p1
OG0018165: TcSYL_0113680.t1-p1
OG0018166: TcSYL_0113700.t1-p1
OG0018167: TcSYL_0113710.t1-p1
OG0018168: TcSYL_0113720.t1-p1
OG0018169: TcSYL_0113730.t1-p1
OG0018170: TcSYL_0113740.t1-p1
OG0018171: TcSYL_0113750.t1-p1
OG0018172: TcSYL_0113760.t1-p1
OG0018173: TcSYL_0113770.t1-p1
OG0018174: TcSYL_0113780.t1-p1
OG0018175: TcSYL_0113790.t1-p1
OG0018176: TcSYL_0113810.t1-p1
OG0018177: TcSYL_0113820.t1-p1
OG0018178: TcSYL_0113840.t1-p1

OG0018179: TcSYL_0113850.t1-p1
OG0018180: TcSYL_0113870.t1-p1
OG0018181: TcSYL_0113890.t1-p1
OG0018182: TcSYL_0113910.t1-p1
OG0018183: TcSYL_0113940.t1-p1
OG0018184: TcSYL_0113980.t1-p1
OG0018185: TcSYL_0113990.t1-p1
OG0018186: TcSYL_0114000.t1-p1
OG0018187: TcSYL_0114010.t1-p1
OG0018188: TcSYL_0114030.t1-p1
OG0018189: TcSYL_0114100.t1-p1
OG0018190: TcSYL_0114130.t1-p1
OG0018191: TcSYL_0114140.t1-p1
OG0018192: TcSYL_0114160.t1-p1
OG0018193: TcSYL_0114170.t1-p1
OG0018194: TcSYL_0114180.t1-p1
OG0018195: TcSYL_0114190.t1-p1
OG0018196: TcSYL_0114200.t1-p1
OG0018197: TcSYL_0114220.t1-p1
OG0018198: TcSYL_0114250.t1-p1
OG0018199: TcSYL_0114310.t1-p1
OG0018200: TcSYL_0114350.t1-p1
OG0018201: TcSYL_0114360.t1-p1
OG0018202: TcSYL_0114380.t1-p1
OG0018203: TcSYL_0114390.t1-p1
OG0018204: TcSYL_0114410.t1-p1
OG0018205: TcSYL_0114420.t1-p1
OG0018206: TcSYL_0114430.t1-p1
OG0018207: TcSYL_0114450.t1-p1
OG0018208: TcSYL_0114460.t1-p1
OG0018209: TcSYL_0114470.t1-p1
OG0018210: TcSYL_0114480.t1-p1
OG0018211: TcSYL_0114490.t1-p1
OG0018212: TcSYL_0114500.t1-p1
OG0018213: TcSYL_0114510.t1-p1
OG0018214: TcSYL_0114530.t1-p1
OG0018215: TcSYL_0114540.t1-p1
OG0018216: TcSYL_0114550.t1-p1
OG0018217: TcSYL_0114560.t1-p1
OG0018218: TcSYL_0114570.t1-p1
OG0018219: TcSYL_0114580.t1-p1
OG0018220: TcSYL_0114600.t1-p1
OG0018221: TcSYL_0114730.t1-p1
OG0018222: TcSYL_0114800.t1-p1
OG0018223: TcSYL_0114810.t1-p1
OG0018224: TcSYL_0114830.t1-p1
OG0018225: TcSYL_0114840.t1-p1
OG0018226: TcSYL_0114850.t1-p1
OG0018227: TcSYL_0114970.t1-p1
OG0018228: TcSYL_0115040.t1-p1
OG0018229: TcSYL_0115060.t1-p1
OG0018230: TcSYL_0115070.t1-p1
OG0018231: TcSYL_0115110.t1-p1
OG0018232: TcSYL_0115120.t1-p1

OG0018233: TcSYL_0115160.t1-p1
OG0018234: TcSYL_0115190.t1-p1
OG0018235: TcSYL_0115270.t1-p1
OG0018236: TcSYL_0115280.t1-p1
OG0018237: TcSYL_0115290.t1-p1
OG0018238: TcSYL_0115300.t1-p1
OG0018239: TcSYL_0115320.t1-p1
OG0018240: TcSYL_0115330.t1-p1
OG0018241: TcSYL_0115610.t1-p1
OG0018242: TcSYL_0115620.t1-p1
OG0018243: TcSYL_0115630.t1-p1
OG0018244: TcSYL_0115640.t1-p1
OG0018245: TcSYL_0115710.t1-p1
OG0018246: TcSYL_0115820.t1-p1
OG0018247: TcSYL_0115840.t1-p1
OG0018248: TcSYL_0115860.t1-p1
OG0018249: TcSYL_0115870.t1-p1
OG0018250: TcSYL_0115880.t1-p1
OG0018251: TcSYL_0115890.t1-p1
OG0018252: TcSYL_0115930.t1-p1
OG0018253: TcSYL_0115990.t1-p1
OG0018254: TcSYL_0116060.t1-p1
OG0018255: TcSYL_0116080.t1-p1
OG0018256: TcSYL_0116120.t1-p1
OG0018257: TcSYL_0116240.t1-p1
OG0018258: TcSYL_0116340.t1-p1
OG0018259: TcSYL_0116380.t1-p1
OG0018260: TcSYL_0116400.t1-p1
OG0018261: TcSYL_0116410.t1-p1
OG0018262: TcSYL_0116430.t1-p1
OG0018263: TcSYL_0116440.t1-p1
OG0018264: TcSYL_0116460.t1-p1
OG0018265: TcSYL_0116470.t1-p1
OG0018266: TcSYL_0116490.t1-p1
OG0018267: TcSYL_0116500.t1-p1
OG0018268: TcSYL_0116530.t1-p1
OG0018269: TcSYL_0116540.t1-p1
OG0018270: TcSYL_0116550.t1-p1
OG0018271: TcSYL_0116580.t1-p1
OG0018272: TcSYL_0116590.t1-p1
OG0018273: TcSYL_0116610.t1-p1
OG0018274: TcSYL_0116620.t1-p1
OG0018275: TcSYL_0116630.t1-p1
OG0018276: TcSYL_0116640.t1-p1
OG0018277: TcSYL_0116650.t1-p1
OG0018278: TcSYL_0116670.t1-p1
OG0018279: TcSYL_0116690.t1-p1
OG0018280: TcSYL_0116710.t1-p1
OG0018281: TcSYL_0116730.t1-p1
OG0018282: TcSYL_0116740.t1-p1
OG0018283: TcSYL_0116760.t1-p1
OG0018284: TcSYL_0116780.t1-p1
OG0018285: TcSYL_0116790.t1-p1
OG0018286: TcSYL_0116810.t1-p1

OG0018287: TcSYL_0116830.t1-p1
OG0018288: TcSYL_0116850.t1-p1
OG0018289: TcSYL_0116860.t1-p1
OG0018290: TcSYL_0116890.t1-p1
OG0018291: TcSYL_0116910.t1-p1
OG0018292: TcSYL_0116930.t1-p1
OG0018293: TcSYL_0116950.t1-p1
OG0018294: TcSYL_0116960.t1-p1
OG0018295: TcSYL_0116970.t1-p1
OG0018296: TcSYL_0116980.t1-p1
OG0018297: TcSYL_0117000.t1-p1
OG0018298: TcSYL_0117020.t1-p1
OG0018299: TcSYL_0117030.t1-p1
OG0018300: TcSYL_0117050.t1-p1
OG0018301: TcSYL_0117060.t1-p1
OG0018302: TcSYL_0117070.t1-p1
OG0018303: TcSYL_0117110.t1-p1
OG0018304: TcSYL_0117120.t1-p1
OG0018305: TcSYL_0117130.t1-p1
OG0018306: TcSYL_0117140.t1-p1
OG0018307: TcSYL_0117170.t1-p1
OG0018308: TcSYL_0117180.t1-p1
OG0018309: TcSYL_0117200.t1-p1
OG0018310: TcSYL_0117210.t1-p1
OG0018311: TcSYL_0117230.t1-p1
OG0018312: TcSYL_0117240.t1-p1
OG0018313: TcSYL_0117250.t1-p1
OG0018314: TcSYL_0117260.t1-p1
OG0018315: TcSYL_0117270.t1-p1
OG0018316: TcSYL_0117290.t1-p1
OG0018317: TcSYL_0117310.t1-p1
OG0018318: TcSYL_0117320.t1-p1
OG0018319: TcSYL_0117340.t1-p1
OG0018320: TcSYL_0117360.t1-p1
OG0018321: TcSYL_0117370.t1-p1
OG0018322: TcSYL_0117390.t1-p1
OG0018323: TcSYL_0117410.t1-p1
OG0018324: TcSYL_0117420.t1-p1
OG0018325: TcSYL_0117440.t1-p1
OG0018326: TcSYL_0117450.t1-p1
OG0018327: TcSYL_0117460.t1-p1
OG0018328: TcSYL_0117480.t1-p1
OG0018329: TcSYL_0117490.t1-p1
OG0018330: TcSYL_0117500.t1-p1
OG0018331: TcSYL_0117510.t1-p1
OG0018332: TcSYL_0117520.t1-p1
OG0018333: TcSYL_0117540.t1-p1
OG0018334: TcSYL_0117550.t1-p1
OG0018335: TcSYL_0117570.t1-p1
OG0018336: TcSYL_0117580.t1-p1
OG0018337: TcSYL_0117610.t1-p1
OG0018338: TcSYL_0117720.t1-p1
OG0018339: TcSYL_0117750.t1-p1
OG0018340: TcSYL_0117780.t1-p1

OG0018341: TcSYL_0117790.t1-p1
OG0018342: TcSYL_0117800.t1-p1
OG0018343: TcSYL_0117810.t1-p1
OG0018344: TcSYL_0117830.t1-p1
OG0018345: TcSYL_0117840.t1-p1
OG0018346: TcSYL_0117860.t1-p1
OG0018347: TcSYL_0117880.t1-p1
OG0018348: TcSYL_0117890.t1-p1
OG0018349: TcSYL_0117900.t1-p1
OG0018350: TcSYL_0117910.t1-p1
OG0018351: TcSYL_0117920.t1-p1
OG0018352: TcSYL_0117930.t1-p1
OG0018353: TcSYL_0117940.t1-p1
OG0018354: TcSYL_0117950.t1-p1
OG0018355: TcSYL_0117960.t1-p1
OG0018356: TcSYL_0117970.t1-p1
OG0018357: TcSYL_0117980.t1-p1
OG0018358: TcSYL_0117990.t1-p1
OG0018359: TcSYL_0118000.t1-p1
OG0018360: TcSYL_0118010.t1-p1
OG0018361: TcSYL_0118020.t1-p1
OG0018362: TcSYL_0118030.t1-p1
OG0018363: TcSYL_0118040.t1-p1
OG0018364: TcSYL_0118050.t1-p1
OG0018365: TcSYL_0118060.t1-p1
OG0018366: TcSYL_0118070.t1-p1
OG0018367: TcSYL_0118080.t1-p1
OG0018368: TcSYL_0118100.t1-p1
OG0018369: TcSYL_0118110.t1-p1
OG0018370: TcSYL_0118360.t1-p1
OG0018371: TcSYL_0118450.t1-p1
OG0018372: TcSYL_0118460.t1-p1
OG0018373: TcSYL_0118470.t1-p1
OG0018374: TcSYL_0118480.t1-p1
OG0018375: TcSYL_0118500.t1-p1
OG0018376: TcSYL_0118510.t1-p1
OG0018377: TcSYL_0118520.t1-p1
OG0018378: TcSYL_0118530.t1-p1
OG0018379: TcSYL_0118540.t1-p1
OG0018380: TcSYL_0118570.t1-p1
OG0018381: TcSYL_0118580.t1-p1
OG0018382: TcSYL_0118590.t1-p1
OG0018383: TcSYL_0118600.t1-p1
OG0018384: TcSYL_0118620.t1-p1
OG0018385: TcSYL_0118630.t1-p1
OG0018386: TcSYL_0118640.t1-p1
OG0018387: TcSYL_0118650.t1-p1
OG0018388: TcSYL_0118660.t1-p1
OG0018389: TcSYL_0118670.t1-p1
OG0018390: TcSYL_0118680.t1-p1
OG0018391: TcSYL_0118690.t1-p1
OG0018392: TcSYL_0118700.t1-p1
OG0018393: TcSYL_0118710.t1-p1
OG0018394: TcSYL_0118720.t1-p1

OG0018395: TcSYL_0118730.t1-p1
OG0018396: TcSYL_0118740.t1-p1
OG0018397: TcSYL_0118750.t1-p1
OG0018398: TcSYL_0118760.t1-p1
OG0018399: TcSYL_0118770.t1-p1
OG0018400: TcSYL_0118780.t1-p1
OG0018401: TcSYL_0118790.t1-p1
OG0018402: TcSYL_0118800.t1-p1
OG0018403: TcSYL_0118810.t1-p1
OG0018404: TcSYL_0118820.t1-p1
OG0018405: TcSYL_0118840.t1-p1
OG0018406: TcSYL_0118850.t1-p1
OG0018407: TcSYL_0118860.t1-p1
OG0018408: TcSYL_0118880.t1-p1
OG0018409: TcSYL_0118900.t1-p1
OG0018410: TcSYL_0118910.t1-p1
OG0018411: TcSYL_0118920.t1-p1
OG0018412: TcSYL_0118930.t1-p1
OG0018413: TcSYL_0118940.t1-p1
OG0018414: TcSYL_0118970.t1-p1
OG0018415: TcSYL_0118990.t1-p1
OG0018416: TcSYL_0119000.t1-p1
OG0018417: TcSYL_0119020.t1-p1
OG0018418: TcSYL_0119030.t1-p1
OG0018419: TcSYL_0119040.t1-p1
OG0018420: TcSYL_0119050.t1-p1
OG0018421: TcSYL_0119060.t1-p1
OG0018422: TcSYL_0119070.t1-p1
OG0018423: TcSYL_0119080.t1-p1
OG0018424: TcSYL_0119090.t1-p1
OG0018425: TcSYL_0119100.t1-p1
OG0018426: TcSYL_0119110.t1-p1
OG0018427: TcSYL_0119120.t1-p1
OG0018428: TcSYL_0119140.t1-p1
OG0018429: TcSYL_0119150.t1-p1
OG0018430: TcSYL_0119160.t1-p1
OG0018431: TcSYL_0119170.t1-p1
OG0018432: TcSYL_0119180.t1-p1
OG0018433: TcSYL_0119190.t1-p1
OG0018434: TcSYL_0119200.t1-p1
OG0018435: TcSYL_0119210.t1-p1
OG0018436: TcSYL_0119230.t1-p1
OG0018437: TcSYL_0119240.t1-p1
OG0018438: TcSYL_0119250.t1-p1
OG0018439: TcSYL_0119260.t1-p1
OG0018440: TcSYL_0119270.t1-p1
OG0018441: TcSYL_0119280.t1-p1
OG0018442: TcSYL_0119290.t1-p1
OG0018443: TcSYL_0119300.t1-p1
OG0018444: TcSYL_0119310.t1-p1
OG0018445: TcSYL_0119320.t1-p1
OG0018446: TcSYL_0119330.t1-p1
OG0018447: TcSYL_0119340.t1-p1
OG0018448: TcSYL_0119350.t1-p1

OG0018449: TcSYL_0119360.t1-p1
OG0018450: TcSYL_0119380.t1-p1
OG0018451: TcSYL_0119390.t1-p1
OG0018452: TcSYL_0119400.t1-p1
OG0018453: TcSYL_0119410.t1-p1
OG0018454: TcSYL_0119420.t1-p1
OG0018455: TcSYL_0119430.t1-p1
OG0018456: TcSYL_0119440.t1-p1
OG0018457: TcSYL_0119450.t1-p1
OG0018458: TcSYL_0119460.t1-p1
OG0018459: TcSYL_0119470.t1-p1
OG0018460: TcSYL_0119480.t1-p1
OG0018461: TcSYL_0119490.t1-p1
OG0018462: TcSYL_0119510.t1-p1
OG0018463: TcSYL_0119520.t1-p1
OG0018464: TcSYL_0119530.t1-p1
OG0018465: TcSYL_0119540.t1-p1
OG0018466: TcSYL_0119550.t1-p1
OG0018467: TcSYL_0119560.t1-p1
OG0018468: TcSYL_0119570.t1-p1
OG0018469: TcSYL_0119580.t1-p1
OG0018470: TcSYL_0119590.t1-p1
OG0018471: TcSYL_0119600.t1-p1
OG0018472: TcSYL_0119610.t1-p1
OG0018473: TcSYL_0119620.t1-p1
OG0018474: TcSYL_0119630.t1-p1
OG0018475: TcSYL_0119640.t1-p1
OG0018476: TcSYL_0119650.t1-p1
OG0018477: TcSYL_0119670.t1-p1
OG0018478: TcSYL_0119700.t1-p1
OG0018479: TcSYL_0119710.t1-p1
OG0018480: TcSYL_0119720.t1-p1
OG0018481: TcSYL_0119730.t1-p1
OG0018482: TcSYL_0119740.t1-p1
OG0018483: TcSYL_0119750.t1-p1
OG0018484: TcSYL_0119770.t1-p1
OG0018485: TcSYL_0119780.t1-p1
OG0018486: TcSYL_0119790.t1-p1
OG0018487: TcSYL_0119800.t1-p1
OG0018488: TcSYL_0119810.t1-p1
OG0018489: TcSYL_0119820.t1-p1
OG0018490: TcSYL_0119830.t1-p1
OG0018491: TcSYL_0119840.t1-p1
OG0018492: TcSYL_0119850.t1-p1
OG0018493: TcSYL_0119870.t1-p1
OG0018494: TcSYL_0119880.t1-p1
OG0018495: TcSYL_0119890.t1-p1
OG0018496: TcSYL_0119900.t1-p1
OG0018497: TcSYL_0119910.t1-p1
OG0018498: TcSYL_0119920.t1-p1
OG0018499: TcSYL_0119930.t1-p1
OG0018500: TcSYL_0119940.t1-p1
OG0018501: TcSYL_0119950.t1-p1
OG0018502: TcSYL_0119960.t1-p1

OG0018503: TcSYL_0119970.t1-p1
OG0018504: TcSYL_0119980.t1-p1
OG0018505: TcSYL_0119990.t1-p1
OG0018506: TcSYL_0120000.t1-p1
OG0018507: TcSYL_0120010.t1-p1
OG0018508: TcSYL_0120030.t1-p1
OG0018509: TcSYL_0120040.t1-p1
OG0018510: TcSYL_0120050.t1-p1
OG0018511: TcSYL_0120060.t1-p1
OG0018512: TcSYL_0120070.t1-p1
OG0018513: TcSYL_0120080.t1-p1
OG0018514: TcSYL_0120100.t1-p1
OG0018515: TcSYL_0120110.t1-p1
OG0018516: TcSYL_0120130.t1-p1
OG0018517: TcSYL_0120160.t1-p1
OG0018518: TcSYL_0120170.t1-p1
OG0018519: TcSYL_0120180.t1-p1
OG0018520: TcSYL_0120190.t1-p1
OG0018521: TcSYL_0120200.t1-p1
OG0018522: TcSYL_0120230.t1-p1
OG0018523: TcSYL_0120240.t1-p1
OG0018524: TcSYL_0120280.t1-p1
OG0018525: TcSYL_0120290.t1-p1
OG0018526: TcSYL_0120300.t1-p1
OG0018527: TcSYL_0120310.t1-p1
OG0018528: TcSYL_0120320.t1-p1
OG0018529: TcSYL_0120330.t1-p1
OG0018530: TcSYL_0120340.t1-p1
OG0018531: TcSYL_0120350.t1-p1
OG0018532: TcSYL_0120360.t1-p1
OG0018533: TcSYL_0120370.t1-p1
OG0018534: TcSYL_0120380.t1-p1
OG0018535: TcSYL_0120390.t1-p1
OG0018536: TcSYL_0120400.t1-p1
OG0018537: TcSYL_0120410.t1-p1
OG0018538: TcSYL_0120420.t1-p1
OG0018539: TcSYL_0120430.t1-p1
OG0018540: TcSYL_0120440.t1-p1
OG0018541: TcSYL_0120450.t1-p1
OG0018542: TcSYL_0120460.t1-p1
OG0018543: TcSYL_0120470.t1-p1
OG0018544: TcSYL_0120480.t1-p1
OG0018545: TcSYL_0120490.t1-p1
OG0018546: TcSYL_0120500.t1-p1
OG0018547: TcSYL_0120510.t1-p1
OG0018548: TcSYL_0120520.t1-p1
OG0018549: TcSYL_0120530.t1-p1
OG0018550: TcSYL_0120540.t1-p1
OG0018551: TcSYL_0120550.t1-p1
OG0018552: TcSYL_0120570.t1-p1
OG0018553: TcSYL_0120590.t1-p1
OG0018554: TcSYL_0120600.t1-p1
OG0018555: TcSYL_0120610.t1-p1
OG0018556: TcSYL_0120620.t1-p1

OG0018557: TcSYL_0120650.t1-p1
OG0018558: TcSYL_0120670.t1-p1
OG0018559: TcSYL_0120680.t1-p1
OG0018560: TcSYL_0120690.t1-p1
OG0018561: TcSYL_0120700.t1-p1
OG0018562: TcSYL_0120710.t1-p1
OG0018563: TcSYL_0120720.t1-p1
OG0018564: TcSYL_0120730.t1-p1
OG0018565: TcSYL_0120740.t1-p1
OG0018566: TcSYL_0120750.t1-p1
OG0018567: TcSYL_0120760.t1-p1
OG0018568: TcSYL_0120770.t1-p1
OG0018569: TcSYL_0120780.t1-p1
OG0018570: TcSYL_0120790.t1-p1
OG0018571: TcSYL_0120800.t1-p1
OG0018572: TcSYL_0120820.t1-p1
OG0018573: TcSYL_0120850.t1-p1
OG0018574: TcSYL_0120870.t1-p1
OG0018575: TcSYL_0120890.t1-p1
OG0018576: TcSYL_0120900.t1-p1
OG0018577: TcSYL_0120910.t1-p1
OG0018578: TcSYL_0120920.t1-p1
OG0018579: TcSYL_0120940.t1-p1
OG0018580: TcSYL_0120950.t1-p1
OG0018581: TcSYL_0120960.t1-p1
OG0018582: TcSYL_0120970.t1-p1
OG0018583: TcSYL_0120990.t1-p1
OG0018584: TcSYL_0121000.t1-p1
OG0018585: TcSYL_0121010.t1-p1
OG0018586: TcSYL_0121020.t1-p1
OG0018587: TcSYL_0121030.t1-p1
OG0018588: TcSYL_0121050.t1-p1
OG0018589: TcSYL_0121060.t1-p1
OG0018590: TcSYL_0121070.t1-p1
OG0018591: TcSYL_0121080.t1-p1
OG0018592: TcSYL_0121090.t1-p1
OG0018593: TcSYL_0121100.t1-p1
OG0018594: TcSYL_0121110.t1-p1
OG0018595: TcSYL_0121120.t1-p1
OG0018596: TcSYL_0121160.t1-p1
OG0018597: TcSYL_0121170.t1-p1
OG0018598: TcSYL_0121190.t1-p1
OG0018599: TcSYL_0121360.t1-p1
OG0018600: TcSYL_0121370.t1-p1
OG0018601: TcSYL_0121380.t1-p1
OG0018602: TcSYL_0121400.t1-p1
OG0018603: TcSYL_0121420.t1-p1
OG0018604: TcSYL_0121440.t1-p1
OG0018605: TcSYL_0121450.t1-p1
OG0018606: TcSYL_0121470.t1-p1
OG0018607: TcSYL_0121500.t1-p1
OG0018608: TcSYL_0121510.t1-p1
OG0018609: TcSYL_0121580.t1-p1
OG0018610: TcSYL_0121620.t1-p1

OG0018611: TcSYL_0121630.t1-p1
OG0018612: TcSYL_0121660.t1-p1
OG0018613: TcSYL_0121670.t1-p1
OG0018614: TcSYL_0121700.t1-p1
OG0018615: TcSYL_0121730.t1-p1
OG0018616: TcSYL_0121800.t1-p1
OG0018617: TcSYL_0121920.t1-p1
OG0018618: TcSYL_0121930.t1-p1
OG0018619: TcSYL_0121940.t1-p1
OG0018620: TcSYL_0121960.t1-p1
OG0018621: TcSYL_0121980.t1-p1
OG0018622: TcSYL_0122000.t1-p1
OG0018623: TcSYL_0122030.t1-p1
OG0018624: TcSYL_0122040.t1-p1
OG0018625: TcSYL_0122050.t1-p1
OG0018626: TcSYL_0122060.t1-p1
OG0018627: TcSYL_0122070.t1-p1
OG0018628: TcSYL_0122090.t1-p1
OG0018629: TcSYL_0122100.t1-p1
OG0018630: TcSYL_0122110.t1-p1
OG0018631: TcSYL_0122120.t1-p1
OG0018632: TcSYL_0122140.t1-p1
OG0018633: TcSYL_0122150.t1-p1
OG0018634: TcSYL_0122160.t1-p1
OG0018635: TcSYL_0122180.t1-p1
OG0018636: TcSYL_0122190.t1-p1
OG0018637: TcSYL_0122210.t1-p1
OG0018638: TcSYL_0122270.t1-p1
OG0018639: TcSYL_0122370.t1-p1
OG0018640: TcSYL_0122390.t1-p1
OG0018641: TcSYL_0122400.t1-p1
OG0018642: TcSYL_0122410.t1-p1
OG0018643: TcSYL_0122420.t1-p1
OG0018644: TcSYL_0122440.t1-p1
OG0018645: TcSYL_0122450.t1-p1
OG0018646: TcSYL_0122460.t1-p1
OG0018647: TcSYL_0122470.t1-p1
OG0018648: TcSYL_0122480.t1-p1
OG0018649: TcSYL_0122530.t1-p1
OG0018650: TcSYL_0122540.t1-p1
OG0018651: TcSYL_0122550.t1-p1
OG0018652: TcSYL_0122560.t1-p1
OG0018653: TcSYL_0122580.t1-p1
OG0018654: TcSYL_0122590.t1-p1
OG0018655: TcSYL_0122600.t1-p1
OG0018656: TcSYL_0122610.t1-p1
OG0018657: TcSYL_0122620.t1-p1
OG0018658: TcSYL_0122630.t1-p1
OG0018659: TcSYL_0122640.t1-p1
OG0018660: TcSYL_0122650.t1-p1
OG0018661: TcSYL_0122670.t1-p1
OG0018662: TcSYL_0122690.t1-p1
OG0018663: TcSYL_0122700.t1-p1
OG0018664: TcSYL_0122710.t1-p1

OG0018665: TcSYL_0122740.t1-p1
OG0018666: TcSYL_0122750.t1-p1
OG0018667: TcSYL_0122790.t1-p1
OG0018668: TcSYL_0122810.t1-p1
OG0018669: TcSYL_0122820.t1-p1
OG0018670: TcSYL_0122830.t1-p1
OG0018671: TcSYL_0122840.t1-p1
OG0018672: TcSYL_0122850.t1-p1
OG0018673: TcSYL_0122860.t1-p1
OG0018674: TcSYL_0122870.t1-p1
OG0018675: TcSYL_0122880.t1-p1
OG0018676: TcSYL_0122890.t1-p1
OG0018677: TcSYL_0122900.t1-p1
OG0018678: TcSYL_0122910.t1-p1
OG0018679: TcSYL_0122960.t1-p1
OG0018680: TcSYL_0123110.t1-p1
OG0018681: TcSYL_0123120.t1-p1
OG0018682: TcSYL_0123140.t1-p1
OG0018683: TcSYL_0123160.t1-p1
OG0018684: TcSYL_0123180.t1-p1
OG0018685: TcSYL_0123190.t1-p1
OG0018686: TcSYL_0123230.t1-p1
OG0018687: TcSYL_0123240.t1-p1
OG0018688: TcSYL_0123250.t1-p1
OG0018689: TcSYL_0123260.t1-p1
OG0018690: TcSYL_0123280.t1-p1
OG0018691: TcSYL_0123290.t1-p1
OG0018692: TcSYL_0123300.t1-p1
OG0018693: TcSYL_0123310.t1-p1
OG0018694: TcSYL_0123320.t1-p1
OG0018695: TcSYL_0123330.t1-p1
OG0018696: TcSYL_0123340.t1-p1
OG0018697: TcSYL_0123360.t1-p1
OG0018698: TcSYL_0123390.t1-p1
OG0018699: TcSYL_0123400.t1-p1
OG0018700: TcSYL_0123410.t1-p1
OG0018701: TcSYL_0123430.t1-p1
OG0018702: TcSYL_0123450.t1-p1
OG0018703: TcSYL_0123530.t1-p1
OG0018704: TcSYL_0123540.t1-p1
OG0018705: TcSYL_0123570.t1-p1
OG0018706: TcSYL_0123580.t1-p1
OG0018707: TcSYL_0123590.t1-p1
OG0018708: TcSYL_0123610.t1-p1
OG0018709: TcSYL_0123620.t1-p1
OG0018710: TcSYL_0123670.t1-p1
OG0018711: TcSYL_0123680.t1-p1
OG0018712: TcSYL_0123790.t1-p1
OG0018713: TcSYL_0123820.t1-p1
OG0018714: TcSYL_0123840.t1-p1
OG0018715: TcSYL_0123850.t1-p1
OG0018716: TcSYL_0123860.t1-p1
OG0018717: TcSYL_0123870.t1-p1
OG0018718: TcSYL_0123880.t1-p1

OG0018719: TcSYL_0123890.t1-p1
OG0018720: TcSYL_0123910.t1-p1
OG0018721: TcSYL_0123920.t1-p1
OG0018722: TcSYL_0123930.t1-p1
OG0018723: TcSYL_0123970.t1-p1
OG0018724: TcSYL_0123980.t1-p1
OG0018725: TcSYL_0123990.t1-p1
OG0018726: TcSYL_0124000.t1-p1
OG0018727: TcSYL_0124010.t1-p1
OG0018728: TcSYL_0124020.t1-p1
OG0018729: TcSYL_0124030.t1-p1
OG0018730: TcSYL_0124050.t1-p1
OG0018731: TcSYL_0124070.t1-p1
OG0018732: TcSYL_0124090.t1-p1
OG0018733: TcSYL_0124100.t1-p1
OG0018734: TcSYL_0124110.t1-p1
OG0018735: TcSYL_0124120.t1-p1
OG0018736: TcSYL_0124130.t1-p1
OG0018737: TcSYL_0124140.t1-p1
OG0018738: TcSYL_0124150.t1-p1
OG0018739: TcSYL_0124160.t1-p1
OG0018740: TcSYL_0124170.t1-p1
OG0018741: TcSYL_0124190.t1-p1
OG0018742: TcSYL_0124220.t1-p1
OG0018743: TcSYL_0124240.t1-p1
OG0018744: TcSYL_0124250.t1-p1
OG0018745: TcSYL_0124260.t1-p1
OG0018746: TcSYL_0124270.t1-p1
OG0018747: TcSYL_0124280.t1-p1
OG0018748: TcSYL_0124290.t1-p1
OG0018749: TcSYL_0124300.t1-p1
OG0018750: TcSYL_0124310.t1-p1
OG0018751: TcSYL_0124330.t1-p1
OG0018752: TcSYL_0124340.t1-p1
OG0018753: TcSYL_0124350.t1-p1
OG0018754: TcSYL_0124360.t1-p1
OG0018755: TcSYL_0124370.t1-p1
OG0018756: TcSYL_0124380.t1-p1
OG0018757: TcSYL_0124390.t1-p1
OG0018758: TcSYL_0124400.t1-p1
OG0018759: TcSYL_0124410.t1-p1
OG0018760: TcSYL_0124420.t1-p1
OG0018761: TcSYL_0124430.t1-p1
OG0018762: TcSYL_0124440.t1-p1
OG0018763: TcSYL_0124450.t1-p1
OG0018764: TcSYL_0124460.t1-p1
OG0018765: TcSYL_0124470.t1-p1
OG0018766: TcSYL_0124480.t1-p1
OG0018767: TcSYL_0124490.t1-p1
OG0018768: TcSYL_0124500.t1-p1
OG0018769: TcSYL_0124510.t1-p1
OG0018770: TcSYL_0124520.t1-p1
OG0018771: TcSYL_0124530.t1-p1
OG0018772: TcSYL_0124540.t1-p1

OG0018773: TcSYL_0124550.t1-p1
OG0018774: TcSYL_0124560.t1-p1
OG0018775: TcSYL_0124570.t1-p1
OG0018776: TcSYL_0124590.t1-p1
OG0018777: TcSYL_0124600.t1-p1
OG0018778: TcSYL_0124650.t1-p1
OG0018779: TcSYL_0124660.t1-p1
OG0018780: TcSYL_0124670.t1-p1
OG0018781: TcSYL_0124690.t1-p1
OG0018782: TcSYL_0124700.t1-p1
OG0018783: TcSYL_0124720.t1-p1
OG0018784: TcSYL_0124730.t1-p1
OG0018785: TcSYL_0124740.t1-p1
OG0018786: TcSYL_0124760.t1-p1
OG0018787: TcSYL_0124780.t1-p1
OG0018788: TcSYL_0124790.t1-p1
OG0018789: TcSYL_0124800.t1-p1
OG0018790: TcSYL_0124820.t1-p1
OG0018791: TcSYL_0124830.t1-p1
OG0018792: TcSYL_0124840.t1-p1
OG0018793: TcSYL_0124850.t1-p1
OG0018794: TcSYL_0124860.t1-p1
OG0018795: TcSYL_0124870.t1-p1
OG0018796: TcSYL_0124880.t1-p1
OG0018797: TcSYL_0124890.t1-p1
OG0018798: TcSYL_0124900.t1-p1
OG0018799: TcSYL_0124910.t1-p1
OG0018800: TcSYL_0124920.t1-p1
OG0018801: TcSYL_0124950.t1-p1
OG0018802: TcSYL_0124960.t1-p1
OG0018803: TcSYL_0124970.t1-p1
OG0018804: TcSYL_0124980.t1-p1
OG0018805: TcSYL_0124990.t1-p1
OG0018806: TcSYL_0125000.t1-p1
OG0018807: TcSYL_0125030.t1-p1
OG0018808: TcSYL_0125040.t1-p1
OG0018809: TcSYL_0125050.t1-p1
OG0018810: TcSYL_0125060.t1-p1
OG0018811: TcSYL_0125080.t1-p1
OG0018812: TcSYL_0125090.t1-p1
OG0018813: TcSYL_0125100.t1-p1
OG0018814: TcSYL_0125110.t1-p1
OG0018815: TcSYL_0125120.t1-p1
OG0018816: TcSYL_0125130.t1-p1
OG0018817: TcSYL_0125140.t1-p1
OG0018818: TcSYL_0125160.t1-p1
OG0018819: TcSYL_0125170.t1-p1
OG0018820: TcSYL_0125200.t1-p1
OG0018821: TcSYL_0125210.t1-p1
OG0018822: TcSYL_0125220.t1-p1
OG0018823: TcSYL_0125230.t1-p1
OG0018824: TcSYL_0125240.t1-p1
OG0018825: TcSYL_0125250.t1-p1
OG0018826: TcSYL_0125260.t1-p1

OG0018827: TcSYL_0125270.t1-p1
OG0018828: TcSYL_0125280.t1-p1
OG0018829: TcSYL_0125290.t1-p1
OG0018830: TcSYL_0125300.t1-p1
OG0018831: TcSYL_0125310.t1-p1
OG0018832: TcSYL_0125340.t1-p1
OG0018833: TcSYL_0125360.t1-p1
OG0018834: TcSYL_0125370.t1-p1
OG0018835: TcSYL_0125380.t1-p1
OG0018836: TcSYL_0125390.t1-p1
OG0018837: TcSYL_0125400.t1-p1
OG0018838: TcSYL_0125410.t1-p1
OG0018839: TcSYL_0125420.t1-p1
OG0018840: TcSYL_0125470.t1-p1
OG0018841: TcSYL_0125490.t1-p1
OG0018842: TcSYL_0125500.t1-p1
OG0018843: TcSYL_0125510.t1-p1
OG0018844: TcSYL_0125520.t1-p1
OG0018845: TcSYL_0125530.t1-p1
OG0018846: TcSYL_0125540.t1-p1
OG0018847: TcSYL_0125550.t1-p1
OG0018848: TcSYL_0125560.t1-p1
OG0018849: TcSYL_0125570.t1-p1
OG0018850: TcSYL_0125580.t1-p1
OG0018851: TcSYL_0125590.t1-p1
OG0018852: TcSYL_0125610.t1-p1
OG0018853: TcSYL_0125620.t1-p1
OG0018854: TcSYL_0125630.t1-p1
OG0018855: TcSYL_0125640.t1-p1
OG0018856: TcSYL_0125650.t1-p1
OG0018857: TcSYL_0125660.t1-p1
OG0018858: TcSYL_0125670.t1-p1
OG0018859: TcSYL_0125680.t1-p1
OG0018860: TcSYL_0125710.t1-p1
OG0018861: TcSYL_0125720.t1-p1
OG0018862: TcSYL_0125730.t1-p1
OG0018863: TcSYL_0125740.t1-p1
OG0018864: TcSYL_0125750.t1-p1
OG0018865: TcSYL_0125780.t1-p1
OG0018866: TcSYL_0125790.t1-p1
OG0018867: TcSYL_0125800.t1-p1
OG0018868: TcSYL_0125810.t1-p1
OG0018869: TcSYL_0125820.t1-p1
OG0018870: TcSYL_0125830.t1-p1
OG0018871: TcSYL_0125840.t1-p1
OG0018872: TcSYL_0125850.t1-p1
OG0018873: TcSYL_0125860.t1-p1
OG0018874: TcSYL_0125870.t1-p1
OG0018875: TcSYL_0125880.t1-p1
OG0018876: TcSYL_0125890.t1-p1
OG0018877: TcSYL_0125930.t1-p1
OG0018878: TcSYL_0125940.t1-p1
OG0018879: TcSYL_0125950.t1-p1
OG0018880: TcSYL_0125960.t1-p1

OG0018881: TcSYL_0125970.t1-p1
OG0018882: TcSYL_0125980.t1-p1
OG0018883: TcSYL_0125990.t1-p1
OG0018884: TcSYL_0126000.t1-p1
OG0018885: TcSYL_0126010.t1-p1
OG0018886: TcSYL_0126040.t1-p1
OG0018887: TcSYL_0126050.t1-p1
OG0018888: TcSYL_0126060.t1-p1
OG0018889: TcSYL_0126070.t1-p1
OG0018890: TcSYL_0126080.t1-p1
OG0018891: TcSYL_0126090.t1-p1
OG0018892: TcSYL_0126100.t1-p1
OG0018893: TcSYL_0126110.t1-p1
OG0018894: TcSYL_0126120.t1-p1
OG0018895: TcSYL_0126140.t1-p1
OG0018896: TcSYL_0126150.t1-p1
OG0018897: TcSYL_0126160.t1-p1
OG0018898: TcSYL_0126170.t1-p1
OG0018899: TcSYL_0126180.t1-p1
OG0018900: TcSYL_0126190.t1-p1
OG0018901: TcSYL_0126200.t1-p1
OG0018902: TcSYL_0126210.t1-p1
OG0018903: TcSYL_0126220.t1-p1
OG0018904: TcSYL_0126240.t1-p1
OG0018905: TcSYL_0126260.t1-p1
OG0018906: TcSYL_0126270.t1-p1
OG0018907: TcSYL_0126290.t1-p1
OG0018908: TcSYL_0126300.t1-p1
OG0018909: TcSYL_0126310.t1-p1
OG0018910: TcSYL_0126320.t1-p1
OG0018911: TcSYL_0126340.t1-p1
OG0018912: TcSYL_0126350.t1-p1
OG0018913: TcSYL_0126360.t1-p1
OG0018914: TcSYL_0126390.t1-p1
OG0018915: TcSYL_0126400.t1-p1
OG0018916: TcSYL_0126410.t1-p1
OG0018917: TcSYL_0126430.t1-p1
OG0018918: TcSYL_0126440.t1-p1
OG0018919: TcSYL_0126460.t1-p1
OG0018920: TcSYL_0126470.t1-p1
OG0018921: TcSYL_0126480.t1-p1
OG0018922: TcSYL_0126490.t1-p1
OG0018923: TcSYL_0126510.t1-p1
OG0018924: TcSYL_0126520.t1-p1
OG0018925: TcSYL_0126530.t1-p1
OG0018926: TcSYL_0126540.t1-p1
OG0018927: TcSYL_0126550.t1-p1
OG0018928: TcSYL_0126560.t1-p1
OG0018929: TcSYL_0126570.t1-p1
OG0018930: TcSYL_0126580.t1-p1
OG0018931: TcSYL_0126600.t1-p1
OG0018932: TcSYL_0126610.t1-p1
OG0018933: TcSYL_0126630.t1-p1
OG0018934: TcSYL_0126650.t1-p1

OG0018935: TcSYL_0126670.t1-p1
OG0018936: TcSYL_0126680.t1-p1
OG0018937: TcSYL_0126690.t1-p1
OG0018938: TcSYL_0126700.t1-p1
OG0018939: TcSYL_0126710.t1-p1
OG0018940: TcSYL_0126720.t1-p1
OG0018941: TcSYL_0126730.t1-p1
OG0018942: TcSYL_0126740.t1-p1
OG0018943: TcSYL_0126750.t1-p1
OG0018944: TcSYL_0126760.t1-p1
OG0018945: TcSYL_0126770.t1-p1
OG0018946: TcSYL_0126780.t1-p1
OG0018947: TcSYL_0126790.t1-p1
OG0018948: TcSYL_0126800.t1-p1
OG0018949: TcSYL_0126810.t1-p1
OG0018950: TcSYL_0126820.t1-p1
OG0018951: TcSYL_0126830.t1-p1
OG0018952: TcSYL_0126850.t1-p1
OG0018953: TcSYL_0126860.t1-p1
OG0018954: TcSYL_0126870.t1-p1
OG0018955: TcSYL_0126880.t1-p1
OG0018956: TcSYL_0126890.t1-p1
OG0018957: TcSYL_0126910.t1-p1
OG0018958: TcSYL_0126920.t1-p1
OG0018959: TcSYL_0126930.t1-p1
OG0018960: TcSYL_0126940.t1-p1
OG0018961: TcSYL_0126950.t1-p1
OG0018962: TcSYL_0126960.t1-p1
OG0018963: TcSYL_0126980.t1-p1
OG0018964: TcSYL_0126990.t1-p1
OG0018965: TcSYL_0127000.t1-p1
OG0018966: TcSYL_0127020.t1-p1
OG0018967: TcSYL_0127030.t1-p1
OG0018968: TcSYL_0127040.t1-p1
OG0018969: TcSYL_0127050.t1-p1
OG0018970: TcSYL_0127060.t1-p1
OG0018971: TcSYL_0127090.t1-p1
OG0018972: TcSYL_0127120.t1-p1
OG0018973: TcSYL_0127150.t1-p1
OG0018974: TcSYL_0127160.t1-p1
OG0018975: TcSYL_0127180.t1-p1
OG0018976: TcSYL_0127190.t1-p1
OG0018977: TcSYL_0127200.t1-p1
OG0018978: TcSYL_0127210.t1-p1
OG0018979: TcSYL_0127220.t1-p1
OG0018980: TcSYL_0127230.t1-p1
OG0018981: TcSYL_0127240.t1-p1
OG0018982: TcSYL_0127260.t1-p1
OG0018983: TcSYL_0127270.t1-p1
OG0018984: TcSYL_0127290.t1-p1
OG0018985: TcSYL_0127300.t1-p1
OG0018986: TcSYL_0127310.t1-p1
OG0018987: TcSYL_0127330.t1-p1
OG0018988: TcSYL_0127340.t1-p1

OG0018989: TcSYL_0127350.t1-p1
OG0018990: TcSYL_0127360.t1-p1
OG0018991: TcSYL_0127370.t1-p1
OG0018992: TcSYL_0127390.t1-p1
OG0018993: TcSYL_0127400.t1-p1
OG0018994: TcSYL_0127420.t1-p1
OG0018995: TcSYL_0127430.t1-p1
OG0018996: TcSYL_0127440.t1-p1
OG0018997: TcSYL_0127460.t1-p1
OG0018998: TcSYL_0127470.t1-p1
OG0018999: TcSYL_0127480.t1-p1
OG0019000: TcSYL_0127510.t1-p1
OG0019001: TcSYL_0127520.t1-p1
OG0019002: TcSYL_0127530.t1-p1
OG0019003: TcSYL_0127540.t1-p1
OG0019004: TcSYL_0127550.t1-p1
OG0019005: TcSYL_0127560.t1-p1
OG0019006: TcSYL_0127570.t1-p1
OG0019007: TcSYL_0127580.t1-p1
OG0019008: TcSYL_0127590.t1-p1
OG0019009: TcSYL_0127600.t1-p1
OG0019010: TcSYL_0127610.t1-p1
OG0019011: TcSYL_0127650.t1-p1
OG0019012: TcSYL_0127660.t1-p1
OG0019013: TcSYL_0127680.t1-p1
OG0019014: TcSYL_0127690.t1-p1
OG0019015: TcSYL_0127700.t1-p1
OG0019016: TcSYL_0127710.t1-p1
OG0019017: TcSYL_0127740.t1-p1
OG0019018: TcSYL_0127750.t1-p1
OG0019019: TcSYL_0127760.t1-p1
OG0019020: TcSYL_0127770.t1-p1
OG0019021: TcSYL_0127780.t1-p1
OG0019022: TcSYL_0127800.t1-p1
OG0019023: TcSYL_0127820.t1-p1
OG0019024: TcSYL_0127840.t1-p1
OG0019025: TcSYL_0127850.t1-p1
OG0019026: TcSYL_0127860.t1-p1
OG0019027: TcSYL_0127870.t1-p1
OG0019028: TcSYL_0127880.t1-p1
OG0019029: TcSYL_0127890.t1-p1
OG0019030: TcSYL_0127900.t1-p1
OG0019031: TcSYL_0127920.t1-p1
OG0019032: TcSYL_0127940.t1-p1
OG0019033: TcSYL_0127960.t1-p1
OG0019034: TcSYL_0127980.t1-p1
OG0019035: TcSYL_0128010.t1-p1
OG0019036: TcSYL_0128020.t1-p1
OG0019037: TcSYL_0128040.t1-p1
OG0019038: TcSYL_0128050.t1-p1
OG0019039: TcSYL_0128060.t1-p1
OG0019040: TcSYL_0128070.t1-p1
OG0019041: TcSYL_0128080.t1-p1
OG0019042: TcSYL_0128090.t1-p1

OG0019043: TcSYL_0128100.t1-p1
OG0019044: TcSYL_0128120.t1-p1
OG0019045: TcSYL_0128130.t1-p1
OG0019046: TcSYL_0128140.t1-p1
OG0019047: TcSYL_0128160.t1-p1
OG0019048: TcSYL_0128180.t1-p1
OG0019049: TcSYL_0128190.t1-p1
OG0019050: TcSYL_0128200.t1-p1
OG0019051: TcSYL_0128210.t1-p1
OG0019052: TcSYL_0128230.t1-p1
OG0019053: TcSYL_0128240.t1-p1
OG0019054: TcSYL_0128250.t1-p1
OG0019055: TcSYL_0128260.t1-p1
OG0019056: TcSYL_0128270.t1-p1
OG0019057: TcSYL_0128280.t1-p1
OG0019058: TcSYL_0128290.t1-p1
OG0019059: TcSYL_0128300.t1-p1
OG0019060: TcSYL_0128310.t1-p1
OG0019061: TcSYL_0128320.t1-p1
OG0019062: TcSYL_0128330.t1-p1
OG0019063: TcSYL_0128340.t1-p1
OG0019064: TcSYL_0128350.t1-p1
OG0019065: TcSYL_0128360.t1-p1
OG0019066: TcSYL_0128370.t1-p1
OG0019067: TcSYL_0128380.t1-p1
OG0019068: TcSYL_0128390.t1-p1
OG0019069: TcSYL_0128400.t1-p1
OG0019070: TcSYL_0128410.t1-p1
OG0019071: TcSYL_0128430.t1-p1
OG0019072: TcSYL_0128440.t1-p1
OG0019073: TcSYL_0128450.t1-p1
OG0019074: TcSYL_0128460.t1-p1
OG0019075: TcSYL_0128470.t1-p1
OG0019076: TcSYL_0128510.t1-p1
OG0019077: TcSYL_0128520.t1-p1
OG0019078: TcSYL_0128530.t1-p1
OG0019079: TcSYL_0128540.t1-p1
OG0019080: TcSYL_0128550.t1-p1
OG0019081: TcSYL_0128560.t1-p1
OG0019082: TcSYL_0128570.t1-p1
OG0019083: TcSYL_0128580.t1-p1
OG0019084: TcSYL_0128610.t1-p1
OG0019085: TcSYL_0128620.t1-p1
OG0019086: TcSYL_0128630.t1-p1
OG0019087: TcSYL_0128640.t1-p1
OG0019088: TcSYL_0128660.t1-p1
OG0019089: TcSYL_0128670.t1-p1
OG0019090: TcSYL_0128690.t1-p1
OG0019091: TcSYL_0128700.t1-p1
OG0019092: TcSYL_0128710.t1-p1
OG0019093: TcSYL_0128720.t1-p1
OG0019094: TcSYL_0128730.t1-p1
OG0019095: TcSYL_0128750.t1-p1
OG0019096: TcSYL_0128760.t1-p1

OG0019097: TcSYL_0128770.t1-p1
OG0019098: TcSYL_0128790.t1-p1
OG0019099: TcSYL_0128800.t1-p1
OG0019100: TcSYL_0128810.t1-p1
OG0019101: TcSYL_0128820.t1-p1
OG0019102: TcSYL_0128830.t1-p1
OG0019103: TcSYL_0128840.t1-p1
OG0019104: TcSYL_0128850.t1-p1
OG0019105: TcSYL_0128860.t1-p1
OG0019106: TcSYL_0128870.t1-p1
OG0019107: TcSYL_0128880.t1-p1
OG0019108: TcSYL_0128890.t1-p1
OG0019109: TcSYL_0128900.t1-p1
OG0019110: TcSYL_0128930.t1-p1
OG0019111: TcSYL_0128950.t1-p1
OG0019112: TcSYL_0128980.t1-p1
OG0019113: TcSYL_0128990.t1-p1
OG0019114: TcSYL_0129000.t1-p1
OG0019115: TcSYL_0129010.t1-p1
OG0019116: TcSYL_0129030.t1-p1
OG0019117: TcSYL_0129040.t1-p1
OG0019118: TcSYL_0129050.t1-p1
OG0019119: TcSYL_0129060.t1-p1
OG0019120: TcSYL_0129080.t1-p1
OG0019121: TcSYL_0129090.t1-p1
OG0019122: TcSYL_0129110.t1-p1
OG0019123: TcSYL_0129120.t1-p1
OG0019124: TcSYL_0129130.t1-p1
OG0019125: TcSYL_0129150.t1-p1
OG0019126: TcSYL_0129170.t1-p1
OG0019127: TcSYL_0129200.t1-p1
OG0019128: TcSYL_0129210.t1-p1
OG0019129: TcSYL_0129220.t1-p1
OG0019130: TcSYL_0129230.t1-p1
OG0019131: TcSYL_0129240.t1-p1
OG0019132: TcSYL_0129250.t1-p1
OG0019133: TcSYL_0129280.t1-p1
OG0019134: TcSYL_0129300.t1-p1
OG0019135: TcSYL_0129310.t1-p1
OG0019136: TcSYL_0129320.t1-p1
OG0019137: TcSYL_0129330.t1-p1
OG0019138: TcSYL_0129340.t1-p1
OG0019139: TcSYL_0129370.t1-p1
OG0019140: TcSYL_0129390.t1-p1
OG0019141: TcSYL_0129400.t1-p1
OG0019142: TcSYL_0129410.t1-p1
OG0019143: TcSYL_0129420.t1-p1
OG0019144: TcSYL_0129440.t1-p1
OG0019145: TcSYL_0129450.t1-p1
OG0019146: TcSYL_0129470.t1-p1
OG0019147: TcSYL_0129530.t1-p1
OG0019148: TcSYL_0129540.t1-p1
OG0019149: TcSYL_0129560.t1-p1
OG0019150: TcSYL_0129570.t1-p1

OG0019151: TcSYL_0129580.t1-p1
OG0019152: TcSYL_0129590.t1-p1
OG0019153: TcSYL_0129600.t1-p1
OG0019154: TcSYL_0129610.t1-p1
OG0019155: TcSYL_0129620.t1-p1
OG0019156: TcSYL_0129630.t1-p1
OG0019157: TcSYL_0129640.t1-p1
OG0019158: TcSYL_0129650.t1-p1
OG0019159: TcSYL_0129660.t1-p1
OG0019160: TcSYL_0129680.t1-p1
OG0019161: TcSYL_0129690.t1-p1
OG0019162: TcSYL_0129700.t1-p1
OG0019163: TcSYL_0129710.t1-p1
OG0019164: TcSYL_0129720.t1-p1
OG0019165: TcSYL_0129750.t1-p1
OG0019166: TcSYL_0129760.t1-p1
OG0019167: TcSYL_0129770.t1-p1
OG0019168: TcSYL_0129830.t1-p1
OG0019169: TcSYL_0129840.t1-p1
OG0019170: TcSYL_0129850.t1-p1
OG0019171: TcSYL_0129870.t1-p1
OG0019172: TcSYL_0129880.t1-p1
OG0019173: TcSYL_0129890.t1-p1
OG0019174: TcSYL_0129910.t1-p1
OG0019175: TcSYL_0129920.t1-p1
OG0019176: TcSYL_0129930.t1-p1
OG0019177: TcSYL_0129940.t1-p1
OG0019178: TcSYL_0129970.t1-p1
OG0019179: TcSYL_0129980.t1-p1
OG0019180: TcSYL_0130000.t1-p1
OG0019181: TcSYL_0130020.t1-p1
OG0019182: TcSYL_0130040.t1-p1
OG0019183: TcSYL_0130050.t1-p1
OG0019184: TcSYL_0130070.t1-p1
OG0019185: TcSYL_0130080.t1-p1
OG0019186: TcSYL_0130090.t1-p1
OG0019187: TcSYL_0130100.t1-p1
OG0019188: TcSYL_0130110.t1-p1
OG0019189: TcSYL_0130120.t1-p1
OG0019190: TcSYL_0130130.t1-p1
OG0019191: TcSYL_0130140.t1-p1
OG0019192: TcSYL_0130160.t1-p1
OG0019193: TcSYL_0130170.t1-p1
OG0019194: TcSYL_0130180.t1-p1
OG0019195: TcSYL_0130190.t1-p1
OG0019196: TcSYL_0130200.t1-p1
OG0019197: TcSYL_0130210.t1-p1
OG0019198: TcSYL_0130220.t1-p1
OG0019199: TcSYL_0130240.t1-p1
OG0019200: TcSYL_0130250.t1-p1
OG0019201: TcSYL_0130260.t1-p1
OG0019202: TcSYL_0130270.t1-p1
OG0019203: TcSYL_0130280.t1-p1
OG0019204: TcSYL_0130290.t1-p1

OG0019205: TcSYL_0130310.t1-p1
OG0019206: TcSYL_0130340.t1-p1
OG0019207: TcSYL_0130350.t1-p1
OG0019208: TcSYL_0130360.t1-p1
OG0019209: TcSYL_0130370.t1-p1
OG0019210: TcSYL_0130380.t1-p1
OG0019211: TcSYL_0130390.t1-p1
OG0019212: TcSYL_0130400.t1-p1
OG0019213: TcSYL_0130410.t1-p1
OG0019214: TcSYL_0130420.t1-p1
OG0019215: TcSYL_0130430.t1-p1
OG0019216: TcSYL_0130440.t1-p1
OG0019217: TcSYL_0130450.t1-p1
OG0019218: TcSYL_0130460.t1-p1
OG0019219: TcSYL_0130470.t1-p1
OG0019220: TcSYL_0130480.t1-p1
OG0019221: TcSYL_0130490.t1-p1
OG0019222: TcSYL_0130500.t1-p1
OG0019223: TcSYL_0130510.t1-p1
OG0019224: TcSYL_0130520.t1-p1
OG0019225: TcSYL_0130530.t1-p1
OG0019226: TcSYL_0130540.t1-p1
OG0019227: TcSYL_0130550.t1-p1
OG0019228: TcSYL_0130560.t1-p1
OG0019229: TcSYL_0130570.t1-p1
OG0019230: TcSYL_0130580.t1-p1
OG0019231: TcSYL_0130590.t1-p1
OG0019232: TcSYL_0130610.t1-p1
OG0019233: TcSYL_0130620.t1-p1
OG0019234: TcSYL_0130630.t1-p1
OG0019235: TcSYL_0130640.t1-p1
OG0019236: TcSYL_0130650.t1-p1
OG0019237: TcSYL_0130660.t1-p1
OG0019238: TcSYL_0130670.t1-p1
OG0019239: TcSYL_0130680.t1-p1
OG0019240: TcSYL_0130690.t1-p1
OG0019241: TcSYL_0130700.t1-p1
OG0019242: TcSYL_0130710.t1-p1
OG0019243: TcSYL_0130720.t1-p1
OG0019244: TcSYL_0130730.t1-p1
OG0019245: TcSYL_0130750.t1-p1
OG0019246: TcSYL_0130760.t1-p1
OG0019247: TcSYL_0130770.t1-p1
OG0019248: TcSYL_0130780.t1-p1
OG0019249: TcSYL_0130790.t1-p1
OG0019250: TcSYL_0130800.t1-p1
OG0019251: TcSYL_0130810.t1-p1
OG0019252: TcSYL_0130820.t1-p1
OG0019253: TcSYL_0130830.t1-p1
OG0019254: TcSYL_0130840.t1-p1
OG0019255: TcSYL_0130860.t1-p1
OG0019256: TcSYL_0130870.t1-p1
OG0019257: TcSYL_0130890.t1-p1
OG0019258: TcSYL_0130940.t1-p1

OG0019259: TcSYL_0130950.t1-p1
OG0019260: TcSYL_0130960.t1-p1
OG0019261: TcSYL_0130980.t1-p1
OG0019262: TcSYL_0130990.t1-p1
OG0019263: TcSYL_0131000.t1-p1
OG0019264: TcSYL_0131030.t1-p1
OG0019265: TcSYL_0131040.t1-p1
OG0019266: TcSYL_0131060.t1-p1
OG0019267: TcSYL_0131070.t1-p1
OG0019268: TcSYL_0131080.t1-p1
OG0019269: TcSYL_0131090.t1-p1
OG0019270: TcSYL_0131130.t1-p1
OG0019271: TcSYL_0131140.t1-p1
OG0019272: TcSYL_0131160.t1-p1
OG0019273: TcSYL_0131170.t1-p1
OG0019274: TcSYL_0131180.t1-p1
OG0019275: TcSYL_0131190.t1-p1
OG0019276: TcSYL_0131210.t1-p1
OG0019277: TcSYL_0131220.t1-p1
OG0019278: TcSYL_0131230.t1-p1
OG0019279: TcSYL_0131250.t1-p1
OG0019280: TcSYL_0131260.t1-p1
OG0019281: TcSYL_0131300.t1-p1
OG0019282: TcSYL_0131310.t1-p1
OG0019283: TcSYL_0131330.t1-p1
OG0019284: TcSYL_0131340.t1-p1
OG0019285: TcSYL_0131370.t1-p1
OG0019286: TcSYL_0131390.t1-p1
OG0019287: TcSYL_0131430.t1-p1
OG0019288: TcSYL_0131460.t1-p1
OG0019289: TcSYL_0131470.t1-p1
OG0019290: TcSYL_0131500.t1-p1
OG0019291: TcSYL_0131510.t1-p1
OG0019292: TcSYL_0131520.t1-p1
OG0019293: TcSYL_0131530.t1-p1
OG0019294: TcSYL_0131540.t1-p1
OG0019295: TcSYL_0131550.t1-p1
OG0019296: TcSYL_0131560.t1-p1
OG0019297: TcSYL_0131570.t1-p1
OG0019298: TcSYL_0131590.t1-p1
OG0019299: TcSYL_0131610.t1-p1
OG0019300: TcSYL_0131620.t1-p1
OG0019301: TcSYL_0131640.t1-p1
OG0019302: TcSYL_0131650.t1-p1
OG0019303: TcSYL_0131670.t1-p1
OG0019304: TcSYL_0131680.t1-p1
OG0019305: TcSYL_0131690.t1-p1
OG0019306: TcSYL_0131710.t1-p1
OG0019307: TcSYL_0131720.t1-p1
OG0019308: TcSYL_0131730.t1-p1
OG0019309: TcSYL_0131740.t1-p1
OG0019310: TcSYL_0131750.t1-p1
OG0019311: TcSYL_0131760.t1-p1
OG0019312: TcSYL_0131780.t1-p1

OG0019313: TcSYL_0131790.t1-p1
OG0019314: TcSYL_0131800.t1-p1
OG0019315: TcSYL_0131810.t1-p1
OG0019316: TcSYL_0131820.t1-p1
OG0019317: TcSYL_0131830.t1-p1
OG0019318: TcSYL_0131840.t1-p1
OG0019319: TcSYL_0131850.t1-p1
OG0019320: TcSYL_0131860.t1-p1
OG0019321: TcSYL_0131870.t1-p1
OG0019322: TcSYL_0131880.t1-p1
OG0019323: TcSYL_0131890.t1-p1
OG0019324: TcSYL_0131900.t1-p1
OG0019325: TcSYL_0131920.t1-p1
OG0019326: TcSYL_0131930.t1-p1
OG0019327: TcSYL_0131940.t1-p1
OG0019328: TcSYL_0131950.t1-p1
OG0019329: TcSYL_0131960.t1-p1
OG0019330: TcSYL_0131980.t1-p1
OG0019331: TcSYL_0131990.t1-p1
OG0019332: TcSYL_0132000.t1-p1
OG0019333: TcSYL_0132020.t1-p1
OG0019334: TcSYL_0132030.t1-p1
OG0019335: TcSYL_0132040.t1-p1
OG0019336: TcSYL_0132050.t1-p1
OG0019337: TcSYL_0132070.t1-p1
OG0019338: TcSYL_0132080.t1-p1
OG0019339: TcSYL_0132090.t1-p1
OG0019340: TcSYL_0132100.t1-p1
OG0019341: TcSYL_0132110.t1-p1
OG0019342: TcSYL_0132120.t1-p1
OG0019343: TcSYL_0132130.t1-p1
OG0019344: TcSYL_0132150.t1-p1
OG0019345: TcSYL_0132160.t1-p1
OG0019346: TcSYL_0132170.t1-p1
OG0019347: TcSYL_0132190.t1-p1
OG0019348: TcSYL_0132200.t1-p1
OG0019349: TcSYL_0132210.t1-p1
OG0019350: TcSYL_0132220.t1-p1
OG0019351: TcSYL_0132230.t1-p1
OG0019352: TcSYL_0132240.t1-p1
OG0019353: TcSYL_0132250.t1-p1
OG0019354: TcSYL_0132260.t1-p1
OG0019355: TcSYL_0132280.t1-p1
OG0019356: TcSYL_0132290.t1-p1
OG0019357: TcSYL_0132310.t1-p1
OG0019358: TcSYL_0132320.t1-p1
OG0019359: TcSYL_0132330.t1-p1
OG0019360: TcSYL_0132350.t1-p1
OG0019361: TcSYL_0132360.t1-p1
OG0019362: TcSYL_0132380.t1-p1
OG0019363: TcSYL_0132390.t1-p1
OG0019364: TcSYL_0132410.t1-p1
OG0019365: TcSYL_0132420.t1-p1
OG0019366: TcSYL_0132440.t1-p1

OG0019367: TcSYL_0132450.t1-p1
OG0019368: TcSYL_0132460.t1-p1
OG0019369: TcSYL_0132480.t1-p1
OG0019370: TcSYL_0132490.t1-p1
OG0019371: TcSYL_0132500.t1-p1
OG0019372: TcSYL_0132510.t1-p1
OG0019373: TcSYL_0132530.t1-p1
OG0019374: TcSYL_0132540.t1-p1
OG0019375: TcSYL_0132550.t1-p1
OG0019376: TcSYL_0132560.t1-p1
OG0019377: TcSYL_0132570.t1-p1
OG0019378: TcSYL_0132580.t1-p1
OG0019379: TcSYL_0132590.t1-p1
OG0019380: TcSYL_0132610.t1-p1
OG0019381: TcSYL_0132620.t1-p1
OG0019382: TcSYL_0132640.t1-p1
OG0019383: TcSYL_0132660.t1-p1
OG0019384: TcSYL_0132670.t1-p1
OG0019385: TcSYL_0132680.t1-p1
OG0019386: TcSYL_0132700.t1-p1
OG0019387: TcSYL_0132710.t1-p1
OG0019388: TcSYL_0132730.t1-p1
OG0019389: TcSYL_0132740.t1-p1
OG0019390: TcSYL_0132750.t1-p1
OG0019391: TcSYL_0132760.t1-p1
OG0019392: TcSYL_0132780.t1-p1
OG0019393: TcSYL_0132790.t1-p1
OG0019394: TcSYL_0132850.t1-p1
OG0019395: TcSYL_0132870.t1-p1
OG0019396: TcSYL_0132980.t1-p1
OG0019397: TcSYL_0132990.t1-p1
OG0019398: TcSYL_0133020.t1-p1
OG0019399: TcSYL_0133050.t1-p1
OG0019400: TcSYL_0133060.t1-p1
OG0019401: TcSYL_0133070.t1-p1
OG0019402: TcSYL_0133090.t1-p1
OG0019403: TcSYL_0133100.t1-p1
OG0019404: TcSYL_0133110.t1-p1
OG0019405: TcSYL_0133120.t1-p1
OG0019406: TcSYL_0133130.t1-p1
OG0019407: TcSYL_0133150.t1-p1
OG0019408: TcSYL_0133160.t1-p1
OG0019409: TcSYL_0133170.t1-p1
OG0019410: TcSYL_0133180.t1-p1
OG0019411: TcSYL_0133190.t1-p1
OG0019412: TcSYL_0133200.t1-p1
OG0019413: TcSYL_0133210.t1-p1
OG0019414: TcSYL_0133220.t1-p1
OG0019415: TcSYL_0133230.t1-p1
OG0019416: TcSYL_0133240.t1-p1
OG0019417: TcSYL_0133250.t1-p1
OG0019418: TcSYL_0133270.t1-p1
OG0019419: TcSYL_0133300.t1-p1
OG0019420: TcSYL_0133320.t1-p1

OG0019421: TcSYL_0133340.t1-p1
OG0019422: TcSYL_0133370.t1-p1
OG0019423: TcSYL_0133380.t1-p1
OG0019424: TcSYL_0133400.t1-p1
OG0019425: TcSYL_0133410.t1-p1
OG0019426: TcSYL_0133430.t1-p1
OG0019427: TcSYL_0133440.t1-p1
OG0019428: TcSYL_0133450.t1-p1
OG0019429: TcSYL_0133470.t1-p1
OG0019430: TcSYL_0133480.t1-p1
OG0019431: TcSYL_0133500.t1-p1
OG0019432: TcSYL_0133510.t1-p1
OG0019433: TcSYL_0133520.t1-p1
OG0019434: TcSYL_0133530.t1-p1
OG0019435: TcSYL_0133540.t1-p1
OG0019436: TcSYL_0133550.t1-p1
OG0019437: TcSYL_0133560.t1-p1
OG0019438: TcSYL_0133580.t1-p1
OG0019439: TcSYL_0133590.t1-p1
OG0019440: TcSYL_0133600.t1-p1
OG0019441: TcSYL_0133620.t1-p1
OG0019442: TcSYL_0133630.t1-p1
OG0019443: TcSYL_0133670.t1-p1
OG0019444: TcSYL_0133680.t1-p1
OG0019445: TcSYL_0133700.t1-p1
OG0019446: TcSYL_0133720.t1-p1
OG0019447: TcSYL_0133730.t1-p1
OG0019448: TcSYL_0133740.t1-p1
OG0019449: TcSYL_0133750.t1-p1
OG0019450: TcSYL_0133760.t1-p1
OG0019451: TcSYL_0133770.t1-p1
OG0019452: TcSYL_0133780.t1-p1
OG0019453: TcSYL_0133790.t1-p1
OG0019454: TcSYL_0133800.t1-p1
OG0019455: TcSYL_0133810.t1-p1
OG0019456: TcSYL_0133820.t1-p1
OG0019457: TcSYL_0133830.t1-p1
OG0019458: TcSYL_0133840.t1-p1
OG0019459: TcSYL_0133850.t1-p1
OG0019460: TcSYL_0133860.t1-p1
OG0019461: TcSYL_0133900.t1-p1
OG0019462: TcSYL_0133930.t1-p1
OG0019463: TcSYL_0133940.t1-p1
OG0019464: TcSYL_0133950.t1-p1
OG0019465: TcSYL_0133960.t1-p1
OG0019466: TcSYL_0133980.t1-p1
OG0019467: TcSYL_0134000.t1-p1
OG0019468: TcSYL_0134010.t1-p1
OG0019469: TcSYL_0134020.t1-p1
OG0019470: TcSYL_0134040.t1-p1
OG0019471: TcSYL_0134050.t1-p1
OG0019472: TcSYL_0134070.t1-p1
OG0019473: TcSYL_0134080.t1-p1
OG0019474: TcSYL_0134090.t1-p1

OG0019475: TcSYL_0134110.t1-p1
OG0019476: TcSYL_0134120.t1-p1
OG0019477: TcSYL_0134140.t1-p1
OG0019478: TcSYL_0134150.t1-p1
OG0019479: TcSYL_0134160.t1-p1
OG0019480: TcSYL_0134170.t1-p1
OG0019481: TcSYL_0134180.t1-p1
OG0019482: TcSYL_0134210.t1-p1
OG0019483: TcSYL_0134220.t1-p1
OG0019484: TcSYL_0134230.t1-p1
OG0019485: TcSYL_0134240.t1-p1
OG0019486: TcSYL_0134250.t1-p1
OG0019487: TcSYL_0134260.t1-p1
OG0019488: TcSYL_0134280.t1-p1
OG0019489: TcSYL_0134300.t1-p1
OG0019490: TcSYL_0134310.t1-p1
OG0019491: TcSYL_0134330.t1-p1
OG0019492: TcSYL_0134340.t1-p1
OG0019493: TcSYL_0134350.t1-p1
OG0019494: TcSYL_0134360.t1-p1
OG0019495: TcSYL_0134390.t1-p1
OG0019496: TcSYL_0134400.t1-p1
OG0019497: TcSYL_0134410.t1-p1
OG0019498: TcSYL_0134420.t1-p1
OG0019499: TcSYL_0134430.t1-p1
OG0019500: TcSYL_0134440.t1-p1
OG0019501: TcSYL_0134450.t1-p1
OG0019502: TcSYL_0134460.t1-p1
OG0019503: TcSYL_0134480.t1-p1
OG0019504: TcSYL_0134490.t1-p1
OG0019505: TcSYL_0134500.t1-p1
OG0019506: TcSYL_0134510.t1-p1
OG0019507: TcSYL_0134530.t1-p1
OG0019508: TcSYL_0134540.t1-p1
OG0019509: TcSYL_0134550.t1-p1
OG0019510: TcSYL_0134560.t1-p1
OG0019511: TcSYL_0134570.t1-p1
OG0019512: TcSYL_0134580.t1-p1
OG0019513: TcSYL_0134590.t1-p1
OG0019514: TcSYL_0134600.t1-p1
OG0019515: TcSYL_0134640.t1-p1
OG0019516: TcSYL_0134660.t1-p1
OG0019517: TcSYL_0134670.t1-p1
OG0019518: TcSYL_0134680.t1-p1
OG0019519: TcSYL_0134720.t1-p1
OG0019520: TcSYL_0134740.t1-p1
OG0019521: TcSYL_0134750.t1-p1
OG0019522: TcSYL_0134760.t1-p1
OG0019523: TcSYL_0134770.t1-p1
OG0019524: TcSYL_0134780.t1-p1
OG0019525: TcSYL_0134790.t1-p1
OG0019526: TcSYL_0134800.t1-p1
OG0019527: TcSYL_0134820.t1-p1
OG0019528: TcSYL_0134830.t1-p1

OG0019529: TcSYL_0134840.t1-p1
OG0019530: TcSYL_0134850.t1-p1
OG0019531: TcSYL_0134860.t1-p1
OG0019532: TcSYL_0134880.t1-p1
OG0019533: TcSYL_0134900.t1-p1
OG0019534: TcSYL_0134910.t1-p1
OG0019535: TcSYL_0134920.t1-p1
OG0019536: TcSYL_0134960.t1-p1
OG0019537: TcSYL_0134970.t1-p1
OG0019538: TcSYL_0134980.t1-p1
OG0019539: TcSYL_0134990.t1-p1
OG0019540: TcSYL_0135010.t1-p1
OG0019541: TcSYL_0135020.t1-p1
OG0019542: TcSYL_0135030.t1-p1
OG0019543: TcSYL_0135040.t1-p1
OG0019544: TcSYL_0135050.t1-p1
OG0019545: TcSYL_0135060.t1-p1
OG0019546: TcSYL_0135070.t1-p1
OG0019547: TcSYL_0135080.t1-p1
OG0019548: TcSYL_0135090.t1-p1
OG0019549: TcSYL_0135100.t1-p1
OG0019550: TcSYL_0135110.t1-p1
OG0019551: TcSYL_0135120.t1-p1
OG0019552: TcSYL_0135130.t1-p1
OG0019553: TcSYL_0135140.t1-p1
OG0019554: TcSYL_0135160.t1-p1
OG0019555: TcSYL_0135170.t1-p1
OG0019556: TcSYL_0135180.t1-p1
OG0019557: TcSYL_0135190.t1-p1
OG0019558: TcSYL_0135200.t1-p1
OG0019559: TcSYL_0135210.t1-p1
OG0019560: TcSYL_0135220.t1-p1
OG0019561: TcSYL_0135230.t1-p1
OG0019562: TcSYL_0135240.t1-p1
OG0019563: TcSYL_0135250.t1-p1
OG0019564: TcSYL_0135260.t1-p1
OG0019565: TcSYL_0135270.t1-p1
OG0019566: TcSYL_0135280.t1-p1
OG0019567: TcSYL_0135300.t1-p1
OG0019568: TcSYL_0135310.t1-p1
OG0019569: TcSYL_0135320.t1-p1
OG0019570: TcSYL_0135330.t1-p1
OG0019571: TcSYL_0135360.t1-p1
OG0019572: TcSYL_0135370.t1-p1
OG0019573: TcSYL_0135380.t1-p1
OG0019574: TcSYL_0135390.t1-p1
OG0019575: TcSYL_0135400.t1-p1
OG0019576: TcSYL_0135430.t1-p1
OG0019577: TcSYL_0135440.t1-p1
OG0019578: TcSYL_0135450.t1-p1
OG0019579: TcSYL_0135470.t1-p1
OG0019580: TcSYL_0135490.t1-p1
OG0019581: TcSYL_0135500.t1-p1
OG0019582: TcSYL_0135520.t1-p1

OG0019583: TcSYL_0135530.t1-p1
OG0019584: TcSYL_0135540.t1-p1
OG0019585: TcSYL_0135550.t1-p1
OG0019586: TcSYL_0135560.t1-p1
OG0019587: TcSYL_0135570.t1-p1
OG0019588: TcSYL_0135580.t1-p1
OG0019589: TcSYL_0135590.t1-p1
OG0019590: TcSYL_0135600.t1-p1
OG0019591: TcSYL_0135620.t1-p1
OG0019592: TcSYL_0135640.t1-p1
OG0019593: TcSYL_0135660.t1-p1
OG0019594: TcSYL_0135670.t1-p1
OG0019595: TcSYL_0135680.t1-p1
OG0019596: TcSYL_0135690.t1-p1
OG0019597: TcSYL_0135700.t1-p1
OG0019598: TcSYL_0135720.t1-p1
OG0019599: TcSYL_0135730.t1-p1
OG0019600: TcSYL_0135740.t1-p1
OG0019601: TcSYL_0135760.t1-p1
OG0019602: TcSYL_0135770.t1-p1
OG0019603: TcSYL_0135790.t1-p1
OG0019604: TcSYL_0135800.t1-p1
OG0019605: TcSYL_0135810.t1-p1
OG0019606: TcSYL_0135820.t1-p1
OG0019607: TcSYL_0135830.t1-p1
OG0019608: TcSYL_0135840.t1-p1
OG0019609: TcSYL_0135850.t1-p1
OG0019610: TcSYL_0135860.t1-p1
OG0019611: TcSYL_0135880.t1-p1
OG0019612: TcSYL_0135900.t1-p1
OG0019613: TcSYL_0135910.t1-p1
OG0019614: TcSYL_0135930.t1-p1
OG0019615: TcSYL_0135940.t1-p1
OG0019616: TcSYL_0135950.t1-p1
OG0019617: TcSYL_0135960.t1-p1
OG0019618: TcSYL_0135970.t1-p1
OG0019619: TcSYL_0135990.t1-p1
OG0019620: TcSYL_0136000.t1-p1
OG0019621: TcSYL_0136010.t1-p1
OG0019622: TcSYL_0136020.t1-p1
OG0019623: TcSYL_0136030.t1-p1
OG0019624: TcSYL_0136040.t1-p1
OG0019625: TcSYL_0136050.t1-p1
OG0019626: TcSYL_0136060.t1-p1
OG0019627: TcSYL_0136070.t1-p1
OG0019628: TcSYL_0136090.t1-p1
OG0019629: TcSYL_0136100.t1-p1
OG0019630: TcSYL_0136110.t1-p1
OG0019631: TcSYL_0136120.t1-p1
OG0019632: TcSYL_0136140.t1-p1
OG0019633: TcSYL_0136150.t1-p1
OG0019634: TcSYL_0136170.t1-p1
OG0019635: TcSYL_0136190.t1-p1
OG0019636: TcSYL_0136200.t1-p1

OG0019637: TcSYL_0136210.t1-p1
OG0019638: TcSYL_0136230.t1-p1
OG0019639: TcSYL_0136240.t1-p1
OG0019640: TcSYL_0136250.t1-p1
OG0019641: TcSYL_0136260.t1-p1
OG0019642: TcSYL_0136270.t1-p1
OG0019643: TcSYL_0136280.t1-p1
OG0019644: TcSYL_0136290.t1-p1
OG0019645: TcSYL_0136300.t1-p1
OG0019646: TcSYL_0136310.t1-p1
OG0019647: TcSYL_0136330.t1-p1
OG0019648: TcSYL_0136360.t1-p1
OG0019649: TcSYL_0136370.t1-p1
OG0019650: TcSYL_0136380.t1-p1
OG0019651: TcSYL_0136410.t1-p1
OG0019652: TcSYL_0136430.t1-p1
OG0019653: TcSYL_0136450.t1-p1
OG0019654: TcSYL_0136460.t1-p1
OG0019655: TcSYL_0136480.t1-p1
OG0019656: TcSYL_0136500.t1-p1
OG0019657: TcSYL_0136510.t1-p1
OG0019658: TcSYL_0136540.t1-p1
OG0019659: TcSYL_0136560.t1-p1
OG0019660: TcSYL_0136570.t1-p1
OG0019661: TcSYL_0136580.t1-p1
OG0019662: TcSYL_0136590.t1-p1
OG0019663: TcSYL_0136610.t1-p1
OG0019664: TcSYL_0136620.t1-p1
OG0019665: TcSYL_0136630.t1-p1
OG0019666: TcSYL_0136660.t1-p1
OG0019667: TcSYL_0136670.t1-p1
OG0019668: TcSYL_0136680.t1-p1
OG0019669: TcSYL_0136690.t1-p1
OG0019670: TcSYL_0136700.t1-p1
OG0019671: TcSYL_0136710.t1-p1
OG0019672: TcSYL_0136720.t1-p1
OG0019673: TcSYL_0136730.t1-p1
OG0019674: TcSYL_0136740.t1-p1
OG0019675: TcSYL_0136760.t1-p1
OG0019676: TcSYL_0136770.t1-p1
OG0019677: TcSYL_0136780.t1-p1
OG0019678: TcSYL_0136790.t1-p1
OG0019679: TcSYL_0136800.t1-p1
OG0019680: TcSYL_0136810.t1-p1
OG0019681: TcSYL_0136820.t1-p1
OG0019682: TcSYL_0136830.t1-p1
OG0019683: TcSYL_0136840.t1-p1
OG0019684: TcSYL_0136860.t1-p1
OG0019685: TcSYL_0136880.t1-p1
OG0019686: TcSYL_0136890.t1-p1
OG0019687: TcSYL_0136920.t1-p1
OG0019688: TcSYL_0136930.t1-p1
OG0019689: TcSYL_0136940.t1-p1
OG0019690: TcSYL_0136950.t1-p1

OG0019691: TcSYL_0136980.t1-p1
OG0019692: TcSYL_0136990.t1-p1
OG0019693: TcSYL_0137000.t1-p1
OG0019694: TcSYL_0137020.t1-p1
OG0019695: TcSYL_0137030.t1-p1
OG0019696: TcSYL_0137040.t1-p1
OG0019697: TcSYL_0137050.t1-p1
OG0019698: TcSYL_0137060.t1-p1
OG0019699: TcSYL_0137080.t1-p1
OG0019700: TcSYL_0137100.t1-p1
OG0019701: TcSYL_0137130.t1-p1
OG0019702: TcSYL_0137150.t1-p1
OG0019703: TcSYL_0137170.t1-p1
OG0019704: TcSYL_0137190.t1-p1
OG0019705: TcSYL_0137230.t1-p1
OG0019706: TcSYL_0137240.t1-p1
OG0019707: TcSYL_0137250.t1-p1
OG0019708: TcSYL_0137260.t1-p1
OG0019709: TcSYL_0137270.t1-p1
OG0019710: TcSYL_0137280.t1-p1
OG0019711: TcSYL_0137290.t1-p1
OG0019712: TcSYL_0137300.t1-p1
OG0019713: TcSYL_0137310.t1-p1
OG0019714: TcSYL_0137320.t1-p1
OG0019715: TcSYL_0137330.t1-p1
OG0019716: TcSYL_0137340.t1-p1
OG0019717: TcSYL_0137350.t1-p1
OG0019718: TcSYL_0137360.t1-p1
OG0019719: TcSYL_0137380.t1-p1
OG0019720: TcSYL_0137400.t1-p1
OG0019721: TcSYL_0137430.t1-p1
OG0019722: TcSYL_0137450.t1-p1
OG0019723: TcSYL_0137470.t1-p1
OG0019724: TcSYL_0137480.t1-p1
OG0019725: TcSYL_0137490.t1-p1
OG0019726: TcSYL_0137500.t1-p1
OG0019727: TcSYL_0137520.t1-p1
OG0019728: TcSYL_0137530.t1-p1
OG0019729: TcSYL_0137540.t1-p1
OG0019730: TcSYL_0137550.t1-p1
OG0019731: TcSYL_0137560.t1-p1
OG0019732: TcSYL_0137580.t1-p1
OG0019733: TcSYL_0137610.t1-p1
OG0019734: TcSYL_0137630.t1-p1
OG0019735: TcSYL_0137650.t1-p1
OG0019736: TcSYL_0137670.t1-p1
OG0019737: TcSYL_0137680.t1-p1
OG0019738: TcSYL_0137690.t1-p1
OG0019739: TcSYL_0137710.t1-p1
OG0019740: TcSYL_0137720.t1-p1
OG0019741: TcSYL_0137730.t1-p1
OG0019742: TcSYL_0137740.t1-p1
OG0019743: TcSYL_0137750.t1-p1
OG0019744: TcSYL_0137770.t1-p1

OG0019745: TcSYL_0137780.t1-p1
OG0019746: TcSYL_0137790.t1-p1
OG0019747: TcSYL_0137800.t1-p1
OG0019748: TcSYL_0137810.t1-p1
OG0019749: TcSYL_0137820.t1-p1
OG0019750: TcSYL_0137900.t1-p1
OG0019751: TcSYL_0137980.t1-p1
OG0019752: TcSYL_0137990.t1-p1
OG0019753: TcSYL_0138000.t1-p1
OG0019754: TcSYL_0138010.t1-p1
OG0019755: TcSYL_0138020.t1-p1
OG0019756: TcSYL_0138030.t1-p1
OG0019757: TcSYL_0138050.t1-p1
OG0019758: TcSYL_0138070.t1-p1
OG0019759: TcSYL_0138090.t1-p1
OG0019760: TcSYL_0138100.t1-p1
OG0019761: TcSYL_0138120.t1-p1
OG0019762: TcSYL_0138130.t1-p1
OG0019763: TcSYL_0138150.t1-p1
OG0019764: TcSYL_0138160.t1-p1
OG0019765: TcSYL_0138180.t1-p1
OG0019766: TcSYL_0138200.t1-p1
OG0019767: TcSYL_0138220.t1-p1
OG0019768: TcSYL_0138230.t1-p1
OG0019769: TcSYL_0138240.t1-p1
OG0019770: TcSYL_0138250.t1-p1
OG0019771: TcSYL_0138320.t1-p1
OG0019772: TcSYL_0138480.t1-p1
OG0019773: TcSYL_0138590.t1-p1
OG0019774: TcSYL_0138640.t1-p1
OG0019775: TcSYL_0138650.t1-p1
OG0019776: TcSYL_0138660.t1-p1
OG0019777: TcSYL_0138680.t1-p1
OG0019778: TcSYL_0138700.t1-p1
OG0019779: TcSYL_0138740.t1-p1
OG0019780: TcSYL_0138770.t1-p1
OG0019781: TcSYL_0138780.t1-p1
OG0019782: TcSYL_0138790.t1-p1
OG0019783: TcSYL_0138800.t1-p1
OG0019784: TcSYL_0138830.t1-p1
OG0019785: TcSYL_0138880.t1-p1
OG0019786: TcSYL_0138890.t1-p1
OG0019787: TcSYL_0138900.t1-p1
OG0019788: TcSYL_0138920.t1-p1
OG0019789: TcSYL_0138940.t1-p1
OG0019790: TcSYL_0138960.t1-p1
OG0019791: TcSYL_0138970.t1-p1
OG0019792: TcSYL_0138990.t1-p1
OG0019793: TcSYL_0139000.t1-p1
OG0019794: TcSYL_0139010.t1-p1
OG0019795: TcSYL_0139040.t1-p1
OG0019796: TcSYL_0139060.t1-p1
OG0019797: TcSYL_0139070.t1-p1
OG0019798: TcSYL_0139080.t1-p1

OG0019799: TcSYL_0139280.t1-p1
OG0019800: TcSYL_0139340.t1-p1
OG0019801: TcSYL_0139350.t1-p1
OG0019802: TcSYL_0139380.t1-p1
OG0019803: TcSYL_0139510.t1-p1
OG0019804: TcSYL_0139760.t1-p1
OG0019805: TcSYL_0139800.t1-p1
OG0019806: TcSYL_0139820.t1-p1
OG0019807: TcSYL_0139940.t1-p1
OG0019808: TcSYL_0139950.t1-p1
OG0019809: TcSYL_0139970.t1-p1
OG0019810: TcSYL_0139980.t1-p1
OG0019811: TcSYL_0140000.t1-p1
OG0019812: TcSYL_0140010.t1-p1
OG0019813: TcSYL_0140090.t1-p1
OG0019814: TcSYL_0140100.t1-p1
OG0019815: TcSYL_0140110.t1-p1
OG0019816: TcSYL_0140170.t1-p1
OG0019817: TcSYL_0140190.t1-p1
OG0019818: TcSYL_0140210.t1-p1
OG0019819: TcSYL_0140260.t1-p1
OG0019820: TcSYL_0140270.t1-p1
OG0019821: TcSYL_0140280.t1-p1
OG0019822: TcSYL_0140290.t1-p1
OG0019823: TcSYL_0140310.t1-p1
OG0019824: TcSYL_0140330.t1-p1
OG0019825: TcSYL_0140340.t1-p1
OG0019826: TcSYL_0140370.t1-p1
OG0019827: TcSYL_0140490.t1-p1
OG0019828: TcSYL_0140580.t1-p1
OG0019829: TcSYL_0140600.t1-p1
OG0019830: TcSYL_0140610.t1-p1
OG0019831: TcSYL_0140620.t1-p1
OG0019832: TcSYL_0140630.t1-p1
OG0019833: TcSYL_0140650.t1-p1
OG0019834: TcSYL_0140670.t1-p1
OG0019835: TcSYL_0140810.t1-p1
OG0019836: TcSYL_0140820.t1-p1
OG0019837: TcSYL_0140850.t1-p1
OG0019838: TcSYL_0140860.t1-p1
OG0019839: TcSYL_0140880.t1-p1
OG0019840: TcSYL_0140890.t1-p1
OG0019841: TcSYL_0140910.t1-p1
OG0019842: TcSYL_0140920.t1-p1
OG0019843: TcSYL_0140930.t1-p1
OG0019844: TcSYL_0140940.t1-p1
OG0019845: TcSYL_0140950.t1-p1
OG0019846: TcSYL_0140960.t1-p1
OG0019847: TcSYL_0140970.t1-p1
OG0019848: TcSYL_0140990.t1-p1
OG0019849: TcSYL_0141000.t1-p1
OG0019850: TcSYL_0141010.t1-p1
OG0019851: TcSYL_0141020.t1-p1
OG0019852: TcSYL_0141040.t1-p1

OG0019853: TcSYL_0141050.t1-p1
OG0019854: TcSYL_0141060.t1-p1
OG0019855: TcSYL_0141070.t1-p1
OG0019856: TcSYL_0141090.t1-p1
OG0019857: TcSYL_0141100.t1-p1
OG0019858: TcSYL_0141110.t1-p1
OG0019859: TcSYL_0141120.t1-p1
OG0019860: TcSYL_0141130.t1-p1
OG0019861: TcSYL_0141140.t1-p1
OG0019862: TcSYL_0141150.t1-p1
OG0019863: TcSYL_0141160.t1-p1
OG0019864: TcSYL_0141170.t1-p1
OG0019865: TcSYL_0141190.t1-p1
OG0019866: TcSYL_0141200.t1-p1
OG0019867: TcSYL_0141210.t1-p1
OG0019868: TcSYL_0141220.t1-p1
OG0019869: TcSYL_0141230.t1-p1
OG0019870: TcSYL_0141240.t1-p1
OG0019871: TcSYL_0141250.t1-p1
OG0019872: TcSYL_0141260.t1-p1
OG0019873: TcSYL_0141270.t1-p1
OG0019874: TcSYL_0141280.t1-p1
OG0019875: TcSYL_0141290.t1-p1
OG0019876: TcSYL_0141300.t1-p1
OG0019877: TcSYL_0141310.t1-p1
OG0019878: TcSYL_0141320.t1-p1
OG0019879: TcSYL_0141330.t1-p1
OG0019880: TcSYL_0141340.t1-p1
OG0019881: TcSYL_0141360.t1-p1
OG0019882: TcSYL_0141380.t1-p1
OG0019883: TcSYL_0141390.t1-p1
OG0019884: TcSYL_0141410.t1-p1
OG0019885: TcSYL_0141420.t1-p1
OG0019886: TcSYL_0141430.t1-p1
OG0019887: TcSYL_0141450.t1-p1
OG0019888: TcSYL_0141480.t1-p1
OG0019889: TcSYL_0141490.t1-p1
OG0019890: TcSYL_0141500.t1-p1
OG0019891: TcSYL_0141510.t1-p1
OG0019892: TcSYL_0141520.t1-p1
OG0019893: TcSYL_0141540.t1-p1
OG0019894: TcSYL_0141550.t1-p1
OG0019895: TcSYL_0141560.t1-p1
OG0019896: TcSYL_0141570.t1-p1
OG0019897: TcSYL_0141580.t1-p1
OG0019898: TcSYL_0141600.t1-p1
OG0019899: TcSYL_0141610.t1-p1
OG0019900: TcSYL_0141620.t1-p1
OG0019901: TcSYL_0141900.t1-p1
OG0019902: TcSYL_0141950.t1-p1
OG0019903: TcSYL_0141960.t1-p1
OG0019904: TcSYL_0141990.t1-p1
OG0019905: TcSYL_0142000.t1-p1
OG0019906: TcSYL_0142010.t1-p1

OG0019907: TcSYL_0142020.t1-p1
OG0019908: TcSYL_0142040.t1-p1
OG0019909: TcSYL_0142050.t1-p1
OG0019910: TcSYL_0142060.t1-p1
OG0019911: TcSYL_0142070.t1-p1
OG0019912: TcSYL_0142090.t1-p1
OG0019913: TcSYL_0142100.t1-p1
OG0019914: TcSYL_0142120.t1-p1
OG0019915: TcSYL_0142130.t1-p1
OG0019916: TcSYL_0142160.t1-p1
OG0019917: TcSYL_0142190.t1-p1
OG0019918: TcSYL_0142210.t1-p1
OG0019919: TcSYL_0142230.t1-p1
OG0019920: TcSYL_0142240.t1-p1
OG0019921: TcSYL_0142250.t1-p1
OG0019922: TcSYL_0142280.t1-p1
OG0019923: TcSYL_0142290.t1-p1
OG0019924: TcSYL_0142310.t1-p1
OG0019925: TcSYL_0142320.t1-p1
OG0019926: TcSYL_0142330.t1-p1
OG0019927: TcSYL_0142390.t1-p1
OG0019928: TcSYL_0142400.t1-p1
OG0019929: TcSYL_0142420.t1-p1
OG0019930: TcSYL_0142430.t1-p1
OG0019931: TcSYL_0142440.t1-p1
OG0019932: TcSYL_0142460.t1-p1
OG0019933: TcSYL_0142470.t1-p1
OG0019934: TcSYL_0142490.t1-p1
OG0019935: TcSYL_0142500.t1-p1
OG0019936: TcSYL_0142640.t1-p1
OG0019937: TcSYL_0142690.t1-p1
OG0019938: TcSYL_0142810.t1-p1
OG0019939: TcSYL_0142870.t1-p1
OG0019940: TcSYL_0142890.t1-p1
OG0019941: TcSYL_0142910.t1-p1
OG0019942: TcSYL_0142930.t1-p1
OG0019943: TcSYL_0142950.t1-p1
OG0019944: TcSYL_0142970.t1-p1
OG0019945: TcSYL_0142980.t1-p1
OG0019946: TcSYL_0142990.t1-p1
OG0019947: TcSYL_0143000.t1-p1
OG0019948: TcSYL_0143020.t1-p1
OG0019949: TcSYL_0143030.t1-p1
OG0019950: TcSYL_0143050.t1-p1
OG0019951: TcSYL_0143080.t1-p1
OG0019952: TcSYL_0143100.t1-p1
OG0019953: TcSYL_0143130.t1-p1
OG0019954: TcSYL_0143140.t1-p1
OG0019955: TcSYL_0143150.t1-p1
OG0019956: TcSYL_0143160.t1-p1
OG0019957: TcSYL_0143170.t1-p1
OG0019958: TcSYL_0143180.t1-p1
OG0019959: TcSYL_0143190.t1-p1
OG0019960: TcSYL_0143200.t1-p1

OG0019961: TcSYL_0143210.t1-p1
OG0019962: TcSYL_0143260.t1-p1
OG0019963: TcSYL_0143270.t1-p1
OG0019964: TcSYL_0143280.t1-p1
OG0019965: TcSYL_0143290.t1-p1
OG0019966: TcSYL_0143300.t1-p1
OG0019967: TcSYL_0143320.t1-p1
OG0019968: TcSYL_0143330.t1-p1
OG0019969: TcSYL_0143340.t1-p1
OG0019970: TcSYL_0143350.t1-p1
OG0019971: TcSYL_0143360.t1-p1
OG0019972: TcSYL_0143370.t1-p1
OG0019973: TcSYL_0143380.t1-p1
OG0019974: TcSYL_0143390.t1-p1
OG0019975: TcSYL_0143400.t1-p1
OG0019976: TcSYL_0143420.t1-p1
OG0019977: TcSYL_0143440.t1-p1
OG0019978: TcSYL_0143450.t1-p1
OG0019979: TcSYL_0143460.t1-p1
OG0019980: TcSYL_0143470.t1-p1
OG0019981: TcSYL_0143480.t1-p1
OG0019982: TcSYL_0143490.t1-p1
OG0019983: TcSYL_0143500.t1-p1
OG0019984: TcSYL_0143510.t1-p1
OG0019985: TcSYL_0143520.t1-p1
OG0019986: TcSYL_0143530.t1-p1
OG0019987: TcSYL_0143540.t1-p1
OG0019988: TcSYL_0143550.t1-p1
OG0019989: TcSYL_0143560.t1-p1
OG0019990: TcSYL_0143570.t1-p1
OG0019991: TcSYL_0143580.t1-p1
OG0019992: TcSYL_0143590.t1-p1
OG0019993: TcSYL_0143600.t1-p1
OG0019994: TcSYL_0143610.t1-p1
OG0019995: TcSYL_0143620.t1-p1
OG0019996: TcSYL_0143630.t1-p1
OG0019997: TcSYL_0143640.t1-p1
OG0019998: TcSYL_0143650.t1-p1
OG0019999: TcSYL_0143660.t1-p1
OG0020000: TcSYL_0143670.t1-p1
OG0020001: TcSYL_0143680.t1-p1
OG0020002: TcSYL_0143690.t1-p1
OG0020003: TcSYL_0143700.t1-p1
OG0020004: TcSYL_0143710.t1-p1
OG0020005: TcSYL_0143720.t1-p1
OG0020006: TcSYL_0143730.t1-p1
OG0020007: TcSYL_0143740.t1-p1
OG0020008: TcSYL_0143750.t1-p1
OG0020009: TcSYL_0143760.t1-p1
OG0020010: TcSYL_0143770.t1-p1
OG0020011: TcSYL_0143780.t1-p1
OG0020012: TcSYL_0143790.t1-p1
OG0020013: TcSYL_0143800.t1-p1
OG0020014: TcSYL_0143810.t1-p1

OG0020015: TcSYL_0143820.t1-p1
OG0020016: TcSYL_0143830.t1-p1
OG0020017: TcSYL_0143840.t1-p1
OG0020018: TcSYL_0143850.t1-p1
OG0020019: TcSYL_0143860.t1-p1
OG0020020: TcSYL_0143870.t1-p1
OG0020021: TcSYL_0143880.t1-p1
OG0020022: TcSYL_0143890.t1-p1
OG0020023: TcSYL_0143900.t1-p1
OG0020024: TcSYL_0143910.t1-p1
OG0020025: TcSYL_0143920.t1-p1
OG0020026: TcSYL_0143930.t1-p1
OG0020027: TcSYL_0143940.t1-p1
OG0020028: TcSYL_0143950.t1-p1
OG0020029: TcSYL_0143960.t1-p1
OG0020030: TcSYL_0143970.t1-p1
OG0020031: TcSYL_0143980.t1-p1
OG0020032: TcSYL_0143990.t1-p1
OG0020033: TcSYL_0144000.t1-p1
OG0020034: TcSYL_0144010.t1-p1
OG0020035: TcSYL_0144020.t1-p1
OG0020036: TcSYL_0144030.t1-p1
OG0020037: TcSYL_0144040.t1-p1
OG0020038: TcSYL_0144050.t1-p1
OG0020039: TcSYL_0144060.t1-p1
OG0020040: TcSYL_0144070.t1-p1
OG0020041: TcSYL_0144080.t1-p1
OG0020042: TcSYL_0144090.t1-p1
OG0020043: TcSYL_0144100.t1-p1
OG0020044: TcSYL_0144110.t1-p1
OG0020045: TcSYL_0144120.t1-p1
OG0020046: TcSYL_0144130.t1-p1
OG0020047: TcSYL_0144140.t1-p1
OG0020048: TcSYL_0144150.t1-p1
OG0020049: TcSYL_0144160.t1-p1
OG0020050: TcSYL_0144170.t1-p1
OG0020051: TcSYL_0144180.t1-p1
OG0020052: TcSYL_0144190.t1-p1
OG0020053: TcSYL_0144200.t1-p1
OG0020054: TcSYL_0144210.t1-p1
OG0020055: TcSYL_0144220.t1-p1
OG0020056: TcSYL_0144230.t1-p1
OG0020057: TcSYL_0144240.t1-p1
OG0020058: TcSYL_0144250.t1-p1
OG0020059: TcSYL_0144260.t1-p1
OG0020060: TcSYL_0144270.t1-p1
OG0020061: TcSYL_0144290.t1-p1
OG0020062: TcSYL_0144300.t1-p1
OG0020063: TcSYL_0144310.t1-p1
OG0020064: TcSYL_0144320.t1-p1
OG0020065: TcSYL_0144330.t1-p1
OG0020066: TcSYL_0144340.t1-p1
OG0020067: TcSYL_0144350.t1-p1
OG0020068: TcSYL_0144360.t1-p1

OG0020069: TcSYL_0144370.t1-p1
OG0020070: TcSYL_0144380.t1-p1
OG0020071: TcSYL_0144390.t1-p1
OG0020072: TcSYL_0144400.t1-p1
OG0020073: TcSYL_0144410.t1-p1
OG0020074: TcSYL_0144420.t1-p1
OG0020075: TcSYL_0144430.t1-p1
OG0020076: TcSYL_0144440.t1-p1
OG0020077: TcSYL_0144450.t1-p1
OG0020078: TcSYL_0144460.t1-p1
OG0020079: TcSYL_0144470.t1-p1
OG0020080: TcSYL_0144480.t1-p1
OG0020081: TcSYL_0144490.t1-p1
OG0020082: TcSYL_0144500.t1-p1
OG0020083: TcSYL_0144510.t1-p1
OG0020084: TcSYL_0144520.t1-p1
OG0020085: TcSYL_0144530.t1-p1
OG0020086: TcSYL_0144540.t1-p1
OG0020087: TcSYL_0144550.t1-p1
OG0020088: TcSYL_0144560.t1-p1
OG0020089: TcSYL_0144570.t1-p1
OG0020090: TcSYL_0144580.t1-p1
OG0020091: TcSYL_0144590.t1-p1
OG0020092: TcSYL_0144600.t1-p1
OG0020093: TcSYL_0144610.t1-p1
OG0020094: TcSYL_0144620.t1-p1
OG0020095: TcSYL_0144630.t1-p1
OG0020096: TcSYL_0144640.t1-p1
OG0020097: TcSYL_0144650.t1-p1
OG0020098: TcSYL_0144660.t1-p1
OG0020099: TcSYL_0144670.t1-p1
OG0020100: TcSYL_0144680.t1-p1
OG0020101: TcSYL_0144690.t1-p1
OG0020102: TcSYL_0144700.t1-p1
OG0020103: TcSYL_0144710.t1-p1
OG0020104: TcSYL_0144720.t1-p1
OG0020105: TcSYL_0144730.t1-p1
OG0020106: TcSYL_0144740.t1-p1
OG0020107: TcSYL_0144750.t1-p1
OG0020108: TcSYL_0144760.t1-p1
OG0020109: TcSYL_0144770.t1-p1
OG0020110: TcSYL_0144780.t1-p1
OG0020111: TcSYL_0144790.t1-p1
OG0020112: TcSYL_0144800.t1-p1
OG0020113: TcSYL_0144810.t1-p1
OG0020114: TcSYL_0144820.t1-p1
OG0020115: TcSYL_0144830.t1-p1
OG0020116: TcSYL_0144840.t1-p1
OG0020117: TcSYL_0144850.t1-p1
OG0020118: TcSYL_0144860.t1-p1
OG0020119: TcSYL_0144870.t1-p1
OG0020120: TcSYL_0144880.t1-p1
OG0020121: TcSYL_0144890.t1-p1
OG0020122: TcSYL_0144900.t1-p1

OG0020123: TcSYL_0144910.t1-p1
OG0020124: TcSYL_0144920.t1-p1
OG0020125: TcSYL_0144930.t1-p1
OG0020126: TcSYL_0144940.t1-p1
OG0020127: TcSYL_0144950.t1-p1
OG0020128: TcSYL_0144960.t1-p1
OG0020129: TcSYL_0144970.t1-p1
OG0020130: TcSYL_0144980.t1-p1
OG0020131: TcSYL_0144990.t1-p1
OG0020132: TcSYL_0145000.t1-p1
OG0020133: TcSYL_0145010.t1-p1
OG0020134: TcSYL_0145020.t1-p1
OG0020135: TcSYL_0145030.t1-p1
OG0020136: TcSYL_0145040.t1-p1
OG0020137: TcSYL_0145050.t1-p1
OG0020138: TcSYL_0145060.t1-p1
OG0020139: TcSYL_0145070.t1-p1
OG0020140: TcSYL_0145080.t1-p1
OG0020141: TcSYL_0145090.t1-p1
OG0020142: TcSYL_0145100.t1-p1
OG0020143: TcSYL_0145110.t1-p1
OG0020144: TcSYL_0145120.t1-p1
OG0020145: TcSYL_0145130.t1-p1
OG0020146: TcSYL_0145140.t1-p1
OG0020147: TcSYL_0145150.t1-p1
OG0020148: TcSYL_0145160.t1-p1
OG0020149: TcSYL_0145180.t1-p1
OG0020150: TcSYL_0145260.t1-p1
OG0020151: TcSYL_0145280.t1-p1
OG0020152: TcSYL_0145290.t1-p1
OG0020153: TcSYL_0145310.t1-p1
OG0020154: TcSYL_0145320.t1-p1
OG0020155: TcSYL_0145340.t1-p1
OG0020156: TcSYL_0145360.t1-p1
OG0020157: TcSYL_0145370.t1-p1
OG0020158: TcSYL_0145390.t1-p1
OG0020159: TcSYL_0145400.t1-p1
OG0020160: TcSYL_0145410.t1-p1
OG0020161: TcSYL_0145430.t1-p1
OG0020162: TcSYL_0145440.t1-p1
OG0020163: TcSYL_0145450.t1-p1
OG0020164: TcSYL_0145460.t1-p1
OG0020165: TcSYL_0145470.t1-p1
OG0020166: TcSYL_0145480.t1-p1
OG0020167: TcSYL_0145490.t1-p1
OG0020168: TcSYL_0145510.t1-p1
OG0020169: TcSYL_0145520.t1-p1
OG0020170: TcSYL_0145530.t1-p1
OG0020171: TcSYL_0145550.t1-p1
OG0020172: TcSYL_0145560.t1-p1
OG0020173: TcSYL_0145590.t1-p1
OG0020174: TcSYL_0145600.t1-p1
OG0020175: TcSYL_0145610.t1-p1
OG0020176: TcSYL_0145620.t1-p1

OG0020177: TcSYL_0145630.t1-p1
OG0020178: TcSYL_0145650.t1-p1
OG0020179: TcSYL_0145670.t1-p1
OG0020180: TcSYL_0145690.t1-p1
OG0020181: TcSYL_0145700.t1-p1
OG0020182: TcSYL_0145730.t1-p1
OG0020183: TcSYL_0145740.t1-p1
OG0020184: TcSYL_0145750.t1-p1
OG0020185: TcSYL_0145770.t1-p1
OG0020186: TcSYL_0145780.t1-p1
OG0020187: TcSYL_0145790.t1-p1
OG0020188: TcSYL_0145800.t1-p1
OG0020189: TcSYL_0145820.t1-p1
OG0020190: TcSYL_0145830.t1-p1
OG0020191: TcSYL_0145840.t1-p1
OG0020192: TcSYL_0145850.t1-p1
OG0020193: TcSYL_0145860.t1-p1
OG0020194: TcSYL_0145870.t1-p1
OG0020195: TcSYL_0145880.t1-p1
OG0020196: TcSYL_0145890.t1-p1
OG0020197: TcSYL_0145900.t1-p1
OG0020198: TcSYL_0145910.t1-p1
OG0020199: TcSYL_0145930.t1-p1
OG0020200: TcSYL_0145950.t1-p1
OG0020201: TcSYL_0145970.t1-p1
OG0020202: TcSYL_0145990.t1-p1
OG0020203: TcSYL_0146000.t1-p1
OG0020204: TcSYL_0146010.t1-p1
OG0020205: TcSYL_0146020.t1-p1
OG0020206: TcSYL_0146040.t1-p1
OG0020207: TcSYL_0146060.t1-p1
OG0020208: TcSYL_0146070.t1-p1
OG0020209: TcSYL_0146080.t1-p1
OG0020210: TcSYL_0146120.t1-p1
OG0020211: TcSYL_0146250.t1-p1
OG0020212: TcSYL_0146270.t1-p1
OG0020213: TcSYL_0146390.t1-p1
OG0020214: TcSYL_0146430.t1-p1
OG0020215: TcSYL_0146440.t1-p1
OG0020216: TcSYL_0146470.t1-p1
OG0020217: TcSYL_0146480.t1-p1
OG0020218: TcSYL_0146490.t1-p1
OG0020219: TcSYL_0146500.t1-p1
OG0020220: TcSYL_0146510.t1-p1
OG0020221: TcSYL_0146520.t1-p1
OG0020222: TcSYL_0146540.t1-p1
OG0020223: TcSYL_0146560.t1-p1
OG0020224: TcSYL_0146570.t1-p1
OG0020225: TcSYL_0146580.t1-p1
OG0020226: TcSYL_0146620.t1-p1
OG0020227: TcSYL_0146640.t1-p1
OG0020228: TcSYL_0146660.t1-p1
OG0020229: TcSYL_0146690.t1-p1
OG0020230: TcSYL_0146700.t1-p1

OG0020231: TcSYL_0146710.t1-p1
OG0020232: TcSYL_0146720.t1-p1
OG0020233: TcSYL_0146730.t1-p1
OG0020234: TcSYL_0146750.t1-p1
OG0020235: TcSYL_0146770.t1-p1
OG0020236: TcSYL_0146780.t1-p1
OG0020237: TcSYL_0146790.t1-p1
OG0020238: TcSYL_0146800.t1-p1
OG0020239: TcSYL_0146810.t1-p1
OG0020240: TcSYL_0146820.t1-p1
OG0020241: TcSYL_0146830.t1-p1
OG0020242: TcSYL_0146850.t1-p1
OG0020243: TcSYL_0146860.t1-p1
OG0020244: TcSYL_0146870.t1-p1
OG0020245: TcSYL_0146880.t1-p1
OG0020246: TcSYL_0146900.t1-p1
OG0020247: TcSYL_0146910.t1-p1
OG0020248: TcSYL_0146920.t1-p1
OG0020249: TcSYL_0146940.t1-p1
OG0020250: TcSYL_0146950.t1-p1
OG0020251: TcSYL_0146970.t1-p1
OG0020252: TcSYL_0146990.t1-p1
OG0020253: TcSYL_0147000.t1-p1
OG0020254: TcSYL_0147020.t1-p1
OG0020255: TcSYL_0147050.t1-p1
OG0020256: TcSYL_0147060.t1-p1
OG0020257: TcSYL_0147070.t1-p1
OG0020258: TcSYL_0147090.t1-p1
OG0020259: TcSYL_0147110.t1-p1
OG0020260: TcSYL_0147120.t1-p1
OG0020261: TcSYL_0147150.t1-p1
OG0020262: TcSYL_0147160.t1-p1
OG0020263: TcSYL_0147170.t1-p1
OG0020264: TcSYL_0147200.t1-p1
OG0020265: TcSYL_0147310.t1-p1
OG0020266: TcSYL_0147350.t1-p1
OG0020267: TcSYL_0147360.t1-p1
OG0020268: TcSYL_0147370.t1-p1
OG0020269: TcSYL_0147390.t1-p1
OG0020270: TcSYL_0147400.t1-p1
OG0020271: TcSYL_0147500.t1-p1
OG0020272: TcSYL_0147510.t1-p1
OG0020273: TcSYL_0147530.t1-p1
OG0020274: TcSYL_0147550.t1-p1
OG0020275: TcSYL_0147580.t1-p1
OG0020276: TcSYL_0147590.t1-p1
OG0020277: TcSYL_0147610.t1-p1
OG0020278: TcSYL_0147620.t1-p1
OG0020279: TcSYL_0147630.t1-p1
OG0020280: TcSYL_0147640.t1-p1
OG0020281: TcSYL_0147650.t1-p1
OG0020282: TcSYL_0147670.t1-p1
OG0020283: TcSYL_0147690.t1-p1
OG0020284: TcSYL_0147700.t1-p1

OG0020285: TcSYL_0147720.t1-p1
OG0020286: TcSYL_0147730.t1-p1
OG0020287: TcSYL_0147740.t1-p1
OG0020288: TcSYL_0147760.t1-p1
OG0020289: TcSYL_0147770.t1-p1
OG0020290: TcSYL_0147780.t1-p1
OG0020291: TcSYL_0147800.t1-p1
OG0020292: TcSYL_0147810.t1-p1
OG0020293: TcSYL_0147830.t1-p1
OG0020294: TcSYL_0147840.t1-p1
OG0020295: TcSYL_0147860.t1-p1
OG0020296: TcSYL_0147880.t1-p1
OG0020297: TcSYL_0147890.t1-p1
OG0020298: TcSYL_0147920.t1-p1
OG0020299: TcSYL_0147930.t1-p1
OG0020300: TcSYL_0147940.t1-p1
OG0020301: TcSYL_0147950.t1-p1
OG0020302: TcSYL_0147970.t1-p1
OG0020303: TcSYL_0147980.t1-p1
OG0020304: TcSYL_0148000.t1-p1
OG0020305: TcSYL_0148040.t1-p1
OG0020306: TcSYL_0148050.t1-p1
OG0020307: TcSYL_0148080.t1-p1
OG0020308: TcSYL_0148090.t1-p1
OG0020309: TcSYL_0148100.t1-p1
OG0020310: TcSYL_0148110.t1-p1
OG0020311: TcSYL_0148170.t1-p1
OG0020312: TcSYL_0148180.t1-p1
OG0020313: TcSYL_0148190.t1-p1
OG0020314: TcSYL_0148200.t1-p1
OG0020315: TcSYL_0148220.t1-p1
OG0020316: TcSYL_0148230.t1-p1
OG0020317: TcSYL_0148260.t1-p1
OG0020318: TcSYL_0148270.t1-p1
OG0020319: TcSYL_0148300.t1-p1
OG0020320: TcSYL_0148310.t1-p1
OG0020321: TcSYL_0148320.t1-p1
OG0020322: TcSYL_0148350.t1-p1
OG0020323: TcSYL_0148360.t1-p1
OG0020324: TcSYL_0148370.t1-p1
OG0020325: TcSYL_0148380.t1-p1
OG0020326: TcSYL_0148390.t1-p1
OG0020327: TcSYL_0148400.t1-p1
OG0020328: TcSYL_0148410.t1-p1
OG0020329: TcSYL_0148420.t1-p1
OG0020330: TcSYL_0148430.t1-p1
OG0020331: TcSYL_0148470.t1-p1
OG0020332: TcSYL_0148480.t1-p1
OG0020333: TcSYL_0148490.t1-p1
OG0020334: TcSYL_0148510.t1-p1
OG0020335: TcSYL_0148520.t1-p1
OG0020336: TcSYL_0148530.t1-p1
OG0020337: TcSYL_0148540.t1-p1
OG0020338: TcSYL_0148550.t1-p1

OG0020339: TcSYL_0148560.t1-p1
OG0020340: TcSYL_0148580.t1-p1
OG0020341: TcSYL_0148590.t1-p1
OG0020342: TcSYL_0148600.t1-p1
OG0020343: TcSYL_0148610.t1-p1
OG0020344: TcSYL_0148620.t1-p1
OG0020345: TcSYL_0148630.t1-p1
OG0020346: TcSYL_0148640.t1-p1
OG0020347: TcSYL_0148650.t1-p1
OG0020348: TcSYL_0148660.t1-p1
OG0020349: TcSYL_0148680.t1-p1
OG0020350: TcSYL_0148710.t1-p1
OG0020351: TcSYL_0148720.t1-p1
OG0020352: TcSYL_0148730.t1-p1
OG0020353: TcSYL_0148740.t1-p1
OG0020354: TcSYL_0148760.t1-p1
OG0020355: TcSYL_0148780.t1-p1
OG0020356: TcSYL_0148790.t1-p1
OG0020357: TcSYL_0148810.t1-p1
OG0020358: TcSYL_0148820.t1-p1
OG0020359: TcSYL_0148830.t1-p1
OG0020360: TcSYL_0148840.t1-p1
OG0020361: TcSYL_0148850.t1-p1
OG0020362: TcSYL_0148860.t1-p1
OG0020363: TcSYL_0148880.t1-p1
OG0020364: TcSYL_0148890.t1-p1
OG0020365: TcSYL_0148920.t1-p1
OG0020366: TcSYL_0148930.t1-p1
OG0020367: TcSYL_0148950.t1-p1
OG0020368: TcSYL_0148960.t1-p1
OG0020369: TcSYL_0148970.t1-p1
OG0020370: TcSYL_0148980.t1-p1
OG0020371: TcSYL_0149010.t1-p1
OG0020372: TcSYL_0149080.t1-p1
OG0020373: TcSYL_0149100.t1-p1
OG0020374: TcSYL_0149120.t1-p1
OG0020375: TcSYL_0149130.t1-p1
OG0020376: TcSYL_0149150.t1-p1
OG0020377: TcSYL_0149160.t1-p1
OG0020378: TcSYL_0149170.t1-p1
OG0020379: TcSYL_0149180.t1-p1
OG0020380: TcSYL_0149190.t1-p1
OG0020381: TcSYL_0149200.t1-p1
OG0020382: TcSYL_0149210.t1-p1
OG0020383: TcSYL_0149220.t1-p1
OG0020384: TcSYL_0149230.t1-p1
OG0020385: TcSYL_0149240.t1-p1
OG0020386: TcSYL_0149250.t1-p1
OG0020387: TcSYL_0149260.t1-p1
OG0020388: TcSYL_0149270.t1-p1
OG0020389: TcSYL_0149280.t1-p1
OG0020390: TcSYL_0149290.t1-p1
OG0020391: TcSYL_0149310.t1-p1
OG0020392: TcSYL_0149320.t1-p1

OG0020393: TcSYL_0149330.t1-p1
OG0020394: TcSYL_0149340.t1-p1
OG0020395: TcSYL_0149350.t1-p1
OG0020396: TcSYL_0149360.t1-p1
OG0020397: TcSYL_0149370.t1-p1
OG0020398: TcSYL_0149390.t1-p1
OG0020399: TcSYL_0149400.t1-p1
OG0020400: TcSYL_0149420.t1-p1
OG0020401: TcSYL_0149430.t1-p1
OG0020402: TcSYL_0149460.t1-p1
OG0020403: TcSYL_0149480.t1-p1
OG0020404: TcSYL_0149490.t1-p1
OG0020405: TcSYL_0149550.t1-p1
OG0020406: TcSYL_0149560.t1-p1
OG0020407: TcSYL_0149570.t1-p1
OG0020408: TcSYL_0149590.t1-p1
OG0020409: TcSYL_0149620.t1-p1
OG0020410: TcSYL_0149630.t1-p1
OG0020411: TcSYL_0149640.t1-p1
OG0020412: TcSYL_0149650.t1-p1
OG0020413: TcSYL_0149660.t1-p1
OG0020414: TcSYL_0149670.t1-p1
OG0020415: TcSYL_0149680.t1-p1
OG0020416: TcSYL_0149690.t1-p1
OG0020417: TcSYL_0149710.t1-p1
OG0020418: TcSYL_0149720.t1-p1
OG0020419: TcSYL_0149730.t1-p1
OG0020420: TcSYL_0149740.t1-p1
OG0020421: TcSYL_0149750.t1-p1
OG0020422: TcSYL_0149760.t1-p1
OG0020423: TcSYL_0149770.t1-p1
OG0020424: TcSYL_0149780.t1-p1
OG0020425: TcSYL_0149790.t1-p1
OG0020426: TcSYL_0149800.t1-p1
OG0020427: TcSYL_0149810.t1-p1
OG0020428: TcSYL_0149820.t1-p1
OG0020429: TcSYL_0149830.t1-p1
OG0020430: TcSYL_0149840.t1-p1
OG0020431: TcSYL_0149850.t1-p1
OG0020432: TcSYL_0149860.t1-p1
OG0020433: TcSYL_0149870.t1-p1
OG0020434: TcSYL_0149880.t1-p1
OG0020435: TcSYL_0149890.t1-p1
OG0020436: TcSYL_0149900.t1-p1
OG0020437: TcSYL_0149910.t1-p1
OG0020438: TcSYL_0149930.t1-p1
OG0020439: TcSYL_0149950.t1-p1
OG0020440: TcSYL_0149960.t1-p1
OG0020441: TcSYL_0149970.t1-p1
OG0020442: TcSYL_0149980.t1-p1
OG0020443: TcSYL_0150000.t1-p1
OG0020444: TcSYL_0150010.t1-p1
OG0020445: TcSYL_0150020.t1-p1
OG0020446: TcSYL_0150030.t1-p1

OG0020447: TcSYL_0150050.t1-p1
OG0020448: TcSYL_0150060.t1-p1
OG0020449: TcSYL_0150070.t1-p1
OG0020450: TcSYL_0150090.t1-p1
OG0020451: TcSYL_0150100.t1-p1
OG0020452: TcSYL_0150140.t1-p1
OG0020453: TcSYL_0150150.t1-p1
OG0020454: TcSYL_0150160.t1-p1
OG0020455: TcSYL_0150180.t1-p1
OG0020456: TcSYL_0150190.t1-p1
OG0020457: TcSYL_0150200.t1-p1
OG0020458: TcSYL_0150210.t1-p1
OG0020459: TcSYL_0150220.t1-p1
OG0020460: TcSYL_0150230.t1-p1
OG0020461: TcSYL_0150240.t1-p1
OG0020462: TcSYL_0150250.t1-p1
OG0020463: TcSYL_0150260.t1-p1
OG0020464: TcSYL_0150270.t1-p1
OG0020465: TcSYL_0150280.t1-p1
OG0020466: TcSYL_0150300.t1-p1
OG0020467: TcSYL_0150320.t1-p1
OG0020468: TcSYL_0150330.t1-p1
OG0020469: TcSYL_0150350.t1-p1
OG0020470: TcSYL_0150360.t1-p1
OG0020471: TcSYL_0150390.t1-p1
OG0020472: TcSYL_0150400.t1-p1
OG0020473: TcSYL_0150410.t1-p1
OG0020474: TcSYL_0150430.t1-p1
OG0020475: TcSYL_0150440.t1-p1
OG0020476: TcSYL_0150500.t1-p1
OG0020477: TcSYL_0150520.t1-p1
OG0020478: TcSYL_0150540.t1-p1
OG0020479: TcSYL_0150550.t1-p1
OG0020480: TcSYL_0150560.t1-p1
OG0020481: TcSYL_0150570.t1-p1
OG0020482: TcSYL_0150590.t1-p1
OG0020483: TcSYL_0150600.t1-p1
OG0020484: TcSYL_0150610.t1-p1
OG0020485: TcSYL_0150620.t1-p1
OG0020486: TcSYL_0150640.t1-p1
OG0020487: TcSYL_0150660.t1-p1
OG0020488: TcSYL_0150670.t1-p1
OG0020489: TcSYL_0150680.t1-p1
OG0020490: TcSYL_0150700.t1-p1
OG0020491: TcSYL_0150710.t1-p1
OG0020492: TcSYL_0150720.t1-p1
OG0020493: TcSYL_0150740.t1-p1
OG0020494: TcSYL_0150750.t1-p1
OG0020495: TcSYL_0150760.t1-p1
OG0020496: TcSYL_0150770.t1-p1
OG0020497: TcSYL_0150780.t1-p1
OG0020498: TcSYL_0150790.t1-p1
OG0020499: TcSYL_0150810.t1-p1
OG0020500: TcSYL_0150820.t1-p1

OG0020501: TcSYL_0150830.t1-p1
OG0020502: TcSYL_0150840.t1-p1
OG0020503: TcSYL_0150850.t1-p1
OG0020504: TcSYL_0150860.t1-p1
OG0020505: TcSYL_0150870.t1-p1
OG0020506: TcSYL_0150880.t1-p1
OG0020507: TcSYL_0150920.t1-p1
OG0020508: TcSYL_0150950.t1-p1
OG0020509: TcSYL_0150960.t1-p1
OG0020510: TcSYL_0150970.t1-p1
OG0020511: TcSYL_0150980.t1-p1
OG0020512: TcSYL_0150990.t1-p1
OG0020513: TcSYL_0151000.t1-p1
OG0020514: TcSYL_0151020.t1-p1
OG0020515: TcSYL_0151030.t1-p1
OG0020516: TcSYL_0151040.t1-p1
OG0020517: TcSYL_0151050.t1-p1
OG0020518: TcSYL_0151060.t1-p1
OG0020519: TcSYL_0151070.t1-p1
OG0020520: TcSYL_0151080.t1-p1
OG0020521: TcSYL_0151090.t1-p1
OG0020522: TcSYL_0151120.t1-p1
OG0020523: TcSYL_0151130.t1-p1
OG0020524: TcSYL_0151140.t1-p1
OG0020525: TcSYL_0151150.t1-p1
OG0020526: TcSYL_0151160.t1-p1
OG0020527: TcSYL_0151170.t1-p1
OG0020528: TcSYL_0151190.t1-p1
OG0020529: TcSYL_0151200.t1-p1
OG0020530: TcSYL_0151210.t1-p1
OG0020531: TcSYL_0151230.t1-p1
OG0020532: TcSYL_0151240.t1-p1
OG0020533: TcSYL_0151260.t1-p1
OG0020534: TcSYL_0151270.t1-p1
OG0020535: TcSYL_0151280.t1-p1
OG0020536: TcSYL_0151290.t1-p1
OG0020537: TcSYL_0151300.t1-p1
OG0020538: TcSYL_0151310.t1-p1
OG0020539: TcSYL_0151330.t1-p1
OG0020540: TcSYL_0151340.t1-p1
OG0020541: TcSYL_0151350.t1-p1
OG0020542: TcSYL_0151360.t1-p1
OG0020543: TcSYL_0151370.t1-p1
OG0020544: TcSYL_0151390.t1-p1
OG0020545: TcSYL_0151400.t1-p1
OG0020546: TcSYL_0151420.t1-p1
OG0020547: TcSYL_0151440.t1-p1
OG0020548: TcSYL_0151450.t1-p1
OG0020549: TcSYL_0151500.t1-p1
OG0020550: TcSYL_0151510.t1-p1
OG0020551: TcSYL_0151520.t1-p1
OG0020552: TcSYL_0151530.t1-p1
OG0020553: TcSYL_0151540.t1-p1
OG0020554: TcSYL_0151550.t1-p1

OG0020555: TcSYL_0151560.t1-p1
OG0020556: TcSYL_0151570.t1-p1
OG0020557: TcSYL_0151580.t1-p1
OG0020558: TcSYL_0151590.t1-p1
OG0020559: TcSYL_0151610.t1-p1
OG0020560: TcSYL_0151620.t1-p1
OG0020561: TcSYL_0151640.t1-p1
OG0020562: TcSYL_0151660.t1-p1
OG0020563: TcSYL_0151670.t1-p1
OG0020564: TcSYL_0151690.t1-p1
OG0020565: TcSYL_0151700.t1-p1
OG0020566: TcSYL_0151710.t1-p1
OG0020567: TcSYL_0151720.t1-p1
OG0020568: TcSYL_0151730.t1-p1
OG0020569: TcSYL_0151740.t1-p1
OG0020570: TcSYL_0151750.t1-p1
OG0020571: TcSYL_0151770.t1-p1
OG0020572: TcSYL_0151780.t1-p1
OG0020573: TcSYL_0151820.t1-p1
OG0020574: TcSYL_0151830.t1-p1
OG0020575: TcSYL_0151840.t1-p1
OG0020576: TcSYL_0151850.t1-p1
OG0020577: TcSYL_0151860.t1-p1
OG0020578: TcSYL_0151880.t1-p1
OG0020579: TcSYL_0151890.t1-p1
OG0020580: TcSYL_0151900.t1-p1
OG0020581: TcSYL_0151910.t1-p1
OG0020582: TcSYL_0151920.t1-p1
OG0020583: TcSYL_0151930.t1-p1
OG0020584: TcSYL_0151940.t1-p1
OG0020585: TcSYL_0151950.t1-p1
OG0020586: TcSYL_0151960.t1-p1
OG0020587: TcSYL_0151970.t1-p1
OG0020588: TcSYL_0151980.t1-p1
OG0020589: TcSYL_0151990.t1-p1
OG0020590: TcSYL_0152000.t1-p1
OG0020591: TcSYL_0152010.t1-p1
OG0020592: TcSYL_0152040.t1-p1
OG0020593: TcSYL_0152050.t1-p1
OG0020594: TcSYL_0152060.t1-p1
OG0020595: TcSYL_0152080.t1-p1
OG0020596: TcSYL_0152110.t1-p1
OG0020597: TcSYL_0152120.t1-p1
OG0020598: TcSYL_0152130.t1-p1
OG0020599: TcSYL_0152140.t1-p1
OG0020600: TcSYL_0152150.t1-p1
OG0020601: TcSYL_0152160.t1-p1
OG0020602: TcSYL_0152170.t1-p1
OG0020603: TcSYL_0152180.t1-p1
OG0020604: TcSYL_0152200.t1-p1
OG0020605: TcSYL_0152210.t1-p1
OG0020606: TcSYL_0152220.t1-p1
OG0020607: TcSYL_0152230.t1-p1
OG0020608: TcSYL_0152240.t1-p1

OG0020609: TcSYL_0152250.t1-p1
OG0020610: TcSYL_0152260.t1-p1
OG0020611: TcSYL_0152300.t1-p1
OG0020612: TcSYL_0152310.t1-p1
OG0020613: TcSYL_0152320.t1-p1
OG0020614: TcSYL_0152330.t1-p1
OG0020615: TcSYL_0152340.t1-p1
OG0020616: TcSYL_0152350.t1-p1
OG0020617: TcSYL_0152360.t1-p1
OG0020618: TcSYL_0152370.t1-p1
OG0020619: TcSYL_0152380.t1-p1
OG0020620: TcSYL_0152390.t1-p1
OG0020621: TcSYL_0152400.t1-p1
OG0020622: TcSYL_0152410.t1-p1
OG0020623: TcSYL_0152430.t1-p1
OG0020624: TcSYL_0152440.t1-p1
OG0020625: TcSYL_0152460.t1-p1
OG0020626: TcSYL_0152470.t1-p1
OG0020627: TcSYL_0152480.t1-p1
OG0020628: TcSYL_0152490.t1-p1
OG0020629: TcSYL_0152500.t1-p1
OG0020630: TcSYL_0152510.t1-p1
OG0020631: TcSYL_0152520.t1-p1
OG0020632: TcSYL_0152530.t1-p1
OG0020633: TcSYL_0152540.t1-p1
OG0020634: TcSYL_0152550.t1-p1
OG0020635: TcSYL_0152560.t1-p1
OG0020636: TcSYL_0152570.t1-p1
OG0020637: TcSYL_0152580.t1-p1
OG0020638: TcSYL_0152590.t1-p1
OG0020639: TcSYL_0152600.t1-p1
OG0020640: TcSYL_0152620.t1-p1
OG0020641: TcSYL_0152630.t1-p1
OG0020642: TcSYL_0152640.t1-p1
OG0020643: TcSYL_0152650.t1-p1
OG0020644: TcSYL_0152660.t1-p1
OG0020645: TcSYL_0152670.t1-p1
OG0020646: TcSYL_0152680.t1-p1
OG0020647: TcSYL_0152690.t1-p1
OG0020648: TcSYL_0152700.t1-p1
OG0020649: TcSYL_0152710.t1-p1
OG0020650: TcSYL_0152720.t1-p1
OG0020651: TcSYL_0152730.t1-p1
OG0020652: TcSYL_0152740.t1-p1
OG0020653: TcSYL_0152760.t1-p1
OG0020654: TcSYL_0152770.t1-p1
OG0020655: TcSYL_0152780.t1-p1
OG0020656: TcSYL_0152790.t1-p1
OG0020657: TcSYL_0152800.t1-p1
OG0020658: TcSYL_0152820.t1-p1
OG0020659: TcSYL_0152830.t1-p1
OG0020660: TcSYL_0152840.t1-p1
OG0020661: TcSYL_0152850.t1-p1
OG0020662: TcSYL_0152860.t1-p1

OG0020663: TcSYL_0152880.t1-p1
OG0020664: TcSYL_0152890.t1-p1
OG0020665: TcSYL_0152900.t1-p1
OG0020666: TcSYL_0152920.t1-p1
OG0020667: TcSYL_0152930.t1-p1
OG0020668: TcSYL_0152940.t1-p1
OG0020669: TcSYL_0152960.t1-p1
OG0020670: TcSYL_0152980.t1-p1
OG0020671: TcSYL_0152990.t1-p1
OG0020672: TcSYL_0153000.t1-p1
OG0020673: TcSYL_0153010.t1-p1
OG0020674: TcSYL_0153020.t1-p1
OG0020675: TcSYL_0153040.t1-p1
OG0020676: TcSYL_0153050.t1-p1
OG0020677: TcSYL_0153070.t1-p1
OG0020678: TcSYL_0153080.t1-p1
OG0020679: TcSYL_0153090.t1-p1
OG0020680: TcSYL_0153120.t1-p1
OG0020681: TcSYL_0153140.t1-p1
OG0020682: TcSYL_0153150.t1-p1
OG0020683: TcSYL_0153160.t1-p1
OG0020684: TcSYL_0153170.t1-p1
OG0020685: TcSYL_0153180.t1-p1
OG0020686: TcSYL_0153190.t1-p1
OG0020687: TcSYL_0153200.t1-p1
OG0020688: TcSYL_0153220.t1-p1
OG0020689: TcSYL_0153230.t1-p1
OG0020690: TcSYL_0153250.t1-p1
OG0020691: TcSYL_0153260.t1-p1
OG0020692: TcSYL_0153270.t1-p1
OG0020693: TcSYL_0153280.t1-p1
OG0020694: TcSYL_0153290.t1-p1
OG0020695: TcSYL_0153300.t1-p1
OG0020696: TcSYL_0153310.t1-p1
OG0020697: TcSYL_0153320.t1-p1
OG0020698: TcSYL_0153330.t1-p1
OG0020699: TcSYL_0153340.t1-p1
OG0020700: TcSYL_0153350.t1-p1
OG0020701: TcSYL_0153360.t1-p1
OG0020702: TcSYL_0153370.t1-p1
OG0020703: TcSYL_0153380.t1-p1
OG0020704: TcSYL_0153390.t1-p1
OG0020705: TcSYL_0153430.t1-p1
OG0020706: TcSYL_0153440.t1-p1
OG0020707: TcSYL_0153450.t1-p1
OG0020708: TcSYL_0153460.t1-p1
OG0020709: TcSYL_0153470.t1-p1
OG0020710: TcSYL_0153480.t1-p1
OG0020711: TcSYL_0153490.t1-p1
OG0020712: TcSYL_0153510.t1-p1
OG0020713: TcSYL_0153520.t1-p1
OG0020714: TcSYL_0153530.t1-p1
OG0020715: TcSYL_0153540.t1-p1
OG0020716: TcSYL_0153550.t1-p1

OG0020717: TcSYL_0153560.t1-p1
OG0020718: TcSYL_0153570.t1-p1
OG0020719: TcSYL_0153580.t1-p1
OG0020720: TcSYL_0153590.t1-p1
OG0020721: TcSYL_0153600.t1-p1
OG0020722: TcSYL_0153610.t1-p1
OG0020723: TcSYL_0153620.t1-p1
OG0020724: TcSYL_0153630.t1-p1
OG0020725: TcSYL_0153640.t1-p1
OG0020726: TcSYL_0153650.t1-p1
OG0020727: TcSYL_0153670.t1-p1
OG0020728: TcSYL_0153680.t1-p1
OG0020729: TcSYL_0153690.t1-p1
OG0020730: TcSYL_0153700.t1-p1
OG0020731: TcSYL_0153710.t1-p1
OG0020732: TcSYL_0153730.t1-p1
OG0020733: TcSYL_0153740.t1-p1
OG0020734: TcSYL_0153760.t1-p1
OG0020735: TcSYL_0153780.t1-p1
OG0020736: TcSYL_0153800.t1-p1
OG0020737: TcSYL_0153810.t1-p1
OG0020738: TcSYL_0153830.t1-p1
OG0020739: TcSYL_0153840.t1-p1
OG0020740: TcSYL_0153850.t1-p1
OG0020741: TcSYL_0153860.t1-p1
OG0020742: TcSYL_0153870.t1-p1
OG0020743: TcSYL_0153880.t1-p1
OG0020744: TcSYL_0153890.t1-p1
OG0020745: TcSYL_0153900.t1-p1
OG0020746: TcSYL_0153910.t1-p1
OG0020747: TcSYL_0153920.t1-p1
OG0020748: TcSYL_0153930.t1-p1
OG0020749: TcSYL_0153940.t1-p1
OG0020750: TcSYL_0153950.t1-p1
OG0020751: TcSYL_0153960.t1-p1
OG0020752: TcSYL_0153970.t1-p1
OG0020753: TcSYL_0153980.t1-p1
OG0020754: TcSYL_0153990.t1-p1
OG0020755: TcSYL_0154000.t1-p1
OG0020756: TcSYL_0154010.t1-p1
OG0020757: TcSYL_0154020.t1-p1
OG0020758: TcSYL_0154030.t1-p1
OG0020759: TcSYL_0154040.t1-p1
OG0020760: TcSYL_0154050.t1-p1
OG0020761: TcSYL_0154080.t1-p1
OG0020762: TcSYL_0154090.t1-p1
OG0020763: TcSYL_0154100.t1-p1
OG0020764: TcSYL_0154110.t1-p1
OG0020765: TcSYL_0154140.t1-p1
OG0020766: TcSYL_0154150.t1-p1
OG0020767: TcSYL_0154160.t1-p1
OG0020768: TcSYL_0154200.t1-p1
OG0020769: TcSYL_0154210.t1-p1
OG0020770: TcSYL_0154220.t1-p1

OG0020771: TcSYL_0154230.t1-p1
OG0020772: TcSYL_0154250.t1-p1
OG0020773: TcSYL_0154280.t1-p1
OG0020774: TcSYL_0154300.t1-p1
OG0020775: TcSYL_0154320.t1-p1
OG0020776: TcSYL_0154330.t1-p1
OG0020777: TcSYL_0154350.t1-p1
OG0020778: TcSYL_0154360.t1-p1
OG0020779: TcSYL_0154370.t1-p1
OG0020780: TcSYL_0154380.t1-p1
OG0020781: TcSYL_0154390.t1-p1
OG0020782: TcSYL_0154400.t1-p1
OG0020783: TcSYL_0154410.t1-p1
OG0020784: TcSYL_0154420.t1-p1
OG0020785: TcSYL_0154440.t1-p1
OG0020786: TcSYL_0154450.t1-p1
OG0020787: TcSYL_0154460.t1-p1
OG0020788: TcSYL_0154480.t1-p1
OG0020789: TcSYL_0154500.t1-p1
OG0020790: TcSYL_0154510.t1-p1
OG0020791: TcSYL_0154520.t1-p1
OG0020792: TcSYL_0154540.t1-p1
OG0020793: TcSYL_0154550.t1-p1
OG0020794: TcSYL_0154560.t1-p1
OG0020795: TcSYL_0154570.t1-p1
OG0020796: TcSYL_0154610.t1-p1
OG0020797: TcSYL_0154620.t1-p1
OG0020798: TcSYL_0154630.t1-p1
OG0020799: TcSYL_0154650.t1-p1
OG0020800: TcSYL_0154660.t1-p1
OG0020801: TcSYL_0154670.t1-p1
OG0020802: TcSYL_0154700.t1-p1
OG0020803: TcSYL_0154710.t1-p1
OG0020804: TcSYL_0154720.t1-p1
OG0020805: TcSYL_0154730.t1-p1
OG0020806: TcSYL_0154740.t1-p1
OG0020807: TcSYL_0154750.t1-p1
OG0020808: TcSYL_0154780.t1-p1
OG0020809: TcSYL_0154810.t1-p1
OG0020810: TcSYL_0154820.t1-p1
OG0020811: TcSYL_0154870.t1-p1
OG0020812: TcSYL_0154880.t1-p1
OG0020813: TcSYL_0154890.t1-p1
OG0020814: TcSYL_0154900.t1-p1
OG0020815: TcSYL_0154940.t1-p1
OG0020816: TcSYL_0154950.t1-p1
OG0020817: TcSYL_0154960.t1-p1
OG0020818: TcSYL_0154970.t1-p1
OG0020819: TcSYL_0154980.t1-p1
OG0020820: TcSYL_0155020.t1-p1
OG0020821: TcSYL_0155040.t1-p1
OG0020822: TcSYL_0155050.t1-p1
OG0020823: TcSYL_0155080.t1-p1
OG0020824: TcSYL_0155100.t1-p1

OG0020825: TcSYL_0155110.t1-p1
OG0020826: TcSYL_0155130.t1-p1
OG0020827: TcSYL_0155140.t1-p1
OG0020828: TcSYL_0155150.t1-p1
OG0020829: TcSYL_0155160.t1-p1
OG0020830: TcSYL_0155190.t1-p1
OG0020831: TcSYL_0155210.t1-p1
OG0020832: TcSYL_0155230.t1-p1
OG0020833: TcSYL_0155240.t1-p1
OG0020834: TcSYL_0155250.t1-p1
OG0020835: TcSYL_0155260.t1-p1
OG0020836: TcSYL_0155270.t1-p1
OG0020837: TcSYL_0155280.t1-p1
OG0020838: TcSYL_0155290.t1-p1
OG0020839: TcSYL_0155300.t1-p1
OG0020840: TcSYL_0155320.t1-p1
OG0020841: TcSYL_0155330.t1-p1
OG0020842: TcSYL_0155350.t1-p1
OG0020843: TcSYL_0155360.t1-p1
OG0020844: TcSYL_0155370.t1-p1
OG0020845: TcSYL_0155380.t1-p1
OG0020846: TcSYL_0155390.t1-p1
OG0020847: TcSYL_0155400.t1-p1
OG0020848: TcSYL_0155410.t1-p1
OG0020849: TcSYL_0155420.t1-p1
OG0020850: TcSYL_0155430.t1-p1
OG0020851: TcSYL_0155440.t1-p1
OG0020852: TcSYL_0155470.t1-p1
OG0020853: TcSYL_0155480.t1-p1
OG0020854: TcSYL_0155490.t1-p1
OG0020855: TcSYL_0155500.t1-p1
OG0020856: TcSYL_0155510.t1-p1
OG0020857: TcSYL_0155540.t1-p1
OG0020858: TcSYL_0155570.t1-p1
OG0020859: TcSYL_0155590.t1-p1
OG0020860: TcSYL_0155600.t1-p1
OG0020861: TcSYL_0155620.t1-p1
OG0020862: TcSYL_0155630.t1-p1
OG0020863: TcSYL_0155640.t1-p1
OG0020864: TcSYL_0155650.t1-p1
OG0020865: TcSYL_0155660.t1-p1
OG0020866: TcSYL_0155670.t1-p1
OG0020867: TcSYL_0155680.t1-p1
OG0020868: TcSYL_0155690.t1-p1
OG0020869: TcSYL_0155700.t1-p1
OG0020870: TcSYL_0155710.t1-p1
OG0020871: TcSYL_0155740.t1-p1
OG0020872: TcSYL_0155760.t1-p1
OG0020873: TcSYL_0155770.t1-p1
OG0020874: TcSYL_0155780.t1-p1
OG0020875: TcSYL_0155790.t1-p1
OG0020876: TcSYL_0155800.t1-p1
OG0020877: TcSYL_0155810.t1-p1
OG0020878: TcSYL_0155820.t1-p1

OG0020879: TcSYL_0155830.t1-p1
OG0020880: TcSYL_0155870.t1-p1
OG0020881: TcSYL_0155880.t1-p1
OG0020882: TcSYL_0155910.t1-p1
OG0020883: TcSYL_0155930.t1-p1
OG0020884: TcSYL_0156010.t1-p1
OG0020885: TcSYL_0156070.t1-p1
OG0020886: TcSYL_0156120.t1-p1
OG0020887: TcSYL_0156160.t1-p1
OG0020888: TcSYL_0156170.t1-p1
OG0020889: TcSYL_0156220.t1-p1
OG0020890: TcSYL_0156250.t1-p1
OG0020891: TcSYL_0156270.t1-p1
OG0020892: TcSYL_0156300.t1-p1
OG0020893: TcSYL_0156340.t1-p1
OG0020894: TcSYL_0156350.t1-p1
OG0020895: TcSYL_0156360.t1-p1
OG0020896: TcSYL_0156370.t1-p1
OG0020897: TcSYL_0156410.t1-p1
OG0020898: TcSYL_0156420.t1-p1
OG0020899: TcSYL_0156440.t1-p1
OG0020900: TcSYL_0156450.t1-p1
OG0020901: TcSYL_0156560.t1-p1
OG0020902: TcSYL_0156580.t1-p1
OG0020903: TcSYL_0156590.t1-p1
OG0020904: TcSYL_0156600.t1-p1
OG0020905: TcSYL_0156610.t1-p1
OG0020906: TcSYL_0156700.t1-p1
OG0020907: TcSYL_0156770.t1-p1
OG0020908: TcSYL_0156790.t1-p1
OG0020909: TcSYL_0156800.t1-p1
OG0020910: TcSYL_0156810.t1-p1
OG0020911: TcSYL_0156820.t1-p1
OG0020912: TcSYL_0156840.t1-p1
OG0020913: TcSYL_0156850.t1-p1
OG0020914: TcSYL_0156860.t1-p1
OG0020915: TcSYL_0156890.t1-p1
OG0020916: TcSYL_0156960.t1-p1
OG0020917: TcSYL_0156990.t1-p1
OG0020918: TcSYL_0157000.t1-p1
OG0020919: TcSYL_0157010.t1-p1
OG0020920: TcSYL_0157040.t1-p1
OG0020921: TcSYL_0157060.t1-p1
OG0020922: TcSYL_0157070.t1-p1
OG0020923: TcSYL_0157080.t1-p1
OG0020924: TcSYL_0157100.t1-p1
OG0020925: TcSYL_0157120.t1-p1
OG0020926: TcSYL_0157130.t1-p1
OG0020927: TcSYL_0157180.t1-p1
OG0020928: TcSYL_0157220.t1-p1
OG0020929: TcSYL_0157240.t1-p1
OG0020930: TcSYL_0157250.t1-p1
OG0020931: TcSYL_0157260.t1-p1
OG0020932: TcSYL_0157270.t1-p1

OG0020933: TcSYL_0157280.t1-p1
OG0020934: TcSYL_0157290.t1-p1
OG0020935: TcSYL_0157300.t1-p1
OG0020936: TcSYL_0157310.t1-p1
OG0020937: TcSYL_0157320.t1-p1
OG0020938: TcSYL_0157330.t1-p1
OG0020939: TcSYL_0157340.t1-p1
OG0020940: TcSYL_0157350.t1-p1
OG0020941: TcSYL_0157390.t1-p1
OG0020942: TcSYL_0157400.t1-p1
OG0020943: TcSYL_0157410.t1-p1
OG0020944: TcSYL_0157420.t1-p1
OG0020945: TcSYL_0157430.t1-p1
OG0020946: TcSYL_0157440.t1-p1
OG0020947: TcSYL_0157450.t1-p1
OG0020948: TcSYL_0157460.t1-p1
OG0020949: TcSYL_0157480.t1-p1
OG0020950: TcSYL_0157490.t1-p1
OG0020951: TcSYL_0157500.t1-p1
OG0020952: TcSYL_0157520.t1-p1
OG0020953: TcSYL_0157530.t1-p1
OG0020954: TcSYL_0157540.t1-p1
OG0020955: TcSYL_0157560.t1-p1
OG0020956: TcSYL_0157580.t1-p1
OG0020957: TcSYL_0157590.t1-p1
OG0020958: TcSYL_0157600.t1-p1
OG0020959: TcSYL_0157610.t1-p1
OG0020960: TcSYL_0157620.t1-p1
OG0020961: TcSYL_0157630.t1-p1
OG0020962: TcSYL_0157640.t1-p1
OG0020963: TcSYL_0157660.t1-p1
OG0020964: TcSYL_0157670.t1-p1
OG0020965: TcSYL_0157680.t1-p1
OG0020966: TcSYL_0157690.t1-p1
OG0020967: TcSYL_0157700.t1-p1
OG0020968: TcSYL_0157720.t1-p1
OG0020969: TcSYL_0157730.t1-p1
OG0020970: TcSYL_0157740.t1-p1
OG0020971: TcSYL_0157750.t1-p1
OG0020972: TcSYL_0157760.t1-p1
OG0020973: TcSYL_0157770.t1-p1
OG0020974: TcSYL_0157780.t1-p1
OG0020975: TcSYL_0157790.t1-p1
OG0020976: TcSYL_0157800.t1-p1
OG0020977: TcSYL_0157810.t1-p1
OG0020978: TcSYL_0157840.t1-p1
OG0020979: TcSYL_0157850.t1-p1
OG0020980: TcSYL_0157860.t1-p1
OG0020981: TcSYL_0157870.t1-p1
OG0020982: TcSYL_0157880.t1-p1
OG0020983: TcSYL_0157900.t1-p1
OG0020984: TcSYL_0157910.t1-p1
OG0020985: TcSYL_0157930.t1-p1
OG0020986: TcSYL_0157940.t1-p1

OG0020987: TcSYL_0157950.t1-p1
OG0020988: TcSYL_0157960.t1-p1
OG0020989: TcSYL_0157970.t1-p1
OG0020990: TcSYL_0157990.t1-p1
OG0020991: TcSYL_0158000.t1-p1
OG0020992: TcSYL_0158020.t1-p1
OG0020993: TcSYL_0158030.t1-p1
OG0020994: TcSYL_0158040.t1-p1
OG0020995: TcSYL_0158050.t1-p1
OG0020996: TcSYL_0158060.t1-p1
OG0020997: TcSYL_0158080.t1-p1
OG0020998: TcSYL_0158090.t1-p1
OG0020999: TcSYL_0158110.t1-p1
OG0021000: TcSYL_0158130.t1-p1
OG0021001: TcSYL_0158140.t1-p1
OG0021002: TcSYL_0158150.t1-p1
OG0021003: TcSYL_0158160.t1-p1
OG0021004: TcSYL_0158170.t1-p1
OG0021005: TcSYL_0158190.t1-p1
OG0021006: TcSYL_0158200.t1-p1
OG0021007: TcSYL_0158210.t1-p1
OG0021008: TcSYL_0158220.t1-p1
OG0021009: TcSYL_0158240.t1-p1
OG0021010: TcSYL_0158250.t1-p1
OG0021011: TcSYL_0158260.t1-p1
OG0021012: TcSYL_0158290.t1-p1
OG0021013: TcSYL_0158300.t1-p1
OG0021014: TcSYL_0158310.t1-p1
OG0021015: TcSYL_0158320.t1-p1
OG0021016: TcSYL_0158340.t1-p1
OG0021017: TcSYL_0158350.t1-p1
OG0021018: TcSYL_0158360.t1-p1
OG0021019: TcSYL_0158380.t1-p1
OG0021020: TcSYL_0158390.t1-p1
OG0021021: TcSYL_0158410.t1-p1
OG0021022: TcSYL_0158420.t1-p1
OG0021023: TcSYL_0158430.t1-p1
OG0021024: TcSYL_0158440.t1-p1
OG0021025: TcSYL_0158450.t1-p1
OG0021026: TcSYL_0158470.t1-p1
OG0021027: TcSYL_0158480.t1-p1
OG0021028: TcSYL_0158490.t1-p1
OG0021029: TcSYL_0158500.t1-p1
OG0021030: TcSYL_0158510.t1-p1
OG0021031: TcSYL_0158530.t1-p1
OG0021032: TcSYL_0158540.t1-p1
OG0021033: TcSYL_0158550.t1-p1
OG0021034: TcSYL_0158570.t1-p1
OG0021035: TcSYL_0158580.t1-p1
OG0021036: TcSYL_0158590.t1-p1
OG0021037: TcSYL_0158600.t1-p1
OG0021038: TcSYL_0158610.t1-p1
OG0021039: TcSYL_0158630.t1-p1
OG0021040: TcSYL_0158640.t1-p1

OG0021041: TcSYL_0158650.t1-p1
OG0021042: TcSYL_0158660.t1-p1
OG0021043: TcSYL_0158670.t1-p1
OG0021044: TcSYL_0158680.t1-p1
OG0021045: TcSYL_0158690.t1-p1
OG0021046: TcSYL_0158720.t1-p1
OG0021047: TcSYL_0158750.t1-p1
OG0021048: TcSYL_0158760.t1-p1
OG0021049: TcSYL_0158770.t1-p1
OG0021050: TcSYL_0158790.t1-p1
OG0021051: TcSYL_0158800.t1-p1
OG0021052: TcSYL_0158820.t1-p1
OG0021053: TcSYL_0158830.t1-p1
OG0021054: TcSYL_0158850.t1-p1
OG0021055: TcSYL_0158860.t1-p1
OG0021056: TcSYL_0158870.t1-p1
OG0021057: TcSYL_0158880.t1-p1
OG0021058: TcSYL_0158890.t1-p1
OG0021059: TcSYL_0158900.t1-p1
OG0021060: TcSYL_0158910.t1-p1
OG0021061: TcSYL_0158930.t1-p1
OG0021062: TcSYL_0158940.t1-p1
OG0021063: TcSYL_0158950.t1-p1
OG0021064: TcSYL_0158960.t1-p1
OG0021065: TcSYL_0158980.t1-p1
OG0021066: TcSYL_0159000.t1-p1
OG0021067: TcSYL_0159020.t1-p1
OG0021068: TcSYL_0159040.t1-p1
OG0021069: TcSYL_0159060.t1-p1
OG0021070: TcSYL_0159070.t1-p1
OG0021071: TcSYL_0159080.t1-p1
OG0021072: TcSYL_0159090.t1-p1
OG0021073: TcSYL_0159100.t1-p1
OG0021074: TcSYL_0159120.t1-p1
OG0021075: TcSYL_0159130.t1-p1
OG0021076: TcSYL_0159140.t1-p1
OG0021077: TcSYL_0159170.t1-p1
OG0021078: TcSYL_0159190.t1-p1
OG0021079: TcSYL_0159210.t1-p1
OG0021080: TcSYL_0159230.t1-p1
OG0021081: TcSYL_0159250.t1-p1
OG0021082: TcSYL_0159260.t1-p1
OG0021083: TcSYL_0159270.t1-p1
OG0021084: TcSYL_0159290.t1-p1
OG0021085: TcSYL_0159300.t1-p1
OG0021086: TcSYL_0159310.t1-p1
OG0021087: TcSYL_0159320.t1-p1
OG0021088: TcSYL_0159330.t1-p1
OG0021089: TcSYL_0159370.t1-p1
OG0021090: TcSYL_0159630.t1-p1
OG0021091: TcSYL_0159650.t1-p1
OG0021092: TcSYL_0159680.t1-p1
OG0021093: TcSYL_0159750.t1-p1
OG0021094: TcSYL_0159770.t1-p1

OG0021095: TcSYL_0159840.t1-p1
OG0021096: TcSYL_0159890.t1-p1
OG0021097: TcSYL_0160020.t1-p1
OG0021098: TcSYL_0160030.t1-p1
OG0021099: TcSYL_0160040.t1-p1
OG0021100: TcSYL_0160050.t1-p1
OG0021101: TcSYL_0160060.t1-p1
OG0021102: TcSYL_0160070.t1-p1
OG0021103: TcSYL_0160080.t1-p1
OG0021104: TcSYL_0160090.t1-p1
OG0021105: TcSYL_0160100.t1-p1
OG0021106: TcSYL_0160110.t1-p1
OG0021107: TcSYL_0160130.t1-p1
OG0021108: TcSYL_0160140.t1-p1
OG0021109: TcSYL_0160150.t1-p1
OG0021110: TcSYL_0160160.t1-p1
OG0021111: TcSYL_0160170.t1-p1
OG0021112: TcSYL_0160180.t1-p1
OG0021113: TcSYL_0160190.t1-p1
OG0021114: TcSYL_0160200.t1-p1
OG0021115: TcSYL_0160220.t1-p1
OG0021116: TcSYL_0160240.t1-p1
OG0021117: TcSYL_0160250.t1-p1
OG0021118: TcSYL_0160290.t1-p1
OG0021119: TcSYL_0160300.t1-p1
OG0021120: TcSYL_0160310.t1-p1
OG0021121: TcSYL_0160330.t1-p1
OG0021122: TcSYL_0160340.t1-p1
OG0021123: TcSYL_0160350.t1-p1
OG0021124: TcSYL_0160360.t1-p1
OG0021125: TcSYL_0160370.t1-p1
OG0021126: TcSYL_0160380.t1-p1
OG0021127: TcSYL_0160390.t1-p1
OG0021128: TcSYL_0160400.t1-p1
OG0021129: TcSYL_0160410.t1-p1
OG0021130: TcSYL_0160420.t1-p1
OG0021131: TcSYL_0160430.t1-p1
OG0021132: TcSYL_0160440.t1-p1
OG0021133: TcSYL_0160450.t1-p1
OG0021134: TcSYL_0160460.t1-p1
OG0021135: TcSYL_0160470.t1-p1
OG0021136: TcSYL_0160480.t1-p1
OG0021137: TcSYL_0160490.t1-p1
OG0021138: TcSYL_0160500.t1-p1
OG0021139: TcSYL_0160510.t1-p1
OG0021140: TcSYL_0160530.t1-p1
OG0021141: TcSYL_0160540.t1-p1
OG0021142: TcSYL_0160550.t1-p1
OG0021143: TcSYL_0160560.t1-p1
OG0021144: TcSYL_0160570.t1-p1
OG0021145: TcSYL_0160590.t1-p1
OG0021146: TcSYL_0160600.t1-p1
OG0021147: TcSYL_0160620.t1-p1
OG0021148: TcSYL_0160630.t1-p1

OG0021149: TcSYL_0160640.t1-p1
OG0021150: TcSYL_0160650.t1-p1
OG0021151: TcSYL_0160660.t1-p1
OG0021152: TcSYL_0160670.t1-p1
OG0021153: TcSYL_0160680.t1-p1
OG0021154: TcSYL_0160690.t1-p1
OG0021155: TcSYL_0160700.t1-p1
OG0021156: TcSYL_0160710.t1-p1
OG0021157: TcSYL_0160720.t1-p1
OG0021158: TcSYL_0160740.t1-p1
OG0021159: TcSYL_0160750.t1-p1
OG0021160: TcSYL_0160760.t1-p1
OG0021161: TcSYL_0160780.t1-p1
OG0021162: TcSYL_0160790.t1-p1
OG0021163: TcSYL_0160800.t1-p1
OG0021164: TcSYL_0160810.t1-p1
OG0021165: TcSYL_0160820.t1-p1
OG0021166: TcSYL_0160830.t1-p1
OG0021167: TcSYL_0160840.t1-p1
OG0021168: TcSYL_0160850.t1-p1
OG0021169: TcSYL_0160870.t1-p1
OG0021170: TcSYL_0160890.t1-p1
OG0021171: TcSYL_0160900.t1-p1
OG0021172: TcSYL_0160930.t1-p1
OG0021173: TcSYL_0160950.t1-p1
OG0021174: TcSYL_0160960.t1-p1
OG0021175: TcSYL_0160970.t1-p1
OG0021176: TcSYL_0160980.t1-p1
OG0021177: TcSYL_0160990.t1-p1
OG0021178: TcSYL_0161000.t1-p1
OG0021179: TcSYL_0161020.t1-p1
OG0021180: TcSYL_0161040.t1-p1
OG0021181: TcSYL_0161050.t1-p1
OG0021182: TcSYL_0161100.t1-p1
OG0021183: TcSYL_0161110.t1-p1
OG0021184: TcSYL_0161120.t1-p1
OG0021185: TcSYL_0161140.t1-p1
OG0021186: TcSYL_0161160.t1-p1
OG0021187: TcSYL_0161190.t1-p1
OG0021188: TcSYL_0161200.t1-p1
OG0021189: TcSYL_0161210.t1-p1
OG0021190: TcSYL_0161220.t1-p1
OG0021191: TcSYL_0161230.t1-p1
OG0021192: TcSYL_0161260.t1-p1
OG0021193: TcSYL_0161270.t1-p1
OG0021194: TcSYL_0161280.t1-p1
OG0021195: TcSYL_0161290.t1-p1
OG0021196: TcSYL_0161310.t1-p1
OG0021197: TcSYL_0161330.t1-p1
OG0021198: TcSYL_0161340.t1-p1
OG0021199: TcSYL_0161350.t1-p1
OG0021200: TcSYL_0161360.t1-p1
OG0021201: TcSYL_0161370.t1-p1
OG0021202: TcSYL_0161390.t1-p1

OG0021203: TcSYL_0161400.t1-p1
OG0021204: TcSYL_0161410.t1-p1
OG0021205: TcSYL_0161430.t1-p1
OG0021206: TcSYL_0161440.t1-p1
OG0021207: TcSYL_0161450.t1-p1
OG0021208: TcSYL_0161460.t1-p1
OG0021209: TcSYL_0161480.t1-p1
OG0021210: TcSYL_0161490.t1-p1
OG0021211: TcSYL_0161500.t1-p1
OG0021212: TcSYL_0161510.t1-p1
OG0021213: TcSYL_0161520.t1-p1
OG0021214: TcSYL_0161540.t1-p1
OG0021215: TcSYL_0161560.t1-p1
OG0021216: TcSYL_0161570.t1-p1
OG0021217: TcSYL_0161580.t1-p1
OG0021218: TcSYL_0161590.t1-p1
OG0021219: TcSYL_0161600.t1-p1
OG0021220: TcSYL_0161610.t1-p1
OG0021221: TcSYL_0161620.t1-p1
OG0021222: TcSYL_0161640.t1-p1
OG0021223: TcSYL_0161660.t1-p1
OG0021224: TcSYL_0161680.t1-p1
OG0021225: TcSYL_0161690.t1-p1
OG0021226: TcSYL_0161700.t1-p1
OG0021227: TcSYL_0161710.t1-p1
OG0021228: TcSYL_0161730.t1-p1
OG0021229: TcSYL_0161740.t1-p1
OG0021230: TcSYL_0161750.t1-p1
OG0021231: TcSYL_0161760.t1-p1
OG0021232: TcSYL_0161790.t1-p1
OG0021233: TcSYL_0161800.t1-p1
OG0021234: TcSYL_0161810.t1-p1
OG0021235: TcSYL_0161820.t1-p1
OG0021236: TcSYL_0161830.t1-p1
OG0021237: TcSYL_0161840.t1-p1
OG0021238: TcSYL_0161850.t1-p1
OG0021239: TcSYL_0161860.t1-p1
OG0021240: TcSYL_0161870.t1-p1
OG0021241: TcSYL_0161880.t1-p1
OG0021242: TcSYL_0161890.t1-p1
OG0021243: TcSYL_0161900.t1-p1
OG0021244: TcSYL_0161910.t1-p1
OG0021245: TcSYL_0161920.t1-p1
OG0021246: TcSYL_0161930.t1-p1
OG0021247: TcSYL_0161940.t1-p1
OG0021248: TcSYL_0161950.t1-p1
OG0021249: TcSYL_0161960.t1-p1
OG0021250: TcSYL_0161970.t1-p1
OG0021251: TcSYL_0161980.t1-p1
OG0021252: TcSYL_0161990.t1-p1
OG0021253: TcSYL_0162000.t1-p1
OG0021254: TcSYL_0162010.t1-p1
OG0021255: TcSYL_0162030.t1-p1
OG0021256: TcSYL_0162040.t1-p1

OG0021257: TcSYL_0162050.t1-p1
OG0021258: TcSYL_0162060.t1-p1
OG0021259: TcSYL_0162070.t1-p1
OG0021260: TcSYL_0162080.t1-p1
OG0021261: TcSYL_0162090.t1-p1
OG0021262: TcSYL_0162100.t1-p1
OG0021263: TcSYL_0162110.t1-p1
OG0021264: TcSYL_0162130.t1-p1
OG0021265: TcSYL_0162140.t1-p1
OG0021266: TcSYL_0162150.t1-p1
OG0021267: TcSYL_0162160.t1-p1
OG0021268: TcSYL_0162170.t1-p1
OG0021269: TcSYL_0162180.t1-p1
OG0021270: TcSYL_0162190.t1-p1
OG0021271: TcSYL_0162200.t1-p1
OG0021272: TcSYL_0162210.t1-p1
OG0021273: TcSYL_0162220.t1-p1
OG0021274: TcSYL_0162230.t1-p1
OG0021275: TcSYL_0162250.t1-p1
OG0021276: TcSYL_0162270.t1-p1
OG0021277: TcSYL_0162280.t1-p1
OG0021278: TcSYL_0162290.t1-p1
OG0021279: TcSYL_0162300.t1-p1
OG0021280: TcSYL_0162310.t1-p1
OG0021281: TcSYL_0162320.t1-p1
OG0021282: TcSYL_0162330.t1-p1
OG0021283: TcSYL_0162340.t1-p1
OG0021284: TcSYL_0162350.t1-p1
OG0021285: TcSYL_0162360.t1-p1
OG0021286: TcSYL_0162370.t1-p1
OG0021287: TcSYL_0162380.t1-p1
OG0021288: TcSYL_0162400.t1-p1
OG0021289: TcSYL_0162410.t1-p1
OG0021290: TcSYL_0162420.t1-p1
OG0021291: TcSYL_0162430.t1-p1
OG0021292: TcSYL_0162450.t1-p1
OG0021293: TcSYL_0162460.t1-p1
OG0021294: TcSYL_0162470.t1-p1
OG0021295: TcSYL_0162480.t1-p1
OG0021296: TcSYL_0162490.t1-p1
OG0021297: TcSYL_0162510.t1-p1
OG0021298: TcSYL_0162520.t1-p1
OG0021299: TcSYL_0162530.t1-p1
OG0021300: TcSYL_0162540.t1-p1
OG0021301: TcSYL_0162560.t1-p1
OG0021302: TcSYL_0162570.t1-p1
OG0021303: TcSYL_0162590.t1-p1
OG0021304: TcSYL_0162610.t1-p1
OG0021305: TcSYL_0162620.t1-p1
OG0021306: TcSYL_0162630.t1-p1
OG0021307: TcSYL_0162650.t1-p1
OG0021308: TcSYL_0162660.t1-p1
OG0021309: TcSYL_0162670.t1-p1
OG0021310: TcSYL_0162680.t1-p1

OG0021311: TcSYL_0162700.t1-p1
OG0021312: TcSYL_0162720.t1-p1
OG0021313: TcSYL_0162730.t1-p1
OG0021314: TcSYL_0162740.t1-p1
OG0021315: TcSYL_0162750.t1-p1
OG0021316: TcSYL_0162760.t1-p1
OG0021317: TcSYL_0162770.t1-p1
OG0021318: TcSYL_0162790.t1-p1
OG0021319: TcSYL_0162800.t1-p1
OG0021320: TcSYL_0162810.t1-p1
OG0021321: TcSYL_0162820.t1-p1
OG0021322: TcSYL_0162830.t1-p1
OG0021323: TcSYL_0162840.t1-p1
OG0021324: TcSYL_0162850.t1-p1
OG0021325: TcSYL_0162860.t1-p1
OG0021326: TcSYL_0162880.t1-p1
OG0021327: TcSYL_0162890.t1-p1
OG0021328: TcSYL_0162900.t1-p1
OG0021329: TcSYL_0162920.t1-p1
OG0021330: TcSYL_0162930.t1-p1
OG0021331: TcSYL_0162940.t1-p1
OG0021332: TcSYL_0162960.t1-p1
OG0021333: TcSYL_0162970.t1-p1
OG0021334: TcSYL_0162980.t1-p1
OG0021335: TcSYL_0163000.t1-p1
OG0021336: TcSYL_0163010.t1-p1
OG0021337: TcSYL_0163030.t1-p1
OG0021338: TcSYL_0163040.t1-p1
OG0021339: TcSYL_0163050.t1-p1
OG0021340: TcSYL_0163060.t1-p1
OG0021341: TcSYL_0163080.t1-p1
OG0021342: TcSYL_0163100.t1-p1
OG0021343: TcSYL_0163110.t1-p1
OG0021344: TcSYL_0163120.t1-p1
OG0021345: TcSYL_0163130.t1-p1
OG0021346: TcSYL_0163160.t1-p1
OG0021347: TcSYL_0163180.t1-p1
OG0021348: TcSYL_0163190.t1-p1
OG0021349: TcSYL_0163200.t1-p1
OG0021350: TcSYL_0163210.t1-p1
OG0021351: TcSYL_0163220.t1-p1
OG0021352: TcSYL_0163230.t1-p1
OG0021353: TcSYL_0163240.t1-p1
OG0021354: TcSYL_0163250.t1-p1
OG0021355: TcSYL_0163270.t1-p1
OG0021356: TcSYL_0163280.t1-p1
OG0021357: TcSYL_0163290.t1-p1
OG0021358: TcSYL_0163300.t1-p1
OG0021359: TcSYL_0163310.t1-p1
OG0021360: TcSYL_0163320.t1-p1
OG0021361: TcSYL_0163340.t1-p1
OG0021362: TcSYL_0163350.t1-p1
OG0021363: TcSYL_0163360.t1-p1
OG0021364: TcSYL_0163370.t1-p1

OG0021365: TcSYL_0163380.t1-p1
OG0021366: TcSYL_0163390.t1-p1
OG0021367: TcSYL_0163410.t1-p1
OG0021368: TcSYL_0163420.t1-p1
OG0021369: TcSYL_0163430.t1-p1
OG0021370: TcSYL_0163440.t1-p1
OG0021371: TcSYL_0163450.t1-p1
OG0021372: TcSYL_0163470.t1-p1
OG0021373: TcSYL_0163480.t1-p1
OG0021374: TcSYL_0163490.t1-p1
OG0021375: TcSYL_0163500.t1-p1
OG0021376: TcSYL_0163510.t1-p1
OG0021377: TcSYL_0163530.t1-p1
OG0021378: TcSYL_0163610.t1-p1
OG0021379: TcSYL_0163620.t1-p1
OG0021380: TcSYL_0163660.t1-p1
OG0021381: TcSYL_0163730.t1-p1
OG0021382: TcSYL_0163740.t1-p1
OG0021383: TcSYL_0163760.t1-p1
OG0021384: TcSYL_0163770.t1-p1
OG0021385: TcSYL_0163830.t1-p1
OG0021386: TcSYL_0163840.t1-p1
OG0021387: TcSYL_0163870.t1-p1
OG0021388: TcSYL_0163880.t1-p1
OG0021389: TcSYL_0163890.t1-p1
OG0021390: TcSYL_0163900.t1-p1
OG0021391: TcSYL_0163910.t1-p1
OG0021392: TcSYL_0163920.t1-p1
OG0021393: TcSYL_0163950.t1-p1
OG0021394: TcSYL_0164010.t1-p1
OG0021395: TcSYL_0164020.t1-p1
OG0021396: TcSYL_0164040.t1-p1
OG0021397: TcSYL_0164050.t1-p1
OG0021398: TcSYL_0164060.t1-p1
OG0021399: TcSYL_0164070.t1-p1
OG0021400: TcSYL_0164080.t1-p1
OG0021401: TcSYL_0164140.t1-p1
OG0021402: TcSYL_0164160.t1-p1
OG0021403: TcSYL_0164170.t1-p1
OG0021404: TcSYL_0164180.t1-p1
OG0021405: TcSYL_0164190.t1-p1
OG0021406: TcSYL_0164200.t1-p1
OG0021407: TcSYL_0164210.t1-p1
OG0021408: TcSYL_0164220.t1-p1
OG0021409: TcSYL_0164240.t1-p1
OG0021410: TcSYL_0164250.t1-p1
OG0021411: TcSYL_0164260.t1-p1
OG0021412: TcSYL_0164270.t1-p1
OG0021413: TcSYL_0164290.t1-p1
OG0021414: TcSYL_0164320.t1-p1
OG0021415: TcSYL_0164330.t1-p1
OG0021416: TcSYL_0164340.t1-p1
OG0021417: TcSYL_0164360.t1-p1
OG0021418: TcSYL_0164370.t1-p1

OG0021419: TcSYL_0164390.t1-p1
OG0021420: TcSYL_0164400.t1-p1
OG0021421: TcSYL_0164410.t1-p1
OG0021422: TcSYL_0164440.t1-p1
OG0021423: TcSYL_0164450.t1-p1
OG0021424: TcSYL_0164460.t1-p1
OG0021425: TcSYL_0164470.t1-p1
OG0021426: TcSYL_0164480.t1-p1
OG0021427: TcSYL_0164490.t1-p1
OG0021428: TcSYL_0164500.t1-p1
OG0021429: TcSYL_0164510.t1-p1
OG0021430: TcSYL_0164520.t1-p1
OG0021431: TcSYL_0164530.t1-p1
OG0021432: TcSYL_0164560.t1-p1
OG0021433: TcSYL_0164570.t1-p1
OG0021434: TcSYL_0164580.t1-p1
OG0021435: TcSYL_0164590.t1-p1
OG0021436: TcSYL_0164600.t1-p1
OG0021437: TcSYL_0164630.t1-p1
OG0021438: TcSYL_0164640.t1-p1
OG0021439: TcSYL_0164670.t1-p1
OG0021440: TcSYL_0164680.t1-p1
OG0021441: TcSYL_0164690.t1-p1
OG0021442: TcSYL_0164710.t1-p1
OG0021443: TcSYL_0164730.t1-p1
OG0021444: TcSYL_0164750.t1-p1
OG0021445: TcSYL_0164760.t1-p1
OG0021446: TcSYL_0164770.t1-p1
OG0021447: TcSYL_0164790.t1-p1
OG0021448: TcSYL_0164820.t1-p1
OG0021449: TcSYL_0164830.t1-p1
OG0021450: TcSYL_0164840.t1-p1
OG0021451: TcSYL_0164850.t1-p1
OG0021452: TcSYL_0164860.t1-p1
OG0021453: TcSYL_0164870.t1-p1
OG0021454: TcSYL_0164880.t1-p1
OG0021455: TcSYL_0164890.t1-p1
OG0021456: TcSYL_0164900.t1-p1
OG0021457: TcSYL_0164940.t1-p1
OG0021458: TcSYL_0164970.t1-p1
OG0021459: TcSYL_0164980.t1-p1
OG0021460: TcSYL_0164990.t1-p1
OG0021461: TcSYL_0165000.t1-p1
OG0021462: TcSYL_0165010.t1-p1
OG0021463: TcSYL_0165020.t1-p1
OG0021464: TcSYL_0165030.t1-p1
OG0021465: TcSYL_0165040.t1-p1
OG0021466: TcSYL_0165060.t1-p1
OG0021467: TcSYL_0165080.t1-p1
OG0021468: TcSYL_0165090.t1-p1
OG0021469: TcSYL_0165120.t1-p1
OG0021470: TcSYL_0165130.t1-p1
OG0021471: TcSYL_0165140.t1-p1
OG0021472: TcSYL_0165150.t1-p1

OG0021473: TcSYL_0165170.t1-p1
OG0021474: TcSYL_0165190.t1-p1
OG0021475: TcSYL_0165210.t1-p1
OG0021476: TcSYL_0165220.t1-p1
OG0021477: TcSYL_0165230.t1-p1
OG0021478: TcSYL_0165240.t1-p1
OG0021479: TcSYL_0165250.t1-p1
OG0021480: TcSYL_0165260.t1-p1
OG0021481: TcSYL_0165270.t1-p1
OG0021482: TcSYL_0165280.t1-p1
OG0021483: TcSYL_0165290.t1-p1
OG0021484: TcSYL_0165300.t1-p1
OG0021485: TcSYL_0165310.t1-p1
OG0021486: TcSYL_0165330.t1-p1
OG0021487: TcSYL_0165340.t1-p1
OG0021488: TcSYL_0165370.t1-p1
OG0021489: TcSYL_0165380.t1-p1
OG0021490: TcSYL_0165390.t1-p1
OG0021491: TcSYL_0165400.t1-p1
OG0021492: TcSYL_0165430.t1-p1
OG0021493: TcSYL_0165450.t1-p1
OG0021494: TcSYL_0165470.t1-p1
OG0021495: TcSYL_0165480.t1-p1
OG0021496: TcSYL_0165500.t1-p1
OG0021497: TcSYL_0165530.t1-p1
OG0021498: TcSYL_0165550.t1-p1
OG0021499: TcSYL_0165560.t1-p1
OG0021500: TcSYL_0165570.t1-p1
OG0021501: TcSYL_0165600.t1-p1
OG0021502: TcSYL_0165610.t1-p1
OG0021503: TcSYL_0165620.t1-p1
OG0021504: TcSYL_0165650.t1-p1
OG0021505: TcSYL_0165660.t1-p1
OG0021506: TcSYL_0165670.t1-p1
OG0021507: TcSYL_0165690.t1-p1
OG0021508: TcSYL_0165700.t1-p1
OG0021509: TcSYL_0165720.t1-p1
OG0021510: TcSYL_0165730.t1-p1
OG0021511: TcSYL_0165740.t1-p1
OG0021512: TcSYL_0165760.t1-p1
OG0021513: TcSYL_0165770.t1-p1
OG0021514: TcSYL_0165780.t1-p1
OG0021515: TcSYL_0165790.t1-p1
OG0021516: TcSYL_0165800.t1-p1
OG0021517: TcSYL_0165810.t1-p1
OG0021518: TcSYL_0165820.t1-p1
OG0021519: TcSYL_0165830.t1-p1
OG0021520: TcSYL_0165840.t1-p1
OG0021521: TcSYL_0165850.t1-p1
OG0021522: TcSYL_0165870.t1-p1
OG0021523: TcSYL_0165890.t1-p1
OG0021524: TcSYL_0165920.t1-p1
OG0021525: TcSYL_0165930.t1-p1
OG0021526: TcSYL_0165940.t1-p1

OG0021527: TcSYL_0165950.t1-p1
OG0021528: TcSYL_0165960.t1-p1
OG0021529: TcSYL_0165970.t1-p1
OG0021530: TcSYL_0165980.t1-p1
OG0021531: TcSYL_0165990.t1-p1
OG0021532: TcSYL_0166000.t1-p1
OG0021533: TcSYL_0166010.t1-p1
OG0021534: TcSYL_0166020.t1-p1
OG0021535: TcSYL_0166030.t1-p1
OG0021536: TcSYL_0166050.t1-p1
OG0021537: TcSYL_0166080.t1-p1
OG0021538: TcSYL_0166090.t1-p1
OG0021539: TcSYL_0166100.t1-p1
OG0021540: TcSYL_0166120.t1-p1
OG0021541: TcSYL_0166130.t1-p1
OG0021542: TcSYL_0166140.t1-p1
OG0021543: TcSYL_0166150.t1-p1
OG0021544: TcSYL_0166170.t1-p1
OG0021545: TcSYL_0166180.t1-p1
OG0021546: TcSYL_0166190.t1-p1
OG0021547: TcSYL_0166200.t1-p1
OG0021548: TcSYL_0166210.t1-p1
OG0021549: TcSYL_0166230.t1-p1
OG0021550: TcSYL_0166240.t1-p1
OG0021551: TcSYL_0166250.t1-p1
OG0021552: TcSYL_0166270.t1-p1
OG0021553: TcSYL_0166290.t1-p1
OG0021554: TcSYL_0166300.t1-p1
OG0021555: TcSYL_0166310.t1-p1
OG0021556: TcSYL_0166320.t1-p1
OG0021557: TcSYL_0166330.t1-p1
OG0021558: TcSYL_0166340.t1-p1
OG0021559: TcSYL_0166350.t1-p1
OG0021560: TcSYL_0166360.t1-p1
OG0021561: TcSYL_0166380.t1-p1
OG0021562: TcSYL_0166390.t1-p1
OG0021563: TcSYL_0166400.t1-p1
OG0021564: TcSYL_0166410.t1-p1
OG0021565: TcSYL_0166420.t1-p1
OG0021566: TcSYL_0166440.t1-p1
OG0021567: TcSYL_0166480.t1-p1
OG0021568: TcSYL_0166490.t1-p1
OG0021569: TcSYL_0166500.t1-p1
OG0021570: TcSYL_0166510.t1-p1
OG0021571: TcSYL_0166520.t1-p1
OG0021572: TcSYL_0166530.t1-p1
OG0021573: TcSYL_0166540.t1-p1
OG0021574: TcSYL_0166560.t1-p1
OG0021575: TcSYL_0166570.t1-p1
OG0021576: TcSYL_0166580.t1-p1
OG0021577: TcSYL_0166590.t1-p1
OG0021578: TcSYL_0166600.t1-p1
OG0021579: TcSYL_0166620.t1-p1
OG0021580: TcSYL_0166630.t1-p1

OG0021581: TcSYL_0166640.t1-p1
OG0021582: TcSYL_0166670.t1-p1
OG0021583: TcSYL_0166680.t1-p1
OG0021584: TcSYL_0166690.t1-p1
OG0021585: TcSYL_0166700.t1-p1
OG0021586: TcSYL_0166710.t1-p1
OG0021587: TcSYL_0166730.t1-p1
OG0021588: TcSYL_0166760.t1-p1
OG0021589: TcSYL_0166770.t1-p1
OG0021590: TcSYL_0166790.t1-p1
OG0021591: TcSYL_0166800.t1-p1
OG0021592: TcSYL_0166810.t1-p1
OG0021593: TcSYL_0166830.t1-p1
OG0021594: TcSYL_0166850.t1-p1
OG0021595: TcSYL_0167030.t1-p1
OG0021596: TcSYL_0167130.t1-p1
OG0021597: TcSYL_0167230.t1-p1
OG0021598: TcSYL_0167270.t1-p1
OG0021599: TcSYL_0167280.t1-p1
OG0021600: TcSYL_0167310.t1-p1
OG0021601: TcSYL_0167360.t1-p1
OG0021602: TcSYL_0167370.t1-p1
OG0021603: TcSYL_0167380.t1-p1
OG0021604: TcSYL_0167400.t1-p1
OG0021605: TcSYL_0167410.t1-p1
OG0021606: TcSYL_0167430.t1-p1
OG0021607: TcSYL_0167440.t1-p1
OG0021608: TcSYL_0167470.t1-p1
OG0021609: TcSYL_0167490.t1-p1
OG0021610: TcSYL_0167500.t1-p1
OG0021611: TcSYL_0167510.t1-p1
OG0021612: TcSYL_0167530.t1-p1
OG0021613: TcSYL_0167540.t1-p1
OG0021614: TcSYL_0167550.t1-p1
OG0021615: TcSYL_0167560.t1-p1
OG0021616: TcSYL_0167580.t1-p1
OG0021617: TcSYL_0167590.t1-p1
OG0021618: TcSYL_0167600.t1-p1
OG0021619: TcSYL_0167610.t1-p1
OG0021620: TcSYL_0167620.t1-p1
OG0021621: TcSYL_0167630.t1-p1
OG0021622: TcSYL_0167640.t1-p1
OG0021623: TcSYL_0167650.t1-p1
OG0021624: TcSYL_0167670.t1-p1
OG0021625: TcSYL_0167680.t1-p1
OG0021626: TcSYL_0167690.t1-p1
OG0021627: TcSYL_0167710.t1-p1
OG0021628: TcSYL_0167720.t1-p1
OG0021629: TcSYL_0167730.t1-p1
OG0021630: TcSYL_0167750.t1-p1
OG0021631: TcSYL_0167760.t1-p1
OG0021632: TcSYL_0167770.t1-p1
OG0021633: TcSYL_0167790.t1-p1
OG0021634: TcSYL_0167800.t1-p1

OG0021635: TcSYL_0167810.t1-p1
OG0021636: TcSYL_0167820.t1-p1
OG0021637: TcSYL_0167830.t1-p1
OG0021638: TcSYL_0167840.t1-p1
OG0021639: TcSYL_0167850.t1-p1
OG0021640: TcSYL_0167860.t1-p1
OG0021641: TcSYL_0167890.t1-p1
OG0021642: TcSYL_0167900.t1-p1
OG0021643: TcSYL_0167920.t1-p1
OG0021644: TcSYL_0167930.t1-p1
OG0021645: TcSYL_0167940.t1-p1
OG0021646: TcSYL_0167950.t1-p1
OG0021647: TcSYL_0167960.t1-p1
OG0021648: TcSYL_0167970.t1-p1
OG0021649: TcSYL_0167980.t1-p1
OG0021650: TcSYL_0167990.t1-p1
OG0021651: TcSYL_0168020.t1-p1
OG0021652: TcSYL_0168030.t1-p1
OG0021653: TcSYL_0168040.t1-p1
OG0021654: TcSYL_0168050.t1-p1
OG0021655: TcSYL_0168070.t1-p1
OG0021656: TcSYL_0168080.t1-p1
OG0021657: TcSYL_0168090.t1-p1
OG0021658: TcSYL_0168110.t1-p1
OG0021659: TcSYL_0168120.t1-p1
OG0021660: TcSYL_0168140.t1-p1
OG0021661: TcSYL_0168150.t1-p1
OG0021662: TcSYL_0168170.t1-p1
OG0021663: TcSYL_0168180.t1-p1
OG0021664: TcSYL_0168200.t1-p1
OG0021665: TcSYL_0168210.t1-p1
OG0021666: TcSYL_0168220.t1-p1
OG0021667: TcSYL_0168230.t1-p1
OG0021668: TcSYL_0168240.t1-p1
OG0021669: TcSYL_0168250.t1-p1
OG0021670: TcSYL_0168260.t1-p1
OG0021671: TcSYL_0168270.t1-p1
OG0021672: TcSYL_0168280.t1-p1
OG0021673: TcSYL_0168290.t1-p1
OG0021674: TcSYL_0168300.t1-p1
OG0021675: TcSYL_0168320.t1-p1
OG0021676: TcSYL_0168330.t1-p1
OG0021677: TcSYL_0168340.t1-p1
OG0021678: TcSYL_0168350.t1-p1
OG0021679: TcSYL_0168360.t1-p1
OG0021680: TcSYL_0168380.t1-p1
OG0021681: TcSYL_0168400.t1-p1
OG0021682: TcSYL_0168410.t1-p1
OG0021683: TcSYL_0168420.t1-p1
OG0021684: TcSYL_0168440.t1-p1
OG0021685: TcSYL_0168460.t1-p1
OG0021686: TcSYL_0168470.t1-p1
OG0021687: TcSYL_0168490.t1-p1
OG0021688: TcSYL_0168500.t1-p1

OG0021689: TcSYL_0168510.t1-p1
OG0021690: TcSYL_0168530.t1-p1
OG0021691: TcSYL_0168540.t1-p1
OG0021692: TcSYL_0168560.t1-p1
OG0021693: TcSYL_0168570.t1-p1
OG0021694: TcSYL_0168580.t1-p1
OG0021695: TcSYL_0168600.t1-p1
OG0021696: TcSYL_0168610.t1-p1
OG0021697: TcSYL_0168620.t1-p1
OG0021698: TcSYL_0168640.t1-p1
OG0021699: TcSYL_0168650.t1-p1
OG0021700: TcSYL_0168660.t1-p1
OG0021701: TcSYL_0168670.t1-p1
OG0021702: TcSYL_0168680.t1-p1
OG0021703: TcSYL_0168700.t1-p1
OG0021704: TcSYL_0168710.t1-p1
OG0021705: TcSYL_0168730.t1-p1
OG0021706: TcSYL_0168760.t1-p1
OG0021707: TcSYL_0168770.t1-p1
OG0021708: TcSYL_0168780.t1-p1
OG0021709: TcSYL_0168800.t1-p1
OG0021710: TcSYL_0168820.t1-p1
OG0021711: TcSYL_0168830.t1-p1
OG0021712: TcSYL_0168850.t1-p1
OG0021713: TcSYL_0168870.t1-p1
OG0021714: TcSYL_0168890.t1-p1
OG0021715: TcSYL_0168910.t1-p1
OG0021716: TcSYL_0168920.t1-p1
OG0021717: TcSYL_0168930.t1-p1
OG0021718: TcSYL_0168990.t1-p1
OG0021719: TcSYL_0169010.t1-p1
OG0021720: TcSYL_0169040.t1-p1
OG0021721: TcSYL_0169070.t1-p1
OG0021722: TcSYL_0169100.t1-p1
OG0021723: TcSYL_0169190.t1-p1
OG0021724: TcSYL_0169250.t1-p1
OG0021725: TcSYL_0169280.t1-p1
OG0021726: TcSYL_0169290.t1-p1
OG0021727: TcSYL_0169300.t1-p1
OG0021728: TcSYL_0169310.t1-p1
OG0021729: TcSYL_0169320.t1-p1
OG0021730: TcSYL_0169330.t1-p1
OG0021731: TcSYL_0169340.t1-p1
OG0021732: TcSYL_0169360.t1-p1
OG0021733: TcSYL_0169370.t1-p1
OG0021734: TcSYL_0169380.t1-p1
OG0021735: TcSYL_0169390.t1-p1
OG0021736: TcSYL_0169400.t1-p1
OG0021737: TcSYL_0169410.t1-p1
OG0021738: TcSYL_0169420.t1-p1
OG0021739: TcSYL_0169440.t1-p1
OG0021740: TcSYL_0169450.t1-p1
OG0021741: TcSYL_0169460.t1-p1
OG0021742: TcSYL_0169470.t1-p1

OG0021743: TcSYL_0169480.t1-p1
OG0021744: TcSYL_0169490.t1-p1
OG0021745: TcSYL_0169500.t1-p1
OG0021746: TcSYL_0169510.t1-p1
OG0021747: TcSYL_0169520.t1-p1
OG0021748: TcSYL_0169530.t1-p1
OG0021749: TcSYL_0169540.t1-p1
OG0021750: TcSYL_0169550.t1-p1
OG0021751: TcSYL_0169560.t1-p1
OG0021752: TcSYL_0169570.t1-p1
OG0021753: TcSYL_0169580.t1-p1
OG0021754: TcSYL_0169590.t1-p1
OG0021755: TcSYL_0169600.t1-p1
OG0021756: TcSYL_0169610.t1-p1
OG0021757: TcSYL_0169620.t1-p1
OG0021758: TcSYL_0169630.t1-p1
OG0021759: TcSYL_0169640.t1-p1
OG0021760: TcSYL_0169650.t1-p1
OG0021761: TcSYL_0169660.t1-p1
OG0021762: TcSYL_0169670.t1-p1
OG0021763: TcSYL_0169680.t1-p1
OG0021764: TcSYL_0169690.t1-p1
OG0021765: TcSYL_0169700.t1-p1
OG0021766: TcSYL_0169710.t1-p1
OG0021767: TcSYL_0169720.t1-p1
OG0021768: TcSYL_0169730.t1-p1
OG0021769: TcSYL_0169740.t1-p1
OG0021770: TcSYL_0169750.t1-p1
OG0021771: TcSYL_0169760.t1-p1
OG0021772: TcSYL_0169770.t1-p1
OG0021773: TcSYL_0169780.t1-p1
OG0021774: TcSYL_0169790.t1-p1
OG0021775: TcSYL_0169810.t1-p1
OG0021776: TcSYL_0169820.t1-p1
OG0021777: TcSYL_0169830.t1-p1
OG0021778: TcSYL_0169840.t1-p1
OG0021779: TcSYL_0169850.t1-p1
OG0021780: TcSYL_0169870.t1-p1
OG0021781: TcSYL_0169890.t1-p1
OG0021782: TcSYL_0169960.t1-p1
OG0021783: TcSYL_0170010.t1-p1
OG0021784: TcSYL_0170080.t1-p1
OG0021785: TcSYL_0170090.t1-p1
OG0021786: TcSYL_0170100.t1-p1
OG0021787: TcSYL_0170110.t1-p1
OG0021788: TcSYL_0170120.t1-p1
OG0021789: TcSYL_0170130.t1-p1
OG0021790: TcSYL_0170140.t1-p1
OG0021791: TcSYL_0170150.t1-p1
OG0021792: TcSYL_0170160.t1-p1
OG0021793: TcSYL_0170170.t1-p1
OG0021794: TcSYL_0170180.t1-p1
OG0021795: TcSYL_0170190.t1-p1
OG0021796: TcSYL_0170200.t1-p1

OG0021797: TcSYL_0170210.t1-p1
OG0021798: TcSYL_0170220.t1-p1
OG0021799: TcSYL_0170240.t1-p1
OG0021800: TcSYL_0170260.t1-p1
OG0021801: TcSYL_0170270.t1-p1
OG0021802: TcSYL_0170280.t1-p1
OG0021803: TcSYL_0170290.t1-p1
OG0021804: TcSYL_0170300.t1-p1
OG0021805: TcSYL_0170310.t1-p1
OG0021806: TcSYL_0170330.t1-p1
OG0021807: TcSYL_0170400.t1-p1
OG0021808: TcSYL_0170490.t1-p1
OG0021809: TcSYL_0170500.t1-p1
OG0021810: TcSYL_0170510.t1-p1
OG0021811: TcSYL_0170530.t1-p1
OG0021812: TcSYL_0170540.t1-p1
OG0021813: TcSYL_0170550.t1-p1
OG0021814: TcSYL_0170570.t1-p1
OG0021815: TcSYL_0170630.t1-p1
OG0021816: TcSYL_0170660.t1-p1
OG0021817: TcSYL_0170680.t1-p1
OG0021818: TcSYL_0170730.t1-p1
OG0021819: TcSYL_0170740.t1-p1
OG0021820: TcSYL_0170750.t1-p1
OG0021821: TcSYL_0170760.t1-p1
OG0021822: TcSYL_0170770.t1-p1
OG0021823: TcSYL_0170790.t1-p1
OG0021824: TcSYL_0170810.t1-p1
OG0021825: TcSYL_0170830.t1-p1
OG0021826: TcSYL_0170840.t1-p1
OG0021827: TcSYL_0170850.t1-p1
OG0021828: TcSYL_0170860.t1-p1
OG0021829: TcSYL_0170880.t1-p1
OG0021830: TcSYL_0170890.t1-p1
OG0021831: TcSYL_0170900.t1-p1
OG0021832: TcSYL_0170940.t1-p1
OG0021833: TcSYL_0170970.t1-p1
OG0021834: TcSYL_0170980.t1-p1
OG0021835: TcSYL_0171010.t1-p1
OG0021836: TcSYL_0171020.t1-p1
OG0021837: TcSYL_0171030.t1-p1
OG0021838: TcSYL_0171040.t1-p1
OG0021839: TcSYL_0171050.t1-p1
OG0021840: TcSYL_0171090.t1-p1
OG0021841: TcSYL_0171110.t1-p1
OG0021842: TcSYL_0171180.t1-p1
OG0021843: TcSYL_0171200.t1-p1
OG0021844: TcSYL_0171210.t1-p1
OG0021845: TcSYL_0171230.t1-p1
OG0021846: TcSYL_0171240.t1-p1
OG0021847: TcSYL_0171250.t1-p1
OG0021848: TcSYL_0171260.t1-p1
OG0021849: TcSYL_0171270.t1-p1
OG0021850: TcSYL_0171290.t1-p1

OG0021851: TcSYL_0171300.t1-p1
OG0021852: TcSYL_0171310.t1-p1
OG0021853: TcSYL_0171320.t1-p1
OG0021854: TcSYL_0171330.t1-p1
OG0021855: TcSYL_0171340.t1-p1
OG0021856: TcSYL_0171350.t1-p1
OG0021857: TcSYL_0171360.t1-p1
OG0021858: TcSYL_0171370.t1-p1
OG0021859: TcSYL_0171380.t1-p1
OG0021860: TcSYL_0171390.t1-p1
OG0021861: TcSYL_0171410.t1-p1
OG0021862: TcSYL_0171450.t1-p1
OG0021863: TcSYL_0171460.t1-p1
OG0021864: TcSYL_0171480.t1-p1
OG0021865: TcSYL_0171500.t1-p1
OG0021866: TcSYL_0171530.t1-p1
OG0021867: TcSYL_0171540.t1-p1
OG0021868: TcSYL_0171550.t1-p1
OG0021869: TcSYL_0171560.t1-p1
OG0021870: TcSYL_0171570.t1-p1
OG0021871: TcSYL_0171580.t1-p1
OG0021872: TcSYL_0171590.t1-p1
OG0021873: TcSYL_0171600.t1-p1
OG0021874: TcSYL_0171630.t1-p1
OG0021875: TcSYL_0171650.t1-p1
OG0021876: TcSYL_0171660.t1-p1
OG0021877: TcSYL_0171670.t1-p1
OG0021878: TcSYL_0171680.t1-p1
OG0021879: TcSYL_0171690.t1-p1
OG0021880: TcSYL_0171700.t1-p1
OG0021881: TcSYL_0171710.t1-p1
OG0021882: TcSYL_0171730.t1-p1
OG0021883: TcSYL_0171740.t1-p1
OG0021884: TcSYL_0171750.t1-p1
OG0021885: TcSYL_0171760.t1-p1
OG0021886: TcSYL_0171770.t1-p1
OG0021887: TcSYL_0171780.t1-p1
OG0021888: TcSYL_0171800.t1-p1
OG0021889: TcSYL_0171810.t1-p1
OG0021890: TcSYL_0171830.t1-p1
OG0021891: TcSYL_0171840.t1-p1
OG0021892: TcSYL_0171850.t1-p1
OG0021893: TcSYL_0171880.t1-p1
OG0021894: TcSYL_0171900.t1-p1
OG0021895: TcSYL_0171910.t1-p1
OG0021896: TcSYL_0171920.t1-p1
OG0021897: TcSYL_0171950.t1-p1
OG0021898: TcSYL_0171960.t1-p1
OG0021899: TcSYL_0171990.t1-p1
OG0021900: TcSYL_0172010.t1-p1
OG0021901: TcSYL_0172020.t1-p1
OG0021902: TcSYL_0172040.t1-p1
OG0021903: TcSYL_0172050.t1-p1
OG0021904: TcSYL_0172060.t1-p1

OG0021905: TcSYL_0172070.t1-p1
OG0021906: TcSYL_0172080.t1-p1
OG0021907: TcSYL_0172100.t1-p1
OG0021908: TcSYL_0172110.t1-p1
OG0021909: TcSYL_0172130.t1-p1
OG0021910: TcSYL_0172140.t1-p1
OG0021911: TcSYL_0172160.t1-p1
OG0021912: TcSYL_0172180.t1-p1
OG0021913: TcSYL_0172200.t1-p1
OG0021914: TcSYL_0172210.t1-p1
OG0021915: TcSYL_0172230.t1-p1
OG0021916: TcSYL_0172240.t1-p1
OG0021917: TcSYL_0172250.t1-p1
OG0021918: TcSYL_0172260.t1-p1
OG0021919: TcSYL_0172270.t1-p1
OG0021920: TcSYL_0172290.t1-p1
OG0021921: TcSYL_0172300.t1-p1
OG0021922: TcSYL_0172310.t1-p1
OG0021923: TcSYL_0172320.t1-p1
OG0021924: TcSYL_0172330.t1-p1
OG0021925: TcSYL_0172350.t1-p1
OG0021926: TcSYL_0172360.t1-p1
OG0021927: TcSYL_0172380.t1-p1
OG0021928: TcSYL_0172390.t1-p1
OG0021929: TcSYL_0172410.t1-p1
OG0021930: TcSYL_0172420.t1-p1
OG0021931: TcSYL_0172440.t1-p1
OG0021932: TcSYL_0172460.t1-p1
OG0021933: TcSYL_0172480.t1-p1
OG0021934: TcSYL_0172490.t1-p1
OG0021935: TcSYL_0172500.t1-p1
OG0021936: TcSYL_0172520.t1-p1
OG0021937: TcSYL_0172530.t1-p1
OG0021938: TcSYL_0172540.t1-p1
OG0021939: TcSYL_0172560.t1-p1
OG0021940: TcSYL_0172580.t1-p1
OG0021941: TcSYL_0172600.t1-p1
OG0021942: TcSYL_0172610.t1-p1
OG0021943: TcSYL_0172620.t1-p1
OG0021944: TcSYL_0172630.t1-p1
OG0021945: TcSYL_0172650.t1-p1
OG0021946: TcSYL_0172660.t1-p1
OG0021947: TcSYL_0172710.t1-p1
OG0021948: TcSYL_0172740.t1-p1
OG0021949: TcSYL_0172750.t1-p1
OG0021950: TcSYL_0172760.t1-p1
OG0021951: TcSYL_0172780.t1-p1
OG0021952: TcSYL_0172790.t1-p1
OG0021953: TcSYL_0172800.t1-p1
OG0021954: TcSYL_0172820.t1-p1
OG0021955: TcSYL_0172840.t1-p1
OG0021956: TcSYL_0172850.t1-p1
OG0021957: TcSYL_0172860.t1-p1
OG0021958: TcSYL_0172870.t1-p1

OG0021959: TcSYL_0172880.t1-p1
OG0021960: TcSYL_0172910.t1-p1
OG0021961: TcSYL_0172940.t1-p1
OG0021962: TcSYL_0172950.t1-p1
OG0021963: TcSYL_0172960.t1-p1
OG0021964: TcSYL_0172980.t1-p1
OG0021965: TcSYL_0172990.t1-p1
OG0021966: TcSYL_0173000.t1-p1
OG0021967: TcSYL_0173010.t1-p1
OG0021968: TcSYL_0173020.t1-p1
OG0021969: TcSYL_0173030.t1-p1
OG0021970: TcSYL_0173050.t1-p1
OG0021971: TcSYL_0173060.t1-p1
OG0021972: TcSYL_0173070.t1-p1
OG0021973: TcSYL_0173080.t1-p1
OG0021974: TcSYL_0173090.t1-p1
OG0021975: TcSYL_0173100.t1-p1
OG0021976: TcSYL_0173120.t1-p1
OG0021977: TcSYL_0173130.t1-p1
OG0021978: TcSYL_0173140.t1-p1
OG0021979: TcSYL_0173150.t1-p1
OG0021980: TcSYL_0173160.t1-p1
OG0021981: TcSYL_0173170.t1-p1
OG0021982: TcSYL_0173180.t1-p1
OG0021983: TcSYL_0173190.t1-p1
OG0021984: TcSYL_0173210.t1-p1
OG0021985: TcSYL_0173230.t1-p1
OG0021986: TcSYL_0173240.t1-p1
OG0021987: TcSYL_0173250.t1-p1
OG0021988: TcSYL_0173260.t1-p1
OG0021989: TcSYL_0173290.t1-p1
OG0021990: TcSYL_0173310.t1-p1
OG0021991: TcSYL_0173320.t1-p1
OG0021992: TcSYL_0173330.t1-p1
OG0021993: TcSYL_0173340.t1-p1
OG0021994: TcSYL_0173360.t1-p1
OG0021995: TcSYL_0173370.t1-p1
OG0021996: TcSYL_0173390.t1-p1
OG0021997: TcSYL_0173410.t1-p1
OG0021998: TcSYL_0173430.t1-p1
OG0021999: TcSYL_0173440.t1-p1
OG0022000: TcSYL_0173450.t1-p1
OG0022001: TcSYL_0173460.t1-p1
OG0022002: TcSYL_0173470.t1-p1
OG0022003: TcSYL_0173480.t1-p1
OG0022004: TcSYL_0173490.t1-p1
OG0022005: TcSYL_0173520.t1-p1
OG0022006: TcSYL_0173550.t1-p1
OG0022007: TcSYL_0173570.t1-p1
OG0022008: TcSYL_0173590.t1-p1
OG0022009: TcSYL_0173620.t1-p1
OG0022010: TcSYL_0173630.t1-p1
OG0022011: TcSYL_0173640.t1-p1
OG0022012: TcSYL_0173650.t1-p1

OG0022013: TcSYL_0173660.t1-p1
OG0022014: TcSYL_0173670.t1-p1
OG0022015: TcSYL_0173680.t1-p1
OG0022016: TcSYL_0173690.t1-p1
OG0022017: TcSYL_0173700.t1-p1
OG0022018: TcSYL_0173710.t1-p1
OG0022019: TcSYL_0173720.t1-p1
OG0022020: TcSYL_0173740.t1-p1
OG0022021: TcSYL_0173750.t1-p1
OG0022022: TcSYL_0173760.t1-p1
OG0022023: TcSYL_0173770.t1-p1
OG0022024: TcSYL_0173780.t1-p1
OG0022025: TcSYL_0173790.t1-p1
OG0022026: TcSYL_0173800.t1-p1
OG0022027: TcSYL_0173810.t1-p1
OG0022028: TcSYL_0173830.t1-p1
OG0022029: TcSYL_0173840.t1-p1
OG0022030: TcSYL_0173850.t1-p1
OG0022031: TcSYL_0173860.t1-p1
OG0022032: TcSYL_0173870.t1-p1
OG0022033: TcSYL_0173880.t1-p1
OG0022034: TcSYL_0173890.t1-p1
OG0022035: TcSYL_0173900.t1-p1
OG0022036: TcSYL_0173910.t1-p1
OG0022037: TcSYL_0173920.t1-p1
OG0022038: TcSYL_0173930.t1-p1
OG0022039: TcSYL_0173950.t1-p1
OG0022040: TcSYL_0173960.t1-p1
OG0022041: TcSYL_0173970.t1-p1
OG0022042: TcSYL_0174000.t1-p1
OG0022043: TcSYL_0174020.t1-p1
OG0022044: TcSYL_0174030.t1-p1
OG0022045: TcSYL_0174040.t1-p1
OG0022046: TcSYL_0174060.t1-p1
OG0022047: TcSYL_0174070.t1-p1
OG0022048: TcSYL_0174080.t1-p1
OG0022049: TcSYL_0174090.t1-p1
OG0022050: TcSYL_0174110.t1-p1
OG0022051: TcSYL_0174120.t1-p1
OG0022052: TcSYL_0174130.t1-p1
OG0022053: TcSYL_0174160.t1-p1
OG0022054: TcSYL_0174200.t1-p1
OG0022055: TcSYL_0174230.t1-p1
OG0022056: TcSYL_0174250.t1-p1
OG0022057: TcSYL_0174280.t1-p1
OG0022058: TcSYL_0174290.t1-p1
OG0022059: TcSYL_0174300.t1-p1
OG0022060: TcSYL_0174340.t1-p1
OG0022061: TcSYL_0174350.t1-p1
OG0022062: TcSYL_0174380.t1-p1
OG0022063: TcSYL_0174410.t1-p1
OG0022064: TcSYL_0174510.t1-p1
OG0022065: TcSYL_0174670.t1-p1
OG0022066: TcSYL_0174710.t1-p1

OG0022067: TcSYL_0174770.t1-p1
OG0022068: TcSYL_0174790.t1-p1
OG0022069: TcSYL_0174910.t1-p1
OG0022070: TcSYL_0175020.t1-p1
OG0022071: TcSYL_0175030.t1-p1
OG0022072: TcSYL_0175040.t1-p1
OG0022073: TcSYL_0175050.t1-p1
OG0022074: TcSYL_0175060.t1-p1
OG0022075: TcSYL_0175070.t1-p1
OG0022076: TcSYL_0175080.t1-p1
OG0022077: TcSYL_0175090.t1-p1
OG0022078: TcSYL_0175100.t1-p1
OG0022079: TcSYL_0175110.t1-p1
OG0022080: TcSYL_0175120.t1-p1
OG0022081: TcSYL_0175130.t1-p1
OG0022082: TcSYL_0175140.t1-p1
OG0022083: TcSYL_0175150.t1-p1
OG0022084: TcSYL_0175160.t1-p1
OG0022085: TcSYL_0175170.t1-p1
OG0022086: TcSYL_0175180.t1-p1
OG0022087: TcSYL_0175190.t1-p1
OG0022088: TcSYL_0175200.t1-p1
OG0022089: TcSYL_0175210.t1-p1
OG0022090: TcSYL_0175220.t1-p1
OG0022091: TcSYL_0175230.t1-p1
OG0022092: TcSYL_0175240.t1-p1
OG0022093: TcSYL_0175250.t1-p1
OG0022094: TcSYL_0175260.t1-p1
OG0022095: TcSYL_0175270.t1-p1
OG0022096: TcSYL_0175280.t1-p1
OG0022097: TcSYL_0175290.t1-p1
OG0022098: TcSYL_0175310.t1-p1
OG0022099: TcSYL_0175320.t1-p1
OG0022100: TcSYL_0175330.t1-p1
OG0022101: TcSYL_0175350.t1-p1
OG0022102: TcSYL_0175360.t1-p1
OG0022103: TcSYL_0175370.t1-p1
OG0022104: TcSYL_0175380.t1-p1
OG0022105: TcSYL_0175390.t1-p1
OG0022106: TcSYL_0175400.t1-p1
OG0022107: TcSYL_0175410.t1-p1
OG0022108: TcSYL_0175420.t1-p1
OG0022109: TcSYL_0175440.t1-p1
OG0022110: TcSYL_0175450.t1-p1
OG0022111: TcSYL_0175460.t1-p1
OG0022112: TcSYL_0175470.t1-p1
OG0022113: TcSYL_0175490.t1-p1
OG0022114: TcSYL_0175500.t1-p1
OG0022115: TcSYL_0175510.t1-p1
OG0022116: TcSYL_0175520.t1-p1
OG0022117: TcSYL_0175530.t1-p1
OG0022118: TcSYL_0175540.t1-p1
OG0022119: TcSYL_0175550.t1-p1
OG0022120: TcSYL_0175560.t1-p1

OG0022121: TcSYL_0175570.t1-p1
OG0022122: TcSYL_0175580.t1-p1
OG0022123: TcSYL_0175590.t1-p1
OG0022124: TcSYL_0175600.t1-p1
OG0022125: TcSYL_0175610.t1-p1
OG0022126: TcSYL_0175620.t1-p1
OG0022127: TcSYL_0175630.t1-p1
OG0022128: TcSYL_0175640.t1-p1
OG0022129: TcSYL_0175650.t1-p1
OG0022130: TcSYL_0175660.t1-p1
OG0022131: TcSYL_0175670.t1-p1
OG0022132: TcSYL_0175680.t1-p1
OG0022133: TcSYL_0175690.t1-p1
OG0022134: TcSYL_0175700.t1-p1
OG0022135: TcSYL_0175710.t1-p1
OG0022136: TcSYL_0175720.t1-p1
OG0022137: TcSYL_0175730.t1-p1
OG0022138: TcSYL_0175740.t1-p1
OG0022139: TcSYL_0175750.t1-p1
OG0022140: TcSYL_0175760.t1-p1
OG0022141: TcSYL_0175770.t1-p1
OG0022142: TcSYL_0175780.t1-p1
OG0022143: TcSYL_0175800.t1-p1
OG0022144: TcSYL_0175810.t1-p1
OG0022145: TcSYL_0175820.t1-p1
OG0022146: TcSYL_0175830.t1-p1
OG0022147: TcSYL_0175850.t1-p1
OG0022148: TcSYL_0175860.t1-p1
OG0022149: TcSYL_0175870.t1-p1
OG0022150: TcSYL_0175880.t1-p1
OG0022151: TcSYL_0175890.t1-p1
OG0022152: TcSYL_0175900.t1-p1
OG0022153: TcSYL_0175910.t1-p1
OG0022154: TcSYL_0175940.t1-p1
OG0022155: TcSYL_0175950.t1-p1
OG0022156: TcSYL_0175960.t1-p1
OG0022157: TcSYL_0175970.t1-p1
OG0022158: TcSYL_0175980.t1-p1
OG0022159: TcSYL_0175990.t1-p1
OG0022160: TcSYL_0176000.t1-p1
OG0022161: TcSYL_0176010.t1-p1
OG0022162: TcSYL_0176020.t1-p1
OG0022163: TcSYL_0176030.t1-p1
OG0022164: TcSYL_0176040.t1-p1
OG0022165: TcSYL_0176050.t1-p1
OG0022166: TcSYL_0176060.t1-p1
OG0022167: TcSYL_0176070.t1-p1
OG0022168: TcSYL_0176080.t1-p1
OG0022169: TcSYL_0176100.t1-p1
OG0022170: TcSYL_0176110.t1-p1
OG0022171: TcSYL_0176130.t1-p1
OG0022172: TcSYL_0176140.t1-p1
OG0022173: TcSYL_0176150.t1-p1
OG0022174: TcSYL_0176160.t1-p1

OG0022175: TcSYL_0176180.t1-p1
OG0022176: TcSYL_0176190.t1-p1
OG0022177: TcSYL_0176210.t1-p1
OG0022178: TcSYL_0176220.t1-p1
OG0022179: TcSYL_0176230.t1-p1
OG0022180: TcSYL_0176240.t1-p1
OG0022181: TcSYL_0176250.t1-p1
OG0022182: TcSYL_0176260.t1-p1
OG0022183: TcSYL_0176270.t1-p1
OG0022184: TcSYL_0176280.t1-p1
OG0022185: TcSYL_0176290.t1-p1
OG0022186: TcSYL_0176300.t1-p1
OG0022187: TcSYL_0176310.t1-p1
OG0022188: TcSYL_0176320.t1-p1
OG0022189: TcSYL_0176330.t1-p1
OG0022190: TcSYL_0176340.t1-p1
OG0022191: TcSYL_0176350.t1-p1
OG0022192: TcSYL_0176360.t1-p1
OG0022193: TcSYL_0176370.t1-p1
OG0022194: TcSYL_0176380.t1-p1
OG0022195: TcSYL_0176390.t1-p1
OG0022196: TcSYL_0176400.t1-p1
OG0022197: TcSYL_0176420.t1-p1
OG0022198: TcSYL_0176440.t1-p1
OG0022199: TcSYL_0176450.t1-p1
OG0022200: TcSYL_0176460.t1-p1
OG0022201: TcSYL_0176480.t1-p1
OG0022202: TcSYL_0176490.t1-p1
OG0022203: TcSYL_0176500.t1-p1
OG0022204: TcSYL_0176510.t1-p1
OG0022205: TcSYL_0176530.t1-p1
OG0022206: TcSYL_0176540.t1-p1
OG0022207: TcSYL_0176560.t1-p1
OG0022208: TcSYL_0176570.t1-p1
OG0022209: TcSYL_0176580.t1-p1
OG0022210: TcSYL_0176590.t1-p1
OG0022211: TcSYL_0176600.t1-p1
OG0022212: TcSYL_0176610.t1-p1
OG0022213: TcSYL_0176620.t1-p1
OG0022214: TcSYL_0176630.t1-p1
OG0022215: TcSYL_0176640.t1-p1
OG0022216: TcSYL_0176650.t1-p1
OG0022217: TcSYL_0176660.t1-p1
OG0022218: TcSYL_0176670.t1-p1
OG0022219: TcSYL_0176680.t1-p1
OG0022220: TcSYL_0176690.t1-p1
OG0022221: TcSYL_0176700.t1-p1
OG0022222: TcSYL_0176710.t1-p1
OG0022223: TcSYL_0176720.t1-p1
OG0022224: TcSYL_0176730.t1-p1
OG0022225: TcSYL_0176740.t1-p1
OG0022226: TcSYL_0176760.t1-p1
OG0022227: TcSYL_0176770.t1-p1
OG0022228: TcSYL_0176780.t1-p1

OG0022229: TcSYL_0176800.t1-p1
OG0022230: TcSYL_0176820.t1-p1
OG0022231: TcSYL_0176880.t1-p1
OG0022232: TcSYL_0176890.t1-p1
OG0022233: TcSYL_0176900.t1-p1
OG0022234: TcSYL_0176910.t1-p1
OG0022235: TcSYL_0176920.t1-p1
OG0022236: TcSYL_0176940.t1-p1
OG0022237: TcSYL_0176950.t1-p1
OG0022238: TcSYL_0176980.t1-p1
OG0022239: TcSYL_0176990.t1-p1
OG0022240: TcSYL_0177000.t1-p1
OG0022241: TcSYL_0177010.t1-p1
OG0022242: TcSYL_0177020.t1-p1
OG0022243: TcSYL_0177030.t1-p1
OG0022244: TcSYL_0177050.t1-p1
OG0022245: TcSYL_0177060.t1-p1
OG0022246: TcSYL_0177080.t1-p1
OG0022247: TcSYL_0177090.t1-p1
OG0022248: TcSYL_0177100.t1-p1
OG0022249: TcSYL_0177110.t1-p1
OG0022250: TcSYL_0177120.t1-p1
OG0022251: TcSYL_0177130.t1-p1
OG0022252: TcSYL_0177150.t1-p1
OG0022253: TcSYL_0177160.t1-p1
OG0022254: TcSYL_0177180.t1-p1
OG0022255: TcSYL_0177190.t1-p1
OG0022256: TcSYL_0177200.t1-p1
OG0022257: TcSYL_0177210.t1-p1
OG0022258: TcSYL_0177220.t1-p1
OG0022259: TcSYL_0177230.t1-p1
OG0022260: TcSYL_0177250.t1-p1
OG0022261: TcSYL_0177260.t1-p1
OG0022262: TcSYL_0177270.t1-p1
OG0022263: TcSYL_0177280.t1-p1
OG0022264: TcSYL_0177290.t1-p1
OG0022265: TcSYL_0177300.t1-p1
OG0022266: TcSYL_0177330.t1-p1
OG0022267: TcSYL_0177340.t1-p1
OG0022268: TcSYL_0177360.t1-p1
OG0022269: TcSYL_0177370.t1-p1
OG0022270: TcSYL_0177380.t1-p1
OG0022271: TcSYL_0177390.t1-p1
OG0022272: TcSYL_0177400.t1-p1
OG0022273: TcSYL_0177420.t1-p1
OG0022274: TcSYL_0177430.t1-p1
OG0022275: TcSYL_0177450.t1-p1
OG0022276: TcSYL_0177460.t1-p1
OG0022277: TcSYL_0177480.t1-p1
OG0022278: TcSYL_0177490.t1-p1
OG0022279: TcSYL_0177530.t1-p1
OG0022280: TcSYL_0177540.t1-p1
OG0022281: TcSYL_0177550.t1-p1
OG0022282: TcSYL_0177560.t1-p1

OG0022283: TcSYL_0177570.t1-p1
OG0022284: TcSYL_0177580.t1-p1
OG0022285: TcSYL_0177590.t1-p1
OG0022286: TcSYL_0177600.t1-p1
OG0022287: TcSYL_0177610.t1-p1
OG0022288: TcSYL_0177620.t1-p1
OG0022289: TcSYL_0177630.t1-p1
OG0022290: TcSYL_0177640.t1-p1
OG0022291: TcSYL_0177650.t1-p1
OG0022292: TcSYL_0177660.t1-p1
OG0022293: TcSYL_0177670.t1-p1
OG0022294: TcSYL_0177680.t1-p1
OG0022295: TcSYL_0177690.t1-p1
OG0022296: TcSYL_0177700.t1-p1
OG0022297: TcSYL_0177710.t1-p1
OG0022298: TcSYL_0177720.t1-p1
OG0022299: TcSYL_0177730.t1-p1
OG0022300: TcSYL_0177740.t1-p1
OG0022301: TcSYL_0177750.t1-p1
OG0022302: TcSYL_0177760.t1-p1
OG0022303: TcSYL_0177770.t1-p1
OG0022304: TcSYL_0177780.t1-p1
OG0022305: TcSYL_0177790.t1-p1
OG0022306: TcSYL_0177800.t1-p1
OG0022307: TcSYL_0177810.t1-p1
OG0022308: TcSYL_0177820.t1-p1
OG0022309: TcSYL_0177830.t1-p1
OG0022310: TcSYL_0177840.t1-p1
OG0022311: TcSYL_0177850.t1-p1
OG0022312: TcSYL_0177860.t1-p1
OG0022313: TcSYL_0177870.t1-p1
OG0022314: TcSYL_0177880.t1-p1
OG0022315: TcSYL_0177890.t1-p1
OG0022316: TcSYL_0177900.t1-p1
OG0022317: TcSYL_0177910.t1-p1
OG0022318: TcSYL_0177920.t1-p1
OG0022319: TcSYL_0177950.t1-p1
OG0022320: TcSYL_0177960.t1-p1
OG0022321: TcSYL_0177970.t1-p1
OG0022322: TcSYL_0177980.t1-p1
OG0022323: TcSYL_0178000.t1-p1
OG0022324: TcSYL_0178020.t1-p1
OG0022325: TcSYL_0178030.t1-p1
OG0022326: TcSYL_0178040.t1-p1
OG0022327: TcSYL_0178050.t1-p1
OG0022328: TcSYL_0178070.t1-p1
OG0022329: TcSYL_0178110.t1-p1
OG0022330: TcSYL_0178140.t1-p1
OG0022331: TcSYL_0178160.t1-p1
OG0022332: TcSYL_0178170.t1-p1
OG0022333: TcSYL_0178180.t1-p1
OG0022334: TcSYL_0178190.t1-p1
OG0022335: TcSYL_0178200.t1-p1
OG0022336: TcSYL_0178210.t1-p1

OG0022337: TcSYL_0178220.t1-p1
OG0022338: TcSYL_0178230.t1-p1
OG0022339: TcSYL_0178240.t1-p1
OG0022340: TcSYL_0178250.t1-p1
OG0022341: TcSYL_0178260.t1-p1
OG0022342: TcSYL_0178270.t1-p1
OG0022343: TcSYL_0178290.t1-p1
OG0022344: TcSYL_0178300.t1-p1
OG0022345: TcSYL_0178310.t1-p1
OG0022346: TcSYL_0178320.t1-p1
OG0022347: TcSYL_0178330.t1-p1
OG0022348: TcSYL_0178350.t1-p1
OG0022349: TcSYL_0178370.t1-p1
OG0022350: TcSYL_0178380.t1-p1
OG0022351: TcSYL_0178390.t1-p1
OG0022352: TcSYL_0178400.t1-p1
OG0022353: TcSYL_0178410.t1-p1
OG0022354: TcSYL_0178420.t1-p1
OG0022355: TcSYL_0178430.t1-p1
OG0022356: TcSYL_0178440.t1-p1
OG0022357: TcSYL_0178460.t1-p1
OG0022358: TcSYL_0178470.t1-p1
OG0022359: TcSYL_0178490.t1-p1
OG0022360: TcSYL_0178520.t1-p1
OG0022361: TcSYL_0178530.t1-p1
OG0022362: TcSYL_0178550.t1-p1
OG0022363: TcSYL_0178580.t1-p1
OG0022364: TcSYL_0178600.t1-p1
OG0022365: TcSYL_0178610.t1-p1
OG0022366: TcSYL_0178630.t1-p1
OG0022367: TcSYL_0178640.t1-p1
OG0022368: TcSYL_0178650.t1-p1
OG0022369: TcSYL_0178670.t1-p1
OG0022370: TcSYL_0178680.t1-p1
OG0022371: TcSYL_0178690.t1-p1
OG0022372: TcSYL_0178700.t1-p1
OG0022373: TcSYL_0178740.t1-p1
OG0022374: TcSYL_0178750.t1-p1
OG0022375: TcSYL_0178760.t1-p1
OG0022376: TcSYL_0178770.t1-p1
OG0022377: TcSYL_0178790.t1-p1
OG0022378: TcSYL_0178800.t1-p1
OG0022379: TcSYL_0178820.t1-p1
OG0022380: TcSYL_0178830.t1-p1
OG0022381: TcSYL_0178840.t1-p1
OG0022382: TcSYL_0178860.t1-p1
OG0022383: TcSYL_0178870.t1-p1
OG0022384: TcSYL_0178880.t1-p1
OG0022385: TcSYL_0178890.t1-p1
OG0022386: TcSYL_0178910.t1-p1
OG0022387: TcSYL_0178930.t1-p1
OG0022388: TcSYL_0178940.t1-p1
OG0022389: TcSYL_0178950.t1-p1
OG0022390: TcSYL_0178970.t1-p1

OG0022391: TcSYL_0178980.t1-p1
OG0022392: TcSYL_0178990.t1-p1
OG0022393: TcSYL_0179000.t1-p1
OG0022394: TcSYL_0179010.t1-p1
OG0022395: TcSYL_0179030.t1-p1
OG0022396: TcSYL_0179040.t1-p1
OG0022397: TcSYL_0179060.t1-p1
OG0022398: TcSYL_0179070.t1-p1
OG0022399: TcSYL_0179080.t1-p1
OG0022400: TcSYL_0179090.t1-p1
OG0022401: TcSYL_0179110.t1-p1
OG0022402: TcSYL_0179180.t1-p1
OG0022403: TcSYL_0179230.t1-p1
OG0022404: TcSYL_0179290.t1-p1
OG0022405: TcSYL_0179300.t1-p1
OG0022406: TcSYL_0179310.t1-p1
OG0022407: TcSYL_0179320.t1-p1
OG0022408: TcSYL_0179330.t1-p1
OG0022409: TcSYL_0179340.t1-p1
OG0022410: TcSYL_0179350.t1-p1
OG0022411: TcSYL_0179370.t1-p1
OG0022412: TcSYL_0179380.t1-p1
OG0022413: TcSYL_0179400.t1-p1
OG0022414: TcSYL_0179410.t1-p1
OG0022415: TcSYL_0179420.t1-p1
OG0022416: TcSYL_0179430.t1-p1
OG0022417: TcSYL_0179460.t1-p1
OG0022418: TcSYL_0179470.t1-p1
OG0022419: TcSYL_0179480.t1-p1
OG0022420: TcSYL_0179490.t1-p1
OG0022421: TcSYL_0179510.t1-p1
OG0022422: TcSYL_0179520.t1-p1
OG0022423: TcSYL_0179530.t1-p1
OG0022424: TcSYL_0179560.t1-p1
OG0022425: TcSYL_0179570.t1-p1
OG0022426: TcSYL_0179580.t1-p1
OG0022427: TcSYL_0179590.t1-p1
OG0022428: TcSYL_0179600.t1-p1
OG0022429: TcSYL_0179640.t1-p1
OG0022430: TcSYL_0179650.t1-p1
OG0022431: TcSYL_0179660.t1-p1
OG0022432: TcSYL_0179680.t1-p1
OG0022433: TcSYL_0179690.t1-p1
OG0022434: TcSYL_0179710.t1-p1
OG0022435: TcSYL_0179720.t1-p1
OG0022436: TcSYL_0179740.t1-p1
OG0022437: TcSYL_0179760.t1-p1
OG0022438: TcSYL_0179780.t1-p1
OG0022439: TcSYL_0179790.t1-p1
OG0022440: TcSYL_0179810.t1-p1
OG0022441: TcSYL_0179820.t1-p1
OG0022442: TcSYL_0179840.t1-p1
OG0022443: TcSYL_0179860.t1-p1
OG0022444: TcSYL_0179880.t1-p1

OG0022445: TcSYL_0179910.t1-p1
OG0022446: TcSYL_0179930.t1-p1
OG0022447: TcSYL_0179940.t1-p1
OG0022448: TcSYL_0179950.t1-p1
OG0022449: TcSYL_0179960.t1-p1
OG0022450: TcSYL_0180010.t1-p1
OG0022451: TcSYL_0180030.t1-p1
OG0022452: TcSYL_0180060.t1-p1
OG0022453: TcSYL_0180070.t1-p1
OG0022454: TcSYL_0180090.t1-p1
OG0022455: TcSYL_0180100.t1-p1
OG0022456: TcSYL_0180150.t1-p1
OG0022457: TcSYL_0180160.t1-p1
OG0022458: TcSYL_0180170.t1-p1
OG0022459: TcSYL_0180180.t1-p1
OG0022460: TcSYL_0180190.t1-p1
OG0022461: TcSYL_0180200.t1-p1
OG0022462: TcSYL_0180210.t1-p1
OG0022463: TcSYL_0180220.t1-p1
OG0022464: TcSYL_0180230.t1-p1
OG0022465: TcSYL_0180250.t1-p1
OG0022466: TcSYL_0180260.t1-p1
OG0022467: TcSYL_0180270.t1-p1
OG0022468: TcSYL_0180280.t1-p1
OG0022469: TcSYL_0180290.t1-p1
OG0022470: TcSYL_0180300.t1-p1
OG0022471: TcSYL_0180310.t1-p1
OG0022472: TcSYL_0180320.t1-p1
OG0022473: TcSYL_0180330.t1-p1
OG0022474: TcSYL_0180340.t1-p1
OG0022475: TcSYL_0180360.t1-p1
OG0022476: TcSYL_0180370.t1-p1
OG0022477: TcSYL_0180390.t1-p1
OG0022478: TcSYL_0180400.t1-p1
OG0022479: TcSYL_0180410.t1-p1
OG0022480: TcSYL_0180420.t1-p1
OG0022481: TcSYL_0180430.t1-p1
OG0022482: TcSYL_0180440.t1-p1
OG0022483: TcSYL_0180450.t1-p1
OG0022484: TcSYL_0180460.t1-p1
OG0022485: TcSYL_0180470.t1-p1
OG0022486: TcSYL_0180480.t1-p1
OG0022487: TcSYL_0180490.t1-p1
OG0022488: TcSYL_0180510.t1-p1
OG0022489: TcSYL_0180530.t1-p1
OG0022490: TcSYL_0180540.t1-p1
OG0022491: TcSYL_0180560.t1-p1
OG0022492: TcSYL_0180580.t1-p1
OG0022493: TcSYL_0180590.t1-p1
OG0022494: TcSYL_0180610.t1-p1
OG0022495: TcSYL_0180620.t1-p1
OG0022496: TcSYL_0180630.t1-p1
OG0022497: TcSYL_0180660.t1-p1
OG0022498: TcSYL_0180670.t1-p1

OG0022499: TcSYL_0180690.t1-p1
OG0022500: TcSYL_0180700.t1-p1
OG0022501: TcSYL_0180720.t1-p1
OG0022502: TcSYL_0180730.t1-p1
OG0022503: TcSYL_0180750.t1-p1
OG0022504: TcSYL_0180760.t1-p1
OG0022505: TcSYL_0180770.t1-p1
OG0022506: TcSYL_0180780.t1-p1
OG0022507: TcSYL_0180810.t1-p1
OG0022508: TcSYL_0180820.t1-p1
OG0022509: TcSYL_0180840.t1-p1
OG0022510: TcSYL_0180850.t1-p1
OG0022511: TcSYL_0180860.t1-p1
OG0022512: TcSYL_0180870.t1-p1
OG0022513: TcSYL_0180880.t1-p1
OG0022514: TcSYL_0180920.t1-p1
OG0022515: TcSYL_0180960.t1-p1
OG0022516: TcSYL_0181000.t1-p1
OG0022517: TcSYL_0181010.t1-p1
OG0022518: TcSYL_0181020.t1-p1
OG0022519: TcSYL_0181030.t1-p1
OG0022520: TcSYL_0181040.t1-p1
OG0022521: TcSYL_0181050.t1-p1
OG0022522: TcSYL_0181060.t1-p1
OG0022523: TcSYL_0181070.t1-p1
OG0022524: TcSYL_0181080.t1-p1
OG0022525: TcSYL_0181090.t1-p1
OG0022526: TcSYL_0181100.t1-p1
OG0022527: TcSYL_0181110.t1-p1
OG0022528: TcSYL_0181120.t1-p1
OG0022529: TcSYL_0181130.t1-p1
OG0022530: TcSYL_0181140.t1-p1
OG0022531: TcSYL_0181150.t1-p1
OG0022532: TcSYL_0181160.t1-p1
OG0022533: TcSYL_0181170.t1-p1
OG0022534: TcSYL_0181180.t1-p1
OG0022535: TcSYL_0181190.t1-p1
OG0022536: TcSYL_0181200.t1-p1
OG0022537: TcSYL_0181210.t1-p1
OG0022538: TcSYL_0181220.t1-p1
OG0022539: TcSYL_0181230.t1-p1
OG0022540: TcSYL_0181240.t1-p1
OG0022541: TcSYL_0181250.t1-p1
OG0022542: TcSYL_0181260.t1-p1
OG0022543: TcSYL_0181270.t1-p1
OG0022544: TcSYL_0181280.t1-p1
OG0022545: TcSYL_0181290.t1-p1
OG0022546: TcSYL_0181300.t1-p1
OG0022547: TcSYL_0181310.t1-p1
OG0022548: TcSYL_0181320.t1-p1
OG0022549: TcSYL_0181330.t1-p1
OG0022550: TcSYL_0181340.t1-p1
OG0022551: TcSYL_0181400.t1-p1
OG0022552: TcSYL_0181450.t1-p1

OG0022553: TcSYL_0181500.t1-p1
OG0022554: TcSYL_0181510.t1-p1
OG0022555: TcSYL_0181520.t1-p1
OG0022556: TcSYL_0181540.t1-p1
OG0022557: TcSYL_0181550.t1-p1
OG0022558: TcSYL_0181570.t1-p1
OG0022559: TcSYL_0181580.t1-p1
OG0022560: TcSYL_0181590.t1-p1
OG0022561: TcSYL_0181610.t1-p1
OG0022562: TcSYL_0181620.t1-p1
OG0022563: TcSYL_0181630.t1-p1
OG0022564: TcSYL_0181640.t1-p1
OG0022565: TcSYL_0181660.t1-p1
OG0022566: TcSYL_0181670.t1-p1
OG0022567: TcSYL_0181680.t1-p1
OG0022568: TcSYL_0181690.t1-p1
OG0022569: TcSYL_0181700.t1-p1
OG0022570: TcSYL_0181720.t1-p1
OG0022571: TcSYL_0181730.t1-p1
OG0022572: TcSYL_0181740.t1-p1
OG0022573: TcSYL_0181750.t1-p1
OG0022574: TcSYL_0181770.t1-p1
OG0022575: TcSYL_0181780.t1-p1
OG0022576: TcSYL_0181790.t1-p1
OG0022577: TcSYL_0181820.t1-p1
OG0022578: TcSYL_0181830.t1-p1
OG0022579: TcSYL_0181850.t1-p1
OG0022580: TcSYL_0181860.t1-p1
OG0022581: TcSYL_0181870.t1-p1
OG0022582: TcSYL_0181880.t1-p1
OG0022583: TcSYL_0181890.t1-p1
OG0022584: TcSYL_0181910.t1-p1
OG0022585: TcSYL_0181920.t1-p1
OG0022586: TcSYL_0181930.t1-p1
OG0022587: TcSYL_0181940.t1-p1
OG0022588: TcSYL_0181950.t1-p1
OG0022589: TcSYL_0181960.t1-p1
OG0022590: TcSYL_0181970.t1-p1
OG0022591: TcSYL_0181980.t1-p1
OG0022592: TcSYL_0181990.t1-p1
OG0022593: TcSYL_0182010.t1-p1
OG0022594: TcSYL_0182020.t1-p1
OG0022595: TcSYL_0182040.t1-p1
OG0022596: TcSYL_0182050.t1-p1
OG0022597: TcSYL_0182070.t1-p1
OG0022598: TcSYL_0182080.t1-p1
OG0022599: TcSYL_0182090.t1-p1
OG0022600: TcSYL_0182100.t1-p1
OG0022601: TcSYL_0182110.t1-p1
OG0022602: TcSYL_0182120.t1-p1
OG0022603: TcSYL_0182140.t1-p1
OG0022604: TcSYL_0182150.t1-p1
OG0022605: TcSYL_0182160.t1-p1
OG0022606: TcSYL_0182180.t1-p1

OG0022607: TcSYL_0182190.t1-p1
OG0022608: TcSYL_0182200.t1-p1
OG0022609: TcSYL_0182210.t1-p1
OG0022610: TcSYL_0182220.t1-p1
OG0022611: TcSYL_0182230.t1-p1
OG0022612: TcSYL_0182240.t1-p1
OG0022613: TcSYL_0182250.t1-p1
OG0022614: TcSYL_0182260.t1-p1
OG0022615: TcSYL_0182280.t1-p1
OG0022616: TcSYL_0182290.t1-p1
OG0022617: TcSYL_0182300.t1-p1
OG0022618: TcSYL_0182340.t1-p1
OG0022619: TcSYL_0182350.t1-p1
OG0022620: TcSYL_0182360.t1-p1
OG0022621: TcSYL_0182380.t1-p1
OG0022622: TcSYL_0182390.t1-p1
OG0022623: TcSYL_0182410.t1-p1
OG0022624: TcSYL_0182420.t1-p1
OG0022625: TcSYL_0182430.t1-p1
OG0022626: TcSYL_0182450.t1-p1
OG0022627: TcSYL_0182460.t1-p1
OG0022628: TcSYL_0182470.t1-p1
OG0022629: TcSYL_0182480.t1-p1
OG0022630: TcSYL_0182490.t1-p1
OG0022631: TcSYL_0182500.t1-p1
OG0022632: TcSYL_0182590.t1-p1
OG0022633: TcSYL_0182600.t1-p1
OG0022634: TcSYL_0182620.t1-p1
OG0022635: TcSYL_0182630.t1-p1
OG0022636: TcSYL_0182640.t1-p1
OG0022637: TcSYL_0182650.t1-p1
OG0022638: TcSYL_0182660.t1-p1
OG0022639: TcSYL_0182670.t1-p1
OG0022640: TcSYL_0182680.t1-p1
OG0022641: TcSYL_0182690.t1-p1
OG0022642: TcSYL_0182700.t1-p1
OG0022643: TcSYL_0182730.t1-p1
OG0022644: TcSYL_0182740.t1-p1
OG0022645: TcSYL_0182760.t1-p1
OG0022646: TcSYL_0182780.t1-p1
OG0022647: TcSYL_0182790.t1-p1
OG0022648: TcSYL_0182810.t1-p1
OG0022649: TcSYL_0182820.t1-p1
OG0022650: TcSYL_0182840.t1-p1
OG0022651: TcSYL_0182850.t1-p1
OG0022652: TcSYL_0182860.t1-p1
OG0022653: TcSYL_0182870.t1-p1
OG0022654: TcSYL_0182880.t1-p1
OG0022655: TcSYL_0182890.t1-p1
OG0022656: TcSYL_0182900.t1-p1
OG0022657: TcSYL_0182910.t1-p1
OG0022658: TcSYL_0182920.t1-p1
OG0022659: TcSYL_0182930.t1-p1
OG0022660: TcSYL_0182940.t1-p1

OG0022661: TcSYL_0182950.t1-p1
OG0022662: TcSYL_0182960.t1-p1
OG0022663: TcSYL_0182970.t1-p1
OG0022664: TcSYL_0182980.t1-p1
OG0022665: TcSYL_0182990.t1-p1
OG0022666: TcSYL_0183000.t1-p1
OG0022667: TcSYL_0183010.t1-p1
OG0022668: TcSYL_0183020.t1-p1
OG0022669: TcSYL_0183040.t1-p1
OG0022670: TcSYL_0183050.t1-p1
OG0022671: TcSYL_0183060.t1-p1
OG0022672: TcSYL_0183070.t1-p1
OG0022673: TcSYL_0183080.t1-p1
OG0022674: TcSYL_0183090.t1-p1
OG0022675: TcSYL_0183100.t1-p1
OG0022676: TcSYL_0183110.t1-p1
OG0022677: TcSYL_0183120.t1-p1
OG0022678: TcSYL_0183130.t1-p1
OG0022679: TcSYL_0183140.t1-p1
OG0022680: TcSYL_0183150.t1-p1
OG0022681: TcSYL_0183170.t1-p1
OG0022682: TcSYL_0183180.t1-p1
OG0022683: TcSYL_0183200.t1-p1
OG0022684: TcSYL_0183210.t1-p1
OG0022685: TcSYL_0183240.t1-p1
OG0022686: TcSYL_0183250.t1-p1
OG0022687: TcSYL_0183260.t1-p1
OG0022688: TcSYL_0183270.t1-p1
OG0022689: TcSYL_0183280.t1-p1
OG0022690: TcSYL_0183290.t1-p1
OG0022691: TcSYL_0183300.t1-p1
OG0022692: TcSYL_0183320.t1-p1
OG0022693: TcSYL_0183330.t1-p1
OG0022694: TcSYL_0183340.t1-p1
OG0022695: TcSYL_0183350.t1-p1
OG0022696: TcSYL_0183360.t1-p1
OG0022697: TcSYL_0183370.t1-p1
OG0022698: TcSYL_0183380.t1-p1
OG0022699: TcSYL_0183390.t1-p1
OG0022700: TcSYL_0183400.t1-p1
OG0022701: TcSYL_0183410.t1-p1
OG0022702: TcSYL_0183420.t1-p1
OG0022703: TcSYL_0183430.t1-p1
OG0022704: TcSYL_0183450.t1-p1
OG0022705: TcSYL_0183460.t1-p1
OG0022706: TcSYL_0183470.t1-p1
OG0022707: TcSYL_0183490.t1-p1
OG0022708: TcSYL_0183510.t1-p1
OG0022709: TcSYL_0183520.t1-p1
OG0022710: TcSYL_0183530.t1-p1
OG0022711: TcSYL_0183540.t1-p1
OG0022712: TcSYL_0183550.t1-p1
OG0022713: TcSYL_0183560.t1-p1
OG0022714: TcSYL_0183570.t1-p1

OG0022715: TcSYL_0183580.t1-p1
OG0022716: TcSYL_0183590.t1-p1
OG0022717: TcSYL_0183600.t1-p1
OG0022718: TcSYL_0183610.t1-p1
OG0022719: TcSYL_0183620.t1-p1
OG0022720: TcSYL_0183630.t1-p1
OG0022721: TcSYL_0183770.t1-p1
OG0022722: TcSYL_0183780.t1-p1
OG0022723: TcSYL_0183800.t1-p1
OG0022724: TcSYL_0183810.t1-p1
OG0022725: TcSYL_0183820.t1-p1
OG0022726: TcSYL_0183830.t1-p1
OG0022727: TcSYL_0183840.t1-p1
OG0022728: TcSYL_0183870.t1-p1
OG0022729: TcSYL_0183890.t1-p1
OG0022730: TcSYL_0183900.t1-p1
OG0022731: TcSYL_0183920.t1-p1
OG0022732: TcSYL_0183940.t1-p1
OG0022733: TcSYL_0183950.t1-p1
OG0022734: TcSYL_0183960.t1-p1
OG0022735: TcSYL_0183970.t1-p1
OG0022736: TcSYL_0183990.t1-p1
OG0022737: TcSYL_0184000.t1-p1
OG0022738: TcSYL_0184010.t1-p1
OG0022739: TcSYL_0184020.t1-p1
OG0022740: TcSYL_0184030.t1-p1
OG0022741: TcSYL_0184040.t1-p1
OG0022742: TcSYL_0184050.t1-p1
OG0022743: TcSYL_0184060.t1-p1
OG0022744: TcSYL_0184070.t1-p1
OG0022745: TcSYL_0184090.t1-p1
OG0022746: TcSYL_0184110.t1-p1
OG0022747: TcSYL_0184130.t1-p1
OG0022748: TcSYL_0184140.t1-p1
OG0022749: TcSYL_0184150.t1-p1
OG0022750: TcSYL_0184160.t1-p1
OG0022751: TcSYL_0184180.t1-p1
OG0022752: TcSYL_0184190.t1-p1
OG0022753: TcSYL_0184210.t1-p1
OG0022754: TcSYL_0184230.t1-p1
OG0022755: TcSYL_0184240.t1-p1
OG0022756: TcSYL_0184260.t1-p1
OG0022757: TcSYL_0184280.t1-p1
OG0022758: TcSYL_0184290.t1-p1
OG0022759: TcSYL_0184300.t1-p1
OG0022760: TcSYL_0184320.t1-p1
OG0022761: TcSYL_0184330.t1-p1
OG0022762: TcSYL_0184350.t1-p1
OG0022763: TcSYL_0184360.t1-p1
OG0022764: TcSYL_0184370.t1-p1
OG0022765: TcSYL_0184380.t1-p1
OG0022766: TcSYL_0184390.t1-p1
OG0022767: TcSYL_0184400.t1-p1
OG0022768: TcSYL_0184410.t1-p1

OG0022769: TcSYL_0184420.t1-p1
OG0022770: TcSYL_0184430.t1-p1
OG0022771: TcSYL_0184440.t1-p1
OG0022772: TcSYL_0184450.t1-p1
OG0022773: TcSYL_0184460.t1-p1
OG0022774: TcSYL_0184470.t1-p1
OG0022775: TcSYL_0184480.t1-p1
OG0022776: TcSYL_0184490.t1-p1
OG0022777: TcSYL_0184500.t1-p1
OG0022778: TcSYL_0184510.t1-p1
OG0022779: TcSYL_0184520.t1-p1
OG0022780: TcSYL_0184530.t1-p1
OG0022781: TcSYL_0184540.t1-p1
OG0022782: TcSYL_0184550.t1-p1
OG0022783: TcSYL_0184560.t1-p1
OG0022784: TcSYL_0184570.t1-p1
OG0022785: TcSYL_0184590.t1-p1
OG0022786: TcSYL_0184600.t1-p1
OG0022787: TcSYL_0184610.t1-p1
OG0022788: TcSYL_0184620.t1-p1
OG0022789: TcSYL_0184630.t1-p1
OG0022790: TcSYL_0184650.t1-p1
OG0022791: TcSYL_0184660.t1-p1
OG0022792: TcSYL_0184680.t1-p1
OG0022793: TcSYL_0184690.t1-p1
OG0022794: TcSYL_0184700.t1-p1
OG0022795: TcSYL_0184710.t1-p1
OG0022796: TcSYL_0184720.t1-p1
OG0022797: TcSYL_0184730.t1-p1
OG0022798: TcSYL_0184740.t1-p1
OG0022799: TcSYL_0184750.t1-p1
OG0022800: TcSYL_0184760.t1-p1
OG0022801: TcSYL_0184770.t1-p1
OG0022802: TcSYL_0184780.t1-p1
OG0022803: TcSYL_0184800.t1-p1
OG0022804: TcSYL_0184810.t1-p1
OG0022805: TcSYL_0184820.t1-p1
OG0022806: TcSYL_0184830.t1-p1
OG0022807: TcSYL_0184840.t1-p1
OG0022808: TcSYL_0184850.t1-p1
OG0022809: TcSYL_0184860.t1-p1
OG0022810: TcSYL_0184870.t1-p1
OG0022811: TcSYL_0185020.t1-p1
OG0022812: TcSYL_0185040.t1-p1
OG0022813: TcSYL_0185050.t1-p1
OG0022814: TcSYL_0185070.t1-p1
OG0022815: TcSYL_0185080.t1-p1
OG0022816: TcSYL_0185090.t1-p1
OG0022817: TcSYL_0185100.t1-p1
OG0022818: TcSYL_0185110.t1-p1
OG0022819: TcSYL_0185130.t1-p1
OG0022820: TcSYL_0185140.t1-p1
OG0022821: TcSYL_0185150.t1-p1
OG0022822: TcSYL_0185160.t1-p1

OG0022823: TcSYL_0185170.t1-p1
OG0022824: TcSYL_0185190.t1-p1
OG0022825: TcSYL_0185200.t1-p1
OG0022826: TcSYL_0185220.t1-p1
OG0022827: TcSYL_0185230.t1-p1
OG0022828: TcSYL_0185250.t1-p1
OG0022829: TcSYL_0185260.t1-p1
OG0022830: TcSYL_0185270.t1-p1
OG0022831: TcSYL_0185290.t1-p1
OG0022832: TcSYL_0185300.t1-p1
OG0022833: TcSYL_0185320.t1-p1
OG0022834: TcSYL_0185330.t1-p1
OG0022835: TcSYL_0185340.t1-p1
OG0022836: TcSYL_0185350.t1-p1
OG0022837: TcSYL_0185370.t1-p1
OG0022838: TcSYL_0185380.t1-p1
OG0022839: TcSYL_0185400.t1-p1
OG0022840: TcSYL_0185410.t1-p1
OG0022841: TcSYL_0185420.t1-p1
OG0022842: TcSYL_0185440.t1-p1
OG0022843: TcSYL_0185450.t1-p1
OG0022844: TcSYL_0185460.t1-p1
OG0022845: TcSYL_0185470.t1-p1
OG0022846: TcSYL_0185480.t1-p1
OG0022847: TcSYL_0185490.t1-p1
OG0022848: TcSYL_0185500.t1-p1
OG0022849: TcSYL_0185510.t1-p1
OG0022850: TcSYL_0185520.t1-p1
OG0022851: TcSYL_0185530.t1-p1
OG0022852: TcSYL_0185540.t1-p1
OG0022853: TcSYL_0185560.t1-p1
OG0022854: TcSYL_0185570.t1-p1
OG0022855: TcSYL_0185580.t1-p1
OG0022856: TcSYL_0185590.t1-p1
OG0022857: TcSYL_0185600.t1-p1
OG0022858: TcSYL_0185620.t1-p1
OG0022859: TcSYL_0185630.t1-p1
OG0022860: TcSYL_0185640.t1-p1
OG0022861: TcSYL_0185650.t1-p1
OG0022862: TcSYL_0185660.t1-p1
OG0022863: TcSYL_0185670.t1-p1
OG0022864: TcSYL_0185680.t1-p1
OG0022865: TcSYL_0185690.t1-p1
OG0022866: TcSYL_0185700.t1-p1
OG0022867: TcSYL_0185710.t1-p1
OG0022868: TcSYL_0185720.t1-p1
OG0022869: TcSYL_0185730.t1-p1
OG0022870: TcSYL_0185740.t1-p1
OG0022871: TcSYL_0185750.t1-p1
OG0022872: TcSYL_0185760.t1-p1
OG0022873: TcSYL_0185770.t1-p1
OG0022874: TcSYL_0185780.t1-p1
OG0022875: TcSYL_0185790.t1-p1
OG0022876: TcSYL_0185800.t1-p1

OG0022877: TcSYL_0185810.t1-p1
OG0022878: TcSYL_0185820.t1-p1
OG0022879: TcSYL_0185830.t1-p1
OG0022880: TcSYL_0185840.t1-p1
OG0022881: TcSYL_0185850.t1-p1
OG0022882: TcSYL_0185860.t1-p1
OG0022883: TcSYL_0185870.t1-p1
OG0022884: TcSYL_0185880.t1-p1
OG0022885: TcSYL_0185890.t1-p1
OG0022886: TcSYL_0185900.t1-p1
OG0022887: TcSYL_0185910.t1-p1
OG0022888: TcSYL_0185920.t1-p1
OG0022889: TcSYL_0185930.t1-p1
OG0022890: TcSYL_0185940.t1-p1
OG0022891: TcSYL_0185960.t1-p1
OG0022892: TcSYL_0186010.t1-p1
OG0022893: TcSYL_0186020.t1-p1
OG0022894: TcSYL_0186030.t1-p1
OG0022895: TcSYL_0186040.t1-p1
OG0022896: TcSYL_0186060.t1-p1
OG0022897: TcSYL_0186070.t1-p1
OG0022898: TcSYL_0186080.t1-p1
OG0022899: TcSYL_0186090.t1-p1
OG0022900: TcSYL_0186100.t1-p1
OG0022901: TcSYL_0186110.t1-p1
OG0022902: TcSYL_0186120.t1-p1
OG0022903: TcSYL_0186140.t1-p1
OG0022904: TcSYL_0186160.t1-p1
OG0022905: TcSYL_0186170.t1-p1
OG0022906: TcSYL_0186180.t1-p1
OG0022907: TcSYL_0186200.t1-p1
OG0022908: TcSYL_0186210.t1-p1
OG0022909: TcSYL_0186220.t1-p1
OG0022910: TcSYL_0186230.t1-p1
OG0022911: TcSYL_0186240.t1-p1
OG0022912: TcSYL_0186250.t1-p1
OG0022913: TcSYL_0186270.t1-p1
OG0022914: TcSYL_0186280.t1-p1
OG0022915: TcSYL_0186290.t1-p1
OG0022916: TcSYL_0186300.t1-p1
OG0022917: TcSYL_0186310.t1-p1
OG0022918: TcSYL_0186320.t1-p1
OG0022919: TcSYL_0186330.t1-p1
OG0022920: TcSYL_0186340.t1-p1
OG0022921: TcSYL_0186360.t1-p1
OG0022922: TcSYL_0186370.t1-p1
OG0022923: TcSYL_0186380.t1-p1
OG0022924: TcSYL_0186400.t1-p1
OG0022925: TcSYL_0186410.t1-p1
OG0022926: TcSYL_0186420.t1-p1
OG0022927: TcSYL_0186430.t1-p1
OG0022928: TcSYL_0186440.t1-p1
OG0022929: TcSYL_0186450.t1-p1
OG0022930: TcSYL_0186460.t1-p1

OG0022931: TcSYL_0186470.t1-p1
OG0022932: TcSYL_0186480.t1-p1
OG0022933: TcSYL_0186490.t1-p1
OG0022934: TcSYL_0186500.t1-p1
OG0022935: TcSYL_0186510.t1-p1
OG0022936: TcSYL_0186520.t1-p1
OG0022937: TcSYL_0186530.t1-p1
OG0022938: TcSYL_0186540.t1-p1
OG0022939: TcSYL_0186550.t1-p1
OG0022940: TcSYL_0186560.t1-p1
OG0022941: TcSYL_0186570.t1-p1
OG0022942: TcSYL_0186580.t1-p1
OG0022943: TcSYL_0186590.t1-p1
OG0022944: TcSYL_0186600.t1-p1
OG0022945: TcSYL_0186610.t1-p1
OG0022946: TcSYL_0186620.t1-p1
OG0022947: TcSYL_0186640.t1-p1
OG0022948: TcSYL_0186650.t1-p1
OG0022949: TcSYL_0186660.t1-p1
OG0022950: TcSYL_0186670.t1-p1
OG0022951: TcSYL_0186700.t1-p1
OG0022952: TcSYL_0186710.t1-p1
OG0022953: TcSYL_0186720.t1-p1
OG0022954: TcSYL_0186730.t1-p1
OG0022955: TcSYL_0186740.t1-p1
OG0022956: TcSYL_0186760.t1-p1
OG0022957: TcSYL_0186770.t1-p1
OG0022958: TcSYL_0186780.t1-p1
OG0022959: TcSYL_0186790.t1-p1
OG0022960: TcSYL_0186810.t1-p1
OG0022961: TcSYL_0186820.t1-p1
OG0022962: TcSYL_0186850.t1-p1
OG0022963: TcSYL_0186880.t1-p1
OG0022964: TcSYL_0186900.t1-p1
OG0022965: TcSYL_0186920.t1-p1
OG0022966: TcSYL_0186930.t1-p1
OG0022967: TcSYL_0186950.t1-p1
OG0022968: TcSYL_0186960.t1-p1
OG0022969: TcSYL_0186990.t1-p1
OG0022970: TcSYL_0187000.t1-p1
OG0022971: TcSYL_0187010.t1-p1
OG0022972: TcSYL_0187020.t1-p1
OG0022973: TcSYL_0187040.t1-p1
OG0022974: TcSYL_0187050.t1-p1
OG0022975: TcSYL_0187060.t1-p1
OG0022976: TcSYL_0187080.t1-p1
OG0022977: TcSYL_0187090.t1-p1
OG0022978: TcSYL_0187110.t1-p1
OG0022979: TcSYL_0187120.t1-p1
OG0022980: TcSYL_0187140.t1-p1
OG0022981: TcSYL_0187160.t1-p1
OG0022982: TcSYL_0187180.t1-p1
OG0022983: TcSYL_0187190.t1-p1
OG0022984: TcSYL_0187200.t1-p1

OG0022985: TcSYL_0187210.t1-p1
OG0022986: TcSYL_0187220.t1-p1
OG0022987: TcSYL_0187230.t1-p1
OG0022988: TcSYL_0187240.t1-p1
OG0022989: TcSYL_0187250.t1-p1
OG0022990: TcSYL_0187260.t1-p1
OG0022991: TcSYL_0187280.t1-p1
OG0022992: TcSYL_0187300.t1-p1
OG0022993: TcSYL_0187310.t1-p1
OG0022994: TcSYL_0187340.t1-p1
OG0022995: TcSYL_0187350.t1-p1
OG0022996: TcSYL_0187370.t1-p1
OG0022997: TcSYL_0187380.t1-p1
OG0022998: TcSYL_0187390.t1-p1
OG0022999: TcSYL_0187400.t1-p1
OG0023000: TcSYL_0187420.t1-p1
OG0023001: TcSYL_0187430.t1-p1
OG0023002: TcSYL_0187440.t1-p1
OG0023003: TcSYL_0187450.t1-p1
OG0023004: TcSYL_0187480.t1-p1
OG0023005: TcSYL_0187490.t1-p1
OG0023006: TcSYL_0187500.t1-p1
OG0023007: TcSYL_0187520.t1-p1
OG0023008: TcSYL_0187530.t1-p1
OG0023009: TcSYL_0187540.t1-p1
OG0023010: TcSYL_0187580.t1-p1
OG0023011: TcSYL_0187600.t1-p1
OG0023012: TcSYL_0187610.t1-p1
OG0023013: TcSYL_0187620.t1-p1
OG0023014: TcSYL_0187630.t1-p1
OG0023015: TcSYL_0187640.t1-p1
OG0023016: TcSYL_0187680.t1-p1
OG0023017: TcSYL_0187690.t1-p1
OG0023018: TcSYL_0187700.t1-p1
OG0023019: TcSYL_0187720.t1-p1
OG0023020: TcSYL_0187740.t1-p1
OG0023021: TcSYL_0187760.t1-p1
OG0023022: TcSYL_0187780.t1-p1
OG0023023: TcSYL_0187790.t1-p1
OG0023024: TcSYL_0187810.t1-p1
OG0023025: TcSYL_0187820.t1-p1
OG0023026: TcSYL_0187840.t1-p1
OG0023027: TcSYL_0187910.t1-p1
OG0023028: TcSYL_0187930.t1-p1
OG0023029: TcSYL_0187940.t1-p1
OG0023030: TcSYL_0188000.t1-p1
OG0023031: TcSYL_0188010.t1-p1
OG0023032: TcSYL_0188030.t1-p1
OG0023033: TcSYL_0188050.t1-p1
OG0023034: TcSYL_0188070.t1-p1
OG0023035: TcSYL_0188090.t1-p1
OG0023036: TcSYL_0188110.t1-p1
OG0023037: TcSYL_0188130.t1-p1
OG0023038: TcSYL_0188140.t1-p1

OG0023039: TcSYL_0188150.t1-p1
OG0023040: TcSYL_0188180.t1-p1
OG0023041: TcSYL_0188200.t1-p1
OG0023042: TcSYL_0188210.t1-p1
OG0023043: TcSYL_0188220.t1-p1
OG0023044: TcSYL_0188230.t1-p1
OG0023045: TcSYL_0188250.t1-p1
OG0023046: TcSYL_0188260.t1-p1
OG0023047: TcSYL_0188270.t1-p1
OG0023048: TcSYL_0188290.t1-p1
OG0023049: TcSYL_0188300.t1-p1
OG0023050: TcSYL_0188310.t1-p1
OG0023051: TcSYL_0188320.t1-p1
OG0023052: TcSYL_0188340.t1-p1
OG0023053: TcSYL_0188350.t1-p1
OG0023054: TcSYL_0188360.t1-p1
OG0023055: TcSYL_0188370.t1-p1
OG0023056: TcSYL_0188410.t1-p1
OG0023057: TcSYL_0188420.t1-p1
OG0023058: TcSYL_0188430.t1-p1
OG0023059: TcSYL_0188440.t1-p1
OG0023060: TcSYL_0188450.t1-p1
OG0023061: TcSYL_0188460.t1-p1
OG0023062: TcSYL_0188470.t1-p1
OG0023063: TcSYL_0188490.t1-p1
OG0023064: TcSYL_0188500.t1-p1
OG0023065: TcSYL_0188510.t1-p1
OG0023066: TcSYL_0188520.t1-p1
OG0023067: TcSYL_0188540.t1-p1
OG0023068: TcSYL_0188550.t1-p1
OG0023069: TcSYL_0188570.t1-p1
OG0023070: TcSYL_0188590.t1-p1
OG0023071: TcSYL_0188600.t1-p1
OG0023072: TcSYL_0188610.t1-p1
OG0023073: TcSYL_0188620.t1-p1
OG0023074: TcSYL_0188630.t1-p1
OG0023075: TcSYL_0188640.t1-p1
OG0023076: TcSYL_0188650.t1-p1
OG0023077: TcSYL_0188680.t1-p1
OG0023078: TcSYL_0188690.t1-p1
OG0023079: TcSYL_0188700.t1-p1
OG0023080: TcSYL_0188720.t1-p1
OG0023081: TcSYL_0188730.t1-p1
OG0023082: TcSYL_0188740.t1-p1
OG0023083: TcSYL_0188750.t1-p1
OG0023084: TcSYL_0188760.t1-p1
OG0023085: TcSYL_0188770.t1-p1
OG0023086: TcSYL_0188790.t1-p1
OG0023087: TcSYL_0188800.t1-p1
OG0023088: TcSYL_0188810.t1-p1
OG0023089: TcSYL_0188820.t1-p1
OG0023090: TcSYL_0188830.t1-p1
OG0023091: TcSYL_0188840.t1-p1
OG0023092: TcSYL_0188850.t1-p1

OG0023093: TcSYL_0188860.t1-p1
OG0023094: TcSYL_0188880.t1-p1
OG0023095: TcSYL_0188920.t1-p1
OG0023096: TcSYL_0188930.t1-p1
OG0023097: TcSYL_0188940.t1-p1
OG0023098: TcSYL_0188950.t1-p1
OG0023099: TcSYL_0188970.t1-p1
OG0023100: TcSYL_0188980.t1-p1
OG0023101: TcSYL_0188990.t1-p1
OG0023102: TcSYL_0189000.t1-p1
OG0023103: TcSYL_0189020.t1-p1
OG0023104: TcSYL_0189050.t1-p1
OG0023105: TcSYL_0189060.t1-p1
OG0023106: TcSYL_0189070.t1-p1
OG0023107: TcSYL_0189090.t1-p1
OG0023108: TcSYL_0189100.t1-p1
OG0023109: TcSYL_0189110.t1-p1
OG0023110: TcSYL_0189130.t1-p1
OG0023111: TcSYL_0189150.t1-p1
OG0023112: TcSYL_0189160.t1-p1
OG0023113: TcSYL_0189170.t1-p1
OG0023114: TcSYL_0189190.t1-p1
OG0023115: TcSYL_0189200.t1-p1
OG0023116: TcSYL_0189210.t1-p1
OG0023117: TcSYL_0189220.t1-p1
OG0023118: TcSYL_0189240.t1-p1
OG0023119: TcSYL_0189250.t1-p1
OG0023120: TcSYL_0189260.t1-p1
OG0023121: TcSYL_0189280.t1-p1
OG0023122: TcSYL_0189300.t1-p1
OG0023123: TcSYL_0189310.t1-p1
OG0023124: TcSYL_0189320.t1-p1
OG0023125: TcSYL_0189330.t1-p1
OG0023126: TcSYL_0189350.t1-p1
OG0023127: TcSYL_0189360.t1-p1
OG0023128: TcSYL_0189370.t1-p1
OG0023129: TcSYL_0189390.t1-p1
OG0023130: TcSYL_0189400.t1-p1
OG0023131: TcSYL_0189410.t1-p1
OG0023132: TcSYL_0189430.t1-p1
OG0023133: TcSYL_0189440.t1-p1
OG0023134: TcSYL_0189450.t1-p1
OG0023135: TcSYL_0189470.t1-p1
OG0023136: TcSYL_0189480.t1-p1
OG0023137: TcSYL_0189500.t1-p1
OG0023138: TcSYL_0189520.t1-p1
OG0023139: TcSYL_0189530.t1-p1
OG0023140: TcSYL_0189540.t1-p1
OG0023141: TcSYL_0189550.t1-p1
OG0023142: TcSYL_0189570.t1-p1
OG0023143: TcSYL_0189580.t1-p1
OG0023144: TcSYL_0189600.t1-p1
OG0023145: TcSYL_0189610.t1-p1
OG0023146: TcSYL_0189620.t1-p1

OG0023147: TcSYL_0189630.t1-p1
OG0023148: TcSYL_0189640.t1-p1
OG0023149: TcSYL_0189650.t1-p1
OG0023150: TcSYL_0189660.t1-p1
OG0023151: TcSYL_0189670.t1-p1
OG0023152: TcSYL_0189680.t1-p1
OG0023153: TcSYL_0189690.t1-p1
OG0023154: TcSYL_0189700.t1-p1
OG0023155: TcSYL_0189710.t1-p1
OG0023156: TcSYL_0189720.t1-p1
OG0023157: TcSYL_0189730.t1-p1
OG0023158: TcSYL_0189740.t1-p1
OG0023159: TcSYL_0189750.t1-p1
OG0023160: TcSYL_0189770.t1-p1
OG0023161: TcSYL_0189780.t1-p1
OG0023162: TcSYL_0189790.t1-p1
OG0023163: TcSYL_0189800.t1-p1
OG0023164: TcSYL_0189820.t1-p1
OG0023165: TcSYL_0189830.t1-p1
OG0023166: TcSYL_0189840.t1-p1
OG0023167: TcSYL_0189860.t1-p1
OG0023168: TcSYL_0189870.t1-p1
OG0023169: TcSYL_0189880.t1-p1
OG0023170: TcSYL_0189890.t1-p1
OG0023171: TcSYL_0189900.t1-p1
OG0023172: TcSYL_0189910.t1-p1
OG0023173: TcSYL_0189920.t1-p1
OG0023174: TcSYL_0189930.t1-p1
OG0023175: TcSYL_0189950.t1-p1
OG0023176: TcSYL_0189960.t1-p1
OG0023177: TcSYL_0189980.t1-p1
OG0023178: TcSYL_0189990.t1-p1
OG0023179: TcSYL_0190000.t1-p1
OG0023180: TcSYL_0190010.t1-p1
OG0023181: TcSYL_0190020.t1-p1
OG0023182: TcSYL_0190030.t1-p1
OG0023183: TcSYL_0190040.t1-p1
OG0023184: TcSYL_0190050.t1-p1
OG0023185: TcSYL_0190060.t1-p1
OG0023186: TcSYL_0190070.t1-p1
OG0023187: TcSYL_0190090.t1-p1
OG0023188: TcSYL_0190100.t1-p1
OG0023189: TcSYL_0190110.t1-p1
OG0023190: TcSYL_0190130.t1-p1
OG0023191: TcSYL_0190140.t1-p1
OG0023192: TcSYL_0190150.t1-p1
OG0023193: TcSYL_0190160.t1-p1
OG0023194: TcSYL_0190170.t1-p1
OG0023195: TcSYL_0190180.t1-p1
OG0023196: TcSYL_0190190.t1-p1
OG0023197: TcSYL_0190230.t1-p1
OG0023198: TcSYL_0190260.t1-p1
OG0023199: TcSYL_0190280.t1-p1
OG0023200: TcSYL_0190300.t1-p1

OG0023201: TcSYL_0190310.t1-p1
OG0023202: TcSYL_0190320.t1-p1
OG0023203: TcSYL_0190330.t1-p1
OG0023204: TcSYL_0190350.t1-p1
OG0023205: TcSYL_0190360.t1-p1
OG0023206: TcSYL_0190380.t1-p1
OG0023207: TcSYL_0190390.t1-p1
OG0023208: TcSYL_0190400.t1-p1
OG0023209: TcSYL_0190410.t1-p1
OG0023210: TcSYL_0190430.t1-p1
OG0023211: TcSYL_0190440.t1-p1
OG0023212: TcSYL_0190450.t1-p1
OG0023213: TcSYL_0190470.t1-p1
OG0023214: TcSYL_0190490.t1-p1
OG0023215: TcSYL_0190510.t1-p1
OG0023216: TcSYL_0190520.t1-p1
OG0023217: TcSYL_0190540.t1-p1
OG0023218: TcSYL_0190560.t1-p1
OG0023219: TcSYL_0190570.t1-p1
OG0023220: TcSYL_0190580.t1-p1
OG0023221: TcSYL_0190590.t1-p1
OG0023222: TcSYL_0190600.t1-p1
OG0023223: TcSYL_0190610.t1-p1
OG0023224: TcSYL_0190620.t1-p1
OG0023225: TcSYL_0190640.t1-p1
OG0023226: TcSYL_0190650.t1-p1
OG0023227: TcSYL_0190660.t1-p1
OG0023228: TcSYL_0190670.t1-p1
OG0023229: TcSYL_0190680.t1-p1
OG0023230: TcSYL_0190690.t1-p1
OG0023231: TcSYL_0190700.t1-p1
OG0023232: TcSYL_0190720.t1-p1
OG0023233: TcSYL_0190740.t1-p1
OG0023234: TcSYL_0190750.t1-p1
OG0023235: TcSYL_0190760.t1-p1
OG0023236: TcSYL_0190770.t1-p1
OG0023237: TcSYL_0190780.t1-p1
OG0023238: TcSYL_0190790.t1-p1
OG0023239: TcSYL_0190800.t1-p1
OG0023240: TcSYL_0190840.t1-p1
OG0023241: TcSYL_0190860.t1-p1
OG0023242: TcSYL_0190870.t1-p1
OG0023243: TcSYL_0190880.t1-p1
OG0023244: TcSYL_0190940.t1-p1
OG0023245: TcSYL_0190950.t1-p1
OG0023246: TcSYL_0190980.t1-p1
OG0023247: TcSYL_0190990.t1-p1
OG0023248: TcSYL_0191000.t1-p1
OG0023249: TcSYL_0191010.t1-p1
OG0023250: TcSYL_0191020.t1-p1
OG0023251: TcSYL_0191030.t1-p1
OG0023252: TcSYL_0191040.t1-p1
OG0023253: TcSYL_0191050.t1-p1
OG0023254: TcSYL_0191060.t1-p1

OG0023255: TcSYL_0191070.t1-p1
OG0023256: TcSYL_0191080.t1-p1
OG0023257: TcSYL_0191090.t1-p1
OG0023258: TcSYL_0191100.t1-p1
OG0023259: TcSYL_0191130.t1-p1
OG0023260: TcSYL_0191140.t1-p1
OG0023261: TcSYL_0191150.t1-p1
OG0023262: TcSYL_0191180.t1-p1
OG0023263: TcSYL_0191200.t1-p1
OG0023264: TcSYL_0191210.t1-p1
OG0023265: TcSYL_0191220.t1-p1
OG0023266: TcSYL_0191230.t1-p1
OG0023267: TcSYL_0191240.t1-p1
OG0023268: TcSYL_0191250.t1-p1
OG0023269: TcSYL_0191260.t1-p1
OG0023270: TcSYL_0191280.t1-p1
OG0023271: TcSYL_0191300.t1-p1
OG0023272: TcSYL_0191310.t1-p1
OG0023273: TcSYL_0191320.t1-p1
OG0023274: TcSYL_0191330.t1-p1
OG0023275: TcSYL_0191350.t1-p1
OG0023276: TcSYL_0191360.t1-p1
OG0023277: TcSYL_0191370.t1-p1
OG0023278: TcSYL_0191380.t1-p1
OG0023279: TcSYL_0191400.t1-p1
OG0023280: TcSYL_0191410.t1-p1
OG0023281: TcSYL_0191420.t1-p1
OG0023282: TcSYL_0191440.t1-p1
OG0023283: TcSYL_0191450.t1-p1
OG0023284: TcSYL_0191610.t1-p1
OG0023285: TcSYL_0191640.t1-p1
OG0023286: TcSYL_0191730.t1-p1
OG0023287: TcSYL_0191740.t1-p1
OG0023288: TcSYL_0191750.t1-p1
OG0023289: TcSYL_0191760.t1-p1
OG0023290: TcSYL_0191780.t1-p1
OG0023291: TcSYL_0191790.t1-p1
OG0023292: TcSYL_0191800.t1-p1
OG0023293: TcSYL_0191810.t1-p1
OG0023294: TcSYL_0191820.t1-p1
OG0023295: TcSYL_0191830.t1-p1
OG0023296: TcSYL_0191850.t1-p1
OG0023297: TcSYL_0191860.t1-p1
OG0023298: TcSYL_0191870.t1-p1
OG0023299: TcSYL_0191880.t1-p1
OG0023300: TcSYL_0191890.t1-p1
OG0023301: TcSYL_0191900.t1-p1
OG0023302: TcSYL_0191910.t1-p1
OG0023303: TcSYL_0191920.t1-p1
OG0023304: TcSYL_0191930.t1-p1
OG0023305: TcSYL_0191940.t1-p1
OG0023306: TcSYL_0191950.t1-p1
OG0023307: TcSYL_0191960.t1-p1
OG0023308: TcSYL_0191990.t1-p1

OG0023309: TcSYL_0192000.t1-p1
OG0023310: TcSYL_0192010.t1-p1
OG0023311: TcSYL_0192020.t1-p1
OG0023312: TcSYL_0192030.t1-p1
OG0023313: TcSYL_0192040.t1-p1
OG0023314: TcSYL_0192060.t1-p1
OG0023315: TcSYL_0192080.t1-p1
OG0023316: TcSYL_0192090.t1-p1
OG0023317: TcSYL_0192110.t1-p1
OG0023318: TcSYL_0192120.t1-p1
OG0023319: TcSYL_0192130.t1-p1
OG0023320: TcSYL_0192150.t1-p1
OG0023321: TcSYL_0192160.t1-p1
OG0023322: TcSYL_0192170.t1-p1
OG0023323: TcSYL_0192180.t1-p1
OG0023324: TcSYL_0192240.t1-p1
OG0023325: TcSYL_0192250.t1-p1
OG0023326: TcSYL_0192260.t1-p1
OG0023327: TcSYL_0192270.t1-p1
OG0023328: TcSYL_0192280.t1-p1
OG0023329: TcSYL_0192290.t1-p1
OG0023330: TcSYL_0192300.t1-p1
OG0023331: TcSYL_0192310.t1-p1
OG0023332: TcSYL_0192320.t1-p1
OG0023333: TcSYL_0192330.t1-p1
OG0023334: TcSYL_0192340.t1-p1
OG0023335: TcSYL_0192350.t1-p1
OG0023336: TcSYL_0192360.t1-p1
OG0023337: TcSYL_0192370.t1-p1
OG0023338: TcSYL_0192380.t1-p1
OG0023339: TcSYL_0192390.t1-p1
OG0023340: TcSYL_0192400.t1-p1
OG0023341: TcSYL_0192410.t1-p1
OG0023342: TcSYL_0192420.t1-p1
OG0023343: TcSYL_0192430.t1-p1
OG0023344: TcSYL_0192440.t1-p1
OG0023345: TcSYL_0192450.t1-p1
OG0023346: TcSYL_0192460.t1-p1
OG0023347: TcSYL_0192470.t1-p1
OG0023348: TcSYL_0192480.t1-p1
OG0023349: TcSYL_0192490.t1-p1
OG0023350: TcSYL_0192500.t1-p1
OG0023351: TcSYL_0192510.t1-p1
OG0023352: TcSYL_0192520.t1-p1
OG0023353: TcSYL_0192530.t1-p1
OG0023354: TcSYL_0192540.t1-p1
OG0023355: TcSYL_0192550.t1-p1
OG0023356: TcSYL_0192560.t1-p1
OG0023357: TcSYL_0192570.t1-p1
OG0023358: TcSYL_0192580.t1-p1
OG0023359: TcSYL_0192590.t1-p1
OG0023360: TcSYL_0192600.t1-p1
OG0023361: TcSYL_0192610.t1-p1
OG0023362: TcSYL_0192620.t1-p1

OG0023363: TcSYL_0192630.t1-p1
OG0023364: TcSYL_0192640.t1-p1
OG0023365: TcSYL_0192650.t1-p1
OG0023366: TcSYL_0192660.t1-p1
OG0023367: TcSYL_0192670.t1-p1
OG0023368: TcSYL_0192680.t1-p1
OG0023369: TcSYL_0192690.t1-p1
OG0023370: TcSYL_0192700.t1-p1
OG0023371: TcSYL_0192710.t1-p1
OG0023372: TcSYL_0192720.t1-p1
OG0023373: TcSYL_0192730.t1-p1
OG0023374: TcSYL_0192740.t1-p1
OG0023375: TcSYL_0192750.t1-p1
OG0023376: TcSYL_0192760.t1-p1
OG0023377: TcSYL_0192770.t1-p1
OG0023378: TcSYL_0192780.t1-p1
OG0023379: TcSYL_0192790.t1-p1
OG0023380: TcSYL_0192800.t1-p1
OG0023381: TcSYL_0192810.t1-p1
OG0023382: TcSYL_0192820.t1-p1
OG0023383: TcSYL_0192830.t1-p1
OG0023384: TcSYL_0192840.t1-p1
OG0023385: TcSYL_0192850.t1-p1
OG0023386: TcSYL_0192860.t1-p1
OG0023387: TcSYL_0192870.t1-p1
OG0023388: TcSYL_0192880.t1-p1
OG0023389: TcSYL_0192890.t1-p1
OG0023390: TcSYL_0192900.t1-p1
OG0023391: TcSYL_0192910.t1-p1
OG0023392: TcSYL_0192930.t1-p1
OG0023393: TcSYL_0192940.t1-p1
OG0023394: TcSYL_0192950.t1-p1
OG0023395: TcSYL_0192960.t1-p1
OG0023396: TcSYL_0192970.t1-p1
OG0023397: TcSYL_0192990.t1-p1
OG0023398: TcSYL_0193000.t1-p1
OG0023399: TcSYL_0193010.t1-p1
OG0023400: TcSYL_0193020.t1-p1
OG0023401: TcSYL_0193030.t1-p1
OG0023402: TcSYL_0193040.t1-p1
OG0023403: TcSYL_0193050.t1-p1
OG0023404: TcSYL_0193060.t1-p1
OG0023405: TcSYL_0193070.t1-p1
OG0023406: TcSYL_0193080.t1-p1
OG0023407: TcSYL_0193090.t1-p1
OG0023408: TcSYL_0193100.t1-p1
OG0023409: TcSYL_0193110.t1-p1
OG0023410: TcSYL_0193120.t1-p1
OG0023411: TcSYL_0193130.t1-p1
OG0023412: TcSYL_0193140.t1-p1
OG0023413: TcSYL_0193150.t1-p1
OG0023414: TcSYL_0193160.t1-p1
OG0023415: TcSYL_0193170.t1-p1
OG0023416: TcSYL_0193180.t1-p1

OG0023417: TcSYL_0193190.t1-p1
OG0023418: TcSYL_0193200.t1-p1
OG0023419: TcSYL_0193210.t1-p1
OG0023420: TcSYL_0193220.t1-p1
OG0023421: TcSYL_0193230.t1-p1
OG0023422: TcSYL_0193240.t1-p1
OG0023423: TcSYL_0193250.t1-p1
OG0023424: TcSYL_0193260.t1-p1
OG0023425: TcSYL_0193270.t1-p1
OG0023426: TcSYL_0193280.t1-p1
OG0023427: TcSYL_0193290.t1-p1
OG0023428: TcSYL_0193300.t1-p1
OG0023429: TcSYL_0193310.t1-p1
OG0023430: TcSYL_0193320.t1-p1
OG0023431: TcSYL_0193330.t1-p1
OG0023432: TcSYL_0193340.t1-p1
OG0023433: TcSYL_0193350.t1-p1
OG0023434: TcSYL_0193360.t1-p1
OG0023435: TcSYL_0193370.t1-p1
OG0023436: TcSYL_0193380.t1-p1
OG0023437: TcSYL_0193390.t1-p1
OG0023438: TcSYL_0193400.t1-p1
OG0023439: TcSYL_0193410.t1-p1
OG0023440: TcSYL_0193420.t1-p1
OG0023441: TcSYL_0193440.t1-p1
OG0023442: TcSYL_0193450.t1-p1
OG0023443: TcSYL_0193460.t1-p1
OG0023444: TcSYL_0193470.t1-p1
OG0023445: TcSYL_0193480.t1-p1
OG0023446: TcSYL_0193490.t1-p1
OG0023447: TcSYL_0193500.t1-p1
OG0023448: TcSYL_0193510.t1-p1
OG0023449: TcSYL_0193520.t1-p1
OG0023450: TcSYL_0193530.t1-p1
OG0023451: TcSYL_0193540.t1-p1
OG0023452: TcSYL_0193550.t1-p1
OG0023453: TcSYL_0193560.t1-p1
OG0023454: TcSYL_0193570.t1-p1
OG0023455: TcSYL_0193580.t1-p1
OG0023456: TcSYL_0193590.t1-p1
OG0023457: TcSYL_0193600.t1-p1
OG0023458: TcSYL_0193620.t1-p1
OG0023459: TcSYL_0193630.t1-p1
OG0023460: TcSYL_0193640.t1-p1
OG0023461: TcSYL_0193650.t1-p1
OG0023462: TcSYL_0193660.t1-p1
OG0023463: TcSYL_0193670.t1-p1
OG0023464: TcSYL_0193680.t1-p1
OG0023465: TcSYL_0193690.t1-p1
OG0023466: TcSYL_0193700.t1-p1
OG0023467: TcSYL_0193710.t1-p1
OG0023468: TcSYL_0193720.t1-p1
OG0023469: TcSYL_0193730.t1-p1
OG0023470: TcSYL_0193740.t1-p1

OG0023471: TcSYL_0193750.t1-p1
OG0023472: TcSYL_0193760.t1-p1
OG0023473: TcSYL_0193770.t1-p1
OG0023474: TcSYL_0193790.t1-p1
OG0023475: TcSYL_0193800.t1-p1
OG0023476: TcSYL_0193810.t1-p1
OG0023477: TcSYL_0193820.t1-p1
OG0023478: TcSYL_0193830.t1-p1
OG0023479: TcSYL_0193840.t1-p1
OG0023480: TcSYL_0193850.t1-p1
OG0023481: TcSYL_0193860.t1-p1
OG0023482: TcSYL_0193870.t1-p1
OG0023483: TcSYL_0193880.t1-p1
OG0023484: TcSYL_0193890.t1-p1
OG0023485: TcSYL_0193900.t1-p1
OG0023486: TcSYL_0193910.t1-p1
OG0023487: TcSYL_0193920.t1-p1
OG0023488: TcSYL_0193930.t1-p1
OG0023489: TcSYL_0193940.t1-p1
OG0023490: TcSYL_0193950.t1-p1
OG0023491: TcSYL_0193960.t1-p1
OG0023492: TcSYL_0193970.t1-p1
OG0023493: TcSYL_0193980.t1-p1
OG0023494: TcSYL_0193990.t1-p1
OG0023495: TcSYL_0194000.t1-p1
OG0023496: TcSYL_0194010.t1-p1
OG0023497: TcSYL_0194020.t1-p1
OG0023498: TcSYL_0194030.t1-p1
OG0023499: TcSYL_0194040.t1-p1
OG0023500: TcSYL_0194050.t1-p1
OG0023501: TcSYL_0194060.t1-p1
OG0023502: TcSYL_0194070.t1-p1
OG0023503: TcSYL_0194080.t1-p1
OG0023504: TcSYL_0194090.t1-p1
OG0023505: TcSYL_0194100.t1-p1
OG0023506: TcSYL_0194110.t1-p1
OG0023507: TcSYL_0194120.t1-p1
OG0023508: TcSYL_0194130.t1-p1
OG0023509: TcSYL_0194140.t1-p1
OG0023510: TcSYL_0194150.t1-p1
OG0023511: TcSYL_0194160.t1-p1
OG0023512: TcSYL_0194170.t1-p1
OG0023513: TcSYL_0194180.t1-p1
OG0023514: TcSYL_0194190.t1-p1
OG0023515: TcSYL_0194200.t1-p1
OG0023516: TcSYL_0194210.t1-p1
OG0023517: TcSYL_0194220.t1-p1
OG0023518: TcSYL_0194230.t1-p1
OG0023519: TcSYL_0194240.t1-p1
OG0023520: TcSYL_0194250.t1-p1
OG0023521: TcSYL_0194280.t1-p1
OG0023522: TcSYL_0194290.t1-p1
OG0023523: TcSYL_0194300.t1-p1
OG0023524: TcSYL_0194310.t1-p1

OG0023525: TcSYL_0194320.t1-p1
OG0023526: TcSYL_0194340.t1-p1
OG0023527: TcSYL_0194480.t1-p1
OG0023528: TcSYL_0194490.t1-p1
OG0023529: TcSYL_0194520.t1-p1
OG0023530: TcSYL_0194530.t1-p1
OG0023531: TcSYL_0194570.t1-p1
OG0023532: TcSYL_0194580.t1-p1
OG0023533: TcSYL_0194590.t1-p1
OG0023534: TcSYL_0194600.t1-p1
OG0023535: TcSYL_0194610.t1-p1
OG0023536: TcSYL_0194620.t1-p1
OG0023537: TcSYL_0194630.t1-p1
OG0023538: TcSYL_0194640.t1-p1
OG0023539: TcSYL_0194650.t1-p1
OG0023540: TcSYL_0194660.t1-p1
OG0023541: TcSYL_0194670.t1-p1
OG0023542: TcSYL_0194680.t1-p1
OG0023543: TcSYL_0194690.t1-p1
OG0023544: TcSYL_0194700.t1-p1
OG0023545: TcSYL_0194710.t1-p1
OG0023546: TcSYL_0194720.t1-p1
OG0023547: TcSYL_0194730.t1-p1
OG0023548: TcSYL_0194740.t1-p1
OG0023549: TcSYL_0194750.t1-p1
OG0023550: TcSYL_0194760.t1-p1
OG0023551: TcSYL_0194770.t1-p1
OG0023552: TcSYL_0194780.t1-p1
OG0023553: TcSYL_0194790.t1-p1
OG0023554: TcSYL_0194810.t1-p1
OG0023555: TcSYL_0194820.t1-p1
OG0023556: TcSYL_0194830.t1-p1
OG0023557: TcSYL_0194840.t1-p1
OG0023558: TcSYL_0194850.t1-p1
OG0023559: TcSYL_0194860.t1-p1
OG0023560: TcSYL_0194870.t1-p1
OG0023561: TcSYL_0194880.t1-p1
OG0023562: TcSYL_0194890.t1-p1
OG0023563: TcSYL_0194900.t1-p1
OG0023564: TcSYL_0194910.t1-p1
OG0023565: TcSYL_0194920.t1-p1
OG0023566: TcSYL_0194940.t1-p1
OG0023567: TcSYL_0194950.t1-p1
OG0023568: TcSYL_0194960.t1-p1
OG0023569: TcSYL_0194970.t1-p1
OG0023570: TcSYL_0194980.t1-p1
OG0023571: TcSYL_0194990.t1-p1
OG0023572: TcSYL_0195000.t1-p1
OG0023573: TcSYL_0195010.t1-p1
OG0023574: TcSYL_0195020.t1-p1
OG0023575: TcSYL_0195030.t1-p1
OG0023576: TcSYL_0195040.t1-p1
OG0023577: TcSYL_0195050.t1-p1
OG0023578: TcSYL_0195060.t1-p1

OG0023579: TcSYL_0195070.t1-p1
OG0023580: TcSYL_0195080.t1-p1
OG0023581: TcSYL_0195100.t1-p1
OG0023582: TcSYL_0195110.t1-p1
OG0023583: TcSYL_0195120.t1-p1
OG0023584: TcSYL_0195130.t1-p1
OG0023585: TcSYL_0195140.t1-p1
OG0023586: TcSYL_0195150.t1-p1
OG0023587: TcSYL_0195160.t1-p1
OG0023588: TcSYL_0195170.t1-p1
OG0023589: TcSYL_0195180.t1-p1
OG0023590: TcSYL_0195190.t1-p1
OG0023591: TcSYL_0195200.t1-p1
OG0023592: TcSYL_0195210.t1-p1
OG0023593: TcSYL_0195220.t1-p1
OG0023594: TcSYL_0195230.t1-p1
OG0023595: TcSYL_0195250.t1-p1
OG0023596: TcSYL_0195260.t1-p1
OG0023597: TcSYL_0195270.t1-p1
OG0023598: TcSYL_0195280.t1-p1
OG0023599: TcSYL_0195290.t1-p1
OG0023600: TcSYL_0195300.t1-p1
OG0023601: TcSYL_0195310.t1-p1
OG0023602: TcSYL_0195320.t1-p1
OG0023603: TcSYL_0195330.t1-p1
OG0023604: TcSYL_0195340.t1-p1
OG0023605: TcSYL_0195350.t1-p1
OG0023606: TcSYL_0195360.t1-p1
OG0023607: TcSYL_0195370.t1-p1
OG0023608: TcSYL_0195380.t1-p1
OG0023609: TcSYL_0195390.t1-p1
OG0023610: TcSYL_0195400.t1-p1
OG0023611: TcSYL_0195410.t1-p1
OG0023612: TcSYL_0195420.t1-p1
OG0023613: TcSYL_0195430.t1-p1
OG0023614: TcSYL_0195440.t1-p1
OG0023615: TcSYL_0195450.t1-p1
OG0023616: TcSYL_0195470.t1-p1
OG0023617: TcSYL_0195480.t1-p1
OG0023618: TcSYL_0195500.t1-p1
OG0023619: TcSYL_0195510.t1-p1
OG0023620: TcSYL_0195530.t1-p1
OG0023621: TcSYL_0195540.t1-p1
OG0023622: TcSYL_0195560.t1-p1
OG0023623: TcSYL_0195580.t1-p1
OG0023624: TcSYL_0195610.t1-p1
OG0023625: TcSYL_0195640.t1-p1
OG0023626: TcSYL_0195650.t1-p1
OG0023627: TcSYL_0195660.t1-p1
OG0023628: TcSYL_0195670.t1-p1
OG0023629: TcSYL_0195690.t1-p1
OG0023630: TcSYL_0195700.t1-p1
OG0023631: TcSYL_0195710.t1-p1
OG0023632: TcSYL_0195750.t1-p1

OG0023633: TcSYL_0195760.t1-p1
OG0023634: TcSYL_0195780.t1-p1
OG0023635: TcSYL_0195790.t1-p1
OG0023636: TcSYL_0195800.t1-p1
OG0023637: TcSYL_0195820.t1-p1
OG0023638: TcSYL_0195830.t1-p1
OG0023639: TcSYL_0195840.t1-p1
OG0023640: TcSYL_0195850.t1-p1
OG0023641: TcSYL_0195870.t1-p1
OG0023642: TcSYL_0195920.t1-p1
OG0023643: TcSYL_0195930.t1-p1
OG0023644: TcSYL_0195950.t1-p1
OG0023645: TcSYL_0195960.t1-p1
OG0023646: TcSYL_0195980.t1-p1
OG0023647: TcSYL_0195990.t1-p1
OG0023648: TcSYL_0196010.t1-p1
OG0023649: TcSYL_0196020.t1-p1
OG0023650: TcSYL_0196030.t1-p1
OG0023651: TcSYL_0196050.t1-p1
OG0023652: TcSYL_0196070.t1-p1
OG0023653: TcSYL_0196080.t1-p1
OG0023654: TcSYL_0196100.t1-p1
OG0023655: TcSYL_0196110.t1-p1
OG0023656: TcSYL_0196130.t1-p1
OG0023657: TcSYL_0196140.t1-p1
OG0023658: TcSYL_0196160.t1-p1
OG0023659: TcSYL_0196170.t1-p1
OG0023660: TcSYL_0196180.t1-p1
OG0023661: TcSYL_0196190.t1-p1
OG0023662: TcSYL_0196220.t1-p1
OG0023663: TcSYL_0196240.t1-p1
OG0023664: TcSYL_0196250.t1-p1
OG0023665: TcSYL_0196260.t1-p1
OG0023666: TcSYL_0196270.t1-p1
OG0023667: TcSYL_0196280.t1-p1
OG0023668: TcSYL_0196300.t1-p1
OG0023669: TcSYL_0196330.t1-p1
OG0023670: TcSYL_0196340.t1-p1
OG0023671: TcSYL_0196350.t1-p1
OG0023672: TcSYL_0196360.t1-p1
OG0023673: TcSYL_0196370.t1-p1
OG0023674: TcSYL_0196380.t1-p1
OG0023675: TcSYL_0196400.t1-p1
OG0023676: TcSYL_0196410.t1-p1
OG0023677: TcSYL_0196420.t1-p1
OG0023678: TcSYL_0196440.t1-p1
OG0023679: TcSYL_0196450.t1-p1
OG0023680: TcSYL_0196460.t1-p1
OG0023681: TcSYL_0196500.t1-p1
OG0023682: TcSYL_0196520.t1-p1
OG0023683: TcSYL_0196530.t1-p1
OG0023684: TcSYL_0196540.t1-p1
OG0023685: TcSYL_0196550.t1-p1
OG0023686: TcSYL_0196580.t1-p1

OG0023687: TcSYL_0196600.t1-p1
OG0023688: TcSYL_0196610.t1-p1
OG0023689: TcSYL_0196650.t1-p1
OG0023690: TcSYL_0196660.t1-p1
OG0023691: TcSYL_0196670.t1-p1
OG0023692: TcSYL_0196690.t1-p1
OG0023693: TcSYL_0196700.t1-p1
OG0023694: TcSYL_0196710.t1-p1
OG0023695: TcSYL_0196720.t1-p1
OG0023696: TcSYL_0196740.t1-p1
OG0023697: TcSYL_0196750.t1-p1
OG0023698: TcSYL_0196770.t1-p1
OG0023699: TcSYL_0196790.t1-p1
OG0023700: TcSYL_0196800.t1-p1
OG0023701: TcSYL_0196810.t1-p1
OG0023702: TcSYL_0196840.t1-p1
OG0023703: TcSYL_0196860.t1-p1
OG0023704: TcSYL_0196880.t1-p1
OG0023705: TcSYL_0196890.t1-p1
OG0023706: TcSYL_0196900.t1-p1
OG0023707: TcSYL_0196910.t1-p1
OG0023708: TcSYL_0196920.t1-p1
OG0023709: TcSYL_0196940.t1-p1
OG0023710: TcSYL_0196950.t1-p1
OG0023711: TcSYL_0196970.t1-p1
OG0023712: TcSYL_0196990.t1-p1
OG0023713: TcSYL_0197030.t1-p1
OG0023714: TcSYL_0197040.t1-p1
OG0023715: TcSYL_0197050.t1-p1
OG0023716: TcSYL_0197060.t1-p1
OG0023717: TcSYL_0197070.t1-p1
OG0023718: TcSYL_0197090.t1-p1
OG0023719: TcSYL_0197100.t1-p1
OG0023720: TcSYL_0197110.t1-p1
OG0023721: TcSYL_0197120.t1-p1
OG0023722: TcSYL_0197130.t1-p1
OG0023723: TcSYL_0197140.t1-p1
OG0023724: TcSYL_0197170.t1-p1
OG0023725: TcSYL_0197190.t1-p1
OG0023726: TcSYL_0197200.t1-p1
OG0023727: TcSYL_0197210.t1-p1
OG0023728: TcSYL_0197230.t1-p1
OG0023729: TcSYL_0197250.t1-p1
OG0023730: TcSYL_0197260.t1-p1
OG0023731: TcSYL_0197280.t1-p1
OG0023732: TcSYL_0197290.t1-p1
OG0023733: TcSYL_0197300.t1-p1
OG0023734: TcSYL_0197310.t1-p1
OG0023735: TcSYL_0197320.t1-p1
OG0023736: TcSYL_0197330.t1-p1
OG0023737: TcSYL_0197350.t1-p1
OG0023738: TcSYL_0197360.t1-p1
OG0023739: TcSYL_0197370.t1-p1
OG0023740: TcSYL_0197380.t1-p1

OG0023741: TcSYL_0197390.t1-p1
OG0023742: TcSYL_0197400.t1-p1
OG0023743: TcSYL_0197410.t1-p1
OG0023744: TcSYL_0197420.t1-p1
OG0023745: TcSYL_0197430.t1-p1
OG0023746: TcSYL_0197440.t1-p1
OG0023747: TcSYL_0197450.t1-p1
OG0023748: TcSYL_0197460.t1-p1
OG0023749: TcSYL_0197480.t1-p1
OG0023750: TcSYL_0197490.t1-p1
OG0023751: TcSYL_0197500.t1-p1
OG0023752: TcSYL_0197510.t1-p1
OG0023753: TcSYL_0197520.t1-p1
OG0023754: TcSYL_0197530.t1-p1
OG0023755: TcSYL_0197540.t1-p1
OG0023756: TcSYL_0197550.t1-p1
OG0023757: TcSYL_0197560.t1-p1
OG0023758: TcSYL_0197570.t1-p1
OG0023759: TcSYL_0197580.t1-p1
OG0023760: TcSYL_0197590.t1-p1
OG0023761: TcSYL_0197600.t1-p1
OG0023762: TcSYL_0197610.t1-p1
OG0023763: TcSYL_0197620.t1-p1
OG0023764: TcSYL_0197630.t1-p1
OG0023765: TcSYL_0197640.t1-p1
OG0023766: TcSYL_0197650.t1-p1
OG0023767: TcSYL_0197660.t1-p1
OG0023768: TcSYL_0197670.t1-p1
OG0023769: TcSYL_0197680.t1-p1
OG0023770: TcSYL_0197690.t1-p1
OG0023771: TcSYL_0197700.t1-p1
OG0023772: TcSYL_0197710.t1-p1
OG0023773: TcSYL_0197720.t1-p1
OG0023774: TcSYL_0197730.t1-p1
OG0023775: TcSYL_0197740.t1-p1
OG0023776: TcSYL_0197750.t1-p1
OG0023777: TcSYL_0197760.t1-p1
OG0023778: TcSYL_0197770.t1-p1
OG0023779: TcSYL_0197780.t1-p1
OG0023780: TcSYL_0197790.t1-p1
OG0023781: TcSYL_0197800.t1-p1
OG0023782: TcSYL_0197810.t1-p1
OG0023783: TcSYL_0197820.t1-p1
OG0023784: TcSYL_0197830.t1-p1
OG0023785: TcSYL_0197840.t1-p1
OG0023786: TcSYL_0197850.t1-p1
OG0023787: TcSYL_0197860.t1-p1
OG0023788: TcSYL_0197870.t1-p1
OG0023789: TcSYL_0197880.t1-p1
OG0023790: TcSYL_0197890.t1-p1
OG0023791: TcSYL_0197910.t1-p1
OG0023792: TcSYL_0197920.t1-p1
OG0023793: TcSYL_0197930.t1-p1
OG0023794: TcSYL_0197940.t1-p1

OG0023795: TcSYL_0197950.t1-p1
OG0023796: TcSYL_0197960.t1-p1
OG0023797: TcSYL_0197970.t1-p1
OG0023798: TcSYL_0197980.t1-p1
OG0023799: TcSYL_0197990.t1-p1
OG0023800: TcSYL_0198000.t1-p1
OG0023801: TcSYL_0198010.t1-p1
OG0023802: TcSYL_0198020.t1-p1
OG0023803: TcSYL_0198030.t1-p1
OG0023804: TcSYL_0198040.t1-p1
OG0023805: TcSYL_0198050.t1-p1
OG0023806: TcSYL_0198060.t1-p1
OG0023807: TcSYL_0198070.t1-p1
OG0023808: TcSYL_0198080.t1-p1
OG0023809: TcSYL_0198090.t1-p1
OG0023810: TcSYL_0198100.t1-p1
OG0023811: TcSYL_0198110.t1-p1
OG0023812: TcSYL_0198120.t1-p1
OG0023813: TcSYL_0198130.t1-p1
OG0023814: TcSYL_0198140.t1-p1
OG0023815: TcSYL_0198150.t1-p1
OG0023816: TcSYL_0198160.t1-p1
OG0023817: TcSYL_0198170.t1-p1
OG0023818: TcSYL_0198180.t1-p1
OG0023819: TcSYL_0198190.t1-p1
OG0023820: TcSYL_0198200.t1-p1
OG0023821: TcSYL_0198210.t1-p1
OG0023822: TcSYL_0198220.t1-p1
OG0023823: TcSYL_0198230.t1-p1
OG0023824: TcSYL_0198240.t1-p1
OG0023825: TcSYL_0198250.t1-p1
OG0023826: TcSYL_0198260.t1-p1
OG0023827: TcSYL_0198270.t1-p1
OG0023828: TcSYL_0198280.t1-p1
OG0023829: TcSYL_0198290.t1-p1
OG0023830: TcSYL_0198300.t1-p1
OG0023831: TcSYL_0198310.t1-p1
OG0023832: TcSYL_0198320.t1-p1
OG0023833: TcSYL_0198330.t1-p1
OG0023834: TcSYL_0198340.t1-p1
OG0023835: TcSYL_0198350.t1-p1
OG0023836: TcSYL_0198360.t1-p1
OG0023837: TcSYL_0198370.t1-p1
OG0023838: TcSYL_0198380.t1-p1
OG0023839: TcSYL_0198390.t1-p1
OG0023840: TcSYL_0198400.t1-p1
OG0023841: TcSYL_0198410.t1-p1
OG0023842: TcSYL_0198420.t1-p1
OG0023843: TcSYL_0198440.t1-p1
OG0023844: TcSYL_0198450.t1-p1
OG0023845: TcSYL_0198460.t1-p1
OG0023846: TcSYL_0198470.t1-p1
OG0023847: TcSYL_0198490.t1-p1
OG0023848: TcSYL_0198500.t1-p1

OG0023849: TcSYL_0198510.t1-p1
OG0023850: TcSYL_0198520.t1-p1
OG0023851: TcSYL_0198540.t1-p1
OG0023852: TcSYL_0198550.t1-p1
OG0023853: TcSYL_0198570.t1-p1
OG0023854: TcSYL_0198580.t1-p1
OG0023855: TcSYL_0198590.t1-p1
OG0023856: TcSYL_0198600.t1-p1
OG0023857: TcSYL_0198610.t1-p1
OG0023858: TcSYL_0198620.t1-p1
OG0023859: TcSYL_0198630.t1-p1
OG0023860: TcSYL_0198640.t1-p1
OG0023861: TcSYL_0198650.t1-p1
OG0023862: TcSYL_0198660.t1-p1
OG0023863: TcSYL_0198670.t1-p1
OG0023864: TcSYL_0198680.t1-p1
OG0023865: TcSYL_0198690.t1-p1
OG0023866: TcSYL_0198700.t1-p1
OG0023867: TcSYL_0198710.t1-p1
OG0023868: TcSYL_0198720.t1-p1
OG0023869: TcSYL_0198730.t1-p1
OG0023870: TcSYL_0198750.t1-p1
OG0023871: TcSYL_0198760.t1-p1
OG0023872: TcSYL_0198770.t1-p1
OG0023873: TcSYL_0198780.t1-p1
OG0023874: TcSYL_0198790.t1-p1
OG0023875: TcSYL_0198800.t1-p1
OG0023876: TcSYL_0198820.t1-p1
OG0023877: TcSYL_0198830.t1-p1
OG0023878: TcSYL_0198840.t1-p1
OG0023879: TcSYL_0198850.t1-p1
OG0023880: TcSYL_0198860.t1-p1
OG0023881: TcSYL_0198880.t1-p1
OG0023882: TcSYL_0198890.t1-p1
OG0023883: TcSYL_0198900.t1-p1
OG0023884: TcSYL_0198910.t1-p1
OG0023885: TcSYL_0198920.t1-p1
OG0023886: TcSYL_0198930.t1-p1
OG0023887: TcSYL_0198940.t1-p1
OG0023888: TcSYL_0198960.t1-p1
OG0023889: TcSYL_0198970.t1-p1
OG0023890: TcSYL_0198980.t1-p1
OG0023891: TcSYL_0198990.t1-p1
OG0023892: TcSYL_0199000.t1-p1
OG0023893: TcSYL_0199010.t1-p1
OG0023894: TcSYL_0199020.t1-p1
OG0023895: TcSYL_0199030.t1-p1
OG0023896: TcSYL_0199040.t1-p1
OG0023897: TcSYL_0199050.t1-p1
OG0023898: TcSYL_0199060.t1-p1
OG0023899: TcSYL_0199070.t1-p1
OG0023900: TcSYL_0199080.t1-p1
OG0023901: TcSYL_0199090.t1-p1
OG0023902: TcSYL_0199100.t1-p1

OG0023903: TcSYL_0199110.t1-p1
OG0023904: TcSYL_0199120.t1-p1
OG0023905: TcSYL_0199130.t1-p1
OG0023906: TcSYL_0199140.t1-p1
OG0023907: TcSYL_0199150.t1-p1
OG0023908: TcSYL_0199160.t1-p1
OG0023909: TcSYL_0199170.t1-p1
OG0023910: TcSYL_0199180.t1-p1
OG0023911: TcSYL_0199190.t1-p1
OG0023912: TcSYL_0199200.t1-p1
OG0023913: TcSYL_0199210.t1-p1
OG0023914: TcSYL_0199220.t1-p1
OG0023915: TcSYL_0199240.t1-p1
OG0023916: TcSYL_0199250.t1-p1
OG0023917: TcSYL_0199260.t1-p1
OG0023918: TcSYL_0199270.t1-p1
OG0023919: TcSYL_0199280.t1-p1
OG0023920: TcSYL_0199290.t1-p1
OG0023921: TcSYL_0199300.t1-p1
OG0023922: TcSYL_0199310.t1-p1
OG0023923: TcSYL_0199320.t1-p1
OG0023924: TcSYL_0199330.t1-p1
OG0023925: TcSYL_0199340.t1-p1
OG0023926: TcSYL_0199350.t1-p1
OG0023927: TcSYL_0199360.t1-p1
OG0023928: TcSYL_0199370.t1-p1
OG0023929: TcSYL_0199380.t1-p1
OG0023930: TcSYL_0199390.t1-p1
OG0023931: TcSYL_0199400.t1-p1
OG0023932: TcSYL_0199410.t1-p1
OG0023933: TcSYL_0199420.t1-p1
OG0023934: TcSYL_0199430.t1-p1
OG0023935: TcSYL_0199440.t1-p1
OG0023936: TcSYL_0199450.t1-p1
OG0023937: TcSYL_0199460.t1-p1
OG0023938: TcSYL_0199470.t1-p1
OG0023939: TcSYL_0199490.t1-p1
OG0023940: TcSYL_0199500.t1-p1
OG0023941: TcSYL_0199510.t1-p1
OG0023942: TcSYL_0199520.t1-p1
OG0023943: TcSYL_0199530.t1-p1
OG0023944: TcSYL_0199540.t1-p1
OG0023945: TcSYL_0199560.t1-p1
OG0023946: TcSYL_0199570.t1-p1
OG0023947: TcSYL_0199580.t1-p1
OG0023948: TcSYL_0199590.t1-p1
OG0023949: TcSYL_0199600.t1-p1
OG0023950: TcSYL_0199620.t1-p1
OG0023951: TcSYL_0199630.t1-p1
OG0023952: TcSYL_0199640.t1-p1
OG0023953: TcSYL_0199650.t1-p1
OG0023954: TcSYL_0199660.t1-p1
OG0023955: TcSYL_0199670.t1-p1
OG0023956: TcSYL_0199680.t1-p1

OG0023957: TcSYL_0199690.t1-p1
OG0023958: TcSYL_0199700.t1-p1
OG0023959: TcSYL_0199720.t1-p1
OG0023960: TcSYL_0199730.t1-p1
OG0023961: TcSYL_0199740.t1-p1
OG0023962: TcSYL_0199750.t1-p1
OG0023963: TcSYL_0199760.t1-p1
OG0023964: TcSYL_0199770.t1-p1
OG0023965: TcSYL_0199790.t1-p1
OG0023966: TcSYL_0199800.t1-p1
OG0023967: TcSYL_0199810.t1-p1
OG0023968: TcSYL_0199820.t1-p1
OG0023969: TcSYL_0199830.t1-p1
OG0023970: TcSYL_0199850.t1-p1
OG0023971: TcSYL_0199860.t1-p1
OG0023972: TcSYL_0199870.t1-p1
OG0023973: TcSYL_0199880.t1-p1
OG0023974: TcSYL_0199890.t1-p1
OG0023975: TcSYL_0199910.t1-p1
OG0023976: TcSYL_0199920.t1-p1
OG0023977: TcSYL_0199930.t1-p1
OG0023978: TcSYL_0199940.t1-p1
OG0023979: TcSYL_0199950.t1-p1
OG0023980: TcSYL_0199970.t1-p1
OG0023981: TcSYL_0199980.t1-p1
OG0023982: TcSYL_0199990.t1-p1
OG0023983: TcSYL_0200000.t1-p1
OG0023984: TcSYL_0200020.t1-p1
OG0023985: TcSYL_0200030.t1-p1
OG0023986: TcSYL_0200040.t1-p1
OG0023987: TcSYL_0200050.t1-p1
OG0023988: TcSYL_0200070.t1-p1
OG0023989: TcSYL_0200080.t1-p1
OG0023990: TcSYL_0200110.t1-p1
OG0023991: TcSYL_0200120.t1-p1
OG0023992: TcSYL_0200130.t1-p1
OG0023993: TcSYL_0200140.t1-p1
OG0023994: TcSYL_0200150.t1-p1
OG0023995: TcSYL_0200170.t1-p1
OG0023996: TcSYL_0200190.t1-p1
OG0023997: TcSYL_0200220.t1-p1
OG0023998: TcSYL_0200230.t1-p1
OG0023999: TcSYL_0200240.t1-p1
OG0024000: TcSYL_0200260.t1-p1
OG0024001: TcSYL_0200270.t1-p1
OG0024002: TcSYL_0200280.t1-p1
OG0024003: TcSYL_0200290.t1-p1
OG0024004: TcSYL_0200300.t1-p1
OG0024005: TcSYL_0200310.t1-p1
OG0024006: TcSYL_0200320.t1-p1
OG0024007: TcSYL_0200330.t1-p1
OG0024008: TcSYL_0200350.t1-p1
OG0024009: TcSYL_0200360.t1-p1
OG0024010: TcSYL_0200370.t1-p1

OG0024011: TcSYL_0200390.t1-p1
OG0024012: TcSYL_0200410.t1-p1
OG0024013: TcSYL_0200420.t1-p1
OG0024014: TcSYL_0200440.t1-p1
OG0024015: TcSYL_0200450.t1-p1
OG0024016: TcSYL_0200460.t1-p1
OG0024017: TcSYL_0200470.t1-p1
OG0024018: TcSYL_0200480.t1-p1
OG0024019: TcSYL_0200490.t1-p1
OG0024020: TcSYL_0200520.t1-p1
OG0024021: TcSYL_0200530.t1-p1
OG0024022: TcSYL_0200540.t1-p1
OG0024023: TcSYL_0200550.t1-p1
OG0024024: TcSYL_0200560.t1-p1
OG0024025: TcSYL_0200570.t1-p1
OG0024026: TcSYL_0200580.t1-p1
OG0024027: TcSYL_0200590.t1-p1
OG0024028: TcSYL_0200610.t1-p1
OG0024029: TcSYL_0200650.t1-p1
OG0024030: TcSYL_0200670.t1-p1
OG0024031: TcSYL_0200730.t1-p1
OG0024032: TcSYL_0200750.t1-p1
OG0024033: TcSYL_0200810.t1-p1
OG0024034: TcSYL_0200870.t1-p1
OG0024035: TcSYL_0200930.t1-p1
OG0024036: TcSYL_0200940.t1-p1
OG0024037: TcSYL_0200960.t1-p1
OG0024038: TcSYL_0200970.t1-p1
OG0024039: TcSYL_0200980.t1-p1
OG0024040: TcSYL_0200990.t1-p1
OG0024041: TcSYL_0201020.t1-p1
OG0024042: TcSYL_0201030.t1-p1
OG0024043: TcSYL_0201040.t1-p1
OG0024044: TcSYL_0201070.t1-p1
OG0024045: TcSYL_0201080.t1-p1
OG0024046: TcSYL_0201090.t1-p1
OG0024047: TcSYL_0201100.t1-p1
OG0024048: TcSYL_0201110.t1-p1
OG0024049: TcSYL_0201120.t1-p1
OG0024050: TcSYL_0201130.t1-p1
OG0024051: TcSYL_0201140.t1-p1
OG0024052: TcSYL_0201150.t1-p1
OG0024053: TcSYL_0201160.t1-p1
OG0024054: TcSYL_0201170.t1-p1
OG0024055: TcSYL_0201180.t1-p1
OG0024056: TcSYL_0201190.t1-p1
OG0024057: TcSYL_0201200.t1-p1
OG0024058: TcSYL_0201220.t1-p1
OG0024059: TcSYL_0201230.t1-p1
OG0024060: TcSYL_0201240.t1-p1
OG0024061: TcSYL_0201270.t1-p1
OG0024062: TcSYL_0201280.t1-p1
OG0024063: TcSYL_0201310.t1-p1
OG0024064: TcSYL_0201400.t1-p1

OG0024065: TcSYL_0201420.t1-p1
OG0024066: TcSYL_0201440.t1-p1
OG0024067: TcSYL_0201490.t1-p1
OG0024068: TcSYL_0201520.t1-p1
OG0024069: TcSYL_0201570.t1-p1
OG0024070: TcSYL_0201580.t1-p1
OG0024071: TcSYL_0201590.t1-p1
OG0024072: TcSYL_0201600.t1-p1
OG0024073: TcSYL_0201620.t1-p1
OG0024074: TcSYL_0201630.t1-p1
OG0024075: TcSYL_0201650.t1-p1
OG0024076: TcSYL_0201660.t1-p1
OG0024077: TcSYL_0201670.t1-p1
OG0024078: TcSYL_0201690.t1-p1
OG0024079: TcSYL_0201700.t1-p1
OG0024080: TcSYL_0201710.t1-p1
OG0024081: TcSYL_0201720.t1-p1
OG0024082: TcSYL_0201740.t1-p1
OG0024083: TcSYL_0201750.t1-p1
OG0024084: TcSYL_0201840.t1-p1
OG0024085: TcSYL_0201850.t1-p1
OG0024086: TcSYL_0201870.t1-p1
OG0024087: TcSYL_0201890.t1-p1
OG0024088: TcSYL_0201900.t1-p1
OG0024089: TcSYL_0201990.t1-p1
OG0024090: TcSYL_0202000.t1-p1
OG0024091: TcSYL_0202170.t1-p1
OG0024092: TcSYL_0202180.t1-p1
OG0024093: TcSYL_0202190.t1-p1
OG0024094: TcSYL_0202200.t1-p1
OG0024095: TcSYL_0202210.t1-p1
OG0024096: TcSYL_0202220.t1-p1
OG0024097: TcSYL_0202230.t1-p1
OG0024098: TcSYL_0202240.t1-p1
OG0024099: TcSYL_0202250.t1-p1
OG0024100: TcSYL_0202260.t1-p1
OG0024101: TcSYL_0202280.t1-p1
OG0024102: TcSYL_0202300.t1-p1
OG0024103: TcSYL_0202310.t1-p1
OG0024104: TcSYL_0202330.t1-p1
OG0024105: TcSYL_0202340.t1-p1
OG0024106: TcSYL_0202350.t1-p1
OG0024107: TcSYL_0202390.t1-p1
OG0024108: TcSYL_0202400.t1-p1
OG0024109: TcSYL_0202450.t1-p1
OG0024110: TcSYL_0202480.t1-p1
OG0024111: TcSYL_0202560.t1-p1
OG0024112: TcSYL_0202580.t1-p1
OG0024113: TcSYL_0202600.t1-p1
OG0024114: TcSYL_0202610.t1-p1
OG0024115: TcSYL_0202620.t1-p1
OG0024116: TcSYL_0202630.t1-p1
OG0024117: TcSYL_0202640.t1-p1
OG0024118: TcSYL_0202650.t1-p1

OG0024119: TcSYL_0202660.t1-p1
OG0024120: TcSYL_0202670.t1-p1
OG0024121: TcSYL_0202690.t1-p1
OG0024122: TcSYL_0202710.t1-p1
OG0024123: TcSYL_0202720.t1-p1
OG0024124: TcSYL_0202730.t1-p1
OG0024125: TcSYL_0202740.t1-p1
OG0024126: TcSYL_0202750.t1-p1
OG0024127: TcSYL_0202760.t1-p1
OG0024128: TcSYL_0202770.t1-p1
OG0024129: TcSYL_0202780.t1-p1
OG0024130: TcSYL_0202790.t1-p1
OG0024131: TcSYL_0202800.t1-p1
OG0024132: TcSYL_0202810.t1-p1
OG0024133: TcSYL_0202830.t1-p1
OG0024134: TcSYL_0202840.t1-p1
OG0024135: TcSYL_0202850.t1-p1
OG0024136: TcSYL_0202860.t1-p1
OG0024137: TcSYL_0202870.t1-p1
OG0024138: TcSYL_0202880.t1-p1
OG0024139: TcSYL_0202890.t1-p1
OG0024140: TcSYL_0202900.t1-p1
OG0024141: TcSYL_0202910.t1-p1
OG0024142: TcSYL_0202920.t1-p1
OG0024143: TcSYL_0202930.t1-p1
OG0024144: TcSYL_0202940.t1-p1
OG0024145: TcSYL_0202950.t1-p1
OG0024146: TcSYL_0202960.t1-p1
OG0024147: TcSYL_0202980.t1-p1
OG0024148: TcSYL_0202990.t1-p1
OG0024149: TcSYL_0203000.t1-p1
OG0024150: TcSYL_0203010.t1-p1
OG0024151: TcSYL_0203020.t1-p1
OG0024152: TcSYL_0203030.t1-p1
OG0024153: TcSYL_0203040.t1-p1
OG0024154: TcSYL_0203050.t1-p1
OG0024155: TcSYL_0203060.t1-p1
OG0024156: TcSYL_0203080.t1-p1
OG0024157: TcSYL_0203100.t1-p1
OG0024158: TcSYL_0203110.t1-p1
OG0024159: TcSYL_0203120.t1-p1
OG0024160: TcSYL_0203130.t1-p1
OG0024161: TcSYL_0203140.t1-p1
OG0024162: TcSYL_0203150.t1-p1
OG0024163: TcSYL_0203170.t1-p1
OG0024164: TcSYL_0203200.t1-p1
OG0024165: TcSYL_0203290.t1-p1
OG0024166: TcSYL_0203340.t1-p1
OG0024167: TcSYL_0203450.t1-p1
OG0024168: TcSYL_0203460.t1-p1
OG0024169: TcSYL_0203470.t1-p1
OG0024170: TcSYL_0203520.t1-p1
OG0024171: TcSYL_0203540.t1-p1
OG0024172: TcSYL_0203550.t1-p1

OG0024173: TcSYL_0203560.t1-p1
OG0024174: TcSYL_0203570.t1-p1
OG0024175: TcSYL_0203580.t1-p1
OG0024176: TcSYL_0203590.t1-p1
OG0024177: TcSYL_0203600.t1-p1
OG0024178: TcSYL_0203610.t1-p1
OG0024179: TcSYL_0203620.t1-p1
OG0024180: TcSYL_0203640.t1-p1
OG0024181: TcSYL_0203660.t1-p1
OG0024182: TcSYL_0203690.t1-p1
OG0024183: TcSYL_0203720.t1-p1
OG0024184: TcSYL_0203730.t1-p1
OG0024185: TcSYL_0203740.t1-p1
OG0024186: TcSYL_0203750.t1-p1
OG0024187: TcSYL_0203780.t1-p1
OG0024188: TcSYL_0203790.t1-p1
OG0024189: TcSYL_0203800.t1-p1
OG0024190: TcSYL_0203810.t1-p1
OG0024191: TcSYL_0203840.t1-p1
OG0024192: TcSYL_0203850.t1-p1
OG0024193: TcSYL_0203860.t1-p1
OG0024194: TcSYL_0203870.t1-p1
OG0024195: TcSYL_0203880.t1-p1
OG0024196: TcSYL_0203890.t1-p1
OG0024197: TcSYL_0203910.t1-p1
OG0024198: TcSYL_0203920.t1-p1
OG0024199: TcSYL_0203930.t1-p1
OG0024200: TcSYL_0203950.t1-p1
OG0024201: TcSYL_0203960.t1-p1
OG0024202: TcSYL_0203990.t1-p1
OG0024203: TcSYL_0204000.t1-p1
OG0024204: TcSYL_0204010.t1-p1
OG0024205: TcSYL_0204030.t1-p1
OG0024206: TcSYL_0204040.t1-p1
OG0024207: TcSYL_0204050.t1-p1
OG0024208: TcSYL_0204060.t1-p1
OG0024209: TcSYL_0204080.t1-p1
OG0024210: TcSYL_0204090.t1-p1
OG0024211: TcSYL_0204100.t1-p1
OG0024212: TcSYL_0204120.t1-p1
OG0024213: TcSYL_0204130.t1-p1
OG0024214: TcSYL_0204140.t1-p1
OG0024215: TcSYL_0204160.t1-p1
OG0024216: TcSYL_0204170.t1-p1
OG0024217: TcSYL_0204180.t1-p1
OG0024218: TcSYL_0204190.t1-p1
OG0024219: TcSYL_0204200.t1-p1
OG0024220: TcSYL_0204210.t1-p1
OG0024221: TcSYL_0204220.t1-p1
OG0024222: TcSYL_0204230.t1-p1
OG0024223: TcSYL_0204250.t1-p1
OG0024224: TcSYL_0204260.t1-p1
OG0024225: TcSYL_0204270.t1-p1
OG0024226: TcSYL_0204280.t1-p1

OG0024227: TcSYL_0204290.t1-p1
OG0024228: TcSYL_0204310.t1-p1
OG0024229: TcSYL_0204320.t1-p1
OG0024230: TcSYL_0204330.t1-p1
OG0024231: TcSYL_0204350.t1-p1
OG0024232: TcSYL_0204360.t1-p1
OG0024233: TcSYL_0204370.t1-p1
OG0024234: TcSYL_0204380.t1-p1
OG0024235: TcSYL_0204390.t1-p1
OG0024236: TcSYL_0204400.t1-p1
OG0024237: TcSYL_0204410.t1-p1
OG0024238: TcSYL_0204430.t1-p1
OG0024239: TcSYL_0204440.t1-p1
OG0024240: TcSYL_0204460.t1-p1
OG0024241: TcSYL_0204510.t1-p1
OG0024242: TcSYL_0204530.t1-p1
OG0024243: TcSYL_0204550.t1-p1
OG0024244: TcSYL_0204560.t1-p1
OG0024245: TcSYL_0204570.t1-p1
OG0024246: TcSYL_0204590.t1-p1
OG0024247: TcSYL_0204600.t1-p1
OG0024248: TcSYL_0204610.t1-p1
OG0024249: TcSYL_0204630.t1-p1
OG0024250: TcSYL_0204640.t1-p1
OG0024251: TcSYL_0204650.t1-p1
OG0024252: TcSYL_0204660.t1-p1
OG0024253: TcSYL_0204670.t1-p1
OG0024254: TcSYL_0204680.t1-p1
OG0024255: TcSYL_0204690.t1-p1
OG0024256: TcSYL_0204700.t1-p1
OG0024257: TcSYL_0204710.t1-p1
OG0024258: TcSYL_0204720.t1-p1
OG0024259: TcSYL_0204730.t1-p1
OG0024260: TcSYL_0204740.t1-p1
OG0024261: TcSYL_0204750.t1-p1
OG0024262: TcSYL_0204760.t1-p1
OG0024263: TcSYL_0204770.t1-p1
OG0024264: TcSYL_0204780.t1-p1
OG0024265: TcSYL_0204790.t1-p1
OG0024266: TcSYL_0204800.t1-p1
OG0024267: TcSYL_0204810.t1-p1
OG0024268: TcSYL_0204820.t1-p1
OG0024269: TcSYL_0204850.t1-p1
OG0024270: TcSYL_0204860.t1-p1
OG0024271: TcSYL_0204870.t1-p1
OG0024272: TcSYL_0204880.t1-p1
OG0024273: TcSYL_0204890.t1-p1
OG0024274: TcSYL_0204900.t1-p1
OG0024275: TcSYL_0204910.t1-p1
OG0024276: TcSYL_0204930.t1-p1
OG0024277: TcSYL_0204940.t1-p1
OG0024278: TcSYL_0204950.t1-p1
OG0024279: TcSYL_0204960.t1-p1
OG0024280: TcSYL_0204970.t1-p1

OG0024281: TcSYL_0204980.t1-p1
OG0024282: TcSYL_0205000.t1-p1
OG0024283: TcSYL_0205020.t1-p1
OG0024284: TcSYL_0205030.t1-p1
OG0024285: TcSYL_0205040.t1-p1
OG0024286: TcSYL_0205050.t1-p1
OG0024287: TcSYL_0205070.t1-p1
OG0024288: TcSYL_0205080.t1-p1
OG0024289: TcSYL_0205090.t1-p1
OG0024290: TcSYL_0205100.t1-p1
OG0024291: TcSYL_0205110.t1-p1
OG0024292: TcSYL_0205120.t1-p1
OG0024293: TcSYL_0205130.t1-p1
OG0024294: TcSYL_0205140.t1-p1
OG0024295: TcSYL_0205150.t1-p1
OG0024296: TcSYL_0205170.t1-p1
OG0024297: TcSYL_0205190.t1-p1
OG0024298: TcSYL_0205210.t1-p1
OG0024299: TcSYL_0205220.t1-p1
OG0024300: TcSYL_0205230.t1-p1
OG0024301: TcSYL_0205240.t1-p1
OG0024302: TcSYL_0205250.t1-p1
OG0024303: TcSYL_0205260.t1-p1
OG0024304: TcSYL_0205270.t1-p1
OG0024305: TcSYL_0205290.t1-p1
OG0024306: TcSYL_0205300.t1-p1
OG0024307: TcSYL_0205310.t1-p1
OG0024308: TcSYL_0205330.t1-p1
OG0024309: TcSYL_0205350.t1-p1
OG0024310: TcSYL_0205360.t1-p1
OG0024311: TcSYL_0205370.t1-p1
OG0024312: TcSYL_0205380.t1-p1
OG0024313: TcSYL_0205390.t1-p1
OG0024314: TcSYL_0205400.t1-p1
OG0024315: TcSYL_0205420.t1-p1
OG0024316: TcSYL_0205430.t1-p1
OG0024317: TcSYL_0205440.t1-p1
OG0024318: TcSYL_0205460.t1-p1
OG0024319: TcSYL_0205470.t1-p1
OG0024320: TcSYL_0205480.t1-p1
OG0024321: TcSYL_0205490.t1-p1
OG0024322: TcSYL_0205500.t1-p1
OG0024323: TcSYL_0205520.t1-p1
OG0024324: TcSYL_0205530.t1-p1
OG0024325: TcSYL_0205540.t1-p1
OG0024326: TcSYL_0205550.t1-p1
OG0024327: TcSYL_0205560.t1-p1
OG0024328: TcSYL_0205580.t1-p1
OG0024329: TcSYL_0205590.t1-p1
OG0024330: TcSYL_0205600.t1-p1
OG0024331: TcSYL_0205610.t1-p1
OG0024332: TcSYL_0205620.t1-p1
OG0024333: TcSYL_0205640.t1-p1
OG0024334: TcSYL_0205650.t1-p1

OG0024335: TcSYL_0205660.t1-p1
OG0024336: TcSYL_0205670.t1-p1
OG0024337: TcSYL_0205680.t1-p1
OG0024338: TcSYL_0205690.t1-p1
OG0024339: TcSYL_0205700.t1-p1
OG0024340: TcSYL_0205710.t1-p1
OG0024341: TcSYL_0205720.t1-p1
OG0024342: TcSYL_0205730.t1-p1
OG0024343: TcSYL_0205750.t1-p1
OG0024344: TcSYL_0205760.t1-p1
OG0024345: TcSYL_0205770.t1-p1
OG0024346: TcSYL_0205780.t1-p1
OG0024347: TcSYL_0205790.t1-p1
OG0024348: TcSYL_0205800.t1-p1
OG0024349: TcSYL_0205810.t1-p1
OG0024350: TcSYL_0205820.t1-p1
OG0024351: TcSYL_0205830.t1-p1
OG0024352: TcSYL_0205840.t1-p1
OG0024353: TcSYL_0205860.t1-p1
OG0024354: TcSYL_0205880.t1-p1
OG0024355: TcSYL_0205890.t1-p1
OG0024356: TcSYL_0205900.t1-p1
OG0024357: TcSYL_0205910.t1-p1
OG0024358: TcSYL_0205920.t1-p1
OG0024359: TcSYL_0205930.t1-p1
OG0024360: TcSYL_0205940.t1-p1
OG0024361: TcSYL_0205950.t1-p1
OG0024362: TcSYL_0205960.t1-p1
OG0024363: TcSYL_0205970.t1-p1
OG0024364: TcSYL_0205980.t1-p1
OG0024365: TcSYL_0205990.t1-p1
OG0024366: TcSYL_0206010.t1-p1
OG0024367: TcSYL_0206020.t1-p1
OG0024368: TcSYL_0206040.t1-p1
OG0024369: TcSYL_0206050.t1-p1
OG0024370: TcSYL_0206060.t1-p1
OG0024371: TcSYL_0206070.t1-p1
OG0024372: TcSYL_0206080.t1-p1
OG0024373: TcSYL_0206090.t1-p1
OG0024374: TcSYL_0206100.t1-p1
OG0024375: TcSYL_0206110.t1-p1
OG0024376: TcSYL_0206120.t1-p1
OG0024377: TcSYL_0206130.t1-p1
OG0024378: TcSYL_0206140.t1-p1
OG0024379: TcSYL_0206150.t1-p1
OG0024380: TcSYL_0206160.t1-p1
OG0024381: TcSYL_0206170.t1-p1
OG0024382: TcSYL_0206180.t1-p1
OG0024383: TcSYL_0206190.t1-p1
OG0024384: TcSYL_0206200.t1-p1
OG0024385: TcSYL_0206210.t1-p1
OG0024386: TcSYL_0206220.t1-p1
OG0024387: TcSYL_0206230.t1-p1
OG0024388: TcSYL_0206240.t1-p1

OG0024389: TcSYL_0206250.t1-p1
OG0024390: TcSYL_0206260.t1-p1
OG0024391: TcSYL_0206270.t1-p1
