# Supplementary material for: Assembly of highly repetitive genomes using short reads: the genome of discrete typing unit III Trypanosoma cruzi strain 231
Source: Microb Genom. 2018 Feb 14;4(4):e000156. doi: 10.1099/mgen.0.000156 (PMC5989580; doi:10.1099/mgen.0.000156)
Supplement: Supplementary File 3 [file mgen-4-156-s003.pdf]

>scaffold1.size104391
TTTTTTTTCTATTTTTTGTTAATGTTGATTTACCTGTAGGGACGCACATTCTTGTATGCGTGATGTGT
GTGATGTGGGCGATCACCAGAACGGGGTATTC
CATCTTTTGGTAACGACAACAATAGACGACGGAAAGAGAGAGAGACAGAGAGAGAGAGAGAGATAGAGGA
GAAAAAAAAAGAAAAGATAAGAAGTGGGACTG
AAACATACATAAGTCGACATAAATATATATTTTTATGCCCAGTACTTTATTACTTACGCGCGGTGTAC
TTATGTATACATACATGGATGCATGCATGCAA
TGCAGTGGGTGGAATGGGGATTACTTAGGCATACTTATAAAGTTATAAACTTATAATTAAGTGAGGAA
ATATAAAAATGAATGTTCTGACGTTAGGAGTG
GTATTCAGAATTATTTATTGAATTGGGAAAGTGAAATAGATGAACAGAAACGTGTAAATACACATCGA
GTCCTACTTGTGTGAGACTACACTAAAAAAAA
AAAAAAATTTACAATCCGCTAACGAAAGAGAATTGTAGGAAGGTGTGTTTTCAGCTTGGTGGCTTTGA
GGGAGGCAATATTTCCTTGTGTTTTTTCTTTT
TTGTTTGTGTACGTCATTGTTGTACGATTCACATGGGTCTTTTGAATTTTTTTTTTTTCTTTTTTTTA
GCTTTCGTACGTGTGGTGGGCAAGAAGACTCC
CACCCTCCCCTTTTTTTTTTTTTCTTTTGATTCAGTTGTGTTCTTGTAATTATTGTTGTTACTNNNNN
NNNNNNNNNNNNNNNNNNNNNNNNNNNNNNNN
NNNNNNNNNNNNNNNNNNNNNNNNNNNNNNNNNNNNNNNNNNNNNNNNNNNNNNNNNNNNNNNNNNNN
NNNNNNNNNNNNNNNNNNNNNNNNNNNNNNNN
NNNNNNNNNNNNNNNNNNNNNNNNNNNNNNNNNNNNNNNNNNNNNNNNNNNNNNNNNNNNNNNNNNNN
NNNNNNNNNNNNNNNNNNNNNNNNNNNNNNNN
NNNNNNNNNNNNNNNNNNNNNNNNNNNNNNNNNNNNNNNNNNNNNNNNNNNNNNNNNNNNNNNAATTT
CTGCGCATATTTTTTTTTTATTGATTTGGGAA
TTCCCGTAGCCGTGAGTGACCATGCGGCTCAGCAAAGGACGGTGCCGATCACGTGCTCTGCCTCACGG
GAACAGTGCGTAAATGGATAATTGTCTCTCTC
TCTCTCTCTCTCTCTCTGTGTGTGTGTGTGTGTGTGTGTGTGTGTGTGTGTGTGTGTGTGTGTGTGGG
TGGGTGAATGGTTGCACTGTGGATGTTTATTG
GATGTCTACTCATTGCGCCGTTTGTTTGTTTTTTTTGTTTTTTCCCTCTTGTTATTTCTTCTTACTGG
NNNNNNNNNNNNNNNNNNNNNNNNNNNNNNNN
NNNNNNNNNNNNNNNNNNNNNNNNNNNNNNNNNNNNNNNNNNNNNNNNNNNNNNNNNNNNNNNNNNNN
NNNNNNNNNNNNNNNNNNNNNNNNNNNNNNNN
NNNNNNNNNNNNNNNNNNNNNNNNNNNNNNNNNNNNNNNNNNNNNNNNNNNNNNNNNNNNNNNNNNNN
NNNNNNNNNNNNNNNNNNNNNNNNNNNNNNNN
NNNNNNNNNNNNNNNNNNNNNNNNNNNNNNNNNNNNNNNNNNNNNNNNNNNNNNNNNNNNNNNNNNNN
GAAGCGGGCACATTGGCGGTGTGGCGAGAGGA
TTAATGCAGGAGGAACACACGTTTGATGATCCAGAAGCGGCATTTTTTACCCCAAGCCTTTCCATTGC
TACTGGTGACACTCAAATTTATGCCGCGCCGC
TGGGGAATTACAGCAGAGAGAATGAGGCGCAGCATAACGAGCTAAAGACTTTTCTTGGCGAAACAGAG
GAGTTGGAAGATGAGAAAGAAGCGTACGCCTC
TTTGTTTCATGAGGGTATGAATGACGATGAGGAGGCGACATATAAACAGCAGATGGGACAACAGGCTT
TGAGTGGGATGGAAGGCGACGAGGCCACATTT
GATGCNNNNNNNNNNNNNNNNNNNNNNNNNNNNNNNNNNNNNNNNNNNNNNNNNNNNNNNNNNNNNNN
NNNNNNNNNNNNNNNNNNNNNNNNNNNNNNNN
NNNNNNNNNNNNNNNNNNNNNNNNNNNNNNNNNNNNNNNNNNNNNNNNNNNNNNNNNNNNNNNNNNNN
NNNNNNNNNNNNNNNNNNNNNNNNNNNNNNNN
NNNNNNNNNNNNNNNNNNNNNNNNNNNNNNNNNNNNNNNNNNNNNNNNNNNNNNNNNNNNNNNNNNNN
NNNNNNNNNNNNNNNNNNNNNNNNNNNNNNNN
NNNNNCGGTGGTGTTGATGCTGCAGGACTTGACAAGGCTGGCGGCGGCGGAGGCTGAGCGACAGAGGC
GGAGGACAGTGACGTACGCGGATATTGCGCGG
GTGGTGCATCACTTTGACCGCTTCTCATTTTTATCGGACATCATCCCACCCGTTTCGGTAGTGGCGGG
CGGCGCAAAAGCGGTGGCTGTCGGTTCTGCCA
CTTCTCTTGCGGCCGAATCGAAGACAAGCCGCAGCGCCGCTCGTGAGGGAGCGGTACGAGCGCAGCTG
AAATCAACGGAGACTGGGGGCGGCGGTGCAAG
TATGCGACAGACGACACTTCGCTTTTGAAGTTGCAGCCAACGGCAAACGGCTTTTAATTTCTTTCTCT

CTTTTTTTATTATTACGTTCTCTTATATTACA
GGCCGGTTTGGGCTTCAACTGCCGTTACTGTCTGCCCGTGCGTAATATCCCTCACTGCTTGTGTCTTT
CTTCTTTGACTTTTGTGGGGAATAGGCTTCAT
AGTCCCCTCTGCATCACATCCTTGGGGGTGGGGCGCAAGGGAAGGGCACGAGGCAACATAATTCCGTT
TAAAAAGTAAAAACTGAAGGGGGGGAAGAAAA
GAGCAGAGGGTGAGAGAAACCAACAGCAAAAACGAACAAAGAAAAGGAAAAGAGAAAAGGAAAAAAAA
AAGAAGGCGAAAAAAAAAATTACGCATATATGA
TGAGCGTCTCTGTTCCCACCACACCGCACCACGAGAAGGATCGTGTACCACATTTGATGTCCGATGAG
GAGCTGCGGAATGAGTACGTGCAGCTGCGCAA
GAAGAATGCCTCACTAAATGAGGAGTGCGCCTCTCTTCAGATGGAAATAAACTCCATTTATGAGGAGT
CGGTTCGCCTTCAACAAAGCACGGAGATCGAG
GAAGAGAAGTTGGCAAACCAGCTGCTCCGTCGTCTTCAGTCGGAGGAATTGCAAAAACGTCGGTTTCA
TGACCTTATCCGCCGCGAAGAAGTCGCCCGCC
AAAAGATCATGAACCAAATCGCCCAAATCCGGACCGAGAAAACAGATTTGGCAGGACAACTGGGGAAG
CAAGAGTCGCTGATGCTTCAGCTGCAGAAGAA
GTTAATGGAAGTGGTGACGAAGAAAAGTGAGGTGGAACGTGAACTACTGAAGGAACGTCGTAGATACT
TGGATGTGCTCTCCACGCAATTGGGTCTTCTT
ATAGAAGGATCCATCAATGAAAAGAAGGGGGAAAAGGCCTCTGACGCCGGCCAAAGCGTTGACGCAAG
CGAGCAGAAACCAATGGAATCATCATCAATGG
ATGCGGACAGTAAGACAATCAATTGTGCCACTTCCATCCCTGCATTTGAGGCCGCTACAACTGATACT
CATGTAGCTATCAATCGTCTAGAGAAGCAGCT
CAACCGTCTTCTCTGCGAGCACGCTGCGGCGGTTCAGAATTTGACGAGTAATGAGGTGTGCTGTGCGG
AACTGGCAAAGAAACTGGACACCATACAGCAG
GCCGCTTTCCTAGACCGTGCACGAGCGTCCAAGCTGAAGGAGGAACTCAAGGAAGCCAAATCTCGCTT
AGCAGGACTAGAGCGTCAGATGTCACAGATGA
GCAGCTCGGCTATTTCAGAAGAGAGTTCTGGTATACCAACACCAACGCTGTGTTGCCTTAGAAGCTTC
TCACTGCGTGGCCGAACAAGGGGGGCTCTGTC
ATCACGTCAACGTGAGACAAGTGTCAGTGTCCAACCTTCCGTCGATGCATCGGATACAGGAGATGACT
CACGCTCTTTTGCATGAGGGGAGTTTTGTATT
CTTATTTATTTGCTTTAGAAAAGGAGGAACATTTACCACATCTGCCTGCACGAGAGAGAAACTGCGAG
GGAGAATACGAAGGAGAAAGAGAAAAAGCAGG
CAGAAGCAAAAAAAAAGGAGGAAAGGAAAGATCTGCATTGTTACGGCAAATGATAGGGGGAAAAAAAA
AGAGAATTCAAAGGGCGAACAACAAAAGAAAC
GAGAAACGGCGAAAACAACAAATAGGGGCATGAGTGCTGTACCCGGTCATGTCATCTTTTTTTATTTT
ATTTTTGTTTCACCCAGCGACAAAAGAAATAC
GAGAAGGAAGTGAGGGGTTCCTCCCACAATAATACTTCAAGGAGCGAAGGACAGAGAAAGAAGGACGG
GGAGGGCATCTAGAAGATGGACGAACCTCTCC
AGCAGTGGCGAGACCAACGGGAAACGGGAGAGAGACACCGGAAACCTACCATTTGTTTTTTGGTGAGG
TTTGACGCCGCTCCGTTGGAGGCTGCCGACGG
CGTGTTTCGCGGCGCTCAGGCATTTCGATCCCCTTCGGAGATTGTGGTGCCGTTGAAGATGGCACTTG
AAGAGGCCTCTTTGACTGCTTTACAGTCTGAC
TTCATGTCTCGCACATTCCGTCGATTTCCACAGCTCTCGAATCGTACTGTGGAAGCCTTTTTCGTTAG
TGCTGAGGAACATTTTATGGCGTCGAGCACAG
GCTTGAGCCGCCCGTACTTGTTGCGTCTAGATGCGTGCATGAACGGGGATGATACACTGGATAGCTTT
TTTACCCCTGTAGCTACAAATGCTCCTATTGT
GTATGAAGTCATTGCACACTGGGCCCCTGAAAAACTGACGGACACCGTCTCCAGTCACATCACCTTGG
AGGGAGAAGAAGAAGAGGAGAATGGAGACACG
TTACCCAGTGACACGGTCTTAGAGGATACCATTCCGGCACCTGTTGTTTACCCTTTTGCACATCGTTG
CACCTATGCGAGATTGATACCAATTCAGCCAG
AGACTCCGGTCACAGATGGTTCTACGATTGATGAAGTGAAGGCAGAAGCCCCTCCTATTCTCAACGCC
ATGAGGAGGAAAGAAGCCGCACGAATGGCGCG
ACGGGAGATGGAATTTGTGGAGGGCACTGAACGAAGGAAACTTCTTGAGGAAGAACGTAGCGCACGCG
GTACGGTTGCTGAATTAGAAGAAGAGAGCCGA
ATACAAACAGTAACGCTGCTTCGTTTGATGTATGATGAAGTTCCGGAATGTGGCGTCAATTACATTCA

ATCAATTGCACCAAGACAGCGCTACCCGGTGG
ACACGTTGCCGCTCGCTCCCTTTCCGAGTTCAGTGTATATGAAGGGATTATCGAAGTGTGCGGAAGAA
ACTGCCCGGCTTTCTCATGTCTTTGCGACAAC
CGCGATATCTGGCTCGGCGGCGGCCCCCACCAAACATGAAGATACTTTGGTTGCACCTGTTCCGGCTG
ATAAATTAAACAACGAAAAAATTCAAGAAGAA
ACCCAGGAGGCTATTCCGTGTTCCGACAGCGGGGAAGAGCTCGTCCGGCACTATGCCGATTTGCGCCG
CGACTTATTATTATGGGAGCGTACGACGTTTC
GGGCTCTTACGACGGAAATGCGTCGCATTCTTGAAAAACAGAAAAAAATAGATGAGGAAATTGTGCAA
AAGGCTTTTTTGGAGGAGGTGGAAAACGCATC
TATACTTGACTTTGACAGCTATGTACATCATTACAGCCATGAACATCTTCTTGAGGGGCGCAGCGACA
CTCCGGACACGGTGACAAATTACATTCCTGGT
GAATGCAAACGTTCAAGCCATGCGGCGTCTCTCGTCTTCAGGCGACCTACAGAATCGCCTAAGTTTCC
AGCGGCGCAGAAAGAGGTGGAGACCACTGTAG
GAGCTGAGGTGGGGGCAACCCCCACAACTTTATCCTTACCAGGGGCTATTAGAACACCTTCTCCTTCT
CTGCCAAGACCACGTTCTCCTCGTTCCCTTTT
GGCGGAAGAGAAGCAGTTTCGTGAGGCACTGGAGCGAAGCAAAGCGCGCGCCAAGGCGGCTTTGGAAG
CTATTGCCCTTGTTGACAGGCACTGGCATGAG
CCTGTTGCGTTTAAGCTTGCGAAGCATCGACTTTTGCTTGATGAGAAGGGGTCGAGAAATGCGATCCT
TGCAGAGGAGATGGAGAGCTGGGAGAATCATG
TTAAAGAAGTCAAAAAAGTAGAAATAGGTTGGGTAATGAAAGCCATTTCTGCTCGAAAGCATCTTTTT
GGTCCAGTTATTGGGGAGGAAGAAAAGTTTAG
AGCAGATCTTGATGAGCAACAAGAGCGGCATTTTGCAATGCTTCTTTCCTTGTATTATCTGGGCATGG
AGAGATAAGTAAATCTTGTATGATCAAAACGG
GCTGCAGATGTATAGTCAGTATTTGTATTGTTTCCATTATAAAGATGATGATGATTATTGTTTTTCCA
GTGATTCTTGTAAAATGACTGTTATGCGATTT
ATTTCTGGCTGAGGCAGTGCTTTTTGTGCATACATCATGTAGACCTCCTTGCTTTATGTGTTCTTTTT
CTTTGTTGTTGTTTCCTCTTCTTCTTCTTCTT
TCTTTGTTCTATGTATTTTGTTGTTGCTGATTTCTTGAAGCGGTCAGTGGCCCATTTCTTTCGGTCTT
TCGCCCACTTTGTGTGCGCACGCGTGTAAGAG
GAGGAGAGAAGAGAGAACGAAACACATTTTACCGTAAGGAAAGAAAGCACCGATAAAAATCCCTCTTT
TTTTGCCTTTCCCTTCTTTCCGGAAAGAGGAA
GTTACAATAAAGAGCGGGACGACAAGAACGAAAAAAAAAACACGCACACGGAAAAGAAAAGCAAAACA
AAACAGGCCAAAAACAAAAACGAGCTTCAGAC
TAAAGACACCCCGTTCGCCGTGTGATTTATTTGTTTTTTTTGTTTTTTTGTTTGCTTGTTCCACACCT
CATTTTTTTTTTTTTTTTTTGCCCGCCTGTTT
GTGTGTGTCTGCGTGGTCGTGGGCGCATGTAGTGGTTGACAGCTGTGGTGTTTGAGAGAAGGGTGTGA
CTTTGTGAAAATGGCCTTTCGACTTATTTTTG
AACGTCGGTTGGCGCCACTCTCGAACAGTTGTAGGCGTTGCTGCCGCACGATGTTTTCTGGTCCCACG
CCGCGAATTACTTCTGTGACGCGTAGTGGACT
GAGAGGATATGTATTGTGGCAATCTCCACCTCATGGGAGTTGCATCTGCCCTGCGCTTTCCTCTTCTA
GAGCTTTTATTCACTTGCCAGAAAGGACAACT
ATGGCTTCTTCGGAGGAATTGCTTGAGAGCCTTCAACGGATTGTTGGCTTAGATGAGAAGAACTCCAA
GGACCTGTCCACAAAACCGGAACGCGTGCAAG
ACCTCCTTGGCTTCTTTGCGAATCAAAACATGACACCAGAGTCCCCTCGCGAGCAAAAAATGATGCTT
TTTAACTTGTGGGCCAAGGTTAAGCTTCCCAA
GCACCGCGATCTTCTTGCAGACTACGTGAAGAGGAAAAAGCTGGATTCAACCCAAAAGGTGGATGCCG
CCATACGCTTTGTGGATGGCATGAAGGAAGAT
GCTCTCTTTGATGCTGCAAAATTTGAAAAGGAATGTGGCGTGGACGTGGTTGTCTCGAAAGATGATAC
ATTGCGGCTCGTAAAGACGGCATTGGCAGAGG
AAGACCTCAAAGCATTGAAAACGTCATGGGTGAAAAACCCGAACATGCTGCTTGGCAAGATGAAGCGG
GTACCGGGTTTGCAATGGGCAGACTTTACTCT
GGTGAAGACCATCCTTGAGGAGTTGGTACCGCCCATGATTAAGGATGTTGTCATTGATGAGGCCGAAC
CCAAGACCGAAGAGAAGACCGCCACTCCACCT
CGCACGAAAGGCGCTGAAGTGGCAGCGACGCAAAAGTGCCCACGCAACGCGGATTGGAAATGTGTTGT

GGATCTGCATTCACTCCATCGCATCGGTGATT
TATCGTCTATGCAGGAAGGTACAAAGGTGCGTGTTGTGGGATGGGCACACCGAGTTCGTCATCAATCG
CGTCTTTCATTTGTGGTACTACGTGATGGATC
TGGGTACATTCAAGCCGTATTCAATGGTGAAACAGAACCCTTTCACCGCGAAACGTCTTTGGCAATTC
GCGGTACACTGAAGTACGAACCCAAGGCTGCT
ACTGAATTGCAGCCACCATACGAGCTTCATGTGGATGAATACGCCATTATTGGTAACTCGGATGGCAG
TATTGAGAACGCTATTACCCCTGAAAGCTCCC
CTGACAAATTGTTGGATGATCGTCATTTGGTGCTTCGTGGAACCTATGGCTCAACAACCATTAAAGTT
CGTCACGAATTAATTCGAGCTTTTCGTGAATA
CTGTTGGTCCGTAAATATGTTTGAAGTAACACCACCAACTCTTGTACAGGCTGAGTGTGAAGGGGGAT
CTACGCTTTTTGATGTCATTTACTACGGAACG
ACGGCTCACCTTACACAGTCTTCCCAGTTATATCTTGAGACATGCACTTCTTCAGTGGGAGATGTGTA
CTGTATTCTTCCCAGTTATCGAGCGGAGAAAT
CCAAGACGAGGCGCCACTTGAGTGAATACACACATTTGGAGGTTGAGTACAGTATCTGCGACTTTGAG
CTTTTCCTCTCAAAACTGGAAGATCTTATTGT
GGGAGCATTCAACCGAACAATTGAGCGGGCGGGAGATTTGATCGCCTTTATGAATCCAGAACAGTTGG
TGGATCCAAAGGCAAACCCATGTGATCCCAAC
AACTGGAAGTTTGCACCCAAGAAGCCGTTTAGGCGTCTCAAGTATGCTGACGCCATTAAATTATGCAA
TGATAACGGCATTTTGAACCCAGAGACAAATG
CACCATTCCAATTTGGTGACGACATCACGGATCAGCCGGAGCGGGCCATGGTGGCGATATTGGGGGAG
ATGGTGTTCATGACACATTTTCCTGCGGTAAT
GAAGTCTTTCTACATGGCCCGAGACCCAGAGGATCCGACACTTACAGAGTCCGTGGACGTTTTGGCTC
CTGGTATTGGGGAAATTGTCGGCGGCTCTATG
CGTATGTGGGATCATGAAGAACTGATAGAGGCGTACAGGCGTAAGGACCTTGACCCAAGTGTGTATTA
TTGGTACACGGAGCAGCGCAAGTATGGCTCCA
CCCCACATGGGGGCTTTGGTTTGGGAGTGGAGCGCTTCTTGGTGTGGCTGCTGAACTTGGACTCGGTC
AGGGAGGCATGTCTTTTCCCGCGTTACATGGG
AAGATGCAAGCCGTAGCGAGGAGGACAGAACGACGAAGAAGAATAATTCATAGGTAGTAATATCAGCA
TACATAATGAAGCGAAGAATCCGCAAGAAAAC
AGAAAAAAAGAAAGAAAGAAAGAGAGAGAGAGAGAGAGAGAAAGAGAAAGAAAGAAAGAAAGAAAGAA
AGAGAGAGAGAAAGAAAGAAAGAAAGAAAGAG
AGAGAGAGAGAGACGAAAAGGGAAAAGAGAAATAGGAATGAAAGATACATAGGGATAAAGCGTTAGTC
CCATTGTTAAAACGAAATTAGGAAAAGGTTTA
ATGTATTTGTCGTGGTTTACGATGAGCGTCTTCAATGGGTATTATTGAAGTGAATTAAAAAAGAAGGA
CATCTTATACTATTACATTCTTTAGGTGTGTA
TACAAAAATACAAATGTAAGCAAATAAATGTTTACTTGTCGTTACATAAACAAATACATATAAAATAT
ATTGAAACATATGTGGTATATCTGCATGTTCG
TTTCACCCCATTTCTCTCTCTCTCTCTCTCTCTTGACTCTTGTGAATTTGACGAGGAAATAATTTTTT
TAAATGTTTTTTGGAATGCTCGCAGGGGCGAG
TGGAGGTTTCTCCGTCTTATGATTATTTGAATTCTCATTTCCTCCCCCTCCTCCGCATTCCTTTGTGC
TCTTACTGTGCTACTTTCTTGTTGTTGTTGGA
TCCATTGACGAAAATTTGTAGTTTTTTTCTTTTTCTTTTTTTTTCCTTCTGCTGCAGCTGCACTACTT
GGGAGCGCAGCACACGGGCAAGACGGCTCTCA
CCCCCACCCCACCACACCCCGATACGCGCAGAAGCAAATTAGGGGAAGGAGTGGGGAAGAGTGGGACG
GTGTGTGTGTGTGTGTGCTCAGGGGGGAAATG
ATACATTTTATCCTTTTGCAGAACCGGATGGGCAAGACAAGGCTTGCCAAGTTTTACGTTCCATGCGA
CGACGCCGAACGGGAGAAAACAAAGCGAGAAG
TGCATGCGCTCATCAATGGCCGCGATGCGCGGATGTCCAACTATGCCGTCTGTGGCTCCCTAAAACTT
GTCTACCGCCGTTATGCCGGATTGTTTTTCTG
CTTTGGCATTGAAAAGGACGACAATGAACTCTTTTACATGGAATTTATTCACCTGATGGTGGAGGTGC
TTGACATGTTCTTCAGGGACGTTTGTGAGCTT
GACCTTGTGTTTAACTTCCACAAAGTCTACCTGATCATAGACGAGATGATTCTGGCGGGAGAGGTAGA
GGAGGTGTCACGCGCTGTCATCATGGATCGTC
TCAGGACTCTCGACATCACAAACACGTGAATGGGAGCACAACGATGATTGGCTGCCGCTTGAGTGGGT

AATTTGCTGGAGAAGAACACTTTGGCCGCGAA
GGTGAAATGTAAAGTCGGAAAAAAAAAAATAATTAGAATGCTGGCGGATATGTTAAGGTGTCACCGAAA
AGTGGTGAGAGAGGCCGCAATGCGGGCGTTCG
AAAGAAAAGAGAGCTGAGGGAAGGAAGAAAAGGGAAAAGACCCTGGCCCCTCTTACTGTTTTCTTTCT
TTTTCCTTTATTTTTTTTATGTCTCCTTTTCC
CCTTTTTGCTTCGGACTGGAAGGATTTGTGTGTGCGTGTACAGTTTGTCTGCTTCTTATTTTTGGGAC
GACGCGAGGCGCCTTGTGTTGCGGCCTTCGAT
GCTCTCTGTGCGCCGTCGCAGGGGCCGATACGCAAATCACTTTTTTTTTTTCTTCCCCATAATCTTCT
CCTCAAGTGGTCTTTGGGCCCTTTCAATAGCC
GCTGCCTTTATTTTTGCCGTTGTGTGTAGTGTCGCTCACCTCTTTTTTTATTTATATTTATTTTCGTA
ATGCTCTCATGGGACCGATTCATATTTCCTGC
GGGCCTTTTGCTCCTCGCCCACAGCGTCTACCTTGCGCTGTGCATTCGTGAGCAGCTTCAGGAGCACC
ACCACAGTGGCGGCGGCGGCAGCGTGACTTCC
GCATACTTCCCCATGTCATCGTACCCAACAGGCGTCATGGCTCATATGAATGCGTCCACTTTACCCAT
TTTTGTAGAGCTGTTGGTTGGTGTTGTCGTCG
CCATTGTTGGTTTTGTGAAGCAGTCGACATTTAAGCGAGCGCGTCTTATTGACAAGAACTGCCACCAG
CGTTACGACGATACAATTTTTACGGGAGTAGG
CTTCATGCACTTCAACCATCGTGGTTCTGTGATGGGTAACCACTCGGGCGGGGCCGTAAACGGTGACG
CCTCCGTCTCAACATCAAAGAAGAAGGTGTGA
AAGGATGGTGGCGAGGGAAATGTGAAACACACGCACAGAAACAGGAAAAAAAAAAAGAATGAGAGAAA
ATATTTATGCATGCATATGTGCATACCATAAA
AACTAATGAAAGCTGCGAAAAATAATGGGGTTTGCTGCTTTCTGATCTTTTTTTTTTTCTTGGCATGT
TTTTTTCTTTTGTCTTGGTCATTGTTGCTGCC
TTTTTTTTTATATTTACTTCTGTGCTGCCATTTTGTGCGTATCTTCCACTTTCCTTCTTTCTGTGTGC
GTGTATGTGTGTGTGTGCGGTCAATGAAGGCG
GGTGATTCCGAGGCCAAAGTGTGACGCATGCGAGGGCCGTGGGGTGCGTGCAAATGTGCCGGTTACTG
GTATATTTTTAGACAGAAGAAAACAGAAGGTG
AAGTGCGGAAGAGGAGGATTGGAAGAGAAGGGATAAGAAAGAGAGGAAAACGCGGTGGGTGTGGGTGG
GTGCACCCGAAGAGATGACGCAAACCATGCAG
TTATTTGTACATATGCATGCAGACACGCAATAGCATCATTGGGTAGCCTCCAGCCACCCCGCGAACAA
CCGAGACGGAGGAAGCGAATACAAGAGTAATT
TTTGCGCTGAGAGCGACCGGTCCGAAAGAAGAAGGGAGAGAAAAGTCTGCAATTGCTTGCGTAATGAG
GCGCCTGCTGCAGGGATTGCATATATTCACCT
TGTGCTTTTTCTATCCGTTTCTCTTGGGGATTTTGAAGGAAAAGGGAAGAAACGACAAAAGAGACGCA
AGGGGCGTGGGGGATCAAGAGAGGAATTGTTT
CTCGGTTATTGCGGCTGTTGATCCATTATAACCTTAGTCCATATACCCTTTCTTAATATACACATACG
GAAGGGGGGGCGAGAGAGGGGAGAAACAACTG
ATTTTAGTTTACTTGGACGCTTGTCATTGATGTTGCGCAGGAACACGTTGGCGTTCTCTTTGATTCGC
CAAACGCTGCCGCTTCTAGGCAGCAAAGGTGG
AACGCCGAAGCGAAAGAAGAATCCCATGCAGCTGCGTCGGAAGGTGTACGGCCTGCACTTCAAGGAGA
AGTACTTGAAGACGGAGGAGTGGTATTACTGT
CCACTTTGTGCTGAGCCAAAGAAACAGGGGGAGTGGTGTCGCCGTGAGGACTGTCGCCAAATCAAGCC
ATAGGCTGGGCGGTATTTGAGAACGAAGGGAG
TTTCTGGTGTGGGATAGCGAAGTGTTAAAGGAGCATGAAGGAACTTCTTTGCGTTTGTTTGTTTTTGT
CTTGTATATCATTGTTGCTGTCTCTTTCTTCT
GGACGTTGTTGTTTTTCTTCCATGGGCCTTTTGTGTCACATGTTGGTTTTATTTCTGCTTTTAACGTA
TGTACTTTTGTGTGTGTGTTCAATTGAGGAGG
AAATAATGGATTTGATTTTTGCTGAGGGAACACGGACGCCTTCTGCAAAGCCCTTTGCGATTCCTTCC
GAAGTGAAATCCCGTCTACGTGCGAGAAATCC
GGCAGAACGAGAACATGTAGGAGGAACGGAAGAAGCGGGGAAAAGAAAAGAGGGTGAATTTAGTGGCG
CAGTGGCGGCACGAGGCCTGCCAGGATCCCTA
GTGAAGTACATTCAACGATGCATCGAGTACGGTCAGAACTCTACACACCTGGAGGAGGAGTTACAGGC
AGCAAACGGATTGACAGATTCTACGCGACGGT
TGGCTGCGTTGCAATCGGCTCTTTCTATTCTATGTCACATAGCCGCCATGGACAAGTCCTATTGTTTT

ATTTCCAGTCGTTCAACAGCGCCCCGGAAACA
ACCACTCGCCAGAACAAGCAGGAACCGAAGCCAAACCACGAAGGTCATTTCAGATCCATTTTTCTCAC
TCACAGATATTTACACGACCGTCCGAAGTCTA
AAGTTGGAGTATGAGGATGTACTGAAACAACAAAAGAGTGGGATTGGTAAGCAGGAACATATGAGTGT
AAATTCGGTAGCAGCCTCTGTTTCCACTATCG
AGGCACGAGTGGATGCCTCACTGGCTTTATTGGAGTTTACCGTATTGCAGATGCTTCTCAACTTTGAT
CGAGCACGTGATATTTCGGTTGGGTACGTTGC
TGACATGCTGCGAACGATGCCGCAGATTTTTGCCACGGAGAAAAGGCCTCTCACAGTTCTGCAGGGTG
ACGATGGTAAAAATAGAGAAAGAAAGCGTTTT
CGCGATGGTGGAAATGTCGTTGACGATGTGGCTAATGCGGCAAATTCAATTGTTTTGAAAGCACTTCA
AGCGCTGAAAAGTAAAGATTGCCTTCGCACCT
TGAGCCGGGCAAACAGCGGTAATACTGTTCGAGCATCGGATGGTTCGCTGCCGGTGTCATTTGATGGG
ATGATGACCAACGCACTGGATGGCACATACAG
AGGGCGTGGTATTTACGGTGCATTGCATGACATGAAGATGCTTCTGTATGGATTATATGCCACCGCTC
CCTCTGAACAGGAGGAGCATATTGCATTAGCC
CGTCTTCAGTTTGAGCATTCATTAAGAGAGGAATATGATCTTTTTTTGCGCAAGACAGGGCTCCGTGG
AGAGGTGGAAGATCCGCTATTGCCAGAGAGAA
CCATGTTTGACGTGAATCCACTTTTTGCAGACGCCGACTCCACATCCTGCACTGATACACAGCTAAAA
TCTTTGAAGCTGTTAGATTTGATTCGTGAGGT
GGATAACGAGAATTGGTTTGAGTACCCCGTGTTCGACATGGCCAATATAGAGCTAAGTAGCATCGAGA
AATGGGTACGTGGCTCGATATTTGGTATGAAG
ACAAACCGTGAAGCCTTTTTGGCTCTCCGAGACGTTTTGGAGCAGATGGTGGATAACTGTGTGCTGAG
CTATGGGCCAACCAGCCCGTTTTCTCAAGTCA
TCACATTGATGCGGCAGCGTTTGGTTGATGCCGCCCGCGAGGTGGGCCTGTTGTGATGACATTTGCGA
CGAGGAAGAGGAGTGCGGAGTATTCTTCAGGA
AGCGGGTTCGAGGTGGCCACGTTTTTTTTTTTTTTTTTTTTTTGGCAACTGTGGCACACATAAAAAT
AGATGCATTTTGAAGCAACTCTTTTCCCCTCT
CAAAATAAATAGATGTAAGAACGGCAGCTAAGGAAGAAATAAAAAAAAAAAAAAAACGAAGAGGGCAG
AAAGATGATTCCTTGCTGGTGATATTACTTTT
TTCTTTGGGTTGTATTCTCCNNNNNNNNNNNNNNNNNNNNNNNNNNNNNNNNNNNNNNNNNNNNNNNN
NNNNNNNNNNNNNNNNNNNNNNNNNNNNNNNN
NNNNNNNNNNNNNNNNNNNNNNNNNNNNNNNNNNNNNNNNNNNNNNNNNNNNNNNNNNNNNNNNNNNN
NNNNNNNNNNNNNNNNNNNNNNNNNNNNNNNN
NNNNNNNNNNNNNNNNNNNNNNNNNNNNNNNNNNNNNNNNNNNNNNNNNNNNNNNNNNNNNNNNNNNN
NNNNNNNNNNNNNNNNNNNNNNNNNNNNNNNN
NNNNNNNNNNNNNNNNNNNNAAGCCAACGAACGTACACACACACCAAAAAAAAAAAAAAGCAACGACA
AGAAGAAAAAGAAAAAAAAAATAATCCAAGAA
GAGACTGGGCGAGAGATTTGGAGGAAGCAACAACAAGAAAGGACAAGAGCAGACAAGGAAGAAGGGAA
TTCAGCATGCTCACTGTGCATTTTCGTCTGGA
TTCCACTCTACAGGAGGCTTTTGCAACCTTTCGGGAGGCGAATGCGCCGTTTCGCGCTCTTTTGGTAA
CGGTGGAGGACGAGGTCATGCGGCTCCATGGA
AAGATGCTTGAGTCCAGCGCAAACATGGACAATGACCTTGCCAAGGTTAGAGAGAACATTACGACGGA
GAAGCTGGAGGCCGCTTTTATTATTGTAAAAA
TCAGTGAGGGAGAATTTGCTCTTGTGTCGTTCTGTTTGGATAGTCTAAAGCCAAGGGTCAGGATGTTG
TATGCGTCAGGTGCTGGGCATTTGGCAGATGT
CTCCGGATTAAACTATATTCTAAAGGAACACGCGATGCGTGTGGAGGAAATTACACCATCGCTTTTTT
CAAGGGAAAATTCTGCTAGTCGTACAGAACTT
ATGACGGAAAAGGAAAAGCTCTACGCCGCTATTGAAAAGATGGAAATTGCACCAATGCAAATGACAAT
GCCGGGTGTCACAATGCCAATCACGGAAGAAG
GGGTGAATCTACTGAAGGAGCTCTGCCGGGGTGATTTGAATGCAATATCTTTTGCGGTTGACACGGAC
AAAATCGTAGTGGATAAAGTGCTTGAGGCTTC
AGAGGATTTATTATCGTCATTTTCATTGTCAGAGTTTGTGGAGACTGTGAAAGTTCTCCTCCCGGAAG
ACCACCCACGTTTCGTTCTGCTCCGTTTTCCA
TCAAACGAAACTGCAAATATAATAATGGTGTACGTTTCCCCCTCAGCATCCAAACCGCGAGAGAGGAT

GATATATGCCTCCTCCAAATCCTCCTTCGTGT
TGCAGGCAGAACAGCATGGAATTAATTTTTTGCGACGGTCGGAGGTGGGCAGTGTGGATGAGTTGCAG
GAAGTTGTCGCGGACGCATTTGGCATGGAGCA
GAAAGAGGGAAGCGAGGAGTGACCAAAAAACCCCACGCCCTCACAGTGAAAAAGAATGCCTCTTTAGG
GAACAATCAAGAATACATCTACATGCGTGTGTG
AAGGAGAGTCTAGATTGTGGAAGGCCTTCAGTTTGAGCCAACGTATCACAACAAATTGGGTTTGGCTT
TCTTCTTTCCCTTTCTTTTTTTTTTTCTTTTT
CTGTTTTCTTGCGTGTGTTCGTGAAGTCCTTCCCATCGTTGTTGTTTTTTGTTTGTTTTTTCGCTCTT
TTATCGCCAATTTTTTTTTTCCCCGTCTTTTT
TTTTATTTTTGAATTCCACGAAGGAGGACGCGCATGAAAGAAGCCCAATTGAGGGTTATGTTTTTCCC
CACCTTTTATTTATTTGATTTCTTCTTTCTTT
GCGTGTCTTTACTGTCGTATTTGTTGTCACCGTCGTTATATTCTTTTTTTTTTTTTTTATAATTTTGT
TTCTTTTTTTTTTTTTTTTTTTTTTTGCCGGAG
CGTCTGTTAATTGGGAAGGGCGGGTCCTCTGGAAAAAGTAACAACCAAACAGCAACAGGACAGGGGAG
GCAAAAGAAAACACGATGTACTTCCGGCGTGT
GATCCATCCAAGGCGTCATCTTCTTTTTTCTGCGGCAGTGTTGTCATGTATGCGTTGCGTGAGCAGCG
GCTCGGCAAGCGCGAAGCCCCTGTCACCGGTG
AATGTTGACAGCTTCACGCTAAATGAAGAAGTTGTAAAAAAGGACATGGAGGCGCTGCAGCGCTGGAA
CGAACTCTCGCAGATGATAGAGAGGTCACGGG
CCGAGAAAAAAAAACGAGAGTATTCTAGCGGCTGTGGAAAAAGGACTGGCTATGCTTTCAGAAATGGGC
GCCGTGAATGCACCAATACAATGTGAGACACT
CCTTTTGTTGGAGGCGTCTCAGGCGCATTATAACCTGCAGCAGTTTGAGGCCGCATTGAAGAGCGCCC
AAAAGGCGAAAAATATGCTTTTAGAAACCCCG
TCAGCCGTGAGAGACGCTGCGTTGTTGGCGGAGGTGAATCAATTGATGGCGTATGTTCTCCTTGAAAG
TGGAAAGACAGAGGAGGCAACCAATGCGTTTA
CGGAAATATTACGTTGGATTGATGTGGATTCAAAGGCAGCGATGCCGATGCAAGCTGTGGCTGCTGTG
AACATGCGCCGGTCTGTAGTCACGGGTATTGG
TTTTTGTTATCAGAAGCGTGCCGAAAAGGAAGCTGCGACAGGTGGCGATGGGAAAGAATTGTACGGTA
AGGCACTTGATCTTCTCATTGAGGCGTTGAAT
GCTCACATTGATGAAAATGACTACGACCTTGTGAAGAAAACTCTTTCTGGCGTGTTAAGGTGCTTTGA
GGGAGTTAATGACATCTCGCAGGCGATATCGA
CGGGCGAGAAGTATGTTAGTTGGTGTCGTCGGCACTCGGACGAGGCGGGAGTTGCTCAAGGCACTGCG
TGGCTGAAGGAGCTGCAGGAGAAGCACGGAAA
AGGAGGTGACACAATTCTGTGAAGGGGAAAGGTGGGGTATTACATGCACACATAAATGTACTTTTTTC
CTCGTTCAGGCCCAGACATTCGGTTTGGTTGG
GGGGGAATGTACGTCCGCTTGAATAATGTTGAAGTGTGGCGTTTCAAATGGGCAAAGGCATGGGAAGT
ATGTGTAAAATCAAATAAAAGGGAAAAAAGGA
GCGATAAACCGGGAAGTCTGTTTTTTTTTTTTTTTTTACTTTGCTAAAATTATGATACTCGTCTTTTG
TGATGGCAGAGCCGTAATGCAAGGCGTTATGC
AATGCGTCCTTTTTTCTCTCTTAATTAATTTTTATTTTATTTATTTTTATTATTTATTTATTTAATTT
TTTGAAGTCCACGGTGTGATTACACCCGCACG
CGCGAACCGAGCCAAAGGGAGGTTGTAAAAAGTGGGCGAGAAAACCAACACAAAAAGAGAAGTGTGTG
GAGGGTAAAAAGAGACGGAGGAAAGGGGAGGT
CGGAGGATTAGACCGGCGACTGATGGACGAGGAGGATGAGAAATGTGTGGACCTTCGCCACGCATGGA
GAACTTTTATGGTTCCTCACAGCAATAAGAAA
GAAGGGCCTCCATGTAGTCGATTTGATGGGAAGTACACACTCCTTCGTTCCTACCGTTTGGTCGGAAC
GGATACGGACGATCAGCCGCTTTTCTTTCTTT
CTCTTATTAGTCCAAATCGAGAACTGATCAGCACGACAGCGCACCTGTTCGTTCAGTTCAATGGCCAG
TTCCGTGACTGTGTGGCAGAGAACGAAAACAG
AATGATGGCGATGCGTGCCCTTCACGAACGGCAGCTTGCGGACCTGGTGGAAGCAGAAAACCGAGATA
CCAGAGACATGGTGGTGGGTTCGGAATTAACG
CGGAATTTTGTTTTGAAACACCAGAGGGAACTGGAGGAACTGCAGCGGGAGTGTCGCGAAAGGGAAAT
ATCTGTCGAGTTGGAGGTGCGGCAAAGTCTAC
TTGCCTTTTTGAAAACTTCTGCGCCCGATGCCTTGACTGCCCTTGCGAGGGAAGCTGGAAGAGAGAAA

TTGCGTGCAGATACTGCTCAGTTAACTCGTAT
CACAGACATGGCACACTTTGTCGCAACACCAGTTCCTGTGCGGGCAGTGGAACTGCCCAATCCTCTCT
GCCACGAGAAGGTTGGGGCTCACTCGAGGGAT
GTGTCACTACGGCCGGTTGTTGTGGACGTTTCACCGACAACATCTTTGCAGGCTTGTCTCACTTCCTT
GTATTCCGCAGAACGGGAAACGGCGCCTGCTG
TGGCGGAACATCTTCTTCGTTTATCACAGCAGACTCACGCTGTGCTGTTGCTTATTGGTTCGCAGGCG
GAGGCGGAATCGCTTCTGTCGACTCCAGCTGC
TGAACTTTTTCTGAAGAACTCGTTTTACTCCGGCGGATCCCCGCATTTTGTTCCGTTAAAACGTCACT
GTCACCTGAAAATGATGCTTTCCACACGATTT
TGGGGAGCCAATATTATTTTTCTTTGGACGCCACCCAAACAGGAGGGGCATCCTTCAGGATGGCCACT
TGTCGTGGAGGATGCACTTCAGTTGGCATTCT
CATGGAACGCGGACATGTTCTCCGTGGCCGTAATGAAATCTTCTAGTTATTGGCAGGGGACATCCTAT
CGGAGCTCTATCGAGCAGGCACAGGATGTCTC
CATGGCGGTTCTGCGTCAGCTGCAGCGTGGTGTGGCGCGCCTTGCGCATGAAGCAGCGGGTGTAAAAC
TCTCCACAACGTGGGGGATGCCAGAGGCACCT
GATACACACACGTTACCGCAGCCGCAGCGATGGGAGGATGGTTATGTTGTTAAATCTCCCGCCTCAGT
TGGGAAACCACAAGTGAAGTTTAATACACATA
TTGCTATTCGTGCATTTTTTCCGTTTTCTGTTTCTCAAACAGAGGAGGAGGAAGGATTGTCATTGCGG
GATAGCGCCCCTGTAATCAATTCCTTTTCTTC
TGCTACTGTTGCTGCTGATAACTCAACGGACCGGGGTCGACGGACGGGACGGCGGAGACGTAGCATGT
CTACTGCATCCCTTTCAAGCGGTGGAAGTTTT
TCAAGCGAGTTGGCGGTATCACATAAAAGAAGTTCTCGCCTGCAGCAGCCAATGTCTTTTAAGAAGTT
GCTCGTGTACACGTTTGGTAATGCTGTTGTGG
TTCATTGACTTCTCCGTTCTCTCTCTCCGTCTCCTCCTTTGGCTTCTTTACCTCTCGAACTGTAAAGC
AACCTGCACGTGGCATCGGAACCCCTCCCGTT
TTGGCTTCAAACGATGACCCGGAGGGGACATGGTGGTCATTACGGTCACATGATGATGGCTCCGAGGT
TACTTTTTTGGCTTCCTTCTCGAGCTTTTTTT
TCTTCTTTTTTTTTTTTTTTTGTTTTCTCTTATTCCCACAGTGCGTGTGAGGAGGAGATCTCTTGGTGG
GTTCATACACATACAGTGTGAATATTTTAATC
TTGACGAGTGAGGCGACAACGGAAGGGACACGGTGGGGAAAGCAAGAAGAAAAAAGCGACTGAAGGGC
ACAAAAAAACACAGGGAATCATATTAAAATAG
GAATAGCAAAAGGAAGGGGGCATAAAATAGTTGGCGGATGTCATCGAAACAAATTGCGAGCCCGCAAG
TGAACCTAACGCCGCGCTCAGCGGATGAATCA
TGGGACAAACATATTGATGCTGTGCTCTCGGAGCTGCGACGAATTTATTTTGAACGTGTTCGTCCTGT
TGAGACGAAGTTTCAGTACGACGTGTTTCGAC
CCTCGTGGTTTGCCGAGTCAATTGTACAAAAGAGGCCGTTTGTAACTTTCCTGGGACCCTTTTCATCA
GGCAAGTCCACCTTTATCAACTATTTGTTACA
AACCAACTATCTCTTGACGGGACCCCAGCCACTCACCGACAAGTTTACTGTTATCATGCATGGTGATG
AGTTTCAGCAGATCCCTGGACGTGTACTTATG
GCGGATAGTTCCCAACCTTTTCGTGGGCTCAGTCAATTTGGCGAAACATTTGCGGAAGTCTTTCAAGG
GCTAATTGTGCCGCACCCCCTTTTGCAGTCCG
TGTCACTTATCGATTCTCCTGGCGTTCTCGAGGCGGCGGGCGATATCCATTCACGGCGGTATGACTAT
GTTAAAGTGTGCCGATGGTTTGTAGAAAAAAG
TGATCTTGTGTTTTTTCTCTTTGACCCGACGAAGTTAGACGCCGGGACGGAGCTTCGCATGGTGTTTA
AGCATGCCCTGCGGGGTCATGAAAGTAAAATT
CGCATTGTGTTAAATAAAGCCGATTCCGTTGGGCCACAAGAACTATTGCGTGTATACGGTGCTCTTTT
TTGGAATCTTTCGGGTCTTATCAGTACGACGG
AACCACCACGTGTTTACATCTCAAGTTTTTGGGACCAACCATATCAAAAAGACACCGATCACTGTCTT
TTCACGGATGAAAAGTCCGATCTTCTCTATGA
TCTTACGGAATTAGTACCGCTACAATCCCTGGATCAGCGGGTGAGTTCAGTGATGCTGCGAGCTAAAC
ATGTTTTGATCTTTGCACTTGTGTGTGCCAGT
TTCAAGTCACGAATGCCTTGGCTTTTTGGAAAGGAAAAGGCCCGTGCAGAGTTCTTTGCGCAGCTGCC
GCAAATTTCAGAGGATCTTGCGAATACGTTCC
GTCTTGGCCTCACGGACTTCCCCACGCGCGAAGAGATGTCGGATTTCTTGTCAAGGGTTCAAACCGAC

GACTTCTACGGTATGGATCACCTACGAAAGAA
GGGGTGGCCACAACTGTTAAAGCGGACGATTGAGGAGGATCTTCCCGCGCTTCTCAACCCAATTAAAC
ACACGGAGGTGGTGGATCCGCGCGATCGCAAG
CATGCCATTATGATGCAACGCAATTACATCAAGCAAATGTCGAACCAGTTAGCCGGCCAGCGTGGGAT
ACAAGGGGGTCTGGGACAGCAGCAGGCACCGG
GACACCGCGTTCAAACAATGCCAAATGCTGCTTCATTGAACACGGGACTTAATTTCCCCACGCAACAG
ACACAACAAACTTTTCTTTCTCAAATGCCGGC
GGATCAAATGTCAATGATGATGCAGATGATGCAAAACATGATGTTGCAGTCGCAGCAGCAGCAGCCAC
AATTGCTCCAGCTAGAACAAGCACCCACACAA
CAGTTAACACCAGATCAAATGTCAATGATGATGCAGATGATGCAGAACATGGCATCCAATAATTCTTC
TTAGTAGGATTGTGCTAGTGCATATCTCGAGT
ATATATATATATACTCGTGTCCGCCTTAATTTTTCATTATCCCTGTATGTTACCTGTTTGTTTGCCGT
TCTTTCCTCCTTCGATCCCTCCACTCAACCCC
CCAGATTCTTTTAGTGCGTCCCTTTTTTTTTTTTTATTGTCTTGTTGTTGTTGTTTTTAGTGAGCTAT
GANNNNNNNNNNNNNNNNNNNNNNNNNNNNNN
NNNNNNNNNNNNNNNNNNNNNNNNNNNNNNNNNNNNNNNNNNNNNNNNNNNNNNNNNNNNNNNNNNNN
NNNNNNNNNNNNNNNNNNNNNNNNNNNNNNNN
NNNNNNNNNNNNNNNNNNNNNNNNNNNNNNNNNNNNNNNNNNNNNNNNNNNNNNNNNNNNNNNNNNNN
NNNNNNNNNNNNNNNNNNNNNNNNNNNNNNNN
NNNNNNNNNNNNNNNNNNNNNNNNNNNNNNNNNNNNNNNNNNNNNNNNNNNNNNNNNNNNNNNNNNNN
NNGGACTGGATTTGGGGGGGGGGGAGATGTCT
TGGTAACAAAGTCAAGAGAGGGAGGAAGAAAATAACATGAGAAACAAATAGAGCAACCCACATACACA
AAAGCACAGGCACAGGCACAGAAAAAAGGCAG
AAATAACACTGTGCTTGTCGCCGGCACCTGTCGTCTATCTGAGTGCATTCACATGTACTCCATTGACT
TATGGCGCGACACCGCATTTTTAATTCTTTAG
CAAATGGATGTTATTTGATGCCAACATTCCCATTTCCTTAAATCTGTTTTCTTTTGCATTTCTTTATT
CTCTTGTATTCTTCCCTCCTTGCTTGTGTATT
GTATCGCATGTAAAGGAACAACATTTTTTATTTGTTGTGTTTTCTTTATTTATTTCTTATATATTTTT
TGTTTGTTTGTTTGGGCCTGGCCTCCTGATGT
ATACATGTCCACAGCTATTGTGAACTATCTGTTGTGTGTAATGTGGTGGGCTTCTTCCGACAATCCTG
TTTCTTCAAGAGCAAACCGAGGCCTCTGGAAG
TAGTATATATATATATATATATATGTAGCGTGTCGATGCGGAGAAAAAGGGAGGAATATGTAAACAACGG
TGAGGAGAAGTGTTTACGCATGGCACGGCTGG
AGGGCGTAAATAATGCGAATACTGTCTCAGTGGAAAGCGAGATAAATCCGCTCACAAATAAGCCGTTT
TCAAAACAATACTACGAGATTCGTGCCCTTCG
GGCCACGCTTCCTGTGTATGAAAAGGCCGAATTGCTCAAACGGTATGTGCGTGATCATCAAGTTGTTC
TGCTTGTGGGAGAAACAGGGAGCGGCAAGACC
ACTCAAGTCCCACAGTTTATTGCGGAAATGGGTTATGCAGGTATTATTGCCTGTACTCAACCTCGAAG
AATTGCCGCCACATCCGTAGCGGCGCGTGTCG
CCACGGAAATGGACGTTAAGCTTGGGGAGGAAGTTGGCTATCATGTTCGGTTCCAGTCCATGATGGGT
GAAAAAACGAGGGTGCTGTACATGACAGATGG
TATACTTTTACGGGAGGCCTTTTCGGATCATAATTTGTCACGCTTTAGTGTTGTAATTGTAGACGAAG
CACATGAACGCACAATAGATACCGACGTTCTT
CTTGGCACTTTACGCCTTCTTATGGAACGCCGTTCTGAGTTTCGACTGATTGTGATGTCTGCAACTCT
TGAAATGCAAAAATTTCAGGCATATTTTTCTC
AGGCACCGTTGTTACAAGTGACAGGTCGAAAGTATGAGGTCCAAGTCAAATATACTACACAACCCGTG
AAGGATTATGTGGAGACCTCTGTTCAGCGCGT
GTATGATATTCACATGAATGAGCCACCTGGGGACGTCTTGTGTTTTCTCACAGGCGAGGCCGAAATTG
AAAAGGCGGTGACGTGGACAAAAATGAAACTT
GACGCATTGCTGGTCCGAGATTCAGAGGCGACACATGATAGTCGTCAGGGACCAATGTGGGTGCAGGT
ATTGCCCCTGTATGGTTCACTGAGTATGGAAG
AACAGAAGCGTGTGTTTGCCTCTCCTGGTCCTCATACAAGAAAAGTGATATTTGCTACGAACATCGCG
GAGACCTCACTCACCATTGACGGTATTGTTTA
TGTGGTTGATTGTGGGTATCATAAGCAGAGTTTATATAACTCAGAGGTTCGTGTGGATTATTTACTTC

CTGCGCTCATTAGCAAAGCCTCAGCAGAACAA
CGTAAGGGGCGTGCCGGCCGAACTCGGCCAGGCAAATGCTTCCGTTTGTTTAGTCCGCAGGACTTTGC
TACTTTTCCGGATCAGACGCATCCTGAAGTAC
TGCGGTCGAGTTTAGTCAATACCGTGTTGTTGTTACTTAAGCTCGAGGTGGAGAATCCATATCAATTT
TCATTTATTGATCCACCCTCCCAGCAAAGCAT
CATGGATGCATATTACCAGCTGAGTTTTTTTGGTGCTGTCGATGAGGATTTACATCTGACAGAGTTTG
GGAGATTAATGGCAGAGTTCCCAGTGGATGCC
TGTCTTGCTCGTATGTTAATACGCTCTGCGGAACACCGTTGTGGCGCAGACGCGGCAGTTATTGCTGC
CATGTTGGAGGCTGGTAACGTCTACGTGCGTC
CCTCAGGGCGTGGAAATGAGGCAGATCAACAACACACCACGTTTCAGCATCCAGATGGTGATCACCTG
ACACTCTTTAATGTGTTTCATGCATTTTGGCG
CAACAATCAATCGCCACAGTACTGTTTTGACCATTTTCTGCGCTATCATGCCCTGTTACAGGCTACTC
GTGTGTATGCGCAGCTAACAAAACTAATGGGG
AAGAAAAGTATTCCTGTTGTCTCTACGTATGATGATAAGACGAGATTGTTTGACTCCGTGGCGCTGCG
GAAGACGGTGCTTGAGGGATTCTTTACACAGG
TCGCCTACAAGCCGCCCTCTGCAGAGCATTACAAGACCGTGAAGGATTCTCAATGGGTAGTATTACAC
AATCAGTCGGTGTTGTCAAAAAAGAAGCGTCC
TCCATGGATTGTGTACGACCGCCTCGAGGTACATGGAAAAGAGGGAACGTTTATTCGTTTTGCTTCAG
TGGTTGAACCGGAGTGGCTGCTTGTTGTTAGT
GACTTTTTCACGGACCCTGCGGAGTTGTCTGACGGTGAGATTTGTCACATCTTGCAACGTCTAAAAAA
CAACGACGAATTAAATTTGTCTTCTGCTCACC
CATAGTTTTTGGTCGAATCAGTTTCTCATCACGATGCCAGCAGTCACGGGTGGGGAGACAAGAAAAGA
AAGCAAAAAGAAAAAAAAAGTGGAAAACCAAG
AAACAAATAAAAAGGGAGATTTTGAGGTGCCATATGTTTCTCTCTTCTTGCCTGGTTGGGTCCGAATA
TTGTGATCCCTTCGGGTGTATCTTTTTTTGTT
TTTTTGTTGACAAATTTTGTTTTTCTGCTCTGTTCTTTTTGTTCGCCTTTTTTTTTTGTGTATGTTGT
TGTACAATCATGAAGCTAGCAAGGCACACACA
CACACACACACACACACACACACACACACACACACAAACACATGGAGTTGCAGCAGAACGGCGACGGG
AAGATTGAGCTCGAGGTTGTGAGAAAAGACGG
GCGTCCCATTGTCTGTCGTCCAATCGATGTGAACGAGTTGCTCAGCAAACAGGGCCACACCATCGACC
TGGGCGATGGTGTTCGGGTTCGGCTAGAGGAA
TACAGCAGCGACACCTTCTCCTCCTCGGAAGAAGGAACGGAGGAAACAGACAGCGAGGCGAGTGCAAG
TGATAGCAGGACCAGCGACAGAACAAACGTCC
CACCGGGACGCCGCAGGTTGACTGGAGACGGTGCTGCTTTTGACCTTCATGGAGGCGAGGAGAAGGCC
AAAACGCAAAAGCTGAGTTTTCACGATCCGGA
GCGTAACAAGGGAATGGCAACGCAGCACGGGACAATCAGCGGCAGCAACGTTTCTGAAAAAGTGTTGC
ATTCTCGTCGAGATATTTGGGCCTCCTTTGCG
CCCCCTATTGACCCGGAAAGAAATCATAATTGGCTCCAATCTACATCCATAATTCCAGGTGCAACTTC
TAGAAGGTTCCGTTTACCCCCACTTATGTTGC
ATGCCACTTCGTCTTACCTCGGCCCGTCCGACCAAGGACAACGAATTCTCGTGTACGGAGGAATTACC
AACTTTGGTAAAAAAGTCGAGAAGGAGCTTTA
CGAATTTTCTGTCTTAACGGGCCATTGGCGACGAGTTGAGGGTAAACATTTTTTTTTCCCTGGTAATT
ATGGCCATGCGGCGGTGGTTGTGGAGCGTCTT
CAGCGTCTCGTGGTGATTGGGGGTATGAGTCCTGGTGGACAGCCGGTTCAAGGAGAAAGATATTCGGA
TGAATTCTCCAAACTACGAAGCCGCATCCTTT
TTCCATGCCAAGATAAAACCAAGACAAAGCGACCACCATCTGTGCGAATGGAATTTAAAGAATCCGAG
AAACCGACGCTCTGGTCGCTTGCAATGCGGGC
AGAAGAGGAACCCCCGATTGGGTTTATGCCGGTATTATTTGAGATGAACTTGAAGACTCTTCGTTGGC
GGGTCGTCCAGACATCCCCTGAGATTCCTGTG
GCGCTCCACACAGCCGTGGCAACTTCTAACACGATTTATTTGTTTGGGGGTGTGACGAATCGATTACT
TGTTTCAGCACAGCTTATAGGGATCCATGCGG
AGAACAATAGTATTTCGCTTATTCGTTCCTCTGCAGTGTCACCAAAGGCACGTTATCTTCACAGTGCC
GCCATTTATGGGAACTGGATGATCGTTTACGG
GGGATTTGATGCCTACAACGAACCACTAGCTGATGTGTGGGCATTTGATTTGGTAAATGAGCGTTGGG

AACAGCTGGAGTGTCACGGTACGCCTGCCCGT
GGTGCACATGCCTCTTGCATTCTGGGTTCAAGTTTGATTGTCATTGGTGGATTCGAAGGTGCTATAAC
AACTGAGACAAAGCCAACTAGTAGTATTTATG
AGCTTCAGCTAGTACCAACGAGTACGGGACAATATCTCTGGAAAAAAATAACCGTCCGACCTGCAATA
CCACCACTTGCTTTTTCGGCTGCCTGTCATTG
CGCCGATGATGTTTCCTTTATTGTGTACGGTGGTTGCACCGTCACTGGAATAAAGGCGAAGAAACGTC
CGCCTGATTCCAAGAGAGTGGGTCAACTTGCA
TTCACCAAAAGTAGAGACGCAAGGAATATTTCCAGGCATGACAGTGATGGCAATGTTTACAATGACAA
TTTCTGGCAGGGGTTGGTTCCTTTGGATGACG
GCTTTGCGTTTACGTTTCCGTTGAAAAAGAATGATGCGACAGGCTCTCATGGGGATGGTAGCGGCGCA
TCGATACATGTTAATGAATTGGGAGTGGTGAC
TGACCCCGAGGAATTGACGCCGGAGTTTAAGGCATTCGTGCGACGGCAGGAAGAATTCATCCTCAAGA
AAAACGCATCCCGCTCTCAGGCAATGAAAAGG
ACAATACTTGACGAACTTGAGAACATGGAACCAAATTTATATTTGACGTCAGAGGAGATTGAGTTGCT
CCTGGAAAAAAGCAACCTCTTGTGCGAGAGCC
TTACGGGATACAATATGGAAGCGATGCCGCACAACATTCCTGAAAGAGAACAACGACTACAACACACT
GAGAAATGCATCTCGCTTAGTCGCCAGATACG
GGATGTCCTCATGTCGATGAAGGGATACGCCCCTGGTGTGACGGCTGTGAAATCAAAGACTCATCGAA
TAAAATCCGGGGAGAAGTTCGAGGATCATTCA
GTTGCCAAACCATTTCGTCGTGTAATTGTCATGGGTCTTGTGAGGGAAATTAAAACAGAACTTAAACG
TATTCATAACTTGAATATTGTTCTAAAAACTG
TGGAATGGCCTGAAAAAAAGGAATACTTGGATGCCGTGGAAACGATGCAGGAGCGGAAAACGGAGTTT
GTGCAAGCTGTGAAGGATATTCTTGAAAAATA
CATTGAGCGGCGCGTAGAGTCTTTGATTTCCGCTGCGGATAGACAGAAGGATAACATGCGGCGATTGG
CTGAAATTGTGGCGAAGATTCGGCATGAAAAG
ATATTTCAAACGGAAGGACCAGATCCACAGCGTGAGCGTGTGCGGCAGATGTGGAGCAAGGGAGAAGG
ACAAGAAACTCCCCAGACAGATACCAAGCGCT
CTGTCTCACGTAGTCATCATCATCACCGTCGCTCTCGCTCCACCGGGGCTGGCTATTACAAGGATGAA
GCCAAGGCTTTCATCTCGCTGCAGCCGAAGGA
ATTGCAAAAGCTACTGAAAAAAATACACCTTGTGCATAAAGTTGCTACATCATTTTCTTCTTACTGCC
AGTCGAAGAAATCTGATGTGCAGAAGTCATCA
AGTGAAATGCCGCCGCCGCCGTCACCATCAACGGCGCCTCCTGCCCTTTCCAACCCAGAATCTTCATC
CCTTGCTGTTCCTGCCGCACTTGCCATTGTGG
AAACGGTGCCCCCTTCCAATGTTTCTCCAGCCACGCATACGGCTGATGCAGATTTTGGGACGGCTTCC
CCGAGTGAACTTGTTATACGTCAATCGGATGC
CATCCGAGAAGAGGCCATAGAAATAGCAACTTCTGTGGGCCAAAGCGTCTGTGCATTTGCGAAGGAGC
TGGAAACTTCTTTTGAAGCGGATACTAAGCCG
AACTCCCAAACTGATGTCAATAAGGATGAGAAGAAGCCGACGCTTTTGTTTCTTTCTTCACTACGTCC
TCTTCTGTCTTGCAAGATGCAGCTGGCACAAC
TCCGTGAAAAGATTTCTAGAATTCGCGTAAATCGTTGGGCCGTGGATGACGTTGCTGGCGCCCCTCAG
GACGTGGAGGCTCAGAAACGCTGTGAGAAGTT
ATCAAAGTGTTTGGCCGCTCTTGTGCAACGCCTCACGGCGAGTTTTTTATCCAAAGCGGGAGCGAGAC
CGCCTCGCTTGACCTTCAGAAGAGCGCATTCT
TCAGGAGCTTTGAAACCCCCATCGTCTTCTTTGGCTTCGCCCCCGCGCTCATCACCCTCCTTATTCTC
ATCGTCATCATCGCCGGCAACAGACAAGCATA
GACGAGTATTGCAGGCCATTTCTACAGTGGATTCAAGTGACGTTGCAGCGTTGGAAAAGGGCGACGAT
TCTTCTCCGCAGGCGCAAGAAGAAACAGCAGA
GCAGGAACCAGAAAAGCAGTCAACTTGCGTCTTTGGACCGCTCTCTACACCATTGATGCTTCTGGATA
CACCTCCTCTTTTAAAGGCTGCTGCGATGCCG
GAAATACCCAGAACCATTTGCCTCACCTCAAATGAAGCGCAATCGCTTGTACCTCCATTTTGCACAAC
GAGTTCCATTTGTAACGAGGGTGCTATCATTC
CATCTGTGAGGGATACAAAGGTCATTTCGAACACTCATCAGGAAGCATCCACCTGGTCGAACAAAGAT
TCTTCCATTATGGTTCCCCATAAGGAATTCTT
TACCGCTGTGACTTGCAAGGATTCATCCCAATCGCCACCGCCACGCATTTTTGTTTCAAAGGTCCAC

AGACGACCACGGCTGCTGCGGCCATGGCTATA
CCAGCGGAGTTGCCACCATTCGGGGAGTCGTTGTCTGCCATTCATATCCGGTCAAACACAGATGGTGA
TAAAAATACTTCATCTTGTATGGTAAGTCTTT
CCCCAGGATGTTTTCATGGATCTAGCACATCTGGTGTTGCGGTGGACCCAATAAATCGCGTTGATCCC
CAACAAGAGGCGTTATGTCTTGAAGAGGATTA
CTTTTTGTTTGGTCGCCGTTACGCAGCAGAGCCTGTGGCTTCCCCGTGTGTTGTGGAAGATGCACGGA
ATCGACAGGTTCTTGTGGCGAAAGATTCTCCA
TTAGTGGTCAGGAGTATGCATGCTGTGAAAGTATCACCACCTGTGGGTACGCCTGCGGCTCCCACTGT
GACAGCACCGCGGAAGCGAGTAGTGAATTCGA
TTTTTCGGAAGCATTTTACTTCTGCTTCTCAACCTGGCGCGCCCGAAGATGTCTCTGCGAAACCCCTG
AAGAAGGGATTGTTTTCTGGCAAACTAACGCC
CGGTGAGATGCACATCATACAGGCAAGGGAGCGTCTGCGTACCTCCTCATCACGGCGTTAATTAACAC
ACATAAAAAAAAAAAAAAAAAGAAAAGGGGGGA
AAAAGTAACTTCCTTTTGCTGTCCTATAGAAAAGAAATAGAAACAGGCAAGCAAGAAAGAAGTACAGC
TGTGGAATAGGCTGCGAATGCGAAATAGAAGA
GCATAGACACAACACAGGGAGTAAATTCAGTAAGAAGGAGCGAAGNNNNNNNNNNNNNNNNNNNNNNN
NNNNNNNNNNNNNNNNNNNNNNNNNNNNNNNN
NNNNNNNNNNNNNNNNNNNNNNNNNNNNNNNNNNNNNNNNNNNNNNNNNNNNNNNNNNNNNNNNNNNN
NNNNNNNNNNNNNNNNNNNNNNNNNNNNNNNN
NNNNNNNNNNNNNNNNNNNNNNNNNNNNNNNNNNNNNNNNNNNNNNNNNNNNNNNNNNNNNNNNNNNN
NNNNNNNNNNNNNNNNNNNNNNNNNNNNNNNN
NNNNNNNNNNNNNNNNNNNNNNNNNNNNNNNNNNNNNNNNNNNNNNCGTTGAAGGGGGGAAAAAACACA
AAAAGAGGAAAGAAAAAAAAAAAAAAAGTGAAG
AAGAACAGCAGCCGTTACCGAATGGGGAATTGGACACGGGGGATGGTAACAGTGATTCCTGATAAAAG
CCACAAGAATTAGGATAAAAGGAACCATCCCA
ATGGGTATTGTCCCCCACACACACACTTTTTTTTTTTCCTTCTCCCCTTTTTGTACGTATCCCAACTT
GCTTCTTCGTCTTCTTTTTCTTTTTTCTTTTT
TGGGGGGTTGACTTGCAGCTTTTCTCTTGCTGTTCTAATTGTCGACTCCATTCCCCACCCCAGTGCAT
TCCCTGTAAAAAGAAGTGAGAACAATACGTAA
AAAAGGAAGCATAGAAACGGAAAGGCTCTGGAGGCAAGAGGGGTAGAATAAATTGAAGTTTATTTCTT
TTATAGCGGAGTAAAAGAAGGGAAAAAAAAAA
AACAGGAGAAGAGAAGAGAAAATAAACACAAAAATACATACACTGAGAGGAACCGAGACGAAAAGGAA
AAAAAAAAAAACAAGTGAAAAGAAGCAGAGGA
CTGTGGGTTAATTGTTTATTACAAAAGGGCGAGGGACGAAGAAAAACACATGCCGTTTTGGCGGCGGA
GTCCATTGGCTCGTGAGTACGAGGTCACAAAA
CGACCCATTTTTTTGCTGGATGGCTGGAATATAGTGCAAGGAACGGATGCTGCCGCATATGTCTCCGG
TAGTGTAACAGACGGTGTGATGTATGGCACCA
ACGGATATCTGGGTGTTCGGCACCGTAGTGAATGTGAGACAGTCGGTTTCGAATCAAATGTCTTTGTG
AATGGAGTATACCACAACGTTCCAGTTGATCG
TCAAATTGATAGCGAAGCCCTACCTTACGAGATGCAGATGGGGGCCACTGTTTGTACCGTCAATCTTG
ACTGCCTAATTGATGGCGATTGCGGTACCCTT
CGGTCGGTGCCACCCCGACGGCTTGATTTGCGTAATGCAACGTATCACAGTGAATGCCGTGATGTAAT
GAGTGGAGATGGTGGATTAATGTTTGATGGAG
ATTATCGTCGTGTCGTTTCCATGAAAGAACTGGACTTATGGGGAGCGGATATTGTTTACTCCAATTTT
ACTTTTGGTTCATCGCCATATGATCTTTACCT
CGGTGAATCCCCCAATATTTTTTACGCAAACAATATGAACTCTACGAATCCGGATGCATCTCCCTCAT
ATCTTTTGGAGTTTGTTGTGACAATCACCGTG
GATTTGAGAGAGTGGGCTATTCTGCGAGGTGACAGTGTAGATGATATTTTGCTGCAGTCCCGCACCTC
CGGCGTCGTGGAGAGCATCGTTACGCAATCCA
CCTGTGCTGTGGATTATGAAAACAGTTCCGTGAAAATTACCCCGGAGCACCAGTTACTGTGCGAGACG
GGAGACCACGGTTTGATGGAGCAAAACTTTTC
TGCGGCAGCCCTATCGTGTCCAAGTGACAGCGAGAGTGATCCCGGGTTTGGTGGTCGCATTATTTCTT
CCTTTGAGAATCAAAAAAAGAAAACTTTATCT
TTTAAGAAGCGATTTATTCTTTCCATTACTCCGGACACACTGCCAACGAGGGTGACGGTGACATATTT

GGCCCGGCACCACAGCGGCGGTATCCCCTCCG
CACGTTTATTTCCCACCATTTCAGAAATAATGGAAGATCAACGTTTGATCTTGAATGAATTCTGGGAT
CATTTTTTTATTGACTTGCAGCAACAAGAAGA
ATTAACACCGGATCGGCTACGTCTCTCCCTATTGTTCAATGCATTTCGACTGTTTTGTGTTTCCCATA
ACTTACATAACGGGCTACCACGTGGCGGTTGC
TCGGCTACAAAAACGAGCCTTTTATATGATCTTCATCAGTACTTGTATCATGGTATATTTTACACATT
GACTTCACCACCCCGCGCCCTGGCATTACTTC
GGGGCTTGTATTCGCTTCTTCCCCAGGCAAGAGTCAACGCTCATCTTATTTCTCTCAAACATGGCGCT
ATTTTTCCGCGTAGTACAATTAAAGGAACGGA
ATGTGAGCAACACAATACCGTAAGCCAAGCACGATTACATGTGAATGCTGAGATTGGGTACATCATCA
ACTTTTTCTTTGCTGCTGTAGAGACTGTCGAC
CAAAAAGACCGTCTATGGCTTTTAGAACTTATGTTAGAAACGGCACGTGTGTGGCCTCAGTTAGGTGA
ATGGGTAGAAAGTGAGGGTGTGTTTCGTCTGG
ACAATATTGCGGGACCGGATGAATATAACAGCACGGCTTCTGGAAATTTTTATATTCATTTGTCCGCC
AAAATGCATATGCGATATGTTCTTGATCTCTA
TGAACAACAACTGGCATTGCTTGGAGAAGAAGCTATGACAAATCTGCTAAAACAAATTGACATGGACA
AATCTGAGCTTGAAAATTTTCGAGCCACATCT
GATGGTATTGTTATACGTCGTAATGATCATTTAGGAGTCTACTTGGTACATGATTACTTCCATACACT
TAAGGAGTGGAAGGGTGAGCGTCCTAAACACC
CATTGTCAATGAATTATCATCCTCTTGCAATATTTTCTCACATGCTTGTTGATATTCCCGAAGTATTG
CTGGGGATGCTTCTGTATCAAGAAGAATTTGA
AAAAAAGGACTTGGTACGAAATCTGGCCCATTACGAGGCGCTTTGCACATATGATTCACCCGAGTCTC
TTGCTATTGTGGCGGCTGTGGATTTTCAATCC
AATGGATCATTTGCTCATGGTCTGCCGTTATTACGCTCATTGATGAGTCTGGATGTGGATAATGTCAT
TTACACCGCCGATGAAGGACTCCATTTTGGCG
TCATGTCTTCTTCTTGGATTGCCATTGTCAGTGGCATCGGTAGACTCAAATTGGGTAAACGGACTCTT
TATTTAGATCCATGCTTTCCTGCAGGATTATC
GATTGTCAAGTTTACAATTTGTTGGCGTGGGTCAACATTAAGTACCACGGTAGACAAGGATTACGTTA
CATATGAACTTATTGCAGGGGATAGTATTCGT
TTTGTGCATGGCGATGGCCATCGTATTCATCTTCACACGGGATTTCACCGATATACCGCCAAGAGGCA
AGTGTCTGTGCCACGGTTGATAAGAAGTGGCG
AGGGGGAATTTGACGGTGCCGTTTTTCTTTGCGAGACGTTATTTGATGAAATTACAGATATACACTAC
ATGGCTTGGTATAAAACGCTTGAATCCCTCTT
TGAGAATTACCGAGCACTGCATCTTCGTGAAATTCAGCCCTTAACCACAGAAGAATTCTTTGAAAAAG
TTATTTACCAAGCAGAAGAACGTGAAATTGCA
TTCAGTGGCATACACAACGTCTTATTGGACCGTGGGATTGATCTCGAGCTCGGTACCCCGGACGACGC
CGAGATCGTCGAGACACGATATGGCCTGGCGA
ACGCCAAAGTGGCAGAAATGGCGGAAATACTCTCTCGCATGACCCCTCGAGTGAACGCTGGCATGCAC
GCATTGTTGAAGGATCTCTCCAATAGCGGTAT
TGCGCTTGCGGTCGTGACATACTCACGTAGTCTCAAGACGCTGATGCACTACAATCCTGAGGTTATGC
CACTGTTTCTCGCTCACATTGACGGTGAGGAG
GCATACGATCGGCACATCAAAAGCCGTCCTCATGTTGATATTTTCCTTCGCGCCGCAGAGAAAATTCA
TGTGAATCCGGCGCGCTGCGTTGTTTTCTCCA
TGTATCTTGATCGCGGCTTTAAGGCCTCCAGCCTTGCAAACTTTCGCATGTTTTTTGATGTGGAGGGG
ATATTTGCGACAAAACGGATGTCGCAGCAGAC
GCAGCCGTATCCCACGCTCTCCAACGATGCAGCTGCCGCAGTGGGGCGCGATGGTGTGCCCCTTATTT
TGCGTCTCTCGCGGGAAAACCTGCCAACGACA
ATTGACGCCCTGGAGGACATGTTGTACGGACGCTGCAACGCGGTAGACGAACAGCATGTTGCTTCTTA
CACGAAATGAAACGCTAGTTTGAGAAATAGGT
CAGGGAAAAATGACAGTGGGAGTCTGCGCATGGGAACAGGAGAGCCACAGGAATAAGAGAGAAAATAA
ACGGTCCGTTATCTACAAAGTGATGTGGGTGT
GCATTTGAGAGTTAAGTTATTTATCGATATTAGTATTATTATTCTGCTGTACACTTAGATATTTGCAT
AGATTGGGAGTCATTTTGAGAGCGCAAAACCC
GTAGCGTCTTTTTTTTTCTTTTTTTTGCCTTTTTTTCCGCCACTGTTCGGTTTAGCTTTTCGCTGTGG

TTATTACCCCGACAACGATGACCATGTTGCGT
GAGAATTGCTGGCTCGTTATGAATTTTCTTGGCTGCCACCGAGGATTTTCATTGCTGTTTTGCCCTGC
TGTCCTTTTGTTGTTGTTTTTGCTTTCTGCTT
TTTGCATGCAATATGTTTATTTAAATATTTGGGGCGGTAAGTCGTCATGTCCGTTACCGATACATTAG
GGCCTTTGTTCACATTCCTCATCATTATCAAT
GTTATAGGCACGGCCCTTCCACTGCTACGGCATCGCTTTGTTTTGTATCCTTTGGCATTTGTACTCTT
TGGTTTTGCCGGTCTTTTTCTCATACTGGCCG
TTATTTTTCTTCTTGGTGATGCAACGGTTTCGGTCTACAACGTGAATGCCAAGGGGCAGGTGAAGGTG
CAGGCGAACGTTCCTTTTGACCTCCCGGCTTT
TTTTCGGCTCAATTTGACGATGACCACATGGATGCTCTCATCCGTTCTGGCGATGGTGACTTGGCTCT
GCTACCAGTCTCGCAAGGGCTGGCATGTTTAC
ATCGTGCCAATGCTGCAAGACCTGAACACACTCGAGACGTCATGTTGGTCCAATACGCTGGATTTGCG
TAACCGTGTTGCCCCAAACACAATGTGACGCC
ACACTTTTGCCACAGAATGAGAGATGGGACGGCGAGAAAAGGGAACTTGCCCCGTTCACATAATAGAC
GGTGCACAACAAACAGAAAAAGAAAAAAAAGA
CTGAAGTTTTCTTTTTGTTGTTTTCTTCTCTTTTTTTTCTTGCACTACTTTTATTGAGTTTATTTTTC
TTTTTTTTTCTTTTTTTTTTTTGCCCTGCGGG
TGTTGGTAGAACACATATATATTTTTTTTTTACTCACTCATTCGCTCGCCTTACCCGTCTGCATTTTT
ACGTGACTCTGCGCTTCGCTCCACCGCTCAAG
ACGATTCGACATACCCGCACTTTGTACAGGGCCATCAGGTGCGCACTGGGGCCCACTCACCACTTAAA
TTGCCAATTTTTTGGGCAGAATACCGGTGCCA
CAGTCGGTGCGCTTCGGACAAAGTCGAGTATTGCGCGGATGGCGCGGCAGCCCAACACTGTCATTGTG
TTGCATCCTGTGAACAGCGCCATTATTACATT
AGATATCTGTTTGCAGCTTGATGACTCTTTGAGCTCCATATGGCGAAAGAACATGGCGAGAAGTGAGG
ACGAGGTGGCAATGAGGACAATTGAACGGCTT
CAGATTTTACTGGTTCAACAGAAGGAAGGGAAGAATAGCCGACGAATGAAAAGGGAAGATTTACTCAA
AGACTCTCAGCCCATTCACGTATTTGGTTTGG
AAATTGATGAGCATGAAGCGGAAGGTCAAGCAACAGCGCCAACTTCCAACACCCTTAAGCCTTTGGAC
ACATCCATAACTAACAAATTATTTTGGAAGCG
GGCCAAAGAGATGTTAATTGGTTCGACTGTTGTGAATATAAAGTACAACGTTCCCACCATTACATCCA
TTGTCCCCCCATCTGCGCTTTATGTGGGATTG
CCGGCTCTTTGCTGTGGCATAACCACATTGTTTACATCAGAAGTTGGTATTCTTTATGAATGGTGCTT
ATGCAGCACGGATTCACGCGAACACACAAACA
TAAGTGTCATTTCGACGGATGCTGTTTTTACGCCTCTTGAGGAGCATCTAGGGAAAACTTTGGTGCTC
CGTGTATCACCTGAAGGGGACTCTGGTCTCTG
GACACAGGTTGATTTACCCCCTGTTCGTGCTGCATTGACACCTGTGGATCGGTGGAAGCACACATTGG
CGCCAGCAGAGGCACCTGCTTTTAGGGTAGTA
ACGTACAATGTTCTCCACGATGAGTTTTGCAGCACAAGCGCGGCAAAGAGACGTATTTACCCCTTTGC
GACCGACGACATATTGTCATTGGAGTACCGCC
AAGTGCGCATATTGCAAGAACTTCTCGCATACAGAGCCGACGTGATTTGTCTTCAGGAATGTGGGGAA
AAAGTTTACCGTCAGTTCTTTGAACGCATCTT
ACATCACAGTGGCTATGATGGGCGCTACACAAACAAAAATGGGGGCGTAAAAGAGGGTTGTGCATGCT
TCTGGAAAAGAACTCGTTTTTGTATGAATGAG
ACCCTTGTTTTCCCGTTGAACTGGACAACTTTACAGGAAGATCATCCCGATTTAGTGGCACGTGTGTC
ACTTTACCCCGAATTTCGGGAAGCACTTGAAA
AAGTCACGTCTATTGGGGCGTTGGTTCTCTTGAAGGATCTTCAAACCAAAGAGGAGCTAATTGTTGGT
AACACACATCTCTTTTATCACGCTAACGCTTG
TCATATACGTTTGCTGCAGGTATATATGTTGTTGTATAAGCTGAAGATTTTTGCTGTGTCGCAGCCTT
CTGTCGTTCTCTGTGGAGATTTTAACTTTACT
CCCACAACGGGCGGGTACAGGCTTGTGACGAGGGGTCAGACGGAGGCGGAACATCATTCCTGGAAGAA
AGGTGAGCTTTTCTATTGGGGATGTGATCGCA
TGCTGGGTGTCGGCACAGAGGAAATGGAGGGGGTGGAAGAAGCGGCTGAGGAGGGCGCGTCATCGTCG
GTGCCGGTGACTCAGCCGGACAAACGTCAGCC
GCCATTTGGGGCATTTCGAGAAACCCTTAGTGCCCCATTGCAGCTGAGGGATGCGTACAGCGAGACGG

GGCAAGAATTGCCCTGGACAAATTATGCCATG
ACTTTTCGCGAGGTCATTGATTACATATTTTTTTCACCGACGCGTCTCTCCGTTCTGCGCACCGTTCC
GATTCCGCCGGAGAGCGAACTATCGGAAAATG
TGGCGTTACCAAACAAGCAGTACCCATCCGATCATCTCGCGTTAATTGCTGACTTGGTGTACACGTCC
TCTTCGTCGTCGTCATCGTCGTCATGCTGAAC
GCCGTTCGTCTGCGAGGAGCTGAAGAAGAGCCGCCGGAGTTTCCCTTTTTTTTTTTTTTTTTTCTTTT
CTGGTCTCTCTGTGTGTCTGTTTGTGGGCTAC
ACCATTTGTTTCCTTTTTGGGTATTTGTGGTGGTTCCTTCCGTAGCATTGTTGCTTCTTTTTTTTTGT
TTTTCTCCATTCAATTTTGAACGGCTGTCGG
CCCGTGAGTCCGCTGATCGATGGATTTGGCACACCGCCACCCAAAATACGAGGAAAAAGGAAGAGGGA
TTATGTTAATGGCGTGAGAAAATATGTTTTTA
TTTGTTCGTTCCTACTGTTGTTTTTCTTCTTGAAAAGCGCCTGTTTTTTTTTTTTTCTTTTTACGTGT
GTTTTTACGTATGCATGTATTTTTCAATGTAC
GATATTTCTTCTTTGCGTTTGAGGCTGCTGTGGGGTGTGGGGGAATATACATACATGTGGATGTAGAA
GGGAGAGTGTGTGAGGGAGGGAACAAAGAAAA
CAACACGGGACACCTCCACAGAGAAAACAAGACATTTTACTCCGTTGAGTAGTGAACTGACGGCGGCA
CGAGATAACTCCCCCGCCCCGTCCCCTTCCCT
TATGTTGTCCATCACCCGTCGCCGCGCGGATTTTGGGAAGGAGTTGTACGGCAAATTATCGACACTCC
CGGGGGATTTTTATGAGGCCTGTCGTAGTCAC
TTTGGAATTACCCTGCACCAAGACACATTCATTGGAGTCCGTTCGTCACAGGTACGAGGTTTGTTTTT
GTCTACCAGCAGTACACCGCTGGAGGCCAATA
AACCAATTGCCACTATTCCGCTGACAAGTCTCTACACGGTTTCTAACATTCAAAGTAAACCCAACACA
CTTCGTCACGTGACATTAGATGGTGTTCGTAA
TGCCGTGCGAGAGGAGGAGTTTAAGATGATGTCACCTCAGTTTTATCTGGGTCTTCAGTTCAGTGCCA
TCATCTCTAGTATTCCCGACATCACTCATGCG
CGGGACGCAGAGGAAGTGGAACGAATTACGCAAATTCTCCGTGGTGGAGCCATGCCATGGGCTAGGTT
GATTGATGATGAGGATTTTAACGAGCAGTTCA
TTTTTGGTATGTACGGTATGGCGCTGGATATGTGGCAGCGAAAAAGCTACGAGGAAATGACGAAGTTG
TTTCATCACAATATTACAGCCATTCATGAGAA
ATTTCAGCCACCGTTTTCAGTAGACACGTTTAGACGCATCGCACGTTTAGTTCTGGCGCGTGTGGAGC
ACATGCCTCCACCGGACTATTACGACGGTTCA
GCCGTGTGGCGTCGTGTGCGTCACTACATGCGCCAGTGGATAAAAAAGCCAGACCCGTCAGAAGTGTG
CATGGTGCCACTGCTTGACCTGGTGAATCATT
CTAATCGACCCAACTGTGGCTTACGCGTGGGTCCGAGTTCTGTTGTGGGCGGCAAAGGTGCCATAACA
CTTTATTCCATTGCCCGCATCAACCCCGGCCA
AGAGATTTGCCGGCATTACAACTTTGCCATTAATCGTCCCAATGCCCTTTTCCGCTATGGGTTTTTGC
CGTTTGACTTGATTTCCATCGTGGAGCACGAC
GCCATTGACGAGTACCTTGTCAAGAATCAGCACATGCTGCGAGAGGAGTCGGAGGAGGTGCGAATGAA
GCAGCAAAAAGAGAGGGAAGAGATTCAGAAGC
TGGAAAAGATTTTCCAACACGCACGCTCGGGAAGATGATCCAATAATGCAGTAAAGAATTAGAATCTG
AAAGGGCGCTTTTTGTTGATATGTAGTTGGTT
GGTTGCTGCAAGAGCATTTCCTTTTTGTTGTTGTTGTTTTTCCGCCGCTGTGGGCATCGTCGCAGGGT
TTTATTGTTATTTATTATTGTCGCCGTTACGA
TGTGTTGCTGCCCTCTTCACTTCCGCGGTGGCTCCCACATACAACTTTTTGTTTTACCACCCCTTTTT
TTTCTCTCTCTCTCTTTTGGTGTCAGCACATT
GGGCTGGTGGCTGGGAGGGAACCGTTTCCGATACGAAAGGAGGGGCAGCCGTTGGTGTTATTATTCAT
TTATACGCCAATACATGATCCCGCCCCAACTC
CACTTTTGGTCCCGCAGCTCTCTCATGCGTCGCCTTTACGGAATTCATGCGGCTTCCCTTCCAGCTTA
TTGTGTTTTCACCGGGCAAAGGTGTGAGAAGA
GACACAAGAAGACGGACTGTACCATGGGACGTGGGAAAAATGCTGCTGGCGTTGACGAGATGGCTGGT
GATCCCTGCAAAGAGAAGCCTGTGCCTGGTGA
AAGGCAGCCGAAGACGGGCATCGGTGAAAATGTGCGTTCAGATGTGTATTCTTCATTAATTACTTTCA
CGGAAGTCTCTACAGGAACAGGTGTGCGATGG
TTGGATGTTGCCGGACGAACCCATTCGTGGGCCCATGACTCGTTTGTAGAAGAGGGTGAGTTTGTGGC

TCATGCCATGGACGCACTTCGTCGTGGAGAAC
TGCCACCGGATATGATACTCAAAGTCAGTCATAATGTACGTCCGTTGCCATTTGTTTACGTTGAGGAG
AGTTGGGCTTGTTTGACGTTGCGTTGTGCGCG
ACAAAACCCTTTTTGGTTTTCTATGATTTTGCCCGAGAAAGTAAAAGACTTTTCCGCGCCCGCATTGG
ACGATATACGGCGGTCAAAATATTCCAAATTG
CAGGAGAGTGGAGAGGATGAGGATGACACTCTGGACTTTAGTGACGCGGACGATGATCAGGTGCGTCG
TTATCAAGGAAAGGCATTGGGCAAGATGAAGA
ATGTTCGACGTAGAATGTCGTCCCAAGTCACGATAACAGAGTCAACAGATCGTCTTCATATTTTTCTG
TTGAAAGAAAGCCAGACATCAAATGAAAAGAG
CGACAAACCGAACAGGTTCCATTTGGGCCCCCATCAAGCGGGAAAAGTCTCTCAAAAGGGCGGTGGAG
ACAGTAAATTGAACCCGTGTGCCGAACAGTGC
TGGATAGTTACGGTCCACCGACACCGTTTGCCGTTTTTGGAGGAATTGTTGAACCAGTGGGTGAGAAG
CAGACGACATGGGGATACATGGATCGACCTAT
TACGGCGAATTGTTCGCGGCGCTACGATGAGTGTACAGACTCTGGATCACAAAGATGCAGAGCGTCTT
GATGAACTTGAAACGGTTTTGTTTGGATCTGC
CACAAAATTGAATGAACTTTCAAAAATTCTCACGCAGTTGCATATTATTAACAGACGATCGAAGATAC
ACGCTAACCTCCTTCGTGAGACCCAACGCTGT
TACATGAAGCTTTTGACAGTGTTGAGCCTGCCCGTACAAATTGAAGACCAAAGGGAGTTAGTTTATCT
CTCCACTGTCTCATCGCTGGCCGATGAACTTC
ATGATCAGTCGCGTTCTCTTCTCGCGCTTCAGTTTTCCGTTGCAGTTTACGTGCTTGAGGATCATCTT
CGTATTCTGACCCTTTTTACAACTTTCTTTAT
TCCCCTTGAATTAATCACCTCGTGGTTTGGGACGAACTTCGTTGGACTAGAAGACTTTATGGAGGATC
CATGGGGTTTGTTCATTGCAGGGACCATGCTG
GTGGTAACTGCAATTATCACGAGCGTGTGGATGCGTCGCCCTGCCGTGTCTTTGTCCTGAAAACAGTT
TTTTTTTTTTTTTTTTTTTTTTTTTTTTTTTT
TTGTGGCGCGGAACTTATTCCCATGGCAGTAGTGAAATCCCTTCTTCTTCTTTGATTGAAGTGAAGCG
AAGTGGTGGTATGACTTGTGCAAGTTTTGCAC
GTCTGTAGTGGTGGGGGGAGGGACTCAGGGATGCCGCGTGTTGATGAGGCGCGTTCTTGCTTTACTTT
GTTTCTTCTATGTTTCTTTTTTCTTGTTGTTT
TTCCCATGTATATGCGCCGTGTACCAATGCTGTGGTTTTCCTTTTTGGTTGATGTGTTTGCGGCTGCG
CCCGCGTGTGTGTGTGTGAGTGTGAGTGAATG
GAGGGGCGCCTTGCTTTCTTCCCCTCCTTTCTTTCTTTCCTTTTTTTTTTTTTTGTTGACTACATGTT
GTGCGTGCCTCGTATTTTCTGTTTTTCTTGTC
TTTGTTGTTGTTGTTGTTGTTTTTGTTGCGTTTTTTCTTTTTTTTGTGCACTTTATTTTGCCTGAGGA
GAAGAGTTAACGTAATACGGGGCGTGAGCATC
ATTTCCAGACAATAGAACGAACTTCAAGGAACCATTACTTTACTTTTCTGGTTCTTGCCCCCGCACAA
ACACAAACACCAGCGATCTATTCAATATATAA
TTAATAGGAAGGAAATAGAGATAAGGAGAAAAGGCCAATGAAGCCGTTGAATTCAAGACGTCGGACGC
TGAAATCTAAATGCCCTTTGGCGGCCATTTTT
CTTTCGATACCGCTTTTTTTTATGGTATTACTTTTAACGAGCATTGTTTTTGCGGAGGCATTTCCGTT
TACGAAATTCTCTGGCGTTGTGGAACTAACGC
CTGCAACCTTTAAGAATTTTGTGTCTTCCCACAAGCCAGTGTACATTCTTTTTTATGCCCCGTGGTGC
GGTCACTGCCGCAGAATTCACCCAGAGTGGGA
GAAATTTGCTCAGTCTGCCTACGGCACCGTCCGCGTGGGTGCCATCAACGCCGACGAACACTCGCAGA
TTGCAGGGCAGTTTGGCATTCGTGGTTTTCCG
ACCATAAAGTACTGGAACGTGGGTGAAAAGGACATCAACAAGCCACAGGAATACAATGGGCCGCGACA
GGCGAAGTCATTTCAAGCCAACGCGATGAATC
AAATCACTTCCTCTGGGATAAAGACAATTACGAGCAGTGATGCCTTGCGGGAGGCAGTACAAAAGGCA
CCTGAGAAGAAAATTGTGGTTCTCTTCTCTTC
CAAGCCACGTATTCCCGCCATTTTTGCTGTTTTGTCACATTCCCCACGTTTGAAGTCTATGCCGTTTT
ATTTTGTTGGTGAAAATGCGAAAAAAGAAGTC
TCGGAGGAATTTGGAGTGCAGGAACGCCCGAGCATCGCTGTTCTCAACGCCACGGAGGGCGACATCAA
GACTGTAATATACCCGGGAAAACAGATCGCCT
ATGAGCCCATTGCGAAGTTCTTGTTGGCATGCGCAACAGATGATGCCGATAATTTGGATGCCGCGAAG

GAAAATGCAGGAGAGCATCCACAAGAAAACGA
CAGTGGCAAGGGCAAGAATACCGCTGCGGCTGGTGGTGGGACACCCACGCCAGCATTACCCGTGCGCC
CCGTACGACTAACCGCACAGTTGTTGGCGGAC
TTCTGCTTACCGGCGACACTGCGCGTACATGAACGAGCACCGCTGTGTGTGTTGTCGTTTACGTCCGC
GTTTACTCTTGAGCGTGTTCACGAGATGTTTC
ACAACGAACCCCTTTTATTCTTCGAGGCAGATGCCGAGCGTGATGTCATTCTTTCTGCCCTTCGGAAT
GATCTTGGTCTGGAAGAGGTGGCCAACAGTGT
TGAGGGTGGTGCCTCTCATCAGATGCTATTGTTACGGGCAGCGAAGCGTGACACGATCCGCTACAAGC
TTCTTGACGGGATAGAGACAGTTGATGGCGCA
AATGAAGTACTGCAGAGGGTGCTTTCGGGAGATTTGCAGCTCGGGAAAAAAATTGCTGCAGTGATGACT
TTACGGAGTCCGGTATTCTTTCGGCGTGGGTA
GGAGGCATGTTTTTTTTTTCTTTTGAACTTGCGTTTGCGCATCTCTTTTTTTTTTTTTTTCCATGAAC
TCCCACGATTTGCATTTATTTCTAGAACCGCC
ATGATATTCTGTGTCTTTTTTTTCGTTTTGTTTTCTTCCTTTTGCACGTCCTTTCGCTTGTTGATATG
GACGCGTAGGAGTGCGGCGAGTGTTTACCAAC
AGGGGGGAAAGGAGAACGAGAAAGAGAGAGAGAGAGAGAGAAGGAGGCGGCCGAGCGGGCAGTAAGGT
GCCTTTGAGAGCGGTAGAGGGGGATATAAACA
AAGGGCAGAGGAGGATCTTCTTTGGCGACGGAGTAAAAGTTGACAAGGACATTAGCGCGGACGAATTC
CACACAGAGTAAGAGAGGCGACCAAGGGAAGG
AAGAAGAATGGGCGCATGTATTGCCCCTGGAATAATCCACGCAATGTGGGGCGCGAAGCAGCGGAAGT
CAAAATTGATGGAGAAGCATTACGAATTGCAG
AATGGCGGCGATAATGGTAGTGCAACTGGGCTGTTGCCTGCATTGCTGACAGAAAAATTGGGGCTTGT
GAGCTCTCAATCACAAACATTTCTGGAGGATT
TTATGGAGGAGCTTTGCCAGGGTCCATATGCGTCACTTTTTTTGGCTCATCCGGATTTGTTATTTTCT
AATCGAGCAGCTGTCATGTGCACAGGAGAATT
TGCTCCGGGGGAGGTTGTGGGAAAATACTCTGTCCTGGGTTCCTTGCCAACAGGAACGGTGGGTCGAA
GTTTTCTTGTCAAAGCACCACCGGAGGAAAGA
GGAAGGGGACGAGGCGGAACGATGGGAAAAACAGGGAAAAATGTGGGGTCCGAAAACAATGTGAGCTA
TCAGCTTAGCAGCAGTCTCACCTCACAGTCTC
CAGAAAACGTTTTTAAAGTGGTCACGTTTGTCACCCGCATGAACTTGGCCGAGCGTGTCTTGGATGAC
CATAAAGTGCTGACGAACTTGATTCACGATAA
CGTTCTGCGCTGCGTGGATGTACTGGATGATGGACAGCATGAGAACTTTGTTTTTGTCTCTCCGTATA
TTGAAAACGGCAGTTGTGAGGCTCTCGTAGGC
AAACTGGAGACGAAGGAACGATTACTCTCCCTGCTGCATGATGTTGCCGTCGGCCTTCGTGTCCTGCA
CTCTCATCGAATCTACCACCACAACCTCAAAC
TGGACAACATTCTCATCAAGAACAGTGGCACGGCTTGTATCGCGGACGCTGGATTTGGACATATTTTT
GCCTCTCAAAGTTCTGAAGGCCTTGTTTTCAA
TGGTGAGTTGGCTTGTATGCCGCCCGAGGTTTTTCAAACCGAAGATGTTTCTGCAATAAATCATTTTA
AGGCGGATGTATGGGGCTTTGGAGTTTTAATG
TATCGCCTCGCCTACGGCCGAAAACCGTTGGATGTTGAAGGCAAACCTTTCGGAACGATACGGGATTC
TGTGTTGCATGGGAAAATAGAGTGTCCGGATG
CGGATTGGGTTTCGGATGTCAGTTTCCAAGATGTTGTACTGTGGTGTCTAGATCGTGACCCCAATAAT
CGGCCTTCCATGATCGAAGTGCTGCGTCACCC
TCTTTTCGGGGAGAATGTAAGACAGAGCATTAGTGGGAGCACAATTTCTCCCCCCGCTGCCACAAATT
CTCATTATGGCGGAAAAGTTGGCTCAATTGCT
AGTCTCAGTACCATCGGGGCACGATTTTCGCGGTCTCAGAAGCGAGGTGGCTTGCCAATAAGTGAAGC
TTTTGTCAGGGGGCGAAACTGTGAGACGTTTC
TTGTAAACTCGGCTCGTAACATGACGAAGATTGTTTTGAAGGTCATGAGGCGATCGGTAATGAAGAAG
TTGGAGAATTTGTCCTATGCAGGCAACATTCT
TCATGACTTGGCCTTCTGCCGTGTGGCCCGTCACCCCAATATATTGCATCTTGTGGAGATTGTGGAGA
ACAAAGATGGCTGTTTTGCGACGCAACCCTAT
GCGGAAGGGGAATTTCTTAGCATGAATTTCCCTCCGCTTACAAATATTGAGGATCCTTATTTCACTTT
AAAGGGGATGCTTGTGGATGTTCTCAGAGGGT
TACATTTTCTTCACACAAACGGTGTCTCACACTTATGTCTAACGCCGTCTAATATTTTCTATCAAAGA

GGCGTTGGTTTTGTGTTGGCAGACTTTGGCCC
CCTGTTTTTAACCAGGGACGAGGCATTAACAAAAGATGATATGGGGAATTTTTGGTACTCATTTCCTC
CATGGGTTATTGAAGATCTTAAGAAACCTTTA
CATAAGACAAAACATGCCGTGGACACTTTTTGTGTCGGATTACTTGCGGCCTCAGTGATACCAGTGGT
GTATAAGCAAGTCTGGCAATCCTTTTATAGTG
GAAATGAGGGCGGTACTATTGCGCAAAATGTCATTAAGAAGGTTGAAGAATTTTTAGATCTTCTTACC
CAACCAGTTTTGAGTTTTATTTCTAAGGCGAT
CACGACCAAGACGTCTGCAAAGGAGCTGTTGTCTCACCCGTATTTGGCGGATGCGTTTGATGATTCCC
AACTTCTTGGTGTCAAACCTTTGCAGCTGTCA
TCTGCTTCTATAAAAGGTGCGGTATCTCTGGATTTTAGTTTTCGTGAGGAGCGCCGACTTATGGACGT
GGTGGGTCAAGATCCCATAATTGATACCGAAT
TTTTTGGTAATTCGAACTTTGTGGATTCCATTGTCCATGAGGAAAGTCGAGCCAGTGTGTTTGACCCC
AAAGAATTGGATTCTTCTGTGCTATTGCATTT
TAAAGATAAAATTGTCTGTGGACTCTGTCATGTGGATTTGGCAATTGTGGTATTTCGATGTTCCGTAT
GTCCTTCGTATATTCGATGTGGAAAATGCGCC
CTGAATGACGACCATGAGGAAGGTCATGAGATGAAGCCGTATCTTATTCACACCATTGAACATAAAGG
GGATGGAAACCAGGAGGCCATGCTTCTTCCAC
CAAGCAGTATTCACAATGCACATGTCTTGGAAGAGGTAGAGATGCAGGCCAACTTGCCGGCTGGATCT
ATTGTGGATATCACAAATTGCCTTTCGTCACC
TAAAGGAATCTCATTGAGTCATTTTAAAGTGCAGTGTAGAAAAAATAAAAGTAAAAAGAAGCTTCCCA
AAGCCTGTGAGATCGATGATTTGTCGTGGGAG
GAGGAGATAAACAAATGCCGTGAAAATGGTAACCCAGAACTGTTGTTGTATCGTTTTGATCTGACCTC
TGTACCGAGAGAAGTGTTTGACCCGCCGCTAC
TTCACGTGGTAAGTTTGGATCTTAGCTACAATAAATTAACGAGCATACCGCATGAGTTATCGTTTTTG
CACAAACTTCGTAGTTTGGTGATTGCAAGCAA
CGAGCTATCGGAACTCCCAGATAGTTTGGGTAACTTGAGCCAACTGGAGCGACTGGATGTAAGTCATA
ATCAGCTTCAACAGCTCCCGCAGTCTTTTATG
TATCTGTACAACCTGGCTACTCTTTCCATGGACTACAATAACTTTGCCGGTATTCCGGAGAGCGTTTT
GGAAATTGTGACTACCGCCCCGTCGATGGCAA
TGTTATCTGTCATTTACTTGGCGGAGAATCCGCGCATTACCGAATTCCCAGATCAAGAAGTTCTTGAG
AAATTTCCCAAATTAAAGCTAGCACTTGATAA
TGAGCCGAATGTATACCGAACGTACATTTCAGAGAAATTGGAAGAAAAATTGCCCAATGTCAGCATCA
TGTGGAACAAAATTTACCCTGATCGCATTGTT
GAGCATGTGTACTGCGGTAGCCTGCGGAGTGCACAGTCTCAGCTGGTGTACGATAAACTTTCCATTAA
AAGTCTTTTAACCGTGGGCCGGGAACTTGTCC
CAACACCGCCAATTGGCGGTGATCATTTGACGCTCAGTATTGATGACATTGAGGGTGCAGATATCAGA
CTTACCTTTCAGGAATCGGTTGACTTTATTGA
AAAAAGCGTAAAAAAAGGAAGGGGATGTCTTGTACATTGCTTTGCTGGTATGTCGCGCTCTGCGACAA
CCGTCATTGCATATCTTATGATGAAAAGAGGT
ATGCGTCTTGATGAGGCGTATCTTAAGACTAAAGAGGGGCGTCCTGCCATTTACCCAAATCAGGGCTT
TTTTAATCAGCTTTTACAGCTAGACGCCGAGT
TATTCCCACAACAACGCCCACTGAATATGGCGGCGATGGAGAGAGATGATGTTTCTAGGCCATGACAA
AGAAGAAAAATTAAAAAAAGAGAAAAAAGCAATAT
GTGTATATTTGTTTATAGGTCAATTTTATTTTATTTTTTTCCTTCTTGACGGATGCTGTTTATTTATT
TTCTTTGGATTTTTTTCTTTTTTTGTTTGTTTG
TTTGTTTTTGTTTTCACCGTTCATATTTGTTTTGTTTTTTTCCACGTACGCGGCCTTTTTCCATGTAA
TGAATCTTTTATTCTCTGTATATATATGTAAA
TACGTACATATTAGAGCGGAACCAAAGAGAAGGAAATGTGAGAAGGGGCGGGATGAATTTTATTCTCT
TGTGTATTTTTGGTTTGTTTTGTCTTGTGTCC
TTTATTTATTTATTTTGTGAATTATGCACTTTTAAATATTTTGACTGCTATTATTTGGTTTGGCTTTT
TTTTTTTTGTTTTTTTTTTTTTGTTTTCATGGA
GATGGGTTTGTCTCTGATGACATGAGGAAGAGCAGGCAGCCACGCCACCGCAGTACGTCGCCCCCTCC
ACCCAACGTGCGGAATTAAATGGTGACAGACG
TCTGTCTCTCCGTTTGTGTGGGTGGGTGTGTGCAGGACGCCGGCGACGCGAGGAGGGGGAGGAAAGTG

CGGCGCATCCTTCATGACAGAAAAAAAGGAAA
AAAAAAGGCTGTATGAAAGAGCGGGGACGCGTATTCTGTTGCGTTTTAACAAATTATCCCACCGTGTC
TTTGGGACGCAAATTAATTCTGTGGCGTCCGA
TCACGTTCACGCATGGAAACTTCTTTCCTGACCGTGTTTTTTATTTCTCTGAGAGGCGGAATTCCCAT
CTTTTTTTTTTCTTCTTGCTGTTGTTGTTGTT
GTTGTTGTTTTGAAGTCTCTTTTTGTTACAATCGTCTCCGTGGTGCCTCATGCAGCTTCTGGATGTAC
AGCCATACGAGACGGCTTTGCAACGATTGCTG
CGCGCCGGAGTCTGCAGTGAAGATCAGTTGCACGATTATCATCTCTCTCAGGCATTCCGTAGCTTGGC
ATATGCTGTGAACCGTGCGTCTCTCTCCGTTG
CTGCTCTGCTGCCCTCGCACGGTCCTCCGATATCCAATGAAGCACCGCTTTCACCCCCGTCGTCCGGT
CTAACATCCTCATCCACAACCAATGCGGATGC
AACAGGCAGTGCAGTCGTACTGTGTCGCATGTGTACATGTGCGTTGGCCTCCCAGGCGAGTTTAAGAA
CGCTGATATACGTATCCACACGTTTACTGTGC
GATGAGGCTTCGGCATTTGGTGAGGGGAATGAGCGGCCGTGTTGCACAACAGCGTGGGTCGAGGCACT
TTGTTCCCCTGGTGTCAGAAGATCATTCCTAC
ACAAACAAATGGGTGTTCATCGTCGTCTCACGTTGGGCTATTTGCTTCTTCTTTCCGGTACAGTCCGC
GCTGTGACTCATGGCTGTGCCAAGGGCAAGCG
ACTTCATAGCCATTTTGTGCAATTTGCCCAACAATTACTGGCTGACGTGGTGCCGTATGTGGCACTGA
TAGCGGACACAAGAGAAGCCACGACACTGGCA
GACGCTGCTTTTGTGGACGGTGATGACGCATCATTACGGGATGGGACAAAAAAGGAGTTGCTCGTGCC
TTCTCTTATGACAGTGGAGGCGAACAATACAC
TGCTGCATTTATTTCATGGGATTGCGCTGGATGTATTGGAGGTTCATCAACATGATGAGAAGCAGACA
ACGACTAGTCGTCGTGCGGTGTTGGATCGTGA
TGCTCCGTCCCGTATTTGGTCTGTGGTGTCTATTTTACAGAGGGCATTACTCCGTGCGCTTGAATGGG
TTCAGAGTGAAATTATTTGGGAAATGAGTTCC
ATCAACTCTGCGGATCCTTCAACGAAAACGCAGGCGGTGAATGAGACCTTCGCAGTGGTCAGAATTGC
TCTTGAAGATGGATTTGCACAACTGATTAAAA
AACAAGAAACACTTGAATGTTCTCGGCGAGCTGTTGAAAAGCAATTTGGGCAAAATTCAAAGGACTAT
CACGCCATCAGTGGCATGATGTGGACAAACAC
GTTGCGACTTTTCTTTTTTTGTCGGAGCTTATCAAATGCGTGGACATACATTTACGGCGGCCTTGGTG
AAATGGGGGTGCAACACACCGATGAACAAATA
CTCGCTCTTATTGCCTCCTTTGTGTCTTCTGTGCCATCATTTGAGGGAGAAGAAAAGGAGCATGACGT
GTTTCTTGCCACCACAATGCTTCTTTCAGGAG
CGCTTGCGGCTGTAGCGGCGGCCCCTGCTCCATCGATGCAGGCTGCACTTTGGTTCAGTGAGTTGAAT
TTAACCCAACAAATTCATAATACGATTGTTCA
ACGAGGACTTCGATACCGGGACGAGAATGTGCAAAAGTTGAGCGCCTTGCTGTCGCGGCTAATATTGT
ACAGCTCATCTAATAACTCCGCCTTTCGCATT
ATTGCAGACTCTCCCGATCAACTGTTGGAACTTCTTTCGAATGGCCGCGATGGGGCGTCGCAGTGCGT
CATTGGAGTTATGATACGTGCGGCCGTTGAGC
GACCCCATTTCATTCTTCCTTCACTTTTTCGCTTGTTGCAGCATGGGGACACCAACACGAGACGCAAT
GTTCTGGATGTACTCTCCGCGCTTCCAAAGGC
CTCCGATGACTCTGCATACGGAGGAGCCATTGCACCCGAGCGGATGCGGCCTGTATTGCGACTTCTTT
CGGAAGAACTGTTATTGCGTATTCAAGATGAG
GAACTGCAAATCCGATTGCAGAGTTCGAAACTGTTTGCAAAAGTATGGCCGGAAGATGTTTGTCGCTC
ATTGTTGAAGCTGGCGACGCAACGCGACCGGT
CTGTTAAACGAAAATCTTCGGCGCAGCAGGCTTTACGCTTAGTTGTTGAAGCCCACACTAGCGATGGT
AGTGTTGTGTTACTGCTAATAAAAGAGTCCTT
GACTTTGCTGGGAGAAAACGTAACATCTATCTGTGCCACCCCCTCCACACCAGCTGACATTTTGGCTT
TTGCAACATTGCACTGCGGCACACTCCAAGAT
ACCGTTGAAAGTCAACAAGTAACCAATTCCTCTACTGAGGAAGAGGGAAATATTAATGAGCGAATTAT
GCGACTTGTTGCATTGTTAGGACGACACTGGG
CTGAGGGGGTAATAAACTGGACAAAGGCATGTATTCTCCCACTGCTTCAGCGTGCTATGGAGGCACGA
GCTCCGTTGGAACAAGAACTTCTAGTTCGCGT
GATGTCACAGATAGCTTATGTATGCGGTGCGACCAATGAGGGCTTAGTTGCACTCTCTTTGAGTTGTC

TCCATATCATGAAGCCCACAGAAGAGGGCACA
AGCAATTCAACTGTCTGGCTTGAAAATCTCACTGGGCCTTCAACGGCACTGAGCTCGGACGCGTCAAT
TTATCAAATAATAGCACCATTGCTTTTTCTTC
GGGGCTGCCCCAGACGAATTTTTGCCACATTTGTCACGGTGACGCAAATGCCACCACCCGTTGCGGAG
ATATGGGAATTCCTGTGGAGCGTTCTTCTTAA
TCCAAATTATCGCGAAGAATTGTCACTCGATGCTCAGCGCCTAATACTTGAACTGGTGTGCAAGTACA
ACGCCTCTGTTATGATGGAGCAGCTACGCCAC
CTTGGGTCACGATACATGAAAGAGGGTTTTTTTTCACTTGTTGATGGTAGCATCGCCTTCTTCATATG
TCGTGTCGGTTTCTTTTGTATTGGTTTCCTCC
TGTCCCAAAACCAGGCATTATTTTGTCCGAAAGACATGAAATCTAATCTTGGCTTGAATGAGCATGAA
GCTGAAGAAAGAGAAATGGTGGGAATGGTCGC
CACTGCGGAAAAAGAAAGGGAAGAAGAAATGGAATTTACTGAGCTTGTGAGTGGGGCACAGATGGTTG
GACGTTGGGCCGAAGATGTTGTGTTGCCGTGG
CTTTTGAGCGAGGACGACGCAGGGATCTGCGATGTTCCTTTTCCTGTGGATTTTCAGCGCCTAAGCCG
GTCTGCAGTGGAGTGTTTAGCGATTGTTATGA
TCGCTGCGTTAAATCGCCAGGCGTTTAGTGATACCGTTACCCCTTTTGTCATGCTGCCACTCACTCAA
CTGGCACAGCACTGTAGGGAGCGGGTCAATTT
TTGGAAGGAAAATTGCAGCGCCACCGATGCACTTGTTGAATGGAGTCCCTCCCCTCACTTGCTCGGAC
GATTTGAGTTTGCCATTCATATTCATCAGAGA
GCCCTCAATCTGCTGCGGGCACACCCTTCAGGGGGGACGACGATAGGCTTATATTTTAAACACTACAT
GCCGCCTTTAATTGAGCTTGCCAATGCAATCT
GTACCGCAGTTTTGCGTCTTGGTAAGAAGGATATTGCTCAGGTAGCCGTTGAAAGTTGTCACGCCCTG
TTTTTGGCTGTGATGGCCTCAACCGGCGCATC
TCCAGGATTATCTGGCGGCGAGACACCGAAAGGGCGGGCAGAAGCATCAAAAAGTGTACTGATGTTTC
TTGATGTCACCGACCGAAACGCGCTAGTTACC
TTTGCCGTGGGGAGCGCCAGGTACTCCGATCTTCCACAGGTGCAGTCCGAGGGAGTGAAGTTACTATC
GGCGCTTTTGGGTGCAGCACCAGAAATCTTTG
TGACGGAAGCTAACATGGATGAGGCACCGTTAGGAGAAGCTCTTACCGTCTTGGGGTCGGTGGCGTTG
ATGCATCCCGACGCCAAAACCCGCGTCATTGC
GGAACAAGTTTTGGGGATTTTACAGGCGAACTGATGCCTTTTCAAAGGTGTCTGCTTTCAGCGTCCCG
GTGCAAATTTATTAGGTTCTCTGTTTTTTTGC
CTGGGAGGGGAGAAAAACGTTATCTATCTGTGAGTTCTTTCTCTCTCTCTCTCTCAAGACAGAAATAA
AAAAATATCAACAAGCCAGCGACGAGAAACAT
TCATGTATTTTGCTATGCAATGGAGTAAATCTATTATTTATGGTTATTTGTTGTTGTTGTTGTTTTGC
TTGCGTTATTTTTTAACTGCTTTTTTTATAGA
TGCATATGCGACCATGATGCACGCTTGGTATGCGCTACCATCCCCGGCAGCGGTGAATCTGCCAATGA
CACTCTGTGGTGGACAGTGTTTCCGGTGGCGT
CGGACACCACGAGGCACATGGGTTGGTGTGGTGGAACGTGGAGCGTACGAACTCTCTGATGCTGCCCA
TCCGCCCGAATTCCAGGCAGTCTATTCACGTG
GGGAAGAAAAGCGGGGAATGAGGCCCTCTTTGTCCCATCCTTCTGACGACCTCTCTGGGGATGTTCTT
TGGTTTCGCTGTCTTCATCGTGAGCCAAAGAA
TGCATCGGATTTGTCTACAGAAGCCTGTTTTTTGCGGCATTACTTGGCGTTGGATGTGGACCTGCAGA
AATTATGGCGGCGGTGGACACGTGACAATCCC
ATGAGAAACCATCCTTTGGTTCGTTACCTCACCTCCAATGCTGGCAAAGGGCCTTCTGTGAAAATTCG
TCATTTGCGACAGAACATTCATGAGACCCTTC
TAGCATTTCTTTGCTCACAGAATAATAACGTACAGCGCATCACAGGACTTGTGGAGAAATTGGCCACC
TCGTATGGAGACCACTTGTGTGATTACAACCT
TGAAACAGGCGATGTTCGAAACGTGGGTTATTTAAATCACAGCTCCACGAGGTCTACAAAAAATGCAA
AAAGGGCTGATACGGGAGATGGAGATTGGATA
CCTTTACATACTATTCCCAGCATGGATGAATTAGCTAGACGGTCGGAAGATGAACTCCGCGCGTTGGG
TTTTGGGTACCGCAGTAAGTACATCGTTCAAT
GTGCCTCCATCATTCAATCCAGTGGTGCAACCAGGCGGAAAAAGGAGGAAGGCGTGAATTGCTTTTGT
TCCTCCATGCAGTCTTACAAGTGGTATGACGA
TCTTTTGGACCCTTGCCTTAGTTTGTCTGATCGGCGAGAAAAACTTCTGTCCCTTCCCGGAGTGGGAC

GAAAGGTTGCAGATTGCATCCTTCTATTTGCG
GTGGGTCATCATGAAATTGTACCTGTCGATACACACATGGCGCAGGTGGCAACAGAGTACTTGGCTGG
GACAGCGACATGCGGCAAAAAGGTCTTATGCA
ACGGAATGGGCGAGAAACGAAAACGCAATTCTGAAGGGAAATCTTCCCTGACGGCAGTATCAACGGAT
GGATCTTGTTGGGAAAAGGTTTTGGCAGATTG
GTACAGAAAAGGAAAGGAGCGGGACATGAAAATGCCGGCGCTTTTGCACAAACATCATGATGCCATTC
AACTGGGGTTTTGGCATCTTTTTGGTGATTAC
TGTGGTTGGGCACATAGCATTCTCTTCTACGCACGCATGCGAAGAGGGCAAAGAGACAACTGAATTTA
AATTTCTATCCTACGATGCTTTGAGAGCTTTG
GAGTGCGGCGTGTTAAAACTCTCTTTCACCAACATAAATAAAGGAATAAAATACATTTATTTTATTTC
CCCAGAAGTAGAATGTGTGGCACATTCTTTGT
TTCATGCGATGGGGAGGAGCATTATTTGAATAATTAATGTCTTAATTGTGGTGTATATAGACAGATAT
ATATATGTGCATACATGTCTATTGAATAAGTC
CGTTGTGGTGTGGGGATGAATTTGATTTTATTTCCATTTTTTTCTTCTTATTCTCTCTTTTGCACGTC
TCGATGGGGTATGGAGATGTCGTGGAGTATGT
GAGGCACACACACACAAAAAAAAAAAAAAAACATGTATATTGTAGAAATGCATTGTGGTGTGGGTTTGT
CTGGCGGAATGAGTCTGCCTTGCCGTACGGTT
GAAATTCACATCGTCAAAAGGTCATGACAAGGATNNNNNNNNNNNNNNNNNNNNNNNNNNNNNNNNNN
NNNNNNNNNNNNNNNNNNNNNNNNNNNNNNNN
NNNNNNNNNNNNNNNNNNNNNNNNNNNNNNNNNNNNNNNNNNNNNNNNNNNNNNNNNNNNNNNNNNNN
NNNNNNNNNNNNNNNNNNNNNNNNNNNNNNNN
NNNNNNNNNNNNNNNNNNNNNNNNNNNNNNNNNNNNNNNNNNNNNNNNNNNNNNNNNNNNNNNNNNNN
NNNNNNNNNNNNNNNNNNNNNNNNNNNNNNNN
NNNNNNNNNNNNNNNNNNNNNNNNNNNNNNNNNNGGATACACACAGTTACAGGAGGTCCTGCTAAAAA
AAAAATGACATCGTCATTATCCTCATTAACTC
CGGCGTCACGTAATGCGGTTGTGACGTGCGTATCATCCCCCCGTCCAGTCAAAACGAAACATATTCCA
CAGTGAATGGGGTTATACGGGTTCTTGCAGGA
AAGAATGAGTTCTTTTTCCGCTTGAAAGCACTTTCTCTGTTGGAGGCGCACTTTCTTGCATATTTCTC
GCATCCAGTGCACATATTTTTTTTTTTGTGAA
AACATACTCAACGGGATGAAAATGGCGATAACGTGTTTGATACCGTCGGCACGTAGGGTAAATATCTG
AGGATGTGGCAAGGTTCTGGCGGGAGCTGCCG
GATGGTGTGACGGAGGAAAAGTTCAATGCGTGGATGCGATCGATGCCACTGCGTAAATATCAGGGGAG
AGGATATCGCATCATGTGCCGTTTCTGGGCAG
GACTTGTTTGTTTGGCAATTGCCTTCTCTACATGCCTACGAGTATTATTGGCGGCTCGATACAGATTC
TTATCTAACGAGAGATATTTCATCTGATGTGT
TCAAGCTGATGCAGGCAAATGACTGCGTGTATGTCTATAGACGCATTCGCGGTGAAAATCCGTGCGTT
GTATGGGATCTATGGTCAACATTTTTGAGGAG
GGCCAATATGACGTTGTCTTCGGCATAGTTACAATGAGTGAAGAGTGTTGCCATAAATAATATTAAGA
AAGAGTACGAAGGGAGGATGTAATACAATAAA
TTTGAAATTGGCGCGATGAAGTTAAAACGTCATTCGCTGTATCGTTTTATTTTTTATTTTCTGGACAC
GGAGGAGCCGCGTGGCATCATGCGTTATCGTT
GGGGTTATGTACCGGTACACGCATTTGGTGTGGAAGCGGTGATGCAGCGTGAATGCTGGAAGAAGTGC
CAATTTGACCAAAGTTTTGCTGGGTACAGGAG
TGGGAAGCAATAGGGTATTAATATTGTGTTAATTGGCTCTTTATCGCTTTTATTACTAATGTTATTAT
TGTTGGTGTGGTTCTTTGTCGCTTTTCGTGTT
TCCTGCGGCATTGAAGTGTGAATATTTTGCCGCATGCTCAAAGAGAATGTGTTAAAATGTCTCCTTAT
GGAATGCAGGCGTTGTCCTCTTCTCACTGTTT
TGGGTTATGCGGTGCATTTGCCGTGGAACTGATTGTTGTTTCTTTTTTTTTTTGTATGCATTTATTTG
GAAGAATTGCCTGGTTTATTTACCTGTTACTT
TTTAATGCATTCTACTCCTGCTGTGTTCCTTTTATTTTTATTTTTTCTTTTTTGTGTGCAGGGGGTAT
TTAGGTGGGTGGGGGAGGCATCTGTTGAGATG
GGCGAGGCGTTTGAGCTTCTGCGCTTTGCGGCTCTCTCTTCTGCCGTTCATGCCGACGCTGTTGAGTG
CCTTGTGGATGACACTCGCTCTGTCTTGTCGC
GGATTGCCATGGATACGGAGCGGCTGGAAGCCCTCGAAGAGCAGCTCGCCGGACTGCTGGAGGAGCAG

CGGCGCATTCATGCCGTCCGTGACGTGTTGAG
TCGGTCGAGAAGCACGCGGAATACGACGGAACCCACAAAGCGCGGAAAACACCCCACAGAAATAAGAT
CAAAGTTGGATACGCGAAGTCCGGGGGCGTCT
CCCCCTGGAACTTCCCGGCTAGGCACCGAGAATGTTTTGCGATTTGCCTCCAGGGCCGTGCCTGAGGA
AGATGTTCCCACAATGTCACGGGCGCCGCGGC
GCGAGCATGTGGCGACGGTGGAAATATTGACAGACGACTTGCGGGAGGAAAAAGCGACGGAGGGGGAG
AAATGCATCGATGAGATGGTGGAGCGCGTGAA
GTCAGAGATACGTTCACTGGAGAGTATGTCCGTCAAGACAAAGCTGCGTGAGTTGGAAGCACTCAAAC
GCCGGATAAAAGGTTTGGATGCGAACGCCATC
ATTGAACGGTATGTGGAGCCCTTCCGGTTTGGTGACGAGGTGGAGAGGCTTTGGGATGCCCCGGATGC
TATTTTGGACAATACAAACATGGACCTGTTCA
TTTCCCGGGCATGCCAACGCTTCTCGGGTCGTTACGAAGACGCTCTGGGTGCCATTAGCGACTTTTCA
TCATATGAAGATGGTGATGCGGCGCAGGTATT
TGGTTCTTTAATGTACCTTGAAGCACTAACCTCTTCGTCCAATTACGACGCGGCTGCCGTGAGGACGC
TGCAGGGCTTAAGTAAAGAATCGCTCCTGGAA
TACTACAGGGAGAAGAGACGTGCCGCTACAGTTAGTACCACCGTGAGACGTAGTCGTGTGGAGACGGT
TCTGGGACCGGAGGAGTTTGGATTTTTACCGG
AAGATTGGTGGAGTTCGCGGGCTCTTTTTATTCCTGCAGGCATTCAGTTGCCACCGGCGCGGCACATT
CTGACGGCGCGATTCTCCTTCGCCGATGCCCA
TGAGCTCAACACTCTTCAGTCCGTACGGGTTGATTTTCAACGGAGCGTGGTGGATGCCTTTATTCGCA
TGCCGAAGGCGTTAGCAGAACTTCGGGAGCAG
ATAGAGTTTCAATCAAAGCACGACGAAGAAAGAGATGTTAGCGCCTCTTTGCTCCGTCTTTTGGTAGA
AAATGAGAAGGGTGGTGGTGGTGACATTTGGA
CGACGATAGCGCGCTGCAGCTGAACGAGTTGACTTTAGTGTTTTATTTATTTACTATTATTATTATTA
TTCCCTTTGCCTTTGCTGCATGCACTGTTGTG
TGATTTTTTTTTTTTTTTCCCTTTGCCTTTGCTGCATGCACTGTTGTGTGATTTTTTTTTTGGGGGTT
TGGGTTTTGCTTCCTGCTGTTGTTTTTGTTCG
TTCCTTTTTGCAATTTCTCCCACCTTGTGCGTCTGCACACACACACACGGAAGCGAGCAAACACAAAA
ACAGTTAAGAAGGAAAAATTTTTTCTGTCAAG
ATACACGTGGAAAGCAAGCAAATAATTAGGGAAAAAGAAAGTCTAAAGGCTCACATTCATCATTGTGA
GGCTTCACCGTACATATTCATTGGGACCAGCG
GGTTGGCACAGTCGATCGCACCGCAAGTTTTACAAATCAACATTCGGGCAAACTGACAACTCAGGACA
TCCTTTCTTTTTTTTTTCATCTTCATTTTTTT
GTTGGTTTTGTTGTTGTTGTTGTTATTATTGTTGTTTTACGGTGGAGGGAGTTTACCACTTCTTTCTG
TTCTTGTAGACGGCGTTTTCTATTGGCGTTTC
TGCGCGTGGTTGGTCAAACTGCAGGTTAGTATCGCCTCCCTCTACGGCTATCCCTGTGTTTGTCCCTG
CGTGTGAGAATTACACTGGAGGGTTGTGCCTC
CTCTGTGGCATTTTTCCCTAAATGCCGGATGCCTCTATTCGTATTGGGCTCCTAATCCTGTGCATATT
GACCCTTGGGCTCTCGTTCGGTGTGGGTAGCA
CGACTCTCCTATCTGTCTCCGGAGACCAGGCTCATGCGCAGGATGCTAATTTGCTGAAAAATGTGGAG
ACGGAAAGCGCTGGCGATGAAATTTCTGCTTC
CCGTCCGGACAACAAGGAATTGGTGCCGTCTTACCGCCTCCATCCCTCAAACAGACTACTGCTGTTGC
ACAAGGATGGAAAGATCTCATCTTACGGGCTT
GATAATGGGGTGCCAATGTGGACTGTAGATACAGGCGGAGATATGTTGCGTGTAGAGATTGAGAAGCA
TCCGACTGAACGAGTAATTCGTCAAGATCCTC
TTGCGCTCCCTTTTCTACTGGAGGGCGGCATGCTTTTTACGAGACGTTGCGTGGCGCCATTTGGGTGC
AAGAAGCAGATGCCAGAGCGTTGTTTTGACAG
AAAAGGGATAGAGGTGGAAACGCGTCCCGAAGCAAGGCCTGGTGAAGCGAAAACTAGAAGCGGCTCAG
GGGATTCATGTGAAATACCCTTTTCATCAACG
CATCCACGATGCTTTATGAATCTCTCAACGCTAATAGAAAAGAAACATGTACAAGTTGGCGAAACGGA
TATATTTGTTACTACATCAGCGCACATTATGG
ATGTGGATAGTAATGATGGAAAACCGGTACCGGAAACAAACTCACTTTTCTCTTATCTACATGTTGTA
CGATATGACTTTACTGTCCATGTGCGACGTCC
CGGTGAATACAGTTGGCATATGAGTATTGCTCAATACAAGCTCTCTGAAAAATCTCCTCTGCAAAGTT

CCGAAGAAAATTTAAGTGACCATGATCCACTT
TTTGTCTCTCGTGTGCTTCAGAGCCTGCTTCAAAAGGGGGATTGCGGCGACAATGAATATGTCCCTTC
AACGAATGAAGAGAACACTGTTGACAAGGTAA
ATATTGAGCCAGTTGTGGAAGAGGGTTCTCATAACGATATTCGTAATAAGCAATTGATGATCCGAGAA
GTTGATAGGAATACGTACGTCCTTTGGAGCAA
ATTAAAGCATGCTCCTGTTTGGTCATCCGTCGTAAACCCTCTCAGTACGGTCATGTCGGCTTTTGTGT
GGTTCTCAGACAGGGGCAAGGTGCATTCTCTT
CCTGTTTACCGCCTTGAAACACCAAAGAGCATCGCGGATACGGTTGAAATTTTAGAGCCGGAAAACAC
GGACTCCCTATATCTCCCATCTTCTCTCAACA
GCGACCAGCACCGGAGCAAAATGTCCTTTAATTTGCATGGATTTCCCAATACGTGGGAATTCGACATT
GTTGCCGATGAGCTTGAACTCCGTGAGCTTCA
AAACTTATATCAACAGTGTTCTTGGATTCATGGGTGTGATGGCTCATCTGGGAATATACCAAATCATT
CTCCCAATCAAAGAAATCCTGGAATGTCCTCA
CAAATGTCGCTTGTAAGCCGCTTCCCGTTGTCTCACACAGATACAGGGAATACATTGCTCCTACTGTT
TAACGTCTCCTGCTATGTCGTTTCGTTTCTAT
CAGCATTTCTGGCCGTACTCTTCCGCTGGTACTTGTGGAATACGACGGGCGTTGGGCCAGACGCCTTT
GTGGATCGCATCAGTAATTCTTCTAGGCCGGG
CTCGATAAAAATGAACCCACTGGATCCCAAAATCTCTGCGTCCCAAAGTGTGAAATCCATTGACAGCG
CATACAAAGAATTGAAGGCATCTCATGCCACG
GAGGGCGGGAGGAGTTTTGATTCCATCACTCCACTTGCGGAAGCTGAAATGCTCCCACCGAGCACAGT
AAACGTGGTGGGAACAAAATCAATGCATCATG
TAGATTCATGGTGGCGATACTCGGGAGATGACGAATTTTCCATAAGTGACAAACCCAGCAGTGAGTGT
CGCATTCAACCGAATTCAACTGTGTCATCTCA
ACTTTTTCAGCAGCAGTTCCAAAAGCCCGAAAAGATTGGATCCGGAGCAGAAGGAAGCGTTTTCCGTG
TGAAGCACCAATTTACAGGCGTTTCCTACGCC
GTCAAGGCTATTCGTATACCAGATGACAATGAGATATACATCCAAGAAGCCGTGCTGCATAGCACATT
TGATTCGGTTAATGTTGTGCGCTTTTTCAATG
CCTGGATTGAACGTGTGCCCCGGAACTTTGCGGAGACGCTTGGCATTCTTCATCGTGATGACACCATG
GACAACATCTCAGCGGAAACGCTGGAAAGTTT
TCACGAAACACCTTTTACGGAATCCGCCTCCAGTGATGCCTGTTATACCGTGCTGTTTATTCAAACGG
AGTGGTTTGAGCGCGGCACTCTGGCAGACCAC
TTTGTTAGGAGAAAAGGCTTTACTCGTTTGGAGAATTTAAAACATCTTCTTCAAATTTCTGAGGGACT
TCAGTATCTTCACTCGCAGTGCGTGGTGCACT
GTGATCTCAAGCCGAGAAATATTTTTATGTCTGACTCTGGGATTATGAAAATTGGGGATTTTGGACTT
TCGAGAAAAAATCGTAAGCGGCCCCATAAATT
GCGCGGGGATGCCGATGGGTTTACATCCAACTCGGAAGCAGGTGACGAGGGACACGCCATTGCCGCCT
TTACTCCTCTTTACTGCAGTCCGGAACAGAAA
CGTGGGGATGCTGCAACCACCGCGAGCGATATTTACTCTTTGGGGTTGATCGCTCTTGAGTTTTACTG
TGTTTTCACCACGCAGCATGAGAGGTTTTGCA
CACTGGGCGAAGCACGCCAGGGTGTATTTCCAAAGGAGTTTGCGGATACGTATCCGGTGGAGAGGGCC
CTCTTTCAGAAAATGTTGTCGGAGGACGAGAG
TTGTCGTCCATTGATGAAGGATATCGTTAAAGCCCTGCGACAGGGAATTAAGGAGGAGGAGGAGGTGA
TGGATGGGTTGATCATGCGGTCTAAGACGCTT
AGCCCAATTGACGAGTACGAATCCTCAAATCCCGCGTCTCCACTGGAAAAATTCAAAGAGAAGGGAAA
ATTAACACGGGTCGCTCCAAAATTTACCAGAT
TATCTTTGAGCTCGCTGGAATCCACAGAGTTTACTCAAAACACATGACGCGCAAGCAATTAAAAAAAA
AAAAAAAAAAAGGAGGGGATATATACATACGG
TTATATGTACATACAANNNNNNNNNNNNNNNNNNNNNNNNNNNNNNNNNNNNNNNNNNNNNNNNNNNN
NNNNNNNNNNNNNNNNNNNNNNNNNNNNNNNN
NNNNNNNNNNNNNNNNNNNNNNNNNNNNNNNNNNNNNNNNNNNNNNNNNNNNNNNNNNNNNNNNNNNN
NNNNNNNNNNNNNNNNNNNNNNNNNNNNNNNN
NNNNNNNNNNNNNNNNNNNNNNNNNNNNNNNNNNNNNNNNNNNNNNNNNNNNNNNNNNNNNNNNNNNN
NNNNNNNNNNNNNNNNNNNNNNNNNNNNNNNN
NNNNNNNNNNNNNNNNAAATATTTATATATTTATTTTGGGTTGCTGCTTTTATACGGACACTGAGGGT

TATTGCTACGACAACAACAACAACAACAAAAA
AAGAAAATGAAAGAAAAAAGAAAAAAAGAAAGAAAAAAAGAAAAGTGACACGTACCAAAAATGAAAAG
GAAAAAAAAAAGTTTGAAAAAAAAAAATAATGCA
AAGCAAAAATGATAACAAAAGGAATGAGTGTATGTGCATGTTTTGATGGACACCAATGGAAGGCATAT
ATATATATATATATACCGGGCCTCCACTACCT
GTGAATTCGTTTATTTATTCCGCCCCTCTTTTTTTTTTTTTTTTTCATTGACTTGTGTGGCTCACGCA
AATTCACTTTTCTTCCATTTTTTTCTTCACGT
AGGCCGTCACAGCCACTCACGGAGGAGAAAAGAGAAAAAAAAAAATTTATGTATATCTTGACAGAGGT
TTTTTTCTTTTTTTCTTTTCCGGTTGTTGCTT
TTTTTTTTTTTTCCGTTTTTTGTTTGCGTGTGTGTGTGTGTGTGTGTGTTGAATGGAGGAGCGAAT
TTGTATGAATTTCAAAGATCTCTGCGTCCAAT
GTGTCAAATTGGCCTCCACGCCGTACACCCTACCGACAGGATTTCAATTGCAGAGTGAAGTGGGGACT
TACTTGTTGTGTGCAGCTGTTGGCATTGGTAT
CGATGAACTGCGATGATTCTGTTTGGTGCATTTGGCGTGTGGTGTCGCAACGATGACAAGGATTTGGA
AAGGAGGAATTCGAGTTTATTTTGCTCTTTTT
TTTTGGGTATGTGGAGAGGAGGAGGAGTTGGCGAAGAAAAAAAAAAATTGAGGAAAGAACGAATGTNN
NNNNNNNNNNNNNNNNNNNNNNNNNNNNNNNN
NNNNNNNNNNNNNNNNNNNNNNNNNNNNNNNNNNNNNNNNNNNNNNNNNNNNNNNNNNNNNNNNNNNN
NNNNNNNNNNNNNNNNNNNNNNNNNNNNNNNN
NNNNNNNNNNNNNNNNNNNNNNNNNNNNNNNNNNNNNNNNNNNNNNNNNNNNNNNNNNNNNNNNNNNN
NNNNNNNNNNNNNNNNNNNNNNNNNNNNNNNN
NNNNNNNNNNNNNNNNNNNNNNNNNNNNNNNNNNNNNNNNNNNNNNNNNNNNNNNNNNNNNNNNNNAG
GAAAGAACGAATGTAGGTCTTGGTAAACATAT
TTGTTTATTTTTCTGCTTTTTTTTTTTTTTTTTGGCGTGCGTATTGTTGTTTGTTTCTTCTTTTATTCT
GTGGGTTTTCCTCCACTCCTTTGGGGACGCAG
ATGGAACAGTGGAAAGAAGGGAAACCTGATCTTTGTTGTGATTGCATCCAAGTTTTTTTGTTTTTTTT
CTGTTTGTTTGTTTCTCTTTCTTTCTGTGTGT
GCGTGCGTGTCTGGTCGTCATGCTTGTGTGTTTCATTTTGTCGTTTTGCCTCCTTTTGTTTTCTTTTT
CATTTTTGGTTTTTTTTTTTTTCCTTCTTTGTG
CCTCGCCACCCCCTTGTCGCTCACACTTCGCGCTGGATCCCCGGCGTGCCATTTTATCGACGGTTTGT
TTCTTTTTTTTTTTTTTTTTTTTCGTGTGTGTG
TTGTCGTTGGAGGGGGGATGTGAGCGGCGCAATGAGAAGAGGACGAACGGCAGATGAGTGGGGTGGGAG
GAAAGAAGGGCAACTCGAGTGTGTTGTGCGGC
CGGTAAAAAAAAAAAAAAAACAGAAATAAATAAATAAAAGATGTCGGGTGCTTTGAGAGGAGAGCACGG
CCTTGGGGAAAAAAAGAGAGAAAAAAAAAAAA
AAAAGAGGAAAAAAGAAAGAAGTGATTGGATGAATCGGATGGGATCGACACGCAATAAACAAAAGAAG
GAGGGATACGTGTGTGCAATACATTTTTATTA
CAGGAGTTATTTGTCCCTCATTATTATTATTATTATTATTGTGGTTGCCGTGATGTGTTTTTCCCCCT
AGTCCTCCGTGCTTAACGTGAGGCCAGCTGGT
GCTTGCTGCTGTTTATTTATTTGGTGTATTTTTTATTTTTATTTTTTGCTTTGNNNNNNNNNNNNNNN
NNNNNNNNNNNNNNNNNNNNNNNNNNNNNNNN
NNNNNNNNNNNNNNNNNNNNNNNNNNNNNNNNNNNNNNNNNNNNNNNNNNNNNNNNNNNNNNNNNNNN
NNNNNNNNNNNNNNNNNNNNNNNNNNNNNNNN
NNNNNNNNNNNNNNNNNNNNNNNNNNNNNNNNNNNNNNNNNNNNNNNNNNNNNNNNNNNNNNNNNNNN
NNNNNNNNNNNNNNNNNNNNNNNNNNNNNNNN
NNNNNNNNNNNNNNNNNNNNNNNNNNNNNNNNNNNNNNNNNNNNNNNNNNNNNNNCATTTTTTTACTTCA
AAGGGATTGTTTTTAAAGAAAAAGAAGGGGGA
CCAGTGAAATCGCACCTCGTTATTTTTATTTGTGATGCACAGTGGTGGGGTGCAGAAGGCGGCGGCAG
GAGCACCGCAGCTGCCGCCGACCGCCGCCTTT
TGGGAGGAAATGGAGATGCAGAAAAAAGAATCGCAGGAGCTTCAGGCGAAAATTGAGGCGTTCTGCAG
TCTTTTTTGCCCGGAGCAAATGCATCTGGCGC
CGTTTTTATCCGTTGAGTTTTGCGGTCGTGAGGAAGAACTTGAAGGAATATTGAGTGCCATGTACGGT
CACTCACTCTCGAAGATGCCTCGCGCGAAAGG
CGATGAGGAGAAAGAGAAGGGTGCGCACTCAACTGGTGGCGCGGTGACCGATTCAGATAACGATTTTA

TGGCGCAGGCCGTGGCTCTTTCATCTTTTTTT
GGTCTCGGTCGTCATTTTGTGCCGGCAGTGTATGCGCAGACGAAGCGTACGAGGAATCGAAGAAGAAT
GTCGTGGGGAGCGTGGCTGCCAAAGGATGAAG
AGGAGTTGGGTGCAGTGGAAGGCGTAGGAAAAGAAGAGGAGCGTCACATAAAACAAACCGTTCTGTTT
GAGGTGATTGCTGCCCTTTTCCCTGACTTTTT
ATGCACTGTTTTGGAGGAGGGCGACCAGCGTACTGCCTTTGTGGCTAAACGGCTAGCCGCCTTTTTAT
CGTTTCATGAACCTGAGAGTGACGTGAAAAAA
TATCTTGTGTCGCACGGCGGACGCACATATCTTGGCAGGAGTTTCCGTAACGTTTTTATGAAGCACTA
TTTCTCTTTTCTCAAGGAGTGCTCGGGAACAA
ATTTGTCTCTTGATGACCTTGACGAAGTGGGTTCTCTCTCTTCCAGTTCTGTTGTGACAATCGAATCT
GCGGGTGGTGGTGAAGACACAACGAGTCGACG
CCGTAAGACGAAGGCGGTATTGCGTGAGTGGACTACAGCATACGGGTTGCGTGCGCACTCCACTCCAA
TGCCCTCTAGATCGTGTCCTGAGCTTCATAAC
GTGGCAGTTGACACGGAGGAGCTAAGTGCGATGGAGCGAATGAGGGCAATTAAGTTGCGATTGAGCCG
CACGGAAATGGGAACGATGACACAGTCGCAGC
CACCAGCATTTGGCTTTGATAGCACCAAACCCGCAACGCGTCGTTTCTACGGCACGGGAACACCATTT
TGGGGTGGTGCTCACAATGAGATACATGCGGC
CGCAGCTCGATGGAAACAGAAGTGGCTTCAGAATCCAGAGGAGCACATATTGCCAAATCCTTCTATTG
TCTTGGAGACCAACAGCTGCCAAAAATGTCTC
TTTTTAGAGGCACAACTAGCGGAAACCAAAGCGGCCTTTCAACGTGCCTCCAGGAAGTTTGTTGCAAA
TGAGGAGCAGCAGTATTATAACGAGGCCTCAT
GCTATGTGAAGCAGAAGGAAATTCTTTATCCCTTTGATTTATCCGAGGCGAATGGCTCTTCCTCAAAA
AAAATTATGCCGCAACATTTTGTTCAACCGTC
TCATGGGTGTCTCATGTGTCAAGATTTGCGGCAACGCATTAAATGTCTTGAGCAAAGGCTGCGCGAAA
GCTATGATAATTGTCGTGAACTTATTGTCAAG
TTGCGCCGCAGCCAACTCCCACTACCGCCTCATATGTGTGTAATTTCTTCTTCTGTTAACGAGGCGAA
GAAAAGGGATGTTTCCACAATGACAGAACTGA
CCGGACAACAGTTAGATGAGTGTATTGGGTACATGATCCTGGAATCTTTGTAGGAGTGTCAGTGGGCC
CAGTCGCTTTTTACTTCTTGTGCCGTCAGCCT
ATAATACTATTATTTTCCTTTGGTGTATTTATTTATTTATTTATTATTATTTTTTTTTTTTTTGGGGG
GGGGGNNNNNNNNNNNNNNNNNNNNNNNNNNN
NNNNNNNNNNNNNNNNNNNNNNNNNNNNNNNNNNNNNNNNNNNNNNNNNNNNNNNNNNNNNNNNNNNN
NNNNNNNNNNNNNNNNNNNNNNNNNNNNNNNN
NNNNNNNNNNNNNNNNNNNNNNNNNNNNNNNNNNNNNNNNNNNNNNNNNNNNNNNNNNNNNNNNNNNN
NNNNNNNNNNNNNNNNNNNNNNNNNNNNNNNN
NNNNNNNNNNNNNNNNNNNNNNNNNNNNNNNNNNNNNNNNNNNNNNNNNNNNNNNNNNNNNNNNNNNN
NNNNNGGGGGGATGTGATAGAGTTTTTTAAAT
CTTGTTGTTGTTGTTATTATGGCCCACATTTATTCCTTTTTTTTCTTCTTTTGCCCCCCACGTTGATA
CGGCTGTCTTTTACTTACTTTGTAGTCGTTGT
GATTGGGAGGAAAAGGGGTATAAATAAACATCGAAAAGGAGTGTCCTCCGTGCCTCTCTATTTATTTC
TCTCTCCCTCCATCTCTCTCTGTGTATGTGGC
TGTCGATCCCTCCCTCACTTTCTGTCTAACTTTTTGCCGCTGGCATGATGCGTCATTTGTTATTTGCT
GCTCGCCAGTTATTGTTTTGGTTTGTTTTCCG
CAGCCCCCACTTCATCCTTTTCTTTTCTGTTTTGCGGTGCTATGCATCGCGGTCAGTTGCAAACTGAT
CGGTGCGGCCCCCCACGTTTCCCCACCAAGGC
ACAGACACCGAAACTAAAGGGAGAAGTGTGCAAAGAAATTGGGAAATGAGTCGTAAAGAGCAGGAAAG
TGTGACGACGACGGTTTACGCTGTGCGTTTAG
TGCACGCTGTGGTGCTGTGGTTGGCGGTGGTATTCTTTGGGATATCTGTGGTGTTCCTTAAACGTCGT
CTGCCGCGGGCTACTCCGATTTCTTTTTACTT
CTGCTGTCTGTTGGGCATCGGCGGTGTTTTCGTCTCCGCCCTCTATCAGCTGGCCGGTCCCCGGTCTG
CGCGCGGTGGTGGTGGTGGTATTTTTTGGGTG
ACTTGGGCGGGTCTTGTCTGCTGGCTTTTGCTTTTGATGCGCTCTGTGCGTCACGCAAATCTCCCAGA
CGTATTGTTCGTCTTCCTGTTGCTTGCGCTTG
TGGGTATGGAGTGCACCGGAATAGTACTGCAGTTTATCTCCACTCGTTCTCGAATTGCGGACATTCTC

ATGGATGCGTTTATTGCGGTGCCGCACATGCT
TGTCTTTGTGACGCTTGTGGGGTGGGATTGGCTGATGGTGAATGACATTGCCCTCGTGTTTCGCGAAA
ACACCCCAATTGAAGGAGGCAGATACGAGGGA
AATAATAAGGATGATGATGATGATGATGATGGCGGCCTTGGCCATGTCCATGACAAAAATCCCCATGC
GTTTTTTTTGCTTCCTCCTAGTCAAGACATTT
TCATACAGAGCTCGGCCGCATCCTCTTTGTTTGGTGAGAGTTTGCGCTTTGATGGGCACACGTCCATC
ATCCGAACAATCGTGCGTCACTGGGGTCCGAC
GTACTTCCTGCTCGCACTCGTGCGTCTCTTGTATGATTTTGGTGGACTTCTTCCGGCGTATTTTCTTC
GTCAATTGGTGGACAATCTTATGCAGGCGAAA
ACAAGTGGAAAGAAAGGGAAAGATGGAGGAGCAGACATCCACATGTCTATTATCAGTGTTCTGCTTAT
TGTTATAATTACCCTGACCTGCACGTTTCTTC
GTGTGCATTACAACATGAAACTACAGAAGATGTCGCTTTACAACCGAAATCTTCTAACCATCGAGCTT
TTTCACAGCGCAGTCAGATGTCGTCGACACCA
ATTAGGAAACCAGAGAGAAGGGGAAATTATGAATTACCTTTCCGTGGATGTACAACGTGTTGCCGATG
CTGTGCAAACAATAAATGATCTTTGGGCCTTG
CCACTTCAACTGGCATTTGCCCTTTATTTATTGTATGTTCAGGTATCTTTTGCTTTTGTCGCCGGGGT
GGTTGTCACAATTATACTCATTCCTGTAAATA
TGCAGCTGGCTAAGCGCATTAAACACGTACAGACCAGTCTTATGCGTGAGAATGACGAACGTGTACTG
ACTATCACGGAGATTATGAACAATATTTTATG
CGTGAAAATGTGCGGTTTGTCACATATCGTGCAGCGATGGGTGGAGATTCCACGATCACGATACATGA
AACATCTTTGTTGGCTTAAGTTTTTGGACGCC
TTCTGTGTCTTTTTCTGGGCTGTCACACCCACACTTGTGTCACTTCTTACTTTCATCACGTTTATCTG
GATGGGAGGAAAACTAACGCCTGGCAAAGCCG
TCACCGCCTTGGCACTTTTTGGCTCCCTCATTCTCCCATTAAATGCGTATCCCTGGGTGATCAATGGC
TTTGTGGAGGCGTATGTTTCTTGGCAGCGTTT
AAAGCCTTTTTTTTCCAGTTCAAAAGATCAGGGCTGTTTTTTTGATGGAACCACATCGGTTATACCTG
GTGCCGACGACGAAGTGGTGCTATGTGACGTG
AGGGAAAACATAATAAATACAAATTTACTAGAAGAGGAACAGAAAAGTCTGCACATGCCGGGGGAAAA
TCAACCCCTTTTGCAGGCAACGAATCAACTCA
TGTGTGGTGTGCATCCCGTGGAACAGCATCAGTTAAATAGTCCTAAACAACGGCAAGAGATTAGCTCG
GATGTTTTGGTCGACATTCAACAAGGGGATTT
GATTTTAGCGGCATCCAGTGGAGATGATGTTTTGGAGCAGTTGTTTACGTTGCGTATTTCAAGGTTTC
AGGCGTTGCGTGGTCAATTAATAGCAGTTGTG
GGACACTCTGGATCGGGAAAAAGTGCATTTTTAAGTAGTCTGGCAGGGGAACTTATTATGACACCAAC
GTTTTCACTTACCGTTGCACGGAGTTCCATGG
CGTTTGTAGAGCAGGCACCTTTTTTAATGACGGGGACAATACGTGAGAATATTCTTTTTGGGCTCGCA
TTTGATTCTGCAAGATACGAGTCAGTGATTAG
GGCGGTGGCTCTTGACGAGGATCTCCGTCATAACTTTGCCCCAGATTTGGATCGTACATTTGTGGGCG
ATCGTGGTAGAAAACTGAGTGGTGGTCAAAAA
GTACGTGTGGCATTGGCCCGCGCTTTGTATGCTAATAAGGATTTGTATCTTGTGGATGACTTTATGGG
TTGCCTTGACACGACGGTGGCTCACCACATTG
TCCAAGAGGTTTTTGTAACTGCAGCACAGCAAGGGAAGTGTGTCATTGTTGTGACTCATAATGAGGAA
CTTATACATTACGCGGATGCCGTGTATGAGTG
CGCAGGTGGATGGCTCGCCATCACGGAGCGATCGAAAGCCACATCTGCCTCATCCCCCACATGGATGC
CACGCGAAATGGATCATAAAGGACAAACGGAA
GAAGGAATAGGTAAAAAGACGGAGATTGATTCTATTGAGGACGCAAAACCTTCTTTTAGGGCCCTGGA
GGTGAGTGAGCATGGGGTTATTGCCTGGAGCA
CAATGAATTGTTACCTTCGCCGCGTAGGTTGGGGATTAACGGTGTTTATTGTCATCTCCGTTGCCGCC
ATGCAAGTTGCTCGGAACGCGAGTGATCAGTA
CGTTGTGGTATGGTCGAAAGATGGCGACGGGGATACCGCCGCATTTATACATGTTTTAAGTGCACTTG
CTGTAATTAATAGTTTGTTGGCATTTATCCGT
GGATTTTCTTTTGCAATTGGAGGTCTACGCGCCGCACATCGTATCCACAACGAGCTTTTGCATCATGT
TATGTCCGCTACGTTTCATTTTTTTTCTAATA
CACCACCTGGACGAATTATCAATCGATTATCAAGCGATATTTACACAATTGATGATTCACTTCCTTTT

ATTATTAATATTCTTTTGGCACAGACATTTCT
TCTTATGGGCTCAATGATGATTATTATTCTTAATTCCACGGGAATAATTTTATTGACCCTCATCCCAC
TTTCCCTGTTATATTACCGCGTTCAGCTTCCA
TACCGTATCGTTTCACGCGAACTGCGGCGTTTGGAGGCTGCAGCCCGTGCGCCAGTGCTTGATACCAT
GCGTGAAGTGCTATTGGGTGGAGTGGTGATTC
GAACTTTTGGAGTTTCTGTAGCTCGATTCTACATGCATCGGGTAAATGAGCAAGTTGGGGTTCTGCTA
CGCACACAATACAACGCACTGATGTTAAATTC
ATGGTTTGCTCTCCGGCTTGAATTGGTGGGTATTTTGTTGCTTATTCTTGTTGGAGTGCTTGCTATTT
ATTACCATGGAAACTCGCACGCTCCCATGCTG
GGATTGGCACTTGCATATGTTCAACCCCTGACATCATATGTAAATGGTGTTATTGGTGCGTTTAGCTC
CACAGAAAGGGATCTTATCAGCGTTGAAAGAG
TTTTGCAGTACTTTTACTTGGAACGGGAAGATGTCATGGACAGGGATGTGCCTTTGCTTTTTTTTGAT
TCGTGGCCCACTCGGGGAAAGATTGACTTTGT
GGGTGTATCTATGCAATATGACGATTCCGGCCCCAAGGTGCTGCAACGCCTCACGTTTCATGTAGAGG
CAGGAGAAAAATTGGCCATTTTGGGGCGAACA
GGTGCTGGTAAGTCGTCTATTTTTCTTGCCTTGCTTCGTCTTGTAAAATTGACGGAGGGATACATCGC
CATTGATGATATCGACATCTGTCAATTGCCGC
TGGATGTCATTCGTACCCGTTTGAATGTGATCCCACAAAATCCAGTCATTTTTCACGGCACACTTCGT
AAGAATGTGGACCCACTGGGTTTACACTCAGA
TGATGAAATCCGTGCGGCTTTGGCTTGTGTCAACCTTGCCGATCTTAGTCTGGATTCCAACATAGTTG
ACGGTGGCAATGTGTGTGGCGGAAAGCAACAT
CAAATTGCAGTCGCCCGCGTGCTGCTCAAACGATCGTCTCTTCTTCTGTTGGACGAACCCACATCTCA
GCTAAATTCTGAGGCGGAATCGTTGCTTTGGC
ATGTTTTGGGCACCCACCTGAAAGAAACGACGCTGCTGTGTATTACGCATAAACTATCACACATTGAT
TTCTTTGACCGTGTGATTGTTATTGACAAGGG
CCGCGTTGTGAATAGCGGCTCGCCTTCGATTCTTCGCCGTGATGGTGCATGGCCGTTTGCATAAAACG
AGGCCTCTTCCCCATTTCTTAGCGTTTATCT
TGGGCTGAAGTCGCGGAACGTGTGGAGCACACGAAGGGGAATAAAGGGGAGGGTGGAGGTCATTGTGG
TCGCTCGTCTGAAATAATGGCCAGTGGATGAA
GTCGTGTATTGTGTTTCACGCTGACAGCTCTCTCTCTCTCTCTTTTTTTTTTTTTTTTTTTTTTTTTT
TTAGGAAACATTTGTTTGTGTGTTTTGTTTTG
CTGTTTTTGTTGCTTTTGCTTTTTAGCTCTGAGTGAATATGAAGCCGCGGCGTCAGATGGTGACCATC
TTGACGGGTGCTGGCATCTCTGCAGAGTCCGG
CCTATCGACATTTCGCGATAAGAACGGTTTGTGGGAAAACCACCGCGTTGAGGAAGTTGCGTGCCAAT
ATGCATTTTTTCGTAATCCGCTGCTTGTTCAG
CGGTTTTATAACGAGCGGCGAAGAGCGCTTTTTTCAGATTGCGTCGCACCCAATCCAGCGCATTTGGC
TCTCGCCAAGCTTCAACGAGAGTACCGTGGAG
GCCGTGTTGTGTTAGTGACGCAGAATGTTGATAATCTTCACGAGCGTGCGGGATCTGTATCGGTGCTG
CATATGCATGGGGAATTGCTAAAGGTGCGTTG
CACAGCCACGGAGGAAGTGTTTGATTGGACCCAAGATGTGGTGCATGGTACGAGCCGATGCAAGTGCT
GCAATGCAGTGGATACGTTGCGGCCCCACATT
GTGTGGTTTGGTGAAATGCCGTTACACATGGATGAAATAAATGCGGCGATGGATGAAACGGATCTTTT
TGTCGCTGTTGGGACATCCGGCAACGTGTACC
CCGCTGCGGGATTTGTGACGCGGGCGAGAGAAAAGGGGGCAAGGACGTTGGAGTTGAATTTGGATTCG
GGAACGAATTTCAGTGAATTTGATGAATCCAT
CTATGGGAAGGCATCGGTGATTGTACCGGCATGGGTGGATAGGCTGCTTCAAGACGCGGTGTAGGAGT
CTGTGGGACGCATGCCCCTTTTTCCTGCCACC
ATTCACCCTTTTCCTTCTCTTCACTAATTACTGAGGCCTTTGCCACTTTGGTTTCATTTACCTCTGAT
ATATATCCCTTTTTTATTATTTGTTTTTGTTT
TATTTATGTTTTCTTCCCTTCCGTACATTCTGTGCTGATGTTTCTAACGTTTTCTTGGTTGGTTGCTT
GGGTTTTTCCTTTTTTCTTTTCTTTTCTTTTC
TCTCTGTCTTTTCTTCGGCGCCCTTTTGCCTCCGCTGCAAGAAGAAGTGAAGTGAAGCTAAATTCAAC
CGAAAAAGAAGAGTAACGCGGAGCGTCTCTCC
TAGGAAATTATTAAAGACAAAGGAAGAAACCCCGAGGATAAGAAAAAAGGAAACATATACATATATTT

ATCTACTCTTTGGCACCAGAGCCCCTTAACCA
CGGCCTGATGACGTTGCGCTCCGCCATCCGTTATATTGGGTTGGTTCAAATCCTTCTCATTTTCATTG
CATCGGGATTTCAACAGATCTCCAACCCCGCG
GTGAGTGCCGCCTTGTTGGCGAAGAGCAACTTTCCCACGATGTTGAAAATGGCCGGCGTTGGGTATCG
ACTTAGCGCCTCCGAATACACAACTTTAATTC
AGGCGTCTGGTGCACTCTTTCTTGGTTTCTCTCTCTTTATTTTGTTGGGGGTGGGTCGGTGCTTCTTC
GCCTTCATGTTGGCGTTGAGCACCGTCCTTGT
TACAGTTGCCTTCCACGTGGACCCTGAAAACCCGGGGAGGATAAGTAACACGGATTTTTTTCACGTGT
TGAAAAATATTTCCATTGTTGGTGCCCTTCTG
TTTGTTGCAGGTAGCGGCCAGCGGACCCACCGTTTTTCGAGGGCGCACCAGGAGGCAGAGACAGAAAA
GAAAAACCGTTAACCGCAATGGGAGTTTTTCT
GTTGTTGTTGTTGTTCTGTTGACCCTCGTTGGGTGCACAAAGTGGAAGGGGAAGGGGAAGGGGAAGGG
GGAGAGATAGAGGGATTTATTTTCATCTTTTT
TTTTTATTTATGTGTGCGTGTGTGTGTGTGGTTGCTTTCTTTAATACTCGTATTTTTCCCGCCGCCTT
GTTTTATTTCCATTTACACATTTCTTTTAATT
CCAATGAACATGGAAGGCGCTCTGCGCGGAAGTTTTTTTGTGGCGCAAACAAAACGCACCGCGGGAGC
CTCACGAACCACCGACAGGGGCTCGGAGGTGA
AAAGGGGGGAGATCACAGTACGAGAGAAAAGAAGGGTGCGACGCACGGGACGTAAAAAAAAAAAATTT
TTTTAAGGGTTTCTGCTGTTGGCATAGGGCAG
GGGACAAACACTGCAATCAAGAACAGAAGAAAAAAATGAAATGAAATGAATGAATGAATGATTTGAGG
AAACAAAAGACGGAAGTGCGTCGATGGAAAAG
AAGAAGAAAAAGAAATGGAAAGAATGTAAGAAAAAGAAAAAAAGGGGGGATGTGACATGGTAAGAGCA
GTGGAAAAGAAGAAAAGCGGCTGAAATAAAAA
AAAAAAAGAGGTTGGAAGAAAGGGTTAGAAAATAAAGGCGTGGAAGGACCCTTGTGGTCTCCATGACT
GCACGTGTGTGTATGTTTGTGTATCTGTGCTC
ATTTTCGACCCTTGTCTCCGATGAAAAAATGTGAAATGGTCTGTTTTTCTGCCAGATCCATTTGGAGG
CTTTTCTTTTTCTCCGCCCTTTGGAGCCTGCG
GCCTCCAATGACCCCAACAGGTGTTGTCTTCTTTTCCCCATTTTCTTTTTTTGCCTCTTGCTTTCACG
ACTTTTAATTGTGGTTTAACCTCTCCCATCAA
AGCGGGATCGTGCAGGTGAAGGGAGGTTGTTTTAGGGAGGGGGGGAAGAGGGGAAGGGGATCCAGCCA
AGGACGAAGAGAATATAAATAAGAGAAGGGAA
ACCAAAAAGAGGAGAACAGAATTGAGGGAGAAGGCCTCTTTCTTTTTTGTTTTTTTATTCACCCCCAA
AGGAAAAGAAAAGAAAAGAAAAGAAAAGAAAA
GAAAAGAATAAGTAAAATTTTATGCTCGTTAAGGTGAAGACGGTAAGCAACAAGGTGATTGAGGTCGC
ATCGCTCAATGAAGAGAACACCATCGCAGAGC
TCAAGGAGAAGTTGGAGGAATCAGAGGGCATTCCGGGCAAGATGATACGACTTGTGTACCAGGGGAAG
CAGTTGGAGGATGGAAAGCGTATTAAGGAATA
CCCCATCGCAGCAGGCGCAACTCTGCACATGGTGGTGGCACTGCGCGCCGGAACGTGACGTGAGGTTT
GTTTCTTATTGCCGTTTGGGTGTCAGGAGGGA
AATAAAACACGAAACTCACAAAGTGAAGTTTTTTTTTTGGTGAAAAAAAGTGTTAATAAAAAAGCGAA
GCATCAGGGAGTTTACGTCTCAATGTGGCGCC
TTAATCAAGAGCGTAGAATGGGACTAGCGTGACTGCAGTGTCTTTTACTCCCTTTATTATTATTATTT
TCTCTCTCTGAGTCTGTATCTGTGCGTCATCT
CCTTTATGCGGTGTCGTAAAGGGTATTTTATTGTTTGGTTGTTGTTGTTGTTGTGTTTGTGTGTGCGG
TTTGGCTGAATCACTCTTTGATGATTCAAGTG
ATTTGGGAATATCTTCTTCTGCGAGTTGTTTTTGTCTTTTTTTTTTTTTTTNNNNNNNNNNNNNNNNN
NNNNNNNNNNNNNNNNNNNNNNNNNNNNNNNN
NNNNNNNNNNNNNNNNNNNNNNNNNNNNNNNNNNNNNNNNNNNNNNNNNNNNNNNNNNNNNNNNNNNN
NNNNNNNNNNNNNNNNNNNNNNNNNNNNNNNN
NNNNNNNNNNNNNNNNNNNNNNNNNNNNNNNNNNNNNNNNNNNNNNNNNNNNNNNNNNNNNNNNNNNN
NNNNNNNNNNNNNNNNNNNNNNNNNNNNNNNN
NNNNNNNNNNNNNNNNNNNNNNNNNNNNNNNNNNNNNNNNNNNNNNNNNNNNTCTTTTTTTTGTTTTTG
GCTGTGTTTCTTATGTCTTTTGGGGAGTTTCT
CCCTGTTTTCTCTACTGCGTTGGTGCGTGCATCATGAAAGGACACTACTTTTGTTGTTGTTTTTGTCG

TTGTTGTTGATCTGAGGGAGGGGTGAGGGGGA
TTGAATAAATAATTGTGTATGGGTGAGGCAAATGTCATTTTGGCCTTTCCGTACAGGCAATGATGATC
GATTTCAAGTGAGCAGCAAGGAGGAAACAATA
TACGATGCAATCAAGGGTGAGATGCCTCCACACCGCTATGAAACAGAAGTCAGTAATTATTTAGAGTG
GCGCTGGCGTAGGGGTTTAATCCAAAGTGTCT
TTCCAGTGACATGTTATAGTGCGGTATTGGGTTTATCACTGGGTTTTCGTCAAGCACGCTTGGAAGGA
CGATATATCGGTCGCTATAGGGTGTTATGGCG
CTACAGTTCCACATTTGCTGCGGTAGGACTTTTGACCACAGCGTTCCATCATTTGCTTGTAGTGCGTA
ATAAATATAACGATCGGATGCATTATCCTATG
ATAGCTGGAGCATCGGGTGCCGTCGTATTGACAATGGCATCACAAATGGGAACCATTTCCCAAGGCAT
GCTTGCTGGCTCCTTTATCGGGGTTCTGTACA
CAGCTGGTTGTTATGGCATGACGTACTATCACAAGCGTCGTTTGAAAATTTTTTTGTCTCAGCAGCAA
CTGCAACAAGTGCCTATTCACAGGGTATCGCC
CGAGTTGCAGCCCATGTACAGGGCATACCTTTATGACAACAGGCCATTGGAGGAGGAAGATTTTCTCA
AACGACAGGCCGTTGTCTTATCGTTGGGCGAA
GAAGACGTGGGGTTGGACGCAAAACGCATAGTCAGCAACATGGTTCCCGAGATTGTGGACTGGGTGAA
CTTCCCCGATTGGTGGCCGCTGAAGTTTCCTA
CACAGACAGAGGAGGAACGAATGATTCTTGAGCGCAAACGCCACGAAGAGGTAGAGCGGCGGAAACGC
CTTATTTTGGAGTCGGAGGATGGTGCCTTGTT
GAAGCGCGTCAACAGAGCCAAAAATTACCGAGATGCGTAGCGATTTCTGCGGGTTTGTGTGGGAAGGG
AAGAAAGATTGACCGTCCAGGTGGAGGAGAGG
AGCGGTTGATGTGAGTGAATGTATTTTGGGTGGCACAATGACTGCAAAAGGAAAGCCTTTTCTTATGG
GAAGTTTTTTTTTTTTCCTTGAGGAAAATAAA
GAGGGAAAAGACAAAAGGAGCAACAACGACTCTTTTTCGTTGTTGTTGTTGTTTCTTAGCTATTGGTG
CACACATAGAAATAGATAAAATACATTCACAG
AGAAGGTGAGAGAGAAGGAGGTGCAATTCCATCGATTGTTTGTTTTTCCTCTGCCGTTTTAGGGGAT
GCCAACGTCTGGTTGTAAATTTCGTGTAGCGC
TTCAGCGCTTAAATATTGAGAGGCTCCTCCCTCCTCCCTTGCGTGAAAAGAGAGGCAGCAATACAGTT
GGCCGTATTAACAACAATAGCGGTGGTGTCGA
GTTGCGTGAATCTGTCAATAAAGATTTTAAGGACGTGTGTTCCGCTGTTGGGAATGGTGCTCGTGGGG
TGTATTGCAATGGGTTGTCCTTTCATGTGCGG
TGGGACCGCATTCAAAGAAACCTGGCAGTTTTAGGGCGTGTTTCGAATGGGAACACGGTGGTTCCTTA
TCGTGATAGTGCTGAGGCCTTTCGAGTCATGT
GGAAGGCTGTGGATGAAGCCAAGCGAAGTGTTCTTTGGCAAACATACATTTGCAAGGATGATCTTATT
GGCCAGATGACAGTAGAAAAAATAGCGGCTGC
GCAGCGCCGTGGGTGTCAAAGCGAGTTGCTGTACGACTGCGGCGGAAACATCACGGGACGACGTCGTC
TCATTGACAAGCTCAAACAGTCTGGGGCCAAT
GTTATTGAACATCGCCCTGTTTTACATCATGTTTTGCCGTATTTGTTTGGGGGCATGAAGTGGGAGCG
CAGTCCTGCCATCCGAAACCACCGCAAGATCC
TCATTGTGGATGACAAAATTGGTTTTTGTGGTGGACTGAATATTGGCAATGAATACTGCGGTAGGAGC
CAGGGCGGGACGGGTCGCTTTCGCGATACACA
TTGCTCGGTAGTGGGCCCGGCTGTGGCACACCTTCGTGAGGCGTACGAGGACACGAAGGCACCAAAGC
CGTGGCAGTGGAGCTGGACACGTTGGCGAAAA
ATTGCGTCCACGACGATGGCTCGTCGCTACAAGCAAGGAAGAAACGCGACAGATGATCTTCTTGGACC
CATGACAAAATCTGTGCAGCAAAGAGGAAAGT
TGTACCTGCAACAACAAATGGAAAGGGCCGAGGAGAAGAGGGTAAGATTTATGGAACGCATTCGATGG
AAGCGGCAGCGCGAGCGATTAGAGTTATTTAT
GCGGTGGTATCCAAAAAAAGCCGCGAAGTTGTTACCACAAAATTTGGTTCATGTTGACGTTGAAGATG
GCACTAAATTTCCTACGGAACGCGCAGTGGAC
ACCGGGGCATCAACAGTTATGAAGCAAAGGATGGAGCACTATCGTATGCGTGCACTTAACAGAGCCAA
GGCGGCGTTGGAACGACGGTTAGCTGAAAGAC
CACTGTCCAAGGCGCCACTGAGTGATGCGGATCCTGTTCCTGGGGCGGAGGAATATGGCAAGATACGG
TCCAATGCTACTCAGATCATATGTTGCAACCC
TCACACACGGGACTGGTCGATTCCGTATGCCTTTTGGCAGGTTTCTCAAAAGGCGCACCGACGATTGT

GGGTAACAACCCCTTATTATTTGCCGCATCGA
AAATTGTTGCGTGCTATTCTGTATGCTGCTCGTCGTGGTGTTGATGTACGCATTCTTGCTGGAAGTAA
TTGTACAACCGACCCTTGGTTTATGTGGCACG
CGTCGAATTACATTACCGAACGACTTCTAGCCGCAGGTGTTCGTATCTATGAATACAAGGGGGATCAG
ATTATGCATGCCAAGACGGTGGTGGTTGACAG
TATTTGGTGTTCCGTGGGAAGTTACAACTGGGATATGATGAGCAACAAGAACATGGAAGTCTGCCTCT
GTCATTTGGGGTATGAGATGGCACGGGAAATG
GAGGGCCATTTCCTTAAAGATCTGCAGACAAGCATCGAGGTAAAATATGAGGAGTATCAGCGTCGTTC
CTTTTGGCTTCGATTTACTTCTTGGTTTTTTT
ATGTGGGGCTTCAAATTGCGGAGAAACTGACGTTTCGCACCTTTTGGGATGCGGACCTCTCTTCAAAG
GTTGACGATCTGGACTAAACGTGCGGTGCGCC
CTCTAACACCTTTCCTACGCACAGACACACAAACAAACAAAAAACGATATCAATGGCACGCAACAACA
GCCAATAAAATAAAAATGGATGGCTGAAAAAT
ATGCAGGAATGTGTTTATTGTTGTTGTTGTGTACATTTGATATCTTTTCTCTCTCTCTCTCTCTCTCT
TTCTACTTAATTGGTTGGGTAATATTTCTTGG
CTGCTTTTCGTTTTCCCTTTTTTTTATGTTTTTGGTTTTTCCTTCTGTTTGTTCTTCTAGAAAAGATG
TGCCTTCGATTGTGGAGCCTCAGGAAGCCTGC
AATAGAAATGTGCATTGATATATACATGCAGACAACGGCTTCCTGTTGTTGCCGTTGATGTTCTTTCA
CGGCACTGTTCCACATGATTTTTGTTCGAATT
GGTGAATGAAGAACGACCGGCTTTTTTTTGTTTTTTTTTTTTCGTTTTGGGAGAAGGAGGAAAAAAAA
ACAGGAAAGAAACGAAAACGAAAATTAAATAA
TAATAAAAGTAATAATAAAAGTAAAAAAAAAAAAAAAAGGGAGGGGTCAGTGGGGGGAGGGGCGGGTAC
AAAAATTAATAATAATTATTATTTTTTTTTTC
CCCCCTTCTTTCCGTCATTTTATCCTTTTCCGTGACGATATTTGTATAGATATGACTTTGTGTGTGGA
ACGCAGCGAAATTTGTGCGTTTTTTGGGAACT
GCGCCGGATGTTATTTGGTGTTGGTGATGCATTGAATGCCGGTCATCTTTTCTTTTTATTGTGTGTGT
GTGTGTGTGTGTGTGCCAGGGCGGTGTGAGCC
TTTTTTTTTTTTTTTTTTTTCTTCTTTCTGCCCTTTTTCTCTTTCTCTTTCTTGTGGCTGCGGTTGGCG
TGCGCAGGTTTGAAAGACAAAAGGAAGAAATT
TAGTCAACGGTGCCTCTTGACAGCGTCTTTGACTTTACTTTTTTATTTGTGTGTTTGTGTGTTGTTTT
ATTTCCCATTCTTTTTTAAATAAATAAATAAA
ATTTTGCTTTGGGTACTTATCATCACAATTATCGTTGGTCACTGCCACGGCGGCTGCAGCTTTGTATC
GGGACAGTTGGCGCCCCTACTGAAATGAAAGG
TGCCGGAGAAAGGAGCTTCCTCTCTCATTTTTTGCGTGCAAGCGGCCATGGCGGGGCTGTGGACGACG
TGTATCTGCTGCTACTGACAGCAATGAGACGA
CTCACACGCGATCTTCGGGACCTCGTTTTCAGAGCGGGCATGTCGCCTGCACGTCGTGAGGCATGTGA
CAAACTTGTTGAGGACCTGAAGGATGTTATCA
AAGAAGTGGCGGCTGTTCCTGAAGACCGCGTCGTGGGGACCACTGGCGAACTTAAGACTCTACACGTC
TTTAGTCACGGCAGCTTCGTTATTGACACGGC
CCTTTATGACTCGGCTGTTGACATTACGCTCGTAAAACAAGTGCATCCCTCTATAGACTACTTTCTGC
GAAGGAACGTTGGCAATAACGGTGCGCAGAAG
TTAACACAAAGGGACTGTTAAGAGCTTCTATAGCGAACACGGAGGCCTCTATTCTTTTGCCGTATCCC
TCATGCTATTCCTTGGCGCTGCCATTGATGCA
GGAGCAAGACTTACTGATCAGCATCAGGGATGTTTTTGTCGGTCGACTGGGGCTGCAAATAAAGCAAA
ATGCCGCATTTTGTAGTAATAATGCACGGACA
CTTCGCTTAATTTCCTCGGCAAACCACATTGACGTGAATGTAACGGTTGACCGAACGGAAGCCGTAGA
GGCATCGGCAGTACTGCAAGAACTACTGAACG
ACAGCAAATCAGCACACCCCGTTATTATTTTTATGAAGGTGATTCTAAAACAATTTAACGTTCATTCT
TCGCAACTCACCCCGTATGTCTTAACATTGAT
GGTTGTTGCCTTCTCTAGGTTTTGCTCTTACATGTCTCCGTTGTTCAGTTTTAATTATTACCCGCATA
CACGCGAGACAGTTGAGCCAGGATATCTTTTA
TTTTCCTTTCTTAGCTTCTTCAGTCCTCTTCCGCGGGGGCAATTTGACCCAACACAGATGTGTCTTGT
CCCTATTCACCCGCACGGTGTTGTTTACGAGA
CGAACGAAGGGGATCATTTGTCACAATTGGGTAAGAACGTTGGCGGTATTTATTGGCGTGTCATGGAG

CCAACAAGGCGTGGTGACAACGCCGCAGCCTC
GTGTGTATTGATTGATAAATGTCGTGACTTGTTTGAGCGTATTCTTGTCGTTTTGCTTCACCATTACA
CTGGTGTTGTCTTTACTTGTCCAGTGTTTGTT
GACGATGCCCATGCACAAATTTTAGGTGCGGAGGAAGTGCCGAAGACGGAGAAGATTCCCATCGACAA
TCACCTCATATCTCTTTTGCTTCGGGATTGGT
GGCTCAAGAAACAGAGTGAGTTTTTCTAGTCACTTACTTCTTTTTTTTTTTCTTTTGATTTTTTTCTT
CACACGGGCGTGGGTTTTCGGTGGTGCTGATC
GGATAACGTGAACTGCTCTGCCTCCCATCTCACTTCTTTTCTTTTTTTTTTTTTTTTTTGGCGAATTA
TAAGGCCGCTTTTATTTTTTTTTTTTTTTTTT
TAAGGGGTTTGACGCTGGGAGGCGACATGACAGCAGAGCACTGGGAGAGGGGAAAGAAGATCAGGGGC
GCATGTACGGTTTTCCCGTTGCTTGGAGTGTT
TGCCGAAAAGAAGTGCGAGCCAAGGCATGACTTTTGTGTGCGTGTGTGTGCCGTCACTTACGGCGATC
ATATATGCGGGGGGCGTCTTTTTTATTTTATG
TTGTTGTTGTTGTTGTTGTTTTAAAAGTATATTTTTTTTTTTTTTGCCATTCTCCTGCTCCCAGTCTC
TACAGTCACGCGCACTCTCTTTTTTCTCCCTC
TCTTTGCGTGTGTGGGAGTTAGGAAATAGAAGGCATGTGGAAAATCCGAGTGTGGAGTGCATTTTCCC
ACCGCACACTCACCCTTGCTTTACAACGGCTT
GACGTGGTGTCTGCGGCGTCCGCTCACTGTTTCTATCCAGCATGGCATGTTGCAAGGCGAGAATGCAC
CACAGGGAGTGAAGGGCATCCACTCCAATCAT
TGAAGGATGCCGACGCACCACCACAAACAACAACAACAACAACAAAAATATCAACTACAGCATCAACG
AAGAGCAATGATATCGACTGGGACGACAACAC
TGTTGTGGAGGAAATTAGAAGCATCCTGACTTTGGAGCTTTTACCAAGAAAAGGGTCTTGTGATGAAG
GGGGTGACAATACCGTGCCTCTTTATGCGCTG
TTTCGTGCACTGGGGCCGACTCTACGGTCGCAGCTGCAGAATTCCTCATTTGGGGCACTTTCCAAGGC
AGTGAAAAGCAGGCCCGATTGGTTTATTATTA
GTGCAGATGGCGCCAGAGTGGGACTCACAGACGCTGCGTACTCATGCCGTGCGACCAATCACGATGCG
ATTGAGAGCAACAAGAAGGGCATTGATGGCCT
TCGACAAGACAAAAGTTGTCGTGCGAGCACCGCCGCTTCAGGTAATGTCATGCAAACGCCTTGCCTCG
AGAAGCGCGCGATCACGCGTTCAAAAAGTTAT
GTGTTGGTGCGCGGATGCTTTGGATCGACCCCGCTGGATGTCTTAAAGCCACCTCTCACGTCCGCGGA
GATTGAGGAGTGGAAACTTACACGCATTCTTC
TCCCTGTCACCCTGTATTTTCTTATGGACAAGGAGCCGATAACGCTTTTTCCGTATGGTGAAAAGCCA
CGACGTTTGTCACCCGATCTGGAGGAAGCCGA
GTCCCTTTATGTATCGAAGGGACTTTTTCCACCCTCTGAAGTGGCAGCCGCATTTGTGCACATCACAC
CCACGTTTTTTGTGGAAACTCGTCACGTTCTT
GCTGCGATGTCTCCGGATGTGGCTCAGTGCTTTGAAAGTCACATTTCGAAGGATCGCGTCGTGGATCT
TTTCTTTAAAAAGTATCCCATGATATTCAAGA
TTAAGACGGGCAAGTTTCAGAAGACGGCGGTGAAACTAAATCTGGATTTCAGTTTTATTCAGAATTAC
CCGGGGTGTGGGAGGGCGGATCGTTTATTGCG
TCGCCACAACCAGGAGTACCGAGCGACAGTAGGCGGAGTTGTCTCGCGACAAATCACCTCACCCTCAC
GTGAACAAGAGAAGCAGGGTCGTTCAGGAGAG
TCTCATGTTGACGTGAAAATTTTCGAGATTCTTTTACGAAATTTGCCTCGGTTTCCGAGCGACAAATC
ACTTTCAGGAGCAGAGCTGGAGGAAAGACGTT
GTGCGCCAATTCCCCTGGTGAAGTGGATTGACGGTTTCCCTCAGGAGGATCTGGACGTACTGAATAGT
GTCCCACAGCGGCGGGTGCTAACATTACTAAC
GCGATACTCACATATCTTTCAGTTGGTGTGCCGAGGCGAGGATTCAAACCTGTTTGCGGAGCATATGT
ATCTGAATTCGTCAAAGAACTCCAACACACCA
CGACGCGCTCAACAGGACGATGGAAGTAAGAATAGTTTTGTGCGGCCTTTGGATTCAACGTCCGTCTG
CGAGTTAAATGAGATTCCTCACGCAGATGAAT
CTACTGTTGAGGAGGAGAAGGCGGATAAATTGGAGGCAGACACACCGCTTCCACCTGAAACCGAAATG
AAACGACGGGAGGCAAACATAGCGGCGGCGCT
ACGAGATGACTTGATTGGTGTGGATGACATTCTCAGCTCTGATGCGGAATCGGTACCGACATTGACAG
ACGAGGGAGAGGATACGGAGCTGGGCGACAAG
TTAAATACCGATGAAGATGAGGTGTATTCCGCGGAGGGTGAAGAGGGGGGTGAATTCTTTGACGCGAA

CGAAGTTAAGGAGGAGGAGGAGGATGTTGATG
GTGGCGAATCGCAAAGGAATTTGGAGGAACGTGGAGGAGATGGTGTAAAAAAGGATCCACCGCTCACG
GACATGTCATACGCCGATGATGAATCTGACGG
TCCTTATATCCCCTCTCAGTACGATATCATTTATGTCCGACGTCTTCCAAGAAGAATTGCGCCACGAA
GTCTGAGTGACTACAATGCCGCCAATTCCCCG
GAGCCAGAGTTACTGCAGCATATTGCCTCGTTTCTTAACCCACCCCCTTCCTTGCGGGAAGCAAAACA
ACAGATTATGCAGTCGAATAGAAAAGCACGCA
AAGTCCCTCTTTTTACCAGCGGAAGTGCTGTCGGCCCGTGGCGGTGGGTGCCGATTCAGCGCATTTAT
GCCTCCCTAAGTAAAGAGCAAAAACGTCTTTT
GCGACCCTTTAAAGGACTTGTTCACTTCATGCGACTTCATGGTGAGATCTTTGAAATAAGCATGGACT
TTTTGCATGTTATTGCCCATGATCCAGAGGGA
CAAATTGCACCGTTTGTCCCAACACAACTGGTTTTCCATAGCGAGGATCGGGTGCTTCTTCCACAGAA
ATTTGATGATGATGAGAAATCAAAGGCGTCGT
TGATAGCGGATTTGGAGCGAAATAAATTTAAGAGTATTTTGGGTGCCAGTCAAATTCCAACGGATCGT
CCACAATTGTTACTGCTGGATCCATTGAATCC
CGTACTTAATCATGAGATATTATGTGAGGAGGTTTCTCTTTTCATGCCGGATCACCCTGTTCCGCTTC
ATCAGTTGATTAATCGTCTACCACCAATTTTG
AAGGCGGCACTTTCTTTGCGACACAAAAACAACTTTAAGACCAGTAAGCATCTAAAGGTGTGGAATGA
GGGCAACCGCACGATGCTGCAAAAGAGTCAAT
TGGCCGTGCCGGAGTCGGCTTTGATGGGCGAGGAAACTATTTCTGTAGAGGATGCTATTGAATCGGTA
CGTGAGGTGATACCAGATGAAGGTGTGATGAT
AGGACATCTGCACCGTATGCTTCCCTATGCAGCTAAAAAGACGTTAAATACACACTTTGGAAATGTCT
ACAACGCCATCCTTGGGTATCCACAGTACTTT
TATATTGATAAGGTGGAGGAAAACAGATCAAACAGCACGGTTTTTCTTGTTGAGCGTCTTCAAGAAGA
GATACAATGTTGAGAGCCAAGTAAGAAACCTT
TGTTTTTTGTTTTGGGTGTATTTGTTTTTCTTACTGAGGCGCGTTTATGCTTGCGAGAATGTCCTGTT
GGAAAATGGAAGAGAAAAGGATGTTCACCTTG
CGTGCTCTCCCCTTTTTTTTCTTTTTTTTTCTCCTCATTGATGTGTAGTTTTATTTGTGTGTTGTTGT
TGTTGTTGGAATACGAATCTTCACATCTATTT
ATTTTGTTGTTATTATTAAACTGGATTGATTCCTGTCGTGGCGATATCGTTATTGCTTTGAGGGGTTG
TAAAGATAAATTGAGCGAGTATGTGTGAGAGT
CCACGCGCTGGAGTCAAATTTGAGTTTTATCGCGTTCTTATCCTAACCCGTTTTTGTTTTTTTTAAAT
ATATATCCCTCTTGGCCTTTGCTTGGGGGTGA
ATGTGGGCGGGTATTTCTTTGCCTTTCACACTTCTTAGGGGCTTCGTGAAAGGAATATTCGTTTGATG
GACGAGGTGCTCGATGAAGAGGTCGGCGTACC
GTACTTAACTCCCGGACAGGTCCCTTTGCAAGCAGCAGCAGTCTTACCTTTTTTAACACCACCGGCTG
GGGCGCCGACCTTCCCTCGTTTGTACGCGGCA
TCACTGGGGGAGGAATACATCGATACAGCGGAATGCGTTGAATATGCATCGTCCAATGACGCTGGATT
TATGGTGAAATCTTGTTTTGTTCTTGTGGGTT
ACGGAGGTGTTTTATGCGGATGTTTGGCAGACAGTCTTGAGCCCAATGAACCCACTGCGCAGCGCAGC
TTCACTGAACTACTTGAGGCCAAGATAAATGA
AACAAGAAACACGGATTATTCTTTAGATACCATAACCTTTATTCCATTCTTTCCCGATGATACGCTGG
ATCCTGCCTTGGGGTCTTTGTTGGGAATCTTC
AGTGTGGTAGAAGATCCAACGTGGTGTCTTTTTGTTAGTCATGCAAAAAGAGAAGAAAATGGAAGTGA
TTGTATTTTACATGTCAAGATATTGCGCATTC
CCATTGTTGAAGAAGCAGATCGACTACACAGGGCTTTGAATTCCTTGGTTCTCCATCATTGTGGTGGG
CCAAATGGAAATAGCATTTGTCGGTCTCTCAT
GTCAGTGGAGGAACTTTTTGCCACTGCGCGCAAGTACTCAGATTGTGTTGTTGCGGGGCCTGCATGGG
ACAGACTATTTTCTTTGCGTTTGACAAATGAA
GAAATTTTTTGCATTGCAAAAAAAATGATATTGAGTCAGCCGACTTTTTTAATGGACGTCAATGGAAC
AAAATTGGAGGAGATTCCGCCTTTGGCAGTTG
CCGTGAGGATTGAAGCGTTCACGAATGTACCGAAATCGGAAGGTGAGTCGGGAGGAGTTGAAAAAGCC
GTGGCATTGGAGGAGAAAGGGACCGCGGCGCA
ACAACGTGAGGTGCTGCGGGAGTTGGCATCCGTGCGGGCGCAGTACCGGGCCGTTCTTCGTGAGGGAA

ATGATCTGATGGCTTGCATCGATGAGGAAGAG
CACAGGTGGAATAAGATTGAGATGCATCGAGAAGAGGAGCAGCGCAAGCGGAAGGAACACCCGGCTGC
GCCGCCAAGTGAGCACGATGAACACATCAGGA
AGAATGTATGCGCATTAGAGACTCTGCAGCAGAAGAGGGAGAAGGAGGAGGGGGAGGAGGAATCGGCA
CAGGCGACCGCGCGGGCGTTTGTGGCAGAGAA
GAAAAGGAAGAAGACGGGAAAGGGCAGTCGTGGCGGTGGTGGCAGTGACAAGAAGTTTGCGTCTACCA
GCAATTTTACCAGCAAGGAGGCTGAAATACGG
AACGTCAGAGCTCACAACACGGCCCTTCGCAGACTTCTTGAGAAATTAGCCCACGAGCTGAAGGAGGA
GGAACAGACAATGGAGAATTTGTCTATTGACC
TCCGTGGATTTATGAGCGACATCAACAAACTAGAAAAGGCAAAACTCCTCCTCACAGTTGAGCACAGT
ATGCTCATGAATACCACCGCCCGTCTGCAGCA
GTCCTTACGGGATCAGAAGGAAAAGCTGCGCCAGGAGCAGGAGGCCGCCGCCGCAGAGCAGAGGGAAA
TTTCAAGGCGTCTTCTGGTACAAAAGGAGCGT
ACGAAAAAAATCACTGCATGTGAAGAACGGCACCACGGAACTTCTTCATCGACTAAAAACAAACCTGAG
CAAATAGGTGGATGTGGTTTACAAGGCTCCAT
CCTTTTTTTTTTATTTGTTGCTGTTCTTTATCGTCAATTGTCTTTTTATTATCGATCACATATTTTGC
CTTGACGCCGTATCTTCCCCTGCATCGCGTGC
CTCTTTTAATTAAATAACGGCACAAACGCCATTTGATATGAAGTATTATCAAAACAAAATGGTGTTTG
CCCAGACATGTATCCTCACTGCATTTTTCTTT
TAGTCACGTTACCCGCTTCAATTATCTCTTTTGTATTCTTTTTTTTCCCCCTTTTTTTTGATGTGTAC
TCCTCCCTGCCACGTCCAAACTACACACACGC
ACGACACTTTGAATGCAGAGGCTGTATGAAAAGGGATTTGAACCCCAACGCTGGTGAGTTTCACCCGA
CGGGTGGGCGACTCACCCCAAAGCCCTCATTA
AATGAGGGGGGTAGTCGTTCTTCATCCTTGCAAGGTGGCTCCGCACCCGCCGCAGCTTCTTCGTCCCA
ACAGAATAACGCAAATCCTAATGATAGAACGC
CCTTTCAAGTTTCCGAGGGCCTTGTAGAGGCATGCTACTTTGTGCCAGCGACACGATTCTTAATGGAT
TGTCGCGCCGCATTCTATGAACACCCGGAAAC
CACTAAATGTAAGCACATGGAATTTGAACGTCTACTGCGTCGTTGCTTTTTTGGAGCACATGAAGCCA
ATGAACTGAGCATCTCCTTGGCTTCTGCGTCT
GGTCAAAATAGACCATTCTGGGTGTCGTATTCTGCGGCCTATTTTCGGATGGCGCACGACGTCGTACA
GGAGGTTGAACTTGGGACGCCCTGTCCTTCCA
CTCAACTGTTACCCCCGGCGGCGGCAGGCGCTTCACTCGCAATGCCGATGATTCCACCTAACTTGACA
AGCTTCACAAATAACTCGACGGAGGAAGAAAG
GCAACGTGAATCTCTTCGTGAAGTTTATCTGTCAAGGCTTCGACCTGTCGTTCCGTATTCTATTCGCA
AGCAACTGGCTTTCCGTGCCAAACGCCCTGGC
GTCGATTTACAAGAGGATGTGACGAGGGCTATGACTTACGCTCTTGACATCTTTTTTTTCTTTACGCG
GTTGCAATTTCTTCGGTTTTTAGATGCATCTT
CTGGACTAAGCTACTCTGCATGTGTTCAATTTTTAACCAGACTACGCGAGCGTTTGATGAAGCTGGAA
GTGGCCGATTCCTTTCTAAGTGTTGCCCTCGT
CCTGATGTTAGTTTCCACCGCTCACATAACGTTTGCACCACGTGCGGTGGAAGTTGCTCTCAACGAGA
CAAATTCTTCAAAGTTAAAAGCGCAGTGGCTC
TTCTGCAGGTATTTTTCGGCATTGGTTGTGGCATTCCTTTCTGGATCATCGTTGCAGTCTGTGGAAGA
GCTCCGGCATTCGTTTGTCTCTACAAAGCAGG
ATGATTCGCTGCCGGTGCTTCGAGCATGTCCAGGGGCTCTTGCTGCGATCCGGGAGGTCATATCTTAC
AACAACAAAACAGCGATGTTGTGGAGTATCAC
ATTGAGCGAGGTGGTAAAATATGCTCTACGACACTTGGGACAGACGGACCGCACAAAACGCCCCAACA
GAGCCGTTCCTTTTTTTCTTTCAGAGTCCACG
ACGCTTGACGATCACCTGCATGTAAAGTTCCCCACAGTGTTGGCCGCATTGTTGGGCTCTACCAACAC
CGTAGCGGAGGAGAAAGAATCACAGCATGTAA
CGCAACAAAAAGGTGGGGTTAGCTCACGTTCCAAGGAGTTTCATGCTTTGGCAACGAAGGCCGCTTCA
ACATCAGGTGGGAGCGAGTATGAAACTGTTTA
TTTCAGTCTTCTTGCTCTGACAAACATGGGGTGTTTTAGTCCGACTGGGCTTTCATGGTTGTTTAAGG
CGCTGGAAGACTCTTACAATTCCGACAGCCAT
CCCAGCGCACTGGATGCATTTCTTTCTTCCACCGTGTGCGCGACGGTGTCGCCAACAGCACGGGAGCT

TTTGGATGTTCTTCGCACGCTGATGCGGGTGG
GCCACAAGCCGCTGCAAcTTCAGGCTCTCATAGAATTTAAGGGCATTCTCGAAAGGCAACAGAACGCA
CTGCCATCCGACGGCACCCCAACGAAGCAACC
AACAAAACCGTGACGCGAAGCGAGGCGAGGCAAGACAGCAGCAACTCCCCTCCCCCCGAAAAACGAAA
GCACAACAACAAAAATAATAATAATCAAAAAT
AAAGAGAAAAAAAAAGAAAAAAAGGGGCAAATCACCTTTAAAACAATGCGTTGCACTCACTCACATAT
AAAGAACAACGAAGTAAGAAGAAGAGGGAAGA
AATGAAAATAAAACAACAAAAGAAAAAGAAGAAGGAAAGAAAAGAAAAAAAAAACGGAAATTGGAGGG
AAAAAAAGCGAAAATAAAAACGGAAGAGGACGA
AACTAAAAAAAAAAAAAAAAAAGTGGGGGCGGATGGTTTAGAGTCCGTACGATTTAGTACACTTATTCT
CATTGTCTTGTTGTTGCTGCTGTTGTGATGGA
TGATTGCCATTTTTAGTGCTTCCTTTTGGTAATGCGGAAAGAGGAAATAATTGCAAGTATGAGCCTTA
TGAGGGGAGAGGAACACCGCCGATGGCCACAG
AAAAAAAAGAAGGAATGATGAAGGGGAATTAGCGGATGTTTTAGAGAGTAGTGGATAAGGTAAGAAGA
GTCAGGTGTATTCCTGACTCATGTTTATATAC
ATACATACATATATACATGTGTCTGTGTTTGAGTCTGCGGGGTGCCTTTTTAATATTGTGAGAAATGG
GAGTAGCGGAAGAAAAAAACTCTCTGAGCAAA
TTTTTTGGTTGCGTGATTTTTTGGTTCTTTTTTTTTTTTTTTTTTATGTGAGGTTTGTTGTGTTTGCT
TTGCCGTCGTTTTCCTTTTTTTTTTTTTTTTT
TTTTTCTGTGGGTATGTGTGTGTGTGTGTTTCTTTCAAACCCTCCATTTCACGGAGGGAGGAGAATTC
ATGCACGCGGATTAAATTTACATTCCCTGTAC
GGCCGCATATCACTAGCGCCATCTTTAATGTTGGTGCTTTTCTTCTTTTTCTTTTTGTCGTCGTATTC
ACATCATTCTTTTTTATTATTTTTATTATTCT
CCGTTTTTTTTTGTTTTTTTGTTTTGGCGTGTATGTGTATGCGCTTGACTCGTTTGTGACCCTTTCGG
TGTTGTTGTGCCTGTCTCTTTTGGTGGGCTTT
GTTTCCTTCTTTCCTTCTTTCTTTCTTTTTTTTTTTTTTTTTTAATTGTGTGTCCCTCTAGTATTATT
TTTTGGATGGTGCCGTTGTTTTTGTGAGGGGT
GTGTCTATTTGTTTGTTTCTTCCCCTTCTTCATCCTCTGGTGTTGTGTACATGCATCCGTTTGTGTGT
GTGCGTGCATGACGGGGGCGATATTCGTGTGG
ATGGGAGGAACGGGAGGGTACAGGCGTGTCCACGCACGAAACACTTCGGGCTTGCACAAACATATATA
TATATATTTGTGTGTGTGTGTGTACACATGCA
CCAGCGAATCTATACATGCTCCACTGACCCACTCACAGTTTTTTTTTTTTCTAAAAAAAAAAAAGAAG
GAAATGAAGCAACACATGGCGGNNNNNNNNNN
NNNNNNNNNNNNNNNNNNNNNNNNNNNNNNNNNNNNNNNNNNNNNNNNNNNNNNNNNNNNNNNNNNNN
NNNNNNNNNNNNNNNNNNNNNNNNNNNNNNNN
NNNNNNNNNNNNNNNNNNNNNNNNNNNNNNNNNNNNNNNNNNNNNNNNNNNNNNNNNNNNNNNNNNNN
NNNNNNNNNNNNNNNNNNNNNNNNNNNNNNNN
NNNNNNNNNNNNNNNNNNNNNNNNNNNNNNNNNNNNNNNNNNNNNNNNNNNNNNNNNNNNNNNNNNNN
NNNNNNNNNNNNNNNNNNNNNNNTGTCTTTCTC
CTACCCAGAGGGTAGGGTAGAGTTTTTTTTTTTTTTTTTCCTCCCCCCTCTGTGTCTGTGGCGTAACT
CCCGTTACCCTGTATATACGTCTATAGTAAGG
AACATAAATAAACATGTATGTATGTATGATTATTTGTATTTTCCTTTTCAGCTTCCTTTCTTTTTTTT
TTTCTTTTACTTCCTTTGACAGCAATTTGGCG
TTAACTGTGACCCTTGTTGCTGGTTCTAGGGAGTAAGGGGGGAAGGGTGGGGTAACGCTTGAGATACC
ACTTAGTCATCGTATTTGTTGTAAAATCAAGT
GAAGGAATTGCAATGCCGCCGAAACTCTCTCCACTCCCGCGCAAACCGACTGTTCAGCCAAGTTTTCT
CTCTGCCGATGACGACATCTGGCTTGGTAACA
TACACGTGGGGCCAGGTGGAGTGGAGTTCACTGGAGAAGGAGATCTGCACGTCCCTTGTCAGGACCTC
TCCCTCGACTCGCTGGATTGTGTGAAGTGTTT
GGGAAAAGGCACCCAAGGCAAAGTGAGTAAGTGCATCAATCAAAAGGACGGCGCTGTTTATGCGGTGA
AGCGGCTCCTTATTCCCAGGACGACGGACCGA
ATGAATAAAAGCACCGTGGCAGGAGAATTACGTAACATTTTCTCTCAGGGAACCAATGCATACACCGT
GGAGCTTTACAACGCCTTTTATAGGGACGGTA
CACTCTCACTTGTTATGGAGTATATGGACTGGGGAAACTTGGAAGAACTGATCGCTATCTGCCCAACT

ATTCCGGAGCCGGCATGTGCCTACATTGCATC
ACAAATTCTGCATGCCTTACAGATCCTTCATACCAAGGCGAATCACCCCACGGAAACCGATCAAAAAG
GACGCCGTCAGATTCATCGTGATATCAAGCCA
GCCAATGTAATGCTTTCTAAAAACGGTGTTGTGAAACTCACGGATTTTGGCATTGCCGCAAGCACAGA
AACAATTGGCGTAAATTCTTTTGTTGGTACTG
CTACATACATGAGTCCAGAGCGGATTCAAGGACGGACTTACAGTACACCAAGTGACATCTGGAGCGTG
GGAGTATTAACTGCCCAACTGCTGCTTGGACA
TTTTCCCTTTTCAGCCGCAGAAGGAGGATTTATGGCTTTGTTACGCCAAGTGACGGAGTGTTGCTCAC
TCTCTTTAGTTGATCAGTGCGCATGTTCTCAA
AAGGCGGAGAATTTTATAAATCATTGCATTTGCCGAAACCCTGATGATCGCAGCACTGCGACTGAGTT
GTTGCAACATGAGTGGATTGTCCAAAATGCCT
CAAACGGGAAGGACGTTCTTACAGAGCTGCTGGGCACAGTGGGTGAGCCGGTGGCTTCTGCGGAGTAT
GGCAATTCCTAACATGAGGTTTACCGCACATT
CCCACACGCACAGACACGCTATCTTTCTCTCCCTCTTCACAAGTGTTTGTGAAAGCGTATATATATAT
ATATATATACAAATATATACATGGCGGGATAA
ATCAGGCGAACGGATTCTTTACCATCCCATTCCATTTACTCACCCCCTATTCTGGACACGATCAACAA
TTATTGTTTTTTCTTTTCTTTTTTTTTTTCTT
TTCTTCCCCTGAAAGTGTGTACAAATTTATGCATAATTGTTACTGTGTGCATATTACGTCTTGAAGCA
TGGCAGGGAGTGAGAGGGAGGGAACATGCAAT
GAAGCAAGAAATCATTAATTGATTGCTTGACGTTCCTCATGCATTTGTTTCTCTTTTTTTTTTTTTTT
TATTGAATTCCAAACCAGAGGGCGTCCATACG
TTTATATTTTCCGCCGTGCAATTCTGTGACGACGGGAAACTCCAGGTTTTATCGAACTGCGCGAGAGG
GTGGAGGGGAGTTTTGGTTCCTGTTGTTTTTC
GTGTTGTTTTCCCCCAATTAGTCTTTTCCAACTGGCCGGAGGATGTATTTTTTTTTTTTTTTTTCTCT
TGTCTCAGCGTGGGGTGGGTGGACGGGGGGAA
AAAGGGAAAAAGAAATTAATAAATAAATAAAAAAGGAGAAGAAAAAAGGAAGGAGTCTTTTCAGAAGC
ACGGGAAGAAAAAGGACGAAAAAAAAAAATGCA
TCAGAAGAAGCGCTTTTTTGAAACATTTGCATTGGAGGTTGAGGACAAAGATGTCAACGCCATCACCA
GCCAGCTTGAGAAAAAGGGAGGTTGCGGCACA
TTGTCTCCCGATTCTGGGAAGCACAAAAAGCTTAACACGACGTCTTCTTTTACTGGAGGCACACATGT
ATCATCCCCGTTTATGCAGCAGAATGACAGCA
ACGTCATTGAACCTCTACGCATGCTTCATAACTGCATTCGTCACTTTGCAAGTCATCCCGAAACGTAC
GGAAGTAATCTCCATGCCCTGGTGGATCGGGA
AATCTGCCATGTCATGCGTGTAAAGGCTTCCGAAGCGGCGCTTCTTACACCAGGGATGGTTCCTGTCG
CTAGTTACCCGCTTCCCGCTATCAATAATACT
AAGGCAAGGAACAATAAATACCAACACCCTGCAATGAACAAATGTGCGAGTTTAATGTTCTGCCCGCG
TTCCATCGAGGACGCGGTTTCTGCAGGCACGG
TGGCTGGGGTCGATGGGATGTTTGGATTTGGCGCCACAACCTTCGTGGAAGGCACGCCCGGCTTTTTT
TCCCTTCTTGATAAGGCTAGTCGACTTCGGAC
TCTACACGAAGAGCCTAATGCGCTTGAGATAGCTGATACAGCGGGCGACGTTTCTGCACTGGCTTCTA
GTGCCACGGATTTAGTGACTTTGGGGAAGGAC
TCCTCTTTACAAGACGGCACGCCACAACAAGAAAGCACACGAGTCAGATTTGTTGCTTTTTCTGATGG
ATTTTCCGGAAATACTCCCTCTTCCTCCGCAT
TACGTACCGGTGCCGGACCAAAGCTTCTCGTTGATGGAACAAGCTATGAGGTGTCGTTTTTGCGATCG
CAGCTCCAACAAGTCGAGGCGGCGTACAACGC
CAAATGTATTTCCCACCATGAGCTACAGCAGGAAAATGCTCAACTGAAGTCGGAACTAAGCAAGACAG
AAAAAAAGCTGCAGCAATACACGACGAAGAAT
CAAGAGATGCGGGTACAACTAGAGAGCTTGAAACGAGAACTGGACGCTTGGAAGCAGCGTAGCAGTGA
GTTCAACTTGGCTGGACACGCTGGCAACTCTC
ATACCTCGTCCGAAGGATCACGGCGTGTGCAGGCCATGCAGCTTGGTCATGCAAAACGTGAGTTGTCG
CACATGTCACGTCTTTGGCGAGAGACGGAAAA
GTCTTTACAAGAAACCAAACGAACCTTGGAAAGCGCAGAAAAGGAGACGCAACTTTCGCACCGTTATC
TAGACGATGCGTTTTTCTTAATTGAACGCCTG
GAGAGACGAGTTTTGCGGCGTGACCATTATATTGAGATACAGCAACGACGTCAGAGCTCCTTGGAGGA

AAAATATGAGAAACTCATGTGGTGTTTTGAGG
AGCTGCGAACAATGAATGGGGGAAGCTCCTACGTAGACTTTCTCCTCTCCCAAGATAATGGATGGTCA
TTATTTCTTTTTGTGCGTCTACAGCGACGTTC
TATGGGATATGCCCAGATGGAAGAACCCTTCCCGGCTAACCCTGGACCCCTTTTCTTCCGTCGCACAC
CATTTGGTGGCAGATTTTGTGATATGTACAGT
CCGAATCTTGTTTTGAAACTTGTAACGGATTACACCCAGAGGAAACTTGGAAATACGGAGGGAATGAC
AGAAATGGCATGGGGGGTTTCTATTCCACGCT
GGCGTGGAAATTATATCATTGATTACACTCCTCCTTCTGTCGGTGGAAATGGTGAGGGTATTAATACA
GAGAACGTAATGACATCTTGGGAAGGTTTGTA
TCGTAGCTTTCCAGTTCTTTCATTGGCATCTGTTCTTCTTCCGCAGCCAAGCACCGCATTGACGGGCA
ATGTACCCATAGAAGAAGATATACCCAACAGC
GCACAATACGATCAGGCAACCATTCGTTTAATAATATACTGTTTTTGGAGTGAGCGGTTACTTCAGTA
TAAAAAAGAAATGGAAAGGCGTGTGAGGGTCT
TCAAATCATCACGTAGGGAGCCCAATTCGGGTCAATTGCAAGGAGAAGCAAAGGGTGAGGGAGACGTT
ACAGATAGACCCATTTATCCATCAACCTTCTT
GGCTGCACTGGTCGATTTTGTGCAGCGTACCTTTCCTCTCAACACGAAGGTGGCAGAGGAAAATGCTG
AGGAGGAAACCAAGAGACTCGTGGTGCGAGGT
GTGGTTGGTAAAAACGAGAAAGCCGTTGATAGTGTACGAATGCTCTTTTCTACTATTTTTTATATCAT
GAAAGATGGTAAAGAAGTACCCGTGGTACTCT
CATCACAGGCCCGTGAGTTACTATTTTCATTATACTATTATGCTGAGGAATACAAGGAGGTGGACGCT
GACTTTCGACTATTTTATCTTGTTGCCCATCA
GCAGGTTCCAGAATTGGTAGCGATTAATTTCTGGGCCAGCCTAGAGGCTTTGCGCAAAGATTGTGAGG
AGCTTCTCTGTCATCATATTGGTCCAAGCTCG
TCGTTTCGATCTAGTTTGAGTAAAATGCATGATTCTGACACAGCGTTCTGCAAGAAGACACCTCAAGA
GGAGTTGATTCATGACACCCCTTTCAATGCTC
TAAAAAGGGCAGTGGAAGTCATTGACACCGCCCCGTTGGTGGAACAGAGTACTAGTATTGAACCTGAC
AGTGATGGGGAGGGATATGGTCTCATCCTGCC
AGGGAGCAACGTCAGCAGTTCTGAAAAAACATCTTCACATGACCGGGCTGCTGAGCCTGCAAGGTCCG
ACTTTTATACACTCAAACACAACATTCATGAG
TATTTGTTTCGTTTCAAGGAGGAGAGCGCCGTATCCTCTGCAGTACCGGAGAAACCTGAAAAGGGAGA
CACTCCATTCAAGGATGTAGCTAAAGACAAAT
CCGCGGAGAAGAAAGAAATTCCGACGGCGTGGAGAACTCTTGATGAAAGAATCGGAGCTGTTTTAGGA
CCACATGCTTCCTTTTTGGAGGCATTTAAAGC
GAATCAGGGCCCAGATATATCCAAAAAGCAAAGAATTAAGCGGCAGTCTTTCAATAAAGAAACGACAT
CATTTTCAGCCTCACGTGGACTCCTTCCTCTA
GATGAGGTTTTAGAATTAATGTACAAACACTGTTTTGCGACGTATGCCGTTTCCTGCTGTGGTGCGGC
ACGTTTTTCGCTCGTAGAGACTTTAACACGAA
CTGGAGACAGTTCTACCGTGGATGGTATTTTAAATTCGCTGGAACATCTCACAAAGGTTGGCTGGCTT
CCGCCTACAGAGACACAGATGCGACGTTTGCG
ATTTGCCATTTCGATGGATCAACCATCGACACTTGTGCGCTTCACGGATTTATTTGATGTGGATTCCC
GTTACGGTTTACCCTCGCATTTTCATGATGAG
TATCTTCAGCTAACATTAGATGCATACATGGAGCATCAAGAAGCATGGATGAATACCATTCTGAATTT
TGTTATTCCAGTGGATGATGGTTATGGTGTGA
GAAATAGTGATGAGATTGATATGCTTGTCCCCTTTCCAATCATTCGAAGGGCCGTCTTAAACGTCTTT
CATCAATTATTGCAGAAGCCTGAGCAGGGCAA
TACTCTCGCAAAACACTTTCTTCTTTACTGTAATTTGCTACGGGAAGATGAGAATGTTCGTCTTGGGC
AATTTACAGATGCTCCATTTTTTATCAATGCC
CTTCCATTATCTCCGGCGGAGGCTGAGAGCAAATTTGCCAAGATCGAAAAACGGCGATGGCCTGTGGA
GGAAGAGTCCGCTTCATGCTCGCTGTTGCACC
TTTCCTTAGCAATTCGCATGACCCATATTATATGGGGCTCCTCTTCCACTCCATCTGCAAGACTGGCT
GGGTTGGTACTTTCTTCCACCGCCAGCGAGCA
GGCGAGGGGCGTGGAACTTCCCGGTGGGCATGGCACTCCCTCCAAAGGGATGAGAGATGGTGCATTTT
TAAATAATGTCAAGGAGGAGTTTTCGGATGCT
CTGCTCCGAGTAAAGACGGGGGTGATGCAACACAACGTACTGCGTCATTTGGCTGCAGAATTTACGGG

AGCAATGAGAGAAATGGAGGAAACGCCTTCAC
CGTTGGATCTTACTGTTGATTCTAAAAGTGCGCTTGAAGCCAGTTTCCACATTCGCCGTCTTTATGCT
CCCTGGGATGATGACCAACGACGGGCGTTACC
ATCACGAGGAAGCGTGCCCCGCGGTGGAAGGCGATCCTATACACCGCGGCGAATGACGTCTGCTTTTT
CTGGATTAAATTTACAGCCAGAAACGACAGTA
GACCGTCATTATACACCGGAAGTGGCGTCGCTGACCCGATTCTTGCGTCTCAGCCGCATAGGTAAATC
CAGGGGTGATAAAAAGAGGAAGTCGAAATCAC
CGAGAGGTTTTCAAGAAACGGAAACAATCTTCTTGGCGCCTTGTGCATCTTATGAGACTGCGTATATG
TCTCATCAGGCACGACTTGTGAGACGACTCGT
AGAGGCGGCGAGTACGCCACAACCTCTCCCTGCAGTTTCAGCCGAGGAGACGAGTGCTGGACCGCAAC
TGCGGACCGACAGTGAAACGGCAATAGCTTCA
TCCTGCTTTGCGCCGGCTGCGGATCGTTTCAATTCCATTGACAGCAACATCGTAGAAGCCGCTGTCGC
TGGGGCTGCGAATATTAGTTCTATGAGCACGA
CTGATGATTCGAATATTGCACTCCCAGATGTAATTTTGGATGAAAATGGCAGCACTCAAAAAGGAAAA
GAGACAATAGGGGGAAGAAACGCGGTTTATCA
GAGGCATGTATCGGTGCGGCAACTGCACGCCGGTTGTGCAACTTTGAGTCTGATGTAAACAATGCAAG
CAAGAACCATTAAAAAAAAAGGAAGGAATGAA
GGAAAAGAAAATGAATATTAAGGAGAGGCATATGCTCGGAGGAATTGAGAACAATTATTTTTGACGGA
GTTTGGGGTTGTTATTCTGTTTCTGCTGATAA
TAATGTACAGGATTGATTGAGTTTCATATACGTCCGAATTTGCGATTTTGTAATGCTTTCATGTAAAT
ATTTATTTGAGTTTCCCTGTATATTTGTGTAT
CTTCCCGGGATTGCCTGCCATCTGTTTTTTTTTTTTCTGGTTTTATTTATTTGGATTTATTTGTGTGT
GCGTCTGTTCTATATGTGGTTTTTAATATTAT
TTTGAATGAAGCTTTAACAATGAGCGGCATTTTTCGCTAAAAATGTTTTGGGTTCCGTAGTGCATGCG
TTCCTGTGTTATGGCAGAGATGCGAAGGTTGG
ATGATGCTGACTGGAGTGCGCCGAGATTTTTTTTTTTCCTGCATTTTGCTGTGGTTTTTGTTGTTTTT
GTTGTTGCCGCTTAAATGTGTGTTAAACGATG
GCGTGCCCTTTTAATTTTACTCCTTTAAAAAAAAAAAAAAAATCCCCTATTGTGTCCATGCGCATGCA
TGCCCCATTGATGATTGGGCATGGAAAAGTAA
GGAAAGGGAAAGAAAAAACAAACGGCACGGTAATATATTATAGGGCTCCGGCAAATGGCACAAACGAT
GGACCGCGGTGCAGTTTCGGACCCAAACTCCA
TTTTAAACGTGGAGTTGGAAGCGAAGAGTGAAAGCTGGATGGCATTCATGCGGCGTCTTTGCGCGGAG
CGGAAAAGGATTTTTCCCATGACTGCTGTGTT
TATGAAAAATGCACTTATGAACGCAAAGGCCCACAAAGTATCATACTGCATAAGTTTTTCTCCGTAT
TTTTGGTAGTGTTTCTGTGTGTGATATTAATG
TCGACGATTTTTAACCTTCCAATCGTTTTTCTTCGACTTGGTGAAGTACACAACGGGAAGAACGATCT
CGCCATCACAACCGGAGGAGAGCTGCATTCCG
CATCGTCGTTAAACTATTCCATTGTTGAGGAAATATTTCCACTAACGGATCCTCTGCGCGGTTATCAT
TCTCCTCGGATCAGATTTGGGGGATACATTTT
GAAATATTCCACCTGTTCAGGGATAACAAAGCCGCAGGATTTATGGTACGATAAGAAGAAGGAATTAT
GTCTTCCTGCTTGTTTGAGTGAATATTGCGAT
GGCACCGAAACTCCGACTGTGATTTTTGCTATCAACGAGGAACGTGAAACTCGAATGGGTTTTGGGAT
ATCATGGAAGGGGCGAAAATTAAAGAAAGGGG
AAGTGATTTTGTCGATCGGTGTGGCTGTAGCTGCAAATAACACAAGGGTGGGGGATAAAGTTCTGCTT
CTTGCATCGCTCGGCAATCCTCTACATGAATT
TTTTAAAAGTTATCCTTCACTGATTAGAAATACACGTACGATTGTGCCTTTAAAAGTGGTGGAAATTG
TCGTTCCAGACCGTTCCAAGTTTGATTCGGAT
GATTTTGTCGTTCTGGACTACGATACATTTGTTTCTATGGTTTCTGAGGGACTTTTTCCAAAGCACGA
CCAGGAGGATGTTAAGAAATTTGGGGAAAGCA
ATCCAAAGTCGTGTGCGACCTCCATTCATTTTAACTTGGGCCCAAGTGAGCGTACGAACACATACAAG
ACCACGGACTATGCAACTGTACGCCTACGAAT
CTCGAGTTGGGTTAGTTCAATTGCCGAGCCACTTGGTTTTAATCAAATAACCGTGAACACGCCTATAT
TGAATTTTTTGTACACGGTCCGATTTTTCTCG
CTATTTGTGGAATTGATCGTCTCCATTATTTTACTATCACTGGCTTTTTTGAGTATTATGCTCATATA

CTCGCTGCTTAATTTAAGTATAGAGAATCAGG
TATATGAGCTGGGTGTTAGGCGGATGATTGGATTTTCAAGAGGGACTTTGGTGTTCATGCTTTTTGTG
AATGCATACGCCTTTACCATACCCGCATGGAT
ACTTGGACTTATTGCAGGACAGGTGGTGTTTTTAGGGTTGCGCTCTGTGTTACGTGATATTATTGGTG
TCAATTTACCTTACATTGTTCCAGGACAAGCA
TTTGGTTGGGCGACATTACTCGGATTGTTGCTTCCTCTTTTTGGAGCCTTACTTCCTGTTATTAATCT
CCTGAGACAAAATCTTCCGGAAGCTCTGAATG
CCAGTCGTGGACGTAATGTAGGTGTTATATACAAAATCGAGCGCGGGGGCTATTCAAGGTCAGTAAAT
TACATGGTGTTTGGTATCGGAACCGCTTTTGC
AGTCTTTGGTTTTCTGTTGCATTACTTTTTCCCTATTGGTTTGATGCACATGCGGCTTGGGCTGCTTT
TCTACATATTCTTTGGTATTTTGATTGGCATG
CTAGCGGGAATGGTGCTGTTGTCGCTTAACTTTGAGAGGACGCTTCAAACGGTTTCATCATACATCTT
TTTTTTCTGGGAATCAGCTGCCGTATTCCGGG
CTTTTCAGAATTGTCTGGTGGCTCATCGAAAACGGAACCGCAAAACGACATTGATGTACGCCTTTTCT
CTCGGTTTTATTATATTCATCACCATTGCCTT
TCAAGTTCAACTGAGATCGTTTCAGTACGGCATCCGGAGATCGATGGGGGCGGAGGTGGTGGTTGACT
TTGAGGGCCTGGACTTGTTTACGTTTTCCAGG
ATGTCACATTTTATAAAGGCGGCTCTCCCTCAAGTCTCAGCGGTGACGTATCTTTCCCATACGGTCAC
TGCAGATTTCAACATTCAGAATGTGTCATTGT
ATTCGCCCGGGCGTTATCAGGGGGTTTTCATTAATTCGCTCGTTGCGATATCACCCAATTACCTCGAT
GCTCTAAATAAGGATTTTTTGTTGGTGAAAAA
GTACAATAAAGAAAGTCGAAAATATTCCATCTCCGGTGCTGTATATACGGAAAAGAGGAATCAGATTA
TTGTATCTAGCTCTGTCTTTTCGACGCTTCAA
TCCAGCTCTCTTGAGGATCCTGAAATGCTTGTAACGGCTATAATGACGGATGCAAAGGAACGATCCGT
CGAGAATGTGCGTCATGCCGTAAAGCCATCTG
CAGTGCTGGATGCAGCTCCCCAAGTATCCATGACAAAGTTTCGTGAAATGGATGGTGTTGTGTTGACT
TCATATCCTTCCTTGACGCGTTTCAGCGGGGT
GGAATATTTAAGTGTTCGCAGCATCAAGATATCCACCGTGAGTCTACGAGTCCCAGACTCTCGTTACG
TCAATTATGTCTCCCGGGTAATTGAAAAATAT
CTTCGACGCCGCCGCATTACCAAGACTTCTGTTCGTGACGACGGCAGAACAGCACAGTATTTGGGTAT
CGCAGATCGTACACTTGGTTTCTTTTTTATAT
TTGTACAAGTCGTGACGCTGGCAATCTGTTTCTTTTCTTTGCTTTCTAGCATGACGGCAAATATATCC
GAATCAACTAAAGAAATTGGGATTTATCGCTG
CATTGGTATGACTAGATTTCAGATCTACAGAATTTTCATATGGGAATCGTTTGTTGTTGTCATTGCCT
CCGGCATTGTGGGTATCATTGTGGGCTTGACG
ATAGGATACAGTATGCAGCTTCAGAATTATCTCTTTACGCAACTGGAGATCCCGTTTGCACTTCCCTA
TACACAATTGCTAATTGTTCTCTTCATGGCGA
TTGGAGCGGCTCTCTTGGCGAGTTATCCCCCAGTTGCATTTCTCCTTCACCTTCCATCCATCACTCAT
ATTTTGCGTCGAACTACATGACGTGCGCATTC
GGTACGCATGGAATGTATCTACCCCTTTTTTTCTTATCCATTGCCTACATGCCTTATAAGGCTTACAG
AGAATTTTTAACATTTGCCTTTTTGTGGTGGT
GGTTTTTTTTTTCTTTTTTTTTCTTTTTTTTTCTTTTCTGGGAGCGCGTGAAAATAATGTTTGCACGA
GGTACATTGGCTACCTAACCAATTATATATGT
ATATATACACTTTTCCTGAGAAAACCAATATATCTCTGTATGACTGTTCGTCCCTATTTTATGAAATT
TTGGTTGACTGCGATTGAAGGAATCCGGGAAC
CGATGGTGGTAAGAAGAAATCATTTATTTTTATTTTTATTTTTACACTCACGCCCGATTTATTCATCT
TGACATAAAGAAATTGCAGTGGTTTCTTTCAT
AAGACGACGGAGTGGTGCCAAATCGGCGCCCGCCTGTTGGACTTTGTACGTCAGTGAGGCGATATTCA
GGTGCAGTTTATTTTTATTGGTGGGCATGGGC
CGTCGGTGCAACAATGACAGGCCTACGCTTGGTTTCAGGAACCATCGCCAAGCACTGGGAGGATGCGA
TGTATTGCGGGACGTATTGCGAGTCTCCGGAC
GCCTCAATTGTCAAATTTCGAGGCTCAGTTGCTCCTGCCCATCGTGTGCGGGCTGCCCGGGGGTTGTG
GGTCCAGCAAGGGCTGACCATTGCTGGAAGAG
CGGAGACTGAACACCGACCACGCCAGCGCCGTCGCCGACGCTCGGAATCGGCGTGCGCAGCTTTACTC

GCAGGTGGAAGAGGCGCATAATTACTTGTCGG
CGCGCGAATGTCACATTACGGTGGCCGGCGGTCCTCTTGCGGGACGCGAAGACGCCGTGCCAACGAGA
AGCGCCCACCTCTCGGAGAGGGCCGCCAGATG
AGCCCCCACGCATGCATGCGCGACTATGGCGTCAGCACACGGCCACACCAACCGCATCGTTTGATGGC
TGGGGGTGTGCCAGCATCCACGTTTCCCCAAC
GCACTCTCACGGGAGAGAGACGGCGGGTCAACACTCAAACGTCTCAGGGCAATGTGGGGAGGCCACAC
CTCCCTCACTCGATGCCTTTGCCCTCCCGCGC
TTCCCCCTTGGAAGATGACTCCGGTCCAGAGAATCAGTTACTGCATTTTCTTGCGTTCGACGTCACCA
CTTGGTTCCCGCGTATATGGAACGAGGGCGTC
TCCGGTGTCCTCCTCGTCGACCCCCGATGCGCATACAGTTATTATTGGCACTAAATGGACGCTCAGGT
TCAAGTTGCCTTTGACGGTTTGGTATCGTGCA
GGCGCGCGCTGGAGATAGCTCGGGATCCCACCGATCAGCTCATGGATTTTGGCAGGGCCCTACTGCGG
ACCTTCCAGATCCAGATCATGATGGATCCGAC
CCGGCATCCCGCTCCCNNNNNNNNNNNNNNNNNNNNNNNNNNNNNNNNNNNNNNNNNNNNNNNNNNNN
NNNNNNNNNNNNNNNNNNNNNNNNNNNNNNNN
NNNNNNNNNNNNNNNNNNNNNNNNNNNNNNNNNNNNNNNNNNNNNNNNNNNNNNNNNNNNNNNNNNNN
NNNNNNNNNNNNNNNNNNNNNNNNNNNNNNNN
NNNNNNNNNNNNNNNNNNNNNNNNNNNNNNNNNNNNNNNNNNNNNNNNNNNNNNNNNNNNNNNNNNNN
NNNNNNNNNNNNNNNNNNNNNNNNNNNNNNNN
NNNNNNNNNNNNNNNNNCGAGGGCAACCCAGCCTCTCGTGTAACGGCGAGCTACCCAACAACAACAAA
AACGACTGTTGCGATGCCGATTGTGTCGTATT
TGTGGACACGACGCATCTGCATGCAACGTGCGCAGCGCAGCCAGAAGAAATGACTTGCAGCACGGACG
GCCGCCAAAAAACGGCGAGGGGCTGCCGGCCG
CTTATGAAGGCGGAAGAAAGCAGCCCCATCCCTTCCTGTATGGCGTCGCACCGCACCGCCGATGCTCC
GCCCGATCTCTCAAATTGTCCGACGCAATCGT
CGACAATGGCAATGGAGCTTATCCGCCAACGACGCTCGACTGTGGTACGAAGAATCACCGACATCTCC
GCTTTGAGCCGGCCATATGGGGAGCGCGAACG
AGTAGGCTTAGAGAAATGTGTGCGCTGCCAAGGAAGGAGCAGAGAATGTCACTAGCGGGGCAATTTTC
AACTTTTTGTGCTTTGCTGCACGCACGAACAG
CCGATGAACGCGGAGAGCTGCGCGGCCTTTTTGGTGGCAATATTAGATGTGGCGCCATCGACCCGACA
GCAGCACTCCAGGATGCTGCGGTCCATAGTGG
AGATAAATTCAACCCCGCTGGACATGGTGATTCTGGGGTCACGAAGAATTGCGGCGCGATCCGAGACG
AAGCAGGCGCGCCCCTTGACAGAGGAAGAGAG
GGATGAATTTATCCGCGGCCGAGTCGATTGGAAGCAACGTGTTGTTTTTTGAAATGCGTGGATAACGA
CAAGCCGTTGTTCCGAGATTGCGTCCTTGACA
CCCAAAAAATTCCACAGTGGAGCCGGACGGGACCGTAATTTCTGATTGGTCCGTGGCGCCGAAGACGG
CGAGGGCGGATCCCCACCGCGCC
>scaffold10.size64778
TTTTTTTACGGAAGAGGAATGATGCAGTTGCAAATGCTCGGAATATATATATATATATTGTTTTTGTT
GTTGGATTAGTTTTGTTTTGCCGCCATTTCCTC
CAATATGATTGCGAAAAACTGCAGCTTCATATGTCTCTTTATGTACTTAGGTTTCTTCATGTGAATTT
AGGAAGCGTGTGTGATTCATAGATTTGTTGTT
GTTTGTTTGTTTGTTTTTCAGTCTTGACTCTTTGAAGCGTTTGATATTTATTGCTTTGTTATTTCTTG
CATATTTTTTATTTTTTCCTATCTGAGTCGTT
GGGTGGCGTCGGCCTGCAAAGCGAGGCAGCGCTATGACCCACATGAGTGTTGAAACGGCATCATGGAA
TTGTTTTTCAACGTTCCGGCAGGAATGACAAG
GAAGAAACAGGAATTCGGCCGAGGCATGTGTTGTTTTATTTACCATCCGTTTATGTGGGAACTTTCTT
TCTTTTTTTGAAGTTTTTTTTTTGTGTGTGTG
TGCCTAAAGGAGGGGGTTCGGGAAGAACATGTCGAAGCAAAAGAAGGAAGTGGTGATTAACCTTGACG
AGGTGGATGAACAGACGGGGTATAAATCCGCC
GAACTGTTTGTGTGGCTGAAGTTTGATGAGACGTATCTTCAGAAGCCCGAGGACGATCGTGAGGCGGA
CGTGGATGCGTGGGTGGATGCCTTCTGCGAGC
AGATGGAGGAAGAAGGAATGAATTATGACCGCAACTTTGTTCGTTCTGTCTGTGTGCTGGGCATTTAC
GCTGGTGCACGCGACGAGTTTCAGCAGCGCAT

GGGAAGTGGAAAGGCGGTTTATCCCAACGGGGATCGCTATGACGGGGAGTTTTTTGAGGGAAAGAAGC
ACGGCACGGGGCGCTACTGCTTTGTCAGTCGG
GGAAAGAGCGAGTGCGACCGGATTGTTGAGAAGGAGATGCAAAAGTTGGGAGGTGCGGTGCCGGACGA
GCAGTTCATCACAAACGTTGCTGAAAAGTACA
AAATAAGTCGTCAAATTGTCTCGTGGATTATTGAGTATGGGGTGTATCCCTGCTACCATGGCGACTAC
GTTCGTGGCGTAAGAACCGGACAGGGGCTGAT
GAAGAACAAGGACGGTACCGTTTACAAGGGGGATTTTCTGGCGAATAAACGCCATGGCCAGGGAATGT
TCTATTATTTGAATGGCGACATATTTTCAGGA
ACGTGGAGGAATGGGCTGAAGCATGGCTATGGAACATATCACTTTGCAGGAGGCAGTGAGTACCGCGG
ATCTTGGGTGGACGGTGTCTTTACACAAGGTC
AATGGATCTTCCCGGATGGCACGTATTACGAGGGATTGTTTAACAAGAAGAACCGACCTTGCGATGAT
GCTGCGTCCATGCACTTTCCGAGTATCAAAAT
GGCCCAGACTGGTGTTTTTAGACGTGGTGTTTGGGCACCCTCTAGTGCTCTTCAAGTTTGCGAGGAGA
CACCCGTGGATGGCATGTCATGGATAGATTGA
GTTCTTGATAGAGATGTATCTTTTTTATGTATATCAGGAGGATTGCTCCTTCTTCCTCGCCTGCTTGG
CGCCTCATTTCTGCAAGAGAGTGAGAAGGAAA
AGAATAATTATAAAGAAAGAGGGATGGCACGAGTGGGCATTCCAATGCGTTGACTTTACTGCCGTGTT
GGTTTCTTATTTGTTTTTATTTTCTGCTTGTGTACCTT
CCTTTCTTCTTATTGTGCCTTTGAAAAATATAATAAAAACAAACAACAACAACACAAAAGGAAAAAAA
ATTAGAGATTTTACGGACTTTAATCAAAAATG
GTGTACACACGCTGGAAGTGTGACCGGCTGCCAGTCTTTCAGTTGAAGTTGTTTTCACAGGAGTACCC
ACTCCATACAACCGTTGGAATTCTGTGCATGA
TGTTTCTTTGGAAGCACATGGGGCACTGCTCGGAGGAAACAGAGCGCAAGCACGGTTGGTGGGCGGGA
TACCCGTACTGGCGTGACCCCATTGCCCGCAG
GAATGAGGCCAAATACAAGCAACTGATTAACAAGAACGATGTGGACATCACAGACCCAAAGTGGACTG
GATGTTCTTTTGAGCAGCTTGAGAAGCTAAAG
AAACTGATTTAAGGCATAGTGGAATGGTGGATGGTTGTGTGGCTTTTTTCTTCCTTTTTCTTTTGCTG
TTTCTCTGTGCGGCTGGCGCTTTTTTCTTTTA
GTTTTTTTTGTTTTGTTTTTCCCTCGTAAAGGGGATTAGTGGATACCTACAATTTAACCCGTGAAGGT
TATGAAGTGAGAGAGAGGGGGAAGGAGTGATG
AATTTATTGCCACCTTTCTTTCCATCTCACCGCGACACACACACACACACACANNNNNNNNNNNNNNN
NNNNNNNNNNNNNNNNNNNNNNNNNNNNNNNN
NNNNNNNNNNNNNNNNNNNNNNNNNNNNNNNNNNNNNNNNNNNNNNNNNNNNNNNNNNNNNNNNNNNN
NNNNNNNNNNNNNNNNNNNNNNNNNNNNNNNN
NNNNNNNNNNNNNNNNNNNNNNNNNNNNNNNNNNNNNNNNNNNNNNNNNNNNNNNNNNNNNNNNNNNN
NNNNNNNNNNNNNNNNNNNNNNNNNNNNNNNN
NNNNNNNNNNNNNNNNNNNNNNNNNNNNNNNNNNNNNNNNNNNNNNNNNNNNNNNCCTTTCTTTCCATCT
CACCGCGACACACACACACACACACATAAGGC
ACATGTATGTGTGTATGTATCGCTCTGCTCGTGCGTGTGCGTGCATGTGCCTGCCGCTTTATGTTTAC
TCGGTTTTTTCCTTTGACTCTTTTTTTTTCTT
TTTGCCCCTTGGCAGCCTTACTCTTTAGGAATATTTTGTCCTCATCCTCCTTTTTTCTTCGTTTCGTG
TTTTTAAGGTTGCGCTGGGTGCAGTACGCGTG
AAGGGAGGAACAAATAGAACACATAGAGAAGAGCCTTGCCAACCCTTCAAAAGGAGAGAGGAAAAAAA
AAAAAAAAAGGATTCAATTAAATTTATTAACAC
AAATATTTTTTTTTCTTTAAATTTTCTTTTATTTATTTGTTGTTGTAGAGAGGTGGGAGGCTGCTTCT
AGATGACACGACGATTGGCTTTTTGTTTCTTC
CTGCTATTGCTGGGGGTCGCGATGCCGGGGAGCGGCAGGGAACTGGCACCGCTGAGGGAAGTCTTTCC
TTTGCATGGAAGGACCGTTGTGGATCTCAGAG
TACGCGCGTCAGAGGTGTCTGTTCCTTTGCGTGGCTACGGCGAGGGGGCGGTGTTCACGTACACGGGA
CGGTTGTACGTTTACGATGACGTTGCCTCCGT
TCCAGGCCCTGTTATTCACGTCAAACCCGGGGGGAAACTGGTCATGCGGATGTTCAATGATTTGGGTG
GGCAGACGTATGCGCACGGTGAGGATGAGTTT
TACAATTTCCACGAGGTCAATACCACAAACGTTCATTTCCACGGCGTGCATGGGGATCCCAGAGTGGA
TGACGTCTTTAAGACAGCGTCCCCTGGACATT

TTTTGAAATACGAATTGGACATACCGAAGAAACACCTTCCTGGTTTTCATTGGTATCACACCCACGCC
CACGGCTCTGCATATCTTTTGCTCATGGGAGG
ATTGTTTGGGCCTTTTATTATAGACTCAACGAAAAATGACCCGATCGCTTCATTGCCTTCATTTATTT
TGATGGTGCATTTGTATCGCTTAGGCAAATCA
AACCTTTGCGATGGAATGACAATGGAGGAAGTGGATATCGCCATTAGAACAAACATGTCATCGAGACC
CCGTATTTTGAGTAAAAGTGGGAAGGTCCTAC
CAATGCCTTTAGACTTGTTTCTCGTCAACGGACAACATAAACCGACTATGAACATCAATCGTGGAGAA
AAGACGCTTCTTCGTTTGGCTTTTGCGGCTGG
ATCTTGTCATTTGAACATCAGCTTTCCGAAGGAGTGTCGTTTCCACGTTGCTGCCATTGATGGTGTTC
CATTGAAGTGTACACGTGAGTTGAAGGATAAC
TGGTTATACTTTACAACTGCTACCCGTTACGATGTGGTGGCGGTATGTGAAGATGATGAAAAGCGGGA
GCTACCGGTGATTTCTGGAAGGTTGGAGGAAC
CGATTTTTTATATAGCAGTCCGCTCACATCGATCGAAGCAACGTCGTCCTCATGAAATTACGTTTCCT
CTTTGTTTTCCATTAACTAATGTAAGCTACTT
AACCGCGGACAATCCAACCATGTGGCGCGACATCTCTTTTTCCCAGGAACAAATTCCAACGCCAAAGC
CGTATTATGTCATTGGATACGGCACAGATTGC
TCTTCGCCTGTTCATTCTGGAACCTGCTTTCGTGAGCATTTCTCAGGAGAGAAGGGGGAGTTACGTGA
AAACTATCATGGATTTGTTGTACCTCTTGGTG
CTACTGTTGAGGCTCGTGTGTTTGGGGACCCCACGGATATCGTGCCGCATCCACTTCACCTACATGTG
AATCATTTTGTATTTGTAAGTTTTACTCCGCG
CAAGGGAGGCCTACACGAGAACTTTTCAATGTCTGAATTTGGAGTGTTCTCGGGGGAGTTGCGTGATA
CAATTCCTATACTCGACGGTGAAACTATTATT
CGTTGGCGTGCGGCAACGTACACGGGGGAGGTGGTGTATCACTGCCACATTCTCACCCACGAGGATCT
TGGCATGATGACCTCCTACTTAGTCTACTCCA
GCGCAGCCGGAAAGACTCGCACAGAGACTGTGGTTGCCGCAGATAAAATTGTGGGTGAGGGTGACGCA
GGCCGGTTTTTTTTTCTTGTGCTTGCCATTAT
GTTTCTTCTTTGTTGTTTGCTCTGCGGTCTTTACAAGTGGTGGCGCTCTAACCCGCCACATCCAAGCA
ATTGGGCTCGCAGTTGCAGAGAATTTTTTGCC
GCATTCCCCTACCGTGGCGACGAGGGTACTGAGGCGGAACTTCAGCCCCTGAGGATGAGTCACTGACC
GTGACGGCTCGCACTGAGTGTTATTATTACGC
TTTTTTTTTCTGTTTTTTTTTTTTTTTTTATGTATCTCTCTCTCTCTCTCTCTTTCTCTCTCTCTCTNN
NNNNNNNNNNNNNNNNNNNNNNNNNNNNNNNN
NNNNNNNNNNNNNNNNNNNNNNNNNNNNNNNNNNNNNNNNNNNNNNNNNNNNNNNNNNNNNNNNNNNN
NNNNNNNNNNNNNNNNNNNNNNNNNNNNNNNN
NNNNNNNNNNNNNNNNNNNNNNNNNNNNNNNNNNNNNNNNNNNNNNNNNNNNNNNNNNNNNNNNNNNN
NNNNNNNNNNNNNNNNNNNNNNNNNNNNNNNN
NNNNNNNNNNNNNNNNNNNNNNNNNNNNNNNNNNNNNNNNNNNNNNNNNNNNNNNNNNNNNNNNNNGG
AGCTTTCTTCAGCTTTTGTGATGCTGCCCTTG
GGATGACCGCATCTTATGTGGTGGTTATGAGTGGATGCTGCTTTTTGCCGAGGTGTTTTTCTCAATAC
ACGTTGGCTAGAGCTTTCCGTCTCCCGCTGCT
ATTTCTTATCCATGTACTTGTGCATTAACGATTCCTTTTCCCAGGAAAGAGGATTCTCCCAGTTTAAA
ATGGAATTAAAATAAGTGTGAAACTGAACGTG
CGAAAAAAGAAAGTAAAAAAAAAAAAAGGAGAGAGACGCCCAGTCGGGATGTGCAGTGAAAATGCAAC
GGCGCCTTCGTTTTTGACCGCCCACGCCTTTA
TTTNNNNNNNNNNNNNNNNNNNNNNNNNNNNNNNNNNNNNNNNNNNNNNNNNNNNNNNNNNNNNNNNN
NNNNNNNNNNNNNNNNNNNNNNNNNNNNNNNN
NNNNNNNNNNNNNNNNNNNNNNNNNNNNNNNNNNNNNNNNNNNNNNNNNNNNNNNNNNNNNNNNNNNN
NNNNNNNNNNNNNNNNNNNNNNNNNNNNNNNN
NNNNNNNNNNNNNNNNNNNNNNNNNNNNNNNNNNNNNNNNNNNNNNNNNNNNNNNNNNNNNNNNNNNN
NNNNNNNNNNNNNNNNNNNNNNNNNNNNNNNN
NNNTCAGTGACGTTTTCAATTCTTTTTTTTTTTTCTTTTTTTGAGGTGCTTTTTTTTTGTGTGTGTGT
CTTTGGCCCCACTTCCCCCACCTGCTGAAGCC
GAAACGGGTGATGTCTTTACTGATTGAGTGCTTGACATCTTTTGCGGCCCTCCCATGGAAATTCTCTT
AATGCTCTGTTACAATTGCCAGACCTCAAGGA

AGAGGGGTCTTTTCGGTTACAAAAAGGGAACCGGAGAATATTGTTTATCAGAGCCCAAAGAAACAAAA
AGTAGGTGACTAGCATACTTTATTTTTTATTT
ATATTCACACATGATTCAAACTGAGAGCGAGCAACCACTCATTTCTCGTGCCAATGAGGGGGGTCGCT
TTCCCTGTTGTATTACTCTAGAGGACATCAGT
GTGGATGTCTGCACCAACTTCACCTCTGCTCTATCGTCATGCGTGGAAACCCCATGCTGGGATGAAAT
GCGTGTGGTCTTTTTTAGGGATGGTGTCACTC
AGACCACACGGTGGCTCTCTCCTGCAGTGGCAATGCATCAGATGATGAGTGCAAGTGCCAAAAGAATT
GGTATCGACAAAGGATCTTCTGAAAGGCTGCC
TGGTATCACTGAGGTGAAGTGGTCCCCCATCAATGCAGCGGCCTATACAGTATACTTTAGGTTTCTTG
ATAAGGATGCCGACCACTCGCTCGATCCGGGA
TATGAACGCCCAGTGTTTTCCACAAACCTCAAGTATTTGGATTCATACAACACGGATGACGCCGACCG
ACTTCGTTCAAAGGTATCGCCGAAGGTAATTC
ACCCTTTTTATTTTAGCATCGAGTGTCGTAATCCCAACAGAGCCTTTGTATACACCGGAGGTGAAGGA
GAGCGTCCTACCAGTGCAAAGGTTGAGGCAAT
GTCTGCCTACTATTCCCGGTGCGTATCGAAGATATCAACTGTGTTTAGGAAGGCTTCCATCAAGCTTT
CTTCAGGATCCACAGAAGGAAGCCGTTCTGTT
GAGGGAGAAGCCGCAGATCGTCCTCAGACGAAGGGACATGGCCATTGCAGCAATTATTCCTCTTCATT
TTACGTGTGTGATAAGTATGGGGTGGACCCTA
GTGAAGCGTACAACGCTCCCGGGGGAAGGCGATGTATTTCATTGCCACTTATTCCTTCGCATTCGAAA
TTACCCCAAAACAAAACAGGCGTTCCTCTTAC
GCTTCACCTTACCATGGTGGCTCACCTTAACGACCCCTTGATGCGCCCTGTACGGGATTATCATTCGA
TGGTGGTTTTGGTCGATCAAGTGATTTTAGAT
GTCATGAATGTCGAGGCGGAGACGGGAGGGAAGGATGCTTCTTCTGCCAGACGAGTAGAGGCGTATGG
CTTTGGTTTGACATCGAATACTTCCCAAACCG
CGGAACTACGGTTTTCTTCTCCGTTCTTGGCTCCGCCCGACATTCCATTAAATCAAGAGGGGGGAGTA
CGACTGGTGGACTTCTCCGTTTTACATCCCTC
ACAAAATGAGCGTGAATTAACGGCATTGGATCTGGCGAAGAACCCGACGTCCATATGTATTGTCTCTT
CTGGATGCAAGGTGGGAAACAAGAGGCGCATT
GGAAACCGTCCCGTGCTGGTGTCTCGATTTAAGACGGGGGTCGATGGTCTTTGCAAATCGTCATACAA
GTCTCGGTTGGTGATGGGATTGATTCGTCGCT
ACAATACTGGTGAGGTGGAGGTGGAGCGATGCTCGCATCCTGTCGACCTCGCTTCCATCCTTAACCAG
GAGGAATATTCGGACAAAATCGGAGCCATGAT
TTTCTCCCGAGGAGAATATATGAGTTTCAGACTTCGAAGGCGCTGCTTTGCGGAACCGGAGCGATACC
GCACGGAACTACTCATGCCGTATGATTACGAC
AACAGCGGTGTAGAAATCAGTGATGTTGTGGACGATGACGATATCGTCATGTTCTCTGCGCAAGGTGC
CAGAATATTACCTGAAGAAAATGCCAGCAAAT
CTGGCGGTGGCGACGATGTTCCTAACGGTGATGGCGGTCGCCCGGATGAGAGTCGGATGACTCCACAA
GCCGCCACTCCCACGGGTACCACGGGGCAATT
GGTGGAGAAGCCGATGCCAAGTGTAACGGATAGTGTGATGACTTCGTACAGCCATGAATATCAAAGTT
ATGTCCCCGCACAGAAGAACGAGAAGGAGGCG
GCAACGGGTTCATCAAATCGTTTTATTATAGGGAAGCCACAGCGGGATGGAGATTCACCCGTCGTAAA
AACTTCTCGCGACATTCATGCAGAGGACTCTG
AGTCTTTGCGGGGTAAAGGGAATGCGGAGGGGGCTGAATCAAATGCGGATGGTCGAGTGTCTTCACGC
GACGTCTCTCTCTTAAGGGAAACCGGCATGGC
GGTTGAAACGGGACAAACCAATCCTTTTGAAAAGACGACTAGCAATTTTTTGTTTGATTCCGATGCCC
CCAAAGAGGGGGCAGCGCATGAAAGTTTGTCA
CTTGTCACTTTTGAATTAAATGCTATGGGAGGGATAAATGGAAATGACGATACCCAATTTGCCCTGCA
AGTAGAGCCGTATATATCAGAGACCATTGATG
CCCCACGCGCAACAGAGAACCCACGTGTTTACTGGTGGGAGAGCTAACATGCTTGAGGGATTTTAATG
GTACGATTGCAGTTTTGTTTTGTTTTTTTTGC
AATTTCTTTCTCTCCCTTTTTTTTTTTTTTGCCTGTCTTTAACTGCTAGCGTTGCCATTATTTTATTAT
TATCAATTATTATTATTATTAACGCTTCTTTT
GGCATTTGCCATAGTATATGCAGCACTCATGCGAAGTGTTGGCGCTCCAACATGGATTTTTTCCACGA
CTGAACCCAAAGACATTTGCCATGGAAAGAGT

GAGTGTGCGAAAGGGAGGGAAAAGTGGCCCGCAGTGCAGACACTTAAATTGTAGTGAATGAACATTGA
AAATAAATGCATACACACACACAACAATAATA
ATAATATCTTTTTTTTTGTTAATTTTTTTTTTTGTTTTATCCAGTTGTATGCGTGCGCGTATGTGGGA
TGTTTCTAATTTCCTTTTTTTTTTTTTGACGG
TTTTCCTTTATGTTTTGTTGAGTATATCTGCCTTTTTTATGTTCCGTGGTATTTTGGGCGGGGCAGGC
CGACAGTGGCGTTCCGAGCGTAGGCTCGCGAC
CGTAATGAACGCCACGAGTAGGAATCCCGCGCAGAAGTCGACGTACCGCATCAGTAGAACAAATCTGG
CAAAACAGAGCGCTGTGCGAATGAAAGAGGAC
GCAAAGCGAAGGCAGAAGAGTGCCGTGGCGCAAAGTGTCAGCCGCGGTGATCTTTCTCATGCCGATGC
GGAGTTTTTGCGCCATCAGCAGGATGAGGCGC
TTGCAGAGCATGACGCTCGTGTGAGAGCCTTTACGAAGGATACAGGCAGTGCGCATCATCATCGTCAT
GCTCCAAATCCCCCGCGGCATTTGGAGTACGA
CAGTGTGGATGCGAATAGTGGTCATAGTATCATTCAACAGCGCTTTGATGCGTTGGTGCACGACGCGG
TGCTAAAGTATGGACCTCCGCTTCCTGCTGAA
TTCGTCTCAGAAAATGACATTGTGTCGCCATTTGGCTCTAGGGGAGATTTTAACGCATCCTCGCCTTT
TCAGGAAGAGAATCCTAATGCTCACTACTTTT
ATGATGAGGTGCAAATTAGACCTCAGGAGCTGTTTGGTATCATTCGAAAGTTGGACCCTTCGTTCAGT
GTACGTACACACGCCGACGGTGTCCCTTTCTC
GTTGCTCATCAAGAACTGTTTGTATTTTAGGGTGTGGGGAGGGAAGGTGCACTTTATGCGCTGCCTTA
AGCGTGTGCCGTTGCGCACGGGTGAAGCGGGG
GAAGATGATGAAGAGATGCGTGGAGAAGTAACGACTACACTCTCAAAGTACAAGATGAGGGAGTCCCC
GTCGTTCGATGGCCTTCGTCAAGGTCCCCAGC
CTTGGCTGTATAATTATCGCATGATGTGAGGGAACCGAGCCTCTCTCTCTTTTTTTTATGTTGACGTT
TTCTGTTTGTTTTTTTTTGTTGTTGTTGCATT
ACTTTAATTAGATGTGAATGCGTGAAGGAATGCACCGCAAACCCACTTTGTGAAGATGCCGTTTAGTT
TGCTTCTCTCCTTTTGTACTATTATTATTGTT
TTTCTTATTTTCTCTTTTACTTTTTTCTTTACCCCTTTTAATTTATCAGATTCATCTGTTTTTATGGA
TCCTTAAGGGCTCATAGAGAGCAACCGAGAGA
AGAACTGAAGTCACGCGTGGGATTCTTTTCTTTAGTTGCAATGAAGGAGGAGGGGAAGGGAGCGACAT
GTACTTTGAACGTAAACGCACGTCACGTGGTG
GAAGGCATATCTGCGCTACTCAAACCTGCTCTTGGTGTTGAGCACATTTCACGGAGCGTTCTTTTGCA
ACAGCAACGCCTTCTTCGGGAAATTGAGTCTC
TAAATGATGAGTTGGAGAATAACCGAACTCATTTTGAAACCCTTAACGAGGCCCAGCGAGAGAAATTA
GATATCATTAGTGCCAAGATTGCTCGATGTCG
CTCAAGTGCAGCGCTTTTATTTCGACGACTCTCAGAAATAAAGGAAAGGCTTTTACGGGTGCATGACG
TGATCCTGCAGCGGAGACTGAAGAAGCCACTA
GAAAAAGTGGCGCCGGAGGAGGAGCCGGTTACCACTCCCTCCGAACAGGGTAACGTTGCTGTGGTTCT
TTTCGCTACGGAGGAAGTGGAGGCTTCCAGGC
AACTCAACAGACACCCCGAGGACGTGGACAATCAACATGAAGAAGCAGTTCTTCCTGCCGAGGGGGAG
GCACATGATGAGTAATATATCCATATCTATAT
GCATAAAAAAGGGTGGACCTTACCGTCTATGGTGTTGGTGATTTGTCTAACACGAGAAGGAAGTGGAT
TCCACTTTTTGGGACGAAATACATCCACGACT
AGCGGTGCTGGATGTGGCGGGGGGTCACCGTCTCCTCCTGCTTTAAGGATGAGAAGTAGAGGGGATGG
GCCGGTGGGGCAATAATGCCTCATTGATCACC
CTTTCCATTTATCGATGCCAATGCTTTTACAGTGTCTCGCTCTTGCGATTAGTAACCTCCATTGCATT
TCAGTTTGGGGCCTCATGCTCCGACGGAAAGC
CTACCGTACCTCATTTTGAACGAAGAAATACTTTAGAGTTGACAGATTTTTTTCCCTATCACCGGTGA
ATAGGCGAATGCGCCTCTTATTACGATTATGA
AATGGAGCTTGCATGGAATGAGAGCACATGACGTCTCTCTTTTTTTTTTTTTTCTGCTTTTGCTCCTTT
TTTGTTTTTATGCTGATTTGGGCGGTACGGGG
ACTCTTTTCTCTCCTGCGCGTGGGTTCGGCTGCTGACGCCCTGCTTGGATGGCCCTGGCGTGACGGTG
GTAAAATTCCACTTCTTCTGCTTTATTTATGT
ACACACCTTTATGCGGCCTTTTTGCAGCAGCAGAAGCAGTTTTTTGTTTGCCTAGTGAAAAAGGTTCA
CAGTGTTGCGTCACAAGCTGTCATCGGGAAGC

CGCATTCCGACCGGCTCCACGGGTGGGGCAGGGCTCACCGGGCAGGCAGAGGATGGCATTGGCACCGT
GTTGGATGGCGCAGGCCGCTACTGGGACAATT
TTATGCATCAGGCGTGGAATCTCGTCAGGGACGAGCCTCCAGAGGAGGTGGAAGTTATGGAAATGCCA
CGGGAGAATGGAATCATGGGTGATCTGCAAGA
AATGTCGTCTCTTACATACCACCAACGTTTTGTAGGTTTCTTCGCCACTCTTGGTATGGGTTTATGCT
TCATTGCGATTGCAACAGTATTTGCTCCAAGC
GTTGCCGTTTTTCCAAAGAAGTTTGCCTTTTTTCTTACGGCAGGAAACCTTTTCTGTTTGGGGTCGAC
AACCTTTCTGGTGGGCATCCAACAGCAGATTC
GGTCTATTTTTGATGCCAAGCGTATGGAGGCAGCAGTTATGTACGCTGTTTCCGTCATATTGACGCTT
GTTTCTGTGCTCCACTGGAAGTCAAGTGTTTT
AGCTATTGTCTTTGCCGTTGCTCAGGTATGCTGTTTGTTGTGGTATGCGCTGAGTTATGTTCCCTTTG
CGCGTCGCACCATTGGAATTGTTTGGTCGTAT
GCGTGGTACATTATTTGGCCTGTATTTTGCGCTGTGTCTCAGGCACTGCGGAGGTGCTGCGGACTTTT
GATCTCGAGCGTAAAATAGTTTTCTTTCTTTC
CTTGGGATTTCACCCGAAAGGGGCGCCCATCAACCAGCTACGAAGAAGCAAAAGGAGCGGAGTTTATA
TCCTTATTCGCACGGCGGGTGGAAGGCACCTG
TGGGTGCGTATCCGACACCGGCGTTTTTTTTTTTTTTGTCTTTTCCTTTTCTTTTTTTTTTTTTTTTG
GTTGATGTTTGCTGTTTTTGTATTTGAATGAT
TTTGTTTTTTGTTTTAGTTTTTCTTGGGTGCGTTTTCGGGTCGTTCTTGAGAGGCGCGGTGAAATGCG
TGNNNNNNNNNNNNNNNNNNNNNNNNNNNNNN
NNNNNNNNNNNNNNNNNNNNNNNNNNNNNNNNNNNNNNNNNNNNNNNNNNNNNNNNNNNNNNNNNNNN
NNNNNNNNNNNNNNNNNNNNNNNNNNNNNNNN
NNNNNNNNNNNNNNNNNNNNNNNNNNNNNNNNNNNNNNNNNNNNNNNNNNNNNNNNNNNNNNNNNNNN
NNNNNNNNNNNNNNNNNNNNNNNNNNNNNNNN
NNNNNNNNNNNNNNNNNNNNNNNNNNNNNNNNNNNNNNNNNNNNNNNNNNNNNNNNNNNNNNNNNNNN
NNTACCCGCATTGTTCGGTGCGCTGCTGGGCT
TAGCCTGGCGCTGCCAAGGTGGAGACGGCAGGAAGTTGGGGCCCCAAGCACGCATGCATGGCGCATGT
TCTTCACCAGCGACTCGGTTCCGGTGACGGAG
GCACGGACGGAACCCGCGATGCCGACGACGGGGATGCGCTCTGCGTGGCATGATCTCAGCCTGCATCC
TGCCGCGGATCCATCCATCGTCACGTTTGTCT
GTGAGATCCCAAAGGGAACTCGGGCGAAGGTGGAGTTGCAGAAGGAGGAACCGCACAACCCCTTTGCA
CAGGATGTTCACAAAAAGAAGGAGGGGAAACC
GCTGCGTTTTTACACGTACGGTGACATTCCGTTCAACTACGGCTTTGCTCCACAAACATGGGAGGATC
CGTTGTTGGTGGACGCGGACACAAAATGCACT
GGCGATGGAGATCCCATAGACATTGTGGAAGTCTCCGATTCCCCTCTCCCTATGGGCTCGATTTGGGC
TGTGCGCGTGCTTGGCGTGTTGGGGCTTATTG
ATGAGGGTGAGACGGACTGGAAAATCATAGCGGAAACGCTGCGACCTGAGGGCAAAATGTACGAAAGC
CTTGAGAAAATTCCCCAAGAACTGAGGGACAC
GATTGTGCGGTGGATGCGTGATTACAAGACAACTGATGGAAAGAAGCGGAATGAGTTGGCGTTTAATG
GTGAACTGCGTGGAGCCGAGGAGGCCTTACAC
GTGATTAGGGCGTGCTCACGCCAGTACGCCACTCTTATCGATGGCAACGCTCAAAACCCCGGCTATTG
GCTGCGTTAGTGCCAGGATGCGTGACAATGGT
CTGCAATTTCGCATTTCCAAGTTTTAATTTGGCAAGGATATTATTGTAAGAAATCTAAAATAAAATGT
TGTTTTGTGTTGTTATTGTTTTTGACGCAAAT
CGGGGTTCTTTCAAGCTCTTTGGTTGTTATATATATATATATATATATAGTTTATTTCATGTTTTTTT
TTTTTGCTTTGACGGTAGTGGCGGACGAGAGT
TTCTAGCAGGTTGCGTTGGCAGGGAACAGTAGTCATTGTAAAGATAACACCGACAAATACACACCTCC
AGCCACACACAAACACGGACTCACGTTATGGG
CAAGTCTAAAGTGATGCCGGGGACGGTGGACACCGCGGAGGCGGACAAGGCCACACCGAAATCTATTA
TTATTTACAGAGGTGAGGTTGGAAACAACGTT
CGTGCTCTCATGCATGATTGGCGTGCCGTCTTCCTTCCCTGGAGCAGCAGGAATCTCCACGGCAAGAA
CAGTTCTCTTAAAGACTTTCTGGCCATTGCCT
CAACATTTAGTGTCTCCCACCTACAGTTATTTACGGCACCTTTGCATGGAACGTCACTACGTATCATG
CGGTTTGCGAACGGTCCGACGTTGTACTTCCG

TGTCCTTTCCTTTACGTTGCGTCATGAGATTGTCGCGACACAACGCCGTCCCATTTCACTCAACCAAG
CCGTCTGGGAGGCGGCACCTATTGTCGTCTTA
AACAACTTTACCCACCCGGATGCACGGCGGCGCCCTGAAGTGCCGTTGTTGGAGGCAACTTTTAGGGG
CATGTTTCCAACGGTGAACATTCAGTTGGTGA
AGAACTCCGACATTCAACGCGTGTGCCTCTTTCACTACGACCATGTGGAACATCTTGTGGAGGTGCGA
CACTATTTTATCAATGCGAAGGCTGTGGGCGT
CAGTAAAACAGTTAAAAAGCTTCTTGAGCGCCGTTGCCCAACGAAGCTTGGGACTTTGGAGTCTATCG
ACGATGTTCTTCACCGTGAAGATGTGTGGTCC
GACACGGACGGTGAGGGGGAGGAGGTGCCACTCGCCCAGCCATTTCGTCAGCACCGCGAGCAATGCCG
CATTAAACTGCACGAGATTGGCCCTCGCCTCA
CATTGCAGCTTGTGAAGGTGACAAACGGGTTTGCCGGTGGCGAAGTGCTGTTTCATAGGTTTGAGAGC
AAGACACCGACAGAGGTGGCAGAGAATGCCGT
GAAGGTTCGCGCAAGGCGAAGCGAAAAGGCCAAACGAAAGGCTGAACAGGACGAAAATGTGCGCCGAA
AACAGAACATGAAAGAAGAACGCCTGCGAGCA
AAGCGGCTGCGACGCGAAGAGACCATGCGGCAGCAGGCGGAGAACCCCTTGGAGGTTGTGGATTCCGG
TTATGGCGCCGACGCGGAGTAATGCGGGGATG
GTTCCCTCTCCTATGCGACAAGAGCGCGACGAGCGAAGAGTAAAGAGGGGAAAGAAAGAATGCAACGG
ACAAGAAATTTGAAGGGAAATATTCCTTGGAA
AAGAGAGAGTGAGAATGGAAGATGCATAAATGACGGTTAAAAAAAAACATGAATACATGATTCTTACG
GTTGAAAGAGCCACATGAATTCATGGAATTTT
ATGAGATGTACCATCACATTTTTATTTTCTGAAATCTGTTATTATTATTTTTTTTTATTCTAGAGGGT
GGATACACAAAGTAATATCTGCGTTTTAT
ATAATAGTTACTTAAATATAAATATTTAGTAAACCTATAGGTTTATGTGGCCAACACGGGGTCATATG
AAACGGTTAGTTAAAAGTGTCGTTCCTTGTAT
TGTTTGTTTCTTTCAGGATGCTGTATTAGACTGTGTGGTGGCATGGAAAAACATCCATAAGACGCTTT
ACGTACTCTTTTGCTTCTTCACCGGACGAAGT
CATCTTGCCTATCCCCACCCTTTGGTGCAGCTCCGAAAGAAAAAGGCGGAATTGGCGTTGCATCGAAG
CCGCAGTACAAATCCTACCGTATCCCATGCTT
CATCAAGCAGCTGTACAACGCTAGAGAGGTTGGGGTCGTCTTGGGATGATGCTTCACCGAGAGAAAGT
AGCGGCAAGGAGGGCAATTCTCTGTTGATGGG
GCGAAAACCCGCGTTATCGATGGCCGAAGGAGATACCGCGGAGAATGAGTGTTGGATTCAACGTCATG
TTCGGCGTGGTCACAAGGGTGAACTCTCTGAG
CGGGTACTGAAGACACGTGCGTCACCCCTTGTCTCGCCGGCGTATCATCTTTGTCACTCCGAAAGCTT
GCACCGCCTTGTGAGCAATATTATTGGTCCCA
TTCAACAACCGACAGTTTCGGCGTTACCAACGAGACACACCGCACCCCAGTGGCCACCACATTCATCG
GGCCGCTCCCTTGCACGACACGATGGCCGCGG
GGATGATTTTTCCATCACCACTGATGAATACAGTAGCACTACCAGTAGTCAGTGCCACCCGTTGCGTA
GTGTTGGCGTTCAGTCCAACACGACCTCTGGC
TCTGTGGATGCTAGAAGTAATAGCAGCTTCCGGGCTGTCCCTTCTGTTGTATATCGACGGGAAAGTAG
CAAACAGGTGCAGTTTGATCGCTCAAAGGGGT
CAAGAATGCCGCTGCAGCCCATTGCCTCCATCACATCCCATCACGTGCTTTCGTACCAAGCCACGCGT
CAGAAAGTACTGATAATGGATCTTGACGAGAC
ACTCTGCTTTGTATCTACAAAAAGTACCTCCTCCTGTTCTCCCCCGACTTTTTCGGAGGTCATTCCGA
CCGCCTCTGGAGCGGAACTCTTTTACGTTTGG
GAAAGGCCCCACGTACGTTTATTCATTGGAACTTTGGCGAAGCTGTTTAATCTTGCCCTTTTTACTTC
CGCCACGAAGCCGTATGCGGATTCTATTCTTC
GTCGACTGGACCCGGAACGCCTCATTAGGCGTCGCTACTACCGTCATCATTGCAGACAGATCCCACGA
TCTGCGTGTGGAGTGTCGCCCGGAGGGCGTAG
CTTGGCTGAAAGTGTGGGATCTTCCACTAAAACGGCTGCGCAGAATGTGACAAGTTTGACGGGACAAA
CAGAGAGAAGTTGTGGAACAGCCGCCAACGAT
GTTGTGGCTGGTCGCATGGGTGACACTGTTCCGCCTTCTTGTGTTCACGAGGGCGCCAGAAAATCTGA
TGGAGGTGAGGGTGTGAGTGGCACGGTACTTG
TGAAGGATCTCAGGATCTTAAAAGTACCACCAGAGTTAATGATTATGGTGGACAACACGGAGGAAAGT
GTTTTGGCCAACCGAGAGAATGCCCTTGTTGT

ACCCCCCTTTTTTCCACCCGCAGTCATGGGTACAGGAGACAATGCTGCAAGCGATGATGTTTTGCTAT
CTCTCATTCCCTTGTTGGAGGCCCTGCTGGTT
GTTCCGGATGTGCGTTCTGTACTGCGACACGGGCGAGTTTATTGGCCGAACTTAAATGAAAAATGAGT
TTTGAAAGGAATCAATGGACATTTTGTTTTGT
TTGTTTGTAGTTGCTTCTTTTATTAAAATGTTTGGATTTGACTTGCGCGTTGGACTCTTGCGACACCC
GTTTGTCAAGTTTGAGCGCGCGGTTACGGGGC
GGTGGGAGGTTGTAACTGGTGTTGTTGATTTTACTGCTGTTGTTTTTTTGTTTTTGTTTTTTTCGCGT
GGTACGTACTTTTTTTTTTTTTTTTTTGGGGG
GGGGGTATTTTTTTCGTTGTTACTGCATTGTTTTTTCGTGTGTTGCACTCTGTCTTTGCTCCTCTTTG
CCTTTTTTGTTTCTGTGGATGATTTGTGCACG
ACACTCGTCTGGCATGCATCCTTCCAAACGGACCGTAGGTAGAGAAGTGAAGGGAAAAGAGCGGGGTT
GGCTGCACGTGTGTTTGGTAGAGGCGAGGGAA
ATATGCTTTTTAGGCTTGGCCGGCACGTCGTGTCAAGAGTTGCGCTCGTGGCGCTTGTCCCCGGCGGC
AGTGTCCGCCTGCTAAGCAATTCCGCCCTGCA
ACTGGCACCCAAACGACGAACGAAGACATCAGAACCCGGAAAGGCAGCAGCATTGGGAGCTTCTGCAT
CACCAACACAGAAGAATGACCCGCCGCCACGA
CGCCGGCAGCGAAAAACCAAAGGGGACGGTCATGACACTGAGGTTTTGAATCCGCGAGGTTCACTGGC
CTCCTTGCCACGTGCAAAGAAGACGAAGGGCG
TGCAGTCTGTGGCAAAGGAAGTAAGAGGGCCCACTCCCACTACAAGCGATGAGACGAGTGAGTCTGCA
ACGCCGAGCTTCACAGTTGATGTTCTCCGCGT
AGTGGAGGAATGGGGTGCGGCGTGCGCAAACGTGCAACCGGAGGATGTCGAGAGCGGGGAGACCCAAG
TCGAAAATAAAAATGCCGCGTCTCACGTGGCG
GTTGCAGCTGGCACAAAAGGAAAGCCTCAACTTGGGGCCACTGCCCTCGCTGCGCTTCTCGTCCGTGC
CGTGAAGCTACCAGAAGAATATCCAACACTTC
ACGGCGTGCTGCAAGTTTACCAACAGACGCCACTCTCCCGAAGTGGCGTTGTTGTTCTTCACGCAATT
GAACTTGTCCGTAACGTGGATCATCTCTCCAC
TCTTGTTGCTTTTTTGCGTGTGTATCGCACGAGTGCCTTTAGCAGCACTCCGGTATACGAGGCAGTGA
TGGAGGAGCTTGTGCGAGCGACGACGATGTGG
TGGCATCAAGATCAGAATAGCACCAAGAGAGGAGAAAGAGATGAGATTGTAATGCGGACTACACTGAT
GTGGATTTTATTGGAGTTTCGAAGGAATTATT
TTGAGATGAATGCCACTTCCACGAGGATATCCGATGCGTCCAAAGGAAATAAGAAGAAAGTGGGAGAT
AAAAGAGGTGCCTCTATTGAACACAGCAAATG
GTTAGAAGAGACCACCCAAAAATTACTTGCAGACGCTGCCGTACCCGCTGCCGATCACATAGCCCAGG
AGGCAGGCGATCGTAAACTACAGACACTAACA
GAGGGTGTGGTGAATATGGCTCTTTTCCTTCTCAGTTCTGCCATGGCAACCGAATTACAAATGTACTC
TTCGCACAAAGAAACATGGGAAAGGGGCACCT
CTCAAGTGCAGACGGCCATAGACGTATATCTGAGTCACAATTCATCTGAGGATTCTCTAAATGCCAGT
ATGAAGCATCTCCGAGCGCACGGGTTCGACCT
GTTGGACGCACTGAGGCTTTTATCAATGCTTGACACGGCTCTTTCCCCTCTTTTGCGGGAACGCCTGA
AACGCACCGCTTCTGAATGCTTTTTAAAATTG
TGTGGTGCTGTGGGTGCACGACCAACCCTATTTACAGAGGTCTTGTGCACTCCGGAAATGGCACAGCG
TGTACGGGCATTAACTTCAAACGACATTGGCG
ATTACGTTAAGGCAGCTCTTTTTACGGGTGCGGCTCTCTTTTGCGAGCGTGCAGATGACGCTGCGGTC
AACCGGATAGCGCAACAATTGCAGGCGGTGAA
GAGGGTATCCGCTAAGAAAGAAGGTGATCTTTGCGTTGATGCCGACGAAAAGGAAGAGGATGACGCGG
AGGTGACTTCTCCGCGATCGAGACGCGCAACC
ATCGCGTATGGACTTTTTTCTGCGGGGTTTGATCCTGAGGCGGTGCTCAGCAGTGGGTTATGCGTTGG
ACACAACGCGATGGAGGTGCTTGAGCTGTTAC
AAGCCGCTGCCCTCTTCTCACGGTCATTTGCCCTTTCCCCATTGCCCGCGCGGCTCTCCATGTTGCTT
ACTACGGAGGTCGCAGCGCTTATCGAAGCGCG
TATTAGGGCAGAGAACCGCGCAGGTGTGACACCAACAGATTGCATTGAGGGGCCGAGGCCACTTGCTG
AGTTGTTGGAAAATTTTTCGCCTGTAGAGAAA
AACACGAGCGGGAAAAAGGATGGCCTTGTGCCCCTTGAAGTTATTTTTCATTTGGCCTTTGGGACTGC
CGTGGAGCTTCTTGTGCGGGGAGAAGAGAAGA

AGCTCAGCTCACAGCTTGTGCGGCTGCTCACGCTACTGGAGTGGGAAGGATCGTATAAGTACCTCGTT
GATGGGCTGTCACTGTTGATTGACGGATTTGT
TGAGCGCTTCCGCACCGCTGGGCGGTGCCAACATGATCGGCACCAGCAAAAATCGAGCATGACGACAT
CTACTGAGGTTGAACAATCCTTGTCGGAAGTG
ACAGCCTTTTTTGTTCCACGTGTTGTGGAGGCTGTGCAAAGACGATTGGCGAAAACAAAACGTGCCAG
CGCTATGAGTGACGCGGAGGAAAAGGAAGCGG
CGCAACTGCTACAGCTGGTTTTACACTTCACCGTGTATGTGGCCGCATGCGGTATGAATGCGACGACG
GCTAAACAGCTCGTTGGATTACATACCATGGT
TGTAAAGGGACTAACATCCGGCGGCTCTGACACCCATCTTGCCTCGCTATCGGAGACGGTCATACGAA
TGAATATCCTTGTTTCATATTTGACAGCCAAA
CAGCGAGAGTTTGGTGTTGCGCGTCATCCCGAATCGCCTGGATTGCGACATTGTAGTATTTTTCTCAA
TGACGTTGTTGCCGTCCACTTGAAGCCGATTG
CCGCCGTTGTTCGTCGCGGTGGGACTATTGTGGTGGCCCAAGAGCGTTATCAGGTGCTTTCCCTTGTG
AGTCTCCTTTTCCGACTTGTTTCCGAGTACAC
TGTGGAGGCGGAACCAGCCGAGTTAAGGGCATTCCGCACAGACGTATTGGACCCCACACTGACGCGCG
TACTGCAAGAACAAAATAACGGGGATGTTCAG
TGCAATGAAACCCTTGATCTTGACGCACTGAGCGAAATCACAGTGGTGATGTGCGTACCACACACCAT
TCATCACCTTTCTAGCCAACTGTTGGATCAAC
TCGCTGCTGCTGTGGTGGTGGCGGCACCGAAGCAGCTTCAATTGGCCCGTACAGAGGATCAAAGGCGG
CACTCTAGTAAGACGGCGACGCGTTTGCTCGT
CTCTGCTGCAGCTGTGTGTCAACTCCGACAGGACAAACTGACGGCGCGGTATCTCTCTTTGGTGCAGG
CATTGGGACCGTTGTTGCTTCCAGCCGAGGTG
TCAGCCGTATTGTGCTCTTTTGTTGCTTTTAATGTGCCATCCGATACCGATGCCGTTGTTGAACTACG
AAATCGCCTCACGGAAGAGGCAGTATCAACAA
ATTCATCGTTGTCTGTACCAGAGACAATTTCATCTTTGCAGGTTCTTTGCGAATTGGAGGACACAACT
CGGTTGGCCCATGCAGCAATCCTGCGACGCAT
TATTTATTCCGGACATCGTTTAACACCGGGAGAAGTTGGCATTCTTGTACGCATTACTACAAGGCTGC
AACAACTAAACGGCACTCTTCCTGAAGATGAC
TTGAAGTTTCTTCAAGATCTTCCATTCTCTCTTGCGGAAGAAGTGTTACGAGTTCGTCAGCACTGCTC
AGCTTCGGATCTTCCTCTCGTATTACAGGGGT
ATTCACAGCTGGAGGAGGATCGTTACGGTGAACTAAACGACCGTGTAATGAAAGCGTACATTCTTAGG
ACCATTCAGGTCCGCCCCCTTCTGGGCCCCAA
GGAGGTTGTTGAATGTCTAGAGAGTTATGCGGCAGCACGAGTGAGTCATCAATATTTATTTGGCGTGC
TCCTTGCACGTGTGGCGGACGTCAAGTTAAAA
TTTTCATTGCCCCTTGCGGTGCGTCTTGTTCGGTGTGGAGTGAACGCCTCATGGGATAAAAACATTCA
GACGGCATGCATTACAGCCGCGCAGCCGGTAT
TTATTGGGCTTGTGCGTCATTTACTGCAAAATGATCCCGCCTCGCTTCCTTCTGTTTCAAGCAACGCC
GTGGTTGTACTCCAATGCCTTACCGAAGCTTT
TCCCAACGACCCCACCAGTGATCTTTTGCTGCAGAACATGGCGTCGCACCACACCGCCCTGTCACTGT
CGACGATGATGGCGGTGCTTGAACTGATTGCA
AGGAGGAGTTCAACCGAATACAACATTTTGAGATGCATGACAGAACATGCTGCGTCCGCGCTTTTTCC
ACAGACTTCTCCCAGTGCATTTGCAGAACTGA
CGCGTTTATTTGTTCGTTGCGGCGTGCGCTCCCGCGTCTTGTTTACGGCTTTATCGCGGCGATTCAAG
GAAATTAGCAACAAATGTGATGGCAATGTGTT
GGCAACTCTCGCGGAGGCCTTTGCTGTTGCCCATGTTGACCTGGAAGAAGGTATCATTACAACTTTTA
TTGAACGCATAGTTGCTCTTTCAGAAGGAAAG
CAGCTTCTTTTACAGTTAAATCACTGCATCAGTATCCTTAAGCTGTTTTCTCACATAGACGCAGAGAA
GGTTGAACAGGCCACCAATCTTCTTTTAGAGT
CTGCCAGAAAATCTCTGGAAGCGAAGGAAATGGTTCACGATGGTGTGAAGATACAACAGGCTCAAGAG
ATTAATATTCTTTTATCTGCTTCTCTTCGGAC
AGCCCCCTTGCGGCATCGCGCTCTTGCAAAGCAATGCGCCGACGCACTGCTGGGAATTGTGGGGGCAG
AAGGCGTTGGGGAGGAAAAGAACTGCGGCGCT
TGGCAGACTGACCTCATGCTTATTGTCCAGCTGGCCAGCAACATAGTGCGATTGGGGCTAAGAAATCA
CCCTGTAGTTCCTTACCTGTTTGATGCCTTGT

ACGAAAAACGAAGTGCACTCATGCGGCGCCGGCTGCTGCTACAAATAACCGCCGAGACCATCCAGATA
GCAGGGGAACACACACATAAACAACTCTACGC
CCTTCTTGTGCAGGGTAAACTTGTGGCCTGAGAATCTCGGATGCAAAAAGATGTGGTTGCCGTGTCGT
TTAACCCTGAAGTTCTTTTGGCTCACTCGTTT
TCAGTTTATTATTATTCATATATATTTTGGCGCCGTGAGAAAACGATACTGTGGGTAAAAGAGCTGGT
CATCATTTAAATAAAGAAGAGCAAGGGGCTTT
TTATGAAAATGAGCATATTTTTTATTAGAGTAAGCTTTTTTCTTCTTCTGCGCAATTTGCTTCTGCTG
GCACTGCTTCTTTTCCTTTTTTTTGACAAGGT
TAATTTGGCACGCGCGCTGAGCACCCAAATTCTTTTTTTTGTGTGTGTGTGTGCTTTTGTGTGTCT
TTTTTAGTGGACATCCCTTTCGTGTGCTGCGC
GCCCTTGCTGCGATTTGAACGAGAAAATCGCCACCCTTCCGTTGGATCTTGCAGCTGCGAGGGCGGTG
GCCTGGTGTTCAAACTTTGGCGAGCGTTGCGC
AGAAACCCGCCATGGGCCGAGAACAATCGGCAGACTTCGAAAAAGCATTCGCAGATTTGCTGCTTGCG
GGGCTTCGTCGGCAGGGAGAGGTGCATGGCGA
CGAGCGCGTCCGCTCCATCATGCGACGGGTGCTGGTCGAGCGCGGTGGCGACGGGAGGGAGGAAACTG
GGACGGTGGCCTCAGCAGTTTTTTCTTTGGCT
GGAGCGGGCCTCGGCGACAAGGGGGCGATTGCGGTGGCGGAGGCCCTTCTGAAGCTCGGCTCGCCGCA
GATGGTGAAGTCCCACGGCGTGCGCCGCTGGA
CGGTTTTGCTCAATGACAATGGCCTGACGGACCGGTTCTCGGCGCACGTGTCGCTGCTGGTGTCTCGC
TGCGCGCCTCCCGTGGTGCTGCTTGATGCGCG
GGGCAATGCGATGACGCCGGCGGCCATTCAGCGCATAGAGGAGGCCCGCGAGCTGTCCCCCGCCCCCT
GCGCTCTTCGGCTGCCTCCGACTGCGGGGACG
GCTGGGGTGGCTGCCGCGGCGTCGCTTAGGGAAAGCGCCTCTTCTGCCTCTACGGCGGGCGGGTGGCG
CCGCGGTTTGGATGCGGAGGCGCTGCGGAAGC
GTCGTTCCGCGCTGTTTGCGAGCGGGGCTGCTTCCTCCACCGCGGTGGGGAGCGAGCTGGGCGGGCGG
GGTCGACCGCGGAGCCGCCAAAGCGAGGGCGA
GGCCCCGCATGACGTGGCAGCGGTCGAAGTCGCTGCACGGGCGCCGAGCTCGGCGAGTGGCCCCGCAC
CTGAGGAACGGAGCCGCCGAATGGAGGCGAGT
GGCCGGCGGCATTCGGCTGGCGCGGGGAAGAGCGAGAAGAGGGAGGAGGAGGCTCCTCCTCGCGGGGG
CGATTGCGGGGAGCCCCTCGACGTGACGGCGA
ACCTCAGCCCTCTCTTGGCGGTTGTGCTGGATCTTTCGAATGCGGTGCCCACGGGTCGTTTGCAGGCG
CTTTATGGCACCGACGATATCTGGAGCGTTGT
GGCGTCGTCTGGCGAGGCGGTGTCGCACGCGTTTCCGCCCAAGCAGACGCCATTTCTTCGGGCAGGGA
TTAAATCAATGGTTTCTTTTCACTCTTTGACA
GTTCTTTGCATTCGCAAAAACCGTCTGGAGTCATTAAAAATTTTGCCACCGTCGCTTTTGCGTCTGGA
TGTGAGTGAGAATGAGTTACGAGAGCTTACGG
GTGTGGGGCAGTGCAGAATGCTTACACTTCTGAATGCACGTAGAAACCGCTTACAGTCTATTCGTGGG
TTGGAGCGAAACCTCGCTTTATCTCATCTTTT
TCTTGCCTACAACGAAATCGCCTTTGTTGAGGGCATTGCACATCTGCTTCTGCTGGAGACGCTTGACT
TGGCGCATAATAGACTTAAAACGCAGGCTTCC
ATCCGCCCTCTGTCACTGGCAAGAGGATTGCGACACTTGCTGCTGCGTGGCAACCCAGTGGTGAAATG
TATAAAAGGTAGTTATCGTCCTGTGTTGCGTA
ATCTCTGCCCGTCGCTGCTTTCTATTGATGGTGAGCGCCTTACTTTTTCACGATTTGCGGCAAAGGCA
CAACAAGAGTGCAGCGATATTCGTATACCATA
TGCTCCCGGGGATAGTATCGGTGGTATAAACGTTTCTTTGATCTCTGCAGAGGATGGGAAAGCGACGA
AGGTAACACAGTGTGCCAATTTCATGCATCTG
CTGACGCGTGGTACTGCAGTTGGAGCGGGATCTGGATACAGTGATGCTGCACGGGCTGTGCGCACGGC
AAAGGCGTTGCAAGCACGAGGAGCAGCAAATG
ACAAAAAGGGGAAAGAACAGCGGCGTCCGGCATACCTCGCGGATGGGGAGCCTCTTCGAAAGGAGCTG
CTAAAGCGTCTTGCCCAAGAGTCAAGGAAGTA
CCTGGAATCGATGCTTGGGGAGCGACTGTCCAACATACAGGCTTCGCATGTTTATGACAACTTACCTG
GTAGTGGTGAATCCAGGGATGGGAACAATGAA
TCTGAAGGGCTCTCTAACGACACGTCTGCACAACGTGAGAGCTCTGGACAGGGGAGCGGGCCACACCT
TTTCAGTGGTGACCGCAATCGGAAGGCCCCTG

AATTTACCCAAGGGCTTTTCCCCGTGGAAGTTGCTAGTGAAGAAAACATCGAGAAGCATCGAACAATT
CCACTCGAAAAAAAGATGCCAGCAGTGCACCG
CGATGCGACGCTGGATCATGAACAGTGGCTACGTCTCCGTGAGTGTCGGATCCGCCGCAGTGCACCAT
TTGGAGATACACCTCAACGTATATCGCCGAGT
CCAAAGACTCCGGATATTTCTTACACTAAATCTGAGGAAAAAGAAGTGTATATCTCACCTATTCGTAA
GGATCACGATGAATGCCAGGCTGCTTCAATAA
ATGTCACGGAAATCCTACCTGATATGGAAATGCTGGATTCTCGTTTCCGTGCCGACGGGAAGGTTTCC
GGGGAGCCAAATGGTAGGCCTCGTTTGGTGAA
AAGTGATTTGGGGCGACCTATGCTTTTGTCTCCATCTGCTGAGTGCTCACCTGTTAAGGCGCATAGGG
TTTACTCGTCTCTTCAGTCGCAGAAAGGCAAC
AAACCTATTTTATCATCTTCCGCGACTCCCTTACATGGCGTAAAAGCAGCGAATGAGCCTGTTATTTT
TTCTCCACCACCGCCTTTGTTGTCACCATGCT
CTCTCCATTCCAATGCAAGAAGTTACGCCGTAAAGAAATGGGTAAATCAACTCCGTGAAGACGCAGAG
GCTGTTCAGGAAGCTCTAGAAACCCTTGTTTC
TATTCTAGACTCGCGGTGGTGGAGTAAGGCTGCAATTACTGGTAAGCCTTCTTTTATTCCTCCTGAGG
TGTTAGAAGAGCGAAAGCGCTGTGTCGATATT
CTTACCGAAAGCGGAATGCTCTTGGATACCGAGATTCCTCTTGACGTTGTAGAGTATTATCATTTTAC
CCCCGATGAGTTGAGGCACACTGGTGAGGAGG
ATTATGGTGAGGAAGGTGATTGCGCTGCAAATCCACTTTCTGTGGTAACTGCGTGTGACTCCTGTCGA
GAAAAACGTGAAATTTTGCACTGTATTCGTGT
CATTGGAGACGGAAAGACATGTTTGCGATACCTGGTGCTACTTATAGAGGAAATGAAGGAGGAAGAGC
TGCAGCAGTATGTGAAGGAACTTCAGTTGCTC
ACAACGTGCGAGTAGCCTGGGCGATTGGGGAACCTGGGAGAAGGGAAAAGAAAGGGATCGAGACGATA
ATTTTAAAAGGCGAAGGAAAAGTGAAAACATG
CAAAATGAAACATCTATTTTTTTATAGAACAGAGATACCCAGTGTATAGATAATATATATATGGATAG
AGGTTTTTTTTTCTCTCTCTCTCTCTTTCTCC
ATTTTTCGATTGAGCTCCATGATGGTCACTGTAGTTTTGAGTGTGGAATAATGGAGAACAGGGCATCT
AGAGACCCTTTTGCTAGACTTATTCCGTGGGG
CGCGGTTTGTAGTGGGTGCCTCCTTTTGTTGTTGGGTTTTTACTGACATGTATATATATATATTGAAC
CGCCTTGCCTTGTAGTTGTGTTCTGCCTTTGT
TTTTTTTAAGTCTTTTTTTTTTTTCTATCTTCTTCCTTTTCCTTAAATTCCTTCTCGCTACCTTTTTT
TCTCGCTGCGGGCTGTGCGGCACATCATTATT
TTATTCTTCCGAGGGTGTTTTTTTTGTGTGTGTTTGGCTGAGTAACCAGCGAGTCAGTTTGCTGGGGG
AGGGGGGAATTTCTACTGGGTTGTGTTCGTCG
TTGCACTCGTCACATTCATCTGACACCCGCGCCGGTGACGCAAAAAGCGACAACGCGCATCAAGAGAC
AATGAAGATCACCGATTTTTCAGCCAAACTGC
AAGGCCCTCCGGAGACGGAAACGTTGCTAAACTGCTTGGTAGGTGTTCTTCTCGGTGTCACACGAAGA
AAAGCAACTGAAGGGCCGACACGAAAAGGCAT
TTCTACCTCGCCAGATGGAAACAGTGAGGCGGGAGTTATTTCCTCTCTCCGTAGCGTGAATCCCTCTT
CGGAAGATGACTCTTTTGCCCAAGACGTCTAT
CTCAATGAATTATGTTCTTATTTATCATCGGGGACATCTTCGAGAGAGACGGGCGAAACTCCTGGAGC
GTCTCCGCCGATTCCGTTGCCCTTGCCTAAAA
TTGGATCCTTTCAGAAGGCTCGGACACTTTTCACGCGTCTCGCTAAGTCTCATTACTGGCGTCGCAGG
TTTTTGATTCCATTACTGCGCCGTTGGTGGCG
GGAAGAGGGTCATTCTGTTTTTGGTTTTCTTCTCCTTCAAAGTATCACCGACGCGGTTGCGAGGGAGT
TGGTGTGCATCCAATCGGATAGTGGGGAAGCA
GTCATGGGGAATGAAGGACAAGAGACGTGTCATGATGATGGGATGGGAAATAACAAAAAGCTTCTTTG
TCCAGGAGCCATGCAGGATTTAGAGGAGTTGC
TGAAGTCTACAGGATCCTTTAGGGGTTTGTGTATTGGCGTTGATGCCATCATTCGAAATAATTTGAGT
AATCTGCTGCGGTGTGTGAGAAAACTTCTTGC
GGGAGGCGCCTGCAAGGATGAAAAGGATGCACTCACTGGGAGCGCTCGAGCTGAAATGGAGTGCCTAG
TAGACGGTTCGACGAAATCCGTATTAAAGGAT
TTGTTCGCTTTTTTTTTTCGGGACAATGAATGATGTGGAAAAGGCATTTCGTGAATCTGTTTGGACATT
TTTAGTGGCTCTTCTTTCAGCTTACACGGGTG

CTCTTATTCTGCTTAAGAGGGATACCAATTCCGATGCCCAGGACAATCTTTTCTTCTTGGAGGCTCAA
CGTATGTTTATTGGAAAAATGCAAGAACACTT
GCGGGGATATCTCACGGTTCGTCTCTCGTCCGACAGCGTGGCCATGGGTAACTCCCCCGTGACGGTGC
ATAATGTTGTGTGTCGACAGTCCTTTTTCTGG
AGGGCTTCAATGGAAGTGGAAATTGCAATGGAGTCGTTGCCACCTGCTTTCATATCAAATTCGTTAGT
GGTGTCCTGGGCACGTAAATTGGTAGCTGCTG
CATGGGGAAATGCGTTTTGTCTCGTACAGTCACTTGTGCGTGCACAGGAGGCTACTGAAGAGGTGCCG
GGCACGGTGGAAACCACGGATTCCATTATTTC
CGCTATACGCCGACGGCACCGTGAGCTGTCGGAGGAGCATTTGGAACGGCTGGTGTCGGCAGAGTTGA
GATGCTTTTCCGGTACAAAGCGTCCGCGCATG
CTTGAAACGGACGCTGTTGGTGACAAAACAAAGGGAGGATCAAGAACAGTGCCCACGACAGAGGAGAC
GCCGGCATTCGGGTTTTCATCATATGCCACTG
AATATTTTCTAAGAACGTATCATCCAAGTTGCTTTGCCGTTCTGTCGTTGGTAGAACAGGACACCCTT
GTTCTTTTGTTGCGTCAACTGTATGCTGTTAT
TGTCAACAAGGAACGTGAGGCGATACGGCGTTCTTTGCTGACTGTTACTGTGCCCGATGGCAGTAACG
TGACGGATACTCAGAAGTCGCTGTTGCAGTGG
CATTTACATCATGTTCACACTCAAGTTGTGCAGGCCCTTGGGGAAGATGCCTTACTGCTGATGTGTTT
GTTGCATGCTGTATTTATCTTTCTTGAACGTA
TTGGGAAGACAAATCTGAATGTGTGCGATGTTCTAAGCGACACACTTCTACCTATTCTGGCTATGCTG
TGTTCAGAACGCCACGGCGGCTTTGCTGAACA
TGAGGATGGGGAACAATACTACGAGATCCAGCAGTGTATTATGATGCTCCTTGCATCAGGTTTTCTTG
TGCTACCTTGGTGCCTTCGTGGGATTCGAATT
CGACAGGTTCTATACAATTATATTTGGGCGAATTTACGGGATTCTTTTTCTGCCGAAGAGGCTTTCAC
GGAGGTGAGTCATATTTTTGTGCAATATGCCG
ATGTTCTTAATCCAGCAGAGGATGCAGCGGCAGCTCTGTGTGGAGATCTTTCGGACGATCCTTTTTTG
CGGATGCTGGCGTCTGAAGGCGAGGCGGCAAC
GACCACAATGGAGGTGATGGATGATTTATTGCGTTTAGAGACGCCTGTGCAATTTCCTGCACCCTGCG
GCGCGTTGTTGCTCATCTCCATTTTTCGCACT
TTGTCGCTTAACTTTTCGCGACTTTTTGCAGAGTGGGAGCAACAGAAACAGCATGTGCGGGAAGGTGC
AATGGATTATTTGGGGCGGGAGGCTACATTCT
GTACGATAAGGGATATTGGTGTCTTTCGAGTTCTTATCCAGTTGGCACATCGTATGGTACTAGGCTTT
CAGTTGCAACCAGTTGGCTTACTGCGATTATG
GCTATCATTTTTGACTCATCTCTTTACCCATTTTGTACCTCATTTGACTGAAGATGATGAAATTGTGC
TGATGACATTACACACAAAGGATGGATCCCGG
TCTCGTGCCGAGGAAGAACCACCACGACGCGCATATAAACGCTCGCCGAATGAAACCTCTCAGGGCGA
GCTACGTGCCATTTTGGGCGAACTTCTTGCGG
ACCTTGCATTGATGCCCCTTGTGGATGCTGAGGCGGTTATGCAGTGTCCTGTGGCAGTTCCACACCGT
CCTGGTAGCGCACAGTGGATACAGAAGCAAAG
TGAGCGGTCACAGGATGTCCTCATACTGCAACGCGTGCTTCCCGTTCTTGTGCTGGAGGAATTGTGTT
TTGCGCTGAGAATCCCCATCGAGGATTTGGAC
GAGAGGGAGGCGGATCAGGCGTTTGTACGCCAAGTGGGAAAGACGGTGGAGCAGTCTTTCTTGGGTGT
ACTTGGCGTGTTTGCATTCTGCCAACGCATGG
AGCTTTTTGTGGTCCCGGGAACTTTTACGGACAAGCGCTTTGCAGAGTTGTTGCAAACTCTCTGGTTG
GCGGTGGAGGCAGGTTCCATGGTGATGGCGCC
CACGTGATGTCTTTATCATCTGTTTTTAACAACAAAAAATTATTATTATTTTTTTTTGGCGTCGCTGT
TTTTTTTCCACTTTGTTCCTTACAATGAAGG
CATTGCATGGGAATACATGGCAGTTGAGGCGGCACGAAATGCTTGGCACCGCCTGCGCCAAAAATGGA
ATAAAAAGAGGGGTGCAATGTGGACTCAATGT
TCTTTTTTTGCTTTCTAGCGTTTTTGCTCATTTGTTTTTGCACCTATTTTTTTTTTCTTTCTTGCCCC
CTTTTCCCCTTTTCCATTGGCTCTACTACGAA
AATAGTGTTGTGGGGGACCGATGAGGACGGGTAGTGTGCAGCTTGGGCGTCTTTTTTCGCCATCTCTT
ATCGGGAGAAATCGTTACTGTACGCCGCATCT
GCAGCAGGCAAATTATCTCCTGCCTTTTGTTGCGGGGGCCTGTTGTAGCGTGGAGGATGCGAGACGGT
TGTTTGCTCGTTGCTGTGGTTGCTATCATCAC

TATCGTGCGGAGGGAGAGGACGAGAGTCACGGTGGTGACAACTCCACCGACACGGAGTCCGTGATGGA
GGAAAGTGTGATGTCCTTGCAGCGCCTTGGCT
TGATCTCGCACACACACGAAGAACAGGCTGTCAGGGGAGAGTTGCAAGTTCGAAGGATTTATCGTGAG
CCCATTATCACGATCAATTTGCATTCCATCAA
TGTGACCTCTTTACAGAGAGGTGAGAAGGAGACGGAGGCGCTCTTTCAGATGTTGCACGGTGTTATCA
TTCCTCTGATGTTCTCTCGTCTTGCGTCGTCC
TCAGCCACTTCTCCAACAAATTGCAGTCCGCATTTCGAGGAAGAAGTTTCAGCGCAGTTGTGCTTTTC
TTTGCTGCAACAGTCAAGAGGAACCTGCTCAA
AAGTGAACGACACGACGTGCCGATTTATGGTGGATTTGGTGAATGAGGCTCTGCTTCTTGGGGTTCGT
GAGCGGGTACTGTTCCCATGTGGGGGTGGTCT
TTTCCGCTTGCATCGCGAAATGACGCAGCGGGTTTCACCGTGTTTGTTGACGTTTCAGTGGTGGTGGT
GGCGGCGAGTAGATTCATTTTTGCGACATGTT
GTAGCAAGCCGTGCGGCAGGCAAGGATGTGGCGTCTGAAGCCATGATTGACGCGGCGCAGGAGCGTGT
TCGTTATTGGATTAAAAGGCGAGATGAATATC
TCCCACATTTTTTTTTACACGCATCCATGGACGATACCATGGCGTTGGTGGCGGATGCTGTGCGCTGT
TGGGGTGTGCCTCAATCTCCCGTGCAAGGGCA
AAAGGCATTTTCTGTGCCCAAGTGGAGGCTGCAGTTGTCCGATGAGGCCAAGGCATCACTGGCGTTGG
AGACGTTGAATTCCATCAGGTCTTGTCTCGTT
CGGCGTCCTGCAGGCATCTCTGTAGATGAGCTATCGACACTTATTGCCTGGTCTACAATATCGAGCCT
TCTTCAAAAGAAAAGCATCTTGAAGACATTGA
ATTCGTTTCCTTCTAACTTTGTTGTAAAGACGGTCCAAGGGGTCACGGTGGCGCAGCTCCAGCTTTGT
TTGGGTGAGACCAATACTATTAACGCCACGGA
GGCAAGAACGTGGGTATTGGGCAGAAGAAATGAGTCCTCTGATACCACTGTATCGTTTCTTCAAGAAG
TCGATTTTGTCATACGATCCGTGTTGTTTCTG
CGGAAGCGGCACCTGCTTGAGAAGAGAGTGACATTTGAAGATTTGCGTGCGGTTCTTCTGCCTGAAAA
GGACATGGAGTCTACAGACGCTTTTTTAGAAG
TTCTGCGCCGTTATGATGTTGTGACCGGCTTTGAAGCAGAGAAGCCTGTCTCGTGGCTTTCTCTGCAG
CAGGAGAGGGTTTTTCTGTCTTCACGAGAATC
CGTCGCGCTGAAGGCGATTGAGGCCTCTAGGAAGAGAAAAGGAAGCAAATATCAGCTCTATGCGGAGG
CAATTAGTCCTTTCCTTTCCCGGTGTAGTGAA
GAGGCTACCGCAGCCGGAATTTCTGACGCGTCTATGCCCATTGCTCTCTTGGAACGTTGGTTACAGTC
CGATCGGCTCTTGTTAAATCGAGCAGATCTTT
TTAAAATGCTGCGTGAATGTTCTTTGCCATTTGTCATGGATGAGAAGGCGCAGTCCATTTATCTCGGA
CGTGGGAAAATGGCAGATTTGTCTCAATGTGC
TGTGCCACCGTCTCCAGTAGATCCGATGCCCTCAACGTTACCTCCACCCCCACCGCCGCTGCCGACGC
CCGTAACTCCATTACAGAAATTAACGTTTGAT
TCGCAGCTTTCCCGTGCATTAAAAGGACAGCATCCAGACGCTCTGTTCCGTTTTGCTGAGGCTACAAT
ACATGCGGTGTTTGGGCTTATTTTGCCTCTTG
TTTCTGTTCCTTCTGGAATGCGGCTTCAGAACCTTATTCGCCGTGTGCGCTGGGGATCTGTGGCAGCA
ACGCTTGGCGTCCTCCCATCGTTTTTAGAGGC
ATTTGATGGCCTTTTTTTTGATTTGTTTGCTGATACCAGCGGGCCAATGGAGGATATGGTGGTGACAG
CGTATCGTGGCCATGTAGGGGCTTGGCTTCTT
TATGCCCGTCTGATATCACGCCTATTCCCAACTGACGTGAACATCCCGCTACGACTTGTTGCCGAGGG
TCTATCGTGGGGCGTTTGGTTTGCCCCCCGGT
TTGGTGATCTTCCCACGCTTCTACGGCGTGTGGGACGCTCTTGTCGAGAAGGATATCTTCTGGCAAAG
AATGAGTGTACTGTTGTCCCATCCGCGGAGGA
CGATGCAGCGCTGTGGGAAATTTTAGAAAATGTGAGACGAGAAGAGAAGGATGAGCAAAAACCGCATA
AAGTCTTGACGGAGATTCGGTCGTACTTATTA
GTCAAGCCGACATGTCTCATTCGATACATGCGTGGGAATAAGGAAACTGACAGCAGTGGCGATGACAT
GTGGATGCAATATGCAGAAACCGCCATTCGAC
GTTTTCCGTTGTTTTTTCAGTGGAATACTGCTTTGCAGGGTGCGACACCACTTCTGCGTATTGCGTTG
AAATGCGCCCCCGCACCTGATGGACTTGCCGC
TTTAGTGGAAGAATACGTTTGCCCCCTTCTGCGACAGCGCCCTCAGGATGGTACCAGCATTGCGGAGT
TGGATGATCGACTTGGGTGGTCCCGCGGCAAC

TTTGATACGCATCCCGCGAGTTCACGTGCTCTTGGCGTTGTTGCCAGTGGAACTTCGCTGTATGGTAT
TTTGCAACGGTACGTGGAGGCCGTAGATCCGC
CGCGGATTTTGCTACAGCGGGGATCAACACGGGCAACGGAGCATGAGAGTGTGATGGTGCGTCCCAAT
ACTTGTATTTATCGAAGCCCAGAGGACATGTT
ACTGGAGGTAAATGGCCTAGAATACATGACAAATGAGAACGCGACGCTTCGTCCATCGGGGAGACCCG
TCTCTCTTTTTGAATTGTTGGCGGAAGAGCAA
GAACTGCAGGGAGGGGAAAATGTCCCAAGCCTGCCCCGAACAGAACAGTGGCGCGTCTCGTTGCAGTG
CAATGGAGAAAAAGCCGCTGAGTGGGAAGTGG
AGTTGGCAAATGGCAGAGTTGACGTTCTGGTCTGGTGTGAGTCGTGAACACACGACTTTCTTAATTCC
TTACGAAGGTTTGTATGTGTGTGCCTGAGGGA
GGGAGGGAGAGGAACCATCAGGTACGATAAAACTTTTTTCTTCTCGATCGTTGTGCAAATGATACGGT
TGATAATATTGTTGCCCTTAATGTCATGTTTT
ATTTTTTGGTTGGTGTTTGCTGTCTCCGCAGGAAGATAGTGGGCGCAAAAGGAGGAAGGGGAAACGGA
GGGCAACAATAGGGAGATAATTGAGGGGGAGT
GGGAGGGAGGATGTCCTCCAAGAGTGGAAATGGCTCCCGGGCGCCAACAACCCGAGCATCCACCTCGA
TGCCTCTTTACAACACCGCAGTGCACTCTTCT
CTGAACCCGCGACCGGTGATGTTGCGTGTACCTTTGCTCACACCGAGTCGCAATGAGGCGGAGGACGA
AAATGCGGATGTTGGTGAAAGCCCCTTGCATG
CGGCGGATCCGCAATGTGGCCCTGCCACAGCAATGGACAGGAGTAGTTGTTGTACGAAGGCAGGACGG
GGTGCGGATGTTTGTGAAGGAACCGGCTTGAA
CAAAGGCGATGCAAGAATTTCCTTAATGTCACCGCGATCCCTTTGGCCACTTTCAGCAGGAGTGGGCG
TGGCAGAAGGCCGTGATCAACGCCGCGTCACA
AAATCAACGCCACCGGTTTTTTGTAGGCGCCGTAGTACTCTTGTGCTATGCTCACCACGACCATCCCC
GCCGAGCAAGGGCGGCGTGGGTGTGGAGCGTC
CTGATGAAAAGGAGCTCCTTCCTGCCACCGTGATTACTGATGCTCCAAAAATACGGCTGAGAACAGGT
GGTGACAATGATCGTGTGGAGATAAAGGATAT
TCTCCCTGCTGCCCCTCCCCGCCGCAGGGTAACAAGTCCCCTTCAGCATCTCCTGGTCTACCAAAATG
AAGCACAGCGACAGATGCAGGGCTCTTCTGTA
GGTCATATATGCGGTGTTCAAGGGAGCTCGACACCCCCATATGGCGTTGCTGCATCTACGCCGTTCTT
GGAGGCTGAACTACAACGACCATCGCCTTTTG
TACAAGGAGGCGATGTGCATTCAGCTAGTGATAAATGCCGTATTTCGCCGACTTCTCTTCAGTTTTCC
CTGCCTCGCGACGACAAAAGCGATTCTTCCAC
TGATACTTCCACTTTTGCGGCGCAAATGCCCTTTATTAACACTCGACGAAGCCCTCAGCGGAGCTGGA
TGTCTCCCCTCCCCAATAGCAGGGCTGTGTCT
TCTCTGGCAATTGGAAATGAGGACTGCTCTACATCAGAAGAAGATAATGGAAACAGCAATAAAAAGGC
GACATCACAACCGGTTCCTTGCTTTGGTCCGG
TGCCATCCGGCATGATGACTCAAGTTGCCGGTAAGGCAAGGGATAACGCTCGATCGTCTCGGGCCTTG
CGCGGTTCCCTTGCTCCTTCCGCAAGACCCTC
TGACACGGTGGCGAAGCATCGAACTCGGCCAGCAATTCCGGCAAAGACCGATATCATGAGGGCATTTC
ATGTCTTGGAAATACAGAAGAAAACGCTGGTG
CTGATGGAATGCGAAGATAAGCAGCGTCGTAAAATCATTAATAAATACGTGCACGCCACACGTGGTAT
ACTCGTGAATTATACCTTTCGACGTTTTTTGA
TTCTCGCACAGGATGAGACAGCGTCGTCATCTCTTTCACGTAGTCGCAAAGTGGCATCCTCTTCTTCA
GAGGCGGAAACAAGAAGGCGAACAAGCGAGAA
TGTGGCGAAGAAGGAAGGAATGAAAGTGACGACTGGTTTAGAGGTGCCTGGTCCACTGTCATATGCTT
TGATGCGAGAAGGATCCGGCTCCTCTTCATCA
AGGTCATCCAGATGGCGTAGTGCGGCTTCTGGTGCCAATATAAATGCTCGGGAGTACTCAACGCCTTT
TTCTACAGGTGGCAGCCCTAACGTGCAACAGT
ATCGACGAACGGGGAAAGAGTTAACTGCGAAGGGGTGTGTTTCTTCACGTTCCACCTGTGAAAATAAT
TTTTTTTCTTGTGCGGCGCCTACCCTTCCTCT
AGAAGTTGGTGAAAGTATGAAGAATATCCACCATGCCATGCGCCGTTTTGAGGGGCAAATTCTCCGTC
TGCTTTCGGCAGAGATAAGATCTAGACAACGA
GTGGTAGAAGCAGAGGGTGAAGCGTTTCATTTCCTTTTAATTTATCATCGTCTTTTTGAGACGGCACT
GCAGCCTTCTTTTATTTACTTACAGGAGGCAT

GCCACTGGCAGAGCATCGTGACGGAGGCAACTAATGCATTTCATCGCCTACAATGTGTTCGACATGAT
TGTGAGCTTCAGCAAAATATTCGGAGTGCTCG
TGTTTATGCCCGAAATGTGGCAGCGGTGGTAGAAGAAGAAACTCGCATCCGCCTCATGCTTATTCAGC
AAACAGAGTCCCAGGAATATCATCGTTTGACA
CAGCAATTTCATTTGGGTTTGGCAGCACTTTTGGAGCTGAACATGTGCAGCCAGCGACGCTTTCTGCA
GGAAGTACGTGAAGAGATGGAAACCACCGCAA
TCCAGGAGATGCGGGGTCGAGAGGCGTTGTGGACGGAAGAGCTCCGGCAGAGAGGAATATTGTACCGA
TGTTTTGACGTTGAATATGGAAATCATAAATT
GTGTCATTTGGATATGCCACTCTCCACGCACAGGAGGAGGGAGGAATTGGCTGTGAATGCGTCGATTG
AGTTAAGCTCCATAATGGGTGGGAACGACGAC
AACTCGTGTCTCAGCCACGTGGAGGCTTCTTCTGCAACGCCACTAGAGGTCACGATAGCAAATGTAAA
AACATCCTCAACCGTGCAATCTGTCAAGTGCG
GTGGTGCTCATCTTTTTGCTATTGCCACTGGGGAACACGACACTGCCCGAGATGCATTCTGTTTGAGG
AGTGGGGAACAGGCTGCACAAGTGGAACGCGA
GTTTTTCCAGTGGAGCTCGACATCAAACACTTTACCCACCACGACGAATAATACGACGACAGGCTCTG
CCACAGTTGTTGTGGGCGGTGGATCACATTCT
TTACACAACACTTCAATAGGTGAAAATAGGAAGCCGCAGACAGCATCATCGCCGCAGCCACAGACTGT
TTATTGCCCCAGTGCTACTTTGCACTCGGGTA
CATCTTCTCTTCAGATTAATTCCGTTGCGGGGGAAAAGGGTGATAGGGGTGAAAAAAAAAGTGTCGAT
GCATGTGTTGACGTTCCCGTTCGAGTGCATGG
AGGAGATGTCGATGCGTCCATAGAAGTATTGGATTTCCCTGTTGATTCATCGGGGTTGCAGAAATCAG
TGTCATCTAATTCGATTTCTAAGTCGTTGCGC
CGTGACGTTGCTGTTTCGGTCAATATGCGTTCACTTGACACCTCACGAAGTGGAATGGAAAGTCATGN
NNNNNNNNNNNNNNNNNNNNNNNNNNNNNNNN
NNNNNNNNNNNNNNNNNNNNNNNNNNNNNNNNNNNNNNNNNNNNNNNNNNNNNNNNNNNNNNNNNNNN
NNNNNNNNNNNNNNNNNNNNNNNNNNNNNNNN
NNNNNNNNNNNNNNNNNNNNNNNNNNNNNNNNNNNNNNNNNNNNNNNNNNNNNNNNNNNNNNNNNNNN
NNNNNNNNNNNNNNNNNNNNNNNNNNNNNNNN
NNNNNNNNNNNNNNNNNNNNNNNNNNNNNNNNNNNNNNNNNNNNNNNNNNNNNNNNNNNNNNNNNNNC
GTGCAAGAGAAGAGTCGTCAAGGGTATGTAAA
GGAAATGGGAGGCGGTGATGAAACAATTGAAAGAATTGAGAGCATTGGCGACACCTGTCACACTGCAG
TAGGGGTAGATGAACACGCAACACATGGGGGT
GAGGAAGCATTTCTTGTTGAAAATAATTCAACATCCTCTCATAGTTCCAAAGCGGTTCACCCGACCGA
GAAGGCTGTAGAACTGCAAGAAAGGAGGAAGG
GTACATCTTCTACGGGAACTGCACGGCGTGAGGGTGACACTTTTCCTGAATATTCTCTCTCCACCATG
TTAGAGAACACGGCAGCTGCGAGCGTAGATCC
TTACGAAACGGATGGTCGAATGGTAAAGGAAAATCCCGCTCTCACGGCTGTGGAAATAACCACTGTTT
CCTCTCCATCTTCATGCAGTCACAGCGTGAGC
GCAACTACGGGGTTGAATGAATTATCTGATGGGGATGCTAATGGCGATAATTGTATAAATGACGGAGA
AGACTGTGGTGGTGAGTCTTATATGTCTCCCA
TTGTGGTACAAGCCAATGCTTTTCAGGAAACAAGGAGAAACACTGTGGATGCCTACTACCCTCCGCTT
AATTACATGCGTCCTACGATGTCGTGGCGGAG
ACAACACGAAGGACTTGAATGGCCAACAAGGCTTCGTCCTTCGCAAGGACGTAGCCGTAGCGTGACAT
CTAATTATAGCCTGCGATCAGGGAATGAACGA
AACTGGAAAGAAAATACTCTAGTTCACTTGATACGTATCCCTTTCGAAGAAGGTCCAGGTGTATGGGG
AGATGTCGAGATGCCGCGTAGGCCATTTGGCT
ATGATGCTTTGGGAAACAAAGCCTACGAGATGACAAATGCCGTTTGTTGCCGAGCACCCGTCACTACC
ACCATCTCCTCCTCGGCTCGAAGAAGAGATGC
GGCTTGTCGATTCATGGCGGAGGGATCTACAAGCCGCAGTCCTTCACGTGCAAGAAGTTCAATTTCGT
GCCACGCGAATGGCACCCCAACAGCAGAGAAG
GAAAACCAGGAGTTTTTTGAAGATCCCACGACGCTGGAGAATCTTTGTGGAATAGTTGGGCGGAAATT
TGTCGCACCACAGCATAGAATAACTGCTACGA
ATGCTGACAGGATATCTACCACTAGAAGATTGAGGTCGATGACGCCTTCGTATGTTGATGCTTTTTAT
AAACTTGACCGTGAGGTGGCGGGACGTAAGCG

TTTTCTTGCTTCTCTGCGTCGTGGTGCGACACTGCGGGAGTCCTCTCGGAGAGCAGCGACAACTCCAT
GTCGTCAGCATTCCCAACATCCGTATCGACGC
AGTGATGGTGGGAACATTAGTGGTCGTGGGTTGACTTTGTCTCCAGGTACTTCCTGCATGAACTTTTC
TTCTTCCCCGTGGAGCCCGAAACCGAAACCGT
GGCGTTGAAAGGTTATATACAGCTATAAGTACATGTATCTTTCCAGATGCACACACAGAGAGGGAGAC
ACACACATACACACGCACACACTTGCACTATC
ACGTACAATGCGTTTTTTTTCATAAATATACATTTGTCAATTGTCCATAATTTTGTGGCAAGCCAAGA
AATGTGGAAACTTTTATTTCACTTTTGCAATT
TTGGTTTGATTTGCAATACAAAGTCCGTTTTATACTCGGGCGTTTAGAGGCGGGAAGTGGTGGGGGAT
TATTGGAGTGTTCAGCCCCCTGTTTTGCCGTA
CAGCTGCTACAATTTGCCAGGAATAAACCGTTCAAGGGTAATAATAACGTGGAATTTGAAGAAAAAGT
GCCTTTTTTCTTTCTTTTTTTTATTTTTTTCG
TTTTTTTTGTGCGTGCGCATTTGCGGGATTTTACGGGTTTTTTTGTTGTTGTTGCCACACGCTGAGG
GAATTGTGCAGCGTCGACGAATAAGGTAAGGA
GGGAAACGAGATATTAAAAAGGAAGAAAAAAGAAGGGAGGGAAAGGTAGTGATAGACATGTCACCTGG
AGAGAAACTCTGTGACGGCTGCGGCAATCCTG
CTTTCTCTTTTCAGTGTCCACTTTGCCAGGCGGAGATTGCCACTGACCGTGGTTTTTTTTGCGGTCAG
GAGTGCTTTGCGAAGAATTGGCGGAATCACCG
CAACACATTCCATAAAAAAGGCGTTGTTCGGGCCAAACAGCCGCCAGGACCCGCTTCGGTCGAGGCAG
CCGGGGACTGCCAAAAGCGGTTGACGGGGGGT
ACGAAGGAAGCCGGTGGATCCATCAAAAAACGGCGGAAACTGGAAAAGGAGGTGGGAGTGAAGACGAA
GTGTCCAGGTGCCGTGCCTTGGCTTCCGCCGC
CAAGGGGGAGCCCTGCTACGGCAGAGGTGACACCCCCGTTACCCCCCGAGACTCCCCGTGCAGTAGTC
GGATGTCCATCGGCAGATCGTCGCATCGCATT
CTGGTCAGCAGCTGTTGCAGCGTCGCACCATATTGCCGCTGCTTTTTCTCAGGGGGAAGGCGTCGCAC
CGTTTAGCGTGCTAGTTGTCACAAGCAGTCCA
TTGGCAGCGCATGCCATGGCCTGGGCAGCGCGTTGTGCTGGTCTTGCGGGTGTTATGCGTCTTGTTGT
GAGTGCACCGAGCGAAGCTGTGGGAGCTAACC
TATTGAAGCACACTTCCGACGAACGACGGGTTATTATCACGACACAGGAGATTGTTCGGTGTGTGGCC
GACGGATCCGTGGCCGTGTGGCTGCCGAGAGC
AGAAAGTCTTCTTGTGACGCTGCCAGGTGTTGTAGATGCGTGCGACCTTCAGGCTGTGCACTTACGTG
CTGTCTTTTTTGTTGGTCGCACCGCTGGTTCA
GATTCAACGGAGGACAATTGGTGTGTGGAGGAGGAAATTGTTCATCATGTCTCACCGCCGCTGAGCCC
TCCTGCTGCCCTACTTCATCAGGTTGATGTTT
TTTCAATTCCAACGGAGGATTCCTCAGCCACTGGCAACAAGGAAGCAACGGATACCACACCCAAATTG
AAATTACTTCGCTTTGATAATGATGTGTCAAC
CTCACTTTCACGTGGTGACCTTTCGGGGGCTCTTCAGCATCTTGTGCGCCTGTACAGTGCAAGTCGGG
GCATGTTTGAAGAGGTTTTTTTAAGCACCTTC
TTGTCGGCGCTGGGTGCTGCAAGCGTCACTCACGCCCATCACATGCTGGCGTACGCCGCACGTTATCT
GGTGGACCACTCCGCGCGGTTCGCTGGTCCGC
CGGGAGGCACACTAAAGGAAATGACCCTCCGTGCAGTGACAAAGGTGGTCCGCCTATTCCCACCCATC
AAGGAAGCAGAGGAAGGAAAAGTGGCGACAAA
GAAGAAGGGAGATGCCGTCACCACTGACGCCACTGGGAATGGTGATTTAAGTGAATTTCTCACTCAAC
CTTCTGCCATAGAGAAGAGAAAGTTTTTGAAC
TACTACGAGACGCTTCCCAACCTTCAGCTACAGGCTACCATTTGCTACCTCTACGAACATGCCCCACC
TGGGACTGTAGACGCCTTTGGTGCGGCTTGGG
GCGTGGATAAAGTTGTACCAGGACTTGAAGCATTGAAGTTGGCAGATAGAAAACGTGAGGATGCGGTG
CAACGGTTACGGAATCGTTACGGATCTAAGCT
TAGTTCAGCATTTGCCAATTATCTTGTGGTCCTGATGCACTTTATCTACGACGCAGTGGCTGGGTATT
CTCTCGCTGTGGAGGAGTTAGAGAGACGCACC
CTATGGTCTTTGACACTTGAGCCAGAAGTGGGATCTCTCCGTAGTTTTCTTTCGAATTGTTTTCTGCT
GCAAAAGGAAGACACATCTTGCGCTGAATCAA
AGAGGGGGGAGACGGCAAGGCTTTCAAGCCGAAAGGTGTCGAATAAGCCCCCGAGCCGCACGGAAATT
CTTCGTTCTTTTCCGGTGCGAACTTCTGTTTT

TGAGGAGCCACTCTTTCTTTCGTGGTTGACGCTTCCACTTGTGGGGAAACGGCAACGTGATATGGAAC
GGTTGCAACGTCAAGCTGTGGAGGAGATTCTT
ATGGCAATGCCAAGAAATCCCCGGCCCATGTACATTGGTGACGTTGGCAATCTTATTGGTAAATGGAA
CCATTTCAATGCTCGCTTTGAAGGTGCTCTTG
GTGTAAGTCTCATGGAATTTCTTATGCAGCATCCTGAACATTTTCGTGTTGTTGGCAACCTTGTGACG
CGGCGTTCTGCAGGTGTGTCTGAGCAAGTGAG
GATGCGATTTGATAACGACGACAGCGGTGACGATGATGATAATGGCAAAGAATCGGATCAGGAACGCA
AGGCAAAGGACCGTGCGTTGCTTACAGGTGTA
AAGGGCAGCGGTGGACGTGGATTGAATGCGAAGGATCTGCCCGCGCGAGCACGGAAGAAGGCGACTGT
GAAAGCGTTCAATAAAGCACGCTTTAACCGCA
ACTACAAGCCAATGGATCCAGCGGCAAAGGTGCCCGGATATGTCAAGCATGGCCCGCGCAAAGTAAAG
GGCAGGGGCCGCAAGGTTAACAAGCGTGTCAC
GAAGCGTGGCGGTTAGTGACTTTGATGCACAGCCACAACGAGGGAGGGAATCAGAAAAGTAATGGAAA
CACAAACAAACAAAAAACAGTGGGAGAGATAA
AAAGAAAGTGAAAATAATAGACATCCGTCAATCATCTTTCTGAGCCTCATGGTGGTGCAAAAATCAGG
CTCTGCTGATTTAATGCGGCTCCAATACACAA
ACACGTTTAAACAAAGATGGATAAGGTAAAAACGGAAAAGTAGAAAATAAAATGTGTGAGAGTGAACG
GTGGCTGCGGCGGCTTTGGACCATATATATTT
CCACATATATATGTGGGGGGGACTCAATACATGAAGTCTCTTAGCCTCTTAACCACCACCACCACCAC
CGTTCCAACGTTCCAAATGTTTCATTGAGGGA
GAGATAGATAGAGGGAAAGGACAGATTTCTCGGCTTTTCTGCGTTTTTTTTTTTCTGAAAAGGAAGGC
GCAGAATTTTCTTCAATCGATGATGGCCTCCC
TTTTTGTCGTTTCTCTTGTCTTGACACAGTGAAGGAAGTAAAAGGGTGGAGGACCCAAATAAACACAG
AGGACGGAGGGGAGAAATCAATTAAGAAGAGG
TGGTAGGATTGTTGTGCGGTGATGAGGGCATCGCCCCGCTTGTCTGCATCTTTTTCTTCAAGTGCCAT
TTCTTCCACTCCTACTGCGGTGGCCGCTGCCG
CTTCTTCTTCTTCGTTGGATGGTACACTGCATGAGGTGGAGCGATGTTTCTTGCGCCATGTGATTCCA
ATGCGTGTACAGGGCGCAGGATCTGCTGCGGC
TCTTTACCGGCATGCTTTTCTCCATCAGGAGAGCCTTATCCAACGTGTGCTTGGTGCACTGGAGTCTG
TTCCACTTCCCGCTCTTCCCCGCATGCTTCTT
GCGGAGGGTTTTCAGCGGATGTTGGATGGGGAAACGCCACAACGGGATATTCGAGCTCTTTTCGAGGA
AGCGGAGAATGAGGCGCGACGCGTTTTGATTC
AAGCCGAATTCCATTCTTCTTTGAATTCTCAGCATTGTGACGGACATTTCTATGCGGCTATCAAAACC
AACCCATTGATGCGGTTGTTGGCGTACGAACT
GCGAGCAGCTTGTGGGTACTATGCGCAAATAATGTCAATTCGAAGCCAACCTGAGGATGGAGCTGCGA
TGCTGCTACGCACACTCATTGCGGCCGATATT
CGTACTGATCCTTTTTTAGTTGACGTTATGCTAAAGTTTGCCGCGCTGCGCCGAAATGTGGATACGGG
AGAAAGACTGGAAGCCTGTCCATCGCCCGTCG
AGGAGTTTGTTAAGGAATGTCTGTTACTGGAGCGTGATGCCTTTGGTAGATTTCGTTTTGATGCCCGT
GGTGATAACCGCCACCTCTTGCACTGCATTAA
ACTTTCTGATATTACGAGGGCCCCGAGGGATTTTGCAGTTTTACTGGATCCAGTGCTGCGGCAGTACG
GCAACTTTTGCATTCAAAGCACATCTGTGCAT
CAGGGGAGGTGGATGGAGCACCGGCTGTCGTGTGCGGAGGAGTCACACCGCGTCGATCCGCACCTCCC
ACTGCTTGAAACTGTTGAGGTTCCTTCTAACA
AAGAAAGTGAACTTTCTGTCGGTGACGCTGTTGGTGTTGCTCACGGAGTATCTGGCAGGCCGCCGCTA
GTCTTTTTTGTGCGGTACAACGATCCCATGTG
TCGCCGCCATAAGGAGACAACGCGGGAAAAAGGGGCCGAGAAGGACCACCTCGAAGTGTTTGAACTGG
CCGTGGAGGACAAAAGTCGCACATTTTGGGAG
AAGTGGTTTATGGATCGCTGAGAGGCAGGGCGGGGGGGCTGACATTTGGGAAGGGGACACGAACGCAA
GACCACAACAAAGCCATGACATGTCTTTGAGC
AAAAAGAAGGTGATGTTTATATTTTTTCTTTAACAGGTTGTACTTACTATTACCATTATTACTATTAG
TATTATGGTGTGAATTACAGAATATTTACCCT
TACGTACTCTTCTTCCTTTTTCGTTCTTTTTGTTGTTGTTCCTGCGTATTCAAGGCGTCTCTTTGACA
CGACGTTCTCAGTGTGGAGACCACTGAAGTCC

CTTTAGCGTAAGAACCACAAATAATAGCGGACACTTTTGCTTGCCTGACGATTCCCCCTTTTTTTTTT
TTTCGTGTGTGTGTGTCTGTGTCTGTGCATAG
AGACAAAAAACGACAGCATTTATTCGATAAAGTACACCATCCCAGCGCACGGGCATATATACATCTAT
ATATCAGGAGGGGAGGAAAAAAAAAAAAAAAA
GGTGGACGGCATATTGCGAGTAATAATATTGACAGGAATAATAATACAGGGCATGCGGGACTGTATAA
AGAGAAATAAAAAAGTAAAAATAACATGAATA
ATTCTCAGAAGCCGGAAAATGTGAGGGGAAACGCCTCTTTGCGTATTGATCAAGTTTCCTTCGGATCA
TACCTCCCTGAGAATGCGGAAGAAAGGATGCG
GGCGTATGTGTACCGTGGGACGGACAAAAGTCTACTTTACAAGTACGTGTGGCGACCATTGTGTGCCA
GAATAGTGACGTACTTACCCGTATGGCTATCG
GCAAACATGATAACATTTGCTGCTCTCATTTTCGTCTGCTCCACACATGTATTGCTTGCCTTTTATAT
ACCCAAGTTCTCTGTTGCGAGTGGTGGCAATG
GCGTTGGAGGCTTTTGTGAGATTGGGGAGGAGGGCAACGAATTTATGAAGGAGAATTTTCCACCACCA
CCGTCTTTTGTATTTATTTTGTGCGCCCTCTC
ACTTATGGCGTATACTTTCCTTGACAATCTTGACGGTCATCAGGCGCGCCGAACACGCATGGCATCGC
CGCTTGGACTAATTATAGATCATGGCTGTGAT
TCATTTAACTCTATTATTCTTTCTCTCTCTATATCCGCGTCTACGTTGGCAGGACCAACATGGAAAAC
CTGGACGTTTTTACTTTGCATGATGACAGGCT
TTTTTATGAATACATGGGAGGAATATTATCTCGGGGAGCTTCTTTTACCTCTTATTAATGGTGCAAAC
GAAGGAGTGATGGCTGCCGTTGGTGTTTATTT
ATTTACAGCATGGATGGGTGGACCTCAATGGTGGTTTATGAATGGGGTGGAATTGCCAGAGCGACTTA
TTCCAACTGTGTTGACTTTGTCACCTCCGGTT
GCCACTGTGACAATTGAAGCAAGTGTGTTGCGGACTGTGTGCCCATGTATAAGTGTACCTTATTTTTA
TGTCCCTAATAGCACTCTCTCGCTCCTGCCAC
TGTTATTTGGCCTCAACTGCAGTGACGCATACATTTCTGCACCACCCATACCTATTAACGCTGCGAAT
CAAACGTCTCATGCTGTATATATAGGACATAA
TACGCTGCAGAACACTATTCTTGGACTGTATGATCCCAGCCATCCCAATTTGTTTTGGGTGCGATTTA
ACACTGTTGTTGTTTTATTTTTTGCATGTAGC
ACACTGGTTACGGTTCTCGGGAACATTATCCGCGTGTACCGTGCGCTTCACCGTCACAATGATATGAG
TATCAGTACATCAGGAAGTATAGTTTGTCGAA
TTTATTTTGTACATGCTCTTACGCGTTTAATGCCACTTGTTTCAATTATGTTTTTTGCCACATTGTGG
TTGTTTACTTCCCCCAGTAATATATTTCGGCG
ACATCCACGCATTTTCTGCTGGACATTTGGGCTACTTTTTGCAAAATCCTCCATCCATTTGATGATAG
CGCACATATGCAACGTTAAATTTCATCCGCTT
CACCGCACTTTCTGGCCATTTTTCTTCCTGGCTATCCATTTGACACTGACTTACTGTCGCAATGTGGT
TGAATGGATACAACATTACAATCATGGCGATA
CTTTAGCTTTGCTTAGTGCCAGGAACGTGTGGGGTGTTGCGTTGGACGAGGAGCAGGCACTGTGGTGT
CTCTTTGTCGTGTGTGTGTGGAATTACATTCA
CTTGGTGTGTACTGTTGTGCGTGACACAGCGAAGAGGTTGGGCATCCCTGTTTTTACGGTGCCCCGCA
GCAACCAAGTAACGCTGCGAAGGCAGATTGAG
GAAGAGCGAGCTGCGAATGAAAAGGCGGGGGAGAAAATTTTAGGGGCGACCCATCTTTTGAAAGACGA
CGGCACCCCAATGGATGAGGGGGGGAAGAGGAA
AGAAAAAGGGGTCTGTTTCAGGGGAGTAAGGAGGTTAGGCAGGTGTAACGAAGGAATTAAAAGAAAAA
GAAGAGGGTGGGGCTCTGAAAAGGAGAGGGGA
GCAGCTGCGCCTTTTTTCTTTTTTTGTCGGCGTGCCCTCTCCTTTTTATTCTCCTACTCCTCTATTGT
CCCGCAAACACTTTTATCCCTTTGTGCCTCAC
TGAAGCAGAAGCAACTAAAAGAAGGAAGAGACCGTTTTATTTTTCTCTTTTTCTTATTTTTATTTTTT
TTTTACGTTTTCCTTCTTCTCCCAGCGTGCAG
AAGCGAACGGTCAACACAGGCCACGTTTGCGATTTTGGTTTGTGTGCCTGTTGCCGCGGAAGGAAGGA
TTTTATAGACCAGTGATAAGAGTCATAGAAGT
ATGTTGCAAACTGCACGCATTCGCACAGAAAAACCTCCGGTTTTTCGTCGCCGTCATCGTCAGGGTGT
TAGTTCAACGACGACTACGCCATTTCCGGGTT
TTATTCCTGGTGCCTCGTTTGATGCCACCGCCCCTTCCAGACGGGAGGCGTTGCAACAGTTCCACCGT
CTCGAGATGATGTTGGAGGTGGAGGAAAGATT

GCGCGCGGAGGACGGACTTGTCCATTCCGACACGGGGGGCGGTTCTGAGGATGGCCGCTCAGAATGGG
GGGACAAAACCGGAGATAAATCAAGACTGGAG
TCTGCTGCGAAGGCCGCTCTGCATGACGCTCGGGAAGAACTTGCGGGCCGCACACTACATGCGGATCC
ATATTGTGTTGAAAATGTGTGGTCTGAGGCTC
AGCGGGAGTGTCAAGTGGAGGAGGGGCACAGAATGGGTGAAGATGAGGCCATGTTCTCTGCTCGTAAG
GGCATTGATTTTGAGCCAAGTAATCTCCTTGA
AGCTGATACCCATGCGGCTATCATGGAGGATCTTGAAAAGCTGCATTCTCGCATTCAACAGGCAAGAG
CTCAAAAGTCTCGCCATTCGTCTCCTCTTGGA
GGTGACGCTGAAGGGGCCTCATCGACCAGTGAAAGCTCCATCCATTTAAGTGAAGAGGTGGAATTTGA
CCCAGAACGTTTTGTGGCACCGTTGCTACGCC
ATTCTGATGTCATTGACGGTGCAGAGTTGTGGACGCCGCAATTTGGAATTGATGGCGGAGGGGTGGTT
TGTTTCCGGACCGCTCATGACATTTTTTCTGA
CACCACAACTGTGCAGGAGGCTTTGCAACTCCTGCAGTATAGTGTGCATTCGAACCGCCCAGATGTGG
TGGCGTGCGTGCAACGATGTCAGGAACACCAG
CGGCAGCATCAACCGATAACAACAATACCCAAGCTTCCGACGGGGGAGACAGTGAACCCCCCCAGAAG
AAAGAGGAAGGTAGTGATCCCGGCAACGAACG
TGGGTAAACAACATGAGGAGGAAGCGAAGTTGTATCGACCTCTCCCTTCAGAGTCGCCGCCGTCTGCA
GAAGAACTGGCGGCGATTTTGTCCAAGACCCT
CCCAGCCATACCTGACGAATTAAGGAACGAACTTATCGATCAGCGGAGACTCCTTGAGCGACTCGCTG
CGGCGCAAGAGGCGAGCTTTCGTCAACTAGCG
CGTGAGCAGCAAGAGTCAGCACGGCGACGCATCATGGCACCACGTGCCGACACCACCATGCCACTACT
TGCTCCATCAAAATTGCAGTACTCGGAACCGT
TTGAGATTGCTGAGACACTCCAAAATCTTCAAGCAGACCCAAAGCGACCGCTTCCTCCTGTCATAACG
ATCTCAACGGTGCGCCCACAGCCAACAAAGAT
TCTTCCGCCATCACCCCCTTTATTGCCTCCGCATTACATTGAAGAAGAGCTGAGGCGGCTTCACCGCC
ACTATGAGAAAATTTTGCGGGACGAGCGTCGA
GCATGGACAGATATTATGCAGAAACAAAAGGCGACTTTTGAGATGGAAAAGAACGCTGCAGTACAGAC
ACTACGGAGTGCTGCGGAGCAGCGACAAAATG
AAAATACGGAGGAAATTAAACGGGCAATTGCTTTACAAGAAATAAAACGGCGTCGCTGGCAATACGCT
TCTGAATCAAAACAGCGTATAGAATTCCAGAA
AATGTTATACAAGACATTTCCTTTGGGCGATGTTGGGCATGGGCATGGAGGAGGGAAAATAGGGGTGG
CTGCTAGAATCACGCCAGGTATTCGTCAGCGG
CAAACGGACATCGCGGAAGGGGCGAAATTTTTCTCTTCTAATAAACAAATCCATTCACCAGAAGCAGA
GAGGGAGGAGGAAGACGAGAAGAAGAAGAAGA
AGAAAAACGAAAAGAAAAGAGATTTGAAACGTCCCTTTGTCCGGGGCCCTTTTTCTGTTGTGCTGCCG
GAGGAGCCGGGAGTTGAACCGACTGCAACGGC
CCATGATGCTTCATTGGAGCGAGTGGATGAAGTTGTGATGTTGTCTGATGGTGAGGAGAAGAACGGAA
TTCAACAAGCGCGTGGGGAGGCAAGTGTCGGC
TCACGTGCGCTCACTGATAAACTACGAATAGGATTTTCTTCACCTGCAGGAAAACAGAGAAGGGTCTT
AGGACCACAACGCTCACCTTATGCGGTTTCAT
CTAAGCCATCCGTATTGAGGGGAACTGCTAAGACTAGTAGTGGTGGCGGTGTTAAATCTGCTGCTGCT
GCTGCTTCTGGCATAAAACACGCACGTGCCCC
GTCACATTCTCGGGGTGCAAGACCACAACATCGGCTTTTGAGTAAAGGATCCCAGGAGAAGTCTCCGC
ATGCCTTTGCAGTTCATGACGCTCGAATTGGC
GATGAACGGAATTTTTACAACACGACGGAGACGGTTCTCACCGAGTCGGTTCATTCACTAGACGCCAC
TCTAACCCCTCCTCCTGTTGTGGGTGGTACTT
CACACCTTCACGAAAACACGCTTGATATAGGTGGGAGTATTGCTGGGCGGGCGGTATTCCCACGTAGC
ATCTTGTTGCTTTCCGATCCGCTGACAAGTGA
AGAAATGGAGCGGCAGCACAGTCTGAATTGTGCTTTCAACGCTCTCCTACCACCCCCACCTCGGGCTA
CTAGTAAGGAGGGAAGTGGACAAATTCAAAGA
CCATGCGACATTTATTATTTTGGGGAAGAACACGGTGCCTTGACGCCACGCCGACGAGCAAAACTTCG
GGCTGCGGAGGCCCAGACACGCATAGAGGCAA
GGCGCCGCCTAGAGCATATAAAGGCCCTTTCATTGACTTCGAAAGGTCAGAAACTCGGGGAGCCTGTA
CCACATTTTCCAGAGAGTCAGTTATTTGAGGC

AAGGAGTGAGCAACGCCTGGCGGATCTTTTGGCCAATGAGACCATTCATAGCATTATCGGCGGCGCAG
CTGAGGAAAGCTTATTTGCGGATTTGTTTAAA
GAGCTTGAAGATGAGCTGGTGAGCGCCGAGTTGCGCCGTCTCGCATTCAATAATCTGAAGCAGAGAGA
TGAAAGTGGACAAGAGCAGAAACTGATGGAAG
AAGAGTACGAAGGAGATACGAAGCCACCTGACATGGTATCGATGGAACGAGCTGTGGCAAGTGCAAAA
ATTTTTGATGCCCCTATCAATGAGGAAATTTT
TTTGGAGGAGACAGTCTTACGCCTTCTTGAAGAGTCTTTGTTGCATTTTCTTTTACAAGAGAAATCGG
ACGAGAAGGAGGAGGAGAAGACAATCGATAAT
GAGAACATAGAGGGAGGGGAGGCGACGATGGTATCCTCTCAAAGAGGACAAACAGTATTGATGTCGGA
CGGTGCATACGAGACGGAGACGGGCCCGTTGT
GGGTGCGTGAGGTTGTTTCAGCCACCACAGATCCTCTATGGCATGGAGCAGAGGAAGAGGTGAATCCG
CCCCGTGAGTCGTCCATTCAGGCGACGACAAT
GGTGAGCAGTGAAGAACATGCTCCAGTACTATCAGCACCACCGCTGTCAGCACCACCAACATCAAAAA
AGGCACCGTTGACGCCTGCTTCTACTGCGCAG
ATGGGCGTTGTTATTACGATGGAGGGAGCGAGAGGGAATGTTGTACCGGATACCGCTGGTACCGTTCG
TATTGTTCTCGATATTGCACCTGTCATGGAGC
ACCTTACTGCGATTGCACCACTGGATGCGGTGCAACCTACACGCCCCGCACAAGTTGATGCTATTTTG
AGAGAAGACCCTCGATTGCCGTGTGCCAAAGA
GGAAGAACTAATTTCTTGTGAGGTTGTGGATGCTCCAACTATCACAGGCACCCAACGGTTAACGGTGA
GTACGGAGGTAAAAAGGGACGAAATGCAGGAA
GTGAGGCCGCCTCTTCTTGTTTCTGATATTCAACTCGGAGAGTCCCATCCACCCGCGCTGACCAAAGA
GGAGAAGACAAATGTTCCGCAACCGTCCCCTC
TTCCTCTACAGGGTTATGTGACAGTGACAGAAGTACCCAAGGCACGTCAGTCGCAGCATATTGAGATT
GAGCGTCAAAAGGAATGTGCTCGATTGTGTGA
TAATGAGCTGATTCAACGACAGATGCTGGAAGAACAAGAGGATACACTGCGAGCCTCCTGGCACATGA
TGTGGGACTGGCAACAGATGAATGTTGCGATG
CTAATTCAGCATCAATATCAAAAAAATCCATTCGATGTGGCGTCACAACCCTTTTCTGCTGCCGTGCC
TTTTACAGAGACGCAGACAGTGAACAAGGTGG
AGCCGAGTGGTAATAACGTTTTGCCGCCAGCGCCGTTGGACTCTACAATTCGTGATCCCATCACGAGA
TTTATCTTTGAGTGGATGCACGATTATGACGA
AAAGAAGAAGGAGGAAACAGCACGAAAAAGGCTTGTGGAATTGCCTCACGAGGCAAGTTGCGTGCCCG
AGGGAAAAACATTGCAAGAAGGAAGTAGCGGC
ACGTGTACCACTGGGACGGAAGTGCGTCGGCGGTTACTGTTCGATATCGACAACGCGGAGTCGTCCTC
GTGGCTTTCTTCAACGCACCTGAGCAGTTTTG
CTGAGGATGGTTTCCCGCGGTTCGCAAATCGTCCACAACCAAGGGTGGTACGGCCACAGCGCAAATAC
CGACGCAGTGGTGATGAAAATTTTTCATCTGA
AACGACCGAGACTACACGCTACACTAACTCCTCTTCGATTTTGTGGCTTGCGCAAGAGGCCTGGAGTC
GTGGGGTTTCTCCGCCACCCGTTTTCCCTACA
GCGGCAGTGGGCAACGCCACAAGCACAACGGTGACGACGTCCAGTTCCAGTCGTACATGGCCTTCGAC
GAGCTCTTCAGAGAAGCCACACCTTAGGGAAA
CCATGCTGCGTCACCTCCGCCGAATACAGTCGTCGGATCCGTATCAGCAGGGAGAACAGGAGAGCCAA
AGGGAGGAATACCCCTCTGTGGAAGAGCTGAG
GAAGGAGGTGGAAAGGGTCCAGGCTTCCGAGCAAAGAGCTGCCACACATGATGCCAGTCGTTTGGAAG
GGGCAGATCAAGAGTCACGCCACACGGAGAAC
TTTGTGGCTTTGCTACCGTTGCTTCAGCGTCCCCAAAGTTGGTAGAAAAATACCGACGTGCGAGTTTT
TTTTTGTCATTGGGGAGTGGAGGATTTGGAGA
AGCATACTTTCTCACATCCCATTTACGTACACGGGAAAGCATTCCTGCGTGTTTTCTTTTTTCTCCTG
ATGTATATTTATTTACTTATTACTTATTTCCC
TCACTTGCGGACGTATTTTTGCTATTTCTTTTAGTACGTTGTTTTTTGTTTTTTTTAAACAAGCCGTA
GCTACATGCTGTTACTTTGTGTCCACCAAAAA
CTGAAGCAAACAAGTACACAGGCGATGGTCCTCTGAAAACTTGGCATACGCTGGAGACTTAATTACCG
CTATTTTACATGGCTTGACTGCAGAAGGCATC
CAGCACTTGCTGGGGTGATATTGGAATGACCTCAATTATTACGGGGAATCTTCAAGAGACGAACTTTG
GCTATAAAACAGCTGAGGTCTTGTCTCCTTTT

ACAAAATCCACGTGCCTTTCGTCCCTTTTTGTGGTCGCTGCATTTGCAGCATATCATGAGGACGAGCG
TGTGATTGGGGAAAAGGTTGCGAAAAGAGTGA
ATGAGGTCTGAAAGACGTTTATTTGCCTTTTTATGGCTGGACGCAACTTATGCATATATGTATGCTTC
CAGTTTTTTTGTCTAATGCTTTAAGTGTATGA
ATGTGTGTGTTTTTGTGGGCGTATCGGCTTTTGTCTTTTGGCTGCTGGTTTTTTCTTTGCTCTAATTA
CTTCCTCTGTTCCCCCATCTCTTTTTGTTGGG
CTAAAAGAATAAAAGAAACATAGCGAGACGAAAAAAACGCATATATATATATATATATAGGGGCGATA
AATATATAAAGAAAAGGAGAGAATTAAACAAT
AATAATAATAATAATAATAATAATAATAATAATAATAATAATAATTAGATAAGGATAATTGGAGT
TGGACGTGCATATACGGTTATATCAATATATA
TATATATATACTACCGGTCTGTACGTGTGTGTGCAGGAGAGGACGAGGGAGTAGCCGACCGTGTGGAG
GGAAATTTAAAAGAAACATACAAATACACTAA
ATATATATTTTATATTTTTTTTTTTTTTTTTTGCTTTTGCATTCAGCACTGGACTAAAAGGGTGCAGCG
ATTTTTTGTTTGTTTTGTTTGCCATATAATAG
TCTACGGCTGGAGGGACACCTACGGCACAGCAAAAAAAAAGAGAGAGCGAGATTCTTTACCACCCTCA
CCTGTCCCCCTCTTTTTTCCCCCCCGCTTCTG
CCGGTAATACACGTGGTAGGCCGCTATTTACGGGGAATTCAGTAAGCACTTTGAAACAGCAGAAAAGG
CATCAAGCACCGTGCGTGTAACCCACGAAATT
CCCGTCGAGCCGAGGAAATATATGAACTCGGTGACTGGAGCCCGTGGTTCCTCGGAGACTGCACCACA
AGCGGCGGATGACGACAGCGGTGATGCAACCG
CGAGTGGTAATACGAACGGAAATGTCGCAAGCGGAAACTCGGCTCAAGCAAAGGCGACATCAGAGCAA
GCAGTTGAAACAGAAATCACCACCACCACCAC
CACCACAAAGACACCAGCGGCGGCGGCGGCGGTAGGTGTTCATCATCGGGGATGCCCTGGGAATTCCC
GCGGCGGTGAGTGCGGCTTTTGTTTATTTGAA
GAGGTCGTGGTGGAGGCCTCACCGCCATCGTCATCGTACTTGTTTCCGCAAAATGCAAGCGCATCGCC
CGGCGTCGATTCCCTGCAGCAGTGCCGCCCAC
TGGACTCTTTTTTGGACACCGCGTGTGGAAAGGCGTCAGGCCACTTGAGTTCTTCGACGCGAATTTGC
CCGTCAGAAACGGAATCGACGGCTACTTTTGT
GAACGGTGTTGTCCAAAGTGATCGCAATTTGAAGGAATCCCGGAGAATCTGGGCAACGTCATTTCCCT
CATCGGAATTTTTTTCTGCGGCTGAGACAGAA
AAGGCGCTGCAACATCAAATGCAGCATGAAACCGCGGCCAGTTTCCCGCTTTCTGATAAACCCGCTCC
AGAGCGCCGCCACGCTTTGCCTCTTCTGCAGG
TTCCTGCTCAAGATACTAATGCCGATTCTGTCATAAAGAAACAAAATATTGCTGCTCTCATTTGTGTA
CTGGAGCGGCAAAAATTTGCTTTACAGCGACA
ACTGAAAGAAATAAGGAAAGAATTGTCGGCTCAGAACATATTTTCCCTGGTGAGGGCGAGCAACGCTT
CACAGCTTCAGTATCTTCTTGAGAACAAAATT
TGTGACGTCAACAAGCGGGACTATAATGGATGTACTCCATTGCACGTTGCAGCGCTGGAAGGCAATGA
AGTGATTGTGCGCGTGTTAATTTCGTTTGGTG
CGGATCTCATGGCTGTTGATAATACGGGACGGACCCCACTTGATTGGGCTGCCGCCAACCGTCACAGC
AGCGTTTGTCACTATTTGATGAATGTTATTCA
ACGTCTTATGCTTACGCAGGAACAGAAAAAGCCACAAATACAAAGGAATAATGATGCAGAATCAGTGG
AATCCACTGAAAAAAATGTTCAACGAAATACG
AGTTTTGGTGTGAGCTCCACAAACAACTCAATGATGCAGTCAACGCATTTGACGTCAGCATTGCGAGA
AAGTACATGTTCACTGCCTACGCCATCGTTAC
CACTGGATTCCGAGATTGGAAATGAACAGCAAAATGAGCATGGTCTTTTATCTTCATTTTTGACCTGT
CTCCCGCCTGACATGATCTCATTGATGCGCTC
ACGATCTGCATTATCCGCTAATCCTATTGAAGCTCCCCTGCACGAAGACGAAAGTGAATTTGAGGAGC
AGGGCACATTGTCCAATTTTTCAAGCTACACA
ACCATTTCCGGTACGGTGCCTCTTGTTGTTTGCATGGTAGGTTTACCGGGACGCGGTAAATCGTTTAT
TGCCCAACGTATCTCACGTTACCTCAATTGGA
AGGGTGTTCCTTGTCGAGTCTTCAATGCAGGCAATTATCGCCGACGGCTGCTTGGTGTTGAGGGAACT
TCGGACGCCGGTTTTTATGATCCAAACAATGC
AAACGGAAAGCAAATGCGTGAGAAAATGGCAGAATTCGCATGTGAAGACCTCGTGGAGTTTATCTCCC
GCCATCGTATTGCTGTGGGTGTGTTTGATGCC

ACCAACACCACAAAGGCACGGCGAGAACATCTGGTGGAATTTTTTAACAAGGCTTTCAAGCAACGCCA
TATGGAGAGTCGTGTCATGTTCATTGAGTCGA
TCTGCAATGATGACACGATTATCACTGAAAATATTCTCCGTGCAAAGTGCGACAACGACGACTTTAAG
AACGTTGGAGACACAAGCAAGGTGATTGCTTC
CTTTCGTTCTAGAATTTCAGAGTACGAGAAAGTGTACGAACCACTGGACCGCGCCGATGATTTAAGTT
TTATTCGTATAATTGACGTGAAACGACACGTT
GTGATGAAAAATATACCGTGCGGTCTGGCAAGCAGTATTGCGTTTTTCCTAATGAACCTGCATCCAGT
TGCCCATCCCATATATATTTCCCTCCCTGGTG
AAACGGAAGGTGAAAAGAATCATATGTATGGTGGTGATGAGCATCTGACGCCGCTTGGAAATAAATTT
GCATTGGCACTGAAGCGTTTTATTTTGGAGCG
ACATTTCCCACATATGATCGTGCTGCACGGTACAAACCACAGTGTGCTGAACACTCTGAAGCCACTTG
CACAATCCTTGCAAGAGGACAGTGGGGATGGT
GCGGATAAAGTGGAAGAAGAAGAAAACAGCGCCTTTAGCGGGATTACATGTCAGGAAGAACTTCTCTG
CCCATTACCGGGTCTTGATAGCATAAACTACG
GCTTGTTTAGTGGACGGACAGTACAGTGGGTACGAAAGCGGTACGCAAAACTTTCACGGCTTCTGTAC
GTGGCATCTCCGGTGTGTGGTGATGAATCTGC
GCTTAATTTATATAACTCGAAAAAGTTGTACGCTGTTGATGTAGATGATAAAAAGAAGGGGATAATTG
AGACACATGAGTATCCAAAGAAGCAGGATCAA
GGGGAAAAAGACGCAAGAAAACAGCATTTTGACAAAAGTGCTCGGGCAACTAGCCGTTTCCTTCATGT
ACCGAATGGCACAGACCCTCGCTTATCGTACT
GTGTGCAGTTTCCGAATGGTGAAAGTTGTAGACAGGTGAATGTTCGACTAGAACCAGCGCTTATGGCG
GTGATGCGGGTTCAAGGGCCAGTCTTTGTTGT
CGCAACCTCAGTACCTGCACAAGGCGTCCTTGCTTTTTTCACGGATACCATGCCAGAGGTGTCGCCAA
CTCTGCGTTTGCCACAGCACGCGGTGGTAGAG
ATTGGTGTCAAGGGAGAAATCACAGTGCATCAGCTTGTAGAACCCGTTGATCATGTCCCCCAACCTGG
ATCCATAAGAGAGGGCCGATAATGAGATAAAT
ATGTATGTTTAAATGCAGGCATGTATGTACACACACGTGAGGGTGATGATTGGGTAAGCGTGAATGGG
GTGTTAATTTGAGAGGCGTAGGAAAGGAAAAA
AAAAAAAAAAAAAGAAACAGTTGTGCATCCAAAAGCACANNNNNNNNNNNNNNNNNNNNNNNNNNNNN
NNNNNNNNNNNNNNNNNNNNNNNNNNNNNNNN
NNNNNNNNNNNNNNNNNNNNNNNNNNNNNNNNNNNNNNNNNNNNNNNNNNNNNNNNNNNNNNNNNNNN
NNNNNNNNNNNNNNNNNNNNNNNNNNNNNNNN
NNNNNNNNNNNNNNNNNNNNNNNNNNNNNNNNNNNNNNNNNNNNNNNNNNNNNNNNNNNNNNNNNNNN
NNNNNNNNNNNNNNNNNNNNNNNNNNNNNNNN
NNNNNNNNNNNNNNNNNNNNNNNNNNNNNNNNNNNNNNNNNTGAAACGCGCTTTTTGTTAGTTTTTGTTG
TTGAAGTCTTTTTTTGTGTGTGTGTGTGTGTT
GTGTGTTGGTGGTGGGAGTAGGTTTATTTTGTCTTTGTTCTCCCATGGGCATGCTATTCCGCTACTGT
TTTTTTCCCTCTTTTGTTTTTCTCTTGTCATT
TGATTGTGTGCCTTTCCTTTTTGGCGTTTTGGTTTGCTGTTGTTATTCATTTCCTTGTCTTGAAATTT
CTTCTCTTGTTTCCGGTGCAGGCTTCACTCTT
CCAAATCAGCTGCCTTCTCTGAGGCAATGTTGGCACGATCCAAATAGGAGGAGCGGAGGCTGGATGTT
TCCAGTTGTTTCTCTCTTGTTGTTGTTACCTT
CCGCTGTGTTTGTTGTCCTGCCACACCATGTCGACTTCTCCCCATTTTGTTGCTTTGAATATTTGCCT
GCGCCCATATTTTTCTTTTCCTTTTTTATCTT
TTTTTTATTATTTATTGTGTGTGTTTGCGAGTGTGGTTGTGGTTTGTCGCGGTTCTTAATTGTGAATA
GTTGATTGCCTGCACCTCGTATCCAATTAACT
CCACTGACGCGGCGTTATTTTTCTCCCTCTCTCTTTTGTGGGAGCGACCACAGGCACACGGTAAGGAG
GCTGCAGCGGTCATGATCATATTGGAAAGGAC
TCCCATGACAAATGGCGAGGTGTTTGCGCTGCTGCGAAGACGGCGCGACGAGCGAAATGCGAAGGCGC
AATTTCCATTTGGAATGCAGTTTGTGGGAAGC
AGCAACATGTCGCAGATGCAGCAGCAACGGGTGTCAGCCTCTATGGCAGCGAATGCGTCGGCACACAC
GAGTCTTTTTTTCCCACCGGAAGCACTTGTGT
CAGATGCAAAGTTTTTCATTGTCAATGGAACAATTTCTGCAACTCCTCTTCCGAATGCGAATAATCAT
CTTCTCTTCTCTTCGCCAGCGGCTTCGCACCT

TATGGTGTTGTTGACAGAGGTACGTGCACTACGATACCTTAGCCGGTATGCCACGATCAGCGGCCATA
ACAGTTTGAACGCTCTCTACGGCCCCACCAGT
GTTCATACTCGGCGCTATCAGGAACTCGGTGCATTGGGTTCCGGCGAAAACTTGCCGCGTGCTCAGCG
GCAGAGTGAAGAGAATGGATTCGATGCCTTGC
CACCGCAAGCAGGCGAGGCCCCTCGGGAAATCAATGCTGTCACAAAAACGCAAGAACAGGCGTTGACG
CAGCTGGTGAGGCGGTATCGCCCGGGCACTGT
GGGTCATGTGCGTGCGGTCTTGGCTCTTCTTGGAATTTGGGAAACGAACGGACGCGAGATGGAGCGTC
AGGGCTCAACAAGAGTGAAGTTGGTGTTGCGT
GCAGTGCGACAGAGGCTCAGCGACTCCGCTCGGGGAAACGAGGAAATCCCCATTGTGCACAATAACGA
TAAGGACAATCAGAATGGGGAACAACAACAAC
AGCATCTCCCTTCAGAGCAACTGCGCCCGTTGTTTACCCCGCCTTCTCTGCCGCTTGCAGTATTTCTT
GCAGAACAACCGCCTGTGGTCACCGCTGCTGA
CACTTCTACAGAAAATTATCAGTGGACGGAACAGGATGTCATTCGGCTTGTTATGGCGCGGCCGCAAA
GTAGCCTTGATGTGCACCGCGTCGTGGAGAAC
ATTGAGGAGATTGTCGGCCATAACGAGGAGTTGTTGAACTTTTTCGAAGAGGAACTTGTGAAGGTCTT
TCTGTAGGCTGCCGTTCCGTGTCTAGTGGGCG
GTGGGGACGATAGCACGAGCACTTTTTGTTGCCGTTGTTTTTTTATTCTTCCACTCCCCCGTCCACAC
ACACACGCAGAGAGACCCACAACACAGACAGA
GTTTATCCGTTGTTTTGAGCTGCGTTGCTTTTCACCGATGAGAAAATTTATTGACATCCGTTTTATTC
GAGATGAACCGCGGTGTTTTGTTTTTATTTTT
TTTTTTTTGTTTTTGTTTATTTTCCTTTTTTTGTTTCTTTCCGCCTGTCTGCCTTTTTTATCTCTTCA
TTGTTCTTTGGTGCTTGCTGTCTCTTCTTCGA
CCCTTTAGTGTTGTTATTATTATTAGGAAATAGGTGGCTATTTATTTTATTTAACGTACATTTTTGTA
CAGTTTTCTTTTGCTTTTTTTTTTATTTTTTT
TTTTCCCCCCTCTTTCTCTCATCCACCAATCCGTTGAATTTGGAAAGATTGCAAAATATTTATATTTA
CTTTTTTATTTTTATTTTATTTTTTTTTTTTT
TTATTTGGNNNNNNNNNNNNNNNNNNNNNNNNNNNNNNNNNNNNNNNNNNNNNNNNNNNNNNNNNNNN
NNNNNNNNNNNNNNNNNNNNNNNNNNNNNNNN
NNNNNNNNNNNNNNNNNNNNNNNNNNNNNNNNNNNNNNNNNNNNNNNNNNNNNNNNNNNNNNNNNNNN
NNNNNNNNNNNNNNNNNNNNNNNNNNNNNNNN
NNNNNNNNNNNNNNNNNNNNNNNNNNNNNNNNNNNNNNNNNNNNNNNNNNNNNNNNNNNNNNNNNNNN
NNNNNNNNNNNNNNNNNNNNNNNNNNNNNNNN
NNNNNNNNGGGGTGGGGTGGGGAAAAAAAAAAAAAAAAAACTGGGTATCCGTGCCGTCACTTTAGCGG
CCGACGCCGACGGATAATCCAGGAGGGGGAAA
GAAGAAGAAAAAAAAAAAAAAAGGAGTGGAGAAGGGGGGAATAACAAAAATAACAGGAGAAGAAGATAC
AAACCAACATATACATACATACATACGGATAC
GCATATATTTCACAGCGTTTTTGTATTTGGAAGCTGCTGCAGAGGATTCCTGATTTTTGTTCCAGCTA
TACCTCGTGTTGGTGCCTTACGCTCCCTACCT
ACGGTCAATTCACCACGGAAGACACACACGGAGGCGTAAAATATACATATTTTACTAGCTGCTGTGTG
TTAGCTTTTGCCTTTCAGAGGGTTCTACTTGT
TTCATAATCCTTTATAGAGGATGTCTGAGACAAAAATGGTAAATAAGAGCAAAAATGGTATTCGTGAA
AAACTCCACAGTGCCATTGCATGGCTTTTTAC
TCCCGGACACAAAAGTATTGAATATTGCAAGAACCAGGGCGAAGGAGATCATCAGGTCTCTGCTGCCG
TGGATGGGTCCTCATCGTTGGCAGGTGATGGG
TGTCCGCTGACGACGCCACGCTCCAATTTAAATGTAAAAATCAATCGAGAGGGTCTCCAGCAGGCGGC
GGTGTCGGCAGCGACATCCCAACATGCAGGCG
AACTCTCCACGCGAGCTGCTGGTAACAATCTCGATTTGGATTTGGATGCTACCTCAATTGATCACAGC
GAGTTCTTTTCGTTTCTTGAGGACGTTATTGG
AGGCAAACACGTTGAAATGCTGAAGGAGCTAAACCAATTGCACGAATCAAACTCCAATAGCAACGTGG
AAGCCAACGCTGTTGACTCACCTTCTTCGGCT
CCCGTAGAAAGTGGAGAAGGTTTTAGGGAAAGTGTTCTGAGGGCGTCCTCCTTTCTTGCTCCTTGTGT
AGAAGCACAAGGACAAAGTGAGAGGATTTCGG
AAGTAAAACAAGACACCTTAGCTAATAAAAAAGAGAGAGACGGCGCTTCTTCGGCTGCTCGCTCGCTC
AAGGGCTATGAGCTCATTGCATATCTTGGTAA

AGGCGCATTTGCCGAGGTTACTTTGGCACGGCACAAAGCAACTCAGAAGCTCTTTGCGCTAAAGAAGA
TATCAAAGCGAAAGGTGCGAGAAGAGGGTTGT
GTGCAGCGTACCTTCACGGAACGTCAGTTGCTTGCGTCACTTAAGCATCCCTACTTGGTGAAGTTGTA
TCAGGCATTTCAGAGTCTTACGCATCTATATC
TTGTTCTTGAATTTGCTCAGGGAGGAGATATGTATTTTTTTCTCGAATCGAAACCATGGCTGCGGGAG
ATGCGTCGTAAAGTTCAAAAATTGAGGAGGCT
TTCCTTTTGCAGTGACACGGCATGCCCATTTCCAGGCGGCGCGCAAGACGAGTCTGATCCGACGTCGA
ATCACTCTTGTCCTCCTAAGCACTTGATTTCA
ACCGTTTCCTCTTTCAGTAGCACAATACAATTGGCCCCAGACCAGAGCCGTGCACCAATTCGTTTGGT
CGCCTTTTACGCTATTGAGCTTGCGTTAGTAC
TGCAGTATCTGCATGATCACGGCTTTGTCTATCGAGACCTTAAGCCGGAGAATGTTCTTATTGCACGG
GACGGCAACTTGATGCTTACCGATTTTGGTGT
CGCGAAGTATTACGGCGGCACAAAATTGAAGAAATCGGGTGCTGAACGAACGAATAGTTTTACCGGTA
CGACACCATACATGAGCCCGGAGATGCTTCTT
GGGGAGCCACAAGATGCCCGCATGGATTGGTGGAGTTTTGGTTGCATCCTTTTTGAAATGGCAACGGG
ACGACGGCCCTTTGAAGGGGAGAGTCAATATG
CTGTCGTGCAGTCTATTGTCGAGCGTGATGTAGAAGTTTGCCAAGATGACTTTCTTCTCACATCGCTG
GAAATAGAGAGACGTGTAATGCAATTGCAGGA
ACGGTACGAAGCATACTTGGTTCGAGTAGTAGAGCAGATTAAAAAATGCAAGGAAAATGGGAGTATGA
CAGAGGAAGAACGGACACAATTTAGTGGTTCC
CTTGAGGACGCACAATTGACGTTTACTGTAGATGCAGCTTCCGCGCTACATGATACCAGCTCTTGTGG
TAGCAAAAATTTACTTGGCGATGGTACACGAA
GTTCGCTGGGTGAAGCATTTTGCCGCACCTTTGGAAGTGAAACACTGAACGCAAAATCTTCCATGACT
ATTGAAAATCAATCCCTGTTTATGAATTTTGC
CGTGGGAGAGTTGGATGAGGCGTGTGATTTGTTAAAAGACCTTATCTGTTCACTGTTGGAGCGCCGTA
CCGACCGGCGCTTAAGTGGACCAGCGGTGCTC
GAACATCCATTTTTCTCGTGTCCGTATGTCTGTTCCCAGATATATTACAAACATGATGCTCGCGTGGA
TCCCGGCGACATTTCTGGCTTTTACGTCCCGA
CTAAGTGGAGAAACAGTTTTTCTTCAGGCGGTCACTTTGCAGAAGATGAGAAGAAAAATGAAAATGGT
GAGAAAAATGACGGACATGATGCGGAAGATGA
TGATGGGGGCACAACGGAGCTTCAAAAGCATCTTGGTGCTGCGCGCTTCCTCGCCCAGTCCCCGACCC
AGCGGCCGGACAACTGGCGCGAGCTCTTTTTG
GAGGGAGTGGTGTCATCGTTGTATGTTCCCCGCCTCTTGTCGGCGGACGATCTGCGTTACTTTCCACA
TGCCGTGACGGCGACGGGGGATAGTGTTGTCA
CTCAGCAACGTGCTCTGCGGGAACAGATACGGAGAAAACACGGTGCGCTGCATCCACGCGGAGGTGAA
GGCGACCACCAGGCTTCTCCCCTCAGCGTCAC
CCAGAAAGGAGGCCATCCGTTGCATCAAGAAAATCAAATGAGTGAAGAAGACGAGGATGTTGAGACTT
TGCCACAGTCGGCTTCACAGCGAAGCTCCTTG
GTAGAGTCATTGAGGGGGAGTAAACATGCGGCTTTCGCACATGAAGAGCCATTATTTATGGGAGAAAC
AGTCAGCAGCCTGGCATTTCCACACCATGACA
ATTTCAGCGTCTACTCAGTTTCGAGTCTGGAGGTGTCACCTGCAAAATATCCGTCTTTTCTGGCAAAG
TCATATGGCAGGTCATATAACAAAGAGTGTTG
TAATGAAATTGGAAAAGAAACTGTAACGGAGCCAATGGAAGAGTCCTCTAATCGCCTAAAATTGTCAG
CATTGGACCTCTCTTATTCCACCTCAGCAGAA
GCAGCAAAAGCAACAGCAAAAACACCGGAAGCAACGCTTGGAGGGGTTTTAGCGGAAGTGGCACCTTC
CCTCATTTCTCATAGCTTTCCTTTGCAATTAG
GAGAGTCACAGACTGCTGCCCACCAAGAGTTGCACCCCGACTTGGGTAGAGCATACACAGCTCCATCG
GAAATATTTACATCTACGCCCACCAAATCAAG
GGATGCTTTACCTTCATTTAATTTATCCACTGGGAATTTAGGGGCAAGAGAAGTCCTATCGTCTGACA
CGTCTTATGTCACCACTGAGGATGAAGACAGC
ACTGGGAATGCATTTGACGATATCAAAGGTCAAACACAGGGGACACTTCATGGTATTGCGTACGATGT
GGACGATTATTTTCCAAGTTGTACAAAACAAA
AAAGACTGTTGTTAAAGAGTAAAACAGGTATTCCGACCCAAAGGCCATCTAAGACAAATAGGAGTATC
TTTAGTGTACAGCGATTGAGGCGAAGTTTATT

AGATAAAAGAGCAACAAAATTTTCTCGACAGTTGGTGGCGAGCGCGAGGGCATCGCCAACTGTTCCTC
AGCAGCTCCCTATGTGTGCTTCATATGAAACA
TTTGCGTTCAAGAGTCCCAAAAAGAACTCCCACAAGACTGACAGCGAATATGATAACAGGAGATGCAG
TGAAGACATGGAGGGCCGCCGCTTATCCCTTC
TTTCAGTGTATTCCCCAATGTTGTACACCGCCACCGTCTCGCCAAGGTCACCAAAGAAAACGGAATTT
TTAAATAGTGACTCTTTGGTCCACGAAACAAA
TATACTCCCAGGCCTTTGCAGTACGTCTCGGACGGGCGCGAATAGCGATTCAGTTGAGGAGCGCCCTA
ATTCTCCCACAATGGAGAGCTCTTCGCATCCT
GGAAACGGTGGTGCTGGTACTAGCGTGCAGCACTACTTGGGCTTCACATTTGACTCAAGAGCCGGCAA
CGCCTTTCTCCTGGGGAATACATCCAACGGCG
GTAGCAACGACAAGTGGTGTTTTTGAAAGAAGGTCCGTTAGTCGATGAAGGAGTGGGGTGAACAGAAA
GGGGTGCACCGGCCTGGAAAGGGAGAACTTGA
GGGAGTAGCGAAGCAAAAGATATATAAAGGCGAGCGGATTTTGTATCGTTTGTGTAATTGTGTCCGTG
GAATTCGGACATGCAACCAATTAAAGACAGGC
AAAAATATGACAGCTTGGCAGACAAATAGGCACATTTTTTTGAGGCAAGTCTCTTTGCTTCTTCTTCT
TCCATATCAACATGACGGATCTAAGGATCCTT
CCCCCCCAACCAGGAGAACGGGGAAAAAGGGGAACGCTCTGTTCTTTTTTTTCTTTTTTGCTTTTTTT
TTTTTTTTTTTGTTTGCATGCGTGTTGTTGTA
TCCCTTTATTTTTCTACCATGAATTTATTTGAGCTCAACGATTTTTATTTATGTATTCCCATTTATCT
GTCGTTTTTTTTTTTCTTTTGGCTTTTCGTGG
ATCATCTTCCGTCATTGTTTTTCAAGTCGTTTTTTTTTTTTTTTTTTAGCGGGAGTTTGTATACGTGG
AATGGCTTTTGTTTTGTGTTGACGTGTGTGTG
CCGTTGTTTGCTTGTTTCTTATCTTGCTATGCCATGAAAAAAATTTTTTTGTTGCTATTGACGCCATT
TGGTGTTTGCACCGCGATGATCAACAACCCAC
CATGAATGTAACAAGACGAGGGGAGTGTGGGAAAACGATAACAAAAGGCCCGCTACACTGCCCATGCC
TCTCAGAGTGTGCGTGTGTATTTGAGTATATT
TATTTCTCTCTTTTTTTTCTTTCTTTTTATTCCATGTATGTACGCATATATATATATATATATATGCA
TGCTTCTGTATGCACTGACTTTAGGTTTTTGC
GTGTATACGGCACCGGATGTGAGCTCCGTGGGGTGTGCTTTTACTGATGGCGGCAATATTCCTTTTTA
ACGGCGTCATCGAGAGAAATGCTACGTAAATG
CAAGGAGCAGTTGTACAAAGCGTGAACAAATGCACCACGAGATAAGGGAAGGGCGAGGGGTTTGGGGG
GTTCACGATAAACTCATCAATCTCCTTGTTGC
TGCTATTTGTGCGTGTCCGTGTGATCGTTTGTTCCATATTTTATTTTATTTTATTTTATTTGTTTTGT
TTTTTTTTTTGCTTCGAGCGCGGTGGATATTG
TTTTTTTTTTTGTGTGTGTGTGCGCGGAGGTATTTTGCCGTTGGCGATGGTGGGAGTTGCGTCATTGC
TGGAGAACAGCGCTTTTTTTCTTTTTTCTTTT
CTTGTCTCGTGTGCGTGTGTGTGTGCGAGGGGTGCACCCCGCACGAGGCCTCAAACAGACTGAGGAAG
CGGAATA
>scaffold100.size32916
CACCGTGTAGTCTATGTATACAAAGGCTTTCGATGGGGATTCACGGCAGAGGTTGCCGCTCAACTGCA
GCCAGGAGTTGTAGTAGATGTAGGAAAGAAATT
TTGAATAAAAAACAGCCAAGCTTCTCTGCATGTCGTTGCCCTGAAACACAACCGCCGAAAAAGACTCC
ACAATTAAGGCCCAACGCAAAAAAATGACGCA
CTGCACCTGACTCGACACAACGTTGCCTCGGATCACCACGGCAGAGTTGTTCAGTACACTCAGCGACA
AAGAATGTCTAGAGCCCCCAATATATATCAGA
GAACCGTCACCGCCGGCCATATGAAACATGTTGCCCAGCACCGCAAACAGGGAGCGCCACAACACCCT
GAGCGGAGATCCAACAACGTGGATGGCGCTTG
ACGATGCCGTCACGGTCTGAAACACATTGCCACTGAGCACCACCGCGGAGTCGTTGCTGATCACCAGC
CCATTCATCGCAACACACGACGGGCTGTCAAC
GTCCAAACCCGGCCGAGAGATCAGCCTCGTGACCGTGAAGCGGTTCCCGCTGATCGTGATTTTCGTGT
GCGGTGGAAACCTACCCTCCGGGCAGAGCAGC
GCGTCATCGAACGTGTTGCCGGTGACAACAATGTTCAGCGACCCCACAGCGGCATACGCGTTGCCCAA
CAGCACCAGCACGGCGCCGTCCCTCCACGTGC
ATCCATCAAACGTGACGTTCGCCACGTCGCCCGTCATCGCCCCAATCAGCACCGTCAGGATCATGCCG

CCCCCGCTGAACGTCACGTTGCGGTAGCACAG
CCACGACAGCCCGCTGCCCACAGCCTGCGCAACCTCGGGGTACACCATGTCGCTGATGCATCCACCAG
CCGGCGGCGGCGGTGGCGGTGGTGGGAGCAGC
ATTGGTGGTGGCGGTGGGCCAGCAGGCACAGGCGCTGGGACGCACACATCGCCATGCCCTCCAGCAGC
GCACTGGCACTTGCAGTCAATGACCGCCGTCG
TCAGCGGAGCAAAGCAGTCGCCGTCCCTCGTGCACTCCCCGCACGCGGCAACAGTCGTCACGTTGGTG
ATGCCATTCAGCTCCAGTTCAGCTGCCGTCGT
CAGCACACGTCCCGCAACCGTCAGGCACCCAGCAACAAACCGGTAGCCAACGTTAGCACGCGATAGCA
GTGGTCCCGACAACCCCATCGAGCCCATCTTG
CAGCCCGTCAGCGTCACAACACTGCTGCCGTCAATGCTCACAAAAGCAGAATCCCATACTTTGAAAAT
CGCTCCCAATCCCACGTCGTTGTCTCGCCAAT
CCAACCAGATTGACCGCTGGACGGTAAAAAAGCTTAGTGAATAGATGGCGTAGGACACGGAACCGCTG
TTCCCCACCACGCGCAGCACGCTGTGATCCGA
CACGCTAAACATTTGCTTCTGATACAAGAAGCTTGTCCCGGAATCCATCGTATTGTTGAGCAGCGCAA
AAACAGAGCGATCACTGACACTGAGGATTTTC
ACGTAAAACAGGTGTTCGTACCCATCAAGGCTGCAGTACCGCGTGTACAGCGAGCTGCCGCCCACCAG
CGTCAGTGTCCCAATAGAGTACAAACCAAACG
CAACGTAACCAAATGCACACGCTTTGACGTTGCTAACCAACAGCGAGGACGCACTCAACAAAACGTCC
CTCAGCATCATAACGCTCCACAAGTTGTTCAC
AGATTCCAGCAACTGTACCGCCGAGGACTGTACCGCGTCAACCTCAGTGACAGCAATGTCACAGTGCT
GCGGGAGGGCAAGTGCAAACACAACAACGGAG
GACCGCATCACCGTCCGGCTCACGCGGATCAGGACGCCGGCTGACTCCGCCGCCTCAGCACTCCCAAC
AAACTGCAGCGCGGCTCCGTCACGGAATGTGC
ACCCAGTGAGCGTGATGTTGATGGGGAGATGCAGCGGCATACTGTTGAGGAAAAACGTCAGCACAGCA
CCCACGCCTCTGAATGTCACACCCACGTAGCA
GTGGTGCGTCTTCCACATgNNNNNNNNNNNNNNNNNNNNNNNNNNNNNNNNNNNNNNNNNNNNNNNNN
NNNNNNNNNNNNNNNNNNNNNNNNNNNNNNNN
NNNNNNNNNNNNNNNNNNNNNNNNNNNNNNNNNNNNNNNNNNNNNNNNNNNNNNNNNNNNNNNNNNNN
NNNNNNNNNNNNNNNNNNNNNNNNNNNNNNNN
NNNNNNNNNNNNNNNNNNNNNNNNNNNNNNNNNNNNNNNNNNNNNNNNNNNNNNNNNNNNNNNNNNNN
NNNNNNNNNNNNNNNNNNNNNNNNNNNNNNNN
NNNNNNNNNNNNNNNNNNNCTGCCGTCAATGCTCACAAAAGCAGAATCCCATACTTTGAAAATCGCTC
CCAATCCCACGTCGTTGTCTCGCCAATCCAAC
CAGATTGACCGCTGGACGGTAAAAAAGCTTAGTGAATAGATGGCGTAGGACACGGAACCGCTGTTCCC
CACCACGCGCAGCACGCTGTGATTCGACACGC
TAAAGATTTGCTTCTGATACAAGAAGCTTGTCCCGGAGTCCATCGTATTGTTGAGCAGCGCAAAAACA
GAGCGATCACTGACACTGAGGATTTTCACGTA
AAACAGGTGTTCGTACCCATCAAGGCTGCAGTACCGCGCGTAAAACGAGCTGCCGCCCACCAGCGTCA
GTGTCCCAATAGAGTACAAACCAAACGCAACG
TAACCAAATGCACACGCCTTGACGTTGCTAACCAACAGCGAGGACACACTCAACACGACGTCCCTCAG
CATCATAACGCTCCACAAGTTGTTCACAGATT
CCAGCAACTGTACCGCGGAGAACTGTACCGCGTCAACCTCCGTGACGGCGATGTCGCAGTGCTGCGGG
AGGGCGAGTGCAAAAATAACGACAGAGGACCG
CATCACCGTCTGGCTCACGCGGATCAAGACGCCGGCCGACTCGGCCGCCTCAGCACCCCCAACAAACT
GCAGCGCAGCTCCGTCACGGAATGTGCACCCG
GTGAGCGTGATGTTGATGGGGAGATGCAGCGGCATACTGTTAAGGGAGAACGTCAGCACAGCTCCCAC
GCCGCTGAATGTCACACCCACGTAGCAGTGGT
GCGTCTTCCACATGTTCAGCGTGAGGTTCTTCAGCGTCTGGTTCACCACGCAGCTCGTGTCGCCGTCG
ACGGCTCTCTCCGGCAAGGGCGGCACAACCGT
GTCGGGCACCTCGAAGGGAAGACACGACGCGCCATGCCATCCGTCCTTGCAGCTGCACGAGCACGAGC
CGCGGTCCACCGACTCCGTCCCGGGCATGTAG
CACGCAGCATCTTCGTTGCACGTGCCGCACCCAAACACCTCCACTTCCTCCGGAAACACACCGTCGTA
CGACACCTCCTCATCGCCCTGCAGGTTGCACC
CCACCATAAACCGCGCGGGTGAAACCACAATCATGTTCGACAGAGCCATTTTAACAAAAGGACAGCTA

TCGTCCACCAGACGGTTGTGTGCAAGTGCCAC
GGCAGTGCCGCTGCCCAACAGCCTCATTTTGTACAGGGATTTTGGCATACCCATTACTGAGGCTGCAC
TTACGTTGTTGCCCTCCACACGCCACTGCGCA
CCATCCTCGAGTGTCACCAAGCCGTCCAAATGCACCAGCGCAGGTTCAAAAAACAGTGTGATGCCAGC
AAAGGTACAGTCCGCCACTCGCAACAGCCCAG
CGGAGCTTACGATTATGTCCCCATAAAAATGAATGCCACTAACTGCGCTCATCGTGTTGTTCTCCACG
TCAAAGTAGCCGTCGTTCTTTAAAATAGCAGA
TTCAAAAAAAACAGCGGATTCCAACGGAAATTCCTCCTCCGCCCCCCTCAGCGTATTTCCCTTCATAA
TGATCCCGCCACCATCAACAACAACAAAGGAT
AAACGGACTGCGTAACTTTCCCCTTCAATGAGGTTGTCGAGCAACAGCAGCGTCGAGTTCGCACCAAG
AGAAAACCTCTGGAATTGAACGGCGTGGCGAG
ACCTCATCAAAAGCGTACTGCCAACCACCAGTATCTGGGAACTCGCGCCAAAATCTCCCTCAAACTCC
AATGCACCAGAAGCCAGCATCGAGGATGTCAC
GTTCACGTGCACGCGCGCACCACTCCCCTTGATCGACAGGCCCATAAGGACGCACTGCAGCAGCGTGA
TGTTGAGTGTCTGCTCTGGTGCGGCAAAATAC
AACAGGTCCAGCACGATCGCGGCGGTGAAGGTCACGTTGACGAAGCACAGACCACGCACACCGGGATC
AAGATAGTCCACGGAGCTGATGCTGCCACCGT
GCACGCAGCGGACCTCCGTGTCCTTCGCATCGGGGAGCGGGAGCGGCCCAAGGCCGTCCGGAACCGCA
GCTGGCAGACACGTGTCACCGTAGCCGCCAGC
AGCGCACACGCACTCACAGCCGCGGATGCTGCTCGTCCTCGCAGCAAAGCACACGGCGTCCACGGTGC
ACGCACCACAGTCCAGTGCCGTCACGGGTGCT
CCGATATTGAGGTCATCTTGGTAATTCGTCACAGGTGAGCCCCTTATCTCGTTGCACACGCCGTAGAT
GATCGGGCGAGAGTCGGATGGTGGCCACCAGT
TGCTGATCATGTATGTTAAGACCGAGTAAAAGCCGTATTTAAAGCTGTTGTAAAGTATCGACCACACA
CTCTGATCGCGGAACACCACGCCGTTCTCGTT
GGCTACGTACACAACGTAGGCGGTGCGGAACTCGTTGTCGCGATGCACCAGCCAGCTGCCACCAGTCA
CAGTCAGCGTGTTTGCCATGAGCATGGCGAAG
CCGAGACGGAACTCGCTGGACACAACCTGCAGCACGCTGCCGCCATCCACCGCAACGTCCCCAAACAC
ACACGCACCTTCGTAGAAGCCAATGCTCGGCG
CACTCCAAGAGCTGTTCTGTATGGAGAACACGGAGCCGCCGCTGACACGCAGGTCCGACGCAAGGGCG
TACATCACGTGCGACTGCGACAGGACAGCGCA
GTTGTCCATGTAGAAGACGCTGGACAGGTTCGCCACGAGGTCGCGCTCCACAAGGATCGCAGCGCACG
AGCCCCCGCCACACACCAGCGTAGACCGCGTC
ATGACAAACCGCGTCGACAGAAGCCGGACACCGTCCAGGACAAGCGCGGGGCCGTACCGGGACCCCGC
ATGGCCACGGGTCGTCGGCACGTACTGCGACC
CGCCAACCGTCGCGCGCAGCGTTGAGTTCGCCAGCAGCACACTCGAGTGCAGCGGCATCGTGCCATGC
AGCACAATCGTGCCCTCCAGCGACGTCACATT
CGTCATGTTGACGAGGGCGTGCGGAACCAGGCGCGCCGTGCTCTCGCTGAGGCCGCCAATCCGCAGCT
GCGCGCCGCCCACAAGCACACAGTGGCGCAGC
GTCACGTTCAGCGCGTCAGCGGACAAGTCCATCGAGCGCAGGTCCACCGCCACAGTGATCGGCCCGCT
GAACACCACCGAGTCGAAGCACGCCGTCGCAC
GCCCGCCGCCAACCGTCACGGACTCCGTCAGCGTCACGCCACTCACGCAGTCCTGCGCACCGCCGCCG
CCGGCCCTCGCAGGCGGCACGTCGAACGGCAG
GCACGCGTCGCCCACACCGCCGGCGCGGCAGCTGCACACGCAGTCGGAGAACGACGCCGTCAGCGCAT
CGAAGCACGCCAGCGCTGCCGCACAGCCGTCG
CACGGCACCACGCTCACGGAGGGCAGGCCGGCCATGCGGTAGTCGCCGCTGCTCTGCAGCACGCGGCC
GCCCGCACGGTTGCACTGCACGGACACAGCGC
CGCTCGTGATCGTCAGCCCGGAGACAAGCGTCGCGCCGGTGGCAGCGCAGCGAGCAATCGACACGGCG
CCGCCGCTCAGCGTCACACCCGACAGCGACGC
CACAGACGACGCCTCGCCCACCGCCCACACGTCGCGCAGCTCCAGCCACCCGCCGACACCGACCGTCC
CACCGTCGCAGCGCACCAGCGACGACGCGACA
GACCCCTCGACGCCCACGAAGCGCAGCACCGAATCAAACACCGCGAGGCGCTCGCCAAGCACAATGCC
GCCGCCGCTGCTCACTACCGACACGTTCGTCA
CGGAGAACACCGAGTGGCTCCGCAGACTGAGACGCGACGAGGACGCCACATGCATCGCGGACGCGGTC

GAACCGCCCGACGCCTCCAGCAACACGCCGTC
AAGCACCACCGCGCTCGACAGCAGGTCCACACCGCCGCCAACGTACACCGCCGACCCGCCCGCCTGCA
ACGACCTCAGCACAGTGCTGCTCACACGCAGC
TGCGTCTGCAACAGCGACGTCGCGTGCAGCACAAGCGGCGACGCCACCGCGTCCGCCGGCCCGCTCAG
CTGGGAGTAGCGCATCGGTCCCGGCGTCACAA
TCGTCGAGTCACGGACCGTCACGTTCGTGCGTGCCGGCAGGTTGTGCACAAACACGAGCGAGCAGTAG
CTCCCCGACTGCCCGCTCACGCTCACCTCCAG
GCTCCGGTCACTCGCCGGCTCGCCGCTGTAGCCCCGCACGTAGATCTGCGCACCGCCGTCGCACACGC
AGTTCCTCACTTCGATGCGCAGCGCGCCGGGA
AGCATCGCCGCAACGTCGAACACCACAGCCACGCCGTTCGTGATGCACACGCCGTCCATCAGCACCGT
GTCCACGGCGCGGCCAACCGTGATGGCTCTGT
CCACCGTGCCGCCCCGCAGTCGCAACACCACCGCATGCGCCGCGGGCACCCACGCCGTCGCCGCAACC
AGCGCCAACGCCACAAGCAGCGCCACACGCAC
AGTCGCAATGGCCCTGCGCCGCACACGGTGCCGGCTGCGCGGACACGCACCTTCCGCAGACCGCATCC
ACCGACACAAACAGGCACCAACAGGGCTGTCG
TGGCGTGTCGCACTCGCACCGTCAACCGTAACTCCTGTCTAACAAATCCCCTTTCTTCAGATGGCGAA
TACAGATACACAATTCCTATATATCGCTATCC
CACTAATTCTGCTATTCCTAATACAGCAATTAATAATTAACACCTTGAAACGATAGTTGTGCTGGTGA
AGCCAAAGTAACTCTAAATGCTCGGTCAGAGG
AGAAGGAAAAAGCAGGAACAACAGCAAATAAACACAGTGGCACGCAGAAGCATCCGGCCACAGCCCGA
CCGCCAACATACAGACAAGAAGACGCGCAGAT
GCAATCACAACAGTGACATCATCGCCCTCCCTCATCGAACAGCACCGCACTAAATCACGCCGTGGATA
CTTTGTGAACTAGCGGCAATGGGGCAGCGTAC
ACGGAACGGCATCAAAATTCCATCCTATAATTGCTAAAATTTATGTTTCTCGTACTTGTTGGCAACAA
CACTAAAAAGACCACAGAAGAAAAAGCTTCCA
TCCTTCCTCAAAATCAAAAACACAAGAAAAAACCTCTCTACCCTCTCCCTTTCCACGGCCTGAGTGCC
GCAAGAGGCTGTCAATAAAAACACGTGTGTGG
AGGCCCCAAATGTGCGGGCGGGGTCGGCATCCACCCACACACCCGCGCTCCCTCCACTCTCCCCCACA
CGGGGGCGGTGCCATGCGGGAAAGAAGTCCGC
CACACGACGCATGCAAAAAGCACTCGGTGAAGCAACACCACCCATGTCGCACCACAACAAAGAGCCAT
GCGCTCAGCACAGACCCACGGCCGCAACGAGA
GGAACGGGACGCACCCAAGTCCACGGCAATGTCCTTTACTTTTCTCTTCCACGACAAATTTAAATTGC
ACCCCACAACAGGTTGTGGCGGGTGGCGTCCC
ACACCTACGGTGACGCACGGCACACACCGTTCGCTCCCCTCCGAGTGCACTCAGCAGGCCGGGGCAGG
GCGCCCCGTCATCCTCGTGTCATCGCAAAGGG
GAACAAAAACAATCGTCGTCGCTGGCCACCGGCGCCTCTAGCAGGGAAGATGAAAGAATGGGGGCATT
TCAACAGCAAAGCCGACAGAGGGACATCCAAC
GGGACAAAAGACACAAAGAGACAGTGGGAACAAAACATGCAAAAACGAGCGAAGGGATTTACAAAAAA
ACAAAACACAATCAATAAAGGGCAGAAAAAG
AATGACGGTCCAGCAAAAATCACAAAAGAAAAAGTGATCAGGCAATACTTCGATTTCGTGCACCAAGC
AACAAAAAACGACCGTGCGTTTGGGTACACAC
ACATTGCCCACGCCAAAGAAAAGTAACAAGAAAAAGATTGTACTTTTCCACGAGACCACACAACGCCC
CTCACATTACATGTGGCACACGATCTGCTCCT
CCGCTCCTCCTTTTGTGCAGTCCGTGGGAATCACAGGAATGAGTCCGGCACAGCGTGTGGGCAACAGA
AGCAAACAGGAAGAAGAGGGCGCGCCACACAT
GAGGACGTGTGTGAATGATCAAAGAAACTTCACAAAAGCAAGAATTCAATGGATAAACAAACACAAGC
ATAAAAAAAAAAAATTACTCTTCGGAAGGCAAT
GGAATTGGAAGTGGTGCTGCGGATGTGTTCCAACGATTGCAGCAACCCCACACTCTCCCAGACCCCAA
CAGAAAGCCGCAGTGCCCCAAAGCCCCAAAAG
CGGGAGCAAAAACACCCGAGACACACACCCATGCACGGTGCTCTCACCCCTTAAATCCCTTGCCCTCA
AGTTATTGCTGCTCTCGGAATAAGAAGAAACG
CTGTCAGAAATGGCCTCACTGCCAGCTCACCCACTCCTCAGGCAGCGCTGGTAGCGGCTCCATCTTTG
GCCCTTCCGGCCCTTTTGATTCTCCCCAAGCA
AGAGTACTAGACACTGGCACAACGTTGAAGTGGCCGGCTGCGCCAGGGTGCAATGTACTTCCGGAATT

CTTCCCTACTCAAGGGTCGGTTGTACAGAAAG
ACGTTTTTCGTCGTCACACGCCTCTCACTCCGTGTGTCGTCTGCGCTGCCTCCATCCCCTTCAATGCT
AAAGCGTCCGATCTCCTGTGAATTCCTTCCAG
TCGCTGATGTCGATGCTGAATTCTTGAAACGCTGGCCAGTAGTGTAGACAGAGACCCGACTGCCAGTC
CAAGGCACAACCGCCACCTGCCATTCCTTACC
CAGTCCCAAAGTGATTCCGTTTGTATCCTTCTTTCCGTTGAACATTGTTTCCCACCTCATTTCATGTG
TTTGCGACTGTTCAAGAAATGCAGCGCTGCCT
GTTTCATTTACCCCCACACCCATCAAAAAGATGCTGCTGCCGCAAGACAGTGACAACTTGTAAATAGA
AATCGTCTCCACAAAAGGTGAACCCGATGATT
GCAAGGCAATAGATCTGATTCTTCCCCCAATTCCCAACGGACCGCTACGCCCAGAAACCAACGCCCTT
GGAAGCCGATCCACTGGAAAGCCCTTGTTCCA
CAGCTGCCCCACCCGTCTCCGTAGCGTTCAGCCCCCAAGTCCCTCTTCCCTCCATGTAGTCTCGAATA
ATTTGCCGGACAAACATCCAACCAGCCCTGCT
GGAGGCATGAAAGTGTCGCAGCCCTCGACAGACAGAAGATTTTTCTTGACATTTGAGTAAGAGCAGAA
CTGGAAGACACAAATGCACAGCTCCCTCAAAG
CCCTCACCAAATTCTTCACCCGTTTCAGCTTCGCAGTCAGGCACACAGACAAATGGCTGCGTGCCCAT
TCCCCACCGTCCTTCTTCCCGCACAGTAAAAT
CAACTCNNNNNNNNNNNNNNNNNNNNNNNNNNNNNNNNNNNNNNNNNNNNNNNNNNNNNNNNNNNNNN
NNNNNNNNNNNNNNNNNNNNNNNNNNNNNNNN
NNNNNNNNNNNNNNNNNNNNNNNNNNNNNNNNNNNNNNNNNNNNNNNNNNNNNNNNNNNNNNNNNNNN
NNNNNNNNNNNNNNNNNNNNNNNNNNNNNNNN
NNNNNNNNNNNNNNNNNNNNNNNNNNNNNNNNNNNNNNNNNNNNNNNNNNNNNNNNNNNNNNNNNNNN
NNNNNNNNNNNNNNNNNNNNNNNNNNNNNNNN
NNNNNNTCCTCATCATCCTCGCCTTCACCACCTTCGTAAAAAAGGGAGCTGGCGGCAGCAGCATACAC
ATTACGGGATGCCAGCCAAATACCCACAATGT
GAACATTGTCCGCCAGCCAGAGAGAAGGCCGACCCTTTTCCTTTTCGCTCGCTTTATAACACACCGGT
AGGGTAACGCGCAGAACATTCCCTTCTCCAAT
CACCGCAGTGATGAAGCCGCTCAGAACGCCTTTCCCCTGTCCATCATGCCAGTTGGCCCACAGGCGGG
CAATTGAGTGTCCCCAGGGGCTCCGCCCACGC
CTCCCCATTTTCACTGGACGCGTCCACCCTGTGGCCGCCATCGGACGCAGTGATCATGCAAAGATTTC
CACATTCCCACACCACGACAAAGGGATCACTT
CACCCCTCAGCAGATATCGCCCTCGACAGCATTTATTTCCCAAGCTTTGCCAAGCACGCGATCGGTGG
GGCGGGATTTCCCCAACACTCACAGTTTTTAC
AGGGATCACCAATCCTCCTTCAGCGTCATCAACTGCAGCGGCTACGCCGCCAATAATTTGTGTCAGAG
ATCTTTTTTTAAGCTGTTCTTAGCGCAAAGCT
CCAAGGACGGTTGTCTTCGCAAGGTAATTTTCTTAATTCCTCCTTTTCCCTGCTTGTCTCCGTGAAAT
TCACCAACAACCAGCAGTGGTTCGCGGCACCT
CCTCCGTTCGACTGCTCATTCCAGGCGTATTTATTGCCGTGGCCTCCCAAAAGAAAAATTACCTTATT
TCTCCTTTGCAATCGCAGTAGGACTCAGGGCA
CAAGAAACTGGAATAAATCCGGCTATGGAAGCCGGCAGTTTTGTCCTCCAATCCTTCCCATCATATTC
ACTGGCAATACCGGCCACCCCGGCGTGGTGCC
TGGGTCAATTGCCCGTATTGTCGGGGGAAGCGAGCAATGCTTTCCTCACGGAATTTTTGATGCTGGCG
CAGGGCTGATGGCAGCAACCAGTACACGGGCC
AGCCGCGTATTGCCGGGCACATTACAACATCACGAGGTCTGGAGGCACGCGTTGGCGTGCCGTCAGCA
CTGCCATCCGTACTGTGTCAGTGCTAAAAGGA
GATGTGGAAGAGAATCCCCGCCCCTTTCTGCAGAAGTTACGGTGAAGCGCTGCGGGTCTCACTCTGGC
AAAACGCTTGGCGCTGAGCGTGATGTTGTATG
ACAAAAACGCGTTGTTCGCGCGTTGGGTGTGAAGAAGATGTCCCCACCCTGAAGCCACTGGTTCCAGC
CCGCCAGGGTTCCAGCACCATGGAACTGCTTG
CATCTGTCCAGGCGGCGGCGTGTCCATTCTCGTCCGTGAGGGGCTGCGGGCGGAAGCAGGACCGGCCC
TTTTCGCCGGCATCGGGCTTGCGCAAGCGACC
AGCCACCCAGAGGCAGGAATAAAACTGACGGTGTCGTCGGCGTATCTCCTTCCCTCCTCTGCCAAGAG
TATAAAGGTGGAGGAGTCGGATGGGCAGCTCT
GCGAGGACGGGCCACAACTAATAGGGCAAAGACACCAACGCACACAGTGAGGCTTGGGAACGCAGCAT

CCCATCAGGCTCCCGTGGCGATATCGTTGCGC
AGCGGTGCATTGAGAATGTACCCCAGTTCATCAACGCCGGAGAGCGCACGAGGTACACCGAACAACAG
GGCAGTTTAACATCCGACATAGCCCTGCCTGA
AGACTGTGTCGTCGCCCACTGGGCAGCACAACCCTCACCGGACAGTGATCCCTGCATGACCCTCCTTG
ATGCTCTGATCGGAGACGACGCACCCGCAGTG
GTTCCGAGGCCTAACCGGAAATAATTAACATGGAAGAAGGCAGATTGGAATAAGCAAAGCGGGAATAC
CAAACAACTCGGCAGAATCCCGCAAGAATTAA
ACAAAATGAGCGGCACAGAGAAGCACATTGCATCCGGAATACAGAAATCCACCAAGGCAACTGTCCAG
GCAGGAAACCGCCAGTCGTCCCCCACTCTGGA
CCCCAGAGCTGGCGGAGCCGGACTGCGACATCGCCGCCGCACCCCACGCAGAACGCAGTGAGCGGCTC
AAACTCCGTCACAGAGATGTGCTGCACGAAGT
ACCCAAAAGCCGCTGGCGCACGCTCCTCTCCAAACTCGAGTCCGCGGACGGTAACAGCCGGCAGATAG
TACGCCGTGCGTATGCCACTCGCTCCCTGAGC
ATGCCAGCCATGAAAACAGATGGCCCAATGCTCAGCAGACACCGGAACGTCGGTCACACAAAGCCGTG
GGCGGCAAAAGCAACAGGACACCCAAAGCCAT
GCCCGCCGCCACCGATCCTGACGAATGCGAGAAATTTCACCCCAACGAACATGGCAGAGCTGAACCGC
ACCCTACGACTGTTGCCCTTCGGACCGGCGCC
TGTCCCCGACAGAATGAATGGAGAGTCGCTGCGCCATCTACGCCGTGTAGCAAAGCGTGCTGTCCCCG
GTTGTCCAATATACGCCTACGCACAGAAGCCG
TCCGGCGGAATTGGCGACGTGGAGTCACGACACCCTTCCCTCCATTCTGGGGAGAACGGCATCAAAAC
ACCCACTCACACCGACCTGTCGCGCTGAGCAG
CTGCCTCGGATCGCTCATGTAACGTATTTGTGCCGCACGCAGCCGAGACATCATCGAGTCCCAGTTGT
CTCCACAGCCATCTGGCCTTCGGCCCAACAAT
TCCTCCCTCGACCAATTGCTGCACCTCAGGGCTGCCATGCACTGCAACGCTCCACAACACCGCACGCT
GGCTGTCTTTGTGGACCACGCCTATGCCTTTG
ACACAGTAAACCATGACAAAATCGTTTTGGAAATGCAGCGACTGTCGGCAAACAATCACATCGCCGTG
GTCTCCATCACGCTCGCAACAACCCCCTGCTG
CAATGTTCTTGTATTCAGGCAACGCCCCGGCACCGCGTGAAGGCGCGACTTCATCGCCGGGGAGAAGG
ACGCCGCCGCAGTTCCCCAGTAGCAAATTCGT
GCCTACGTGTCTTGCTTAACGCAGCCGCAGAAACAATACGGAAATAGAAGACATGCAGGCACACAGAC
ACAAGAAACGCAGTGGGCGCGGTACAGAAGCA
CAGCCAGCAGTGAGAAACCCAACAAACCAAATACAAGCAAAAAAACATCAAAAGAGAATATTCCTAAA
GACTCCCCACAAATGAATTTCTGCGGGTGTGT
GTTCTACGGCTATGTTTATATATTTCACTACACACAAAAAAAGCATACGCTTCAGCACACAATATCAC
GTCCTACGTTACGGTACAACAGAAAATCGAGC
GCAACAAGGGCGACAGCTAAATCCCCTCCCACACAGTCGTCGGGAAACGGGAAGAGTCGAACGAGCAC
TCGCTCAAAAATAGAAAAAAGTCTATAGAAAC
TACGAACAACGACACGGTCGGTGCAAGGGCAACGAGTGGTTGTGTTGCGCAGCAGCCATGGCGTCGCA
CCCTTCCCTCATCGACCAGGAACACACGCCTG
CCAGTTCCAAGTAGCGCACTTCAGAGAACGGCGTGATCAATCAATACGGCTCGCAATGGATGCGCGGT
CCAGCAGGCTCAGGGCGTCTGAGCGAATGTCA
CAGGCCACAGACAGCAAAGACGGTGCGGGTGGTCGGTACGACGACGCAACGGTGGTGCCTGAGGCGTA
CGACGATGGCGCCATGTCATGGAGCTTCTGCG
TCTCCTCGTCGCTCTCCTCGTCATCGCGCAGCAGCGCCTCCAGCCCGCCACGCCGCGGCTCGCGCAGC
TCCTGCCAGTGGCGGTCCTCCGCGTGCCACAC
AACGACGCCGTACACAGTGACCGCAAGCAGCACAGTCGTCAGCAGCAGCACGATCGCCGCGTACGCCC
TCGCTCCGCCGTCGCTCGGCGCAAGATGGTTG
GCCGCACTCACCACGCACAGCGCCGCAAGCAGCACAAAGCTCGCAGTCTGCATCACCGTCAGGAAGGC
CGAGCGCATCATGTTCGTGAAGGCAACCACGC
CCGCACCCGCAAGCAGCACAGCCGCCATGCAGAAGTACAGGACGTGGCAGCCTCCCACGGGCGAGTGC
ACAGCAGCAATCAGGCCCACCACACACAGCAC
CGACAGCTGGAACACGCACCAGTACACGCGGCTGCCCCTCATGCTCGTCAGCATGCCGCCGTACATCC
GCTGCTGCGCCGCGGGGTGCCAATACCCGACG
GGGTACAGCAGGCGCTCGTGCAGCGGCTNNNNNNNNNNNNNNNNNNNNNNNNNNNNNNNNNNNNNNNN

NNNNNNNNNNNNNNNNNNNNNNNNNNNNNNNN
NNNNNNNNNNNNNNNNNNNNNNNNNNNNNNNNNNNNNNNNNNNNNNNNNNNNNNNNNNNNNNNNNNNN
NNNNNNNNNNNNNNNNNNNNNNNNNNNNNNNN
NNNNNNNNNNNNNNNNNNNNNNNNNNNNNNNNNNNNNNNNNNNNNNNNNNNNNNNNNNNNNNNNNNNN
NNNNNNNNNNNNNNNNNNNNNNNNNNNNNNNN
NNNNNNNNNNNNNNNNNNNNNNNNNNNNNNGGCGCTGGCGGCGGCGGGCAGCGTGTCGGACGCGATCGTG
AAAGACCCAAGCAGCACACCCTTGCAGCCGCC
AAACACCGCCACAGCGTCGCAGCGAATGACTATCGTCTCGTCGGCACCAATGAAGTAACCGCGGTGCA
CGGGCACAGCCAGCTCCAGGACGGTGGACGGA
TTCGTCGCGTTGCGCACCCACGTCGCGTTGCGCAGCATCGCTCCCCACGGACCGCCGAACCCGCGCGG
CTGCGCCGTCGACACAGGCACGAAGCTCAGGT
GCGTGCCGAGCTGCGGTTCGCCTGCCCACCGAAACGGCGGCGGCAGCGCCACCATCAGCCGTGTCGGG
ACGGCGCTCCCACCGCCGCGAATGTCGTTCTG
CGTCAGGCTCCCACCAGCCGCCGCGTCGTCACGGCGAGTGTCGGGCACGCCACGTCGCTCCACCACAA
AGTGCTGCTCACCGACGCCGTCGGGGTCCGCG
TGGGTGACAGCGCTACCGTCTCTGTCACCTGCAGCGTCTCCGTCGGACTGTAGTGCGTCGCGCTCATG
CCCGGCGTCCACGTTGGCGTCGGTGTCAGCGT
CTCCGTCGGCGCCGCCGTGTGGCTCAGCAGTGGCGGGGCGAGCGTGCGGTTGCACCCGTCCACGTGCG
GCAGGTACACCGGCAGGCAGTCGTGCCCGTAC
CCGTCCTCAGCACAGCGGCAGCCGCACGACCCCGACATCGCCCTCGTCGCAGCAGCAAAGCAGCGCAC
GTCGGCGTCGCACACGCCACACGCCACGGGGC
GGAATTCCCCGATGATGCCGGCGGACCTGTAGTCCATCGGCTGCAGCGCCTGCCCGCTCAGCGTCAGG
CACCCCACGACAAACTTTCCGCCGGCGCCCCC
AAGCGCAACGGTGCCGTCCATCACAACGGGCCCGCTGCCAGCGTTCCCGTGGAGCGTCAGCGTGCTCT
GCACAAGCTCCGCACTGCGCGGGTACGACGGC
ACAGANNNNNNNNNNNNNNNNNNNNNNNNNNNNNNNNNNNNNNNNNNNNNNNNNNNNNNNNNNNNNNN
NNNNNNNNNNNNNNNNNNNNNNNNNNNNNNNN
NNNNNNNNNNNNNNNNNNNNNNNNNNNNNNNNNNNNNNNNNNNNNNNNNNNNNNNNNNNNNNNNNNNN
NNNNNNNNNNNNNNNNNNNNNNNNNNNNNNNN
NNNNNNNNNNNNNNNNNNNNNNNNNNNNNNNNNNNNNNNNNNNNNNNNNNNNNNNNNNNNNNNNNNNN
NNNNNNNNNNNNNNNNNNNNNNNNNNNNNNNN
NNNNNGTTGCCGTCCCTCGTGCACTCCCCGCACGCAGCAACAGTCGTCACTTTGGTGATGCCATTCAG
CTCCAGTTCAGCCGCCGTCGTCACCTCCCGTC
CCGCAACCGTCAGACATCCGGCAACGAACATGTAGCTGGCCTCTACCAGAGATCGCAAAGGCACCGAC
AACCCCATCGAGCCCATCGTGCAGCCCGTCAG
CGTCACCACACTGCTGCCATCGATGATAAGAAAAGTGGATTCGGGTTCGTGGAACATCGCACCCGCTC
CCACGTTGTTGTCACGCCAATCCAACCAGCTT
GACTGCTGGACGGTCCACGAGCCTTCTGCATAAATGGCGTATGACACGGAGCCGCTGTTCCCCACCAC
GCGCAACACGCTGTGTTCCGACACGCTAAAGT
CCTGCTGCTGATAAAGGAGGCTTGTCCCGGAGTCCATCGTATTGTTGAGCAGCGCAAAAACAGAGCGA
TCACTGGCAATGAGGATATTTAAGTGGAACAG
GTGTGCGTATCCATCTAGGCTGCAGTAACGCGTGTACAGCGAGCTGCCGCCCACCAGCGTCAGTGTCC
CGGTCGAGTACAAACCATACCCGCCGCGCCTC
GACGTATGCGCCTTGACGTTGCTGACCAGCAGTGAGGACACACTCAACACAACTTCGTGCAGAGTCAC
TACGCTCAATTTTTTGTTCACATTGTCCGGCA
AATGGACCGCGGAGGACTGCACCGCGTCAACCTCCGTGACGGCGATGTCGCAGTGCTGCGGGAGGGCA
AGTGCAAAAATAACGACAGAGGACCGCATCAC
CGTCTGGCTCACGCGGATCAGGACGCCGGCTGACTCGACCGCCTCAGCACCCCCAACAAACTGCAGCG
CAGCACCCTCGTGGAATGTGCACCCGATGAGC
GTGATGTTGATGGGGAGATGCAGCGGCATACTGTCGAAGGAAAATGTCAGCACGGCACCCACGCCGCT
GAATGTCACACCCACGTAGCAGTGGTGCGTCT
TCCACATGTTCAGCGTGAGGCTCGTCAGCGTCTGGTTCACCACGCAGCTCGTGTCGCCGtCCACGGCT
CTCTCCGGCAAGGGCGGCACAACCGTGTCGGG
CACCTCGAAGGGAAGACACGACGCGCCATGCCATCCGTCCTTGCAGCTGCACGAGCACGAGCCGCGAT

CCACCGACTCCGTCCCGGGCATGTAGCACGCA
GCGTCGTCGTTGCATGTGCCGCACCCAAACACCACCACGCCCTCCGGAAACACGCCGTCGTACGACAC
CTCCTCATCGCCCTGCCTGTTGCACCCCACCA
CAAACTGTGCGGGTGACGTCACAATCGAGTTTGACGGAGCCAGACCAGCAAAAAAAGAAGTATCTTCC
ACCTGACGGTTGTGTGCAATTGCCACAGTGGT
ACCGCTNNNNNNNNNNNNNNNNNNNNNNNNNNNNNNNNNNNNNNNNNNNNNNNNNNNNNNNNNNNNNN
NNNNNNNNNNNNNNNNNNNNNNNNNNNNNNNN
NNNNNNNNNNNNNNNNNNNNNNNNNNNNNNNNNNNNNNNNNNNNNNNNNNNNNNNNNNNNNNNNNNNN
NNNNNNNNNNNNNNNNNNNNNNNNNNNNNNNN
NNNNNNNNNNNNNNNNNNNNNNNNNNNNNNNNNNNNNNNNNNNNNNNNNNNNNNNNNNNNNNNNNNNN
NNNNNNNNNNNNNNNNNNNNNNNNNNNNNNNN
NNNNNNGGGCGCCCACGGTACACGTGCCACAGTCCAGTGCCGTCACGGGTGTCCTAATATAGAGGTCT
TCATCGTAATCAGTCACAGGAGATCCTCTTAG
CTCGTTGCACGTGCCGTAGATGATCGGGCGTTCGTCGTCTTGTGCCGACCAAAAACTTGTCATGTATG
CAGCGGTCGACGAATAGGAGCCGTAACCAAAG
TCGTTGTAAAGTATTGACCACACACTCTGATCGCGGAAAGCCACGCCGTCGTATTTGTCCACGTGCAC
AACGTAGGCGGTGCGGAACTCGTTGTCGCGGT
GCACCAGCCAGCTGCCACCAGTCACAGTCAGCGTGGTTGTCATGAGCATGGCGAAGCCAAGACGGAAC
TCGCTGGACACAACCTGCAGCACGCTGCCGCC
ATCCACCGCAACGTCCCCAAACACAAGCGCGCCTTTGTAGTACTCGGTGCTCGGCGCACTCCACGAGC
TGTTCTGTATGGAGAACACGGAGCCGCCGCTG
ACACGCAGGTCCGACGCAAGAGCGTACATCACGTGTGCCCGCGACATAACGACGCAGTTGTCCATGTA
GAAGACGCTGGACAGGTGCACGCCAAGGCCGT
GCTCCACGAGGATCGCAGCGCACAAGCCCCCGCCACACACCAGCGTAGACCGCGTCATGACAAACCGC
GTCGACAGAAGCCGGACACCGTCCAGGACAAG
CGCGGGGCCGTACCGGGACCCCGCATGGCCACGGGTCGTCGGCACGTACTGCGACCCGCCAACCGTCG
CGCGCAGCGTTGAGTTCGCCAGCAGCACACTC
GAGTGCAGCGGCATCGTGCCATGCAGCACAATCGTGCCCTCCAGCGACGTCACATTCGTCATGTTGAC
GAGGGCGTGCGGCATCAGGCGCACCGTGCTCT
CGCTGAGGCCGCCAATCCGCAGCTGCGCGCCGCCCACAAGCACACAGTGGCGCAGCGTCACGTTCAGC
GCGTCAGCGGACAAGTCCATCGAGCGCAGGTC
CACCGCCACAGTGATCGGTCCGCTGAAANNNNNNNNNNNNNNNNNNNNNNNNNNNNNNNNNNNNNNNN
NNNNNNNNNNNNNNNNNNNNNNNNNNNNNNNN
NNNNNNNNNNNNNNNNNNNNNNNNNNNNNNNNNNNNNNNNNNNNNNNNNNNNNNNNNNNNNNNNNNNN
NNNNNNNNNNNNNNNNNNNNNNNNNNNNNNNN
NNNNNNNNNNNNNNNNNNNNNNNNNNNNNNNNNNNNNNNNNNNNNNNNNNNNNNNNNNNNNNNNNNNN
NNNNNNNNNNNNNNNNNNNNNNNNNNNNNNNN
NNNNNNNNNNNNNNNNNNNNNNNNNNNNNNGCTCACAGCCCCGCGCACAGACACCGCGTGCTTGCACAGC
AGAAAGTCGCTGTCAACAAACGCCACCACGGA
GCCGCCGCTGGCCGCGACACCGCCCTCCACCGACACGCACAGCCCCAGCGCCGTCTGCACCGACAGCC
CGTCCAAGTACAGCGCACCGCGCGACGACAGC
GCCATGCCGCCAACAACCAACACGCCAACAGCGTCGTCGAACACCGCCGCCACCTGCGTGCGCGCAAT
GGTCAGCACGCTCCCCGTCAGATTCACGTTGT
GCACCACAAGGCACGCGGACGCATCGCCAAGGTCGTAGGCGCCGCGGTACGCAACGCGCTTCTCTGCA
ATCAGCACCGAGTCCACCACAGTGACGCGCGA
GCCCGGCGGGTAGCGGTTCGCCACAACCACGCCGCCGCCGGAGCGCGACTCGAGCCCGCTGATCAACA
CATCGGCACGCTGGCCAGCAGTCGGGTCGGAC
AGCCACCCGACAACGTACAGGCTCGCTCCGCCCACAAGCGTGCAGTTCACCAGCGTCACGTTGCGCAC
GCTCCCCGACATCAAGCCCACGTCCACCACGA
CGTCCGCCGCAAAGGTCACACACACGTAGCACACCACCGACGTCCCGAAACCGGCGTTCACCTCCACA
CCCACACTCACGTCGCGCACGCACGAGTCGGC
GCCGTCGGTGCTCGGTGGCTCGGGAACAGCAGCGGGCAGGCATGCATCGCCGTAGCCGCCCTCAGCGC
ACGTGCAGGCGCAGCCATCACTCGACGCCGTC
GTCGTGTACGCGGGGAAGCACGATGCCGCAATAGTGCATGGGCCGCTGCAGGTCAGCAACGTGGCGTT

GTACACGGACGGGATAACGTAGTTCGCTTCCT
CCTTGCCATTCACTGTGTTGCACGCAGCCATTATCGCTCCGTTCGTGATATCGCGAGACCCTGAGTAT
ATCCATAGCACTGTCGGCTTGACCGCGCTGGA
CATGAGTTGGTTGCCGGACACAGACACAGTGGAGTCGCGGAGATTCACTGTGAAGCTCAAAACAGCAA
AGGTATCCGATGGGGATTCACGGCAGAGGTTA
CCGCTCAACTGCAGCCAGGAGTTGTAGTAGACGTAGGTATAGTAGTTGGTGAAGAAAACAGCCACACT
TCTCTGCATGTCATTGCCCTNNNNNNNNNNNN
NNNNNNNNNNNNNNNNNNNNNNNNNNNNNNNNNNNNNNNNNNNNNNNNNNNNNNNNNNNNNNNNNNNN
NNNNNNNNNNNNNNNNNNNNNNNNNNNNNNNN
NNNNNNNNNNNNNNNNNNNNNNNNNNNNNNNNNNNNNNNNNNNNNNNNNNNNNNNNNNNNNNNNNNNN
NNNNNNNNNNNNNNNNNNNNNNNNNNNNNNNN
NNNNNNNNNNNNNNNNNNNNNNNNNNNNNNNNNNNNNNNNNNNNNNNNNNNNNNNNNNNNNNNNNNNN
NNNNNNNNNNNNNNNNNNNNAAAAACAACCGC
TGACAGAGACTCAACACGCAAAGCCCGAAAAAATAAAATGAAGTAATGCACCGGCCTTGACACAACGT
TGCCTCGGATCACCACGGCAGAGTTGTTCAGT
ACATCCAGCGACGAGGATTGGAAAGACCCTCCAAGATGCATCAAAGTGCTATCACCGCCGTCCATATG
AAACATGTTGCCCACCACCGCAAACAGGGAGC
GCCACAACACCCTCAGCGGAGATCCAACAACGTGAATGGCGCTTGACGATGCCGTCACGGTCTGAAAC
ACATTGCCACTGAGCACCACCGCGGAGTCGTT
GCTGATCACCAGCCCATTCATCGCAACACACGACGGGCTGTCAACGTCCAAACCCGGCCGAGAGATCA
GCCTCGTGACCGTGAAGCGGTTCCCGCTAATC
GTGATTTTCGTGCGCGGTGGAAACCTACCCTCCGGGCTGAGCAGCGCGTCATCGAACGTGTTGCCAGC
GACAACGATGTTCAGCGACCCCACAGCGGCGT
ACGCGTTGCCCAACAGCACCAACACGGCACCGTCCCTCCACGTGCATCCATCAAACGTGATGTTCGCC
ACGTCGCCCGTCATCGCCCCAATTCAGCACNN
NNNNNNNNNNNNNNNNNNNNNNNNNNNNNNNNNNNNNNNNNNNNNNNNNNNNNNNNNNNNNNNNNNNN
NNNNNNNNNNNNNNNNNNNNNNNNNNNNNNNN
NNNNNNNNNNNNNNNNNNNNNNNNNNNNNNNNNNNNNNNNNNNNNNNNNNNNNNNNNNNNNNNNNNNN
NNNNNNNNNNNNNNNNNNNNNNNNNNNNNNNN
NNNNNNNNNNNNNNNNNNNNNNNNNNNNNNNNNNNNNNNNNNNNNNNNNNNNNNNNNNNNNNNNNNNN
NNNNNNNNNNNNNNNNNNNNNNNNNNNNNNTG
ACGAAGCACAGACCACGCACACCGGGATCAGGATAGTCCACGGAGTTGATGATGCCACCGTAAACGCA
GCGGACCTCCGTGTCCTTCGCATCGGAGAGCG
GGAGCGGCCCAAGGCCGTCCGGAACCGCAGCTGGCAGACACGTGTCACCGTAGCCGCCAGCAGCGCAC
ACACACTCACAGCCGCGGATGCTGCTCGTCCT
CGCAGCAAAGCACACGGCGCCCACGGTACACGTGCCACAGTCCAGTGCCGTCACGGGTGTCCTAATAT
AGAGGTCTTCATCGTAATCAGTCACAGGAGAT
CCTCTTAGCTCGTTGCACGTGCCGTAGATGATCGGGCGTTCGTCGTCTTGTGCCGACCAAAAACTTGT
CATGTATGCAGTGGTCGACGAATAGGAGCCGT
AACCAAAGTCGTTGTAAAGTATTGACCACACACTCTGATCGCGGAAAGCCACGCCGTTGTATTTGTCC
ACGTGCACAACGTAGGCGGTGCGGAACTCGTT
GTCGCGGTGCACCAGCCAGCTGCCACCAGTCACAGTCAGCGTGGTTGTCATGAGCATGGCGAAGCCAA
GACGGAACTCGCTGGACACAACCTGCAGCACG
CTGCCGCCATCCACCGCAACGTCCCCAAACACAAGCGCGCCTTTGTAGTACTCGGTGCTCGGCACACT
CCAAGAGCTGTTCTGTATGGAGAACACGGAGC
CGCCGCTGACACGCAGGTCCGACGCAAGAGCGTACATCACGTGTGCCCGCGACATAACGACGCAGTTG
TCCATGTAGAAGACGCTGGACAGGTTCACGAC
AAAGCCACGCTCCACGACGATAGAAGCGCACGAGCCCCCGCCACACACCAGCGTAGACCGCGTCATGA
CAAACCGCGTCGACAGAAGCCGGACACCGTCC
AGGACAAGCGCGGGGCCGTACCGGGACCCCGCATGGCCACGGGTCGTCGGCACGTACTGCGACCCGCC
AACCGTCGCGCGCAGCGTTGAGTTCGCCAGCA
GCACACTCGAGTGCAGCGGCATCGTGCCATGCAGCACAATCGTGCCCTCCAGCGACGTCACATTCGTC
ATGTTGACGAGGGCGTGCGGCATCAGGCGCAC
CGTGCTCTCGCTGAGGCCGCCAATCCGCAGCTGCGCGCCGCCCACAAGCACACAGTGGCGCAGCGTCA

CGTTCAGCGCGTCAGCGGACAAGTCCATCGAG
CGCAGGTCCACCGCCACAGTGATCGGCCCGCTGAACACCACCGAGTCGAAGCACGCCGTCGCACGCCC
GCCGCCAACCGTCACGGACTCCGTCAGCGTCA
CGCCACTCACGCAGTCCTGCGCAGTATCGCCACCGCCGCCGCCGGCCCTCGCAGGCGGCACGTCGAAC
GGCAGGCACGCGTCGCCCACACCGCCGGCGCG
GCAGCTGCACACGCAGTCGGAGAACGACGCCGTCAGCGCATCGAAGCACGCCAGCGCAGCCGCACAGC
CGTCGCACGGCACCACGCTCACGGAGATCAGG
CCGGCNNNNNNNNNNNNNNNNNNNNNNNNNNNNNNNNNNNNNNNNNNNNNNNNNNNNNNNNNNNNNNN
NNNNNNNNNNNNNNNNNNNNNNNNNNNNNNNN
NNNNNNNNNNNNNNNNNNNNNNNNNNNNNNNNNNNNNNNNNNNNNNNNNNNNNNNNNNNNNNNNNNNN
NNNNNNNNNNNNNNNNNNNNNNNNNNNNNNNN
NNNNNNNNNNNNNNNNNNNNNNNNNNNNNNNNNNNNNNNNNNNNNNNNNNNNNNNNNNNNNNNNNNNN
NNNNNNNNNNNNNNNNNNNNNNNNNNNNNNNN
NNNNNCTACAATCAGCACCGAGTCCACCACAGTGACGCGCGAGCCCGACGGGTAGCGGTTCGCCACAA
CCACGCCGCCGGAGCGCGACTCGAGCCCGCTG
ATCAACACATCGGCACGATGGCCAGCAGGCGGGTCGGACAGCCACCCGACAACGTACAGGCTCGCTCC
GCCCACAAGCGTGCAGTTCACCAGCGTCACGT
TGCGCACGCTCCCCGACATCAAGCCCACGTCCACCACGACGTCCGCCGCAAAGGTCACACACACGTAG
CACACCACCGACGTCCCGAAACCGGCGTTCAC
CTCCACACCCACACTCACGTCGCGCACGCACGAGTCGGCGCCGTCGGTGCTCGGTGGCTCGGGAACAG
CAACGGGCAGGCACGCATCGCCGTAGCCGCCC
TCAGCGCACGTGCAGGCGCAGCCATCACTCGAGACCGTCGTCGTGTACGCGGGGAAGCACGACGCCGC
AAGGGTGCATGAGTCGCTGCAGGTCAGCAATG
TGGCNNNNNNNNNNNNNNNNNNNNNNNNNNNNNNNNNNNNNNNNNNNNNNNNNNNNNNNNNNNNNNNN
NNNNNNNNNNNNNNNNNNNNNNNNNNNNNNNN
NNNNNNNNNNNNNNNNNNNNNNNNNNNNNNNNNNNNNNNNNNNNNNNNNNNNNNNNNNNNNNNNNNNN
NNNNNNNNNNNNNNNNNNNNNNNNNNNNNNNN
NNNNNNNNNNNNNNNNNNNNNNNNNNNNNNNNNNNNNNNNNNNNNNNNNNNNNNNNNNNNNNNNNNNN
NNNNNNNNNNNNNNNNNNNNNNNNNNNNNNNN
NNNNACGTCCCTGTGTTGGACATGAGTTGGTTGCCGGACACAGACACCGTGGAGTCGCGGAGATTCAC
CCGGGGGGACAAGTGGACAAAAGCATTCGATA
GGGAATCACGGCAGAGGTTACCGCTCAACTGCAGCCAGGAGTTGTAGTAGACGTAGGTATAGTAGTTG
GTGAAGAAAACAGCCACACTTCTCTGCATGTC
ATTGCCCTCAAAAACAACCGCTGACAGAGATTCCACACGTAATGCCCGAAGAAATAACATGAAGTACT
GCACCGGCCTCGACACAACGTTGTCTCGGATC
ACCACGGCAGAGTTGTTCAGTACATCCAGCGACGAGGATTGGAAAGACCCTCCAAGATGCATCAAAGT
GCTATCACCGCCGTCCATATGAAACGTGTTGC
CCATCACAGCAAACAGGGAGCGCCACGACACCCTAAGCGGAGATCCAACAACGTGAATGGCGCTTGAC
GATGCCGTCACGGTCTGAAACACATTGCCACT
GAACACCACCGCGGAGTCATTGCTGATCGCCAGTCCATTCATCGCGACACACGACGGGCTGTCAACGT
CCAAACCCGGCCGAGAGATCAGCCTCGTGACC
GTGAAGCGGTTCCCGCTGATCGTGATGTTCGTGTGCGGTGGGAACACACCCTCCGGGCTGAGCAGCGC
GTCATCGAACGTGTTGCCGGTGACGACGATGT
TCAGCGACTCCACAGCGGCGTACGTGTTGCCCAACAGCAACAGCACGGCGCCGTCCCTCCACGTGCAT
CCATCAAACGTGATGTTTGCCACGTCGCCCGT
CATCCCCCCAATCAGCACCGTCAGGATCATGCCGCCCCCGCTGAACGTCACGTTGCGGTAGCACAGCC
ACGACAGCCCGCTGCCCACAGCCTGCGTAACC
TCGGGGTACACCACGTCGCTGATGCACCCACCAACCGGCGGCGGCGGTGGTGGGAGCGGCATTGGTGG
TGGTGGTGGGCCAGCAGGCACAGGCGCTGGGA
GACACACATCGCCGTGCCCTCCAGCAGCGCACTGGCACTTGCAGTCGCTGACCGCCGTCGTCAGCGGA
GCAAAGCAGTCGCCGTCCCTCGTGCACTCCCC
GCACGCAGCAACCGTCGTCACATCAGTGATGCCATTCAGCTCCAGTTCAGCCGCCGTCGTCAGCACCC
GGCCCGCAACCGTCAGACATCCGGCAACGAAC
CCGTAGCCGGCGTCAATCCGCGACAGCGGAGACTCCGACAACCCCGTCGATCCCATCTTGCAGCCCGT

CAGCGTCACCACACTGCTGCCATCGATGTTCA
CGGAAGCGTAAAAGGAAAAGTAGTAGAACATCGCACCCGCTCCCGCGTCGTTGTCGCGCCAATCCAAC
CAGCTTGAATTCCGGACGGTCCAAGAATCATA
TGCATAGATGGCGTATGACACGGAGCCGCTGTTCCCCACCACGCGCAGCACGCTGTGATTCGACACGG
TGAAGTCGTGGTACTGAAACAGGAAACTTGTC
CCGGTGGCCATCGTATTGTTGAGCAGCGCAAAAACGGAGCGATCACTGGCAATGAGCCTATGCATGTA
CAACATGTATGTGTATTTATCAAAGCTGCAGT
ACCGCGTGTACAGCGAGCTGCCACCCACCAGCGTCAGTGTCCCGGTCGAGTACAAACCATACCCACCA
TGATCCAATAAACGCGCCTTGACGTTGCTGAC
CAGCAGCGAGGACGCAGTCAACACGACGTCGTCCAGCATCACCACGCTCAGCCTGCTGCTTGCAGTGC
CTGAAAGCTGGATATCGGAGGACTGCACCGCG
TCAACCTCCGTGACGGCGATGTCGCAGTGCTGCGGGAGGGCACGCCTAAATAAAACAACGGAGGACCG
CATCACCGTCTGGCTCACGCGGATAAGGACGC
CGGCCGACTCGGCCACCTCAGTGCCCCCAACAAACTGCAGCGCGGCTCCGTCACGGAATGTGCACCCA
GTGAGCGTGATGTTGATGGGGAGATGCAGCGG
CATACTGTTGAGGAAAAACGTCAGCGCAGCACCCACGCCGCTGAATGTCACACCCACGTAGCAGTGGT
GCGTCTTCCACATGTTCAGCGTGAGGTTCGTC
AGCGTCTGGTTCACCACGCAGCTCGTGTCGCCGTCCACGGCTCTCTCCGGCGCGGGCGGCACAACCGT
GTCGGGCACCTCGAAGGGAAGACACGACGCGC
CATGCCATCCGTCCTTGCAGCTGCACAAGCACGAGCCGCGGTCCACCGACTCCGTCCCGGGCATGTAG
CACGCCGCGTCGTCGTTGCATGTGCCGCACCT
GAACACCTCCACTTCCCCAGGAAACACACCGTCGTACAACACCTCCTCATCGTCCTGCAGGTTGCATC
CAACCACAAAACGCGCGGGTGACGCCAAAAAC
GTGTTCGATGAAGCAATTCTCGCAAAGGCTTTACTCGAGTCCGCCTGACGGTTGTGTGCAAGCGACAC
GATGGTGCCGCTGCCCGATAGTTCAATACTGT
GCCAGGAATACGGCATAATGAATACTGAGGCTGCGCTCACGTTGTTGCCCTCCACACGCCACTGCGCA
CCATCCTGAAGAGCAACCGAGCCGTACAAATA
CAACAGCGCAGAATTCAGAGCCTCCGTGCTGCCAGTGAAGGTGCAGTCAGCCACCCTCAGCAGCCCCG
CGGAGCTCACAGTGGTGTCCCCGTAAAAATAA
ATGCCGATGGCTGCACTCATCGTGTTGTTCTCGACGTCAAAATAGCCGCAGTTCTTCAAGTCAACAGT
GTGAACGTATACGGCCGATGCCGAACCGTAAT
CCTTCTTCTTTGATCTCAGCGTGTTTCCCTTCATAATGATCCCGCCACCATCAACAACAACAGCAACG
GGGAAATAAACTGCGTAAATATCCCCCTCCAT
GTTGTTGTCGAGCAGCAGCAGCGCCGAGTTCGCACCCAAAGTAAACCTTGGAAAGTGAATGGCGTAAC
CCAAGTTCGTCACAAGCTTACTGCCCGCCACC
AGCATCTGGGAACTCGCGCCAAAATCTCCCTCAAACTCCAATGCACCAGAATCCAGCATCGAGGATGT
CACGTTCACGTGCACGCATGCACCACTCCCCC
TGATCGACAGGCCCATGAGGACGCACTGCAGCAGCGTGATGTTGAGTGTCTGTTCAGGCGCGTTAAAG
TACGACAGGTCCAGCAGAATCGCGGCGGTGAA
GGTCACGTTGACGAAGCACAGACCACGCACACCGGGATCAGGATAGTCCACGGAGCTGATCCTGTCAC
CGTAAACGCAGCGGACCTCCGTGTCCTTCGCA
TCTGAGAGCGGGAACGGTCCAAGGCCGTCCGGAACCGCAGCTGGCAGACACGTGTCACCGTATCCGCC
AGCAGCGCACACACACTCACAGCCGCGGATGC
TGCTCGTCCTCGCAGCAAAGCACACGGCGCCCACGGTACACGTGCCACAGTCCAATGCCGTCACGGGT
GTCCTAATATAGAGGTCTTCATCGTAATCAGT
CACAGGAGATCCTCTTAGCTCGTTGCACGTGCCGTAGATGGTCGGGCTCGTGTCGTCTTGTGCCGACC
AAAAACTTGTCATGTATGCAGTGGTCGACGAA
TAGGAGCCGTACGTGAAGTTGTTGTAAAGTATTGACCACACACTCTGATCGCGGAAAGCCACGCCGTC
GTATTTGTCCACGTGCACAACGTAGGCGGTGC
GGAACTCGTTGTCGCGATGCACCAGCCAGCCGCCACCAGTCACAGTCAGCGTGGTTGCCATGAGCATG
GCGAAGCCAAGACGGAACTCGCTGGACACAAC
CTGCAGCACGCTGCCGCCATCCACCGCAACGTCCCCAAACACAAGCGCGCCTTTGTAGTACTCGGTGC
TCGGCGCACTCCACGAGCTGTTCTGTATGGAG
AACACGGAGCCGCCGCTGACACGCAGGTCCGACGCAAGAGCGTACATCACGTGCGTCTGCGAATTGAC

AGCGCAGTTGTCCATGTAGAAGACGCTGGACA
GGTGCACGCCAAGGCCGTGCTCCACGAGGATAGCAGCGCACGAACCCCCGCCACACACCAGCGTAGAC
CGCGTCATGACAAACCGTGTCGACAGAAGCCG
GACACCGTCCAGGACAAGCGCGGGGCCGTACCGGGATTCCTCGTGGTCAGGGGTCGTCGGCACGTACC
GCGAACCGCCAACCGTCGCGCGCAGCGTTGAG
TTCGCCAGCAGCACACTCGAGTGCAGCGGCATCGTGCCATGCAGCACAATCGTGCCCTCCAGCGACGT
CACATTCGTCATGTTGACGAGGGCGTGCGGAA
CCAGGCGCGCCGTGCTCTCGCTGAGGCCGCCAATCCGCAGCTGCGCGCCGCCCGCAAGCACGCAGTGG
CGCAGCGTCACTTTCAGCGCGTCAGCGGACAA
GTCCATCGAGCGCAGGTCCACCGCCACAGTGATCGGTCCGCTGAACACCACCGAGTCGAGGCACGCCG
TCGCACGCCCGCCGCCAACCGTCACGGACTCC
GTCAGCGTCACGCCACTCACGCAGTCCTGCGCAGTATCGCCACCACCGCCGGCCCTCGCAGGCGGCAC
GTCGAACGGCAGGCACGCGTCGCCCACACCGC
CAGCGCGGCAGCTGCACACGCACTCGGAGAACGACGCCGTCAGCGCATCGAAGCACGCCAGCGCAGCC
GCACAGCCGTCGCACGGCACCACGCTCACGGA
GATCAGGCCGGCCATGCGGTAGTCGCCGCTGCTCTGCAGCACGCGGCCGCCGGCACGGTTGCACTGCA
CGGACACGGCGCCGCTCGTAATCGTCAGCCCG
GAGACAAGCGTCGCACCAGTGGCAGCGCAGCGAGCAATCGACACGGCGCCGCCGCTCAGCGTCACACC
CGACAGCGACGCCACAGACGACGCCTCGCCCA
CCGCCCACACGTCGCGCAGCTCCAGCCACCCGCCGACACCGACCGTCCCACCGTCGCAGCGCACCAGC
GACGACGCGACAGACCCCTCGACGCCCACGAA
GCGCAGCACCGACTCAAACACCGCAAGGCGCTCGCCAAGCACAATGCCGCCGCCGCTGCTCACAACCG
ACACGTTCGTCAAGGAGAACACCGAGTGGCTA
CGCAGACTGAGACGCGACGAGGACGACACATGCATCGCGGACGCGGTCGGACCGCCCGACGCCTCCAG
CAACACGCCGTCAAGCACCATCGCGCTCGACA
GCAGGTCCACACCGCCGCCAACGTGCACCGCCGACCCGCCCGCCCGCGACGACCTCAGCACAGTGCTG
CTCACACGCAGCTGCGTCTGCAACAGCGACGT
CGCGTGCAGCACAAGCGGCGACGCCACCGCGTCCGCCGGCCCGCTCAGCTGGGAGTAGCGCATCGGTC
CCGGCGTCACAATCGCCGAGTCACGGACCGTC
ACGTTCGTGCGTGCCGGCAGGTTGTGCACAAACACGAGCGAGCAGTAGCTCCCGGACAGCCCGCTCAC
GCTCACCTCCAGGCTCCGGTCACTCGCCGGCT
CGCCGCTGTAGCCCCGCACGTAGATCTGCGCACCGCCGTCGCACACGCAGTTCCTCAGTTCGATGCGC
AGCGCGCCGGGAAGCATCGCCGCAACGTCGAA
CACAACAGCCACGCCGTTCGTGATGCACACGCCGTCCATCAGCACCGTGTCCACGGCGCGGCCCACCG
TGATGGCTCTGTCCACCGTGCCGCCCCGCAGT
CGCAACGCCACCGCATGCACTGCGGGCATCCACGCCGCCGCCGCAACCAGCGCCAACAGAACAAGCAG
CGCCACACGCACAGTCGCAATGGCCCTGTGCC
GCACACGGTGCCGGCTGTGCGGACACGCACCCACCGCAGACCACATCCACCAACCCAAACAGGTATCA
ACACGACTGTCGTGGTGTGTCGCACTCGTCTC
GTCGACCGTGCCTCCTGCTCCGCAAATCCCTTCCCACAGATGCCAAATACAGTGACCCGATTCCGCCA
GGTCGTTATCCCACTGCTATTTCTACTACTAA
TACTACTACTGCTGCGAGTAATTACTAAAACCTTGAAACGCTAGTTGTGGTGGTCAAGCCACAGTATC
TCTATCTGCACGGTAAAACAAGGAAGAGGCAG
CAGCAACAGTAACCAGACACAGTAGGACGCAGCGGCATCCGGCCACATATATTCCACATCACAGCCAG
CGACAAAAAGACGCGCAGACACAGTCAGCCCA
GTGATGCCATCGGCCTCCCACCGCCCGCGCAGCGCCGCACAACCGCCGTGCAGAGTGGCGGGACCCCG
GCCAAGAGGCACATGACCATTGGCACCGCCCT
CCTCACCAAATGCCTCCAACACATGACGACCCCGATGCCGTGGACAACACGCAAAAGCCGAGGCAGAT
ACACGTGGCATTGTTGCAACCGCCAGCCATCG
AGCGCGTCCGTGAACGTGGTGGTGGTGGCGCGGAAGAGGGGTGGTGAGTGCGTCAGTTGCCAGTAGTC
AAGGCGTCGTTTCTCGCTCACGTGGGTCGGCA
TTGCAGAACAAAGAGCGGCATCATGCGCGCACGGCATGCACAAGGGCAAAAGGGAGGAACAAAGAAGC
ACGGTGGCGGCACTCAGCCACGGGGGACAAGG
GTCCACATCGACTCTGCGGTCGGTTGACCTTCAGACGTCCGGTTTGTAATCACGCCGCCGCGCAGTTC

GTGTGTCTGCAGGAGACTGGGGGATGGAGGGA
GGGAAGAGAGAGGCGGTGCGGCCCGCATCGGTCCCGGTTGGGTGTGACAACGGTGAGGTCATCTCGTC
AGGTCGCCGGCAGCAAAGACGGTCGCCGTCAG
CGGCGGTCAGTGCCTTCCTTCTCCCACCGCAGCGCCACTCCACCGACGCCGTCACCCGCCTCCTCCCC
ACCGACACAACGAGGAGACAACAGCAGGAGAT
GCAACGCCCGCAACGATCAGGCTGCCGGAGAGTCTCGTTGCAGCCGCAATGACCAGACGAGTGGGATG
CAAACAAAAAGAAAAAACAAAAAACAATTGCCC
CCTCAAGCATCTCAGCACAACGTGGAAGCACAGCAGCCGGCACACAAGGCGCAACGCCGTTCAGTGGC
TACCAGCACTCACCCTCAGTTACGCGGCAACG
TGAAAATGAACGAGCTCCTCCCACACAGCCAACCTGCAACACGAGTGAGTCACACGAGCAGTCGCACA
AATTAACACCACGCCACGCAAACCAGCAATCA
AAGACACCGCCGGCGAAGGAAGCGATGGATGGCTGCGGCGCGCAGCCGCAATAATAAAACATGTGTCA
CCTTGCTTGGNNNNNNNNNNNNNNNNNNNNNN
NNNNNNNNNNNNNNNNNNNNNNNNNNNNNNNNNNNNNNNNNNNNNNNNNNNNNNNNNNNNNNNNNNNN
NNNNNNNNNNNNNNNNNNNNNNNNNNNNNNNN
NNNNNNNNNNNNNNNNNNNNNNNNNNNNNNNNNNNNNNNNNNNNNNNNNNNNNNNNNNNNNNNNNNNN
NNNNNNNNNNNNNNNNNNNNNNNNNNNNNNNN
NNNNNNNNNNNNNNNNNNNNNNNNNNNNNNNNNNNNNNNNNNNNNNNNNNNNNNNNNNNNNNNNNNNN
NNNNNNNNNNCGGACACCACAGTCAGCCACAC
CACTCCCGTTGCATGTGCGCCCTCTCGTCCCCATCATCCCTTCCTCCCTCCTGCCGGCATTCACAGAA
GAATGCTGCGGATGCCGGAGAAGTGTTCCACG
TCGGCATCTGTGACGCCAGCCCGCCGCGCCTGCCACCCCGACAAGACGTGCCGCACAACGACATCCGC
GGCGAGGTCCTTTACCTCGGCGCTGTTCATCT
CACCCCTGCTGTACTTCGACGTCACCTCCGCGAGGAGATTGTCGTCGGGGCAGAAGAACCGAATGAGT
CGGTACGCCACGTCTGCGTGCAGGTTGACACC
CTCCGCACGCATGTCCTCCAGCGTGCCACCCCCACCAGAGAAGGCCTTGCGCACCTTCTTCCGCACCT
CCTGCTCCGTGTCGTGTAGTGTGATGACTCCG
TTCTCCCCGGCGCTGCTGCTCATCTTGAACTGCGCTCCCTTCAGTGCGGGGAGGAACTTTGTGTGCAG
CAGTGCGGGGGCGCAGCACTTCATACGCGGCG
CGACGTTGCGTGCCAGGAGGAAAAAAGCGTCCTGATCAATTGCACAGGGAATGATGCACTTCAGCGGC
GGGTCCCAGCTAGCCGTCGTAGCACACGGGGG
AAGGCACTGCTAAAACACGGTGCGGCCTGCACCGCTGGAAAGGCTGCCTTTCCAACATTGTCGCTATC
CTTCAGGCCAAATGCATTCTTCACAGCGCTCA
GCGTCAACATGCGCTGCACCTGCACGACAGTTGGGTACATGTCCCCCATGTACACCGTGTTGCGGAAT
ATAAATGTCTTGCGTGGATCGAAGCCAAAGGC
AATGATGTCTTTTATGTTCTCCACCACCAACCCGCTCGCCCTCTCCCCGCTGACGGGAATGTCGCGGA
AGAAGTGCTTCTCATCATCGGTTATCTGGATC
ACCAACGGAAGACCGAGCGCATCCTGCAGGTACCGCGTCAATAAAAACGGTATGACATGGCCAATGTG
CATCGACCGCGCGCTCGGTCCACGACCAGTGT
ACAAGTACGCGCGGTGTGTCCCCGTCGCAAAGGACGCACGCACGCAGTCAATGGCGGCTCCAAAATCC
CTGTGGGAGAAGACGATGCCGCGGCGGAAAAA
ATGGTGCAATGCCATTCGTCCCGCCTCCTCTTGTGCGGTGCTGGTTGCCTGCGCGTGCTCCCCCCGTC
CGTTGCATGTTTCCTCCAGCCGCTGCAGAAGG
GGCTCATCAACGGGCTCCGACCTGAATCGTGCGAGGACGCGGCCATCGTCCCCACCCCTTGGGCCCGT
GGCGGCAACACCCCACGGCGTCACAACGTCCT
CCTTGCGCTCTGTGCGGCTGGCATCATGCCCACGCGTCGTGGCACAACTTCGTCCCCCACAAACCAAC
GGCGCAGCAAAGCGTGAGACGCGACGCACCAC
AAAAGGATCCAAACGACTACTTCGCCCGTGCACCGCAGTGCCACACCGGAAAGAAAAAAACAGGCAGG
AGAAGAGAGGCAGGACTTCGGGACAGCACACA
TCACCTGATGCATCACA
>scaffold1000.size11471
CGTGTTGGGCATGTGTTTTTCCGGAGCGTTGGGCGGCTGCCATCTGTCTATGGCGGCTTGTGTGAGTG
GGCACGTGCGGATGAGGGTCAGGGCGTATGGAA
TGAAGGAATGGCGTGAGGAAAGGGGAATTTGATTTGGAGGGTATTTGGACCGGATTCATTTGGCTGCG

GTGGACACATGCACGACGGATGTGTTTCCGCA
TAATGGGGGAGGATGCGACAGGATTTTCCGAAGTGGCCCGTCAATGCAGTTTGGGTTTTATAGCACTG
GGGCACAGTGGTCTTCCTGCAATGGCTGGTGA
CGGTCCCTAGTGGACCGGTGTATTTATGCTGCTGCTGCTGCTGCTCGAGGAGGCGTAGAAGTGCGATT
TGAGGTGAGTGGCAGAAGGGCCGCACATCCCG
CAGTGACGGTCGTCGTTGGCCCAGTGGGTGCCTCTGAGGGTTTTGTTGGTTGCAACGTCCTTATGCCC
GTCTCCGGATGGCGGCATCCCTGATGCAGCCA
ACGAACGGGGCTGGGAGCGACGGTGAGACGCAAGCACGTGTGGATGTTTGGAGCGGCCGGCGCACCGC
ACGCTGAGAGGATGGCACGGTGAGGACCGGAC
CTAAAAAAGGGACACCGCGTGGGACGTCAATCTTCACTGCAGCGACCGCCACCGAATTCGGATGTGCT
CGCACCTTCTGCGTTCCCTCTTCCTCCCCTCA
CTTCTGTGCCGAGGACTATGCCGCGGGATCCAGCAGCGCGATGTTTGGCCGCAGGAGCATGGCCCGAA
CAAATGATTTGTGGGAGGGGATGTGCACTGCG
ACGAAAAGAAGGAAGAAAATGTTGCGAGACGACCACCACGACAAGACCGGAGTGAAGTTGTGTTTTGT
CCACTTGTGCGAGGGCGGCCTGCCACCTGGTC
GGTTGGGAGGATTATTATTTTTTGCGTCGTTGCGCTCTCTTTTTATTCCGCTGCGTTTTCTTGCGGAC
CTGCGAAGGGAGATTTGCAAGAAATTCCTGCG
AGGCGTCCGTGCCGTCGTCGGAAAAGCGGATCCCCCTTGAAAAACGCTACTTTTTAAATTTGTTTTGG
GCGGGGAGTTCCTGTCGATTCAACGCGACTTT
GTTTTGCGGATTTGATGCGTGTGTTTCCGTGTGGTTCGATCTGTGCTCCCGGCCCACTCGGATGTGGT
GTGCGCGTCGACGTGAAGGTGCTTGCCAGCCG
TTTTATTGCCTCCCCACTGTGGCTGCCGCCGCTGCCTTCCTCCCCTCCTGAGGACCTGCGGCTTCTTG
ACAAGATGCTTTGGAGAAGGCGTTGCCGCTCT
TGTCGTCCATGGTATTTTGATTCTGGTGTCGTGGTCAGGTGACACTGAACGAGAGGGTTTGCTGCCCT
CCTGTTTTATTTGTCCTCTCTGCCTGAAGGCC
AGTTCTACACAAACGTGCTCCTCGGCACCTCAGTTGGTGCTGGCGGGTTGCTCACAGTTGTCACTGGC
AATCGCATGCGCCAACTCTTCTCTGCTCCTTT
TCAGTGTCGCATTTTTTTATTGATTGACGGACGCCATTCTGTCCGGCGTGCCACTTTGATTGGATTGT
GTGGGACTGCTCGCCGACGTCATCGGAGAAAA
CAGGCCGACCTTTTCACGTATTTAAATGCCATCTTGCGCGCAGTTGTTTTTTTCACATGTTCTGTTGG
TGTGCACTCAAACAGCGAGGTGTCCGGAGCTA
TGCGTGCGTGGAATTCTTCCTGCCATGTGAACCGCGGGCAACAACGAGAGGTTGCCACTTTGACTCCG
CAGAGTTGCTGTGTCTCTTTGCGGTGCTGGCA
GGGAAATTCCATGCAGCCTGCCACTGCTGGTGTGTCCCCAAATGTTGTCACTCCATGCGAATAATGTG
CTTCAGCGAGTGAGGGTGTTGATTGGATTTGT
GTGCGCTCTAATTGTGTTTGCTACTTTGTGCGGGCACGCAACACTTGAGTGGTGGGAAAATGTAGCGG
GGCGATGCATGGCTGCGTCTCGCCCATTGTTC
ACTGTACGATGGTGCGGTGCGCATTGTGGTGCTGACTCTGCGATGTCACTGCGGTCTCCTGTGCGGCA
ATGGTGGTGGGTGGAGGAGAGTGTTGACCCGC
GGCGTGACGGTGGGATCGTGTTTTTTTTTCTTTTTGTTCCTTCCATTTTGTGAAGGTGCACTTATTTC
TTGTGTCGGTGATGGGGCAAACAGACCGACAA
AGTAAATAAAACAAAATAAAATAGAAAAAGAATATAATGTGGCGGTGTTGGGGTTGGTTGCACATTGC
GTTGCTGTAGGGGTGGCGGGCCTCGGCTGCTT
CACCGACAGGCGTTGCAGTGCGGCTGCATGGCGCACCGACCGTCCCGCCGTGCGAGTGCCACGCCCAA
CGCCATTGGGACAGCGGGACGAAACAGCCGCG
AGTGTCCTTTGGAGCCAGCGGCGCCTGCTGGCCGCAGTTGGGCGGTGTGAGTGGGATGCCTCACCGCA
CGGGTGTTGTGATGGCACCTCGCCGTGGCTCT
TGTGATGATTCCGACGCTGCAGCTCAACATGTGGTGGAGAGTAAAGTGTGGCCGCAGTGGATCATGAG
CAGCACCAACATGAGGCTGAGTGATTTCCTTT
GGAATTATGTTGGCGGCAGGGCGGCCGTTGATGAAAGACCACAATGTGACGATGGAGGTGTTTGCTCA
GGAGCCGGATGATTACGTACACGACAAGTGGC
TTCTTGAAGAAATTATTAACTTAACTGCGTACCAAGCGCTGGAGGCCATTACTAAGCCTCTTCACGAA
GGTGTGTTCTTTCTCGGGCAACGTGGGGATAA
TGAAAGAAAAGGATACGATCACTCCTCTTGCAAGGGGAAAACCAACCGGGGTACTCACGCAGGTACCG

ACAGAAGCGAGGCGGGAGGCAGAGGAAAGGCT
AAGAGGAACGCAAGAAATGAAATTTACCATTTCCACTAATATCGAAGAGGTACTGTTCAAAGGAAGAG
TCCGCGTCAATGAAATGAAGCTGAACGACTTT
CTTACGAGGGAACTGGACGGCAGGGGCGTTGTGGACACCAATCGGGATGTCTTGCTGGAGGAGTTTTC
AAGGACCCCACGAAGTATATCCGTGATGAAGG
AGCGTTGAACGAAATGCAGGCATCAGGTCATTATTTGGGCATGAAGCGAGCTGTAAAGGGTGAAGTGA
CCTTCGGTGAGGATATACGCGGGCTTTGCGAT
AGGGGTTTCAACAACCTACCGGGGTGGTCATTGGCTGCTGCGGGGGTAAAAGCAACTGTTCATAACTC
CAGTAAACATTTCTTGGATGCAGCTGCCGAGG
AGGCGAGGAACCCAACGACGACGAGCGCACCGGAGAAACTGGAGGGAGTGTACGAGTCTGTGCACAAT
GCGAGGTGGAGCCATGCGGAGGAACTTCCGGA
CGGCGTGGAGCGAAAGAAAACGGTGACGGGAATGGGGGTGCATGAGGGGAGGCCAGAACAGTCATGGT
CGTACAGGAAAGCTGACGACGCTATTGAAGAG
AATGACGCTGTGCAGCAATCCGGTGCAGCACCTCCCGTGCTGATGGTGCTCACCTCGGAGAAGGGATG
GCCGTACTCGTGGCATACGATACAGGATCTTC
CTAAGGACGTTTTTGTAAACTGTGAGGTGGAGAGAGTGTGGCAGATCGTCAAGGGCGATGTAACCGCG
TGGTTCAGCCCTCACGGTGGGACCGACTTTAC
GCCCAAGCGACGTGTGTTGATTGGGACGCCCGGGATCGGGAAGTCGATGGCTGCCGGCTCGTACCTCC
TCTACCGGCTGCTGCACTGCGATGCCGAGAAA
ATCCAAGTGGTAGTCCACTGTTTTGGTGGAGGAGGTGCGTACGTGTCTGACAAGACCACCAGAGCGGT
GACAAGATGCAGCGATGAGGACATGTGCATCT
CGGAGTTGAGAAGTTTGAGGGAGCGTGGGAGGAATGTGTATATTATCCACGGTGTGGCAAAGGAGGGA
ACGCCGCCGCCGAGACATTTTGCGCCTACTTT
TGGATGGGGCATGATTGCGGTGCCATTCCCAAAGGTAACCAACTATGATGAGTGGGCGAAGCAACTAC
AGGCCGCGCGAATCATCGTGAATTGCCCCGAC
GAGACGGATGTGAAGGCGATGTGCGTGGATAACGCGTGATGAGGCTAAAGAGAAACAGGCGGAGTGCT
GGAAAGAGGTCAAAAAACACATGTATCTTTTG
GGACCGATTCCACGTCACGTCTTTGATGAGGAGGCGTTTACGGAACGCTGTGGAGCGGTGAAGTTTGC
ATTGCAGTCGATCAACGAGGTAACTGCGAAAG
AGCACTTTTCACGGGGAGGTGAGTTGCCATGGTATTCTGAGGATCCGTCCCACAAGCTTGTGAAGGTT
GTTCGGGAAATATACGCAAGCGGTGTGATCCT
TTTCAATGCACCGATATCTGCTTGCCTCGAGGAACGGACATTGGAACGCCTCCCAAGTGTAGCAGAAT
AAAATGGCTTTTTGAAGCTGATGTTGGCGTCG
AGGGATAATATTTTGCCAATATTTTTGGATTGTTTGCTTTGAACTCATTCGCGGAGAGTGCCTTTGC
CAAGATACCAGCGACGAAACTCACGGAGTTTG
AGGCGCCAACAGGACGCCAAGCAAAGCCCTGCGTCTTGAAGTTGGCCCCTGAATTGCATCCCAAGGGA
GATGAGGGTTTGCTGCCGAGTGAATATGAAAA
ACCGATCCGTAAAATAAAGTGCGGGGGGCTGCACACACCGTGGGCCGCAAACTTTCCGCTGGTGGACG
GCTTTTTTTCTCAGAGTCCAATCCAACGACGG
TGGTTGCGCTGCGGATGACTACGACGGGTGGGCGCCACATCACCGCCAGCACTGTGCGGCAGCTCACT
GAGTGCGTGGCGGCATATTTTAATGGTTGGGA
GGAATCATCCCGAGACATGTCGTGGGAGATTATTTATGTGCAGCAGACAGACAGCACGCCGATGAATG
ACTGGCAGAGATGTGATTTTGTCAATTCCAAT
AATGTGAGCGACGAGAGCCGTGGAATAACGGCGTTTTGGAAGGAAGAGGTGCGCCAGTACCAAGTGTC
AATATCGTACAGAGACTGTAGAAGTGACGAAG
CCCTCCGAAGTGTGCAATAATAATAATAATAGGAAAATAGATTAGTAAATTGAAAGTGAACGAGTCAT
TTTGATTTATGGTAAAAAATATTATGGGATTT
ATTTATTTTTGGATGCTTGTGCCTGAGCATGCGGTGCGGGCGCGCCATCTCCATGCGGCAGCACCGCA
CACAAATGCCAAAAATGTCCAACGCTATCGAA
CATAACAGTGACAGAAGAAATTAGGGAGGGAAAGGGGTTGTTTGGCTTCTGGTGGAAAAACATCGGGT
GATTTTTATATGTTATTTTGATTGTGTGTGGG
TACGAGGCGTAATTGGAGAATTGCCCCGGTATGAGGGAATCATTCTGTTTGGCGGTGTCGCGGCGTTA
CCGCATTGTCCATTTCCTTCTATTGTGCTCGA
AGGCCCATCACTAACAGAAGCATCTTAAAGGGATGGACGACGAATAAGNNNNNNNNNNNNNNNNNNNN

NNNNNNNNNNNNNNNNNNNNNNNNNNNNNNNNN
NNNNNNNNNNNNNNNNNNNNNNNNNNNNNNNNNNNNNNNNNNNNNNNNNNNNNNNNNNNNNNNNNNNNNNNNN
NNNNNNNNNNNNNNNNNNNNNNNNNNNNNNNNN
NNNNNNNNNNNNNNNNNNNNNNNNNNNNNNNNNNNNNNNNNNNNNNNNNNNNNNNNNNNNNNNNNNNNNNNNN
NNNNNNNNNNNNNNNNNNNNNNNNNNNNNNNNN
NNNNNNNNNNNNNNNNNNNNNNNNNNNNNNNNNNNNNNNNNNNNNNNNNNNNACCGCATGGAGTGAGGTGCC
ACGTCATGGATGGATGAGGGTCCGCTTCCGCC
CGTGGCTCTTTGGGTGATTGTTTGCTCCGTGTGCGTTGTGTGCCGGCAGCTGGCTGTGGCCATATCCC
GTTACCGTGGGATTGAACTGGAGGCGGTGGCG
ATGTTTCTCTTTTCGTTTTTGTCCATGTTGGGTGACTGTTGGCGATGTGTGCGTGTGTTGTGTGGAGG
CCGAATCTTCTCACCTGCGGACTGACGACATT
GCGATGGTGTGTGTGTGTGTGAGGGCTGGTGTGGAGTTGCCGGCCAGGGTGAGGGCCTTTGGCGGTGC
TTTTCCCGCCTGTTGTTTGTTTCCTCCGTGTG
TGTTGTTGCTTCTTGCTTTTCTCCCTCTCACCATTTTCTCCTATGTTCCTGCGCGGAGTGGCCGCATG
CTCACGGGCCATTTTGATTGTGAGTGCCTCTT
GACGGCATCCATCTCCCTCTCTCCCTCTGTCTTTCTGTCTGTCTTTCCCTGTTGGTTATGAGGCGTGT
TGCACCGCGTGAGGGCTGGCCCGCACACACCG
ACGGCCCGCCGCGACGCCCACGATGGCCGGTGCGGAGTGGCGGTGCTCGCTGTCTCACCTCTTTACTT
GGCTTGTTTTCCCCTTTCCCTCTACTTCTTGA
ACACACACGCTAGGAAAGCAAGCAAGCAAGCGCTAAACGCTACTGTGAAATTCGAAGGCTCTGAAAGG
ACCAACAGCACACACACCGCCCACACACACAT
ATTTGTATGCTCTCACGTGTTGCTGCAGTGAAGGCACCCCGCACACACAACCGTCGCCGCGTGACCGG
ATCCAGCGGAAGGAGGAGGGAAGGAGGAGAGA
GTGAGCCGCAGAGGCCCAACATGTCGCGGCGTGTGTTTACTTTCGCGGTGCTGCTCCTCCTCGTCGTG
ATGATTTGCTGCGCCACCTGTGTAGCTACGCC
TGCAAGGGAGAATGATGGCAACAGTGATTTAAGAACTGTTGAGGGGCTGCAATGGGTCGATTTATTTG
TGCCGAATCAGACGCAGGTGCTGCCGAAAGGT
GGAGGTACTCCAGGGAAGAAGCGGGATTCATTTGTTTCACCCTCTCTTGTCAGTGCTGGTGGAGTAAT
TGCTGCTTTTGCCAGAGGTAAAATAAATCCCC
AGTATGCCGCTGATGTTAAATTAATTAAGCCACTTTCTTCTGATATTGTGGCGGGGTACATTGACTCT
TCGTGGGAATGGTCCACTCTTGTTGAAAAGGT
GAGTGAAAGTACATGGAAGGCACACACTGTGCTTGGTACAACTGATGGAACGAATCGTGTGGGCGATG
TGCTCAACCCCACAACAACAACGAAGGGTAAC
AAGGTGTTTCTTCTTGCGGCAGGCTTTGAAGTACTCAATCAGAGTGATGGGAATTGGAAACGGGACAG
ACNNNNNNNNNNNNNNNNNNNNNNNNNNNNNN
NNNNNNNNNNNNNNNNNNNNNNNNNNNNNNNNNNNNNNNNNNNNNNNNNNNNNNNNNNNNNNNNNNNNNNNNN
NNNNNNNNNNNNNNNNNNNNNNNNNNNNNNNNN
NNNNNNNNNNNNNNNNNNNNNNNNNNNNNNNNNNNNNNNNNNNNNNNNNNNNNNNNNNNNNNNNNNNNNNNNN
NNNNNNNNNNNNNNNNNNNNNNNNNNNNNNNNN
NNNNNNNNNNNNNNNNNNNNNNNNNNNNNNNNNNNNNNNNNNNNNNNNNNNNNNNNNNNNNNNNNNNNNNNNN
NNTGGTGGCTCAGGTGTTGTGATGGAGGATGG
CACGCTTGTGTTTCCTATGATGGCGAAGAGTGAAGAAGAAGACGTTTACTCCATGATCATTTACTCGA
AGGACAACGGCAGTGCCTGGGCGCTCTCTGAG
GACATGTCCCCTGCGGAATGCCTTAACCCCCGCATCACCGAGTGGGAGGGATCACTTCTCATGATTGT
TGATTGCGAAGACGGCCAGAGGGTGTACGAGT
CGCGTGACATGGGGACAGCGTGGACGGAGGCAGTCGGGACACTCTCAGCCGTGTGGGTCAAATCACGA
TCATTCCTTTGGGATCTGAGCTTGCATGTGGA
GGCCCTCATCACCGCGACCATTGAGGGAAGGAAGGTCATGCTGTACATTCAGAGAGGGAACTTCTCGG
GGAAGAATAAGGCCAATGCGCTCTACCTTTGG
GTGACGGACAACAACCGCACATTTTTTGTTGGACCGGTTGGCATGGACAATGCTGCGAATTGGATGCT
CGCCAGCAGCCTGCTGTACTCGGATGGGAGTT
TGCATCTTTTACAACGGAGTATAAATGGTGAGGGCAGTGTCATTTCACTTTCCCGCCTGACCGAGGAG
CTGAAGACGATCAAGTCCACCCTCAGCACTTG
GTCGCAAAATGACGTTTTCTTTTCCAGCCTTTCTATACCCACGGCTGGTCTGGTTGGATTCCTGTCCA

ATACGTCGTCCGGTGGCGACACGTGGATCGAC
GATTACCGCTGCGTGAATGCATCCGTGACGAAAGCAGCGAAGGCCCATGACGGGTCCAAATTCACGGG
GCCTGGGTCCGGTGCGATATGGCCTGTGAACA
GCCGGGAGGATAATAATCAGCACAGATTTGTGAACCACAGATTCACCCTTGTGGCGACGGTGACCATT
CACCAGGTTGCGAAGGGGAGCACTCCTCTGCT
GGGTGCGGGTCTGGATGCACCCGTCAGCACGAACTTCATTGGGCTGTCGTACAGCATGAATAAAACGT
GGGAGACGGTGTTTGATGGGAAGAAAACGACA
TCGAACACCACGTGGGAGCTGGGGAGAGAATACCAGGTGGCGCTCATGCTGCAGGACGGCAACAAGGG
CTCCGTGTACGTGGATGGTAAGATTGTGGGGA
GCCCAGAGAAGATACCAACACTTGAGACGCAGGGACATGAAATCACACATCTCTACTTTGGAGGCGAG
GGAGACATCAACAGCAGCGTGACAGTGACGAA
CGTATTTCTGTACAACCGCCCACTGAGTGTCGGTGAAATAAAAATGGTCAAGAAGAGCGACGCCAAAA
AAGGGAAACGGTGACGGCTCCATGCGTGGGGG
CGTGTCGCAGTTGCTGCTGTGCGGCATTGTGACCCTTTACTGAGAAATTGCAGTGAGGACTTCCTGCT
TCCAGCGGGAACATATGCATCTGTCCACGAAC
TCTTTTAGGTAATTGCCGCTTTCTGTGATGCACCGTTCATTTTATCTGGTTTTAATTTTATTTTACAT
CCCCTTCGCTGCGCAGAGCTCTTCCTCCATGT
GGAAGCATCCTCTCCCCAGCGCCAGCACCGCGTTTTTCGTGTTTTTCCTTTTTTTTGGTCCGACAA
TTACGGGACCTACTCAATGTTTTTTTCCCCCT
TTTAATTGATCATGGGCGCTCTCGGCATTGTGTGCCACGCCGATGGCGCCGGCTAATAATTGGAGCTT
GTGGAAAGAATGATCGCGGGAGAGCTGGCTGC
CCTAATGAATGTATGTGTATATCCTGATAAATGAATGCATTCTTTATGATACTTTCTACCGTATGAAT
CCTTTGGGAAGAATGCCACTTTGTAGGGGCAG
GGAACCGATAGAGGCCAGACAAATTTTTATTTTTATTTTTTACAGACACACAGCCAGGTAAGTTTAAG
GAAACGGCTTTGGTCCCTTACACACCCTCCGA
GTGGTGTGATATTGGCGCCCGTCTCTGGGACATGGTGTGCCAATGTGGCGAGACCCAGGTGCAGATGA
CGTTCATTGATGGTAACCAGCTGTCGGATGAG
ACGTTGACCGTGGCCCCATCAGCGACAGGCTTATGCCTGTTTCATGGACCTGTCTCTAAAGCATGGCA
GGATTCTACTCTCGTTGGCCATATTGCGAATC
TCCGGGCGCCTCAGATCCCTAATTTTGAGACACACGTGCTCCTGCCCGCTATGGGGGTAGCCCGGGAG
TTGGGGCACCAACAAGGGCTCATCACTGCTGA
AAGGGAACGCCTAAATTTTGATTGCGCAAACGCCGTAGCGGACGTGCGGAATCGAAATGCTTACCTTA
ACTCGCGGGCTCAGGAGGCGCATCACAACCTA
TCGGTGAGGGAATCCACTATCGCCGGAGCCGGCAACCAGGTCCTAGAACTACAACAGCAGTTGGAGGC
CGCTCACGCCCAATTACGGGAGCGCGAATTCA
ACTTGGGAGTCCTCAATGCTCAACTCCAGGAACGGGAGGCACGCCATAATGCACAACAACCCGCGCGG
GATGCGGAGACGGCGGGGCGGGACGAGTCCCC
CGCTACTCGACACGTTGCCAACGATGGCGTAACTACACTACCGCATCAAACGGATATCTTGATGGCAG
GAGGCGTGCCTAGCAGTGCGGCCCGACGCAGT
TCGGCGGCTTACAGACGCCCTCACGGCAATGTTGGGAGACCACATTTCCACCCTTCCACGCCGTTGCC
CTCCCGCACTTCTCCCCTGGACGCCCAATTAC
CCATGGAAGAGGACCGCGGTTTCTGCGCATTCGACGTCACTACGTGGCCCTCGCGCATATGGAATGAG
GGTGCCTCCGGAGTCGTTATCGACCTCCGGTG
CGCATACCAATGCTGGCAACAGGACGACGAGCAGGTTAAACTTGCCTTTGACAATTTGGTCGAGTGGA
TCGGCGCGCTGGAAGACTGACAGAATCCCACC
GATCGAATCATGAATTTGGGAAGGTCCCTACGGAATACATTCTGGATGCAGCTCACGATGACATCCGA
TCCCGGCATCCGGCTTTCCAAACTGCGCGCCC
GTCTTTACACCGCTGTTCACCAGACGGACGCTTATGCGAGGGCGACCCAGCTTTTCGTCGATCGGCGA
GAAACCCAACGCACTGTGCGACGCCAACGTTG
TCATATTTTTGGACACGAGGCATCCACCTGCAATGTGCGCCCGAGAGGGAATTACTCACAATACAGGC
GCCCTTCAAAAAACGGCCGAGGGGCTGCTTGC
TGCTTTTTATGAAAGCGGACGACGGCAGCCGTATCCAATCCGCCACCTCCCACCAATCCGCACAGGCG
TCGCACCGCGCCGCCGATGCTCCATCCGATCT
TTCAAATCGCCCGAAGAAATCGTCGAGAAGGGCGATGGAGCTTATCTGCCAACGACGCCCGACTGTGG

TACGACGAATCACCGACATCCCCGCTTTGGGC
AAGTTATATGAGGAAAGCGAACGAGGTCGCTCGAGAGAAGTGCGCGCCGTCAACGTGGGAGCAGAGAA
TTTCCCTAGCGGGGCAATTCTCGACTTTTTGC
CGCACGCACGAACAGCCGATGAAcGAAGAGAGCTGTGCGGTCTTTTTGATGTCAATCGAAGTGGCGCC
GTCGACCCGACCGCAGTACGCCAGGATGCTGC
GGTCCATTTTGCAAATGGATCGAACCCCGCTGGATATGGCGGCATTGAGGTTACAAAAGATTGCGGCG
CGATCAGAGACGAGGCGGGCGCGCCCCGTGAC
AGAGGAAGAGATGAATCAAGTTATCCGCAGCCGGACCGATTGGAGGGAACGTGTTGTCTTACGACTTG
CGTGGATAACGGCGAGTCGTTGGTCCGGAATT
GCGGCCTTGACTCCCAAAAATTTCACACTGAAGCCGGACGGAGCCTTAATTTTGGATTGATCCGTGGC
>scaffold1001.size7002
AAATTTTCTTTCACGGGGGACGGTGGGGAGGCGACGGCAAAAAAAAAGAAAAATGTATTTTCCCGAAT
TGCTCTGAGAATTCTGAGTGTTGACCCGCCGTC
TCTCCCCCGCAAGATTGGGTTGGGGAAACGCGGATGCTGACACACCCCAGGCCATCAAACGATGCGGT
TGATTTGGCTGACACAAAGGCGGTAGCGGCAC
GTACGCACCCCCCATCTGGCGGCCCTCTCCGAGAGGTGGGTGACTCTCGTTGACACTGCATCTTCTCC
TTCCGCAAAAAGGCCGCCAAACACCGCAATGT
TAGTTTCACGTGCCGAGCAGTAGTTGAGTGCCTCTTCCGTCTACAAGTAAATCTGAGCAGTCCAATTC
TGTACGTCCGCAACGGCACGGGTGTAGTCCGA
GCCTAATATCTGCCTTTCCGCAATGACGAGCCATTGCTTGCCCCCAACTCCCGGGCCGCCCCCACACG
ATGGGCAGGAGCACTTGAGCATCGAAATTTGG
TACTTGAAACGCCCGGCGATTCCCAAAGTGCTCCGCAACGGTCGAGCCCTCCAACTGCTTGGCGATAC
TTCCTGAAATCAATCGCAAACATGTCATGGTT
GCAGCGACGGCTACTGCCTCATCCGACATCTCGGCTCCGTTATTGAACGTTTTCTGCACCTGGCTTTC
GTCACATTGACGTACAAATTCCGAAAAACTTT
CACCAAATCGCTCTACTCGGATGTCGTGTAGGGAATCAATGCCGTTTCCCAAAACCAACCTGCCCCTG
TCAGTAAATTTAAAAATAAAAATAAAATTGCC
TATTCGCTGTCTCCTCCCCGCTTCCCCTAAAGTCGTGTTATTCCCACGTTCATAATATAGGAAAAGTA
ACATAAAGAAGTGCATTCGTTTTATCAGGATT
TAAATTAATTAATTTAGCCAGCTCTCCGGCGATCATTCTCTTCACAGGCACCTATAGCAACAAAAAGT
CGTACGGAGGGTTTAATTAAACCGATTGGCCC
CAAGCACCACCCTCCTCTATTTAAACGACAAACCCTCTAATTTAGCGCTACACCGTGTGTGCTATTCG
TTCGAAGTGTTGCGAGTCTCATGGCTCTACTA
AGGAGCTCCTCCAGCCACGACTCCGTCAACTGTGTCTCATGCGATGAGAAAACTCCCTCAATCTCCAC
CTCATGCATCAATACTCCCTTCTCCGTCACCT
GCGTCATGTCATAGCGGAACATTCCATCTGTGATGCTGAGCCGATCCTTCAGACGTGTATGACCAGCT
CGTGGGCCAGCACCAGAGAAACGCAAATCCCC
TGGAGTTTCTGTTGACACGGCGAATCGAAGATTATACGCGCCTAATGGAACCACAATGTTTTTCACAA
GTATACGTTTCTTCCGCACAATTGATACGCAA
CAAGTAGGGTTACCATCCTCATCTGTGGCGTATGTATGGCGCCATCCACGTAATGAAACGTCGTGCGT
CACGGATCTTGTCATAGAACTATTTCCCATCT
CGGTCATTAAATAAGTCTTGATACGCTCGTAATCCTCCGTGGAGACACAAGCTGTAAAATTTGCATCA
ACAATCCGCAGCCCATTAGTTTGTGCACCGTT
CTGGTCGCCGGTATTTTCTCGCAAAGCATCTTCCCCTACCTTAAACTGGCCCAGACGCATTTCCACTT
CAATGAATGGGTTGGATAAGTGTTTATTTACG
GCGTCAAACAACGCCTTGGCCACGGCACGCTGCTCAGCATTGCCCATACTCACCACCTCGTGCCGCGA
AATCCCCTGCCTATCGGAAAGCATCTCCCCCT
CTTCTTCCTTTCTTACTCTTTGTTTTATTTGTCCATGTTACACGGCGCATTTCCACGAGAACGATAAG
GGTGGGGTGGGGGGGAAATATCGAGAGCGAGC
AGGGACGAAAACAAGAAACAACAACAGTAACTTGGAGCATATCTTGGGAAAGGATCCACCACATTCTC
CCTGTCCTATTAGAGCATACACAGTTCCGGTT
CCATCTTTCTTATGTTCTTTACAATTTTTCCCTCCCAACAGCCCCAAAAATCCATGGCAACATCCCAG
CGTCATTCTTTTTTCCCTTTCTGTTACTCGCA
GAAGAAGCAGCACAGGATCGCAGTCGTGACACTTCAGTCGGTGAAATCAATTACGAAGTACGAGATCT

TTTACGGCGCAATAGAGGACACTCCGCACGGG
TGTGTCCAGCTTCATTGCAATAGCCACAGTGCATGTATGATGTCGCATGCGCACGGCTACGATGCCCT
ACAGGTACGTTTGCGGAATCCGTAACCCTCTC
GGTACTTTGCATTTCGCGAATCTTTTGTATTTCTTCTTCCCTCTCCCGCTGCTTTTCCTCTGCCGTTT
TCCGTAGATCCATCACAAGTTGTTCCACGTCT
GTCATCAAAGACCTGGCCTCATCGTGGTTGGGATTCAGCTCCAGACATCGACGCAATGCCGTGTATGC
CTCATGCGGGTGTTTTAAGTGATGCTGCGCGT
GCGCAATGCGAAAGAAGGCTCTTGCATACATGGGATTCACCTCCGTTGCCTTCATTCCGTCTTCCAGT
GACTCGAGATGACGGCCTGCCCGACTATATGC
CGCACTTCTATTGCAGAACAAGACTGCTAACTCAGCAGACTTGCTGCTGCTAACATCGGTGGAGTTGT
CCTTATTCCTTTCATCACTTCCTGTAGAACAC
CTTGCGTGCAATTCAATGGCCTTTGAAAAATGTCGCACGGCACTCAGGTATTCACCCTTTCCCATGGC
CTCCTTTCCTTGTTCAATACCTTTACGATAGT
AATATGCATCCTTCGTCTCCTGTGTGGCAATGCCTTTGCCGTTGCGTATGAGCTCCAGCACCACCTGA
TGCTGCTCCACGTACAAAGCGTTCGTAGGGTC
CAATGTATGCGCCCGATGATAGTGCTGCGCCGCCTCTTCATAATGTTTAAGACCATGATATGCCGTAG
CCATGCGCCCGTGTAATTTGCTATTATTTTCA
TCTAACTGCAGCGCCTTTTTTGCGGCATCTAGAGCACCCGCATACTGATCACGTGCCGCAAGGCATGC
ACTCATATTGGAGTATGCCCTCACCTTTTGTG
GTGTTGTGGCGCGAGCGTCCTCGACAATTTTAGCATACACACCAATGGCGTCATTGTGACTCCCCTCA
CGCACAAGAGTTGTCGCCATTGTGAAATCTTC
CTCAAGAGTTCCCGACATGCCGTGTATCTTTCTTCATCCGCCCTTTCTTTCACTGAAAGAGAAAGAGA
GACAGTGAGAGAGAGAGAAAAAGGACGGGAAA
GATAATAAAGAGAACATGGGTAAAGGAAGACATGCAGAATTGTTGGGAAGTTCAAACTCATGGGGGAG
CAAACAAATCCCCATTACAGGCATTGCAACAA
AAGTGCGGCAGCAGAAAAAAGGGGGAAAAAATAGCTCACGACAATGTCATTTGCTGTACTGAATGTGT
GCATGCTTACTACACATTGCTGAATGCAACTT
GAACTGTCAAAGGCTCATTTTTTTGCCATGAAATATATTATAACGATAATGGAAAGGAGAATTAGACA
GCAGATAAGGTACACAGCCATCATGGGGCGAT
CCATCCTTTCAAGCGCCTTCTTTAGCTTAACGTTTAATTTGCCCAATCTGCCATGAACATCGTCCATC
TTCTTTTCCGTTTCATCCAGCATCTGTTCCTG
TGTGCAAAGCTCCGAAGTGATAACCGTCGCATTTTGATTAAGTCTTTGCACACCAACGTAAATGCAAT
CCAGTGACTTGTCAATCACCTCGTCATTTTTT
TGTGTTTCATAAAAAAATTGCAAGAACTCCACGTCCTCATGAAGATTGTAGGCATTGCCGGGCTTCGG
GACAACGTTACCGGAATTCGAAGCCAGGAAGG
CACTCTGGTTCACGGCATATTCCAGGTCAGTGAAGGGGAGTGGTTCCCTTTCAAAGGTGCTACCGCCT
GGCTTAGCAACAGGAAGTCCCCCACCGTAGCG
CTCGCGGACAATGTCAAGTGTATCGTTGAAGTGACGGGACAACAACTCAAAGTCCTGCATTCGTCCCT
CACTCTGTGCCACCCTCCTTGCATTCTGCATC
TCAACCTTGGCCTGGAGCTCCTCCTTGCGAATCTGTTGACTCACCTCGACACGAAACTTCCGGCTCGC
GTCGGCGGACAATCCATCTAGTTGCGTCACCA
ATTCTCGTATGCGCCGAATACGACTGGCAACCTCGAGTTGTGCCTTCTCGTACGGCGTCATCGTCTTC
TGCGGTTGCATGTGTAATTCCTCACGGAGGGG
CAATAGCGGTGCACCCTTGGTGTTTTCACTGGTTCCATTGATAAGCTGTAGCCGCTTCATGATGCCTT
GCATCGTCCCACACCGCCCCTCAATACTCGCC
GCACAGCTACAAGACAGGGGCTGACCTTGTATCCTTGAGGAAAAGTTGAACGATTTTGAAAAAAAAAA
AAAACAATAACAAAAGCAAAAAATAACAAGTG
ATTAAATAACAAATCAACAAATCATTAATTCAATTCATGTATAAAAAACAAAAGCAGTGAATAGCGGC
CACGAGCATCGACGTTATGCCGCATCTGCGAG
GGACTTCTGTGCCTCCTCGCCGCGTGGAGGTGCTGTTGGCAGCAGAGCCTTTCCACCCCGAGGAGAAA
CTGCGCCAACCCTTTTCATGCAGCACGGCTTC
CTCACGTCTCCAGCGTTCGCTCCGTCTTTTTTTTATAGCCACCGGCAAAGCCGGGCAGCGGAGTAGAT
GCGGACTTGTCTCGTCGCACAGTTCATGCTGG
CTGGGGTTTTTCGCGACGTCCGGTCAATGCCTGTGGCATGCCGTGCGGTGGCGATTCAGTATACATAC

ATATGTATATATACAATTGTATATATCCCGAT
GAACAATCTGGTGACACGCCGCACTTCATTCGGATTGGAGGTACTTATAACCTAAAAACTGGAAGACT
TTGAATTCCTCCAGCAACAACACCCCGCCGCA
AGAGCGGAAATCAAAAAAGGACCGGGTAGAACGACCAAAATATAAGTGAAAGTAACATTCTTCTTACC
GCTATCGGTTCCTTCACCCCTTCCATTGTGGC
CTTCCAAATGGTCAGAAAATGATAATAATAAAAAAAAATTCTATTTAAATAGCTTCTTCTTATCAGAA
CATATATTAATTTGGGTATCCAGCATTCTTGC
AGACATTCGCTTCACACGCTCTCATTATCTGTGCTCATAATTCGTAACGAAAGTAATAACAAACAGTG
ACAAAATAATAATAATCCAATTAACATGCCTT
CCTCAAAAAATAAATAAATTAAATAAATAAACCATTGCGCATTAGACTGCATTGTGGTATACCCGCCA
CAAGTCCTTCTGCCTCACGGGTAAAAGAGTCG
CCATCTCGCGAGCCAACTGGACCTCCGCATCTGCCTCTTCGTTCATGGGATTACTAGACGCAAAAGCA
ATGGCCTTTTCAAACATTACAATACTAGCAGA
AATGTTGTCATACTCTTCTTTTGGATCCTTGAAAAACCGCTTCATCTGCAAATGCGCCAATCGCATCA
GCGACCTGAAGAAGGGCAGTCTAGAGTCTATG
TCTATTGTCTCGGGGATCTTGTTTGTCTGTGGTTCTTTCCATGTATCACAGAAACGCCTGTAAAACCC
TTGTACCTTATCTGTAAATGCATTTATTTTCT
TATCGGACAGCGGTTTCTCCCCCTCCATGGGACTGTTCTTCTGCTGAATATAAACCTCCAGCATCTCT
TCATGCATGCCTCCCAAATCAAATAGCAACTG
ACGGACAAGTGTAGGGTACGCGTTAAAGTTCAAGTCACTCGGAAATAGTTCGATAAGCGCTATCCTTC
GTTGATGCATAGCAATGATGCGATCACGATTT
TCCTCAAAAGGAATAAGCGCCGCGTACAACTGCGCAATATCCTTCTTAATATCAATATGCTCTGTGCA
ACATCCATCATACACATAATAATTTAGCGCCT
TGTTGAACCAAACGTTTGCCTCCTTAAAGCACTCACGAGCGTCTTCAAAAGTTTCAATTGCCGGAAGT
GGCTGTGGTGGAGGAATATCAAGTGGGAAATC
AACCCACCATCCTTCAAACTCATCCGGAAAATGGCCACGCGGTGCACAATTACGTCGCACTTCGGCGT
AATAATGAAGCCGATGTAAATAAAACTTTCCA
AACGCCCATGCCACAATCCCAAGAGTCTCCTCGTTCGCGTTTTCCTCCGGCATAATGTACTCCCCCGC
ACGAAGACAATGTAACGCCTTCCCATATTCAA
AGCAGCTGCTATAAAATGCACTAAGGTGCAATGCGTTCGTTGCCCAGTCCTTCTTTGAAAACTCCATT
TTGTTTAGCAGTTGGTGATACAACGTGTGGTG
ACAGTAACGTGAGGCCAGTGCCGTCATCTTGCATGCCGTGTAAACTTGGGCCAAGAAGAACAGCGTGG
ATGTAAACGCCGTATCCATTTCAAACCGAACT
ATGTAAATTGGCAGTGCGTCCGGAGAGATGCTTGCCCGGTCAAGAGTACCGTTTTCTAACACAGGGAT
GTCCTTCATGAGACAATGCCCAGGGAAAGACT
GATGAAACCAATCATTCCATGCTCTATATGCCTTCTCTGCCACAATAAGAACCTGCTTTGCGTCGTTG
ATACGCAACTCAACATCGCGGTTTGAGAAATA
AAACCCCAACGCGTTATGCACACGCATAAATTCCGTGACAAACGCATACTCCTTTTCAGCCGTGACAA
ACGGAACCTCATTCCTGAGAATCTCCTCGAGC
GGGTGAGCCGCGATGTCCACAACATCGTCCAGTAACTCTTTCTTCGCTCCAAAGAGAGTCAAAGTGTA
CGCCTTGCAAATTTCATTTACTCCCTCCGTCT
CTTCATCAGTTTCCAACATATTGAAGCCAATGAAAACGTAACACCGACAGATTTGGCTCATCGCCGTC
TGTGAAGGAGATTCTTTGTACGCATTTCTCAC
AGACTC
>scaffold1002.size7804
GCTTCTTCAGGACAGGAGCCCGTAAAGCAACCGACATCTGGAACTTCTTCCAGTGGAAACAAAAATGC
GGATGGCACTCCCTTGTCTGGTGGTGACAAAGC
TGTGACAGCAGGGAGTGGAGAAACGGTTCAGGGAGATGGATCACTCCAAACTGCCGAGGTGAGCGTGA
GCTCTGGTGCGGATGGGGAGACGGCGGGAGGA
ACGGATGTGCAGGAGGTTAATTCACAGGACAGGGGAGTAAATGCTGCGGCACTCAGCAGCAGCCTCGG
AAATGTGTCGCAGGGGAATAACACCGATGCCG
GCACTGTGCGTGGGAGCGGACTGCTGCCGTTGCTTCTTCTGTTGGGGCTGTGGGTGTTTGCGGCTCTG
TGAGGAGTGACTGGCTGCCGCGTGGGACAATT
GCGACCTCCTCTGCACCGATACACACGGTAATTAATTTATCTTCTTTAATAACTTTTTTCACTTTTTC

CCTGTTTGCTTTTGCTGTGCGTTATTATATCC
CTTTTAATTGCTTTTGGGGCAATCACTATGGACGTTTTTTTCTTTTCTACTCTTCCCTCTTTAATTGT
TTGTTGCTGCACTCTTCCTGTGAGGGGTGCCG
TGTGTTTCCCGCCCAACTGCTCCACTCGCACACCCACCGACACGCTCATGACGACGGCCCTGTGCGCC
TCGCCTGCTGCACGCATGAGGAGGACCGCGAC
GAACGGCGCGCTGAGGGAGCCGTGACGTGTGGGCGTGCGACGAGGGCCTGTGGGGCGGCGCACACATT
TCTTTCCTCTTGTTTTGTTTTCTTTGCGGCCA
GGGCGGGCAGACACCCGCAGCGCCACACACACAGCCACACCCTGCCGCCGTGTTTTTCTTTAAGGATT
TGTTTTGCTTTGCGTGTTTATTTCATGCGGTG
GCGCACCCTTCGGTGTCGGCCTCGTGAGCATTTCTCTCCCCTCCGATGATTTCTTTTTCTTTTGCTGC
GCTCACTCTCCCGATGGAGTGACGCGACTGCC
GTCATGTCTGCGTGCTTCTCTCAGTCCCCACACACACGAGTGACTGTGCTCTGCGGGCCGCACTCACG
CGCCGATGAGAGGGCTGAGGGGAGCAGCAACA
CGCGGATGACCCCATGAAATATAAACGATGTGCGGACACACGGAGGGTCGGACGGTCGAAGCACACGC
AACCATCTTTTTAATTTTCTTTTGTGGGCAAA
ATACATACAGACAGTTTGGTGTGGCCACACGGCAGCCAGGCGATTTTTTTCTTATTTTAAATTTGTGT
GGAGCAGTAATGGACGATTTCTTTTCATTTAT
AATTATTTTGTGTGATGGGGTTTACATAAGACGAAGGGGGACGTTTTTTGTTTTCAATAAATATTGTG
TGAGTTGCCGCTGCTTGGGCTGCAGTGCAGTC
ATGAGTTTGAGCGGTCTTCTTCTTCTTGTTGTTTCCGTTATGCGTCTCCGCTGTACATTTTTGTGCGT
CCCTCCGCATACTCTGCTCCCTCTTTTCTACG
TCTCTGCTTTGTGTTGCTGCAGGCACACACAACCACACACACAAGATGCACATTGCGGTGGATTTGAA
TGCACTTTTTGTAGTGCATGCTGGCAGTTGCG
TGTGGCGGCTGTTGTCTTCTTCCGNNNNNNNNNNNNNNNNNNNNNNNNNNNNNNNNNNNNNNNNNNNN
NNNNNNNNNNNNNNNNNNNNNNNNNNNNNNNN
NNNNNNNNNNNNNNNNNNNNNNNNNNNNNNNNNNNNNNNNNNNNNNNNNNNNNNNNNNNNNNNNNNNN
NNNNNNNNNNNNNNNNNNNNNNNNNNNNNNNN
NNNNNNNNNNNNNNNNNNNNNNNNNNNNNNNNNNNNNNNNNNNNNNNNNNNNNNNNNNNNNNNNNNNN
NNNNNNNNNNNNNNNNNNNNNNNNNNNNNNNN
NNNNNNNNNNNNNNNNNNNNNNNNGTGCATGCTGGCAGTTGCGTGTGGCGGCTGTTGTCTTCTTCCGT
GTGCGTGACGGCGACAGGAGACGATAATAGTG
ATAACAGCCGTCCTCCCAGCCCTGAGCTAGGGAAACCGGATCCTACAAAAATATGCCTGGCGCATTAC
AACCATATGGTGTGGAGACCGGTGTGCCACAG
ACAGAGAGTGGGAGAGACAGCGAGGAGGGTTTGAGAAGTCCAGGGCCTTCGTTGCCAAAGGAAGCAGG
TACAGCCATCACTGGacaGACACGATTTCCGC
CAGAGTCACTGAATTCGGCTAGCGATAATCCAGAGCCACCAGGTTTGGCAACTCAGTCTCCACACACA
CACAAGGTGCGACCGGCATGTCAGATGCACAA
AATAAAAGGCGCTGTAAAATACACGAGGCAATGAGAATCATAAGGCAAAGATATCAAGTCAGAAGATC
AAAATGCCAAATAGTACCACCAATAAGACTGC
AACAACGTTCTGGTCAGCCGGCCACCTCAACGTGGTGCCAGGGTCTAGTACTCTTTGCTAGGGAGGAG
GCCAAGCGCCTACTGCCCATCCGCTGCGCGCG
GAGAGGTGAAAGGCGCCGAACAAGCGGGTCGGAAGGGCACCAGATGGAGCCATTTGTAATGGTTGTCC
GCTGAGCGGTATTTCTATCCGCTGCTGAATTC
CATCGGCGCTTATCAGCGCTATACATACCGTTTGCGTGCCGTATGTGACGCGCAACGACAAAAGCTAC
TGCTAAGCGGAGACGTTGAGCAGCACCCAGGT
CCCATAGCAGTACTCCAGATGAACGTTTCTTGCCTCACGCCGTCAAAACTCGCAACATTAATGGCGCA
AGGAGCAGACATAATACCATCCAGGAGACTTG
GAAGTCGTCGGAGCAGATCGCCAGCATGCACACTGGAGATTATGTGCTCTATGCACAGTCGCGCATCG
GCAAGGGAGGCGGNNNNNNNNNNNNNNNNNNN
NNNNNNNNNNNNNNNNNNNNNNNNNNNNNNNNNNNNNNNNNNNNNNNNNNNNNNNNNNNNNNNNNNNN
NNNNNNNNNNNNNNNNNNNNNNNNNNNNNNNN
NNNNNNNNNNNNNNNNNNNNNNNNNNNNNNNNNNNNNNNNNNNNNNNNNNNNNNNNNNNNNNNNNNNN
NNNNNNNNNNNNNNNNNNNNNNNNNNNNNNNN
NNNNNNNNNNNNNNNNNNNNNNNNNNNNNNNNNNNNNNNNNNNNNNNNNNNNNNNNNNNNNNNNNNNN

NNNNNNNNNNNNNCACAGTGGGAGCCATTCTT
GGAGACTTCTCCAAGCGAGGTTGCTGCAGAATTTTTAGAACTGTGCACGGATGCGGGGCTCACCTTGG
TTAGCACCCCTGGTGAGATCACGTATGCCCGT
GGCACAAGAGAACGATCCTGTATCGATCTGACATGGTCAAAGCATTTGACTGTGTCGGATTGGTCAGC
TTCCGTGTCGCCGCTTAGTGATCATTATATGC
TGACATTCACGCTGCATCAGGCGTTTAAGGATACCATACCTTCGGCACCTAGGATATTCCACAGTTGG
GGGAAGTGCAAGTGGGATTTATTCGTCAAGGA
CCTCGACGCACAACTTCCGGCATACGACTATAAAAAGCAGTCCACCGGCATTAAAGCTTTCACGAGAG
CGCTTATAACTTCTTATCGACGACATTGCCCC
CGCGGCATGCACAAGGACGGTCCCAGGCTTTGGGACGACACTCTCATGGAGGCAGAGCGGATCGCTAN
NNNNNNNNNNNNNNNNNNNNNNNNNNNNNNNN
NNNNNNNNNNNNNNNNNNNNNNNNNNNNNNNNNNNNNNNNNNNNNNNNNNNNNNNNNNNNNNNNNNNN
NNNNNNNNNNNNNNNNNNNNNNNNNNNNNNNN
NNNNNNNNNNNNNNNNNNNNNNNNNNNNNNNNNNNNNNNNNNNNNNNNNNNNNNNNNNNNNNNNNNNN
NNNNNNNNNNNNNNNNNNNNNNNNNNNNNNNN
NNNNNNNNNNNNNNNNNNNNNNNNNNNNNNNNNNNNNNNNNNNNNNNNNNNNNNNNNNNNNNNNNNNC
CCGCTATCTACAGTCACCGACGCCCGACCGTG
AGGCAGAAATGCAACGGACAAGGAGCCAATTCTTCCTCCTACTCCGAGAGCGCTTGCGCAACACGTAT
CCACGTCGCATCAGCAAATTAAATCCAGGCGA
GCCACTCGCATGGAAATACATTTCCGGACGAAAAAAGGCATCACTTCCATCTCCCACATCAATGTTAT
TGGGAGATGGTCAACACACTTATAAAACAGCA
AGGAGAGCAGCGAATGCTCTCAATCGCATCTTTCTTCCATTTCACCCCTCTCACAAGGCAGTAAGGCC
TTCCAAAGGCAACAACAGACAGCGTGCATCTT
TGAACATTAATGCTTCTTTTCTTTTTGGGCCGCAGAACAGCAACGAGTCTGCCGCTACATTTACATCA
GGGGTTTTGTCACTGCCCTCTTGATGCACCTT
TTAATCGTACGGAACTGCTTGCTGCGCTACGTACTACGCCGTATGGCAAGGCCCCGGACCGGATGAAG
TCTACAGTGAGGCACTGCGACATATTTCGTCA
AAGGGCCTCCGATTCCTTCTTCGCTGCATTAGCCACAGTTGGACGACCGGTACGATTCCGGCTGAGTG
GAGACGCGCCACCATCGTTCCACTCTTAAAAC
CCGGTAAGTCGCCGGAACTGCTTGAGTCATATCGACCCATCAGCCTTACTTCCATTGTGAGTAAGGTT
GCTGAGAAAATGGTACTGAAGAGATTGCTTTG
GGTGTGGACGCCGCACCCCCACCAGTATGCATATCGTAGTGTGCGTACCACGACGATGCAGCTGGCAC
ACCTGACACACGAAGTGGAGCATAATAGAAAT
CACTATTTCCAAGTGAGCCTTCCCAAGAAGAGCGGTATTGGCAATCAACTCCACTACAGACCCCATCG
GACCCTGCTGGTGTTGGTTGACTTCAGCAAGG
CCTTTGACTCCATAGACCATCGAGTCCTCAGTCGCCTGCTGGCTAATATTCCGGGGGTGAATTGTAGA
AGGTGGCTTAGAAACTTTCTATGTGGTCGCCA
CGCGAAGACACGAGTTGGCCACAGACACAGCGATCGGCGTCCCATGCTGCGAGGAGTTCCTCAGGGAT
CCGTGCTGGGACCGTATTTGTTCTCCCCTTAC
GTACACCCACTTCTCAGTCTGCTGAACAGCTTTGCGGGTGTCACAGCAGACATGTATGCGGACGACCT
CTCTATTATCGTTAGGGGGCAGTCCCGGGAAG
ACGCCATTCCCACCGCCAACATGGTTCTTGGAAAACTGCATGCGTGGATTNNNNNNNNNNNNNNNNNN
NNNNNNNNNNNNNNNNNNNNNNNNNNNNNNNN
NNNNNNNNNNNNNNNNNNNNNNNNNNNNNNNNNNNNNNNNNNNNNNNNNNNNNNNNNNNNNNNNNNNN
NNNNNNNNNNNNNNNNNNNNNNNNNNNNNNNN
NNNNNNNNNNNNNNNNNNNNNNNNNNNNNNNNNNNNNNNNNNNNNNNNNNNNNNNNNNNNNNNNNNNN
NNNNNNNNNNNNNNNNNNNNNNNNNNNNNNNN
NNNNNNNNNNNNNNNNNNNNNNNNNNNNNNNNNNNNNNNNNNNNNNNNNNAAATGTGGCGGCCTCCAA
GCAATGCGCTGCCACTTCGCAACGGATATCGC
AGCCACGCTGCATAGCGCACAAAGAGGCGGGGCCATCTCCACATGACCTACGCACTTTCGTCATTGGA
TACGGTGCTTCCAAATTACGCTATGGCAGCGA
GCTCATATGGGCAGTAGCGACGGATTCAGCGGAGAATGAGATGCAGAAGACGTACGCAACCCTAGCAC
GCATTGTCAGCGGAGTTCCGAGCACTGTCGAC
CCGGAATCCGCGCTACTGGAGGCTAATATGCCCCCGCTCCACGTCCTCTGCCTGCNNNNNNNNNNNNN

NNNNNNNNNNNNNNNNNNNNNNNNNNNNNNNN
NNNNNNNNNNNNNNNNNNNNNNNNNNNNNNNNNNNNNNNNNNNNNNNNNNNNNNNNNNNNNNNNNNNNNNNN
NNNNNNNNNNNNNNNNNNNNNNNNNNNNNNNN
NNNNNNNNNNNNNNNNNNNNNNNNNNNNNNNNNNNNNNNNNNNNNNNNNNNNNNNNNNNNNNNNNNNNNNNN
NNNNNNNNNNNNNNNNNNNNNNNNNNNNNNNN
NNNNNNNNNNNNNNNNNNNNNNNNNNNNNNNNNNNNNNNNNNNNNNNNNNNNNNNNNNNNACACGCGCATGTC
AGACGGACTGGATGCGGAGACCCCCGCCTGAG
CCACCGCCTCGCGCCGGTTTCCGCATCTCGCCATTATCTCGGGACGAGCTATATGCCTTCGTAGACGC
ATACACAAAGGACTACGGCATCACCGAGAGCT
CACCACGCGAGGAGCGGTTCTTTCGCAGCTCCATTCCTCCCTGGTTTGCGGCCTCCGCTCACCGGGCC
ACCATCGGTGTGGAACTTCCGATAGACCACTC
AATAACTGACGAAGAGGAGCTGATACGGGAAAAGCGCAGGGTCAGCGAAGAGGCTCTAGCGCTGCACA
GCCATCGTTCGTGGATACTTGCGACCGGTGGC
GGTGTCGACGTTCCCAAGTCAGCAGGGGTTGGAATACTGCTCTCATCCCTCAACTCATCGGAGATAAT
AGAAAAGGCCAGCATAAACTGCGGTGCACGCC
CATGCAGCTACAGGACGGAATCCCGTGCGCTGCTTCTAGCCCTAGAGAAGCTGATGATCTCTCGTATC
CAACACAGGCATAAAACCCTGCTTGTGGTTAC
GGACAGTCAGTCTCTTCTAGCGGCTCTAAGCAAGGGCCCGCTCAGTCAGACCGACTGGACGGAGGATC
AGATCTGGCAGCGTCTCTTGACACTGACGTGT
GCTGGCTGGTCCGTGCACCTGCAGTTTTGTTACGGACATTGCGGAGTACATGCTAGCGAGCCTGCAGA
TCAGTATGCGACGCAGACTATGGCAGCTGGAC
AATACACGGAGCACGGAATCGCAACTTCATGGCATACGGATCTGCTGACATGTTTTACTACCCAGCTC
ACCAACAAGTGGCGTAGTACCATTTGTCAAGA
CACTCATCGCTACTTGCTTTGCGGCACAAGGCCATCCGATCTCAGCGGTAAGGACCTGATCACTCAGG
AAGTTCTACATCGTCAGGAACTGGTTCACCTC
GCAAGGGCAAGGTGCGGGGAATCTGAGCTCTGGGGCCGACTATACTGGGCTGNNNNNNNNNNNNNNNN
NNNNNNNNNNNNNNNNNNNNNNNNNNNNNNNN
NNNNNNNNNNNNNNNNNNNNNNNNNNNNNNNNNNNNNNNNNNNNNNNNNNNNNNNNNNNNNNNNNNNNNNNN
NNNNNNNNNNNNNNNNNNNNNNNNNNNNNNNN
NNNNNNNNNNNNNNNNNNNNNNNNNNNNNNNNNNNNNNNNNNNNNNNNNNNNNNNNNNNNNNNNNNNNNNNN
NNNNNNNNNNNNNNNNNNNNNNNNNNNNNNNN
NNNNNNNNNNNNNNNNNNNNNNNNNNNNNNNNNNNNNNNNNNNNNNNNNNNNNNNNNAGATTGCACGAACCAA
TGCCGTTTCTGCAACATCTCACCGGAACAGTC
TGCATATATGCGCTCTAACAACGATCCAACTGCACCGGGGACGGACACTGTTCCTCTGCCGGCAAGGG
ATGAAGACGTCTCTCCAGTAAGGAGACGGACC
GTCACACGCCGTCGGAAGGAGAAATGTCCGCACTGTGATTCTACATTGACGGGATTCTCAGGTCTCGT
CAGTCACTGTCGGTCATTTCATCCGGAACATC
CTCCACCGCTTCCCGAGCTCAAATGTGATTTCTGTGACACGGTCTTCCCCACACGGAGAAGCACCGCA
CCGCACAGAAGTTGCTGCGCACACAACCCAGA
CGC
>scaffold1003.size8453
AAAAGAAAATAATAATAAGAATAATAATAGTAATTTGCTCTCCGGACGGAGAACACCCCGCCCCCCCC
CCCAAAAAAAAAAAAAAAAAAAAACGGCATGCA
CACGCAGGACGGCCACCAGCATGTGGAGGGCAACAGAAAAGGAGGGAAGGAATTTCATATGCAGGTGG
TTGTATATGTATATATATTAGCAGCTGTAGTG
TCTCCTTTCTTCACGGGAAAAGAAAGTGAAAGAAGAAAAAAAAATAATCCTCTTCCATGTGGTGGATA
ATCGCCACACACAGACACGATTTCAATGCACC
TCCTCCCCCTCAGTGGAGCACCATCAGTGGCAAAAGCAAGCACAGACACACACACACACACACGTAGG
CACATAAACCCACTAAAGTTTAGGTGTCACCG
GGGTGGCCAAAGACAAAACACTGCCATCCACTTCCCTCACACCTTCGATCTATTGGAAGTCCATCCAG
GCACGGAAACGAGGTGTTCACCGGGACACACA
AGAGCAGTGAGCTCCCCGCCATACACACACCGAGTGTCCAATCCGATCGCATGAGGGTAGCGTTGGAG
GCCTCGTCTGGCGTCGTGGCCAAAGACAACAG
TCTCCGGACCCTGCCACAGCGATGCCCACGGCACACCGTCAGTTGTATCCTCAGATGCCCTGTACAGC

CCGTCATCCTGCAAAATGAGGTTGCGCATCGT
AGTGACCTCCTTAACCAATTGCCGCTCGAGCGGGTGTTGAACGTGAAGGCCCGCATGTACCACCAGAA
TGTTGTGTGCGGGAATGTGGAGAATATGCGGC
AGTTGTAATAAATACTCTTCAACATCCTGCGGTAGCGACATGGCAAGCGGGGCCAAACTCGATTGGCT
ATTCTTCCCGTGCAGCGTCCCATGTTTTCGTA
TCTTTTTCCGAAGTTTGAGAAGCTTGGCGTCATGATTGCCAATCACACCGCGTGCTCCAAGGCTCTTC
AGCAGACGCACAACGCCAAACGAGTCTGGCCC
TTTGTTCACCATGTCCCCAGCGGTGATTAACTGATCCCTTCCCCTCACAAAGTCTGTGACTGATAAAA
TGTCCTCCAGTTGAGCAAGGCAGCCATGAATG
TCGCCCACAATGACAACCCGCCCATTCACGTTGTGGAGAGTCTCTAACTGTGCGTATTTTCTTCCCAT
CGTGATGCTAACAGCACACACATACACACACA
AACGCCCTAGTGCAATAAAAAAAGGCACCAGACTATTGAGCGTCACAAAGAGAAAAATCACAGAGAAA
TAGTAACATTGCAAATCCATTACAACCGCAAC
TGCCAAAACCACCACTACCATCAGCAGCGGCGGCAAAAATGCCCCGACTAAAATTCAGAAAAAAGAGA
AATAAAAGAAAAAAATATCTGAGATGGCAGTA
ATGTCCATAATATATATACAACCCCTCCAAGCGTCTTTTGTTCTACCGAGTTTCCTTCAGTCTATGGC
TCTGCAAAACAGGCATGACAATCTCACTGAAC
CTCCTTCATATGTGCACACATGCACCAAAATAACAATAATAATAATAATGATATTACGTGGATAAGTA
AAAACAGACAAACGGTATAATTGCGTGTGTGT
ATGTGTGCTCCTAGAGACAGTGATACCATACCACCACCTCCGCCTCTACCGGGTCAATGCAGACATAG
CAGTAGTTCAGGTTACTGGGCTCCGCGTCATC
GGTTCGAGTAAAGAGTGAAAAACCGCTCACTGCCTGCACAGAAACAACAGTTGAAACCACTTCCGGTG
TCAATACGGTACCGTCCACATATGCGGCATGA
TCGGGCTCGTCCATGCCACCCCCAGCAGATGGTGCCTCATTCTTCTGCACCGCACGAATGGCGACAAG
GCCCTTATACCATGCCCGCGCCACATCAAGGT
AAACTCCCAAGTCGTCATCCCACTGGTTGTTGCTGCCGCCACCCGCCACAAGGCGCCATAAAATGTGA
TACAAAAATTCGGAACGCTCCGCCTCCGTAAA
AACACGACGAAGTGCGGCAAGTGAAAGTCCTGCACCAGAGCCATAATTAATCGCGTCCCAAGCCTCCT
CCTCGGCATACTCATCTCCCTCATGGCATATG
GCGCGATTGTGTGCAAGCAAGAAGAGTGCCCGCAACTCATCCGTCACGGCAATGTCATGTGGAAGGTA
AAAATCCGGGCGTCGGACGACGGGCAAACGTA
CGCAGCCGTCAAATATGGCCGCTTCTGCCTCATCATGACATACATCTCCCTGCACCTCCACATGCCGC
ACAACTCCTGCCTTGACGAGACGAGTGAATGG
CTCAAGTGTCGTCCAACTACAGTGCAGCTCGGTGTATTCCACCGCGTCACGTGTCAGTCTCAGTGCAG
GAGAAAGCGCAAGGGCATCACGCACGGCGGTG
GAGTCAAAGAGAGACACAAGAAACTCCTGAAGCACCCCTTTGTGAAATCCAGAAAACCGCATCGCACC
AGCCACAACGGCACCGTTGCCAACACCACCGC
CATGAGACGCACCCTCCTGCGCGATTCGGCTGTTATTCTCCTGCTCAAACCGGAGGGCACACACACGA
GTCTGACTGTGCAGCCCCCACCTTCGCTGTGC
CTCCTTATAGGCGGGGTCCGTCAAAACAATTTCCAAACGCCGATGAGTTAACGGCTCAAAAGAGAAGC
GAGTTTGCGGCCCCATTCTTCCCCCCGTTTCT
TTCTTCCCCTTCGACTGCCCCGCACGCTACAGCGACACAAAGAACAAACAAAAAAAAAAAAAAAGGGTG
CGAAAAAAAAAAAACATAAAGCAAAAAAGCAT
AAGAACATGCGCAGCTAACAAAGCAAAAAAAAAAAAAAACATCGCAGCGCTACCACACACCGGCACAT
GGNNNNNNNNNNNNNNNNNNNNNNNNNNNNNN
NNNNNNNNNNNNNNNNNNNNNNNNNNNNNNNNNNNNNNNNNNNNNNNNNNNNNNNNNNNNNNNNNNNN
NNNNNNNNNNNNNNNNNNNNNNNNNNNNNNNN
NNNNNNNNNNNNNNNNNNNNNNNNNNNNNNNNNNNNNNNNNNNNNNNNNNNNNNNNNNNNNNNNNNNN
NNNNNNNNNNNNNNNNNNNNNNNNNNNNNNNN
NNNNNNNNNNNNNNNNNNNNNNNNNNNNNNNNNNNNNNNNNNNNNNNNNNNNNNNNNNNNNNNNNNNN
NNTCAATTGAGCGACAGAAACACAACTCACAC
AAACACACAAATATACGCAGTAACGTGGGCAAAGATTTATTTTTTTTTTTTAAGGGTTAAAAAGCACA
ACAACAAAGAACAAAGCCCATAAAAACACCCA
ACTATTATTTTTTTTTTTTTTTTTTTTAGGTGGAACCTGTTTTGGCGTATCTCGCTTCCTCCCACAAA

ACGAGTCTTGGCCGCATCACCAACAAAATTCA
TACATCCCTATTCAATCCACGTAAAAAAAAAAAAGGCCTTCGCTCCCAAAAACAGTAATATATATATA
TATATATATATTTCATTCTCTCTAGTTTCCGC
CGTCTGTTTTTTATTCCGTCCATTTTATTCACTTCTCTCTTATTGGGTGTTTACCCTTTTTCTTTTCT
TTCCTGAAGCTTTCATATCGACCCGAAACTCC
GGTCCCCACACTAATGGATACCCCGCACTGCAGTGGAGTGACGCACATTGACCGAGCCCCAGCAGAGA
GGTGTTGGCGGTGTATGTTGCCTTCCATGACA
TCTCTCTCCCCTCCTCACATCAAATCACACAAACGACTTACGCTCTAAGAATCGCTTCAAGAAGTACA
GCTGGAAGCAACTCATGATAACAAGTGTAAGA
ACCTGAAACACACTCCATATGAGCAGGCGCTCGCTCGACTCCTCCACCGTCGCCCGATGCTCCTTCTG
CTGTGTACGCAGGAGCATCTGAAGCGACTCCA
CCTGACGCATCAAGGAAGAAATATGCTCCACTTTTCGCTCCACCGGGTCCAGCTCCGACTGCTTTGCC
ACAGATGTGTAGTCTACATGGTAACGGAAGTG
AAAAACAACAAGTTTCGGGGTGTAATGTGCGTAGGTGTTGTCAAGACAAGCAACGATGGCCTTTGTGC
CGCTCTGGGCAAGAACTTCATACAACCCGTCT
GACGTCTCCCCCCACTCTTTAAGAATATGCCCCTCCTGATCAGCAACCGAGGCGCGTATGTCGTGGTT
TCCGCCACCAACAACCTGAAACTGGAACGTCA
CCCTGCTCTGCGGCGGCACCACCTCCCGCAGACATAACTTCTCGCCACTGCCGACAGTAGTCGTGGCG
GCCTCGACGCCAATTAAACACACAGTCAGTGA
CAACAACGCTGTGGACAAAAGAAACAACAGAAAAGAGAGCTGTGGTGGTGATGACTGCTGACCACGCA
AACGCTCCCTCAAGTTGGGCATTTCCCTTCCG
TCTTTCTTCTCTATCCTTTTCAATGTTCCCCGTTTTGATTGCAAACACGCACAATCGCACCGCCGCCT
CCTCTGTCCCGGCTCCTTTTTCCCTGTTTTTG
TTATTTCCTATTCTCCAGTTTCCACCAACAAAAATTTAAAAGTTGTGCAAAACACCAGAGAAAAACGT
AAGTCTGTGGAAGCCCATCCACAGGAAAAAAA
AGAAAAATACATGCGTGCGGGCAGGGACAATGTGAAGGTGGAAAACAATGCCATCCACCGAAGAGCGG
CGCAAAAGAAAAGACAAGGAAAGTGGCGGTGA
CTGCACCCAAACACACACGCAGAAAAGAAGAAATCATAAACGCGCACCTCTCTTTCTTACCTTCACTT
GTTATTTCCCTCCCCTCCTCCCGCCGTTGTGG
CACACAACACACACACGATTCCTCGCCACCCGCCGCAGCCGCAATGCCCCATGTCCGCAGCCAAAAGG
GCGTGCACGCATATGACGCCGCGCCGGCATAA
AAGGAGAGGCTGCAAGAAACAAAAATAATGCCTGGTCGAACAACAAAGCATAGCGCATGCAAATAAAC
ACGTATTATCCATACAAGTGCTGTCAACAAGA
AGGAAAAACCATCCCGCATTCTATCTGGAAAGACAGTGGCCGTCCAAAAAGCCCTGAATGTCGCTCAA
GACCCTCCTCCAGGTTACTTCCGTGAGAACCT
CGTGCATCAGCCCCTCGTATTCAATAAGTCTCTTGTCCGTCGAGACTGCCAACTCCATAAACCGACGT
GAGCCGCCCACGGCACACAAAAGGTCCTTTGA
GCCGTGTACAATAAGAAGCGGGAAGGTGCTTCTCTCCATATTTTTCCACACGTCGTCAATCGCACGCA
TCATCTCCGCCCCATAACGTGCCGTGAGAGGG
ACAGAAACCCTAAATGGGTCCTGCTTCGCAAGTTCCACAACCGGGCGGTTCCCACTGATGAGGTCAAT
ATCAAGTGACCTCACAGCAAGCTTCGGGAAGT
GCCTTGAAACCATGGGCGCAATCCGGCGCAACAGGGGCGTCGTCAATTTGGGATCCAATTCGAGTGCT
GGCCCGCTGAGAACAACGGCGGCCCACGCGCC
TGGGTCACGGAACGCCACGTGCGCTGCTATGAGGCCGCCCATAGAGTGTCCCAAGAGAAAGTGTGGCA
GGACTGCATAGCCCGGGTAACGGGAAAGTATG
ATTTTCTTAAACAGCAAGAAGTCATCCACGAAATCGTAGAAGTGTTCCACATGGAGGCGTTTTCCCTC
GCTTCCGCCGGCCCCCTGATTATCCATGCAGA
ACACATGATAGCCCTCGCGCGCAAACGTCAACGCCACCGGATCGTACCGTGCCGTGTGTTCGGCAACA
CCGCTGACAAGAAACAGCACCGCACGCACGGC
GGGCACCTCGCGTGGCGGCTCCCACTCCGCAAAGCGCAGCCACAGGCCCTGCTTGTTCTGAAAATACC
GCGGGAAAAGTTCCGGGTCGGGCGGAACACGC
GACGGCTTGGAAAACTTCAGTGCGTGTGACGCATCGCAGCAAGAGCAGCACGAGCCCATAGCCCGGTG
CCCCCACAATTGAGCTGCGGAAACGGAGGAAG
GAGAAAAAGAAAGTGTGGGAGAACTAATGTCGCTTCCCAGCCAACGCACGTCCTTCGCCCTACAACGT

ACACGTGCAGTGGGGTTGTTTGCAGCATCGGA
GGAACAACAACAAAGAAAACAGAAGAAAAGAAACAACACAGGAATAAGATGAGACACGTCGGAGCGGA
GAAAGAAAGGAGCCAACAGAAATGAGGCCTAA
AACACACAGGGGACTTTATTCGCAGNNNNNNNNNNNNNNNNNNNNNNNNNNNNNNNNNNNNNNNNNNN
NNNNNNNNNNNNNNNNNNNNNNNNNNNNNNNN
NNNNNNNNNNNNNNNNNNNNNNNNNNNNNNNNNNNNNNNNNNNNNNNNNNNNNNNNNNNNNNNNNNNN
NNNNNNNNNNNNNNNNNNNNNNNNNNNNNNNN
NNNNNNNNNNNNNNNNNNNNNNNNNNNNNNNNNNNNNNNNNNNNNNNNNNNNNNNNNNNNNNNNNNNN
NNNNNNNNNNNNNNNNNNNNNNNNNNNNNNNN
NNNNNNNNNNNNNNNNNNNNNNNNNCACGCAGAGGAAATTGATGCGTAGAAACACATGTTTAAAACAG
AGAGAAACAACAAGAGCAGAAGTGGTGGCAAT
AACGCACACACGCACATGCACATACTATATATGTGTATAGGGACAGGGATTCCATGCCTGTGTACGTA
AGATACAAAAAAGAGGTGCAGAGAATAAGAGG
GGCAAGTGGTCGCTGATAGGCTCGCTCTCACACACACAGAGAGAGAGAGACGCTCTCTCCTCCTCTAT
CTTTCTCCTTCCATCAGAGGCCCCGTAAAGCG
GTCGGAGGGCTGCAGTATCACCTCCAGCTGCTTTGCCATCCGCACCACCTGCGCCCGCAGGGCTTGAC
GATCCAAATCAAGTTGTTGAAGCACAAGAAGA
CGCTGCCGTAGCTGCGCCTCTGCCTGCGCAATTCGTGTCTCCACTCCCACGCAGTTTCGCCCCTGGGT
AGAATACAGCCGAAGCTTCTCACGAAGCATAC
GAGCCTCATCACGCTCCGCCGAGGCAGCAGCCAACATTGCACGGTACGCCAACAGGAGTCTCTTGCCT
TCCGCAACGGTAATGGAGGGGTCACTACAGAC
AGTTGCCATCGATGCGTTCAATGAGACGGCGGGAGTGGGAGCTGCGAGATGAAGTGGCGGGGAAACAT
CTCCACCGAGGGCGCATGAATGCGTCGAAGAG
GATATCCCCGCTGCGTCCACACGCGAGGCTGTGTCAATTCCACCAGCAGCACGCCTCGGCGCTGCAGC
AGTGCCCATGCGCACATGACGCACTGCAGGAG
GAATGGCGGCGCAACGGGCAACAAGACGCCGACGAAAGACCTCCACAGAGCCATCGTTGACGGCTCGT
TCGCAAACTGTTCCCTTGTGAGGCTCAACACA
AACAACAGCAGAAGGGGAAGCAAAGGATGTCTCGGCGTCTGCGGCCGTGCTCGCCATCATAACCGCAC
CCGTTGCTTTTGTTGACTGCGGGTCCTCATGG
AAAGACTTCTCGTCGCCTGCCGACCGCCAATATGTCATGAGGACGCTGCTCACAAGGCGGCTGACGGT
GAGATGCCGCAGAAGTTCCCGGCGGCGCCTTG
GTGCTGCCTGCTCCAACGCCCTATACCCACACCGTGACTGCATGCGTTGAAGCCACCTTTGAAAGGAG
AGGCGGGGCAGGCGACTTCGTAATGAGGCATC
GTAACAGTTCCCTGCCTGAGCAACGCGGTGCTTACACAGCTGTCTCCACAGTGTGCGCTCCCTCCAGT
GCTGCAGGACGTCGGATTTACAACGGCAGGCC
GCGTCACTCCGCCGACGCTCACACACCGCCTGCCGCTCAGAGAAGAGAAGCCACCGATAGAAATACGC
ACAAAGAATGACAAGAGCGCTTCGTCGGCTAT
GGAGCGTGCTGGCAGTGCGCATAAGCCATCGCTGGTAGCCATGGGCCATGGCACGCCGCTCTCTATGA
CTCTCTACTTGCTCAAGTGCCGTTTCATGCCT
ATGCAAACGAATAGCGCGCCGCCGTGCAGTGCCGCCAAGCCACCGAATCCAGTAGAAACGCATCGCGA
GCAAACCCCACGCCAGCTTCTCACGAAGACAA
GGAGTATGGGCGGAACATGTTGGAGGAGCACAGCTACTCGTTGCAGCCACCGGCGCGCCACCCTCCAT
CCAGGCCACGGGTACGTGCAGAAGGAGCGCAG
CCACATGTAATCGACGAGCTCTTCGTTTCCAAGTCACGTGTCTCCAGACCACCCAACACGACTGCTGC
CGCCAAATCGCCGCCGCAGTGGTCAGCTTGTT
GCACGCAATGCACCGCTGGGCGTGATGATGCCAAGCGTTCCAACACCGCAGAAGAAGCGACTTCATGG
TTCTCAAGCCCATCGCAGTCACATGTGCCCTC
AGTTCACTTGCCGCATGGGACCACGTCTTCCAGGCCTCCACTGCGCGTCGAAG
>scaffold1004.size7626
GATTTAAAAACAAAAACGCCTGCAGGGACTCCACCGCTCAGCAATCAGGCAAGTAAGGAATCATTACC
CACAGTAAAGCCAGAATCCCCCACCACATCAGA
CTCAACAGTTACGGTGAAGGGTACGGATCAGATTTCAGAAGAAAAGCTTGCTGTGTTACAGGGACCAA
GTACGGTCAATGCTGGAGAGGATGGGAAAAAA
GCCGTTACGGAAATAAAACCAAATACGACCACCAATACGCCAGACGCAGCACCGACACTGTCCTCAGA

GGGTGATGTGACAGAGCAACACGGTAAGGACA
CAGACTCATCGAATTTCATGAATAATGCAAATACAGGCAGTTCGGCGGAAACAACAGCATCATCATTC
TCCAAAAGTGGCGGTATTGACGCCCCGAAAAA
TGAGAGTGAGGATGAGGGCGATGCTCAACGGCCTAACAGCAAAGAACCACAAGAAAAACCCGAGGACA
GTAATACCAACGATNNNNNNNNNNNNNNNNNN
NNNNNNNNNNNNNNNNNNNNNNNNNNNNNNNNNNNNNNNNNNNNNNNNNNNNNNNNNNNNNNNNNNNN
NNNNNNNNNNNNNNNNNNNNNNNNNNNNNNNN
NNNNNNNNNNNNNNNNNNNNNNNNNNNNNNNNNNNNNNNNNNNNNNNNNNNNNNNNNNNNNNNNNNNN
NNNNNNNNNNNNNNNNNNNNNNNNNNNNNNNN
NNNNNNNNNNNNNNNNNNNNNNNNNNNNNNNNNNNNNNNNNNNNNNNNNNNNNNNNNNNNNNNNNNNN
NNNNNNNNNNNNNNCGCTCAAACGAATGATAC
ATCTAAGTCTGGCGACAATGACGGCAGCACCGCGGTCTCCCACGCCATCTCCCCTCTTTTGCTTCTTC
TTCTTGTTGTTGCGTGTGCTGCTGTTGCTGCG
GTGGTGGTCGCGTGAGAGTGAGTGTGAGAGAGCCATGCACCGCCCGCACACACACTCTTCCTTCTCTC
TCTGTGTGTGTGTCCCCTTGCATGGACTCTCG
CCCTCACCTCACCCCACGAGGCACACACATAATGTGCCCATTGTATATATGCATGCACACCCATGCAG
GCCCGTTGGTGCTCTCTTTTGCACGACACGCA
CCGCTGCCGTCCTGCCTGGCTGCATGGGCGGAGCACCGTAACTGTTCTGTTTTGTTTTTATTGCATTG
AGTGGGCGACCTCTCTCTCTCCCTCTGTGTGT
GTGTTTTGCTTTACGGGAGCAATGAGAGTGTGTGTGCGCTGGTCGTCCCACGCTTCGGTTTTTTTTCT
TTTTGTTTTTTGCTGCACTCTTCCTGTGAGGG
GTGCCGTGTGTTCCGCCCAACTGCTCCACTCGCACACCACCGACACGCTCATGACGACGGCCCTGTGC
GCCTCGCCTGCTGCACGCTGAGGAGGGGCCGC
GACGAACGGCGCGCTGAGGGAGCCGTGACGTGTGGGCGTGCGACGAGGGCCTGTGGGGCGGAGCACAC
ATTTCTTTCCCTCTTGTTTTGTTTTCTTGCGG
CCAGGGCGGGCAGACACCCGCAGCGCCACACACACAGCCACACCCTGCCGCCGTGTTTTTCTTTTTTT
CTGCTTTTTTTTCTGTGGATGGGTGTGTGCCG
CTGCGACCGTGCGGTGATGGAGGGCGGAGAGCTGTGCCGGACGTATTGCCGACGGACCACTGTGCNNN
NNNNNNNNNNNNNNNNNNNNNNNNNNNNNNNN
NNNNNNNNNNNNNNNNNNNNNNNNNNNNNNNNNNNNNNNNNNNNNNNNNNNNNNNNNNNNNNNNNNNN
NNNNNNNNNNNNNNNNNNNNNNNNNNNNNNNN
NNNNNNNNNNNNNNNNNNNNNNNNNNNNNNNNNNNNNNNNNNNNNNNNNNNNNNNNNNNNNNNNNNNN
NNNNNNNNNNNNNNNNNNNNNNNNNNNNNNNN
NNNNNNNNNNNNNNNNNNNNNNNNNNNNNNNNNNNNNNNNNNNNNNNNNNNNNNNNNNNNNNNNNGGA
CGTATTGCCGACGGACCACTGTGCATCCTCAC
TTTCTTCTTTACTTAATGGCTGTCTGCTGCACTCTGTAATGAGCGAAGTGTTTCCCGCTCTGTTTTTT
TTTTGCCCACGCGGTGCCGGCCCTGTGGCTGT
GCGTCCCACGGACTGCATGAAACGACGATTGGAAGAACAGGCCGGATGCCGCGTGAAATGTTGGGGAT
GCTGTGTGGGCTGGTGGAACAGTAAAGTCTTT
TATTGACTTCTTTTTATTGTTGAGGGCAATATTTATACACAAACACACGGTCATATTGTACTCCTTGG
CACATGAAATAAACGTCTTTCCGAATCATTGT
TTTTACTTTTTTATGTTGCTTGGGAGTTCTTCTGGACTCTCACCTTTTTTGTTTTTGGTAAATCACTG
CGGTTCATTTCTTTGTTTTTGCTGTGTTCACT
GTCGCTGAGTGTCTCCGCGTCCCGTAGAGTGCCCCACTGCTGGCCGTGCTGTTGACATGTCCTCAttt
cttttttcctgctagagacNNNNNNNNNNNNN
NNNNNNNNNNNNNNNNNNNNNNNNNNNNNNNNNNNNNNNNNNNNNNNNNNNNNNNNNNNNNNNNNNNN
NNNNNNNNNNNNNNNNNNNNNNNNNNNNNNNN
NNNNNNNNNNNNNNNNNNNNNNNNNNNNNNNNNNNNNNNNNNNNNNNNNNNNNNNNNNNNNNNNNNNN
NNNNNNNNNNNNNNNNNNNNNNNNNNNNNNNN
NNNNNNNNNNNNNNNNNNNNNNNNNNNNNNNNNNNNNNNNNNNNNNNNNNNNNNNNNNNNNNNNNNNN
NNNNNNNNNNNNNNNNNNaaaGCATGCGTTG
TGCGGCGGGCTTTTCTTTTCCATGTCTTTGCCCTTTTGTGTGTGGAAAAGCTGCGGGAAAGGATATGT
GTGCGGGAGAGTGAGCGTGGGTGGCTGCCAGC
CTCGCCCTGCGCATTGGAGAATTCCATACACGTGGCTCTGCTTGACGGCGTTTCGCGGCACCCAGGGT

GTGGCGAGGAGGAGGGTGGGGAGGATCTGTTG
TGGGCAGAAGGTGGATGGACAGGACAGTCCCCTTCCGTGTCCTTCCCTGTGTCGTTGGCAGTACATAC
TGAGAGCGTAAACTTGAGCGAGTGCGGGATGA
AAGGTTTATGCCGCTTTGTGTGTGCTACTCAATCGTCGTCATTGCAGGCTGTCCTCACGGTGCGTTGC
GGTGCGGTGCTGTTAGATGAGGGAAGGACGCG
GTGCCACGGCTGCTGCGCACCACGACCATTTATTGCCTCTGCACCGTCATTGTTGTTTTTCAGTTTGT
GTGAATTTTTTCTGCGCTCGCATAATGTTTTC
TTTTGCTTAACGATGAATTTGTTGTGGGAGCTTTGGTTTTGGTGCCTGCTTCACGGCGCTTCGCCTCG
TGTGTCGGTTGCGGTGCTTCCACGTGGTGCCA
AAGCGTTTGATGGGGTGTTTGCCCTTCTTTTTGCGCCCTGCAGCCGACCTTGTTTGGCCATGGCGGCA
GCAGTGGGGCTCTGCGACTGCGTGCTTGCCGT
GCGTGTCTCATCTTTTGCCGCTGCTCCCTCGTTGTGTTGGTGGGGGAGGAGGCGAGCGACTGCGTTGG
TGGAGTTGCGCCCCAGTGGGAGAGGGAAGGCA
CCGACCGCTGCTAACGGCGACGGTCTTTGCTGCAGGCGATGTGACGGGATGACGTCACCGGTGTCACA
GTTAACCGAGATGGATGAGGGCCGCACTGCGT
CTCCCACCCCTCTCATCATCCTCTCTGTCCACTGCAGTCACACGCCACACGCACCGCAGAGCAGCGGC
GCGACCACCATCCGGATGTCTGTGAGCCAATT
GGAATGCAAAATCGATGTGGACCTTTTTCCCCGTGTTTGAGTGCCGCCGTGCCTCTTTATTTGTTTCT
TCTGTTGTGTATGTCGTGCGCGCATGTTGCCG
CTGATTGCTGCGCAATGCCGACTCGCGTGAACGAGAAACGATGCTTTGACTGCTGGCAACCGGCGAGC
TCTCCACTCCACTTACGCACCACCAACACCAA
CAACCTTTCGGACGCGCTTAATGGCTGGCGGTCCTAACGCTGCCACGTGGTCTGCCTCGGCTCTTGTG
TGTCGTGCACGGCATCGGGACCGCCATGTGAT
GCAGTTATTCGGTAACAGGAGCGGTGCCAGTGGTCATGTGGCTTTTTGCGGGGATTCCGCCACTCGGC
ACGGTGGCTGTACGGGGTTGTATGGGCGGTGG
GAGGGCGATGGTGTCACTGACTTGATTGCGTCTGCGCTGCTTTGTGTCGGCGGCTGTACTGTGATGGT
TATTGTGTATGACCGGATGCCTCTGCGTGCCA
CTGTGTTTGATTACTGTTGTTGTTGCTGTCTTTTTTGAGCGTGCAGTTAGAGATACTTTTGCTTGACC
AGTACAACTATCGTTTCAAGGCGTTAGGGATT
ACTTGCAGTATTAGGATTAGCAGTATTAGTATAACGATCTGGCGGAATCGTGTCACTGGATTCGCAAT
CTGTAGGAAGGGATTTGCTAGGCAGGAGTTAT
GGTCGACGGGGCGAGTGCGACACACCACGATAGCCTTGTTGGTACTTGTCTGGGTTGGTGGATGCGGT
CTGCGGTGGGTGCGTGTCCGCGCAGCCGGCAC
CGTGTGCAGCGCAGGGCCATTGCGGCTGTGCGTGTGGCGCTGCTCGTTGTGTTGGCGCTGGTTGCTGC
TGCTGCGTGGATGCCCGCGGTGCATGCGGTGG
TGTTGCGACTGCGGGGCGGCACGGTGGACAGAGCCATCACGGTTGGCCGCGCTATGGATACGGTGCTG
ATGGACGGCGTGTACATCACGAACGGCGTGGC
TGTGGTGTTCGACGTGGCGGCGATGCTTCCCGGCGCGCTGCGCATCGAACTGAGGAACTGCGTGTGCG
ACGGCGGTGCGCAGATCTACGTGCGGGGCTAC
AGCGGCGAGCCGGCGAGTGACCGGAGCCTGGAGGTGAGCGTGAGCGGGCTGTCCGGCGGCTACTGCTC
GCTTGTGTTTGTGCACAACCTGCCGGCACACA
CGAACGTGACGGTCCGTGACTCGACGATTGTGACGGCGGGACCGATGCGCTACTCCCAACTGAGCGGG
CTGACGGATGCGGTGGCGTCGCCGCTTGTGCT
GCATGCGACGTCGCTGTTGCAGACGCAGCTGCGTGTGAGCAACACTGTGCTGAGGTCGTTGCAGGCGG
GCGGGTCTGCGGTGTACGTTGGCGGCGGTGTG
GACCTGCTGTCGAGCGCGGTGGTGCTTGACGGCGTGTTGCTGGAGGCGTCGGGCGGTTCGACCGCTTC
CGCGTTGCATGTGTCGTCCTCGTCGCGTCTCA
CTCTGTGGGGCCACTCGGTGTTCTCCTTGACGAACGTGTCGGCTGTGAGCAGCGGCGGCGGCATTGTG
CTTGGCGAGCGCCTTGCGGTGGTTGATTCGGT
GCTGCGCTTCGTGGGCGTCGAGGGGTCTGTTGCGTCGTCGCTGGTGCGCTGCGACGGTGGGACGGTCG
GAGCCGGCGGGTGGCTGGACCTGCACGACGTG
TGGGCGGTGGGCGAGGCGTCGTCGGTGGCGTCGCTGTCGGGGGTGACGCTGAGCGGCGGTGCGGTGTC
GATTGCGCGCTGCGCTGCCACCGGCGCGACGC
TTGTCTCCGGGCTGGCGATCACGAGCGGCGTCGTGTCGGTGCAGTGCAACCGTGCTGGCGGCCGTGTG

CTGCAGAGCGGCGGCGACTACCGCTTGGCCGG
CCTGTCCTCCGTGAGCGTGGTGCCGTGCGACGGCTGTGCGGCAGCGCTGGCGTGCTTTGATGCGCTGA
CGGCGTCGTTCTCTGACTGCGTGTGCGGCTGC
CGCGACGGCGGTGTGGGCGAGGCGTGCCTGCCGTTCGACGTGCCGGTTGCGAGGCCCGGCGGGAGTGG
TGGCGGTGCGGAGGACTGCGTGAGTGGCGTGA
TGCTGACGGAGTCCGTGACGGTTGGCGGCGGGCGTGCGACGGCGTGCTTCGACTCGGTGGTGTTCAGC
GGGCCGATCACTGTGGCGGTGGACCTGCGCTC
GATGGACGCGTTCGCTGACGCGCTGAACGTGACGCTGCGCCACTGCGTGCTCGTGGGCGGTGCGCAGC
TGCGGATTGGCGGCCTCAGCGAGAGCACGGCG
CGCCTGATGCCCCACGCCCTCGTCAACATGACGAACGTGACTTCGCTGGAGGGCACGATTGTGCTGCG
TGGCGCGATGCCGCTGCACTCGAGTGTGCTGC
TGGCGAACTCAACGCTGCGCGCGACGGTTGGCAGGTCGCAGTACGTGCCGACGACCACTGGCCACGCA
GGATCCCGGTACGGCCCCACGCTTGCCCTCGA
CGGCGTCCGGCTTCTGTCGACGCGGCTTGTCATGACGCGGTCGACGCTGGTGTGTGGCGGGGGCTCGT
GCGCTGCGATCCTCGTGGAGCGCGGCCTCGGC
GTGAACCTGTCCAGCGTTTTCTTCATGGACAACTGCGCCGTCAATTCGCAGATGCACGTGATGTATGC
TCTTGCGTCGGACCTGCGTGTTGGTGGCGGCT
CCGTGTTCTCCATACAGAACAGCTCGTGGAGTGCTCCGAGCACCGAATACGGCAAAGCCGCGTGTGTG
TTTGAGGATGTTGTTGTGGCTGGCGGCAGCGT
GCTGCAGATTGTGTCCAGCACTTTTCGTCTTGGCTTCGCCATGCTCATGGCAACCACGCTGACTGTGA
CCGGCGGCAGCTGGCTGGTGCACCGCGACAAC
GAGTTCCGCACCGCCTACGTTGTGTACATAGTCAAGGAAAGCAGCGTTGCGTTCCGCGATCGGAGTGT
GTGGTCGATACTTGACAACAGTTTTACGTACG
GCTCGTACTCGTCAACCATTGTAAGTATGACAAACAGTTGGTCACCACCATCCGACGCGCACCCGATC
ATCTACGGCGTGTGCAACGCGGCAAGGGGCTC
TCCTGTGACGAATTACGGAGTCGACCTCAATATTGGAGCACCCGTGATGGTGTTGGACTGCGGCGCGT
GCACCGTGGGCGCCGTGTGCTTCGCAGCGAGG
ACGAGCAGCATCAGCGGCTGTGAGTGCGTATGTGCTGCTGGCGGCTATGGTGACACGTGTCTGCCAGC
TGCGGTTCCGGACGGCCTTGGGCCGCTTCCGC
CCCCCGACGCGAAGGACACGGAGGTCCGCTGCGTGCACGGTGGCAGCGTCAGCTCCGTGGACGTTCCT
GATCCCGGTGTGCGTGGTCTGTGCTTTGTCAA
CGTGACCTTCACTGCCGCGATTGTGCTGGACCTGTCGTATTTTGACGCACCACAGCAGACACTCAACA
TCACACTGCTGCAGTGCGTCTTCATGGGCCTG
TCGATCAAGGGGAGTGGTGCATGCGTGCACGTGAACGTGGTCTCCTCGATGCTGGATTCTGGTGAGCT
GGTTTTTCAGGGCCATTTTGGTGCGAGCTCCC
AGATACTGGTGGCGGGAAGTACGATTGTGACGACGTCGGGCCACGCCATTTTATTTGTGAAGTTTGCT
CTCAGTGCGAATATGACGCTGCTGCTACTCGA
CAACTACATTGAGGCGAGTAGGTACGCAGTCTATTTCATCGATGCTGTTGTTGTTGATGGTGGCGGGA
TTATTGTGAAGGGAAATACGCTGAGCACAACG
AAGGAcgatgagggtgtgg
>scaffold1005.size7579
CACGCTGTTGCCCTCCACACGCCACTGCGCACCGCCCTCGAGTGTCACATAGTCAGGCACACTCACCA
GCGCGGGATAAAAAACTTCCGTCCTGCCAACAA
AGGTGCAGTCCGCCACCCGCAGCAGCCCCGCGGACCTCACGATGGTATTCCCAAAAAGAATAACGCCA
TTGACTGACCTCATCGTGTTGTTCTCCACGTC
AAAGTAGCCGCCGTTCTTCACATCAACAGCAAGAACGCAAACGGCTGACTCCGCACCGTCATCCTCCT
CCTTTGTGCTCAGCGTATTCCCCTTCGCAATG
ATTCCGCCACCATCAACAACAACAGCATTGATGAAATAAACTGCGTACCTATTCCCCTCGATGCGGTT
GTCAAGTAGCAGCAGCGTCGAGTTCGCACCAA
GAGTAAATTCCGGAAAGGCAATGGCGTGGCTCGACGTCGTCACAAGCGTACTCCCAGCCACCAGTATC
TGGGAGCTCGCGCCANNNNNNNNNNNNNNNNN
NNNNNNNNNNNNNNNNNNNNNNNNNNNNNNNNNNNNNNNNNNNNNNNNNNNNNNNNNNNNNNNNNNNN
NNNNNNNNNNNNNNNNNNNNNNNNNNNNNNNN
NNNNNNNNNNNNNNNNNNNNNNNNNNNNNNNNNNNNNNNNNNNNNNNNNNNNNNNNNNNNNNNNNNNN

NNNNNNNNNNNNNNNNNNNNNNNNNNNNNNNNNN
NNNNNNNNNNNNNNNNNNNNNNNNNNNNNNNNNNNNNNNNNNNNNNNNNNNNNNNNNNNNNNNNNNNNNNNNNNNNNN
NNNNNNNNNNNNNNNNNTCACGTGCACACGCACA
CCACTCCCCTTGATCGACAGGCCCACGAGGACGCACTGCAGCAGCGTGATGTTGAGTGCCTGTTGTGG
CGCGTCAAAATGCGATAGATCCAGAACAATCG
CGGCGGTGAAGGTCACGCTGACGAAGCACAGACCACGCACACCGGGATCAGGATCGTCCACGGAGCTG
ATGCTGCCACCGTGCACGCAGCGGACCTCCGT
GTCCCTCGCATCTGAGAGCGGAAGCCGTCCTAGGCCATCCGGAACCGCAGCCGGCAGACACGTGTCAC
CGTAGCCGCCAGCAGCGCACACACACTCACAG
CCGCTGATGCTGCTCGTCCTCGCAACAAAGCACACGGCCTCCACGGTGTACGCACCACAGTCCAACAC
TGTCACGGGTGCTCCAATGTTGAGGTCGACTC
GGTAATCCGTCACAGGAGAGCCCCTTAGCTCGTTGCACACGCCGTAGATGATCGGGCGCTGGTCGGAT
GGTGGTGACCAGTTGCTTGTCATATGCGCAAT
GGTCGACGAGTACGAGCCGTACGTGAATTTGTTGTGAAGTATCGACCACACACTCCGATCGCGGAACG
CCACGCCGTTCTCCTTGACCACGTACACAACG
TGGGCGGTGCGGAACTCGTTGTCGCGGTGCACCAGCCAGCTGCCGCCACTCACAGTCAGCCTGTTTGC
CATGAGCATGGCAAAGCCGAGACGGAACGTGC
TGGACACAATCTGCAGCACGCTGCCGCCAACCACCGCAACGTCCTTGAAAACAAAAGCACCTTCGTAG
TATTCGATGCTCGGCGCACTCCACGAGCTGTT
CTGTACGGAGAACACGGAGCCGCCGCTGACACGCAGTTCCGATGAAAGAGCGTACATCACGTGCGTCC
GCGAAATAACGACGCAGTTGTCCATGTAGAAG
ACGCTGGACAGGTTGGCGCCAAGGCCGCGCTCCACGAGAATCGCAGCGCACGATTCCCCGCCACACAC
CAGCAACGACCGCGTCATGACAAACCGTGTCG
ACAGCAGACGGACGCCGTCCAGGACAAGCGCGGGGCCGTACCGGGATTTCTCGTGTCCACGGGTCGTC
GGCACGTACTGCGACCCGCCAACCGTCGCGCG
CAGCGTTGAGTTCGCCAGCAGCACACTCGAGTGCAGCGGCATCGCGCCATGCAGCACAATCGTGCCCT
CCAGCGACGTCACATTCGTCATGTTGACGAGG
GCGTGCGGCACCAGGCGCGCCGTGCTCTCGCTGAGGCCACCAATCCGCAGCTGCGCGCCACCCGCAAG
CACGCAGTGGCGCAGCGTCACGTTCAGCGCGT
CAGCGAACGCATCCATCAAGCGCAGGTCCACCGCCACAGTGATCGGCCCGCTGAACACCACCGAGTCA
AAGCACGCCGTCGCACGCCCGCCGCCAACCGT
CACCGACTTCGTCAGCGTCACGCCACTCACGCAGTCCTGCGAACCACCACCGCCGCCGGACCTCGCAA
CAGGCACGTCGAACGGCAGGCACGCGTCGCCC
ACGCCGCCGGCACGGCAGCTGCACACGCAGTCGGAGAACGACGCCGTCAGCGCATCAAAGCACGCCAG
CGCAGCCGCACAGCCGTCGCACGGCACCACGC
TCACGGAGGGCAGGCCGGCCATGCGGTAGTCGCTGCTGCTCTGCAGCACCCGGCCGCCGGCACGGTTG
CACTGCACCGACACGGCACCGCTCGTGATCGT
CGGCCCGGAGACAAGCGTCGCGCCGGTGGCAGAGCAGTGCACAATCGACACGGCGCCGCCGCTCAGCG
TCACCCCCGACAGCGATGCCACCGACGACGCC
TCGCCCACCGCCCACACGTCGCGCAGCTCCACCCACCCGCCGACACCGATCGTCCCACCGTCGCAGCG
CACCAGCGAGGACGCAGCAGACCCCTTGACGC
CCACGAAGCGCAGCACCGAATCAATCACCGCGAGACCCTCGCCAAGCACAAGGCCACCGCCGCTGCTC
ACAACCGACACGCTCGTCACGGAGAACACCGA
GTGGCTCCGCAGACTGAGACGCGACGAGGACGCCACATGCATCGCGGACGCGGTCGGACCGCCCGACG
CCTCCAGCGACACGCCGTCAAGCACCACCGCG
CTCAACAGCAGGTCCACGCCGCCGCCAACGTGCACCGCCGACCCGCCCGCCTGCAACGACCTCAGCAC
AGTGTTGCTCACACGCAGCTGCGTCTGCAACA
GCGACATCGCGTGCAGCACAAGCGGCGACGCCACCGCGTCCGTCAGCCCGCTCAGCTGGGAGTAGCGC
ATCGGCCCCGCCGTCACAATCGTCGAGTCACG
GACCGTCACGTTCGTGTGTGCCGGCAGGTTGTCCACAAACACGAGCGAGCAGTAGTCGCCGGACAGCC
CGCTCACGCTCACCTCCAGGTTCCGGTCACTC
GCCGGCTCGCCGCTGTGGCCCCGCACGTAGAGCTGCGCACCGCCGTCGCACACGCAGTTCCTCAGTTC
GATGCGCAGCGCGCCGGGAAGCTTCGCCGGCA
CGTCGAACACCACAGCCACGCCGTTCGTGATGGACACGCCGTCCATCAGCACCGCGTCTACGGCGCGG

CCAACCGTGACGGCTCTGTCCACCGTGCCGCC
CCGCAGTCGCAACACCACAGCATGCACCGCGGGCATCCACACAGCAGCAGCAGCAACCAGCGCCAACA
CAACAAGCAGCGCCACACGCACAGCCGCAATG
GCCCTGTGCCGCACACGGTGCCGGCTGTGCGGACACGCACCCACCGCAGACCGCATCCACCGTCCCAA
ACACGTACCAATAGAGCTGTTGTGGCGTGTCG
CACTCGCCCCGTCGACCATAACTCCTGTCTAGCAAATCCCTTCCTACAGTTGGCAAATCCAACAACAC
TATTCCGCCAAATCGTTATCCCACTACTAAAC
CTAATACTGCAAGTAACTCCTAACACCTTGAAACGATAGCTGTGCTGGTCAGGCAAAAGTATCTCTAA
CTGCACGCTCAGAAAAAGAATAAGAGGCAGCA
ACAACAACAGTAACCAAACACAGTGGCACGCAAAAACATCCGGTCATACACAATCCCCGTCACAGTAC
AGCCACCGAGACAAAGTCGCGCTGACGCAATC
AAGTCAGTGGCACCATCGCCCTCCCACCACCCACATAACCCCACACAACCACCATGCAAAGTGGCGGA
ATGTCGGCAAAAAGCAACATGACCACTGGCAC
CGCTCCTTTTACCGAATGGCTCCATCACAGGGCAATCCCGATGCCGTGCACGACACACAAGAGCCGAG
GCAGACCACGTGGCAGCGTTACGACCGCCAGC
CATCAAGCGCGTACAAAAGGTGTTGGCGTTGGTGGTGCGTAAGTGGAGTGGAGAGCTCGCCGGCCCCC
AGCAGTCAAAGCGTCGTTTCTAGTTCACGCAA
GTCGGCATTGCACAGCAAGCAGCGGCACCATGAGCACACGGCATTGCACAAAGAAGGGAACAAAGAAG
NNNNNNNNNNNNNNNNNNNNNNNNNNNNNNNN
NNNNNNNNNNNNNNNNNNNNNNNNNNNNNNNNNNNNNNNNNNNNNNNNNNNNNNNNNNNNNNNNNNNN
NNNNNNNNNNNNNNNNNNNNNNNNNNNNNNNN
NNNNNNNNNNNNNNNNNNNNNNNNNNNNNNNNNNNNNNNNNNNNNNNNNNNNNNNNNNNNNNNNNNNN
NNNNNNNNNNNNNNNNNNNNNNNNNNNNNNNN
NNNNNNNNNNNNNNNNNNNNNNNNNNNNNNNNNNNNNNNNNNNNNNNNNNNNNNNNNNNNNNNNNNNN
ACGGTGACGGCAATCAGACACGGGGGAAAAGG
TCCACGTCGATTGTGCGACCTGTTGACTCATAGACGACCGGTTTGTAATCACGCCACTGCCCTGCAAT
TCGTCAGTCAGCAGTGGAGTGTAAGGATGATG
AGAGGGGTGGGAGACGCAGTGCGGTCCTCATCCATCTCTGCTAACTGTGACACCGGTGACGTCATCCC
GTCAGGTCACCTGCAGCAAAGACCGTCGTCGT
CAGCGGCGGTCAGCGCCTTCCCTCCCCCACTGGGGCGCAACTCCACCGACGCAGTCGCTCGCCCACTC
CCCCACCAACACAGCAAGGGAGCAACGGCAAA
AGATGCCACACGCACGGCAAGCACGCAGTCGCAGAGTCTCACAGCTGCCAAAAAAAAAAAAAAAGACT
CTCTTAGATTTCGTAGTACCCACGTATTCTCG
CACAGTTTGGGTGGTCTTGTCAAACAGGAATGCTCAATTACAAACAAAGCGCGCAACCGCAGGGAGTC
GCTCCGCATCGTAGTGCAGCAGCTGGTAAAGG
AGGTACGAACCGGCAGCCATCGACTTCCCGATCCCGGGCGTCCCAATCAACACACGCCGCCCAGGCGT
AAAGTCGGTCTCACCGTGACTGCCGAACCATT
CGGTTACATCGCCCTTGACAATCTGCCACNNNNNNNNNNNNNNNNNNNNNNNNNNNNNNNNNNNNNNN
NNNNNNNNNNNNNNNNNNNNNNNNNNNNNNNN
NNNNNNNNNNNNNNNNNNNNNNNNNNNNNNNNNNNNNNNNNNNNNNNNNNNNNNNNNNNNNNNNNNNN
NNNNNNNNNNNNNNNNNNNNNNNNNNNNNNNN
NNNNNNNNNNNNNNNNNNNNNNNNNNNNNNNNNNNNNNNNNNNNNNNNNNNNNNNNNNNNNNNNNNNN
NNNNNNNNNNNNNNNNNNNNNNNNNNNNNNNN
NNNNNNNNNNNNNNNNNNNNNNNNNNNNNGACGTGCTGCACCGGATTGCTGCACGCCGTCATCCTTTT
CGAGGGTGTCGCCAACTGCCCTGTAAGTCCAT
AACCTCGGTGTTCCCCTCATAAGCCTCACTTCCATTCCCGTCCACTCGCCGCCAGGAACCCCCACAAC
ATGATGCCACCTCGCATTGTACACAGACTCTT
ACAATCCCTGCAGCATGTATCCTTCCACCCGAATGTAAATAGCCCACATTAATCGCCGCACCTCATTC
GCCCTTCTGCGAGTCGTAGCGGGTTGATCCGT
GTCGCTGTGGGTCTCCGGCCTCGCTCCCCTGCGACCATCTCCCCGCCGCACAGTGGACGCAGGGCCCT
CCTCATGATCGCCGTGCACTCTCTTGTGCTTT
CCACGCATTGTTTCCCCTTGTCCCCGCAGTTGGCTCGAGTTAAAGCCGAACACTGACCACCAATCAAA
GGAGAATAAGAGAGAGATGGGGTAACTAGGCA
CAGAAATGACGGGCAGCCACAACAAGAGAGTCATCCACACAGTATTCGATAAAAGCCANNNNNNNNNN

NNNNNNNNNNNNNNNNNNNNNNNNNNNNNNNN
NNNNNNNNNNNNNNNNNNNNNNNNNNNNNNNNNNNNNNNNNNNNNNNNNNNNNNNNNNNNNNNNNNNN
NNNNNNNNNNNNNNNNNNNNNNNNNNNNNNNN
NNNNNNNNNNNNNNNNNNNNNNNNNNNNNNNNNNNNNNNNNNNNNNNNNNNNNNNNNNNNNNNNNNNN
NNNNNNNNNNNNNNNNNNNNNNNNNNNNNNNN
NNNNNNNNNNNNNNNNNNNNNNNNNNNNNNNNNNNNNNNNNNNNNNNNNNNNNNNNNNAAAACAACTG
CCTCGTTGTGGTGTCATCGCGCAGCCATCCGC
CTGGGTAAGAACAAAGGGAAAATCAGGCGTCCGTCCGCCTGGAGGGAGGGAGAGACTCCACCGCAGCG
TCCTTCAGGACGCAAACACTCCGCGGCCAGTG
CCCGGTGTCGTGTGCGGGCTCCGCGAGAATGTTTTGGGCAGGCCGCTGTGCCACGCGTCGTGTCCCCT
CGGAACGTAACAGCAGACATGTTCTCTTCGGG
TCGCACTCGTGGCGGTGGTGGCATCCGCTGCCTCGCTGCGTGCTGGTGGCGTCGAATACGATGCTGCA
GTGTGAGTGAGCGAGGGTGCCCGACAGGAGCG
GTGCTGCGTTGGGTGGGCAAGGCCCTCCGCGGCGAGACACCCAGTGCGACCCTTGACCGCCGCGTTCA
CGCACTTCCCTCCCGCTCCTCTCCCTCCATCC
GGCAACCGCACAGCCACAGGCGCATTGTCCCCGAAGCAACACACAAGTGAAGGCGTCGTGGCATATCT
CTCGAGAGAACGGAGTGACAGCTACGCGCTGC
GGTCATCGTGCCAACAAGTGTGCCCTCGGACAACTTTGTGTTGGGGCCACACGCACACCACGCAGGGA
TCAACCACAGCAACGCACAAAAGTGCAATCAA
AAACATTCGCCCACCGTCACCGCCACCGCACATCTACAGAAGAGACACAAACACGCAAAAGGCCCAAC
TCCGCCGCAGACGCACAATCACGTGCGCCAAC
AGCTGACAAGAGGAGTCTCTCACCTCGTGCCCCTTCGATGTACCGGGAGATACCGCATTTCGAACCAA
TCATGCTGCACGCGGTGCGCTGCACACAAAAT
GAGAGAGAACCGCAGTGGACCCCCTCCCGGTGTCTCTCCCTTCTTCATGTCCACAGAAGAAAGCGGCA
AGCAAACACCAGTAAAGAATAAGAGTGAGAAT
CATGCTTGGGCACCGCAATATATTTTGTGCATTGTGTGCGTGTGCCCGCACGTAAGATGGGAACACAC
ACGGTGCGAGTCGGATGAGATCATCGTTCCAG
CTCGCATCTCCGCCAGGTGAGTCATTGACGGCTCCATCCCGCTTCACGACAGGTCGAATAACAGTGCA
CGCGTAAGTAGCTATAAGTTGCATTCACATGT
GGTTCCCCTTTTGGGTCCAGCGTCGTCAGGTGGTGGAAAGAGAGGAGTCGCATTATCATTCTTCCATT
TGCTGCTGCC
>scaffold1006.size7006
CGATGGAATGTGAACCAGCGACTGGTCGACACTCTCTTTAAGCGCGAACAGGATTCCTTGATGCAGCT
GGTTGCAGGCGGTGGATCGGGTGTTAAGATGGA
GGACGGTGCGCTTGTGTTTCCCGTGGAAGTAACGAAGAAGGGTGAATCAGAAAACGACGGAGAGGAAG
GAAAGAGTGTCTCACTGCTCGTATACACTTCG
GATACTTCGGATGATTCGAGTTGGAAGCTGTCGAAGGGGATGTCTGACGGTGGCTGCGGTGATCCCTC
CGTCGTGGAGTGGGAGAAGGAGAAACTCATGA
TGATGACTGCGTGCGATGGCGGCCGCCGCAGGGTGTACGAGTCCGGTGACAAGGGGGGGTCGTGGACG
GAGGCACTCGGGACACTCTCGCGCGTGTGGGG
CAACAAACACGAGGAACAGACGAAACGTGTTGCGAGCGGGTTCACCACAGCGACTCTTGGAGATGACA
ACAAAAAAGTGATGCTCGTTACTCTGCCGGTG
TATTCTAAGGAGAAGGGCAGCGAAAACAAGGAAAAGAGTGAACTCCACCTTTGGCTGACGGACAATAC
GCGCATTGCTGATATTGGGCCCGTGTCCGAGA
AGGACGAAGACGAAGTTGCCGCCAGCTCCCTGCTGTACGAAAGTGCTGAAGGTGAAGATAATAATAAG
GAGGAGGATAAGTTGATTGCACTGTACGAGAA
GAAGAAGGGCGGTGAGAAGCCATCACACGGTCTTTGGTCTGTGCGTCTGACTGAGCAGCTGAAGCGGG
TGAAGGAGGTGCTGGCGACGTGGAAGAAAGTG
GACGAAGCTGTCTCCAAGCTGTGCCCCACTGCAAGTGCTGCGAAGAGTGCCTTGACTGAGAATGACTG
CAGCGCTGGGGAGATCACGGCTGGGCTGGTTG
GCTTTTTGTCCGACAACTTCTCTGGGAACACATGGAGGGACGAGTACCTCGGGGTGAACGCCACGGTG
AATCATAATGGTGGAGCTGAGAAGGCGAAAGA
CGGCGTGAATTTCCGGGGAGCGTGGGCGGAGTGGCCCGTTGGCAGGCAGGGGGAGAATCAGTTGTACC
ACTTTGCGAACTGCAACTTCACTCTTGTGGCG

ACGGTGTCCATCGAGAAGGCGCCGGAGGGAGACAACCCCATTCCATTGATGGGTGTGCGTGCGGGCAG
TAAAGGAGAAACCAAACTTATGGAGTTGTCGT
ACAACAGCGGAAAGAAGTGGAAATTGTTTTGCGGTGGCGACACGACCAATGAGCTCAGCAGCCACTGG
GAAGCAGGGACAGCACAGCAGGTGGCCATTGT
GCTACAGAACGGCACCCAGGGCTCTGCGTACGTTGATGGTAAGCGCGTGGGTGCTGCGCCATGTGAAT
TGGAAAATGCGGATTCGGAAGAGATCTCGCAC
TTCTACATTGGAGGGGTTGGAGACAGCGCAGAGGTCCAAGAAGTCGTGTCTGTGACTGTGAAGAACGT
CTTTCTGTACAACCGCNNNNNNNNNNNNNN
NNNNNNNNNNNNNNNNNNNNNNNNNNNNNNNNNNNNNNNNNNNNNNNNNNNNNNNNNNNN
NNNNNNNNNNNNNNNNNNNNNNNNNNNNNN
NNNNNNNNNNNNNNNNNNNNNNNNNNNNNNNNNNNNNNNNNNNNNNNNNNNNNNNNNNNN
NNNNNNNNNNNNNNNNNNNNNNNNNNNNNN
NNNNNNNNNNNNNNNNNNNNNNNNNNNNNNNNNNNNNNNNNNNNNNNNNNNNNNNNNNNN
NNNNNNNNNNNNNNGCCGTTCCTCAATCGG
TGGAAGGGGATCAAAAGGCGATGCCTCCTGCGGGTAAGCCTTCTGAAGCACCAGGGGAGCAAGCGACC
TTGCAGCAGCCTCCAGAAGAGCGTGAAACACA
GGAAAATACGACGGTGGGGGAATCTGCCTCCACACAGCAAGTGCCGGCGAATCGATCGCAGGGAAGTG
TGGGGAGTGCGGCGGCTCTGAACTCCCATGCC
GTTGGAGGAGCAACGGGTGATGGCGGCACTGTGCGTGGGAGCGGACTGCTGCCATCGCTGCTGCCGCT
TCTGTTGGGACTGTGGGGGTTTGCGGCTCTGT
GAGGAGTGGCTGGCTGCCGCGTGGGTCACTTGCGACCTCCGCTGCGGAGAATTACTTTTCCCCTGGAA
GGAATTTCTTTTCTCATAAAACATCTTTTTTG
TTGTTGTTTTTATAAATTCTTTTGTTTTTGTGCACTTTTTTTGTTTTTCTTGTGTTTTAATGTTTTCT
TTTTATTTTGCTCCTCCCATCCCCCATCCATT
TCTTTTTTGCTTTTGGGGGCAGTCACTGTGGACGTTTTTCTTTTCTACTCTTCCCTCTTTTAATTGTT
TCTTCTGCACTCTTCCTGCGAGGGGTGCCGTG
TGTTCCCGCCCAACTGCTCCACTCGCACACCCACCGACACGCTCACGACGACGGCCCTGTGCGCCTCG
CCTGCTGCACGCATGAGGAGGGGCCGCGACGA
ACGGCGCGCTGCGGGAGCCGTGACGTGTGGGCGTGCGACGAGGGCCTGTGGGGCGGAGCACACATTTC
TTCCCTCTTGTTTTGTTTTCTTTGCGGCCAGG
GCTGGCAGACACCCGCAGCGCCACACACACAGCCACACCCTGCCGCCGTGTTTTTCTTTTCTTTTTTT
TTTTTTTTTTTGCGCCGGCGCACCCTTCGGTG
TCGGCCTCGTGAGCATTTCTTTTCTTCTCTGATGTTTTTTTCTTTTGCTGCGCTCACTCTCCCGATGG
AGTGACGCGACTCCCGTCGTGTCTGCGTGCTT
CTCTCAGTCCCCACACACGAGTGACTGTGCGCTGCGGGCCGCACTCACGCGCCGATGAGAGGGCTGAG
GGGAGCAGCAACACGCAGATGACCGCATGAAA
TATAAACGATGTGCGGACACACGGAGGGTCGGACGGTCGAAGGACATTTAAATATCTTTTAGTTTTAA
TTTGGGCAAAATATACGGACAGTTTGGTGGGA
CTACAAGGCAGCCGGCTGCTTTTTTGAATTTGTGTGGAACAGTAGTGGACGATTTCTTTTTCTTTTAT
AATTATTTTGTGCGATGGGGTTTGTGTAAGAC
GAAGGGGGTGGTTTTCTTTTTGTTTTGAATAAATATTGTGTGAATCGCCACTGCTTGGGCTGCGGTGC
AGTCATGAGTTTGAGCGGTCTTCTTCTCGTTG
TTTCCGCCATGCACCCTCGCTGTACATTTTTAGTTTTGTGCGTCTCTCCGCAAACTCCGCTCCCTCTT
TTCTAATTCTTTGCCTCGTGTTTCTGCAGGCA
CACACAACCACACACACAAGACGCACATCGCAGTGGATTTGAATGCTCCTTTTGCAGTGCATGCTGGC
AGTTGCGTGTGGCGGCCGTTGTCTTCTTCCGT
GGCCATGAGGAGAAGGGAGGATGGAGGGAGTGTGCAGCCCGAGTCTATTTTCCTGCGGGTGTGATGCA
CCGCGTGTGGCTGCGTTGAGTTGGGCTCCGTG
TCGCTTTGAGGGCGGGATGGCTGGAATTTTGTTTCCGCCTCGTGGATGGATGAGGGTTCGCCTGCTCA
TTGTGTGACTGGTGTGGTGGCCGCGGGTTTTA
GCCACTTCCCTGGGGCTGCGGGAATGAGCTGGGGGAGATGGTGGTGGATGTCTTCTTCTCCTGCATGG
TGGGTGGCTGCTGGCGTTGTTTGCCTTCCTTG
CGTGGATGCCGTCTCGTGGTGTGTGAACTGTGCGGCTGGGCCTGCTGTCCTCTCCTCATGTTGTCTGC
TCTCACTCGTTGTATCCGCTGTGCCCCGTCCC

TCTCCCTCTCGTTCCATTCCTTTTCCTTCTGCCTCACGGATCAGCTGACTGACTGCAGCGACTCCACT
ACGAGTGATGACTGCGATGGTGACGATGGTGA
CGGTGCGGCGCCGTGTTGGTCCTTGCGCCCTTGTGCTGCTGCTCGTCCGTCCGTGTGCGTGACGGCTG
CTGGGAGTGCTGGAGATGTATCTGGTGCTTCG
GATGCGGTGGTTGTGCCGGTGGATGTGTCGTGTGCCCTCAGAGTGACGGCAGCCTGAGCTGTCGCTTG
TGTGTGCGCGTGGAGGAATTGTTTCGCTGCCA
GTGCCGAGGCTCAGTATGATAATTATACTTTCAGTGGCTTTGATGTGCACATGCATGGCAGGAATGGT
GAGCTGGGTGCTGTGTGCCATGTGGCCAATGT
GGTGCACACATGTAGCAACTGTGCTGCCAGCTGTGCAGCGGAAGGGGCCACCGTTGCCTTCACGATGA
ATGTGACGACACATGAATACGCCGGCCTTATG
AATTGTGGCGGCGTCAATTTCCCCCCGATGGAACCATCCATCCGCCTGCACGGAGCAGCGATGCTGAC
CAGACGGGCATCTCTCAGTTGCCGCCACCCCG
TGAAGAGAAGGAAGACACAAAATCTGGTGGGACAACTGCGCCGCAAGTTAATGCTGAGCCGTCACGTC
AACACGAGGATTGGCGATGTTCTGTGGCGGCT
GACGGACCCAACACCGTCGATCCGAGTCCCGCATCCAACACTGCGACCAACACGTCCGAGACGAGGCC
TGAGTTTGCCACCAATCCGAGCGAAGTGATGG
TCCCGAATGCTTCCAAGAAGAGGCCTTCCTTCGCCAACGGTCCGAGAATATTAGTGGCGTCGAATGCA
TCCGGGATCACGTCTGTCACTTGTGGCGGCAG
GGAACACGTTGCCGCGGATAACACGGACGAGGTGAGTGAGCGCAGCACCACTGGACACAAAACTCCTT
TCTTCAGAAAATGGCGCTGCAGGCAGTGGACT
TGTTGCTGAGGGTGATGCGGGCGGATTGACTCAACGCCATGCCTCTGGACACGAAAGCAAACCCCTAT
CACAAAAACTCACCGCGCAGTATTCCGGGAGC
GGTGTGGGTCTACACGACTGCTTTGCCCAGCGCTGCGACGCGAAATTGACACTGCGAGAGTTAACTTT
TTCCAGCAGTGCGCGTTTTTGCGTGTATTGGG
CGTTGCTGCTTTTTATTGTATTGTGTGGGGTTGTGGCAGTCTTGTGAGCCAAAGAGTGTGGAATTGCG
AATTCTCACGGAGAACATGTGCCGCGTTCCCT
GCGTGATTTCTTTTAATATTTACATCTTGCTGTTGTTTTTTGTTGTTTTCCTGGAATGATAAGCATCC
TTGCTGTTCCTTTATTTAATAGTTCGTTCCTG
CACACCGTCATGAGTGACGTGATTCCCCTCTTTCTGTTTGCTGTTTTGTTGCCCACACGCGGCGCCGG
CCTCATTGCTGTGCGTCCCACGGACTGCACGA
AATAATGAGTGGAGGAGCAGGCCGGATGCCGCCTGAAATGTGAGGGGTGTTGTGTGGGCTGGTGGAAC
AATAACGTGGTTTTCTTTTTTCTTTTTATTGC
TGCAGGCATTATGAACATACAGAAGCACATGGTCGTTTTGTGCTGCTTGGCACATGAAATAAAAGTCC
TGTCGAAAGAATTTTATTTATTTATTTTTGGG
AGTCCCGCTGGAATCTAAACCTCGGTTGTTTTTTATACTTCTGTTTTGCGGGGCGTTGTGTGTTTTTG
CCCTTTCACTGCTTTCAACTGCCTCATGGTGT
GTGTGCGCTGCCGCTGCTGGGCTGCTGCCATTTGTGACTGTGCGGTGGCTGTGATGACACACCGCAGG
CTTTACTTTCTGGCTCTGGTGGCGGCACGGTT
GTTGGTACTGGTGGTGGGAGAATACTGAGGACGGCTCTGGTGTGTTGGGTTGTGCCACTGAGGCCAAA
GGGATGGGCATCGTTTAGGGGAGACGGACAAT
AATGCGGCAGCGGTAACGGGGGAACTGTCCGAAACACGCATCCCCGAGTCGTCGATGGAAGAACCGTC
CTTGCCACCACCGCTGCAGCCGGACTCGGAAC
TAACGTCTCCTCAAGGGCCGGATGAAGCCGCACCAGCGAAGGGCATAACCCCCGCAGTGCAGCGGATG
AAAAGGGTGCGGCCGATTTGGGCGGCACGCGG
ATCGCATCCCGTGAGGCTCCCGTCACGCAGTCCACATCATCGGGCACTCCAACTGGCCCCACTGCTGC
TGCAATGACGCCTGCAAACAGACAGGGTGACG
ATTCCGCTGATACTCCAACAGCACGTAAAAAAGATTTGAAAGCAACCGAGCGCACCGCCAGCAGTGAT
GCGCTGAATACTCGGGAGAGTGACAGTAGCCC
GCAGTTATTCCAGCCACACAGCCTTACGCCAATACGGGCAGGACGGGGGTCTTTGACGTTGAGAGCAG
CTCCGGGAGAGTTGAACCCGCCGCCTCCACAG
ACGACGAAGAGAATGATACGAAGTCATAACTTCAGAGCGCATCCGACGGCACGGGCACCGCGGCAGAC
AACAGTGAGGGGTTCACAACAATACCAATAAC
GCCACTAATAAAATGAACGTCTGACGACAGTGACGGCAGCACCGCGGTCTTCCACACCACCTCCCCTC
TTTTGCTTCTTTTTGTTGCGCGTGTGCTGCTG

CGGTGGTGGCTGCGCTTCCCGTGGACCAACAATCCACGTGATGTGCGGGGATCTATTCTTGTCATGGT
TAATTTGCTTTTGTGTTGCTGTGTGCGCTGCA
CCTATTCTCGTGTGTGCGTGCCGCTCCTGTTTGCTTCTCCCTCCTCCATCTGTGTGTTGGTGTTGCGG
CAGGGCTGTGTGTGTGTGCCTGCTGCCGTGGA
TTGGAGTGTGTGTTGTGCGTGGTGTGCGGCGTACGGTGCATGCCTGGCCGCTGCCCTCTCCTCATGTG
TGTGTTTGCCCTCTGTGTGTGTCTGCCGCACC
TCTCGTCTCCCCTGTTGCTCTCCATGTCCCTCACTGTGCAGATCAGCTCCACACCACTGAACGACCAC
ACTCACGATGATGACGTGCCGTCTGCTGTGCG
CCCTGTTGGTGCTTGCCCTGTGCTGCTGCCCGTCCGTGTGCGCGACAGCGACTGGTGGAGAACAAAAA
GGGGCAAACGGTTCAACGCTCCCTCAGCAacc
ggaaaca
>scaffold1007.size7109
GAGATGGCGGAGCCGATGCGCTGGTGAAGGAATTCGTGCTGTGCGCTGCTGCAGAGGAAGTGCTCCGC
ACCGAACGTGAGTGGATATCCCCACATGTTGTG
CGAAACGGGAGTTCCTGCCAGACGCTGCACGTTGGATCGGTATTCGAGTGGTTGGTGTGTAGACGGAG
TATCCATGAAGGGTCAGCAAAACGCTCGATGG
ACAGTTGCCCCATCACTGGTCCCGCACTGGATGTGAGGGATGCTGCCGCTGTTATTTGGAGGGGAGAA
TATTTGTCTGCGGTGCCTGGGGACAAGCCCTC
TTCGTGGTGTCTGGACACGCTTCCCACCAACACGACGGATGCGAGGGACCCGGAAGAAACATACATCT
CGGTGCTGTCATGCATGCGGCATTGAAGTGCC
TCGGNNNNNNNNNNNNNNNNNNNNNNNNNNNNNNNNNNNNNNNNNNNNNNNNNNNNNNNNNNNNNNNN
NNNNNNNNNNNNNNNNNNNNNNNNNNNNNNNN
NNNNNNNNNNNNNNNNNNNNNNNNNNNNNNNNNNNNNNNNNNNNNNNNNNNNNNNNNNNNNNNNNNNN
NNNNNNNNNNNNNNNNNNNNNNNNNNNNNNNN
NNNNNNNNNNNNNNNNNNNNNNNNNNNNNNNNNNNNNNNNNNNNNNNNNNNNNNNNNNNNNNNNNNNN
NNNNNNNNNNNNNNNNNNNNNNNNNNNNNNNN
NNNNTGAGAGTGACTGAAGAGGGTAACGCCTGTTGTGCCCCATGCACTGACGAGGCGAAGATTGCGTG
GGCCGCTCCGCGCTACGACGAGGGAAAAGCTA
TTTTCCGTGACCACGCGCAGGTGTGCACGATATCCGCCACCAGCGGCAGGATACTTCCTGTGGTTCCG
TGGGGCGGCGATGTGAGAGGGTGTGGAGCAGG
ACGGTCTCACTGGCATATACTGAACAAGCTTTGCTACGGGCATCCGTCCCCGATCCAGAGAGCATTGG
GACCGGCCGTGGCACACCACCGAAGGATGTGG
ACACGGCTGCTCCCCAGAAGCATCCGTCGTCAGAGGAGAAGGAGGTCGCCTCCGGTGCCACGACGGCA
ACGGTGTGCTCTTGCCCAGCGACACCGCGGGT
ACGCCGCAGGAGGCGGAGAAGGAGCGCGATCTTCCGGACATCGACGATGTGGCCGCCGATGGTGTGAC
GTCTGTTGTCGGACAATCATTAGGAGATGAGG
CCGTCAGCGACAAGGCCGGTGGCGGTGCCACAGCCACGGGACCCAAGGGCCCTGATGCCGCTGATGCC
GCGCAGGGCGCCGTGGACCCTCCGCCGAGGGA
GGATGAAGCGGCGGTGGTGCGCGGGGTTGGGAGGCGGACATGAAGGACGCCCCGCCCCGGTCCACGGC
AATAACACTGCGGCTGCGGCTTGTCCCTTTCA
GCTGATTCTTCTGCTTGCCGCTGCCGCCGCGGTGCCAACACTCAGCAATGGGTGCAAGAGAGAGAGGC
CTATATCAACTGGCATGTTCCTGTCAGTATAT
ACATGCACTCTGCTTGTCTGTATACTTTGTACCAGGGACATGCTGTATGCACTCTCCCTCTATTGTCG
GTCGCTAATCTGGCGGCACCACGCGTGTATTA
TGGGTTGGGATAAGAAATTGTTTGACTTCTTCTGAAGAATACTGTGCTGTGTTTGTGTGCGTGTGCGA
GGGATCTGTGTGGTTTTGATTTCCCTATTTTT
GTTTACCTGCTTTGGCATTGTGTAATGATGCTTCGCTTCTCCATTATGTGTGTTCTGAAGTGTTGCTA
GGGCACCGGGAGTGGATGCCTGTGGACGCACG
CCGTGCATTATGCGCTACATTGGTGGGAGGGGAAACTGACATAACTGGCGGGATTCAGTAAGGCGCAT
TTTGCCCACAGACCCTGCAGCCGCAGCAGCCT
GAGCGGCGCTTTGCATATGGACGTGTGGATTGTTTCTCTCTTGCGAGGCCTTTTTTACTGCGTGTCAT
TTTCCGCTCGGGTACCGTGCCCGTGGCAGCAG
CGGCGGCATCAAAAGGGCTGTGTGCGTGGAGAGTCCTTGATTGAGTGCCTGTCTTACTTCTCTGTTCT
GTTGATTGATTGTACCGTGTGCCGGCCAAACG

GTTGACATGACGTTCTTCCGACGGCGGGGTCCACGTGACACGATGTTGCGTCGCGTGCTTTGTTGCGG
GTGTGTGATTTCAGCAACGGTTTTTGGGCGGT
TGACTGTTGGGTCGTTGAGGGCGGCGTGGCGACGGGTGCGGATGGACCCGATTGTGTGCCGCGGCTTT
TCGTGCGGGGGAGTCGCGGGAAGGAGGAAGGC
AGCGGAGCGATGGACTCACGAGGAGTGCGGTAGCGTGCAGCCATGAAGATTGGCGACAGGGAACTCAC
TGCCGCTGTGAGTTGCCGTGTGTGGTGTTTGT
TTCTGTTATTATTACTTGCGTTGTTGGATGTGGCCGCTGTGGGGAAGACTGTTTTTTGTTTCTTTTNN
NNNNNNNNNNNNNNNNNNNNNNNNNNNNNNNN
NNNNNNNNNNNNNNNNNNNNNNNNNNNNNNNNNNNNNNNNNNNNNNNNNNNNNNNNNNNNNNNNNNNN
NNNNNNNNNNNNNNNNNNNNNNNNNNNNNNNN
NNNNNNNNNNNNNNNNNNNNNNNNNNNNNNNNNNNNNNNNNNNNNNNNNNNNNNNNNNNNNNNNNNNN
NNNNNNNNNNNNNNNNNNNNNNNNNNNNNNNN
NNNNNNNNNNNNNNNNNNNNNNNNNNNNNNNNNNNNNNNNNNNNNNNNNNNNNNNNNNNNNNNNNNCA
GGGGGGATCTCAGCAGGCGGTCAAGAGCAGCC
AAATTTATCTTCTGGTGCGGAGGGAGCATCGAATAAAACAAATCCCAACAGCACAACAACAACTGGAG
ACGATGATCCAGCAGCTGATGTTGCAGGGACA
GCAGAGGGAAAACAAAATGAAAATAAAGATGCCAATCCGAAAGAAACACCAGTCGAAGCCACAGCCAT
GAACACTGCAACGGCGACGACTGGCGACAGTG
ACGGCAGCACCGCGGTCTCCCACACCACCTCCCCTCTTTTGCTTCTTCTTGTTGCGTGTGCGGCTGCG
GTGGTGGCCGCGTGAGAGTGAGGGAGAGAGCC
GTGCACCGCCCGCACACACACTCGTCCTTCTCTCTCTGTGTGTGTGTCCCCTTGCATGGACTCTCGCC
CTCACCTCACCCCACGAGGCACACACATAATG
TGCCCATTGTATATATGCATGCACACCCATGCAGGCCCGTTGGTGCTCTCTTTTGCACGACACGCACC
GCTGCCGTCCTGCCTGGCTGCATGGGCGGAGC
ACCGTAACTGTTTCTGTTTTGTTTTTGTTGCATTGAGTGGGCGACCTCTCTCTCTCTCCCTGTGTGTG
TGTGTTTTGCTTTACGGGAGTAATGAGAGTGT
GTGTGCGCTGGTCGTCCCACGCTTCGGCTTTTTGTTTTACTTGTGTTGTGGTTGAGTGCACCCATTAC
ACTTTTTATTCGATTATTGGCAGTTTGCTTTG
ACTGCGGACGCCTAAATTCTCTTTTTCATTTTTTTTTCCTTTGAGATATTGTCAGTGTTTCTTATTCT
TTTCAAACCATGAAGTATTTTCTGTTACGTAC
AGCATGTGTCGGTTGGTCCACGTCACCTCACAGTCTGCGGTGCTGCTGGTGCTTCTGTTTGTGATGTG
TTGTGCTGGAGGGTGCCTTGCTGCCAGCCTGA
CGTACTCCTGCTCCTCTGACAAAGCGATGCGTGCCCACAGGGCGAAGTCGATCGCGGCTGTGGTGAGG
GACGTGCTTCTTGGATCTGAGGGTGCGTCGCA
GACATGCACCGTCATTGGGAGGAATGGATGGGCGCCCATCCGCATCAATGTGTCGACAAGGGACCCGG
ATGATGCCAGGAAGTATTGGATCTCAGAGAGG
TACTACACAATGAATTCATTGCGGCAATTGCGAGTGTGAAGGTGACGTTTTTCTATGCTGAGAAAAGG
GAAGTATTTGTTTCGTTATACTTTGCTGCCAC
TGTTCGTTAATGGCCCGCGGGCTGTCCGTAGCGATGTGGCCAGTTTTTCCTTCTTGAACGGTGCTGTC
GTGCGAGAGGTTGTGTTCCCAAATAGTCGGAT
GCTCAGTTGTCGAGGACGGGGGCATTCCCACAGCGTGTGCGGCAGGCCTTCCCGTGGATTGTCGAGGG
CTCAGCGGCTGGTGGGGATTGCAGCAGAGGAG
TGGCTGAAGGGCTACGGCTGTCATTGGAGCTTGCAGAGGTGCGTTGTCCGTTTTTTGCTCGTTGATAC
GCTTGACATTTTCGGTAATGACCTGCGCGTGA
GGCACTTGTTGGGGAGACCGTGGAGCTGTCGCAGAATCCACATGTGAGGCCTTCTGCAACGATTGGAG
TGCCAGGGTTAGTGCGTCGGGCGTGTGCCTCC
ATATGGTGGGCGGACGAGAAGACGCTGCTGCAGCTTGGGCGGGGCGACGTTGATCTGCTGCTCGTCGT
TCTACGAGGAGGTGCCGTGGACACGCCAGGTG
GAGGTTCCGGAGGAGGTCGGGGCGGTCCTGCCTCTTCAGTGTGAGGAAGGGCNNNNNNNNNNNNNNNN
NNNNNNNNNNNNNNNNNNNNNNNNNNNNNNNN
NNNNNNNNNNNNNNNNNNNNNNNNNNNNNNNNNNNNNNNNNNNNNNNNNNNNNNNNNNNNNNNNNNNN
NNNNNNNNNNNNNNNNNNNNNNNNNNNNNNNN
NNNNNNNNNNNNNNNNNNNNNNNNNNNNNNNNNNNNNNNNNNNNNNNNNNNNNNNNNNNNNNNNNNNN
NNNNNNNNNNNNNNNNNNNNNNNNNNNNNNNN

NNNNNNNNNNNNNNNNNNNNNNNNNNNNNNNNNNNNNNNNNNNNNNNNNNNNNNNGCAGGGCGCGTGCCGG
TCAGCCGCAGGTTGGGCAGGAGGACGTCAATC
AGCCGCTTGCTGCTCGGCAAGGAGTGGCGGTGCTCACGTCGGGAAGAGGTTACGGCGGAGGCAGAGCA
CAGACGCGGTCCCAACAGCACCGCAGTACAGC
CGATCCCAGAGGACAGGGACGTGGCTCCGTCATGCCTCGGGGCACCACAGGCCTGGGATCGAGAGGGC
ACCGAACTGTTTCGCACGGACTGCTAGCGGCA
CGATGAAAACACGGCGAAGGGCATGATTGCGGTCCTCCCATTTGCGGAGCAATTCAGCTCGGGGCAGG
CTGCCGTTGCAACACACTCGGCCTGCTCCATT
CCGTCCGTCGTGATTCCTCTGGTCTGTCACGACAGCCGCACGGTATGGTATATTTGCGCATCCCCAAC
CATGTGGTCACAGAGGCCAGAGGTGTTCCACA
CGCCGAGGTGGAAGCATTCCAGTCGGCATCACGCAGGACAAAGGCGACATGTTTCCGTTGTGGCAGCA
TGCCTGCCAGTCGTGGGATGCCGTCCTCCGTC
CCCGCAGTGTGGGATTGCAGATGGTGATCCGAGTCCATGTAAACGGGTGGACGTACAGTGGGAGACAC
GAAATACGCTTTGCAAGTCACGTCGGGAAACG
AGACGCTGCGGCCGCTAATCCGCGTTGCCTGTGCCGTGTCGTCGGACAGCTCAATCTCCATGCCGCTT
GGGGGGGCGGCGATGTCCTCTCCTGCCGTCGT
TCATGGTGAGCGACCGTCCCACAGCTCATGGTGTGAATTCAGGTCAACCGCAACAGGCGCTTCAGGAC
CAAGACGACGAATGTCGCTCGCAAAGTGAGCA
GTTGCCGGGAAGGCTGCAGAGGTTCACCACGCACATGCTGGGGCTGGCCGGTGGGCACACGCGGAGGG
CGGTGGTCTCCAGGCAGTTGGTTCCAGCAGCG
ACAGGCGCTCGCACGGAAAGGTGTGGTGCACCACGAAGGTCACGCCACCGGCACGCCCACTGCAGGCC
TGGTGGAAGTCAATGCAGCCAATGAGCCTCGT
CGCTGGCACTCTGGTGCCTGCACTCCTGCAGAAGAACGATGTCGGGCTGGTGCTGCGCCAGTCAAGCA
GCAAGGGCAATCCTGAGGGGGAACGGCTTGAA
CTTCTGAGATGCGGCTCTTGCGGACACCACCGCAGCAGGTGATGCCGGCAAAGTGGCCGCAATGAAAC
GACTGTGCGGTTGTCAGTCGGGCTGCAGGCAA
ACCGCCTCGCCGCTCCACAACCAACAGAGCGTGGGATCCTCTCGAGGGGTGACGCCGTCGTGGTCCCC
GTCACGCCCGCAGCAATGCCCTGCTGCAATCA
CTCCCACAGAGAGACAATAAAGAGTCTTGTGGATGTGTGCCGAGAGCACAGCGACTGTCATTGCCAGA
GAAGGCCATCGTGGCACCGTCCGTCACGCGTC
AGCACACACCGAGCAACGCACGCACCCGGCAAAGCAAACACACAGGAGAGTGGAGAATGACCCCAAGA
GCAGGAAACACCTTCACACATGGCGGGCATCG
TGCAGCCACGTCAGCCGGCCTCCAGCACCATGCAACAAACGTACCACACGCATCCATCCAATGACACC
GTGATTCCCACTGCTAACGGTAGACACGCAGC
GACCATACTATGTCAGCACGGGATGCAGGCCAACGTCCCACCGTCACCAAGACATTCCTGCAGAGCAC
CGTGCAACTAAGTATATATGTGTGTGTGTGTG
CTGGAGGAGCAGAAGAGAAGAGGAGCACAGGAAGGCACATCAACACAGCAAGAGGGACAGCTGGATGG
CAGCGACAAATGCAGTACGGTCCCGACATCAA
CAACCACACACGATGGCAACAAAAACAAGAGCAAAAAAACAAAACAAATCCTTAAAGAAAAACACGGC
GGCAGGGTGTGGCTGTGTGTGTGGTGCTGCGG
GTGTCTGCCAGCCCTGGCCGCAAAGAAACAAAACAAGAGGAAAGAAATGTGTGCGCCGCCCCACAGGC
CCTCGTCGCACGCCCACACGTCACGGCTCCCT
CAGCGCGCCGTTCGTCGCGGCCCCTCCTCATGCGTGCAGCAGGCGAGGCGCACAGGGCCGTCGTCATG
AGCGTGTCGGTGGGTGTGCGAGTGGAGCAGTT
GGGCGGAAACACACGGCACCCCTCACAGGAAGAGTGCAGCAACAAACAATTAAAGAGGGAAGAGTAGA
AAAGAAAAAAACGTCCATAGTGATTGCCCCAAA
AGCAAAAA
>scaffold1008.size8326
CGTCGACACGCACCAATGGAGGTGCAGCCCAGAGGGGGTGAGCCACCGTCGTCACCACCGCAATTGCC
GAGGTAATAATAATCTGGAGAATTTCTGGGCTG
GCCTTGTATCCCATTGAAAGTCGTGTCAGCTCCACCAGCGTGCCGTCCTCCACGCGGCATCGAAAGAG
ATGCCGAGTCTCCCGCGGCAAAGAGACTATGA
AGGACGACTTTACATCCAGGCAGGAAGCGGCCTCAGCCATCACAGGCGGCAAATAATGGGAAATGAGC
AAAAGAGGTGCATTAGCCTCGTAAGGGTCGTC

GCTGTTCCTGTCGCGTGGCCACGCGATCCGTCGTCGTCGTAAACCGGTGGTTTTCTCCTCCACAACCG
AAAAGGGGCATTATCCATCCGCCTGTTACTGT
GGACGAAGCGTCCTCGATAATTCCTGCTTTTCGTAACACTCTCGCGTCAGCGGTTTGAATCCTCCGTG
AGCCTTCGCGCGCTTTACCGGAGGAAGACGGA
GGGAAAGGGGACCGCCCGACGAGTCCCCAAACTTGCATGAGCCTCTCCAAGGTCGCCGGGTTCAACCG
ACTTTTGATCTATTCTAGATtCAATACGGGCA
TGTTGACTCGCTGTAACGGGATGGTTCGTTCATCTTGTGGAGTGGCCAGACGCGTCCTTGTCGCGCGT
CTCGCTATTGTAGCAGCATGATTGTGATAAAA
TCATGGAGAAAACGCTCCGCTGCTCTCAGCCAGGGTTCTTGCCCTTCCGCCGTTTCTCCCTTCACATC
AATGCGACCAGCGACGACACCTGGGTCAGCAG
TGTGGTGTACCTCCCCAAATATCGCACTGTGTTCTGGGGAGGGTCGAACGGGTCGAAGTGCTTCGCCA
ACTGCGGGATCACGTGTGGATCCAAATTGTAT
GTCTCCAAGATTTGAGCCACGTGCCGCAACGCACCCCGTTTTGTGGGATGCGCCGATGCATTCCAGGA
GGCCAGAGCTCGTTCGACGTGGGCGGTCGTAA
GATTCGTGAGCTTTTCGTTGTGTTGAAGTGCCCTGCATAAATTTATGGTGTCGAACGCATCCTGCCCT
CGTATCCTCACGAACCGCGAGGCGCGGTGGGG
ATCCGCCCTCGCCGTCTTCGGCGCCACGGACCAATCAGAAATTACGGTCCCGTCCGGCTCCAGCGTGA
CATTGTTGGGTGTCAAGGACGCAATCCCGGAC
CAACGGCTTGTCGTTATCCACGCATTTCAGAAAACAACACGTTGCTTCCAATCGGCTCGGCCGCGGAT
AAATTCATCCCTCTCTTCCTCTGTCAAGGGGC
GCGCCTGCTTCGTCTCGGGTCGCGCCGCAATTCTTCGTGACCCCAGAATCACCATGTCCAGCGGGGTT
GAATTTATTTCCACTATGGACCGCAGCATCCT
GGCGTGCTGCTGTCGGGTCGATGGCGCCACATCCAACATTGCCACCAAAAAGGCCGCGCAGCTCTCCT
CGTTCATCGGCTGTTCGTGCGTGCAGCAAAGC
ACAAAAAGTTGAAAATTGCCCCGCTAGTGACATTCTCTGCTCCTTCCTTGGCAGCGCACACATTTCTC
AAGCCTACTCGTTCGCGCTCCTCATATGGCCG
GCCCAAAGCGGAGATGTCGGTGATTCTTCGTACCACAGTCGAGCGTCGTTGGCGGATAAGCTCCATTG
CCATTGTCGACGATTGCGTCGGACAATTTGAG
AGATCGGGCGGTGCATCGGCGGTGCGGTGCGACGCCATACAGGAAGGGATGGGGCTGCTTTCTTCCGC
CTTCATAAGCGGCCGGCAGCCCCTCGCCGTTT
TTTGGCGGCCGTCCGTGCTGCAAGTCATTTCTTCTGGCTGCGCTGCGCACGTTGCATGCAGATGCGTC
GTGTCCACAAATACAACACAATCGGCATCGCA
ACAGTNNNNNNNNNNNNNNNNNNNNNNNNNNNNNNNNNNNNNNNNNNNNNNNNNNNNNNNNNNNNNNN
NNNNNNNNNNNNNNNNNNNNNNNNNNNNNNNN
NNNNNNNNNNNNNNNNNNNNNNNNNNNNNNNNNNNNNNNNNNNNNNNNNNNNNNNNNNNNNNNNNNNN
NNNNNNNNNNNNNNNNNNNNNNNNNNNNNNNN
NNNNNNNNNNNNNNNNNNNNNNNNNNNNNNNNNNNNNNNNNNNNNNNNNNNNNNNNNNNNNNNNNNNN
NNNNNNNNNNNNNNNNNNNNNNNNNNNNNNNN
NNNNNCGGAGTCATCTTCCAAGGGGGAAGCGCGGGAGGGCAAAGGCATCGAGTGAGGGAGGTGTGGCC
TCCCCACATTGCCCTGAGACGTTTGAGTGTTG
ACCCGCCGTCTCTCTCCCGTGAGAGTGCGTTGGGGAAACGTGGATGCTGGCACACCCCCAGCCATCAA
ACGATGCGGTTGGTGTGGCCGTGTGCTGACGC
CATAGTCGCGCATGCATGCGTGGGGGCTCATCTGGCGGCCCTCTCCGAGAGGTGGGCGCTTCTCGTTG
GCACGGCGTCTTCGCGTCCCGCAAGAGGACCG
CCGGCCACCGCAATGTGACATTCGCGCGCCGACAAGTAATTATGCGCCTCTTCCACCTGAGAGTAAAG
CTGCGCACGCCGATTCCGAGCGTCGGCGACGG
CGCTGGCGTGGTCGGTGTTCAGTCTCCGCTCTTCCAGCAATGGTCAGCCCTTGCTGGACCCACAACCC
CCGGGCAGCCCGCACACGATGGGCAGGAGCAA
CTGAGCCTCGAAATTTGACAATTGAGGCGTCCGGAGACTCGCAATACGTCCCGCAATACATCGNNNNN
NNNNNNNNNNNNNNNNNNNNNNNNNNNNNNNN
NNNNNNNNNNNNNNNNNNNNNNNNNNNNNNNNNNNNNNNNNNNNNNNNNNNNNNNNNNNNNNNNNNNN
NNNNNNNNNNNNNNNNNNNNNNNNNNNNNNNN
NNNNNNNNNNNNNNNNNNNNNNNNNNNNNNNNNNNNNNNNNNNNNNNNNNNNNNNNNNNNNNNNNNNN
NNNNNNNNNNNNNNNNNNNNNNNNNNNNNNNN

NNNNNNNNNNNNNNNNNNNNNNNNNNNNNNNNNNNNNNNNNNNNNNNNNNNNNNNNNNNNNNNNNNNNNNNNNNNNNNNNNNNNNNNNNNNNNNGACGA
ACAGTCGTACAGAAATACATCTCCGTTTCATC
AGGACACATATACATTGGGGACGCCAGCTTGCCCGCGAACATTCTCTTCACAAGCTTCTAATAAAACA
AAGACATATTTCCGTACTATTCCCTCCTATCG
CTTCGAGTTCCTTTATTAGACAGCGATGGAAGGAATCAAGGAACCGATAACGTCTGTACGGTTTTCTA
GTTCTTTTGTTGTCCCACCCACCCGCTTCGAT
TCCCACCATTGCAGCGGGGTTTTTGTGGCTGGAAGAGCCCAAAGTCCGCCACTTCGCAAGCTATAAAT
ATATATGCATCCAGTCCGAATAGAGCGTTCAT
CATGTCGCGAGAGTGGGGCCACCAGAATGTACGAATAAATACATACGCGGAGCAAACAACGAGGATTT
GGCGAACCCACTGCCTTTCCATTGACTCATTT
TTCTCTTCCGTCGTATTTTATTTGTATGGAAAAGTTTGTTGTCCGGNNNNNNNNNNNNNNNNNNNNNN
NNNNNNNNNNNNNNNNNNNNNNNNNNNNNNNN
NNNNNNNNNNNNNNNNNNNNNNNNNNNNNNNNNNNNNNNNNNNNNNNNNNNNNNNNNNNNNNNNNNNN
NNNNNNNNNNNNNNNNNNNNNNNNNNNNNNNN
NNNNNNNNNNNNNNNNNNNNNNNNNNNNNNNNNNNNNNNNNNNNNNNNNNNNNNNNNNNNNNNNNNNN
NNNNNNNNNNNNNNNNNNNNNNNNNNNNNNNN
NNNNNNNNNNNNNNNNNNNNNNNNNNNNNNNNNNNNNNNNNNNNNNNNAGTCAGCAGGCCGGCGCCCAGT
CGCCCTCGTGCCATCGCAACGGGGGAACACAA
ACAATTGTCGTCGCGGACCATCGGTGTCGCTAGCAGGAAAGAAAGAAGAAGAAATGGAGGCATGCCAG
CAGCACAGCCGGCAGAGGGACAGTCAACAGGA
CACGGCGACACAAAGTGACATGGGACACGGCACAGACGGAAAAGAGACTTTAGTGATTTACCAAAACA
AAAAGAAATGAAAAATGAAAAAAGAAAAAAAC
CAAAAAAAAGCAAAAGAAAAAACAATTGACTCGGCAGATATGTCATTTCATGTGCCACGCAGCACAAT
GCAACCGTGCTTTTGAGTTTGTACATACTGCC
CGCACACACAAAAATAAAAAGGAATTGTACTCTCCCACCAGCCCACACAACATCCCTCACATTCCATG
CGGCATCCGGCCTGCTCCTCCAATCCTCCCTT
CGTGCAGTCCGTGGGACGCACAGCAATGAGGCCGGCACCGCGTGTGGACAAAAAAAGCAAACAAAAGA
GGGAGAAGCACGTCACGCATGACGGTTTGCAG
CAGGCGACCGTTTTTTTGGGGGACAACACACAAACAGGGGTGCTGATTATTTCAGCAGAGCACCAGCA
AGAAAAAACACCAAAAAACAAAAAACAGGAGA
AATGCAGAAAGCAATAAATCAACAGAAAAAACAAAAAACAGAAAAAAAAAGAAAAAAACAGAAAAAAA
AAACAGAAAAAAAGAAAAGAAAAAAAGAAAAA
GAAAAAGAAAAAAGGAAAAAAAAGAGAAAGTATCCTTTACAAAACTCATTGAGTATTGCTGGGATGCC
GCTCAGTTGTTGCTCCACAGAATTTTGCAATT
TTTCTTTCCCAATTCATTCAACAAAGAGCCGCAGTGCCCCACAGCCCCAGAAGCATCAGCAACAACAC
CCGAGACACACACCCATGCATGGTGCTCTCAG
CAAATAGAGCCATGCCCGCCAACTCCTCGAAATGCCCGTTCTCAAGCAAAATGCCGGTACCCGAGGGT
GCTGTTGCGTTGGACCCCGATGGGATCTTGGT
TTCCCCAGGCAGGGTGCTTGTATTGCCCGATGCGGTGCCAACGTCCTCACTGTCTTCATTTTCAACCA
AAGGCGCAGTGGTTTCTTGCGTCTGCCGCTCA
GTGGCCCCTTCCGGAGAGGTCGCTTCTTCTTCGTGGATCGGTCGCTCCACGTCGGCCTTCGGAATGAC
TTCAGTCCCGGGATGTGGTGGCACTTCTTCCT
GCAGAGAGGTGCTTTCACGGACACCCATCCCGTCGCTGCCCAAATGAGAGGCAGTGCTCACACTCGAT
AAGGCCGGTGGAGGCGCCAATTCGCCGCTGCC
ATCCCTCTCCGTCTCCTCCTCCCTTGTTCCGTGCGAGCCACGGCCCCCAGAAGACACTGCGGCAGCAT
GACTGCCTTCAGAAGCTTTCACGGATGATTGT
GGCCATGGCGCCTCCTCAACTTCCTGCGTTTTTTCTTCCTCTTCGTCTTGAGATGCTTTCCTAACCTC
AGGTGATGGTGCCTGGGCAGCAGAATTTGTTG
TTTTCTCCGGCACCGAGCGTGACTCCTCNNNNNNNNNNNNNNNNNNNNNNNNNNNNNNNNNNNNNNNN
NNNNNNNNNNNNNNNNNNNNNNNNNNNNNNNN
NNNNNNNNNNNNNNNNNNNNNNNNNNNNNNNNNNNNNNNNNNNNNNNNNNNNNNNNNNNNNNNNNNNN
NNNNNNNNNNNNNNNNNNNNNNNNNNNNNNNN
NNNNNNNNNNNNNNNNNNNNNNNNNNNNNNNNNNNNNNNNNNNNNNNNNNNNNNNNNNNNNNNNNNNN
NNNNNNNNNNNNNNNNNNNNNNNNNNNNNNNN

NNNNNNNNNNNNNNNNNNNNNNNNNNNNTGTGAGTTGCCCCACACGCGAGTAAGTGTCTCACCATATG
GATACCAATCCACCCCACTCACAGTGGATCCG
TACACGTCACGGTAACCTTGCTCACAAGAGGCCATCATGAGGAGTCTGTCTTCTTTTCCCCACTCCAC
GATGGTCGGGTCCCTGCAGCCATCAACATCCG
TTTGGTGCGAAAGCTTCCATTTCTTCTCTGACTTATCAAAGCGCATGGACAGCAATGAAGTGTTTTCG
TTCTTTGTGGCCTGCATGGGAAACATAAGTGT
ACCGTCCTTTGTCTTAATCCCCGATCCTCCACCAGAAACCAACTGGGTCAGGAATTCAGCAGCATATT
CATCGTTTCCAAACGTCACCGCATGGGTCTCA
TTCCACGTGATTCCATTGCCACCACTGACACTTCCCTTTACCAGCAAAAGTCCCCAGTCACGTGCAGT
AGCCGATTTACCCACCGACGTCGTAAAGCTCT
GATTTCTCAGAAGCATGTAGACACTGTCGCCAGCCACAACTGTCGTTGGCCGCATAGATTCTTTTGCC
TTGGCGGTACCTGTAGCAGCACCCTTTGGAAA
CGGCATCAACAAACTTGTGTTCGTCGTCAATATCTCCGTTGGCTTATCATCAGTTAGATTGAGGCGTC
TTGACGCAATCCCAGCACGGCTGCAGCCTTCG
TCGTCTTCCTTGCACTGCGCTTCAGCAACCACGAACACAGCACTGTTCACCTCAACAAGGCTGGGAAC
GCGGAGCGAGAAGAATTTTTCATTACCTTCTG
TAGAGCCTTCCCATTGAGGGGATGTCCATCCAATTCCTGTAAAAGGATCAACGTCAGTCTTCGGCTGC
ACAGCGTGTGCAGCCCCACTGCCGCATAACAC
GACGAGGAGCAGCAGCACCGCAGAATGGAAGAGATGCCGGGACATGTGGGGCCTCTGCTGCTCCCTCT
CTCTTCCTCTCCTCCTCCTTCCGCTGGATCCG
GCCACGCGGCGACGGTTGTGTGTGCGGGGTGCCATGACTGCAGCAACACGTGAGAGCATACAAATATG
TGTGTGTGTGCTGTGTGTGCTGTTGTTCCTTT
CAGAGCCTTACAATTTCACAGTAGCGAGTAGCGCTTTTTTGCTTTCCTTCCTAGCGTGTGTGTGTTCA
AGAGGCGGAGAGGAGCACAGCAACAGCGGCAA
CACATCAGACACTTCAGTGGGGAGCAGAGGAGAGCACCCACGCGGAGAGCGGAGCACGAGACAATTCG
GAaNNNNNNNNNNNNNNNNNNNNNNNNNNNNN
NNNNNNNNNNNNNNNNNNNNNNNNNNNNNNNNNNNNNNNNNNNNNNNNNNNNNNNNNNNNNNNNNNNN
NNNNNNNNNNNNNNNNNNNNNNNNNNNNNNNN
NNNNNNNNNNNNNNNNNNNNNNNNNNNNNNNNNNNNNNNNNNNNNNNNNNNNNNNNNNNNNNNNNNNN
NNNNNNNNNNNNNNNNNNNNNNNNNNNNNNNN
NNNNNNNNNNNNNNNNNNNNNNNNNNNNNNNNNNNNNNNNNNNNNNNNNNNNNNNNNNNNNNNNNNNN
NNNTGTTGCGTTGGACCCCGATGGGATCTTGG
TTTCCCCAGGCAGGGTGCTTGTATTGCCCGATGCGGTGCCAACGTCCTCACTGTCTTCATTTTCAACC
AAAGGCGCAGTGGTTTCTTGCGTCTGCCGCTC
AGTGGCCCCTTCCGGAGAGGTCGCTTCTTCTTCGTGGATCGGTCGCTCCACGTCGGCCTTCGGAATGA
CTTCAGTCCCGGGATGTGGTGGCACTTCTTCC
TGCAGAGAGGTGCTTTCACGGACACCCATCCCGTCGCTGCCCAAATGAGAGGCAGTGCTCACACTCGA
TGAGGCCGGTGGAGGCGCCAATTCGCCGCTGC
CATCCCTCTCCGTCTCCTCCTCCCTTGTTCCGTGCGAGCCACGGCCCCCAGAAGACACTGCGGCAGCA
TGACTGCCTTCAGAAGCTTTCACGGATGATTG
TGGCCATGGCGCCTCCTCAACTTCCTGCGTTTTTTCTTCCTCTTCGTCTTGAGATGCTTTCTTAACCT
CAGGTGATGGTGCCTGGGCAGCAGAATTTGTT
GTTGTCTCCGGCACCGAGCGTGACTCCTCACCAGGGTTTGGAATAGGGACTTTGCTGGTACTGAGTAG
CGCAATTTCATCCGTACTGATTGTGCGGTTGT
ACAGCAGGACGTTGCTCACTGTCACGTGGCTGCTTTCCGAATGCTCCTTATTCTCCGCGCCAAAATAG
AAGTGCGAGATCCTGTGATCTTTAAACAGATC
CCCGTTGTAGTGCCAAGAATAAACCCGCATTCCATCAACGTACACATCCCACTCATTGGCATCCATTT
GCAGCATTACTTGATGTGTTGTGTTTGGTTGC
CAATTATACCTACCCTCATAAGCTCTATTGGGAAGTGTGAACTTCCACTTCTTGTCGTGGGTGTACGA
CAGACCAAAGAGGACCGTGCTGCTGGTGTCAT
TCATCCTCACGCCAATCAAAGGAATGCTGCCACTCTGCGGCACCTCGTGGATGGAAACCGTCGCCACA
AGAGTGAACTTGTTGTTAGCAAAGTAGTACGG
CACAGtctgccccatgtcgccaacagg
>scaffold1009.size6765

GAAAAGGAAAAAGGAAAAAGGGAAAAGGAAACACAATGATACACCTTTGGAGCCTACAGACATACATG
CACGCACGAAAGTACATAATAATACACACACAT
GGGTATGTGTATCCAAGAAGAGAGTATGAGTGGGAGTGTTCAACGGATACCAATCTACGCATGCACAC
ATAAGTGTCCCTTTTTTTTTTTTTTTTTGTCTT
GTCTTGTTTACACACACAAATCCATTCCATATAAGAAGCAAACACACACACACACACACACAGATA
TGTTTGTTTCCTCTCATATGTATCATAATATG
CAAAGACTGAAATGGTCATTCATCTCTCACCCTTCTTTTATTCCACTTGTGTGCATACATAATTTTGG
TCTCCTCTTTAAACGTTTTCTTTTTTCTTTTC
TTTTCTTTTCTTCTTTTGAAATTCTTTCATTTTCCTCGTTATATTTATATTTATTTATATACTTTCTT
TCTTTGTTGTGTGTGTGACTTCCCCTCCTGTT
GTTTTTCCTCCCACACGTGATGGCTGCTTAATTCGTCACAAAAGAAAAGGCGTCTGCCTCCCGCTCGA
GCAGCTGCCGCGTCCGTCGAGTGCCAATGATC
CTGTGAAGTTCGTGCGTGTCAAATCGACAGAACAGCGTCAGCGCAAATGCGTTTGGAGATGTGGCGAC
AAGGCGGCCGACGAGGGATGTGTAGAATGCCG
GGAAGAGGGGCGGGCTGTAGAACACCATCACCTCCGCCAGCTTCACAAAGTAGCGCTTAAAGAAGTAG
AAGCGCTCCGTCACAAGCAGCAGCGGCCGCTC
GAGATCCGTAAATTGCCCCAATGCCTTGCGCTGGTGCTGCAGTGAAGTGTACTCACATATGGCGGCGA
AGGAGTCGCGGTGCTCTCGGATCATGTAATTC
CGCAGCCGCACAAAGTCAAAGTAAGAAGGCACAAAAATAATCGTGCGTACATCCCGCTCCACAAGTGG
GCTGATTTTCTTCGCGTACACTTCACGTGTGA
AGAAGTCAAAGCGGGTGTCGTCGCACTTCTCCAGCGAGGTTCCCGGATCGAAGCGCAGAAAGTGCTGC
CGCACAGGCACCATCACTTGTGCCAACACCCC
GGCCTCCTCGCGGTGCTGTAACAACACACGGCCGGAGTTGTTCACACACGAGCGAAAGGTCGCAAGGA
GGGTTGCGTTCGTCAAGTTTGTGGACATAATT
GTCTGGCGATGGCGGCCACTGTGCCCCGCAAGCGCCCATGCGTACACACGGCGCAGATCGGACAGTCC
GTGTGTTGTGTCTTTCGGCCGCTTGTTGAGTA
GCCGCAGCACCTCTGTCATGTGCTGCCAGTTCTGCATAAGCAACACGTGTGCCTCATCCACGATGCAC
ACTTCAATGGAGGCAAGCGACACCAACACATC
ACCGCTGCGCTCCAGGCGACGCCGAAGGCCGAGCGGCGAGCAGATGATGAGATCGCTGTTCAGTGGAT
GGGCGTAGACCCGCACACGGCTCGGCTCTAAC
CGGACACCGACACAAAACGAGTCATCAATGTTCCCAGCAAACTGCCGCTGATAATCTCGTGGACGGCG
TTTGAAGGTGGGATCCACCGCCTCCGCCAGCT
CCGTGAAGTCTTCATTGAATGTGGACAACTTTGCACATTCATCAGGCGTCGCACGCAGCAGCTTCACG
AGCGTTGTCACGTAGCGATGGGCAATGTTACG
CATGGGCAGCATGATAAGGACCCTCGTTTTGCCAAACCCGCGATCACGAAATTCATATTCCTCTTCGT
CTTGGTCGACATCAGCATCGTCATCCCCCTCC
TCCTGTTCCTTTTCCCCTCGATTTTTCTTGCTGCTCTGAATCTTCCGCTTCTTCTGCCTCCCTTTTTT
ATTCTTCCTACGCTCTTGCAGAAGGGCATCAT
GCGCTAACACCACAGACCGTGCCTTAAACCAGTGGTTGAGTGCGTGAAGAATGAAGATCTCACGCCGT
GCATCCGCATTTTCCCACGAACGACAGCAGTC
CATGACATCCGGGTACCCCTGCAGCAGATCCAGCAGACCACGCTCCTCCGCCGTCATCGGATTTCTAG
ACTCCGCGGCGCAGTACTCCAGCCACTTTGCC
CACAGCGTCTCATGCATGTACGGCGGCTTCTGCTGCTGTTTTTTGCCAAAAACAAACGGCGGGTGAAG
AAAGTGACGCACGGCATGGGCAGAGGCGGCCA
CAGCGATGTTGGCGACACGACTAACATCCTCCATTTTTCCGTCACCTGCCGCCTGCTGTTGACGCCAG
TGCTCCTCTCGTGACTGCATGGGCGTCTTGTC
GATACCGCCGTGCTTGTCCTGGTGGTACTTCACAAACCACGGGTCGTTGGCGGTCACGACATGCCGCA
GGGCCGTCACGCCCGTCGTGCCGGGACGGAAT
TGCGGCAGGAGCTGCACCGCTTCTTCCATCCGCTGCTGCCGTTGTTTTCCCCCTTCCTTCTCGACATC
ATCATCATCATCGCTGTAGTCTACAACACTCT
CATCGTCCTCGTACTCGTCCTCCTTCTCAGCATCCTCCCCTTCCTCTTCTTCCTCCTCCTCCGGCTCA
TATTCATCGTCATCGCCATCATCACCAGGCAT
TTCATCCAGCGACATTTCCTCGTCACCATCCGTCTCATCCTCTTCTTCTTCTTCTTGTTCCTCCTCAT
CCTCCTCGGAAAGATACGCCTTCCAATCATCA

TCCTCACTCAGCTGCTCTGCCATCTCCGTTGCGTCTGTACACTTGTCGTCCTGATACTTGCGACTGTT
ATGGCCGTTACCATTTGGCTTGTTTGTGCTAT
CATCCATGCGACTGCGTTTCGAGCCGCCAGCAACACCGATTAGGCGTCTTAGTCGCAGTAATGCGTGC
TCCTGTCTGCGCGCCTCCATCTCTGAGTCACT
CAAGACCGCGTCATTGTCGTCATCACTGCTGCTGCTGCTGATGGCTGCGAGCGCCTCCGCCTCCCGCT
TGGCGGCATAATAGTCAACACGTTCGTTGTGT
TTGACTCTTCTCTCCTCTTTTGAGGCGCCTTTCGTGCCGCGACGCCTTCTTGAGGCGCTGCGCTTCTG
TTTGGCTTTCAGTCTCTTAAAGAAGCCGCCCG
CTTTGCCCATTTCAAAGACTCACACGCACCCCCTTCTACACACAGTCGCCGGGGCTTTCCGACATGGA
CACGTGTGCAGCGTGAAAGAGAAAAGTAAATG
ACACAGGAGACAAAATACGGCGAAAAGGCACGGCAACAAAGCCCAAACATCCACATACAACGTCCCAC
GAGGAAATAAAACAAATTAAAAGAATATGAGA
GGAAGAGAGAGAGAGAGAGAGAGGGAGAGGGAAGGAGAAAAAAAAAGGGGATTTTAATTTGCATCCATTT
TCTTTCCGTTCCTGTTGACCTTCGTTCGTTGA
TCCCCCATCGAAGGCCACCGCAAATAACACACCGTCCACTTGCCTGCCGTGCAAAAACACGTCCCTCC
AGGGCAGAAACCATCATCATCATCAACAACAA
AATCAACAAGTAAGGAAATAATTTTGTATGCAGGGAGAAGCTTATGGCAAAATTATTTCATTCGCATC
AATTATTCTACAGCGCCGCCACGCGATAGTTT
TCTGCCGCTGCCAGTGCTCTCTCCTGCGCCTGCACAACCTGCACGCATGCGTTCAGCACCTCACGAAA
TTGCTGTTGACAGGCACGCAGCCGCTTCGAGG
GGACGGACGCGAACCAACGCGCCGGATCTTTTCCCTCACCGGCTGCCATTTTCACCACGGACAACTCA
TAACGGTCGTGGCTCCCTTCTCCCCCTTCCTT
GTCCATCTCCTCCGTCGATTTGGCACGCCACGCCACGCCCAGCTGTGGACACACTGCCCCCTCAAGCG
CCGGGACGGCCGCCAAGCCGAGGACAAGTCCC
GCACGGTCCATCTCCCACTGAACATCACTGTACAGAAAGTGCGCTGCCTGGAGCAAGCTGTGGAGCAG
CGAACGACGTCGCTCCAGCTCCGCAACGGCCT
CTAACGCCGTGTCGAACAGAGAATCCAGCCGCTCCTCACTCATGTATGATATATGCACAGGCCGTAGC
TATAAACACCGCACACATAAAGGAAAAACAAA
ACAAAAAAGAAGACAAGTGACGTGGCAGGTTGTTGGATGGGCGGGGAGGCAAACGACATCAACACTGC
CCTCTCTTTTTTTTTTTCTTCTTTTTGTGGAT
GGGAAGTGGAGGGGGAGGGGGCGGCGGCGGGGCGGGGGGGAAAAAAAGGGGCAAAGCGGTGAACACT
CCCCCAGGAATAACAACAACAAAAACCAAATA
TAAATATAAATAAATACATGTATGCATGAGGCAAAAACAAACAAAAAAACAAAAAGGCGGAAAATAAT
CATCAAAGAGTAAAGGCAGTAAAAAAAAAAAA
AAACACACACAAAAAGGATCCATAAAGAAATTCAAACAAAACATTCGTGCCTGCGTGCGCAAGTGAAG
AACACACACACACACAGAGAGAGAGAGGGAGA
CATAAAAAAGAAAACCATCATGTACATAAAACGGTTTTTTTTCTTTCGTTTTTCGTTTCGCCCCATAT
TTTGCTGTCATTCCCCCTATTCTCTCTCTCCC
CATCCCCCGCCATCGTAGTGGCTTCACTCCTTCCTTCCCTCTTCCTCTCTTCTCTTCTCTTTCTATTT
GCCTTTTATCATTTCCAACGCCCTCATGCCTC
ACTCACTCACCCTTCATTTCCATCCCCCCCTTTGGAAAAGCCCATAAGCCCACATGCACACACACACA
CACAATATATATATATATATATATATATGGATAG
GTACTTACATGCATATATACACATATGCGCACATACACGTCCGCTTCGTATCTTCTCCCACCCCACCA
CCACTTTTTTTTTGTGTGTGTTCGGGACGTGT
TGCGCTTCCATTTCGTGCCCCCTGTAATGCAACATTTTTTTCATTCTTCAAGGAAAAGAAAAGGAAAA
TAAATAAATAGCCCTGTCTCCTCCTCCCACCC
CCATCGTTTCCACCGCCCCCTTCTCCCCACTTCCCGGGTGTATCCACTCTGCTCCAAGGAAATACGGG
CAGCAACGGCGGGTAAACTTCTTTTCTTCGTT
TAATCATCGCCGTGAAACACCACGTACTCCTCCTTCTTGTCGCCAATCTGTACAGTCCGTGGCGGCCC
CGCTTGACCGCCCTCTCTCAATTCACTCTGCT
GCTGCTGCTGCTGCTGCTGCTGCTGCAACTGAGACCCGCTGAGTGCGTTGAGTGCATCGATCGGGTTA
CCTCTGCAGATAATTTCAACCCGCACCTGCAA
ATCCCCATCATCCCCGAGTGCGTCCCACAGAACTTCCTTCGACACCGTCAACGTCTGTACGCCGGGTG
TCTCCTTCCTGTCGAGCTTCGCGATGCTCATG

CCTCTGCTGTCGAGCAGCTCGATACTGTGCGGTGGGACATCCCTCTTGCGAGAGAAGAGGAAAAAGAC
AAGATCTTTGATCTTCCGAACGTCGTACGGGG
CAAAGGCGTCGTTGGCGCCGGCCTCCTTTTTGATCTCAAGCCTCAAACCGATGAGAACGCCTTCGTGC
ACGAACCCATTCGAGGAGTACGGGTTCAGCAC
TTGCCTGAATAGATGTGGCACGGTCCACGTCACCACCAGATTCTTCGGCGACACACGTAACAGCGCCA
GACTGTCGTTATCAAAGCAGAAATTCACAAAG
AGCACATCATCCCCGCCAATAAATTCAAGAATGTCAGGCCGATAGCATGTGTTCCAGTGACCACACCG
TTCCGTGTTGTAGGGAATTGTGTGGGCCGTGT
GCCGCCGCATTATCTCACCCTTGCTGTTAGAAAAGTAGTACGAGTACTTTGGAATGCCGGTTGCCTTG
TAATGCAAATACAGCCCTATGTTACCATTTGT
GTCAATCGTCATGTGGAGGTGAAACTTTGCCTTTGTGAAGGCAACAACGTTCATACCGTCTAATGTGG
TGCCTAACTTGAAGCTAGAGAAATTTTCCAGC
CGCCACGTGAAGCAGCGGCCTTCGTCATCCGTGGCCAACATTTCCCCATGGCGTTTCTTTGAAAAAAT
CCCCATTTATTGTTTTCTTCCTCTTGTAGAAA
ATATCTAAAAATGTCTAAAAATATAAATAAATATATTTGCACGTACGTCTTACCACCTATTGTCTCCT
CTTTAGTTTATATTATCGTTTGGCTTTCCTTT
GAAACCTGCTTAATTCTTCACTCTCCCTCTCACTTCTCCCCTGGTGTTTCCCTCCACACACACACACA
CAAACGTACGGGTAGAAGACCGGTACCTCCCC
CCCTTTTAACGTGAGAGAAAAAAGAGAGATGCCATGAGAAGAGCAGTTCCAATACAAACACACACACA
TACACACATATAAAGAAAATGCATGCACCCAC
CCTCCCCCCTCGTCCTGGAGGGGAGGAGAAAGAGGAGAGGGGGGTAGAAATACACAGACTATATATAT
ACATATATAAATATATATATATGTATCTGTAT
GCATAGGGATAATTAAAAAAAAAAAACAGAAAAGTACGGCCCACAAAGCACAAACAATGTACATACGT
AAATTGGCCGTATTATTTTGATATCACCGAAG
ATAAATTTGGCTTATTTTGTTCCAAATAATAAAATAAAAGCATCCATCCATTATAAATCCACACAACA
CACACACACACACAGAGAGAGAGAGAAAGATA
GACGCATAAATAACGATCATAGCAAAGAAGCAAAAACAAAAAACAAAAAA
>scaffold101.size29135
ATAAAAAAAACAGGAAAATTTAAAAAATAAGTAGTAACAAAGTGCTTTTCGTTCTGGTGAAATATATT
GGGAGTATTTTAACGATTTTTATATGTGTGTGT
GTGTGCCTACGGGGTCTAGACAGTGAGTGCCTTTCCTTTCAGAGAACCGTTGTGATCGGCAGTGCCAC
ACTCACCCACTCTCTTTTGATTTTATTTTATT
TTTTCCACCGGAGGCGCATCGTCACTGGAGGCTGTTGCGCACCACAGGGGCCGCACAATTTTTTTTTA
AATTTTTATGGGATTTTGTGAAACGAATGCTT
GAGAGGACGGCTGGCTGCCCAAATAAATTCATATCTCGCGCAAACGAAGATATGTCTTTACAACTTTT
TCCTCTTCTATCGCACTGTCTTTTGGGAGACC
GCGATTGAGGGAGTCAGGGAGCCGACAGCTGCCAGACGAAATTATTATTTTTATTTTTTATTAGGAGC
TTGTGAAAAGAATGATCGCGGGAGAGCTGGCT
AACATAATTGATGTATGTGTTTATCCTGATAAATGAAGGCATTCTTTATGATACCTTCTACCGTATGA
ATCCTTTGGGAAGAACGCGACTTTGTAGGGGC
AGGGAACCGATAGAAGCCAGTGTACTTTATTTTTTACAAACACACAGCCAGGTATGTTTACAGACATG
TCATTGGTCCCCTGCACACCCTCCGAGTGGTG
TGATATTGGCGCCCGTCTCTGGGAAATGGTGTGCCAATGTGGCGATACGCAGGTGCAGATGACGTTCA
TTAATGGTAACCAGCTGTCGGATGAGACGTTG
ACCGTGGCCCCATGATCCACAGGCTTATGCTTGCTCCATGGACCTATCTCTAAAGCATGGCAGGATTC
TACTCTCATTGGACATATTGCGAATCTACGGG
CGCCTCAGATCCCTAATTTTGAGAAACAAGTGCTCCTGCCCGCTATGGGGGTAGCCCGGGAGTTGGGG
CACCAACAAGGGCTCATCACTGCTGAAAGGGA
GCGCCTGAATTTTGATTACGCAAACGCCGTCGCGGACGTGCAGAATCGAAATGCTCAGCTTAACTCGC
AGTTTCAGGAGGCGCATCGTTAACCCATCGGT
AAGGGAATCCACTATCGCCGGGGCCGGCAACCAGGTCCTAGAACTACAAAAGCAGTTGGAGGCCGATC
GCGCCCAATTACGGGAGCGCGAATTCAACATG
GGACGACTCACTGCTCAACTCCAGGAACGGGAGGCACGCAATAATGCACAACAGCCCGCGCGGGATGC
GGAGACGGCGGGGCGGGACGAGGCCTCCGCTA

CTCAACACGCTGCCAACGATGGCGTAACCACAATACCGCACCAAACGGATATCTTGATGGCTGGAGGC
GAGCCTAGCAGTGCGGCCCGACGCAGTTCGGC
GGCTTACAGACGCCCTCACGGCAATGTTGGGAGACCACATTTCCACCCTTCCACGCCGTTGCCTTCCC
GCACTTCTCCCCTGGACGCCCAATTACCCATG
GAAGAGGACCGCAGTTTCTGCGCACTCGACGTCACCACTTGGGACCCACGCGCATATGGAATGAGGGC
GCCTCCGGAGTCATTATCGACCTCCGGTGCGC
ATACCAATGCTGGCAACAGGACGACGAGCAGGTTAAACTTGCCCTTGACAATTTGGTCGAGTGGATCA
GCGCGCTGGAAGACTGGCAGGATCCCACTGAT
CGAATCATGAATTTGGGGAGTTCCCTACTGAATACATTCCGGATGCAGCTCACGATTGCATCCGATCC
CGGCATTCCGTTTTCCAAACTGCGCGCCCGTT
TACACACCGCTGTTCACCAGACGGACGCTTATGCGGGGGCAGCCCAGCTTTTTGCCGATCGGCGAGAA
ACCCAACGCACTTTGCGATGCCAACGTTGTCA
TATCTTTGGACANNNNNNNNNNNNNNNNNNNNNNNNNNNNNNNNNNNNNNNNNNNNNNNNNNNNNNNN
NNNNNNNNNNNNNNNNNNNNNNNNNNNNNNNN
NNNNNNNNNNNNNNNNNNNNNNNNNNNNNNNNNNNNNNNNNNNNNNNNNNNNNNNNNNNNNNNNNNNN
NNNNNNNNNNNNNNNNNNNNNNNNNNNNNNNN
NNNNNNNNNNNNNNNNNNNNNNNNNNNNNNNNNNNNNNNNNNNNNNNNNNNNNNNNNNNNNNNNNNNN
NNNNNNNNNNNNNNNNNNNNNNNNNNNNNNNN
NNNNNNNNNNNNTGACAAAGGAAGGGATGAATCAACTTATCCGCAGCCGGACCGATTGGAAGGAACGT
GTTGTTTTTCGACTTGCGTGGATAACGGCGAA
TCGTTGGTCCGAGATTGCGGTCTTGACTCCCAAAAATTTCAAACTGAGGACGGACGGGACCTTAATTT
TGGACTGGTCCGTCGCGCCGAAGACGGCGAGG
GCGGATCCCCACCGCGCCCCGCGGTTCTTCAGGATACGAGGGCAGGACGCCTTAGACACAACGAAAAG
CTCACGAATCTTACCACCGCCCAAATCGAACG
AGCCTTGGCTCCTTGGAATGCCACGGCGCATCCCACAAAACGCGGTGCGTTGAGACACGCTGCTCAAA
TCGTGGAGACAGACGATTTGGATCCACACGTG
ATCTTGCAATTGGCGAAGCACGTCAACCCGTTCGACCTTCCCCAGAATACTGTTTGATATTTGGGGAG
GTACTCCACAGTGCTGACCCAGGTGTCGCTGC
TGGTCGCATTGATGTGAAGGGGGAAACTGCGGAAGGGCAGGAACCCTGGCTGAGAGCAGAGGAGCGTT
TTTTCCATGATTGTAATCACAATCATGCTGCT
ACAATAACGAGACGCGCGACAAGGACGCGTCTGGCCACTCCACAAGATGAACACGCTATCCCGCTACA
GCAGCAGGTCAACGTGCCTGTATTGAACCTAG
AGTGGGTTAAAAGTCGGTTGAACCCGGCCATCTTGGAGAGGCTCATGCAAGTTTGGAGACTCGTCGAG
CGGTCCTCTTTTCCTCCGTCTTCCTCCGGTAA
AGCGCGCGAAGGCTCACGGAGGATTCCAACCGCTGACGCGCGATTGTTGAATGAAGCGGGAATTATCG
AGGACGCTTCGTCCACAATAACAGGCGGATGG
ATAATACCCTTCTCAGTTGTGGAGGAGAAAACCACCGGTTCACGACGACGATGGATCGCGTGGCCACG
CGACAAGAGCAGAGACGACCCTTACGAGGCTA
ATGTTCCTCTTTTACACATTTCCCATTATTTGCCGCCTGTGATGGCTGAGGCCGCTTCCTGCCTGGAT
TTAAAGGCATCCTTCATAGTCTCTTTACCGCG
GGAGACTCGGCATCCCTTTCGATGCCGCGTGGAGGACGGCACGCTGGCGGAGCTGACACGGCTCCCAG
TGGGATACAAGGCGGGCCCAGAAATTCTCCAG
ATTATTATTACTTCGGCAATTGCGGTGGTGACGACGGTGGCTCACCGCCTCTGGGCTGCACCTCCATT
GGTGCGTGCCGACGTATGGATCGATAATATAC
GCATTGCAGGGTCGAAAAGCGATGTGACATTGTGGGAAGCCCAAGTACTTCGTAACGCGGACAGTTGT
CGCGCATCCATGGGGGAGGACCGCGAATCGGG
CGCCACGCAGTACACCTTCCTGGGGGTGCACTTTGATCACACTCAGCGTGCGGTATCCCTGAGTGAAA
GGTTTGTCTGCTCTGTGCGCGCCATGCCGGCG
CTTAATTCTTCAACCATCGCGGAAATGGAGGCAACGGTGTCACGCTTTTTATATTTGGCTGCCATTTT
AGGCACGCGTTTATGTGACTGCTACTTTTTCA
TCAAGGCAGTGCGACGACGATTATCCGCACTTCACCGGGGGATTGTGCTGGAGACATCCCCGGCGAAC
CTTCCGCCGTCAGCGGTTGGTTTGGACGAGAG
ATTGCAACACATCATCGAGAATATAATCGTAGGCGAATCATCAAGCCCACGAAAAGGCATCTGCCGCC
ATCATCACGAACGCATCGCTCCATGGATGGGG

AGCCGTTTTCATTCCAGACTCCGGCGACGTTAAAATTGCCGGAGGGAAATGGGAGAGGAAGTCTTTTC
TTATCATGCAGGCCGAGGCGCGCGCGGTACGC
TTAGACTTATTGGCCTTTCCCGCCATCTTGCCATCCACCACTGACGTTTGGGTGGACAGCACTTCGCT
GCAAGGAGCTGCGAATAAAGGCAGCCCAAAAT
CACACGCTACGGCGCGGGAGCTGCAACGGATATATGAGTTTTTGGACTCTCGCGGAGTGCAGGCATTC
TTTGGTTACGTACGGCCAGCGGAAAACCCCGC
AGACGGCATATCACGCGGTCGTGTTTTCACACTTGAGGAATTGGCGAAGGGGTGGAACTTGCGAAGGG
GATCGGCGGGATCTTGTGGCTGAAGGACCCCG
AAGTCTGCCACCCAGTAATAAATAAATAAATAAATAAATAAATAAATAAATAAATAAATATTCCATCG
GAATGGAGTGAGCAGGGCCACGATTGAGGCTA
ACGTATGGTATAATATATACATACACTTACTAGGTGTTAAACTTTGGGAATGTGGTGAATCCACTCTT
ATCCCACTTATTCCTTTCTTATTCCTATCTGG
TTTGGTTTCTATTATCGATAAACTCATGATCCGGCCAGCTGCCCAGAAGATCATTCTAATTTTTTTAT
CGATCTACCTCTCTCTATGTGTGCTTCTATAT
TTCCTGTGAAAGGATTCTTTTCTGTAGAAATGCCCTTGCGAGCGCTTGCCAATTTTTTTTTCATTGGT
TACTTCTTTACATTTTGTTTTATTATTGTTTT
TCGTGTCTAACTCTGCCGCCGGGTTTTATTACGGGAAAAGAAAAAGGCTCAGACGGATGGAGACCCCT
ATGGTGGTGCTGCTTTTTTTTTGTTTGCGCCCA
ATAGGCCAATAAAGAAAGAAGGCTTGGCCATTACGCCAGGGTGGTGATTGAGGTACTGCCTCGTGCGG
TTGTTTTTTGCGGTGGTGATGGGGGAGGTGAT
TGGGGAAACACTGGGCAAGAAATGTGACTTGAAATCCTGAACCGCATTTTCAGAATGTCTGGATGGCT
GTCGCCCCGAGCTACAACAATGCCCTGCATTG
AAGAATAGTTATTTTATATTTTTGTGCCTGCGACGTCTTTATGCTGAACTGAAGTTTCTTTTATTATT
CGCGGGTGCCGATAGGACAGTTTAAAACACTT
TTGTTGTTTTTAGACATGTAGGATGTCTGGTGACTGTGCTTTTTTGGGGGGGAGTTTACATGAGAGGA
ACGGGGATTGAGCGGTGCGGGATATGTGGTGG
GTGCCGCAGGCTTGTTGGCCCGCGATTTGGGTGCCCTCCAAAATTCATGGCATTTTGGGCTCCTCAAC
GAACACTTTTATTCTTTATGGAATCATGTGGT
ATTACAAATGGCACCCTGGACTGTTGTGTTCCGCCATCCTTTTTTTTTTTACTGGATGTATGAGAGCT
TTGCATGCTGCGGCGGGAACTGCATTTGTTAC
TGTGCCATTGGTCTCTTTTTTTTTTGTTTCCATTCTTAGCCATTTTTACTTTTGTTGCTTATTTTGTTG
CTGGTCATGCTGAGACTCCTGCGTGCTCCGTG
CCTCCACTTCACTCACCAAATGGAGGTGCACGACCATTGCGCTGGAAGGAGTCTTTTTTTTTTTTTGT
TCACATTGACACAGAACCATCATTGAGCACAT
TTTTATTTTATACTTCTTTTGCTTTCTTTCAGGAGATGCTCCCTTGGCTTTGCCTCCAACGGGATATG
CGCGGAGTTACACGCGCCTTAAACACCGAAGC
CACGAGCCCTTTTTTGGAAAGGATGCTTTATCTCTCTTCCAACATTAATTTGTTGTCCATGTTGATGT
TTTTCTTTGAGTGGAGTTCCTACTTGGCGACG
ATCCCACGTATGGTTTGGTGCTGATCCGACCAGGGCGGACTATATCCACTGCGTCGGGGAAAGGCTTG
TGGTGGATCCAACGACACGGCGGCTGGGGGGA
AATAATAACGGGACGGACGTTATCCCGTTGATGGTGGCCCCGCTGATGCTCGATCTGATGGACTTTCG
GAGGATGATGTGCAACATCAGTGTGCCCATCC
GGCTGTTGGTTTTGGTGCAGAACGGGCGGGAGGCGATGCTGTCGTTGTGCCTGCAGGAACTCGGGAGG
GTCTATGGGTGGTCCGGACGCCTGATTGTGAG
TCGTCACCCGGAAAACATCGGGTACAGCGCTGCGGTGAACATCGGGTTACGACTTGCACTCTCGCTGC
CGCGCGAGGAGGTGCCTTTTGTTTTTGTGACA
AACAGCGACGTAAAGTTTTTACCCGATCTTCTTCCCAACCTTCTACGCGATGTGCATGAAATGACGAG
GCATGGTGCCGCTCGCATGGATGAGTTGGCGG
TGGAAGCGGCGAATGAGCCGAGCGAGTACAGTTTTGTTTTGCGACGGGGCTTGAGGGTGCTGCGCTCC
ACGGTGAACGATAACCGATTGTCGACGTCTGC
GCTGCTGCCTGACCGCATTCGTTATGCCTCTGCGAAGGAGCGTGAAAAGGCGTTTTCGAAGCATTACG
GGCACTTCTGCGCGTATTGCAAAAGCAGTTGT
TTTGTGTCTGTTATGTTGACACGCCTGGCGATCAGTACGGTGGGATATTTTGACGAGAACTTTTACCC
AGCTTACGTGGAGGATGTCGACTACAGCTTGC

GTCTCCGTTTGCTTGGATTTCGGGGGCGGAATGTTTTATACGGCAAATTTGTGCATCGCGGCAGCTCG
AATATTCGTCTCTCGGAGCAACTGGAGCTACC
GGACGCTCTCTGGTACCGTCGCGTCAAGTCTTTGATGACGAACCAACCATATGCCGTGATGAAGTGGA
ACGGTCTAAAAGCATGCTGCGATGGGTGCAAG
GGGCCATATGACGGCATGGTCCCCCTAGACGTTTGGGTGAAGGATGAGGCACGCATACAGCGCATTCG
GGTCTACGGGCATGATGAGGAGCAAGGGGTCC
CAAAGGTTGACTATGACAGGACTCTTTTGTACCCTGTGAGGACTAAAGGAAGATGATTCGGCAACAAA
CACAAGTTTTGCTTTCTTATTTTGTTTTGCTT
TAATTTTTAAAAACTTACACTTCTGTTTGGTTATTTACACGATTAATATTTAAGCGAGTACGCCATGG
GTTTTAAGGAGCGCAAAAACATATGTTTGGCA
TGACGGGGGGGGGGGAGGGTGCAGCGTCCAAACCTGGCCGTGTAGCCGAAGGCAGGAGAAGGAAGTAG
CAAGCAGGAAACATGATGATGATGGATGAGGG
AGAAATAAAAGGGGAGGGAAGCAGAAAACAAGTCAAAAGTATTGTGAGCACAACGCTGTTTCTTATCG
TGGGCGATCGACACCGCAATTGGCCACTAATT
TACCCACTGAAGGTGACACCCGTTTCCGCTGGATATGCTGCGTGCATATCTGTCGGGCTCTTTGTTTG
TTCTGTTGTTTTGTTTGTCTCCCGCACATTTA
TTTCTTCGTGCCGCCTGACCCACTCGTTTATTATTCCCCTCCCTGTTTTTGTGTGCGCACCGAGAGAA
GGGCTCGGGGACATGAAGTACGCTCCCCTTCT
GTGACATGCCACCAAAAACAGGAGGAGAAAAAAAAGAAAAGTGAAGCGGGGAGGGGAACCATCGCCAC
GCGTCATTTTCTCCGAGAAGGCTGCTCGCCAC
GCCGCTGAGGCGCGCCGTGTGCTTCACGAGGCCCGCTGGACTCGATTGGGGAGCCGTGAATGATGGTG
CGTGTGAGTGTGGCGGATGCGTCTCCCAGGCG
TTCCCAGAGGACCCTCTGGCCATAGGGACTGCGTGGAATGCGGGATTCCGCGGAGCCCTTTGCCCGTT
TTGTTTTTCCTTTTCTATCCGCCTCTCCCAAA
CTTCAACACGGCGGCTGAGCCAACGACTGCGACGTGATCCATTGCGTATTTCTGGATCTCTCTTGCGT
TGCAGCGGCGGGCCTGGTGTGTCCTGCTCGGC
AGACGCGGTGGACGGAAGTGGGCTGCCTTATCGGGAAAACGATGGTCACGGCGGAGCGTGTTGCGTAC
GTCGCAATTTGGCGTGTTTGTGGCGATCCTTG
CGGATGGTGTGACGGCGCCTCTTTTACTTGCGGGGATAGAAGCGATGCTCCACGGTGGTGCACACGTT
GCGGGTGGAGGCTTCTCTTGCTGTTGGTTTGT
GAATGTTTATTTTTTGCTTTTGCCGGAACACCACAGAGGACGGTGTCTGCGGACAGCAGAGCATTGCG
AGTAGGGGCACCACACGATTTTTGGCGCGATG
AGGCGGCTTTTATGACTGTACGGTGTTTATTTTCAGGATTGAGAGGCTACCCTTTGCTGGAATGTATT
GTGGGAGACCATCCGCGTTTTTGTTTTTCTTA
ATTAATCCCTTCGCATTTGGTGCCTGCCGTGAAATGGATGTTGATGGCCGTTTTATGGCGCTGAGATG
CATTTTTATTTATTTAATTGGCGAGTGGGTGA
ATTGTGTTGCGTTCTGCCATGCGGCTCGTTGCTGACGGCACGTGGCCGCAGGGAGACGTTGTGGGCGC
CTCCGACACGAGGTGCGATTGGGCAGTCACCT
CCTCCGCTCGCCGGTACACGCTGTTGAGGCGGCGTGGTGGTAATGAATCGGCCTCTCAGAAAAGCAAC
GAGAGAACGTTTGCTCTTCCTGCTGCGGTGTA
CGAGTGGACTCTACAATCCGTGGGCGGGGTCGCCCTGTGTGCCGTATTTCTTTGAATTCCATGAGTTT
TTTACATCGCCATCCACACGCACATGCACGCA
GTCTCCCTGTTTGCCCTCCTGTGGGCGGGAGACCGGGATGCAAGGGAAAAATGTGGCCCGTACCAGCA
CGTGGTAAAGTCTCTCTTGGGAGGGATTGCGA
CTGTGATGCACGTCCTGGCAGGCAGGAATGGGATGAGAGGAGAGCGGAGGGGCGAGTAACAACGATCG
TGTGCGGGGCAATGCGCAGTGACTAGGGCGAA
TTGGTATTTGTGTTGACTTTTCAAAGCATTTTTGTCTTTTTCTTTTGTGGCAATGGAAAGAAACTTTG
GATGACGTGTCGTGGATTATTTGCAGGTGATT
CATCGCGGCAACACCATTTGCGGAGAAAATGAATGACATCTGCGTGAGGCTTTTTTTGTGAGCGCGAG
ATGGTCGGGGAGGTTTTCAACGCCCCGAGAGG
CCAGGAGCAAAGAGGCCTGCACTGCTGCAAGCAGAGGGCTGCACGCTCCTGTTGGTGGCGTTGCTGTG
GAGCCCTGAAGGTCCTGCAGGGAAGTTGTTTT
TCCACCGCCTCATTATTTTGTTGATTGATGTTTGTCGCGGCCTTTTGAGGCGGGGCGTGGGATGGACC
GAGGGAGTGAGTTTCGTGTGACTGTGCTGTGA

ATTGTTGCTGCCCACGTTTGTGCAGATACTTCCGTTTGTGTCCGCGTCGTTGCCGCGGCGGTGCGCTG
GGAGCGTGTGACTGATCACTGTATCACTTGCA
GACTGTTTCGGCAGGACTTTGAATGGCGTCGCCGAATGATGAATTTCCGCGTGTACGTGTGTGTGTTG
GCTTCCGCCGTGTTCCGCCGCNNNNNNNNNNN
NNNNNNNNNNNNNNNNNNNNNNNNNNNNNNNNNNNNNNNNNNNNNNNNNNNNNNNNNNNNNNNNNNNN
NNNNNNNNNNNNNNNNNNNNNNNNNNNNNNNN
NNNNNNNNNNNNNNNNNNNNNNNNNNNNNNNNNNNNNNNNNNNNNNNNNNNNNNNNNNNNNNNNNNNN
NNNNNNNNNNNNNNNNNNNNNNNNNNNNNNNN
NNNNNNNNNNNNNNNNNNNNNNNNNNNNNNNNNNNNNNNNNNNNNNNNNNNNNNNNNNNNNNNNNNNN
NNNNNNNNNNNNNNNNNNNNNTTGTTTTTTTT
TTTGTTTTCTGTGTGCAATTGTTTTCCATGCTGTTCCTTCTGTGTTCTCTCGAGTCCCGCACTTTGCG
TGAGTGATGTGCTCTGCTCCTCACTGACTTGT
CAGCTGCATTGCCGCCGCTGTTTTTTCCTTACCGCATGCACACAGACACGCTATGAAAGCAAGCAACC
AAGCGCCAATCGCTACTGTGAAGTTGGAAGGC
ACTGATAGGACCAACAGCACACACACCGCCCACACATATTTGTATGCTCTCACGTGTTGCTGCTGTGA
AGGCACCCCGCACACACAACCGTCGCTGTGTG
ACCGGATCCAGCGGAAGGAGGAGGGAAGGAGGAGAGAGTGAGCCGCGGAGGCCCAACATGTCCCGGCG
TGTGTTTGCTTCCGCGGTGCTGCTTCTCCTCG
TCATGATGATCTGCAGCGACCGTGGAGCTGCGGCCGTTGAACAGGCAGGGGCCGCTTTTGACCCGTTT
AAGGGGACGACGTCGNNNNNNNNNNNNNNNNN
NNNNNNNNNNNNNNNNNNNNNNNNNNNNNNNNNNNNNNNNNNNNNNNNNNNNNNNNNNNNNNNNNNNN
NNNNNNNNNNNNNNNNNNNNNNNNNNNNNNNN
NNNNNNNNNNNNNNNNNNNNNNNNNNNNNNNNNNNNNNNNNNNNNNNNNNNNNNNNNNNNNNNNNNNN
NNNNNNNNNNNNNNNNNNNNNNNNNNNNNNNN
NNNNNNNNNNNNNNNNNNNNNNNNNNNNNNNNNNNNNNNNNNNNNNNNNNNNNNNNNNNNNNNNNNNN
NNNNNNNNNNNNNNNAAGGAAGGGAGCCGGCT
GCTGTGCCGGGATTGTGTCAAAGCACCTGGATATAAAGGGTGACTCAATGGATATTTTGACGTCGGAT
ATAAGTTTGCTTTGCATGCAACATGGGGACAC
TGCCGCAAATAATTTTGGGACAACGGAGTTGTTGCGGCCAACGACACTTGTACTTGGGGACAGTGTTT
ACGTGCTCCTGGGAAACTACAGCCATACAGGG
CCGCAGGTTGGGGGCACGAATGAGCGGGGCCTTTTACTTTTGAAGGGAACTCTCACTGATGAGGGTGG
AAGGAAGAAAATCAAATGGAATGAGACCCATG
TGGTGGAGCCTCAAGGCAGAGGGGATTCTCGTTCCTTGACCGAGTTAGTTGGTGGCGGAGGATCGGGT
GCTGTGTTGCGTGACGGCACGCTTGTGTTTCC
CATGCAGGCCAAAGACAAGGATGGAACGAGCGTTTTGCTGTCTATGCGTCTCTCCAATTCAGAGAACA
AATGGAAGCTTTCATCCGAGACGCCTGGTAAC
GGCTGCAGGGATCCAACCCTGGTGAAGTGGGAAGAAAAGAAGGACGACGAAAGGCTCTTCATGATGGC
TCATTGTGCTGGAGACTACTATGACGTTTACA
TGTCCACTCCGCATGGAGTCAATTGGTATACGTCGGGTCAGCCAATTACCCGCGTGTGGGGCAACTCA
CACAATCGAAAGGGTACGGCGTCCAGAGCGGA
TCCACCACCGCAATCATTGAGGGAAGGAAGGTGATGCTCATCACCGCGCCGGTGTATCCGAAGGAGGA
AAATGAAGGAGGAAAGGGTCGGCTCCATCGTT
GGGTGACGGACAAGGCACGTGTGCATGATGTTGGGCCGGTATCTCGTGAAGATGATGACGCCGCCGCG
AGCTCGCTGCTGATAAAGGATAAAAATAAAGA
GTTGATTTCACTGTACGAGAACAGGAAGAAGAGCGATGGAGCATACAATCTTGTTGCTGTGCGTCTGA
CCGAGAAACTGGAGCGGGTAAAGGAAGTGGTG
AAGACATGGAAGGATTTGGACAGCGCCTTGAAAACATGCCGTTCCGGTTCCAGCGGCAATGTTGACGC
GCTCAGAAAGGGGCATGTGCAATGGTCGTGTT
CCCACCAAAGGGCTTGTTGGCTTCTTGTCTGGTAGCTCAACTGTGACCGAGTGGCGAGACGAGTACCT
CGGCGTGAACGCCATTGTCCATGGCCCAGCGG
AAAAAAGGATAGGGGTTCCCAATGGAGTGACGTTCCAAGGGCCCAGAGCAGGGGCGGAGTGGCCTGTT
GGTAAATTGGGGCAGACTGTGCCGTACTACTT
TGCGAACAACAGGCTCACTCTTGTGGCGACGGTGTCCATCCACGAGGTGCCACAGAGTGACAGCAGCC
CCGTTTCTTTGATGGGTGTGATCATGAATGACACCAAA

GGCACGATCCTTTTTGGTCTGTCGTGCACCCATGACAAGAAGTGGCTGGCCATAGCGGAGAAACGTGC
TATTTCGAAGAGTGTTGATGAGTGGGAATCAA
ACAAGACGTATCAGGTGGTACTGCAAATGGATTATGATGAATGGACTGTCATTGTAGATAGAAAGGAA
ATCGACCACAAGAGCTATGATAAGAGTCTGTT
TAACTTCCACAGGATTTCACACTTCTACATCGGTGGGGACAGTAAGCACCAAAGTGCAACGGGCGGCC
ACGCGACGGTGACCAACGTCATGCTGTACAAC
GAGGAACTGTTGGAAGGTGATCTGCATAAACTCAATGCCAGCAAAGTCActattccatcactgggtGT
TGAGGAACATCCCACAGGACAGGTGACCAGCA
AAGACGTCTCATTTGCTTCCGAGTCAAAGAATGAAGAAATCACCGCCAGCTGTGCGGAATTGAATGAG
GATGATATTGAAAAGCAAGAGGAAGAAGCGGC
GTCGATGATCCGGCGCCTGCAGCGCCCCCTTCCACGGTTGACGCGGGATCATCCGTTTCTGAGCCTGC
CATCGCAGCGGAGAGCGCAGGGAATGACCGTC
CATATGACAATGCCCAGTTTCACCAGGGCGAAACGGCCCAGCAAGCCACGCCGAATGAAGAGAATAAA
TCGATGCAACGGGGCTCAGACCTGCAGCCACG
AGACCCACAGCCAGCGGAATTAACGGAGGTTACCGATGTTGAAATGTCCTCTGGAAGTTATGCTGAAG
AAATGCCAGAGGAGGAGGGAGAAGCGGATGGC
AGGAGTGGCGGATCAACGTCTTCAGTGGGTGCATCATCGGATATGGATACGGCCACCGAAACAGTTGA
CAGTGAGCGCCAATTGCAACAAAGCACTGAGC
TTTCCGCTGAAAATGAAGGCCTGCGGTCCACTGGAACCGGAACCACTGGCGCGGAGCAAAGCCTCGGC
CTCGAGGCGGGGGACAGAAATTCCGAGAGAAC
AATGGGCTCAGACAGCAGCCTCACTCCTTCAGGGATTGACGCCGAACCGACATTCGCGGAGGACACCG
ACGACGTCTCTCGGACTGAGGGAACCGAAGTT
TCTTTTGAAGACGGCAAGGAGGTGCCACAGACGGTCGACACCAAACCGGAGAATACAAACACCACGCC
TGGAGGCGAAGGGATCCCCTCAACAAAAGGCG
CGGCACGACACTCGGACAACGACACCTTCACCGGGGAAATTGCGGAGTTGCTGTCCATGGGTCTGAGC
CGTGACAGCACCGTGCATGTGTGTGTATTTCG
GGTGTTGTTGCTGCTTCTTCTGCCGGGGCTGTGGGGCATTGTGGCTCTTTGAGGAGTGATTGGGTGCC
GCGTGGGACACTTGCGCCCTCCCCTTCACCAA
CACACACACACACCGCTAGGAATAAATTTTGCTACGCAGAGAACAATTTCTTTTCATTTCTTAAAATT
GTTTTCTTATCTTGCTTGTTGCCTTGGGGCAG
CCACCATGGATTTTTTTTTGCTTTTATTGTTTCTTCTGCACTCTTCCTGCGAGGGGTGCCGTGTGTTT
GCCGCCCAACTGCTCCGCTCGCACACCCACCG
ACACGCTCATGACGACGGCCCTGTGCGCCTCGCCTGCTGCACGCATGAGGAGGGGCCGCGACGAACGG
CGCGCTGAGGGAGCCGTGACGTGTGGGCGTGC
GACGAGGGCCTGTGGGGCGGAGCACACATTTCTTCCCCTCCTGTTTTGTTTCCTTGCGGCCAGGGCGG
GCAGACACCCGCAGCGCCACACACACACACAG
CCACACCCTGCCGCTTTTTTGGTATATTGCTTTTTTGTGTGCATTTCTCGCGCTGGCAGTGCCGTCAG
TATTTTTCTTCCCCTGATGATTTTCCTTTTGC
TGCGCTCACTCTCCCGATGGAGTGACGCGACTCCCGTCGTGTCTGCGTGCTTCTCTCAGTCCCCACAC
ACACGAGTGACTGTGCCCTGCGGGCCGCACTC
ACGCACCAATAAAAGGGCTGAGGGGAGCAGCAACACGCAGATGACCGCATGAAATATAAACGATGTGC
GGACACGCAGAGGGTCGGACGGTTGAAAGACA
TTTAAATACCTTTTAATTTTCTTTTGCGGGCAAAATAAATGCAGACAGTTCGGTGTGGCCACACGGCA
GCTAAGCGAATTTATTACTATTATTATTACTT
TTTTCCTTTTATTTTTAAATTTGTGTGGAACAGTAACAGAGGACGTTTTTTTTTTAATGACGGGGTTT
ATATAAGAAGGGGTGGCTAGGGAAGGTTTTCT
TTTGCTTTGAATAAATTTTCTGTGGTTTGCCGCTGATCGGGCTGCAGTGCAATCCTGAGTTTGAGCAG
CCCCTCCATTCTTCTTTCTTTGGACATGCGCC
TCTGCTGTGCATTTGTGTTTGTGCGTCTCTTCAGATACTTTGTTTCTTTTTTCTGTCTTTTTTCCATT
TCGCGTGGCAGTGGGCACACACAACCACACGC
GCAGGACACACACATTGCAGTGCAGGCGGATTCTTCTTTTGTACTCGGTGCTGGCAGTTGCGCGTGGC
TGCTGCTCCCCCGTTGTGTTGGTGAGGGAGGA
GGCGCGTGACGGCGCCCGGTGGAGAGAGAAGGCGCTGACCGCTGCTGACGGTGACGGTCTTGGTTGTA
GGTAATCTGACGGGATGGCGTGACTGGTGTCA

CAGTTAACCGAGATGGATGAGGACCGCACCGCATCTCTTTTTCGTCTCATCATCCTCTCAGTCCACGG
CAGACACACACACGCAAAGTGCGGCGGCGGCG
CGACCACCGTCCGTACGGCTGCGAGCCAATCGGACTGTGCAGTTGATGTGGATCCTTTTTCCCCGTGT
CTGAGTGCTGCCGCCGTGCTCCTCCATTCTTC
TTTTTCTTCCTGTGTATGCCGTGCGCGTATGAGTTCGCTGTTTCTTGTGCAGTGCCGACTCATTGGTC
ACACCAGCGACGCCAGTGGTCATGTGTCTTTT
TGACAGGATCGTGCAACTTGGCACCGCGGCTGTGTGAGACTGCGCGTGCGGTGCGAGGGTGATGGTGC
TACTGAATTGATTGCGTCTGCGCGGTTTCCTG
TTGGCGTTTGTACTGTGTTTTGGATTTTTTGTGGCCGGATGCTTCCGCGTGCCACTGTGTTTGGTTAC
TGTGGTTGCCTCCTTTTATTCCATTTCTGACC
GCGCAGATAGAGATACTTTGGCTTTGCCAGCACAACTATCGTTTCAAGGTGTTAGGGATTACTCGCAG
TATTAGGATTAGAATTATTGGGATAACGATTT
GGCGGAATCGTGTATCTGGATTTGCCATCTGTAGAAAGGGATTTATTAGGCGGGAGGAATGGTCGACG
GGGCGAGAGCGACACACCACGACAGCCCTGCT
GATACGTGTCTGGGTTGGTGGATGTGGTGTGCGGTGGGTGCGTGTCCGCGCAGCCGGCACCGTGTGCG
GCACAGTGCCATTGCGGCTGTTTGTGTGGCGC
TGCTCGTCGTGTTGTCGCTGGTTGCGGCGGCGGCGTGGATGCCCGCGGTGCATGCGGTGGTGTTGCGA
CTGCGGGGCGGCACGGTGGACAGAGCCATCAC
GGTTGGCCACGCCGTGGACACGGTGCTGCTGGACGGCGTGTACATCACGAACGGCGTGGCTGTGGTGT
TCGACGTGCAGGTGATGCTTCCCGGCGCGCTG
CGCATTGAGTTGAGGAACTGCGTGTGCGACGGCGGTGCGCAGATCTACGTGCGGGGCTACAGCGGCGA
GCCGGCGAGTGACCGGAGCCTGGAGGTGAGCG
TGAGCGGGCTGTCCGGGAGCTACTGCTCGCTTGTGTTTGCGCACAACCTGCCGGCACACACGAACGTG
ACGGTCCGTGACTCGACGATTGTGGCGGCGGG
GCCGATGCGCTACTCGCAGCTGAGCGGACTGACGGACGCGGTGGCGTCGCCGCTTGTGCTGCACGCGA
CGTCGCTGTTGCAGTCGCAGCTGCGTGTGAGC
AACACTGTGCTGAGGTCGTTGCAGGCGGGCGGGTCGGCGGTGTACGTTGGCGGCGACGTGGACCTGCT
GTCGAGCGCGGTGGTGCTTGACGGCGTGTCGC
TGGAGGCGTCGGGCGGTCCGACCGCGTCCGCGATGCGTGTGGCGTCCTCGTCGATTCTCAGTCTGCGG
AGCCACTCGGTGTTCTCCGTGACGAGTGTGTC
GGTTGTGAGCAGCGGCGGCGGCATTGTGCTTGGAGAGCGTCTCGCGGTGCTTGATTCGGTGCTGCGCT
TCGTGCGCGTCGAGGGGTCTGTCGCGTCTTCG
CTGGTGCGCTGCGACGGTGGGACGATCGGTGCCGGCGGGTGGCTGGACCTGCACGGCGTGTGGGCGGT
GGGCGAGGCGTCGTCTGTGGCGTCGCTGTCGG
GGATGACGCTGAGCGGCGGTGCCGTGTCGATTGCGCGCTGCGCTGCCACTGGCGCGACGCTTGTCTCC
GTGCCGACGATCACGAGCGGCGTCGTGTCGGT
GCAGTGCAACCGTGCCGGCGGCCGGGTGCTGCAGAGCAGCGGCGACTACCGCATGGCCGGCCTGCCCT
CTGTGAGCGTGGTGCCGTGCGACGGCTGTGCG
GCCGCGCTGGCGTGCTTTGATGCGCTGACGGCGTCGTTCTCCGACTGCGTGTGCAGCTGCCGTGCCGG
CGGTGTGGGCGAGGCGTGTCTGCCGTTCGACG
TGCCGCCTGCGAGGGCCGGTGGCGGTGGTGGCGGTGCGCCGGGCTGCGTGAGTGGCGTGACGCTGACG
GAGTCGGTGACGGTTGGCGGCGGGCGTGCGAC
GACGTGCTTCGACTCGGTGGTGTTCGGCGGGCCGATCATCGTGGCGGTGGACCTGCGCTTGATGGACG
CGTTCGCTGACGCGCTGAACGTGACGCTGCGC
CACTGCGTGCTTGCGGGCGGCGCGCAGCTGCGGATTGGCGGCCTCAGCGAGAGCACGGCGCGCCTGAT
GCCGCACGCTTTCGTCAACATGACGAATGTGA
CGTCGCTGGAGGGCACGATCGTGCTGCATGGCGCGATGCCGCCGCACTCGAGTGTGCTGCTGGCGAAC
TCAACGCTGCGCGCGACGGTTGACGGGTCGCA
GTACGTGCCGACGACCCCTGGCTTCGCGGGATCCCGGTACGGCCCCGCGCTGGTCCTGGACGGCGTCC
GCCTTCTGTCGACGCGGTTTGTCATGACGCGG
TCGACGCTGGTGTGCGGCGGGGGTTCGTGCGCTGCGATCCTCGTGGAGCGCGGCCTNNNNNNNNNNNN
NNNNNNNNNNNNNNNNNNNNNNNNNNNNNNNN
NNNNNNNNNNNNNNNNNNNNNNNNNNNNNNNNNNNNNNNNNNNNNNNNNNNNNNNNNNNNNNNNNNNN
NNNNNNNNNNNNNNNNNNNNNNNNNNNNNNNN

NNNNNNNNNNNNNNNNNNNNNNNNNNNNNNNNNNNNNNNNNNNNNNNNNNNNNNNNNNNNNNNNNNNNNNNN
NNNNNNNNNNNNNNNNNNNNNNNNNNNNNNNNNN
NNNNNNNNNNNNNNNNNNNNNNNNNNNNNNNNNNNNNNNNNNNNNNNNNNNNNNNNNNNNNNtcgctcctgctc
gtcgtagtcCGGCTGTGAATGTGTGTGCTCTG
CTGGCGGCTACGGTAACACGTGTCTGCCAGCTGCGGTTCCGGACGGCCTTGGGCCGCTCCCGCTCCCC
GATGCGAGGGACACGGAGGTCCGCTGCGTGCA
CGGTGGCAGCATCGGCTCCGTGTACGTTCCTGATCCCGGTGTGCGTGGTCTGTGCCTTGTCAACGTGA
CCTTCACCGCCCCGATGTTGCTGGACCTGTCG
TATTTTGACGCGCCACAGCATACACTCAACATCACGCTGCTGCAGTGCGTCCTCATGGGCCTGTTGAT
CAGAGGGAGTGGTGCGCGCGTGCACGTGAGCG
TGACATCCTCGATGCTGGATTTTGGTGCATTGGAGTTTGAGGGCGATTTTGGCGTGAGCTCCCAGATA
CTGGTGGCAGGCAGTACGCTTGTGACGACTTC
GAGCCACGCCATTTTATTTTTAAAGGTTTTTCTTGGTGTGAACTCGACGCTGCTGCTGCTTGACAACA
ACTTGGAGGGGAGTAATTGCGCGTTATATATT
TCCAATGCTGCCGTTGATGGTGGCGGGGTCATTGTGAAGGGAAATACGCTGATCACAACGGAGGAGGG
TCAGGGTGTGGAATCATCTGTTTGCGTTAATG
CTATTGATGTGAGGAACGGCGGCTACTTTGACGTTGAGAACACCACGATGAGTGCTGCTAATGGTGTT
TATATTTTTTGGGATACCACCGTGAGCACCGC
GGGGCTGCTGCGAGTGGCGGACTGCACCTTTATTGGCAGCACGAAGGTTTTGACTTCTGCGCTGTCGT
ATTTGTCTGGCTCGGTGACCCTCGAGGGAGGT
GCGCAGTGGCGTGTGGAGGGCAACAGCGTGGGTGCAGCCTCGGTATTAAATATTCCTCATTTTCAGCA
CAAAATTCAGCTGTCGGGCAGCGGCACCACCG
TGGCGCTTGCACACAACCGTCGGGTGGACTCGAGTGTTTCCTTCGCCAAATTTCTCCCATCGAGCATT
GTCGTGAAGTTACCCGCGCGGTTTGTGGTTGG
GTGCAACCTGCAGGGCGATGGGGAGGTGTCGTACGACGATGTGTTTCCGGAGGGCGTGGAGGTGTTCA
GGTGTGGCACATGCAACGACGACGCGGCGTGC
TACATGCCCGGGACGGAGTCGGTGGACCGCGGCTCGTGCTCGTGCAGCTGCAAGGACGGATGGCATGG
CGCGTCGTGTCTTCCCTTCGAGGTGCCCGACA
CTGTTGTGCCGCCCTTGCCGGAGAGAGCCGTTGACGGCGACACGAGCTGCGTGGTGGACCAGACGCTG
ACGGACCTCGCGCTGAACATGTGGAAGACACA
CCACTGCTACGTGGGTGTGACATTCAGCGGCGTGGGTGCTGCGCTCACGTTTTTCCTCAATCGTATGC
CGCTGCATCTCCACATCAACATCACGCTCACT
GAGTGCACGTTCCGTGGGGGTGCCGCGCTGCAGTTTGTTGGTGGTGCTTCTGCGGCCGATTCAGCCGG
CGTCCTGATCCGCGTGAGCCGGACGGTGATGC
GGTCCTCCGCCGTTGCTTTCATACGTGCCCTCCCGCAGCACTGCGACATTGCCGTCACGGAGGTTGAC
GCGGTGCAGTCCTCCGAGGTCCAGCTGCTTGA
CANNNNNNNNNNNNNNNNNNNNNNNNNNNNNNNNNNNNNNNNNNNNNNNNNNNNNNNNNNNNNNNNNNNNNN
NNNNNNNNNNNNNNNNNNNNNNNNNNNNNNNNNN
NNNNNNNNNNNNNNNNNNNNNNNNNNNNNNNNNNNNNNNNNNNNNNNNNNNNNNNNNNNNNNNNNNNNNNNN
NNNNNNNNNNNNNNNNNNNNNNNNNNNNNNNNNN
NNNNNNNNNNNNNNNNNNNNNNNNNNNNNNNNNNNNNNNNNNNNNNNNNNNNNNNNNNNNNNNNNNNNNNNN
NNNNNNNNNNNNNNNNNNNNNNNNNNNNNNNNNN
NNGTTTTACGTGAATATCCTCAGTGTGAGTGACCACTCCGTTTTTGCGCTGCTCAACAATACAGTGTC
CTCCGGGACAAGCCTTCTTTATCTGCGCCACG
GATTCAGCGTGTCGGATCACAGCGTGCTGCGCGTGGTGGGGAACAGCGGCTCCGTGTCCTATGCCATA
TATTCACTAAGCTTTTTTACCGTCCACCATTC
AAGCTGGCTGGATTGGCGTGACAACGACGTGGGTGTGGGTACGATGTTTCATTACTCCCTAATAACTA
CTGTGAACGTCGACGGCAGCAGTGTGGTGACG
CTGACGGGCTGCACGATGGGCTCGACGGGGCTGTCGGTACCTCTGCTATCGCAGGCTGCCGCCGGCTA
CCGGTTCGTTGCTGGGTGCCTGACGGTCGCGG
GACGGGAGGTGACGACGGCGGCTGAACTGGCGCTGAACGGCATCACCAACGTGACGACGGTTGCTGCG
TGCGGGGAGTGCACGAAGGAGGGCGACTGCTT
TGCTCCGCTGACGACGGCGGTCATTGACTGCAATTGCCGGTGCGCTGCTGGAGGGCACGGCGATGTGT
GTGTCCCGGCGCCTGTGCCTGCTGGCCCGCCA

CCGCCGCCACTGCGGCCACCACCCACGCCGTCACCGCCGCCGGTTGGTGAGTGCATCAGCGACATGGT
GTACCCCGAGGTTGCGCAGGCAGTGGGTGGCG
GGCTGTCGTGGCTGTGCTACCGTAACGTGACGTTCAGCGGGGGCGGCATGAGCCTGAAGGTGCTGGTT
GGGGCGATGACGGGTGACGTGGCGAATGTCAC
GTTTGATGAATGCACGTGGAGGGACGGCGCCGTGCTGTTGCTGTTGGGCAACGCGTACGCCGCTGTTG
GGTCGCTGAACATCTTCGTCACCGGCAACAGA
TTTAGTGACGCGCTGCTCAGCCCGGAGGGTGTGTTTCCGCCGCACACGAACATCACGATCAGCGGGAA
CCGCTTTACACTGACGTGGCTGGTCCCTCGGT
CGGGTTTGGACATTAGGAGGCCGTCGTGTGTTGCGATGAATGGACTGGTGATCAGCAACGACTCCGCG
GTGGTGCTGAGTGGCAATGTGTTTCAGAGCGT
GACGACATCGTCAAGCGCCATCCGCGTTTCCAGATCTGCGCTGAGGGTGTCGTGGCACTCCGTGTTTG
CGGTGGTGGGCAACACGTTTCACATGGCTGGC
GGTGACGGTACGCTGATACATCTTGAAGGACCTAGATACTCCTCATCGCTGAGTGTACTGAACAACTC
TGCCGTGGTGATTCGAGGCAACCTTGTGACGA
GGCCGATGAAGTGCTTCGTGTTATTTATTCGGACCTTAGACGTGGAGTCTTTTTCAGCGGTTGTGTTT
CAGGGCAACGACATGCAGCGAAGCTCGGTTGT
GTTTATCTCAGGCGACTCCTCCAACATCTACTACAACTCCTGGCTGCAGTTGAGCGGCAACCTCTGCC
GTGAATCCCCATCGGAAGCGTTTGCCTTTCTT
TATCCCAAAGTGAATCTCCGCGACTCCACGGTGTCTGTGTCCGGCAACCAATTCGTGTCCAGCTCGGT
CACGCCGACAGTGCTGCGGATACCTCAAAGTT
CCCGCGATCTCACGAACGGAGCGATTGTGGCCGCGTGCAACACCGTGAACGGCGAGGAGGGGGCGTAC
TACGCCATCCCGTCCGTGTACAATGCCACCAT
TATGACCTGCAGCGACCCATGCACTCTCGCGGCGTCGTGCTTCCCTGCGTACACGACGACGGTCTCGA
GTGAAGGCTCTGCCTGCAGGTGCGCGGAGGGC
GGCCATGGCGATGCGTGCCTGCCCGTTGCTGTCCCCGAGCCACCGGGCACCGACGGCACCGACTTGTG
CGTGCGGGACGTGCGTGTGGAGGTGGAGGTGA
ACGTCAGTTTTGGGACTTCGGTGGCATGCTACGTTGGCGTGACCTTTGCGGCGGACGTCGTGGTGGAC
GTGGAGTCGATGTCGGGGAGCGTGCGCAACGT
GACGCTGGTGAACTGCCTGCTTGTGGGCGGAGCGAGCCTGTACGTTGTTGGGTGGCGGTCCGACCCGC
CTGCTGGCGAGCGTGCTGATGTGTTGATCAGC
GGGCTTGAGTCGTGCTCCGGCGGCGGCGTGCTGGTGGCGAACCGATTTCCGCCGGGCTCGCGCGTCAC
TGTGGTGGACTCGGTGCTGATTGCAGAGGCGC
GTGTTGCGTACCGCGGCGCCTACGGCCTTGGCGACGCCTCCGCGTGCCTTGTGTTGCACAACGTGAAT
CTGACCGGGAGCGTGCTGACCATTGCGCGCAC
GCATGTGGCGGCTGTGTTCCGTGACGCTGTTGGCGTGTTGTTTGTTGGCGGCGTGGCGCTGTCGTCGC
GCGGAGCGCTGTACGTGGACGAGCTGTTGGTG
CAGACGGCGCTGGGGCTGTGCGTGTCGGTGGAGGGCGGTGTCGCGGCCAGCGGCGGCTCCGTGGTGGC
GTTTGTTGACAGCGACTTCCTGCTGTGCAAGC
ACGCGGTGTCTGTGCGTGGGGCTGTGAGCGTGTCGGGCTCCGCTGTGGCGTTTGTGCGGAGCGAATTT
TTGTCGACGGAGGACTACGCGGTGGCGTTTTA
TTCGACGGTGAGCCTGGATGGCGGGTCGATGCTGCTGGCGAAGGGCAACGTGCACGACGGCGCCTCGA
GGGAGATGCTCTATGCCGCTGGCGCTGTGACC
GCTGCTGGGAGCACGCTGAGCTTTGTGCGCAACCGAGCGCTGCTGCCGCGGATGCTGTCGCTGAGCCT
GTCGCTTGCGGCTGGGGCGCATGTGAGGGTGG
CGTGCAACGACGCTGGTGACCGTGTCCTGTCGACGGCGGAGGAGTACGCTGCGGCTGGTTTTGGCGAC
GCTGGGAGCATCGACGTTGCTGGGTGCGACGC
CTGCGACAGGGACACGCACTGCTACGCGCCCGGGACGGCGTCGGCGAGCATGAGGAACGGTGTGTGCG
CGTGCTTGTGCGGCAGCGGCGGGCACGGCGAG
GCGTGCGTGCCTGTGGGAGCTCCCGCGCTGCCGCCCGCTGTGGGCACTGCGTCGAGTGTGTTCGTCCG
CGAGGGCGTGACGGTGCGGTCTGTGTTCGTTG
TGCCTGCCGGCGCGAGCGAGGTGACGCTGCGCCACGTTGTGCTGGACGGCGTGAGTCCTGTGCTCTAC
GTGCCGTGGATGGCGCGGGACGGCGTGCGGAT
TGTTGTGCAGAACGTGTCGCTGCTGAACGGCGCCCGTGCTGTACGTGATGGGCGGTGGAGCGTTGCGCG
GTGCGGTGTCGGCGGGGAGCGACGAGGGCGGG

CCGGTGGAGCTGTCCGTGTGCAATGTGGAGGCGCTGAACGGTGCGCTTGTGCTGACCGGCACGTTCCC
CGCGGGGTCTGTGCTGACGGTGACGGACTCCC
TGCTGGTTGCCGCGCGGCCGACGCCGCTTGTGTATCTTCCCGGCTCGCAGTCCTCGCCGTACGCACCG
GTGCTGGTGCTGTCGGGCCTGCGTCTCGTGCG
GTCCGTGCTGATTGTGTCCGGCGTGGCCCTTGTGACCGTTATGACTGGTGGGCGGACGGTGGTGGTGG
ACGGCGCCGTGCTGGAGCTTGTCGGTGGCGGT
GTTGCACTGGACGCGGCTGTGTTCGGTGGCGAATATGCGTTGTACGCGAGTGCGCGCGTGGTTGCGTC
GTGGGGCGCTGTGCTGCGCGTGTCGGGGAGCC
AGGTGTACGCCGCGCATGGGTTGGTGTTTGGCAGCGGGGTGGAGGCCAACGCGTCCGCCGTCGTAGTG
AACGACAACGCCGGTGCGCTGACCGATGGCGC
GCTGCTGGTGCTGCGGGGGTCGGCGTCGTTTGCGTCCGGGTCGTGGCTGTCGGTGCGCGGCAACAGCA
TCAGCGGCAGGCTCCTGTCGGTGCCGCCGTAC
CCACGCCGTGCGGAGCTTGTGCAGAGCACACTGACGCTCCACGGGAACGCCGGCAGTGGGCCCGTCGT
GATGGACGGCACCGTAGCTCTTGGGGGCGCCG
GCCGAAAGTTCGTCGTGGGGTGCCTGACGCTCAACGGGCAGGCGCTGCAGCCGATGGACTACAGGTCC
GCCGGCATCATCGGGGAATTCCGTCCCGTGGC
GTGTGGCGTGTGCGACGCCGACGTGCGCTGCTTTGCTGCTGCGACGAGGACGATGTCGGGGTCGTGCG
GCTGCCGCTGCGCTGATGGCGGGTACGGGCGC
GACTGCCTGCCGGTGTACCTACCGCACGTGGACGGGTGCAACCGCACGCCTGCCCCGCCGCCGCTGAG
CCACACGGCGACGCTGACGGAGACGCGCAGCC
CGACAACAACGTGGACGGCGACGCCGACACCGAGCCTGAGCACGGCGCACTACAGTCCGCCGTGGTAC
GGTCCGACGGAGACGCTGCAGGTGACGGAGAC
GGTGGCACTGCCGCCCACGCGGACCCCGACGGCGTCGGTGAGCAGCACGCTGTGGTGGAGCGACGTGG
CGTGCCCGACGCTTGCCGTGACGACGACGTCG
GCTGGTGGGAGCCTGACGCAGAACGACATCCGCGGCGGTGGGAGCGCCGTCCCGACGCGGCTGATGGT
GGCGCTGCCGCCGCCGTTTCGGTGGGCACGCG
ACCCGCAGCTCGGCACGCACCTGAGCTTTGTGCCGGTGTCGACGGCGCAGCCGAGCGGGTTTGGTGGT
CCGTGGGGAGCGATGCTGAGCAACGCGACGTG
GGTGCGCAACGCGACGAATCCGTCCACCGTCCTGGAGCTGGCTGTGCCCGTTCACCGCGGTTACTTCA
TTGCTGCCGACGAGACGATAGTCATTCGCTGC
GACGCTGCGGCGGTGTCTGGCGGCTGCAAGGGTGTGCTGCTTGGGTCCTTCACGATCAGGTCTGACAC
GCTGCCCGCCGCTGCCAGCGCCCTCTCGGCGA
TCACCGGCGTTGTTGCGTGTGCGACGGCTGTTGCGGTGGTGGTGACCGGCGGGCTGGGCTCCGTCCTG
GAAATGCAGGCACTCGGCGTGTTCGCGAGGAT
GTCATGCGCCTCGGCGCAGGAGCGTGCGAGCACCGTTGCGCTGCCGTACTTCCTGTCGGTGTTCGCTG
CCCTTGACCCGCTGTGGATGGTGGTGGGCAAC
GCGCTGCTTGCCGCTGCGTTCGGGTGCGTGCACTGCGGTGTGACGGCGGCCTTCCAGCGGTGGCGCGG
CGTGGACGCGGCGAGTGCGTGGGCGGCGATGC
GCTTCCCGAGCCTGACGTACGTTGTGGCGCACGCGATGCACCTCGGCATTTTCTTCGGCTCGGTGCTT
GCACTGGCGATGCCCGGCGCCCGCGTGCTGCA
CCGCGTGATCGGTGTCGTTGGCGTGCTGTACGGTGTGGTGTTCCCTGCTGGCGTGTGCTACTTGATTG
CCCGACACGCGGGCGCGAGCTTCACGAGGTAC
TGGCAGTTTTCGAGGAAGCCGCTGCACGAGCGCCTGCTGTACCCCGTCGGGTATTGGCACCCCGCGGC
GCAGCAGCGGATGTACGGCGGCATGCTGACGA
ACGTGAGGGGCAGCCGCGTGTACTGGTGCGTGTTCCAGCTGTCGGTGCTGTGCGTGGTGGGCCTGATT
GCGGCTGTGCACCCGCCCGTGGGAGGCTGCCA
CGTCCAGTACTTCTGCATGGCGGCTGTGCTGCCTGCGGGCGCGGGCGTGGTTGCCTTCACGAACATGA
TGCGCTCGTCCTTCCTGACGGTGATGCACGCG
GCGAGCTTTGTGCTCCTTGCGGCGCTGTGCGTGGTCAGTGCGGCCAACCACCTTGCGCCGAGCGACGG
CGGTGCGAGGGCGTACGCGGCTATCGTGCTGC
TGCTGGCGACTGTGGTGCTTGCGGTCACTGTGTACAGCATGGTTGTGTGGTACGCGGAGGACCGCCAC
TGGCAGGAGCTGCGCGAGCCGCGGCGTGGCGG
ACTGGAGGCGCTGCTGCGCGATGGCGAGGAGAGCGACGAGGAGACGCAGAAGCCCCACGAAGTAACTA
CATTTTCGTACGTCTCAGGCGCCACCGCTGCG

TCGTCGTACCGACCACCCGCACCGCCGTAGCAGCCCATGGCCGGTGACACCCGCTCAGACGCCCTGAG
CCTGCTCGAGTGTGCATCCAGTGCGAGAGGCA
AAATTGGTTGTGCTACGCCGGAGAAGTAATGAATTTGTGCTGCTCAAGAATGTGGGCGCAGTTTTACC
TCGAGTTGGAAAGTGTCTGCCCAAACAGTACC
TCCACTTGGCGAGAGTGTACGAGGCTCCTCATGGATGCGGTATAACGTAGGTGCTTTTGGCAATGTGT
TTACTTCTTTATTATTATTATTTATCGGATGC
TTGGTTGATTGAGTCGTTGCTTTCGTTATTTTATTTTTATCGATTTTGTCAGGAACGGATTATCGAAA
TATCGCGCTAGCCCAGGGCGGCTGTGTCACGG
GTGTGTGCTGCACGCTCGCCGCTGCAGCGCCCTCTCCGGATTTCGGGGTTATTATTTAGATTTTTACT
CATTAACGAGGACGTGTTCTGCGATGGTGGCT
GTTGTGCGGAGCATTGAATTTATTTGAGGAAAACTTAGGGACAAAAAACGATAACGCCCTTCTGTGGC
TTTCAATAAATGAAATTTGGACGTGTGCGTTG
CTGGTCGATGAGGGAGGGCGTGGGGCCACTGCTGCTGCGCACCACAGCCACCCATCGGACTCCGCAAC
GGCATTTTCGCTGTTTTTCTGTGCGTTGTTTC
TATTTCTGTGCGGGTGCCTGCAGTGACTCTCTCCTGCTCCCCGATGACTGTGGGGGGGGGGGGGGGGG
GGGGCGTGCCTGTTTTTCTCGTTGGTTATGAT
GTTGTATGAAATGCGGAAAAAGAAAATGAGCGGAAAAGTATGCAGGCTGATGTGTTTTTTTAGGAGCT
TGTGAAAAGAATGATCGCGGGAGAGCTGGCTA
ACATAATTGATGTATGTGTTTATCCTGATAAATGAATGCATTCTTTATGATACTCTCTACCGTATGAA
TCTTTTGGGAAGAACGCGACTTTGTAGGGGCA
GGGAGCCGATAGAGGCCGGATAAATTTTTAATTTTATTTTGCCACCCCACCCATCCCCTTTTGGCTCC
CACCACGCGGCGCGGTCTGGTGGTTGGAGGAC
CCCAAAGTCTGCCACTTCGTAAGTAATAATATTTCAAATCCCAACTGAGGACGAAGGACCATGCTAGT
GGTCCACAGAATTCTATATATATTGAATGAAA
ATAAAACATTGAAGCAATTAGCTAAAGAACCTTTTTTCCTAACTTGTTTGGTTTCCATAAATAATTTC
AGGATCCGGCCAGCTGCCCGGAAGATTATTAT
GTATCGATATGTTTTTTTTGGATTACTCTGGTGGTTCCTCCTGTCTCCTTTTGCGTTGCTCCACACGT
GTCTCAGTGTTTGCTTTAGGACGGCCACACAC
GGGCAGCTTGAGCCGCGGATATCTTTTTTCTTTTTGACGCCATTTCGTGTTAGGCACCAACGCGGTCA
CGTTCGGCAAGCATCCACTCTCCACAGCGTGG
TGCCTGATATTTTGGAGTCATCTCAGATGTAACGCCGGCACCACCGCCGTGGCAGTCATGTGAGGTAA
GATGGAACGCGGGCAGCGAAGGATATTGTGCT
GCACATGTGTCTGTTGCATTTGTTCTGTCGTGGTGGCTTCAATGTAGAAGGAGAGGGATGAACATTGC
AAGATTCCGTGGCTTGGAGGAGTTATTACCCA
CTTTTTGTATGATCTTTGGTTCCCGCCCGTGTCTGCCGCGATTGCTTCCCCTGAGTGGAGCTGCGCCA
TGGCCATCCTTTTTTGTTGTTGTTGTCCTTTT
ATCTCAGGGCACGGCGGGATTGAGGAGCACTGCCCCACGAAAGGCATTGCGCACATACACACTCTCAT
GCCGCTGTCGCCCCGAGGTCGGGTGCAAATCC
AATTTTTGCTGCTTTTGCATTGGCGCAATTATTTTTATTTATTATTGGCTGACGCCTTCCTGTCCAAT
GTGCCACTTTGATTGGATTGTGTGGGACTGCT
CGCCAACACCATCGAACGAAGCAGGCCGACGACTTCCCGTATTTAAATGCCATCTTGCACGCACGTAT
TTTTTTACTTGTTCAGTTGACGTGCACTCAGG
CAGCGAGGGATTTGGAACTATGCGTGCGTGGAACTTGTCCTGCCATGTGGACTGCGGGCAACACCGAT
GGGCTGCCACTTTGACTCCGCAGAGTTGTTGT
GTCTCTTCGCAGGGCTGGCAGGGAAATTCCATGCAGCCAGCTACTGCTGGTGTGTCCCCAAATGTTGT
CACTCCATGCGAATAATGTGCTTCAGCGAGTG
AGGGGGGTTGATTGGGTTTGTGTGCACTCTGATTGTGTTTGCTGCCTTGTGCGGGCACGCAACACCTG
AGGGGTGGGAAAATGGAGCGGGGCGATGCATG
GCTGCGTTCCGCCCATCGTTCACTGCACGGTGGTGCGGTGCGCATTGTGGTGTTGACTCTGCGATGTG
ACTGCGGTCTCCTGCGCGGCAATGGCGGTGGG
TGGAGGAGAGTGTTGGCCCGCGGCGTGACGGTGGGATCGTGCCTCCTTTTTATTCTTTTGTGAAGGTG
CACTTATTTTTAGTGTCAGGGATGGGGCAAAC
AGGCGGACAACGTGAATAAAACAAAACAGAAAAGGGCAATAATGTGGCGGTGTTGTTGCCAATTGCAC
GTTGTGTTGCTGCGGAGGCGGTGGGCCTTGAC

TGTTTTGCCGACAGGCGTTGCAGTGCGGCCGCATGACGCACCGACCGCCCCGCCATGCGAGTGCCACG
CCCAACGCCATTGGGACTGCGGGACGAGACAG
CCGCGAGCGTCCTTTGGAGCCAGCGGCACCTGTGTTGGCTCCGATGCTGCAGCTCAACATTTGCTGGG
GAGTAAAGAGTGACCGCGTTGGACTCTTGACG
GTCTGGTGGAAGATCTAATGCTGGACGGAGATACTCTGAGCACCAGCTTTAGGCTTAAATTTTTTTTT
TTTTGAATTGTATTGGCTCCAGGGCGACCGTT
GATGAAAGACCACAATGCGACGGGGGAGATGTTTATTCGGAGACTCGATGCTTACGTAAAAGTCCGGC
AGATTCTTGAGGGAATTCTTAATTTGACAGAC
TACCAGGTGTACAAACTTCTTCGCGAGGGTGTGTTCTTTCCTCGTCAATGGAGGGACTATGGGAGGAA
GGACACGATCAATTTCCGGGCAATGCAAAAAC
TTGATTCAGCTCTGGAAATGCCAGACGGATACTCGGAGCAACAACAAAAAAATCCACCCTTGTAACTA
CGATTGAAGATGTACTGTCTAAAAGAAAAGTT
CGTGTCATGGACCGAAAGCTGAATGAGCTTCTTAATAGGAGATTTGATGGCAGGGGCTTTGGACGCCA
ATCGGGATGTCTTGCAGGAGGGGTTTTTAAGG
ACCCCGAGAAGTATATTTGCGATGCGGGAGTATTGGGCGAAATGCAGGCATCCGACCGTTATGTGATG
ATGGAGAGGGCTGCAAGGGATGAAATGGACAT
GGCAGAGGATGTACACAAGCTTTACGAGCATTGCATGGACAAT
>scaffold1010.size6656
CGTCGTGTCGGCACCCCTCACGGAGAGTACGCACACACACAAGAAGGTGCAGCGCACACAGCAACACA
ACAGCAAAGTAACAATAAAAAAGGAGATCCCCG
CACACCACGCGGATTGTTGGTCCACGGGAATTGCAGCCAATATTCACCACAAGCAGTTCAGTTGCCGC
ATGCCTTCGTCCACATTCATCGCACTCAATTT
CATGCAGGCCGGACACCACCGCAACAGGAGCTGCTGCACACGCAACAAGAAACAAGAAAGGGAGGTGG
TGTGGGAGACTACGGTGCTGCTGTCACTGTCG
CCATGCGAAGCTGTGTTTATGTTGTTAGTGGCGGTGGCGGTGTCCCTCTTGAACCCCTCGCTGTGGGC
TGCGGGGTAACTGTGCCGGCTGATGTGCCTTC
GGTTTTGTTGTTGCATTCTTCTTCTCCACTTCTTCTGTGGAGGGTCCGGCGTCCTCTCCGTTCAGGTG
GCTCGCATTATGATTGGTCGTGGTGGTGCTGG
CGGCGGCGTTAAACTCTGCAGCTGGAAGAAATGCGGTGCAGTGGTCAGCCTCGGCAGCAGGCGCTTTG
TCCTCACGCCGGTACGTTTCTGTTTTCTCTGC
AGCGACAGAAAGTGGAGGCTGTTTGAAAATCTCCTGCACGGTGCATTCCTCTTTCCAATCTAATGCAG
CAAAGCCGCCATTTGAAAAAGACGATGACTGC
GATGACCTCTCGTGAATGGGAGATGGTGCATGCTGCACGGTGTCTTGGTGTGCTGCAACTTCCTTCCT
TTCTGCCTCATCTCCACCAGCTGCTTCTACTG
CAACTTCTCCTCCCGATTCTTCTTGTGGTGCATCACCCGGTACTTGTGCAAAAGTTGTGTTTGAACCC
GCATCGTGTTTTGGCGCTACCTTCGCCATTCT
CTGGCCATGAAACTTCATGTCGGCTGTCAGACCCTTCTGCCCCTTGCGGCGGTTCCTGCCCCTGCTGT
TTGTCTTATTTGTTCGCCCGGTACTGCGGCTG
GTTTTGTTGGTAGTGATCCTTTTCGCCCCCCAATGGCAGGCTGTGATGGGGGCTGGGCTCCTCCCTCT
GTCCCACCGCCCCCGATGACTCAGCGACTTTC
AACTTTTCATCCTGAGCGGATTCGTTTTCCCGACTGCCGGTGGCGCGACTGCCATCCGAAGAGGGCAA
TGCTCGCTCCCTTTGGGGCACGAGTTCCAGGC
GCCCCACAGTGACAGGAGCCATCAGCATCTGAGCCTTGCCTTCTGAACCATTCACAGTTGACTCCTCC
CGCTGTCCAACACCACTCAATGCGTCGTGTGA
CTCCGACACATTCGTTTTCATTTCGGGCGGCAAATAACGCCATCCCGCTGATCCTCCACATACTGCCG
GTGCCGCTGGTGGCGCGCCGCCTTGTCTTCTC
CTCGTTCTAACGGCAGGCGATCTACGCGGCTGGTGACGCATCTCGTTGAGTGTTCATTGGATTGCAGC
ATACCCCCTTATCCTCAGCGGTGTCAGCAGCA
ACGGCAATCCCTTGGCGCCTCCTAACGACGACGGCCATCCAATTACGCACGCATTCCTCCACGGCGGA
CGCATTCGCCCTGCCTCCTGTCCTTCTGCTGC
CATCCATCGCGCATTCCCTCCGCAGCTGAGCACGCCGCCGCAAGAGCTAATACTCACCCGTACCGCTA
TCGCCACCACAGCACGCAGAGGGCACACACCA
GAAGCACACGGCCAGTCATCATCGCCACCACTCCTGTCACTTGCCTGCAGCCTTCAGCAAAAGTAACA
CCACGTCAACAGAGAAGGAAAGCAAAAAGATA

GACACAGCCGCTACACCACGCACAGCCACCGCACAGCAACAAACGACAGCAACAAACGCCAGCAGCCA
AGCAACGCACACACCACGAGACAGATGGAATC
AGTACGATTGCAGAGCTCGCACACCGCCCCGCAAACCACAAGTTTAAAAAGCAATAAAAGCAATAATA
AAAAACAGAAATTAAAAAAGAAAAACAAACCC
AGGGCTCCAGTGCGCATGCATACGAGTGTGTCCGGGATGCGTTCAACGTGACGGATGCGGGGCACAAC
GCGGAGCACACAGCACGGCAGCAGAAGCAAGT
GAAGCAAAACAAGATCATTGTTTTCTCTTATTTTATACAGTGAACCACAACACACACGCACAAATCCC
CGCGGTTGAGTGCTGGCACGCACAGCCCGCAC
ACACCCCTTCAGCCCACAGTGGTGTACGCCAGCGCGGATGCGGCGAGCACCAGCGGGGCACACATCCA
CGCAGAGCTGCTGAGGCTGCCGTCGATTTCGC
GAAGACGTGACGGTGCGCGGGTGGTCGACACAGCTGGCGCCTCTGTAGTCGTAGTACTTGGTGCCTCT
GTAGTGGTGGTCGTCGTGGTCGTCGCGGCAGC
ATCTTTGGTTTCTCGATCTGTTTGTTCTGCGGATTTAGTCGTGCGGCTTTGTGGCCCTGTCGTACTCC
CATCTTCTTCGTGTACAGTATCGATAACATCG
CCTGACTGCCGTTTCGGAGCTGTCAAACTTTTTCCACCACCTACGTCAGCCTCTCCCTGTTCCACAGT
GGACCCCTTACTTTGCGACTTTGGCGCTGTTG
TATCATTTTCATCTTCTGTCGTACCTTTCTTTTCTCCTTCCTGTACCGGCATTTCCGCCTCATTTGAA
TGCGTTACGGCACCGGAAGAACCTGGGGTCAC
TGGCACTTTTGTTTTCCCTTCCTCAATGGACCCCGTACCTCAACCCCGTTCCTCATCGGAATTGTCCT
CCACTTACGACTCAGAGAACAAAGTACTCGAG
CTGGTGGGCTCCTTTACATCATCAACCTCACGAACAACAACGGTCTCCTCCGTAGAATGTGCCACGCA
CACGGACGGGCAGCAGCACAGGGCAAGCACCA
ACAGGGCGCACAGCAGACGGCACGTCATCATCGTGAGTGTGGTCGTTCAGTGGTGTGGAGCTGATCTG
CACAGTGAGGGACATGGAGAGCAACAGGGGAG
ACGAGAGGTGCGGCAGACACACACAGAGGGCACACCACATGAGGAGAGGGCAGCAGCCAGGCATGCAC
CGTACGCCGCACACCACGCACGACACACACTC
CAATCCACGGCAGCAGGCACACACACACACAGCCCTGCCGCAACACCAACATACAGATGGAAGAGGGA
GAAGCAAACAGGAGCGGCACACAAGGCTCCAC
CGCATGTACATACGGCGAGAGCGGTCCGTCGTGTCGGCACCCCTCACGGAGGGTGCGCACACACACAA
GAAGGTGCAGCGCACACAGCAACACAAAAGCA
TAGTAACAATAAAAAAGGAGTTCCCCGCACACCACGCAAGTTGTTGGTCCACGGGAGGTGCAGCCAAT
ATTCATCACAAAGCAACACTTCAGTTGCCTTT
GGTCTTCGTCCACATTCATCTCACTCAATTTCATGCAGGCCCGGCCACCACCTCAACAGCCGCACACG
TAAATATAAGCGCAAGGGATGGAATAATGCAA
GCATTCATTTATTTGTGTTTCTGTTTGGATGTTGGCAGATGTCTGCATATGTTGTTTTGTTTTTGCAG
ACGGCAAACCTTCATGCCTGGATCGTGTGTTG
GGGATTTCCGCAGGGTTAAAAATTATTTTCCTCTTGTGAATGTTTCTCGTGTTGGTGCAGCCAATAAT
TCGGTTTTTGTTTTGTTCCTGCTTCCCTTTGT
TACAGTGCTGCAATAAAGAATAATCTTCCGGGCCGCTGGCCGAATCCTGAGTTTATCAATAATAGAAA
CCAAAAACGATAGAAATAAGTGAGGGATCAAC
GGAATAAGAGTGGATTCACCACACCCTAAAACACCAGGCAAGTGTATGCTTATATATACGTATACATT
CTTTAGCCACAATCGTGGCCCGCTCACTCTAT
TCGGATAGGATGCATATATTTATTTGGTACTTACGACGTGGCACACTTTTGGGATCCTGCAGCCACAA
GACCCCGCCGCGTGGTGGGAACCAAAAGGGGG
TGCGTGCGATGGTAAAAATAAAAATAAAGAATTATCCGGCCGCTGTCGGCTCCCTAAAGTCGTGGCCT
TCCCAAAACTCATACGGTAGGAAAGTGTCGTA
AAGAAGTGTACATTCGTTTTATCAGGGTATATATATATATACATTCATCAAGGCAGCCAGCTCTCCCG
CGATCATTCTCTGCACAGGCTCCTAAAAGGGC
TCAAACACACCCACAGGACTCCCAGCAAGAAAAAAGGAAACACGGCGTGACTGACACGACTCAAAGTA
ATGCTTCTAAAACAAAGTGTGGATGATTCCGC
CGACGCCACACCCGCAACACATGCCATCAACTCCTCCACGGCAATAATCCTGCAGCGTGCGGAGAGGA
AGTTTCACGAGCGCATTATTGGCGGAGCCAAG
CAACTGTACCGTCAGCAGAGGGCAGCGGGCGGCGAGGGAAGTGAAGGTGGTGGGCCTGTTGGGGAGGG
TCGATGCGCAGTGTCCCTCACAAATACGAGGC

GGATGAAGTGAAACACTCGCCTGAACGTCACCGGCCACCAGATGCATGGCTCCCTGCCGTGAATATTG
ATGAGCTCGAGGCTGGAGTAACTACTCGCAGT
GCGTGAAAGCGTCTTCCTGCACTGACGCTGCAATTCACATTAGACGGGCAACTTCAGCACAGTCGATC
CCCAGCAGTAAAATGCAGCTGGCAGTGACGCT
GTGGATTCTCGATTCCCCTGGCAACGGAGGGCGGAGAAGACAACTGGTCTCCCCAGCTGCGCGGCATC
CATCCATTCACTCTGTGCGTCCGGAGCGCCCC
GCAGCTGCCTTGAGAGATGCAGCGGACAGACCTCCCTTATTTTCGCCTCGATCCGTCGACGAAGACTT
CCGCGGTGCTGTTGGCACACACCGAAGTCTCC
CTCGTGCCCACTAAACAAAAGACACACACGTTGAGCATCGTCACGCGCGTCGACGGCCACGCAAATAA
ATACCCACCGCAACGCAAAGTGTTTCTTATTG
GGCACGAAAAGAAAACTGGAGGCGGCGGCCCGGAAAACACACATCAAATCATCCGCCAGGCAACGCTG
AAATACATGCGAAAGAGCCAACGCGGGACCAG
CAGCACAACCACTCGAGAGCTGCTGCGTGAACTCCACGGTAAATGGATCAGCCTCAATGCACGGGGCA
CTAATTACGGCGGGTGACAGCGCGAAGGCCAA
CATTTCCGCAAGGCCGGGAATTGTGGGGACGGCATCCAAACCCTCCCGTTACTCGTGTGCCAAGACAC
GCGAATAATCCGCCCCTCGGTGCAAGTCGTCA
CCATAATCCTCATCGCTCTCTTTGCCGTCATTCGTATTGTCACACTCAGAGCCGTTGAGGGAAGGTGC
TGCCATGATGATTGTGACATCACACTAAAAAA
AAGAACACAATGCCTCAAAGGGTGCCGAGTGCAGGCATCCCGTCAGGCGGCGACACGGCGCACTCCTA
GCAATGTACGAAGCAAAGGCAACAAAACGGCT
TTCACCCGCCATCGTAATCTCGTCCAGCGGGTTCGGCCTCTTCTACTCCTTCGGCGCCTCGTCCTTCT
CCGCAGCACCAGCTGCCGCTGCCGTAGATCAC
GCTCCTGCCGCAATCACTCTCACAGAGGGACAAGGAAGAGTGTGTGGATGTGTGCCGAGAGCACAGCA
ACTGTTGTCGCCGGAAAAGGCCATCGTGGCAC
CGTCCGTCACGCGTCAGCACAAAAACGGCAACGACCGCAACCAGCAGAGCAAAAACAAAAAGCTGATA
AAAGAATGACCCCAAGAGCAGGCAACAACTTC
ATGGCGGGCATCGTGCAGCCGCGTCATCATTTCAGCACGGAACGTCAGCCAACGTCCCTCGTTACCAA
AAACATTCCTGCAGAGCACTGTGGAAATACTC
ATTTGCGTGTGTGCTGGAGAAGCTGCAAAGAGAAGCACAGGGAGGCACATCAACACAGCAAGAGGCAC
AGCTGGATGGCAGCAATACATGCGGCACGGCA
CAACCTCAACAATCACGCACGGTGGCAGAACACGCCCATCCAGAGAAAAGAAAAGAAAAACACGGCGG
CAGGGTGTGGCTGTGTGTGTGTGTGGCGCTGC
GGGTGTCTGCCCGCCCTGGCCGCAAAGAAAACAAAACAAGAGGGAAGAAATGTGTGCTCCGCCCCACA
GGCCCTCGTCGCACGCCCACACGTCACGGCTC
CCTCAGCGCGCCGTTCGTCGCGGCCCCTCCTCATGCGTGCAGCAGGCGAGGCGCACAGGGCCGTCGTC
ATGAGCGTGTCGGTGGGTGTGCGAGTGGAGCA
GTTGGGCGGAAACACACGGCACCCCTCGCAGGAAGAGTGCAGAAGAAACAATTAAAAGAGGGAAGAGT
AGAAAAGAAAAAACGTCCATAGTGATTGCCCC
AAAAGCAAAAAAGAAAGAAATGAAGAAATTGAGAAATAAAAAACAAAAAGGAAAAACGCACAACCAAA
AAAAAAACGCAAAAAAAGTTTAAAATTACGCA
CATAAAACAAAAATAAAAAAACAAAAAGAAAGTAGCAAACAGGAAATTAAATTTAAAATAAGCAAAAA
CTGAGGAAAAATTATTTGAAAGGGAAAGAATT
AGTTCTTGGACGCAATTCGGTGCTGGGGAGGTCGCAATTGTCCCACGCGGCA
>scaffold1011.size7481
aaacacgaGAAGGCCACACGCAAGTGACGGGAGTGATGGCGATGATGATGACTGGCCGTGTGCTGCTG
GTGTGTGCCCTCTGCGTGCTGTGGTGCATCGTT
GCTGCGGTGAATGGTGGTGCTGTTGCCGCAATGGAAAGTGAAGAAGAATCCGAGCGTTTACCGTCTGG
ACGTCAAGACGGCGATGGAAGGGGTCACCAGG
AATCAGAGGATTCAGTCCAGCAAGAGTCCGAGACCGCAAATGAATCGTTGGGTGCCGGAAAAGGGAAG
AGTCCTAAAAAGCAGGATGGAGTTGTCTCCAC
ACAACCAATAAATTCCGACAGAACGGTCATAGAAACGGAAACAGAAAGAGGTAAAAGTAAAGAAGAGG
GGAGTAAAGAAACAACGGGGAGGATTGTAACT
CCAAAGAGGGATGAAAAGATAACCGAATTAACATCAACGACAGAAGAAGATCGAAAAAAGGCATCTCC
ACCGGCTGAAAGAGCACAAACAAAGGAAGAAA

TCACGCCGGGAGTAACGCCACCCGCAAAAAATGAAAACTCAGCAGGAATTTTAACTGCAACGCCAGGA
AAAGCAATGGTCATCCCTGAAGGTAATTCCAA
ACAATCAGAAGGAGGAACAAATCCAGCAAATGGATTGGCAACAGCAATGCCATCAACAAAAAAAGGGG
AAGCTCCAGAGGCACTAACATCACCACCCACA
AAAAATGAAAAAGCAGAAGGAGATTCAACAGTAAGAAGAAATACTGAAAACCCCATCCCTGAGGTTAC
CGCCTCCGTGCAAAAAACAGAAATTCTCCTGG
ACGACGAGGAGGTAACAAGCAATCAACTAAATGAAGACCTTGCATCAACCACCGGCAACCAAGAGGGT
GAACAATCGAACTTTCATGCCGAATCGACACC
AAAATCAATCTCAGCTGCCAGTAATGCTGCCAATGATGACGCTGACACGGATACTGATCAAGGGATTC
CAAATAATGATTCAGCAACCGGTGGTGCAGTC
ACAGAAGGAAGACACCAAGATGGAAAAAAAGAAGAGAGAGCGGAGGAAAAAACACCTCTCAAATCTGT
CTCTACCATAAATGATACGGCGATGCCTGGCA
ACAGTGACGGCAGCACCGCGGTCTCCCACACCACCTCCCCTCTTNNNNNNNNNNNNNNNNNNNNNNNN
NNNNNNNNNNNNNNNNNNNNNNNNNNNNNNNN
NNNNNNNNNNNNNNNNNNNNNNNNNNNNNNNNNNNNNNNNNNNNNNNNNNNNNNNNNNNNNNNNNNNN
NNNNNNNNNNNNNNNNNNNNNNNNNNNNNNNN
NNNNNNNNNNNNNNNNNNNNNNNNNNNNNNNNNNNNNNNNNNNNNNNNNNNNNNNNNNNNNNNNNNNN
NNNNNNNNNNNNNNNNNNNNNNNNNNNNNNNN
NNNNNNNNNNNNNNNNNNNNNNNNNNNNNNNNNNNNNNNNNNNNTGGACTCTCGCCCTCACCTCACCC
CACGAGGCACACACATAATGTGCCCATTGTAT
ATATGCATGCACACCCATGCATGCCCGTTGGTGCTCTCTTTTGCACGACACGCACCGCTGCCGTCCTG
CCTGGCTGCATGGACGGAGCACCGTAACTGTT
TCTGTTTTGTTTTTATTGCATTGAGTGGGCGACCTCTCTCTCTCTCCCTCTGTGTGTGTGTTTTGCTT
TACGGGAGTAATGAGAGTGTGTGTGCGCTGGT
CGTCCCACGCTTCGGCTTTTTTTTTTGTTTTCCTTTTTGATTTTGTGGTTGAGTGCACCCATTCCGCG
GGCTGCGCGTGTGTGATGATATGAGTAATTTG
CGTGTGTGCTATCTGCCTCTCTCTGCCGTTGCTTTCTTTGAACTCTCTTGTGGCGCACAGCTGCTCCG
CAAGTATATCCATTGACAAACACACAAGCACA
CACGCCATTATGCATACGCTGTAGAGCGGCGTGATTTCTGAGAACGGATGCACGCACAAGATTCTGGC
TGCGAATTCCCTTGCGAGGAGGGAGGACGGGA
TCCTGCAATTTATTTTTTGACACGTTGCTGGCGTGCAGTCGATTGGGTCGTGTGGCTTAAGGTGCCTG
GGACAAGTGCGCATTGCTTTTCGCCCAGCTGC
TGCACGTGCCAATGTGTATCTCCTATGCTGTGTATACTTTGGTCAGGGATTCTTCGCCTGTGTGGGAT
GATGTGCATTGGAGAAGACAACAGTAAGATGA
CTGGCGACAGCGCGTCGGTGCTGCGAAGGACTGTCTTGTTCCCTTTTGCATCCCGTTGGTTGTTTTTT
GCTGGGCGTTGCTCGGTGTATGCACACGCGTG
ACGAATAAGAAACCCACCGTGACTCCTGCTGCTGCTGCTGGTGGCAGTCGCTGTGTGTTCCCGGTGCA
CTGTTCTTCCTTCTCGTTCCGTGTGTCTGTGT
GCGTGAGTTGTTGTTGTGGGGAGTGACTGCGGCGGAGATGATGGTGCCACTCACTGTGGATGGGTGGC
GGTGCCCACTGTTTCCTTTTTATTAGCCTGTT
TTTTCTTCATTTATTTCACTCTCTTACGAATAAAGAAAACAAGCGCTTAACGCCACTGTGAAATTCGA
AGGCACTGAAAGGACCAACAGCACACACACCG
TCCACACACACATATTTATATGCTCTCACGTGTTGCTGCAGTGAAGGCACCCCGCACACACAACCGTC
GCCGCATGACCGGATCCAGCGGGAGGAGGAGG
GAAGGAGGAGAGAGTGAGCCGCAGAGGCCCAACATGTCCCGGCGTGTGATTACTTCTGCGGTGCTCCT
CCTCCTCGTGATGATGTGCTGCGGCACTGGTG
GGGCTGCGCAAGCTGCTGAGCCAACGATACCTGAGGCACCTGTACCGAAGTCATCTTTTGGTTGGAGA
GATACAACAGGTGATGAAACAGTGAGTTCGCT
TCGTGGTCCCAGTCTTGTTGAGGTGAATGGTAAAGTGTTTGCCGTTGCCGAGGCGCAGTGCAAGAATG
GTGAAACCACCGTCCTTACTGGCATTGCATCC
CGACTACTCACGATGGAAAAGGGTAACGAACCGAAGGATGTGCTGAAAGAAGCCAAGGTGAAAACACA
AGTTCTGGAGGAAGGCGGTTCTGGAAAGGGAAAGA
AGAAAGTAGATGTGAGCCGACCAACAACAGTTGTGGAGGGAAGTGACATTCACATGCTTGTTGGAAAA
CACAGTCATGAAGATCTCGCTAACTGTCAGGT

TACGACCGAAACCATCAATTCGGGAATATTGCTGGTGAAGGGCGAGGTCGTTGAGGACGGTAACAAAG
TAAATTGGAACGATACTGACGGTGTACCGTGC
ACGCTAGGCCCGAAACACGAATCCTTGTCACAACTGATTGTCGGCGGTGGATCGGGTCTTAAGTTGAA
GGACGGTACACTTGTGTTCCCCGTGGAAGCCA
CGAAGAAGAAGAATGAGGAGGACAAAGATGTGAAAGCCGTTTCGCTGATCATATACTCCTTGAAGGAT
GCTACGAATTGGACGCTGTCGAAGGGGATGTC
TGACGATGGCTGCAGTGATCCCTCCGTCGTGGAGTGGGAGAAGGACAAACTCATGATGATGACTGCGT
GTGATGATGGCCGCCGCAGGGTGTACGAGTCC
GGTGACAAGGGGGAATCGTGGACGGAGGCACTCGGGACACTCTCGCGCGTGTGGGCCAGCAAACAAAA
GGGAAAGGTTGAAATTGTTGGAAGCGGGTTCA
TCACAGCGGCTCTTGGTGATGGAGATGATAAGAGAAACGTGATGCTCGTTACTCTGCCGGTGTATTCC
AAGGAGGAAGGCAATGGAAAGGGCGTACTCCA
TCTTTGGCTGACGGACAATACGCACATTGTTGATATTGGGTCGGTGTCCGGGAAGGAAGACGACGTTG
CCGCCAGCTCCCTGCTGTACAAAAGTGGAGAT
AATGAGAAGGAGTTGATTGCACTGTACGAGAAGAAGGGCGGTGAGCAACCATCACCCGGTATGGTCTC
TGTGCGTCTGACTGAGCAGCTGCAGCGTGTGA
AGGAGGTGCTGACGACCTGGAAGAAAGTGGACGACCGTGTCTCCAAGCTGTGCCCCACTGAGAATGCT
GAGCATGATTCCTCAACTGACGATGCCTGCAG
CACTACTGATAAGATCACGGCTGGTCTGGTTGGCTTTTTGTCCGGCAACTTCTCTGATGGCACATGGA
GGGACGAGTACCTCGGCGTGAACGCCACAGTA
AATGAGAATGTTGGGGCGGAACCGGCGGATAATGGCGTGAAGTTCACAGGACATGGTGCAGGGGCGGA
ATGGCCCGTTGGCAAGCAAGGGGATAATCAGC
TGTACCACTTTGCGAACTACAACTTCACTCTTGTGGCGACGGTGTCCATCGACGAGGCGCTGAAGAGT
GGCAGCCACATTACTTTAATTGGTGCGAAGAT
GGATGACAATGAGAAGAGTGTACTCGTGGGACTGTCGTACAACAACAAGGAAAAGAAGTGGATATTAC
TGTGCGATGGCGGAGAGAACAAGGAGCACAGC
AGCCATTTGGAGACAGAGAATTCAGAACACGTGGTAATTTTGCTACGGAACGGCAACCAGGGCTCCGT
GTACGTTGATGGTCAGCGTGTGGGAGATGCAC
AATTAGACTTGAAGGATATTAAAGACAAAAAGATCTCTCACTTCTACATTGGAGGGGATGAAGGCAGC
GCAGAGGGCCAAGAGGGCCAAGAAGGCGTGTC
TGTGACCGTATCAAATGTCCTGCTGTACAACCGCCCGTGGACATCCGAAGAGATTGCAGGGCTTGCCA
AAAATAAAATTACTATTTCGAAGCCGGAAGGT
CCGAAGACATCGATGGATACTCAATCATCAGCGACAAGTGGATCACCTGTGCAGGGAGCCGTGTCCTT
GTCCAATTCTGCCAGGCAACTGCCGTCGGAAC
AGGAAACGCTGAAAGAGAATATTGGTGCGGACGGTGTATCCGGTGCAGCTTCCGTTACTACTCCTTCA
GCAGATGCAAACACTCCTGCCTCTAATGGCGA
GGGGAAAGTTGAACCTGCAGTGAAAAAGGAGATGAGCGTGAGCTCTGGTGAAGATGCGGAGACTGTGG
GAGGAACGGATGTGCAGGAAGGAATCCATCCA
CAGGCCGGGGAAGTAAATGCAACGGCACTCAACAGCAGCCTCGGACATTTGTCGCAGGGGAATAACAG
CGATGGCGGCACTGTGCGTGGGAGCGGACTGC
TGCCATCGCTGCTTCTGCTGTTGGGACTGTGGGGGTTTGCGGCTCTGTGAGGCGTGACTGGCAGCGGC
GTGGAATACCTGCGACCTCCCGAGCACAGAAT
TACTTCCTCGATAACTAATATCTCTGATACTTTTACTTTTTTCTTGCGTACTTTAAATTTATTTTCCT
GTTTGCTTCTTTCTTGTTTTAAAGTTCTTTTT
TTTCTCATCCCCCATCCCTCTTTTTTGCTTTTCGGGGGCAATCACTATGGACGTTTTTTCTTTTCTAC
TCTTCCCTCTTTTAATTTCTTCTTCTGCACTC
TTCCTGTGGGGTGTGCCGTGTGTTTCCCGCCCAACTGCTCCACTCGCACACCCACCGACACGCTCATG
ACGACGGCCCTGTGCGCCTCGCCTGCTGCACG
CATGAGGAGGGGCCGCGACGAACGGCGCGCTGAGGGAGCCGTGGCGTGTGGGCGTGCGACGAGGGCCT
GTGGGGCGGAGCACACATTTCTTTCCCTCTTG
TTTTGCTTGCTTTGCGGCCAGGGCGGGCAGACACCCGCAGCGCCACACACACACAGCCACACCCTGCC
GCCGTGTTTTTAAGATTTCTTTTTTCTTTTGC
TGCGCTCACTCTCCCGATGGAGTGACGCGACCGCCGTCATTTCTGCGTGCTTCTCTCAGTCCCCACAC
ACACGAGTGACTGTGCTCTGCGGGCCGCACTC

ACGCGCCGATGGGAGGGCTGAGGGGAGCAGCAACACGCAGATGGCTGCATGAAATATAAACGATGTGC
GGACACACAGAGGGTCGGACGGTTGAAGGACA
TTTAAATATCTTTTAATTTTATTTGCGGGCAAAATATATACAGACAGTTTGGTGTGGCCACACGGCAG
CCGGCAGCTTTTTAATTTTGTGTGGAACAGTA
ATGGACGATTTCTTTTTCATTTCTAATTATTTTGTGTGATGGGGTTTGTGTAAGACGAAGGGGGAAAG
CCTTTTTTGCTTTGGATAAATCTTGTGTGAAT
TGCCGCTGCTTGGGCTGCGGTGCAGTCATGAGTTTGAGCGGTCTTCTTCTTGTTGCTTCCGCCATGCA
CCTCCGCTGTACATTTCGTGCATCTCTCCACA
TACTCTGCTCCCTCTTTTTTACTTCTTTGCTTTGTGTTTCTGCAGGCACACACACACACAAGATGCAC
ATTGCGGTGGATTGTTCTTTTGCAGTGCATGC
TGGCGGTTTCGCTCTTGTGCTGCTGCTGCTCGTCCGTGTGTGTGAGGGCTGCTGGGAGTCGTGGACAT
GTATCTGGTGATGCGGATGAGGTGGTTGTGCC
GGTGGATGTGTCGTGTGCTCCGAGTGACGGCAGCCTGAGCTATCGCGTGCGTGTGTGTGGACGTGGGA
GAAGTGTTCCGCTGCCGGTGCTGAGGCTCAGC
GTGAGAATTACACTTTCAGTGGCTTTGGTGTGCGCATGCATGGCAGGGATGGTGAGCTGCGTGCTGTG
CGCCATGTGGCCCATGCGGTGCACACACGTAG
CAACTGTGCTGCCACCTGCGCAAGGAAAGGGGAAGGGGCCACCGTTGCCTCCACGATGAATGTGACGA
CACATAAATACGCCGGCCCTTATGATTTGTGG
CGGCGTCAATTTCCCACGATGGAACCATCCATCCGCCTGCACGGAGCAGCGATGCTGACCGGACGGCA
TCTGTCAGTTGCCGCCACCCCGTGAAGGGAAG
GATGACACAAAATCTGGTGGGACAACTGCGCTGCAAGTGAATGCTGAGCCGTCACGTCAAAACGAGCG
TGCGCGACAGTCTGCCGCAGCTGACGCACCCA
ACACCGTCGATCGGAGTCCCGCATCCAACACGGCGACCAACACGTCCGAGACGAGGCCTGTGTTCGCC
TTTTGTGGAAGCACATGGTGACTTGGACACCA
CCCGAAAGAAGGCCCACGCCTGTCACCGAGCCGCAGGGGAATCCTGTTGGTGATGCGATGATTGGTTT
AATAAATGTTGAGTTGCCGCAGGGAGTCGATC
TATTTGTGGCGCAGACGACGCTGGTGCAGCCGAAGGATGGGATTGTTCCAGTTACGGCGAGGGATTCG
TTTGTTTCACCCTCTCTCGTCAGTGCTGGTGG
AGTAATTGCTGCTTTCTTTGAAGGTTGCATGGATGCCGAATATCAACGTGGTCAATTAATTATGTCCT
TTTCTTCTGGTTTTGTTGCGGGGTACATTGAC
TATGCGTGGAATTGGTCCGCTCTTGTTGGTGAGGTCAATAAAAGATGCATGGAGGGCACACACTGTGC
TTggtgca
>scaffold1012.size21197
TTTTTTTCGTTTCCCTTCACCCCCGTTCTCTTTCGCCGCTTTGCAAAGGCAACGCACACGCACGCACA
CATGCAAAGCGCATTTCATTCAGCTCAATCTAA
CCGCACATAACCCTGAGCAGGGAGAAAAATATTTTACGCTAAATGCACACAAACTGCAGGGAAGGTGT
TGGGAAGGTGGGGAGGAGGAAATCAACAACAT
TTAATCGGTGTTGTTGTTCTTGCTTTATTCGCACCTGCCCATTTCTGTTATTATTCTCATACCATTTA
CTCTATTCCCGCAGCTTAAATCCAAGCTGCAC
CACCTGAATCTCCGTGTCCGAGAGAGACGCCGCCTCCCCTTCCAGCACCCGCCACCAGTCGTACAGGT
ACAGCTGTAACGGCTGAAGTTTTGGGGCATCC
TCCGGCCGCTCATCCTTGGCGGCCCCCGTCTTGTAAAAGATGTACTGGCAGTACCGGTCGATAAACTC
AAAGCGGGGGTGATACGTGGTGACGCGCTGGG
TGTCGTTAAATTTGAAGTAGCGTCGGTCCTTCCCTTCTGGTGTTCGCCGAAAAGCGGGGTCCGGCACC
ATGCCCCGCTCAAGGTGGCCCACATGCTCCAT
TTTCCAGTCGACGTTGAAGTCCAACGGTTCACCAGGTGGAGGGGTGGTCCTCTTCGGCACGGGCTCAT
CGTAGCCGCCCCGTTCTTTCATGCGGTACCAC
GAATGATTTCGATACAACTCTAGTGCCGTGTCACGGAGAAATCCGGGCTTTGTCTGGGAGGCATAATA
TTCATACCGCTCCGGGTTTCCAGGGTACGACA
CAATGTAGCCAAGAAAGACGGAAGGCCCAGGGTTGCGTAAAACAAGGCGACCGAGAAACTGCGATTGA
AGTAGCCCATCCCCCCAATGCTCTGTGTTGCC
AAAGTGATCCACATAGGTATGGATGGTGATACCAAACACATCAAGCTCGGCGTGAATCAGACACGACA
GTTCACCCTGAGGTGAGGGGGTAATGTACCGC
CGGACATTTAAAATGGGGTACTTTGATATCAATGCGCACCCATACGTGTTGTCCAGCGCGGTGGGTCC

GTAACTGGTATACCGATACCCAAGATGATACG
ACAAGAATTCGACTATGTCACGATTTCCATTCGCTAAGTGCATGGAGTCACTTTCCAGCAGCCCAATC
ACACCCGCTCCAGTCAAATCCGCCTGCTTCAC
CATTCTTGTGAGAGAATCCGTATTTGCGTTGTCAAGCGCAAAATGGACGGTCCATATCATGCCCGAAA
AGAACGTCATTTCCAACGCGGCCTCCCAAACT
TCCACTCTTTCTTTAAAGCTTAAAAGTGAGAGTAAAATTCTGTGTTTTTCCTCTGGAATATATTCGAT
CAATTTCGAGTTGTTGCTTTCCCCCTCCATCA
CCGCCTCTTTCAGGTACTTCAATTGGGTCCCATAGTGTTCCTCATGAAATTTAACCTCATACTCATTT
AAAAGAGGGTCAGTGATGACATTCTTCTGTGT
CTTTAGCCTCGTTATAGCCTCGTGTACATGCACCACCTCACGACTAAAGCGACGGGCCGCGTCCTTCC
CATAGGGAATACTACGATTATTGGGCTCCAGT
GCAAAGCGGCCAATAACAATAGAAAAAACAGCAAAGCAGAGGAATAGAACCATCATAAGCGGAATACG
CCGTTGCCGCTTCATTTCGTAAAAATCTTTCC
CCGCCACAGAGACCGCATTCCCCACACGGCGAGACAAGTACAGGGCAAGAAAGAACACGCCGGCCACC
GTGCCAAAGTGCAGTGAAACGGAACGCTCACG
CACGAGCCATGCCATTGGAACAAATGGGTACGAAATGCATAGAGAAAAAAGCAAAAGAAAACCGATAC
TCGTCAAGGCCCATACTAATTCAAAGGATGTC
ACACGTGCCACCGGAAGAGCGTGAGGCTCGTGTGAGTCTTCTGTTTTCCCTGGAGCAACGTTCAAAAG
ACGACGTGCTCGCTGATGGACAAAGACCAGCT
CTACGATAAGTGGATACAATACGCCAATGCAAAACGTCATGGCAATTCCTCCACAGAAGGCAATATAT
TTGTTACCAGAAAAATCCTCTTCTACCGCCCA
AAAACGAATGTCCACATGCTCCTTCAAACTCTTCATTGTTGTTGGATAGTGCTCATGAGAAAGTTCGT
AGGAGCGGTTTGTTTGACGCGTGCCAAAAAGC
ATTGTGAGACATCCTCCAAGAAGGCAAAAACCCAAACGGCCCCACAGAAGCCAACCTTGTTTTCTATT
TGATACAACGGGTAGAATGTCCTGCGAGTAGT
AAAGGCCCAAAAAAAAACTCAGGGCAACCAACAAATTGGCTGGATACGGATCAATTGCCAGAAGCCGT
GGAATATAATTGGCGCATGAGAAAAAAGTTAA
GGAAACGGTGCCGAGAAAACCAAACAACAGACCCAAAATGGCAGGAGAAAACGGCTTGTAGGCCAATG
CTAGAGTTGCCATAGCGTTTGTCTGTGGTGTA
CGAGAGAAGTGCACATGTCGTTGCTGATTCCCGTTGTTCGCATATGTTAATTCAGGCTCACTTTGGCT
AGAATCGATCTCTGAAAACATCACATACCGGT
ATAGAATGGTTGTACATGTGATACCAAGAATAATACCAAACAGCCCAGCGTACATGGCATCCGTAAAG
ACGGGGTCTTGTGAAATGTACAGAATCCGAAC
AAGCATGCTCACAACAAGGCCCAATGGAAAAGAGTACAGCATACGACAGTTGTCATCGCTGGTGTCTC
GCACAAAGGATGATGGGTAAAGGTAGCGACAG
AAGGTTGCCAAGGAAAGAAAAAATGGTCCAATCATTGTCATCAACAGCTTCAATCGTTCACCGTTTTT
CACCTGGACATGGACGTGGGAAAGGGAGGCAA
CCAGGTAAAAAAAGAGATACATGGGAACATGTTGCTGGCTATTCCCCTTCCCTCCTACAAGAGATGAT
TTTATAAACGGCGCTGAACCAAGTGCCCATCG
ACGGAATGCTGCAAATTTCAAAAGCACCGGTGAGCAATATGCGAAGCCACCAAACAGCTCCCAGGAAA
TAGACATAGCCACCAAGGGCATAAAGTACATG
TGCTCCACCACGTGGACAGTCATCTCCCAAAAAAGATACGCCCAATACGTGTCCACCAGCCACATCGT
CGAGCTCGACGGAGCACAGCAGAATGTGAATT
CGTGTATCTTCACATCGTCCTCGGGTTTATCATCCTCTGGGAAAAAATTTGACCTCATGTGAAGTGTGA
TATTCCCCCGGTCTCTGCTCGCTACCGCTACT
AGCTTCCCCCACTGGGTCATGCATAATTTTCGAGGCAAGTTCCTTGCACTTCCCTGGTGTCATGCCAA
AAGCCCACGAAGTCTTCTTCAGTTCAAACCAC
AACGTGGCGTCAAAACAAATATTGCACGCCGCAAAAAACCACTCCGACATTGTGGATAGACTGTATGC
ACCAGGAGCGCATGTGTTAATGTGACGAACGT
AAAAGATAACGTGGGAAAGAGCACCCGTCGTCTGTCCAATGAGAAAGACACGTTTCCAATAAGAAGAC
AAGCGATAATCGTCCGATGAGGCATAAATTTC
AGGATTGTGCGCGTTTCGCCGCACGACAATGTCGATAATCACCTGCACCAGAAAGCTCAGAAAGACGT
AAAACGCAAAACCCACATTGTGTCGATCGAGG
TTCTCAATGGACGATATCATAGTCCACGTTAGCGCACCGAGAAGACGAGTTATATCGAAGGCAAATAT

CGTCAGTGTCAAAAAGGCAGAAGATCTAACCA
AACTGATGGGGGAGCGTCGCATGCTACTCAATTCACTCATACCTCCGCAGGCACGTTGCCAAAATACC
AGAAGGAGCGAAATGGCCACTCCAAGACGAAA
CGGTGTGCATAGAACAAACGCGGTCCGCCATATCTGTCGCTCCGGCTGAAAGTCTCCGGTAAGCGTAC
TTATACTTGGCCAATACTCCGGCACCCTGCAG
TGCGTGTATAACAACTCATCATGGTGCTTCCAAATCCCCACAAAGAGAGTAAACAATAACCCCGTGCC
CACAATAATGACATAGATTAGCGCTGCGTACG
AAGAACGAATAACAAATACATGTTGCCTCATTGTGTCCGCTGTTTTGCGCCTCCCTCAAACGACGTTG
TCCTTCCGCGTGTTGAAGGTATATATATAGAT
AGATAGATAGATGTATGCGTGTGTGTGTGTGTGCAGATAGAGAAAGATAGATTTATTGCTGTATGGTC
CGTATTACACACACAGAAAAAAAAAAAAAAANN
NNNNNNNNNNNNNNNNNNNNNNNNNNNNNNNNNNNNNNNNNNNNNNNNNNNNNNNNNNNNNNNNNNNN
NNNNNNNNNNNNNNNNNNNNNNNNNNNNNNNN
NNNNNNNNNNNNNNNNNNNNNNNNNNNNNNNNNNNNNNNNNNNNNNNNNNNNNNNNNNNNNNNNNNNN
NNNNNNNNNNNNNNNNNNNNNNNNNNNNNNNN
NNNNNNNNNNNNNNNNNNNNNNNNNNNNNNNNNNNNNNNNNNNNNNNNNNNNNNNNNNNNNNNNNNNN
NNNNNNNNNNNNNNNNNNNNNNNNNNNNNNAA
AAAAATAGATAAAACTAAAAAAAAAATAAAAAATAAAAATAAAAAATAGCACCTTTGATGGCTTCCCA
CTAGCACTGTGACCCACCAAAGGTTCTCCTCG
TGTGTGTTTATGTTTTATTTTCGTGCTGTTGTTGTTGTTGTTTTTTTGCTTGTTTGTTTTATATTTTC
AAAAACAAAAACAGAAATAACTAAAAAAACAC
GCCACAGACCAACACAAAAAGACAAGGAACGTACAGGCATGCGGACAAACACACATGCCCAGAGTGGC
AGTGGAGAGCAAAAAAAATAACGCAGAGAAGA
ATACATGTATTTTACGGGTGGCGACCCTCCAGCTTCACCCACAGAGTGCATCCCCCCCAACCCCCCTC
CAACAACGGAAAAAAAGACAACAACAACAAAA
AACCTGCAATTATCCCCACGCGAACTTCGTAGTGAACGGGTCTTCACCAAATGGTTCTGCAAAGTTTT
TTTTTTTCTTTTTTTCTTTTTTTTTTCTGTCA
GACGCGGTCTCTTCTCTCCTTTCATTCTTAGCCATCGCACTCCTCCTCTTTTGGGCCTAGTAGTTGCG
GTGATGCAGGTGTTCTTCCCCTCCTGTGCTGT
GGCCTGGTGTCGAGAGGTATTTTGGCACGTGCGCCAGCGGTGTGTCGAGGCCAATTGCCGTCTTTGGG
TTCGCCTGGTACCACTGCTCCTTGAACGGCTC
CTGAATAGCCACGTAACGGAAGCAGTCACGGCCGCCGTAGCTCCACATGAAGACCTTGTACGTCGACA
CCAACCCAACGATGGTGGCAATGGGCCAAACC
ATGCAAATGGCGTAGACTTCAAAGCGACGGACCCAGCCGGGAAGTGCCATTACAACGCCCGACCAAAT
AATAAATAAATAAATAACACGCTTCCACGTGA
GTACTTATTGTGGGTCTGCGTCCCAGTTTTGCGGCTGCGACTGCTCTTCAACCCCACCACCACCTCCA
CGGCCCGTGTAACGTCTGTTTTCTAGACAGCC
GGGACTTACAATGCCGTCGTTAAAAAGTCACAATAGCAAAAAGCAAAACAAAAAGGAAATAATAATAG
ATATCCACCCGACGGGAAGAAGAGACATGTGT
TCAATTCGTGGAGCACAAGAAATTGTTCCCGGTAGAACAAACATGCGCTCCACCAACTGTAAGAACAA
GCCAGTAAAAAAAGACAGTGGCATTAGACTTT
GTTCGCAAATTTCTCTTCACCATACTCAAAGAAGCCCGTGGCATGCGCCTTTCGTTACTCCCGTTTCA
ATGGAGGTGGGTCCCCTCCCCTTTCTTTCCAC
TTCCTTGTTATTATTATTGTTGATTTTTTTTTTTCGTTGGTTGCCGTGTTGAACAACACAAAACCCAC
ATAACGTTCTCTTTCGCTTTAAAACAAAACAA
AAAGAAAAAAGAAACTTTTCCCGTTAGGCCCCCCTCAATTCTTCTGCCTACCTTTATTTGCCTCATTT
TTTTTTTCCTTTTCTTCTTTTCTTTTCCTCTC
CAACGCCTCCCGCCTTCTCATTGCCACTGCATCACATACCAGGACCCCATGCAGAATGCCGACGTCAA
CTACACTCCTTTTCGACAGTGTGTGTGTGTGT
ACATGCGCGTACATTATTTACATTGTTGTTTGCGATGGCAACATATTTCTGCATGAAAGCCAAAGCCA
GTTGTTCACGACGCCTTTTATTTTCTTGATTA
TTTACTATGCGTACTTGTGTTTGTTTTCCCCTCCCTTTTTTCTCACCACTTTTCTTTATTTCCACCCC
ACCCCCACCATTCCCCCCACGCTCTTTTGCCA
TGAGAGCTATCCGTTTATGTTAAGGCAACAAAAAACCACCAACAATAACGGCCTATACATCTGTGCGG

ATACCTATTTACATATATCCACAAATATGTCA
GTAAAATACGCTGGCAAAAGCAACAACAAAAAAAGGGAAATTGAGTGGCGCATCGCCCTTTCAGTGGT
CACGCGGGATTGTAAATAGACATTGCAAAAGT
CTGCAGCAAAAGTGTCACGCCGAACCCAGTAGCCTCATCGCGTGAAACTGGTGCCGCCATGTCTATAT
GCGCCCAGCTGCCCTGATACTTGGGGTCAATG
TGGGTGCCAATAAAATAGGCTGCACAGCTCGAAGAGGCGTTGCCGCGGTTCGTCACGCTGTTGCGACT
GTCCGCGACGCTGCTGTTGAACTCCAAGGAAT
GAAACTCTGGGCAATGTAAGATTGGGAAGCAAAGATCACCACAAATGCGGGACGCCTTTAAGAGGCGG
TTTTCAATTTCTTCGTTATTGGCGTAAAGGGC
CGCATGGAAGCGGCCTGTCGCGACGCCCTGCGTCCCTGTCAGAGTCCCCATGTCGACAATGATGTCGG
GTGTGTAGGGCAACAGGGCGGAGGCGTGGTAG
ACACCATCGCCGAGAACAAGTCGTCCTTCTGCATCGGTGTTGTTGATTTCAACCGTGTGACCGGACTT
CATGCGGATAATATCGTCATTACGCTGGGAGC
GTGGCCCCACGGCATTGTCAGCGAGGCACAAAACTGTGGATACCTGCTGCGGTGCCTCAATCATGGCC
AGACCGAGAAATCCACAAAAGACGGCCGCTGC
ACCCCCCATGTCATGCTTCATCGCACACATACCGCCAAGCGTCTTGATGGACAAGCCACCCGTGTCGT
ACACGATGCCTTTCCCAACAAACGCCAACTTC
TCTTGCGGTTTGATGCCATTCGACGGCTGGTATGAGAGTGTAACAAGGTGCGGTGGAAACTCTGCCGC
CTTGCCAACACCGTACATTCCACCGTAGCCCT
TTTCTCGCAGTTCCTCGCCAGCGATAATACCGATGGAAACGTCACGTCCCTCTCCCTTTAGTTTTTCC
ACGTGCCCCGCGGCAATTTCTGTAAAGGTGAT
GGTGTCGAGCAAATTCGTCGGGGCGTCTACGAGCCGTTGACACAACTGCACCACCGTCACTGCCACCT
CCAGTGCGCGACGACTCCCACACGCGAATCCG
TCCCCATCCACTGCCGCACCAGGCTTTGTAGTATTGGCCGCAAAAACAACGCGGACAGGAAGCCCACG
TTCCCAATAACCCTCCTCCGCACGACCGCGTT
TTGCGGAAAAGCTGCGATTTGCAGCACGTGCTATGGCTGTGGCAACGCAAAGTACATTCTGCGACTCA
CAGCAATAAATATCCACAGACCTTAAAACTGA
CTTCTCACAATCTCCTGTACGAGCCTGATGAACTGCATCCTTAACCAAGGTGGAAATCATGTCTGGTC
GCAAGGGGCAGTTGTGACGAGAAGCCTTGGAA
GAGACCATGCCAACAAGAACACGTGCATGTGGTGTAATCTCCGCCAAATAGTTTTTACCGCTAGCAGC
AACGTTGATTGCCGACTCCACCGCCTTGCTGT
AGTACGGAAAAAAGGTGCTCAATGCCTTCTTTTCCTGTAGCTGCTTCTGATTGCCAAGAAGCAGCACA
ATATTTGGTTCCTGCGGATGATTCTGCAGGGC
CTCGTTCGCTAATTGCATTCCCTCGGCCGCAGTGTGAAACGTCAGGTTTGTCCTGAAGTGAATGCAGC
TCTCAACAAACGCTGACACATTGTGCTCCTCA
TACGCGGATTTTGTACGTTGCCGCTTGTAGGCAGTAGACATGATGGCCTCTTACTCTGCTTATGTATC
TAAAGAAGATATGTGAGAGCAAAATAAAATTT
TTTTTAAATGTATGTGTATGTATGTGTGTTCAGGATTGTGATCAACAAAAACAACAACAAAAAAAAAC
GCAAATCGCCTCTCGCCCTCTCGAGTCGTATT
AGTGAGATGTGAATAGAGAAGCAGGAAGAACGCACCGATACAGCAACAGGCAAAGGGCCACTACAATG
TCGACCGCACATAAACCAGCAGCATCAAAACA
CACACACACACACACACTAAAAAAAAGGAAAGCAAACCTTTAAAAAACAACAACAAGCAAATAAAAGA
AGGGAAAGAATTCCTGTTGGCGGAGTATATTT
GTGTTGGGTTTCTCCTGCAACGCACGTAAATACATGTAAACACATTTCATCGATTCTCGGCAAAGAAA
AAAAAACGGATTATAGAACCACCGCGCCGGCC
GAGGGGCTCCCCTTCACACCTCCACCCAGCAGGACTGGACGTGCTTTTCGATGGCTGGCGTGCACACC
ATCAATTTCATTCAAGAAGTTACACATAAAAA
AAACAAAAAAGGAAAAATAGAAAGGCATATAAACCCCACAGGGGCACTCGCATCTATTACTGAATACT
TGATGAAAATAGCACGCACGGAGCACACACGG
CTAAAACATTTTAGTTTCCCTATCGATCTTTTTTTTTTNNNNNNNNNNNNNNNNNNNNNNNNNNNNNN
NNNNNNNNNNNNNNNNNNNNNNNNNNNNNNNN
NNNNNNNNNNNNNNNNNNNNNNNNNNNNNNNNNNNNNNNNNNNNNNNNNNNNNNNNNNNNNNNNNNNN
NNNNNNNNNNNNNNNNNNNNNNNNNNNNNNNN
NNNNNNNNNNNNNNNNNNNNNNNNNNNNNNNNNNNNNNNNNNNNNNNNNNNNNNNNNNNNNNNNNNNN

NNNNNNNNNNNNNNNNNNNNNNNNNNNNNNNNNN
NNNNNNNNNNNNNNNNNNNNNNNNNNNNNNNNNNNNNNNNTTTTTTTTTTTTCTCTTCTTCTTCCTTCAA
TTAATTTATTGGTGCTCACCGCATTTCCTTTT
TGTGCCATTTTCGTTTCCTTTTACGCCTGTGCCAGCACGTCTACAAGACGGTCCATCGCCCGACACTG
CCCCGCACCGCTGCCACGTCGCCGCGCTTTGT
TCATCGCATGCAGCGCCTCCCTCACCCCGTGCAGCACATCGTCAGTCAACAAGGAGTCCTCGCAGACG
CGACGCACAGATGCTTCCACATCAACGCACTC
TGTGCGGTTTCGTACAAACGGTATAACCATGTTGTCGTCCTCTTCTTCCATCAATGCGTCAATGTTAC
TTCTCCCCTCAGCACCACCAGAAAGTGTGCGG
GAGGTGCTGCTACGCGCCGTCATTAATGCAAACTGCTCGCGTTGTCTTATTTTCTCCAGCGAGTTAAG
ATTCTCGTCAACATCATGCCAATACACCTTGT
CGCCATGAACCTCACTCGCCTTCTCCCCCTCTTCCATAAAATCCCGGGAGCACAAAATGCTTTCCTCG
CACTTGACACCAGGACTCTCGTAGACGAGGTT
GCGCACGACGTGGCGCAAGCCATTGTAAAACTCCTCGTCGTCATCAAGGTCGTGGAATGTGATGAGGA
GGGAGTGGTGCACCATGCGAATAAGAGCACGG
CTTCTAGCCGAGCTGATTATCCACGCCGTGGACCCCCAAAAGTGGCTCTTGCCAACCATAGACGTCTT
TTCCTGTGATGTGCCAGCTCTGCGTCCTTCCA
TGGTGCCAACGCTAACATTCCTGGAGTCATGATCTCCAAAGGCATGACAGATACTGCCGCGGATTGTA
ATGTTGCTGCAATCAACACTCACAAGATTTTC
CACCGGAACCAGACGGGCACTGACAGGGCCGATATATATTTTTTCCGCAAAGTGCGCCACGCGACTCA
TGACCCGCGCAAAGAAATTGTGCGGTACAATG
TTGAGCGTGAAGCGGTAGAAGGGGCCGAAAAGGAAAGAGGGCAGATGCAAATTAAATGCCGATGGAGG
TTGACACGTAAAGCAGGCGGGAATGAGCATAA
AAAACTCTGACGATAAAACGGATGCCCCTGCTGTTTCTTCTTCCTCCTCCTCCTCCTCTTCCTCATCA
TCAACATCATCATCATTCTCCACAGGATCATT
CCCGCGAAGCACACTAGTGTCCATCATCCCGTGGGATTTATTCTCGCGCAGCTCACCCATGAAGGAAG
AGAACTTCAGGCGGCTTCCCATAATAATAAGG
TCATAACCTCGCAAAAGCTCCAAAATACTGGTAAGATGACAAGAATCCAAGCCCTTCTCGCCGAGAAC
ACCGCGAAAAAGGGACATGGCTGTCTTCATAG
AGACAACGCCTTGGTTGAAAACACAGCACAGATCAGAGGCCATAACTGTTTTAACGTCAAAAGGAATT
ACATTTTGCATGTCTTCCATTGTCTTCCGGCA
AAACAATAAAGGCAGAGGCTGCAACAAGGAATTGTTCTCCGTGCCATAACCACGTGAATATGTACAAC
AGGCCAATACGGCCACCACGCGGAAAACCCAT
TGAACATCTATCACTACATACTTCTTCATAGTAAGGTGCCTGAAGTGATGATGCAAAACTCCCCATCC
CACCAGAAGCTGTGTATGGCGATGCAAATCAT
GTCTGCTCAAACCATAGCGTGCATCCACAGCCTTCGCAATGGCCTTATAGTTTGCCGAAGTGAGATAC
CATTGGCCTTCCCGATTTATCTCACGAATCTT
CTTTGAAAGAGCCACCGCCCGCAAGGGTACCTGGGCATTTGGGAATTCCACATCATTGCGGCAGCGCT
GCAGAGCCTGATCGCTAAACCAAGACAAAAGC
TCCGGAAACTTTTTGAACTTAGAAAATCCAGGAGAAACTACAACACGGCCAATGTTATCAACTGCAAA
GCGGCCCAGTATAGAAGGACGTTTCTCCGGAA
TGGCCTGTACAAGGAAAAAATAATTATTGATCTCCGTCTCTATAATGGCAAATAAATGAGCTAACTTG
ATCTCATCAAGAGAGATTTCGTCCAGGTGCGT
CCCAATGAGAATCACCGTCGCATCTCTATTAAAGGTTCGACAGAGAATAAACTCCAGTAGAGGTTGCA
GCGTCTCCAATGTGGGCAACTCCCTAAGCGAG
AAACACAGGACATATGAAACATGCCGCGTGGGTAAAAGGCTCTCAAAAAAAGAAAAACAACGATTGCC
ACTCAACTCCATCAAATTCATCTGCACAGGCT
GATTTCCCGCGAAAGTCTCAATCAAGGAGGAATCCACGCCAAAAGTTGGTCGCACATGCAGTTTGTCA
TTCGCGAACATCGAATACCACCTGTCGGGTTT
TTGCAAGTTTTCACGAAGGGCGCTCTTCCCAGAATTGCAGGCACCAAGAAAAAGAAATGTATGACGAT
AAATAACCGTAAGTTCATCGCTTGGGGAAATA
ACTACCTTCCCGGCCGTCCGTGTCGTCCGTGGGGTGATGCCCACCACAGAAATACCGTGTGGGGCACT
CGTCAAAGAGGTTCCCGTTGGGGTCATGGGGT
CCCGATGCGCCTTCTTCGTACGTGCACTGTTCGATTGCATGCGTTGCAGTGCCTCCTTCAGTTTTAGG

GCCGGTACAGGCGGATGGCCAGCATTCCCACT
GCCAGCACCAGTAAGGAGTGGAGACGACTTCAAACTATGGTTGCTGTTTGAGGAAGATGACTTGGCGT
CAATACTCAATGATTGCATTGGAATACTTTCC
GAATTTCGCACAGTACGACGCCGCACCGCCGACTGTGGTGCAGAAGGATCAATAAATTTAAACTGCAC
GTATTCATCGCCCAGGCACAGCACAGTCCCAC
TCTTCATAGGAGTATCCCCATCTACGGGGTCTGGATCATCGTCAATGTAGGTCCGATTGCCACCCAGC
GGCCGAAGCAACACTTGGCCACCATCTTGTCG
CACAAAACAACAATGTTGTGGGGCAATACCAGCTGTCTGACTCCCACTCACCACAATATAACGCGCAT
CATTACCAGTCGCAGATGCTTCAACTCCCAGA
TATGTAATACGAGCAACAATGTACTCAACTACCCAATCCCCGGCTCCATCCAGATTAAGAATAAAAGG
ATCATTACCGTTCACTCTGATTGTAGACGTAT
TCCGATGAAAAGCAGGCATAATGAAAGATTCCAGGGAAATCCCCTTATTGGCTACAAGATTTTGAACT
AAATTCTCTGCGCGATGTAATTCTCTTTCCAA
TACCGCCGCATCTCCCGCCTGCCTCGGTGTCTTAGCGTTGGCGTAGACCATATCATCTTCGACAATGG
CCTTTGATTCCCCATCTTTACCAATTTTATCT
CGCAACTGTGGGGAGCCACTGATGGACATTTGACTCATCACTTCCCCACGTGCAATCATTTCCTCATA
GTGTCGCATTTTCTCTCGTAGCAGACGAACCT
CATCCTCTAATTCGCGTATTTTCTTATCCCCTGCCGATTCATTGATGACCGCTCGAGTCACAATGGCC
TTGGCACGGTCAGCGTATCGCAGCGTACTCAA
TGTCTCATCGTAATTTGCCGCCGCAGGACTAAGAGTCGACAGCATAAATGTTTTACTGTTACCACCAA
GAGACTCTTTTAAAATAAAGGTGAGTGTAGAA
TCCCGATATGGCACATGCTTCAAGCCTGCCGCGTCCGACTTTTGTTTGCTTGCTTGTGCGAGAGCACT
GATCACTTGACCCAGGACTGTCAGGGACCTGT
TAATATTGGCCCCCTCCCGGAGTGTGTCGCCCTCCGCCCCCGTCGATTTTGCCCGCTCACTCCCCGCA
AGGTCCACCAGATTAATTTTGGCCCGAAGCAT
TGTTGACGTCACCTTGCCGTCCTTTGTGATGCTCTTTCGCTCTTGAATGAGTGAAATGGCAAAGATGG
CGTGAGACCTGCTGGAACGATCATTCATCTTT
GTTGCGGCTGTGTGACGGCATTGATTGCCTCTTTCCATGATCTGCATAATCTCCTCCTCCGACGACAC
CTTCACTTCCCTGAGTCCCTCCACGTATACAC
CAAGCACGGGGTGTTCGCGAACTTTGAGATTATCAAGTTTGCTGTTCAGTAGACAATTCACACGTTCC
TGATAGATCTCCAAATAACTCACCAAAACGAT
GACGTTTACCTCAGGTGGAAGGGCATGTTCTTCCACGGCATTCGCACGTGCTGCCTCACGATCGCTCT
GATTCTTCGCCTGCCGTTCCCGCAGCATGGCA
AAGAGTCCTTTTGACAAACGTGGAATAATACCTGGGTCCACAAACACGTCACGGCCGCCAGAAGGACC
CATCATAGAGTAACTCTTCCCGCTTCCCGTCT
GACCATACGCAAAGATGCAGGTGTTAAACCCTTTGAAACTGGAATCCAGCACGGGCAACCCCAAGTCC
TCATACAGAGAGGACTGTGGCACGTCAGTGTC
ATAGGCATGATCAAAGGCAAACACCTTTCCCACGAAAGACTCATCCAGCCCCTCTACACCATCCGCGG
CAAAACCTTTCCTAAAAATTTGTACTTTTTTC
CCCTCATGAAACGTCAGACATGACGGGGATTTTAAACGTTTTTCACGCTCATTGAGTGGACGAGCTCG
CACACAAACGGTGATGCGTCCTTCCTCTGATT
GTGCGCTCTGCGGCATCCTGATACAAGTTTCCCCCTTTTATTTGTGTAAGTTTGAATATTTCTTTGCC
GGAAACTGTGTTTTCTGGTGCCACCCTCCCCT
TGTTCTCTCGCCCCTTCCTCCTACTCTTCTTCACTTCCTATTCTACGCCACAGCTCTACACACACACA
CACACACACACACACACAGTCAACTTCAAAGT
GCTTGTGTTCTATCTTACCGAGTCCCCACCAATGAAGACTAACAAACGCACACATTTAGCAATAAATA
AATAAATAAGAATAAATGCCAAAAACACAGAA
AAGTCCATAAGGACAAAAACAGGCCCTAGGGCACAGAAGAAGCACAAAACATACAAAAATAAAAAAAT
AAAATAAAATAAAAAGCAGCTCATGCAATTTC
ATTCAAAATCTCCGCACCTCAATTGCCAATCCTAATGATCCCTCACAAACAAAACACAATAAAAATAC
CCACCCGCAATAATGATAATAATAATAAAAAC
AACAATAAGAAAAAAAAAAAAGAAGCACGCATAAAAATTATTTTTTATCCCCTATCGTCGCGATAGCGG
CGCTCAAGATGCCTTCGATAGTGTTCAGGATT
CAGAGTCTCCCCAGTGGCCTGCCTGAGCAGTTCGTCAGTTGTCAGCGAAGAGCCATGCTGCCAAATCT

TCTCGTTCTGCTTCGCAAGAATCTTACCGAGG
TCACCCTTCCGGATGCATTCGTCCACAACCTCCTCACCGAGCTCCCTCCGGACACAACTCATCAGCTG
CGCAGCCACCATTGCACCTAGCGAGTACGTCG
GGAAGTACCCAAACATACCTCCTGACCAATGCACATCCTGCAGACAACCCTCCTTGTCGTTGCCAAGA
GTTTCCAGGCCAAGGTAGCTCTTCATCTTCTC
ATTCCATACCCTCGGTACTTCCTCCGCTTCAATATTCCCGTCCATCAGATCGCGCTCGATTTCATAGC
GCAGCATGACATGCAGCGGATAGCAAAGCTCG
TCGGCATTAATTCGAATCAGACCAGGGGAAACACGTTGGATCACCCTCTTCATGTTGGAAGAAGTAAA
GGCCGGCTGGTCGCCAAAGTACTCTACAAGCC
TCGGGGCAAGAAACTCCATAAAGGCACCGGAGCGGCCGATCTGCATCTCGGCAAACAGCGACTGGCCC
TCATGGACACCAAGTGAACGTGCCATGCACAC
CGGCTGAGTCTCAAATCCCTTGGGACCGCAATTCTGCTCATACTTGGCGTGCCCGGTCTCATGAATAA
CACCCAGCAGGCTCGTGACAAACTGGGTCTCA
GTGTAATTTGTAGTGATGCGCACATCCTCCTTTGAGTTCCCACAAAACGGGTGCGCGCTGATGTCCAG
TCGCCCGCCATCAAAGTCAAACTTCCACACGT
CCATGAAGAACCGGCAGAGTGCCTCCTGCTTTGAAACGGGGAACGGACCTTTGGGTTCCAAAACGGTC
TCGCCAAGTGTCTTCTGTTTCTCCTGCACCTC
CTTCAGGAGTCCCGGCAGCCACGACCTGACATTGCCAAAGATCTCGTCCAGTCGCTGGAGCGTCATAC
CAGGCTCGTAGATGTCCACCAGTGCCTCGTAC
GGGTGCTTGCCGGAGTTGCCAGCGCGCAATTTCCCCTCCTCGCGGAAAAGTGCAATCAGCTCCTTCAA
TGTCGGGAGAAATCCCGCAAAGTCATTTTTCT
CGCGGCACGTCTTCCACAGCTGGTGAGCCTTTGTCGTGAGAACCGTTTTTCGCTCAACAAACTCTTCA
GGGAGAAGATTTTCTAGCTCCCAAGCTCGGCG
CATCTCACGGAGGTTGGCACGTTGTAGCTGCTCAAGATCTCCCACCGACTTTTCAGCCTCTTCTATAA
GAGCACGCATCTTGGGAGCGGTGATTGTGTCG
TGCATATGCAACTGAAGCTCCGCCATTGCAGCGCCACGTGCGTCAGAACCCTTGCAGGGCATCATTGT
TTGCGAATCCCAATCCGCCAACAACAACATAT
GTCCATAGCGATATAGTTTTGTAAAAACCCGCTCCAGCTCTTTGTAAGGCTTCATTTACTGCGTATTT
CTCCTTTTTCTTTCAAACAACAATTGAAATTC
CTTGTGTGTTTTTCCGCCTTCTTTTCGTCGGATCCTTCCCTAAATTCTCCCTTGCCTTTTCTGTTACC
GTAAAACAAGTCTAAGAAAAAAAAAGTCGCAAG
TGAAAAAAAAATGAAGAAAAAAAAAACATGCAACCATATACATGTACATATATATATATATTTATTTATG
ATTCATTNNNNNNNNNNNNNNNNNNNNNNNNN
NNNNNNNNNNNNNNNNNNNNNNNNNNNNNNNNNNNNNNNNNNNNNNNNNNNNNNNNNNNNNNNNNNNN
NNNNNNNNNNNNNNNNNNNNNNNNNNNNNNNN
NNNNNNNNNNNNNNNNNNNNNNNNNNNNNNNNNNNNNNNNNNNNNNNNNNNNNNNNNNNNNNNNNNNN
NNNNNNNNNNNNNNNNNNNNNNNNNNNNNNNN
NNNNNNNNNNNNNNNNNNNNNNNNNNNNNNNNNNNNNNNNNNNNNNNNNNNNNNNNNNNNNNNNNNNN
NNNNNNNGCAAAGTCATTTTTCTCGCGGCACG
TCTTCCACAGCTGGTGAGCCTTTGTCGTGAGAACCGTTTTTCGCTCAACAAACTCTTCAGGGAGAAGA
TTTTCTAGCTCCCAAGCTCGGCGCATCTCACG
GAGGTTGGCACGTTGTAGCTGCTCAAGATCTCCCACCGACTTTTCAGCCTCTTCTATAAGAGCACGCA
TCTTGGGAGCGGTGATTGTGTCGTGCATATGC
AACTGAAGCTCCGCCATTGCAGCGCCACGTGCGTCAGAACCCTTGCAGGGCATCATTGTTTGCGAATC
CCAATCCGCCAACAACAACATATGTCCATAGC
GATATAGTTTTGTAAAAACCCGCTCCAGCTCTTTGTAAGGCTTCATTTACTGCGTATTTCTCCTTTTT
CTTTCAAACAACAATTGAAATTCCTTGTGTGT
TTTTCCGCCTTCTTTTCGTCGGATCCTTCCCTAAATTCTCCCTTGCCTTTTCTGTTACCGTAAAACAA
GTCTAAGAAAAAAAAAGTCGCAAGTGAAAAAAA
ATGAAGAAAAAAAAAACATGCAACCATATACATGTACATATATATATATATTTATTTATGATTCATTCA
TCTTGTTTCTTTGGTGTTCACGTGGCATATCC
TTTTTTTTTTTTTTTCGTTCTTCTCCCTTTATTTATTCGTTATCATGTGAACATCCAATTCTCTTTTA
TTTACCGGATTGCTTACACGTTTTTTTAGCGC
ATTTCTTTCAGTGTGGAAATATATATATGTTTGAGGGACACCAACTCCGGATGAGTTCTCTCATTTTG

TTGTTGTTTTTTCTCTTCTTTTGCACTCAGAG
CTCTTTTTTTTCAAGTAAATGTGGTTGAAACGTCACTCGGCGGTCGTCGGAGCCCGTCCCTCCACGCT
GGCCTTCAATTTTCACTCTTATTCTTGGCGCG
CCTGGCAAGTTTGGCCTTGTTGCCTTTCATGTGCGGGTGGGCAACCGTCATGAGTCGCTGCATCGCCT
CCCGCTGGTGTCGTTTGTTCATGTACTGGCGG
TTGAGCGGGATGTAGGCGTATGGTGCCATGTTGCCGCGCCGCACGTCACCGTCGCCCCTCCCCTTAAA
CTCCTCGCCCTTGCGGATATCCTCTTCGACCC
GCATCCGCTTCAGTTCCCGCTTCTCACGCTGCTGGTCACGCAGTTTTTCAATTTGGTTCGCTCCGGGA
CCGACGCGGGACTGCGCCGCGGCTATCGTGCC
TCTAGCCGACTCGTTGCTTATTTTGTTGCCGTACCGAAGCAGGAGCTCGTCATTCTCAAAGTCTTCTG
CCTCCACCCGAAAGCGCTTGCCGCGCATGCCC
AACGCTCGCTCGTTGAGGGAATCAGCATGGGCAAGATGACGCCCGCGATGCAAGAGACGATCCGCCAT
TTCCTGTCGACGTTGCCGTTCAACTTGTTGGC
GCTTTTCTTCAATGCTCATGATGTGCAGTTTACCGTCTTGAAACTCGAGGTGCATGTTGTCGAGTTCA
TCACCGCTGTCCTCTGCATCCTCTGCGATGCC
GAAAGAGGGCAAGTCGTATGTAGACACCAGGCTTGTCAAGGCACCAGCCTGCAGCAGGTCGCGCTCCG
CATCATCCTCGGTTCCAGCCTTCATCGCAGCC
CCAAGGAAGAGGCGGTCAAACTCATTTTTCTTCTCGGTCGCATGTTTCATGAGTTCGCGCTCTTCCTT
CTTTTGCTCCCGTCTCATCATCTTCTGCGTGT
AGCGAAGAAAGTTCTTTGAGCCAACTGGGAAAATTGGCTCCAGACGCTCAAAACTAAAACGCTTGATG
CATTTCTCAAGCAATACCCGCATCTGCAGACG
AATCTTGCTAGATACACTTGGTTGGGATGTTGTGAGAGCAATGGCAAGAAGCAGCTTTGGTAGCGACC
TCTCCACTGCCGCCGCAACGCGTCGACATCGC
TTCATGAGTTTCAACACCATCCGTGCAAACATTGCTGCCGCTGACCTGACATCCGGTAAGGCGGACTC
CATCATGCGGAACCCAAGAGAAATGGCGGCGT
CAAGGTCAGCCTCCGGCAATTCTTGATGGGTGACGTACACAACTTTCGCCATGGCCACCACAGTGCTT
GAAACCATCCACGGCGTCTTTCCTGAAAACCC
GGCAAGCACCAGACGCAAAAGCATATTCACCGGATTGCCAGCACCGACAAGGTAACGCTGCATCTTTT
CTAAAAGATCCATAGAGGTGGCCCGAGTCTCG
CTAGAACGCTCGCGTGAAAGCATGATGGTTTCACCCACAATGGCCCGGATCATGGAAACAATCTGTTC
TGGGTAATACATCTTGCAGGCATCCAGGGCCC
ATACCAGACAGCGCAGGCGCATTTTTATACCTGAAATTGTGATATGTTGACGTCCAACCGTCAAAAGC
CCGGCAATGCGGGGGAACAATGGAAAAATATC
TTTCATACGGTGTTCAAACATGGCAAACAAAAGCTTGTACGCCTTTTTCTGCAACAAACGAGACTCTG
GTACCGGGTCCATCAACACGGGCTCAATAAAG
TCATTGAAAAGCGTCTCGACATGCACCGCAGGAAGCTGAGAAATCACCGCACATGATATGTCAAGTAC
CATGCGACGTTTTCCTGTCAAAGGCGTATGGT
TTGACTTCGTCACAGCTGTAATATCCTTCCCCTGCGTCTGTAGGGCAATGTTTGTACCAAGATCCAGA
ATGCCCTTAAGAATAACCTGCATGACGTCCGG
TGTGCAAACACGCGAGAAGCTCTGAATTGCCAACAAAATTGCCGTAGAATCATGACCCTCAAAAGTGT
TACATAGCTTCGGCATGATATTGGAGCTAAAC
CTCGCAATGACGTTTTTGCAGACATCCACAGCTCTTTCCTGCGAAATGCCGTGATAAATGTGACGATT
CCATGTAGGGTCGTTGAGAGCAAGGAAGTAAT
TATCTTCTTCAAGGCCACTTTTCTGCGGCAGTGCCCTATTTCCCTGTGTAAAAGTTTCCCGTTGATCA
TCATCAAGACGCTCCTCCTCGTTGCCTTCGTT
GTTGCCCACAATTTCATCCATGTCATCTTCCAGAGACTCCGTCGTCGCAAGCTCGTGGTAGCCGTGGC
AGAGGACGTGCAAGGCGGTCGCACTGGTGTCC
ACAAAGAGTGGATGCGACATTAGACCTACCAGCTGCTTAGCAACCTCTCGAAACGAGTCCTTTGTTAT
AATTCGTGGGTAGTGGCAAAATCCGACACCCA
CACGCCAATACTGCTGCAGCAGCGCCGTCCATTTGTTCGCTAATTCCATACGCTGCTGTTTTTCGTAA
TCCAAGGCAATATTTCGGACAAACTGAATAGG
TGGAAAAAAGTTCTCGACAAAGAAAGGCAGAGAATCGTGCGACGCAATTCTTCGAAGAACACCAAGCA
CATAACTGGTGGCCCATGCATCCTCCTCTGTA
ACAGCGTCGGCGGCCTTGAGCTCCGCGTCATGTGCCCGCCACGGGTCAAACGAGATGATGCCCACAAA

CTCCTGAACAGACATGCCCTTCCCAAGAGCCA
CCATCACCCGCTCAATCTTCCCGTTTAGTTTCGGGTCGTCCATAACCCGCAGCTTGTTAAGCACCCGG
AGAAGAAAAGGGAAGGAAGGAAAACGCTGCGT
AAAAGACATTGCATCCCCAGACGATGTGCGTTTCAGATAATTCAACCGGCCAAAAAGAGATTCCATAA
TTTCCAGTGCGTACCCCCAGTAAAGTTGGCAG
TGCGGTTTTAGAAGCTCCAACAGGTGCGTGGAGACAACCCCCTCAATCATCTCACGCCCCTCCAACGA
CTCCACAATGTAGGGCGAAATGGTACGCTGAA
AGAGTCCAGCAAGCTCCGACGTCGTCGTCGATACAACATTAACAGGAGAGTCAGTGAAGTTGGCAACA
CACACCTTTACAAAACGCGGTAGGGCCTCCAC
AAAAAATCTTTGGTTCTCACTCAGTACGTAGGCTCCGGTTTTTGCATCACGAACGGAATGTGCACTTG
GTGGCGGAGATGTCACGCACAGAGCCGCACGT
TCCAGCAGTTTGCGAAAGGCCACTATGTACATTTTCATGGTCTCCACCTGCAGTTTACTCGGCTCCAA
GCGAGAGAATGCCGAAAGTAGTCGCATGGACG
GAGGTTGAAGGGAGAGCATGAGTTGTTGAGCAAATTTCATTGCCTTGGCCACAACAAGATGATTAGTG
GCCACATTTTCCGCTATGATTTGCTCCGTCAG
TGTGATCGCAGCATCGAGCTGATGCATCAATATACCTTGACCAAGGCGGGGCGCGCAGGCTGACACAA
TCGCAAGCAGGTATGAAATGTGGTGTTTCTCT
GGAGACCGTGAGAGGGCCGTAAAGGCGGCCATAAAGAATGACAGCGGAGTGACGGCCTCTCCCAGGAT
TTCAATGGACTCCACCAGTCCGTCCTGCACGC
ATCGCAGAATGTCGCGATCAAATGTCTTGTGCGTCTTTTTGTTGTGGGCCGACTGAATGCGCCTTAAG
GCCGTTTTCTTCGTCTCCACCTTCTGTAGCTC
CGCCTTCTGCCGCCTCACCGCCATCGCCTGGCGCCGCAGCTCCAAAAGTTTCTGGCGGCTCATGTTTG
CACTCTTCCGCGCATGCTTGAGGAGCGTCCTC
CCTCCCGCAGCCTCCTTTCGATGCTTCATTTTACCTTCACTTTTATTTGTTTGTGTGTGTGTGTGTGT
TCCCTTTAATTCCTTTTCTCCTCCACAA
>scaffold1013.size15167
GGTCACCAATTCCTGAACATCCTCAGCCGGGGTCACCAATTCCTGAACATTCTCAGCCGGAGCCACCA
GTTTCCGAATATCCTCCGCCGGAGCCACCAGTT
CCTGAACGTTCTCAGCCGGGGTCACCAATTCCTGAACATCCTCAGCCGGGGTCACCAGTTCCTGAATA
TCCTCCGCCGGAGCCACCAGTTTCCGAATATC
CTCAGCCGGAGCCACCAATTCCTGAACATTCTCAGCCGGGGTCACCAATTCCTGAACATCCTCAGCCT
GAGCCACCAATTCCTGAACATCCTCAGCCGGG
GTCACCAATTCCTGAACATCCTCAGCCGGGGTCACCAATTCCTGAACATTCTCAGCCGGAGCCACCAG
TTTCCGAATATCCTCCGCCGGAGCCACCAGTT
CCTGAACATTCTCAGCCGGGGTCACCAATTCCTGAATATCCTCAGCCGGAGCCACCAGTTTCCGAATA
TCCTCAGCCGGAGCCACCAATTCCTGAACATC
CTCAGCCGGAGCCACCAGTTCCTGAATATCCTCAGCCGGGGTCACCAGTTCCTGAACATTCTCAGCCG
GGGTCACCAATTCCTGAACATTCTCAGCCGGG
GTCACCAATTCCTGAACATTCTCAGCCGGGGTCACCAGTTCCTGAATATCCTCAGCCGGGGTCACCAG
TTCCTGAATATCCTCAGCCGGAGCCACCAGTT
CCTGAATATCCTCAGCCGGAGCCACCAGTTTCCGAATATCCTCCGCCGGAGCCACCAATTCCTGAACA
TTCTCAGCCGGGGTCACCAGTTCCTGAATATC
CTCAGCCGGGGTCACCAGTTTCCGAATATCCTCAGCCGGAGCCACCAATTCCTGAACATTCTCAGCCG
GGGTCACCAGTTCCTGAATATCCTCAGCCGGG
GTCACCAGTTCCTGAATATCCTCAGCCGGAGCCACCAGTTCCTGAATATCCTCAGCCGGAGCCACCAG
TTTCCGAATATCCTCCGCCGGAGCCACCAATT
CCTGAACATTCTCAGCCGGGGTCACCAGTTCCTGAACATTCTCAGCCGGGGTCACCAGTTTCCGAATA
TCCTCCGCCGGAGCCACCAATTCCTGAACATT
CTCAGCCGGAGCCACCAGTTCCTGAATATCCTCAGCCGGAGCCACCAGTTTCCGAATATCCTCAGCCG
GAGCCACCAATTCCTGAACATTCTCAGCCGGG
GTCACCAGTTCCTGAATATCCTCAGCCGGGGTCACCAGTTCCTNNNNNNNNNNNNNNNNNNNNNNNNN
NNNNNNNNNNNNNNNNNNNNNNNNNNNNNNNN
NNNNNNNNNNNNNNNNNNNNNNNNNNNNNNNNNNNNNNNNNNNNNNNNNNNNNNNNNNNNNNNNNNNN
NNNNNNNNNNNNNNNNNNNNNNNNNNNNNNNN

NNNNNNNNNNNNNNNNNNNNNNNNNNNNNNNNNNNNNNNNNNNNNNNNNNNNNNNNNNNNNNNNNNNNNNNN
NNNNNNNNNNNNNNNNNNNNNNNNNNNNNNNN
NNNNNNNNNNNNNNNNNNNNNNNNNNNNNNNNNNNNNNNNNNNNNNNNTTTCCGAATATCCTCAGCCGGAGCC
ACCAATTCCTGAACATTCTCAGCCGGGGTCAC
CAGTTCCTGAATATCCTCAGCCGGGGTCACCAGTTCCTGAACATTCTCAGCCGGGGTCACCAATTCCT
GAACATTCTCAGCCGGGGTCACCAGTTCCTGA
ATATCCTCAGCCGGGGTCACCAGTTCCTGAATATCCTCAGCCGGAGCCACCAGTTCCTGAATATCCTC
AGCCGGAGCCACCAGTTCCTGAACATTCTCAG
CCGGGGTCACCAATTCCTGAACATCCTCCGCCGGAGCCACCAATTCCTGAACGTTCTCAGCCGGAGCC
ACCAATTCCTGAACATTCTCAGCCGGGGTCAC
CAATTCCTGAACATTCTCAGCCGGGGTCACCAATTCCTGAACATTCTCAGCCGGAGCCACCAGTTCCT
GAACATTCTCAGCCGGAGCCACCAGTTCCTGA
ATATCCTCTTGCTGCTCGACAGCCTGAATGTCCCTAGTTTTTTCCACGTTCTTCCCGGGATCTTGTCT
GTTTTTCGGGCTCGATGTTATAGAGTCATCGT
TTTCTGTTCTTTTTTCCTCAGTAGTCACTGCCATTTCCTTAGGAGGAATAAACTTACGCAGTTTTTTG
TTTATATCAATCCTATCTTCAGCCTCTAGCCG
AATCATTCTTTTCCTTACTTCTCTTTCATCGTCATACACACCGACAATAATCTTTTCGTTGTAGAAGG
CCTCACACAAATGACGGAACGCCAAACGTTCC
TCCATTTTGCGCTCCAAAAAACTGTAGTAATGCTGGGAATCCAGTAAATTACGCTCATCCATGTTTCT
TTGAAATTGACGGTCAACAAGCCAACGGCGTT
CCGCAATTCGCATGTCCCGCAGGAAACGTTGTTGCATCTCGTCTCGCTCTTTCCATTTTTTGAGGAGA
GCCTCGTGCCGAGCAGCGTTGAGCACATCTAA
TTTTGCCTCAGACGTCTTTGTAATCGCCTTTTCCAATTCGGGGATGAGAGAATCAAGCATCCGAGAGA
TCCGGCGTATAGTGCGATCTCGCTCCGCAGCC
TGACGCTCTTTTATGCGCTCCTCCACTTTCTCGTCTAGTAGGGCCTGTTGCTTGCTGTGTTCGTAGTT
GGTGAATACTTCTTGGAGGCGGGAGGCACGCC
ATAGACGTATGCGCTCATTTATACTTGCAATTTCTTCTTCAACCGAAGGACTGTCATTAACCACATGT
CTTTGACTAAATAACAGCGAGTTAAGCACTGG
GCGTCCGGCATCGGGCAAGGATTCGGAGACCCCTGTGTAGAATGTCATGTCGTGTAATGCATCGCCTA
CCAAAAGCCCCTTTGAGACATCTTCTGCGGCT
GCGGCCCCTGGATAAATCGCGGGACCCATGGGCTTCCGTACAAGAGACATGGGAAAGTAATCTGGCTT
GGTGGGTTTTGTCGTAGAGGGCCTCGTTGGCC
GCAATGGTTGCAGCAAAGTTTCCTCTGCTTTTACTGCTGGTGCTCTCGAAGAGAACATCCGTCGTTTC
TCAATTTTTCTCCTTTTTACAATTCTGTTTTC
TTTACTAGTTTTTTTTTTTTTTTTTTTTAATCCTCACTTCCTTTTTACTTTATGCGTTCACTCTGCCCT
GTTCCTGTCCGAAAGCTGCGGGTTTGAGCCCC
GTCTCAAACCGAGCAAAGAGCTAGAGAATTGAAAACAGGGGCATTTGGACACATCAAATGCAATGTTA
ATGAATGATTAAAGAATATATATATATATATG
CACACACGCACACCATTAGGGGGAAAAAGCCTTCAAGGTGTAATTTACAGTTTCTTATATTGAAAAAA
GGTTATTCTCCCTTCCCATTCGCTTCTGCAGT
GGTTGCACAAACGGTATCGCTTGCAGGTGGCAACCGAGGATTAGGGTGATATTTGGGGGCAAGCGTGA
CTACTGGTTCATGCGGGTGAATCCCATCCGTG
AGAATAAACACCTCCGGGTACCGCATGAGGTATTCTTTGGGACCACCAGAGCCCCGAGGAAATATTTT
GCGCCGCACATCGCTGCTGATAAATTTTGGTA
TATGTGACGGAAGCAATTTCCGCGATCCACATGTTCCCAAACGCGCGGCCACAATGGAGACCACTACT
CGCAATTGCTCCTCAGGATCATCTAAAAGCCG
CTGCTGATATTCACAAGACGCTTTCAAAGGCCCCGTCGCAGTGTCTTGCTCTTCTTCTTTGAACTTTT
GGGACTCATCTTGTTTGTCGTCTTCATCGCTG
TTCGCGGCAGAGGCTTCCATCCGTTGGACGCAGACCTCAGGAGGAGTCTCCACCAAGCGGAAAAGGTG
TGGATATCCTCTCAACCACGCACGAGAACGCG
CGTCCGTAATGCGCCGCATAGTTTCAGGGAGCAACTTGGGCAAGTGGCCGGTAAGTATAAGCTGGCCA
TCCATGGGAAGGATATCAAAGACATAGTACGC
CAGAACATCAGGATGGCAAATGGGAGACACGCCCAACTCGTGCAGGGCAATGCAGCGCTTTAGCAGAC
GTTTTCTATCAATAATCTTCACACGCTTATGA

TTGGATAGTTGGTTCAGATCTTTAGGGGTTATTGTCTCCAACTCAGACAGGTCCTTCTCTAGTTCTGC
GGGTGACATACTACGGTACTGCGGCGGCAGGT
AGCGTTCCTCGTCAAGCCAGAAACGTACCCCCACAATGCGAGTCGAGTCGTCGGGGTCACAATCAAAT
TCCAACAAGGAGCGTTTTCCATCCACACCATT
CAGCCCTACAGTCGTCTTACTCTCTTCCCCCGCTATTGCCAGGACGGCTGCTAAAGGCTGCGTCAACC
ATCCCTCCACGATATCCTGCAGCTCCGGGAGT
CGCATAAACGTTGTTGTACTCAACGCACGGCACATGCGGTAAAAATTGTACTCCTCAATCCCATTTTC
TAAACAGTGCGACGAAGTCGTGGCATCCATTG
GGAGAGGCGCAGTAGGGACTAGGAAGCGAACAAAAGTGGAACTCCTTCTAAAAGTGGTTCCAGAGGAA
ACTTCACCATGTGAAGCTCTCTTCTCCCCCAC
GTCGTTTCCCAGTGAATCGCTACAGGCTTCACTGCGTAACATGGTCACTGCCTGATTTAGTTTCTCCC
TATTATCAAGATGCACCCACCGAAACGCCTGA
AGGACCTCTTTTCCTTCCATCACCCCTGACATTTCGGGGTCAACAACAGTCCTTTTCTCAACTCCAGT
CACTGTCACCGCTGCTTCTGCTGATGGAAACT
TGCTTAATTTATATTCTGCTGTGAGTAGCCACAATTCCGCGCTCTTCACGGGGTGCACATACCCATGA
AAAAAGTGCTTCATCATTTGAATATTCTCATC
CTTTTCTTTATGAGAGGCATTAAAATCAACCGTCCCCGACGGGCGTGCCGCGTAATTGAACCTCATAA
AACTCTCGAGAGTGAGAAAGCGGCCCGGGACA
ATTGGAATTGACAATGACACTTTCTTCTTGCCCTGAGTCTCTTCCACCTTGCCCTCCTCTGTGCTCTT
AGTACCATTGTTGCCGGCGGGTGTCACGCACT
GGGCATGGAGAAGCCCGTTCGTTCCACGGAAAATACGGTAGCGTGCAGGAAAATTTTCCAGGTGGCTC
TTGAGCCCATTTTCTAATTTCTCAAGCACCTC
ATCCGGAAGCAGTTTGGCCAGTTGCGTCAGCGGCATCGTACTGTTTGGCAGCAAAAAGGGTAAAACAG
GATCATCGTCCTCAGTGACATCGGAGGGCAGA
GGACTGCCACAGGGTGGCGACGGCCTCGAAGGCAAAGAGCTCGCATAACGAAGCATAACATGCCATGG
CGTTGACCACGCAGAACAAAATGTGATGGGCC
TTGCGCACACGGGAAAAAAACCGTGGGAATAGGAAGGAATTAGGGGGACAAAGCGCCTCCAAAGCAGC
GGGGAAAAGACACGGTGAGAACTCACTTCTCC
CGGCGACATATCAGATCCTCCCGCTTTCTATGAAACACCCGCACAAAAGACCCCGTCTTCGTCACACC
TCTACCCTCCAAGAGGCAGCACGACAGCAAAT
ACACAGGACTATTGAACCGGTTGCCAGTGAGAAAGAAGCCACAAAGCCAAGTGACAAATGCGCCACGT
GTGATTTCATTTGAAACAAGTCAAACCAAACA
ATAAAAAGAAAAAGAAAAAGTCGAAGCGTAAAAGACTGTTGAAAGACACAAATTGAAAAAACAATAAA
AGAAAAAATGAGTGACAGCAAACGACTTTCCC
CGTGTGTCTCCCTGACTATAGACGTGCCGAAGTGTTTCCTGTCGATCAATGATGCTTCCTCGGCAAAG
GAATGACAAAGAGGTAAAATACCCACACAGCA
CAAACATAAACGCAAACAAAAAAGAAATAAGTTACACAAATTTATTGACTAATTCATCGGTTCTCTGT
TTCGCACAATAACCCCACGGGTGTTTACTTCC
CCAATGTCCACAACAGAGGGATTCGGGTAATGTGGCCTTTTTCCATTCACCCAAATCCCCAGGCGACG
TCTGTTGTTTTTTTCTCTCTCCTTCGGTCTGT
TTACCCCGTCACACACACAAAAGAACTTGCTCCATCGTTCTCCTTACCACAATGCACCTCACAACACA
CATCCCACGCGTGAATAATCCTCAGCGTCAAA
ATTTGCGTCGCTCTGGAACCCATTGTTTCACACACACCGCATTGGGGCAATGCCTAGAAGGGGGAAAA
AAGAAAAAACAAACGGACAAAACGCGTGTAAA
GAACGCTGGACGTCCGCGCCCTGAACAGTGGGGCGTAACCAACCGACACTGTCATGCACCACATCGCA
TTCCGCCGCCCCGTATCTTCCCGTCGGGGCAT
AACCTGTTTCATCACAAAAAAATTTGGCAGTGGCCACACGACAACGGTCGTGGTCGCAGCCCGTCTCT
GTCAGACAGCTCAAAGCTGCATACCCGCACGA
GAAACACACGTTTGACACTCGTCAAAGGCAACCCACATTTTTTCGTCCAATGGTCAACGGAAGCCAAT
TTACTTCCCTTCAAAGAACTCATTCACATCTT
TAGTTTGCGACTTCGCCGGCATTCGATTGTAGTTTATTCCACCCGCCGTAACTCGTGCGTGACGGAGG
AGCGACAATACGCGGAACTCTTTGGCGAATAA
AATGACGTGTAAAAAGGTGATCGTTTTACAAATAACGCGCGCCTCCGCGCGTCGTCTCGCAAATGAAA
AAATTCCCCATATTTAGTTCTGAAGTCAGCAG

CGTGAATTAAACACCGAAAATGTCATCTCGTCAAATGGCATGATAATTCAAATGCATAGTGCAATCAA
ACCGTTCCTACCATTCACCCCTCCCACCCTTC
TCAAGCTGTCTCGGAAACACTAATGAAAGTGTATCAATTCCCATAGACTCAAAGAACTGCTTGCCCCT
CTTCTTCTTCCATTCGCTTCACTCACTCCCCC
ACGATAGTGAAGAAAAAAATAGGACGTCATCGCAACAGGTGTGAGCTCGAGCAAAAAAATCTCAGGTGA
AAACAGGCCTAAAGAGGCCCCTGACAAAAAGT
CGTGAAGACGCGATAAGGCGGATTGAACCCTGTCACCGTGGTCCTCAGCGGAAGAAACGTGCAGTCTG
CACCGCCCGCCCCGCAGTGCGCATGCCTCAAT
CTGCATCAATTTTCACTTCTCGCGTTACGCTCCCGTGCAAAGGGTGTGGTGTCACTGCCAGAGGCGAT
GCGGCTGATTTTTTTTACCCGTCGCACTAAAT
GACTTTGTGGCAATCAAAATCACCGCCACGCAGAAATACATGAGCCGTCCATCCAACCGCTCATCGTC
AAGGCAGCGCGGTCAGCAACGGGGAAACTCAA
AAGACGCCCCACCAAAGTATGGATTCCGCCTAAGGTCACACTGTTGTTGTTCTTGTCACGTCACTGTC
ACAGGAATGTCTTGAAGACAGCCCCACACGGC
GAGCACCAGCCGTGCCGAGAAGAACGACCTCTACCGGAAGGCACGTGTCGCCATCGCACAACATCGTC
TCCATCGCCCGCCAACTCTTCATCTTGGCGGA
GACGACGGGATCACCCGTTCTCAACCTCTTCGTGCCATGTCTGTGATGCCAGCGTTGCCGACTTACTT
CGCCCTCAACAGGACAAGAGGAAGCAGAACAG
CCAGGAATGCGAATGGGAGAATTCTTAAGAAACGGTACAAGAAATGAGACCTTTTCAGCGGCCGCGAG
AAAAAAGCAGATATTGCTACCGCTCCCTGCAC
CGCTATGCTCGGTAGCACCACCCCTCTTCGGCCCTTCATGAAAAACTTCTGTTCCTTcGCGGAAAACA
TTGCGCAGTGAGAACCGCGTATAATACTTTAC
CAGTTCCCATCCAGCGTCATCCCATACCCACGCGTATGCCATACGAGTGCCAAAGCGCATTGAAAAGA
ATGGCGCCGAAGTAACTGCCCCAACGTGAAAC
AGCCCGGCAGGGCTTCCTGGTTTCAAAACAAAAAGAGCACTTCCCTCACGTTGGTGCTCTCGACCACC
CCTCGCCGCGAATTTCAGCAGGACGCGTTGAT
GTGCGTTTGATGGAGAGCAACGCAGTCGAGCCATACGAGAGATCTGAAAAGACGCATTTTTACCGCTC
ACATCGGTAAAGGCAGGACACCCAATCAGAGA
CAGCAATGTTTCTCTTTCCTCCTCGCCAATTATTCGGACGTTGCGTCTGTACCCAAGCGATTGACAAA
GACGAAACGCCCACGCAATCTTTTCGTCGTCA
TGGCGCATCGTTCTCCACGCGGTGGCGGTGGCGTCAGTCAGAGAAGCAAAAAGCTTCCGGGCGAACTG
AAAATGACACATGCGATCCCGCCGCACGGCGC
TGCCGGTTGTGTCCAATTGGGGAAAAGACTCTTTGGAAAAGACAAGAATAGACATAACACAGCGATGC
ACAGGAAAAGAGGTATGATCAATGGTTTCGAG
GTTTGGTGGAATTGACGGGAAAACGCACACCGTCGTTCTTTTAATGAGTGATGATGAATTGTTGTTTG
TTTCATATCCCTCTTGTTGTGCAAATGAGGAA
ATCTTATTCACTCTACACCATCTTCCCACAGTGGCATGCACTCGCCCCCTTATCCATTCTAGGATGAA
TGAGCCATCCCTCGTCGACGACATATCGGCTC
CCTTCACGCTGACTGCATCAGCACAACACCAGAGTTCAAACATGGAACACATGGGTTTGGGTGCACCA
CAGGGATTCCAATGAGAAACAAGTCGCATTTC
TAATCCTCGTCTGGCACGAGCCCCAATGTGCACAGGTGTGGGGGCCACAAGATGGTACGTACCGCCAT
CCACATCCTCATTCCGCAGAAGTATAACGGGC
ACAACTATTGGTGTTGTGTcGGTGTCAAATAATTCCACGCCTCCCCTCAACAACCATTTCGCTTTGCT
AATAGCCTTTCCCAGAGACCACATGTACCCGT
AAAAAACGCCACTCTCAGTACGCCGCCAACACGTTTCATACGGCAAGGAATTAACCGGTGTTTCTGCC
ATCGTCGCCAGATCTCTCGTGTGAAGAGTAAC
CGGGGGTGCTTTACAGGTTTGTATCATCAAAATGTTCATCACCTCCTCGGTAGTTAATGGGTGCCCGT
TTTCCCGTGGTTGGATTGCCCAGACACACATA
TGAGAGCGTTCGGCCGCAAAACACAAGGGCGGTGGAACAGCCATTGGCTTGCTTTTAAGCCGTCGGGG
AGGTTTTCCCAGTGCACAACATGATGGCAGCC
GTGCAGCCCATCGCTGTAGGACACGATGCTGTTTACGCTGGCTTGTCGTCGGCACAGCAATCCGCACG
GTCACAAGCCTCGAAGAAAAGTCGTCATCCGG
TGATGAGGAACAGCCGTTAATACGCGTCTTGAGAACACTGTAATGGAACCGACGGCAAAATCGCCAAT
GGGTGGGCAGCCAAAGCACTGCCGTCCGGCCT

CCACCAGAGGAAAAGGCCTGCGTAGAGAGACACTGGGGAAGAGAAGGTTGAGGTGCAGCAGCAGGAGG
TCCACAGCTGCAAAGGGGAAGTTGCGTCTTGC
CGTAACGAAGAGCAATAATCCGACGTCGAATTCGACGACGTGTCGTCGCTCGTCGCCTCAACAGAAAT
CTACGGCGTCGCCTTAAAAGCAAGCACCACGC
CCTATAAGTTAGAGGACGTTTTTTCTTCTGCACCCGTCCTTTCTCTTCTTCGACTGTTTTCGGTGTTG
CTGGTGGAGGCTCCTCGGCTGCCAACAGTCGC
ACAAAACGTTTAGACCTCCGTGAGCGAAAATTCGCCAACCGCATCACGTCTGACAGCGGCACATTCGC
AGAAGACGGACGGCGGTAGTGAACAACAGAGC
GTGCTGTACGAGAAAAGGCCCTCTCATTCTCATGACGAGGTCGTTTACGAAACAACAAGGCAGAAAAT
GCAGCCAACTCTTCCACACGGCCGGTTAAAAA
CTGTGCGACATTCATCAAGCGGGCTTACTATTCTTGGAAGGTGTTTCCATGTGCAGGACCATATTTTT
GTTAAATGGTAAAAAAACATCAAAAAAAAAAA
AGAAAGAAATTTATTTATTTATTTATTTGTAAAAAAAAAAAAAAAAGGGAGGGGAAAGGGAAATGACG
ACGCAAAGATTTTCCCCAACCCCTCCAATAAG
TTCCAACGGTGGAAAGAGATATATATATATATATGCATATATATCAATATGCCTCCCGTCCCAGACAA
AACAAAAAAAAAAAAGCCTTGTTTATGTCGTTT
CGATAGCAACAGTTGGAAAATTCAAAAGAATGACGGTAAACCTTGAGACTCCCATTCTTGCAACGCCC
GTTCCATTGCCGCGTGTCTTGGGGCAGCCAAA
AACTTGCCAGCAGTTGTGACGATAAACTCTTTTGACAGACGACTTAGTTTCTCTACAAAATGCTCTTT
TACGCCTTGGAGACAGCGCTGCTGAAGGACCG
CATCCCAGCCCTGACGCCCCTCTCTAATTAACAATGTGTGCATTGTGTACACGAGGCACCGCCGCAGA
CCCTCTTCGCCAATGGCCTCAAGTTTGTCCGC
ATCACTCACAACGTCTCGAACAAAAACCCAGTCGGACGGCAATTTCGCTTCAAACCACCGCATGCATT
GCTTCTTCTCCTTTGAGAAAGAAATCGCTTCT
ATGGTGGTGAGAGCGTGACTGAATGCCTCTTCACCGTCCATCCCCGCAGCTGTTATTGCCCCCTTTTC
TGCCCCTAGAAACTCTCGAACCTGCTGGATGA
GCCTGCCTGCCGCATCGTACTTGTGATCAGCAACGTCATGAAGCATGGCGACAAGAATAACTCGCGAG
ATTGTCAACGAGGGGCAAGAAGCCGCTCCGTG
TTCCATGTGAAGAATGAGAAGGGCGTTCCCCGTGACAGTCTCCATGTGCGCCAGCCCATGGGTGGCGT
CACGGCCAGCACACACATCGCCTACAAACCTT
CGGCACCGCGCCCAAAGCAGGAGGTGACCCGCCTCCATCGTTGCACCACCCCTCCCTTGAAGAAAAAA
ATAAATAAATCAAACAGTGGTGAAGCAGAGAC
GTGAAAAAAAAAAAAAAAACTGTAAAGGCAATCCACCACACATTCATCCTTCACTGTTCCTAAAGTCCC
ACTTTTTTTACCCTCTTCTTCTCGTGAGTGGG
AGTGAAATGCATGCCAGATGGTGCCCCCCAGTCCCGCCCAATGGTTCAACGAGGTCCCTAAATGACAG
CCCAAGCAAAAGCAGTCTCAACAGCTATACAT
CACACGCACAAACTCACATATGCATCTTTTACCCGCTGCCGCCCTCTGCTGCATCAAACATCAGCGAC
AACATGTTGAAGTGAGACAATTCCTCCACACC
ACATGCAAACAGTTCCGCTTCGTATCGCCGTAAAAAACTCTGAAGAATTTCCTCGTCGTTGGCCTCTG
CAAATCGCCAGCCCGTGTAAACATAATGCTCC
TGGAAAGACCGCACCACGCATTCGCGAATGATAGCATCACGCATAAATGGCAAACGCATCATTTCAGC
TTGCTCACGCACACTTCCGCAGCACCCTTCGG
GATACCCGTCTGCTGCAGGGTCGTTGATGTCTGTTGTGGCGATCAGGTACCGGCTGCGTGCAAGAGCA
TAATGAAGACGGATACCCGTCAAGAGCGGATA
ATTTCTCGGCAGCAGCACCTCTAAATCCTCTAACAGTTTTTCGCATAGTTGCATCAGTCGTGTGACGT
CATTTGGCGTCATAATGGCATTCAAAGAAGTA
GCCAAGCATTCCATCCGCGCCTGCAAACGGTAATGATGTGTCGATGGAAGTCCAGAAACCAATGTGTC
AAGATCCATTAACTGACGCACGGCTTTCTGAG
CGCGCTGTGCATATTTTTTGTCATCATCATCTTCTTCTTCTTTATACTTTTCCGTTTCTCCAGCTCCA
AAACCTTCCTGCATGCAATTTCCATGTTCATG
CACGGCATCCGTAATAGACTTTCCCAAAAGCTCGCAGTGGATGTTGTCAATGATTTTCTTGAACCCTG
ATTTCATTGGCCCGGCGAGATCCTTGTAGCTA
CTGTATCCCGCGTATGCACAACCAGGCAGGTGTGCGTACGGTTTCACCCGCCCATTTGCTGTGTTATT
GGCCCTCACAGAGGAAACTACATCAACCGCTT

TAGATGACACCTCTTCCTTGTCGTTGTGATCATCGTAAACAAGCTGTTGGCACGTAGGGCACACCACA
CCCTCCAATAACGCAGCGTAAGAAAGACAAAA
GGTACAGCAACAGTAGAAGCCATTCCGCTCTCGTTTTAACTCCCTTGAAAGAAGAAGTGAGGAAAGAT
ATGTGACTGTTATTTCCTCACCTGGCTCAATG
TCACAAAGAGCTACAACACGCAATACACCGCCTCTCGAGGAACAACAGGCACCTTCTTCTTCACCAAA
GACAAATAGCGCGTTTGGTGAACAACTGTGGT
TTATAAAACCGGCAAACGGATACAACCCCTTCCCCTCCCCAGGGGCTCCCTCCAACTCAATCCTAAAT
TCTTCCACGGGGCAGAATGGAATGGCGCGTTC
CGTCCAGCAGCGCACAAGGTCGGCAATATCGCGCGCAAAACCATCCTCCGTTCCAATACTGTTTCTGT
CCGCCGGTTGCATGGTGGCAATAAGATGCAAC
GCATTCCACATTTCACCAGCCTGTGTTGGCTCCCCAAAAAAAGCACCACTTTGAAGAGATTCCACTAC
TTGTGAAGTCACCGCATCGCTTATGAACGTCA
TACCGCCACGCCGAAACAATTCAAGTGCCGCACCCGTGACAGTTGACATCTCGTTGTTTTGCATCTCC
GTCTTAGCCGCGGTTTGATCTGGTGAGCTATG
AAACAAAAGAGACCGTTCCTGCAACATGACACTGCCTTCAGAAATCTTTCCTGTGGCCACAAGTCCCA
CGCCATGTGTAACACGACACGCATTCTCAGAC
GGTTCAACAGAAGGATGCACGTAAAAAAAGTCTTCACAAGATGAAAAAGTGCCATCTGTTTCACTCGC
CACTTCCTTCACGCCAAATAATGCCCGTCGAA
CTTGTTGTCGCAAACCCCCACAGGATGCACCAAAGTGTATTGTGGCCACTTGCACTGCAGTTATCGAA
GAAATAAGATTCTTCCATGCAAGGGATAGGCA
TTCACGCGAAACCCACAATAATTCGTGGCTACTGCCCCCGCCGGATGTTTTTCTACTCTTCTCTGTGT
AACGAGGTTGAGCAGCATTCTTTAGAAATGCG
AGACAAGTCAGCAGATGAGCTTGATGCAGCAAAGCTGTCGCCTTCATTGTGAATAGACGTTGCAATAC
GCCACGACTCACTGCTATGCGGTAGAAAAAAA
AACTTGCAGGTAATGTAAATAAATTATGCAAAAGCTCTTTAGCAGTGGCTAGCTCTGTACGAGGAGAC
TCTGCAATACACTGTTGAGACACCGTCGCATA
AACCTCCTGAAAAAGTGAGCGTGCTGCCGCCGCAGTTGTCTCACTTAGAGAGTTTGAGTTTGCAGAAG
TCATCGCGTCGGGGCGCCACCACCAATCGGTG
GGTGTTGCCACGCCGTGAGATGCTCCATAACGCCATGCCGCTTCATCAGCTGGCAAGAGTGTGAGATG
CGCCTCACACCAGCGCCAGGCCTGCTCTGCAA
CCTCCTCGTGGTCCCACCAGTCGCACAGCTTTTCTGCGAGGTCTTCTTGTCCCTTCTCCCGAGAGGAT
GCTGCCATCAGCTCACAGCACACCGCCTGCAC
CGCGTTCTTGGTTCCTGTCCACTGGATGCAGGAGTCCAAAATAGCAAGAACCACCTCCACAAGGAGCT
GTGTTAGCCGCTGGTGGGGTAGTGCGGTCTCC
GTCCCCTCCTTTGCATCCTTGTCGTCATCGGCCATCTCGTCCTCCTTCTGTTCCATTTGCAACAATAA
CTTCAGCAGCGCCATTCCAACAGATGCCGCCT
CCGCAACTGCCGCCTGCGTTTGTGCTTGCCGTTGCTGCCCCAACCCCCTTGTTTGCCCGCCATTAACC
GAATTCCCGGAGTCGCCGCAGGCCCCTTCAAG
ACGTTTCGCCCAACGCAACACCAGACACGATGCGTCTTCCATCAACAACTTCAGCGACCTCGCCGTCA
CTTATTGCATCCAACACTTAAATTAAAATTGA
AGTCAAAGCACACATACAGAAAGACGCCACTCACATGCGACACGATGTGACTCACGCACATACACAAA
CACACAAAGAAAAGAAAAAAAAGATCCAACTG
CACTGACAGGTTTCAAAAAAATAAAAATAATAACAATAAAAATAAACGTCATCAAAATGGCTACAAAA
CTGGCCTTTAAATGAAGTCTTTTCCCAAATTC
CTACCAAAAGACACGGCGAACAAAAAACAATAAAAATGCTATTCTTTCGGAACACGTCAATACCTCAT
AATTCCACTGTCACCGTAAAGGTCTGTGGATG
CATTATGATAAAATGGAGGTAGTGTAGCGTTAAAAACAAAAAATAAAAATAAAAAATAGAGACAAAAC
AAGAAAGTAACCAAACGGAAAATTCAAGACGC
AGTCGGTCTGATATATTTGTGCCTAAATCACTAAAAAAATATAAAAGGGCATTTTATTCACTAAATGA
TATCCTCTACTCAGCTCGCCACTCACATTTAT
CAGTCTTAATTTCCTCTTTAACTCATTCAAAGCCCTTTCACCTGCTTGTTCGTCAAAAATTCTGCCCT
TTCATGGCCTGCATTGAGAAGTTGCAACTCCA
AGGAAATTATTTCTTCGTCGGCTTTCACCGCCGCCGCGCGGAACCGGAAGTACATGACAACAACAACC
ACAATCAGTGCAAGAAGAACGGTGCCAAGAAC

GGCAACGATGATAATAACAACATACTTATTATTTCCACCAGCTACTGGCACAGATGGGGGCACATTGT
CGGCCTTCCACCCAGGAACATCCGCCCCAGCC
CCCAACACAAGAAAACCTGCACTTTGTTCTGCGCCCCACTCCGTTTCCGACACACGGACACGAACCTG
GATTGAGTACTTGCCATGTTCCTTGCCTCGCG
GAATGCTGAGAACACAGAAAAGCTCCTTCTCCGTGGCATTAGTCACCCTGCAGAGAATAGGGTT
>scaffold1014.size7313
CCAGCGCCATTAGTGGTGTTTTTTTTACCTTCAGGATTATTGCCACCTTGAGACGCACCACCCGAAGG
ATCTTGAGTTGGTTCAGAACATCCACCACCGCC
GGCACCGCACCACAGCACGCAGAGGGCACACACCAGCAGCACACGGCCAGTCATCATCATCGCCATCA
CTCCTGTCACTTGCGTGTAGCCTTCAGCACAA
ACTAACAACACGTCAACGCAGAGTGAAAGCAAAAGGAAGAGACAAATCACAAAGTAACACGCACCGTA
AACAACGGCAGCAGCCCTGCAGAGGAACGCAC
ACTGACCTGCACACCAGAAGTAAAAAAGCAATACCAAAACAAAAAGAGTAGTTGGGTGCCCAACAGGA
GTCTCCTCCTCAAAAAAAAAAATACACTTCAGC
AGAATCATCATTTCATACGCCATGCAGCAAAGCAGGACTGTGTTTATATGTACATGCGTACAGTACCT
GCACAAGGAAAAAATTATTACTGTTCCACAAG
CCCACACAACATCCCACACATTTCATGCGGCATCCGGCCTGTCTCTCCACTCCTCCTTTTGTGTCGTC
CGTGGAACGCACAGCCGTGGGGCCTGTACCGC
GTGTAGGCAACAGAAGCAATCAAGAGGGAAAACAATTCGCTCATGACGGTGCGCAGCAACCGACCATT
AAGTAAAACAAAAGAAAAAGGTACTCCCATTG
TTCCATTAAACAACATTAAAAAGAACATCAAAAAAAGAAAGCCCGCAGGGAACACGGCACATATTCTC
CGTGAGAAGTCGCAACGCCACACTCCGTGGCC
CAGAACACTGCCACAACCACACACAGTCCCAAAAGCAGAAGCAGCAGTGGTGTGTCCACACACGAGGT
GATACATTCGCCACCGCTCACCGTCGGCAGGG
ACATGTCACCTTCGAACTAGACATCTCTACTCTCTTTATTTTTTTGTTTAAAGAAGATGTGGTCTGTG
TGGCTCTGCTGCACGCACCGCTGTTTGTGGGC
TCCTTAGTGGCGGGGGCGTGTGCTCAAGGACCATCGATACAGTCCCATCGCCCGTACACATCGGTGGT
ATTGGACGTAATGAGGTCATTATATTTTTTTT
CCTACTCTCACCAGCGCGTCTAGCTATACAATGCCATCTGGGTGCCGAAGAGGTGGTCGAGCAGCAAA
ATGAGTTTCCTGCTATGAAGCAACTGCCATTG
AGAGAACTTTCCGAGGCGAAGGTCCCGTTCAATGCCCAGCCGTTGACGCACAGCCAAGGTGCCTGGAC
ATTCTAGAAGCAGATGGTGCAAGGTTTCCTGC
GGGCCAATTGGCGTGCTTGTGGAAGCTGGCTGATCCTGCGGCAGCAGAGATCGCCTCCTGGCACTGCT
GTTTCGATGTCGTGTGGCGTCTGGGTTGTGTG
CGCAGCGACTTCTGTGCGGTGCGGTGCTTCTCCGTGTGGGGAAAACCATGTCACAGAAATCACATTTG
AGCTCGGGAAGCGGTGGAGGATGCTCCGGACG
AAATGACCGACAGTGACTGACGAGACCCGAGAATCCCGTCAATGTAGAATCACAGTGCGGACATTTCT
CCTTCCGACGGCGTGTGAGGGTCCGCCTCCTT
ACTGGAGAGACGTCTTCATCCCTCGCCGACGGGGGAACAGTGTCCGTCCCCGGTGCAGTTGGATCGTT
GTTAGAGCGCATATATGCAGACTGTTCCGGTG
AGATGTTGCAGAAACGGCATTGGTTCGTGCAATCTCTCACAGTCCAGTATAGTCGGCCCCAGAGCTCA
GATTCCCGCACCTTGCCCTTGCGAGGTGAACC
AGTTCCTGACGGTGGAGAACTTCCTGAGTGATCAGGTCCTTACCGCTGAGATCGGATGGCCTTGTGCC
GCAAAGCAAGTAGCGATGAGTGTCTTGACGAA
TGGTACCACGCCACTTGTTGGTGAGCTGGGTAGTGAAACATGTCAGCAGATCCGTATGCCATAAAGGT
GCGATTCCTTGCTCCGTGTATTGTCCACTTGC
CATAGTCTGCGTCGCATACTGATCTGCAAGCTCGTTAGCATGTACTCCACAATGTCCGTAACAAAACT
GCAGGTGCACGGACCAGCCAGCACGCGTCAGT
GTTAAGAGACGNNNNNNNNNNNNNNNNNNNNNNNNNNNNNNNNNNNNNNNNNNNNNNNNNNNNNNNNNNNNNN
NNNNNNNNNNNNNNNNNNNNNNNNNNNNNNNN
NNNNNNNNNNNNNNNNNNNNNNNNNNNNNNNNNNNNNNNNNNNNNNNNNNNNNNNNNNNNNNNNNNNNNNNN
NNNNNNNNNNNNNNNNNNNNNNNNNNNNNNNN
NNNNNNNNNNNNNNNNNNNNNNNNNNNNNNNNNNNNNNNNNNNNNNNNNNNNNNNNNNNNNNNNNNNNNNNN
NNNNNNNNNNNNNNNNNNNNNNNNNNNNNNNN

NNNNNNNNNNNNGCACATCGTTTATATTTCACGGGGTCATCTGCGTGTTGCTGCTCCCCTCAGCCCTCC
CATCGGCGCGTGAGTGCGGCCCGCAGAGCACA
GTCACTCGTGTGTGTGGGGACTGAGAGAAGCACGCAGACACGACGGCAGTCGCGTCACTCTATCGGGA
GAGTGAGCGCAGCAAAAGAAAAGAAATCATCG
GAGGGGAGAGAAATGCTCACGAGGCCGACACCGAAGGGTGCGCCACCGCATGAAATAAACACGAGAAG
CAAAACAAATCCTTAAAGAAAAACACGGCGGC
AGGGTGTGGCTGTGTGTGTGGCGCTGCGGGTGTCTGCCCGCCCTGGCCGCAAAGAAACAAAACAAGAG
GGAAGAAATGTGTGCTCCGCCCCACAGGCCCT
CGTCGCACGCCCACACGTCACGGCTCCCTCAGCGCGCCGTTCGTCGCGGCCCCTCCTCATGCGTGCAG
CAGGCGAGGCGCACAGGGCCGTCGTCGTGAGC
GTGTCGGTGGGTGTGCGAGTGGAGCAGTTGGGCAGAAACACACGGCACCCCTCGCAGGAAGAGTGCAG
CAACAAACAATTAAAGAGGGAAGAGTAGAAAA
GAGAAGAACGTCCATAGTGATTGCCCCCAAAGCAAAAAAAGAGGGATGGCGGATGAGTAAAAGATTAG
GAAACAAAAAAAGAAAAAGTTAAGAAAATAAA
CCATTAAAAGATGAAAAAAACTCACAAAGCCCCGAAGGAAGCAAAAAAAACAAGGGAAATAAACACAA
AAAACAATTAACAACAACAACAGTAGAAAAAA
AAAGAAAAAACAAAAAAGAAAGTAAAAACACAAAAAACAAAAAACAAGAAGCTGCAACGAATGGCTCC
TGTGTTCATGCAATTACACACAAAAATGCGTC
CACGCAAACAGTTGTGGGCGTGTCGTGCATACGAGTGTGTCTGGGATGCGTGCCAAGTGACAAATACG
GGGAGCAGCAACGCGGAGCACACAGCAGCGCA
GCTGAATCAAATGAAGCAAAACACAACCATTATTTCCCCTTATTAATTACNNNNNNNNNNNNNNNNNN
NNNNNNNNNNNNNNNNNNNNNNNNNNNNNNNN
NNNNNNNNNNNNNNNNNNNNNNNNNNNNNNNNNNNNNNNNNNNNNNNNNNNNNNNNNNNNNNNNNNNN
NNNNNNNNNNNNNNNNNNNNNNNNNNNNNNNN
NNNNNNNNNNNNNNNNNNNNNNNNNNNNNNNNNNNNNNNNNNNNNNNNNNNNNNNNNNNNNNNNNNNN
NNNNNNNNNNNNNNNNNNNNNNNNNNNNNNNN
NNNNNNNNNNNNNNNNNNNNNNNNNNNNNNNNNNNNNNNNNNNNNNNNNNTAACCAAGGTGAGTCCCG
CATCCGTGCACAGTTCTAAAAATTCTGCGGCA
ACCTCGCTTGGAGAAGTCTCCAAGAATGGCTCCCACTGTGGGTGATGCATGTTGAAATCCCCGCACAG
CAGGAGCGGCGACGAGGCTGTAAGGCAGTTTA
ATAACCGCCTGAGGGATTGCGTTACTTGCGGTGGTGGTCTCATATAGGCACTCGCTACAATAAGATCA
CGGTTCTGGTCCAGAGCAGCCTGGACCACCAC
CACTTCATGGCTGGTGTCATGCTGGGGGATGGTGAGGGGTATACGCTTGGAGCGGAGAGTTTTCCGCA
CCAGCACCGCCACACCGCCTCCCTTGCCGATG
CGCGACTGTGCATAGAGCACATAATCTCCAGTATGCATGCTGGCGATCTGCTCTGACGACTTCCAAGT
CTCCTGGATGGCTATTATGTCTGCTCCTTGCG
CCATTAATGTTGCGAGTTTTGACGGCGTGAGGCAAGAAACGTTCATCTGGAGTACTGCTATGGGGCCT
GGGTTCTGCTCAATGTCTCCGCCTCGCAGTAA
CATTTGTCGTTTCGCGTCACATACGGCACGCGAACGGTATGCATAGCGCTGATAAGCGCCGATGGAAT
TCAGCAGCGGATGAAATACCGCTCAGCGGACA
ACCATTACAAATGGCTCCATCTGGTGCCCTTCCGACCAGTTTGTGCGGCGTCCAGCACCTCTCCGCGC
GCAGCGGATGGGCAGCAGGCGCTTAGCTTCCT
CTCTAGCAAAGAGTACTAGACCCCGGCACCACGTTGAGGTGGCCGGCTGAGCCAGAGCGTTGTTGTAG
TCTTATTGATGGTACTATTTGGCATTTTGATC
TTCTGATTTCGTGTCTGTGCCTTATGATTCTCATCGCCTTGTGTATTTTACTGCGCCTTCTATTTTGT
GCATCTGACATACCGGTCGCACCTTGTGTGTG
TGTGGAGACTGAGTTGCCAAACCTGGTGGCTCTGGATTATCGCTAGCCGAACTCAGTGACTCTGTCGG
AACTCGTGTCTGTCCAGTGATGGCTGTGCCTG
CTTCCTTTGGCAACGAAGGTCCCGGACTTCTCAAACCCTCCTCGCTGTCTCTCCCACTCTCTGTCTGG
NNNNNNNNNNNNNNNNNNNNNNNNNNNNNNNN
NNNNNNNNNNNNNNNNNNNNNNNNNNNNNNNNNNNNNNNNNNNNNNNNNNNNNNNNNNNNNNNNNNNN
NNNNNNNNNNNNNNNNNNNNNNNNNNNNNNNN
NNNNNNNNNNNNNNNNNNNNNNNNNNNNNNNNNNNNNNNNNNNNNNNNNNNNNNNNNNNNNNNNNNNN
NNNNNNNNNNNNNNNNNNNNNNNNNNNNNNNN

NNNNNNNNNNNNNNNNNNNNNNNNNNNNNNNNNNNNNNNNNNNNNNNNNNNNNNNNNNNNNNNNNNNNNNNN
tttatttgcgtctcttaccttattacgtAGCA
ACCATTTATTTGTGTTTCTGTTTGAATGTTGGCAGATGTCTGCATGCGTTTTTTGTTTTCGTTTTTGT
TTTTGCGGAGGGCAAACCTTCATGCCTGGGTC
GTGTGTTGGGGATTTCTGCAGGGTGCACAACAATTAGTTTTTCCTCTTGTGAATGCTTCTTGTGTTGG
TGCAGCCAATAATTCGGTTTGTGTTTTTCCTG
CTTCTCTTTGGTAAAGTGCTGCAATAAAGAATAATCTTCTGAGCCGCTGGCCAAGTTCTGAGTTTCTC
AACAATAGAAACCAAACACGATGGAAATATGT
GAGGGATCAACGGAATAAGAGTGGATTCACCACACCCTCAAAGGCTTAACACCAGGTGACTGTATTTT
TATATACCATCCGTCAGCCACAATCGTGGCCC
GCTCACTCTATTCGGATGGGATACATGTATTTATGTATTACTTACGAGGTGGCAGACTTTTGGGGCCC
TGCAGCCACAAGACCCCGCCGCGTGGTGGAAC
CAAAAGGGGGCGGGTGAGACGGTAAAATAAAAATAAAAATTATCCGGCCGCTGTCGGCTCCCTAAAGT
CGTGGCCTCCCAAAACTCATACGGCAGAAAAG
TGTCGTAAAGAAGTGTATATTCGTTTTGTCAGGGTATATACACATTCATTAAGGCAGCCAGCTCTCCC
GCGATCATTCTCTACGCAGGCTCCTAAAAGGG
GCTCAAACACACCCACAAGACTCCCAGCAAGAAAAAAGGGAAACACGGCGTGACTGACACGACTCAAA
GTCATGCTTCTAAGACAATGCGTGAATGACTC
CGTCGACGCCACACCCGCACCATATGCCATCAACGCCTCCACGGCAATAATCCTGCAGCGTGCGGAGA
GGACGTTTCACGAGCGCATTATTGGCGGAGCC
AAACAACTGTACCGTCAGCAGAGGGCAGCGAGCGGCGGGGGAAAGTGAGGGTGGTGGGACCGTTGGGG
AGGGTCGATGCGCAGTGTCCCCCACAAATACG
AGGCGGGTGAAGTGAAACACACTCGCCTGAACGTCACCGGCCACCAAAATGCATTGCTTCCCGCTGTG
AATATTGATGAGCTCGAGGCTGGAGATGCTAC
TCGCAATGCGTGAAAGCGTCTTCCTGCACTGACGCTGCGATTCACACTAGACGGGCAACTTCAGAACA
TTCGATCCCCAGCAGTAAAATGCAGCTGGCAG
TGGCGCTGTGGATTCTCGATTCCCCTGGCAACGAAGGGCGGAGAAGAAAACTGGTCTCCCCAGCTGCG
CGGCATCCATCCATTCACTCTGTGCGTCCGGA
GTGCCCCGCAGCTGCCTTGAGAGATGCGGCGGACAGACCTCCCTTACTTTCGCCTCGACCCGTCGACG
AAGACTTTCGCGGTGCTGTCGGCACACACCGA
ATTCTCCCTCGTGCCCGCCAAACAAAAGACACACCCGTTGAGCATCGTCACGTGCGTCGACGGCTACG
CAAATAAATACCCACCGCAACGCAAAGTGTTT
CTTATTGGGAACAAAAAGAAAACTGGAGGCAGCGGCCTGGACAACACAGACATCAAATCACACGCCAG
GCAATGCTGAGATACATGCGAAAGGGCCAACG
CAGGATCAGCAGCACAAACACTCGAGAGCTGCTGCGAGAACTCCACGGTAAATGGATCAGTCCCAACG
CATGGGGCACTAATTACGGCGGGCAACAGCGC
AAAAGCCAACGGTTCCGCAAGACCGGGAATTGTGGAGACGGCAGCCAAACCCTCCCGGGATTCGAGCA
ACAAGACACACAAGTAATTCGCCCCTCGGTGC
AAACCGTCACCATACTCCTCATCGCTCTCTTTGCCGTCATTCGTATTGTCACACTCAAAGCCGTTGAG
GGAAGGTGCTGCCATGATGATTGTGACATCAC
ACTGAATGAAACAACGCAATGCCTCAAAGGGTGCCGAGTGCAGGCATCCTGTCAGGCAGCGACACGCC
GCACTTCTTAGCAATGTACGAAGCAAAGGCAA
CGAAACAGCTTTCATCCGCCATCGTAATCTCGTCCAGCAGGTTCGCCCTCTTGTTCCTCGGCGCCTCG
TTCTCCGCAGCACCAACAGCCGCAGCCGAAGC
CCACTCTCCTGC
>scaffold1015.size9041
TTTGGGTTGAGGGGCGGGGAATTTGTGCACACACACACACACACAAGGGTTACTGGAGAAATTTGTTT
TTGTACAGTTGACTTTTGTACTCCTTCTGCTTT
TGCTTTTCTTTTTTGCTTCGTTTTCTTTTTTCTGAGGGAGGACTTTCTGTTTTTTTTTTTTTTTTTTT
ATTGTCTCTTTACTGCCCGGGGGTGGTTTTGT
TGTTGTTGTTGTGTGTTTTTTTTTTTTTTTTTAATATTAATTTTTTATTATTACTTTTTTTTTCGTTTT
TGCCGGTGCAAACGATAAATGGAGGTGATTGG
CACGTTATCCGCAGCTGTGGCCCTATCCAAGACCACCAATACTTTGAATCCTGCCACGGAGTCGTCGG
GCTCATATGTTTTAGCGAGCATCAGTCATTTA

AGGAGTGACTTTGTGCGGCCGAACGGGTTGCGTGTTTGCGTGCATAGTGGCGAAATGTTAATGAGGAC
GCACATCCTGTCAGACCCAACGCCTCATTTGG
AGAAACGGCAGCAGGAGGAGGAGGGAAGTGATCACCAAACGCATCAGGGAGAGGGCAGCGGTGCCATG
CCAAATGGTGAGGTGTTTGGTCGTCTTCTTTC
GAGAGTGGGAAGCGGCAGTGACATGTCAAATTTAGGTTCGCGGCATGAAGTGACATCTGCGGTGTTTT
CGTGGCGGTGGCTGGTGGAGAGGAATGACGTG
GCGCCGTTCAATCACGAGTTGAAATCAACAATATCCAGCGGTAAAAGGACGGACGATCGCATTTTAAC
TCCGCCTGGGCCTATCCACACGCCGCGTTGTG
CAGGTTCGGGGCGGATGGATGATGAGGCCTTGATTGCCATTGATATGCTGCGCGACTGGGGCGAAAAG
GAAAAGGAGGAACTTTTTTTACGTCACCCGCG
CTTGGAAGCGTACGCCACAAGAGAGCTCCACACGCCGCTCTCTGCTGTCCTGACAGGATTTCCGCTTG
CTGGCACTTCAATGAACGATAGAAATGCGAGG
CCGGTGGCAATGTTGGCTCCATTACCCAATCTTCCGTCTCAGGGAATGACGACTACGAATAAACGTCA
TGCCCTAGCATCCCATCCATCATCGACAGATT
TGGGGCTTCTCTTGAACCCCAGGGTCTCGACAGAAAGGAATTATACACCTCAATTGGGTAAGTTGCTG
TCTGGGTTTGCACAGGAGGGAAGGCAAGTGCC
ACTGGAGGTTGCATCCGTCAGTCACACCGATGACATACCCGAGGAGGACGATCTGTTGAGCCGGGGAC
TCTGCGGTCGTGGCGATCCGATCTTGGGGGAT
CGCGGGAGGGTGTGGCCCTGGCGGAAGGGCGGGAGGATAGACAAGCGTTCTCTGCCTTTGAACGACGT
CCTGCTGGAGTGCCATTTTTTTGGGGTGACAG
ACATTCTTGAAGTTTTGCGGTATGACTGTGAGGGTGACGGCAGTGATGATGTGCTTTTGACCAATTTT
CAAGATAACAGTGAACTGAGCAGGTCAGTTAT
AGGCCGGGTTGTGGCGCTGGCGGACCGATCTCTGAGACGGCATGCGGGAGAGGATGAATATGGGGAGA
TTAGCACCAACACAGATGTACACTTCTCTACA
GCGGGAATGCAGGAGAGGAGGAATGACGCTCTTCAGGCCTCACGCATTTTAGTAGATTCCTATCAGTC
TGCCGAGGATGAACTTGGCTACGTGGTGGATC
TGTGGAGGATGGCAGCGGAGTGTGCGGAACCTTCATCGTCGTTGACACCTTCTACAGAGACGGAGGTT
ATCACCTGAGAAGGTAAAACATATATAAATAA
ATATATATATTTCTCCACACTTATTTTAANNNNNNNNNNNNNNNNNNNNNNNNNNNNNNNNNNNNNNNN
NNNNNNNNNNNNNNNNNNNNNNNNNNNNNNNN
NNNNNNNNNNNNNNNNNNNNNNNNNNNNNNNNNNNNNNNNNNNNNNNNNNNNNNNNNNNNNNNNNNNN
NNNNNNNNNNNNNNNNNNNNNNNNNNNNNNNN
NNNNNNNNNNNNNNNNNNNNNNNNNNNNNNNNNNNNNNNNNNNNNNNNNNNNNNNNNNNNNNNNNNNN
NNNNNNNNNNNNNNNNNNNNNNNNNNNNNNNN
NNNNNNNNNNNNNNNNNNNNNNNNNNNNNNAAAAAAAATAATATAAAATATAAAATATATATTTCTTCC
ACACTTATTTTAAAACGACAGAAGTTAAAAAT
ACTGAAAAATAAAAGCGTCTCAGAAGAGATGTGGTAACCGGCACGATAAATAACGATAATGCCAAATA
ATAACAGTAATGATAAGATGTATTGCCTGTTT
AACTTCTCAACGCTTATATGTACATAAATATAAATAAATATATAAATATATATTTATATAAAAAGAGG
GTTTTATGCATGCAAGATGCATTGAAAGGACG
GTGTGATCGCTGCTGTTTTTTTTTTCTTTTTTTTTTTTTTTGACAGATTTTCTGCTGCGTTGATGATT
ACACTGGATTTAAGTTTTTCCTGTTTTTTATT
TATGAGTGTGCGTGTGTGGGCGCATTTATTTTATTTTATTTTCCGTTTCGGCTTTCAGCGGCGGCTGT
GGAGATGCGACATGATGGCAAAATAGAATTCC
TTCCATTCCCAACACCAAAAAGGAAGAAAAGAGAAGAGAACAAACAAACAAACAAACAGGAATAGAAG
AGGGGAAAAAAGTGAAAAAAAAAACGTGAGAA
GTGGGGTGAAGTGGACGGACCTTTGGAGTCAGTTGCGGTGTATCTATGTATCTCTTTACGGAAGGAAT
ACCACTGTCGTTGTTTCCCGTAATGCGTGTCG
TTTAAAAAAACAAACAAACAAACAAAAACTTGTGCAGAGACGTGGTGATGTTTACATATATATATATA
TATCTTTTTTCCTGCTTGCGCCTTGAACATCA
CGACTACCCGAAGAGAGGGCACTGAGGTGCGGCAACCCCTTCCCATGCCATTTAATTTAGTGCCATGT
GTACGGTCATGGCGTTAATTCCCCCGTATGTA
TTTAATTTGACACACACACACACACACTCTCTCTATATATATAAATATGTGTACAGCATGCATACATG
TATTTCTTTTGCCTTTTCTTTTGCTTTCTATT

TCTTTTTTGTTTTTTCCCTCACGGCGAATTCACGGTGCGGACTGGTTTGCCGCGGCCCTGAGCAGTGA
AACCAGTGGGTTAAATCAAGAGAAAAAAACAC
AGAAGAATATTCCTGGAACGAAAGGAAGGACGCGCGCACACGCAGGGGAGGCCCGAAAAAATTTCAAA
GGGGGAAAGAAGGCGTACTTATTAGCCTCAAC
TGTAATTCAAAGGGGAAAACAACATCAACAACAACAACAAGAACAAACAAATAAATCAAGGGGAAAGA
GAGAGAAGTCATCCGACGACACAAGGGTGGAT
ACATACATACTTACACAGAGAGAGAGAGAAAAAAAAAAAAAGGGCAAACGAAAAAGCATGCCAAAAAAT
GAAGACGATGAGGTGGCGACACTCATAGCGTT
TATGCCTTGGCTGCAGCGGGAGGAGGCTCTGAAACTGTTAAAAAACTATGGTGGTGACGCAGAGAGCG
TCATGCGGTTTCTCGAGGAGGCAGCCTCTAGC
CAAGGGGAGCCGCAAGCGTCACCATCACCAGCGGATCAGAGGAAGAAAACGGGAGCCAAGAAGGCGGA
AGGCAAGAAAATGACCGAAAAGTATTTTGTTG
GCGGAGGTTCGTCGTCCGGACAGGAGGTAATTGGCGGGGATGGAGGGGTAGATAACGTCATCGAGTCT
CTTTTTGACAAGGCGCGTCAGCAGTTGGGTGA
GGAGGGTGCTGAGGCGCAGTTCTTTTTTGGCCGTGGACGACGTCTTGGCCATACAACGTCGGAAAGTC
CGTTTATTGAGTCCACGCTGAAACCACGACGA
GAAATTGTGCTCACAGTCTACCGTGACAGCTATCGTGTTGACGATGGACCGCGAATGCCGAAGGAAAG
CGAAGAAGGCATGGCGTTTTTTAAGGCGCTGG
AGGCGGGTGTGGTGCCTGAAGGAATTGCCGCAATTTACCCCAACACAAGCATTTCTGTACGACTGGTT
GACTGTACCCAACAGGATGCTCCGCCAACTTT
CTTACCCTTTGCCGGGGAGGGCCACAGACTTGATTCCAATACGTCAACGACTGCACCAGCCGCCTCTT
CTGTGGCGAAAACAGGTGCGGATGCCACTCCT
GTGAATATGAGCGACAATTTCGAGCTTCATGAGGACGAGGAGGTGACAAAGCTGGCGATTGTGAATCT
TATTGGGGAGCGCAAAGAGTTTAGGGTAAACC
CCAATCGCCACACCGTTGGGGATGTTTTCTTTTTGGCGGCGCGGCACGCACAACCGGGGGCGTTGGCC
TTTCAACTCATTGCCCGTGACGTCCCTCCGCG
TCCATTGACGGATCGTTCTCTGACGATTGACGTTGCTAATTTGAGGAATGCCACGGTGATTATGCGCC
GAACTTGAAGTAACTTTGCAAGGCGACCATAG
TGAAACCCATAATGAGCATTCAATTTTATTTTATTTTATTTTTGTAGAAGCCACTGGAATGCTTTCTT
GCTACCGTTTTAGACTGCCGCATTTGTTCTTC
ATATGTGTATACATATACATGTACGAGGTGAGGCTTTCTCTGTTCCTGTTTTTCTTCTGTTTCTTCTT
TTTTTTTTCTGCCTTTCTTTCACATCCATGAA
TATGTGACGCTAAAAATGAAGTGGCTGCACATAAACACACTACTCACGGAAAAGAAGCAGTGAAGAGA
AAATAAATAAATAAAAAAGAAAACAGCAACAA
CAATACGCAGGAGAGAGGGAGGGTTGGATGTTACGCCGATCGTTTGTGCTGTGCATTGCGACAGCTCC
AATTGCAGTGCCAAAGGACATCCACGATTGGT
CACGACTACGGCAGTACTTGGAAGGCTTCACAGTAGGAGCCTGCACAGACACCGGAGTCCTGGATGCC
TCCATCATACCAACGCGGCACCTACAGCGAAG
TCTTTTTCGCCTTCCCATTCCAAAACTAAAGAGCACATGCTCCCGCTACCTGGACGCCGTCAAGCCTT
TGGTAACACCCAAACAATTTGATACAACGTCC
AAAATTGTAAAGGACTTTGAGTCGGGCGAAGGACTTAGCTTGCACGAGGAGTTGGTGCGTACGGACAA
GGCAAACAAGCACACCTCTTACATTTCCGCGG
ACTGGTTTGATTTGTATCTATTGGATCGAAAACCGTTACCGGTGAATACGAATCCATGTCTTATTACA
CGACGAGACCCGGATAAAGGGGATATGCTTAC
TCGTGCTACGTTCTGGATTACGAGCAGTGTGGCCTTTTACCGCATGTATCTTGAAAATACCCTGAAGC
CTGATATATTCAATTTTGGTTCAAGAGATCAT
TACAGCAGGAAAGATTGGTTTGAACGCACGGTGGCGCTTTCGCCAAGGAGCATTTCCGCAAAAGTGTG
CGTGTTTGGCTCTAATTTTCACGCCTTTCCAC
TGGACATGTCACAGTACGCTAATTTGATGAATTCTACTCGTATACCAGGATTGATTAAGGATGAGGTT
AGGGGATTCGGTTTTGCACCACATATTATTGT
GCAGTTCCGTGGCCACCAGTACGTCGTGACTGTTGCTGACAGCGAGTGTAGACCGCTTCCAGAGGAGC
AGATTTATGCGAGACTGAAAGCCATCATAGAT
TTGAATCCAATGCCTCCAAGGGTGGACATTGGGGTCTTTACGTCTACTGACAGAACAACGTGGCATTC
CGTGCGCACGGAAATGTTGCGTGATAGCGAGA

ATCAAAAAAGCTTTGAACTGCTGGATTCAGCTATGTTTGTATTGAACCTTGACGATGATGTGGAAGTG
GACTTTTTGACTTCTCGTGGCGCTGTCGAAAC
GATTCGTGTGATGCTTGTAAGAAACGTGAATCGCTGGTGGGACAAAAGTCTGTCGGTTATTGTAACAA
AAGATGGTTTTCTTGGTATCAACTTTGAGCAT
AGTTGGGGGGATGGGGTTGCGGTGCTACGGTACACACAAGACGTTTTTAATCATTCGATCATGCGCAG
CCGCAGAAGCATGCGTCACGATGCAGCTCCTA
CAGAGGGAGTGAGGCAGTTAAAATGGTGCTTGACACCCAAACTGGAGCAGGAGGGTGCCAAGGCAAAG
AGACGCATCGATGCGGATATTCGCCGGATGGA
CTGTTGCTCATGTCTTGTAAATGAGATTGGATCATCCGATCCCGTATTCCGCAAGGGTCCAATAAAAG
TTGACCCGTTTTTGCAGTTGGCAATGCAACTG
GCTTGGTGGCGCCTGTACAAGAGTACAGTCAGCACATATGAGAGCGCCAGTACCGCGGCATATCTTCA
CGGCCGTACGGAATGTATTCGTTCCGCGACGA
TTGAAAGTCAGGCCTTTACAATTTTGATGGATAACCCGCGTGCCACCACCAACGAAAAATTTGAAGCG
CTTTTAAAGGCGGCGAAGAAGCACTTGCAGAT
TTCCAAAAATGCCAAAATGGGTGAAGGTTTGGATCGCCATTTGTTCGCTCTACAGAATGTGGCGCTGC
GCCGCAACCCGAATTCTATACCGGATTTATTT
TTAGATCCCTCATACAAAACACTTGGTAGCAACATGATGAGCACGTCGACACTTCGCTCGGACGCCCT
TGTTGGCGGTGGTTTTGGCCCTGTAAGTCCCG
GATATGGTATTGGCTACGCGTCGGAAAGAAAATGTTTGTGTTTTAATGTTTCCTGTTGGAAAGAGTTT
GGACCGCGGTACTCGGCCGACGAGTTTACGCA
TGCTCTTTACTCCGCTGTCGTGGACATGTACAATATTATGCTTTCAGTCAAACAGAATGAGTGAGATA
TTTGGCTCCAGGCATGAGTGGCAGCCAAAAAG
GCGCTGCTGGGGCGGGGGAATAAAGCCATTGGGTTTGGCTGGTGGGTTAGCGGTCTTTTGGGCTACGT
TCTTTTTTTTTTCTTTTTGCTTTTGTAAGTGC
GCGCTCTGGATGCTTGTGTTCTACCTGAATCCACACATGTCCGTTATTTATTGTCCTTCTTTATGGTG
TGCATGTGTGTGTGTTTTGGTCTGCTGTCTGC
GCCGCCGGTCTTTAGTACAGCTTCTTTTTCTTTTAAATTTTCTTTTACTTTGCCTTTGTGTGGCTGGT
CACCGCGGTGGTGTGGCACGTCGTTCCCATTG
CCATCCGGCCCGGGAGGTTTACTTTGATATTTAATCCAAAGGCGAGGAATTTCTCCTTCTTTTATCCC
TCCACACATCCACCGTCGCGTAGGATTTTTTT
TTTACACTTGCCATGGCCCCCAAAAAGAAGAACGCTTCTGCTGCTGCTGCGGCAAAAGAGGAGACAGA
AGAATGCGCCCCGGCTGAGGTGACAACAACAA
CACCCCCCACATCCCCGCAGACTGAATCATTGCCCTCCGCATGCAACGCTGTGGAGTTGTACGCAACC
CTTCAGGCAGCTGTTTCGGAAGATCCTGTAAA
GCTGAATGATTTTGTGCGGGAATTACTCCAGGCGCTGAGCGATGCGGAGTGCCCGGATGCACCGCTTC
TGGTCCGTGTGCGTACCCTTGAGTTTATTAGC
AAATACGGACAGCATGTGCGTGACGGCAATGCTCTTAAAAAGATTGTTACATCTCTTGTAAAAATATT
GAGTGGCTCAGAGGACACGCCGCAGCTTCTTG
TGGCTGCTGTTCAGGGGCTTTCATCGCTAGGGCCTGTCTCTGTACTGGACAAGAAGTGGGAATATTTG
TCCCGCGAAGGGGCAGATGTTCTTATGCAGGT
AATGTTGGACGAAGAGGGATTTGCGGAAAGTGTGCGTCAAGCCGCTTCCAAGGCGTTGGACTCCCTCA
TTCAGACCGCATTTCGTCCCGTTGTGACGAAG
CTGCTACACTGGATAAGCGACAATCGTGAGACGGAGGACGAAGATCAACTGAAGAAGGAGCGGCGCAT
GGCTCTGACGCGACTGAGGAAGCTGGCGCACA
CACCGTCCCTGCAGTGCCAGTGGACGGAGGAGGTGCAGGAACATGTCTTGAGCCTCATTGTTCGTGTG
CTTCCAACGGTCACTGTGCAGGAATTTGTGCA
ACTCACGAGCATTGCTGCATCATTGCCGATAGTACGAGCAGAGGCGGGAGTGCCTTTCCTCAAGGCGT
TTTTGGCGCAAAATGCAATCAACACTGACCGT
GTGTTGGAGTCCCTAAGTATTATTGGAGAGCATATTGGCGCCACACCGTACGACATTGTTCCCGTTCT
TGAGGATGCAGGGCTTCTTACAACACCTGTTG
AGAAGAATACCGCACGGGGCATGTGGCACGCGAAGGTGTTGTTGCTGGCTGCTCGACTTGCCACTCCT
GATAACATTGATAAATTGTACAATGCGGTGTT
GGAACAGCTGGCGCATGTCATGCATGATGGGAGTGCGTTGCCGGAGAATATGACGACTCTCGAGGTGC
TACTCTTTGCCGTTATGGCGGCAGGACAAAAA

AAACCAGTTGAGATGTTGAAGTATTTGAACGATGATGCGTTCGTTGCGAAGTGCATGGGTCTGGCGGA
GCTCGTCGCCCAGATGGAACCACTCCTGATTT
ACGCCGTTAAGAAACGTATGCAGAAGTCATCGGCGGGCCAAAAGGATGCGGAGGTAATTGGATGCTGC
CATAACGTGCGAGTCATCTTGAGCGCCTTCTC
CGCCAAACACATGCCCACTGGTACCCTCACGGAGAGTTGGCTCCACAAGAATAAGTTGCCAAGTGTGA
AACACGCCCGCGACCCAGCGGCGACGGCAAAG
GCAACAGAGACTACAGCGGGGATTGCTGGGGCCGCCGCCGGACAGTTGGGTGTGCTGCCGCCTCCCCC
TGGCACGGAGGAACATGCTAAGAAAAGGTCTC
GTGGTCAGGAGTCCGGTTACAACAAGAATGTTAACAGGAATAAGGGTGGTTGGAATGGCAACGGCCGG
TACAAGAGAGCACGTCGTGGCGGAGTCTACTA
ACTGTGGGGAAGAGTAAGAATTTGCCAAGAATGAAAAAAA
>scaffold1016.size9001
TCCAAAAATCCCTTCTGCCGCCGTCATCGCCGTCGTCGCTGCTTGCGCCGCGAATGCGTGCACAGAGC
TCCAGAAAAAGCACAATTTGTCGCACAGACTGC
TTCGCCTGCTCTGCCATGTAGCTACGGTTGGCAAGGAGCATCAACAAAACAAGTTCAGCCTTTAGCCG
CTCGATATCTCCATGAAACGGATTGACCGCCC
GCAAATCTGACGCCATATCGCTGCTCAGCAACGCACCCAAAAGCCACTCCAGTGGAACAGCAGACCGG
CAACCTTGCATTCGCTGCAGCCGGTACTCCTG
CACACGCGTCTCAACAAAATCGTCGACTTCGTTAGCCGTAGGGCCTGTATGGGTAATGTCTCTGTCGT
CTTCCTTCTTGATGGGAGCAAAAATATCATTG
GCGAGGTGCAAAACCGATTCGGTGAGTTCCCGCACGTCTGCCGCGTCCGCTGCACTTAAGGTGGACAA
GGCGGCAATGCATTTCTGCAGTTCCTCCGTCG
ATGTCATGAGTGCCTCCTGGACAGCAGACATGCAGTTGTCACAAAGAATCAAACGCAGACGATCTCGG
GTAAGAAATGACAGGGAGTAGTGTACCTGCTC
TCGCTGCTGTTGCGCAAAACGATCCGCATCTTCTTCAAGTCGCGATAACAGGTGCTTTGCAAGTGGGG
TGCTGCACTGGGCATGCTGATAAAGATCAAAA
GGTAGTCGAGTACGACCACCGCTACATGTAAAAGCATCAGGATGGGACCCGCTCTCCTCGGTACGCAT
GGAGATGTTCTCGCTGCAAATAAAGGAAGACA
ACATCTCCACCTCGCTGAGGGGTGCACCAAAGAGGGCTGTTAATCGCTCCCGCATCCTCAGTGCAGCT
GGTAATTTATGTTGCGCCTCTTCTTCCTCAGG
CCTCACCCACGCCCCTTGCGTCTTTGCATGGAGAGAATTAAACGACAGCTGCAGCTGCTCCATACTTG
TGTTCGCAGGAATAGCGCGTGGCTGCAAATGA
TCCGCCATACAAACATACATCTCGCGGTCCATTGCAGTGTGGCTCTGACTCAGTTGAATCCGCTCCCG
ATGCCGCATCACCGCCTCATGCCGTTCAAAAA
CCCTGCGCGCCACTTCATTCACGGACTGGTAGAACTGCACGAGAACGTGCGGCGTTGTTTGGAGCCTG
CCCTCCCCTGTTGACTCCGTCAGGCCTACGCC
CTGACGCATACGGTACTGCACTCGCGCCTCTGTCACCGCATCCGGCCAGCCAGCAGAAGGCACAAGGT
CAAGCAGCTGCAGCACTGTGCTGCACCACGAG
GGCCGCATACTTGTCTCTCCATCCACCTTCGTCTCCCGTCCCCAGCGAGCGTGTCGCAGATGAAACCA
TCGGCTAAGAAGCTGCCCCAGCGTCCCACAGA
CATCCTCCCCAAAGAGCTTCGTTGATAAAATGCCATTTTTCAGGCAGTACAAAAAGTAAAACCCCAGT
TTGCTATTCGCCCCGCTCTCTTCATCAAGAAG
TAACTTATCTGCCCAGAGAAACTCCACCAGGGCTGTCTCCTGCAGACTATCCGCTTCCGGTGTCTCGT
ACGTGAGACGCTGTAGCTTTTGTGGCTCAATA
AGCGACCATGGATACAATAGAAGGCGCTCCCACGGGAATCCACAGCGAACCACCGCCGGTGCCTTCAT
CTTCACAGATACAGGGAAATCCATAGCCAAGC
GCGATGTTGTTCGCCTCCGTTCTTCCTTCACCAAGGCGGCCCACAGCTGCAGTTGGGCACGAACAATA
GCTGGCGCCTTGGAACAACGTCGAAGAAGGTC
CAGGATTTCATCACGCGTCAGCTGACAAGCCCGTGAAACATGTCGTTTCTTACACAAATACCCGGCAA
GATCAACGTGTAGGGGCCATGGAAGTTCCTGC
GTGCTGTACTGAATGGCGTGTGCCAGTTTGAGCCGTACGGCGTGGGCATCGGGAAATTTGTCCGGAAG
CGTCCGCTCCATCAGCCCAAACATGAACGACT
CCTCCACGCTGAATGTGCTGTCCGTGTAGCATGATTCCAACGTTCGCATCGCTCCTGTATAGTGTCGT
GTGGCGCACTGCACAACGGCGTAGTACAACGC

GGCACCAAGCGTGGGTGGTGTCAAAAAACTCCCACAAGGATGCACCAAAAATAAATAAAAAGGAGACG
CCACATTTTCCTGCCAGCGCAAAGAGGAACGA
TCGAATAGCAGCAGTGGGCTAAATGGGTCATCCCGCACATCCATTGCGGTAAATTCATGGTTCGGCAC
CATAAGCGCAAACCCATTGGTGTTGTTGCAAA
GTGTCAGACACTCCTGAAAGGGCCGCCGAAGATGCTGCAAAAAGCCGCCAACGCTTTCATCCCGCAGA
TTTCGTGGCATGTCATGCGCTTCAGCCACATA
CCAACCGGGGTGATCGACAAGATGCAGGCGCAGCTGGCCCGTGGCGGTGTCAACTTGTGGTTGAAACT
TCGCACGAAGTCGTGGAAGTTCAATGGACACG
ACATCCGCCAAAGTCGACGTATCCTCCTCGTCCAGGCGCTCCTCGTCATCTTTGAGCGGTGGCTGCTG
CGATGACACGCGGCCCCACACCAAAATATGTG
AACAGTCCTCCAACTTCGTTAACAGCTGCAGCAAAGGGGAAAGCGCGGGATTCTGCAGGAGATTCAGC
AACACGATATGTTCCGCCTGCCGGCCTTCTCC
TCTAAAGTCCTGTTGCGGGTCACTATCTCTGAAGGCTCCCTCCTTCTGTTTCTCCCCCGCATGAACAT
TCGGGCCCAGTCCTTCTGCTgCCTCTTCATTC
CTGGTCACCTCCTCATTACCCGCGGCCCTTTGTTCTTTCTCCTCAATGGCGGTAATACTGTCGTAGTT
GGTGGGATCAGACGCCCACTGAATTGCCGCCG
TAACGTCCCCATGCACATGCCGCAGCAACCAGCGACATGTCGCGTCCGATAAGGACAACAATGCACTT
CGCACCAACGCCACCTGGGCGCTGCTGATGTC
TTCATCGCCTTCCTCTTCATTCTCCTCAACCGTGTTCCTCCCCGTCGCACTCTTGTCGCGACTTGTGA
GGTGATGTGATGCGTCATCCAGGCGCATGACG
ATGCAGTCGCCCTTCCCAGGCCGTAGTCGCACCTCCAGGGCGTAATTAAACCAGTAGTCCTGTGCCTC
ACCCATGGTGTAGCCACGAATGACAGTGTCCT
CGTCCTCGTCCTGCCAGAATAGAAATGCCTCAAGTAACGCACTGGGCAACAGGCCCTGGAGCAATCGC
GCCGGCAGGTACATTTCCCGCAGATGAAGACT
GGAGTTGTAGCGATGGATTTCTATACAACCCTCACACCGTACTCGCCGCATCAAGTCCCCTGCCTGAA
ACGCCGTAAGGTGCAGTTCTTCACGCTGTCGT
GGCCGCACCACAATCGGGAGACTGTGCAGACAGTAGCGCTGATTGGATGTGAACACAAGGGAGCGGTA
CATCTTGCGTGCGTGGGAAATGAGGTTAAACA
CATGAAAATGCGGAACAGGATGCTTGTACGCAATCAATTCCTTCCACGTTGCCCGATCCCCTTCTTCA
TATTGAGGGGAGTCGTTCATGATAAAGCGCAT
CACACGCACCTCACTTTCTACTCCCTCGTCCTGCCGTTCCTGCCGCGCATTGACACGTGCCTCTGCGA
CGGGAAAGTACTTCATGCCACCCGCCGGAAAC
ACCTCTCGTAGCACCGCCCGCACAATGTCCACAGCCCACCGCTCCGCACGTACCGGCCATGGTGCCTC
ATCGTACACATCCACAACGCGGTCAAATATCA
CGTGGTCGTACGACGCCGAGAGAACCGGTTCTATTGGAAGATTCCCTTGCAGCACAAGCGGGGAGTAA
ACGCCCTGATCCAGTGGGCTTGGCGCTGTCCA
CTCAATGATATCAAACGGCGTACCGACAATACGCACCCAGTGACGGTGTTCGTGACGCATCACAAGGC
CACACTGCAGCACATCGTGACCAAGGATTGTC
TCAAAGTCCGTAAAGTGCGACATGCTATCAGGCACCGGCTTAAGCTCATCGTTGCGCCAGAAAAGCTC
ACATGTGTGCACATAAAAAACAAGGCCAGTTG
AAGCAGGTCCGCGGTAGACACCAGGCGAACACAATTCCCACAATGTTTCTGTTGTTTCCTCGTTGGAT
AGATGCGGGCAAATTGCACCGGGGCCTCTGTG
AGTCAATAACGACAACTTATTACTATCCATGCTCTCAGCAGCACCATCGTTTGTGACGTTCTTGTTGT
CGTGAATGTGATCGCGGAGCACCGTCCGTACG
ATACGCTGAAAAAGGGTAGAGAGTTCGGTCGCGGAAAGTGATTGCAGGTATCCAACAATGGTGTGCAA
CTCCTCCAGTAGGTAACTAAAGACGACATTCT
CCGGCACATCCGCTGGCGGTATTTTGAGCCGCCGCGACTCCGTAGTACTGCGGGCGTCGCTACGAAAA
AGATTCGGTAGCCGCACCGTGTCGTTATGGTA
GCGGCCGCGGATCTGCACAAAGACGGCGCGGCGGCGACCACCGCTAAGCATCAACTGCTTCCCCTGCT
CCAGTAACTTTCTCGAATTTCTGGAGGTGTCA
AGACCTTGGGCCTGCAGGAATCGCTGCAACTTCTCCTCCGGGGACAAAAGACTGTCCCCCTCCTGTGC
GGTGAGTTGTGTGCGCTGCATGCCCATACCAA
AGCTGTGTCGGGCTCGCACAAAAGCGCACGACTCAAGAAGAGACACCAAGTGCTGATGGGATGCCATT
GTGCTAAAAGAAAAAGGCGAGATGTCCGCACC

ACGCTGCTGACCACTCTCCCCATCAACATCGTGTTCGTTTCGAGAGGGTGAAGACAAACACTCCGTCG
TCAGTGCCATCTTCCACGCCGCACGATAAATG
ACGCCCAAATAACTCTGCAGCACGCACTGCGTCGGGGTGTCATCATTCTCTTCGCCCTCACGGCACCA
CTCCTTTAAGGTGGGCTGTACTGTCGTGAGAA
GGAACCGCAGCATCGATGTCTGGTAGGCCGTAAGCGTCTCCAGTTGCGCCGAACCCTCTAGCGACGGC
CATCCACGCACAATATTACCAAAGGCAAGCAC
ATCACACACGACACCTACAATAAACAATACATATGGTGCGTTCGCGCTGTAGACAGAAGAGGGGCCAA
TTTCCAAAACACTTTCAAGGATACGGCGCAGC
GGATCAAGCACAGACGCGGGGCTATGTCGTAATTCGTTCAGCAGCAGGCCGTACGTGGTGCCGAGACA
CGACTCAAAGTTCTCCACCGAGAAGTCGCCAC
GCAAGTGACGCAGCTCAATGACCTTTCGCTGCTGCTCAGTGAGCCGCATTGGCACCATAGTCGGATTT
GCCTTCTGCAGCGTCTCCGGCGCAACAAACAC
GGAGGGTTCAAAAATCACCGCACGGAACACATTCTGTAGGGCGGATGTAAAGAGATACGCATAACGGT
CCTGTGAAGCAAAAAAGTCAAGAACAAGCGGC
AGTCGCACATAGGGCACCGTCAGACATGACAACAAAATCTCAGACTCCTCCTCACTCATTGTGCCATC
AAAGGTTGGCATAGAAGTGGCATGCAGCACAT
CGTCCTCGGTGGGGTGCTCCACGGAGAGAAGGATACTGACGTCGATGGGGGACTGTAAAACAAGACCC
TCACCGTAAAGCAACTCGCGATCGCCAAAGCC
CTTTACGACAATGTCGGCAATGTCACTGTCACGGCCACGTACCAGAACACACTCCCACTCCGGAGGGG
AGGGGGTTGTACGCCAGCCGGCGCTCATCTGG
CGCCCGGGTGCATCCTCATCGAAGGTAAGACGCCAAGTCGCATATTCGTCCGTTTCTTTGCGGCGACG
CAGTAGCTGCGCATCACGCATCTCCATGGTGG
CAAGAAATTTTGTGAGTAACACCATGTCCCGCAGCCGCGCAAATTCAGGATGCTCACGTGCCAGTGGA
GTACGTGTGCTGCAGAGCCACTGCATCAGAGT
GTCCATCTCCGAACCCGCAGCGGCAAAACCGCCTAAGTCATGCAAACTGTAGGAGCAGTGGTCAAGAA
AGCGGCGAAGAAACTCCACAGTGACGCTCTCC
TTGCGTATCTCCAAATTTTCCGACATGCGCATATCAAACAACTCATGGGCGTAGTGCTGCTGCTGAAA
TGCAATGTAGCGCAAAATGGCGGAACGTGCCA
GCAAGAATTGCGGTCGCACCAACTCCAGAGTCGCACTGATAGAAGCAAAAGACAGCGATTGTCTGCCA
ATGGAGGTGGAAAGATAATAACCGCCGTTACT
CTCTAGCAAGGATGCAAGAAGACTGCATCGTCCACCGCCTCCTTTGCGTCGGACCGCTGCGTCATACA
CGATAAGCATAGCCATGGCAACCAGGCACCGT
TCTGAATCAAACGCACGCGTCGGCACCTCCACTGCCTGCCAAGCAGTAGTGTACGATAGCACAAGAGA
GTAGATGCTTTCTTGTATGCATTCCTGAACAT
CACCTCTTCCACCTCCGCTGTTGTTGTTGTTCGTAGAGTTCGGCTCAGGAAGTGGCTCTTCCGTGTAG
AAACTCGACACAATCGGCTGCTCTTCAATCGG
CACCGCGCATGCGTCGGTTCCACGTGGAAGTGGCAGGGGCACAATTTCCAAAAAAAACCGCGAGACAA
GCTCAATGATGTGCATTTGAATCGCCACGCGT
GATGTTGTGCCACTGTGACGTGCACGCAACCACAACGCCTCCACGGCCTCCACTGCGCGCCTGAGTGC
CACATCGAGCTCCGACCATCCCGTGGCACGGC
GCACATCACTGATGTCCTTCAGCTCCGGCACAACCGCCTGCACCGCCTGCCCATAATACGCCGCTGTG
GATGCGGACGGCAGAAGACTTGCGTTGGAGAA
CGTGCTTGCCTCCACCGTCACAAATCGCTGCTGCGAGAGCTGAAATGACTGATCCTCTCCCTCTTGCA
TCTGCGTGTTGTTTGAATACACATCCGCTGGG
CCATTGGCGGAGAGGCCGCTTCTCGTTGCTTGCGACTCTTTGGGCAGCGCATCCACCATCCGCTGCAC
GGCACTAAGCAGATCCAAAACACGCGTGGCAT
CCTCCGAGGTGAGTGTGCCGCCATCAACAGAAAGGCGTTTATTTGTGTGGTGTGCAAGTTGGGCGGCG
CTGCAGCGAAACATCGCCACGTCTACTGCCGT
CAGGGGCGTGGCGCCACTCCACGGGCCCCGCGGAAACAATGTGGCTTCCGGGTCTGTGCGAAGTGCGA
CCACCCCACGCCGATGCACAAGTCGACCATCG
GAAGAAAAAAGAAGAAGCTCCGGAATTTGGCTGATGTCCAAAAGTTTTAGAAGTTCCGTGCAGGGATA
GGCGGCGCGGGCAAATGTAAACATTGATGCAT
GCTGTTCATAGGCATGAAGATCGCGATCGCAGCTAACAAAAAGAACCTCAACACCCGTTAATGCACCA
AACAGTCGTGGTGATGCCTCCGCAAGAAGAGT

GGAAAACTTCTTGCATGACGACTCATGTATCCCCCCGCAGTAGACAAGCACCGTCTTCCCTCTAAAGT
GTTCCCGCGTGAGGATGACGTTTCTGGCATTT
TTCAAGATGATGTTCGCTGCTCTTGCGGTGCGCCAGACATCGTCCATGTTGGCACTAGACATCGTGTC
GTCAACCCCGGCCAGTCGCAGCTGCCGCATCG
CCCGTCGGATGCGTAAAAGTCGAGTGTCATCACTGCCGGCCACAGCGTCCGTACGCGTCTCATCATCA
AAATTTGCCTCGGGACTGCTGTTGATGTCAGT
TGTATAGGAACGCATAAAGCGACGAGCAAGCGCGCTGAGATCCTCCGCTGCACGCATCATAAACTCGT
ATTTGAGCGCGTACTTTATGGATTTTTGTACA
GAGCGTGCACGGACACCATACTGCTCCAAGAGGTACACAAATGCCGTGACGGCTCCTTTGATGGCGCT
CGATGCGCTGCGCACCGGTGTCGCTCGATGCT
CGTGAATGGCGGCAGAAAAACAATGCCTGGCGGCGGTATCATCATCCTCGTCCGTTCGACTATCACAA
GTGCCAAGCAGCATCTCCGTCGCAAACCGCGG
CGGGTCCGCGTGTTTGCCTCCCTTCTCCACGAGCCACGGCAGCAGCACGTCGTAGAACACCTCCGCGC
GGATAAACTCCGACCGAGCACCCGACCCGTTG
CTACGCATCCACAGAGAAAGCAACAGCAGCCAAAAGGTGTGGTCTAGCAGACGTGCGCTTGCTACACC
CTCAAAGCGGAGAATTGGACACGACACAATTT
TCTCCGCTGTTTGCCACGACGGGTGGTACGGCATCCCCTCGTGCCCACGATTCACGACAACGACGTCA
AAGTTCTCTGGCCCACGCCGGTGAATAAGCAG
GTATATGGCGTTGGCAGCATTCAACCCGTTCCAACCACCTGGAAGCAGAAGCCACTCCCCTACCGCAA
GTCCATCTACAGATGACACGGCCTTCTTCACA
ACAGGAAGGACCTGCTCTGGTTTTTGCGCCTGTTCCGCCTCCTGCGCAAAGTAGTAGCAGCGGCGCAG
CGCACCGTGTGCTCTCTGAACGGCCTGACGAT
CTGCCGCACTTAGTTGCCGCAGCAGCGGGTGGGAAATGGTCGCCTGCTCCTCGTTTCCCAAAGACTGC
AAGAGTGCAGCGGCGGTCCCACCACCGCTTGC
TGTGGCCACGCCCAGCGTGTTGATACGCGTGAGCGCCACGTCAAGTGTCAGAAGCGCCACCCCCACCC
CGCCGCCCTCCAGTGAAAAGCCCTTCCCCTCC
ACGTAGGGATCCGCCATAAAGCCGGCAAACGATGGAAGCAGCCAAATATTCAGCGGAAGATTTCGTAG
CAGTTTCTCTCGTTCCTCCTCCTGCTGCTTCT
GTGCGGCGGAAGTGGTATTCACTGACACCACCTCCGGCACGGAAGAGCCAATAAGCATCAACCTTGCG
TTGTTGCGTACGCCCATTTGCTCAAGTGATTT

>scaffold1017.size7196
AGACGTCCAAATCATGGCTCAGAACCCCTCAGCAGTAAACAACAAAAAGGAAGTGCCCGACAGGCGAA
CATGCATTCTTGACACCGTCCTCGACGCCATCG
GCTCCACCCCCTGCATCCGACTCAATCACGTGCCGAAGAAACACGGCGTGTGCTGCGATGTGGTTGCA
AAGTGTGAATTCCTCAACCCCGGTGGCAGCGT
CAAGGACCGCATTGCTCGGCAAATGATTCTGGACGCAGAGGCGAGTGGCCGACTGCGCCCCAATCAAA
CAATTATTGAGGCGACAAGTGGGAACACGGGC
ATCGGGCTCAGTCTGGTGGCTGCGGTGAAGAAATACCCCATGATCATCACCATGCCCAAAAAGATGTC
ACATGAGAAGGAGGTGACCCTCCAGGCCCTGG
GAGCAGAGGTGATTCGAACGGAGACCGCATTGCCATGGGATCACCCGGATAGTCTCATTGGCATGGCT
CGGCGTCTGGAGAAGGAGAAGGGCTACGTGTT
GCTGGACCAGTACCGGAACCCAAGCAATCCGAAGGCGCACTACGAAGGCACGGCTCAGGAGATTTACG
ATCAGTGCGGTGGAAAGGTTGACATGGCGGTG
TTCACCACTGGGACGGGCGGCACAATGGCCGGAGTGGCAAGGCGATTGAAGGAATTGCTGCCCAACAT
TGTCATTGTCGGCGTCGATCCGTACGGGAGCA
TTTTGGCCGATCCCTCGGTACCGCTGGATCCGAAGCCGTACCTCGTTGAGGGCATCGGGTACGATTTT
GTGCCAGATGTCTGCGAACGGAAGTACGTTGA
TCGGTGGGTGAAGTCCGCTGACAAGGAAAGCTTTGCGCTGGCGTCGGAGGTTCACCGCGAAGAGGCGC
TCCTCGTCGGAGGGAGCAGTGGTGCCGCCATG
TGGGGTGTGCTGCAGGCAGCGAAAGATCTTCGTCCTGACCAGCGGTGCGTTGTGGTATTCCCGGATGG
CATCCGCAACTACATGACAAAGTTCCCGGACA
AGAATTGGCTGGTGGAGAACGGCCTGGAGGAGGGCGAAGTGACACGCCCAACTTACGGGTCGCTGGAG
GCGCAGCTGGAAGAGGCAAAGAAAAAACTGTC

GGAGTATGAGGCGAAATGCGGCGGCAGCGTGGAGGAGGGCAAACGTTGCTGTGAAAAAGCAAAATAAT
ATATGAATACATACATATATATAAATAAATAA
AACATAAACATACATAACGGGGCAAAACGAGGTAAATTTTGAGTGAATTCTGGGATCAGGGCGAATGC
AAGGCAACGATAATTGCATGGGTACGGCAGCA
TTGTTAGGGAGGGAAGTGGGATGTTTGGGGGGCACTGCGGGCGAGCGAAGACAAGAGCAAAAAAAAAA
AAAAAAAAGCGTCCGCCATGAGAAATACTGGG
GAAATATGTGCAGGGAGACAGCTGGTGGGATGATGTGTGCGGAGGCGAAACGGAAAACCGGCAAAGCA
TAAGAGGAATTGCGGGATGGGTGCTGGGGATG
ATTGATTACCGCAATTAACCGAATACACTACTAACATAACGCCAGAAATGGAGGGAGGACCTCGTGTC
TTCTACGGGTCGATGCAATTCGGGTTCCCAGT
GGTGAACCGATGATGCATGCCCTGCTTTGGATCCGAGAGGAAGGGCAAAGCAAAGGGGAGGGGCGCGA
TGTTTTTTTATTTTTTATTTCCTATTTATCTT
ATTTTTTTTTTGAATGTGTTTTTCTTGAATATGTTTGGTGTGTGTGTTTTTATTATTGGGGGCGTGCG
AAAAGAATGTCCGCGAGAATGCGGGCGACCTA
ATTTAATATGTTCTGATAAGACGAATATGTTTCTGCGCGGTCCTTTCCCATCTTAGGGTTCCTTGGAA
GACCGCGATGGGAGGAGTCAAGGAAACGATGA
CGGCAGGATGAATTTTATTGTTTTTGCCGTCTAACCCCCTTTTTTGACTCCAACCCTGCGGCGGGGTC
TTGTGGTTGGAGGAGCCCAAAGTCTGCCACGT
NNNNNNNNNNNNNNNNNNNNNNNNNNNNNNNNNNNNNNNNNNNNNNNNNNNNNNNNNNNNNNNNNNNN
NNNNNNNNNNNNNNNNNNNNNNNNNNNNNNNN
NNNNNNNNNNNNNNNNNNNNNNNNNNNNNNNNNNNNNNNNNNNNNNNNNNNNNNNNNNNNNNNNNNNN
NNNNNNNNNNNNNNNNNNNNNNNNNNNNNNNN
NNNNNNNNNNNNNNNNNNNNNNNNNNNNNNNNNNNNNNNNNNNNNNNNNNNNNNNNNNNNNNNNNNNN
NNNNNNNNNNNNNNNNNNNNNNNNNNNNNNNN
GTAAGCAATAATATTTCATACACTCCCTGAGTGCAAAGGCCACGTGAGCGGTCAACAGTATATATATA
TATATATATTATAGGAGGGAAACACGGAGGCA
ATTAACTGCATCTTCGATACTCCCCTTTTTCTCTATCTTGTTTGATTTTTGTAAATAATTTCAGGATC
CAGCCAGCGGCCCAGAAGATTATTCTTATTTC
TATGTTGTTTGTTTTTTATTATTGATAAGCTCAGGATTCTTCCTGCGTCTCGGAAGATTATTTTTTTT
CAGTAAGACTAAGCACAAAAACATTTTCAGAA
ATTGTTGTAGTTGCGTAATCAAAGTGAAGCAAAATTTTTTTTACCGATTTGGTGCATTGGTTTTATGC
CATGAGCTAATTTTTGGTTTGCACCGTCCCAT
GGGTGGCTCGTGCAAAGTTACGCATTGGCTGTTTTTTTTTCCGATTTTTTTTTTTTTAATGTCTATTG
AAGTGGTAGCTTCAATGTGGCGGAATGCTGAA
TGCCCCGCGGCAGACGAAGGTTCCGTGTTATTGCAAAAGATATTTCCCCCAGCAGACGGGGTTGCGAC
AGCCAAAAGAAAGAGGAAAAAGAAGAGGAGGA
GGGTACTTTATGATCAATATCTTGCGGATTCGGGTAGTGAGTGCTCTACCGGGTCAAGTGAGGGAGGG
GGTGAGGTTCTGAATGCGTACGAGTTTGAGGA
GTTTGAGCCCGGCATGGACATCAATGTTCTTATTCAGCAAACTGTGCAAAATGTTGCACAAAGCGTTA
GCTTGGAGGATTATGATGGCCTGTCGCAATTT
GGTGATTACGACTTGATATGTTTTGCTGCTGCGGTGAAGCACAACAGCTCCATTCTTTCACTTCAGAT
AAGGTACCTTGACGTTTCTGATGTGTCGCTTG
TGCCACTTTGCCGTGCATTGGAGGGTCATCCCTGCATCCGGGCCTTGGATCTATCAGGCACGCGAGGT
GGAAACGCCACCATTAAGGCTGTGTTTCGTCT
TGTTTGCACCAACCCGAATATTTTGTTCGTCCGCTTGGACGATACAATGGTAGCTTCCCATGATGCTG
GGGAAATAGAGCTTGCAACTCGATATAATGCA
CTAGGGTGTCCCGATCCGACGAATAACCCATTTCACTTGGGACTTTTGCGCAAGATTGGTGACATGGA
GGAGGAAAAGCAGAAGTATAAGGAGCAGCTGT
CGGCGCACTCCTGGCTTTTTTCGGATCATACTGGTGGCGCTGCGAATCAAGCGACGAAGAGGACGAAG
AAGTTTCTATTTGCTGAGAAAGTAGCTAAGTC
ACGTATCGGTGCCGATGTCTGTGCACAGTTCATGAGTGGTAGATGTAACTATGGCTCGCGTTGCAGGT
ATATTCATCCTGATAAAACAGCGGCACTCCAG
AATGCCATTACTTTAAGTCAATATAAAATATCGAAGGAATTGGAAGGGGATGTGAATTCTCTGAGGAC
CTGCGCCGCTCTTCTTAGTTCTGGTTCCAAAA

TGAGACTACAAAGCAGATTGCGTCCTGCAAAGTTTGCATTGAAAGAGCATGCATGTTCTCGCATTGTG
GCTGAACCGAAAGACGTCGATTCTTGCAGTGA
AGTGGATGATTTTTCTACGCCAAACGTTGTGTGGGCGCATGCAGTTTGGATTTTTTCTGTTACTGTTG
TGGTTTGCAGTGTTGTCGTGGCTGTAAATTAT
TGATAGGGTGGCAAAATTTAGTGAATGAGTCTTATTGGCTTGGAGCCAGCGGCGTGTGTTTTGTTTTA
TAGAATGATTTTTGGCCAGTGGGTATGGAATA
GGTGTGTGGACGTGTGCGTGTGCAATGCGTCTTTGTTGGTGATGTATCCGTACATATATATATAAGTT
GCGATATCGCATTTCCTTTGAGTGGGTCTATT
TAATCCTTTTGATGATTGTCTTTAAAATACCCAACAATATCACGAATAGCCGCGGTGACGGACTGCAA
ATTTATACACAACAAACAACGAACGAAACGTT
AAACGTACTAAACGTCCATGAAGAAACATTTCTTCTTGACAACCACCGATGTTTCTAAGCTATAATCT
TAAGTAAGGCACAAGGCCAATGACAGGCGTTT
TTTTGAAGTCATGCGATTCACCACCCGCCAACGCTTGCGGCATTCCCATGGTGGATGTGCAAGGCGGG
TGCCAACTATTAACAAATTCACAGGTTTTTCA
GCAGAATTGCATCTCCCCATCTTTTATACCAATATTGAAAGAGATGATTCCTCGGTACGAAGCAATGC
TGGGGTTTGGTGGCAGTTGGTTTTGGAATTGC
TATTTAAATGCGCTTCACGGAGAAGATATATTTCTGGATTTGAAAGGAACTGGAGTTAGTTTGTCAGA
AGATTCTTTGGAAGCGCAAGTATCTCTACGGC
GAAAGTTGGCTTCTTTGGATGATGCCAACATGTTTGAAGTTGCTGCTGAACACTGCTGCTCAAAAGTT
GAGGCTCTCTGCGCCATGTGTCTTTCTATCGT
TCTGCATGTATCACATCGGCCGATGCTGGAGAGTGAACAAGGACCCCAAGCTTTGGTGCTTTGTGCCA
CAAAGGATCAGTGTGATGAGTTATACGTATTG
CTTGATCGTTTTGGATCTGCTCTTCATTTAGTTACGCACAACTTGTTTGGGGCGTACCCTCCTATGCC
AAGTGGAAAAAGGGCGGATATTGTTGTGGGCA
CCGTGCCGCTCTGGGAGAGCGTGGCAAAATTGAGTTTACTTGGCACTCTTGATGGTCTTGAAGAAATT
CTCTATCAGGTCTCGCACGATTCGCGTTGGAC
GGCGTCATCAACGCGGTGGAGACCATATTCGCTAGATTTTGTTGTCCAACTGTTTTTTGTGGATCTGG
ACCTGCAGCATGCTTTGGGATACGGACCAATG
CTGCAGCGGTTATTTATGGAACACAAAAGGCCTGTGTTACCGAGGCCCAAGCCAGTAAGGAATAAAAT
TCTTCACGGAACTAGACATGGCTCTATCAGTT
TTAGTGGGCTCCCACAAAGATGTCAGATGTATTGTTTGGTTGGTGGATGTGGGAAAGGAAAGGATCTC
TTTGAAATACTGCTTGCTTTACGTGACAGGCG
TGGAGATGGCAGGAATAAAAATGAGGTTGTACGAATTTCCATGAAACAACCCCTGGTTCACCCTGACA
AAAACGGCGATCTTGTGACCGACGCTCCTCGC
AAGCGTGTGAGAGATGAACATGATCATGGTGTAGCTATTGAGAAGGAATCCAAGAGGGAATGTCCTCG
ATTGGTGGTGCACAACGCCCTTTCACACTCTC
GGTTAATGCTTGATGCAGCCGCTTTTGAAGAATTTGCAGCAGAGACAAAACGACGTGCTCAAAATTTT
TGGTCAAGGTTTGAAGCAAAATCATCTCCTAA
CGCAAACGAAAACCACAGAACTTTTGTCATAGACGTACAGTTGGCATACTTATGCATAGGCCCGGACC
AACACTCTGTAAAGAATGAGAGGACATGGTTC
ATGGCGGAGACAGTAGCCATCATTACACCCATAGGCGATGGTGCCGCACATCCGTCAAGGACTGCACT
GCTGATGCGTCACTTGGAAGATGAGCTCTCCG
GGCAGATATTTGATGGAAGTGTTGTACAGTGTCTTTTCCAGCGCGGTTCAATCGATCGTGTGGTTTCT
GGTGCTGTGATGTTAAATCGTGTTTTTTCTGC
CACGGCAAAGTCTATTTCTCAGGAACCGCTGTTTTTGCGCTTTGCTACTGAGGATGGAGAAGAGAAAG
AGGAAGCAGGGGAAGCAAAGAAAGAAAACACG
GATCTGGAAATGGTGGATACACTCACAAACCTGGCACACCTTCGTCAGCTTGCTCAAGAAGCATCGTC
TGAGGTGGAAGGGTCATCATCTTTTCCACTGC
ATAAATTTCTTGCCGTTTCTAGATGTCAGTCACGGCGCACTGTTCTCTCCTTTCGCCAGATTGCTCCA
ACCATCTCGTTGTTTTGCGACGCTCAAAGTAA
CAGCAAATCAATGGCGCTTCTGTACTTGGAGGAGTGTTGCCAGTACGGGAAGGTTGTTTCTTACTTTA
TATTTGGACAGCGGCGTGGTGGGAAGGACGGG
AAGGGCTGCGTACATTTCTTTGTGGAGTTTGCGGGACATGAAGGGGCAGCAGAGGCTGTGCGGCAGTT
TTCATTGCGTTTCGCAAGCGAAGGCAACGAAA

CGGAAAATCACATGCGCGTGAAGCTTTTTCCCAACGAGTTGTATTATGAGGGGGTCACGGCCGAACTA
CAAAAAAGACGTAAAACAGGCAAAATAGATAA
CACGAGTGATAATAGTAGTGATAGTGATGATGATGAATTCATGGGGGTTTCACTCCTCGGTGAGTAAG
GATAAATATTGGGGGTAAATAATGCCGTGTTA
TAATTGCTCGTGGTAGTATCGAGTGGGATGATGCTGGTGGTGTTGGTGTCGCAGCTGCCGCTAGAGCC
ATTGAAAATGGGGAGTAGCACCATTTATTTGC
GTGGTTTTGTTATTTTCTACTAATTTGACGAGCTGACCAAAGAAAGCCGGCATTTCTTTCTGCGTGCC
GCTCTATTTGTCTTTATCTACAGTAGATTTTT
TATTATTCCTTCCAACATTATTATTATTATTAGCTTACGGATTAAAAATAAGCGAAATGAAACATACA
CGTACATATATACATGTATGTATATATTTGCA
ATACAATTACTTTCAAAAATAAAAATTTCTTTATTTTTTTTTCTTGATGGAACTGGGGCTCGCTATTC
TCCAACTGCGTGGCTCATGCGTCGAAGTGTTT
TTTATCCGATAAAGAAAAACATTCTTTTTCTCCCCCTTTAAAATTTTTTTTTTTTTTTCTGATTTATG
CACAAATGAAACAGGTCATTCCGCTAC
>scaffold1018.size7617
GTGAGTTGTTGTTGTTGGGAGTGACTGCGGCGGAAATGGTGCCACTCACTGTGGATGGGTGGCGGTGC
TTATTCTTTTACTTCTTTTATTAGACTGTTTTT
TTTCATTTCTGCCACTCTCTTACGAAGAAAGCAAACAAGCACTAAACGCTACTGTGAAATTCGAAGGC
TCTGAAAGGACCAACAGCACACACACCGCCCA
CACACATATTTATATGCTCTCACGTGTTGCTTCTGTGAAGGCACCCCGCACACACAACCGTCGCCGCG
TGACCGGATCCAGCGGAAGGAGGAGGGAAGGA
GGAGAGAGTGAGCCGCAGAGGGCCAACATGTCCCGGCGTGTGTTTACTTCCGCGGTGCTGCTTCTCCT
CGTCGTGATGATGTGCTGCGGCAGTGGTGGAG
CTACAGCCACTGATAATGGGGGTTCAACCCCGAGAACTTCACCGGAGAAACATTTTGTTTGGAGAGAT
AAGAACGGAGAAGAAACAGTGAGTTCGCTCCA
TGTTCCCAGTCTTGTTGAGGTGGATGGTGGCGTGTTTACCGTTGCCGAGGCGCAGTTGAAGGAGGGAG
AAGGCGATTTCTTTACTGGGATTGCCTCGGAG
CTCCTGGAGTGGACCGATAAAGAATCAAAAGAATTGGATGCAACTAAACTGAAGACACAAGTACTGGA
GGAATGTCCTGCCGGAAATAACAGTTGTGCCT
CTCCAAAAAGAGCTCGGGAGGCTTCTGCTTCCCAAAGCCGGACAAAAGTACGTGTGAGACGGCCAACA
ACAGTTGTGAATGGGAGTCGTATTTACATGGT
TGCTGGAAACTACAGTTTTGAAGTTACTGATAGGGCAGCTGGTAACACAGCCGCAGCAGCTAAATGGG
GACTTTTGGGGGCTGTGGGAAGAGTCACTAAT
GATGGGAGTAGCGGTAAAAAAAAAATTTATTGGAAAGACGCTTTCGTTATCTCGTTGACTGATTTTGA
AAAACACGAATCCTTGACACGGCTGATTGGCG
GCGGTGGATCGGGTGTTAAGATGAAGGATGGTACGATTGTGTTTCCCGTGGAAGGCACGAAGAAGGAT
GGAAACGCCGTTTCGCTGATCATATACATAGC
CACTGAGGATGGGAAGCTGTCGAAGGGGATGACTGACGGTGGCTGCAGTGATCCCTCCGTTGTGGAGT
GGAGGGAGGGAAAACTCATGATGATGACTGCG
TGTGACGATGGCCGCCGCAGGGTGTACGAGTCCGGTGACAAGGGGGGGTCGTGGACGGAGGCACTCGG
GACACTCTCGCGCGTGTGGGGCAACAAACACA
AGGGAAATGAGAAGGGCGTTGGGAGCGGTTTCAGCACAGCGACGATTGATAACAGGGACGTGATGCTC
GTTACTCTGCCGGTGTATTCAAACAGAAGGGG
AAACGGAAAGGGTGAACTCCATCTTTGGCTGACGGACAATACGCACATTGCTGATATTGGGCCGGTGT
CCGGCAACGATGACGTTACCGCCAGCTCCCTG
CTGTACAAAAGTGCTGGAAGTGGTGATAATAATAATGAGAAGTTGGTTGCACTGTACGAGAAGAAGGG
CGATGGGAAACCATCACTCGGCATGGTCTCTG
TGCGTCTGACTGCGCAGCTGAAGCGGGTGAGGGAGGTGCTGGCGACCTGGAAGGAAGTGGACGACCGT
GTCTCCAAGCTGTGCCCTTCAACTGCTGCGAG
GAATCCCTCAACTGGCAATGACTGCAGCGCTGGTAAGATCACGGATGGGCTGGTTGGCTTTTTGTCCA
AAAACTTCTCTGGTGGCACATGGAGGGACGAG
TACCTCGGGGTGAACGCCACAGTAAAGAAAGGAACGAATGGGGTGGCAACAGGGTATGCGGATGGAGT
GACGTTCCGGGGAGCGTGGGCTGAGTGGCCCG
TTGGCAGTCAGGGGGAGAATCAGCTGTACCACTTTGCGAACCACAACTTCACTCTTGTGGCGACGGTG

TCCGTCCATGGTGGGCCGAAGGGAGACAACCC
CATTCCATTGATGGGTGCGACGATGAATGACAAGAATACTGTGCTCCTGGGACTGTCGTACAACAAAG
AGGGGAAGTGGCAGGTGCTGTGCGGTGGCAAA
ACGACCAAGGAGCTCCGCAGTAATTCGGAGCCAGGGACAACACACCAAGTGGCCATTGTGCTACGGAA
CGGAGAACAGGGCTCCGCGTACGTCGATGGTC
AGCGTGTGGGAGATGCGTCATGTACATTGAAAAATGCGGATTCGAAAGGAATCTCACACTTCTACATT
GGAGGGGATGGAGGCAGCACAGGGAGGAAGGA
AGACGTGCCTGTGACCGCGACGAATGTCCTGCTGTACAACCGCCCGTTGGATGACAATGAGATCCGCG
TCCTCAACGCAAACAAAATTTCTATTCCAAAG
CTGACGGATCTGAGAACAGTGGCGGCAGGAGCAACGGGTGTCGGTACCGCTCGCCACTTTGGGGCAAA
TGGTGATGGCAGCGCTGTTTGCGGAGGCGGAC
TGCTGCCGCTGTTCCTTCTGCTGGGACTGTGGGGGATTGCCGCTTCTTGAGGAGTGTGTGGCCTCGGC
GTGGAACGCCTCAGCACCGAATTACTTCCCCA
TGGGAACAAATTTCACTCATAAATCTTTTTTTTTGCGTATTTTAAATTTATTTTCCTGTTTGCTTCTT
TTTGGCTGATTTTACTTTAAAAAGCTTTTTTT
GCGTTTTTTTATTATCTCACATCCCGCCATCCCTTCTTTTTTGCTTTTTGGGGCAGTCACTATGGACG
TTTTTCTTTTCTACTCTTCCCTCTTTAATTGT
TTCTTCTGCACTCTTCCTGTGAGGGGTGCCGTGTGTTTCCCGCCCAACTGCTCCACTCGCACACCCAC
CGACACGCACATGACGACGGCCCTGTGCGCCT
CGCCTGCTGCACGCATGAGGAGGGGCCGCGACGAACGGCGCGCTGAGGGAGCCGTGACGTGTGGGCGT
GCGACGAGGGCCTGTGGGGCGGAGCACACATT
TCTTTCCCTCTTGTTTTGTTTTCTttgcggccagggcgggcagacacccgcagcgccacacaNNNNNN
NNNNNNNNNNNNNNNNNNNNNNNNNNNNNNNN
NNNNNNNNNNNNNNNNNNNNNNNNNNNNNNNNNNNNNNNNNNNNNNNNNNNNNNNNNNNNNNNNNNNN
NNNNNNNNNNNNNNNNNNNNNNNNNNNNNNNN
NNNNNNNNNNNNNNNNNNNNNNNNNNNNNNNNNNNNNNNNNNNNNNNNNNNNNNNNNNNNNNNNNNNN
NNNNNNNNNNNNNNNNNNNNNNNNNNNNNNNN
NNNNNNNNNNNNNNNNNNNNNNNNNNNNNNNNNNNNNNNNNNNNNNNNNNNNNNNNNNNNNNNGTGCAC
ACGTGTAGCAACTGTGCTGCCAGCTGTGCAAG
GAAATAGGAAGGGGCCACCGTTGCCTCCACGATGAATGTGACGATACATAAATACGCCGGCCCTTATG
AATTGTGGCGGCGTCAATTTTTCCCCCGATGG
AACCATCCATCCGCCTGCACGGAGCGGCGATGCTGACCGGACGGGCATCCGTCAGTTGCCGCCACCCC
GTGAAGGGAGGGATGACACAAAATCTGGTGGG
ACAACTGCGCCGCAAGTTAATGCTGAGCCGTCACATCAGAATGCGGATTGGCGACAGTCTGCGGCGGC
TGACGGACCCAACACCGTCGATCCGAGTCCCG
CATCCAACACGGAGAGCAACACGTCCGAGACGAAGCCTGAGCTTGCTTACGTTTAGAATATCAATGTG
GCTCCGACACCATCCGAAAGAAGGCCCACGCC
TGTCACCGAGCCGAAGGGGAATCCCATTGATGATGCGATGAGTGGTTTGATAAATGTTGAGTTGCCGC
AGGGAGTCGATCTATTTGTGGCGCAGACGACG
CTGGTGCAGCCGAAGGATGGGATTGTTCCAGTTACGACGAGGGATTCAATTGTTTCACCCTCTCTCGT
CAGTGCTGGTGGAGTGATTGCCGCTTTCGCCG
AGGGTCAAATGGATGCCGAATATCAATGTGCTCAATTAAGTAAGCCCTTTTCTTCTGATTTTGTTGCG
GGGTGCATCGACTCTGCGTGGGATTGGTCCAC
TGTTGTTGGTGAGGTCAATAAAAGGATGCATGGAGGGCACACACTGTGGTTGGTGCAGCGGAGGGAGA
GGAGAGTTTGGGTGTTGTGCTCCGCCCCACAA
CAATCACGAAGGGCAACGGAGTGTTTCCTCTTGCGGGAGGCTCCGATGTGTCCTACGAAGGTGGGTGC
TGGAGAGAGGGCGGCTTGGAGCCGAAACTGGT
TGTGGGTGATGTCGCGAATCCCACGGGTGGGAAGCAGAGTGGACGGATCGAATGGGGCGACCCCAAAC
CACTCTCGAGGCGGATCTCTTCTGCTTCTCAC
GAGGGTGAGTGGAGGGAGTTCATTGCTTCTGGTGGCGCAGGTGTTGTGATGGAGGATGGCACGCTTGT
GTTTCCTATGATGGCGAAGAGTGAAGAAGAAG
ACGTTTACTCCATGATCATTTACTCGAAGGACAACGGCAGTGCCTGGGCGCTCTCTGAGGACATGTCC
CCTGCGGAATGCCTTAACCCCCGCATCACCGA
GTGGGAGGGATCACTTCTCATGATTGTTGACTGCGAGAATGGCCAGAGGGTGTACGAGTCGCGTGACA

TGGGGACAGCGTGGACGGAGGCCATCGGGAAA
CTCCCAGGTGTGTGGACCAATTCACAACCGGAAGTCTCTCAGGATGAAAGCCTGCATGTGGATGCCCT
CATCACCGCGACCATTGAGGAAGGAGGTCATG
CTGTACACAGAGAGAAGGCACGCCTTCGGGGGAACGGAAAGGGCCACTGCGCTCTACCTTTGGGTCAC
GGACAACAACCGCACGCTTTCTGTTGGACCGG
TTGCCGTGGACAATGCTGCGAATTGGACGCTCGCTGGCACCCTGCTGTACTCGGATGGCAGTTTGCAT
CTTTTACAAGGGAGGGGCAACGGTGAGGGCCG
TGTCATCTCACTTTCCCGCGTGACGGAGGAACTGAGTGCGGTCAGGTCCGTCCTCAGTACTTGGATGC
AAAAGGACATTTTCTTCTCCAGCTTTTCTATA
CCCACTGCGGGACTGGTGGCAGTTTTGTCCGATGCGTCCAGTGATGGCAGGTGGAACGACGAGTACCT
TTGCCTGAATGCAACGGTGACAAGTGCAGCGA
AGGACAACGACGGGTTTCAATTGACGGGACTTGAGTGCAGGGCAATATGGCCTGTGAACACTTGCAGT
GATAACGTGCGTCATGTATTTTTGAGCCACGA
CTTCACACTTGTGGCGTCGGTGACCATCGAAGAGGCTCCGAGTGGGAACACTCCTCTACTGACTGCGG
TGCTGGCGAACACTGCGTCCAATCATACCGTG
GGACTGTCGTACAGCCATAATAAGAAGTGGGAGACGAAGTTCGAGGGCAAGACAACAACACGGAGCAG
CACTTGGGAGCCGAAGAAAGAACACCAAGTGG
CACTCATGCTGCAAAGCCAAAAGGTCTCTGTGGACGTTGATGGCGAGTCGCTGGGGAAAGAAGAATTA
CCGTTAAAAGGTGAGAGTACACTTTGTCTTGT
ACGCTTTTGCATTGGGGCGTGCGGTGGGCACTAATCTCATGTGACGGTGAAGAACGTTTTTCTGCACA
ACCGCCTACTGAACTCCACTGCGATGCGTGCA
CTCAAGGACAGGGTCCCCGCTCCGACGAGAGAAGCGGCTCCGACACCACCCGAGAAGGTTCGAGTGGT
TGACAGCGTCAGAGAGCACATTGCTGAGGATA
TTACCGGAGAGAAATGAATGGGCACAACGCAATGCCACTGGACATGAATCCCCATTACAAATATTCAC
CTCGCAGGGTGTGGAGCGGCGTGAGCCCCGAC
GGCCGCCTCGCCTCCCCCAACGGCGTGAAATTGATACTGCGTGATTTGAGCGGTAACAACACCGTGCG
TGTGTGTGTCTTGTGTGTTGCTGCTTCTGGGA
TTGTGTTTGGGGGTGGCAGTCTTCTGAGCCGCAGAGTGTGGATTTGCGACTCCTCACGGAGAATACGT
GCCGCATTTCTTGCCCTTTTTTATGCCACCGG
TTTTATTTATTTTTTAACGTTTTACTGTTTGTTGATTCTTTCACTGTTTTTACTTTTCAATGTTACCT
TTAATGCAGTCTATCTGTTATAATCAGTACTG
TTGTTTTTTTCTTTGCGCAGTGTTCGGTTGATACATACCGTGATGAGTGATTTGTTTCCCCTCTTGTT
TGCTTTTTTTTTTTTGCCCACACACGGTGCCGG
CCTCATTGCTGTGCTTCCCACGGACTGCACGAAAGGAGGAGCGGAGGGACAGGCCGGATGCTGCAATG
AAATGTGAGGGATGTTGTGTGGGCTGGTGGAA
CGGTACGATCTTTTTTTTTCATTTTTTTTTGTTGCGCAGGCAATATGTATATACAGAAACACAAGGTC
GTTTCTATTGCGTTGCATGGCACATGAAATAA
AAGTTCTGCCGAATTAATTTTCCCTAATTGTTTCTTTTGAGGGTCATGCCGGACTCTCACTTTTTCCT
TTTCTTTGGTAACTCACTGCAGCGTGCTTCCA
TTGTTACCGNNNNNNNNNNNNNNNNNNNNNNNNNNNNNNNNNNNNNNNNNNNNNNNNNNNNNNNNNNN
NNNNNNNNNNNNNNNNNNNNNNNNNNNNNNNN
NNNNNNNNNNNNNNNNNNNNNNNNNNNNNNNNNNNNNNNNNNNNNNNNNNNNNNNNNNNNNNNNNNNN
NNNNNNNNNNNNNNNNNNNNNNNNNNNNNNNN
NNNNNNNNNNNNNNNNNNNNNNNNNNNNNNNNNNNNNNNNNNNNNNNNNNNNNNNNNNNNNNNNNNNN
NNNNNNNNNNNNNNNNNNNNNNNNNNNNNNNN
NNNNNNNNNGGCCGTGGATCGAGAGGATGTGAGGGTCTTCCCCAAGCACAAGGAGGACAACAAGATGC
AGTTCATGACGGCCAACCTGATCCGCGGGTGG
TTCCAGCGCATGGAGCACGTGCGCAGGTACGCGTTCGGTGTTGCACCCATGATTGGGGAGGAGAGGCG
GCACGACGTTGTGGCGCTGTGGGTGTTGTGTG
GCGGTGTCATGCCGGCGGTTGTGAGGATGTGGAGGACACGGAGCTGTTTGACTGGGAGGAGGTCGCTG
ACGTCGCGGCGCAGCGGGAGTGCATGACGGAC
TGCCTGTGCTGGGAGGGCCCGACGATCCTGAGGCCTGTGCTGGAGGGGCGTGTGTTGAGGTGAGCGTG
GCGTGCTGGTGAGGGACTGCGGAGAGAGAATA
ACGGTAATAGTAACTGTGGGGGTGTGAGGGACGGTGCACGGGCGAGGAGCACTTCTTTTTTTTTTTTT

CTGGTTTGATTGTTTTTGAGCGGCGCTCACCC
AATTGTTTTTGTGCATGACTCTCATTGCTGGATGGGACGGTGCGTGGCTGTTTGACGTGTCTCTCTTT
GCTTTGACTCTGCGTTGACGTGGAGTTAGTTT
GTGCTTAAGGCTGCACGCAAGTGACGGGAGTGATGGCGATGATGACTGGCCGTGTGCTGCTGGTGTGT
GCCCTCTGCGTGCTGTGGTGCGGTGCCGGCGG
TGGCTACGCGTGGGATTTT
>scaffold1019.size11140
CCGCAACCGCCGGACTCCCCTCCGCGTGAGGGCTAACAGCGGGGGAGGCGGGACAGAAAAAAACTGCC
CTCGCTGCACTCCAGCACGGTGTCATCGGGGGA
AAAATTCAGTTAAGAAACAAAAAAAGGCGGGAAAGTTCGTCAAAACACAATATCACCAAGATAAACAC
CCTCGTCTTCGCACGTTCCTCTCCACGGGCCC
CCATCATACATCAAAAACATGGTATGTATCCCCGCCACCGTAGAGAGTCAAAAGAAATGAAACCAAGT
AGATACGAGAAAAACAAAGTCAGGCGCTCCTA
CCCCTCCTGCTGTGTCATTTCGTGCTCACGCACCCATGAGATACATACGTGGTGACACCTTCGCACTC
TCCTGGCAGCTCTAACCAAATACACACGGGCA
TAAATTGACACCATGGAAAGAAAAtAGAAAGGAAAACAGAAAAAAGAACACATACACACAAGGCGCCT
CGTAAATTAAGGGATTTGAAAATAACGTGTGG
CAAGCACACCACGTGGATTTCTTTTCCTTCCATTGGTGTTGGGTGCCGCCTCTGCTGCTCCCCTGGTG
GTGGTGCCCGCTGTCTTCGCCGTGGTGCCAGG
GTCAATTGCCCCGTATTGTCGGGGGAGCCAGGAGCCAGCAACGTTTCACTCACGGGGCCATTGTTGTT
CGCGCAGGGCTGACGGCAACAGCCAGCACCCG
GGCCTGCCGCGTCTTGCAGGGACACAGCCGTAACGCCATGAGGGCTGGGGGCACGTGTCACCGCAACG
CGGGCCATCAAATCTATTTTAAAAAACACAAA
ACAAAACAAAAGGCTCTGCCGCCACTCTCTCCTCTCGGATCTCACACGCACACACGCGAGGCCAAGAG
AAAAAAAACAATGGCAGCAGCAGAGACGGCAG
TGTCAAGCGCCCATCTCTCTTGTGATGAGTGAACACGACCGACGCCGTTCTCTTCACATTCATCCTCG
TGGAAAACGGTATCGTGTGCCGCAGCACCCAC
CCTCAAGCANNNNNNNNNNNNNNNNNNNNNNNNNNNNNNNNNNNNNNNNNNNNNNNNNNNNNNNNNNN
NNNNNNNNNNNNNNNNNNNNNNNNNNNNNNNN
NNNNNNNNNNNNNNNNNNNNNNNNNNNNNNNNNNNNNNNNNNNNNNNNNNNNNNNNNNNNNNNNNNNN
NNNNNNNNNNNNNNNNNNNNNNNNNNNNNNNN
NNNNNNNNNNNNNNNNNNNNNNNNNNNNNNNNNNNNNNNNNNNNNNNNNNNNNNNNNNNNNNNNNNNN
NNNNNNNNNNNNNNNNNNNNNNNNNNNNNNNN
NNNNNNNNNGGCCGTCACGTTGAATCCAAGCATCACAAGCAACGGGAGCTGCACACAAGCACGATCCA
AACAAACATCAACACCCCGCCTTCTTGCCTCC
CGCGTGTACCACTCGCCGTACAGAAGGCGGTTAAGCAGCACGGAGGAACACGTGGTGGCAAAGAAGCA
GAAGTTGTAGTGGAGTCCAGGATCCTTCGCAT
AGATGGTATTAATTACGAGAATTGTTACCGCTGCAATAAATCCATTGCCCAGTGCCCCAATAACGTAC
GGAATCGGCAAAATATCCTTATTGCTGATTGT
GAGAAAAAGGACTGCCATCACGATGACGGAAATGGTTGGTAGAAAAAGTGACAACGTAATGGGAATAC
GATCTTCCGCCTTCCTTTTCTGCGTCCAGACC
TCAAGTGCGCTCATTAATAAGCGACCCGCCGCACTTCCCACCCCGTTCAGCACCGTTAGTAAAGTATT
AAGGGAATTGTCGACGTCCTCCCCAGACATGG
CGGCAAAAATAAAACGAGAGTTGGTTAGTACGACAAACTCTGTACCAAGTGTGCAGAACAACGACCAA
AAGATGGCCCAAAGCTTCACCGTGCAAAGACT
CTGTAGGAACCGTGTCTGATATTGCGGCGCAACGTAGTCCATCTCCAACGTCGGTAAAATTGCTGTTC
CATCGTTATCTGACACTGGTTCTTCAACGGGA
GTCACGGCGTCGCAAGAAGAAGAGCGCCGAATTTTCCAGCTTCGGTCCAGAAAATTGAACGGCATGGC
GACAATGGGGTAAATGCCCAGAGAGACAATTG
TCACAATGGCAAACGCATTTCGATACCCCCATGACAAATTCAAGTACGCAATCAACGCACTCTGTAAC
GGAAGAAACAAAATAAGAAAAACGACAATCAC
AAATCCAATGGCAAAACGCATGGATGGcGGTTGCTGCTTTAAATATATGGCCTTCATCATGATGCGTT
TTTCGACCTCCGCGTCTGTCAAACGACTTCGC
TCGTAATCCGTCAATAAATAGGGAGGCTGGCGTATCAGTATTAACGCCAAGACACCAATAACACCACC

AAAACCCATTAGGAAGAAGAAGTAGTTTGTTG
GACTACCCTCAAAATAGGCCAACTGAATCGCCCCGATAATCGCGGATCCCAGCCCAATGTATGTCTTC
ATTACGGCCACAACTACGCCCTTTCGCGTTGG
AAATAAACCGATAACCGTTACGACGCATGCAAGGTCATAAACGCCCGTGCCAAAGTTAAAAATACCGT
TGAATATGCAAAGCCGCAACACACTGGCAGCA
ACGGTCCCATTGAATGTCAGCGCCATGAGCAACGAGCCCACAGTGATCAATACGAGTCCAATCACAAA
CACGGGCGTCACACCAAGGTAGTCGTAAACAA
AAGCATATGGTATGCCAAAGTAGGCAAAGACAATACCCACTGTGCTTATTGTTGACATATCTGCCTGA
GAAAGTTCATAAGCCTTCTGCAGATCACCACT
GAATATATTGAACCCATAAGAGAGAGACACGCAGAGACAGCCATAGGCACCTGCCACAAGCATGCCAA
ATCGTTTCGGCTCACTGATCCGTTGAGTTTCA
CGGGGAAGTTCGCCACTGACTCGTAATTCCTCTTCCCCCATCTATTGTGGTCGTGTCCCGTAAGTATT
TGTATGTATTTATTTATTTATATATCTTGGAA
GAGTTTCAATTCCCTCGTTAACTCAAAGCCTAATTTCTAATTATTATTTTTGTTTTTGTTTTTGTTTT
TGTTTTGTTTTTGTTTTTGTTTTTTTTTTTTTT
TGTTTTTTTCCTTTTCAGTGGCGGTCCGTGTAAGATCCAAATGCTGTGGTGAGTGCGAGAATAAATAA
AAATTTATAAAAAGTTTAAAGGTGAGGGGCAA
AAAATAAATAAATAAATAATAATAATAGAGGCGCATGAAGTGAGATGAACAAAAGGAAAACAGAAGGA
GAGAAAACCATAAGGAACGAATTGACAGTGTT
GCTTGTGGCATAAGCCCTTCCGCCTGCCTTGCCCGTCAGATAGCCGACATGAGAACACCACACAACTC
CTTCAGTATCGCCGTGGCCGTCGCACCGGAGC
CGTCATCGACGGTTTTTTCCTTCTGCTTCTCCATCTCTCGTAGGCATTCGGGCAGCAAGACGTCTGAC
ACCGGAGCGTAACAAAACACACCCAAAGGAGC
TAGAAATCGCAACCAGCGACGAACACGCCACACAAAACGACCCACAACTGCTGCTTTTAGTCCGCTCT
TTGCCTCATGCAAAAGCCACCGCTGCAATGCG
TAACACTGACGATATAAAAACACAGACATAATAACGCAGCATACGTTGCACACCATCAATGAACATGG
CGAAGAGGAAGAAAAGGCAGAAAAACACCCCA
AGAAGGACTTCATGTGCGGAGGAATCAAAAGCATTAGGCGTGTCTTGTTTCTTGAGAGTTGTCCGTCC
GACGTCGCAGTAAACGTTGAACCTGGTGCCGA
GGAATGAACCAAAGTAGACGGCTGAACGATTTCCTCCGTCGCAAAATCAATCAATGCGTTTCGAAGCA
ACGAAGCAGCCAAAGAAATATCTGAACTTGTG
GGAACCATCCGTGTCTGGCTCTTGGACTTCACAGTGTCCTTTCTGCCGTCGTCCGCGGTTTGGGGAAT
CTGCCCGTCGGCGCGTGGACGCTTAAGTCGCA
GCGTCAGACGCGGGCCATCTTCTCGTTTCTGTGATCCCTCTCCTAACGTCGCCACGGTCGTATGGACT
GGTGGTACAGAAGTGCCTCGTCCGCCGTGGGC
GACCACCAGTGGAACGTCACTAAAGGCAAAGCTGTTCAGCAAAATCCGCAGTCGGGCGTCAAGCTCCG
CCGTCTGTCTCTCACGCGCCTCCACCTCGCCC
GGTGCCAGCATTGTTTCCAAGTGGCGCCAGACACGTTCTGCGATGTACAGCTGCGCAGCGGGTATTCT
AAGCGCCAACGCAGCAAGAAACGCCGAAATAC
TGCAAATTCCCTCGTCACCACCCCACTGGGCCCCTTCGCTCAGCTCCCCGTTTAGGTCCTCAAAGGAA
GGCACAAAAAGTGTTACATCCGGGTGGTCCAG
AGCGCTACGCACATACTGCGAGGCCATGCTGTGGCCATGGTAGAGCGAAAGAAGCAACGCATACGCAG
CTTGAACTACAGCAGCTCGCCCACGCTGCGGT
TCCTCGTGAGAACCATATGCCTCGTCTTTTTCCACCAAACGCAGTCCATGAATCAATGTGCGACGCCA
CAGAGGATGCACCTCGTCCGTCGTCATTGACA
AAAACGACGATGATTTTGATGATACAAGTGGCGCCACCAAAAAAGAAGAAGTTTCCTTCCTCAGCTCC
GCATTTCCTGAAAGTGCCTCCATCCGCCCCAT
ATCATAATCATGCCTGCTCTCGCTCAACCACAAACACAGCAATGCATGCGTCAAAAACGTTGAAGGTC
CCTCAAAGTTCGCCAACAACTTCGCCTCTGGC
TCGGTGTCGCCCACAGTACCGGTGAGGGGATGCACCAACGCACGCAGTGCTGACGGCCAAAAATTAGC
ACAGGCCCCCTGGCTCAGTAGCATGTGCATTG
TGTCAGCGGCGGCGTTGGCATCTGCAGCAAAAAAAAAAATTTGTCGCCACAAATCGCGCCACGTCTGT
GGCACTGACGGTCCCACTATCGTCGTTCGTAG
AAGTTGCAGCGTGCGTGCAGGATAATGCAGCGTCAAGTACGTGGCAAGACCCGGAGGAAGTTCTCGAA

GAGACAATGAATGCGGCAGTGACGCAGCATTT
GAAGAATTCCACGACTGAAGCAGCGCAGCCAACAACTGTTCCGCGTCTTCTCCATGAAAATCTACAGG
CGGTTGGTTCCTTTCCATTTCCCCGCTCTTCC
TCAAGAGAGAGAGAAATGGCAAAGAGCACTTTTTCCGTCGTGACAGCATTGTTGTATTCTCATTATCG
TCAATCCTCGCCATCCGTTCGGCGGGTCTGAT
GTAAAGCTCGTGACTCTGCCGCACAATAGCAGTAGTGCTGTACGGCGGGCTGAGCTCTTTAAAAAGAA
ACTGACGGCAGACGTCGTGTTGGAGGCCGCCT
GCAGAAATGAGCTGCAACGTCAAAGCACCCCTTTCCATGGCAGACATATGAGGCAAAGCCACACGTGC
CGTTTTCTGCACTATAAAGGAGAGAACGGATT
CAATTAGAGTCCCCGCTGGTCCGTCTTCTACTGCACAGACCGCACAGAGCTCGAGAACAGTGTCCATT
CTCCCAGCCAGTGAACGCCGCCGCCCCCAAAC
CCCCTCCCCCCCTCTCCCTTTTGCACTTCGCCTTCACCATTGAGGAGCCATTTCGATAAAAATAAAAA
TAAAAATAAAAAATAAAAGTCACATGAAAAGA
AAAAAAAAGTACACCGAAAACACGTCAATATTTATATCCATGCATGAGATACGGCAGCACAAATTTAT
GTATTTATTTGTACATTCACAACAGGATTCAC
AGGGCAAAAAAAAAAAAAAAGTAAAATACAAAATAAAATAAAATTAAATAATAAATAAATAAATTCAT
ACTATTATAGAACCCAACATTTTTTTTTTTTT
TCTTTCACATTGATGTCATGTCGCATCCAATCCCCACACTGTATCCGTTGGTAAATACATGCGTAATA
AAATGCTGCTTTGGTATTCCATGCGATTTACA
TCCACGTAATATAATCCTCTCTGCGTCCTGCGGAATAAATACAACAAACCGTAAGTAAACCAAAAAAA
AAAAAAAAAAAAAACGAAAGAAAGAAAGAAGA
AAACAAAAAACACCACAACCAAAAACCTGGAAAGAAAAAACAAGCAAACAAAAAAAAAAAAAGATAAA
AATTTGGCTCAATTTATCCCCTATAAAATCCC
AACATGTCCCCGCACGGTATTATCTATTAACTCCGAAAAAACATCGCACCAAGACAAGCCGATAACAA
TAACAATTAGTTGGTGTTTCTTCATTTACCCT
TTCCTACAACATTATCGTAAATTACGAAGATATTCCATCAGCAGTGCTGGACCAAAGCCAGTGGCCTT
GTCATTTTCAAATGCGGGAAAGGCCATATCCA
CGTGGATGTGCTTGCCTTTGAAGTCAGGGCTGAGGTGATTAGCGACAAAGTGTCCTGCGCACGAGACA
CTGGCGTTGTTTACCTGCTTCACTGAATTGCG
CATATCAGCAACGGGGCTCCTGAACTCTGTTACATGATACTCCGGACAGTACAGCACGGGGAAGCACG
TCTCGCCGGAGACCCTGCCGGCCTTCAAGAAG
GAAAATTCTTCTTCTTCATCGTTTACAAATATGCCCGCGTGCCTGTGGCCGGTGGCAATGCCCTGCGC
ACCCGTTAAAGTAGCCATGTCGACAAGAACAT
CCGGCTTAAAGGAAATCTCATGTGTTGCGTGAAAAACTCCGTCCCCGAGTACAAGGCGACCCTCAGCG
TCGGTGTTATTCACTTCCACAGTCTTCCCCGA
CTTCAATGTCAAAATGTCATCATTGCGGTACGCATCGGGTCCAATCGCATTCTCAGCCAAGCACAATG
TACAACTCAATTCGATTGGCATCTGCAGACGC
ACAGCAGTGAGGAAACCACAAAAGACTGCCGCAGCACCCCCCATGTCGCGTTTCATCCCTGTCATAAA
ATTGGTTGGCTTCAGCGACAAACCACCTGAGT
CATACACGATACCCTTACCAACCAATGCGACCTTTTTTCTCGTCTCATCCTTGGGTTTATATGAAAGC
GTTACAAGCCGCGGTGCTTCTACCGCACATTT
TCCCACAGAATAAATACCGCCATAACCTCGTTCGCGTAGTTCCTCGCCACAAATGACATCCACAGCGC
ATCCCAGCTCCTTGGCGTAACTCTGTGCAACC
TCTGCAAAGGTGGCAGTGTTTAACAGATTGGTCGGTGCATCCACAAGACGTTGGCAAAGCTGTGTCGA
AGTGGCGACAACAGCCAGTTCGCTTGGTGACG
GATTGGGGGCTTCCGGGAAGACCACAGTGAGACGAACACAGTTGTCACAGTACCCTCTCGCGGCCAGA
CCCTCCTTTGCAGTAAAGTTACGTTTTGCGCT
GCGCGCGGCTGCCACCGCAATTGCGAGAGCCGCAGTTGAGCGGACAAATACGTCGACTTTTGTTTCAG
GTCCTTTTATTTCCTCAAGAGCCGCAGAAAGA
AGGGATGCAACCACATCGGGTCGGGCAGAACAGTTTGTGCGGGAAGCCTCAGAGGGCACCTCGCCAAC
CAACACACGCACAAACCTCTCATTTGCCTCAT
TCTTCACGCCCTCCGCAAGTACCGCGTATGTTTCACCCTTTTTCACGCGTTGAATGGCCTCAGCGACG
GCAGGGCAATAAAATGGAAGCTCTTTCAGCGC
ATCCTCCCGCAGTTGGGCATGTGTGCCGAGAATGACGACAAAAGTTGATGCAAGGCCCGCTGTGTGTG

TCTTTTTGTATGCGTCAACGTCAGTAAATTCA
ACGTTTGTTTTGAAGTTGACGCATGATCGCACAAACTCCGACGCCCCTGAATCTGTAGCAGGAGGTCT
GTTCATTTATGTATTATTGTTCCTTCTTTACC
TTTCCCTTTCCTTCACGTCACAAGGGCTCCTGTAACCACACCGTCAGTGCTTCACGATAAAGTACACT
GCACCACACGTGTGGCGCCATCACAACAAAAA
CGGAAAAAGAAGAAGAAAAAAAGAAAAAGTAACAAACAACAACAACAACAAAACAAAATAATAATAAT
AATAACGAAGAAACGACGTTTCAACTCATCC
AGGCCCACAAGCGCACATCACCATTCTCCCCCTCCCATTGGCAACAAAAAAAAAAAAAAAAAAAAAAA
AATTTGTTCGCCCCACAAGACAAAGAACAATG
TGAACAGACAATACACGACGCAGGGTGTCATGTTAACTGCCTGCAGCCAATCTCTTTTTTTTTTTCTT
CTTTTTTCTTTTCTCTCACACATACGTTGGAA
GACTATTCCCTCCTACTGACACCGTTGTTGCTCATCATCACATTCCTTCCAGTTACCCTCCCTCCCAT
TTCTCTGGGGTTGAAAATGTGAAGAGAGAACA
CAAACACAGGGAGCGAAAAATAATAATAATAATAAAAGTAAAAGCAAAAATAAAAAAGAGAGAAACAG
ACAGAAAGACACGGAGAGAGAAAGAGACAAAA
CAACAAATATATATGTATATGCATATATGTATGTaTGTATATATTGATATATATATATATTAATATAT
ATTTGAGATGGCAAGGAGTAAACGACACCGCA
CAGACATTTCTGGCCACCACCGCCGCCCCCCTACTGGTCCTGCACACGGAGGAAGGGAGGGCGGAGGG
GAAGGGAATAAACAAACAAACAAAAACGGTAT
CAAAAAAAAAAAAAAATCAATCTTTTCTTCTTTTCTTTTCTTTTTTCATGTCTCTTTTTTTTTGTGTGT
GTGTGTTTCTTTGGGTGAAAACAATAACAATA
AGCACATTAAAACTGATTCGCCACCACTACCCTTCCCCACCCTCCTTTGGACATGGGCGTACGTCCTC
CCGTGCAGACGCACATGTTTTTTTTTTTTGTT
TTTTTATTTTCTTTTCGTTTAAGGAGGAGGGGGAAAAGAAAGAAAGGGAGGAAAGAAGCGCCAAAGAA
AAAAGAGATTAACAAAAAATGTGTAAAATGAA
AAACAGACAAAAAAAAACAAATCTGCGGAGAGCCGGTTGGCACAAATCAACAAAGACACACGCAGGCA
CCACCCCACACACACACACTCCCACACATATA
TATTTATATTTATATATTTATTTAATGCATCTGCCCCACCCAGTGGAGTTTATTACAAAAGTACCCAC
AAAACAAAACAAGAAAAAACAAGAAAAAGGCA
GCAAGACGCATGGATAAATACAATGAGCCCACCACTAACAGAACAGGAGCAATGGTAAAAAAAAAAAG
GATTTCAACGTCGCAACGCCTTAAAAAAGACG
ATAATGGAAGAAAAATAAAAGATGGAAAAAAAAGAAGCAATAACAGTTGATGCTGCAGTCTCTTCTTG
AAGGAAAACAAGCAGCAGCCGCCCCCCACCCC
GTCCCCCCGGCTGCGTAGAATGTGTTATTATTATCATTTTTTTCTTTTCCTTTTTTGCCGTCTCTTCT
TTGCCTGATTACATGTGAGTTATTCACTTCTC
ACATGACCCAACATGCCCACATGATTATATAAATACACATTTCCGTGTGGATTTCTTCTTTTTTTTTT
TCTTTTTGTTTGTACAATGAGACGGAAAGCGA
AATCAAATTTGAGTGCTTTCCGTATTGTTTCCCAACACAAACGGTGAGGCACGGAAGTGCCATCGTTC
AACGCACTCCTTTTTTTCCCACATCCCCAATT
TGATTTTCTTTTTCCGTCTTTCATGCCGGCTTCTCACCGCACCACTTCTCAGCCTCAACCCGTCACAA
TGCGTGTGTCCCCCCCCGCTGCGTCACTCGCA
GCCAAACGACACGGAAATATTCCTTCACGCGGTGAAGCACAACAACAATAATAATAAAAATTTAAAAA
ATTTTTTAAGACAAGTCGTGACTCTGCATTTC
GCCCACGCACCCCATTCAATCCGCGTCCACCGCCCCATTTCCTTCCGGTTCTGTCATTTCTTCTAACG
TCCCGGCACGCCGCCAGTGACGGGGGAGGGGG
GCCGCGTAACACACACACAAAACAAAAAAAAGAGGGAAAAGGAGGAAGATGAAGGGGAGAATTAAAAA
AGAAAAAGAAAAAAAGACCGATACTACATGTA
ACAAGCGCAATGCCGCCGCACAAATAAGACATGTGCACCCACCACCACCACCAAAAAAAAAAAAAATG
AAAAAAAAAAAAAATTTATTGGGAGACGTGAA
AAAATTATTCTCCGACGGGTCAGGGCAGCGAGGACAAGAATATATAAAAAGTGAAAAGAAAAAACCCA
CTTTTTCTTTTCTTTTTTTTTTTTTTCGGTTTT
GTTGTCATCCATTTCTAGTCGTTTTGTTTCTCAACCTTCTTTTTTTCCCTCCCCGCTCCCTTGTTGTT
GCTGCCGCTGCTGCCTCCTCGCCTGTCATGCT
CGGTGGCAAGAAATTCCCGGCGGGGAATGTGGCAACAGGCATCACATTATTACCCAACACCTCTTCCC

CTCCCGCAAACACATTTCAACAAAGAAGAAGA
AAACTGCGAAAATGAAGCGCGGTGTCGCAAAACAACAAAAACGAAAAACAACTTACATAAATGTTTTT
AATCCCCCATCCCCTTCAAAAAAAAAAAGGGA
AAATAATCCTCTTTTCATACCGTCCACTCTCTATTCAACCGACACTCAGTGCGGTCGAACTGAGATCC
AGAAGTGACCCTACTTCAAACACGTTCTATGG
CGCAGCTTCATGCGATTACGTTCCATCGTTGCGATCGGCGCCACCTCGTCAAATGATGGCGGGGCCGG
AAGAGACGTCACGGACGCCGCGTTCACACTCC
GCGACAGCTCCACCACCAAAGGCATGCACGGCAGCCCGTCCGTCTGAAAGTGGCGTATCTGCTGCGGC
AGCCGTCGCACACTCTCGCAGTACTCCTTTAG
TCGCAGGTACTGCTCCATGTTCTCGGCGTACCAAAAACCATGACGGTCCATCAACACACGACAGCGCA
GTTTGTGGATCAGATGCCAAACGTCGTTTGTG
CGAATAAGAGTGATAAACGACGTCTCGGAGCGTGGCTCAATACCCACAACGGTAGCAATATCAGGTCC
ACATGTCGCCTCAACCACACGGTTTAGGACAA
TAATGGTGCGATCCCGCAATTCACAAGGAAACATTTGAACAAAG
>scaffold102.size30567
GTCTCCATCCATCCATCCATCCAAAACAAAACAACAAATAAAAAGGGGGATGAAAAGGAAATGCGTCC
TAAAGGATTGGACCCCTCGTGCTCCCTTATTTG
GGTTAGTTGGTGTCGATGTAGTCTTCACTTATTGTGCAGGAACGGCTCTACAAAAAAAAAAAACAAAA
CCAAAAAAAAAAAACAAAAACAAAGCAAAAAGA
AAAAAAAAAAAACGGTTTTTTTTTTTGCCTCTTTTTTTTTTTTTTCACGAGCTTCTTTGGCATACAATA
CAATACATATGGATATACATTCCCTGGATGGG
TTGTAACAGATTTCCTCACGCAATTGCCATTCTTTTCCCTCCCTCCTCCTCCTCCTCCTCCTCCT
CCTTTTCGTTCATCGCCCCCCCCNNNNNNNNN
NNNNNNNNNNNNNNNNNNNNNNNNNNNNNNNNNNNNNNNNNNNNNNNNNNNNNNNNNNNNNNNNNNNN
NNNNNNNNNNNNNNNNNNNNNNNNNNNNNNNN
NNNNNNNNNNNNNNNNNNNNNNNNNNNNNNNNNNNNNNNNNNNNNNNNNNNNNNNNNNNNNNNNNNNN
NNNNNNNNNNNNNNNNNNNNNNNNNNNNNNNN
NNNNNNNNNNNNNNNNNNNNNNNNNNNNNNNNNNNNNNNNNNNNNNNNNNNNNNNNNNNNNNNNNNNN
NNNNNNNNNNNNNNNNNNNNNNNGACTCGCTG
TAAAACTCAAAGATGGGCAGCCACCTCTTCCTTTTTTCCTTTCTTTTCTTTTCCTTTTTTTTTTTTTT
TAAATGACTCCTCCTTCTTATCCTTCTCCTCA
TTCTCCCCCCACAACCACCGGGACTCTCCCCACTCGCGCGGAGTGGCAGCCGAAGCGGGGCGGGTGGA
ATAAAAAAACGAAACCGAAAACAAAAAAAAAA
AAAAAAACCAAAAAAAATTTGCGCCTGCGCTGCCCCTTTCCAAAACGCCGTTTTAAAAAAAAAAAAA
AAAAAGAGAACAACAACAAACAACGGCAAACC
AAACAAAAATTACCCAAAAACGAAATCCACAATATATATATATATATATAAACAAAAGGGGGGTTTTC
TCCTCTTCTTCCTTTCTTCTCCGCTTCCTCGT
TTATTTATCCTCATCTGGTGGCATTATTTATATATATAAATATATAAATATGTAACGTCATGTCATGT
CATGTATATTCCCTTTTCCGCGATTTTCCAAA
CCAGCATATATATGTATATATGTATGTAACGAAAAGCAGCAAAAAGCAGTGCAAGTGACAATAATAAG
AAAAAAAAAAAGAACAAAAACAAAAACACACA
CAAAAAAAAGAACATGTTGCATTTACTCCGTCCATCTCTTTACCCTTCGGCGCAAACACACAGAAAGA
ACCACCCAAGCAAAACCAAAAACACATACAGA
CATATGACTTGCTTAAGACATGGTGGTGTGATTGAGATTAGGAGGGGGAGGAAGGCCGGTTTTTTCTT
TTTTTTTTTTCTTTCTTCTTTCCACTCCCTTT
CGCCTATTATTTACAACGTCATCCGGAGAGAGAGGGAGAAAAAGAGGGAGAAAGAGGGAGAGAGGTGT
GTGTGTGTGGAAGAAAAAACAAACAAGCAAAC
AAACAAAAAGATAAATATGTATGTACGTATGTATAATGTATACATACAGCCCAAATCATGTGAATAAG
TGGCGTTTTTATTGGGCAAAACAACACATTGG
CCTTGTGATTACATGACTCAACTTCATACCATTTTCTTTTTTTTTCTTCTTCCTCTTGTGCGTGTTTG
CTTGTATGTTTCTTTATTTGCACTTACTTCTT
TAGATCATATATGTATGTATATGTATATGTATATGTATGTACACATGTAAGGGGTAATACGCAAAGGA
AAAAAGAAGAAGGAAAGGAAAAGACATTTTTT
TTTTTTCTTCTTTCGTTTTATTCTTCTTATTTCGTTTTCCTTTGGATCATATAGAAAGGGAGAGGCAA

GAGGCAAGAGGGGAGGGAGAGGCAAGAGTAAT
GTTGCACCACACCCACACCCTCAAACAACCCAAACAAAACAGCAAACAAACACACACACACAAAAAAA
AAAAACAAAAGAAAAGAAAACAACAACAAAAA
ACATAAATATGAATACAAACAAAAAACACATGCGGAAAATAAAATTTTTTTTTCTTTTCTTTTTTTT
TTTTTGTTTTAAGGAAAAATTTATATATAAAT
AAATATAAATATATAAAAATGTTTTTTCTTTCTTTCTTTCTTTCTTTCCCTCCCCCCCACCACCTCTT
TTTTTTTTTTTTTTTTTTAAGGCGTTCTCTTT
TGCTCCTCCCTTCNNNNNNNNNNNNNNNNNNNNNNNNNNNNNNNNNNNNNNNNNNNNNNNNNNNNNNN
NNNNNNNNNNNNNNNNNNNNNNNNNNNNNNNN
NNNNNNNNNNNNNNNNNNNNNNNNNNNNNNNNNNNNNNNNNNNNNNNNNNNNNNNNNNNNNNNNNNNN
NNNNNNNNNNNNNNNNNNNNNNNNNNNNNNNN
NNNNNNNNNNNNNNNNNNNNNNNNNNNNNNNNNNNNNNNNNNNNNNNNNNNNNNNNNNNNNNNNNNNN
NNNNNNNNNNNNNNNNNNNNNNNNNNNNNNNN
NNNNNNNNNNNNNCCCTCTTTCTACTCCTCCCATCACCTCCGTTTCTTTTCTTTTATTCCTTCTTTTT
TTAATGTCCAAATTTCTTCTCCGAGGCGACCA
CCACCCCAATTAATTACAAAAAAAAAAAAAAAAAAAAAAAAAAAAAAGAATCCGGCCTGCCAGACGACCA
GAACGACTGACGAACAGCCAGACAGGCAGGCA
GGCAGGCGGAAACACACACGCACACACACACACACACATGATACATGCACCGACACAAAAGTCAAGAA
ACAAAACTACAGCAGGATGATAAAACAAAACC
ATCAGGAAAAAGAAAAGGAACACACACACACACAAACAAAACAGCAAAAACAAAAAACAAAACGGAAA
GGGCAAGAAAAAAAACCAAAAAAAGAAAAAGA
AAGAGCAAAAATCACAAGCAAAAAAAAAAAAGAAAGAAAGAAGGAAAGAAAAAAGGAATGGAGTGCTG
AAGGAAGAGAAACACGTCTCTCCTCAGTCCCT
GTTCTTCAAGTGTTCCAGTAATTAATTGGTGTCACGGGTACGGAATTCAATACCCCAATGTTTCCATC
CGGCAGCAGAACATAATTCCCTATTGGCTGCA
CGGATCGTGACTGTAACCCCATTGGTCCGTTAAGAACACCGGTAAAAGTTGCCCCAGTCGGGACAAAA
GGAGTTGAAGGAATGGATGGAAAAAGAGTCGA
TGTAGCTCCGTTTTCGGTGGTAATATTTCCAGCATTTCCACTGACACCATCATTTTTGCTGCTTTCCA
TGGTGGTCAAGTTGGGGCTTGTGGTAGTAGTT
GTGGTACTGGCACCTGAGTTCAGGGGTACGATATTCTGCTGCGGTACTGCAGGGCCTGACGGTGTGAG
AACAACATACGGTATTCCAAATGATTGCATTG
GGAGAGGTGCCGGTTTGGGTGGCTCTGAATAAAGAGGAATAAAAGTTGGACCTACAGACGCTGAGCTT
GCGGGAAGAGAAAAGGAGCTTGAGGCAGGATT
AAGCGACTGTGGAAGCATCATAACAGAGGAATTTTGTGGGGCCGTAATAGAAACGTTTCCATGGGGCA
GCACAGTGGCAGGCAAGCTTACAGGACAGATT
GTCGATGGCCCCGAGGGTAAAAATGGCGACGGCATCATGGAGTTGACAGCTTCCTCATACTCGGGTGG
CGGTCGTGTGGCGTGCGGTTGCGATGCCCCAC
CCACACGTACTCGGCTGACTGGGGGAATATCGTCTCTTTTTTTATGCTCCTCAGCGATATTCAATAAC
GGGTCCAATGTCTGATTGGATCTGTTGCCTTC
AGCACCACTGGTCGTCCCATTTTTTTTATCCCGCTTCACCTTTACAGTCGTGAGAAACGGACCGCAGA
GAGGGGAGTCAAAGATCCGGTATATTTTCCCA
TCATGGCTGCATTCTTGCTCTGCACTCCAGACGCTCAGGTCCTCCGTTGGGGTTTCACGGGCTCGAAC
CACTTCACTTAGCAAAGTCTCAAGGTGTTTTT
TCACCCGCGATGTAAATTCCACACCACCCACAAACATGTTTACATCGGGGCTTGTCTCCAACCAGTTG
GCTATCATTTTTTCCTTCTTGTGCATCGCCGT
GTCGTCCATGCCATACTGTTCCCACATCCCGCGTACCGCGGAGGGGTTGATGGAGGAACACGCCCAAA
AAATAAATCGCAACAAAAGAGACCCCGGCATT
TCCATGACAAGACCACTTTCTCCACTTTCCGGTACCTCATAGAAGCGGAAGCTTGGGGCATCATTGTG
CTCCTGCACATTGCCCACGCTCCAAGACCAGG
CGGCGGTGTCGTACAGGAATTTCAGCACCGGGTAGCTATTCCAAAACGTGCTGCTGACGAGAGGCAAA
CGGGAAATACGGCGTAAAAACATCTCCTTCGC
ATTCGCTTCCCTGTTAACTGTCGTAACCGCTCGAAAAAATTCCTCCTTGAGTGCCATCGCGGCAAGCG
TAACCACTCTCTCCAATGTACGATCTCCCGCA
GCCTTTAAAATGGCTTTTTGTAGGTAAAGACACGGTTCGTCGTCCTTTTTATCTTCGTACCGGTGACA

GCCAAACTGCCCAATAACATTTATGCGCCGAC
CATAGAATAAGAAACAGTCTGTCCCCTCTCGACCATTCACATAGACAAAACAAACAGCACAAGGCACA
GGACGCAGTGGGGCATCCTCATCTTCGACCAT
TTCCTTTAAAGAATCATAAAGAGTTTCCTCAATAACATCAAGAAATGTGTCAAGATCCTTCTCTGTAT
ACGACGCTGGTGGTAGTTCCTTCATGCTATAC
TTTGGACTACGCATGAAATTGTAGCGCACACTTGGATCATTACGCATACGAACAAACTGCTTCCGCAA
TTCCTTGTTTTCATTGCAAAAGTAACGACGAA
GTAAAGGAATCAGTGCGATTTCATGCGACCCATAACGAAGAGAGAAAAAGGTCGGATACGCATCGCGA
GAGGGAACCCCGCCCATGCTGATAATAACACT
GGTGGATGCTTCCTCGTCTTTGCACCTTTCCTTTTTTTTTTGTCTCGTCTCCCTTTTTCCCCTTTTGA
CTGCAGGAAAAAAAAAAAAAAAGAATAGTCTAC
GCTTTACCTGTCCCTGCCAAAAGATTAAAAAAAAAAAGAAAAGGCAAAAGAAGGCGAGAGAGAGAGAG
AGGGAGGGAGGGAGGGGAAGTGTGCGAGACAC
AACAAGTGGAACCATCGCATCAATCATACATGCATAAAAGGACTGAGAGGAGGAGAAGGCGACATGTA
ATGTAACGGAGGTAAACATACAGCGAGAGAGA
AAAGAAAAGAAAAGAAAAAAACAACAACAACAAACAAACAAAAACAAAAAAACAAAAAAAAAAAGAAA
GGAATAAAAAAGAAATCCATAAAAAATGGTCG
TCACATTCGCCAAAAAGGAGTACAAACAAACAAACAAACAAACAAACAACAACAAATAAATAAATAAA
TAAGTAAATAAAAGAGAGATACGGCAGAGAAA
GCAAAGGCAAATGCATATATATATATAGATAGGTATATATATATATATATAAACGGATGCAGACATCC
AGCACAAAGCACCACCAAACAAACAAACAACA
AAAACAAAAAGAACATATGGAAGGGGGAAGATAAAATTCGGTCCCACCCGTACAAATAGCCGCACACT
CCATTTATTGTAATTAATAATAACAAGAAAAT
ATGAAAGGGGTTAGGGGAGGGAAGGAAGGAAGAAAGGAGGGGAAAGTGGGTTTATAAAAGAAGAGCAG
GTTGTGGCAGAGAGAGCATACAAAAAAAAAAA
AAGAAAAAAAAAGAAGAAAAAGAAAAAAGAGGAGGAGGAAAGGGAGACGGGGTCCATTTGCCACTTAC
ACCGCCTTCCTCCTGCCTTCACCGCAGTCGTC
ATCTTCCCGTTGTTGTTGTCGTTTTTACCCACTCAATGGATACATGTATGTATATATACATAGATATA
CATACATGCCGCTGCCTCCCTCTTCCCCAAAC
TTTCTCCACCCAACACAAAGTACTCAAGGCGCTCTCAAAAATGAGTCGTAAAAGAGAGAGAGAGAGAG
AGAGAGAGAGAGAGAGTAAACCAATCATTAAT
TAATTCATTAAATAATAACATATAAAATAAACAAAAAACTAACAAAAATCAACCCATTCAATTTCATT
TCCCCCAAAATTCCCTTCTGTGCCCCCTTTCT
CTCTTTCGGTGTGTGTGTGTGTGTGTGGACGAGATGAGGAGTGAAGAAGGAGGGGAATGAAGCAGGCA
TGGCGGTGGCGACACAAAATGTTGATCAAAAG
CAAACAAACAAACGAACGAAGATCACCACAAATAAAAACACCCTTCTTTTTTTTTTTTTTTTTTCCCA
TTACTATTATTACTATTATTATTATTCCTTCT
TGTGTNNNNNNNNNNNNNNNNNNNNNNNNNNNNNNNNNNNNNNNNNNNNNNNNNNNNNNNNNNNNNNN
NNNNNNNNNNNNNNNNNNNNNNNNNNNNNNNN
NNNNNNNNNNNNNNNNNNNNNNNNNNNNNNNNNNNNNNNNNNNNNNNNNNNNNNNNNNNNNNNNNNNN
NNNNNNNNNNNNNNNNNNNNNNNNNNNNNNNN
NNNNNNNNNNNNNNNNNNNNNNNNNNNNNNNNNNNNNNNNNNNNNNNNNNNNNNNNNNNNNNNNNNNN
NNNNNNNNNNNNNNNNNNNNNNNNNNNNNNNN
NNNNNCCCCCCCCCTTTTTTTTATTATTTTTCCCTCCTTTTTTTTAATTTTTATTTTCTTTTAATTTT
GTTTTTATTTTCGTCTTCATTTCTTTTCCTTC
TGAAATTTTTATTTATTTTTTTTTTTAATTAATTAATTTATTTTTATTATATTTTAATGGCAATGCAA
AAATATCTCCGTGCACTTCAAAGACTTTTTTT
TTTCTTTTTATTTATTTATTTATTTTTTTCTTTCCGTTTTTCGTTTCGGTTCATTTTTTTTGTTTTTT
TTTTTTGTTTCCTCCGTTTTGAGTCCATTCCT
CCTTCCGCAAGCAAATTTCACCCAAATCCGCACATACACGAAAGAAAACAAAAAAAACTCCACACGCC
ACCGCTCTAAAAATGCATGAAACAGGCACCCG
TGCAACTACACCCTCTTGAGTTGCATACACATAAATATAAATATAAATGTGGATATATGTAGGGGCAC
GTTTCTCTCTCTCTCTCTCTCTCTGTGTGTGTGT
GTGTGTGCGTCTGTGTATTTGCTGGCGCGTTTCTCCTCCCCTCCTTTTTCTTTTCTTTCTGGTGTTTG

TATTTGTGTTTGGGTAAAAGGCCGCAAATACA
CAGACGCACATATATATATATATATGCAGACATAGAGGAAGAGAAAGCGAGGGATGCAGAAATACCCA
ACGCAGAAACACAGTTCTCCTCCAGGCAAGCT
CTCATGCTCGATTCATTGACGTCTCTGCCCTCGCTTTCGTACTTCCCTTCTTGCATACACATTCTGGG
CTCACGGTGATCACTGCATTGCCCATGCAAAC
GCCGACCACGGCAGTATCGCCATTTTAATAATGGGCGTCACTTTCAATGTAGTTCTTTCTTCTCATTC
CGGTGGTGCTGGGGCTGTTGCTGGCGGCTGGG
AGAACATGTATGAACTATAAGGAACTCCATACGTGGGTGGCTGTGCAAAGTACATTGGCGGGGCGGCG
CCAGGTGGGTAGGCGGCGGTTGCGGTCGGGGC
ATGTGGCATCGCGGATGACTGGGCAATTGGACCACCAGGCAAGACATATAAAATCTGCTGCGGTGGCA
CACCACGCGCAGCCGCCACACCGTAAGATGAA
GTTGCAGGAGAGAGAACGTGCAGACCGGGAGGCGAAGAAAAGGGAGAGGCGCCTGGAAGAGTCGTCTG
TGGCGAGGCGTATTGCAACTGCGGTCGGCTTC
CGGGGAACATAAATTGCTGCTGCTGACCTGGCATGAGTGGAATTGACGTTGCCGCGGCATTAAACGGC
ATAACGTTTGTTGGCCATCCGGGAGGCGCCCC
CATAAACATCGTAGGCATATGCGACCAAAAATTAGCAGCAGTTGTAGCGGTACCGGATGTAGGCGGAT
ACATGATCATTTGTTGGGCGGCAGCGAGTTGA
GCTTGTTGTTGCAAGGCCGATTGAGACCCCTTCAAAAGCCATTGTTTAAGTGTAGATGCAAGCAATGA
GCCACCATACAGACGTTCATCTTCTTCCTCCT
CCTCCAAAAACGCCCCACTGTCATGCCGTTCCTGCATTAATCGTTGGCGTTCTTGTAGAAAATCAATG
GTATTGCAGCGGATGTGTTGACCCTTGACAGA
ATCCACCCGCATTTTGTTCTCCACGTATCGTAGCCACAATCCTAAAAACTTGTTGCTGAACGTCACTT
CCAAACCGTACTCTCCGAGGTGATTTTCATAC
ATGGAAAAATGTGGAGGCGCGGTTTCGTGCATACGGAGTTCTTTGATCATGTCTAACAGAGCATATTG
GGAGTAAAACATACGTAACTCCCGGTAATTAT
TATGCTGCAGTATAATGCGTAGTTGTAAACGTTCCATATAATAGTCAAAGGAGGCCTCCACATCTCTA
TCCTCAGCAGGATAATAAAGGGCAGAACTATT
CACAAATTGTTTTCGAAACGCTAAGGAGGCTAGAAGCACAACGGACTCTGTAAGGTACCCATGCGCCT
CAACGTCGTCAAATATAACATCTAACACCACA
GGAATGGATGGTTTTTTACTATCCCCTATACCTGTATGTGAAGAGTACGCGCTACTGCCTCCGCCACC
AAGAGCCTGCGTCTTGGTAAAGGTGGCAGGGT
TAATAAGATGCACTGGACGACCCAAGCAACATGAGATAAACCCCACAATTTGCAGACGATGTCCCTTG
ATAATTAATACATTGCCGCTATTCGCCGCAAT
ATTTTGTTGTGCCCGGTTCAAATGTGAATGCGATTTTGATGTGGCTGACGAAGACTCATTACTAGGGT
TGATGGGGTCCAATATACAAACAGCAAACGGC
ACTGGACGCAACAGTGGAACTGGAGAAGAGTGCGATGTGGATTGCTGTTGATGGTGATGAGAGGTATT
CATTTCCGTTGCTGAGCCCGCTGCTGACGCTG
ATGGCAAATCTGGTGTGGTTAACTTCTTCTTTATATGCGGCTTATTCCCAAGCAAGGCATCACTGCTT
ATGATGGCGTCCTTCACCTTGACGAGAAAAAA
TGGATCGTTGGAGGCAATGATGCAGTCCACAATAGGACCCCCGCTCGTTGTCTCTGTACGCCTCGAAT
TTGCGCTCGCCGTCGCACTTCCGGGTGTCGTC
GTCCCTGACGGTGTCGCTGCTGCTGGGGGTGGGTCGTTCCCCCTGCGCGACAGAATGTCATTTAGTTT
CCGCTTGGCCTTCTTGTGCGTGTCATTGAAGA
AACGCGGCAGCAGAGGCACAAGGCAGACCTCAACTTGGGTCTCCGTGGAGGGCCGAGGCACAAACGTC
GTATAAAACAACTCTGGAGTGTCGTGCGACGG
GAAAGCGCCCATGTTCTCGCTCTAAATGGCTTTGCACCACAAGGAAGAAAGAATACACACACTCACTC
CCGCACTCCACAAAAAAAAAAAAATATTCGCAA
TGAAGTCCAAAGGCTTCGTTGCCTCCCCTTTTCACTAAACCGATAGGCGCTGTTTCTTCTTCCTCTCG
TGTCCCTGTGCAATGTTTGTGTGTGTGTGTAC
GTATGAGAGAGTCCTCAAGTTATGCCACCTCACCCCCCCTTGCCTTTCCTTTTTTTTTTTTTTTTAAA
TATTTTGTGAGATCCGCTGCTGTGAGTTTTTG
TTGGCTTCGTTCCCCCCTTCAGCAAAACTTCGCCAGTGACCCACTCCTTGCAACGGTCGGTCCCTCCT
TTTTAAAAACAAAAAGTTTTTTTCTCTCGCCG
TATCCCTCACTCTCGTGTGTGTGTCCCCCCACCTGCCTGTTGCTTCGAAATAAAAACAATTGCGTCTC

CGTTAGTGTTGCTTCCTCTCTCTCCAATTTTT
TTTTTTTTAATCGGTATTTCTCCCCTTCCCCTCTTTCTTAAAAGTTCAAATATATTTATTAATTTTAT
TGGATTTGACTGTGCGTGCCGCCTGCCGCCTG
CCGCCACAACCACCACCGCTTTTTCTTTCTTCCCCCCTCCTCCCCCTTTCGGCTCCTGCTGTTGGACT
GTTGAGTGTATTTCGTTCTCCCCTCCCCTCCC
CCTTCCCTCCCTTCTCTTCTCTTCTCTTCTCCCTCTTTTGGGTTCGGTGTTTTGCCCCTCCTCAACTC
GGGGTTTTTTTCTTTCGTTCCCTGTTGAGCGA
CACAACCAACAACCAACAACGAAAAACAGAGATAAAAACGCAGCAATAAAAAACAACCAAACAAACAA
ATAGCACACAACAAGAAAAAAAAAAAGAAAAAA
AAAGAAAAACAACAATAAACCTTTTTCTATCTGTCTGTGCCTGTGTGTTTCTGTGCCTGTGTATGTGT
GTATGTGTTTGGGGACACTTTCCCCCTCCTCC
CCCCTCGCCCCGCCGCACAACAACAAAAACAAGAAAAGAAAAGAAAAGAAAAGAAAAGAAAAGAAAAA
ACAGAAGAGAAGACACAAAAACCGGTTAAATT
CTCAGCAATAAAGTGAATTACATTTATAAATACATACATACATATATACATATACGCACATTTACACG
TATGTCCCCGCATCTCCTTGCCATGTGTACAA
GGAGAAGGAAAGACGAGTCATGGTAGGGGGGGGCGGATGACAAAAAATCAGAATAAAGAAAAAGAAAA
TAAATAAAATACAATATTAAATAACAAATGTTTC
TTGAGCCCGTGTGACGCGTCCTCATTTACTTCGCTTTCCCTCTCATTTTTTTTTTTTCTCTCTCTCTC
TCTCTCTCTCTCGTCCACGCTCTCTCAACCAC
ACCTTCTCATCCCGTAATACCTGCAGTCAAGAAGAGAGCGGACACAACCAAACAGAAGCAGCGAGGGA
CATAAAATGTATATGTTGCACATTATGCCATC
TCTCACACGATCTATGCATCCGTACAACGAGCAGCAAAAATTCCCGGGATTTTCTTCTTCTTATGGCG
TTTCACCACTTCCCCCCCCCCNNNNNNNNNN
NNNNNNNNNNNNNNNNNNNNNNNNNNNNNNNNNNNNNNNNNNNNNNNNNNNNNNNNNNNNNNNNNNNN
NNNNNNNNNNNNNNNNNNNNNNNNNNNNNNNN
NNNNNNNNNNNNNNNNNNNNNNNNNNNNNNNNNNNNNNNNNNNNNNNNNNNNNNNNNNNNNNNNNNNN
NNNNNNNNNNNNNNNNNNNNNNNNNNNNNNNN
NNNNNNNNNNNNNNNNNNNNNNNNNNNNNNNNNNNNNNNNNNNNNNNNNNNNNNNNNNNNNNNNNNNN
NNNNNNNNNNNNNNNNNNNNNNAAAAAAAAAA
AAAAAGAGAGAGTGAGGAAGTAAAAAAGAGACGCCAAAGAAGCGCGAATCGACAAAGACGAGCAACGA
GCGGAAATGAATTGTAGGAAATGCCTGCCACT
CAATTCCAGAAGGATGCGTCTTTTCGCATCTTCGTCAACACACGCTCCCGCCAGCGGCTCACAAAGGA
GGCGGGAAGATACACTGTTTCTTCCGACTGCG
CATCCGTTGGATCCGCAGACAAAACCCGGCCGTATGACATGACAAAATTAAATAAATCTTCCACAATA
CGCTCTGCCATTGCCAGTCTTGTCGCTGCTTG
TTCTTGCTGCAGCGGCTGCTCCTGCGTTGCGCCCAGGTTCGCCACATCTTCCTCCTTCTCTTGGCTGA
TACCAATCAACAGCCGCACCGGGAAGTCGACG
TTCAACAATACCGGCGGCACACGCAGGATAGTGCTAGGCCGCATATTTGACAGGTGACCCACATACAA
AAAGGCACCATCGTCCTCGCGGGCAAGATAGA
TACCAAGACAAATGCCGGGCGCAAGAGGCGCTGGTGATGTGAGAAAGACCACGATAGACTCCGGGGCG
TTGCCTAAAGGTGCAATCCAACGAACACCGTC
CAGGCAATTGAATTCTGTTACGAGGGGAAGGCCGGGTAAAATCGCCGCAAACGGCGGCAAAACATTCG
GCGCAACGGCCATTTTCCGCTTGTTATTTTTA
TTCCCACTCCCACTCTCTGTCTGTCTGTGTTGTTTTTTTCTTCTCTCAACGGCAACGGCGTGCCGATA
TGTTTTGTGTTAAAAGGGGGATGGGGAAGGGG
GAAAAAAAGAGAAGTCGCGCTATAACTGTGGGAGTGTGCGCCAGGCGTAATACTGCAAGAGCTCCGCG
GCGCAGGATCCTCTCGTAAGGGTCCGCCCACA
CCTCACCACTTTTCCTCCTCTTCTCTTCGCCAAGCAGCCAGTGGGCATAAAACAAATAACAAAAACAA
AAAAGGAAAATAAGAAAAATAAAGATTGAAGC
CGCAGATAAAGACAGGAAAAAAAAACCACACACACACACACCAAAAAATTCATGTCTTCCACAATCAG
CATCCAGCCACATCCCACAACTTTAAACTCCA
AGTATCGTTGCCACTACTATTCCTGTCCTTCTTTTCTTCCCCCACCTCCTCCGTCTTCCGTGCAATGC
CATCCCCACGCCACGTTCACCGCCCCACCCTC
CGTCACGTATGGTTTCGTTTGCACGAATGCGGGGTCAAACTGTTGAATCTGCGCCACATTAGAGATGA

GAATACTCTGTAACGCCAAAATTTTATCGTCT
AATGCCATTCGACGATTTTCATCCTCCTCGCGGCGCAACTTTTCTGCCTCCACCGCCTCAAGAGACTC
CTTTTCCTTCTTGCGTACCTCATCCGCGCGGA
CCATCTCCTGCACCTCCCGGACACGGCTTTGATGAATGTAATAATAATACAAATTGTTTGTCTGATTC
TTCTCTGCGTAGACCCACCGCTGCAGCTTGGC
AATCTTCAGCACCAGGCGAAGATGCTTTGCGGAGTCGAGAGGGACGTCATCGTGCTCCTTGCACACGG
ACCATAGATCTACAAGCCGTATGGGTTGGTGG
AAGAAAAGAAGCTTAATGACGTAATTACGCGGTATCTCGCCCAGGTCACGAGGAATCGCCTTGATGTT
ATTCTGCATAAGGGGCTTTTTAAAAAGCTCGC
GAATATTTGAGTTGAAGGCACGGTGATTGGCGCACCGCAGCATGGTCACCTCACTGCGGTAAGGGATG
GTGAGGCGGTACTCTTTGCCTGGCCGCAACCC
ATGCGGACCTTTGGGGCGATTCATAAAACAAAATGACCGGCGCAACATGACGTGGGACGCGTTTCCTG
CAGCCACCACAGTCCTAAAAGAGGAGTGAGAA
GACCCCCTGCACAGAGAGAAGGGAGAGAAAGAGAGAGGGAGACGAGGAAACCGGTGCAAAATCGTCTC
ACACAGGGGGGAGGTTTGCTCGCCCACTGA
TGGACACCGTCACAGCCAAAAAGTGCATGCATCATGGCATTGCAACAATCAAACCACGGATAGAGAAA
CAAACAAACAAAAACAAAGAAAAAAAGAAAAA
GAAAATACATGTGAGATAAAATGGCTTTCACCAAATAACATCATCCTGCCAACACGAGCAGCAACAGC
GACGGCAGTCCGCGCGTAGAAACAGCGATGCC
AAACGACCAAATAAACTTCCACTTATAAAGAATGACAGACGAGGCAGAAACGGCAATCGATTGCCTTC
CCCTCCCGGCCAGAAGTCATCTTTGTAGAGTC
ATTTTCCGCAGAACCAGCGGGATAATGGCAGACAAACTCATCACACATTACAGCCATGTCCATTATAA
TGAAATGATTTCTACCATTTCTCCATTCCCAT
CACCTCCAATTTTCTTTTTATTCTTCTTGTCCCCATGCCTCCTACTGTGCACTACGGTCTATCTGGAG
AGAGAGAGAGAAAAGAAACAAAAACAAGAA
ATCAAAAAAAAAAAAAACTATCCGGTCCTTTCCATTCAAGTAAGCATATATTATATATACAGCTGTAA
TGCCATACACAAATAGTAAGTAATTCATTCAT
TCAACAACAAAAACAACAAGCAGAAAGAATGAGGGAAGCACAAACGCAAAATGAATATTGCTCTCTAT
TTCCAAATGAGACCCGCAACTTCTTTCCTCCT
GCCCTGTCACTTCACATTCTGCCATCAAACCGAGCAACGCATTACTCATCCGCCCATACAAATCCTCC
ATTCTTGCCTCACTTCTTCTACTTCTCATGAC
TCTATCTATTTTCAAATCAATCAGTCTTCTGATGCGACAAACCGTACGTCAACATCCGCGTCTTTTCG
CAACCGACTTATAAGCGGCTCAGTGTCACTAT
CTAACAGTCGTCTGCCCTTTTTCAGCGCCAAAAGTGCCTTGGCACAGTTAAGACGCACATTCGGCACA
CTGTCTTGAGCAAATAGACGCAACATGGGAAG
AATGTGTTGTGAGGCGGTGCCGCAGTCCAGGCTTTGGACACTCGCCAGTGATGCATAAAAGTGTGCGA
GTGCAACGCGGTGAAGATAGTTGTTTGTGGAC
GCCATAGAACTAAGCCGAGGGAAAAGCGATGCTCGTGACCATGCCGTCCCATTCTCCGCCACCAGCGT
GCAGCAGGATCGCACCGCCGCTTCGCGAATAG
TCGCCACACGGTCGACAAGGTGCTGCACACAGAGCTCCACCACATCTTCTGCGTTTTTGCCTAACGAG
GTGATGAGAAACGGAACCTGTGATATAACGAC
TTCACGAATACGCCAATTCGGATCTCCCGCAAGTGCCACAACAACGGGAACGAGTTTGGGTGCCATTT
CTCTCGCCTCGGGGGTATTTCCCATGGAACTA
AATCCAGAAACAAGAGCCAAGCGAACATCGGCTTCTTGGTCTTTTAACAATTGGGTGCAGGTCGGTAC
AATTGTGGTCGCCCACAGCTCTTTTGCAACGT
GTGCCACGGACCGCAAAACCGCACTTGCCAAACACATCCGCACATGTGCATTTTCGTCGTTCACAAGT
CGACATCCGGCCGTTAAGAGTTCCCTCTTTGC
AGACGGGTCACGAAAAACGGCCAATAAACTGGCCATACTAAAAACTGCTGAGGCCCTAATTTCCGCCT
CGGCATCCTGCGAAAGGGAACGAAAGAATGGC
AGAATATTCTTCACCTCGGCCGGTGACATGGCCTCGGCGAGCTTACCAAGCCGGTCCGCCGCCATATG
CCGTACACGCCAAGAATAATCGCTCATAAGTG
CCTTAATCGAAGAGAAGACATTTGCATGTAGCTCGTACGGTAACGCTTTGAGAAGTGCGGCGGCAGCC
TCTACCGTCTGAAGGCGGACGGCATCAAATTT
GTCCTGTGAGAAAGCAGTCAACGCGTTAATCAACTCAGTACTTCGATTTCCAAGAACATGTACCATGT

GTTTTCCTAGCGAAATACACGCAGCGCGACGC
ACCATAATTTCTTTATCGTTACAAAGTTCAATAAACAGGCTCATTAACTGCCCTTTAATTGCTGTGGA
CACGTGAGGGTAGGCAGTGGCGATAAGGTACG
ATGCACTTGATCGACATTGCGTAGTTGCTTTGCCAAGTCGATGTACCAAATCAAGAAAATCACGTTGC
GCCTGCGCAGCCGCTTCGCCTGTCTCACGAAA
TACCTGCCCACCAATTGTCTGCAGCGACGTTACAGCTTCGTCACGAACGGTAATTTCATCTAAACTGC
TCAACAACTCCAGGGGCGTCAAAATGCTCTGC
AAATGGTCGGCACCACCCACTTCTTGCAGCATCGTCCCCAATGCATTGGCAAAAACCCGTAACACCAC
TTCATTCTCGTCCAGATAGTCGGTGAGGTACG
GCAATAACTCGTCACGAGTGCGCTCAGGTCCAAGAGTAGAGGCAATGAGGTGAATGCCGCGCATGCTT
GTCAGCCGTTCCTCCGGATCCTCCGAGCGCAG
GTTATTAATCATTGACTTGACAGAGTCAAAGTGTTCAGTCATCTCCTTTCTCTTTTGCTAATAATAAT
AATAATAATAATAATAATAATAATAATGCCGT
TCTTCCCTTGTTGTGCAATGCAAGCCACTCTACGGATGGTCTCCTACCTGCCTCCTTGTTTCTTCCTT
AGAATGTTTTGTTTTTGGGCCTGTCCTCCGTG
CAGAAACAAGCGGATCCGTTTTTCTCTCTTCTTTTTTGTTTCTCGCCGTATTTATGTCGTCAAAACAA
CACCAGCGCAAATAACGCAAACACGTGTTT
TTTTTTTTTTTTTTGCAATATCACCACTGTGGCCTCTTCTTCTCTTTCGCTAACAACACGCAGAGAAA
AAGAGAAAGACGTGCAGCACAACTGAGAACAG
ACGCAAAAAAAAAAAAAAAAAAATAGATAAATAATCCCTTTCCCCCTCCACACACATAAACACAAAGAC
ATATATATATAAAAGAAATAAAAAGATATTCC
CACCTCATTGCTCTTCGTATTGAATTTGTTTCTTTCATTAAACTCTGCAAAGGGACATGAATGTCTTA
TCAGTTTGTATGATAGAAGAGGCATGTAGGAT
CACTCCTTACACCGGTAAAAGTGGAAACCATACCCCATATTTCTCTTCCATTCCTTCCTTGCAGGTAT
AGATATATCTGGAGGTCATTCTCTTTGCTCTG
GTCCCTCCTCCACAGACATCTCTTCCACCACATGTCTTCCCTTTCAAAACAAAACAACAAAACAATAA
AAAACAAAAGGCAAATATTTTTACCCCTACTT
CATTTTTCTGCCTAATTAATCGCTGCGTTTGTTACAGGAAGAGTCTGAGAGATGTACACACACACACA
CACGCAGACACACAAAAGAAGGAGAGCAGAGT
TCTGCTTTCGCCCATTCGCCGTGCCGCCCCACACTCCCATTTCCTCTGCGCGCTACTCATCCGTTTCG
TCAGTGACGCCGGAATCTTTCTTTTTGGTTTT
ATTGCCGTTATTATTCTTTGCGCTCACGTGAACGGCGGTGACAATGCCGTCAGCCCCATCCCCCACCG
ACGCATGGATTTTCGCCTCCACATAACTTCTC
GCACGGGTCTTGACCTTTTCAACATCCGCTGGCGAGCCATATGCCCGCTCAAATGACATGAAACGTGT
GAGGAACTGCTGCATCACCTTTGCTGAGAAGT
TCGTGGCAACTGCACGTTCAAGTAAAGCCCGCACGAGGGGCACCGCGGCGGACTCGTCACGGCGCACA
AGGAGAGCCATTTCCTGATCCAAGTACGCCGA
CCACACATCCGACTTCTTCGGCATGCGCACAACAAGACCTTCAAATAACGCACGACCATTCTCAACAC
TGCCACTCTTGTACTCATGGACGGCAAGGCGC
ACAACCGTCAGAGAATACGCGTCACGACGTAGAGCACCGCCCATGTCTTTTACCATGCGCCTCAACTG
ATCGCGTTTTTTCTGGTCTATAAGCACCTTAC
CAAGTCGCTCCCACGTGCGTTGCTCATTGCGAAACTTGGACACCATGTTGCGGCACAGAGCCAGGAGT
TGATTTGGCTTGCGTGTGGCGGAAAAGATGTC
GGCGAGACGTTCATACACGACAAGTTGGTCAGGAGCACGTTGCAGCGCTCGCTTAAACACTGCCGAAA
GAGACTCCACCGTACCATGAATATTCTCAAGA
TTCATGTAGGCCACCCAAACATTCAGACGTTCCTGCGTCTCACGAACACCAATGGTACTCAGCGCCTT
TTCGGCCACAAGACGGGCTTCTTCCTGCTGCT
GGAGAGCAACATGATGTGCCATCCATTGGACCCACAAATAACTGTTGTTTGGAGCAGCCAGAAGTAGC
CGCCGGAACTCATCCGGCGAGGATGGCACAGT
CGTTTCCATAGAACGCTCGTATGCATCAATAGCCTCCTCAATTTTTCTTTTGCGCAATTTCTGTTCCC
TACCTCCATTTTTGTCGGTGGTGCCATCATTA
TCATCAATGCTATCGTTTTCGTCGCTCTCAGTTTCAGGATGTCGCTGCTGCCGCTGCTGCTGTTTCCC
TTCAAGTGAATGTTGTCTCTTGCGGTCACGCC
AGGGAAAAAGACCAAGGAGTCCTTTCATTCCCGGTGGCAATGGTGCCCTGCGCCAATGCGGCAAGGTG

AAGACGGTACGGTTGCTGGAAAGCACAAGAAA
CGGGAGAACCTCCTGCAGCAGTGATCCGTGCGGCACAAATTCAATCACGAGTTGCATAGAGGCCCCCA
CGACGGGTGGCTCGCCGGACGCGGCGGTCCCA
TGCCAATTGTAAACATAGTACCCAATCAGACTGCTTGCACCACACGGGACCTCAACAATGGCGCAGTG
ATCGTCCGTGAGTGAAAGCAACACTGTGGCTG
GCACCACATCACCTGCCTGCAGACCTGAGAAAATTGTCTGCATGGCGATCACATCGCTAGGGGTGCGT
TCCACCACCTCCGTCCGTGCGGTGACATTGGC
AACGCCAGTCTGAACGTCATAATTCAGCAGGCGCACCTCCACCGCACCGGCATGCGCATCGACATGTT
CAAGCGCTGCCAGGGCAGTGAGACCACCGGCA
ATCGACAGCACCGCACCACGCTCAGGGGATGCCTTCACAACACGCGCCATGGTGGTGGTGCCTAGACC
AATAAGTACGTTTTTGCGAAACAAATTGGGGA
GTAACGTGGCCACTGGCGGCGGGTACGTTGCGACATCGCTCCACAGCTCACGCAGTTGTATGCTAAGG
ATACGATACTGCTGTGCATCACGCGTGCGTCT
TGCCACGGCCCGCACCTGTTCGCGCGACGCAAGGCGTTTGAGAAGTTTCTCCGCAAGGAGATCACCTC
CTAATGTCTCAACCGTTAATCTGCCAAAACCG
CCATTGCCCGGGAGTTGCACGACAATGCCGTCTGGCGCAGCGTGATGCACGCGATAGTCGCGAAACAA
CATAAAATTTAAATTCCTCTTTTCTCTGTGTG
TCTGTGTGCTCGCGAGTGCGTATCTATCCTCCGTGTGTTTCCCTCCTTTCAACTAACAACCACGACCA
CCACAACAAGAAGACTTCTTTCAAACCAATAA
GGATGAGTGTAAAAATATATATATATATATATATTTGGTAGGGCGGGTGGGGAAAGACAAGAGCGTAA
TATATACATATATTTATGCATGTACTCCCTTT
CCCTTTTTGTTTTAGGCCGATAATTCACCTCCTTTTTTTTTTTTTTTCCTCTTCTCTCCATTCATTCA
TTCATTCAATGACTGTTTATTGTTTTCCTATT
ATTATTATTATTGTTTTGTTTACACGGAAGATCAGGAAAGACATTTTCATAACGTCATGTTTAAAACT
TCTCCCCGTCACGTATCCACACATATCCCCCT
CCTCCCCCTCCTCCCCCTCTCTCTCTCCCTTTATGTATGCACATATAATATATGTATATGTATGTGTG
TATGCATGCAATACTTTGTATTTATTTATTTA
TATTTATAGAAATAAATAAATATAAAGAGTGAGAAACAACTTTCCCCCATCTTCAGAAAGTTACTAAC
GGCTGCGTGAGAAAAGAAAGACAAATAAACAA
ACAAAACGACCAAAAAAAAAAAAAAAAGAGGGAGTGATATGAGTGAGTCTAAAAAATAACACCAAAGA
CTTCCCAACGCATGCAAAACGATAAAATATCA
CATGAACGCAGCCGCCTAACATCTGTCCGTGTTTCTCCCTCCCTCCCTCTCTCCTGGGTATTCATGCA
CTTACTTCTCTCTTTTATTTAATTTTGTGTTA
TGGACTTTTTTTCTTTTTTTTCGTTACTTCCCTTCTTATTCTTCCACCACCACCACCACCTCCCCCTC
TCATTCCGCACTCCGGCGGCAAACAAATGTGT
GCATGTCATTAAAGCAGGGCGTACAGACGTATGTTTCAAGTACACCCCGTGCTGAATCAATCAATCAA
TCAATCAATAAATATGAGCAGCATTACACACA
ACACGCACTTCTCCACTTAATTATATGCATGTATACATGTGTCTATTTAAATAGAAACTCCCGTTTTT
TTCTCCCCCCCCCCCCAAAAAAAAAAAAAAAA
AAAAAAATCCACACAAATGATAGGTAAAAGATAGAGCACGTTTGCCGTCAAACTGGCTGATGTCAATT
TATCAATTTTATCTTTCTCTTACCTTTACTTT
TTCCCTTTGCATGCAATGGAACCNNNNNNNNNNNNNNNNNNNNNNNNNNNNNNNNNNNNNNNNNNNNN
NNNNNNNNNNNNNNNNNNNNNNNNNNNNNNNN
NNNNNNNNNNNNNNNNNNNNNNNNNNNNNNNNNNNNNNNNNNNNNNNNNNNNNNNNNNNNNNNNNNNN
NNNNNNNNNNNNNNNNNNNNNNNNNNNNNNNN
NNNNNNNNNNNNNNNNNNNNNNNNNNNNNNNNNNNNNNNNNNNNNNNNNNNNNNNNNNNNNNNNNNNN
NNNNNNNNNNNNNNNNNNNNNNNNNNNNNNNN
NNNNNNNNNNNNNNNNNNNNNNNTCTTAATGGCTATCTATCCATCTATCTATCCATATATACATGTGT
GTAAAATAATGGTGCAACAGAGTACGCACCAA
TAAACAATAAAAAAAGGAAAATAACACTGGCGAAGATCACATTATCACACACATTGTTCTGTTTGCGT
CTGTGTGTTGATGTCACCTCCAAAAAAAAAAA
AAAAAAAGAAAAGAAAAAAAGAAGCGGGCAACGCAACACACCACAAAAGCAAAAAAAACAACAACAAC
AACAACAACAAAACAAACAAACGAAAAACAAC
CCCGTGATGCTGTCATCTTTGTTGCAACAGCAATATTTGCACGATCAAACTCACAAGATTCTGCTGCA

TTAAAAGCAGTGCCATACACCCTCACGTGCAC
ACGGGGTGAATTCCCCCCCCCTTTTTTTTTTTTTTCTTTTTACTCATTGAGAATGGAGGAGGGGAAAG
AGGAACACAAATACATGCGTGTCCATTGAGGG
GGAAAAGAAAGGAAAATAAATAACAAAAACAGACATACACACACGCCGAAGTAGAAAGAAATGGAGAG
AGAGAGAGAGAGAGAGAGGGTGGGAGTGAACA
TAGAAAGAACACAACAACAACAACAACAACAGGGAAAACAATATAAAAAATCATAAAGGACATAAAAA
AACAACTCCTGCAGTTACTTCTGTGATAAACA
GGCCATGCACGACGACCGACACACACACGGATTTTCAACGGCGCCTTTCAGAATCGCTCACTCGCTTT
ACGGTTCAAGTACTCCTCTCACCCCGCCTTCA
AAACACTCCTTCCAACAGAAATTACACAGTTCAGTTCAACTCCTTTTGTCCCCCACCGCGAATCGGCT
TCACGATAATCCCATGGCCCATGGCAACGGGG
GAGTCTGCAGGTGAACTGGGTTCCCGTGACGTCAGCGCGGCTTCTAGCGTCGGTGTGCTGCTCCTGTG
TTCATTACGGTGCCTCCCGTTATGTTTTCTAC
TGCGGGTGTGGGAGTTATTAAGCAGATCATCGTATAGCTGTCGCCAGTGAAGAGCATCGTTGCGTGCG
TCCTGAAGCTGGGCTTGATGACAAACCCGTTC
AGCTTCCAGTGTACGCTGCAGCTCCTGTTTTGCCGCACACTCTTGCCGCAGACCGTGTTTAAGGGTTT
CAATACGTTCATGGCGGATGACCAACTTCTTC
TCTGCGAGCTGCAGCTCCAACGTCAATGCCCCGTTCAAATTTCGCAACTTCTCCACCTCATCCATGAC
AGCCTCCATGCGATTCAGCATCATATCCTCAT
TTTGGTCCCCATCGCCGCTATGGTGGCCGATCCAGTCATTGTCCTCCATTTCTCGAATACTGTCGAAG
ACTGCTGCGGTGACATTGTCGCCACGCCGTAA
ACCCGTCCTTTCGTAGCGTGGCGGGATGATAACAAATGGTTGACTCTCCACTGGGCATGATTTCCTGT
CACTCGTTGATTTTTTATTCCCCCTTGAGCCC
TCAAGAATACCCGTGCCTGCGTGTGGAGTTATGGGCGGTGATTCACTCTTATTTGTGTTCTGTGACAA
ATCACGACTAAACGGCGACCGCCGAACGTGGC
GCTGCGGCAAAAAAGATCCATCTTCTACCATTCCAAGGATTGTGTGCAGCTGCTCCATATATGATGTC
AACTGCAGTGGCACAACTTCCGCCTCCTCTCG
TGCCTCATGAAACAACATTCGGTAATGTTCCGCGTAGCGACGTTGCGCGTACAACTCCCGTGTAACGT
TGGAAAACTCCTCCTCCCATGCAACACACTCC
TGTTGTAGAATCTGCACCACCGAGGCCTGTGCTGCAGACTGCGCATAAAGAAGATCCGTCGTGTACTG
CGCGTCACGTAACGCGTCACGCAGGCGCTCCA
TTTCGGCCCGCATATCCTCCAAAGAAAGTTGCTCACGGACACCTTGCTGCAGCAAAGCATCCACACTA
TCCAATTCAAAAGACTCCTTGGTACGACGTGA
CAGACCCCCTGCCTGCGCCTCTAAATGACCTCGAGAAGTATATGTTTCCCGTGTCAACTCACTACATG
CCTTTCCTCGCAGCAGACACCTCAGCCTTTGT
ATCTCCACCTTCGCCAGATCTAACATGCCTTTAAGTTCCTCAGTGGTAAACTGCCGATTGACGACCGC
CCGGTTACACACGTTCTGTGCCCGTGCACCAA
ATCGCAGTGTCGAGAGAGTCTCATGGGCCTCCAGTTGAGACGGACTGCAGCACAAAACGAGTGCCGTG
CGTGAGTTACCGCCAAGAGAGTCCTTTAGAAT
CCGTGTAAGTTTGCTATCACGGTAGGGTACATGCGCCCTCTTTTCCGCCAGGCAGATGATAACATGTC
CGAGGGTGGTGAGAGACTTATTAATCAGTTTT
GCCTCCTCTAATCGAAGACCGTCGGCCAATGTCTTCGACACCTTTTCACAACCCGCAAGGTCCACAAG
GAATAGTTTCCCAATGGTTGCCTCGTGCTTCG
CCCGATTGACACTCTTCACTACAATGTTCAGAACGCAGTGACTACGGCTACTGGCGTCGTTAATGGTT
GTTGCAGCGGTTACGCGCCGACGCAGCCCACT
ATGAACGAGTTGCAGCACTTCCTCCGTACTTGTCACGTACGGCATTTCGCAATTTTCAACGTAGAATC
CAGCCGGGTCCTCACGCACATGTAAACTACAT
CCTGTCGGATTAAGAAGGTCCCGCACACGTTCCATGTAGATTTCTACAAACTGCACACGTATCTCAAA
CTCCACCACTTCCTCGGCACTGTGAATGGCGT
AAAAAAGTTGTCGCACCGCACGAGGTACGATACCAGCGGCGTTTTCATGCATATTAAAACTCAACGAC
CCGGATTCCTCACTTAGAGGGGAATGCAAACC
AAACATTGTATGCGTTTTTCCAGCCCCCGTCTGTCCATAAACCAATAGTGTGCCGTTGTACCCATGCA
TGACATCGTCCACAATGGGGCAGGCAACATCG
TCATATAACTGTTTTTGCGTCGTGTCGGGTTCATACACGCGAGAAAAGGCAAACTTGTAATAATCTGA

AGCCACCACAGTCCCACTCAGCGAAGAGGCGT
CATCTGTCAGCGTGCCACGAGGACTTGCCACCAGCTGCTGGGTCCAACACGGACTCCTTGCTGTCCTA
CAGATCACATGTCTGGGTCCTTCAAAGGCCAC
GCACAGGCAGGCATTTTGCTCCGCNNNNNNNNNNNNNNNNNNNNNNNNNNNNNNNNNNNNNNNNNNNN
NNNNNNNNNNNNNNNNNNNNNNNNNNNNNNNN
NNNNNNNNNNNNNNNNNNNNNNNNNNNNNNNNNNNNNNNNNNNNNNNNNNNNNNNNNNNNNNNNNNNN
NNNNNNNNNNNNNNNNNNNNNNNNNNNNNNNN
NNNNNNNNNNNNNNNNNNNNNNNNNNNNNNNNNNNNNNNNNNNNNNNNNNNNNNNNNNNNNNNNNNNN
NNNNNNNNNNNNNNNNNNNNNNNNNNNNNNNN
NNNNNNNNNNNNNNNNNNNNNNNNNCTTCTTTTCTTTTATTTGTTTTGAATTTCTGTTTCTTTCTTCCT
CCCTTTTTTTTTTTTTGTTTTGTTTTTATATAC
CAGCTCTTCACGTGCTTGCGCACACAGACAAACACAAAAACACCGGCAAAAAAAAAAAAAAGGAAGTA
TTGCAGGAAGCCGGGGTCCGGAGCCCATTTAC
TCAGGCCGTAGTGAACACCGTCAATTTCTTTTGCAAAGCAATTCAATCGCGCTCACGCAAATGCCGAT
TATATAAAGAAGCAGCAAATAGAGGGACGGAC
AGGGTGAAAAAGAACATGCGCACTTTTGTTCCCCTCACTTTCTCTTCTCATCCTCATTCCACTCGGTG
GTATATTTGGCAACCGCCTTCCAACAACAACA
ACAACAAAAAAGTAAAAACGGCAAGCCGTCATTATCGTCACCGCTAAACAACAACAACAACAACAAAG
CGGATGTCGTCTCAACATTATATACGAACAG
TCTTTGCCATTTGAAGAAGCCCATCCCTTTGTCAAATGGAAGGCAAACCGTCCACACGGCAGTGGTCC
TCCTACTCTACAATTAAAAATAACAATTATTT
ATAATATACCATTACATACCAAATACACCCCAGTTATATGCGTACAAACACACCATGAAGGTGCTCAA
CAAGATCCTTGCACGGGATCTACACAAGCTCC
TCCCCCTCTTCATGTATATTTCTCTCCCCTTCCCCGTCAGGCAAAAACACCAAAAAAATAAGACCAAA
TAAAAAAAAATAATGACAATAAAAAAATAAATAT
AGAATGAAACAACAAGCTTTATGGTACCTCAACCGTAATTGTTCGCACTCTACAAATGTCTTTGGGTT
CGGTCGCAAAATCGATAGATGCGCTTCTTCCA
ACGCAGAATCTACGCAGCGTAAATTTTTCCCTTGTCGCTTTGTTTTTTCGTTCGAGGAGCCAAAGCTA
TGGGAATTCCCTTGAATGGTCCTTTTTTTGGC
CTTCTCACGTGTCCCATTCTGCCTTCCGTTATTTTCCCCAGAAGGACCTTTGAACTTGCCGTACAAC
GCTCGCCCAAGTCTGGGAGCTGCACGGCGTCA
CCCGATGGATGCACACTGGAAGCCACCGCCACAACAACGTCATCGTTTGACGACATGTCGTTATCTTG
CAGGCCAACAACCGTAGGGATGGGACTGAGAA
CATCACCACGTGAGTCGTCCACCACACCAACGGCACCGCCGTACCTGTCATCTTGCGCTTTTCCTTTA
TGCCCTCGCCAGTCTGGCATTTCCTGTTTCTG
ATGCCGCTGCTTTCCGCTAGCATGTCTATTTGCCCCTTTTCTGGATGCTCCGCCCGTCGCACTCACTT
GTGACAAATCCAACGCCGCACGCGATAAGTCG
ACGGGTGTGCCTAATTCTTCAGCCATGTATTTTACAGATCCATGACGATCACCCTCATAACCGACAAA
TTCTCCTTTTTTCGTCGCCACCGCGTTACACG
GTTCAAAAGCAAGCGTCTCCACTGACGTTTTGCTTCGTACGCGTTCTGAAAACGATTCGCCCCCAAAA
TGAAGCTTGCTGTTGCTCAACGAGTTCATTTC
CGGTTTCATAGGCACCGATCGGTCCTGCGCAGGCTTCAGAATGGATCCCGGCAGAATATTACCCTTGG
GGTTTTTACCAGCATCCGTCCTCGGAGCCTCA
GGGCCTATTTCATGCGGAGACGTGGCTTCTGTTTGTAATAATTTTCCTGAGGCAGGGCACTGAGCCAA
AATGTAACGCTCCAACCGTTCAATGGTTATCT
TCTGCGACTCTAACCTCACGTGCAGCTTCTGGCATTCCTTCTCCGTGATGCGTTGCTCGGTGCGTAAT
TCATGCACAAGGCCTGCCAATGACATTAAGTG
CGAGTGAATGGTGTCACTTGAAAGCATTTCTCTCTCCGTGTGCTGCCCCTTCACGCAGCTACCCACAA
GTCGGAAGGGACATACCACGACGGACTCATCC
TCATACTCCACGCTACCATCATCATCATTATCATCACCGCAACCATCATTTCCGTCAAGAATTACAAG
CCCAGCAAAGACACTCAACTCATCTTCTGTTA
ACAGGCTTTTCCCCGTTTGTTGCCGACGCTGCTGTTGAAATTGTTTGGGGTCTATTCCTCCTCGATGG
AGAATCCATGCTGGAGACAACGAGGGCGGCGA
CTCCACTTTAGACGGCAGAGACTTTCCATTAAAGCCCTTCGTATTAGTCGCCGCTGCTGCCGTCCAGT

TTTCGGATTTTGCATCGTTTGAGAAGTTGTGC
CCAGCCTTCTTCTTTTCGTCTGGTTTTCCTTCATTGAATTTTGTGCCGCCAATCGAAACCAACGTGCA
CAGCGAGTCTGGCGGGTTCACACGTCTGGTCC
TATTTCTTGTCGCGACTCCTGCCTCGCACTGGATCTCGGTAATTGGATCGGGTGAACACGACCGCTTC
ACGTCCTTTTTATGACTCAAACGCCTCAGCCC
TGAAGAAGTGACGGTGCGCGCTGTGTCTTGTGTGTTTAAACCCTTTCCTACCGGCGATCTTACGTCGT
CTTCCGTTCGCTGCTTTTCAGCTCCTCGTTTC
GGAGGGTGCTGCTTTCTTTGTTGCCCGCTCGTTCCTTTTTTCGAATTCGCAACGGCGCCATCCCCGGT
GGTGTTTTGGTCTACTGGCGTTTTAACCCCGT
CACCGTTCATATGCTTCGCCTCGTTTGTTTAGAAGCCCACGGTGGGTGGATCTCTATGCGTTATATAA
TTCTGCCCCTTGCATTACACACACAAACACAT
AAATGAGGGGAGGGAAAAGTCCTGAACAGGCGCATACAACACATTCACTTGTCCTGTAGAAAGAGACA
CACACGTTGAATTGCACAATAATACAAAGATA
AAAAAAAGGAAACAACAACAATAACAACAACAACTTTGGTGTCTTGTCATTCCGATAACTCTGCATCC
ATCTGCCACAGTAATTCCCACTCCCGCTCTGT
GTAACCCGCGGCACACAACATCCGAGCCGCAAAACGCCGTTCTTCTGGAGAGAAAAGAGCTGTCGGGT
CTGCGTAGCCTTCGGCTTCAAGCAATGCAGTG
CCCAATTGTAGCATCAACTCCTCCTCCCTTTCATCCATTTCGTCAACGAAATGGGCCCCACCTTCCGC
GGTGATGCCGAAGCTACTACGCGTCTCGTTCC
ACCCATGTATACCATCACCACCAGCAAAGTTCAAGCCGCGCGAGACAGCCAGAGGCACGGAACTTTGT
TGTGCTGAAAGGAACAGTTCGCTACCCACGGA
GGAGATCACAATCTCATCCAGGGTCACGCACACAAGGCAGACACCGCGGCCGCTGGAGGCCCGAAGAA
GACTCGGCTGTTGTTGTTCTTGCGGAAATTCT
GTAAAATCACGCACTAAAGCAGAAGATACGCCAGTAGGCATGACTGTCGTTGCGCAGTTGGAAGAAGC
CCAAGCTGGGTGCACGGAGTTCCCCATAAATC
CACCCTTCAAGATTGTAAACGAGGATGCATCTGCTCCCAGGCGTGACTCAGAAATCTCGCTGTTGCTG
CCCACAAGGTAGCAGTTTCCCTCTGTGGAGAG
AAATACGGTACAAAACTCATCTTGTCGGACACAGAAATTACAAACACACGAAAGAAACCACTGTGGCT
TCAAACAGCCCGACGCTAGGCACACAATAGTG
GCGGAAAACCATTTATTAGGAGGGTATTCATTTCCATTTTCATGTTGTTTTGTACTTAACGTCCTTGG
GACCGCCCCCATAGAACCCTCACCGCACGCAG
GCGACATCTCAAGGATAAGCCAACGCCCATCTTCGCTGACAATGGCAAACTGCAATTTATTTTTACTC
ACCGCCACATCCACCGCAAAAGAAACAGGCAA
ATCATTCTCAAGTAAATCCAATGCCGTCTCACTATCCTCCTTGTAATTTAATAAGGCGCCTGCAGCGG
CGGTGTCGCAGACACCACGCTGTAACTGCTGC
CTACGTGGCGACAGCAACAATTCTTCACGCGGTACCACGTTACAAGTATCTGGTGAAGAAACCGGAAG
GCTCACTGTCGACAGGTCAAAAAACCCGCTCT
GTGTGTGGCCCTCATCCATGGAGGACTTCAATGGCCACCGCTGTCGTTGTTGCATTTCTGAGGTTCGA
CTCATGGAGCATTGCCATAATGGCTGCAAAGG
TGTCCTCCGAGTACCCGTCACCTCATAAGGATTTAATACGTCCGTGGTATAAAAGGAGGAGGAATAAG
CCGTGTGAATATTTGGACCACCTGTTAATGGA
AAATCCCCTAATGGACACTCCTGTGTTGCAACACACCGCAGGTTGCCTGAGCCTCCCCACTCCTTTAC
ATCTTGGCTGGATGCGTTCAATAAAACAAGCC
GTTCACCACAACTTACCAGGAGCAATGTTGCACCTGCTACGGTCTGGGTGGATGATCCAATAAACACT
GCATCATTTCTGGGAGTAGAACCGTCGAAAGT
TGTCTGCGGTGTTGCCGCAGCATTCCTACAAGGGAATAGAGGAGCGTTGATGATGCGCAAATGACCTT
CTGTAGCAAACGGCACAGTAAAACACTTCATG
GAGGTTACGGGTGCCTGCAGCAAGAGAGATCCAAGACACTCCCCGTCTGTTGTGATGGAGTACACATA
TATACAATTGTGGGCGCCAAGAAATATCGTCG
TGCCACCCACTTCTGCCGTAATATCGCCGCACGTGCAGTTTGGGACGATACAAAAGACATGTGAAGAA
GTGGATGGGGGGTTTGACTTTTTTCCCTCGTC
GTGACCACCCCAGCCGTGATTCATGTGATTGCTGGGCTCCAGAGAGGGTGGCAATCCCCAGTCACACT
GGCCCTTGCTTTGGAGTGCGTTGCTGGAGGCG
GATGGTGGGACTTTGCAACCGCCTGATAGAAACCCCTCCGTGACCTCGCATGTTCCTTCCAGCGTCAC

CAATACAAGTCGACGGTAGGTTGCGCTATACA
ACACAATGGAGATGGTTGCGGCAAGCGTGCGGGAGAAGATTGGCATGCGATGGTCCGGCTTCACCACA
AGAATTTGCCCCTCCGTGGTACCAACAACAAT
TTCTTCCACGCCATCGCCATCCACGTCAGATGCGACAAGCGCATTCGGCATGACGGAGCCTTCCCGCG
CGTGTTTACGGAACTCCCATGAATCCAACTGA
ATCGCCGGTAAACGCCGCATTCTATTAAGCCCACGGGATCCACCGGTGCTGCTTGCATTCGTCTCCGC
CTTCAGCGAGTAAATACCCACGGACATGTTTG
ATCTCGCGTCGCCACACCGCCCCGCCACCACACCGCCACCTGCTGCCACAATAGATAAGAGGAGAAAA
AAAGCAAAACAGTCCAGAAAAAAGCACCAACA
AAAAGAAAACAACAACAACCAACAACAAACCGTCCCATCGTTCTTTCTTTCTTTTCCCCTTTATATGT
ATATAATTTTTTTCCCCTCCTTTCTCGGCCC
GTTGGACTCATTCCCATCATTACAAGTGGCGTGAGACGGCTGCCGCAACACCACAACCAACACCAACA
ACAACAAGATTATCAGAAAGAAGATGACACCA
CCCCGTTCATGTGTATTTGTGTGTGTCTGTACAAAGGAGCTGAGGATCGAAAAGCGACTACCGCGCGC
GGAAGGTTTTAACGCGCGGATCCCTTGCACCG
ACACCCTCAAAGCCATGAAAGCGTGGCACAACATCGCTTTCACGGAAGACCTCCTCAAAATCCAATTC
CTCTTCTTTGTCCACCAGCATCTCGTGCAGAC
GGTTAAACTCCTGCTGCACATGTTGCACGCGTGTCGCCTGCACACGACGACCGCAGTTCACATAATTC
ACAAAGTCCTCCACGACAAAAGGTGATGTGCA
GGGGCGCTGCGTTGCCTCATTTTGCATCGCTGCGGAGAAATCATTTTCATCAAATGCCCCATCCTCCT
TGTGCTCTGCAAGATGTTTCTCCCACTCAATG
CGCTTCTTCACCCCCCAGCCGCTCGAGATACTGTTTCACCACCTTTTCATAGACACTGCGATGCCGGCG
CGGGTGGATTAAACTCACGGTACCAGAGCGAA
AGTCATACTTCGAGTAGTACTCCGCAAAGCGTTGCAGGCAAAAAAAGACAGCCTCACCAAGTTTCTCA
TCATCCCCTTGTAGCGAGTCGTAGATGGCGGG
TAGCTTAAAACTCTCCAACTGAAGCTGCACATCAGGCAGCGTCAACTCGCCAAAGTCACCCGTTGGTT
TTGAAAAGACAGGCAACAGTCCAAGTTCCTGA
AGGACCATAAGTGCCATGGTCGTCACGGTGAAGCTGTTGAATGTGGATCGCTCCGGTGCAATCACCTC
CCGTGCTTTACCCCACTCTTTCACAAGGTGAA
TGTAGGCCCGGTAAAAGTCAGGGAACACCTCACGGATGGCGGCAAGGATCTTTGAGTTTTCCACCCCA
CCCACGTTTCCAATGCTAAGATCGCAGTGAAG
CCCTGAAAGGCTGTCGATGAACTGCACCACAGGAATGCGAGCACGAATGTAACGTACATCCTCC
>scaffold1020.size7244
CTCAAAGACACACACACAAAAAAAAAAAAAAAAAGAAAGGCGGAACTGTAGAATAATGCCGGTGTATG
AAAACAGGCACCTCCTGCGTCTTCTTCTCACCC
CGCAAGAGCAGGCAAGGCAGTTTGTGGAGTACTGTCGAGCCCATCAGGTGATCACGCGGGGTCTTTGT
CGCATAGGCACAACAAAGCGTGGCGGCTTGCG
TGGCCTTATTGCCAACAAAGAAATAAAGAAGGATGAAAACGTCGTGATGCTTTCCGTACGAGCCTCTC
TTCACCCTGGAACGGCACTGCGGGACAAACAA
TTTTTTGATTTGCTCCCGCTGCGGGAGAGGGAGATGTATTTTCTACGGCGGCCTTTCATTGAAAACAG
CCGTCTGGCAGATCGCTCTATTATTCGGCATA
ACCAATTCCTTCTTGCCACCTACATGTCGTATCTTATCCTCCGTAACGATGTGAATGAGGGATCTCAG
GCACTTAAATACGTGAATTTTATGCCCCGCAA
TGAGGGCGATTTTGAGCAACTGATGTGCAACATGAGTGGCGCTCTAGACAGTGTGGAGGTATGTCAGG
TTTGTCAATCGGCTCTGGCATCGTTCTTTAAA
GTGACGCAAGCCGAAGTGAGACCGCTGCTTCTGTGGTCATTGTCGATGATATTTAGTCGCATGGCCCC
AATTGACCATCGTTGGACGTTGCGGCACGCTT
TTAGTGGGACACCTTTTGTGGATGCCTTTGACATCACTGGGCCGGATGACGATGAGAATCTTGTGAAG
GAGCCCATTGTGTTTCTCTGTCCTGTGATTGA
TATGTGTAATCACAGCGATCATGAGAATGTGGCGCTGATGGTCCCGGAGAAAACCCCCACTGCCCACG
AACCTGTGATTTGCCTGCGCTCCTTGCGGGAT
ATCGAGAATGGGGAAGAACTCACCATGACGTACGGAGGATCACCGACGGAGCTGAAGGTCATCTGGGG
GATGCAACATGTGCTGTGAAACAACGATGGTG
ACATGATTATTATTTTGTTGTTGTTGTTGTTGTGATCCATATCCTTTTGTAAATATGCGTACCGT

CTGGTTGTTATTATTTTTTTTTTCTTCTCTCT
TTGGTGTGGCTTTGGAGAGTGACTGTTCTTCATGACGCTCCTTGCGGAGTAGGCGAGAGGCCACTTTG
GAATGGAACCGCCCAACTCATACGATGACGTA
CGAGAGTGACGTGGAATCCCGCCTCCATTGCGCAAGTAAAACTCTTTTATTTTTATTTTTTTATTTTT
TCTTTCCCTTTGTCCTTTTGGTGTGCAATTGG
ATATGTGTGTACTTCTGTGTGCCAGGTGGTTGCTTGGAGGAGGGCCGTCGTTCACTCACTCACGCGCG
TAGGCATCGCGGAGGGAGAAATCACACAATTA
CTTTACTTTCCTGGGCATCCCTTCGGTGTGCGTGACGCCTGCAAGCACACAAAGTGGGAGGAGACTGA
CACACAAAAAAAAAAGCGATAATTGCTTTATG
CTTCATGGGAATTGGAAGTATTTGCGCCAGGGCGGGCGGCAGTGGATGGAGAAGCAGGCCCGCGATCC
GTTTGTGTTGGAGGCTCGCCGACGCGGGTACG
TCGCCCGTAGTGCCTTTAAACTTCTAGAAATGGACGACAAATTTGGGCTGTTTGCCCGAAATCACACA
CGCGTTGTGGTGGACATAGGCTGCAGCCCCGG
TGGCTGGCTGCAGGTGGCACGTGAACGCTGTGGAGACCGCTGTGCACTTTTCGGTGTGGATCTGCTGC
ACGTTTCCCCCACAGTAACGGGTGCGGTGTGC
ATTCAGGGCGACTTCACAGACATGGCGATTCAGCAGCAACTTCTTTCGCGGATGGAAAATGTTCACGC
CAATGGGCTGGTGGATGTGGTGATGTCAGATA
TGTGTCTCAATCGCGGATCTGGCGGCACTGACGATCGACAGAAGCAAACGCAGTTGAATATGGAGGCG
TTGGCTTTTGCCACTAGTCAGCTGAGAACAGG
TGGGAATTTTGTTTGCAAGGTACTTGGTACTCCAACGGCGTTTGAGGAATTACGCGGGGAACTGCAGC
GGTGGTTTGTGCGTTCGACTCTCTGCAAGCCT
TTTTCGAGCCGCCGTGGGAGTGACGAGGCGTTTATGGTATGCCTTGCAAAGAGAGAGACGCCACGGCC
AAGAGGCTGCAACATCGCCGAGGGTGAAACAA
GAGACTTTGGACTGGATGACTGGCCAGGCTTTCTTCGCAGACCACGGCAGCAAAGATGATTACGATGC
GATTGATGAAAGTTTATCCCTTTTTTCTTTAT
TTTTGTTTTTGTCGTGGTAAAATGGGATGGTTGTATTGTTTGCCCTTCACTCCGCCCTGCTTTTTTGG
TTTGTCGTGGGATCATCAGCTCTGATAGCATC
TCCTTTTCTATATTTTTTTTTTTTTTGCGTGTGCCTGCTTTCTGTCTACTTTTTGTTTTTCCTTTCTCT
CTCTCTCTTTCTCTTGTCTGTATTGTAAAATA
CATGCAATGAAAGGGGGGAAAGTTGATTGAAGTGGGCCTTCATCGAGCGGGAGGGACGGGGAGGGAGA
TTGGCCTCCATCATGTGGCTCATTATCTTTGT
AATTCTTACTGCAGTCCTTCCCGGCTGCGCGGAGGCCTCTGCTCATTACATGTATGATGGGGAATACG
ACGTGTTGAGAGTGGGGGAGGTGCGATTTAAT
GACACCATGGCATTTCCCGCAAGTATGGCCACCGAGGTGCGACGACGGTACGTGTTAACCGGGGTGGA
ACTGGGCACGCGGTATCTCGTTCATCTCTCGC
ACTTAGGCTCACCGTCCATTGAATACCGGATACACGTGGGGCGTGTGCACAGATCTGCCATCAAGGGA
AAAATATGGCCTGCGAAGGAACTCGCCCCGGG
CCGGCGGAAGCTTGCGGATGTTATCATGTTGCACTTTAAGACACACGTGGATGAGCTGCTCTTTGAGT
ATATTGACGGGGATGGCGATGTCGACAACGGT
GACATCGAGTATGGGGTCAAGAATGACGAATTGGTTGTGTTGGAGATTCGCGGTACACGTAATGCCTT
TCCAGTTCAACCTGACAAATGGCGGAAGTTCT
CCTACAACCTGCGCGTGGAGGCCATAGGGAGCGGCAGTGGAATCAACCGGCTCATCATGTGGCACGCC
ATGTTGGTGATCACCGCAGTAGCTGTCGTTGT
GGTGCTCGTGCCACGACTCATTGGAAATACCATTGCCTGTGATGCCCCGATGCCGACACGTCCACGGT
ATGATTTTCTTATTTTTTTAGTAAATAAAAGA
ACAAAGACAAAAGCATAAGTGGATGTAGACATACTGCTGATACGAGCAAAGCGTTTATTTGCATGTGT
GTGTGTGCGTGTTTGTGTGGCTTAGAATGCAG
CTGGGGGGGGAAGAAATAATGCGCCTTTTATGTTCCAGTTTATCAATCATCCTTTTGCTCTTATTTGT
CTTTTTTTTTTTCTTTTTGCCCCTTTGCCATA
TCAGTACACACACGCAGACACAGACACAAACAGCACTGTCGCACATAAACAACAATTCAACCAGCACA
AGGAAGAATAGGAACAACAATATATTTATTTA
TTTATTTATTTATTTATTTGTATACAAAAATAAAAAATAAAAACAATAAAGTGACATGGAAAACCTCA
CAGTAGAGGAGGCCCGTCATCGGCATGATAAA
GACGTTGTTCAGCCTTATAAGGCAAATAGTGCTAAATCGTATGTAGGAAAATCCATTTCCGATCGCCA

GGCCCTGTACGCCGAATCCAAGAAGGATCCCG
TCCTTTTCTGGAGCAAGATTGCAGCAGATAACTTTTACTGGAAAAATTTATGGCCCTCAGAAAAGCCG
GCGATGGAGTTCAATTTTCACAAGAGTAAAGG
AAAAGTCTTTGTGAAGTGGTTTGATGGAGCAAGGACTAACATTTGCTACAATGCTCTTGACCGCCACC
TTGCACAGCACAAAGACCGCGTGTGTTACTAC
TGGGAAGGAAACGCAGAGGGGGAAACCAGCTCGGTGACGTACGGCAATATGCATGAGGCTGTTCTGCA
GTTGGCAGCCGTGTTACGGCACCAGTATGGCG
TCCGGAAGGGCCAGGTGGTCACTCTGTACCTCCCAATGATCCCATTCACAGTGCAGCTCATGCTGGCA
GCAGCCCGCATCGGAGCCGTGGTCTCCGTTGT
ATTTGCCGGCTTCTCTGGAAAAGCGCTTGCCTCTCGCATCATTGACTCCGCCAGCGAACTTGTCATTA
CGGCTGACTCTTCTTTTCGTGGAGAGAAGCCC
ATTCCTCTTAAGGAGGTTGTGGATGTGGCCATTGCGGAATGTGAGAGCGAAGGTCATCATATCCAATG
CCTCGTGTATGAGCGACACGGTCGGGAGAATA
TTCCAATGAAGTCAGGCCGTGATCGGTGGTATGGCGATGTGGTTGCCGCCATGACAGCCAGCCAGCTG
GCGGATTGTCCCATAGAGTGGGTGGACGCAGA
AGACCCACTCTTTATGTTGTACACCTCCGGCAGCACCGGTAAGCCAAAAGCAATTCTTCACACCGTTG
GAGGATACATGGTGTTTGCGGGAACCACTTTC
AAGTACAGCTTTGACTACCACGAGGACGACGTGTACTTCTGCACCGCGGACATTGGTTGGGTCACAGG
CCATTCGTATGTGGTGTATGGTCCCATGTTAA
ACGCCGCCACGTCCGTGCTTTTCGAGGGCACACCAACATATCCCACACCCTCCCGTTGGTGGGAGCTT
GTGGATAGGTATGGTGTCACGGTTTTTTACAC
CGCACCAACAGCTATTCGATCGCTCATGCAGTATGACGATGAACCCGTAAAGAGGACGAGTCGAGCGA
CACTCCGCGTTCTTGGTAGCGTCGGGGAGCCA
ATCAACGTGGAGGCTTGGAGGTGGTATTATGGGGTAGTCGGCGAAGGCCACGTGGACATCAGCGACAC
GTGGTGGCAAACAGAGACAGGCGGGCACCTCA
TTACACCGCTGCCGGGCTGCACGCCGCTGAAGCCTGGAAGTGCAACGCTGCCGTTCTTTGGTATCGAG
CCTGCCCTGTTGGACCCAATGACAAATGCGGA
AATTGACGGTCCCGGTGAAGGGCTCCTTGTGATCAAGAGTCCGTGGCCGGGGCAGGCCCGCACTATCT
TTGGGGATTCTGAGCGCTACGAACAGATGTAC
TTCCCCGTAGACGGGTACTACATGACAGGAGACGGTGCACGCCGCGACGAGGACGGCTATTACTGGAT
CACCGGCCGCGTGGACGACGTGTTGAACGTCA
GTGGCCACCGCATCGGCACCAGCGAGGTGGAGGACGCCGTAAACACGCACCCCCTTGTTGTGGAAAGC
GCCGTTGTTGGCATCCCGCATAATACGAAGGG
GGAGGGCATTTATGTCTATGTCACATTTCACCAGAACACCATAATCGACAAGGATCTCCTCAATGCCG
TCAGGGCTACTGTCCGTAAAGTGATTGGGCCG
CTGGCAACACCCGATCACATCCACCCGGCGCAGTGTGGACTTCCAAAGACGCGGTCTGGTAAGATTTT
GCGTCGCATCTTGCGCAAAATTGCCGCCGGCA
TTTTTGATGGCCTTGGCGACATCAGCACCCTTGCGGATCAAACAATTGTGGCGAAACTTATCGAGGAA
AGGAATGGAGTCTCTATTTGATGTAGAAGGAG
GAGGAATATCTCCAAATGCAAGATGCATGATATAATCACCTTGCGTGGCTCCCCCCTTCTTTTTTTTT
TTTTTTTTTTTTTTTTTTTTTTGAGGGGGGAGG
GGAATTTGTCATATGCGCAGATNNNNNNNNNNNNNNNNNNNNNNNNNNNNNNNNNNNNNNNNNNNNNN
NNNNNNNNNNNNNNNNNNNNNNNNNNNNNNNN
NNNNNNNNNNNNNNNNNNNNNNNNNNNNNNNNNNNNNNNNNNNNNNNNNNNNNNNNNNNNNNNNNNNN
NNNNNNNNNNNNNNNNNNNNNNNNNNNNNNNN
NNNNNNNNNNNNNNNNNNNNNNNNNNNNNNNNNNNNNNNNNNNNNNNNNNNNNNNNNNNNNNNNNNNN
NNNNNNNNNNNNNNNNNNNNNNNNNNNNNNNN
NNNNNNNNNNNNNNNNNNNNNTTTTTTTTTTTTTTTTTTTTTTTTTTTTTTTTGAGGGGGGAGGGGAAT
TTGTCATATGCGCAGATGAGTGGATTTTATTA
TTGTTTTGGTTTTTATTTAACAATTATCCCTGCCGCTTTGCACGTTGCATTCCGCTGTTTTTTCCCCT
GCGGACGGGACGCGTTGCTTTGGGGAGGTGGG
GGGACTGATGTATCTTAACTCAATTTGCAAAATGTTTGGCAAAAAAAAAGGCCCTTTTTCTTCAATTT
CTTTTTGTTGTAGTTTTTCTTTTTTTTTTCTT
TTGTACTGTGTGCGTTTTTAGTGTTTTTTACTGTGGGCACTGAAACCTCCCGTTGGACTGTGCTCCTT

CTCTCTTTTTGTCTCGCCCCTCCTTCCCTCCC
CTCCAAAATCTTCCCTTTTTGATGATGCGTTGTTGTTGTTGTTGTTGTTGTTGTTGTTTTTTATT
CTTTTTTGTTGTCTTTGTTTTGTTTTCTTCTT
GAGGGGAGAGGAGAGTGGAGTTGCGACACCGCCACTCAGTGTGGCTGTACGTGTGTGGGTGTGTGGAT
GTCTACATGTACATTTTGAGTGTGGGTGTATG
CATGTGTGCTTTGTTTCTCTCCCCCTTCCAGTGGTCCCACTCGTCCACGTGGATGTGTGTGTGTGTGT
GTGTGCGCGTGTCTGTTTTTTGTTGTTGTTGT
TGTTGTTTTGGCTTCTGTGAGTGTGAATTCCAAAGTTGGATGCGTGCAACTACAAAGACACAAACGCA
TGGGCGTCTGCAAAAAAGAAAAAAGAAAAAAA
AAAAGAAGAAATGAAATTAAGAATGAGAAAGCGAGACGTCGAATTAATTTTATGGGGATTTTTTTTTG
TGTGTGTGTGTGTCTGTATGTGCGTGGGTTGT
GAGCAGGCGCAGAAACGGGTTTGTGATGACCCCTCCTTCCTCTCTAATTTTATATTTATTTATGCCTC
TATATACACAGAGACCCCCCTTCGTAAATCTG
GCTATTGTTTTCGTTGTCGTTGGGTTTCTATGCTTTCACCGCTGCTCTTTTGTTTGTTTGTTTGTTTT
CATGCACGGAAAGAGGAGGATGAGGTCTGTGT
GTGTGTGTGTGTGTTTTTGTTTGGTGGGTGGGTGGGTGGGTGGG
>scaffold1021.size29173
ACAAAGATGTCGCAGCGCTCCACACTGAAAAAAAACAGACAGAAAGACAGAAAAAAATTAATTTTAAG
AAAAAAAGACATCATCGCTAATTTTACATCGCC
GCTCCCGTGATGCCAAGAAGCAAAACAAAAAAAACAACGCCTCATTCATCCTCCTCCTCTTTTTCTGC
GCGGCTCGCGGCTATTTGTTGTTGTTGCTGTT
GCTGCTGCTGTTGGCTGTTGTGGTGCGCCGCAATGACATCTCGAAAGTCCCGCAGACAATCGCCAATC
ACGTGCAGCTGGAACAATACATCAGCCTCCCT
CACATCAAACTTCCCCCGACCAGAGTACGGCGCCGTCCACGCAATGCAGCGGTCGTAGAGATTTTTGC
ACGCCATTCGGACGTGACTTACCGTGCGAGAG
GTGGAGCTTCGCTGGTTGATGCATCGTTCGAGCTCATCCTGCTTAATTTTTATGCTCTTCTGCATCGT
CTCGTAGGTCGCCTGCAGCTCATTGAGTTGAT
TCTGCAGTTCGACGCTCTCGTTGTTTTTCTTCTGGCGCTTCAGGTTCAGAGAGTTCTTATTGCGCAAC
AGTTCCTCCTCCAGTTGCGTCTTGCGCCGCTG
CAGCACCTTCGTGTTATCCTGCAGCGTGTTCCAGCGCTGAATGATGTCGCGGGGACTTTGGTACTCGT
CGCAGTCGTTGCGCTGCAGCACCCCCTCAAGG
TACTGCTGATACTTTGCGTAGGCATCGTACTGCTGCCGCAGCTCCTCCCGGCGTTTATTCAAACTGTC
CAACTTTGCCGTAAGCTCCACGATCTGTTCCT
CCCGCTCCCTTTTAATGCGTTCCTCTGTGGCGGCCTTTCGCACCGCCCGCCCCCGCCTGATTTCATTT
TCTTGAATGAATTTGTAGTACTGCACAAGTTT
GTCCTGCAGCCTGGAACGATCCGCCGCGATGACATCCTCGACCTCCCGCACGCGTGCCATGCGCACCA
TATACAACTCTCTTTTTTTGTCCAACTCCTTC
TGGACCTCCATCACAGCCCGACGCAACTGCGTCGCCGCCGTGATGCTCTCAAGTGTACCCGCCATCCT
GCGTGCTCTAATGTGTTGTAATGTGTCTCCTT
CGTCACCCCTTCTTTGGCAAGGCTACCACTGCCACCAATACTTCTCACTGCTGAAGACGGCAGAGAGA
GTGCAGCGAGGCGATACGTGGTGTAAAGGAAA
AAAAAAAATGACAAGGGATCCAGCAGAGCTTATGCATGCATACATGCGGCTGTATGTGTGTGTGTGTG
ATGTGCTTTGATATGATGTGCATGCCCGGCCC
GCAGAAAAGAAAAAGTACCGACCAGATGAACAGAAGTCCGTGGTTTTGTGGCAGGCTCTCACATGTCG
TCTTGTTCACTTCCCTCAAACACTCTCGTCGT
TTCTCTTATTCCTCTCTTGCATTTCGTTGTACTCTTTTTTTTTTCCTTTTTTTTCCNNNNNNNNNNNN
NNNNNNNNNNNNNNNNNNNNNNNNNNNNNNNN
NNNNNNNNNNNNNNNNNNNNNNNNNNNNNNNNNNNNNNNNNNNNNNNNNNNNNNNNNNNNNNNNNNNN
NNNNNNNNNNNNNNNNNNNNNNNNNNNNNNNN
NNNNNNNNNNNNNNNNNNNNNNNNNNNNNNNNNNNNNNNNNNNNNNNNNNNNNNNNNNNNNNNNNNNN
NNNNNNNNNNNNNNNNNNNNNNNNNNNNNNNN
NNNNNNNNNNNNNNNNNNNNNNNNNNNNNNNNNNNNNNNNNNNNNNNNNNNNNNNNTTTTTTTTCCTT
TTTTTTCCTTTACCTGCCTCATTTTCATTCAA
TGTTTCCTTTTCTGCCTTCCCGCTCTTCTCCGTGCCTCCCCCCGCGTCCACACGCGCAGCCACCATCC

ACCCATCCACCTCGGTGGGGCGGCCGCCGGGC
CTTCAATGCTACGAGTGGTACGAGGGCATAAATATCACCGACTGGTCCGACGCCTCCACCGCCGCCAG
CAGGGAGCCGCGGGTGAAACCGTCAAACACCG
CCAAGTGCGCCGCGTCAAGCTCCGCCACGCCTACAGGGATGAGTGTAAACGCGCCGCCACGGCGGCCG
TAGTGGCACACAAGCAAATGACCTGTCTCCAG
CGCAATGTAAAGACGTCTGTCGCTACACGCCATTCGCCGCACGTTGCCGCCGATGCCACCGGCAATAC
CCGTCGACACAAGAGCAATGACTTGCACGCCA
ATGAACGGGTTGGCGTGCGCTATAACAACCCCCTCCACGTGAGCGCACTGGAGGAAAAAGTGATTTGG
CCCGAGTGCCTGCGCCTTCGTCATCTTCAGCA
CCGCTGCTGCGGTGGGAACGACAGACTCTACGCAACTCGGAAAGGCGATCATCACAACGACGCTGGCG
CCGCACACGAGCTTCACGGCAAACGTGTCATC
ATCGGCGAAAAGAACGTCCTCCGTGGCACCCTCAACCGACACGTTCTTTCTAAGCGAAGAGTTGGACG
GTGGCAAGCGTATATCGTGAAAGTGTAACGCG
ATGTGCTCCGTGCTGCCGCAAGCCAAATATCGCCCGTCGCGGCACGAGAAGCTAATGCACGTCGTCGG
CACATGCGTGGTGCAGCGGTAAAATAAACAAT
GCGCCTTGCGGTCCACGTCATCCTCTTCTCCTGCACCCAACCCCTTTTCACTGTGACCATTATTGCTC
CTGCCGTAGACGGTGACGACGGGAGAGACGCC
AAAGGAGAGGGACCAAACCAACGCACCGCCATCGCAGCCGACCGCCAGCACATCACGCGAGAGCGGCT
TCCACGCGACGCAGCGCACGTGGCGCTGAAAG
GCGTGAGTGAGCACACATGTCTCCTTCCCTTCCGCGACGTCGTATACGACGACCCGCGAGCACCCAGC
GCCCTCGTCAACAGCGGCTGCCAGCACCATAC
GATCGGGGTGGAACGCAACAGCGGTGACGCACTCCGGGCAGTCGGCAGCATTCAACAGACGCGGCGCG
CAGTTTGCAGACGCCGGCACAACAAACGGAAA
TACAAACCGCAAAAGCGGCATGACCGATACAAGAGGACGGAGTAACCACCGCACCACCCCAAATGTCT
GCTGCTTCTTCCACCACGACAACGAAAACACC
GATGACGACAACGATGATGGCTTCTCACGCCCTGCACGACGGCCAAGAACGTTTCTATTTGCGAGCTT
CCATAACGGATACACTGCACCACCTTGCGCCT
CAAATGAGACACGTTTGCTGCTGCCGTACAAAGACCCCGCGGCCTCACCAAGCAAACGGTCCCCGGCG
ACAAACACGGCGGCGGCCTCAGAAGGCAGCTC
AAGCAGCGGCTGGTACATTTGGAGGCTTTAATATGAAGTGTTATGTGTTACCAGGCAAAGTGAGCACA
AATGAAAAAAAAGAAAAAAAGAAATAAGCCAA
ACCAAACGCAGTACAAACATGTATATATAGAAGGAGATGGATAGCGATGCATAAGCACATGGATGCAT
TAGAAAAGAATTGGCTTAGTGGCAGTCACAAA
AACGACATACAGACAAAAAAAAAAGGGAGATGTTGTTGTGATTTGACCAGCGGCAGACGCCCGCCACC
GCTATTGTCCGTAATTGGAGTAACTGCGCATT
AATTCTGGCGTTAATTGCCTTTTTCACTTTTTCGTCTTGCCCCGTTTGTTCCCACCCTTCGACTGCTG
CTGCTGCTTCTGCTTCTGCTGTCTCCCCTTCC
CCGACTCTTTATTCGACTCCTCCAAAAGCTCAGCCACAAGCTTAAGCTGCTCCTTCTCCAAGTCTGCG
GTGTCATTCTCGCCCCGCTGTCCGCCCACCGA
GGATGAGGACGGCGGTGGCGGCGGTGGCGCTGTCCCTCCGTTCCGCTGGAGCGCAGCCATTCCAGTTA
CTGCGGCCGCGGCTGAGTTTTGTTGGCGCACG
AGCTGTCGGTTTGCCGCCACATATAACCCCGGATTAGGCCCTTTGAAGAACTTCTTCATGTGGAACGC
GTCCTCTTCGTCCACGTAGTACCCCTTTTCTA
CGTTGGCGCAGCGGAATCCGAGGGTCTCTTGGTACAAGTGTAGGGCCGCGTCGTTGGTCTTCCGCACG
TGCAGGGAGCAGTAGTTGGCGTCATACTCCTT
TTCCATCTCCTTCATTGAGGCCCGCATCACACGTGACGCGATGCCAAGGCGACGGTGAGTCCGAAGCA
CCGCTATGCTCGTGATGTGGCCGTGaATGTTT
GCCGGCTTTTCCTCATCCTCCATCTTGGCAAGAACGTACCCGACAATGTTACGATTGTAATCCTCCTG
CACGTACAGCAGCTGTGGCCATGAAAGTATGT
GGTAGAAGTAGTACCGCAGGTTGTAGTTCTCGGGCAGACACCGCAAGTTGCAGTGCTGCATCTGGTAC
ATGTCCTCCATCGTGGCGCGACGGATCTGCAT
GCTGGACAAAAGGAAAGGCGTGCTGGTTCAAAAAAAGAAAAAAAAACAATCTTCATCCAATGGAATTC
GCAGAAAAAATAGATGGTGAGTAGAAGGCTCG
TGTGGGAATGAAAGCACACACGCACACACACAGAGTAAGACCGCAGCACACACCACAATGGCCAAAGT

TTTAAAAAAAGCCAAAAAGGTGTTCGCCGACAA
AATAAACAAGGGGCATACAAGCGACATAAGGCGTCGGTCAACTCCCATACAAGCAAACGTACCACCTC
ATACGAAGAGAGAGAGAGAGAAAGAGAGAGGAAA
ACGACAAAATAAAAGTGTACAGTCAGCCAATGCCTCCCTCGCCCCTCCAATGCACGCCCACACACACA
CACACACATACACACGTCAACCAGTTATTCCT
TACAACTCCATGATGCGCCACGGGCCATCGCGGGAGACGGTGCAAAAGTAGCACTCACTGTATTTACA
CGCCGAACCGGAACTACTGAACGGACTTTGAA
CCTCCCCGTCACACGGCACCAAAACATGACGCGCCACAATCACCAACGATGCCTTGAGAAACTCCAGC
GCCACATGACACGATGCATCGCTTACAAGTCC
GCTCACCTTTAGTCGTTGCACCAGCTGTTCCTTGGTGATACCAGGGAAATGATGCACGAGACTATAGA
GGGCAAATAGAAAAGAGCGCCACACAAACTCA
TGAAACGAACCATCCACATGATGGAAAATGCTCGGAAATCGTGGCAAGGGTAATCCTTCATTCTTCGG
TGTTGCGCTCGCAGAGCGCGCCATTATCACCT
GTTCTACGGAAGGGGCGGAATCAACAGGGATGTCGCGCAGCTCGAGATGGTTCAAATGCCGCCATGGA
CGAAGAAGAGCACGCGCAGGATACGGCTCCGT
TTTGGTTCGCGTTAATGTCGCTGCCGACACCGCTTCAGGAATCACAGCCGCATTAGCTGCAGCCACAC
CAGGCGGAAGTGTTGTCTTTGGTGCAGCAGCT
GGGTGTGAGGGCCATGCAAAGCGTGGCAGCCGCAGCACACGGAGTCCCTTCTGCTCCCGAATCTCCTC
TGCAAATGAGTCCAAGTTCATTGACGGCTGCT
CAACAAGGCGTGGAGCCTGGCAAAGCGAGTCCCTCCCAGTCGTGTTTTCCGTCACTGCAAACGGAGGT
ACACACCATCGCTGGCAATTCGGCGGTGATGT
GAAGAGCAGAGAGGTGGTGATTTCCTGCAAAAAATTAGAGGCCCTGCTCTGGCAGTGAGTAACCGTTG
TCGCCAATGTCGCCGGGACAAATGTGAAAATC
GGCGACAATTCAATGCGCGGTATTCGGCCGGAGCCACCCGACCGTGTTCGGAAGGAGGGGAATTTGAA
TAGCCAGTCGATGCAGCGCAACTGCTCCTCCT
CTGGAAACTGTGCAAGTAAAGCCTTTGCCGCAATGGCATCGTAATGTGCCTCGTCAGTTAAAAGGATC
ATCCGCAAGACATCCTGCAGTGCCTCACATGG
TACCCCGTGGGAGGCGTCCATGGCGCTGTTTTCCTGTATGGCAGGGCCAGTGGGTGCACCATCTTCCT
GCTCAGAAGCTCTTTGCTCCCCATCGTCGGGT
GCCAATGAAAAATTGGGCGAAAGCACCGACGTCATCACTGAGTGCACCGCAGGCGGCATCCCGCTTTG
CTGTTGTCTCTCGATAACATTCATGAGAACCT
GTATCAATGTCTGGATACTACCATGAACACGTTTTCGTTGCGGGTCGTAGCGGGAGGAACACAATTCT
TTACTGTAGCCTCGCACGAGCCTCTGCAGGTT
TGCAAGGCCAAAGTATGGTTGTTCCGACTGGAGAATAGCTCCGAGTGCCTCGGCCATTGTCACACCAT
TCACCTCGATGCGACGCCGCGTGCAAGAGGCA
CGATGACCCTCGCCACGTCTCTTGTTGGATTCTTCCACCTGTATCACGTCACGGAGGGTGCGTTTCCG
CGCGCGCACAACTCCTTGACGCTGCAGAACGT
GTTCTGCGAGGATGACAGGGTCTATGCGTAGCAGTCGTGAGAGGGCAATCAGCTGTGCATTCGACAAA
TTCGGTTCAGTTGCCACAACCTCACTGATGAG
CTCCATAGCGCGAAATGGCTCATGCGTCACTGCGGCAGTTGTCCACGACTCGCTCCAGAAATTCAGCA
CCGCGCGACACGTGTCTTGTGGAGTCATGTCA
GTACCGTCGCAAAATATGTGCCGGCGCCGTCGCTCATCCAAAGCGGTGGAAACAAGTGCGATGGAAAC
CTCCGCAAGCGGCACGTCCAAATAAGCCACGA
GAGACCTTTCACACACCAGGAGACCTTTTGACTGCAAACCGGCGAGGGAATGCAGCAACGGCTGCACG
CCAGACCGACGACAGCGAGTCTGCAGCGAGGG
GGGAAGCCGGCAAATTGGTGTTCCCCACAAACATGACTCCATTGATCTTCCGCCCACATACTCGCCCA
CATCAACATCCGCAACGCCGACGATGATGCAA
AAAGAACTAAAGCTCATCTCATGCAGTACCCGCTCAACTGTCATCGGGGTACTGGGTGTCTGCCTGAG
GGCAAGCCAAAGACGCCAAAGCTCCAAATGCA
GTCGCGACAAGCGAAATAGGCCAATCCGGCTATATCCGTTGCGCACCATTGTGACCTTGGCGACCATG
CGTACGCTTCTTGAGCAGGGCTTCGTAGCGAC
GGCAGCAGCGGCGGAGTCATCCTCATCACCCTTTTTAGGATCTGGCAGGTGCTTTATCAGCGCAGTCG
ATCGTTTCCGCTTCTGCCCACGAACAGAAGCA
CCATCAAGGTGCGAATGCATCAACTTCGCCCGCAACTCAGCTGTCAACTCCACACCGGCAATGGTAAC

GACGCCAAGGCGTCTGCCTTTAGGTGTACTTA
AGCCTGTCGTCGCAACAAGGCCTTTGTCACGCAGAAATGGAATTACACGACCCAAGGTGCGGCGGTCA
ACGATGCGTAAAAGATCATTCAGCAATAATGC
CTGCAGGGGTGCCACATGCAGAGCCTCCATGACTTTATTCACGGCCCAACTCGACACGCCCTTTACCA
GATCCAACTCCTTCCCTTCCATGCTACTCATT
TTATCGGCGCTACCATGTCGATCGTCACCCCACCCTTTTGGTCGCAGGAGACGGGTGAAGGCCTTGCT
ATGAGCACTCAACACGTTGGTACTCTCAATGG
TTTCCCAGCGCTCTTCGAACTGCCGGGCAAAGCGGAGCAGTTCGTTCCCCGTCGCCCTCAAATCGTAC
AGAGTGACACGTGGTAACACCGATTCCACCAC
CAGTGGCCGCCGCTCCGCCTCCTTTACCAATTGCAGCGACAACGGGTACGACGGGTCAACGCCGAAAG
ACTCTTTGTTCCTCTTAGCATTCGCATTTAAA
TCGGCTGCCGCACCACCACCATGTTCTTTACTATCATCATTACCCTCATCCGCGTCGCTATCATCCTC
ATCACTGGAGTTCGATGCTCCACTCATATTGC
CATCATCGTCCTCACCATCATTATCATTATCATTATCATTATCATCATCATCATCATCAGCATCAGCA
TCAGATGCGTCCGGAGAACTGTTAGCAACCAC
AACACGCGCATTGCGCTTACTTTTCCTGATAAAAATGGGTGCAATGGCAATGCGCAGTCCCCCGGCTC
GAAGCATGCGTCTTAGGGCCAAATACATCCTT
CGACCGTTTATAACCGGCACTTCGTGACCGTGTGCCGCAAATATGGCGTGTATGGCATCCTCAAAAAC
CAGTCGACGCTCCGGTGATGATAAAACAATCT
GGCGTGCCACCAGGGGATCTATGACAATAAGTGGCGGCTGCAGTTCCCCACGACGCCTTGCCAAATAT
GCCTCCAAGGGCCGAAGGTGTTGTTTTGGTTG
ATTCGGTGGCGGTGGCGTAGCAAAGTGTGGAAACAGATGCGGCACAACCGGGGCTCCCTCGTCCACGC
ACACATAAAACACGCGCAACCACTGCTCTCGT
AGCAGCCGCCGGATGCCACCTCCCAAGCCTGCCTCGGATGGGCGTCTTTCTTCTTCACCGGCTGCAGA
ATTCTGAAAGCGGCGGACCCCTGTCACTAAGT
TATCGCACGCGTAGTTCACAGCGATCCGTGTGTTCTTAAAACGGAGAGATACACCTAAAGCCTTTTCC
TGCAACGCCATGCTTGGATAAAAGGTGCACTG
CTCGTATGGCACTGTGCCATTCAATAGCTCGGACGGCACCACAGGGTTCCCATCCCGTTTGACGCTAA
CTTCGCTACTTTCGCTTAGAAGAAGGTCTAGC
ACCATCTGGCGCAGAGATGCATGGTCCACAAGGCCGTGGTACGGAAGTGCGTCTTTGGAAACAAATGA
GAGCAGCTCCTTCCCGCTGCAGCCGTACTGCT
CGCTGTGGGAGCCAACAAACTCCAGCACGCACTCAAGGATCTCCCTAAACATAAAAAACCAACTAACA
ACTTCTGTTGTTTCCGTTGTTGCCTTTTTTTT
CTTTTCTCGTCGATTTCTCTTCCACGAGAATCGAGAAATGCCGCACGCTGCACCAATTCCGTAAGTAA
TGATGATGCTAACAACAAAAACAAAAATTACT
ATTATTATTATAGTACGGTACCAAAAGAAAAGAGAAAAAGAAAACGCAAAAATGAATTTCATAAAATA
AAAGACCGGGCAAATTGATAGTATTCAGAAGG
AGGGTAGACGGTGGGAATGCTCCAGTGTCTCCTCGTATATCTGCTATGCCCAACCCAATGTGAATCCT
CTCTCTTCTGTTTATGGAAATCTTTATCGTTT
CACTTGTACTTCTGTTCTCACTCTTTTTTTTTTGTTCGTTTTTTTCGTTTTTTGAGAACAAAAAAAAA
AACCGTGGGGATATTTATTCATTCGCCCCTTG
GCTCTTCTCTACTGGCAGAGGGTAAAAGGAAAGGATTTTAAAGGCTCCGTTGCAATCGCCGCAGTGGC
GAAGAACGTTGAAACAGTGAAGCGAAGACGAG
GTGTAGCGATGGTGATCGTGGTGTACATTGGCTGACCCTTCATTCCCTTCCGGTGGAGCAGGTCCCCT
CCCCATCGCTGCCACAATCGCTCCTGCCGTAG
AAATTATCAGAAGAGGAGGAGGAAGAGGAGAAAAAGGAAGAACCGCTACTGCTGCTGCTGCTTGAATT
CTCCTCTTCTTCCTCCTCCTCAATCATCCGAC
GACGACGCCCCTGTTTTAATCCCCGCTGCTGCTGCTGCTGCAGTTGTTGTTGGTATTGCCGCTGTCCC
GTTTGTTTGTCGTCTTCTGCCGCTGCCGCGGA
GTGTTTTCCTCGTGAGCCCCTCGACTTCCGTTGTGTGCTGCCTCGGCTACCGCAGACGTTCTCGTTTT
CGTCAGTCTCCGCCTCCATATCGGCCTCCCGC
ATGAGCCTCGAGAGGGCGGCACAAAGCTCCTCCAGTGCGCGGCGCCGACTGTTAGTAGCCGCAGCACT
AGCAGAAGGACCCTCCCACaGCGGAGCCACCG
TCTGCAGAGCGGCTGCGATGCTCTGCCGGAACGCCACGGAGGCTGCGCTCTGCGGTTCACGGCGGGTA

AGCGGCTTTGCCTGATGTGTGTCGCCGCCACT
GCGTCGCATTGCCAAGCGCCGCAGCTTTGCCCGATGCAGGCCGTACTCATTACAGAGAAAGGCGCGCA
GCTGGAAAATACACAGAATTCCAAAGCACAAC
CACAGCTCAGCAGAAATATCCGGCTTTGCCGTGGCTTCACTCCAACGATCAATTTTTCTCCTCTTCGC
CAAATGTGTGGAGCGTTTCTCTGGCTGCAGGA
GAGATCGCTGCGCCTCTGCAAAGTCAAGACAGCTCTGTCCAACCAAGTCCACAGCTGTGCGACATCCG
TCCAGTACATGCAGCACATCGCTTTCTTGCGT
GTAGGGGACGTGTGCCATGACAATTGCCAAATGACCCAAGAAAGGAAACGGGCTTCGCGCCGACTCCC
CATCCTTCTCGCGGTGTTCCCTTAGCCACTGT
GCCGCCCGGCCTTCTTGGTAAAAGTACCGCAGCAATGATGAGAGAAATGCATCACGGTGACGCTTACG
AAATGATGTGAAGAGGTGCGCATGCACACACT
CTACCGCCTCCGGACCAGTGTCGGAAAGGACGGCGCTCCGCAGCACGTCCACTCCACATGCGTCGTGA
AGATCAAATGCCTTCACTACACCAGTGGCAGC
ATTGGCGGCAACGATGTCAGCGTAACGCTTGGTCTGTGATGCCATACAGTCCACGGCAGCCTTCCGAA
TTACTTCGTTCTGTGGATCTGCAGCAAGAACT
ATCAAGGCGCTTGCATACTTGGACGGTGGAAGTAGTCCCCCCTCCGCACAAAGCTGCAGAACCTCAAA
AGCCAGCTGGCGCACCGCCACAGAACTAATCT
CGCACAGTTGCAGAATCTGCACGTGAAATTTCTGCAACACCCATGTCGCCATGCCACTGTTTTGGTCA
TCTACGCATGGGGCAATTTCAACATGCAGCTC
ATCGCGCTTCCTTCTTTTCCTCTCAGGAGGAGACGAAGAAGGAGATGAAGAAGACGGCAGCGGCACAG
GAGCGTTTGTGGATGCCGCATATTCGACGCGA
GCGTCCTCGTCTTTTAGAAAATCTCGAATAAGGACAAGGCCCTGAGCCTGGAGAAGCATATCCCCGGC
AGGATCAAGAGCACAACGTAAATATGGCTCGC
AACGATAAAAAAAAACGTTGGGCTGCAAAATGCAAAGTGACGCAAAGACACGCAACGCAATGACCGTT
GTTCTCTTCTGTGCTTCCTCTGAGCATTCCTT
TAGTACCTGCTCCACGGCCGCGTACACATTTGCGCAGATGCCATCCCCGAGCGCCAATTGATTCGGCA
CTCCTGACCCCCTCACGGCAGTCTCTTCGGTG
AGCTCCTGATGATGCCGCTTCCACTCAGGATATTGGCGCAAAAACTCCGAAAGAAGAAAAAGAAAGCG
TAGTGTGTACGCCACACTCTGTGGATCCGTGC
GAAGTGACGAAATAAGCGACATAGTACGCACGTAATAGGTATTCATAAGGGAGTAGCAGAGGAGTAGG
TACTTTGTATTGGCATACTTTGAAAGCTTTCC
CCCACCACACGTGATAGTAGCCGTTAGAGCGCCACTAGCTGACAAAAGAATTTGTTGTTGGTGTGGTC
CGACATATCTTGACAATAGGGTGGTGAGAGCA
CGTGCAAGCGTGTCCAGTGAGAGCGGTGGGGCGCGTGGCGCCAACAACACTGTCCGAAAGATCCGACA
CACATGCAGTAATGTGACACCCATCGCCTCCC
TTTCCACCGCCTGCGACGATGGTGATGATGGTGGTGACGGGAGCGTTATAGACTGCAGAAGGATCTGT
GCAAGTACCTCTAGTAGTGGTTCTATCCACTC
ACTACATGCCATTGCAAGAGCGTTAATGGCGGCGAGTGCAGCTGCGGCCTCTGGTGACGGCATCAGTC
GTTGGTGCTCCGTCCACAGCACCTTGGCGGCT
CCAATCATTACGGCACAGAGCTCCAGAGAGTCTACCCCCAAGTTTTTTGTGGTACGCCCCGACATCTC
ACGCCTCTCCGTCAAACGACACATTCCGCGCA
GAAGTTTGACAAGGTGTAGTGACTCCGGTACCTCGTACGGATACGCCATGTTTGTTACAGTGAGGCGC
ATCAGTTCCTTTGCAACGCGCACACGTGTGTG
GTGGGCAGCGATGTCGACGCTCTCCTGCCCCACCCAGCGGTGCAGGAAAAGTCGCATCACACAGGTTT
GATGTCGATTCTCCTTCGCCGAGGCAACCATC
GCCAGCAGTTTGGACTCTGTAAAAGTCAGTAGGTTTTCTCCCCTCGCTGACGTCTCTAAAAAACGTCT
AAACGAAATGTCTGTAAGTATTGTATCAAGAG
CCGTGATGGCACGCGTGACAACGGCCACGCTGCGATCACTAATGAGGTGTAAAAGAGAGCTGACGACC
TCAGTGGCCGTCTCCTCCACGCGGGAAGAGGC
ATCACCGCTCCCGCACATTTCCGAGAAGAGCGTGAGAAAAAGCGGAATGATGGCCTCACGCACGCGCG
CGTTATCGTCGCGAACGCAACGGCGGGCGATG
GGCCAAATCTTTGCAAGTAGTGGTGGATGCAGTCTGGCAAGTTTCGCCATGTGTTGGAGAGCCTTTTT
GCGTGATGCCTCAAGTCCCGCAATGTGTTGCT
CCTCTCGCAGTTGGAACACAGAAATCAGCCACGACGGCAGTCGTTCTTTCGCCATGGCACTCAATAAA

GACTTTTCAAAGGCGGCACACAACTGCGCACT
GCACGCATGTAGGACGCCCCAGTCAACAGAAACTCCACCATCTGCCACGTGCATTCTGTGGAGTAATG
GCTCCAACACGGCATGCGGGAAGCGGGCTTCA
TCTTGAAGGGCCCATGTGAAGATGTGCGCCGCGCGCAAGTGAAGAGAAGCGGCTGCAACATGAGGCGC
AGGATCATCAACACGGTGCGAGAGAGCCATGT
AGAGCAGTGCACGCCGCATTTCATCATCGGGAAGTGGTAATTCCGTATCTATCGTCGCCTCGCCGGGG
TGTGTGAGATGGCGCCACTCTCGCAGTTGAGC
ATCCGTCAGCACAGAAAAATCTGGAAGCGCAGAGTGCAGCACATTCTGCTGCGTGGCGCTGAAAACAA
CACAAGACACACTAAAGACAACATCCAGGAAA
AGTGCTCGTAGCCCCTCTGTCGCGGAGGAAGATAGGCAATACTGCGCAAAAGATACTAGAAGGGAGCG
CAACAGCATGTCTGCCACAGGGTACTCCGGTA
GTCCCACCATGTTTGCCAAATCTTCACAGAATTGCAAGGCAATTGTGCTGCACATGTCGCGCTCCTTC
TTTTCCTCCCTATCCACTAGAAGAAATTGCTG
GAAAAACATATTTGACCACGCGCAACATTGCTGCTGCAAAATTTCTGCAGAACTTTTTGTGTTGTTTC
CAGTGACGATGGGAAGAGACTGCGCTGCGGCC
ACCACAGCCGGCGTGAGCGCCATTGTTTTCACCCCTGACGGAAGCAGATGCAGTCTTAGCAAAATATT
TTCACCATGTGGCAGCCGGGGGAAGAAACGCA
CCCATGTTGTCTCGCGCTGTTTCTCCAGCCTGTTCCAAAGAGCGGAGTAGAGATACAGTGCATGGCTC
AACAAATATGCAGCGTAGTTTGTTACCTCCTG
TTGTTCACAGGCATTTGTAATGACAAAAAGGCACTGGAAACAAAGGTCCTCCAAACGCAGCAGGTCTC
CGTGAACACAAATATTCAGTCGGGTGTCGGAC
ACAATCACTGCAAAGCAGTTCAGCATATTCCGCAGGCATTCCAGCTCAGTCTCACCGCTGGCGTCGTC
AGGGACATGAGCCGAGTGTCTTTTTTTGATGC
GGCGGATTAAAGCGGCCAGAATGGTGATGAGGTGATCAACGGTATCCTCTGCGAGAATAGAAGGCTCA
AAGGAAGGATGGGCAATGCACGCACAGCAGGC
CAATGCTCGCTGCATCACAGACACGGGAATCACAAACTCCGTGCAGGACGTCGCATTCTCCTCTGGTA
GAGCCACTAATGACGACACAAAAGCCGTGAGA
CGATCTGCAGGAATGTTTTTGAAAGGCTGCGGTGCATCTGACAAAACGCCTAAAAGAGACTCGAGAGA
GTGGGCTCCCTGCAGTCCCTTTTCAATGATAT
CTGAGATGGTCGAAGAGCAGGACAACTCTGATTTGCGCTCAGCATTTGAAAACTCGGCATCATGCGAG
AGAACCGGTGCTGTAAGGCTGGGCTTCGGCTC
GAGTTTCGGCACCTTAGCAACTCGTCTTGCTTCTGCTTCACCCTCCTTTTGATCGTGAGAATCAAGGG
CGGAACGCGGGCGTAACTCCATGCGACGCTCC
GTATTGTGTGCCCACCTCGCCTGCTTGGAGGGCGCCTCAATGTTCCTCATACCGCTTATGCTGTGAAT
TTGATCTATCTCTATGCGCAGCAGTCCCTCAT
GAACGCGTCGCGCCAGAGGAGAGAGTTGGGAAGTGCCTTTGTCGTCGCTGTCTTCTTCACACCTCTCT
GCGCTGCGGCGCAGTGTCATGTAACTGCTAGA
TGCCATGAGAGTGCGGGCGGACTTCATGACAGTGTTCTTTATAACATCATCAGGCACCTGTGTCGTGT
CACACGCCACACCGCGGAGCTCTGGAGGTGCC
GATGTCGTCAGTAGTTGTGGCAGCGGAAACGCCGCCAGCACCTCGAAGAGCGGATCGACGGCAGCCAG
TATATNNNNNNNNNNNNNNNNNNNNNNNNNNN
NNNNNNNNNNNNNNNNNNNNNNNNNNNNNNNNNNNNNNNNNNNNNNNNNNNNNNNNNNNNNNNNNNNN
NNNNNNNNNNNNNNNNNNNNNNNNNNNNNNNN
NNNNNNNNNNNNNNNNNNNNNNNNNNNNNNNNNNNNNNNNNNNNNNNNNNNNNNNNNNNNNNNNNNNN
NNNNNNNNNNNNNNNNNNNNNNNNNNNNNNNN
NNNNNNNNNNNNNNNNNNNNNNNNNNNNNNNNNNNNNNNNNNNNNNNNNNNNNNNNNNNNNNNNNNNN
NNNNNGCGGGAGTGGCACTGCGGCATCGCGCG
TGGATGACATGAGAAAACCCCCAACCCTTCCATAAGAAATAAGTGTCCATAAAAAAAAGCAAGAAAAA
AATAACACGAGAAGGATCCATAAAGACATTAA
TATATTTATGGGTGCTTATACATGCACGAATATACCAGACAAATTCAATAAAAAGACCGAAAAAATAA
AAATAAAAAAGAGGAGGTTATGCTTCCTCTGT
AATCGCCGCTGAAACAGAAGCAAGAGGTGGAAGCAAAAAAGCGCACTCCGCATGCAGTTGCTTTTCCA
GTGACGGATCAATGACACACGCGTCTTGCAAG
AACTTCTTGGCAGCGGTCTGTAGCCTTTCATCTGTCTTTGTGGGAATGCGTGGCAATGCCCCACGTTC

GTGCCACGTGAAGACAACAGGGAGCACCTCTT
CAACAACCCGCCGCGACAAAAAGTCTGTCGCGAGGGCAAAGAGTGCTTTTAGTCCCTCCATTGCCGCT
ATCACTGCGGGAGTAGGCTGCACGGCCTCAAG
TACGCGGCGTTCTTGCGCTGATAGTCGCTTCGAGTTGCTCGTGGTGGTAAATCCGGAAACAGGCTCTG
CAAGAATCGCCAAAAAGGATAGATATACCTGG
TACACCGTGCGCAGGTGGTTGCGTGGAAGTACTGCCGGGGTGTTATCTTCCCATTCAAACACAGGTGG
TGGCTGATCGCCATTGCTTTCTGTCTCATTTA
TCCACGTCTCCGCCCTGGGGGTAGTAAGAAATACCGTGAGGCCACGCGTGACAGCAGAAATCGCGAGA
GGCGCCACTGCGTCATTCCGCGTGCAGTATGC
CAATAGCATCTGCACGGCCTCCAGAACAGCAAGTTGAAGAATCTGGACGCGTGGCTCCGCCGCGGTGG
TCATGCAGTCCTGATCCTCGTGGATTCCAAGT
TGTGGGACGGAGCGGTTGAGTGCCGCACTCACGCTGAAACAGTCTAAAAGCAGCGCAACGGAGGCAAG
CGCACCGGTGGTCTCCTCCTGCAGTGTCGAAC
GTCGACATCCATCGGAGGCAACCTTTACGGCGGAAGAAACAAAATCTGAGACTTTGGGAACGGCAGAA
GTTACCAACACAACCGCCGCTGGGTTGCGGCC
TCTCGGAACGTCCACCCACTGCCGCGATGTAATGGCAGTAAACTCCTCCTTCCCGGGAACTGACAACA
GCTTGCTCTGAAGAAAACTGACGCCGCCACGT
AACACGCTGGCTGCGGAAACACGAAGATCTTCTTCCGTAAAGGCACGCGCAGCCTCATCAACGATATA
CCCGCTCTGGCTCAGAAAGAAGGCAAGCGTGT
CTGGGTAAAACCCCGCCTCTCCAAGAGCCTTCATGACTCCCATGGCAGCATCGCGTACAAAAGTTGGG
CCCGTTGCCTTTTCCAGCACAATGTACAACAC
CAGCAAGTTCAATCTCTGTACTGCTCGTTTTCGCTTCGCCCTATGAATCACTGGTTCGTCGCGCAACA
GCCCCGCGGCAGTCTCCAAAAAACACAAAATC
GTCGCCGCAACCGTCTGCCTATGACGCAACTGCTGGTACGAGCAGAGATTTTCATCCTCGGTGATGTT
CCACAGGTGTGGCTGTGCAACAATGCTCCATA
GCTGCTCAAAAGTGTCGGATAGTATAAATTCCTCCGCAGGTCGCCCTTTTTCCTCCTCCTCCGTCACC
CACGCTGGGGTCCTAAACTGCCACAGCACAAG
GCGTGTAAGAACATAAATAGTTGGTGGGTGTAGCATGTAGAGGTCCCAGTCCGCCAGTGTTGCCTCGC
ACTCAGCCATGAAATCCTTCAATACCTGCTCC
CCTACCGTGACGCATCCTGCCGGATCCTCTGATGTTGTTGTTGTCGTCGTCGTCGCCGTTACCGCGTA
CGCAGCGCACTCGATAAACGTATCCCCCACAA
TACACGGGTAACTGCGCGAAACTTTGGCACTGTACAGATCCTCCACGCTCATCTCCGTCCCCACCACC
TGAATGCATCGCTGGAGCACACGCTGCATTAG
ATGTTGGTTGTGGTCCTCCACTGGGAATGCTGTGGCACGTGTGTCCTTGCAACGAAGCAGCGCCGAAG
AAAAGTGCAGCAGCTCCACACCACGCAGTTTC
TTCAACTGTGCTTGAAGCAACTCTACCACCGCGCGTTCCTGAAAAAATCCGCTCGGTATTTGGGTAGT
GGAATCAGTAGCGCCACCAAAAACACCGCCAC
TAAGAATAAAATTCACGTTTGAGAGTACAAACCAACCGACAATGACCTCCCGCATGAATGGTGTGTCA
AGAAATGTGCCGAGTGATGGGAGAAGTAGCAA
ATCCGTGGTAAGATGCAGCAGGGATTGTGCGAATGGAAGCGTCTTTGCCCCAAGTGCCAGCCGGCTGG
GATCCAGCAACCGAGTCATACTATGCCCCAAA
TGGCCCACTGTACTCTGTAACCACTGCGTGTCATCCCCTTGCGACACCAGCGAGGCCTTCAGAGATAC
GCGCAGTAATTCCACCGCCCCGGTGCGAACGG
CAACAGCATCTGTACCGTGATGCGCGCGGTCAATATAATGCACACACGCCGAGACAATCCCAGGAAGA
CAGAGTCGTGTGTTCCTACAACCGATAGCTTT
CACTACAGCTACACATGCGTGGAGGCTCGCTTTCTGCAGTTTTTGAAACCAAATCCGTCTAGAACCTG
AAAGGGAGGCCAACAACTCATGAATGAAGAAG
GCGGCGTTTGTGTGGTACGAATCGCTTTGGAAAAACACTCCATGACGCTGCAATCCCACACTATGAAA
CGCCTCCACAGCCCGAACGACCAACAACTTCT
CCTCCTCTGTGGATGCCAAAGAACTGACTTTAGGCTTCGATGGACTTGACGGCATTTCACCACCTCGA
AAAAGTTCCACAAACTGTTGCTGGAGGATCAG
TGTCAATTCCTGAACATTGTCAATTTGATTGACCTTTTCCGCAAGATTATAAAGAACAGTGGCCGCCG
AGAGACTCATCCGCTCCCCTGTCTATTGTCTA
TTACCCCTTACCCCAACTCCCACAGGAAGCCCTCTTTTTTCGTTCTCCACAGACAAATGCACGCACGC

ACGTAGAAAAACTCGACTCCTTGCTAAATGGC
ACTCTTTAAAAAAAAGAAAAAGCAAAACACATCTCGGGAGAAAACATTTAAAAATATACGCCGAGCGC
AGCAGAACAAAGCCCTACAATAAACAACCCAC
AATATCAAAACACAGAAAGAAAAAGACAAGAAAAAAATGTTACCCACATTTTTCCTTTTTTTTTTTTT
TTTTTNNNNNNNNNNNNNNNNNNNNNNNNNNN
NNNNNNNNNNNNNNNNNNNNNNNNNNNNNNNNNNNNNNNNNNNNNNNNNNNNNNNNNNNNNNNNNNNN
NNNNNNNNNNNNNNNNNNNNNNNNNNNNNNNN
NNNNNNNNNNNNNNNNNNNNNNNNNNNNNNNNNNNNNNNNNNNNNNNNNNNNNNNNNNNNNNNNNNNN
NNNNNNNNNNNNNNNNNNNNNNNNNNNNNNNN
NNNNNNNNNNNNNNNNNNNNNNNNNNNNNNNNNNNNNNNNNNNNNNNNNNNNNNNNNNNNNNNNNNNN
NNNNNTCTCCTCCCCCTATGCAACTGCGGTAA
TCAGACTCACACTCAGCTCTTAAAACAAGTCGTCGTAATCGTCCACAGCATGCCCAAAGAATTTTGGC
GTCTCATCGTACATAGCGTTGAAATAGTGAAC
GTAATGTTTCTCGATTCTCCGTCGAGCAAAACCATATTTGCATGTAATGAAGCCGTTAGCCAGGGTAC
ACTCCTCTATAAAGACGAACCGGCGAACGTGT
GACCGTGATGGCAAACCCTGCGAAACGCGACGCAACTCTGCCCGAATACAGTCTGCCTTTTCGCGCTC
GCTCACAATGGGCACGCCGTCTCGTCTCTCCA
CCTTTCGTAGGTGTGACGCGATGGCCTTAGCATTCGGAACCACTAACGCCGTTAGGTAAGGGCGGTTT
TCCCCGTGCACAAAGATTTGCCTAATAAAAGG
ACTCTTTATGAGTGTTCCCTCCACTTCTAATGGATCCACCACTGCGCCATTGGCGAGCGTAATAAGAA
CGTCCTTGTTCCCCTTGATTGATATCCATCCA
CCCGTCACGGAGCCGTAGTCAGAAGTTCGCACAAACGTCCTACCACCATAAGCAACAAGTGAATTCTT
CGCCTCGTCAGGATCGATATGTATGTCAAAGT
ACCCCTGCATTGCGTGTGGAGCCTCCACAAGCAGCTCACCTATATCTCCTGGTGCGACAATCTCGTTC
TTTTCATTCACAACACGAACCTCGATACCTGG
AAGCGCCTTGAGACGGAGTGGCACGTCAATGTCGGTGGCGATGAGACCACCGGCTTCTAAGCACCCGT
ATGTGCTTACAGTAAACACGTCTAGGTCTGCC
AGCAATTCCATCTGATCACGCGTCAGTTGGCTACCAATAATAAAGGCAATACGAAGCTCCTTTCCAAA
CTGTTCCTTGAACTTTCCAAGCAATACATGTG
ACGCAACTCGTAGCATTTGACCTGGCTTCTGAAGCATTAGTCGATTTTCTGTTATGCGACCAAGTGTA
AACCGTGTTATATCCTTTGTTAAACTACCACG
ATTCATGATATCGTCAACCATATTGGCATAAGCCCTCTGGAAAGGTAGCGCGACAGAAGTAATGACAC
TTGGTTGCACTAAGGGAAGATCCTGAAAACCT
TCGAGTTGTTCAGGTGGGATGCAAATGCAAGCCCCTTTACAAATTGCCTGATACAACTCAACCGTGAT
TGCACCAGCTACACACCAGGAACACAAAGACA
TAAATAAATCGGTGTGCGTCAGCGCCCCGCCAAAGTGCGCATAAATGGAACTAATGTTCGCCACCAGG
TTTCGGTGTGAAAGCATCACACCCTTTGGAGG
TCCAGTAGTGCCCGCGGTAAACATAATCATGGCGGTATCCTCCGCGGTGATGTCATTTCGTGGAGGAA
TTTTTTCCGGCACATCCGATGCTATATTGATT
GCCACAGCGTAGCTACCTTCACCAATTTGGTCTTCAAAGCAAATCACATGTTGTAGCTCTCCCACCTC
TCCAATCCACCCTTTCACAGTGGCGTAAGAGG
ATGTAGACTCAACAAACAACACCCTAGCCTGCGTGCTTTTTAATACCTGTTGTGCCTCCATGGGATTC
ACATTGGTCGGCAAAACTACAAAGTGGCCTCC
AAGTTGTAGCGTTGCAAAGTGAACAACCACCCATTCATAGCGGTTTTCACTGATAACAACAACTCGAG
AACCCTTATCAATCCCCATCTTGTGCAGGAGA
GTTCTCATTGCAGCCATTTCCGCCTCAATCGACTCATATGTGGCCCAAACATACCCCAATTGGCCACG
CACCCACATCTTTGTTCCAAGAAAACGCCGTA
ATGGCCACACCTTCACAGCCTGCTCCCATAATTCAACAAGGGTGCTCTGTGGTTTAATCACAAGCCCC
AAGCGCATATCATTGTTCATTGACCCCCCAGA
AGCCACATCCGATTCGGAGTAGTAAGTACCAGCAACACTTGTACCAAAACGGTAAGATAATTGAAAGG
CAAATCCGCCACATCTGCTGTTGGTGCCACCA
ACGCAGCCAGTAAAAGTCAAGATGCGCAAAAAGAGTCTCACATTTTGCATCCTTTTTTACCCTTATTG
TTCTTGTTTTGTTTTTGCTTTTTGATGGACTA
GCTGTTTTTTCTTCTTATCGTTTCCGTAAAACCTGTGTGGCCTAGTGACGGTAGCAGCAACAGTGGTC

AACACTGCCAGAGGTTGCGTCTGCACACAAAC
CAGGCAAAAGATAAGAAAAAAAAAAAGGAAGTGATGACCATAATTATAATGAGTTTAAAAGAAAAGGTC
AACAGTATTGCCTATTTAAGGCACAGATATAC
ACAATTCACAGGTTGCACTAAATGTCATCAAAAGACGCGGGTGTACCCATCTCTATTTCATTGATTAC
ATGGCGAAAATAACCGGAGAAAAAACAAAAAG
GGGGGAAAAAAACGGTGTTTATTGACATATGAAAATAGAAGAAAAAACGGTGGAAAATTTGGTGATTT
TCCGGTTTTTTTTTTTTGCTGTTGTTGTTGTT
TTTGCTCCTTTTCGGTTCTCCCATTTCTTCTGGCGGTTCTATTGCGTTTATTTTTGGCGTTTCATCTT
CTTTCTTCGTTTTCTACGGCTTTTCTCTTATT
TCCCCCTTGAAAAATATGGGTGAATCAAAAGTCAGCCCACGTAGAAAAAAATACATCTCCAGAAAGGG
GATAAAACCACCAACACACATTCTCCAACACC
CTCCCTATTCTTGTCTTCTCCAGATATTTATATCCGCTCCGATGGATTCTTTACATGTCCCCCTCCGC
CTCTTCCTCTTCTTCTTCAACTTCATCCTCAG
AGTCCGGTTCTGGTGGATCCTCACCTCGGCGGACCAACTTCTCCGTTGCCGGTGTGGGATCATCTACC
TCCGTGATGTCACCACACTCTGGGAGCACCGG
TGGGGCAAGCGTGGGCGTAAAGCTGAAATCTGTCTTATTGAGTCCGTTACCAAAATAGACACAGCCCC
ACTTTTTGCCGCCCTCCGCGGCGAATGCAACA
GCGCCTGGCCAACGCAAAGAACGAACCACTACCACGCGATGCTTCTTGCTGTTGTTGTTTACAACCCG
TACTGTCCAAGCGGCAATCTTTACCTTCATCG
GATCATCGTCGGGTACATCAGCGTCAGTGACGGGCTTTGGCGGCATGTCGTCAAAGTCTTTCTCCGCC
TCCGCCGCATCCTCCGCGGGGTCGTCGTCCTC
TTCTTCCTCCTCTTCATCCTCCTCGTCTACCTTCGGTGGTTCTGGGATGTTAATGACCGCGTACAGGA
AGTCAAGGTTAATAGGAGTAAAAAGTTCCCTC
TCTTCTTCTTCTTCCTCCCCTTCCTCCTCCTCTTCCTCTTCCTGTTGCTGCTGCTGTTGCAGCTC
CTCTGCCTCCTCGTCCTCCTCATCCTCCTGCT
TCTCTGGAACCTTTGTCTGCCTACCATTGGCATAGATGTAGCCTTCCGAGTGAACCCATTGATCAAGC
TTCGCCCACTGTGTGACGTCAGAATCTTCCTC
TGAGGCAAAACCCTTCACTGGTTGGGTGAGTGGGCGGTATTTCGCCTCCCTGGGTTTCTTAGCAGCAT
TCCCCTCATCGTCCTCGGCTTCTTCCTCCTCA
TCAACCTCTTCCTCTGGCTCTTCAAGAGCCCCCATTGGAGAGATGTATGTCCCGCTGGCAATACGGCT
AAGCTGCACACGAAGGTAAACAGCTTCATTCC
ATTCGAACGGGGGGCTGGCGATAACAGCCGCATTTAAATCACCGGTAAACAGCCTCTTGATCCGCCTC
CCTGCCAAGATTTGCTGTGGTGTCACATCTGG
AAGCAACTTCCATTCGGCAGCAGGATGAGATGTTACCCAAAATGAGAGTCGATTTACCCCAACAAAGC
CCGGCTCAGGTTGTACAGGTACTTCCAACTTG
CGTCGCGCAGGACGAGGCATGGTGTTGACTTCTTTGTATACTTTTTCTCCATCTTGAACATAGCGTTG
ACTGGAAACCACGTAGTAGTCAGCTTTTGTGC
CAAAAATTTTTCCAAAGAAGCGAACATCTCTCAGCTTCTTTTCCATTGCCAGCATCGTGATGGATCGA
TCCAGTAAAAATGCTTCTTCTTCCGTCACACC
GCATCCTGCCCAGGCCCAGTAAAGATTGTGCTGAGCAACAGAGTCATATGGGGGAGCGGTGTGTGGCT
TTATCCGTGTTGTTGTAACTGTCGTATATGGA
TCTGGCTGTTCTATTGTTGTCAATGTTTCAGGAGGCGGACGATCGAAAAGGCGTTCATTATCCGCAAG
GCGCGCAAGTTCAGACGGTGGGACAGAACAAG
GCTCGTTACACGCAGGGGTACTTTGTCCATTCACAAAGCTGCTTTGCTGCAAAAGGGTAAAAATTTcG
TTAAATCTCGATGGGTCAGCGGCAATGTCATG
TGGGTTCTCATCCAGTAACTGCTCCATCAATCGGGTGAGTTGATCATAGACGCTGACGCCATCCTTGT
TGGCGTTCATAAGGTACGCTTTTGTTCTCCCA
AACATCTCCTCAACATCTTCTACAAGAGGTCGAACAGACATTTTCCTTTTTGTTTGCCTTCTTCTCTT
TCCCTGCTTGGCCTCTCCGTTCCTTTTATGCT
CACCCCAATCTCTGCAAAAACCGTAGAAAAGAGCAAAAAAAAATGTTTTTTTCTATTATTAATTTTTG
TGCGTGTGTGTTCATTAAGCATGGATCGTGAG
AAAAGAAACAATAAAGGGAGTTAAAAACAGCAAGACATATAGGAACGTATGCAAAAAGCAACAAAAAA
AACGGCAGAAAAAAACCAGAAAGTTCTTATTA
CCCCATTTTCTGTTTCCTTTCAAAGCTACATAAAAAGACATATCACGATGAACCGATTCGCTGCTTGA

TCCTCGTAACATACTTTGCTATTGAAGGCTCG
GCTTCAATTGCATCGCTCTGTTCAAGCTGCGTTAAAGCATCCTCCAACCCCAACCACAAAAACTTCTT
TTTACACTCCTCATATGCACAAAAAGAGCCTA
TAGTAATAAGAGCTATTTTCCTTAGTCCCACCTTGTCTTTCCTTAGGATCCGCAGAAGAGCCTCCACA
ACGCTGTTTCCCATTAATGCAGAAGTGAGTGA
GCCACCATTTCGCACAAAATTGCTGACCGCACCGGCAGCGTTTTGTCTTGTCTTCTCATCACTACTAG
ACAAGAGTTTCACAATGACTGGGATCGATGGC
CTAAGAATCTCATAGAGAAAATCGTTGTGGAAGGCTGCGTTGCCGGCGGCAAAGGCTGCAAACTTTTG
TGTTGTGGGATCCTCATCGCTACACCGCTCGA
CAAGAGCCTCGATCAGTTTGTGTTTTGCCAGGGGCTCGTAAAAATATGGAGAATGTTTGCACAGATTT
CCAAGGAGATTACACATCTTGCTCCGAATACC
GGCATCTCTGTTTCGCATAAGATCTAGAAAGCTTTCATACAAATTTGCGCTATGAATTGGCGGATAAA
AATCCCTGGATATACGAGCCAGTTGTGAAAGC
ACGTTAAGCCCGTCCGAAACAAGGCCAATACTCGCCTTTCTTGAATCCAGGACTTTCCTTATCCGTTC
GGGATGCAAACCGTCTCCTTCTACAAACGCCT
TGGCAAATAATGGTGAACCCATAACTAGCTTTATGATGACCGTAAATGGTGAACCCCAAAACGCTACT
TCTGTGTAATCAAGTGCAGCAATAAGCATTTC
CATAACCCCTTCCTTGTAAAGAATTTGCTGTACAGCAGCAATAGTATCATCATCCGATCTAGAACCTT
GCTTTGCAGGCTGAAACGTCACCAACAGAATC
TGAACAACTATTGTAAGATGGGCATTTACCCCAACAGTTCCCCCTCCCCTATATTCCGGCCACAAACG
AAGCTGCTTGAGATAGTTTTTTCTTAACTGAC
GACAAACAATTGCAATTACAGAAATAGGGCCGCCACCATCTCCATAGTAAGGTTCTACAGACTCTATC
AAAAGACTCATTGATTGCATTCCTTCTTGTTG
GTTCTGCAAAGCCTGCTGAACACAACGAAGTGCGGCCAACACGCCGTTCGGTGACAATTCAACCTTGG
AATCGCTATCACGGAGGAGACCAATCACTGTA
CGAAGTGACCCCCACTTGTCATTGTCAAGAAAATTTCTATTTAAAGAATCCGAATTTTGTTGATACAC
GATTGACTTTGGGTCCGAAAAAACAACCGACA
ACATATGCGTCACACCGTCTAAAAAACCATAATCAGCAAACCCATAACTTGTCCCAAGAGCACGTGAC
ACCATAAGCTGATCCGAAATTCGACGAACACC
CTCTACGATAGCACACAAGCCATCCATCAATATAGAGTCTGGTTCAAAAACAATGGTATCCTTCATCT
CCCGGTGCACAAAGGCTACCAATAAAGCTGCT
GCTGCTGCACAATTAAGCGCAGCAATATCACCACTTGCAGACGGGTCGTAGTAGCAAACAGCTGTCAA
TCCAGTAGACACAAACTCAGAAACCTTATGTG
CATTTATTTGGTCGGGAGAAGACAACTTTATATGCGGGACCAACGCCGAAACTGTGTGAAATGCAACT
GCCGCGTAAAACTGAAAATGACTTCCATTTCT
TACTGTTGATTCTTTTACGGAATCAAGTAACGAAGCCCAAAAAATGGGGAAAATCCTCATTTTGATCA
ATGCAGCTCCAAACCCAGTTCCCCATTTTACA
CATTGAAAAATTACCTTAACACAATCCTCACGAAGATTTTTCCGATTCTCAATGAATTCGTTCATCAT
AAAATCAAGTGTCTCCTGGGGTTTTTCCATGA
GCTCCAAGGCTACTTGCTGTCGTATAAGAAAATCAAATGGTGGAAGTTCACTTCTACTTTGCAAGGCG
CTCATTATAGTTTCCGAAGGATACTGTGATGG
AAATGGAAAAACAGATCCATTTTCAGGAAACACAAGTATCCGAATGAATTCTAATGCATAGCGAACTA
TATCTATCGTTTGCCCATTGTTGATCCGCCTT
AGTGTGGGTCCTAAAATACCCATTTGTGGAATTCTTTCCAGTAAGATAGTACTTTGTGAAGGAGATGA
TGTTAATGTATCAGCTGCTTTCGCAAGCCCCT
TGAATGTTTCAATGATTTCCTGTGAATTCTTTGAAGAAGAAATAATCTCTATCGAAGAAGCAGAAAAT
AAACAACTTTCCTTGGAACCCAAGGAGGACTG
GCGCGAGACCCCGGTAGAGCGAGGTGTATGCCCTGAGGAGTTGGGCTGAAATCTGCTTGGCTGAGCGC
AGAGTCGAAAGCACTCCAGTCGGTCCATATGT
TCCTTCATTACCATTTCGTGTTCTTTAATTTTCTTCTGCCACTTTCCATCCTCCTCACCAAAAGCAAC
AAAAGGATGGTTCAATAAGTGAGGCCAATTCA
ATCTCGCCGAAAAGGACTTTGTCAAAAGACCTTTTAAAAGGGACTTGAAATCCGGTGATATAGGTTCT
TCAAATTTGACGGGATCATTGACAATTTGATT
AATTAGTTTGTAAAGATGATTTGTGCAAAATGGTGGTTTCCCATAGTACAACTGGTAAAGAATACATC

CAAGGGACCACAAATCCGCACAGTGGTTGTAT
GGTTGTTCTTGGACAAGTTCCGGCGCCATATAAAGGGGTGTTCCCTTAATACTTGTAAGCACCATGGT
GTTATAACTCATTGATCTAGCAAAACCAAAAT
CAGCCAATTTAACTGAGCCATTCTGGCCAATAAGAATGTTCTGGGGTTTCATGTCCCGATGCATAATA
CGGTTAGAATGCAGATAATAAAGTGCCTGGAC
CAATTGCTTCGAAATACGTTGAACAACGTCTTCAGGAAGCTTTTTGTCGTCTTCAAGAATTTCAAACA
GCTCCCCTTGGGCGTATTCCATCACCACAACA
AACTCTTGTTGGGTTTCAAATGCCTCAAACAAAGTAATTATATTATCATGATCAAGCTTTGTCATAAT
TTCTATTTCACTACGAAGATTTAGGAGCTCCT
TATCATTTTTACCTTTTTTCACGATGAACTTCATCGCCACAACCTGGCCCGTCCCCTTCCGTCTCGCC
TTATAAACCTTGCCAAACGATCCCTCGCCTAT
TAGTTCAACTATTAAATAGTTTTCCATCTTCAGCACCCCTTCCAATAATAAATAAAAAAATAGAAAAA
TAAATAAGCAAATAAATAAATAAAAAATAAAA
ATAAAAATAAAATAGGATTATAATATTGTAAAAAAATCCATTCATTATTAATATTTCAACCAAGGGCA
ATTCAACTTCTAATCCTTTGAGTTGCATTGTA
TTTCCTATAGCTGCCCCTTCCCTTAATTTCAAGCTTTGTTTCCTTGTCAACAAAAAACAAATGGCTAG
GAGACTAGTAAAAATAATTTAAAAAAAGTCAA
TCACCAGCAAGCCTCTCACTTAGCGACATTTTCATTTCCTTTCTCGAGTTTCTTTGCCTCGCGCACCT
TCTGTTGTTCTTCGTATTTTTCGTTGAACCCA
GGCGCCTTTGTGACAACAATTTCAATCTTGTCGACAGTGTTGTTCCTCGCGCCCTCACCACCTCCTCG
GGAAGTACTGATTTTCTTCACAAGAACCATCT
GCTGGTCCTTTAGCATTTCCACAACAGAAACGGCCTCATTAATAGCCTTTCCAAGAGCGGAGATGCTC
ACCTCTTTTTCACCATCATGTAGCAAATGCTT
GGTAATGTCGACGTAGAGGAACTTGGAGCCATGAAATCCCACACGAACCGCGTTCTTGGGTCGCTCAG
CGTTTTCTGCAGACATTTCAGGTGGCTGTGGA
GTGGATTCTTTCTTTTTTTTTTTTTTTTTCTTTTCTTCTGATTGCTAATTCTTGTTTTTCTTAAGAAT
AAAAAAAAAAAATAGAAAATATAGATGCCCAA
TACTTTAAAAGTTTTAACAAAAAAAAAAAAAGAAAAAAGATAATTCGCCTGGCAAGGAATTCCGTACA
CGAAGAAGAAAAAAAGAAAAAAGAAAAATAAA
AAAGTTCCAGAAAGCCATCCTTTCAATCCGCGCAGGTATTATTTTCCCCTAGACCTTAAATAAACGCT
TCTCTCGTTTTTTCTTTTTTTATCCTTTAGCA
CACATCATTTATATGTATGTAATAAAAACATTTACTGACCTTTTTCAGCACTTTCATCTTTTCTTTTT
TGTTGTTCTTCGTAAATACTGTCAAATTCCTT
CGATTTCACGACCAAGATTTCAATCTTATCAATTACTGACGTGGATGAAGATTTAACATTGCCCCGTG
AAGTAGTAATTTTCTTCACAGTGACGAGGCCT
TGGTTCTTCAATATTTCCACTACGGCAACCGCATCAGAAATGGCGCGTCCCAAACCAGTAATAATTAC
TTCTGGTTTCCCCTCGTGAAGACGGTGCTTGG
TGAAGTCAACGTAGGCGAAATTGCGGCGATCGACGCCAACACGGACCTGAATAGGACCAGAAGGTTCT
TCAGCATCGCAAGACATATGAATAGGCTCAAA
AAAAGGGTGTTTTATGATAATACCTGAGATCCTTAACAAATTAAAAAGACAAAAAAAAGGAAAAAAAA
GTTTGGTATTTAATGAAAGCGCACAGCAAGAA
AATAAGGAATCCAAGATAGTAAACCCAAGCTTCCGTGCATGAGCAATTTTTTTCCCAAAAAAATCCAC
TGTTGGCCAATTTTAGACAAAGGAAAGATTCA
ATTAAAAGAAATAGCCGCATTGGAGTACTGTATTCTTATCATCATCCACCTAACAAAGGTTATTATCC
CCGTCCCATCCAAGAACCATAATAATGTACGC
ATTTTAGGAATAGTTTCAAAATTAAAATCTCCCCCAAATAAGAAATCTGAACGGACAATAAAAAGTAT
TGCCGGTTACTCCGCTGAAAAATGCACGAAAA
TTCAAAAAAAAACAAACCCATGTTTTTTTTTCTTTTGCCTTTGATGTTGTTTCCTTCCTGCTCCTGTT
TTGACGTGTCCGTGCTCTTCCCAAAACTTTAC
TCCATCTCAGAATTTTGACTGAGCTCAAACTCGATTCACTGCGACAGAGTAATCCGCACACCGTTGTT
GGTATATATGCGATAAAGGCATATTTCTTTGT
TGTTGTTGTTTCTTTTTCTTTCAACCGTTCCCGCAGCTTCCATCATATATATATATATTCCATTTTTT
TAAGAGCTTCCGAAGAGAACCCCCACGCGACA
AGCCGGCTATCGGATTTAACGCGTACACCCTGATAAAGCGAACGCTGCGTGGCCTCTTCCCTATTTTA

TGCGTCTTGGCCGGCCGCGATTCTAGCAATCA
GGGAATCAACAACCCCAAGACGATTCATGCTTATTTTTATTTTCACTTCTGCTTGCGCAACCGCTCTC
GATTCCCGTAAGCTCGTGGAAGACGCTGCGAT
GGCCCCCGGCACGGCGGAATGGAGCCCCATCGTGTCTGCGTCGCGGTTCCGGCACTCCGGTTGGGCAA
CGCCGAGGCGCGGCCGGACGCGGCGCCACCAA
CCAAACGGGATTGCGCCAGGGCGCGTTTTCCCCCCAACCCCGCGGGCGTGCGCCCCGGCTTGGGGGAG
GGGg
>scaffold1022.size9441
GCTTGGTTGCTATGGTTTCTGTCTGTCTTCCTTCCTTTTGTTTGCCTTTTGATTGCATGTGGACTCTG
TTTTTTTTGTTGTTGTTTTTTTTTGTCGTTGTT
GTGTGTGTAGGTGCTGCGCAAGGTGGGATTTGGATTTATTGCCTTTTTTTTGTTTTTATTTTAAGCGC
AATCGATCGTTGGATTTCTTTTTGTGGCATTA
GTGCTGTTTCTCATGTCTTCTTATCGGCGCATCACCGTACAGTTTGCCGGGGGCTGTGAATTACTTTT
TGACAAACAAACCCAGCTGCAGCTGGAGGGTA
TCGTTCCACACGGCACAACCATCAACGGACTGGTGCAGATGCTGAAGGTGAATCATATAAAAGAAAGG
CCTGACCTTTTTGTGGATCAATCAGGAACAAC
GCTGCGCCCTGGGATTCTTGTGCTTGTGAACGCATGTGATGCGGAGGTTGTGGGTGGACTCGATTACG
TTCTAACGGATGGTGATACCATCGAATTTATC
TCGACACTACACGGTGGATGAAGGAAACAAAAAATACACGTAAAAGAAAATGATTCCTGCCCTTTTTT
TTTGTCTCTTTCTTTCTCCTGAGGGACGCAGA
GTATGCTCTCCTAGCATTTGCGCCCGTTTCATTCCAGTGAGTTTTTAAGAAGAAGGAAAGTCGCGTCA
AACGTGTCGGTTATTTTTTCGTGTACGCGCTT
TTGTCTCTTGTTGTGACCACTTGTCAACCGACATTTTTTGCGGTGTATTATTTATTATTTATTATTAT
TTTTTTATTTTTTTGGGGTCGGCGGCTGGATG
TGGTAGGGCTAGGCTATTGTTCACCTTGAACTATCTTTTTAATCGATACCGAAGGATGACGGGGGAAG
AGACGCTTCTGGCGAACATTAATGTTGGAATT
TTGGGTCATGTTGACAGCGGCAAAACGTCACTGGCGAAAGCGCTCTCCTCCACTGCCTCCACAGCGGC
GTTTGACAAGAGCCCTGAGAGCCAGCGGCGCG
GTATTACTCTCGATCTGGGTTTCAGCGCTTGCTGCATCTCATCCGCCGTGGTGGCACCACCATTGCAT
GCGGCAGGTATTGAGCAGATCCAATGCACGTT
TGTCGACTGTCCGGGCCATGCATCTCTTATCCGGACTATCTTGGCTGGGGCACAGATCATAGACATCA
TGATTCTTGTTATAGATGCTTGCAAGGGGGTA
CAGACGCAGACGGCAGAGTGCATCGTAATTGGTGAGGTCCTGTGTAAACCGTTGGTAGTGGTGATTAA
CAAGATTGACGCGGTCCAAGGCTCAACCCCCG
AAGAGAAGCGCGTAACACTTGACAAATTGCGGAAACGTCTTCAAAGGACCTTTGAACGCACGCGCTGG
CCCTCCGTACAGATGGTGGAAGTTGCCGCTGC
TCCGCGCAGAGACGAAGCGACATCAGGAGAGGTTTCTCATCCCATTAATATGGAGGAGGTGATTTCCG
CCGTTTTGGGTCTTGTGGATTCAACGGCGTTG
AAGGACCAGTTGACAACACCGCTACGCTCCGAAGATTTTGTGATGATGTTTGATCACTGTTTTGTGTT
GAAGGGTCAAGGCACCGTTTTCACCGGCACTG
TGATTTGTGGCCAAGTCAGTGTCGGGGATAACATTTTGATCCCCGACTCGCACGTGGTAAAAAAGGTG
AAGGGGCTGCAGGCATTTCGAAAGCCTCTCCA
GCACGCAAGAAGAGGTGATCGTGTGGGTTTGTGCGTGGTTCAATTTGATGCAGAGGGGGTGGAACGGG
GTATATTGTGTAGTGCACAACACCAACGAGGG
AATGCAATCTCCAACGTACCTCTCCTGTTTGAGAGCACATCAGTGATTGTTGCTGAGGTGAATCGTGT
GCGTTTTCATCAGCTTTTGTGTGATGCTCACA
CGAAATTTCACATCACCATTGGACACTCAACAGTGATGGGAACGATGAAGTATTTTAGCCGTCCCACA
ACCATTGCGCCTGAGGAGAAGGAAGCATCATT
TGATATCTGGCAGGAAAGTATGTATGTTGAGGAACTGACAGAAGACGCCTTTCTCACCTTTCATAGCA
CGGAAGGACAGCCACAAAGTGGTCAACCGGTT
GCCGTGAAACGCGGTCCCCTGACATATTATGCCATCATTCTCTTGGAACGGCCTATTATGGCCGCCGT
TGGTACACCACTAATCGCCACACGACTCGATA
TGGAGCGGGACAACATGTGTCGCATTGCAATTTCGGGGTCTGTGCGCGCGTTTCTAAGCAGTGGAACC
TCTGCTGACGCATCGATCATTTCCTGGCGCGA

GCTGCCGATTGTGCGCTTTAAATCCAGGGCGCTTGTTGTGGAGCGCGTGGTAAACGAGAAGACTTGTA
TTGCCAGTGGCGTTGTGGGATTTGAGGCCCCT
GAAACACGTGGCAGTAGTATAAACCAGAAATCAAAAGAAAAAGACACGCTGCACGCGGAGGTGAAGAA
GTTCATTGGAATGCGGGTACATTTTCGCCCGA
CAGGGGAAGAAGTAAATGATGCCACCATCCTCACGGGAGTGAAGGAGGGAATAATTGACTCCGCCTTT
GGTAAGACAGGAAAGGTGAAGCTTGTATTCAA
GGATCCCATTTTTGCGGAGAATGAGCCTTCGACCACCGGGCGAACGAAGCGAGGGGGCGTGACATTTC
TCACGGACGGTGTGGTGGAGCTCACGCTGAAA
AAATATCCTCTGGCTGTTCACTGTCAGCTCCAGCAGTAGACGGTTGTGAGAACGGACGAACAGGCCGG
TGTATTTTCACATGAAGCGTATCTTTTGGCTC
TTGGCCCTTACGGTGCACGGTGTCCTTGATTTCTGACTCCACCCACCTCTTTTAAGCTGGTTCACTAC
TCATCTCATCGTTGTTAAGATTATTATTGTCA
CCACGCCTGTTTTTGATTTCTTTTTTTTATTTATTTATCCACAAGGACCAGTTCCTTTTGCGATGAAT
CGACTCGAAGTCATTCGGAAGTCAATTTCATA
TGTTGGCCTCGATCCGGCCATGTTCAAACCTTACCCTTTTAATGTCAGTAGTTCCGCCCTCTCAGAAC
TGATGGACTGCATGTCCTACATTGTATTCCCC
GAATTCAGTGAACCCCCAATGAATCCTGTGACTGTCCCCGAGCAACGCCTTGGTGGCAGTGCACTGCT
TCAATGGACACTGACCCGCATGGCCGACATTA
TCAACTCACAGATTTATTCTGCCCTTATGCTTCAGGGCCACACGAAATGCTCTAAGGATGAAGCAGGT
ATTCCCCACTCGTTAAATGCTGAGAGCGGGTC
AAATGATGATGGTTTTATACCTCAAAATGAAACTCATGCCTTTGGCGAAGTCAGTTCTTTTCATTTGG
AAGCCAAGAAAAAGGCGGAAGAGATCACAGAA
GCTTTTCTAATGCGTAAATTACGCAACATACGGTGGCTTCTTCGTACCGATGCAGAAAGTATCCTTAA
TAATGATATTGCATCTAACTCTCTCTCGGAAG
TGGTTCTTTGCTATCCCGGACTACGCTGCATGAAACACCAGCGCACTGCACATGCGTTACACGAACTG
GGGGCTCCAAGCAATTTTACGCGCCTTCTTAC
AGAAATGGCGCATTCCACGACCGGCATTGACATCCATCCTGCTACCTCTATTGGCCATCATTTTTTTA
TTGATCATGGTACTGGAATTGTGATTGGGTCA
ACCGCCATCATTGGCAACCACGTATCTATATATCATGGGGTCACGCTGGGGACAAGGTCGTTTCCTGT
GGTTGCTAAAACTGGGGAGAGGGTGAGGAACC
TTGCACGCCACCCCATTATTGAGGACAGAGTGACCATCTACGCCAACGCTATGGTGCTTGGCCGAATT
CGGATAGGGAAGGATAGCGTCATTGGTAGTTA
CTGCCTGGTCTTGAAGGACGTACCTCCCAAATCCATGATTCGGCGGACTCCCAATGCAAAACTCCCTA
CTCCATATATAACCATTTAAAAAGGTGGATCG
GATATCTGAGAGCACCACACACAGTGAAGTAATGACAAAATTGAAAGTATTTTATTTTTTTTCGAACT
CGGATAGTCAAAAACACATCCACACACACACA
AAAAAAAAGAAGAAGCAAGAGAATGTGAGGAATAAGGTGAATTGATATTCTTGGGTTACAGCAGTTGG
GTTACTTGTGACGGTCGGATTCAAGGGGACAA
AGCAGGTGGTGGATATATCAGTATATATAAACATCTGATTTTATTCGCTTGTCATTTACTACTTTTTT
TTTCTTTTTTTTTTTTTACTTCTCGAAGTAAA
ACCAGTAATTTTATGAGATGGAGTAAGTTGAGATTAGGCGTATATTTATAGGTTGTTATATTTTCGTT
TGTGTGAAAAGTCTTTACTGTTGTAATGGTGT
TTGAATGACGAACTGATTGAATTTGGGGTGTTTTTTATTTTTTATTTTTTATTTTTGATTTACATGTT
GTACATGCGATGCCTGAACGTAAATATTTTTT
TCTCTCTACCCGCAAACAGGCAGAGTATTGGTATTAGTAGTTTTTGCCTACCACGACGACAACAACAA
CAAGATGTTTAGTGCTTCTGCCCTATGGCTCC
GAGGTCAGTACAAGACACGTCTCAAGAAGCGCATGGTCGGCTTCATTCCCAAAGTGATTCCACGGAAG
ATAAAGAACAATATGGTGGCTCTGCGTAGCGA
GGCAAACACAGGGCACATGGAGGGCTACATGAAGACCGAGGCGGAAAGACTTGACGCTACAGGTCGCA
AGGTTCAAAAGGTGCTATGGGACCCCGTCTTG
CAGAGGCACTGTTTGTTCAAGGAAACAAAGATTCGAGGGCCCTTCATGACGAAAGCGAACATCGTTAA
GAAGATGGATTTCCCCATTGGAGCAGCGGGGA
CCGCTTCTTTGAAGTAAGTGGCCCTGTCAGTGCGAAGAAGAATGGGAAGGGCGGGAAAGAAAAGAGGT
GTGCGGAAAACACAGCGTAGCTGATTTTTCAA

GAAAAATTAAACCTCTTAAAAGATTATTATTATTATTATTTACCGCCCAACAACACGTGGGAATTTGG
ACAGATATCAATTTTCTTCTTTTATTACTTTT
TTTGGACGTTTTTCCTTTTTTTTTCTCTTTTCCGTTCACGAAGGCGATTGTGTGCATCTATGCCCTTG
CAAAAGTCAAGAGTGGCTGCCGTGGTAGATGG
GGAAGACGACTCGGATGACGGAGAGCTTCCTCACACGGCCAACGCAGAAAGAGTTCCTTTTGTGGAGG
AGCTCAAACAGAACGTCACTTTATTGTACCAG
CGTGCGGCAGTTCAGTCGTCGTCACAACAGGCTACCCCACCTCGGAATGACCCACACGTCGTGGAATT
GCCACCACCACCGCCAGCGCCTTCCCGAGAGT
TGCAGGTCCGAGAATCGGACGGAAGCCTATCAGCGCTACAGAGGCAATTAACAGCACTTCAGTCAGTG
CTTAAGAGTAGTCAAGAAAGGTTCCAAGTGCG
TGACGACGCAACGGCAAGACGCTCGCTACCAATTGTTACTGCATCCCAATTCAAACGTCCAATCAGTG
AGGACTTCCTCCATGAATTGAGAGAATGTCGC
TTAACACAATCTGGTGGAGATGAAAATACTGCACATCTAAAAGAGGGTTTCTCTTGTCACTGTGAAAA
CAATTTACGGACTGCTTACAACAGATGGAGAG
GTATTCTTGCAGAAATACACGCAAATGCATTAAAAAGGACATCAACAATTGCCTCCAAGCTAGATTCA
GAAATCAAAGGCCAGAAACAATTCTTGGAGGA
AGCACGGCAGCGTATTAAAGGTACTATTCTCCTAACAAGCACGCTTTCTGATGCAATCACTTGCTTTA
CTCGCTATTCTCCGGAGCATTATGGCGCTCTT
CAGGAAAACCGAAAAACTCCAATCTAATGAACGAAACGAACACAGTGAATTCCAGTTTTTCGGCAAGG
AAGAATTCCAATTGCGCCACCTTTCCACCACT
GCATTCTATTTCCTGTTTGTGGGCATGCTTGTTCTCACTTTTTTATTATTGTTATTATTTTTTTATTT
CTGCCTTGCCACACTGGATGATCTCACTCTTT
TTTTAGGGAATCCCAGGCACACTCAAATGTCTCACTGCAAGTTTGAACACCCACGCCACGGCCATCTT
GGCTTCCTGCCACGCAAGCGCAGCCGACAGAT
TCGCGGCCGCGTTCGCTCCTTTCCTAAGGATGACCCGTCACAAAAGCCGCACCTGACCAGCTTTATGG
TGTTTAAGGCGGGCATGACGCACATCGTTCGC
GACGTTGATCGCCCCGGTTCCAAGGTGAACAAGAAGGAGGTTGTGGAGCCGGTGACGATCTTGGAGGC
ACCGCCCATGGTTGTGGTGGGCATCGTCGGCT
ACCGCCAGACGCCTGTGGGCCACAAGACGATCGGCACCGTGTGGGCGCACCACACCAGCGTGGAATTC
CGCCGCCGCTTCTACAAGAACTGGAAGCAGTC
TGCGCAGCTGGCCTTCACGAAGCGCAAGCAGTTTGCTCGCACGACTGAGGGTCGCCTTGCCGAAGCGC
GCACGCTGAAGGCCTTTGCCAAGAAGGCTGAC
ATCATCCGTGTTGTTGCCCATACCCAACTGCGCAAGCTGCGTAACAACCGTGTTGGTGTGAAGAAGGC
GCACGTGAGCGAGATCCAGATCAACGGCGGCA
CCATTGCCGAGAAGATTGAGCTTGCCAAGTCTCTCCTGGAGAAGGAGGTCCGCATTGACTCCATCTTT
CAGCAGTCCGAGACGTGCGACGTGTGTGCCGT
GACAAAGGGTCATGGGTTCACTGGTGTCGTGAAGCGCTGGGGCGTTGCCTGTCTGCCGCGCAAAACCC
ACCGTGGTCTCCGTAAGGTGGCCTGCATTGGC
GCGTGGCACCCCGCCCGCGTGATGTACACCGTTGCCCGTGCTGGCCAGCACGGTTACCATCACCGCAC
GCACCTCAACAAGAAGATCTACCAGCTGGGCC
GCGCGGTGTCGATGGAGCCGAACCAGGCCACGACCACGTACGACCTCACAGCCAAGTCCATCACTCCG
ATGGGTGGCTTTGTTGGCTACGGTACAGTGCG
CAACGACTACATCATGCTGAAGGGCTCCGTTGCGGGTCCTCGCCGTCGCGTCATCACACTGCGTCGTC
CCATGGCCCCACAGACATCGCGCAAGCTCACG
GAACAGATCACGCTGAAGTTCATTGACACAAGCTCCAAGATTGGCCATGGCCGCTTCCAGACGAAGAA
GGAGAAGAGCCAGTGGTTTGGCCCGCTCAAGA
AGGACCGCATTCGTCGCGAGGAGCGGCTCCGCAAGGAGCGCGTGGCTCGCGCTGCGGAGCGCAAGGCG
AAGGGTGTTGCTGTTGCCGCCGCCCCAAAGAA
AGCAAAGAAGTGATCATCTTAGCTCTTGCGTAAAAGTTTCTTAACTAAGAAGAAACACGTGCTGGGAA
ACACCACCTAATTTTATtTTATTTTATTTTCA
CGTGACTTTGCCTTTTTTTTAATTTTGATTTTGATTTTGTTTTATGCCAGATGTGCTTTTCCGATGGT
TGTTTTATGGTTTGTTTTTGTTGATTAATTTT
CGTTTTTATTTCACCTTTTCTGTTTATTTTATTATTATTATTTTAGTTAGTTTTTGTTGTTGTTGTTG
TTTTGTGTTTATATTTTCTGTTCTCATATTGT

GTGCAATGTCGAGGGGTTCAGCAAACGATGATTCAAAAGTATTTGATTTAACGTTTGGATTTGCTGCT
GGAGGCTGGTTTGGGATGTATCACTTTGGTGC
TTGTCAAGCGATTGTTGATTCCGGAATTATTGATCGATTGACGGCGGAGGGAAAACGTGTCCGCTTTG
CCGGAAGTAGCGCGGGCGCACTTGTTGCTTTG
TGCATGGTTTTGAAGAATTACCGGTTCAAGGAAATGCGGGATTTTCTAATGGGGTGTGCGGAGCATTA
CCGTTATTCATGGCTTAACTTGTTTCGTATAG
ATCGATATCTTGACGACGCTTTGGATCAATTTGGTGGTCATCTGAGAGAATTGGAGATGAAGCAGGAG
TTATTCGACACGTTGAATGATGGAGCACTGGA
GATATTTGTGACAACGCTTCCGAAGATGAAGTCAAAAATTTTTACGGAGTTCCATTCGCATGAGGATG
TCGTGGAAGCCGTGAAAGCGTCTTGTTGTGCG
GCGCCGTTTGTGGGCGTGCCGTTCCGGCTGCGAGGAACAGGGGAATGGGTCTGCGATGGTGGTTTGTC
GATCGTCACACCGCACAATGGCGAGCCCCGAA
CAATTACGGTGAGCCCTTTTTATTGTAGCACGGCAACCGTACATCCTACCATTTTTGTTCCGTTCTGG
TGGGGATTACGCCCACCTGGGAAGGTTGCTCT
CCGGAATCTGTTTGCACTGGGTTACAATGACATGATAGAGGGACTTGTTGTGAACGGTTACATTACAC
CCGAGAAAGGCGAGTCATTGCTAAGGCCTGAA
GTACAATTTTTAAATGGGGGTGGTATGTTCCGGCGTTACGGTTTGTATTTGCTTGATTTTTTGGCTTT
CATATTTGTTCGTCCGGTCATAGTTTTATGCA
TTTACGTCGAACTTGCATTCATGAGTTTTTTCTATTTCTTGAGGGGTTTATTCTGCCTGGATAACAGA
CCAATTAGGAGAATGTATGACAACCTGAGAAA
TATTGTGTCTATTCGAACATTGGGTAGACTTATTTTCGGCGAAAATGTACCGCACAATGTCGAGCGTC
TGGAGAAAAGTTCTCGGGTTTACCGCGTGTTC
AATCCAATACTTTTGGGAGGGAAAAATACCACGGAGAGAGCTGTCATGAGTCCCACAGGAGTTTTCAG
TGGTGTTATGCCTACACTATCTTCCAAAACGC
GGAAGAGGGCAACGAGCGCTGCCCAAGAGAAGTCGGAGAAAATTTATCGCCGCTTGTGATTATGAATG
CTTTTTTCTTTATATATATTCTACATGTCTTT
TGCAGTTTTATATTCTCTTTTTTGGCCTACTTGCGCCTACACCATTCAATATATACACATATCTGTGA
AAGTCAGTATTTTATACGCGAATGTTTAGTAC
TTGTCGGCGGCCGCAGCGAAGCCGGATGAAGAAAAGAACGATAATTTGAATATGTCTGTTTTATTTGC
ATCATTCCCGGAGGTTTCTTTTTCTTTCTTTC
TTTGTTTTTTTTATTTTTATTTTTACGTGTTTTTTTTTTTTAATCCGCTGTTGGGCGAGAGTTTGGTG
GTGCTGAGCAGCAATTCTCATGAACGGAGGGA
GGAGGGAGGGGTTTTTTCTCGGCGGGTGCGTTGCCTTCCCACTTTTATCCGTCATGCGTTTTTGTGAC
GTTTTGTTGTCGGGAATGTAGTGGTTTGTTTC
TGTGTGTGTGTGTGTGTCTGCGATTTCCCCTTCTTTATTA
>scaffold1023.size7233
TCCTCCATTCGGCCGCCTTTCGGTATTCCCGCAACGATTCTCGCACGACCTGTAATGTGCGAATCCGG
CGTGGAGCGGGACGCTGGGAAGGCCACTCCGCA
CCACACTCCGATGCCGTACATCGCCTTCGCCCGCGCCGGTGCAACGCAGAACGCCCGCAGGGTGTCTC
TCATAGTCCCCCACGCGGCGGGCGGAATCGGT
GCCATTCGTGGTGGGCGCTGGTTTGCCTCCTCGGGGAGCGCCGCCACGCGCGCGCCCATTCCACGGAA
TTCCGGAAATTGACGCCCAGGAGGGTCGGCAT
GCGCCCCATGGTGGCTGGGCCCCCTCGCGCGTGAGGTCCGAGAAGGGCGTGACGCCGAAGGTCGCGTG
TGGGTTTGCCGCGGCGTGCAGCCTCGCAAGAA
ACAGGTTCGCCCGGAACACGCTCAGGCGGAACGCCTCCTCCGCGGCGCTCCCGTACACCCTGCCATGC
TTCTGCTTGAATTCTGCGAATTGCGACGCCAG
CGTCTCCTCCGCATGCAGGCTCGCCGTCGCCGCGGGGACGAGGCACGCCATGACGACCAGGACGGCCG
CGAGCGACAGCGCACGCGCCCAGCCAGACATT
ACTGCTTGTGTGGGCGTGTTTCCTTCCTTGTGTGCCTTCAACAGCGTCACCTTCAGACTGTGGTGGTG
TGCGGGCGTTGACGTGGAGAGAAGGAGCATGC
GCGCGTGTATGTGCACGCCGCGGCAAAGGAGCGTTGCCACGCCACTCCGCCCCGCATGGTCGGCTGAC
AGTCGGGGACGGCGGTGGGTTCAGCCACAGTG
GGGAGTGCTCGGGCGCTCAACAGAAGCATATAATAGAAAAAAAGCATGCACAGAGCAAATCAATAAAC
GATCCACTCCACTGGGACTTGTTTTCCCAATA

ATAATAATAACCAATCTTTGCCCGCACCGCGGCCGGATCCCTTAAGTGCCGATGGCGATCCAAAGTTG
TGGAGTTAAAAATGGGCGAAGAAGACGGCGGT
TTTAACCCAACGCCGGCATCAGGCGTATACGAATTTCTTCGGCCCCTTGCCGTCGTGAGACGCCGCGC
TCTATTCTGATCGGGTTCGCGCTTACCGATTG
CGAATTGGTACAATTCGGGCTCTTCCAGCCGCAAGACGCCGCAGTGTGAGGATCACAAAGCGGTCGGG
CGGGACGGCAAAAGCAAAAACAATTAAAATTG
TTTAGAGGCTTCCCTGCTTCCGGAACCGCTCCCGTCGCCGATTCATAAAATAGGGACAAACATTCCAA
TTGTTTTTTGTCTCTTGCCTTTGTGGCGGGAT
CGGGCGGGGCAAAAAGAAGGAAAAGAAGGGCTGCGGCAACGGTTCCGGGATCAGGAACTGCACCGTGG
TGCTTTGCGCCTTGCGCGAAGATTCCTGGCGG
GAGGACCGCGTGATACCCCGGAATGTGCCCGACACGCCCTAAACATGCCGCTTTCGTTTTCCAGAAGA
CAACGCAAATGCGCCCGATTGTTCGAGGAGCA
AAAGCAACACTTTGTAAGCCGTAAATACTTGTATCCAATCCGAATAGAGCGTTGAACAATTAAGCGAG
GAGGGGACGGACGAATTCTTTAGCAAGATTGC
GATCAAAAACTCCCCATTTGGTTTTGAAACTATTTTTACGAGGTTACTTTTTTATTGGGAGCTGTTGT
GTGTATCTTCTCGACTATTTAGCTAAGATCAA
GTTTTTAAACTGTTCTTATAAGAGTAATTCCTGACATGGGCTTCATCCCAGGAATAAAAACCGCTGAT
CTCTCAGGCGTTCTTCCGGAGTTTCGCGCGTC
CGGATGGAGCGTCTCCTTCTCCAAAATTGTGTTCGCTTTTTCCTTTTTTTCGCAAGCCGAAAATAGGC
AAAATATGCGTCTGGACCGCAGGCCGGACTCC
ATCTTTATCTCCGACCAAACATTATAGGTAAGAGGGGCGACGCAGAAGCACGTAGATCCCCCAATGTC
TTCTGACAAAATGCGGATTCTGTTGTCCACTC
GAGTGCCCTTTTGTACTCGGTTAGGATCTGAAATATTATTATTGACGCGGCTTCTCCCGGTCCTCAGC
GATGACGTTGCACCTTGTCGCGGTGTTGGGCT
TTGGTTGCGTATAGGAACTCCGCAGCAGGGCGTTGCCGCGAGCACGACGGAGACCAAGGTGACTTCAT
TCCCCGAGAAGGTTCCACGCCATGCCCGGTGC
GGGGAGGTGAGGCGGTCTGCCTGCAGACTGTCGGTCAGAACTGCACGACCGTTTCGCAGCGGTCACTC
CGTTGATATCACCCGTGGCGGTGACGTTCACA
AGAACCCTGGCCCGAGGAGGATGGCTACATCAATATTTGGCAGCTCAACATTGCAGGCCTCTCGTCCG
TAAAAATATTGCATTTGCGGCGCGATTGGAGC
AGCGTCCACCTGACATCGTTCTTCTGCAGGAAATAAGTAACCGTGGTGGCGTGGCGACGAAATTATGT
GGCCATAATGACTGCCTTCAGGCCTCACTGGG
CGCGGTGGTGGCGTTGCCATTCTGGTGCGCCACTCTCTACCGTGCGAGCGCCTGTCGCTCCCTGGCAC
TGAACTCCTGGAGACCATTGCTGTCCGTGTAT
TTCCGCCGGGCTGTCGCAGCATTTGCGTGGCGAATCTCTACAACCCTCCCACAGTAACTGTTCAAGAA
TCCGCATTTATCAATATCGTTTGTCGCTTGGG
CTCGAAGAGATTATCGCGGGTGACCTGAATTTACATCGTGAACTCTGGGACAGTCATATACACCATCG
ACGACTGGAGGCGATAATTTCGCCGCTGTTCT
AATCGATATGGATTTTGGGCTGGCAAATTACCCGACACAGGCAACACGGATCACACATCGTGATGCCT
CGTCCCCTGACGTGGCTGCGTATTGCGCCCTA
CGCATTTCCCACTGGACATTCGCCCCTTACATGGACTCGGACCACCGCTGAATATCTTACTTGGTAGA
GGTGGACGATGGCACTCCACGACTAGCAAACA
CGCTGCCTTGACGGAAGAAAGCCACCTTTGCCCTGCGTAAAGCGGATTGGCCTGCTTTTTCATCGTTG
TGTGAAACCCTTCTTGCCACCGCGTCCACATG
GTTGGATCCTCGCGGAGGCATCTTACGAGCAGCTGAGCGCCACATCCCGCGCGGCAGCCGTGACAGCT
CCAAAAAGATGTGGACACACNNNNNNNNNNNN
NNNNNNNNNNNNNNNNNNNNNNNNNNNNNNNNNNNNNNNNNNNNNNNNNNNNNNNNNNNNNNNNNNNN
NNNNNNNNNNNNNNNNNNNNNNNNNNNNNNNN
NNNNNNNNNNNNNNNNNNNNNNNNNNNNNNNNNNNNNNNNNNNNNNNNNNNNNNNNNNNNNNNNNNNN
NNNNNNNNNNNNNNNNNNNNNNNNNNNNNNNN
NNNNNNNNNNNNNNNNNNNNNNNNNNNNNNNNNNNNNNNNNNNNNNNNNNNNNNNNNNNNNNNNNNNN
NNNNNNNNNNNNNNNNNNNNNAAATGGAACAAG
CCGAGATCGCCGCGGAAGCAGCATACAACGCACACATTGCCTCACCTGGGGACAGCCTACTTCGAGCC
GAATGTGCCCGAAAAAAGGAGGAACGAAATCA

AGCCTTGCGCCGTTGCTCCGCTGTGCTTCTGGAAGCCCGCGCGAAAGGACTTGGAGCCTCACCCAGCC
CGAGGTGGCGATATCTTCACGGCGTGGCGGCG
CCGCACCCTCATCCTCATCCTTTGGAATCGGTTGTGCTCCGCACTGAACTTGGCCGCGTATGCGCCCT
GCCTCGGCAACAGGCAAATCTGCTTGTCCGTC
ATTTTGTGCGGTTTCACGTGCCATACCGCCTTATACTGCTGCTGCTGCTCGCTGCACTCCTTTGGTTC
CTGGTGGCTGGGAAATGGATCGGCCTTTCCCG
CCGTATGTACTTGACGTGACCATCCGCGACTCTTTGCTTGGCTCAGCCCCTGGCCCCGGCGACATGTT
GAATGAATTCCTGCACCGTCTCGGCCCGTTGC
GCGTGGCACACTTGGAACGATGATCCACAACCCCTTCGCAAACGGCAGCCTGCCTGGCTCCTGGAATA
CGGTGGACGCCATTCCCATTCCAAACCTTGGG
AAGGGCCATTCCGCCCAGAGAGTTACAGACCTATCAAATCACTCTCTGTTTTATTCAAGTTAACCGAA
AGAATGACTCACCGCCATCTGTCGGCGTTATT
GCCGCACCACCCACGTCAATTTCGGATTTGCACCTCCGCGTTCTACGTCGGACGTGGTGGCACCTGTT
ATCGACAAATTACTCGTGGACTGAATGAGTTC
AGCACTGTGGAGTATGAGCGCCCTGGAGACGACGCCCCCACACGACGCCCTCGTCGCCATCGCTTGTG
GTTTTGATTGATTTTCCGACTGCGTTCGACAC
CACTGACCATGGGAATTTATTCGGCATGCTTGACAGACTTCCGCGCCTTGGCCCACGAAACAAACGCT
GGCAGCACAACTACCTGCGGGGCCGCTATGTT
GGGGTATGCACTCGTGAACAGCACTCCCGGAAACAACTCACCTCAGACGGAGTACCGCAAGGCAGCGT
CCCGGGACCCCAACTCTTCCTCCACTATGTGG
GTGATCTGCTGCGCCGCCTGGGCAGCATCTATTCTGCCTCCGCATTCATGTACGCTGACGACCTCGCA
CTTGTTGCCTCAGGTGCTGACATCCATGCCTG
TGCTGCGGCAATGTGACCGCCCTTGTCCCTTGTATCGACATGGGCTGTCGAGCACAACCTCAAAATCA
ACGTCGCTAAGAGCGAAGCTGCTCTATTCCAC
ATCTCTTCACACACACACGGTCTGACGAGGACATGGTCGATCTCCATCTTGTTAGCGGGAACCTGCGT
ATTCAGTCACGCCCAGTGCGCTTGCTGGGCAA
CACGTTCGATCGACTTCTTAATTTTGTTACGCATGCTTCCACCGCTGCAAAGAGGACCATGCCACGCC
GCCATCAGCTGCGTCTGGTCGTAGAGGCGGGC
ACGTCCCATCACACCATGCGATCCTTTTTGATTGGAGGCGTTCATAGTGTGCTATTTCATAATGGCGA
GGCAATTGTCCCGTGACTCGTCCCCACTCACC
TCCACAATATGGAAGTACGGTACCGCGACGGCAGCAAAACATTCTTGGCCTGAGCGCCTCTACGGAAG
ACACGTCTGTCTATTTAGAGGCCAATATTCTT
CCGCTCCGTAAGGTACTCTGGCGTCGCGCACTCACACAGTACGAACGCTATATACGCCTCCATGATCA
CGAGGATTTGCGCTCCCTTATCTACTCGCAAC
CTATGCCTTCGCCCATGCATGGGAAAGCGGCGACATCAATCCGCTTCCGAGAGATGCTGCTATTAATG
GATTGCTACGCGTCTGTGACATTATTGGGACA
CCCCACAACCACATTCGCGCGCCTCTTGCCCAACATCGAATTTTTTCCCTGGATGCTGTGATCTGCAA
CAACGTGAGATTTTATCAGCCACTGTTTGCCA
AAGACGCCTCTGACGATGTCGGGCGCACGGCTTTTTATTCTACTCACTCCTCTTTGGGGGATTGTGAT
TTTGAATTGTGGACGGACGGTTCTTCATCTCT
CGCTGGACTCGCATCTGGATCCGCTGCGCTATTATATGGCTCCACCACGACAAATGATCTCCCTGTGG
AGGTACATCGTGCAGCAGCTGGCAGCCTTGCA
CGCTCATATAGGGCGGACTGTCTTGCGATCGAAAATGTTTTTAGACATCTCATACCTCCTTGTCTGGC
GCATCCCAATCGCCCACACGAATGCTGGAGGA
AACGGACTCCCTCTCGGCAATGGAGGCGCCCCGCGTTGCCCGCCGACCGTGCGAGACCGCGTTGCTGA
GTAAATTTGGATTATGCCGCTTTCCTTGGTGA
AGCGAGTTCACCCCGCACACCTTATATTTTCCCATCACACTGCGGTATTCCACGCAGTGAGACCGCTG
ACGAACAAGCCACGATTGCACGATTCCTCTCG
CAGGACTGCGTCAATGCCTGGCACGTGGGCCCCCACACCGCCGCCAAGAGGTTATACTGGAAGGATGC
CCAGGAGGAAGTTGCACTCACCGGACGTCAGG
CGTTCGTCGGTGCAACAAAACACATCCCTGGTGCCGAGCCAAACGTTGTGCCCAGAGTGGCGCCTCCG
CTCACGGGCGCGATGCGACACCTGCCCACTCT
TCGGCTGACTGGCACGCGCCCTGCGAATCCCAAGCGACCCTCTGTGTCGGTGGTGCCGTCCCTCCCTC
ACTGAAGAGACGGATCCGCCCCGACATCCTCC

GGAACCTCCACCTGGCGTGTCGACGGCGCCTTCTCGTAGAACGACGAGCGGCAAATCAACGTCGCCCT
TCCCAAGCCGCAGCAGCGTCTTCTCGTCCGTC
CACAATATGAAGGCACACGCCCGACGCACTAACCCTGGCNNNNNNNNNNNNNNNNNNNNNNNNNNNNN
NNNNNNNNNNNNNNNNNNNNNNNNNNNNNNNN
NNNNNNNNNNNNNNNNNNNNNNNNNNNNNNNNNNNNNNNNNNNNNNNNNNNNNNNNNNNNNNNNNNNN
NNNNNNNNNNNNNNNNNNNNNNNNNNNNNNNN
NNNNNNNNNNNNNNNNNNNNNNNNNNNNNNNNNNNNNNNNNNNNNNNNNNNNNNNNNNNNNNNNNNNN
NNNNNNNNNNNNNNNNNNNNNNNNNNNNNNNN
NNNNNNNNNNNNNNNNNNNNNNNNNNNNNNNNNNNNNNNCCGACAATCCACAGGAGGCCCTGCCGCAC
ACGCTGTGGGAATGCCCCCGTCCTCGACAACT
GAGCATCCGACTATTTGGGAGCACACCCTCTCGCACGACAGCACCATTCAAGAGAGAAAAACCGGCCA
CATCGCCACGGACAGCCCGCAGGCCATTAACG
AACAGTGGCAGCAAAGTATAACGAAACAGAAAAAGTGGCAGACTTTGAAGTCCTTCAGCCACACGACA
CCGCCGCTGCCCCTCACAAGTTCCACCCTTCG
CCAGGTCCTGAAGTGTAAAAACACGACCGCGTGATATGCCGTCTGCGGGGTTGTCCGCTGACCGCACG
TATGCAAAGGATGCCTGTGCTCCGCGAGAGTC
AAAAAACTCATGTATCCGTTGCAGCTCCCGCGTCAAGGCGTGCGATTTCCAGCTGCCTTTATTCGCCG
CTCCTTGCAGCGAAGCGTTGTCCACCCAATCG
TTATTGGTGGATGGAAAGATGGCGGAAAAAGC
>scaffold1024.size7774
CTTCTCAAGCCAGTAAATGTTTTCTGTTACGTACAGCATGCGTCAGTTGATCCACGTCACACCACGGT
CTGCAGTGCTGTTGGTGCTTCTGGTTGTGATGA
TGGTTGTGATGTGTTGTGCTGGCGGGTGCTTTGCTGCCAGCCTGACGTGCTCCTGCTCCTCTGAAAAG
GCGATGCGTGCGGACAGGGCGAAGTCGATCCC
GGCTGTGGTGAGGGACGTGCCTCTTGGATCTGAGGGTGCGTCGCAGACATGCAGCGTCATTGGGAGGA
ATGGATGGGCGCCCACCCGCATCGATGTGTCG
ACAAGGGACCTGGATGGCACCAGGAAGTATTGCAGCTCGGAGCGGTATTGTGCAATGAATTCATTGTT
GGCAATTGCGAGTGTGAAGGTGACGTTTTTCT
ATGCTGAGGAAAGGGAAGTATTTGTTTCGTTATACTTTGCAGCCACTGTCCGTTAATGGCCTGCGGGC
TGTCCGTAGCGACGTGGCCAGTTTTTCCCTCT
TGAACGGTGCTGTCGTGCGAGAGGTGATGTTTCCAAATAGTCGGATGCTCAGTTGTCGAGGACGGGGG
CATTCCCACTGCGTGTGCGGCACGGCCTCCCG
TGGATTGTCGGGGCTCAGCGGCTGATGGGGGTTGCAGCAGAGGAGTGGCTGAAGGGCTACGGCTGTCA
TTGGAGCCTGCAGAGGTGCGTTGTCCGTTTTT
TGCTCGTTGATACGCCTGACATTTTCGGTAGTGACCTGCGCGTGAGGCACCTGTTGGGGAGACCGCGG
AGCTGTCGCAGAATCCACATGTGAGGCCTTCT
GCAACGATTGGAGTGCCGGGGTTAGTGCGTCGGGCGTGTGCCTTCATACTGTGGACGGACGAGAAGAC
GCTGCCGCAGCTTGGGCAGGACGACGTTGATC
CGCTGCTCGTCGTTCTACGAGGAGGTGCCGTCGACACGCCAGGTGGAGGTTCCGGAGGAGGTCGGGGC
GGTCCTGCCTCTTCAGTGTGAGGAAGGGACGG
CACCACCAACACAGAGGGTCGCTTGTGATCTGCAGGGCGCGTGCCGGTCTGCCGAGGAGTGGGCAGGA
GTCGCATTGTATCTGCGCGAAGTCGTGATTCG
ATGCCCGATGTCTGCCTGCACACCAGGAACACAGCCCTTGCCACACCGACGAGCGCCTGGCGTGCGGT
GTGTGCGAATTCTTCTTCCTCCTGGAATTCCT
TCCAGTACAACCTCTTGACGGCGGCAAGGAATTCCACGTGCCAGATCGGGACGCCTCATTGCTGCAGG
GATCGTGCAATCGTGGCTTCTTCGTCAGCGGC
CTCATTGCGTGGGGCGCTGCAGTGCGATGGGGGAAATATAAGGTGTGCGGGGTGAACTCGCTTCACCA
AGGAAAGCAGCATAACCCAAATCTCTTCAGCA
ACGCAGTCACGCACGGCCAGTGGGGCAGCGCGGAGTGCCTCAGTTGCCGACAGGGAGTCGGTTGACAC
CGGGGTTTGTGTGGACGATTGGGATGTTTCCG
GACGAGGAGGTATGAGATGTCTAAAGCCAATTTTCAGTGACAAGGCTCTCTGCTCCCTATGAGCGTGC
GAGTATGCCGGCTGCTGCACGATGGACCTCCA
CAGGGAGACCATTTGTCTTTGTGGGGCTGTATAATAGTGCAGCGGATCCAGATGCGAGTGCAGCGAGT
GATGAAGAACCGTCTGTCCATAATTCAAAATT

ATAATCTTCTAGAGAGGAGTAAAGGGAATTAAAAGCCGTGCGCTTGACATCGTCAGAGGCGTCGTTGG
CAAAGGGTGGCCGATAAAATTTCACGTTATTG
CAGCCCGCAGTGTCTCAGGGAATAATTCGATGTCGGGTAATAGGAGGAGCATGATTGCGGTTGTGTGG
TATCCCAATAGTGCCACATACACATTCTAATC
CATTAATAACGGCATCTCTCGGAAGTGGGATTGATGTCGCCGCTTTCCCATGCATGGACAGCGGCACA
GGTTCCGAGTAGATAAGGGAGCGCAAATCCTC
GTGATCATGGAGGCGTATATAGCGTTCGTGTTGATTTGTGTCACTGAGCCAGAGTATCCTCCGGAGCG
GGAGAGGATTGGCTTCCAATTAGAAAGATGTA
TCTTCCGTTGGTGCACTAATGCCAAGGTGCGTTTTACAACTGTTGCGGTACAGTAATTCCGTATTATG
GAGGTAGGTGGGGGCAAGGCATGGGACAATTG
CCTCGCCAGAATACAGTGACACACCATGACCGTATCCAATCGAAAAGGATTGCGTATTGTGATGGAAC
GCACCAGCCTGTGCAACCGGTCGCAATTGGTA
GCGGCGTGGCGTTGTCTGCTTTGCAGCGGTGGAAGCGTGCGGACCAGAATTAAGAAGTCGATCGAACG
TGGTGTCCAGCAAGCGCACTGGGCGGGACTGA
ATACGCAGGTTCCTATTGCCAAGAAGGAGATCGACCATTTCCTCGTCAGACAGTGTGTGTGTAGAGAT
GTGGAATAGAGCAGCTTCGCTCTTATCAACGT
TGATTTTGAGGTTGTGCTCGGCAGCCCATGTCGTTATAGGTGACAGGGCAGGTTGCATTGCCGCAGCA
CATGCATGGATGCCCGCACCTGAGGCAACAAG
TATGTGTTCGTCAGCGCACATGAGTGCGGAGGCGGAATAGATGTTGTCCATGCGCCGCGGCAGATCAT
CCACACAGTGGAGGAAGAGTTGGGTCCCGGGA
CGCTGCCTTGTGGTGCTCCAGTTGAGGCGAGTTGTTTCCGGGAGTGCTGTTCGCGAGTGCATACCCTA
ACATAGCGGCCCCGCAGGTAGTTGTGCAGCCA
GCGTTTCGTTCGGTGGCCAAGGCGCGGAAGTCTGTCAAGCATGCGGAATAACTTCCCATTGTCAACGG
TGTCGACCGCAGTCGGAAAATCAATCAGGCCA
CGAGCGAGCGATGGTGACGAGGGTGTTGTGTGTGGGCGCCGTCTCCAGAGCGCTCATACTCCACAGTG
CTGGACTCATTCAATCCACGGTTAATTTTACC
GTTAACAAGTGTCATCGCGTCCGATGTTGAACGCGGAGGTGTAAAGCCAAATTGACGTGGGTGGTGCG
GCAATAACGCTGACAGACGGCGGTGaNNNNNN
NNNNNNNNNNNNNNNNNNNNNNNNNNNNNNNNNNNNNNNNNNNNNNNNNNNNNNNNNNNNNNNNNNNN
NNNNNNNNNNNNNNNNNNNNNNNNNNNNNNNN
NNNNNNNNNNNNNNNNNNNNNNNNNNNNNNNNNNNNNNNNNNNNNNNNNNNNNNNNNNNNNNNNNNNN
NNNNNNNNNNNNNNNNNNNNNNNNNNNNNNNN
NNNNNNNNNNNNNNNNNNNNNNNNNNNNNNNNNNNNNNNNNNNNNNNNNNNNNNNNNNNNNNNNNNNN
NNNNNNNNNNNNNNNNNNNNNNNNNNaccacg
ccgtcaAACGCTGACAGACGGCGGTGAATCATTCTTTCGGTTTAGTAAAACAGAGAGCAGCGTGGTAG
GTCTGCAACTCTCTGGGCGGCATGGGCCCTTC
CCAGGTTGGGGAATGGGAATGGTGTCCACCGTATTCCAGGAGCCAGGCAGGCCGCCATCTGCGAAGGA
GTTGTGGATCATCGTTCTAAGAGTGCCGCGCG
CAACGGGCCCGAGACGGTGCAGGAACTCATTCAACATGTTGTCGTGGCCAGGGGCTGAGCCGAGCGAA
GAGTCGCGGATGGCCACGTCAAGTTCATACGG
CGTGAGAGGCCGACCCATTTCCCGGTCACCAGAAAGCAAAGGAGTGCGGCGGCAGCAGCAGCAGTATA
ATGTAATGTGACACGTGAGACCCGCACAAAAT
GATGGAGGAGCAGATTTGCCTGTTGCCGAGGCAGAGCGCAGTCCGAAGTTCAGTGCGGAGCACCACCG
ATTCCAGGGGATGAGGGTGCGGCGCCGCCACG
CCGCGGAGATATCGCCAGCTCGGGCTGGATGAGGCCCCAATTTTTTCCGCGCGGGCTTCCAGAAGCAT
AGCGAAGCTACGGCGTAATGTTTGATTTTAGT
CCTCCACTTTTTGGATGAATTCGGCTCGAAGCAGGCTGTCTCCTGGCGAGGCGACGTGTGTGCTTTGT
GTGCTGCTTCGGCGGCGGGCTCGGCTTGTTCC
ATTTCGTTCGTCCAGATTGTTTTGGGCTGTCGCGGCTGCCGCGCGGGATATGGCGCTCAGCTGCTCGT
AAGATACCACTGCGAATTTCCAGACATGTGGA
CGCGGCGGCAAGGAGTGTTTCACACAACGAAATGAAATCAGGCCAATCCGCTTTGCGCAGGGCAAAAG
TGGCTTTCTCCCGCCGAGGCAGCGTGTTGGCT
AGTCGCGGAACGCCCTCATCCATCCCCACCGAGTAAGATATTCAGCAGTGGTCCTAATCGATGTAGGG
AGTGGATTGCCAGTGGGTGACACGCAGTACGC

GATACGCAGCCACGTCAGGGAATGAGACATTACGATTTGTGATCCGCGTTGCCTGCGCCGGGTCATTT
GCCAGCTCGAAATCCATGTCAATTAGAGTAGA
GGACAAATCCTCTCCTCCGGTCGTTGAAGGCGTATGACTGTCCCGGAGTCCATGATGTAAATTCAGGT
CACCCGCGATAATCGCTGCGGGGCCTGAGCGA
CGAGTGGCAGTAATAAACGAAGAGCTCTGAACGGTTGCTGTGCGAGGGCTGTAGAGATTCACCACGCA
GATGCTGGGAAATCCCTGTGGAGGTGCACGGA
CAGCAATGGTCTCCAGGAGGTCTGTGCCAGGGAGCGTCAGGCGCTCGCACGGCAGAGAGTGGCGCACC
AGAGTGACACCGCCACCACCGCGCCCAGTGCG
GCCTGAAGAAAGCTATTATGGCCGGTTAGTTTTGCCGCTGTGGCAATTCGAGTATTTGTTTCCTGGAG
AAGAACGATGTCAGGTGGACGCTGCTCCAGTC
GCGCAGCAGATGCAATCTTTTTTGCGGACGAGAGGCCTGCAATGTTGAGCTGCCAATTAGTGATGTAG
CCATCCTCCTCGGTGCCAGAGTTCTTGCGAAC
GTCACCGCCACGGGTGACATCAACGGAGTGGCCGCAGCGAAACGGTCGTGCAGTTTTGCCCGACAGTC
TGCAGGCAGACCGATTCACCTCCCCGCACCGG
GCATGGCGTGGAACCTTCTCGAGGGGTGGAGTCGTCTTGGTCTCCGTCGTGCTCGCAGCAATGCACAG
CAGCGGAGTTCCTATACGCAACCAGAGCCCCA
GCACCGCGAAAAGGTGCGACTTCATCGCTGGGGCTTACCGAATGTTGTAAAGGACCTCATTTTAATTA
TTTTTTTCTTTCTTTTGCTGCTTGTGTTTTAT
TGGAATAATAATTATCTTTGTGTTTTCCCAATGGCTGTTTGACTGCTCTACACATCATTATGAGTGAC
GTTTTTTCCATCTTGTTTTCTTTCCTGTCCAG
TCGCTGTGTCGGCCTCACTGCTGTGCGTGCCACGGACTGCACGTACGAGGACTGAAGGAACACGCCGG
ATGCCGGATGAATTATAAGGGATTTTGTGTGG
GCTGGTGGAGCAGCAAGATCTTTTCTTTTTTTATCAGATTATTTTCTGCCATTTTTGCGGGGAAGATG
TATACACAAACACACGATCGTCTATATTGTGT
TGCATGGCACATGAAATAAAATTGCGACCTAATCTATTTTCTTCATCCTTTCCGGTGTTACTGCCGGA
CTATTAACCCAGTTTTTATTTATTCTTACTTT
AATTTCTGATTTACGGCGCGCTGTGCATTTTTTGACGTTTATTNNNNNNNNNNNNNNNNNNNNNNNNN
NNNNNNNNNNNNNNNNNNNNNNNNNNNNNNNN
NNNNNNNNNNNNNNNNNNNNNNNNNNNNNNNNNNNNNNNNNNNNNNNNNNNNNNNNNNNNNNNNNNNN
NNNNNNNNNNNNNNNNNNNNNNNNNNNNNNNN
NNNNNNNNNNNNNNNNNNNNNNNNNNNNNNNNNNNNNNNNNNNNNNNNNNNNNNNNNNNNNNNNNNNN
NNNNNNNNNNNNNNNNNNNNNNNNNNNNNNNN
NNNNNNNNNNNNNNNNNNNNNNNNNNNNNNNNNNNNNNNNNNNNNTTTTGTTTTTGCTTCACTCTGCGTT
GACGGGAAGTTAGTTTGTGCTGAAGGCTGCAC
GCAAGTGACAGGAGTGATGGCGATGATGATGGCTGGCCGTGTGCTGCTGGTGTGTGCCCTCTGCGTGC
TGTGGTCCGTTCCGGCCGATGGAGATATTGTT
GTTGTTTCTGGTGGGGAGGACAACATTCTGAAAGAATTATTTATTCCAGTTGCGAGATTGCAGTTAAG
ACGAGAACAAGGAGCAGCAGAAGCAGCAGCTG
ACGCAAAGGCAGCAGCAAAAGAAGCAGCAAAAGAAGCATAGGCAGCAGCAGAAGCAGCAAAGGCAGCA
GCAGAAGCAACTGAATCAGCAGCAACAAAAGC
AAAGGCAGCATCAGAAGCAGCAACAAAAGCAAAAGCAGCAGCAACAAAAGCAAAGGCAGCAGCAGAGG
CAGCAGAAACGGCAGCAGAAGCGGCAGAAGCA
GCAAAGGCAGCAGCAGAGGCAGCAGCAGAGGCAGCAAAGGCAGCGGCAGAGGCAGCAGCAGAAGCAGC
AGAAGCAGGAGAAGCAGCAGTACATGCAGATG
CAGCAATAATATTAGCAACATCAGCAGCAGAAGCAGCAAAAGAAGCAGCAGAAAAGGCAGCAAAAGCA
AAAGCAGCAGCAGAAACAGCCGAAGCAAAAGC
AGCAGCAGCAGCAGCANNNNNNNNNNNNNNNNNNNNNNNNNNNNNNNNNNNNNNNNNNNNNNNNNNNN
NNNNNNNNNNNNNNNNNNNNNNNNNNNNNNNN
NNNNNNNNNNNNNNNNNNNNNNNNNNNNNNNNNNNNNNNNNNNNNNNNNNNNNNNNNNNNNNNNNNNN
NNNNNNNNNNNNNNNNNNNNNNNNNNNNNNNN
NNNNNNNNNNNNNNNNNNNNNNNNNNNNNNNNNNNNNNNNNNNNNNNNNNNNNNNNNNNNNNNNNNNN
NNNNNNNNNNNNNNNNNNNNNNNNNNNNNNNN
NNNNNNNNNNNNNNNNAAGCAGCAGCAACGGAAGCGGACGCAAAAGCAGCAGCTGCAAAGACACCAGA
GGCAGCAGCAGAAGCACTGCGAGGTACAGGAG

TCGGAGAAGACGAGGTAAAAAAAGCAACACATGATCAGGATAATTCAGTTGAACACCATTCTGGAGAA
AAACAAGAGCTTATAAAAGAAGAGGAACGACA
AGAAAAAGAACAGCATGAAAAGCAGCAACACCAACAGCGTGAAAATTCCGCAGGAAATGGTGAAGAAT
CCGCGAAAGACAATACTGCTAATGGTACAAAT
GCAACCGCAATTAAGGACTACAGTGACAGCAGCACCGCGGTCTCCCACACCACCTCCCCTCTTTTGCT
TCTTCTTGTTGCGTGTGCGGCTGCTGCGGTGG
TGGTGGCCGCGTTAGAGTGAGGGAGAGAGAGCCGTGCACCGCCCGCACACACACTCGTCCTTCTCTCT
CTCTGTGTGTGTGCCCCTTGCATGGACTCTCG
CCCTCAGCTCACCCCACGAGGCACACACATAATGTGCCCATTGTATATATGCATGCACACCCATGCAG
GCCCGTTGGTGCTCTCTTTTGCACGACACGCA
CCGCTGCCGTCCTGCCTGGCTGCATGGGCGGAGCACCGTAACTGTTTCTGTTTTTATTGCATTGAGTG
GGCGACCTCTCTCTCTCTCCCTGTGTGTGTGT
GTTTTGCTTTACGGGAGCAATGAGAGTGTGTGCGCTGGTCGTCCCACGCTTCGGCTTTTTTTTTTTAC
TCGTA
>scaffold1025.size7371
acgagtgggcggcataagtaggcgtcgCCAACCAGTGTCATTGGATTTGACTCCACAAAGAAAAAGCC
GTCCACCAGCGGAAGGTTTGTGGCCCACGGTTT
GTGCAGCACCCCGTACTTTATTTTACGGATCGTTTTTTCATATTCACTCGGCAGCAAACCCTCATCTC
CCTTGGGATGCAATTCAGGGCCAACTTCAAGA
CGCAGGGCGTTGCTTGACGTCCTGTTGGCGGCTCAAACTCCGTGAGTTTCGTCGCCATTGCCTTGACA
AAGGCACTCTCCGTGAACGATTTCAAAGCAAA
CCATCCAAAAAATATGGGCAAAATATTATCCTTCGACGCCAACATCAGCCTCAAAAGCTATTTTATTC
TGCTACACTTGGGAGGCGTTCCAATGTCCGTT
CCTCGAGGCAAGCAGACAGCGGTGCATTGAAAAGGGTCACACCGCCTGCGTATATTTCCCGAACAACT
TTCACAAGCCTGTGGGACGGATCCTCAGAATA
CCACGGCGACTCACCTCCCCGTGAAAAGTGCTCTTTCGGCATTACCTCGTTGATCGATTGCAATGCAA
ACTTCACCGCTCCACAGCGTTCCTTAAACGCC
TCCTCATCAAAGACGTGACGTGGAATCGGTCTCAAAAGATACATTTGTTTTTTGACCCCTTTCCAGCA
CTCCGCCTGTTTCTCCGCAGTCTCATCACGCA
ACATACGGGCGCACATCGCCTTCACATCCATCTCGTCAGGGCAATTCACGAAGATTCGCGCGGCTTGT
AGTTGCTTCCCCCACTCATCATAGTTGGCTAC
GTTTGGGGAAGGCACCACAATCATGCCCCATCCAGAAGTAGGCGCAAAATGTCTCGGTGGCGGCGTTC
CCTCCTTTGCCACATCGTAGATAATATACACA
TTCCTCCCATGCCCCCTCAAACTTCTCAACTCCGAGATGCACATGTCCTCATCGCCGCATCTTGTCAC
CGTCCTGCTGGTCTTGTCAGACACGTACGCAT
CTCCTCTCCAAAACAGTGGACTACCACTTGGAGTTTCGTGGCATCGCAGTGCAGCAGCTGGTAGAGGA
GGTACGAGCCGGCATTCACCGACTTCCCGATC
CCGGGCGTCCCAATCAACACACGCCGCTTTGGCTTAAAGTCGGTCCCACGGTGACTGCTGGACCACGC
GGTTACATCGCCCTTGACGGTCTGCCATACTC
GCTCCACCTCACAGTTCACAAAAACGTCCTTAGGAAGATCCTGTATCGTATGCCACGAGTACGGCCAT
CCCTTCTCCGAGGCAAGCACCATCAACACGGG
AGGTGCTGAACCACATTGCTGCACAGCGTCATCCTTTTCGAAGGCGTTGCCAACTTCCCTGTACGACC
ATGACTGTTCTGGCTTCCCCTCATGCACCCCC
ATTCCCGTCCCCGTTTTAATTCGCTCATCGTCGTCCGGAAGTTCCACCGCATGGCTCCGCCTCGCATT
GTGCACAGACTTGCACACTCCCTCCAGTTTCT
CCGGTGCGCTCGTCGTCGTTGGGTTCCTCGCCTCCTCGGCAGCCGCATCCAAGAAACGTTTGGTGGAG
TTATGAACAGCTGCTTTTACGTCCGCAGCAGC
CAATGACCGCCCCGGTAGATTGTTGACACCCTTATCGCATAGCCTGCGTGTATCCTCGTCGAAGACCA
CTTCACCCTTTACAGCTCGCTTCATGCCCAAA
TAACGACCTGATGCCTGCATTTCGTTCAACGCTCCTTTATCACGGATATACCTCGTGGGGTCCCTGAA
AAACTCCTCCAGCATGACATCCCGATTGGTTT
CCGCAACGCCCCTGCCGTCCAGTTCCCTCGTAAGAAAGTCGTTCAGCCTCATTTCGTTGACGCGAGCT
CTTCCCTTAAACAGCACCTCTTCGATATTAGT
GGAAATGGCAAATTTCATTTCTTGCGTTCGTCTTAGCCTTTCCTCTGCCTCCCGCCTCGCTTCTGTCA

GTACCTGCGTAAGCACTCCGTTTGGTGTTCTT
CTTGCAAGAGGAGTGATCGTATGCTTTTCTTTCATAGTCCCTACGTTGCCCGAGAGAGAGAGCACACC
TTCGTGAAGAGGCCTAGTAATGGCCTCCAGCG
CTTGGTGCGCAGTGAGGTTAAGAATTTCTTCAAGAGGCCGCTGGTCGTGTACGTAATCATCCGGATCC
TGAACAAACACCTCCATCGTCACATTGTAGTC
TTTCACCAACAGCCGCCCTGCCGCCAGCATAATTCCAAAGGAAACCACTCAGCCTCATGTTGGTGCTG
CTCATGGTCCACTGCGGCCACACTTTACTCTC
CACCACATGTCGAGCTGCAGCGTCGGAACCATCACAAGAGCCGCTGCAAGGTGCCATCACAACACCCG
TGCGGTGAAGCATCCCACTCGCACCGCCCAAC
TGCGGCATGCAGGTGCCGCTGGCTCCAAAGGACACTCGCGGCTGTTTCCTCCCGCAGTCCCAATGGCG
TTGGGCGTGGCACTCGCACGGCAGGGTGGTCG
GTGCGCCATGCAGCCGCACTGCAACGCCTGTCGGTGAAGCAGCCGAGGCCCACCGCCCCCGCAGCAAC
ACGAGGCGTGCAACCGGCAAAAACACCGCCAC
ATCATCGCCCTTTTTCATTTTGTTTTATTTACGTTTTCTGTCTGTTTGCCCCATCACCGACACCAAAA
ATAGGTGCACACATTCACAAAAGGGAAGAAAC
AAAGAGAAAAGGAGGCACGATCCCACCCTCCCGCCGCGGGCCAACACTCTCGTCCACCCACCACCATT
GCCGCACAGGGGACCTCAGTCACATCGCAGAG
TCAGCACCACAATGTGCACCACATTATCGTGCAGTGAACAATGAGCGAGACGCAGCCATGCATCGCCC
CGCTCCATTTTACCACCACTCAAGTGTTGCGT
GCCCGCACAAAATAGCAAGCACAATCAGAGCGCACACAAACCCAATCAACACCCTCACTCGCTGAAGC
ACATTATTCGCATGGAGTGACAGCATTTGGGG
ACACACCAGCAGTAGCAGGGTGCATGGAATTTCCCTGCCAACCCCGCGAAGAGACACAACAACTCTGC
GGAGTCAAAGTGGCAGCCCCTCGGTGATGCCC
GCAGCTCACATGGCAGGAAGAATCCCACGCACGCATAGTTCCGGACTCCTCGCTGTTTGAGTGCACAT
CAATAAAACGTAAAAAAAAAAATACCTGCGCGC
AAGATGGAATTAAATATGCGGAAATGTCGGCCTGCTTTCTCCGATGGCGTTGGCGAGCAGTCCCACAC
AATCCAATCAAGGTAGCACGCCGGACAGAATG
GCGTCCGTCAATCAATAAAAAAATGCGACACTGAAAAGGAGCAGAGACGAGTTGGCGCATGCGATTGC
CAGCGACAACTGTGAGCAACCCGCCAGCACCA
ACTGAGGTGCCGAGGAGCACGCTTGTGTAGAACTGGCCGTCAGGCAGAGAGGGCAAATAAAACAAGAG
GGCAGCAAACCCTCTCGTTCAATGTCTCCTGA
CCACGACACCAGAATCAAAATACCATCGACGATAAGAGCGGCAACGCCTTCTACAAAACATCGTGTCA
AGAAGCCGCAGGACCTCAGGAAGGGAAGAAGG
CAGCGGCGGCAGCCACAGCGGGGGAAGCAATAAAACGGCTGGCAGGCACCCTCACGTCGACGCGCACA
CCGCATCCGAGTGGGCCGGGGGCGCAGACCGA
ACCGCTCGGAAACACACGCATCAAATCCGCAAAACAAAGTCGCAGTGAATCGACAGGAACTCCCCGCC
CAAAACAAATTTATAAAGTAGCGTTTTTCAGG
GGGATCCGCCTTTCCGACGACGGCACGGACGCCTCGCAGGAACTTCTTGCAAATCTCCCTTCGCAGGT
CCGCATGAAAACGCAGCGGAATAAAAAGGGAG
CGCAACGACGCAAAAATAATAATCCTCCTGACCGACCAGGTGGCGGGCCGCCCTCGCGCAAGTGGACA
AAACAAAACTTCACTCCGGCCTTGTCGTGGTG
GTCGTCTCGCAGCATTTTCTTCCTTCTCGTCGCAGTGCACATCCCCTCCCACAAATCATTTGTTCGGG
CCATGTTCCTGCGGCCAAACATCGCGCTGCTC
GATCCCGCGGCACAGTCCTCGGCACAGAAGTGAGGGAAGAAAGACCGCAGAACGTGCGAGCAAATCCG
AATTCGGTGGCGGTCGCTGCAGTGAAGATTGA
CGTCCCACGCGGTGTCCCTTTTCTGGGTCCGGCCCTCACCGCGCCATGCTCTCAGCGTGCGGTGCGCC
GGCCGCTCCAAACATCCACACATGCTTGCGTC
TCACAGTCGCTCCCAGCCCCGTTCGTTGGCTGCATCAGGGATGCCGCCATCCGGAGACGGGCATAAGG
GCGTTGCATCCAACAAAACCTTCAGAGGCACC
CGCTGGGCCAACGACGACCATCACTGTGGGATGTGTGGCTCCTTTCGCCGCTCACCTCAAATCGCACT
TCTACGCCTCCTCGAGCAGCAGCAGCAGCATA
AATGCACCGGCCCACTAGGGGCCCTCACCAGCCATTGCATGAAGACCACTGTGCCCCAGTGCTATGAA
ACCCAAACTGCATTGACGGGCCACTTCGGAAA
ATCCTGTCGCATCCTCCCCATTATGCGGAAACACATCCGTCGTGCATGTGTCCACCGCAGCCAAATGG

ATCCGGTCCAAATACCATCCAAACCAAATTCC
CCTTTCCTCAAGCCATTCCTTCATTCCATGCGCCCTGACCCTCATCCGCACGTGCCCACTCACACAAG
CCGCAAAAGAATGGTGGTGGCCGCCCAACGCT
CCGGAAACACATGCACAAGCGCACGGCACAACGGCGGAGAAGCGCACACCTAAAAGGATGCGGCCCTC
CACAGCAGTCGCAGCCCATCGCGCGCATCACA
CCCGCAGGAAGAAGAGTGACTCGCCGTGCGCTCCCTCTCTCCTCCCCTCGCTTCATGCCCACAGAGGA
AGCAGCAGCCACCCGCAACCGCCAGCACACGC
CACAAAAGAAGCATCCAAATCCACTGCAATNNNNNNNNNNNNNNNNNNNNNNNNNNNNNNNNNNNNNN
NNNNNNNNNNNNNNNNNNNNNNNNNNNNNNNN
NNNNNNNNNNNNNNNNNNNNNNNNNNNNNNNNNNNNNNNNNNNNNNNNNNNNNNNNNNNNNNNNNNNN
NNNNNNNNNNNNNNNNNNNNNNNNNNNNNNNN
NNNNNNNNNNNNNNNNNNNNNNNNNNNNNNNNNNNNNNNNNNNNNNNNNNNNNNNNNNNNNNNNNNNN
NNNNNNNNNNNNNNNNNNNNNNNNNNNNNNNN
NNNNNNNNNNNNNNNNNNNNNNNNNNNNNNNGAGGAAGCAGCAGCCACCCGCAACCGCCAGAACGCACT
AAAAAAGAAGCATCCAAATCCACTGCAATGTG
CATCTTGTGCGTGCATGTGGTTGTGTGTGCCTGCAGACACACGAGGCAAAGAAGTAAAAACAGGAGCA
GAGCATGTGAAGGGACGCACAAACAAAAATGT
ACAGCAGAAGTGCATGGCAGAAATAACGGGACTGCCCAAACTTATGATACTGCACTGCAGACCAAAGC
AGCGGCAATTCATAAAAAATTTATTAAATTTT
AAAAAAAATCATCTTCATTCCACACCTCACGCTCTGGAAAGAAAAAAGAAAACAAGAAAAGATAAAAA
AAGAAAAATAAAAAAGATAATAAAAAAAAAAA
TTGAAAAAAGTCGTCCTCTATTACGGCTACACACAAATAAGAAAAAAGAAGAAAAGAAAAGAAAAACC
CATCACACAAAAATTCACTGGTTACTGTGTGC
GCCCACCAAACTGTTTGTATATTTTGCCCGCACATTAAAAATTAGATATTTAAATGTCTTTCAACCGT
CCGACCCTCCGTGTGCCCGCACATCGTTTATA
TTTCATGGGTCATCGGCGTGTTGCTGCTCCCCTCAGTCCTTTTATTGGTGCGTGAGTGCGGCCCGCAG
AGCACAGTCACTCGTGTGTGTGGGGACTGAGA
GAAGCACGCAGACATGACGGGAGTCGCGTCACTCCATCGGGGGAGTGAGCGCAGCAAAAAAAAAACAA
AAAAAAAACACGGCGGCAGGGTGTGGCTGTGT
GTGTGTGGCGCTGCGGGTGTCTGCCAGCCCTGGCCGCAAAGAAAACAAAACAAGAGGGAAGAAATGTG
TGCTCCGCCCCACAGGCCCTCGTCGCACGCCC
ACACGTCACGGCTCCCTCAGCGCGCCGTTCGTCGCGGCCCCTCCTCATGCGTGCAGCAGGCGAGGCGC
ACAGGGCCGTCGTCGTGAGCGTGTCGGTGGGT
GTGCGAGTGGAGCAGTTGGGCGGGAACACACGGCACCCCTCACAGGAAGAGTGCGGAAGAAACAATTA
AAGAGGGAAGAGTAGAAAAGAAAAAAATCACT
TCCTTAGTGGTTGCCCCAAAAGCAAAAAGAGGGGATGGGGAGTTAAAAAAAAAATGAAAACACAAAAA
AAAATTGATTCGGCAGGAATTCCATGTGCCAT
GCAGCACAAGAAGATCGGGGGCGTATGCATATTCCCTGCACAACAAAAAAGAAAACAAAAAAAGGAAT
TGTACCCTCCCACCAGCCCACACAACATCCTC
ACATTCCATGCGGCATCCGGCCTGCTCCCCCACTCCTCGTTTTGTGCAGTCCGCCGGAAGCGCAGCAG
TGACGCCGGCACCGCGAGTGGGCAACAAAAGG
GAAACAAAAGTCGGAAACACGTCACTCACAACGGAtNNNNNNNNNNNNNNNNNNNNNNNNNNNNNNNN
NNNNNNNNNNNNNNNNNNNNNNNNNNNNNNNN
NNNNNNNNNNNNNNNNNNNNNNNNNNNNNNNNNNNNNNNNNNNNNNNNNNNNNNNNNNNNNNNNNNNN
NNNNNNNNNNNNNNNNNNNNNNNNNNNNNNNN
NNNNNNNNNNNNNNNNNNNNNNNNNNNNNNNNNNNNNNNNNNNNNNNNNNNNNNNNNNNNNNNNNNNN
NNNNNNNNNNNNNNNNNNNNNNNNNNNNNNNN
NNNNNNNNNNNNNNNNNNNNNNNNNNNNNNNNNNNNNNNNGTGATTGGCACCATCATCCCCGCCGCAGCCAC
TCCCAACAGCAACTCACGCACACAGACACACG
GAACAGGAGAAGGAAGAACAGCGCACCGGGAACAAACAGCGACTGTCAACGCAAGGCAGCGTCTACAC
AACAAACGCACACACCGCCAGCAGTCACCCAC
CAATGGACAAGAACGAGAAAAGACCCACCGCCATCATCGCCTCCAGTTCAGTCCCACGGACAACGGGA
TACAGCCACAGCCCGCTGCCGGTACACAACGC
ACGCCGAGCAAGCAGTCACCCAAAGAGCCACTCGCAAAAGCGATTCCGCATCCATCCATGACGCGGCG

>scaffold1026.size6478
ATCGCGGCATCCGCATGCGAGGCTCTCTCAAACCATTGGAACGCCAGGCCGAGCACGTCGGGCATGTG
CCCTCATACTGTGGACGGAAGAGCGCACTCTGC
CGCAGGTTGGGCGGGAGGACTTCAATCCGCCGCTCGCTTGTCGGCAAGGAGTCGCGGTGCTCACGTCG
GGAAGAAGCTCCGGGAGAGGCGGGCCATTCTC
CTCAGTGTGAGAGAGCGGCACCACTGACACAGAGGGTTCCTTGTGATCCGCAGGGCGCGTGCAGGCCT
GCCGAAGAGAGGGCAGGAGTCGCATCGCGCCT
GCGCGCAGTGCCGCCACTCGGGGCACGATTTCCGGGCCAACACCAGGAATAGGCTCCTCGCCGCACCG
ACAAGCGTCTGTCACGCGGTGTACGCGCCTTC
TTCTTCCTCCTGGACATCCTTCCAGCATAACCTCTTGACTGCAGTGAGGAATTCCACTTGCTACATTG
GGATGCCTTCTTGCGGGAGAGAGCGTGCAATC
GTGGCTTCTTCGTCAGCGGCCTCATTGCGTAGTATACCGCAGTGTGATGGGGAAAGTATAAATTCTAC
AAAGTTACTTCGCTTCATTAAGGGAAGCAGCA
CAATCCAAATCTCTTCAGTAACGCGGTCTCGCATGGTCAATGGGCAACGGAACGCCTCAATTGCCGAC
AGGGAGTCCGTTGCCACCAAGATTCGTGTGGG
CGATTGGGATGTTTCTGGACGAGGAGGTATAAGATGTCTAAAGCCACTTTCAATGGCAAGGCACTCTG
CTCTATATGAGCATGCGAGCCTGCCAGATGCT
ACACGATGTACCTCCGCAGGAAGATCATTTGCTGTGGTGTATTCGTACAATATTGCAGCGGATCAATG
TGCGGGTTCAGCGAGAGAGGAGGGACTGTCCA
TCCATAATTCAAAATCACAATCTCCCAGAGAGGAGTGAATAGAATCAAAAGCCGTGCGCCCGACATCG
TCAGAGGCGTCGTTGGCAAAGACTGACTGATA
AAATCTCACTTTGTTGCAGCTCACAGTGTCCCAGGGAAAAAATACGATGTTGAATAAGAGGAGAGCGC
GAAGGTGGTTGCGGGGTGTCCCAATAATGTTA
CAGACGCGGCGTAATTCATTAATAACGGCATCTCTCTGAAGCGGATTGATGTCGCCGCTTTCCCATGC
ATGGACGGCGGCATAGGTTCCGAGTAGATAAG
GGAACGCAAATCCTCGTGGTCATGGAGGCGCATACAGAGTTAGTATTGTGTGAGTGCACGGAGCCAGA
GTATCTTCCGGAGCCGGAGAAGATTGGCTTCC
AAGTAGACAGATGTATCTTCCGTTGCTGCGCTAAATAAAAGAGATGTGTTACAACTGTCGCGGTACCG
TACTTCCATATTATGGAGGTAGTTGTAGGCAA
GGCATGAGACAATTGCCTCGCCACGATATAGTGACACAGTGTGGACGTATCCAATCGAAAAGAATCGC
ATGGTGAGATGGGACGCACCAGCTTGTGCGAC
CAGCTTCAATTGGTAACTGCGTAGCATGGTCTGCTTTGAGGCGGTGGAGGCGTGCGTGCCAAGATTAA
GAAGTTGATCAATCGTGGTGCCCAGCAAGCGC
ACCGGGCATGACTGAATGTGTATTTGCCCATTGCCAAGATGGAGATCGACCATTTCCTCGTCAGACCG
TGTGTGTGTGAAGAGATGTAGAATGCAGCAGC
TTCGCTCTTATCGACGTTGATTTTGAGGTTTTGCTCGGCAGCCCATTTCGATACAAGGGAAAGGGCGG
GTTGCATCGCCGCAGCACATGCATGGATGTCC
GCACCTGAGGCAACAAGTGTGTGTTCGTCAGCGTGCATGAATGCGGCGGCAGAATAGATGTTGTCCAT
GCGGCACATCATCCACACAGTAGAGGGAGAGT
TGGGGTCCCGGGACGCCGCCTTGTGGTACTCCAGCTGAGGTGAGTTGTTTCCGGGAGTGCTGTTCGCG
AGTGCACACCCTAACACGGCAGCCCCGCAGGT
AGTTGTGCAGCCAGCGTTTGGTTCGTGGGCCAAGGCGCGGAAGTCTGTCAAACACGCAGAATAATTTC
TCATGGTCAACGGTGTCGAAGGCAGTCGAAAT
GTCAATCAAGACTACGAGCGAGCGATGGCGACGAGAGTGCCTTGTGGGGGCGCCGCCTCCAGGGCGCT
CATACTCCACAGTGCTGAACTCATTCAATCCA
CGGGTAATTCTATCAATAACAAGTGCCACCACGTCCGATGCAGAACGTGAAGGTGCAAAGCCAAATTG
ACGTGGGTGGTGCGGCAATAACGCTGACAGAC
GGCGGTGAATCATTCCTTCGGTTAATTGTAGTGAAACAGAGTGTAGCGTGATAGGTGTGTGACTCTCT
GGGCTGCATGGACCTTTTCCCAGGTTTGGGGA
TAGGAATAGTGTCCACCGTATTCCAGGAGCCAGGCAGGCTGCCATTTGCGAAGGGGTTGTGGATCATC
GTTCCAATTGTGTGCCGCACGCAACGGGCCCG
AGACGGTGCAGGAATTCATCCAACATGTTGTCGGGGCCAGCGGCGCTCGAGCCGAGCGAGGAGTCACG
GATGGTCACGTCAATTTCATACGGCGTGAGAG
GCCGATCCATTTCCCGGTCACCAccttcgttTGGAGCGCGGCGGACAGCAGCAGCATAAGGTGATTTG

GCACGTGAAACCGCACAAAATGATGGACAAGC
AGATTTGCCTGCTGCCGATGCAGAGCGCAAACGCGGCCAAGTTCAGCGCGGAGCACAACCGATTGCGG
AGGATGAGGGTGCGGCGCCGCCATGCCGCGGA
GATATCGCCAGCTTGGGCTGGATGAGGCCCCAAGTTTTTTGCGCGCGGGCTTCCAGAGGCATAGCGGA
GCCACGGCGTAAGGCCTGATTTTAGTCCCCTC
TTTTTCAAGTAAATTCGGCTCGAAGTAGACTGTCTCCGGGTGAGGCAATGTGTGCGTTGTATGCAGCT
TCGGCGGCGATCTCGGCTTGCCCCATTTCGTG
TGTCCATATCGTTTTGGGGCTGTCACGGCTGCCGCGCGGGATATGGCGCTCAGCTGCTCGTAGGATGC
CTCTGCGGATGTACAACCATGTGGGCGCGGTG
GCAAGGAGTGCTTCACACGAGGTGGAAGCAGGCCAATCCGCTTTGCGCAGAGCAAAGGTGGCTTCCTT
CCGTCGAGGCAGCGTGTTGGCTAGTCGCGGAA
TGCCATCGCCCATCCCTACCGAGTAAGATATTCAGCAGCGGTCCGAATCCATGCAGGGAGTGGATGTC
CGGTGGGAGATGCGTGGGGCGCGATACGCAGT
CACGTCAGAGGACGAGACATCACGATGTGTGATCCGTGTTGCCTGCGCCGGATCATTTGCCAGTCCGA
AATCCATATCAATTAAAACGGCGGCAAAGTTC
CCTCCTCCAGTCGTTGTTGGTATGTATGACTGTCCCAGAGCTCATGATGTCAATTCGGTTCACCCACG
ATAATCGCAGCGGGGACCAAGCGACGGAGGAT
TTTGATAAATGTAGATTCTTGAACAGTTTCCATGGGAGGGTTGTAGAGATTCGCCACGCAAATGCTGC
GACAGCCCTGATGAGTTGCACGGACAGCAATG
GTCTCCAGGAGGTCAGTGCCAGGGAGCGACAGGCGCTCGCACGGTAGAGAGTGGCGCAGCAGAATGGC
AACGCCACCACCGCGCCCAGTGAGGCCTGAAG
AAAGTCATTGCAGCCAGAAATTTTTGTCGCTACGTCACTGCGATTATTTATTTCCTGCAGAAGAACGG
TGTCAGTTTGATATCGCTCCCATCCCGCAGCA
AAAGCAATCTTTTTACGGACGAGAGGCCTGCAATGCTAAGCCGCCGAACAGTGATGTAGCCATCCTCC
TCGGTGCCAGAGTTCTTGTGGACGTCACCGCA
ACGGGTGATATCAACGGAGTGACCGCTGCGAAACGGTCGCACAGATTTTCCCGACAGTCTGCAGGCAG
ACCGCCTCACCTCCCCGCACCGGGCATGGCGT
GGAACCCTCTCGAGGGGTGAAGTCGTCTTGGTCTCCGTCGTACTCGCAGCAACGCCCTGCTGCGGAGT
TCTTATGTGCAACCAGAGCCCCAGCACCGCGA
CAATGTGCGAATTCATTGCTGGGGCCCAGGAGAGGCCGCAGTGCCTCCTGCTGTCATCCGGAATCTTT
GTTGACGCCTTCTTGCGGACAAGCGCCCATTG
TTTCTTACAAACAACGCTGCACACCCGCGACGCATCTTGCGCTGTGAGAGGCACGTCCCTCACCACAA
CCGGAGTCGCTCACGCGCCGTCGGTACGCACC
ACATCGTCAAGAAAAGAGCAGGAATGTGTCAGGGTGACAACGAGGCACCCGCTGGAGAGTCGAATCAC
AAACATCACCAGCAGCGCTGCAAGCCACGGGT
TAACGTGGAACAACTGACGCATGGCGTATGTCGAAGGAAAACTTAGTGCCTTCACAAGAATAAGAAAG
AATAAAACTCTCCCAAAAGAATATGAGAATAA
CAATAAAAGATTGAAGTGCCCGCGATCAAAGCATGCCGCAAAGCAAAGAAAAGTGGAATGGGTGCACT
CAACCACAAAATCAAAAAGGAAAACAAAACAA
AAAGCCGAAGCGTGGGACGACCAGCGCACACACACTCTCATTACTCCCGTAAAGCAAAACACACAAAC
AGGGAGAGAGAGAGAGGTCGCCCACTCAATGC
AATAAAAACAAAACAGAAACAGTTACGGTGCTCCGCCCATGCAGCCAGGCAGGACGGCAGCGGTGCGT
GTCGTGCAAAAGAGAGCACCAACGGGCCTGCA
TGGGTGTGCATGCATATATACAATGGGCACATTATTGTGTGTGCCTCGTGGGGTGAGGTGAGGGCGAG
AGTCCATGCAAGGGGACACACACACAGAGAGA
GAAGGACGAGTGTGTGTGCGGGCGGTGCACGGCTCTCTCACACTCACTCTCACGCGGCCACCACCGCA
GCAGCAGCCGAAACAACAACAAGAAGCAAAAG
AGGGGAGGTGGTGTGGGAGACAGCGGTGCTGCCGTCAATGTCGCCAGGCGTTTGAGTGCCTTCGGTGT
TCGTCTTATTCGGTGCTCCTTCCGGGCCTGAA
GTTGAATTCCTTTTCTCTTGACTGACAGCCGGCATCCCCTCTGAGGTGTTGTCATCATCCTTTTCTTT
ATCCTCTTCATCTTCTTCCACCCCATACTTGG
CTTCTTCTTCCTTGGTGTTTTTCTCACTTTCTTCTCCCTTTTTATCCTCTTCCCTTTCCTCTGCCTTC
TCTCCCTCTTCTGCATCCTCTTGTTCATCGTC
TTCCTTGTGATTTTCTTCTTCCTCTTCGCTGTCATCTTTCTCCCCACCTTCTTCTTCTTTCTCTTCTT

TGGCGTTTGTCTCATTGTGTTTTCCTTGAACT
AAAGTTTCTCTTGCAGGCGGCACAATAGCAGGTGCAGGATTCACTGACGTCTGTTCACTTGCCACTGC
TGTTTTTTTGCCTGTAGATGCTTTTCCTGAAG
TCGCCAGTGTTTCACTTGTTTTTTCATCTTCCACCGTGTTGGGATTCTCTTTTCCACCACCTTCAGCA
GCAGCAGCAACAGCAACGGCGGAAAGTTGACG
CCTCCAACGGCTGCGGCCATCCACAGCAGCATGCAAACTCTCCATCACCTGACGCACGCATTCCTCGA
CGGAGGACGCATTCGCACGGCCTCCTGTCCTC
CTGCCCACCTCCTCCGCGCATTCCGTCAGAAACCGCGTACGCCACTGCAACGGCAAATACTCACCCGC
ACTGCCATCGCCACCGCCAGCTCCATCCGCTG
ACAACACCGCCACCGAAGCACCGCACCACAGCACGCAGAGGGCACACACCAGCAGCACACGGCCAGTC
ATCATCATCGCCATCACTCCTGTCACTTGCGT
GCAGCCTTCAGCACAAACTAACAACTCCCCGTCAACGCAGAGCAAAAGCAAAAGAAGACACGGCCACT
ACACCACGCACAGCCACCGCATGGTAACAATT
GGAAGCAGCCCAGCAGCAGCGCACACACCACAAAAAAGTTGGAAACAGTACGATTGCAGAACTTGCCC
ACCGTCCTGTAAAACAGATGTAAAAAAAAATA
AAAAACATTAATTAGCAAGCAAAAAAAAAGTTTAAACCCACACCCAAGACTCCAGTGCGCATGCATAC
AAGTATGTCCGCGATGCGTTCAACGTGGCGGA
TTTGGGGAAGCAACGCGGAGCACACAGCAGGACATCGGAGCAGCAAAGCAAAAAACGATCATAATAAT
TACTCTTACTTTATACATCGGACCACAGTAGA
CGCACACATTTCCCCGTGGCTGAGTGCTGGCACGCACAGCCCGCACACACCTCTTCAGCCCACAGTGG
TGTACGCCAGCGCGGATACGGCGAGCAGTAGC
GGGCCACACACCCACGCAGAGCTGCCGAGGCTGCCGTCGATTACGCGAAGACGTGACGGTGCGCGGGT
GGTCGTCGT
>scaffold1027.size7251
GGGAAGCACTAACAGGACCGACATGAGGCTGAATGATTTTCTTACGATGGAATTGGACGGGAGGGGCG
CTTTGGACGCCAATCGGGATGTCTTGTTGGAGG
AGGTTTTCAAGGATCCCAAGAAATATATTTGTGATGCGGGAGTATTGAAAGAAATGCAGGCATCGGAT
CGTTACAAGAGGATGGAGAGGGCTGCGAGGGA
TGAAATGGACATGGCGGAGGATGCAGACAAGCTTTACAATAAGGGCATGTACAATCTACTTGGGTGGC
CATTAGCTACTCCGGAGGTAAAGGCAAGTGCT
CATGGAGCTACTAAAAGTTTTTTGGATGCAGCCGCCGAGGAAGCGTGGAACCCAATGACGACGATTGC
ACCGATGTACCTGGAGGGATTGTACGAGTCTG
TGTACAATGCGAGGTGGCATCATGTCGTGGAAGTCCGTGGCGGCGAGGGGACGGGAATGAGGGTTGAG
GTGGGGAAGCCCGAACAGTCATGGACGTACAA
GGCGGTTGGCAACACCCTTGAAAAGGATGACGCTGTGCAGCAATCCGGTGCAGCACGTCTCAGGCTGA
TGGTGCTCACCTCGGACAAGGGATGGCCGTAC
ACGCTGAATGGGCCGCATAGGTGTGGTAATGACTTGTGTGTTAACTGCGAGGTGGATCGAGTGTGGCA
GATCGTCAAGGGCGATCTAAACAAGTGGCTCA
GCAACTCTGACTTGACTCTCAATCCTTCACCTTTGCTTCGTCTGTTGATTGGGACGCCCGGGATCGGG
AAGTCGATGGCTGCCAGCTCGTACCTCCTCTA
CCAGCTGCTGCAATACGACGTCGAGAAACTCCCAGCGGTTGTCCACTGTTTTGGAACCGCGATGTACG
TGTTTGACAAGACCGCCAAAACCNNNNNNNNN
NNNNNNNNNNNNNNNNNNNNNNNNNNNNNNNNNNNNNNNNNNNNNNNNNNNNNNNNNNNNNNNNNNNN
NNNNNNNNNNNNNNNNNNNNNNNNNNNNNNNN
NNNNNNNNNNNNNNNNNNNNNNNNNNNNNNNNNNNNNNNNNNNNNNNNNNNNNNNNNNNNNNNNNNNN
NNNNNNNNNNNNNNNNNNNNNNNNNNNNNNNN
NNNNNNNNNNNNNNNNNNNNNNNNNNNNNNNNNNNNNNNNNNNNNNNNNNNNNNNNNNNNNNNNNNNN
NNNNNNNNNNNNNNNNNNNNNNNAAGTGGTTG
TCCACTGTTTTGGGGATACGGCGTACGTGTTTGACAAGACCATCAAGACGGTGTCGGAATACGAGGGT
AACAAAACATCGAAGAATGTTTTGTATGGTTT
GCGGAGGAGTGGGATGAAAGGGTATATTATTTACGATGTGGAAACGCAAGGAACACAGCCCGACCCAG
GTTTTGCGCCCTCCACCGGATGGGGCATGATT
TTGGTGTCATCCCCAGAGGTAAGCAACTATGATGGATGGGAGGAGCAATTAAAAGTCAGTCGAATCAT
CATGAATTGCCCCGACGAGACGGATGTGAAGG

CGATGTGTGCGTGGATGAAGCGTGACGAGACCAAAAAGAAACAAGCGGAATGCTGGAGGATGGTCGAA
GAACGCATGGAAAAAGTGGGGCCGATTATACG
TCACATCTTTGATGCGGATGAACATGGAAAACGCACGAAGGATGTTATGAGGGCCTTGGAATGGATCA
ATATTGGGGATCGAGGAAAGTACTTTACACAG
GGAGGAGAGAACGAATGGCATTCCGAGGATCCGTCCCAAAAACTTCTGAAGGTTGTTCGAGTGAGAGA
ACATGGCCCAATTGAGGACTTCGCTAATGCAC
CTATATGTGAGTATCTTGGGGTTTTAACAGTATCTCGTTTGGCAAAGGTGCTGAGTCCGCATGATATT
CTTTTCCTCGTATTGGGGATGAAGAATGCTAT
TCAATCTGCAGATTTGAAAAAGTATGCTTTGAACGCATTCTTGAATGAGGACTTTGTCACTTCCATGG
TGAAGGATCTCAAGGAGCTGCCGCCACCATCG
CCTTCCAAACCACGGCCCTCGGTGCTAACGTTAAACCCTCATGGTTACCCCACCGAGGCAGCTGCAAT
AACTGAACTGAATTTAATTGATGGCCTGCAAA
AATTAAAGCGTCGGCTGCTGTACATACCGACGTCCCCAACCTTTCCGCTGGTGGACTGCTTTTTCTTC
ATGGAGTCACCGCGGAGGACACTGGTTGGGCT
GCGGATGACGACGGCGAGTGCGCATCACACCACCGCCAGCACAGTGCGGCAGTTCACTGAGCGTCTGG
CGGCATATTTTGAAGGATGGGAGGAATTTTCT
CAAGGATTGTCGTGGGAGATGATTTATGTGCAGCACGCAGACAGCACGCCGATGAATGACTGGCAGAG
ATGTGATTGCGTCAATCCCAACAATAAAATTG
ACGCTGAGAAAAAAATTGTGGCGTTCTGGGACGGGAAGGTGCACCAATACCGGGTTATGTTGAAAGGT
AAATTTCCAGAGGAAATCAAATTTTCTGATGA
TAGTCTACCAGAGGGGGATAAAAAAAACAGGAAAATTTAAAAAATAATTGGGGAACAAAGTGCATTTC
GTTCTGGTGAAATATATTGGGAGTATTTTAAC
GATTTTTATGTGTGTGTGTGTGTGCCTACGGGGTCTAGACAGTGAGTGCCCTTCCTTTCAGAGAACCG
TTGTGATCGGCAGTGCCACACTCACCACACCC
TCTTTTCATTTTATTTTATTTTTCCCACCAGAGGCGCATCGTCACTGGAGGCTGTTGCGCACTACAGG
GGCCGCAGATTTTTTTTTATTTTTATGGGATT
TTGTGAAACGAATGCCTCTGAGGACGGCTGGCTGCCCAAATAAATTCATATCTCGCGCAAACGAAGAT
ATGTCCTCACAACTTTTTCCTCTTCTATCGCA
CTGTCTTTTGGGAGACCGCGATTGAGGGAGTCAGGGAGCCGACAGCTGCCAGACGAAATTATTATTTT
TATTTTTTATTAGGAGCTTGTGAAAAGAATGA
TCGCGGGAGAGCTGGCTAACATAATTGATGTATGTGTTTATCCTGATAAATGAATGCATTCTTTATGA
TACTTTCTACCGTATGAATCTTTTGGGAAGAA
CGCGACTTTGTAGGGGCAGGGAGCCGATAGAGGCCGGATAAATTTTTAATTTTATTTTTTACAGACGG
CCAGGTAAGTTCAAGGAAATGACTTTGGTTCC
CTGCACACCCTCCGAGTGGTGTGACATTGGCGCCCGTCTCTGGGAAATGGTGTGCCAATGTGGCGAGA
CCCAGGTGCAGATGACGTTCATTAATGGTAAC
CAGCTGTCGGATGAGACGTTGACCGTGGCCCCATGATCCACAGGCTTATGCCTGTTCCATGGACCCAT
CTCTAAAGCATGGCAGGATTCCACTCTTATTG
GACATATTGCGAATCTACGGGCGCCTCAGATCCCTAATTTTGAGAAACAAGTGCTCCTGCCCGCTATG
GGGGTAGCCCGGGAGTTGGGGCACCAACAATG
GCTCATCACTGCTGAAAGGGAGCGCCTGAATTTTGATTACGCAAACGCCGTCGCGGACGTGCAGAATC
GAAATGCTCAGCTTAACTCGCAGGTTGAGGAG
GCGCATATTAACCCATCGGTAAGGGAATCCACTATCGCCGGGGCCGGCAACCAGGTCCTAGAACTACA
AAAGCAGTTGGAGGCCGATCGCGCCCAATTAC
GGGAGCGCGAATTCAACATGGACGACTCACTGCTCAACTCCAGGAACGGGAGGCACGCCATAATGCA
CAACAGCCCGCGCGGGATGCGGAGACGGCGGG
GCGGGACGAGGCCTCCGCTACTCAACACGCTGCCAACGATGGCGTAACCACAATACCGCACCAAACGG
ATATCTTGATGGCTGGAGGCGAGCCTAGCAGC
GCTGCCCGACGCAGTTCGGCGGCTTACAGACGCCCTCACGGCAATGTTGGGAGACCACATTTCCACTC
TTCCACGCCGTTGCCTTCCCGCACTTCTCCCC
TGGACGCCCAATTACCCATGGAAGAGGACCGCAGTTTCTGCGCACTCGACGTCACCACTTGGGACCCA
CGCGCATATGGAATGAGGGCGCCTCCGGAGTC
ATTATCGACCTCCGGTGCGCATACCAATGCTGGCAACAGGACGACGAGCAGGTTAAACTTGCCTTTGA
CAATTTGGTCGAGTGGACCGGCGCGCTGGAAG

TCCTGCAGGATCCCACCGCTTGAATCATGAATTTGGGGAGTTCCCTACGGAATACATTCCGGATGCAG
CTCACGATTGCATCCGATCCCGGCATCCGGCT
TTCCAAACTGCGCGCCCGTCCTTACACCGCTGTTCACCAGACGGACACCTATGCGAGGGCAGCCCAGC
CTCTCGTGGATCGGCGAGAAACCCAACGCACT
TTGCGATGCCAACGTTGTCATATCTTTGGACACGAGGCATCTACATGCAATGTGCGCCCCAGAGGGAA
TTAATTACAACACAGGCGTCCTTCAAAAAACG
GCCGAGGGATGCCGCACGCCACTCATGAATACTGACGACGGCAGCTCCATCCATTCCACCACCTCCCA
CCAATCCTCACAGGCAGCGCACCGCACCGCCG
ATGCTCCACCCGATCTTTCAAATTGCCCGACACAATCGTCGAGAAGGGCGATGGGGCTTGTCTGCCAA
CGACGCTCAACTGTGGTACGACGAAACACCGA
CATCCCCGCTTTGGGCCAGTTATATGAGGAGAGCGAACGAGGTCGCTCGAGAGATTTGGGCGCCGGCA
ATGTGGGAGCAGAGAATGTCCCTAGCGGGGCA
ATTCACAACCTTTTGCCGCACGCACGAACAGCCGATGAACGAGGAGAGCTGTGCGGCCTTTTTGATGG
CAATCGAAGTCGCGCCATCGACCCGACAGCTG
CACACCAGGATGCTGTGGACCATGTTGGAAATGAGTCGAACCCCGCTGGACATGGTGATTCTGGGGTT
ACAAAGATTGCGGCGCGATCAGAGACGAGGCA
GGTGCGCTCATCGACAAAGGAAGGGATGAATCAACTTATCCGCAGCCGGACCGATTGGAAGGAACGTG
TTGTCTTACGACTTGCGTGGATAACGGCGAAT
CGTTGGTCCGAGATTGCGGCCCTGACTCCCAAAAATTTCAAACTGAGGACGGACGGGACCTTAATTTT
GGACTGGCCCGTGGCGCCGAAGACGGCGAGGG
CGGATCCCCACCGCGCCTCGCGGTTCGTGAGGATACGAGGGCAGGACGCCTTAGACACAACGAAAAGC
TCACGAATCTTACGACTGCCCAAATCGAACGA
GCCTTGGTTCCTTGGGATGCCACGGCGCATCCCACAAAACGCGGTGCGTTGAGACACGCTGCTCAAAT
CGTAGAGACGCACAATTTGGATCCACACGTGA
TCTCGCAGTTGGCGAAGCACGTCAATCCGTTCGACCTTCCCCAGAATACTGTTTGATATTTGGGAAAG
CACACCACAGTGCTGACCCTGGTGTCGTCGCT
GGTCGCATTGATGTGAAGGGGGAAACTGCGGAAGGGCAGGAACCCTGGCTGAGAGCAGAGGAGCGTTT
TTTCCGTGATTTTAATCACGATCATGCTGCTA
CAATAACGAGACGCGCGACAAGGACGCGTCTGGCCACTCCACAAGATGAACACGCTATCCCGTTACAG
CAGGTCAACGTGCCCGTATTGAACCTAGAGTG
GGTTAAAAGCCGGTTGAACCCGGCCATCTTGGAGAGACTTATGCAGGTTTGGGGACTCGTCGGGCGGT
CCTCTTTTCCTCCGTCTTCCTCCGGTAAAGCG
CGCGAAGGCTCACGGAGGATTCCAACCGCTGACGCGCGATTGTTGAATGAAGCGGGAATTATCGAGGA
CGCTTCGTCCACAATAACAGGCGGATGGATAA
TACCCTTCTCAGTTGTGGAGGAGAAAACCACCGCTTTACGACGACGATGGATCGCGTGGCCACGCGAC
AAGAGCAGAGACGACCCTTACGAGGCTAATGT
TCCTCTTTTACACATTTCCCATTATTTGCCGCCTGTGATGGCTGAGGCCGTTTCCTGCCTGGATTTAA
AGGCATCCTTTTTTCAAGTCTTTTTACCGCGA
GAGACTCGGCATCTCTTTCGATGCCGCGTGGAGGACGGCACGCTGGCGGAGCTGACACGGCTCCCTAT
GGGATACAAAGCCAGCCCAGAAATTCTCCAGA
TTATTATTACTTCGGCAATTGCGGTGGTGACGACGGTGGCTCACCGCCTCTGGGCCGCACCTCCAGTG
GTGCGTGCCGACGTATGGATCGATAATATACG
CATTGCAGGGTCGAAAAGCGATGTGACATTGTGGGAATCCCAAGTGCTTCGGAATGCGGACAGTTGTC
GCGCATCCATGGGGGAGGACCGCGAATCGGGC
GCCACGCAGTACACCTTTCTGGGGGTGCACTTTGATCACACTCAGCGTGCGGTATCCCTGAGTGACAA
GTTTGTCTGCTCTGTGCGCGCCATGCCGGCGC
TTAATTCTTCAACCATCGCGGAAATGGAGGCAACGGTGTCACGCTTTTTATATTTGGCTGCCATTTTG
GTCAGGCGTTTATGTGACTGCTACTTTTTTAT
CAAGGCAGTGCGACGACGATTATCCGCACTTCACCGGGGGATTGTGCTGGAGACATCCCCGGCGAACC
TTCCGCCGTCAGCGGTTGGCTTGGACGAGAGA
TTGCAGCACATCATCGAGAATATAATTGTAGGCGAATCATCAAGCCCACGAAAAGGCATCTGCCGCCA
TCATCACGGACGCATCGCTCCATGGATGGGGA
GCCGTTTTTATTCCAGACTCCGGCGACGTTAAAATTGCCGGAGGGAAATGGGAGAGGAAGCCTTTTCT
TATCATGCAGGCCGAGGCACGAGCGGTACGCT

TAGTCTTATTGGCCTTTCCCGCCATCTTGCCATCCACCACTGACGTTTGGGTGGACAACACTCTGTTT
CAAGGAGCTGCGAATAAAGGCAGCTCAAAATC
ACACGCTACGGCGCGGGAGCTGCAACGGATATATGAGTTTTTGGACTCTCGTGGAGTGCAGGCATTCT
TTGGTTACGTACGGCCAGCGGAAAACCCCGCA
GACGGCATATCACGCGGTCGTGTGTTTACACTTGAGGAATTGGCGAAGGGGTGGAACTTGCGAAGGGG
ATCGGCGGGATCTTGTGGCTGAAGGACCCCGA
AGTCTGCCACCCAGTAATAAATAAATAAATATTCTGTCGGAATGGAGT
>scaffold1028.size7123
GGGGTGCTGTCGTTTGTTGCTGTGCGTGGTGTAGCGGCTGTGTCTCCCTTTGTTTTTCTCGTGCTATC
GCAGGTTGGCTCCGCAGCGTTAGGCGACTCCAC
TGTTTGTGATGATGACTGGCCGTGTGCTCTGCGTCCTGTTGGCTCCTGCAGTGATGCGCAGCTGCTCG
TGTGTGTGAGTGAGGGCTGCTGCGACGGGGTG
CGTGTGCGGCCGCGGGTGATGGCGCGGTGACTGCTGGGCACGCGCGGGGTGTGATGGCTGCGGAAGGT
GGACCTGTGGCCGGTGGCTCTGGACGAAGGGA
GAATGTGAACTCTGAGGACAATAGCAATCAGGAAGACGAGGGGGGTGGAGGAAACACCGTGGGTGAGG
ACCCCAATCCCACCTCGGCAGGAGCACTCACT
GCTCCGACGAGGCAGGACCAATCCAGCAGCGACCCACTGCGAAGTGGCACTTGCAATCCGTCCGGCAC
TGGCAGCGCGTCTGCTGCTCGGATGCGTGCAA
GTGGACCGGAGTCTTTGGGCGGAGGGCAAGCTGCAGGCACAACATCCCCACGCTCCGACGCATCTCAG
CAGGCAGCGGGTGGAGTGCATGCTGTTGGCGG
GAGGACCTCCCAAGGCAGTCAGGCTTCGGAACAAACCGTCACGGGACAGTCACAGGTGCCAGGGACGG
CGAAGGAAGCACCGCGGGGCGATGAAGGAGGA
GAACCCGGAGCGCAACACGAGGCCGACCAAACCACAAAAATGACTGTGGTGACCTCTTGCGCAAAAAC
AGCGGTGGAAGAGGAGAGCCGCCAGCGGGATC
GCAAGATGCACCACGTCTCTATAGTGGCGGGAATGCTCATTATTATTATTTTTCCCTGCTTTTACCGA
CATGTCTAGCCACACGATGTCATCAGGGTGCC
GAGGAGGTGGTCGAGCAGCGACATGAGTTTCCTGCTATGAGGCAGCTGCCATTGGGAGAAGCTTTCGG
TGCCAAGGTCCTTCTTAATGCCCAGCCGTTGG
CGCACAGCCAGAGCGTCTGTACATTCCGGAAGCAGTTGGTGCAGTGTTTTCTGCGGGCTAATTGTTGC
GCTTGTGGAGGCTGGCTGATCCTGCGGTAGCA
GTGATTGCCTCCTGGCACTGCTGCTGCGGTGCCGATTAGCGTCTGGGTTTTGTGCGCGGCGATTTCTG
TGCTGTGCGGTGCTTTTTTTGTGTGGGGAGGA
CTGTGTCGCAGCAATCGCGGTGGAGGTCGGGAAGCGGTGGAGGATGTTCCGGATGAAAGCACCGACAG
TGGCCGATGTGGTTCGTGTGTCCCACCAGTGT
GGCGTCACAGTGCGGGCATTTCTTCTTCCGACGCTGTGTGGAGGGCCGTCTCTCCACAGGCGGTGCGT
CGGCCTCCCTTGTCGTCGGGGGAACAGCTTCC
GTCCCGGTGTGGTGGGATCGTCGTTGGAGCGCATGAAAGCGGACTGTTCCGGTGAGATGTTGCGGAAA
CGGCGTTGGTTCGCGAAGTCTCTCACGGCCCA
GTATAGTCGGCCCCAGAGCTCGGATTCCCCGCACCTTGCCGTGCAGTGTTTATCCGTTCCTGTTGGTC
GAGGGCCTCCTGAGTGATTATGTGCTTGCCGC
CTGGATCTGATGGCCTGGCACCGCCAAGCAAGTAGTGGTGGGTGTCCTGATGCATGGTGCTACGCCAC
TTGTTGGTGAGCTGGGTGTTAATGCATGACAG
CAGGTCCGTGTGCCGTATGGGTGCTATTCCATGCTCCGTGTGTTGCCCGATTGCCGTGTTCTGCGTCG
CATGCTGATCTGCAGGCTTGTTAACGTGTACT
CCGCAATGTCCGTAACAAAACTGCAGCTGCACGGACCAGCCGGCACGCATCAGCGACAAGACACGCTG
CCAGATCTGATCCTCCGTCCAGTCTGTCTGAC
TGTGCGGGCCTTTGGTGACGGCGGCTAGAAGGGAATGGCTGTCCGTAACCACAAGCAGGGTTTTTGCC
TGTGCTGGATACGATGGACACTCAGCCTTTCC
ATGGCCGGGAGCAGTGCACGGGACTCCGTCGTGCGGCTGCATGGGCGTGTGCCGCCGTTGGTACTGAC
CTTCTCTGTTATCTTCGAAAGTTGCGGGGATG
AGAGCAGTATGCCAATCCCGGCTGACGTGGGAATGCCGACACCGCCATCCGTTGCCAGTATCCACGGT
CCCCAGCGATGATGTTGCACCTTGTCGCGGTG
TTGGGGCTTTGGTTGCGTATAGGAACTCCGCAGCAGGGCGTTGCTGCGAGCACGACGGAGACCAAGGT
GACTTCACTCCCCGAGAAGGTTCCACGCCATG

CCCGGTGCGGGGAGGTGAGGCGGTCTGCCTGCAGACTGTCGGACAGAACTGCACGACCGTTTCGCAGC
GGTCACTCTGTTGATGTCACCCGTGGCGGTGA
CGTTCACAACCCTGGCACCGAGGAGGATGGCTTCATCACTATTTGGCGGCTTAACATTGCAGGCCTCT
CGTCCGTAAAAAGATTGCATTGGCGGCGCGAT
TGGAGCGGCGTCCACCTGACATCGTTCTTCTCCAGGAAATAAGTAACCGTGGTGACGTGGCGACGAAA
TTATGTGGCTATAATGACTACCTTCAGACCTC
ACTGGGCGCGGTGGTGGCGTTGCCATTCTGGTGCGCCACTCTCTACCGTGCGAGCGCCTGTCTCTCCC
TTGCGCTGACCTCCTGGAGACCATTGCTGTCC
GTGTATTTCCGCCGGGCTGTCGCAGCATTTGCATGGCGAATCTCTACAACCCTCCCACAGCCATCGGT
CAAGAATCCGCATTTATCAATATCGTTTGTCG
CTCATGCCTTGATGCGATTATCGCGGACGACCCGAATTTACATCGTGAACTCTGGGACAGTCATATGT
ACCATCAAGGACTGGAGGCGATAATTTCGCCG
CTGTTCTAATTGATATGGATTTTGGGCTGGCAAATTACCCGACACAGGCAACACGGATCACACATCGT
AATGCATTGTCCCCTGACGTGGCTGCGTATTG
CGCCCTACGCGTCTCCCACTGGACATTCGCCCCTTACATGGACTCGGACCACTGCTGAATATCTTACT
TGGTCGAGATGGACGATGGCACTCCACGACTA
GCAAACACGCTGCCTTGATGGAAGAAAGCCACCTTTGCCCTGCGCAAAGCTGATTGCCCTGCTTTTTC
ATCGTTGTGTGAAACACTCCCTGCCACCGCGT
CCACATGGTTGGATCCTCGCGGAGGCATCGTACGAGCAGCTGAGCGCCACATCCCGCGCGGCAGCCGT
GACAGCTCCAAAAAGATATGGACACACGAAAT
GGAACAGGCCGAGTCCGCCGCCGAGGCAGCACACGAAGCACACACATTGCCTCACCCGGAGACAGCCT
ACTTTGAGCCGAATGTACCCGAAAAAAGGAGG
AACGAAATCAAGCCTTACGCCGTTGCTCCGCTGTGCTTCTGGAGGCCCGCGCGAAAGGACTTGGAGCC
TCACTCAGCCCGAGGTGGCGATATCTTCACGG
CGTGGCGGCGCCGCACCCTCATCCTCTGGAATCGGTTGTGCTCCGCACCGAACTTGGCCGCGTATGTG
CCCTGCCTCGGCAACAGGCAAATCTGCTTGTC
TGTCATTTTGTGCGGTTTCATGTGCCATACCGCCTTATACTGCTGCTGCTTGCTGCACTCCTTTGGTT
CCTGGTGGCTGGGAAATGGATCGGCCTTTCCC
ACCGTATGAAATTGACGTGACCATCCGTGACTCTTTGCTTGGCTCAGCCCCTGGCCCCGACGACATGT
TGAATGAATTCCTGCACCGTCTCGGGCCCGTT
GCGCGTGGCACACTTAGAACGATGATCCACAACTCCTTCGCAAACGGCAGCCTGCCTGGCTCCTGGAA
AATGGGAGACGCTATTTCTATTCCCAAGCCTG
GGAAGGACCCATGCCGCCCAGAGAGTTACAGACCCATCACGCTGCTCTCTGTTTTACCAAAGTTAACC
GAAGGAATGGTTCACCGCCGTCTGTCAGCGTT
ATTGCCGCACCACCCACGTCAATTTTGGATTTGCACCTCCGCGTTCTACGTCGGACGTGGTGGCACTT
GTTATCGACAAATTACTCGTGGACTGAATGAG
TTTGGCATTGTGGAGCATGAGCGCCCTGGAGACGACGCCCCCACACGACGCCCTCGTCGCCATCGCTT
GTGGTCTCGATTGATTTTCCGACTGCGTTTGA
CACCACTGACCATGGGAATTTATTCTGCATGCTTGACAGACTTCCGCGCCTTGGCCTCGAACCAAACG
CTGGCTGCACAACCACCTGCGGGGCCGCTATG
TTAGGGTATGCACTCGTGAACAGCACTCCCGGAAACAGCTCGCCTCAGCTGGAGCACCGCAAGGCGGC
GTCCCGCGACCCCAACTCTTCCTCTGCTGTGT
GGATGGTCTGCTGCGCCGCCTGGACAGCATCTATTCTGCCTCCGCTTTCATGTACGCTGACGAACACA
CACTTGTTGCCTTGTGTGCAGACATCCATGCC
TGTGCTGCGGCAATGCGACCGCTCTTGTCTCTTTTGTGGCCACATGGGCTGCCGAGCACAGCCCAAAA
ATCAACGTCGCTAAGAGCGAAGCTGCTCTATT
CCACATCTCTTCACACACACACGGTCTGACGAGGACATGGTCGATCTCCATCTTGTTAGCGGGAACCT
GCGTATTCAGCCACGCCCAGTGCGCTTGCTGG
ACAACACGTTCTATGGACTTCTTAATTCTGTTACGCATGCTTCCACCGCTGCAAAGAGGACCATGCCA
CGCCGCTATCAGCTGCGTCTGGTCGCAGAGGC
GGACGCGTCCCATCACACCATGCGATCCTTTTTGATTGGACACGTTCATATTTTGTTATTTNNNNNNN
NNNNNNNNNNNNNNNNNNNNNNNNNNNNNNNN
NNNNNNNNNNNNNNNNNNNNNNNNNNNNNNNNNNNNNNNNNNNNNNNNNNNNNNNNNNNNNNNNNNNN
NNNNNNNNNNNNNNNNNNNNNNNNNNNNNNNN

NNNNNNNNNNNNNNNNNNNNNNNNNNNNNNNNNNNNNNNNNNNNNNNNNNNNNNNNNNNNNNNNNNNNNNNNNNNNNNNNNNNNNNNNNNNNNNNNNNNN
NNNNNNNNNNNNNNNNNNNNNNNNNNNNNNNNNNNNNNNNNNNNNNNNNNNNNNNNNNNNNNNNNNNNNNNNNNNNNNNNNNNNNNNNNNNNNNTCGCCCC
CCACCCACCTCCACAATATGGAAGTACGGTAC
CGCGACGGTAGCAAAACATTCTTGGCCTGAGCGCCTCTACAGAAGACACATCTGTCTATTTGGAGGCC
AATATTCTTCCGCTCCGGAAGGTACTCTGGCG
TCGTGCACTCACACAATGCGAACGCTATATACGCCTCCATGATCACGAGGATTTGCGTTCCCTTATCT
ACTCGCAACCTATGCCGCCGTCCATGCATGGG
AAGGCGGCGACATCAATTCCGCTTCCGAGAGATGCTGCTATTAATGGATTGCTATGCGTCTGTGACAT
TATTGGGACACCCCACAACCACATTCGCGCGC
CTCTTGCCCAACATCGAATTTTTCCCTGGACACTGTGATCTGCAACAACGTGAGATTTTATCAGCCAC
TGTTTGCCGAAGACGCCTCTGACGATGTCAGG
CGCACGGCTTTTTATTCTACTCACTCCTCTTTGGGGGATTGTGACTTTGAATTGTGGGCGGACGGTCC
TTCATATCTCGCTGCACTCGCATCTGGATCCG
CTGCGCTATTATATGACTCCACCACGACAAATGATCTCCCTGTGGAGGTACATCGTGCAGCAGCTGGC
AGCCTCGCATGCTCATATGGGGCAGACTGCCT
TGCGATCGAAAATGTTTTTAGACATCTCATACCTCCTTGTCCGGGAACATCCCAATCGCCCACACGAA
TGCTGGAGGAAACGGACTCCCTCTCGGCAATT
GAGGCACCCCGCGTTGCCCACCGACCGTGCGAGACCGCGTTGCTGAGTAAATTTGGATTATGCTGCTT
TCCTTGGTGAAGCGAGGTCACCCTGTGGATTT
TATATTTTCCCATCACACTGCGGTATTCCACGCAGTGAGGCCGCTGACGACGAAGTCACGATTGCACG
ATCCCTCCCGCAGGACTGCGTCAATGCCTGGC
ACGTGGACTTCCTTACCGCCGTCAAGAGGTTATAACGGAAGGATGCCCAGGAGGAAAAGGAGGGCGAG
CACACCGCGTGACAGGCGCCTGTCGGTGTGGC
GAGGACTCTATTCCTGGTGTTGACCCGGACATTGGTCCCCGAGTGGCGGCACTGCGCACAGGCGCGAT
GCGACTCCTGCCCACTCTTCGACAGGCCTGCA
CGCGCCCTGCGGATCACAAGGAACCCTCTGTGTCGGTGGCGCCGTCCCTCCCTGACTGCGGTGACCGG
CCCGCCTCTGCCTCCGCCGGAACCTCTTCCCG
ACGTGAGCACCGCGACTCCTTGTCGACCAGCGAGCGGTGGATTGACGTCCTCCTGCCCAACCTGCGGC
AGAGTGCGCTTGTCCGTCCACAGTATGAGGGC
ACATGCCGGACGTGCTCAGCCTGGCGTTATAATAGTTTGCGAGGGCCTCCCATGCGGATGCCGCAATG
GCGCCACCGTCTTCTTAACAGGCGCCTCGCGC
GCAGGTCGCCACCGAAGTTGCCAAGCATGCCAGCAGGCAAGACACGCAGGAAGCACAACCGCGGGTAC
TCGAGATGGACCAGGCTCTTCCGCCACCGCCT
CATCACGGACGCCACGGGCTGCTGATCCCCGACGGACCACGGGAGACTCCGCCTCATCCACTGCGGGA
ATGCCCTCGTCGTTGGGAACTGGGACTGCGAC
TGTGTGGGACTGCCCCAGCCCGCACGACGGAATACTTGCCACTTTTGTACGGACGGCCCTTGCGCTAC
TCACTCGTTGTTGTAGCAGCATACAACCAAAA
CAAGATAACAGGCTGCTTTGTGGTGAATATTGGCTGCACTTCCCGTGGACCAACAATCCGCGTGGTGT
GCGGGGATCTTCTTTTTTTACTGTTAATTTGC
TTTTGTGTTGCCGTGTGCGCTGCACCTTCTTGTGTGTGTGCGCACCCTCCGTGCGGGGTGCCGACACG
ACGGACCGCTCTCGCCGCATTTGCATGCGGTG
GAGCCTTGTGTGCCGCTCCTGT
>scaffold1029.size6853
TTGACAAAGCACAGACCACGCACACCGGGATCAGGATAGTCCACGGAGCTGATGCTGCCACCGTGCAC
GCAGCGGACCTCCGTGTCCTTCGCATCGGGGAG
CGGGAGCGGCCCAAGGCCGTTCGGAACCGCAGCTGGCAGACAAGTGTCGCCGTAGCCGCCAGCAGCGC
ACTCACACTCACAGCCGCTGATGCTGCTCGTC
CTCGCTGCAAAGCACACGGCGTCCACCGTGCACGCACCACAGTCCAGCACCGTCACGGGTGCTCCAAT
ATTGAGGTCGTCTTGGTAATTCGTCACAGGAG
AGCCCCTTGCCTCGTTGCACATACCGTAGATGGTCGGGCGCGTGTCAGATGGTGGTGACCACTTGGTT
GTCATACAGGTAAAGGTCGAGTACGAGCCATA
CGTGAAGTTGTTGTCAAGTATCGACCACACACTCCGATCGCGGAACACCATGCCGTGGTGGGCGGCCA
CGTACACAACGTAGGCGGTGCGGAACTCGTTG

TTGCGGTGCACCAGCCAGCTGCCGTCAGTCACGGTCAGCGTGGCTGCCATCAGCATGGCGAAGCCGAG
ACGGAAAGTGCTGGACACAATCTGCAGCACGC
TGCCGCCAGCCACCGCAACGTCCTTGAACACACACGCGGCTTTGCCATATTCGGTGCTCGGTAAAATC
CACGAACTGTTCTGTATGGAGAACACGGAGCC
GCCGCTGACACGCAGGTCCGACGCAAGAGCGTACATCACGTGTGTCCGCGACCTAATAACGCAGTTGT
CCATGTAGAAGACGCTGGACAGGTTCACGCCG
AGGCTGCGCTCCACGAGGATCGCAGCGCACGAGCCCCCGCCACACACCAGCGTCGACCGCGTCATGAC
AAAACACGTCAACAGAAGCCGGACACCGTCCA
GAACAAGCGCGGGGACGTACCGGAAGCTCTTGTGGCCACGGGTCGTCGGCACGTACTGCGACCCGCCA
ACCGTCGCGCGCAGCGCTGAGTTCGCCAGCAG
CACACTCGAGTGCAGCGGCATCGCGCCATGCAGCACAATCGTGCCCTCCAGCGACGTCACATTCGTCA
TGTTGACGAAAGCGTGCGGCATCAGGCGCGCC
GTGCTCTCGCTGAGGCCGCCAATCCGCAGCTGCGCGCCGCCCGCAAGCACGCAGTGGCGCAGCGTCAC
GTTCAGTGCGTCAGCGAACGCGTCCATCGAGC
GCAGGTCCACCGCCACAATGATCGGTCCGCTGAACACCACCGAGTCGAAGCACGCCGTCGCACGCCCG
CCGCCAACCGTCACCGACTCCGTCAGCGTCAC
GCCACTCACGCAATCCTGCGCACCGTCGCCACCACCACCGGCCCTCGCAGGCGGCACGTCGAACGGCA
GGCACGCCTCGCCCACACCGCCGGCACGGCAG
CTGCACACGCATTCGGAGAACGACGCCGTCAGCGCATCAAAGCACGCCAGCGCAGCCGCACAGCCGTC
GCACGGCACCACGCTCACGGAGGGCAGGCCGG
CCATGCGGTAGTCGCCGCTGCTCTGCAGCACCCGGCCGCCGGCACGGTTGCACTGCACCGACACGACG
CCGCTCGTGATCGTCAGCCCGGAGACAAGCGT
CGCGCCGGTGGCAGCGCAGCGCGCAATCGACACGGTGCCGCCGCTCAGCGTCATCCCCGACAGCGACG
CCACGGACGACGCCTCGCCCACCGCCCACACG
TCGCGCAGGTCCAGCCACCCGCCGGCACCGACCGTCCCACCGTCGCAGCGCACCAGCGACGACGCAAC
AGACCCCTCGACGCGCACGAAGCGCAGCACCG
AATCAAACACCGCGAGGCGCTCTCCAAGCACAATGCCGCTGCCGCTGCTCACAACCGACACGTTCGTC
ACGGAGAACACCGAGTGGTTACGCAGACTGAG
GCGCGACGAGGACGCCACATGCATCGCGGACGCGGTCGGACCGCCCGACGCCTCCAGCGACACGCCGT
CAAGCACCACCGCGCTCGACAGCAGGTCCACG
TCGCCGCCAACGTACACCGCAGACCCGCCCGCCTGCAAAGACCTCAGCACAGTGTTGCTCACACGCAG
CTGCGACTGCAACAGCGACGTCGCGTACAGCA
CAAGCGGCGACGCCACCGCGTCCGTCAGCCCGCTCAGCTGCGAGTAGCGCATCGGCCCCGGCGTCACA
ATCGTCGAGTCACGGACCGTCACGTTCGTGTG
TGCCGGCAGGTTGTGCACAAACACAAGCGAGCAGTAGCTCCCGGACAGCCCGCTCACGCTCACCACCA
GGCTCCGGTCACTCGCCGGCTCGCCGCTGTAG
CCCCGCACGTAGATCTGCGCACCGCCGTCGCACACGCAGTTCCTCAATTCGATGCGCAGCGCGCCGGG
AAGCATCGCCGGCACGTCGAACACCACAGCCA
CGCCGTTCGTGACGTACACGCCGTCCATCAGCACCGTGTCCACGGCGCGGCCAACCGTGATGGCTCTG
TCCACCGTGCCGCCCCGCAGTCGCAACACCAC
CGCATGCACCGCGGGCATCCACGCAGCAGCTGCTGCCAGCACCAACGCCACAAGCAGCGCCACACGCA
CAGCCAAAATGGCCCTGCGCCGCACACGTTGC
CGGCTGCGCGGACACGCACCCACAGCAGACCGCATCCACCGACTCAGACACGTATCAGCAGGGCTGTC
GCGTTGTGTCGCTCTCGCCCCGTCGACCATTC
CTCCTGTCTATCAAATCCTTTCCTACAGATGGCGAATCCAGTGACACGATTCCGCCAGATCGATATCC
CAGTACTACCAATCCTAATACTGCGAGTAATT
CCTAACACCTTGAAACGATAGTTGTGCTGGCAAAGCCAAAGTATCTCGAACTGCACGCTCAAAAAAAA
ATAAATAATAATAATAAAAGGCAGAAAGAACA
ACAGCAACCAAACACAGTGGCACGCAGAGGCATCCGGTCATACACAATCCCCACCACAGTACAGCCGC
CGAAGCAAAGCCGCGCAGACGCAATAAAGTCA
GTGACACCATCACCCTCCCACCACCCACACAGCCCCATACAACCACCGCGCGTCGTGGCGTGGATACA
GACTGGCGTCGGCGGTGTGCCAGCAGTGCTGA
CAGGCGGCGGTGGACCAGTCCTTCGCTCACCCCTAGCAGGACAGAGAGCAGCGGGATTGGCCAACAAC
TCTCGCCGTGCGGCATGTGTCCTTCCCGTGTT

CGCAGATGGGAGTGGTGTCCCCGTTTCGCAGGAACTTCGCAGACTGCCGTCAGCGAGGGAGGTATTGA
TCATCGTTCTGTGCGAGGAGCGGGCAGCGGGT
GCAAGATGGCACAGAAACACATTCAGTGCGCCGCCGCGGCCAGGGGCCAAGCAAGGCAAACAGTGGCG
GATGGTGAAGTCAAGCGCATACGGCGTGAAGG
ACCCATCCATATCACAGCCACCAGGGGACACGCAAGCACAAAGTGTGCAGACAGCACTGGGGATCGCA
GCACGTGCGACGCGGACAGTGTGACGGACAAG
CAAATTTTCCTGCTGCCAAGACAGAGCACAAACGCGGTCCCAAGAGTACCGCGGCACGGCCGATCCCA
GAGGGCAGGGACGCGGTGCCTCCATGCCTCGC
GGCATCAGAGGCGTGGGATCGAGAGGGCACCAAGCTCTTTCGCACGGACCACAAGCAGCATGGAGAAG
TTACGGCAGAGGGCATGATTGCGGTACTCCCG
TTTGCGGACATCTTCAGCTGAAGGCAAGCTGCCTCCCGGTGAGGCTGTGTGCGCGTCCCATGCTGCCG
CTGCAACACACTCGGCCTGCTCCACTCTGACC
GTCCAGGTTCATCAGTGGCTGTCACGGCTGCCGCGCGGAATGGTGCGCCTTGCCGCTCGCACGGTGCA
TATCCAACCATGTGGTCACAGTGGCGAGAGGC
GTTGCACACACCGAGGTGGAAGCATTCCAGTCGGCTTCACGCAGAACAGCGACGGCATTTTTCCGTCG
CGGCAGCCTGCCTGCCGGTCGCGGGAGGCCGT
CCTCCGTTGCTGCGGTATACAACGGCAGATGTTGGTCCGACTCCATGCAAAGGGGTGGACGTCCAGTG
GGAGACACGCAGTACGCGTTGCGCAGTCACGT
CGGGAAACGAGACGCTGCGGCCGCTGATCCGCGCTGGCTGTGCCGGGTCGTCGGACAGCTCACACTCC
ATGCCGCTTGAGGTGGCGGCGAGGTTCTCTCC
TGCCGTCGTGGATCGTGAGCGACCGTCCCGCAGCTCATGGTGCGAATTCAGGTAGCCCGCAATAGGCG
CTGCAGGACCAAGACGACAAATGGTGCCCGCA
AACTGGGCAGCGTGAGCGATTTCCAGAAAAAGGCCGCAGAGGTTCACCACGTACATGCTGGGGCAGGC
CGGTGGGCACACGCGGAAGGCAGTGTTCTCCA
GAAAGTTGGTGCCACGAGCAGCAGGCACTCGCACAGCAAGGTGTGGCGCACCAAGAGGGCCACGCCAC
CGGCACGCCCACTGCAGGTCTGGTGGAAGACA
CCGCAGCCAACGCGCCGCGTCGCTGGGGCGCCCTGGTGCCTGCAATCCTGCAGAAAAGCGGTGTCAGG
TCGGTGCTGCGCCAGTCGAGCAGCAAGGGCAA
TCCTGAAGGGCGAAGGGCTGGAAGTTCTGAGCTGCCAGACAACTACTAGGCCATCCTCATCGGGGCGG
CGGCCCCTGCGGACACCACCGCAGCGGTTGAT
GCCGACGGAGTGACCcCAATGAAACGACTGTGCGGTTCTGTCAGTTGAGCCGCAGGCAAACCGCCTCG
CCGCTACACAACCAACAGAGCATGGAACCTTC
TCGAGGGGTGACGCCGCCGTGGTCCCCGTCACGCTCGCAGCAACGCCCTGCCGCAATCACTTACACAG
AGAGACAAGAAAGAGTGTGTGGATGTGTGTCG
AGAGCACAGCGACTGTTATCGCCAGAGGAGGCCATCGTGGCACCGTCCGTCACGCACCAGCACACACC
GAGCAACGCACGCACCCGGCAGAGCAAAACCA
AAGAGAAGAAAAGAATGACCCCAAGAGAAGGCAGTGCTTTCACGTATGGCGAGCATCGTGCAGCCGCG
TCAACCGGCCTCCAGCAAAATAGAAAGAGCAT
TCCTGCAGTGCACTGCGCAAATAATTATGTGTGCTGGAGGAGCAGAAGAGAAGAGGAGCACAGGGAGG
CAAATCAGCACAGCAAGAGGCACAACTGGATG
GCAGCGATACATGCGGCACGGCCCAACCTCAACAATCACGCACGGTGGCAACACACGCCCAACCAGAG
AAAAGAAAAGAAAAAAAACACGGCGGCAGGGT
GTGGCTGTGTGTGTGTGGCGCTGCGGGTGTCTGCCAGCCCTGACCGCAAAGAAAACAAAACAAGAGGG
AAAGAAATGTGTGCTCCGCCCCACAGGCCCTC
GTCGCACGCCCACACGTCACGGCTCCCTCAGCGCGCCGTTCTTCGCGGCCCCTCCTCATGCGTGCAGC
AGGCGAGGCGCACAGGGCCGTCGTCATGAGCG
TGTCGGTGGGTGTGCGAGTGCAGCAGTTGGGCGGAAACACACGGCACCCCTCAAAGGAAGAGTGCAGC
AACAAACAATTAAAAGAGGGAAGAGTAGAAAA
AAGAAAAAAGCGTCCATAGTGACTGCCCCCAAAAGCAAAAAAGAAAAGGGATGGGGGAGATAATAAAA
AAAAAANNNNNNNNNNNNNNNNNNNNNNNNNN
NNNNNNNNNNNNNNNNNNNNNNNNNNNNNNNNNNNNNNNNNNNNNNNNNNNNNNNNNNNNNNNNNNNN
NNNNNNNNNNNNNNNNNNNNNNNNNNNNNNNN
NNNNNNNNNNNNNNNNNNNNNNNNNNNNNNNNNNNNNNNNNNNNNNNNNNNNNNNNNNNNNNNNNNNN
NNNNNNNNNNNNNNNNNNNNNNNNNNNNNNNN

NNNNNNNNNNNNNNNNNNNNNNNNNNNNNNNNNNNNNNNNNNNNNNNNNNNNNNNNNNNNNNNNNNNN
NNNNNNGTATAAAACAGCAAATAAATTTAAAA
TACGCAAATAAAAGTTATTTGTGAAAATAAATTCGTTCCCATGGGAAAGTAATTATGTGGTGGGGAGG
GCAAGTGTCCCACGCGGCAGCCAGTCACTCCT
CACAGAGCCGCAAATCCCCACAGTCCCAGCAGAAGGATCAGCGGCAGCAGTCCGCTTCCGCAAACAGT
GCTGCCATCACCATTTGCCCCAAAGTGACGAG
CGGTACCGACACCCGCTGCTCCTCCCGCCAAGGATTCAGAAGAGCGCTTCGGGCGAGACACAGCAGGT
GAAGAAGTACGCGCAGCCACTGGCGTTGGAAT
ATTAATTTTATTTTTGGCAAGCCCTGTGATCTCTTCGGAGCTCAACGGACGGTTGTACAGCAGGACGT
TCGTCACAGCCACAGATACACCTTCTTCACCC
CCTGTGTTCTCTGCATTGCCTCCGTCCCCTCCAATGAAGAAGTGAGAGATCCCTTTCGATTTTGTATT
TTCCAAGTTACTTTGCACACTCTCACACACAC
GCTGACCATCGACGTAAACGGAGCCCTGGATGCCATTCTGTAGCACAATGGCCACTTGGTACGTTGTC
CCCGGCTCCCAATGGCTGCTGTGCTCCAACGT
CTTTTCGCCACTGTGCAACAATTCCCACCTCTTTTCTCTGTTGTACGACAGTCCCAGGAGTACAGCAT
TCTTATCGCCATCATTCACCTTCGCACCCATC
AATGGAATGGAGTTATCTCCGTCCTTTGGCAAACCGTGGATGGACACCGTCGCCACAAGAGTGAAGTT
GTAGTTCGGAAAGTGGTACAGCTGATTCTCCC
CCTGACTGCGAACAGGCCACTCCGCCCACGCTCCCTGGAACCTCACGCCGT
>scaffold103.size41559
TGTTGGCGACGGAGCCGTTCCACTTCAAGGAAAAACCGCGCCAACTTTTTCGTGAAGCGCGGCTTCCT
CGCTTCCTCAGTGTTCAGTCCATGTGTTCCCCG
GGCGCCCGCAGAGCCGGGCATCGGAGTAGATGCAGAATTGTCTCCTCGCGCTGTTCATGCGGGCCGGT
TCTCACAATGTCAGGTCGACCTCCGTGGCGTG
CTGTGCGGTGGTGGGTCGGTCCGTCACCGCTGCGGCGCCAAAAGCCGCATGCTGCACAGCGAAATTCG
ACCGGTGGTGATCCCATCGGGGTGTCGTCGCC
GGGTGAGTTGCGCCTTCTCGGCGGCCTGACCTCTTTGCCATCATGGAATCGGCGACATTGCGTTGCAA
ATTGGTTACATCGCTCCTCTTCCAAAAAAAAA
AAATTCTCTTTTTTTGGGGCGGTGTATCCGTTGGCACTGTAAACATTACGACAGCACGCACCCCCACA
CCCCAAGTTGCAACAGTGAGTTCCAGCAAGAT
ACAGCTGGAGTGGAGCTTCGCCTTCGGTATAGGGTCTGTGCTAGCCATGATCCCGTAAAGAAGAATTT
GCCGTCTCTTGGCTCGCCTGGCAGCATTGAGT
ATTGCGTTCACAAGGCATGATGAAGTAACCTGCACTGCCAGAGGACCCAATTTCATCTTCTCACACAG
TAACTGAACGACATCATTACGCCCCATGCGTA
CCTACCTCGCTCAAAACACTCGCAGCAAGAACGAATAAAAAAAAATTAATAAAAAAACAAGAGTAAAA
AAAAGCCACCCCATGGTAAAACCCGTGACGAT
ATTTTCCTCAGAAAGCGTTCGGTTTTTAATTTTGTTGTTGTTGTTTGTTTCTTCTTGTATTCTTTTTT
TTTTTCTTCTCAGTCCATCCAAAACGCCCTAC
TTTTTGCGATTCTATGAACTATGCATGCATACATAACCATAAATACAAATATATAAACACAACAGGAC
ACAAATAAAGATATTACGTTTTTTAAAAAGCA
ACTAGAAAATGCCATAAGGATCGTGTCGGTAAACACGAGCACGCCTCATAGCAGCCCGCCCACAGGCG
GAACCAATCATTTGTACGCCATTCGGTTCGCG
GCACTTCAATCCGACCAATGGCTCCAGCGTTCCCTCCTCACCTGATGAGCTGTGCCGTGGTCCCTCGC
TGCTTGCTTCTGAAGGGCCGTTCAGCCCCGCC
CACGAATTCAGACCCTCAGCATTAACAGTAGGCGCCATCACCCCGTCGCCACGGCTGTTCCTAGCATC
AATTGTCACTGGAATCTCTGGCGGCACACCAC
ACTTTTTACGTTGACGAGAACACTGGAGTTGACTGTCGCTATCGGCGCTCTCAACAGACGACCTCAAA
GCACCTGGTACACGCTGGCGGTGCGAACGACC
CGCTGCACGCTTGCAAACCTCAGATATAGTTGGGTCAACATAAATTGCGTGAATGAAACTGCAGTCTT
CGCCGCGGCTGCACAGGTAATTGAAGTAGTAA
TGTGCACAATGCGAAAGTGTTTGTGGTCTCCCTTTCGAAAATGAACGCGCACCCCGTGTCGCAAGGAC
ATACTCACTTGAGATCATCACTGCAGGATGCT
TGCAATTCGGCAGCAACACTTCCAAAACATCCCCGGGAGGCAGCCGCTCATAAATGCAAAGCTTCTCA
TCACGCCAAATGTAGTTCACATGAATGGGCTG

TCCCTCCAGCGTTGAATAGTCCACGTCCGCATGTACAAACTTGCAGTTTTCGCCCATATTACATGAGT
CCGCCTCACCGCACCTGTAGTTCCGGCAAATA
ACAAATCGGGAAATATTAAAACGTGCCGGGGGTGTGTGGACCACCAAATGGGACGGCACCGTGTATAG
ATGCGTAGGTGCAAAACTAATAACGAATATAA
AAGTAGCTTTTTTATTTCCGCGCGCGCAACTTTTTGCCTTTTGTTTCTAAGTATTCCATTTCCTACTT
GTGTGTATTCGTTGCGTAGCTAGAAAAAAATTA
AATTAAAGTATAAAATGAAGTATTTGTGACAGCAATAAACACAGTAAAAATAAAATTGTCTCTCTTCT
TTTGTTTCATTTGTAAACTTCTTGTGCAGTAA
AATAGAGATGATTCATAAGAGGCAGAACATAAATGTGGCAACAAATTGGTAAGAAACAAATCAGAATT
TTACTCTGGCCCTGAGCTTCTTCCCCCATTCT
CACAACAACAAACAAACAAACAAACATACAAACATACATAAATACATACAAAGAAAAGGAAAAAAAGC
AGCACCATTAGGGAGATGACACCTTCGTCCAG
TGCAAAAAAAATATATATGCATATATTATTCTTCCCATTTTAGGGAAAATACATACAACACCCGTACAC
AAATAAACAAATGTTTGCATAACTCATTCTGC
CACAACCTAATTTCTTAGAAACCCACGAAATGGCAGTCTTCAAGAGCCCCGCAACAGCTGGAGCTGCC
CCCTTCCCTCCGCTCGGTGGGGAATAATGGGG
TGGGGGTTGGATGGGAAGGAGAACAAACTGTCCTTGCCACCTCCAAGCTCGCTCCCCCCATCTCCACG
AAACAAATCCATCAGCATGCAAAGAGGCCAAA
GTATATATTACAAAACACCTGTTCATTTATTTATTCTCATCTGGTCGCACATTCCTTCCATGTGTTTC
TGAAATCGATGTGCACCTCTCCCTCCTCCACC
GGCTGCTCAAGATGAGCAAGTGAAAAAAGAACAAGACGCCAAAGCACAAAAAAATGTGTGCTCAAAAA
ATATTCAGGTTATTATTATTATTATTATTTCC
CATTTTTCCCCCTCATCATCTTTTTGTTTTTTTTCCTCTTCACTATATATTTCTATAAATAAATATTT
ATATATTTGTAAATGTATACATGTATGTTTAC
CTATGTGGATATAAATACATGGAAGAGGAAGGATCAAGTTAAATATGGCAGTCTCAGATGCGTGGCAT
ATATATAAAAAAAGGGGCATCACTGTTCATCAA
CAACCGCATAAATAACTTGTCGACCTACACAGAGGGAAGAAAAAAAAAAAAGAAAAAACAAAAAAAAA
ATTGCGTCTCACAATTATGATTACGCCAATCC
CACACTCACGTTCAGTTTTTATTGCCGTGGTTACTTTTTCTTGCTTGTCTGTTTGTTAGTTGTTGTTG
TTGTTGTTTTTTCCATGATGGAAATTCTCGTT
TATTTGTTTCATTGGCCTTAACCACAAACAAAAAAACACACAGAGAGAAAAAAAAAAGTACCAATAAT
AATAATAATAATAATTTTTCNNNNNNNNNNNN
NNNNNNNNNNNNNNNNNNNNNNNNNNNNNNNNNNNNNNNNNNNNNNNNNNNNNNNNNNNNNNNNNNNN
NNNNNNNNNNNNNNNNNNNNNNNNNNNNNNNN
NNNNNNNNNNNNNNNNNNNNNNNNNNNNNNNNNNNNNNNNNNNNNNNNNNNNNNNNNNNNNNNNNNNN
NNNNNNNNNNNNNNNNNNNNNNNNNNNNNNNN
NNNNNNNNNNNNNNNNNNNNNNNNNNNNNNNNNNNNNNNNNNNNNNNNNNNNNNNNNNNNNNNNNNNN
NNNNNNNNNNNNNNNNNNNNTTAATGCAGACG
TGCATACATGCCTCTAATTAAACAACCATATACAGCCCACTTTTAGTTGGCCCTCACAGATAAAAAAT
TAAAACAAAATAAAATTTCAAAAAAAAAAAAA
AAGAAAAAAAAAGAGAGAAGATCTTTCCGAACATTGTTGCTTCACCGCACACATGCACACACACGCGC
TCTCTTTCTCTCCCTCTGTTATCGTTGAAACC
CATTGCCCCTTTATTATTATTTTTATTACGACAAGCCTCAAACCACCGACTTCGCGGAGCTGCGGCAA
TTGACTCTCTAAGATTGCAAACACGAATAAAA
ACCCACAACAAATGAAAAATGCATAAATAAAAATAAAAAAAAGAGGCAAAAACAGTAAAAAAAAACAA
AAAACAATACTCTTCTCTCAGAGCCATACGGC
GCCCCAGCGCTTTATTTATGAGAGCTGAAGAGCTGATACCCCGTCTGACAAAATTGAATCCTCCGAAT
TATCCACAAATGTCCATTCAATCGGCCTTGTC
TCCCCACTGGTGCTGCTGCGCGCACTGCCGTCACGTACGGACAATAATGTGTGGGAAAAGGAGTCCGT
AACGTGCTTTGGTATTGCAAGGTCAAGGTTCT
GATTTGCGGTTGGCACCGTGTCGCCATCTATGTCAAAATCCTCAGAAAGGATATTGTTGTGCCCCCGT
GATGGTTGCATCAAAACTGGTTTCTTCTTGCT
CACGGAGGCACAACCATTAGCAGCCACATCGGATCGTGATGAAGCAAATAATTTTGCCCGTGATGCCC
GCTTTTTGGTCGGTGGAATAGTTCTGGAGGCC

GGGGCACGCTTAAATACACCCTCCACACTTAGATCGACATAAACTGTGTGAATGAAGTTGCATTTTTC
ACCCCGGTTGCAAAGACAGTTGAAGTAGTAGT
GAGCGCAACGTGAAAGTGGTCCAATATGGTTTTTCCAGTTGATGCTACCACGTGTAACCAGAATGCGT
TCACTCGGAAAAGTCTCCAACGGTTGTCGACC
ATTTGGTGCCAAAACATGAATCATTTCGCCTGCTGGGAGACGCGGGTAAGTGCAGCATTCTGCGCTCC
GCCAAGAGTAGTTAACATGAATGGAATATCGT
GGTGCACCTGTCGTATCGGCATGCACAAATTTGCACATGGCACCCTTCCAGCAGCTATCAGGGCGACC
AGGGAAATATTTTCGACAAAGAACCAATCGAG
AAACACCCAATGAAGGGTGTGGCTTGTGAAAGATTGCGGAAGGTGAAAGAGATATAATACAGCTAAAG
TCAGAAGAATACACATCAACGCGGAGCCCATC
ACCAGCAAAATCACCCTGATTTTTAATACTAATCGTACCCTTGTTGTTGGTGGTTGTTGGATTTCTCC
TGTAAAAATCACTTTTTCTAATACTGTCAGGT
CCTGGCGGCAAAACATTGTTCCCATTTTGAGCCAATGGTGTCGCTGACGGATGATACGCAAAAGACAA
CGGCCTTGAAGAAACATGTGTGACAGGTGACA
TAACCGAGAAAACCGTGGCCCGGGAAGTATTTATTGCCTGTTCATCCAAAAAACTCGTTGATTTTAAT
GGGGATATGACTCCTTCTCCAGCATCCCTTGG
ACCGCCTTCAGCCGGAGAGCTTACACCACTGCTGCATCTTTTGCTCCCTTGGAAATCCTCGCTCGTAA
GCAGTGAGGCAGGATTTAAAGGACCCGGTCTC
GCCAAAACTAATTGCTGGACGGGCATGCATGAGATTGGCTGCCCATCGGCCAGCTTGACCAAAAGAAC
TGCCTGCCGTTGAAGCCGCTGAGACAAAGAAG
TAGCCACTGTATTGGTGGAACCATGGGAAGCTGGTGGTATTACAGCAGTGGGGGTAGCAATACTAACA
GAGGCAGTATTAACAGCCAAGGGAAAGGAAGT
CGCAGGTACTATATCTTGATTTGCAGTAGATGTGGGTGAGCCTCCGTTGTTTGTGCTCCCCATATAGC
CGTACGGCAGAAGCGAGACGGAATTCCCAATC
GAGTTACATGATGCGGAATCCCCATATGAGCCAAAAAAACGTGACTTCGTAACTCCAATAAGGCCATT
TTCACGCTTGTTTCGGATGCAAAAGTGTGTCT
GATACATGGCATCCCCATAAAACCCATTGCCATTTGGAGTGCACATCCTTGAGATTCGTTGATTTCCC
TCGCTATCCTTCCCTATCCCTCTTATTCTCTG
GTTTTCGTTGCTTTTATTATTATTATTATTATTATTGATGTCGCTTATTATTTCTATTTGTTCTCTTT
CCTTCACTCCTTCTCTTAGTGTCGCTTCCGAC
TTTGTGCCCTCCTCCTCCTTCTCCTTTCCTTCGGAAACAGGGGGAGAAGCGACGCGTCGTAAAATTCT
GGTGCCTAAACACACCCTCGCCAAAAAACCTC
CACACCGCAAAGTAACGGAGGCTCCGACGGGTCCCGGTGTCGCTTGCAGAAGCGCAACGCAAAAAAAA
AAAAAAAAGGCAACACTGAAACCGGAATATTT
TCGGATGTAAACAAATGGGGGTGGCACAGCACTGCCGATCATTCCTTCCTGCGCCCCTCCCTTAGCCG
GAACGGGCTGCCCACGCGCTGTCAACACGTGT
TCCTCTGCCACCCACAACGCCACAAGGCAACGAAAAACGCACAGAAGAAGTCGTGAGTCAGAAGGGTT
GCATAAAAAAGCAACAGCGCAGGAACAATTCA
TGGCCCCCCTCCCACACACACACGCACAATCTGGGAAAGAGAAAAGAAGAAGAAAAAAAAACGTAAAT
TCACAACTGAAACAGGATGATAAAAGCAGACA
ACAGTTTCTCTGGAAAGTCAGAGACAAAAAATATATATATGCGTCAACATCTGACGCTTGCGATTCCT
TTTCTGTCGTCTTTTTTGGTGATTTTTTTTTT
CTTTCTTTCTTTTGCGTACCATATCGAAAGGCAGAAAATAAATTGGGAGAGAAGAAACCCAGCCATTT
ACAATAGTTCTTTCTCCTACTTGAACTCCTTC
ATTGCGCCTCTCCTTATATATATATATTGTGCTGTGCTGGGTGTGAATTCGTCGTTTAATTATTGTTT
TGTTCAATCCTGTACATCCGGCCTCGTTCCCT
GAGACACAACACCTTTTAAAAGGGCGATGTACACAGCCCCTGGATGCATAATTGCGGTTGTCAAATTT
GCAGTGAACACAAATTGCCGGTGTCCATCATT
TGAACTGAAGCGCCTTGGCTCTCCTCACTAAAACCTACAGCCATGGGAAGAGTCATGATCCATCCCTC
ACGACAGAGCGTCCTGAGGACTTGTCCTTCGCC
ACCACATCCGCGTCCGTGGCCGCACGAATACGCGCAATCAACACGCTTGTGGAAATGCCCTCCGTGCG
AGGGGCTGTTCGAAGAATCCCAAGACGCCGTG
GAACGGCGTAGAACGTATCATCGGGCCGGTCGTACTCTTTGCCGCAAACAACAACATGAATGTTGTGC
CGTTTGATCATTTCCTCCGTAACACCAGTTAC

CGGACTGTTTGAAATCACCTGCGAGACAAACTTGCACATACGTACCTCGTTAACACGCTCCTCTGTTG
TCATGATTGGTCGCCGCTTGTAATTCTCACAA
TCTTCGTCCCCGCAGACTCCAACAATAAGGCGGTTGCCGTACTGCAACGCATTTTGCATAAAGTTTTT
GTGCCCCGCATGACACaAGTCAAAAACACCGT
CGCAAAAGACGTTCCTTACCGGTACAAACAACGGAATACGGGTGGAATAAGAAAGATCCACCAACATA
CCAATGATGTACAGAACGGAAGCTACAATGGA
AAAGATGTCGTTAAACACAGAACCAATACTAATGCACAACACTCCCGCATGCACACGACGCCTGGCAA
CGTTGCTAACATGTACCTCTATCGAAAGAAGT
GCGACCACCAAAGCTCCACTAATCACGCTGAGAACACTGTAATTGTTAAATAATAAAATTTTATATAG
AGGAACTACGCGCGCAACCAAACAAAGCGCGA
TTCCACTCTTGTACTTCCGCTTCATCTTCAGTAATGCCACATTAACAAAGCTCACCACCACCAGCAAT
AAAAAACTGTATTTTGGAAAATTTGACGTAAA
CAAAACGTAAAAGCCCGTAGATGGATACACAAGACGTGTAATTAAGGCGCAGAGGATCAAAAAGGACA
ACTCCGAATCCCGAAAACAATACGTAAGATGA
TACGCAAGATTCTTCGCCTTTTGAGGTTTGAGGTTATCCGCATTAATACGGCTAAGTACCGTGTTGAG
CTCTACTAGCTGCATCGCCATGAGCAAATACC
ATTTCGTATGAAGGTCTCTCACGCTAAATGCCTCCATAAGTGTCAAAGCAAAAAATATACGAGAAATA
CTGCTGCACACGCGGGAAAAAATATCACCAAG
AGGAGATGCCGATCGACAGCGCTTTGCGTGAACACCATCCAAGGAACCGCAGACAATGGCCACAAGGC
ACAGCAAGCACGATAACAAAATGGGAGTCTGA
GCTTCGATATTGTTTGGGTCACTTTCGCTTCCATCATAATGATCACTAATAATTTGATACGACTGCAT
GGAGCTCACCAGACCCGCAAGTGTAATAGCGT
TAGGCGCCACCATCTCCGGCACCAAATGCCGACTCATCGCTTGCCAAAGTGGACTGAAAATAACCGTA
ACGAAGCTGCTTGGACGGGTGTCAACAAACGT
CTCCGCGTGAGCGATGCGGTCCCGCTCAAGGGGCGTAAAAAAAAAACTGTGCTCAATGATATCCGACG
ACATACTCGCCCGGCAAAGCGATCACGTACAA
GAATAAGAACGAAAGTGCAGCACACACCCACAAGGACGTAATCAGAGAAAAACGACAGAAATAAAGAG
ATGCCAACAAACGACAACAACAACAACAACAA
CAGAGAGAGAGAAAAAAAAATTGAAAAAACAAATACAGCAGAAATAAGTCAAGAAACTATGTGTATTA
TAACACGCACCGCCTACACAAACACACAGAAG
GCAAGAAAAGAAACAAAACAAAAAAACTCCTTTGTAATGTTGTGTCTGAAAGTGCGGGTGGATTCAGC
AATCCCGTCTCTTCTCGGTGTTTACAAAATCC
GCAGTAAAAAAAAAAAAAGCCTGCCAGGTAAATTAATTACAGATGATAAATGAAAGTCTGCGCAATGT
TTGGCGTCCTTGTTTTTGTATCTTTGTTGTTG
TTGTTGCTTTTGTTTTTCCCTTTGTTTTGTTTTTTTTTTTTCTTCCTTCACTTCCCAATAAATTTTTT
GATTGAAAACAACACCCACAAACACAAAAAAA
ATAAAATAAAATGCCTAAGCCACCCACCCACCCCCGTTCTGCCCTAATCCCCTTTCTATCTGTGTGCG
TGTGCGTGTGCGTGTCCCCGCGCCAGCCCCAC
GCCGCTCCGTGTAACACCTGCAGAGACGCGTGCGGCGCACGGAATGGCAGGATGACAGGCGAAGACGA
TGGCAAAAAGAGAGAAAAGGAGAGAAAAAGCG
ATTGTGTGGAAACACAAAAAAGACTGCCACCAGAAGCCAAACACACAAAGTCATCCACACAAGAAGGC
AGAAGCAGCAGAAACAGAAAAAAAAAAAGACA
GCAAAAGAAGAAAATTCCTTCTTGGCATCCTCACGCCACTCCCGCTTGCACCGCAAGCCCTCCAAAGA
GAAGAATAGTAAAAATACATCAGCCCCCGCAA
CACCGGCAGCTTCCGTCTCCGAGTGTAGCGCACAAAAACGAAATGACGAGGGATGAGGGTTGGCAAAA
ATGCATACCAAAAACAAGGCAAAAATGAGGGT
ATACATCTCCACAAACGCAGAAGTCAAGAAGAAGAAAAAAAGCAAGCAAAAAAAAAAAATTCCCAGCA
AAAGAGGAAAATGCTGCAGAATAAGAATAAGA
ATTACATAACAAAAGAACAACAACAACAAAAAAAAAAAAAAAAAATNNNNNNNNNNNNNNNNNNNNNN
NNNNNNNNNNNNNNNNNNNNNNNNNNNNNNNN
NNNNNNNNNNNNNNNNNNNNNNNNNNNNNNNNNNNNNNNNNNNNNNNNNNNNNNNNNNNNNNNNNNNN
NNNNNNNNNNNNNNNNNNNNNNNNNNNNNNNN
NNNNNNNNNNNNNNNNNNNNNNNNNNNNNNNNNNNNNNNNNNNNNNNNNNNNNNNNNNNNNNNNNNNN
NNNNNNNNNNNNNNNNNNNNNNNNNNNNNNNN

NNNNNNNNNNNNNNNNNNNNNNNNNNNNNNNNNNNNNNNNNNNNNNNNNGGACAAAAAAAAAAAAAAAAAA
GTAAAATGCATCGTCTCCTCACCCGTCCAATT
TTTCCAAGGACAAATGAAGTTGTTTCCTGATCTCCGCAATGCATCAGAGAGAAGAAAGCCAAGAAAAA
AAGAAAAGAAGAAAGGAAAGAAGGGAAAAATA
TGTTCTGATTCTGCAGCACATTGAGCGCTACCCACGTTCTTCCAGATTCGTCTTGTTACGGTAACTGC
TGTACGTGTGGTGGTGGTGGTGGTAAAAATAT
AAATAAATGAGAGCAAAAAGAAAAAAAGAACTGGTGCGGAACGCTTTCGCCACCTCAAAAACTGTTCC
TCTCAACGCCGCCTAATCCTCACCATTGCTGT
CACCGCAAACTGCAGTTCCTGCGCTGGACGCTCCCAGACAGATCCAAAGGTGCAGCCATTACCCTCCA
CCAATTGCACGAACCCCTCCTTTTCGGTTCCG
CGCACGCGATTGATGGCAGCGACAGAAAACCCCGCCACTCGATGAATCGGCCGTGTGTCATACCCCAG
GAGCGTCATTGTGACCGGTTTCCCATTAACCG
TCTCGTCGCAGCAAGCTTTTATAAAAGATCCTGAAATCGTGACATCCACAGCATTGGCTTCAAGGGGA
ATCTCCAGCCGTTCAAAGGCCGTGTTGGCGAG
GGCAGCAATGTGTTCACTTTCCAATTCGTTTCTAAGCACGTTGTGGACGGAGAAAGATGAAAGCGAGT
AAAGAAAATCCTCCGCTGAAAATTGTACAGAC
TGACCCGTCGTTGAGTCCCCACGGCTCATCTCGGATGCGACTAGGGCAAGACAGTGAAACGCAGGCCA
CACGCTCGGTTTGAAGGGATGATGCACAATTG
AATGACGCATATCATAGCCGTAGCAATCAAGATACATATCGCGCTGTGATGTGGTCGTAAGCATAAGA
GTACCTCTAGCACGCAGGGCGATCGAATCTAC
AGCTGATATAGCATCAAATAGGACGTCCAATGGACACACCCAGGTTCTGTAAAAAGATCCATGGTTCG
AATCACTGAGTGTCACAAGAGAACTGGATTCA
TCAAAGTGAAGCACAATCGCATAAGATCCGCCGTTGCTGCCATTCCACACCGCCTTATTAGCATGTAT
GATGGAAGTCTGAAAATTCACAATTAGACAAC
GTGACTCTGGAAATTGTAAGGCAAATCGAATTTGTTGAAGAAGGCCTTGTAAGGTGGAACCTGTTGCA
CCCTTGTCCATGTGTCTTGTAGCAACACTTAC
ACCATCCTCTGCATGCGTGTGAGAGTAATTGCGTGCCACGGTGGCCGCGTTCTCTAAAGGCAAGTGCT
CTGAAAGCATAAACGAGAGATGAAACCCACTG
AAATTTACAACACTCTCCACATCCGCTTTAAATCCCAGACGAGCAAACGCATCCGCGAGGATCGTAAC
AACATAATTTTTTTTCGGGTATTCAAACCGCA
ATAATCGCTGCGTTGGTGGAAGCGCATAACGAGCCTCACTCAGAACGGATTGTTCAAACCCCGTTGCG
TTAAGTTTTTCAATGGTTTTCCGTGATTTGGC
TGCCACAATGTAGCCGTAACCAATGACAGCGGAATAGAGCCGTGTTACCGGTAGATGCCAAGTCTTAC
GGTATTTTTTCACATTCACATCAGCCAGCCTC
ACTATTCCACGCTCCTGATCCATGCCAATAACAATGGCAAAATGGCACGGTTCTGGTCGGTTGTCCAC
CTTGACGTTGAGGGCAACATTTACATCAAATC
CCATAAGCATCACACTGCCACTTTCCACGGCAAGCTCAATATGCCTCATAAACGTATCATCCGAAATG
TTTACAGGTCCATCTTCAGCCCCACCACGACG
TTCTACAAGTACAATCCCCAGTGCAATGGCATCCTCGATTTTTAGTTTCTTCAAGAGGACGCGATACC
AGCTAAAGGCAGAAGGGACGGAAGCCTTGTTA
CTTCCCCCAACAATTGTCGCAATACCTAGGCCGAGTTGTTGACAAACCAGAGTAACCGGAATACCTCG
TAACATCACATCACAAAAATTAGTGGCATTTT
GTCGCTGTCGCTCTGTGTCGTCAAGGAGAACGTTGGTCACGAGGTGATGCATGATTGACAGTCCAAGA
ATATGGGGAGAAATCGAAGTATCAAGTGCAGT
CGTTAGCAAACCGCCAGTAAGAGAGCCGTCAAGAACGTTTAGAGGAAATATAGATGGTACCGTTTCAA
CAGGTTGATCACTGATAAATACACGCACAAAT
CCACGAGAACGATTACAGTAATTATCCTTGACGCAAAGTGCCTTGTAAAGAACTTGCACAGGGACCGT
GTGTTCTTGAATCACAATACGGCCATTTTCCA
ATAGGATCGGTGTCCCCACAGTAACAGTGTGCAACGCAGGACTGAAGTTGAGAATCAAACCAAACGTT
CCTTGATTTTTTGCCGGCCACCGTGGAACAAT
TGTTTCCATCGGATAATCTGTCTCATTTAACTCCATGTAATTAAGCCGCATCCGTATTTCGTGCTGTT
CAATCAGATACGGGTCAAAATTAAGAACGCAG
ATGGAATTAGGATCGCAGTTGGAGAGGTCTTTACGAAACTGAGAAAGTGTCATGATGGGCAGGCGTTC
TCCCGTGCCCATCACTTCCCCCAGCTCATCCA

CCGTCTCGGAGTCAAATGTTGCCACCTCACAATGTACCCTCATCTCCCGTAGCTTGGGGTGGTCTGTC
AGAAACTCATTTGTGACATCAAAAAGCTCGGC
AAGGGTCACGGAGGGAAAAGAAACGTAGTGAAGCCCAAGACGGTTTTGTCGAAGGATGTCTTCAATGG
TGATACTTTCACCAGAAACGTAACTGCATGCA
AGCGCAATGCTCAACAACCACGCGCAAGGGGCACTGCGCAGCTCCGCAACATTTTCTTCACCTGGAGA
CAATATCATTGGCTTTACAAAGTCTTGCAACT
TCTGCCTTGCTGCGTCCGCCTTGCGACGAGTATTTAATGAATTTGTTGTCTCCGCCCCATGCCCTTTT
TCTTGAAAGCCAACGCGTTTCGGTGACCCTCC
CCCTGATATAACTGGAAGAAAAAAAGAAGACGGAGTGGTGTTTGGGGATGCTTCAGCGGCAGGGCTTG
CTGCATTTAAGCGCGATCCACGCCCGATGGGT
ATCGTTGGCATTTCCATAACCGCAGCACCATTTGTGAACGATTGAACAGGCGTGGAACGAGTGACGGA
GTTGTCTTCGTTGAGGAACGAAACACTATGGG
GAACAATTTTTTTGATGTCTGCATGCATGGCTTCCAACTTGCTCTGGAGAGCCTCCAACTTGGCCTCC
ACTCCTCCTGATTTAGTCAACATTGGTTACGC
AGTGTGTGTTTTCCTTGTACTCTTCGTCCTTTCTCTCAATAGCTGCCCAAAAGACAAGAATGAAAAAA
GAAAGAAAAAGAAAGAGGGATTGCAGCACAAA
ATTTGCCACAGGAATTATCATTAGACAAAAATGCGCGTCATCCACATTCCACTCGTCTCGAATCCAGC
ACTCCCGTGGGATCCCCAACTCCCGCTCCAGC
TCTCGCAATTCCAGACGCCATTTCCGGAGAAAACAAACACGGCGCAAAAAATTCACACGCGCCTGACA
CGCACGCAAAGAAAAACAAGAAGAAAACACAA
AGAAACAGGGAGAAAGAAAAGGCAAAAAAGGAAAAAGAAGAAAAAAAAAAACAAAACATGATCCCATA
CTCAGTAAGAAGCAGAAAATTTTGTCCGAATC
CCCACGCCATAACTGAAGCAAAAGAAAAAAAACAAAAACATCATAGCCACCGCCTCCATAAATTGCAC
CAAAGCACTTGACTAAGAGGCCAGCAGGTCTG
AAAAGAAGGCCGTTTTTCCCACACTGGACCTCCACTTCCGACGCAGCCCCGCTAAATCCGTCATAAGC
AGCAGATCCACAATCTCTCCCTCGCCCGAGTC
CTCTTCACTCACTGTTGCCGGAAAAACAGCGTTGCCGCTCCTCAGCCTGAAACCATGCGAAAGTCCTT
TCGCAATAAAAGTCTCTTGACCCGCCCCATCC
TCTAATTTTTTTCCCGTGGCACGTGCTGAATTAAGATCATGTAAATCTATCACCGCCCCATCTTTTCT
ACAAACAAGAAGAGCTCCTTCGTGATAACGAC
CACCAAAAAAAATAGAAATTTGGTGCTACAAGAACGCGGGAAAATCCACGTTTCATATAGTACAGAAGC
AGCTTTGTGTCCGCTGCTATACAGTGCAGCCA
CACCTCCCCCACACCGTACTGAATCCTACCACTTTGACTGTCTACCGTGGGGTTCACGGAGTCACACA
GTTTCTTATCTTTCAACGTCTCTTGAGGGACA
TGGTTCGTGCCTTTTCGATCCCTCCCAAACCACCACCGAGACATCCAGTCAAATAACGTGGGATATGC
GGAGGAGACCAATCCCCACAAACCCTCGCACC
CGAGATATGGAGGAGAGTAGTCAAGAAACAAGTGAGGTGGAACTGATACGGTAAAAAACATGTACGAC
AAGAAGCGATCCAAAAGAGCTCCCCCCACACC
ACAACGCTGCATCCTACTGTCCACCGCAAAAGACCCCAAGTACCCAGTCGGTGACGCATCAATCCGAC
CATCACGTCGTTGTCCATATGCGATCTGTCCA
ATGCAAAAACCAACCATGACAGAGTAAAGTGAAGAGGATCCATCATCTTCTACATCACCAAAAGAATT
TTCTTCATCCCTTTCCGTTTCAGTATTTTGCT
GATGCTCAGCAGCCTTCAGCAAAGACAGGATGCAGTTTTCGGTCGCGAATGCAAGTAATGCCAGGCAT
GAGTCCGTCAGCATCCAGTCGTAGTATTCGGA
TTCGTACTTCACAGGAAAAGCAGCGTCGTGCAGAACCCCCAACGCTGTGAGATCTTCCTTTTGAATGC
CCCAACGCAGCTTTATTTCTATTGGCACCCCG
CCCCGAGGCGGTAGTCTCACAATCTCCGAGCCACTCATCACTTCGCCTTCCACTTCTGTTTTTTTCTT
TTCCCCCTTCTTCTTCTTTCCTTCCTTCCTTC
CTTCCAAAGAGACCCTCCTTTCGCGAAGAAACAAAAGAAAAAAACAAAAACTCGTTTCTTTTTTCCTT
CTTTTTTCGGGCAGTTACACAGAAAAGAAACG
CATAAAAAAACACGTGAAAAAAAATGGCAATGCGTTGAGGCATGACCGTTGACCCGCCACAACACATA
AACACACATATTTATATTTATATATAATTAAG
GGGAAAGGGCCATATATATTTGAGCCGGTGATATCAAATCAAAAAATAAAAATAAAAATAATAAAAAT
AAATTAGTTCTTCATGAAGAAAAAAAATAGAA

ATACCAGATTTCCATGTACAAACACTATGAAGTAACAATTCCGTGAATACGCAGAAACATACACACTT
ACACACACACGCACCTCTATATGCATTTATGC
ATATGACTATTCCTTTAAATGGCTCCCTTTGACGTACCTCACCCAAAAAAATTTTCCCCTTCCCGGAA
GAAATGGGCGCAAGAAATGAGTACACTTTTTC
CTTCTTCCTCCCCAATTCTTCATTGCTTGGGTAATAATTCCATTTTAATTCAACCATTATCGGTCTAA
CGCCCAGCCCCTCATCCCCTCATCACCCACTT
CATCAACGGGTAAATTATTTATTCATTTACTTATTTTAAGCGATGCATTTAAAAATTGTGTATGCGCT
TTTATAATTGTATGCGAATTTGTAGTTTTCTT
TTTTTTTCCTCGCCTTTTCCTCCTCGTAATATCGCGATTTTTCGTTTTAATTTAATGGGCTTGCAACT
CCATCCATCCTGGTATCGTGTTGTGCTCTGAG
TCATCCCTTGTCCTCTGTTATTTTCGGTCTATGGCCGTTGGTTGATCACTCACTTCTGCGGTAAAGGA
AAATTGAATAAAACGGACCAAATCTCAAAATT
CTGCGGGTAACTTCCTCCCTCATCCGCTCCATTCGCCCAATCCGACGCAAACAAAAAAATTATTTTGT
TCTTTGTTTCCCGCCCCAGCCGTTCATCCCTT
TTCCCCACTCACGCAGGCACGTCAACACGACGAGACCCAAAAGAGTTCTCCTTCGTTTGCCTGGAACT
GCGCCACAATTTTGCGTTTCGCGTTAAACACT
CAATCACAAAGAAACAAACCACTGTATGGGTTGCACAGCGGCAAAAGTACGGAATAAAAGAGTCATAG
ATTGTCAATACACCTTCCTCACGGGCAATGCG
GGATATTGCCTGCAAAACATTTTTATATTTCAATTCAGCAGCTTGACCGGAGGGCAGTTTGCCCTTAC
GAGCTTCGGCTGACATGCGTACACGTGCCATC
TCGACAGGCATCGTGCAGAAGGCATAGAAGAGGGCTGTAAAAATACTGGCAGTATTCACAACAAGATT
GTCACCGTACGGAGACACGCCCTTCTGTTCTG
CTCGGCATTTGTTATGACGACGCAGGAAAGTAAGCATCTGATCGTGCACAGCGACCTGACACACACCC
ACAAGGGCAGCACGTGAAGCCAGGGCTCCCAC
TCCGCGCCAGTAGCCGGTAATTCCACTCGCCGTGTACACTTGCGTTGCTGCATTGAGGATGGAGACCT
TCATCGGAGAGGTCGTCTGCAGCACAAGGCAC
ACCTCCACTGGGGAACTCAAGACACTCGCAAAAGCCCCGGAAAGGGCTCCAGCAAGTGCCCGGTCTGC
GATGGATGGGGCGGTCTCTGGGCTGGCACCCT
TCAGCATGATGAGAGCGTCGCGAAAAATGGGATAACATCCAAGACGCGCGGGGGCGTATACCACCTGA
CGGAGGAGCCCCGCCGAGAGCCCCTTGAAAGC
CCCTCGCTCTCGTACTTTTCGTAGGACATCTAAGTGCGAATTGCCGGGGGGTGCCATCATAACTGTGT
TCTTCCACATCTCAAATGGGTGTGTAAAGACC
CATCCAAACATGCCGGAAAATCCGGCAATGCCGATTGTCCAAGACTTCGGCAGTGGGGCATCCCTCCG
TATCTTTTTATCGGTCGACATGCGTTCCGAAA
CCCAAAACAAATACCAACTCTCGGCGGCCAAATTGCTATACAAAAATCAGACATGAGAAGGCAAACAA
AAAGGAAAAAAAGCACCGCGTTTTTGACATCC
GCCTTAAAGCGAACCACTCACACAAACACACATATAAAAGTGCACGCAATTTTCTGCCCCCTTCATTT
TATTTTAATTATTGGAAAAAATATAATAAAAA
AAAAAAGAACGGGTGCCCTGTCTGTGTCTTCCTCACCCTCATCCAGCCTCACACACTCACTCTTCAGC
ATATATACACGAAGAGATATTTCCTATTCCAT
TTCGCTTTTACCCCCACGTGTCGCAATGCTGTTTTTGCCCCGATAGCCTCCCTGTTGAAGAACAGCCC
GTTGCCGCAAAGTTCCCCGCCAGTGATCATGC
AGCTGCGTTGCTGTCCGATCACGATGCCACACGCCACGGTAGGCATAAAGTATACTTTCGAACCTTCC
AGGCCCCTTTGCAAAACGCGCCACCCCCTGAA
GTATTGCCTCGTCCTCTTCAGGGGTAAACTTATGCCGTCGCTGCTTTGGACGCAGCCCAACAGGGGCA
ATATCACTCCTAGAGGTTCCTTTGGGTTCCAA
ACCCTGTTGTCTAGAACTGTGGGATTCAAGCCCTCCTCGTTCGCCGTCGTCCACAAGACCCGCCACGG
CATCAAATGAGTTCAGGGCACAAGTGGTACCG
GGCAATGCATGTATATGCGCCAAAACCTCCAACGCATCGCTACACCCGGCCCCATCAACACCGTCGAC
GGGTTCAAGATAACGGCTCTTTGGAGGGGCAA
CTGCAGCCGACTCTGTCGCAATAACACCCAAATTTTCCTCGGAGCCATCCGATTCACGTGCCCGCTTC
AACGCCTGGGGACCGATGGTGGCCGAAGGGAT
AGTTGTTCCCTGCGATTGGCTTCTCCGCTCATCAAGGCTGCAAAAACGCTCGTGTAGTACGGCAAATA
ACTCATCCAAATTCACCCGCATCGCTTCGGAC

GGAACGGAGACAGCTGCATCAAGCTCTTTGCTTCTGCCGTTGCTGGCGTTGTTCTCCTTGACTGGATC
CATTTCCAACTGATGAATGACCCGGCAAAGAA
GGGGGTCTTTACCGCACACTCGTCGCAGCATAAGATAAAGCTCGTTCTCTGTCAAAAATTCTTGATTC
AAATTTGGTGCGATCTCCTCTGTCTGGCATCT
GCTTTCCTGCGGGGGATGTGTTTTCACTCTGTCGTTTCCAACGTAATTGGCAATTACAGCGGACGCGA
TTCCATCGTGATATGTACACGTCTGCCAAAAC
GCTGGATCCTCATGAACGACAGTGAGGGCCAGTATTCCCAAAACTGCAAAGTGGTTTCCTCGGCGTAT
GTGGTCACGCATGATCTCGTGGAGTACTGTGA
AATCCATCGGAGGAGGAGACGTGGAGACCGGGCACTGCGTGTAAGACATCTTCCCACAGTATTTCAAT
GCGGTGGATCCAAACAAATAAAAGCAATTGAA
ACAATGGAAAAAAAAAATAATAATTCAAAACGCAAACCTCACCTCCCCTCCCTTATATTCTTTTGTAAT
TCCCTTTTCTGCTTTGGTTCTCCCCTTCCACA
GGCCAATTTATCTTTGTCCTGCTTGGTTTACGCAAAGAAAATATTCTATTCCACAAGTATATATGTAC
AGAGAGAAAGAGGGAAAGAAAGGTTCTCTCCA
ATTGGTCCTTCCTCGCTTAATTTAGCAAAAAAGAAAACCAAAAAAAAAAGAGCATGTGTCGTTTTTGC
CTCCACCTTACACCTCACGTCTTTACGCCTCA
TATTCTCACTCCCTCGCCTCTCCCTTGCATAATTAGGGTGAATTCCACCCCTTCCCCTCCCTGGATTT
TTTCTTTTCATTTTCGTTTGTTGCCCCTTGGA
GTCAGAGACTCCCCACTCCCCACGCCTCCACTTCACTCACCGCAAGAGAAAAAAACTATCGTCCATAC
ATACATCAGGAAAAAATAATAACAGTAATAAT
AATAATAATGAGTGCATCGTCACTTAAGTCTCCAAGAACAGATGCTGCACTGTATGGCCGCCCCCTAT
TCCTATTTTTTGTTTGTTGGTTTGTGCATGCT
TAGTCTACTGTAAAGATGTGTCTATTACGAAGCGGGAGTTCAGCCATGCGCTCGGCAAGCTTCACCGC
CGCCGCCTCCTGAAGCCTCGCCGCCTCCGCTC
TTCTCCTCAAGCGCTCGGCCTCACGGCGTGCTTCGCGATCAGCAATAAGCGCCTTCTCTCGGTCCTCA
TCATGCCTCGCCTCAGTGCGTCGGTGTATTTC
TCTTTGCCATGCATCCTGCCTCTGCCGCAGCAATTCCAGTCCTGCGGCGTCCTCCGCAGTCTCTGTGC
CATCCCCAACATGTACAGCACCATCCGCACCT
GCCGTCGCCGACACCTCTGTGAGCGTGAAAGTTGAAGTTGAAGCTGCTCCCTCCAACGCGGTGGATCC
AGTTACTACTTCTCGTTGGGGTTCATGCTCAG
GATTTCCGGCAGGCTCTTTCGCGGGGTCATCATCATTTTCCTTTTCCTGCGGATCGGATCCTGTTTTT
GCAAAACTTGTCTCTTGTTCTTCCGCTGCGGC
GGCGGCCTTCTCACGCTCCAGTCTCGCTGCCTGCACCTTCTCCATCACGATTCGTACACGATCCGGAT
CCCCAACGCAAGAAAACTGCGGAACGGATGGC
GTCTTTTTAGGCAACTCCACCGCAGTGGGACGGGCCTCCGCCGGCAGTAAATCGCTGACGGTGGAAGT
TTCATCATTCGCAACGCAACCCGCAGTAGCTG
AGTCCTGTGCGGAATCATTGTTTTTCCTCTCGGTGACCACATCTTCGGCGTGCTCCGTCACCGAGTCC
GGTGGGTTTCCAGCAGAAAGTAATTCTTGTCG
CATATCTTCCGCAGTTATTCCTGTTGGTGCTGGGGGCACGACGCGCAGCACCAACGTGAGCCGGGATT
TTCGTCGCTCAAACTTCGCCTCCAATGGCGTT
GACTCCACCGAAAAGGGAAGCGTCACGCTCCCATAATAGGGATGTGAGGAACTCTTCGCAATGATTAC
GTATGTGCTTTCTACCTCAATGTTAAGATCCG
CCGCGGACTGCACGCCTGTAAAATCCATCTTGACGACAAGAGCCTCAGGCACACCAACGCGCCGATCC
ACAATTTTCCATCCCCAAGTATCGGTGAGATC
AATATGTCCTTGGTGAGCAATTGTAAAGTGAGGAATATGTTCCTTCTTCTCCACTTTATCACCAGCTT
CTGCATCGGTTTCTTTCTTTGCCTGAACCCCA
CTAGTTTCTTTTCCCTTGTTACAGGGCGTCGTCCTCCCCTTCTCGCTGGTGGAGGTCAAAGGTGGGAG
CACTTTTGTCGGATGCGTCAGTGTCTGCTCGT
TCATCGCAGCCGCAAATGGCGACTTCCCCTTCTCGCGGCTAATCGTCTGGTTCTGTGGGGTTCCCACC
GATTGGATGCGGCTAGGCAGACGACGAAACTC
AAATCCATGGCAATCGCCATAACCGCTATTGATATGTTCTACGGCGATGTTCACTAAAAAACAACAAA
AGCGATCGCTGCGATCTGTTAACTGCAACGTG
TTTGGATGAAACACGGCGTCATATACAACGACGGGGTTTGTTCCCTCCCCGCTTTCATGAGTTTGTTT
TTCTCCCGTCTTCCCCGACGTTGCCGCTTCTT

CCGAAAACAGCTCCGTTCTCGGTTTTGATACAGACACCGGCACAAGCCAATTGCTCTTCTCCCTGTCT
CCTGTGGACTGCTCAGAAAAGGGGTCAATCTT
CTCGCTACTACACATGTTAACGTACACCCGAGGGACATGTTTCTTCTTTTTGCTCTCCGCTTTCAATG
ACGGGTGCGTCACTTGATAAGACGTAGACGGC
TCCAGCAGCTCCACCACAAACCCCGGTTTGGGAAAGACAAAGTCAAAGCTGTAGTCGCCGCCCGCCTT
TGCCTGGTGCTCCACTTGCTTGAGATAAGCCT
CCTCCTCCCGGCGTGTCTCGGGGTCCTCCATGGACTTCATGTAACTCTGGAAAAGCTCCACAAACTTG
GGGTCCTTCATCTTCTCCTGGATGGTGCGCAG
CTCCTCGGGTGTTGGAGTGAATTTCTCCCCATCCTGGGCATCCTTTCCAGAGAGGTTTATGGTCTCCA
GCGGCATCCCCTCCTGCCCCCCTCGTTTATGA
CGTCCACTTCGCAAATGTCACTTCTCAAAGAAAAAGAGAAAGTGGTGGGGAGAGGAGAACCCCAAGAA
GAAGAAAACACCGATAGGAGCAGCAAAAAAAA
AAAAAAAAAGGACTCAACAAACACACACACANNNNNNNNNNNNNNNNNNNNNNNNNNNNNNNNNNNNNN
NNNNNNNNNNNNNNNNNNNNNNNNNNNNNNNN
NNNNNNNNNNNNNNNNNNNNNNNNNNNNNNNNNNNNNNNNNNNNNNNNNNNNNNNNNNNNNNNNNNNN
NNNNNNNNNNNNNNNNNNNNNNNNNNNNNNNN
NNNNNNNNNNNNNNNNNNNNNNNNNNNNNNNNNNNNNNNNNNNNNNNNNNNNNNNNNNNNNNNNNNNN
NNNNNNNNNNNNNNNNNNNNNNNNNNNNNNNN
NNNNNNNNNNNNNNNNNNNNNNNNNNNNNNNNTCCCCACTCCACTCAAAATGGAATACGCATTTTCATT
ATTTCCCCCCACTTCACCCCCATCCCCGAGTA
AAATTACCGAATGGAACCCCAATAAGTAGGCGGCAATGTCAACAATAATAATAATAAGAGAGAGAAAG
AAAAAAGAAACTGGCATACAAAAAAAAATAAT
ATATAGATATATATATGGAAAGCAAAAAATAAATGAGAGAATGCTATTACAGATTATTTGCGTGCTTC
CCTATGTGTGCATATACATGCGTATACGCTTT
CGGTGTAGACCTCTTTTTACGTTTGCTTTTCAGTTTGATCCCATTTAATCTCATTCCACCATGTCATT
CGGAATTCGTACGAGTATTCCTTCAACTCCTG
TCGACCACCCCTACAAAATAAAAAATAAAAATAAAAAAATTGCCTCCTCTCTACTCTATTACATGTAC
GCGCAGACGCATAAAAATGTTTCATGTATAAA
CTGGGCAAACCCAACTTAATTACACCGAAGAATACGCAGGATTACGTCTTTTAAAGGCAAACCTATTG
CGCCCCTCAACCCTCGCTGCGGTCAGTGGGGG
CCGAAATAATGGCATCCTCGGCGGAATCACAGTAGCACCGTGGCCAGCATCGTTTTCCTCCGCTTTTT
CTTCTTCTGTGTCGCTAAAATCGTGTAAAGTC
TGATTAACCACAACGGCTGAACTCGGTTGCCGTACCTCCGCCGTCAGCTCCCTAATTTTGCTTGGCTG
CGTGGCATCCCTCTGCTGAAAAGCGTAGTTAC
GAGCAGATCCCGCCACGCGTGTCGCAGGTCCCCATTTTTTATCCGTACTCCCTGAGCTGTGCAACCGT
GGGGCTTGATGTTCCATCACACCTCGTGATCG
GCACTCACCCTCGCGCTGATCGCGTTGTGGTTGATCTGAGGTTTCACGAACGAAATGCGTCTTGCACC
ATTTTTTCCAGAACTTTTCGTACAGCCGAAGG
GGGACATATCCCGCTTTCCCCGGCGATGGGCGACGGGCGCAAAAAGAATCTGCTGGAGTGGATACCAA
TGAGGGAGAGCGCAACCGTGTTTCCCTCCTGG
GCGAGGTGCTGCACATCCTACGGGTGTTACCATTCAACGATCCCACGTTCACACGGTTGGCTGGAGCA
CGTTTTCTTTGCGGTGACCACACCAAATCTTC
TCCCTCCTCCCTCTCCTCATCCTTCCCACGACCACAAAATGCCCGTTTTTGTGCCTCAATGCTAGAAT
ACATGCCAAGCTTCTCCATCAGAAACCTCCTT
AGAGTTGGAACTTCCCATGGCCATACCTCTTTCGATGAGACATTACCTTTTTCAACGAGTCGTTCACT
TGGCGACTCTCTTCCAGCCAAAAGAGGCCTTC
GACGAGGACCCAACTCTGATTGATAAGGAAATAATTCCGAGAAAATCCTGGTATTGGACTTTTTCGTC
GCCAGTGAATTTCGAACCTTCTGCGCTTGTGC
GGTGCCAGTGTGACTTGAAGAAACAATCGTACCCTTCGATCTCCGACTCATGAGACTCCGCAGCTTCC
TTTGACAATCAATAACTTCCTGCTTCATCAAC
AATTGACGTCGTTGGCACACGCCACGACGCCTGCCGCCAGCAAAAATGCAAAATTGTCGCCCCCCCTC
TTTTAGGCCCGGTCTGCTAGTTGGCGTTTCCT
CATCTGTCGCAAAAGACACTGAAAGTGGCCGAGGTGCTTCCGTGGAAGCAACAGTCATTTGAGGGAAG
GGTTCCAAAACTATTTCCTGTTTGCGATCAGG

CAGCCCAATTGCTGCCACTCCTGATATTTTACGACATCTCTTCGTGTGAAAGTGAACCCCATTCAGGG
AAGGGAATTCAGATGGCGATCGAAGAATACCC
ACATTATTTTGCGCACATGCATAGGGTGAATTGCCGACTTCAGGCAGTGACAGTGAGGCCCTCTTGGC
CACAGTGATACCACGGCCTTCCTTTTCGCTGC
CGCTGTAGGAGGTGAGACTAGTGGAATCACTGCTCTCCTCTTCAACTAAACTCAGGTAGCCTTGTTCT
GGGCATGTATGTTCTCTCCACGGTGGTGAATC
CTCGCATAACGAAGTTGGCGTCAAAGCTGCGCCGCCCGACAACAACGAGGTGACCGACTTCCTGGGTG
TCCAATGCACATTGACGGAGTGGTTTTCCAAA
TCCTGCACAGGAGGAGAATTGACAAAATCCGAGGTCATGAAAGAAACAGTCGACGAGCAAACCGCATT
GGCATCATCCTCATCCTTATTTTTTGCCACGT
CGGGGAATACCAGTGAGACCCCCGTGCCTCGGCAATATCCTTCGTAAGAACTGTAGACGAGAGAGGCG
TCCTTCAGCGGTTCCTCATTGCTCTCCAGAAA
ACAATTGTCAGTCATCCACATCGTTGCCAGCTGCTTGAGCCTCTTCTCCTCGCTCCGACCGGACACAT
AGGGCAACACGAGACCCCAGGTGTGTGAAGAT
TTGCCTCTCTCTGGCAATCTCGCCCTCCGCACATCGTCGCCTTCCATGGACCACCCAAACACAATGGT
GTTTTCTACCGCATAGAAGGTAATGGAATCAC
GTTTCGGCACACTCCGCAGCAGTACGCCAATGACGGAGCTCTGCCCGTTAGACTCCACTGTGAGTGGC
AGTCGAAAAACACAGCGGGAGATTGTCGCCTC
ACTTCGCCCTCCCAGCAACACATGACAGAGTCCAAAATCCTTCATCTTTTCACAGGTGTCGGAATCAG
GAACGGGGTCGGGACCTTTAATTAACTCCTGT
GAATTGATTGAGTCCTCGCCATTCCGCCAAAACAATTCACATAAACTCAACGACACGTAAGAATTATC
ACCCGCCACCACAATGCACCTACCAATCGACT
GTGGGCCCGCAGCCCAAGCTGAGAATATGCACTTGTGGGCCAAGCAATGCCCGCCATCTGAGAGAACA
AGACCGCTTCGTCCATGACGCACCAGTTCAAG
AGAAGACTCCCGGATATCAAGCTTTCCATTGTCCTTCACCCACACACCGCTGTCGTTACGATCAAAGA
GAGCATGAGAAACGATTGCGCTACTCTTATCC
CTCAAGACAAGACTGTGACCGCCACAGCGGACAACAGAAATATAATCCGCTTCAAGCCTAGCGGTATC
AAATAAAGCCAGTGCGGTGGACACCACACCTG
GACCCGAAAAACTACTGTTCAAAATATGAAGCTTTGTAAGGCCGGAGGCTGTTACGGCAGGCACCTCA
TTATTGCCCTCCCATCCCGCCACACCGCAACG
ATAATCAAAGTCACAGTCAAAGAAAAAGCAATCCCCATCCATTGCAAGATGAACACTTGCAAATTGTG
ACGATCCTGGCAATCCCACTACATTGCCACCG
CATGTAGACGCTCCAAAAAAGCACTGCGAAAAGAAGACTGGACCTCCTCCCACCACCTCTACAAAGTT
CATGAACCAGACTCTATGAAAAACAAGGGAGG
CGCGCGAATAAATCTTCATCGGCTGCTGAAACACTGCCATAGATGAAAATCCATCCTTTCCCACAAAA
CCACTTTCACCCTCTCTATCCGTCAACGAAAC
AACTGTATCTCTCACACTAGATGCGCCAGCAACTAAACATGAAGCGGTGTTTTCGATAATCTTACAAG
CGTACGTGCCCGGAGATAAGCAAAGACGCATA
GGCTGACCACTCGAAAATTTCTGTAGCGCAGGTAACGAAGAGTCAGGCGTAAGATCTGTTGCTGGCCA
ACTTAAGGCATTGTGCATAAATTTGTTGAGTT
CCGACAGAGGAAAAGAAGTGCTTTGCAATGCATCAGCGGCGGTTTCCTTTTCTCCCGAATCCATCTCC
CTTCTACTAAAGCAAAGAAAGCAGGCCGGATT
ATCTGTGACGCCCATAAATTCCAACAAAACAAAAAAGTTAAGGGAGAAGAAAAATAAAATAAAATAAA
AGAACATGCACAAACACAAATAATAATAATTA
ATAATAAAGAAAGGATGCCACAGAATACACATTTCATTCGATCCACAGATAGGGACAGATACCCAGCT
TACAATTCCACAGCCCAGAACGCTGTTTTGTT
GTAGTTTTTTGCCACCCATCTCTTCTCATTTCGCTCTCAATTTTACTTCCCAATCATTTATTTTTCTC
TTGTTTACTTCACCCATCTGGTGGGAAGTACT
GGCGCCGGAGGTTTACTCGACGCTCCGCCACAATGGCGGCCACTTCTTTTAAATCCGTGGATGAATAC
GGAGAATCATTCTCTGGGAAAAAACCCCACTG
TAGTTGCCATGCAGCTCGACCTTCCTGAGAGGTCACAGACATGCCCCCAGCACTCCTTGAGTCACGGT
CATTCGGGTTTATTGTTGGAGACGCGTAGCAA
AGCGTCAGTTCCTCCCCTTCCTGTATCTCCCGAATACTAAACGTATGCAGAAATGGTTCGTATAACAA
ATCAGAAGGAACACCAGCTAAGAGATTGTCAA

AAACATCAATTCCGCAAAAAGTCTCTTCTGTAGCCATCGTGTAATAGCAATTGGGCTCCGGATGATGA
TTGAGTTTGTCTACCAGAGGAATTAAGGTCGC
AGCCCCAGCACGTCGTGACAGCACCAAACTCACCCACCAAGCATACTCCTCTTTGGATAGGCGCTGAC
GCAGATAGGCACTCAGCGGTAAACCTTGCATG
TCGTTATGAAAATACTCCCACAACTCAAGCGCGGGGCTCTTGTTACTCACGGTATGACTTTCCCCTAT
CGCCCCTAATAATGGCTTGGTTTTATCGCTCT
GCAATATCCGTGCAATCTCCTCCCTTTCCAGGAAAGGCAGGTTGTTGCTCGGTATCACCGATTTCTTC
AGGTAATTCACATAGCCCGCATGTGAGGGACG
CCAATCATTCTCCGATACATCGTCGCCCGCCACCACCAACTGTCCCACCAGCTCCTCTACATACATAA
AAAACGACGTTATAGGGTCAGTAGCAGGCGGC
GGGCTCGCAAAAAAGATCGCCTCCACGGGTAAACTTAAAAGATACACGCCAGGTAATAAGCGCCGGGT
AGCCATAAAGGAGCTTCCACGCACCGCACTTT
TCCGCCGCGGTTTCGTCAAGGTCAGAAAGGGTGAGACCACTGCCTTCAGATCCCAAATAGCACTCTGC
ATGAACAAGACATCATCCGTGTCGGTGAATAA
CGAAGCATCTGCAGCATTCTGTGAGGGAAGTTTTCCCCCACGCCACCAGCGTCGGTGTGCTGCTTTCG
TCAAACCGCTTCGCCAGTGACGCAAAGAAGAG
GCCCCCATACCGAGCTCACGGCAAAAAAAGTCCAGCACAGCTTCCCCATCGTTTGTCGTGGAAACCGT
CAGTGGAAGAGAAGAGCAAGACGGTATCCCAC
GGTCACCCTCCACCACGCCGCCGGTTCCAACACCCGCAGCCTCCTTGTAGAGTTGGGCAATAGCGGGC
AATGAGCAGTGGAGATTACGAAGAAGTAGCGA
CGGAACGGAGTGAGACATCTCTCACTTCATCCCTCACATAAAAAAAAAAAAAAAATTCTCACAGCCGTC
TGCGTCGTCACTCGGCGCCGACCCACACACCT
TGTCCCTCTTCCACAAAAACAAAAAAATAAAAATAAAAAAAAAATATCCAACAAGAAATAAGGGAAAA
AAATAAATAAAAATAAAAAATGCAAATTGAGG
GTAAAATAGCAATGGCACACACATGCGTATGCCATGCAAACTCAAAAAAGGAGCCAGACAGGCATGAA
ATAAATGGCACACCTCAATATAGCACCAGTCA
CGCAGATATAATCACCTAAACAAAAATGTGCGTCAAGAGAAAGGCAAGGAAACAAGACAAAACAAAAA
GCCTATTTAAAAATACAATCCGGTGTTCTTCT
TAATCCATTTGCTGTAGATTTAAAAAAGAAGGATATTCAGATAGTGGTAAAGTGGTGAAAGGAGTAAA
ATGTTATATATATATATATACGTTACGGTTTG
ACAGCAGTTGAATGACGCAGCCGTACAATAAAAGTAATGAATTCGACCCTGAGCAAATAAATCACCCT
CAAAACCCTTTTCCGCCCTCATCATTCCCCAC
ATTTTACTTTCTTAAAAGAGTAAGAAAAACAAAAAATAAATAAAGTTTAAGAAAGTTAATATCATTTT
GAGGTGGCATCATACAAAAAAATTAGCATGCAA
TTGTATTTGACTCAGGAGATCCTTTTTTTTGCTCTCATTCAAACTTGCCATCAGCTTATTGGTGGTGG
CATGCCAATCTTCTCTCGTTGGTTGCAAAATT
GTATTCTTACTGGTAGTAGCACTACTGGCAATAAAATTAGTATTATTCTCAGCTTCAAAAGCAACAAG
ACCTGTTCGAATGGACCACGAAGCTAAAAGCC
ACTCCAGAGCCTCCAGCTCTTCCGCATCCCTCTTGTGTGACGTGCGAGTAGCAGAGGGACGGTCTAAA
GGGAAATCTCCTGCAGCAATAAAGTGGCATAA
CTTCTGTAGTCCCGCTTCGCCCCTGACAAGTTGGGTGAAGCTATAAGCTGTGTGTTTGTATACAGAAT
GTAAAAAAGATTCTCTTGTCTCGTCCTTCGCT
TCCCCGGTGTCGTGTACATCCTTCGCCTGGTCTCCAAGGCTCCTCAGTGCAAGAACAAAAAAACGCAG
CGCCCACTTCTCCTTAAAGGGTGGGGCATAAG
CCGTCGAGGCATCAGGGTTACGCTGGACCTGAGTGAGACTAAGTTTGAGACGGTTTTGTACCGCATGC
GGAAGCGTTTCATGTGGTGCTGTCAATGGCAA
AAAGAATGTACATGGTGAAAAGGGGGATGAAGTAAAAAACAACTTCTTCATGCCGCATAAAAAATGCG
CGTCAAACGCATTCTCACTTAGGATCAAACGT
GGAAAGACAACTACATTCTCCGGCACAAAGGAATGGGTAGAAGGGGAGGGGTAAATCACCTTACACAA
CACCCCAAGAATACCCGCTTCTCTCCCGGCCG
CCAACGACGCCACACAAAACAGTGAAGCTGGATCATAACAAGAGGAAATTCGCCTGGCAAAAGTACGT
TCCGCGGAATCAGACACCGTGCCAGCCACGAG
ATCTAGAGCAACATCGGAAACGTGTGTGCCTGCCTCCACGGCATTCCTCACATCCTCCACATTACTTC
CCAATGCAGGAACAGAAAAGGCCACGGTCGCC

GAGCATCCATCCCCCACATAAATTACAATGGCCCATGACAAAGATGTCTCCTCGCCCTCAGGAGAGAG
TGAGGCGCAACTTCTGGGGAGGTACACATGTG
TCACCCCCGCTTGATCACCATTCTCGCAGCGCGGCAAGTTCCTCTTGCCATTCTCGTCGTTATCCCTC
GTCACTCCCTCCTCTTCCTCCACAAAAAACCG
CACCACGGCACCTTTTCTATCCTCAAGCGCGGTTAATTTCACTTTACAATGTGCAATGACAGCTAATG
ACCCAGATGGTTGTTGATTCTTTTCACCATTG
GGGCCTTCTGTTCGTGGAGTCCCTAAAACCGACTCTGCCCAATGAAGTTCTTTACTGATGGCAGACTG
CTCTGAGATAGCAACAAGTTCCAGTTCCGGAA
ACACCTCCCCTGCACGAGCCCAAACCCGGGCAGTCACAACGGGTCCATTTAATCCTCGTGAAATTTTT
TTGCTCCTCCTCTTTTGATTCCTCCTTGCGCG
TGCAAAAGGCAATTGCATTTGTAACAAATGTCCCCAACAACCTCTAAAAGGGCCCTGAATAACGCCCC
GATCTCCGTCCTCGTAGTACTGTACTGTCTCA
CTACCGAATGCACCCTTCTTGCCTTTAATTTTCGAATTTCCATACTTCTTCTTTTCTCCTTTGGCATC
CCCAGCAGCAGGGGCATAAACAAGAAAAGGAC
CGTTCACCATCTCTATTGTGGTCGGCGTGGGGACCTTCACCTTGCCATTGCCCATGAGAAACTCAACC
GCGGACCTCCCACACGCCAGTCTTGCCTCCGC
AACGCTGCTGCCGTACAGTTCCTTCCCAAGTGCTTCTCGAGAAGCCGCAAACGCCCCAGAATCAAAAA
AGCTCGGCAGTCGGGAAAAAACTTCGTGCATA
CCCGACTCTGGACACCAAATGGTACACTTCTTATTAGCCAATCTTTTGCCATCATGAGCCACAGCAGG
AGCGCACGTTGAAAACAACCGAATTGTGTTGG
GCTGGCCACTCTCAGAAACCATCATCGTAGTCAACGGCAAAAACAAAGTGGAATAGATTAGAGGTGGA
ACAAATGGGTAATGCTGGGAAAAGTCAACCAC
AATGTGGAGCAATTGCCCTGCGAGCGGGCCATCAGAAGGAGAGACATACACATTCCACCTCGCCAGGG
TGATGCTCTCGCTTTCAGCTTCAATTTTCGCA
TGTGAGCATTTACCCTTTTCTAGTCGTAGTTGAGAATTAATAACCGTGACATTTGGAATAGGGTGCTG
CAGGATGTACTGAAGCTCCTTTTCCACTCGGC
GAAGTACCGCAGCGGTCCGTGGTTCTAACTCTTCCATATGAGCACTCTGTTCTCTTCCCTCAGAAGCC
TTGCACCTATAAAAAATCGGTATGTGCAACTG
AATAAATCCAAGTTCTTCCCTCGTGATATGCGATGGCATCTCCAACACTTTTTGCTTCGCCATTTCAC
GCGTCTTTTGGTCAGTGCCAATAAAAGCGGAT
TCATCATCGGATCGAATCAAACCTTCACTCTTCTTCCCATCATTCTCTCGTTGCTTCTGCTGCTGTTG
TTCTTGCTCTTCCTTCTTGTTCACCTGGTTAA
AATGTAGCGCCTTTATTTCTGGTCGCGAGCCATTCTTGGAGAAATTGAAATTAGAGCAATCAACCAAA
GGATTATAAATGCGGTCCCTCTCCCTATCGCC
AGTGCTACTCGGTCCAACCTCGTGCGTAGCTCGTGGAGAGACAACCACACCCCTCACTGCGTTATTTC
CACAGAGGCTTGTATGACCAGAAAGAGAAGGA
TCTATATTGGCTTCTCCCTCTGACTTGAATTCAGTCTGCACGGATGTACTGTTTTCGTTTCCACTGCT
ATCCACCACTCCACGCCGGATTAAAGCAAAAT
TGCCCTCCAGTTTTATTGCGGAGTCCGTCTGCTCTGCAGCAAGCGTGTTAACAATTGGTAACTGCATT
TCTACGTGTGTTTCCGTGATTTCCTCCACGGT
TGAATTATCAGGTGCAGTTTCCCCGTTCGCAGAAGTTTCAGTGGCCCCGTTTTCATCATCCCTCTTTT
CATCCTCGTTTTCCAGTAAAACAATTGAGGCG
GGCTGTTTCCCCCACTCCTCTTCCTCCTTTATTTCCTCCAGGAGTTGCAGAAAAAACTTCATCGCCCG
CCTTCCCTCCATCTTCGCATGACGCTTGAGCC
GGCTTAGTGTGCGACGAAGCTCCGGCAACCCTTTGATAATTTCCAGTTGTGACTTGGAGGGACAACTG
ACGTCCCCTGAAATCATTTTTTGGCGCAGATT
GTTTGCTGATTCAAACCGTTTCTTTAAAATTCGCAACTCATCGGCATCGTTAATCTTTCGAAGATATT
TGCAAATGAAGGTTTCTCGCGCGGCAGGTGGA
AGGGATCGAAGATGTTCAACGACACGCTCCTTCTCGGACTCAAAGTTGCCAACAAAACCGTTCGCAAC
CACCTGCTTCTTCGCATTACCCCTCTTGTTTG
TCTCCGTGCATCTAGACTGCTGTTGTGACGAGCCTCTCTCGTCCATCACCTCCTCCTCTTCGTTCCCA
TTCTTCTCTACCGTAGCAGAGCCTTCTTCACT
GCGCTGTGCAGCGACATCATCAGCAAGTTCCAGCTCATTTCTTGATTCGGTTCCCACCACCACGGCAG
TGTCTCTCGCAGGTAACTCCTCTTGGCTCGTC

GCCCCATCCGGTGCCTTAATACTTGCGTTTGGCAACTTCGATGGACGTAAGGGCGGTGGACGACCCTG
CCTCAGATGCTCCAGATCCTCAAGCCAGAGTG
GATACATCTCGACCTGCCGGCTCTGCGCTTCACTCAATACCCTCAGCTCACCACTTGCGTGTTGCTCA
AAGACAGGCCTTGCACGGTCGATGCGGCGGCA
AAGCTGCTGAATCCGCTTCGAAAACACAACCTCCTTCTCCATTGTGCGACTCAATACCCTCCAACAAA
ATGTATTCCCCAAAATTTGCAATCCGCTGCGG
AAACAAAGCTGCCCAAATCGCACGTACCGCCTCCTTCCTACTCCCCTCACCCCAATCCCTCAACTCGA
CACGCGTATCAGCAATTGAGAAAATAAAATCC
CCCCTTTACGTAAGAAAAAAAAAAAAGATAATAATAATAAAATGTGAACACTGAGCCCGCTTCCTCCAC
GCCGCGAGTAAACCCCAGCAAAGATAAACCAC
CAAAGTGACAAACTCAAGCCTTTAAAGGATTAAAAAAAAAAAAAAAGGAAAAACAGAATTCAACTACA
AGAAGAAGCAGTAAAACAACAACAACAAAAAA
AAACGCAATGCTTTTTGCGTTTGCGCCTTTGAATTCAACGCCGGTGCATAGTATTGGCACCCCTCACG
CCCGTCCTTTCCATTCGGTGCCCAGTCAAAAG
AAATTTCCTCTTCGACTGACAACTGGCAACAAAAAAAAAAAAAAAAAACGCTCTCAACAGCTTCGTGG
CGCCAACCACACCTAAATAAACGCGGAGAAGA
AAACTAAAGAATCCTTCCCCACAAAAGACAGGAACTCACAAGCTCCTCGCACGCTTAACTCATTAATT
ATTATAAATTTATCGTTTTTTTCCCCTGTTTT
TGTTGTTGTTTCCTTCCGATCGCCTTTTAGCACAACCCACCGGTTTATTCACGCGACCCGCATTATTT
TCTTCGGTGGAAAGAACACGTACGACCATTCA
CGTCAGGGAGGTTGTATTCGTTTCATTATCAGAAATTCATTTTCCTTCTCACAATCATCCCCTTTTTC
TTTTTTCTTCTTCCTTTTTTTCGTTTCTGACA
TGCTTTCGTTTTCGTTTTCGTTTTTTTTTTTTTCTCAAACTACAAATATTGGTGGAGCACCTTTCCC
TCTAAAACAGCAGGTGGCATTTCCCCGAAGCT
CAAGGAAAAACACACCCGGACAAAAAAAAAATGGTACATGCCGGACGCAACCCATCATTAAGACATAC
AAAAAGAAGGAAAAAAGGAGTATGCAAGCACC
TCCAACACNNNNNNNNNNNNNNNNNNNNNNNNNNNNNNNNNNNNNNNNNNNNNNNNNNNNNNNNNNNN
NNNNNNNNNNNNNNNNNNNNNNNNNNNNNNNN
NNNNNNNNNNNNNNNNNNNNNNNNNNNNNNNNNNNNNNNNNNNNNNNNNNNNNNNNNNNNNNNNNNNN
NNNNNNNNNNNNNNNNNNNNNNNNNNNNNNNN
NNNNNNNNNNNNNNNNNNNNNNNNNNNNNNNNNNNNNNNNNNNNNNNNNNNNNNNNNNNNNNNNNNNN
NNNNNNNNNNNNNNNNNNNNNNNNNNNNNNNN
NNNNNNNNAAAACAACGCCATATAATTACAGTCATATTTAGCATGCAAAAAAAAAAAAAAAAAAAAAGG
GCTTTCCCCTTTGGGTCTGCATATCATAAAAG
CCGCATATCACCGCAGTTTACGTGTTGCCATTCTTTTATTATTTTTTTTTTCCTTTCATCTCTCCCAC
ATCATTCTGATTTCCTTCCCGCGAGAATTCTC
TCCGCAATTCATCAGCCTTGTCATTTATTTCCCAATGCCTTTTCCTATAGCCTTACCTGCTCACATCC
CTGGCTAGTTAGTAACTGCGTCCAAAGAGCAC
CCAGTGCTTCGCAGGGCGGCTGCAGCTCTTGCAGATACATGTTTTGCCTTCGACAGACTCCGGCTGAT
CAAACGGGATGCAGAGCGTTTTAGCACCCATG
CTTGGGGCACGTGCATCATCCTTCATCTCCTCGCTCTGCATTGCCTTTGACTCCTCCGCACTGTCCTT
TTTCACCTGCTCCTCGCACGACATCTCACCGC
ACCACGGTGCCAACACCAAGCACTTCCTGTTGAGTGCAGGGGTAAAGTCACTCCACTCTGTCAATTTC
TTTGTATTTTCCTGACGCTCCCTTTTGGCCTT
TGCAAACATGCTATTATGAATTTCGTCCATCAACAATGTCATTGCAGTGGGCAACTCCTTGTCCCATA
AAAGAGAGCGACGGCTGCCATCGTGGCGCAAG
ACAAGTGATATTTTTAATGATGCGAGCTCCCTCGGCCCCAATTCGACACGCACCGGCACGCCCTTCAC
CTCCCAGTGGTTGAACCTCCATCCAGGGCTGT
AGTTATCACGAAGATCTGCCTTGGCACGGATGCCACCGCTTACCAAAACCTTTTCAAGGGTCTTGCAT
CCCTCCAGCAAGGACTTTCGCTCCTTATCCGT
AGTGTTCTTTGTAATACCCACAGGAATAATGACAACCTGCACCGAAGCGACGCGTGGAGGCAGCACTA
TCCCCGAGTCATCCCCGTGGACCATTGTCATG
ACGCCAATAACGCGAGTAGAAAGTCCCCACGAGTTCTGCCACGGAATAAGAGTGGAACCATCATTCTT
CTCGGGATCCTGGAAGCTGATGTTAAACATCT

TTCCAAAATTCTGGCCCAAATTGTGGCTCGTCCCACCCTGGCAACCGCGACCAATCGCGGCGATATAC
GTCTCCACAGTTGTTGTGTAATAGCCGCCGGC
AAACTTCTCCTTCTCTGTCTTCCTTCCCTTCACCACCGGAACGGCCAGCAGTTCTTCGTAGACAGCAG
CGTAATGATCAAGGATTTCAAGTACCTCCTGG
GAACACTCCTCCTCCGTCTGCCACGCGCAGTGTCCCTCCTGCCAAAGGAACTCTCGGGTACGAATAAA
CGGTGTGGGATGTGAAAATTCCCAGCGAATAA
CGTTATTCCACATGTTCAGCCTCACTGGCAAGTCACGGTGTGAGCGGATCCACTTTGCATAATATGGG
TACATCACTGTTTCGCTTGTTGGACGAATAGC
AACAGGCACCTCAAGATCCGTCTCACCTGCCTTCGTCACCCATGCCACCTCCGGCGCAAATCCTTCCA
CGTGATCCTTCTCGCGTTCCAGGCAAGACTTG
GAGACAAACATGGGAAAGTAACAATCCTCAATTCCCATCCTTTCAATGCGGGCCCCAAAAAATTTTTG
GATGCGGCTCCAGATAAAAAATGCCCATGGGC
GAATGATGTAGCAACCCGAAACATCGTAGTACTCGATCATCTCAGCCTTCGTAATCACCTCGACGTAC
CACTGTGGGAAGTTTTCCTCACGCTTCACCAG
AATACCCAATTTTGTTTCACCAGTAACATTAGCGCCGCCGGTAGTTGCGGCTGCGGCAGGCTGGGAAG
TTTTTTTCCCCGTCTCCTTTAGAGACGTGGAA
CTCTTTGCCCTTGTGTTATCGCCAGACGGGGTACCTCCCGTGGCTTCCGCCGAAAAGTCCACCACCAC
AAATGGATACTCCAGCTTCCTAAGAAAATCTT
GCAGCTGTTTGGGCGTCATCAGACAGCTCTTGTCATTACGGCACGGGTGCAAGACTACAGGGACGGTA
CTTTCCACCAGCGCCTTGTCTAGCACCAATTT
AATGTTCTTTGATTCGTTGTTTACGAGTGCCAGCGGGGTAACACCCCCCTGCACAACGCCCAGATGAG
TCCGCAAAACGTCCTCCGGGGCAAAGCGAAGA
TCTTTCAGAGACAAAGCATCCTGTATCACTTTCATGTTCGTCCTGGTGGTGTGTAATGCCGTCACAAG
AACCAGGTCCTCCTTCTTACTCTTCAAAAAAA
TGTTTTTGGTGCCCGTGCACGGTACGCCAAAGCGCCCAAGCTCCTTGTTGGCTTCCTCCACAGTATGC
TTCTCCTCGTGGGAAATTGTAGGCAGATCCAT
ACCAAGAGCTCGGAGAATCTTCAGCATCTCCGACTCGCCCCTGCAATCACTTGCTGACATCGTTCTGG
TAGAATTCGTTACCTGCTCTTTGTTGAGAGAA
GAATAAAAGAAGAAGCCGGAGGAAAGATCTGCACCAGACACACTCGCCTCACAGTGACGGCGGTAAAT
CCATCGTGCTGAAGAACAAAAGACAGTACGGT
TACTGAAATAAGTATTTTCCAAAGACGATGCCTTCCTCCTTGAACTGAAAGGCAACGTGCACCAACAC
CGCAGCGCCGGTGGAGAGTGGACGCTGTAACT
CATTTTCATGATCAGGACTCTCCTTTGCAGCGCAACAATGATAACGACAACGACAATAATAATAGTGA
CAACACAAAAAACAACAAAAACCCCCACACTC
CAATTCACGCGAAATTTAGAAATGGTGCTCTAATAAAACCCATGCACATGCGCTTATATTTACCGCAC
TATACATCCAACGGTGTTGAAAAAAGACACAC
GTACAAAAAAAAAAAAAAAAAGAAAAAAAAAAGGACCAAAATAAAAAAATGTTATAGGAAGACAAGCA
AAGTAGTCGTACTCCCTTTCCTCGAGAAATCC
CTTTTTTGCTGTTGCCGTTGCTGTTTTTTTTGTTGTTGTTTTTGTTGTTTTCTTGTTTTTATTTTTTC
CTCCTTATCGTTTGGAGAAACAGAGAGATGGA
AAGGTGCAAACAACAACAAGAAAAAAAATATATAATAATAATTATTGTGTTTTACTTCACAAGAAAAAA
TTTCGTATCAGGTCGCATGGTGCGCAGCTCC
TCGAGTTTGTTTTTTCCGTGATGCAAATGCCATTCAATGCGAGTAATACACCTGCGTGGGATATCACG
ATTGCTGTCAAACTCACTACGAATGAACTTTC
GCAGGTCTTTTCTTGTCCGCGGGTCCTCCAACCGCTGCAACGCCCTCAGCATATCACGGTAAAGGTTC
AAAATATCCACTTGAACGCCACTTCTTCTTTG
GGAACGAGGCATCTCACTGGATGCGGACGTCGAAGCACCAGTTTGATCCCCTTTTTGTATTTTACCTA
ACTCAGCCACAGTGGAAGCTGAAGCCGGCGGC
GAACGCGCAATACCAGCTGCAGTTGCGCTTGTCTGACGTTCCGGGTACATGGGGTTCCCGGCGTATCG
TGCATCCTTGGCATACACCGCCTCCCGGTACA
CCTTTGTCATGATGTCAAACGCAAAATTGTGCGCATTTCCAGCGGCGTCACGCAGCTTGTGACGGAGG
CAAAAGGAGAGATTTACCATGCCTTCTTTACT
GCCTCTTCCCCTTCCAACGCCCGACAAGGACCAAAGTCAACAAAAACAACAATAAAAAAAAAAAAAAA
AGAACGACAGAGAAATAGGAAAAAAAAAAGAAA

AAAAATGAGATAAAAAAGAGGCAGAACCCACATCTCTAGTTGGGGCCAATAATTGTTATTTGAATTTG
TTGTTCTTTTATTATCACTCGTGGAAAAACAA
ATAAATAAACTGGATGAAGGCGTAACCGCAAACATTTGACGCCTCCTCATTACTGTTTGCTTTAGTCG
AGGCCGTCATCGTCGTCAACCTCCATTTCCAT
CAAAAAATCCCGGCCTCGACTCCGCCGACACGCCTCAGGGTGAACAACACTGACACCGGAGCCCAATA
ACAACTCTGGGGTAAAAGCCATTTCACGAAAT
AACGCCGCCTCCTCTGTGAGACCCACAACCCGCACCACGGGTCGATCCTCCAGCTGAAAAAGCGCACT
GAGATTGGCGTCAAACGCGGAGGCCGCTGCTG
CTGACTGGCCGCTCTCATCGTACAACCTTAGAGAATGACGACTATGTGCCACCGTCGTCCATGGAAGA
AGATAGCCGGCGTAGTGCCCACCAGACCCAGC
AGGCATCGTTCCAGTGACCGTGGTCGTGTTATTAGCCCCTGCGGAGACAACTTCCCATACGGCTCGTG
GCGTCCCCAGCCAGCGCCACAGGCCCCTACCA
ACACCACTTCCAAGAGACAACAACTCCTCCTGTAGCTCCCATCGCGTCGTACTTCCGCCAATAGAAAA
ACCTCCCATTGCCGAATAAGCAACGCATTTTG
GCAACGACTTGGGTGTCTCATCTCTTTTCTCCTCTTTTACTTCTGGCATTGCCTCTGCTTCAGCTGTT
GACAGTACTTCCACATCCTGGAGAGGCAAAGA
TGAACCAAAGCTACTGAACTCCGTACATGTACCATGTAATGTAAGCAGATCTAAGAGTGAACGAAACA
CGGCACAGATAGAAGCAGATCCGACATTTATT
CCAAGTTGGCTCTTCATAGATGCATCACACGAGCAGCAACTTTGATGAAGCAGCAGCATCGTCCAGAG
CAGCCTCCATGATACAAGATTTTCCGACTCCA
TAAATACAGAAAATGATTCCTCCTCCTGGTTCAATCCTCTCATATTACAACTAAGTGATTTTTTCATT
GTGCGATGCCACACATCAAAAAAAGCAACGGC
AATCGCTGTGGCAAAGTTCGCCCGCAGCCACTCCCTGCATTCCGGCAAACGCAACAATAGGGCCTGTA
TCGCCACAATCCGCAAAGCCTCTATGACAGCT
GTAACGTCATGACGAGAGGGGTGCTGAAGAAGATGACGTGCAGTGAAGAAAGTCGATCGGCCTCCCGC
ATGATGTTCAATTTCAGTCAGTGCGTCTGCCA
CATGATCGTTGTTCCTAGCATCACCACCAGTGGATAAAGACGACCACTTTTCAAGGGCGTCGTAATAT
CGAAAAAGAGATGTTTCCAGCACGCGGAAGGC
TGTGTTGCGGAGGATGGTAGTCACATCGTTGTGCTTCACGACAACACCACCTGCCTGAAGAGCGGATC
CTTCAGGCCCCTCCTCGAGTAGACTGGCTGTC
TTCTCCGTCTGCACAGCCCTCACAAGACGACGTGCGGCATGAGAAAAGGAGCGAAGAATGTTCACTTG
ATCCTGCAACGGCACCCCAGGAAAGGTTGCGT
CAGCACCAGAAACTTTCAGCTTTCGCTCCGCATCGCAAAGCAACCTGTCGCAGAGAGCCTTAAGCAAG
TGAATCCGCGGCTTGTGAGGCAATAATGAGAA
GCAGGAAGGCGGTGGACGCTTCTGTAGTTGCTGTAACTCTCCAATGATGCTATAAATCAATGCCCTGA
TGCCATTAAAAGAAAGTCGAAGTGGCGAAGAG
AAAACACCATCCAAGACGACCGACACAAATTCCGATAATGGCGTGATAAAGCGGAAAATAATTGAAGG
ATCCTCCAGCTTTAGCAAAGGCTTGACCAGAA
CCAGATAATCTAAAGAAATCGCCGCCGTCTCAGACGCTGCACTGCAAAAAGAAGTCGGCATTAGACGT
AGGAGGAGACGCCTAAACACATCGTCATCCAC
AACAGGAAGTATCAAAAAGGTTTGAGATTCCTTTCCCACAAATCCTGCATGAGGAAAATACTCATTCG
CCTCACATCGTGTTGAGTGACACAAACGCACA
TAAGCTTCACGGACAAAATTGTTTCGCAAAATCAATGGGATTTCACCCTGCCAAAAAATGCAGTCAAT
CAAATCGTTACTCGTGGAAACAGAACGTGAAC
CATCCGACAACGTTTTTTCCGGTGTTAGAGCAACACCATCTCCCGTAGCAAGTAACTGCACCATTCTG
TCAAGCACCTGACGCAAATCAAGAGACACCTC
ACGCTGCGTATCTACCGCCTCATGTAGTAATTTGGGAGTCATGTACTGCAGAAAAAACCCCGGCGAAG
GTCCCATAAAGCATCGGCCGTTGGTTTTCAAA
GCGGCAAAATCATCCAAGTACAGTGACAGTTGTGGGAGCCATTCGCTGAGAGGAATATCAGAAATGCA
AACCAGCCACTCGACAATTACGGGAAGAAGCG
ATCGCACTCTTGGGTGTCCGATTACGTGAGGAAGTAGCGGTTGCAAAACGCATCTCGCGACCTTGCCA
AGATCCTCCTTGGAGATACCGCTGCGCCTGGC
CCAGCCACCGCCACCACCAATTGTGCCACCCTTCCAGACTGCGAATCCCAGCCCTTTTAAAATCTCCA
CGCCCAGTAAGGGGTGCAGAATGTCACTATTT

CCTCCATGACTACCGCGTTGGTGCCCGAGTAGAATCGTCAAGACAGTGTCAACGCTTCCAGGCGTCTC
CCAGAACCGAGTCGCTGAGTTTTCAAGTAGAC
GTCGAACATCCGTCGACAGGGCATTGAGGGTCAAACGGGCCGTTGTCTTTGACGGCCCAGACGGCCTG
CTTTCCGTGCATCGACTGCCATCCGCCGAATC
GCCGTCCGCAGGGAGATGGACGCAACGACCACCACCGGTGCTGCTGAACGGGGCAACAACGCCAATCA
AATGCAAGCACCACCAAGCAGCACGCCTCATA
TTTTCCCCTGAGGCTGAAAGAGAGAGAGTATATATGTATATAAAGAAAAGAAAGAAAAACAGCACAGA
AGAGAAGAAGCCCGCAGGTGGGGGGGTCACCA
TTCGCAACCACAACAAAAAGAAAAAGTAAAAAAAGAACGTTTCAATCGAGTTTCCTCAAAACGCAAAA
GACGCCCATACTTTGTGTGATAAAACCAAAAC
TGTCAATCTATCGCACTCTTCTTCTCTCATACCCTCCGCAGAAGAGCACAAGAAAGAAAAAAGAAAAA
AAGGGCAAGAAAGAAGAAGAAAAAAAGACACACAA
ATATCTAACAACAGGCAAAATAAAAGTACCGAATGGCTATACCCCCCTGGTGAAGAAGATGATTCTAT
TTTAAGAGGCGCATAATCATACCGCATCACTG
TTCTCAAAGGATTCATTTATATAAATGTCAGTTTTCTGTCTGCAGTGAAGTTGTCCGGTAGGACGATG
CACCATACTGGCTTACATCAGTCAATATTCCC
ATCATTTCGGCTTGGCGCATCCATACCTCTTGATGTTTGCGCATTACACCCCTCTGAACTCTATTGCT
GCAACTGTGATGCTCATCCGTGGCTTCCCTTG
GGAGCGAATTGGAAAAGCGGCCAGATGACGACGAAGAGCTTGAAACAATTTCTCCAGATGGCAACAAA
GAACGAAATTGCGTCCGAGGCATATGCTGAGA
ATGAGAACGATGATGCCGTGGTTGATTCGGTTTTCCGTGCCCAGAGACAGTAGCAGGCACAAACGACG
ATAACGTCCCTCGTATCATACCCGTTCGGTCG
GCACTGTGGAAGTGCTGCACCCCATCCTCCGTTGCGCATAGAGCACCTCCGCTGCGAGGAGCAGTCTT
GGGCGAATCCAAAACAGGACCATTAGAAGTAT
CGTCACCAAATTTCTCACCACCCAAAGATGTTGACGACGTCCGAACTCGGGAAAAAGACGCTGGCGAG
TTTCCCAAAGATCCAGGTGTCTTCGGTACCGG
TTCGTTGCGCAGCTCCTGATGCAATTTTTCTCCTTGACGCATTTGTAACCGCAAGAGCGTGCGTATTG
CACTTTCCTTCTCAGCAAGCTCCACTCGCACA
CTTTTAAGTTCCGACCGCAAGGCGTCAAGAGATTCATCCTGAGCTCGCTGGTTCAGATCACGCTCTTT
GCGAACACCTCGCCGTCGTTCCGGAGACGCAG
TGTTACGCCACTCTGCCTGCGTCAAGTGATTCTCATGTGCTTTTTTCACTGCTTCCAAAGTCTCCATA
AGCAACGCGTCTAGCGAATTCCCAAATATTTC
CGTCTGCTGCTCCATGTGCTCTATCAAACGTTCAAAATCGCAAGTGCGAGCCCCATGCAAAGTGTCAA
TATCATCCCCGGTGGCCTGTGTCGCCACAAAA
GGCCCTTCACTGGCCCTTCTTGCCGGCTCCTTGGATGAATCACCGGCAGAACGCCATTCCTCCTTAAG
TTTCCAAATGGTTTCATTGCGCTCCCCTAAAA
TAGCCTTTAACCGCGTCTTTTCCTCTTCAGCTGAAAGCAGCAGCTTCTGAAAATGTTGCGCTCCCTTG
TTTTGACGCCCCACAAGTTCATTCCGATCGGC
ACGAACGACCTCTAGCTCCTCCGTCAGCTCAGAATGTCGTCTCTGAAGTGCACTTGCCCACTCTACCA
ACTGAGACACACGCTCTTGGGCGCGACTCACA
CAATCAGTTATGGAGTTCCCGTCGTTTATAGCAGTCAACTGCCCATCAAGCTGCTCATC
>scaffold1030.size6510
TTTTTTTAAATGTTTTTCTTTTTTTCAAAATTCTTTTATTATTATTATTCTTCTCTTCCCTATTCTCT
TTTGCTTTGGGGCAATCACTATTGAATTTCTTT
TTCTTTTCTACTCTTCCCTCTTTAATTGTTTGTTGCTGCACTCTTCCTGCGAGGGGTGCCGTGTGTTC
CGCCCAACTGCTCCGCTCGCACACCACCGACA
CGCTCATGACGACGGCCCTGTGCGCCTCGCCTGCTGCACGCATGAGGAGGGGCCGCGACGAACGGCGC
GCTGAGGGAGCCGTGACGTGTGGGCGTGCGAC
GAGGGCCTGTGGGGCGGAGCACACATTTCTTCCCTCTTGTTTTGTTTTCTTGCGGCCAGGGCGGGCAG
ACACCCGCAGCGCCACACACACAGCCACACCC
TGCCGCCGTGTTTTTCTTTTCGTTCTGCTTTTTTTTCTGTGGACGGGGTGTGCCGCTGCCACCGTGTG
GTGATGGAGGGCGGAGAGCTGTGCCGGACGTA
TTGCCGACGTATCTCTCTGCATCCTCACTTACCTCTTTACTTAATGGCTGTCTNNNNNNNNNNNNNNN
NNNNNNNNNNNNNNNNNNNNNNNNNNNNNNNN

NNNNNNNNNNNNNNNNNNNNNNNNNNNNNNNNNNNNNNNNNNNNNNNNNNNNNNNNNNNNNNNNNNNNNNNN
NNGCAGCGCTGTGGAGT
TGGATGCGTCCCTCTTGTTGCGGCCGTGGGTG
TGCGGTGCGCATGGCTCTTTCTTGCGGTGCGGCGTGGATGGCGGTGCTTCTGCATGCGTTGTGCGGCG
GGATTTTCTTTTCCATGTCTTTGCCCTTTTGT
GTGTGGGAAAGTTGTGGGAAAGGATATGTGTTCGGGAGACTGAGTGTGGGTGGCTGTCAGCCTCGCCC
CGCGCATTGGAGAATTCCATACACGTGGCTTT
GCTTGACGGTGTTTCGCGGCACCCAGGGTGTGGCGAGGAAGAGGATGGGGAAGATCTGTTGTGGGCAG
AAGGTGGATGGGACAGTCCCCTTCCGTGTCCT
TCCCTGTGCTGTTGGCATTACATACTGAGAGCGTAAATTTGCGCGAGTGCGGGATGAAAGGTTTATGC
CGCTTTGTGCGTGCTACTCAATTGTCGTCATT
GCAGGCTGTCGTCACGGTGCGTTGTGGTGCGGTGCCATTTGATGAGGGAAGGACGCGATGCCACGGCT
GCTGCGCACCACGACCATTTATTGCCTCTGCA
TCGTCATTGTTGTTTTTCAGTTTGTGTGATTTTTTTCTGCGCTCGCATAATGTTTTCTTTTGCTCCAC
GATGAATTTGTTGGAGGAGCTTTGGTTTTGGTG
CCTGCTTCACGGCGCTTTGCCTTGTGTGTCGGTTGCGGTGCTTCCACGTGGTGCCAAGGCGTTTGATG
GGGTGTTTGCCCTTCTTTTTGCGCCCTGCAGC
CGACCTTGTTTGGCCATGGCGGCAGCAGTGGGGCTCTGCGACTGCGCGCTTGCCGTGCGTGTCTCATC
TTTTGCCGCTGTTCCCTCGTTGTGTTGGTGGG
GGAGGAGGCGAGCGACTGCGTCGGTGGAGTTGCGCCCCAGTGGGAGAGGGAAGGCACCGACCGCTGCT
GACGGCGACGGTCTTTGCTGCAGGCGATGTGA
CGGGAGGACGTCACCGGTGTCACAGTCAACCGAGATGGATGAGGGCCGCACTGTGTCTCCCACCCCTC
TCATCATCCCTCCGTCCACTGCAGTCACACGC
CACACGCACCGCAGAGCAGCGGCGCGACCACCATCCGGATGTCTGTGAGCCAATTGGAATGCAAAATC
GATGTGGACCCTTTTCCCCGTGTCTGAGTGCC
GCCGTGCCTCTTTTTTTTGTTTCTTCTGTTGTGTATGTCGGGCGCGCATGTTGCCGCTGATTGCTGCG
CAATGCCGACTCGCGTGAACGAGAAACGATGC
TTTGACTGCTGGCAACCGGCGAGCTCTCCACTCCACCTACGCACCAACAACACCAACAACACCTTTTG
GACGCGCTTGATGGCCGGCGGTCGTAACGCTG
CCACGTGGTCTGCCTCGGCTCTTGTGTGTCGTGCACGGCATCGGGACCGCCCCGTGATGCAGTTATTC
GGTAACAGGAGCGGTGCCAGTGGTCATGTGGC
TTTTTGCCGGGATTCCGCCACTCGGCACGGTGGCTGTACGGGGTTGTATGGGCGGTGGGAGGGCGATG
GTGTCACTGAATTGATTGCGTCTGTGCGGCTT
TGTGTCGGCGGCTGTACTATGAAGGTTATTGTGTATGACCGGATGCCTCTGCGTGCCACTGTGTTTGA
TTACTGTTGTTGCTGCCTCTTATTCTTTTCTG
AGCGTGCAGTTAGAGATACTTTTGCTTGGCCAGCACAACTATCGTTTCAAGGCGTTAGGGATTAATTG
CAGTATTAGGTATTACTTGCAGTATTAGTATA
ACGATCTGGCGGAATTGTGTCACTGGATTCGCAATCTGCAAGAAGGGATTTTGAAAGCTGGAATTATG
GTCGACGGGGCGAGTGCGACACACCACGACAG
CCCTGTTGATACTTGTTTGGGTTGGTGGATGCGGTCTGCGGTGGGTGCGTGTCCGCGCAGCCGGCACC
GTGTGCAGCGCAGGGCCATTGCGGCTGTGCGT
GTGGCGCTGCTTGTGGTGTTGGCGCTGTTTGCTGCTGCGGCGTGGGTGCCCGCGGTGCATGCGGTGGT
GTTGCGACTGCGGGGCGGCACGGTGGACAGAG
CCATCACGGTTGGCCGCGCCGTGGACACGGTGCTGATGGACGGCGTGTACATCACGAACGGCGTGGCT
GTGGCGTTCGACGTGGCGGCGATGCTTCCCGG
CGCGCTGCGCATCGAACTGAGGAACTGCGTGTGCGACGGCGGTGCGCAGATCTACGTGCGGGGCTACA
GCGGCGAGCCGGCGAGTGACCGGAGCCTGGAG
GTGAGCGTGAGCGGGCTGTCCGGCGGCTACTGCTCGCTTGTGTTTGTGCACAACCTGCCGGCACACAC
GAACGTGACGGTCCGTGACTCGACGATTGTGA
CGCCGGGACCGATGCGCTACTCCCAGCTGAGTGGGCTGACGGATGCGGTGGCGTCGCCGCTTGTGCTG
CATGCGACGTCGCTGCTGCGGACGCAGCTGCG

TGTGAGCAACACTGTGCTGAGGTCGTTGCAGGCGGGCGGGTCGGCGGTGCACGTTGGCGGCGGTGTGG
ACCTGCTGTCGAGCGCGGTGGTGCTTGACGGC
GTGTTGCTGGAGGCGTCGGGCGGTTCGACCGCTTCCGCGTTGAGTGTGTCGTCCTCGTCGCGTCTCAC
TCTGTGGGGCCACTCGGTGTTCTCCGTGACGA
ACGTGTCGGTCATGAGCAGCGGCGGCGGCCTTGTGCTTGGAGAGCGCCTCGCGGTGGTTGATTCGGTG
CTACGCTTCGTTGGAGTTGAGGGGTCTGTTGC
GTCGTTGCTGGTGCACTGCGACGGTGGGACGATCGGAGCCGGCGGGTGGCTGGACCTGCACGACGTGT
GGGCGGTGGGCGAGGCGTCGTCTGTGGCGTCG
CTTTCGGGGATGACGCTGAGCGGCGGTACCGTGTCGATTGCGCGCTGCGCTGCCACCGGCGCGACGCT
TGTCTCCGGACTGGCGATCACGAGCGGCGTCG
TGTCGGTGCAGTGCAACCGTGCCGGCGGCCGTGTGCTGCAGAGCAGCGGCGACTACCGCTTGGCCGGC
CTGTCCTCCGTGAGCGTGGTGCCGTGCGACGG
CTGTGCGGCAGCGCTGGCGTGCTTCGATGCGCTGACGGCGTCGTTCTCTGACTGCGTGTGCGGCTGCC
GCGCCGGCGGTGTGGGCGAGGCGTGCCTGCCG
TTCGACGTGCCGGTTGCGAGGTCCGGCGGCGGTGGCGGTGCGCAGGGCTGCGTGAGTGGCGTGATGCT
GACGGAGTCGGTGACGGTTGGCGGCGGGCGTG
CGACGGCGTGCTTCGACTCGGTGGTGTTCAGTGGGCCGATCACTGTGGCGGTGGACCTGCGCTCGATG
GACGCGTTCGCTGACGCGCTGAACGTGACGCT
GCGCCACTGCGTGCTCGTGGGCGGTGCGCAGCTGCGGATTGGCGGCCTCAGCGAGAGCACGGCGCGCC
TGATGCCGCACGCCCTCGTCAACATGACGAAT
GTGACGTCGCTGGAGGGCACGATTGTGCTGCATGGCGCGATGCCGCTGCACTCGAGTGTGCTGCTGGC
GAACTCAACGCTGCGCGCGACGGTTGGCGGGT
CGCAGTACGTGCCGACGACCCCTGGCTTTGAGGGATCCCGGTACGGCCCCGCGCTTGTCCTGGACGGC
GTCCGGCTTTTGTCGACGCGGCTTGTCATGAC
GCGGTCGACGCTGGTGTGTGGCGGGGGCTCGTGCGCTGCGATCCTCGTGGAGCGCGGCCTCGGCGTGA
ACCTGTCCAGCGTTTTCTTCATGGACAACTGC
GCCGTCAATTCGCAGATGCACGTGATGTATGCTCTTGCGTCGGACCTGCGTGTTTGTGGCGGCTCCGT
GTTCTCCATACAGAACAGCTCGTGGAGTGTGC
CGAGCACCGAATATGGCAAAGCCGCGTGTGTGTTCAAGGATGTTGTTGTGGCTGGCGGCAGCGTGCTG
CAGATTTTGTCCAGCACTTTCCGTCTTGGCTT
TGCCATGCTCATGGCAACCACGCTGACTGTGACCGGCGGCAGCTGGCTGGTGCACCGCGACAGCGAGT
TCCGCACCGCCTACGTTGTGTACGTGGTCAAG
GAAAGCAGCGTGGCGTTCCGCGATCGGAGTGTGTGGTCGATAATTGACAACAACTTCACGTACGGCTC
GTACTCGTCAACCATTGTAAGTATGACAAGCA
AGTGGTCACCACCATCCGACACGCGCCCGATCATCTACGGCGTGTGCAACGCGGCAAGGTGCTCTCCT
GTGACGAATTACGGAGTCGACCTCAATATTGG
AGCACCCGTGATGGTGTTGGACTGCGGCGCGTGCACCGTGGACGCCGTGTGCTTTGCTGCGAGGACGA
GCAGCATCAGCGGCTGTGAGTGCGTGTGTGCT
GCTGGCGGCCACGGCGACACGTGTCTGCCAGCTGCGGTTCCGGACTGTCTTGGGCCGCTTCCGCCCCC
CGATGCGGACGACACGGAGGTCCGCTGCGTGC
ACGGTGGCAGCATCAGCTCCGTTGACTATCCTGATCCCGGTGTGCGTGGTCTGTGCTTTGTCAGCGTG
ACCTTCACTGCCGCGATTTTGCTGGACCTGTC
GTATTTTGACGCGCCACAGCATACCCTCAACATCACGGTGCTGCAGTGCGTCCTCATGGGCCTGTCGA
TCAAGGGGAGTGGTGCATGCGTGCACGTGAAC
GTGGTCTCCTCGATGCTGGATTCTGGTGAGCTGGAGTTCAGGGGTGTTTTTGGCGTGAGCTCCCAGAT
ACTGGTGGCGGGCAGTACGATTGTGACGACGT
CGGGCCACGCCATTTTATTTGTGGATTTTTACCCCGGGACAATCTCGTCACTGATTCTAATTAAAAAC
ACAATTGAGGGGAACTGGTACGCGGTCTATTT
CTCCAGTGGTGTTGTTGTTGATGGTGGGAGGATTATTGTGAAGGGAAATACGCTGAGCACAACGAAGG
ACGATGAGGGTGTGGAATCATCTGTTTGCGTT
ACTTCTGTTGCTGTGAGGAACGGCGGCTACTTTGACGTGGAGGACAACACGATGAGCTCGGCTAACGG
CGTTTATCTTTTTGGGGATACCACCGTGAGCT
CCGCGGGGCTGCTGCGAGTGGCGGACTGCACCTTTGTCGGCAGGACAGAAATTTTTGATTCCGCGCTG
GTGTATTTGTCTGGCTCGGTGACACTCGAGGG

TAGTGCGCAGTGGCGTGTGGAGGGCAACAACGTGGGTGCAGCCTCAGTATTAACTATTCCGTATTCCT
GGCAAAAACTTCAGCTGTCGGGCAGCGGCACC
ACCGTGGCGCTTGCGCACAACCGTCAGGTGGAGGGAAGCGCACTCTTTGCCAAGCTTTCTAAATCGAA
CAGGATTGTTGCTTTACCCGCGCGGTTTGTGG
TGGGGTGCAACCTGAAGGGTAATGAGGAGGTGTCCTACGACTATGTGTTTCCGGAGGACGCGGTGGTG
TTCAGGTGTGGCACATGCAACGATGACGCGGC
GTGCTACATGCCCGGGACGGAGTCGGTGGACCGCGGCTTGTGCTCGTGCAGCTGCAAGGACGGATGGC
ATGGCGCATCTTGTCTTCCCCTCGAGGTGCCC
GACGCGGTTGTGCCTCCCGTAGCGGAGAGAGCCGTCGACGGCGACACGAGCTGCGTGGTGAACGAGAC
GCTGACGAacctcacgctgaagatgtggaaga
cgcacca
>scaffold1031.size7104
CATTGACCTTCTCATTGCCGAGTGGGAGCAGGGACAACTTCTCGTGATTACTCATTGCTATGGGAGCC
GGTACTGCAGAGTGCACGAGTCACGTGACAGGG
GGACAATGTGGGTGGAATTACCCGGGGCAATTGCAGGCGTTTGGGCCAATTCACAATCGGATCTTTGG
GAAAAGAGCTTGCGTGTGGGAGCCATTGTCAC
TGCGACCGTTGGAGGAGAGAGCGTCATGCTGTAGGCTTGGGAAGTGAGCTCCCCGTGGTGGGAGAGTG
AGGCCCATGCGCTCTGCGTTTGGGTGACTGAC
AGCAACCGCGTGTTTTGTGTTGGACCGGCTTACATGGACACTGTTCTGAATAAGCTGATTGCCAACGC
CCTGCTGTACTCGGATGGTGCGTTGCACCTTG
TGGGAGCGAGGGACAATGAAGAGAAAAGCAGCCTGTTTCCTGCCCCCCTGACGGAGGAGCTGCAGATG
ATCACTTCCATCCTCCGTAATTGGGCGGACGT
GGACGCTTCCTTCTCCGCGTCGCCCGTAAGTACGGTGGGTCTGGTTGGATATCTTTCCAACGCAGGCA
CTGCCAACACGTGGATCGACGAGTGCCGTTGC
GTGATTGCAACGGTGACGAATGGCAAGAATGTCACAAATGGGCCTCAATTCGCAGGGCAACATTGCCC
CGTGAACCGTTGGGAGGATAATAATCACTGTG
GCTTTGTGAATTACGACTTCGCTCTTGTGGCGACGGTGGCTATTTGCGAGACACACGCAGGCGTCAAG
AGTATCCCTTTGATTGGCGTGAAGTAAGCAGG
CCGCACTGTGCACTTTTGACTGTCGCACAGCCATGAAAGGAGTTGGGGTGTGATGTTCAACGCAAACA
AACACACCGGCTTTTGGGAGTTGAACAAACAA
TAATGAGTGGTGCTCACGCTGCAGTACCGCCAACAAGGCCTTCGTATAGATCAATGGTGAGTATGTGT
GTGTGGGATGCTGAAACAACATCATCATCGGG
AAAGGGGCCACGCGCGAATTTACACTTTTACATTGGAGGGGATGGAAGCAGTGCAAGCGACACCGGGA
GCCAAAACAAGTATTTGACAGTGACGAGTGTC
TTTCTGTACAACCGCATATTGACCCGGGAAGAAGCCGCAAAAGTTGGTGCCAGCAACCTTGCTCTTCC
AGAGTCTCTTTCTGGGAAAGTGTTTGAAGGAC
CGGATGATCCAGCGGGAACTGGCGGTGGCTCCAAAGATAGAAACGCCTCTCAGCTCACGAACAACACT
TTTTTGGAGAAGGACTCTAGTGGCTACGGCAC
GGTGTGTGTGTGTGTGTGCCTCGGATCTTGTTGCTTCTCCTTCTGGGGCTCTGGGCATTGCAGCTCTC
AGCTGTGTGAGAAGGTGGGAGTGGAGCCGCGA
CTCCCCGTGGAAAAGGGTGTGCAGCTTTTCAGGAAGACTCAATGCACTCCGTTGAGGACTTTTTATTT
ATCCCTTTTGTTTTCACCTCTGGATATAATGT
GCTGCTGGTGCCTTGTTGGAGGGATCAGAACCCCTGCCTTTTTACTATTTTTTAATTTATTTTTCGTT
TGCCGCTAGCTGCACACTCTCATGAGTGACGT
GTTTCCCCCTGTTTTATTTGCTTTTGTTGCACCGCACGCGGTGCCGGCCTCATTGCTGTGCAGTCCCG
CGGACTGTACAAAACGAGGGTTGGAGGAAGAG
GCCGAGTGCCTCATGAAATGTGAGGGATTTCTTGTGGACACGTGGAACACTGCGATCTTTTTTTCCCC
TCTCTGTAAGCAACATATGGAGATGCACAATT
CCTGCTGCGCTGAGGGATGCACGAAATGAAAGTCCACTGAGGTGATTTATTTCTTTTTTTCCTTATTA
TTATTATTTATTTATTTATTTTTGCTGAACCT
ACGCCCTTTTTCTTGGTTTTCTGTATTTGGCAATTTGCTGCGGCTTGTTTTATTATTTGCTTTGCTGA
ATGCCACTATGTGTCTTTGTGTCCCATTGGGT
ATCCCACTGCTAGCTGTGCAGTTGACACGCTCTCATCACTTTTTCTTTTCTGCTAGAAGCATCGATAG
TCCGCGACGACGATCGTTTGTGTTCCCCCATT

GCGATGGTGCGAGGGTGACTGGGCGCCCTGTTGAGTGCGCTCGGAGGGGAGCGGATGGTGTGCGCCGT
GCCTCACTGTGAGTGTGGGGCGCCACCGCCAC
GACCTGCTGTGAGGCGTAACGCTATTCCTTCGGGGCAGAGGTAAGTAAAGGACTGTGGTGAACTTGGA
TGCGCCTCGTTCCTCTTGTTGTGGCTGTGGGT
GTGCTATTCGCATTGCTGTTTTGCGGTACGGTGTGGATGGCGGTGCTTCTGCGTGTGTCTTCTTGAAT
GCACTGTGCGGCGGGCTTCTCTTCTCCTTGTC
CTTGCCCTTTTGTGAGTGGGGGGTGAGTGTGGGTGGCTGTCGACCTGCCCCGAGCATTGGGGTCCACA
CACGTGAGGCTCATCTTGGCAGCCTCTCGCAG
CTTCCATGCCTCGTGACAGAGCGAGGGAAGAGTGGCGTGTTTCCTCCTACTATAGACCCATCAATTCC
GAGCAGTTGGAGTGAAATTAAAGGGAACAAGC
GTGTGTGTCTTCACCTTCTTGCGGGAGGACGCGCAGAGGTTGGTTTTCTTTTGTTAATCTGTTCGTTC
ACGGTGTCTGTGTTGACGTTATTATTATTTTT
TTTCCAGCGGCATTCATTTTGCGATTTGTGCGTCTTTGGTGTCCAAAGTTTTCCGCGCCGCCAAATGG
GATGTTGCGCAGGATCGGCATACATGGATGTG
CAAGAGCATTGTGTCGCTCGCCCGTAATAACACACACGCGCACACAGTTTCAGCGCGTCTGACACGTC
ACGTTCTTCAGAACGGTCGAAATCACTTGCGG
GACGTCACTTCATTTGCGGCGTCGGGACAGCGAGGGAAAATGGAGAGATATGCCGAGAACGTAATGCC
CCTCCATAATACCGCAGGCTGGCGGCGACAAG
GATCGGAGAGCTTCTACTCAACATTGAGAAGAGTGCAGCACTCTGATGATCCGTCTTTTGCGCTTTAG
GAACTTCGAATGTAGTGTGCACCGTGTGGGTC
TTTTTGCTGCGGATATGCAGAAGGGTGATGTGGGGTGGAATTATTGTTGAGGGCAGCACGGACGCCGC
CTGACCTTTATGGGAGGGATGAGTGGTGTTAT
TCCGTGCTTCCTGGCCTGCGTTGTCGCACTCATGCTGTGTGTGCGGATGGCCACAGTAGTGGAGCTGA
ATACGGTTCAATGTGTGTTGCTCACGGGGACT
GCGCGACACATGTTTTTTGTTGGGCTTCCCAAAAGGAGGGGCGCATCGCCATTGCGTGATTCCCGCGG
CAGTTCATTACTCTGAGACTGTGCGTGCCGCC
GTCCGCTGCGCCACTGCAAGCCTTTGCCTTTGTGAGAAATGGGGAGCGTGACTGCTGTGGCGGTGGTC
GAATGTTCCCGAATTGGTACTTTTGGTGATCC
CACTTGGAGCGCCGAGCAGCGAGGGGCCGCACACGCCCAGGAGGTTGGAGTGTCAGCTGCAACCGCAC
TTGCGATTTGCAGTTGGGATGTGGCATGTTTC
TTTGTTATTTTTATTTTTGGTGATATGTGGGGCAGCGGTGTGTTGGCGTGTGCGGGCTGCTCATGGCT
GCCGGTGTTGTCTTTCCTGCTGTTCTCCCGCT
CCAGTCCTTTGCGTGCGACAGACAAAACGTTTTTACGGGCGTGTGGTGGGGCCGCGTTCTTCTTTGGA
AAAAAGGCCTTCTGCAGGCATCATGAGACTGC
CGCCCGTGCCACGCGAGGCAGTGGGGAATTTCCTGGGATTTATTGGCGGGGTGGCACCGCACTCTTGC
CGTGTGGTTGATTTGTATTCCGCAGTGCACGG
TGAGGGGGATGTGCGGTGACAACTTCCTCAAGTGGAGCGACACGAGCAGCACGGGGAGCGGAACGGAG
GAGTGGTGGCTGCGTGCTTTCCAGTGATACGT
GAATGGCGAATGCAATATGAAAGGCAGCGTCACGGCAATCTTTTGCCAATGCACACCGCAGATATTTT
CCACAAAGCATGCAGGTTGCACTCTTTTCTGT
TGTGTGGGGAATTACCGTGATGCATCGCTGTTGCGGTGTGTATGATTTGTCCTCCCTGCATGTGGCCG
TTGCTGCACCGAATTCTCCTCCCACTTTTGTG
TGTGTGTCAGGGTTAATGTGCACAGGGGTGCGGGACCGTGGCGAGCGGTATGCGTGGAGGAGCCCTCC
GTGCGCACCACGTTGTTGTTATTGTTGGCACC
CACCAACACTGTGCTGGGTGGATGACATCCACATATCGTTGTGGTGGTCCTCGTGCATTTGGTGTGCA
CCGAATACTGTGCCGTCACACTGTGTGGGTGA
GTCTCTTGTTGTGGCTTCGAGCCATGACTCTGCCTATTGTATTTCTTTTTCTCATTCTCCGCGTTGGG
TGGTTATTGTTTAGATCTAACTCAAACTAACT
AAGAAAGCAAGGAGAAACAATGTCTGGAAGGCCCGAGGAAGGACTTTACGGCAATGTGGAGAGTCAAT
CGTCCAATGTCTCGCAGGGAGGTCGGAGAAGA
ACGAGGTCGGAATTTGAGGGTGACACAGATTACTCCTCTACGACTCGCATAAGATTGGAGGGAATGTA
TCGACCGCAGTGGACCATGAGCAGCAGCGTAA
AGGACATTCTGCTGGAGGGAAGCACTAACAGGACCAACATGAAGCTGAACGATTTCCTCCGGAGCAAC
TGGGGCGAGGAGTGGGTTGTGGAAAGAAATGG

GAATGTTATTATGGAGGCGTTTGTTCGGAGACCCAATGCTTACGTACAAGACCAGCAGCTTCTTgNNN
NNNNNNNNNNNNNNNNNNNNNNNNNNNNNNNN
NNNNNNNNNNNNNNNNNNNNNNNNNNNNNNNNNNNNNNNNNNNNNNNNNNNNNNNNNNNNNNNNNNNN
NNNNNNNNNNNNNNNNNNNNNNNNNNNNNNNN
NNNNNNNNNNNNNNNNNNNNNNNNNNNNNNNNNNNNNNNNNNNNNNNNNNNNNNNNNNNNNNNNNNNN
NNNNNNNNNNNNNNNNNNNNNNNNNNNNNNNN
NNNNNNNNNNNNNNNNNNNNNNNNNNNNNNNNNNNNNNNNNNNNNNNNNNNNNNNNNNNNNNNNNAAG
GCGTGAGGAACAAATCAATTTTAACTTAACTC
TCAGCATTAAAGACGCAATTTTTCAAGGAAGAGCTCGCGTCTATAAAATGAAGCTGAATCGTTTTCTT
AAGATGGAATTGGACGGCAGAGGCGTCGCAGA
CACCAATCGGAGAGTTTTGCTGAAGGAGTTTTTCAAGGACCCCACGAGGTATGTCCGCGATGCGGGAG
TATTGAACGAAATACAGGCATCCGATGCTTAT
TCGAGGGCCGAGAGGGCTGTACGGGAGGAAATGGATATGGAAGAGGATGTAAGGAAGCTCCACTATAA
ACATTTAAGAACTCTGTTGGGGTGGTCAATAG
CTTCTGCGGAGATAAGAGCAAGTGTTCGTGAAATCACCAAAGGTTTTTTGGATGCAGTCCTTGAGGAG
TTGAGGAAACCAACGACGACGATTGCACGATA
AAAATAGAGGGATTGTACGAGTCTGTGTACAATGCGAGGTGGCATCATGTGGTGGAAGTTGTTGGCGG
CGAAAGGACGCAAACGGGGACTGGAATGGAGG
CGAAGGAGGGGAAACCGAAAAAGTCATGGACGTACAGGGCAGTTGGTGACACTCTCGAAAAGGATGAC
GCTGTGCAGCAATCCGGTGCAGCACCTCCCGT
GTTGATGGTGCTCACCTCGGACAGGGGATGGCCGTACACGCTGAATGGGCTGCATAGGTGTGGTAATG
ACTTGTGTGTTAACTGCGAGGTGGAGCGAGTG
TGGCAAATCGTCCTTGACGATCTCACCAAGTGGCTCAGCAACTCTGATTTGACTCTCAATCCTTCACC
TTTGCCGCGTGTGTTGATTGGGACGCCCGGGA
TCGGGAAGTCGATGAATGCCGGCTCGTACCTCCTCTACCAGCTGCTGCACTACGATGTGGAGAAGCTC
CAAGTGGTTATCCACTGTTTTGGAAGTGCGAT
GTACGTGTTTGACAAAACCACCAAAACCGTGACAAAATACATGGGTAACAAAACATCGAAGAGTGTTT
TGGGTGGTTTGTGGCAGCGTGGGATGAAAGGG
TATATTATTTACGATGTGGCAAAGAAAGGAACGCCGCCCGACCCAGATTTTGCGCCCTCCACCGGATG
GGGCATGATTGTGGTGTCATCGCCAAACCTGG
ACAACTATGATAAGTGGGAGACGCAACTAAAAGCCAGTCGAATCATCATGAATTGCCCCGACGAGGTG
GATGTGAAGGCGATGTGTGCGTGGATGAAACG
AGGTCTGGAACAAGATAAGCAAGCGGGATACTGGGAGAAGGTTGAAAAACGCATGGAAAAAGTGGGGC
CGATTCCACGTCACATCTTTGATGAGAATGAA
TATGGAAGACGCTTGGCTGCCGTTGATGATGCCTTGTCAGCGATCAAAGATACTGATGTTGAAGAGTA
CTTTTCCATGGGAGGAGAAGAGAAAAAATGGT
ATTCCAAGAATCCGTGTCACAAACTCGTGAAGATTGTCCGGGAAAGAAAAGTTGAAGGTACTGAGGTA
TTTCTCAACGCATCGATATGTACTGATATCGA
GTTTCGAATTGCAGACCGTTTGGCAAAGAAAAAGGGTGCGAAGGATCTTTTGTTGCTAATATTGGCAT
CGCGTGGTACTCTTGCTTCCCAACTTGTGGAA
CAATTAGGTTTGCGCGCCTTCATGTATGGGGAGTTTGTCATTGCATTATTAAAGGAACTCAAGGAGCT
GCCGCCACCATCGCCTTCCGAACCACAGGATT
CAGTGCTGAAGTTAAACCATCAAGGGTACCCCACTCGTGCCGTTGGATTAGCGGGACTGGAAGGCGGT
GTCACGAGGATTCCGATGGAGTACAGGGTGCT
GTACATACCGGCGGTCCAAAACTTTCCGCTGGTGGACGGCCTTTTCTTTGTTGATTCGCCGCGGAAGA
CGCTGGTTGGGCTGCGGATGACGACGGCGGGT
GAGCACCA
>scaffold1032.size7058
TTGTTGCCCTCCACACGCCATTGTGCACCACCCTGGAGAGTCACAGAGCCATCCAGATGCACCAGCGC
CGATTCAAAAAATTCCGTGCTTTCAACAAGGGT
GCATTCCGCCACTCGCAGTAGCCCTGCGGAGCTCACAGTGGTGTCCCCGTAAAAATAAATGCCATTAA
CGGCTATCATCTTGTTGTTCTCCACGTCAAAG
CAACCGCCGTTCTTCAAGTCAACAGAGTAAACATAAACAGATGATTCCGCACCGTGATCCTCCTCCGT
TGCCCTCAACGTGTTTCCTTTCACAAAGATCC

CGCCACCATCAACGACAACAGCACCGGATAAACAAACTGCGTACCTATTCCCCTCCATGTAGTTGTCA
AGTAGCAGCAGCGTCGAGTTCATCCCAGGGTA
AAAATCCAAGAAGCCAATGGCGTGAGACGACGTCGTCACAATTACACTGCCCGCCACAAGTATCTGGG
AGCTAGCACCAAAATCACCCTCAAACTCCAAT
GCACCAGAATCCAGCATCGAGGATGTCATGTTCACGTGCACGCGTGCACCACTCCCCCTGATCAACAG
GCCCATGAGGACGCACTGCAGCAGCGTGATGT
TGAGTGTCCGTTCTGATGCGTCGAATCTCCACAGGTCCAGCACGATCGCAGCGGTGAAGGTCACGTTG
ACGAAGCACAGACCACGCACACCGGGATCAGG
ATAGTCCACGGAGCTGATGCTGCCACCGTGCACGCAGCGGACCTCCGTGCCCTTCGCATCAGGGAGCG
GGAGCGGCCCAAGGCCTTCCGGAACCGCAGCT
GGCAGACAAGTGTCTCCGTAGCCGCCACGTCGCGTGTGTGACACTCACAGCCGCGGATGCTGCTCGTC
CTCGCTGCGAAGCACACGGCGTCCACGGTGCA
CGCACCACAGTCCAACACCGTCACGGGTGCTCCAACGTTAAGCTCTTCTTGGTAATTATTCACAGGAG
AGCCCCTTAGCTCGTTGCACACACCGTAGATG
ATCGGGCGCGATTCGGATGGTGGTGACCAGTTGCTTGTCATACTTACAATGGTTGACGAGTACGAGCC
GTAAGTGAGTTGTTGTGAAGTATCGACCACAC
ACTCCGATCGTGGAACACCACGCCGTTCTCGTTGGCCACGTACACAACGTAGGCGGCGCGGAACTCGT
TGTCGCGGTGCACCAGCCAGCTGCCGCCAGTC
ACGGTCAGCGTTTCTGCAATGAGCATGGCAAAGCCAAGACGGAAATTACTGGACACAACCTGCAGCAC
GCTACCGCCATCCAAAGCAACATCCCTGAACA
CACACGCACCTTCGTAGATTTCAATGCTTGACGCAATCCACGAGCTGTTCTGTATGGAGAACACGGAG
CCGCCGCTCACACGCAAATCCGACGCAAGAGC
GTACATCACGTGTGCCCGCGACATAACGACGCAGTTGTCCATGTAGAAGACGCTGGACAGGTTCACAC
CGAGGCCGCGCTCCACGAGGATCGCAGCGCAC
AAGTCCCCGCCACAAACCAGCGTCGACCGCGTCATGACAAACCGCGTCGACAGCAGCCGGACGCCGTC
CAGGACAAGCGCGGGGCCGCGCCGGAATCTCG
CGTAGCCAGGGGTCGTCGGCACGTACTGCGACCCGCCAACCGTCGCGCGCAGCGTCGAGTTCGCCAGC
AGCACACTCGAGTGCAGCGGCATCGCGCCATG
CAGCACGATCGTGCCCTCCAGCGACGTCACATTCGTCATGTTGACGAGGGCGTGGGGCATCAGGTAAG
CCGTGCTCTCGCTGAGGCCGCCAATCCGCAGC
TGCGCGCCGCCCGCAAGCACGCAGTGGCGCAGCGTCACGTTCAGCGCGTCAGCGAACGCGTCCATCGA
GCGCAGGTCCACCGCCACAGTGATCGGTCCGC
TGAACACCACCGAGTCGAAGCACGCCGTCGCACGCCCACCGCCAACCGTCACGGACTCCGTCAGCGTC
ACGCCACTCACGCAGCCCTGCGCACCGCCACC
GCCGTCACCGGCCCTCGCAGGCGGCACGTCGAACGGCAGGCACGCCTCGCCCACACCGCCGGCACGGC
AGCCGCACACGCAGTCAGAGAAAGACGCAGTC
AGCGCATCGAAGCACGTCAGCGCAGCCGCACAGCCGTCGCACGGCACCACGCTCACGGAGGGCAGGCC
GGCCATGCGGTAGTCGCCGCTGCTCTGCAGCA
CCCGGCCGCCGGCACGGTTGCACTGCACCGACACAGCGCCGCTCGTGATCGTCAGCCCGGAGACAAGC
GTCGCGCCGGTGGCAACGCAGCGCGCAATCGA
CACGGCGCCACCACCCAGCGTCACCCCCGACAGCGACGCCACCGACGACGCCTCGCCCACCGCCCACA
CGTCGCGCAGCTCCAGCCACCCGCCGGAACCG
ACCGTCCCACCGCCGCAGCGCACCAGCGACGACGCAACAGACCCCTCGACGCGCACGAAGCGCAGCAC
CGAATCAAACACCGCGAGACGCTCTCCAAGCA
CAATGCCGCCGCCGCTGCTCACAACCGACACGTTCGTCACGGAGAACACCGAGTGGCTCCGCAGACTG
AGACGCGACGAGGACGCCACACGCATCGCGGA
CGCGGTCGGACCGCCCGAAGCCTCCAGCGACACGCCGTCAAGCACCACAGCGCTCGACAGCAGATCCA
CGCCGCCGCCAACGTGCACCGCCGACCCGCCC
GCGTGCAACGACCTCAGCACAGTGTTGCTCACACGCAGCTGCGACTGCAACAGCGACGTCGCGTGCAG
CACAAGCGGCGACGCCACCGCGTCCGTCAGTC
CGCTCAGCTGCGAGTAGCGCATCGGCCCCGGCGTCACAATCGTCGAGTCGCGGACCGTCACGTTCGTG
TGTGCCGGCAGGTTGTGCACAAACGCGAGCGA
GCAGTAGTCGCCGGACAGCCCGCTCACGCTCACCTCCAGGCTCCGGTCACTCGCCGGCTCGCCGCTGT
AGCCCCGCACGTAGATCTGCGCACCGCCGTCG

CACACGCAGTTCCTCAGTTCGATGCGCAGCGCGCCGGGAAGCATCGCCGGCACGTCGAACACCACAGC
CACGCCGTTCGTGATGGACACGCCGTCTATCA
GCACCGTGTCCACGGCGCGGCCCACCGTGATGGCTCTGTCCACCGTGCCGCCCCGCAGCCGCAACACC
ACCGCATGCACCGCGGGCATCCACGCCGCAGC
AGCAGCCAGCGCCAACAAAAAGAGCAGCGCCACACGCATAGCCGCAATGGCCATGCACCGCACACGGT
GACGGCTGCGCGGACACGCACCCACCACAGAC
CACATCCAATGAACCAATCAGATACCAACAGGGTTGTCGTGGTGTGTCGCACTCGCACCGTCGACCGC
ACCTTTTGTCTAGCAAATCCCTTATACAGATG
GCGTATCCAGCGACACAATTCCGATAAATCGATATCCCACTACTACTAGTCCTAATGCTGCGAGTAAT
TCCTAACACCTTGAAACGATAGTTGTGCTGTT
CAAACCAAAGTATCTCTAACTGCACGCTCAGAAAAGGAAGAGGCTGCAACAACAGTAACCAAACACAT
TGGCACGCAGAAGCATCCGGTCACAGACAGTC
CCCATCACAGCACAGCCGCCGAAGCAAAGCCGCGCTGACGCAATCACTTCAGTGACACCATCGCCTTC
CACCTCCCACGCAGCCCCACAAAGCCCCCGCG
CAGAGTGGCGGAATCCCGGAAAAAAGAAACATGACCACTGGCACCGCTCCTGTTACCGAATGGCTCCA
TCACATGGCAATCCCGATGCCGTGAAAAAAAC
ACAAGAGCCGAGACAGACCACGTGACAGCGATACGACCGCCAGCCATCAAGCGCGTCCGAAAGGCGTT
GGTGTTGGTGGTGCGGAAACGGAGTGAAAAGC
TCGCCGGTCCCCAGCAATCAAAGCGTGGTTTCTAGTCCACGCGAGTCGGCATTGTGCAGCAAGCAGCG
GCACCATGAGCACACGGCATTGCACAAAGAAG
GGAACAAGGAAGCACGGCGGCAGCACTTGTTCACGGGGAAAAGGGTCCACACCGATTGTACATTCCGA
GTGGCTCACAGACATCCGGATGGTAGTCGTGC
AGATGCCGCGCGGCGGGTGTGACTGCAGTGGACTGAGAAGACGAAGAGAGGGGACAGAGACGCAGTGC
GGCCCTCATCCACCTCGGTTGACTGTGACACC
GGTTACTTCATCCCGTCACATCGCCTGCAGCAAAGACCGTCACCGTCAGCGGCGGTCGGTGCCTTCCC
TCTCCCACTGGAGCGCAACTACACCGACGCAG
TCGCTTGCCCACTCCCTCACCAAGACAACGAGGGAGCAACGGCAAAAGATGCCACACGCATGGCAGAA
ATGCGATCGCGGAGTCTCACAGCTGCCAAAAA
AAAAAAAAAGACTCTCTCAGANNNNNNNNNNNNNNNNNNNNNNNNNNNNNNNNNNNNNNNNNNNNNNN
NNNNNNNNNNNNNNNNNNNNNNNNNNNNNNNN
NNNNNNNNNNNNNNNNNNNNNNNNNNNNNNNNNNNNNNNNNNNNNNNNNNNNNNNNNNNNNNNNNNNN
NNNNNNNNNNNNNNNNNNNNNNNNNNNNNNNN
NNNNNNNNNNNNNNNNNNNNNNNNNNNNNNNNNNNNNNNNNNNNNNNNNNNNNNNNNNNNNNNNNNNN
NNNNNNNNNNNNNNNNNNNNNNNNNNNNNNNN
NNNNNNNNNNNNNNNNNNNNNTTGTAGTACCCACGTATTCTCGCACAGTTTGGGTGGCTTTGTAAAAC
GGGAATGCTCAATTACAAATAAAGCACGCAAC
CATTGGAGCTTCTCGACATCGCAGTGCAGCAGCTGGTAGAGGAGGTACGAGCCGGCAGCCATCGACTT
CCCGATCCCGGGCGTCCCAATCAACACACGCC
GCCCAGGCGTAAAGTCGGTCTCACCGTCAGTGCTGAACCACTCGGTGAGATCGCCCTTGACAATCCGC
CACACTCGCTCCACCTCACAGTCCACATAGCA
GTCACGAGTGGATTTGTTCTCCTTCCATTTCCACGAGTACGGCCCTTCCCTTGTCCGAGGCGAGCACC
ATCAGTCTGGGACGCGGCCATGCACCGGATTG
CTGCGCAGCGTCATTCTTTTCGAGAGTCATGCAAACTGCCCTGTACGTCCATGACTGCGGTGTTTCTC
CTTTCCCTCACTTCCATTCCCGTCCACTCGCC
GCCAGGAACCCCCACAACATGATGCCACCTCGCATTGTACACAGACTCTTACAATCCCTGCAGCATGT
ATCCTTCCACCCGACTGTAAATAGCCCACATT
AATCGCCGCACCTCATTCGCCCTTCTGCGAGTCTTGGCGGGTTGATCTGTGTCGCTGTGGGTCTCCGG
CCTCGCTCCCCTCCGACCATCTCCCCGCGGCA
CAGTGGACGCAGGGCCCTCCTCATGATCACCGTGCACTCTCTTGCGCTTTCCACGCATTGGTTCCCCT
TGTCCCCGCAGTTGGCTCGAGTTAAAGCCGAA
CACTGACCACCAACCAAAAAAGGAAAATGAGAGAGATGGGAACAACAGGCACAGTCCCGACGGGCAGC
CAAAACAATAGAGTCATTCACACAGTGTGACG
GCACAGTGTGCGATGCACACCACATGCACGAGGACCACCGCAACGACGGCAAGTGGGTGCCACTCACC
CAGCACAGTGTTGGGAAAAGCTAACAACAAAA

CCAACAACACCGTGGTAGGCGCGGAGGGTTAGCCCATGCATTCCGCTCGCTACGGTCCCCGCACCCGT
GTGGCACCGCCCTTACCGCAAACACTCTGCAG
AAATACATGTAGGCATAGCCCCTAACACGAAAAAGTGGGAAGAGAATTCGGTGCAGCGACGGCCACAC
GCAGGTATCACAAAACATACACGCCACAGCAG
CAATGCATCATGAAAAGCTCCCACACAAGGAATACATATAGAAAATTTCCGCAATTCACACAGCACAA
AAAAACCATGACGCTCCTTTTCCCATTGCATC
CGTCATTCACATTCCCAATGCGTAACCCTGCAGACATTACCCCTCCATGGTGAAAACAACTGCCTCGT
TGGGGTGTCATCGCGCAGCCATCCGCCTGAGT
AAAAACAAAGGGAAAATCAGGCGTCCTTCCATCTGAAAGGAGGCACTCCAACAAAGTGTCCTATTTGA
CGCAAACACTCCGCGGCCAGTGCCCGGTGTCG
TGTGCGGGCTCCGCGAGAAGCTGTGGGCACGCCGCTGTGCCACACGGAGTGCCCCCTCCGAACGCAAC
AGCAAAAGTGTCCCCTTCCGGTCGCACTCGTG
GTGGTGGCGGCATCCGCTGCCTCGCTGCGTGCTGCTGGCGTCGACTACGATGCTGCAGTGTGATAGAG
GGTGCCCGACACTAGCGGTGCTGCGTTGGGTG
GGCAGTGCCCTCCGCGGCGAGAAACCCAACGCGACTCCTGACCGCGGCGCTCACGCACCTCCTTCCAC
TCCTCTCCCTCCACCCGGCAATCGCACAGCCA
CCAGCACATTGTCCCCGAAAAAACGCACAAGTGAAAGCGTCGCGGAATATTTCCCGAGTGAACAAAGT
GACAGCCACACGCTGCGGCCATCGCGCCGACA
GGTCCGACGCAGCCGCGCCCACACCCACAGCAGCAGACACACAAAAAAAAAGATCTTCGACTCCGTGC
CACGTCCTCGTGCGTGATGGTGCGGCGCCTCA
AAACAATACCGCCACACGCCGTGCAGCATTCCCACAGGGAATGAGAGCCGCAGCGCACTCTCACCGCC
TCCCCCTTCCTTCTCTTCATTTCCACTGAGTG
AAGCAAAAAACACACGCAGCAGCCAGCAACGAGTGTAGGCGGAACATCCAACTGCACCGCAACATGTA
CGCCGTGCGGTTGTGTGTGTGCCTGCAGTCAC
TCAAAGAATAAAAACGAGAAGAAAAAAAGAGAGCCACGCAAGTGAAGGGGACGCACACAAACACGCAG
CAGAAGTGCAGGACGGAAAAAAAAGGGAGGATG
AAAGCCGCTGAGGCTCGAGATGGCACGGCAGCCCAACCAGCGGCAACCCACAATAC
>scaffold1033.size7204
GGCGGCAGGGTGTGGCTGTGTGTGTGTGTGGCGCTGCGGGTGTCTGCCCGCCCTGGTCGCAAGAAAAC
AAAACAAGAGGGAAGAAATGTGTGCTCCGCCCC
ACAGGCCCTCGTCGCACGCCCACACGTCACGGCTCCCTCAGCGCGCCGTTCGTCGCGGCCCCTCCTCA
TGCGTGCAGCGGGCGAGGCGCACAGGGCTGTC
GTCATGAGCGTGTCGGTGGGTGTGCGAGTGGAGCAGTTGGGCGGGAAACACACGGCACCCCTCACAGG
AAGAGCGCAGCGGGAAACAATTAACAAAAGTA
GAAAAGAAAANNNNNNNNNNNNNNNNNNNNNNNNNNNNNNNNNNNNNNNNNNNNNNNNNNNNNNNNNN
NNNNNNNNNNNNNNNNNNNNNNNNNNNNNNNN
NNNNNNNNNNNNNNNNNNNNNNNNNNNNNNNNNNNNNNNNNNNNNNNNNNNNNNNNNNNNNNNNNNNN
NNNNNNNNNNNNNNNNNNNNNNNNNNNNNNNN
NNNNNNNNNNNNNNNNNNNNNNNNNNNNNNNNNNNNNNNNNNNNNNNNNNNNNNNNNNNNNNNNNNNN
NNNNNNNNNNNNNNNNNNNNNNNNNNNNNNNN
NNNNNNNNNNAAATTGAGGTGGAGAATTTGCCCACAGGGCGTCAGGCAGAATAGACACACACATACAG
GGGACGAGTCGTACCTTTCATCATTCGTGTGT
GGCTGCTCCGCACCGCTGCCCAGCGGCACATGCAAGGGCGGTCAGCAGCAGCAGCAGTGGTGTGTGCA
CCCACGCAGTGGCGATGGCGCTGCTGTCCGAC
GTGCCGGGATCCTGCGCTCCAGTGGTGGCACTGTTGTTGGCCTCCTTCGTATATGTCGGTTTGCCGGA
CTGTGAGGCAGCACCGGCCGTGGCTGCACCGG
AGGCCCCACCATTGCCGCTGGCGGGAGTGGCTGAGGGTGTTTGCGTCGTCCCGGATGCATCCGTTCTG
CCAACCCCTTCGCCTGTTGGCAGCTGCGAAGT
GTTNNNNNNNNNNNNNNNNNNNNNNNNNNNNNNNNNNNNNNNNNNNNNNNNNNNNNNNNNNNNNNNNN
NNNNNNNNNNNNNNNNNNNNNNNNNNNNNNNN
NNNNNNNNNNNNNNNNNNNNNNNNNNNNNNNNNNNNNNNNNNNNNNNNNNNNNNNNNNNNNNNNNNNN
NNNNNNNNNNNNNNNNNNNNNNNNNNNNNNNN
NNNNNNNNNNNNNNNNNNNNNNNNNNNNNNNNNNNNNNNNNNNNNNNNNNNNNNNNNNNNNNNNNNNN
NNNNNNNNNNNNNNNNNNNNNNNNNNNNNNNN

NNNCACCACTGCTCGCAACAACAGTGTAGCCATGAGCACCCAGTGAGCTTACACACTTATTCCATTCT
GACCGGCCAGGGAAGCGCCATTGCAACGTATT
CTCACTGTCCGCACACGAGATCTCCACCGACACGTTCACGGCAGACGCTCCGGAATAAAACTCAGTTG
ATTGTACAGCAGCCCCCGTCGCAGCCGCAGCC
GTCACGAAAATGGAAGCGCAGCAGCACAACAGCGCGAGGGACAACAGGGCGCACACTTCACGGCGCAG
CGCAGTCGCCTTCATCACAGTAGTCATCATCC
TTCCTAGTGGTGCTGGGGTCAGTCCGTTGATCTGCACACGGAGTGGGGAAGTGAATGACAGAACGGGA
GAGAGGGAGAGAGGCGCAGTATGGGCATAAGC
AAAAAGAGGGCACACAGTATGGCAAGAANNNNNNNNNNNNNNNNNNNNNNNNNNNNNNNNNNNNNNNN
NNNNNNNNNNNNNNNNNNNNNNNNNNNNNNNN
NNNNNNNNNNNNNNNNNNNNNNNNNNNNNNNNNNNNNNNNNNNNNNNNNNNNNNNNNNNNNNNNNNNN
NNNNNNNNNNNNNNNNNNNNNNNNNNNNNNNN
NNNNNNNNNNNNNNNNNNNNNNNNNNNNNNNNNNNNNNNNNNNNNNNNNNNNNNNNNNNNNNNNNNNN
NNNNNNNNNNNNNNNNNNNNNNNNNNNNNNNN
NNNNNNNNNNNNNNNNNNNNNNNNNNNNNCGGAGTGGGGAAGTGAATGACAGAACGGGAGAGAGGGAGA
GAGGCGCAGCATGGACACACGCACAAAGAGAG
CACACAGCATGGCAAGAGGCAGCCACAGCCCACAGACTGCGAGACCGCACACACACACACACACAC
CAGTCACGCGAGGAAGTTGGTTCTCAGCCATC
CATAAATGGGCGCCAAAATTCCACTGCCATCCCACACTCAACGGCATGCGGCGCCTCACTCCAACCGA
CCGTACGGAGTGCATTGCAGCTGCAGGGAGCA
AAACGGCATGACACTCTCCCCGTGTCTCTCTCTCCTTCACGGCCAAGAAAAAATAGAgCAGCCACACT
CAACTGCCAGCCCCGGAGACAAAAAAAATCTT
CCACGTGCACTCTGGTGTACATGACGTGTGCGCGAAGCCGTAACTGCCTGCGGACACGCACAAACGGA
AGGGGTGGAAAAAAAGAAGACCACCACGACTC
AAGCTGAGACTGCAGGCTGCCAGAGGATCAAAAAAGTCAAGAGGGCCGCCTCCCGTCGGCAATCGCTA
CACCACTTGCTTGCGGACGAGATGTCGTGCGC
GATTGGTGTAGCTCACTGTGACGCATCCACCCTCGCAGCCCCAGAGGGCGGAACAGCTACCGCCAACC
CAAACCGCTGTCAACACGTCATCGCCACAATC
GTGAAGAGCCGGTGGCATCACACACTCGAGAAAGGGACACCAAGACACGCCATGCAACAAAGGGGCAC
ATCGAGTCCGCAAACAGTGCACTGCATTCCAA
CACCGCAGGGACCCTCCCTTGCCCTCCTCTGCCGCGCACCGCAACATGCATCCCCATGGCCCTCCCGC
ACGNNNNNNNNNNNNNNNNNNNNNNNNNNNNN
NNNNNNNNNNNNNNNNNNNNNNNNNNNNNNNNNNNNNNNNNNNNNNNNNNNNNNNNNNNNNNNNNNNN
NNNNNNNNNNNNNNNNNNNNNNNNNNNNNNNN
NNNNNNNNNNNNNNNNNNNNNNNNNNNNNNNNNNNNNNNNNNNNNNNNNNNNNNNNNNNNNNNNNNNN
NNNNNNNNNNNNNNNNNNNNNNNNNNNNNNNN
NNNNNNNNNNNNNNNNNNNNNNNNNNNNNNNNNNNNNNNNNNNNNNNNNNNNNNNNNNNNNNNNNNNN
NNNGCAGGAGAACACAGAGGCGGCCATACACA
CCGCGTGCGCACCGACTCCACCAATGGGACGGATCGTCAATCCGTCGTCGAGGGAGCGTCCACCACCG
TTGCACAGAGTCTTCATCGGCCGCAACGCTCC
CTACACGCGGAGTGCTTGGTCCTTTCTCCTTGTGGTGTCGCAATATTTGTGTGACCGGCCACTCAACA
GCCACCACTTGCGCAGCGCCCGTCCCAGGTGC
AGGAGCATCGCACAGTGCTATCCCAACAGCCCATATCGCATGGCATCAGCGCACTCCGCTGGCCTGCT
TTCATGAGGCATTGTCGCCTTGCGTGCACTGC
CAGGCACCCAGCGTCACACCCCACCCGCCGCATATGTCACATTGAATAGCGGGCTCGAACGCAGCCGT
GCTCTCCGTGTTTCCGCGTTTCAGGATGCTGC
ATCGGTTGTGCGAAAAAAAAAAAAAAACAAAGAACTGTCTCCACTTGAGCCGTAAGTTCGCAACGATT
GCACTGCAACGGTGAGTCTTCGATCTGCTTCT
CTTCTTNNNNNNNNNNNNNNNNNNNNNNNNNNNNNNNNNNNNNNNNNNNNNNNNNNNNNNNNNNNNNN
NNNNNNNNNNNNNNNNNNNNNNNNNNNNNNNN
NNNNNNNNNNNNNNNNNNNNNNNNNNNNNNNNNNNNNNNNNNNNNNNNNNNNNNNNNNNNNNNNNNNN
NNNNNNNNNNNNNNNNNNNNNNNNNNNNNNNN
NNNNNNNNNNNNNNNNNNNNNNNNNNNNNNNNNNNNNNNNNNNNNNNNNNNNNNNNNNNNNNNNNNNN
NNNNNNNNNNNNNNNNNNNNNNNNNNNNNNNN

NNNNNNTTTGTTTTGTTTTGTTTTTGCTGTTGTTTTTAGGTGTGGAGATTCTGCGAATACAAAAACCT
CGCCCCATACCTGTTCTTCTTTTATCCCGCCT
CCTCCCCACTGCAGTGGTGAACGCTCTGCTCCCACCATCACCCTTTGCGTTTGTGAAATGAGCAAACA
GTCACGGAGGAAAAAGAAAAAGGCAGAAAGAA
ATCCCACTGCGAGTGTACCCGCCACTCCCACGACGGTTCAAAAGGCTTTTTTTTTTTTTTTTGCGTGT
GTTTTTTTTTGTTTGTGTGTTTTTTTGTTTTT
TTTTTGCCATCGGCCCGCCGTCACCGCATGCGCCAATGCGGTGATGAGTGCTGAGTGCCTGAAAAGAA
GTAAATAAATAAATAAATAAGAAAAAAGAAAA
AAAAAGGCAACCAAAGTTTTTTTCCAGCGATGCGGGTTGGAACCACCCACCCACCGTCGCCCCCTGGC
GCCTCTTCTCCTTTTCCCTTTCAATCTCNNNN
NNNNNNNNNNNNNNNNNNNNNNNNNNNNNNNNNNNNNNNNNNNNNNNNNNNNNNNNNNNNNNNNNNNN
NNNNNNNNNNNNNNNNNNNNNNNNNNNNNNNN
NNNNNNNNNNNNNNNNNNNNNNNNNNNNNNNNNNNNNNNNNNNNNNNNNNNNNNNNNNNNNNNNNNNN
NNNNNNNNNNNNNNNNNNNNNNNNNNNNNNNN
NNNNNNNNNNNNNNNNNNNNNNNNNNNNNNNNNNNNNNNNNNNNNNNNNNNNNNNNNNNNNNNNNNNN
NNNNNNNNNNNNNNNNNNNNNNNNNNNNGCGA
CGCCGGTGTGTTGTTATCTCCACGTGCCGGTGAAAACTTCTGGCTTGACTCGTCACGCGTGTGATATC
TAAGCCCAAAACGGAGGCAGCGCAGCAAAGAC
AAGACAAGAGAAAAGAAAAGAAAAGAAAAGGAAAGAAGAGCGACACCCCCAATACAACCAGACGCAAA
CAAAAAAAAAAAAAAAAAAAGCTCCCGTTCATA
CGATAGCACCCGACCCTTCGCGTATCGTCAAGACGGACTGCGGCAGGAAGCATGCACTGATGAGATCG
TTGTGCCTCACGCTTACGGTCCAATCATTGCG
GATGCACACAAGCAGAAGGGGAAAAAAGAAAAAAGAAAATAAAGAGAAAATAAACGGCAACAAACATC
ACTTTCCTTCTTTATTCATGTGGTCCCTCCTC
CCCCAACGTGAAGGCCGCGCCGTCCTTTGCCGCTCTTCACCCATCCTTGTTACTGTCTGCAATGGCTG
AGTGAATTATTTCCTTCATGTCTCCTCACCCC
TAAATTTAACAACAACATTTTTCTTTTCTTTTTTTTTCTCCGCTCATCTCATTATTATTATTATTATT
ATTTTCCTTTTTCGATTTGGCTGCGGAGGAGA
AGCCGGTGCCCTCTCCGCCGCGGCGTCGCCCTCCTCATGGATCATCCGCCGTTAGCCGTTACACGTGT
AGAAGAAAGAAAAAGAGAGAGGGGTGGCGAGA
GGACAAGAGAAAGTGGCAAAGCAAACCAAAAAGACGTTTGTCTCACCCCCATTGTCATTCCTTCTGCT
GTTCCTTTTCATTGGCATTGAAATCCGCTGCG
GCATGTAACCCCTTCTTCTTTTCTTCCTTGTCTGCTTTCTTCTCCTGCTGCTTCTCCCCTCCCTTTGC
AAAGACGAGGGGTTCGGATAACACGGCAGCGA
TGTCATCTGGAAGAGGCGGAGGTCGCACGTCGTCCGGCACAACCAACGGCTCTGCTGGTGGCAGCCCA
CACAACACCGTCACGCGCCTCTCAGAGACAGC
CACCGTGGCGGAAAAACGTGCGGTGATGTGACGATCCCCTTTGATGCGCAGGACAGAAAGAGGGTCAA
TAACGCCCTGCCGCCGCAACATCGCCACGGCC
ATGCGTGCCTTAAGAGGGTTCTCCAAGTGTTTGAGGCTAAACGGGAAACACTGGTGCTTCTCTGTAAT
TTCTTGTAGCGAGGCAAGTACAGAGCGCACGC
GGGCGTCACTCTGAAATTCCACTTCGTTACGACGGTAAATTGTTGTCACACCATCAGCAGGTGACACC
TTGTACGAGGGATGATCGGTAAAGGCTACATC
CAGCGTCCACACCTGATTTGCACCTATGGTACAATCTGCAACGTCCTGGTGAGGGTCCTCCGCTGACA
CACGTCGTGTGATGATGCATTCCATGCCATCT
GGAACCCATCGTTTTGTGCGGTTTGAGAGTACGCCCTCTAGTCCCTGGACTTCAAAGTAATTTCCCAC
ACGGTGTATAAAGTCTGTGATATCCGCGTTCA
ACATGCCTGGCTGCAGGAGATGAATCATTCCGTGCAATGCCACACGTGCCGCCTCCACCGCGTTACTT
GCCCCTTGAGTGAGTTGCCGCACCTCTTCATT
TGCAGCAGTGACGTCATGTGGCACAAAAACCGTTCTTGCTGCAGTGACCGGGTAGCCGTCAATGTGGC
ACCCCATATGAATCGTCACCACATCGCCACCG
CGCAGCACGACGGCCTCTTCTTCTACCAACGGTGCATGATTGCAGAGGACGTAATTCACCGAGACATT
TGTTGGATACGAGAGACCGCGGAGAATGCGGT
TACCGTCCGCGTCCTTCGCCTTGGAGAACATCGCTCGCACCCGCTGCAGAAGTTCTTCATCACCACGA
TCACACAATTGTTTTGTGGTTGCCCCTGGCAC

GCACGCAGCTGTGAGGATGTCCATCACCTCGTCAAGAGCACGCCCGGCAGACTTGTATTTTGTCCTAA
CGTCCGGCTTCACAATTGTCTCTGCCACCCGC
TCTGCCGGCTCCAACTCACCCTCGAGTTCCTTCTCCATGAGCGCGAGCTTTTGACGAATCGTGGACGC
AGACCGCACCCTCGCACTGCCGCCTCGCTGCG
GCGTTGGTGCCCGCTTTGCTGAGGTTTTGTGTCCCGCATCTACCGTTGGGGTTTGGGCCTCCGGCTTT
GGGACGGCAGCGACTTTCTGGCGCGTCGTGTG
GGCCGGTCGCACGGACCCTCTCAATTGCTGCAGCGAAAGTGACCGCCGGCGCTTCATCCGCAGGGTCG
GCCGCGCACTCTTGCTGCGGCTGTTGCTGTAC
CCACGCCCCATCCGACAGCTTGAGGCACCTCAGACTTATCCGCACAGCGCAAAAAAAAAATAAATAAA
TAAATAAAAGGCCCAACAATCACACACACACA
CAG
>scaffold1034.size15126
GTGCTGAGAATTTGCTATACGCAGTATTGAAAACGCGCTCACGCTGTTGCCCTCCACACGCCACTGCG
CACCTCCCTCGAGTGTCACAGAGCCGTAAAAAT
GCACCAGCGTGGAATCAAAAACCTCCGTGCTTTCAACGAAGATGCAGTCCGCCACTCTCAGCAGCCCC
GCGGAGCTCACGGTGGTATCCCCGTAAAGATA
AACGCCATGGACCGCGCTCATCGTGTTGTTCTCCACGTCAAAGTAGCCGCCGTTCTTCACAACAGCGT
AAACGCAAACAGATGATTCCGAACCGCTACCC
CCCTCCGTTGCCCTCAGCGTATTTCCCTTCACAATAATCCCGCCACCATCAAGAAGAGCAGCTTCGGA
AAAATAAACTGCGTAACTACTTCCTTCGATGA
AATTGTCAAGCAGCAGCAGCGTAAAATTCGgtgacttcatttttaggtttttacgttaccgcaccgag
ctgcagctgtgttagaataacgggtggtggtc
atagaccctcgagcgaggttttgCACCCTCAAACTCCAACGCTCCAGAATCCAACATCGAGGATGTCA
CGCTCACGTGCACGCGTGCACCACTCCCCTTG
ATCGACAGGCCCATGAGGACGCACTGCAGCAGCGTGATGTTGAGTGTCTGTTGTGGTGCGTCAAAGCT
CCACAGGTCCAGCACAATCGCGGCGGTGAAGG
TCACGTTGACAAAGCACAGACCACGCACACCGGGATCAGGATCGTCCACGGAGCTGATGCTGCCACCG
TGCACGCAGCGGACCTCCGTGTCCCTCGCATC
GGGGAGCGGGAGCGGTCCAAGGCCGTCCGGAACCGCTGCTGGCAGGCACGTGCCACCGTATCCGCCAG
CAGCGCACACGCACTCACAGCCGCTGATGCTG
CTCGTCCTCGTAGCGAAGCACACGGCGTCCATGGTGCAAGCGCCACAGTCCAACACCGTCACGGGCGT
CCCAATATTGAGGTCTTCTTGGTAATCCGTCA
CAGGTGAGCCCCTTGCCTCGTTGCACACGCCGTAGATGGTCGGGCGCGAGTCGGATGGCAGTGACCAG
CTGCTTGTCATACTTGCAATGGTCGACGAGTA
AAAGCCGTACGTGAAGTTGTTGTACAGTATCGACCACACACTCCGATCGCGGAACGCCACGCCGTTCT
CGTGGACAACGTACACAACGTAGGCGGTGCGG
AACTCGTTGTCGCGGTGCAACAGCCAACTGCCACCAGTCACGGTCAGCACGTTTGTGATAAGCATGGC
AAAACCGAGACGGAAAGTGCTGGACACAACCT
GCAGCACGCTGCCGCCAGCCACCGCAACGTCCCCAAACACACACGCGCCTTCGTAGACGCCAATGCTC
GGCGCACTCCACGAGCTGTTCTGTATGGAGAA
CACGGAGCCGTCGGCAACGCGCAGGTCCGATGCAAGAGCGTACATCACGTGCGTCCGCGACATGACAG
CGCAGTTGTCCATGTAGAAAACGCTGGACAGG
TTCACGTCGAGACCGCGCTCCACGAGGATCGTAGCGCACGAGCCCCCGCCACACACCAGCGTCGACCG
CGTCATGACAAACCGCGTCGACAGCAGACGGA
CGCCGTCCAGGACAAGCGCGGGGCCGTACCGGGATCCCGCGTGGCCAGGAGTCGCCGGCACGTAATGC
GACCCGCCAACCGTCGCGCGCAGCGTTGAGTT
CGCCAGCAGCACACGCGAGTTCGGCGGCATCGCGCCATTCAGCACAATCGTGCCCTCCAGCGACGTCA
CATTCGTCATGTTGACGAAAGCGTGCGGCATC
AGGCGCGCCGTGATCTCGCTGAGACCGCCAATCCGCAGCTGTGCGCCGCCCGCAAGCACGCAGTGGCG
CAGCGTCACGTTCAGCACGAGAGCGAACGCGT
CCATCAAGCGCAAGTCCACCGCCACAGTGATCGGCCCGCTGAACACCACCGAGTCGAAGCACGCCGTC
GCACGCCCACCGCCAACCGTCACCGACTCCGT
CAGTGTCACGCCACTCACGCAGCCCTCCGCGCCATCGCCACCGCCGCCGGCCCTCGCAGGCGGCACGT
CGAACGGCAGGCACGCCTCGCCCACGCCGCCG

GCACGGCAGCTGCACACGCAGTCAGAGAACGACGCCGTCAGCGCATCGAAGCACGCCAGCGCAGCCGC
ACAGTCGTCGCACGGCACCACGCTCACGGAGG
GCAGGCCGGCCAAGCGGTAGTCGCCGCTGCTCTGCAGCACCCGGCCGCCGGCACGGTTGCACTGCACC
GACACGACGCCGCTCGTTATCGCCAGCCCGGA
GACAAGCGTCGCGCCAGTGGCAGCGCAGCGCGCAATCGACACCGCGCCGCCGTTAAGCGTCACACCCG
ACAGCGATGCCACAGACGACGCCTCGCCCACC
GCCCACACGTCGTGCAGGTCCAGCCACCCGCCGGTACCGACCGTCCCACCGTCGCAGCGCACCAGTGA
CGACGCAACAGACCCCTCGACGCCCACGAAGC
GCAGCACCGAGTCAACCACCGCAAGGTGCTCGCCAAGCACAAGGCCGCCGCCGCTGCTCACAACCGAC
ACGTTCGTCACGGAGAACACCGAGTGGCTCCG
CAGACTGAGACGCGACGAGGACGCCACATGCATCGCGGACGCGGTCGAACCGCCCAACGCCTCCAGCG
ACACGCCGTCAAGCACCACCGCGCTCGACAGC
AGGTCCACGCCGCCGCCAACGTGCACCGCCGTCCCGCCCGCCTGCAACGACCTCAGCACAGTGTTGCT
CACACGCAGCTGCGTCTGCAACAGCGACATCG
CGTGCAGCACAAGCGGCGACGCCACCGCGTCCGTCAGCCCGCTCAGCTGCGAGTAGCGCATCGGCCCC
GCCGTCACAATCGTCGAGTCACGGACCGTCAC
GTTCGTGTGTGCCGGCAGGTTGTGCACAAACACGAGCGAGCAGTAGCTCCCGGACAGCCCGCTCACAC
TCACCACCAGGCTCCGGTCACTCGCCGCCTCG
CCGCTGTAGCCCCGCACGTGGATCTGCGCACCGCCGTCGCACACNNNNNNNNNNNNNNNNNNNNNNNNNN
NNNNNNNNNNNNNNNNNNNNNNNNNNNNNNNN
NNNNNNNNNNNNNNNNNNNNNNNNNNNNNNNNNNNNNNNNNNNNNNNNNNNNNNNNNNNNNNNNNNNNNN
NNNNNNNNNNNNNNNNNNNNNNNNNNNNNNNN
NNNNNNNNNNNNNNNNNNNNNNNNNNNNNNNNNNNNNNNNNNNNNNNNNNNNNNNNNNNNNNNNNNNNNN
NNNNNNNNNNNNNNNNNNNNNNNNNNNNNNNN
NNNNNNNNNNNNNNNNNNNNNNNNNNNNNNNNNNNNNNNNNNNNNNNAGTGCACGCACCCGAACACAGCG
GCGAGCAGCGCGTTGCCCACCACCATCCACAG
CGGATCAAGGGCAGCAAACACCGACAGGAAGTACGGCAGCGCAACGGTGCTCGCACGCTCCTGCGCCG
AGGCGCATGACATCCTCGCAAACACGCCGAGT
GCCTGCATCTCCAGGACGGAGCCCAGCCCGCCGGTCACCACCACGGCAACAGCCGTCGCACCCGCAAC
AACGCCGGTAATCGCCGAGAGGGCGCCGGCAG
CGGCGGGCAGCGTGTCAGACCTGATCGTGAAGGACCCAAGCAGCACACCCTTGCAGCCGCCAGACACT
GCCGCAGCGTCGCAGCGAATGACTATCGTCTC
GTCGGCAGCAATGAAGTAACCGCGGTGCACGGGCACAGCCAGCTCCAGGACAGTGGACGGATTCGTCG
CGTTGCGCACCCACGTCGCGTTGCTCAGCATC
GCTCCCCACGGACCACCGAACCCGCTCGGCTGCGCCGTCGACACCGGCACGAAGCTCAGATGCGTGCC
GAGTTGCGGGTCGCGTGCCCAACGAAACGGCG
GCGGCAGCGCCACCATCAGCAGCGTCGGGACAACGCTCCCACCGCCGCGGATGTCGTTCTGCGTCAGG
CTCCCTCCAGCCGCCGTCGTCGTCACGGCGAG
CGTCGGGCACGCCACGTCGCTCCACCACAGCGTGCTGCTCACCGACGCCGTCGGGGTCCGCGTGGGCG
GCAGCGCCACCGTCTCCGTCACCTGCAGCGTC
TCCGTCGGCCCGTACTGCGTCGGACTGTAGTGCGGCGTCCACGCTGGCGTCATCGTCGGGCTGCGCGT
CTCCGTCAGCGTCGCCGTGCGACTCAGCAGCG
GCATGCCGGGCGTGCGGTTGCACCCGTCCACGTGCGGCAGGTACACCGGCAGGCAGTCGCGCCCGTAC
CCGCCCTCAGCACAGCGGCAGCCGCACGACCC
CGACATCGCCCTCGTCGCAGCAGCAAAGCAGCGCACGTCGGCGTCGCACACGCCACACGCCACGGGGC
GGAATTCCCCGATGATGCCGGCGGACCTGTAG
TCCATAGGCCGCAGTGCCTGCCCATTGAGCGTCAGGCACCCCACAACGAACTTCCGGCCGGTGCCCCC
GAGCGCAACGGTGCCGTCCATCACGACGAGCC
CGCTGCCGGCGTTCCCGTGGAGCGTCAGTGTGCTCTGCGCGAGATCCGCACCGCGCGGGTACGGCGGC
ACCGACAGGAGCCTGCCGCTGATGTTGTTGCC
GCGCACCGACAGCCACGACCCGGACACGAACGACGCCGACCCCCGCAGCACCAGCAGCGCGCCATCGG
TCAGCGCACCGGCGTTGTCGTTCACTACGACG
GCGGACGCGCTGGCCTCCACACCGCTGCCAAACACCAACCCGTGCGCGGCGTACACCTGGCTCCCCGA
CAAGCGCAGCACAGCGCCCCCCGACGCAACCA

CGCGCGCACTAGCGTACAACGCATATTCGCCACCGAACACAGCCGCGTCCAGCGCAACACCGCCACCG
ACAAGCTCCAGCACGGCGCCGTCCACCACCAC
CGTCCGCCCACCAGTCATCACGGTCACGAGGGCCACGCCGGACACAATCAGCACGGAACGCACAAGCC
GCAGGCCCGACAGCACAAGCACCGGTGCGTAC
GGCGAGGACTCCGAGCCGGAAAGATACACAAGCGGCGTCGGCCTCGCGGCGACCAGCAGGGAGTCCGT
CACCGTCAGCACGGACCCCGCAGGGAACGTGC
CGGTCAGCACAAGCGCACCGTTCAGCGCCTCCACATCGCACACCGACAGCTCCACCGGCCCGCTCTCG
TCGCTCCCCGCCGCACGCGCACCGCGCAACGC
CCCGCCGCCCATCACGCACAGCACGGCACCGTTCAGCAGCGACACGTTCTGCACAACGATCCGCACGC
CGTCCCGCGCCATCCACGGCACGTAGAGCAAA
GGACTCACGCCGTCCAGCACAACGTGGCGCAGCGTCACCTCGCTTGCGCCGGCAGGCACAACGAACAC
TGACCGCACCGTCACGCCCTCGCGGAAAAACA
CACTCGACGCGGTGCCCACAGCGGGCGGCAGCGCAGGAGCTCCCACAGGCACGCACGCCTCGCCGTAC
CCACCGCTGCCGCACTCGCACACGCACACACC
GTCCTTCATGCTCGCCGACGCAGTCCCGGGCGCGTAGCAGTGCGTGTCCCTGTCGCAGGCGTCGCACC
CAACAACGTCGATGCTCCCAGCATCGCCAAAT
CCAGCCGCTGCGTACTCCTCCGCCGTCGACAGGACTCGTCCACCTGCGTCGTTGCACGCCACCCTCAC
ATGCGCCCCAGCCGCAAGCGACAGGCTCAGCG
ATAGCATCCGCGGCAGCAGCGCTCGGTTGCGCACAAAGCTCAGCGTGCTCCCAGCAGCGGTCACGGCG
CCAGCCGCGTGGAGCATCTCCCTCGAGACGCC
GTCGTGCACGTTGCCCTTCGCCAGCAGCATCGACCCGTCGGCCAGCCTCACCGTCGAATAAAACGCCA
CCGCGTAGTCCTCCGTCAACAAAAAGTCGCTC
CGCACAAGCGCCACAGCGGAGCCCGACACGCTCACAGCCCCACGCACAGACACCGCGTGCTTGCACAG
CAGGAAGTCGCTGTCAACAAACGCCACAACGG
AGCCGCCAATGGCCGCAACACCGCCCTCCACCGACACGCACAGCCCCAGCGCCGTCTGCACCAACAGC
CCGTCCACGTACAGCGCACCGCGCGACGACAG
CGCCACGCCGCCAACCACAAGCACGCCAACAGCCTCACGGAACACCGCCAGCATGTGCGTGCGCGCAA
TGGTCAGCACACTCCCAGTCAGATTCACTTTG
TGCACCACGAGGCACGCGGAGGCGTCGCCAAGGCCGTAGGCGTTACGGTACGCAACACGCCTCTCTGC
GATCAGCACCGAGTCCACCACAGTGACGCGCG
AGCCCGGCGGGTATCGGTTCGCCACCAGCACGCCGCCACCGGAGCGCGACTCAAGCNNNNNNNNNNNN
NNNNNNNNNNNNNNNNNNNNNNNNNNNNNNNN
NNNNNNNNNNNNNNNNNNNNNNNNNNNNNNNNNNNNNNNNNNNNNNNNNNNNNNNNNNNNNNNNNNNN
NNNNNNNNNNNNNNNNNNNNNNNNNNNNNNNN
NNNNNNNNNNNNNNNNNNNNNNNNNNNNNNNNNNNNNNNNNNNNNNNNNNNNNNNNNNNNNNNNNNNN
NNNNNNNNNNNNNNNNNNNNNNNNNNNNNNNN
NNNNNNNNNNNNNNNNNNNNNNNNNNNNNNNNNNNNNNNNNNNNNNNNNNNNNNNGCACACGGTGCC
GGCTGCGCGGACACGCGCCCACAACAGACCGC
ATCCACCGACCCAGACAGGTACCAATAAAGCTGTCGTGGCGTGTCGCACTCGCCCCGTCGACCGTAAC
TCCTGTCTAACAAATCCCTTCCTACAGATGGG
AAATCCAGAGACACAATTCCGCCAGATCGTTATCCCACTACTACCAATCCTAATACCGCAAGTAACTC
CTAACACCTTGAAACGATAGTTGTGCTGGTCA
AGCCAAAATATCTCTAACTGCACAGTCAGAAAAGGAATAAAAGGCAGCAACAAAAGTAACCAAGCACA
GTGGTACGCGGAGGCATCCGGTCACAGACAGT
CCCCACCAAAGCACAACCACCGACAAAGAAAAGCGCAGACACAACCACGCCGTTAACACCATCGCCCT
CCCACCGCCAAAGCAGCCCCACACAACCATTC
AGAGTGGCGCCATCCCAGCAAAAAAGGCACATGGCTATTGGCACCGCTCTTGCAGGCAAATGGTTCTA
CCACAGAGCAGTCCCGATGCCGCGAAAGACAA
ACAAGGGCCGAGACACACCCACGTGGCAGCGTTACAACCGGCGACCATCAAGCGCGTCAAAGAAATAA
ATGGTGACACGGGAGAGGAGTGGAGGATTCGC
CATTCGCCAGCAGTCAAGACGTCGTTCTTCCTCGCGTGAGTCGGCATTGCACAGCAAGCAGCGGCACC
ATAAGCGCACAACACACACAAGAATGAAGGAT
GAAGAAGGGCGGTGACGGCACTCAGATACAGGGGACAAGGGTCCACACCGATTGTGCGGTCGGTTcca
cccctctcccacNNNNNNNNNNNNNNNNNNNN

NNNNNNNNNNNNNNNNNNNNNNNNNNNNNNNNNNNNNNNNNNNNNNNNNNNNNNNNNNNNNNNNNNNNNNNN
NNNNNNNNNNNNNNNNNNNNNNNNNNNNNNNNNN
NNNNNNNNNNNNNNNNNNNNNNNNNNNNNNNNNNNNNNNNNNNNNNNNNNNNNNNNNNNNNNNNNNNNNNNN
NNNNNNNNNNNNNNNNNNNNNNNNNNNNNNNNNN
NNNNNNNNNNNNNNNNNNNNNNNNNNNNNNNNNNNNNNNNNNNNNNNNNNNNNNNNNNNNNNNNNNNNNNNN
NNNNNNNNNNNNAAGGGTCCACACCGATTGTG
CGGCCTGTTGACTCATAGACGTCCTGTTTTTAATCACGCCACTGCCCCGCAGTTCGTCAATCTGCAGG
AGACTGAGGGATAGAGAGAAGGGAAAAAGCAA
AGGTGCGGCCCACATCCATCTCGGTTGGATGTGGCACCGGTGACGTCATCCCGTCAGGTCACCCGCAG
CAAAGACCGTCACCGTCAGCGGCGGTCAGCGT
CTTCCCTCTCCCACTGGGGCGCCATTCCACCGACGCCGTCGCTTGCCTCCTCCCCAAAAAACACAACG
AGGGAGAAGCACCCACACGCAACCGCCAGCACA
GAGTACAAGAGGAGCAACCAACTACACTGCAATAACTATATCGTGTAACTGTCTCGTGTGTCTGCCTA
CGGCCACAAAAAAATAAAAAAAGCACCTGAGG
ACAAATACAACCACAGCAGAGCGCCAGAAAAAAACAAACGGGATGAATGTTGAAACGCAACACCGCAC
GGCTGCCCACGCCGTGGCGAGTCGCGGGAAAT
ATGCTGGAAAGTAAGAAAGGATAAATCCGTCTTCCATTACGCATTCACTCAAAAAAAAAAACAAGAAA
AAACTGCACGCTGCTGCGTGACATCCAAATCG
GTGCGCATGTCCCGCCTGCAGATAATGAAGAAATACGTCACACTCCTTCCAGTCGTTTAAACCCCCGG
TGCCCACACATTATTGATATTCCACTGGGACA
CTCGGCGTGGTTTCGTGCACCCCATTCCATTGTCTCCTTTATTAGCCGGCGCCATCGGCGTGGCACAC
AAAGCCGAGCGCGCCTCTGATCAAACAAACAA
ACAGAAACAAGAACACTGAGTAGATCCCGCCGTTGTCGGAGGACCACAACACGGGGGGGAAATAAAAA
ACCAACCAAACAAAAACAACGCGATGATGGCC
CCGGGGGGAGCATGCTCCCACATGGAGGAATGGACCTGCGCGGCGAGGGGGGTACAAAGTAAAATAAA
ATGGTTAAAACAAGGCGTCACAGAAAGTGGCA
ATTAATTTAAAAGAGTGCGTGGCCAGACGCATATGTTCCCGCTGGAGGCAGGAAGTCCTCACTGCAGT
TTCTCAGTAAAGGGCCACAATGCCGCACAGCA
GCATCCGAGAGATGCCCCCACGCATGGAGCCGTCACTGTTACCTTTTTTGGCGTCGCTCTTCTTGACC
ATTTTTAGTTCACCGACACTCAGTGGGCGGTT
GTACAGAAAGACCTTCGTCACCGTCACATTGCTGTTGATGTCTCCCTCGTCGCCCCCAATGTAGAAGA
ATGAGACTTCAGCCCCCCGCGTCTCAGGTGTT
CGTAGCGTCTCCGGGCTCCCCACAAGCTCGCCATTCACGTACACGGAGCCCTTGTTGCCGTCCTGCAG
CATGAGCGCCACCTGGTATTCTCTCCCAGGTT
CCCACGTGCTCCCCGGCGCCGTTTTTGTGCCGTTGAACACCTCCCACTTTCTATTCATGCTGTACGAC
AGCCCAATGATCTTCGTGCCGGCACCGTCCCC
CAGACCCGCACCCAGCAGAGGAGTGCTCCCCTTCGGAACCCGGTGGATGGTCACCGTCGCCACAAGGG
TGAATCTGTGGTTCACAAAGCCGTACTGTTTA
TTAGGTCCCCACCTGCTCATCGACCACATTGCCCTGGACCCAGGCCCCGTGAATTTGAAACCATTTTT
GACCTTTGATGCTTTCGTCACGGATGCATTCA
CGCAACGGTAACCGTCGGTCCACGTGTTACCACCGGACGGTGCGTTGGAAAGGAATCCAACCAGACCG
GCCGTGGGTGTGGACAAAGCGGAGAAGGAGGC
GTCCAACTGTGTCCAAGTCCTCAGGACGAAATTGATCATCTGTAGCCCCTCCGTTAGTGGGAAAAGTG
AAATGGTGTTGCTTGCTTTAATAATCCCATCC
TGTAAAATGTGCAACGCACCATCCGAGAAAAGCAGGACGCAAAAACGTTCCAAGTTATTATCCATGGC
AAAAGGTCCGATGTGAAGCGTGCGGTTGTTGT
CCGTGACCCAAAGGCAGAGCACATTGGCCCTTTTCTCCCCCGAGAATTACACTCTCTGAGTGTACAGC
ATGACCTTCCTTCCCTCAATGTTCGCAGTGAT
GAGAGCATCCAAATGGAAGCTGATATCCAAAATGACTGTTAATTGTGGCTTGGTCCACACGCCTGGAA
GTGTCCCGATGGTTCCCGTCCACGTTTCCCCC
ATGTCACGCGGCTCGAACAACCTCTGACCATAAAAAAGATCAGTAACCATGAGAATTCGTCGCCCCTC
TCCCAATTGGTGATGCGGGGGTTAAAGCATTT
CGCGGGAGGCATTCTCTTCGAGACATCCCAGTTTTTGCCGTCGTGCGTCAAATAAGTAATCACGGAGA
GAAATTCACCATTGTTATTCCTCCCCTTACAC

GCGAAACACGAGCGTGTCGCTCTCCACCACAACACTTGGGCCACCGGAAGCAACGCATCCCCTCAACT
CACATTTCTGAAAAGCTGAATAGATGTGGTGC
AAAAACGATTTCGGATCGCCCCATTTGATTCGTTCACTCAGTTTGCCACCCGTAGAATTCGTGACATC
ACCCACAACCAGTTTCAGATCCAAGCTGCACT
GTCTCCAATTCCCGCCACTTGCTTTGTGCACATCAGAGCTTACCGCAAGAAGAAACATCTGTTGCCCT
TCGCGGTGGTTGTGAGGTGGCATAAACCGCCC
ATACGTTCCTTTCCATCTCCCCCACCAAGCACAGTGTGTGCCCTCCATTTACTCTTATCGACCTCACT
AAAAAGAGTAGAACAATACTACGCAGAGTCGA
TGCACCCCGCAACCACATCAAAAAAAAAGCTTATTTAATTTACCACCTTGATATTCGGCATTCATGTG
ACCTTCGGTGAAAGCAGCAATTACTCCACCAG
CACTGACGAGAGATGGCGAAATAAACGAATCCCTCGTCGTACCTGGAACAGTCCCATCCTTCGGCTAC
ACCAGCGCCGTCTGCGGCACAAATAGGTCGAC
CCTTTGCAGCTCATGAATATTTATTAAATCATTTCTGCCACCTCCCCGCTCCCCCCACAAATGGTGCA
GCACATCGACGTCACACAAGAAGCAGCACCGC
GGAAGTGACGCCGGGACATGTTGGCCCTCTGCGGCTCACTCTCTCTTCCTTCCCTCCTCCCTTTCCGC
TGGATCCGGTCACACGGCGACGGTTGTGTGTG
CGGGGTGCCTTCATTCCGGTCACGCGGCGAGACGGTATTGCGTGCGGGGTGCCTTCACTGCAGCAACA
CGTGAGAGCATATAAATATGTGTGTGTAGGCG
GTGTGTGTGCTGTTGGTCCTTTCAGAGCCATTGAATTTCACAGTGGCGATTAGCGCTTGTTTGCTTTC
CTAGCGTGTCTGTGTTCAAAAAGTGGAAAGAC
AGCAACAGCAGCAATACATCAGACACGTCAGTGGAGAGCAGAGCAGGGCACCCACGCAGAGAGCTGGA
AAAAAGACAATCCGGAAGGGACGAAATGAAAA
AACAACGCCCAAAAGAAGCAGAAACACCCACATCGCAATTCTGCGGGAGAAAAGATTCAGACCTCCAC
ACAACAAACCCCCACACCGCCAGCAGTCATCC
ACCAATGGACGAGGAAAAGAGACACACCGCCATCATCGCCTCCAGCTCATTCCCGCGGCCAACGGCAT
ACGGCCACAGCCCCGCAGCCAACAAACACAAC
GCACGCGGAGTAAACAATCTCCCAAAGAGCCACACGCAAAAGTAATCCCTCATCCATCCATGACGCGG
CGCCTCACTCCATGCGGTCACACGGAGCGCAC
TTTTTCCTCAATAGAACTTACCCTTCGTCCATCCCTTGAAGATGCTTCACTTAATGATCGGTCCTATA
ACAAAATAGAATGAAAAGGAAAATGTATTAAC
ACTGCGATACCGCCAAAGACAGTAATTCCTTAATAACAGAGCAATTCTTTTATTACGTCTTGTCTACA
GACAGAGGAATTCGCATAAATAAAAATCCTTC
AATATTTTCTTACCATTAACCAAAACGCCCCTGGTATCTCTGATTTGTTTTTCTGTCGTTGTGAAGGA
ATATCGCGTTGTAATTTTTTGGCATCCGTGCG
TATTACTCCCAAAGGGGATGATGCACTGATGTCGTATAAAAAAAAAAAAAAAAAACACTCACATGTAT
TAAACAACTCGAAATATGTTTTTCACCAAAAA
TATAAATTTCCCTTTCCCCACTTAATTTCTTCTGTCACTGCTATTATCACTCGCGTTGAACATTCGTA
ACACTTTTGTGCCGTGCTGCCGCCTGATGCTG
GTGCATCTGCACTGTATGCTGACACAAGTGTTTACATATATATATTTCAAAAAAAAAAAAAATACCAC
AAATCAAAATTTCTCATTCACTTTTCAGTTAC
TAATCTGTTTTCTTTTTTCTGTTTTTCTTCCACACTTCGGAGAGCTTTTTTTCGTATGTCTCTGGATG
ATACAGAAACTTGGTACTGGCGCACAATTTCC
TTCCAGAACGCCACAATCTTCTTTTCAGCGCGGTCCAAATCATTGGCATTGACGGGACCGCATCTTCG
CCAGTCATTCATCGGCGTGCTGTCTGCGTGCT
GCACATAAATAATCTCCCACGACATGTCTCGGGATAATTCCTCCCAACCATTAAAATATGCCGCCGGA
CACTCAGTGAACTGCCTCACAGTGCTGGCGGT
GGTGTGGTGCTCACCCGCCCTAGCCATCCGCAGCCCAACCAGCGTCCTCGGATTTGACTCCACGAAGA
AAAAGCCGTCCACCAGCAAAAAGGCTCCAACC
GCCGGTATGCACAGCAGCCGATGCTTTATATTAGTATTTGTATGGTATTCCATTGCAAGCAATACGCA
TAAGACTCGGCGGGGTGCCCTCAGAATCGCCT
TCaGCTCTGCGCACTGTCCTCCACGGCGTGCTGGTGGCCTCAGCTCCTTCAGGTCCTCTACTAATGCA
CTGAAAAACTCCCCAAAAAAATTAATGCGCGC
AAGCCAAACCTTCCACACATTCAGACGAAACAGTATTCCTCTTCCCCAATACTAGCAACAGAACTGAA
TCCCCATCACTCATCACGTCTCCCAAAATAGT

GCGGTATTCGACGCCAGAGGCAAACAGATATCGGTGCGTTGAGAAATCCCTCAGCACCGACTTCTCCT
CTTGCTCGGGCAACCCTCACCAGCTTTTGAGG
CGGATTCTCAGAATATCACAGTCTCACTCCCCCATGCGTAAAGTGCCTTTCACCATCCCGGAAATTGA
TCCCATCCAAGGCATCCTCAGTGGCAGCGGAG
TGTGCAATGAACTCATTCGCATCAAAGATGTAACGTGGAATCGGCCCCACTTTTTCCATGCGTTCTTT
GACCATCTTCCAGTATTCCGCTTGCCCATCTG
TAGTCCCATCGCGCTTCATCCAGGCGCACATCGCCTTCACATCCATCTCGTCGAGTACTTCACACTGG
TCCGCCCGGCTTTTACTTGCGTCTCCCATTCA
TCACAGTTGCCCACGTTTGGGGGTGACACCACAATCATTCCCCATCCGGTGCTAGACACAAAATATTT
CGCTGGCGGTGTTCCTTTCTCTGTCACACAGT
AGATAATATGCCCTTTCATCACACAATCCCACAATCCCACAAAACGTCCGTGGATGCTCCTCTACCCA
CGTATCTTGTCACCGTCTTGATGGCCTTGTCA
CACACGTACGTCGTACTTCCACCAAAACTGTAGACAACCACTTGGAGTTTCTCAACATCGCAGTGCAG
CAGCTGGTAGAGGAGGTACGAGCCGGCACCCA
TCGACTTCCCGATCCCGGGCGTCCCAATCAAGAGATGCTGCTTGGGCTTAAAGTCGGTCCCACCGTCA
GTGCTGAACCATTCGGTTATATCGCCCTTGAC
GATCTGCCACGCTCGATCCACCTCACAGTTAACACACAAGTCATTACCGGCCCTATGCGGCCCATTCA
TCGTGTACGGCCATTCCTTGTCGAGGTGAGCA
CCATCAGCCTGGGACGCGGTGCACCGGATTGCTCCACACCATCATCCTTTTCGAGAGAGTCGCCAACT
GCCTTGTACGTCCATGACTGCGGTGGTTCCCC
CTTCTTCACACCCATTCCCGTCCCTTTGTCACCAGGAACTTCCTCGACATGATGCCACCTCGCATTGT
AAACAGACTCGTACAATCCCTCCAGGTATATC
GGTGCACTCGACTTCTTTGGGTTCCTCGCCTCCTCGGCGGCTGCATTCAAGAACCATTTAGTGATTTC
ATGAACATTTGCCGTTACCTCCACAGTAGCCA
CCAACCACCTCAGTAGATTGTCCACACAATGCTCGTAAAGCTTGTTTACATCCTCTTCCAAATCCATT
TCATCCCGTACAGCCCTCTCCATCCTCTTGTA
ATGATCCGATGCCTGTATTTCGTTCAATACTCCCGCATCGCGAATATACTTCTTGGGGTCCTCAAAAG
ACTCCTCCAGTAAGCGAATCTCATTGGCGTCC
AAAGCACCCCTGCCTTCCAATTCCATCGTAAGAAAATCATTCAGCCTCGTTTTATTCACGCGAACCAT
TCCTTTAAACACCGCATACTTAATCCTAGTGG
AAACGTCAATCCCCAGTTCTTGTCGTTCTCGCCTTTCCCTTTCTTCTGCTTCCCGCCTCGCTTCGCTC
AGTACATAAGAGTAGGCTTTGTTTATTTGTGC
CCTTGCAAGAGGAGTGACCGTATCCTTTCTTTCAAAGTTACTCCAATGCCTGAAAGTGACAGTATATT
TTTTGTTAAGCTTATTTGAGGCCTCCAATAGC
ATGTAAAACTCCTCACGCTTTTTTTTCAGCTCTTGGCATGGCGGCGATGCCTCTATTGTCCTCAATAA
CACTTCGTCTTGGATGAAGTCATTGGGGCTAC
TTAAAAAGTCCTTCATATGGGCGTTCTCACTGAATGCCTCAACGCCCCTGCCGCCAAAATAATACCGA
AGGAAATCATTCAGCCTCATGTTGGTGATGCG
TTCCTTTCCCTCCAGCAGCACCCTTTCCAGCCAACTTTCAAGAGTCCACTGCGGTTGCCGTGGTTCCT
CCACTCGTATGTGGGTTGCAGCGTCGGAACCA
TCAGAATAGCCACGGCGAGGTGCCATCACAACACCCGTGCGGTGAAGCATCCCACTCGCACCGCCCAA
CCGCGGCCAGCAGGTGCCGCTGGCTCCAAAGG
ACACTCGCGGCTGTTTCGTCTCGCTGTCCCAATGGCGTTGGGCGTGGCACTCGCACGGCTCGGTGGTC
GGtgcgccacgcagccgcactgcaacgcctgt
cggtgaagcagtcgaggcccaccac
>scaffold1035.size6778
TTTCCCTCTCTCTCTCCCTCTAAAACAAAACAAACAAACAAAAAAAACGCGTTTTATTTACTCTCCAC
CTTCTTCTCATGTTTCCTCATGTCTACTCTGAA
TATAACGCACGATGCGTCCAAACACTTTTGGTGGTCAGACCGAATGTTACATCCCCCTCCAACATCAT
GTCAAGAATGACGGAATTTTTTTCTTTGGGTC
GCCGCTAAGCCACAAAATGGAGCTAAAAAGAAGAATTCGCGCAATATCCAACAATCCCATTCCTACTG
TTCCCACGTACCAACGCATAAATTCCATATGT
GTACTATACAGTGCCTCTCCTTCCTCGAGTAGCATGAAAAAATACAAGACGGAGCAGCCAAAAGAGAA
TACCAATTGCGTAACATAAAGTGCACTTAACC

ATTGGCTTAACACCACCCCGACAGGGGGCAAAGAGGCACTACATAAATCAAATGCACGCAATGTAGCC
ACCACGTGGACGGGTGAATATAATGACAGTGG
CGAGGACAGCCGTGAAGGGCGTCGGGCACATAACCATCCAAGCAGTTGCAACAAAACCCCCACATCAC
AACCCAGAACATACAACGTTCCACAACACGCC
AGGTACCCACTTATTGTCCCCACCCAGAAAAATAAATGGTGAATATGAAAAATAACTAAGAAAAACAA
CATGGCTGTATTTAGAACAACTATAAAGAGAG
CACGTCGACTATTAACGCAATGCCAATACCTGAAAATGCTTCGTAGATTCCCCCGTCCACCGCCGGAT
TCCCCCCTCCAATCAGCCTTCTTAATTCTCGA
ATAATGACTCGGGGCATACTCACCCTCCATGTATGCATATGCCTTCTGTTCGGTATTGAAGGGTGGAT
TTGTTTTTGGAAACTTCCCCGGTCTTACCTCG
GCGCATCGGTCCGACAGTGTGGCAGGTAAACAACCCTTCTCACCAACTCGTAGATTGGCTACTGCAGC
TGTGCCCCCCTTTTGCGTGGCAGTATCCACTC
CCGAATGACGTAAATTAGATGACACAGAACAACCGCCCCTTCCTCTTCGATTCAAACGGCCTTCCATA
ACGTGCAGCACCATGTCACAAATACAGAAGAA
AAAGGCATACAAAACCTGTGAGGGACGCAATAAATCGGTAAGACAATTGGATATAACTGAGAGCCAAC
CCACGCCCACCATAACACGTTGGGTTAATGGC
AGCGAAAATAAGCTCACTATAGGATCTTCGTCATGGTCGTCATCGGTGTCAGACGCATCGGTGACTTC
CACGGCTGTCCCCACAGTATCATCTCCACAAG
AGGATGCAAATTGTTCGTTACCAAAAGGAACAGAAGGCCTGCCGTTGTATCCATCCGTGCCCAAATTA
TTTTGCAACTGAGATGTGCCCCCCATGACTGT
CAATGATGTTGCCATCACATTAACAGGTGAAGCAGCAGGATGTGCATTAGGGGGTGTTTGTAAAGAAG
CGTAAGAAGTTTGTACGAAACGCTCCGTATCT
GTCGAGAAAAAACCGGACACGATGTTACCATCCCTCTGGGTCTGCGTAAACACCTTCACCCTACAAAA
GACAGACACCATGCAACACACTAGGATACCGA
CATCCGCAGAGCCCACTTTCATTCCAAAAGGTTCATTCATAAAACTCATCCTTACTTTTTGTCACCCC
CCTGCCTCCCCCCTCCTCATCTCCCCTCGGTG
CGATGTACATGTACACATTTATATAGAGAGACAGAGAAAAATAGGCAAAAAAAAAAAGCAGATGGAAA
TGTGAAGGAACAAAACAAACAAAATAAACAAA
AAACCATGATGGATGTATCGTCCTTGACTCCAGTCTCCCCCTTCATTTATTTCCCCTCCTCTCCCCCT
ATCAATGACTCACCCCCACCCACCCCGACACG
TCAAAAAAAAAAAAAGAAAAGAAAAGAAAAAATGACATATATGATCTATATGATGGCCGTTACCGATA
GGGATAAAAAACATGCATGCGTACACGCATTT
ATAGCCCCTAAAAACCCCCCGTGGCAAACTTCATCAGAATCTATATAACTGCTTTATTATAACCGAAA
ATATACTCCCCCCCCCTCCACAGGACACTGGA
AATGGAGAGTTAGGGCAAGCGATAAACAGTGGGATCAAACAACCCCAAGAAAAGACATGTATTAACCT
TTTTTTAATAATAATAAAAAATTTCATCATAA
TGACAATTCAAGAAGAACAACAACAAAAATAAAGACGCCGGTTGAAATTTGCAATCCCATTATGTTTC
CTGCACTGTCAATTCGGATATTTCCAGTATAT
CCGAGGACAACATGGACAAGGAAGGATGATTCAATCTTGACAGCTGGTCGTAGTACTGCTCAGCATGG
GCCAAAGAAACCACCCTTTGCCACACAGTGCC
AACCGTAGGCATCGACAGCGCGACCGCGGAAAGAACTTCCTCATTACTATTTTCACTCGCCTTCTCTT
CATCTTCGGTATTGTCCCATTCACTCCTACGG
TCACAACTGTCACCACGGCCGCTATAAATTTGCTGCGGTAATACGTAAAGAGGGGAAGACGAGGGTAC
ACCAGCAACAGCAGAAGCGAATGCATCAACAA
ATGCACCGCCATGTTGCGAAGACGGTGTCACATCCGGCCCCTTTTGCCCATTCGTCACTGGCTTTGAG
GGTAACGCTGGTTCCGCCAAATTATGCAGATG
TTCCACAGAATCGGCATTCGATAAGGGTATTTCCATTCCTCTTTTTGTAGAGGAAGATGCACTTCTGG
CAGCATCAGAAGGCACTGGTGACAATTGTCCT
TGCATCCGCTTTGGTGTCTCTGGGGAGTAAATGAGTCTTCTCTCCAGAGAAGAAGCCTGCAAAGGAGG
AGATTCATTTATTTCACAACATTTTTCTTGTG
TCGTTGTCGCTGTCGACCCCATGCGGAGATCGGGGACATTCGGTGGAGTTTTATCCGTTGTCGCCGGT
TTCCCTCCACGGATAATCCCTTTTTCCGGCCT
GCACGGCTCATGAATAAATGGCGACCAGTCATGACGGCATGTACTGTCCATTGCGAAGTACTTCTGTT
CACTGACGGGTGTCATTGTGGAAAAGGATGGC

GTAGGCTGAAAAGAACTTTGATTTTGTAATTTATATAAGCAACAAGATGCCTCACGTGGCTGCATTCC
CTCCACGTGTTGCCAATCACCATCAACAACGG
TCTCACCGAGAAACAAACAAGCAGAAGGCATTTCTGCACCGTCAGGTGTGAGTCCCACACTTTCCCCA
CTTTCCACTGCCGCGGTGTCCGCGGTGGGTGA
CATCCTTTTCGCGACGCCGACATGCTCACAATGAGAATCAAACACCTCTCCTCCCTCGATGGACACGT
CTTCGTCGTCACACATGGCATGCTTCCCCGCG
GGTTTCCCTCTTCCTTTTTTCCCCTGTCTATTGGGGGGGAAAAATGATTGCCCATCACGACAACACTC
CGCCGCCTTCTCACGGAGGGCCGTCACGGTGA
GATGGAGCCGTTTGAACTCGTCTTCCACGCCATCATAAATGTCATTGTACGTCGTTGTTGGAGCCTCC
ACCACGGCTGGCTGAGGCACCGGCGTCTTCTT
CAGCAGCAGCACAGACTCACAGCGACTCAAGCGGCTTTCAAGATTGCCCAATTTCCGGTGTAAATGCC
GACGCCACATTTTCATTTGCTGATCCAAATTT
AGAAACCCTTCCGTGATGCGGCGTAACGCCACAGCCGACCGAACATCGTTCACAGGATGGCACGCTGG
CCTCGCTGAATACAGAAGCGGCAATCTCCCCG
AGGATGTTCCATCAACTGGTACCTGCTGCTGCTGCTGCCGCTGCTCTTCGAAAAAAAGATAACGAACT
GATGGGTCATGACTGTCGACTGCCATGGTCAT
GATCCACAAACAAAAATCCTTTTTTTTTGTGTGTGTATAAGTCCAGCAGTAGGTTGTTATTATGTGTT
CAACACGGTTCGAGCGTAATAACGACCCGACC
TTTCTATCTATGTATATATGCGTGTATCTTTTTATCTATGTATTTGTTTCTGTATGGGAGTAACGTCC
TTTCCTTCCTTCCATACATTCCAAAAATTAAA
TAAATAAATAAATAAATAAATCATTACAGAAAACATAACGCAACACGGTAGTTAATTGTATCCAATCT
ACCTTGAATCATCTGTGTAATTTGATACAGCA
ACAGCAACAACAAAAATAACAAAATGCATATACACACGGTGAAAGAGAGACAGTCTGTCATTGCGTGT
GTGGATATATATATATATATATATATGTTTCT
GTTAAAAAAAAAAAAAAAAAAGGTATCAAATAACAAATATATGAAACATGTACAGTGAAATTTATGTCC
TGCCAGACATGTAAATGTTACGTCAGTCCATT
TCAATACATCACACGCATAAAGGAAAACTAAGATGGGTACCAAAAGGAAAATAGAGAAGAATAGCAAC
AACAAGAGAGGGAATAATGATGCCTTCAACAG
TAGCGATAATTTTCAAGTGTGCAAAGAATACATTTCAAAGCAATATATATATATATATATATATGTTG
GAACATCCTTCTTTTGGTCCCACTTTAAAATG
CTCTGTTTGCCTCAATTTTCCTTTCTTCTTTCCTCTTTGATTCCTTTTGTTAGTGTGCTCTTGTTTCT
TTTCAAAGATTGACTCGAATCATCCATCCATC
CCTCTTTCCGTTGGTGTGTGTGTTTCTTGTTGTTGTTTTTTCCTTCGCCCCCTTCGAAATTATTCTCA
ACATCCAGAACGGTCAGACGTCCGCTACAAAC
ATCCCTCCTTTCCCCCATTTACTTCAGCTTCGTCATCCACGTCGTTTCATCTGGGTATTCACAGTTGA
TGTTCGTTGTTGCATCGTATGGCTTGCAAACC
TGATGCGTGGCCCGTGCGGGGTATCGAATACCAAACGTAAAGCCTTTTCCCTTCCGTGTGCTATCGGC
GATGGTGTCATTCTCAACAACATACGAAGGAC
CGCCGTCTCCCAATCCATGCAAATTCTTACTGCGTCTGTAGTCGCCACGGTAAAAGACAGTAGCACGA
GTCGCTTCGGGTGGCTTGACATCATAACAAAG
TGGACCACCGGCGTTTTGAACTAAAAAGCGTTCGCCAAGGCCAAAACCAGCCACAGCCCGGTACCTCG
AGGGCCGCACAGCGTCCAAATTCGGATAATAC
ATATTAGGCCCCGAAGCGGCATTCGATGCGGCCGCCGCTGCCTTCTCTTCCATTTCCGCAAGAGCGAT
TGCATCGTACGGCTGGGCTGCAAAGCGAAACC
CATTGGATGTCATCGGTGGCTTCCCCAGATCATACAATCCAGGGCCAACCGCATCACTTGCCGCGGTC
TTGGGTTGATAAAATACACGCGCCCCAAGTGA
AACGCCTTTGTTGTAGTCCCGGTCAAAATCCGATGGCAGGTAATAAGTATATGGACCCTGTGATAATT
CCATTGTACCTCGTACGGTCTTGTGGGGTGGC
ACAGTCTGTACCGGTGGATGAAACTTAGGTGTACATCGTATACTCTTTCGCATGATTCGGCTCACATC
ACCGTATTCGGCTGGTCCAGGACCAGTGGACA
AGGAGCAATTAGACTTGGAATGGCGACCGCCAAAAAGTGGACCTTCATATAGACGTAATTTTTTTGGT
GCGAGGCGGGCAGCGATTGTGGTGGGATCGTC
GTAGTTCCCGGGTCCAGGCCCATTTTCTCTTTCAGGAACCCTTCGGACAGAAGTAATGTATTCCTTTC
TTTTTGACGTGTCATCACCAAAACGAGGCAAC

TGATATGCCCCAGGTCCTGGAACTATATCACGAGTCGTCAAGAGCTTCGTCCGCTCACCAAAGTGCGG
CCCCTTGCCGCTTGAATCAAAGGAAGAGGAGA
TGACGTACTCGCCAGGGCCCACACCACTACCTAAAGGCCGATCTCGAAGTCTACCGTAGATGGAGTAC
GCTGGTGTATCGGGTGACAATGCAAGGCTGTA
CTGCTTCGGAAGTGGGGATAGCTTGCGTCCTTTTTTTTTTCTTAGATTTTCTTTGCTGCTCTTTTCAC
TCCCCGCTTTGGCATCTGCAGCCGATTCTGGA
CCAGACGCTGGGCCGAACACGGTTGAGCGGCACTGACCAACAGTTGATGGTATTTGGTATTCCCCAGG
GCCTACGGTCGCCTCTGCTGGTCGCGCGGGGT
ATTTCTTCAGAATGGATCTCTCCGGAACGTCATTCCGAACCGGAATGTAACTCATCGGCCCCGGACGA
TGGTCACGCAGCCGCAGATGCGCCCACTCCGT
GAATCCACCAATCAACACCATCGTTCCTTCTTTTTTGTTTTTTTTTAGGCCCTCACCTCCTTTCTGCC
CACCAAGACGCCCATCGAAATGCGCAAACAAT
TAGGAAAAAAAAGGCAGAAGTAAATATAAAAACACATGTAAGCATAATATATATAAAGAAGCTAATAAA
TTCGATGAAAAGAGGATGCCACGCAACAACCTCCTC
TAAATCCGAGAAAGAGGACCCGCTCTCTGCTTTTAATATATATATGCGGAATCACCGTTTCAAAATAG
AAAGAAAAAAGAACAAAGAGCTTTCACATCAC
ACATCCTTCCTCCACATGGAAGGCTTAGGCTGTGGCCTCAGCGGCGGGAGCCTCTTGGGCTTCCTCGA
CGACGCGTGCCTCACCCTCAGCCGAGGCGGGG
ATCTCGCCGTGTGCATTCAGAAGCGCCTGGGCGTCAAACCGGGGGGTGCGGACCACCTTCACCTTGCG
GATGCGGAG
>scaffold1036.size6789
TTTATCCTGATAAATGAAGGCATTCTTTATGATACTTTTCTACCGTATGAATTTTAGAAAGGCCACGA
CTTTAGGGTGCCGATGGCGGCCGGAGAACCCTG
ATTTTTATTTTACCTTCCCACCCACCCTTTTTTGATTCCCACCGCGCGGCGGGGTCTTGTGGCTGGAG
GACCCCAAAGTCTGCCACTTCGTAATAAATAA
ATAAATATATATAGATATCCCATCGGAATAGAGTGAACAGGCCACTATTGTGTCTAAAGGATTTATGT
ATGCAATTACTTGGTGTTAAACTTTGAGGATA
TGGTGAATCCATTCTTATTCCATTCATTCCTTTCTTATTCCTATCTGGTTTCATTCCTATTATTGACA
AACTGCAGGATTCGGCCGACGGCCCAGAAGAT
TATTTTTAATATATCGGTTTGATTTTGTTTCGGATTGGATTTTCTGTGGTTTGATTCTGCTGTGGATT
ATATATATATATATATAATAGGATGTGTGTGT
GTGTGTGTTCTTTGTTGTTGCATATTTTAAATAAAGATTGAAAGTAGAATGTCGAAGATTACGTCGAA
GGGGTGGTGCTATGCCCGTTGTGGCCCGATTT
CCCGTGTCCTGAAACATGTGACGTTCGAAGTTGTACCAAAAAAGGAAGAAGTTATAGTTGATGTACTG
CAAGCGCCGTTGCATCGTACAGATGCAGCCGt
GGTAAATGGCACGGCTTTGGGGCGGAGACGGTTTTCTTCTGCTGGGTTTCCAAGAGTGGGAGGCTGCG
AGGGGGTGGGGAAGGTGGTTGAGGCCGGTGCT
TCCCAGAATGTGAAGGATGGTGATACGGTGTGGGTGGCGCCGCTTCATGGTACGTGGGCAACGCGTGT
TGCAGTTGATCACAAAATGGTGCATAAAATTG
ATCCCAAACACGTTTCCCTTGCCGTTAACGCCTCCAATTATGTTGTCGCCCAGCATCTCCTGAACGGG
TTTGCAAGCCTTCGGAAAGGGCAAGTCATTGT
GCAGAACGGTGGCAGCAGTCCAACTTCGTTGGCGGTCGCAGCACTGGCGAAGCCGCTTGGTGTAAAGG
TTTTCACGGCTTGCACCCCTGGAGAGCGCTTT
AAAGGGGCGAAGGAGCGTCACGCCAAGTACAGCAGCGAAGTCTTTGAGTACAACGGGAAGGGAGCCAG
AGCCATGCGGCAGGCAATAGGAAACGTTGGTG
CGACATTGTATCTCAATGGTGTTGGCGGCCGTCACTTTGATACCTTTTTGGGACTTGTTGGTTGTGGT
GGGCATGTAGTTTCGTACGGTGCACAAAATGG
TGTGGGATTGATGATTTCTGGTTCAAATCTTATTTACAACGAGGTAACAATACAGGGATTATTTCTCC
CTTCATACCTTGCAGGTATTTCCTACAGCGAA
CGTCAAACACAACTTGATTTTGTACTTCAGCAGCTTTCTTCAATGGGTTTTAGCTATCCGACTTCCGT
GGCCGCCTCGCTGGAACAGCTGCCAAATGTGT
GGGATGACGCGTTTGTACATGGAGGGAAAAAAGGTGTGGTTATCGTAGGAAAATAAATTGAGAGAAGT
GTTTGTTGAGCGGTGTGGAAGTGTTATATATA
TATATATATATATAATGTATCTGCGTGTGTGTGAGTGTGTGTGTTGCTGTCATTTTAGATTTTTGTTT

TTTAGCTAGCAATGCTCTTTTAATGCAATTCT
TTTATTATTATTATTATTATTTTAATGGGAGGGGATTGGGTGGGTGGTATTGGAGGGGAGGAAACAAA
TGGTGATAATGGATTCTTTCTGACTTATAGGC
GAGCAATGTATCACAATTTCGCTGGCATGTCATTAAGTATTTGTGTAACAGTGGCTTTTTTTTTACCA
CTGTCGGTTGTTTGTTTTTTGCGTAGGTTTCA
GGTGGTCATGGGTAACAGTCGCATGTATATTGCACGTGGGTAGTAAAACAAAAGCCAATGATAAGGGG
GTTTGTGCGTACATTTTTTCGTTTGTACGTTT
GAGTGGGTACATCTTCTCTTTGAAGAAAAACGTAATGTGTTTATTTGTTTCTTTGATGCCTCGATTTG
TTTTCTTTTGCCATAAGACATCATTAAAGTCA
TACTGTAATCTCACTTCTACCACGCAATTGCGCACTCTTCCTGTGGCCGATTATTATTATTTCCTTTT
TCTTTTTCTTCCTTTTCCTTTCTTTTTTCTTA
TATATTTTTTTTTTTTTTTTGTATGTGTGTGTGCGTATATGTATTTGGTTTTGGTATTGTATGTATTC
AGGTGGTCATTAGAAGTGAGGGGGTAGGGAGA
AAGGTTTTCAATTGGCATCATGCCACTCTCGGATGGCGCCAGAGTGGCCATGCACGTTGACGCCCTTG
CCGTGCTGGACGATATACGGCGAACAGGGCAA
CTCATAGAAGAGTTCCGCTGGCTGAAGAAGCACGGTGGAAGCCGTGTGGCGCTGGAGCGACTGCGATC
AGAAATCAACAAACTTAAAGCGTCTCAAGCCA
GTTTACTCGAATCTCGAGGAAAAGGGAAAGATCCGCACGGTGCACAGTACGAAACCTTCCATGCATCG
CCATCATATCAGGGTGATGGATTGATTATTAC
TCCGCGATCACGCCCTGACAGACTCCAGCAGGAAGGTTTAGAGGCCCCTATGCATGTTGGTTGTACGC
CTGATGTAGACAGAAGGGGTGTAACGGAAAAA
CATGGAACAATTGCATTAGACCATAAACAGTATGATAAGCCCAATCAAGACAAAGAGAGGGTCCATTT
AAAACCACTTTTGTCTTCGACTCCTTCTAACC
AGGAATACAAGAGATATTGCTCCGTTGTATTTGCAGTTGTTCTGGCAGAGGCACATATAAGACGTGAA
ATTATTCACAAATACGAAAAACGTCGATTAAA
GCTTATTAACAACTTCAAAGAAGGTTGCATCATCATTGCATTGTCAAATAGACCAACATACCTCCACG
GTGTATCAGGAACATCAAACAGCAATGTTGGC
CCACGTCACGAGTATTACCTTGTCTCAGAACCAAATAAAGAATGCCATTCAACATCAGTAAGAACCTC
CATAATTCAGCACACCCACCGTACTAATTCAT
TTTCCTACATCAAGCAACAGGAGTTGTTTATCCCGCCAATCCATTCTATGAAAGAAGAAAATCCTTGC
AAAACGAACTACGAAACAACGTACGAGAGCAA
TACCCCATACGAAATAAGTCACACAAAAGCACAACAGAAACAGTGGGCCATAAAAAATGCCAACGAGG
AAACCGCCACACGGAAAGCTGAGGAAGAAGAA
GCTGCCAGACAGAAGGCTGCGGAGGAAGAAGCTGCCAGACGGAAGGCCGCGGAGGAAGAAGCTGCCAG
ACGGAAGGCCGCGGAAGAAGAAGCTGCCAGAC
GGAAGGCTGAGGAGGAAGAAGCTGCAAGACGGAAGGCTGAGGAGGAAGAAGCTGCAAGACAGAAGGCC
GCGGATGAAGAAGCTGCCAGACGGAAGGCTGC
GGAAGAAGAGGCTGCCAGACGGAAGGCTGCGGAAGAAGAAGCTGCCAGACAGAAGGCTGCGGAAGAAG
AAGCTGCCAGACGGAAGGCCGCGGAGGAAGAA
GCTGCCAGACGGAAGGCTGCGGAGGAAGAAGCTGCCAGACGGAAGGCCGCGGAGGAAGAAGCTGCCAG
ACGGAAGGCTGCGGAAGAAGAAGCTGCCAGAC
GGAAGGCTGAGGAGGAAGAAGCTGCCAGACAGAAGGCCGCGGAGGAAGAAGCTGCCAGACAGAAGGCT
GCGGAAGAAGAAGCTGCCAGACAGAAGGCTGC
GGAAGAAGAAGCTGCCAGACAGAAGGCTGCGGAGGAAGAAGCTGCCAGACGGAAGGCTGAGGAGGAAG
AAGCTGCCAGACGGAAGGCCGCGGAGGAAGAA
GCTGCCAGACAGAAGGCTGCGGAGGAAGAAGCTGCCAGACGGAAGGCTGAGGAGGAAGAAGCTGCCAG
ACGGAAGGCTGCGGAGGAAGAAGCTGCCAGAC
GGAAGGCTGCGGAAGAAGAAGCTGCCAGACGGAAGGCCGCGGAGGAAGAAGCTGCCAGACAGAAGGCT
GCGGAGGAAGAAGCTGCCAGACGGAAGGCTGC
GGAAGAAGAAGCTGCAAGACGGAAGGCTGAGGAGGAAGAAGCTGCCAGACGGAAGGCTGAGGAGGAAG
AAGCTGCCAGACAGAAGGCTGCGGAGGAAGAA
GCTGCCAGACGGAAGGCTGAGGAGGAAGAAGCTGCCAGACGGAAGGCCGCGGAGGAAGAAGCTGCCAG
ACAGAAGGCCGCGGAGGAAGAAGCTGCCAGAC
GGAAGGCTGAGGAGGAAGAAGCTGCCAGACGGAAGGCTGCGGAGGAAGAAGAGGCTTTTCTGAGTCGT

GATCGCGAGCTTATACGGAGTGCGACCGCCCA
TTGTATCAGCGGTTTTGCAGATAATTTTCTGTCAGAGTGTGTTGCGAGCGCTGCTGGCACCTTCCGCA
CTGAGCTGGATAGACGAGTGGCGGAAGAAGAG
GCTGCCAGACGGAAGGCTGCGGAAGAAGAGGCTGCCAGACGGAAGGCTGCGGAGGAAGAAGCTGCCAG
ACGGAAGGCTGAGGAGGAAGAAGCTGCCAGAC
GGAAGGCTGCGGAGGAAGAAGCTGCCAGACGGAAGGCTGCGGAGGAAGAAGCTGCCAGACGGAAGGCT
GCGGAAGAAGAAGCTGCCAGACGGAAGGCTGA
GGAGGAAGAAGCTGCCAGACGGAAGGCTGCGGAGGAAGAAGCTGCCAGACGGAAGGCTGCGGAAGAAG
AAGCTGCCAGACGGAAGGCTGAGGAGGAAGAA
GCTGCAAGACAGAAGGCCGCGGAGGAAGAGGCTGCCAGACGGAAGGCTGCGGAGGAAGAAGCTGCCAG
ACGGAAGGCTGCGGAGGAAGAAGCTGCCAGAC
GGAAGGCTGCGGAAGAAGAAGCTGCCAGACGGAAGGCTGAGGAGGAAGAAGCTGCAAGACAGAAGGCC
GCGGAGGAAGAAGCTGCCAGACAGAAGGCCGC
GGAGGAAGAAGCTGCCAGACGGAAGGCCGCGGAGGAAGAAGCTGCCAGACAGAAGGCTGCGGAAGAAG
AGGCTGCCAGACGGAAGGCTGCGGAGGAAGAA
GCTGCCAGACGGAAGGCTGCGGAAGAAGAAGCTGCCAGACGGAAGGCTGAGGAGGAAGAAGCTGCAAG
ACAGAAGGCCGCGGAAGAAGAAGCTGCCAGAC
AGAAGGCTGAGGAGGAAGAAGCTGCCAGACGGAAGGCTGCGGAAGAAGAAGCTGCAAGACGGAAGGCT
GAGGAGGAAGAAGCTGCCAGACGGAAGGCTGA
GGAGGAAGAAGCTGCCAGACGGAAGGCCGCGGAAGAAGCTGCCAGACAGAAGGCTGCGGAGGAAGAAG
AGGCTTTTCTGAGTCGTGATCGCGAGCTTATA
CGGAGTGCGACCGCCCATTGTATCAGCGGTTTTGCAGATAATTTTCTGTCAGAGTGTGTTGCGAGCGC
TGCTGGCACCTTCCGCACTGAGCTGGATAGAC
GAGTGGTGGAAGGAGAGGCTGCCAGACAGAGGGCCGCGGAGGAAGAAGCTGCCAGACGGAAGGCTGCG
GAAGAAGAAGCTGCCAGACGGAAGGCTGCGGA
AGAAGAAGCTGCCAGACAGAAGGCCGCGGAGGAAGAAGCTGCCAGACAGAAGGCCGCGGAGGAAGAAG
CTGCCAGACGGAAGGCTGCGGAAGAAGAAGCT
GCCAGACGGAAGGCTGAGGAGGAAGAAGCTGCAAGACAGAAGGCCGCGGAAGAAGAAGCTGCCAGACA
GAAGGCTGAGGAGGAAGAAGCTGCCAGACGGA
AGGCTGCGGAAGAAGAAGCTGCCAGACGGAAGGCTGAGGAGGAAGAAGCTGCCAGACGGAAGGCTGAG
GAGGAAGAAGCTGCCAGACGGAAGGCCGCGGA
GGAAGAGGCTGCCAGACGGAAGGCTGAGGAGGAAGAAGCTGCAAGACGGAAGGCTGAGGAGGAAGAAG
CTGCAAGACAGAAGGCCGCGGAAGAAGAAGCT
GCCAGACAGAAGGCTGCGGAGGAAGAAGCTGCTAGACGGAAGGCTGCGGAAGAAGAAGCTGCCAGACG
GAAGGCTGCGGAGGAAGAAGCTGCCAGACGGA
AGGCTGAGGAGGAAGAAGCTGCAAGACGGAAGGCTGAGGAGGAAGAAGCTGCAAGACGGAAGGCTGAG
GAGGAAGAAGCTGCCAGACGGAAGGCTGAGGA
GGAAGAAGCTGCCAGACAGAAGGCCGCGGAGGAAGAAGCTGCCAGACGGAAGGCCGCGGAGGAAGAAG
AGGCTTTTCTGAGTCGTGATCGCGAGCTTATA
CGGAGTGCGACCGCCCATTGTATCAGCGGTTTTGCAGATAATTTTCTGTCAGAGTGTGTTGCGAGCGC
TGCTGGCACCTTCCGCACTGAGCTGGATAGAC
GAGTGGTGGAAGGAGAGGCTGCCAGACAGAGGGCCGCGGAGGAAGAAGCTGCCAGACGGAAGGCTGCG
GAGGAAGAAGCTGCCAGACGGAAGGCCGCGGA
GGAAGAAGCTGCCAGACGGAAGGCTGAGGAGGAAGAAGCTGCCAGACAGAAGGCTGCGGAAGAAGCTG
CCAGACGGAAGGCTGCGGAAGAAGCTGCCAGA
CGGAAGGCTGCGGAAGAAGAAGAGGCTTTTCTGAGTCGTGATCGCGAGCTTATACGGAGTGCGACCGC
CCATTGTATCAGCGGTTTTGCAGATAATTTTC
TGTCAGAGTGTGTTGCGAGCGCTGCTGGCACCTTCCGCACTGAGCTGGATAGACGAGTGGTGGAAGGA
GAGGCTGCCAGACAGAAGGC
>scaffold1037.size6391
AAGCAGAAGCCAAAGAGAAAGAAGAGACAACCGAAAAACAAACCGTTGCAGCCAAAGGCCTTAACCTG
AACAACACGGCAGCAGCAGGCGACAGTGACGGC
AGCACCGCAGTCTCCCACACCACCTCCCCTCTTTTGCTTCTTCTTGTTGTTGCGTGTGCGGCTGCTGC
GGCGGTGGTGGCCGCGTGAGAGTGAGGGAGAG

AGAGCCGTGCACCGCCCGCACACACACTCGTCCTTCTCTCACTGTGTGTGTCCCCTTGCATGGACTCT
CGCCCTCACCTCACGCCACGAGGCACACACAA
TAATGTGCCCATTGTATATATGCATGCACACCCATGCAGGCCCGTTGGTGCTCTCTTTTGCACGACAC
GCACCGCTGCCGTCCTGCCTGGCTGCATGGGC
GGAGCACCGTAACTGTTTCTGTTTTGTTTTTATTGCATTGAGTGGGCGACCTCTCTCTCTCTCCCTCT
GTGTGTGTGTTTTGCTTTACGGGAGTAATGAG
AGNNNNNNNNNNNNNNNNNNNNNNNNNNNNNNNNNNNNNNNNNNNNNNNNNNNNNNNNNN
NNNNNNNNNNNNNNNNNNNNNNNNNNNNNN
NNNNNNNNNNNNNNNNNNNNNNNNNNNNNNNNNNNNNNNNNNNNNNNNNNNNNNNNNNNN
NNNNNNNNNNNNNNNNNNNNNNNNNNNNNN
NNNNNNNNNNNNNNNNNNNNNNNNNNNNNNNNNNNNNNNNNNNNNNNNNNNNNNNNNNNN
NNNNNNNNNNNNNNNNNNNNNNNNNNNNNN
NNGTATGTTTTTTCTTTTCTACTCTTCCCTCTTTTAATTGTTTCTGCTGCACTCTTCCTGTGAGGGGT
GCCGTGTGTTTCCCGCCCAACTGCTCCACTCG
CACACCCACCGACACGCTCATGACGACGGCCCTGTGCGCCTCGCCTGCTGCACGCATGAGGAGGGGCC
GCGACGAACGGCGCGCTGAGGGAGCCGTGACG
TGTGGGCGTGCGACGAGGGCCTGTGGGGCGGCGCACACATTTCTTTCCCTCCTGTTTTGTTTTCTTGC
GGCCAGGGCGGGCAGACACCCGCAGCGCCACA
CACACAGCCACACCCTGCCGCCGTGTTTTTTGTTTTCTTTCTTTTTTTTTTTTATTCGATGGGTGTGT
GCTGCCACCGTGCGTGATTGTTGAAGTTGGGG
ACGTACCGCATGTATCGCTGTCATCCAGCTGTGCCTCTTGCCGTGTTGATGTGCCTCCCTGTGCTCCT
CTTTCTGCTTTTCCAGCACACAGAAAATAAGT
GTTTGCACAGTGCTCTGCAGTAATGTTTGGCGACGGCGGGGCGTTGGCTTGCGTCCCGTGCTGAAATG
GCAACNNNNNNNNNNNNNNNNNNNNNNNNN
NNNNNNNNNNNNNNNNNNNNNNNNNNNNNNNNNNNNNNNNNNNNNNNNNNNNNNNNNNNN
NNNNNNNNNNNNNNNNNNNNNNNNNNNNNN
NNNNNNNNNNNNNNNNNNNNNNNNNNNNNNNNNNNNNNNNNNNNNNNNNNNNNNNNNNNN
NNNNNNNNNNNNNNNNNNNNNNNNNNNNNN
NNNNNNNNNNNNNNNNNNNNNNNNNNNNNNNNNNNNNNNNNNNNNNNNNNNNNNNNNNNN
NNNNNTTGACTGCTCTTGGGATCATTCTTCTC
TCCTCGTTGTATTTGCTCTGCCGGGTGCGTGCGTTGCTCGGTGTGTGATGACGCGTGACGGACGGTGC
CACGATGGTCTCCTCTGACGATGACAGTCGCT
GTGCTCTCGGCACACATCCACACACTCTTCCTTGTCTCTCTGTGGGAGTGATTGCGGCAGGGCATTGC
TGCGAGCGTGATGGGGACCACGACGGCGTCAC
CCCTCGAGAAGGTGCCACGCTCCGTCGTTTGTGGAGCGGCGAGGCGGTTTGCCTGCAGCCCGACTGAC
AGAACAGCACAGTCGTTTCATTGCGGTCACTC
CGTCGGCATCACCCGCTGCGGTGGTGTCCGCAAGAGCCGCAGCCCCGATGAGGATGGCCTCGTAGTTG
TCTGGTGGCTCAGAACTTCAGGCCCTTCGTCT
TTCAGGATTGTCCTTGCTGCTTGACTGGCGCAGCACCATCCCAGCATGGTTCTTCTGCAGGAGTGCAG
GCACGAGAGTGCCCCAGCGACGAGGCTCATTG
GCTGCGGTGACTTCCACCAGGCCTGCAGTGGGCGTGCCGGTGGCGTGGCCTTCTTGGTGCGCCACACC
TTGCCGTGCGAACGCCTGTAGCTTGTGGCACC
AACTGCCTGGAGACCACCGCCTTCCGCGTGTGCCCATCTGCCAGCCCCAGCATGTACGTGGTGAACCT
CTGCAGCCTTCCACGGCAATTGCTCACACTCT
CACTTTGCGAGCACCATTCGTCGCTTGGTCCTGCAGCGGTTATTTGCGACCTGAATTCACATCATGAG
CTGCGGGACGGTTGCTCACCATCCACGACGGC
AGGAGAGGACCTCGCCGCCACCTCAACCGGCATGGAGATTGAGCTGTCCGACGACCCGGCACAGGCAA
CGTGGATCAGCGGCCGCAGCGTCTCGTTTCCC
GACGTGACTGCGCAACGCGTACTGCGTGTCTCCCACTGGACGTCCACCCGTTTACATGGACTCGGACC
ACCATCTGCCATCCCACACTGCAGGGACGGAG
GACGGCATCCCGCGACAGGCAGGCATGCTGCCACGACGGAAAACATGACACCTTTGTCCTGCGTGAAG
CTGAATGTGATGCTTCCACCTCGGCGTGTGGA
ACACCTCTGGCCGCTGTAGTCACATGGCTGGAGATGCACCGAGGCACCGTACGAGCGGCGGCCGTGAC
AGTTCCACATGTATCTGGACGGACGGAATGGA

GCAGGCCGAGTGTGTTGCAGCGGCAGCCTGTCACGAGCTGAATTGCTCCGCAAACGGGAGGACCGCAA
TCATGCCCTTCGCGGTGGCTTCTCCATGCTGC
TAGAGGTCCGTGCGAAACAGCTTGGTGCTCTCTCGATCCCAAGGCCTGTGATGCCCCGAGGCATGGCG
GCGCCACGTCCCTGCCCTCTGGGATCGGCTGT
GCTGCGGTGCTGTTGGGACCGCGTCTGTGCTCTGCCTCCGCCGTGACCTCTTCCCGACGTGAGCACCG
CCACTCCTTGCCGAGCAGAAAGCGGCTGATTG
ACGTCCTCCTGCCCACTCCTCGGCAGACTGGCACGCGCCCTGCGGATCACAAGCAACCCATTATGTCG
GTGGTGCCGCCCTCTCACACTGAAGAGGCAGG
ACTACCCCGACCTCCTCCGGAACCTCCACCTGGCGTGTCCACGGCACCTCCTCGTAGGATGACGAGCA
GCGGATCAACGTCGCCCTGCCCAATCGGTGGC
AGCGTCTTCTCGTCCGTCCACAATATGAAGGCACACGCCCGATGCACTAACCCTGGCACTCCAATCGT
TGCAGAAGGCCTCACATGTGGATTCTGCGACA
GCTCCACGGTCTCCCAACAAGTGCCTCACGCGCAGGTCACCACCGAAAATGCCAAGCGTATCAACGAG
AATAATCTTCCGGGCAGCTGGCCGGATCCTGA
AACTCTATGGAACCAAACAAGTTAGGAAAAAAGGTTCGTTAGTTAATTGCTTCAATGTTTTATTTTCA
TTCAATATATAGAATTCTGTGGACCATTAGCA
TGGTCCTTTGTCCTCAGTTAGGATTTGAAATATTATTACTTACGAAGTGGGAGACTTTGGGGTCCTCC
AACCACAAGACCTCGCCGCTCCCCTTCGCCAA
GTTCCGAAGTGTAAAAACACGACCGCGTGATATGCCGTCTGCGGGGTTTTCCGAAGACTGTGCGTACG
CAAAATTTGCCTGCACTCCGCGAGAGTCCAAA
AACTCGTATATCCGTTGCAGCTCCCACGTCATGGCGTGTGATTTAGAGCTGCCTTTATTCACCGCTCC
TCGCAGCGAAGTGCTGTGCGCCCAAACGTCCA
TGGTGGATGGCAAGATGGCGGAAAAGGCCGATAAGGCCAAGCGTACCGCTCGTGCCTCGGCCTGCATG
ATAAGAAAAGGTTTCCTCTCCCATTTCCCTCC
GGCAATTTTAACTTCGCCGGAGTCTGGAGAATAAAAACGGCTCCCCAACCATGGAGCGATGCATCCGT
GATGATGGCGGCCGATGCCTTTCCGTGGGCTT
GATGATTCGCTTACGATTATTCCCGATCATGTGTCGTAATCTCTCGCCCAAACCAACCGCTGACGGCG
GTAGGTTCGCCGGGGATGTCTCCCGCACAATC
CCCCGGTTAAGTGCGGATAATCGTCGTCGCACTGCCCTGATAAAAAAGTAGTGGTCGCATAAACGCGT
GCACAAAATGGCAGCCGCGTACAAAAAAACGT
GACGCCACAACTTCCATTTCCGCGATGGTTAAAGAATTGAGCGCCGGCATGGCGCGTACAGAGCGGAC
AAACTTCTCACTCAGGGATACCGCCCGGTGAG
TGTGATCAAACTGCACCCCCAGGAAGGTGTACTGCGTGGCGCCCGATTCGCGTTCCTCCCCAAGGATG
CGTGACAACTGTCCGCATTACGAAGCACTTGG
GCTTCCCACAATGTCGCATCGCTTTTCGACCCTGCAATGCGTAATATTGTCGATCCAAACGCCGACAC
GCACCGATGGAGGTGCAGCCCAGAGGGGGCGA
ACCACCGTCGTCACCACCGCAATTGCCGAAGTAATAATCCGGAGAATTTCTGGGCTGGCCTTGTATCC
CATCGGGAGCCGTGTGAGCTCCACCAGCGTGC
CGTCCTCCACGCGGCATCAAAAGAGATGCCGAGTCTCCCGCGGTAAAAGGACTTGAAAAAAGGATGCC
TTCACATCCAGGCAGGAAGCGGCCTCAGCCAT
CNNNNNNNNNNNNNNNNNNNNNNNNNNNNNNNNNNNNNNNNNNNNNNNNNNNNNNNNNNNNNNNNNNN
NNNNNNNNNNNNNNNNNNNNNNNNNNNNNNNN
NNNNNNNNNNNNNNNNNNNNNNNNNNNNNNNNNNNNNNNNNNNNNNNNNNNNNNNNNNNNNNNNNNNN
NNNNNNNNNNNNNNNNNNNNNNNNNNNNNNNN
NNNNNNNNNNNNNNNNNNNNNNNNNNNNNNNNNNNNNNNNNNNNNNNNNNNNNNNNNNNNNNNNNNNN
NNNNNNNNNNNNNNNNNNNNNNNNNNNNNNNN
NGGCGGCAGATAATGGGAAATGTGTAAAAGAGGTGCATTAGCCTCGTAAGGGTCGTCTCTGTTCTTGT
CGCGTGGCCACGCGATCCATCGTCGTCGCAAA
CCGGTGGTTTTCTCCTCCACAACCGAGAAAGGTATTATCCATCCGCCTGTTATTGTAGACGAAGCGTC
CTCGATTATTCCTGCTTTTCGTAACAATCGCG
CGTCAGCGGTTGGAATTCTCCGTGAGCCTTCGCGCGCTTTACCGGAGGAAGACGGAGGGAAAGGGGAC
CGCCCGACGAGTCCCCAAACTTGCATGAGACT
CTCCAGGGCGGCCGGATTCAACCGACTTTTGATCCACTCTGGATTCAACACGGTCACGTTGACCTGCT
GTAACGGGATAACGTGTTCATCTTGTGGAGTG

GCCAGACGCGTCCTTGTCACGCGTCTCGTTATTGTAGCAGCATGATTGTGATAAAAATCATGGAAAAA
ACGTTCCTCCGCTCTCAAACAGGGTTCTTGCC
CTTCCGCCGTTTCTCCCTTCACATCAATGCGACCAGCGACGCCACCTGGGTCAGCAGTGTGGTGTACC
TCCACAAATATCGAACAGTATTCTGGGGAAGG
TCGAACGGGTCGACGTGCTTCGCCAACTGCGAGATCACGTGTGGATTCAAATTGAGTGCCTCCACGAT
TTGAGCAGCGTGCCGCAGCGCACCGCGTTTTA
TGGAATGCGTCGTGGCATTCCAAGGAGCCAGAGCTCGTTCGACTTTGACATTAGTAATATTCGTGAGC
TTTTCCTTCTCTTGAAGTGTCCTGCATAGCTT
TATAGTGTCGAAAGCGTCCTGCCCTCGTATCCTCACGAACCGCGAGGCGCGGTGGGGATCCGTCGTCG
CCGTCTTCGGCGCCACGGACCAATACAAAATT
AAGGCCCCGTCCGCCTCCATTGTGAAATTGTTGGGAGTCAAGGCCGCAATCTCGGACCAACGACTCGC
CGTTATACACGCAAGTCGAAAAACAACACGTT
CCTTCCAATCGGTCCGACTGCGGATTACTTGATTCATCTCTTCCTTTGTTAAGGGGCGCGCTTGCTTC
GTCTCCGATCGCGCCGCAATTTTTTGTAACCC
CAGAATCGCCATGCCCAGCGGGGTTCGATTCATTTCCAACATGGACCGCAGCATCCTGGCGTACCGCA
ATTGGTTCGAAGGCGCCACA
>scaffold1038.size14001
ACGACCTCTTGGGGAATCCAGCGTTACTTTGTTTTTGGAACTTCCAAACTCTGGGAGGGTAATAAAGT
GCTTGAATATCTTGCGAGGATTGTCCGAGTACG
AGGAGAGCGCAATGGCGAGTCGCCTTTCAATGCACCGATAACTGACCACCTCGCAAGTAAAACACTGT
GCAAGTTGAAAACGTTAATGACGCAGGCTGAG
TTTAACTTATTTGTATCGAGGATCAGGGATTGTCTCATGCACGCAAATTTTGGGAAGTGTGCCATGTT
TGCATTTTTGAATGTGGCCTTCATGGCTGCAA
TAAGACGTAAACTGAAGGAGCTGAGGCCACCAACACGACGTCCGTCACACCGCTGTGCGCCGGACGTG
CACTCACAGGAGGGCCCCACCAGCCACTATTT
TTTGCCGTCGGTGGAACGCATTGGCAAGAAGACTTGTATAAACCACCGGCTGCTGTACATACCGGAGG
TCGAGAATTTTCCGCTGGTGGACGGCTTTTTC
TTCATGGACTCAAATCCAATGACGCTGGTTGGGCTGCGGATGGCTACGGCGGGTGCGCATCACACCAC
CGCCAGCACTGTGCGGCAGTTCACTGAGCGCC
TGGCGGCATATTTTAATGGCTGGGAGGAATTATCCCGAGACATGTCGTGGGAGATTATTTATGTGCAG
CACGCAGACAGCACGCCGTTGAATGACTGGCA
GAGATGTGATGTTGTAAATTCCGATAATGTGAGCAAGAAGGAAAGTCAAAAAATTGCGGCGTTCTGGG
AGGAGAAGGTGCGCCAGTACCAAGTGTCAATA
CCATCCAGAGACGCTTGAAGAAACGAAGCACTCCGGAGTGAGGAATAACCGCCGAAATAGGAAAACAA
ATTGGGGAGGGAAAGGGGCAGTTTTGACTTTT
GGTGAAGAGATATGGTGAGATTTATTTATTTTTGGATACTTGTGTGTGTGCGTACGGTGCGGGCGCAC
CAGCACAAGACGGCAGTGTATCGCACACAAAC
GCCAAAAGTGTCCAACACTATCGAACATAACAGTGACAGAAGAAAATTAGGGAGGGAGTGGGGTTGTT
TGTTTCTGGTGAAAACGTTGGGTGGTTTTTAT
ATAGTATGTTGATTGCGTGTGCATACGAGGCACAATTGGAGAATTGCCCGGTATGAGGGAATCATTGT
GTTTGGCGCTGTCGCAGTACTACTGCATTGCT
TGTTTTCCTCTATTGTGTTCTAAGGCCCATCACGAACAGAAGCATCTTAGCGGGATGGAGGACGGATA
AGTTCTATTGAGGAAACAGTGCATTCCCTGTG
GCTGCATGGAGTGAGTGCCGCACTCCATTGATGGTGAGACGGCATTTTCGTGCTGGCGCCAACATTCC
TTCCCCCTGCGATTTCATGCTTGACTGCAACA
TGGTGCCAGGGCCGGTTGCCCTGTATTATCCGGGGAAGCCAGGAGCCAGCAATGCTTCCCTCATGGGA
TTGTTGTTGCTGACGCAGGGCTGACGGCATCT
ACCGGCACCCGGACCAGCCGCGTACTACTGGGGCACACGGTTGTAACACCACGAGGTCTGGGGGCAGT
TGTTGCCGTGCCGTCTGCTGTCCATCGGATTT
GTTTGAGAAAACGCAAGAAAAAAGAAAAACATATTGTGTACGCATCCTTCGCGCGTGAGACAGGTTTT
TTATGGGTGGTTGTTTTTTTATTATTTTGTGT
TTGGCGAGTCACATATTGGAAGCTAATGCAGCGTCCAGAACGGCTTTATCATTGCAGTAATTATTGAA
AGGCGGAGTGCACTTCTTACCACGCCGGCGTA
TTCGGACGGAAATACGAAGCAAAAGGTTGCGTGCATGGTTTTCAACCGACATGCAACGCATGTATGAT

TTTGACTCTCAATATTTACTGCGGGTGGTGAC
GTTATCTCCAGTGTCCGGCTTTATGGCGTTGGCAACGAATTTATTTCAGAGTTCGAAAGGAGGTCTGA
TGGAGTATCATACATTCTTGCTTCTGTGCGCC
TGACTGGGCAGATGAACTGGATAATGGATGTGGAGAGGAGCCGGAAGGAAGTGGACACCGCCCTACGG
ACTTGCAGGCCCAATGGCAAGGTTGATCTGCG
TACAAAACGTGTGCGTAATTGTCCTGCTTCCAATGAGGGGCTTGTTAGCTGTTTGGTAACTTAACCGG
TACCGAGCGGAGGGACGAGTACCTCTGCGTGA
ACGCAACCGTTCATGGGGCATTGACAGGCGCGGAGCGGGGTGTGACGTTCGAATTTGCTGGGGCAGGA
ACTGAGTGGCTTGTCGGCAAACTGGCGCGTAA
TCAGTTGTGTAAATTTGTGAACAGCATTTCTACTCTTTTGGCGACAGCGAATATCCACGGTGTGTCAA
AGGAGGGCAGCAGTACTGTGCTCTTGGGCCTC
TCGTAAACGAAGGACAGGAGGTGTGAGGTAACATTGAACGGTAAGAGTTCCGGCGTTGTTAGACGATG
ATGGTCGGTAATTTGGGAACATCAAACAGAAG
GTAAACAGGGTGGCCGTGAGGGAAATGTGTGTAAACGACGAATGGGTATGTGTACGTGTTGGTGGGAA
GACTATTTGCGAAGAGGTCTATTCTGGCTTTT
TCCCCAATTCGAGTAGGAACTCACACGCCTCCACATTGGTGGGGAACAGCAGGATGACGGGAGAGAGC
AGTCCTTTGACGGTGCGCAACGTTTTTTTTTG
TACATCCGCGAACTGGCGGAGAAGGACATCGAGAGACTCATAGCCAGCAACGTCACGCTTCTAATACC
ACCTACTGCTGGCAAATCAACCACGGAGTTGG
AGGGTCACACGGTATCCGGCGGTGCTGTGAAGGCTACTTCCGAGCGGAGCGTTTGAGGCAAATTTTCT
TTAACGATATGTGGGCGGGTGCCTAAATGTGT
ATCCATCCGCGGTTACAAGGTCTGCTTCTCAGGCAGGAAGGCACAAGGGGCACTATTTCTGGGAATGT
CTCCACCCAGGATACGGTAAATCCACCATCAC
TTTATGCACCCAAATTCCCCAATACCTACAGAGGAACAACTGCAAACCGACGAAGACGAATCAGCAGA
CAAAAAATGCACGAACACCGCCTGTAACGGCA
ACGAAAAGCGATGAACTCATTGATGCTGGATTATCCTGTGGGGATCATATCTCTTCCACGACGAAAGA
TGAAAAGAAGGAAAAGAGAAGGTGGTGGCGAA
AGAAGAGTCGACTGGTGATGGTGAATCGAGGTCGCCGCAGCACGAGGCCACTGTGCGCCAACTGCGGC
AAGGCAATCCACTTACGGCTGAGAGTGAAGAT
GTGCCGTCCCATGTACCTGAGACCAGCAGTGAAAAATATACGACTGACCTCAAAAGAAGAGGACGCTG
CTCGCGAGGCAACAGTGGGTTCAGATACCAGT
CCTGCTCCACACAGTGGCGCCGAACGGAATTCCACGAAGGACATCCCCAAGACTTCTTAGTCTGGTGC
AGCTGAAATTCCTCTTGGAAACGATAAGGAAG
GGCGCCACAGGGAGTTGACACTGCACCGACTAATAAAAGCACCACATTTGAAACGGATACGATTTACT
GAACGCACAATGCAGCACCGTCCTCTGGTGAC
TGCAACGTTAATTTCTGGAACATTACTGGCTTGAGACTCCATGGAGGAAACGGTGACACCACCGTGCA
TGCGTGTGACTCGCGTGTTACTGCTTTTCCTG
CTGGGGTTATGAGACATGACAGCCGTCATTTGAATAAAAAGGGAGGGGTGAATTTGCGAGGCCTCATG
AAAAATGACAAATTTTTGTTTCCTGGAGCTAT
TCTTTGCACCTTGAAGAATCCTTTTTTTATTTTAAAATTTTGTTGCGGGGTTGAGTTTTTTTTTCATT
AGAGTTTATTCTACTGCAAAGTGCAGCGACTT
GTTTCCGTTTGTGCCGTGCTCCACTGCCACTTTGTGTTACCGTGTCCAGTTGAGTGTCCTTCTGCCGG
CTGTGCTGCTGACATGCCTCTGTTTCTTCTTT
TTTTCTTCCCTGCTATAGGCACCGATTGTCCGCAACGATGATTGGTTGTGTTCCCCTCTGCGATGGCA
CTGGAGCGATGGAATGCCCGACTCGGCCTTCT
GCTTCTTCTGCAAAGGGAGGGGACGGTTTGCGGCGTGCCTCGTTGTGAGTGTGCGGGGCACTATCCAT
TGCAGCCTGCCGTGCGGCATGAAGCCAATCT
GTGAGGACAGAGCCGTGGCCGACTGTGCCGTGCGCTTGGGTGCGCCCCGTTCTTCTCGTTGCGGCTGC
CTGCGTGCTGTGCGCACGGCAGTTTCTTGCGG
AGCAGCATGGATGACGCTGCTTCTGCGTGCGCTGTGCGGCTGACTCCTTCTCCACGTTTCTTCACCCT
CGCACGAGGGGGCGTCGTCCGTCACCCGCCCG
TGGGAGGCTGTCATTCCCAGTATCTCTGCATGGAGGCTGTGGTGCTTGCGGTCACTACGTACTGCATG
GTTCTGTGGTACGTGGAGGATTGTGACTGGCA
GGAGCTGCGCGAGCCGCGGCGTGGTGGGCTGGAGGCACTGCTGCGCGATGGCGAGGATGGCGCCGAAG

AGACGCAGAAGTCCTACGACATGACGTCATCG
TCGTACGCTTCAGGCAAACACTGTTTCGTCGTCGTACCAAACACCTGCACCGCCGTTGCACCCTATGG
CTGGTGACACCCACTCAGACGCGCTGAGCCCG
CTGGACCGTGCATCCAGTTCGAGCCGTATGATCGATTACGCTGCCATGTGCCGTGTAACACGCGGAAT
TCGTAAGTATGCGGTGATGGTTGGTATGAGGG
AAGGGTGCGGGGCCACATCTGCAATGCGCAGCACAGCCATCGATTGTTCACTGCACTGGCAGCCTCGT
TGTTCATTTTTTTCTTTTTTTTGGTTTTCTTT
TATTTTTTTGCTGAAATGTACATTTTTTTAAAATATTGCTTACTAGAGGCCACTTTGTGCTGTCACAC
TGTGTGGCGGGCTCTCTTGTTGTGGTTGTACA
GTCATGTCTGTGCGTATATATATATATTTTCTCTTTCTCTCTTTATTCTATTCCTTCTTTCTTTTCTT
GATTTTTGTTTAGTGTTCAGCTTTAACTCAAT
CCAACTGCGAAGACAAGGAGAAACAATGCCTGGAAAGCGCAAGAGAGTGCACGGGGATGATAATACCG
AGAGTCAAGCGTCTGCTGTGCCGCAAGGAGAT
GGTCAGACGAGAGCAAGGCTGTCGTCCCATGGCGACACGGATCAACCCGCTGCGACCCGCATAAGAGT
GGAGGAAACGCGGCAACCACAGTGGACTCTTG
ACAGTTGGCTGAATAAAGTGCTGCTGGAGGAAAAGGAACGCACTATGGAAATGTGGCTGAATGATTTC
CTTCGGAATTATTTTGACGGCAGGGGCGTTGA
GGAATTCAATGAGAACGTCACTATGAAGGAATTTTTAGGTAATCCCAATAAGTTCATAAAAGACGAAG
TGTTATTGGACACAATAAAGGCTTCGCCGTCA
TACCAAGAGATGAAGAAAGAGTGTGAGGAGTTTTACATGCTATTGGGGCCCTTAAACAAGCTTTCACA
CGAATGTATTCTCACTCTCAGGCATTGGAGGG
ACTTTAAAAAAAAGGATACGGTCATTCCTATTGCAAGGGCACAAATAAACAAAGCCTACTCTCATATA
CTGAGAGTAGAGAGGTGGAAGGCAGAAGAAAA
GGCAAGGCGAGAACGACAAGAACTGTTATTTACCATTCACAGTAGGATTGATGATGCGGTGTTTAAGG
GAAGGGTTCGCGTGAATAAAATGGATCTGAAT
GACTTTCTTACGATGGAATTGGACGGCAGGGGCGCTTTGGACGCCAATCGGGATGTCTTGTTGGAGGA
GTTTTTTAAGGACCCCGAGAAGTATATTTGCG
ATGCGGGAGTATTGGGCGAAATACAGGCATCCGATCGTTACAAGAGGATGGAGGGGGCTGTAAGGGAG
GAAATGGATATGGAAGAGGATGTAAACAGGCT
TTACGATAAGGGCATGTACAATCTACTGAGGTGGTTGGTGGCTACTGCGGAGGTAAAAGCAACTGTTC
ATGAAATCACCAAAAGGTTCCTGGATGCAGCT
GCCGAGGAAGCGAGGAGCCCAAAGAAGTCGAGCGCACCGAGGTACCTGGAGGGATTGTACGAGTCTGT
GCACAATGCGAGGTGGCATCATGTCGTGGANN
NNNNNNNNNNNNNNNNNNNNNNNNNNNNNNNNNNNNNNNNNNNNNNNNNNNNNNNNNNNNNNNNNNNN
NNNNNNNNNNNNNNNNNNNNNNNNNNNNNNNN
NNNNNNNNNNNNNNNNNNNNNNNNNNNNNNNNNNNNNNNNNNNNNNNNNNNNNNNNNNNNNNNNNNNN
NNNNNNNNNNNNNNNNNNNNNNNNNNNNNNNN
NNNNNNNNNNNNNNNNNNNNNNNNNNNNNNNNNNNNNNNNNNNNNNNNNNNNNNNNNNNNNNNNNNNN
NNNNNNNNNNNNNNNNNNNNNNNNNNNNNNgc
aGGAGGTCCCTGGCGGCGAGGGGACGGGAATGGAGGTGCATGAGGGGGAACCACCGCAGCCATGGACG
TACAGGGCAGTTGGCCGAACCCTTGAAAAGGA
TGACGGTGTGCAGCAATCCGGTGCACCGCGTCTCAGGCTGATGGTGCTTACCTCGGAGAGGGCATGGC
CGCACACGTGGAAATGGAATGAGAATAATTCC
ACTCGTGACTGCTATGTGAACTGTGAGGTGGATCGAGTGTGGCAGATCGTCAGCGGCGATCTCACCGA
GTGGTTCAGCCCTCACGGTGGGAATGACTTTG
AGCCCAAGCGGCGTGTGTTGATTGGGACGCCCGGGATCGGGAAGTCGATGGCTGCCGGCTCGCACCTC
CTCTACCAGCTGCTGCACTACGATGTGGAGAA
ACTCCAAGTGGCTGCGTACATTTTTGGTGGAAGTACGGCGTACGTGTTTAACAAGACCATCATGACGG
TGACAAGATACGTGGGTAGTCAAACATCCAGG
AATGTTTTGCGTGATTTGGAGCATTTGAATATGAAGGGGTATGTTATCTACGACGTGACAGAGAAAGG
GATGCCAGCGAGTTGTTTCGCGCCCTATGGAT
GGGGCATGATTGTGTTGTCATCGCCAAACGTGCGCAACTATGCTAAATGGGAGACGCAAGTAAGAGCC
GAGCGAATCATCATGAATTGCCCCGATGAGAT
GGATGTGAAGGCGATGTGTGCGTGGATAAATCGCGATGGGATTgCAGAGAAACAAGCGGAATACTGGA

AGATGGTTGAAGAACGCATGGATAAAGTGGGG
CCGATTCCACGCCACATCTTTGATGCGAATAAGTTCATTGCACACTCTGCTGCCATTGAGGATGCCTT
GGATGGGATCAAGTCCCGGGATGGTGAAAAGC
ACTTTACGCATGGAGGAGTGAGACTGTGGTATTCTGAGAATCCGTCTCAAAAGCTCGTGAGGGTTGTC
AGAGGAAGAGGAGAAGTCGGTGCTGAGGTATT
TCTCAACGCACCGATATCTTTTTGCCTTGGGCGTCGAATACCGCACTATTTTGGGGAACGTGATGAGT
GAGAAAGGTATTTTTCTTTCCTGTTGCTGGTA
TTGGGGAAGAGGAATACTCTTTCGTCTGAATGTGTGGAAATGTTTGGCTTGCGCGTGTTTATGTTTGG
GGGGGTTTTCGGTGCATTAGTAGAGGACATCA
AGGAGCTGAGGCCACCAACACGCCGTGGAGGACAGTGCGCGGTTCTGAATGCGAACCCTTAAGGGCAC
CCCAACGATTCATATGGATTGGTTTCATTGAA
GAATCCTTTTGAGGGGGGAAATTTGGAGTATCGGCTACTGTGCACACCGGAGACGAAGAACTTTCCAC
TGGTGGACTTTTTTCCCTTTGTGAAGTTACCG
TGGAAGACGCTGGTTGGGCTGCGGATGACTACGGCGGGTGCGCATCACACCACCGCCAGCACTGTGCG
GCAGTTCACTGAGTGCCTGGCGGCATATTTTA
ATGGCTGGGAGGAATTATCCCGAGACATGTCGTGGGAGATGATTTATGTGCAGCACGCAGACAGCACG
CCGTTGAATGACTGGCAGAGATGTGATGTTGT
AAATTCCGATAATGTGAGCAAGAAGGAAAGTCAAAAAATTGCGGCGTTCTGGGAGGAGAAGGTGCGCC
AGTACCAAGTGTCAATACCATCCAGAGACGTT
TGAAGAGACGAAGCGCTCCGGAGTGAGGAATAACCGCCGAAATAGGAAAACAAATTGGGGAGGGAAAG
GGGCAGTTTTGACTTTTGGTGAAGAGATATTG
TGAGATTTATTTATTCTTGGATACTTGTGTGTGTGCGTACGGTGCGGGCGCACCAGCACAAGACGGCA
GTATATCGCACACAAACGCCAAAAGTGTCCAA
CACTATCGAACATAACAGTGACAGAAGAAAATTAGGGAGGGAGTGGGGTTGTTTGTTTCTGGTGAAAA
CGTTGGGTGGTTTTTATATAGTATGTTGATTG
CGTGTGCATACGAGGCATGATTGGAGAATTGCCCGGTATGAGGGAATCATTGTGTTTGGCGCTGTCGC
AGCACTACTGCATTGCTTGTTTTCCTCTATTG
TGTTCTAAGGCCCATCACGAACAGAAGCATCTTAGCGGGATGGACGAAGGACAAGGTCTATTGGAGAA
ATGGCGCGCTCCGTGTGACCGCATGGGGCGAG
GCGCCGCGGCGCCTGGATTGTGCGACGTCACCGTCCGGCTGGCATCTTGCGTTTTCCCCCACGCGACC
TGACGCGCGACCGCCCCAGCGCAGCGTCAGGG
CCCCGCACCCCGTATTGCCAGGGGAAAAAAGAGCAAACAACTTCTCCACATGGAGTCCTTATTGGTGC
GGCGCTGCTGAGCAGGGATGTGGAACAGAACC
CAGCCCCAGTCTGCAGGGGGTTGCGGGGGAACGCCGCCGGCATCACTCTGGCGACGCCTTGCCGATGG
GATCTTTGAATGTCGGGTTTCGTCGCCTGGCG
CGCGGCTTTGGGTGCTGCGAAGATGGCGGGCGGACCCTGCCCTTCCTGTAATAATTTAAAAAAAAAGA
CAAAACGGGGAGGGAGTGGGAAAGGCAAGCGG
GACGCGCCCCAAAAAAAAAAAAAAAAACAGCCACGGAGGCCCGAGGCGGAGCGGCAGAAGCGAACTGA
CAATCACACACGCGCGCCCCGGCGTCTTCCTA
GCGCGTTCTCGCCTGCCCGCCTCGTCCGCTGTCCTTCGGCCGCCGCGAAATAAGAGGCAAACACGGCG
CGAGCCTCGTGTTGCTCTTCGGCGCGCGGGAG
AAGCAGCGCCACAACCGCGCCGCGCCCGGCTGGCTCTCCGCTCGTCCGCGAGCCGCCGGCCTCCATCG
TCGCCAGAAATGCGTCCCCGTATGGAGGCCTG
CGGCCCGCCGCCGCCGTCTTTTCCGCGGACCCCCGCACGGCGTTCCAGAACAGGAGCCCCGGGGGGAG
ACTTTCCGTTGGTCCCCGCCCTTCTCCCCCAT
GCCCTCCGCCCACCACGCCCGCGCGCCCCCGCCCCGAGGCCGGCAGGCATGCGCCGCCCATTCGCTAT
AACACTTTTCTCGCAATGAAGCCCGAGGACGC
ACAAAAGAAAAGGGGGGAAAAAAGTGAAGGAAAAACAAATATACGCAGACAATGCGGAGAAGCAAAGA
AAAAAAAAAGCAGGCGCGCGTGCGGGATGCTC
CTGCCTCCGCTCTTCTCTTCGGCGCCGCCGTTGGCGTATGAGCGAGAGCTGCCGAATGAAAGAAAAGC
AACATCAAGGGCCGCCCACGGCAGCAATCGCG
ACAAAAACGCTTTGCACGGTGCGGCGCGGCACCGACGGGCTGGGTTTACTTCTCCTTTCCGTCCTTGC
TCCGCTTGAGGCGGGCCTCCGCGCAGACGGCG
CAAAACGGGTCTGGGTGATATAAATTCCCAAGAAAACTATTTCCCCACTCCGCCGCCCGTTTGGCACG

ACGCCACCGAAGCAGCCGCCGAACGGACGCCT
CGTCTTCTTCTAGCCGCCGCAGTTTTTCACCAAACCCGCGAAACGCGTCTTTCGCATTCGCTGTCGCT
CTCGCATCGTCACCCACTTGCCCGGGAAGGAG
CCGTTCCAGTTCCACAATGCTGCCCCGGCCTGACGGAAGAAAATCCTCAACGTTCGGAGCGCCCCAAT
ACAATGGAATCGCCCCGCTCAAGAGAGCGTGG
TAAACCTTTTCCGTCACATAATCCGTCTCGTTTGAATTCTCAAGTGCCATGGCGTACTTGTATTTTTG
AAAGACGCACAACTTCTGCGGGTAGCGTCCGG
TCCGGCGAGCGCATTCCGATGGAATCGCACGATTTCTGGCGCAAGCACCAAAACTATGAACCGGGTAA
TGCTCCGACAGGCGGCGAATAAACTCTGCACG
GCGCGCCTTGCAGCGAGACACGAACACTGCCACCGCAGAGTGGCGTTGCTGCCACTTCCCCCAGTCAA
ACGCATCTTTATTAGCGAGGGGTTGATTGTTG
CCATGAAGAATTTTCAGGAACTGTTGTTGTTGACGATGGGCCCATGTGAAGTAGTAGGGCCGATGGTG
ATGAAAAGAAATCACAACATCATACAGACGCT
GGTAGGCCTCCGTGCTCCGTTTCTTGTCTGTGTGGTTGCTCTCCCCTGTGTAGAAAAGGGTGAGGTGT
GGCCGTCGTTGACCGAATGGACACGATGGAAC
AGGCCCGTTGTGGTTGATTGCGATGTCTGCACGCCGGAACTCCGCCACGCGGTGTCGCGAATGCACGT
AACAGTTAAAATCTTTGCCATTTTCCTGCCCA
TCGTTTCCACATGACTGGACACAGCCCTCTGGCAGCAAGGTTGCATTCGACATGTACTCGCAAAGATA
AGTGCCTGGCCGCGTGGGTACGACAAGAACAT
CGTTGACTCCATTGAACGTTCTTACATGTCGCGGATGGCCATCTGATGATTGAGGCGACAAAACCCCC
TCAACATTCGCCGCAAAGGAGTCTACATCGCC
TCCACCCGCGTTCCACAGGGAGAAAATGGCAATGGTACAAAAAAGGAAAATGATACCACGACGATGAC
GACTCCTCGTATGCATGTGACCCACTTCTATC
TCTCTGCGCTACTCACAAACAAAAGAAGAAAACAAACAAAAAAGAAAAAAAAAAGAAAAAAACGGCAG
TTATACACACATCAACAGCGAAAAAAAAAAGAA
AGCAGATAGAGATAAAAATGTGTGCGTACACATACATGCAATTTGCTTACATGATTGAATTCATCGAC
AACCCGTCGATAATGGAAAGAAGAACCATAAC
GTCCCCCCTTAATTCTGCACCCTTTCACCCCTCAGTGCATGGAGGCATTTCATATTAATGATAATAAT
AATAATAATTTATTTCTTCCTATAGACAATGA
CGATGCGTCTCACCCTCCTTCCAACTGCAACATCATGGGCCTTTTTTTTCTTTTTTTTTTCAAGCAGC
AGAAACGCCCCCATGCCTTATTTGTTTGTTGT
CTTTTTTCCCCCCTCCCCTAAGGCACACACATCCCCTCATATNNNNNNNNNNNNNNNNNNNNNNNNNN
NNNNNNNNNNNNNNNNNNNNNNNNNNNNNNNN
NNNNNNNNNNNNNNNNNNNNNNNNNNNNNNNNNNNNNNNNNNNNNNNNNNNNNNNNNNNNNNNNNNNN
NNNNNNNNNNNNNNNNNNNNNNNNNNNNNNNN
NNNNNNNNNNNNNNNNNNNNNNNNNNNNNNNNNNNNNNNNNNNNNNNNNNNNNNNNNNNNNNNNNNNN
NNNNNNNNNNNNNNNNNNNNNNNNNNNNNNNN
NNNNNNNNNNNNNNNNNNNNNNNNNNNNNNNNNNNNNNNNNNNTTACTTTTTTTTTTTTTTCTTTCGTT
CTTCCCAGACCTCGTGCTCACTCGTACGATGT
GACAGGGGTTGCCCGGAGACCCTGAATAGCAATACGGAGCAGCGGGGATGCGCAAAGAAGCGCCCAGA
AAACAAATGCCGCGCAGATAATCCAAAACGGG
ACAAGTGTGGGGTATTCATCGCGGTTAAACGGCACTTTGCAGTTCATACCGAAAACTGAAATGACAAG
ATGAATCGGGAGGCAGATAGCGGCCAGCTGAT
TCAACAGAGACATCTTGAAATCGAGCCCCGCCGATGCCTGCGCCATCCGCATCGAGACGCCAGTGACA
AAATTCAAATTCGCCTGATTGAGCATGTCACG
GGCGTCATCAAGTCGCTCTGACACCTGCCGCACCTTCTCCAACGTCTCTTTATAGTCCCGCACGGTGT
CTGGCTCCTTTGCCACAAAGGACAAACGCATC
GTTGGCGCAATAAGTTCCTGTAAAAGTTTTTCCTTCAAGTACAACCCCGTACGAAAGTTTGATATACG
ACGGCGTAAGAGCGCCACGCGGCGAAGAAGAT
CCCTTTGGTCCTGCTCGCCAGGGGAGATGAGGAGCACCATTTCGTCAATGTTGCCCACCTCGGCGAGC
AGAGACGTGGGATCGGGAAGGAAGGCCTCGCA
GGTGAAGCACACCAACAGGGAGAGAATAGCGGCACTTGTGATGCCCCGCAGTGATGGAGCCGGGGCGA
TGGATCTTTGCATCTGCGGCGATGCGGTGTTG
GGGGCTGCAGACGCGCCGCCGCGATGCCCCCCAGATCCCTCCACTTCGACGTGTCGCAGCAGCTCCGC

GAGTCCGACAAACGGGGCTGCATGCATCGTCA
CCAAAAAACGGTCGTTGCATGCAAAGGTGCAGACGACTGTATTCTCCTTTTCCACCTCCGACGCGGCG
GGGGCAGAGAGGGAGGGACCGCGATTGGGAGG
ACATGCGAGGCTCCCAATGAGGTACTCTCCCTTCACCGGCTGCAATTCCACCGTATCATATGTGTCAC
ATCGGATCATCCATTCTATCTGACTTTGCTGC
AGGTCTGGGAAGAGCAGCTGGAGAACATTCCTGTTCTGTTCATCGGTGCATCCCTGCATGTCCACCCA
CACGCAAGGCCCATCTGGGGAAACCTCTCCAT
CCTGCATGCGTTTCCTCAGATCATCCACCACCCTGTCTGGGTTGCAAAAGACATTGGTGGAGAATGGC
ACTGCCGTTGCCGTGTGAACCACAAGGCAGCG
CTGCATCGGTTGCACAATGCCCACGGCAGCTGACCCCACCTCCTGCGCATCCATCGCAAGCCCGTGAA
CCTTTGCTCCCTTTTCCCCCTCACGCATCCTT
CCTCAAAAAAATTAAAAAAAGACAAAAATTTTTGAAGCCAAAGAATCCTCTTCTCACTGTCTTTTTCC
TTCTCTTTGTTGCTGTTATTTTTTCTTTTTTT
TTTTTTTTTCTGCTGTTTGTCCCTCCACTCCGTCTCTCGGTCGTTTCGCCGCCAAAAGTCACCAACGT
TCTTTGCTGGGAAACTCCCCCACGTTGCGTTT
GCCTGTTAAACCGCACTGACGCCTGCGAATGGCGAGGAGACCGAAACAGAAACAGCCGAATTTCCTTC
GGCGTGTTGCTACGATGTAAGTCCCGTCAGGC
GATGTCTCCCGGCTGCTTGGCGGGAAAATGTATCGGGACGTGCTACGCAAAGCTAAAACCACACAAAC
ACACACACACACACACAAAAAAAAAAAAAAAA
AGCTCAAATAAATTTGTTATTGTTTTCTGGGCTGTTGCTGTCAGTCGTTACTATTTCATGCTGCATTC
GCAAACACGTACACAAAGAAAAAAAAAAAAAA
AGAAAACAAATGGCAAGGGGAGTGTGAGAGAAATAAAAGAAAAACAGACACATGCATGTTGGATGCAG
AAATCATTTACAGTCGGATAGACAACGTACAT
GTATCTATTACAAAATAATTCGGCGGTGGTCACCTCCACCTCAGCCAACGTCCGCGTCCCGCAAAAAA
CGAATCTTCGCGCCCCACTGGGTGCGCTCAGT
CAAAAAAGGCTGCGCGCAGATCCAATCAGGGGGATGCCCCGTGAGCATCCACTACCGCCAAGAAGCCC
CAAGATGCTTATTGCCCAAATAACCCATACAG
c
>scaffold1039.size6794
GAGGACAAGATAACAACGGAAAGCAGGCCTTGGGAGCCGAAGAAAGAATACCAAGTGGTACTCATGCT
GCAAGGCAACAAGGCTTCTGTGTACGTTGATGG
CAAGTTGCTGGGGGAAGAGGAGACGATGTTAACAGGTGAGACACCACTTGGGCTTGTGCGCTTCTGCT
TTGGCGCGTGCGAAATTCACAACTTTCCTGTG
ACGGTGAAGAACGTCTTTCTGTACAGCCGCCCACTGAATCCCACTGAGATGCGTGCAATCAAGGAAAG
GAGGCATATCCCTCGGATTGAGGGTGTGCGAG
CCAAAGCGCCTGCTGGGAGCGGACTGCTGCCGCTGCTTCTTCTGCTGGGATTGTGGGTGTTTGCGGCT
CTGTGAGGAGTGTGCGGCCTCAGTGTGGGACA
CTTGTGCCGTTCCCTGCACCGACACTCACACACACCGCTAGGAATAAAATTTACTACGCAGAGAATAA
TTTGTTTTTGTTTTCTTTTAATGTTTTCTATT
TCCCCCTTGCTGCTTTAGGGCAACTATTATAAGCGTTTTTTTTTTCCGGCTCTTAATTTTCTTTTCTG
TTTGGAGCATAATTTCTTCATGTTTTTTTATG
TTTGTTTTTAATCAATTTTGTTTGCTTTTTAAAAAATTTTTGTTTCTGTTTTTTTTTTTTTTTTTGCT
TCACTCTTCCTTCTTTTTTTGCTTTTGGGGCA
ATCACTATGGACGCTTTTTCTTTTCTACTCTTCCCTCTTTAATTGTTTCTTCTGCACTCTTCCTGTGA
GGGGTGCCGTGTGTTTCCCGCCCAACTGCTC
CGCTCGCACACCCACCGACACGCTCGTGACGACGGCCCTGTGCGCCTCGCCTGCTGCACGCATGAGGA
GGGGCCGCGACGAACGGCGCGCTGAGGGAGCC
GTGACGTGTGGGCGTGCGACGAGGGCCTGTGGGGCGGAGCACACATTTCTTCCCTCTTGTTTTGCTTT
TCTTTGCGGTCAGGGCGGCAGACACCCGCAG
CGCCACACACACAGCCACACCCTGCCGCCGTGTTTTTAAGATTTTTTGCTTTTCGTGTGCATTTCACG
CGCCGGCAGTCCCTTCGGTGTCGGCCTCGTGA
GCATTTCTTTTCCCTCCTGATGATTTTTTCTTTTGCTGCGCTCACTCTCCCGATGGTGTGACGCGACT
GCCGTCATGTCTGCGTGCTTCTCTCACTCCCC
ACGCACACGAGTGACTGTGCTCTGCGGGCCGCACTCACGCGCCGATGAGAGGGCTGAGGGGAGCAGCA

ACACGCAGATGACCCCATGAAATATAAACGAT
GCGCGGACACACAGAGGGTCGGACGGTTAAAAGACATTTAAATGTCTTTTAATTTTATTTTACAGGCA
AAATATATACAGACGGTTTGGTGTGGCCACAA
GGCAGCCAGCTGTCTTTTGTTAATTTGTGTGGAACAGTAATGGACGATTTCTTTTTCATTTATAATTA
TTTTTTGTGATGGGGTTTGTGTAAGATGAAGG
GGAAAGGTTTTTTTTTGTTTTAAATAAATCTTGTGTGAATTGCCGCTGCTTCGGCTGCAGTGCAGTCA
TGAGTTTGAGCGGTCTTCTTCTTGTTGCTTCC
GCCATGCACCTCCGCTGTACATTTTATTTTGTGCTTCTCTCCACATACTCTGCTTCCTCTTTTCTACT
CCTTTGCTTTGTGTTGCTGCAGGCACACACAA
CCACACACACAAGATGCACATTGCAGTGGATATGAATGCTTCTTTTGCGGTGCATGCTGGCAGTTGCG
TGTGGCGGCTGTTGTCTTCTTCCGTGGCCATG
AGGAGAAGGAGGAGGAGGGAGAGGGAGTGCGCAGCCCGAGTCTCCTTTCCTGCGGGTGTGGAGCACCG
CGTGTGGCTGCGTTGAGTTGGGCTCCGTGTCC
CATTGAGGGCGGGATGGCTGGAAATTTTGTTGCCGCGTTATGGATGGATGAGGGTTCGCTTGCGCGTG
TGGCTGGTGTGGTGGGTGCTCGCTCCGTGTGC
GTGANNNNNNNNNNNNNNNNNNNNNNNNNNNNNNNNNNNNNNNNNNNNNNNNNNNNNNNNNNNNNNNN
NNNNNNNNNNNNNNNNNNNNNNNNNNNNNNNN
NNNNNNNNNNNNNNNNNNNNNNNNNNNNNNNNNNNNNNNNNNNNNNNNNNNNNNNNNNNNNNNNNNNN
NNNNNNNNNNNNNNNNNNNNNNNNNNNNNNNN
NNNNNNNNNNNNNNNNNNNNNNNNNNNNNNNNNNNNNNNNNNNNNNNNNNNNNNNNNNNNNNNNNNNN
NNNNNNNNNNNNNNNNNNNNNNNNNNNNNNNN
NNNNCACTACAACAACAACACAGGCACGAGGTAGTACGGCTACGACTACAGAGGCGCCAACCACTATG
ACGACCACCCGCACACCGTCACGTCTTCGCGA
AATCGACGGCGATCTCAGCAGCTCTGCGTGGGTGTGTGCCCCGCTGCTGCTCGCCGTATCCGCGCTGG
CGTACACCACTCTGGGCTGAAGAGGTGTGTGC
GGGCTGTGCGTGCCAGCACTCAGCCACGGGGATTTTTGCGCGTCCACTGTGGTCCGATGTAAATAAAT
AATAGAAAATAATGATTGTGTTTTGCTTCACT
TGCTTCCGCTGCCNNNNNNNNNNNNNNNNNNNNNNNNNNNNNNNNNNNNNNNNNNNNNNNNNNNNNNN
NNNNNNNNNNNNNNNNNNNNNNNNNNNNNNNN
NNNNNNNNNNNNNNNNNNNNNNNNNNNNNNNNNNNNNNNNNNNNNNNNNNNNNNNNNNNNNNNNNNNN
NNNNNNNNNNNNNNNNNNNNNNNNNNNNNNNN
NNNNNNNNNNNNNNNNNNNNNNNNNNNNNNNNNNNNNNNNNNNNNNNNNNNNNNNNNNNNNNNNNNNN
NNNNNNNNNNNNNNNNNNNNNNNNNNNNNNNN
NNNNNNNNNNNNNGAAGGCTGCACGCAAGTGACAGGAGTGATGGCGATGATGATGACTGGCCGTGTGC
TGCTGGTGTGTGCCCTCTGCGTGCTGTGGTGC
GGTGTCGGCTGTGGTTTTGCGGACAAGGTGGTGGAAGCTGCTGCCGGTGTTGTTGCCAGTAACAGAAC
GGATGAGGAGAATTTGATCTTGAATTGGTATG
TTCTGACGCAGGGAGAATGCGCGAATGAAAGCACGACAGGAGGGAAGTTGAATGTGACAGCTGAGAGG
ATCTGCATGCACAAAGTGATGAAGGAGGTTTG
TGACGCTTTTTATAATAAAACGTCGGGTGTAACTCATGACCGTGACGTTGAGTTTATTTGTACGTATT
ACGCTACCATTCCAGAGGAGCCCGTCGAGCCC
CCAACACCGCAAGGGCCACCATCACATTCAGCTGTCAGCAATGCCACCAGTGATGAGGGGACGCCGAA
GAATGCACCGGAATCCGATGTTGCAGGGAGAG
GAGAAGGAAAAGAAGATGACAAGGAGCACGGCAATACGAAGCAAAAAGCAGTCGAAACCGCCGCTGTG
GAAAGTATCACGAGGAGTACCGACAGTGAGGT
CAGCACCGCGGTCTCCCACACCACCTCCCCTCTTTTGCTTCTTCTTGTTGTTGCGTGTGCGGCTGCTG
CTGCGGTGGTGGCCGCGTGAGAGTGAGGGAGA
GAGAGCCGTGCACCGCCCGCACACACACTCTTCCTTCTCTCTCTGTGTGTGTCCCCTTGCATGGACTC
TCGCCCTCACCTCACCCCACGAGGCACACACA
ATAATGTGCCCATTGTATATATGCATGCACACCCATGCATACCCGTTGGTGCTCTCTTTTGCACGACA
CGCACCGCTGCCGTCCTGCCTGGCTGCATGGG
CGGAGCACCGTAACTGTTTCTGTTTTGTTTCCATTGCATTGAGTGGGCGACCTCTCTCTCTCTCTCCC
TCTGTGTGTGTTTTGCTTTACGGGAGCAATGA
GAGTGTGTGCGCTTGTCGTCCCACGCTTCGGCTTTTTGTTTTTTGTTTTACTTGTGGTGTGGTTGAGT

GCACCCATTCTATTGCCCTTTTTTTGATTTGT
GACTTGCTTTGACTGTGGACTCCTAAATTGTTTGCTTTTTGTGTATTTTTATTCTNNNNNNNNNNNNN
NNNNNNNNNNNNNNNNNNNNNNNNNNNNNNNN
NNNNNNNNNNNNNNNNNNNNNNNNNNNNNNNNNNNNNNNNNNNNNNNNNNNNNNNNNNNNNNNNNNNN
NNNNNNNNNNNNNNNNNNNNNNNNNNNNNNNN
NNNNNNNNNNNNNNNNNNNNNNNNNNNNNNNNNNNNNNNNNNNNNNNNNNNNNNNNNNNNNNNNNNNN
NNNNNNNNNNNNNNNNNNNNNNNNNNNNNNNN
NNNNNNNNNNNNNNNNNNNNNNNNNNNNNNNNNNNNNNNNNNNNNNNNNNNNNNNNNGGCGGGTGTCTTG
CTGTCAGTCTGACGTATCCTTCGTTCCTTGAC
GATTTGTTGCGTACCGACGGTGCGTGGGCGTCTCTGGTGATAGTGAGGGACGCGGGTCTCAGAGCGCA
GGATGCGTTGCGGGTGTGCGGCGTCGTTTGGA
AGTACCGATGGGTGACCATCCGCATCCAGGCGTCATGACGGACTGTGAGGACAGCATAAGGCACTGCG
GCTTCTTTTGGTGTTGCACAAGGAGTTTATTG
GACGGTGCGTTTGTTTTCAATCTGTTGCAGCTTTTGTGGGGGAAGGTGGGAAGTTGAGGGCATTAGGA
CTGAATTCATTGAGTGGCTGCGTACCGTTGAC
TTGCGGCCAGTCGTGTCACTCTTTTGGGGGTTGGCCGCTCGTCTCTTTTGTTTTCTTCCCACCGCAGC
ACGTGGATAACACTGATCCGAGGAACATTTGT
TTGGCCGCGTGTGAGCTGCTGCCGTGCGTGGTGCATGGACTATGTGATTGTGTGGGGAGGTGCGTTGG
GGTGACGAACACTTCTGTTTGGCGTGTCGCGG
TGTGTTGGAATGATGTTCGGATGACATGTGGAGGGTGTTGTTTGTGGTTTGTTTTTCTTCTCTGGTGG
AGCCGACATGTGTGCGGGATTGTGGCATTGCT
GGCTGACAGCTCCACGTCACTTTTCGGCATTCCTGGATGTGCTGAGGAATGACGGCTGGCGACCAGCG
TGTTGGTGCTGCTGGTGGGGATAGGTACACGA
GAAATAGGCCGTTTTCTCCTTTCCGTGCTGGAGCCGATGAACTGGCACGAGGGCACCGGCTGCAGGCT
GCCTGCGAGCACGTGTGAGCAGCGGATCCATG
CACACACCGACGCTCATCGCAAGAGCATGTGCGATCACAGTGGCCTTTTGTGGCGTGCTTTTGTGGGC
GTCAGCCCATCGAAGAATGTGTGAAGCAAACG
GCTGACTGCACACGAGGAAAATAAGTGCGTGCACTGTGCCGTGTTTCAGCGTCGATGGCAGAGCGACA
GGAGGCCCGTGCTGGCAACTGAGATCTTTGCG
TCGCGAGGGATGTCGGTATTGATTTTGGATCGTCGAGGGTGAATTGATTTTGCTGCTGCCCGTGTGTG
TGGCCACTCCGGCGGAGGGCGAACACAAAAGG
GAGGGATGCAGGCGCTGCGTGTGTGTGTGCAGGTGCCATGCAACAACGTCGCAAAAAACGTGCTGCGA
CTCGGTGACCCTCACATCGCTGTGGTCCTCAC
GAGCAGCCGAGGCACGGCCCACACCGATGCACTTATTGGACGGAGTGAGGCATCTTTTGAGGCGCAGC
AGGGAACAAGATGTGTTGACGCAGCCACGACG
TGCATGGAGTGTCACGATGGCACAGTCAGCTGTGGCTGGTGGAATAATTACTTCTCGGAGGCAACACA
GAGAGAGAGAGAGAGAGCTGCTCCTGCTGTTG
GCGTCGTTGCTGCCTTTTGTGGCCTCCCATGCCGATTATTGTCTGTCCGCTGATCATGGGTGGTGTGC
TCCACTAGTGACGTCGTCTGCAGGCATTCCAC
AGCACCAGTGAAACACACATCACGTCGCATGCTTTGGCTGTGGATGTGGTGTCGTGAGTGCAGCCGGC
ATTATATTGCCCGACTCCTTTTAACGGCCTTG
CGACGAATAGATGCGTCGCCACCCGAGTCATTCACTGCAGCTCCGTGTGTTGTACTGCCAGCACAACA
CTCTCTTGTTGTCTGGCGCCTGGCTGACAGTC
GAACACCACAGCGTTGGGACATGCAGGGATGTGGACTGCCGCTCACGGTGTTGGGCACCGCTGTTTGG
ATGCGGCGGCCACACGAGCGGATGTATTTTGG
CACAATCAAATGCGTGAAGTACGCGGAATCACAGCGGCTGCAATAATATCAGCGGTGTGGCACCACGG
AATGTGTGAGTGGGTGATGCATGATGAATCAC
GGTGGGCGGCATGCGAGGGAAGTGCACCGCGACGGAGCGTCATCCCGCCCTCAGCCTGCAGGGGAATG
TACGCCCACAAAATATATGGACGAGACACCAC
AAAACACACATGCCCTTCCATGCAACAATAAATAGGAGCGAAGGTGCGTTCTGGTGTGTGGGAGTCCA
CTCACATATTTTCATTTCCACCGCAATAAACG
GTGAGGAATCAGGTGCCACAGCAGCGAGAGGAAAAACAAACATCTACTGCAACACTTTACCGGCGAAA
GGCAGGAACAACACAAACCGAATTGTTGGCTG
CACCAACACAAGAAACATTCACAGGAGGAAAAATAATAATTGTGCACCCTGCGGAAATCCTCAACACA

CGATCCAGGCATGAAGGCTTACCCTATGCAAA
ACAAAACAACGCATGCGGACGTCTGCCAACATCCAAAAAGAAACACAAATAAATGATTGCTTGCTTTA
TTCCATTCCTTGCGCTTATTTTC
>scaffold104.size35768
CAAACATCTGCAAAAAAAAAAAAAAAAAAAAAAAAAAAAAGAAAAGAAAAGGGGAAGAAAGTCCAGAAA
AGGAAAAAGGGCGGTGAAAAGTGGATTCCTTCT
CATCCCTTCACACTCGTGCCATCACCAACACCATCACCCCTCCCCTGTCGGCGCTTCGTTTCCGCATG
TAATTTTTGTGCATTTCTCTCATTCTCTCTGC
GTGAATTCTTTTTTTTCTTCTTTTTCCTCCCCCCTCCTCGGGTGGGAATGTGTGATGCCGTCGTTTTT
ATTGCCCCCTCCCCTCCTTCCCCTCTCGTATT
TTGTGTCTGACTCTCCATCCTCCTCATACATGCGGGTGATTCATGCCTTCGTTTCCTCCACTGTGTTG
TGTTGTGTTGTTTTATTATTATTATTTTTCCT
GCTTCCCCATTTCCCAAATGGCATTACATTCCCAATCCTACTCCTCCTCTTCTTCTTCTTCCCCCTCT
TCTTGTCTGTGCGTGCGTGCATGTATTTGTAC
GTGTATGTGTGTATAATATGTATGTACGTGTGAGAGCTTCCCCTCCCCTTCCTCTCCCCTACACCCAC
TTCGCCTCTTCCTCCACCTTCCAGAGAAAAAA
CACACACACGCACACGTATATATATATATATATATATATACGCATGCATGGATATTGAATGTGTGCAT
GTAAGCCCACTTCATTTCACCTTCTTTCATTC
CCATCAAACCACTTGCCTTCATTTCTCTTCGCTTCTATACACTATAAGACACAAACAAACACACACTT
TCTCTCTCTCTACGAATAAATACAGACATA
TACATATATATTATATCATGTACGTCCCCCATTGCAAATCTCTGTTTGTTTCTGGTCACTACCGCTGT
TGTTTGGGTATTCCCCTCTTTCTATCTTCTCA
CTCCTGCTCTTTCTTTCTTCCTTTTTATTCGTCTTGCTCTCCTTCTCTCCCCGTCCCTGTCTTCACCT
GTCACTTCCCTCTTCATCCTCCTCTAGTAGGC
CTTTTTTTTTCTTTTTTCTTTTTTTTTGTTGTTGTGTCTTCTGCTCTTCTGTTCCTTTTTTTTTCCCT
CCCTTCTGTGTCCGTCCTCCATGCAGTGCAGT
GCAGTCCTTTTCTTCACTCTCACGCTTCCCCCCTCTCCCCATCACAATACTTTTTGTTTTCTTCTCTT
CTCCTCCTCCGCCTTTCGTTTGTTTTTTTGCT
TTTTTTTTTTAGTTTTTTCTTTTTTGTTCCTCCTCACATCACCCACCACTCGTTGCATTACTTTCATT
GAAGGCCATCACTCCACCCACACCCCTTCCGG
CCTCTGAGCTTTTCTCTTCTTCTTCTTCTTCCTCGATTCATTCGTTCTTTTTTCGTTTCTTGAGTGCG
TGTGTAATTCCGACGTTTTTTTTTCTTTTCTT
TTTGCCTTTCCCTTTTTTTTTTTTTTTTTTTTTATGGGAGGGAGGGGAGGGGAAATTTTTTTCTATTTT
TTTTTTTTCGTCGTTTTTCTTTTCATTGTTTG
TTTGTTCTTTCCTTCCTTCTTCTCGCTGTCATTTCAGTTGGTTGGTTTTCTTCTTCCTCTTCTCCTTT
TATTCTCCTCTCCTTCTCTCTCTCTCTCACCG
TGCTCCCTCCCGCCCCGGCTTTCTTTGCCACCGCCTCCAGGCCTGCGCCTTATGCGGCGTAGAGTTCA
GGGTCAATCTCACCCGGAATGGCGTTTATTAC
CGTGTCGAGCTCCTTCTCGATGCGGTAGACGTTGTAGCGGTCATCATACGTCACAAAGTTGATCGCAA
GGCCCAGGTGTCCAAAGCGACCGGAGCGGCCA
ATACGGTGCAAATACGTCTCAGCATATTTGGGGAAGTCAAAGTTAATCACGACGTTAACCGCCTGAAT
GTCGATACCACGAGTAATGAGGTCGGAGCAGA
CAAGGTTGCGGCAATGCCCCTCACGAAAGTCGTGGAAGACGCGGTTACGGTGTTGTTGCTGCATACGG
GCGTGAATGTAGTAGCAGCTATAACCGAGCTG
GGTGATCTTCTTCGCCAGCAGCTCCACCCGATTAACGCTATTGCAGAATATGATGGATTGGTTGATCT
GGAGGCGGTTAAAGAGTGTGTTCAGGCAATGA
ATTTTCTGCCGTTCTTCCACAAAGACATAATACTGCGTTACACCTCGCAGAGTCAGTTCCTCCATGAG
GTTGATCTCATACGGGTTGCGCAGGTAACGGT
CTGCAAAGTCCTTCACAGTCACAGGGAAGGTGGCGGAAAACAGAAGAGACTGGCGATCGGCAGGCAAG
TAGGTGTAAAGGTCCTCTATCAATTCCGTAAA
CTCCTGTGATAGGAGTTTATCTGCCTCGTCAAGCACAATGATGCGGCAGCGGTCAAGCTTCGCCGTGC
GCTTGCTCGCCAAATCCACCACACGACCTGGG
GTCGCCACAAGCACATGGACAGGGTTTTGAAGACGCAAGATGTCATCACGCAGGGTCGTTCCACCGGT
TGTAACCATCACCTCAAGTCCTGAAATGTGTT

TCCCAAGTTCCTTTGTTACTTGCGCCGTCTGCAGTGCCAACTCACGGGTTGGAACCATCAAAAGTGCC
TGTATATGTGGCTCTCGTGTGTCCACTTTCTC
CAAAACGGGAATGACGAAGGAAGCCGTCTTCCCTGTACCATTTTTGGCACGTGCTAGCACATCTTTGC
CTTGCAGCGCGACGGGGATTGCCTCCTCTTGC
ACAGGGCTCGGCCGCTCAAATCCCTTTTCAAAGATGCCCATCTGCAGTTCACGCCGTAAACCATATTC
TTCAAAGGTGACGTTGCGCCGAGACTCAACAT
CTTCTGTCTTCTTGCGTGTATCCTTCTTGGGTGCAAGGAGGTTCTTCTTCCAGTCGTCTTCCGAAGAC
ATTATTCTTCTCCTTCTTGTGCCTTTAAAGAT
GTTGTTTGCTGTTTCTTCCTTCTTTTCCGCTGAGGGGATGTGCAATCCTTTTCCCTTCTTTGCTTGAG
TGGTTCTCTTCTTATTATTTTGGTTGTTTTTC
CTTGCAGTTTACTGCAGATGACGTCGGGTAAAGGCACCAGAGAGAAAATTAAAATAAAATGGAAGATA
ACGATGACAACAAAGGGGGATAATAATAAAAA
AAAACAACGAAATATGACGGGCGGTAATAAAAATTTCTCTCAAACTTCTTTTTTTTTTTTTTTTTCCT
TCTCGGCACGCACGTGATGTTTGATTTTTGTT
TTTGGTTCCTTTTATTTTCCACCCCGTTCACTTTGCTCTCACGCCCTCCCTATTATATATATATATAT
GCANNNNNNNNNNNNNNNNNNNNNNNNNNNNN
NNNNNNNNNNNNNNNNNNNNNNNNNNNNNNNNNNNNNNNNNNNNNNNNNNNNNNNNNNNNNNNNNNNN
NNNNNNNNNNNNNNNNNNNNNNNNNNNNNNNN
NNNNNNNNNNNNNNNNNNNNNNNNNNNNNNNNNNNNNNNNNNNNNNNNNNNNNNNNNNNNNNNNNNNN
NNNNNNNNNNNNNNNNNNNNNNNNNNNNNNNN
NNNNNNNNNNNNNNNNNNNNNNNNNNNNNNNNNNNNNNNNNNNNNNNNNNNNNNNNNNNNNNNNNNNN
NNNACACCTCCCTCTCCCTCATTTCCCCATGT
ATTTTGGCGGTCGCTTCTCCGCGAATGCCCGCAGCCCCTCCACACGATCTTCCGTCTGCAGCACAACG
TCGTACTGCGCCTCCTCGCCCCGTAGTGCCTC
CTCACGCGGCCGCCCAAACCCGTGACGCACCGCCGCCTTCGCCGCGGCCAACGCCACTGGCCCATTGC
GTGCAATACGTTCAGCCAGCGACAACGCGGCC
GCGGCCGCCCCGCCCTCCGGGACGACTTCATTCACAAGACCAAACTGCAGCGCACGCTCGGCCGTTAG
CTGCTCCGCGGTCAAGATAAAATACAGCGCGC
GACTGAGACCCATTGCCATTGGGGCACGGTACGTCCCACCAGCACCGGGAATAATCGCGAGACTCGTC
TCCGGCAGCCCAAACATCGCCGCCGCACCGGC
CACACGCATGTCGGCACAAAGTGCCAATTCACAGCCACCACCAAGGGCTTTGCCCTCAACCGCGGCAA
TTGTTGGCATCGGCAGATCCTCAAACGCGGTG
AAGGTGTCGCGAAGGTCCTTCACAAACGCACGTGACGCCTCAAGTGACATGGTACGACGTTCCTTCAA
ATCTGCCCCGGCAGAAAAAATACCGTCGACCT
GGCTCTCAAGCAGAAGGCAGTGGATGGCATTTGCCGGGTCGCGGCACATGCGGATGCACTCGCTTAGT
TCACGAAGGAGTGTCTGACCAATCGCGTTTTT
TCGCGAGGGACGGTTGAGTGTAAGGACGCCAACATGATCGGTCATCCGTGTGAGGCGGCATTCGCCAT
CCAGGAGAAGATGCGCCGCCGTCGTGTGAAGC
CACATGGTACGCCGCATGACTGAAATCCCCAAAAGACAGCCCAGCACGCAAATAAAAAGTCGCCCCCC
CCCCCCCCCCGTGTTTTTTCTTTGTGTGTCTG
TGCCTGCGCTTTTGCACCGTCCCCTCCTTCCTCAGCACCGGGATAATATCACAGGGGCTTCTCCCTCC
CGTCGCCACAGACAGGTAGGCATGCCCATTGC
GGTGCAATAAAGAAAGTGTAAAAAAAAAAAAAGGAAGCAACGGGCTCAAGACAAAGAAAAACTCGTCA
ACTGCGGCTCATTATCTCACACCCCAGTCCTT
TCCCTCTTCTCCAATCCCGTCTTTTTTCACTCAGTCCCTTTTATCAAATACCTCTCATGCACACGACA
CGGCCGCCGCCGCCGCCGACGAAAAAGAACAC
ACACCCTTCGTGAAATCGACCACAAATGCACCCATTTCAAGTCGTGCAGCGTCGAGAAAGACGTCAAT
GTGATATTTTCCCGCCTTACGTTTCAGCACCG
GTCCATCCACCTGCACGGCCACCGATCCATCCGCATTACGCTTCGCCGGCTGCTTTACAAAGAGTGGA
TGACGCTGCCCAAGTGAGACGTGCACGCCAAA
TTGACGCTCAAACGCCGTCACATCGCTGACAATGCGGCCCCTGCCCACATGCGGACTGAGACCAATAC
CAAGTTCTCGTACGTAGCACGCTCCCTCTGCC
TGTCGCACCAGACTGACCAATTCCACAAGCTCCTCCGGCGCATTGGGCCCAATGTCCGTGACGATCCC
ATTCTCGATGCGCATTGTCACCGGATTTAGAT

CCGACATGGTGACTTCCTTAAAGAGGCTCGGGTATGCAAAGACGGAGCAGGATCCGTTCAGTTTGCTC
AGGTCAAAGGACTCACTGATGACCTCTCCAAC
AGGGAATGTTCCGCCAATACTGCTTCCTACACACGCCCCGCAATCATTGCCACGACGGCGAACGCCAT
CGTCATTCTCATCAGATGGGGAAGAAGTGCGT
CCCGCCGTATTTTTCTTCCCTGTACGCTTTAACATTGCCAAATACTCCTTCTTTTTAAACGCCCTCG
TTTCTCTGCATCGCTGGCCTTGCGCATAGATG
CAATGTCTCCCTCCTCTTTTTCCTCCGCCCCTTCTTCCACTTTATTCGCCTCACCGGCATCACGCACA
GGAAAGCGCTGACGGTGACCCTGAACACAATT
ACGCTCCGGTGCGTGTGCGGCGGCATAGTAGCCGGTGTTGAGCAAACACTCCTCCAGTCCGCCTTCAA
AACTCAACACTTGACCGTCTTGACATATGATA
CGAAGTGGTGCCCCTGGACCGCTAATATTCCATGACGCGGCGGAAGGATCCATCCCCGTGACCGTGTT
CTTCTCGTAGCAACTTCCCTCCCAACGGAGCA
CTCCCGCGTTGTAGACGGACAACAAATCGGCGTTGGACAGTGACTCATAGAAGGATGCGGGCGGTACC
ACGTCCAATTTGACGCCATCGCTTCCATCAGC
AGCAACGGTTCGTCTCGACCAGCTCAATGCATCAATGGAATCGGCAATGGCGCGTCCCATTGACTCCG
CCACATGTGGTTGCAACGAACATGATTGCATG
TAATAAATAATCTCTTCTTCCTCCAAGCATCGTTCCCTGCTTCCGCCATCCTTCCCGCTGGCGAGAGT
ATCTGCCGTGCGACTCATACACTCCAGGTGGT
TATGTTCGGCGACTCGGAGACCCATTTTAAAGAGGTCCAGTCGCAGGCGGTATTTCTCCAGTTCATTC
TGAAAAGCATTACGTTGAATGAGTATACAGCC
CAGCAACTCACGTGGCTGCCCCGCACGCAAAGTGTGAAGAAGGGCATCTTTCCACTCAACAGAGCCAT
CGGCATTGTCGTCCGCACGGTATGGCGACAAA
ATGGGTGCCAGTGAGTCAAGGACACCTTTCATCTTCTCTTCTGACCACTGTCCACCAATTCTCCCTTC
TTCCGTATCAACGGGCGCACCACACTGACGCA
AAAACGCCTGCAATGTTTGCACATCCTCCAAGAAGGACCCTGCGGAAAAGGGCAATACACCGTATGTC
ACCTTCCAGTCCTTGTCCAATCCGTTGCTGTA
GGAGGGCGTTGTTGCAAGAAGGTCGAGATCAAGACGAGGCGGGCCTTCTACCGGGTTGGACGACCCCA
TGTTGATGGCGTCCAGTTCCTGCATATAACCC
TCAAGAAGCACCTTCTGCAGCGTACACGGCAATCGGTCTACCACGAGCAGTACATGTGGGACGGCGCC
TTGAAAAGCAGAGAGACGGAGACTTTGCCACA
ATATCGTCCGTGCACAGGCGCGTGCTGCCAACACCGCTGTTGACATGACGATAAAAACAAAAGGGAAA
AAGAAAAAGAAAAAAACAACAACCCTAAATAC
TCAATTCCTTCCTTCTTTGGTTTGACGTGAATGGTGGATAAAGTTCAACCTTTCCAAGGTCCAACCAA
CCACCAACACAGAAACCAAAAAAAATAAAAA
TAAAAAATGAGGAGGCGACGGACGCATGAACACAACAAAAGAAAAAAAAAAGCAACAAATAGAATTGA
CCACTTGGAAGAATGAAAAACAACAACAAAAA
CAACTCCGTGAAGGGAAAAGAAATAAAATTACAAAGAGCTGCAAAAAAAAAAAAAGCAGAAAAACCAC
AAGAAAAAATCCCAGTTTCCCCACTATGCCAA
TAAAACCCAGGCACACACACACACACAAAGCCACTGCGTATGCACGTCCGTATTTATCCACCAATGGC
AACAACAACAACAACAACAGGTCATGACGATC
CCACCCTCACCAGAAGCGCCACCAAAAAGGGTAGTGGCCTGCAAGATCATACGGCAACGCATGCGACT
TTTGCAGTGCCTCCGCCCGTCTATAGAAATGA
CGACGTGAAATGGGCACGCCGTCGACCATTTCACTCCCAACGACGCGTCTTGCAATTACCACAACATT
GGCGGAAGCCGGCACGGGTATCTGAAAAACAT
TACCGCGGCCAAACACTCGACGGCATCTGACCATAAACTCGGGATCAGGTGCTGGCAGGTTAAACGCC
GCCACACCCACCGACGATAACACACCATGACA
CAACTGCAAAAATGACGAAGAAGCGAGAAACGCGGGGGGCGTGCTGCCCACAAAAAGATCAACAAAAA
GAACATCATAACGCTGTGCGCGCTGTCGACCC
TTGACAACTACAGGGGCCTCCTTGACGAACGGTGGTGAAGCGCCGGAGTAAAAAACAGCGGCTGCGTC
CTCACTCAGCCCCGTGACGGCCCCCTTTAGGA
ATGCGGCTGCGTCCGAGACATGGCACGACATCGCACCCCCTCGCATCGTCTCGCGGAGGCCGAGGTCT
TCCACTGCTGCCCGGAAGCATTGCGGCTCTGC
GTCGACCAGATCCAGCCGCATGATGTCCCGTGAAAAGTGTTGCTGCAGAAATGATGGGAGGGAACCAC
CACCGACGCCAAGCACCGCCACACGTAATCGC

GGCAACGGCGAAGGCAGGAGAAGGAAAGCACTCAACATCATGCGAAGATACGGCGCCCCCAAATAACT
CGCATCGGCCACACAGTTCATGGATGGTGAGG
CAAAGGGGTCGCAGCGAATAAGCGACTGGTTTGTCGTTCTATCCGCCAAACGTCGCATCACGTCAACG
GGTTCACCCGCAGCTGAAAAGGAAGACGACGG
CTCCATTGTCATAGCCGCGTACTTCGCCGTACCGAGTGGCCATGTCGAGGAGGCAACAAACGGAGAAT
TTACTGCGGTGGTGGAAGTGTCCGTGTCTGGC
AGCGGCGCCCGTGAATCCCCCACCACCTCGCGCACGAGATGCAACAAATTCTCATCGGCCGAAGAATC
CCCGCGATCGGCGGCGGTGTGCGTGTCATTTG
AAAACAAAGAAAGCAGCCTTTTGAGGAGCGTTTCGCCGCCGTCGCCGTTTCTGTGCACGTCATCCTTC
TGCACGCGGGTGTAGTAGTTCGACACCACAAC
GGAGTAACCGGGGAAGGCCTGGCAGGGGTATCGGGCGTATTCCTCCACCCGTTCGATAGCGCTGGCAT
CAAACAGGGGAACGGTGTCTTGCCGTCGTGCA
CGACGCAGCCACCCCGCCTCTCCATCACTAAAGCCGCCAAATGCAAATAAAACTGCTGTCGCCGCTCC
CGCAAGAAGCCCACACGCACGACCACTCAACG
AATTCACGAAGTACGGCATGCCTCCGACATACAAAAAAACAAAAAATAAATAAATATAGTGGCGGTGT
GTTTATGTGCAACCGCCAAAATTAACTAGCGC
AACAACAACAACAACAACAACCAGTAAGTGGGGGGATGGGAAGAAAAAAAAAGTCGGTGAATCAACAA
ACGCAAGCCAATGCACGGCACAAAACGACACC
CAAAGGCAACAAAAGTAAGAGGGAAAGAAAAACGGCCGACAAATTGGGGTACCAAAGTGGCAGTGATG
CATTCATCGAAATTCCCACATAAAAaAAAAAA
AAAAAAAGATGATGTTGATGATGATAATAATAAAGACACAGAAAGAGGAAGAGAGGGCAAAAGAATTT
GCCACTGAATTTATGCCTAGCCCATGTCCATG
CGCAGAGGGGAAGAAACAATCAAACAAGCAGCACGCTTCCACAAACGTGACGTTCATACCAGAGCCAA
ANNNNNNNNNNNNNNNNNNNNNNNNNNNNNNN
NNNNNNNNNNNNNNNNNNNNNNNNNNNNNNNNNNNNNNNNNNNNNNNNNNNNNNNNNNNNNNNNNNNN
NNNNNNNNNNNNNNNNNNNNNNNNNNNNNNNN
NNNNNNNNNNNNNNNNNNNNNNNNNNNNNNNNNNNNNNNNNNNNNNNNNNNNNNNNNNNNNNNNNNNN
NNNNNNNNNNNNNNNNNNNNNNNNNNNNNNNN
NNNNNNNNNNNNNNNNNNNNNNNNNNNNNNNNNNNNNNNNNNNNNNNNNNNNNNNNNNNNNNNNNNNN
NAAAAAAAAAAAAAGATGTCGATACATAGGCAC
AATAAACAACCACCACTTCACCCGCATAAGGAGGAGGTGGTGGAGTGATGATATCACGTATCCTTCTT
ATGCACTGTGGTGGATTCCCTTCCACGCACAC
GCCGAACGATTTCATAGTGCACCTTGGAGTAGGCTGCCATGACTAGCCGCAACCACCCAGAGAAGAAA
AACTGGAGGGTGAGCTCCAACAAACTTGTCGC
CGGCTCGATACCAGCAGATCCCAAACCCAAAAGCACGGCATCCATTCCCAGGGATACTTCAAAGCGAT
ATCGTTTAATATTCTTTACGATGCATTTTGCC
ATAGCCTCGGCTGTGTAGGGGACATTGCCACCAAAAGAAGAAATTGCCTTACACTCAGGACTCTTGAG
AAGGTTTTCACTCTGTAGCCCTGGCGTCTCAA
TATCCGGCGGGCAAACAACTTGGCATCGCACCCCTAAACAACTGTTCTCCATGTCGAGACTGTGGGCA
AAGGCACGCACGGCGGCTTTGGAGGCGGCGTA
AGACGAATATCCGGCAATTGCACAGCGTGAAGCTAAACTGCTCACCAGTACAATGCGTCCATAATGAC
GTTCAAGCATCGCCGGCAGCACCACACGTACC
ACATGCACACATCCAAAAAAATTCACCTCCATCATTTTGCGGACATCCTCAGACGATGTATCAACAAA
ACGTGCTGGAATTGAAAATCCGGCGTTGCACA
CAACGAGGTCAAGTCCTGCACCACCAGTCATCGTTGTCTCAATCTCCTCCACAGCGTGAGCAACAGAC
GCCTCATTGCTCACATCCATCTCCACGTAAAA
TACCTGCGTGCCGGTCTTCCCTACCTCCCTACGGAGCTCCTCAGCCGCCGCCCGCAAAAGATCCACCC
GACGCGCCGATATGACAACAACTTTCGCACCC
TGACGAACCAATTGTCGGGCGGTCTCTAAACCAATACCCGCGCTGCCGCCAGTCACCAACGCACGGCA
GCTTTTGATGTTGAAGGGGGGCACGTGTTGCA
CACTCCACGCCGCGAGAACCGCCACAGCTACAACCAAGGCAAATCCAATGGTGACAAGTAGTGAAAGC
AGCATATTTCAAACAAGAAACAACTAAAAGTC
GCATAAAATACAAAAGAAGTAAAAGAACGGAGTAGACCACGCACGAGATAAAGAAAAGTCAAATACAC
ATATAATATATACACGGACACGCACACACAAA

GGAAAAAAAAAGGAAAGAAGAGCGAAAAAAAACAAACATTGTACAATCACCACTTACAATTACGCATA
ACTGAAGGCAATTGGAAATATAAATAGATGCA
TTCAAAAAAAAAAAAAAACATAAAAAAAAAAGGAAAAGAAGGAAATTTGCGCCTACCGTCTTTTTACA
TCACTTCTGCCTTTACTTTTGAGCGCAGAGGA
CCTCCATACCTGCGTCCCACCCACCCACCAATCCACCCACCCCTTTAAAACAAATTAAACTCCCTTGT
AATGCGGACCGCACATCGCACGGTGCATTAAA
GACGGCACGTTGATGAATACCCAATTTTTGCCCGTGCCAGAAGTAACAGCCAAGCGTCCCATTCCTCT
CTCTTCTTTCTCCCCTCTCTCTCTCTCTCTCT
CTAACTTTCTCTTCTCACGTACGCACTCGGGTGTATTATTTGGTGGCATTACGATATCATCTTAACTT
CCATTCATACCCCACCCACCCTTTTATTGCAC
GCATTCGTCCATTTCCCATTTTTCATCTAAAAAAAGGAGTACTTCCGTATCCTTTTTTTTTTCCTTTT
TTCTCCCTTCTCTTCATATAAATTAAAAGCAA
AGACACACACAACAACAATAAAGAAAAAAGAAAAAAGACATTGGCGTAGCACTCCTAACAGGAGACAC
AAATAATTATTTAAAGTAAAAGAAATAATAAA
TAATAATAAATAAATAAATAAATAAATAAATAAATAATAAAACAACAAAAACAAAAACGCAAAAAAAA
AAAAAATGGGNNNNNNNNNNNNNNNNNNNNNN
NNNNNNNNNNNNNNNNNNNNNNNNNNNNNNNNNNNNNNNNNNNNNNNNNNNNNNNNNNNNNNNNNNNN
NNNNNNNNNNNNNNNNNNNNNNNNNNNNNNNN
NNNNNNNNNNNNNNNNNNNNNNNNNNNNNNNNNNNNNNNNNNNNNNNNNNNNNNNNNNNNNNNNNNNN
NNNNNNNNNNNNNNNNNNNNNNNNNNNNNNNN
NNNNNNNNNNNNNNNNNNNNNNNNNNNNNNNNNNNNNNNNNNNNNNNNNNNNNNNNNNNNNNNNNNNN
NNNNNNNNNNTGAAAAGAAAAAAACTGCTTGA
CCTTTTTTTTTTTTTTTTTTTATTTCCTGCCATCAACAACCTCTTACTACTAATAATACCATTTTATT
TTTCTTTTTTCATTTTCTTCTTTTCTCAGCCA
CACACCACATGAAAACAATAAAATTACTTTTATAATTTTGTGTTATAAATTACGGCTATTTCATTCTC
CTCTTCATCTACCTCCCCCTCCCACCCACCTC
GTTTTTTTTTTTCCTTTTCGCTGGTCCCTTGTTCCCTCGCCCAGGCACATGTACACCATGCATGCATA
CATGTATACGCCCCCATGTCTCTTAAAGAGCG
GTGCGGATACTCGGCTTGATGCCATCGTACCAGGGCCATGCCGCTAAAAAAAAAAAAAAAAAAAAAAA
GATACATACCATAAATACATACATAAATACAG
ATATATTAAAGGGCATCGATACAAAAAGACCGGCAGTTAAGACATTTTCATTCCCTTTCTTTTTATTT
TTTATTTTTCTTTACACAAACTGTTTTTATTA
TTTTTTTAATTTTTTCTATTTTTTTTTTTTTTGGAGGGGGGCTGTAAAAACCTCTGAAATAAGAAGAG
GTGCTACACACAATACAAGAAAGAAATAAAAA
AGTCAGCCCCAGAAACAAACAAACAAACCAAATTTAAAAAAACTATAATGACAGGGGCAAAGAGTACA
ACAGAAAGGAAAATCACTATATATATATATAT
ATATACAGACGATACTGAGATCTTAGTATTGGTTATTCATTACTACTCTTGCAAGGACTTTGGAATCG
GGCGAAGCTGAAGCTGCACGTGCTTAGACATG
TCCTTAATGACATCCAGCACTAATTGGAGATCATCACGGGTGTGTGCCGCGGAAACGCAGAAACGTAT
GCGTCCACCCAGAAGTGGTGTAGCCGGATACC
CAACAATGACAATTGCAAGGCCTCGTCGCATGCATTCCCTCGCCATCACTGGCATCGCACCCGTGCGG
TACAACATAACTGGGATCACCGGTGAGGAGTC
GTCCCCAAGCACAATGAGGCCTTGGGCGATGAGACCCCGTCTTAAAAATTGGGAATTCTCTTTGAGTT
GATGAATGCGTTTTCGACCGATATCCGTCCCG
TCACGACCAGAAATCACTTCCAGGGCAGCCAACACCTGCTGTGCACAAGGAGGGGCAATCGTGTCGCA
GTGAAGAGAGATGGAGCTGTGTATACGCAGGT
AATCAATTACCCTCTGTTCCCCAGCTATATAACCACCAATGGCGCCAAAGCTCTTTGTGAAGGTACCC
ATGAGAATATCCACATCCTTCGGATCAACGCC
GCAGTGTTCCGTTACGCCACGACCCGTACGCCCAATCGCACCAATGGAGTGGGCCTCATCTACAAACA
GCAGGGCTCCGTACTTCTTCTTGAGTGCCACA
ACCCGCGGCAAATTGGCAATTTCGCCCTCCATACTATAAATACCCTCAATGATGATAATGATCCGAGT
CCACGGCAAATACTCCCCGCGGGGGTTTTGCC
CAAGAACCACCGCCTCTCGCAGAATTGTTTCCAATTGCTGGAAGTCGTCATGCTTAAATACCTTTGTG
CGTGCGCCGGACATGCGTATGCCGTTCACCAG

CGATGAGTGGTTGAGGCTGTCTGAGATGACCAACGATTCCCTCCCAAAGAGGGCGGGAAGCCCACGAA
AGTTTGTACCGAACCCCATGCCACAGACAATA
GCATCATCCTTGCTCAAAAAATCAGCAATGGCGCGTTCGAGCTGCACAACGGGTTGGTGTTGCCCTGC
CAGCTGCGGGGGACTGCATGATGCAATTCCAT
ACCGGTCAATACTGTCAAGCACTTGGCGCGTAATACTCGGAATATCAGACGCAAAGCCAAGATAGTTG
TAAGAAGAAAGATTTACACAAGGTAAAATATT
CCCAGTTAAGGAGAAGTTTTCATTGTAATCGTAAGAAACACGCTCCATGACGCCAATGACACGTGATG
GGCGGGAATCAATCGGATAATTAAAGCAGTCA
CGGATACGTTGATAAAAATATTTCTGCCAATATCGGTCAAAACCCTTCAACAATGGCGCATATCCATC
CCGCTCCTTTTCCTTCTGAAAAAGCCTGTAAT
AGAATTCATAAAATAAAGAAAATAAGGTCAACATGCCAAACGACATGTACACCCCAATTTTGAACAGT
AAAGAACAGGACTCCATTTCCTCACCATTCTT
CCTCGGCTCTTGTAGCGGTTGTTCAGCTTGCTGACAGGTCTTCACAACCTCTTGCTCCTCTTGACGTG
CATCATCCGTCTCTTGCGGTGAAAGAGGGGGT
GTTGTGGTGCTCTCTTTGTTGCATGTCTCCGAGGGGGAGCCCATCCAAATGTCGGTCTTGGCCGTGAT
TTGCTCCACTTCCCCCTTAACATCAATAAGGG
TCTCAGTTGTCACGTATGAAGGCATTGAAAAATAGATATAATAACAATGTAAATATAAATACAAATAT
AAATATAAAGTAACCCTTTTATGTTCTAGTTT
TAGTTGTTGTTTTTTTGTTGTTGTTGCTGCCCTATTTTCTCCTTTTCCTTTCTGAGCGCCTTCTCTTC
GTTTCCTTTTTTTTTTTTTTCCTTTTCTTGACT
TATTTTTTATTTTTATTTCCCTTTTCCTCAACTGTCAGCTCACGTCTCTCTATATTTTTCCTTCTCCT
TTTCTTTGCCTCTCTGTCTGTCGGCTTGTTTG
TTTGTTTTGCCCTTATGACCTCAATATTCCCCTGCGCCACGTAAACAAAAAAACACCAATGAAGGATT
AAAACAGAGGTAGAAACAAAAACAAGTAAAAA
GAGTATGACTACTGTGGAGGAAACCATTGGAGACAAATGTTTTTTTTTTTTTAAATGCGCATCGCGTC
ATTAACGCATGCCCAAACGGTAAAAGGTCAAA
AAGAAAGAAATATATCGATGGGGTGNNNNNNNNNNNNNNNNNNNNNNNNNNNNNNNNNNNNNNNNNNNN
NNNNNNNNNNNNNNNNNNNNNNNNNNNNNNNN
NNNNNNNNNNNNNNNNNNNNNNNNNNNNNNNNNNNNNNNNNNNNNNNNNNNNNNNNNNNNNNNNNNNNNN
NNNNNNNNNNNNNNNNNNNNNNNNNNNNNNNN
NNNNNNNNNNNNNNNNNNNNNNNNNNNNNNNNNNNNNNNNNNNNNNNNNNNNNNNNNNNNNNNNNNNNNN
NNNNNNNNNNNNNNNNNNNNNNNNNNNNNNNN
NNNNNNNNNNNNNNNNNNNNNNNNNGGGGTCTCATACCAAAGACCCCCCCCCCAAAAAAAAAAAAAA
TTGCAGTCAACTTTTAACTTTTGCTTTTTCCT
TTTTTTTTCCTTCTTCCCCTGTCTCTTTTCTGCTCATTCACTACCGCCCACACGATGGAGGTCGGGTG
CCTGACGCATATTTTGCCGACAGTGCGCTCAG
GTCCATTCCGGAGAGCCAACTTGCCTGCAGGCGATCATACGACCAGCGAAGGAACATGTCATCCACAC
AACCACAATATCCCTTTGGTCCCTTCAGGCGG
CAGTGACACAAGTCCGCCTCCGCAGGGTTGCTGCATGGCTTGGGGCGGTTCTGAAGGGCAGGAATGGG
CGGTGCCAGTCCGGGGTACGTGGTGTTCTCAG
GCAACATTGTGACACAGCTTCCAATGCAACGAGAAACACCTTCCGACAACAACCATTCCACCACCTCC
GCATTTCCTATCGCCAGGGCGGCATGCAACGG
GGTACGACCCCAGCGATCATACACGTTAATACTGCCTCCAAATTCACGCAAAAGTGAAATGACATCTA
GTCTTCCTGCGATGACGGCGATAAACATCGGG
CTGTAGCCGTGCTGCATCTGATTTACAGCGACATCGGGGTCGCTAAGAAAACTCATATTATTTGGGTC
TGCCCCAAGCTCCAGACAGCGCCGTGCGATTT
CCACACTCCCTGTAACGACAGCCAACAATAGATAGGTGGCCCCCCGGTTCATCGAGTCTTTCTTCTCT
CCACAAAGTTCCGGGCAGAGCTCCTCAAAAAA
CTTAGCAATGGATTTCACAGGCTCCGCGATGCTGTTTTGGGCTCCCGCCGCATTCGTGTTGTTGTTGT
TGCCACCGGCACCGCTGCTCCGATTGCAGTTG
CTACCGGATAGCTTTGCAAGGTTTTCCGTAATGCCCTTTGCAGAAATTTCGTTGGGCAATGCGATATT
CCCTCGAATGGGGTTGAAGGCGCTCCATGGGA
TGGCAGGGGCGGTGCAGAAGGAGCGCAACAGATGTCTGTCGGGGTAGTCGTAATCGGCAAGACTAAAC
ATGCAGTTTTGCTCCATTTCAACGCCACCCCA

CTCGGAGACTTTGCCATCGCAAGCCCCACACATTTCTTCTGTGGGTGACATCTTGTGCGACCGCGGCA
TGCAGAGAACAGACCACCGCACGCCGTGTCCT
CTTCGCCTCCCTTCGTTTATCCCTGTTTATTTTTATTTTCTTCTTCCGCACTCCCTATAGAAGGTGGA
AAGGGATAAAAGCAACAAAGAAGAAAGACAGT
TACAACGAGAACAATAATCGCCAAAAATATTTCACGAGGAATGATGACGGAAAGGCGCCAGAAGAAAA
AAACAAAAACAAGAAAAAGCACCATGCGGCTA
TCAAAAGGTTAAAAAAAAAAAAAAAAAAAAAACGCAAAGAAGCAAACAAACGACAATGCGCCTGCACGC
ATACACANNNNNNNNNNNNNNNNNNNNNNNNN
NNNNNNNNNNNNNNNNNNNNNNNNNNNNNNNNNNNNNNNNNNNNNNNNNNNNNNNNNNNNNNNNNNNN
NNNNNNNNNNNNNNNNNNNNNNNNNNNNNNNN
NNNNNNNNNNNNNNNNNNNNNNNNNNNNNNNNNNNNNNNNNNNNNNNNNNNNNNNNNNNNNNNNNNNN
NNNNNNNNNNNNNNNNNNNNNNNNNNNNNNNN
NNNNNNNNNNNNNNNNNNNNNNNNNNNNNNNNNNNNNNNNNNNNNNNNNNNNNNNNNNNNNNNNNNNN
NNNNNNNAGAATTACACGCGAGCAAATACGTG
TATACAGAGGCACCGTCACAATCCCGCACAGAGCTATATGAGAAAAGGAAGGGATAAAACAATAACAG
TGACCCCCCCCCCCCCAATCAAAACGCATGGA
CGCATAGGAAAAAAAAAAAAAAAAAAAAAAAAAGAGAGAATTAAAGAGGGAATATAAGTACAAATATTGA
TCTAAACACTAAAAGTTTTCGGGAATTGTAAC
AGGACCCGCAGAAACACGGGTGTGGGCTCCCCCCTCTACATTGGAAGCTTCTCCCTGATTCCCATTTG
CTGAGCGACGGAGAATCTCGTTTTTCAACTCA
CCGTAACGAGTGTATTCCGCTGTCCATACCCGTCGGTCATTTTTCGTCGGGGGGCGACCATTCTTTTG
CTCAAAGTCACTCTCAAACCGATGAAGGATGT
ATTTTAAATACCGCTTCTCCGTTGCAAGATCATCGTCAGACATCTGACTCGCTGGTTTCCCTTTACGA
ATAGGCCGTGCCGGGGAGTTCTGCCGCGGCAC
ATTGATGGAAGACGTTACGGAAGCCGGTGCTGTAAATTGTTTACTACCAGCAATATCCATTTGGCTGC
CCCTAATTTGAGAAGGCTGCACCCCAACCGTT
CCGCCCCTTTCATCCCTACCGGCATCTCCGTTATTCCCACCGCCCTCATACACCGCCTGCTGCGGTTG
ATATGGTTCATGTTTTTTTTCATAATATTGCG
GTTGGTGAAGCTCGGAGGTATCTTCTACTTTTGGCTCAAAGACGGTTTTCCGTGGGGACTCCGTCTCC
TTCAGCCGATTTTTCGCCGCTTTGTAAAGTTC
GTATACATGATGCAACGACGCCTTGTCATGCGCTGTCACGGCGACTCCGTGTTCCTCACGGAACGCGC
GCTCCCACTCATGAATACGCCGCTTCATTTCC
TTCTTGAAGACCTGGCACTTGCTATGTGACATGTTTGCCATAAAGGCCGGTGGCACACCACGTTCGTT
TGCTTCCTTCATGAGTTCAAGTGACGTCGTCT
CCCCGCGGTACGTGGCCCTCAGCTGCGCCGCGATGGCGTCCGAGGAGACGGCACGAGGGCCATAATTC
TGCTCAATGCGACTGCGTTGCTCACGCTGCAA
AGAGCGAACGGAATTAATGGTGGCTTTACTATGGGCGCGGAAGTCCTCATCAATGACATGGCTTTGTG
TAAAACTAAGCGCATCAATCGTCTGCGTAGCG
GCCTGAAACAATAAATCATTCATATTCATTGCTCTCACTTTTCCTTTTTCCACCCCCTTTTTGTCCCT
CTCTCTCCTCTTCCCTGCTGCTGAGCCCCGCA
AAACAAGGCAGGAGAGAGAGAGGGCGTTGAATAAATAAATAAATATAAATATATAAATATATACAGATAC
GTAGATGGTTATGTATGTATATATATATATAT
ATACGTATCCTTTGCAACAAAAGTAAGCCAAATACCTTTCTCCAGTTTTGTTCCGCTTGCCTGCGCGT
TACCAAACAACACTCTCTGTCTCTATCGTCAC
ACAAACACACAACAACAAAAAAAAAAAAAAAGACTATGAACACAGACATAATAATGTAAAATAATATTG
CAACACATTTTGCCGCAAACGTCAACACATTG
TACAATTGAACACAAAAACTAACACAGGAAAACAAATAAACAGACAAAAGAACATTGCGCTATTCCTC
ACCCCAAACATGCAAAAAGAAGAAAAAAACAA
GACGCCATTGAAACACCTGCCGCACTCGATGGGGGCCAAAACAAACATCACATGCGTGCGGATTGGAT
CATTGCATCAGACAATGAGGCAAATGACACTT
TGGAATGTGCCATGCATTTCAGCAGTGACGAGGGACGCAAGTGTGGGCCGTGCCAGTTTGACAGTTCC
TCCATTTTCTGCACAAGTGCAGAAATGCCGGC
TTTATCCGCCAGTGCAAGAGCGCCGCCAGTCGTCTCACGAAAACCGATTGCGGCAATCGTCAAGACAT
TTGCGTCCGTGGCACTCCGCACACAACCGGAC

AATATCATCTCGCAACATTCATTGACCACTACCGCAAGCAGTCGATCCGCCACCTCGGTGTCAGACAA
AGGGCGAGAAAAGTAACGGCGTAATACCTCGG
CGTTCGGTGCGATTGCCGTGCCGTCCTCACGATACGAGAAAAAGCCCCCACGACGTGAATCGACACCC
AAGTATCCATCGTTAAGCATGCCATTAAGTGC
GTGGTGAGCAATGGCAGGAAGCAAGTCAACCAGCAATGGATTGCCCTGTGTGTTCATGTTGTTTGTCT
CTTCCAGCATTCGAATAACCATTGCTGGGCCA
AAGTAGTCAATCAGATGAAAAGGTCCTACGAATAGGCCAAGCACATCCGTAGCCACGCGCTCAATGCA
CGCCACATCACAGTGCTGAGTGAGTCGACAAG
CCTCCGTCGCTAAAGCCGCAATAAGCCGTTTAGAAGAAGATCCACGGCTCACAACATAAGGGCTGTCT
TGAGTCTGCAAATACGTGAGCGCCGCATTCAA
GGCGGTCTCAGAGGCCTCTGATGAGCAGCTATCCTGAGCAAAGACACGAACTTCCTGCAGTCGTCCAA
GAGCAGAAACACGATAGGGTGAGGCACATGAT
ACAACAACGCTGCATGGTAGCGTTGAGATAATAGGACGAGCAACGTGCTCCTCGCCAATAAGGACCAC
ATTGACATTCACCAAAATGTCTTGGTGGGAAG
CTACAATCTGTGCGGTGGCATTAATAAACCTCTGTGAGCAGTCCAGTAATACTGCCGCGCAGTCATCC
TTTGCGGAGGACAATGCTTTCATTTTTTCATC
CCACTGCTGCATCTCCTCTCCACGAAATGTTACAAAGCGCCGACTGGGCAACGAGGGAAGAGCACGGC
GCTTCATTTCACATCCTAAACTGCACACATTG
GCTGCGTTTATCGCAGGAGTGGTGTATAACATCTCACTAAAGATACTCTCAGACCTCATTTCCTGTGT
ACGCACTGCTTTGCCATCATTGTTAGTAGCAC
ATTTCCCCTGCACGATGGCGGCCATAGCATACCGTCCCCACGCTTTCAGCAGTGGGTTGGGACTCACC
AGGACTGCAGATGTATCAAAGCCACTGGTGAA
AAACAATTTGCGGAGCAGCAGTTCTTTCTGTGTGGCTCTGGAAAGAAACTCGAGCATGGCGCACTCAA
GACGGAGAAGAAACGAGGGAGCAGGCGGGGAG
GAATGACCTTCCAGAAGGCCAACATCCATGATGCTCTCCACACTGTATTCATGGAATCTCGGTGCCAT
CTCAATAGTGCGCTGTGAACCAAAAAAGCAGC
TTAGCCGGCGCACGGTGCTTGCTGCAGGAAAATCCCCTACTTTAATCGATGGAAACCCAAAAGTTGCC
TGGGCGGGATCCAGCAACACACGGTGATGACA
CGCCAAAAACAATTCCGCTCCAAAGTCTTCAATAGCACCTCCATGAACATTTGCAACAAAATGAATTT
GTTTCGAAGCATTTTCCATGCGTGCAAAAAGA
CTTTCCTTGGCCCGAAGCAACTCCACACGCTCACCCGACGTCAACTCCAACGGGCCATGTTTGGCGTT
AAAGAAACCTAGCGTAGGGAAAGTCGTGAACA
GTACTTCCGTCTTCTTACCAGGAGCTATGTGTTGATTTTCAATGAGTGTAATTGTCCGATGCAAAAAC
TTCACAAGCGACATGATATTTGTTGGTTGGGT
CTCTAACAGGATGGTTGTTACCGGTGACCCATCGTTCTTAAAAGACACTTTCGCGCAAGACGCGTCTT
CTCCATCCTCCGGCCTAACGCTTGGCATCCAT
GGAGCCAACGGTGGTCGTTCCACTGGCATCTTTGTTGCCGCATTCCTTGGTGACGGAGTTGCAGTTTC
ATTTAGTGGAAATGATGATGATTCCTCCATCG
GTTTCAAGGAAGCTGTCGTCGTGGAATGCTCTGTTTTTTCATTATTTTTAGAACTGTCCTTCGTTGGC
ATTGGCGGTGTAAAGTCCGCCCGTTCACTGAC
AACGTCTGGAATTTCATGCCGCCCAATCTCCGCAGCCTCCTTATGAGGTAGCGGATTCAACTTATTCT
TGTCAAACTCCATATCAGTCATGGACTGTTCG
TCACCATTCACAACGTTTAGCCTCAGTTTCGCATTTGTTTCCTGTGCAACATCAGGAGAAGGCACAAG
TGGACGTGTTGCACGCGCAGTTTCAATTTCTT
CATCCACTGAGAAGCCAACGCTTTTGGGCCACGTCCTTCCCTGAAAACCTTCCTCTGACGTCCGTGGT
GCAATGGTGAAAAAAATATCATCCTCACCTTC
CTGTGAGGTGCCGACGGGAGCTGAAAAAAGACTCTCGCCAGTTCCACCTGTAGATGGAATGGGTGTAC
TGAAAGAGGGCAATGTCTGTTGCGGTAGTGAT
GGAGTAGGTGATGGCGGTAATGGTGCCGAAGGCGCAATAGGTGGTGAGGGAGGAGACGCAGTGTGAGA
AGGCTTATCGGTCCCACGCTGCGCAAACACCA
CCTTTATTTCTCCCACATCATCAATAATATCATTAAGAGAAAGGCGTTTACTTCCCACTTTTGCCCCA
GAGCCGGTGGGTTTACCCCTGAATTGAAAGGA
CTCACCACGCTTACTCGGATGACGGTCCTCACGTTCTCCGCCACGCTTCCTATGACCACGTATTTTAT
TATCCTCAGTACCCAAACGAACATTCTTACGC

GCTGAGACAAACGACGAAGAAGATGCTGTCCTTGGTGGTTCTGGTGCAGCAGGTGCAGCCCTTGGTGG
TTCTGGTGCAGCAGGTGCAGCCCTTGGTGGTT
CCGGTGCAGCAGGTGCAGTCCTTGGTGGTTCTGGTGCAGAAGGTGCAGCCCTTGGTGGTACCGGTGCA
GCAGGTGCAGTCCTTGGTGGTTCCGGTGCAGC
AGGTGCAGTCCTTGGTGGTTCCGGTGCAGCAGGTGCAGTCCTTGGTGGTTCCGGTGCAGCAGGTGCAG
CCCTTGGTGGTTCCGGTGCAGCAGGTGCAGTC
CTTGGTGGTTCCGGTGCAGCAGGTGCAGCCCTTGGTGGTTCCGGTGCAGCAGGTGCAGTCCTTGGTGG
TTCCGGTGCAGCAGGTGCAGCCCTTGGTGGTT
CCGGTGCAGCAGGTGCAGCCCTTGGTGGTTCTGGTGCAGCAGGTGCAGCCCTTGGTGGTTCTGGTGCA
GCAGGTGCAGCCCTTGGTGGTTCCGGTGCAGC
AGGTGCAGCCCTTGGTGGTTCCGGTGCAGCAGGTGCNNNNNNNNNNNNNNNNNNNNNNNNNNNNNNNN
NNNNNNNNNNNNNNNNNNNNNNNNNNNNNNNN
NNNNNNNNNNNNNNNNNNNNNNNNNNNNNNNNNNNNNNNNNNNNNNNNNNNNNNNNNNNNNNNNNNNN
NNNNNNNNNNNNNNNNNNNNNNNNNNNNNNNN
NNNNNNNNNNNNNNNNNNNNNNNNNNNNNNNNNNNNNNNNNNNNNNNNNNNNNNNNNNNNNNNNNNNN
NNNNNNNNNNNNNNNNNNNNNNNNNNNNNNNN
NNNNNNNNNNNNNNNNNNNNNNNNNNNNNNNNNNNNGCAGCCCTTGGTGGTTCCGGTGCAGCAGGTGC
AGCCCTTGGTGGTTCCGGTGCAGCAGGTGCAG
TCCTTGGTGGTTCCGGTGCAGCAGGTGCAGCCCTTGGTGGTTCCGGTGCAGCAGGTGCAGTCCTTGGT
GGTTCCGGTGCAGCAGGTGCAGCCCTTGGTGG
TTCCGGTGCAGCAGGTGCAGCCCTTGGTGGTTCCGGTGCAGCAGGTGCAGCCCTTGGTGGTTCCGGTG
CAGCAGGTGCAGCCCTTGGTGGTTCCGGTGCA
GCAGGTGCAGCCCTTGGTGGTTCTGGTGCAGCAGGTGCAGTCCTTGGTGGTTCTGGTGCAGCAGGTGC
AGTCCTTGGTGGTTCCGGTGCAGCAGGTGCAG
TCCTTGGTGGTTCTGGTGCAGCAGGTGCAGCCCTTGGTGGTTCTGGTGCAGCAGGTGCGGTCCTTGGT
GGTTCCGGTGCAGCAGGTGCGGTCCTTGGTGG
TTCCGGTGCAGCAGGTGCAGCCCTTGGTGGTTCCGGTGCAGCAGGTGCAGCCCTTGGTGGTTCCGGTG
CAGCAGGTGCAGTCCTTGGTGGTTCCGGTGCA
GCAGGTGCAGCCTTTGGTGGTTCTGGTGCAGCAGGTGCAGTCCTTGGTGGTTCTGGTGCAGCAGGTGC
AGCCCTTGGTGGTTCCGGTGCAGCAGGTGCAG
CCCTTGGTGGTTCTGGTGCAGCAGGTGCAGTCCTTGGTGGCGCAGATGACGCCGACCGTGCCCTCTGA
AGTTGTAGATGGGACTCACATATAATTCGCAT
TCGACCCCTGGCGTGCCAGCTGGTTGCTGCAATCATAGTGCGCCAGACACTACGCATTTTGCACGGTG
TTACAAGTGCCTATAAGATCACTCACAGAAAA
AGAAACCAAAAACCCTGCTGCTGTTGCTGCCTTGCGCACTTTACCTCCGGTGCTTTCTATCTCTTTTT
CCTTTGTTTTCCCTCCGTGCCGCCGAAGGCGG
TTCCACAAATTTCTTCCACCGACTATGTGGTCAAGCTATACTCTAAGAATATATAAGGAGTGAAGGGA
AAAAAAGGATatatatatatatatattccgtt
ggaaaaggaaatagaccgttatcttgttgcaaNNNNNNNNNNNNNNNNNNNNNNNNNNNNNNNNNNNN
NNNNNNNNNNNNNNNNNNNNNNNNNNNNNNNN
NNNNNNNNNNNNNNNNNNNNNNNNNNNNNNNNNNNNNNNNNNNNNNNNNNNNNNNNNNNNNNNNNNNN
NNNNNNNNNNNNNNNNNNNNNNNNNNNNNNNN
NNNNNNNNNNNNNNNNNNNNNNNNNNNNNNNNNNNNNNNNNNNNNNNNNNNNNNNNNNNNNNNNNNNN
NNNNNNNNNNNNNNNNNNNNNNNNNNNNNNNN
NNNNNNNNNNNNNNNNNNNNNNNNNNNNNNNNTCGTTTTGTTTCGTACGCACACAACCAAGTGCAATG
CACAGACCCACGTGACCCCATCGTTTTTTTTT
TTTCTGTCGCCCTGATTTCACTTGTGCCAACCCCGTCGTTGGATAAGCGGCGCCATCGTGACCGATCT
CAGTCGTAGACCTCAATGGCCACACTTGAGCC
TTTCGGTCCCACCGTGCGGATGACCTCGTAGCACCCCCACACACAAATCGGCAGAAGCGGCAAAAGAC
ACACCGTCGAAAGCGGCACACCGTACAACTTA
TCTTGATATTGTGTCAGATTGACTGGGTCATTCGCACGAAGTGTGTAGAACGTCAGTGTGGTGACGAT
GAGCACCCAAAGTGGTAGTACCTTTGGCGTCA
AGAACATATGAATTTGTGTATGTATGTAGTTTCACTTTCTTATCCTTTCCTTGTTATCGCGTATTAAT
AGTGGCAGAAAAAATCTTGCTTTCCTTTCCTT

TTCTTTTCTTCTTTTTTGTGTCGCAAAATGAGAAACCGTTCTTCTACAGAAAATATATATATATATAT
ATATATATATATATATATATNNNNNNNNNNNN
NNNNNNNNNNNNNNNNNNNNNNNNNNNNNNNNNNNNNNNNNNNNNNNNNNNNNNNNNNNNNNNNNNNN
NNNNNNNNNNNNNNNNNNNNNNNNNNNNNNNN
NNNNNNNNNNNNNNNNNNNNNNNNNNNNNNNNNNNNNNNNNNNNNNNNNNNNNNNNNNNNNNNNNNNN
NNNNNNNNNNNNNNNNNNNNNNNNNNNNNNNN
NNNNNNNNNNNNNNNNNNNNNNNNNNNNNNNNNNNNNNNNNNNNNNNNNNNNNNNNNNNNNNNNNNNN
NNNNNNNNNNNNNNNNNNATATATATATATAT
ATATATATATAATATAAAATCTATATAAAATAAGGGGCAATGAATTAGAGGAATATAAAAAAAAAAAA
AAAAAAAAACTCCTCATCATGGGCATGTTTCA
TTTCACCACCAAACGTTTCAGTTTCAGTTTCACAGTACCCCGAGACCGTTGTCGTTGCTTTTATCGTT
TTTATTTCTTTTTTCTCCGCTGTTTGTTCCCT
CCTTCTCATTAAGCGCAAATGCAAAATTCCTTCAAACCCATCCGAGACATGTACACTAGATCACCCGT
TAGCACACCCCACCATCTACACCCACACCTCG
CGCAGAAAAGCGTGGCTGTGAGAGGCCAGTCGGGCGGGGGGAGGGAATCACCCGAAATGAGCAAACGA
GACGGTGATGTACAACAGCGGTTGGTGCCACT
TTTCAGCGCCCCGCTTCCGGTGACGACGTCCCCCGCCAAAGGGATTGAAGGTTGGCCACGTAGATCCC
TCCGCAGGCAAACACCAAAACAACGGCACCTT
CACAAGTTGGGGCTCAGAACCGGGAAGGGCAACACCATCGGGAATTCGCCACTCCGCGGCTCCAAGTA
ACTCATCGCCGCTAGTGTACAGCGCCATGAAG
AGCGGGGCAGTGACACTGTACACACCCAGCTGAAAGGATTCCTCCCAACACGTACATGCATGGCAAGG
GTTGTTCTTAGCAGGACCGCTAGGAACCACAC
CGGCACGACTACGCACCTCTTGGAATCCGTTCGTAAGCGATAACACGGCACCGCTGCTGCGGATACCC
TCCACCTCACGAACATTTATATACGCCACACC
GATATACTGCGGCGGGCCATCATGATTCAGGGCCCCGTGCTTTGATATGTCCAAATGACCCTGCACGT
CTCGATCGGGTGGCGGTAGCATGGCCTGTAGA
ACATGTGGATTACGTTCCCTCGTAAAGGCTCCTTCATACTTCAACGTGATGTACTCTTCCCGCACCTC
ACGCCGGGAGGAACTACCCACCGGCTTGTATA
CAGCAACTCCGCTGCCTAAAAAAAATCCAGCATACTCAAGTTCGTCATTCACAATGTCGTTACCACGT
TTGGCCATAAAATCAATCTCTGCTGCGTCCCA
ATTGTCCAATTGCACTTCGCGACAGCAATGCGGCCTAAGTTGTCGGTGTACGCCGACACAATGAATAC
AAACAAAGCAACCAAATGGCACCAGTACGGCC
CACGTGGGCCACTTTTCACCACAATCGGCACATATCTGGTTCGACGGCCTATCCGCAATACAGGGCGC
GTAGAGGGGCGATTGCATGAGGCGCTCGGGCA
CCTCCATCTTATCATCGTCACAAGTCACTACGGATTCACGGAGACTCTCCTCTTCGTCATTATTCTCT
TCCTCTTCTTGTTTTAACCGCTTATTTTCCTC
ATACAGACGCAGGACGTCCTCCAGCTGTTCCACCTCATGCATCTCCAGCGGGCCGTCGCCCGTCACGT
CAATAATTCTCGACGGACGCTCTAGTATTCGA
TATATGCCCCTGCTGTCGTCGTTTTCATGAGATCCGCAGCTTGCGGAAAGCGCCACGTTCGGCTCTTG
CTGTTGCAGTGCCAAAAATGGTGGCGAAGCTG
ACGACGCAAGGCGCAGGAGATGAATAAACCTCCACTTAAGTAGTTTATTGCCAACAAAGAAAAAAGCT
ACAAATATCTTGCTCAAAGATCCCTCATTCTT
CAATGGACTTACCGGGGCGGATGTCCGTAACGAGGTAAAAAGGCCAGAAGGACGCAGTTGTTCCACAC
TCGAAGCGTCCGCAAGCTCCAAACCAAAACAA
AGGCGGCCACGGTGCGTGCCACAGTCCACCACACGCGAACCAAAAAGGTATAACCGCTCCCTTAGATT
TTTCGCCATGGAATCTTTGGGTTCATCAAAGC
AGTAAAGCACGCCATCCGTGTGAATCTCAATTGTCCGCATGACTTTTTGTCGCTTACGCCAAATAAAC
CGGCACGAAACTGCCCCCGAAAGCAACAGTGT
TTCTAGAGTCGCCTCCATCATTCCTTTGCAGGCAACTGTATATGTACCTATTTATTTATTTATTTTTT
TTACAGAAAACCACACGTGAAGAGCAGAGGAC
TTTACGGGTCAATTGTTGTTGGTGAAGAAATTAGAATGACCCCTGTGTTTAGGATCCTTCAGAAATAA
AAAAGAATTATGGCGGCCACATATACAAAAAA
AAGAAAAAAATAATTTGTGGAACACAAACCGCATCTCATATAAAAAAATTGGTGAAAGTCTTAAGGTAT
GTGTTTGTTTCTTCTTTCTTTTTTTTTATTAT

TTGCCAGCTGGGAGTGAGTTCCTGCGCCTTTAGAACTTTTAAAGGCATTACTCCTCCTGGGAATAACG
CGGACGCTTGCGGCCCTGCGCGGAGTTGTCCC
CGTCTCCTGCTGGGACGGATGCGCCGTTCGAAAACACCTCCGTCATTATGCGTTGCAGCATCGCATCC
TGCTCGGATTCCTTCTGGATCTCTGCGGCACG
CCGCTCAGCCTCCATCTGACGATACACCGCTTCCTCCACCGCCACCGCATAGTGACCAAGACGATCGC
TGTTCACAATGCTAGCAATGACAAAGGGGTGT
AATCCCGCATCCATCGTCATTGTCTCGGGCGTCTGGTGATCCGTAGGACTCTCGGTGGCGTGCTTGAG
GACGGTGATCGTGAACAACACCGAAGGTGATG
ACGATGACGGCAGAAGAATGTACACCGGATTCTCCGTGCTGGGCGTGCGAGGAAGCCAAACGGCACGA
ATTCCGCTAAATGAAGTGCCCACGGATAGGGT
CTTCGTGTCGTTAAAACCGCCTCCTGCCCCATCCTTCGCACCAGGGGTCGCAGCTGACACAAAAAAGG
CAGACGCACGCCGCATATCTGCCTCACGTTTT
GAAGCAACAAGTCGCAAGGCCGCTTCACTACCGCCGACTGCTGTGGCGTACAGGAGTGTCACTGTCAT
ACGTTCCCCGGGAACGGCAAAGTCCTTCCAGC
CCATGCCACCTTCACAGGGAACAAAAATGGAGGGTACAAATACCGGCCATTCGCGAGACGGCGTATTC
GACGTCGCGTGTGGCGGCAATATCGTCCGCGC
GTACAATCCGTCGTCCCCTACTCGTTGGATGCAGACAGACACCACGGAAAAGGCATGAAGTCCTTGTG
GAAAGCTGTGCAGCTGCTCTCCCGCATTGTCT
GGCTCTGTCTTCCGTGCGTGCAACGCCTGATAGAGTTTCCCGGCGAGAGCCTTCTTGGAACTCGCAGC
GGTAGAAATCGACGAAGAGGAAGAAGAGGAGA
CACCCGCTTGTCGTAATCGCTGAAAAAGGACTGTAGGCTTTTGTGCCTCAGCAGTGTCAGCTGCTGCG
TTTACAACACCGCGCAACTCCGCCTCAAGGTT
CTCGCTTGCCTCACCATCAGCGGATGTCAGTCGCACCTCAACAATGTCCCAGGGAGACACCTTGACAA
AGTTGGTTTGTGTCTGCAGAAAAACCGGTCGG
CCTTTCCGTATCAGTCCGGCGTCTACCTCGCATTTGATCATACGATTGCCACCACCACCACCACCACC
ACTACCACCGATATTGCCGCTCTCGTGCTGTG
GGAGGACGAACCCACGCAACACGCTTCCCCTCGCCACATCCTTCGTCGATCGCCGTGTTAGTAGAATG
TGCTTCAAGGCATGCTGAAGCGCCGCGTCGGG
CCCCACATGCACCACATACATTTCATTGGATGTCAAAAAAGGCATCTGATGAGTGGTGAGAAACTCGT
TCGCGTCATGAACGGTGGCAAATTGCATCAAC
ACAGCAAACCGACCAATGGCGTGGGCTCGGACAGACGACAATGCCTTAATAAAGACATCTCTGGCCCC
AGCATTTTTGCTTCCATAATCCACTGCTCCCT
CCTCAGCAGTTGTCGACCCAATGCGTTTGGCTCCTGGAAAAGATAATAACTTCTCCTCGATTTTCACA
CCCTCCCCGACGAGTGAGCGCAAGTACGACGT
GTCAATGGCCTTCGGAGACGTCACAAGCAATTCCGCCCCGACATGAACAATAGGTGGGAGAAAGCTAA
GAAACCACCGCCGCCCTGTTTCCTCAACGTCG
TTTTGCAACTTTTCCGAGGCGGAAATTCGCTGGAACTGAATAATGGCTGCGCCGCTGTGAAAGGCACG
ATCCTCCTCAAAGCACGCCTCTTCAATGACAC
CGTAGCTTGCAAACGTACGAAACAGCTCCGCCCGCAACACAGCGTCCTCCGGCTCGACTCCTCCTACG
CGAACGCGGCGACTGAGAGAAGACCCCTGCAT
CGCTGTCAGAGAACTGTCGTGCTTAGATCCCCGCGCAAACGAAAGTCAGACCACTCACCCCCCACACA
CACACAAAAAAAAAAAAAAAAAAGAAGCCACA
AGATGTGAACTCAACTTACGTACATATATAAACATATGAAAAAATCCGCACACAACAAGACTCTGTAA
TAGCCAACCCCCCACTCACCCACACAGAAAAA
GAGTATTGGAAAAATGATGAAAAACATGAACGAAATAAATAAATGCAACGTTTGGGATCCCTGCTTAC
CAAAGAAAAAAAAAATATATTTTTTTCTTCCA
TCCACTCACGCGGAGGTGATGTCAGAGTCACCAACGTCTGTGATGGAAAAAACGCAGCTACGGAAGTG
CCGGCCGCACGCTGTTCCGAGGTCAAGGTTGT
TTCCAGCGTAGTGGTGCATGGGGGTCTTGCTCAGCGTGCAGTAATACTCAATCTCTGCCTTGCGGATG
GGAGGGCAGTTGCTGGAGATCACGACAAGCTT
GCTGCGGCCCTGGCGCAGGGTCTTAAGAGTCTGCTTCGTTCCAAGCACATACTTGCCGGACTTCATCA
CAAGCTGGATCTTGGTGTTGATCGTGTCCACC
TTCGTCTTGTTCTTCTTGGCCATTGTGCTGCGTGGATGGATCCTGCATGTGGGGTGTCAAAATAAAGT
CATACAGTGTGAGGGGGAAAAAAAAAGGAAGA

AGAGACAGATGACCAAAAAGGATCAAAGTAAAGTTAAAGACAAGAGAAGTTGTACCGGTGGCAGAGAG
AAAAACATGCAAAGGCCGTCAGATTGAAAATC
TCAGCCAATAGAAAAGAAAAAAAAAAAGAAAAGCAGTAATAAGAGAGCCTCCTCTTTCATTTGATGCC
GCGAATTTCTTCCATCCACTCACGCGGAGGTG
ATGTCAGAGTCGCCAACGTCTGTGATGGACAAAACGCAGCTACGGAAGTGCCGGCCGCACGCTGTTCC
GAGGTCAAGGTTGTTTCCAGCGTAGTGGTGCA
TGGGGGTCTTGCTCAGCGTGCAGTAATACTCAATCTCTGCCTTGCGGATGGGAGGGCAGTTGCTGGAG
ATCACGACAAGCTTGCTGCGGCCCTGGCGCAG
GGTCTTAAGAGTCTGCTTCGTTCCAAGCACATACTTGCCGGACTTCATCACAAGCTGGATCTTGGTGT
TGATCGTGTCCACCTTCGTCTTGTTCTTCTTG
GCCATTGTGCTGCGTGCAAAATATGCAAAGAAAAAGGCTAACACACACAAAAATAATAATAATAATAA
TAGCAATAATGCTAATAACAATAAGAATAAAG
CAGTTGCAAAGTGTAGAAAGAATATTGCAGGCAAAGTTCCATTAAACATGAATACAAGGCAATTTTGT
CTCACTTCACTCTCCTCTCTTTCTCACACTTA
CACACAATTCGTGCATATGTGCAGAGCAATGACGCCCGAACAAACAAACAACAAAAAACCACAGGAAT
TCCCCAGCGCTTATTTTTTTATTTATTCTTTT
TTTTCTTTGTTCGTGTGTATGTTTTGCTTTCCCTCTTGACTACTTTCAAGGGGTGTTTAGTAAGGGTA
AAAGCGTGAGTAACATCTAATACATGTAGATA
AAATAAATTTTTTTTTTTTTTTTTTTGTGTNNNNNNNNNNNNNNNNNNNNNNNNNNNNNNNNNNNNNN
NNNNNNNNNNNNNNNNNNNNNNNNNNNNNNNN
NNNNNNNNNNNNNNNNNNNNNNNNNNNNNNNNNNNNNNNNNNNNNNNNNNNNNNNNNNNNNNNNNNNN
NNNNNNNNNNNNNNNNNNNNNNNNNNNNNNNN
NNNNNNNNNNNNNNNNNNNNNNNNNNNNNNNNNNNNNNNNNNNNNNNNNNNNNNNNNNNNNNNNNNNN
NNNNNNNNNNNNNNNNNNNNNNNNNNNNNNNN
NNNNNNNNNNNNNNNNNNNNNNNNNNNNNNNNTGTATGTATACAAAACAGATCTAAAAGACATCTTCGTC
AACGTCGCCATTCCCTACATTTCACTGTTCCC
TTTTTTTCCTTTTTTTTTTTTTTTTGCCCCTGCGTGGCATGTATATTGCTGCGCTGAAGGGTGGGGACC
ACTGTGCAGGTAAATTCAGGCAAAAGGATGCG
GGATGCAAACACCGCCATTATTTGGAGAATAATAATAACAATAATAAATAAAAATAAGGCTCATCGTC
TCCTTCAAAGAACCGCCAAATGGTGCACCGTA
CGCCACAACTCCGTGCCAGCATCCCACCCACCTAAATCCACAGGCAAGTCAGAAAAAAACTAAGCACA
TGAAAAAGTCATGACGAAAACACGCACAAGCT
GCGCCTAAACATTTCCTTTACTTTAATTCATTCTAAACGTTTTTCCCACACACACACACACACACCCT
TTCTTTTTTTTGTTTTTCTGTGTTTGTGTTTT
GAGAATATTTCTTTCCTTCCCTTATTGCTCTCGCAGCCCTCACCAGATGCTGTCCTCAGGCAGTCCCG
ATTGTGTAAGTGACATCATATCCGCGCTGGAG
GGACCGTTAGCTATACGCATGGTATTGGTGGTGGCTTCCATGTTGATTGAGCGAGAGGAATTCGCCGA
TGACAGGGTGGCCGTACCCCGTGTCAAATCCT
TACTGCAGCTGATGTTGCTACTCCAGAAGGCAGAGACTGTGCGGTCGTACTCGCTACGGAGCACGGGA
TGGCCAAGGACATTGCGGGCCTCAACGACGAT
GGTGTCATGGGCGTCTGCGCCCTGTGGGTCAACTTGTTTCATACTTTTGTACTCCTTTTGCCAGCGGC
GGTAACTCGCAATAATTTCTTCCTGCGTCGCG
GTGCGTTGGATGCCAAGGACATCATAATACGTGCCGCGCGTGTTTCGGTGTGGGTAACGGCAGCGCGG
TGGAAGGCGAATGCGAAATGCTTCCATGCCCT
CATCCCGGCGCGTCCCCTTTTGCGGTGAGACGTCCTTTGAGTCGTGTACGCTGCCGAGGAGGAGGAGC
GTGTCGGTGCTGGAGGCAAGACTTGACAAGGA
GTTATCACACAAATTCGTGTTGCTGCCGCTGATATCATACAGGCGAGCAGTCCGCTTACTGCGACGAT
TGGTGCTCGTTTTCTCCTTCTCAACGTGCTCG
CCACCCAGTGAACGGTGGGCAACACCACCGTCTACGGCAGCCGTCGCGGTGTAATTGGCGGCGTTTCC
ATCCGTCGCGTAAAACGTGCGATCGTTTTTGT
TGTTTCCGCTACTGCTGTTGCTGTTAAGTGGCGCCACAAAGTGCTTCGGGATGCTCTCCACCGGCACC
GTCTCAAGGAGTTTGTTTTGCGTGTATCCGTT
CAGCTCGGAGAAGTGATGCGCATGTTCACAACGGCTGTCAAAAGGGCACTGAGCCGCCTCGTAAAAGG
CCACGCAAACACGTGTCTTGCCTCCAGCGTTC

ATCAACGGTGGTACAGCGAGATGCAGAAGCTCGGCAGCTGCGGAGGTGTCTGCCGCCTcCACACCGCG
GCGCATGCCATCAGACGGAACAAACCCTGACT
CGATAAATGAAAGCGACATGTTTGCGTCAATATTTTTCCCCTCGCCGTGCTAACTTATTTACAGTCTC
ACCCTCCCGTTCTTCTCTTCTTTATGACTCCG
TCTTCTTCTTCCTACTATAGTATGTACACACACACACACTAGTTTTGATGGGACTTGGCTTCACCGGC
CAAAAGACTTGTGCGTGGGGAGGGAAGGAATA
AAGAAACAAAAAAAACACATGTACATTACACGCACGCGGTGATTCACAAATGAGGACCGCAATAAAAC
ACCACCACAACCATCATCACCACATATTCCTC
CACAGCACGTGGAAAAACAACAACAACAACAACAAAAAGAAATAAACATATATACGCACGCAAAGGAA
GTAAATAACTTCGTCTGTGCGAAAGAAGCCGC
TACATAAACTCCTTCCAATAATAATTCGTAATTCCAACACCGTTACGCACACACAAAACCCACACGCA
TGGTGTGGCAGGAAAGCAGCAACGCACACACC
GGCCACCCACCCAACATACAGACATACAAACACATAGACACCAACCCGTCCTTTCCTTCTCCTCCCCA
GGCATTCATTACCGTCCAGCCGCAACGTTTTC
AACAACAACAGCAACACACTCCTCTAAAATACCACGCGGTGTTTCCGGTGACGTCATTACTTGACACT
GGGAGGAAGTGAGCAAAAAAATGGCAAGAGAGA
AAAATTACCCACAAGAGTCCATATATCACCAAGACGCGAAGCAACCATCACACCCATTGTAAACGGAT
ATATACAGAATTCGTCCCTTTCTGTCTTTCCA
GAAATAAACCCACATATTTGCACACTCCCTCCACCTTCTCTCTCTCTATATATTATCAATCACTGTCA
CATTCATTCATTCATCACTCACTCCCTTGTTC
ATGTGTCTACTACCTACACTCACACCCCTAGCTTCCCACCCAATCTCCCATCCAAACTCCAATCCTTT
CCCGCCCTAGATCCAAACCCCCATAAGTGTTA
GTCCTCTTACTGTTTCATGCCATTTTTTTTAAATTTTCGCCTCCCCTTTTTTTCCTCCTCTCCCCTTC
TTTCTTTCTTTCCCCCAAGTCCTCTTCAAACC
TTCTCACCCTCAACTCCATATTTTATTCCTTTCCACATATGCATAAAAAAATGCAACAAACACAGGGA
CGGCCAGCCTCTCCAGTCAACTGGATGAGCAC
ACACAGCAAAAATAAAAATAAAGCAGTGAATCATGAATAAAAAAGCCGCGGTAAATGCAGGAGCAAGA
GACCACACCAAATAAAATGTTCTAAGTGATGC
ATATCTAACCAGAGATTTCAAGTTTCACTTCTTCGGATCCTGCTTCTGTGGATTGTTTTGCACCGAAG
ACTTGAGCTCGGCGATGAGGTCCTCCGAAGTT
TTGCCGCTGTAACGCTTGCCCGGCTGCATCCGCTGGAGGTCCTTGTCCACGGCAAAACGCTTATTCAA
AAATTTCTTCAGCATGTCACCACGTGCCTTCT
CCAAGTCATCGCGAATGCGGTCTTTGCGTTCCTTGTACGCGGCCGTCTCTCGTGCCATGCGTTCTGTG
TCCATGCGCTTCAGTTCTGCCATCTCTTCCTG
TGTTAAGGCCTTCTTGCGCTTGTCAAAGTCCTCCCGGACAACTTGTTCCCAGGGGCTGTTGTAGCCAA
GTCCTTCCCTACCGATATTCTGGTCTTCCCGC
GCAACCTTCTTAACCTGCTCCGGGTCCAGCTCCCAAAAACGCCACTCGTTCCACTTTTGCGCTGTCTC
CAACTCCCTCCACGTCACCTGGAGAATGTACT
CCACGGGCCACCCCCAGAAGTACCGCTTCTCCAAAGTGCACCAGTCATCCTGGCACTCCTTGATTCCG
TGGTTGATGCGTTTCACGAGTGTCATGGCGAT
ATTGTAGTACGTCGACTCGGGCGGAATGTCGGAGGTGGTCAAAAACGCCTGCAACTCTGCCGCCGCGG
CAAGCAGCTTCGGCTTCCATAGCGGTTCTACT
GCAACACCTGCCAAGCCCGTTGTGGACTTCACCGGAAAGGCACGCGGGATATATTCATCGCTGATTGT
GAACAGTGCGGCTGTCCGACGCAGTGCGGCCC
GGAGCGAGTGTGAGCCCCGCATGCGTTTCTCGCTTCCTCGTAATGGTTATTGTTTATTGTTTTTTTTT
TCTTTTGTTTGTTGTTTGCTTGCTTGCTTGCT
TCCCTCCTATTTCCACGGCGGCAACGATGCGTCAAGACAAATGTCACCGGCCCTTTATAGGCAAATTC
CACACCCTTATATATGTATGTATGTATGTATG
TATATTTATACACCCTCTTTTTGCCACCGAGAGATCAGGAGAGAAACAAACAAAAACAAAATGGAAAG
GGGGCATTTTTCATGAGTCTGCAGAACAAAAA
TTGGGTGGAAGGAAAAACAGGTGACAGGGGCGTGTGATGAATTTAAACACACGCCGGCAACAACAACA
ACAAATAAGGAAAAGAGAAGCGAAGTAACAAG
AAATACATGAACAAAGCCAGGAGCAGCCTCTACCGATCGCAAACAAACAAACATCAAGACCCTTCTTC
TTGACACCACAGACGCCAATACCAATCAGAAG

TCCACACCACCACCCCCAGTAGCACCAACAAGTATAAAAGAGCAGACAGTGGTCACGCATCGCACATC
ATAAACAAGATGTTGGCAAAAAGAACAACACC
AACGTCTATCTCTATATACACCCGCACCACCAGCTACGCTCTTTAATAAATAGAAAAAAAANNNNNNN
NNNNNNNNNNNNNNNNNNNNNNNNNNNNNNNN
NNNNNNNNNNNNNNNNNNNNNNNNNNNNNNNNNNNNNNNNNNNNNNNNNNNNNNNNNNNNNNNNNNNN
NNNNNNNNNNNNNNNNNNNNNNNNNNNNNNNN
NNNNNNNNNNNNNNNNNNNNNNNNNNNNNNNNNNNNNNNNNNNNNNNNNNNNNNNNNNNNNNNNNNNN
NNNNNNNNNNNNNNNNNNNNNNNNNNNNNNNN
NNNNNNNNNNNNNNNNNNNNNNNNNNNNNNNNNNNNNNNNNNNNNNNNNNNNNNNNNNNNNCCAATCT
TTTCCTTCCCTCTGGTGTATTTAGTACACAAA
GAGCATCCTCATCGCAATAACAAAACAGCCAAGCGCTACCCACAAAGAAAGTCTATCGCCAATGACAC
TACACTTGTAAAGAAACTTGCGGTAAAGCTCA
TTATGATCACGATGAAAAAGTGTAGGCCGATACTGTCGCAGGTAAGTCGCCGTGCAAATGACAAGAAG
AAGCACATAGGCGATCGTTTCAAAATCAAAGA
ACGCACTCATGCCTGCTATGCCCCCTTCCACCCTAGTTTCTAATAGAGACAAAGGCACTTCCTGTTTC
ATATGTAAAATAGGGGGGTAAAAAAAAAAGGA
AAACAATGAAAACCCATAAAACAACATTGAAACAAAAAGAGAAAGAGAGACAAAAAAAAAAAAAA
>scaffold1040.size12319
CCTCCCACTTGTAGAAACGGACATACACACACACACACACACCAATAGCACTTTTGCATGCATGTATA
CATTGCATTCCTTTCTCTTTATATATTTACAAG
CAATGAAGCACAACACATGAAATGTACACAACTCTACCGGCGCTCTATAGGCGCAACTCCTCCAGCAG
GTATCGATAATCGCACAACACACCCCACAACA
ACTTTGGAATTCCATGCACACGGCGCCGTGCAGCCGCCTCCTCACGAGATTCGGGCGGAGGCGCCACT
ACTCTAGCAGAAGCAGCAGTGGCAGCGTTTTT
TCTTCGTTTCGGTGCATTCTTATCATCATCGGCATCATTGTCGTCATCAGCGGTATCAATCATCTCCC
GCTCAAACGGTTTGCGGCACACTCGCACGCAG
CGGTCCATGACCCACGGCGGTATGACACGTTTTGCGTTACGGAATAATTCCACAAGACATTTACGTAA
CACCTTCTCCTCAATGAAGCCGGTGCGAATAT
CATCGTAAGCAAAGAAGGCATCGCGCAGTCGATGGTAGTCACCGTCACAGGATCCAAGCAGCTCCTCT
CGCAGTAGTCGCTTCATTTTTATTCTTCCCTT
CCGTACACGCTCCTCAAGTTTAAATTCCTCGGCATTTCCCATGGCACTTTGGATCGTAACGCGTGGCG
TGGCCTCCTTTATCCATGGCTGTGACACGGAC
AACGCTGCTGCGGGACGACTTGACATCTCCACTGATTTTTCCCTCCCCGCACCAACCTTCTTCTTAGG
GGTCTCTTCGTCAGACGTAACCGCATCTGGGA
GACCCACGTAGCTCTCCTGCACCAAAAGCGTCCCAACAAGCGACGTGTAGTGGCAGCGATTGCCATCG
CAGCCATTGATAATTCCAGCACGTACTAACTC
GTTGATTTCACCTTCTGTTAGGTCTGGCGCAACCGCGTAAAGCGCGCGGCCAAGGTCTTTGGGCGACA
CAAAGCCATCCTGCGACCGATCGTAGTTCGTG
CAAAGTGAGAGCAGCTGAGAGCATTTTCCGTAGAGTTTTTCCAAAATCTCTTGCGATAACTCCGGGGG
GTCGGTCTTGTTGGCATCGCTTGTATCACTTT
CCGACGCATTCGAGTGAGTTGGTGGGCGCGGTGGAATCCCAGGCATTGGGGGATTATGTCTCGTTGCA
CTCGTGTCTGCGCCCTCATGTGTGCTCTGTGT
CCCTTCAGAGGGGAATCCTGGTCGGCGTCGCACACCATCTAAAGAGGGAACCAGCGAGACACGCACTA
CACCAGGCCTCGTCCCGCCCAAGCGATCTGAA
GAGGATCCATCTTCTGCGCCGCCGGGGTGATTGCGATGCATGCGTGCCGATACACCGTTTCTGGGTGA
GACAAACGGCTGCACACTCCGAATAACTCCAG
CCCGAGGCACAGTCTTGTAGGCAAAGACAGAGGCTGCAATGGACGAGTTCTGTTTTTGTTGCGGCTCC
TTGCCAACAACTTGTTTTTTTTCACTCATGAA
AGTGGGTTCCTCACCGCTGTTGGAGTTCCTTCTCCTGGGCGTGATGTCTGTTAAGATGTTTCCATTTC
CGTCCTTCGTGTCCGGCTTCCGAATGATTGCA
CCTCGACCTTGGCTCAAGGACACAGCACGCTTGGGTGGATAATATTTAAATTTTTTCTCGTGCTCATG
TGCCAGCGAGGAGGCGGTTGGCTTCAACAACC
TTCCCCCCACATTGCTATTACCAGCTCCTTCTCCACCCGATGGGGATGCATTTCCCAACGAGGAAGAG
GATGATCTCGTCCTCGCTTCCCCGGAATTGCT

CACCACTGCGCCACGCATGACGGAATTTAACGGCTTCTTTTCGCTGAAATCGACCGGTTGATTCATTG
GGGATGGGGAAGGTTCATCCTCCGCTCGCTCA
GCACTATTCTCATTCATGAGATCACCGTACTTCTCCTGAAATTCCTGTTCCAATCGCCTAAGAAAGTC
TTCTTCTTCAATGCGTTGTCTCTGCCGCGCCA
GTATTTCTCTGTGTAGCATCGCGTAGTCTTGCTTAGCCAGACCTGTCGCCTCGCCGGCTATTTTGGCA
TCTGTTGTGGATGAAGCAAGCGGCACACTCGG
GCTAGACATCTGTCGTATTCTACTTCTGTTCATGGTTGTTTGTGGATACGGCATTGGCGCACTTAACT
CAATTTCATCTTGAAGCCCTCGTTCTATAGGC
AGATCATACGGATGTGGACTCTTCGATCTTGAGGCGGTCATTACAACAGATTTCTGTTGCCTCTCCAC
GAAGGATTTCGTCTCCGATTCCCTAACGGTAT
TTGAGAGATGAGGGGATGGCGATGTGTTTGCACGGCGTGTGGGTGTCACCCGTGCTAATAAGGGTCTC
TCTGAGTCAGGTGAGGGGGAAACGGAGACGGA
GGAGCCTGATGTTAGATGATGTTCTACTGCGGCCACTGCCGTGCTACGCCCTTGCGGTGTGCTGGCCA
AACTGGACTCTATCGCATGGCTGAAAGCCAGG
CTTGTATGCACGCTCGGTGGGGGGCTGAAACTATTGACGTCCATGTGCGACACCGGCTGCTGCTTGGG
GCGGTACATTACTTCAGTGGGCGGCGAGGATG
GTGGTGGGGGTGACAATGGCGACTGAGAGGGTGACATGAAATCGTATGACGTTGGGTTCATCAGTGGC
AACGGTTTACCCGGAGTCGCCACGTCTTTCTC
TGTATTCTGACGTTGCCGTACATCAGTCCTATCACGTCCGCTATCTGTGACACAACGTGCACTGGACA
CCAGCGGGGACGATGGCTCCGTTGCAGCCGCA
ACCCAAAGCGCCGACCGACGAATACGCTCAATGAGGCTGGAACGCATGCGTGATACAAGCATACAAAA
GTCCACCATGTTTAATATTCTGTCACGGTCAT
CGTGAGCAGCAGCATTTGTGCTTGAAACATCAGACATCTGAGAGACGTCCAGTTCCAAGGAGTCTGCA
AGGGCAGCCAGCTCCAAGAGCGAGATATCAAT
ACCACGTCGGTGGAATGCGTTCCACACCTCTTGTAGTGAGACGTTACCATCTTGATCCGCGTCCACTA
AACGGAATACTTCCCGAAGAGAAATCAACCCT
CCTCGGCTGGCTAACCCTGAAATGGAGGTTCCATGACTTACTCTCGATGTATTGTGTGGCAGGGAAGT
GCTTGTCAAGTGTGTGTCCTGAACCAAAGACC
TGTTTGTGTCAATCATGGTTGTCGTTTTTGCCGCTGCGCTCGGCCCTTTTAAGGATGCTCCTTTAGTT
TCCATCTTCCCCGCATCCATCATGTCTTTTGA
ATCATTGTTTGGTGGTCGCAGTGCTCCTCTGTCCCCTCTCCCACTCAACTCCTCGTGCGACGCTTCTT
CCTTCAGCCCTCCGGATGACAGTTGAGAGGTA
CTTCTTGAAAAGGCACACTCCTTTGCCTTCTGCAGCATCCGCTCCACAGAAGAAGTACGGCTTGACGA
AAAGGAGACGCGTTCCACGGGACGTTCAACAA
ATTCGGATTTATTTGCGCCCCCGCTTGTGACAAGTGGGGCTTTATGTTCCCGCCCATTCGCTGCCAAA
GCGGTGAATCTACTTCGCGGAATGGCAAAACG
ATGACTTGAAGCAGAACTCTGCAATAAAATCCCGGTCGGCTCACGCGCATCGGGAGAAGCACATGAGG
AGTAAAACGTCTCGTTTCTTTTGTCATCGCCA
CCGGACTTAAATGCGCCATGATGTGAATCATAATTCGCGGATTTCTCCGCACACGACGCCACCTGGCT
AATGACATATGGCTGCGGTCGTTCATGTGAAA
TTCCGGCCGCTATCGCACCTTTTTTCATATCACTTTCATCAGAGAAGCGGGACGCAGAGCGGCGGCCG
AAGCTGTCATCATCGTCCCGCGCCAGCAGCGA
TGAATTCATGCCCTCTGCTAACTGTTTCATGCTGTCCAGGAGAGCTGCGTAGCGGACACTCCCTCGTC
CTTCCACCTTGAACTGCTCAGTGAGCGCCTCC
ACCTGCGCCAGGGATCCACCGTTGCGCAGCAGCACCACTTTGAAATCCTCACACGGAATAAAACCAGA
CCTTTCTCTATCTAATGCCCTCAGGTCCGACT
CTAATTGCTCAATAGATTCCGTCATCTCTTGCCGAGACATCGCGGCATCAATGGAATCCCTAAACGGA
ATAAGAAGTCTATGCCTTCCTTTTTACCAAAA
TGGAAGGGTGAGAATAGAGAAAAAACAACAACAACAACAACAAAAACGATATGCATATTATTCTT
CGGTGGTAAATTTATTACACGGCGTGTATACG
TGCATTCCAACAAAACTCTCTCATAGGCTTCCGAGGTGATTTTTTTTCAACTTATCCTCAATAATTAA
AAAAAAAAGACACAAAAAGTACCCCCGTCTCC
TGTGCTGGGCGGAGAAGTCTGTTTTGTTGAGTGAGCTTCCAAAACGTACCCCCTTTGCGAGAATATTG
ATACTGAAGGAATTGAGGAGTTCACATGATGC

ATAATTAACAAACAAACAAACAAAAAATCAAACCGTGAGGCCAAACCATTTGCTCTCTTAAGCTAACA
ACAACAACAACAACAACAAAAACAGGGATGAG
ACAGCACTCACAGAGACCAGCGACAGCAGGCGGAGGAAAGGGCACACAAAACAACATACACACCATCT
CCCTCTGTATATGTGCTGTAGAGGCCCTCTTA
CCTCTGCAAATATTCTTCTCTATGCAATCAAAAAAAGGAAAACAACAACAGAAAAAAAAAAAAAGGAGA
AAAAAAAAACCGTTTGTGATCTGGTTGTCGTC
TTTTTATGCCTCTAACATTCTCTCCTCCTCTCCTCCCATCGTCTTCTATTTTCTCACTCTCGATATAT
TTATACAGAGATAAACACAGAGATAAATAAAT
AAAGCGGAGAACAAACTTCTTTTCATCAACTCAAACCATAACCCCCCCTTTCCAAATGGATTCCTCAT
AACCTTTTCTCATCCAACTTTTGGATTTCTCT
CCCACACACACACAAAACTAAAAATTTCGAATGTAATCCGAACTCTCGCATGCCTTGTATTCACTCAC
CAGGCTCTCCAGAACTTCCCTAGAGTGTTTAA
ATTCGTCAAGGTTCTCCTTCATGGGGCCGTACCGTTTATACTGCTCCAGGAAAACACCGCGGTTAAAC
AGCAAGTCAAACTGCTTGAGCGTTCGTTGAAA
GAGAGAATTTATACTGGTGTGATTTGCCATTACAAGTCCACTCACACGATGCCGTGTGTCTAAGTAAG
GTGACTTCTTCGATAGAATCACTTGAATAGAA
GCGGGTCCCCATGGGATAAAGTTAGGTGAACGCTCGCGAATCCGCTCAAGTGAACGATGTACTTGTGC
CGGGTCTACATCGCCTTGAATGAGATTCAACA
GGGAGATGTAACAGCCACTCTTCATAGAAGTTGAAACCATCATGTTCTTTGGCATCAAAAGCCTTCGC
ATAACGTCGTGCACGGATGTTTTCTGTACAGA
AGACTGAATGTTCGAGGTGTCTAATGTTGTGGGTGTGTAACCGGTGCATATAAAGTGGCAGCGTGGAG
TAGGAATCAACGAAGCAAGCATACTCATGAGA
TCATTATTCATATAACCCGGATAACGTAAAGTTGCAGTAGAGGCTGCCATAACGGTGCTCACTAATCC
ATTCATCTGTTCAACAGTGGGTGAAGAAATAT
GTAAATTATCTGTGGCAATACGATTCAGAGCCGTGTTGTCCAGAACAACGACACAATCAGCATGCAGC
GTCAGACGTTTGATTGCTAACAAACTATTGTA
CGGCTGTACAATGACGTCGCTGTCTCCACCACGGGACTGATTTGGGAAAACACTATAAGTCTGAATAA
GTTTCTTCGGAAACCGATCGTTGAGATGCTCA
AGAAGATAACTTCCCATTCCACTGCCTGTGCCCCCAGCAATGCTGTGTGTAAGCACAAACCCCTCAAG
ACTGTCACTATTCTCTGCCTCGCGTTCAATCA
TATCAAACAGTGTTTCTTGCACGCTATCGCCCATTTCATATCCATGGGCCCAGTTATTCCCGGCACCG
CCTCCCTCAGAATGGATGTACACATTTTCCGG
GTTAAAAAGACGCTGCATGCTACCGCGTTGAATGGCATTAATAACGCGCGGCTCCATGTCAATAAGAA
GCGCACGAGGAATATAGTGATCGTCGTCAGCC
TGGTAAAAGAAGACGTCTTTCCGATCGTCCCCAGCACTTGCAAAGGGCTCCACAATACCATCATGACG
AATACCATGTTCGGCGCAGAGCAACCGCCAGA
ACTCAGAGCCGACTTGGTTGCCACATTGCCCTGCCTGAAGTGTAATGATCTCTCGAGGCATCTGATAT
TCTGCTCTCCTATGGGTCTACCCTTCTTTTCG
TTATTTTAACGCAAGTGTGAGTTCCCCGAAGTTGGGTGTTTTTTTAATTGTTTGTGAATTCCCTTTTT
TCCCTTTTCCTTGTTGCTTCTTCCCTCCTTCG
TTGCTTCTTCCCTCCTTCGTTGCTTCTCACGCAAAAAGCACACGAACACACCTCTGTCACACTCACGC
TGGAACCGAAGGACAAACAAAAAACAGAAAAA
AAAGGGATGATAGCAACAATGTTCTTCCTCCCCGTTTGGGAAAGTGGGGTTAAAGGCAAATTCCGCTC
CTGACTACTCGCGATTTTTTCAAGCAACAACA
ACAAAATAAGCGTAAAAAATGTCCCAGTAAAAGGGCAAAAAAAAAAAAAAAAANNNNNNNNNNNNNN
NNNNNNNNNNNNNNNNNNNNNNNNNNNNNNNN
NNNNNNNNNNNNNNNNNNNNNNNNNNNNNNNNNNNNNNNNNNNNNNNNNNNNNNNNNNNNNNNNNNNN
NNNNNNNNNNNNNNNNNNNNNNNNNNNNNNNN
NNNNNNNNNNNNNNNNNNNNNNNNNNNNNNNNNNNNNNNNNNNNNNNNNNNNNNNNNNNNNNNNNNNN
NNNNNNNNNNNNNNNNNNNNNNNNNNNNNNNN
NNNNNNNNNNNNNNNNNNNNNNNNNNNNNNNNNNNNNNNNNNNNNNNNNNNNAAAAAAAAAAAAAAAA
AAAGATAAAGAAAGTAATAAATGACGTATTTG
CTCAAATGAATCCCCAAGTATAGGTTACTTTGCATGTGGCTCAGATGGAGGTGTCTGGGTATAGACAA
GCAGCTTACCAGACTTTTGCTTCGCAGCCTCC

AGCCACTCCAACCATTCTTCTTTGAGCGAATGTAAATAAGCAATATCGGCATCTTCCTCAAACTTCCT
TAGGAGTCTCCTGGCCTCCAATCGATCCTTGC
GCACCATACCCTTTTCTTCTTCCAGTACGGCTATTTGCTCCATGCATGAGGCGATGTCAACTATAAGG
CGGGTAGCGACACACATCACCTCCGCAGGAGC
CTTTGCAAACTTCTTTTCTTGAAGAAAAAAATCATTTTTGTGTTTTTCGGCAACTCGCCTCCGAGCAA
TCTCTTTCCGTAATACCTTGACCTCTGCACCA
CCGCAATCGATGTGCTCGTAACCAACAAACAGTTCCGCCTCCACCACGGCACAATCTTCTTCAATTTG
AAGGAGAGATAGCTCACGAGCCACTTGAACAA
GCGAAGACCACGCCCATGACCGGCCTAAAGAAGCTGGAGGCTGGGAAATTCTCTCTAATCCCAAATCT
CCATGTCCTCGTCTTGCACATACACGTCCCAA
TACAGCCAGATATAAAGGGGATTTTACAATCTCCCACACATCACCGGTGATGCAAGTTGGTGAGGCCT
CTTTATCCAAGGAAGGATCATTCTTTGGACCA
CCATCATCCGTAAGCCCCACAATAAAACGTAGTAGTGGTAACAGCTCTTTTCCCCCCTTGAAATCATC
GTCGGTTTTTTCTCGGCACGGGGTCGAATAAG
CTTCAAGAAGGATACACACCTCTTCCCTCGCTTTTATTAGGCCATTTACCTCGCTACCACTAAAATGC
AAAGAAGAATGCTCAGTATCATGAACAAAACC
TAATGTCTCTAACCACTCAATGAACTGAAGTATATGGAAGGACGGAGTGAGATAGCGTTCCCACATCA
CGGAAGCCATATTTATTCTTCTGTACTTCGCT
ACTGAAGGGTGCGTAATAATGTTTTTAAAAATCATATCAAGGATTTCAAGAGCTTTTGTTCGGCATTC
GGCCGATGCCTGACGGTGCATAAGGCAAAGCA
ACAGCGACAGCCTATCCAGCATTCATCTGCTGCTTTTACCCGTTCCCTTTCTCTCTTTCTCTCTCTCT
TTTTCCTTTGAAAGTGGAACTCCGAGCTGTTA
TCGATCAAAACCGAAATAAAATAAATGGGAACAAAGCCAGGGCACACTAAATAAACAAATAATAGCTC
AAATGGGTTGCATAAAGATTTGTTCAAGCAAG
TTAAAAAAGAGGTTTTGACAATATTATTTTTGTTACTTGTAGTGCATTAGACATTCTCCCTTTTATTA
TCCTCATTCTTGACCCCAGAGGAAAGCAACAA
CTCCAAAATATGACGACGATCAAGCAATCTTGCTGTCAAAAACTGATACGGATCAGCACACTCCTTCC
TCAGTTTTCCACTACTCAACGCATACCGTGAA
AACGTCTGTTTTTCATCGGGTGCCAATAAAGGGGTCGATTGCAAAGACTCTGAAAGTTGCTTTTCTAC
TTCAATAATTGGTTTTCCAATATCATGTTTCC
ATCTAGAAAAGGAAAAGGACAATTCCACTGAACGAGAAGATTCATAAAAATGTCCTGGAACACAATGT
TTTTTTGAACCGTGCACTTCTTCCGATGGATT
TCCGCGTTTTCGACGGGTTGGCCTTTTCACAGCAACAGAATTCTCACGTTCCATGCGCTCGTAAAGGC
CATGAATATATATTTTATCCTCAGCAAGACGC
AGTAGTGTTTCTTTACAACTTTCTTCCGTCGTATCCTGTTTTAGGTTCTGGTACAAAAGGGGCTTCAA
GAGCCAGGAGCGGATACGCTGCAAAGATTTGT
CTTCGGTCAGGGTACTAACCGTTGCATTCTTAAGCTTACGGGGAACTGTCTGAGGCTTAGTTTCTAAT
GAACTACTCGTGTTTTCTTCCTTCTCGGGCCT
GCAAGAGAGACAATGAGAGGAATTTTCGGATTTTCCTGCTGCCAAATTGTCTCCCCCTATCTCAACAA
CTTGTTGACTCATATTTTCATTTTCACCCGCC
CCATTCAATTCCTTCAAGGGAATTTGGCTCACCAATTGGGGAGGTATGGCATTCACTTCCAATGAAAC
CGTTGGCGCAGCAGAACTTAATAACACGTTGT
TCGAAATATCCAGTTCGACCAAAGGATTCGTCATATTCCCTAAATCTGTTGCAGCGCCATTATCCGTG
CTCATTTGGGTTGGTACATCACACATATATTG
AGCCCAAAAAAAAAAAAGAAATTTCTTTTCGGAAGAAAAATTAAACCTAAACCGTATCATAAAAAAGA
AAAAGAGGGAAAAGTAAATAATGGCAACAATA
ATAAACGGTAGCAATGAACCAAAGGAAAGAAAACTCCCGAATATAATTTCTTTTTGCAAACAAAACTG
TTTTGATTCGCGATCAACTAACCGCCGTCACA
TGGAAAATAAGAGAAATCTCTCAATCAAGATACATCCTAGCGTCAATCATTCTTCTTTCATTCATGAA
ATAGCCACTAGAGACTGTGGAAGCCACTCACG
ACACAAAAGAAGATTCAAATCTTTCTTTACTTCCAGCTGCTGTAGTGTCATGGCATTTTCACAGTTTG
CCTCAGCTTGGCGTCTAGTGCTTTCAAAAACA
AAATCTGCACTATCATCCCCATTTGACAAAAACAAAATTACAACGGCCTTATGTCCACAACAAGCGGC
AAGGTCCAATGCTGTGCTTCCTCTAAAATCCC

TCACACTAAGAGAGCAACCGGCTTTCTTAATCAGAAACTCGACCATTTCAACATCCCCTCGCACGGCG
GCAAGCATTAAAGGTGTGCGACCGGTATATGG
GTGAGGAAGATCCACGAGTGACCTAGACCCAGGTCTGAATTGCCGATTTTTAAGATGCGCAAGGCATA
AACAATTTAAATCCGCACTTTTGCATCCAGAA
CAAGATGTAGAATAGTATAATATTTCCAACATTTTTACATCGGAAGACCATGCTGCGTAATGAAATAC
ATTAAATTGAAACATGTCGCGAGGATACATTG
AGGCCCCAAGATTAAGCAACATTTCAGCAATAGAGTGCTTTCTAAGGGCAACAGCCCACATAAGCAAA
CTCATTCTTCTTTCCGTATCAACATAATTAAC
ATTTTGTGGTGTGAGGTTTTTGGAAAGAAATTCAACATTACCATGTTCCACCGCCAGAAGAATCTCCT
CAACACGACTAGCCATTTTCTTTTTATAGTAT
GCGTAGACTTCAAGAAAAGAAAATAAAAAATAAAAAGGCAATATCCACAAAGAAACCTATATACTTAT
TTTCTATACGAGCAAGACAAAAAAAACCAGCG
AATCCCTTTTCTGGAATCCACAATCCCACATTAAATATATATATATATATATAAGTCCAAATTCTGCA
CACATAGAAATAAAGCAATTAAAAAAAAAAAT
CTTTGTATTATAGTCGGTCTGTAAGTATCTAAACAACAACAACAACAACAAAAAAAAAACAATAAATA
AGAGATCTATGGATAAAACGTAGGTAGGATTT
CTACTCCTCGGAAAACAATTTCTGAGAAACTGGATCTATGAGTGAATGCTCATTTTCACTGGCATTCT
CAATTGACTTTGTATTCCCAACAAGCTTCTCC
GTTGACACACTTGGGGAAAACACGTTAATTTTAGTAGTGGAGTAATAAGATGGCTCATTATGGTTTGT
ATAAAGATTGTGTTGTGATTTTGCGGCATCCC
CAAGTTTCCTTTCCTCTCGTACACTCCGAAGAACCGTAATCCGTCGAGGAACTGTCGCAGGTGTCCTT
AGGCGATTCAAACCTGAATGTAAGACAGGAGC
ATTCGAGGTTTGAGGTGCACAGGCAGGAGCAGCAAAAATAGAATTCCATTGTTGTACCGGTGGTTGAA
ATAATTTGACACTCTGCATGACAGGTTGTGGG
TGAGAAAAACCAACAACAGGCACCGGGATTGGATCATGATGTAATTCTGATGGCGCATTCCACGAAGG
TGTTCTGTATCCGGGATAAAACTGACAGCTCG
TACCTGATGGTGAACAAGTATGCATGCTGCTTGGTGAAGTGTCAGCACGAGCTGGTGTGGCAGTGTTG
AAGAGAAAATGCTGGGGTGATTCCTGATGAAA
TACTGCTTCGGGTTTTTCGGGCGTAGAATTTTGCTGGTTATGCCCAGAGGGTGTAAGATATATTTTAA
TACCGGCATCCAGGGGAGATATATCAGTCCCT
CTTATAAGTGAAGAGGCAGATGGAGGTACCACATTAGTTACTGGCTCGTAAAAATCTTCACTTCGAAG
TATAATTTTCAAGCTTTCTTCAACAGGAAGTC
TCTTTGGAACATAGATGGAATTACGTGCCTCTTTTATTTTTTCTGTCGAAAGACGTACACGCCTTTCT
TTTGGTTCTTGTGTAAAAAGTGGACTCACCCA
TTGCAGCCGTTGGTTTTCTATAACAGATTCCAAAGTGGTTGGAGATGCATCTTCGTTTTCTATAAAGA
CATTGTCAAACATATGCAATGCCCGACGCCAT
TTTCGCAGTGAAACGTCCCATGTACGCTTACTGCCTTTATTTAAATCAGGCGGTGACAGTGGAAGAAT
ACCACCGCTTTTCAACAGAGGGTCACTTTCAA
CCAATTTTACCATGTTGCGGTACCCTTCTGTTACAAAACCGTATGAAATTTGTTTTTCCCGTTGCGCA
GTCCGCCTTTCCTGTTCAAGGGAATTTTGACA
AGGGCGCCTACCTGAGGAATGCTTGCCTGCCATCTTTTTCTCTGAAAATGCATTCTCAATAGGAACAT
TTTTTTCATTGCCCAAAGTTGGCCGAACAGGC
GTTTGCATACAAAAATTATTGCTCATTTTTCTCTCCAACCAGCTCAAGCAATCTACCCTTGATTTTGC
TAAACCGCACAACAGATTAAAAAGGAAGTGGG
TAATAAATTTTTTTTGAGAACCACAGTGATGGTAATAGCAATATTAATAAGACAGTTAAGAGAAAATG
ATTTTCATAAAAAAAAAAAAAAGAAAAAAACAAC
AACAATGTACGAGAGAAAGCAAACGCTGTTACAAACAATAACGCCGCCTTCTTTCGCGCCGCCTTCTG
TTTGCACATGTTAGTTGTTCTTTGTTCGCGCA
TGGCGGTTGTTGTTTGTT
>scaffold1041.size6938
AACTGCCAACCTATCGTCCGCTGAAAGAAATCAGCATTGGGGAGGGGTCAGCGGCTCGTATGCATGTT
GAAAGAACGCCAGAGAATCATCGTGAACACAGA
GTCATCGCTGCAGGCCGCAGAAACAGCACGCCAGAAATAAAAAAACTGATCATACGTCAAAGTTTTGG
ATATATTTCCATCAAAACACTGCGTCATCACA

AAACGCCAATCGTCACTGCTACCGATGAGAGTACCTTCTACCTTCTCAGGGTGAAACAATGCCAACAC
ACGCTCCACTTCAACGTGGTCCTGTTTCATTG
TAAGACCCTTCACATTTTTAAGTTGATCAAAAAGTTGCCGGACAAGAGCCTCCCGGTTTGCTGGAAAT
CTACCAGAAAGCAGCGACACCAGACGTTCTCG
ATCCCATCCCTTGAACCGCAGCCACTCAAGTTCAACGTCACTCACGGCCTCCGGAACACCCGCCACAC
GAAGCACATCAATGAACCTTGCGACAGAAAGT
GGACCACCATGAAGCGCAAGATGTAAACGTGGAAGGGCCAACATACCACCAGATTTCACCAAATAGCG
CCGCACTGCCTCTTCAACAATTTGAAACAGAG
ATTCACTCACAGGCACAAAATGTCGTTTAAATTCACAAAGGAGTGTGTGAGCAGAGAGAAGCTCTTCA
GAACTTTCTCCTCTTAAATAATAGAATAGCTG
ATGCACTTCCTCTAACAACAGACCAACACGCAAATACCTACATAAATCCTCAAAAGACTTGCGTGTCA
GAGAATAATGCTCCAGCTCTCCTCCAGTATCG
ATATTAACGTCAAAAACTTCCTCACGATGCATGCCATGATTTGGTTTTGGCGACAAACGATGCAGTCC
CTCAATAAGGGCAAGCGTACCCAAAGGTGCAC
CAATATTCGGATCACAAATCGCCTCAGCAAAGACGGACATCAGTAATGGATACTTCACATCAGAAGGC
AACGGCCTTTGATACAGAAACGCGTAAAGGTA
AGAACCATCAATAGCTACTGTGGAGGAAGACGGAGGGAGTCCCATAGAACAATAGTCAGTTAGAAAAA
AGGCCTCGGAAAGTGTCATATCATAAGATTCA
GTCAGGTAATCAAGAATCGCGGCTCTCTCAAAAACACAAGCATCTCCTCTACCATCTCGTGCGGACAC
TAAGAGTGCCTTACCACAAGCTGATTCATATG
GGTGGGGTCGTTTAGGCCTAGTGTCAATCCTCCGAAGCATTTCTAACACTAAAGAAGCTTGCACAGGA
CACATTTTCCGCAGGCATAACAACAACACCAC
TGGGCTAACTAAAACATCATCACCTCCGTTCCCCGTGCCATTTCCCCCATAGTCCTCAAGCGGTAACA
TCGCAAGAATGATTGAGAGTTCTCTATCGCTC
ATAACGCTCACTGGCAGAATCTGACGTAGGATGCCTTTCAAGGACCATACTTCAATTGCCTGCTCACG
AGAGCGCACACGATATCTTGTGTCAATCTGAT
TAAATGACGAGGAAAGAGAGCGGGCAAATGAAGAAGGGTCACATTTCACAAGATACTGAATGACTTTG
TGATATAGTCGCAGCAAGTACTGCGGGTCTCG
CAAAAGAGACTCTGCGGTAGTATCAAGCAACAACATAAAAAGAACGCTATATTTTTTTTTGTAGTAAT
GTTTTACCCCCTTCGCTTTGACTTGAACCGC
TTTAACTCACAGAGAAAATATAGAAAAAACAATAATAATAGCAATGCACTTCACGCTTCATTCATATT
ACCAAATGTACACAACCAATTCTTTTGCCCGC
GCACAATTCTATGGCTTGATGGGATACGAGCAGCAACCATTCACACACTTTCTTTTTTTGCTGTTGCC
TCTGCCTTGCATTCCCATCGCTGCCATCTTCG
CCTTGCCGTGAATAAACTCTCAAACTATCGGTATCAGATATGTTTTTACACTTATTGCCGCCACCTTC
CCAACCCCACTCATGCCTCTACTAATTATTCT
TTCTTCTGTCAAAATCAAATATTTATTTTCTACGTCACAAACAAATTATATTTTCATCCCCAACCATT
CCGGCCATTTTTGTTTTCCCTTTTTTTTTTTT
GCCTCGAGCAGCTACCCGCTTGCACTGTGAATTCAGAGCCCCACACTCCACGGCGTCCTCCGCGAAAC
TTAATTTTGGGAGAAAAGGAGCGACAGCATGC
CTCTCCTTCAGCAAAAGTAAATGTGTCCTTTTGCCATAAATTTTGTCAAACAATTGAAAAATACACTT
TCGCAGAAAACGTAATGCATTGCCAATGCCTC
CTTCTTCTGTTCAAGCATTAAAAAAAAAAAACGTCCAAAAAAATAAAATAAAAAAAACAAGATTTATC
ACCTCTAGTAGCTAAAGTAACTCAAGGGGCGC
CTGCGGCCCGTCCTTTTCCCCATGCACCTTCCAAGAGCGAATTGTTTTCCTGTCATGCAGCTGTCTAA
CGACGCGTCTACAGCAAATTATAAAGTAAAAG
AAAGTACAAACCGCTCACATTGCTGGTTCCTTTAGGAAAAAAGTTTCAGTGCAGGAAGACGGTTTAAA
GAGGGCATTTCAGAGAAACGGAAATTTCGCCT
CATTCCACAATCAAAGTCAGAATGCGTTCCATCGCCATCGTCTATACGTATGAATATGTTAAAAGTGT
GCCAGAGTGAATTGCAGACCTTCTCCTCATCC
CTCTACACCCTCCTCTTGTTCCCGAGCATGGCTTTGGTTATAAAGTATTATTGGAAAAATAGGTAAGA
ACAGTGATGCTGCAAATGCAATGTAAATGATT
TTCAGCTGGCACTCCAATTGCAAGAAAGAGGATAAGGAAAATAATGAAAATCTTAAAAGGCATAAGCC
CCTTTAAAAAGGACTCAATTTTTTTTTTTCTG

ATATAAGTTTTTATTTCCTCTCGTTGTAAGACGCAAAGTTAAAAAACAAGAAGCGTTTATTAACGCTC
CTTACTGCCTTTTTCAGTAGGCTTCACACAAA
GATTTGACGGCGGGTGAGCCTTTGGCAACAAAGGATATAGTGCACCTGCTGCCAGCGCCGTCACCATT
GCCACCCACTCCATCATAGTAAAATGGCGATA
ATGACCAAAGTAAGCAACTGACAACGAAAATGTACATAATTTACGCAGAGTCAGCGTCAAAATTAATG
TAAAAGCAGAGGTTTTGTCATTTAAAATATAA
ACTCCAGTTATACAAACATATTGCGTAAGGGCATTCAGTGCAAAATGCATGTAACTATCTGAAGAAAT
AGAGGCAAATTCTCGGAAAAGGCGCCCGGATT
GCAGGAAAAACAGAGGTATAGAAATGATATGGGAAAAAAAAAGAGCTTCTGCCCACATTGGCGGCGGT
GATAATGATGATTGGACCGACTCATCTCTCTT
TTTTGTTTCTTCTTCTCGACGACGTGCCGCTGCATACATGTACTCTTGAAATATCCCAAGACCAGTGG
AAAAGGCAGTAGTGCAAGCGAGAACTGTCAGT
CCTAAAAACCACCAAAGATTCCCTTCAGATGTCTTCATACCATTCTCAGCATTAAGGTTCTGAACCTT
TCGCGACTTCTCCATTGCAAGAGCAATAAGGC
CACCCGATATAGCTGTGGCACAGAGCACCTGTGAGCGTGTGTAGCGTTTCTCCAAGAAAAAGTAGCCG
GCCAACATATTCAACAAAAGTGACGATGACCT
AAACGTTGCGTGAAGAGGCACAGAGATGTATAATCCAAATACCAAGTTGCTGCTCACAGACATGAGCC
AGGAGGAGAATGCAAGAATGAGTTTGTGTGAT
GTCAGTATTCGCATTGGTCGTAAACGTATGCGTAACCAGCCTCCACCACGCGAGGAGTCAAAAAAGTT
GGACACAAGAAAAAATGGAAAGAAGTGAAACAA
CTACGTACTGGACAAACGTCATGGCATACAAAGTGTTGGGGCTTCTCACTATAATAAGTTCTAGTAAA
AATACATTTGCCTGGCAGCCACCCAAAACACC
ACATATTGCCAATGCGGCGTCTGACATGGTGTTAGCTTTTCTCGTTGAAGCATTTCAAGGAAGAAAAA
AAAAAAAAAAAAGGTCGCTCTGTGGGGAGTAAA
AGAGAGCGAGTGGCACGCAAAAACAAAAAAAGACTCCCGCAAGAAGCGAACGTGAGATGTTCCAAACA
ACTGGGCCCGGAAAAAAAAAAAAAAAAAAAGAA
AAAACAATAAGCACACACCCACAAATACGCACAATAGAGCAAGAAGAGATGTAACCGTAGAACTTTCA
CACACACACACAAAAAAAAACGTCAACGAAAAC
TAAAACTTCTTAATTTCGAGTATCCCACTTCATTTCATTCCTAGTCAAAGAAAAATGCTTCGGCAGAG
ATGACCACAACCACGTGAAATGAGGAAAAAGA
GAACCGCTCCCCCCATTCGTCTACATATTACGACGTCGCGTCCTATCAATGATAAAAGAAGGGATCTC
TGCAACGACAAAATTCGAAGAGCGAAACAGTG
GCTTCGATCTCAGATCGTGTGGTTTGTGCAGCATTGTCCTTTTGCGACGACGGCTACCGCTGTGATGT
GTGTGACTACTACCAAAGGACTCTTCCCGTGT
TCTCGCAGTCCCGTTGAAAGATAGAGAGGACGGACCACAAAGTCGAAACTGTTGAGACACTTCATTGC
GGAGGCGTTGTACGCGATTGTTTAGAGGCAGA
GGCTGCACGGCGGCGCCTCGAACACGCTGTGCAGAATCAACACGCTGCATTAGTGCCTTTAGCAGAGT
GTCAGAATTACGGCGAATGGCTTTCCGTTTAA
TAATATCACCATGATGATTGTAAATATTAGCCTCTGACGCCACATGCTGCTGCATTCGATCCGCAATT
CCGAGTCGTCGAGAAGCTTCATCGCCGATGTG
CTGTGTGGCGGTCAACTGACGGTGCAGGCCAGCTAATAACGCATCCTGCTTCTTCACCTCCTTCAAAC
GATCTTTAAGCTTGTTGTTGTCAACACCCTGT
GCCCAAATTTCCCCTTTTGCCTGCTCAAGTTTTGCCGTCAGCTCCACCACCTCACGTTGCGCATTCCG
ATGCTCTTCATTCAACCTCTCCCACTCCGACT
TTAACTCACAAAGTTGGCGTTCAGAAACTTGCTGTTTCGCGTTGTGTTCCTCAATGTAGGTAAAAAGA
TACGCAATCTGCTGCTCCATATGTTCACGTTC
TTCTCTATAATCCTGCTCAAGAGAAACAAGACTCTTATACTCATCCTCGCAATTCTGAAGCCGTTTCT
TTAGAAGTTCATTTTCCTTTTCCAAATCTTCT
ATTCTGTACTCCAGCTCCTTGTTTCGCAAGAACTCTTCTTCTAGTTGTCGTTTCACAGTTCGTATTTG
ACTCCGCAACTGTACGTTCTCGTGGCCCTGAT
GCAGTGAGGAAGAGGGTGTACCGTTCGCACCCTCACTCTGATCCCCAAACGACATAAATCGCGCCTTC
TGTTGTGGAGTAAGAAAAAGCCAAATAGTCCG
AAATGCTTCGTCCTTTTTTCGCATTTCCTGCAGAAGTGCACCTTGGTGTTTACTGCTGCGATCAAGTT
CCTGCTCTGTGCTATTTAAACGACTTTGAAGA

ACGTCACAAAGATTTCTCTGATCCTCAAAGGCCGCTCTCAATCGCTCACACTGCTCATCTCTTTCAGT
CAATGCCTTGAACCAAGACATCGTCTCCTCAC
GAATGGCACGCTCGGCATACTCACGTAGAAGCTTTAAATTTAGTTCATCAATTATACCTTTCAATTTC
GTATTTTCAGCAAAAACATCGTTAAACTTCCG
AAATGATTCCTCTTGGGCATTTTCGAGCTGCAAAACGTTCTTTCGGGCCTCCAGAAGATCCGTCATAA
TCCGGTGCTGTTCACATCGCATTCCTTCCATT
GCTTTCATCATGAACTTTTTCTGTTGACATACACCATCCAGCTGCTCCTGCATCCACACAATCGTGTC
AGTCACCTTCCGGAGTAATTCACTTGTTTCTA
ACTGAGGTGCCGCCTCTTCAAGAGCTCGTTGCGCTTCTTCTCTTTCCCTCGCCTGTTGCTGTAGTTCC
CGACGTACTTTCTCTGCAATATCCCCTCGTGA
TTCAAATGCGTTTAACTCATTTTCCCGATCACGCAAAATGCTCTGAAAAACGGCACACTGACGCTCCA
ACTCCAGGACGTGCCGGCGCATTCTCGCAGCC
TCGGGGTTTTCCTCAACTAGCACAGAATTATCATCGGCCTTACATCTGTTGGCTGCCTTTGCGGGCGT
CTTATTGAGACTGTCATCCTTTGATACAAGAG
CAGAGCGCTGCTTTTCAATATTTACTTCCTCCTGAAGTGTGCACACACGACGACGTAATGTTTCCACC
TCTTTCATCAAATGCGTGTAGGTTTCCTCAAA
ACTATTTGTTCCATTCATAAGAAAATCTCTTGCGCGGCTTTTGCTCTTTGTCAGAGCACATCTTTTTG
CCGTGGTTGCAAAGTCAAGAGAGTTCTTTGTC
TCAGCATAATCCCTTTTCTCCACGGATACAGTTGCAATAAGGAGAAGATAGCAATCAGTGAGAGTCGA
AGCAAGATAGAGAGTTAGGATGGATTCTCGTA
CGGGCAACACATCCGCATCATCTGCTGTAGCCCTAATGACAGCATGTAGCACTGAAAGAGATTTATTT
ATTGCAATGGCCTCCTCCAGCTCTTCCCCTAC
AACACCCGTCTTCTTTACGCGCTCGGAACCTGCAAGATCGCCAAGTGTTAGTCGACTTTTTAGTACAG
GAATGCCCTCAATGCATTTTGTCACATGAAAG
GATAGGAGAGCATGAGAACGAGAGCTACGTGCATTCAATACATGAGAACCCACAAATCGTTTGCGTTG
CGCCTCAGCAATTTTTGATTTTGCATCTGCGG
CATCACGCAAAAGATATCGCGGTTCAGTTACCAACGt
>scaffold1042.size9938
ATCAACATATCTGCCTGTCCCATGTGGCTTCGGCTTCCCACCGCACCGCTTTGCCTTTCACCCCCGCT
TATTTGTGTTGGTTGTTCGTGTTTTGGCGTCAA
AGTGGCGTTCTTTGGTCTCCCCCGCCTCGGAATTCCATTTTCCTCCACTTATTTACCGCCACAACTAC
GAATTTCTTTCCAAAAAGGCGCCGTGCACATC
AACACACTCAAGAGCCCGGAACGAAGGTCACGCCCCCCCACCCCACCCCCCCCAATACACACAGGCAT
GGACAAACAGGGAAATGCGTGTGTGTGTGTCC
GTGGAAGAAAGAAGGGAGAAAGAAACAGATATTTAATCGCAACAAAACACAAAAAAAAGAAAAAAAAA
CAAATAAAAAAAGCAAATAAGTACAGAGCAAA
AGAGAAAAAAAAAAAAAACATCGATGCGCGGTAAGGGAGCCGTGCCAATTCATATACATAAGGATACA
TGGGTATCCATTGTACCTAAGTTCAAATAATG
GGATAAAAAAAAAAAAAAAAAAAAGAGTGAAAAAAAAAAAAAAAAAAGACCGAAGCATCCCCTCGTAA
CGCCAATTGCAAGCGTCCAACATCAAATTATA
CCCACACCAAAAACCTCTCAACCGGTTGTTCAAGAAAAAAAAAAAGGACGCACGCACACACACACACA
CACGCACAACAACATATATAACAGAAAGCATT
TAATAAGTAAATAAATAAGGAATAAGGAAAAAAGAAAAAAACCTCACATCTGTGCACTGTAATTCGGA
CCGCCACCTCCCTCCGGCACACTCCAATTGAT
GTTCTGCGTGGGATCCTTGATATCACAAGCCTTACAGTGCAGACAGTTCTGTGCGTTGATGACTAATT
TCCCATCCACGAATTCGTACACCTTTGCAGGA
CAATACTTCCCCTCAGGACCCTCATACACAGCAAGATTGACTTTTTCCGGCACTGAAGAGTCCTTCAG
CTTGAGGTGCGCCGGTTGGTCGGCGTTGTGCG
CAGTGCCGCTACGGCTATGGTTGGTGAGAAGATCAAAGGTGAGTTTCCCGTCAGGCTTCGGGTACTCG
ATTGGCTCACAATCCGCGGCGACTTTGAGGGC
CTCGTGGTCCGGCTTGTGATGTGAAAGCGTCCATGGCTCCCAGCCGCGCGTGATCGTTGTTGTGATCC
CTGTGTACAAAACACCCTGGAAGAAGTTGCGT
GCAAACACCTGGCGCACATTGCGCACCGCGTAAAGTTCCTTGTGCAGCCAACTGTTGCGGAACCGATC
GTCGTAGCTCCTGCACTCCACCCCATCCCGCT

TTTCCTTTCCGGAAGCAAAGGCGTCATCGTATACCGCCTCTGCTGCAAGTATACCAGACTTCATTGCC
GTGTGGGTCCCTTTAATCTTCGGCAGGTTCAA
AAATCCGGCGCAGTCACCAACGAGCAGGCCACCGGGGAAGTGTAATTTGGGCAGAGAGACGTAACCGC
CCTCAACAAGGGTTCGTGCGCCGTACAGCAGG
GGTTTTCCATCCTTCAGTTGCGACGAAATCAACTTGTGTGTCTTCCACTTCTGCATTTCCATGTACGG
TCGGATATGCGGGTTGCTATAGTCCAGACCAA
TAACGAAGCCAAGTGAAATCAATCCATCGCCGTAGTGATACATGAAGGAGCCACCGTATGTGTTGTGA
TGTCCCTTGTCCGTGAGGGGCCATCCAATGGT
GTGCATCACCGTCCCAGGACGATGCGCCTCCGCTGGCACCTCCCAAACTTCCTTGATGCCGAGCCCAT
ATGTCTGGAAATTTCCCTCACCACGGAGACCA
AACTTTTTCTCTAGCTGCTTTGTACAGGATCCGCGGCATCCTTCGGCAAATATAGTTTGCTTTGCCTT
GAAGATCATGCCAGGTTCGTATTGGGGTGTTT
TTTCACCCTTCTTGTTGATACCAACGTCATTCAGCTGCACCCCTTCTAGCGCCTCGCCACTGCCGTCA
TACACTGGCTGCGCTGCGGCAAAGCCTGGGTA
TATCTCCACACCCAACTCCGTCGCCCGCTCGCCTAGCCATTTACACACGGAACCAAGAGACACAATGT
AGGAATGGCGATTGTGTAGTGTGGGCGGAAGC
AACGGCGACTTCATGCTTCGCTCCTTATCCCGAAGAAGATATAATGCATCATTCTTGACAGCTGTCAT
TGTCGGCAGATCTGTGGCTTTGCGCCAGTCCG
GTAACAACTCATCTAGCGAGTGTGTCTCAATACAGGCGCCAGAGAGCGTGTGCGCCCCAATCTCACTT
CCCTTCTCGACGAGTCCAACCCGAAAGTCGTC
CTTCGCATCGCCGGCAAGCTGTTTCAGGCGGATGGCCGCAGATAATCCCGCGGGGCCACCCCCCACAA
TGACAACGTCAAATTCCTCCACCATCCGTTCC
ACAGGCGCGCAAAAAAATGCGGCAGGAACGACGAAACATGAATGAATATGGTACGCTGGCAGTGTATTT
AATTAATAATGTACTATCCACCTAGACAAATC
TGAATGTTCGCGGTAATGATTCCTGCAGAGATAGGAAAAGATGTAAAAAGTGAAAATAAAAATAAAAA
TCATAGAGAAAGAGAAGCAAAGGCACATTTAT
TTTATTTTATTTATTTTTTTTTTTTTTTTTGTGGGTGTGGGTGTTTGTTTTGGTGTCGTTTATGAATAA
TATTTCGTTCAATACATGTGTGTTTATTTGTT
TGTTTGTGTGTGTGTGTGTGTTTATTTATTATTCTCTGTCTCTCTTCATCTTCTCGGTGGCGGGGGCA
CAAAAAATTCAAACGAAGGGGTAATTCCTACA
TATCCCTCATTTCCCCGGCCCAACGAAAAAAAGGCGTACTCAAACACGTTAAAAATAATAATAAACGC
CAAGCAAAAAAAGGACAGTAACTTAAAGAGAT
ACAAGAGGGAAGGAGATAAATCAGTGGGCATGCGCACTCACGGCCGTTCCTCAAACTTTCCACAGAGG
CACCCTTATGTATATATATATATATATATCCC
TAGTAACACGGGCATTCAAACATGCCCCGTGTTATTGAAATAACAGAACACGAGTAGAATAAAAAAAA
AAAAAAAAGAACGTACATTATAAGCAGAAAAT
CAGCATGTTATTTCATCTGTACACTCCTCTCCTTCTTTCTTTATCTATCCCTGTCTTTTTTTTTTTTT
TCCCTCCGGCATTCCACTTTCTTTCGTTTTTT
TGTTTTTTTAGTTTTTTGTTTTGTTTCTTTTCTTTTCACTTCGTATGTTTCCCCTCCACCTGCTCAAT
CACGCCACATGGTGGCGTAGTAGCAGTAGTAA
AAACAAAGTTGTTGCGCTGGCCGCAAACGTGTAGTCATCCCACCGCCACTCATGCGACACCACATCAG
GCAATGTTTCTGACCCAGTGGAGAGCAGCTGC
TTCGGCCCCTGATGTTCCTGCCTTCTTTGTTGTTGTTGTTGTTGTTGTTCATGAAGCTCCGGCGCCAT
TGACGCTTTTTCAGTTTCATTTTCCGCACCAC
TGCCACCGTCACCCCCGCCGCCACCACGCGCCATCGGCATCGGTAACCGCAGCATCCAGGAAAAGCCG
AACTGCTGGTGCGAAAAGTACAGCCACCACCA
CTGGAGGTTGAGGGTCATTAACGCCAGCTTCACAGCGTAGCCGCTCCCGCCTGAGCCAAACTCCATGG
CACGGTCGTAATACCGCTTTGCCAAGTACAGC
CGCATCTCCTCCGCAAGAAGAGCGTCCTTGATATTCTTCGTACTCGTCGTCCTGCTAAAATTGGCTTC
ACTTCCACTTTTGCCTCCTCCTTCTGTCTCCT
CCTCTAGTAAGGTGTTGGTAACGATGCGGTAAAGGGCATCGGAGACGCTGGAGACTTGGTCCCTGCCA
GAAACGCCAAGCCCTAATTGGTACATAAATCC
CAAATTAAAATATGCCTTGGGGTCTTGTTTCGCCGCAGCAGCCTCGTAATGCAGCAACGCCCGCGACA
TGCTTACGCCAACAAGTTGGCTCTCACCGTAG

TAGTAGAAATCTCCTAGTCGCAAGTTGGCCTCCGCACTGCCAGAAAGAGCGGACAACCCGTACATTCG
CAGTCGCAACATGTTAAGCGCCTCTCTCTGCG
ACAAAAGTCGTGCGTCCGCAAGTGGAATAATTGATGCTTCCCCAGTGGGCACGGGACGCATGGAGTTG
TGATACCACTGCAGCCACACGACGCCACCGTC
CATACGCAAGGCGCGCTCAGGAGGGCGACCACTCTTTGCAAAGACGCCGTCCTCAAGTTCATCGTCTG
TCTCTTCAATGATGGGCAATTTATCCAACAGT
TGCGCGACCTGGTACCGAGACGCGGCATGCCCGGTCTCTGCCATTGCAAGCATTTCTAAAAGCTGCGG
GAAAATGTTATCTGCACTTGAAGCAAACGATG
ACGAATACAGCGAAGACGACGACGACGATCCATCGCTGAGTGCAAAGTACGGCACGTCGTTGCTGTGG
TCGAAAATAGAGTCGAACCCCGCCACACGACT
GTATAATTGCATCGCCTGTTCGCAGTTTCCCTTTCTCTGCTCATGGCGTGCGAGTTGCCAAATGGAGA
GAACATTTCCTCGCATCGCTGCCAATCCAAAC
TGCTTGATCGCGGCGGCTGGATCATACGGCACCCCACGACCGTGCAGAAGCAGCACCCCAAGGTTGTG
CGTTCCCTCCACGCTTCCACGGTCAACACTCT
CGGCAAAGAACTTTGCCGCCTTCTCAAAATTCGGTTTTCGCCCCTTCGCTGCTGACGAGTTCCCGTCG
TTAAAGAAACCGATCGCGTGCAGGTATCCCAT
GCCGTTAAGTGAGACGGCGTCCTTTTTCTCTGCTCCTTGGGAAAAATAAGTCGCGGCGGTTGCAAGAT
CTCGCCCAATTGCCGGATTTGCAGTACTGTCG
CCCGTGGCGTACAGTCTCCCCAATGCGCCGTATGCCGCCACATCACCCTTGGCGAGGGCGTCAAGGAA
ATAAAGGCGTGCTTGATGCCAATTGCGACGCA
ATCCATAAATTCCCTTAAAATAAGCGTAGCCAAGGGAAATGATCGCGTCCGTGGAGCCCTCGTCTGCT
CGGAACATCAGCATCTGTACCATCTTTGAATT
AGAATGCCCGCCATGTTTTAATGCGTCCGCGCTGAATTGCCTGGAAAACCTTTCTGTGGGATTAGGCA
TGCCCTCATAATTCGTAACGACGACGTCAGCG
GCCTCGCGATAGTGCTCCAATGCGTCCTGGCAACTCTTCGCGACGCCCAGTCCATCTGTATACCGCCG
CCCAAGAGCCATGTGAGCCTCAAATACTTTCT
CCAACGCTGCAAAGGTATAATGCAAGACCGCCTTGGCATCCGATTGCGGAACGCCAATTCCATTCGCG
TGTAATACACCCAGTAACCAATGCAGACGACC
GTGACCATGCAAGGTTCCCTCCTTAATAAGCTCAAAAGTAGACGAAAGGTTGCCGTTGTAGATGGAAC
GCACAACATTTTGGTAAAGCTCCGACGGCTCC
ACATTCGATAATTGGCTTGGTACAAACGAGTACGCATCCGCCCCTTCCGTCTCGGTTTCGGTTGTAGG
GAATTCTTTCGAATAAATTTCCTCGATACCAA
ATTCGGCTGCCGCATCAACCGCATTATCGCGATTCATATGATGGTTCGTTTCATCAACGTCACGGTCA
TTTACACTATACTCCGCGGCAGAAACTGAAGT
TGTGAGCGTATCCACGTCATTCACGATTGGCATAACACCATCGTCCTCCTGTTGTACAACCAACGCAA
ATTCCCTCATCGCATCATTTCTGCGTTTCTCA
TGCCTTTTTTCCTCCGACGCCTGCAGGGGATTCTTCTCTTGTTTCTCCTCCATCACATGGGCGAGCGC
AAGCGAATCGGATGGCTCAACCACACTCCGGT
CAGCCTCCTTTATTCCTAACGCGTCTTTTTTCGACTGAGTTAACTTCTCTGACGCCGCGGAAATTTCA
TTCTCCCGTTGCAGTTCGGGATATTCCTTCAC
GGGTAATGCTGCCCTCTCTGTGGCCGTTTTCACCGCATCTCGTGGATCCTCCTCTGAATCACCACTGG
CCGTTGACACGGACGAGGATGGACCAGAGGAG
GAGGAGGGAGAAGAAGAAGAGGACAGGGCCGTAGAGGGCAAAAAAAACAAAGAAATTATTAATACTAA
AAATACCGGCGTCACCGACCCCTGTGGCCGAA
GGAGCCGCAGTTGCGGCAGAAACAGTCCCGACATGCAAAGAATAAATGCCGTCGCTACGACAAGCCAG
GAATGTTCTGCAAAACACACACACACAGAGAC
AAAAAAAAAAAAAGCCTTCTAATGCTGAACAAGAAGAAAGAAAAAGCACCTAACCTCCCCCCCTTCCTT
GCAGTGGACGCAGCACACAAAAGAATAGATGG
GGAGAAAAGCAAAAAAAAAAAAAATTTTTTTTAAGAACAATTAAAAAAAAAGATCTTAAGACATCCAA
ATAAAAACACAAGGCACACCACAAACATACGT
ATTGGCTTGCCGCTGTTGTATTTGTTGTGTTTGTTGGGATGGGAGTTTATCGCAATGAGCCTTTATTA
ATCGAAACGGTATCATACTCCGTTTTTTTTTT
CCTTCCCAATCGCGAGAGAAGAAAGAGAAAAAAAAAAGTTGACTTTTAAGGAATCCTTGTTGTTTTGTG
TTTTTTGTGTGCCCCTGGTCCTCCCTCTTTCA

TTCATTTACTTTTTTTTTTATTCCTTTTTTTTTCNNNNNNNNNNNNNNNNNNNNNNNNNNNNNNNNN
NNNNNNNNNNNNNNNNNNNNNNNNNNNNNNNNN
NNNNNNNNNNNNNNNNNNNNNNNNNNNNNNNNNNNNNNNNNNNNNNNNNNNNNNNNNNNNNNNNNNN
NNNNNNNNNNNNNNNNNNNNNNNNNNNNNNNNN
NNNNNNNNNNNNNNNNNNNNNNNNNNNNNNNNNNNNNNNNNNNNNNNNNNNNNNNNNNNNNNNNNNN
NNNNNNNNNNNNNNNNNNNNNNNNNNNNNNNNN
NNNNNNNNNNNNNNNNNNNNNNNNNNNNNNNNNNNNCCTCTTCTTCTCTCTTCTTTTGACGCCCTCACTT
TCCCCCTCCATCGTCCCCGTAAAAGTAGGCCT
CCAGCGCCGCGGTATCGGTGCAAGCGGCCAGTGTGCACGCGAGGGAGACTCGAATCATCTTCGTGTCG
TCTTCTTTTGACAACATGCCGCGCACAACGTC
GCGGTGGTACGGCAGGCGTATCTTCACCTCCTTTGAAACGCGCATCTCCGCCTCGGTGCTCTCCACGA
ATCCAAAGCACGAGAAAGAAAACCCTTTGGGC
ATGTCCATTAAACTACGGTAATTTCCCAACGGTCCATTTGACGCCTCCTGCCGCTGGGTAACACACGC
GAGAGGTGAAGAAGAGGGGACAAGGGAAAACG
AAAGCGCCACAACACTGTGCTCCAGTGCCGCGCACACCATATCGTCCGTCATATCACGGGCATCGTCC
ACGCCGGCAAGCACAATCGACTCAAAATGCCT
CAGTTGACCCCTGAACAACGTCGTGGACTCATCCGCTTCTGTCAGTTGCAACTCGTTCCCACCATTCT
TCCGCCGCTTCTCATCCAGGGTCTCGTAATAG
CAGAATATGGGCGCCAAATGCTGCTGCCAAATGGCACTACGAATAGCAGAGGCTTTAGGCTGTATACC
CTGCTGCAGTGATCCCTCGCTCCGCGTTACGT
TTACCGTATGCACTCTCCCAGACCACTCAGCGGTAGTAGCATACTCCGGTCTATTCATGACGGAATGC
GGGTACGGGAGTCGCCCAATGACGTCGTTCAC
TTTCCTTTTCGTGTTTTCCAACTCGGCACGGCGCACAAAAAACCTTTTGTGCAGAACCTCCGAATTGA
GGCCTTTGGTAGGACACATGAGCAACTCGCTG
TCGCGCGCCCAGCGTGTCTCCCCTTCCTTCACCAAGTGAACAATGTGCTTGGGTTGTAGGCGGCGTAT
CGCCTCCACCGTCATGCGCCGTCCCGTGCTAA
GCACCCAACCGTGTGTGTTCACCACGACAGGAAATCGTCGCTGCAGTGGCCCAACAATGGCACAAAGC
TGCTCAATGGCGATCGCTGTCGCTACTGGGCA
GCGCGGACGTGACCCGCCAAGGAAAAATCCCTTCACAAGTTTAACCTTCCTTGTGTCACGTGGGCGAA
GAAGAGGTTGTTGGACGCAGTAGAGAGAAAAT
ACCCCAGGGGGAGAAAACTCCGGCTGTCCGAGATCCAAATCAAGCCAGTAGCAGACACCATGCTGTGA
AAATAGGGCATTTGCAATAAACCGCGCAAGAG
TCGACTTGCCGATGTTCTGTGAACCCAACACAACCACCACACCGGTGCCTTGCTTCACAATGGCAGGA
ATTACCTCCGGTAACAAGACCGCATCAATACC
AACATCATTTCCACGTGCAACAAAGCGTGGAACTTCCATAAAAGCGTCGGTTAATTTCTTCCCTTTCC
CTTTAAGACGATGCCGGCGCGTTGGGGGCAGA
CCGTAAGGGTCATATGGCTGCACAACTAGCACAATGCTTGGCATCTTGTTCGCAAATCGTCCCCGCCA
AGACCCAATCGTCGTCTCCACCCAAACCCAAT
CAATGGCGCCAAAAATCTCCACGTCATTATCGTCCGCCGCATCCTCATAACTTTCTCGTATCGTCGGA
GGAACGACAGTGACTGGGTCGTTTTTTGCCTT
GAAAGAGAATGGCACTGGCACCGGAAGAGACTCCAACCCCGCATTCTTGCTTTTTAGGCGTTGCACAG
GCGTCAAAACAACCCTATCGGCTTCACTGTAA
AGATTAATTTGTTTCTTTCCGAGAAAGAAACCACCAACGGCAACCTCACCAATACCAAACCCAATGAT
GCCACAAGGGCCCTGAACAGTGACGGGCCCGC
TCAGACAAATAAGTGTGTGGTGGCATGGACGCATGGCCTCTCGGGCAATGTGCATCTCAAAGAACATC
GAAGTGTAGCGGTAACGGCGGTCACTAGTGTG
GTTGGTCGCAGCATCCACTCCACCCTCATCCTTGACCGCCCCCATTTTCCCTTTTTGCGATTGTTGTT
CCCTTTGCTGATGACGCCGGCGTTCAAGAAGA
CCAAATCCAGCTCGGTCTCCTTCGTTCTTGGGCGGAACATTATTATCCTTATCATCTTCACCTCCATC
ATCATTTTCAGAGCTCTCACTCCCGCTGGTTT
CTTCCTCCTCTTCCTCTTCCTCTTCCTCTTCTGAAAGAGATTCAATGTTTTCGCTCTCATAGCCGGCA
AAGTGTCGATCGATCACCCTCCTTTGCTTTTC
CGTACGCGCATCCGCATTGCCGTCATCATCCTGTAATTTTTCATCCGACCAAGATGAGATTAGAGAAG
AAGAGTCACTGTTTTCAGCCCGCCCTGTAGCG

GCAAAGCGAATAAATTCATCATTGCTATTGGCAAAATGCTCGGCTGGTGGATTCAATTCTCCACTTCG
CTGCTGACCCTCGCCTTGTCGAGGTTGAGGTC
GAGGAATTCGGCGCACCGAAACCAGCGCTCTCAAATCATCACCTGCCTCAATATCCGACGCTGACGCG
TGATGTTTTGCCGCAGCCAGCTTTTTCGAGTG
AATTACCTCAGCGACACCTTTTGCCGCACTGTCCCCCTCAACGTTCCCCATACCGGATGCGGGCGGTG
TCTTTCCGAGGCGTGTCAGCACTTTATCCGGT
AGTTTTAGCCGCTTTACCGGTGGCGCCGTGTCCATATCGTCCCTGTTGCCCCCTTCCGGCAATGCGGT
GACGGCGTCGGACGGCGCCCATGGGTGGACCA
AAGGAATTCCCCCGCCGCGAGTCGTGGTCTCCTCTGCCCCTGCAGACACCTCGGTGGTGCCAGTGAAC
AGAAGCCGCCTCGTCCTCCGCATAGCCACTAG
GATCAATAGAAAAAAAAAAAAAAAAAAAAAAAAAAAAAAAAA
>scaffold1043.size7008
TTTTTTTTTATGGAACAGAGAATTGAAGGAGTAAATACCACGGAAACGAAAGGGAACGGCAAATATGA
AAGGAAGAAGAAGAAAGGGGGGGAGGAATGTGA
GAGAGAGTGAGAGAGAGACAGCAAGAGAGAATTGGATACGTGAATATAATAAAGAAATGGTGGATAAT
GGATTGTTGTTGTTGTTGTTGTTGTTTTGGCT
GTGGCACAATTGAGCTCTGCCCAATGCAACACGTGAATTCAACTCACCGTCGTTTCATGCATTCTGCA
CAATATTCGAGAGTTTTGTGTTTTGTTTTTTG
TTTGTAATTTTCATAGAGAGAGAGAAAGAGAAAATTAAATTGCGACGGTGTGTGGGCTTCTTACTATT
ATTATTATTATTGTTGTTGTTGTTGTTGTTTT
CTTCTCTTTTTTTTTTTTTTTTTTTTTTTTTTGTGTGTGTGTGTGTGTTTTTGGGGGGTTTTGTTGTCT
TTTTTATTATTATTGGATGGAGCCCATGGAAG
CAAAGCTGTCTTTTGTGCCGTGTGTAGAAGACGACTGGCGGCGGGCTCGTCACAGGGAAAGTGATGCA
AATTCCCTGTCGTTGGCGCGTCGCCTGGCTTT
TCTCGAGGGTCATGTGGAAGAGGCGTATAACCTGCTGGAGACGGGACGTAGGCAGGGGGACTTTTCTT
CTTCTTGGGCACGACGGGAGTGGAAAGAGGCA
GCCTTGACACTTCTTTCACTCCACAACACGGAGGACGATGCATCGCGGTGGGTTGCGAGTGACCTCTT
GCCATTACCATCCAAAGCGGCTCGCACGGCGC
TTGGAAAAATGGACAATGTGATGTACCATTACTGCATCTTGTACTGGGTGAAGCTTGCAGTTACGGCA
ATGATGCATCGAGAGATGTCTTATGACTTGTC
ATTGAGGGCAGCAGCAGACACGCTGCTTTTTGTACTTAAAGATATCTGTTGCATGCGTCTCTCCTTGT
CCTCACGTTGTTTGCCCACATGGCGATTGTTG
GAGCATGTGCTCCTGGAGCTGTTGCGTGTGGGTGAGAGCAGTGCGGATGCCTCTGTGTCCTCCGCTCT
GGACGTGGTGCGGCGGATGCACCAGTACCTTG
GCCAGTATATTATCCGCTACCTGGAAATGAGGGATCGTCCGATGGAGGGACAGCGTGGCCTTCTTACG
TACCGCGGCGTATTTGACCGCCAGTCCGGGGG
CATGAATGGGGGCGACGATGAGACGACGGGCAAGCGTGAGGAAGAGGGCGTGGAACGACTTCCCACAA
GGTACTTGCCTCGTGAAGTGTGCGGGGCGTTG
CGCACACTGTGCAGGAGCGGGGACACATGCAGGAGTCTTGAGCTCTTGAAGGATAGTCACCCCACTGA
TAGTGCCGCTGCCGCTGCTGAGGATAAAGATG
AAGATGATAAGAACATATTGGCGTTTGAAGCGTTTTTGGACCACTACTTTTTCCGCAGTGCACCGGTG
GCGTTTGAAAGGCCGTTATGTAGCTCGGAAGT
TTAGTAGTAAAGAAAAAAAGAACAAAAAAAAAGTCCCTTCAACGGGGGGGGAGGAGATGTCTTGCCAT
GATGATGATGATTATGATTACTATTAATTTTC
TCTTTACTCACGCACACAATTCTCCCATTATTTTTTTTTCTTTTTTTTTTTTTTTTTTTGGTTTCAC
GACTTCATTCCATTTCTCTTTCAGGGTGACGA
GCCGTGTGGTGTCTATTGGCGCTTGTCTTTTTTTTCTATCTTTTTCTTGTGTGTGTGTGTGTGTGTAG
CGCGTCGCCATGTTCACCGATTCCGCCGCGCA
CACTTCACTCGCATTTGAAGATGCCCTCCCCGATGAACCGCATGCCACCGTCAAGCGGCGTGAGATCT
GTCAACCCTTTCAACACGGGAAGTGTCGGCAT
GGTACGGCCTGCCCGGAGCGTCATGTCATCTCACAGTTCAAGACGATGCGTCTGGAGGTGTGTAAACA
TTGGCTTCGTGGTGCCTGCGTCAATGGTGAAA
ATTGTCTGTACCTGCATGAATATGACGACCGGTATGTGCCGTTGTGTGCCTTCTATCAACGCCTAGGA
GAATGCACGAACCCGGAGTGTCCTTTTCAGCA

TGTCATACAGGTGGAGAAACAACCGGAATGTGCCGCCTACCGCCGTGGATTCTGTCCACTTGGACCAA
AATGTCATCTCCGGCATGTTTTTCGTCCATCC
TGTCCATTCTACATGGCAGGGTTTTGTCCGCTCGGACCCAAGTGTACTATGGGGCATCCTGTCCAAGA
GTTATATAACCGTAATAGCGTGTCTGAGCGTC
TTCGACAACGCATGCTTATTGAGCGTGTGGATGACCCAACATTTAACAAAAATGCAACATGTTATCGC
TGCTTTGATCCTGGCCACTTGTCACCCAACTG
TCCAGGTGTGCAGAGTGGCCTCCTCCGACGGATGCTCATGGCAGTACAGGAGCCGGGAGAACAACTTT
ACTTCCAGAGCGATGGGCGGTCAGCACGAAAA
TGTTGTTTTTTTTGTGGAGATGAGAGCCATGAGGTGCGGGATTGCCCAAAGAAACCAAAGAATCATCA
ATGGCACACTCACAGACGTGCATAAATGTCAA
GAAGGTAGACGCAGAAAGTCGTCATGGTGGTGTAATTTTTTTTGTTGGACACAGGAATTCTAGGCGTT
GAGGACGAAATTTTTAAATATGCATACCCGTG
TGTGTGTATGTACGAGTGTGTTTGTGTGTGGATTTAAGATTGTGTTTGTCGCTGGTCGATATTTACTT
GATTTGCCGCATTCTTCCAGCGTGAGGGTGAA
ATATTGGCTTCTATTTCTTGACGAGTAGTCATCAGAGTGTTTTTGTTGTTGTTGTTGTTGTTGTTGTA
TCCTCTTCTGTGTGTGTGGCGCCTCACTTCTC
TTTAACCCCGTCCGCCTTCCGCACTTTGTTACTTATTTGAATGCCTCGCTGTTGCTGCGAGGCGCGTC
AAGCCTAAAGAAAGGAATGAGGTTTTCTCGTG
TCATTTGTGGTCGGCTGCGACGGCGCACGCATGGCAACGTGCCGGTCATGCAGCAGCAGAAGCAGCAG
TGTGCATGGGAAAGAGAGGAGACGTTGGCCAC
CCTTCCTCTGAGTAATGCAGAGAGGACGGCAAGTGCGCTTGCATTTCAGTTGTTAGAGAGGCACCGGC
GTCGACACGCATCAGACTTACATGGGCGGGCC
GCTGTGGGTATTGACGGTACGGCAGAAGTTGGCTCGCATCCGACGCAGGAGGAAATTCAAGAAGGGCC
ATTAAACAACTCCCGGACTCGGCCATCGCATC
TGCCCGCCGTAGATGCTTTTACAGGGTACGGCAAAACTGCTGTCACGACAAACTTGGCGCACATGGCG
GATCCATGGGTTGTGGATACACGGAATCTGCT
GGAGTCCACAGACCGAGAGTGGGAGGCGCTGACGGCACGAAGCAAGATGTCAGCCACCTTAACGGATA
CCGGCGACCGACCCACAATGCGTGCGTGGGTT
CGATATTCCCGCGGCAACGCCTCgGCGCACCTCCCTGAGAAATTCGGTGAGAAAATTATTACACCGTT
TGATGCCGTGATGCGACGGTACAACTCGGTGG
TGACAAATCATAGATTCCGCGTTGTAACGGTGGATGCTTTTTGTAGCTGGCGTGTAAACATGCCTGCG
AGCCTGACGCAGCGGGGCACCATGCGTTGTGT
TTTGGCACGAAAAGAACAGCTGGATCGCCAAAATGAATGGCGTAAACTGAGGAAGTCTCAGTTGCCGC
TAGGAAGTGACATTGGCGATGGTAAATTCGAT
AAGGAAAGGAGAAATGTAGAGAAATTATCGTCTAGCCGAGATTTCAGCAGTATGCCTTTTGCCTCTCC
CGTGTTTCGTCGGTACTGTTTGCAGTTTGGCA
GTCATACACTGCGGCGAGTGGCATTCTTGACGGACCCTACGTTGTATTTTGTTCTGTGTAACTGGCGT
TCAACCGGTAATGAATCCCTTATGGAAGTACG
CCAGCAGGCGGAGTGCTTGAGCCACCGTTATATGTCGATGGCGTATCTTCCCTGATGCGGGAGCAAAT
TGTCAAACAAGCAATTCACACTTAAATGGGTC
CAATGGAAAAGTCATTCTTATGGTACTTTACCCGCTATTCTATTATTTTCTCTCTATAGAGTGTATGC
ATGAAGGGGAGAAAAAGGGAGTCATATACACA
TATATATATATATATATATATAGGCGCCCCGAATAGAGCCTTACCACATGCAAAAATAAAATAAAAAATT
TTTTTTTTCTCTCTCTCTCTCTCTTATACATG
AAAGGGTGGGAAAGTGTGGAATGTATAATACATATGGCAATAGAAGAAATAAACACCTAAGACGCCCT
TACCTCTCAGTTTCCTTTTTTCTTTTTCTTCT
CCCTTTTTTATACATAAGATCTCCATCCCTCCCGTCATCCCACTCATTTGTCACATCAACTGCAACGA
CATCGGCATCCGTTGCAATTTTTTTTTTTTTT
TTTTTTTTTGCAAACGGCATTTCCCTTTGGTTTTTCCCACACACACACACACACACACAAAAACGGCT
AGTAGTCCACACGCAAACACAATGATATATGA
TGACATGGTCTCCGGTAAGTTGTCGGAGGGAAGTATGCCACAACGTGAACTTGGAGACATTCAACACT
CTATTGAACGCATTGAGAAACTGCTGCATCGT
GGTCAAAATTTATTATATGATGAAGGTAGTAGGGAAGTGGGACCACCACATGGTACCATGCATCGTAA
AGGAGACGCCAGTATAAATGAAGGGGAAAAAT

TAAATTCCCTCCCCTCCATGAAAAGAATTCGACGAAGCGTAACACAAAGATCCCCAACTGCTCACGTT
ACACGGAAAATGTTCCCGAATACCACCTTCCA
ACGCACAGCAACTGAATTACTTTTGCTTCAACGGGAACACGCAGCAGATGGAAAGCAAAAAAAAACAG
GCGTACACGTGCGGCATCGAGAAACAATGCCA
TTTGCTAAGGGAACACAATACTCCACAAACTATGGTGTGGCAACGAATATAAAATGGGAAGCGGCAGA
TAGTAGTGACTACGAGGATGTCTCTATTGCCG
AGCTTCGAGTACGTATTCGTAAGGAATTGGAGGCGTACCGTCTCAACGGACCACTGACGGGGGTGAAG
CAAAAAAAAGCTGATACTATCCCACAGCCGGT
GACACGTCTTTACCCGCGACACAAAAGAACCTCACAGGAGGAGCCAATAAAAATGGTGTCACCGCAAT
CGAAGCGACGACTTATCACAGGGAATCAAGCA
ACCGGAACACCGCAGTCTTATGGACACGAGGGGCATCGTAAAAGGACAACATCCCCTCAAATATCTCC
AGAAAAAAGTGCTTCCCGTTCTCCCTTGGAAG
GGAATATTAAGTTACATGAAAAACACCACATCTCTCCATGGAAGCGGTTATATAACGATGCCATTAGA
ATGCAAGAAAAAAGGCAACATCATCAGATGGC
ACCTCCGTCTTGGGAGGAAAATTCCCCTGCCCACAAAAAGAATCCATGGGAACGTGGTTTTCTGATAA
AGCGGTTTTCAATGAAACCTACGCCAGGAGCA
CCACCCCACAGTGTGCAACAGGCGTCAAAGGCCTCGTATCATAGAAGAGAGACCCACGACGCAACACC
GAAAGCGACATTAGTGGGGGAATCCCGAAAGC
CGTCGGAGAGCGTACGGTCGCTACAGTTCCCCGCAACTGTACTCGCACACCGTTTGCTGAAGCCACAG
AAAAATCATTTGTTAGGGGCATTACGGGACCG
GAAAAAGAAGGAAAGGGAAGGAATATCACAACAACATCACCCCGAACCCAAAGTCAACACGATTGGGT
CGTTACCTTTAGCCGCTGAGGAAAAGAAAACT
GATGAGAAGCCGCTGGAGATTGTGAATGATGAAAATGCTGCAGGGAACGCAGGGATAGCGAAACATGA
AACATCGGAGTCTGCATCAACCTCCAATCGTA
TGCAGTGGCCGACGATCACGCCTCCTTCCACCGATATCTCAGAAGGTTGGGGAATGTTTTCACATGCT
ACAAACGACACCCCATTGGCCGTGGTATCACA
AGAGTCGTCTCCCACAAATCAACTCCATATTCCACTTAGTATACGCCCACTCGACTTGAGTGCGCTTC
GCAAATAGAGATGCGATCTCTTTTCCTTTCTT
TCTCTCTCTCTTCTTTTTCTTTTTTTTTTTGCTTTGTTCGCATGTTTGTTGTTTTTTGGATCTTATCC
CCCCCCCCAAGAAGCTCCATGCGAGCACATAC
AAGTGCAAATTGGACAGACGCNNNNNNNNNNNNNNNNNNNNNNNNNNNNNNNNNNNNNNNNNNNNNNN
NNNNNNNNNNNNNNNNNNNNNNNNNNNNNNNN
NNNNNNNNNNNNNNNNNNNNNNNNNNNNNNNNNNNNNNNNNNNNNNNNNNNNNNNNNNNNNNNNNNNN
NNNNNNNNNNNNNNNNNNNNNNNNNNNNNNNN
NNNNNNNNNNNNNNNNNNNNNNNNNNNNNNNNNNNNNNNNNNNNNNNNNNNNNNNNNNNNNNNNNNNN
NNNNNNNNNNNNNNNNNNNNNNNNNNNNNNNN
NNNNNNNNNNNNNNNNNNNNNGAATGTTTATAATATATACATATATATACACCTTTTTGCGTAAACAT
CACTTCTCCATCTTTCATTTAAAACCCCATTT
TTTTAAAAAAAAAAAATTTTTATTTGTTTTTCAGAAGGGGAGAACAGACATGTTTGCCCCTTTTTTTT
TCTTTTTTTTGTTTCCTTTGCCAAATCGGTAA
TAAAAGCGGAAGCACATCCCGTAGCTATGTGGAAATGCAAAGCTGCAGAATTACCGTCTAATCCTCTG
TAGAAGTAGTGCATGGTTTCAAAATTAGTAAT
AATATTTGATGTGCAACTCGGTATGGAAGAAGGACGCGTGTGTGTGCGGGCGGTGCATGGCTCTCTCA
CAATCACtctcacgcggccaccaccgcagcag
ccgcacacg
>scaffold1044.size11402
TCTGCATATCCGCAGCAAAAAGGCCCACACGGTGTACACTACATTACAATTTCCTAAACAGCAAACCG
GATCATCAGAGCGCTGCCCTCTTCTCAACGTTG
GGTAGAAGCTCTCTGATCCTTGTCGCCGCCAGCCTGCGGCATCATGGAGTGGCATTGCGTTCTCGGCA
TATCTCTCCGTTTACTTCACTGTCCCCAGGCC
AAAAATGAAGTGATGTCCCACAAGCGATTTCGACCGTTCCGAAGAACGTGATGTATCAGACGCGCTGA
AACTGTGTGCGTATGTGTTATTACGAGCGAGC
GACACAATGCTCTCGCACATCCATGTATTCCGATCCTGCGCAACACCATATTCGGCGGCGCGGAAAAC
GTTAGACACCGAACACGCACAAATCGCACAAT

GAATGCCTCTGGAGAACAAACCCCCCATTAACGTCAACACAGACACCGTGACAACAAAGTAACAAAAG
AAATCCAACCTCCGCGCGTCCTCCAGCAAGGA
GGTAGAAGACACACACGCTTGTCCCCTTAGTTCCACTCCAACTCCTCGGGTTTGATGGGTTTAGCAAG
AGGAATCACGCTACTCCTCCCTCGCTCCCTGT
CACGAAGCATGGCCTTGCGCATATCCAATGCATCTTCGTCATCCTTAAACGTCCCTTTGGGGCCATCG
CCTCGGTAGTAACCAAGCACGCGTCGGTAGAC
AATGTATCCCAGTTTATGGTACATTTCTTGGGCGACCTTGTTGCTTTGCCGCACAAACAAGTCCACAA
AGTACGCATTGTGAACAAGTGACGTTGTGGCC
TCGAGCTCCTTCATGAGTGCCGCTCCCAAGCCAAGACGACGAAATATCGGCGCCACTGTCACTGCAGA
CACGTGCCCGTGGAAATCCTCGCCCTTCCCCT
CAGCCTTGCCAAGTGCGTACGCCATACAAATGCCGGTCGTCGGGTGGACACATATACGCTGATACTCG
GGCCAGTGTGTGAGATACTCACCGTAGAACGA
GGTGCTGTAGGTCTCCGTCAACTGATCCAGATTTACAAAGTTGAATTCCATGATGTCCGTGAGTTTGA
AATCCCGGTATGTCGTCATGGTTCTTTGGCTC
CCTTCAATCAATAAAGTACAAAACAATCAACTGTGAAACATGTTTGAATTACACATACAAAAATAAAA
ATAATAATAATAATAATAaACAAAGAATAAGA
ATGACGCAAGCGCAGTTAGCTTCCATGGGAAAAATGACAGCTCATGCCATAAAAAATTAATAACAATG
TTAACAATAACTATATATATATATATATATTAAA
TTGGGTGCAATATGAGTCTTCTTTTTCTTGGGTTTGTAATTGCGCTATTCATTCCGTGTGAATTTCGT
TCCTATTCCCATCTTCAAGCGTATTACGCGTC
AAATTCCGTAAATTGAAGAGTTTTATTTTTCGGTACCCCGTCTTTCTTCCCTTGAAACCAGTCACGCG
TCACCTTGCGCTGCTTCTGCACAATCATACTT
CGGTACCGATCATCATGACGTACTTCCACCTGAAGTCTCCTGCGAACTCGAGAGCCATCACACCCTCT
TACCCTCTCTTGTGTGTTGAGAGAAGACGCCT
CTCTCGCGATCTTCCTGTCCTCCATAATAAGATTCTGCAGCTGTGCCCGAACTTCACTGGGTCTCTTG
TCAAGACCAAAACTGTGTGCAATGTGGCCAAG
GTGAAGCATATCCAAATTAAAAAATCGAGTTTTAAGCTCTCGGGAGTGCCCTGCGTAAGCTCGTATGT
AAGACTGGTATGCGAACAGTGCAAGTCGAGTG
AGATCATCGTTTCGGCTCGCATCTCCAGCGGGCAACTCATTTACAGGTTCCTCTCGATTCATGACAAG
TCGACTGATGGCGCGCTCGAGAGTAGCCGTAC
TTTGCATCCAAATGTGGTTCGATTTTGGATCCAACTTTGCCAGGTAATAAAGAAACGACTCGTAATTC
CTCTTTTCCATTGTTGCCACTACTGCTGCCGC
CTCATTTCCCGTAAAAGAGCTCCCGCTCTCCTTCGCAATAAATTTACTTAAGTATGGGACGTATCCTG
CCTCGTGTGGCATGAGGAACAGCAGAGAGTCC
CCAACGTTGCCAATGCGAGCGGTGCGTCCAATACGATGAACGTAACACGCTGGATCTGTCGGAGGATC
GTAATGCACAATCCAGTCCACTCGTGGCATAT
CCAATCCTCGGGCAGCCACATCTGTACAGAACAGGACACCCTTTAGAGAACGCCGAGCAGCTCCACCG
ACGTGTTTGAAGGCTTTAAAAACAGAGGCCCG
GTCAACCTGCGACATGTTGCCATGCAACTTCAATATGTTGACATCAAGAAGAGCGTTTCTACCTGAGA
GCGTCTGCCCCTCATCATTATCATCCTCACTG
CCCGAATCATCCTCAAAGGTGACAATGGCCTCGCTCTGGTTTTGTACATGTCTGTTGGCCTCCTCTAC
CCGCCGCTTCACACCGTATTGGCGAGCATTTT
GCTTGGATGCACCTTTAAATAAAACTTCTTTTCTCCTGTCGCAAAAAGGGGATTGTAGGCGTGATAGC
AAACGGTAATGAAATTCTGCGCTATCAGCGGT
GGAGACAAAAACAATAATTCTTTGAGCACCAGCATCAATTTGGGAGCGAAGAAAGCCAATGAGGGTTG
AGAGTCGATGCTTAATCGGTACCAATGCGTAG
TGCTGGCGAAGTGAGGAAGGGATGGAGAAGGTATCCTCTGTCTCACCTACACGAACAACGTTGCTGCG
AAGCGCAAAATGTGAAAGCCTCTCAACCTCAG
CAGTTATCGTTGCCGATACCAAAACGCGCTTGAGAGTATATTTTTCCCTTGTCTCTGTAAATATTTCA
TCACATGAACAAGCTGAGTTCTCTGTCTTCTC
CAATAATAGCCCCATAATCTCCTTTATAGCCCGCTCAAACCCCATATCCAGTAATCGATCAGCCTCAT
CAAGGACAATTGTCTGCGTTGAGGCAACGCAG
AAAGACACGGTCGCCCTGAGATGATCAAGAAGTCTCCCGGGTGTCGCTATCAAGAGGGGAACCCCCTT
ACGCAGTCTTGCCTTTTCCTTGTGCCGATTTT

CACCACCGTGAATACCACCCACCGTCAAAAAAAGCGCACATCGAGTGAGAACACTAAGCACATCTGTC
ACCTGCACAACCAGCTCTCGAGTGGGGCAAAG
CACAATAATAATACTTCCAATCTGACGCTGAATTGGTCGCGCATCACACTCACACAGCAACTGATGGA
GCAGAGGAAGAGCATACGCCAGCGTCTTCCCA
CTCCCCGTTTCGCTCCGTAGAAGAACATCACGCGTCCTGTCAACCATGGGTGTCCAGCTCTGCTTTTG
AATGCACGTTAGGGATGTTATGTGAAGACACT
CAGTTAGCGGCCGCAGCAACTTGCTATGCACAAGTTCATTTAAAGGAGGCAGCGATTCGGCATCCACG
CACGCCTTATCCGCTTCAAACACCGTGGAAAG
TGAAGAAGAAGGTGGTATGTTTTTGTTTTTCAAGGGGGCATCCGCAAAAGCCGGAAATTGGCCGCGGA
AAGCCCGTGACTCCGCGGTTGCTTTCGAACGG
AACTCGTCTTCTTCCGCATCCAAAGTCGACGTCCTTTTCCGTTTTAAACGTTTCGGTTCGCTCTTGTC
CTCCATGACTGGGGCTGCGCGGGCCGCGGGCG
ACGGTTGGGAAGGCGCATCAACGCACAAATCGCACCCATCACCCCAATCATTGAGGTCTACAGATGGT
CCCCGTTCTGCTCCGGCCAGCAGCCTGTCAAG
GGTGTCATTTTCACTGCATTTTGACAAACCAAGAACGGCAAGGTGTTGTTGTGCATCCATCTCGGCAA
GCGACATGTTATCCTACTTTCCCACTCTTTCT
CTTTACTTCACACCTTTATTCCGGCAATTAACATCAGAAGATATCATTTACAAAAAAAAAAAAAAAAG
TGCGCAGAGTTCTCGTGTAAAAAGGCATCACA
TTATATACTCAGGAGAGTTATTAACGTCCTCTTCCCTCTTTTCTTTCCCTGTTGTTGTTAATGGTGGT
GGTGGCGGTAGGGCTTTTAAAGACAAAAATAA
AAAGTAAAATTATAAAATAAACGCAACGCAACCAAAAATTGCAGTAAAAAAACTGCCAAAACAAAAAC
GGCCCTGCAGATCCTCTCATATATATATGTGC
AAACACATAAAGAGACATAAAACCATGCGCTCAGTCACTCCCGGTGACGTTTGCTTAATATTTTTTGA
TAATTCAATTCCGAAAGAAGACCTTCTCGAAG
TAATTGCCGCTGCTCCTCACGCTGGCGTACATTGTACATCCTGAGACCAAGTATAATCACTCCCATGA
TAAAAAAGGGGAGCCATCCTTGTTCTAGAAAA
TAAACCAGCCAACTTTCCTCCACTACTTCTGACGACATATTTCCTCCTCCTCCTCCTCCCTTTCT
TTTTCTTGTGGTGGTGTTTTGTGTTATCATTG
GAAATGTAAAAAGTGAAAAAAGATAACAAAAAAAAAAAAAAAGGGAAAANNNNNNNNNNNNNNNNNNN
NNNNNNNNNNNNNNNNNNNNNNNNNNNNNNNN
NNNNNNNNNNNNNNNNNNNNNNNNNNNNNNNNNNNNNNNNNNNNNNNNNNNNNNNNNNNNNNNNNNNN
NNNNNNNNNNNNNNNNNNNNNNNNNNNNNNNN
NNNNNNNNNNNNNNNNNNNNNNNNNNNNNNNNNNNNNNNNNNNNNNNNNNNNNNNNNNNNNNNNNNNN
NNNNNNNNNNNNNNNNNNNNNNNNNNNNNNNN
NNNNNNNNNNNNNNNNNNNNNNNNNNNNNNNNNNNNNNNNNNNNNNNNNAACAGTGGTGCGCGGGGGGA
GGGGTAAATTATTATTATTATTATCGTGAAGT
TAAGACCCACAAATCCATCGCACACTTCGCTGGAGTGTCTGGGACAAAAAAAAATCTAAAGGCAAAGA
GTGCGGCACGTCATTGCAGAAGCACAAACACC
TCCTCTTTCTTTCCCCATATGGTCCTAGAAGTAAATTGGCCGCTCCATGACACAATCTCTATTTGGAC
TTTATTTGTGTCTGGCGGCGCACTAACCGGAT
GACAATCGCACCATCCCGTGCATTTTGTTCCATGACATGTCCCTTTTCATTGGGTCTCCCTCTGGCAC
TGCCCAGCCAAGGGTTTCGGTCCTTGTTTCCC
CTCATTTCCTTTTTTATTGCCCCCTGTTGGACTTCAAATGCCCCGTTCATCTCCGCGCAATGCCTTGT
CATATTTGGCACATGAAATTGCACCTCAACAA
ATTGCAAGGATTCCTTCATCCTCCAGTGGTCGCTTTGAATTCCGTAAGTGCAGCCACCAAGCAGACGA
ATAAACACATCCCGTGATGCCGTTTGACATTC
CCGCAAATCACTCTTGGGATTTTTGAGAAGAACTCCAGTATGCTCCGCAAGCTCCGTCGTCTCCATCG
TGCTCCCCAACCCAGGACGATGCGGTGTGTTC
AAAGTGTTCGATTGCCCCCCGCCGCAGACGGTGTACAACGGCGGCGTCAACTCCCACGCATTTGTGAT
GAAGGCATGCAACCCGCCGGCGTGCAGGATGT
TCTCCCCCTCGTCTGCGGCCGACTCCAAATCAGGACCCGCGAAGGTCTCGTGAATCCAGTCCACCGTC
ATCGTCCCACCGTCCACTGTGAGCGTACCTGC
GACAATAAACGGATCACACGGTGAGTGCACCTCAACGCCCCAATCTCCACTCATCCGCGACACAACGG
AAAAGGGCAGCTGGACCCTCCCACGATTCCCA

TGAACACACAGGGACACGCACAGTAAAAAAAACAGTGAGGCAACCCAGTGCAATGTCATGCTCATTCC
TTCACGGAAGAAGAATATATATATATATATAT
ATATAAACTCCCTTCTTAAAGCTTCTGACCAACACTAGTGGGAGAAAACAACAAACAAACACCACAAA
CGAGCAAAACAGAACGTGTAGTTAAATAATAA
GTCACGCAAAAAAAAAAAAAAAAAAAAACTATCAAATGGAGAGGAAGTGAAACAAAAAATCGGGCATA
AAATAACGCCCACGGAAGTTTCTCAACAAAGT
ACGACCGCCAACCCCGGAGCCGCATATGTTTCCCTTTTGTCACTGCTACTGTTCACCACGGCAACAAG
AAAAAAAGAAGAATATGCATGCAAAAGGAACG
TCAACTCCCCCGTAAGCGCCTGCAAGAGCCAATTGTCGACAGGACTCAAAGATCATCATCATAATTAG
GCTTCAAGTACACGTTCCACACGGCACTCAAT
AGGTCCACCTGGTACTCCAGCTTCACCGCCAAGTAAACCACAAGCAGTCCAATCAGTAACAAGAAACA
GTCAAAAAAACGGAAGAAGAAAACTCCCGTTG
GTGCCGGTGCCTCACATCGTTGACCATGCATCTCACCACACACACATACACCTCCCCTCAGTAGCGCT
AACAATAAGGTAGAGAAAAATAAGGAAATAAA
TAAGGCAGCAAAAAAGAAGAGAAAGAGAGAGGCACAGCATGTCTTTACGTTCCCTTTACCCGGTCACA
TAAGGCAAAAATATGTGTTTCTTTGCAGCCTC
ATTTAAAACACAGAGAATATATATATATATATATTGAACTGGCTTTGCAACCAGTGTGGCACATTCCA
TAGCGACAGCAACACGGCACACTAGTCATCTC
GTTTGCTTTGATGCGCTCTTCTTTCAACCATCCCAAGAGAAACACCAAACAAGGCATCTGCCTTTTCC
TCAACCTTTTTAGCAACACGCACCTCCTCTTC
AATCGCACGACCCAGGGGCTCTTTTACCAGTGGTGCATCTGTAAAGCCAGGTTTCAGCGCTAAAAAGT
AATTCCGATCCTTTTTCCTGCGTGGGTTCCAC
TTCTTGGGACGACGCATGGACTTTTGGAAAAGCCCAGGAAATGGGGACTTTGGTTCTGGCGTGATCGG
GTGCCATGTGGCGTGTCGGTACCCCATACAAT
AGAGATGGTCATTGTCCACCTCACATGCGGCTACCTGAAAACACCACCTCATATGTTGTCTGACCGCA
GAGATGACAGGTTTGTCCTGTGGCCTCACACA
AAAAGGAAATCGTAACAGCAACGCACCTGAAGGCCGAAGAAGGTGTGTGCCCAATGCCATTACATTCA
CAAGGGAATCCCGAATGTGCATATCTGTAAAA
AAACGCTCGGGTGATGTTCCCATTGATGATGTTTCCCCCACAAAATCCACAGCAAGAAGATCGGCCGC
CTGTCGAGGCAAAAATGCCGATCTTAAATCGC
GTAGCAAAGTGTGACTTCTCAAGTATTCACCAGACAACTCATTTACCGATGATCCCATACACGTCGAC
AGCTCGGTTGAGTTCCCAGTCCAGATTTTTTG
TTTATCGTGCCTAATGTATGACGCCGCCTCCGTCGTGGTAAAAGGAAATGTTACCACTTCTCCATGGT
CATCTTCCCTTTCTAGCAGAGGCATTCTCGGG
TTTGCATCGTCGAATACGTAGAATGCGTTCATAGGGGTAGAGCTGCCTTGCGGGTCACTGACCGTCAT
TTCAAGAAACTGCCTCTTGGAAGCGAACCCGT
CTTTCATCTTCGCCCAATCCCTCGCGGATTGTGTTTGCGGTGGCGTAGGCCGTCCTCCATGTTTGCGT
CTTTGCTGATGTCGGGTAACCCATCTTCGCAC
ATCGTCCTGCCAATTCCCAGCGAGGAAGAAGACGTGCGCATCGCTTGCGAGCCAGCCATGCTTTTCCT
GCAGGAGGTGCAGGATCTTCCCTACGCCGTAC
CTGCCCCCTAACGTCCCTTTCATCCGCGCTGTGCGCCAAAACAACAACAACAAAAAAAAGCACTAAAA
TGAAGGGCGTCAAGCAGGCAAAACAAAACAAA
ACCAAAAAGAAGAATAAAATGGCACGTCGCGGATGCCAACGGGCGGACAGGCACGTCCCTCCAAATAG
AAATAATAATAATAATGATAGAAGAAAATGAG
CAGAAAAACTTCTATTTTCATTCTTTAAGAAGCCCTCACTTCTGTCGTACCCACCCATCATTCCCTTC
CTCAGATCAACCGACACGCTCCCACCCCGCCA
ACACTCTCCGTTCCCTCACGAAACGAACGCCGATCGCCCACCGCACCAAACGTTAGCCGTGATGCGTC
CAACTCCCACCCCGTGGGCACGGAGATTTACT
CAAGAACCAGAACGTGAGATACCCAGTGAACATGCATACGTATTTGGAAGAAATCAAGCCCTTTTCCT
CCACTCACATACACACAAAACAAACAAACATA
CGTAAAAAAAAAAAAAAAAAGATTTTTAGCTTCACAGTCTTCGAATCGTCAATCCTAAGCACACCCAT
CAGCCCCACCGCTGGAACAAATGTACTCAGAG
GCCTCTTTGAGCATTAATGAAAGAAGACAATATGTATGGTTTCACTCCGGTGGCGTTGCTACCCCGTC
CACCCAGCCCTAAACGCGTAGAAAAGACTAAA

TGCCCCTTCACCTCCCTCCCTCTCCCCCAGATAATAGACGCACATATACAAAATAGCACCCAAACCAG
AACACACACAAAGACAAAAATATATATATGAT
CTCTGCCGGCTAAAAAAAAAAAAAAAAAAAAAAAAAAATTGTTGTTTTTCTCCAATTCATTCAACTTT
GTAAACGTGTGTCTTCAACAAGTCAAATAATT
GTGAATGTGAATAAGTGGGAAAGACCACAAGCGTGGCAAAAACGCAGCTACATTTCCTTCCAAAAAAA
AAAAAATACCAACTGTTGCTGTCATGCAGTTG
CATGGCTTCACACTGCGTCCCCTATCCAGGCCGTCCCAAAACATTTCTGTGTTCTTGTTCATATTTGG
TTCTTAGTAACTTTCAACAGCGGGACCTCCCC
AATACGTGTGGCGTCTAAGCATTACAAATAATTTCAGTTCATTACCAGGAATTAACTTCACCCCCCCC
ACACACCCCTCTTCCCAGTGCATTAATGAGAC
TCTTTAAAAAATGCATTTCCCCTGACCCCAAACCCCCTCCCCTCCAGTTAATGCCTCTTGTCTCTTTC
GGGGTGGAAAAGATGACGGGTCGCACACGTGC
CACAACGTCCACGCCCCCCTTATCTCCCCCTCCCCATACACACATATAAACAAAGCGCACTGCGCTAA
AATCCAAAACAAGAAGATAAGTCCAAAACCTT
TTTTTTTTTTCTTAAGCCAATTCCCATAAAAAAAAAAAAAAAAAACTCGTCCTTCGTTTTTCATTAAA
AAAAAAACTCATTTTTTTCTCATCGCCCCCTC
CACCTTTAAATCACTTTTCTCCTCCACACATTTTTTTTCTTTTTTCTTTTTTTCTTTCTTTTTGTGGC
GACCTTCGCTAGCGCTCCACATTCAGATGCGG
GGCGTCCCCACCACTTTGAGGCCATTTTGGGGTACAATGGGAAACATAAAAACGTGTGGGCGCCCAAC
CTCTTCCTGAAATAAGCGCTTCTCCACATCCG
TTGCGCCAGCGTCAGCACCGTAGAAACGCAAATCCCAGTTCAGAAATAGGTACGCTAGTGCCACCTCT
GTCTCCATCAAAGAAAGATGTTGTCCAAGGCA
GTTGCGCGGACCGTTAATAAAGGGAATAAATGCATACGGGTCAACGGTGTCCTTGTATGCCTCATGAT
TCGGGGTGGTAAAGTTATTTTCAAGCTTCACG
TCAAGGAAACGTTCTGGTTTAAATGCATCAGGATCTGGCCAGATATCCGGGCGCCGGTGCACCGCCTG
GATCCCTACAGCCACCGAGCAGCCTGCGGGAA
TAGTGACGTCCCCATCAAGCCCAGTTATGGAGGCAGGCCATACGTCGTTTTTCAAAGCACGCCGCATG
ACCAAGGGAACAACCGAGTGCTTTCGTAGTGT
TTCACGAAGACAAGCCGGAGACCATCGTAGTGTGTTGCGTACATCCGATGCGGTGGGAATGCCCCACG
TCTTTCCTTCTGGTGTCACAACCTTCTCTTCG
CAGCGTGCCGGATCAAACAACACTCTTGCCTCTTCCACAATTTTTTCACACGCATCAGGGTGACTGAT
CACCTCATAGGTTGCCCATGTTAGCAACGCCG
CTGAAGTTTCGTGTCCCGCCAATAAAATGGTTTTGACATCGTCGCGCAGCTGCACCACCATTGCGTTA
TCCATTTTATCAATTTGAGATAAACAGAGTGA
GATAATGTCACGTTTATGCGGCTTGTGGCGATCTTCCCAACGTTTCAAAATAAGCTCGCAAATTACAT
GATTCAACTCCTTCAAACAGCGGCGACGCTCT
TGAAATCCTTCCAAGAAGGGCATCACGACACGCCACGGCTGCCATACACGTCGATTGCATTCGTGAAC
AACAGGTAAGTAAAGAGACGGAAAAATACGAT
CTGTTTCCTCTGGTTCCAAAGAAAGCGTCGTTTCTCCAATCACTTGCAGTGTCATGTGACGGAACGCC
TCATTCAAATCCACAAATGGATTTTTGTCGTC
AATAGCGGCAATTTGATCCATGACTCTTCCAACTGCCTTGATTGTCATTTTGGGAACTTCCTCCAATA
CATCTATGCGCATAGCATGGGAAAGTAATAGG
CGACCCTTTTTCCAATGCTCTCCCTCCGCTGTCACAAGCCCAGTTCCAAGTAGACAGAGGAAATGTTT
GTATGACGTTTCAACATCCTTGGCGTAGTTTC
GCTGGTTTGTCAGAAGGACCCGCTTCAATAATAAAGGATCATTGATGTACACAACGCGCTGATAAAGA
ACATTAAATCTGACAAGTCGATTTGTACGCGG
AGCATCCTTCTTTTTATTACTATCCGCATGGGAAAACGAATCAAGACTCCAGTCTGCCATTTTTCTCC
ACGGTGATCCCCCCATAAGAATAAGGGCGTGG
CCGACCACTGGGATTCTCCCCGGTGCCTCCGGAATATTTCGGAGGTGCCTGTCCATGACAAAACCCCG
CAGAAACAAGAAAAAATAGTACAGAATGATGG
CCCAAATCACAATAAGAATCCCCATTAAAAATAACAATCCATAAGAAGAATTTGCCAACAACTCTGCA
CCGCGCATGACGCCACTTTCCCACACCGGCCG
CACAGCGTTCATAATTGATGCCAACAACAGGTATGACTTTTGGCTGCCAACTCCAAAACAAAATCAAA
TATATATAAATATATAAATATATATAGAGAGA

GAAAGAGAGATCGCAGATGCAAAGACCCTCTTGTAATTTCTTTGGCTGCTGCTGTCTCCCCCCCCCTC
CCTCCCTTCCCACTAATTGCTGCACCTCCCTC
AGCGGGTATTTCTTTTTGGTTGGTAAAATTGTCAATATATATATATATATATATATTCACAATGGAAACA
CCCTTTTTTATATTCTCAATACTGAATGTGGA
T
>scaffold1045.size10349
GTAACTTGTGGAGGGAATTTTTGACTAGATCCGGCTTAATATATATATATGTATGAAAGAGAGAGAGA
GAGAGGGATGGCTTGTGTATCTACTTTGTTTTC
CTTAAAATTTTACTGTTTTATTTTTGGTTTCAAATATACCACGCGTGACAGTAGCGCGGTTGCAGTTT
CTTTATACCTTCTACCCTATACAACCTTTCTC
TTTGCCCTCCGCGCGGAGAGTTTCGGTGGTGCGAGAGAGGGAGTAAAGACTGCCAATTCGTGCGATTA
TAATAAGTCAGTTTGTTTTAGGTTTATTGCTG
AGCCAACGGATTTGGTGGCGCGTGAGTCTGGCAAATGATCCGAATGGAGAGGTTTGGCGCGTTGGGCC
AGACTATGACCTTTTTTCTCTTTTTCTCCCCT
TGTTGGAGGAAAGGTGAAGGTTTCATCCAAGCGTATATTCCTTTTTTTTTTTTTTTTTTTTTTTTTTT
NNNNNNNNNNNNNNNNNNNNNNNNNNNNNNNN
NNNNNNNNNNNNNNNNNNNNNNNNNNNNNNNNNNNNNNNNNNNNNNNNNNNNNNNNNNNNNNNNNNNN
NNNNNNNNNNNNNNNNNNNNNNNNNNNNNNNN
NNNNNNNNNNNNNNNNNNNNNNNNNNNNNNNNNNNNNNNNNNNNNNNNNNNNNNNNNNNNNNNNNNNN
NNNNNNNNNNNNNNNNNNNNNNNNNNNNNNNN
NNNNNNNNNNNNNNNNNNNNNNNNNNNNNNNNNNNNNNNNNNNNNNNNNNNNNNNNNNNNNNNNNNNN
TTTTTTTTTTTTTTTTTGGTGGGGGGAGAGGA
AAAAGGGAGGTGGCAGAAGAACGTTTTTTTTTTTTCCTTACTTTACCCAGCGAGCAAAGCAATGACGG
AGACGAAAATTCGAACAGAGGAGGCACGTGAC
CTCACGCGTGTTGAGCGCATTGGGGCGCATTCGCACATCCGCGGCCTGGGCCTTGACGATGCTCTTGA
GGCGCGTATGAGCAGCCAAGGAATGGTGGGGC
AGGTGGAGGGTCGTCGTGCTGCCGGCGTGGTGGTGCAGATGATAAAGGAGGGAAAGATTGCCGGTCGA
TGTGTTCTTCTTGCCGGTGGCCCTGGAACCGG
CAAGACGGCGATTGCAATGGGAATGGCACAGGCACTTGGTGCCGAGACCCCATTTACCATGATTGCTG
GCAGTGAGATCTTTTCGCTTGAGATGTCAAAG
ACAGAAGCTCTCACGCAGGCATTTCGCCGAAGCATTGGGGTTCGCATCAAGGAAGAGACGGAAATCAT
AGAGGGTGAAGTGGTTGAGATAACTATTGAGC
GACCGTCGACAAATCCCGCAGAAGCAAAAGGACGCACCGGTCAATTGGTGCTGAAGACATCAGACATG
GAGTCAACGTTTGATCTCGGATCTAAGATGAT
TGAGAGTCTTCAAAAGGAAAAGGTGCAGGTTGGTGATGTTATTACCATCGATAAAGCCACCGGCAAAA
TTAATAAACTAGGCCGAAGTTTTGTGCGAAGC
AAAGACTATGACGCCATGTCGTCCAACACGAGATTTGTTCAGACGCCAGAAGGGGAACTGTCAAAGAG
AAAGGAGGTGGTCCATACGGTAACGCTTCATG
AGGTGGATGTCATCAACTCAAGGCAGCAGGGTTTCCTTGCCTTGTTCGCTGGGAAGCAAAAGGACGCA
CCGGTCAATTGGTGCTGAAGACATCAGACATG
GAGTCAACGTTTGATCTCGGATCTAAGATGATTGAGAGTCTTCAAAAGGAAAAGGTGCAGGTTGGTGA
TGTTATTACCATCGATAAAGCCACCGGCAAAA
TTAATAAACTAGGCCGAAGTTTTGTGCGAAGCAAAGACTATGACGCCATGTCGTCCAACACGAGATTT
GTTCAGACGCCAGAAGGGGAACTGTCAAAGAG
AAAGGAGGTGGTCCATACGGTAACGCTTCATGAAGTGGATGTCATCAACTCAAGGCAGCAGGGTTTCC
TTGCCTTGTTCGCTGGTGATACGGGCGAGATC
AAGCACGAGGTGCGTGAGCAAATTGATCAACGTGTGGCTGAATGGCGAGAAGAAGGCAAAGGTGAAAT
TGTGCCTGGTGTGTTGTTTATTGACGAAGTAC
ATATGCTTGATATTGAGTGTTTCTCGTGGCTGAATCGTGCATTGGAAAGTCCTCTTGCACCCGTTGTA
ATTATGGCCTCCAATCGCGGTATTTCCCGTAT
CCGTGGGACGCACTATAAAGCGCCACATGGCATTCCCATTGATCTGCTTGATCGCATGGTCATTGTCA
CGACGAAACCATACTCTGAGGCGGAGCTTAGC
AAAATTATTCGCATTCGTTGTGAAGAAGAAGACGTGGAAATGGATGAGGATGCCATAGCATTGCTTAC
CATGCTTGGTAAGAGCACATCATTGCGATATG

TCCTCCAGCTCATTACAACGGCAAATTTGGTTGCCCAGAAGCGTCGGGCCTCTACAGTATCTATTCAC
GACATCAAGAAGGTGTACTCTCTTTTCATTGA
CCTGCGTCGCAGCGTGGAGCTTCTGCGGGAACACGAGAAGGACTTTCTTTTTGGGGAAGCAGATGAAA
ATCTTGAGTCGGGTCACCGTACAAATTGCCGA
AACGAAGAAGAGGATTAGATCTTTTTTACCCTAATTATTTCTTTCATTCCTTCCTTTTTTTTCTTTCT
CTATTTGGCAATTCATGTATACGCCTGAGAGA
AATGGCTCCTCAACTAGCAGCAAGCAAGATGGATGGGATATAAACATACGTATACATATATATCTATA
TATACGGATTCCTGTGCGCACTGTTAGATCGA
TTGTGTGCTTTATTTATGTTGTAATTGTTGTCCCTTCAGCGTTGGAGGAAGCAAAGAAACAAAAAGAT
ATAGATTGAGGTGAAGCGGGTGGATAGGGCTT
GCTTCGCTTAGGCACTCAACACACTGTTTGACAGGTCCATTATGAACAGAAAATGAGAGTGATGAAAT
TACATACACAAACGCACACACACATACACAGG
CACAAGAACGGCAATAAATAATAATAATGGTAATAATAAAGAGAGAGAAGGAAAGGAGAATATATATT
TTTTATGATAAAACAAACACAAACAAAGAAGA
AAAAAGAAAAGAAAGAAAAAAAAAAACAAAAAAAAAATGTTTGTTGCGCCACGCTTGGTGAAGGATAAG
CAATATTTTTCGATCCTTTTGTTGAACACCGT
TACTTCTACTGTTAGAGTTGCCCAAAGCAATGCGGCATTGGAATGATACGGGGTGATATTTGTTCCTT
TCTTTTAACTTGACTACATTCTTTTGATGTTT
ACACGTTGACTGTCTTTCTTTCTTTTTTTTTTTTTTNNNNNNNNNNNNNNNNNNNNNNNNNNNNNNNN
NNNNNNNNNNNNNNNNNNNNNNNNNNNNNNNN
NNNNNNNNNNNNNNNNNNNNNNNNNNNNNNNNNNNNNNNNNNNNNNNNNNNNNNNNNNNNNNNNNNNN
NNNNNNNNNNNNNNNNNNNNNNNNNNNNNNNN
NNNNNNNNNNNNNNNNNNNNNNNNNNNNNNNNNNNNNNNNNNNNNNNNNNNNNNNNNNNNNNNNNNNN
NNNNNNNNNNNNNNNNNNNNNNNNNNNNNNNN
NNNNNNNNNNNNNNNNNNNNNNNNNNNNNNNNNNNNNCTTTGTTTCTGTGCGCACAGACTACCGCTTTG
GCACGGAGGAAAGGGTCACGTGGGAAGACGGG
CGTGAGGCAGGAGGGTGGATGTTTTGCTGAGGAGCCGTGAGTTCCAACAAAATCATGAGGAGAATTCA
GGTGGCGTTTTTAAAAAAACTGCGGCAACGAA
GGAATTGTGGCTCTTTTAATGAACGGGCGCTGGTGCAAAGGACATGTGAGCATATTTATTGTAGCAGC
TCTGCTCGGGAGCAGTTGCGCCAACTAATCCA
CGAATCATCCGTAGAAAATGCCCTGCGTCATTTGGGCGAGTTTTCCAATGATGCTTCTGTGTTGAAAG
GGTCTGCTTGGCTTGGACAATTATCAAAGGCT
CCCGATGTTGTTGGACGCATTGCTGTTATCTTGCCGCAGTTCGAGAAATGGTATGTAGCCCGGAAAGC
TCCAGAAAAGGTGACGATGCATGAGCCCTGGG
ATTGGTATCCAAAGGCTCGCTTTATGCGACGTCGATTTGTTTTTCACTATGGCCCCACAAATAGTGGA
AAGACGCATGCCGCGTTGGAAACTTTGGTGAA
GGCGAAGAGTGGTGTTTATTGTGCACCTCTTAAAGCACTCGCAGCACAGGTTTGGAAAAGGATTGACG
CAAGCGTACCATGTGACTTGTTGATCGGCGAC
GAGCGTCGATTTGGTGGGGGTGCAGAACATGTCTCTTGCACGGTGGAAATGACGCCCATCGATTATCA
GATTGATGTGGGGGTAATTGATGAGGTGCAAA
TGATTGCTGATAGGGACAGGGGATGGGCGTGGACACGGGCACTTTTGGGATTACCGGCCCGCGAAATT
CACCTTTGTGGCGAGGAGCGTGCCATTCCGCT
CATTCGCAAGGTGCTTTATAAAACGCATGAGTTGCAGCGACTGGAGGTTGTGCCACATAAGCGGCTTG
TTCCACTTGAGGTTTCCCCCTCCTTGGATGGG
GATCTGAGACGGATAGAGAACGGTGACACTCTGGTGTGCTTTTCGAGGAGGGCCATTTTTGATATGAA
GAAGAAGCTTGAAAACATTTCGGGGATGGCTC
CGCATTGTATTTACGGCTCAATGCCCTTTTCTGTTCGTGAAGCCCAGGCAGACGCATTTAACAACGGT
ACACGGGACGTCATTGAAGGAAAAAGTGGGAA
AAAACACGTTCTCATCTCCACGGATGCCATCGCGTACGGACTGAATATGAGTATTGAGCGCATTATTT
TTACTTCCATGAGAAAGTTTGACGGGAAACAG
ATGGTTACTTTACCGCAGGCAACAATTTTGCAGGTGGCTGGTCGCGCAGGTCGTTTTGGCGTATTGCG
CTCTCATAAATTTGGTCGATGCACAACTCTTG
ATTCGAACGACTTTGCAGTTTTGTGTGAGGCGGTCAACAGTCGTCTACCTTCGTTGCAAAAGGCAGGG
CTTCTCCCCACAGCTGAAGTACTCGAGCTGTA

CATTAGACTACGTGAGGCGGAGGAAGGCGGTAGGGTGACGGAAAATAATTTAGCATCATTTTATCAAC
GCATGCGGGAGTTTTCGGAATGTTGTCAGGCT
TCTGATTTGTTTTTTCCTTGTGATCTCTCGCGCTCATTGCTTCAGGTTGCAAAGGAGCTGGAAGCGGT
CACCGATCTTTCCTTAAGCGATCGGATCTTGT
TCTGCTACGTACCTCTGAATGATCGTGGGAAAGAGACTTATGACCTCCTTCGGGCGTTTGGGCATGAC
CATGCCGCCGGGCGACACGTCGTTCTTCGCAT
TGACGAAGAATTTGAACGACTGGTGAAGCAATGTGATCTTGTCTGTAGTGGAGATACGAGGAGAGCTC
ATCAGGTCTTGACAAGGATGGAACATTTATAC
CGAGTGGCAGAGATGTACTGCTGGCTTGCATGGCGCTTTGGTAAGACGTTTGTTCATCTTGAGACGGC
TACGGCGTTGAAGGAAAGGGCTGCGTTGAAGA
TGGAAGAAATATTGCAACATCTTTAAATACGTTGAGCGTGAGGGCTCATATGCGGGTCCATCTTTGTC
TGCTTTTTTTTTTGTGTGTGTGTGTGTTTGTG
CGTCTTTGGAGGGTTGGGGAATCAATGGGGGACTATGACGTCCCTTCTCGAATGGCGTTTCTTCTTAT
TTCCCATCTCTCTCTCTCTCTCTCTCTCTCTC
TCTTTATTATTAATTATTTATAGTCATTGATCTGATGGGATTTATGGGCGTTCTTTGTTTCTGGCGCT
CGAAAAAAAGGGGGAAAGACGATGAGATACGG
ATTGTGCATGCTGGTCGCCCATCCATGGAGTACTGTGACAATACAGTGAAGAGCTCCAAGTACACGCT
TCTTACATTTCTTCCGGCGAACCTCTTTGAGC
AGTTTCATCGTCCGATTAATCTTTACTTCCTTTTACTGACAGCGCTCCAGTTTACCCCCTCTGTGGCC
CCAGTGAGTCCGTTTACCTCACTTGTGCCGTT
AACCATTGCCTTTTTGATCACCGCCATCAAGGAGGGCATTGATGACTGGTATCGACACAAGCTGGACG
ACGAGTGTAACTCCAGGTGGTACAGTATAATC
GATATGGAAACCCTAAATGTGGAGCGGGTACAATGTAGGGACATACGTGTTGGAGCTGTGCTGCTCTT
GTTGCCCATGGAGGTGGTTCCGTGTGATGTTG
TGTTACTGCTTTCCTCCGGTGAAAATGGAGAGGTGGTGATTTCCACCGAGGCGTTGGACGGTGAAACG
GTTTGCAAGAAACGCTATGCAATATTAAGTGA
CATGTACGCCCAACGTCCGACAACAAGTGTGGCAACTGTGGATTCCATGCAGGAGGATGAAACAAATA
TACGTGTGGCAGCAGAGTTTATTAGGGATACA
GAATGTGTTATAGAATCAGAGGGACCCAAGCCTTTTCTCAATAGTTTTACAGGTGTTGCCAGAGTACA
AGTGCCCCATGATACGGAAGAGGAAGTAACTA
TTGGCGTCAATGCCGAGAATGTTGCCTTAAACTCAAGCGTACTAAGAAATACTCGCTTTGCTCTGGGT
GTGGCTGTGTTTACAGGCAACGAGACACGGGT
TTCGATGACACGCACTACACCACCCACAAAGTGGTCTTTGATTGATCAACGCTTTAATCAAATGGTCA
TCGTTGTTTTTGTTATTCAATTTATTTTAGTG
ACGGTGTACGTGGGTTTATCAGACGCAGCGTTAGAAAAGGATCAACACGCCCTTTGGTATCTTGGCAT
GTATGAAGAAAAGATAGCCTTGAAGGAGTACG
TCATTCTGTTGCCATTACGGTTTTTTTTATTGGTTTCCCCGATGGTGCCGCTTTCTTTTAAAGTGATG
GTCGATGCATCCAAAGCATTTATCAGCATTCT
CATCAAGTGGGATGAGGAGATGTCCGATGAAAACATTAAAACCGAAGTTCATAATTCCTCTTTGATGG
AAGATCTTGGGCAGGTGGAGTATTTGATGACA
GAAAAAACGGGAACCCTCACACGTAACACATTAACTCTCAAGAGTGTTGTAGCGACGAGTGGAAAAAT
ATTCAACCCAGGGACTCCACATGAGGCGTCTG
TCGTCAGACGAGAAATGAGGCAGGACGAATCGTTATTGCAATTGATGCGAGTATTGGTTTATTGTAAT
ACTGTGGAGAGCACACGAGGAATAACATTGAA
TGTGGCGCAGACATCAGCGCCAAAATCTCCACCATCTTCCGCATGGGTGTCGCCGTCACCGGATGAGG
TGGCACTGGTAGAAGGAGCCACTTTTTTGGGT
GTCAAGTTACTTGAGCGAAAAAGAAGACGAGCTTTTGTCATGTTTGGCAACAATGTTGAGTCTGCAAC
CGTGCTGAAGTTTCTTCCGTTTACTCCAGAAC
GTCGTCGGTCAAGTATACTGCTTCAGATTGATGGGGATACGCAATTTCTTCTTCTGACGAAGGGCTCA
GATGAAAGTGTTTTGCCGCGGTGTATTAAAAC
CTCTTATAACCGTCAGAGGGTTTTACACAGACTGAGTTGCTTGAGTAGTGAGGGGCTTCGTACGCTTG
TGAGTGCCTACCGCTACTTGACAGATGAGGAG
GCAGAGATATGGCTGGCAGATGTACGAAGGAAGGAAGCGATAGTCACTGCCGCTGCACGTGCTGTAAA
TGTCGGTGAGGCATATGAGTCTTTGGAAAAAG

ACCTTATTTACTGCGGCATCACTGCGGTGGAGGATCAACTACAGGACGACGTTTGTCGCACCATCACA
CAATTGCGATCCGCTGGTATACGGGTGTGGAT
GTTGACGGGGGACAAAAGTCAAACGGCGAAACAGACAGCCATAGCAAGCGGGCTTGTTGGCCCTGACG
ACAGAGTTATTGCGTTGACCCCTGAGGACGTC
GAATCCCTGTCAGTCCAGGAACATTTCGAGTACGTCGAACGGCGTGTGAATTATGTATTACGTAAACG
GAAATTCCAAGAGGCTGAGAAGAAGCATAATA
TTCACCGTTCCTGGTACGTGCGTTCTGGTCTTCGTGACTTTGTAGGGGAATTTTTTCACCATGAATCC
ATTTTATCTTTGCTTTACCACCCTTCGAGTAA
GAGTTTTTCACAGGACTGTGATGAATACTTCACGCGTGAGGATGTGGCAGAGAATGAACAGGAGGATT
TACTGAGCAACTCCGTGACAATTCAGAGTGGG
GGCTATGTGATTCTTTTTTCAGGAAAAACGATTGAGTTTTTAAAAGAATGTGGCAATAAGGAACTCTT
TGTAAACTTCAAACGAGTGTTATTGCATGCCT
CTACAGTCATTTGTAGTCGTATGGCACCAGAGCAAAAATCTTTTGTTGTGCGAACTGTGAAGAAAGCA
GGTCATGTGACGTTGGCTATTGGGGATGGTGG
AAATGATGTGTCTATGATCCAGTCTGCCCATGTGGGTATTGGAATGAGAGGAAGAGAAGGCACTCAAG
CGACAGCAGCTTCTGATGTCGTTGTGTCTCAG
TTTTGCTTCTTGTCCAGATTGCTTTTGTTTCACGGTCACACAGCATACCAGCGTTCGGCGATTATTGT
ACAACAAAGTTTTTGGAAAACCGTTGCTTTAG
CATGGATGCAATTACTCTTCAACATATACACGAAATTTTCTGGTGTTTCTTTTTGGGATTCTTTTTCA
CTTATGATGTACAACTCGCTTTTTACTGTCCC
CGTGACTTTTCTCTGTGCACTGAATATACCACTTTCAGCTTCCGTGCTTCTCAACAACCCCCAGCTGT
ACATCCTCTGTCAGAGGGGTAGGTATTTGAAC
ATGGTAACTTTTTATGGATATGTTTTTAGAGGATTCCTGCATGGAACGATTATCTTTTTCCTTGCGTT
CGGTTTTCTCAATGACAGTTCCAGTGGAGTCA
TGCGGTTTGGTCACCCAATGGACCGTGCGTGCGATTTTTATGTGAGTTGCTTTGCTTTAGTGACGCTT
CACTCATGCATCATCCTGCTGGAGACGCACTA
CATCACATTCTTTCATTGGTTGGCACTAATATGGTCAGTGATGGCGTTGTTTTTGTGCCTTTCGTTGT
ATGGATTACTGTCTGAAAATCATTCCCTCTTG
CAGCTGTTGAGAAATGCATCTTTTTATTTGCAGTGGAATATTGTGGTGGCAGTACCTTTTATTTCCCT
GTCCGTGTATTCGGCAATTAAGCTTTTTTTCT
TCCCGGATGTTCTGCAACTTCAACGACTTATCATTCATAAAATGTCGAAAGTCCCGTTTCTCTCTGGT
TTGTGGCCATTTTGGGTAGATGAAAACCCCTC
CCGATTTTGGGGTTCCTTCTCGGTGCGTCACTGGTGCCACGATAAGAGTCTTACCCCTTGTGGCTCCC
AATCCGTAGGCGGGATCACTAGTCTGGACATG
CAGGCACAAATTAATGCCGAAGACCATTGTGGCGAACCCCACATTGTAAAGATGGCTTTGGATAGAGT
TCGAGATTCCACGGCTCTGTCGGAGCCCTTTA
TCAAGAGAGTGTAGGGCACTCACAACAAGCGTTGGCCTTCTAAAAAATGAAAAGTAAATGAGACTCTA
TACGCATGCGTGTGTGCCTGAGTGAGGAGGAT
AGAAAGAGTTACGTGAACAACGATAAGAATGGGGACCATATGAGGGGAATGTTGTATTTATAATCGCA
GGGTGTTTTACCTTTTTGTGCTGCTTTTGTGT
GTGTGTGTGTGTTTTTTTTTTTTTCTTTTACCTGTTTTGGAGTGAATTTTTTTCTTTTTTTTTCCTTTTT
TATATTTACTTTGGAAGGCTGTGAAGAGCGAA
GGTATGACCAGAAGTGTCCCATAAAATAGCACTTCGTCGTCTAATAGCTCCGTTTCTTTATTTATGAA
TTGATTTCTTTTTGTTTGTTTCCCTTCAGAAT
GATACCGCGCATTGCAATGTGGCGAGGGTGTTTTTGTTTTTCTGTGTCACCAGGAATTTCCTTGATTT
TCCACGTGGAGAGGACGCTGGAAGTTGTGAAG
CCCTAAACGAGTGCTTATTCTTGACTTTACGACGAGGTCGGCTATCATAACATGCGGCCTTTGAAATG
AATATGTTTATCTGTTTGTTTATTTAGAATTA
TTCTCATATAAAGTGGTTTTTTCTTCTTTATTTTCTTTTTTCTTTGGAAACATTGGACTAAACTGTCA
TATCCCGCCATTGTGCATACTCTTTATGGGAA
TAGAGCCTCATGGAGACGGATATTCTTTACTCTTTTTTTTTTTTTTTT
>scaffold1046.size5948
TGTGTGTGCGCATGTCTCCACCTCTTCCTCGTATTACTCGCTTCTTTTTCGTTGTTGTTGTTGTTGTT
GTTTCATATTATTATTATTATATTTATTTATTT

ACTTTTATTCCTCATCTCCTCCTTTCTTATATAAATATATATATTCCGTTTGTTTGTTTGTTTTTTGC
TTTTCTTATGTGCATCCCGCCACCTCTTTATC
TATCTCTCTCTATTTCTCTCACACTCACTCACCGCAGTCGGAAGTCTTGATCGTAGTGACTTGACTGC
AGCTTTCCGTCAAAATCAACGTGCAGCGCAAT
GTCAAGATCGCGCGGGTTGCCTTTATTCGGGCAGCAGCTCATACGAAACTTTGCCTTTTCACCTTTAT
TCATGTGTATTGAATTAAAAAGATAGAGCACC
GTCTGTCGCCAGTGCGTCGGGGCCTCCAGCGGCGTCGTCGTGAGCACAACCACCTCATGGCCGGCAGC
GAATGGGGTGTCAAAGTGCACGCTGATCGCAT
CCACACACCCTGATCGTTCTGCGATAAGTTCAAACTCAGTTGTAAACGCAAGTTCCGCCTCCTGCACG
GTATTAATATTAAATGAGAAAAAATTAGACAT
GTTGGTTAGGATTTGATTGCGGTCCACTGTGTCTATGAGCGGTTCAATGTAGCTCAGCCGCTTGAAGT
AGGAAAAGTCAAAGCCTTGCACATCGTCCCAG
ACGTCAAACCGCTCTTCTTTGTACTGCTGGTCGCTAATGCCACAAGCGTACATGTTCGCACGGTCCGG
AAAGAGTTTCACGTCGGGTGTACCCCATCGAT
CACGCGCGTAAAGAACCGTGTTTAACATTGATTCGTACAACAAAAAGTACCCCATCCACTCACTAATG
ATGATATCAACCTTCTCGTCAAGTTGTAAATC
CTCCATCTTTCCTTGAATAATGGTGATAACATCTTCAAATCCATTATCCTTTACAATCTGCCGCGCCT
GTACTGTTACGCTGCTGCAGTCAACACCAATC
ACTTTCCTGGCGCCTGCCTTTGCAGCAAACATGGAGAGAATACCCGTGCCGCAACCAACATCCAGCAC
CACCTTTCCCTTAAAAAGATATGCATTGCGCC
AAATGGCATCACGGTAGGAAGTCGTGCGGTGATAATCCTTCAACATTTCCATATGAATACCATAATGA
CTGTAGGAATCAAAGTAGTAATCCCCTGTTGC
GGATCCTGCCACAGGAGCTTTTGTCGGCGCCGGTGTTTCTGTTGTCGTCATTTCCTTCTGTGATGCTA
ACAGTGCCGTTTGTGGAGTTCTCTTTCGTGCC
GTGGCAAAAGGAAATGACGCGCGCTGAATACGACGCAAGAGAGAAATGTCTGCAACTGAGAGGGGAGG
GAAGAAAGAAAAAAAAAAAAAAAAAAAGAACAG
AAAAGAAAAGAAACTGAAAGAAAATGTGCCCCCTCCCACTCCCACTTTTAAAAAACAGAAAACAGAAA
AGGGGAGAGCGCGTGATGACTTTTCGTGGGCG
TTTTCTGTTCTTTTCTTTTTTTTTTTCCTTCGGCTTTTCATCCTCCCTTTTATCCCAAAGAGCATCCA
ATGGCCCGCTTCATGCAGTCAAGCGCCTCCTC
GCCAAAGACATGACCCTCCGACCAGACCATCACCGTCTCACGTAGAAATGCGTCGACTGGGGAATTCT
TCGGCACTGTCCCTGCAAGTGCTTCTACCATC
CGCTGCATGTAAAATTCAAATACAGATAGATATCGCAAAGGCCTCTCCAAAGCGTCGTGCTGCAGTAA
TAACATGTTGGCAATAAATAATAACGTCCCTG
CTGCCTGTAATGTCTTTTCGTCTGAAGCCCCGTCCGCAGAATCTGTGACGCAGGCACCACTACTACGA
CGTCGCGTCTCCCTCCGCACCTCGTGTATACG
TACATCACACATGTAACAGAGTACATACAAGAACACCTCCGTCATAGGGAGGCGACGCAGCATAAACT
GTACGACATCCTCAAGAGAGACATGCTCTCGT
TGGGCGCCGTCCAACATGTCAACAAAGGCAGTAAGATCCGCCTCCGTACGAAACATGTGCGGCATGTG
CGGTCCACGTACACCAGGCGTCGAGGAAAAGT
AATCCAGCAACGACATCCACAACACCGCCGTGCCGCCGTGCGTCATTAAAGAGGAAGTTCGTTCTGGG
GCAAATGCCGGCGCCACGACTGCTGGTGTTTC
TGCCCTCTTCTGGTGGCCTCGCCCCTCCAGCAGCCTCACGTACAGCCGCCTCTCCGCCTCGTCTTCGT
TCCGTGAGAAAAGAAAACGATGTTTTGGATCC
GTCACGTGTGCCACGCAGTACTCCGCAAAGTCAGGGCTTGTCAGCTGCATCCGCACAACCCGTTCCAC
AAGTTCACTTTGCCCCTCCATTCTCCCCTCCC
AGTTTTGACGCCGTTGGAGATGTCCCCCGTTTCTTCTCCTGCCTCCACTCTTTTTTTCTCGCGGCCCA
CACAGAACACACAATGAGAAATCAAAAAAAAA
AAACAGAGAAACAAGAATAAAAAAAATCCCAACACCACGCCAATGATAAACACTGTTGCAGCACCTGT
TAAAAAATATTTATTTAAGAACAACACACATA
ACAAACACGTAAATGCGCCCACAAAAAATAAATAAATATAAATATAAATATAAGCACTCAAGAACGCA
GGCCACTGTTGGGGTATTAACACATGCAATAT
TGTTTACTCATCACCCAATTACTACAAAAATAAGAAAAAGAAACAATTACATGTATATATATATATTA
TGCCTATATATAAATATATATATATATATATA

TACATGTGGATGACTGATGGTCAATGCACGTCCATACATAATCCCATTTATACTAAATAAAATTCATC
CGTACCTCTGCCGGAAAACCATATATATATCC
ATATAGATAGATAGATAGATAGATGGTGGCTTCCCGTCCGTCCTCTCCAAACAAGCAATAAAAATATT
CCTTCCTTGTTGTATTGCCTGTAAAAATGGAA
AGTTAGCCAATGAAACAATAATAATAATAATAATAAAAGGGGATAGTAACAACTTGTTTCCATAGCTT
ATTGGCAACGTAGAGCTCCTCCCAATATGCCT
AATTACTCATTAAAAACAAATTCCTTTTTGTACGTGTGGGTCGAAACGCCCTGGTCGCAGTTCCACCA
GAAATCTTCTCTTTGGATGTCTCCATCACCAC
CACCAAAGCCCCCCATGGTAGTTTATTTGGGTCCTTGCATTGCGCCTTACGCATGTTGATGAACGCCC
ACAGCCACGGCCTCTGGGAGCCGTACACGTAG
TGCACACGCACACCACCCCCGGTACGTTCATCATCATCATCATCATCCATGTCAAAGAACACATTTCC
GTTCAGTTTTTCTACCAATTCGGGGGCCGCAT
CCGCACTGACGTAGACTGCGACGCTGCTACGACCACCCCCGTCGACATGCGGCTGAGTCTCAATACGA
TGGATAGTGACCTCCGGGTGTGTGGCGTGTAG
CAGACGCAAGACGCTCGTTGCATAGTCCGTCTCTGGTAGTAATTGGTCAAAAATGAGTTTTTTGTAGG
GCCCAGATTGGCGCTTACAACGCATGCACGAC
TCGCACACCAAATCATGAGCCTTCCCGCAGGAACGGCATGACCAGAAGCAAGATTTCTGCAGTCCAGT
ATTGGGCGCGTCAAGCAAGGCCTGTTCATGTG
CCGCCTGCACCTGAGAAAGATGGCAACGGTATATAATTGGGTACATGTTCCCGCACCTCAGAAATCGC
TCGGTGATGTGCAGTAAATTATTGTCTCCACA
GCTGAAAGCCACCTCCACCATGTGCAACTGCGCCCACAGACGCTGAGAGGTGGTGAAAACCGTCTCGG
CATGTTCCCCCACGTCGCTTGAATACAAATTA
CTACCACCAGAGGAGGAAGCACCGTTAAGGCTTTTCGTGCCGCACGTGCCAAGAGACACAGGCGTCCC
TACATCTTCCCCTAGTGACTCACTTTCTGTCT
TCTTTTTCCGACGCTGACGACGGTGTGGGCGGCGACGCGACTGCGATGGAGCATTCGCAGAGTTCACA
TCGTTTCCCGTGGAGGAGGCCGAGGAGGCGGC
GTTCCCATGCGGATTTTCCCCTGCATGATCCCCCTCGCTTTGCTCCGCTGGTTTCACCTGCAGCCTGT
ATGTGTTCTGTCGACCGAGGGTGGCTGGCACG
CCCCAGTCAAGCTGCTCCTCCTCCGCCACATCCCGCATTGCCGCAAATGACACCAGCTGCTGCAGCAG
CGTAGGACGGGGACAGCTTGTGATGATTGTGC
CAACCTCGTCGGAGGGGCCAAACTCTGACGAGTTGGTGAAGAGTGTGAACAGGATTGCCAGCATAATA
AAGACGGGGCTTCTGCTGCCGAGAGGACCCAA
CGCCTTCCCCTCAACCGTGAGACATGAACCGCCACGCCGCATTTTATTTGCCTCGTTGTTGTTTTTTG
TTTGTCTTGTTTGTCTTCCTTCTCTCCCTTTT
AACTGCGGTTTTAAATTCCCTCCCAGTGCTTGGATAAAACAACAGCAGACACACACACACACAAAAAA
AAAAAAAAAAAAAGTTATTTTCACGCGGGGAGA
AAAAGTCATCTCCGCTTCCAAAAACAAAATCCGTCGGGATGCGTGGAAGGTGACATCCCCTCCCCCCT
CCCACAGAGAGAGAGAGNNNNNNNNNNNNNNN
NNNNNNNNNNNNNNNNNNNNNNNNNNNNNNNNNNNNNNNNNNNNNNNNNNNNNNNNNNNNNNNNNNNN
NNNNNNNNNNNNNNNNNNNNNNNNNNNNNNNN
NNNNNNNNNNNNNNNNNNNNNNNNNNNNNNNNNNNNNNNNNNNNNNNNNNNNNNNNNNNNNNNNNNNN
NNNNNNNNNNNNNNNNNNNNNNNNNNNNNNNN
NNNNNNNNNNNNNNNNNNNNNNNNNNNNNNNNNNNNNNNNNNNNNNNNNNNNNNNNNNNNNNNNNNNN
NNNNNNNNNNNNNNNNNCCCACACACAAACAA
ACAAACAAACAAACAAACAAAAACTTCTTTTTTTTCTTCTCTTTTTTCCTAATAAAATACAACACACA
CAAAAATTACATGTAAAAGAAGAAAAAAAAAA
AAAATTTTGTTATGCGTACGCAAAGGCACACAAATATAAATATATATTTATATAAAGAACAGAGAGAA
AAGAAAAACCAAAATAAAAACAGATGTATACA
AACATACAAGCACACCAATAAATAAATAAATAAATAAATAGGGGGAGAGAGAGAGAGAGAGAGAGAGA
GAGAGAGAGAGAGAGAGAGATATAAATAGATG
CAAACAGCCAGGGATGCACGTATGTTGTANNNNNNNNNNNNNNNNNNNNNNNNNNNNNNNNNNNNNNN
NNNNNNNNNNNNNNNNNNNNNNNNNNNNNNNN
NNNNNNNNNNNNNNNNNNNNNNNNNNNNNNNNNNNNNNNNNNNNNNNNNNNNNNNNNNNNNNNNNNNN
NNNNNNNNNNNNNNNNNNNNNNNNNNNNNNNN

NNNNNNNNNNNNNNNNNNNNNNNNNNNNNNNNNNNNNNNNNNNNNNNNNNNNNNNNNNNNNNNNNNNN
NNNNNNNNNNNNNNNNNNNNNNNNNNNNNNNN
NNNNNNNNNNNNNNNNNNNNNNNNNNNNNCCCCCCCCCCCCCCTTCTTCTTCCTTTTTCTTTTTTCTT
TTTCTTCTTCCGTGGTGGTGGTGGTTGACTTC
AATGATTTCATATTAATTTCCCTCAATCCTGCAAACCAAACACACACAAACATATGTGTACACATATA
CACGCACCCAAGCAGACAGATGCACGCAAAAA
CAAAAGCAAAAGAAAAAAGAAAAAAAAAACAAATATTTATATAAATATATATCTAAACGCACATTCGTG
GAATTATACCCAAAAAAAATGCACGCATGACT
GTGGCCCTAACCCCCTATTCCTACAGAAACAAAAAAAAAAAAAAAAA
>scaffold1047.size6998
AAGCGCCGATGGAATTCAGCAGCGGATAGAAATAACGCTCAGCGGGCAGCCATGTAAATGGCTCCATC
TGGTGCCCTTCCGACCCATTTGTGCGGCGTCTG
CCACCTCTCCGCGTGCAGCGGATGGGCAGCAGGCGCTTGGCTCCCTCTCTAGCAAGGAGTACTAGACC
CTGGCACCACGTTGAGGTGGCCGACTGACCCA
TAGCGTTGTTGTAGTCTTATTGGTGGTACTATTTGGCATTTTCATCCTCTCGTTTGATATCCTTGTCT
TATTATTCTCGTCGCCTCGTGTATTTTACTGC
GCCTTTTTATTTTTTGCATCTGACGTACCGGTCGCACCTTGTGTGTGTGTGGAGACTGAGTTGCCAAA
CCTGGTGGCTCTGGATTATCGTTAGCTGAACT
CAGTGACTCTGGCGGAACTCGTGTCTGTCCAGTGATGGCCGTGCCTGCTTCCTTTGGCAAAGAAGGTC
CTTGACTTCACAAACCTTCCTCCCTGTCTCTC
CCACTCTCTGTCTGTGGCCCACCGGTCTCCCCACCCTCTGGCTGTAATGCACCAGACATTTTTTTTGT
AGGATCCGGTTTCCCTAGCTCAGGGCTGGGAG
GACTGCTGTTATCATTATTACCGTCTCCTGTCGCCGTCACGCACACGGAGCGAGCAGCAGCACAAGAG
CGCAAGGACCAACACGGCGCCGCACCGTCATC
ATCGCAGTCATCGTCACCCGTAGTGGAGTCGCTGGAGCCAATCAGcTGATCCGCGAGACGAAAAGGAA
AAGAATGCAACAAGAGGGAGAGGAACGGGGCA
CAGCGGATACAGCGAGAGAGAGCAGACAACATGAGGAGAGGGCAGCAGGCCCAGCCGCACAGTTCACA
CACCACGAGACGGCATCCACGCAAGGAAGGCA
AACAACGCCAGCAGCCACCCACCATGCAGGAGGCGAAGACATCCACCACCATCTCCTCCCAGCTCATT
TCCACAGCCAACGGGAAATGGCAAAAAACCCG
CGGCCACCACGCAACGCACACGGAGCGAGCACCCACCACACCAGCCACACGCGACAGTGGACCCTCAT
CCATCCATGACGCGGCACCAAAATTTTCAGCC
ATCCCGCCATCAATGGGACACGGAGACCAACTCAACGCAGTCACACGTGGTGNNNNNNNNNNNNNNNN
NNNNNNNNNNNNNNNNNNNNNNNNNNNNNNNN
NNNNNNNNNNNNNNNNNNNNNNNNNNNNNNNNNNNNNNNNNNNNNNNNNNNNNNNNNNNNNNNNNNNN
NNNNNNNNNNNNNNNNNNNNNNNNNNNNNNNN
NNNNNNNNNNNNNNNNNNNNNNNNNNNNNNNNNNNNNNNNNNNNNNNNNNNNNNNNNNNNNNNNNNNN
NNNNNNNNNNNNNNNNNNNNNNNNNNNNNNNN
NNNNNNNNNNNNNNNNNNNNNNNNNNNNNNNNNNNNNNNNNNNNNNNNNNNNCCCGCAGGAAAGGAGA
CTCGGGCTGCGCACTCCCTCCCTTCTCCTCAT
GGCCACGGAGGAAGACAACAGCCGCCACACGCAACTGCCAGCATGCACTGCAAAAGAAGCATTCAAAT
CCACTGCAATTTGCATCTTGTGTGTGTGTGCC
TGCAGAAACACAAAGCAAAGAAGCAGAGAAAAGGAGAAGAGTATGTGGAGGGACGCACAAAATGTACA
GCGGAGGTGCGTGGCGGAAGCAACAAGAAGAA
GACCGCGCAAACTCACGATTGCACCGCAGCCCAAGCAGCGGCAATTCACACAAGATTTATTGAAAACA
AAAAAAAAACTTTCCCCTTCAACTTACACAAA
CCCCATCACACAAAATAATTATAAATAAAAAGAAATCGTCCATTACTGTTCCACACAAATTTAAAAAA
TCAGCCGGCTGCCGTGTGGCCACACCAAACTG
TCTGTATATATTTTTCCTGCAAAAGAAAATTAAAAGATATTTAAATGTCTTTCAACCGTCCGACCCTC
TGTGTGTCCGCACATCGTTCATATTTCATGGG
GTCATCTACGTGTTGCTGCTCCCCTCAGCCCTCCCATCGGCGCGTGAGTGCGGCCCGCAGAGCACAGT
CACTCGTGTGTGGGGACTGAGAGAAGCACGCA
GACACGACGGCGGTCGCGTCACTCCATCGGGAGAGTGAGCGCAGCAAAAAAAAAAAGAAATCTTAAAA
ACACGGCGGCAGGGTGTGGCTGTGTGTGTGGC

GCTGCGGGTGTCTGCCCGCCCTCGCCGCAAAAACAAAACAAGAGGGAAGAAATGTGTGCTCCGCCCCA
CAGGCCCTCGTCGCACGCCCACACGTCACGGC
TCCCTCAGCGCGCCGTTCGTCGCGGCCCCTCCTCATGCGTGCAGCAGGCGAGGCGCACAGGGCCGTCG
TCATGAGCGTGTCGGTGGGTGTGCGAGTGGAG
CAGTTGGGCGGGAAACACACGGCACCCCTCACAGGAAGAGTGCGGAAGAAACAATTAAAGAGGGAAGA
GTAGAAAAGAAAAAAAGTCCTTAGTGATTGCC
CCAAAAGCAAAAAAAAAGAAAAGATGGTGGAGCAAAAGATTAGGAAACAAAAGAAATTTAAAAAATTA
AAGAAAATAAACCATTAAAAAAATGAAAAAAA
CTCACAACGCCCCGAAGGAAGTAGAAAACAGGGAAAATGAACACACCAAAAAAGTAACAAAAAAAAAG
AAAGTAAAAAAGAAAAAGTAAAAACAAAAAAC
ACGCAAAAAAAAAACAGCTGCAACAGATGACTCCAGCGTACATGCATCCACACACAGACATGCGTCCAC
GCAAAAAGCTGTGGGCGTCTCGTGCGTACGAG
TGTGTCTGCGATGCGCTCAACGTNNNNNNNNNNNNNNNNNNNNNNNNNNNNNNNNNNNNNNNNNNNNN
NNNNNNNNNNNNNNNNNNNNNNNNNNNNNNNN
NNNNNNNNNNNNNNNNNNNNNNNNNNNNNNNNNNNNNNNNNNNNNNNNNNNNNNNNNNNNNNNNNNNN
NNNNNNNNNNNNNNNNNNNNNNNNNNNNNNNN
NNNNNNNNNNNNNNNNNNNNNNNNNNNNNNNNNNNNNNNNNNNNNNNNNNNNNNNNNNNNNNNNNNNN
NNNNNNNNNNNNNNNNNNNNNNNNNNNNNNNN
NNNNNNNNNNNNNNNNNNNNNNNCGAGCACCAACAGGGCGCACAGCAGACGGCACGTCATCATCGTGA
GTGTGGTCGTTCAGTGGTGTGGAGCTGATCTG
CACAGTGAGGGACATGGAGAGCAACAGGGGAGACGAGAGGTGCGGCAGACACACACAGAGGGCACACC
GCATGAGGAGAGGGCAGCAGCCAGGCATGCAC
CGTACGCCGCACACCACGCACGACACACACTCCAATCCACGGCAGCAGGCACACACACACACAGCCCT
GCCGCAACAACAACACACAGATGGAGGAGGGA
GAAGCAAACAGGAGCGGCACACAAGGCTCCACCGCATGCACATACGGCGAGAGCGGTCCGTCGTGTCG
GCACCCCTCACGGAGGGTACGCACACACACAA
GAGGAGGTGCAGCGCACACAGCAACACAAAAGCAAAGTAACAATAAAAAAGGAACGATCCCCGCACAC
CACGTGGATTGTTGGTCCACGGGAAGTGCAGC
CAATATTCATCACAAAATAACTTTTTATATTGTTTTTCTCGCGCCGGAATGAGCAAATAGCGCAAGGG
TTCCCCGTACAAAAGTGGCAAGTATGCCGTCG
CAAACAGGCCGTCGTGCAGGCTGGAGCAGTCCCAAACAGTCACAGTCCATATTCTCAATGACAGGACA
TTACCACAGTGGATGAAGCGGAGTCTCCCGTG
GTTCTTCGGGGAATCAGCAGCCCGTGGCGTCTGTGATGAGGCGGTGGCGGAAGAGCCTGGTCCATCTC
GAGTNNNNNNNNNNNNNNNNNNNNNNNNNNNN
NNNNNNNNNNNNNNNNNNNNNNNNNNNNNNNNNNNNNNNNNNNNNNNNNNNNNNNNNNNNNNNNNNNN
NNNNNNNNNNNNNNNNNNNNNNNNNNNNNNNN
NNNNNNNNNNNNNNNNNNNNNNNNNNNNNNNNNNNNNNNNNNNNNNNNNNNNNNNNNNNNNNNNNNNN
NNNNNNNNNNNNNNNNNNNNNNNNNNNNNNNN
NNNNNNNNNNNNNNNNNNNNNNNNNNNNNNNNNNNNNNNNNNNNNNNNNNNNNNNNNNNNNNNNNNNN
NNNNCTGTGATGAGGCGGTGGCGGAAGAGCCT
GGTCCATCTCGAGTACCCGCGTTTGTGCTTCCTGCGCGCCTTGCCTACTGGCATGCTTGGATCTTCGG
TGGCGACCAGCGCGAGAGGTGCCTGCTAAGAA
AACGATGAAACCATTGCGGCATCCGCAATTGAGAAATTCTCAAACTATCGGAACGCCAGGCCGAGCAC
GTCGGGCATGTGCCGTCACGCTGCGGACAAAC
GGGCGCACTCTGCAGCCGATTGGGCAGGAGAATGTCAATCCGCCGCTCGCTGGTCGACAAGATGTTGC
GGTGCTCACGTTGGGAAGAGGTTCCGGCGGAA
GCAGAGGCGGGCCGGTCTCCTCCGTAAAGAAAGGGACGGCACCACCGACACAGGGAGTTCCTTGTGAT
CCGCAGGGCGCGCGGGCCTGTCGAAGAGCGGG
CAGGAGTCGCATCGCGCCTGTGCGGAGGTCCGCCGATCGAGCACGATGTCCGGGTCAACACCAGGAAG
TGAGTTCCGCCACACCGACAGGCGCCCGTCAC
GCGGTGTGTGCCCTTCTTTTCACCCTGGAATTTCTTTCAGTACGGCCGCCTCACTGCGGTGAGGACGc
CCACGTGCCAGGCATTGACGCAGTCCTGCGGG
AGGGATCGCGCCATGATGGCATCAGCGTCCGCTATCACGCTGCGTGGGACTCCGCAGTGTCATGGTGA
GCATTTGAAGCCAACAGTGCGAACTCGATCCA

CCAGTGAAAGCGGCACGGTACAAATCTCCTCGGTGACGTCGTCGTGCGCCGCCAGCGAGCCAAGACGG
AGCGCCTCCATCGCCGAGAGGGAGTCCGTGGC
GGCAGGAACTCGTGTGGGCGATCGGGATGCTGCTGCCATGCGGGGAACTGAAAACTGTCTGTGGCCAA
GCTCAGTGGCAGGACACTCCGCACGGCAGGAG
CACGCGAGGCTGCCAGCAGCTGCGCGGTGAGGCCCCACAGAGAGCGCGGCTGTCCGGGGAGTAAAGTG
GCAGCGCAACAAACCCAGACGCACGTTCAGCG
AGAGACGACGAGCCGTCCACCGCCGTGGGCACGGATAGCGGCCCCGATTCACTCAGCAACAGCGATCT
AATGCCCTTGGATGGTGCAACAGCCACGCCTG
AGCCCAACACAGCCCTAGCAAATGCTGAACGAGTGATAACGTGTCGTGTGGCGGTGATGCCGCACAGG
ATTATTTGCGCACAACGTCTGGGAAACCAGAG
ATCCCATCGGAGCACAACGCAGCATCACTTGTGAGCAACGGTTTTTGTGAGCACGGACATTTCACGGA
TTTGTTGACCATGGCCGAAATTGGTGACATCA
CCGTGCATGGGTGTCTGTCTCGCGTGTTGTTGCTTCTACTTCTGGGGCTGTGGGGCACTGCGGCACTC
TGCTGAGGTGTGAGGGAGGGTGGAACTGCGGC
TTTTCGTGGAGCAATAACTGTGCAGCTTGCCAGTATTACCCACTGAGTTTTGTCAATAATTTAAGCCT
TTTAATTTTCTTTTATTTATTTATTTTTGCTG
CCTTTGCCCTATTGGAATAATCAGCATTCCCGTGTTTCTCCCAAATGGTTGCCTGCCGCGCACTGTCA
GGAGTGACGTGTTTCTTCCCCTTGTTTCCCTC
CGTTTGTTGCCCATACGCGGTGCTCCACACCCGCAGGAAAGGTGACGCGGGCTGCGCACTCCCTCCTC
CCTTCTCCTCATGGCCACGGAGGAAGACAACA
GCCGCCACACGCAACTGCCAGCATGCACTGCAAAAGAAGCATTCAAATCCACTGCAATGTGCATCTTG
TGTGTGTGTGTGTGTGCCTGCAGAAACACAAA
GCAAAGAAGCAGAGAAAAGGAAGCAGAGTATGTGGAGGGACACACAAAATGTACAGCGGGGACGCATG
GCGGAAGCAACAAGAAGAAGACCGCTCAAACT
CACGATTGCACCGCAGTCCAAGCAGCGGCAATTCACACAGTATTTATTGAAAACAAAAAAAAGCTTTC
CCCTCAACTTACACAAACCCCATCACACAAAA
TAATTATAAATAAAAAGAAATCGTCCATTACTGTTCCACACAAATTTAAAAGAGACAGCCGGCTGCCG
TGTGGCCACACCAAACTGTCTGTATGTATTTT
GCCCGCAAAATAAAATTAAAAGATATTTAAATGTCTTTCAACCGTCCGACCCTCTGTGTGTCCGCACA
TCGTTTATATTTCACGGGGTCATCTGCGTGTT
GCTGCTCCCCTCAGCCCTCCCATCGGCGCGTGAGTGCGGCCCGCAGAGCACAGTCACTCGTGTGTGTG
GGGACTGAGAGAAGCACGCAGACATAACGGGA
GTCGCGTCACTCCATCGGGAGAGTGAGCGCAGCAAAATAAAAAATCATCAGGAGGGAAAAGAAATGCT
CACGAGGCCGACAGCGAAGGGACTGCCGGCGC
GTGAAATGCACGCAAAAAAAAAATCTTAAAAACACGGCGGCAGGGTGTGGCTGTGTGTGTGGCGCTGC
GGGTGTCTGCCCGCCCTGACCGCAATAAAAGA
AAACAAAACAAGAGGGAAGAAATGTGCGCTCCGCCCCACAGGCCCTCGTCGCACGCCCACACGTCACG
GCTCCCTCAGCGCGCCGTTCGTCGCCGCCCCT
CCTCATGCGTGCAGCAGGCGAGGCGCACAGGGCCGTCGTCATGAGCGTGTCGGTGGGTGTGCGAGTGG
AGCAGTTGGGCGGAAACACACGGCACCCTCAC
AGGAAGAGTGCAGAAGAAACAATTAAAGAGGGAAGAGTAGAAAAGAAAAGAAAAACGTCCATAGTGAT
TGCCCCCAAAAGCAAAAAAAAGGGATGGG
>scaffold1048.size6860
CTGTTGTTGTCTGTTTGTCTGTATATGGTTGGCTGCCTTTTTTTTTTTTTTTTTTTTTAACTGTTTG
TTGTTGTTTTCTTTGATCCGATTTATTTATTTA
TTTATTTATTTTATACACTCATGCGTCTCTCTCTCTCTTTTTTTTTTTTTTTTTTTTTTTCCTTTTTTG
CGTGCTTTCAATATGTCTCTGTTTCTCCCCCC
TTTTCTCTAAACAAACAAACAAACAAACTAATAATAATAATAATAGCAGGTACATCTGTAGGGGTGTA
CACATGGCGGTTGCATACGTACGTACGTACGTG
TGTAAGCAGAGGCATATATAAATATAAATATGTCTTGTGGATGCATTTATACTTTTTTCCTTTTTATT
TTTATTTTTATTTTGTGTTTTGGGTGCCTTTT
GCTCGTTCTTGTTTGTTAATTTATCATTATGTTGTCTTTACTGTTCTATGTGAGGAAAAAGGGCGTCG
TGCATATACGGCTTTCTTTTTTTTTCCATTTT
TTTCCTTTTTTTTTTTTTtNNNNNNNNNNNNNNNNNNNNNNNNNNNNNNNNNNNNNNNNNNNNNNNNNN

NNNNNNNNNNNNNNNNNNNNNNNNNNNNNNNN
NNNNNNNNNNNNNNNNNNNNNNNNNNNNNNNNNNNNNNNNNNNNNNNNNNNNNNNNNNNNNNNNNNNNNNNN
NNNNNNNNNNNNNNNNNNNNNNNNNNNNNNNN
NNNNNNNNNNNNNNNNNNNNNNNNNNNNNNNNNNNNNNNNNNNNNNNNNNNNNNNNNNNNNNNNNNNNNNNN
NNNNNNNNNNNNNNNNNNNNNNNNNNNNNNNN
NNNNNNNNNNNNNNNNNNNNCGGCTTTCTTTTTTTTTCCATTTTTTTCCTTTTTTTTTTTTTGGTTTGT
TTGGTTTTCTGCTCTTTTTATTTTATTTTATT
TTTCTTGTTTGATTGTTTGCCGACGCGTCTTTTTTTTTTTTTTTCCCGCTTTTACGCCAGAAGGAGGA
GGAGGAGGAAAAGGGAGGGCAGGGGATGCCAC
TAACGTCAGACGACGCCGTGCAAGCAGACATGTCACGCACCACCAGCGTTGCTGCACCGCCTTCGTCG
TGCCTTTTTGTTTTCAGAAGCATGTGGGAGGA
ATGTGCTTTTTTTTTTTTTTTTTTTTTTTTTTTGGAGGGGAGGAGGGGAAGGAAGGGGATGTTGGGTGA
AATGAAACAAAATGCCGTCACCCCGTCTTGCT
GTACGTTCACCCTTTTTTTGTGGGGGTCTGTGGGGGAGAAGACCTCATCCTTAGCGCATATTTCTTTT
TTTTTCTTTTGTTGTTGTTTGGGGGGAGGCGT
GGAGACCTTTTGTTTATTTTTATTATTATTTGTTGTTGCTTTGGTTTGTGTTTGTTTGTTTGGAGAGG
GAGGCGGGCACGCCTGCTGCTGTCGCGTTAGA
CCGCCCCCCAATGCAACAGGGGCGATGGGGCGGTGAGGCGGGGATGGAGGGGCCGTAGGAGATGAAAC
AACAACAACAACAGGGGGAATTAAGTGGCCGA
GGAGTGGGGGGATTTTTTTTTATTTATTTAATTTATTTGGCCCCCTCTTTTGTTGTCTGTCTGTCTGT
GGGTGGGTGGGTGGCAATTGTGATGTGCTCCT
TGACAGTCTTCCCTTGTGTATATATACGCCGTGGGTAAAAGCGTGTTTACATGGTGCTCTTTTCACGT
AACCCCCTCTTTCCTTTATTTATTTATTTTAT
TTATCTGTATGGCCGCTTGCATGCATGTACTTATTTGCAGCTCTTTTCTGTGAGAATGCGGGGGGCCG
CGGTGTGACTCCTCCTCCCTCTCCCCTCTACT
TCTTGTGTTTTTCTTGCTGGCCATCATGGGTGTTTGTCTGTCTGTCTGTCTGTCTGCCTGCCTTTCTT
TGTGTGTGGAGCATTTTTACTCCCACTCGGCC
CCAAAATTTTATTTTATTTTATTTTATTTTATTATTTATTTATTTGTTTGTTTGTTTGTTTTCcCCTC
CTCCCCTCCTCCCCTCCCATCTCTTTCTTTCC
CATTCGCAGACGGACAATGATCACGATGCATGTGGTGTACACACACACACACACACACACATATATAT
ATATTTATATAACGTAATACATGCGTGCTGAT
CTAAATTGTCTTTATTATTGTAAATATTTTGTGACGTTTGACGGTAAAAAAAAAAAAAAAAAGGAGGG
ACGACAGAAATACAAAACACACGCGTGCACAA
CAAACAAGAAGGTCCCCTCCTCCTCCTCCTCCGCTCCCCAAAGAAGAAAAAAGGAAAAAAGGTGG
GTGGGTGGGTGGNNNNNNNNNNNNNNNNNNNN
NNNNNNNNNNNNNNNNNNNNNNNNNNNNNNNNNNNNNNNNNNNNNNNNNNNNNNNNNNNNNNNNNNNNNNNN
NNNNNNNNNNNNNNNNNNNNNNNNNNNNNNNN
NNNNNNNNNNNNNNNNNNNNNNNNNNNNNNNNNNNNNNNNNNNNNNNNNNNNNNNNNNNNNNNNNNNNNNNN
NNNNNNNNNNNNNNNNNNNNNNNNNNNNNNNN
NNNNNNNNNNNNNNNNNNNNNNNNNNNNNNNNNNNNNNNNNNNNNNNNNNNNNNNNNNNNNNNNNNNNNNNN
NNNNNNNNNNNNNTCGCCATTTTTCCTTTTTTT
TTTTTCTTCCTTTTTTTGTTACTTATCCTTCAGTGTTTGCACACATAACCGCAAGCCAGAAATCAGGG
GAAATAATGTTTGGTTCATTAACGCACGATTT
GTTTTTTTTTTTTTGTTTACGTGAATGTTTTGTGTTTGTGTCCGTCTTTCTGCAAGGGGTGAGGGGGTC
CAAATTTAATAATAATAAAAATAAATAAATAA
ATAAATAAATAAAAAAATAAAAAACGTCTTGGCACCAATGGAGGGGATTTATTTACTGATTTTATTTA
CTCATTCACAAAGAAAAAAAAAAAAAGAAAAGA
AGCAGCAACAAAACATGTGTATTGACTAAATATACAAATGTGTGTATTATCATCATTGCTGCTGCCGC
TGCCACTGTTTTTAAGTGTTTCTGAACTCTTT
TCTTTTTTTTTTGCTTTGCAGTCTTAGGGTGTGTGTGACTCCGCGTGTTTGGGTGGGTGGGTGTCGTA
CCGGTGCGCACACGGAGTGCGTATACACCGGC
CCTTCGCTCTGCGTTTCCCGCCTTAAAGGGAGAAAGAGGGAAAAAAAAAAAAAAAAGGGAGTCGAAAA
GCAATTGGAAGTGTAACGGGAGGGAGAATGAG
CAGCGACAGGGTGGCGTCCGTAGGGGAGGCGACCGCGTGCCACGTGGCGCGGATCGTGAGCCGCCTTG

AGGGTCGTTCTTTTGATTCCACGCACCGCGAC
GAGGGGGGCACCGGGGAGTGTGGGCCGTTTATGCAGCGGCTTTTGCCGGTCGTCGAGGAGACGCGAGT
CGATAGGCGCTTGGACGACGTCTTCAGGCGCG
CACACTCAATTTTTGAGCCGCTTTCACAGGCGACTGTTGCGGCTGGGGACGATGCGGAGGCGTTGATG
GCTGACGTTGCGTCGAGGGCAAGTCGTCTGGT
GGTTGAGGAGGTCTTGCAGCCGTGTGACTCAACGAAGTCATCACCATCATCATTCTCAGAGTCACCAT
TGCGCACTCAGCAGGCCGCATGGTGCCGCACT
GGGCAGTTGTCTCTGGGGATTATTCGAGAGTCCACGTGTGGTTTTTATTTTGCGCCGAGCACTGGCGC
CAACAGCGCGCCGCAACGCGTGCCCGTTTTGC
TGCAGCGACGAATTCCATGGCGGTGTAAGGCCAATGATGGGAGCGGGAGCCATGACGCGGTGGGCCGA
GACTGTGATGCCGATAGCGGCCGCGAGGAGGG
AGACGCACCAATGTTGTCGGGCGTCACTCTGCAGTGGGGCGCTGTCTTGGGTTTTCTTCAGTTGCTAT
CACGTGCTGTCGGCTTGACGACGTCGGTGGAT
GGAGAATACACGGCGGTCTCCTCATTAACCGAGGGCGAGCACTCGCTACTTCATATTGTTGCAGACTC
CATCATCGTCATGATGGAGCGCAACCACGCTT
TATTACAAGAGGCACGCGATATGAATCAACTCGGCATGGCTGCTGAGGTACTAATTATTGCCGCGAAT
AATTTTGCATGGATTCGGCAGCGTTGTCAGCG
GATTCCGATGGCAAATGCACGGGAGGTGGAGGAGATTCTCACGCAAAGCCTGGCGGGTGTTTTGCAAG
ACATTATTGACGGTTTTGTGGAGGAAATACAG
TACATTCTTTCCATGTATATGACGTCGTCTGGTGGGGGCGGCGGTGGACGGCAGCGTGTCGTAACGTA
CAGTTTTTTGGCGACACGGGTAACCGCAATTT
GTAGACTTTCCAATGGTCTGGTGCATTTGTTGCCGCGGCAGGAGGTCATGTCAATTGTGCTGGAGCAG
TCTTTGCGGCCCGTCGTGGAGCAGCTGCTGGT
GGGGGATAAACAGAATGAAGTGGAGAGGGGCGGGATCACGACGGGCGTCTTAAAACGGGTCGGCGACA
TTTTTCTTCCGGCGGATGCGTTTTTGAGGCAC
GATGCGGCACATGTTGAGTCGGGCGTCGATGAGAACGGCAGCGGCACTTTGGCACAGCGGCTTTCAGA
GGTGCTGCAGATTCTGGAAAGTGAGGAGGCGT
TTGGGGCGGCGATTTGCCAGCGAAGCCCTCCGTTCCGCACCCTCCGGCGTCTGCTTCCAACAATGACC
TTCTAAGCAGTCACTTTACAGACAAACACACA
CACACACACAGACACAGAGAGACACATGTAACTGACTAGCTGCTTTGCGGAACGGACGACATGAGCGG
GAGGCGAGAGTTCATGTCGTTTTTCACCCTCT
TTCTTTTTTTTTCTTTTGCGTTTGTGTTTGTGACAAAATTTACTGTTCTTTTTCTTTTTTCTTCTCTT
TTTCTCATACCTCCACACACAAACGTATGTGT
GTTTCCCTGCCAATTCATTGAGTGGGAGAAAAGAAGGAAGAGGGGGTGCAAAGCCGTCTGCATAAATC
CCTCTGGAGGTAAACATGTCGAGGGAGCGCCG
TGACGCCGCGTCGTCGGATGCGGGAGATGACGTCACTCCAGCCAAATTTAATTTTTCGGAGGGAACGG
GAACACCGCTTGCTGAACTGCCGGCAGTGCGG
GCGGTGGGGAACCGTGGTGCAGCATGGAAGCAATTAGAAACACGGTTGGAGCCGTTGCACCGAATGGT
GTTTGGTCGTCCAGGAAGAACTGGCGAGCGAC
GCCGTGCGTTGGAGTCATTTTGTGGCTTTCCAGAGGCGATGGGTGAAGTTGTATTGCAAAGCGTGCAA
AAACTGAGGAAAGATAAGCTGCGGGAACTTAT
CCAGGCGCTTGGCATGCCTGTTCAATTCACCATTACACATGCGCGGATGGCGGATGGCGTAGCGGCGT
TTCTTATGCAACCTCATGTCGCCGGGACAAGG
GTGAGTTCTTCCGGCAAAACGCCTCAGCCCCAAAAGAAGGCCGTAAAACGGGAGCGAGCAACGACAGT
GAGTGAAAGTGGTGGGGCTGCGAAAACACCAA
AAAGAGAAGTGGACCCGGCAAGCACGGGGGCTGAGAAAGTGAAGGAATCACTGAGGCGAATGAAGAAC
GAGGTACCCGTTGCGGAGAGTTCGTCTGCGGT
GCAGCCCCCGAGTGATGACACCATTCGTGTCGCCGTGTACAAGCGGGTTCTCGCCACGTCACGCGCGG
AGAGGAGTCATCTCACCACAAAGGCACTGCGT
TTGGAGCTGGAACGACAATTTAGTGATCTTGAGGGGCGTAAGGTGGTCATCCGGGAGGCGGCATCGGA
GTGCCTTGCGGCGTTGATCGCAGCAGAGGAAG
CCGCCATCATTTCTGCCTCTCTTGGAAATGCAGCTGCTGTTGCAAGTCTCAACGGTGGCGACCGCACG
GTGGAGCCTTCCCCGGCCCCGCATCCTCCTCA
GTTTACGGAGCCACCAACTGTGAATGCTTCCCGCAGTCAGGCCCCACCGCCGGTGACACCAGACGGAT

CCGTCGCACTGAATTCATCCGTGCCGACCTTT
CCTGCACACCCGCCGACAACACTGCCGACAGTTTCCAACCCGCCGCAGCTACCGCCAGGGGCACCCAT
GTTTTGAGCCGCGCGACCAAAGAAAAAATGAA
TAATAATAATGATAAAACGAACGCATATAAATACACATGTATAAATATAAGTGTGCATATGCGCACTT
TAAAAAGAGTCTAGTAAACACACAACGGCGAG
AAGAAAGGAATTAGAAAAACAAAATAAAAAAAAAAAGGAGAAAGAGCGTGTGTGTGTGTGTGAGAAGC
GTGAGGAAAATGAAGGCGGCTTGTGGTTTCTT
ATGTTCCTTATGAGAGGGGAGAAGGAAGAGATGTAGGCTACTTGTATCCGCAGTAAAATCAATAGATG
GATAACAGGAGTGAGCCACATGTATCTTTTGA
TTTTGTCATCATAGAAGAGGCGATTGAGGTGCGTGTGGATGTTTGTAGGGGTGAGTGGGGGAAGACAA
TACAATAGAAGCGAATTATCAAACAAAGAGTG
GAATGATGTGAGGATGATGAAGAATGAACAAGAAGGAAGAAGAAGAAGAAGAAAAAAAGGGACAACAT
CTGCGGAGCTTTGGATGGGACCTCTATATTCC
CTTTGCTTTGTTATTATATATACATATTTGTATGGCGTGTGCGTTTGTGTACACACGGGACGCCTTGC
GCCTTGCTTATTATTTATTATTATTTATTATT
ATTGCTATCATCATCATCATGGTAGCTCTCCGTGCCATTTTGCCCTCTTCCCTCCCTCACCTCACCCG
CTGCCTTAATAAGCCTCACTGCCCTTTCATTT
CATCTTTGTCTTTTTTCCTTCTTTTCTCTCTCTCTCTCTCTGTGTGTGTGTGTGTGTG
>scaffold1049.size6829
CGCGATGCCTGCAGGGGAGCACCACGACTGCGACGACATTGATGGCATGGAGCTGCAGGATGACGATG
GGAGCGGGAGGACGATGGAGGCGCACTTATCGC
AGCGCCACGCGAGGGATGAGTGAATGGCACCGATTGAGAGTGCCGGTTACTGCAGAGGAAGTGCTCCG
CACCGAACATGAGTGAATATCGCCACATGCTC
TGCGCAGTGGACGTTCCTGCCAGACGCTGCGCGTCGGACCGGTATTCCAGCGGTTGGTGTAGACGGAG
TATCCTTGAAATGTCAGCAAAACGCCCGATGG
ACAGTTGCCCCATCAGTGATCCCGCACTGGATGTGAGGGATGTTGAGGCTGTTATTTGAGAAGGGAGA
ATATTTGTCTGCGATGCCTGGGGATGAGCCCT
CTTTGTGGTTTCTGGACACGCTTTCCACCAACACGACGGATGCGAGGAACCCGGAGGAGACACATCTC
AATGCTGTAGCGCATGCGGCATTGAGGTGCCT
CGGCATGCAGGTGCATTTGGAAGTGACTGAAAAGGGCAACGTCCGTTGGTCCCATGCACGGACGGAGC
GACGATTGCGTGGGCCACTCGACCCTGCGCTG
GGGGAAACGCTATTTGCCGTGACCACGCGCAGGTGTGCACGATATCCGCCACCAGCGGCAGCCTACTT
CCGGTGGTTCCGTGGGACGGCGAGGAGAGAGC
ATGTGGAGCAGGACGGCTTCACCGGCATCTACTGAACAAGGATTGCCAGGGGCATCCGTCCCCGTTAC
GGGGAACATTGAGACCGACCACGGCACACCAC
CGAAGGATGTAGACACGGCTGTTCCCCAGAAACATCCGTTGAAAGAGGAGAAGTGGATCCGGCCAGGA
TACCCCGACAGACCGCGAGAAGCTTCTCTCGG
CGGCAGGGATCGAAGCAGCGAGACGGACGGCGAGCAGGCCCGTCCCCGACTCCCGGTGATGACACACT
GCTGCTGCTTGTCCCTGTCCGCTGCTTCTTCT
GCTTGTCTCTGCCGCCGCCGTGGCAACACTCTATCGATTTGCGGTGTGGTCGGCGTTTCGCTGCAAGG
GGACTGCGCTTCCTTCTGCTTGGCCTGCAATC
AGCACTGGAAGCCCAATAGAAAACGATCCATTTCAATGGGTCCCTTTCTCCATATGCATGTGCTGTGT
GTAATCCGTCTGACTTTGCGTTTGAAATTGGT
GGTACTTCCGATGAGCACTGCTCTTCTTGTGTTGGTTGCCGATGTGGGCCACCGCATGGTCGTATGGC
TTTGCTTGTGTGCTTGTTAGATTTTCTGAGAC
GCACATACACAAAAAACGGTGCGAGTCAAATGTGCGCACACAAGGCACACACACGCATATCCAAGCAC
ATTGTCTGTGTGACTTCTACTTGACATGGTAT
GCGGGGCGGGTTGGTGTGAGCAAGGCCGACGGAAGCGGGCAGCCGCGTGAGTCCAGCAATGGCTTTTG
TGGTTGTGTGCGGGATCATATGTTCCATCAGA
TGCTTGACGCCGCTCATGGATTGTCCAAAGTTTTTTACTGCGATGCCCATGCTGCGTCTGTGCTAAAC
TGCTCAATGCGTCTCCATGATCTCATGGAGCA
CGGCGTGACGCTGCTCGGGGACCTCATGACGCCGCGGCAGCCGGTTATCAGCAGCCCTGCGCGCTACT
TTTTGCCGTGGAAGATGCGTCTGTGAGCCGCG
TGGCTGAGGATTGGATGGCAAAGGTCCCGCACAGGGATGCACACATCTTTGCGTTGGGGTGCACCCCG

CATCGCCGCCTTCAGCAAcTGGTGCGGGCTCG
GATAGCGCCGAAGGCGATGCGTTTGAAGGACATTATGTTAGACTTCGTCGCCACTGAAGTGCTGGTGT
TTCACCCCAGCATGCAGAATGGGTTCCCTCAG
CTGCTGTCGCCACTGTCGCCGCCGACTCGAGAGTCCGTTTTGAACGTTGCTGAGTCACTTATTGTGGC
GGCTATTCACACCATGAACAACGGTGTGCCTG
TTATTCGCCACCAAAACAGAGGCAGCATCTGCCATGGGTTTGCCAGGACCTTTTTTGAGGGCTTTTCA
GGCTCTGCTACGACGAGCCGGATTTCGCTTAG
GGGCGCCGACAGCCGCGGCAACCCAGTGCGGATCATCGTTGATTGGGGCTGTGTCACGGTGGCGCCGC
TGATGCACCAACGCACATGTCAGTGTCTGCTT
GATGAACTGATGCCGCTGGAGAAGAAAGTGTATGAGCAGACCTGTAGGAATCGTCTGGGTGTGGGCTC
GAAGCGGCAGTAATTGATGAGGAAGATGTATA
CTAGTGCTCCTACCGTCATTGTTTTCTTGCGCAGTGCGTGGAGGAGCTTTCGGTGGCGCTGGAAAAAC
TCCACGTGTATCACCCGGGATTGGCGCAGGAC
GTGGAGCCGAAGGCCAACCTTGCGAAGCTGGGCAGTGCGGTGCGGGCTCCGCCGGAGTTTTGGGAGAG
GCAAGCCGGACCGTCGCTGCACATCGATTTTT
TGCGCGAGGCTTGTGGCACAGTACTGGGAGAAGCGACTGGCGGAGGTGCGGGGGGTGGAATAAGACAC
CGCCGTTGGGCACAAACTCCTCAGGAAATACC
TGGATGGCGTGCGGCACCTGACGAGGGATGCGGCGATGCCCCAGCCAGTGCGTCTGTGTCTTCTCCTG
TTGCTTGCGGCTGCGAGCAGCACCGACGAGTT
GACGGAGGCCAATAAGCAGCAGCTGATCCGAGACGGTGGACTGACGTTAGACGCCCATTTGTTTGCGA
CCCTCGAGCATGTGACGAGGAGGGCCGGGAGC
GTGCATCGATAAAGCACAGCGTCGGCGCAGAGCGGACGGAGCACGAGTGATTCTGCGAGCAACGCCGC
GACGGACGAGGATCCGTTTCTGAAGCAGAACT
GTATGATGATGGAGACGGCGGGCAGGAATAGGACGCTGCGGGTTGAGCTGTCGTTGGCATCGATGGAG
CGCGATCATGCGATGGGGCCAAATGGCGCGAG
TGGTGGTCAAAGCAGTGGAGCATGAGGGAACAACGCCGGGCCATTTCAAGGGTGTGGTGGCAAGTGGG
AGAATCATCCTGATCTGTGTGGCGGTGGTGGT
GGGACGATTGCCCTCACAACTCCTCAGCGCATAGTGCTGCTTGTTTTGGGTGGTGTGGCCTGCAGCGA
GAGGCGCTCGGTCCATGAGATATCCAAAAGGT
ATGGCCGTGGGGTTTTCATTGGGGAGCCTCGCTGCCGCGTCCTGAGGTGTGTTTGTGCAGGGACTTAG
CACCCTACGGCACTGTGACTCACGGGCGAGAC
ACGCACAGGAACGCATCGAGGGCCAAAGAGTGGAACAGATGCAACCACACACGATTGCGGGTGTCTGT
GGAAATTGTGTGCGCTTTTTCCTGCATTCCGT
TCCTTTCCAAGAAGCGTTTCATACGATGTGCGTATGTGTGTTTCAACGGCTGCGCTGCTTGCCTTTTA
CCTTTGTATTCTTGCGTGTGCGTTTGCGGGCC
ATTTCCATGCAAATAAGAAGGCGGCTAAAAAAGGAAAGACATTTGTGGAGCATGCACCCACGGTTTCA
GTGTTCATCCACTGGTGCTGACCTTTTTTGCT
GGGGCATTTGTGCGCGCTTTCCCTCCATTGTCTATTTTTTTCGCGCAGGAATCAACAGGTGGGCATTC
GTGAGTGCTGCGGCGGGCTTTTGTTGCCCTCG
CCGGGACGCGCTTAACGGCGGCAGTTCCATCACTTTGTGCACAAAAGGAATGGGACTTTCTCCGGATC
CTCACCGCAGGCGCATGCCGTGGACGGCCGCA
AAGGAGTGTGTGCCCGGTGTTGTTCGTAGTTCTAAGGAAAAGATGGTGTTGGATGGAGCACAGCGAGT
GGATGTGGAATGTGTCGACCGTGCATCGCAGG
TGTATCCGCTGGAGGCATTGCGGGCTGCCGTCGCCACATGTGAGTACAATATATTTAGAGGAAAGAAC
ATTTTTAATTGGTCTCTCAGGTGGAATCCAAT
GTGCTCAGGCAATGCTTTACGGCAAGGAGTGGCCGGAGGGGACGCACGACGAGGACGCCACTCGCACC
GCCATTTATTTCCGTCGCCGCGAGACCACAAC
GCTGTCCGCGCCAATACGCCTTGCGGATTTTCCGAGGCGGCATGCGCTGTTCTGTTTGTCGCCACTGT
TCCCCCATCCCCGAGCGTCGGCGCAGAAGTCC
CTGTCGATGCAACTTCCTATCGATTGCGGACACATCCATTGATTGATGCGGCCGGCACCGCCCCCAGC
AGCTGGTGCAGTGGGACTGGGGACGAGAACAC
AGCAGACCAAGTTCAGTCAGATGGTGCTGGCAGCCCTGTCAGCGTCTCGCCTACCACAGCGGCGAGCA
CAGAAATCGACGGGAAAGGAGAGGAATCAAAT
GACTGCCAGGAAAACAATTGTCTTTTAACATCGCTCCCTGTCAAACAGCCGGAAGAATGAACGACAAC

ATCAGCCACTAACAATGACGAAGGAACAACGA
CCACCGCCGATAGCGACAGCAGCACGGCGGTCTTCCACGCCACCTCCCATCCTTTGCGTCTTATTGTA
ACGTATGCGTGCTGCTGCTGCGGAAGTGGCCG
GCCTGCATGAAGTGGTGTGCGCTGAATATGGACGAAGGCCGATGACAACCGAATTATTTTGCGATGCA
TGTTGGCTGCATTTCCCGTGGACCAACAATCC
ACGTGGTGTGCGGGGATCTCCTTTTTTAATTGTTCCTTTGCTTTTGCGTTGCTGTGTGCGCTGCACCT
CCTCTTGTGTGTGTGCGTATCCTCCGTGAGGG
GTGCAGGCACGACGGACCGCTCTCGCCGTGTGTGCATGCGGTGGAGCCCTGTGTGCCGCTCCTGTTTG
CTTCTCCCTCTTCCATCTGTGTGTTGGTGTTG
CGGCAGGGCTGTGTGTGTGTGTGCCTGTTGCCGTGGATTGGAGTGTGTGTCGTGCGTGGTGTGCGGCG
TACGGTGCATGCCTGGCTGCTGCCCTCTCCTC
ATGTGGTGTGCCCTCTGTGTGTGTCTGCCGCACCTCTCGTCTCCCCTGTTGCTCTCCATGTCCCTCAC
TGTGCAGATCAGCCCCACACCACTGAACGACC
ACACTCACGATGATGACGTGCCGTCTGCTGTGCGCCCTGCTGGTGCTCGCCCTGTGCTGCTGCCCGTC
CGTATGCGCCGCAGAGACTGAGGAGATCACTG
TTGATCTTGAGGCTGAAGATGAAGTGGATGGAAGAGACAACCGTCAATCGCCCCATGCACCAGGAATA
CCAATGAATCAGAATCAAACTCAGGCTGAATC
TCCTGAAGCCGCCCCCAGTCTACCGCCCCCTCCATCAGTGGCACAATCACAGGGGACCGGTCAGCCAG
GGTCGTCAAGTAATGGGGCCGCTGCGAAACAC
GGAAATAATGCGGGAGATACAGGAACCTTGCCTGGTCCGCATTCGCAATCAAGCGGTGCGCATAATAC
GGTACAAAGAGAAGGTACGGAAGACAAGTCAG
GGGAAAAAAGTGGCGCGAGTACATCCACAGAACAAGCAATTAAGAAAGTCGACGATTCTGCCGAGACG
ACGATGACGACCACGACCACTACGACAAAGGC
ACCAACTACGACCACTACAACTACGACCACAGAGGCGCCAACCACGACGACCACCCGCGCACCGTCAC
GTCTTCGCGAAGTGGACGGCAGCCTCAGCAGC
TCTGCGTGGGTGTGTGCCCCGCTGCTGCTCGCCGCGTCCGCGCTGGCGTACACTGCTGTGGGCTGAAG
AGGTGTGTGCGGGCTGTGCGTGCCAGCACTCA
AAACCACGGGGACATGTGTTTGCCCACTGTGGTCCGATATAATAAACAAAAGGAGAAAATATTAAGTG
TGTTTTGCTTCACTTGCTTCTGCTGCCGTGCTAT
GTGCTCCGCGTTGCTTCCCCAAATCCGTCAGGTTGAACGCATCGCGGACACACTCATATGCATGCGCA
ATGGAGCCCTGGTTAGTTTTTTTTTTTCTTTC
TTTAATTATTGTTTTTTATTGCTTTTTAAACTTCTTTTTACGCGGTGGTGCGCGAGTTTTGCAGAAGT
ACTGATTTCAACTGTCTTGTGTTTTGTGCTCA
CTTCTGCTATGTGCTGCTGTCATTATTTGCTTTGCGGTGGCTGTTGCTTTGCTGTGTGACTTGTCTCT
CGTCTTTGCTTTCATTCTGCGTTGACGGGGAG
TTGTCTTTTGCTGAGGGCTGCACGCAAGTGACAGGAGTGATGGCGATGATGACTGGCCGTGTGCTGCT
GGTGTGTGCTCTCTGCGTGCTGTGGTGCTGTG
GTGCGGCGGTGGTATCGTCTATCCCTGACGTTACCGGTGGTGAAAATGTTCCGCGTGGGTATTTGGTT
GTTAATTGGGAAACGTTGCTAAAGAACGAATG
TGCGACGGACGAAAATTACATTAATAAGGATGGGACGGTGAATGAGTCGGCGGTGAAAGACTGCATAC
GCGATGCGATGAGGGTGGTTTGTAACGTTTTT
TGTCGCAAGATGTCAGGAGAACCTGACGACTCTGAAGGTGAGCGTATTTGTGAGGAGTACGTTGGCAG
CCGAGATGAGGCCGGCCAATCTTCTCATGTAA
TAACACCGGCGGAAGCAAAAACTCTACAAGCACCAAGGAGCGAGCTGGAGGAATTGGGAAAAACACCG
GAAGAGACACCGAAGCCATTATCGGGAGGACC
AGCAACCGAAGGTAAAGCAGCAAAACCAACAGGAGGACCACCAACACCGCCAGTGGAAGCACAACGCA
ACGGTGAAGAAAACATGCCGGTTTTACTCTCA
GAAGATGACCCAGGGACCACAGAGTCAA
>scaffold105.size32495
TTGTCACCGCTCTGGTGGTCTTGTCAGGCACGTACGCATCTCCTCCACCAAAACAGTGAACAACCACT
TGTAGTTTCTTGGCATCGCAGTGCAGCGGCTGG
TAGAGGAGGTACGAGCCGGCAGCCATCGATTTCCCGATCCCGGGCGTCCCAATCAACACACGCTGCTT
GGGCGTAAAGTCGGTCCCACCGTGAGGGCTGG
ACCACGCGGTTACATCGTCAAGGACGATCCGCCACACTCTCTCCACCGCACAGTTTACAAAAACGTCC

TTAGGAGGATCCTGTATCGTATTCCACGAGTA
CGGCCATCCCTTGTCCGAGGCAAGCACCATCAACACGGGAGGTGCCGCACCGAATTGCTGCACAGCGT
CGTTTTTTTCGATAGCTTCGTCAGCTTTCCTA
TACGATCATGACTGTTCTGGCTTCCCCTCATGCACCCCCATTCCAGTCCCCGTTTTATTTCGCTCCAC
GCAGTCCGGAAGTTCCACCGCATGGCTCCACC
TCGCATTGTGCACAGACTCGTAGCATCCCTCCAGTTTCTCCGGTGCGCTCGTCGTCGTTGGTTTCCTC
GCCTCCTCGGCAGCCGCATCCAAGAAATGTTT
GGTGGAGTTATGAACAGCTGCTTTTACCTCCGCAGCAGCTAATGACCACCCCGGTAGATTGTTGACAC
CCTTATCGCAAAGCCTGCGTATATCCTCATCG
AAGATCACTTCACCCTTTACAGCTCGCTTCATACTCAAATAATGACCTGATGCCTGCATTTTTTTTTT
TTTTTTTTTTTTCTACTCTTACCAGCGCGTCT
AGCTATACAGTGCCATCTGAGTGCCGAAGAGGTGGTCGAGCAACGACAAAAGTTTCCTGCTATGAAGC
AACTGCCATTGAGAGAACTTTCCGAGGCGAAG
GTCCTGTTCAATGCCCAGCCGTTGACGCACAGCCAAGGTGCCTGGACATTCTAGAAGCAGATGGTGCA
AGGTTTCCTGCGGGCCAATTGGCGTGCTTGTG
GAAGCTGGCTGATCCTGCGGCAGCAGAGATCACCTCCTGGCACTGCTGTTTCGATGCCGTGTGGCGTC
TGGGTTGTGTGCGCAGCGACTTCTGTGCTGTG
CGGTGCTTCTCCGTGTGGGGAAAACCATGTCACAGAAATCACATTTGAGCTCGGGAAGCGGTGGAGGA
TGTTCCGGATGAAATGACCGGCAGTGACTGAC
GAGACCCGAGAATCCCGTCAATGTGGAATCACAGTGCGGACATTTCTCCTTCCGACGGCGTGTGAGGG
TCCGTCTCCTTACTGGAGAGACGTCTTCCTCC
CTCGCCGACGGGGGAACAGTGTCCGTCCCCGGTGCGCTTGGATCGTTGTTAGAGCACATATATGCAGA
CTGTTCCGGTGAGATGTTGCGGAATCGGCATT
GGTTCGTGCAATCTCTCACGGCCCAGTATCTTCGGCCCCAGAGCTCAGATTCCCCGCACCTTGCCCTT
GCGAGGTGAACCAGTTCCTGACGGTGTAGAAC
TTCCTGAGTGATCAGGTCCTTACCGCTGAGATCTGATGGCCTTGTGCCGCAAAGCAAGTGGCGATGAG
TGTCTTGACGAATGGTGCTACGCCACTTGTTG
GTGAGCTGGGTAGTAAAACATGTCAGCAGATCCGTATGCCATAAAGGTGCGATTCCTTGCTCCGTGTA
TTGTCCACTTTCCATAGTCTGCGTCGCATACT
GATCTGCAAGCTCGTTAACATGTACTCCACAATGTCCGTAACAAAACTGCAGGTGCACGGACCAGCCA
GCACGCGTCAGTGTCAAGAGACGCTGCCAGAT
CTGATCCTCCGTCCAGTCTGTCTGACTGAGCGGGCCCTTGTTTAGAGCCGCTAGAAGAGACTGACTGT
CCGTAACCACAAGCAGGGTTTTACGCCTGTGG
CGGATAAGAGGGATCATCAGCTTCTCTAGGGCTAGAAGCAGCGCACGGGATTCCGTCCTGTAGCTGCA
TGGGCGTGCACCGCAGTTTATGCTGGCCTTTT
CTATTATCTCCGATGAGTTGAGGGATGAAAGCAGTATTCCAACCCCTGCTGACTTGGGAACGTCGACA
CCGCCATCGGTCGCAAGTATCCACGATCGATG
GCTGTGCAGCGCTAAAGCCTCTTCGCTGACCCTGTGCTTTTCCTGTATCAGCTCTTCTTCGTCAGTTA
TTGAGTGGTCTATCGGAAGTTCCACACCGATG
GTGGCCCGGCGAGCGGAGGCCGCAAACCAGGGAGGAATGGAGCTGCGAAAGAACCGCTCTTCGCGTGG
CGAGCTCTCGGTGATGCCATAGTCCTTTGTGT
ATGCGTCTACGAAGGCATATAGCTCGTCCCGAGATAATGGCGAGATGCGGAAACCAGCGCGAGGTGGT
GGCTCAGGCGGGGGTCTCCGCATCCAGTCCAT
CTGACATGCGCGTGTGTTCTCAAATATTGAGAGCCGCGCGCGCAGGCAAAGGACGTGGAGCGGCGGCA
TATTAGCCTCCAGTAGTGCGGATTCCGGGTCG
ACAGTGCTCGGAACTCCGCTGACAATGCGTGCTAGAGTTGCGTACGTCTTCTGCGTCTCATTCTTCGC
TGAATCCGTCGCTACTGCCCATATGAGCTCGC
TGCCATAGCGTAATTTGGAAGCACCGTATCCAATGACGAACGTGCGTAGGTCATGTGGAGATGGTCCC
GCCTCTTTGTGCGCTATGCAGCGTAGCTGCGA
TATCCGTTGCGAAGTGGCAGCGCATTTCTTGGTGGCCGCCACATTTAGCGTCAGTCGTGGATCGAGAT
CCATGCCGAGAAGCTTCGTATTTCGCGATGCC
CCCATGGTCATGACTGGGATTTGACATCCAGCCACTACCAGGGGCCACCTTCCTTCACGATCATAATC
TGACTCCGTGTGCGTGGATAGTGTGAACCAAG
CAGCTTCACACTTTGACGGGTTGATGGCCAGGCCATTTTCCTGACTCCACGCATGAAGCTTTTTGAAG

AACCATGTTGGCAGTGGGAATGGCGTCTTCCC
GGGACTGCCCCTTAACAATAATAGAGAGGTCGTCCGCATACATGTCTGCTGTGACACCCGCAAAGCTG
TTCAGCAGATTGAGAAGTGGGTGTACGTAAAG
GGAGAACAAATACGGTCCCAGCACGGACCACTGAGGAACTCCTCGCAGCATGGGACGCCGATCGCTGT
GTCTGTGGCCAACTCGTGTCTTCGCGTAGCGA
CCACATAGAAAGTTTCTAAGCCACCTTCTACAATTCACCCCCGGAATATTAGCCAGCAAGCGAATGAG
GACTCGATGATCTATGGAGTCAAAAGCCTTGC
TGAAATCAACCAGCACCAGCAGGGTCCGATGGGGTCTGTAGTGGAGTTGATTGCCAATACCGCTTTTC
TTGGGAAGGCTCACTTGGAAATAGTGATTTCT
ATTATGCTCCACTTCGTGTATCAGGTGTGCCAGCTGCATCGTCGTAGTACGCATGCTACGATATGCAT
ACTGGTGGGGGTGCGGCGTCCACACCCAAAGC
AATCTCTTAAGTACCATTTTCCCAGCAACCTTACTCACAATGGAGGTAAGGCTGATGGGTCGATATGA
CTCAAGCAGTTCCGGCGACTTACCGGGTTTTA
AGAGTGGAACGATGGTAGCGCGTCTCCACTCAACCGGAATCGCACCGGTCGTCCAACTGTGGTTAATG
CAACGAAGAAGGAATCGGAGGCCCTTTGACGA
AATATGTCGCAGTGCCTCACTGTAGACTTCATCCGGTCCGGGGGCCTTGCCATACGGCGTATTACGTA
GCGCAGCAAGCAGTTCCGTACGATTAAAAGGT
GCATCAAGAGGGGAGTGAAGATGAGCAACTAAACCAGATGTGGTGGCAGCTTCGTTTTTATCCTGTGG
CTCAGAACTAGATGAGAGTGAAGAACCTGAAG
TAAATGTAGCGGCAGACTCGTTGTTGTTCTGCGGCTCAGAAATACAAGAGATAAGAGAGGTGGAAGAA
ACTAAGCTAGAAGTAGCGGCAGACTCGTTGTT
GTTCTGCGGCTCGGAGCTAGAAGAAGTAGAAGAAAATGAAGTAAATGAGGCAGCAGACTCGCTGTTGT
TCTGCTGCCCAAAAAGAAAGGAAGCATTAATG
TTCAAGGATGCACTCTGTCTGTTGATGCCTTTGGAAAACCTAACTGCCTTGTGAGAGGGGTGAAATGG
AAGAAAGATGCGATTGAGAGCATTCGCTGCTC
TCCTTGCTGTTTTATAAGTGTGTTGACCATCTCCTAATAACATTGATGTGGGAGATGGAAGTGATGCC
TTTTTTCGTCCGGAAATGTATTTCCATGCGAG
TGGCTCGCCTGGATTTAACTTGCTGATGCGACGTAGATACGTGTTGCGCAAGCGCTCTCGGAGCAGGA
GGAAGAATTGACTCCTTGTCCGTTGCATTTCT
GCCTCACGGTCGGGCGTCGGTAACTGTAGATAGCGGGCCTTGCTGTCGGTAGCCATCCGCTCTGCCTC
CATGAGAGTGTCGTCCCAAAGCCTGGGACCGT
CCTTGTGCATGCCGCGGGGGCAATGCCGTCGATACGAAGTTATAAGCGCTCTCGTGAAAGCCTTAATG
CCGGTGGACTGCTTTTTATAGTCATATGCCGG
AAGTTGTGCGTCGAAGTCCTTGATGAATAAATCCCACTTGCACTTCCCCCAACTGTAGAAAAACTTAG
GTGCCGAAGGTATGGTATCCTTAAATGCCTGA
TGCAGCGTAAATGTCAGCATATAATGATCACTAAGCGGCGACACTGAGGCTGACCAATCCGACACAGT
CAAATGCTTTGACCATGTCAGATCGATACAGG
ATCGTTCTCTTGTGCCACGGGCATACGTGATCTCACCAGGGGTGTTAACCAAGGTGAGTCCCGCATCC
GTGCACAGTTCTAAAAATTCTGCAGCAACCTC
GCTTGGAGAAGTCTCCAAGAATGGCTCCCACTGTGGGTGATGCATGTTGAAATCCCCGCACAGCAGGA
GCGGCGTCGAGGCTGGAAGGCAGTTTACTAAC
CGCCTGAAGGATTGCGTTACTTGCGGTGGTGGTCTCATATAGGCACTCGCTACAATAAGATCACGGTT
CTGGTCCAGAGCAACCTGGACCACCACCACTT
CAAGGCTGGTGTCATGCTGGGGGATGGTGAGAGGTATACGCTTGGAGCGGAGAGTTTTCCGCACCAGC
ACCGCCACACCGCCTCCCTTGCCGATGCGCGA
CTGTGCATAGAGCACATAATCTCCAGTGTGCATGCTGGCGATCTGCTCTGACGACTTCCATGTCTCCT
GAATGGCTATTATGTCTGCTCCTTGCGCCATT
AATGTTGCGAGTTTTGACGGCGTGAGGCAAGAAACGTTCATCTGGAGTACTGCTATGGGGCCTGGGTT
CTGCTCAATGTCTCCGCTTAGCAGTAGCTTTT
GTCGTTGCGCGTCACATACGGCACGCAAACGGTATGTATAGCGCTGATAAGCGCCGATGGAATTCAGC
AGCGGATAGAAATACCGCTCCGCGGGCAGCCA
TGTAAATGGCTCCATCTGGTGCCCTTCCGACCCGTTTGTGCGGCGCCTCCTGCCTCTCCGCGGGCAGC
GGATGGGCAGCAGGCGCTTAGCTTCCTCTCTA
GCAAAGAGTACTAGACCCTGGCACCACGTTGAGGTGGCCGGCTGAGCCAGGGATGCCTGCATTTCGTT

CAACGCTCCTTTATCACGGATATACTTCGTGG
AGTCCTTGAAAAACTCCTCCAGCAAAACATCCCGATTGGTGTCCGCAACGCCCCTGCCGTCCAGTTCC
CTCGTAAGAAAGTCGTTCAGCCTCATTTCATT
GACGCGAACTCTTCCCTTAAACAGTACCTCTTCGATATTAGTGGAAATGGTAAATTTCATTTCTTGCG
TTCCTCTTAGCCTTTCCTCTGCCTCCCGCCTC
GCTTCTGTCAGTACCTGCGTGAGTACTCCGTTTGGTTTTCCCCTTGCAAGAGGAGTGATCGCATCCAT
TTCTTTCATAGTCCCTACGTTGCCCGAGAGAG
AGCACATCCTCGTGAAGAGGCCTAGTAATGGCCTCCAGAGCTTGGTACGCAGTTAAGTTAAGAATTTC
TTCAAGAGGCCGCTGGTCTTGTACGTAATCAT
CCGGCTCCTGAACAAACAACTCCATCGTCACATTGTAGTCTTTCATCAACAGCCGACCTGCCGCCAAC
ATAATTCCAAAGGAAATCACTCAGCCTCATGT
TGGTGCTGCTCATGGTCCACTGCGGCCACACTTTACTCCCCGCCACATGTCGAGCTGCAGCGTCGGAA
CCATCACAAGAGTCGCGGCAAGGCGCCATCAC
AACACCCGTGCGGTGAAGCATCCCACTCGCACCGCCCAACTGCGGCCAGCAGGTGTCGCTGGCTCCAA
AGGACACTCGCGGCTGTCTCGTCCCGCAGTCC
CAATGGCGTTGGGCGTGGCACTCGCACGGCAGGGCGGTCGGTGCGCCATGCAGCCACACTGAAACGCG
AGGCGTGCAACCAACCACAACACCGCCACATT
ATATCGTCCTTTTTCATTTTGTTTTATTCACGTTGTCGGCCTGTTTGCCCCATCACCGACACAAAAAA
TAAGTGCACACCTTCACAGAAACAAAGAGAAA
AGGAGACACGATCCCACCGTCCCGCCGCGGGCCAACACTCTCCTCCACCCACCACCATTGCCGCACAG
GAAAACGCAGTCACATCGCAGAGTCAGCACCA
CAATGTGCACCACACCATCGTGCAGTGAACAATGGGCGGGACGCGGCCATGCATCGCCCCGCTCCATT
TTCCCACCACTCAAGCGTTGCGTGCCCACACA
AAGTAGCAAGCACAATCAGAGTGCACACAAACCCAACAATACCCCCCACTCGCTGAAGCAAATTATTC
ACATGGAGTGACAACATTTGGGGACACACCAG
CAGTAGCAGGCTGCATGGAATTTCCCTGCCAGCCCTGCGAAGAGACACAACAACTCTGCGGAGTCAAA
GTGGCAGCCCGCGGTGTTGCCCGCAGTTCACA
TGGCAGGAAGAATTCCACGCACGCATATTTCCGGGTTCCTCGTTGTCTGAGTGCCCAACAGAACATGT
GAAAAAAATACATGCGCGCAAGATGGTATTTA
AATACGTGAAAAAGTCGGCTTGCTTTCTCCGATGGCGTTGGCGAGAAATCCCACACAATCCAATCAAA
GTGACACGCCGGACAGAATGGCGTCAGTTAAT
AAATAGAAATAATTGCGCCGATGCAAAAAGCAGCAGAGATTTGGGTTTTACTCCCGACTTCGGAGACG
ATAGGTGGCATCAGAGTGTGTATGTGCGCAAG
ACATTTCGTGGGACGGTGTTGTCCAATCCCGTCGTGCCCCGATACAAAAGGGCAAAAAAAAATAAGAA
CACACCCTCCAGAAAAGGAGTGACGGTTGGGC
AGTCAACCCCCGCTTCGTCTCACATGACATCCACATATGTGCACTCCACCTCAAATACGTACCTCCCT
GGACGCCGCATACATGACGTGTGGTTTCGTCA
CTCGCAATGTGTTTTTCCTCCCGCAAACAACCCAAAAAATAATAATAATAATGGCCATGGCGCAGCTT
CACTCAGGGGAAGCAATCGGAGCAGACACGTG
TGGGGCCAGGGATCATACCAACAAAAATATATCTAAAAAGGGGGCAACAAATCCTCTAAGCCACAGAA
TCGTCCAATGTTCATCCCTCTCCTTCTACATT
GAAACCTCCTCAACAAATGCAACAGAGACATGTGCAGCACAAAATCTCTCCCTGTCCCGCGTTCCATC
TTACTTCCCATGAGCAACACGGCAGTGGCGCC
GGCGTTACATCTGAGATGACTCCAAAATACCAGGCACCATGCTGTGGAGAGTGGGTGCTTGACGAATG
TTTCCGCGTTAGCGCCTAACACGAAATGAAAT
CAAAAAAAAAACGACCCGCGACTCAAACTGCCCGTTTATGGATGTACTAAAGCAAACACTGAGACGCA
TGTACAGCAAAAAAAAGGAAGAAAAACATGAC
AATCCAAATATATATATATATATATGAATTTATGCATCTANNNNNNNNNNNNNNNNNNNNNNNNNNNN
NNNNNNNNNNNNNNNNNNNNNNNNNNNNNNNN
NNNNNNNNNNNNNNNNNNNNNNNNNNNNNNNNNNNNNNNNNNNNNNNNNNNNNNNNNNNNNNNNNNNN
NNNNNNNNNNNNNNNNNNNNNNNNNNNNNNNN
NNNNNNNNNNNNNNNNNNNNNNNNNNNNNNNNNNNNNNNNNNNNNNNNNNNNNNNNNNNNNNNNNNNN
NNNNNNNNNNNNNNNNNNNNNNNNNNNNNNNN
NNNNNNNNNNNNNNNNNNNNNNNNNNNNNNNNNNNNNNNNNACACACAGAAACACTTGTTTTCCCCTC

AATAATTATTTAGAAAAAAACTTTGTGTGTGT
CATCACTGTCCTCTTCTTTTTTTTTTTTTTTTTTGCTCTAAGTTCAGTGTCTACGTTAATTTTTATTA
CCTAGTAACCGTTTATTGTGCGGGTTCCGCAG
CAGTGTGTTCTTGTTGCTCGGCTGATTGCTGAAAATCGTCCAAGTGAGAAGGTGAGTTTTCATCATCC
GCATTGCCATTACTCTTCTTTGCCCGCGGTGC
ATGTCCTACTCGGCACTGAGCTCCAAATTGGCAGGGACGTTTACTATCGTCTTCATGAAGAAAACGAC
AAGAACCAACAAACTTACAGTTACCTTGCAGA
AAAAAACAGCAGACCGTTTGTCTTTCCCACTCACGCTGTGTTTCCAATGAGCCACCAACAAAGGGCTT
CTTTCGGTGAGAGCGAACTCCAAACGCACCTC
GGCCGTAGTCACCCCCCATGACCATAGTGTAAGCACCACGTCCATAGCCCCCACCTCCACGCCCCCCA
GCGTAGACGGATCTTCCACGATTTACTTGACC
TGAAACGGTGCCTTGGAAATTCTGAAAAAGTGTTGGGTTATACGTTCCTTGTGGATACTGAGAATAAT
AACCACTTTGCGCATGGTACTGGGCAGGAGCA
TGTAATTGTTGGGAAGGATAGCCCGAATGGCTATAAGCATTGTAGCCGCCATACCCACGATTCTGCAC
AATCTGTGGCTGACCAAATGGTTGCATGGTCT
TGTTTGATATTATATATATATATATATATATAATTATATATATAAGCCTATACAAGACTATATTATAT
ATATATATTATATATATATATATATAGTCACTTT
CGGCAAGCAAAAATACAAGGAAATGCAATCAAATATAAAATAAAAATAAAAATTTTAACATACGTAAA
CCTCATCAAACAAACAAACAAAAAAAAAAAGAA
CGCCAAAATCGAATAATTAAGAACCATAAATTATAGAAAATAAAGGAAACACGGATTATATATATATA
TACATATATATAACATTGTGAGGTATTTTTCC
TCATAATGTATCATCCAAAATATTTGGGGCACATGTCAAATTTAGGTGAGGGATACCGCAAATGAAAC
AGTTCTCTATCAACTGAAGCCCTGTGTTACAT
CGTGAACAGAAAAAAACATCTGTGGGTAAATTCTGATGAAGACATTCAGTGCATACCCACGAAGAACA
CCGTACTCTGCTCTCATACACACTTTGCGCAC
GCCCCCCACCACAAAAACGACACTTACTCAGCCAACTATAATTCCAATGGCCGCAGACATGGGAACTT
ACAAGCGCTGTCTGAGACACGATTTCTTGACT
GCACTTCCAGACGTAAACATCATCAAACTGTAAGTCTGATCGTGGGGTCAAACAGGAGACACAGTGAA
AGCCGGTCTGTATGCTTTTAGATTCACAAGAA
TTACACTTAAAAGGATTCAGTTCATTGTAACGAAGAAATGCATTACAAAACTCACAGTGGCTATCCAA
AGGCAGATTCGATCGAAGGTATTTTTTTCCGC
ACTGATGACATTGGCGTTGAAGGTATGGAGAGCAAAGAACATTTTGATCCCGTGGGTGAATATGACCA
CAATGTTGACACGCAATCACACGTATCTTTAT
GGATTGTAGAGTCAAATAGAAAAATGAATGTTTACTTGAGACAGATTTGCAGAAATTGCACGTATATG
CACCATAAATGGCACGGGAACATGTTTCACAG
CCAGTGATGCAAGGATGATTTCGCCTACGACATCCACATACCCACGGCTCCAGTGGTGTAACAATGCA
GCCAACTCTTCGTCTTCCAGGACACCGTTGGC
ATTTGGACCATGTCCAGTGATTATACTCTTCACATTCATCACAACGCCATAAAAGGGGTTTCTTGTCT
TCATTCGCCGCATAGCATAACGGACAACAGAC
TGCTCGTCGACTGTATGATTTTCCACAAAAAGAGCACTCCCTTGTTGCAATTTCCTCACAATATGTAT
TCCCACATTTGATGCAACCACTAGAAGATAAT
ACTACATCACCACAATTCATACACAATTCAAGACCCATGCTAGCTGCTTTGAGTCGTCGTGGATTTTC
TGAACCGCACATACAACTCTTTTCTACATCTG
TACCTCTGTACTCCGCCCCACATGTAACACAGGTCCAAGCCGCATTGCGTTTTCGCAAGCACATTAAA
CACTTTTTTGCACAAGCCGGAATTTGCGTTCC
ACAACCGCATTCCCACCAAAACTCCGTCTTATTTACAACGCAAAACTTTTCTAGCAAAGGACGGATTT
GTGGATTATCACAAAGTTGCACCCCACTCAAA
CGCATGGAAGGGAAATAATCATCTTGGAGGGCTTTAATAAATTCCTGTACAATAACGGATGCAATTTC
CCCATGAATTTTCCATATTTCGCTCAATAAGC
TTAGAAGACTGTCCAAGACCCACAAAAAATTGACACTTGGATGCTCCTTCAAAAAACAATAAAAATTA
CGCATGGAAGCAGACGTCTCCTCCAGGGAAAG
ATGGTTTATAGAGCCAACCAAAAAACGAACAACCTGAGAAAAGGCCGCCCTATCCAATGTTAACGGAT
TCCCTTGAAATGCACAAACATGATAAGCTTCG
AGAACTCGTTCGTAATTCAAATAAGTTGGTTTGGACGTCACAGCCGCTTTAAAAAATGGCTTCCATAT

AGATTGAAGCCCTCCCTGTGAGTTGCCATGAA
GTCGTACTGCAAGGGATGCTACGTCAATTATTGTGCGCCACAGACTACGCGTATCTTCCCTCTCTGCG
CATACGTCGACCAGGCTGCCTAAAACTCTAAC
AAGATACGCCAATGGCATTTTAGTTTCCAAAGCAGGTACCATTTCAAGAAGTTTAAAATGCAAAATGG
CAAGAGACTCCCCTTCATATTGCCGCATATAA
ACACGACTTAACTTGCTCAGTAAAGGAGTCCATTGAATTGATAAAAAGTCAAAGGATGGACCAATGTT
TTTTACGATATTCATCGCAATGGCATGCATTT
CTTGGAAGTGAACAGACGAAAAGAAATGATCATTGCACAAGAGCCCACTCAAGCACTCAACAAAAGGA
CCGGGACCAATGCCTTTTCCAGAGTATTTTCT
CAACTCGCAATCCATGATATAATCAAGAATGAAGGGGGTAGCAGACAACGACAATATCAACCGCAAAA
TATAGCCCGGAATGGGGCGATGAAAGTTTCTT
ATTTCAACCAAAAAGGAGTCATCCAAACATAAAGGGGAGGCCACAAGCGACATCACCAACCGTATGCA
TGTAAAATCCTCGTGGAACGACATCGTTGAAG
AGAGTCTTTTAAGTCTATCAACAACAATTCGAACACAATGGTATCCCATAACAGAAGTACCACTAAAA
AGATGTGCCTCTGATAAACTAAGAGAAACATC
GGTAGTGTGTAACGTCAATAGCAATAAAGGCCAAAGAAACTCCACAGTGTCAGAAGAATACTTACTTT
GATGTAGCGTAGCAACGATAAGAGGATAATAA
CAGTTTGAAGGAGCCAAAAAAGAACCATCGCCGCCAAGAAAAAAAAGATTTGGCCCACTGGATAACTC
GTGTTGAAGCAAAAAACATTCTTCAAACGCAA
TTGATGACATCGATGAGAGATCCGTCCGCATCTTGCGTCTCGGTAGGATTCTCTTCGCAATGCTGGGC
CATCGCCGCATTCAAACTCTACCGAAGGACGC
CACTAACCATAAAAATAGAAAAGAGGCACATGTATATTACATGTACATACCAAATTAAGAAAGAAAGA
AAAATATAGAGACAAAAAAGAAAATAAAGAAG
ATGAAAATCTACACTCCAGTTGGGACGATTGTAAGGCCGCTGCTGAAATGGCAGCTGGATGTAGTTTT
ATGCGTGATACACACAGAAGAAAAATACCATG
AAACAAAATAATAATACAATATATATATATATATATGGCTCTAAGAGAAAATCCCCGCACTCGCATCA
AACAAGCCGTTCATCATCTCCTGCGCTTCCGT
CGTGTTGTAGTCTGTAGAGAGTACCTCAGCAATGTAGGGATCCAGCACACTAGTACCTCTCTTCTCAT
AGTACTCCTCTGCTTGAACCCTATTGTGCGCA
CCCGAATCAATAAATTCTAAAAGATGGAACATGGATGGAAAAGTTCCGGCGATGGTGGAAGCCCGTCG
TTCGGAAACAGATGATATCTCCAAAAGCATGT
CAAGATAAAGAGCGTGAAAGTCCGTCGGATCGCATTTTCGTCTTCCTTCCGACCGAACGATTGGATCT
CCTTCCAAATTTTGTTGTTTAATTGCCTTGAG
ACCATGGGCCACAGCAACCTCGGCCAATTGCTCAACAGAAGAAACAATGTCAAATCCAACTAAAATGG
ACAAAAATGAATCATCAGCACATAGGCAAGAA
ACCTCTTCCCAAAAACGCGACTGTCTATGTCCTGTTAACTGTCCAAGTATTACCACATACACCGGACA
ATTTGATGCATCGTATACGACCTTCAAATAAT
TACAAACACAATTGTGGCGCACCATCAGCATCCAATCGTCAAGCGTAATGAGAAGTAGAAGCAGAGAG
GTCGTGCTGTCATTATTTACAAAAACCGTACT
CGGAATGATATTGTGATCCTCCCTGGCATGTATGCACTTGGCATCCAAAAACATTTTACGCAGTGCAA
TGGTAATCACCTCATACAGGCGCTCGCACGCG
GCATCCACAACCAATTCAACAGCCATATACAACACTGTGCGCCTCAATAAAGAAAGTTCCGGAGAAGT
GCTTCCCTAAAAAAATGAACCAAAAAAAAAAA
AAAAAAAAGGGAAAAATGAGGCTTTTGCAGATTGCAGCAATTTTTACGCAGCCAATATTTTAAAAATC
AATAATAAACGGGAAATCACCAAATAATCCTC
TACAGCAATATCGAACAAAGTGAAGACTGCTCTCCCACAACTTACATGCATGCCGCAAAAAAAAAAAA
AAAAAAAAAAATTCCAAGAAAAAAAAAATTTT
AAAGACAAAACTTTTAGAAAATCTAAAAATAACTGCATGCCACAAAGCAACAAACAATTGAACGCACA
TGGAGGTCTCTTCATGCGGAGAAATGGTTATT
GTACCTACACAATAAAGCAGGAAAATACCGACGTAAATTATTTTCAAAGAGCTGAACAATTCTCTCAA
TTACACAAGATAATGCCTTGACGTCAAAATGT
CAAGGGTTCACCGACTTTGTGTCCCTCCTCGTTCAAAAATTTATGTCACTGAAAATAAAAGTTTTGAG
GCACAACAATCCAAGTACGGCGGTTTAATCAT
ATACATAGAATGAATATCAACAAAAAAGTGCAATCCATAGAGTTCCTCTTTACAGCAGTTCCGTGGTC

ATACAATGTTATTGCTATCATCTAAGAGTCTT
ATTGCTTCCTTGGCATCGGCCTCTGTGAGACCAGTGAATGCATTTGTGGTAATAGCATGACGAGCAAA
AAAAGCTCGATTGGGCGCCGTGGCAGGTAAAT
TTATGTCATCAAGTGCGACAAAGTTTACAATATTGTACCGTTCCTTGTTGGTCTCAATCCACGATAAT
ATTTCCACCGCCCGAGACTTGTCATGAAAATA
CGGCGTGCACGAAATATAGTTCGGCATATTATTCGTGGCCAGTTGCCTTGCCACTTCTTCCCGCCCAA
ACTCCGACATCCGCCACGAGGTGCTCAGGACT
ATTTTGGCCCCTGTCTCCGCAATAATAGTTCGCAATAGGGACATTTGCTTGGGCGCAAACATCTTCCC
GCCAAAGGGGGACACAACACCGTCGCAGTCAA
GAAATAAAATCTTGGTACCATCAATTGGCACTCTGTTGGCTACCGTCAAATGCTTCTCTTCTTTTTTC
CTGCGGCTCATTCGTTTGCTTTCCTTTTTTTT
TTGGCCCTTAGAGTTGATTTTCTGATGATTTCCTTTAAAAAAAAAAAAAAAAAGATTCATAAAAAAAT
CCCCCACTCACGCACACACACACACAAAAAAA
AAAATTTGCACCCACAAATTAAGCTCAATTTCAAAGCAGAAATAATTTCGTGAAGAATTCACCCGCCC
AACCCCGCGCGGCAAAAAAAAAAAAAAAAATGC
AAATGCAAAAAAGGCCACTAAAATTCTTTATTTGTGTATTTGCTTCTTCATTTCCTCAAGCTATTCCT
GTAACAGATATAACAAAAAAAAAAAATAAAAAA
ATAAATTGAATTTCCCATTAATTCGAAAGAAGGGGCACTGAATTTCCTCGCTCGTAAATAAATAAATA
AATAAATAATTAATTAAATAATAATAACTGTT
GGTGTGCTTTTGAGTGGGAAGGAGAGAAAGAAGAGGTCATCAGTTCCGAATTACTCACGGGATCTTTT
TGGCATAAATTTGTTCATGGGGAGAAAGAGAT
CGTCTTTGACTTCCCCAGAGGCAAGTTTCCGTACATGCAGCACCAAATCATAGGCAATCCGCTCCGTT
AATCTAGAATCATGCCCATAGTCAGGATGCTG
CATAACAAAATTTCTCAGGTATTCTGCTGCTGTGGGAATTTCTCCTGCAGCTCGCATGGAAAGGAAGC
AAAGGTAACCTTCGAGTGTGGAACTAAGCATG
TTTTCCTCCTCCATGTATCGTCGGACAAGAGGAATCAACCCATAATATTCCCCATCTTTTCCATTGAA
GACTTCATTAACGGTGAATTCACTGTTCTCAG
AGGGATCAGTAGAAATATTTTTCGCAAATATATCGCGTCGCATAACGTACTTTTGCCTGCAAGGACTG
CGATTGTGCGCATAACCCATGTTTTCATCAAC
AATGCTCATCTTGGTGTAAAACAATGGATTGTATTTGATAATAGCACGGGCAAGCAAAGGAACAAATA
CAGAAAAGGCTGCATTTTCAAATGGTGTTGGC
ATCACGTCCATAATGCGAAATTCTACACGCCAGCCAGTATTACCATCAAGGGAAGGTGGCTTAAGACG
AACAGACTGCCAGTTTGTGCTTTGAATGTTCT
CAAAATGTTCAGTGTGTGTAGTATCATCAATATCAATCATCTGATCGTAGACCACCAGGGGGTCTCGA
ATAAAAAGGTGCGCAACATGCTTCGCAAGACG
CGTGTCTACACCCGCACTTTTGAGAACCTCAAGATAAGTATCATTAATGACAAACTCCTCGTCGTTAA
ACTCCTCCAAGTTTGGCAACGTTAAGCTAACA
AAAACCGAAAATGAGTCATAGCGTGATTTGATGATGTGTGGAACCTCCTCCCTTTTGCGGTCATCAAC
TGAGGCGGCAATCGTTAGCCAGCGAACGTCTG
AATCGCATAGAATACCCTTTTGAAATGGTGTCGATGAACTCATTGCGAGAAACAATGGACATAAAACC
CCTAACTGATCGTATACGTGACGCGCCTGGGT
TTCATTTTCCAATTGCATAGTGACTTGCAAACAATTGCAACCCATACCAAAAGCCATGCAATCCATGT
AGATACATGGATGGCTTACTGAAGGTACTGGG
CACGGACATGCATCATAACGTTGCTTCACAAGTTCCATCTGTGACTTCTTAAAATACTGTGCATAGTA
ATAATGTGTGGCAGGAGTATAAAAATGGGTCA
TTTCCTCTGCCGATGGATATTTTGAACTAATATTACTGTCTTTATTATTATTATTATTGCTGCTGTTG
TTTTCCTTCTCTTTCGAAATTACCGAATCATT
AATAGTGAATTTCTCTCCCTTGGCTTTTGTAACTTCTGTCGAACTGCAGTCAATATCAATATTATGGG
GTGTGCGGTCTATATTCAGTCGAGGATCCACT
GTTTTCTCCATCGTATAAGTATCAATATAAATTGGAACTTGAATGCATACCTTGTGACCGCGTCTCAG
ACGAATATTCCGCGTGAGATTCGTAAAACGTG
GATGCGACTGATTGATACAAACATCCGGTACAAAAAGTGAGCGGGAATATGGAACCTCTTTACTATCA
GAAGTTATAAAATCGCCAACGCCCATAAGAGG
AAAGGTCACTAATGTCAAGGAGAATGTGTTTTCACCAGCGACACTGTCCAACAGCTCACATCTCCGTC

GAATATTCCCTTCAACAGAGCACAAAGAATCC
ACTTCAAGGGTGTACGGTACACCAGGGACACTTTCCACCATGAAGCTTCCATATTCATGATGCCACAC
CGCATTATCCTTTTGAGACGATTTTGTAAGTT
GCTCAATCACATCCGCAGCATTCAATGAAAGTTTTACTTTCCCTCCCTCCACACGCACAAGTTGATGT
TCCACTTCCTCTCCCCAATAAAAAGGGAAATT
TTTCTCTTCTTTTTTTCCCGCTCGCATTGTCAAAAACTGTTGAATTCCATGCGAGGATACATGTGGAA
TGGCGCGGTTATTTTCGTCGGTTCCCCACCGC
ATAGGTTTGCCGCCCGTTGTCAAGAGACCCATTCTTTTCCAAATTGCGTTGATAACTTGCGAGTGTAT
CACGGTACTTTTGTTTATTTATTTATTTATTT
ATTTTTTTTGAAACACCTGCAATTATTTCCGCAGAAAAAAAAAAAAAGAACGGCAACTGTTTTGATGCA
GCTTCTGCTCCGCCAATGAAAGACACTTTTCA
TATCCGTCAACAGGAGTCAAGAGGAAATATATATATATATATAGAAAAGAGCGACAGCAAGAAAAAAT
TGCATCGCCCCTACCACGGATTCGTTTCAAAC
AACAAGCAAAAGAAGAAAGCAAGCCACAACAAGAGTATGAATATAAACGCAAAATTATGCAGGAGCCA
AACAAGGTTGCTAACCACCAACAATACACATG
AGTTCTTCATAGGCTGCCTTTAAAATTTTAAACTTTTCTTCAGCCTCCTTTCGCTGTTGATCAGACGC
ACTGCACCACTTATCTGGATGCCACCGCATTG
CCAAGGCACGGTAGCTCTTTTTCACTCGCTCCGCACTCGCGCCTGTGGGAAGTGACAATTGTGCGTAC
AATTGCGTGACGACATGATGGTCCGGTCCCCT
CGTGTGTTGTTTTCGTCTTTCATTTTCCCCTGACCCCGATGCATGAGTTCGGGGGAAGCCATTTCTCT
CTGGAGCACTTCCATCCTTTGAATCTTTTCCC
TCTTCTCCCTCCTCTCCGTCTTGATATCCTGAATGGAACCTCTGCTTTTGCTGTTTTCGCCACTCGTA
CTCCTTTTTTCGATCACAGAGACGCTTCCAAG
CCTCGACAGTCTCGATGGTACGATGCTCCCTCATAGCTAGTTCGGCACTTCGAATCGCATCCGACCAT
CGAAGAAGCTTTTCTTCAGAAAGAGCCTTAAG
AAGCAGAAGTTCCGCACGTACATTACCGGATGTTATGTTTCGTATGGCAGCGTCAATATCATCCATGC
ATTCATAATAATGCTTGATTTCATAAAATCCA
CGCGCACGCTTAGCCAAAATCCCTGCACGTAATCGATCACCAATAAATAGGGTCTTTACGGATTTTGA
ACACAACTCAATGAGACGCATCCATTCACTCT
TGGATACCAAGGACTCCATTTCCCTGCATAAATCAACGCAATACTGAACACGCTGAAGTACTTGGCGT
AAGCGTCCCGTAGAATCAGTAGAGCCAGTTAC
ACTACAAAAAGGAAGAACAGCCTGTAGTTCTGCCATGCTGGCACTCTCAAGGAAGCAGAGTTGTGCGC
GCCAATACAAAAGTTCTGGAAAGTCCGTGTAG
ATGCGCAGGGTGTTATTTACATGAGCCAAAGCCACCTCATTACCTTCCTCAATGGCAAGAAGCTGGAC
CTTTAGCACTTCAAAAGTGGCACATGGAACGC
GTTCCAGCAACTCATTCAAATGCATTAAGGCTGTCGAATGTTCATTCCCCTCTAAAAAGGCACCGTAC
TTCTCATACAAGGAGATATCAGTCATTTCGCG
CTCAAAGTTGTAACCGCATTTGTTTCGCCTAAATTTTTCGATAGTTTGTCTTGCTTCTGCAACCCTAC
CGCAGCATATGAGAGATCTTAAGGCTCGGCAA
ACACCAAGGATATAATCATTATCCATTTCGTAAGAACGTATAGCATCGCGAAGAGAAAGTTTGTAATT
CTCCACACGCAAGTAGCACGCGGCACGGTTAC
TTATAAGTGCGGCATTCAACATATTTGGCAGACAAGAGTCGATTACGGAGGAGAATATCTCGGCAGCC
TTGCCATAACTTCCTCTTTGATAGGCCTCGCT
CGCTTCCTGGCATTTCCTTCTCTTCTCCCCATCGAGAGGAAGTAAGTGCATTGCGGGATCCAGTAAAA
ACAAATCCGGAACTTCATTTGGTGAATATACA
AAGTCATTTTCCCCATAATGAACGCCCGCTTTTGTTGCAGAATGAGCGGATAGAGCAGCCGTAATATT
TTCCAGTTGCTTCGTCAGAAAAAAGAATTCCT
CATCACTGAACAACCCTGTTTTACTTGTGGAGGGAACGTCAACGCATGTGGGAATTCGCGCCTTTTTT
TGGAACGCAATCTCTTCTTGTTTTTTCATTTG
CCACTCCTCCACGGCGGCATTCCAATCTTGGCAGACGGAAGCATCAAGAAACTGCACGAGGCGATCAT
CGTGCGCCAGGAGCACTCTAGCCGGATAGTTG
CGTACATCCTCACCGTTCATTTGGCACAACCCGTCCCCGCATGGAAAAGAACCCCTCACGCAAAATGA
ACCAACAAAAGCTATCATCATTAGAAAGAAAA
AAGGAAAACGAACAATAAAACGCCCGTGATCAAAAGAAAAAAAAAAAAATGGGTAAAACCAGACAGAGG

AAAAATAAACAGCCTCGTCGTCCACATAAATT
AGAAACAGAAAGAAAAAAAAAAATGAGTGACAGGTAGCCCAATGGGACATCATGAGATCTCGCTCTATT
TGACCCCGTAAACCCTTTATTTCCACTATATA
CATATATAACTATTTTTCTTGTTTTCTGATTCCCTCCACCACCATCTTTCACGAAATAAAATATTTAT
GGCAAATGGTGACATTATACATCTCTCATTAT
TCAAAATGCAGGGAACACAATGAAATAACTACGCACGAACAAAAAAAAACTTGTGCGTGGCCTTGTCA
TAATCATGTACGTTCGCATTGCTGCCGCACAG
CTTCCACATACCCTCCTCCTCCACCGCATCGAGTAACGAAACAAAAAGTTGGGACTGACCAATATCTT
CGCTGGCAAAATGGGTCTGAAGTTCACCGTAC
CAATGTGGTGGTTCCTCCTTTCGTACAAACGCAAAATGAACTTTTCCGGTGTTCGTCGTGGTGCAAGC
ACCCGGAAGCACATCATTGAGCTTCTCAATGG
TACTTTCTTTCACAGGGCCAAGAATAGAAATGACAGGTGGATTGCAGGTCACATTTATAATTAAATTC
TTCTCCATTGTTCTTCTTTTCTTTTCTTTTAT
TTTCTTTTTTTCAATTTTCGGTGTTTATTATAATCGCACGTGTTTCTTAAACGCCGCACAACTGACCT
CCCGGCGCCCATACACAGAACAACAACAACAA
CAACAACAAAAAGAAAAGAATGTACTGCAACTTGTAATAGGAGTAGAGTGTACGGAAAAAGTAGTAAG
GCTTTCCCAGTCACGAACGAAAAACAAAAACA
AAAATAGATACGTCCATATATATTTAAAGATAGTTAAAAACAAATAAATAAATAAACGTACATCTACA
TTTCAAGGCAACAGCATCGCATCGTCCGAAAA
CCTCTTCTCAGATACCTCCTGCTGTCGTCGTCGTTCAATCTCCTCCAATGCTTTCCGTTCCAACTCTT
CCTTGGTAGGTGGTATATATGTACCCTGAAGA
CGCGCAGCCAATTCCACAAGACTGCACCTCGGCGGAATTATTGAACGCAGCTGTTGCACCTCCACGGG
ATTCAGCACCGTTTCGCCATTGGTTGTCACAA
TCCGAACTAGATCATCGACTGTCATTTCTCCTGCTCCCTCCTTATCAAAGACTTTCAGCGCCCCCAAA
ACTTCCTTCTGCTCGGTTGCTGCATCACGGTA
AATCTCCTCTGCACATGATATAAAAATGTTCCAATCAATGTTTTTATCTCTTCCACGGGAACGATTT
TGACATTTTGTTGGGGCACTTGACCTTCCGTA
GCGCCTACCTGCATAGCTCGCTCCACGGTAGACTTTCGCGGCTTCTTTTGTGAGTATGCCGAAGAAGA
CTTTCTTTTTTCCCGAAGTTCCTCGCGGCGAC
GCTCCTTCTCCCGCCTAAGCTCCTCTTCACGACGCCATTGTTCAAGGCGCAAAACAGGATCTGCCATT
CGTTCGCGGAGATAGTCCATGTCGGTCTGAGT
AGGATTCATTCCCATACCACGCAACAGAGTCATGCAGTCAAGAAAAGAAACCAACTTCTCCTTGTTTT
CAAGCATTGTAAATATCGTCTCTGCCTCGTTG
TAGACCTCACTCGTGAGAACAGAGGGCGGACGACGGGCCCGTTCCTCCATCGCGGCGGACAATACACT
CCTCCCCCCACGCTTTGTCAGTAGCACAAATC
AAAAGAAACTAAGGGAAACAAAGGGAAAAAGACAAAAAAAATTCTTTAAAATTAAAAAAAAAATAAAA
AAATAAAAGGGTCAGTGCTCCCACAGCAGTGG
GGGAAAAAAAAAATGACGCCAATTCATTCATCACAATAAGGACCAATAATAATAATAATAATGTAAAAA
GAAACACAAGATATAAGTAAGACCTGTAAAAAT
TTTGTCTTTGGTAGAAGTTAAGTAGTGCTCAAAAAAAAAAAAAAAAAAAAAAAACCACTAAAAATAAT
AAATAGACCATCGCAACACACGCACATATATA
TATATGGGTATATATGCATATATTTATATTTGTATCAAAAACGGCCATAAACGAAAAACACATTGAGA
AAAAAAAGGCAAAAAAAAAACCCTATACAAAT
GCATAAATCTCGAAAGGGAAAAAAAAAAAAAAAAAAAAAGACAAACGTAAAAGTATAAAACATGTTAA
CGTACTTTATACTGTGAGTCAGTTCGATTGTA
TACAACTGCCTCCAATTCAACCTCCTTTTTTACAGAAACATCACTGTCGTGCTCGCTACTTTCGTCGT
TCTGGAAAACACTCTCCAGTACAACAAAGTCT
TGCAGAGATGATAAAACTTGAGCCTCCTGTGCCAGAACCCTCCAATCCCATTGAGACATCAGGTAATA
CAAGAGCGGAATTTCAAAGCAAAATCCAGTCA
AAAGGCCACCGAGAACACCTCTTACGCCCCAATTGAAAACAAAAACGTAAAGCGCTGACGCTGGTAAA
CCGACGAGCCATAATGTAAAGACCACACCACG
CGCTGCTTGCTCCTGTCGTCCAGCACCACGAAAAACGCCTTGTAAACAATACTGAAGAGAATCTCCAA
TATGACACAATATGACAAATGGCATCACAGAC
TCCACCGCGCTTGCCATCTCAGGAACATTTGTGTAAAGACGTGCAATGTGTCCTCCAAAGTAGCCCAT

AGCCACTGCAGTGCATATACCAATCATCACAT
CGCAGATCAAGATAAACCTGGCGTACTGTGATGCAAATAAAGGTCGTTGACCCCCCAGCGCATTTCCA
ATAATGGTACTTGCTGAGACACTCAGCCCAAG
TGCTATTGAAAACATCACGGATATTATGTTCATTGCCACACCAAAAACTGCCAACGCAAGTGGTGAAA
TAGCAATAGCAAATAATGCCTGCACTTCAAAG
GCCCACCACTCCGAACACACTGCGATCAGAGAAGGTATCCCAACTTTTAAAAAATGTATCCAGCCAGA
TTTATGACAAAGTGCCGGGGAGGGCCACTGCG
CCTCCCGGACGACAACTTCAGGGTGAAAAAGGCAAAGCAACACATCGGTAACAGCACACATCATTCCA
GTCAACCCCTGTGATAAAGCTGCTCCTTCAAT
TCCCATAAATATTAGAAAATAGTTAGCTACAGGAGACGTAAGTGCCGCTGCGGCGTTAGAATAAACAA
CGAGGGAAGAAAGACACTGAGCATATAGCGTC
TTGCTTATAATTCCAGTAAGAATCTGTGTCAATAAGTACAGTGGGCACATCCGCAAAAACCTCGCAGC
ACCATCTCCAACGGCTTCTCCAAAAACGTAAA
CCATAATGGGTTTGCTGTTAAGGAAGGCAACGCTCATAATAATCCACAAAACCATATTAATGATGAGT
GCACGCTGCAGTGTTTCGCCAATCACAGAAGA
TCGTGGATTCCGTCCAAAAGCCTGAGAACTTATCGTATCTATAGCCGAATTCAAACCACTAATAATAG
ACATGGCCGTAACGTTAAATACCAAAATCCCA
GCACTGTACTGTGACAACCCATCCTCGCCAAGGCAGTGTCCCACAAAAATCATGGTAACCATTTGTGA
AGCCATCCAAAGAACACTGCTGAAGTCCGTCG
GCAATGCCACTTTTATTGTTCGACAAACTAATGCATGAATGCTGACTCTGCCATCGGCATTTCCACTG
AGCATGCAAGCATTGTCATCATCGTTTTCCCA
GCGATGTTGTGGATTCATGTTCTCCACCTTATGACCCTGAACAGTCGTTGCCTTTCGCTTTTCTCTCT
CTCTTTCTCTTTCTTTCGTTTTTTTTTTTTTT
TAATAAAGAAACCCTTTAACAAACAGCAACCCATAATAAAGGCAAATCCCATTTATTATTATTATTAT
TAGTAGTAGTAGTGGTAGTAGTAGTAGTATTT
TAGAATATTTACAGGAGTAAAGAAAAAAGGAATATATGAAGTTCAATAAATGGAGCAATCCTCTCCCC
CACTCACATACACACAGACAAAAAAAAAATGA
TAATAATAATAACAATTTAGATAAAACACTCCCCTCCACCACACAAAATTAGTACAACATTTATTTAC
ATAAGCAAGGCAAAATTTGCCCAATGACGCAT
TCCGTTTTTGTTTTTGTTGTTGTTGTTGTTTTTCGTTTTTTTGCCTTTCTTGTGTGGGTACGCGTTTT
AAAATGACACATTTACATATACTCGGGATGAT
AAATAAACGAGCAGGCAACAAATAAAGATAGTCTGTAATGACTCATGACACCAGCAGCAATGACGCCG
CCAAGCTCGCCTTAACATCACCTACCGTGGAT
AACACCTCATCCAATGGCAGCGCAATTAATGAATTCTCTAAATCCTTTGGTGTATTCTGAAGTTTCTG
AAGACTCTCAGGCGTCATTTCCAGAGTGACAG
AATGCACCTTGTATGGCGTAGTCCCTTCATTCCCTATTGTATACCGTTTTGAGCTGAGTGCATGCGTT
TTTTGAAGGTCAACACCTAACGGTATAAGTAA
TTCATCATTCGGTATCGTGAAGCTCCCGTCATTGTTTTCAACGCCACATAAAGCCTCCACAATAAAAT
TGCAACCAACGGAAACCTGACGTTTTCTGACA
AGTATAGCGTACCGAATACCATTCACGGTGGCAACAGGAAAAATAATAACAGGATTGGAGTCACAAAT
ATCTACCGTTTGAACAATTTTTCCACTGCGTG
TAACACATTCCACTTCCATAATCAACCCCACAATGCGCTGAGCAACCCGTCTAATACTACGAATAATG
AAGGTGGAGGGCACGGGACCGTGCTCTGCACA
GGTAGCCACATACTTACAAAAAGGCAAATAACTTGCCACAGCCCTCACGCCGCCTTCAAGATCATCAC
GGTAGGTCTTATGCGTCGTAAGCAATGCCACC
TTTTCGCCCAGCTGAATTTCCATCCTCTTATCCTCAATCTCCTTTTCTTTCCAAGCACTTACACACTC
TCAAAAAAAAAAAAAAAAAAAAAAAAATGTTTT
TTTGAAAAACACTACTGGAAGAAGCATTATTATTATTTGTCTTTGTTATTCCCCTTCTTCTTCTTCGT
CTTCCACTTTGCGAACAGGCCCGCAAGTATAT
TTTTCAGCAAATCCCAATTCTACCACGACACTTTACCAAACAGCCTCAAACGTTCCGGCTACGGTGGT
CCTGCAGACCCAATAAAGTTAGCAGACGCTGA
ACTGGCTTCGAGAGCATGATGGGGCCCAGAAGTGGAACCGACTCTAAAACGCGAGCTATCATCGGAAA
AAAATTGCCAAAAAGATTCAGCGCACCAAATC
CTTGAAGCAAAAGACCAACGAAACAAAAGCCCGATATAATGAGCAACATGCCCAAGAAAAAGCACCCA

GAAGCTCGACGACGGGAGAAGAAAAACGACTG
AAAGCGTTTGGGACCCATTACCAAAGCTACCCCGGCAACAAAAAGAATATTTCCCATTGTCAGAAGAG
CACTATCCAAAAGCATTATGACACCCAAAAAC
GTGAAAAGTGCCCCCAGGCCCGTCAAGGCAACGCCAATCTTTGTGGAGTCATTCCACTTGTACAGGGA
GTCCATTCGACTTTTCTACAACACCTCCTACG
CTAGAGGTCAAAAATCTAATCACAAAAACAAAAATAAATGCAAATAGAAAATGGGGGTCAAATAAAGA
ATAAAAATTGCAAAAAAAAAAAAAAAAAATTCT
GCAAAACAAAAAATACACACACACACACACACGTGTTTGCGTAGTATTCATAAATATATATGGAAAAC
AACATCTAATCTTTAGTACCGCCTAATCACAG
TTCCCGATCTTTTTATACGAAATGTTAAATCAACTCTCCTCATTCTCTCCCACACTCACTCTCTTGTA
GAAATTCGTTTCCCTAGATGTATAGAAAATAG
ATGAATGCGTTACCCTTTTTTCTTCGGAATACAGTTGCTAATTACTTGTTTTTTCTTGTTTTTTTTTT
TTTCGTCTTTTGACTTGATTGCGATTCCCTCT
TAAACTTCAAAAGCACGTACCGAACGTTTACGGCTTTTTCTCTCTCTCCCTCCTTCCATTATCGCCTC
TAACGTCGTGCGCTCCCCTCCATTTGCATTCT
TCTGCTTCGTGGCAAATACTTCAACTCGTTGCTGGCGAAGCATTTGATGATATGTCGACAACGGATTT
CCAGCAGCTTTTAATTCATCCTCCAGCTGCTT
CACCGCTGCCAGGTCGCATGGACCATTGCCCGCCTGATCGCGTCGTGCTATAATGCGTTGAGTTGTTG
TCTGCATGTGACGAAGAAGAATGGTCAATTGT
GAGTGAATCTTCGGTATTTTCACCTCGCTACGCAACCGTTTAAGAGTCGTTTCCACAACCCAGAAAGC
CTCTGGAAAACCAAGAGTGTGGCTATGCGTTG
CCAAATGCTCTGTAAGGAGATAAAGGCACTCAAAAAAAATATTTTGATGATAACTTGAACTACGCGAC
TGTGCCTTTTTCATACGAATTGTGAACTGCAA
GTCCACACGTTCAAATTTTTCCCCTTCTCCTTGGGTAGGACGATTCAAAGCAATACTCGGTGAAGTTA
GCATGCGCAAAAGGTACGGAGCTACAGGAATA
AAAACCCCATCGGCACGAAGAGAAATATGATTAAGAACCTCTATGACCTGTAGATGCATAGGAAACAT
GCGAGGGGATGAAAAAAGATCCATTAGTCCAA
GTGCAATTTGAACAATAGGATGAATAAGTGGACCTAGTTGTGACGGCTCGGAATATTTTGAGACGACG
GCGCCCCAAGTACGTAATGCGTTCAGATATTG
CCAGTTTACCACAGCTCTTACGTTACTCTGAGTTTGCTGATGTAGTGCAGAACGAAGGTAAATCGCCA
ATTGACGAAGATAAACAAATGTGTGTTGGTAT
GCAGCCTCCATGTCAGTACCATACAAATCAACAACACAGTTTATAAGGAAGGCTATCAATGACGCATT
GTGGATGTTATAGTGATATGCTTTCTTCACAA
GAGTAAGAAATATGCCCTTAAAGGCGGCAGACTGATACATGGATCTTGTCCCCGTAGCACGTGTTGCA
ACCGCTCGGACAACAACGTAGGCTGCTATACG
TACCGATTCCTCTGAGGCTGAGCATAGTGAAAGTGACGCTCTCAAAACAGACTTCGTAATACCTTTTA
TGTAATGTAAAATGGGCACAAAGGGTGTTAAG
GCGTGAAGTAAACCAGCTGTTAGTACGGCGTCCACGTTCCCTCCCTCTGACACAATTGTCACAAGAGA
AATAAGCAATCGCCTCATCAAAAAACGAGTCT
TGTATGATTTGAATGAGCCTTTCCCGGTAATGAAATGGCTGAGGTTTGATCCAATGGCGCGTGCAATG
CGAAACACAGACTCTTTCACCAACGACGGGTC
GTCAAACTTACGAGTTGAACTTATCTTCGGATTCTCCTTAACGTTATAACCCAACTCCCGCACGGCAC
TCACAAAAATATCAACGCATGCCTCAATGCTT
CTCCCCTTGACGAGATGTTCAATTTCCTTTCGAGTAATGCGAGATGAGGAGAAGCACTTCGCACTCTT
CTCAAGATAACAGTCTCTCGCCTTATATTCGT
CGTCGTCGTTATTGTCCTCATCCTCACCATCCATCTCAGTCTCGGCATCGCTGCCCACACCGTCATCC
ACAACCTCCATTTCTTCCTCGCCAAACTGCAG
GAGTGTCGGGTCCTCCTCCTCCAGGTAAGAATAAAACTCCGGATCGATTTCCTTTAAATGATCTAATT
GCTCTTGATGTTCACGCTCCTCACGCTGAACT
TCTTTGACTTCACGCATTTTTTTCCGCTCAACACGCTCCAATTTTTCCTTCTTGGCTTTCGCGCTCTT
ACGGCGGTTCTCAATCACCGTTGCCAAATGTT
TCTTGGCATATTTCTTAAACGACTTCTTATCTTTCGTTGTCATGTGCTTCCTCCCTTCTTGTGTGCGT
GTGTAGAGTCAAATATATCTTCTTCACTCCCT
GACTTCCTCCTTCTGCGGCCCTCCGCCTCCTCCTTTATTCCCTCGACCGACCTGATTTGACGCCCACA

AGACTTACTCCGCTTGCTATCCAAATCAATGG
CAGCGGCAGTTTATGGTGGCAAGAACGACCTTATTCCACACGAGACACCAACCACGCCCTTCACTCAC
CTAAAATGACTCAAACAAGATAATAACAAAAT
AAAGTTGAAGCTAAAGACTGTATTGAAATGACAGTTCAGAAAATTATTTGCGTTCAAAAGTAAACCAT
ACACAAATACAAATTAACAACAGTAACAAGAA
AAATTTTTTAAATAAATAGATGCCAAAAATTTGGCACTCGTCAGCAAAAAAAAAAAAGGCAGTCAACA
CAATAATTGTGCCATGTTTTACTTGTCTTTTT
CCAACTCCCCCTACATCTATATATCTTTCTCTATAAAACCACGCAGTTGTGGCACCCCTGAAGCACAG
GTTTCCGCTTTCTATGCAAATTTTTCTTTTTC
TTTTTCTTTTCATTTTTCTCATATATCCTCTTCTTTTCTGGAGGAGAAACCGATGCAGTAGCAGAACA
GTCTGCGCAGTCGTCCTTTTTATCGCTTTTGC
TCCGCTCTCGAGCAATAACAGCATATGCTGGAAACTCTTCAATAGAGTAGCAATTTGTTTGCGGCGCG
TGTGGCAGATAAGGCCTATTGTACATATCCAC
GTGCACGTCAGCAGGGGAAACGGAAACGCCAAATTGATTCGCGCCATGAACCTCTCTATCAATGCTTT
GTGGCATACCCACACCCTTTTCAGCATCGCAT
TCAGTTTCGCTGGGGCACTCTGAAGACATATGGGTCCTCGAAATTTCTTGACATTGGCCTTCATCTGC
AACTGCCGGAGAAAGCAAGGAGAGCTTCGACC
CTAAAATTACAGAGGAATCCGACGATGATTCGGATGAAAAAGACGATGATGAAGCATAAATCACATGT
AATTTATGGTCTAAATGAATTATTGTCGGTTT
TTCGTTGGAGAACGCCTCCCCTGAATTCACAGACGCTGACTCCTGGAACGGTGAGCAAACCTGCAGTT
TATTCAACGCTTGTCGCTCAATCTCTTCTGCA
AGTGGCGCCGCTGAGAAACTTTCAGTATCACTCGAAGAAGTAACCTCTTTAACGTAATCATCACGCCA
CTGCGTCATGGCTTTCAATAAATTTAATTTAG
GATTTTCCATAGCGCCGTCGATGCCAGTATGCATTGGGGTGAGCTGCGATAGCGAAGATGATCCACCA
ATGGAACTCTCAAGTGCCCCATCCTGCTCACC
TTGAAGTGTAACCAGGTGTTGCAAAACATTTTCCTTCAGTTCGTCCGATGAATCCAGTGGAGTGCACA
ACACTATCGCCAAGGGCACTAAGGGTTCAAGG
GTTAAAAGGCCACCACAGCATTCAAAAGCGTCATGAGTATCACCCATTATCTCCACCAGGTGCTCATT
TATCTCAAAAAATGCCAAAACAAAAACCCTAT
TGACTCCATCGTATTCATGAGCCTCCCACGTGTTGCTCTCGCCACAAGTCAATCGGTGGAGACCGACA
CATGCCGGAATCCCAGCATCAAGCATAGATAA
ACCCATAGCAATGTCCCCAACGTCTAGAAACGCCAAACCGCCCATTGCTTCGTTGACATCACGGGGCG
CCATACATGTCATCAAAGAAATATAACCAACC
ACTTGCACTTGAGAAGGCAAATAAGTGTCAAAGTAATCATCAAACCCCACAGAAAGCTCTGGAAGTTC
ACTGAGAATCCGACTAAGAAGCCAACCAGCCA
CGCGCATTGCATCTTTATAACGCAGTAGGCGCTGCCGATGCCCCACAATAGACGCGGAAATCATGGGA
GAGCTTTCTTGCAGAGCAGATTCATGGTTCTG
AAATAAAGAATCTGAGCAATGATCCTTCGGTAATCGGCCAATCACAGTTTCTTCACTAACTTTTCGAA
ATTCACCATCGTAATAATGATATTCTTCCCGT
TGTTCATGTTGCCTTCCCACAATTCTTCTCAAAATCATTAACTCCTCCAACAATTTTTGATTTTCTCC
TAATTGAAACAAACTCATATCCTCGTTTGGCT
TCTTTGGAGCATTTTTAGTATTGATACACTTTAGACGCTGTGTGTATCGCATAGTGCTCATTGTTTCG
TAGTAAAACGCTGTATCGGGATGAATACATGC
AAGAAATACCGTCATAGAAGCACCGGAAAGATATCGTTGAAGAAGCACAGTCAGTGTGGTATCGCGAA
AGTTCACGTACGATGTGCTACCGTCTGATTTC
AGTCTTGCTGAGCTAAGAGCCTCCAACACCGTACTAAGACGCGACAAGGACTTGTTAATGGCAATACT
TTCCTTATTCAGTTGTGGTGAGTATGTGGTCT
GTCTCTCACTTCCCGCAAGATCTACTAAGCATAGACGCATACCCCTGGTTTTAATAGTAAATATCGCA
TGACTACGACTACTGCGAGAATTACGCTCCGT
TGGCGCCGTTCGCCTTGCCAGCATCCCAAATTCCGAGAGCGCCTTCCATTCCTCCATCGACGTTAGGG
AGCAAGATGTCAAATTTTCTACCATTATTACC
GTTCCGTCAGGGCCTGCGCGACGCCGAATCATGCGTCGCTCACCTTGGGCTAGAAGGTCGTGTACACT
CTCTCCATACACTTCTAGCATTGCAACCTCAG
CCACACCGTACGTGTGCAGTAACGCCTCGGCGGAAAGTGTCAATAACCCCGGTTCTTTGTCTGTTCCA

GCAATTGAGTGAGTTTTACCGCTGCTGGTATG
CCCATATGCCAGAAAACATGAGGTGTCAGGTTCATCAACCGAGGAACGAATGCGTCCAAGTACAAGAC
TACGAAACAGCTCTTCATTGCCGCAATCTTCT
TGAACGACTTGATCAACATGAAATTCTCGGGGGGTGCTGCTCGCATGTGTTCCTGATAGATCCACCAC
CGTAATTTCCTTTTTATTCACACCAACACTgA
CGCATGAGGCTCCGCCCTCCCCTGGACGGAGACGGATGACGAGTTGGACTCTTCGTCCCATTCAGAAG
CCTCACTTCTTTTTAATGTGTATATCGAAAAA
ACGAAAAGTGCCTTTAAAAAATAAACGAATAAATAAGGAGGAAAAAATAAAAATAAAAGACTTTTTAA
TGGGTTTCCAATTCCCTTAAAAGAAACATTCT
ATACACACAAATGGCCACATGTCTGTGGGTATGGATAGTGAAGCCAAAGACTGAAGATGGAAAGACAG
GTGTTGATGTGCGTGGTCAATCACCAAAATAC
ACCTTTGAGTGGCTGGGTGTGGACGGAAGGTAGTAGTGAAAAGACCACAATATTATCAGCCTCGGACA
GAAAAGGGGAAGGGAAGGGAAGGGGAGGAAAA
TGAAAGACAGATAAGAATGAACCATGCATCGGCAGCACATTCAGCACCCCCTCCGCCCCCAATGAACA
TTATCATTGAAATGTTGCATCTTGTGGATCCT
TCAAAAGGAAACAAACAAACAAACCATGAAGCATATTTCACCAAAACGTTGCTATGCCAAAGCGGCAT
AGTAAAATATGCCACAAAACAAAAGTAAAGTT
ACAGCAGCGATAAAACAAATAATGAGAAACCGCCGCGTTTTTTCAGTATCACGCACCCCTCCTCCCCT
GGAAGACATGGAGGAGCCTGTTTTTGAATCAA
TGCCCGCCATGACATCTTCAAAGGTGCGTCGTACAGGTGCTGTTCTTCCAACACGCGACACGTTCATG
CTACGGGAGGAGTTCGCGGTTGCAAATACTTT
CTTTGTGCGCTGAAGGTATAAATCAAATTTGATAAAACAGTACGGTCGAGTCACCGCCTCCACCTGAG
ATCCATACTGCTGCAGGAACTCCCGAGCCACA
TCCTCTAAAAAAGCCACAGCGATACCGGCTGGCATAGCCGTGTCACACATTGTAAGAAATGACACAGC
CGATTCGGAAAGAATGTGGAACACATAATTCT
TCCACACAATAGTAAGGAGTGGCGGAACGCACAATGATGGATTACTGTGCTCCGCAGCCAACTTTCGG
AGAAGTGTTTTTGCCGCCACCTTGCATTGCTC
CAGTTCTGCACTGGACGTATCCGTATCCGCCGCCAGTGCCAGCGCATCTCGCTGGCGGACGATAAGAG
TAAAAAAAAGCATGCGCGAAACGTGAATGCTC
AAATTTATTCTTCCTTTCCTTGTTGTTGCTGTTGTTTGTATGTATTCCACTCTATTGGAGGATGTGTC
CAAAACCACTAAAGGCAGCGTATAGCCACAAA
TAATAATTAATAATAAAAATAAAGGAGGAAAAAAAAAAGAAAGAAGGGGGTAAAAGAATGTACGGCAA
GTTCAAACCGTAAGAATAAATAAAATAAAACA
TATTCCGAAAAACAATAAAAAAATCGATGCACAACAACAAAAACAACATCGTGGCATAATAAGAATGA
GCGACAAAACACAGAGAAAAGACACAGTCGAA
AGAAGAAAAAAAAAAGCAACACATCGACAGAAACCTGGTGATGATTAACTCAAGAAATAAACGCAAAT
TGACGTCTCAAAATATCCCGCCGACAAACCGG
GCAAGATTGCATATTCCGCGAGCACAGCCAGCATACACACAAGTGACGACATGGATCAAACAATGTGT
CCCGCCTATGAAGCATGCACACAACGCACTCC
TCTCCCTCATGTCGTGCAGTGGTTGGCGCGTACTCTGTCGGCGGCAAATCGCAGCCCCTATGCAAGGA
TGTCAACAGGACACATTTTGTGAGAAGAAAAG
AGCCATATACCCCCAGCGCCACCAATATGGAATACGTCACCAAACGCGCAGAACCCGTTGCACTTTCT
GTCTGCAGAAGTCGGTCCAACTCTTCTCTTTG
TTTGCCTATGGTTGTCTGGAGTCTACTAATGAGTTCCTGCTGTTTGGCCTGTCTTTTGCCGAGAGCCC
ACAACGCGAGGCCTACGGGTACAAGAGCAATG
GTCTCTAGCATATCGTTTTCATGCATAGCGTTCCTAATTAAAGTAAAAAGCAGCTGAAAGGGTAAGCA
AAAGCTCACACATTATCCCCAAATTTGCGGTA
AAAAGAGGTAGAAGAGAGAAAAGGAAAAAAAACTACCTTCTTCAAAATTAAAAGTTGTTACCGTTACA
AAGAATAACATAAATTAAGACTCAACACAGAA
ACGCATACACACACACACACACACAAAATTAATAAAAAAGACTCAACAATACAAAAAATTTATCCTAT
CGAGATAGCAACAGCAAAAA
>scaffold1050.size12074
TCTACTTCTTCGGTTTGCTTTTCGGCACAGGGACACGCTCCTGGGGCTGAGTCATGAAGAGCTGCACA
TGGCACGGACTGCACATGTATGGCGTAATACGG

CCGTGAGCGCGGTAAGTGCGGCGGCGCATGCGGGCGGCCTGATCCACCTGCACGTGTTTGATGACCAT
CTTGTTGGGGTCAAGACCCTTCTCGATGGCGT
TTGCCTCGGCATTCTTCAGCAGTGAGAGCATTGCAACAACAGACTTGCGTGGCCAACGTCCCTTTGTC
TGTCCCCACTCCTTCGCCTGAGCGGTACGGCC
AATCTTGCCATTGTAGCGCTTAAAAGGAATGCAGCGTGTCTTAGCAAGAACCTGACGGTACAGCTGCT
GTGCCTTGCGAAGGGGCATGCCGTTGATCACA
TTCGCTGTCTCAAACGTGTTCTTGAAGTGGCAGCGGAGATCACTGACCTTCGCCTTGGCGCTCTTCGA
GCTCACCTGAGGCTTCCTTGAGTAGTGCACCA
TGACCCCAATCCTGCCCTTTCTTTGTTGATTTGTTGTTTTGGGTGTGTTAACAGAGAACCCAGAAGGA
TTGCAACGAGCCAGAACAAACACAACGACAAG
AAAAAGACTTCATTATTATTATTATTATTATTATTATTATTATTATCTTATTTTGCTTCTCGCGC
CAAATCACAAGGTCATCCCTGCTCCTTCTCCA
ACCTTCTTTTTCACCATTCTTTGGCAAATTCAAAAACAAAAAAACAAAGCGGAAAATTAAAAAACGGT
ACCACATCGTCACATCGCAAAGTGCTCAGTCG
CGTCCTCTCTGACGTCTCGAATCATCCCGACGACGGGCAGAAAAGGCGGCAGCCTTTTCCTCGCGTTT
TTTAATGTTTTTCTCCCTTTTAGCGCACAGTT
CCGAAGCCATTGTGGAAAGTGAGACAACCACTGTCCCGTTCTCTTTTCGCACAATAAAAAAATTACTT
TGTGATGCAGTAAACTGTGCTAGGCCACCAAA
AGCCACCAACGCTTCTTGAATCTCCTCGGGTATGGACGAGGCCCATTTGTTTATGGGCATCCACGACT
TCCCACTTGGGTAGAGCTCCAGTAGCTTTGCC
ACGAGCTGGGGCTCGTTTAACGAATTGCGATAGCCACCTTGTTGAAAGTTCTGCGGGGGCCTTCCCCT
CAGAGGCCGACCATCGCGAGGACCACCAAATC
GTTGTTGTTGTTGCTGCTGTTGCTGCTGCTGCTGCTCACGCTGTTGGTACCGACCGCTGCCACTACCG
GATTGTGACCGCGCTAAGGCGGCGACAGTAAT
CAATCTATTTAACCGGAGGGATCGCATAATTGCCTCCTTTACTTTATTACGTTGCTTGCAAACTAATA
AACATAGCAAAAAGGGAATTTCCTCACCAATA
TCAACATAGAAAACATTTTAACAGACAAACAAAGCCACACGGGGAGAGAATCGTGCAGATTACCGTAA
GTACTTATATATATATCCCTCTCCCAAAGAAA
AAATTATACCAATTGTTAAACAAATCTCACAACCCGTGTTCCATGAACCTCAGTAACACCCAGATCCC
TGGCAATTTTCTCAGCATTCAAACTAGTAACG
CCACCTCCGGGCATTATAGATATTCTATTTTTTGCATATAAAACGAGTTCCTTCAGCCAATCCAAATT
TTCTTCAATATTGGTACTTGCGGATCCTCCAT
GGGTTAGAATCCGGTGAAATCCGTAGGCCGTTAACGCATCAATGGCCTGAAATTGCTTTTCGGTAGGG
ATTTCATCAAAAGCCATGTGAAACGTCAGAGA
CATCCCTTTGGCAGCAGACAATAGGATGCCAATTCGGGGGTCCACATTGCCAAGGACGTCAATGGCGC
CAAAAACAACACCATCTGCCCCTAAACGCCTC
GCCAATATCACGCTTTTCAACATTACCTGGAATTCCTCATCACTGTAATAGAAATTGCCCTCCCTACA
GCGTATAATAACCATTACCATAGCATTTCTAG
AGTGGCAATATTCCACTGTCTTTTGAATTGTATCAGCGGACGGGGTGACGCCGCCAACATCCAAACGG
GCACAAATCTCAACACGCTGCGCCCCCGAAGC
TATGGCCTCTGGTATCCGTTCATAGCCCTCTGCACAAAACTCTTTTAAAATCATTGATTTCCTTCCTC
GAAATGAAGGTATTAGTAACAATATTTCAAAT
AATTTACTCACACACACAAACAACAACAAAAAAAAGTTAAAAAAAAAAGGAAACGCCTTCCCGCCTCA
AAAGAAAACAAAAACTGAAAAACTTTTCCACT
AATCTCTCTTCTCTTCTCAAACATTAATACTCTTGCAATTACTAGTAGCAATAACCAAAGCGTCATGA
CAATACTTTCTCGGTTTTCACTCAAAAGCTGT
AGGTATCACTAAATGTTATTGGCATGCCAACCCTTTTGTTCTTCATTATATAATTCCTTGAAAACGTT
TTCATTGCCTTGTTTTGGAATCGCATCAGTTG
CTGAGGAATAAGTTCCTCCCCATACGGTATGAGACATTCCATCTGCAACTGGAGCCGCAGTTGAGTAT
TGTACGGATGGGGTACCTTCCCAAGATACGGG
GGCACCGGTTGACCACCCATCATCAATTACCGTGTTTAAAGCAGCATAGTGATCCCCAAGATTTTCCG
GCCCAATATTTTCTTCAGATTGAGGACTGAAC
AACGGAAAATGCTGGTTATCCACCAAGGGATCGGCTGGAGCAGAAGGAACCGCCGGTCCTGATTCTGC
TGCGGGTGCGCTGACAAAAAGCTCATCCATAG

AGAATGCCGCCTCTTTCTTTTCAGTAGCAACAGGTGGTATAGGAGGGGACTCTTTACCACTTCGTGGT
TGTGCGTTCACTGAGGGTGATAGTGGAGGTGA
TTTTTTTTCGACCGTTTCTCTCGGCGGACTCTTTATTTCACTTCCACATATTCCCAATTTTCCCAACG
TCTCAGTTACATCTTCCCTTGGAATGCTACTC
AAGCGAACATCGTCCTCCAATTGCCCCAGAAAAGAATCTGGCAGAGGCTTTAGCTGTGGTGGAGGTTG
CATAAAAACTTTTGATGATCTGAGGATCGAGT
CAAATAATAAGCGCAGTTTTTCCACTACCACACTGTACTGCCGGTAAAGCTCCAGCCAAACTTTTTTT
AGAGGAAGTGAAAAGTCGTCGAATCCGTCAAG
TACAAATATGACTCGATTAGAAAGCAGTTGATACAACATTTTCCCATCACATACAAGCCTTTGATACG
CTTCCAGTGTACAAAAGTTATCTACTATGGCT
TCTTGGTATTCCACAGCAACGAGGGCAGCGAGCTGACCCAACAGCGCTCCAAAGAAAGGGGTTAGGGA
ATCCACATCCATTGAACGCAAATAAGAGCTGA
AATCAACACTCTCCAAACGATTATCAACACCAATTGCCTCCTGTGAAATTGCACGCTCTTCAAGATAA
CTTAAATATTGTCGAATGAACAATGCCTGCGC
TGCGCCCTCCACACTTTCAGAAAGGTCCTTCAGATTCCGAGCGCAAAATATACTTCGATTTCGTTTTA
TACAGGCATTGAATTCATTCGACCCATCTGTC
ATCAAACGGTGCAATGTCACCAGAGTCTTGAGTACCACAATCCAATTGTGTGTGTGCATTCTTTTTTC
AAGTTCTGCCACGATATGCCCACAGATGCTTT
TCCCATCGCGAGTATCATTTTTATAATGCCCATAAGTCGTAGCCAAGAGTCTTTGCATGTGTTTCTCT
TTTGGTGCCTTTAGCATATGGCTTGTTACCTT
CATAATTGCCCGATCCAATTCATCTCCTGTCACACGAGCCAATCCAATAATAGCCTTTTCCTTGAGGT
ATCCTGCGCCTCTCTTCAGTTCATTAGAATCT
TTCACATTCATGGCTCAAGCTCCTACCCAATAAAAACAAGCAGAAAGATAAAAGGCGGGAGAGAGAGC
GTGTGTGAGTGTCTTTGAGTGTATATGTTTTT
GGATTCTCCCCACCCATACAAAAAAAAAAAAAAAAAAAAAAAAAAAAAAAAAAAAAAAAAAAATGATAAA
ATAAAAATGCATTTTTACAGTAAATTTTGAGA
TAATACACGGCCCTTCACAGCGCTCTAGGTAAGTATACAGCCTTTGCTTCGAGTGAGGATTTTTGAAT
CCCTTTTTTAAACGAGAGAAAAGCTTTTGAAG
ATGAGGAGTTAGGTTGTCTTTCGTGATTATTTTCAGCAAAATGAAGCTCGGCGTACAGCAACTCAATC
GTTTGCTTGGCCCCCAGCAATTCGAGACGGAG
AGCATTAACAAGATCCTCGGTTTTTTGCAGCTGTTTCCGAAGGGCTGTATTCTCATCCTTAAGAATGA
GTCGACGCCTTTTACTTTCAAGAAGCATTTTT
TTCATGCTTTCAATGTTGGCACCCTCCTCCTCAAGAGTCTTCTTTAGTCGTGAATTAGCCTCAGCCAT
TTCCTTTCGTTCCTTTTCTTGCTCTAGCTTTA
GAACACTTAGGGTCTTGGCGTTCATATCCTCAGTGGATCTCACAGTTTCCCTCAGTTTGCATTCTAAT
TCATTCAACTTCGACGTACTGGAAAGTGAGGC
ATTTATTACATCCTGCTTCGTTTTTAATAACTCGTTACTAAGCAAATCCTTTTCGCCCTCTAACGTGG
AAATCATGGATTTCAAATTTGTTATTTCAGCT
TCCTTTTTGCGTTGTTCTTCTTCCCGAAGCTGCAACTGCATTTCAAAATTTTCCTTTAGTGTATCGAC
AAGTCGGCTTTGGTCGCATTCGTATCTTAAAA
CTTTGGCATTCGTCACAATTTCTGACGGAGAAGCTAATACTGTTTTGGTGTGTAAATCCTCCGACACT
TGAACAACGGACTTCAATGTTTCCAAGAGCTG
CTTTGATATAACCTTTACCTTTTCAAGTGAATGTCTTGAAGAATCCCAAGTATCTCGGATTACATGTA
CAGAAAAAGCAAAGGCGTTTTCGCACGTTTCA
TTTTTATCAGGGTACACCATTGAGTCAAAATTTTTTCGTTCCCTTTCAAATTTTTTCATCTCTTTTAA
ACGTTCCCTCTCCATTGTCGGAGGTCTATAGG
GAACACCTGGATTTGTCATTTGTGCGAGTGCATCAATGCCACTATTTAATGATGTTTCTATCCGATCC
TCAATATCCCACAATGACACATTGAACTCCAC
AAGCCATTGCCGATACAGCATCAGAAGAGAAACTGCCTCACGCAAAAAGGACTTTTGAGAGTCATCGA
CCGAATTGTCAGACAACTGCTGCACCCTCTCA
CGTGTAGATGATAATTCCAATTGATATTGAGCCACTTGTTCTTTAAGAAGAAGAACTTCAGCTAGAGC
TTTAGAATATTTCCCCTGCGTTTCTTCCAATT
CAGACGCTGTGAGACTGTCCGTTTTTACCATTCTATTTAGACGTTCAATTTCACGCTCATACTCTCTG
TTACGATAATCTAGCCCATGAATTGTCTCATC

TTTTCTTTGAAGCTCCTCAACTAATTTTGTCAGTCTCCCATCAACCTGCCCTCTTAGGCTATGCAAAA
GATCCGAGAGGCGCTTACGTTCAGCGAGGGCC
TCTGCAAGTTCCCCTTCTAAAGAGGTTCGACCAGACTGGAAACGTGCTATTTCCATTTCATGTGCATG
TTTTTGATTCTGAAACTGGCGTTTGGCTTCCT
CCAAATCTTTGTTTAAGCGTGCCACTTCTACCGCGCGTTCTTCCGCAACAGAACAAAGGCGTTCATTT
TCTTCTTTCACAATTGCCAAAATTTCTTTCGT
GACAGCTAAATCCTGACGAACGTGTGCTATTTCGCGGTGCGATGAAAATTCAATTTTTCCACCAACTG
GAGTTGTGGACATTCCACCGACTAATTTAAAA
GGGAAAAAATAAATAAATCTAAAATAAATGATAAATAAATAATTAAATAAATAATAATCAAACAAATAA
ATAAATATAATAATAAATAAATATAACAATAA
TAATAAATAAATAAATAAATAATAGTAAATAAATAAATAAAAATAAATAAATAAATAAATAAATAAAT
TAATTAATTAATAATAATAAATTAACAATAAT
AAATTATTAATAAATTAATAATAATAAATAATAAATAATGTTAATAATAATAAATAAGAATAATCTTC
CGGGCAGCTGGCCGGATCCTGAAATTATCTAT
GGAAACCAAACAAGTTAGGAAAAAAGGTTCTTTAGTTAATTGCTTCAATGTTTTATTCTCATTCAATA
TATAGAATTCTGTGGACCACTAGCATGGTCCT
TTGTCCTCAGTTAGGATTTGAAATATTATTACTTACGAAGTGGCAGACTTTGGGGTCCTCCAACCACA
AGACCCCGCCGCGTGGTGGGAATCAAGGGGGT
GGGTGGGATGGCAAAATAAAATTAAAAATATTATCTGGCCTCTATCGGTTCCCTGCCCCTACAAAGTC
GCGAAGAAGGGTTTTCTAAGTATGGGTAGAAA
GTATCATAAAGAATGCATTCATTTATCAGGATATACACATACATTAATTAAGTTAGCCAGCTCTCCCA
CGATCATTCTTTTCACAAGCTCCTAAAAATGG
CAAAAATCGGCCTCGTTATCATGGCCGTAATCCGCTCGTAAATTCCNNNNNNNNNNNNNNNNNNNNNN
NNNNNNNNNNNNNNNNNNNNNNNNNNNNNNNN
NNNNNNNNNNNNNNNNNNNNNNNNNNNNNNNNNNNNNNNNNNNNNNNNNNNNNNNNNNNNNNNNNNNN
NNNNNNNNNNNNNNNNNNNNNNNNNNNNNNNN
NNNNNNNNNNNNNNNNNNNNNNNNNNNNNNNNNNNNNNNNNNNNNNNNNNNNNNNNNNNNNNNNNNNN
NNNNNNNNNNNNNNNNNNNNNNNNNNNNNNNN
NNNNNNNNNNNNNNNNNNNNNNNNNNNNNNNNNNNNNNNNNNNNNNNGTAAAAATAAAATAAAAACGGC
AAAAATCGGCCTCGTTATCATGGCCGTAATCC
GCTCGTAAATTCCTTCCATACGCTTAAAAGGTGGATAACCGAGAAAAGACGCACTTACAATGGCGCCG
TCCCAAGTCTAACTTTTGCCCTTCCATTTTTT
GTGTCCCGATTGGTCGTGGTAAGACCCTTTCCCGGAATTCGACTCCCCCTGCGCCCCCGTAGTCCCCA
CTTCGTTCGTAGCAAAAACCGACATCGGGTGA
GCATCGTGGAACTGCGTGCGCCCCACGTCTTCCATGTAGTGCAGCTGTCTCCGACGATAAACAACCAG
TATAACAATGCCCAAAATCAGAAGTGCCAAAA
CAATCACAACAATGAGAATGGTGATAAATAATTTCCTTGACGGCTCCTTTTCAATTGCCTCCACACAG
GCTCTGATGTCACCCCACGCATCGGACACCAT
GCTGCAGGCAATTACCTTCCGGCACCACGAATTACACTTCCCACCGGGGAACAATTTATCACTTCCCA
CGCCGCCACCACACAACCTCAGATATTTTGCC
CACAATTTGTCATCGGTAACGAGCCTCATTGCAGATGCCCGAAGCGACGCGGTATCGGTCAAATCACC
AAATAGGTCTTCCACACGAACCCCATTAGTCC
AATCGTAATCGAAATAGCGCTGAGTGAGGCTTTTCAAGGAGAGGCTTTTGTTGTCCAGTTGTGCAATG
AAATAAGAAGGGGCATTCCCGAAAAGGGGTGA
TATTGCCGGAACAATAAAAAACGGCACACCCCCTGAAAGTGCCTGATAGTAAAATAGATGTGTATGCC
CATACAGTTGAACCGTCACTGTATCTGAGTAC
TCCCTCATCAACTGGTTAAAGCGCTTCTGATACAATGGCTTCCAGTACATATCCTCTGCGACGGAGGT
AAAATTCCCACCTTCAAGCACTTTCCATATGT
TCGACATTGGGGGAATGTGGCTCATGATAATGACCTTTGAATTTGCTTTCCGTGCATTCTCCAGCTCA
GTGGTCAAAAATTTAAACTGACTGCACGGATC
TTCGTCGCCATCTGGAATAGCGGGCACAATGGTGTAGCACCAAAGAAGAGTATGAAGTACGATGACTC
GCAATTTTGGAGAAACAACACGCAAATAATAT
CCGCATTGCTGGAACTGTTGGGCTTCGTCTGCCATGAGCACGCCATTTTTCTGCATGACGGAAGCCTC
TTCCAAAATAACCCGGGATGGAATTCTTGTTA

CATCGAAGTAGTAATCCGGCACCACGTCATTGTTCCCCAGGGCAACCAACACCGACGGTCTCCCACTG
GGGGTCTTGTTCGGCGAAACTGCAGCAAGTCT
TTCAATGACGAACCCAAATGTAGCAGTAACAGTAGTTTTTAGGGAAGGAAAATCGTGCCTTTGCAGGT
CACCCGTCACAAATGTGCAGGCCGATTTACGT
GTTGCCATATCTGAGGTCCAATATTTGACAAAGTCAGCCGAAGATTCGCATCCTCGCTTAACAAGGGG
CAGGCCTGCGGACCGACAGGCCGTACCAAATG
CGTCAGGCCCCCCATAGAAAGGATCGTAGTGTATATCCGATGCAAAAGTAACTTGCATGCCTCTGCAG
CATCCGGGAAACAGGAAAAACGATAGTAAAAA
CAAAAGTAAAGACAACTGTGCCATTTTCGCTTGCCTTCCTTTGACTCCTTATCAATTCAGCACAGAGA
GAAAAAAGGCAACAGTCGTTTCGCAGGCGTCT
GCCTCTGTTGCCCCGGCCTAGTCAAGTGGCCTCCTAAGACCCCGCCCGCACTCAGGTTTATTCCACAC
CCACCGCGTGGAGGTGCACTCAGACAGGGGGG
CGCTCAATGCAAAGCTGCCCCTCGCAAACACTCCCTTATTTGTTTCTCCTCCATTTTTTCACCATTTT
CACTCGTTTTTATTTGGTGGACAATGGCTGCA
CCCACAGTCACTCATTTCCCTCTCGGGGGCGGGTGAGATCCCACATAAACACACATGCACCAAATAGA
GAACTGAGAAAACAAACGCAAAAAAAAAGAAA
AGCGCATCCTTTTGCTCACAAGAGACACCCACTTCTGACCGACGTTTATCAAAAACGACGCGGCGCAC
GCACGCGAGAAGCGGCGCGGTCGGTGGGGTGG
AATAAAAAAAAATTGCGCGTGTGGCAAATACAAAAAGATGAAATCGACGGCCATCGAGCAAAAAGGGT
GAGTGACGGCAAAAAAAAAAAAAAAAAAAATTA
AACTGGCACAACAGAAAGCCGCATCAATCCACAGGGCGGGCCATTCCCAAATATCCTCTCTCGTCGTC
TACGCATGGAAGGAAGTGCGACCACTGTGAAG
GCGGTTTCCGCTAACTGAACTCCTCCTCTGTGTGTTCCAAAAAAAGCCTGTGTCGCCCCTTTCCCGCT
CCCACAACAACAGGTGGGTGAAATATAATCTT
GTAGTGCGATATCATGTAGCCAAAGGAGAACGTAATCATCAGCATACCAAAACAAGTAAAAACAATTG
CCAGGATTAACCCAAGACGCTGCGAGGGCAAA
TCTATGGATGCACAACGCCCAAAACTGCTGCAATAACCACAACGCCGGCCATAAAAACCACCTGGCAG
CCGGTAGCAACAAGCGAGGTGAAGAGCAAAGC
GTTTGGGGGAGCGCGAAACACATCTCCACGCACAAGCTTCCACCCGCTTTCCTCCGAATTCTCTTCAG
CCAAGTCGGCATCCGCGTAGCTCAGAAGGTCC
TTACGAACAGTCCGCGTCAGAATAACCATGACAATTATTCCCAAAAAACTGAGTAACGCAAGGGAATT
CATAATCGCCATCAAGTGACCCCTCTTTGATG
CCGGATGCCCAGCACGGGCGTACACATCCCATCGTGTCGCCACAAACTCAATTTTCTCCGTGTCCAAT
TCCCAAGACACAGAATAAAAGTACtTCACATC
CATTGATTCAGGAGTCGCAGGTTGGATCGACCCACCTTCCGTAGTTTCCGGATCCGGACAGCCAGTGA
GTACATTCACTGAGCTTGGGACGACGTAAAAC
CCAGTAATTGAGAATCCGCCTTGCTCCGCATTTGTGTACGTCACTTTAAAATGCAGGTAATTGTGTAT
GGTCGTCTTTGTTGATTCATCGCTTGCCGCAG
GCACGCCAAGTTGGTAACCAACCCGGAGATGTTGTTCGGTTGAGGCATTCAAGGGCCTTTCCAGCAGC
GGGACACCATCAAGAAACATGTGGGCGCAATA
TTGCTGTTCAATGAGTTTTTGGAGTCGCTTCATTTCAGACTCAGAATACGCCACGAAGCACACCTCTC
GGCAGGTAATGTTCTCCCCAACCTTCACGGAA
TACATAGAGGGCAAGATGCGGTTTCCAAGAAGGATCTCGCCAAGATTCTCGTGGATTCGCTCGTCCCG
CATCCGCCGTTCATCGGGCTGACAGGTTTTCA
CGGAGTAGAAATTGTACGGAAGCAGGCCCCTCTTGGAAGTCAGTGAGTTCACGGTGATAGGAATCTCG
GTCCCGGCCTGGTATATGGTGGGGGAGGAGAG
ATGGTCCAAGAGGGCACATGATACACCGGGCGCTGCAAAAAGCAGCAGCAATGACAGCAAGGCAAAAT
GACCCCAGCGAGCCATCAATCTGTGTTTCTTC
CTTCCCTCCTTTTTGCCCCCTCTTCCTTCCTTTTCGTGGTTTGTTTCTTAGTTCTCTTTTCCTACTAT
TTTTGCCTTTCCGTAAGCGTGTGTTTTTATCC
CTCCACTCCACTGAGCCCCGTGGAAGAACAGCAGGGGAGGTGGGGAGGAAGCAACGACAGAAGAAGGA
AAAGAAGAAGGCAATGATTTCAACGGCGTCAA
AAAAAAAAATCACGACAAGAAGACCAATCAAGTCGCCGACACAGCCCGCCATTTCTCGGGGCCACACC
CCAGCAACCTCAATAAGGAAGCAAGGTTTAAA

AAGTGAAAAACAAAAATAAAAAACAACAGCCTCAGCGACAGATGGTTTTCAGTCAGTACGGGATCATG
GCCTCGTACTCGGCAGCACGGCGTGCCAGCAA
CTCCACTCTCTTCGCGCGAACGATACCGCCGTTGCGGGAGCTCCTCTTCAATGGAGGAAGAGGCTTGC
CGGCCTTCTTCGCCGCCTGGAAGGTCAAGAAA
TCCTTCTGCTTCTGCTTGAAGGCCTCCTGGCAGCGCGATTTGCGCACGTGTTCGAAGCGCACGCAGAT
GCGCTTGCGGACTGTGCGGTTGCGAACCGGCT
TGTTGACAATCACACCAACACCACGAGGAGTAACGTTCCAAACAATACCCGTGCGGCCGTGGTAGTAC
TTGTGTGGCATACCAGCGCGCACGGCGGAGTC
CGCCACAACATCAACGTAGTCCCCGACCTTGAAGTTCGTCAGGATGGTGCTCACAGATGGAACGCCGT
GCTTGCGGAACTTTTTCGCAAAAAGGTGGCGG
GTGCCACACTTGTATCCGTGGGAATGAACCATCTTCGGCTATACTTCCAAAAAAGCAGTGAGATGGGA
ACAGAAATCAAGAGAGTGGCCCGTAACGTATA
TGGATGTGCATAAATAGGACACGCTGGCAAACTCATTCCTTATGGAGCGCGGCGCGCAAACAATAGGA
CCAGCCACGATCCCCACGGAGTGCCACAACCA
CTGTGCCCCGTCGTCTTCTCTCAGTTACACGCCGAAATTAATGGCCATCGGCAAATCATCAACCAATA
CAAATGATTTAAAGACAAGTGGTCCACGAATT
CTTAACGGCATTTAATTACAAACAACAAATCCTTCCCATCAATAATTTCTTACCTCTTTCCTTCTCCA
CAGTTTCCATTAATTCCACGTTTCACAAAGTA
CGTCCACACACAAAGAAAGCGTTTTTCATCGGACAAGAAACAAAAATAAAAAAGTCATGCGCCTCATT
CCGGCCCTGTCTCCGTATTCTCACAAGGCAAA
AGTGAGGCACGAGGTTATCACAGTTTGTCTCGGTGTGTCCAACACCGAAACCCCAGTGGAATCAGTCA
ATTGCTCGTGTTAAAAAGAACAAAAAGCCTTT
TTTATTATAATTTGAAAAGACACAGGAGAAAGGCAATGCATTCACCGCCTTTTCAACGCGGCATTAAT
AAAAACA
>scaffold1051.size7132
GGGCGACAGTGGCATGAGAGTGTGTATGTGTGCAAGACATTTCGTGGGACGGTGCTCCCCAATCCCGC
CGTGCACTGACACAAAAGGGCAACAAATCAAAA
AGGGGAGCGCACTCTCCCGAAAAGGAGCGACGGTTGGACAGTCAACCCCCGGTTCGTCTCACATGACA
TCCACATATGTGCACTCCATCTCAAATACATA
ACTCCCTGAACGCCGCATAAATGACGTGTCGTTTCGTCACTCGCAATGTTTCTTTCCTCCTGCAAACA
ACAAAAAGGATGGCCATGGCGCAGCTCCACTC
AGGGGAGGCAAATCGAGGTAGACACGGGTGGGAACCAGTGATCATACAAAAAAAAGGGGGTAATAACT
CCTCCAAGACTCGGAATCGTGCAATGTTGATC
CGTCTTCTTCCACATTGAAGCCACCACGACAGAACAAATGCGACAGACACATGTGCAGCACAATACCT
CTCCCTGCCCCGCGTCCCATCTTACTTCCCAT
GAGCGCCACGGCGGTGGTGCCTGCGTTACACCTGGGATGATTCCAAAATATCAGACAGCATGCCGTGG
GAAGTGGATGCTTGACAAACGTGCCCGCGTCA
GCGTCTAACAGAAAATGACATCGAAAAGAAGAAAACCATCCGCGACACAAACTACCCGTGTGTGGCCG
TACTAAAGCGAAACTGAAACACGTGTGGAACA
ACGTAAAAAAAAAAAAAAAAGGAAAATGATGGAATAACACAAAAGAATACACCAATATATACATACAC
AAACAAGTACATCATTCAAAATAAAGGGCAAA
AAATTTGACCTCTTCGCATCCTACGCAACAACAGCAGTAACAAGAGTGACGAGCACGCCCTTCCACAC
AGTCATCGTGCAACGGAAGAAAGTCATGCAAG
CACCCGCAAAAAAAGGCACAAGACACATAAAAGAAATCAGTGCCACTGCCGGTACAGTGAGCGAGGAG
TGGCTGTGATGCGCAGCAGCCGTGGCCCCGCG
CCCTCCCCTCATCTACCAGCACCGCACGCCTCCAAATCCTACGTCGTAAAAATCACAGGAGAGCGCTA
TCGTTTTTTATCCCCAACGTTTTATTAAATAA
ATCCTATGATCCGCAAGCAAGCCACCGCCGTCAAGCACCTCCTCATTACTGAGTAGAGATATGAAAGA
GAGAGCACCAGCCCCCATATTCGGTGCTGCAG
CGGTGGCAGCAAAAAACAAGCACACACACGCACACGTTACGCATCCGCCCCGGGCTGGCGTAATAGTG
CTATAATCCGTTGTCGTCGACAATAAATAAAT
AAAATAAAAACAATCACCCAACCAATCTATGAACAATGAGAAAAGGTATCTGCCAGAAGCATCCACGT
TATGCCGCATCTGCGAGGGGCTTTGCGTCTTC
TCGCCGGGTAACGGTACTGTTGGGGACGGGATCTTTTCCACCTCAAAAAGGAGAAAACGCGCCAACTC

TTTCGTCGAGCAAAGCATCTTCACCTGTCCAGC
GGGGCATAATTAATGCTGCAGCTCGCAATGGATGAACAGCCCAGCAGGCTCAGGGCGTCTGAGCGGGT
GTCATCGGCCATGGGCTGCAACGGCGGTGCGG
GTGGTCGGTACGACGACGCAACGGTGGTGCCTGAGGCGTACGACGATGGCGTCATGTCGTGGAGCTTC
TGCGACTCGTCGTCGCTCTCCTCGTCATCGCG
CAGCAGCGCCTCCAGCCCACCACGCCGCGGCTCGCGCAGCTCCTGCCAGTGGCGGTCCTCCGCGTACC
ACACAACGACACTGTACACAGTGACCGCAAGC
ACCACAGTCGTCTGCAGCAGCACGATAGCCGCGTACGCCCTCGCACCGCCGTCGCTCGGCGCAAGGTG
GTTGGCCGCGCTGACCAGGCACAGCGCCGCAA
GCAGCACAAAGCTCGCTGTGCGCATCACCGTCAGGAAGGCCGAGCGCATCATGTTCGTGAAGGCAACC
ACACCCGCGCACGCAAGCAGCACAGCAGCCAT
GCAGAAGTACTGGACGTGGCAGTCTCCCACGGGCGGGTGCACAGCAGCAATCAGGCCCACCACGCACA
GCACCGACAGCTGGAACACGCACCAGTACACG
CGGTTGCCCCTCATGTTCGTCAGCATGCCGCCGTACATCCGCTGCTGCGCCGCGGGATACCAATACCC
GACGGGGTACAGCAGGCGCTCGTGCAGCGGCT
TCCTCGAAAACTGCCAGTACCTCGTGAAGCTCGCGCCCACGTGGCGGGCAATCAAGTAGCACACGCCA
GCAGGAAACGCCACACCGTACAGCACACCAAC
GACACCAATCACGCGGTGCTGCACGCGGGCGCCGGGCATCGCCAGTGCAAGCACGGAGCCAAAGAAGA
TGCCAAGGTGCATCGCGTGCGCCACAACGTAC
GTCAGGCTCGGGAAGCGCATCGCCGCCCACGCACTCGCAGCGTCCACGCCACGCCACCGCTGGAAGGC
GGCCGTCACACCGCAGTGCACGCACCCGAACA
CAGCGGCGAGCAGCGCGTTGCCCACCACCATCCACAGCGGGTCAAGGGCAGCGAACACCGACAGGAAG
TACGGCAGCGCAACGGTGCTCGCACGCTCCTG
CGCCGAGGCACATGACATCCTCGCAAACACGCCGAGTGCCTGCATCTCCAGAATGGAGCCCAGCCCGC
CGGTCACCACCACGGCAACAGCCGTCGCACCC
GCAACAACGCCGGTGATCGCCGAGAGGGCGCTGGCAGCGGCGGGCAGCGCGTTNNNNNNNNNNNNNNN
NNNNNNNNNNNNNNNNNNNNNNNNNNNNNNNN
NNNNNNNNNNNNNNNNNNNNNNNNNNNNNNNNNNNNNNNNNNNNNNNNNNNNNNNNNNNNNNNNNNNN
NNNNNNNNNNNNNNNNNNNNNNNNNNNNNNNN
NNNNNNNNNNNNNNNNNNNNNNNNNNNNNNNNNNNNNNNNNNNNNNNNNNNNNNNNNNNNNNNNNNNN
NNNNNNNNNNNNNNNNNNNNNNNNNNNNNNNN
NNNNNNNNNNNNNNNNNNNNNNNNNNNNNNNNNNNNNNNNNNNNNNNNNNNNGACGTGATCGTGAAG
GACCCAAGCAGCACACCCCTGCAGCCGCCGAA
CACCGCCGCAGCGTCGCAGCGAATGACTATCGTCTCGTCGGCAGCAATGAAGTAACCGCGGTGCACGG
GCACAGCCAGCTCCAGGACGGTGGACGGATTC
GTCGCATTGCGCACCCACGTCGCGTTGCTCAGCATCGCTCCCCACGGACCGCCAAACCCACTTGGCTG
CGCCGTCGACACAGGCACAAAACTCAGGTGCG
TGCCAAGCTGCGGGTCGCGTGCCCATCGAAACGGCGGCGGCAGCGCCACCACCAGCCGCGTCGGGACG
GCGCTCCCACCGCCGCGGATGTCGTTCTGCGT
CAGGCTCCCACCAGCCGCCGTCGTCGTCACAGCGAGCGTCGGGCACGCCACGTCGCTCCACCACAGCG
TGCTGCTCACCGACGCCGTCGGGGTCCGCGTG
GGCGGCAGCGCCACCGTCTCCGTCACCCGCAGCGTCTCCGTCGGCCCGTANNNNNNNNNNNNNNNNN
NNNNNNNNNNNNNNNNNNNNNNNNNNNNNNNN
NNNNNNNNNNNNNNNNNNNNNNNNNNNNNNNNNNNNNNNNNNNNNNNNNNNNNNNNNNNNNNNNNNNN
NNNNNNNNNNNNNNNNNNNNNNNNNNNNNNNN
NNNNNNNNNNNNNNNNNNNNNNNNNNNNNNNNNNNNNNNNNNNNNNNNNNNNNNNNNNNNNNNNNNNN
NNNNNNNNNNNNNNNNNNNNNNNNNNNNNNNN
NNNNNNNNNNNNNNNNNNNNNNNNNNNNNNNNNNNNNNNNNNNNNNNNNNNTGCAGCGGACTGTAGCGC
GCCGTGCTCATGCCCGGCGTCGAACTCGGTGT
CGGGCTGCGCGTCTCCGTCAGCGTCGCCGTGTGGCTCACCAGCGGCATGCCGGGCGTGCGGTTGCACC
CGTCCACGTGCGGCAGGTACACCGGCAGGCAG
TCGCGCCCGTACCCGCCCTCAGCACAGCGGCAGCGGCACGACCCCGACATCGCCCTCGTCGCAGCAGC
AAAGCAGCTCACGTCGGCGTCGCACACGCCAC
ACGCCACGGGGCGGAACTCCCCAATGATGCCGGCAGACCTGTAGTCAATAGGCCGCAGTGCCTGTCCG

TTGAGCTTCAGGCACCCCACGACGAACCTTCC
GCCGGCACCCCCGAGCGCAACGGTGCCATCCATCACGACGGGCCCATTGCCGGCGTTCCCGTGGAGCG
TCAGTGTGCTCTGCACGAGGTCCGCACTGCGC
GGGTACGACGGCACCGATAGAAGCCTGCCGCTGATGCTGTTGCCGCGCACCGACAGCCACGATCCGGA
CGCAAACGACGCCGACCCCCGAAGCACCAGCA
GCGCGCCATCGGTCAGCGCACCGGTGTTGTCGTTCACTACGACGGCGGACGCGTTGGCCATCACCCCG
CTGTCAAACACCAATCCATGCGCGGCGTACAC
CTGGCTCCCCGACACGCGCAGCACAGCGCCCTCCGACGCAACCACGCGCGCACTCGCGTACAACGCAA
CGTCGCCACCGAGCACAGCCGCGTCCAGCGCG
ACACCGCCACCGACGAGCTCCAGCACCGCGCCGTCCACCACCACCGTCCGCCCACCAGTCATCACTGT
CACGAGAGCAACGCCGGACACAACCAGCACGG
ACCGCACGAGCCGCAGACCCGACAGCACCAGCACCGGTGCGTACGGCGAGGACCGCGAGCCGAGAAGA
TACACAAGCGGCGTCGGCCTCGCGGAAACCAG
CAGGGAGTCCGTCACCGTCAGCACGGACCCCGCAGGGAACCTGCCGGTCAGCACAAGCGCACCGTTCA
GCGCCTCCACATCGCACACCGACAGCTCCACC
GGCCCGCTCTCGTCGCTCCCCGCCGCCACCGCACCGCGCAACGCTTCACCGCCCATCACGTACAGCAC
GGCGCCGTTCAGCAGCGACACGTTCTGCACAA
CGATCCGCACGCCGTCTCGCGCCATCCACGGCACGTAGAGCACCGGGCTCACGCCGTCCAGCACAACG
TGGCGCAGCGTCACCTCGCTCGCACCGGCAGG
CACAACGAACACCGACCGCACCGTCACGCCCTCGCGGAAGAACACACTCGGCGCAGTGCCCATAGCGG
GCGGTAGCACGGGAGCTCCCACAGGCACGCAC
GCCTCGCCGTACCCGCCACTGCCGCACGCGCACACGCACACACCGTACCTCATGCTCGCAGACGCCGT
CCCGGGCGCGTAGCAGTGCGTGTCCCTGTCGC
AGGCGTCGCACCCAGCAACGTCGATGCTCCCAGCGTCGCCAAAACCAGCCGCTGCGTACTCCTCCGCC
GTCGACAGGACACGCCCACCAGCGTCGTTGCA
CGCCACCCTCAAATGCTCCCCAGCCGCAAGCGACAGGCTCACCGAGACCATCCGCGGCAGCAGCGCTC
GGTTGCGCACAAAGCTCAGCGTGCTCCCAGCA
GCGGTCACGGCGCCAGCCGCGTGGAGCATCTCCCTCGAGACGCCGTCGTGCACGTTGCCCTTCGCCAG
CAGCATCGACCCGCCAGCCAGGCTCACAGTCG
AATAAAACGCCACCGCGTAGTCCTCCGTCGACAAAAAGTCGCTCCGCACAAGCGCCACAGCGGAGCCC
GACACGCTCACAGCCCCACGCACAGACACCGC
GTGCTTGCACAGCAGGAAGTCGCTGTCAACAAACGCCACCACGGAGCCGCCGCTGGCCGCAACACCGC
CCTCCACCGACACGCACAGCCCCAGCGCCGTC
TGCACAAAAAGCCCGTCCACGTACAGCGCACCGCGCGAATGCAGCGCCACACCGTCAACCAACAGCAC
GCCAGCAGCGTCACGGAACACCGCCGCCACCT
GCGTGCGCGCGATGGTCAGCACACTCCCCGTCAGATTCACGTTGTGCACCACGAGGCACGCGGACGCG
TCGCCAAGGTCGTAGTCGCCGCGGTACGCAAC
ACGCGCCTCTGCAATCAGCACCGAGTCCACCACTGTGACGCGCGAGCCCGGAGGAAATCGGTTCGCCA
CCAACACGCCGCCGCCGGAGCGCGACTCAAGC
CCGCTGATCAACACATCAACGCGCTCGCCAGCAGGCGGGTCGGACCGCCACCCAACAACGTACAGGCT
CGCTCCGCCCAAAAACGTGCAGTTCGCCAGCG
TCACGTTGCGCACACTCCCCGGCATCGACGCCACGTCCACAACGACATCCGCCGCAAAGGTCACACCA
ACGTAGCACGCCACCGACGTCCCGAGACCGGC
GTTCACCTCCACACCCACACGCACGTCCCGCACGCACAAGTCGGCGTCGTCGGTGCTCGGTGGCTCGG
GAACAGCAACGGGCAGGCACGCATCGCCGTGG
CCGCCCTCCGCGCACGTGCAGGTGCAGCCATCACTCAAGGCTGTCGTCGTGTACGCGGGGAAGCACGA
CGTCGCAAGGGTGCATGGGTCGCTGCAGGTCA
GAATGGCGGCATTGTACACGGACGGAATGGCGTATTCCGCCTCCTCCTCGCCGTTCAGAGTGTTGCAC
GCAGCCACAATCGCTCCGTTTGTGAGATCTTG
GGACCCTGTGAATATCCAAAGTATCTTCGACATTCCTGTGCTGGAGATGAATCGGTTGCCGGACACAG
ACATCGTGGAGTTGCGGAGATTCACCGTAGGA
TTAAAAAAGAGGAAAGCTTCCGATGGGGATTCACGGCAGAGGTTGTCGCTCAACTGCAGCCAGGAGTT
GTAGTAGATGTTGGAGAAGTAGCATGAAAAAA
ACACAACCAAGCTTCCCTGCATGTCATTGCCCTGAAACACAACCACTGACCGGGACTCCACACGTAAG

GCCCAAAGAAATGACATAAAGTACTTCACCGG
CCTCGTCACAAGGTTGCCTCGGATCACCACGGCAGAGTTGTTCAGTACGCTCAGCGAATCCTCGTTGC
TAGGCCCTTCAAGATTTATCAGCGTACCGTTA
GCGCCGTCCATATGAAACGTGTTGCCCAACACCGCAAACACGGAGTGCCACGACACCCTCAGCAGAGA
TCCCACAACGTAAATGGCGCTTGACGATGCCT
TCACAGTCTGAAACACATTGCCAATGAGCAc
>scaffold1052.size6637
AAACAAAGAAATTGACGGTGGTAAAGAATATGATATAATGAAATAGAGAAATAAGAGGCAGACACCGA
TATTGCAAACAACATATATACAGCGCAAGCTCA
CAAATACACACATGCATACGACAAACACATAAACGTTATCAAATGCGCCGCTCTGGGTGATGTGGAGA
GATACAGAGAGGTTCGTCACGATGCGCCGACG
AGCCGCATCCTGTCTCGCGCTGCAAAAGTTGCAAAATCTCCCGCACAGTGTCACCCACTTTAAGGTCA
AAGAATTTCACAGTGTGAAGCTGCTCGTTGAG
AATTAGTGCATCCATCTGCTCCGTTTCTGTCATTGTGTGGTACTCCTCCCGCACAATCTTATCCATCA
AATTCCGCCAACGGCGTCGTTCCGCCGTGATG
CCTGTTGATCCCAGCGTCGACACATCGTAAGAGGCGATGCCACTCTCTCCTTTTGCGTGGGCAAGGTA
TGCCTTGTAGAGCGGGGCATACTCCTCAAATT
CCTCGGGTAACTCAATGGCGGAGTGTGAGGAGTTGCGGAGGCTTCCAGAATCATCCACAACACCAGCA
GGCAAAAATTCCTTGTCTGCTAGAGCGGGTGG
GTGCGTTTCGTTCTTGACGGATCCGCCTTCGTCCACCTCCGGTGTATCCTCAAAAAGCGAAAGCTCCG
GAATGGAGGCAATGAAGGCGGCATCATCGTCA
AGGGTAGTTGAAGAATTCCCTCTTGATACCGGCAAAACGGCATTGCCCTCCCCCTCTTTTTCTTCACT
CTGCTCTAGCGCCTGCCGCACGAGTTTCCCTT
CTGCAATGCGATGACGCAGCGCCTCTTGCTCCGCCTCAAGGGATTCCAGGTCGAGGGAAAGAAACATG
CGGAAGTACACCTCACGTGCGGCATGTGTTTC
GCAGCCGTAAATCACTGCGCAAGCCTCCATCAAAGAAAAAAGGTAGTGCTTGTCCTCTCCGCCCTTTC
CACCATCTGAACTCTGCGCCGCATGTCCCTCC
AGACGGCTTTGCAGAAAGTTCTGAAATCGGTCGCAGAATTGCATCCGCTTCTCATCAAACAAGTAACG
TGCCGTTAATACCTCTCTTGTGTCGGGGTCAA
CGTCTGTCTGCATGTGCAGCTGGTCGCTGGGCGCCCCTGCGGCACCAATAGCCACCCCACGCCGCTCA
CGGTTCATCACACCCGCAAGGTGTTGTCGCCC
GCGGCGCAGTTCCACCTCGCTGTACGCCAGCGCACCCAACTCAAAGCTCAGCTGGTCACGATGCCGCT
TCGAAACAAACTCACGGTGAATCATCAAGTTG
TGGCCAAAATCCTCTTCGTACTCCACTGTCTGTGGGAGTGGCATGGGGCGGTAGACTATCTGCAGCGC
CTCCTGTGCGCTGACGCCATTTGTCGGGGCGG
CGCCGCCACTTGACGCCGTCGGCGACCATGTTCGTTGCCCCCACTTGCACCGAGCCGCGTAGCACAAA
CACCTCCGCATCATACCTAATTTTCCTGTTCG
TGTGTCTCTCTGCGTCCCTGCAGTCGTCGTGGTCGGTGCTAGGGGGGAAAAAAGGCGGTGCGGGAAGA
AGCTAAAAGCCGTAAAAAAGAAAAACAACTCT
TTATCTCGGGTATGGAAACAAGGAAAGAAACAAACAAGCAAAAAACGCCTGCAAAAAACACACAGAAA
AGCAGAGATATCGCCCCCATCCACATCCGTTC
ATCGCGCCCACACAAATAATTACAATTTCTCATACACCCACACAAATGCAAATACAAAAATAAAATAA
AAAAAAAACCTCCGTTTATTTCTTATTTTTTT
GACCTTTTGGGTCCTGCATTTTTACTCTCGTCCCCCTCCCTCAATCTCCACCTAAAACAGGTAAGGTA
ATTTCCCCTCACTCAAGAAAGAGGGAATTGGA
TAGCACCTTTTTTCTATTCCTCGTGTAAAAATCAAGCTCCATTTCTGGGTAACCGGCTGCTGCACGAT
GTTTGTAGAAGAGGGTTCGAAGTGACGCAAAA
TATACCTCGGCGAGATCGGCAACATCTTTTGGATTATCGGCGCTCGCACCAGCAGGGATGGGCAGAGG
GGACCCCACGACAATCGTTAGCGGCGTACGAT
GTGGCAGCGGTAACAGCCAACGACCGTACGGCAAAATTGGTAAAGGAAATCCCAGCACCTTCAGTGTG
GCATGATGCAGTCTTGGTGCTCGAACAATGCT
CAGAACATTCTGTTCCGCAAAGCTTATCAGCGGCACAAGTGGCACCTTGTGCTTCAGTGCCAGTTTTA
CAAAGCCGTGGTGGTACGTAATGAGCGACATG
ACCTTGTCACTGCGCCTCGTTAAAATTAACTCCGCCTGCCCACCAATGACAATGAGCGGGCTGTTTCC

TCGAGCAAGAGTGGATGCAATGGAAGAGCGCG
TTACAGAGAGCGCCCCTAATCTCAAATTAAAGTCTCGTATAATTGGAATATTGAACAAGACACTCGCT
TCATGTATGGTGACGTGAGTCTTTGAGTTGTA
CCCAATACTTTGCTTCCACGCGTCTGAGAAAACGCCGCACAGAGCAGTGCCCGCATAGACCCCATGAG
GATGAAATGCGAAGATGTATTGATGACTGCTG
TCCCGCAGATTCAGGGATTCATCTTCCCGAATAATCCGGTAGTTAAAATAACGATCAATGTCTCTAAA
TATGTACTTCTGTGTCCATTTCACAAACTCCA
CACTATGGTACGCGCCCTTCTCCTCCGGTTCTCCATCATACGAGGGAAGGTACCAGAGCCACACCAGC
AGTACCACCGCATCGACGGCATAGTACCTCAG
CGTGGATAGTGCAGCAACTACTGTACCGCTGTCGAGTACACGAAGATATTCCAGGTAGCTCGCATACC
CGAGCATTGCATACACACCAAACCTTAGCAAC
ATGCGGAACTGACGAGAAGGAGCAGCCACACCCAAGGAGAAGTACATAAATCCCGTGTAGTACGCTCC
AACAGAATGAAAGGGTAAAAAAAATGCCGGTC
CCAACGCAATCCAGAAAGAAATGATTGGCAATGAATACAAACCGCACGTTACAACACACTCCCTAAAT
GATACCAATTGCTCCAACATACCAATATCCAT
GACATGTGGCACAAGAGAAATCAACAAGGCAAGTGTGCCTGCCATGAGCGCAAAAGGTGTCCAATACT
GTATGATGTGCAAGAGTCCACTGTGAACGCCG
TTAACAATATAGTACATTACAAAAATAAATGCCATGGGTGTAACCATTCCAAGCATCAACATCATAAT
TTCTGATAACCGGAGAGTAATATTCAAAAAGA
GTGATTTGCGACGTCCCAATGGCACATCTTCCTCAGTGTCAATATCACGGCGCGGGGAATACGCCACC
GTCTCCAAACGTCTGGCGGAACGACTGAGTGT
GATCAAAATAAACGGCACGGCAGTCATAATGCCAAGTGCCATAATAAAAAAAGGAACGTGCGATCGCC
GTGATGAGGCAGTGATGTCTTCTTCAACACCA
GAAGTATCATCCAGAACCCGAAAAAACTCCTGCGCACGCAGATAGAGATGACCTATCAAGAACGCACC
AAGGAATACCAACATCGCCACAACTCCCTCAC
CATTCTCGTCCAGGTATGTCAGACGCTTGGGCCGCGAGTCAAATTTAGGTATGGAGAATAGAATAAAA
ATTTGACTGAATACTGAAAAAAATGCCCCGGT
AAGAAGTTGGACGCACCCCCCCTTATCCTCCAAGGTGTAAATAACAGTCAAAACAAATGTGCATGCGT
AGCTCACCCAACCAGCAATTTGCAGTGCCACA
AACACTGAGCCTCCTTTAAATGGCATAAAAAATGTATATTCCCCGGTGCGATCATGTAGCAATGCTCC
ATATCCACAGTGCGTACTCACACACGAAATGG
TACACACGGTAAGGCCACAAAGAGCAAATACAAACCCAACGAGCGGTACCGTGTTTGCCCAAAGTGCG
GCAATGATATAACTCGAGACCGAGGAAAAGCA
CATCATCACCACCAACAGGGTTGCTGCACCATGCCGGTTCTCTGCCTCTGTCGAAAAGAGGGGTAAAA
TATCATCCTCCTCCTGCGTTCCGTCAATAATC
TCTTCTGCAACTTCTTTTATTGCTGTACACTTTCGTTTGGGTTTGACACTGAGACAAAAACGAGAACT
GTCGTACTGCATACCGTGAGCTGAAAGGGTGG
ACGCGGCCAAAATCCGCATGGACATTTGCAACGCCGCCCTTTCACTCTCCTCTTGTGCATCGACGCGT
TCATCCAGTGCCCTTTTCACGCGTTCAAGAGC
CTCCACAACCATTCCTTGACGTCGTCGTCGTGAAACAGAAAAGCGACCTTCCATGACCACGGAAAGCA
CAATCAGTAAAAGCGGAACCACCTGGAGAAAT
CCCTCCAAAGTGAACCAAAGAAAAGGGGGACGGTCGGAAGAAAAATACACGGACGCTAAGCCATTCAG
AGCAAGCAGAGAATCCAGAATATTCTTCAGTA
CAAATAATGCATACAAAATCCATCCTAAGGCTTGCAAGGCAATATACGTACCACTACCCCGAAACGGT
GCAAAGAAGGGAATATCTTTGCTTTTCCCAGA
ATAATGAGCCAGAGGTGCACTCACCAACGAAGCCAGTGAAGAAAAACCCATCAGCACGTGCGTCGGAA
CAGCTGAAAACTCTCCGTCTTGTGTTAATGAG
ATGTCTACCACCAGGCGTAAAATGATAGAAGCCAGTTGAAGAAGAAGGCTAAGAATCAGTTCACCGCT
GATGATACTTTCCTTTTCAGATTTACTCTCAC
TTCTTTTCCCCAAGCCTAATCCCTGCTTTGCCTCCGTACAAAAACCCGAGGCAAAGGCTTTGATAGAA
AATTGAATACAAATTTGTCCCATAACGCCAGC
AGCCGCAGCAGAAAGAATCCATCGAAAATTTGTCGCTGTCATATGAAGCGTCATGCAGAGAATGCAGG
CGGTAAAAAATGCCCAACCAAGCGCCTGAAGA
AACACAAATCCCACAGAACCACGAAAAGGCTGAAAAAAGCTGAAAGCCGCCCCGTAAATCACACGCCC

AATTAGATGTGTCACTGGAGTCAATGCAAGTA
AAAAACACACTGACAAAGTTTGGCAAAACAACAAGCCATCCATTAATGCCCGTCCATTCCTAGACTCC
ATAAGGAAATGTTTCTGGACCTGTACGTAATA
CATAATGCCGATTGCAATAAATGCCAATGCGATCGCCATGGCTGAAAGAAGAAATCGAGCATACCTCG
CCACGACAAAGTGAAAAGATACATGAGACTCT
TTAATCCGTGCGGTTCTAACTAGCGCCAACATGGATAAGATCCCAGTAAAAGCCGCCAACGCTGTCAC
TCCAAAAGCCACCTGTGGAGCTTCTGTCCCTG
TGCGAATCAGAGCAATTCCTCCCACAAGTGCCACAAAGTACACCAACAAAGAAAGCAAATAGAAAAAA
GTCTCCACGGGTCCTCGCAGCATTTTGAATCC
CTCCATCTGCCTCCATTCAATCAACAAATGAACAAGGATACCACTCAATAAATGAAAGATTGTAAATA
CAACCATCGCCGGTGAAGAAATAATTTGAAAT
AAAGCCCCAATATTACAAAAAAACAACTGAAACAAAACAAAAATAATCACCAACGCCGTTTCAGAGTT
TGGGCTATGTAGTATCCACTTCAGAGGGGACT
CTCTGGCTGGCTGGCCTGTTGGATGCTGCGGAGTTGCTTCAACAATATTCCGAGATTCCGAAGGGCTA
AAGTAGCGAAGTGACACGGCCAACAAAATATT
CCCTATGGATGCAAATCCTTCCAATATCGTGAGCGCTCCGTACCTATACATGGGTGGCTCCAACTGAA
TAAGATACAAAACAATGAAAGATGAACAAGAG
ACAACGAGTGTCCAACCACATACTTGGAGCAACACATACCGTTCTCCACCTGCAAATGGCTGATATAT
GCTAAATGAATCCCACAAACTGCATCCAAGCA
GAATGGAGAAAAGAGCACAAAGACCATTAAGTCCTACTGCCAAGCATGCGGTTGGAATAAAAGGGAAA
ATTGGCGCATTTCCTTGAAACGCATCCGCTAT
TGCGCCCGTAGTCATTGCCACAACAAATCCAAGCGTCCAAACCACCATAAGCGAGACAGTCGAAAAGT
GTGGCATAGGACTGAATCCACCAAGTCTTTGA
TCCAAGAACATGACCAAGAGAAATACAATAATCGCAATATCACCTTTAAAACAAAAATAAAGGGGGAA
AAAAAAAACATTTCTGCCTCTCAACGCAATGC
TTAAAGGAATATTCACCTGGGTCACTGTCCAAATCATGAGTTTATCAATAACAGAAACCAAACAAGGC
ATGAATAAAAAAGAGATAAATGGAATAAGAGT
GGATTCACCACACCCTCGAAATTTAACACCATGTATGTATATATATATATATTTCTTACAAAGTGGCA
GACTTTGGTTTCCCCCAGTCACAAGACTCCGC
CGCGAGAAGGGAACCAAAAGAGGGGTGGGTGAGATAAAAAAAAAACACAACCGCGTGATATGCCGTCT
GCGGGGGTTTTCCGCAGACCGCACGTATGCAA
AGGATGCCTGCACTCCGCGAGAGTCAACAAACATAT
>scaffold1053.size7735
GTTATTGGGTCATTATTTTTATCTTTTTCATTGTCTATTCGGAATTGTCACTGATTTACTTTTGTCTC
AGCTCTTCATTACTTTGTGGATCTCATTAATTA
GCAGAGAAGGGAGCCACGCTTTGACGAACAGAACGACGAAAAGGAGGAGGGCTTATTTTTACTATGCG
TCGATTCTGTTTTCCGGTTGCTACCTTTGCAC
AGGCAGCGTTGCGCCACCGCGGAATCCGCTGGAACACCACGATGGCCGATAACGAATCTCACACAGGG
GCGAAAGCCTCTGCATTTTCGTCAACTCCTTC
GGAAGCCAATGAAATTTCAGCGATGGAGCGGGCGTCCGAGGCTCGCAGAGAAATTCACGACTTGTGGA
TGTCTACAGAAACGATGCTGGATCCGGAGAAT
CGCTTGCGGTCTGTCGCTTCACTCATTGAAAAGTACAAGCTTGATCCTTCGACCCCCCGTGAGAATGA
TGTGAGCCGTGGATTGGGTGATGCGTTTGACC
GTCTGCTGCTTCTTTGTATCCCGTTGGGCAAAGATTCCTCAAAAGGTACGGATGACCTGGAACGTCTC
ATGAATCTCGCTGGGCGAAACGGCAGGGAAAT
TTCAGTGCGCACCATCCAGCATCTTTTTGCTCGTACTGACTCTTTTAGCGAGGCTCTGGCTGTTTTCT
ACGCCATGCGTCGATGTCATGTGGCAATGAAC
ATGGAGGCTTATTACGCCATGTTGTACTCATTGCAGCGACTTGAGGAAGAGGGGTGGGCTCAGCGTTT
TCGCGAGGAGTGTGAGGAGAAGGGGGGGGTTT
CGGAACAGGCGATGGACTTTGTAGTGAAGGGTATCAATAATGCTCTTCTTCCCGAGAATAAGCCTTGG
CTTGGTCGGGTGATGTTTGGGGACAGAGACGC
ACCGGCGCAGCGACGCGAGGCTCGTGATTATGATGAATTGAGTGCCATGTGGACTGAACGTTACAGAG
ATGGCAGTGCATTTCCCACATCACCCTGATGC
AGGACACACACACACACACACACACAAGGAACTAAAGCAAATACAACCACAAGCAATTTGGAGATGGG

AAAATTGGAGGGACTGGGAAAACTTTGTGTGA
GGGAAGTGCGTTGTCCATAATCCCATGATAAATATCTATAGAATGCATTGATTTTTTTACTTTTATTT
TTATTTCACTTCCTTTGCATAGGTTGTCATTT
TAGGAGCCATGGTTTTTTTGACACTGCTTCGCACGGCAGCCTACTTTATCTATGGTGCATACCAGCGT
CGTCGGATGCTTAGTACCAACCCACAGGCTGT
TATTGAGCCAATGAAGAAGCACTATACTTGCGTCTACGCCGGAATACGAATGGCTGATATCTTTGGCC
ACGTTAATAACTCCAAATATCTTGAGATATGT
GAACTGGCCCGTTGGCATTACGGAGGCTACGTCGGTATGATGAAAATACTGTGGGAACTAAGGGCAAG
CTTTGTAGTTGCCTCTGTGAACGTGCAATATG
TGAGAGAAATTTTGCCGTGCCGTCGTTATCTGGTAACAACAGAGGTGCTGCGCTTTGAAGACGCAGGC
TCTTCATCAGATAAACCAATCTCGGGGAAAGA
CGAGAGTGGGGTGGCAAGAAACTTACCACCCGCCGGGCGTATGGTGATCATGCACGAGATATGGAGTA
TGGACAAGAAAACACTTTATGCTGGTATTTTG
TTACGAGCTGCACTGGTAGGTGATGCAAAGTCACCCCTGGCTATCCCCTCTGGAGGTAAGCGACGCGG
CAGATATGCCCTGTTGGATTGCAGGGCGGTTt
ACGCCGCGATTAGAGGCGCTGATGGTGCGAATGAGCTGGCCAACGAGAACATGAGGAATTTCTACAAC
CGTCTCGGCTTCCAGCCAAATCAGGAAGATTT
CAATGGCGATCCATGGATTGAGGCTTCGATGCGTTTGGAAAAGGAATGGCGCTCAAAGCTACAGAGTG
TGAGGGAACCCTAATGTCGTAAAAATTCTCTT
GAAATCACATGAAGTAATAGTTTTTTGGCTTTACTTACAGCGTTGTGAGATTTGCTAGTGATTTCCTT
GGAGTCGTTCAAACAATGGCTTGGTACAGAAA
ACAAGAGATGTTGCGCGAAATGTGGTGTTTCACTGGTATTTCCCACAGTAGCGGATGTTTTCTCCCGC
ACCAACTTGCTCCCTCCCCACTTTTTTTGGTT
CAGGACATATAGAGGGAGGATACATACAAGGAAGAGGGAAAAGAGATAGAGACAGGGAGGGAGGGAGA
GAGAGGCAAACCCAAAGAAAAAAATCTCGA
GTGTGGGCTTGCTTACTTTTTCTCTCTCTCAGTGTGTGTGTGTGTGTGGCACGGGTGCTGTCAGATGG
CCGCGTTGGCCCCGAAGAATGGATCGTTTCTT
CACGCTTTGCAGCTGGAAAACGATGCATTGCGTCGTCAACTGCGGAGGGCTCATGAGGATATGGAGCG
TGTGCTTCAGCAGCTTCAGCGCGAGTCGAAGG
GAGGCCGTGGAACTGCAGAGGGAGGGAGTCATGGTGGTCGTCATGAGGGTGGTGGCGACGCATGGCAC
GTCGAACGCCTCGAGCGGCGGTGTGAGCTGTT
GGCGACTCGACTGGTGGGAATTGTGCGGGACAGGCAGTTGTCAGAGTGCGCAGAGATTCACTTTCTCC
TTCGAACACAGGAGGAGGAGCGCCGGTTTCTG
CACGATGCCCTTGTTACACTTCTGGAGAGGCAAAAGGATCCTCTGGCGGCGATGAATATGGGGCTCA
GACGGAAAAAGAGATTCATCCAGGCATGTTGA
GTGCATTAATCATGAGGGTGGCTGAAAATATGCAAAGTTCCCGTTCCCAGTTGAAACTGGAGCAGACC
GAGCGTATTATGCTTTTGGGGCAGACCATCGA
TCTTATCTTAGACATACAGAAGGCGATCGAAAAGGCGGCACGGTGGGTGTACGGCTACCACTGCGATT
TCCTTCACGCGGGCTCTCTCCAAGAGCTGACG
TTATGGAGGGAGGCTCAAAACTCTGGCATTGTTGGGCTGCGTTCGCTGGCGGAGGTCACCCCGGATGT
TCCACCGCGTACTTCTTTTCGCAAAAGAAGCA
GCAACCATTCAGAACGGCGTTGGCGGGAGGAAGTTGACTGTTCCATTGAATTTCCCTCAAGCTCTGTA
CACCAAACCGAGGCCGCAGCGAACTTTTCTTC
ACGTAGGGGCGATTTGCTGGAGGCGTGTGACGTACTGCGAGCATGTCACCAGGCCTTAACAGGCGTTC
GGACTCTTCTTGGTGATGCTATGTCCAATGTC
ACTTCCTCGTGCTCCTCACGAAAGGGGAAACCGACGAGCGTAGAGGAGCTTTTGAAGGGCTTTCATCG
GGATCTGCAAGAAATGGTACGGAGTAACGAGA
GATGCCGAGAGACGTTGGCGCGGCGATTGGAGGCTGAGATTGAGGCTCACCGACGAACAGTTCAGAAG
TTTGAGGCACGCCTGAAGTTAGCCGAGAGGGA
GTTGATTGATCATCTCACCTCTTCACACAAATTTCAGAAACCGTTGGGATCCAATGACGCAGACGCGC
CCTCCTTTTCTAATTACCCCAGCAAGGTGAAG
GCATATCATGGCGAAACGGGGGCCACAACGCCGCCCACACCAACAGGAACAACCGCAACGGGAAACCG
ACGTACTGTGAGGGTCAACAACAATACTCGGT
TTCAGTTTGCTCCAGAAAAGACTGTGCCGCTTTCTCCCCGGCGGGATTTGCCATTTGTTTTGGAGGGG

CAAAATGACAAGAAACAAAAGAGACGGGGGGA
TACCCGTCCTGGCACTCCCCATAAGGGCAGCGCTTGTGATGTGGTTTCTTTTTTTCCCCCTTCTTTGC
CTTCTTTTGCCGCTCTTGCAGGGCCTCTACCC
GAGACGCGGGGGCGGCAGCATGAGGATTTGCAGTCCTTTTCGTCCCGAGACAATTTTGAAGACGGGAC
GCGCAGCAACTCTACCGGGGAGGGTTCGGTGG
CGCACTCTTCGCCTCCACTGCCTGCATCAAGGAGAGAGCGACACCCTTCGAGTGGCAAAATCCAGGAG
GGTAGGGAGGCGGTGTCTTCGCAACGATGTGT
GCGATGGCCGCGGGGGGCGGTCTTGCTCAGTCCTTCAGCTTTTTGAAACCCACCACGTGAAAAGCGAA
CCTGTGGGAAACTTGCGTGTGGTTGGTGGATG
CTCTGGCGTAGCGGCAGTGTGTCTAGATGCACATCCCCTTTCTTTCTGTTTTTACTCAATTTATTTTT
TCACCTGCGACCATACATATACACTCATACAT
ATATAAGTATACGTGTATTCTTATTGAATTGGGGTTTGTGAGTATTTTTCACCGCTGCATCTATTTTT
TATGCCGTTTTTTCTTCTTTCCTTCCCTATCG
CCGAAATCTAAAGATGGGGTTTACCCCCATGTTTTTTTGGCCGGAGATTAATGAAGCTGATACACGTG
CCTCTGGAGTTGCTGCGTCTCTCTCTTTCTTG
ACATTGATGGTAAAACACACCAGAATTCTGCGTCAGCCTGTGTGTAAGCAGAAGAACGGTGTGCCTCT
TATTTGGCAGCGACGATGGCGGCGCTACTACC
CAATACAACTGAAGTGCAGTTGAAGGCGTGAGATGATTCTCCATTCTGGCGACGAGGAATGATGCGCA
CCCAAGTGCCCTGAAGAGACGCCAAGGTATGA
TCTCCTCTATGGCGAGGGAATTTTTTCTAGCCAACGGAGTCACTACGGATTTTCCCACGTGTAATTCC
TTTATTCCTATTTCTTTTCTTATTCGCCAGAA
TGTTTCTCCAATATACTTCCGGATGCGACGGCTTTTTAACGCAACCCCGACTTCTTTTTTGAACGATG
CTTTAACACGCTTGCAAGAGATGCTGAGAGCA
AAACATTGCGCTCCGCATTCGTTGAGAAGCGAATAATTTTGGCAGATGTGGAGGCCACTCAGAAGGGC
GTATTCTGCGTGGGACATTCTCGGATGCTTTT
ACCATTTCTTTCATTTCCGTATGGGCTTGCCGCCGCATTTTTTTTTTTCTGAATGGAATTTTATCTGC
CATTTTTTTGATTGTTGGTTTTAACACGTGTT
TTGCTCGCTGCACTGGGCAGTTAGGATAGCGCCAGAGGTGTATAAGCCGTGTAGTCTCTCTGATGTAT
ATGCGTGACAGACGTCCTCTATTAGCGGACGA
TGCCGCGAATAAAAAAGTAGTAATCAATAAATTTTCAGGAAGGGAAATGCAGCACCGTTTGCATGAAG
AAACTCCTCCACCCATGACGATTAAGAGAGCG
TCCGTTTGTGTTCTTTTTTTCCTTGTCGTGCTCGGGATTCCATTGGGAGTCATCTACTATTTTACGCC
GTTCTTTGTCGTTGAGAACTTGGGTTGCAGGG
CTCTGGAGAAACTCTGGGGGGATGACACAGTGCGCGTGCTAGAGGAGGGTTACCACAGCTACAAGACC
TCACCGCCGCGGTTTCCCTCCCTCGAGTACAA
CAAAACGATGCTCTGGGGAACATATGAGCCTGGTCTCCTTTTTGCCATGAAGACACGCACCCCAAAGC
CTGTGTACATTGGAGTTGCCTGGTACGACGAG
GCAGGCCTACACCCCGTCCGGCATAAGATGGTGGACGTGCTGTCGGTAGAGCGACGTCGGGACAGCAA
TGACACCATGGTCTCTGACGAAAAGACAGGTG
CATTTGAAGCAGCCTGGTCACTACACGATGGCGTCCATTACGGGCATCTTTTTGTGCGAGATGGCCCA
GCCAAGATAAACATTGAATTTCTCAAGAGTCC
TCCAGGTGACAGTTGGCACGTCCGTGTTCATGGCCAGATGGATGGAGCGAAACCTATTGAGTTTGTTG
TTTACGTTGTGAACGAGGGGGGTGACGCCCTC
GAGCCTGAGCTGGCAACAACCGACGGTTCATGGGATCCGCAGATCAAGAGCGAGTTCGAAGATGCAGA
TGGGCGTCGAGAAGGCTTTAATTTGCTTCTTC
GCGATGATCATAACCCCCTCTTTACGGTCGCCTCCTGGCAAGTGTATGGGTGGCGGTCTGCCGAAGGC
GAGTTTCTTCGTGGCACCAATCTTCGTAACGT
TCATTTAAATGCTCAGTCGTGTAAAATTGACTTCTCTGGGGTAAAGCGATCGCCACTTCTCAAGACTG
GGCACGAAACACACAACGTGATGATATTTCGT
AAGAGATATGAGTCAGACTTTCGGGTCGAACTTTCAATGCGCCACGCCTCGCATCTTGTGGCAGCTGT
CCCTGATGCGACAGCACACCAACGATTTATGG
CAGATTACGAATCGTTGACAACATGCCAGCTGACCAACGCTTTCCGTGCTCGGGAGAAAGAAGTTTTG
TATCAGATGCGCCAAATGCTAATTCCATGGAC
AAAGGGGATTCATGTCAATCGCAAGCTGTACATCAACAGAGCTTCTCGGGTTCTCAGCGGGGCGCTAG

GCTCGTGGGGCTTTTGGTATGGGCGATACCTT
TACCTGGATCCAATTCTTGACGCTTCATGGGACGAGCAAGGAAAATTGACAAGAGTTCAATCGGACGG
ACAACTACGGGAAGCAGCGTTTGATGTGTCTG
CTTTTGGGGCAGTGGCTTCTCGAGTGGGGAGTTCGTACGGTGACATGACTCTGACAGGATTTCATCTC
CTTTTTATTATACGCTGGAACAAGGAGTGGAC
AAAGGATGCCATTGCTTCTTGGCTTGTTGGCGCTCAAGAAAGCAATAGCGGCTTTATTCCACATAGGG
CAATCCTTACGGCGGCATCGCGTGCATTTGCT
CCACCCTCCGCACGCTACGAATGCCTGACGTTTGCTGCACCCCCCACCATTCTTCTTGTACTTGATCA
ATTACTACGCAGTTACCAGGGAGAGGCAGCTG
ATAAGGAGTTTCTTGAGTTTTTACTGCCACACCTGCGTCGTTGGCGGACATGGTTTCACAAGACGCAG
AGCTCAGGGGATACTGAAATGAGCTTAGAAGC
ACTTGCTGCCGCAGAAACAACGAATGGGACCGAAAAAAGCTCACCGCCAAGATATCGATGGCGTTCGC
GTGATGAATATCGCATCCCTTCCAGTGGTATG
CCAGACTACCCCCGACCCGTTTGCCCCGGCTACCACAGAATGGAGGCGCATGTGGATCTTTTTTCGTG
GGTTGCACTGCTGAGCTCGATCATCAGCGATG
TTGAGCTGCATTTGGGAGCTGCTGAGACTGTTCCCAAGCGGCTTTGGACGGTTTGGTTGGACACGGTG
CACTGGGATGCTGCAAACCAGCGCTATGCCGA
CCGTGCTGGCTGCCCTGGTGACTCTTTCTCGCCGTACATAGGGTATGTCAACCTCTACCTTTTTCTTC
TTGGGATCATCGACAACAAGGGGCGTGCACTG
ACGATCGTTGAACTTGCCAAGACGGAGCTGATGACCCGCTACGGCCTGATGTCTGTTTCGTACGACTC
GGTGCGCGCGGCACGTGATGCGGGTCTGCGCC
ACGAGAACCGCTGGATGGGTCACGTGTGGCTCTCAGCGAACGTCCTGATGCTGCACGCTCTGAGAACG
AAATACATTGACATTCTCGGGGACCCGGCAGG
GGAACTATTCAAGCGGCTGCGGTTGTGTATGTTGGAGATCAGCGGTGGGTCCCCGATGATGCAGGAGG
CCTATAACCCTGTGACGGGCGCCGCAGAGAGC
ACCGTCTCGCTCGTGGGGTACCGTGCCATGTTGC
>scaffold1054.size7254
GCCCACGCGTGTTTGCGTGTGTGTCTGTTCGCATCGCATCGCACTGCCGCCCCCCCTTTCCTCACATC
CTCCACCCAATTTTTTTTTCTTTTTTCTTTTTT
TCTTTTCAGATTTTCCTTCTTTTGGTTTTGGCTTTTTTTTTTTCTAAAAAAAAAAAAGAAAAAAAAG
AAAATTCGACGCCCCGCCACAATTCCCAAACT
GCCCCACACAAACAACCAAAAAAAAAAAAAAAAAAAAACGCGCATGCCCCCTCTTCCATCCCCCAACA
AACGTCCACACACACACACACACAAATATATA
TTATATAAATAATGGGTGGATGGGGGTAAGAAGGAGGAGGAGGATGTGGAATTAACTCGCAACGCACC
ACTCGCATACATTTCACTCACACTCACAAACC
AAAAAAAAAACAAAAAACAAAAAACAAAAAAAAACACACGCCAAAGTGGGAGTTAGAGAGTGGGGAGA
GAGACAGAGAGACAGAGAGAGAGAAAGAGAAT
AGCGGAGTGAATAGACATGACGAGGCCAACACAAATATTGGAAATGCCAGCCACATCACCACCACCAA
ATACATATATATATATATATGTGTGTGTGTCA
AGGAGTAAAAAAGAAAAAAATGGGAGAGGGCAGGGAACAGAGAAGCATAAACACAACGCGCCAACAAA
AAACAAAAAAAAAAAAACTAAACCGCAACAGAA
AAGAAAAAAAAAAAAAAAGGAAGCGCATGCGTCTGTAGACATTAGAACAATGCTCCCGTCCAGCAAGA
AGCACGGAGGGGAGGGAAACAACAACAACAAC
AACAAAAAAGCAGCAACCCACACAAATGGAAGTAGTATACAAACGAATTTGCAAACTGAATAATAATA
ATAATAATAAAAAAGCAAGAAGCAAAAAAAAG
TAAATGAGCAACAAGAAGGAATTACATCAGAAAAGGGGGTAAAAATGACCCAACTGCCCCTACATGCG
TAGAAACAAAACCCTGCATGTAACTACGACTG
CCTCTCTCTCTCCCCCCCCTTTTTTTTTTCTTTTTGTTGTTTGTTACTGTTTGTTTCTCCCGCAAACA
CGCAGAGAACAGGGGGGAAAGAAGGAGCCACG
CCGTTCAATGTCTCACGTTCAACAGCCTTGCAGTTTAAACACCACCACCGCCCTTCTCCCCTCCTCGG
CACGCAAAAATTAACGAAACGCCTACACTCCG
GTGTTCATTACGTGCAATTCCTGACCACTCAAGTACATAAATACACATGTACACATCATATATATGCA
TACACACGTATAGCTGACTTTGGTCGCGGGCG
GGTATTATGTGTGGCACAATGGATGAAGACAAAAAAAAAAAAAAAAAAAAAAGAAAAAAAGGAAGAAT

ATCATTATAAATTTTTTTTAAATTTTCTTCTT
GTTGTTTCTTCATCACCTCTACGACGCGGGAAATCCACAACAAGAAAAACAAGAATAGCAGAAGACAG
CGTCGTAATATTTCAATTACATGAAGATCGTA
TACACCCACCGTGTTGCAGAGCACATGGAAGAGAAAACAAACAAACAAAAAAAAACACCCCATACTAG
CCCCCCTCCCCAAAAGAAAAACTAAATAAAAA
ANNNNNNNNNNNNNNNNNNNNNNNNNNNNNNNNNNNNNNNNNNNNNNNNNNNNNNNNNNNNNNNNNNN
NNNNNNNNNNNNNNNNNNNNNNNNNNNNNNNN
NNNNNNNNNNNNNNNNNNNNNNNNNNNNNNNNNNNNNNNNNNNNNNNNNNNNNNNNNNNNNNNNNNNN
NNNNNNNNNNNNNNNNNNNNNNNNNNNNNNNN
NNNNNNNNNNNNNNNNNNNNNNNNNNNNNNNNNNNNNNNNNNNNNNNNNNNNNNNNNNNNNNNNNNNN
NNNNNNNNNNNNNNNNNNNNNNNNNNNNNNNN
NAACACATAAAAGAAATGCAAAAAAAAAAAAAAAAAAAGGGGGAAGAAGGGTGTCTTCGTTCGAGGGGA
AAAAAAAGTGATCGTGGAAGGAAATGAGCAAC
ACGTGGGAATCAAAGGACTCTCGTACCTCAGAAATCACCATGCATGGATGCTTCTAAGATTCAGTTAA
TTCCCCCCCCACTCACTCACTGTTTCCCATCG
CCATTTTTTTAGGTTACACTGGCCGTTGTGCACCGCCACTTCTCCCCCTACTCACCCATGCAACGTTT
GCGGCATCCCCTCCACTCTCCAACTTTCAACC
CTCTCCACTGCCGGTAAAAATTGCGTCAACTATACGCATCCACATGGATAGAGAGAGAGAGAGAGAGA
GAAAAGAGAAAACCAAATAAAAAAATTTAACT
ATATGAGAGGCAGTGGTGCGGCCCCCCTTCGAAGGAAAAAAAAAAAACACACAAAAAAACAAACGAAA
CCCCAGAAACACGCTCACATATAGCCCTCTTT
CCTTTATTATTTTGTTCTTTTGGCTTTAAAAATAAATCAACATAAAAAACTAATATTGCTGTTCCCTT
CAAATAGACCCATCCCTCTCCCTCCCCATCAC
CCCCGCTGTTCCCCTGGTTCATGCACGCTGGACGGAATACTCCACTCTCCACTGTCCCTTTGATTTTC
TCCAGAAATTACAAAACAGGCATGCGCATGTC
CATGTGGATATTTCCATATATTTTTTTATCTTCTTTATTTTGTTATGTTTATTATTCGTATTTCCTTT
TTTTTTGTTTTGTTTTAATTTTCTTAAAAAAA
TTTTTTTCTTTAAAAATAATTTTTAAGGGATGCACACTACAGCACGGATCTCAACTTCCCCATTTCCC
TCGCTTCTTAAAATTAAGCACAAATAAAGCCA
CTCTCACTCACTCGATCTCTCTGTCTCTCTATCTCTCTTCTTCTCCACTTCTTTTCATTTTGGTTCTT
TTTTTTTCTTTTTTTTTCCTCCGGTCTCACCC
TCACCCCTATTGTCACATGGGGAATAGGGGAGTTGCAGTGAATTATGTTTCACTGACTTTCACTGCCG
CAGTTACTGCGGTCACTGTTGCTCTCAATGTG
GTTCTGCTCTGTGCGTCGCCCCAAGCTGCGCATCCATTCCTTGAAGAGACCGGCAAAGTGACCCATCT
GCCCTTGCGCAGCGCTTGTGGCACGTTCCACA
ATAAGATGCTTCAACACGTCTAGTTGTGCGACTCGTGCCTCCCACGCCATATTTAAATTTGTAAACGC
CTGTTCATCATCCCTTAGCACATGAAAGCGGC
GGCGACCATCGCTAAGGACATCGCTGTAATTCCTCTCGATACGGGCGCGCAGGCAGATGAGATAACAG
CAATTACAAATACGGCACCATGGGGCAGAGCG
TACTTTGAGTCCACCACGTCCACGCCCCGATCCCCGTGTGGTGTGAGACGTCGCAACCTTCGAACGCT
GGGAGAACAATTTGTCCTCATCACCTTCTTCT
TGCCGCCAGTTCGTGCCATCTGTGAGTTCATACGTGTCAAACATCCAATCATATTCCTTCTTTTGGTT
ATTGGTGCTTTCAACAGCCACAGTGGCCTGAA
TCTCCCCGTTATCATCCACAACAACAACATCCTCCTCGTCCTCCTCCTGCTCACTCACCTTGTCATGT
TGTAACGCATACATATCACGCCCTACCTGCGA
ACAACAATCATTACATAGGAGGATACCACAGCGGCGGCAGTGATGCTTCCTTACTGTTATGGAGAAAA
GTTTGCCACAGCGTGTACAATGAGTGACGGAC
TCATTGCTTTGCCAGTAGTGTCTACCGGCTTGTGAGCGACCGTGGCGAAGTGTTTCCATCTGCAAAAG
AATGCGCTGCTCTGCCAGCATTAGTTGATTCT
CAGCGTCACGGAATGCCCTGCGTTTGACAGCGTCGTCCTCGTCAAAAACAAATTCCAACATCCCTGCA
CTGCCAGTTTGCACTAAACTAAGGGAGTAATA
AGAAAAATCCAATTCCCCGTCGTGGGTTTTCCACCCATTAACACCGCTTTTCTTATCCACACACGGGA
GAGAAGTCTCCTCTTCACCGCGGTTATCCCCA
GTAGGAGCATCATCATTGCCATGACCATCGGCAGCAGCAACAACAGTATAAGTGGATTTCCTGGCACG

TCTTCCTCCCAACCAACACTTTCCAGCCTTCG
GAAAATCATAAAGATTGGGCGGGTTAAAGCGAGACGCCAAAGAGCCACCATCATTTCCCAAACTAGTG
CATTTGCCAAATGGATCGGAGGGGTCAGCAAC
CATCCCGTTCCTTGTCGCCCGAGGTCTTCTACCACTGAACAAAAAATTGTTGTCGTTATTATGTCCGT
GAAACGGCTGATCCAACACAGCAGCGTTTCCC
GTGTCCTTTTCTCCATCGTGTGCCCCTTCATCCTCAGCCGGGGGCGTAAGGATTCCATTGCATATGGC
CTTCGTATTACATATCACGCTGGAAGTTACGT
CAGATCCATTCGATTTGACCACAGTCACGTCATCGACAGTTGATGCTGCCTTCCTATTTCGGTTTTTC
CTTCCTCTCGACGTACAAAGAGGTGAAGATAG
TCCTCTTGTCACCTTCGGATTTTTCTGCCTCCACACCTCGTAGAGTTGCTGTCGTCGTTTTTCCTCCT
CCCTACGGAGCTTTAAGAGTTCTTTCCTCAAG
TTTGCAAGCTCCGTCTCCAAGTCAACGATGCTGACATCAGCTACCCCATTCGTTGTGAGGTCACCGCC
ACGTTCCCCGTAGCCTAAAACCACAAATGGAG
ATGGGGGTCGCAGCATTTCTTCCTTTCCACCTCCCTCCTCAGTGGCCCTCAGGTGGTTCTGCGCCACC
ACCTTGACCTCCTCTAGTCCTTCCTCGTTTGG
TATGGAATTCATTATCTCCGCCATGGAGGAGGCTAAACTGACCTTGCTATTCCCATCTTCTCTGGAAG
CATCGGAGGAGTGTGAAGTGGGGTCTCCTCCG
CGGGGCCCTGAGCAAGAGGGAAAGCATTCCAAATAATCCGTTTTGCTTACTGATTGAGAATAGTTCTG
CTGCGAAAAGGACGGGGATGAAATGGCACTCG
GACCATGGTGAGAGGGCGGCATATACGCCTCTCTCTCTCACACTCCCTCCCTCCCTTCCTATATATAT
ATATATATTTGTTTGACTTCAGGACAAAAAAT
AAATAAATATCACAGACTCCTGTTTCCTCCTTTCCCCCTCCCCCTCCTGGAAACAATCGGTTGGCAAA
AATAAATTCCTCCTCTTTCGTTGCGTTTCACA
CCGTAATATGGATGTGTATATAAATATTATATGCGGCCACAGACCGATATATAATAACTTTTTTTTTT
TTAGTGTGTATGTGTGTTTTTGATTTTATTTG
CTTTTGCCTGAGAAAAAAGCACACAGACACAAAAAACTGGGCCAATTGTACTCCTTTACAGATATTCT
CGTCACTCCTCTATCTTTGCTTTCCTTTTCTT
ATTGTTTTTCTCTCTTTTGTCTCCTTCCTCTACAATTCTCCTTGTGCTGCTGGCTGCCCAAGGGACAA
GCAATTCCCCGCTATTCTCCTGCAGGACCAAA
ATGAAGCAGGCACTGACGACAGCTGCCCCAGGGATGGAAGAAGTCACTCACCACCACCACCACCTTTC
GTCTTGACGGTCTTTGTTTGCCCCCCGGCAG
CGAACCACAAAAGTGAGTGAGAGTGAGTGAGTGAGAGAATCACAAGTGATGGCCTCTCTCCGTGTGGA
AGAGTTTCCTGGGCGGTAAACAGACAACAGTC
CTGAAACAAAAGAAGTCGATGCAAATGAAGAAACAAGCAGAAATCCTCTGAACGCTGAACACGTCTAC
TTCTTTTTCTTTTTCTTTTTTCCCTCTCCTTC
CTGCAGAATACCTGCTACCACCTCCCTCCACCAACCTTTTTTTTTTTTGTTTTCTTTTTTTCTTGTTG
TTGTCCTTTTTTTGTTTTTCTCTTGTTTTCCT
TTTTTGTAGCCTGACTTGGATGCCCAGGCCAGGTAGGTATAAATAATGGCCAACCCTTTTTGCAGAGA
TCAATGCCTTGCGGGATATTCGTTGTGATTTT
TTATTTTTATTTTTATGTGCGTGCAGAGTGCAAAGCGCACATAATGATAATAATAATAATAATAAAGA
AATTGGAAAAAAAAAAAAGAAAAAAAAAGCgN
NNNNNNNNNNNNNNNNNNNNNNNNNNNNNNNNNNNNNNNNNNNNNNNNNNNNNNNNNNNNNNNNNNNN
NNNNNNNNNNNNNNNNNNNNNNNNNNNNNNNN
NNNNNNNNNNNNNNNNNNNNNNNNNNNNNNNNNNNNNNNNNNNNNNNNNNNNNNNNNNNNNNNNNNNN
NNNNNNNNNNNNNNNNNNNNNNNNNNNNNNNN
NNNNNNNNNNNNNNNNNNNNNNNNNNNNNNNNNNNNNNNNNNNNNNNNNNNNNNNNNNNNNNNNNNNN
NNNNNNNNNNNNNNNNNNNNNNNNNNNNNNNA
TAATAATAATAATAAAGAAATTGATAAAGAAATTGGAAAAAAAAAAAAGAAAAAAAAAGCGAGAGAGA
GAGAGCGGTGAAAAAGGGGACATGCGGTTTTG
GGCTGTCAAGGGAGACAACATCGTTCAGTGCAGGAGAAGTACAGAAAGGGGGGAAGGGAAAAGGCGAG
GAGGAATCCCATTCGCCAAACATCTCGTGTAT
TCGTATGCACATAAACATTCACAGAAATAAATAAAGACAAACAACAACAACAACAACAACATAATCAC
AAACAAACCATTAATATCAAATAACAAAAGGA
AAGGAACAACACGCACAAAACAAAACGATTTAAAAAAAAAACCAAAACAAAATAAATATGACTCATAG

TATTTTTGAGACCGGTAAATATGCTTTTGACC
GTTCAAAGATTTGCTTCCGCGTCATACGCGGCGTGCCTTTCGCATGCACCACGGATGCTGCAAGGAGT
GCCTCCGCTTCCTTCCTTGTCGTTATTCGCCT
GCTCTCCACGCTCTCGACACTAACACGCCACACAGTTGTCGCGGCCGGTGGTACGTTTTCTCCCCCAT
CCGGGCCGTACACCTCCTCCGGTGCGCATACG
ATCTCCCACTTGTCACCTGTGCACATCAGCGACACAACTTTTTGAAAGCACAGAAAAGATTGCTGCAA
ATTATGCCCTTCCGCTTTCTTTCCATTCACAT
AAGTTGAAAAGAACCGGGTGCCATCGGAAAGAAAACCCACAATGTGAAACCGCAATTTCCCTTCTTCG
CCAACACACCTCAGTTCATTTCTAAGCTCACG
CCACTTGTCGTCTGTCTCGTCTTCGTCGCCGCGCCAACGGTTGTCATTGTC
>scaffold1055.size7594
TTTTTTTTTTCTTTTTCCTTCTTTCACACAAAATGGATTTTGAATTTTTTCGCACCATTCCTCTCGTG
ACGAGATCCTTCCTCCTGTTGTCAGTGGCTTCC
GTCATGCTAGTTTCCTTTGGTGTGGTTCATCCCGTAGAAGTTGTATTTAGTCCTCTTCTTGTGTTTCA
AGAGAGACAGTACTGGAGGTTGATCACTAACT
TTTTCTACTTTGGGCACCTTGATCTAAATAGCATCTTGGAGCTCCATTGGTTATGCGTGGTAAGTAGC
GGCATCGAGTTGCAATACTTTCGGCGAAGAAA
AGTAGATTATTGCATCACCCTTTTTGCAGGAATGTCTCTTCTTCTACTTTTTCGGTGCCTTAGGGTCG
TGGATACTCCCTATTTGAGCTTCTCACTTTGC
AATGCTTTGGCATATCTTTTTTCTCGTCTAATGCCTGAACAGGAAGCGAACATCTTTTTACTGGTGAC
GATTCCAGTACGACTGTTGCCGCTTTTTTTTC
TCGCTATCGCCATCATTTTTGATATGCAGAGAAGCATCAGGCTTATTGTTGTGGAAAACCTTGTTGGA
CACATTCTTTGGTACTTCCTCGAGATATTTCC
ATGCATTACAAGAGTGCATCCATTGCGGTTGCAAGAAATGTTTATGCAGTAAGGCAAAACGAAAGAGA
AAAAAAAAAAAAAAAGGGGNNNNNNNNNNNNN
NNNNNNNNNNNNNNNNNNNNNNNNNNNNNNNNNNNNNNNNNNNNNNNNNNNNNNNNNNNNNNNNNNNN
NNNNNNNNNNNNNNNNNNNNNNNNNNNNNNNN
NNNNNNNNNNNNNNNNNNNNNNNNNNNNNNNNNNNNNNNNNNNNNNNNNNNNNNNNNNNNNNNNNNNN
NNNNNNNNNNNNNNNNNNNNNNNNNNNNNNNN
NNNNNNNNNNNNNNNNNNNNNNNNNNNNNNNNNNNNNNNNNNNNNNNNNNNNNNNNNNNNNNNNNNNN
NNNNNNNNNNNNNNNNNNAAAAAAAAGGGGACC
TCTTCAGCGCCGTGTGTGATGTGGTTGTTCTGCGGGTATTCAAGCTTTTACACGTTGTCATATACATC
TGTCTCTCTCTCTGTGTGTGTGTAGATGCACC
ATCGATGTGCTGTTTGTTGCTGCGGGTTAACTTTCCACGCGGAGGGTGCCGTTGTTCTTGGTGTAGTT
GTATTTCCCACCATCTGTGGTTTGTGCGCCTT
TGTTCATTGCATTTTGGATATGCATCTTGGTTTTGAATTTTGAAATAAATAATGATCACTCCCCCGGC
AGAAACCCAGCAACAACAACTACAAAGGGAGA
AACAGTCCCGGGAGCAGGGGGGTGGACGAACACGCACAGCAACACTAAGAAAAGATAGGAGCTCAAAA
GAGAAGGGAAGTGAGGGCGAGTGTGTGTGACA
AGTTATGAACACGCTATCGGTTTGGTGCGAACGACAGACTGCATCCTTCACTAAAGCCTTTAAGAAGC
GGTATGTTACGTTTGATCCGGAGTCTCGCAAG
ATATCGTACGCCGAGAACGAACAAAAACCCCCGAAGGGTGTCATCATTGTCACAAAGATAGTGCGGTG
TTGTCAGGTGATGGAAGTGAAAACATCTGATT
TATTTACACTGATGGTCGACGGGAGCCATGAAGATGGGAAAGAAGATCAGTGGACTCTCCGCATGCCA
AATCGTCAAGTTTTTGAGGAGTGGACAGAAGT
CATCGGAAATGTATGTGCAGCCGCTGGACTGATGCAGCCGTTGAACTTTGGACTTCCCTCTATCGATC
CACGAACAAATCTGCCTTTCGCTCAAGTACCG
CTGGAGTATCTATTTAAGTTTGCAGTACTGGAGAAGGCTGTCATTCACTTCTTTGGTACTGTCACGCT
TGTGACATCCACAACGGGTAAGGATAATCATC
CGGCCCGTCATAATCTCGTGGTAGGGGATAAGGCAATTTACATTTTTACTCAGAATGCAACTATTTTG
TATTGCAGCCTTATCAGTCATATTCGAAGGAT
ATATCATGGTCCTGAAGCAACCTTTTTTGCCGTCCACGTCAAATCACCAGCGCCAGACATTGTGGTGA
AGGATTTTTCAGAAGGTGCACAAGTGGCTTTT
ATCCTTGCTAGGTTGTTTCATGTGACGACTGGTAAAAGTCTCAGCGTCATGCCTATCAATGTTCCCAC

CGCTGAGGTACTTGTGGAGTCGCAACAGCTGC
GGCTGAATGGGGATGAAGGGTATAAGCTTCACGTTGTTTCACCAACTACCAAGTTGCGTCTTCGTCAG
GTGATTGATACGAACAAGGATGGAAGGAGTCT
CAGCGGCAGTGTAAACATGAATGCAAACGGGGGAGGTCAAAAACAGGGCGTTGACAAGGCTCTCGCCG
TTGAACCCACCGTCGTTGTGCCCACAGATCCT
CTTACAACACTTCTCCATAAAATTGGACTGCCAGAATACGCTCCAAAACTGTTGAGTCAACATGTCGA
TTTAGATGTTTTGCAGTGTATGGATGTATCCG
ATCTAATGACCTTTGGTGTCTCAAATAAATCCCATTGCCAAATGATTCTCGACGCCGCTTTGAGTTCA
GACACGGCGGATCCCGAAAATGTGGACGATGA
TGCGTTGGATGCTTTTGACCCGAAGACTGGATATCCCTCTGGTAAACCCGTGGGTGGTGTAACACTAA
GTGACGATGAGGACGATGAGATTGATCTTTTC
GTCCCACCTCAGAGGACAGTGACGCTGGACGAGGACTCGGATGAAGAGCTTCTCCCTTCAAGAACCCC
TGCTGTAAACGTGAGCAAACCACCAGCTATCG
TTATTGATGATGATGACCTTTAATATTACTAGCGGAAAAGAAATTGTTTTGGATATCTTTTGATTTTG
TTTTTTACATTTTACTGAACTAGTTGTACGTA
CACTCATATCGTTTGCTATTTTTTTTTTTCAAACACGCACATACAATCTCTGTGTGCGTATAGAAAAA
GGTTTGTTACCCCAAAAACAAAACAAAACAAA
ACAAAAAAACGACAAAAACAACTTTTTCGCGGTAGGCTCATGCGTGGGCGACCAGAAGTATTTATTTA
GCTTGTTTTTCTGTGATTGTTAGTTGGTTGGG
GGTGGGGTTGCGCTGACGGACGTTAATTGACCTTAAGGACAATTGTTTATCTCTTTGGTTGCCTACAC
TAATTTATTTATTTATTTATTTATTTGTTTTT
TGGAATATTAATTTAAGGTTGTTTTGTAATACGGATGTCACGCGCGTTCCTAGAGCGTTGTCCTAGAA
GGCATTTGGTGATACATATGGACATCAACAGG
ACAATTATTCAGGTTGATTCCGCTGGCCAAAGAACAATGGAGGATGCCCTTAACGGCAACATTGCAGC
AAATGTATGGGGACGCTGCGAAGAAGACAAAT
GGGTGGCTGTTTTGGGGCCTGGAGAAGAGGGGGATCGTTCTGGCTTGGTAACCTTCGATAAGTATGTA
GATAATTCCTATACCGAGCCTCCTTTGATGCA
AGAACTTCCAAAAGCCGAGAGAGAGTGCATATGGCGTGATATTTCAGCGAGGAGGAGGTCGGTTGTAC
GGACATTTACTCACGCTGGTCAGCCAGGCGAA
AATTACGCGCAGCACGTCGAAGAACAGAGGAAAGTGTTGACCGCAGCGTCTAATTGTTCCATGGTTCC
ATCCTTTTTCCAACTTGTCAACACACTCTCGG
AGTTAAATTGGTCATTCACCATGATTTTTCGAACATTCGGTCATGATTTGGCTAATGTGCTGCAAGAG
TGGCGACAGTTTCTTTTTGGCGAACATGCTCA
TAAGCCGCAGGGTGCTTTGCTTAGGCGGATGAAGGAGAAATACGTTCCTGAGATGACGGGGTGTATTT
TTCGTGCAGAAGATCATATTTTTTTCTGTGTT
GGGCCAGACAAGGCTGCCGTGGTGCATCATCCAGAGGGGGTAGAAAAAATGTCTCCTTCTGAGGTCCT
GGCGCAGCTATCGGCAATGCCTTCCTGCAAAG
AGGTTCATCAAACAAATTTTATGCAGCTTCATGATCAGATCTTGGAGTACACTTCGGCATCAAACAAT
GTTGGTGGAATAGTGGACTACTATCCGTTTTG
GGCCCAAGGAGCCGAACGGCGTTCTGGAGGAAAGGTGTTTCCCGTTGCCATTACCTCTTCTTCCTGTG
TTACGGCCCCTGTCACGCCGCGGTTCTACGTT
TTCTTTGATGACAACATCTTTATTGGGGAGGAAAAATCAATAGTGGACCTGAGAGATATAGTGACGGG
GAAAAGTATCACTGATGCTGCAATAGAGCGTA
AATATTGCGTTGCTGTAAATCCATACAAAGCTACTGTTGATAAGGAGTACTTTGTGGATTGCTTGGCG
GGGAGAATCCGATTGCAGCTAGGGGAGGATGA
GATATGCATCGATTAATCTGTTCCTTGAGGGAAGTTAAGTCTAAATGAATTTTAATGGGTGGAGTGGA
ACGAATTGTGTAACTTCGTCGTTGACGTACAC
ATTGTAATTCTTTTCTTTGCATACTGGCTCTTTTGCTGATTCACGCATTAGGAGGGGAAGGAGAAGTT
GACAGCGTTGTTTGTGGAGGCGCGGAATTTTG
AAACCAGCACCACCACCACCACCAAAACGCTCATTCCAATCATGACGGAGTATAAAAAATTGTGCGCC
ATTCTGGCGCAGCTTCGGGAGGAAGTTACATC
GCTGGTCCGTGCTTTTGAAGGTGGGGAAGGACGGGACACCGTTGCGTTGCATTCATTATCGACTTCAA
TTCAAACACTAGTTACAAATGCACAGCCTCGT
TTGTTGAAAATACTTCGCAAGGCCACTGAGACGGACCCTAATCGCCAAATCTACAACGAGGCTATGTG

TGCAGCGATAAAACAGCTATTTGACGACTTTT
GCGAGTTGTTGGGTTGTCTCTTTGGGGTGCCGATGAAGGAGATGGTTCTCTCGGAGGGTAAAATAAAT
TTTGAGGATTCCCCATCCATTTCGTGGACGGA
GGATGTGCATAACAACTATTTGCTACACCTTGCGCAAACAGAGGCATGGAAAAAACGTATTGCGGCGA
ATATTGCGGACTTGGTGTTTTTTGAAGAGGAA
ACGCGCGCCGTCTATTTTGCGGAGGAAAGGAAAGCACGTGAAACATTGTTGCAGACCAAAAGAAATGA
GAAGACTAATATTCTGCACATGTTGAAGGAGA
GAGAGGCGGCAAAATGGGAGGCTGAGGTGCGGCGTCGCAATGATGAACACAAGGGGCTAATGAATGCC
TCTAGTTTTTACGGGGTTCAAAACATTGCGAC
GGTATTGTTACGTGTTCCAGAGCCGTTTCGGAAGCTTCTGGCGGGAAACATGGCTCAGCTTGTACGAG
CTCTCCGCACAACACCAGAGGACCCCAACATT
CGACGAATTCGATGTAACAATAGGAGGGTAATGATGGATTATAGTCATATTGTCTTCTGTGTTGAGTG
CGAAACGTGTCGGATACTAGTCGCTGCAGCTG
AAATTTTGTGGTATATAATGGGATATCGGGTGGAGTACTCGACCGCACCTACATCTTCTCTTCGCACT
GTCATTGACAATAACCCACCGATTCTGTTGCC
ATGTGGCCGTTACGCATCAGAACATGCCATCGCGGTGATTGGTTTTGAGGAGTACTCCGAGCGTTTCT
TCACCCTCCATGAGCCCGATCCCATGCGGGAT
TCCGATGAGTGGATGGTTTGGTACGCAACGCTGGAGGCGTTGATAGCACGTCTTGAGGATTGTCTAGT
TTAGTCCAACGCCGAGAGAGAGAGGGCCGACG
TTTTTCTGAGGTGGAGTTTGGGGTTTTTCTGGGGGCTGGGGCGGTGAGTTATCAGACATATGTTTCGA
TTGCAGGGATCACGTGCGTTCTTTTTTATTTC
TTTGTGATTTTTAAGGGAATTTAAGGTGTAGGCTACATTCTTTCCCCTCCCCTCTCCTGTAAAAGATA
AAACTCTGTAATTTGATTTCCATACACTTTTG
AGGTGTGAGTCATAATAATAGCAGTAGCCACGGCTATCAACAGTACACACCTCTCACAACCTAAACAA
CAACAAGAAGAGGAAGAAGAAAAGAAAAAAAA
AAAAGGACGGAAGGATCAGTCTGTTAGCGGGGTTATGTATGGCACAAGCAAAATTCAGGGAGGGGGAT
TAAGAGGTTCTCTGCATTATTGCACCCAGCAG
CGATTTCTTCCTTCAGCGAATAGATCTCCCGGCTTCGTAAATGCGCGAGGAAAGGTAACGGCCAGCAA
AGCGGCAACTTCCTCCGAATACCACGCCGCCT
ACTTGACAGAGTACGAGAAAAGCGAGATTCGGGGATACAAAGATGTATACTACGTGGGACAGAATTGT
GAGCGGAAAAATTCAGGCCCCAGTGGAGGGCGG
CTACAACTGCGGCTACGATGACGAACGAGGGGATTACACAATAATGTTGCATGATCATATAGCCTACC
GCTACGAGGTGCTGGGCACTCTGGGGAGTGGA
TCCTTTGGTCAGGTCGTTAAGGTCGCCGATCACCAGAATGGCACTACAGTTGCACTGAAGATTATAAG
AAACAAGAAACGCTTTATGTCACAGGCAAAAA
TTGAAGTGCAAATTCTGTCACATCTTCGAAGGGGCGACCCTAATGGAATTTACGGAATTGTTCAAATG
CTTGACAGCTTCACTTTTCGGTCTCATGTGTG
CATTACATACGAGCTTTTATCTATCAATCTATATGAGCACCTAAAGCAACGCAATTTTCATCCGTTAT
CGTTGAGCGCCGTCCGCAAGATTGGGGCCGGA
GTATTGGTCTCGCTCTCTTATATATGGCGTGAGAATATTATCCACTGTGACTTGAAGCCAGAGAACAT
CCTCCTTAAAACGCCTGATAGGGCAACGGTGA
AGGTGATTGATTTTGGATCTTCCTGCTTTGAGAACGCTCGCATCTATACATATATTCAGTCGCGTTTC
TATCGCGCACCCGAGGTTATATTAGGCTGTTC
ATACAACAAACATATCGACCTTTGGAGTTATGGCTGTGTGCTGTGCGAGCTAGCCACAGGAGTTCCCA
TTTTTCCGGGAGAGAGCGAACAAGATCAGCTG
GGCTGCATCATGGAGTACCTTGGAGCCCCTCCACATGAGCTGATACTTCAATCCTCACGCAAGCAAGA
GCTATTTGATGTTAATAACGGTTATGCCCCAA
AGTTAGTTCCCAACAGCAGGCGGAAAATACGATACCCAGGGACAAGGTCCCTCGCGGCGTTTCTCGGT
CTTCCTCAAGACGATGGCTTTGTTAGTTTTGT
CAGGCAATTTCTGTGCTGGATCCCGGAAGAGCGGGTTTCCCCGCGTAGGGCGATGCGTCATCCGTGGA
TTGCCGACATATTTGAAGGACCGCCACCGCCC
TTGTCGCAGGTGAGCAAGACCCAAACAAATTTGGCAGTACGTGCGGCCATTTCCAATGCCCACCGCAT
CACAGGGCAAGCTTTGAAAGTGCCGCATCTCC
CAAAGATTGGTGAGCACTGAGGCGAAATAAAGCTCTTATTTGGGGCGTTTCTTTTTTTCATTCTTCCC

CTCCTAACCTTGTTGTTATTATTTG
>scaffold1056.size7115
CGCCCTCGCACCGCCGTCGCTCGGCGCAAGATGGTTGGCCGCGCTGACCAGGCACAGCGCCGCAAGGA
GCACAAAGCTCGCAGTGTGCATCACCGTCAGGA
AGGACGAGCGCATCATGTTCGTGAAGGCAACAACGCCCGCGCCCGCAACCAGCACAGCCGCCATGCAG
AAGTACTGGACGTGGCAGCCTCCCACGGGCGA
CTGCACAGCCGCAATCAGGCCCACCACACACAGCACCGACAGCTGGAACACGCACCAGTACACGTGGC
TGCCCCTCATGTTCGTCAGCATGCCGCCGTAC
ATCCGCTGCTGCGCCGCGGGGTGCCAATACCCGACAGGGTACAGCAGGCGCTCGTGCAGCGGCTTCCT
CGAAAACTGCCAGTACCTCGTGAAGCTCGCGC
CCGTGTGGCGGGCAATCAAGTAGCACACGCCAGCAGGAAACGCCGCACCGTACAGCACGCCAACGACA
CCAATCACGCGGTGCCACACGCGGGCACCGGG
CATCGCCAGTGAAAGAACGGAGCCAAAGAAGATGCCGAGATGCATCGCGTGCGCCACGACGTACGTCA
GGCTCGGGAAGCGCATCGCCGCCCACGCAGTC
GCCGCGTCCACGCCGCGCCACCGCTGGAAGGCCGCCGTCACACCGCAGTGCACGCACCCGAACACAGC
GGCGAGCAGCGCGTTGCCCACCACCATCCACA
GCGGGTCAAGGGCAGCGAACACCGACAGGAAGTACGGCAGCGCAACGGTGCTCGCACGCTCCTGCGCC
AAGGCGCATGGCATCCTCGCAAACACGCCGAG
CGCCTGCATCTCCAGGACGGAGCCCAGCCCGCCGGTCACCACCACGGCAACAGCCGCCGCACCCGCAA
CAACGCCGGTGATCGCCGAGAGGGCGCTGGTG
GCGGCGGGCAGCGTGTCGGACCTGATCGTGAAGGACCCAAGCAGCACACCCCTGCAGCCGCCGGACAC
TGCCGCAGCGTCGCAGCGAATGAATATCGTCT
CGTCGGCAGCAATGAAGTAACCGCGGTGCACGGGAACAGCCAGCTCCAGGACGGCGGACGGATTCGTC
GCGTTGCGCATCCACGTCGCGTTGCTCAGCAT
CGCTCCCCACGGACCGCCAAACCCGCGCGGCTGCGCCGTCGACACCGGCACGAAGCTCAGGTGCGTGC
CGAGCTGCGGGTCGCTTGCCCAGCGGAACGGC
GGCGGCAGCGCCACCATCAGCCATGTCGGGACGGCGCTCCCACCGCCGCGAATGTCGTTCTGCGTCAG
GCTCCCACCAGCCGCCGTCGTCGTCACAGCGA
GCGTCGGGCACGCCAAGTCGCTCCACCACAGCGTGCTGCTCACCGACGCCGTCGGGGTCCGCGTGGGC
GGCAACGCCACCGTCTCCGTCACCTGTAGGGT
CTTCGTCGGCCCGTAATGCGTCTGACTGTAGTGCGTCGTGCTCAGGCTCGGCGTCCACGTTGAAGTCA
GGCTGCGCGTCTCCGTCAGCGTCGCCGTGTGG
CTCAGCAGCGGCATAGCGGGCGTGCGGTTGCACCCGTCCACGTGCGGCAGGTACACCGGCAGGCAGTC
GCGCCCGTACCCGCCATCAGCACAGCGGCAGC
CGCACGACCCCGACATCGCCCTCGTCGCAGCACCAAAGCAACTCACTTCGGCATCGCACACGCCACAC
GCCACGGGGCGGAATTCCCCGATGATGCCGGC
GGACCTGTACTCCATCGGCTGCAGTGTCTTCCCGTTGAGCGTCAGGCACCCCACAACGAACCTTCGGC
CGGCGCCCCCAAGCACAACGGTGCCGTCCATC
ACGACGGGCCCGCTGCCAGCGTTCCCGTGGAGTGTCAGCGTGCTCTGCGCGAGGTCCGCACTGCGCGG
GTACGGCGGCACCGACAGAAGTCTGCCGCGGA
TGCTGTTGCCGCGCACCGACAGCCACGACCCGGACGCAAACGACGCCGACCCCCGCAGCTCCAGCAGC
GCGCCATCGGTCAGCGCACCGGCGTTGTCGTT
CACCACGACGGCGGACGCGTTGGCCTCCACACCGCGGTCGAACACCAACCCATGCGCGGCGTACACCT
GGCTCCCCGACACGCGCAGCACAGCGCCCCCA
GACGCAACCACGCGCGCACTCGCGTACAACGCATATTCGCCACCGAGCACAGCCGCGTCCAGCGCAAC
ACCGCCACCGACAAGCTCCAGCACAGCGCCGT
CCACCACCACCGTCCGCCCACCAGTCATCACGGTCACGAGGGCCACGCCGGACACAACCAGCACGGAC
CGCACGAGCCGCAGGCCAGACAGCACCAGCAC
CGGTGCGTACGGCGACGACTGCGAGCCGGGAAGATACACAAGCGGCGTCGGCCTCGTGGCAACCAGCA
GAGAGTCCGTCACCGTCAGCACAGACCCCGCG
GGGAACGTGCCGGTCAGCACAAGCGCACCGTTCAGCGCCTCCACATCGCACACCGACAGCTCCACCGG
CCCGCCCTCGTCGCTCCCCGCCGCCACCGCAC
CGCGCAGCCCTCCACCGCCCATCACGTACAGCACGGCACCGTTCAGCAGCGACACGTTCTGCACAACA
ATCCGCACGCCGTCTCGCGCCATCCACGGCAC

GTAGAGCACAGGACTCACGCCGTCCAGCACAACGTGGCGCAGCGTCACCTCGCTCGCACCGGCAGGCA
CAACGAACACCGACCGCAGCGTCACGCTCTCG
CGGAGAAACACACTCGACGCAGTGCCCGCAGCGGGCGGCAGCGCGGGAGCTCCCACAGGCACGCACGC
CTCGCCGTGCCCACCGCTGCCGCACAAGCACA
CGCACACACCGTTCCTCATGCTCGCAGACGCCGTCCCGGGCGCGTAGCAGTGCGTGTCCCTGTCGCAG
TCGTCGCACCCAGCAACGTCGATGCTCCCAGC
GTCGCCAAAACCAGCCGCAGCATACTCCTCCGCCGTCGACAGGACACGGCCACCGGCGTCGTTGCACG
CCACCCTCAAATGCGCCCCAGCCGCAAGCGAC
AGGCTCAGCGATAGCATCCGAGGCAGCAGTGCTCGGTTGCGCACAAAGCTCAGCGTGCTCCCAGCAGC
GGTCACGGCGCCAGCGGCGTGGAGCATCTCCC
TCGAGACACCATCGTGCACGTTGCCCTTCGCCAGCAGCATCGACCCACCGGCCAGTCTCACCGTCGAA
TAAAACGCCACCGCGTAGTCCTCCGTCGACGA
AAATTCGCTCCGCACAAGCGCCACAGCGGAGCCCGACACGCTCACAGCCCCACGCACAGACACCGCGT
GCCTGCACAGCAGGAAGTCGCTGTCAACAAAC
GCCACCACGGAGCCGCCGCTGGCAGCAACACCGCCCTCCACCGACACGCACAGCCCCAGCGCCGTCTG
CACCAACAGCCCGTCCACGTACAGCGCACCAC
GCGACGACAGCGCCACGCCGCCAACAACCAACACGCCAACAGCGTCACGGAACACAGCCACCACCTGC
GTGCGCGCAATGGTCAGCACGCTCCCAGTCAG
TTTCACGCTGTGCACCACGAGGCACGCGGAGGCGTCGCCAAGGCCGTAGGCGCCGCCGTACACAACGC
GCTTCTCCGCAATCAGCACCGAGTCCACCACA
GTGACGCGCGAGCCCGGCGGGTATCGGTTCGCCACCACCACGCCGCCGCCGGAGCGCGACTCGAGCCC
GCTGATCAACACATCGGCGCGGTGTCCAGCAG
GCGGGTCGGACCGCCACCCAAAAACGTACAGGCTCGCTCCGTCCAAAAACGTGCAGCTCGCCAGCGTC
ACGTTGCGCACGCTCCCTGACATCGACTCCAC
GTCCACCACGACGTCCGCCGCAAAGGTCACGCCAACGTAGCACGCCAACGACGTCCCGAAACTGACGT
TCACCTCCACATCCACACGCACGTCCCGCACG
CACAAATCAGCGCCGTCGGTGCTCGGTGGCTCGGGGACAGCAACGGGCAGGCACGCATCGCCGTAGCC
GCCCTCAGCGCATGCGCAGGCGCAGCCATCAT
TCGACGCCGTCGTCGTGTACGCGGGGAAGCACGACGCCGCAAGGGTGCATGGGTCGCCGCAGGTCAAA
ATGGTGACATCGTACACGGACGGGATGATGTA
TTCCACACCCTCCTCGCCGTTCACTGTGTTGCACGCAGCCACAATCGCTCCGTTTGTGATATCGCGGG
ACCTTTTGGGTATCTTTATCAATGTTGGCGTG
CCTGTGCCGTACATGAATTGGTTGCCAGACACAGACACCGTGGAGTCGCGGAGATTCACCGTGGGGTT
CAGGACTGCAAATGCATCCAATGGGGATTCAC
GGCAGAGGTTGCCGCTCAGCTGCAGCCAGGAGTTGTAGTAAATGTAGGAAGGGTAGCTCGGGAAGAAC
gcgaccgagCTTCCCTGCATGTCGTTGCCCTG
AAACACAACCGCTGACCGAGACTCCACACGTAATGCCAAAATTAGTAATAAAAAGTACCTCACGGGCC
TCGACACAAGATTGCCTCGGATCACCACGGCA
GAGTTGTTCAGTACGCTCAGCGATGAGGATTGGGCAGAACCCTCAAGATGTATCAGCGTGCCGTCACC
GCCATCCATATGAAACGTGTTGCCCATCACAG
CAAACACGGAGCGCCACAACACCCTCAGCGAAGATCTGACAACGTAGATGGCGCTTGACAATGCCGTC
ACGATCTGAAACACATTGCCGCTGAGCACCAC
CGCAGAGCCGTTGCTGATCACCAGCTCATTCATCGCAACACACGACGGGCTGTCAACGTCCAAACCCG
ACCAAGGGATCAGCCTCGTGACCGTGAAGCGG
TTCCCGCTGATCGTGATGTTCGTTTGCGGTGGAAACCCACCCTCCGGGCTGAGCAGCGCGTCACTAAA
CGTGTTTTCGGTGACGACGATGTGCAGCGCCC
CCACAGCGGCGTACGCGTTGCCCAACAGCAACAGCACGGCGCCGTCCCTCCACGTGCATCCATCGAAC
GTGACGTTCGCCACTTCGTCCGTCATCGCCCC
AACCAGCACCGTCAGGATCATGCCGCGCCCGCTGAACGTCACATTGCGGTAGCACAGCCACGACAGCC
CACCGCCCACAGCCTGCGCCACCTCGGGGTAC
ACCAAGTCGCTGATGCACTCACCAATCGGCGGCGGTGACGGCGTGGGCGGCGCTGGCGGCGTGGGCGG
TGGCGGCGGGCCAGCAGGCACAGGCGCCGGGA
CACACACGTCGCCGTGCCCTCCAGCAGCGCACCGGCACTTGCAGTCAATGACCGCCGTCGTCAGCGGA
GCAAAGCAGTCGCCGTCCTTCGTGCATTCCCC

GCACGCAGCAACCGTCGTCACGTTGAAGATGCTGTTCAACTCCAGTTCTGCCGCCGTCGTCACCTCCC
GTCCCGCGACCGTCAGGCACCCAGCAAAGAAC
CGGTAGCCGGCGTCGGCCTGAGATAGCAGAGGTCTCGACAACCCCGTCGAGCCCATCGTGCAGCCCTT
CAGCGTCACAACACTGCTGCTGTCAATGCTCA
CAAAAGCGGACTCAGTGTCGTAGAACATCGCACCCACTCCCACGTCGTTGTCGCGCCAGTCCAACCAG
CTTGACTGCTCAACAGTCCACAAGTCATCGTT
ACAGATGGCGTATGACACAGAACCGCTGTTCCCCACCACGCGCAACACGCTGTGATCCGACACGCTGA
ATACTTGTTGCTGATAAAGCAGGCTTACCCCG
GAGAACATCGTATTGTTGAGCAGCGCAAAAACGGAGTGATCACTCACACTGAGACTGCACAAGTAGAA
CAGATGCATGTACCCATCGAGGCTGCAGTACC
GCGCGTAAAGCGAGCTGCCACCCACCAGCGCCAGCGTCCCGATCGAGTACAAACCAAACGCATCACGC
TTCGTTGCACGCGCCCTGACGTTGCTGAACAA
CAGCGTCGACGCAGTCAACACAACGTTCTTCAGCATCACCACACTCACCGTTTTGCTCACAGTGCCCG
GTAATTCAACCGCAAAGGTCTGCACCGCGTCA
ACCTCCGTGACGGCGATGTCGCNNNNNNNNNNNNNNNNNNNNNNNNNNNNNNNNNNNNNNNNNNNNNN
NNNNNNNNNNNNNNNNNNNNNNNNNNNNNNNN
NNNNNNNNNNNNNNNNNNNNNNNNNNNNNNNNNNNNNNNNNNNNNNNNNNNNNNNNNNNNNNNNNNNN
NNNNNNNNNNNNNNNNNNNNNNNNNNNNNNNN
NNNNNNNNNNNNNNNNNNNNNNNNNNNNNNNNNNNNNNNNNNNNNNNNNNNNNNNNNNNNNNNNNNNN
NNNNNNNNNNNNNNNNNNNNNNNNNNNNNNNN
NNNNNNNNNNNNNNNNNNNNNNCTGCACCGCGTCAACCTCCGTGACGGCGATGTCGCAGTGCTGCGGG
AGGGCAAGTGCAAACACAACAACGGAGGAACG
CATCACCGTCTGGCTCACGCGGATCAGGACGCCGGCTGACTCCGCCGCCTCAGCACCCCCAACAAACC
GCAGCAAAGCACCCTCAAGAAATGTGCACCCA
GTGAACGTGATGTTGATGGGGAGATGCAGCGGCATACTGTTGAGAGAGAATGTCAACACAGCACCCAC
GCCGCTGAATGTCACATCCACGTAGCAGTGGT
GCGTCTTCCACATGTTCAGCGCGAGGCTCGTCAGCGTCTGGTTCACCACGCAGCTCGTGTCACCGTCA
ACGGCTCTCTCCGGCAAAGGCGGCACAACCGT
GTTGGGCACCTCGAAGGGAAGACACGACGCGCCATGCCATCCGTCCTTGCAGCTGCACGAACACGAGC
TGCGGTCCACCGACTCCGTACCGGGCATGTAG
CACGCAGCGTCGTCGTTGCATGTGCCACACCAAAACACCACCACGCCCTCCGGAAACACATCGTCGTA
CGACACCTCCTCACCGCCCTGCAGGTTGCACC
CAAACACAATCTTCGCGGGTAACTCAACAATCGTCTGTGGCAGAGAAAGATCAGTAAAGGAATAATTA
TCGTCCATCTGAAGATTGTTTGCAAGCACCAC
GGTAGTGCCGCTGTCCGACAGCTGAATTTTGTGGTGGGCATGTGCTATAATTAATACTGAGGCATCGC
CCACGATGTTGCCCTCCACACGCCACTGCGCG
CCTCCTTCGAGAGt
>scaffold1057.size7204
ACACGAAATGGAACAGGCCGAGTCCGCCGCCGAGGCAGCACACGAAGCACACACATTGCCTCACCCGG
AGACAGCCTACTTTGAGCCGAATGTACCCGAAA
AAAGGGAGGAACGAAATCAAGCCTTAGGCCGTTGCTCCGCTGTGCTTCTGGAAGCCCGCGCGAAAAGA
CTTGGAGCCTCACCCAGCCCGAGGTGGCGATA
TCTTCACGGCATGGCGGCGCCGCACCCTCATCCTCTGGAATCGGTTGTGCTCCGCACTTAGCTTGGCC
GCGTATGCGCCCTGCCTCGGCAACAGGCAAAT
CTGCTTGTCCGTCATTTTGTGCGGTTTCACGTGCCATACCGCCTTATACTGCTGCTGCTGCTTGCTGC
ACTCCTTTGGTTCCTGGTGGCTGGGAAATGGA
TCGGCCTTTCCCGCCGTATGTACTTGACGTAACCATCCGTGACTCTTTGCTTGGCTCAGCCCCTGGCC
CCGACGACATGTTGAATGAATTCCTGCACCGT
CTCGTGCCCGTTGCGCGTGGCACACTTAGAACGATGATCCACAACTCCTTCGCAAACGGCAGCCTGCC
TGGCTCCTGGAAAATGGGAGACGCTATTTCTA
TCCCAAGCCTGGGAAGGATCCATGCCGCCCAGAGAGTTACAGACCTATCACGCTGCTCTCTGTTTTAC
TAAAGTTAGCCGAAGGAATGATTCACCGCCGT
CTGTCAGCGTTATTGCCGCACCACCCACGTCAACTTTGGATTTGCACCTCCGCGTTCTACGTCGGACG
TGGTGGCACCTGTTATCGACAAATTACTCGTG

GACTGAGTGANNNNNNNNNNNNNNNNNNNNNNNNNNNNNNNNNNNNNNNNNNNNNNNNNNNNNNNNNNNN
NNNNNNNNNNNNNNNNNNNNNNNNNNNNNNNN
NNNNNNNNNNNNNNNNNNNNNNNNNNNNNNNNNNNNNNNNNNNNNNNNNNNNNNNNNNNNNNNNNNNN
NNNNNNNNNNNNNNNNNNNNNNNNNNNNNNNN
NNNNNNNNNNNNNNNNNNNNNNNNNNNNNNNNNNNNNNNNNNNNNNNNNNNNNNNNNNNNNNNNNNNN
NNNNNNNNNNNNNNNNNNNNNNNNNNNNNNNN
NNNNNNNNNNTCCATGATCACGAGGATTTGCGTTCCCTTATCTACTCGGAACCTATGCCTTCGTCCAT
GCATGGGAAAGCGGCGACATCGATCCGCTTCC
GAGAGATGCTGCTATTAATGGATTGCTACGCGTCTGTGACATTATTGGGACACCCCACAACCACATTC
GCGCGCCTCTTGCCCAACATCGAATTTTCCCC
TGGGACACTGTGATCTGCAACAACGTGAGATTTTATCAGCCACTGTTTGCCGAAGACGCCTCTGACGA
TGTCAGGCGCACGGCTTTTTATTCTACTCACT
CCTCTTTGGGGGATTGTGATTTTGAATTGTGGACGGACGGTCCTTCATACCTCGCTGCACTCGCGTCC
GGATCCGCTGCGCTATTATATGACTCCACCAC
GACAAATGATCTCCCTGTGGAGGTACATCGTGCAGCAGCTGGCAGCCTCGCATGCTCATATAGGGCAG
ACTGCCTTGCGATCGAAAATGTTTTTAGACAT
CTCATACCTCCTTGTCTGGGAGCATCCCAATCGCCCACACGAATGCTGGAGGAAACGGACTCCCTCTC
GGCAATTGAGGCACCCCGCGTTGCCCACCGAC
CGTGCGAGACCGCGTTACTGAGTAAATTTGGATTATGCTGCTTTCCTTGGTGAAGCGAGGTCACCCTG
TGGATTTTATATTTTCCCATCACACTGCGGTA
TTCCACGCAGTGAGGCCGCTGACAAACAAGCCACGATTGCACGATCCCTCCCGCAGGACTGCGTCAAT
GCCTGGCAAGTGGGCTTCCTTACCGCCGTCAA
GAGGTTATAATGGAAGGATGCCCAGGAGGAAAAGGAAGGCGCGCACACCGCGTGACAGGCGCCTGTCG
GTGTGGCGAGGACTCTATTCCTGGTGTTGGCC
CGGACATCGTGCCCGAGTGGCGGCACTGCGCACAGGCGCGATGCGACTCCTGCCCACTCTTCGACAGG
CCTGCACGCGCCCTGCGGATCACAAGGAACCC
TCTGTGTCGGTGGTGCCGTTCCTCCCTCACTGCGGTGACCGGCCCGCCTCTGCCTCCGCCGGAACCTC
TTCCCGACGTGAGCACCGCAACTCCTTGTCGA
CCAGCGAGCGGTGGATTGACGTCCTCCTGCCCAACCTGCGGCAGAGTGCGCTTGTCCGTCCACAGTAT
GAGGGCACATGCCGGACGTGCTCAGCCTGGCG
TTATAATAGTTTGCGAGGGCCTCCCATGCGGATGCCGCAATGGCGCCACCGTCTTCCTAACAGGCGCC
TCGCGCACAGGTCGCCACCGAAGTTGCCAAGC
ATGCCGGCAGGCAAGACACGCAGGAAGCACCACCGCGGGTACTCGAGATGGACCAGGCTCTTCCGCCA
CCGCCTCATCACGGACGCCACGGGCTGCTGAT
CCCCGACGGACCACGGGAGACTCCGCCTCATCCACTGCGGGAATGCCCTCGTCGTTGGGAACTGGGAC
TGCGACTGTGTGGGACTGCCCCAGCCCGCACG
ACGGAATACTTGCCACTTTTGTACGGACGGCCCTTGCGCTACTCACTCGTTATTGTAGCAGCATACAA
CCAAAACAAGATAACAATCTTCTTTGTGGTGA
ATATTGGCCGCACTTCCCGTGGACCAACAATCCGCGTGGTGTGCGGGGATCTCTTCTTTTTTTATTGT
TACTATGCTTTTGTGCTGCTGTGTGCGCTGCA
CCTTCTTGTGTGTGTGCGCACCCCTCCGTGAGGGGTGCCGACACGACGGACCGCTCTCGCCGTATGTG
CATGCGGTGGAGCCTTGTGTGCCGCTCCTGTT
TGCTTCTCCCTCTTCCATCTGTGTGTTGGTGTTGCGGCAGGGCTGTGTGTGTGTGTGCCTGCTGCCGT
GGATTGGAGTGTGTGTCGTGCGTGGTGTGCGG
CGTACGGTGCATGCCTGGCTGCTGCCCTCTCCTCATGTGGTGTGCCCTCTGTGTGTGTCTGCCGCACC
TCTCGTCTCCCCTGTTGCTCTCCATGTCCCTC
ACTGTGCAGATCAGCTCCACACCACTGAACGACCACACTCACGATGATGATGACGTGCCGTCTGCTGT
GCGCCCTGCTGGTGCTTGCCCTGTGCTTCTGC
TCGTCCGTGTTCGCGTCAGAGTCTGAGACGGCTCAGGTTGAAGATATTGTAAGCGGAATCTATTCACC
GTCTCATGTACCAGGAGGACCACCGGGGGTCA
GTGAGCCGCTGCCACCAATTACGTTGTCCACTGCGCGAGGGGCACCAATGACTGTGATCGGTCAATCA
ATGTCGTCAGGTGAGGTGTCCGGTTTGGTACC
GAGAAAAGGTGGCGGAAGGAGAGCAGATGTGGCTGATGCGTTACAGAAAAAAGATAGGATTGATATGA
CAGCGCCAGAGACTTCAGATACAAGTCCGGCA

GATGGTCTGCTAGGGAATCAAGATAGAGAGGGCTTGGAGCCTAGTACAAAAGAGCAAACAGTTCTGGG
GACCGGTGAGGGCGGTACGACAGTATCACAAA
GTCGCACGAGACAGCCGGCAGAAGAAACAATTGAGAGCGTCGGAGGCCTTGCCAAGACGACTAAGACC
ACTAAAATAAAGTCACCAGAGGGAGCATCACA
GGGGGATTTGCCAGCGAAAGCAACACATCTAACACCATCACCGCCAGTGGAAGGACCACTCCCCAATG
CAGATAAGGCGCCGGTGCCGCAGCCAGAGGAA
GACTCAGTGAGCATGAAGCCAAACAAAGATTTGGCAGAAGAGGATATCGCCACCACCGTAGACACAGA
TCAGAATGAAACATCCAGCGGTAGAGCCACAT
CAACCACAACAACAACCCTCGTTGCCAGCAATAATGCAAATAACGAAGCAGACAGAAGCACTGGAGAA
GATATTCCGAACAATACACCGGATTCCGATGT
GTCAGGGACGGAAGAAAGGCGGGATGAAAATAAATATAATAATACGAAGAAAACACCGGTCCAAGTCG
CGGATATAAAAAGTGTCACGGTGGCATCTGGC
AACAGTGACAGCAGCACCGCAGTCTCTCACACCTCCCCTCTTTTGCTTCTTCTTGTTGTTGCTGCTGC
TGCGGTGGTGGCCGCGTGAGAGTGAGTGTGAG
AGAGCCGTGCACCGCCCGCACACACACTCGTCTTTCACTCTCTGTGTGTGTGTCCCCTTGCACGGACT
CTCGCCCTCACCTCACCCCACGAGGCACACAC
ATAATGTGCCCATTGTATATATGCATGCACACCCATGCAGGCCCGTTGGTGCTCTCTTTTGCACGACA
CGCACCGCTGCCGTCCTGCCTGGCTGCATGGG
CGGAGCACCGTAACGGTTTCTGTTTTGTTTTTATTGCATTGAGTGGGCGACCTCTCTCTCTCTCCCTC
TGTGTGTGTGTTTTGCATTACGGGAGTAATGA
GAGTGTGTGTGCGCTGGTCGTCCCACGCTTCGGCTTTTTGTTTTGTTTTCCTTTTTGATTTTGTGGTT
GAGTGCACCCATTCCGCGGCTGCGTGTGTGTG
TGTGATGATATGAGTAATTTGCGTGTGTTTTATCTGCCTCTTTCTTCCATTGCTTTCTTTGGACTCTC
TTGTGCCGCACAGCTGCTCTGCAAGTATAGGC
GCTGCCAAACACACATGCACACANNNNNNNNNNNNNNNNNNNNNNNNNNNNNNNNNNNNNNNNNNNNN
NNNNNNNNNNNNNNNNNNNNNNNNNNNNNNNN
NNNNNNNNNNNNNNNNNNNNNNNNNNNNNNNNNNNNNNNNNNNNNNNNNNNNNNNNNNNNNNNNNNNN
NNNNNNNNNNNNNNNNNNNNNNNNNNNNNNNN
NNNNNNNNNNNNNNNNNNNNNNNNNNNNNNNNNNNNNNNNNNNNNNNNNNNNNNNNNNNNNNNNNNNN
NNNNNNNNNNNNNNNNNNNNNNNNNNNNNNNN
NNNNNNNNNNNNNNNNNNNNNNNACACGCCATTATGCATACTCTGTAGAGCGGCGTGATTGCTGAGAA
CGGATGCACGCAGAAGATTCTGGCTGCGACGT
CCCTTGCGAGGAGGGAGGACGGGAGCCTGCGATTTATTTTTTGACACGTTGCAGGCGTGCAGTCGATT
GGGTGGTGTGGCTTAAGGTGCCTGGGACAAGT
GCGGATTGCTTCTTGTCCAGCAGCTGCACGTGCCAATGTGTATCTCCCATGCTGTGTATACTTTGGTC
AGGGATTCCTTGCCTGTGTGGGTGATGTGCGT
TGGAAGGGACAACAGTAAAATGACTGGCGAGAGCGCGTGGGCTTTGCAAGGGGCGGTCTTGCTCTTTT
GCATTCCGCTGGTTGTTTTGTGTGTAGGCAGT
CGTCGCTCGGTGTGTGCTGACGCGTGGCAGAAGCGCCCGCCGTGACTCCTCCTGCTGGTGGTGGCAGT
CGCTGTGTGTTCCAGGTGCACTGTTCTCCCTT
CTGCTGTTCCGTGTGTTTGTGTGCGTGAGTTGTTGTTGTTGGGAGTGACTGCGGCGGGGATGATGGTG
CCACTCACTGTGGNNNNNNNNNNNNNNNNNNN
NNNNNNNNNNNNNNNNNNNNNNNNNNNNNNNNNNNNNNNNNNNNNNNNNNNNNNNNNNNNNNNNNNNN
NNNNNNNNNNNNNNNNNNNNNNNNNNNNNNNN
NNNNNNNNNNNNNNNNNNNNNNNNNNNNNNNNNNNNNNNNNNNNNNNNNNNNNNNNNNNNNNNNNNNN
NNNNNNNNNNNNNNNNNNNNNNNNNNNNNNNN
NNNNNNNNNNNNNNNNNNNNNNNNNNNNNNNNNNNNNNNNNNNNNNNNNNNNNNNNNNNNNNNNNNNN
NNNNNNNNNNNNNNTCTTTTCTTGGCTTGTTTT
CCTTTATTTATTTCTCACCCTCACAAAGAGGGAAAAAAGCGCTAAACGCTACTGTGAAATTCGAAGGC
ACTGAAAGGACTAACAGCACACACCGCCTACA
CACACATATTTGCATGCTCTCACGTGTTGCTGCAGTGAAGGCACCCGCACACACAACCGTCGCCGCGT
GACCGGACACAGCGGAAGGAGGAGGGAAGGAG
GAGAGAGTGAGCCGCAGAGGCTCAACATGTCCCGGCGTGCGTTTACTTCCGCGGTGCTGCTCCTCCTC
GTCGTGATGATGTGCTGCGCCATCTGTGGTGG

AGCTACGCAAGCTGTGGAGCCGTCGTCTGATCCGAAGTTTGAATGGAAGGACGCTAATGAGGAGGGCG
TAAGTGTGAAATCGTTGGGTGTTCCCGGCCTT
TTGAAAGTGGGGAATGTCGTATTTGCCGTTGCTGAGGCGCAGTGCAAGAATGATGAAAAGAACGTCTT
TACTGGCATTGCATCCCAACTTCNNNNNNNNN
NNNNNNNNNNNNNNNNNNNNNNNNNNNNNNNNNNNNNNNNNNNNNNNNNNNNNNNNNNNNNNNNNNNN
NNNNNNNNNNNNNNNNNNNNNNNNNNNNNNNN
NNNNNNNNNNNNNNNNNNNNNNNNNNNNNNNNNNNNNNNNNNNNNNNNNNNNNNNNNNNNNNNNNNNN
NNNNNNNNNNNNNNNNNNNNNNNNNNNNNNNN
NNNNNNNNNNNNNNNNNNNNNNNNNNNNNNNNNNNNNNNNNNNNNNNNNNNNNNNNNNNNNNNNNNNN
NNNNNNNNNNNNNNNNNNNNNNNTCGTGGACG
GAGGCACTCGGGACACTCTCGCGCGTGTGGGGCAACAAACACAAGAGAGAAGCGAAGGTCGTTAGAAG
CGGGTTCATAACAGTGACTCTTGACGGTGATG
GAGATGATGAGAAAAATGTGATGCTCGTTACTCTGCCGGTGTATTCCAAAGAAACACAAAATGAAAAG
GACAGACTCCATCTTTGGCTGACGGACAACAC
GCACATTGTTGATATTGGGCCGGTATCCGAGAAGGACGATGACGCTGCCGCCAGCTCCCTGTTGTACA
AAAGTAGTGAAAGTACAACTGATGAGGAGAAG
AGGGAGGAGTTGATTGCACTGTACGAGAAGAAAGGCGGTGGGGAAAAACCATCAAACAGTCTTTGGTC
TGTGCTTCTGACTGAGCAGCTGCAGCGTGTGA
AGGATGTGCTGGAGACCTGGAAGGAAGTGGACGAACGTGTCTCCAGGCTGTGCCCTTCTGAAAGTGCT
GAGAAGGATGGCTCACCTGACAGTGCCTGCAA
AAA
>scaffold1058.size6979
CGTTCCGGATGAAAGCACCGACAGTGACCGATGTGGTTCGTGTGTCCCACCAATGTGGCGTCACAGTG
CGGGCATTTCTTCTTCCGACGGCGTGTGGAGGG
CCGTCTCTCCACAGGCGGTGCGTCGGCCTCCCTTGTCGTCGGGGGAACAGCTTCCGTCCCGGTGCGGT
GGGATCGTCGTTGGAGCGCATGAAAGCAGACT
GTTCCGGTGATACGTTGCGGAAACGGCGTTGGTTCGTGCAGTCTCTCACAGTCCAGTACAGGCGACCC
CAGAGCTCGGATTCCCCGCACCCTTCCCGTGC
AGTGTTTATCCGTTCCTGTCAGTCGAGGGCCTCCTGAGTGATCAGGTGCTTGCCGCCTGGATCTGATG
TCCTGCGCCGCCAAGCAGGTAGTGGTGGGTGT
CCTGATGCATGGTACTACGCCACTTGTTGGTGAGCTGGGTGTTAATGCATGTCAGCAGGTCCGTGTGC
CGTATGGGTGCTATTCCATGCTCCGTGTGTTG
CCCGGTTGCCGTGGTCTGCGTCGCATGCTGATCTGCAGGCTCGTTAGCATGTACTCCGCAATGTTCGT
AGCAAAACTGCAGGTGCACGGACCAGCCAGCA
CGCGTCAGCGACAAGAGACGCTGCCAGATCCTCCGTCCATTCGGTATGACTGCGCGGGCCCTTGGTGA
CGGCGGCTAGAAGGGAATGACTGTCTGTAACC
ACAAGCAGGGTTTTACGCCTGTGCTGGATACGATGGACGCTCAGCCTTTCCATGGCCGGGAGCAGCGC
ACGGGACTCCGTCGTGCGGCTGCATGGGCGTG
TGCCGCCGTTGGTACTGACATTCTCTATTATTTCCGAGGAACGCGGGGATGAGAGCAGTATGCCAATC
CCGGCTGACGTGGGAATGCCGACACCGCTATT
CTTCGTAAGTATCCACGGTCCCCGGCGATGACGTTGCACCTTGTCGCGGTGTTGGGGCTTTGGTTGCG
TATAGGAACTCCGCAGCAGGGCGTTGCTGCGA
GCACGACGGAGACCAAGGTGACTTCACCCCCCGAGAAGGTTCCACGCCATGCCCGGTGTGGGGAGGTG
AGGCGGTCTGCCTGCAGACTGTCGGTCAGAAC
TGCACGACCGTTTCGCAGCGGTCACTCCGTTGATATCGCCCGTGGCGGTGACTTTCACAAGAACCCTG
CCACCGAGGAGGATGGCTACATCAATGTTTGG
CAGCTTAACATTGCAGGCCTCTCGTCCGTGAAAAATATTGCATTTGCTGCGCGATTGGAGCAGCGTCC
ACCTGACACCGTTCTTCTGCAGGAAATAAGTA
ACCGTGGTGAAGCAGCGACGAAATTATGTGGCCATAATGACTACCTTCAGGCTTCACTGGGCGCGGTG
TTGCCACTCTGGTGCGCCACTCTCTACCGTGC
GAGCGCTTGTCGCTCCCTGGCACTGACCTCCTGGAGACCATTGCTGTCCGTGTATTTCCACCGGGCTG
TCGCAGCATTTGCGTGGCGAATCTCTGCAATC
CTCCCACAGCAATTGGTCAATAATCCGCATTTATCAATATCGTTTGTCGCTTGTGCCCGAAGAGATTA
TCGCGGGTGACCTGAATTTACATCGTGAACTC

TGGGACAGTCATATACACCATCAAGGATTGGAGGCGATAATTTCGCCGCTGTTCTAATCGATATGGAT
TTTGGGCTGGCAAATTACCCGACACAGGCAAC
ACGGATCACACATCGTAATGCCTCGTCCCCTGACGTGACTGCGTATTGCGCCCTACGCATCTCCCACT
GGACGTTCGCCCCTTACATGGACTCGGACCAC
CGCTTAATATCTCACTCGGTAGAGATGGACGATGGCACTCCACGACTAGCAAACACGCTGCCATGACG
GAAGAAAGCCACCTTTACCCTGCGTAAAGCGG
ATTAGCCTGCTTCCACCACGTTGTGTGAAACCCTCCTTGCCACCGCGTCCACATGGTTGGATCCTCGC
GGAGGCATCTTACGAGCAGCTGAGCGCCACAT
CCCGCGCGGCAGCCGTGACAGCTCCAAAAAGATATGGACACACGAAATGGAACAGGCCGAGTCCGCCG
CCGAAGCAGCACACGAAGCACACACATTGCCT
CACCCGGAGACTGCCTGGTTTGAGCCGAATGTACCCGAAAAAAGGGAGGAACGAAATCAAGCCTTACG
CCGTTGCTCCGCTGTGCTTCTGGAAGCCCGCG
CGAAAAGACTTGGAGCCTCACCCAGCCCGAGGTGGCGATATCTTCACGGCATGGCGGCGCCGCACCCT
CATCCTCTGGAATCGGTTGTGCTCCGCACTGA
ACTTGGCCGCGTATGCGCCCTGCCTCGGCAACAGGCAAATCTGCTTGTCCGTCATTTTGTGCGGTTTC
ACGTGCCATACCGCCTTGTACTGCTGCTGCTG
CTCGCTGCACTCCTTTGGTTCCTGGTGGCTGGGAAATGGATCGGCCTTTCCCGCCGTATGTACTTGAC
GTAACCATCCGTGACTCTTTGCTTGGCTCAGC
CCCTGGCCCCGACGACATGTTGAATGAATTCCTGCACCGTCTCGTCCCGTTGCGCATGGCACACTTAG
AACGATGATCCACAACTCCTTCGCAAACGGCA
GCCTGCCTGGCTCCTGGAAAATGGGAGACGCTATTTCTATCCCCAAGCCTGGGAAGGATCCATGCCGC
CCAGAGAGTTACAGACCCATCACGCTGCTCTC
TGTTTTACTAAAGTTAGCCGAAGGAATGATTCACCGCCGTCTGTCGGCGTTATTGCCGCACCACCCAC
GTCAATTTCGGATTTGCACCTCCGCGTTCTAC
GTCGGACGTGGTGGCACTTGTTGTCGGCAAATTACTCGTGGACTGAATGGGTTTGGCATTGTGGAGTA
TGAGCGCCCTGGAGACGACGCCCCACACGTCG
CCCTCGTCGCCATCGCTTGTGGTTTTGATTGATTTTCCGACTGCGTTCGACACCACTGACCATGGGAA
TTTATTCTGCATGCTTGACAGACTTCCGCGCC
TTGGCCACGAACCAAACGCTGGCTGCACAACTACCTGCGGGGCCGCCATGTTAGGGTATGCACTCGCG
AACAGCACTCCCGGAAACAACTCACCTCAGCT
GGAGCACCGCAAGGCGGCGTCCCGGGACCCCAACTCTTCCTCTACTGTGTGGATGATCTGCTGCGCCG
CCTGGGCAGCATCTATTCTGCCTCCGCATTCA
TGTACGCTGACGAAAACACACTTGTTGCCTCATGTGCGGACATCCATGCCCGTGCTGCTGCAATGCGA
CCGCTCTTGTCTCTTGTGGCCACATGGGCTGC
CGAGCACAGCCTAAAAATCAACGTCGCTAAGAGCGAAGCTGCTCTATTCCACATCTCTTCACACACAC
ACACGGTCTGACGAGGACATGGTCGATCTCCA
TCTTGTTAGCGGGAACCTGCGTATTCAGTCACGCCCAGTGCGCTTGCTGGGCAACACGTTCGATCAAC
TTCTTAATTCTGTTACGCATGCTTCCACCGCT
TCAAAGAGGACCATgccNNNNNNNNNNNNNNNNNNNNNNNNNNNNNNNNNNNNNNNNNNNNNNNNNNN
NNNNNNNNNNNNNNNNNNNNNNNNNNNNNNNN
NNNNNNNNNNNNNNNNNNNNNNNNNNNNNNNNNNNNNNNNNNNNNNNNNNNNNNNNNNNNNNNNNNNN
NNNNNNNNNNNNNNNNNNNNNNNNNNNNNNNN
NNNNNNNNNNNNNNNNNNNNNNNNNNNNNNNNNNNNNNNNNNNNNNNNNNNNNNNNNNNNNNNNNNNN
NNNNNNNNNNNNNNNNNNNNNNNNNNNNNNNN
NNNNNNNNNNNNNNNNNTTGATTGGATACGTTCATAGTGTGCTATTTCATAATGGCGAGGCAATTGTC
CCGTGACTCGCCCCCACACACCTCCACAATAT
GGAAGTACGGTACCGCGACGGTAGCAAAACATTCTTGGCCTGAGCGCCTCTACGGAAGACACATCCGT
CTATTTGGAAGCCAATATTCTTCCGCTCCGTA
AGATATTTTGGCGTCGTGCATTCACACAATACGGACGCCATGTACGCCTCCATGATCACGAGGATTTG
CGTTCCCTTATCTACTCGCAACCTATGCCGCC
GTCCATGCATGGGAAAGCGGCGACATCAATCCCGCTTCCGAGAGATGCTGCTATTAATGGATTGCTAC
GCGTCTGTGACATTATTGGGACACCCCACAAC
CACAATCGCGCGCCTCTTGCCCAACATCGAATTTTTCCCTGGGACACTGTGAGCTGCAGCGGGGTGAG
ATTTTATCAGCCACTGTTTGCCGAAGACGCCT

CTGACGATGTCGGGCGCACGGCTTTTTATTCTACTCACTCCTCTTTGGGGGATTGTGATTTTGAATTG
TGGACGGACGGTCCTTCATACCTCGCTGCACT
CGCGTCTGGATCCGCCGCGCTATTGTATGACTCCACCACGACAAATGATCTCCCTGTGGAGGTACATC
GTGCAGCAGCTGGCAGCCTCGCATGCTCATAT
GGGGCAGACTGCCTTGCGATCGAAAATGTTTTTAGACATCTCGTACCTCCTTGTCCGGGAACATCCCA
ATCGCCCACACGAATGCTGGAGGAAACGGACT
CCCTCTCGGCAATGTAGGCACCCCGCGTTGTCCACCGACCGTGCGAGACCGCGTTACTGAGCAAATTT
GGATTATGCTGCTTTCCTTGGTGACGCGAGGT
CACCCTGTGGATTTTATATTTTCCCATCACACTGCGGTATTCCACGCAGTGAGGCCGCTGACAAGCAG
GCCACGATTGCACGATTCCTCTCGCAGGACTG
CGTCAATGCCTGGCACGTGGACTTCCTTACCGCCGTCAAGAGGTTATAATGGAAGGATGCCCAGGAGG
AAAAGGAAGGCGCGCACACCGCGTGACAGGCG
CCTGTCGGTGTGGCGAGGACTCTATTCCTGGTGTTGACCCGGACATCGGTCCCCGAGTGGCGGCACTG
CGCACAGGCGCGATGCGACGCCTGCCCACTCT
TCGACAGGCCTGCACGCGCCCTGCGGATCACAAGGAACCCTCTGTGTCGGTGGTGCCGTCCCTCCCTC
ACTGCGGTGGCCGGCCCGCCTCTGCCTCCGCC
GGAACCTCTTCCCGACGTGAGCACCACAACTCCTTGTCGACCAGCGAGCGGCGGATTGACGTCCTCCT
GCCCAACCTGCGGCAGAGTGCACTTGTCCGTC
CACAGTATGAGGGCACATGCCGGACGTGCTCAGCCTGGCGTTATAATAGTTTGCGAGGGCCTCCCATG
CGGATGCCACAATGGCGCCACCGTCTTCTTAA
CAGGTGCCTCGCGCGCAGGTCGCTATCGAAGTTGCCAAGCGTATCAACAAGTCAAACACGAACAACGC
AACTCTGCAAGCACCAATGGCAACCATAACCC
TTCAGGCACTACTCTACCGCAAGCCCCTACCACCCGCTGAGCCGCCCGACGGTCCACGGGAAACTCTA
TCGCACATGTTGTGGGAATGCCCTCGTCGTAG
ACCACTGAGCATTCGAATCTTCGGAGAAAATCTATGTCGCATGAAAGCGTTATTCGAGAAGGGAATGT
TGGCCACGTTTCGACGGACAGCCCTCGGGCGA
TCAATGAATAGTGACAATGTCGTATAACAAAATAAATAAATAAATAAAAAAGAATTTTATTCTGTGTT
TTATCGATGCTTTTACTGTTTTCTTTGCTCTC
CACAATTATGGGTTGCACTGATGCTTCTCTAAACCTAACCTTTGCACAACACCTAAATGCCACGGAGG
AACACCGCACGGAAGCAGGCAGTGGGCGCAAC
CAAAAAAAAGCATAACAACACAACAAACAGATATCGCAATGCGAAACAAAAAGGCACGGAAACGTCAC
GTACGGAGCGGAACAGGCACTATTTGTGTGCG
CGGCGGCTGCCGACATCCTCGCTGTCCTCTTCTCCCGCCTCACTCCTTTCTCCCCCGGTGTGGGCGAG
CGGCTCGCGGTGACGCACGGAGGGTCCGCTTG
CTATTAAAGGGGTTGGCGGGCACGGTCTCGTGGAGGTTGTTGGGCTGGGCGGCATCAGCGTCACCATC
TAACGATTTCCCCGTTGGCTCGTCAGGGGCGC
GCTCAGATTTCCCGAGTTACATACATAAGAAGCTCCTCATCACGTGCTGCCGCGGTGCGAAAAGACCG
CCCATTTCACATGCGCACAACGCCCGCTCAAC
CACAATCACGTGACTCAGTTTACGGCCACAAACGAGCAACAAAGGACACATCAAAGAGACCCAAGCCA
AATACAATAACAAAAATAGAGGCCTCTAACAG
CTTCAAGCGCACACGCNNNNNNNNNNNNNNNNNNNNNNNNNNNNNNNNNNNNNNNNNNNNNNNNNNNN
NNNNNNNNNNNNNNNNNNNNNNNNNNNNNNNN
NNNNNNNNNNNNNNNNNNNNNNNNNNNNNNNNNNNNNNNNNNNNNNNNNNNNNNNNNNNNNNNNNNNN
NNNNNNNNNNNNNNNNNNNNNNNNNNNNNNNN
NNNNNNNNNNNNNNNNNNNNNNNNNNNNNNNNNNNNNNNNNNNNNNNNNNNNNNNNNNNNNNNNNNNN
NNNNNNNNNNNNNNNNNNNNNNNNNNNNNNNN
NNNNNNNNNNNNNNNNGTCGCACCGCGACCGCCGCGCCATTCTTTGAGCGACGGACCGTCCGCTGTTT
GTTGCGACGCCATGGTGGCACGCGCTGTGGTC
ATGAGACTCTCAACGGCGCCGAATCTTTCATGAGCGATCCTCGCGGGCGGCGCAAGGCGGTTCCTTGC
ACACGGGACGTTGGAAGCGATTTGCCGTCCCT
TGCGAATCGTGTCGTTCACACCCACGAAAGGCATGGTCGTGTCGTCTCTCGGCTGGCCTGTCATGACG
GTTTTTTCGCGCCGGCCTGGGCGTGCCCGCGG
GCGGTCCCAGCGGTGAGGAAGAGACCAGCCAACTCGAGCGCGGCCGCCGTGTCGGTCTCATCCCGCCC
GAGATT

>scaffold1059.size15216
AATGACAGTGGCGGTGTCGTTGACGGCGACACACGCTTAGCTGCAAAACCACGGAATCTGCCCTGCTG
CGGACGACTTTTTTCGCCTCCTCGCGGGGTGCT
TGGCGCGGAGTTTACTTTTGCACGCTGTTTCGCCGCTGAACTCGCCGTGCTGCCAGTAAAAGCGCCCC
GCGGCCGTTTCCATTTGAATGAACTACTACGA
AGAGAAGAGGTCACACTGGCAGCAGTGGCGATGATGCTGGCGGTGGGAGATGTCACGGAAATTTCACC
GGCGGGATGAGCCCCCGTCTTGGGCGCCTTCC
TGTCGGACACTGCCGCCTCGTTCACAATTTGGCCTTCGCTCAAAATAATACCGTCGGCCTCTGCAGCC
TCCACACACTGGGCGGGCTGCTGCTGGCACTG
TCTCCTCCGGCGCTCCACCATCAGCCGGTACTCCTCCATGACCGCCGCACGTTTTGTGTGGCTATCCA
TCAGTCGCTGGTATTCTGCCCTGGAGGTGCTG
TTGCTGCTCTGCCGGTCGATGCTCTCGTCGTGCAGCCGCATCACCGCGTTCTCCAACTCGTTGACAAG
CGAGAGCGCACTCTGGTCATCCATGTCCATCG
ACAGTGAAATCAAAGAGACCATACCCTCAAGGAGGAACCGCGCCGGATCGACACGCAAAACAAAACTC
CCTTCCTGAATACGCCACGTGCTCCTTTGTGG
ACCGGTGCCTTCTCCGCGCAAATCACTCACGCAGCACCCAACGCACAGCAAAAACCAGCAgATAAAAT
TATATAAAAATAAAAAGAAGAAAAAAAAGACG
ATTCCACAAAAATTGCACCGCAGTCAATAAACAAACAATATCAGCCAAGGTGCAACTCATGCAAAGAA
TCACCACAAACACACAAACACACACACACACA
CAGGCACAACTCCGTGCATCCATGCCTTCACCGAGGGCACACAAAACGCTCCTTACATCACATCTAGT
ACTACTACGAACAATACCACTACTAATGCTAC
CCTCCTCCACAGACATACATATACACACAATTGCACCGCCCGCCACTTTCCAAGCATGTCGACTGCCA
AAAGAAAGAAGAAAAAAAAAAAATCCCCCCCC
CCCCCCCAATACCATAGCCCCTTTCACTCTACAAATCCCCACCGACCCTCAACCACCACAACAAACAC
CACCATCATTGCTACTCCTACTACATTCAATA
AAACGAGAAATACAACTGCACACCATCTCTACACCATCCATTAGCATCCCTTACTGAAATCTTTGCAC
GTTGTACAAATGATAGCCACTCTGATGCTGTA
CTTTTCCCATGATGCGGTCCCAGTCCCACCCCAACATCGAGGTCCTGCTACGTGAGTTTCGCTCCGCC
GCGTAGAGAGCACCCTTCAGATTGTCGTCAAA
CGACTCACACCACTTCTCAAAGTGTGGGTCAACATCGCGATCAAATACAGCAAGGAAACTACAAATAG
ACCACAGTGAAATGCAACCAAAGATGGCGATA
GTAAAAATACGAAGATGTGGCGAGACATCCATGTCTCCAAAGCGATCGGACATCTCTCCTGCCCCGGC
GTACATTGGGTGATGACCCGTCCAGCGATGCA
GCTTTGCGTCGTCATACCACATCACCTCCGGGTGTGTCCAGTGGTCGTACACCTGCGACCCGTCGGGG
TGAAACATGGGGTTCGCAAGGGTATGCGTCGA
CCACGGCGAGGTATTGTTGCGACGGAGAAAGTAGAAATTCGCGTTTTCACGCAAGTATTTCTGAGTTT
CACGCACCATCATGTCACACATGGCAGGATCC
GTGACGTAGCGATACATTTCAAATCGCCGGCGCACTACCTTGTAGGCAAGATTAAACTTGTGTGCATT
CAGCTGCACAAGCCACATCTGTAGCTCTTTAA
GTGCCCACCTGTAAAGTCGGCAAACGCGTTGTTTGTGTGTGAGCCGTGGATCGTCAATGATGGGGTCA
AGGTGGCGGACACTACCAATAAAGTCGTAGAT
GTACGCTTCAGACTTACTCTCGTAGCGTGGGTTGTCTAGAATGCGCCAGCCCTCGTAGTTCTGCAGGA
GTGGCTCATGGAGGTGCCACTCGTGGTACTCC
ACCATGCCGGCGCTAAGCAAAAAACAACTACGGCGCATCATTCTTCCAGGTTTCTACGTGTGTTCCTC
CTCAACTGTTGAGATATCTGTTCCCTTTCCCT
TCTCTCCCTCTCTCACGAGGGTTTAAAAAAAAAAAAAAAAAGCAGCAGCAATGGCAAGTGGTAAAATG
ACTTCGAAGAAGTCCCCCCTCCTGTAAGCATG
TGACACCCAAAAAGGGGGGAAAAACCACGAGGGCTTTGCGTACTCAAACATCACTTCACGCAAAAGCC
CAAGCAAAGACAAATGCAGACATGCAGGCCAG
AAACTCGTCCATGACAATCTGTCAGGAGTGAAGTGAACAACTCGTTGACGTTCACCCGCCCTTTCTCT
CTCTCTCTCTCTCGCTCACTACTCGGCTGCTC
CTCTTGCATTCTCCACTACAATCACTCCACACTCGTACCGCAGATGCACAACAGCAAAAAAAAAAACA
CCTGCAGAAGCAGCAGCCTTCTATCACTCTTC
AACAAACGGCATTCCGCCAAAAAGGTATGTACAAAAACGTCTCCACATTAACCCCCCATAATCCCTGA

ATCCCCGCATTTCGTTATCCCCTCCACATTAC
CCAAATTATACACCCCCCTTCCCCTCCCAATTCAACAGCACACATTTCCGCTAACTACTACCCCGCCC
CCCATCCCTATTATAACATCAGAAAACATCGA
TCTACTAGCCATCGCTGCAGTGCCCTGATGCGCATGCGGACAAATACGAACCCTTTTCCTTTTCTTCT
CCATCTTCTTAAAACCCCAAACATATCCACAT
ATATATAATTTACAAGAAACACACGCACACACAAATAAGAGAAACAGAAGCAAAACGACTCTTTTTAT
ATACGGTTGTTGCTGTTGTTTATTTGGTGTAT
TTGTGTGGTTTCTCTTTTTTACTTCCTTCCCTCCTTCCACTGCAAGAAGGTCCTACCAGAACTTCATC
TCGTAGTACTCCTGCCCTGTCGCCCGTTTCCA
GTGGCGCTCTGCCCAGTGCCAGTCCACCACATTAAAGTACTTGGCAAGAAATGCGGCGCGGTTATTTT
CATAGTCCACGTAATACGTGTGCTCCCACACA
TCGACGCACAGCAGCGGCACAACATGCGTATTCGTCAGCGGGCATCCCGCGTTCGACAGTGAAAGTAT
ATCAAATTGACCCAAACGGGTGTCGTAAACCC
AATAAACCCAGCCACTACCAAACAGATTCAGCGCAGCATCCGTGAAGATCCTCTCAAACTCCTCCACG
GAGCCATACTGTTTTTCAACAGCTGCCTTCAG
GTCAGGAGGAATGTTACTGCCGTAGGGTTGAATGCACTTCCAGAAGAAACAGTGATTGTAGTGCTGGG
CGGCATTGTTGTAAACGCCAGTGAGCATCACA
TCATCGTGTGTGCGGAGGATGATCTCATCCAGGTTAGATCCGTACAACGGCGTGCCCTCAATCAGCTG
ATTCAGCTTCTCTACATAGCCACGATGATGGC
GACCATAATGCAAACGAATCTGGTACGATGAGAAGAGTGGCAAGCAGCCCCTGTACCACGGAAACTCT
AATGTCGGCAGATAAAAAAATCCTTGTGATTG
GATGTAGTGCTCTGCTCGGCGTATGTACTCGTCCACGCGCCAATACGTGTCTCGGTACTTTTCCACGT
AGTAGTCGGGGGCTCTACCGGAGCGGTTACGA
AACTCGTTGTACTGATCCATAAAACTGAAGCGGCCCAACACAATATCCGACTTTGGCGTGTGCATCAC
AAGCCCACCTCTGCGAGAAGCCTGCAGGGCTG
CTGGAGACAAAATTGCCGCAATCGCAGGCACAACGCCTGTTGAAAAAGCCGAACGACGCATTTCTCAG
CACGACACACACACAAAAGATAAATCAACGTATA
AAAAAAAACACGGTAAAAAGAAACAAGGGATAACTTACAACAATCGGATGCAAACGTTTTTCCCTCGT
GCCCGTCCCTTTCCCTTGTGAGACCGAACCTC
CCGAAGCAAATAACAGAAGTGAAAATAATAAATAACGGGTGCATGGACGGGAAGACACGCAGTTGTAA
GAAAGAGAAAGTTGAGTCCGCTCCCTCGGAAA
CGCACATGAAAAAGACACAGACAGATAGACAGGGAGATGGCGGAAGAAAAACCCCCTCCAACACAAAC
AGTAAAAAAAGAAAAAAAGGAAAAAAAAAACGG
ACAGGCCGTCAAGCACCACCAGGCGTTGCGTGACTATCTCAACTCCTCCATTTCCTCCTCCAGCTGCT
GCACGTCATGTTTGACTGCCTCCAACAACGGT
GTTACCGCCCGCACTTGATCGCTCAACTCCCGTGAAAACTGCGCTAGTCGTTGGGCCTCGCGCTCTGC
CAGGGCCGTAAAGCGTCTCAACTCGGCGCTCT
CCGCCTCCATCCACTCCGCCTCCTTCGCAAGCCGTTGCTGCTGCATTAAATGCCGCAGGCTCTTCTTT
CGGTTCGCCACCTCACGCTGCAGGTGCGAAAG
CAGCAGAGATGACGATGACGGTGGCTCCTGCAGCGTGTCTTCAGCTGCACCCATCGCACTCTCGGCCA
CTTCCTCCACAAGTCGCTGGTGTTGCTGCTGC
AGCTCCGCTTTACGCTGGCAAACAGCCTTTAACTCCGCCTTCATGTGGTCTACTTCATCCTCCCGCAT
CTTCAGGAGCTCCTTGAGACGCACTTCCTCAT
GCAAGGCGTCCTCTAATTGACGCTCCTCACGCTGAAGTTCCTCCCGCAACGACTCCTCAACCGCCTCC
TCGCGCTGCAAATCCCCCAGTGCCTCTGCATG
TTCCTGACGAAGCTGCTCCAGGCGTTTCCTCAGCGACTCAAACATTCCCAGCTCCTCCTGTCGCTCCT
GTCGCAATGCCTCCAGACGGGCCTGAAGCTGC
TGCAACTCCGCCGCACTTTCCCTCCGCTTTCGCAGCTCTTCCGATACAATCAACTCAAGGCTCTGCTT
CGCATCAAGTAGCCGGCTTTGCTCCTCCTGCA
AAGACGCTGCTTCCACCTCTGTCGGCCCCTGCAGGATTGTGATCTCGCTCTCGAGCCTTGCCTTCTCC
CGCTCCAGCTCAGCCATGCGTTGCATCAGCGT
CTCCTCCTCCTCCTGCCACTTTTTCGCCTGCGCCTGCCGCTCTTCGGCTAGGCTCTGCAGTTTCTTCT
GCTTCCTCAACTCCATCTCCTCCGTGGATTTC
TGCTGTTGCTGCAACCTGGCAAGCTCCTTCGCAAGGTTCCAAACAGAGACACGTGTCTGAACATTGGC

GTAGTACGTCTTGCCGGATTTCGGGTCCTTCT
TCTCGCGCCACACGCCGCTCTCCAGCGCCGCCCGTACCGCCTCGTCCTGCATCCGCGATACTACACAC
CCGCACACACCGTATGTACACAACAATAAAAA
ACAAAGACCAAAACCGCATCGGTTTAGAGAGATCGCAATAAAGGAAAAACAGAACACGCAGCAAGGAA
GGTTAAGGAAGACATCACGGACCGAGGCACCC
ACAAATAAACACACACACACACACAAAAGCAAGTTTATTTTTAAAAAATTAAAAATCTTTTTTATTTA
CTTTTACATGATCTATTTATTCTCCATAAGGT
GCTGACGGGTGTACTCGTACATCACCAGGGCACCAGTGACGTGTACATTAAGCGAACGAATCAATCCA
AATTGAGGAATTTCCACGCACACATCCAGTAC
GGGCAGCAGCTCCGCTGGCACGCCGTGTCCTTCCGCGCCAAGGAGAATGGCAGCGCGCTCAGGGAAGC
GATACGAAGCGATAGACACGCTTCCAGCCGTC
TGCTCAATGCCAATAAGTGTGTATCCGTCTTGCCGCAGGGAACCCAAATATTCCGGCAGATTCTTTGC
CATTACTGCATCCCACGGCATCCACAGTTCAG
CACTGCGCGCGGCGGCCACGAAATGCGGATGCGCAAAGACGGCCTGGTCTGAAACGACAACCTTCTCA
ATTGCGAAGATTTCGCCGCAGCGGAAGAGGCC
AGCGACATTGACAGGGTTTTGGAGTAAGGAACTTAACACAATGAGCGGTTGCCGCTTTTCTTGCTTTA
GTGCCCGCGGGTGGAGTTCATTGTACACCAAG
CCATTCCACCACGGTGTCACCTTACGCTGAATGTTCTCATCCCCGTCAATCATCCTGTTGTTTGTGTT
GTTGTTATTGTTGTCTGCTGTCAGAAGTGCCA
TGGCTTCGGCAGTGTAATCGACGCAGTACGTCGTGTGGCTCTCCGTGTGTGGGATGTACGGAAAGCTT
TTAAAGGGTTCGAGTATGCGACGGCTTTCAAA
GCGATCTGATATTATCCGCACGCGAAGCATCTCCACCGGGCTTAGCGCACCAATAAGGCAGCGGAATT
CGTGATCAAGAAACCGCATTCGATCAAAGGCA
GCGGCTGGTATACTCTCCGTAAGGATGTTATTTCCCTCCTTTCTCTTAACGCAGAACAGTTGTCGTGG
GGTGCAGGCCTCCGCCAGATTAAAGAAAAGCT
CCTCGGCATGTTTTTCACGAAACTTCACATGCTCCGGTGCTCGAGCAATGTAGTCAAAGACCATCTCC
TCCGTGGGAAGGAATTTAACCCCCTGCTTCGT
ACATGTTTCATGGATTTGATGGAGCCCAATGTGACAGATGACACGCAGAAGGTGTTGATGCGAGGTGC
TCTGCTGAAAAAGACGATGAAGCAGCACATGA
AAAAGACCGTCCACTCGCTCCACTTCCCCGCGGAGCTGTTGAAGCATCATGTGAGCCATGATCAAAAC
ATAACTCCCACAAACCTGAGGCCGCAAGTTGT
AATCTGTCATCGCCGCATCTAAAAGTGTGTATAACGCGGGCTGGCGCTCCAGTATCTTTAATGCATAC
AACTCCATCAGGCGCCTTACACTGCCCATGTT
GGTGAGGGGAAGACACGTCTTTATTACCCGCCCCAAAATACTTTGCTGAGCGGCTGCATCGTCAAGCA
TGGGAAGCAGCGCACAAAGCAACTGCCACAAA
CGAATACGACAGCGGTGGGGCCTGGAGTTTGGCATGCACGGCTCATGCTTCACAGCGGCATGAGTGGT
GTTCCAATCCAGCAGCTCCAACGAAATCTCCG
TCGCCCAGTCATTTCTTAAACGACAGCACCACAAAATGGTAGAAATCGCAAGGGCGCGTCCGACCGCT
GAGAGTCGCACCGTTgGTGGGTACTGTACACG
CAGAGCTTCTGGAAAAAGAAGAATCAAAGGCTCTGTTGCAGCCACCATCATCTCCGAGGCTTCCTCGT
CTCGTCCAGAGTTATACAACACCGCGACATGC
AGAAGAAGATCTTTCAGACGTGGCCAGTTCGCCAAGGGATCACGAAGAACCTCCGTCGCTACTGTCAC
CGCCGCCAAATACACAGAACGCTCCGAATCCG
CCTCGTCAAGTAACGCCCTTGATATGTGTGATTTAAGCAATTCCTCATGCTCCACCGCACCGCAGCGG
AGAACGTTAAACGCGAGAACAGAGACTCGCCC
ATGGTCTCTTGCCCCAACATCCTTCAGGTGTCCAAACATGGCGTTGATAATGGCACGATGATCCACGC
ACTTCTCACGTACCGCGTTATTCTCCGTAAAA
GTCCCACTATTAGCCACCCAGGCAAGAATATCGTAAACGCTTGCAAGGTTTGTTCCCCAGCAACGCTC
CACGTGATCCAGAGCGTAGTCCTGAACTTCCA
AAACCAAATCTGGGCTCTGCTGTGGGTCCACACAACTGCAAAGGACAAACACGGCGTTGTAAACACCA
CCGTAGTACTGCGCCAAGAGGGTCGCCCAGGA
GAGACTGGTCAGTGGACATGGAACATCGCATGTCAATAGTCGCAAAGAAGGAAATCGCATGAGGCTTC
GAAGCCAGTGAAGCAGGTCTGCATTTTCGATA
CGAAGACGACACGTAATCTCAGCAGCGTTCAAACCTTCCAACGTGTTTCCTGGTGTGCCCAGTGTAAT

CACGCCGGACAGTACGCTCTGCAGTAAACGGG
ACGCATTCCTGAACACAACCATTTTGGCAAAGTGCCGAGTCACTTTGGTCGTATCACCCACAGAAGCA
GCAGCAAAAGTCCCATCGGCATCTAAAAGAAG
CAACTGCAACACGTCAATAACGTGCTGTCGTGTTTGGAACCACGTATTCCCACCAGTGGAGTCATGGG
CAACAGCGATTTGCCCTGCAGACAAGACCGCC
GCAACAACTACATCCCACTCCGAGGTGTGTTGCAACGCCCACACGTCCGTGGAAAAGTCATCAAAGGA
GTTCACTTGAGTCATCGCCCGAAATACCTGCC
GAAGGGCACTGAGCGCCACCGTGTGCAAAGCCTCGCGCAAATCAACGACCGTTGCCAGCGAGTAGTAC
CGCTTGCAGGCCACCGCTCCAACGGAGCGATG
AAACTCTACAAACGCGGCCAACGCCGCAAGAAAAGACTCAGACGGACTGTACGGCCTCTTCGCAAAGG
AGAGAAGTGTTGTCCCAATCTCCTGCGCTACA
TCCGCAAGAAGCCGTCGGTCCTCGGACTCCGCTATGTCCAAGGCTCCGCAAAGAAAGAAGAGCTGCTT
TACACGCGCCTCATCAAAGGAGGCAAAACGAA
GAAACTGCCGCACATCCTCAAGCAGACGATCTGTCCGCAACAGGATGCGCGCAAACCTGAAATTGCTC
CCCCCAATCGGTGTAGCAATAGACATCCGTAT
CAGTTGTTCGTTTAAAAAACGAGGGTGAATACATGTGCCTAAAGCACCAATGTGTCCCACGGAATCAA
TGGCAGAGGCGTTGCCCCCACAGGAATTCCCA
AGAGGGCCACAAACACAGAGGAGTTTCCAGAGGCCGTCGCACTGATGCACCAAAGTGGGATCAATCCG
GGCAAAGTGCAGAAGAGCACTAAAGGCAACAG
CACTGAGACGAACATCAAGCCAAAATGGAACACCGTCATGTGCCGCATCCCTCAAGAGCAGCAGCAAC
TCCTGCAGGACATTCGGATCCGTGATCATCTC
CAAGGTTGCCTCTCTGCTACGCGCTTCAGTATCGAGCGACAGCGTCTCTGCAACACCGTGAAGAATGC
AAAGGAGCATCGTCACGCCGTACACGCTAGGC
CTCTCCACAACACCATGCAGGAGGCGGCACATGGCCTCCTTGCGTTTATGAGCCGTGCATAAAACATC
CCGAAAAATGCAAGCGTAAAACCGTTCGAGTT
GATCCGCCATTGGCCCCGCCATACCCTCCAGGGCACGCACCTCGGCCCCCTCAAAGCCCTTGAACATG
GAGAGGTGTGTCGCATGCGGTGTGCGGTCAAC
ATCTGCGCAAAGCCGCGAGTCCAGAGAGGCCTCCACAGCACTCGTGAAGAGAAAAACACTGGAAAAGA
GCCTCAGAACCGATGTGCTCAGAGACCACAGT
CGCCGCAACCCAGTCTTTCGAACGGCCGTGTTTGGATGCAGCAACACCTTCAAAAGGAGCAGCTCCAC
CCATGCCGGGTGAAGGCTACCAACAAGCGAAG
GGCCCGACACCGCCGCCTCCGTATTGCCGCCATCCTTTATGGCGGCCCAGGCCACGGCGTCACAAGTC
AAAGAGTCCACAAGGACGTCAAGCTTCAAGAG
TGCCGGCTGAACGATGTGAAGCCCAAACTCGTTGGTGGCCTCAAGAACAAGAAAAAATAATCCCCACT
GATTGTGGCTTGCTGCGGGAACCCACACAAAT
TGCTCATGAAAGAAAACAGTTTTTTCCCACTTTTCGTGCGTCTCCTGCCCGTGTGTGAGGTCAACAAT
GCGCTTCAAAAGATACTCCACACGCAACTGCA
TATTCCCGCGTCGTCCTGCATCGGCGTCGCACGTGAGAGCATCCCAAAATGCCGCATAGAGTGTGTTC
CACAGATGCGGGGAGCACCGGTAGTCCCTGTC
TTTGTCGCCATCCGTAAAACGAAACAGATACTCAAAAAGTGCCGTGACAAGGACGACGACCTCTCCCT
GCCGCGCTGTTTCCTTTGAGATCGACTGCAGC
AGTGACTGAACACGCGGATGGTGGCTTTCAAGCGACTGGTGGCCGCCGGAGAGAAGTAGTGGTATCAA
CACACGACAAGAAAGAAGAGAACTCAGTGCCT
CAGCCCCACGTTTGCACGAGAGCGCATCCAGTGCGTACCCAATCAACGCATCCCTCAGCGCCGCACGG
CGTGAAGAAGATTTTCCTCTCGGGTCCTTTCC
CTCATTCGTGTCTCCTTCCTCCTTTTCCTCCTCTTCCTCCCGATGCCGCCTCACAAGAACGTCGGTAC
ACTCCGCCAGCAGCTCACTCGCAACCTCCTCC
GGCAAAGATTCACCCAAAGCAGTGTTCTGTAGCGATGCCCCAGCCACTCCAATCAACGCAACGGAGTC
GCCGCACACTGCCATCTGCGCAACGCAAAAAC
AGACGGACGAGACGACATCCCTCGAAAAAGGTGGGTGTGACACAAGTATCGAATGCATCGCTCGCTCC
CATCGCCACACGTCGTGCTCGAGCAAAAGGCC
GCATGCACGCACCAAAGACCCCGCGGAAACAATTTGCCCCACCGTGTAGTCATCTCGATTCCCCTCAA
AGAGCAAAAGGAGACGCTGCAGCTGACACCGA
CGCTCCGTCGCAGCCAGGGCGTTCACCAACTCCCACGCAATACCGTAAGCATGCGATGGATGAGAGAA

GGACCCATTTTTATCAAATCGTCCATGCAGAG
CTGCGAcAACAGCCTCCAACTTCGCCTCCATTGCTTTGCGACAGCACCTCGCAATAGACACGCAAGAA
AAAAAAAGAAAGACTTAACATTGGTATCCCCG
GGGTTTTTTTTTTTAAGAAAAAAAAGGAATGCTGCATGTATGAATGATTACGCAAGGCCATCCACATC
AACAAACAAAATAAACAAACTCCGTAAAGCCA
GAAATCCACACACACACACACACACACAGAGAGAGAGAGAGAGAGAGAGAGAGAAATACGATAACTGC
GATAATAAGCCGCAAAAACAAATATTGTCCTC
CAATTCCTAAAAGAAGAAAAGGGAGTTTGTTATGAGAGCGGAGAGCCTAATCTACCGCTAAGAAGTTC
ACAAACAAAATAAAGAAATAAATAAAATACTC
ATATGCATATGCATATATATATATATATATACATGTATAGCCTATTAATAATGTGGGTAATCCCACAC
CCCAAAGGTTGCCTTTTCTAAGCCTTCAAAAC
ACATGACGCCGAGGCCGTTGGGTACAATTGGAGGATTAAAATATTCAAAAAATGAAAAATAAAGTCTC
ACATACCTGCGCCACCACTATTTCACTGGTCC
TACCATAATGAGAAAACACGCGCAAAATTCCCACACACACACACAAAAGAAAAAAAAAGCGTCCCAAT
AGGTAAACATTAGAACAGGTCGTTTCCAAAGC
CATTGCTTCTCTTCGCAGACGCAACAAAAACTTAACACCTCCTGCGCACCTCATAAAAGTCAAGGAAA
AAGGCAACGACACGGAAAAGCATGCGGCTAAA
GCCCTGTCCACAGCAAATGCAGCGTCCACTGGACCACCGCCAAATTCACAGGGTCTTGGCACGTCACA
TGAGCAGTCTTCATACTTGACACTTTCCGTGG
GCTCTTTGGCCTCCAAGTGCACATAAAATGTATCAAATCATCCGTTCGGCTTATACGTCGCAAATCCG
CTAAGGCGGCAAAATCCACCAAAGACATCATC
GCTGTTCCAAAGCCTCCTGTATCCAACTCCTCCTCCATTGACTGAAGGCTTTCCCATGCAAATGCGTT
TCTCTCCAGGCGCAGATCATCAGGAAAGAGAA
AAGAAGGCAGAGCTTCCGTCCTGGGCGTTGTGAAAAACACGTATTTCGTAGCTGGTACGCCAAACGCC
TTCTTGTCAAAAACCATTCGACTTCTCAGGCG
GTGAAAATATCTGGTTAGACCACCAAGGCGTTCCTCCCCACGCTGCCACCAATACATGAAGGAGGCCA
AGTGAACGGGTGGACTCCTTATGCGAAAGGCA
TCCTCACGAAAATCATTCACAAGAGGATCCCACAAATATAAGAAGATAGCAACAACACGCTCACTTTG
TAAAAAGTGGGCACAGGAGGTTTTATTTTTCA
TATGTCCCTGGTACCCGGGGAAGTATGTCTCAATCAGGGCTGCATGGACGCAGGTAAAGGAAAGAATC
AGCTCGTCACGATCCTCGTAAACAACATACAG
CGCGTCACGGTGAAATTCGTGCTCCGCAAACACTTCCTCTCCACGCAAAAAAAACATCAGCCTGAGCA
ACAACTGCCGCAACATTGAATCGCCAGTAACA
ATAATGCCGTGTCCGTTGCGACCGATACTCGCATGGTGCAAGATCGACAGAGCCTCTAACGGAGAAAC
GTATCGGTAGCTCGGGCACAACGCCGGCCGTA
CCTCAAACGCGAAGCTCGACCCGTTCCCAACGTCGACGGCGTGAAGCGTCGAGTTGCTCTGCACTCCG
TCGTACATCCGGCGAGAGGCACACGCCTCAAA
ACGCCCGTCCCCACGCAGTTGCTTTAACTCGGACAAGCCCAGCAGCCTCAGCGGTTGTTTGAATTTTC
CCCCAACAAAAAATCCACAAGGTGGAGGACCA
GGACGAAGAATCCTATCGCAAAACGCAAGAGACACAGACCCTCTCCTATTGGCAAATGTACGGCAACG
TGATAGCAATGACTTATGTATCAATCTAGCGC
CCGGGCCACATGTCCTCCCGCTCCAACCCAGTTGAAAACCCTGCGAACCGTTAATCTGAGCACCCAAT
CCATGAGCTGCTTGATGAATTTGTGTCGATCG
TTTACCCTGCTTATTCGCATTGTGCCCAGGCCAAAGCACAAAGAAACAAAAATAAACTGCCACAAGCA
CAAACACAGATTTGAATAGCAGGCCGCGGGAA
TTGTAACAAAGCGACATTTCTAAAGAAGAGCAGCCAAAAAAAAAAAAAAAAAAAAAGGTGTTTATTTGT
TTATAGTTACACACATGTGTCATACATATGTG
CAATTGAGTGAAGGGGGGCTGCAGAGACTCACATTCACCGTCGCTCCCGAAAGGGAAAGTACATCTCT
TCCACCAGCAAACCAGCAAAGGCCACTCCAGA
CGGCAGTGCTGGATTAGAAGTCGAATGAAAACACGAGAATGAGAATGCCCCAAAAGAGGGAGAGGGGA
AAAAAAAAGGGTACGTGGAGGAAGGCACCCTC
TGTGCAGGAGGAAAATGTGGTGGTAGAGGTTAAATGGTGCGCAAGATGAGAAGAATGCCAAAAGACAG
CGCCCTGCCAGCATGACGGCAGGAGGTCAGCA
CGAGAAGAGGAACGGCAGATGGACGCAGCGGCGTGGGGCACGCGAGTACGCCCAGTCGCACTGAAACC

AAACAACGTCAGGCACGCATTGTTGCTGCAGG
CACCGTTTTCCACTCCAAAAGAAAAAAAAAACAGGCGGCCCGGCCCACACGCGGGCCTTTCGCCAATC
ATCCACGACACGCCAAGAGCGGTACGGAAGAA
ACAATCGTCTCCCCTCCCCCCGTGGCATCGGGCAGCCACAAAAAGTGCTCATCCGCAAACTGAAGACA
TGTACGGCATGCGATGCATGAGTGTGTGCTGC
GTGGTCGCCTAGCTGGCATNNNNNNNNNNNNNNNNNNNNNNNNNNNNNNNNNNNNNNNNNNNNNNNNN
NNNNNNNNNNNNNNNNNNNNNNNNNNNNNNNN
NNNNNNNNNNNNNNNNNNNNNNNNNNNNNNNNNNNNNNNNNNNNNNNNNNNNNNNNNNNNNNNNNNNN
NNNNNNNNNNNNNNNNNNNNNNNNNNNNNNNN
NNNNNNNNNNNNNNNNNNNNNNNNNNNNNNNNNNNNNNNNNNNNNNNNNNNNNNNNNNNNNNNNNNNN
NNNNNNNNNNNNNNNNNNNNNNNNNNNNNNNN
NNNNNNNNNNNNNNNNNNAAACGGCGTGTGCCAAAGAGAAAAAAAAAAAGAGAGAAATGAACAGAAA
AGCGACGGGAGAGAGCACACAGGCACAATAAA
CGCTGCCACACAAAAGAGGAAATAAATAAATAAAAATGTATCAAAGGCCCATAAATATTTCCCAATTT
CGTGCTACCGCATCACCCCCCCCCCCCCTCTT
TTACCGCCTGTTCGCTACGACGGGGAAAGAAGATGATTGTAGGGCAGAAGGCATAAAGAGAAAAAAAA
CCTTCTCCCCACCGCAATAAGCTCAGAGACAC
TTCCTGCCCATAGACTTACCCCCCGCATACTACATTAAACTCAGCTAACAAGGGAACCAGCGAATAAC
AGCATGGAAAGCGAAAGTAAAAAGGAAAAAAA
AAAAAAAAGGACCCGCTGCCTCCTCACGCTGGTGTGTTTTCCTTCTACAAACGCACACACACATAAAA
AAAATTCAGCTAAGGCTACTTCGCTTATTAAC
ACCTCGTTACCACCTCCCCCTCCTCTCCCCAAAAGCAAAAATGAATGTATTCCTCTTTCGCTCCATTC
TCTCTACCATGCCCTTTCATGCCTTACAAACT
GCAACGAATTTACCACACACATACACTCATGCGGAAATGTATTTTTTATATTTATACACGCGTCTCAT
CACTGCCCTCAGCGCCCGTCTGCTCGTTTGCC
AGCTGCTTCCGTTGGATTTTCCCTCTTTTTTTTTTTTCTTGGCTCCCCCCATTATTTCAACTGGCGAG
ATGGCGGTGCTTTGGGCAGGTCAGAGTGCCGG
TGTGCGTTAGTGACATGAGGGCAAAAAAAATGACACACAGCGCCGCAACATGGGCGCAAAAACACAAC
GCAACACAACACAAAATAAAGCATGAATTATG
TGAATCGAATGGAATACAACAAAATGGTGGAGTCCTCGTCAACGAGGGCAGTGGCTTTCCTTCAGGTG
CGCGCACGGGGAGAGGGCCCCCCTCCCCAAAA
AAAAAAAGAAAGCGGGACAACAAACGCACGCCCCTCAACTTCCCTCCCTCCCCGCGCACACAAATAGA
AAGAGCGGTGTCTGTCTGTGCGTGTCTGTGTG
CTTGGATATGGCGTATTCCACAGCTTGTACGCGTGTAATTATCCACCGCATCCAAAGATGCACACAGT
TGTGCGTGCGGTCATTACACGCAGGTGGtttt
cagcacctccacacc
>scaffold106.size30277
AATTTCACAGTAGAGATTAGCGCTTGGTATTAACTTCCTTCTTGGAGGGGAGCAACGGACAACAGCGG
CAGAAAGGCAGCACAACAGCAGTACACACCGCC
ACCACTCACAGCGAACCACAAAATCACCATCCCCGCAGCCATTACTCCCCAGCACCTCTTAACAGCAC
AAAAAAATATAAAGTATTTAAAAAGCAAAAAG
CCTCACAATGAGGTACCGCAGGCCACGGCCCTGCAAATAAGGCAAGCGACAGGGGGCCACGTGTGCAG
TGAGGAAAAAGAGAGTTGCGCTGCAACGGCAC
GGCGGCGTGATACCCCGTTGGGAGCATCGATGCAGACCAGCAACACCGCCTGCAAGAAGAGTTGCTTC
TCCTGCTGAGCAAAAAGGAAAAACGGCCTCAG
CAGCCCAATATTGAGAGGTCGCCTGCACCTCCTTGCAGAACCTGCAAGCTCTGCAGACAAAAAGATGC
AGACGACGCTGCGAGACGACCACGCGTGACGG
CCGTTGCGCCCTTCACAACGGCAATCGCATCCTGTCGCACCACACGCCGCAGTCGATGTGCACAGTCT
CCGTCTCACTCCCTCCTGCCCTTCTCACTGAA
AACACATTGGTGGCCGTGGCACACCCACCGACATGTCAGGAAGCACTTGTTTTTCTTCTTCCCTCGCA
CATCGCACAGCAGCGCCGCCAAACACATGCCA
CTCCTTCTTTTGCGGATTCTCCTCAGACATGCCTCCCGCCACATTCATCAACCCACAGGCATGCAGTC
CGTAAACGTCACGCAGTCGCCCCGCATGATGG
AGCCGCACACACACCAAAATGGCACCCCGCTACTCGGTGCAAGCAGTGTCTGGACACACAAAACAGGT

CGCCCCAGTCAACAACAGCTCTCTCCAAGCAG
TGAGGCATGACTCGTCACCAACAATCAAACAATCAGCCCCTCGTCTCCTCCTTCCGCCATGCACAAGG
TTCTGCCTGCCTCATACATCCTCCGTGGCGCC
CGGACTCTCAATGAGCAGAAAACACACTATGGTCCCCTCGGCTCAATGAGAAGCCCATTGACAATTCT
CAGCCGGTCGTCTCCCCGGGCTATGCATGGAG
AGCACCACGGCAAGAATGCACAGGAGCTTATATGCAGAAACCCTCAACTCATTCCACGTTAATAAAAA
GCATGGCATCATACATGGGCTTCATAACAATT
TCAACTTGCTCTTCCCTGAGAAGTCTCCAATACACATCGCACGTGATCAGAGTAAAGAAAAAAGCAAC
GCCTCCAGTCCGACCGTCATTCCGCGGTGGag
atcaaagcacaatccaaaaaaatgccgcaaatcatcatgtattccacacagaccatctgcgactcatg
aggcaaatgcacagcacggaagaagagtcatg
ctcacaaatacgcaacgcacagacaaagagggagttgggagaagcagcactagaGCAACCTCAGCCAA
CGACAGAAGAGCTCGTGGGTGCAGCACCCAAT
GCCATTCTTTGTGGATGCAAATGACAATAACAACAAACAACCTGAAGTAACAGTCGGGCACCACAATA
TGGGCCACAGACACCACCCACTCACCCATTCA
CTCTTCTCCCTCCCAATGGTGTGGCCCTCCTTCACCTCCACAGAGACGTTTGCCGCTCAGTCATGCGA
GCTTCCAACGCAAGCACTCAAGGTGCGCTTCG
GTAATCCAAAAGCCAACACCTCTGACCAACAGAGGAGATGCTCGCAGAGGCCACACGGCTCCTCAACG
CACCCTCCAAGCCCTCCTCACGACTCGTTCAT
GCGATGGAGAGGAATAAAAGCGACTCTCGCGTCACAAAACGTGGCCAATGGGAGCGGTGCCCACATGC
AGAGAATGCCAAGTTGCGAAACAGAAGAGGCA
AGAGGAGTGCTCGCAGTGGACGTCACCTGCCACTTCTGATGTGCGCCGTCTCATTCGTGCAACAGAGA
ACCAACGACACGGTTGTTGCGGTGCCTGCCCG
TGTGTGCACCTCACTCTCGGGCCAATGAACGCCTCAAGGAACATCCTATCGAGAGCAAGCAGCTGGCC
GTGCAAACAGCACCAATTCTTGCCAACGTCCC
AGCGGCCGCTCTTTTTGGCGAGGCTCCGCTCTCCTTCTTCCTTGTCTCCCGTGCACCATCCTCGTATG
AAATGAAAACGCTCTCCACGACAGGTGTCGTC
AATTACCTCCAAAGCATGAAAAGAATATCTACAGGGGCCGCTGGCCGCATCCTAAGTTTATAAATAAC
AGAAACCAAAAAAGATAGGAATAAGAAATGAA
TGAATGGAATAAAAATGGGGATTCACCGTATCCTCAAGGTCTAGCAACAAGTAAGTGTACATTTATGT
ATGCATACTTTGTTAGTCACCATCGTGACCTA
ATCACTCTATTCGGATTGGACATGCATATATATGTATGTGTGTGTATTCATAACTTACGAAGTGGCAG
ACTTTGGGGTCCTGCAGCCACAAGAACCCGCC
GCAAGGGTGCGAACCAAAAAAGGGAACGGGTGGGAAAATAAGAAATAAAAAGTTAATAAAAAATCCTT
CCTGCCGTTATCGTCTCCCTGCTCCCTTCAAT
CACCGTCTTCCAAAAGTATTCATGCGATAGGAAAAGGATCGTACAGAAATATATCTTTATGTTTTTAT
TAGGAGCTTGCGAGGAGGATGCTCACGGGAGA
GCCTGGCCGCCTAAATGAATATGTCCTGGGAAAACGGAGATATTTCTGTACGACCCTTTTCCTATCGC
ATGAATACTTTTGGAAGACGGTGATTGATGCA
ACCAAAGGAACCGATAACGTCAAGGCGAATGTTTTAATTCTTTTTATTTTTACCATCCCAGTAGCCTT
CTTTTTTACGTCCCCACCCTTGCGACGGTGTC
TTGTGGCTGGAGGACCCCGAAGTCTGCCCCTTCGTAAATAAGAAATATATTTATATATTTATATATGC
ATCCTATCCGAATGAAGCGTTCAACATGTCGC
GGGAGTGGGGCCACCATAATGTATAAATATATAAATCTGCGGAGCAAACAGCGAGGATTTGGCGAACC
CACTGCCTCTCCATTGACTCATTCTCCCTTTC
CGTCGTATTTTATTTCTTTAGGAAATTTGAGGATCCGACCAGCAGCCCAGACGAGTATTCCTTCCTTT
CGCTTTCCTGTTTGCGTGTGTGTCACAGGCAC
GGTTTGATTCAAGTGGCATTGGCGCCTCCACGCAATCACATCCTTCTGTTGCCGCATTTGCGGGGCAA
TTGGGTTTGGGTATCCAGAGCACACACACACA
CACACACACACANNNNNNNNNNNNNNNNNNNNNNNNNNNNNNNNNNNNNNNNNNNNNNNNNNNNNNNN
NNNNNNNNNNNNNNNNNNNNNNNNNNNNNNNN
NNNNNNNNNNNNNNNNNNNNNNNNNNNNNNNNNNNNNNNNNNNNNNNNNNNNNNNNNNNNNNNNNNNN
NNNNNNNNNNNNNNNNNNNNNNNNNNNNNNNN
NNNNNNNNNNNNNNNNNNNNNNNNNNNNNNNNNNNNNNNNNNNNNNNNNNNNNNNNNNNNNNNNNNNN

NNNNNNNNNNNNNNNNNNNNNNNNNNNNNNNN
NNNNNNNNNNNNNCGCAATCACATCCTTCTGTTGCCGCATTTGCGGGGCAATTGGGTTTGGGTATCCAG
AGCACACACACACACACACACACATCCGCAGCAA
AACCATAACTTGCATCCCTCCCATCCGCAGCCAAAAAGGCTCCTCCATTGAGCCACACAGAGACAGGG
TCAGGGGGGAATTAGTTTAAGTCCTCTCTTTA
TTCGTGGCTGCCACGCGAAGCAACAGCAGTCGGGAAAAAAAAGCAGGGACCAAAATTGAACAAATAGG
TATGACAAGGAAACACCTCATTTTTTCCTTTT
TTACTTCGTCCGCCAACGGTGCACGTTGCCTGCGAAATTTGGTGCGCACGACGGCAGGGGGTGCGGCC
CCCGTTCGGTGAGAAGAGCATTGAAGGCCGTG
TCGCGCACTCGCGCCGCCTCAACTCCACACCAGACTCCAACGTGAGGACCCCAGCAGCACTAGACTCC
GGAAATGAATTCACAACAGCCTCTACTATAAT
TGGGGTGGCCTTTATCCGACGTTGACGCATGGAATACGTTCCATGCGACCACAAGAAAAATAACGGAG
GCGTTTGCCGACGCGTAGTTGAAGGCAATGCC
ACCGCAGAGCAACTCCTCCACTATAAGACGGATGGGACCCTGTCGAGTGGAAGTGGAACTCGTCAGGC
TGGGGTACGTGGGCACATCAACAGCCCGGTAA
AAGACTCCTCAAAGACAACATATTATACACGTGAGGAGAGAAGGACACGTGGCAGGGCCACGTTGTAC
GATAGGCCTATCCCTCGAAGCCGCTCTCGTGC
CAAGTAATCAAAGGAAAGATTGCATTGACACCGCTCCATTGTTTTGCCGCAGTCCACAACGGCCCGCA
CGTCCCTCGTCACAAGCGAAGAGGGGGATCGC
AACCAAATTGAAAACATGCACCGTGCCGCATCATACAACACGTTCATATCATCGTCGATGTTGAGCAC
AGCTTCGGTGCGGAGGAGAGAGGCGACAAGCC
AGTGATTTGCCACGGAATTGGATGGTTGCCGCACCTCGATTTCAACGCAAGCAATTCNNNNNNNNNNN
NNNNNNNNNNNNNNNNNNNNNNNNNNNNNNNN
NNNNNNNNNNNNNNNNNNNNNNNNNNNNNNNNNNNNNNNNNNNNNNNNNNNNNNNNNNNNNNNNNNNN
NNNNNNNNNNNNNNNNNNNNNNNNNNNNNNNN
NNNNNNNNNNNNNNNNNNNNNNNNNNNNNNNNNNNNNNNNNNNNNNNNNNNNNNNNNNNNNNNNNNNN
NNNNNNNNNNNNNNNNNNNNNNNNNNNNNNNN
NNNNNNNNNNNNNNNNNNNNNNNNNNNNNNNNNNNNNNNNNNNNNNNNNNNNNNNNNNNGCAAGATCCCC
GGAAAAAAAAATTAATTTGGCAAACACTTCAT
GTGCCAAGCAGCACAAGGCAATCGGGTGTCTGTGTATATAAATATTGCCCGCACAAAAAATAAAAATA
AAAATTGGAGCAAAAACAGATCTTGCTGTTCC
ACCAGCCCACACAACATCCTTCACATTTCATGCGGCATCCGGCCTCTTCCTCCAATCCTCGTTCGTGC
AGTCCGTGGGACGCACAGCCGAAGGGCCGGCA
CCGCGCGTGGAGAAAAAAACAAACAAAAAGATGGAGAAACACTTCGCTCATTACAGTGTGCAGCAACC
GGACATTACGTAAAGGAAAACAACAGTACTTA
TTACTCCAGCTGAACTACAGTGAAAAGAAATTAAAAAGAAAAAACATTGAAGAAATAAAAGATAATTA
AAAAGGCGCGGGCAATTCGGCATGTATTCTAG
GTGAGGAGCCGCGGATCCACACTCTGTGGCTCAGAAGTCTGCCACCACCCAAATACAATCCCATTAGA
AGCAGCAACACacaagaNNNNNNNNNNNNNNN
NNNNNNNNNNNNNNNNNNNNNNNNNNNNNNNNNNNNNNNNNNNNNNNNNNNNNNNNNNNNNNNNNNNN
NNNNNNNNNNNNNNNNNNNNNNNNNNNNNNNN
NNNNNNNNNNNNNNNNNNNNNNNNNNNNNNNNNNNNNNNNNNNNNNNNNNNNNNNNNNNNNNNNNNNN
NNNNNNNNNNNNNNNNNNNNNNNNNNNNNNNN
NNNNNNNNNNNNNNNNNNNNNNNNNNNNNNNNNNNNNNNNNNNNNNNNNNNNNNNNNNNNNNNNNNNN
NNNNNNNNNNNNNNNNNNTCCAGGGGCATTGCG
TTGTGCCCATTCATTTCTCTTCTCCAATATTCCCAGCAACGTGCTCACTGACGCTGTCAACTACTCGA
ATCCTCTCGGGTGGTGTCGGGGCCGCTTCTCT
CGTCGGAGCGGGGACCCTGTCCTTGGGTGCACGCATCGCAGTGGAATTCAGTGGGCGGCTGTACGGAA
AGACGTCCGTCACCGTCAAACGAGATTCGTGC
CCACCGCACGCCCCAAAGCAAAACCTCAGTACCTCAGGTGGCCTCTCGCCTGCTAACAGCACCTCTTC
CTCCCACAGCGACTCACCATCAATGTGCACAG
AGGTCTTGTTGCATTGCAGCATGAGTCCCACTTGGTATTCTTCCTTCGGCTCCCGATTGCTGCTCCGT
GCTTTTGTCTTGCCTTCGAACGCCGTCTCCCA
CTTTGTATTATGGCTGCACGACAGTCCCACGGTATGATTGGACGCAGTGTCCGCCAGCATCGCGGTCA

GCAGAGGCGTGTTCCCACTCGGAGTCACTTCG
ATGGTCACCGACGCTACAAGTGTGGAGTCGTGGCTCAAGGATACATGACGCACTTTATCACTCCGAGT
GTTCACAGGCCATATTGCCCTGGACTCAAGTC
CCGTCAATTGTAACCCATCGCTGTCCTTCGTGGCGTTCCTCACCGTTGCATTCAAGCAAAGGTACTCG
TCGTTCCACGTGTCATCACTGACCGCATCGAA
AAACACTGCCACCAGACCCACCGTGGGTATAGACAAGCTGGAGAAGAAAATGTCCTTTGGCGCCCAGG
TACTGAGGACAGAATCGATTGCACTCAGTTCC
TCCATCAGGCGGGAAAGTGAGATGACACGGCCCTCACCGTTGCCCCTCCGTTATAAAAGATGCAAACT
CCCATCCGAGTGCAGCAGGTTGCTGGCGAGCG
TCCAATTCGCAGCATTTTCCACGGCTGCCGGTCCAACAGAAAACGTGCGGTTGTTGTCCGTGACCCAA
AGGCAGTGCGAAGTGGCCCTTTTCTCCCCGAG
GCATGCCCTCTCTGAGTGTACAGCATGACCTCCCTTCCCTCAATGGTCGCGGTGATGAGGGCATCCAC
ACGCAGGCTTTCCTTCTGAGGGACTCCTGATC
GGGCGTTGACCCACACGGCTGAGAGTGCCCCGATGGCCTCCGTCCACGCTGTCCCCATGTCACGCGAC
TCGTACACCCTCTGGCCACTCTCGCAGTCAAC
AATCATGAGAAGTGATCCCTCCCATCCGGTGACGCAGGGGTTAAGGCATTTCGCAGGGGAAATGTCCT
CAGAGAGCGACCAGGCACTGCCGTTGTCCGTC
GAGTAAATGGTCATGGAGCAAACGTCTTCCGCCTCATTCACCGCCATCAGAGGAAACACAAGCGTGCC
ATCCTCCATCACAACGCCTGTGCCACCAGAAG
CAATGAAATCCGTCCATTCACCCTCGTGAGAAGCAGAAGAGATCCGTTTCGGGAGTGGTTTGGGGTCG
CCCCATTCGATCCGTTNNNNNNNNNNNNNNNN
NNNNNNNNNNNNNNNNNNNNNNNNNNNNNNNNNNNNNNNNNNNNNNNNNNNNNNNNNNNNNNNNNNNN
NNNNNNNNNNNNNNNNNNNNNNNNNNNNNNNN
NNNNNNNNNNNNNNNNNNNNNNNNNNNNNNNNNNNNNNNNNNNNNNNNNNNNNNNNNNNNNNNNNNNN
NNNNNNNNNNNNNNNNNNNNNNNNNNNNNNNN
NNNNNNNNNNNNNNNNNNNNNNNNNNNNNNNNNNNNNNNNNNNNNNNNNNNNNNNNNNNNNNNNNNNN
NNNNNNNNNNNNNNNNNACACCTTCGTAGGACA
GATCAGAGCCTCCCGCAAGAGGAAACGCTCCGTGGACCTTCGTAGTTGTTGTGGGGCGGAGCACAACA
CCCAAACTCTCCTTTCCCTCCGCTGCACCAAG
CACAGCGTGTGCCCTCCATGTATCTTTTATTGACCTCACCAACAAGAGTGGACCAATTCCACGCAGAG
TCAATGCACCCCGCAACCACATCAGAAGAAAA
GGGCTCACTTAATTGACCACCTTGATATTCGGCATCCGTGTGACCCTCGGCGAAAGCAGCAATTACTC
CACCAGCACTGACAGGAGAGGGTGAGGCAAAC
GAATCCCTCGTCGTAACTGGAACAATCCCATCCTCCGACTGCACCAGCGTCGTCTGCGGCACAAATAG
ATCGACTCCCTGCAGTAACTCAACATTTATTG
AACCACTCATCGCATCATCAACGGGATCCTCCTTCGGCTCGGTGACAGGCTTGGACCTTTTTTCGGGT
GGTGTCGGAGCCACTTTTGTGTTCCGAACAAA
GGCAAACTCAGGCCTCGTCTCGGACGTGTTGCTCTCCGTGATGGTGCGGGACTCGGATCGACGGTATT
GGGTGCGGGGTGCCTTCACTGCAGCAACGCGT
GAGAGCATACAAATATGTGTATGTATGTGGGCGGTGTGTGTGCTGTTGTTCCTTTCAGTGCCTTCGGA
TCTCACAGTAGCGATTGGCGCTTGGTGGTAGC
TCCCTTCCTAGCCTGAGTGTGTTTGTGTGCAAGAAATCAAATTAAAATATAAAAAGCATCAAAAATGA
ACGTGGCAGAGGAGTGGGTGGCGCCACCATTC
ACGGCGTAACACATCCGCTTCGTCGTAACCGCCACAGTAATTACTCTCACCGTCCACAACGGACCAAA
AAATGTGAGGCCACACACAGAAGCAGAGAAAT
TGACGGTTGCGCACGGGCCAAGACGCCCTCCGGCAAACGGCACCAACCAACAGACAGAGGAGAAATTA
AAGGCAAGCAGAATCCCAAGTGCTGCGGGATC
CTTGCAAGCAGCGCTCCCTCACTGTAGAACCTTTAAAGCGTTCCTCGCTGCAACATCCCCCACCACCC
CCTCGGCGTGTCGGTGGATTTTCCCGTTCACC
GGAAGAAAAACGGCGGGATGCTTCGGCAAGGGTCGAAGACACACGGAGCGCGCCGTCGACGCGATCAA
CGAGCGCAGACACTCGCGAAGTGAGATTCCAC
AACCACGATCCCTCCATTTACTCTCCTGGACGTGGAGAAGGCATGAAATGTCCTCCTCTCTACAGTAG
TCCTGAAACGCAAAAAAATCCGCAGGACATCC
CGCGCAGCCACGGCCTCCTCAGCAGCCCGACAGTCCACGCCGTCGCACCGCTCCTCAAGAGGTCGCCA

GGCCCGGTAAGGCAAGCAAAAGGAGAACAGCA
TTTTGGTCAGGGCGTCCAGCAAAATGAGGCGCAGCAGTCGGGTGAGCGCGGCCCATCCACGTCCTCCC
CTCAACGGACCCACCACACAGTCGAATCACAC
CCCTGAAAGGAGCCGTGGCGTCTAACGAAATAGCATGGTCAATAGCACCTCGTGCTCTCCTCAATGTG
GTGTCATGGCACACACGAGGCATCACAGGAAG
AGGCAAATAATTGCATTCCATCGGCATAAGCACGGAAGTAAAGCGCGCATTCTGCCCCTGTGAACTTC
AATCCAAATCGTCTCGGCCACAGAGTTTGTGT
CGCCGACCCTCAGGAAGGGCTGATGCACCAACTGCCGCTGACCGATCACTCAGCGATGCAACAAAAAG
TGCCACCGTCTCTCGAGGTTAGTCATCACCCA
TTCCCCCCATTGAGGGAAGGCAACGCAATGTGACGGTGTGGGCGTGTCTAGCTGGCAGAAATGGAGGA
ATTGACTCCAGCAGCAGAACAAATTGAACTGA
TCCTCCTCCCGAATCCTTCAAAGAGACCGGTCGGTCAGCTGCCGAGGTTGACGGCGCGGAGGACAACA
CATTGAATGTATGGAGCACCGCAGCAACCTGA
ATATATTCTGCAATATCCGCGGTCAGTCCATGAACGCACAACAAAGAGACAGCAAGTTCAAAGGGGCG
TGTGAGCCCGTCAACAATCACCCGCGCCGGGG
AATCGCCACTCCCACCCTCTGGGGGCACCTCAATGAAGTTAAATATTTCCACCGCATTGCATAAAGTA
GTGCCTTGCAGCACCCCATGCAGTGCAGCAAT
AGAAAACCATCTCGAACACGGCGTGCGACAAATATACCAAAGGAAGANNNNNNNNNNNNNNNNNNNNN
NNNNNNNNNNNNNNNNNNNNNNNNNNNNNNNN
NNNNNNNNNNNNNNNNNNNNNNNNNNNNNNNNNNNNNNNNNNNNNNNNNNNNNNNNNNNNNNNNNNNN
NNNNNNNNNNNNNNNNNNNNNNNNNNNNNNNN
NNNNNNNNNNNNNNNNNNNNNNNNNNNNNNNNNNNNNNNNNNNNNNNNNNNNNNNNNNNNNNNNNNNN
NNNNNNNNNNNNNNNNNNNNNNNNNNNNNNNN
NNNNNNNNNNNNNNNNNNNNNNNNNNNNNNNNNNNNNNNNNNNNNNNCATCTCAGCAGTTCCAATCAT
CCAGTAAAAGGCAGTAGAGTCATACAGCACCC
ACAGACTCAGCAAATGTGGCGTCAAAAGGCAGCGAGATGTCAGATGCCGAAAGGCCCGCATTTCCCTC
CGTGACACTTCCACGGATGAATACAAACGCCG
TGTTCTGCGGCCTCTCGACCTCTTCGAGGCAACGTTGCGGCCACATGGCGATGGCCCAACGTGGCTGG
CAATCAGATCCAGTCGTCGCGGCAAAACAGTA
CAGAAAGTAGGGTGGTGGGCCCGCACATTCGCCTCGTCGATGCCTGCCTCAGCTGCAAGAGTGAAACT
CGTCTTCCCAACAGCCACGCTTCGGGTGGCTG
AAACACCTGCCCTCACACCGGCAACAACATCTGCAAGCGCCTGTAATTCCTCGACACAGCCCGTCAGA
AAAAACCAACGGCGCTCAAACTCTGCAGCCAC
ACATCCCTCGTAACCATTTGCAAGCTCGTTGTCCTCCAGAAACACCCGAACAACATAACAGGAAGACC
GTCCAATGCTGGCAAGGGTCAAGAACACGTCG
CTTTCATCCTGCTACAGATGATTGTGCCTCCTGCGTAACGCGGCCACAAGTACGCGGTGCGCCAACAC
CGAAGCGGTGGGAATGCTGCCCCACGACCCTC
CCCTCACCACAGAAGTCCAAGAAGTGCAACAGACCATCCTCTCACCGAAGAAAAAGCCCAATGGAGCG
GACACTACATTGTGCGGACCACACAGCAGAAA
ATCCCTCAGGGAACGAATGCTCTTGAATGCCCTTCAGAAGGGGCCAGCGGGTTGGCCGTGAAGCCAAC
AGACACCGGAGAGCCACCTGCTCTGCACGCAT
CCAATGACTCAATTGGGAGCGCAGTGTTACATGGCACATCAAAAATCCCATTCTCGTCTCCGTCTGTG
CGGGCATGACAGTCCTGCCATCATCCCTCGTC
CTCACAAACTATTCCACAGGGTCCTTTGACCAAATACTGCCACACTGCAATATGCCACCGGATAAGTG
ATCCCCATACTGCACAATAATAAAATGTTCCT
CACGTCGAGGACCGCAGTGAAAACGAGCACAACGATGAGCAACACGAGGGTTGTCTGTGCTCGAATTC
ATCACAACACTCTACCCCCCGTTGGTTCAGGC
CTCTGCCACACGAAGAGGCACAGATGTAACATTACTTACACGCAACAGAGTGAAATCCGCAAGAACAT
CGTCAGAGTCATGCAGGACGCAACAAACACCA
GCCCGCTCAGCAGCTGCTGTGGTCATATTCTGTAAAACATGATCCACGCCCACAGAGAACTTTGTTGT
CCAGTTGCACAAATAATTGATGGGCCAAGCAA
CGTGGGCCCAAGAACGACGACACCAGTCGATATTCGGCCGTGGAATCGCCAGTGAGAGCCATTGATTG
GAACAAACACCAAAGCCACCAACGGCCAACAA
GAACTGCAACAAGGCATTCGTACTTGAATCAAAGGATAGATTCGTCTCGTCCAGGAACAAAAAAAAAA

CATTCTTCCTTCTCACAGAGCCTTCCAGCAAT
AAAACACCATCATGCAGTCCACCCTTATCGGCATTACTCAGGAGCACAAGAACTGTGCAATTTGTGGA
GGGCGCTGCAATCAAGCAAATAAGATGCTCCA
CGGTGGCGGACAGACACCTCACAGGCCAAGCATCAACCGGTCGCGAGCAGCATGTCTCTTCATGGGCT
CTTCGTTTTGGTTCCAACCGAGACGGAGGGCT
TTCGTCGCTCAGCACCAACCCCGCGTTGCGCCGTGTCGTCAGATAAAACAAGTGAGGATGACGGAGCA
CATCATCGGGGAAATTGCCCTCATATGTGGGA
AAAGGCATCGCATCCGCCGGAAACAGGTGCGCATGGTGTCCCTCCACGTCAGCTGCGCAGCCTGTCAG
CTGAAGGAAAATACGGTGAAACGGAAGTCAGC
GCACTCACCTCCACCCCAGGCAGTTTAATCAGGACGATCAACGCACCAAGCTGGAGGCTCCACACTTG
TTGAGGGATATCGTGACGCTCTGACGCGTCCA
CGTGCATGTCCACACAATGTCCTACTTCCTTTTCCCCCGCCGCACACGCCCGCACCGTCTCTTAAGCC
ACATCCAGGAGGGATCGGATGGCGCGTAGCAC
CTGGTCAGCCAGCAGCGTTCCAGTCCTTAAGGCTCCGAATACCGAAGCCCCAAAATCACCGGCCATCA
ATGATGCCGTTGACACAATGCCTCCTCTGGCC
AACGCCACACACTTGTTCCGGAGCACATGTTATCCCATGCCTCGCTGACGCTACCCTCAGCATCCTCT
ACGGGTACACCGTGCATTCACTCCCAAAATAC
GGGTGTGGAGTACACCTCAATAATAACTGCACAATGGGCCTGCAAAGTGCTCGCTGAAAGTTGTCATT
GTGTTGGGTGGCAGGGGCAACGCAATCACACC
TCGCGGCTCCTCAGGAGATCAAACAAGATAGCAGNNNNNNNNNNNNNNNNNNNNNNNNNNNNNNNNNN
NNNNNNNNNNNNNNNNNNNNNNNNNNNNNNNN
NNNNNNNNNNNNNNNNNNNNNNNNNNNNNNNNNNNNNNNNNNNNNNNNNNNNNNNNNNNNNNNNNNNN
NNNNNNNNNNNNNNNNNNNNNNNNNNNNNNNN
NNNNNNNNNNNNNNNNNNNNNNNNNNNNNNNNNNNNNNNNNNNNNNNNNNNNNNNNNNNNNNNNNNNN
NNNNNNNNNNNNNNNNNNNNNNNNNNNNNNNN
NNNNNNNNNNNNNNNNNNNNNNNNNNNNNNNNNNGGGGTGCTTCAATGCATATGAAACCTTGACCGCG
GGACGGAATGCCTATGCGCTCTAAAAACGTAA
GCATGAACTCACGGGAGAAGAATACATAACATAACGGAGGCAAAATGCCACAAATAAGAAAAAACGAT
GGAAAAAATGTATGAAGCAATATAGATATGAA
GATAAGCATCGATCAATGAATAGGCATAACGATATGGGTATGACTACAAAAGCGATAATTTATTTACT
GATGATGTGTATAGATGTGGATATCTCCCTTC
CTTCCCCTAAAATTCGACAGATTGCTTGGTTTCTTGGTTTATTGAATTGCTATTCTATTGGCTGTATT
TGTATATACAGATAGGTATATAGTTGTCGATG
TTAATATCTATTCAGTGCTGTTTATATATATTGTTACTATTCCTCGTGCCTTCTCAAGTAAATACAAA
GACCCACACGCCAGCCACCATCGCAGAGCACG
TCGGCCCAATGATTAAAGATGTGAAAGAGAGCCCCGGCACCCAAAGTGGGAGCCGGACAGCGGAGTAA
ATGCAGACCTGCCTCTCCGCACAGTTCCAGTC
GACCGGTATTCACAATGTCCGGCAGATGCCCGTGGCGTGCCGTGCGGTAGCGGGTCAGTCAGGCATTG
CTGTGGCACGGAAACCCGCACGCTAAACGGCA
AAACCAAACTGGTGGTGGTCGCAGCGGGATGTCGTCGCCGAGCGACGTGCGCCTTCTCGGCGGCCTGA
GCCCTCTGCCGCCGGGAAATCGGCAACAGCGT
GACTTTAATCAATGCATGTGTGCGTGCCATTTTAGACACACAAGGTGCACTGTTTCTCGGTGGCATTC
TCCTCCTGCGCAGAGGCGGGATTCTGCGTTGG
TTTGATGCTCTTCATGCGAATGTTCGAGCTTTGCAGCGCGTGGTTATTCGTTCTCCATTTTTAGTGTC
ACAACTTTCTTTCTGTCAGGTGTTTGTGCCGT
GTGTGGGTGGTCAATCCCCCGGCACTGCCCGCCTGCCTGTCGCATCAAGGGCATTTACGAAGTGCCGC
TTTCTTCGGTGCCGACGGTAGCGCTGTTCTGG
AGTGTTCGTGTGCCCGTATGCGGGATAGTGGTCCCGCTTTCGCTTGAGCTCCGAATTCACAGTACGTG
CATTCGAGCGCGGTGGATGCCGTCAGACCGAC
ATATTCCCGGCAACGCCTCCTATTTGTCGGGGGAACTCTTGCTTGTGGAAGTGCCAAAGATGAGATAC
GGTTCCGGCTTGCCTTTCGTACGACACCGTGC
AAAACTGCATGGGAGTGGCTTCGGCATGGGCTCTGCAGTCTGCGACGGAGGTGCGATCGGCGTGCCGT
CCCTCGCTCTCGTCCTCGCTCAAGTGCTGGGC
GGCGGTTCCGGTGGGATTTCTGGCGGCTCCGACTCACCGCGAAAGCCAAATTCCCGCAGCGCCTGGGA

GCGCGTAATGCCATGGGCGTTAACCATGTGCG
TCACGACACTAGTGCGGCAGCTGCAGTGCTTGCCGCACACCGGGCAGTCTGTCGGGCATCGGATCGTG
GCGGTGTGGGGTCCAGCAGGAGGAGATGGCTT
TTCCGTGCAAAGTTCTTTGCGTGCCTGCTGTGCGGCATACGCGCCGCGCCAACGGCATGTGCGTGGAT
GGCGGGTTGCAGTGGCCGTAGCAGCCATCCGT
ATTCGTGAGAAACACCGGTGCGGGAGCGGGCCGGTGCAGTCTCCTCCCTGCGAGTGACAGTGGATCCC
GTGCGCGTGGGCTCCCAGTCGCCTGCTGCAGT
GGAGCGGTGAGCTCCTGGCCTGAGCTGTCGGACACCAATAATACGTCCGGCCAATGCGACGATGCCGG
TGCTCCATGTGTCTGTGAGCTGTGTGAGGTCG
GATGCCTTCTCGGCTTATGCATCAAATTCTTCTTTCTTGGGTACGCCGCAGTGGCCAAGGACAAACCG
CATCCGTGTGCGCGCTCCTCGGTGCTGCTGGC
AAGAAAAAAGGGTACGTCCACAATGTGAGTCACCAGGTGATCCAGAACCGTCAACGTGTGCTCGCGTG
CTTCCACGGTTCGACGAGAAGGCGCATAAACG
CACGCTGCCGTGGAAATAGTCTCGGTGAATATCGTACTCTCCATGCTAACACCACACCCCTCTGTGTC
TGCTGTGGTGCATGACTTTGCAAAGCCGGNNN
NNNNNNNNNNNNNNNNNNNNNNNNNNNNNNNNNNNNNNNNNNNNNNNNNNNNNNNNNNNNNNNNNNNN
NNNNNNNNNNNNNNNNNNNNNNNNNNNNNNNN
NNNNNNNNNNNNNNNNNNNNNNNNNNNNNNNNNNNNNNNNNNNNNNNNNNNNNNNNNNNNNNNNNNNN
NNNNNNNNNNNNNNNNNNNNNNNNNNNNNNNN
NNNNNNNNNNNNNNNNNNNNNNNNNNNNNNNNNNNNNNNNNNNNNNNNNNNNNNNNNNNNNNNNNNNN
NNNNNNNNNNNNNNNNNNNNNNNNNNNNNCGC
ATCAAAAAGCACCTCACACTTACCAATTCCACGTGGCGTACGTCACATGGCGGCGTAATCGATCATAC
GGCTCGCACTGGATGCACCGTCGAACAGGTTC
AGGGAGTCTGAGCGGGTGTCGCCGGTCACAGACTGCAACGGCGGTGCGGGTGGTCGGTACGACGACGC
AGCGGTGGTGCCTGAGGCGTACGACAATGACG
TCATGTCGTGGAGCTTCTGCGCGTCCTCGTCGCTCTCCTCGTCATCGCGCAGCAGCGCCTCCAGCCCT
CCACGCCGCGGCTCGCGCAGCTCCTGCCAGTG
GCGGTCCTCCATGTGCCACACAACCATGCTGTACACAGTGACCGCAAGCAGCACAGTCGTCAGCAGCA
GCACGATAGCCGCGTACGCCTTCGCACCGCCG
TCGCTCGGCGCAAGGTGGTTGGCCGCGCTCACAAGGCACAGCGCCGCAAGGAGCACAAAGCTCGCAGT
GTGCATCACCGTCAGGAAGGACGAGCGCATCA
TGTTCGTGAAGGCAACCACGCCCGCACCCGCAAGCAGCAAAGATGCCATGCAGAAGTACTGGACGTGG
CAGCCTCCCACGGGCGGGTGCACAGCCGCAAT
CAGGCACACCGCACACAGCACCGACAGCTGGAACACGCACCAGTACACGTAGCTGCCCTTCATGCTCG
TCAGCATGCCGCCGTACATCCGCTGCTGCGCT
GCGGGGTGCCAATACCCGACGGGGTACAGCAGGCGCTCGTGCAGCGGCTTCCTAGAAAACTGCCAGTA
CCTCGTAAAGCTCGCGCCCACGTGGCGGGCAA
TCAAGTAGCACACGCCAGCAGGGAACGCCACACCGTACAGCACGCCAACGACACCGATCACGCGGTGC
TGCACGCGGGCGCCGGGCATCGCCAGTGAAAG
CACGGAGCCGAAGAAGATGCCGAGGTGCATCGCGTGCGCCACAACGTACGTCAGGCTCGGGAAGCGCA
TCGCAGCCCACGCACTCGCCGCGTCCACGCCG
CGCCACCGCTGGAAGGCGGCCGTCACACCGCAGTGCACGCACCCGAACGCTGCGGCCAGCAGCGCGTT
GCCCACCACCATCCACAGCGGGTCAAGGGCAG
CAAACACCGACAGGAAGTACGGCAGCGCAACGGTGCTCGCACGCTCCTGTGCCGAGGCGCATGACATC
CTCGCAAACACGCCGAGCCCCTGCATCTCCAG
GATGGAGCCCAGCCCGCCGGTCACCACCACGGCAACAGCCGCCGCACCCGCAACAACGCCGGTGATCG
CCGAGAGGGTGCTGGCAGCGGCGGACAGCGTG
TCAGACCTGATCGTGAAGGATCCAAGCAGCACACCCTTGCAGCCGCCGGACACTGCCGCAGCGTCGCA
GCGAATGACTATCGTCTCGTCGGCAGTAATGA
AGTAACCGCGATGCACGGGCACAGCCAGCTCCAGGACGGTGGACGGATTCGTCGCGTTGCGCACCCAC
GTCGCGTTGCTCAGCATCGCTCCCCACGGACC
ACCAAACCCACGCGGCTGCGCCGTCGACACAGGCACAAAGCTCAGGTGCGTGCCGAGCTGCGGGTCGC
GTTCCCATCGAAACGGCGGCGGCAGCGCCACC
ATCAGCCGCGTCGGGACGGCGCTCCCACCGCCGCGGATGTCGTTCTGCGTCAGGCTCCCACCAGCCGC

CGTCGTCGTCACGGCGAGCGTCGGGCACGCCA
CGTCGCTCCACCACAGCGTGCTGCTCACNNNNNNNNNNNNNNNNNNNNNNNNNNNNNNNNNNNNNNNN
NNNNNNNNNNNNNNNNNNNNNNNNNNNNNNNN
NNNNNNNNNNNNNNNNNNNNNNNNNNNNNNNNNNNNNNNNNNNNNNNNNNNNNNNNNNNNNNNNNNNN
NNNNNNNNNNNNNNNNNNNNNNNNNNNNNNNN
NNNNNNNNNNNNNNNNNNNNNNNNNNNNNNNNNNNNNNNNNNNNNNNNNNNNNNNNNNNNNNNNNNNN
NNNNNNNNNNNNNNNNNNNNNNNNNNNNNNNN
NNNNNNNNNNNNNNNNNNNNNNNNNNNNAAAGAGTGTGCGGTTGCACCCGTCCACGTGCGGCAGGTGC
ACCGGCAGGCAGTCGCGCCCGTACCCGCCCTC
AGCACAGCGGCAGCGGCACGACCCCGACATCGCCCTCGTCGCAGCTGCAAAGCAGCGCACGTCGGCGC
CGCACACGCCACACGCCACGGGACGGAATTCC
CCGATGATGCCGGCGGACCTGTAGTCCATCGGCCGCAGCGCCTGCCCGTTGAGCGTCAGGCACCCCAC
GACGAACTTTCGGCCGGTGCCCCCGAGCGCAA
CGGTGCCGTCCATCACGACGGGTCCACGGCCGGCGTTCCCGTGGAGCGTCAGCGTGCTCTGCACGAGG
TCCGCACTGCGCGGGTACGACGGCACCGACAG
GAGCCTGCCGCTGATGCTGTTGCCGCGCACCGACATCCACGACCCGGACACAAACGACGCCGACCCCC
GCAGCACCAGCAGCGCGCCATCGGTTAGCACA
CCGGCGTTGTCGTTCACTACGACGGCGGACGCGTTGGCCTCCACCCCGCTGTCGAACACCAACCCATG
CGCGGCGTACACCTGGCTCCCCGACACGCGCA
GCACAGCGCCCCCCGACGCAACCACGCGCGCACTCGCGTACAATGCAACGTCGCCACCGAACACAGCC
GCGTCCAGCGCGACACCGCCGCCGACAAGCTC
CAGCACAGCGCCGTCCACCACCACCGTCCGCCCACCAGTCATCACGGTCACGAGAGCAACGCCAGACA
CAACCAGCACGGACCGCACGAGCCGCAGGCCC
GACAGCACCAGCACCGGTGCGTACGGCGAGGAATGCGAGCCGGAAAGATACACAAGCGGCGTCGGCCT
CGTGGCAACCAGCAGGGAGTCCGTCACCGTCA
GCACGGACCCCGCAGGGAACGTGCCGGTCAGCACAAGCGCGCCGTTCAGCGCTTCAACCTCGCACACG
GACAGCTCCACCGGCCTGCCCTCGTCGCCCAC
CGCCGCCATCGCACCGCGCAACGCTCCACCGCCCATCACGTACAGCACGGCGCCGTTCAGCAGCGACA
CGTTCTGCACAACGATCCGCACGCCGTCTCGC
GCCATCCACGGCACGTAGAGCACAGGACTCACGCCGTCCAGCACAACGTGGCGCAGCGTCACCTCGCT
CGCGCCAGCAGGCACAACGAACACCGACCGCA
CCGTCACGCTCTCGCGGACGAACACACTCGGCGCAGTGCCCGCAGCAGGCGGCAGCGCGGGAGCTCCC
ACAGGCACGCACGCCTCGCCGTACCCACCGCT
GCCGCACGCGCACACGCACACACCGTCCTTCATGCTCGCCGACGCCGTCCCGGGCGCGTAGCAGTGCG
TGTCCCTGTCGCAGTCGTCGCACCCAGCAACG
TCGATGCTCCCAGCGTCGCCAAAACCAGCCGCTGCGTACTCCTCCGCCGTCGACAGGACACGGCCACC
AGCGTCGTTGCACGCCACCCTCACATGTGCCC
CAGCCGCAAGCGACAGGCCCACCGACAGCATCCGTGGCAGCAGCGCTCGGTTGCGCACGAAGCTCAGC
GTGCTCCCAGCAGCGGTCACGGCGCCAGCGGC
GTGGAGCATCTCCCTCGAGACGCCGTCGTGCACGTTGCCCTTCGCCAGCAGCATCGACCCGCCATCCA
GGCTCACCGTCGAATAAAACGCCACCGCGTAG
TCCGCCGTCGACAAAAAGTCGTTCCGCACAAGCGCCACAGCGGAGCCCGACACGCTCACAGCCCCACG
CACAGACANNNNNNNNNNNNNNNNNNNNNNNN
NNNNNNNNNNNNNNNNNNNNNNNNNNNNNNNNNNNNNNNNNNNNNNNNNNNNNNNNNNNNNNNNNNNN
NNNNNNNNNNNNNNNNNNNNNNNNNNNNNNNN
NNNNNNNNNNNNNNNNNNNNNNNNNNNNNNNNNNNNNNNNNNNNNNNNNNNNNNNNNNNNNNNNNNNN
NNNNNNNNNNNNNNNNNNNNNNNNNNNNNNNN
NNNNNNNNNNNNNNNNNNNNNNNNNNNNNNNNNNNNNNNNNNNNNNNNNNNNNNNNNNNNNNNNNNNN
NNNNNNNNAAGCCGCTGTCAACAAACGCCACC
ACGGAGCCGCCAATGGCAGCAACACCGCCCTCCACCGACACGCACAGCCCCAGCGCCGTCTGCACCGA
CAGCCCGTCCACGTAGAGCGCACCGCGCGACG
ACAGCGCCACGCCGCCAACAAACAACACGCCAACAGCGTCACGAAACACAGCCGCCACATGCGTGCGC
GCGATGGTCAGCACGCTCCCCGTCAGATTCAC
CTTGTGCACCACGAGGCACGCGGAGGCGTCGCCAAGGCCGTAGGCGCCGTGGTACGCAACGCGCTTCT

CCGCAGTCAGCACCGAGTCCACCACAGTGACG
CGCGAGCCCGGCGGAAATCGGTTCGCCACCACCACGCCGCCGCCGGAGCGCGACTCAAGCCCGCTGAT
CAACACATCAACGCGCTGTCCAGCGGGCGGGT
CTGACAGCCACCCAACAACGTACAGGCTCGCTCCATCCACAAACGTGCAGTTCACCAGCGTCACGTTG
CGCACGCTCCCCGACATCGACTCCACGTTCAC
CACCAAGTCCGCCGCAAAGGTCACGCCAACGTAGCACGCCACCGACGTCCCGAAGCCGGCGTTCACCT
CCACGTCTACACGCACGTCCCGCACGCACAAG
TCGGCGCCGTCAGTGCTCGGCGGCTCGGGGACAGCAACGGGCAGACACGCATCGCCGTGGCCGCCCTC
AGCGCACGTGCAGGCGCAGCCATCACTCGAGG
CCGTCGTCGTGTACGCGGGGAGGCACGACGCCGCGAGGGTGCATGGGTCGCTGCAGGTCAGGATGGTG
GCATTGTACACGGAGGAAATGCTGTATTTCAC
TCTCTCACCGCCGTTCACAGTGTTGCAAGCAGCCACAATCGCTCCGTTTGTGAGATCGCGGGGCACCG
TGGATATCCGCAGAACGGTCGGCGTGCCCGTG
CTGGACATGAATCGGTTGCCGGACACAGACACCGTGGAGTCGCGGAGATTCACCGTGGGGTTCAGAAA
GAGAAAAGCTGCCGATGGGGATTCACGGCAGA
GGTTGCCGCTCAGCTGCAGCCAGGAGTTGTAGTAGATGTAGGAAGAGTAGCTTGAAAAAAATACAACC
GAGCTTCCCTGCATGTCGTTGCCCTGAAACAC
AACCGCTGACCGAGACTCCACACGTAACGCCAAAATTACTAACAAAAAGTACTTCACCGGCCTCGTCA
CAACGTTGCCTCGGATCACCACAGCAGAGTTG
TTCAGCACCTTCAGCGATGAAAATTGGCCAGACCCTTCAATATTTATCAGCGTACTGTCACCGCCAGC
CATGTGAAACGTGTTGCCCAGCACCGCAAGCA
CGGAGTGCCACGACACCCTCAGCGCAGATCCAAAAAATTTTACGACACTTGACGATGTCGTCACGCTC
TGAAACACATTGCCGCTCAGCACCACCGCGGA
GTCGTTGCTGATCACCAGCTCATTCATCACAACACACGACGGCTTCCTAAGGTCCAAACCCAACCAAT
GGAACAGCCTCGTGACCGCAAAACTGTTCCCG
CTGATCGTGATGTTTGTGTGCGGTGGAAACACACCCTCCGGGCTGAGCAGCGCGTCAATAAATGTGTT
GCCGGTGACGACGATGTTCAGCGCCCCCACAG
CGGCGTACGCGTTGCCCAACAGCAACAGCACGGCGCCGTCCCTCCACGTGCATCCATCGAACGTGACA
TTCGCCACGTCGCCCGTCATCCCCCCAATCAG
CACCGTCAGGCCCATGACACCCCCGCTGAACGTCACGTTGCGGTAGCACAGCCACGACAGCCCGCTGC
CCACAGACTGCGCAACCTCGGGGTACACCATG
TCGCTGATGCACTCACCAACCGGCGGCGGTGGCGGTGGTGGCGGTGGCGGTGGCGGGCCAGCAGGCAC
AGGCGCCGGGACGCACTCATCGCCGTGCCCTC
CAGCAGCGCACTGGCACTTGCAGCCGCTGACCGCCGTCGTCAGAGGAGCAAAGCAGTCGCCGTCCTTC
GTGCACTCCCCGCACGCGGCAACCGTCGTCAC
GTTGGTGATGCCGTTCAGCGCCAGTTCAGCCGCCGTCGTCACCTCCCGTCCCGCGACCGTCAGACACC
CAGCAACGAAACGGTAGCCGGCGTCAGCCCGA
GATAAAAGGGACACCGACAACCCCGTCGAGCCCATCTTGCAGCCAGTCAGCGTCACCACACTGCTGCC
GTCAATGCTCACAAAAGCAGAGCCACTGTCGT
AGAGCATCGCACCCACCTCCACGTCGTTGTCGCGCCAATCCAGCCAGCTTGACCGCTGAACAGTCCAC
ATGTCATCGTTACAGATGGCGTAGCGCACGGA
GCCGCTGTTCCCCACCACACGCACTACGCTGTGATCCGACACGTTGAATCCCTGCTGTTGATACAGCA
GACTTGCCCCGGAGACCATTGTATTGTTGAGC
AGCGCAATAACGGAGTGGTCACTCACACTGAGCCCGTACAAGTAGAAAAGGTATTTGTATCCCTCAAA
GCTGCAGTACCGCGCGTACAGCGAGCTGCCAC
CCACCAGCGTCAGCGTCCCGGTCGAGTAAAAACCAAGCGCATCACGCTTCATTGCATGCGCCTTGACG
TTGCTGACCAACAGCGTTGACGCACTCAGCAC
GACGTCCTTCAGCAACAATACGCTCAGCGTCTTTCTNNNNNNNNNNNNNNNNNNNNNNNNNNNNNNNN
NNNNNNNNNNNNNNNNNNNNNNNNNNNNNNNN
NNNNNNNNNNNNNNNNNNNNNNNNNNNNNNNNNNNNNNNNNNNNNNNNNNNNNNNNNNNNNNNNNNNN
NNNNNNNNNNNNNNNNNNNNNNNNNNNNNNNN
NNNNNNNNNNNNNNNNNNNNNNNNNNNNNNNNNNNNNNNNNNNNNNNNNNNNNNNNNNNNNNNNNNNN
NNNNNNNNNNNNNNNNNNNNNNNNNNNNNNNN
NNNNNNNNNNNNNNNNNNNNNNNNNNNNNNNNNNNNNATTGATGGGAAGATGCAACGGCATACTGTCGA

AGGAAAACGTCAACGCAGCACCCACACCGCTG
AATGTCACACCCGCGTAGCAGTGGTGCGTCTTCCACATGTTCAGCGTGAGGCCCGTCAGCGTCTGGTT
CACCACGCAGCTCGTGTCGCCGTCAACAGCTC
TCTCCGCTGCGGGCGGCACAACCGTGTCGGGCACCTCGAAGGGAAGACACGACGCGCCATGCCATCCG
TCCTTGCAGCTGCACGAGCACGAGCCGCGGTC
CACCGACTCCGTCCCGGGCATGTAGCACGCCGCGTCGTCGTTGCATGTGCCGCACCTGAACACCTCCA
CCACCTCCGGAAACACACCGTCGTACGACACC
TCCTCATCGCCCTGCAGGTTGCACCCAACCACAAACCGCGCGGGTAACTCAACAATCGTTTCTAGCGG
AGAAAGATCAGCAAAGGGATAACTATCGTCCA
CCTGACGGTTGTGTGCAAGCGCCACGGTGGTGCCGCTGCCCGACAACTTAATTTTGTATTGGGGATAC
GGAATAGTTAATACTGAGGCTTCACCCACGCT
GTTGCCCTCCACACGCCACTGCGCACCTCCCTCGAGGGTCACCGAGCCAGACAAATACACCAGCGTGG
AATCAAAAACTTCACTCCCGCCAAAGGTGCAG
TCCGCCACTCGCAGCAGCCCCGCGGAGCTCACGGTGGTATCCCCCAAAAAAATGCCATTTACTGCGCT
CATCGTGTTGTTCTCCACGTCAATGTAGCCGC
CGTTCCTCACATCAATAGCATTAAAGCAAACAGATGATTCCACACCGTCATCNNNNNNNNNNNNNNNN
NNNNNNNNNNNNNNNNNNNNNNNNNNNNNNNN
NNNNNNNNNNNNNNNNNNNNNNNNNNNNNNNNNNNNNNNNNNNNNNNNNNNNNNNNNNNNNNNNNNNN
NNNNNNNNNNNNNNNNNNNNNNNNNNNNNNNN
NNNNNNNNNNNNNNNNNNNNNNNNNNNNNNNNNNNNNNNNNNNNNNNNNNNNNNNNNNNNNNNNNNNN
NNNNNNNNNNNNNNNNNNNNNNNNNNNNNNNN
NNNNNNNNNNNNNNNNNNNNNNNNNNNNNNNNNNNNNNNNNNNNNNNNNNNNATAAACGGATGATCCT
ACACCGTCATCGTTCCTCGTTGCCCTCAGCGT
ATTTCCCTTCACAATGATCCCGCCACCATCAACGACAACAGCATTGGTGAAATAGACTGCGTGCCTAT
TCCCTTCAATGTAGTTGTCGAGTAGCAGCAGC
GTCGAGTTCGCACCAAAAACAAAATCCAAAAAGGCAATGGCGTGGCTCCACGTCGTCACAATTGCACT
GCCAACCACCAGTATCTGGGAGCTCACGCCAA
AAACACCCCTGAACTCCAACGCACCAGAATCCAGCATCGAGGATGTCACGCTCACGTGCACTCGCACA
CCACTCCCCCTGACCGACAGGCCCATGAGGAC
GCACTGCAGCAGCGTGATGTTGAGTGTCTGTTGTGGCGCATCGAAATACGACAAGTCCAGCACAATCG
CGGCAGTGAAGGTCACGTTGACGAAGCACAGA
CCACGCACACCGGGATCAGGAACGTCCACGGAGCGGATGCTGCCACCGTGCACGCAGCGGACCTCCGT
GTCCTTCGCATCGGGGAGCGGGAGCGGCCCAA
GGCCGTCCGGAACCGCAGCTGGCAGACACGTTTCGCCGTAGCCGCCAGCAGCGCACACACACTCACAG
CCGCTGATGCTGCTCGTCCTCGCTGCAAAGCA
CACGGCGTCCACCGTGCACGCACCACAGTCCAGCACCGTCACGGGTGCTCCAATGTTGAGGTCGTCTT
GGTAATCCGTCACAGGAGAGCCCCTTGCCTCG
TTGCACACGCCGTAGATGGTCGGGCGCGTGTCGGATGGTGGTGACCAGTTGGTTGTCATATTTGCGAT
GGTCAACGAGTACGAGCCGTACGTGAAGTTGT
TGTCAAGTATCGACCACACACTCTGATCGCGGAACAACATGCCGTCGTGGGCAGCCACGTACACAACG
TAGGCGGTGCGGAACTCGTTGTCGCGGTGCAC
CAGCCAGCTGCCGTCAGTCACGGTCAGCGTGGTTGCAATGAGCATGGCGAAGCCGAGACGGAACGTGC
TGGACACAATCTGCAGTACGCTGCCGCCAGCC
ACAACAACATCCCTAAAAAAGAACGCGCCTTTGTAGTATTCGGTGCTCGGTACAATCCACGAGCTGTT
CTGTATGGAGAACACGGAGCCGCTAACAACAC
GCAGGTCCGACGCAAGAGCGTATATCACGTGTGTCCGCGACCTAATAACGCAGTTGTCCATGTAGAAG
ACGCTGGACAGGTTCACGTCGAGGCCGCGCTC
CACGAGGATCGCAGCGCACGAGCCCCCGCCACACACCAGCGTCGACCGCGTCATGACAAACCGCGTCG
ACAGAAGCCGGACGCCGTCCAGGACAAGCGCG
GGGCCGTACCGAAACCCTTCGTAGCCAGGAGTCGTCGGCACGTACTGCGACCCGCCAACCGTCGCGCG
CAGCGTTGAGTTCGCCAGCAGCACACTCGAGT
GCAGCGGCATCGCGCCATGCAGCACAATCGTGCCCTCCAGCGACGTCACATTCGTCATGTTGACGAAA
GCGTGCGGCATCAGGCGCGCCGTGCTCTCGCT
GAGGCCGCCAATCCGCAGCTGCGCGCCGCCCGCAAGCACGCAGTGGCGCAGCGTCACGTTCAGCGCGT

CGGCGAACGCGTCCATCGAGCGCAGGTCCACC
GCCACAGTGATCGGTCCGCTGAACAACACCGAGTCGAAGCATGCCGTCGCACGCCCGCCGCCAACCGT
CACCGACTCCGTCAGCGTCACGCCACTCACGC
AGCCCTCCGCAGTATCGCCACCACCACTGGCCCTCGCAGGCGGCACGTCGAACGGCAGGCACGCGTCG
CCCACACCGCCGGCACGGCAGCTGCACACGCA
GTCAGAGAAAGACGCCGTCAGCGCATCAAAGCACGCCAGCGCAGCCGCACAGCCGTCGCACGGCACCA
CGCTCACGGAGGGCAGGCCGGCCATGCGGTAG
TCGCCGCTGCTCTGCAGCACCCGGCCGCCGGCACGGTTGCACTGCACCGACACAGCGCCGCTCGTGAT
CGCCGGCCCGGAGACAAGCGTCGCGCCGGTGG
CAGCGCAGCGCGCAGTAGACACGGCGCCGCCGCTCAGCGTCACCCCCGACAGCGACGCCACAGACGAC
GCCTCGCCCACCGCCCACACGTTGTGCAGCTC
CAGCCACCCGCCGGCACCGACCGTCCCACCGTCGCAGCGCACCAGCGACGACGCAACAGACCCCTCGA
CGCCCACAAAGCGCAGCACCGAATCAAACACC
GCGAGGCGCTCTCCGAGCACAATGCCACCGCCGCTGCTCACAACCGACACGTTCGTCACGGAGAACAC
CGAGTGGCTCTGCAGACTGAGGCGCGACGAGG
ACGCCACATGCATCGCGGACGCGGTCGGACCGCCCGACGCCTCCAACAACACGCCGTCAAGCACCACC
GCGCTCGACAGCAGGTCCATGTCGCCGCCAAC
GTGCACCGCCGACCCGCCCGCCTGCAACGACCTCAGCACAGTGTTGCTCACACGCAGCTGCGTCTGCA
ACAGCGACATCGCGTACAGCACAAGCGGCGAC
GCCACCGCGTCCGTCAGCCCGCCCAGCTGCGAGTAGCGCATCGGCCCCGCCGTCACAATCGTCGAGTC
ACGGGCCGTCACGTTCGTGTGTGCCGGCAGGT
TGTGCACAAACACAAGCGAGCAGTAGCCGCCGGACAGCCCGCTCACGCTCACCTCCAGGCTCCGGTCA
CTCGCCGGCTCGCCGCTGTAGCCTCGCACGTA
GATCTGCGCACCGCCGTCGCACACGCAGTTCCTCAGTTCGATGCGCAGCGCACCGGGGAGCATCGCCG
CAACGTCGAACACCACAGCCACGCCGTTCGTG
ATGGACACGCCGTCCATCAGCACCGTGTCCACGGCGCGGCCCACCGTGATGGCTCTGTCCACCGTGCC
GCCCCGCAGTCGCAACACCACCGCATGCACCG
CGGGCATCCACGCAGCAGCAGCAGCCAGCACCAACGCCACAAGCAGCACCGCACGCACAGCCGCAATG
GCCCTGCACCGCACACGGTGCCGGCTGCGCGG
ACACGCACCCACCGCAGACCGCATCCACCGACCCAAACACGTATCAGCAGGGCTGTCGCGTTGTGTCG
CAGTCGCCCCGTCGACCATTCCTCCTGCCTAG
CAAATCCCTTCCTACAGATTGCGAATCCAGTGACACAATTCCGATANNNNNNNNNNNNNNNNNNNNNN
NNNNNNNNNNNNNNNNNNNNNNNNNNNNNNNN
NNNNNNNNNNNNNNNNNNNNNNNNNNNNNNNNNNNNNNNNNNNNNNNNNNNNNNNNNNNNNNNNNNNN
NNNNNNNNNNNNNNNNNNNNNNNNNNNNNNNN
NNNNNNNNNNNNNNNNNNNNNNNNNNNNNNNNNNNNNNNNNNNNNNNNNNNNNNNNNNNNNNNNNNNN
NNNNNNNNNNNNNNNNNNNNNNNNNNNNNNNN
NNNNNNNNNNNNNNNNNNNNNNNNNNNNNNNNNNNNNNNNNNNNNNGCAGAGTGGCGGAATCCCGGCA
AAAAGCCACATGACCACTGGCACCGCTCCTGT
TACCGAATGGCTCCATCACATGGCGATCCCGATGCGTGGACGTCATACAGGAGCCGAGGCAGACCACG
TGGCAGCGATACGACCGCCAGCCATCAAGCGC
GTCCGAAAGGTTGTTGGTGTTGGTGCGTAAGTAAAGCGGAGAGCTCGCCGGTCGCCAGCAGTCAAAGC
GTCGTTTCTCGTTCACGCAAGCCGGCATTGCA
CAGCAAGCAGCGGCTCCATGCGCGCACGGCACACACAAAAAATAATAATAATAAAGAGGCACGGCGGC
AGNNNNNNNNNNNNNNNNNNNNNNNNNNNNNN
NNNNNNNNNNNNNNNNNNNNNNNNNNNNNNNNNNNNNNNNNNNNNNNNNNNNNNNNNNNNNNNNNNNN
NNNNNNNNNNNNNNNNNNNNNNNNNNNNNNNN
NNNNNNNNNNNNNNNNNNNNNNNNNNNNNNNNNNNNNNNNNNNNNNNNNNNNNNNNNNNNNNNNNNNN
NNNNNNNNNNNNNNNNNNNNNNNNNNNNNNNN
NNNNNNNNNNNNNNNNNNNNNNNNNNNNNNNNNNNNNNNNNNNNNNNNNNNNNNNNNNNNNNNNNNNN
NNGCAAAGCTCCACTGCTTCCGCCATGGCCAA
ACAAGGTCGGCTGCAGGGCGCAAAAAGAAGAATAAACACCCCACCAAACGCTTCAGCACCACGTGGAA
GCACCGCAACCGACACACAAGGCGAAACGCCG
TGAAGCAGGCACCAAAACCAAAGCTTCCACAAAAAATTCATCGTGAAGCAAAAGAAAATATTATGCGA

GCGCAAAAAAATTCACACAAACTGAAAAACAA
CAATGACGGTGCAGAGGCAATAAATGGTTGTGGTGCGCAGCAGCCGTGGCATCGCGCCCTTCCCTCAT
CAAACGGCACCGCACCACAACGCACCGTGGCG
ACAGCCTGCAACGACGACGATTGAGTAGCACACACAAAGCGGCATAAACCTTTCATCCCGCACTTGCT
CACATTTACGCTCTCAGTATGTACTGCCAACG
ACACGGGAAGGACACGGAAGGGGACTGTCCTATCATCCTCCTTCTGCCCACAACAGATCTTCCCCATC
CTCCTCCTCGCCACACTCTGGGTGCCGCGAAA
CGCCGTCAGGCAAAGCCACGTGTATCGAAGCCCTCAATGCGCGGGGCGAGGCTGGCAGCCACCCACAC
TCACTCCCTACACATATCCTCTCCCACAACTT
TCCCACACACAAAAGGGCAAAGAAATGGGGAAGAAAAGCCCGCCGCACAACGCATGCAGAGGCACCGC
CATCCACTTCGCACCGCAAGATAGTGCCATGC
GCACCGCACACCCACGGCGGCAAAGAGAGGGACGGAGCGCATCCAAGTCCACGGCACCGTCCCTTACT
TCTCTCTTCCCCGACAAATTGGAATTACGCCC
CACAGCAGGTCGTGGCGGGTGGCGTCCCACACTCACAGTGAGGCACGGCGCACACAATCCGCTCCCTC
CGAGTGCACTCAGCAGGCCGGCGCCTGTCACC
CTCCTGCCATCGCAATGGGAACCACAAACAATCGTCGTCGCGGACCATCGGTGCCTCTAGCATAAGAA
AAAAAATGAGGGCGTGACAACAGCACGGCTGC
CAGAGGGACAATCAACGGGACACGTAAGCACTCAGCACCAGTGAAAACACAAAGAAAAAAAATGAACC
GCAGCGATTTAACAAAAACAAAAGAAAAAAGG
GTGAGAGTCCAGAAGAACTCCCAAAAATATGGAAAAAAAGTATAAAATTGATTCGGAAAGAAGTTCAT
TTCCTGTTCCAAGGAGTACCATACAGTCATGT
GTTTGTGTATGCATACTGCCTGCAACAAGAAAAAGAAGTCAAAGAAAAGACCTTACTGTTCCACCAGC
CCACACAACATCCCTCACATTTCGTGCGGCAT
CCGGCCCGTTTTTTCAATCATCGTTTGGTGCAGTCCGTGGGACGCACAGCCGCGGAGCCGGCACCGCA
TGTAGGCAAAAACAAAAGCAAAACAAGAGGAG
AAACACGTCACCCATGACGGTATGTAGCAGACACCCATTAAGTAAAGAAGACAGTGAAAATGCGCAGC
TGGACGGCAATACAGCCAGCACAGCTCCCCAC
CCTCCATCACCGCACGGTGGCAGCAGCAAACATCCGTCCACAGAAAAAAAAAGAACAAAAGAAAAACA
CGGCGGCAGGGTGTGGCTGTGTGTGTGTGGCG
CTGCGGGTGTCTGCCAGCCCTGGCCGCAAACAAAACAAAACAAGAGGAAAAGAAATGTGTGCTCCGCC
CCACAGGCCCTCGTCGCACGCCCACACGTCAC
GGCTCCCTCAGCGCGCCGTTCGTCGCGGCCCCTCCTCATGCGTGCAGCAGGCGAGGCGCACAGGGCCG
TCGTCACGAGCGTGTCGGTGGTGTGCGAGTGG
AGCAGTTGGGCGGAAACACGCGGCACCCCTCACAGGAAGAGTGCAGAAGAAACAATTAAAAGAGGGAA
GAGTAGAAAAGAAAAACGTCCATAGTGACTGC
CCCCGAAAAGCAAAAAAATAAAGGGATGTGGGGAGCAAAAGATTAGGAAACAAAAGAAAAAAAAAGAA
ATTAAAGAAAATAAACCATAAAAAAATGAAAA
AAACTCACAACGCCCCGTAGTATGTAAACAGCAAGAAAAACAAACAAAAAACAAGTAACAACGAAAAA
ACAGTAGAGAAAAAAAAAGAAAGTAAAAAAAA
AGTAAAAACACAAAAAACAAAGAAGAAGGAGCTGCAACAAACGGCTCCAGTGTGCATGCAGCAGCACA
CAGACATGCGTCCACGCAAACAGTTGTGGGCG
TGTCGTGCATACGAATGTGTCTGGGACGCGAGCCAAGTGACGGATACGGAGGCAGCAACGTGGAGCAC
ACAGCAGCGCAGCAGAAGCAAACAAAGCAAAA
CACACAATCATTGTTTTCTATTATTTATTAAGCAATAAAACAAAAGAAGCTCACAAATATGAAAAAAA
CTCACAACGCCCCGTAGTATGTAAACAGCAAG
AAAAACAAACAAAAAACAAGTAACAACGAAAAAACAGTAGAGAAAAAAAAAGAAAGTAAAAAAAAAGT
AAAAACACAAAAAACAAAGAAGAAGGAGCTGC
AACAAACGGCTCCAGTGTGCATGCAGCAGCACACAGACATGCGTCCACGCAAACAGTTGTGGGCGTGT
CGTGCATACGA
>scaffold1060.size7398
GGTGCGTGGTGTAGCGGCCGTGACTCTTTTTTTGCTTTCACTCTGCATTGACGTGTTGTTAGTTTTTA
GTGAAGGCTGCACGCAAGTGACAGGAGTGATGG
CGATGATGATGGCTGGCCGTGTGCTGCTGGTGTGTGCCCTCTGCGTGCTGTGGTGCGGTGCTGGCGGT
AGATGTGACGGGGATGAGGTATCTTCCTCTGC

TTTGTCTGGGCCTCGAGGGGGTGTTGATGGGCCGCAGAGTTCATCGAAGTCAGTTGCTGGAGTGTCTG
AAGACGCGAGGCAATTGTTGTATGCACAAAAC
GGAAAGAATGAAAACCAAGGAAGAGGTTCAGTTATAGAACCTTCACTAGATGATGTCTATGAGGAAGA
AAAGGGGGAAGAAACAGAGACACGCGAAACAC
CACCACCAGCAGGCGGAGGAATAAAAGCACCATCTGCTTCTGCTGGTCCTGGTGTCATTCCTACTTCG
GGGTCTTCTTCTCTTTCTGGCGTTGATTCTTC
ATCCCGCATTGGTGGCGGAACAGGGCCTACAGTCAGTACTTCATCCAATACAGTTGGGAAAGCTTCGA
AAGAAGATATGGCACTACAAGAAAAGGAGAGT
CAACACACCCAGCTAGGCGACGGAGAGGCAGCAGGAACACACAACAACGGATCGCAACAAAACAGTTC
TGCTCCACCACCAACGGGGGGCCAAAATGACA
ACGACGCCGTCACGTTGAGTGCCGATGGTTTACGCACTTTGGGAGTACAAATAGCGGTAAAAGGGGAT
TTAGAAGCTTCCCGATCGGAAGAACCATCAGA
TGGTCCAAGTACAAACTTACCAGAAAGTGGAAGGGCTCCGCCGGAATCATCACAAGCAGCGGCAGCAC
CGGAACACACGCCTCAGCACAAAGTAATGACG
TCAGTAAAAGAGGAGACGGAGAGAATGAACATGAATGCATCCGCAAATTTACCTGGGGCGCCTAAAAG
AAGTAACGAGGATCCTGCATCCACAGCCACCA
CAATGCAGAGTACATCAACTGGCGGTCAAGAAGGAGAAGCTACGCCGTCTTCAAATGGAATTCCTAAA
ATTCAGGAGGAAAAACTCACTGGAAACAATAC
CACTGAGAATGTTCAGCCTTCGGATACTGCAGAGACTGAAATACGCCAAAGTGTAAATACATCTAAGG
TTGGCGACAGTGACGGCAGCACCGCGGTCTCC
CACACCACCTCCCCTCTTTTGCTTCTTCTTCTTGTTGTTGCGTGTGCGGCTGCTGCGGTGGTGGTGGC
CGCGTGAGAGTGGGAGAGAGAGCCGTGCACCG
CCCGCACACACACTCNNNNNNNNNNNNNNNNNNNNNNNNNNNNNNNNNNNNNNNNNNNNNNNNNNNNN
NNNNNNNNNNNNNNNNNNNNNNNNNNNNNNNN
NNNNNNNNNNNNNNNNNNNNNNNNNNNNNNNNNNNNNNNNNNNNNNNNNNNNNNNNNNNNNNNNNNNN
NNNNNNNNNNNNNNNNNNNNNNNNNNNNNNNN
NNNNNNNNNNNNNNNNNNNNNNNNNNNNNNNNNNNNNNNNNNNNNNNNNNNNNNNNNNNNNNNNNNNN
NNNNNNNNNNNNNNNNNNNNNNNNNNNNNNNN
NNNNNNNNNNNNNNNTCCTTCTCTCTGTGTGTGTGTCCCCTTGCATGGACTCTCGCCCTCACCTCACC
CCACGAGGCACACACATAATGTGCCCATTGTA
TATATGCATGCACACCCATGCAGGCCCGTTGGTGCTCTCTTTTGCACGACACGCACCGCTGCCGTCCT
GCCTGGCTGCATGGGCGGAGCACCGTAACTGT
TCTGTTTTTATTGCATTGAGTGGGCGACCTCTCTCTCTCTCTCCCTGTGTGTGTGTTTTGCTTTACGG
GAGCAATGAGAGTTTGTGCGCTGGTCGTCCCA
CGCTTCGGCTTTTTGTTTTGTTTTTCTTTTTGATTTTGTGGTTGAGTGCACCCATTCCGCGGCTGCGT
GTGTGTGTGTGTGATGATATGAGTAATTTGCG
TGTGTGCCATCCGCTTCTCTCTGCTGTTGCTTTCTTAGGCTCTTTTGTGCCGCACGGCTGCTCTGCAA
GTGTAGACTTTGGCAAACACACACACAGACAA
TCGCCATGCCGCTCACGCTGTGGAGTGGCGTGAGCACTGAGAACGCACGCACAATATTTTGTCTGCGG
TTGCTCTTGCGAGTTAGGAGGTCGTGAGCCCG
CAATGCGTGCCGGCGACGTGATACGGATGTTATTTTGGCGGCGTGCAACAATTGAATGGTGTGGCGTT
GAGGATGCCTGGGACGAGTGTATATCGACTCT
CTTCGAACAGCTGTACCTGCCGACGTGTCTCTCCCGTCGTGGTTTTGTCGACGAGGGATTCTCCGGCT
GTTTGGGATGATGTGCAGGAGCGTTAGGGGAG
ACAGCAGCAATACGACTGGTGGCAGCGCGTGGGTGCTGTGATGTGCGGCTGTGCCCTTTTGCGTCCCG
CTGGTTGCTTTGTGTGTGGACGTTGTTCAGTG
TCTGCTGACGCGTGACGAATAGCAACGCACCCGCTGTGGCTCCTGCTGCTTCTTCTGGTGGTGACCGT
CGCTGTGTGTTCCCGGTGCACTGTTCTTCCAT
CTCCTGTTCCGTGTGTCTGTGTGCGTGAGTTGTTGTTGTTGGGAGTGGCTGCGGTACGGATGATGATG
CCACTCACTGTGGATGGGTGGCGGTGCTCACT
GTTTCACCTTTTTCTTGGCATGTTTTCTTGGCATGTTTCTCTATTTCTCAATCACACACGAAGAAGCA
AACAAGCGCTAATCGCTACTGTGAAATCCGAA
GGCTCTGAAAGGACCAACAGCACACACACCGCCCACACACACGTATTTATATGCTCTCACGTGTTGCT
GCAGTGAAGGCACCCCGCACACACAACCGTCG

CCGCGTGACCGGATCGAGCGGCAGGAGGAGGGAAGGGAGAGAAAGTGAGCCGCAGAGGTCCAAAATGT
CCCGGCATCTCTTCTATTCCGCGGTGCTGCTC
CTCTTCCTCGTGATGATTTGCTGCGGCACAGGGAGTGCATCCAGTAAGGAGAAGTCGCCAGTACAGAT
ACCTCCACCAAAGACATATTTTGATTGGAGAG
ATGTGAAGGTTGAAGAAACAGTGAGTTCGCTCCGTGTTCCCAGTCTTTTGAAAGTGAATGGTGATGTG
TTTGCCGTTGCCGAGGCGCTCTGCACGGGAAA
AAAAGATACTGGTGAAGGCAGTTTTACTGGGATTGCCTCGGAGGTTTTGGAGTTGACTGGCGAAAACC
CAAAAGAGGAGTTGGGTAAAAATAAACTGAAG
ACACAAGTACTGGAGGAATGCACTTTTGAGGGTGGCAATTGCCCCTCCCAAAACGCTGCTGAGGAAGG
TGGTTCCCGAAGCCAGACGACAGTACATGTGA
GCCGGCCAACAACAGTTGTGGAGGGAAGTGATATTTACATGCTTGCTGGAAGTTACAGCTGGACACTT
ACCGCCGATGATCAGGCAGGTGGCTGGGGACT
CATGCTGGTGAAAGGGAACGTCAGTACTGTTGACAGTAAAAAAAGAATTTATTGGAGTGACACTTACG
GTCTCCCGTGGAGTTATAAAGTCAAACAAAAT
GAACCTTTGATGCGGCTGGTTGGCAGCGGTGGATCGGGTGTTCAGTTGGAGGACGGTACGCTTGTGTT
CCCGCTGGAAGGCACAAAGAAGTTGAATGGCG
CTGCAGAGGATGGAAAGACAACGACCGTTTCGCTAATCATTCACTCCGTGAAGGATATTGCGAGTTGG
AAGCTGTCGGAAGGGATGTCTGCCGATGGCTG
CGGTGTTCCCTCCGTCGTGGAGTGGGAGAAGGACAAACTCATGATGATGACTGCGTGTGATGGTGGCC
GCCGCAGGGTGTACGAGTCCGGTGACAAGGGG
GAGTCGTGGACGGAGGCACTCGGGACACTCTCGCGCGTGTGGGGCAACAAAAAGCGTGAAAAGGTTGA
ACTCGTTGGGAGCGGGTTCATCACAGCGACGA
TTGATAAGAAAAAAAGTGATGCTTGTCACTCTGCCGGTGTATTCGAGTAAGGACGGCAATGGAAAGGGC
GTACTCCATCTTTGGCTGATGGACAACACGCA
CATTGTTGATATTGGGCCGGTATCCGAGAAGGATGATGACGAAGTTACCGCCAGCTCCCTGTTGTACA
AAAGTGTGGAAGGTAAGAAGGATGAGCTGATT
GCACTGTACGAGAAGAAGAAGGGCGTTGAGGAAACATCATCCGGTATGGCCTCTGTGCTTCTGACTGC
GCAGCTGGAGAAGGTGAAGGAGGTGCTGGCGA
CATGGAAGGAAGTGGACGAGCGTGTCTCCCAGCTGTGCACCTCTGAGAGTGTTGGGAGGGATACCTTA
CCTGGCACTGCCTGCAGTGCTGATATTAAGAT
CACGGATGGGCTGGTTGGCTTTTTGTCCGGCAACTTCTCTGAGAACACATGGAGGGACGAGTACCTCG
GGGTGAACGCCACATTAGATAATGAAGGGGTG
GAGCAGGTAGAAACTGGCGTGAAGTTCAAAGGGCGTGGCGCAGGAGCGGAGTGGCCTGTTGGCAGTCA
GGGGGAGAATCAGCTGTACCACTTTGCGAACT
ACAACTTCACTCTTGTGGCGACGGTTTCCATCGACGGTGAGCCGACGGAGGGTAATATTCCGTTGTTG
GGTGTGCTTCTGGACGGAGAAAAAAAAACCAC
ACTCATGGAGTTGTCGTACGACAGAGAAAAGAAATGGATATTGCTGTGCGGTGGCGAAACCCATAAGA
AACACAGCAGCACTTGGGATCCGGAGACAACG
AAGAAACACCACGTGGTAATTTTGTTAAGGAATGGCAACCAGAGCTCCGCGTACGTTGATGGTAAGCG
TGTGGGTGAGGATGTGCCATGTGCACTGGGAA
ATACTGATTCGAAAGAGATATCGCACTTCTACATTGGAGGGGATGGAAAGAGTGCAGTGAGCACAGGG
AACGGAGAAGACGTATCCGTGACCGTGACGAA
CGTCCTGCTGTACAACCGTCCGTTGACTTCCGAAGAGATTACTGCGCTTAACACAAACCAATTATCTA
TTCCAAAGCTGGAGGATCTAAAAACAGAGGCG
AGGGATGCTCCTCCACCAGCTGGAACTGAACCACATACGCTGGGAGCCGTCTCCTCAACTATTCATGG
CGGGCATCAGGACACCGAAGAGAAATTGTTGA
AGATAAGTAATGATGCGGGTGGTGGTGGTGCATCCAATTCTGCAAAGACCACCATGACGACTCACTCA
GTGGGGGGTCAATCTGCAGAGCAGTTGGCGTC
GGGAGGATCTCACGATGTAAATAAAAATGTGAACGTCGATTCCTCGTCTAATGGTAACCCGGCGGTGG
GGACAGTGGGTGGAGACACGAAGCAGGGAAAT
GGATCACCGCAAATTCCTGTGGGTATCTCCGACACAGCAGATGCAAATACTCCCACCACTGAGGGCGA
GGGGCAATATGGACCCACAGTGAATCCTGAGG
CGGGCGTGAGCTCTGGTGAGAATGGGGAGACGGTGAAAGGAACGAATGGACAGGAGGAGGAAGAGATC
CATCCACAGGACGGGGAATTAAATGCCACGGC

GCTCAGCAGCAGTCTCGGAAATGTGTCGCAGGGGAATAACAGCGATGCCGGCACCGTGCGTGAGAGAA
GACTGCTGCCATCGCTTCTTCTGTTGGGACTG
TGGGGGTTTGCGGCTCTGTGAGGAGTGACTGGCTGCCGCGTGGGACACTTGCGACCCCCGCTGCCCGA
CACACACACACACCTGGCAATGAATTTCTCTT
CTCAAATAACTCTTATTTACTTTTTTCCCTGTTTGCTTTTTTGTGTGTTTTTATTTTTATTTTTTTGC
TTTTTTTAAATGATTTTCTTTTTTTCAAAATT
CTTTTATTATTATTATTCTTCTCTTCCCTATTCTCTTTTGCTTTGGGGCAATCACTATTGAATTTCTT
TTTCTTTTCTACTCTTCCCTCTTTAATTGTTT
GTTGCTGCACTCTTCCTGCGAGGGGTGCCGTGTGTTCCGCCCAACTGCTCCGCTCGCACACCACCGAC
ACGCTCATGACGACGGCCCTGTGCGCCTCGCC
TGCTGCACGCATGAGGAGGGGCCGCGACGAACGGCGCGCTGAGGGAGCCGTGACGTGTGGGCGTGCGA
CGAGGGCCTGTGGGGCGGAGCACACATTTCTT
CCCTCTTGTTTTGTTTTCTTGCGGCCAGGGCGGGCAGACACCCGCAGCGCCACACACACAGCCACACC
CTGCCGCCGTGTTTTTCTTTAAGGATTTGTTT
TGTTTTTGTGTTCATTTCACGCGCTGGCAGTCCCGTCAGCATTTCTCTCCCTCCTGATGATTTTTTTC
TTTTGCTGCGCTCACTCCCGATGAAGTGACGC
GACTCCCGTCATGTCTGCGTGCTTCTCTCAGTCCCCACACACGAGTGACTGTGCTCTGCGGGCCGCAC
TCGCACGCCGATGAGAGGGCTGAGGGGAGCAG
CAACACGCAGATGACCCCATGAAATATAAACGATGTGCGGACACATGGAGGGTCGGACGGTTGAAACA
CACGCAACCATCTTTTTAATTTTCTTTTCGGG
CAAAATATATACAGACAGCTTGGTGTGGCCACACGGCAGCCAGGCGATTTTTTTTTATTTTAAATTTG
TGTGGAACAGTAATGGACGATTTCCTTTTTAT
AATTATTTTGTGTGGTGGGGTTTAAATAAGACGAAGGGGACGGTTTTTTGTTTTTTTTAATAATTCTT
TTGTGGATTGCGGCTGCTTGGGCTGCAGTGCA
GTCATGATTTTGAGCGGTCTTCTTGTTGTTTCCGCCATGCGTCTCTGCTGTACATTTTTTTGCGTCCC
TCCACATACTCTGCTTCCTCTTTTATGCTTCT
TTTTTTGTGTTTCTGCAGGCACACACACACACACACACACAAGATGCACATTGCAGTGGATTTGAATA
CTCCTTTTGTAGTGCATGCTGGCAGTTGCGTG
TGGCGGCTGTTGTCTTCTTCCGTGGCCATGAGGAGAAGGGAGGAGAGGGAGCGCGCACCCGAGTCTCT
TTTCCTGTGGGTGTGATGCACCGCGTGTGACT
GCGTTGAGTTGGGCTCCGTGTCCCATTGAGGACGGGATGGCTGGAAATTTTGTTGCCGTGTCATGGAT
GGATGAGGGTTTGCTTGCGCGTGTGGGTGGTG
TGGTGGCCGCGGTGTTTTTGCCATTTCCCTGGGGCTGTGGCGATGAGCTGGGAGGAGATGGTGGTGTG
TGACTCTCATTGCTGGATGGGACGGTGCGTGG
CTGTGTGACTTGTCTCTCCTCTTTGCTTTGACTCTGCGTTGACGTGTTGTTAGTTTGTGGTGAAAGCT
GCACGCAAGTGACAGGAGTGATGg
>scaffold1061.size8950
GAGGAGCTTGCCGAAAACCTGCGGGCCACGGAGGACGCGAAGGCCGAGGTGGAGAGGAACCTCGAGTC
CGTGACCGCTGAGCGCGAGGAGCTTGCCGAAAA
CCTGCGGGCCACGGAGGACGCGAAGGCCGAGGTGGAGAGGAACCTCGAGTCCGTGACCGCTGAGCGCG
AGGAGCTTGCCGAAAACCTGCGGGCCACGGAG
GACGCGAAGGCCGAGGTGGAGAGGAACCTCGAGTCCGTGACCGCTGAGCGCGAGGAGCTTGCCGAAAA
CCTGCGGGCCACGGAGGACGCGAAGGCCGAGG
TGGAGAGGAACCTCGAGTCCGTGACCGCTGAGCGCGAGGAGCTTGCCGAAAACCTGCGGGCCACGGAG
GACGCGAAGGCCGAGGTGGAGAGGAACCTCGA
GTCCGTGACCGCTGAGCGCGAGGAGCTTGCCGAAAACCTGCGGGCCACGGAGGACGCGAAGGCCGAGG
TGGAGAGGAACCTTGAGTCCGTGACCGCTGAG
CGTAACGATGCCGTGTTGTGTATAAGTAAGTTGGAGAAAAGTCGTCGTAAGTTGAGGGATGCTTATGT
GAATCTTCAACGAAAGAGCGCGGAAAATGATG
TTCGTGTACGTGTTAGACATGATCGTCTGTCTTTTACGGATGGTTCTGCGGGGAGTTCTGGAAATGAG
TTTTCTGTGACGAAGAAATTATTAGGTGCTCG
TGGTTTATTTGGAAAGGAGAATAATGCTCAATAGATGATGATTGTGTGTATGCGCATTGTTTTTTTAT
TTGTTGCTATTTTTTTGATTTGTATGGTTGCGT
TAAGGAATGTTATATATATGTATATATATATATATATAGATTTTGTATTGTATTGATTGTACCTAGAT

TGATTCGTGTTTTTTTTTTTTTTNNNNNNNNNN
NNNNNNNNNNNNNNNNNNNNNNNNNNNNNNNNNNNNNNNNNNNNNNNNNNNNNNNNNNNNNNNNNNNNNNNNNN
NNNNNNNNNNNNNNNNNNNNNNNNNNNNNNNNNN
NNNNNNNNNNNNNNNNNNNNNNNNNNNNNNNNNNNNNNNNNNNNNNNNNNNNNNNNNNNNNNNNNNNNNNNNNN
NNNNNNNNNNNNNNNNNNNNNNNNNNNNNNNNNN
NNNNNNNNNNNNNNNNNNNNNNNNNNNNNNNNNNNNNNNNNNNNNNNNNNNNNNNNNNNNNNNNNNNNNNNNNN
NNNNNNNNNNNNNNNNNNNNNNNNTTTTTTTTTT
TTTGTATTCCTTATTTATCCATTTATTTATTTTCGCTGCTGTTATGTGTGTTTGTGTTGCTGTGTTTT
AATGGCATGTATTTGACTTTAGATGTTGTGGC
TCGTTCCGTTTTTTCTTTTGTTTTAATGGATACGGGTTTAGTGTTTTTGCGTGCTTGTCAGGAGCTTG
TGAAGAGTATGTTCACGGCAGAGCTGGCTTGC
CCTAATGAATGTACATACATACATACATGTACATTATATATATATAAAGGTGTGTGTGTGTGTGTGTG
TATATTTCCTGTTAAAAAGGAATATAAATTTA
TTTACGACACTTTTTTATCGTATGAATTTTAGGGACGCACGCGACTATGAAGGGAGGGAGGAGGGAAC
CGATAGCGGCCAGAGAATTGTTTACTTTTATT
TTTTTAAATTTTACCATCCCGCCGGCCTCTTTTTTTTTTTTGATTCCCACCGCTCGCGGCGGGGTGTC
TTGTGGCTGGAGGAGTCCTGAAGTCCGCTACC
TCGTAAGTAATCAATAAATAAATAAATATATCCATCCGAATAGAGCGAGTGGGCCACAGTTGTGGCTA
ATGGGTGATATATTTATATGTATTTATAGACT
TACTGGGTGTTACGCTTTGAGGGTATGGTGAATTCATTCTTTTTCCATTCATCCCCTTCTTATTCATA
TCTGGTTCGGTTTCTATTATTGAAAAACTACA
GGATTCGGCGAGAGGCCCAGAGGATTATTCTGTTGTTGTATTATGCTTTTTTCTTTTTTCTTTTTTGG
CTATATTTTGTGGTGGGTACGGTGTCACGCGA
CCGCTGCCGCGATGGTGGGTGGATGAGGAACAATTGTGGAGGCCTTTCTCAGAGGCATAAGGGCGAGT
GTATAGGGGGCGGACGGCTGCGGGTGGACGAG
TAGAGGCGGAGAAACAACAACAACGGAAAAAGAGAAGGTAGGATGGATTTGGCGGAGAAGCGTCGACA
GTTGGATGAAATCCGCGCGGCGCGGGAGGCAA
AGCAGCGTTCTGTTCAGCAGCAGCTCCAGCAGCAACAGAAGAAGGGCAGCCAACATCATGTGTCTTCC
TCATCCCTTGCGCGCAGCGGCACTCCGCAATC
GCCGATGGCGTCGTCCGTGGGCGGGGATCCTTTGAATCGTGTGGCCTCGACGTTGTCGGCGGTGAGTG
TGCGGGAGGAGCAGCAGACGGGGGATTCAAGG
ACGACGTCGGCCGCAGCGTCAAGACGACCACCGGTTACACTGTCTACAAGAATTTCTTCTGGTGATTT
GCGTTCTAAAGTCAACCGTCGTGGCACGGGGA
GTGGCACTACCGAGGCTGTGGGAGGGGAAGGCAGTGCGTGGAAGGTGGAGGTGGGCGTTGGGGCTTTT
GCGCTGAAGCCAACTGTAGAAGAGGGGGAAAG
TGACCATAGCGTTCGAGATGGCATGATGGCTGCCTCCGTTGTGGGTGCTTTGTCGGCCCTTGAGGTGG
AGGATGCAAGTGCGTGCGCCACAGAACGAACT
CAGAATTGTGAGGCGAGCGGACAGCAGCGTGTTGTGCCCCCGCCTGACGTCGCAATGCAGCTGGCACA
CTCGTTGCGTGCAGAATCGTTTTGCAACTGCA
AGGACCGCTGTGTGTTGGATGTCGTCATGTTGGACCCAAAGATTTCTGGTGGAAACACAGGTGTGAGT
GACGATTGCAGCGGCTTTCTTGTTGCTTTTGC
CCTTTCAGAGCCGCATCAAGCGGATGAACAGAGCGACAGTGCCCCCAAATTGCACTCTGGGCGTGGGA
TTGACGCACAGTCGCTGTTACCACTACCGTTG
TCCACCTCCACAGCAACGACCGCAGCAGCCGTGGCGGAGGTTCTCGGTGAGCTTCGTGGTCTCGTGCT
TCTTTGGTGCATCTCTCGCGGTAAAACTGGTG
ATGCTAGTTACAGTGTTATACCCCTGTGTTTTGACAGCGAAATACGCGTTTTGACGTACACACGACAT
CGACAACATTTATTGTTTGGTGGCGCTGCGAA
CGGCTGTGTGGTTGGGTGGAGGATTGATCATGCTTTGCAGGGACACTCTTGTGCTGTGATTGCGAATG
GTTTGGAGCGTTTCTCTGTAGCTCCGTCTTTA
ATGCCGTTGCCTGCGGGGGGTGTTGCCGCTGTGGAACCGAGCGAACGGTCATTTCCATCACCACATAC
GCATCAGTCTCGTGTGATTGGCATGGCAGTTC
ACGGTGATGTCAACTATCACCATCTTTACACTATCAGTCAAGATGGACGTGTTTGCATGTGGGCGCCG
CCACAGTTGGGGCTGCCTACGTCGAGTCGTGA
CGGGATGGTGTTAGGACAGCCAATTGGATGCGTCGGGTCTTGCGCTGCATTTGTTGATAAGGCGGCCG

ATGCGATGAGCAAAGTTGTTGTTGGCTGCCTT
GACGGTCGCGTACTGGAGGGGCGTACGAAAAGTTCGAGTCTCGTGGAGATGTCTCCAATCAATGCCAT
GCCGGCCTTCAGTATCACAAACGGCGTGTTGC
CGCGTTCCAACTCGACGCAAAGAACATCCGTTGATGGCCCCGCGCATCACGCCACTGTTGTCGCGGTG
GCGCCTCACCCGCTCCATGCCGACCCCCGCAT
TAGCGATACCGTGTTGACCGCTGCTGCTGACGGGAGTTGTATTCTCTGGTTGGGCTCCCGGCGTATCG
CCATTGATGGATTCACCTCGCAGGTGAACTGC
TTGCGATGGTCTCCGACTCACCCGGCGGTGTTTGTTGCGGGGGAAAGCAACGGCCGCGTCGCTGCGTG
GGATTTATCGCGGAACTCGTTTTCCCCCGTGG
CAGTGGTGTATTTGCGAGCCGTCCCATCTGCAGCGGGGTGGGCGAGCACGCCTTCCGCGACAAGCGCG
GTGAGTGCAGGCTCGTATCGCAGCGCAGCCGT
CACCAACCTTGCGTTTTCGGACGACGGTGCGTGGCTTTCGTGCGGCACCGCCGCTGGCGAGATCCATC
TGCTGCACCTGCGATCGGATCTTACGGTAGAG
AATGCGGCGACGACGGCAGCCAAAGAAGTAAAGGCGGGCCCGCTATCTCGCGCGCGCAACGAGGAGGT
AGCATTCAAGGATGGCTGTGATGACGCGACCG
TGACTGCAGCAGCCGTTGGGCGTCATGCCACTTCCTCTTGGATTGACGCGCGGTTTGGCTTTTAGGTG
GGACGAAGAAGTCGGGAGGAAATAAATGGAAT
CCAGAAAAGGGGGAGAGCACCCGTGACGTTCCTTTTTTTTTTTTTTCTTAGTTTTTGTCGGCGTTTTT
TTGCTTGCACTTTGTGTCGTTGAGGAAGATCA
TGCGGGTGCGTCATGTAAAGCAACTGCCAATTTTCTTTTTTTTCTTTTTCTCTTTCTTCTTTTACAAT
TATTATTATTATTATTATTATTTTTACCTCTT
TGATGTTTGCGGCCTTCTCTTTAGCGGTTTTGTGTTGCCCCTTACAATCAACACATATCGGCTTCGTT
GTTTAGAGTAAATAGGACTTTAGGCGTGCATC
CACCCCTGTCGTTTTTCTCCAGCCCCGTCATTTGTAGCTTCCACTCATGTTCCGTCTTGCGGGATGGC
GACTTGCCGCCGTTGCGTCCATGACGCCTTTG
TTGCAGAAGGCAAGTATCAGCAAGAAAACAGAAGAAAATGCAACTGCCCAGCCTGCGAAGCGACGAAT
CGGCACAGTTGTGTCGTTTATGCATCGCCGTG
GGTATGGCTTCATTCGCGAGTTGGAGTCTTCTCCGGGGAAGGAAAAAAAAACTCACAAGGCCGCTGCC
GTGGCTCCAGCGGACGCGGACGCGCCAGGGAG
TAGTGCGGCATCCAATTTTTTTTTCCCGCGATCATCACTTGACGGCGGGTTTTATGTGGCGGAAGGAC
AGACTGTCGCGTTTGATGTCCGGTTGACGGAG
CAAAGCAAAAATATAAATGCGCACCTTGCAGAGGCGAACATTTCTTCTTCCGCTGCCTCTGCCTCCTC
AACACCGCTACGCACAGACGGTACCGACGAGG
AGGTGGATGGCGAGCAGTCAAAGAAGCTCACTTTTGCTCACCGCATTCGTCTGTACGATGCTCGTGGC
GGTAAAGAGAAGCCCGTGACACCCATAATGCT
CGTTGGCTACGTTGAGAGCTGGGATCAGCTTGCAGGCAAAGGTGTCATTGCGGAATTGGACCTCAGCG
GTCGTCTAAATGAGGATGCCCCTCGTTTCACT
GTCGGTCTTGAGGACTTGGACCTTGCGGGGCACTCGGAGTTGCGACGCGGCCGCTACGTGCGTTTCTG
TCTCGAATCTGGTGATCGCACTGCTGCGCGGC
GTGTCATCATTGACCGCGGCGCGGAGAGGAAGCGTGCGGCGCAGTTGTCGGCTGCTGCGGAGGACCAG
GTGGATGGGAATGATGGACGTCAATACGGCAC
CGTGCGGGAGATTGTGGAGGGTCGATTTGGGTTTATTCTTCTCGATGAGACGGGCGAGTCCGTCTTCT
TTCACATGAGCAACGCTGAGGGTGGAGTGGTC
AAGCAGGGCGACACCGTCTCCTTCATCATGCGTGAGGTGACGCAGGGGAAACACGCAGGGAAGAGGAC
ATGCCTCCGGGTGAGACGATGCGCCGCGCGAA
ATTCCACCTCGTCCGCGGGGGCGGCGGCGGGCAACGGACAGAAGTCGAGGGCCGCGCAGGATTTTGAC
GACGACGACGAGTTTGAATTGCTGGAATAAAT
AAACAATATAAAAAAAAGTAAATGAATAGAGCAATAAGGAAAGGGACCGAGGCCGATTCGTAATGCAG
GGGTGACTGTACAAAACGGAGGCAAACAA
ACTGGAGGGGCGAAGAGTAAAACAAACGCCTTGCGTCACCAAATACGTACATCTATATATCTATATAC
ATATTTATTTAAAGAAGAAGAAATAAAAATAA
CGACAATAAAAACAACAACAAGGTAGGTTTGAGAAGCAAAGCGTAAGCAGACGTACATACATATACAT
ACATATATAGGGTGATAGAGGGAAGGCTGG
GTGGCGGGGAAAGATCCTCCCGGATGTGTTTTTACCGTTTCTTTGGGGGGTTTTATGATTATTATTAT

TTTTTTCTCCCTGATTTGTTGATACTCGTCACC
ACAGGCCTTCTTTTTGTTTTTGTTTTTTGTTTGTTTGTGTGTGTGTGTGTGTGTGTGTTTCTGTGCAT
GTGTTTATTACAAAATCAAGCAAACTCCCCAC
AGACACACAAACAAAAAACCGGGTGGGGTCTACATGCACACTTCTGCATACATGTGCTTGCACGGTTA
CTCCACTCTCTCCACACTGCGTTTCCCAAGCA
TTTGGCAGACGACAACAGCAACGACGACATATGGTGCCAAAGAGAGAGAGAGAGTGAAAGAGAGAGTA
CACGTGTGCATCAACAGTTTTGTTTCTTTTCC
TTTTTCTCTTTACATACAATATATACATACATATATATATATCTGCGTAGATATGTACATGCGTATAT
GCGTGAGAGTGTCTGTCCCAAACAATTTTTTT
TTTTTTTTTTGTGTTTTCCCTTGATTCCGAACGGGGCAGGAAAAGCTTCTGTTTGACCAAAAATATAA
AATTATTAAGGACGAGAAAAGAAAAAAAAAAT
CAAACCGGAGAGAACAACAACAACAAAAAGGGAAATTATGCGATTTAAGAAATCATTGACATGCATCG
ACATGCATACGGAAGGTGAAGCAGCACGGATT
GTGACGAGTGGTTTGCCACACATTCCAGGTTCGAATATGGCGGAGAAGAAAGCATACCTGCAGGAAAA
CATGGATTATTTGAGGCGTGGCATAATGCTGG
AGCCACGTGGTCATGATGATATGTTTGGAGCCTTTTTATTTGACCCTATTGAAGAAGGCGCTGACTTG
GGCATCGTATTCATGGATACCGGTGGCTATTT
AAATATGTGTGGACATAACTCAATTGCAGCGGTTACGGCGGCAGTGGAAACGGGAATTTTGAGCGTGC
CGGCGAAGGCAACAAATGTTCCGGTTGTCCTG
GACACACCTGCGGGGTTGGTGCGCGGTACGGCACACCTTCAGAGTGGTACTGAGAGTGAGGTGTCAAA
TGCGAGTATTATCAATGTGCCCTCATTTTTGT
ATCAGCAGGATGTGGTGATTGTTTTGCCAAAGCCCTATGGTGAGGTACGGGTTGATATTGCATTTGGA
GGCAATTTTTTCGCCATTGTTCCCGCGGAGCA
GTTGGGAATTGATATCTCCGTTCAAAACCTCTCCAGGCTGCAGGAGGCAGGAGAACTTCTGCGTACTG
AAATCAATCGCAGTGTGAAGGTTCAGCACCCT
CAGCTGCCCCATATTAACACTGTGGACTGTGTTGAGATATACGGTCCGCCAACGAACCCGGAGGCAAA
ATACAAGAACGTTGTGATATTTGGCAATCGCC
AGGCGGATCGCTCTCCATGTGGGACAGGCACCAGCGCCAAGATGGCAACACTTTATGCCAAAGGCCAG
CTTCGCATCGGAGAGACTTTTGTGTACGAGAG
CATACTCGGCTCACTCTTCCAGGGCAGGGTACTTGGGGAGGAGCGAATACCGGGGGTGAAGGTGCCGG
TGACCAAAGATGCCGAGGAAGGGATGCTCGTT
GTAACGACAGAAATTACTGgAAAGGCTTTTATCATGGGCTTCAACACCATGCTGTTTGACCCAACGGA
TCCGTTTAAGAACGGATTCACACTAAAGCAGT
AGATCTGGTAGAGCACAGAAACTATTGGGGAACACGTGCGAACAGGTGCTGCTACGTAAAGGGTATTG
AATGAATCGGTTTTTTTTTTGTTTTTTTTTTT
ATTAGTGCANNNNNNNNNNNNNNNNNNNNNNNNNNNNNNNNNNNNNNNNNNNNNNNNNNNNNNNNNNN
NNNNNNNNNNNNNNNNNNNNNNNNNNNNNNNN
NNNNNNNNNNNNNNNNNNNNNNNNNNNNNNNNNNNNNNNNNNNNNNNNNNNNNNNNNNNNNNNNNNNN
NNNNNNNNNNNNNNNNNNNNNNNNNNNNNNNN
NNNNNNNNNNNNNNNNNNNNNNNNNNNNNNNNNNNNNNNNNNNNNNNNNNNNNNNNNNNNNNNNNNNN
NNNNNNNNNNNNNNNNNNNNNNNNNNNNNNNN
NNNNNNNNNAGTGCATTATTTTTTTTTTTTTTGTTTTGGGGTTTCAACGGTACCACGTTGGGAGCAGG
GAAACGATAGCGGCCGGACAATTTTTTACTTT
TATTTTCATTTTCACCTTCCTACCCAACCCCCTTTGGTTTCCACCGCTCGCGGCGGGGTGTCTTGTGG
TTGGAGGAGTCCTAAATTCCGCCGCCTCGTAA
GTAATAAACATATTTCAATTCCTGAGTTCAAAAGGTCATTTGGGCGGTCAAAAGAATATACACATATA
TATATTTAACCAGAAGAGAACATCTAGGCAAT
TAACCGCCTCTGCTTTACCTTACTTTTCCCTATCTTGTTTGGGCTTTATTATTGCTAAACTGCATGAT
TCGGCCAGCGGCCCAAAAGATTATTTTGTTGT
TGTTTTTCTTGGATAGTGTTTTTGGCGTTGGGTTGAGGGAGTCGTGTGCGTAAGTGGGGTGGGGGGTG
GGTCTGAGTAGATGCTGCGACGGACGGCCTGC
ATGCGCGGGGGCGCGGCGGCGGAGGATCCCCACCGCTACTCGGAGCGGGCGCAGCGCATTCGGCGGCA
GGAGTTTTTCTTCCGCTGCCTCTGCCTCCTCA
ACACCGCTACGCACAGACGGTACCGACGAGGAGGTGGATGGCGAGCAGTCAAAGAG

>scaffold1062.size6911
TATCGGCTGCTGTGCATACCGGCGGTTGGAAATTTTTTGCTGGTGGAAGTCTCTTTTCTTTTAGAAGC
CAAATCCAATGACGCTGGTTGGGCTGCGGTTGG
CTGCGGCGCATGCGCATCACACCACCGTCAGGACTGTGTGGCAGTTCAATGAGTGCCTGGCGGCGTAT
TTTAATGATTGGGAGGAATTGTCCCGAGACAT
GTCGTGGGAGATGATTTACGCGCAGCACGCAGACAGCACGCCGATGGATGACTGGCGGAGATGTGATT
TTGTCCACCCCGAGAGAGTGTGAGCGACGCGG
AAGGCCGTGAAATGGCGGTGCTTTGGAAGGAAAAGTTGCGCCAGTACCAAGTGTCAATATCATTCGGA
GACTTTTGAAGAGACGAAGCACTCCGAAGAGG
GGAATAACCGTCGAAATAGGAAAACAAATTGGGGAGGGAATGAAAGGGGGTAGTTTTGATTTCTGGTA
AAAACATTGTGGCATTTATGTATTTTTGGATA
CTTGTGTGTGTGCATACGGGGCACAGTTGGAGAATTGCCCCTGCATGAGGGAATCATTGTGGCTGGCG
GTGTCGCAGTACTACTGCACTTTCCATTTTGT
CCTGTTGTGTTCTAGGGTCCATCACTGACGGAAGCATCTTGAAGGGATGGAGGGCGAATAGCTTCTAT
GGGGAAACGGTGCGCACTCCGTGTGACCGCAT
GGGGCGAGGCGCCGCGTCGTGGATGGATGAGGGCCCGCTTCCGCGTGTGGCACTTTGGGTGATTGCTT
GCTCGGTGTGCGTTGGCTGCGTGGAGTGGCCG
CATGCTCACGGGCCGTGTGGATTGTGAGTGCCTCCTGACGACATCCATTCATCTTTATATTTCTCTCT
GTCTGTCTGTCTGTCTGTCATTCCCTGTTGGG
TGTGAGGCGTGTTGCACCGCGGTAGTTCTGGCCCGCACACACCGACGGTCCGCTGTGACGCCTGTGAT
GGCCGGTGCGTGGCGCTGGTGTTTTGCGGAGC
GAGTGGGTGCGGTGTCTGCGAGAGGGTCGGTGCTCTCCTCGCGCCTCGCGTGTGTGTTGCTCTTTTCT
GTGGGCGATGTTTCTTCATGTCGTCCCTTCCG
GATTGTCTTGTGTCCCGCTCTCTGCGTGGGTGCTCTGCTCCCCACTGACTTGTCAGCTGCATTGCCGC
CGCTGTTTTTCCTTACCGCATGCACACAGACA
CACGCTAGGAAAGAAGGAAAACAAGCGATAATTACCACTGTGAAATTGGAAGGCACTGATAGGACCAA
CAGCACACACACGCATATTTGTATGCTCTCAC
GTGTTGCTGCTGTGGAGGCACCCCGCACACACAACCGTCGCCGCGTGACCGGATCCAGCGGCAGGAGG
AGGGAAGGAGGAGAGAGTGAGCCGCAGAGGCC
CAACATGTCCCGGCGTGTGTTTGCTTCCGCGGTGATGCTGCTCCTTTTTTCGTGATGACGTGCTGTGG
GATTGGTGGAGGTGCTTCTGCTGGAGGGAGTA
ATGTGAGGAAAGCAGTTGATGCGTTGATGGGGATTGGATGGGAAAAGCTTGACAACTGGGAGGATTTT
TCTGACGCTGGTGGTAAATATGGCTCGCTTCG
TGGTCCCAGTCTTGTTGATCTGCAGGGTCATGTATTTGCTATTGCTGAGGCCCATTGCAAGGATGGAG
GCGACTGCAGCGACGTCGGCTTTACTGGGATT
GCGTCAAAGTACCTTGATTTAAGTGGTGTTGCTGATCCAACGGAGATCTTGACGGCAGATGCGAGTAT
CTTTGGCGCAGATCTTCTAAAGGAAGGGTCTG
AGGTCATCAGCGCCAGAAACGGCATAACGCGACCAACGACACTTGTACTTGGGGACAGTGTCTACGTG
CTCCCGGGAAACTACAGCCATACAGGGCCGCA
GGTTGGGGGCACGAATGAGCGGGCCTTTTACTTTTGAAGGGAACTCTCACTGAAGAGGGTGGAGGAAG
AAAATCAAATGGAATGAGACCCATGTGGTGGA
GCCTCAAGGCAGAGGGGATTCTCGTTCCTTGACCGAGTTAGTTGGTGGCGGAGGATCGGGTGCTGTGT
TGCGTGACGGCACGCCTGTGTTTCCCATGCAG
GCCAAAGACAAGGATGGAACGAGCGTTTTGCTGTCTATGCGTTTCCCCAAGTCGGAGAACAAATGGGA
GCTTTCATTCGAGACGCCTGGTAACGGCTGCA
GGGATCCAACCCTGGTGAAGTGGGAAGAAGACGAGGACGACGAAAGACTCTTCATGATGGCTCATTGT
GCTGGAGACTACTATGACGTTTACATGTCCAC
TCCGCATGGAGTCAATTGGTATACGTCGGGTCAGCCAATTACGCGCGTGTGGGGCAACTCACACAATC
GAAAGGGTACGGCGTCCGGAGTGGATCCACCG
CCGCAATCATTGGGGAAGGAAGGTGATGCTCATCACCGCGCCGGTGTATCCGAGGGAGGAAAATGAAG
GAGGAAAGGGTCGGCTCCATCTTTGGGTGACG
GACAAGGCACGTGTGCATGATGTTGGGCCGGTATCCCGTGAAGATGATGACGCCGCCGCCAGCTCGCT
GTTGATAAAGGATAAAAATAAAGAGTTGATTT
CACTGTACGAGAACAGGAAGAGCGATGGAGCATACAATCTTGTTGCTGTGCGTCTGACCGAGAAACTG

GAGCGGGTAAAGGAAGTGGTGAAGACATGGAA
GGATTTGGACAGCGCCTTGCAATACTGCAGTTCCGGTTCCAGCGGCAATGTTGACGCGCTCAGAAAGG
GGCATGTGCAATGGTCCTGTTCCCACTGACGG
GCTTGTTGGCTTCTTGTCTGGTAGCTCAACTGTGACCGAGTGGCGAGACGAGTACCTCGGCGTGAACG
CCATTGTCCATGGCCCAGCGGAAAAAAGGATA
GGGGTTCCCAATGGAGTGACGTTCCAAGGGCCCAGAGCAGGGGCGGAGTGGCCTGTTGGTAAATTGGG
GCAGACTGTGCCGTACTACTTTGCGAACAACA
GGCTCACTCTTGTGGCGACGGTGTCCATCCACGAGGTGCCGAGGGAAGACAGCATCCCTTTGATTGGT
GTGAGGATGAATGACACCAAAGGCACGGTGGT
TTTTGGTCTGTCGTACACCCATGACAGGAAGTGGCTGGCCATAGCGGAGAAACGTGCTATTTCGAAGA
GTGTTGGTGCGTGGGAACCAAACAAGACGTAT
CGGGTGGTACTGCAAATGGATTATGATGAATGGACTGCCTTTTTGGATCAGAGGGAATTTGACCGCAA
GAGCTATAATGAGGATCTGTTCGACTCGCACA
GGATCTCACACTTCTACATCGCTGGGGACAGTAATGACCGAAGTGCAACGGGCGGCCACGTGACGGTG
ACCAACGTCATGCTGTACAACGACGATCTGTA
TAATAATGAGCTGTATAAACTCAATGCCAGCAAAGTCACTATTCCATCACTGGGTGTCGAGAAACATC
CCACAGGACAGGTGACCAGCAAAGACGTCTCA
TTTGCTTCCGAGTCAAAGAGTGAAGAAATCACCGCCAGCTGTGCGGAATTGAATGAGGATGATCCTGA
CAAACAAGAGGAAGAAGCGGCGTCGATGATCT
GGTGCCTGCAGCGCCCCCTTCCACGGTTGACGCGGGATCATCCGTTTCTGAGCCTGCCATCGCAGCGG
AGAGCGCAGGGAATGACCGTCCAGAGGACAAT
GCCCAGTTTCGCCAGGGCGAAACGGCCCAGCAAGCCACGCCGAATGAAGAGAATAAATCGATGCAACG
GGGCTCAGACCTGCAGCCACAAGACCCACAGC
CAGCGGAATTAACGGAGGTTACCGATGTTGAAATGTCCTCTGGAAGTTATGATGAAGAAATGCCAGAG
GAGGAGGGAGAAGCGGATGGCAGGAGTGGCGG
ATCAACGTCTTCAGTGGGTGCATCATCGGATATGGATACGGCCACCGAAACAGTTGACAGTGAGCGCC
AATTGCAACAAAGCACTGAGCTTTCCACTGAA
AATGAAGGCCTGCGGTCCACTGGAACCGGAACCACTGGCGCGGAGCAAAGCCTCGGCCTCGAGGCGGG
GGACAGAAATTCCGAGAGAACAATGGGCTCAG
ACAGCAGCCTCACTCCTTCAGGGATTGACGCCGAACCGACATTCGCGGAGAACACCGACGACGTCTCT
CGGACTGACGGAGCCCAATTTTCTTTTGAAGA
CGGCAAGGAGGTGCCACAGACGGTCGACACCAAACCGGAGAATACAAACGCCACGCCTGGAGGCGAAG
GGATCCCCTCAACAAAAGGCGCGGCACGACAC
TCGGACAACGACACCTTCACCGGGGAAATTGCGGAGTTGCTGTCCATGGGTCTGAGCCGTGACAGCAC
TGTGCATGTGTGTGTATTTCGGGTGTTGTTGC
TGCTTCTTCTGCCGGGGCTGTGGGGCATTGTGGCTCTTTGAGGAGTGATTGGGTGCCGCGTGGGACAC
TTGCGCCCTCCCCTTCACCAACACACACACAC
ACACCGCTAGGAATAAATTNNNNNNNNNNNNNNNNNNNNNNNNNNNNNNNNNNNNNNNNNNNNNNNNN
NNNNNNNNNNNNNNNNNNNNNNNNNNNNNNNN
NNNNNNNNNNNNNNNNNNNNNNNNNNNNNNNNNNNNNNNNNNNNNNNNNNNNNNNNNNNNNNNNNNNN
NNNNNNNNNNNNNNNNNNNNNNNNNNNNNNNN
NNNNNNNNNNNNNNNNNNNNNNNNNNNNNNNNNNNNNNNNNNNNNNNNNNNNNNNNNNNNNNNNNNNN
NNNNNNNNNNNNNNNNNNNNNNNNNNNNNNNN
NNNNNNNNNNNNNNNNNNNTGGCGTTTTGTGATCGGAGTGTGTGGTCGATACTTGACAACAACTTCAC
GTACGGCTCGTACTCGTCGACCACTGCGTACG
TGACAAACAACTGGTCACCACCATCCGACACGCGCCCGACCATCTACGGCGTGTGCAACGAGGCAATA
GGCTCACCTGTGACGGATTACCAAGGAGAGCT
TAATATTGGGACGCCCGTGACGGTGCTGGACTGTGGTGCGTGCACGGTGGACGCCGTGTGCTTTGCAG
CGAGGACGAGCAGCATCAGCGGCTGTGAATGC
GTGTGCGCTGCTGGCGGCTACGGTGACACGTGCCTGCCAGCTGCGGCTCCGGACGGCCTTGGACCGCT
TCCGCTCCCCGACGCGAGGGACACGGAGGTCC
GCTGCGTGCACGGTGGCAGCATCAGCTCCGTGGACTATCCTGATCCCGGTGTGCGTGGTCTGTGTCTT
GTCAACGTGACCTTCACCGCCGCGATGTTGCT
GGACCTGTCGTATTTTGACGCGCCACAGCAGACACTCAACATCACGCTGCTGCAGTGCGTCCTAATGG

GCCTGTTGATCAGGGGGAGTGGTGCGCGTGTG
CACGTGAGCGTGACATCCTCGATGCTGGCTTCTGGTGCATTGGAGTTTGAGGGCGATTTTGGCGTGAG
CTCCCAGATACTGGTGGTGGGCAGTACGCTTG
TGACGACGTCGAGCCACGCCATTTTATTTTTAAAGTTTTTTCTTGGTGTGAACTCGACGCTGCTGCTG
CTTGACAACAACTTGGAGGGGAGTAATTGCGC
GTTATATATTTCCAATGCTGCCGTTGATGGTGGCGGGATCATTGTGAAGGGAAATACGCTGATCACAA
CGGAGGAGGGTCAGGGTGTGGAATCATCTGTT
TGCGTTAATGCTATTGATGTGAGGAACGGCGGCTACTTTGACGTTGAGAACACCACGATGAGTGCTGC
TAATGGTGTTTATATTTTTGGGGATACCACCG
TGAGCACCGCGGGGCTGCTGCGAGTGGCGGACTGCACCCTTGTTGAAAGCACAGAGGAGTTTGAATCT
GCGCTGGTGTATTTTGACGGCTCTGTGACACT
CGAGGGAGGTGCGCAGTGGCGTGTGGAGGGCAACAGCGTGGGTGCAGCCTCGGTATTAAATATTCCTC
ATTTTCAGCACAAAATTCAGCTGTCGGGCAGC
GGCACCACCGTGGCGCTTGCACACAACCGTCGGGTGGACTCGAGGGCTTTTCTTGCCAGGTTTCTCCC
ATCGAGCATTGTCGTGAAGTTACCCGCGCGGT
TTGTGGTTGGGTGCAACCTGCAGGGCGATGGGGAGGTGTCGTACGACGGTGTGTTTCCGGAGGACGTG
GTGGTGTTCAGGTGCGGCACATGCAACGACGA
CGCGGCGTGCTACATGCCCGGGACGGAGTTGGTGGACCGCGGCTCGTGCTCGTGCAGCTGCAAGGACG
GATGGCATGGCGCGTCGTGTCTTCCCTTCGAG
GTGCCCGACACGGTTGTGCCGCCCGTGGCGGAGAGAGCCGTCGACGGCGACACGAGCTGCGTGGTGAA
CCAGACGCTGACGAGCCTCACGCTGAACATGT
GGAAGACGCACCACTGCTACGTCGGTGTGACGTTCAGCGGCGTGGGTGCCGTGCTGACGTTTTTCCTC
GACAGTATGCCGCTGCATCTCCCCATCAACAT
CACGCTCACTGAGTGCATATTCCGTGGGGGTGCCGCGCTGCAGTTTGTTGGGGGAGATGAGGCGGCCG
AGTCAGCCGGCGTCCTGATACGCGTGAGCCGG
ACTGTAATGCGGTCTTCCGTCGTTGTGTTTGCACTTGCCCTCCCGCAGCACTGCGACATTGCCGTCAC
GGAGGTTGACGCGGTGCAGTCCTCCGAGGTCC
AGCTGCCACACATTAGGACCAACATGCTGAGCGTATTTTTACTGGTGAGCATCATGTTTAGTGCGTCC
TCGCTGTTGGTCAGCAACATCAAGGCTCATTC
CTTGAGGTACGGTGCGCTTGGTTTGTACTCGACAGGGACGCTGACGCTGTTGGATGGCAGCTCGCTGT
ATGTGCAGTACTGCAGCTTCGCTGGGTACATG
CATATGTttta
>scaffold1063.size9661
TCGGTGTTTCGTCGTACCACAGTCGAGCGTCGTTAGCGGATAAGCTCCATTTCTCTTCTCGACGATTG
CGTCGGGCAATTTGTAATATCGGATGGAGCATC
GGCGGCGCGGTGCGACGTCTTTGCGGAATAGCGGGAGGTGGTGGAATGGATGAAGCTGCCGTCGTCCG
CTTTCATAAGAAGCAGCAGTCCCTCGCCGTTT
CTTGACGGTCGTCCGCGCTGCGAGCAACTTCCTCCGGTTGCGTCTCGGACGTTGCTTTTAGATGTTTA
TGGACACGTAACATGACATAAGTGGCATCGCA
ACGATATTTGCGTACCTCGTCGTTCCAAGGGAATCTGGATTGCCCTCGCAAAGGTTTCAGCCTCATGA
ACAGCGGAGTAAAGACGGGCGAGAACGTTGAA
GAGCGGGATGCCGGGGTCGGATGCCATCATGAGCTGCATCCGGAATGCCTGCAGTAAGATCCCGTCCA
AAATTCATAAAGCGATCGGTGGGATCCCGCTC
CGCTTCCAGCGCACCGATCCACGCAACCAAACTGTCAAATACAAGTTGGGCCTGGTCCTCCGCCCGTT
TCCAATAATTGTATGCGCCCCGGAGGTCTAAA
AAGACTCCGGAAANNNNNNNNNNNNNNNNNNNNNNNNNNNNNNNNNNNNNNNNNNNNNNNNNNNNNNN
NNNNNNNNNNNNNNNNNNNNNNNNNNNNNNNN
NNNNNNNNNNNNNNNNNNNNNNNNNNNNNNNNNNNNNNNNNNNNNNNNNNNNNNNNNNNNNNNNNNNN
NNNNNNNNNNNNNNNNNNNNNNNNNNNNNNNN
NNNNNNNNNNNNNNNNNNNNNNNNNNNNNNNNNNNNNNNNNNNNNNNNNNNNNNNNNNNNNNNNNNNN
NNNNNNNNNNNNNNNNNNNNNNNNNNNNNNNN
NNNNNNNNNNNNNTCTCGTTCTGCAAGAAGGACGCCGGCAACCGCAATGTCAGATTCGCGCACAGATA
GGTTGTCATGCGCCTCTTCCACCTGCGAATGA
GACTGAACGCTTCGATTCTGCACGTCCACGGCGGCGTGGGCGTATTTCGTGCTCAGGCGCTCCCCTTC

AGCAATGACGAGCCCTTGCCACGCGCCCCAAC
TCCCGGGCTGCGCCCATGATGGGCAAGATCAATTGATTCTCAAAATTTGGTACTTGAAACGCCCGGAG
ATTCGCAATATGTCCTGTAAGGCTCGAATCCT
CCCAGGGCTTGGTGACGTTTCCCAAAATCAGGCATAAGCCTGTCGATGAGGGAGCCACAGTTAACGTC
TCGTCCGACATCTCGGTTCCATTAATGAACGT
TATCTGCACCTCGATATCGCCACGTTGGCGTACCAGGTCCCAGAGACGGGCGCCAATATCACACCACT
CGGAGGCCGTGTAAGGAACCAATGCCGTTTCC
TTAAACCAAACTGGCTGTGTCTGTAAGCTTAAAATAAAAAAGTATCCGGCCGCCATCGGTTCCCTGCT
CCCTTAAGGTCATGGCCTTCCCAAAATTCATA
CGATAGGAAAAGTCTCATAAAGAAGAGCATTAGTTTTACCAGGATACATATACATCAATTAAGTACTC
CAGGTCTCCCACGATCATTCTCTTCAGAAGCT
CCTAAAAAAAAGAAATGTAGTAACACCGTAACACCACCAGCCACAATGGTTTTCTCATACCGGGGCAA
TTCTCCAATTATGCCTCGTATGCACACACAAC
TAACACACTATGTAGAAATCATCCAATGTTTTTCACCAAAAACCAAACAACCCCTTTCCCTCCCTAAT
TTATTCTGTCACTGTTACGTTCGATAGCGTTG
GCCATTTGTGGCATCTGTGTGCCTTACTGCCATCTGGTGCTGGTGCACCCGCACCGTATGCACACACA
CACACGTATCCAAAAATAAACAATTCACCAAA
TACTTTTTACCAATAATCGAAACTGCCCCCTTCCCTCCCTAATTTGTTTTCATATTTCGGTGGTTCTT
CCACACTGCGGTGAGCGTCTTATCCTCTAAAG
TCTTAGAATAATATTGACACTTGGTACTGGCGCACCTTCTCCTTCCGGAACTACGCAACGTTTCGGTT
TTCCTTCCCGCTCAAACTATCGGTATTGACAA
CATCACATCTCCGCCAGCCAGTCGTTCATCGGCGTGCTGGCTGCGTGCTGCACATAAATAATATCCCA
CGACATGTCTCGGGACAATTCCTCCCAACCAT
TAAAATATGCCGCCAGGCACTCGGTGAAGTGCCTCACAGTGCTGGCGGTGGTGTGGTGCCCACCCGCC
GTAGACATCCGCAGCCCAGCCAGCGTCACTGG
ATTTGACTCCATGAAGAAAAAGGCGTCCAACAACGGAAAGTCCTCCACCTCCGGTACGTACAGCAGCC
GGTGGTTTATGCAAGTCTTCTTCTCAATGTGT
TCCGCCGACGGCAAAAAATAGTGGCTGGTGGGGCCCTCCTGCGAGTACACCTCCGGCGCACAGAGGCG
TTACTGACGTCGTGTTGGTGGCCTCAGCTCCC
TCAGTGTACAAATTATTTCAGTCATGAAAGTCCCATTCAAAAATGCAGACACGGTGCTCTTCCCAAAA
AATTGCGTATATGAGATGATCACCAATCCTCG
ATTCAAATAAGTTAAACTCAGCCTGCGCCATTAACTCTGCCAACATGCACAGCGGTTCACTTGCGAGG
TGGGCAGTCATCGGTGCATTGAAAGGCGACTC
GCCATTGCGCTCTCCTCGTACTCGGACAATCCTCGCAAGATATTCGAGCGCCTTATTATCCTCTCACA
TTTTTGGAAGTTCCAAAAACAAAGTAACGCTC
GATTCCCCAAGGGGGCGTCCTATCCACAAAGCTGTGGCATCCTCCAATCCAGCAGTTGTAATGTCCCT
CATTAAAGATGTAGCGAAGAATCGGTCCCACT
TTATCCATGCGACCCCTCAACTCTCGCCAGTATTCCGCTTGCGGCTACACGGGTTGATTGCGCTTCAT
CCAAACGCACATTGCCCTCACATCGCTCTCAT
CAGGGCAATTCATTATGATTCGTACGGCTCTCTTTNNNNNNNNNNNNNNNNNNNNNNNNNNNNNNNNN
NNNNNNNNNNNNNNNNNNNNNNNNNNNNNNNN
NNNNNNNNNNNNNNNNNNNNNNNNNNNNNNNNNNNNNNNNNNNNNNNNNNNNNNNNNNNNNNNNNNNN
NNNNNNNNNNNNNNNNNNNNNNNNNNNNNNNN
NNNNNNNNNNNNNNNNNNNNNNNNNNNNNNNNNNNNNNNNNNNNNNNNNNNNNNNNNNNNNNNNNNNN
NNNNNNNNNNNNNNNNNNNNNNNNNNNNNNNN
NNNNNNNNNNNNNNNNNNNNNNNNNNNNNNNNNNNNTCCAAAGGGAAGCGTAAAGTATCCAAGAACGAT
GCCCACAGTTGAAACGGTGGTGATATCGTTCT
GGGTAAAGCCGTAGTCATCCGTCATTTTGCCCGATATAAGCGAAAAAGAGTAGACGGCACCGGATGCG
ATCGCACAAAACATGCTGCACACCATAAACCC
AAAGCGCTGTGACTCGTTAATGGAGTTGGGTGCCTCAAGTCGGTCATTGAAACCAAGCACCATTTTCC
ACGCCTTCAACTTGCTGTTTGTCCCTGTTAGC
TCCAAGCTAAAACTTATTTGCTGTTGCCTTTTTTTTTTTTGCTTGATTTCGCAAAACGTTAAGTCTTT
CCCAAAAATAAAAAAAAGATGGGGAGAAATCG
CCACAGCCATGTCACCGTACGAGTGGCAGAGAAGGTTGTGACAAGTCTGGCCATGCAAGTGATTGCAA

TGGCCCTGTTTTTACCTGCACCATAAATACAT
GTCCACAAACAAGTATATATGTATATGTATGGATATATATATATGTATTTACTCTTGTGCCCAAGAAC
AACAATAAAAAAAGAGCGAAAGCCGTGCCATG
CACTCTCACAAAAGTTATAAACAAATTTCCCCGGCACCGCCTTCCTCGGCGGGGTCCCCTGCGGCATC
CACAGCACACCCACTCGGATCGTCGATTTGCA
CCGCGATGTCGTCAGTGGCCACTTCCAGACAGCCCTCTTCGCCGCGGACGGACGTTAACGCCACGTCA
TGCCGCAGCGCCATTGCACTCTGCTCTAGCAA
TTCCCTCTCCGCATCCGTAAGACGAAAGAAAGCAGCACGAATACTCGCGGTATCCTCGAAGTACTTGT
ACGACGTCGTCGTCCTCCGCCGCTCCAAAGCA
AAGAGCTGCAGCGCAGTCAGGCACAGGCATACCCCACCAGATCGCATCCGCACGCCCCCAGCAGAGTC
ACAATATTTGTATTCACGTGCACTAAATACCC
TCCCCTCTATCTGAGGGAAAAAAACAAATACAACGCAAAAACATGTATGGCTGCAAATGACACAAACA
AACAAGCAGGAAAACTTACAGCAGCGCGAGGC
CAAGTAGAGGAGAAACAGCAAAAAACAACAACAATTAAATACATCCACCCACAAGACACAGAATTTGA
TGGTGTATAGTCACCAAGAAAAGGAAAGAAAC
AAAAAAAGTACGCATCGCATCCGCATGTGGGCATACGCTGTGAAGAGAAAAAGTAATAGAAGAAAAAA
GAAACCAGAAAGGGAAAAAAATGTCCAGAAAA
AAGAACCCAAAGAGATGTATTATCCTGAAACGGTCAGCGATAGGTCGTCCATTGAACCAGGCAGCCTC
CGGGAGAGCGCTAAACTCAAAAGACGCGCGGG
ATCGTTGGTCATGGCAATGTTTACAAGATGAAAAATGCCTTCCGCGCAAACTGTGAGTGGCGTCACAC
CACGACGGACGCCAATGCCATTCTTCCTTGCG
CGTCTGGTGGTCAGCCAAATGAAGCTGTCCATGCACCCATATGAAATGGCGCTCGCCTTCATTTGCGT
TGCGATGCCCGGGGAATAGCGCCTCCCACAGA
AATTAACTGGTTGGTAGCGGCGGCAGTTATCAGAGTTAAACACCTCTGGACGGCGAGTGCCACATACG
TTGTAGTACGTCACGCAGGAAGAACCGTTACT
TGCCCGCAGCGTCACGGCGTTCTCTCTGTTCAAGAACAAAGGATCGAAGGGGCTATCAAAAATAAGTT
CCGCCTCATCCACAGTGGCCCAGTAGCGAGAG
TGCCCTGCCGAATGGTGTCGTCCAAAGCGTGCGTGCACCTCCTCCACAAAGACTGGAGGAAGAGGCTC
ACCATTTGTGTTGAGTGGGTGTGATGCATCAA
AGTCCGACTCGCACGCCGCCGGACGACGGGTCGCTGGCACGGACATTTTGGAGACCCGCCCCCGTACT
TCCTCCTCTGTGCACTCCAGCTGCGACACGTT
GTAGAAGAACCGCCGGCCATTGGCCGCGTTGACGCCAACAGGGACGGCAGTCGTGTGTCCGCGCTCCG
TGAGCTGCAGACGCTCGACAAGACTCTGTGGC
ACCCACACACTTGCCGCGTAGCCCCGCGCCATGGCGTGGGTCCAGAGAATTGCCTCGTTCTCCGGCGA
AAATGTGGCTCCTGTGATGCAGACGGTGGACC
CCATGGCAACTCCGTTGCGGCGTCCTCCTTCAATAACACCGAGAGGCCACGCGCAGCCGTCGGAACAA
AAATAAAAAAGGAAAGCAACACAGAAGGAATG
CAACATTTTTTTCCTTTTGCCAAACACCAACACCATTAAAGAGAAGACCCCCCATTCACAACGCATGT
TATTAGGATGGGGGGTCAGTAGTAAATTTTTA
AAAAGGGGAGAGCAGCACAAAAGCACCAACAAGAGAAAAATCAAAATAAAAGAACATGGAAAACACTT
CAGACGTCGTAGTCTGCTCCTGTTCTTGATGA
AAAATGCCGAGTGCCATACACCACTGCAGCTGTACAATGATAACGATAATAATAATAAATTGCAAAAA
CACACATGGAGGCATACACATGTTAAACAATT
GATATTATGCTACATACGGTGTGACTGCCGGTTGTGGATGGCAATATATATATTCAGAACAAGTACCG
CCAACGCAAGCACATGAATAAATGCCATTGCC
GCCAATATGTGAGGGGATATTTGGACGGAAAACACGGAGGGTAATTCTTCATGTTCCTTAATCATTTC
ACGGCATCTTGCAATGACTTCGCTGCCCTCCC
CACCTGGACCTTGCCTCATTCGATGATATAAGGACGCCACATCAAGAAGATAAAGATGCACCTTGTGC
TCCTTTACCGTCTCAACACAAATGTAAAGATA
TCTCAAAAGCATTAAGCCCCACAGTGTACATAGTACAATGGCGTAAGAATGTCCTGTGGAAAAATGCA
TGCCGCTGTGTAAATAACCAGCCAACAGAATG
TGGACAATATCAAGAATCGTCGCTAAGAATCCAAACAACGCAAATGCCAGGTAGACACTCATGGCGGA
ACTCCGAGCCGCATGATTGGAGCACGTGGAGG
CGTAGAACACAAGAGAACGCACAACACACCATCCAGCCACACCCCTAAACACATCATGTGGATTCAAC

GGTTCATCAAAGTTAAAACGTTGGTTCAAATA
TTCACTGGGTCGCATTAAACTTCGGCCTTCCAAATACGCCAGTCGGGAAATAACTCGCATCTGCTCCA
ATATTTGTCGTTGCATGTCGGTCATCTTCAAC
ACGTAATATGCAAGTATCGTAGAAAACAGCATGTTTGAGATAAAAACCAAAACTGCCGCCGCCATGTC
GTACGTACCCGTTCCAAAAAATGGGTGTCTAA
AACCGACACGCGTGAGAGGAGCTGCAAGCCCCATGACAATACCAACAACAACAGAGCATGCTAGCCAA
CGATAGCGGTAGTGCTTGTTTGTCACCATCCC
TCGGCCCTTCTCATCCCAGGTATAAATACATACTTTTGCCTTTGTGTCCACCAGACCCGCAGTAAATG
CAGAAAGCCGTGGTGTCAAGCGACGTTCAAAC
CGTTCCAAAGCGTGTGCTACTCGCATGTAACTATTATAGGCGGCGTAAAACAAAGACTGCAGCACCCA
AAAAACACATGGAATCACAATCTCCGCCAGTG
TCAGACGTCCATCACTCACTGCGCCCAGATTCACCTTCGATGCAGCAATCACAACTGCTGAAAAACTA
CAACACATTGCAAGCGTCACAAAGTGCAAGGA
AAGAAAAGGCGGGTCGTATTTTCTTAGGAGCGAGGCGCCACTCACAATAAATTCCCGCTCCATCTCTA
GCCTGGCCTTACGAATGGGGGAAAATGGAAAC
TGCGGCAACGACAGGAACGGGAAAAGAAATATGAGGCAATACTTCACGTAATGACTAAATGAGAAAAA
CTCATTGGATGCAGGTGCGGCACATGCAAATA
TCGCATAGGCGGAGGCCCCCATGGCAAGTGCATTTTTTGTAGCCGGTTTCTCTTGCATCTGTATCTCA
GCATGTGCCGTTTGCCGCTGCGTCGTTGCCGC
CGTCAGAGACTCCTCCGTATCCGACAGTGATGCTTGTCGGTCCACAAAACTCCGGGAAATGATTGCGC
GTGACGGAATTCTCGTGGAGGAGCAATTCACA
ATGAAATATTCGGGCTTCTCACTGTTTGATAGCATGGTGTTCTTTTCCGGGGAACACTCCCTGCAGAG
ACGTACTTTTGGCTCACTGATGCGACTGTTGA
CATACAGATGCTGCCAGTTCGGCGCTCGAGTCACCGCCGTGAGTTTCTGCAGCTCATCACACAGAAGA
AAGGCAATGCGCGTGAAAAGTTGCGCGCGAGC
TCCACCGCCCAGGTTATCCTCCGCCTGCTCCACCATAAATCCCTCATGCCACCTGCCACGCGTCAGAC
GGCCATCCTGCACGTACAATCCAGAGCCGTGC
GCCGCAGAACTCTTGAAGCTGCCAGAAAAAAGTGCACCGGTGGGAAGCAACACCTCcGCGTTTCCGTC
TAAATGTCCGTGCGAAAACCTCCCGGCGCAAA
TAACCCCTGACGGAAAGACGCACACGCCACGTCCCTCCGGTTTGCCCTCAACCAACGGCCCTTCGTAC
AGGGCACCGTCGCTGAGGCACACGCTCGCCAC
GTGTTGCATGGCGTCCAAGGCGGCAGGTGCCTGGTAATTTCCGCCGTATGTACCCAGCGGCTCCCCAG
GCATTAGAGCGAGCCAAACGTCCAGGCAACGC
GAGCCACAACACACAAAAAAACACCGTCAAATACAAATATTTGTTTATATATATATATATATACAAAC
ATAGATACAGGCATATATTCACATATTGGCAA
ATAAGTTAATACATTAATATATAAGAGAGGACTAAAAATATAACAAAATAGTGCACAAATTTAAATAT
TTTATACAAAGAATAATCTCCTGGGCAGCTGG
CCGGATCCTGAAATTATCTATGGAACCAAACAAGTTAAGGAAAAAGGTTCGTTAGTTAATTGCTTCAA
TGTTTTCTTTTCATTCAATATGTAGAATTCTG
TGGACCACTAGCATGGTGCTCTGTCCTCAGTTAGGATTTGAATATTATTACTTGCGAAGTGGCAGACT
TTGGGGTCCTCCAACCACAAGACACCGTCGCG
TGGTGGGAATCAAAAGGGATAGGGCGGGATGGCAAAATAAAAATCAAATCCTATCTGGCCCCTATCGG
CTCCCTGCCCCTACAAAGTCGTGTTCTTCCCA
AAAGATTCATACGGCTGAAAGTGTCATGAAGAATGCCTTCATTTATCAGGATGTACATTCATTAGTTA
AGGCAGCCAGCTCTCCCGCGATCATTCTTTTC
ACAAGCTCCTAAAGAAAAAATAAAGAAAAAAAAAAAGGAGGACCAAAATAAATTAAACAAACAACAAAA
AAAAGTATCCATTTTTCTTTTTCCTTTTTTCT
TTTTTTATTCCTGGAGCTTACTGAACTTCTTCGGNNNNNNNNNNNNNNNNNNNNNNNNNNNNNNNNNN
NNNNNNNNNNNNNNNNNNNNNNNNNNNNNNNN
NNNNNNNNNNNNNNNNNNNNNNNNNNNNNNNNNNNNNNNNNNNNNNNNNNNNNNNNNNNNNNNNNNNN
NNNNNNNNNNNNNNNNNNNNNNNNNNNNNNNN
NNNNNNNNNNNNNNNNNNNNNNNNNNNNNNNNNNNNNNNNNNNNNNNNNNNNNNNNNNNNNNNNNNNN
NNNNNNNNNNNNNNNNNNNNNNNNNNNNNNNN
NNNNNNNNNNNNNNNNNNNNNNNNNNNNNNNNNNAGTTACCCCAGCCATCTCCAACGGAACATTTCGT

CCCGCCTCTTCCTGCGCACGCCTGGAGCGTAC
AAAGCGCATGTAGACAAAATGCATCAGCAGACTTGCAGTGAAAGCCAATGTGCAGAGGCACGCAAGCA
CAGTGAATGATGTCCGCACACACTTGCTACGT
CCCAGGCAGTGGACCCGACCATCCGAAGCCCGAACGGAGTTCCGCGTCATAAGTTCACCAAACATAAA
ACGATTGAAGACAATAACACCAATGAGATCGA
AAACAAAGATACTGTTGTAATGCTTCGCAACGTCAATGCTGAAAATGGTGCGGACAGTCAATACGAGG
GTAGCAGCGTAGGAGCCGTTCGCAAATCCTCC
GAGAAGCATTGGTACGATCAGCGCCCGTGCTGGCACAATGAGAAAAAAGATGCAAGAAAGGCACATCA
TGAGGGACGGGATGCAGTACGTGATGGTAATG
GGCGTTCGCTTCTCAAGTGGTCGGCTTGCATTCCATGCTTCAAAGAAACTGACACCAAGAC
>scaffold1064.size6932
CTGTCCGAAGCGCAGGGCGCAGCGCCATGACGAAGCCGTGCGGTTTTCTGGCGGGGTGCTGCGGGTGG
CGGAAGATGGGGGCACTAAAACGCAAAGCATTA
CCACGCACCTCGCATCGGGGAGGGGCTGCAGCTTTGCCTCCAACACGGCATCCTCCACTCTACCGCCT
CTCGGGGCTCACGCAACTATTTTGCTCGCCTG
CTACTTTGCGGTATCCGGCGTGCAGCGGGACGTCGGGAGGCGTCCAACACCATGCGCTGATGCCGTGC
GTCGCCTCCGCCCGCGGCAGCGCTATGCACAA
CGCCCCCAGTGTGTCTCTCCTAGTTCCCCATGCAAAACACAAAATCGTTGCCAGTTGGAGTGGGCGCT
GATCCAATTCCTCACGGCGATTCGCCACGTGT
GCCCATTTCACAAGGATGCTGGAACTTGATGCCCAGAAGAGTCGGCATGTGTTCCATAGCGGCCGGGG
CGTCCTCATAAGCGAGGCGCAGTGGGGCCGTG
TTGCCGCTGCCAAGGAGAGTGTAACTAGTCTTTCTGGCATTCGCCTCCACGAAGTATTCTCTGGTCCA
CTCCGCCACGGCACTCAGGCCTTCTTTTGAGG
CCGAGTTGCTCGCATCCCTGTTGCTATGCTGTGCGATGGTGGTGGGGGCGTCAGCAATGAACCCTCGG
CGAAGTAGGGCACGTTCGAAGGACAAGGACAA
CGAGACTGCATGACGACGACGAACGTGATGGGTTCCAACACTGTTCCATGCGGTGCGCCTCGTGTGGG
TGTCCTGGAGGAGGGTGTTTGTCGATGCAAAC
CACAGCGCTTGTGCTATTAAGGCACGCTTCTGACCAGCGTACGATGCGATTGGGAATCGACAGTCGCC
GCATTTCCAAGATTACCGCGTCAGGGCCCACC
GTGCCAAAGGCATTGGCGTGGTCCACAAAGTAAGCCGCCGTGCGGTGTTGTGGAGCATTGCGATGCAT
GGCAGGCCTGAGGTGCAGCAATTGGTCGGGGG
CGGAATTGCCCGGCAAGAAGCCAGATGGCCGCAGAGCCAACTGGGACTCGATGACGTCCCGGATGCGT
GCGGCGGGGGTGCGTTCCATGAGCTTGCTGAG
GCAGCTGCTCAGCGTTACAGGTCGGTGGGGAGTCGCGGTTGGATGCCGTTCTCTCCGGATTGGGCAGG
AGTAGCACAGCCCCGCGTTGCCAATTCCGTGG
GACGGCCCCTGCACGTAGGCGGATACCAAACAGCCAGGGGACGGCACGCCTCGCCATACGACCTAGAT
GGCGCAGTGCCTCTCAATGTATTCCGTCGGGT
CCGGGCGCCGGTCCCAAGAGCAACAGCCGTATGGTGCGGCCGGGCTCTGCCATATTTATTGGGTGAGG
TCCATCGCATTCGTCGGGATCGGTGGCGGAGG
GTGGGGCTTTGGGTGCCGTGTTGCTTTCGCCGCCTACAGCTTCATGAGGTGGCTGACCTTCAGGTGTC
TGCTGAGCATTGGGCCATCGAAATTCATGGCT
GGCGTGCTCAAGGAACGAGGGGCTTAAGCACGGGCGTACTACCTGCTGCCTGTTGCCGTCCGTGCCCT
CGAGCTCGGAGCGGAGTGTGTGCCAGCGGCTT
TCGGATGCTTCGCGCAGCGCCTCCCTGCGACGGAGGTCCAGCCGCTGACCGCCTTCTGCGTGGGGTGC
GGCGGCAACGGCGCGGTAAAATTTCTCCAGCC
ATGGAGCCCAGAATGGCGATAGCTGGGGGTTCCTCTCCGGGATTGTTGCCGTGACGGCGTTAGCTTTC
TTGATGTGCTTGTGGGAAATTTTTGATAGGAG
CTTGTGAAGAGAATGGTCGCGGGAGAGCTGGCTAACTTAATTAATGTATGTATATCCTGATAAATGAA
TGCATTCTTCATGATACTTTTCCTACCCTGGC
TCAGCCGGCCACCTCAACGTGGTGCCAGGGTCTAGTACTCTTTGCTAGAGAGGAAGCTAAGCGCCTGC
TGCCCTTCCGCTGCCCGCGGAGAGACAGGAGG
CGCCGCACAAACTGGTCGGAAGGGCAGATGTTGGAGCCATTTACATGGCTGCCCGCGGAGCGGTATTT
TCATCCGCTGCTGAATTCCATCGGCGCTTATC
AGCGCTACACATACCGTTTGCGTGCCGTATGTGACGCGCAACGACAAAAGCCACTGCTAAGCGGAGAC

ATTGAGCAGAACCCAGGCCCCATAGCAGTACT
CCAGATGAACTTTTCTTGCCTCACGCCGTCAAAACTCGCAACATTAATGGCGCAAGGAGCAGACATAA
TAGCCATTCAGGAGACTTGGAAGTCGTCAGAG
CAGATCGCCAGCATGCACACTGGAGAGTGTGTGCTCTATGCACAGTCGCGCATCGGCAAGGGAGGCGG
TGTGGCGGTGCTGGTGCGGAAAACTCTCCGCT
CCAAGCGTATACCTCTCACCATCCCCCAGCATGACACCAGCCATGAAGTGGTGGTGGTCCAGGTTGCT
CTGGAGCAGAACCGTGATATTATTGTAGCGGG
TGCCTATATGAGACCAACACCGCAAGTAACGCAATCCTTCAGGCGGTTAGTAAACTGCCTTCCAGCCT
CGTCGCCGCTCCTGCTGTGTGGGGATTTCAAC
ATGCATCACCCACAGTGGGAGCCATTCTTGGAGACTTCTCCAAGTGAGGTTGCTGCAGAATTTTTAGA
ACTGTGCACGGATGCGGGACTCACCTTGGTTA
ACACCCCTGGTGAGATCACGTATGCCCGTGGCACAAGAGAACGATCCTGTATCGATCTGACATGGTCA
AAGCATTTGACTGTGTCGAATTGGTCAGCTTC
CGTGTCGCCGCTTAGTGATCATCATATGCTGACATTTACGCTGCATCAGGCATTTAAGGATACCATAC
CTTCGGCACCCCCTTCGGCACCTAAGTTTTTC
TACAGTTGGGGGAAGTGCAAGTGGGATTTATTCATCAAGGACTTCGACGCACAACTTCCGGCATACGA
CTATAAAAAGCAGTCCACCGGCATCAAAGCTT
TCACGAGAGCGCTTATAACTTCGTATCGACGGCATTGCCCCGCGGCATGCACAAGGACGGTCCCAGGC
TTTGGGAAGACACTCTCATGGAGGCGGAGCGG
ATTGCCACGGACAGCAAGGCCCGCTATCTACAGTTACCGACGCCCGACCGTGAGGCAGAAATGCAACG
GACAAGGAGTCAATTCTTTCTCCTGCTCCGAG
AGCGCTTGGGCAACACGTATCCACACCGCATCAGCAAGTTAAATCCAGGCGAGCCACTCGCATGGAAA
TACATTTCCGGACGAAAAAAGGCATCACTTCC
ATCTCCCACATCGATGTTATTAGGAGATGGTCAACGCACTTATAAAACAGCAAGGAGAGCAGCGAATG
CTCTCAATCGCATCTTTCTTCCATTTCACCCC
TCTCACAAGGCAGTTAGGTTTTCCAAAGGCATCAACAGACAGAGTGCATCTTTGAACATTAATGCTTC
TTTTCTTTTTGGGCAGCAGAACAACAACGAGT
CTGCCGCTACATTTACTTCAGGTTCTTCACTCTCATCTAGTTCTGAGCCACAGGATAAAAACGAAGCT
GCCACCACATCTGGTTTAGTTGCTCATCTTCA
CTCCCCTCTTGATGTACCTTTTAATCTTACGGAACTGCTTGCTGCGCTACGAAATACGCCGTATGGCA
AGGCCCCCGGACCGGATGAAGTCTACAGTGAG
GCACTGCGACATATTTCGTCAAAGGGCCTCCGATTCCTTCTTCGTTGCGTTAACCACAGTTGGACGAC
CGGTACGATCCCGGTTGAGTGGAGACGCGCCA
CCATCGTTCCACTCTTAAAACCCGGCAAGTCGCCGGAACTGCTTGAGTCATATCGACCCATCAGCCTT
ACCTCCATTGTGAGTAAGGTTGCTGAGAAAAT
GGTACTGAAGAGATTGCTTTGGGTGTGGACGCCGCACCCCCACCAGTATGCATATCGTAGTATGCGTA
CCACGACGATGCAGCTGGCACACCTGATACAC
GAAGTGGAGCATAATAGAAATCACTATTTCCAAGTGAGCCTTCCCAAGAGAAGCGGTATTGGCAATCA
ACTCCACTACAGACCCCATCGGACCCTGCTGG
TGCTGGTTGATTTCAGCAAGGCTTTTGACTCCATAGATCATCGAGTCCTCAGTCGCTTGCTGGCTAAT
ATTCCGGGGGTGGATTGTAGAAGGTGGCTTAG
AAACTTTCTATGTGGTCGCTACGCGAAGACACGAGTTGGCCACAGACACAGCGATCGGCGTCCCATGC
TGCGAGGAGTTCCTCAGGGGTCCGTGCTGGGA
CCGTATTTGTTCTCCCTTTACGTACACCCACTTCTCAATCTGCTGAACAGCTTTGCGGGTGTCACAGC
AGACATGTATGCGGACGACCTCTCTATTATCG
TTAAGGGGCAGTCCCGGGAAGACGCCATTCCCACTGCCAACATGGTTCTTGAAAAGCTGCATGCGTGG
AGTCAGGAAAATGGCCTGGCCATCAACCCGTC
AAAGTGTGAAGCTGCTTGGTTCACACTTTCCACGCACACGGAGTCAGATTATGATCGCGAAGGAAAGT
GGCCCCTGGTAGTGGCTGGATGCCAAATCCCA
GTCATGACCGTGGGAGCGTCGCGAACTACGAAGCTTCTCGGCATGGATCTCGATCCACGACTGACGCT
AAATGTGGCGGCCACCAAGCAATGCGCTGCCA
CTTCGCAACGGATATCGCAGCTACGCTGCATAGCGCACAAAGAGGCGGGACCATCTCCACATGACCTA
CGCACGTTCGTCATTGGATACGGTGCTTCCAA
ATTACGCTATGGCAGCGAGCTCATATGGGCAGTAGCGACGGATTCAGCGAAGAATGAGATGCAGAAGA

CGTGCGTAACCCTAGCACGCATTGTCAGCGGA
GTTCCGAGCACTGTCGACCCGGAATCCGCACTACTGGAGGCTAATATGCCGCCGCTCCACGTCCTTTG
CCTGCGCGCGCGGCTCTCAATATTTGGGAACA
CACGCGCATGTCAGATGGACTGGATGCGGAGACCCCCGCCTGAGCCACCGCCTCGCGCCGGTTTCCGC
ATCTCGCCATTATCTCGGGACGAGCTATATGC
CTTTGTAGACGCATACACAAAGGACTATGGCATCACCGAGAGCTCACCACGCGAAGAGCGGTTCTTTC
GCAGCTTCATTCCTCCCTGGTTTGCGGCCTCC
GCTCACCGGGTCACCATCGGTGTGGAACTTCCGATAGACCTCTCAATAACGGACGAAGAAGAGCTGAT
ACGGGAAAAGCGCAGGGTCAGCGAAGAGGCTC
TGGCGCTGCACAGCCATCGTTCGTGGATACTCGCGACCGATGGCGGTGTCGACGTTCCCAAGTCAGCA
GGGGTTGGAATACTGCTTTCATCCCTCAACTC
ATCGGAGATAATAGAAAAGGCCAGCATAAACTGCGGCGCACGCCCATGCAGCTACAGGACGGAATCCC
GTGCGCTGCTTCTAGCCCTAGAGAGGCTGATG
ATTCCTCGTATCCGCCACAGGCGTAAAACCCTGCTTGTGGTTACAGACAGTCAGTCTCTTCTAGCGGC
TCTAAACAAGGGCCCGCTCAGTCAGACCGACT
GGACGGAGGATCGGATCTGGCAGCGTCTCTTGACACTGACGTGTGCTGGCTGGTCCNNNNNNNNNNNN
NNNNNNNNNNNNNNNNNNNNNNNNNNNNNNNN
NNNNNNNNNNNNNNNNNNNNNNNNNNNNNNNNNNNNNNNNNNNNNNNNNNNNNNNNNNNNNNNNNNNN
NNNNNNNNNNNNNNNNNNNNNNNNNNNNNNNN
NNNNNNNNNNNNNNNNNNNNNNNNNNNNNNNNNNNNNNNNNNNNNNNNNNNNNNNNNNNNNNNNNNNN
NNNNNNNNNNNNNNNNNNNNNNNNNNNNNNNN
NNNNNNNNNNNNNNNNNNNNNNNNNNNNNNNNNNNNNNNNNNNNNNNNNNNNNNNNNGTTTTGTTACGG
ACATTGTGGAGTACATGCTAACGAGCTTGCCG
ATCAGTATGCGACGCAGACTATGGAAAGTGGACAATACACGGAACATGGGATCGCACCTTTATGGCAT
ACGGATCTGCTGACATGTTCTACTACCCAGCT
CACCAACAAGTGGCGTAGTACCATTCGTCAAGACACTCATCGATACTTGCTTTGCGGCACAAGGCCAT
CAGATCTCAGCGGTAAGGACCTGATCACTCAG
GAAGTTCTCCACCGTCAGGAACTGGTTCACCTCGCAAGGGCAAGGTGCccGGAATCTGAGCTCTGGGG
CCGACTATACTGGGCCGTGAGAGATTGCACAA
ACCAATGCCGATTCTGCAACATCTCACCGGAACAGTCTGCATATATGCGCTCTAACAACGATCCGACC
GCACCGGGGACGGACACTGTTCCCTCGTCGGC
GAGGGAGGAAGACGTCTCTCCAGTAAGGAGACGGACCCTCACACGCCGTCGGAAGGAGAAATGTCCGC
ACTGTGATTCTACATTGACGGGATTCTCGGGT
CTCGTCAGTCACTGTCGGTTATTTCGTCAGGAACATCCTCCACCGCTTCCCGAGCTCAAATGTGATTT
CTGTGACATGGTTTTCCCCACACGGAGAAGCA
CCGCACAGCACAGAAGTCGCTGCGCACACAACCCAGACGCCACACGACATCGAAACAGCAGTGCCAGG
AGGCGATCTCTGCTGCCGCAGGATCAGCCAGC
TTCCACAAGCACGCCAATCGGCCCGCAGGAAACCTTGCACCATCTGCTTCCGGAATGTTACAGCACCT
TGGCTGTGCGTCAACGGCTGGGCATTGAACAG
GACCTTCGCCTCGGAAAGTTCTCTCAATGGCAGTTGCTTCATAGCAGGAAACTTTTGTCGTTGCTCGA
CCACCTCTTCGGCACTCAGATGACACTGTATA
gctagacgcgctggtaagagtataaaaaaaa
>scaffold1065.size7492
AAGCTTGACAACTGGAAGGATGTCTTTGACGCTGGTGGTAAGTACGGCTCGATTCGTGGTCCCAGTCT
CGTTGAGGTGCAGGGTCATGTATTTGCTATTGC
TGAAGCCCATTGCAAGGATGGAGGCGACTGCAGCGAAGTCGGCTTTACTGGGATTGCGTCAAAGTACC
TTGGTTTAAATGTTGCTGGCCCAACGGAGATC
TTGACGGCGAATGCGAGTATCTTTGGCGCAGATCCTCTAAAGGAAGGGTCTGGGGGCATCCACACCAC
AAACGGCATAACGCGACCAACGACACTTGTAA
TTGGGGACAGTGTTTACGTGCTCCTGGGAAAATACAGTCGTGCAAAGCCGCCGGTTGGGGGTAAGAAT
GAGCCAGGCCTTTTACTCGTGAGGGGAACTGT
CGCTGATGAGGGTGGGACGAAGAAAATCAAATGGAATGAGACCCATGTGGTGAACCCTCAAGGAAAAA
GAGAATCTGTCTCCTTGACCGAGTTAGTTGGC
GGTGGAGGATCGGGTGCTGTGATGCGTGACGGCACGATTGTGTTTCCCATGCAGGCCAAAAACAGTGA

TGAGGAACGCATTTTACTGTCCATGAGTTTTA
CCCCGTCAGACAAAAAATGGGAGCTTTCACACACGGCGACTGGTAAGGGCTGCAGGGATCCAACCCTG
GTGAAGTGGAAAGAAGACGAATACGGCGCAGA
ACTCTTCATGATGGCTCATTGTGCTGGAGGCTACTATGACGTTTACAGTTCTACCTCGGATGGGTACG
GTTGGAATACGTTGGGTGAGCCAATTAACCGC
GTTTGGGGCAACTCACACAATCGAAAGGGGTACGGCGTCCAGAGTGGATTCACCAATGCAATCATTGA
GGACAAGGAGGTGATGCTCATCACCGCGCCGG
TGTATGCGAAGGACAATGAAGGTGGAAAAGGTCGGCTTCATCTTTGGCTGACGGACAAGGCGCGTGTG
TATGATGTTGGGCCGATATCCCGTGAGGCTGA
TGACGCTGCCGCGAGCTCCCTGTTGATAAAAGATGAAAATAGGGAGTTGATTTCACTGTACGAAAACA
AGAAGAGCGACGGATCATACAATCTTGTTGCT
GTGCGTCTGACCGGGAAACTGGAGCGGATAAAGGAGGTGGTGAAGAAATGGAGGGATTTGGACAGCGC
CTTGAGAACATGCCGTTCCGGTTCCAGCGGCA
CTGTCGACTTGCCAACGAAGGGTATGTGCAATGGTCCTGTTCCCACTGATGGGCTTGTTGGCTTCTTG
TCCGGTAACTTCTCTGAGAACACATGGAGGGA
CGAGTACCTCGGCGTGAACGCCATTGTACATGGACCGGCGGAAAAAAGGATAGTGGTTCCCAATGGAT
TGACGTTCAAAGGGTCTGAAGCAGGGGCGGTG
TGGCCTGTTGGCGACATGGGGCAGACTGTGCCGTACTACTTTGCTAACAACGAGTTCACTCTTGTGGC
GACGGTTTCCATCCACGAGGTGCCGCAGAGTG
GCAGCCCCATTCCTTTGATTGGCGTGAGGATGAATGACACCAGCGGCACGGTGCTTTTTGGTCTGTCG
TACACCCATGACAAGAAGTGGCTGGCCATACC
GGGTGATGGGGAATATTTTGATGATTGGGAGCCAAACAGGGCGTATCAGGTGGTACTGCGAATGGATT
ATTATTATTGGACTGTCTTTGTAGACGGAGAG
GAAATCCACCACACGGAATACAATACGAGTCTGTTTGACTCGCACAGGATTTCACACTTCTACATCGG
TGGGGACAGTAAGGACCAAAGTGCAACGGGCG
GCCGCGTGACGGTGACCAACGTCATGCTGTACAACGAGGAACTGTCTGAAGATGATCTGAATGAACTC
CCAGACAGCAAAGTCACTATTCCATCACTGAG
TGTTGAGGAACAGCCCACAGGACAGGCGGCCAGCACAGACGACTCGGTTGCTTCCGGATCAAGGAGTA
AAGAAAGCGCCACCGCTGAGAAATTGACAGAG
GGTGATCGTGATAAACAAGATGAAGAAAGCGTCCTTAATTTGGTGCCTGTGGCGCCCCCTTCCACGGT
TGTCGCGGGATCATCTGTTCCTAAACCTGCCA
CCGCAGCGGAGAGCGCAGGGGCTTCTCTTCCAGATGACAATATCCAGCTTTCCGGAGGAGAAAATTCT
CCGCAAACCACAATGAATGAAGCGAAGGAATC
GATGCAGCGGGATTCAGATGTGCAGACACAAGACCTACAGTCAGCGGAATCAACGGAGTTCAATGATG
TTGAAGGATCCTCTGAAAGTAATGATACAGAA
CAAACAGTGGAGGAGGGAGAAGCAAATGACAAGAGTGGCGGAACAACGTCCTCAGTGGCTGTATCATC
GGATATTGACACGGTTGCTGCACCAGCTGACG
GTGAGCACCAAGTGCAACAAAGCGTTGAGCTTTCCTCTGAAAACAACGACGTGCGGTCCACTGGAACC
GGAACCACCGGCGCGGAGCAAAGTTTCAGTCT
CGAGGCGAGGGACGGAAATTCCGAGAGAACAATGAGCTCAGACAGCAGCCTCACTCCTTCAAAGAGTG
GCGCCGAAACCACATCCGCGGAGAACGCCGAC
GACGTCTCTCGGACAGAAGGAACCGAATTTTCTTCTGAAAAAGGCAAGGAGGTGCCACAGACAGTCGA
CATCGCACCGGGTAATACAAACACCACGCCTG
GGGAGACCAAGATCCCATCGAAGTCCAACGCAACAGCACCCTCGGACGCTGGCATTTTGCTTGAGAAC
GGGCATTTGGGCGAGTTGGCGGGAATGGCTCT
ATTTGCTGAGAGCACCGTGCATGGGTTTGTGTCTCGGGTGCTGTTGCTGATGCTTCTGGGGCTGTGGG
GCACTGCGGCTCTCTGTTGAATGAATTGGGGA
GAGAAAAAATTGCAGAATTCTGTGGAGCAACAACTGAGCGGCATCCCAGCAATACTCAATGAGTTTTGT
CAATGATACTTTCTCTTTTTTATGTTTTTTCT
CCTTTTTTTCTTTTTCTTTTTTCTTTTTTTCTTTTTTACGTTTCTGTTTTTTCTTTTTTTTGTTTTTG
GTGTTTTTTCTTGCTGGTGCTCTGCTGAAATA
ATCAGCACCCCTGTTTGTGTGTTTTCCCCAAAAAACGGTCGCCTGCTGCAAACCGTCATGCGTGACGT
GTTTCCCCCTCTTTTGTTTGCTTTTTTTGCCC
ACACGCGGTGCCGGCCTCATTGCTGTGCGTCCCACGGACTGCACGAAGGGAGGAGTGGAGGAGCAGGC

CGGATGCCGCATGGAATGTGAGGGATGTTGTG
TGGGCTGGTGGGAGAGTACAATTCCCTTTTATTTTTGTGTGTGTGCGGGCAGTGTGTATAAACCCAAG
AACACGGTTGCATTGTGCTGCGTGGCACATGA
AATGACATATATGCCGAGTCAATTGTTTTTGCTTTTTCTTTCTTTTTTCATTTTTTCATTTTTTCTTC
TTTTTGTTTTGGTAAATCACTAAAGTCTTTTT
TTTCCGTCTGTGCCGTGTCCCATGTCACTTTGTGTCTCCGTGTCCTGTTGAATGTCCCTCTGCCGGCT
GTGCTGCTGGCATGCCTCCATTTCTTCTTCTT
TCTTTCCTGCTAGCAACACCGATGGTCCGCGACGACGATTGTTTGTGTTCCCCCTTTGCGATGGCACT
GGGGTGACTGGGCGCCGGCCTGCTGACTGCAC
TCGGAGGGGAGCGGATGGCGTGCGCCGTGCCTCAGTTGTGAGTGTGGGACGCCACCCGCCACGACCTG
CTGCGAGGCGCAGCGCTATTCCGTCGCGGCGG
AGAGAAGCAAAGGACCGCGCCGTGGACTTGGATGCGCCCGTCCCTCTTGTTGCGGCCGTGTGTGTTTG
TGTGTGCTCTGGATGCCCAAGCCCATTGGCTC
CGCAAATGCGGCAACAGAAGGATGTGGTTGCGTGGAGGCGACAATGCCACTGGAATCAAACCGTGCCT
GTGGCACACACGCAAACGGGAAGAGGAAGGGA
AGGAATGTTTGCCTGGGCTGCTGGCCGGACAACAAACTTTTCCATACAAATAAAATACGACGGAAGAG
AAGAATGAGTCAATGGAGAGGCAGTGGGTTCG
CCAAATCCTCGTTGTTTGCTCCGCGTATGTATTTATTCGTACATTCTGGTGGCCCCACTCTCGCGACA
TGATGAACGCTCTATTCGGACTGGATGCATAT
ATATTTGTAGCNNNNNNNNNNNNNNNNNNNNNNNNNNNNNNNNNNNNNNNNNNNNNNNNNNNNNNNNN
NNNNNNNNNNNNNNNNNNNNNNNNNNNNNNNN
NNNNNNNNNNNNNNNNNNNNNNNNNNNNNNNNNNNNNNNNNNNNNNNNNNNNNNNNNNNNNNNNNNNN
NNNNNNNNNNNNNNNNNNNNNNNNNNNNNNNN
NNNNNNNNNNNNNNNNNNNNNNNNNNNNNNNNNNNNNNNNNNNNNNNNNNNNNNNNNNNNNNNNNNNN
NNNNNNNNNNNNNNNNNNNNNNNNNNNNNNNN
NNNNNNNNNNNCAATTGTGGTCATTGGGTGGTACTTATACATATACACCTACCTGGTGTAAACTTTTA
GAATGCGGTGAACCCACTTGTTTTCATCTACT
TCTCATTCCCGTCCTGCTTGACTTCTATAATTGGTAGCCTCAGGATGAGGAAAGCGGCCCGGTGGATA
TTCACTTTTTGTCGCCGCGTGGAGTGACGCAC
CACTTCTCATTGGGTGCGGGATGGCAGTGAAATTTTGTGCGCCGCGTTATGGATGGATGAGGCACCAC
TTCCGCGTGTAGCTTGTGGGGTTGTTGCTCGC
TCGGGGTGCGCTGTGCGTCGGCTGCGGGGATGTGGCCGCATGCTCACGCGCCGTGTGGATCGTGAGCG
CCTCTTGACGGCATCCACCCATCTCTCTCACT
CTCTGTCTGTCTTTCTTTCCCTGTTGGGTGTGAGGCGTGTTGCTGCTCGTTTCTGTGGGCGATGGTTT
TCCCTGCTGTTTCTTCCGGATTGTCTCGTGCT
CCGCTCTCCGCGTGGGTGCTCTCCTCTGCTCCCCACTGACGTGTCTGATGTGTTGCCGCTGTCGCTGT
GCTCCTCTCCGCCTCTTGAACACACGCACGCT
AGGAAGGAAAGCAAAAAAGCGCTACTCGCTACTGTGAAATTGGAGGGCTCTGAAAGGACCAACAGCAC
ACACACCGCCCACACTCACATATTTGTATGCT
CTCACGTGTTGCTGCAGTCATGGCACCCCGCACACACAACCGTCTCCGCGTGACCGGATCCAGCGGAA
GGAGAAAGGGAGGAAGAGAGAGGGAGCAGCAG
AGGCCCAACGTGTCCCGGCGTGTGTTTGCTTCTGCAGCACTGCTGCTTCTCCTCGTCGTGTTATGCGG
CAGTGGGGCTGCACACACTGTGCAGACGAAGA
CTGACGTTGATCCTTTTACAGGAATTGGATGGACATCCCCTCAATGGGAAGGCTCTACAGAAGGTAAT
GAAAAATTCTTCTCGCTCCGCGTTCCCAGCCT
TGTTGAGGTGAACAGTGCTGTGTTCGTGGTTGCTGAAGCGCAGTGCAGGGAAGACGGCGAAGGCTGCA
GCCGTGCTGGGATTGCGTCAAGGCACCTCGAT
CTAACTGATGATAAGCCAACGGAGATATTGACGAGGAACACAAGTTTGTTGATGCCGTTTCCAAAGGG
TGTTGCTACAGGTACCGTCAAGGCAAAAGAAT
CTATGCGGCCAACGACAGTTGTGGCTGGCGACAGTGTCTACATGCTTCTGAGAAATCAGAGCTTTACG
ACGTCGGAGGGTAAATCGGCTACTGCACGTGA
CTGGGGACTTTTGCTGGTGAAGGGAAGTGTCAGTGGTGGCAATGAAATCAAGTGGAATGAGACCCATG
CAGTGACATTTGGAAACGATGAATATGCTGCT
AAATCCCTGACCCAGTTGGTTTCTGGCGGAGGATCGGGGATTAAGACAAAGGACGGTACACTTATGTT

TCCCATGCAGGCCACAAAGAACGGAAAGACCT
CATTGCTGTCCATGCGCTTTGATAAGTCAGAGAAGAAATggaggcttNNNNNNNNNNNNNNNNNNNNN
NNNNNNNNNNNNNNNNNNNNNNNNNNNNNNNN
NNNNNNNNNNNNNNNNNNNNNNNNNNNNNNNNNNNNNNNNNNNNNNNNNNNNNNNNNNNNNNNNNNNN
NNNNNNNNNNNNNNNNNNNNNNNNNNNNNNNN
NNNNNNNNNNNNNNNNNNNNNNNNNNNNNNNNNNNNNNNNNNNNNNNNNNNNNNNNNNNNNNNNNNNN
NNNNNNNNNNNNNNNNNNNNNNNNNNNNNNNN
NNNNNNNNNNNNNNNNNNNNNNNNNNNNNNNNNNNNNNNNNNNNNNNGGCAACTCACACAATCGAACA
GGGTACGGCGTCCAGAATGCCTTCATCAAAGT
GACAATTGAGGACAAGGATGTGATGCTTGTTACCCTGCCAGTGTATTCCAAGGACAACAGAGAAAGCA
ATAAAGAAAAGGGTCGGCTTCATCTTTGGCTG
ACGGACAATGCACGTGTGTATGATGTTGGGCCGATATCCCGTGAGGCTGATGACGCTGCCGCGAGCTC
CCTTTTGATGAAGAGTGGGAATGAAAAGTTGA
TCTCAGTGTACGAAAACAAGAAGAGTGATGACGGGTCATACAGTCTTGTCGCCGTGAGCCTGGGGAAG
CAGCTGGAGCGGATCAAATCCATGGTGAAGAA
ATGGAAGGATTTGGACGGCGCCCTGAAAAAATGCAGTTCCGGTTCCAGCGCCACTGTCGACTTGCCAA
CGAAGGGTATGTGCAATGGTCCTGTTCCCACT
GATGGGCTTGTTGGCTTCTTGTCTGGTAACTCCTCTGATAACACATGGAGAGACGAGTACCTCTGCGT
GAACGCAACTGTGACGAATGGGCAGAGAAGGG
TTCCCAATGGATGGACGTTCAAAGGGTCCGGAGCATGGGCGGTGTGGCCTGTTGGCGACATGGGGCAG
ACTGTGCCGTACTACTTCGCTAACAACGAGTT
CACTCTTGTGGCGACGGTGTCCATCCACGAGGTGCCGCAGAGTGGCAGCAGCCCCATTCCTTTGATTG
GTGTGAGGATGAATGACACCAGCAGCACGGTC
CTTTTTGGTCTGTCGTACACCCATGAAAAGAAGTGGAAGTTCAACACCCATGAAAAGAAGTGGAAGTT
CACACTTCCCAATGGAGCTTATGAAGGTTATA
AATATTACAAATTTAATAATAATTGGCAACCAAACACAACACATCAAGTAATGCTGCAAATGGATGTC
CGTCGGTGGTATGTGTACGTTGATGGAATGCG
GGTTTATTCTGGGCACTACAACGTGAGTCTGTTTGAAGAGCACAGGATCTCACACTTCTATTTTGGCG
CGGAGAATAAGGAGCGTTCGGAAAGCAGCCAC
GTGACAGTGAGCAACGTCCTGCTGTACAACCGCACAATCAGTACGGATGAAATTGCGCTACTCAGTAC
Cagcaaagtccctattccaaa
>scaffold1066.size8852
CGAGCGCCTCGCGGTGTCCGACTCGGTGCTACGCTTCGTGGGCGTCGAGGGGTCTGTTGCGTCGTCGC
TGGTGCGCTGCGACGGTGGGACGATCGGTGCCG
GCGAGAAATTGGGTGCAAGAATCATCCGAGCAGGGGCAGCAAAGTATGTCGGGAGAGCATACCGGCCC
TCCTAGACTTTACTGACGGCTTTTGCAAGGCC
TCACACCGTCACATTATGGATGGTTTACGATCCTTGGGTGTCTCGACGTAACAGCAGAGGTTTATGCT
CGCTTGGCTGACGGATAGACGTGGGCGCACCT
TCCGTATGGGATGTTACTCTGTCTGGGCACTGCTACCGTGCGGTGCTCCTCAAGGGTCTGTTCTTGGA
CCGTGGCCATTCGAAGTATTTCTCGATGGCCC
AATACGTAGCTTTCAACAGGCGCATTTACCGGCAGCCATTGCCCCCCCCGATTGTGCACCTTCCGATC
ATTGATCACACTATCACTGCGGAAATTGTGGC
CTATGCGTGTGACGTGATCGTGAGGGCTACCGTGCCAGGCATAACTCATTTGGATGAGGCGATGCAAC
CGCTCTTTAATGCCATCTCAGAGTGGACAAGA
GCCAAGGGTCTTCCACTCTTGGACAGGATATCCTTTATGGCGATAAACAATGCAGGGCACCACGAAGC
TCCTGTACCGGATTTCTTCAGAATTAATGGAC
GACGGTACCCCTGCGACAGGCAACCCCATAAGATCCTGGGGGTTACTGTGGACTCTCTCCTCACCAGT
TTCGAGGAAGATGTCCGCCGCCGTAGGGCCAG
AATTCAACAGATCATCTTTTGCCGCATTTCTCTGTGTGGAAGACGACACCCCACACGGGTGCGGTCTT
ACGCATGGACGGCAGGACAGATTCTCTTGTGT
GGTTGTGAGGTCCGCGCACTTTTCCAAACACACCTTCAGTGTTGTCACAGTTGTTGCAAGGCTGGACC
TCAGTCATCAGGGCGGTTGCGGAAATACCCAG
CAATGCGGGAACTGCCGACACCCATCTGGACAATTTCCTCTCCACACTGGAGGTCTGCGTGCAAGTAA
GAATCCTGAGGGCCGCAGCAATTTGGACCTCT

TTGCCGGGGGCATTGGTAGAGCTAGTGCTACGAGTCATCTATTCCTTGCAATATTGTGCCAGACCTTT
GCCTGCTGGTATATCGGCTTTTCTCTTACCTG
TACGGATTGACCGCGTCACTTTTTACCTCTTGGCCGCACGAACGACATCGACTGCTACGGCGGAGCCG
TTGGGACTCATTACGGCACGACGTGTCCACTG
TCACATGCTGTTGTCTGAATACAACACCGTCATTGAAGGATGGCCGGCTTGCAGTCTCTCGGAGGAGA
GGAAGGGAGGTGTGGTTATCCTCTTCCGCAAC
GGTGCACAGTTTATAAATGCTCCTTAACCTGTGCTTGTTTGTCACGCGCCCACGCCAAAGGATGTGTG
CACCTACTCTGCGGAAATTGACGCGGGTCTCG
TCTTGCTGGAGGCTGTTCTTCAGTGGCTCCACGAGCACGGAGATTGCAATACACTGCGATCCTACTGT
GCACGGACGGCTTATCTTGGCCGCAAAAGGTA
GCCATTTGCCCATTCCCGTGCGATGCCATCCCAGGCAAGCCTTGTTCCATCCGCACAGACATAACTTT
TTAAAAAAAAAGAGGGTGATAGTATTGCATAT
TTTTGCCCATTGCGAGGATCACTGGGGGGACATGGTGGATCACGAGGTCCAGGAGGCAGCGAAGAAGC
CGCAACGCACAAATTCCTCTCATGGCGCGTGG
ACTACTTGCACAGCCACTGGCGGGCAATAATAGAACGGTGTTTTGTCTTGTTCACATCGTCCCACAAG
TCACATCAGCTCTCAATTATTTCAGACGCTAT
TACGGAGCTTGCCGTAATTCCAACTTCATCACCGTACTTCATCTGTCGCGACGTTATTTGGACTAGGA
GTGGGAGATGTTGGCGTTTGGGTTGCAGGGCG
ATGCTCTACCTGAGGAACGGTGAGAAATGGACGTGTTTACACTGCGGCAACAGCGGAACGGCCCATTA
CTGTGGCACGGCGGCTCACGTATTGCTTTTTT
TTCCGCAGCCCCCTAACATCAATAATCAAGGCGGCAGCATTATGGGGCAATTCGAGTACGGAGCTCGC
AATGGTCATCCAACGTTGCCACTCTTTTTCGA
GGTTTCACGCGGAAGAAGAGGGCAATAACTTAATAATGGCAGCATATTTATACTTTTTTTTTTCGCA
CTTCCGCTCTGGTGTGAATTTTGGTCACAGTG
TTATCTGCCAGTGGAGGGATGCAGTATTTTTACAAGTGCACGAGACAAGAAGAACATGGTGTTGTGTG
CTGGAGCGTCTTCTGTGTCACTGCCGCCCTGT
AGGCTGCCACAACCAATGGCTGTGTGTATTTTGCTGCGTGGAAGTTGTGGGTGCGCGGTGCTGGTTGA
AGGGAGGGCGCGCTCGTCACTCTTTTTACTCC
TAATGTTGTTATAATTTTTTCTCTCCAACAGCGTGCTGAGTGGATGGCACCCAATTGCCGTCGTTGCG
GTGGTTCTCTTGCATGTGTTGGGCCGTCACAC
CGTGTGGATGGCTCTCTTGTTGTGGCTGCCAGTCATTTTTGTGCATATTTTCTCTCTCACTCACTCTC
CTCCTTGTTGGTGGTTAATGTTCAGCTTTAAC
TCCGACTCAAAAAGCGAGGGGAATCAGTGCCTAGAATTCAAGCGTCTGCTGTGCCGCAGGAAGATGGT
CGGAGAAGAGCGAGGCCGGAGTTTGAAGGTGG
CGCAGATCAACCTGCTGCAACTCGCAGAAGAGTGGAGGAGGCGCGACTGCTAATGTGAGCTCTTGACA
GTTGGGTCGAAAGGCATCCATGCAGGGGATTC
TACGAGTCTGTGTACAATGCGAGGTGGAGCCATGTCGTGGAGGTTCCTGGCGGTGAGGGGATGGGAAT
GGAGGTTTATGAGGGGAAACCGAAACAGTCAT
GGACGTACAAGGCAGTTGGCGAATTATTCGAAAAGGATGACGTTTTGGAGCAATCCGGTGCACCGCGT
CCCAGGATGGTGATGCTCACCTCGGACAAGGG
ATGGCCGTACTCGGGGATAGTGGGTAAATACATTCATGACTGCCATGTGAACTGTGAGGTAGAGCGAG
CGTGGCAGATCTTCAAGGGCGACATAACCGAG
TGGCTCAGCAGTGACCGTGGGACCGAATTTACGCCCAAGCGGCGTGTGTTGATTGGGACGCCCGGGAT
CGGGAATTCGATGGCTGCCGGCTCGTACCCCC
TCTACCAGCTGCTGCACCACGATGCGGAGCAACTCCAAGTGGTTGCTCACTGTTTGGGAGGTACGGCG
TACGTGTTTGACAGGACCATCAGTACGGTGTC
AGTATACATGAGTGAGCCCAGGATGATCAAATTTGTGAAGGAACTGTCTTTGAGCAGGGTGAAGGCGT
ATATCATCTACGACGTGGGATGCACGTGGAAA
ATTCTGTCTCCTTGTTTGCCTCCCGGAGAATGGGGCATGCTTGTGGTGACAGCGCCAGAGACAACAAG
TTACATACACTGGGTGGGTATGAAGAGAGAGC
CGCGCGAGCCATAGTGAATTGCCCTGATGAGAGCGACGTGAAGGCAATGTGCGTTTGGAGAAGCGCAA
TCAGCCTCCGCAGCAGCAAGCGGAATACTGGC
GAGAGGTGAAGTGTCCCATGCACAAACTGGGACCGATTCTTCGCTACATCTTTGATGAGAGAGATTAT
AACTATTGGATTGGAAATTGCCACAACCTTGT

GGACAGGACACCTTCTTGGGGAAGCGAGAGTTACTTTGTTTTTGGAACTTCCAAACTGTGGGAGGGTA
ACAATGCGCTCGAATATATTGCAAGGATTGTC
CGAGTACGAGGAGAGCGCAATGGCGAGTCGCCTTTCAATGCACCGATAACTGCCCACCTCGCGAGTAA
AACATTGTGCATGTTGGCAAAGTTAATGCCGC
AGGCTGATTTTAACTTATTTGTATCGAGGGTCAGGGATTATCTCATATCCGAAAATTTTGGGAAGTGT
GCCATGTTTGCATTTTTGAATGGGGCTTTTGT
GGGTGCAATAAGACGTAAACTGAAGGAGCTGAGGCCACCAGCACGACGTCCGTCACACCGCTGTGCGC
CGGAAGTGTACTCGCAGGAGGGCTCCACCAGG
CACGATTTTTTGCCGTCGGTGGAACACCGTTACCAGAAGACTGATGTAGAGTGCGGGGTGCTGTACAC
ATCGTGGGCCACAAACTTTCCGCTGGTGGACG
TCTTTTTCTTTGTGAAGTCAAATCCGAAGACGCTGGTTGGGCTGCGGATGGCTACGGCGGGTGAGCAT
AACACCACCGCCGGCACTGTGAGGTAGTGCAA
TGAGCGTCTGGCGGGATATTTTGATGGTTGGGAGGAGTTATTTCGAGAAATGTTGTGGGATATTATTT
ATGTGCAACACGCAGACAGGGTGCTGATGGGT
GGCTGGCAGAGATGCGGCGCGGTTGAGCACCCGACTGAGGGTGAGTGTGGGGAGGAGGCGGTGGCTGT
TGTTGCGGAATTTGGGCTGAGGAAGGGACGTC
GGTGCCAGGTGGGGGATAGCGGATGCAGAAGTACGTGTCTTCTACAGTGAGTGAAACAGGCGCATGTT
TCTCCCCGAGACTCCCCGTGTTGCTTTGCCTC
AACGAGCACTGCGGTGTGTCTGGGAGGTGGAAGGGATCGCATTTGGGATGGTGCTGGTGGTGGTGGCA
TCGCCACGTGTTTGTTGCGTGGCCACATGGCT
GAGGCGCTGTCTTTGTGTTGCCACCGTCATCGTTGCTGCTTTTTCTCGTGGAGCGGATTGTAATGGAT
GCTGCGTTGTATTGATTTTGTTTGTGAATTTG
TTTTGCACCGCCTTTTGCTCCCGCAGCGCAGCGCTGATGCGGGTGGTCGGCGCTGGGAAGTCTAAACG
AACCCGTGAAACGGGGTGCTGTTGAGTTGCGC
CTCACATGTCATTACTGATGGTGCTTGGGGATGGCATGGGGGGATGCGGGGAGTGAATTGTTTGTGAA
TCTGTTTCGGTTTGTTTTCTGTTTGGGGCGAG
AGAACTGTGGGCACTCCGCTGGGGTGCGCCTTCCTTCTTGGTTTTTTGCCCAACGATCACTACGGTCC
TTTTTCTTTGTGGGGAGATGAGGAGGAGAGTG
TGTGACTTTGTGGCCCACGCGTTGCTGGGGACCGTTGTTGTTGTGTGTCCGTGCATGCCTGCGGGTTG
TTGGGGCTGGCACTGGTCACCGCCCGCCGCCG
TCGCCGGAACACGAAAGACCCAAGCAAGTGAAGTAAAGGACAACAGGAGTCTCCACGTGGCCCTCACC
ACAATTGAGGCACAACCCGCATAGCGACTTTG
CTTCCGAGGGCGGCTGTCTCCGCCGCGCAGGAGGAGAATGCCATCGAGAAACAGTGCACCGTGTGCTC
AAGATGGCGCGCACACATCCATTCATCAACGC
CACACTGTCGCCGATTTCATGATGGCCGAGGGGTCAGGCCACCGAGAAAGCGCACGTCGCCTGCCGAC
GACACCCCGATAAGATCACCACCGGCTGAATT
TCGCCATCCAGCATGCGGCTTTCGGTGCCACAGCAGTGAGGGATTGACCCACCGCCGCACGGCATGCC
ACAACCACCGACTGGACATTGTGAAAACCAGC
CAACGTGAGCTGCGCGAAGAGACAATTCTGCATCTACTCCGCTGCCCGGCTCTGCAGGTGCCCGGACA
AAAAACGTGGAATGGACACTGAAGAAGCGAGG
AGACTGTGCTTCACCAAAACGTTGAGCGCGGGTTATTGAGGTAGAAAGGCTCTGTCCTCAACAGCGCT
CCACGCGGAGAGAAGGCACAATTCCCCTCGCC
GATGCATAAGGGGTCTGCTTTTCACGGTCTCCGCCGCAACTCCCTCTCCGGGGGCAGGGGCTTTCTTT
TACATTCCCACTGAGTGGGGCGGACGTGCTCG
ACGATGGTGGCTGGCGTGCGGGTTATAGAATTTATTTGGGAATAGACGAGGGAAAAGGGTGTTGCTGT
CGCTCCCCATCACGCTTCATCTCCTTCTACTT
GTGGAGCCAATGACGCATGCGGCTGCGGGGGAGATAACGGCAGCCCGAGCTGTGTTTGTGCATGACGA
TGGCACGCCTGTCGTTGGTTGCGCGTCCTCTT
TTTGGACACACACATTCCTTTCACTCCATGTGTGTGGTGGGAGGAAACAATTGCTGTTGCTGCAGCGA
CACTCCGTGCTTTTCAGTGACGTGTCTGATGT
ATTGCCGTTTTTGTTGTCTTTCTGCCGCTGCTGCCTTCTCCACTTCTTGAACAGACACGCTAGGAAAG
AAAGAAAACAGGCGCTAATCGCTACTGTGAAA
TTTGAAGGCTCTGAAAGGACCAACACCACACGCCGCCCACAAACACATATTTATATGCTCTCACTTGT
TGCTGCTGTGAAGACACCCCGCACACACAACC

GTCGCCGTGTGACCGGATCCAGCGGAAGGAGGAGGGAAGGAAGAGAGAGTGAGCCGCGGAGGCCCAAC
ATGTCCCGGCGTGTGTTTACTTCCGCGGTGCT
GCTTCTCTTCGTcNNNNNNNNNNNNNNNNNNNNNNNNNNNNNNNNNNNNNNNNNNNNNNNNNNNNNNN
NNNNNNNNNNNNNNNNNNNNNNNNNNNNNNNN
NNNNNNNNNNNNNNNNNNNNNNNNNNNNNNNNNNNNNNNNNNNNNNNNNNNNNNNNNNNNNNNNNNNN
NNNNNNNNNNNNNNNNNNNNNNNNNNNNNNNN
NNNNNNNNNNNNNNNNNNNNNNNNNNNNNNNNNNNNNNNNNNNNNNNNNNNNNNNNNNNNNNNNNNNN
NNNNNNNNNNNNNNNNNNNNNNNNNNNNNNNN
NNNNNNNNNNNNNTGAGTAGGGCGGAAGCGCTCGACGATGGTGGCTGGCGTGCGGGTTATCGAATTTA
TTTGGGAATAGACGAGGGAAAAGGGTGTTGCT
GTCGCTCCCCATCACGCTTCATCTCCATCTGCGTGTGGAGCCAATGACGCGTGCGGCTGCGGGGGAGA
TAACGGCAGCCCGAGCTGTGTTTGTGCATGAC
GATGGCACGCCTGTCGTTGGTTGCGCGTCCTCTTTTTGGACACACACATTCCTTTCACTCCATGTGTG
TGGTGGGAGGAAACAATTGCTGTTGCTGCAGC
GACACTCCGTGCTTTTCAGTGACGTGTCTGATGTATTGCCGTTTTTGTTGTCTTTCTGCCGCTGCTGC
CTTCTCCACTTCTTGAACAGACACGCTAGGAA
AGAAAGAAAACAGGCGCTAATCGCTACTGTGAAATTTGAAGGCTCTGAAAGGACCAACACCACACGCC
GCCCACAAACACATATTTATATGCTCTCACTT
GTTGCTGCTGTGAAGACACCCCGCACACACAACCGTCGCCGTGTGACCGGATCCAGCGGAAGGAGGAG
GGAAGGGAGAGAGAGTGAGCCGCGGAGGCCCA
ACATGTCCCGGCGTGTGTTTACTTCCGCGGTGCTGCTTCTCTTCGTCGTGGTGTGCTGCGGCCCCTGT
GAGGCTGCAGCCGCTGTGGAGAGTAAGTCCGG
GGCCGTGCAACTGCCGCAATGGGTCGATATTTTTTTGCCGAATAAGACGCAGGTGGTGGCGAAAAATG
GGTCTGCAACTGGAGTGAAATTTGCGTTCTTC
TCACCGTCTCTTGTGAGTGCTGGTGGAGTGATGATTGCCGTTGCCGGGGGCCGCACCAAGCATAATGA
CCCCCACAAATCGGGTTGGCTCGATTCTTCTG
ATATTGTGGCGGGGTACATCAGCGCTGCGGGGACGTGGCCGTCCATTGTCGCTGAGATCACTAAAAAT
GGATGGAAGGCACACACCGCCCTTGGCGGTTG
GAATGGCAAGGATCATGTGAGCGTTTGGTACTGGCCCACAACCATTGCGAAGGACAATAAGGTGTTTT
TACTTGTGGGAGGCTATCAAGAGAAGTACGAC
GATTCTATCGAGCATTGGACGATGGACGAATGGGATATTCAACTGGTTGTGGGTGAGGTCACGCAGGC
CACGGACATTCAGCACAGTAAACCGATCGGCT
GGGGTGAACCCACATCGTTGTTACCACAGATCCCCCAAAAGATTAAAAGCGGCCTGAAGAAATTTGTT
GCTGCTGGTGGCTCGGGCATTGTGATGGGGAA
TGGCACGCTTGTGTGTCCCCTGATGGCGACGAATAGAACAAATCACCCTTTCTCCATGATCACTTATT
CGACGGACAATGGCAACAGCTGGGAGTTCCCA
GGGGGGATTTCCTCTGCCGACTGCCTTTACCCCCGCATCACTGAGTGGGAGAATGGAAAGCTTCTCAT
GGTTGCTCTTTGTTGGAGTGGCCGCAAGGTGT
TTGAGTCGCGTGACATGGGGAAAACGTGGaCGGAGGCCATCGGGACACTCTCAGGCGTGTGGGCCAAG
TCAAAATCAGGAGCTCCTTTGGAAGAAGGTTT
GCGTGTGGGAGCTCTCATCACCGCGACCATAGAGGGAAGGAAGGTCATGCTGTACACTCAGAAAGTGA
GGCATTCTATGGAAGCGAGTGAACCCAATGCG
CTCTACCTTTGGGTCACGGACAACAACCGTACGTTTCATGTTGGACCGCTTTTTTTGGAGGATAATGT
GAATGAGACGATTGCCAACGCCATGCTGTACT
CGGATGGTGCGTTGCACCTTTTACAGGAGAGAGCCAATGAAAAAGGCAGTGCCATTTCACTTGCCCGC
CTGACTGAGGAGCCGAATACAATCCAGTCCGT
CCTCAGTACTTGGGCACAGCTTGACGCCTTCTTTTCCGAGTCGTCCACACCCAC
>scaffold1067.size7200
GCGATGATGACTGCGGTGGTGACGGTGTGCGCCGTGTTGTCCCTTGCGCTCTTGTGCTGCTGCTGCTC
GTCCGTGTGCGTGACGGCGACGGGAGACGATAA
CAATGATAACAGCCGTCCTCCCAGCCCTGAGCTAGGGAAAACGGATCCTACAAAAATCTGCCTGGTGC
ATTACAGCCATATGGTGGGGAGACCGATGTGC
CGCAGACAGAGAGTGGGAGAGACAGCGAGGAGGGTTTGAGAAGTCCAGGACCTTCTTTGCCAAAGGAA
GCAGGCACAGCCATCACTGGACAGACACGAGT

TCCACCAGAGTCACTGAGTTCGGCTAGCGATAATCCAGAGCCACGAGGTTTGGCAACTCAGTCTCCAC
ACACACACAAGGTGCGACCGGTATGTCAGATG
CACAAAATAAAAGGCGCAGTAAAATACACAAGGCGATGAGGATCATAAGGCACCAACATTAAATCAGA
AGATCCAAATGCCAAATAGTACTACCAATAAG
ACCACAACAACGCTCTGGGTCAGCCGTCCGCCTCAACGTGGTGCCAGCGTGCAGTACTCTTTGCTAGG
GATGAAGCTAAGCGCCTGCTGCCCATCCGCTG
CGCGCGGAGAGGTGACAGACGGCGCACAAACGGGTCGGAAGGGCACCAGATGGAGCCATTTACATGGT
TGTCTGCTGAGCGGTATTTCTATCCGCTGCTG
AATTCCATCAGCGCTTATCAGCGCTANNNNNNNNNNNNNNNNNNNNNNNNNNNNNNNNNNNNNNNNNN
NNNNNNNNNNNNNNNNNNNNNNNNNNNNNNNN
NNNNNNNNNNNNNNNNNNNNNNNNNNNNNNNNNNNNNNNNNNNNNNNNNNNNNNNNNNNNNNNNNNNN
NNNNNNNNNNNNNNNNNNNNNNNNNNNNNNNN
NNNNNNNNNNNNNNNNNNNNNNNNNNNNNNNNNNNNNNNNNNNNNNNNNNNNNNNNNNNNNNNNNNNN
NNNNNNNNNNNNNNNNNNNNNNNNNNNNNNNN
NNNNNNNNNNNNNNNNNNNNNNNNNacgtcttaaaaatcttgacacgtgcctacgccGACTCACCTT
GGTTAACACCCCTGGTGAGATCACGTACGCCC
GTGGCACAAGAGAACGATCCTGTATCGATCTGACATGGTCAAAGCATCTGACTGTGTCGGATTGGTCA
GCTTCCGTGTCGCCGCTTAGTGATCATTATAT
GCTGACATTTACGCTGCATCAGGCATTTAAGGATACCATACCTTCGGCACCTAAGTTTTTCTACAGTT
GGGGGAAGTGCAAGTGGGATTTATTCATTAAG
GACTTCGACGCACAACTTCCGGCATACGACTATAAAAAGCAGTCCACCGGCATTAAAGCTTTCACGAG
AGCGCTTATAACTTCGTATCGACGGCACTGCC
CCCGCGGCATGCACAAGGACGGTCCCAGGCTTTGGGACGACACTCTCATGGAGGCAGAGCGGATTGCT
ACCGACAGCAAGGCCCGCTATCTACAGTTACC
GACGCCCGACCGTGAGGCAGAAATGCAACGGACAAGGAGTCAATTCTTCCTCCTACTCCGAGAGCGCT
TGCGCAACACGTATCCACGTCGCATCAGCAAG
TTAAATCCAGGCGAGCCACTCGCATGGAAATACATTTCCGGACGAAAAAAGGCATCACTTCCATCTCC
CACATCAATGTTATTGGGAGGTGGCCAACACA
CTTATAAAACAGCAAGGAGAGCAGCGAATGCTCTCAATCGCATCTTTCTTCCATTTCACCCCTCTCAC
AAGGCAGTAAGGTTTTCCANNNNNNNNNNNNN
NNNNNNNNNNNNNNNNNNNNNNNNNNNNNNNNNNNNNNNNNNNNNNNNNNNNNNNNNNNNNNNNNNNN
NNNNNNNNNNNNNNNNNNNNNNNNNNNNNNNN
NNNNNNNNNNNNNNNNNNNNNNNNNNNNNNNNNNNNNNNNNNNNNNNNNNNNNNNNNNNNNNNNNNNN
NNNNNNNNNNNNNNNNNNNNNNNNNNNNNNNN
NNNNNNNNNNNNNNNNNNNNNNNNNNNNNNNNNNNNNNNNNNNNNNNNNNNNNNNNNNNNNNNNNNNN
NNNNNNNNNNNNNNNNNNNNCACATCTTCTTA
AACAAAAAGATCAAAGAGAGTGGAGACGTCTAGTTTGAAGGTGACTTGTCCTTGCCGACAGTGAGCAG
TGGCGAATGTATCACCTCGTGTGTAGACACAC
CACTGCTGCTTCTGCCTTTGGGACTGTGTGTGGTTGTGGCAGTGTTCTGAGCCACGGAGTGTGGAGTT
GCGACTTCTCACGGAGAGTATGTGCCGCGTTC
CCTGCGGGCTTTCTTTTTTTGATGTTCTTTTTAATGTTGTTTACATGGAACAATGGGAGTACCTTTTT
CTTTTGTTTTACTTAATGGTCAGTTGCTGCGC
ACCGTCATGAGCGAATTGTTTTCCCTCTTGATTGCTTCTGTTGCCCACACGCGGTACAGGCACCACGG
CTGTGCGTTCCACGGACGACACAAAAGGAGGA
GTGGAGAGACAGGCCGGATGCCGCATGAAATGTGTGGGATGTTGTGTGGGCTTGTGGAACAGCAATAA
TTTTTTCCTTGTGCAGGCACTGTACGCATGTA
CATATAAACACAGTCCTGCATTGCTGCATGGCGTATGAAATGATGATTCTGCCGAAGTGTATTTTTTT
TGAGGAGGAGACTCCTGTTGGGCACTCAACTA
CTTTTTTTGTTTTGGTATCGCTTTTTTACTTCTGGTGTGCAGGTCAGTGTGCGTTCCTCTGCAGGGCT
GCTGCCGTTGTTTACGGTGCGTGTTACTGTGT
GATTTGTCTCTGCCTTTTGCTTTCACTCTGCGTTGACGTGTTGTTAGTTTGTGCTGAAGGCCACACGC
AAGTGACAGGAGTGATGGCGATGATGATGACT
GGCCGTGTGCTGCTGGTGTGTGCCCTCTGCGTGCTGTGGTGCGGTGCTGTTTTTGGCTACGCGATGGA
GGATTACTGCGGTGAGGGTGGAGGGAACGGTT

TGAGGCACACGAGTAATGGTGGCGATGACGGCGTGTCTCTAAAGGCGGACTGCGGGTTGTTATCGACT
CGAATGGGATTAATAAAGGCGGTTGAGGCTGC
GGATGGAGAAGAATTGAGTGGAGATCCACTGGAAACATCCGAGGAATCATCACCTGGTAAGAAATTGG
ACGATAAGACTTCGGGCGGTGGAACGCCTGGT
TTAGGAGGCGGTGTTGCAGGTCAACCAGAAGCAGCGGTAGCGCTGGAGCCCGGAGGATCAGGTAGAGG
TGAACCGAAAAGGGAATTGGAAGTAGCGGATA
CAACTGGTGAGGAAAATGCCAACGATAGAAGGAAAGGAAGTGAACGTAATCCCGAAAATCTAAAAAAT
GGTCAATCTTCCTCTTTTTCGTCTGGCAGCCC
TGTTACTCTGGGTGGTGAAGGACTCGCCTCGGAAAAAAATTCCAAGGCTGCTGAACCCTCCGAGGATG
GGCTGAAAGTAACAAAACTATCACAACAAAAA
GCAGAAGGAGACAAAGAAAACAAAAATGATACGATACTCATAGGGGTACATGAAAATCCAGAAAAAAA
CACAGAAATACAAACAGCACCACCACCGCCTG
CAAATAATGGAAAGTCCAGTCCTGAAGATACCGTGCGCATGCAACAGCTACAACAAGTTCTAGATGGC
ATTGAGTCAAACAACAACTCACAGACAAACAG
CATTGCCTCCATCGCAGTCAATAAACNNNNNNNNNNNNNNNNNNNNNNNNNNNNNNNNNNNNNNNNNN
NNNNNNNNNNNNNNNNNNNNNNNNNNNNNNNN
NNNNNNNNNNNNNNNNNNNNNNNNNNNNNNNNNNNNNNNNNNNNNNNNNNNNNNNNNNNNNNNNNNNN
NNNNNNNNNNNNNNNNNNNNNNNNNNNNNNNN
NNNNNNNNNNNNNNNNNNNNNNNNNNNNNNNNNNNNNNNNNNNNNNNNNNNNNNNNNNNNNNNNNNNN
NNNNNNNNNNNNNNNNNNNNNNNNNNNNNNNN
NNNNNNNNNNNNNNNNNNNNNNNNNNGCCACCATCACCCACGGCAAACGGTGATGCCGTCAATAATGA
AGCTGACAATAGCACTGAAGACGGCATGCCAA
ACAATGGCCCAGCAGCTGATGGTGCAGGGACACGAGAAAAACAAAATGAAAATAAAGATGCCAATCCG
AAAGAAACACCAGTGACGGCCGCAGCCATGAA
AACTACAACGGCGACGACTGGCGACAGTGACGGCAGCACCGCGGTCTCTCACACCACCTCCCCTCTTT
TCCTTCTTCTTGTTTTTGCGTGTGCAGCTGCT
GCTGCGGTGGTGGCCGCGTGAGAGTGAGGGAGAGAGAGCCGTGCACCGCCCGCACACACACTCGTCCT
TCTCTCTGTGTGTGTCCCCTTGCATGGACTCT
CGCCCTCACCTCACCCCACGAGGCACACACAATAATGTGCCCATTGTATATATGCATGCACACCCATG
CAGACCCGTTGGTGCTCTCTTTTGCACGACAC
GNNNNNNNNNNNNNNNNNNNNNNNNNNNNNNNNNNNNNNNNNNNNNNNNNNNNNNNNNNNNNNNNNNN
NNNNNNNNNNNNNNNNNNNNNNNNNNNNNNNN
NNNNNNNNNNNNNNNNNNNNNNNNNNNNNNNNNNNNNNNNNNNNNNNNNNNNNNNNNNNNNNNNNNNN
NNNNNNNNNNNNNNNNNNNNNNNNNNNNNNNN
NNNNNNNNNNNNNNNNNNNNNNNNNNNNNNNNNNNNNNNNNNNNNNNNNNNNNNNNNNNNNNNNNNNN
NNNNNNNNNNNNNNNNNNNNNNNNNNNNNNNN
NTTTACTTTTTATTGTTCTTTTCTCTCATTTCCTTTTTTTTTTGTTTTTGGGGCAATCACTATGGACG
TTTTTTTTCTTTTCTACTCTTCCCTCTTTAAT
TGTTTCTTGCTGCACTCTTCCTGTGAGGGGTGCCGTGTGTTTCCGCCCAACTGCTCCACTCGCACACC
CACCGACACGCTCATGACGACGGCCCTGTGCG
CCTCGCCTGCTGCACGCATGAGGAGGGGCCGCGACGAACGGCGCGCTGAGGGAGCCGTGACGTGTGGG
CGTGCGACGAGGGCCTGTGGGGCGGAGCACAC
ATTTCTTCCCTCTTGTTTTGTTTTCTTTGCGGCCAGGGCTGGCAGACACCCGCAGCGCCACACACACA
GCCACACCCTGCCGCCGTGTTTTTCTTTAAGG
ATTTGTTTTGCTTTGCGTGTTTATTTCATGCGGTGGCGCACCCTTCGGTGTCGGCCTCGTGAGCATTT
CTCTCCCCTCCGATGATTTCTTTTTCTTTTGC
TGCGCTCACTCTCCCGATGGAGTGACGCGACTCCCGTCATGTCTGCGTGCTTCTCTCAGTCCCCACAC
ACACGAGTGACTGTGCTCTGCGGGCCGCACTC
ACGCGCCGATGAGAGGGCTGAGGGGAGCAGCAACACGCAGATGACCCCATGAAATATAAACGATGTGC
GGACACACGGAGGGTCGGACGGTTGAAGCACA
CGCAACCATCTTTTTAATTTTCTTTTGTGGGCAAAATACATACAGACAGTTTGGTGTGGCCACACGGC
AGCCAGGCGATTTTTTTCTTATTTTAAATTTG
TGTGGAGCAGTAATGGAGGATTTCTTTTCATTTATAATTATTTTGTGTGATGGGGTTTACATAAGACG
AAGGGGACGTTTTTTGTTTTCAATAAATATTG

TGTGAATTGCCGCTGCTTGGGCTGCAGTGCAGTGATGAGTTTGAGCGGTCTTCTTCTTCTTGTTGTTT
CCGTTATGCGTCCCCGCTGTACATTTTTGTGC
GTCGCTCCACATACTCTGCTCCCTCTTTTCTACTTCTTTGCTTTGTGTTTCTGCAGGCACACACAACC
ACACACACACAAGATGCACATTGCAGTGGATT
TGAATGCACCTTTTGTGGTGCATGCTGGCAGTTGCGTGTGGTGGCTGTTGTCTTCTTCCGTGGCCATG
AGGAGGAGGGAGAGAGGGAGCGCACCCCGAGA
CTATTTTCCTGTGGGTGATGCACCGCGTGTGACTGCGCTGAGTTGGGCTCCGTGTCCCATTGATGGCG
GGATGGCAAGAACTTTTGTGCCGCGTCATGGA
TGGATGAGGGTTCGCTTGCTCCGTGTGCGTTGCGTGGTGGCCGCGGGGTTTTGCCATTTCCCTGGGGC
TGCGGGAAAGAGCTGGGGGAGATGGTGGTGGA
TGTCTTCTCCTCCTGCATGGTGGGTGGCTGCTGGCGTTGTGTGCCTCCTTGCGTGGATGCCGTCTCGT
GGTGTGTGGACTGTGCGGCTGGGCCTGCTGCC
CTCTCCTCATGTTGTCTGCTCTCTCGCTGTATCCGCTGTGCACCGTCCCTCTCCCTCTCGTTCCATTC
TTTCTCTTCTGTCTCACCGATCGGCTGACTGG
CTCCAGCGACTCCACTACGGGCGATGATGACTGCGATGGTGACGGTGCGGCGCCGTGTTGGTTCTTGC
GCTCTTGTGCTGCTGCTCGTCCGTGTGTTTGC
TGCTGGGAGTCCTGGACATGTATCTGGTGATGCGGAAGCGGTGGTTGTGCCGGTGGATGTGTCGTGTG
CTCCGAGTGACGGCAGCCTGAGCTATCGCGTG
CTTGCTTGTTTTGTGGGCGTGGAAGAAGTGTTCTGCTGCCGGTGCTGAGGTTCAGTATGAGAATTACA
CTTTCAGTGGCTTTGATGTGCGCATGCATGGC
AGGAATGGTGAGCTGTGTGCTGTGTGCCATGTAACCAATGCGGTGCACACATGTAGCAACTGTGCTGC
CAGCTGTGTAGCGGAAGAGGAAGAGACCACCG
TTGCCTTCACGATGAATGTGACGACACATAAATACGCCGGTCCTTATGAATTGTGGTGGCGTCAATTT
TCCCCCGATGGAACCATCCATCCGCCTGCACG
GAGCAGCGATGCTGACCGGACGGGCATCTGTCAGTTGCCGCCACCCCGTGAAGGGAAGGATGACACAA
AATCTGGTGGGAAAACTGCGCCGCAAGTTAAT
GCTGAGCCGTCACGTCAAAACGAGGATTGGCGACAGTCTGCGGCGGCTGACGGACCCAACACCGTCGA
TCCGAGTCCCGCATCTAACACGGCGACCAACA
CGTCCGAGACGAGGCCTGAGTTTGCCTTTGTTCGGAACATAAAAGTGACTCCGGCACCACCCGGAAGA
AGGCCCACGCCTGTCACCGAGCCGCAGG
>scaffold1068.size6777
ACACAGACAAATGGAACGAGATGGAAGAACAGTGCAGCGGGAACATCAAAAACAACCACCAGCAGCAG
CAGCAGGAGCCACGCCCGGTACGTCGTTATCCG
TCACGCGTCAGGACACACCGAGCAACGTTCACGCACAAAACAACCAACGGGAGGCAAAAGAGCACATC
CGCCCTTCACAGCACACACGCGCTGCCACCAG
CCTTTTTGCTGTCGTCTCTTCCAACGCACCCGCACATCGCACATCGCCCCGCACAGCCGGAAAATCCT
CCGTCGGTGGATCAACAGCGGGAGGAACACGT
CAAACCGTGCAGCTGCTGGGCTGAAGCCCATCCGCAATAATACCAGACACCAAAATGCCACGCCGCCC
ATGGCTGGATGCCGCCAACATAACAACCGCAG
CACGGCAAAAAAACAAATCGCGGTCATCCCTCCTCGCAGGGGCAGCCGCAGCCAGAATCATGTACGTG
CGTGCGTCCTCAGCGTTCACGCCGCCCCACAG
CGTGCGCAACATGGCGTGTGCATCTGCTCGTGCGCTTGTCAGCGTGTATACTTGCGGAGCAGCTGTGC
GCCACAAGAGAGTCCCAACAAAGCAACGGCAG
AGAGAGGCAGATAGCACACACGCAGATTACTCATATCAATACGCACACACACAGCCCGCGGAATGGGT
GCACTCAACCACAAAATCAAAAAGAAAAACAA
AATAAAAGGCCGAAGCGTGGGACGACCAGCGCACACACACTCTCATTACTCCCGTAAAGCAAAACACA
CACACAGGGAGAGAGAGAGAGGTCGCCCACTC
AATGCAATAAAAACAAAACAAAAACAGTTACGGTGCTCCGTCCATGCAGCCAGGCAGGACGGCAGCGG
TGCGTGTCGTGCAAAAGAGGGCACCAACGGGC
ATGCATGGGTGTGCATGCATATATACAATGGGCACATTATTGTGTGTGCTTCGTGGGGTGAGGTGAGG
GCGAGAGTCCATGCAAGGGGACACACACAGAG
AGAGAGAAGGAAGAGTGTGTGTGCGGGCGGTGCATGGCTCTCTCACACTCCCTCTCACGCGGCCACCA
CCGCAACAGTGCCCGCACACGCAACAACAAGA
AGAAGCAAAAGAGGGGAGGTGGTGTGGGAGACCGCAGTGCTGCCGTCACTGTCTCCAGGCGTCGTCGT

ATCATTTTTTCTGGCGGTAGCTGCTGCTTTCG
CTGTACGTGGTTTTGTCTCACTGGCCGTGGGCGCAACGTTGGTATGACTGGCTTCGGGGTCGTTGTGT
GGCCCTCTGGGGTTGGGCCCTTTGCTGTTGTC
GTCATTCTCATCCGCCCTACTCCGGCCATCACCACTGCCACTTGTAGAGATGGATGATGCCGTTACTT
CCGCCGGGCTGCCGGTAGGTCCACCCTTCAGA
TCCGGTAAAACAGTGCCTTGACCCTGTTGCTCTGCCACACCATCTCCAGAAGTAGGTTCCGTCTCTTC
AGGCTCCTTTTTGTTTGGTGACGGTGGCGTTT
TTGTCCTTTCTTCTGTCAAAGTTTGCGAGTCTGTTTGTGCCGTTGGGCTTCCTCCTGCCGGAACTGCC
ACCGCTGTTGGTCGTTCTTCTCCTACCGTTGC
TGGCGCTGGCGGTGGACATCACGGTGGAAGGACCTTGTGCCTCCGCGTCACTCCGCCGTTTCTCGTCT
CCAGTTGCGGCCCTACCAGCAGCTTCACTTTC
TGGTTTTTCTTGTTTACTGGGAGAGTCTTCTTGTGCTTCGGGTGTTGACTCCGCTGGTGCCGACAATT
CGGGCAATTCGCTCCGTGCACGATTTGCTCCT
GACTGCTCACCTGCTTCTTGAGGTTTCCCTCCCTTTGTGGATGCCGCAGCGCCGGTCTTCTCCAACGC
ATCACTCTCGTCGGTGATGGAGGGTGGGGAAT
TACCACCAGCAACAGGAGCGCTCAAAGATGCATCACCACCTGGACTGCCAACATCAGGTTGACTGCGA
CCACCAGAAGAAAAGGAGCCCAAAATGAAACC
TTTGCTTCCGTCAGAACCTACACTTCCCGTAGGAGCCCCAGGAGAACTGCCACCATCACCGAGAACAG
GAGATGGGGACTTGACATCCGAAGCAGGGGAC
TTCAAAGAGCCATCAACACGTGCTTTAGAACCTCCATTTGAGCCACCACCACCGCCACCAGAAGCTTT
GCTGCCACGAGCTTGACTGCCACTGGCACCTC
CAACGCCACCAGGACCGCCAGCACCGTCGTCTCTACCGCCTCCACCTTCACCTCCGCGACCAGGAGGA
GAACCGGCACCACCGAAATCAGCACCTGAACT
TGGAGCTAAATCCGATTTATTTTCGGCTGTACCAGTCACCAATGGGTCAGTCGAAGCTGCAACGGCAG
TGATGGACAAGACAGATCGTAAAGGCAGCGTC
AGCGCCGTTTTGTCGCATCCGCTCGGCGTATACGATTTATTCGTATTCAACACCCACGACGCCATGCA
GCCACCAACTGCGTCGTTGTCGAGATTACTCG
CATCAGCCCCGCCGGCACCGCACCACAGCACGCAGAGGGCACACACCAGCAGCACACGGCCAGTCATC
ATCATCGCCATCACTCCTGTCACTTGCCTGCA
GCCTTCAGCACAAACTAACAACACGTCAACGTAGAGCGGAAGCAAAAGGAGAGACACGGCCGCCACGC
CACGCACAGCCACTGCACAGTCACAAATGGCA
GCAGCCCAGCAGCGGCAGCGCACACACACCATGAGGgtcaactttcgtcactttcccgtttAACACAC
AACGCCCCGCAGCAAGCAAACAGGAAAACACA
CAAAACGATTACAAACAAAACACAGGTAAAAGAACAGCAATGAAGTTAAAGATGTAAACACACAAAAC
AAAGGGCTGCACAGTATGGCTCCAGCGCGCAC
GCACCCATCCTCAAATGTGACTCCACGCAATCAGTTGTGTGCGTGCCATGCACATGAGTGTGTCTGGG
GTGAGTTCAACGTGACGGATGCGGGGAGCGGC
AAGGAGCACACAGCAGGGCAGCGGAAGCAAGTGAAGCAAAACACAATCATTATTTTATCCTGTTTAGC
ACATCGGACCACAGTGGACGCACACAAGTCCC
CGCGGTCGGGTGCTGGCACGCACAGCCCGCACACAATTCTTCAGCCCAGAGTGGTGTACGCCAGCGCG
GATACGGCGAGCAGCAGCGGGGCACACACCCA
CGCAGAGCTGCTGaNNNNNNNNNNNNNNNNNNNNNNNNNNNNNNNNNNNNNNNNNNNNNNNNNNNNNNN
NNNNNNNNNNNNNNNNNNNNNNNNNNNNNNNN
NNNNNNNNNNNNNNNNNNNNNNNNNNNNNNNNNNNNNNNNNNNNNNNNNNNNNNNNNNNNNNNNNNNN
NNNNNNNNNNNNNNNNNNNNNNNNNNNNNNNN
NNNNNNNNNNNNNNNNNNNNNNNNNNNNNNNNNNNNNNNNNNNNNNNNNNNNNNNNNNNNNNNNNNNN
NNNNNNNNNNNNNNNNNNNNNNNNNNNNNNNN
NNNNNNNNNNNNNNCAACAAAGTCGCTGTGCAACACCAGGAGGAATCGCAGTGCCTCCTGCTGCCATC
CGGGTCCGTTGTGGACGCCATGATGAGTGGAA
ACGCCCATTGGTGCTTCGAAACGACGCTGCACACCTGCGACGCATCATACGGTTTGAGACGCACGTGC
CTCACCAGCACCACAGACGCCCGCGCGCCGTC
GGTACGCACTACATCGTCAAGGAAACAAAGGATACGTCAGGGTGGCAGCAAAGCACTCGCTGGCAATT
CCCGTCGCAGGCATCATCACAAGCAGCTCCAG
CAGCGCCGCAGACCGTGGGGTACCGTGGATCAACTGACGCATGGCGTATGTGGAAAAGAAAGGACGTG

GTGGCTTTACAAGGATACGAGTGGCTGACACT
TTTAGAAGGAGAAAGAAAACAATGTGAGAACACAAAGAGGAGTCAGGCGTCCGCAGTCAAAGCAAACC
AGCAATCAAAAAAGTAAGATGGGTGCACTCAG
CCACAACACAGGCAGAGGATAAAAAATACCAACAACAAAAAGCCGAAGCGTGGGGGCGTCCACCCAGC
AGTGGGGACGCGCAGCAACAGGTGGGACGACC
AGCGCACACACACTCTCATTGCTCCCGTAAAGCCCAATAGGGAGAGAGAGAGGTCGCCCACTCAATGC
AATAAAAACAAAACAGAAACAGTTACGGTGCT
CCGCCCATGCAGCCAGGCAGGACGGCAGCGGTGCGTGTCGTGCAAAAGAGGGCACCAACGGGCCTGCA
TGGGTGTGCATGCATATATACAATGGGCACAT
TATTGTGTGTGCCTCGTGGGGTGAGGTGAGGGCGAGAGTCCATGCAAGGGGACACACACACAGAGAGA
GAAGGACGAGTGTGTGTGCGGGCGGTGCACGG
CTCTCTCTCCTTCACTCTCACGCAGCCACCACCGCAGCAGCAGCCGCACACGCAACAAGAAGAAGCAA
AAGAGGGGAGGTGGTGTGGGAGACCGCGGTGC
TGCCGTCACTGTCGCCAGGTGTCGTCGTATCATTTGCTGGAGCGGTGGTGAGTGGTATTGCTGTTTGT
GGTAGTGCCTCAGTGGCTGTAAAAGCAGGACT
CGTATTTTGAGGCTCGGGGTCTTTGTGTGCTTCTTTATGATCGGGCTGCTGAGCATTATCAGCATCCT
CACTCGCTACACTCTTTGTATCAGCACCGCCA
CTTGTAGAGATGGGTGGAGCTGTTGTTTCCACCAAGCTGCTGGCAGCTTCGTCCTTCACCGAATCCGA
TGCTGCTGTGCCCTCAGTGTTTTGATCTTCCG
CGTCATCCTTTGAAGGGGTTTCTTGTCCCAAAAGGAGCTTTTGGGTTTCTGACGGTGACGTTTGGTGC
ATTTCCTCCGTTGAAATTTGTCTTTTGCTGCT
GGGGTTCCTCCTGCTGAACTTGCCGGTGGTGGTGGTGCTTTCTTGGAGTCTCTTTCTTCTTTTTCTCG
AGTACGTGGGTCTGTTGCGCGTTCTTTTCCTG
CGGGCACTATTATTAGTTCTGTTTTTGGCTGTTCTTGCGGAGCTGCTTGCAGTGTTGTTGATCGTGTG
TCGCTGGAAGGTCCTCCCGTCACTGCGGCCCT
CTGCGTGTTCTCCTTTCCAGTTGCACCGCCATTAACAACTTGATTTCCTGCGGTGTGTGGCTTAATAG
GGGAATGGTTTTCTGCATTAGATGTTGACGCC
TCTTGTCTATCCAAGTTGGGTTGTGCGGGTGCTGGAGGTTCCGCTGATTGTGACTTAACACTTTTTTC
TGAGGAATCCATGCTCAGGGTGGCTGTCGTAC
CCTTGCCGCCCTGCAAAACATCACCGTCAACGGGATTGGGGGGTGAAAGCGTACCGGCGGAAAAAGAA
GCGGATACCATGGATTTAACACCACTTCCAGA
AGAACTTCCACTGGGGCCAACACCAACGGCATTGTCAGCTTCACCACCTTGACTGCCACCGGATACTG
GTGGACCTTCTGGTAAATCCTGTAATTCTGGT
GCTGATGGTTTTGGTGATGGTGGTGACTGCAAAGACAGCGCTTCATTCGACAACACTTGATTGCGTGT
TACCGGAGATCCCACAGGCATCACTGCCAACT
TGTTGTCTAGCCCCACAGTTCCACCGCGAGGGGATGAGTTTATTTGCCCCAAATCCGTCCCCTCCAAA
CGATCCCCCTCGCTCTCCCCATTAGGCTCAGA
AGCAACCAAACCATTGTCAGCTCCACCACTTGGGCCAGCACCAGTACCAACAACCGTGCCACCACCAG
AGCCAGAAAGTAAAGCCTGCGGTGTGTCATCA
CAGCCACCGTGCCGGCACCGCACCACAGCACGCAGAGGGCACACACCAGCAGCACACGGCCAGCCATC
ATCATCGCCATCATTCCTGTCACTTGCCTGCA
GCCTTCAGCACAAACTAACAACACGTCAACGCAGAGAAAACAATACGAGAGACACGGCCGCTACACCA
CGCAAAGCCACCGCACAGTCGCAAATGGCAGC
AGCCCAGCAGCCGCAGCGCACACACACCATGAGAGAGTTAAAAGCAGTGAAAGGGCAAAAACACACAA
CGCCCAGCAGCAAGCAAACAGAAAAACACACA
AAACAGTTAGAAACAAAACACAGGTAAACAAACAGCAATGAAGTTAAAAATGTAAAACACAAAAACAA
AGGGCTGCACAGCATGGCTCCAGCGCGCACGC
ACCCATCCTCAAACGCGACACCACGCAATCAGTTGTGTGCGTGCCATGCACATGAGTGTGTCTGGGGT
GCGTTCAACGTGAAGGATGCGGGGAGCGGCAA
GGAGCACACAGCAGGGCAGCGGAAGCAAGTGAGGCAAAACACAATCATTATTTTATCCTGTTTAGCAC
ATCGGACCACAGTGGACGCACACAAGTCCCCG
CGGTTGAGTGCTGGCACGCACGGCCCGCACACAATTCTTCAGCCCACAGCGGTGTACGCCAGCGCGGA
TACGGCGAGCAGCAGCGGGGCACACACCCACG
CAGAGCTGCTGAGGCTGCCGTCGATTTCGCGAAGAAGTGACGGTGCGCGGGTGGTCGTCGTAGTTGGC

GCCTCCGTAGTCGTGGTGGTGGTGGTAGTTGG
TGCTTTTTTCGTCGTAGTCGTGGTTGTGGTAGTGGTCGCCGTGCTCTTCTCGGCCGCATCGTCGGCAT
TCGTATTTGTTTGGTCTGTGGATGAATTATTG
CCACTTGTTGAGTCTGTCGTATCGTTGTCACTATTTTTCTGTACTGTATCGGCCACCGTACCTGAACT
CCC
>scaffold1069.size5938
cgtctcccgtctgtgttgtccctccctacgtccgcgacgcacacacacacgtGGTGCCATGCAACAAC
GTCACAACAAACATGCTGCGACTCGGTGACCCT
CACATCGCTGTGGTCCTCATGAACAGCCCGGGCACGGCCCGCATCGATGCACTTAGTGGACGGAGTGA
GGCATCTTTCGAGGCGCAGCAGGGAGCAGGAT
GTGTTGACGCAGCCACGACGTGCATGGAGTGTCACGATGGCACAGCCGGCTGTGGCTGGTGGAATAAT
TACTTCTCGGAGGCAACAGAGAGAGAGAGCTG
CTGCTCCTGACGTTGCTGCCTTTTGTGGCCTCCCATGCCGATTATTGTCTGTCCGCTGATCATGGGTG
GTGTGCCCCCACTAGTGACGTCGTCTGCAGGC
ATTTCACAGCACTAGCGAAACACACATCACGTCGCATGCTTTGGCTGTGGGTATGGTGTCGTGAATGC
AGCCGGCATTATTTTGCCCGACTCCTTTTCAC
TGCCCTGCGACAAATAGATGCGTCGCCGCCCGAGTCATTCACTGCAGCTCCGTGTGTTGTACTGCCGG
CACAATACTCTCGTGTTGTCTGGCGCCTGGCT
GACAGTCAAGCACCACTGCGCTGGGACATGCAGGGATGTGGACTGCCGCTCACGGTGTTGGGCACCGC
TGTTTGGATGCGGCGGCCACACGAGCGGATGT
ATTGTGGCACAATGAAATGCGTGAAATACGCGGAATCACAGCGGCTGCAATAATATCAGCGGTGTGGC
ACCACGGAGTATGTGAGGGGTGATGCATGATG
AATCAGAGTGGGCGGCATGCGAGGGAAGTGCACCGCGACGGAGCGTCGCCCCGCCCTCAGCCTGCAGG
GGAATGTACGCCCACAAAATATATGGTCGAGG
CACCACCAAACACGTATGCCCTTCTGTGCAGCAATAAATAGGAGCGAAGGTGCGTTCTGGTGCGTGGG
AGTCCACTCACATATTCCCATTTCCACCGCAA
TAAACGGCGAGGAATCAGGTGCCACAGCAACGAAAGAGGAAATAAAAACATCTATTGCAGCACNNNNN
NNNNNNNNNNNNNNNNNNNNNNNNNNNNNNNN
NNNNNNNNNNNNNNNNNNNNNNNNNNNNNNNNNNNNNNNNNNNNNNNNNNNNNNNNNNNNNNNNNNNN
NNNNNNNNNNNNNNNNNNNNNNNNNNNNNNNN
NNNNNNNNNNNNNNNNNNNNNNNNNNNNNNNNNNNNNNNNNNNNNNNNNNNNNNNNNNNNNNNNNNNN
NNNNNNNNNNNNNNNNNNNNNNNNNNNNNNNN
NNNNNNNNNNNNNNNNNNNNNNNNNNNNNNNNNNNNNNNNNNNNNNNNNNNNNNNNNNNNNNNTGTGA
TGACACACCGCAGGCTTCGCCTTCTGACTCTG
GTGGCACGGTTGTTGGTACTGGTGCTGGCCAAAGTGGTGAAGCTGACAATGCCGTTTGTGGTGTTGGC
CCGAGTGGAAGTTCTTCTGGAAGTGGTGTTAA
ATCCATGGTATCCGCTTCTTTTTCCGCCTGTACGCTTTCACCCCCCAATCCCGTTGACGGTGATGTTT
TGCAGGGCGGCAAGGGTACGACAGCCACCCTG
AGCATGGATTCCTCAGAAAAAAGCGTTAAGCCACAATCAGCGGAACCTCCAGCACCCGCACAACCCAA
CTTGGATAGACAAGAGGCGTCAACATCTAATG
CACAAAACCACTCCTCTATTAAGCCACACACCGCAGGAAATGAAGTTGTTAATGGCGGTGCAACTGGA
AAGGAGAACACGCAGAGGGCCGCAGTGACGGG
AGGACCTTCCAGCGACACACGATCAACAACACTGCAAGCAGCTCCGCAAGAACAGCCAAAAACAGAAC
TAATAATAGTGCCCGCAGGAAAAGAATTACAA
ATTTCAACGGAGGAAATGCACCAAACGTCACCGTCAGAAGCCCAAAAGCTCCTTTTGGGACAAGAAAC
CCCTTCAAAGGATGACGCGGAAGATCAAAACA
CTGAGGGCACAGCAGCATCGGATTCGGTGAAGGACGAAGCTGCCAGCAGCTTGGTGGAAACAACAGCT
CCACCCATCTCTACAAGTGGCGGTGCTGATAC
AAAGAGTGTAGCGAGTGAGGATGCTGATAATGCTCAGCAGCCCAATCATAAAGAAGCACACAAAGACC
CCGAGCCTCAAAATACGAGTCCTGCCTTTACA
GCCACTGAGGCACTGCCACAAACGGCAATACCACTCACTACCGCTCCAACAAATGATAATACGACACC
TGGCGATAGTGACAGCAGCACCGCGGTCTCCC
ACACCACCTCCCCTCCTTTGCTTCTTCTTGTTGTTGCGTGTGCCGCTGCTGCGGTGGTGGCCGCGTGA
GAGTGAGGGAGAGAGAGCCGTGCACCGCCCGC

ACACACACTCGTCCTTCTCTCTCTGTGTGTGTCCCCTTGCATGGACTCTCGCCCTCACCTCACCCCAC
GAGGCACACACATAATGTGCCCATTGTATATA
TGCATGCACACCCATGCAGGCCCGTTGGTGCTCTCTTTTGCACGACACGCACCGCTGCCGTCCTGCCT
GGCTGCATGGGCGGAGCACCGTAACTGTTTCT
GTTTTGTTTTTATTGCATTGAGTGGGCGACCTCTCTCTCTCTCTCCCTATTGGGCTTTACGGGAGCAA
CGAGAGTGTGTGCGCTGGTCGTCCCACCTGTT
GCTGCGCGTCTCCACTGCTGGGTGGACGCCCCCACGCTTCGGCTTTTTGTTGTTGGTATTTTTTATCC
TCTGCCTGTGTTGTGGCTGAGTGCACCCATCT
TACTTTTTTTGATTGCTGGTTTGCTTTGACTGCGGACGCCTGATTCCTCTTTGTGTGTTCTCACATTG
TTTTCTTTCTCCTTCTAAAAGTGTCAGCCACT
CGTATCCTTGTAAAGCCACCACGTCTTTTCTTTTCCACATACGCCATGCGTCAGTTGATCCACGTCAC
CCCACGGTCTGCAGCGCTGCTGGAGCTGCTTG
TGATGATGCCTGCGACGGGAATTGCCAGCGAGTGCTTTGCTGCCACCCTGACGTATCCTTTGCTTCCT
TGACGATGTAGGGCGTACCGACTGCGCGTGGG
CGTCTGTGGTGCTGGTGAGGCGCGTGCGTCTCAAACCGTATGATGCGTCGCAGGTGTGCAGCGTCGTT
TCGAAGCACCAATGGGCGTTTCTACTCATCAT
GACGTCCACAACGGACCCGGATGGCAGCAGGAGGCACTGCGATTCCTCCTGGTGTTGCACAGCGACTT
TGTTGGACGGCGCGCTTGATTGGCGTCTCTTG
GGGCTTTTGTGGGGGGAAGGTGGGAGGCTGAGGGCATTAGGACTGAATTGTTTTGAGTGGCTGCGTTT
CGTTGAATTGCGGCCAGTCGTGGCACTCTTTT
GGGGTTTGGCGGCCCGTCTCTTTTGTTTGCAGCCCAATGCAGCACGCGGACAACACAGGTCCGAGGAA
CTTTTGTTTGACTGCGTGTGGGCTGCTGCCGT
GCGTGGTGCGTGGACTATTTGATTGTGTGGGGAGGTGCATGGAGTGACGAATACTTCTGTTTGGCGTG
TCGCGGTGTGTTGGAATGATGTTCGGAAAAACA
TGTGAGGGGTGTTGTGTGTAGTTTGTGTGTCTTCTCTGGTGGAGCCGACATGTGTGCGGGGATTGTGG
CATTGCTGGCTGACAGCTCCACGTCACTTTTC
GGCATTCCTGGATGTGCTGAGGAATGACGGCTGGCGACCAGCGTGTTGGTGCTGCTGGTGGGAATAAG
TGCACGAGGAATTGGCCGTTTTCTCTTTCCCA
TGCTGGAGCCGATGAACTGGCACGAGGGCACCGGCTGCAGGCTGCCTGCCAGCACGTGTGAGCAGCGG
ATCCATGCACACACAGACGCTCATTGCAAGAG
CTTGTGCGATCACGGTGGCTTTTTTGGCGTGCTTTTGTGGGCGTCAGCCCGTCGGAGAATGTGTGAAC
CAAACGGCTGACGGAACACGAGGAAAATAAGT
GCGTGCACTGTGCCGTGTTTCGGCGTCGATGGCAGAGCGGCAGGAGGCCCGTGCTGGCAACTGAGATC
TTTGCGTGGCGAGGGATGTCGGTGTTGATTTT
GGATCGTCGAGGGTGAAATGAATTTGCTGCTGCCCGTGTGTGTGGCCACTCCGGCGGAGGGCGAACAC
AAAAGGGAAGGATGCAGGCGCTGCGTGTGTGT
GTGCAGGTGCCATGCAACAACGTCACAACAAACATGCTGCGACTCGGTGACCCTCACATCGCTGTGGT
CCTCATGAACAGCCCAGGCACGGCCCGCATCG
ATGCACTTAGTGGACGGAGTGAGGCATCTTTTGAGGCGCAGCAGGGAGCAGGATGTGTTGACGCAGCC
ACGACGTGCATGGAGTGTCACGATGGCACAGC
CGGCTGTGGCTGGTGGAATATTTACTTCTCGGAGGCAACAGAGAGAGAGAGCTGCTGCTCCTGACGTT
GCTGCCTTTTGTGGCCTCCCATGCCGATTGTT
ATCTGTCCGCTGGTCATGGGTGGCATGCCTCCACTAGTGACGTCGTCTGCAGGGATTTCACAGCACCA
GCGAAACGCACATCACGTCGCATGCTTTGGCT
GTGGGTATGGTGTCGTGAATGCAGCCGGCATTATTTTGCCCGACCCCTTTTCACTGCCTTGCGACAAA
TAGATGCGTCGCCGCCCGAGTCATTCGCTGCA
GCTCCGTGTGTTGTGCTGCCGGCACAATACTCTCGTGTTGTCTGACGTCTGGCTGACAGTCAAGCACC
ACTGCGCTGGGACATGCAGGGATGTGGACTGC
CGCTCACGGTGTTGGGCACCGCTGTTTGGATGCGGCGGCCACACGAGCGGATGTATTGTGGCACAATG
AAATGCGTGAAATACGCGGAATCACAGCGGCT
GCAATAATATCAGCGGTGTGGCGCCACGGAGTATGTGAGGGGTGATGCATGATGAATCAGAGTGGGCG
GCATGCGAGGGAAGTGCACCGCGACGGAGCGT
CGCCCCGCCCTCAGCCTGCAGGGGAATGTACGCCCACAAAATATATGGTCGAGGCACCACCAAACACG
CATGCCCTTCTGTGCAGCAATAAATAGGAGCG

AAGGTGCGTTCTGGTGCGTGGGAGCCCACTCACATATTTTCATTTCCACCGCAATAAACGGCGAGGAA
TCAGGTGCCACAGCAACGAAGGAGGAAATAAA
AACATCTATTGCAGCACTTTACCAGCGAAAAGCAGGAACAACGCAAATTGAATTATTGGCTGCACCAA
CACAATAAAAATTCACAAGAGAAGGAAACAGT
TGTGCACACCGCAGGAACCCCAACACACGATGCAGGCATGAAGGTTTACCCTCCGCAAAAACAATAAC
AACATATGCAGACATCTTCCACCCTCCAAAAA
GAAACACAAATAAATGGCTGCTTTATTCCATTCCTTGCGCTTGACTTTGCGTGTGCTGCTGCTGCTGC
TGCTGCGGTGGCTGCGCTTCCCGTGGACCAAC
AATCCACGTGGTGTGCGGGGATCTATTCTTTTTCATGGTTACTTTGCTGTTGTGTTGCTGTGTGCGCT
GCACCTATTCTCGTGTGTGCGTGCCCTCCGTG
AGGGGTGCCGACACGACGGACCGCTCTCGCCGTATGTGCATGCGGTGGAGCCTTGTGTGCCGCTCCTG
TTTGCTCCTCCCTCCTCCATCTGTGTGTTGGT
GTTGCGGCAGGGCTGTGTGTGTGTGTGCCTGTTGCCGTGGATTGGAGTGTGTGTCGTGCGTGGTGTGC
GGCGTACGGTGCATGCCTGGCTGCTGCTCTCT
CTTCTTGTGTGTGTTTGCCCTCTGTGTGTGTCTGCCGCACCTCTCGTCTCCCCTGTTGCTCTCCATGT
CCCTCACTGTGCAGATCAGCTCCACACCACTG
AACGACCACACTCACGATGACGACGTGCCGTCTGCTGT
>scaffold107.size29768
TTCACATTTCTGAGAGCCTCTTTTATTATTATTATTATTATTGTTATTATTTATATATTTACAGCCAT
GTCCTCAAACATCCTTCTCTTTCTTTTTTTCTT
TTTTTTCGTTTATTATTTTGCGGGTTCAGGGTGTCACAGCTTCTATATACCCATCGCCGTTGTCTCTC
TCTCCCCTCCCTTCCCCATGCCATTTTCACCT
CCGAAGAAGTGTCCGAGCATTGAGATGTTTAAGCACAAATGCCTGTTGAAAGGACATCTCACGCCAAT
ACCTGTAGCGATCACTTGCTGAGAAGTGCCCG
CTTTCGAGGTCCATCTTTAGGAGTACTTCGTTGGAGTCCGTCTTGAGGGCGCGCAGTTTGGAAGCCCA
CTTCGCCGGCTCCCAGTAGGCCACACGGGGGT
CATGAAGACCGGCTTGGATCATAAGATGCGGGTAGTCCTGCGCGCGTACATTGTCCACTGGACTATAG
CTATTCATATAGTCAAAGAACTTGTATTCATT
TGGGTTACCCCACTCCTCCCACTCACCTGTTGTGAGAGGAATACTCGGATCACACATCGTCGTCATGA
CATCTACAAACGGCACACCCGCCAGCGCAACA
CGGAAGAGATCGGGTCGCATGTTTAACACGGCACCAATCAACAATCCTCCCGCACTACGCCCTTCACA
TGCTAATTGTGAGGGTGTTGTTAGTCCAATTT
CGATAAGATACTCCGCACATGCGATAAAGTCTGAAAATGTATTTCGTTTTGTGAGGTACTTTGCACCT
ACCTCGTACCACGCCCGGCCCATCTCCCCTCC
ACCTCGGACGTGTGCAATGACATAAATTACGCCACGATCCAAATATGGTAGGTATTTAACACTGAACT
CCGGCTCCACACAGGCACCATACGAGCCATAT
GCATACAAAACTGTTGGGTGTGGCTTACTCATGTCGAGACTGACATCATGCACAATGGAAAGTGGAAT
CTTCGTCCGGTCTGGTGCTGTAGCAAACAGAC
GCCGGCAAGTGTAATTTTTCGCGTCAAAATTACCAAGCACTTCACGAACCTTAACGATCGTGCGAGTT
CCATTTACGACATGCAGGTCAAACCAAGTTGT
GGGGGTTGACATGGAAGTGTAGCTGACGCGCAGCGTCTCCGTGTCGTACATTTTCATGTGTGAAAAAA
CAGGAAAGGCGGTAAAGACGGGTTCGTCAAAC
GATACCTCCTGCACAAAGGCACCACTACGAAACAACCCATCAGAACCCACGGGCATCGCCCAAACACG
TGTTAGTCCCCCACGTCGACCGGAAAGAACAA
CAAACTTTGCAAACACACCAATTTCCCCAATGAACACATCCTCACTGTGCGGTACAATAACGTTTTCC
CAATCCGAAGGGGTGTTACGTGTGGCCGACAC
CACCTTGCCGTTCATGCAATTGTCCTTGTTTGTTAGAATTAACAAAGTTTCCGTGCCATGGAGCTCCA
CATCGTAGCGCACACCCTTTTCACGCGGTCGA
ACCATTTCCAACGTTGTGTGACCGTTGCCTTTGCGTAGATCCAACAGATGCGACTCCGTCGTCTCGAA
CGACCCCGAGCCGATAATCAAGGTGTTACCAT
CTCCTGAAATCGCCATGGCGGTACTGAAGAGCAGATCATTCTCCGTGTATAAGCAGACATCCTCCGAC
TGTGGACGTCCAATAATGTGTCGCCAAATTTT
ATAATTCCGTTCTGCCGCGTCTTTTGTGATGTAGAAGAAACATGATGCATTTGGACCCCATAAAATCT
GGCCATTGGTCCCTTCCACCACGTCGGTCACC

GCATTATCCGTAAAACGGATGGTATACATTTCATTTCCAACGTAGTCAACAGAGTACGCCACAAGATT
GTGATCTGGTGGCGCCGGTTTCACCTCATGCA
CCATACAAAATGATTTCCCCTCTGCCAGTTTGTTCTCGTCCAGTATCACCTGTTCATCTTCACTTTTT
CCAGGCGTCATTCCCAACGGGACACGGCAGTG
AATTTTGTATGACAGGCCTTCCACCTCCCGCGTGTAGTACGTGTAGCCATCGTACCGGTACGGTGCGG
AAATGTCCGTCTCCTTTATGTGTGAGATGTAC
TCTTTAAAAATTGTCTCGGAAAAGTCTTTAATATCCGCCGTGCGCTCTTCAAAGTATGCCTGTTCTAG
CTTTAGATGTGCAATGACCTCTGGATTCTTGC
GTGAGTCATCCCTTAGCCAAAAGAGTGGATCATTTCGACGACGAGGCGGATTCATTGCATTATTCCCG
CGATTCTCCCCTTCAACATAACCAAACACCAC
CTCGTGATCCTTAGGTGCGGCAATGGGACCAGACTTCATTGAACTCACGGTAGCAGTGAGAGTCTTTC
TTTTATTTTATTTTATTTTATTTTATTGCGTG
TGTAGGATTCCTTGCTTTCTTTTCCTTTCTCTGATAATATACTCGTACGAGAGGTGTCAGGATTTTTC
TTTTTTTTTTGGGAGCGGGGGCGGGGCGGGGG
GGACAGAGAATATAAGAAGGAGGAAGGAAAGGGGAAGGACAACATGCTTAACTGCATTAGGGAAACAA
ATGCGTCGAGAAGGAACACACACACNNNNNNN
NNNNNNNNNNNNNNNNNNNNNNNNNNNNNNNNNNNNNNNNNNNNNNNNNNNNNNNNNNNNNNNNNNNN
NNNNNNNNNNNNNNNNNNNNNNNNNNNNNNNN
NNNNNNNNNNNNNNNNNNNNNNNNNNNNNNNNNNNNNNNNNNNNNNNNNNNNNNNNNNNNNNNNNNNN
NNNNNNNNNNNNNNNNNNNNNNNNNNNNNNNN
NNNNNNNNNNNNNNNNNNNNNNNNNNNNNNNNNNNNNNNNNNNNNNNNNNNNNNNNNNNNNNNNNNNN
NNNNNNNNNNNNNNNNNNNNNNNNNGAGAGAG
AAAACGCTGGTAATGGTGATTATTCTGCACATTCATCGCAGGGGGTCTCAAGAGAAGAGAATGAAAAT
GAAAAACAAAAAACGTAAAAAAAAGAAGACGC
AAAATACATTTCATTGCAGGCACACACACACACACAGAGAGAGAGAGAGAGAGAGAGAGAGAGAGAAA
ATACGGACACACCCATATATTATCAGAAAGAG
AATAATATATAAATATACATGCATATATATATATATTTATTTACAGAAATAAAATTCTGTTTCTTTAC
TACCACCATCACTATCACCATATATATATATA
TATATATATATTCATTTCTACTTAATTCTTACGACGTGTTACATGATCGTATATTGTAACTTGTACAT
AATCTGAAGTTTGAATAACGGTACGATTGCTA
TTATCTCCTGGTGTCGGTGCTTTAGGGTCAACATTAATTGTCTTTCTATCCCGCTTATGTTTCATAAG
AAGTCCCATTTCATTATCCGCAAGTACTTGAA
AGGTAAAACTGTTGTTGTAAAAAAGAGTATGTGTTTTCCAGTTATAATAACTCAAATCTGTTCGTTTT
GGTGTAAGTTTCTTGCGTTTTGTAAAGTATTC
TGTAAAGTCTTCTGTTTTTAATCGATGACCGTAGTCAATGTACGCCGATACCTCCTGCCCTCGCACAA
TATCACGGAGGAAAATGATGGTCGATAATTTT
CCATTCCGTACAAGTTCTTCACGCTTCGCCAATGCACTAAGCAACGGGGCATCATCTAACTTTTTCCC
CGCACTGCAGATGACATCCACCGTCTCTTCGC
GCCTTGACGGTCCCTCCAGGGCGAGTTGACATGCCGCCTCAAACTCCTCACGGCTGAGGAAGTCTTTG
CCCTTGTAGCCAAGCTCCACAATTTGCCGGGC
GGCGTCCTGGTTGCCGAGGTAGAAGAGATCCTCCGGCGTCACCTGCTGGTCGAGGTACTCCTCGTATG
AGCCAAAGTCTAACACGTTGCGGTTAAAAAGA
TCATTATCGGCCTGCGCCGCGAGACTCATGTCTGTATCACCGCGTCACTCCCCCCGCTGCCAACGACA
GACATCGAGAAGAAAGGGAAAAAAAAGAAAAA
AAAAAAGAGAGTCAAATGTTACGCCAAAAATCCACATCGACCCCTCTTCAGAAGACAGAAAAAAAACA
ACAACAAAAACAACGGCATAAATAATGTTACC
CATACACCCATCTCAATATACACTGACAGAAGGAACAATATGGTTTTTATTGATGTATGTATGTGTGG
GCGTACGTGTCCTCCTTTGCTTCTTGTAGTTG
TTTAGCAATTGGCATGCCTTGGCTCAATCGGAGCCCATGCACGCCGCAAAGTTTCCCGAGCCAAGAGA
AGTTCCGCAAGAAGGGCTCGCGCCTTCTGTGC
TGGAATGCAGAGCGTGAAATGACCATGCGGCATCCGCCATGCAACCCCAACCTCCATTAGGGGTTTGC
TCTCGATGGAGTCCGATGTTGACGATGACGGT
GGTTGTGATGGCAATTGCCTTGCGTAAATTTGCATGTCGTACAAATGCCCAGCCACACCTTCTTCTTT
CTTCCCCTGTTGTCGTTGCTCTTGTTGCTGCT

GCTGCTGCTTCGGCAAGGCGTCACAGGCACCAAAGCTTGCCTCATGTACAAGATGACGACAGGCAGAT
GTCTCTCCATCGAGTGAACCAAAACACCCAAA
GGAGACACGTGGGGTTTGTCGCAGGAGTGCGCTTGCTAGTTCCATGCGGTGGGTGCGTACGGGGGACA
CAACAGCCGCCGCCGACTCTTCACTCATCCCA
AGGATGCCAAGTTCCCGTACCGCATCGTTCTCCGAGACGTCATACTTCACAATGTTGAGCACGACGGT
GTGTATGGCTGTTAGGGCCTGCGCCACACGTG
CAACGCCAATCTCCATCATCTCCCGCTGTGAAAAGAAAAGTTTGTTTTGTACATCCAATACACCGAGC
CCATCGGAATCCGTTTCCCTCGCATCCTCATG
CGTCGTGAGCAACAGAATGGTGACCGCATGGTCCGACATCTTTGCCGCGGCCACAGGTTCAACGGCAG
CCACCACAGCCAAACGTGCAAGCACACTGTCT
GGGCAGTCCATGCCGCCAAAGAGCCTAAGCTTCATCGTACCGTTCGTGGCACACCTCAGCCACGCAAG
CAAATGTATGCTCACGTAAGGCTCCTTCCACC
ACATACACAGATACACAGAAAAAACAACAACAACAACAACAACATGAGAAGATGTAAAAAATAATGTG
AAGAGAAGCATCCAATTGGCAGGCAGTCTTTA
TATATATATATATATATATTAATGCAAGAGAAGCAAAGCTTCCGAACAATTGCTCCCTGATCCATCAA
AACAGTGAAACACGCACATAAAAAAATCACAC
AGCAAGAAAAAACACAAAAACTACAACAACAACAACAAAAAACAAACAAATCGGCGCTTACGCACCAC
CATCCACCCGCCGTCTTTTCTCCCCAACTTAT
GTGCCGCGTCATTGGAAATTCGGCGCAAAAAACGTCCGTGCCGCCGTTATAAACTGCAAAAATAACGC
CCCGACGCACATCCCAAATGCCACGCGACCCA
CACACACCGCCTGCCCCGCCTGCGTCTCGCCGGTGTCATTGGCACCACTGCGCGTCATCCCTTCCTTC
TTGTTGCCCGACTGTAGGCGGAATTCAAGGAC
CACCGCAGCAACACCCGTCGCGAGCGCCACGCACAGGGAAAGAAGAGTTGTCAACGACAAAGCCGGCA
CCACCACCAAAGGCAACGCCCTTTTCACTGAC
GCCAGGGACCGCATTAACCCCGCCAGAGCGGCCGCAAACGACGATGCGCACAAAATGGCGGTCATGGC
AGGCATCCACGGCAATGAAAAGGGTATAATAG
ATGTAGCCACTTCTGACGGTATATTCGTGAACATCAACCCAAAGAGCGACACCGCCGCCAATGACGTC
AACAGACCAAACTCGCAAAGACGTACAAAAGA
CCCAAAACGAGGCAAAAGACATAACGGCAGTGCGACAATAACGGTGAACAACAAAAAATACGCAAGGA
ACGAAGGGCTGGTAAACGCAAACAGCTTCGAT
GAAAACAGTGACTCAAACTCAATGATAAGAAACAAAACCGCCAAAAGGCAGCCACATGCTATGCAACG
AAGCAAAAAACAAACTTCTACACGGCGCAGTG
TGGCAAACATGGTCCCTGCGTCAATCGCAGAGTGAGAAATACGTGTCGTCCCTTGACAATCGGTGGGT
GGTGGTGGTGGCAGCAGACAGATGGCAAGCGC
AAAAATGGGCCCCACACATGTCAGGTAAACAAAGCCACGTAATACTTCAAGTACTTCGCCCTCACTAA
GACGTAAAGTAATTAAAAAAATGAATATTCCC
GCCGCAATGCTCATTAACCAGAGATAAATTGTAACAAACATAAGAGAAATGGTGCCAAGGAAAGCCCC
CATCTTTGCGCAATTGATAAACAGCATCATCA
TCACAGATAGCACAAACCCCACGGCATGTAATAACTGAGATATCATCATTAACGCAAAGCTACCGGAC
GTATAGCCACGCATAAGACATATTCCACTTCC
AACAGTTGTAATCGTCGCAGTAAAAAGTATAATAAACCGTATAGAGTTCCCACCATGAAAACAAAATG
CTATAAGTGGCATCAGGATGAAAATTGCAGCC
GCGTCAACAAATAAAAGAGTGAGTTTCATCATATTTCGCCTCGTAACAGAGCCTTCAACGTTGGTAAG
GAGATCCATACTGGCAAACAGACCACTTTGTG
CCATAGATGGGAAAAAGATACCCATAGAAAGTATTAAAAAAAGGACCTGACGTGCCATTGCTCTAAAA
AAATCCCGAAAATTTACATCCACTCTCAACAA
CCCTTTCATTTCCTTTTCAATTTCCACGGTAACATCGAAATCCGAATCATCAACGATTTTAAAGACGG
GCAATTCATTTTCGTAAAGTACCCATGCGATG
GTGCGGTCTTCTTCACCAGTTACAAAATTAAAAAATAAAGGGTTGCTTGAAACCTCCCGTGAGTTTAC
ATGACCCAAATGACTATTGGCGTCACCCTGCA
AACCTTCCATCGTACCATATGAAGGCACCAAATGGAAGCGCCGGCTCAGGACTTGTTCACTTCCTTTT
TTTTTTTCTTCTTCTTCTTCTTCTTGTAGATC
TTGCAGAGGTGATGTCACCTGCACGCCGCAGTTTGAGGCCGACATGGGCAACGCGGCGGTTTTCGCCA
AAGGCACCTCCGTATCCTCTTGCTGTCTCCAC

AATCCACACGGCTTCATCGCCCGTGTTTCCGATTCCACGCCATGTACTCGTCGACTACTTGCACTCCA
TGAATTTACGGATCCACACACACTACTTAAAG
GCGAACGTCCATAGAACGCCTTCATCATAGCGTGATGTTCCATGGAAGCATCGTTTTCAATAATACTG
CGCAAAGCCGATTCTTCCCTAATGAGAATCAT
TCTCCCTGCCGTATTGTCGCTTTCCTCGTCTTCAGCGACTTGCTGTAATAAGTACTTTACCATCGCTA
AGGTAGGAACAGATGAAGACGGCGTGCCTATT
TCACTACAGTGAGTTGATGAAGAGATTGGGTTCTCAAGAAACTCAGATGCGATGAGAGTTTGAGGCGA
TAACAATTGAGGTTCCCTCTCCTTCAAACTTC
CTGTGGGATCATTGAAGGGGATATTTGACTTCACTGGGGGTTGAGGTGGATTTGTCTGCTCCGTCCCT
CGCCCCCTTCTTTTACTTCCTTTTCGTGGCAT
ACTACGGCAATAATACCGGCAACCACACCCCCTGGAAGCCCCTCTCTCTCTCTATAGATGTATGTGTA
TTTATTTTACTGATACTTGGAATAGAAAATAC
TATAAAATCAGAATAATAAAACAACAAACAACAGGAAAAAAAACAAAACAAAACAAAACAAACAACAA
CAACAACAAAACGTAGAATACACGTGCCCGTAA
CACACGGCGTGTAATAAAGCATGTGACACACACACACACAAACATGTAGGAAAGCCGTATAGACATCA
ATCATATATGCTCCAATATTTCATCTTGTGCA
GATACGCAACAGCAATAAATATATAAATATATAAATAAATAAATAATTAGGAAGAAATAATAATAATA
ATAAAAATAAAAATAAAATAAAAAAAAAAAGAA
AAAGAGAAATTCATATAAAACTAGTGTCATGTATGTATATATATGATATATATATTCCACAACGCTCC
GAACATTGTTATTACGTGTACAAATCGTGTGT
ACTCTACATACATTTTCCATTGCATGCGTACGTGCCTGCAGGTATGTTATGTATGGAAAGACCAGCGG
TTGACGTACGTCTAAAAAAAAAAAGACAAACA
CGTACAGAGACATAAACACACGGAAAGCAAAAAAAAAAAAAAAAAAAAAAAAAAAAAGAAAGAAAGAAA
GAAAGAAAAGGCAACCCTCCAATTAAACTGCA
AAAAACAAAAGGACAAACAGAAGAACACACATATATATATACACTAACAACAACAACAACAACAACAA
ACACGACATGCTCACAGGTACATCATCAACAA
CAAAACGGCAAGCATTACAATGAAACACACACACACACACACAACACAAACTCGGGTGCGTTACAATC
CATGACTATTCACAGTGACGCACGCAGAATAA
AAAAAAGAAGGTGTGTGGGGTGGTGGTGGTGGTGGTGGTGGAAAAAAAAAAAGAAATGAAGGGAGCAA
AACCTTGTATACATGCACGGCATTTCTCGCCC
AAATTTTCTTCCTTCATTTATTCCCTCCCCTCCACACAATTTCCAAGGACGTTCCGGTCAAAGGAAAA
ACAAAACAAACAAACATATAAAACCCAAATCC
TTGCAATATATAAATAAATTAAATGAAACACGTTTGAAAAGAAAAAGAATGCCTTTACATGTTAATTA
GATAAGAGAGTACCGCTTATACATAAGTGTTA
CAATAACCGCTACCATCAGAACACCACTTGTTAGAGAGAATACTCCAGTTTCTATGGTATCTGTTAGA
AAACCAGCATTCCTTAGACACTTTGCCGCTAA
AAACGGAGAGTATTCGTGTAATGGACGTAAGAAAGTTTCCTCCCGTCCTTGCTGCAATGCATACAGCA
CCAACAAACCACGTCCTAAAGAAGACAACTCC
TCTGACAAACTGTCATGCTTCTCATGCAATTCCTCCAAAGCACGACGTCTCTTATCCTTATCTACTTC
TCCATTCACAACGAATGACAAATTGGCGACAC
GATGAATTCCACTGCTGAGAGCATCAATTCGCGCCTGAATCAATCGCTCTCTCACACTCTGTCCAACC
AGCATCGTTTTCATTTCACATACACGATGCCT
AAGTTCCCGAATCAGCACCTCAAAATTCTCCTCTAAATTCAATGCAGCATCCTCATTCTCACCGTAAC
CAGCATGAATATCAATCAATTCCTGCAAACGA
CGCGTCGGCTCTGTATCCTTACAATCCTCCTCTAACTTCCTCAGCAATTCAGAAAATTCATGCAATTC
CGCCTCAACACAACCAAATTCGAGTTGCACAT
GTCCCAACGCATCCCACCAGTCCTCATTCCTACAATTCCCATTCCAATTCCAACCAAAAACAAAATTC
CTCAGCGAACGCAAAGATTTAAAAACTCCCCA
CAAAAAAGAAACAAAATAACTATCTTTCGCATGATGAAAAGTGCACAACCCATCTTGAAACGACACCC
CATATTCTCCTTCACCCAAACCACGCAACCTT
CTCAAAAACACTACAAACATATCCGTTCCCGTCTTCAATCGTACACCAAGTGAAGGTTTCTCCATGCA
AATCTCGTTAACAGGACCATCAAAAATATTGA
AAGCGTTACCACGCTCCTGTAGCTGGCGCTCCATGGCAGCCAAGGCGTCAACACTCTCACTGGAGCGG
CTCTCCAGCGCAGACTTTTCTCTGCCGTACTC

CTCCAGTCTGTCCCTCAGCGCAGCCAGGGCATCATCACGCTCCTGTAGCTGGCGCTCCATGGCAGCCA
AGGCGTCAACACTCTCACTGGAGCGGCTCTCC
AGCGCAGACTTTTCTCTGCCGTACTCCTCCAGTCTGTCCCTCAGCGCAGCCAGGGCATCATCACGCTC
CTGTAGCTGGCGCTCCATGGCAGCCAAGGCGT
CAACACTCTCACTGGAGCGGCTCTCCAGCGCAGACTTTTCTCTGCCGTACTCCTCCAGCCTGTCCCTC
AGCGCAGCCAGGGCATCATCACGCTCCTGTAG
CTGGCGCTCCATGGCAGCCAAGGCGTCAACACTCTCACTGGAGCGGCTCTCCAGCGCAGACTTTTCTC
TGCCGTACTCCTCCAGCCTGTCCCTCAGCGCA
GCCAGGGCATCATCACGCTCCTGTAGCTGGCGCTCCATGGCAGCCAAGGCGTCAACACTCTCACTGGA
GCGGCTCTCCAGCGCAGACTTTTCTCTGCCGT
ACTCCTCCAGCCTGTCCCTCAGCGCAGCCAGGGCANNNNNNNNNNNNNNNNNNNNNNNNNNNNNNNNNN
NNNNNNNNNNNNNNNNNNNNNNNNNNNNNNNN
NNNNNNNNNNNNNNNNNNNNNNNNNNNNNNNNNNNNNNNNNNNNNNNNNNNNNNNNNNNNNNNNNNNN
NNNNNNNNNNNNNNNNNNNNNNNNNNNNNNNN
NNNNNNNNNNNNNNNNNNNNNNNNNNNNNNNNNNNNNNNNNNNNNNNNNNNNNNNNNNNNNNNNNNNN
NNNNNNNNNNNNNNNNNNNNNNNNNNNNNNNN
NNNNNNNNNNNNNNNNNNNNNNNNNNNNNNNNNNNGCGCAGCCAGGGCATCATCACGCTCCTGTAGCT
GGCGCTCCATGGCAGCCAAGGCGTCAACACTC
TCACTGGAGCGGCTCTCCAGCGCAGACTTTTCTCTGCCGTGCTCCTCCAGCCTGTCCCTCAGCGCAGC
CAGGGCATCATCACGCTCCTGTAGCTGGCGCT
CCATGGCAGCCAAGGCGTCAACACTCTCACTGGAGCGGCTCTCCAGCGCAGACTTTTCTCTGCCGTAC
TCCTCCAGTCTGTCCCTCAGCGCAGCCAGGGC
ATCATCACGCTCCTGTAGCTGGCGCTCCATGGCAGCCAAGGCGTCAACACTCTCACTGGAGCGGCTCT
CCAGCGCAGACTTTTCTCTGCCGTACTCCTCC
AGTCTGTCCCTCAGCGCAGCCAGGGCATCATCACGCTCCTGTAGCTGGCGCTCCATGGCAGCCAAGGC
GTCAACACTCTCACTGGAGCGGCTCTCCAGCG
CAGACTTTTCTCTGCCGTGCTCCTCCAGCCTGTCCCTCAGCGCAGCCAGGGCATCATCACGCTCCTGT
AGCTGGCGCTCCATGGCAGCCAAGGCGTCAAC
ACTCTCACTGGAGCGGCTCTCCAGCGCAGACTTTTCTCTGCCGTACTCCTCCAGCCTGTCCCTCAGCG
CAGCCAGGGCATCATCACGCTCCTTTAGCTGG
CGCTCCATGGCAGCCAAGGCGTCAACACTCTCACTGGAGCGGCTCTCCAGCGCAGACTTTTCTCTGCT
GTACTCCTCCAGCCTGTCCCTCAGCGCAGCCA
GGGCATCATCACGCTCCTGTAGCTGGCGCTCGAGTGTAACGACGACATCAACACTCTCACTGGAGCGG
CTCTCCAGCGCAGACTTTTCTCTGCCGTACTC
CTCCAGCCTGTCCCTCAGCGCAGCCAGGGCATCATCACGCTCCTGTAGCTGGCGCTCGAGTGTAACGA
CGACATCAACACTCTCACTGGAGCGGCTCTCC
AGCGCAGACTTTTCTCTGCCGTACTCCTCCAGCCTGTCCCTCAGCGCAGCCAGGGCATCATCACGCTC
CTGTAGCTGGCGCTCCAGCGTAGCCAGAACGT
CAAAACCCTTTATTGTGTAATCTTTTAGTGAATTCTTTTTGTTTCCGGACAAATCCAGCCTTCTTTGC
ACAGGTGGTGTTCGTGGGCTGTCTTGCGTGTA
AAATTGTTTGATGCGGGTGAATGTTTTAGTATTCGTGTTATTTTCTTCTCTTTCCTTTGTATAGATTT
CTTGTGATAGCTTTTGTCCCAACTGACTTTCA
CCAGTATTTTTACTTCTGGTGTTGATTAAAATTCTTTGTTGAAGTTGGAGCTTCTCTTCCGCATTTTT
TCGAATCTGTTGCACCAGTGCTTTGATGTTCT
GCGCACATTGAATTTGTGTGATTGTTTTTCTTTGCGGTTTCCGTTTTTGCTGAATCCCGTGTGTATTT
GCTGCCTCTTCAACATATGTTTCAGTTTCCGA
TTGAATAATGGGAGGGTACTGACGATTTTTTGCCTGCATGGCGGCCAAAAGATGCAGAGAATTTTGTT
CCTGAAGTATGATCGTCTCATTTCTGTCTGGT
GCTGCCCCCAAGTTACATAGGACACTGTAGATGGAAATGCCACGGCGTTCTGGCTTCATTTTGTAATG
CCATGATCCACTAATCATCTTGATAAGTTCTG
CAAAACGTGGTCTTTCAAAAAGACCCATATGTGAACAACGTTGTCTAGCGCTCTGGTTCTCTCTGGGA
GTGGTGTTGTTGGAAGCACAAAAGTTAGAGGC
GGTTTTCGACTCCACCTCATAAAGACGGTTCCATAACGCATTCATTTCAATATTTAATCTCGCAATCT
TCTTAAAGTCTTCCTGCAGCTTTTGCTCCAAC

TCTTCAAGATCAGAGCTGCGGCGTTCATTTATTCTAAACATCTGATTGCGATGTTCACTTACATTAAT
CATGAGCACGTGTGCCACAACCCATCTGTACA
ACTCCGTAAGAGCAATGCTGCCTTTCATCACCCCCTCATATGTAAACTTTGGTGTATTGATGTACCGC
TGCAACACTTCCTCACGCTGCGCTTGTGTCAA
ATCACTGGAGTCAAAGGTCGCCAAAAGCTCCGTCATTCCCGGACGATGTAAGATGGGCATAATTTCAG
CCCACGTACGTCGCCGCTCGCCTAACACAAGC
ACCACTGCGGCCATCACCCGTACCACGATTTCCGGTGGTGTTCGATATGACACCAACTCGTGCCAGTG
ATGAATGTCAATCTTCTTTACAGCCGTACGGA
CGTCCCCGAGTTCTTCCTGGGCAATTATCACCGCCTCTTCAACCTCCTGCTTCTCCTTCTGGACGGCA
TAGCGTCTCTCCCAAATCTCTTGCGAGGATTG
TTTGGCGTCCTTGATGCACCGCACAAACTCCTTTTCTAGATTCCGCAGTTCCTCAAGTATGGATTTGC
CCTCTTCCGTTGTGGCCACCACGCCGCAGAGA
CTTCTCCGGGCCTCGTGCATCAGTGGCTCAACCAAAATTTTGTTTTCTTCAAGCCTTTCCAACACCGT
CGCCAACCCGCATTGCAACTTTCCTGAGGCTG
TGTCAAAGTACTCGTATGAGATGAGATCACCAACGTCAAAAGACGGAGAAATTACTTGGGCATTTGGC
GATTGTAATTGGAGATGCATCGTCCGGGCATC
CATAGGCGACGGTGAAGAAGACACATTTGCGGGTGTTACGGTTTCTGTTGTCAATTCCTGCCCGTCCA
TGTCCCGAAATGATAAAGTGTTGAGATGTAAA
TTCACTCAACTATACAAACACAAACACAAACACAGACAGCCACAGACCAAAGATTCACTTATAGGGAA
ATTAGACTGAATCAACCAGTGTTGATATATTG
TACTGCTCCCACTCAAGTAAAGGGTCGATAATATACTGCCGAAATAAGGTTTGTATTTGACTCTATAC
AACTAATAATAATAATAATAATAATAATAATA
AACACCTGTATTGTGTTGCAGACTTCCCTTGTAAACCTATATGCATGTGCAATAGCCAAAATAGAAAC
ACACACACACAGAGAGAGAGAGAGAGAGAGAA
AAAAAAAACGACAAAATTGAAAGTGAAAAGCCGCACACGCCATACAGAAATAGTGAATGTGTGCCTGC
CATTCCATTTTCGCTTCCGTGTTCCCCCTTTT
ATGGCAATGTTGTGATGTGAAAAAGTGCCAAGGCTCCACGCCTCAACCTCACGTCAACCACACTCTTT
ATCGTCCTGAATTGCAACAGTTTTTCTCTTTT
CCTTCCACATACACACACAGACACACACAGAGAGAGAACAACAACAAAAAAGAAATTCGCCAGCACTT
GCAGCAAACACGTCCAAGAGTAAGACCAAAGG
AGCGTCGTGGGTCTCACTTCCGCTGCCCCGTCGCTCGATCGTAAAGTTGACTGATGGCCTCCATCACA
AGACGGATGCGATATTTGATACGTAGTGCTTC
TTTACTCAGTCGTGCCGCCCGCTCACGCAGAAGTTTTTCCTTTTTCTGAAGAAACACATCCGCCTCCG
CTGTTGACATTGGAAGAACCACACCACAGCCA
AGATTCACCCACACCTGCGACCCATCCTTGACGGAACACTGCACAAAGAAATAATTGCCAATATCAAC
CATAATTTGATTGCGCTGTGGCCTCTTTTCTT
CATCTTCTTTTTCTCCTTCTCCCGCTGCCATTGTGGCGGGTAAAGAAGGCGCGTCGCCGTCTGTGATG
AATGAGTGTGTTGAGGAAAGTGCCCGCATCTC
CTGAAAGAGTTCCCGCAGCTGGCAGCACTCCGAGGCCATTGTGTACACCGTGTCCCGCTCCCGCAGTA
CCTTCTCGAGGGACTGGCGGAGGACGGCGTCC
AGGAAGTGCTCGAGACGTGCCATTCTGCCAGTCTCCGCGTCAAACGACGAGGGTGGCCCATAAATGCC
GTCTTCCGTGGACATCTTCAAGCCGCAGCAAG
TGTTGTTATCAAACAAGGAGAGAGGGCAACAATGACGTTTGCTCACCGCAAAATAACGCCAATCCCCC
TTCAAAAACAGATAGATTTAAAAAAAAGAAAA
AGAAGAGAACACAAACAACACCACAGTGAAATAAACAAACAAGCTCACATAGTAAAAGACACAATAAA
TAAAAAAAAGGACACCGTGGCACATTTTCTTC
CAATGATTCATCATTGCCGTTTTTTTTTTTTCGTCACGCTTCCGTACCCAGCCTGATGCCCTGACCCG
CACCCAAAGTTCAATTTAAAAGANNNNNNNNN
NNNNNNNNNNNNNNNNNNNNNNNNNNNNNNNNNNNNNNNNNNNNNNNNNNNNNNNNNNNNNNNNNNNN
NNNNNNNNNNNNNNNNNNNNNNNNNNNNNNNN
NNNNNNNNNNNNNNNNNNNNNNNNNNNNNNNNNNNNNNNNNNNNNNNNNNNNNNNNNNNNNNNNNNNN
NNNNNNNNNNNNNNNNNNNNNNNNNNNNNNNN
NNNNNNNNNNNNNNNNNNNNNNNNNNNNNNNNNNNNNNNNNNNNNNNNNNNNNNNNNNNNNNNNNNNN
NNNNNNNNNNNNNNNNNNNNNNNNTGACCCGCA

CCCAAAGTTCAATTTAAAAGACCCCTTTTCTTCCACCAACATATTCGTGCGTGTCTTTCTCTCTCTGC
CTCTCTGCCAGTACATGTCAAATGTGGCATTG
TTAACGACTGATTGTCGTGAACATCATGACTGTGCGTGGTGGCACCACATGCTTGAGTGTCTCACGAT
CGGCAGGATCAGCCCACAACAACACACCGAGA
GGTTGATACATGCTCCATTTTGTTTCTCTAGCGGAGAGACGGACAGGCACATCAGCAATTGTGCCTTC
CTGTAGGAGCCCTTTCAGTGCAACCCACACCG
TCAACGGTATTCTCGCTGTTTCTGGCAAAATCCACACGTGACACGTCGGTTCTGCAGCAGACGTTTTG
AGACGTCCATGCTGACGATGCCGCTGCCTTGA
CGCCATCAGGCCCCTATCCCGTATCATCCAACTCCCAGAAGTATCATTCGCCGCATCTTCTTCAAGAC
GCTCTCGTAGCACATCCACTGCAGAAGTGTCA
TTTTTAATTCCTGAGAGCTCCAGTCTATGGAATGAAATGCAGTTGGCGTACTTTAATTGCCATGGGTC
AAGATAGGCCATGACATGGGCGGGGGTAGCGC
GCCAATGGAGACCAAGAAAGAGACTCTTGCGGTGGAGAGCCCCAAGAAGGGCACTGTCTCCAATTACG
CCAGTCAAGCACTCGCATGGCGAATTGCCCCT
GCGACGCTGCGGGCCAAATCCCCAAAGTTCAtCACCGTGAAGCAGCGTGAAGGCAACGAGTACGGTGA
CAAATGCCCCAACCGTCGCCAGGCGCAAAAAC
ATCCGCCTAAAAGACAATCACGCACACCCACACACACACAGAAAGGATAAAAAAAAATTTTCCACCGC
TGTCGACAATAAAAAAAGAAAAACGCTTAAAA
GATGAACTGAGACTAAGAAGCACCATGAAAAAGCCAAGCAGTCCAACAGAACACGTCTCTCTCCCCAT
CTCTCTGTGTGCATGTATATGTATATGTATAT
ATATATATATGACTTATGGGTCATCTTCACCCTCCTCGGCCATTATTTTTCTCTTTTTACCTTTTTTC
CCTTCTTCTTTTGCTTTTAAGACGGAAGGAAA
ATAGCAAAGGTGGCCACACATTCCACCCATCCCTTTCACAAATCCCCGTTAAATGTGGATGCATGTTT
TCCCTTCTCGGCAACATCTTCTCCCCCTCCTT
CTTTATCTTCCCCAGAGAGAACCGTCCCTTCAGCCTCTATGGCAACGCCAAAAAACTCTTCACTACTC
TTCTCCATGTGGTCGTAGTTCCAACCGGTGCT
TAGAACAATTTTCGCCGCCCGCTCATCAAGGCGAAAATGAGCCCAAACGAATTTGTCGTGCCCATGCT
GGTCAAAGTACTGTAACAACAGCGAGACTAAA
GTGTCCTCCACGGCGCGTCGTAAAGAATGACCATTCTCTTTTCCCTCCCATGTTTCACGGTGACGCTC
CACAAGGAAGGTGACCGCCAAATCCTCAATGC
TGCTCGTTACAACCGCCCCCTTGTTCCAGTACGTTCCACATGGCAGGGCGGCCACGGCAATTCTAATC
ATTTCCTCCGCATCGTGCCTAGAGAAGGGAAG
AAAAAGCATCGTACGTGTAAAGGCAGAGAGGAGTGTCCCATAAAGACCAGCAAACTCATCACGAACCA
GTTGCTCAATTTCCGCCATGCTTTTCCCCCAC
GTGCGTCCCTGCTTCCCAAAGTCAGTGGTCACAATAACAATTAACTGCGCAAGGGATGGCGTACCCTC
TGGTTGTTCCTCAAAACGTTCTGCTCGTCCAA
GGAGTGGCGCAAGTGCAGCCACAAGTTTCGGGTGCATTGCCGCTAGATCATCAAGTAACACAATACCG
TGCGGATACACCTTCTGGTGAGCTGTCACACG
TTGTATGATACGAGTCCGTGCGTCCTCTATCGGCATCCCTTCAAAACCTGTACCGGAGATGACAAGCA
TGTTTTCACCCATGTCGCACGCTGGGCGGGAT
GGGGCACAATGCGGTGATGTGGCTAGAGACAAAAGACGTGCAGTATGCGTCTTGCCAACCCCATTGTC
CCCTGCAAAGTGTAATACAAGTGGATCCCGCG
GGTAAATCAACTTGCGCCTTACCGCCTCTAGAATGACACCCTTCAAATGTTCTTGTCCCACAAGTCGT
GTAAGAAGAAGTTCCTTCACATCCTCCATGAC
GCGATGACGCAATTTCTCATCCTTTTCAACGGAGAACTCCCGCTTCACCAAGACCGCAGGAAGCGAAG
TCAAGACGCAATAACGCAGCCTTAAGAAAAAC
GGTGTGCCACGGGCGGAGCAGATAGGGCAACGGGGGCAGTGCCGCTCCACCTCCTCTCCCTCCGCCGC
GACAGGAAGCGGTCGCAACGAGGACGCGGGAA
ATATCAAAAAAATTACAATACATACGGCAATAGCCATTGTCGCAACCGGAATACCTCGTGTAAAAAGA
CGCCGCGCGGACATTACTGCAAGCAGGAGCAG
AGGCTCACTGTTCTCTTCCTCGCCCCCCCTCCCCCTCGCTCCACAGCAGCGCTCAACTAGTAATGTCT
CCTTCCTTGCGCTGTGTGAGGTGATGATGCTA
CGATATTTGCCTGAGCCCCACAATTATGTGTATATAATTTTTTTTTGTGTGTCATTTCACCAGCCATG
GAAGGGAAAGCAACGGAAAGAAAGCAATAAAT

CCAGCCAGCCAACAGATCACATGTAAAATAGTAATAATAATGCGACAAACAGGCCGGACACACACACA
CACACAAAAAAAAAAATAGGGAAAAACCTGTCA
TTTTGGGGTTTATCTAAATATAAAGATATATATTATATGGGTGTGCCGCTCTTTTATTTGGGTGGCCC
CTGCGGGGGGCCAAAGTCAGGGCCGCCAGGAT
TTGCGCTCTGCGCCGCATTCGCCACCCAACTGGTTGCAATGCCGCCAATGCTGTTCCATACACCGCGC
TCCGCGTCGCGTCCACCAAATTTTGCACGACC
ACCCTTTCGTCTTGCAAGTTGCCCACGGCGGAACCACACCGCTTGCTCCGTTACAGCGTCCATGACAC
GTTGCTGCTGACGCGGCGGCACACCTGCGTGA
ATGCCTGTTGTCTGGGGGTGAAACAACGGGCGCCAGGTGGCATTGTGGTGCAGGTACGTCCGCTCCAA
GTGGGCGGGAATTGTTTTTTGTGTAATGAGCG
AGACGTACGAGCGGGCAATGGCGTTCATCTTGTGGGTGCGCCACTCCGCGTAGCGCGGGTTCATCATG
TGCGACTTTGAGTTGCTCACCCCCTCCAACGG
AATGTTTTTCTTTAAAAAGGCCCGCACCGCAGAAACAATCACGCCCTGATGTTTTGTATAAAGACTGT
ACACACGGCCAAGACGCCCCTGCCGTGCCGTC
CGTCCTGCGCGTGAGAGATACACCAACGCATTTGTAGGCATGTGAAAATTCACCACAACGTCTACATG
CACGTCCAGTCCACGTGCAGCGAGATCTGTGG
CACAAAGAATATTAGTGCGACCGGACGCAAAATCGGCAAACATGCGCCGACGAACATCCCATGGCAAC
GCTGCGTGCAGAAGGGAACAAGTGAAGCCTCG
CCCACGCAGGTGATGATACACCGCAGTGGCGGAATCAATACCACGTGTGAAGACAATAATACGCTGAC
CTTCGCCAAATACCGTGCGGGGAATATGACAC
GTAAAAGGAGCCGCGACTGTTGTGAGATGCTCCCAGCGCAAAGGACGCACCTGCTCCAAGATACGACG
CGCTTCTTCATTTACATCCCGTTCCACGTCCA
GCGACAAGGAATAAGAAGGGGAAGAGGAACAATCAAGAGAATTCACGCTGCCATTGCTCTCACCCTCA
TAATGATGGGATTCCTCCTGTTCCTGCACAAC
TTCCTCTTCAAACTCTGGCAATTCATCAGACTGCTGCTGCTGTCGCTGTTGGTAATTCGGTTGCGGTG
ATGGTGGTGCCGTGTTTCTTCGACGCTCCATG
AACTCTTCGAGTGTCCCCGACAAGTAGTGCGTTTGCGTGTGTGCCTGCACCTCATCTACGTCGGTGGC
GATAACACGTGGATGGTTGCCGTGCTTCTTAA
GCAAGTGCTGCAGCACATCCATCTTCTCCTGCTCCCGCTGAATCGCAAAAAAGCGGTGTGAAAGGGTG
TCCGGCGGGCGATGCATACGTTGCCGCTGCAG
ACATGTTACGGCATTTGGAAACTTCTTACCAATAACAAATTCTAACTTTCGTGTAATGTAGGCGGTTG
CAAATACAGTTTGGGTCTGCACCGGCCATAAA
TATTTAAACATCATCCGCTTTTGCACCTTCATAAGAAGATGAACTGCATCGTGGTCGTGCTGCGATGA
AAGCATCACATCTGCTTCATCCACAGCAACGT
AGCGGATGTCATCAAGGAAAAGTCGACGTGATCGAATAAGACGCAGAACCACCTTGGGATCCATGATG
ACAACATCGGCAAGTGTGTTCTTCAGCAATCG
GCTCAGATGGTATTTGGCCCTTTTCCGGGACGTGAATGAAATGGAGCGCAAACCAGTAGCCGCGTCTA
AAGTGGCACAAATGTGCTGCAACTGTGCAATA
AGTTCCTTCGTTGGCGCCAGGAGTATCATGCGTGGACGGCGCTCGCGCAGTGGGATCTTGTACACATC
CCGATCCTTCATCATGTTCTGATACAAGGGAA
GGAGATAGGCAAGTGTTTTGCCCTCGCCATGTGGGGCCGCCACAACGGTGTCTTTGCCTTGTAAAATC
ACCTCAATGGCCGTTTGTTGAATGGGAGACGG
TGTGTCGACATGTAGACTCTGCGTCAGCGCTTTGCGCAACTCCGGTGCGAGAAGCATGTCCTCGAAGC
TTGTCACTGTCTTTGTCTTCACATTTTTCGCG
AGCAGCGGGGAGTGGGCTGTGCGGGAAGGGGAAAGACTCTGCAGCACAAAGTTCCCGACAAGGTGGTC
GGAGCGCTCCCCAATTGAGCGACTAAATGTGC
CACCATACGCCTTGAGCTGCTGGGGCACAATAATGCCTCGTGCGGGGGTGAGGAGGGCAGTCCTGTTC
CTATTGCTGACGACTCCACCAAAGGAGCGGCA
GCCCGAGGCAACAGTGCACCGCATCCCCGCAGACAATACTCCCACGGAAGGCCACAGCGCCATCGTAG
CTGGGAATACGGATAATAATAATAATAATAAT
AATAATAATAATAATAATAATAATAATAATAATAATAAATATATATATATATATATTTAACACAANNN
NNNNNNNNNNNNNNNNNNNNNNNNNNNNNNNN
NNNNNNNNNNNNNNNNNNNNNNNNNNNNNNNNNNNNNNNNNNNNNNNNNNNNNNNNNNNNNNNNNNNN
NNNNNNNNNNNNNNNNNNNNNNNNNNNNNNNN

NNNNNNNNNNNNNNNNNNNNNNNNNNNNNNNNNNNNNNNNNNNNNNNNNNNNNNNNNNNNNNNNNNNNNNNN
NNNNNNNNNNNNNNNNNNNNNNNNNNNNNNNN
NNNNNNNNNNNNNNNNNNNNNNNNNNNNNNNNNNNNNNNNNNNNNNNNNNNNNNNNNNNNNNNNNNNNNATA
TATATATATATATATATATATATTTAACACAAGC
CTCGCTGCGGCACCGTCCCCTGTAATGTCACACGTGCAGTTAACTCTCCTCCCCTCCTTCCGGCCGTG
TGAACAGGGCATTAGTTTGTGTGTGTGTGTGT
GTGTATTTATAGGAGGTTAGAGAGAAAAAAATGTATATATATAAAAGAGAGAACATTAGAAGACCAAA
ACAGACACAAATTCCCAAAAAAAAAAAACAAAA
CAAAAAAAAAAAAAaCGGCATCTCTCCTTCGCTGCGGCAACACAACACTCCTCCACCCCTATATAAGT
ATTAATTTTTCCTTCTGTTTCTTTTACTCCTC
TTCCTCATCATCCAACGGTGGCTCGTCTCATTAAGATTTAACCCCATCGTATCATTTACCCCAACACC
TGCGTTGCTAATTTTATTTGTTTGGGTTTCTT
TTCCTGTTTTCTTCCTTTTTCGCATTTTAGAAAAAGAAAAAAAAAGTATGTGATTAACGCCTCTCTCC
CCAACCTCACCTCGAAAGAGTTAATACATTAC
ATGTGCATAGGAAAAAAAAAAAAACCTTTAAAAAAGAAAAGAGGAACAAAAAAACTACATATATACACG
TCTGTGGCTCATCATGCGTCCTGCGGGCAGAG
ATGATTCATTCCTGTAAACGTGGATGGGTTTCAATTTACGACGGTCAACGCCTTCACTGCCATCTGCA
ATGCACCCTCGTACGTTTTGTTTCCACCAATA
AATCCAGAGACATGCACAAATATGCCACCTTCAATGCCACTTGCTTGACTCAATTCCTCATCTCGTAA
ACCTCGCCATGGTAACGACTTGCGGTTCTCAA
ATCCTGATGCCTCCTTGGGCACCGCCTGCACGCGCCAGCCCTTCTTGTCCGCAAAGAGAACATACAGG
ACGTGGCCCACTTTGCCATGTTCCTCCTCCAA
CTCCAGCAAGTGTTCCTTCCATGGGCAGAAGCAATCCTTGAAGACGACAATGCGGCCACTAGGATGCA
CATTCATTGCTTCAAGAAATGCCGCCCGGACA
ATCCCACGCGCTGGCATCCACGCGAAGACATGATAATGAACGGAGTCAAAAAATTCAGATGTTGCCAA
CTCCATTGCCTGCAGAAAGGCGGCATTTTCGT
TTTCCACTTTACCGTTCCCTTCTTCATTCCACCATGGCATAAGATTTCCTATGCGAGCTGAAAGCGTC
GTAGAAACATTGTACTTCCTCACACATGATGG
TGAAGACGATGAGACCGGGACGGCGCCCTCCTCGCCCTCCGTCCCTGCAGGTCCGTAGGCATTAACGC
CGTTGTCGATGCCATCAATGTGTTCCACAAAA
TTCTTATAAACAATATCAAACAAAGTATCCAGTTCCTGCTCCGATAGTtTCTTCTTATCTGTTCCCCA
TTTCGTGGCGTCCAGCAGTTTTACGCGATACG
GGGATGCCAATGCAGATTCCACATAACCCTGGATAATTTCACGGCCAAAGTGCTTATACACAAGGCCT
GCAGAGGACAACCGGGTTTGATAGACCGCCTT
TGGCGTTTTCATGGTGTCATGAAAAGACGGCTGATGGTGATCAAACCGCAGTGTCGCCTCATCATAGA
CGCTGCCAACATCCACAACGATATTGCACCGC
TCCAACGCATTCGGGTCACGTGAGCGAAGAATATTCGATTGACCAAAATTTTGACTACAGCGCAGCAA
TCCACATGCCATGGCTTCATCACAGTGAAAGG
AGCCGTTATGCGTCCCAATGACACCCGTAGCGGTGCTGGTTTCCGCAAATGACTCCAACACCCGGACA
CCACAGAGATGTTTCGTCACATACTCGGCAAC
AAACCGTCGAGGCATATGTCCCTGTGTGTAAATGCAAAAGCCGGTATTTAAATTATATATAAATATAT
TGTTGCTGCGTTTTCTATGTAACTATTTAACG
TATTCAAGGATAGGTGGTACGAGGGGATAAAAGTAAAAGTAAAAAAAAAGAAAAACAACAACAACAAA
AATTGCAAAGAGCATACAAGAAGACGATTTCA
CAGCTGTCTCTCTTCCTTTCTTTGCTGCCTTCTCTCTCATAAATATATATATAAATGTATATGCATGT
GTATGACTCTCCTCCACACACGTAGGAACAGG
TGGAATCTCAAAAAATCTCCGCCCGTCCACAATGCTCGGCGCATCCCTCCCTCTCCCTCTCCCTCTCC
CTCTCCCTCTCTCTGTATGTGATGGTCTTTTGT
TCATTTTCATTTTCAATAGAGTTACATGAATGCTGCCACCACATCGTTAAAAAGCGTCTCAAGCATTT
CCGTGACGTGCAGGCCGGTGCACGCACTCGTC
TCGAAGTACTTGTATCCGTGCGCTTCCGCCCAACGCCGCCCATCCGCTTCGCTAACAACGCGCTTCTT
ACCGCCATCAACTTCATTGTCCACTTTGTTGG
CACACACCACAACAATCGCTGGTTTCTTCACAGGGTTCACGCCCAGCGTCTCCCCGCCAGTGTTTCGA
GGCGCATTCACGTATGTTTGCATCTCCTTTAA

CCACTCGTCCAAAGCGGCAAAACTTGAGGCATCGGTGACGTCGTAAAAAAGCAAAACACCTTCCACAG
CGTAATAAAACTCGTTTCGGATCTCAAGAAAC
TCGTCACGGCCAGCCATGTCCCAGAAATTCACCCGAACAAAAAAATTGGGCGGCGCAGGCGACATATG
TGCCGGCGCACGTAAACTCACGCGCTTCACAC
CGTAGTCTATGCCAATGGTGGGAATGTACTTTGACACAAAGCGCCCCTCACAGTATCGCTTGATAACA
CAACTCTTCCCAACACCGGAGGAGCCAATAGA
GATGATTTTAATGCGCGGCTCTGTGCCTTTTATAAGACTGGAGGAAGAGGCGGAAAAAGAAATATTGG
TCTTCGCACTCATCTTTTCTACTTGGACGTGT
GTGTGTGTGATGGTACACGTGTGGTCACCTCTCCGTCCCTGCTCAGCCTACCTTAAAAGTGAGGGACA
CACAGATGGCAACGAAGGCAGTAGCAGCGGCA
GAAAACAAACAAACAAACCAAATGTGTCCATCTTCCTTACCCGACCCTTACTCACCCAAGCAACCACC
CGTAACACATGCAGACAGACACACTGCTCACC
GCCCATCTCCATTTCCGCGGCTTCTTTCCCAATTCTTCTTTCAACATTCCATAAATTTTCTGAAGCCT
ACCAAAAGCCTCAGGCTCGGGTGCAGGCATTG
CATTGTATCCCTCACCCCCGGTCTCTCTCACACCCACAATCCACATCAGAGGGTTTCATTAAGCGTTC
ATTGCCTGCCACGCAACTTTTTCATGTATGCG
TCCGCAAAGCCGTCATCACGAGGCTCTTCAAGACCTAATTCCTTCGCTCGCCACCGCTCACGCTCAAT
GTCATTCTCAATGGACACGTGCCGCCGCCGCC
GTTCCTTTTCTTCCGAATAGTGCACCGCAGCGTAAATGGAGGTGGCGAAAAAGAGGCTACCGAAAAAC
AACTGCTTATCGCGGAGACGATTCCATCCAAT
CCACGACAGGCCTGATTGGTCTCCACAGAAGAAACGCGCATAAGAATTCATGTCGTTCTACTTGTTGA
TGATGCCCTTTTCTTTTTTTTTCTTCTTTTTC
CCCCTTCACCCGTACCGCACGCCTTCCATTAACAACAAAAAACTCAGCAAAAAAAAAAAAAGCAACTC
ATCTCAACGCATGCATTGGAGTGTTATTTCCA
ACACATCGCCTGGCAGGATGTGTCGGTGGCAAAAACAACAACAAAAAAGACGGACATCCACACACAAC
ATACAGGGGCAACTCATTTAGCAGAATTCTCC
GTTCACTGCCATATATATATATATATATATATAGCAATTGATTTCTGTACATGTTTTATGTATTTGTT
TTTCTTTGCTGCGCGTGCTCCGCCAAATGGAT
GGTAAAAAAAAAAGGGGAAAAAGCGTACGGGTCAAATACACTGAAACAAAACGCAGCGCAGGGCACAT
GCAAAGGTAAAAAAAAAGAGAGAGGGGGAAAT
AGGGAGACTTCACAAAACACGTTTGCCCGTGTGCAGTGGGCCATCTAACCCCCGTGGAAAGTTGTAGT
GATCAAACATGACATCCGAATGTTGCGCCTTC
CGTGCGGCGTCAACCCGGCGCCTGCTACTCGGCTTGACGTCACCGTCGGCGGTAGCCATCCAGCCGTG
TATTGTTTCAGGGAATCGGCCCCACATGGTAA
CGGGGAGATCGGCCTCACGTGGCCTCGGCATACGTCGTTCCTTCGTCCATTTTGCAAGTGAATCGACA
GCATTTACATTAATACCGTAGCTGAGGTGACG
AAAATCTTTTGGGGGCGGTGGATGCTGCAACGGGGGAAGCACCGTCCGTCTTGTCGTCCGTCCCGCTG
TGCTGGTACAATGTTTTGCCGTCCATCCCGTC
GTGGACAACGTCGCGTTGCTCTCTATGCCCGTTGCAGTTGTCGCCACCTCCCTTGGGTCACTCGACCA
CGTCCGTGACGCAAAGTGAAATTCTTTTCCTG
TGGAAACAACTCCCGTTATGGGCAGATTCGTCGCTGGGATCTGTGAAGTGTCATTCAAGGGTGGATCC
GTCAGCACGCCTGTTGTGGACTCCCGCTCCAG
TCCGTGAAAGTCAATCGCCAGCTGAGCATACTTCCGCTGAACCTCCTCCGCCGCCCGCCGCTGCGCAT
CCACAGCCGTCTCGCGTCGAGGGGGTGCAGGG
TAACGAATGGTGCAGGTCTGCAGCTGCCCCAGGGGGTCGTACCGCACCTCGTCAATAATGTCACGGTA
AACGGCGTGGCGGCCGCCGAAACGATCGGTGC
GGTTACACTCTTTAAGGAATGCGGTCAACATGTTGTGTCGTAGTAACGTCCGGTGCTCCTCCCCCTCT
ACATCCTGCAGCTGGGCGTCAATAAACGCCTG
CACTGGCAGAAACACATCCTCCTTCCAGGCACGATGGAGCTTCTCCACCACACGCCGCAAGTGCCTCT
CTTCACGGCGCGTCTGGCATGCACAGCGTGCA
TTGAAGTTCTCGTGTTCCTCTACAAATGCAGTAAAGCGGCGCATAAAATCCGCCAGTTCCTGCTCCTG
CGTGTCTTCTCTGGCTCGCCGCTCTTGACGTG
CCTTCGCCGCCTCCGTGTCTCGTGCTGCCTGCAACCGAACCTCTTCCAACTTCATCTGCTCCTGCAGC
TGCCACTCCTCTGCCTCGTGTTGACGACGGGC

CGCAATTCGTTCTCGCTGCTGTTCCCGCTCAATCATGCGGCTCCAGTCTGAGGCATTCATGACATCTG
TGTACAACTCGGCATTGTGCACACGGATACGC
GGCGAAGGGTTCCTCTCGCTTCCCGTCTTCATGTATATATGTATATAACAAATATCAATACAACAACG
TATTTGATATGCCTGGTGCCTGGTGTATTACA
CCGCCTGCGTTCCACACACACACACGCTACCTCCCGCTTTCCCTCCTTCTCTCTATCGGGTTGCAAGG
ACCCGAATTCTTCTAAAAATATTACACAATTA
AATAAAATAAAATCAAAACGATCAAAACATACAGAAAATACAATAGAAACTTACAAAAAAAAGAGAAA
AAAAAGGGATATATAAATATATATAAATATAT
ATATGCATGTAGATATATATATAGAATGGAAAAAAAAAACAACCTCAATTACAACATTCCCAAGAGAA
ATATATAAAAGAGCCGACACGCGCACACAAAC
GCACATGCACTTATGGAGAGCATGTGAGGAGGTGTCAAGCACATTAAATAAATAAATAAATAAATAAA
TAATAATGAATCATACAGATGGTTACAGTCAT
ACATGAAACAAAAAAAAAACAAAAAACAAAAAACAGAAAACGCATGGAAGAAAAGAGGGAAAACACAC
ACACACACACATATATATATATATGTATGTAT
GGAGAAATGTAGATGGAGCCAAACGTGTATTATTTTAAACCCTGCCTTTTTTTTTTGTTTTTTGGAT
AATTTACAGTTACCCCCCGTCAATAATTACAA
GGAATAAACTTCGTATGCATACATGTATATATAAATAAATATATATTTATATGTTGCCATCAACACAC
CCTCTTCTAACAATAACCACGCAATTCTACAG
ATACATGCATGCATGCATACACACACGCACGAAACAAAAACAAACAAACAAAAAATAAGGCTCCTTAA
TCCATCCCCTCTTTCCCCCCTTTTTTGTGCCT
TCTCCCTCTTTTTTCCCTCTCCCTCTCTCTCTTTCTCTGTTTTTCCCTTTGAGCCAATCTAGTCTAAT
CCACTCAACATACATAAAGTAAATGATACAAT
TTGTAAATATCTACACAAATACACAAGACTGTAGCAGTTAACCCGACGTACTTGCACGCACGCACGCA
CGCACGCACACACACACAAAAGAACACACGTT
ACCAACTGCTTCACATGTATATATATATATATATATAACTCAATTATAAATGGCTCTAACAGTACTAC
GCAACAAATAAAGTACTCTTGTTCGCTCGTTT
ATTATTATTACTATTAATATTTTTTTTTTACCTTCTCTGCCTTGTGTTGGTTGCCTTCTTTGTGTGAT
CATTACCCTTTTTGTTGAGGCTGCTTTGTTTG
CTTTTATTATTTTTATGATCCTGCTTCTTCGGCTGCTTTCTCATTGAAGATGAAGTCTTCCTTTTCGG
TGTGGGATGGCCAGTCTTCACTCTCGGCTTAG
GCTGCACCTTCTTCTTGCTGGAAGGCGCATTGTGAGAAACGGTACGCCCCTTTGAGTTGATGCGCTGC
TTTGTCAAAGTCTTCATAACCGCTTTTTTATT
TTTCGCCGGGCTTCGTCCCGTCTCCCTCTTCTTGTTCTTGTTCTTGGTGCTGCTGCTGCTGCTGTTGT
TGTTGTTGCTGCTGCCAGTGCCTTTCTTCGTC
GTTCCTGCCCGCCGCCTTCCACGCCGTCCCACTGCGTCCTTCGTGAGCTCCCCGTTGGTGCTGGCGGT
GGCGGCCTGACGCTTTCCCCCCGCGGTACTCG
TTACAGTGGCGTTGCTTCCGACATGCACCGTCCCCCCCGCAAGACCACCACGTCGTGTTCCTGGCAAC
GACATTTTCAGCTCCATCCCGGATGGTACTGT
CGTTCCGGTTTCGTCTCCGTCCGCATTATCCGCTGCGTCATCACCGGAAAATGAGTTTGACCCTTCTG
ACCGTTCGGCATCATTTACCGCCTCAACCTCA
TTGTTGTCATCCTTCACGCGGCCCTTCCCGTCTAATTCCTCCACCTCCACCTCCATTGCTCCCGCATT
TCCAAGAGTGAGGCGATGGGCCTTTTCCTTCT
TGTTCGCTCTTCCCGGATGCGCCGGTCTGCAGATTTCCTCCGTATGTGCCTCATCTTGCGTCATCACG
GTCGCCCCATGCCCTTCACACTCATCCGTGGC
AGCCACAACTTCTTCGCCTCCCGATTCCACACACGAAATGGCAGTGTCATGCGTACTGTTGATGTTAT
TAGCCACGACATGTGCATGACGTCCACGTCGA
CTTTTTGCGGGAGTACGCGTGATGACGCGACGCACAATTCGTGCTACTTGTCCTTCCCGCACTTTTAG
CCCTTCCCGCAAAGACTGCAATTCGTCTTCGC
AACGTCGACGCATCCGCGCCAATTCCCGCTCTGCCTCCAGCCGCCCCAACGACGCTCGCAACAAGGCC
TCCTCATCCCGCCAAATGCTCACACGCGGATC
GCCCACTAGTGGCATTGTGTCGTTACCAGCACCAACACCGCCACCAGGAAGGCTCCTTACACGTTCCG
TTGCATTCGTCATTCCCGTTGCCGTTATGGGA
GTCGCGTTGCCTGTAGTGGCTACCACAGCCAACGGGTTCGATGTGGATGATGATGACGATGTGTAGGC
TGCCGCAAGGGGCAAGTAAGCAGCGCGAAGCG

TCGCCGTCTGTCGAATAAATGCAGTGAGCATCGTTGTGTTCTTTAAGAAATCCACACGAATCAGACGC
ACAAAAGTCAAATGAACCCTCTCCCTCTGCGT
CGCCTCAATGCAGCACCACAATAAAAATAAAAATAATAAATAAATAAATAAATGTTTATTCTACGGTG
ATGGCACTCCGCCGCTAATGAGAGCAAAACAG
ACGCCGAGACCTCTAAAGAAAAAAAAAAAATAAATAAAAACTAAATAAAATAAAACTACAGTTCAAAAA
CTCCTGGATTGTGAGAGACACAAGGGCTGGTA
AACACCTTTTTTTTCTTTTTTTTTCCTTTTCTTTGTTGTTGTTTGCTTGGTTGCTTGTTTGTTTCTTC
ACTCACTCTAATTGTGTGGAAATTCAAAAAGT
AAAAAAGGAAAAAAAAAAAAAAAAAAAGAAAGAGAAAGAGAGAGAGAGAGAGAGATAGAGGGAGAAGTC
ACAGATATCAGAAACAGCATTCTTTACTAGTA
GTAGTCGTATTATTAATAATATTCTTTTCATATGACGTACATGCATATGCAATAAAAGCATACAGCCA
TATACAGCCATACAGCCATATACAGCCATACA
GCCATATAAAGCCATACAGCCATATACAGCCATACAGCCATATACAGCCATACAGCCATATACAGCCA
TACAGCCATATACAGCCATACAGCCATATACA
GCCATACAGCCATATACAGCCATACAGCCATATACAGCCATACAGCCATATACAGCCATACAGCCATA
TGCAGCCATACAGCCATATACAGCCATACAGC
CATATACAGCCATACAGCCATATACAGCCATACAGCCATATACAGCCATACAGCCATATACAGCCATA
CAGCCATATACAGCCATACAGCCATATACAGC
CATACAGCCATATACAGCCATACAGCCATATACAGCCATACAGCCATATGCAGCCATACAGCCAT
>scaffold1070.size6393
AAACAAAAATCAACTCATCCGTCATCATCATAACAACAACAACAACAAGAGCAAAGCAATCGCAGCAA
AGTGCAATAAACAAAAAAAAAAGGGGGGGTGGG
GATTTATTCCAATTCTTTGCCTTTTCCCACTCCCCTACACTCATCAAGCAATTCCTGCGGGAATGGGG
ATTTGTTGTGTGGGGAAATGGGAGGGGAAGCC
ACAGGAGAAAAATGCCCCCCTTCACTCAACAACGGCAAGTAGAGAATAGAGAAAGCAAGAATAAAAAA
AAAGCAGGAAAAAAAAAAACATCGTTACATATA
AGAAACCACCCCAGCTCTGCCAACAACACTAACAAATAAATGAAACCTCAACCAGAAGCCAATAATAA
TGGAACGACGGAGGTGGCTTCCTCCAAACGTC
ATCATGGCATGGCTTTATCGCAGGCGGCTGCTTCATGCACACTGATGACACCTCCAGGGTTTCTGCTC
GCAAGTATAATTGTGGACAAGGCACTGTTTTC
CATATTTATTTATATATTTATTTATTTATATTTATGTTTGACAAAAAAACCAAAACAAGAGAAAAACA
ACAAAAAAAAAAGACCTCAAGCAGCATCAGGGGT
TTCTCCTACATGTAGACTTTTGCCAAAGAGGCATGGCCACGTCATCATCTTCACTTTTTATGGGTTCC
GGACGGTATCGGCGCAGTCGCCTGCCATTTGC
CGTGCTGAAACGTTTAGTTTGCGGCTCTGTACTATCATCACAGCCGCCTGCATCGCCGTCAACGTCAC
CATTCTCATCGGTTCCCAGATGCACATCATCG
TGACTCTGTAACGCCGTCGTCTCGGTCAATGGGGGGAATTTCACAATTGTCGCCGCACAAGTCTGAGA
AATAGACTGCTCATACCTTCGTCGCCACAACT
CCTCCTCGTGCCGCCGCCATTGCTCTGCCCTATTCTGTAGCCTCCTCTGTCGTAACATCCAACTTGTT
GCAGAACTGTTTTCATCGCATCCGAGCGGACA
GAGTTCAGAGTCGATGGAATTGGCATGAGGGGCAGCAGAGTGCACATCCTCCGCCTGTGCTTCTTCCG
CCGACAACATAACACAAACGCCATCCACCTGA
GACATGCAAATATCTCCATCCCACGCAATCTGCAATCGGAGTGGTTGGTAGGCGGCGTCCTTATTCCC
TGCACCATTTTCAGTGTCCGTACTAACTCCCA
ATGTTGTGCCACCGCCAAAAACCGACAATGTAGGAGTGGGGTTCCGCATCAAAGAATCCACACACTCT
GTGTGGGTCCCAACACGCATTTCAGAAAATGA
GAGTGACAACTGCACATTTGCAGATTCTTCCTCCCTCTGACCCACACCGTTATTCAGCTCTTGACCCA
CCATGCACGTCGTATCATCCTGAACCCTTCCG
CTGTCCTCATTTACACCAGACCTCTTCCCCACGGAATGCATTCCACACACAGACGCCTTCATTCCTTC
TCTATTCCTCTTTAATGCTGCCTCCGTTACAC
TCCCTTCCACTCCCTTCGTACCTGAAATATTATTTTCCAACTGCCCTCTTCGTTGCTTTTCCGCCGCA
CCCACACCCCTCCGCAACTTAAGACCACAAGC
AAAAGAAGCAGTAAACAAAAAAAAAAAAAAAAAGAAAAGAAGAAACATCTCTCAGCAAAAGTTTTCGTT
TCATGTGCCCCAAAGCATACAGGAAATGGGTA

AGTGTATACATATATTGCAAGCACACACAAAAAATATCACTGTTTCACCAGCCCACACAACATCCCTC
ACATTCCATGCGGCATCTGGCCTGCCCCTCCA
ATCCCCGTTTTGTGCGGTTCGTGGGACACACGGCAGTGAGGCCGGCACCGCgtgtgggcaaaaaaagc
aaacaagaggaaacacgtcactCATGACGGTG
TGTCTGTTAGACGGACATTGAGGGAAGAGGATTTTGGAAAAAGGTCAGAAAACAAACACAAAAAACAC
GAAGGTGCTTATTAACCCAACAAAGCACCTGC
GCCAAAAAAATAAAAAGAAAAAAACAGAAAAATAAAATCCCCGGCAAAATGAAGTAAGTGCTGCTGGA
AAGCTGAGCAGTTTTTTCCCCACGGGGAGTCG
CAATTCCACTCCCCATGCCCTCTTCAGCACAGAGCCGTCATGCCCCACAGTTGCAGCACCAAAGCAGG
CAGTAGTAGCACCGGGGACACAGACAAATGGA
CGGTGCTGTTGCCAGAGCTCGGGTCACCCTCGTGCTTCCTCTGCTGTTTGGCAACACCCGATTGTGGA
ATAGGGACTGTGCTTGCATTGAGTTGCTCCAA
CCCTTcACCATACACTGTGTGGTTGTACAGAATAACGTTGGTCACCGTCATACGGCTGCTCTCCGTGC
CTCTTGCACTGCCCCAGCCAATAAAGAGGTAT
GAGATCCTGTGTTTCTCCAGCAGGGTTGCAAAGCTTTCCTCGTCCGCCTTCTTCATGTACAATCCCCT
CCCATCCACAAACACGTAGCAGCCATCCGTAT
TCATTTCAAACGTCACTTGATACGTCGTGTTTGGTTTCCAGTCAAAAAGAGCAGGCTCGTCAGGATTA
GCGTCACTAGGAGTCATAAAGCTCCAATTCTT
TTCGTGGGTGTACGACAGGCCAATGAGCACCGTGCTGTTGGGGTCGCTCACCCCCACACCCACCAAAG
GGACGTTGCCACTCTCCGGCACCTCGTGAATC
GACACCGCCGCCGCAAGAGTGAACTCCCTGTTTGCAAAGTGGTGCGGCACAGTCGGCCCATTCATGTC
GACCGGCCACTGCGCTCCTGCTCCGGCGCCTT
TGAGCGTCACTCCATTAACAGCCGCTGCCGTATTCTTTATTATTGCGCCCACGCCGAGGTACTCGTCC
CGCCACTCGGTCTCAGTTGAGCTGCCGGACAA
AAAGGCAACCAGCCCTTTGGTGGGAACAGGACCAGTGCACATGCCATTTGCTGCCGCCCAGCCAACCT
TGGCAGTACGACTGCATCATTGCAGGGCGCTC
TCCAAATCTGTCCATTTCTTCGCCACATCCTTTACACGCTCCAGCCCCGCCGCCACGCGCACAGCAAC
ATGACCGTCTGATCCGTCCTCGCTCCTCTCTT
TGTTCTCGTACAGTGAAAGCAACTCGTCATTATTCCCCTTCACCAACAGGGAGCTGGCGGCAGCGTCA
TCACCTTCACGGGATATCGGACCAACGCCGTG
CACACGCACAATGCTCGTCACCCAAAGATGGAGCCGACCCTTTTCATTGTCCTTAGAATACGCGGGCA
GGGCGGCGAGCATCACCTTCTCGCCCGCAATG
ACTGCGGTGGTGGATCCACTCTGCACTCCGTACCCTTTTCTATCATGTGAGTTGCCCCACACGCGATT
AATCGACACACGATTTGGATACCAACTCGTCC
CATGCCCATCGGACACGCACAGTTGGTGCTGGCCATCCCCACAAGAAGACATCAGAATAAGTTTTTTG
CCAGCTACCCACTCCACAACGGATAGATCCCC
GCAGCCCCTGCCAGTCGTGCCATACAAAAGCCTCCATTTATTATCCGACGAAGTGAGCCGCACGGCGA
GCAAAACACTCTTTCCATCCCCGCCTGTGGCC
TGCAGGGAAACACAATCGTGCCGCCATGAAACGCAACACCCGAACCTCCTCCACCATTCAAATTCTTC
AGGGAGTTAGTGCGACCTCCGGCATCGGGTTT
AACCGCATGGGCCCCACTGCATCGGATCTTCTTGCTCTCACCCTCTTCAGTGACGGTCCCTTTCACAA
GCAAAAGGCTCCAGTTGCTTGCGTCAGCCGGT
TGTGTGTCAGAACTTGTTTTGCCGTAGTCCCCAAGAGGCATGTAGATGTTGCCACCACTCACAACTGT
TGTGGGCCGCATTATCTCTGTTGCCTCCTTAC
CATCCGTAGCACCAACACCCCCAGAAATCGTGCAAAAGGGACACTGTTGTCCGCCATCGAGATCTCCA
TTGGACCACCACTTAAATTCAGCCGCCTCGCC
ATAATCCCATCACAGCCGCTGACTCCGCCTTCTGCCCTGCACTGAACCCCAGCAGTGGCAAACACACC
GCCCTTCACACCCACAAAGCTGGGAGCACGAA
GCAACGTAAGTTCCACATCATTTTTACCTTCGCCCTATTTAGCAGAAGAAATAATTATCATCCCTTCA
AATTAATCAATGAGCTGCACCGAATTACTTTC
CTCAGCTTTCCCAGCACCACCGGTGCCGCAGCACATCATCACGACAACGAGGAGCAGCACCGCGAAAG
TAGACGCACGCCGGGACATGTTGGGCCCCTGC
GGCTCACTCTCTCTTCCTTCCCTCCTCCTTCCGCTGGATCCGGTCACGCGGCGACGGTTGTGTGTGCG
GGGTGCCTTCACAGCAGCAACACGTGAGAGCA

TATAAATATGTGTGTGTGTGGTGTGTGTGCTGTTGGTCCTTTCAGTGCCTTCGAATTTCACAGTAGCG
ATTCGCGCTTGTTTTCCCTCTATGTGTGTGTT
CAACAAGTGGAGGAGACAGCGACAGCGGCAATACATCAGACACGTCAGCGGTGAGCAGAGCAGAGTGC
CCACGCATAGAGCGGAACACGAGACAATCCGG
AAGGGACGGCACGGAGGACCATCGCCCACAGAAAAGAGCAGCAACACGGCAAGTCACGGCGGGCCGTC
GGTGTGTGCGGGCCAGCCCTCACGCAGTGCAA
CACGCCTCACACCCAACAGGAAAGGACAGACAGTAAGAGAGGGAGATGAATGGATGCCGTCAGGAGGC
ACTCATAATCAAAATGGTCCGTGAGCATGCGG
CCACTCCACGCAGGAACACAGGAGAAAAAGGTGAGAGGAGGAAAAGCAAGAAGCAGCACAAAGCACAC
GGAAGGAACAAAAGACAAGCGAGAAAACCACC
GCCACGGCCCTCACCCTGGCCGGCGACTCCACACCGGCCCTCTCAAACACTAACACCATCGCAGTGCC
GTCAGTTCGCGGGTGAGAAGATTCGGCCCTCC
ACACAACAAACGCGCACACCCCCAACAGCCACACACCAATGGAAGAGAAGAGACACACCGTCAACACC
TCCAGTTCAATCCCACGGCCAACGGGATACGG
CCACACACCGCAGCCAACACACAGCGCACACGGAGCGAGCAATCACCCAAAGAGCCACACGCGGAAGC
GGATCCTCACCCAGCCATGACGCGGTGCCTCA
CCCCACGCGGTCACACGGAGAGACACTCGCCCTGCGCGCTGCCTCTCTCCTCCCTTCGCTTCATGCCC
ACGGGAAAAAGCAGCACCCACACGCTGCTGCC
ACACGCACCGCAAAAGAACCATCCGAATCCACCGTGATTTGCACTCTGTGTGCGTGTGCTTGTGCGTG
CCTGCAGAAACACGAGGCAAAGAAGTAAAAAA
AGAAACGGAGCATGTAAAGAAACGCAAAAACAAAATGTACAGCGGAGACGCATGGCGAAACAACAAGA
AGAAGACCGCTCAAACTCACGATTGCACCGCA
GTCCAAGCAGCGGCAATCCACACAAAATTTATCCAAAACACAAAAAAAGCTTTCCCCTTCGTCTTATA
CAAACCCCATCACACAAAATAATTATAAATAA
AAAAGAAATCGTCCATTGCTGTTCCACACAAATTTAAAAAAGCTGCCGGCTGCCTTGTGGCCCACCAA
ACTGTCCATATATTTTTTGCCCACAAAAAAGA
AATTTAAAAGGTATTTAAATGTCTTTCAGCCGTCCGACCCTCCATTTGTCCGCACATCGTTTATATTT
CACGGGGTCATCTGCGTGTTGCTGCTCCCCTC
AGCCCTCTCATCGGCGTGCGAGTGCGGTCCGCAGAGCACAGTCACTCGTGTGTGTGGGGACTGAGAGA
AGCACGCAGACATGACGGGAGTCGCGTCACTC
CATCGAGAGAGTGAGCGCAGCAAAAAAAAGAAGAAAACACGCAAAACAAGAAAAACAGTCAAAGAGAG
CAAGCAAAATTACTTTTCTTAAAGCGGTAGGG
TGTGGCTGTGTGTGTGGCGCTGCGGGTGTCTGCCCGCCCTCGCCGCAAAGAAACAAAACAAGAGGGAA
GAAATGTGTGCTCCGCCCCACAGGCCCTCGTC
GCACGCCCACACGTCACGGCTCCCTCAGCGCGCCGTTCGTCGCGGCCCCTCCTCGTGTGTGCAGCAGG
CGAGGCGCACAGGGCCGTCGTCGTGAGCGTGT
CGGTGGGTGTGCGAGTGGAGCAGTTGGGCGGGAAACACACGGCACCCCTCGCAGAAGGAGTGCAGAAG
AAACAATTAAAAGAGGAAAGAGTAGAAAAAAA
GAACTTCCATATTGATTGCCCCCGAAAGCAAAAAAAGAGGGATGAGAGATTAAAAAAAAAAAAGAGTA
AAAAAATTGAACCATTAAAAAAAAAAGC
>scaffold1071.size6397
AGTAGAAGAAAATGAAGTAAATGAGGCAGCAGACTCGCTATTGTTCTGCTGCCCAAAAAGAAAAGAAG
CATTAATGTTCAAGGATGCACTCTGTCTGTTGA
TGCCTTTGGAAAACCTAACTGCCTTGTGAGAGGGGTGAAATGGAAGAAAGATGCGATTGAGAGCATTC
GCTGCTCTCCTTGCTGTTTTATAAGTGTGTTG
ACCATCTCCTAATAACATTGATGTGGGAGATGGAAGTGATGCCTTTTTTCGTCCGGAAATGTATTTCC
ATGCGAGTGGCTCGCCTGGATTTAACTTGCTG
ATGCGGCGTAGATACGTGTTGCGCAAGCGCTCTCGGAGCAGGAGGAAGAATTGACTCCTTGTCCGTTG
CATTTCTGCCTCACGGTCGGGCGTCGGTAACT
GTAGATAGCGGGCCTTGCTGTCGGTAGCAATCCGCTCTGCCTCCATGAGAGTGTCGTCCCAAAGCCTG
GGACCGTCCTTGTGCATGCCGCGGGGGCAATG
TCGTCGATACGAAGTTATAAGCGCTCTCGTGAAAGCCTTAATGCCGGTGGACTGCTTTTTATAGTCGT
ATGCCGGAAGTTGTGCGTCGAAGTCCTTGATG
AATAAATCCCACTTGCACTTCCCCCAACTGTAGAAAAACTTAGGTGCCGAAGGGGGTGCCGAAGGTAT

GGTATCCTTAAATGCCTGATGCAGCGTAAATG
TCAGCACATAATGATCACTAAGCGGCGACACGGAAGCTGACCAATCCGACACAGTCAAATGCTTTGAC
CATGTCAGATCGATACAGGATCGTTCTCTTGT
GCCACGGGCATACGTGATCTCACCAGGGGTGTTAACCAAGGTGAGTCCCGCATCCGTGCACAGTTCTA
AAAATTCTGCAGCAACCTCGCTTGGAGAAGTC
TCCAAGAATGGCTCCCACTGTGGGTGATGCATGTTGAAATCCCCGCACAGCAGGAGCGGCGACGAGGC
TGGAAGGCAGTTTACTAACCGCCTGAAGGATT
GCGTTACTTGCGGTGGTGGTCTCATATAGGCACTCGCTACAATAAGATCACGGTTCTGGTCCAGAGCA
ACCTGGACCACCACCACTTCAAGGCTGGTGTC
GTGCTGGGGGATGGTGAGAGGTATACGCTTGGAGCGGAGATTTTTCCGCACCAGCACCGCCACACCGC
CTCCCTTGCCGATGCGCGACTGTGCATAGAGC
ACATAATCTCCAGTGTGCATGCTGGCGATCTGCTCTGACGACTTCCAAGTCTCCTGAATGGCTATTAT
GTCTGCTCCTTGCGCCATTAATGTTGCGAGTT
TTGACGGCGTGAGGCAAGAAACGTTCATCTGGAGTACTGCTATGGGGCCTGGGTTCTGCTCAATGTCT
CCGCTTAGCAGTAGCTTTTGTCGTTGCGCGTC
ACATACGGCACGCAAACGGTATGTATAGCGCTGATAAGCGCCGATGGAATTCAGCAGCGGATAGAAAT
ACCGCTCCGCGGGCAGCCATGTAAATGGCTCC
ATCTGGTGCCCTTCCGACCCGTTTGTGCGGCGCCTCCTGCCTCTCCGCGGGCAGCGGATGGGCAGCAG
GCGCTTAGCTTCCTCTCTAGCAAAGAGTACTA
GACCCTGGCACCACGTTGAGGTGGCCGGCTGAGCCAGGGGCACTGCACGGTCACGACGCCGCTCGTGA
TCGCCGGTCCGGCGACAAGCGTCGCGCCAGTG
GCAGCGCAGCGCGCAATCGACACGGTGCCGCCGCTCAGCGTCACCCCCGACAGCGATGCCACGGACGA
CGTCTCGCCCACCGCCCACACGTCGCGCAGGT
CCAGCCACCCGCCGCCACCGATCGTTCCACCGTCGCAGCGCACCAGCGACGACGCGACAGACCCCTCG
ACGCCCACGAAGCGCAGCACCGAATCAACCAC
CTCGAGGCGCTCGCCAAGCACAAGTCCGCCGCCGCTGCTCACAACCGACACGTTCGTCACGGAGAACA
CCGAGTGGCTCCGCAGACTGAGACGCGACGAG
GACGCCACACGCATCGCGGACGCGGTCAGACCGCCCGACGCCTCCAGCGACACGCCGTCAAGCACCAC
AGCGCTCGACAGCAGGTCCACGCCGCCGCCAA
CGTACACCGCCGACTCGCCCGCGTGCAACGACCTCAGCACAGTGTTGCTCACACGCAGCTGCGACTGC
AACAGCGACGTCGCGTGCAGCACAAGCGGCGA
CGCCACAGCGTCCGTCAGCCCGCTCAGCTGGGAGTAGCGCATCGGTCCCGCCGTCACAATCGTCGAGT
CACGGACCGTCACGTTCGTCTGTGCCGGCAGG
TTGTGCACAAACACAAGCGAGCAGTAGCTCCCGGACAGCCCGATCACGCTCACCTCCAGGCTCCGGTC
ACTCGCCGGCTCGCCGCTGTAGCCCCGCACGT
AGATCTGCGCACCGCCGTCGCACACGCAGTTCCTCAATTCGATGCGCAGCGCGCCGGGAAGCATCGCC
GCCACGTCGAACACCACGGCCACGCCGTTCGT
GATGGACACGCCGTCCATCAGCACCGTGTCCACGGCGCGGCCAACCGTGATGGCTCTGTCCACCGTAC
CGCCCCGCAGTCGCAGCACCACCGCATGCACA
GCAGGCATCCACGCCGCCGCCGCAACCAGTGCCAACGCAAAAAGAAGCGCCGCACGCATAGCCGCAAT
GGCACTGCGCTGCACACGGTGCCGGCTGCGCG
GACACGCATCCACCGCAGACCACATCCACAGACCCAAACAGATATCAACAGGGCTGCCGTGGTGTGTC
ACACTCTTCCCGTCGACCGTAACTACTGCTTA
GAAAAATCCCTTCCTACAGATGGAAAATCCAGAAACACAATTCCGATAGATCGCTATCACACTACTGC
TAATCCTAATAATGCAAGCGATTCCTAATACC
TTGAAACGATAGTTGTGCAGGTCAATCCAAATTATCTCTCTCTGCTCGGTCAGAGAAGAAGAAGAAAG
AAACAACAGTAACTAAGCACAGTGGCACGCAG
AGGCATCCGGTCACGAACAGTCTCAAACAAAGCACAACCGCCGACACAAAGCAGCGCAGACACAATCA
CGCCAGTGACACCACCGCCCTCCCACCGCCCA
CGCAGCCCCACACAACCACCATGCAGAGTGGTAAGATCCCGGCAAAGGACACATGACCATTGGCACCG
CTTTTGCAACCAAATGTTTCTACCACAGAGCA
GTCCCGATGCCGTGAAAGACACACAAGGGCCGGGACAGACCCACGTGGCAGCGTTACAACCAGCAGCC
ATCGAGCGCGTCCGAGGATAAAACGGTGACGT
GGAAGAGGGGTGGAGAATTCGCCAGTTGCCAGCAGTCAAAGCGTCGTTTTTCGCTCACGTGGGTCGGC

ATTGCACAACACACAGCGGCACCATACGCGCA
GACGGCATTACACACAGAAGGAAGGGATGAAGAAGCACGGCGGCGGCCCCCAGTCACGAGAAACCCTA
ACCCAAATCAATTGCAAAGTCCGATTGGCTCG
CGGCCGTCTGGATGGTGGTCGCGTCGCTGCCGNNNNNNNNNNNNNNNNNNNNNNNNNNNNNNNNNNNN
NNNNNNNNNNNNNNNNNNNNNNNNNNNNNNNN
NNNNNNNNNNNNNNNNNNNNNNNNNNNNNNNNNNNNNNNNNNNNNNNNNNNNNNNNNNNNNNNNNNNN
NNNNNNNNNNNNNNNNNNNNNNNNNNNNNNNN
NNNNNNNNNNNNNNNNNNNNNNNNNNNNNNNNNNNNNNNNNNNNNNNNNNNNNNNNNNNNNNNNNNNN
NNNNNNNNNNNNNNNNNNNNNNNNNNNNNNNN
NNNNNNNNNNNNNNNNNNNNNNNNNNNNNNNNCCGTGAAGCAGGCACCAAAACCAAAGCTCCTCCAAC
AAATCCATCGTGAAGCAAAAAAAAAAAAAAAC
AATGACGGTGCAGAGGCAATAAATGGTTGTGGTGCGCAGTAGCCGTAGCATCGCGCCCTTCCCTCATC
AAACGGCACCGCACCACAACGCACCGTGACGA
CAGCGTGCAACGACGACGATTGAGTAGCACACGCAAAACGGCATAAACCTTTCACCCCGCACTCGCTC
AAATTTATGCTCTCAGTATTTGCCGCCAACGA
CACGGGGAAGGACACAGAAGGGGACTGTCCTGTCCATCCTCCTTCCGCCCACAACAGATCCTCCCCAT
CCTCCTCCTCGGTACACCCTGGGTGCCGCGAA
ACGCCGTCAAGCAAATCCACGTGTATGGAAGCCCCCAATGCGCGGGGCGGAGCTGGCAGCCACCCACA
CTCACTCTCCCGCACACATCCTCTCCCACAAC
TTTCCCACACACAAAAGGGCAAAGACGTGGAGAAGAAAAGCCCGCCGCACAACACACGCAGAAGCACC
GCCATCCACGTCGCACCGCAAGATAGAGTCAT
GCGCACAGCAGACCCACGGCCGCAACGAGAGGGACGGAGCGCATGCAAGTCCACGGCGCTGTCCTTTA
CTTCTCTCTGCCCCGACGGATAGGCATTACGC
CTCACAGCAGGTTGTGGCGAGTGGCGCCATACACTCACAGTGAGGCACGGCGCACACCTTCCGCTCTC
CTCCGAGTGCACTCAACAGGCCGGGGCACGGC
GTCCAGCCAATCTGTTGACATCGCAGAGGCGAACACAAACGATCGTCGTCGCGGAATATCGGTGCCTC
TAGCGGGGAAAAAAAGAGGCAATAGGGGCATG
TCAACAGCACGGTCAGCAGAGGGACAAGAAAAGGGACACAAGGACACAAAGTGGCAGTGGACATGGCA
CAGACAAAAAGAGGTCCGCAGGGATTAAAAAG
AAAGAAAGAAAAAAGAAACAAAAGGGTAGGAGTTCAGAAAAACTCCCAAAAACATTTAAAATTAAAAA
AATGTGTTGATTCGGCAGGACTTTCACTTCAT
GTGCCGGTTCAGCACAAGACGACCGTGTGTTCTGTATAAATATTACCTGCACCAAAACAAAAAAATGA
AAATGAAAAAAAGAAAGTAAAACGAAAAAAAT
GAAATTGTACTGTTCCACCAGCCCACACAACATCCCTCACTTTTATTGCGGCATCCGGCCTTTTCTTC
CAATCCTCCCTTCGTGCAGGCCGTGGGACGCA
CAGCCGCAGGGCCGGCACCGCGTGTGGGCAACAAACAACAAAACAAGAGGGGGAACACGTCACTCATG
ACAGTGTGCAGCAACCAAAAATTAAAAAAAAA
AAGAAAAAAAAGAATTACTGAGTATTACAAAAAAAAAAAAAAAGGAAAAAAAAGAACCCGCGGGGAAT
GCGGCACACAATCTCCGTGAGGAGTCGCAATT
CCACACTCTTCGGCTCACAAGACTGCCACAAATGCACACAGTCCCAGAGGTAGCAGCAACGCCCGAGA
CACGCGCACACGCGCAGTGCTGTCACCACGTA
AATCATGCAGGATCAATTTCGCGTCGGTGATCAGGGCGAGGCGGTCGTCTATGCTCATACTTCCATCG
GATGCTGAGTGATTGGTTTCTGGAACGGAAGT
GTCACTTTTGGCGGCAGTGCGTGGACTCAATTCGTCCGTAACATTCTCAGCACTCGATTCACTGCCCG
CAAGAACACGGACAGACCGAAAGTCAGTCGGC
TGTGCAATTGGAGCCTCTTCAACGTCTGTCAGGGAAGTCGTCAGCTGTGTGGCCTTCTGCTTTTCTTC
CCCCGTCGCTGTGACTTCTACCGCAGTGTCCA
GTTCTTCGGCAGCCGGATTTCGAATAGGGACTATCCCGGTGTTGGGTTTTCTGAGGCTATAATGATAC
AGTATGCGGTTGTACAAAAGGACATCAGCCAC
CGTCACATGGCTGCTCCCTTCATCTTCATTATTCCCCGCACCAAAAAAGAAGTGTGAGTCCTGTGTGA
ACCAAACTGATCAGCGTTACgGAGCTGACCC
ATTCCCTCGGCAGCCAGTTTCTCGTCATCCGTTTCGTATATTCTTCTCCCAATAGCGTACGCAAACTA
TTCATCAGCGAAATATTCGTCTGGGCCGCCGG
CCGGATCCTGAAATTATCTCAAGAAACCAAACATGATAGGAAGGAAAAGAAAGGTAAAGCAGAGGCAA

TTAATTGCCTCAATGTTGTTTTCTGATTAAAT
GTATATATATTCTGTTGACCGCTCACTTGACCTTTTGTCCTCAGTTAGGATCTGAAACATCATTAGTT
ACGAAGTGGTGGACTTTGCGGTCCTCCAGTCA
CAAGACACCGCCGCTTCCCCTTCGCCAATTACTGAATTCTAAAAACACGACCGCGTGATATGCCGTCT
GCGGGGTTATCTGCAGACCGCACGTGGGCAAA
GGATACATACACTCCGCGAAAGCCTAAAAAATCATATATCCGTCGCAGCTCCCACGTCAACGCGTGTG
ATTTCGAGCTGCCTTTATTCGCCG
>scaffold1072.size6409
AAAAAAAAAAAAAAAAGAAAAAAGAAAAAAAGCGAAGCCAAAGAAAAAATCCACCCCTTCATCCATTC
ATCATATATAATATATATATCTTCTTCATTTTC
GATTTATAATGTTTCTGTATGCGGGTTACCAGATCTAAAAATCCTTCTCTTTGTTTGCTTGTTCTTGA
ATGAGTCGCAATTGTAGGAATGTGTACAAGCG
TAAGCGACGGTGCATTACGTGTACGTGAGAACGAGAGCGAGAGAGTGTGTGTGCGTGTGTGTGTAGAC
GTCCACTCCAGAGGAACAGGGGAGGCAGTGGA
GTGTCACGCCGCTCAATCATCATCCACAGACACACACAGACACACACACACAAAAAAAAAACTCATTCA
TGTCGCCCACACATCCAGCACACGCGTCGCCT
ACTCTACAAAAGACGGTGGGGTGCCACACACAAGGCGTCTAGACGTCAACATAAAGTTAAGCCCATTG
CAAGCCCGGCATTCGCGGCAGCGCCCATAAAA
GACGGATCCCCACCAACTCACCCCCTCTCTCTCTCTCCTCCTCCCACTCTCTTATCATCTCCCTTT
GTGCCGCAGACCACACTCCGTCCTAAGGAACC
CTCAGTTGCAACGTATAACAGAATAAAGAAATGAAACAAGAAAAAGTGAGTAAACAAAAAAAAAAAAA
AAGAGAGAAAAAAAAAAGGAATCAATAACAAAA
AAAAAATAATAATAATAATTTTCTCTTTTTCCCTCACCTTNNNNNNNNNNNNNNNNNNNNNNNNNNNN
NNNNNNNNNNNNNNNNNNNNNNNNNNNNNNNN
NNNNNNNNNNNNNNNNNNNNNNNNNNNNNNNNNNNNNNNNNNNNNNNNNNNNNNNNNNNNNNNNNNNN
NNNNNNNNNNNNNNNNNNNNNNNNNNNNNNNN
NNNNNNNNNNNNNNNNNNNNNNNNNNNNNNNNNNNNNNNNNNNNNNNNNNNNNNNNNNNNNNNNNNNN
NNNNNNNNNNNNNNNNNNNNNNNNNNNNNNNN
NNNNNNNNNNNNNNNNNNNNNNNNNNNNNNNNNNNNNNNNNNNTTTTACGCCGTTCTTCCTCCCATTTTCA
AACTCTCAATTCCGCGTGCAATTTTCATTAAT
TCTTATTTCTCTTTTTAGCTCTTCACCTCTTTTTCTTATTATTGTCTGTTTTCCATATTCTGTGCCGC
ACCTTTAAACCCTCCGCTGAGAGAAATATACA
TACATACATACATATACGTATATGCATGAATGCATGAATGCCTGTGTTCCTCATCGCTCCGCGTATGG
GTACGGTTGGAAGCAGCCACGAGGAGAGGAAG
AAAGGGATGCGTTCAAGAGGAGACTTTTCGAGACCTTTTTGGACTCTCTTTTTATTTGTTTTTGTTTT
TGTTTGTTTGTTTTTGTCGTTATATATAAACA
AATATATTATTCGTTAATTCCCCTCCTTTCGTGCATCCCTCTTCTCCCTCTCCCTGTTCCCCCGCAGC
CGTCATCTTTCTCACGTTCACACGCCGGCATG
CATCCAATGTAAGCcAGCACCGTCCCTCTCGTCCACAGTCCCGGCGTGTGTGCGGGGGCGGTGAATGC
GAAAGGCGCCTTGAGAGAGTCCCAAGGAATTT
CGTGGGAAAAGAAACCCCTCCAAAAGAATAATAAAAACAAAGCATTCGCACAAAGAACAAAAAAAGAA
AAAAAAAGAAAAAAAAAAAAAAGAATGAAATCA
GCAAGAGGAATAATATTTAAGAGAAAAAAAAAAAAATGGCGAGGCAAAGGCCAAAAAAAGGTAAAAAAA
AAAAAAAAAAAAGCGAAGCCAAAGAAAAAATCC
ACCCCTTCATCCATTCATCATATATAATATATATATCTTCTTCATTTTCGATTTATAATGTTTCTGTA
TGCGGGTTACCAGATCTAAAAATCCTTCTCTT
TGTTTGCTTGTTCTTGAATGAGTCGCAATTGTAGGAATGTGTACAAGCGTAAGCAACGGTGCATTACG
TGTACNNNNNNNNNNNNNNNNNNNNNNNNNNN
NNNNNNNNNNNNNNNNNNNNNNNNNNNNNNNNNNNNNNNNNNNNNNNNNNNNNNNNNNNNNNNNNNNN
NNNNNNNNNNNNNNNNNNNNNNNNNNNNNNNN
NNNNNNNNNNNNNNNNNNNNNNNNNNNNNNNNNNNNNNNNNNNNNNNNNNNNNNNNNNNNNNNNNNNN
NNNNNNNNNNNNNNNNNNNNNNNNNNNNNNNN
NNNNNNNNNNNNNNNNNNNNNNNNNNNNNNNNNNNNNNNNNNNNNNNNNNNNNNNNNNNNNNNNNNNN
NNNNNCATTGCAAGCCCGGCATTCGCGGCAGC

GCCCATAAAAGACGGATCCCCACCAACTCACCCCCTCTCTCTCCTCCTCCTCCTCCCACTCTCTTATC
ATCTCCCTTTGTGCCGCAGACCACACTCCGTC
CTAAGGAACCCTCAGTTGCAACGTATAACAGAATAAAGAAATGAAACAAGAAAAAGTGAGTAAAACAA
CAACAACAAAAAAAAAAAAAAGAGAAAAAAAA
AAAGGAATCAATAACAAAAAAAAAATTAATAATAATTTTTTTCTCTTTTTGCCCTTGCCTTACACTGA
AGAACAGCTCCCTTTTAAGAGCCCAGTCTTCT
CTCCGGTCCTATGTTTGTGCCTTCATCTTGCTCTCTTTTTAATTATTCCTCTTTTGCGGTCACTCAAC
TCCTTCATTTCTATTGTATAATTATATATAAA
TTATATATATATATTCCAATGGCATTTTCTTTTTTTTTTTTTTTTCTCTTGTTCTGGGAATGTCGGAG
GCGCTTTTGTCTTCTCACTCTCTCTTTTTTTT
TTTTTTTTTTTTTCTCTTTCCTTGATTGGTTGGTGTTTCTTCAAAGTTCTAACTTTTTTTTCCTTTTC
TTCTTCTTCTTGTTTTTTTTTTCTTTTTTCTT
TTCTTTTCTTTTCGTTCACGCCGGTTGTCGCTGTAAATTTATACAGAGACATTTATTTATTTATTTAT
TTATTTATTTCCCTCTTTCCTTTTATTATTAT
TATTATTATTTATCTTCTTCTTCCTCCTCGCAGTTTACTTCACTCCACCTCCTTCTTCACTCCTCCCC
AAAGGGAAACAGAGAGAGAGAGAGAGAGAGAG
AGAAAGAAGCTCTGCGGCAATACATACATACCACTATGTATATACCCACACACACAAATACATAGAGA
TATGATCATGTTGCATTTACTGTTTGTTGTTA
CACCTTTAGTTTTTCCTTTTCTTTTTTTTTTTTTTTTTTTTTTTTTTGATTTTATTCTTCTTCTTTGG
GTGCTGTCTCTCCTTCGTTACACGTCCCTCGC
TTCATTCACAGTTTCAGGTCATGCATCAGACGTTCTCTCATACACGCACAGAAAGAAAGACCCGATCA
CCGACACGCATACAGACACACACAAAAAAAAA
AAAAAGAACTAAAAAAAAAGACACAAATAGACACACGCACGCACACACAAAGACGCTTCTAAACGTTCA
TGTGGCGATTCAGCACTTCAAGAGCCTCTTGA
ACCTTCGCATCCAAAAGACCGGGTGTGTCTAGCAGATTAAGAATTTCGCCGTTATCCATCTCCAAAAG
CATGCCAGTCACCTTTGCAGCCGCGGCAGGGT
TGATAGCGACAATGTGGTTGTACAAACGCTCTCCCAAAACATTCTTCTGTTGCTCGGGAGTCAGGTTG
GCAAGCACCGTCGACAGATTCTGTCCCTGTGA
AGCCAACGACTGCGGTTGTGGAGGCGGCGCCATGATCGGTTGCATTGGGGGACGTTGCGGCATGGGGC
GCGCCCGCACTTGATTCATCGGTGTAGGACCC
ATTGGTCGCAACTGACCACCCATTCCCATCGGTGTAGGCATCATCGACATGCGAGGGTTTACAAATGG
AACGCCGTTAAACATTGCTTGCGGTCCCATGA
AGCCAAGTTGTGGCCCCATCATGCCACCCATCAGGCTTCCCATAGGCGGCTGACGCATACGTGGCTGC
TTGTTACGCATGTCTTCCAGTGTCTGGCGACG
AGCATCCTTGCGGAGGGCATATGTCACAAAAAGTGGACGCGGCCCAAGGGCCGTGCTTCCATGAAGCT
CCTGGATGGCACGGCTCGCCGCACTCTGCTCC
TTAAAAGAAACAAAGCCAAAGCACCGGGATACCCTAGTGGCCCGGTCCATCATAACACTGACAGAAAC
AGTCTCGCCACAGCGCTCAAACAGCTCCTTCA
GGCGCTCGCTGGTGACAGTATCGTCAAAGTTCTTGACGTAAAGGTTCGGGTACTTGCTGTGTTGTTGT
TGACGCTCGCGGCGCAGACGTTCGCGCTCGCG
CTGACGTTCATCTCGAGAGCGAAAGCGGCAGACACTCAGTTTCATCCCTTCCTTAGCCAATGGACTCT
CCTTCTCGTCGAGTTCCTCGACAGCCTTGGCG
GCAGCTTCGTGAGTGGCGTACGCAACGTAGGCAAATCCGACTGTCTGGTTTGCGCCAAGGTCACGAAC
CTTGGCGGACGTCACCTCACCAAAAGCAGAGA
ACACTTCCTTCAACTTCTCATCATTGACGGAGGGGTCAAGGTTCTTCACATAAAGATTTGTAAATTGC
TTCAACTGCTCCTCACGATGGTGTGGTTGGTA
GCGTTCCACAGTAATAGCGCAGTCATCGATAACCATTCCGTTTACCTCCTGAACCGCTTGTATGGCAG
ATTCCTCCTTTTCGTAGGAAATGCGGGCATAA
CCACGGGACTCGCCATTCTCGTTTGTTGCGAGACCAATGGCTGTGAGACGCCCGTACTTGGAGAACAT
TTCCTTAAGGGCATAGGTGTCCACGGACGGTG
GAAGTTTCTTCACTACGATGTTCGTCACGCCGCTGCGCCGCTTAGAAGGATCACGTTGAATCTTGGCA
ATGCGGATGTGCTTCGAACCGAGTTTAACGCC
GGCCTGGTCCAGCGCCTTTTCGGCATCCGCAGGGTTCTGGAAGTTCACGTAACCATAGCCAAGTGACT
TCTGCGTGACCGAGTCCACACAGACACGCACG

GAGGCAACTGGGCCAAGGCGTACAAAGTGGTCGTAGAGCTTTTGCTCGTTAAGGTTGGGGTCCAAACC
GCCGACCCAGATGGAGGGACTCGCAGCAGGAA
AGTTCGACATTCTACCTTCTTCCTTTTTTTTTTTTTTTTTGGGTTTTGTTACTGTTGTGTTTTCGGTT
CAAAGTAAACGAATACGGACCTTGTTTTACTT
CTTTTTGTTGTTGTTGTTGTTCTTTTCTGTCAGTTGCTTCCTTCAACTGGATGTATACTATCCTTTTT
TTGTTTTATCCTTATATTTTCTTTTTTTGTTT
GTTTGTTTGTATGTTTGTTATCCCCCGTTCTCTGTGCGTGCGCTCTTTTGTTAGCAACGCAACTTCTT
TCCCTGCGTTGTTGTTTTTGTTTTTGTTTTTG
TTTTTGGTCCTCAGAACTTTTANNNNNNNNNNNNNNNNNNNNNNNNNNNNNNNNNNNNNNNNNNNNNN
NNNNNNNNNNNNNNNNNNNNNNNNNNNNNNNN
NNNNNNNNNNNNNNNNNNNNNNNNNNNNNNNNNNNNNNNNNNNNNNNNNNNNNNNNNNNNNNNNNNNN
NNNNNNNNNNNNNNNNNNNNNNNNNNNNNNNN
NNNNNNNNNNNNNNNNNNNNNNNNNNNNNNNNNNNNNNNNNNNNNNNNNNNNNNNNNNNNNNNNNNNN
NNNNNNNNNNNNNNNNNNNNNNNNNNNNNNNN
NNNNNNNNNNNNNNNNNNNNNNTTTATTTTATTTTATTTTTTTTTTATTTTAATTTTTTTTTTGGTCT
GTTAACGTTGCTCCTGCTGCGTGCTGTGTTAC
AAGACTGCCGACTACTCCCTTTAGTTCCTCACTCTCCTCTCTTTTTTTTTTTTTTTTTTTTGATTTGG
GTTTTGTTTGGTTATAAAAATGGGCTTCAATA
AAAAAAAAAAGGAAGAAAGAAAAAAAGAAACCTGCAAGTAGGAATGACAACGACGAGAGCAGAGAGAA
AGGAAGGAATAAAGCCACGTGCGCGTGTGCCG
GCGTCAATGACAGAAGGAAGCAAAACAAACAAAGTCGGACAGAAGACACACACAAAAAAAAAGTAAAA
TAAATAAACAAATAAATACATGTCATCTACCG
TAGAAAAAGGACGGTGGGAAGGGGGGGGGGGGAGGGGGGAACAACGGTCCAAAAGCACACGGAGCACA
TACACTCAAATAAATGAATACGCACGCAAAAA
TAAAATAAAAAAATAGATTCATGCAAAAACAACATGCTCCCGGCCGAGGCCTTGTCCTTCATTCCCAT
AAAGTGAAAGGACAGCAACAAGAGAGAAACGA
GAGTTTGGTTCAATGCGCCCAGCATCCCCCCGCTTCAAAAACGAGTTAACCCGCACCCACACTCATAC
AGTAGCAAACCGATTCCAAATATATTTGCCGT
GATAA
>scaffold1073.size6351
TGTCTGTGTGTCCAAAAAGGGGACGCGCAACCAACGACAGGCGTGTCCTCGTCATGCACAAACACAGC
TCGGGCTGCCGTTATCTCCCCCGCAGCCGCATG
CGTCATTGGCTCCACAAGTAGAAGGAGATGAAGCGTGATGGGGAGCGACAGCAACACCCTTTTCCCTC
GTCTATTCCCAAATAAATTCTATAACCCGCAC
GCCAGCCACCATCGTCGAGCACGTCCGCCCCACTCAGTGGGAATGTAAAAGAAAGCCCCTGCCCCCGG
AGAGGGAGTTGCGGCGGAGACCGTGAAAAGCA
GACCCCTTATGCATCGGCGAGGGGAATTGTGCCTTCTCTCCGCGTGGAGCGCTGTTGAGGACAGAGCC
TTTCTACCTCAATAACCCGCGCTCAACGTTTT
GGTGAAGCACAGTCTCCTCGCTTCTTCAGTGTCCATTCCACGTTTTTTGTCCGGGCACCTGCAGAGCC
GGGCAGCGGAGTAGATGCAGAATTGTCTCTTC
GCGCAGCTCACGTTGGCTGTTTCTCACAATTTCCGGTCGTTGGTCGTGGCATGCCGTGCGGCGGTGGG
TCAATCCGTCACTGCTATGGCACCGAAAGCCG
CATGCTGGACGGCGAAATTCAACAGGTGGTGATCTTATCGGGGTGTCGTCGGCAGGCGACGTGCGCTT
CCTCGGTGGCCTGACCCCTCGGCCATCATGAA
ATCGGCGACAGTGTGGCGTTGATGAATGGATGTGTGCGCGCCATCTTGAGCACACGGTGCACTGTTTC
TCGATGGCATTCCCCTCCTGCGCGGCGGAGAC
AGCCGCCCTCGGAAGCAAAGTCGCTATGCGGGTTGTGCCTCAATTGTGGTGAGGGCCACGTGGAGACT
CCTGTTGTCCTTTCCTTCACTTGCTTGGGTCT
TTTGTGTTCCGGCGACGGCGGCGGGCGGTGACCAGTGCCAGCCCCAACAACCCGCAGGCATGCACGGA
CACACAAAAAAAAACGGTCCCCAGCAATGCGCG
GGCCACAAAGACACACACTCTCCTCCTCCTCTCCCCACAAAAAAAAAGACTGTGGTGATCGTTGGGCA
AAAAACCAAGAAGGAAGGCGCACCCAAGCGGA
GTGCCCAGAGTTCTCTCGCCCCAAACAGAAAACAAACCGAAACAGATTCACAAACAATTCACTCCCCG
CATCCCCCCATCCCATCCCCAAGCACCATCAG

TAATGACATGTGAAGCGCTTCTCAACAGCACCCCGTTTCACGGGTTCGTTTAGACTTCCCAGCGCCGA
CCACCCACATCAACGCAGCGCTGCGGGAGCGA
AAGGCGGTGCAAAACAAATTCACAAACAAAAACAATacaacgcagcatccactacaatccgctccacg
agaaaaagNNNNNNNNNNNNNNNNNNNNNNNN
NNNNNNNNNNNNNNNNNNNNNNNNNNNNNNNNNNNNNNNNNNNNNNNNNNNNNNNNNNNNNNNNNNNN
NNNNNNNNNNNNNNNNNNNNNNNNNNNNNNNN
NNNNNNNNNNNNNNNNNNNNNNNNNNNNNNNNNNNNNNNNNNNNNNNNNNNNNNNNNNNNNNNNNNNN
NNNNNNNNNNNNNNNNNNNNNNNNNNNNNNNN
NNNNNNNNNNNNNNNNNNNNNNNNNNNNNNNNNNNNNNNNNNNNNNNNNNNNNNNNNNNNNNNNNNNN
NNNNNNNNCAATCCGCTCCACGAAAAAAAGCA
GCAATGATGACGGTGGCAACACAAAAAAAAAACACAAAGACAACGCCTCAGCGGTGCGGCCACGCAAC
AAACCCGTAGCAATGCCACTACCACCAGCACC
ATCCAAAATGCGATCCCTTCCACCTCCCAGACACATCGCAGTGCTCGTTGAGGCAAAGCAACACGGGG
ATTCTCGGGGAGAAACATGCGCCTGTTTCACT
CACTGTAGAAGACACGTACTTCTGCATCCGCTATCCCCCACCTGGCACCGACATCCCTTCCTCAGCCC
AAATTCCGCAACAGCCACCGCCGCCTCCCTAC
ACTCACCCTCAGTCGGGTGCTCAACCGCGCCGCATCTCTGCCAGCCACCCATCAACACCCTGTCTGCG
TGTTGCACGCAAATAATCTCCCACGACATTTC
TCGGGATAACTCCTCCCAACCATCAAAATATGCCGCCAGACGCTCATTGCACTACCTCACAGTGCCGG
CGGTGGTGCTATGCTCACCCGCCGTAGCCATC
CGCAGCCCAACCAGCGTCTTCCGCGGCGAATCAACAAAGAAAAAAAGCCGTCCACCAGCCAAAAGGTT
TCGACCTCTGGAATGTACAGCACCCCGCACTC
TACATCAGTCTTCTGGTAACGGTGTTCCACAGACGGGAAAAAATCGTGCCCGGTGGAGCCCTCCTGTG
AGTACACCTCCGGCGCACAGCGGTGTCACTCA
CTTCGTGTTGGTGGCCTCAGCCCCTCCAGTTTGAGTCCTACTGCAGTCATGGAATCCCCATTTAAAAA
TGCAAACACAGTGTAATTCCCCAAACCTTTTG
GTATTAAATAATCTCTGACCCTCGATACAAATAAGTTAAAATCAGCCTGCGGCATTAACTTTGCCAAA
ATGCACAATGTTTTACTTGCAAGGTGGGCAGA
TATCGGTGCATTGAAAGGCAATTCACCATTTCGCTCTCCTTGTACTCGGACGGTATCCACAAGCTTGT
GGGACACGTTATTAACATCTCACATTTTATTA
GATCCCAAAGTAAGGTAATATTTGGTATCCAATCCAAAGCGGTCATCTTATCAACTGTACTGTTGCAA
GCCTCAATCCGACCACCAGATCATAGCTTATC
AAAGGTGTAGCAAAGAAGCAGTCCCACTTTATCCATGCAACCCTTCACCTCCCTCCAGTGCTCTGCCT
GCTTACCAAAAGGCTGGTCACGCTTCATCCAA
ACACACAACGCCTTCACGTCCTTTTCTTCCGGGTAATTCATTACAGTTCTTACGGCTGTCTCTTGGTT
GTACCATTCCTTAAAGTTACCCTCGTTCGGTG
TTGTTACCATAGCCATGCCCCATTCATGAGGAAGCCGCATCCTAGACGGATCACGACCCTTCTTTGCC
GCGCAGTAGACAACATACCCCTTCACCCCACG
CCAAGAAAAACTCCTCACAACAGACGCATTACCGGAGTCACCTATGTATACTGACACCGCTTGACGGT
CTTGTCAAACACTTACGTCATATCTCCCAAAC
AGTGGAAAACAACCTGGAGTTGCTCCGCATCGTAGTGCGGTGGCTGGTAGATGAGGTAAGGACCGGCA
TTCACCGACCTCCCAATCCCAAGCTTCGCAAT
CAACACACGCGGATGAGGCGTAAAGTGCTCCTCAGGACGACTGTTAAACCACTCGGTTACATCGCCTT
TGACGATCTGCCACACTCGCTCCACCTCACAG
TTCACATGGCAGACACGAGTGGATCCATCCTCCTCCCACGAGTACGGCCATCCCTTGTCCGAGGTGAG
CACCATCAGCCTGGGACGTGCTGCACCGGATT
GCTGCACACCGTCATCCTTTTCGAGAGTTTTGCCAACTGCCTTGTACGTCCATGACTGCGGTGGTTCC
CCCTCCTTCACCTCCATTCCCGTCCCTTTATC
ACCAGGGACTTCATTGACATGGCTCCACCTCGCATTCTACACAGACTCGTAGAATCCCCTGCATGAAT
TCCTTTCCACTCAACTGTAAATAGCCCACATT
AATCGCCGCACCCACTCCACTCTTCTGCGAGTCGCAGCAGGCTGATCCACGTCAGTTTGGGGCTCTGG
CCTCGCCCTCCTCTGACGATCTCCCTGCGGTA
CAGCAGACGCGAGACTCTTGGCATTGTCATTGTCTACACTCTTGCGCTTTCCAGACACTGTTTCTCCT
TGTTTTCCTAGTTGGATTCAGTTAAAGCTAAA

ACTAACAAGCAATCTAAAGGGGAATAATAATAAAAAATACGCACAAAAACGACTAGCAGACACAACAG
GAGAGTCATCTACACAGTGTGACGGCACAGTG
TGCGATACAAAACAAATGCACGAGGACCACCGCAACGACGGCGAGTGGGTGCCATTCACCCAGCACAG
TGTTGGGAAAAGCTAACAACAACAACAAAGTG
GTGCGCACGGAGGGTTCGCTCACGCATTCCGCTCGCTACGGTCTCACATCCGTGTGGCACCGCCGTCA
CCGCACACGCTCTGCAATAATACATGCGCGCA
TATCGTCCCTGACACGTACAAACCCACAAGTGGGAAGAGAATTCGATAAAGCAGCGGCCACaCGCAGG
GAACACAAATCATACACGCCACAACAGCGATG
CATCACGGCAGCCTCCCAGGCAAAATAAAAGAAAAAGGCAAAAGGCAAATTACGTGCTTCATGGAATA
TTCCCGCAATTCGCACAGCACAAAAGAATCAT
AACGCTCCCTTTCTCATTGCATTCGCCATTTATATTCCCAATGCGTAGCACCACAGATATTGCCCCTC
CATGGTGAAAACGAATGCCTTGTTGTGGTGTC
ATCGCGCAGCCATCCGCCTGGTTAAAAACAAAAGGAAAATCAGGCGTCCGTCCGCCTGAAGGAAGAGA
GACTCAGCCGCAGTGTCCTCCACGACACAAAC
ACTCCGCGGCCAGTGCCCGGTGTCGTGTGCGGACTCCGCGAGGGGTTTGTGGGCACGCCACTGTGCCA
CATGGTGTGACCCCCTCGAAACGCAACAGCAG
ACGTGTTCCCTTCCGGTCGCACTCGTGTCAGTGGCGGCTTCCGCTGCCTCGCTGCGTGCTGGTGGCGT
CGACTAAGATGCTGCAGTGTGATATAGAATGC
CCGGCACGAGCGGTGCTGCGTTGGGCGGGCAATGCCTTCCGCGGCGAGACACCCAGCGCGACTGCTGA
CCGCTGCACTCACGCAATTCCCTCCACTCCTC
CCCCTCCATTCGGCAATCACACAGCCGCCGGCATATTGACCCCGAAACAACACACACGTGAAAGCGTC
GCAGCATATCTCNNNNNNNNNNNNNNNNNNNN
NNNNNNNNNNNNNNNNNNNNNNNNNNNNNNNNNNNNNNNNNNNNNNNNNNNNNNNNNNNNNNNNNNNN
NNNNNNNNNNNNNNNNNNNNNNNNNNNNNNNN
NNNNNNNNNNNNNNNNNNNNNNNNNNNNNNNNNNNNNNNNNNNNNNNNNNNNNNNNNNNNNNNNNNNN
NNNNNNNNNNNNNNNNNNNNNNNNNNNNNNNN
NNNNNNNNNNNNNNNNNNNNNNNNNNNNNNNNNNNNNNNNNNNNNNNNNNNNNNNNNNNNNNNNNNNN
NNNNNNNNNNNNCTGCGGCCATCGTGCCGACA
AGTCCGACGCGGCCGCACCCACACCCACAGCAGCAGACACAAAAAAAGATCTTTCACCCCGTGCCACG
TCCTCGTGCGTGATGGTGCGGCATTTCAGAGC
AATACCGCCACACGCCGTGCAGCATTCCCACAGGGGATGAGAGCCGCGGCGCAATCTCACCGCCTCCC
CCTTCCTTCTCCTCATGTCCACGGAGTGACGC
AGCTGCCAGCACCGACTGCAGGAGGAGCATCCAACTGCACCGCAACATGTACGCCGTGCGTTTGTGTG
TGCCTGCAGTCACGCAAAGAATAAAAAAAGAG
AAGAAAAAAAGAGAGCCACGCAAGTGAAGGGGCACGCACAAACACGCAGCAGAGGTGCAGGACGGAAA
AAAAGGGAGGATGAAAGCCGCTGAGGCTCGAG
ATGGCACGGCAGCCCAACCAGCGGCAACCCACAATAATTTTCCTAAAATAAAAATAAGTTAGAAATTT
TCTAAAGCCCCTTTAAAAAAACTTGCACACCC
ATTAAAGAACTAAAAAAAAAAAAAAAATAATCCCATCGTGACGCGGCAAAAAAAGAAAAAGAAAAAAC
TGCTTTTACATTATTCATGCAGAAGTAAAAAC
ACATTTGCTTCGACCGCTCAGCCCTATGGTGTCTACACAACACCCAAATTTCATTGGGTCGCTCAGTG
CGTTTTGTTGTTTGTTTGCATCCCAACTCCTTT
TTTTTTTTTTTTTTTCACTCCGCCATCGTCGTGGCGCCCAATGCCGAGTGCATTTATCATCGGCAGGC
GGAAACATTAAATACAATCCGCCATTATCAGA
AAACTACACCATATAGGAGAAGACAAATATACGTATATATAATGAAAGAGAAAAACAAAAACATATAA
CCAGCGGTGCAGTCCCGGGAGGGGGAGCACGC
CCTTACATAACGACTTTCATGGGGAGAGGGGTATTCAAACCAAAAATAAAAATACATAAAATAAACGG
TGCATCCACAAAAAAGTTGGCAATTACTTCGG
AGGGTGCGTGGAGAGGCACAGATGCTCACGTCCGTggcaggaactccccactgcaatt
>scaffold1074.size6753
AATCAACACACGTCGCTCGGGCCTAAAGTCAGTTCCACCGTGAGGGCTGAACCACGCGGTTACATCGC
CCTTGACGATCTGTCACACTCAATCCACCTCGC
AGTTCACAAAAACGTCCTCAGGAAGATCCTGTATCATATTCCACGAGTACGGCCATCCCTTCTCCGAG
AAGAGCACCATCAACACGGGAGGTGCTGCACC

GAATTGCTGCACAGCGTCATTCTTTTCGATAGCATCGTCAGCTTCCCTGTACGACCATGACTGTTCTG
GCCTCCCCTCATGCACCCCCATTCCCGTCCCC
GTCTTCTTTCGCTCCACGCCGTCCGGAAGTTCCACCGCATGACTCCGCCTCGCATTGTGCACAGACTC
GTAGCATCCTTCCAGTTTCTTCGGTGCGCTCG
TCGTCGTCGGGTTCCTCGCCTCCTCGGCAGCTGCATCCAAGAAATGTTTATTGGGGTTATGAACAGTT
GCTGTTACCTCCGCAGCAGCTAATGACCGCCC
CGGTAGATTGTTGACGCCCCTATCGCATAGCCTGCGTATATCCTCGTCGAAGATCACTTCACCCTTCA
CAGCTCGCTTCATACTCAAATAATGACCTGAT
GCCTGCATTTTTTTTTTCTTTCTACTCTTACCAGCGCGTCTAGCTATACAGTGCCATTTGAGTGCCGA
AGAGGTGGTCGAGCAACGACAAAAGTTTCCTG
CTATGAAGCAACTGCCATTGAGAGAACTTTCCGAGGCGAAGGTCCTGTTCAATGCCCAGCCGTTGGCG
CACAGCCAAGGTGCCTGGACATTCTAGAAGCA
GATGGTGCAAGGTTTCCTGCGGGCCAATTGGCGTGCTTGTGGAAGCTGGCTGATCCTGCGGCAGCAGA
GATCGCCTCCTGGCACTGCTGTTTCGATGCCG
TGTGGCGTCTGGGTTGTGTGCGCAGCGACTTCTGTGCTGTGCGGTGCTTCTCCGTGTGGGGAAAACCA
TGTCACAGAAATCACATTTGAGCTCGGGAAGC
GGTGGGGGATGTTCCGGATGAAATGACCGACAGTGACTGACGAGACCCGAGAATCCCGTCAACGTGGA
ATCACAGTGCGGACATTTCTCCTTCCGACGGC
GTGTGACGGTTTGTCCCCTTACTGGAGAGACGTCTTCCTCCCTCGCCGACGGGGGAACAGTGTCCGTC
CCCGGTGCAGTTGGATCGTTGTTAGAGCGCAT
ATATGCAGACTGTTCCGGTGAGATGTTGCAGAAACGGCATTGGTTCGTGCAATCTCTCATGGCCCAGT
ATCGTCGGCCCCAGAGCTCAGATTCCCCGCAC
CTTGCCCTTGCGAGGTGAACCAGTTCCTGACGGTGTAGAACTTCCTGAGTGATCAGGTCCTTACCGCT
GAGATCTGATGGCCTTGTGCCGCAAAGCAAGT
AGCGATGAGTGTCTTGACGAATGGTACTACGCCACTTGTTGGTGAGCTGGGTAGTAAAACATGTCGGC
AGATCCGTATGCCATAAAGGTGCGATTCCTTG
CTCCGTGTATTGTCCACTTTCCATAGTCTGCGTCGCATACTGATCTGCAGGCTCGTTAGCATGTACTC
CGCAATGTCCGTAACAAAACTGCAGGTGCACG
GACCAGCCAGCACACGTCAGTGTTAATAGACGCTGCCAGATCTGATCCTCCGTCCGGTCTGTCTGACT
GAGCGGGCCCTTGTTTAGAGCCGCTAGAAGAG
ACTGACTGTCCGTAACCACAAGCAGGGTTTTACGCCTGTGGCGGATACGAGGGATCATCAGCTTCTCT
AGGGCTAGAAGCAGCGCACGGGATTCCGTCCT
GTAGCTGTATGGGCGTGTGCCGCAGTTTATACTGGCCTTTTCTATTATCTCCGTATGAGTTGAGGGAT
GAAAGCAGTATTCCAACCCCTGCTGACTTGGG
AACGTCGACACCGCCATCGGTCGCAAGTATCCACGAACGATGGCTGTGCAGCGCCAGAGCCTCTTCGC
TGACTCTGCGCTTTTCCCTTATCAGCTCTTCT
TCGTCAGTTATTGAGTGGTCTATCGGAAGTTCCACACCGATGGTGACCCGGTGAGCGGAGGCCGCAAA
CCAGGGAGGAATGGAGCTGCGAAAGAACCGCT
CTTCGCGTGGTGAGCTCTCGGTGATGCCATAGTCCTTTGTGTATGCGTCTACGAAGGCATATAGCTCG
TCCCGAGATAATGGCGAGATGCGGAAACCGGC
GCGAGGCGGTGGCTCAGGCGGGGGTCTCCGCATCCAGTCCATCTGACATGCGCGTGTGTTCTCAAACA
TTGAGAGCCGCGCGCGCAGGCAAAGGACGTGA
AGCGGCGGCATATTAGCCTCCAGTAGTGCGGATTCCGGGTCGACAGTACTCGGAACTCCGCTGACAAT
GCGTGCTAGAGTTGCGTACGTCTTTTGCATCT
CATTCTTCGCTGAATCCGTCGCTACTGCCCATATGAGCTCGCTGCCATAGCGTAATTTGGAAGCACCA
TATCCAATGACGAACGTGCGTAGGTCATGTGG
AGATGGTCCCGCCTCTTTGTGCGCTATGCAGCGTAGCTGCGATATCCGTTGCGAAGTGGCAGCGCATT
GCTTGGTGGCCGCCACATTTAGCGTCAGTCGT
GGATCGAGATCCATGCCGAGAAGCTTCGTAGTTCGCGATGCCCCCATGGTCATGACTGGGATTTGACA
TCCAGCCACTACCAGGGGCCACCTTCCTTCAC
GATCATAATCTGACTCCGTGTGGGGGGAGAGTGTGAACCAAGCGGCTTCACACTTTGACGGGTTGATG
GCCAGGCCATTTTCCTGACTCCACGCATGCAG
CTTTTTAAGAACCATGTTGGCAGTGGGAATGGCGTCTTCCCGGGACTGCCCCCTAACGATAATAGAGA
GGTCGTCCGCATACATGTCTGCTGTGACACCC

GCAAAGCTGTTCAGCAGATTGAGAAGTGGGTGTACGTAAAGGGAGAACAAATACGGTCCCAGCACGGA
CCCCTGAGGAACTCCTCGCAGCATGGGACGCC
GATCGCTGTGTCTGTGGCCAACTCGTGTCTTCGCGTAGCGACCACATAGAAAGTTTCTAAGCCACCTT
CTACAATTCACCCCCGGAATATTAGCCAGCAA
GCGACTGAGGACTCGATGATCTATGGAGTCAAAGGCCTTGCTGAAATCAACCAGCACCAGCAGGGTCC
GATGGGGTCTGTAGTGGAGTTGATTGCCAATA
CCGCTCTTCTTGGGAAGGCTCACTTGGAAATAGTGATTTCTATTATGCTCCACTTCGTGTATCAGGTG
TGCCAGCTGCATCGTCGTAGTACGCATACTAC
GATATGCATACTGGTGGGGGTGTGGCGTCCACACCCAAAGCAATCTCTTCAGTACCATTTTCTCAGCA
ACCTTACTCACAATGGAAGTAAGGCTGATGGG
TCGATATGACTCAAGCATTTCCGGCGACTTACCGGGTTTTAAGAGTGGAACGATGGTAGCGCGTCTCC
ACTCAACCGGAATCGTACCGGTCGTCCAACTG
TGGTTAATGCAACGAAGAAGGAATCGGAGGCCCTTTAACGAAATATGTCGCAGTGCCTCACTGCAGAC
TTCATCCGGTCCGGGGGCCTTGCCATACGGCG
TATTACGTAGCGCAGCAAGCAGTTCCGTACGATTAAAAGGTGCATCAAGAGGGGAGTGAAGATGAGCA
ACTAAACCAGATGTGGTGGCAGCTTCGTTTTT
ATCCTGTGGCTCAGAACTAGATGAGAGTGAAGAACCTGAAGTAAATGTAGCGGCAGACTCGTTGTTGT
TCTGCGGCTCGGAGCTAGAAGAAGTAGAAGAA
AATGAAGTAAATGAGGCAGCAGACTCGCTGTTGTTCTGCTGCCCAAAAAGAAAAGAAGCATTAATGTT
CAAGGATGCACTCTGTCTGTTGATGCCTTTGG
AAAACCTAACTGCCTTGTGAGAGGGGTGAAATGGAAGAAAGATGCGATTGAGAGCATTCGCTGCTCTC
CTTGCTGTTTTATAAGTGTGTTGACCATCTCC
TAATAACATTGATGTGGGAGATGGAAGTGATGCCTTTTTTCGTCCGGAAATGTATTTCCATGCGAGTG
GCTCGCCTGGATTTAACTTGCTGATGCGGCGT
AGATACGTGTTGCGCAAGCGCTCTCGGAGCAGGAGGAAGAATTGACTCCTTGTCCGTTGCATTTCTGC
CTCACGGTCGGGCGTCGGTAACTGTAGATAGC
GGGCCTTGCTGTCCGTGGCAATCCGCTCTGCCTCCATGAGAGTGTCGTCCCAAAGCCTGGGACCGTCC
TTGTGCATGCCGCGGGGGCAATGTCGTCGATA
CGAAGTTATAAGCGCTCTCGTGAAAGCCTTGATGCCGGTGGACTGCTTTTTATAGTCATATGCCGGAA
GTTGTGCGTCGAAGTCCTTGATGGATAAATCC
CACTTGCACTTCCCCCAACTGTAGAAAAACTTAGGTGCCGAAGGGGGTGCCGAAGGTATGGTATCCTT
AAATGCCTGATGCAGCGTAAATGTCAGCATAT
AATGATCACTAAGCGGCGAGACGGAGGCTGACCAATCCGACACAGTCAAATGCTTTGACCATGTCAGA
TCGATACAGGATCGTTCTCTTGTGCCACGGGC
ATACGTGATCTCACCAGGGGTGTTAACCAAGGTGAGTCCCGCATCCGTGCACAGTTCTAAAAATTCTG
CAGCAACCTCGCTTGGAGAAGACTCCAAGAAT
GGCTCCCACTGTGGGTGATGCATGTTGAAATCCCCACACAGCAGGAGCGGCGACGAGGCTGGAAGGCA
GTTTACTAACCGCCTGAAGGATTGCGTTACTT
GCGGTGGTGGTCTCACATAGGCACTCGCTACAATAAGATCACGGTTCTGGTCCAGAGCAACCTGGACC
ACCACCACTTCAAGGCTGGTGTCATGCTGGGG
GATGGTGAGAGGTATACGCTTGGAGCGGAGAGTTTTCCGCACCAGCACCGCCACACCGCCTCCCTTGC
CGATGCGCGACTGTGCATAGAGCACATAATCT
CCAGTGTGCATGCTGGCGATCTGCTCTGACGACTTCCAAGTCTCCTGGATGGTATTATGTCTGCTCCT
TGCGCCATTAATGTTGCGAGTTTTGACGGCGT
GAGGCAAGAAACGTTCATCTGGAGTACTGCTATGGGGCCTGGGTGCTGCTCAATGTCTCCGCTTAGCA
GTAGCTTTTGTCGTCGCGCGTCACATACGGCA
CGCAAACGGTATGTATAGCGCTGATAAGCGCCGATGGAATTCAGCAGCGGATAGAAATACCGCTCCGC
GGGCAGCCATATAAATGGCTCCATCTGGTGCC
CTTCCGACCCGTTTGTGCGGCGCCTCCTGCCTCTCCGCGGGCAGCGGATGGGCAGCAGGCGCTTAGCT
TCCTCTCTAGCAAAGATTACTAGACCCTGGCA
CCACGTTGAGGTGGCCGGCTGAGCCAGGGATGCCTGCATTTCGTTCAATGCTCCCTTATCACGGATAT
ACTTCGTGGGGTCCCTGAAAAACTCCTCCAGC
AAGACATCCCGATTGGTGTCCACAATGCCCCTGCCGTCCAGTTCCATCGTAAGAAAGTCGCTCAGCCT
CATTTCATTGACGCGAGCTCTTCCTTTAAACA

GTACGTCTTCGATATTAGTGCAAATGGTAAATTTCATTTCTTGCGTTCGTCTTAGCCTTCCTCTGCCT
CCCGCCTCGCTTCTGTCAGTACCTGCGTGAGT
ACCCCGTTTGGTTTTCCCCTTGCAAGAAGAGTGATCGTATCCTTTTATTTCATAGTCCCCACGTTGCC
CGAGAGAGAGCACACCTTCGTGAAGAGGCCTA
GTAATGGCCTCCAGCGCCTGGCACGCAGTTAAGTTAGGAATTTCTTCAAGAAGCCGCTGGTCTTGTAC
GTAAGCATCCGGCTCCTGAACAAACGCCTCCA
TCGTCACATTGTAGTCTTTCATCAACGGCCGCCCTGCCGCCAACATAATTCCAAAGGAAATCATTCAG
CCTCATGTTGGTGCTGCTCATGGTCCACTGTG
GCTACACTTTACTCCCCACCACATGTCGAGCTGCAGTGTCGGAACCATCGTCGGAGATGCCACGGCGA
GGTGCCATCACAACACCCGTGCGGTGAAGCAT
CCCACTCGCACCGCCCAACTGCGGCCAGCAGGCGCCGCTGGCTCCAAAGGACACTCGCGGCTGTTTCT
TCCCGCAGTCCCAATGGCGTTGAGCGTGGCAC
TCGCACGGCGGGGCGGTCAGTGCGCCATGCAGCCGCACTGCAACGCCTGTCGGTGAAACAGTCAAGGC
CCACCGCCCCCGCAGCAACACAACGTGCAATC
GGAAGAAACAACGTCACATTATCGCCCTGTATATTTTATGATTCACGTTGTCTGTCAGTTTGCCCCAT
CACCGACACAAAAAATAAGTGCACACCTTCAC
AGAGGGTGAAGTAAAAGAAAGGAAGAAAACACAATCCCACCGTCCCGCCGCGGGTCAACACTCTCCT
CCATCCTCCACCATCACCGCGCGGGGGACCGC
TGTCACATCGCAGAGTCAGCACCACATTGTGCACCACACCATCGTGCAGTGAACAATGGGCGAGACGC
AGCCATGCATCGCCCGCTCCATTTTCCCACCA
CTCAAGCGTTGCGTGCCCGCACAAAGCAGTAAACACAATCAGAGTGCACAAAAACCAATCAACACCCT
CACTCGCTGAAGCACATTATTCGCATGGAGTG
ACAACATTTGGGGACACACCAGCAGTAGCAGAAGGCATGGAATTTccctgcc
>scaffold1075.size6688
CCGGTCAGGCTGATGGCGTTGGCTGTGCTCTTCCCGAGACTGGCGCTTGTCTTGTGTTCCGCACTCCT
CGTGGGTGCTCTGCTCTGCTCCCCACGGACGTG
CCCGATCTGTTGCCGCTGTTGCTGCTGCCTTCTCCACTTTTTGAACACAGGCACGCGCTAGGAAAACA
AGCGCTACTCGCTACTGTGAAATTGAAGTCAC
TGAAAGGACCAACAGCACACACACCGCCCACACACACACATATTTATATGCTTTCACGTGTTGCTGCT
GTGAAGGCACCCCGCACACATAACCGTCGCCG
CGTGACCGGATCCAGCGGAAGGAGGAGGGAAGGAAGAGGAAGTGAGCCGCAGAGGCCCAACATGTCCC
GACATGTGTTTACTTTTGCGGTGCTGCTGCTC
CTCGTTGTGGTGATGATGTGCTGCGGCAGTGGTGGAGCTGCGGCCGCATCGACGGGGGAGAATTAAGT
GATTGCGCGGCTGCCGTGGGTGGCCTTTATTT
TCGTTCCGGGACGAACGCATTTGGAACCGAAGAGTGGGACTGGTGCAAGTGGAAGGAGGGATTCATTT
GGATCGCTCGCTCTTGTGGGTGTTGGTGGAGT
GACGGTTGTCTTTGATGGGGTTACACGACGCTTAATGATAGCGATGGGGCACCGAGTGAGCTTTCTCA
TACGGATATTGTGGTGGGCTCCATCTTTTTTG
CGTGGAGTTTGTCGTCTTTTTTGGTGAGTTTAATAATGGCCGGAATACACACATCGTGTTGAGCACAG
CGAATATAAAGGGTGGTGTAGGCTCCGCGTCC
AATCCCACGACAATTGAGAGCGGCATAGGGCGTTTCTTCGTGCGGGAAGCTAGCAATGTTTGTTACGA
ATGGTGGGTTCAATTGGGCGTGAGAGACCGTG
ATGTCAACTGGTTGTGGGTGAGTCACGCAGCCCAATGACGGCAGACGGAGCGAGCTGATCCACTGGTG
TGAACCCAAATTGCTGTGATCACAGATCATTC
CCGAGACTCACGAACGCGGCGCGAATGGATTGAGGGTTTTTTGTGGCTCAGGTGCTTTGAATGGCACG
CTTGTGTCCCCGTGATTGCGTACAATGACGAA
AAAGACGATTTCTCCATGATCATCTGTCCGACGGACGGCGGCAGAAGCTGGGTGCTTTCAAGGGGCAT
TCTTCCTGCGGACTGCATTGACCTTCTCATTG
CCGAATGGGAGCAGGGACAACTTCTCGTGATTACTCATTGCTTTGGGAGCCGGTACTGCGGAGTGCAC
GAGTCACGTGACGAGGGGACAATGTGGGTGGA
AGTACCCGGGCAATTGCAGGCGTTTGGGCCAATTCACAATCGGTTCGTTGGGACATGAGCTTGCGTGT
GGGAGCCATTGTCACTGCGACCGTTGGAGGAA
AGAGCGTCATGCTGTAGGCTTGGGAAGTGAGCTCCCCGTGGTGGGAGAGTGAGGCCCGTGCGCTCTAC
GTTTGGGTGACTGACAGCAACCGCGTGTTTTG

TGTTGGACCGGCTTTCATGGACACTGTTCTGAATAAGCCGATTGCCAACGCCCTGCTGTACTCGGATG
GTGCGTTACACCTTGTGGGAGCGAGGCACAAT
GAAGAGAAAAGCAGCCTGTTTCCTGCCCCCCTGACGGAGGAGCTGCAGATGATCACTTCCATCCTCCG
TAATTGGGCGGACGTGGACGTTTCCTTCTTCG
CGTCGTCCATAAGTACGGTGGGTCTGGTTGGATATCTTTCCAACGCAGGCACTGCCAACACGTGGATC
GACGAGTGCCGTTGCGTGATTGCAACGGTGAC
GAATGAAAAGAATGTCACAAATAGGCTTTAATTCGCAGGGCAACACTGCCCCGTGAACCGTTGGGAGG
ATAATAATCACTGTAGCTTTGTGAATTAAAAT
TTCGCTCTTGTGGCGACGGTGGCTATTTGCGAGACACACGCAGGCGTCAAGAGTATCCCTTTGATTGG
CGCGAAGGGAGCAGGCCGCACTGTGCACTTTT
GGCTGTCGCACAGCCATGAAAGGAGTTGGGGATGTTCAACGCAAACAAACACACCGGCTTTTGGGAGT
TGAACAAACAATAACGAGTGGTGCTCACGCTG
CAGGACCGCCAACAAGGCCTTCGTATAGATCAATGGTGTGTGTGCGTGGGATGCTGAAACGACATCAT
CATCGGGAAAGAGGCCACGCGCGAATTCACAC
TTTTACATTGGAGGGGATGGGAGCAGTGCAAGCGACACCGGGAGCCAAAACAATTATTTGACAGTGAC
GAGTGTCTTTCTGTACAACCGCATATTGACCA
GGGAAGAAGCCGCAAAAGTTGGTGCCAACAACCTTGCTCTTCCAGAGTCTCTTTCTGGGAAAATGCTT
GAAGGGCCGGATGATCCAGCGGGAACTGGCAG
TGGCTCCAAAGATGGAAACGCCTCTCAGCTCACGAACAACACTTTTTTGGAGAAGGGCTCTAGTGGCT
ACGGCACGGTGCGTGTGTGTGTCTCGGATCTT
GTTGCTTCTCCTTCTGGGGCTCTGGGCATTGCAGCTCTCAGCTGTGTGAAACGGTGGGAGTGGAGCCG
CGACTCCCCGTGGAAAAGGGTGTGCAGCTCCT
CAGGAAGACTCAATGCATTCCGTTGAGGACTTTTTATTTATCCCTTTTGCTTTCACCTCTGGATATAA
TGTGCTGCTGGTGCCTTGTTGGAGGGGTCAGA
ACCCCTGCCTTTTTACTATTTTTTTAATTTATTTTTTGTTTGCCGCTAGCTGCACACTCTCATGAGTG
ACGTGCTTCCCCCTGTTTTATTTGCTTTAGTT
TCACCGCACGCGGTGCCGGCCTCGTTGCTGTGCAGTCCCGCGGACTGTACAAAACGAGGGGTGGAGGA
AGAGGCCGAGTGCCTCATGAAATGTGAGGGAT
TTCGTGTGGACACGTGGAACACTGCGATCTTTTTTCTCCTCTGTAAGAAACATATGGAGATGCACAAT
TCCTGCTGCACAGAGGAATACAAGAAATGAAA
GTCCACTGAGGCGGTTTCTCTTTTTTTTATTTATTATTATTATTTGTTTATTTATTTCTGCTGAACCT
ACGCCTTTTTTCTTGTTTTTCTGTATTTGGCA
ATTCGCTGCGGCTTGTTTTATTGTTTGCTGTGCTGAATGCCACTATGTGTCTTTTTGTCCCATTGGGT
GTCCCACTGCTAGTCGTGCAGTTGACACGCTC
TCATCAATTTTTCTTTTCTACTAGAAGCATCGATAGTCCGCGACGACGATCGTTTGTGCTCCCCATTG
CGATGGTGTGAGGGTGACTGGGCGCCCTGTTG
AGTGCACTCGGAGGGGAGCGGATGGTGTGCGCCGTGCCTCACTGTGAGTGTGGGGCGCCACCACCACG
ACCTGCTGTGAGGCGTAACGCTATTCCTTCGG
GGCGGAGAGAAGTGAAGGACTGTGGTGAACTTGGATGCGCCTCGTTCCTCTTGTTGCGGCTGTGGGTG
TGCTGTTCGCATTGCTGTTTGCGGTACGATGT
GGATGGCGGTGCTTCTGCGTGTGCCTTCTTGAATGCACTGTGCGGCGGGCTTCTCTTCTCCATGTCCT
TGCCTTTGCCCTTTTGTGTGGGGGTGAGTGTG
GGTGGCTGTCGACCTGCCCCGAGCATTGGGGTCCTCCACACACGTGAGGCTCATCTTGGCAGCCTCTC
GCAGCGTCCATGCCTCGTGACAGGGAGCGAGG
GAAGAGTGGCGTGTTTCCTCCTACTATAGACCCATCAATTCCGAGCAGTTGGGGTGAAATTAAAGGGA
ACAAGCGTGTGTGTCTTCACCTTCTTGCGGGA
GGGCGTGCAGAGGTTGGTTTTCTTTTGTTAATCTGTTCGGTCACTGTGTCTGTGTTGACGTTAATGGG
GGGTTTGTTCTCCAGAGGCATTCATTGTGCGA
TTTTTGCGTGTTTGGTGTCCAACGTTTTCATCACCACTGAATGGTGTGTTGCGCAGGATCGGCATACA
TGGATGTGCGAGAGCATTGTGTCGCTCGCTCG
TAATAACACACACACGCACAGTTTCAGCGCGTCTGATACGTCACGTTCCTCGGAACGGTCGAATTCAC
TTGTGGGACGTCACTTCATTTGCGGCGTCGGG
AAAGTGAAGTAAACGGAGAGATATGCCGAGAACGCAATGCCACTCCATGATGCCGCAGGCTGGCGGCG
ACAAGGATCAGAGAGCCTCTACCCAACATTGA

GAAGAGGGCAGCACTCTGATGGTCCGATTTTTGCTCTTTAGGAAATTGTAATGTAGTGTACACCGTGT
GGGCCTTTTTGCTGCGGATATGCAGAGGGGTG
ATGTGGGGTGGAATTATTGTTGAAGGCAGCACGGACACCGCCTGACCTTTATTGGAGGGATGCGTGCT
GTTATTCCGTGCTTCCTGGCCTGCGTTGTCGC
ACTCATGCTGTGTGTGCGGATGGCCACAGTAGTAGAGCTGAATACGGTTCAATGTATGTTGTTCACGG
GAACTGCGCGACACATGTATTTTTTGTTGGGC
TTCCCAAAAGGAGGGGCGCATCGCCATTGCGTGATTCCCGCGGCAGTCCATTACCCTGCGACTGTGCG
TGCCGCTGTCCGCTGCGCCAATGCAAGCCTTC
GCCTTTGTGAGAAATGGGGAGCGTGACTGCTGTGGCGGTGGTCGAATGCTTCCGAATTGGTGCTTTTT
TTTGGTATTTTTATTTTTGGTGATATGCGGGG
CAGCGGTGTGTTGGCGTGTGCGGGCTGCTCATGGCTGCTGGTGTTGTCTTTCCTGCTGTTCTCCCGCT
CCAGTCCTTTGCATGCGACGGACCAAACGTTT
TTGCGGGCGTGTGGTGGGGCCGCGTTCTTGTTTGGAAAAAAGCCTTTCGCAGGCATAATGATACTGCT
GCCGCCCGTGCCACACGAGGCAGTGGGGAGTT
TCCTGGGATTTATTGGCGGGGTGGCACCGCACCCATGCCGTGTGGTAGGTTTGTATTCCGCAATGCAC
GGTGAGGGGGATGTGCGGCGACAACTTCCTCC
AGTGGAGCGACGCGAGCAGCACGGGGGCTGAACGGAGGAGTGGTGGCTGCGTGCTTCCCAGTGATACG
TGAATGGCGAATGCAATATGAAAGGCAGCGTC
ACGGCACTCTTTTTTGACACGCGCATTGCAGATATTTTCCATAAAGCATGCAAATTGCACTCTTTTAT
TTGTGGGATTACCGTGATGCATCGCTGTTGTG
GCGTGTATGATTTGTGCTTCTGTCAGGGTTTATGTGCCCCCGTCCTCTTGCAGAGTGTGTGCGGTGTC
GGCGGTGCCACACGGGTGCGGGACCGTAGCGA
GCGGGATGCGTGGGCGAACCCTCCGTGCACACCACGTTGTTATTATTGTTTGCTCTCACCAACACTGT
GCTGGGTGGTTGGCACCCGCGCGCCGTCGTTG
CGGCGGTCCTCGTGCATGTGGTGTGCGTTGCACACTGTGTGGATGACTCACTTGCTGTGGCTGCCCGT
GATGTCTGTGCCTAGTTACCCCATCTCTCTCT
TATTCTCCTCCTGTTCGGTGGTTAGTGTTCAGCTTTAACTCAATCCAACTAAAAGAACAGAAGGAAGC
AATGCCTGGAAATCAAGCGTCTGCTGTGCCGC
AGGGAGATATTCGGAGGAGAGCGAGGCCGGAGTCTGAGGGTGACACAGATCAACCCGCTGCGACTCGC
GGAAGAGTGGAGGAGGTGCGGCGATTAATGTG
GGCTCTTGACAGTCGGGTGGAAGGATACACGCTGTGTGAAGACTACAAGTCCGTGTACAATGCGAGGT
GGCATCATGTCATGGAAGTTCCTGGCGGCGAG
GGGACGGGAATGGAGGTGCATGAGGGGGAACCACCGCGGTCATGGACGTACAAGGCAGTTGGCCGAAC
CCTCGAAAAGGATGACGGTGTGGAGCCATCCG
GTGCACCACGTCTCAGGCTGATGGTGCTTACCTTGGGCAAGACATGGCTGTTCTTGTGCAGGGATGCA
ATTACCGACTGCTATGTGAACTGTGAGGTGGA
TCGAGTGTGGCAGATCGTCAGCGGCGATCTCACCGAGTGGTTCAGCAGTCGTCCTGAGGACCACTTTA
CGCCTCATCCGCGCTTGTTGACTGGGACGCCC
GGTATCGGGAAGTCGATGGCTGCCGGTTCGTACCTCCTCTACCAACTGCTGCGCTACGATGCGGAGCT
ACTCCCTGTGGTTGTGTTCGTTGTTTGTGGAC
GTGAGGCATACGTGTTTAACAAGACCACCGAGACGGTGACAAGTTACATGGATAAGTCCATGATGATA
AAAGCTGTGAAGGAACTGTCTCGGCGCGGGAT
GAAGGGGTATATCATCTACGACGTGGGAGGCAGGTTAGATATTCCGTCTCCTTTTTTGCCTCCCGGAG
GCTGGGGCATGCTTGTGGTGGCAACGCCAGAG
AAAACGAGTTACATACACTGGGCGAACGAAAAGAGAGCCGTACGAATCATAATGAATTGCCCCGATGA
GAGCGACGTGAAGGCAATGTGCGTTTGGATGA
AGCGCAATCAGCCTCCGCCGCAGCAAGCGGAATACTGGCGAGAGGTGAAGGGTCGCATGGACAAACTG
GGACCTATTCTTCGCTACATCTTTGATGAGGG
ACGTTATAAATATTGGATTGCAAAGTGCCACAACCTTGTGGATGGAACGACCTCTTGGGGAATCCAGC
GTTACTTTGTTTTCGGAACTT
>scaffold1076.size6512
AAAAAAATACTTTCTTTTTTTTTTTGGGTTGTTATTGTTATTTCTTTGCCTGTATTTCTTAGGCCCTC
GCACATCCAAACCTTCCAAACTTGACACAAGTC
GTTGTCAGCCCTCCTCTTCCTAAGTTTCATTAAGTCCCTCCATTCAAACTGTGGACATTGTTGTGCCC

AGAGACAGACTGCATACATACATGCGCGTGGA
GGGAAAGACTCCCCCTTAAGTACCCATCACCCACCACCACCTACCCGCTGCCTCCCCCGTTATCTCCT
TCTTGAGCAGCTGAACAAGGGGATTAAACTTT
AAAGTAATACAATGACGTTGCTTCCTTCCTCCCTCCCTCCATCCCTCTCTCGTCATTTGCCAGCAGCA
GCCCCGATTCACCAAAACGCCTCACGTCAGTT
CTAGACTCCTTGGCTGGTATAAGCACAGAAATTGAGAAAAGAGAAGAAGTCAGCCAGCCAGGGACGGC
GCGTGTCACATGAACGGCCGCACAGACCTGCC
CTCCTCAACTCCATCGCAGAGCAGACATTTTTCCCTTCTCCACCCAACCGCTATTCTTGTCTTTTTTT
CTGAGTTGTTATTTTGTTTTCCCTTTTTGTTG
TTTTTGTTTTTACTTTCGCTGCTCGTCTGCCCCGTCAGACGCACAATTCCCCAAATCTCCTCTTCTCT
TTTTTTAGCTCTTGTTTTATTTTGTTTTTTTG
TTTTTTTGTTTTGCTTTTTTCCGTTTTTCCTCCGTTTGCCCCATCCCCTTCAGTCCCTTCTTTGAAGG
GTCCCTTCGGGCTGCGTGGCTACTTTGTTCGT
TCGCCATGCTTCCGCAGCGTTGTCTCGATGAGTTCCTTCACCTTGGCCTGGTCCAGCTGGACGCCTTT
CGCCTCCAGCTCTTCCAGCGTACTCACAAGGT
GCGTGACGTTGCCCGCCTCGGAAATTGCGTCTAAAAGACGAAGAAGACCCACCACCTCTGTCTCCGCG
TTGCCGCCGCGTGGCACATCCGAGTCCGTAGG
GATCCCATGAGGGCTGACGCCATAAATGACTTTTCGAAGTGTATCGTACCGCTCAATAATGTTTTCTA
CGCGACTGCGGTCAAGATTTGTGTTGCGCACG
AATGGACGAATTTGCGCATATTGCCGGAAGAAGTGGCGAAGGTCGACGACAAAGTCCTTGATGACTGG
AAAGTTCGGTAACGGCCCCACTGTTGTCACCT
GCTGAGAGAATGTGATACAGGCAAGACTGTTAATGCCATTAATGTTCATGGCGCAACTGCCACAGACA
CCTTCGCAGCAGGAAGCACGAAAAGCTAGCGT
GGGGTCCTGATGCGCCTTTACTGCCGTGATAAGGTCAAGAACCATGTATTCATGCTGCTTGTCGTACT
CATAACTTTCTATTCGTTGCCTGTTTGTTGTG
GGGTCGAAGCGAATCAACCGTAGAATGGCCTTCTTTGACGTGGCTTCCGGCGCCGTTGAGGTGGTGCG
GCGCACGGATATTTTGTACGGAGTGGTAATCT
TACGCAGCATTCGGTATTTATGACCCTGGCCAAGTCTGCTTCTTTTTGTTGCCACGATTGCTGATGTG
AAAGATGATGTTCCTTGAGTGGGTGTCTGTGA
GCGTATGTGAACTCTCGCGTGGGATCCCTTCCCATCGCAGAAATAAGAGAGAAAAAGAAGAGCACACA
TGTTCATATTGCACCAACACCACCAACAACAA
CAAAACTGAATAAAGAAAACAAACATGTCAGACGTCGTTGGATAAAAGACATGTGTTTGCCCGAATTT
TGGGCGAATTCGCCGCTCCAAACCCACAGCAT
CCCCACCCCTTTCAACCCTCTGGCACAAAAAGCGACCAGTCTCGCACCTCAAAAGAAATCAACATACG
CCGCAACACCGCCAGCACCTCCTCCTTCCTCC
GAAGCTGTCGCACCACTCGAGCCTCGCTGCTCTCCGGATGGATCCACTGAGGCGCAGAGATGGAGAGC
AGCACATCGGAAGAGATGGACGGCAAGAAGCG
CAGCACTCCGATTGCCACAAAAACCTCGTTCTCTTTGCCCCGCTCATTTTTGTACTTGGAGATACGCT
GCGTTCCAGAAATAAGACAGCCGTATGCACAC
TCCTGTCCGCTCCCTGGCGCCGCTCCTGGTGGCAGCTGCGCCACCTTTGGGTAATCCGAGGGTGCGAG
TGGCTCCTGTGCGTTGAGTGTGTAGTCCCCGT
CACGGCACTCATTCGCATGTGCGAGGTCCTTAAAGAAAAATACCCCGCACTCCCTGTCACTGACTTCA
GACTGACGGGCAAGAAGCTCAACGATGATGCA
CATACCCGTCTCTGCCTCGGTGTACACCTCCTGTGTGTCGGGCACCTGCCTGAATTCACTCACGTCAA
CGGTCGTGACGGGAAGCAGACAGCGAATGGCA
CCGCCAAACAGTTCCACCACCCTCCCTTCAGTCATATTTGGCGCTACAACTACCAGGAATAAAATAAC
AATGAAATAAAATAAAATGGGCCAACAGAACA
AATAAAATCATCCCACAAAGTGCTGGAAAAAAACAGAACAGTCATCATATACAAGTAACACAACAAAG
AACATGACAATAAACAGGGGGCAACAGACCCA
ATCCAAATACCAGCACCCCTGTTTAAACAGCGAGTATCACTGTTGCTTCATAAATATACGCCGACGCG
ATATCTTCAAGAGACGTGAGATGTCTACGAGA
TGCCGCTGCAACACCACCGACGCGTAGCACAAAAAAAGAAGAATTGCCACAATTTGAATATCAAAAAA
CGTAATGGCGTCACCAGCAATGGGGCGTATTC
TCAGTGCCATTTGGGAAAGGAATGGGGGAACCACGTTGAGCTTTGCTGTTTCGTGTATATACTCAACA

ATTTTCTGCGCACATACCGAGAATCCGAGCGC
TATCACCGCAGCGACTGGAAACAACGCCACGGAGACGCTCGCGTGCAAATGCCATCGTTGACTCCATG
ATGCCACAATCAACACCAATGATACGGATACG
CCACACAAGAAAATAAACGTGCCAGCTGCCAGCATCTCCTGCGAATAATTGACGTTCTCTCTTGCTAC
TTTTCCTTTTGATTATGATTTACTATTATTAT
TATTGGTTGCCCCCACCTGCCCCCAGACGCCTCTCTCATTCACGGTCAAATCGCATAATGCACAGAAA
AGCCGAAAAGAAGGAAAAAAGAAAAAAGTTTT
AATTGAGCGCATCGACTGCAAGAAACAAGCGCCCAGCAAAGCTGCTGGGCCACCCACGGCAGAAGGCC
ATTACTAAAAAAGGAAAACTGCCACGTCAAAC
CGCGCTAGAGTCTCCACCTCCTCTCTTTACCCTCCTTCTCGTCGTCGCCTTGTCTTTTTCTTTGCTTT
TTTGGTTTTTTTCGCCCATCGACACGGCGCTC
ATGGAAGCGGTGATGCCCGTGAGCGCAGCGTCAGTTCGATCAGCTCTCCCTTCTTCTCCCGAATGTGA
TGCTGAAGGAGAAGGTTCGCCTCACGTATGAG
AGCCACAGCGGCGCGGAGTGTGAAGAACTTCACCGATTTGTTTTCAGGTGCATTGGCGTTCTTTACGC
GCTGCAGTAACTCCCCGGGGTTACTCGCCGCA
ATCGCCGCGGGGTCCTTGAAGCCCGCCGCCCACAACGCCCGCGCTCGTGCAGGCTGCAGTCCGCGTAT
CTCCATCAGCGGCACAATGTCTGGTTTCACGC
CGAAGCCAAGTCGCTTCACATATGATGACAGCACGGCCTCCAGCGAAAACCACTCCATCGCCCGACAA
AAGCTCGTGATGGAGCTGCTAAACATGGAGGC
GCTGCGCATAAGACTCTGTAATTGCCCGCGGTTGCACTGATACCGCCGCTCTACGACAGACATCGGTA
CCTCACTGAGAAGATCGGCAAGCATCAGAGCA
ACAAAGAAGCGGCGCGTGGCAAAAGTCCGGCTGCCGCTACCTCCATATGGATCCCCAAGTCCCATAGA
TCGCTGGTGCACAAAAAATTCATCGACTCCCA
CCATGTTTGCAATACGCTGACGGTTCTCGCTCATCTGTGAGAGCACCTCGCAATACGCTGACCAATTA
CATTCTCCAATCTCCCGTAGTGGTGTTAAAAA
GTAACAAAGATGTAGATCATCGGCAAGAATTAGACCTGTGCGCTGCAGATTCTCCAGCTCCCCGCGAA
CCAACAAGGCCTCCTCAATACTGAAACAAGAC
CGTACGGAGCAGGCACCAAAGGGTGTCGCAGACAACGACACTTGTTCATCATCATTTCCGGCAGGCTG
CATCGTGGCGGCATCGCCACCCTCGTCAGATT
CCCTCCGCAGAGAAACACGCTGCACCAAACCACTCCGAATCAACTGGTCCATGGATGATTGCAGAATG
CCCTCCAATGCCACTGGCAATGGCCTTGATGC
CACATCGTAAACCGGATCAAACGGCCCTGCTGCATGATGGCACATGAGGTTTGCCATCCACGCTTGGG
CGGCGGAAGGCGATCCAATGACGCCCACCGCA
ACACACTCGAGCAGGGCCCTTTCAAAGGCACTAGCTTGAGACAGCATTTGACTCGAGCACATCTCCAC
CTCCTGTCGCATCAACTCTCGTCCCCTGTGGC
AGTCTCTGCGGGAGAGAAGAAGAAATGATTCACCAAACTCATCCAGGCCGGCGCGACCCGCACGTCCG
CACATCTGTAAATAACGGGATTTTGTCAAAAA
GTCACGTCCCACGTAAGGTGTCTTGAAGATAACACGTCGCGCAGGTAAATTAACACCGGCCGCCAAGG
TCGACGTACAACACAAAATGTTGATTTCTCTC
TGACGATATGCCCTCTCAATTAACTCGCGCTCCTCCCCGAGCAAGCCACCGTGGTGAAACGCAACTCC
ATGAGGAAGAAGTTGGCTAAGCACGTTCACAT
CCGCTCCAAAGGAGCTCAGTTCATTCTGAAGGGCCCCAAGAGTACTGGTACTTGCGCTGTGGAACCCA
TCTTTCATGCCTTTTCGGTTTCGGGCAATGAG
ACGTGCCGTATCAACGCACTGCTGACGACTCGCGCAAAATATCAACACAGATGCCCCTTGTATCTCTG
TTGCCAACAGCAATAACTGCTCCGTCTCGGTA
GCATACCCAGCCGCGACCAGATTGCGTTCATTCCGCTCACCATCCTCCATCACCATACCATCCACAAC
GGCATATTGCCTCAGTGGAATGGGGCGATACT
GTCCCAAGTAACAGGAAGCACGTAGCCACTTTGCGATATTTGGCAGGTTTGGAATGGTGGCACTCATG
CCAATAATTTGCACCTGGTGAGGTAAACAAAG
TAGTTTGGATAGAAACAGCTCCAGTGTCGCGCCGCGATGAGACTCACCAAGCATGTGCAGTTCATCTA
CGACAACTGCCCCCAGTTCCTCCGTCAAGCCC
TCCTCCAGCATGTGATTAAGCAGCGAATTTGCCTTCTCAATGGTACAAACAAACACGGCAGTACGCTT
GGGAAGGGGCAATCTGCCTTGTGTGCCGTAAT
GACCGTCCACGGCAAATCCCAACGCGTCGCCGAAGGGACGCAGTCCCACTGCCTTCTCCTCCGCTAGC

GAAACAAAGGGAAGAACAAAGAAACAGGACTT
TTTTCGATTCAAAAGACACCGAAGAAGAGAAATCTCCGCAACTAACGTTTTGCCTCCACTCGTCGGCA
AACTGTAGACCAAACTTCCGCCTCCACGCACA
TCATCCCGCATGAGAACTTCATGCTGCCACGGATACAGTTTTTTGATGCCGCGTCGATTCAAAAAGAA
GTTTTGTGCGCTCTGAGGAAGATCGTAAAAGA
CCCCACCACCATCAACAAGAACAACCTCACTACTGCTACTAGCAATATCCCTTGGCTGTACGGAGAGG
TCAATATTATTCGGTTGAGAAGTCACCGGCAC
CGCATCGCTGTTATTGTGAAAAGCCACCACACCTGCGGTATTCGCATTTTCTTTCGTATCATCTTGTT
CTTCCTCTAACAACGGCTGGGAAAAAACAAAG
GGCGGTGTACCAAGACCCGCTGCCGTTTTGCTCGGTGTAAATGGAAGATCCGTGGAGAGTCGCTGATC
CTCTTCCACCTCCCGCTGAAGTTCCTCTTCAT
TTTCAAGCGGCGGCGTCTCCACGGCCGTCCCGTTTGCCGACCGTCCGTGCGGTGGTGGAGCCGTCGTG
TTTGGCGGCAAAGACGACTCTAAAGGCCGTGC
AACAGTCACAGGTGGTGTGTCTCGCGGGCTCGATTCCACGCGTGGACGTTTAATTGCGGCCGTCTCCA
ACGATACGGCAGAGATATCGTCCCCTGCCCCC
TTCTCTTGGGAGACTTCAGCGAACGGTAAGTCATGTGCCGTCGTCACCTTTCGCGGTGCGTGAAACCC
CCCACGGCACACGAACGTCTTCCGCATTGTTT
GTGACTCAGTACCTCTGAGAAAGGTCTAATATGGACTCACCCTGTTTTGTTTGCCTCCCCTCTCTCTC
TCTCTCTCTTTATGTTTCAACAGCAGCATGAG
ATGTGGACGTC
>scaffold1077.size5951
AAGAAGTGCTACTTATCGCAACGGTAGTGGGTGAGGTTCGATTCTATGTGATTCCAAAGGCTTTACGA
AGGGTGGTCTCCCCGTATGCGGTAGTCCGATTG
CATGAAAAACGCATCAACAAAATGAGCATCCTTTTTGATAGTAATGCCCTTTTAACGGCGTCTGATGA
TGGTGTAGTGAAAATGACTTCCCTAGATACAG
GTGTACATCTAAGAACTTTTTTTACTTCGGGATCAGAACAGCACTCCGCCGTGCATGATTTTGCAGTG
AATACACAGCTGCGTCTGCTTGTAACTGTGGG
CCCGGAACGATATGGCATTGTTTGGGATTTTTCGCATGACGCACCGTTAGCCATCTTGGATGCGCACA
ATAGTCCCTGTCGCTGTTGTGCGGTGCACATA
AGACAAAGGCAAATCTTTACGGTGGGAATAGATGGAACAATTTTTATCTTTGACACGCACGGGTATCG
CTTGACGCAGGTGATACATGTACACCATCTTC
ATCCCCAGTGCGTTGTATATGATGAAAAGAAGTTGCGTCTTCTCTGTTTGGCTTCTCATCCTTATTAT
CATGGGAAGCAACTTAGTGCGGCGTTTGCCTG
TTCAAACAAGTACCAGGGTAATATGGCATCGATGGTGGGTATGATATACAACAAGACGTATGATCTTA
TTGTTACTATTGATTTAGAGGGATTTGTTATG
ACATGGAAAAGATCCAACGGTGCTCCTGTTTTTGCATTTCAATTGAAGGATTTTTCCGATTCCGTGGT
TATGAATGCAGCACGGCTTTCTTCCTTTGCAT
TGGATGGATTGGGTCGTCGATTACTGACTGGGTTTCAGAATGGTGCCGTGGCTGTTTGGAATTTGGTA
AATGGTCAGACGACGAATGTCATCACGGCAGC
CACCGAGAGCTTTGCCACAACCACGTTAAGACCAGAGGTGACGTCGCTTGAATCTCTTACGCGGGATG
GCACCACTTTTTTCTTTTTTGCCGCCGCAGGT
CATCTTTTCTCCACGAGGGAGTCTGGCACATTTACAATTGCGAGCGCAAACAAGTGGGAGGTTCCAGC
AGTCTACGGAGAGATCCTTTCCATGCTGCCCG
TGTCACTGCAGATCCTTGTTTGTGGGACTTCTTCTGGTGCTCTTATTTTCTATCATTTCTTGGCTGAG
CGTCAGGTGGGATCGGCATTGTGGGTGACGGA
ACCCACGGAATCCATGCACCGTTCCATTTCATTTTCTTCAGAGAAGCGTCGTAGTGATGTGCAGGCGT
CCGTCCTCACATCGCGTATCATTGCAATATTT
CCCTTGCACGCAGTGGGACCACATATCCTTATGTCTGTGCATGCGGATGGCACGGTAGCGTTATGGCA
CACTTTGCGGCGGCTTTTAATGGATTCTGTTA
GCTTGCGAAGTGCGTTCCCAGCGGGAAGAGGGGAAACGGGACTTGCACATGTGGCAATTGATGAGTGC
AATCAATGTTTTGTATTTGCAGATGATCGAGG
CGATATTCACGTGTGCAACCTTCAACTACAAGCGGTGAATGATCCGGGCTCCATGCGACCAGCGCGTA
CTCCGCCGTTGAAAGGCGATGCAGAAAGACCG
ATGGCTCATTGGCCCGGCTCACCCTTCCGCAACTACGAGTTTGTGGGAAAATCCCCAAGGTCGAAGGC

AAAGGGACAAAATGATGTGGCCGGACAGCCCA
ATGAGAATCAGCAGCAGAAAGGACTAGAGCAAGAGGTGGATGTAGAGAAAACCCTTTATGTATTTCTC
CGATTCTGCCAGGAATATGTGTTTCATTGCGG
TTTCCCCTCGGTGTCAGGGGTGTCCATTGTTAAAGAACCAACACGGCGAGATCCACGAGGAGTAACAA
CAGCATCCACAGCAACAGAGACGCCTTTGCCC
ATCGCGAAAGGGGCGGAGGTGGGGGAGCAAGCGTGTGGGAGTTTAAGTGAGGGAGAAGCAGAGCATTT
TATCGGCAACTACGGTCCGACTTCCGATGCCG
GTTTTTATCTGGACCTCACGTCTTCCACGGCATCAGCAGGCATAATTGTTGTGTCGTCTGGCTCAGAC
AACTTTACCCGAGTGTTTACGCTTACTGGTAC
GATTATTGGGGAGTGTGGAATGAACATCTGGACCCTAGGCAAAGAGTCAACATATGAGTTCCTTGGTG
TAAAACCGACAAGACGACTTCCAGCACGGGGC
TGCTCTGTTCAAGCAGTTGACTTTCTTCAAGAAACACCGAAAGGgGAAAAGAGTCATCCGGTGGGTTC
CCGAGGATTGCGTTTGACGCGTGGTACATCAA
AGGTGGGCAAAATCCTTCGGGATTCTTTCTCAGGAAGTGAGTTGTCTACATGCCATCCCATGGCAGAC
ACGACGGAGCTCTGGACCTCGCCTACAACTCG
TTTTCCTCCTTCCGCGCCCCCTAGTGGGGCAACACCAGAGATCAATCCACTGGAATACCCCTCCATGC
AGCCAACGGGTGCAGAAGTGCGGGAATTTGGG
AATCGAAAAAAAAAAGATTCACACGCAAAAGACCGCTTGCTACGGCGATTTCTCCCGAAGGCAACTAC
ACCCTTTGAGGAAGCCGGTGTCCTATCCACGC
ATATTCACTCACGGCGTTTAAAGGAAGAAAATATTCAGGGTCTTGTCAAGAGTGAATTTATTTCTAGA
CTGCGTGGATTGCCTGTGAACGGCTCTGATGA
AGATTCAGCTGCCCACAGAACACCGCTGGCTTTCGAAAGGAACCCCAGGGGCGGTGTGTATGCTGAGC
CATCAAGTGAATCTTTCTTGTCCGCTTCCGGC
GTCAACAATCACAACAGCATTAACACGAATAATCATTCAGGTTTGACGAATGTTACGGCAGTTGTTAA
TAGTTTACCGGAGGAGACATTCACGCCTCTGG
ATACACGAAACCACGTTCCACCTGTTTTTGTTGTTGGAGGTCTTTCGCACTCCGGTCGAATGGCAAAG
GCGCAGGCTGGAGCAGTCGCATGGAAGTGGGA
TTTACCTGCAGCAAACGATATTGAGGTGACGCAGACTCCTGTCTCATGCGCGCTTCGACAGAATGCTC
TTTCAGCTCTCACCGCATCCCCCGTGGAAGAT
GAAGCTCAGCGGCAGGACCTCATTCAAGAGCACATGGAGGCACAAAGACGCATGTTGCTTGCACTTGG
ACGCAAAGACTCAGTCCTTGGAACAGCGTTGT
CTTGCCGCAGCTCTAGGCACAGCAGTGTATCCCCAGCTGAGAAAATGGGAAAGACTACTGGCACTCAG
TTATTGGAGACACTTCGGATTCAGTCCCTTGG
CATGATGGAAACGAGGGACCGAATCGCGCGGATCACGGCGCGGCTGCACTTAGCCCCTGTCAGTCCAA
TAGCTCCTCCGTCAGTAATTCGTGAGCGCGAA
GGTGATTCTCCGTTGAAAGATGGAAGGAAAAACACGCACAGTGCCGCTCTTCGGTGAGGCGATACGGT
AAGAATTAGGGGGGAAGAAGAGGGGCGAGCAG
ACGCGTCGTCTTGAGCTTTCCTCCCTCATACCAACTGATGTAAACAAATTTTGGAGTTTTGCTTCTTT
TTTTTTTTTTTGTTGTTGTTGTTTTTGCTTCT
TCTTGATTGCCCCATGAAATATATATATTCGTATATGTATGTATGTATGATTCAGTTTAGTCCTGACG
AGCATTTTTGCGCGTGCGTCTCATTTTTATGC
TTTTATCATACCCTTCCCTTTTGGGGAGGGGGGGGGGCTCCTTGTTCTGAGGATGTCGTGAATAGACG
AGGAATGGCCGACTCTGTGGGTTTATGCATTG
ATCACGGTATGTGCTGGAGAGTGTGATGGTTGTATTGTTTTTTGTTTGTTTGTTTGTTTGTTTTTTTC
ATGTGTTTCTCTCTTCATCCTTTGCATTTTTC
TGAGCACCCGGTCCATTAATAATAATCATAATCATAATATATAGGGTTTGGGGGGTTGTGGTGACTGA
GAGGGAAAAGTAGTTAGGGGAACTTTCGGACG
AGGGGTAGAGGGGCAAATGGTGGATGTGGCCAGGCCGATCATTACGTCCTTGCTGGACACAGATGCAT
ACAAGTTGCATATGCAGCAGGCTGTATTTCAC
TTATACCCTTCCGTTTGCACGGCTTTTGATTTTTGCTGTCGTGATAGAGATGAACACTTTGGGGACGC
CGCTGACGCCATACGCGAAGAAGTTCATCTCA
TGCAAAACCTCGCCCTCACAGATGATGAGTATAAATACCTGGCATCGAAACGATATTTACGAAAGGAC
TACCTGGACTGGCTCCGCAAGTATCGGTTCAA
CCCACAGCAAGTGACGGTGCGTGCTGTGCCGTCAGACGACGGAAACGTGGATCTTGCCATCAACATTA

ACGGCCCCTGGGTGGAGACGATTCTGTGGGAG
GTGCCGCTCCTAGCCATTGTGAGTGAGGTGGTGCATCGACGTCGTACGCCGAATTTAGGGGTGGCAGA
GGCGTTGGCACACCTTGAAAAAAAAACTAGAGG
CTTTTTTCGCCCAGACAACTGAAGAGTCGATTGAGACATTTTGCGTCTCGGAATTTGGAACCCGGCGA
CGTTATAGTTTTGGCGTGCAGGAGGCCGTTGT
AAAGGCGCTCCATTCGCATCCAAAGTTTGGTCGATACTTCTGTGGCACGAGCAATTACCTTCTGGCGA
AGAATCTGGGTCTCCCCGCAGTTGGCACACAG
GCACACGAGTGGTTTCAGGCTCATCAGCAGCTGACTCCCATATTACGTGACTCGCAGCGGGTGGCGCT
GACGCAGTGGTTGAAGGAGTACCCGCATGATC
TTCGCATTGCACTCACGGACTGCATCTCCATGGACAGTTTCCTTTGTGACCTCACCAAGGATCTGGCG
GATGCATACACCGGCCTTCGCCACGATTCCGG
TGACCCTATCGAGTGGGGGCAAAAGGCCGTGAAACACTACCGGAAACTTGGCATTGACCCCAAAACCA
AAACGCTTGTCTTTTCTGATTCTCTGGACCTC
GAAAAGGCCGCGATGCTGCATCGTACCTTTTCTAACGAAATCAATGTCATGTGCGGCATCGGCACGCA
GCTTTCCTGCTCCATCCCTGGTGCACGTGGGA
TTAATATCGTCGTTAAGATGACTGACTGTGAGGGGAAACCCGTTGCGAAGCTGTCCGATGATCCAGGA
AAAACTATTTGTCGCGACAGAGACTTTGTTTC
CGAGCTGCGGCGCGCGTTTGGTTTACCAATGATGGCTGTCGCCCTTGTCACAGCGGTCGCATCCGTGC
CGAACGAAGGGATATGTGGCCTACAAGCATAA
TGCGGTGTCTAAACAAAAAAGTTAAAATAAAAAAAAAAACACGAAGAGGGAGAAGGTAAAACAAAAAA
GAAAAGAGAAAATTGAGAGTTATGCGGCTGTA
AAACACATTCATTTATGCGTTTTTGTGTGTGCGTGTATGTGTTCGTTTGTTTAGCACTTGTTTGCTTT
AGATGTCTTTTAGCTGAGACTCAATAAAGAAA
GGGTGTGTGTGTGTGTGTGTGTGTGTGTGTTCTTTTGTGTTTATTTGTGTCGGCCTGTTTCTGATGGA
GAGGGAGGGAATGGAGGAACGAAAGGAGTCGG
TCCCAGAAGCCACAGGTGGAAAAAAAAGAGAACATGAAGGAGGAGGAATCAGTATGTGTACATGCATA
TGTATATTTACTGAGAGGCAATGAAAGAGGCG
GCGAGGGGGGAGAGCAGACTGGAATATTGTACGAGAGGTGACAAGTGAAGGTTTTTTTCCCACCCAAT
GGACGGGAGGACATTTATATACAGCACCCCCA
TCATTTCTAGGGGAAGAAATGGAGAGAAGGAAGAAGAATGAATAAGGGAAAGAAAAAAAAAAAAAAAA
AAAACGGGATAAATGGAAAATAAAGTATTAAC
CAAATTCGTAGCAGAAAAGCAAGTCTTAAGGGCAAAGAGGAAGTTTCCTC
>scaffold1078.size6990
CTCCTCCGGCACCTTGTCGATGGACACCGTCGCCAAAAGAGTGAAGTTGTAGTTCGCAAAGTGGTACA
GCTGATTCTCCCCTTGCTTGCCAACGGGCCACT
CCGCCCACGCTCCCCAGAACGTCACTCCATCCGAAGTCTTTGCTGCCTTTGCTGCGCCACCCTTTACT
GTGGCGTCCACCCCAAGGTACTCGTCCCTCCA
TGTGTTATCAGAGAAGCTGCCGGACAAAAAGCCAACCAGCCCATCCGTGATCTTATCAGCGCCGCAGG
CAGTGTCAGCTGAGGGATCCTTCTGAGCAAGT
AAAGTGGCACACAGCTGAGAGACACGTTTGTCCACTTCCTTCCAGGTCGCCAGCACATCCTTCACCCG
CTGCAGCTGCTCAGTCAGACGCACAGAGACCA
TACCGAGTGATGGCGTATCCCCTTCGCCCTTCTTCTCGTACAGTGCAATCAACTCCTCCTTCTCATTA
CCACTTTTACTACTTTTGTACAACAGGGAGCT
GGCGGCAGCGTCATCATCGGATACCGGCCCAATATCAACAATGTGCGTATTGTCCGTCAGCCAAAGAT
GGAGTTTGCCCTTTACATTTACTTCTTTATCA
GCCTTATTAGCATACACCGGCAGAGTAACGAGCATCACGTTTCTGTTATCTTCAACACCGTCAATTGT
CGCTGTGATGAACCCGCTTCTAACGCCCTTTT
TGTTGCCCCACACGCGCGAGAGTGTCCCGAGTGCCTCCGTCCACGAATCCCCCTTGTCAGCGGACTCG
TACACCCTGCGGCGGCCATCATCACACGCAGT
CATCATCATGAGTTTGTCCTTCCACTCCACGACGGAGGGATCACTGCAGCCACCGTCAGACATCCAAT
TCGAAAGCTTCCAACTCTTAGTATCCTTCAAG
GAGTGAATGATCAGAGAAACGGCCTTTTCATCGTTTTGTGCTTCACCCTTCTTCTTAGCGCCTTCCAC
TGGGAACAAAAGCGTACCGTCCTCCATCTTGA
CACCCGATCCACCGCCGCCAGTCATCCCTGTCCACGATTCTAGTTCGCCAACAGATGCCCGCGAGACA

TNNNNNNNNNNNNNNNNNNNNNNNNNNNNNNNN
NNNNNNNNNNNNNNNNNNNNNNNNNNNNNNNNNNNNNNNNNNNNNNNNNNNNNNNNNNNNNNNNNNNN
NNNNNNNNNNNNNNNNNNNNNNNNNNNNNNNN
NNNNNNNNNNNNNNNNNNNNNNNNNNNNNNNNNNNNNNNNNNNNNNNNNNNNNNNNNNNNNNNNNNNN
NNNNNNNNNNNNNNNNNNNNNNNNNNNNNNNN
NNNNNNNNNNNNNNNNNNNNNNNNNNNNNNNNNNNNNNNNNNNNNNNNNNNNNNNNNNNNNNNNNNNN
NGCGGTTTGTGTCGTGATAATTTGGGATGCAA
TGCCAGTAAAAACATTGTTTTCACCGTTCTTTTTGCACTGCGCCTCGGCAACGGCAAACACGTCACTC
CCCACTTTCAAAAGGCCGGGAACACCCAACGA
CTCCACAGTTACACCATCAGTGATGAATTACTGGATGCGGCCCCACTGCCGCAGCACATCATCACGAC
GAAGAGGAGCAGCACCGCGGAAGTAAACACAC
GCCGGGACATGTTGGGCCTCTGCGGCTCACTCTCTCCTCCTTCCCTCCTCCTCCCGCTGGATCCGGTC
ACGCGGCGACGGTTGTGTGTGCGGGGTGCCTT
GACTGCAGCAACACGTGAGAGCATATTAATATGTGTGTGTGGGCGGTGTGTGTGCTGTTGGTTCTTTC
AGTGCCTTCGAATTTCACAGTAGCGATTAGCG
CTTGTTTGCTTTCCTAGCCTGAGTGTGTGAGAAAGAAATAAAGGAAAAAGAAAAATAAAAAGAGGTAC
AACAATGAACACCGTCACCCATGCACAGTGAG
TGGCATCATCATCCCCACCGCAGTCATTCCCAACAACAACAACTCACGCACACAGACACACGGAACAT
GAGATGGATGGACAGTGCACCGGGAACACACA
GCGACTGCCACCAGCAGTAGCAGGGGTCACGCCTGGCATCTTGTTATTCGTCACGCGTCAGAACACAC
TGAACAACGCCCTTACAAAAAGAAAAAACAGC
AGGATAAAAAAACACAGCCGCCCTTCGCAGCACCGACGTGCTGCCGCCAGCCCCTTTACAGCTGTCTC
CTCCAAAGCACTCGCACATCACCCGCACAGCC
GGAGAATACCTCATCAACAAATCCACAGTGGGAAAGAAACGTCCGCAGTTACAGCTGCAAAGCGGGAA
TCAATCCGCACTCATCCCAGGCACCCCCACTG
CCACACCACTCACTGTCTGCACGCTGCAAACATAACAACAACAACATGTCAACGAAGCAAATCGCATA
CTCCTTCCCTCCTCGCAAAGAGCAACCGCCGC
AGCCACAATCCCGTGCGTGCGTGCATTCTCAGCGTTTTAAACACGGCATTTGTGTGTGCATCTGCTTG
TGTGTTTGGCATCGCCTATACTTGCAGAGCAG
CTGTGCGCCACAAGAGAGTCTGAAGAAAGNNNNNNNNNNNNNNNNNNNNNNNNNNNNNNNNNNNNNNN
NNNNNNNNNNNNNNNNNNNNNNNNNNNNNNNN
NNNNNNNNNNNNNNNNNNNNNNNNNNNNNNNNNNNNNNNNNNNNNNNNNNNNNNNNNNNNNNNNNNNN
NNNNNNNNNNNNNNNNNNNNNNNNNNNNNNNN
NNNNNNNNNNNNNNNNNNNNNNNNNNNNNNNNNNNNNNNNNNNNNNNNNNNNNNNNNNNNNNNNNNNN
NNNNNNNNNNNNNNNNNNNNNNNNNNNNNNNN
NNNNNNNNNNNNNNNNNNNNNNNNNNNNNNNTCTCATTACTCCCGTAAAGCAAAACACACACACAGAGGG
AGAGAGAGAGAGGTCGCCCACTCAATGCAATA
AAAACAAAACAGAAACAGTTACGGTGCTCCGCCCATGCAGCCAGGCAGGACGGCAGCGGTGCGTGTCG
TGCAAAAGAGAGCACCAACGGGCCTGCATGGG
TGTGCATGCATATATACAATGGGCACATTATTGTGTGTGCCTCGTGGGGTGAGGTGAGGGCGAGAGTC
CATGCAAGGGGACACACACAGAGAGAGAGAAG
GAAGAGTGTGTGTGCGGGCGGTGCATGGCTCTCTCACACTCACTCTCACGCGGCCACCACCGCAGCAG
CAGCCGCACACGCGACAACAAGAAGAAGAAGA
AGCAAAAGAGGGGAGGTGGTGTGGGAGACAGCGGTGCTGTCGTCACTGTCGCCTGTACGTGCTGTGTC
ATTTGTTATAGCTGTGGCTTCGACTGGTGTTT
CCTTCCTGTTGCCATGTTTATTGTCACCTCGTTTTGCACCTTCTGTTACTGCACTGTTAGCTGTAGGG
TTATTCTTCGGCAATCCTATTTCAGTGCCCCT
TTCTGCGTCCATATTAGAGGCATTATCAACAGCTAAGTGTCGTGGTTGTGTTGATTCAGAATCACCGT
CCGATTCACCATTCTGTTCTTTTAGTGCGACG
GAAACATTTTCCTTTTTTTGTGAATCATTGTTTGGCTCCACGCCGTCATTGACTCCTTGAGACGTTTG
CTCTGGCACGTCATATTCACTGCTCAGCTTTT
CTTTTTCTGGTTCTGCTCTGGGGTTTTCCACTGTTTTTTCATTTGTTTTCATTGTCGCTGCTGGTGCA
CCTTGCAGTGAAGCTGGTGGTGCTGCTCCATT
TCCCTGTGTTGATGCTGGCGGCAATAAAGATCCTGGCGGTGATGGTTCGCCAGTTCCTCTTCGTTCCC

CTCCTGATGGCGTTGTTGCTGATGAGTGACCT
CCCACTTTCGCCGAACCATTGCCTGCCTGTCTTGCTTGTCCTTCGCCACCTTGTCCATCCAGCGGCGC
TTCCCGTTCTTCAAGTAATTCTCCACTTTCTT
CTCCTGCACTTCCCCCTATTTGTTCTTTCTCTGCTTCTCCTTTGTGGTCCATTGATTTTTCGGCCGTT
GGTGTTTCTATTTTCGGATCCTTCCTCTGATT
ACCAGCCGCTTCTTTTTTTGAATTCAACGCTTCCCCCACGTTTTGGAGCCCTTGACTGACTTGGTCTG
AAGATTCCTGTTTTTCGGTATTTCTGCCTGCG
GGTTCAGACGGTGAACCCTTTGCTGAACCCTGGCTTACATCGGTATTTTCAGAATCATCGGCCATTGA
CACTTCCACCGCACATACACCGCACCACAGCA
CGCAGAGGGCACACACCAGCAGCACACGGCCAGTCCTCATCATCGCCATCACTCCTGTCAATTGCGTG
CAGCCTTCAGCACAAACTAACAACACGTCAAC
GCAGAGTGAAAGACACAAACGCTACACCGCACAACCACCGCACAGTCACAAATGGCAGCAGCTGAGCA
GCAGCAGCTCACACCACGAGATAAATGAAATC
AGTACAATTGCAGAACCTTCAAACAGCCCTGCAAACCAGAAGTTTAAAAGCAAGCAATAAAAAACGGC
AATTAAAaaaagaaaagaaaactacactcagg
gCtcCgctgcgcatGcatatgagtgtgtccAcgatgcgttcaacatgacGgatgtggggcacaacgcg
gagcacacagcagCTGGGCAGCAGAAGCAAAT
GAAGCAAAAAACAATCGTTACTCTCATCTATTTACATCGGACCACGGGGACGCACACATGTCCCCGTG
GTTGAGTGCTGGCACGCACAGCCCGCACACAC
CTCTTCAGACCACAGCGGTGTACGCCAGCGCGGATACGGCAAGCAGCAGCGGGGCGCACACCCACGCA
GAGCTGCTGAGGCTGCCGTCGATTTCGCGAAG
ACGTGACGGTGCGCGGGTGGTCGTCGTGGTTGGCGCCTCTGTAGTCGTAGTGGTTGGTGCCTCTGGCG
CAGTGGTGGTCGTGGTCGTTGTAGTTGGTGCC
TGTGTTGTAGAGGTGGTTGTCTGGGCAGAAGCTTCGGCGTTCTGACCTGCTTTGTGTGGGGATGGGTG
CGTGAGACTTTGTGTCTCTGCCGTACCGTCTG
TCTTTGCCGTTTTTTTCGTTTCAACTGATTGCATCACCGTACTCTTCGAACTTTTTTCCCCTATGGTA
GGTGTTTCCGGTTCATTAACGGGCCCTGAGCT
TGAGGACCCTGAATGACCGGTATCCCCACGTGCTGACTCTGATACAGGGGGCTTTGGATCACCGGTCG
TCGTTGTGGTTGATGGCTCTGGTGGAGGGGGT
AGGGTCCTGGAGTCGTTTCCATCAGCCTGAGCTTGATTCTGCTGAGTCTCTGACGCGCATAAGGAAGG
GTAGCAGCACAGGGCGAGCACCAACAGGGCGC
ACAGCAGACGGCACATCGTCATCATCGTGAGTGTGGTCGTTCAGTGGTGTGGAGCTGATCTGCACAGT
GAGGGACATGGAGAGCAACAGGGGAGACGAGA
GGTGCGGCAGACACACACAGAGGGCACACCACATGAGGAGAGGGCAGCAGCCAGGCATGCACCGTACG
CCGCACACCACGCACGACACACACTCCAATCC
ACGGCAGCAGGCACACACACGCGGCCCTGCCGCAACACCAACACACAGATGGAGGAGGGAGAAGCAAA
CAGGAGCGGCACAAAAGGCTCCATCGCATGCA
CATACGGCGAGAGCGGTCCGTCGTGTCGGCACCCCTCACGGAGGGTACGCACACACACAGGAAAAGGT
GCAGCGCACACAGCAACACAAAAGCAAAGTAG
CAATAAAAAAGAAGGATCCCCGCACACCACGTGGATTGTTGGTTCACGGGAAGTGCAGCCAACATTCA
TCACAAAAACAGATCGGTTGCCATTGGCCC
TCGTTCATATTCAGTGCACACGACTTCACGCAGGCCTGGAAACCACCGCAGCAGAAGACGCACACTCA
AAAATATAGGCACAACTAATGGAATGAAACAG
GCAACCATTTATTTGTGTTTCTGTTTGAATGATGGCAGATGTCCGCATGCGTTTTTTTCTCGTGTTTT
GCAGAGGGCAAACCCTCATGCCTGGATCGTGT
GCTGAGGATTACTGCAGGGTGCACAATTATGTTTTTTCCCTCCTGTGAATGTTTCTTGTGTTGGTGCA
GCCAATAATTCGGTTTGTGTTGTTCCTGCCTT
TCGCCGGTAAAGTGTTGCAATAAAGAATAATCTTCCGGGCCGCTGGCCAAATCCCCAGTTTCTCAACC
ATAAAGACCAAACAAGGTAGGAATGAGAAAAG
GGATCAACGGAATAAGAGTGGATTCACCCCACCCTCAAGGCTCAACACCAGGCAAGTGTATGCTTATA
TATACGCATACATTCTTTAGCCACAATCGTGG
CCCGCTCACTCCATTCGGATAGGATGCATATATTTATTTATTTATTTACTGCTTACGAGGTGGCAGGC
CTTTGGGGTGCTGCAGCCACAAGACCCCGCCG
CGTGGTGGGAAATCAAAAAAGGGGTTGGGTGAGACGGTAAAATTAAAAAATAAAAATAAATCCGGCCG

CTATCGGTTCCCTGATGCCCTTAAATTCGTGG
CCCTCACAAAAATCATGCGGTAGAAAAGTGTCGTAAAGAAGTGTATGTTCGTTTCATCGGGGTACATA
TATACCATTCATTAATTTAGCCGGCCTCCTAA
AGGGCTCATGCACACCCACAAGACTCCCAACAAGAAAAGGAAAACACAGCGTGGCTGGCACGACTCAA
AGTAATGCTTCTAAAACAAAGTGTGGATGACT
CCGCCGACGCCACACCCGCAACACATGCCATCAACTCCTCCACGGCAGGAACAAAGTTGCAAGAAGAC
ACAAAATAAAAACGCCACGGCAGCACTGCATC
CCTCACAACGTCGTCAACCGGCCTGAGGCCTGTCGTCTCGAATACACCTAGCGTACACCGTTCCTCCG
GCATTTTTGTTGAAAAGCGGTCATCCAAAAGC
CGCGATCCGTCGGTCACACGAAGAGTGCAGGCAACAGGCATGTCTGTTGCACCCTCCATCGCCCGCAC
AAAGCGTCGAATTGTCGTTGGCA
>scaffold1079.size10245
TGACTGGCCGTGTGCTGCTGGTGTGTGCCCTCTGCGTGCTGTGGTGCGGTACTCCTGGCGGTAGATGT
GACGAGGATGTTCGGGTTACTGAAGAGAAAAAA
GAATCATCGGGGGATTTGCAGCCTGTAGCTCCTGTTGCTGGTGCAGGAAAACAGCATGCGGAGCCAAC
TAATCAAGATTCCGACCTCGTGAAGCAACCGT
TGGTTAAAGATTTAGAGGGCCCTTCACCAAAGCACCAAACAGATGATAAAGTCTTGGGCATGGTTGAT
AATGAAGACGATGGTACGGATAAAGAAAAGGA
AGAAAGAGATAAAGCACAAGAAGGGGCACCTGATGGTCAAACGCAAGAAGAAGAAGAAGTATCGCCAC
CACCTTCACCTCTTTCTCCAGCACCACTACAA
TCACCACCACCACCAGCAGCATCGGATGGAACTCAACCCGGTGGCGGTCCTGGTGCCGACAGCTCTGG
AGGAGGTTCCAACGGAAGTGGAAGTTCCATTG
GAGGTGGAGGCTCGAATTTGGAGACTCTTGCTCTTAATGGTGGCAGTCAATCTGCTTTTGGGAGTTTA
GGTTACGGTGGTGAAACTGGCTCTTTCTCTGG
TGGAGCGGGAAAAGGCGGAGTCGACATTGGCAGTCCTCGAGGTGATTTCTCGGATTCTTCCCTTTCTG
CGGATGGTACTTTTTTATCTCCCGCCCTCGAT
GCACCATCTTCGCAGGACACGCAATCTTCAGAAAACGTTGAGCGGTCAAAGACAATGCCTGCAGGGTT
ACCATTGCCCAACGTGAATGCACTAAAGGGAC
CAGAATCCAAGGCAGGATCACTGCCCCCGAATACACAAGAGAAATCAAAATTCTCAGATGCTAGTGGG
GGTGCAGCCGGAAACAAGGCAAAGCAAAGTGA
CGTGGAGACGGACGATCCTTCCAGTTCGTTTGCTAACACCGGCGACCAAACAGTAGAAAAAACGCTAT
TATTACCACAAACACCGGCAGTACTACTGCCA
GTTCCATCAGCAGAACTATCCCCAGAAGTGCCACCAGTAACTCCTGTACAGGCAAATCCGGACAAAGA
AGTCTAACAACAGCACCAAGAACCTCAGAGGC
CTCAAAAAGAGAACTGCAGAACAAGGCATCGTCACCCAACAAAACGGGGTCCGAATCACCGGACCGTC
CTTCAAGGGATGGTATGCAGGAGCAAAAAGAT
AAAGTCACAACGCAATTGAATACGACGAAAAAAGCATCTGCCGGTGGTTTAGCGGAAACAACAGCAAC
ATTCATCTCTGCAAATGGAAATGGCGATGTCC
AGAATAAGGAGGATGAGGATGATGCTCGGCAGCCCATACCCAACAGAGTGCAGGACGACGCCGAAGCT
GGCAATACCAACGTCGCTCCTACAGTCAGTGA
GGCAGCACCTCAAACAGTAGAAACAGTCACCACCCGAAAACGTGAGACAAACGAGACTGGTGACGGTG
ACGGCAGCACCGCGGTGTCGCACACCACCTCC
CCTCTTCTGCTTCTTCTTGTTGCGTGTGCGGCTGCTGCTGCGGTGGTGGCCGCGTGAGAGTGAGGGAG
AGAGCCGTGCACCGCCCGCACACACACTCGTC
CTTCTCTCTCTGTGTGTGTGTGTCCCCTTGCATGGACTCTCGCCCTCACCTCACCCCACGAGGCACACAC
ATAATGTGCCCATTGTATATATGCATGCACAC
CCATGCAGGCCCGTTGGTGCTCTCTTCTGCACGACACGCACCGCTGCCGTCCTGCCTGGCTGCATGGG
CGGAGCACCGTAACTGTTTCTGTTTTGTTTCC
ATTGCATTGAGTGGGCGACCTCTCTCTCTCTCCCTGTGTGTGTGTTTTGCTTTACGGGAGCAATGAGA
GTGTGTGTGCGCTGGTCGTCCCACGCTTCTTT
TTGTTTTATTTTTCATTGTGTTGTTGTTGAGTGCACCCATTCCGCCGGCTGCGTGTGTGTGTGTGATG
GTAAGAGTAATCTGCATGTGTGCTATCCGCTT
CTTCCTATCTTTGCTTTGTTGGGACTCTCTTGTGCCGCACAGCTGCTCTGCAGGTGTACACGCTGACA
AACGCACTAGCAGATGCACACGCCATGTTGCG

CACGCTGTGGGGCGGCGTGAACGCTGAGGACGCACGCACGTACACGATTCTTGCTGCGGCTGCCCTTG
CGAGGAGGGATGACGGATGACCGCGAATTGTT
ATGTTGCCGTGCTGCGAGCGTGATTTCCTGTGCTTGCTGCCCATGGGCGGCGTGGCGTTTTGGTGTCT
GGGATTATTGCGGATGGGCTTCAGCCCAGCAG
CTGCACGGTTTGACGTGTTCCTCCCGCTGTTCGTACGCCGACGGAGGATTCACCGGCTGTGCGGGGCG
ATGTGCGGGTGCGTTGGAAGAGACAAGAGTAA
AACGACTGGCGGCAGCGCGTGTGTGCCGTGAAGGGCGGCTGTGCTTTTTTTATCCTGCTGGTTTTTCT
TTTAGTGAGGGCGTTGTTCAGTGTGTGCTGAC
GCGTGACGAATAACGACGTGCCGGGCGTGGCTCCTGCTGCTGCTGCTGGTGATGACCGTCGCTGTGTG
TTCCCGCTGCACCGTTCATNNNNNNNNNNNNN
NNNNNNNNNNNNNNNNNNNNNNNNNNNNNNNNNNNNNNNNNNNNNNNNNNNNNNNNNNNNNNNNNNNN
NNNNNNNNNNNNNNNNNNNNNNNNNNNNNNNN
NNNNNNNNNNNNNNNNNNNNNNNNNNNNNNNNNNNNNNNNNNNNNNNNNNNNNNNNNNNNNNNNNNNN
NNNNNNNNNNNNNNNNNNNNNNNNNNNNNNNN
NNNNNNNNNNNNNNNNNNNNNNNNNNNNNNNNNNNNNNNNNNNNNNNNNNNNNNNNNNNNNNNNNNNN
NNNNNNNNNNNNNNNNNNNTCTCGTTTCATGT
GTCTGTGTGCGTGAGTTGTTGTTGAGGGTGACTGCGGCAGGGATGACGATGATGATGCCACTCACTGT
GGACGCTGGCGGTGTTCACTGTTTCACTCCTT
TTTCTTAACTTCTTTCTCTTACCCACACACAAAGGAAAGAAGTTAACAACAAGCGCTAATCGCCACTG
TGAGATTGTAAGGCACTGAAAGGACCAACAGC
ACACACCGCCCACACATATATTTGTATGCTCTCACGTGTTGCTGCAGTCATGGCACCCCGCACACACA
ACCGTCGCCGCGTGACCGGATCCAGCGGGAGG
AGGAGGGAAGGAGGAGAGAGTGAGCCGCAGAGGCCCAACATGTCCCGGCATCTCTTCTACTCTGCGGT
GCTCCTCCTCGTCGTGATGGTGTGCTGCGGCA
GTGGAGCTGCACAAGTTGTTGTGGAGGAGCCGTCATCTGGTCCTCAGTTTGAATGGAGGGGCATCAAT
GAGAATGGAGGTGAAACTGTGGACTCGTTGGG
TGCTCCCAGCCTTCTAAAAGTGGGGAGTGACGTGTTTGCCGTTGCCGAGGCGCAGTGCAAGAACGGTG
GTGTCAGTTTTACTGGCGTTGCATCCCAACTT
CTCACGAAAGAAACTGCTGACACACCGGAGGAAGTTCTGAAAGAATCAAAAGATGGGACACAAGTACT
GGAGGAAGGTGCTTCCGAAGACCAAAAGAAGA
AAGTGGATGTGAGCCGACCAACAACAGTTTTGAAGGGGAATGATATTTACATGCTTGTTGGAGCCTAC
AATAAAGTTGCCGTTCCTGGACAGGATGCCAA
GCCTGACGCGGCTCAATTGGGACTTTTGCTGTTGAAAGGGAGCGTCAGTAGCGTGGATACAAGTAGCA
AAAAAATTGACTGGAAAGTTACTGAAAGTTCC
TTGCAAGGACTTTTTGGTGCACAGCTCGATTCCTGGACAAGGCTGATTGGAAGCGGTGGATCGGGTGT
TAAGATGCATGATGAGACTCTTGTATTTCCTG
TGGAAGGCACGAAGAAGGCGGCGGAGGGCACTCTAGTGAATGTGAAGACTGTCTCACTGATCATACAG
TCGAAGGATACTACGAATTGGACGCTGTCGAA
GGGGATGTCTGACGGTGGCTGCAGCGATCCCTCCGTCGTGGAGTGGGAGGAGGGAAAACTCATGATGA
TGACTGCGTGTGGTGATGGCCCTCGCAGGGTG
TACGAGATCGGTGACAAGGGGGAGTCGTGGACGGAGGCCCTCGGGACACTCTCGCGCGTGTGGGGCAA
CAAAAAGGGCGTAAGTGGCGTTAGAAGCGGGT
TCATCACAGCGACGATTGACGGTGTTGATGACAGAAATGTGATGCTCGTCACCCTGCCGGTGCGTGCC
GAGGAAGGCAACGAAAAGGGCAAACTTCATCT
TTGGCTGACGGACAATGCGCACATTGTTGATATTGGGCCGATATCCGGAGAAGATGATGATGCTGCCG
CCAGCTCCCTGTTGTACAACAGTGGTGAGAAT
AATGAGAAGTTGATTGCACTGTACGAGAAGAAGAAGGGCAACGATGTGAAACCATCACCCGGTATGGT
CTCTGTGCTTCTGACTGAGCAGCTGCAGCGTG
TGAAGGATGTGCTGACGACCTGGAAGGAGGTGGACGGCCGTGTCTCCCAGCTGTGCACCTCTTTAATT
GCTGAGAAGGATCCCTCAACTGACGATGCCTG
CAACGCTGTAAAGATCACGGCTGGGCTGGTTGGCTTTTTGTCGGGCAACTTCTCTGGGAACACATGGA
GGGACGAGTACCTCGGCGTGAACGCCACAGTA
AATAATAATAAAGAAGGTGCAGAAGCGGCAACAGTGGCCAGCACGGAAAATGGTGTGAAGTTCACTGG
GCGTGGCGCAGGGGCGGAGTGGCCTGTTGGCA

AGCAGGGGGAGAATCAGCTGTACCACTTTGCGAACTACAACTTCACTCTTGTGGCGACGGTGTCCATC
GACAAAATGCCGGAGGGAGACACCCCCATTCC
GTTGGTGGGTGTGAAGATGAATGGCGATGGAAATAATGTACTTCTGGAGTTGTCATACGACGGCGAAA
AGAAGTGGCATGTGCTGTGCGGTGACAAAACG
ACGACGAAGCTCAGCAGCACGTGGGAGACAGGGACACCTCAACACGTGGTGGTTTTGTTGAAGAACGG
CACCCAGGGCTCCGTGTATGTCGATGGTCAGC
GTGTGGAGAATGNNNNNNNNNNNNNNNNNNNNNNNNNNNNNNNNNNNNNNNNNNNNNNNNNNNNNNNN
NNNNNNNNNNNNNNNNNNNNNNNNNNNNNNNN
NNNNNNNNNNNNNNNNNNNNNNNNNNNNNNNNNNNNNNNNNNNNNNNNNNNNNNNNNNNNNNNNNNNN
NNNNNNNNNNNNNNNNNNNNNNNNNNNNNNNN
NNNNNNNNNNNNNNNNNNNNNNNNNNNNNNNNNNNNNNNNNNNNNNNNNNNNNNNNNNNNNNNNNNNN
NNNNNNNNNNNNNNNNNNNNNNNNNNNNNNNN
NNNNNNNNNNNNAGAATGTGCATTGGGCAATGGTGAGTCGAACGAGATCTCCCACTTCTACATTGGAG
GGGATGGAGAAAACGCAGCCAACAAAGAGGGC
GTGTCTGTGACTGTGACGAACGTCCTGCTGTACAACCGCCCGTGGGATGAAGCGGAGATGGCCGCCTT
CAGCCCGAATAAAGCTTCTACTCCATCTGCAG
TGAATGAGGCTTCTATTGAAGGAGGCGCGATTGAGACTGCTCCTGGTGGTGGACGACCGGAGGAACCG
AGAGAGTCGATGGAGAGCAGTGGTGTGAACGG
TGTATCCGCACCAACAGTGTCCAGTGCCAAGAATTCCTCAGGAGAAGAGGGGCCTGCAACCCNNNNNN
NNNNNNNNNNNNNNNNNNNNNNNNNNNNNNNN
NNNNNNNNNNNNNNNNNNNNNNNNNNNNNNNNNNNNNNNNNNNNNNNNNNNNNNNNNNNNNNNNNNNN
NNNNNNNNNNNNNNNNNNNNNNNNNNNNNNNN
NNNNNNNNNNNNNNNNNNNNNNNNNNNNNNNNNNNNNNNNNNNNNNNNNNNNNNNNNNNNNNNNNNNN
NNNNNNNNNNNNNNNNNNNNNNNNNNNNNNNN
NNNNNNNNNNNNNNNNNNNNNNNNNNNNNNNNNNNNNNNNNNNNNNNNNNNNNNNNNNNNNNNGTTGGC
GTCAGAAAAGTCTTTCGATGGAATTAAAAATG
TGGATGGCGGTTCTTTTTCTGATGGCGAGCCAACGGTGGAGACAAGAGAAGGAGGAACGGATAGGCAG
AAGGGGATCCACGCACAGAACGGGGACGTAAA
GGCTGCGGCACTCAACAGCAGTCTCGGGAATGTGTCGCAGGGGAATAACACCGATGCCGGCACTGTAC
GTGGGAGCGGACTGCTGCCACTTCTTTTTCTA
TTGGGACTGTGGGGTTTTGCGTCTCTGTGAGGCGTGACTGGCTGCCGCGTGGAGCAATTGCGACCTCC
CCTGCACGGAATTAATTCCAAGAACTAACTTT
TTCCTTAAGATAATTTTCTTTTCTTGCTTTCTGTTTGTAATTCATAATTTTTTTGCGTGTGTGCGAGT
GTGTGTCCTTAAAAACTTTTTTTTTTCTGTTA
TTAATTTTCGTCTGTTTTTTTTATTTTTCATTCCCCATATCTTCTTTTTTTGCTTTTGGGGCAATCAC
GATGGACGTTTTTTCTTTTCTACTCTTTTAAT
TGTTTGTTGCTGCACTCTTCCTGCGAGGGGTGCCGTGTGTTCCCGCCCAACTGCTCCACTCGCACACC
CACCGACACGCTCGTGACGACGGCCCTGTGCG
CCTCGCCTGCTGCACGCATGAGGAGGGGCCGCGACGAACGGCGCGCTGAGGGAGCCGTGACGTGTGGG
CGTGCGACGAGTGCCTGTGGGGCGGAGCACAC
ATTTCTTCCCTCTTGTTTTGTTTTCTTTGCGGCCAGGGCTGGCAGACACCCGCAGCGCCACACACACA
GCCACACCCTGCCGCCGTGTTTTTCTTTTTTT
GTTTTTTATTTGATGGGTGCCGCCACCGTGCGTGATTGTTGAGGTCGGGGTTGTACCGCATGTATCGC
CTCCATCCAGCTGTGCCTCTTGCTGTGTTGAT
GTGCCTCCCTGTGCTCCTTTTCTCTTCTGCTCCTCCAGCACAAGGACAAATAGGTATTTGCACAGTGG
TCTGCCTTCTGTTTTTGCTTTGCCGGTTGATG
GCGTTGCCTGCTCTTTGTGTGCTGGCGCGTGACGGACGGTGCCACGATGGCCTCCTCTGGCGATGACG
GTCGCTGTGCTCTCGGCACGCATTCGCACGTT
TTTTCCCGTCTCCCTGTGAGAATGATTGCAACAGGAGCGTGGTGCACGGCAGCGGCTGTGGTGTCTTG
GAGAGGGACGAGGCGCCGAAGGAGGAATAAGA
GGGCGAACCCGCTGGATGAGCTGCCGCTGATCCCGTTCTTGCTGGAAGGGTTAAACCGCGAGTACAGT
AAGACGGACACTCGCACGGTTGCTGCTCCGCA
TTTCTTCTAGCACTGCGGTGCCGCCGGCCACATGTCTTTTTGGTGCCACAAGCAGAAGTAGAACAAGA
TGCAGTGCATGACGAATAACCTGATCGGCATG

TGGTTGCAGCGCATGGAGTACGTGCGCCAGTACGCGTTTGGCGTTGCGCTGATGATTGGAGAGGCGGC
ACGACATTGTGGCGCTGTGTGTGTTGCGCGGC
TGGGGCATGCCGGCGATTGTGGAGGACACGGAGCTGCTTGACTGAGGGAGGAAGTGGCGTACGTTGCG
GCGCAGCGGGGGCGCAGGACGGACTGCCTGTG
GTGGAGGGGCGTGCCTGTGAGGGCCTGAGGAGAGGGAATAACAGTGATGGTGACCATGGGAGTGTGAG
GGACGGTGCACGGACGAGGAGCACTCTTTTTT
GTTGTTTTGATTGTtGTTGAGCGGCGTCCACCTAATTGTTGTTGTTGTGGTGCGTTACTCTCATTGCT
GGATAGGACGGTACGTGGTTTTCTGTGACTTG
TCTCTTTTTTGCTTTCACGCTGCGTTGACGAGGAGTTAGTCTGTGCTGAGGGCTGCACGCAATTGACA
GGAGTGATGGCGATGAAAATGTGACTGGCCGT
GTGCTGCTGGTGTGTGCCCTCTGCGTGCTGTGGTGCGGCTTATGACTGGCCGTGTGCTGCTGGTGTGT
GCCCTCTGCGTGCTGTGGTGCGGTGTTAGTGT
GGTGGCGGCCGATGGTGCTGGCGGAGGTGATGGCGGTGCGGATGAGTATTTTGTTTTGCGGTGGCACG
CTCAGCTGCGGAAGGGATGCGCGGAGGCGTTC
GGCAGTAGGTCAGAGGGCAGGAAGGATGTTTTCGCCGTTGATGATTGCGTGCCGTTGTGGATGGCCGT
CTTGTCTGGAGACGCCAGCGTTCTGCGGTGAC
TGCTGGCGTTGACACCACCGATGATGACAGCACCCGCGGATCCTCCTCGGAAGTACAGGCAGAGACGG
ATACTGATTCGTTGGACGGACCACTGAAACCA
TCGGTGAAGCCAATTCCGGGAGAGGTACCAGATGGACAGGAGTCAGGAAAACTTTCAGAGGCTTCTGA
GCCGACGATAAACGGAACTGGAGGGACTTTAG
ATGGTGAAGAAGAGGAGGGGGACGTGAGTCAAGATGAAGGCCAGGACGACAAGGATGTTAGACGAGGT
TGTTGGCGCCGCAGTGGGAAATGGAGAGAGAG
AAACTCCACATACAGAGCACGGAGAAGAGAAGGAAGGAGTCAAGGAGGAAAAAGCAGGAGGCACGACA
AAAGAACGAGGAGGAGATGAAAACAACGCTGC
CGGCCGTGGACCTGCTCAAGGAGCCGCAGCTTCTCGGCCTGCTTCGCCCGTTGACGTCAGTAATCCTC
AAAATCCCGTGTTGGACCAGGACGGCGCTGGA
AGAGAAAAAGGGGGTGCCGGTCCAGTGGTGCTGCCACAAGCAGCAGGATTACGAGAGGCGGAGAGGGC
ACCAGCGAAATTAGCTCCCGGAGGAGGAGCAG
CCGGAAAAGAAGCAGAGAAGCAAAACGGAACGGCGGAGCAGGTGGCTGCAAAGGAAGTGCAAACAGCC
GTCGAAGATAGTTCAGCAGCAGCAGCAGCAGC
GGGAAAAGAATCCAAGGGAGAAAAGGAAGGAGAAAACGTAAGCGAGGCGGAAAAGGAAGGATCGCAAG
GAAATGTTGACGAAGAAGCAGCGGGAAAGAAC
GGAGGCAATGCGACAAAGAAACTACAAAAGGAAGCCACGGACGAGGCAAAGACAAAAAAACGAAGCGA
GCAGAAGGAGGCGGAAAATGCAAATAATACCA
AAGAAGAACCCGACGAAGAAGAAGTCAAAAAAGCCACCCAAGAAGCAGCAGCAGACAAAGAAAACACG
ACCTCAGCCCGAATCCTTACCAAAATCAATAC
NNNNNNNNNNNNNNNNNNNNNNNNNNNNNNNNNNNNNNNNNNNNNNNNNNNNNNNNNNNNNNNNNNNN
NNNNNNNNNNNNNNNNNNNNNNNNNNNNNNNN
NNNNNNNNNNNNNNNNNNNNNNNNNNNNNNNNNNNNNNNNNNNNNNNNNNNNNNNNNNNNNNNNNNNN
NNNNNNNNNNNNNNNNNNNNNNNNNNNNNNNN
NNNNNNNNNNNNNNNNNNNNNNNNNNNNNNNNNNNNNNNNNNNNNNNNNNNNNNNNNNNNNNNNNNNN
NNNNNNNNNNNNNNNNNNNNNNNNNNNNNNNN
GGCGGAGCAGGTGGCTGCAAAGGAAGTGCAAACAGCCGTCGAAGATAGTTCAGCAGCAGCAGCAGCAG
CGGGAAAAGAATCCAAGGGAAGAAAAGGAAGG
AGAAAACGTAAGCGAGGCGGAAAAGGAAGGATCGCAAGGAAATGTTGACGAAGAAGCAGCGGGAAAGA
ACGGAGGCAATGCGACAAAGAAACTACAAAAG
GAAGCCACGGACGAGGCAAAGACAAAAAACGAAGCGAGCAGAAGGAGGCGGAAAATGCAAATAATACC
AAAGAAGAACCCGACGAAGAAGAAGTCAAAAA
AGCCACCCAAGAAGCAGCAGCAGACAAAGAAAACACGACCTCAGCCCGAATCCTTACCAAAATCAATA
CGACACCTGACAACGGTGACGGCAGCACCGCG
GTCTCCCACACCACCTCCCCTCTTCTGCTTCTTATTGTTGTTGTTGCGTGTGCGGCTGCTGCTGCGGT
GGTGGCCGCGTGAGAGTGAGTGAGAGAGAGAG
CCGTGCACCGCCCGCACACACGCGTCTTTCACTCACTCTCTGTGCGTGTGTCCCCTTGCATGGACTCT
CGCCCTTACCCCACCCCACGAGGCACACACAT

AATGTGCCCATTGTATATATGCATGCACACCCATGcaggc
>scaffold108.size54875
TTCGTCTGATTTCCCTTTTTTTTCTCCTTCAACCGCGCGTACACACCACACCCTCCACCGGCGGGCAA
CGTGGTAATAATTAATTGTGTGGCATGTAGATG
CCGCTGCAGTCACCCTTAGAAGCATAGGTGACATGCGTCGTTTCACAGCGGCTCAAAAAATCTACGCA
TGACAAAAAAACGAAAGGCCTGAAGAAGAAGG
CCCTGCTCATTCGCTTTTCGGGATTCCACCAGACGCTCATACAGTGTGGGTGGCGGGGGCGGCCGAGT
GCGAGATCGTGACGATACCCTCTGTTGTGCTT
TCCCCTTTTTTTCCGTTAGGGTGGCGGCGGCAACAGGCGCCAACATTCGGCTGACCTCCGTCGTCGGG
TTTGTTGAGTCTCCCGCGGCGTCGTCCCTCCT
CGCAGACGATTCGGCCGGCGGGTTACCGTCATCCTCCACAATCTCCAAGGTCCCGTCATCCTCCACCA
GGTCCTTTCCTTCGCAAAAAGCTGTCGTGCTG
CCTTTTGTGTTCCTCCCACCACCTCCTGGGGGTGAAGGGATGTCCTCCATTTGTCCCTCATTCTCATT
TATGTTTTTTGTTTTTGCTTTCTCTTCTTCCT
TTTCGCCCTCCGTGCGGTGTTTCTGCCGTGCCGGCTCAAGACGCTCCTCACCCCAAACGGTTTGCCGT
CCAAAGTTCTCGTCGTCCCTCTTGGGGCATTC
GTCAACCCAATGGTTGCCCTTCTCCCCACAGCGGTGACACACGTAGAAGCGAGGAACGGTCTTTGCAT
CACGATATCTGTCACGAATAAGCTGCACCTTC
TGGCCTCGTGTCAGGGTACCGTTTGGGCCAAGAGGAATAATCCGCTTTGGACGGCCGTGGGCTGAAGG
GTTTGGCGCTGCCACCGCGGGATTTTCCTTTA
CAGTAATCCCCTCTTCATGTGGATTTTTAATGGAAACCGGTGGAACATTCATCTCCGTGTTCTCCTCC
TTCTCCCCACTTTCCTCGTCGTTCTGCTGCTG
CTGTTGTTGTTGTTGTTGTTTTTCTTTTTGTCCCTGGTGTCCTTCAACTGCTTTATTTGTTGCGGCAT
TTGCGGGCCGTCGTAGTGCATCCGAATCAATA
TATGTCTCCTCTGTTCGGGGATTTTTCTTCACCAAGACGCCATGCTGGCGCAGCCACCGCCGACGTTC
GTCCGGAATGATAACTCCCCTGGATGCCTTGT
AAAATAGTTCCTCTACCTTTGATTTCACCGTATCTTGTGTGGGAAAACGCTGCCTTCGTTGTGACAGA
TACGCTCCAACGTCCGCAAGATTGGGTGCATC
CGGACGATTGTGAAGCGTCTCCATATGCATCTCCATCTTCCACTCCTTGCCCTTTAGACAAGTAAAGC
GGCAGCCTGGCATGGTACACCAGACATGGTCC
ATCATATGCCCGTCGTATCGCTCTTGGGAAAGAAACCCTTTCTGGCAGGCATCGCAGTAAAAATTGCA
TTCCTCACGATCTACGGGCATCACCTGGGTTG
CCAACCCGGGAAGTCCATTGAGAGGGTTTGCCGGTAAGTCACGCCAAGAACGCAGCTCACGGTTCTTT
TTTTCTCCACGCGCGTTTGTATCCGGAAATGA
TGGCGGAAGAAACATACGAATGCATACATGCCTCCTGTTCTCCTCCACGCCATAATTTCCACGAGAAT
GGGAGGGATGATCATGCGGTAGATCTTGATGT
CGCCCAGTGCTGGGCGCCACGTGTGGGGCGGGCGGAACCAAGTTTTGCGTTACAGGTCGAGGGCAACT
CTGACGAAAATGCGGTTGCTGGTTTTGGCTGC
CCGCTTGTCCCGCACCATCGGGGATGGCGTGCCCCGGGGCAACATACCGGCGTTGTCGCTGGGCCGAC
TCTTCCGAGGGGCATAAGAGGTCCGACGAAAT
CAGTCGAGTCGAGTCGCCCGTGACGCGCGCAGCGGGGCCCGGAGGAGCATCCCACCTTCTTGTCATCC
CGCCCACGCCCACGAATAAATTCAATCAAACA
CACGCAGAGAAAAAAAACAACCAAATGAATAAAAAACAGGAAACAAAATCGAGTGCCACTCGTCCACG
AATTGAAAGTCGCCGGTGGCCAAAACGGGTAA
AAAGGACCCCCGTCACCACAGACGAACGGGGCAAAGCGCCAATTCAGATGGGGGTGTCGTTGTGTCCT
CGCCGCAACAGAAACACTCAGTCGTGCTCGAA
CTGCTGCTACTAAAAGAAGAAAAGGCAGAAGGGAAAAAAATAAAAAAAGAGTGTTCCCAACAGTACAC
ACGCACAAACACAAACACGCATCCCTCCGCAC
CCAACAGAAAGAAATTCTCCTGTTCTTCCTCCGTTCCTTCATTCCCACGGTCGATGCGTGCCTTCGAC
TGGTAGAATGAATGTGACGCAGACAAAAAAAA
GATCCCCTAACAATCCCCCCGGCCGTCTCAGCGTGGTACGCCAGGGCCAATTGCCCCATCTTGTCGGG
GGAAACAGTTCTAACGCCATGAAGCCCAGTGA
CACATCATACCGTCATGCGAGTCCTCAAATATGTTCGGGCAAACATATTTATATATATATATGACAAA
GAAAATACGATAACAATGTGCCCCTTGCAGCA

GCCACGCAGTCCAAAAGGCTAAAAAGCCCAAGGCGCACCCGCGGGAGACGTCGTACGGGACGCCCACA
TCTGTTACTTTTCATTCGCACCCCTCTTTCCC
TTTCCAATAAAAAAAACTCCCCAGTGACAATATCCCCAATACACGCATTTTTTTTCATCGATGCACTT
CCTCCACTACTCGCCGTCGTTTTTAGCGGCTT
ATTTCGTCACCGATTTTATTTGCTTCCGTTGCCCCACACACACACCACCAAACCCCTTTTTTCTCCTT
GGTTTCAAAGCCAAAATGACAATTAATAAAAA
ATAAAAAATAAAAAATAAAAATTAAAAAATAAAAATTAAAAATTAAAAATTTGCCCCTCCCACCCCCA
TTTCTCGATGGATCCTTCGTTTACTCTTTTTT
TGTTGTTTACTTTTTTTCTTTTTCGGTGGAGATACCAGTCACTCTCCCCCCTCCGGTGGCGCAGCACG
TTTCTTCCTTCCGTTCCCTTAATTATTTCAGT
CAGCCCCCGGCGCGGTTCCCCCCGAAACAAAAACAAAGAGACTTACAGCATGCGGACGTGTGTGGGTA
TTTACCTTTCCTACTTCGCTGTGGAAAAAATT
CAGATGCTCAGCAGAATGTCCTCCAGCGTCGCCTGCGTCGCGCTCACTTGAACCACCGGTGGAAGCCC
CTCCAATCCTCCTTCACGGATTTCACTTGCAA
CGCTGCAAATTTGCGAAAGGCTCTGCTGCACCAACAGGGTCACAGTGCGACCGACGACGCCCTCCAAG
ACGCATTCTTCCCCCTCCGTCGAAGAGGAGGT
CCGGAATAACTCACGTAGGCGTTTCATGACGTCGCCGGTGGCATCAATGGCCCCACAACCATCAGCGA
ACACCACGCGCACGGTGTAAACGGAGTCGTTC
GTGTAATGTGTTCTCAACTCCTGTGGCGTCCCAACACATCGCAGCTTCCCTTTTGAGATAAAGGCAAC
ACGGTCTGCAAGAGCCTCCGTTTCATCGAGCA
GGTGCGTTGTGAGAATAACAGATTTTGCCCGCGTTAGTCGTCTGACCACAGCACACGTTTGCCGGCGG
GCGGTGGCGTCCATGCCAGCAGTAGGCTCGTC
GAGCAGCACACTCGTGGTGCCACCAACCAGGGCCACGGCAAGTGAGAGACGTCTCTTGTTTCCAACGC
TCAACGAGTACGCCGGGGTGTTCTTCTGTGAC
GTGAGGCCCAGCGCATCGAGTAGTTTGGGAATCTCTTTACGGATATATTTGTCAGTGGCACCACGCAG
ACCTGCGTAGAGCCTCAGGTGTTCGCCCGGCG
ACAAGTGGTCAATAAGAGCGTCCTTCTGTGGACAGTAACCGACATAGCTGCGCGAGGCGGAGCGGAGA
ACAGAAAGATGATTAATGTACGCTGAACCCGA
AGTCGGGACCACCTCACCAGCCAAAATACCGACCGCCGTAGTCTTGCCGGCGCCGTTGAGACCCAGCA
AGGCCATAATTTCGCCCTTGTGGACACCGAGC
GAGAGGTCGTCAAGAGCAGGCCGGTGGGTACCGCCATATTTCTTTGTGATGTGCTGAAGTGTGATACA
ATCCTCAATGCGGCCTGTCTCGTTGCGGCAAA
CCTCCTCCTCCTCCCGTGCGACGGAAGCATCAATACTCATATTCGCCTCACGTTGATGGCAAATGCAA
GGCGCAGACGGTGGCGCGTCAGCGTTTTGATT
CGACGACTTCTTGTTCTTCCTCTTACTGCATGACATCCGGGTAAAAAACCACTTGACACGCTGTATGC
GTGCGTACTCAAGCAGGGCCAGTAACAAGAGG
GAGACGACAAGGGTGCATGACATGTAAATGAGAGCCGTTCCAATGCCGCCCCTGAACTTGCCTTCAAC
AAACTCAAGTTTGTCAAAGAGATTTGGTCGCT
TTCGTTCAGGAAACATCATATTCGCCAGCTCCACACCTGACAAGGTGAATATACCTTCACCAAAAGAA
ACGGACGGGATGACACGCAGGACGTGTGTCGT
GCGCGTCACAAAAGTTTCCAGCTCTTTGATCCCGCCAATAATCTGCTCTCCCATGACCCACAAGAATC
CAAGAACAAAGTTTGCGATAAGAACAACGCCC
TGGGCGACAAAAGGGGAACTGAAAAAAAAGCTGACAACGTAGCTGAATGGAACGGAACTGATTCCAAA
CACCGTAAAGAGTGTCAAGGATGCCACCACTG
TTTTAGGCTCACCAATAAATTCCGTTCGCTTGAATATGACAAAGATGATCATCGCAAGCGCCTCGGTC
ACAAGGTAGCAGCAGAAGTCAAATAGCATGGA
ACTCAGCCAGAATGCAACAACATTTGCACCCGCAAGCCACTGAAGGTGTCGAGAACCGCTTTGACGCT
CCCTTACCATAAAACTGACAGTGTTACTTGGG
ATAAATGTGAAGGGCAAAAGGATAAAGATCCCAAATATAACACGCCGGAATGCACTTACCATTTTTTG
TTCAAATGGTCCCACGTGCATAGGCACATTCC
GCGCAGTTGTCATTGAGCCTTGACCAAACAACTGGTGATTGACGACATTATAAAGTGCATTAAGGGAC
TGAGGCGCTGAGTGACGGGCGGAGACGTTGTG
CATAAGAATAGAAGTCGGTGTGCCTTTTTTTCCCTGTGCGCTTGTAAGGGCAAGTGCGATATAACGGG
TAGCTGGGTGTGTCCTTATATCAGGGATCAGC

TCAGCAGAGAGTGGCTGACTGCAATTTTCCACATCGCATTTGGCCCTGATAGGCGTGTAATACGGTCC
AAAAGCAGTAGACATATTACTTCTCTCCACAT
CAAATACGTCTGGAAATTTAGATGAGACTGTCCACATGACTTCTGAATACGGCTGGGTGTCGTAACCT
GGATACAGTGACGCGTCAAGGGTGAGTGCCGG
CTGCTTCGGTGGACTCATAAGGTCTGTCAAAAGGGCAAGACTGAGGAAGACCACAGGTAAGAGAATCT
GAAACACGACGAGACGAAGGTCTCTCTTGGCG
CAGTGTACACGTTTCATAAAAAGTACGGCAAAGTGTCTGGCCAATGACTGCCTATGCGGATTGTAGTA
CTCACTCTCATCATGCAAATTGCGGGTCTCAT
TCAGAAGCGACATGCTGCTGTTGTCTCTATTCATCTCGCAGCCAACGGCCGTACCGAAACAAGACCCT
TGGTTCGAGCCCATACCCTCTATCTGGATGGA
AATTCCATGCTCCGTCGCTTTATCAGTTAGTGGCTCATTTGAGGGCGCCGTAGCCATCGTTGATGGTT
GCCTTTTTGTCAGGTACTCCATAACATCCTGC
AGTGTCGTCAGACTCAAGCCAATGCTGCGCAGGCAAAGTGCCTCCCGCTCCTCCTCCAGGGCACGGAT
GATGGCGCTTCCTGAAGCCGAGAGCAAAGATA
GTGGAAAACGAAATGATATCTCGCGGCCACGGCGCTCGATACCCAGCAGCTTGCATTTTGTGGAAATG
GAATGCAGCTCTGTAGACATGTTACGGGAATG
CCCGGGGTATGACTTTTCACGTACAAATTCCGTGAGACGGCTGATGCAAATATTCTCCTCCATCTCAG
AGGAACAGCTCTCCACCACGCATGTCACAACG
TACCCAACGTCCATCTTAGACTTCAAAAACAAGGATGACCCCGCACCACACAGAGCGCCCTCATGCAT
GAGGAGGATCTTGTCCCCAATACGATCTGCCT
CATCCAGCAGGTGGGTTGAAACCATGACCGCACGGGTGTCACGGCCCTTCTGGAGCGCGTCATACACC
GCCTTTCTGCCTCGCATGTCCATCCCGGCTGT
TGGTTCGTCCAAGAAAAGGAGATCAGGTTTCCCAATTAGGGCTATGCCAACGCACAAGCGCCTCCTCT
GACCAGAAGATAGACTTTTTGCAATGCAATGA
CGCTTTTCCTCTAATTGCATGCTAGCGACGATAGCGTCCACTTGTTCCCGTACGTCCCATCCCCCATT
AACTTTCATGCGGGCATAGAAAGTGAGATGCT
CCTCCACTGTCAGTCGATTCCACACAATATCCGACTGTGGGCAGTACCCAATATGCTCTCGGCACTTG
TGCAGTTTATATGCCGCATTGTGGCCGCATAT
GTAAACGTCACCACTCGTGGGTGTCACCATGCCAGTGGCAATACCCATCAGAGTTGTTTTGCCGGAAC
CGTTGGGGCCGAGCAGTGCATTTATCTTCCCA
AAATCCATATTAAAAGAAACATCGCGTACTGCCTCACGGCGATCCCCATCACGGCGCAGCCCCGTAAG
AAAAAGGTACAGCCATCCAATAATGCCCCCGG
TTTCATACACCTTGTGAAGTTTACGAAATACCACCGCATGGGAGTTTACCTCCTCGTCGACGGGCTTC
CCGTCATGCGGCGGAAGTGTCCCTACAGAAGC
AGACGCAAGTCCATTAGAGAATTTCCTCGTGTTTTGTAAGTCTTTTTCGCTAAGTTCAGAACCTTCGC
GGTCACAGCAGCAGAAGCAGCAGGGAAACAAC
TTGCGAACCCACGAAAGGAAAAACAAGGGATGTCTGCGACGACCAATCGAAGACGGCAACACTTGATC
CAAATAAATCCAAAAAATCCACATAATGCCTG
AGGATGCCCACATCAGTCCAATCGCCATGGCAACGTTGTAGTCACCAACCCGTGTATTCTTCCACGTG
AATTTGTAGCCAAAGGAGGCATACTTGGCAAG
CTCATCGAAGGCTTTGGCGTAGGCGACACATGGAACGAAACTCATCGTCACGAGCCGCAGCATGCTCA
TACCATCAGGAAATGAGTAGTACGGCATACTG
CAAAGGAAAATGCACAAAGCCCCAACTGCCCCCGCAATCCGAGGATTGGTGAATAAAGAGCTCAAAAA
CAAGCTCAGGGCGGTGTTCTGCTGCATAAAAC
TGAAATAGAGAAAGAATAGCACGCCAAAGTTCACGTAACTAAAAAAACTGAGCCGGAAAACCATGGCG
GCGAGAAGCGAGGCAACAAAATCCATCAAGAA
TGCAGAGATAAACCACCCAAGTGCCATACATGACCAGCGAAGTCCCATAATTTGCATGGCTTCTCGAA
TTCTGAGTGACTTCTCCTCAACGATCCAACTC
ACAAGCTTCGTCACTGGGAAGAGAGCAGCAAGCATCGCCACAAGCGGCATGAAGTATGCCCAGCGAGA
GTAAAAACTGTTGTCGACATATGGCGGAGCTG
GCATGGGTATCAGCGGTGGTCCTTGAAGGTTAACAAATTCAAAAATGCCTGTGGCATTGGCTCCAGAT
GGAGGTGGCGGGGGCGCCTTGTCAAGTCGATA
ACGCGCGTAGAATTGCTGTACAACATTCTGCAGCGTCATAAATCCGCTTGCCACGTACAACATATAGT
CGTTATCGCCCAACCCCTTGAAAAACTGCATT

TTTGATTCAAACGTCCATGGGGTGGCGGTATAGTTCATGCTAATGGAGAATATGGTTCCTTCCACCCC
TGCCGCTGCTGGCGATCCTGGAAGGGGAGTAG
GCAACTCCACAATGGCCCAGACCCTGTCGTCATGCTTCAGGGCAAACTTCTCCGCTTCGGCTAACGTT
GGGAAAACACGATCCTTATGCACCACCTCGGC
ACAAAGAGCGCTGACATCGCTGCAATATTTGACAAACCCCGAGGCAAAGGATGTGTCCCCGTCTTTCC
CAGCCACCAGAAGGTCTCCATAATGTGACAAA
CTCGCCTGTGCCGAGCGGCTGAAAAACGTGGAACCCGTTCTCCGCGATTCGTGCGACACGAATGCAGA
CAGTGCAAGATAGGCATCCAGGGTAGGGACCT
TAATAGGGCCTGAGGCATAGTACACACCAAACATAACCTTAGAAAAAGTCCCACGGTCGCGCACGCAG
AGAGCCCCATCCCGCACAAAAGGGAAACACAT
GGTACTTGGCCCTGCCGGTCCGCATGGACCTACGTGTAACCTGCGGCCAAACGGACGTGGGCTTGACA
TGTTTTGACAGAAAAAGGCCGGAAGCAACGAC
GACATATCCATCGGTTGTGTTCCAGAATAATCTGTAACGGGTACCTTCGTTGTCGTGGAAAACCAGTA
GCCCAAGGCCAGAAGAAGCGTAAAAACGCAAG
GCAGCAGCAGCTCGGCAAGAGTCGACACCGGTGCCCTCCAACGCTGCAGGAAAACCTTCCAGAGGAAG
GCCCTGAACTGAATAAACCACAAGGAAGCCAT
CGTGAATTCAGTGGCCTCAATCAATGCAGACACACACACACAGAGAGAGAGAGGCAAAAAAAAAAAAA
AAAAGCCGGTATGATATATATATATATAATTC
TAAAAGAAAACCAAAAGCCTTTATTTTCTTGACTTCTTTATTTCTCACTGTTATTCTCCTCCCACTCT
TTTGTTCCTTCCCCCTCTCACTTTTCTACTTT
TTTTGTTTTTTCTTTTCTCTCCCTCTCCCCCTCTATCCTTCAATGTTGATTGTTGGTTCCTTTTGTTT
TTCCTTTTTTCTTTCGTTGAGGTTCAAATTTG
CGGTTAATTTAATTTCCCCTCCTCTCAGTTTCTCCCCAGCGACGGTATGCCCTAAAAAAAGATAACAG
AAGTTTTACTTCCTTCTTTACTCCAATCTGGG
GGAGGGGTGGGGGGTATTTTTTGGTATTTTTTATATGACAGTAGTGAGTTTAGTTGGATTTTTTTTTT
TTTAAAGTTGTGATCGTTTTTTTCTTTTTTCT
TTTTTTTTGTTTTTTTTTTTTTTTTTTTTATTTTCTCCTCCTCCTCGTTGGTTAAAAGTAGTTGAAATA
TTAATTTAATATTTTTGATTTTTAAGTTAAAT
TGCTGCCGCTGCCGCTGCCGCTGCCGCTGCTTTTGTTGTTGCTGTTGCTCTTCGAAAAAATAAATACA
AATAAATAAACAGTTCACTGCTTTATATAAAA
ACGGAAGAATAAAACGCCACCTTTTTGTGTTTTTGTGTTGTTTTTTTTTTTTTCTCTCACCCTATTGC
TGTTCGGTCAGACAAGAAGAGAGTGAAGGGAA
GAGGCAGTCAAACACGTGTTCGTGTGGAGGGCGCATCGGCCTCGTCAAGTGCCCCCTCCCGCACACAC
ACACACACACACACACAGAGAGAGAGAGGGAG
GGAGAAAAAGAGAGAAAGAATGCATGCACGTCAGCTCCAAGACGCATGTACGACATCAGCCAAACTTC
AGCATGAAGTATATCAGCATAAAGACAACAAT
GACAAACACAAACAACACCCACATGTGCGTGACACTCGTCACCCCCGCCACATCCAACTGACGCAGCG
TCCGTCCCAGACGCACGCGTGTGCGGGCAAAA
ACCGCGTCAAGACTCTCAAGTAGTGTGTTCTGGCGCCGCACCTCCTCGCCCATCGCGGTGAATTTGTG
CCGCACGGAGCGCACATCGCCCAAAAGGGCGC
GCATGATCTCGTTGTTCTCCTCCTCCGTTGCCTCGCTGATCACACCCGCATTCGCGCGGCTGTTCTCC
GCCTCCTCATGGAGCCGAGCGCGATGACGAAA
CACCGAGCTCTGAGGCTGCATGCGCCCAGTGCGACGACCCACACGGTCAGAGCGGCAGAGAGACTGGC
GAGGCGAGTGTGGGGGAAAAAAGGCCCAGGCA
CACACACACACACACGGCCCGTCCACCCCTTTCCTTTCCTTTCCCCTCCTAAAAACTGCGGCCGTGTA
AAAAGAAAAAGAGAAAAGAAGAGAAAAAAAAA
ATCCAATGAAAGACGGCACAGCGCCCGCCACAGAAGCAACCATAATTATTTTGTTGTCAAAAAACATC
AACAGCGACGGTCGTACACCCTCCAAATCACC
CCATTGACGGCAAACCATGCGCAATTTCTCGGTACTTCTCCGATGCGGTACCAAAAAAAACGTTCCGA
CTACTCCTGCACCCGCCAAACGGTCATTTTGC
GTCGGTTGAATTCAAAAAGGCGCGGCTCTTGGGACACGTGCGAAGATGCGCCTTCAGCTGCGGCATCC
CCGCCTCGACACGGCCGCACCAAAGACAACTC
AGTTCCTTTTCTTCCATGCGCTTCAGTCCCACCACGTCTTGGTTCAACGTAATGCACTTGTTTCGCTC
CAAGTCCTCCACAACGCTGTCGGCGGGAAGAA

AGAAAGGAGTGGCAAAGCTGTTGTAGTGCTTCTTCTTCTTCAGGTACGGGCTATCAAAGTCCAGGGAA
ATGAGGTGCATGTGCAGATGGGGCAGTGACGG
GAGCGAGTGGAAGCCGGTGGCAAACGACAGCGACGCAAAATCGCTCCCCGAGGCGTCTTTCCGAAGAA
ACTGCACGTAGCGATCCGCGACGTCCATGAGA
TGGCGCAGTAGTGGGACATGTTGAATGCGCAAGGCACTGAGGGAGTGTAAAGAAAGGTCCAACGGCAG
AACCAGACAGTGCAGCCGTGACTTGGGGAACG
CGTCGTTCACGACGATGCACTGCGTGTCCTTGTAAATCACTCCGCTCCTCCGTTGTTTCTCCTCCCCG
GCGCCGAGCGGAGGATACGGCCTGAACGGCGT
CGTGCTCTGATGGGTGTTGGGAGAGCGAAGCGCATCCCGCACCACCTTACGAAGAAACGGCAGCTGGG
TGCGCGGCCCCCCGGCGGCAGCCGCGTCGCCT
GCAGTGCGACGAATAGCACCCGCCAGCCGTGGGGCAGTCACGCCCAACAACATCATTTGATGGCGTTG
TTAGCTGGGGCGCGGCAAAAACAAAAATGTCA
ATTATAACAATCGCGTATCGTGTTGGGGAGCAAACACTCAGAAAACTTGCGGGTCTTTTACTTCGCCG
TCCACGGTGTCCTTTGCTTCGTTTGGTTCTCC
TTGAAACTTGAATTGATGATCCCTCACAGCAACGCCCGAAGGCTGAGAGGGGAACACAGGAAGATGAA
GAGGGATGACGCAGAGGCGGGGAAACAAAGTG
GGGGCCGCCGGCCCTCCCGCCAGCACAAAAGTCAATCGGCGCACAGAGCACACAGCATTCGTTGATAA
GTGCAACACATTCTTCCGCCCCGCACGCACAC
AGTTAGGCGCAGTAAAAGAAGGCCAACAGCCACATGGAGACGCCTGTTGTGACGGCGCTTATCGCGAG
CGTCACCACCCACCCGCAGGCAATCTTCAATA
CGATGCCCCACTCTACACCCCTCGTCCGTGCGTCCAGCAGGCCGCACCCAATGGCCGCACCCACCTGC
GCCTGTGACAGCGAGACGGGAATTCCGATGCC
CGTGAGAATCATCGTGACAAGGGTACTGCCAGCCTGAATACACCACCCACGTGCCGGGGTCACAGTCA
CCAACTCCACTCCGACAGTCTTCATCAAACGT
GTCCCCAGCGTGAACATGCCGATGGCAATGCCCAGCATTGCTGGCAACACAGCCCAGCTGCTGCCAAG
CGGTTCCTCTCCAAGCTCCGACACCGCAAAGG
TTTCGTAAAAAATGACAAAGGCGGCGGTTGCATTCGCCCCAGCCACAGCCCCGTGCACAAAACTAGAC
ATGCTGCCCGCGACAACTTGCAGCGCGGTGAA
CAGGTACTCCGCTCGTGGATTAAATGGTTTAATACCAATTCCACGGAAGTTTATCGGCTGCATCGGGC
GGTCCAACCCCCAGTCCTCCTCCATAAACTCA
TCGTTCAAACCCACGTCAACAGATGCCTTCGGTTCATCTTCCACATTCGCCACCGCGCCGTAGGAGAC
AGTGGACGTCGACGCCATGGACTGCGCCTCAC
CCTTCTTTGGAAAAGACGTTGAATGAATCGAGTTGCGCGTCACGGGTGCGGCCCGGACGCTATGTTGG
GGAAGCAACTGCACCCTAACCGAGCCCAATGT
CGACAGGAAAAAGCTACCGAAATCGTGCATAAGTGCCGCGACCTTCCGTGGCAGGGGCACACTCCTAG
TATTCTCCGCATGGATCTCGGAAACCGCCGTC
CCATGATCGGTAGAAGCAAAAAAAAATTGTGGCATCCGCCCATTGCCGCCTTCGGTCTCCGTGCAGCG
TGCCGCGGCTCTTGACTCGGCATCCTCCTCCT
CCGTGCGCAGCACCTCCGACTCCCAGACAAATGATTTCAATTTCCATGACTCCTCCGCTAGGCGTGGA
AAGACCGCGGAGGAAGCAATCCAGCAGGCCAA
AAGGATTGCCAAAAGAAAAAAAAGATATTGCACATCGACGGGCAGAAATCTTGCAATCGTCACAGTGA
TAACGGGTGGCTGCTCAATCACAACAAACATA
AGGTCAATGAGAAGGGAAAACAACAGACAGTACGGCAGCGACCACAAGGCGTTCTGAAAGGAACGTCC
CTTAACCGTCAATATGTACTTGCGGAGAAGAT
AGAATATACCCGCCGTCACAGCCCCAGCAACCGGAGGAGCGAGCAGCCATGAAACCAAAATTTTTACA
ACGCCGCGTGGCTGCACGTATTGGAAAGAGCC
ACTAGAGAAGACGGCAAATCCAAGGATGGATCCCACCACCGTGTGGGTGATCGACACGGGCAGCCCAA
GTACAGTGGAAAGATAAAGCCATACAGCTGCC
GCAAAACTTGCACATGTCATGCCAATGAGCACCATGTCTCGATCCAGAGTGAACAACGCAGGATCAAC
AATCCCTTTGCGAATCGTCCCACTGACATGCC
CACCCATGAAGGCGGCGCCAAGTAACTCAAATACCGTGCAGTAGAAAACTGCCTGACGCATATTAAGG
GAGCCGCTCCCTACCACCATGGCAAAATTCGC
ACTTAAATCGTTCGCGCCAATGCCGACGCCAGAAAGAAAACTGAATAGTGAGCAGATGCAGACAAGGG
GAAGGGAACGGCTCAAAGGAAGATTGCACTTC

GGATGCATGACTAATTTATATTTATATTATTCCTCCTGTTATCCTCATACCGTATTCTAAGCTGTTTA
AATCTCCCCGTGGCTTCCCACGCACTCTGTGT
GCCCTTCTAGGGTTCTTTCTTGGTCTCTTTTTCCCTTGGTTCCAAAAATCTGCCACACAATAAACCAA
TTAATATAAAAACAATCGCACGCACGCACGCA
CGCAACCGCCCCCTTCCCACTGAAAGAATTAGAAAGTAAAAAACAAAAATGATGCGCCACCTCAACGC
GGCTCCAGATGCACACGCATACACACGAATAG
CAGTGGTCAGCCTCTCCCCAAAGACAAGTAGAATTAAGAAATCCAAGCAGAGAAGCATGCACACACAG
AGAGACAGAAAAAAAAAAAAAAAAAGGTACATA
CATATTACTCCCCAATGTGCTGCAGTTCCTCCGCCCTTCTGTCTTCTCATGCTTAGTAACAGTTCCCC
CTTCACCCTACCGCACAGCGTGGGGGAAATCA
TGATGAAAAAATAAAAATAAAAAATTTAAAAAAAAAAGTCTTCTTTCACCATGAACTATAAACATAAC
AAGTAAAGTACAAAATCCACAGTCGGCAAATA
AAAAAAAGAATCCATTTGTCCCTGTTTCACCCTTTTCTTCCCATTTCTTGTTTCTCTAAGCCTCCTCA
TCCTCTTCGTCGGCAGCGTCCGCCTTGGGAGG
TGGTGGTGCGTACTCCAATTCATCATCCGCCGTTGCATCGCGCTGTAGCACCAATTCAGTCTCGGGAT
ACTCAACAAGGCATCGCGGGGGTAGCTTTGGC
GCGTAGTTCATGCCCTGCAAGCTCTTGAGACCCGTGCCGATATAGACGCTCTGATACTGACTGCCTGG
CTGGCCCAATTCCACCACGGCGTACGTGAAAG
CACCGGGCCAGGTTGCCGACCTGGCAACGTAGGTGCGTGTTTTGCTACTCTCATTGTGAAATGCCTTG
CGGAAGCTCCAGGCGGGTAGTTGACTACGGCT
GTGAGATGCATATGTCAGGGGATCGTCACGGCTGACTTCACACAAAAATGGATGTATCTTCTCGGAGT
GGTCAAACCCTGTATCCTCCTCTTCTTGCTCC
CTCGCAACAGTTCGGCCGGTTTTCAGAAGGGTCGGAGCAATGTGCACCCAATACTTGCCCTGCAGCAA
CTCATCGTCGCGATAGCCGTAGAACCACGACT
TGATCGGCTCGATTGCCTCGGTGTCCTCCTCGTCCGGCACCTCCTGTGGGGTGAGTGGCGGCAGTGTC
GTGTAGGCCCTCACGGATTCGGGAGGCGGCAA
CACGTTTCCCTCTTCATCCTCCTCCTCTGGAACAGCCCCCTCGCTTGTGTACATGTCACGCGGTGCCA
CCCGACACGCACAGCTTATGCGCGCAATTTGG
GCCCGAAGATAGTTCATTTCTTTGCCGGGGAAGTGCGGGTGGGTGTCGACAGAGCTTTCAAGATCCCC
CGTAAAACCGCGACGAATGTGACGTGCAACAA
TCACTTGATTTGGCGTCACATCGGGCAACCGCGTCCAGCTGGTTGGGTCCGCCGTCTGTGCAGCGTAG
TACACCCACTCGTTAAGGCCGCTACCGGCCGG
CTCCACCTCCGTGTCTGCCTGTTTTTTTGCGCCATACGAGTAGAGAACATCCGCAACGTTGTCGATGG
GTATCCCCTCCTCCTCCTCGTCATTTGCCATG
CCAATGTCCTCCTCATCCTCCGGCAGGCGCTCTGGATCGATCTTCGTCTCCACAATGTAGTAGTCGGC
ACCACTGCCAAGGATTTTGCCCCAGAAGCGAG
CAGTCGCGAGCGGCTCCGTCTTGACAAGTTGCTTCATTCCAACCACAAGGCGGAAGGCCTCCTCCGGC
TGCAGCCCTTCTCCCACGGAGTTAAAGATGGC
CTGTTCCCCCACCACGTTGCTGACATCACCAAGCAGCACCTCCTCCTCGTTCTCCTCTGCTTCCTCCT
GTGGCATCTCCTCATCCTCCTCCGTTGGCTTG
CGCCTTGGCTTCGGTGGAGCAAGCACGTTGCCAAAGCTTGACGCCCAACGCAAGTTCTCCAGTGCGTC
CGACGGAACGGCGTTGATGACCCTCGGACGGT
GGTCTGCATAGATGGTGGTGGCGTGTGGCGGCACCGTGCTGCCTGTCTGCAGAACGAAGGAGGAAGGG
ATAATGGCATCCAGTGCACTGCTCGGCTGCTG
CTGAACAACCTGAAGAAGCACCTTCTTCATGTGCTGCCACAGCGGTGACTCGCGGATTTCATTCTCCG
TGAGTCCAGTCATGGCAATAAAAGAACACGTT
GCAGTTATTTCCTTTTCCACGATACCCCGCAATGGTGCGAGCCGCCTTTTTCCTTCTCTATGAACAAT
TCTCTTACTATGCGGGAAAAAAAAAGAAGTGT
TCTTCGTTTTCCATATGTAGGAGAGAAGAAAAACAAAAAAAGAAAATTGGAATAACGTGGGCGGCAGC
ACGCCAAAATAAAAGTAAAACACACCCACGCA
TAGAAACAAATAGAAAATTCATCGGTACCGCCACCATTGCGCCATCCAACCAGCCCAGTTTCCACACA
CACTCTACCAACAGCAGGCCGCTCTCAATAAA
CGGAGAAGGGTCATGTGGAATTGGGGTCCCGCATCACGTCAAACTGACGCTTAGGATGCAGTGGCATC
TCCAATTCACGCAGTGGGGACTTCCAGCCGCG

ATCTTTGTCCAGTAATGTATCACCAATGACGACGCTTGCGTCGGGATTTACACGAGCCTCATAGTGCA
TCTCCGACTCATAGCCAAACAGCGTGTGCATG
AAGCGATCATGGCCTATGGTGAAGTACATCGCAGTCGTCGTGAGAAACCCAATACCAAATATTCTCAT
CGGTGCCCAAAAAAGCGCATTTTTCACTATTT
TATTGCGATCTGCACGCGTGTCGTTGAAGCGTACCTGCATGATACCACGCCAGCTTCGGGTACGGCGA
ATGATGGGGTGTGGACTCGGGGTCTTGCCTAT
GGGACGGCCTGTCACAGGCGGCATTCATTCATCCAATGTATTTACCACAAAACGCACCTCCCTGTCCT
CTCCAGTAGCAAGGTTTCTAAAAAAGGAAAGA
GAAAAAAAAAAGGCAACATTTGCATTTCATCCATGAGAACCACACTGTGGATGTACACATACATTGCT
CCATGCATGCACGTATGGTATATATGCGCAAT
TTGTTGCCCCTTCGTGTTTAGGTCTTTACTTTACACGTTTCACCTTTTTATTATTATTAGTTTTTAAT
TCAGGTCTAAAATCAACGCAATCATCAGTCGG
CAAAAGATGTGGTGTTCACTCAAACGCTTTCTTCACCCGTTTTGCCCGCCTCTTCACCGCAATCAATC
GTTTCTTCGGCTCCCGTCGCCCAGATGAAGCG
GTCACCGGCACAATTACTTTATCCACATCATGAAACCGCTCCTCCTCCTCATCATCATCATCATTGCC
ACCAGCAGAAATGAAGCCTGATGATTTGAATG
CTTCATCCTTCGTCTTCCTTCTCTTTTTTTTGGTGTCAACGGCTGTCTCCCTCGCCCGCTTACTCTTA
TTGCGACCGTCACGCCTCTCCTCGAGCGAAGT
GCCCTTAAAATAGGCAGCCTTGTCCTTCACGCCGTAGTGCGCCATGTAACCATCCTCATCGACCACAT
CCTCACCGTCGTGCGGCGCCAAATCCGACGCC
AGAGGCGCGCTCGGCTCTTCGCCACGATCGACACGAATGACGTGGCCGTGCAAACAAGATCCATGAAG
GTGCTTCTGTGCATAGCTAGCGTACGCGGGCA
GAGCAAAGAGAATACGCGCGATGCCCGTGGGCAAACTTGTGCTTTTATTCACGAGAAGATCACAACTC
AATATCCTACCGCACACACTGAAGTGACAGCG
GAGCTGCTCCTCGGTGATCTCCTCACCAAGGTTGCCGACAAACACCTCCGCTGGCCCCTCCTCTAGCA
AGTTGTACTTCTCTGTTACGCCAGTGATGTTG
CTTGGCTTCAGCTCTTTGGCCACATTCCGCCCCGCACGCCTCGCCTCCTGTTGCGCTGCTGGTGGATT
CACCACCCCCAATGGCAGCACTGGCTGCCCAC
ACTCCTCTTCGTCCGCATCACCATGAAGAATTCCCGCGTGCATGGCCTCACCGTGGCTTTCCTCATCT
GCCGATGCACCGCTCCTCTTGTGCGATAAAAC
GTGGCCCGGAGATTTTGTGTACGGCAGCGAGCCAGGCGCGGCAGCCCGTGCGGCAGTGATGCGGCGGC
CCAACATGGAGAAGCCATCGAGAAACCGTACC
GCCGGCTCCACGTCATCGGCAGACAAAAATGTCAAAAATGCCTTGCGATTCCTTGAGGTCATCTTGAT
GGATTCAAGCCGCTTCTCCAGCTGGGGCGCCA
CATCTCGCAGGATCTCGCGAACAATTTCTGGCTGTGTTATCTTCGCCAGGTCGGACAAAAGGACCTGG
CGGGCAAAACTACTCTTCCCCGCATCTAGGCC
GATGTCCACGTCGTTTGTTGTCATGTCCTTTGGCTTCGGCACCTCCACCCGCAGCTTGCGACCCAGAA
ATTTCCTTCCATTCAGCACACGCACTGCCACA
TCAACGCTGTGCGGAGAGTGAAAAACCAAGAAACCAAACCCCTGGCCTTCCCGCTTGATGAGACGAAC
GGGTTTGCCAACCTTCCATGCCGCAACAAACT
GCATCAACTCCTCCTCAAGAGTGGAGGATCCCGGCGTCGCTTTATGCTCGTAGGGCAGACCATCAATA
AACACCTTGTTGTTGAGCTTCTTTGGGAGCTC
TGTGCCCTCCTCCGTCAACATTGTCTTGACATGACGCTGTTTGTGGAGTGCTGTGGAGCACTTCCCCG
TCGCCAAGTGCACATTCTCCCCCTGCATGACT
CCCTTCTCGTCATCGTCGCTGCCACTATCTTCATGCGACATCACGGCAGATGACTCGGGGCGCTCTGC
CCCCGCGCGCAGTAAATGACCGCCACGGCCAC
GGCCTCTCCCGTGCGCAAAGTCTCGACCTCCGTTGCCGCCGCTACGTCCAAAGCCACGTGCCGCGACG
GAGACGCCAGAAGCACCGCGGCCACCACGAAA
ACCGATTGGCTCTTTTCCGCGTGAATTCATCGTCAGATTTATATGCCCCGTTTTCCTCCTTCTCCTCC
CATCTAAGGGAATGGACTCAAAGAATGAGTGT
GTGTGCAAATGAAATAATGGCCAAAATAAAAAATAAAAAAAAAGTGCGCATACACATCACAACCATCA
GCAGCAACAGGGGAAAGAAAGAAAAAGGAGGA
TGTACACTGAAAAGAAAAGATTTGCAGCGTACACCTGCAAAAGCATACATGTATCTTTCCTGTTGATT
GTTGCCGTTTGCATCCACGCAGGGCTCTGCAC

GAACGAGGAGAAACACCTTCCCTCTCCGCTCCCAAGAAGTAATCTCCGTTCTGCCGTCTGCTGTGAGT
GCGTGGTGATTTACAAAAGAACTCACGCAAGG
GCGTTCCTTGCCTTCGCATTGCCGGTAGAAAAACAAAAAAGCTGCGAAACACAAACACACACAGAGCT
GGACCCACCCCAACTCGCTTATCCCCCACTCC
AGCGCATGGAAGCGCCGTGGTGCAGTCTACTGCCACATTCCCCTCCACTCAAACTCCAAACGCACTTG
TCGTAATTTGAACTGCACCAAAGGACATGTCC
CGCGCGCATCGCTCCTCTTCTCGACCCAAAAGACCCAATAACCCCCCACCCCCAAGGGAATATGATCA
CATAAATGCGACAGCTTCCCCTCCCAAGTATT
TCTTCTATTGCTATGACTATCCCCTTTTTTGCTTTTGCTAATCCCGCTACCAACACGACACCTATTTC
CAAAAATAAAGGGCTTCTACTTTTAAATATTC
CCTCAATCTACCAGTTGCCAGCCTCATGCCACGCGCGGAAATTCGATACATCAAAGAGCAAAGGACTT
GTCGTGTCCTGGTGTGCCCATGGCTCATCCTC
GATTGTGTTCAATGCAATCTTTGTAACACGCTCTACCGCTATGCGGGAGACACGCATTTCGTGACGAC
GGCCTGCAGCTGCCGTCAACAGGGCGTCTGCC
AGCTTCTCCGCAATACGCCAAAACATCGGAAAGCTGATAAATTTCGTCTGCATCTGGCGACGCACAAA
AGGCACCGCAAGAGCCGTCACTGCAGCCTCAT
CGTGGGCCACTCCCAAGGCCTGTTTGTAGAAATCCGCAAACTCTGCCTTAAACTCACTTGTGCAGGGT
TTCTTTAAAGATGGGAAGTCCTTGAAAACAGC
ACCCCACCACGCGGCATCAATGCCGACAACGGTGTCCTCCTCCAGCTCCAGCATCAGCCGTTGTGTCG
TCAGGCCATCACCCTCATTGATGGGCTCCACC
TTGTAACGCGAGACGCCCCGCAGGGGTTGGTAGTGCTCCTTCTCCAGCCGGCTGTATTCCTCCACTGA
AAAAATGTTATTGCCATCATATGCAATGCCTA
AATGATTCCCTGTAATACTTGCGGCTGCACTGGAGGATTTATTGATGGCGTTGCGTGTGTTGTCCAGG
CGTGGAAGCATGTCACCCTGCGCAAACGGTTG
TGTGCGGCCTGTCACAGACGAGCCCCAGTACTCTACGCGACGGCGCTGAAGCAGCAAGGCCGCAGACG
CAGCTGCGGGTGCCCCCCGCGCCCTTGCGTAG
AAGGCCAACATAAACTCCCTTCCCCGCCCTTTTTCTCTATTTTATCCTGCTGTTCTTCGGATTCAATT
CGCATTTTGGTGGGAATTACAAACGGTCATGC
ATGTGTTTTTTGACCACAGCAAAAAAAAAAAAGAAAGCGAGAGAAAGAAGCGCAGGATGATACCACAT
AACGTCGTCCACCGCGCACTCCTCGTTTTCCT
TTCTCCTCTCCTTCCGCCGCGGGCGCATTTATTGCAATGAGTATCAAAGGTGGTTTCTTCGGGCCGCA
ACGGCAACAGCAAAAGAAGAAGTACGGAGGGA
AAAAGGAGAGTAAAAGTGTTTGTGTGCATGTGCGTCTGCACTGACTGCCCACTGCGCATAACAAAGTG
AGCGCACACACACACCAATAAATTAAAAAAGG
TGTAGAGAGCAGCCACATGACCCGCGTTGTTGGTTTACATTGCATCTTCTCACCCCGTACTCATCCCA
TGCGGCGAGAGAGAGAGAGAGAGAAGAGAAAA
GGGTGCCACACGGGAGCAGGAATAAATAAAACATCGCGGCCCCCTTTCTGACGTCTGCCAAAAAACAA
AACAAAATTTAAAAAGTGCGCCACAATCACCG
CAGCAAATGCCCGCCATGGAAGCGCCGTCAGCACTATTCTAATCACCCACCCGTCTGCTTCTTCCTGC
CCCTGTCGTGAGAGGACCACCTCGTCTCTCGC
ATTGCATTTTCTAGTTTTTCCAGGCGTGCCTCCACCTCCTCCAATGAAAACACCGCATCGGCCGGGTT
CTCACTGTGCGACGAGGGTGGTGCCGCAGCGG
CCAATTTGTGGCTTCCCGCCACCCCAGCCAAACCCAAGCCCTGGCGTGTTGTAACTGACCTCCTACTT
TTGTTTGCCAAAGACAAAGATGGGCTCATTTG
CGAGGAAAATGGCTCCGGTGACGGCGGTGTTGAGTCTGGCGGCTTCTCATTCAACTCAAGCTTTCCCA
CCCTCTCGTTCCGCTCGATGCATTTGTTCAAG
CGGAGTATAAAGCCGCGGGTCACTTGCATGTAGCCAGGGCCATCCAGTGCGTTTCTTATGTTGTCCTC
CTCCGCCTGGCGCTCCCTTTCTTTTAAGCACT
CCAGCAACTCATCCTTCTCTCGCTGTAACGCCAAGAGAGGCTGTATGAACTCCTCTTCAAAGGCGCTC
TCAATCGCATCCTCGCACGCGCTCATGAGGTC
ACGTCGCTCCTGCTCTATTTCATACATGTCGTCCCGCACAAATTCCTCAAGCGGCGGCACACAAAAGC
TGTGTATATCCCACTGCACCTGCACCTCATCG
TGTGTCTCGACGGGCTGTGGTTCCGCTAAAAAGGCGCGGAACAGCCTCCAATGCCGAACGACTGAAGA
GGAAAAATAATCGAGTATCTGCGCTACCTCTT

GGAGCGTAAAGTAAGGACCCACCACGATCTCCGTCAAAAATCTCTCCTTTTGTAGTTCCTGTCGTGTA
TAATTAATCCTTGGGGCATTTTCTAGTTTTTC
TTTTAGTGCTGTCCTTCGCTTCTTGTTCTTCTCCTGCGAAATTGCTGTCTCCATTTCGGCCACAAACT
CCGGGTCGGGTATTTCCGACGTCACCGTGCGC
CACGACTTATGCGTTTCCACAACTTTCCTTGGCAGCCCACAGGCCTGCTCAATAAGCAGTTCTTCCAA
CAACTGCGTGCACCGTTTCTCCGCTTCCACGT
CTGTGGCGTCAGGGGAAAGATCCGCCACGACATTCATGAGATGCATCGCTGTGAGCAACACACACCGC
ACCTGCTCCGCCGAGAACCCTCGATAGGCCGT
AAAGTGAATGAGATCCTCACACCATTCACCCAGCGCCACATGCCGCAGCTCCTCCTGCGCCGCCTCCC
GGTAGCGGAGGGGCACGGATGCGGTGTCCTCA
GGTAATGGCGCGGCTGACACAATGCGTCTCCCCAGCGCAAGAGAAACGTCGTCGACAAAGTCATCCAC
TCCTGTTGTGTTAAACCCAAGTGCGCGGAGAA
CGCCAATGCGAAGAAGGTACGAGGCAGATGGGGCACTTTGACGTGCCTCGTCCAATGCCGGGTGCTCA
TTGGGCCAATACTCCTCGTATCGATTCTCCTG
CACGATTCGTGATAACACCCGCGTGCTTTCACGAGACCGTGGTAAAGGCAGGGACAAAAAACTAACCT
CCTGATCCACCCTGCGGGCGTGTCGCAGCAAC
TCCTCGACCAGCGTCAAGGAAAAGAACGTGGCATGATGAACGGGGGCATGCAGTAAAAATGAAATAAG
TTGCTGTTCTTCTGGCGGCGGCGGCCGTGGCT
GCGAAACCTCCAGTGGATCCATTTTCTTCCCTCGACAAAGCACACGGTGTGAAACCAACAAAGAGCCG
CAAAAACAAAACAAAAACCGACGAAGAAAGTA
GTGATATCGGACACACGCGCAAAAATAAAGAGAGAGAGGGAGAGAGAAGGGTAAGAAAGCCTTAGGGC
CAAAAACGACAAGAGATGCGCTCCGCGCAAGA
AACGGCATGGTGTCGATGATGTAAATATCGTCGATAAGGATTAGAAAAACCTTTCTTTTTGTTATTGC
TCTTCTCATTCTCACCCGCAATCCAACCGATC
CATCGATCAAACAAAAAGAACAGGCAGTTTCCTTTACTTTTCATTTTTTTTTCCCCCTTCTTTCTCAT
TCTCTCCCCCCATCTCTATCCCTCACGTTATG
TGTGTCATCCCCCCCTCCCTCGTCAACTGTACTTTTCAGGCCCTCCCCCCCCTCATCACAAAGCAAGA
GGCTATCTAGCCGTGACATCATCTCCGAGGCG
TCACTGCAGTCGGTCAGAGCAGCCAAGGCCTCTTTTGTAACAAGACTCATCTTGGGCTTCTTCGGCAC
GATTTCCACCACGCTGCTGCAGAGGGACACAA
ACGCCTTCTGCAAATACCCCCTTGTGACCATTGCCTGTGCATGCTCTTTTTTCGTCTCATCCGCCTGG
CTGAGTATCCGCTGGTACTCCTTGGGAAAACA
GACCATATCCGTTTGTTGCCCACCCTGCAGCTCCTGAAGGGCCTCAAGGAGACGGAAGCTTGTCAATC
CCTTGTTGCACGCCGCTATGCGTGGGACAATG
CGAGAAAGGTTAGAGCGCACCGCCTCGCTATGAATTCCCACAGAGAAGCTCACCTCTAGCGAGCGTTC
GGAGGCGCAGTAACGAATAAACCGTTCCGTAA
CCATCAAAACCTGCAACAATTCGGTGGTGAACAATGCGCTTCCATCGCCGTACTTAACCTCAAGAGGG
TTTTGGAAAAATGTGTCCTCGTAGCTCAAAGT
AGACATTTTTTCTTGGTACATCTCAGGGGTTCCGGGCAGCAATCTGAATATCCAGCTATGCATGTCTC
TGGCGCTAAAATTTCCCCGCACCTCCATTGTA
GAAAGCACGGGGGACGCATTTCCTTCCGTCACTGCCTTATCGACTCTTCCCACGCGAGCGTAGAGTGG
CAGTGGTCGTAACTGCACCATCTTCACCTGTG
CGGTGCGGGGGGCAGGAGCAGCCAATGCAGTGACCTTCAGCAGTCCCCACTGCCCATCCTCCACCCAA
AATGCAACGGAGCAGCTGTAATTCCGTGTCTC
TAAGGGATGCACAATGGCAATTGTTTTAGTCAAGGGATTATTCCGCGGTATTTCCTCTTGCACAATTA
CCTCCGCAGACTCCTCTAAAAAATGCAGATGC
AACTCACTCTGGAGCACCACGCAGTCCAGCGGAGTATCAACCTCTACTATAAGTCGCAGTGCAGGGCT
GTCTTTTTGTTGCGATAGCGTCACCTTGGTCT
TGAAGGTGGAAGAAACGGCAAGCAATGGTGTGTCGCCTTTCTTCACCCCGCTGTATGCCGCCAGCTCC
CGCGTTCTGGCCTCAATTGACCGCTTAAGGCC
TTCAATTTCCTCGCGGGCTTCGTTGATTTGGGCATCTGTGCACTCTAACACGGCGTCGACAGCCTCCA
TCTCCTTCGGTGCAGCATGCTCCTCTTCCTTT
AGTGTGAAGGTTGTGATCCTGCCACTGAAGGAATGTACCAGCACGCCTGGGCGGCGAGAATCTGTAAT
GAAGCCAGCAGCCATGGACAGGACGCTCTCAT

CCGTGTTCCCCGACCACATGCAAACGGGATGACCGCTATCAGCCCACTCCTCCGCATAAACGTAAAAA
ATCTGCACCGAGCCGTCCTGACGACCCACCAG
CAGCTCCTCTCTCTCGTCCATGTTGATATCATACACAGCAAGGGCGGTTACGGCTGCCTGGTGGTCAT
CGGGAACAAAACTAAAAACCCGTGAGAGGAAA
GAGTCGCCGCTCCCAATATCCAGACAACCCAACGACCCTGCGCTTGTCCCGTAGTACACCCTGCGCAT
CCCATCACTGACACCTAAAGACGTCACGGGAG
CCTCACAACGAATGTCATCTATGGATTTATGACTCCGCAGCATACGAACAGTACGGTCATTGCAGCCA
AGAATACATCGATAATCATCAAAATTGCGTTC
CTCCAGATTCGCGACGGAAGAGACAGCAAAAACCGCCATGGCATTAATTCTGTCTGGTGCCATGTAGA
AACCGAGCTCACTCGCCTCCCTAAACCCAGTC
GCCATAAAGTTGCCGCAGCAAATAATGAATGGAGTGGAAACAAAAAGGGTGTGGATGACATCCGTCAC
GTTGGTGTCAAACGAGAAAAAGCGCCTACCCT
TCCGCGAGTACGCCTCGAGCGTGCTGCCATGAACAAAAAATAGTTGGTCCTCATAGATAGTCGCGCAG
CTCACAGGCCTGTTCTGAGGTGGGGTTTCAAA
GACCGCCACGCGCTCCATCGCCTTTCGCAGTGAAAAGACGGACATGATGCCATTTTTATCCGCAACCC
CTATTCGCTGCCTGCTCTTCTTTCCAAGTGGA
TACACAACCATGCACCTCGGAGATCGCGATGCTACCGTCGTAACCTCACGGCGAACGAGTTGAATGTC
AGGGGCTGCGATCATCGTTGTCGAAAAGCCAC
GCCGTCCGCATGCAAACAAATCCCACACGCTGTCGCGCTGTTGCTCCCGCCAATTCTTATTCCCTTGT
TTGTGTGCCTTTATTCCCCTCCGGCCCCGTTC
CCTGTGGGAGCGGCCGACTAAAAGAAAAAAAGCGGAAAAAAAAAGCGATGAGTTTCTTGTCCCGCCCT
TTAAACAAGCTTCATGCAGGGATGCACATAAC
CCCCCACCATTCGCATTGCCGCTGGCGACGTGAATGCCACCGGAGACAAGTGAACCGCGGAGATGTGC
CCCGCGACAGATTCGACAACCGGCGGCTTCCC
GACCACCCAAAAAACACCTCGTGCAGCAGTCGTTGCGTCACGAAATTCGGATCTTTCCCTCGCGTCAG
TTCCAATGCAGCTGCCAATCGAGAGAAAATCG
ACGAGTCATGGGGCAGCGGCACGCAACGACGGTGCCCCTTGACGACGCAACACATCACCTGCTTTGTG
GAACAGCTTCCCCTCCCAACCGCCCTGAATAT
TACCGCAGCCGTGTGTGACAGTCGGACACGGCAGAGGGATCATGCTGCTGGTAAAAGTCGCACAAACG
CTTCCCAAAGATGATACCACGTGGGCCCGCAT
AATTTATCCGTGTGAACCAAAGAGGAGGGGAGGGAGCCGACATCACCCCGCCTCTTTTCCCATTTGCG
AGCATAGATTTGATTCACCGTCCTTCGCGGTC
CCATTCGCACACATTACACAGAGATTTATTGCACTTCTGGATCAACAGGGCAGAGAAAGAAAAGAAAA
AGAAACGAGACATAATTGACATTCCCCGTGAA
TCCCAATCCAGCCGTTGCGTCTACTTCCTAGTCTCTGAAAGGAAGTGCATTTTTTTAAAAGGAGGCAT
TACCCTCCACGTCCTGGAGTTAATGTTTGAAG
AAAAGCATAACCATCCCCATCACTAATTCTTGAACTCAAAAAGTAACCACTCCCCGTCTCTCCCTCTC
CCTCACTCCTCCTCCTCTTGTCTCCTCTCATA
AACAAAATTTCGCCATGTAACGTTTGTATGTATGTATGTAACAAAGATGTCGCTATTTCAACCTTCAG
GATGCTTGACGGCGTTGCGTGGCTGGGAAGCT
GTGTGTGTTATCGGTTCCTTGCCAAAATCCACCACCAGGCTACTGCTAAAGCAAAGACCATGGTGTTT
CAGCCATTGTCCCACTGTCTCCTCCGTTCCAA
AGAAATTTTGGTGGGCGTAGTTCCCCTCTGCACCACTAGAGGCCACAAAGTGCCTTGCAACATCCGTA
AGCGTACTCGCCTGCACTTCCCTCGTGGGGTC
GAGGCGGCTTACGGCGACAACTCCGGCGATCCGGCCAACATCCGGCACATCCAACGACCCATGGGTGT
AAGAACCGATGGATATGTGCTCTGCCACATGC
GGCGCCAGTCTAAGCGACTCCACCGACTTGATTGTAACATTTTCACCCAAGACAGCGATAGAAGATCG
AATGTCCTCCGCTGTCGCTTCCACAAGGGAGC
GATGCGCTTCCATGGGATTTGTGAGGACATCACCTTTGCTGTCAATGATCTTCCTTTGCAGTGCATCT
TTCACCCTGGCACAGGTGTTCTGGAACTGGGC
GCTACGTGCAGCAAAATCCGTCTCGCTGCAAACAGTAATGACCGCCGCACCGAACAGGCCCCCAACGC
AGGCGACGACAGAGCCATGCTCCGTCACCCGC
GACCGTTTTTTCATCGCCCTTGCCGCTCCCTTCTTGCGTAGCACCTCCATGGCCTTCTCCACGTCGCC
GTGGGTCTCCTTTAAGGCGGCGCCGCACTCCG

CGATCGGAGCCTCAGTGCGAAAGCGAAGATCCTTCACCATTTCCATGAAGGCGACCTTGTTGACGTTG
GCTCGCAGACGCGTTAGGCACAACATCTTCCA
CGCTGTTGGATATATGTATATATATATACAACTGTGTTTGTGAACTCCCCCCCCCCCTCCCCCCAAAA
AAAAAAGGAGGCAGAGCAATAAAAAAATAAAAA
ATAAAAAAGGTTCAACGGATTACACACTGCCAGCCATCCGTGACAAACAAGACTTCATTGACTAAAAA
AAAAACCGCTTGATCTTTTGTTGTTTCTTGTC
ATTTTCAGTTATGATCATCGATATTGAATAGTTGACAAGTGAGTTTTTTTCTTGGGTGGGTGCTGTCT
CTCCCCCACCCCGCTTCCCTTCTCGCTCTTCC
ACCACGCTGGAGAAAAAACCGCAACCGTCACCTGGGAAAAAGCCCAAGGGAGACCCAGTAGTTTAAGC
TGTCATCGAGCAATTCCCTCGTCACCTTCGGA
CAGCTGCACCGAAACCCGAGCGCCCGTAATTTCGACCCATCGACGGCCAGTGGGTTATCGTACAGAAG
CTCGGGCTCCAGGTACGGAGAGAGAGGAGTGA
CGCTGATGCCATGCTCCCTCAGCATCTTCGTCCACGGTTCCATATGACCGTCGTTCGCCTCCTCCACA
AGTTCCTCCAATTTGAGGGATGCCAAATTGGA
CATCAAAGCCCCAAAATATCCCGTCTTTACTTTAAACATGGACTCCAGAACGGCGTTCAGGCTCCCTT
GTGTTGTGTCACCATCATCCACGACGTTATAA
ACCTCGTGAATGGCACCGGCACACACAATATGCCACATAGCTGCAACAACATCTTGCACGTGCACCGT
ATGTATTCGTAGGTCTTTTCCCCATAAGAACT
CCATGCAAGTCCCTGATTTTTCGTACACCGCGGCACAAACAATGCGCGGCATAAGACCACAAATATCT
CCTGGTCCATAAACGATTGGGAGCCGCACAAT
CACCCATGGGAGCTGCGAGATGGATGAAACGGCTCCTTCTGCCTCAAGGTGGTACTTCGCCATTTTTG
TCCAGGGCTTCAATCGAGTGCCTGTCTCCTTT
GACGGAGATTTGTTGTTTGACTCATATACCTGGGCAGTGCTCACCTCCACGTAGCGCTGGCATCCGCC
TTTTCTGGCAGCTTTCTGCGCGCAAAGAGTCC
GTAACTGCAGGATACTTCGCTCGTACATAACGTCCAAATGCCCATACCGTGTTTCACTGGCAAGGTTA
ACGATGATACTAAATGGCTCCCCAGCAAAGAC
ACGATCAACATGTTCCTTTTGGGACAAATCAACCTGGACAACCTCAACGTATGGAAGTGCTAAAAGAT
CCTTGTAAACTTGCGTGAGGAAACACATCTCT
GGAACTTTTTTGTCCGCAACACAAACATATGACGCAAGCTCGTGATCGGCAATGTACTTGAGGAAATT
ACGGCCAATCATGCCCAGGCCGCCAAGCAGCA
ACACACGTGGTCCCTCTGGCATGCGTGGTGTAATGCAGTCTATTCGTGTCTTTTCTGTGTGTAGGGGG
AAGGTACAATTACATGACGCAGCAGCCCGCAA
AAATAAAATAAAAAATAATAAGCGCACAATGAGAACCCGTAAAAGCAAGAGTTTGCCGTGCATATCAG
CATGCATATATATGTATGTATATCGTGCAGCC
ATCGCGTTTCGTGACACGGGGAAAAGGGGTGGAAAGAAAGATTCCAAAGGGTACGGCAGCAACGGAGG
CACACCCCCACGCACGCAAGTAAAAAAAAAAG
AAAAAAAAAACATAACGNNNNNNNNNNNNNNNNNNNNNNNNNNNNNNNNNNNNNNNNNNNNNNNNNNN
NNNNNNNNNNNNNNNNNNNNNNNNNNNNNNNN
NNNNNNNNNNNNNNNNNNNNNNNNNNNNNNNNNNNNNNNNNNNNNNNNNNNNNNNNNNNNNNNNNNNN
NNNNNNNNNNNNNNNNNNNNNNNNNNNNNNNN
NNNNNNNNNNNNNNNNNNNNNNNNNNNNNNNNNNNNNNNNNNNNNNNNNNNNNNNNNNNNNNNNNNNN
NNNNNNNNNNNNNNNNNNNNNNNNNNNNNNNN
NNNNNNNNNNNNNNNNNAACCTTATCTCCTCATTGTTCTATCATAAACCACATAGTATCACATTCACA
CACATAACAGCAACACCATCATAAAATAAAAA
GAAAAAAAAAACATAACGCAAAATGCAAAGAAAAAAAGCTTCGCTCCTGAAGGTTGCGTCCCATCAGA
CACCCTTCCCCCTCTTCAAAAGCAAAAAGACG
CTCAAATCATACACTCTTTTTCGTTGCCAAACTACTTGTCGGATAATATAAAAGCATCCACGCACATT
TAACAATAAAAAAAGATATATTGTGAATGCAC
CATGAAGGAGGTGTGGAAATATACATTTGTGGGGAGAGGCGCCCAAGCACCAACATTGGAAAGCGAGC
TTTTGTTGTTGTTGTTTTTTTGTTTTGTTTTT
CCATGAGCACACAAAAAGCGAACTCCACTGAAACAACTCCTCCCCACCCTTCGCTTAGCGTCTCTTCC
TGGATGCCGCAGTGCCACGCCTTTCTGTGTCC
TTCGGGGCCAAGCTGTACTTTTTCCCCAAGTAGTTCTTCTCCGCTTCCAGTGGGTGGTACAACTCGGT
CAAAAGCAGGGGTCTCCAGCATCCTACTGGAA

AGCCCTCCTCCTGCCATTGCTCGTACGACATGGGAGGGCGAAAAGGCGTAGCCGCGTCATCGGATGTC
GTGAATTTCGAGTTCTGTCCGGCTTTTTGTGT
CTTCTCCCCGATGCCGTCGCCGCGGCTGTCCATGATGTCATCCGGTGGACGCATGGCCCGCAGGCGCT
GCGCAACATcATTCGTACTAAGGGCCAGGAGA
GCGGCTTTCCTCTCACGTACCTCTCGTAACGCGCGAGCACGATTGCGACGATCCTGCACACTGAAGAC
CAATGCTGTTGCGAGCCATTTACGACGAATTG
TTGACGGCATCTTCGGACCCCTCTTCCGTTGTTTTTTCTGTTTTATTCGTCTCTGTGAAGATGTAACT
GAGTTTACTTGGGTCTGTTTCTTCGCGTCCTA
TTACCCCCAATCTGGCACACTCAAAAAGAGCAAACATACACAACGGACATGCGGGAAGAGAAAAAAAA
AAAATTGAATAATATTTGATGATGCAACACCA
AATGTGAAATCGCGCGCGGTGGAACCACAGAATTCCAGCCCACACAAAAAAGCGGTTCTCGGCGTTCC
CTGTTTCTTTTTCCCCTCATGAAAAACGGGCC
CATTCAGACAGGAAAAAGTACCCACACCGTGTAAACACACACACACACACACACACGCACACGATGAA
ATTTACTGCTTAGCCGTAGTTCCTCCCCTACT
TCCCCGCGTCCACGCCATGCGATTATGTTGGCCCCCCACGCCTACTGCTGAACGGGGATGGCAGGGGG
CGCAGGCGGAAGTCCCGGGAGCACTTTATTCC
GTGGCACCCGAACTCGTGGCGGCGGCACAGCGGCAGGGAGTTGTGGCAATGGCGGTGACGGCGTTACC
TGCTGCCCGCTCACCCAGAAGAAAACCCGTCC
GTCCCAAGACGCGGAAGCAAGCGCGTTGGGCACGGCCAGCGACCAATCAAGCCCAACGACAAACTCGC
GATGATGGGCGTAGCGACCAACAAGGGGACGC
TGTGGTTGGTTGAGGTCCCAGATACAGACTCGGCAGTCATAGCCAGCTGATGCCAGCAGCACGCGTGA
GTGAGGTGAGAAGCGCACACGGCGGCAGGCGT
TCTCATGTCCGGGAAGTGAAGCAAGGGGCTGCGTTGGCCGGCGAAGGTCCCATAGCCGCACTGTACGG
TCCACACCACCAGAAGCAAAAAGGCACCCGTC
GTACTTATTCCAATCAATGCTGAGAACAATCTGATGGTTGTGCCCAACCTGAGTCAATACGGAGCGCG
GTGTCCGTGCGTCCCACAGCTTCCAGGTACCG
TCTCCAGAGCATGACAAAAATGATGCGGGATTACGTGTGGAGCAGGCAACCTCATACACCTCTTTCAT
ATGTTCTTGAAATGTCATGCAAGACGCATTCG
GGTTCGCTGCCTGGTAAATCTTCACAGATCCATCCCAGCTGCACGAAAGAAAGTTATCACGATTCCAC
GTCACCCCCGCTACCTCCCCTTGATGCTCCAT
AATGTGCACTAAAGGTGGAGCACCATCAAGATTTAAAGATTGCTGTACGTTGTATAGCTTCACACCAT
CACCGCATGCTACAGCCACAATATTTTGGTCA
ACTTCGCTGAAGCAGGCATCAAACGCTCCGTCGCTCGTTCCCCAACATCCCAAAAGAGAAACAGGAGA
CCCCGGTGGCACCCCCGGCGCCGTGTTTACAA
TATATACTTTCCCTGACCCTACCACACCAAAGTGCTCGGCCGCCGTGAGAATGTACTGCGTTGGCTGC
CATGGATTGCACCGCATGCCGTGACCGGCAAA
TCCGGGATGAAAATTAAAAGAGGGCATTTTCAGCTCTTTTGCTATTTATTTATTTTCTTTTTTTAATC
TTCTGGGTCGGCCTACAGCTGCAGGCTGGCGT
GTGCGTGTGTACCAAATAATTGTTGCTAATCTCTGTCTCTTTCCTTTTGATTTAGTGAGAAAGAAGAA
GGCGGTGCGTGTGGCAGCGTCTGCTTGCACAA
ACGCAGAAAGAAAAACAACAGTAACAAGAAGAAGTCGTGCCTATTGCCGTAAAGACACGCCGCTCCTA
AAAAAAAAAAATCATCATCATCATCCAACTGG
TAAATTACATATGGTTTTTTTCTCTTTTTGAGGCCTCCATGTGGATGCAACACCGAGCCTCGCTCACA
GTACTGTACAGTGACCATGCTTTAACCCGCAC
ACAACGCCACATCATGCAGACGGATCCCCAACACTCTAGAAAAGTTACAAAAAAAAAAAAAACGGAGA
TGTCATCTCTGACGTGAGAAAAATTCTTCGAT
GGTGCGTCTCCGTTTGTTGCCTGCTGTCGTTGGCTTCTTCGCCTTCTTCGCCTTGGACGTCACCGTAT
TTCCCGCGGGCAAGAGATCCGGCGATGCCGAC
CTTTTTTTGTCCCCACTCGCCGATGAAGGACATGGATTGCCGCTCCAAGGACTCAGCAGCTGCACGTC
GCTGTCAGGAAGGCTCCACGGGGTACCAGAAG
TATCCCGCGCCGGTGACTCACCAATGACAAACTCCACATTCTGACGCAATGGCGATGGGCTTGCAGCA
GTCGTAGATTCCGGAGCGTCAAACTGCGAGTC
ATTCGAGCTGCCCAACACGCGTGATACCACGGCGTGACTGTCTGCCACCCACGACGTCGATTGGTCTC
CACGGATCCTGCGCAGTCGCGTCGCAAACTCC

GTCGGCGACAGCACCCGCACAGGCAGCTGCGGTACCAGCCTCACCGCCATCTCTGAAAAGGGGAAATG
TACACTAAAAGCGAGAGCGAGTGGATGACAAG
TGACGTTGCGTGCTCCCGCGCTGTTGCCGCTGGTGACCAGGAACCACTCCGCGAATATACATGCGGAA
AAAAAAAAAAAGAGAGAAGGAGCAAACAAAAT
ATCTCACAACGCCACAGGGCAGTTGCAAGGAGCAGTCACGCAAGGCAACACTCATGGGAACAGTGATG
CCACGCTGTTTTCCGTTTTTCTGCCTCACAAC
ACCAGAATTCGTCGTGGCGCAAATAAGAGAAAAAAAAAATGGGAGCAAAGAAGGTCAACGAGGCAAAG
CAACAAAAAATTCCAACAAACAACAACAAAAA
ACAAATAAAAAGACTCGTATTCCCCTCATTTTTCTCGTTTATTTTCGTCTTTCTCGACCAAATCCCTT
GCGTGTTTTCTCATTGTTCCCGTTTGTAACTC
CCCCTCCCCGTCCCTCCGGCCGTGATGCCTTCTCTTCGCTCCCCCTTTAATTTTCTCTCGCTTAATCT
TTTCTCCCATCATCTGCAGGTTTTCCCTTTTT
TATTATTATTTCAGGGTGCATAAATATATATGCGTATGTATTGTCATGTTAGCCGTTTCGAGATGAGT
CCAGCGCAGAAACGTGTTTGTCTCCTTCTTTT
TTCTTTCTTCGCTCCTGTCTTCGGCAGTCCCACACAATTCAATGCACCCTCCGCTACTGCGGCTCCGG
CAACTGCTGTTTCTTCTTCTTTGCCTGCGCGG
CACGTTTTTCCTTGCGATCACTCGCCGCCTTCGCCTGCTCCTTCTTCGCACGGCGCAGCGCCCGCCTC
TCACGCGGCACCAATTCGTTGATGCCCACATC
CGCGTACTTGCGGTCGGCACTGCGGTAGAGATCCTGCGGCGCAAGTGCGGCCTCGCGGGTGGTAGCAA
AGTTGCCGACTGTCTCCAACGCGATGGCGGGG
GCCTGGCCGTCCACTGCACGCACGCGGGCCGCCAACTCCTCTTGTATCGGCTTTGGTGTGTAGTAGAA
GTTGCTGAGGGCGTCGAGGTGCTGGGCGAGCC
GGCGCCACATTTGAATTGCCCGCAGCTCGTCCCTCTCAATCTCCGTCAGTGGCTCCGCACTCTGCGCA
GCATCACCGCTTGCCTCCTCCAACGCCCGCGC
ACGCTCCAGGTACTCCTTCTCGTACAGGTCCATCAGCGACAGCTTGGACTTCTCCGAGTCGATTGTTG
CGTCGCGGCGCGTCGTGACCAAATCTCCCGGC
GCCGAGAGGGAAGTGCGGCGCTGTACATCGTCGTAGTTGGCGCAAAGGATGCGGCGGCGAATGCGGTC
CTCTAGTTTGGCCGTCACTGCCTCCGTGATGA
CAGGCACCGCCTTCATGGCGTGTTCAAAGTCCAACTCCTCGTCAAGCAGCGCATCACGCGGTCGCTGG
TTCCCGCTCGTCTCACCGCTCATCGCCCACTG
CGTGCTGTAAAGCCTTTCCTGCTCCAGCCGTTCCACAAGACGACGCTCCCGCTGCCGCTCCCGCTGCA
GCATCGTCAGGCTAGGATCGTTGAGCTCCGCA
TCATCGTCGTCATTCTCACCAACCGCATCCCCCCTTTCGTGCTGTTGGTGTTCTTCCTGCGCACCGTC
ACCAGCGAGAATGTCTTCGTTTCCAAAGTACA
TCCCGTCAGACTCCTTGAACACGGCAGTCGGGTCATCCCAAGCAAGGTCCTCTTCGAAATGCGGGTCG
TCATCATCATCTTCGGCCCCGTCATCATCATC
TTCGTCCGGAACAAAGTCCTCACCGTACATTTCCTTCATTGCTGCCAACTCCTCGACGGCGCGCTCGT
CCTCCTCCTCCTCCTCGTTATCATCATCATCA
TCGGCCCCTGCCATCGCCCGAGCATGCCTACGACGGATACGTGCCAAATCACTGTTGCTATCTTCGTC
ATCATCCCCCTCGCCGTCCACGTCAGCATCAC
CGTTTTCGTCGTTGCCGTCGCCATCACCCTTTCCGAGTGCGTAACGCCACAAGTCGTCCCCGTTGTGG
CCATTCGTGCTAGCAGGTTTCGCAAGCTTTCT
TCGCTGCTGCTGCTGCTCAACAAGAAGCTGCGCAATCTCCTCATCCAAGTCACTCTCGCTGAGCTGCG
AATTTCTTGTTGAGAGTACATCTTTGCCCTCC
TCTAAAGCATCGGAATCATCAACATTAACATCGTCTCGTGGCCGCTTCGTTTGTACTCGCGTGCTGCC
GCGTTTCTGTGATGCAGAGGAAGCATTCACTG
TCTTCTGCAGACGTCGCACGTTGTCCTGTAGGCGACGCAAAACGGGACGCAGTAACATGCTCAGCTGT
CCCCATATCTGCTCCACCGTCACATGCCGCGC
CGCACTTGTTTCCAGTGACTTTTTGTAGTAAGGCAGCCGTGGCGTATGCAGCGACGATGCAAGGACGA
AGAGGGAAGAGAGGCACTGACGTGACGTTTCC
TCGCCCGCCTTGCGCGATGAAAAAAAATGGTCCAGGCCGTTGTCCAGTGCTTGCTGGAGGGTGCCCAC
GGAGCCCTTCAGCTTCCGTGGCAGCGCGGCAT
CTTTCTTCGTTGTCGCCATCGCCCCTAACCCCTAACCCTGTTCTTCCACGCGTCCAGTAGTGTGCAAC
TGAGAGCGTATGTGATGCTGCCCGACCACTTC

CCTACACGCGTTCGCTGCAGTGTCAAGAAAAAAAAAAAACTCACTTCAAGTTAAATAATATCAAAAAAA
GAAAGACGTTTTTGCCTCGTCGCCTCCCCGCG
GCGCCGCACACCGTGAATGAGAGCTCAACAAAGGCACCCCTCATCGCACGCAGTGGAAAAGAGAAATA
AAAGGAGGTCGTTGCCAAAAAACAACGGTCTG
AAAAATAACAAGAGAAAGGGCATTGAGGAAAAGTAAAGAAAAGGCAAAAAATGGAAGGAAAGAGCCCC
GCCACCATCACCACCGTAGTTGCAGTCGGTCT
GTTGCCTCAAAAAAAAAAAAAAAAAAAAGGAGAACCAAAAAAGAAAAGAAAAAGACATCCCGATGTGAC
TTCGTTTGCAAATCCAACAAATGTGGCGCACA
TAAAACATACAAACTGCACCACGTCAAAAGTCGTTTTTCCCTCCCTTTCGTCTTCCTCTCCCTCAGGT
GCATGGGTGTGTCTCTGTTTTTGTCGCGTTTT
ACTCCTCCCTTCCCTACCTCGGTCTTCATGCCTTTTTCATTATCCGCCGGTAATATTGAAAAAGCAGC
GTATCTATAAAATAATGGCCAAAAAAAAAAAA
AGCTCAATAGGCATCCTCGTCCCCTGTGGGGAGGTGCAAACCTTACAGATCACCCGCGTCATCCTCCG
TTTTTGTTGGTTTCATTTCTGCCGCGGTTGGC
TCCGCGTCTTCCTTGTCAGACGATTCATCCCCGTCGTTGTCCACCGGAAGCAGCAGTCCTTCGTCGGT
GCCAGTGGTGTCGGGTTTGACGTCTGAAATGT
CGTACTCGTCGTCGTCTGGGGGCAAGATCGTGTCATCAGCCGCAAGACCCACGCGGCCCCTCAGCAGA
CGGTTCACGCGCTTAGCGTACATGGCCTGATT
CGAGACGGGGAATCCCGCCTGCAGGTTGGCCGTGCCGAAAAGGACCCATGCCACGTCAATGGCAGTCT
GGTCGTTTGCGTCCGCCTCAAATCGTGTCAGA
AGATCCCGCACGAGCGGATGGCGGTGGTTAAGTTCCAGCACGCGGGAGTATCGCATCGTGTGGAGGGA
AGAGATTGCCTGTTGATTCATGACGTTCACCA
TGCGAGGTGTCATGTCGTTCTCCTGCGTAGACAAAATGAAAGCCTCCGAGCTCTGTCGCCTTGTCAGA
ATGACCTTCCGCACTTGGCTTTTTCCAAACAG
AGTCAAAAGGCGTTCGGTGAGGGGCTCATATTTCTTCTTGCGCTCCTTTTCAATGGCCTTGTCCCTCT
CCGTCGGCTCGTCAAGGCGCGCGTTGTCTTTT
GCGAGGTTAATAAGCCGTTTGTTGCCGAAGTCTTGCACCTGAGAGACGACGTACTCATCAATAGCATC
CGTCATGAAAAGAACCTCCACGTCACGCTCCA
GCGCCTCTTCAATGTGCGGCGACTGCCTCATCTTCTTAACCGAGTCACCTGTGAGGAAGTAAATGTGC
TTCTGTTCTGGCTTCATGCGGTCTGTGTACTC
CTGCAGGCTAACAAGGGTGCCGTTACTCTTTGTGGATGTGTAACGCAGCAGCTTCGCGAGGCGGCCGC
GGTTGTTGGCATCCTCGAGAACGCCGAGGCGG
ATATGTTTGCCAAACTGAGCCCAGAACTTTGGATACAAGGGCTCCTTGGCTTTATCGTCACCTGATTT
CTTCCCCGTTGTGTTCTCATCCTTCGCTTCAC
CATCTTCCCCTGATTTTTCCTTCTCCAGGCCCTCCTTGAGGCGGGCGTCCTTCTCCGCAATCTCGGAA
ATCATGGACAGAGCCTTGCGCACAAGTTTCTT
CTTGATGACACGGAGAATTCGGCTCTCCTGTAGCACCTCGCGGGAGACGTTGAGAGGGAGATCGTTGC
TGTCAACAATGCCCCTTACAAAGCTGAGGTAA
CGCGGGAGAAGCTGTCGGAACTCGTCAGTGATAAAAATACGTCGTACGTACAACTTGATATTGTCGCG
TGTGTCCTCGTTATTAACGAATATGTCTTGCG
ATGCCTCTTGTGGGATGAAAAGTACAGAACTGAACTCTACCTCGCCCTCAACATTGAAGTGCGAGTAA
TACATCGGGTTTCGGTAGTCGTGGGTGAGGGA
CTTGTAGAATTTGTGGTACTCCTCCTCCGTCACCTCGGATGGCTTGCGTGTCCAGATGGGCTTGTTCT
CATTCACCACCTCCCACTTCTCTCCCTTCTGC
ATGTAGATGGGGAAGTGTACAAACTCGCTGTATTGGTGCACAATATCCCTCACGTTATCCGTGCTAAG
AAATTCCAATGCGTCGGGCTTCATCTCAAGTG
TGATCTCCGTCCCGCGGCCAAGCGTGTTGCCACGTTCGTCCTCGTATATGTAATATTGCCCGTCGCCA
GCAGACTCCCACACCCACTGCACGTCACTGTC
ATCGCTCTTCGAAGCCACCCGCACGCGGTCGGCAACGAGGAAGGCGGAGTAGAAGCCAACACCAAACT
GTCCAATGAGACTATCATCCTTCGTTTCCTTC
AGCTTCTCCATGAACCGCTTCGTGCCGGAGGTGCCAAGGGAGCCGAGGTGCTCCTCCAGCTCCGCCTT
CGTCATACCAATGCCGCCGTCCCGCAAAGTGA
GAGTCTTGCGCTCATTGTCAATTATCACCCGCATTTCCAACGCCGGCACCTCGCCGTCTTTGTTCTTC
GGCTCCTTCGGCGCCGTGAGGTAGAGCATACG

GATCTTATCGAGGGCGTCGCTGCCGTTAGAAATCAACTCGCGCAGAAAGACAGCGCGGTTCGTATATA
AAGAATTAATAAGAATGTCAAGCATCTTGCTC
ACCTCAGCCTGGAATGAGATTGGCCTCCCCTTCTCCACGCTCCCATCATCCTTCACCGTGACCCCAGT
CACCGCAACGCCCAGCACAATAAGTGCGATAA
GTATCGCCTGAATAATGGGGTGGCGTGCCATCACTTTTGTTATTGCGTTACTGTGTTTTTGTTATTGT
TGTTGTTTTTGGTCCCGATTTTATTGTTGTTA
TTGACTTTACTCTTACGCTTGAGCGTAACAAATAACAGTGGGCAGTCGGACAGATAAATTGCCTCCTC
GCCCCCTTCGGTCCCTTGCCTTGTTGTGTTCG
TGTTTTTTTTTTGGTGTTTGTTTGTTTGTTTGTTTTTGTTTGTTTTTTTCTCCTAGCTTTGGCCCACC
TGCCGTCCCCCTGTCGACACCGAAATGACGCT
GATGACTTGCCACAGTCGCTCACTCTGGTGGCGTCACATTGCAATGAAGAGAGAAGGCAGCGGGCAGA
GAGATGCGGGGGGTGGGCAAACAAGAAAAGAA
CGTAAAAAAGAAAAAGAAAATTTTCTTTTAGGTTTCTCAGCTGCAAGCAGTCGGCACGGCGAAATACG
TGCGGGGAATGAAGCCACAGACAGACAGAGAG
AAAGAGAGAAAAAGAAAAAACTTGCCTCTTCGCCGACAGAATGGAGGCCGCGAGGGCACCCCTCCGTC
TTTCAACGCTTTACCGCCACTACTGTTGTTGT
CTCCTCTGTGCCTCCACCTCCTCCCGCTTCCGCTGCGCCATCTCGCACCGCAGCTGCAGAAGACGGTT
CCGCTCCTGCACGGCGGCGTAGTGCGGCAGCA
AGTACTCAACGCCCATCCACCACGCCACGCCAATGCCAAGGAAAAACGTCCCCACCTGTATACGGCGC
AGAATCGTCATCTTCTTCAGCACCTCTTTGTT
CCGCGTCTCCCTCTGCCGGTCAAGGAGACCCTTGTTCATGATCTGCTGCGCCCGCGCCGAACTTTTGA
CCTCAAATGCCCGGCTCGTCTGCTCGCTCACA
AACGCGCGTGGGTTTGACGCCATTTTGCGACGCTGCTGCAGCTCCTCCGCGGAGATCCCCACCTCCTT
CGACGGGTCCCGCCGCGCCTCGTCCACGGCAT
TCCTCATCCACGATGACATTTCGGCCGCCGCCCTCACGTGATCTTTCGGGTTTCCCCCAAATCCCCCA
TCGCCATCTTCCGCATGGCCGCCGCTCCTGCC
GCCCCCACCCGGAACAGCCGACATGTGTGAGTCCAAAGCAGCACCGGACACTCCCCCACGCTACTCAG
TCAAGCACACACAAACGCACACAAACTACCCC
CCGCCAGCCACAAAAAGAGAGGGAGAGCGAGCGAACAGACCCGCACACGCAAAAAAAAAAAAAAAGGAA
AGAAATAAAGAANNNNNNNNNNNNNNNNNNNN
NNNNNNNNNNNNNNNNNNNNNNNNNNNNNNNNNNNNNNNNNNNNNNNNNNNNNNNNNNNNNNNNNNNN
NNNNNNNNNNNNNNNNNNNNNNNNNNNNNNNN
NNNNNNNNNNNNNNNNNNNNNNNNNNNNNNNNNNNNNNNNNNNNNNNNNNNNNNNNNNNNNNNNNNNN
NNNNNNNNNNNNNNNNNNNNNNNNNNNNNNNN
NNNNNNNNNNNNNNNNNNNNNNNNNNNNNNNNNNNNNNNNNNNNNNNNNNNNNNNNNNNNNNNNNNNN
NNNNNNNNNNNNGACGATCACGACAACAGTAC
CAACTGGGTGACAAAAGAAGAAGAAGAAAAAGGAAAAAAAAAAAAAAAAACTTTTTTTTTGTTAAGAA
AAGTCAAAGCAACAAATTTACAACGCACCTCA
TAGAAAACAAATACAACACATACACATACACATGTATGTATAATGTAATTACTATACACGCGGGCCGT
AGGGGTGATAAACCCCTACGAAGATGCCCCGA
GTTCTCTGTCCGCATCAATTGTGGATGCATCCCTGTTACGAGGCATAGAGAGGCTAAATATATATATA
TATATATATATATATAATTTTTTGAAAGGAAT
GACAAAAACAATGGGTCAAACAAACGCCCACAAGATGCATTGGAAGAAATGCAGAGAAAAGAAGTAAA
AATAAATAAAAAAGTCAAGAGAAAAACAAGAA
ACAAAAAATATATATAAAGTGGGCAAAAAATTACTTCGGAGTAAACATGAGAAGCGCGGGTGTGGGCA
CCAGAAAAGGAAAAAGACCCTCCAGTGTCATC
CGCAGTGTTTTATGCCCACCCCCACACACTCAGAGGCGCGTATTCCCCTTTTTACTTCTGCTCCCGCC
CTGCAGCTTGACTTCTCTCTCTCTCCCTCTCC
CACACTTCTTCTCTTATCTTCGTTTCCTTTTGTGCTCCTTGCCTCGTTTGGAAGGATACAGAAAAGTC
ACCCCGACCATCATTAATAATAATAATAATAT
TCTCTTTTTTTCTTCTGTTGCTTCGGCTCTCTACATGCATAAAGCTCATTCCCGCATTTTGCGTCCTG
ATTCCTTACTCACCCACTCCCTCACTTGCGCT
AAATCTTCTATCCGCCACACACACAAACACACACACGTCCTCCTCCCGCCGCGCCAGAGCAATAAAGA
ATCCCGCCCCTTTCTTTTACTTCTCTGTTCTT

TCTTCCCCTTTCCTGGCACCATGCCGTTACTGTTAAACCTTTAATGGCGACAAGGCCTCAAATAACCC
CGCGTTAATTCTCATACTTTTGATTCCAACTT
CTTCTTTGCTTTTCCCTTTTTATAGTTGATTGGTTCCACTGATTTCGTGCTTTATTACGGAGCATACT
TGGCCTGTCGCGGAAATTTACTTCCTTCCTTC
ATCTATATATGTATATGTATATGCAAATCCTAATATTTTCACACCTTTTTTTTTGTGTGTGTGTGTGT
TTTGCCTTGTTTTCTCCCACTTCCCACAAATG
CAAATGTTGACTCCTGTCCACTAAAAGTCGTAGAGATCGGCCTCGTTTTCCTGATTGTATGTGCTCAT
GCTCTGTTGAATCACCCGCTTCATGATGAGCG
CCGCCACCTCCTTGTTGGGCCTCAGTGAATCCGCCGTCAGCGTTTTCCCTGTGATGGGGCACACGGAG
CCGTTCTGCTCGAGCCACGCGAGTATCGCCTC
CTGCTCGAACGTGTGGCCATACGGCGAAACAACAGGTCTCTTCATCACATTGCCGGTGAGGGCGCACA
GCAGGATGGGATCGACACCTTCAATGACGTCC
TTCCGAACCACCTCTATGTACTGTTTCATGTCCACTGGGGCCGCGGCACTTTCAACGGGCACCTTCAC
TTCGGCTGTCTTTGAACGTAGCGGGGGCATCT
CGCCCAGGATGCGATGCCTTCCTCCCGCAGCAACAGGGGCGGGAGACGGCAGCGGCTGGTGCGCCGCC
ACGCGTGCCTCCAAATCGCCAAAGAGCTTCTC
CAGTGTGGTAAAAGTGTGGGTGCTGCGGTCGACAAGTGTGTCCGCCTCCACTGTCTTCAGAGCAGTGT
GCCACGCCCGCGCCCATGCCAGCCCGCCCGGG
GCAATCTCCCGCTGCAAGAAAGCCTCGGTTGTTTTCAAGAGAGCCTCGCTCAGCAATTGTTGAGGGAT
TTCCTCAATGGAGAGATCTTTCTCCCCGCCAA
GAGCATCTATGTTGGCCATGAGGTGGAACATATCCACATACAGGCGCATGAGAACACCAGACGCCGGG
TTATCCTGTCTCACGTGGCGAATTGAAAACTG
TAACATGTCATGCAGGTAAGGACAGAACAGACGCACCCTTCTAGCATGGCGGTGCATACGAAAGGTGC
ACAAAGAAGCAAATTTAAATCCTAGTGACATT
CCGCGTAGCATTTCAATGCTAAGATCCGGGCTCGCTCCCTCCAAACTCTTTTGCAAGGCCTCGCACTG
CATGAGAAGGTACAACGTCACAGAATGATCCA
GTAAGGTGGTGGAAAGTAGAATGTGTTGTCGCCACTGCGTTGACTTTGTGGATGAAAAACGAAATATA
TTCATGACACAACGCATGACACTGCATGTCGC
CACATGGAGCAGTGGAGTCATCACGTTCGTACTGTCCAGTTGCTCGGTCAAGACATCACTGATGACTT
GAACGGCTCTCAGTCGAAGCATTAAGGCCAAC
AGGGAGTCGACCTTCTCCGTAAATAAAAGGCAAGAGGCCTCCTCTACTCCTTTTGAGGAATCAAAAAA
AGCCTGAGGTTGCGTGAAGCCAAGAATGATGG
TTATGCAATTGCCTCGAAGTCCAAATTCGTACGCAGGTTCTTTCAGCAATATAGCGAGCATAAGCTGC
ACAGAAAATTGTGCGTTCCCGTCCGTAAACTC
AAGCACATTCACAGCCTTCAGCTTGTATCCGGTCACACGCATGAGAAGCACGGCTACATGCTCCGTCA
TGCGTCCCAGATCGGGATTAAACGGCAACGTT
CCATCCTCCCTTATCGATTCCTCAAAAATGCCCTCCAGCTTGTCCAATACGTCAAAGATGCAGCCCCG
GATGGGCTTGTGAAGAGCAAGGTTATTCAGGA
TGAGGCACACACGCGACTTGTGATCCAGTGGCACCTCATTCGTGTTGGGGTTGTTCAGGTACTTAGTG
ACCAGTTTGTCTGAGATGCGTTGTGCGCACCG
CTTCGCCTCCTCCAGGCTGGGGCCGTAAAAGACTTGTGCGCGGCCGATTTCACTCTCCCGGGACCAAA
ACGTCATCTCATCCACCCGCAGCGGTACGTCG
GACTCAAGCCGCTCCGCAAGCTTCTGGATGGTCGCCATCTGCCGCCTGCCAAGTATTTTTTCTTTTCC
TTTCGTTCTTTCTTGTTGTTGTTTTAAACGGA
TGTCACCTTTTCTTTTGTATTTGACGTATAACCTCCTGCGTTCGCCCACCCATAAAAAAAAAAAAAAA
AAAAAGTGATCCGGTAAACTCGCGCGGGTGAG
TCAGAGGGTTCGGGATCCCTCGGTGGCTTTATTGCACACGCGCTTGCTGGCCGTGCGTGTATCCAGCA
GGGAAATAACAGCCAAGGGCGAAAAAAGACCG
TCAAATTGGCGCAGCGGCAACTTCATCTCCGAGACCACAGAAGTAAATATATTTTTTTTTAAGTGGAT
TTTTCTTTGCTAGTCGGTATTTCTTTCCCTCG
CCTCGTCGTCCTTCAGGCTGCCCAACGCCTGCGAACACACGTCGGGGAGGTTGCCAAATAAAAAAAAT
AGCGAAAATAAGATGTAAAAGATAAGAGAGTC
ACTCATACCGGATCCGCCAACGGACGCGCCACTCACCCCACCGCTCGAGGCAAAGGGATCGTCTAACA
ATCAACAATGCAGGAACATGCCGTGGTCAAAA

GAAACGACAAAAAGAATACAACAAGTGGACAACGGCGCATAACAGAAAAAAAGAAATAAACCTTCTAC
GGCCCACCTCTTCCCAAAAAAATAGTGAATAA
TCTTCCGGGAGCTGGCCGGATCCTGACAATCTATGGAGACCAAACAATTCAAGAAAAAAGGTTCGTTA
GTTAATTGCTTCAATTTTTTCATTCAATATAT
AGAATTCTTTGGACCACCAGCATGGTCCTTTGTCCTTACTTAGGATTAACATATTGTTACTTACGAAG
TGGCAGACTTTGGGGTCCTCCAACCACAAGAC
CCCGCCGCGTGGTGGGAATCAAGGGGGTTAGGGTGGAATGGCAAAATAAAAACAAAATACAATGGCCT
CTATCGGCTCCCTGCCCCTACAAAGTCACGTT
CTTCCCAAAAGATTCATACGGTACAAAGTATCATAAATAATGCATTCATTTATCAGGATATACACATA
CATTAATTAAGCTAGCCAGCTCTCCCGCGATC
ATTCTTTTCACAAGCTCCTAAAATAAATAAATGAATTGCCCAGTACCATCAAAGAGCCTTTTTCTCAG
ACCATGGATTTATTGTTCATTTGTGCCGCCAG
CAAATCCGTCAGGACCGATTGGCCACCGCTTGGTGGAAGCAGGACTACTTTGTTCCTCCCCGTACTTC
CCACCTCCTTCAGCGAGTCAAAGTACTGGTTC
ATGAGCAACAATTGCACCACATCTCGTGCGCCGACGCCCGGCACGCCATCGACAAACGACTCAATGCT
GCTCTGCAGCCCTTCCATGATGGCCCTGCGCT
CCTCCGCCAGCCCAACGCCAGCAAGACGCTTCTCCTCAAATTCCGCCTCCGCATCCTTAATTTTGACG
ATTTTCTCCAGTTCGGCCTGGTGCTCCGCGGC
AGTCCGGCGGTAGGCATTAAGTTGGGTCTGCGCAATGGCCTGCTGGAGTTCGGTGCTCGGCTCAATCT
GAGTCACCAGCGTTGCCTCAAGCGCAAAGCCG
TATTGaCTGAGGCGCTCCTTTAACTCCTCCTCAACCGCATGCTTGATGTTCCTCGACGCAACAAAAAC
CTCGTCCAGCGTGTACTTTGGCACCTCGCCGC
GGATCACGTTGGCGGCGAAGCTCCCAATCTGCTGCTCCGGGTTCGCAAAACGATAAAAGGCGTTGGTC
GCCTCCTCCGGCAAGACACGGTAGTGGAGGCA
TGCCGTGATGAGAACCAGAGCATTGTCCTTTGTCTTCGTCTCGACATTAACGGAGGCATATTGCAGCT
TCAAAGTCACGCGCCCCCGCACCGTCTCGACG
CACGGAATGAGGCACTGGCAGCCCGGGTTGGCAATGCGCTGAAACTTCCCGCAGCTCTCAACGATGCC
AAGTGACGATGTGGAGACGCAGAAGCAGCAGG
ACATTGCCTGTTCTTCTCCGTTTATGCTCCCCCTTTCCGCTCCGCCCGCTCGCTTGGTTGATGTTACC
TCCCTTTTTGCGTGCTCCTATAATGTTTTCAC
CGCCTGCTTGAGCGTGTTGCTTGCAGCAACATAAAATGAGAAATTAAAAAAAAAAAAAAAAGGAACAG
GAAGAAGCGGCACAACAAACAATCCAAAACAG
TGGTCGTTTTAATTCGTCACAGCCCCACGCAAACGCGCAAGTGGCTTCCGCAAATTGCTCTCACGCAG
ACGCTCTCAATCCTGACCCCTCTCCTCCTCCT
CCTCCTCCCCTTGTAGAGCATGGGGAAACACTGCTTGCCCCACGTGCAGAGTGGGCCGCCATTCTCTT
ATCCCGTTTTGTGCAACGTGTCGGTTCAGCAT
CCGCAGCAATAGAAAAACGTCTGCGGCGCACTTAGACACCATCTCCCTCCACCGCGACGAAATCTCCG
TAAAATATTTTTCAATCGTTCTGGGTGGTGGG
GCTCCATGCACTGCAAGCGTCGCCCACTGCCTATTGTACGGAATGAAGCGAACAGAACCACCATCCGT
GAGTTCAGAAATAACTTCAGGCGTGGCGCCAC
CCTTCTTCAGGCTGTCCTCGTGTGCCACAACGACGAGGTTCCCCTTCTGTGCCAAAACACTTTCACGG
AGACGGAAGAGCAACTGCGGAGAAAGTGTCAC
GCCCTGGTTAACGTAAAGCAAAAATGTCATTCGGCGACGCGTCAGGTTGGCCTGTGCACATGTGTGCG
AAACCAGAGTATATTCCGTGTGGTAGCGGCCC
AGTGGGAATGTCCTGAAAATGACACGACTGACAAACCAAGCGTTGTTGAAGGAGACACGTCGTATGCG
CTCCGCCACACGGGAAATAATGAGCTCCACAG
GAAGCTCCTTCTCCACTTGCAGAAAAAAAGGAAAGGGGAGTTGCAGCGACATTTCCCGCAGTACGGCA
GATGCGCAGGCAAGCTCCTCCGTGGCACGTTC
CACGGCGCTTTGAGTCCCTCTGCTGCTGATGGATACGATTGTCATGGGGCGGCGCTGGTCATCCTCCC
CGAGAAAAATATAAGACGCGGAGGACAACAAT
AACTCCGATGAACTCCGCCGCCGCTTCTGATGATGAATGCCGTTGCCGCCCGGGCCAGAGTCATCAAG
GTACATGGATGCCAGTTCCTCCGCCCCTTCTT
TGTTCCACGCCTCAGTCACGGGCGGCAAAACCTCATCCGGCGGTGCAAACTCCTTAAAGAGGCGCGTG
GCGACATACTGTGACTCGAAGTCCATCCCCAC

GACATTCAATCCAAAGATTGCCAACAAAGAAAGCCACGTTAGAAGAGACCAGTAGGATGACAGAATAT
ACGAGAGAGGCAAATTGAAAAAACAGCGTTGC
TTCATCCTCACCTGCTCCTTAAATGTGAGCTTATCCGCCTGTGAACTGAGGAATCTCCACATGTTGAG
CGTCGTTGCAATCCGCGAACGCGTTGTGGAGA
TGGGGAGCTGAAGATCAATGGCCGCATTAAGAACATATGTGTACCTTGGGTTATTGCCCGCCGACTCG
CTCAGCACCTCGCGCAGAATCATGACACTCGC
CCATGGCGATAGGGTGAGGGGAAGTGATGTCACAAGAAGGCGCAACTCCGCCGGAATTTCGTCACTGT
TTTCACCTGTGTCACCGTCACTGTCGTTACCG
GCGTTACTCTCTTCTTTCCCAAACTTCCCCTCAGTAAAAACATTTACAAGAAGAAACAGTTTTCGACG
GTACTCGTCGTGGATGTTGGGCAAAGGCACAT
GTTTTTCCACAAAGGCAAAGAGCTCATCGCTGACAACCTTGCAGCTTTGTTGGTTTGCCCCAGTCTCG
TCAACAAGTGCCTTGATCAACTCCTCAACAAA
GGCTAAGAAGCGCTCCCGAATGGGAGCTGGTGTGGACAGTCGCAGAGGCCTCATCGTGCCCCCACCAC
AGTCACTGTTAAAATTCCCGCGGTCAACAGCG
GCAGCAGTGGCGGTGGTGGCGGCGGCATTTCGACCCTCCTCAGCAGAGGAAAAGGTGGTGGGAGGAGA
GGCACTAGCAGGGGCAGCCTTATCCCCCTCAC
GAGGCGAGTAACCCCGATTATCATCGTTTGGTGCATCGTATGTCACGGCCTCCACAATATTACGCATC
TGGGCCTGAACTTGCCTTTTCAGCCGCCGGTG
ATCACGAACGAGTGCCCTCAGGATTTCCTTCAGACTTCTGTACGAGAATGTCACCAATGCCCACGGGC
TTCCGTCCATCACCTGCAGCTCCTCCAGTTGC
CCACGCGCCATACTTTCGCAGCTTCCTGTGCCGTGATGCAAAGGAATATTTTTCTGCTGTTGCGTCTC
AAAAAGACGTGCACCAAGGGCAGCCACAAAAC
CCAACTCAACTTTGGAGAAGGAGTGTGCGCCGCGACGGAGACGTTCCTCCATTCCCATCAGCACTTCA
CGAATGACAAGAAAGAAAAACTCCTCATGGCC
GCTACTGTCAAGGAGCGGCAGGATGATCGTCATCACGACACGAAGATCGGTGAACTTTGCCGACAGAA
GACCGCCTTTTTCCAGGAGAATAATTGCATCC
GCCTGGCTGTGCTGAACCACCCGACGGAAATGATCATGGCACACGGAGGAGTTGGGAACCTCCTTCAG
CAGCCAGAGCGTAAATTCCGTTAACTCTGGGC
TATCCGCGGGAACAATATATGTGGCTGCCAAGGACAAGACACTTTGACGACAAATACCAGCTAGAAAG
TTCAAAACATGAGCCAAATCATCCGTTTTCGG
CAATGCCGTAGCAAGAGAAAAAAGTTTCTCGGCGGCGACAAATAAGTAAGTGGGCGGTGTCACCACAT
GACCACCGACTTCATCGGCCTCACGCTTCAGC
TGCTTTAAACATTGCAAAAGCAGTATGGCCCCAAGCACGTGTCGTAGGTTGACCTTTGTTAATTTTTC
CACCTCGCTATTAGATGAATCCTTCCTATATT
CAACCAGTACGTCTCTCAGAACATGAAAAAGATACGGTCTGAGCTCCCTGCTATAAATCGCAAAAGAA
TAGACGGCAGCGGCGTCACACATGCCCCAATC
CACACCGTTCTCCACCATGCGTGCCTCGATACTCTCAAATTGTCGAAGTTCAAGTCGATGTTTTTCAC
CGGCGTGGTGCGCGACGTACATCGACCGAAAC
GAAGGAACACGGGCAAAAGCGGCGGATGAGAGCTCTCCATCCTCGCGGAGCCACTTAACGCGATTGTC
CTCTTCGGGAGTGAGTAGTAGTCGCGCCACCC
AGAGACCTTCCCTTTTCCCCACCCCCTCAGCTTCCTCCAGTTGTGGAAGACATTCGCAAAGCCCGACT
TGTTGCGCCTGGGCCAACGCGGGTCCAACCAA
AGCTTCATATTTCGCCTCGCCCACCAATTCTCGTAACGGCTGCTCAACATCGGCGAGTTTCAGTGCGA
AAACGAAGTCATCCCGTTCCCGATCCTCTTCG
CTGCGAGAAACGGCAAAAGAGGAGTTTTCCGGCTCCTGCTCGGTCACGCGGTAGAGTAGTGCGGCACA
GCAGCAGCGGTTTCTATTTGGCGTGGAGAGAA
TTTCCTTGGGGGAGAGAAGGTTTTTCGCACAGACGCCCGGGGAGGTGTTGCGATTCCTGAGTACCGCG
TACGCCGAGCGACGAAAGGTTTTGTACGTGCC
TCCTTGAGGCATCTCCGCCGCTGACGATGATGGTGGCGGTGGCGGGAGGCGCTGGAGAACAATCGGGA
CGTCGCTCTCAAAGCGGTCAAGACTCTTCACA
GTCAGGCCGCCCGTCGCGCGTCGTCTTAGGCAACTCGTGGAAAAGGCCAAACGCCCCATCGTCATTCC
CGCCTTGAGAAGAAACAGACGTCAAATTGCGG
CACGAACGCCCAAAACAAAACCAAACGCATGCGTTTGTTTTTTTTTTTTTTAAGCGTAAAACGAAAAT
AAAAAGAAGAGCACGGGAATGTCGCAAATTTC

CAGGAATAAAAATAATAATAATAAAAGCACGCGACACATTCAAGAAATAGTGAATAAAAACAAATAAA
ATCGCACGAAGAAAAACCAACAATTCCTCTGG
TATTACAGTCGCTAATGGTGTAATTTATTGGGGTGGGGAGGGTAAGAGAACATCACCCCTTCCTATTC
GTTAAACAACATCATCCACGGCCTTTTTTATT
TGCAGTCCTGGTAGAGCTAGCCGTACCGAGCGGAGCCCCAAATGCAGTCGTACCCCCAGTTCCAGTCG
CAGATGGAGCTGACGCGCCAAAACCAGCAGCA
GCAGTGCTAGCTGTGGTTGTGGTTGTGCCTGCGGTTGTCGCTGCCACAGTAGGAACAGCTGAAGCAGT
GGCAGCAATAGCTGCGGCAGGGGCAACAGGGG
CACCAAACCCAGTGTTGTTGGTACCAACAGACGCCGGGGCGGAATCCTGTCGCTGACGCTCAAGTAAC
ACATCGTATTGCATTCTTCGCTCCCCATTGCT
AAGGGCAGCCAAACGATCCAGACCGACAGAACGTCTATGAATGTCCCCAATTGTATCGTGGTTAGGTT
CTGAAAAAGAAGATGCTTCAGCTGAGCACAGA
GGAAATGGCCTTTTGGGCCCAACGGGCATCTGCCGCTGTTGCTGGGCAAGCAACACCTCGGCCTCAGA
CGATCCATACAAATGGGCAAAGAGATCCCGGG
CGGCATCTGCCCTTGCATGCAGTCGCGAAGTCCACGCAGCCAAATTCAGGAGGGTGGCTAACTCATTG
CTGAGAGTCGTGTTCATTTGAACAACCGGATG
GGAAGCTATTGTAGGCCTTAGCCCGGTCGCAGTATCGAGTGGCTTCATCTCTGCAGTGTATCGTGAAA
AATGCGATGCCGAAGGTTCTGCATTAGTAGTA
TTGGACGTGTTTTTGTCATTATTCGTCGTGCGCTTATGTCGTTGAGCGTTGGGCAAAACAGCGAGCTC
TAATTCCGTCAGGACCGCAGACATCTCTTCCA
TCCGAGTCTGAACTTCGCTAAGACAGCACTGAAAAGGCTCGGAGGGAAGCTCTGCTCGATTACCACCC
AACCGCTGCGTAAAGTCCTGTTCCGCCAATGG
CTCCCAGACGTGCTTTGAGTACTGAACAACCTCCTCCTCACAGTTCTCCAACAGCATGCCGAGATGTT
GCCCCTGAACCTCCCGCTCAAAGCAGTCCACG
CGAATCGCATCCACGGCATTATCACCTGCCGTGAGCTGAACAAGTTTCTTCTGCAGTTCCATATAGCT
TGAATAATCACCGGCACTGGTTCCCATTGAAT
CTGTCAAGAAACTCTCAACAAATCGTTTTGCGTCGTGTTCCGCCTGAATGAAGTTGTGCAGCTCCATT
AGGTGCTGTTGCAATGGCGGGGGCAACTCTTC
AAACAATACATCATCACGAAAAGTGGAGAAATTGACACTCCGCGCCCAGTCGGTGGAGAAGCCCGGAC
CCTTTATGCCCTTGTACGAGGGCACCTGCGCC
TGGCCCCCGATAGCCGCTGCAGTGCCGAAGCCCTTGGCAGCACCAAATCCGCTAAATCCAACGGTGGA
ATTCGCGCCAAACCCACCCGTCGTGGCGGTGT
TTGCGCCGAACCCACCAAAGCCAGAAGAAGCACTTTTCCCCGCAGTCGCTGCTGGCGCGGCCGCAAAT
CCAGTTCCCGCAGATGCCTGTCCAGGTTTGAG
CATGCCCTCACAACAACTAGTAGATCTTTGAGTCAAAGGAAATCAAAGAGGCAATGGTGGGGTGTGGA
CTCGGGGCAAAAGAAACACTGGGAGAGGCATA
AAGATGGTATGATCCATCATCATTCCAAACGCTCAGCGAAGCACCAGCAAGAAAACAAAGGGCAACAA
CGCCACCGTTATGATACAAATGAGGACAGAAT
AGCAAATCTATGATCATTTGGGGGACGCCACACTTCGGGCGGCCCAGTTATTACCATTCCATTCAAAA
GTAATAATGAAACAACGCAACAAAAACAAAAC
AGTTCATCATCGAATAGACCCATTTAACAAAGCAAAACGCCCACCAATCCATTTTTTCCTTCCTACTC
TGACCTCCACGTCTAGCTACACAATGCCATCT
GACTCCCGAAGAGGTGTTCGAGAAACGACAAAAGTTTCCTGCGATGAAGCAACTGCCATTGAGAGGAT
TTTCCGAGGCGAAGGTCCTGTTCAATGCCCAG
CCGTTGACGCACAGCCAAGGTGCCTAGAAATTCTGGAAGCAGACGATTCTGGTGAGAAGTTGCGGGAT
CGGCATTGGTTCGTGCAATCTCTCACACTCCA
GTACGGGCGACCACGGAGCTCACATTCCCCGTATCCTGCTCGTGCAAGGTGGATCCGTTCCTGTCGGT
CAAAAAATTCCTGAGTGATCAGGTGCTCGCCG
CCCGGATCTGATGGCCTGGCGCTGCACAACAAGTAACGGTGAGTGTCTTGACGCATGGTACTACGCCA
CTTGTTGGTGAGCTGGGTAGTAAAACATGTCA
GCAGATCCGTGTGCCATAAGGGTGCGATTCGATGCTCCGTGTGCTGTCCGCTTGCCGTGGTCTGCGTC
GCATACTGATCCGCAAGCTCGTTAGCGTGTAC
TCCGCAATGTCCGTAACAAAACTGCAGGTGCACGGACCAGCCGGCACGCGTCAGTGTCAGGAGACGCT
GCCAGATCTGGTCCTCCGTCCAGCCGGTCCGA

CTGAGCGGGCCCTTGTTTAGAGCCGCCAGAATGGCCTGGCCGTGCGTCACCACAAGCAGGGTTTTATG
CCTGTGTGCTAGATACGAGGGATAGTCAGCTT
CTCCAGGGCTAGAAGCAGCGCACGGGATTCCGTCCTGCGGCTACACTGGCGTTTGCCACGGTTGAAAC
CTAAATTCGCTATTATCTCCGATGAATGAAGG
GATGAAAGCAGTATTCCAACCCCTGCTGACTTGGGAACGTCGACACCGCCATCGGTCGCAAGTATCCA
CGAACGATGGCTGTGCAGCGCTAGAGCCTCTT
CGCTGACCCTGCGCTTTTCCCTTATCAGCTCTTCTTCGTCGATTATTAAGTGGTCCATCGGAGTTTCC
ACAAGAAAGAGGTGGACGCTCGAGTCGTTACT
GCACACAACGGCGGAGGCATCACAAAGGGCTTCATCCGAGACCCTCCAACGCGTGCATGCACCCGCTG
CGCTTTCTTGAAACAAAGCCAAGCACTCCCCA
ACACAAACAGATGATCGATTTCGGCCTCCGCAATGGTATCAGCCAAGACGTCCAGCGGGTTTTTCTTT
CGGCGCTCCTCCAGTCTCTCCTCCGAGCACTC
CCATGCCTCTGCCTCCTCCACAAGGAACCGATAAAGCATCCCATAGTCCACAAACATGCCACCCACGC
GGTAACCATAGCTGCGGGCCACGCGCTCTGTG
GCTAGCCACATCAGCTGATGCAGACCATGAATGAAAAACAAGGGATCCTCATGTTTCCAAATGCCCTG
AAAGCAATGAATGGCAGGAGCAAAGCACATCT
CCTCCTCAACGTCCATGGTGTGCTGCATCGCATAAAAGGAGACAACGCCGGGGCAAACAAAGCGTGGT
GCCTTCACCGCGGAATCAAGAAAAGCGGTTGA
CATCCAAGAAAAACAGCGCCGTTCTTTGGCGTCCGGACGCATGTGCTTGTCCCCGCAGCACGACGGTC
AGCAACAAACAGGACACGGGGCCCCACCGCGT
CCTCACCACCCCGTCGCGCCCCGACACGCAAAACACCCAGGTGAGTGTCCATGTCAGAGTCAAAGCGG
GCGATTGTCGCGTTAAAGCCTGTGTCGTAGCA
CGCCACGAGAACAGGGACGTGCACGGCAACACCCGGCTACAGCTCCGTCTGAAACGCGTCTGCTCGAG
GCTGTTCGGCGCCCACCTCCTCCTCCTGCGGT
TCGAGCGTGACGACCGTCCGCTCATGAACCCTCGTCGCGGCAGTCACGCGCAGCTCTCCCATCGCCGG
CCGCACCAACCTTTGCTCCGTCACGGTCATGG
CGCCACCTACCCTTTAGGAACCAGCTCTACGCTCATCAAACAGAAGACAGAAAAAAAAGGCAAATGTT
CACCGCCGCGCAGTGCTGCAGAGGCCAACAAC
ACAACACAAAAAAAATCGCGTGTCTACTCATGCGGTAAGAATGCTTTCATGCCCAGCTGCTCCACCCC
GACGGCAGCGGCGGAGTGGGGGCTTCTGGGAG
AGGCACCAGCTGCTCCTCCACCCGGCCGTCTACATTCACAACCGTACGCCGCAGAAGTGCCGACTCGT
GCAGCACGCGGGCAACAGCCAACAAGACAGAC
GGCTCCTTCTTCAGCATCGCCTTGATGTCCTGGTGGGTCGCGGGGTCGTCGATTTGCCGCCGCAGCTC
CTTGATGATGCTGTAGATCTCGTCCGTGGCCA
TGCCGTAGATGCTTGACGGCGGCGCTGGCGGCGGCAGCGGCGGCAGCGGCACGTCGCACACGCTCGAC
GCCCCGCTGCTTGGCGACCGCGCCCCCTCCGA
GCCATCATTCACCTCGTTGTCGTCGTCAAAAAACATCCGCTCTTCCTACCTGTACCTGCACAGCACAC
AACTAAAAAAAACGACAAACAAAACGGGAAAT
GTGGGGAAAGTGGCAACCGCTGCACAGGCGCTTGACGCAAAGCGTGACCAACACCAAACCCAAAAATG
CACACATTAACACTTCAGCTGAAGCTTAACCA
CTAATAACATTTTTCAAAAAAAAAAAAAAAAACGCCACCACCACACAGGTGCACCACGACTGTCGACGG
TAAGAGCCACCGTTGCCGCAAAATACCCGCAT
GTTTGGAGTTGCTTGGTCGCACTTCAGCATGCATTTACCTGCAACTCCAACAGGAAATAAATAAATAA
GAACAATCAAACCTAAATACAACATCCCAGCA
GAGAGCCGTTACCAAAATCCCAATGCCGCTTAGTGCCCACAGCCAACCTGCACACAGCGCACCCCAAC
CCAAGGAAAGAAACACTACCCCTCAGCATACA
TGGCGCACATTTGCGTGCAGAGTTTATAGCAAAACAAATAACAAGAAACCAAAACACTCTGGAATTAA
TAAATAAGGAGGGAGCGACACAAGATGGAACC
GCCCACAAAAAAAATTTTATTACAAAATCAAATATTTGCCTGGACTGCTGGCCGGTTCCCTAATTGTC
ATATGAAAAAAACAGGACTGGAAAGAAGAAAG
TAATGAAGTGGCAGTGGGTTCACCACGGCCTCGATGTTTTCTCCGTATAAATATTGTATTCGGGTGGC
CACCGTAGTGAAATGCTGCACCATATTCAAAT
TAGTGATATTTATTACACACAAATTGGCGAAATTTAATATCCCCCAACCACAAAAACCACGCCGCAAG
AAAAGCATCAAAAGGGAGCAGGTTGGATGGCA

AAAAATAAAAACAAATTAAATGCGTCTTGCTTTCATCGGCTACCTTATTCCTTCCATCGCGGCAAGCC
AAAGATCCATAGGATAGAGAGTAGCCGTAAAG
AAATATATTCCACTTATCAAGATGTACATTAATTTGGGCGGCCAGCCTTCCTCGCGAGCATCCTTTCA
CAATATTCTAAAAAAAAAAAAAAAATATTGGCA
AAAGTCCACACTGCTATTAGCTACACTCACACAATCATCAATTACAGCTCGCGACCTCAATGCTCACA
CACCATTCGCTGCCACAGCGAAGCACATTTTA
CACACGCCGGCGAACTTGAATGGTGGCAACCGGGTCCTCCTCCATCCACCACACCTCCTCCGCCGTGG
ACAAGGACGCCTGGCAAAAGCCACTTCGCCGA
TGTAACTCCCCGAGTAGGCCGGCTCTCTCAACAGAAAATCTACGCAACCGCAATGCGCCATCGCTCCA
AATAACAGCACCTTCTGACAAACTCATTGTGC
GCAATCCCCCTTCAATTGAGTCTCCAGAAACACTTTTCTTAATCACACCGCACACCTTGGCCGCAAGC
CGCTCCATCGCCAAACGGCAGACGTCCTGGTC
ACACCCTTCTCCGTCCCCGCTCGGCACCGGGACACTCCAGCGCTGGCACAAAGGGAGCAGCGCCTTCA
GTCGCTGCGGAGCTGCAAAGGTGCCAAACAGT
ACAGCCAGCATGGACGCAACGTAGCCTGAGGGACCGCCCCGAATGGTGGATGCAGAGCCATCACCACT
TGCCAAATCACTCTCATCGTCAAGGTCAGGCA
CCTCATGGTCAAGGCGCAGCGCGGCAAAGGTGCGCGCTGAGCACGGCGTCCAGCTGGTACATCTCCAC
GGGCTTCGGCACGCCGCGAAGCGCCACGGCGC
CAAGTGCAGTCACCTCCACCTGCTCACGCTCAGCCGTGCTCAGCGCAAAGTACGACgcacgcgtcagc
agcacc
>scaffold1080.size8450
TACTGACTGACAGCTATGCCAACTACGTTATTCAAACAGCCATTGTGACCTCTTCGGTGGAAAGTCAG
TTTGAACAATTGCGCGACTCTATTATGCCTCTT
CAAAACTTATTAAAGAATTCACCTTATGGGGTAAAGGTTGAGGCAAAGTTGGCGCGTCGACAACGGGC
TTCTCTTCGAAAACAGGAACATCAGAAGGAGC
AGAGTCGACGGGGGACAGCCCCTTCACATTTTCCTGAAAAAAATGGAGCCGAGAATGTGTTGACCCCA
CAACTCTTGCCTGGCATGCATAGGATACCAGC
ATTGGTTGGATCAGAGCCTCTTCAACAGAACACAGTTGCCTTTTCGGGGATAACTGAGGAAATGACTT
CTGTGATGACTCAACCACACTACATGCCTTTG
ATGCTTCAAGGACAACAGGCTCTTATTGGTTTGCAACAAGGACATTCTCATCAACCCTTTCAAGTCAA
CATAATGAAGGAGACTGCCATGTGGGGGCCTC
AGCATAGCTTTACTCTATTTCAGCAACCCAAGCAGAGAAAAACGGATAGGTGAATATTATTTATTTAT
TTTATTATTATTTTTTTTTTCCGTAATTGCAC
TGTGCTTGNNNNNNNNNNNNNNNNNNNNNNNNNNNNNNNNNNNNNNNNNNNNNNNNNNNNNNNNNNNN
NNNNNNNNNNNNNNNNNNNNNNNNNNNNNNNN
NNNNNNNNNNNNNNNNNNNNNNNNNNNNNNNNNNNNNNNNNNNNNNNNNNNNNNNNNNNNNNNNNNNN
NNNNNNNNNNNNNNNNNNNNNNNNNNNNNNNN
NNNNNNNNNNNNNNNNNNNNNNNNNNNNNNNNNNNNNNNNNNNNNNNNNNNNNNNNNNNNNNNNNNNN
NNNNNNNNNNNNNNNNNNNNNNNNNNNNNNNN
NNNNNNNNTTCCGTAATTGCACTGTGCTTGTGGAGCTTGTGCTGGTGTTTAGTTATGTTTCTATGTGC
ATGCGCATGCTTAGCCTTTTGTTGAGTTGAGT
GGGGCTTGAAGGATAATATATTATTGTTGAACATTTGATAGCGTTCCACTCATATATCTTTAGTGGAT
GCTCAGTATTCTTCACCGAGAACGTATTAGCT
CTATGATGAGTTGAATGAGATTTCCATTTCATATTTGCCTTTTATTTCCTGCTTATGTGAAGAAGACG
ATTTAATTGAATTATATATAGAGAGTGGGTGA
TGTGCTTTTATTGGGATCTTTTGTGTGAACCCTTTGTTTTTTGCGCTCTCAGGATCTGCCGTTCTAAA
GCTTGAAGTGTATTCACGGGGGCCATCCTTTG
TTAATCCAAAGAAGTTATGAAATTTTTGACGATGGCTTTAATTTGTCTTTGTTTTTTTTCACGGGTCT
TTATTTTAGGCTTTGATTGTTACTTACATGTC
TACATCTATATATATATATTTACGGATTTGTTTTCGGGGTACATATATATATGTGTGTGTGTACTTGA
CCCAATTTAAGGGATGCCATTTTCTACCTATGTT
TCAGATATGCTGCTTTTGGAAATTTGATTTTTTTAAATTCTATTCTATATATATGTATATTACGAAGT
TTGTGCTCTCTGCGATGTCAAGAGTTGTTTCT
TTAATTCAGGGCAACATCGTGACTCCTTCGGAGGTCATCTATGATGGTTTTGTGGTTGTCGCAGATGG

TGTTATTGAAAATGTGGGTGGCTCTGCGTCGT
TGCCTTCGGAGAATGATCTACAACAGCGCTACGATAACGCCCAGATTGAGGTTGTGGAGGGGGGCTTT
ATCCTTCCCGGTTTTGTTGACATACACAACCA
TGGAATAGGAGGTGCCGACGATGTTATTGGCTACTGGACGAACCCCGAATATACTCTCAGGGAGCTTG
CACGGTGCGGAACACTTTGTGCTTTGGCATCC
ATTGTCTTTTCAGATGATCACAAACACCTTGTCAAAAAATGTATTGAAGAAGTTGAGAAGCGTGTGGG
TGCTTATATTCCTGATTGTTGCCTTCTTGGAG
GTATTCACGCGGAGGGGCCGGTGATTAACGATCGAGGAGGTCTGCCTGCTTGTGAAAACAGGTGGAAT
CTTGAGCGTTTTAAGAAACTGTGTGCCACCAT
GCCGTCCATGCGTATAATGACGATATCACCACACATTGACGCGCGCAGCAACTATGAGCTCATTGGGT
ACCTACTCCGCCTGGGAGTTCGTCCGTCACTT
GGACACGACCGAGTGGCAACGAAGAAAGAGATTTTGGGTGCCTTGAAACTTGCACCCTCGGAGAGGGA
CAAGCTTCATGTCACACATATGCATAATGTGA
TGTCCTTTGGTCATCGAAATCCATCTCTCGTCAATGTGGCACTTTGCTCTTCATTTCCCAATGCAGAA
ATATACAGAGGTGCCATtCCCCCATATGTGGA
GATCATCGGAGACCTTGTCCATACCGATGCCATTCCTTTGCATGTGACAATTTCTTCCAGAGGGTGTG
ATGATGTTGCTTTGATCACGGATTGTATATCG
GAAAACCTTCCTGGGAAGCGTTTTTCCTACAATGGACGTTTAAGTTGCGTCCGCGCTGAAGGAGGTTG
CTCTCTGTGTGATGCATCCGGCAGGCCGACGA
AAACGCTTGCGGGGGGCACCAGTTCTTTGGCTGATCAGTTTATGAACCTGGTGACTCTCTTCCGGGTG
GACCTTATTTCTGCCTGCAAGATGGTTGCCAC
GACCCCCGCAAAAATTGCCCGACTGGATGGGGTAGGCTCGATTGAGAGGGGAAAGAAGGCAAATATTT
TGATTCTTAATTCAGAACTAAACACAATAACA
AAGCGCATGATTTACGGGAAGTGGACTGCACATGCTGAGTATCGTCTATTACGGCCATCCGTGTCTCA
CATGTGAGTCCAGCAGACTGTGTATTGGGAGC
AGTGTTGATAATCAATATTTTTGGGAGTAAGGGGGAGAGGGAAGGATGAAGAAAATGTGGAAAGATTA
GCAGACACATTTTAAATTATCATAATTTTTTT
TTTCCTTGTGTGTGTATGTGCTCTTGTATGTGTATGAAGGAGACATTCAGCCACATAAAATTGTGTTT
ATTAGGAGAGACTCACGTGTGAATTCCTTACG
AAACACGAAGAGAGTACAAAAGAAAAGTTAACTCAAAAGAATCCAGGGGAATGGTGCGTTGCGTATGA
GGGGAGGGGGACGCAGAGAATTTAAGGCCGAA
GATGATTGAATATTGTTATTGACCAGGTGAAAGGAAAGACTGTTCGGGATAGAATGCTTGTGGTATCC
TTTGCCAAACTACATGCACTTTATATTTTCTT
GTGATACCACGTTTATTCCGTTTGTATGGGAAGGGAACCTTCAATAGCCAAAATAGATACCGGGAAAA
TAAAAAACAAGGCACATAAAAAAAAAAAAAAT
AGCAATATGGGCTGTACGAACACAAAGGAAAAAAAAGATGCTAATTCTTGCGGTGGGAAAGAACAACT
TGCAGCGGGCGTTGAGGAGTTTGGGAAACTGG
CCAAGGAGAATCCTGTTGCCGCAAAGTTGAAGGAAGAATGGTCCGCGTTTGTGTGTTCCTTGTCTCTT
CCTACGCCTCTGGAGGCACCACGAGAAGTCTG
GGCAAATACTGTAGATAATCCTTTAACACACCGCACAGTGGATAAGGTCGGAAAACTTTTTTTGTTGT
ACGTAAAAAACGACCTCACACTGCGCGAATGG
GGCGGCAATTTTGACTATACTGTTGTTGGGTTGGAAAACCAAGGCTTTCTGAAAGCCACCGCCAGCGT
CGACGCCAGCAGTAGCACTGGAAGATCGAAGG
AGGCAATGTGGGAGGTGAAAGTGCACTATCACAGTACAGCAAAAACATCATGAAAGCCTTTTTAATGT
TATTTCTCTGGTATTTATGACCAATCAACACG
TCTGTTTTTTTTTTTTGTGTTTTTTTACGCGGTGCTGAAGCAGGAGAAATCCGACTCTGGGGATTGTT
TTTTTTTTTTTCTTGCATCGTGTGTTTGTGTG
TGCGCTCGCTGATTTATCCTTTTGCCGGACGTTTTGGCATTCAGCGACGCACCTGAAGCTTAGAAAAA
AAAAAATATTTATCTATCTACCTCTCTGTATT
TGTACTTATATATTTCCTGTGAAAGGATTCTTTTCTATGGAAATGCCCTTGCGAGCGTTTACCAATTT
TTTTTTCATTGATTGCTTCTTTACATTTATTA
TTATTATTACAGTTTTTCATTTCTAATTCTGCCGCTGGGTTTTATTACAGGAAAAGAAAAAGGTTCAA
GCGGATGGAGACCCCTATGGTGGTGCTGCTTT
TTGTTGTTTGCGCCCAATAGGCAAATAAAGAAAGAGAATTCGGCTATTACGCCAAGGTGCTGATTGAG

GTGCTGTTTCTTGCGGTGGTGATGGGTGAGGT
GATTGGGGAAATACTGGGCACAAGGTGTGACTTGAAGTCCTGAATCGCGTTTTCAGAATGTCTGGATG
GCTGTCGCCCTGAGCTATAACAATTCCCCATA
TTGAAGAACAGTTATTTTTTATTTTAGTGCCTGCGACGTTTTTATGCTGAACTGAAGTTTATTTTCTT
ATTCGCGGGTGTCGATAGGACAGTTTAAAACA
TGTTTTTTTGTTTTTGGGCATGTAGGATGTCTGGTGACTGTGCTTTTTTTTTTTTGGGAGTTTACATG
AGAGGAATGGGGACTGAGCGGTGCGGGATGTG
TGGTGAGTGCCGCAGGCTTGTTGGCCCGCGATTTGGGTGCCCTCCAAAATTCATTACATTTTGGGCTC
CTCAACGAACACTTTTATTCTTTATGGAATCA
TGTGGTGTTACAAATGGCACCCTGGACTGTTGTGTTTCGCCATCCTTTTTTTTTTTACGAGATGTATG
AGAGCTTTGCATGCTGCGGCGAGAACTGCATT
TGTTACTGTGCCATTGGTCTCTTTTTTTTTATTTTAGCCATTTTTCGCTTTTGTTGCTTATTTTATTTC
TGGTCATGTTGGGACTCCTGCGTGCTCCGTGC
CTCCACTTCACTCACCAAATGGAGGTGCATGACCATTGCTTGGGGAGGAGCCTTTTTTTTTGTTCAAA
TTGACACAGAACCATCATCGAGCACATTTTTC
TTTTATACTTGTTTTGCTTTCTTTCAGGAGATGCTTCCTTGGCTTTTTTTTCCAACGGGATATGCACG
GAGTTACACGCAGTTTAAAAAACCGAGGCCAC
GAGCCTTTTTTGGAAAGGATGCTTTATCTTTCTTCTAATATCTTTTTTTTTTGTTCATGCTGATATAT
ATGTTTGCTTGTTTAGAGGTGGAACCTCTGTT
TGGTGACGATTCCGCTCATGATGTTGAAGTTGATCCGACCATGGCGGACTATATCCACTGCGTCGGGG
AAAGGCTTCTGGTGGATCCAACGACACGGCGG
CTGGGGGAAATAATAACGGGACGGACGTTATCCCTTTGATGGTGGTCCCGCTGATGCTCGATCTGATG
GACTTTCGGAGGATGATGTGCAACATCAGTGT
GCCCATCCGGCTGTTGGTTTTGGTGCAGAACGGGCGGGAGGCGATGCTGTCGTTGTGCCTGCAGGAAC
TCGAGAAGGTCTATGGGTGGTCCGGACGCCTG
GCTGTGAGTCGTCACCCGGAAAACATCGGGTACAGCGCTGACGTGAACATCGGGTCACGACTTGCACT
CTCGCTGCCGCGCGAGGAAGTGCCTTTTGTTT
TTGTGGCAAACAGCGACGTAATGTTTTCACCCGATCTCCTTCCCAACCCTCTACGCGATGTGCATGAA
ATGACGAGGCATGATGCCGCTCGCATGGATGA
GTTGTCGGCGGAAGTGGCGAATGAGCCGAGCGAGTACAGTCCTGTTTTGCGACGGGGCTTGAGGGTGC
TGCGCTCCACGGTGAGCGATGACCGATTGTCG
ACGTCTGCGCTGCTGCCTGACCGCGTTCGTTATGCTTCTGTGAAGGAGCGTGAAAAGGCATTTTCGAA
GCATTACGGGCACTTCTGCGCGTATTGCAAAA
GCAGTTGTTTTGCGTCTGTTATGTTGACACGCCTGGCGATCGGCACGGTGGGATATTTTGGCGAGAAC
TTCTACCCAGATTACGTGGAGGATGTCGAATA
CAGCCTGCGCCTCCGTTTGCTTGGATTTCAGGAGCGAAATGTCTTATGCGGCAAATTTGTGCATCGCG
GCAGCTCGAATATTCGTCTCTCTGAGCAACTG
GAGCTACCGGACGCTCTCTGGTACCGTCGCGTCAAGTATTTGATGACGAACCAACCATATGCCATGAT
GAAGTGGAACCGTCTAAAAGCATGCTGCAATG
GGTACAAGGAGCCATATGACGGCATGGTCCCCCTAGACGTTTGGGTGAAGGATGAGGCACGCATACAG
CGCATTCGGGCCTACGGGCATGATGAGATCCG
AAGAGTTCCAAGTATTGACTACGACAGGAGGCTTTTGTACCCTGTGAGGACTAAAGGAAGATGATTCG
GCAACAAACACAAGTTTTGCTTTCTTATTTTG
TTTTCTTTAATTTTTAAAAATTTACACCTCTGTTTGGTTATTTACACGATTAATATTTAAGCGAGTAC
GCCGTGGATTTTAAAGAGCGCAAACGTATATT
TGACATGACAGGGGAGGGGAGGGCGCAGCGTCCAAACTTGGCCGTGTAGCCGAAGGCAGGAGAAGGAA
GTAGCAAGCAGGAAACATGATGATGATGGATG
AGGGAGAAATAAAAGGGGAGGGAAGCAGAAAACAAGTCAAAAGTATTGTGAGCACAACGCTGTTTCTT
ATCGTGGGCGATCGACACCGCCATTGGCCACT
AATTTACCCACTGAAGGCGACACCCGTTTCCGCTGGATATGCTGCGTGCATATCTGTCGGGCTCTTTG
TTTGTTCTGTTGGTTTGTTTGTCTCCCGCACA
TTTATTTCTCCGTGCCGCCTGACCCATTCGTTTATTGTTCCCCTCTCTGTTTTTGTGCGTGCACCGAG
AGAAGGGCTCGGGGACATGAAGTACGCTCCCC
TTCTGTGAGATGCCACCAAAAACAGGAGGAGAAAAAAAAGACAAGTGAATTGGGGAGGGGAACCATCG

CCACGCGTCCTTTTCTCCGATAAGGCTGCTTGC
CACGCCACTGAGGCGCGCCGTGTGCTTCACGAGGTCCGCTGGACTTGATTGGGGTGCCGTGAAGGATG
GTGCGTGAGAGTTTGGCGGATGCGTCCCCCAG
GCGTCCCCAGAGGGCCCTCTGGCCATAGGGACTGCGCGGAATGCGGGACTCCGCGGGGACATTTGGCC
GTTTTGTTTTTATTTTTTTATCCGCCCATCCC
AAACCTCAACACGGCGGCTGAGCCAACGACTGCGACGGGATCCGTTGCGTATTTCTGATTCTTTTTTG
CGTTGCAGCGGCAGGCCTGGTGTGCCCTGCTT
GGCAGACGCGGTGGACGGAAGTGGGCTGCCCTATCGGGAAAACGATGGTCACGGCGGAGCGTGTTGCG
TACTTTGCAATTTGGCGTGTTTGTGGCGATCC
TTGCGGATGGTGTGACGGCGCCTGTTTTACTTGCGGGGAGAGAAGCGATGCTCCACGGTGGTGCACAC
GTTGCGTGTGGAGGTTTCTCTTGCTGTTGGTT
TGTGAACGTTTTTCTTTGCTTGAACACCACAGAGGGCGGTGTCTGCGGACAGTAGAGCATTGCGAGGA
GGGGCATCACACGATTTTTGGCTTGACGAGGC
GGCTTCTGTGACTTTACGGTGTTTATTTTAGGCTTGAGAGAATATCTTTTGTTGGAATGTATTGTGGG
AGAACATCCGCGTATATATATATATTTCTTCAAT
CCCTTGGCATTTGGTGTCTGCCGTGAAATGGTTGTTGATGGCCTTTTTATGACGGTGAGATGCATGTT
TATTTATTTTATTGGCGAGTGGGTGAATTGTG
TTGCGTTCTGCCATGCGGCTTGTTGGTGACGGCACGTAGCTGCAGGGAGACGTTATGGGCGCCTCCGA
CGCGAGGTACGATTGGGCAGTCACCTCCGCTC
GCCGGTACACGCTGTTGAGGCGGCGTGATGGTAATGAACCGGCCTCTCGGAAAAGCAACAAGAGGACG
TTTGTTCTTCCTGCGGTATACGAGTGAACCTT
CAATTCGTGGGCGGGGTCGCTCTGTGTGCCGTATTTCTTTGAATTTCCATGAGCTTTTTACATTGCCA
TCCACACGCACATGCACGCAATCCCTCTGTTT
GCCCTCCTGTGGGCGGAAGACCGGGATGCAAGGGAAAAATGTGGCCCGTGCCAGCATGTGGTAAAGTC
TTTCTTGGGAGGGATTGCGACTGTGATGTCCG
TCCTGGCAGGCAGGAATGGGATGAGAGGAGAGCGGAGGGGCGAGTAACAACGATCGTGTGCGGGGCAA
TGCGCAGTGACTAGGGCGAAGtggtatttatg
ttgacttttcaaagcatttttgtctttttttcttttgtggcaa
>scaffold1081.size10631
GCCAGGGAAATTTGTTTATAACTTTTGTGAGAGTGCATGGCACGGCTTTCGCTCTTTTTTTATTGTTG
TTCTTGGGCACAAGAGTAAATACATATATATAT
ATATACATACATATACATATATACTTGTTTGTGGACATGTATTTATGGTGCAGGTAAAAACAGGGCCA
TTGCAATCACTTGCATGGCCAGACTTGTCACA
ACCTTCTCTGCCACTCGTACGGTGACATGGCTGTGGCGATTTCTCCCCATCTTTTTTTGTTTTTGGGA
AAGACTTAACGTTTTTCGAAATCAAGCAAAAA
AAAAAGGCAACAGCAAATAAGTTTTAGCTTGGAGCTAACAGGGACAAACAGCAAGTTGAAGGCGTGGA
AAATGGTGCTTGGTTTCAATGACCGACTTGAG
GCACCCAACTCCATTAACGAGTCACAGCGCTTTGGGTTTATGGTGTGCAGCATGTTTTGTGCGATCGC
ATCCGGTGCCGTCTACTCCTTTTCGCTTATAT
CGGGCAAAATGACGGATGACTACGGCTTTACCCAGAACGATATCACCACCGTTTCAACTGTGGGCATC
GTTCTTGGATACTTTACGCTTCCCTTTGGATT
CATCCTTGACTACATAGGGCCGAAGCCGCTTTTTGCGATTGGTATATTTGCGTACGGACTTGGCGCAG
CACTGTTTGCATTGACGTTTTCGGGAAGGATT
GGGGCAAGCGTGGGGAGTCTTGCTGTCATCAATGCCATCATGAACACTGGCTGTGCCATGTTTGATAT
GGGCCCTATCTTGTCTGTAATCAGCTGGTTTC
CAGTTGACCGCGGTCTCTTGGTGGCAGCTGTGAAGTCGATGATTGGATTGGCAAGCTCTGTCATTGCG
ACAATTTACAACACGTATTTTTCAGGCAATCA
CTCCACTTTTATGTTTTTTCTTCTTGCCGTTTTTGTGGCCATTGGGTTCTGGGCGTTTATCTTCATCC
AGATCCCTCCGTACCATATGACAGGCCACAGG
ATCAAGCATTACACCGAAGAGGAGCACGCGATAGCACGTCGTGTGGAGCACATGTATCTCATCAAGAA
GGCCCCCAGACGGCGCTTTCTTATTTTGTTCG
TCATTGTGTTGAGCCTTCTCATCGTGATCACGGTACAGAGCATCGTCTTTGTTTTTGTGGAGGGTGAA
GTGCCATTCAAGACGAAAAACCCACCCGCAAT
TATCATGATTGTGCTCTGCTTTTCGTTATTCCTTGTTGTCCTGCCATTCAATTGCCTTGACAAGCCAT

TGAGGGGAAGCAGAAAATCCACTAGCGGCAGC
AACGAACCCCTCGAGGAATCCAACAAGAAGAACGACAGCAAAGTGAACACATCTGCCGGTGACGCCAA
AAATGAAATAATGGATGGGGCTTTTGAAGGAG
AGGAAAGGCTTGTTTCCAATGATGACAAGAATTTTCCGCAGTACCAGACCGGCTTCTTTCACAATGTC
CTTCACAGTATTCCATTGTGGTGTTTCTGGCT
GAACGCTGTCATTGTGTCGGGTGGCGTGCATATTGTTATGCTCAACAGTCGCCAGCTGTTTGTGGCGA
TATCGGAGGACCCGTCGAGCGAGCAGCTTCCC
GCCCTGTACGTTGCCCTGACGAGTATCGGTAACGCCATTAGTCGTCTTGGTGTCAGTTTCTTTGAAGC
ATGGAATGCAAGCCGACCACTTGAGAAGCGAA
CGCCCATTACCATCACGTACTGCATCCCGTCCCTCATGATGTGCCTTTCTTGCATCTTTTTTCTCATT
GTGCCAGCACGGGCGCTGATCGTACCAATGCT
TCTAGGAGGATTTGCGAACGGCTCCTACGCTGCTACCCTCGTATTGACTGTCCGCACCATTTTCAGCA
TTGACGTTGCGAAGCATTACAACAGTATCTTT
TTTTTTGATCTCATTGGTGTTATTGTCTTCAATCGTTTTATGTTTGGTGAACTTATGACGCGGAACTC
CGTTCGGGCTTCGGATGGTCGGGTCCACTGCC
TGGGACGTAGCAAGTGTGTGCGGACATCATTCACTGTGCTTGCGTGCCTCTGCGCATTGGCTTTCACT
GCAAGTCTGCTGATGCATTTTGTCTACATGCG
CTTTGTACGCTCCAGGCGTGCGCAGGAAGAGGCGGGACGAAATGTTCCGTTGGAGATGGCTGGGGTAA
CTTCCGAAGAAGTTCAGTAAGCTCCAGGAATA
AAAAAAGAAAAAAGGAAAAAGAAAAATGGATACTTTTTTTTGTTGTTTGTTTAATTTATTTTGGTCCt
cNNNNNNNNNNNNNNNNNNNNNNNNNNNNNNN
NNNNNNNNNNNNNNNNNNNNNNNNNNNNNNNNNNNNNNNNNNNNNNNNNNNNNNNNNNNNNNNNNNNN
NNNNNNNNNNNNNNNNNNNNNNNNNNNNNNNN
NNNNNNNNNNNNNNNNNNNNNNNNNNNNNNNNNNNNNNNNNNNNNNNNNNNNNNNNNNNNNNNNNNNN
NNNNNNNNNNNNNNNNNNNNNNNNNNNNNNNN
NNNNNNNNNNNNNNNNNNNNNNNNNNNNNNNNNNNNNNNNNNNNNNNNNNNNNNNNNNNNNNNNNNNN
NCCCACCCACCCACACTGCACGTATGGCCGTG
GGGGCAACGCATGGCGGAAACTGAGATGAAATTCTTTTTTTTCTTTTCTTTTGGTTCTTTTTTTTTTT
CCCCCCCACCACCACACACGAAACCACCAGCA
CAACAACAAGAAGACGAAAAGGGGAAGGGATGGGAAATAAAAGAGGTTGACGTTAGGACGCAAAAAAA
TTATTTATTTATTTGTGTTTCCCTTTGATGGA
AAGTTTTGAGTCGACACACCGGCAGCGTTTGGCTTTTTCTTCTGCATACGCCTGCAGATATTTGCACA
TGACACGTACAAGGATCGGGATCTTCACAGGT
ATACGCACGCAGATGGTTACATGGAGAGAAGGACTCTAAGAAAGTTTTTTTGCTGTTTTTTTCTTAC
TGAAATGTTTTTTTTTTTTTTTTTTGTGTGTG
TGTGTGTATGTTGGATTACATGGGAGGATTTACTTGCAATAATGTGTCGAGGCAAACCAACGAATTGT
TATATTTACATTTGTATTTCAGCCTTTATTTA
TTTTTCCACTTTCCCTGTTTTGGATATATATATATTTATTATTATTTTCTTTTGCATGGTATGCTGTA
GATACCGCGCTTGAGGGGAAAAGGGATTAAAG
GTGTAAATTGCACAAATACATACATACCTATATACATATTCAATTGCGAACGGTGGGTAGGAATATTG
TTCAGTGGGGAGGAAAGAAGAACGGGGAGAGG
GAGAGAGAGTGAGGGAGGAATACAGCGTAACTCATCGTTGATTCATGGGGAAGGGTCGCGACAAGCGC
AAGAAGCATGAGGATCCCTTGAAAGCAGTGAA
GCGTGCGAGTCGCCAGGCCCAGAAACGTGCCAAAGGGATTTACAAGGGAGACGCAGGCGACGACGAGG
TGAAGGGGTTCAACAATGAGGAGGCTCCGGAA
GTTACGTTGAACCGCATACGTAAGCAGGAGGGCAAAATGCGCACCACGTTGGTGGAGGAAAACGTCCC
CGCCCCTAGCCCGCGTGCGAATGTTGTCTTTA
CTGCTCATCCTGAAAGGGATCACGAGCTGATGCTTTTTGGTGGAGAGTATTGGGACGGGGAGAAAACG
GTGGCGTACAATGACCTGTACTTTTACAACAT
CAAGCGCGACTTCTGGTCACGTCTCGTGACGGCGTTGAACCCGCCCCCACGCAGCAGCAGCCAGGGTG
TTCTCTACAAGCACTTTCTTCTTATTTGTGGC
GGGGAATTTGTCTCTCAGTCGCAATCGCAATTTCTGCACTTTAAGGACGTTTGGCGTTTTGACACTAA
AAAATTTGAATGGGAAGAACTAAAAGGATTGA
AAGGGGGCCCATCGTCACGCAGTGGACATCGTATTTGTATCTGGCGTCGTAATGCTGTCCTATTTGGT

GGATTTTATGATAATGCACAGGAATGCCACTA
TTTTAATGACCTTTGGGTACTTTCGCATTTGGACGGGTCGGGAAAGTGGACGGCTGTCAAAACCGCGC
CATATGCTGAGTTGCCTCACCCGCGCAGTGGA
CATTCAATGGCTGTCTGGAATGACACGCTCTTTGTCTATGGGGGATTCTCCAGTGAAAAGTTTAATCG
ATTTAAAAAGTCACAAGCAACCGTGCACCATG
ACCTTTGGAGCTTAAAATTACCGCAGGGTATCGGTGCTGCAGATAATAACAACGCGGAGTGTGAGTTT
ACATTATGGACAAAGATACGTTTGGGCGGTAT
TCCCCCACCCATTCGCTGTGGCGTGGGCAGTGCGTTTAAAGATAACAAAATGTATTTGTTTGGCGGTG
TTGTGGACATTGAGTCTCCTGGCGGAAAGGTT
GTGTCAAGTTTTTGCAGTGATTTATTTGTTTTTCATATGGACACGAAACGCTTTTACCCAATTGTTTT
GCGCGCAAGGAGGAAGACGACTGTATCGGAGT
CGAAGGGAGGGGACCTGCAATCGGAATTGAAGGCTCTTCATTTACAAAATCTGAAAGATGCCTCCAAG
TTTTCAAGTGACGAAGAGAGTGACGAGGAGGA
CGAAGAGGAGGCGGAAAGTCATGGGAACGTTGAGGAAGATGACAAGGAAGAGATGAAAGAGAGTTTTG
AAGTCAATAAAAGAGGGCAGATATATCCACAT
CGTCGGATGAACGCATCCATGCTTGTTCTTGGGAACACTCTATACATTTTTGGGGGTCAATTTGAGAG
TGGCCTGCGGGAAATCACGATGTCGGATCTCT
TTTCATTCAATCTTCATACATGTGACACGTACCGCGTGCTTCTCTCGCAGGATCTTTCACGTTTGGTA
TGGCTTGGTAAGGCGGCAGAGGAGAGTGACGC
TGGCTCATGGGAGAGCGGCAGCACTGTTGTCAGCGCTGCATTTGACATGGAGTACGATGAAGACGACG
ATGAGGAAGCGGAAGGTGCTGAAGAGGCAGCG
GTGGACGGCGGCGTGGACTTTTCCCGCATTCCGCGGTCACTGGAGTGTGGCGACAACGATGATGAACC
ACCGCAGGTTATCCCCGCGGAGCTTGACACCG
CCGTCACACCCGCACGTGACATCGTGGACGGCATCACCACTGTAAAGGGCAAGAAGGGCCTCAAGGTA
CATAAGGAGCAGCTCCTCGCTCAGCTGGGCAG
CGGATCCGCCGTACCAACCCCGCTTAGGGAGGAGACTCCGGTATTATTTTTGAACGGACGAGTGCCT
TTTGGGTGTCGATTGCAACAGAGTCATTTGCG
GATGCCGGTGGAAATATGCGTCAGTCGGAGAAACGGATTTATAGGCGGGCAATGCGGTTTGCCTTTCT
CCGCTACCACGAGGCAAAGAAACTACTTGAGC
AACTGCGACTTGTAGAGGAACGCGAAGAGGAGGAGACGCGGTTCTTCAAGGAGCGCCGACAACAGAAG
GAAAAAGAATGGGAAGAACACGAGAGAAAAAT
TGGTGAACAGGAAGAGGATGATGACAAGAGTGCCTAAAAATGAAGGGGGCGGAGAGCACCGAATCTTG
AAGTTTTTTTTTCCCTCCTCTTGTTGTGCCGT
TTTTGTTTTTTTGTGTGTGTGTGATTGTGGGATGGAGCTGGAGAGTCCGGCTGAATGTTTTTTAAACA
TTTACGTTTCCTTTTTTCTTTTTTTCTGCCTT
CTTTATTTATTTATATATATATTTTTTTGCATGATGGACGGATGTTGCGGTAGGGTTTCTGGGAGGGATG
GCCGGTGCGTCCATCGCCCTTGAAAATGTTGA
CGAGGCACTCGAGGCGAATCGCCGGCTTCAGAAGAAGTTGAAGCGGATGATTCGGCAGTTGTGTGAGG
CACACGATCGGTTTTCAGTCCTTCGTGTAACG
CTATGCATGCCGCCTTCTTCTTCCAGAAGGCGGGAACGTCTTTCCGCGAAACGCGACCGCGTTCGTAG
CGCTCCGGTGACGTATTATTTTAAAGATCGTC
TGCCAAACAGCTCGTCTTTTGCTCCGTTCCGCGATGCCGGTGTTTATGCGGAGGGTGGTATTCCCCGG
TACGCACCACTTCCATTGACGGCGGATGAGGT
GCGCTGGAAGGAGGCCACCGAAACATTTGCTCCACTACGGCATGATGTACTCAACGCAACAATGCGTG
TTGAATTTTCAGGGAAAAAAGAGGAAGGGGAT
GATGATGTTGTGAATGATTATGAATTCATAGCAGCTGTGCGTGACTACATTGGAAACCCATGTGGTGC
GACCTTCTGGAAGGCGTTGGGCGGGCACGGAC
GTCCACAGTTTGACATTGCTAGCAGGTACGTTGCGCTGAGAATCCTCAGGAAGATAAATTCTGTCCCA
TCACATTATGAGGCATACCAACTTAGCGAAGA
AGAAAAAAATGCCGCGGCTAAAGAGGCGGTGCGGTGCCATTTTGGTGATTTGGGGGCAATGTTTGCTG
CCTACGCAGAGCTTCTTGTTGCGGCTGCACGT
CGTCATGCATACATGGCGGAATTAGTTGGAAAGGTGGTTGATCCCTTTCCCCCATATGTATGGGAGAG
TCGTGTCAATTTTATGCGGATGGTGGATTCCA
TCGTGCAGGAGGCAGGCATCTCCGATGGGCATGATGCCCTGTGGGATGGTGAAGATGTAGTGTTGGCG

CAGCGTTTTGTTGGGCAGAGCCAAACAAAGCC
CTCTCTTGTAGACCTCACAGCATGTCTCCTGGCGTTTAAGAGTGAAATCCTTGGAAAGAAAAGTAGCT
TGCAGGAAATTGCACGGATTTTTCTGCCGAAA
TGTGGGGGCCCGTCACTAACTCTGTGGGATTTAAAGCAGTTACGGTTGCCAAAGGAGAGCTANNNNNN
NNNNNNNNNNNNNNNNNNNNNNNNNNNNNNNN
NNNNNNNNNNNNNNNNNNNNNNNNNNNNNNNNNNNNNNNNNNNNNNNNNNNNNNNNNNNNNNNNNNNN
NNNNNNNNNNNNNNNNNNNNNNNNNNNNNNNN
NNNNNNNNNNNNNNNNNNNNNNNNNNNNNNNNNNNNNNNNNNNNNNNNNNNNNNNNNNNNNNNNNNNN
NNNNNNNNNNNNNNNNNNNNNNNNNNNNNNNN
NNNNNNNNNNNNNNNNNNNNNNNNNNNNNNNNNNNNNNNNNNNNNNNNNNNNNNNNNNNNNNNTAAGAA
AAAAAAAAAAAAAAAAAAGGATGGACAGAAAAG
AAAAAGAGAAAAGCAACGAATAAATTCTAATGTAAATGTTCATTTATTTATTTATTTATTTTTTTTTC
TGCATTCATGCGTGGGCGAGTGCGGCAGAGAG
AGAGAGAGAGAGAATATAGATATTTTTTCTGTTGTTACTGTTGCGCCAGTTTCAGTATCATTGGCAGA
TCTATTTATTTATGGATGCATTGAAAACGATG
CGTGAGGGCTTCATATATCTCGTTTGGGTTATTTATTCCGTGTTTTTTTTTCTTTTGCGTCTTTTTTG
TTATACGTATGCTGATTGGTCGTATCATATGT
ATATCACAGCTGCGGAGTGGTGAATGATTCGATCAATGGAAGCCGACAGCGTGGAGCTTGCGGTGCAG
CTTGAGCTGTGCATTCTTCGAGCCGTCACCCC
GAAGGACTACCAGTGCGTCTTGGAGCGGAATGGCAAAATGGACGGGAACACCGTACGCCTGCTGCAGG
CGATTATGCAACAGGATGACAAGGTGCATTTG
GGGCAAACGATACTGTTGAGTGAACATTTTCCGGTTGCTCTCATGGTGGTGGATTTGGCTCCGGCTAC
GGCTGGCGTGTTGTGCGACACGACAACCCTTA
TTGTGCTTCCACCATGCGTTAGTAACGGGGCTACAGATGTGTGCCTCGCTGCACACACGACAGAAGAG
AACCCCAAAGAGGAGGAGGAGGAAGAAGAAGA
AGGGGATATCGATGAAGACCAATGCACTGTTGTTTTGGATGATTCTTTGAGTGGCTCCATCGCGGTGT
CATCCGAGAGGTTTTTTTTGCGCACCCTGCTG
ATGGACGGCGCGGTGGTGTGTGTTGGTGATGCAGAGTACACGACTGCTTTCTCCATTTGCATGAAAGC
ACACGGAGAGGATGGTGCCGATAGTGTTCCTA
CCCTGCACACCTCGCCCGGTGTTTTTCGTAATATTCGGGCCGCCATTCTGCTTGAGTCCTTATCTTCT
GTTGAGGCGACTCGTGTGCAATACTCTGACCA
ACGGCTGAATACTTCACTTTCTGTATTTCGTCCTGCACTCGATTCGGCGTGCATGCCGTTAACTTTGG
GATTGTCGAAAAATGGGTTTAGTGAGTTGCAA
ATGAGTTGTGCCGTTCTTGTGCCTGCGTGGCCTCTCGCCATGTTGAATGCCGTATTGGGAGACACGTA
TGAGAAGATGTTGGTGCTTGCAATAAATGCCT
CCAAGGAGATGTTGTCGGGCCGAATGGTCTCTGAAGGTGACGTTCTTATTTTTCACTTTACGCCTCAA
CTCTTTCTTGAGCGGCAGCTGGATTATACGGA
CGTCACCGAGGCTGTGCTGAGGCATGTTGCACATCTTGTACAGGGCGATGCCGCTGAAACAACCATGA
GATTACGTCAGAACTTTGACGGTACCATTGCG
GTTCCACTCCGCGTTGCCGAGGTGACGACATCTTCAGGTAAACCGTACGGGTTGCTTGAGCTAGATGG
TCAGGGGACGGAGGTCGTTTTATCCATACGCA
GCAAATTGCCTTGTGTGGATGTGACTCCCCTTTATCCACCATTATGTTCAAGCGGAACAGTTTATTTA
AACTTGTCTTTCACTCTGGCCATTCGTGAAGC
GTTGGGTTCCTTTTTTTTACCACTACACTCTGCGGGGACACATGTCTTTGTGGTGCGTGCAATGCCGG
AGAATTTTCCATACGAGTGCATTACGACTGCG
TTGGCAATGAATGGTGTGGCAGCGCTTATTGTTGACCTTGATCAGTGGCGTACCGAGGATGTGCAGCG
TCTTGTTTCAGAGTATATGCAAGGCCGTGGTG
AAGTGGCACTTGTGATAAAGAATGCGCAAATCTTACCTGACAATCCCCACTTGTTGCATTTGTTTGAC
ACGGATGATACCATCAGTGATACCTGTCTTGG
GGTTCGGATTATTTTTCTTGTCTGCGAGGGTATGGAGGCACCACCGCCGACGGTGGCAGCCAGGGCCC
GAAATGTGGAAGGTGTACTAAATTGCGCCAAT
CCATCCGACACGGACCGTCGCATTATTGCAGAAACCCTGTTTCAAGAGGCTCAACGTTCTTTTGGTGT
ATGTAAGAGCCTTCTGTTGTCATTTGATTCCG
TAGCATCATGGACAGTCGGACTGAGTGCTGCCGATGTGGTGGCGTACATGCAGGAATGTCTCTCTGTT

CTGAGATGCATGCGGTTGCCGGAGGGAGTACG
TCCTGTACTTTCGGAGTCATTGTGCGGAAGTGTGCTGCAGAAGTATCAAAAAACGCATGGATACAGTC
TTGTCTCGACTAAACTGCACCCTGTGCGGTGG
AAGGATGTTGGCGGATTGGAGGAGGCGAAGCGAGAGCTGCGGGAGACGATTCAATTGCCCCTGCTGCA
CCCGGAACTTTTTTTAACCGGCACAAAGCGAC
GAGCGGGTATCCTTTTTTACGGGCCGCCAGGGTGTGGCAAGACACTTCTAGCAAAGGCAGTCGCGACG
GAGATGAACATGAATTTCATGGCGGTGAAGGG
GCCTGAGCTGATCAATCAGTACGTTGGTGAGAGCGAAAAGAACATTCGTCTCCTCTTTCAGCGTGCAC
GGGACAATTCTCCCTGCATCATATTTTTTGAC
GAGCTGGACGCACTGGCGCCGGCACGTGGCGCGAAGGGTGACGCCGGCGGGGCGATGGATCGTGTTGT
TGCCCAGCTGCTTGTTGAGGTGGATGGCGTTG
GCCACACACGCACAGACGGCAGTACCACCGCGCAGGTGTTTGTCATCGGCGCCACAAATCGTCCGGAT
CTTCTGGACCCATCGCTGCTACGCCCTGGCCG
CTTTGACAGGCTGTGCTACTTGGGCCTTCCCGCGACGAGGGAGGAGCAGTTGGTCGCCCTCCGTGCAC
TCACGCGGAAGTTTAACCTTGCAGATGACGTG
GACTTTGACGCCCTCCTTGAGCCACTTTCCATGGATTACACCGGCGCTGATTTTTTTGCTCTTTGTTC
TGACGCCATGATGTTTGCTGTCGAATCGATGC
TTCAGCGCACTGCTGCGGAGAGGGGCGACGACACACAGTCGGTGAGGCGTCATGCCGAAGAGGCGCCG
tCTTTTTTTGTTCAGATGCGTGACTTTGTGAG
GGCGCGCGATCAACTCAAGCCGTCGGTCTCGAAGGAAGAACTTCAGCGTTACGAGAGCCTTCGGTCGA
AATTCACCACCTGGTAGATCACTGTGAGGCGT
ACACTCTGGGAATGTGAGAGCGGGTATTTTGTGAGGAACTCGCCTTGAATGAAGCAGCAGCGCGGATC
GGCTTCAATGGCCCCACGTGAAGGGCGAGGAA
AGAAGGGGAATAAGAGAAGAGAACTACAGGAAGAAAGAGAAAATGAAAAGTCATTAAAACATGGTGCA
TTAATGACGATAAATGCACACAAATAACGGAG
AAAACAAAAAAAAAAAAAAAAAAAAAAA
>scaffold1082.size12456
CGCTATTAAAGGGGATGGCGGGCACGGTCTCGTGGAGGTTGTTGGGCTGGGCGGCATCAGCGTCACCA
CCTGACGATTCCCCCGTTGGCTCGTCAGGGGCG
CGCTCAGATTTCCCGAGTTACATACATAAGAAGCTCCTCGCCACGTGCTGCCGCAGTGAGAGATCATT
ATCGGGGATCGCGTGGGTGATCGCACACGCAG
CTGACCCCTGGCGGTGCGGAATGACCGCCCATTCCACATGCGCACAACACCCGCTCAACCACAATCAC
GTGACTCAGTTTACGGCCACAAATGAGCAACA
AAGGACACATCAAAGAGACCCAAGCCAAATAAAATAACAAAAATAGAGGCCTCTAACAGCTTCAAGCG
CACACGCAGTACACGTCCATTGCCCTCTTCAT
CCACCCCGTCGTGGCAGGTCAGCAACCAAAGTATGATCCTTGTTTTTGAACATTCGTTTTGCATTTAA
ATGACTCTCATTGCTCGCATATACATTTTTTT
CCCTTCTTATTTGCATTCATTTCCTGGCACCCCCGCCAAACCCGTTGTTATTTCCTTTCGGAATTTTC
CCTCCGGCCGCCTCAGCGCGGTGCCAGGGTCA
ATTGCTCCGTATTGTCGGGGGAAGCCAGGAGCCAGCAATGTTTCCCTCACGGGGTCGTCGTTGTTGGC
GCGGGGCTGACGGCATCAAAACCAGCGCCCGG
ACCAGCTGCGTCTTGCTGGGAACAACCTCGTCCTCCGTTTGGCAAAGAATGCCTCGCGGCTCCCCCTC
AGAGGCTGCCTTGATGGCGGCGCGGCTATGCT
CGTATGTCGCTGGCTTCTCCAGGCGCGCGGTGAGGTCGACAACCGACACGCCGGGAGTCGGCACACGG
AACGCCATGCCGGTCAGCCTGCCGTCCTGCGC
GGGAATAACCTTCCCAACCGCCTTGGCCGCTCCGGTGACGGACGGGACGATTTTCCACGCCATCCGCG
CCCAGCAGATCGTTAGTGCCGAAGACCGCCCC
GTGACGCCAGAAAAGCGCCCCGCGACGGCCTCGCGGGGGCAAGGCGGGGAGCCCGGGACGGTCGCCCT
GCGTTGGCGGCGACGCCGGCCGCCAGCAAGGG
CCCCTCGCGGGGGAACGTTGCCTGCAAATCCTTTTCCGGGATGGTGTCGCCAAGGGCCGGTTAGCGGG
GATGTCTCTGACGTCCCCGGGGAGCGCGGCCG
GCGCCGGGTGGGCGCGGCCCCTCGCACCAGCCCCTTTCGTGTCGCTACCGACTTCTCTTCGGTTGTCT
CCGGAAGTGTGCATTTCTGTTTTTTTACGTTT
TGTTTTCCTATTTTCCGTTTTTGTGGCGGAGCTTGTTCTAGATAAGGGAATGCTTTCTTCGCAGGAGG

CGTGTCTTTACGAGGGTGCAAAAAATGCCCTA
GCAAAAGTTACAAGAGTCTCTCTTCCGGGCGGCCGTGGGAAACCTTTTGGCGAAAAGCCCGAGGAAGC
ATTAGAAGGCCTCATAGATATGCTGGGTTTAG
ACGCACAGCCGGAGGCTATCGTATGCAAATCCGAAAGCGAGCTTATAGAGGTATCATCATCAACTCCT
CTATTGATGTTTCTTTGCGAATTTGCAATGAA
CCTAAGAAACGAAGAACCCTCACCAATGGAACTTCAAGTCCTTCGCACTCGTTGTACCTTTGTGGCAT
CACTGCCCGATCGAGTAGCGAATGCGTTTCTG
GGTACATTTGGGTCCACAAAAACTCTTGAAACAGACTTGGATCTCCTGGCAACTCGCATATGCCAGTC
ACTGTGTCGTTTTTTGTTTGTCATCCATGAAG
CACCGCCAAGCAAAATGGTGCAAGCAACCTCTACCATGAGACGATACAGGTTCAAAAGGGAGAATGCT
CTTCTTGTATGTGAAGTCCTGATACAGAACTT
GGTTCGGCGTGGGTTTATTCGAACCCTTTTCCAGTGTTTTATCAGCTCTCTAAAAGCGGCATTGCCCT
TTTTGGCCTTCTTGGAAGAAATAGTACAACGT
GTATTTGGGGCTGTTTACAAGTGTTGCTTAAAAAATGGCGAAGTTCCTCGTGCTAGTATGAGGAATAT
TGTGACCGCGAACCTCCACACAAACATACATT
GGTCAGAATGGAATACATGGTTCCTGCGTGTGGCAGAAAGAGACACTGGATTGAGGCCTCTCCTTATA
TCATCTTTGATCAAGATGATCAACATTCCTGA
CAAGAATGAAGAAGCGGCTACGGAAGCACTGGCCATGTCTCGGGCATTAATGAAGGAGTTGTTCGTAA
CAGAAACTTCATATCTACCCACTGAACGGAGC
CTCCTGCGCGTCATTTTTAGAGAGGTTCTTCCAGGGATTGTATCCCACAGTTCAGAAAGGGGAGAAAT
ACCAATTTCAGAGATGGAGATGGGGTTATTCG
CCGAGGCCTTCCAAAGATGGAGTACTCGTGATCTCATTGAAAAGGGCTCTGTTGAACTGAATACCACA
TTGGCGAATTCCATTTTGTACATCTTAATTCA
CATTAAGGAGTATCATGTTAAAGATACAAAAGGACATCTTCCAGGTGCATTACTCCAACCTCTTTTGG
CTGGAATTTCTTTACGCTTCGAGTTGGTTCGA
GCAGAGAGCCTGAAGACTGAAGCAATAACTGTGGCTTCAGTATTTGCGACCTTCTTTGTTGGGAATGA
TGAGGCAAGGAATGCTTTTTCAAGCTTGGAGC
AGTTTCCATCTCTTCTGAATGACTGGTTGAAAGGCGAGGCGGACTCGACGACAATCCCTGAAATGTCG
CCGATCACTCCTATAGGAACTCATCGGGGCGG
AAACAACAAGGTGGATCCTTTTTTCCGTCGTCGGAGCATAGATGATGAAGAGTATCCTCTTGACCCGG
ATGCCGCCTTCATGTTCTTTTGTCGGAGGGAT
GCTGGAACAATGAAAAAGACTGGGACGTTGACAACTGGTGGAACTGTTGTGTTTAACTTGGACACAAT
TGTGAACCATGGGCATTTGCCATCCTTTGGGA
AAACACGGAATGAACAAGATGAACTGGAGGAAAGTGTGACAGTCCTAGTGACGCTGCGTGAATCCTAT
AACGCAATCATTGGGGTTGGACGGAGTAGTAA
TGCCCAAGTTCACGAGGTTCAACAAGCAGTAGAGAGTGGACTTCGGGGTTTCTTTCACGCCCTGAACA
GGCTGAGAGAGAAACTTGGTATTTGGAAGGAA
CAGAAGGTCCATGGAGAGGTTCGAAGACTGCGAGAAAAGGTTGGGATGGAACTTAACCCCATGATCCC
CTCCCTCCTTCCAGCACTGATGTCACTTACTA
TTCATGCACCAGAATCTCGACATAAGGAATTGATGGACCTGCGGTACTCTGTTATTGTATCGCTAGTG
ATTATTGCCCCGGAGAATGCACTGAGTCAGCT
TGGTAAGATGCTGTACTCAAGTCACTATGGTATTTTTCAGCGTGTTGAAATGGCAAAAGCCGTTGCTG
AGGCAGCCAAATACCTATCACAGGTAGAAGTG
CGCGTGGAACCAGAAGAGGGAGGAGGAGCAGCAGCCTCCCAGAAACACGATATTTGGAAGGAAAGACT
TACAAAAAGGCGAATTTATCCTCCTATACGCG
AGGGAAATGCGAAGCCCATGTCGATAGTAATCAGTGAGGGAAGAGAGACTAGACGGTGGGGAAGCGCG
GTAGCAGGAAGAGTTGATCGAAAAGAACGGAT
ATATTGCAGCTTATTGACAAATGTGGTCCCTTTTTTTGTGTCAGCTCTTTTAGAAAAGGCTGAAAATG
ATCATTTTTCTTTTTTGAGTGAGTATGATCCC
TATGTACCAACGGAAATACTTCGGAGCATTGGCACAGTGGTTCAGTGTATGGCTTCAGCTCGTCACAT
TGCTCCAGCCCTTTGTGAAAAACTCATGTCAT
TTGCCATACTGGCTGCAATTGAACATCCACTTTCAGTTGTTCGAAAGCAAGGATGGATTCTCATTGGG
GAAATTATGAGGTGCTGGTGTGGGGCAGGTCC
GCTTGTTCTGGAAGATGGCTCTTCATTTACGTCCCGCGGTGTTTTTGGGGGTTCGTTGTCGTACGTCT

TTTCGCAAGAGTGGTTGGATGCTCAGCGGGCT
TTGGGGACTTTGTTTGTCAAGACAAAGGACGATCCGAGCTGTGCAGAAATCGCACTGCTTGTTCTCGC
CGATATTCAAGACTTGGTAACGGCGAAAAAGG
ATCTAGAAGCAATGGAAAATCGGGTGGTCAACACGAAAAAGATTACCATGACGGCTGATACAAACAGC
GATGTCCGTGTCGCCTGAGGTGTTAGTAACAA
GCCTTCTACCTTTTACTTGTGCATGCTCCTTCTTTCTTTGCGTGTGTGTGTGTGTGTGTGTGTATACG
CTTGTGTCCATATAACGTTGTTGAGATGAATT
GGATTCTTTTTCTTCAAGGAAGGACAATAGAGAGGAGAAAAAAGCAGTACTATATGCGACGTCTCATC
TTGTATCGTCGGGAATTGTGTGGCCGTGAAGC
ATGTGTCTCCTTGCGGCGATTTTTGCGCAGGAGAGGAACACCCAAGAAGGAAGAGGTTGAATCCGTTG
ATTCGGTGGATGTTCCTTCTCTGCGTGTTGGG
GGCATGATGGCGGCACCTGACACTTCTTTAGATGATATCCGCACAGAAATCCTTCGTATGCGTTCAGA
GCATGTGAGACGCGAAGAGAAGCTAAATGAGA
ACGCGGCTTCTTCAGTCAAGGAAGGGTCGACTATGAACGATGCCTGGAACGACGATATCGAGAAGCTC
CGTTCCAACGAAGTGTATGTTAAGGGGCATGA
TGTCCTTGAGCGTGTTAAGGCGAAGGATCGTGCAGTTCGAAAGCAACTTTTTCTAGATGCCGAAGAAG
TCCTGAGAAAATCGAATAAGGAAGACACGCTT
CGGTATCTGCAGTTGGTTTATGGGCGTATTGGATCATACCATCGTGAAGAAGTGATGGAAAGCGTAAA
GGCATTGGTACAGTTAGTTGAAGAGGAGACAC
AAAAAATAAAAGGGAGAATCCAAGAGAATCTTATAGACAGAGACCCCTTGGCGGAATTGGATGCCGCC
ATCTCCTCCGACGAAGTAAAACAGATGGCATG
GGTAAATGCGTTGACAAATATGTCGGATGAAGATTTAAAAAAGCTGTGGAAATGGGGCATACTTGATT
TTGACACGATCGAATCGCTGGCTTGTATGGAC
ATGGATAACGAAACCCGTGTGGTGGATATGAACTTGCAAGAGAACAGTGAAACAGAGAAATTCTTTGT
AAGTACCACAGGTCAACGTCAAGAAGGCGAAG
ACGACAGTTTATTGGATCCAACGATCCCTCAACTCTCCATTGCAGAAAACACTGAGGCGCTTCAGAAT
GTCGGTGAAATACCCGAGTTTACGGCAACACT
TTACGAGAGTCTTTTTCTGGGATTTCCCAATATTGACTTAGCACCTGTCATACAAAAAGTTCAAGAAA
AACGGTATGAGAGCATCTCAGAAAGCGAGCTT
CGTCTATTGGAGCACTACGAAGAAAGTAAACAAAGTATTAACTTGTCGGCACCTTCGACCATCACTCC
CTCCCTGAACAAAACGGAGAAGAGTGAAGAGA
AAGAAGTTGTGAAGCAAGGGGCATTAGGGGAACGCTTCTCCAAAGATACCCATTTCTTGGATACAAAA
ACTCTTCCTTGCAAGATGTCACTGACGGAGGC
GATGATGGATCGGGCGTTTTCTCCTTCCAACAGGTTTTTACATGCGGTTGCTCGCTGCGACCCCGCGT
TGCGCTCATCGCTTGAGGTTATGGAAAGAATT
AAGCGGCATGCGCTGCTTGACCCGGTGTTTCAGGCTGCAGTAGAAGAGGCACATGCGTGGGAGGAGAC
GTCTATACAGAAACATCTTCAAAATGGCGGCG
TCAGTGCAAAAGGCGAGACGCGCAAGCAACGTCGACTGCGGCGTGCCGAGGGCAGCGCCACCCCGATT
CTTGGCGGTCCGCTTGCCAAACCACAACATGC
GGCTGACATCCCATACTATTACGGTAATATCCGCAGTTTTGTGCCACCGCAAGGGCGGTACAACATGC
CAGCGCCCCGTTTGTCGCGGGAAGAGGCTGCC
CAATTGCGTAACCAAGAGCGCAAGCAGAAGAAATCACGAATGCGTTTCAGCCAGTAACAACCGTAGCT
TCTGGAGTAAAAAAAAAAAAAAAAGGAAGAAAA
AGTGAATCTGTGAAAGAAACTCCGNNNNNNNNNNNNNNNNNNNNNNNNNNNNNNNNNNNNNNNNNNNN
NNNNNNNNNNNNNNNNNNNNNNNNNNNNNNNN
NNNNNNNNNNNNNNNNNNNNNNNNNNNNNNNNNNNNNNNNNNNNNNNNNNNNNNNNNNNNNNNNNNNN
NNNNNNNNNNNNNNNNNNNNNNNNNNNNNNNN
NNNNNNNNNNNNNNNNNNNNNNNNNNNNNNNNNNNNNNNNNNNNNNNNNNNNNNNNNNNNNNNNNNNN
NNNNNNNNNNNNNNNNNNNNNNNNNNNNNNNN
NNNNNNNNNNNNNNNNNNNNNNNNAAAGTATATGGAATTTGCGGTTATCCTGAGTGACACACACACAC
GCGCATTACTCTATTCTACTTTACTCGGAGTT
GTGTCTCTTTTTTTTCCTCTCTCTTTTTTCTTGTTTTTTTTTGGGGGGGAGGGGGAGAACTGCGGGGC
GTTTTTGACCTTATTGAAGGACCAAAAACAGC
GCCGTCATCTCTGCTTTCGCTGTGGTGTTACACATTAAGGAGGAAGGCTTACTTAACGTCACATAGAT

ATCTATTATATTTATTTATACGCTTGGTCAAG
AAGAGAGGTATGACCTCTAAAAAGGCTGCCAAAGAAAAGGATGAATACACTCCCCAAGAACGTTTGAC
AGCGGCCGCGCTTGAATATAAGAAGCTTGAGG
GACACAATCAATCCAGCATTGAACGAATGTACGGTGCGCTGTCTTCCCCATTTAGGGTTTCCAACAAG
CCCTGTGAGGTTCCTCGTGCGGCAGTTTTGCG
GTGTTTTGAAGAAATTCTGGGGAAAACAAACAGCGGTGAGAATGGCAGCACTGATAACCTTACTGAAA
CCACAGTCGTTGGTCGCAGTATCAGTGGTGGT
AATAATGCCGATGGGAGGGTTTTATTATCGTCAGACTCACGGTTTCCATCGGTGCATCGCTGCTATGA
TGCAGTTTTGCAGTATGAGTCATGCGTAGTAG
AGAAGACACTTGCGCAGCATCACAGCCTTCTTGCAGAATTTGAAGCCCGTCAATTACAAGAGGTTGAA
TCGACCAATGAAGTGCCGAAAGCTGTCGCGAG
TGGTGCATGAGTATGTGTGCCTGTGTGAGAGAAAAATGGTGAAGAAGAGATGGAAGGGGATACTTAAG
GAGGAGAAACGATCAACAAATTGGATTGATTG
ATCCTGGGGCCAACATTTTCTCAAAAGAAAAGGAATGAAGAGGAACGAGAGGAGTCCTAATGTGCAGT
GTCTCTAACATTTTATTTTATTTTACTTTTTT
CTTTTTTTCTCCCTTTTTTTTGTTTTTATTATTAGTTTTACCCTTTGCCATTTCCCCGGAATGGCACC
CAGAAAGAAGAATGCACCAAAGAAGGAGTTGA
CCGCAGAAGATATGCGGCGTCTTGGGATGTCGGAGGAAGATATCGCCCGCATTTTAGCCGAGCGCGGC
AAGTCAGCCGAGGAGCGACGGCAAGAACAAGA
AGCCGCTGAGGCGCGACGCCGTGAGACGGAGCAGCGCCAAAAGCGTATGAAGGAGCTGGAGAAACAGA
AGGAGGAACTTGTGCGTCTGGAAGGAGGCCCA
CGTGTTGCCATTCGTGACGAGGAGGATGCCGAATGGGAGCAGCTGGAAGAGACACTGACGGTGGAAAA
GAAACGACGGCTGCGGGAGTGTGCAGAGAAGA
TGGTGCAGCAACGCTTTGCAGAGGCGCGTCGCAAGCGCGAAGCGGAAGCAGCGGCGTATCAAGAGACT
CTTGGCCACCTCACACCGGAGGAGCGGGAGGC
CTTCGTCGCTGCGCAGATCGCGGCGGAACACGCCCGCTCCGAGGCAGAGATGCAGGCGAAGGAACAGC
AGCAAAAGCGTGAGGAGCGACGACTTGCACGG
GAGCGGGCGGCACGGAGGGCGCAGCGACAGCGACGAAGACAGGACGGAGAAGAGGACGTGGATGAAGA
AGATGCGGATGATATTGCCACGGTTGGCGGTG
GCGCGAAGGCTTCTTACGAGCGAATAAAATCAATCGATGAACTTTCCGCTGACCTTCACGACAAATAC
GACTAATCAATACAGAGATAAAGTGTGTAGGT
GCACGCATATACCCGCGGAATGGCAGCAGCTGGCGCGAGAGAGAGGGAGTGAGAACAGGAGAGAATGC
GAAAGCGCAAAAGAATGAGAAGGGGAAGAAGA
GAGGGAGGAACAGATGCCGGCAATGATGGTCATGATGATAATATTAATAAACAAAAAAAAAAGAAAGA
AAGAAACGAAGGGAAGTTCTTATTGGTTTAAC
ACGTGAAGTGAGCTGAGATGAGTTTACTAATATGTCACCGCTGCATCATTTGAAAGAGAGACGGGGAT
ATACACACACACACACACAGAATATCTCTCTC
TTACCTTGTCTGTGTGTTTATGTGCGTACTCCCTTTTTTATGCCTATTTTTTCTCCTTTCTCTCCTTT
TCAATTCATGTTTTTGTTGTTGGTTCTTGCGG
GTGCATGCTCCCACCGCACATTGCTGCCTTTTATGTTCATCTCTTTCCCTTGCCAGTCTGTCTGTGAT
TACGGGAGTGGCCTCGCTTGCGAGAACGCATA
TCCCAGTCTGAGACTTGACGGAAAAATTATTTTTCTGTGTAATTTTAAAGGGAAACTAACAAAATTAT
AAAATATTCAGGAGAGAGAGAGAGAGAGAGAG
AGAGAGAGAGAGAGAGAGAGAGAGAGAGAGAGAAAAGAAGGAAATATAAACTATATTTGGGTAGAGACAA
AGACAACAAGTAACTAGGAATAGGACTTTGGT
GTATATCAATTTAATTCTAGAGCAAAAAGCACATACGTGTGGACAGAGAGGAAGGAAGGGAGGGGGGA
AGAACAAATTAAGGAAGGGAAACAAAAGGTGC
TTAAAAAAGGGAAGAACAACGTACAATATAGTGAAGAGAAGCCCATGGAGGGCGTCAGTACGCCTTCT
TCCAGTGATGGCAGCAGTGGCTGTGGTAACAC
GGCCACCGCCGCTGCCGAGGCATCCGCGACGGATGGTAATCCGGGCGGGAGATGCGTGTGGCGACATC
CTCGTAAGAGTGGACGGGCGGGTGGCGATGTT
CCGCCTGCCGCGATTCACCGCATCTCCCAGCCCCTGGCAATACACACTGCAAGGCCCAAATGTGGGGT
CTCCAACCTGATCCACGGCGATACGAGACATT
CACTCCATGTTGCATCGTCTCTGGATATGCACCCGCATGGCGTGTGTGGGCTTCCAATGCCGATTCTG

GACGTCGTGTTTAATTTTCTTTCTGTGGATGA
CTTACGCACGGTGCTCGGCGTGTGTCGGTATTTTTACCGCGCCGTCAGCGAGTCGGACCGAACTGCAT
GGAAAACCGCCTGCTTCTCCATGTGGCATGGC
AAGCAGGGTTTTGCGCGTGTGATGAAGACGTGGGCGGCGACAGAGGAGGCACATCGGCGGGAGGAACT
TGAAATCATCGCCATGCAGCAGCAAATGTTGA
TTGGGACACACATGAACGACAGCACCGCCACAAACACCACCACCGGCAGCACCGGCAGCAACAACAAT
AACAATAACAACAGAATTGCGACAGTAACACA
TGAATGGGTGATGTTTAAATTAGGAGAAACCGAAAGAGACGAGGGGGATAAAAATCATCAGGAACGGC
ACTCGCAGGAGCATCAACAACTGTTCATGCCA
TCATATTGGACTGGGCGTGGCGGGAAGAGCGGGCCCGCNNNNNNNNNNNNNNNNNNNNNNNNNNNNNN
NNNNNNNNNNNNNNNNNNNNNNNNNNNNNNNN
NNNNNNNNNNNNNNNNNNNNNNNNNNNNNNNNNNNNNNNNNNNNNNNNNNNNNNNNNNNNNNNNNNNN
NNNNNNNNNNNNNNNNNNNNNNNNNNNNNNNN
NNNNNNNNNNNNNNNNNNNNNNNNNNNNNNNNNNNNNNNNNNNNNNNNNNNNNNNNNNNNNNNNNNNN
NNNNNNNNNNNNNNNNNNNNNNNNNNNNNNNN
NNNNNNNNNNNNNNNNNNNNNNNNNNNNNNNNNNNNNNNTGGCGGGAAGAGCGGGCCCGCTTCGCTCGC
CACAACACCAAGTGATATTGACAGTGTTTTCA
TAGGGGCAAGAAGTGCGCCGCAACAACTGTGGGATCGAGTTTCACTTCGGAGTACACCGACATACGAT
GCGATCATGAGCACGGTAAACTCGCCCCATCT
ACGAGCGTCGTTGATATCCCCATTGCGGCGCAGCGTCTCGTCTATATCATCCGTTGAGAGTGGAACCG
ATCAAATGTTCAGGACAAAGAAGAAACTGTAC
TGGTGGCAAATGGCACCCGAGCGGCGCCAACAGCAGTTATATTGCATGCAGCAAAAACTCCACCGACG
GCGATTAAGGTTGGAGCTGCGAAATCAGCGCC
GGCAGCGTCATGAGGATTCCTGCGACCTGTTGCTTGACGAGGATGAGGAAGAGACGGACACAAGTCGT
GTCTTTTTGACAGAACGCATCACTGCTCATCA
GAAGAAAGTTTTGATGGGTACTTTCAATTCGCCCCAAAGCCATGAGAGCGGCAGCAGTAACAGCGACC
AACCGTCGCTTTTCTCGCTTCCTTCCGCACTT
GCGGTGACGGCGACAGCGAGAGGATTGCGCTGCACAGGGAAATCGCGAGGAGGGGTTCTGTTGGCGGC
GAACACCGATCAACAGAGATTGATGCATACCA
CCGACGAGACAAAAGAAGGGGAAAAAGAAGGTTCTGAGGAGGATGCAGATGTCTCgGATGACGGGCTT
GGCGATTGCGACAACGACAGCGAGGCCGATGG
GAAGCATGACAGCAATCCATATCAGGAGTTTGAGGAGGATATGGCCCTAACACACTCTCTAAGCGTGC
TGCAGCATGCCCAACAAAAGGCGATGCGTCTC
ATTTCTGAGGGGAATAAGAGGAGGCAGGAAGGGAACGAAGTTGAAAACGATGAGGAAGACATGCAATT
ACCGGTTTCGTGGAAATTTGCATACTACATGT
CACTTCGTGACGCGCGACGCCAGAAGATTACGATGCAAGAGCTCATAGAAGACAAATGGTATGTGTGC
TTTCGCGCCACGGGAAAAACACATCCTGCGAC
GTTTACGGCAAAACGTACCCTAATCATCTACCCTGCCCTGAACAGCTCAAACAACACGAACATGAATA
TTCAGGCCGGTGGGGATGTGGAAAATGACGCT
GCAATGCCTCCTCTCTTATATCACCTACTGAAGGGTGGTTCCGAACTTGTGGTACACAACTTTCCCCC
GCTGAAGGTGTACCGTCGGTCAGCTTTGACAT
CGGCTACTGCTTGTAATAAAGGGGGTGTTGCTCATGGCAGCATCACAACTAACGTTTCTGTTCCTGCT
GTCCCAACGTGGCGTTCAGCCGCCGGTGATAA
TTCCAGAAATCAAACGGCGGCAGAACGTGCGTTCATTGCCGAGCCGGATGCCTCGCTACACCGCCTCA
GGGCCCAGGAGGAAAAACACATACCTCCAGGT
GGAGAGGATGAGGAAGAAATCTATACAGAAGCCGTGTTTAGGCCGGCAGAAGACGACACTCCTGGCAC
GGACTGGGGGTGGGTAATACAAAACTACTTTG
TAAAGATATTCTCCGTGGACACACATATCCCAGTGTATATTCAGCGACTGCGGCGCACATGTGCGCCG
CGGCCCCCCTGATTGTGGCTACACACACTATC
CTGATTTTGGGTCAGTGCCCATGTGCGGCTGAGGGTAGGTTCTGGCTTGTATGCAACTGTTTTAGGTG
TTTCTTCTTTTATTATTATTGTTTTACACGGG
TGGAATGCCAGGGGGGGAGGAGGTGGGGGGAAAGATGTTTATTGCTCTTGTTGATACTTTGTCGCAAA
GTTCCCTTCTTCATTTTTTTTTTTTTAGGATTT
TTTTTTTTTGAGTTTGAACTGTTTTTATTTATTTATTTATTTATTTCTTTTTTTTTTTTTTTCCTCT

TTCTTTGTTACTGCGCAAAAACCTTTTATTTT
TGTACTGCTGGTAGTACGGCGACTGAATTCTTCTATTCATGCAACCCTTTTTTTACCTCTTTTCTGTT
ATTTTATGCACATGCAAGCACGACCTTATTCA
TTTCCGAGGTCGTTTGGTTGTATTCTTCTTTCGTTGTTGCTCCTCCCCTCCCCTCCCCTCCCCTCCTC
TGTTGCTTTCTGCTCTCTCCCTCTTGTAAACT
GCCGAATTCTGATTTTCTTTCTTCTGTTTTTTAAGCGTTAATGTTTTTTTTTTGT
>scaffold1083.size12715
TTTTCTTTTTTTTTTTTTAAATATTTTTTTTTTATATTTTACCCGCCGCTAAAGGCAGAGAGACCCAA
GAACGGGATGTGGCACATCAAATAGAGAATATA
AAAGCAGAAACAAAACAAAACAAAACAAAAAAAAACAAAAAAAAAAAAAAAAAAAAAAAACGTACAAA
CACACACGCAGAAAAATCAAAATAGGGAATAA
ACACAGACGGACACACACACACACACAACACCAACAGCAACAGCAACAAAAGAAAACATGTAAGATAG
AACCACCACTACCACCACCACCACCACTAAAA
AAGCGTGGGGAAAAAAAAAAAAAGAAAGAAAGAAAGAAAAAAAAAAATATATTTGATAAAGTTTTGGTAA
TGTCTTCTTTGGCTTGCCTTTTCGTTTTCCTT
TTGAAAATGCTGCCGGTTAAATATAAATCTTTAATATATATAAACATGCACTGAAAAAAAAACCCCGC
CCCAAATATAAACAAAAAAAAAAAAAATGGAAG
GAAAGAAGGAAAGAAACACACACGCACACACAAAAAAAAAAAGGCAAACAAACAAACAGAAAATACACA
CATACATGTGTAAATAATAATAAAAATAATAA
AAATGTCTCATTTCCTTCGTGATCTTTTTTTTCCCCCATTCCCCTCTCCTTTTTTCTTTTTCCCCGCC
GCAAATACATAAAATATATATATATAAAATAAGC
ACATAGATACATACATACAAATATAAATATAAACATATGTATTTATCACATTTCCAACGTGTTGACAA
AATTTTCTTTTGTTGTCTGGCAAAAAGAAAGA
AAAAAACACCTGATTTTATTTTATTTGATATTATTATCATTATTATTATTGTTATCATTATCATAAAT
GTAAATACGTGTACATACAGCCATACATACAG
CCGTACATACAGCCATACATACATACAGCCATACATACATACAGCCATACAGCCATACATACAGCCAT
ACATACAGCCATACATACATACAGCCATACAT
ACATACAGCCGTACATACAGCCATACATACATACAGCCATACAGCCGTACATACAGCCATACATACAG
CCATACATACATACAGCCATACATACATACAG
CCGTACATACAGCCATACATACATACAGCCATACATACAGCCATACATACATACAGCCATACATACAG
CCATACATACATACAGCCATACATACATACAG
CCATACATACAGCCATACAGCCGTACATACAGCCATACATACATACAGCCATACAGCCATACATACAG
CCATACAGCCGTACATACAGCCATACATACAG
CCATACATACATACAGCCATACATACATACAGCCGTACATACAGCCATACATACATACAGCCATACAG
CCGTACATACAGCCATACATACAGCCATACAT
ACATACAGCCATACATACATACAGCCATACATACAGCCATACATACATACAGCCATACATACAGCCAT
ACATACATACAGCCATACATACAGCCATACAT
ACATACAGCCATACATACATACAGCCATACATACATACAGCCATACATACAGCCATACAGCCGTACAT
ACAGCCATACATACATACAGCCATACAGCCAT
ACATACAGCCATACAGCCGTACATACAGCCATACAGCCATANNNNNNNNNNNNNNNNNNNNNNNNNNN
NNNNNNNNNNNNNNNNNNNNNNNNNNNNNNNN
NNNNNNNNNNNNNNNNNNNNNNNNNNNNNNNNNNNNNNNNNNNNNNNNNNNNNNNNNNNNNNNNNNNN
NNNNNNNNNNNNNNNNNNNNNNNNNNNNNNNN
NNNNNNNNNNNNNNNNNNNNNNNNNNNNNNNNNNNNNNNNNNNNNNNNNNNNNNNNNNNNNNNNNNNN
NNNNNNNNNNNNNNNNNNNNNNNNNNNNNNNN
NNNNNNNNNNNNNNNNNNNNNNNNNNNNNNNNNNNNNNNNNATACATACAGCCGTACATACAGCCATA
CATACATACAGCCATACATACAGCCATACATA
CATACAGCCATACATACAGCCATACATACATACAGCCATACATACATACAGCCATACATACAGCCATA
CAGCCGTACATACAGCCATACATACATACAGC
CATACATACATACAGCCGTACATACAGCCATACATACATACAGCCATACAGCCGTACATACAGCCATA
CATACAGCCATACATACATACAGCCATACATA
CATACAGCCGTACATACAGCCATACATACATACAGCCATACATACAGCCATACATACATACAGCCATA
CATACATACAGCCATACATACATACAGCCATA
CATACAGCCATACAGCCGTACATACAGCCATACATACATACAGCCATACAGCCATACATACAGCCATA

CAGCCGTACATACAGNNNNNNNNNNNNNNNNN
NNNNNNNNNNNNNNNNNNNNNNNNNNNNNNNNNNNNNNNNNNNNNNNNNNNNNNNNNNNNNNNNNNNN
NNNNNNNNNNNNNNNNNNNNNNNNNNNNNNNN
NNNNNNNNNNNNNNNNNNNNNNNNNNNNNNNNNNNNNNNNNNNNNNNNNNNNNNNNNNNNNNNNNNNN
NNNNNNNNNNNNNNNNNNNNNNNNNNNNNNNN
NNNNNNNNNNNNNNNNNNNNNNNNNNNNNNNNNNNNNNNNNNNNNNNNNNNNNNNNNNNNNNNNNNNN
NNNNNNNNNNNNNNNACATACAGCCATACATA
AATACAGCCATACATACAGCCATACAGCCATACATACAGCCATACATAAATACAGCCATACAGCCATA
CATACAGCCATACAGCCATACATACAGCCATA
CAGCCATACATACATACAGCCGTACATACAGCCATACAGCCATACATACATACAGCCATACAGCCGTA
CAGCCATACATACATACAGCCATACATACAGC
CATACATACATACAGCCGTACATACAGCCATACAGCCATACATACAGCCATACAGCCGTACATACAGC
CATACATACAGCCATACAGCCATACATACAGC
CATACATACATACAGCCGTACATACAGCCATACATTACATGTAAATATATAAATATTTTATTATTATT
ATTATTTTTTGTCTGCGTGTTTGTTCATACAA
AAACCACTCAACTTTTGTTTGTTAATTTAAAAAATTAAAAATTAAGCATTTTTTGTTTTATTCTTTTT
TTTTTTTGTTTGTTTGTGGTTGCTGCTGCTTT
TGTTTTTGTTGTTGTTTTTGCGTGCGTGTGAAGAATAATAAAACAAAAAAGGAAAAAAAAAACCAAAT
TCATCGCCGTCTTAAACAAAAAAAAAAAAAAA
AAACATAAAATAATGAAATAAAAACAAAAATGTTTCGACATGTCACCGCCTCACTTCTTCTCTTTTTT
GTTCCCCTGCCCTCCCCTCCATTGGTATCATG
CCACTACAAACAATTGTACACACGCACACATAATCTCTCTCTCTCTCTCTCCCCTTCCCTTTCTTTCT
ATCTTCTCTTATTTTTATTCCTCTGTTAATGT
ATCCTTTTTTTTTTTTTGTTTCTCATGTCTTCTCAAAATAATTCGATGGCTCGCCACACGCCAGCCGCC
ATCGCAGAACACATCCGCACCCCAACCAAGCA
GAGATGTCAAAAAAAAAAAAAAAAAAAAAGGAGAACAGAATAGTAAAAAAAAAAAAAAAAGACCACTGG
CTTCCCCCCTTTCCCTGGAGAAAGGAAATTAC
CGTGTTGTTGCTTCTTAGTCTTTTTCGCTGCGTTTACTTCATTGTGGTTCATTTGATTCATGTTTCTG
TCTCCTCTCGCCCCCGCTTCTTCCCTTCGCTC
CTTCACCTCCTCGACCTCTGTCATCGTTGTTGTTAATCGCTCCCACAGACACTCACCCACACAGATGG
AAAGACTGACATGCGTGCATCCTTTTTTCCCC
CTTTCCGTCTGTGCGTGTCCATGCGTGTGTGTGAGTACGTGGGGCCTGCGGGGGGAGCGTCACGAAAG
GCGCGACAACGTCCCCACCGCCACCATCACCA
TCGCGAGTGTACACGAACGGAGGGAGAAAAAAAAACAAAACAAACAAACAAACAAAAAAAAAAACAAC
CGCTTTGGCTCTTTTTGTTTCAATTCAATCTG
CCGTGCACACATATATGTATGTATATATAAAAAAGATGTTCTTTTTTTTTTTTTTCTCAGTCATTCCG
GCAAGAAGCAAAAAAAAAAGAAAAAAGAAAAG
GAAAACAGGAGGAAAAAACATCAATAAAACACACACACACACAAAAAAAGAATCATGAGCACTAACGA
GCGAGAAGGAACAAAAAGAAAGAAAGAAAGAA
AAAATCCTCAAAAATAAGAAATGATGAAGCAACAAAAACGACAACAAAAAAAGACAATACGACAGAAG
AAAAGGCAAGAGAGAAAGACAAAAAACCAAAA
AAAAAAAGAGGATAAAGAGTAAAACACTTAAGAAAAAATGAAAATGAAAGAAAACAAGAAATATCTGC
CCTACGCTTACACGGGTCAAACGCTCCTTTCT
TTTTGATTTCTTGCAGGTTTCTTGAGTGATTTCATCAATAAAACACGCCACGCCCTTCTACGAAAACG
GAACAACCGTAAAGGGTTGCGACTTCGGTGGC
GGCAGTGGTGAGGCGTACCCCACCTCCGGTGGCCACAGAAGGCTGGACTGTGCGGCAAGGGCAAAGTC
AGATGGACGCAAGTTCACAGCGTTCGAGGCCA
GCATCAGCTGCTGTCCGTTTTCCAGCACAATCATAACGGGCGGGTCATCTTTATTGCTGTTAGCTCGA
CGCACCGGTGGGGACCCTGGAGGACCCACCTT
GCAGGACGCATCCAAAGTATCCATTGGAAGAGGCTGGGACTGCGGAGGTACAGAGAACATGGGCGGAA
GTGCTGCCGAGCCCAACAGTGTAGGCGGCGGT
GGAGTGAACACGTTATCTGGTACCTTGTGAGCCGAAGATGCGGTTGACGAGGTATCACTGCTTAGGTT
TGGGCCCAGTGAGCATGATGTCGAATCCCGAG
AAGGCGTGAGCTCACGAGGACAGGCAGCAACAGTTATATTGCCGTTGTTGGAGAATGCTGTGGCAACA

TCCATACAATTGCACTGATGCACCTCTGGGGC
TGCGGTTTGACGAGGTGGTGGAAATACACAGACACTACGTGGAGGAGGTGCACGGGGAACTTTGCCCT
CTTCGCGGCGGCGGCGGCGCTCTTGTTTGGCC
TCATCCGTGTCAGCGTACTTGACCATCACTGGAGGACCATAAAAGTGTTCTGAACTGCCGTGTAGCCT
TTGGAGGGCATTCTTGGAACAGCTAAGGCAGT
CGTACTCGGCGTAGACAACCCATACAGGGTTACCGTAATGGTCCTCACGCATGGCGCAGAAAGCCAAG
GTGCCAAAGGTCGATAAAAACCTCTCCACCTG
CGCCTGCGGAACCGTGATGGGAATGTTGCGAATGTACAGAGCATTAGATGGAGAGACAGCGGCCCTTC
CACTATGCTGTGAGGGGTAAATTATCAAATTG
AAGTTGTGGCGACATGCTCTAGTGCTCTTTCGGTTAAGGTCATCGTACGCCCTCTTCCCTTCTTCTTC
GGTTCTAAAGAGCACAAAACCAAAGCCTTTAC
TCCTCCCTGTGGAGGCATCCAACATCACCTTTGCGCTGACAGGACGATACTCCGCNNNNNNNNNNNNN
NNNNNNNNNNNNNNNNNNNNNNNNNNNNNNNN
NNNNNNNNNNNNNNNNNNNNNNNNNNNNNNNNNNNNNNNNNNNNNNNNNNNNNNNNNNNNNNNNNNNN
NNNNNNNNNNNNNNNNNNNNNNNNNNNNNNNN
NNNNNNNNNNNNNNNNNNNNNNNNNNNNNNNNNNNNNNNNNNNNNNNNNNNNNNNNNNNNNNNNNNNN
NNNNNNNNNNNNNNNNNNNNNNNNNNNNNNNN
NNNNNNNNNNNNNNNNNNNNNNNNNNNNNNNNNNNNNNNNNNNNNNNNNNNNNNNCCAAAAAAAAAAA
AAACCAAAAAATGCATCCAGGGAGTCAACTCC
GTCGGTTCCATGTGTCTCAACTCCCTCCAACCTGGGCACATCGTGCCTGCGCACAGGGGCAAGAAATT
CCTTCACCGTACATACAAGTGCTTACATCACG
ACCAATAAAACAGCAAAAAAAAAAAAAAAAGGAATAACGGAACAAAACAATAACTCACATAAAAAAAA
AAAAAAAAAAAGAAAGAAAAAAAAAAAAGGAAC
ACTATACTTCAGTATATTATAAATATACTAAAAACAAACAAACAAACACACAGGCGATCGCAACATTT
TTATATTGCTATACATTTGAGTCCGGACTGCG
CACCACATCTCGGGAGATGTGCATGTTAGAAAAGTTATATCATACCCTAACGTGGGACACACACGCAC
ACACAGCAAAGCAAAGCAAAATAATAATAACT
CCCCTTCGTCCCCATAAAATATGCAGACACACACACACACACACAGAAAAAAAAAAAAAAAAAAAACAA
AACAGCGGCGCCAAAACAAGCAAAAGCTAAAA
AAGACACCAACACACACAAAAGTTTCTCTACATAAATAATAATAACTTCACATCAATACGCATTTATATA
TTTACATGTACATATATACATATGTGCATGCT
TATATATATATATAAATGGCAAAAAACAAAAAGAAGAAGAAAAAAAAAACAGAAAGAAAAAAAAATGA
GTCGTTTGAAGATGCGTCGGAGAAATAACGAT
GCGCGTGACAACAACTTCTTCTCCCTTCACTTACACGCAAACTCCGTGTTTTTTTTTTTTTTGTTCCT
CTCACGAAGGAATGGCAGCGCAAAGAACAGAA
CAGCAAGATGCCCAAACAGAGAACAAACGCACGGGGTTGTGGGAGCGAAAAGGGCCGGCAAAAATAGA
GGAAAAACAAAAATAAAGAAGAAAAAAAAAAG
AAAAAAACAATAAAACTCGCAAATAAAAAAAAAAAGTGAGAACCAAGAAAATAAAAAAAAAAAATAAT
AAAAATAACAATAAATTTACGTGTAGAGGCGT
CCAAGCGGTTGGTTTCTTTGAATCCGATCAACTGAGAAGAGCTCCTTCGCAACAACAACAAAACCCTC
CGGTCCGTAGGGATCGTGGCGGAATCGTGAGG
GGTAGGCAGGTGGCAGCATGATTTCCGCCTGGACGCCTTCGGGGTATGGTGGCGGTGGCGGCAGTTCG
GCTTCCTCCGTGCGCACATGAGATTTGTCGGG
CTCACCGTGCGAAAATGCGCAGCGCTCACCAAATGGACAAGTGATCCCCATCGAATAGTTACGGCACA
CCCGCGTCCGGAACTTGCTGTAGTCNNNNNNN
NNNNNNNNNNNNNNNNNNNNNNNNNNNNNNNNNNNNNNNNNNNNNNNNNNNNNNNNNNNNNNNNNNNN
NNNNNNNNNNNNNNNNNNNNNNNNNNNNNNNN
NNNNNNNNNNNNNNNNNNNNNNNNNNNNNNNNNNNNNNNNNNNNNNNNNNNNNNNNNNNNNNNNNNNN
NNNNNNNNNNNNNNNNNNNNNNNNNNNNNNNN
NNNNNNNNNNNNNNNNNNNNNNNNNNNNNNNNNNNNNNNNNNNNNNNNNNNNNNNNNNNNNNNNNNNN
NNNNNNNNNNNNNNNNNNNNNNNNNAGAACCA
AGAAAATAAAAAAAAAAAAATAATAAAAATAACAATAAATTTACGTGTAGAGGCGTCCAAGCGGTTGGT
TTCTTTGAATCCGATCAACTGAGAAGAGCTCC
TTCGCAACAACAACAAAACCCTCCGGTCCGTAGGGATCGTGGCGGAATCGTGAGGGGTAGGCAGGTGG

CAGCATGATTTCCGCCTGGACGCCTTCGGGGT
ATGGTGGCGGTGGCGGCAGTTCGGCTTCCTCCGTGCGCACATGAGATTTGTCGGGCTCACCGTGCGAA
AATGCGCAGCGCTCACCAAATGGACAAGTGAT
CCCCATTGAATAGTTACGGCACACCCGCGTCCGGAACTTGCTGTAGTTTGTGTCTCTGTTTTTGCTGT
GGGTCGTGCTATTGTCAGACATGGTCTTGGAG
CTTGTTGATTTCCACATTTCCCCTTGCTCTGTGTGTATTTTCTGAGCCCTTAATCCGTGGCGTTTCTT
GCCCTGACCTTTTACCTTTTTTTTCTTTTTGT
TGTTCTGTCACCCACTCTCTGCTCCATTGCGGGTGGTGGTGGTAAATGTGTGTGTATGTGTGCACACA
AGAAGAAGAAAAAGCGGAAAAAAAAAAGATAAA
TGAAAATTCGAAAAAAAAAAAAAATCCCAGTGAGTCCATTTCGCTGAGCTCAAGCACATGTCATGCAGA
ACAGCTGAGTGGGGTGCCGAAGTCCCCCACCC
GTCCCCTGACCACAAAGGCGATGGGAGTTATCATCATAGGGGCAACACACAGACACGCATTCCTACCC
ACAGAAACATATGTGCGACCCGCATGTACGCC
TGCCTTCCTGCTGCCCTCCTCTTCGCTGTTCAACTCACTCCTTCGACCTGCAAGGTGGCAACAAAAAA
ATTCAAAAGAAGAAAAAAAAAAAAAACGGCGTT
GGGACAACACACGCAAAAGAAAAACAACAACAACAATTGAGTAGAAAACGAACAAAATAGGGAACGCG
ATGTACCACGGCGCTGAGTTGCTGGAAGGCCG
CACAAATATATAAATATAAATATTCAATTTTCCGTTCGACGTGTGTGTTTGACTGGCCCCACTTCCCT
TTTTTTTTTTTTCTTCCTTCTCTCTGTTTGGC
GCAAAGGAGAACGGGGGGAGGAAAGGTGGGTCCCTACCACCACACGAAAGTCCATCCCATTTTTTTTT
CCTTTTTTTCCCCACTTCTCCCTTCAAACACC
TTTTTTTTCTTTTTCTTTTGTTTAAATATCGTTTCTGCTGCAATGCCCTTTCTCTCTCTCCCTCTCTC
TCCCGCCCCTCACATCCTCCTTTTAGTTTGAC
AAATAAATAAATACTTTTTTTTGTGTTTTTCCCACTTTGTCATTCCCCCGCTTTTGTCCACCCACCCA
TAAATCCACACACACACGCCCCGCACGGCACG
CCGCCCCGCCCGTATTTTTGTCTTCATACATTGTGAGGGCAGTGGCACCGGCTCCGCGGGGGGTGCTG
CGTTGGAGGGACGTGGGCTGTGGGCACATGAA
CCAACGAAGGGGAGGGAGGAGGAAAGAAGGAGAAGAAGAAGGACAAAAAAACAAAAAACAAAAAAAAC
CAGAAAAACAAAACAAAAGAAAAGAAAAAAAC
AACAACAAAAACAACAGGAGAAATCAAAAAAAAAAAAAAAAAAAAAAAAAGAAAAAAGAAAAAGAAAA
AAAAAAAAGGAGAACAGAATAGAAAAAAAAAAA
AAAATGTTAGAAAAAAAGAAAAATAAAAGAAAAAACAAAGGTACGTATGATAGTAAAATGCTACTCGT
TGCATGTATATCTACGAGGGCAATATGCGTAT
AAATATAAATATAAATATATCCGCTGCAATTGCACACCAGACATACAAACTCACGCACACACACGAGT
ACGCGCGTATGTAAGAAAAGAAAAGAAGAAGA
AATTTTTTTTNNNNNNNNNNNNNNNNNNNNNNNNNNNNNNNNNNNNNNNNNNNNNNNNNNNNNNNNNN
NNNNNNNNNNNNNNNNNNNNNNNNNNNNNNNN
NNNNNNNNNNNNNNNNNNNNNNNNNNNNNNNNNNNNNNNNNNNNNNNNNNNNNNNNNNNNNNNNNNNN
NNNNNNNNNNNNNNNNNNNNNNNNNNNNNNNN
NNNNNNNNNNNNNNNNNNNNNNNNNNNNNNNNNNNNNNNNNNNNNNNNNNNNNNNNNNNNNNNNNNNN
NNNNNNNNNNNNNNNNNNNNNNNNNNNNNNNN
NNNNNNNNNNCCGACCAAAAGAAACAAAAAAAAAGAAAGAGGGAGTGGAGGAGGTTTATTGGGGAGGG
AAGGGAAAAATGACGGCGGGAGAAACCCAAAA
AAACAAAAACAAAAACAAAAAACAAAAAAACAAAAGAAAAAAAAAGACAACAACAACAAGAAGATAAA
ACTCTTCAAAAAAAAAAAAAAAAAGAAAAAAA
ATATTTTTCCCGTTCCCCATTTTTTTTTATTTTTTATTTTTTTCCCTCCTCCATTTTGCGTAAAATAA
ATAAATAAATAAATAAATAAATAAATAAATAA
ATAAATAACAAATCACTGCGTGTTTTTTTTTTTTTTTGGTGNNNNNNNNNNNNNNNNNNNNNNNNNNN
NNNNNNNNNNNNNNNNNNNNNNNNNNNNNNNN
NNNNNNNNNNNNNNNNNNNNNNNNNNNNNNNNNNNNNNNNNNNNNNNNNNNNNNNNNNNNNNNNNNNN
NNNNNNNNNNNNNNNNNNNNNNNNNNNNNNNN
NNNNNNNNNNNNNNNNNNNNNNNNNNNNNNNNNNNNNNNNNNNNNNNNNNNNNNNNNNNNNNNNNNNN
NNNNNNNNNNNNNNNNNNNNNNNNNNNNNNNN
NNNNNNNNNNNNNNNNNNNNNNNNNNNNNNNNNNNNNNNNNNNNAAATTAATCTCGATTTTTCTTTTCCTT

TGGGATTGCGGGGGCTTGTGTGCTTGTGCGTG
TGCACACTTACATATTATATAAGTGGCACGCGTCTCCTCACACAACCCACGAACGACGTCTCCGCA
ACACCCCGCCGACGTTCTCGCCCCCTCCGTTT
GCAAAAAAACAACAACAACAACAAACGCTCACGCACACAAGGGAAAGAAAGAGGAAGAATGGAATAGG
GAGAGAGGGAGAGAGTTGGCAAATTTATTTCC
GTCCGGAAGCAAATACGCCTGACTGGACTTCGGGTGCCGCACCGCACAAACAGATGTGTCCTGCGACT
CAACTCCTCCCTCCCTCTCCCCCTCCGCCCTC
ACACACAAACACCCCGCCGTCCATGCACCCACGGCGGGCTCCTCTCTCCGCAAATCGTTTCTTTTTTC
CGTTTCCTAAAAAACAAAAGCACACAAAAAAC
AAAAAACAAAACAAAAAAAAAAAACACGCCGCTCACCTCACTATATACATCCGTAAAAAAAAAAAAAA
AAGAAAACATTTATTTTTTTTTGAGATTTTCC
CGTCGTTTTCTCATTTTTTACTTTTATATAAATATATATATTTATATAAAAGTAAATCTCTTCTCCGT
TGTCGTTCTTGGCGGCTCAAAAGTCTCACTTG
AATACACATCAACTCGCACCTCGAAAAAAAAAAAGAAAAAAAAAGAGAAGAAGAAAATACAAACACAC
ACACAAACACACAGAAATGGATAAAAATAATA
TAAAAAATTGAAAGAACTTTCCCCCATTCCTTTTTTTTTTTTTTTCCCCTCGCCTCCTCCCTCTCCCA
CAAGCGCTCCATTCCCGCAGGCGATGTCGGTG
CCGATGGTGGCATGCATCCCCAGTTTTGCCCGACAAGTGCGCGAGCTCATGAGGTGACGGAAAGGTCG
GGTACAAAGCGTAGACGCATGTGCCCATGCAG
CAGTCCATGACCTCCCGCCTGCTGGCCGCCGCCGCACAAGCGGACGCACGTTGGAGCAATGGGCCCAA
AAATAACGGTAACACACACACACACACACACA
CAGAGTTACCCACAGAAAAATTAAAGCACGAAAAGCGCAATCAACATTAGAAATACACCCAAAGGGGG
AAAATAAAGAAAACAAAAAAAAACAAATGTGG
AAAAAACAGAAAAAAAAAAACAAAACACACACACACAAAAAAAGAAAAAACAAGGGAAAGAAAAAAAGG
ATGGGCTGATAAAAAAAGAGAGCCAAAAATCA
AAGGATGACAACGCGCATAAACAAGCCCCACATTCGAGGGTAATAAATCGAAAGAAAAGATGTGAGGG
ACGGAGCACAAAAATACAAACATCTCTATGAG
CCTAGGGATCACATTGGGCACGGCGGTGCGATGGTTGTCGGCTGGAATACAACAAAGAGGAAAAACTT
CCCTCCCCCCAAAACAAAACAAAAAACAAAAA
CAAGACGACTGTTTGGGCTCCTTGACGAGGGCTGCATTTAATTCACATGACTGCGAGAGGAAGCATCA
GTGCAAGCGTGAGAGAAATCCCTTCACGTCTT
CCTGCGGGATTTTCAACCACTCCAATTCTTTCCCCCCTCCCTCAGACAGTGCGCACATCATCCCGGAC
TCAACATGTTACATTTCTGCAGAGACCCTCGG
TCACTTCCCCTCCCCCTTCCTCCTCGACAAGCCAAATGCAATCCAAACACCCCAACCCGCCCGCCCGT
CCCTCAAAAGAAAATTTTTTTTTTTTTTTTTT
TATGTTCTTCCTTTTCATATTTCCCATTCGATTTCAAACAAACAAACAAATTCATTTTTGTGAACTCG
TACCCAACACTCCAAAATAACTTAGTGTAACC
AAAAATTTACGTCGCTCCCATGTTATATATATATATATATGCAGATTCTTTTTTTTTTTTCGTTATGT
GCACTTTTTTTCCTTCGCATCCCTCTTCATTT
GTTGACCCGCAGTATTCATTTCTTTCGCCCGGCCATGTTAAAGAAAAAGATGTTTTTTTTTTTTTTCC
CTCCTCCTTTCTCTCTTTCCTTTCTCTCTCTC
TCTCCTTTTTGGTAATTCCCTTTTCATTAGACGCTGGCAGAGGCATTTTTTTTTTTTTTTTTTTTGCT
ATCTGGCTCCCCTTCCTCCGTTCCGACGATTT
AGCGCTCTGTGATGGCCTTTGTTTTACTGCGATGACATTCTTCGATTGGCTCGAGGGATATGTTTCTC
AGCCCCCGTTCTGCTCTTCATCAGTTTTCGAA
CAACACTCCATTGAAGCT
>scaffold1084.size8661
AATCCACTCTTATCCCACTTATTCCTTTCTTATTCCTATCTGGTTTGGTTTCTATTATCGATAAACTC
ATGATCCGGCCAGCTGCCCAGAAGATTATTCTA
ATTTATTTATCGATCTACCTCTCTTTATGTGTGCTTATATATTTCCTGTGAAGGGATTCTTTTCTATA
GAAATGCCCTTGCGAGCGCTTGCCAAATTTCTT
TTTCATTGATTACTTCTTTACATTTTTATTATTATTGTTTTTCATGTCTAACTCTGCCGCCGGGTTTT
ATTACAGGAAAAGAAAAAGGCTCAGACGGATG
GAGACCCCTATGGTGGCGCTGCCTTTTGTTGTTTGCGCCCAATAGGCCAATAAAAAAAGAAGATTCGG

CCATTACGACAGGGTGCTGGTTGAGGTACTGC
CTCGTGCGGTTGTTTTTTGCGGTGGGGATGGGGGAGGTGATTGGGGAAACACTGGGCACGAAATGTGA
CTTGAAATCCTGAACTGCGTTTTCAGAATGTC
TGGAGGGTTGTTGCCCCGAGCTACAACAACGCCCTGCATTGAAGAATAGTTATTTTATATTTTTGTGC
CTGCGGCGTCTTTATGCTGAACTGAAGTCTCT
TTTATTATTCGCGGGTGCCGATAGGACAGTTTAAAACACTTTTGTTGTTTTTGGGCATGTAGGATGTC
TGGTGACTGTGCTTTTTTGGGGGGGAGTTTAC
NNNNNNNNNNNNNNNNNNNNNNNNNNNNNNNNNNNNNNNNNNNNNNNNNNNNNNNNNNNNNNNNNNNN
NNNNNNNNNNNNNNNNNNNNNNNNNNNNNNNN
NNNNNNNNNNNNNNNNNNNNNNNNNNNNNNNNNNNNNNNNNNNNNNNNNNNNNNNNNNNNNNNNNNNN
NNNNNNNNNNNNNNNNNNNNNNNNNNNNNNNN
NNNNNNNNNNNNNNNNNNNNNNNNNNNNNNNNNNNNNNNNNNNNNNNNNNNNNNNNNNNNNNNNNNNN
NNNNNNNNNNNNNNNNNNNNNNNNNNNNNNNN
GGAACGGGGATTGAGCGGTGCGGGATATGTGGTGGGTGCCGCAGGCTTGTTGGCCCGCGATTTGGGTG
CCCTCCAAAATTCATGGCATTTTGGGCTCCTC
AACGAACACTTTTATTCTTTATGGAATCATGTGGTATTACAAATGGCACCCTGGACTGTTGTGTTTCG
CCATCCTTTTTTTTTTACTGGATGTATGAGAG
CTTTGCATGCTGCGGCGGGAACTGCATTTGTTACTGTGCCATTGGTCTCTTTTTTTTTGTTTCCATTC
TTAGCCATTTTTACTTTTGTTGCTTATTTTGT
TGCTGGCCATGCTGAGACTCCTGCGTGCTCCGTGCCTCCACTTCACTCACCAAATGGAGGTGCATGAC
CATTGCGCTGGAAGGAGTCTTTTTTTTTGTTC
ACATTGACACAGAATCATCATTGAGCACATTTTTATTTTATACTTCTTTTGCTTTCTTTCAGGAGATG
CTCCCTTGGCTTTGCCTCCAACGGGATATGCG
CAGAGTTACACGCATTTTAAAAACCGAGGCCACGAGCCTTTTTTGTAACGGATGCTTTATCTCTCTTC
CAACATTAATTTGTTGTCCATGTTGATGTTTT
TCTTTGAGTGGAGTTCCTACTTGGCGACGATCCCACGTATGGTTTGGTGCTGATCCGACCAGGGCGGA
CTATATCCACTGCGTCGGGGAAAGGCTTGTGG
TGGATCCAACGACACGGCGGCTGGGGGAAATAATAACGGGACGGTCGCTATCCCGTTGATGGTGGCCC
CGCTGATGCTCGATCTGATGGACTTCCGGAGG
ATGATGTGCAACATCAGCGTGCCCATCCGGCTGTTGGTTTTGGTGCAGAACGGGCGGGAGGCGATGCT
GTCGTTGTGCCTGCAGGAACTCGGGAGGGTCT
ATGGGTGGTCCGGACGCCTGGTTGTGAGTCGTCACCCGGAAAACATCGGGTACAGCGCTGCGGTGAAC
ATCGGGTTACGACTTGCACTCTCGCTGCCGCG
CGAGGAGGTGCCTTTTGTTTTTGTGACAAACAGCGACGTAAAGTTTTCACCCGATCTTCTTCCCAACC
TTCTACGCGATGTGCATGAAGTGACGAGGCAT
GATGCCGCTCGCATGGATGAGTTGGCGGCGGAAGCGGCGAATGAGCCGAGCGAGTCCAGTCCTGTTTT
GCGACGGGGCTTGAGGGTGCTGCGCTCCACGG
TGAACGATAGCCGATTGTCGACGTCTGCGCTGCTGCCTGACCGCATCCGTTATGCCTCTGTGAAGGAG
CGTGAAAAGGCGTTTTCGAAGCATTACGGGCA
CTTCTGCGCGTATTTAAAAAGCAGTTGTTTTACATCTGTTATGTTGACACGCCTGGCGATCAGTACGG
TGGGATATTTTGACGAGAACTTCTACCCAGAT
TGCGTGGAGGATGTCGACTACAGCTTGCGTCTCCGTTTGCTTGGATTTCAGGAGCGGAATGTCTTATA
CGGCAAATTTTTGCATCGCGGCAGCTCGAATA
TTCGTTTCTCTAATGAAATGGAGCTACCGGACGCTCTCTGGTACCGTCGCGTCAAGTCTTTGATGACG
AACCAACCATATGCCGTGATGAAGTGGAACGG
TCTAAAAGCATGCTGCGATGGGTGCAAGGAGCCATATGACGGCATGGTCCCCCTAGACGTTTGGGTGA
AGGATGAGGCACGCATACAGCGCATTCGGGTC
TACGGGCATGATGAGATCCGAAGAGTTCCAAGTATTGACTACGACAGGAGGCTTTTGTACCCTGTGAG
GAATAAAGGAAGATGATTCGGCAACAAACACA
AGTTTTGCTTTCTTATTTTGTTTTGCTTTAATTTTTAAAAATTTACACTTCTGTTTGGTTATTTACAC
GATTAATATTTAAGCGAGTACGCCATGGGTTT
TAAGGAGCGCAAAAACATATGTTTGGCATGACGGGGGGGGGAGGGTGCAGCGTCCAAACCTGGCCGTG
TAGCCGAAGGCAGGAGAAGGAAGTAGCAAGCA
GGAAACATGATGATGATGGATGAGGGAGAAATAAAAGGGGAGGGAAGCAGAAAACAAGTCAAAAGTAT

TGTGAGCACAACGCTGTTTCTTATCGTGGGCG
ATCGACACCGCCATTGGCTACTAATTTACCCACTGAATGTGACACCCGTTTCCGCTGAACATGCTGCG
TGCATATCTGTCGGGCTCTTTGTTGTTGGTTT
GTTTGTTTCCCGCACATTTATTTCTCCGTGCCGCCTGACCCACTCGTTTGTTATTTCCCTCTCTGTTT
TGTGCGTGCACCGAGAGAAGGGCTGAGGGACA
TGAAGTACGCTCCCCTTCTGTGAGATGCCACCAAAAACAGGAGGAGAACGAAAAGAAAAGTGAAGCGG
GGAGGGGAACCATCGCCACGCGTCCTTTTCTC
CGAGAAGGCTGCTCGCCACGCCGCTGAGGCGCGCCGTGTGCTTCACAAGGCCCGCTGGACTCGATTGG
GGTGCCGTGAGTGACGGTGCGTGTGAGTGTGA
CTGATGCGTCCCCCAGGCGTCGCTAGAGGGCCCTCTGGCCATTGGGACTACGCGGAATGCGGGATTCC
GCGGGGATATTTGCACGTTTTGTTTTTATTCT
TTTATCCGCCCCTCCCAAGCCTCAACACGGCGGCTGAGCCAACGACTGCGACGGGATCCGTTGCGTAT
TTCCGGATCTTTTTTGCGTTGCAGCGGCAGGC
CTGGTGTGTCCTGCTCGGCAGACGCGGTGGACGGAAGTGGGCTGCGTTATTGGGAAAACGATGGTCAC
GGCGGAGTGTGTTGCGTACGTCGCAATTTGGC
GTGTTTGTGGCGATCCTTGCGGATGGTGTGACGGCGCCTCTTTTACTTGCGGGGATAGAAGCGATGCT
CCACGGTGGTGCACACGTTGCGGGTGGAGGCT
TCTCTTGCTGTTGGTTTGTGAATGTTTATTTTTTCGCTTTTGCCGGAACACCACAGAGGACGGTGTCT
GCGGACAGCAGAGCATTGCGAGGAGGGGCACC
ACACGATTTTTGGCGCGATGAGGCGGCTTCTGTGAATTTACGGTGTTTATTTTTAGGATTGAGAGGCT
ACCCTTTGCTGGAATGTATTGTGTGAGACCAT
CCGCGTTTTTGTTTTTTTTCAATCCCTTCGCATTTGGTGTCTGCCGTGAAATGGATGTTGATGGCCGT
TTTATGGCGCTGAGATGCATTTTTATTTATTT
AATTGGCGAGTGGGTGAATTGTGTTGCGTTCTGCCATGCGGCTCGTTGCTGACGGCACGTGGCCGCAG
GGAGACGTTGTGGGCGCCTCCGACGCGAGGTG
CGATTGGGCAGTCACCTCCTCCGCTCGCCGGTACACGCTGTTGAGGCGGCGTGGTGGTAATGAATCGG
CCTCTCAGAAAAGCAACGAGAGAACGTTTGCT
CTTCCTGCTGCGGTGTACGAGTGGACTCTACAATCCGTGGGCGGGGTCGCCCTGCGTGCCGTATTTCT
TTGAATTCCATGAGTTTTTTACATCGCCATCC
ACACGCACATGCACGCAATCTCCCTGTTTGCCCTCCTGTGGGCGGGAGACCGGGATGCAAGGGAAAAA
TGTGGCTTGTGCCAGCATGTGGCAAAGTCTTT
CTTGGGAGGGATTGCGACTGTGATGCACGTCCTGGCAGGCAGGAATGGGATGAGAGGAGAGCGGAGGG
GCGAGTAACAACGATCGTGTGCGGGGCAATGC
GCAGTGACTAGGGCGAATTGGTATTTGTGTTGACTTTTCAAAGCATTTTTGTCTTTTTCTTTTGTGGC
AATGGAAAGAAACTTTGGATGACGTGTCGTGG
ATTATTTGCAGGTGATTCATCGCGGCAACACCATTTGCGGAGAAAATGAATGACATCTGCGTGCGGCT
TGTTTTTTGAGCGCGAGATGGTCGGGGAGGTT
TTCAACGCCCCGAGAGGCCAGGAGCAAAGAGGCCTGCACTGCTGCAAGCAGAGGGCTGCACGCTCCTG
TTGGTGGCGTTGCTGTGGAGCCCTGAAGGTCC
TGCAGGGAAGTTGTTTTTCCACCGCCTCATTATTTTGTTGATTGGTGCTTGTCGCGGCCTTTTGAGGC
GGGGCGTGGGATGGACCGAGGGAGTGAGTTTC
GTGTGACTGTGCTGTGAATTGGTGCTGCCCACGTTTGTGCAGATACTTCCGTTTGTGTCCGCGTCGTT
GCCGCGGCAGTGCACTGGGAGCGTGTGACTGA
TCACTGTATCACTTGCAGACTGTTTCGGCAGGACTTTGAATGGCGTCGCCGAATGATGAATTTCCGCG
TGTATGTGTGTGTGTTGGCTTCCGCCGTGTTC
CGCCGCGTTGTTTTTTTTTTTGTTTTCTGTGTGCAATTGTTTTCCATGCTGTTCCTTCTGTGTTCTCT
CGAGTCCCGCACTTTGCGTGGGCGCTCTGCTC
TGAGTCTCACTGACGTGCCTGATGTATTGCCGCTGTTGCTGCCTTCTCCAATTCATGGACAGACACAC
GCTAGGAAAGCAAGCAACCAAGCGCGAATCGC
TACTGTGAAATTGGAAGGCACTGGTAGGACCAACAGCACACACACACATATTTGTATGCTCTCACGTG
TTGCTGCGGTGAGGGCACCCCGCACACACAAC
CGTCGCCGCGTGATCGGATCCAGCGGAAGGAGGAGGGAAGGAGGAGAGAGTGAGCCGCAGAGGCCCAA
CATGTCCCGGCGCGTGTTTGCTTCCGCGGTGC
TGCTTCTATTTGTCGTGTTGGTGTGCTGCGGTATTAGTGGAGCTACGCCCGCAAACGAGGAAGAGGTC

AAGAATGAGTTAATAAATATTCAGAATTTGCA
GAGGGTCGATTTATTTGTGCCACAGAAGACGCTGGTGCTGCCGAAGGGTGGGACTGGCTCAGTTACGA
CGAGGGATTCATTTTTTGCACCCTCTCTCGTC
AATGCTGGTGGAGTAATTGCTGCTTTTGCCGAGGGTCACATGAATGCCGAATATCAAGGTGGTCAATT
AACTAAGCCGTCTTCTGACGTTGTTGCGGAGT
ACATCGACTCTGCGTGGGAATGGTCCACTCTTGTTGGTGAGGTGAGTGAAAGTACATGGAAGGCACAC
ACTGTGCTTGGTGCAACGGATGGAACGAATCG
TGTGGGTGTTGTGCTCCGCCCCACAACAACCATGAAGGGCAATAAGATGTTTCTTCTTGTGGGAAGCT
CTGCCGTTTTCTATGAAAGTGGTGGGAATTGG
AGCTGAAACTGGTTGTTGGTGAGGTCACGAATCCCACGGGTGGCGAGCCAAGTGAGTGGATAAAATGG
GGCGAAGTTCAAACCACTTTCGAAACTGATCT
TTTCTGCTTCTCACAAAAGTGAATTGGTGCAGTATTCTGCTTCTGGTGGCTCGGGTGTTGTGCTGGAG
GATGGCACGCTTGTGTTTCCGTGATTGCGTAT
AATGAAGAAAATGACGGTTTCTCCATGATTATTTATTTGACGGGCAGCGGCGCAAACCGGATGCTTTC
GAATGGAATGTCTCCTGCGAAATGCGAGACCC
CCGCGTCACCGAGTGGTGGCAGGGACAGATTATCATGATTGCTCATTGCGATGGGAGCCGGTATTGCA
GAGTGTACGAGTCACGTGACATGGGGACACTG
TGGAAGGAGGCTGTCAGAAGACTCCCGGGCATTTGGGTCAACGCACCATCAGGAGCCTATCAGAAAGA
AAGCCTGCGNNNNNNNNNNNNNNNNNNNNNNN
NNNNNNNNNNNNNNNNNNNNNNNNNNNNNNNNNNNNNNNNNNNNNNNNNNNNNNNNNNNN
NNNNNNNNNNNNNNNNNNNNNNNNNNNNNNNN
NNNNNNNNNNNNNNNNNNNNNNNNNNNNNNNNNNNNNNNNNNNNNNNNNNNNNNNNNNNN
NNNNNNNNNNNNNNNNNNNNNNNNNNNNNNNN
NNNNNNNNNNNNNNNNNNNNNNNNNNNNNNNNNNNNNNNNNNNNNNNNNNNNNNNNNNNN
NNNNNNNNNTGGGTCAACGCACCATCAGGAGC
CTATCAGAAAGAAAGCCTGCGTGTGGAAGCCCTCATCACCGCGACCATTGAGGGAAGGAAGGTCATGC
TGTGCACCCATAAAACTAGCCATCCCTTGGAA
GCGAGTGAACCCAATGCGCTCTACCTTTGAGCCACGGATAACAACCGCACGCTTCACATCGGACCGGT
TTCTGTGGACAGTGTTGTGGATAAGACGTTTG
ACAACACCCTGTTGTACTCGGATGGTGCATTGCACCTTTTACAAGCGAAGGGAGATCATGAAAGCACA
GACATTTCACTTGCCCGCCTGACGGAGGAGCT
GAAGACGATCAATTCCACCCTCAGTACTTGGGCGAGACTGGACACCTTCTTCTCCAAGTCGTCCACAC
CCACGGCCGGTCTGGTTGGATTTCTGTCCAAT
ACATCGTCCGGTGGCGACACGTGGAACGACGAGTACCGAACGCACTTACGTAGGCACTGCTTCCGTCG
CTCTCAGTTTTTGCCCAAATTCAAGTGCCCCG
GACCCAGGTACCGTTGCACTGGGCAGAACAGCCGGGAGGATAACAGACAGTACGGCTTTGTGAACCAC
AGATTCACTCTTGTGGCGACGGTGGCCATCCA
CCAGGTTGCGAAGGGGAGCACTTCTCTGCTGGGTGCGGGTCTGGGGGACGGTCCCGGCGCGAAGATCA
TTGGGCCGTCGTACGGCATGAATAAAACGTGG
GAGCCAGTGTTTGATGGGAAGAAAACGACATCGAACACCACGTGGGAGCTGGGGAGAGAATACCAGGT
GGCGCTCATGCTGCAGGACGGCAAGGGTTTCA
TGTACGTGAATGGTAAGATTGTGGGGAGCCCAGAGACGATACCAACACTTGAGACGCAGGGACATGAA
ATCACACAATTCTACTTTGGTGGAGGCGAGGG
AGACATCAACAGCAATGTGACGGTGAAGAACGTCTTTCTGTACAACCGCCCATTGAATGTCGGTGAAC
TAAAAATGGTCAGGAAGAGCGACGGTAAAAAA
GGAAAAGATGGCGGCAAAAAGGAAAGGTGACGGTTCCATGCGTGGGGGCGTGTCGCAGTTGCTGCTTC
TGGGGCTTTGCGCCTTTGTGGCCTTTTACTGA
GAAATCGCGGTGAGGACTTCCTGCTTCCAGCGGGAACATATGCATCTGTCCACGCACTCTTTTAGGTA
ATTGCCGCTTTCTGTGGATCCACCGTTCATTT
TATCTGGTTTTAATTTTATTTTACATCCCCTTCGCTGCGCAGAGCTCTTCCTCCATGTGGAAGCATGC
TTTCCCCAGCGCCAGCACGGCGTTTTTGTGTT
GTTTTTTTTTTTGTGGTCCTCCGACAACGGCGGGATCTACTCAGTGTTCTTGTTTTTGTTTGTTTGTT
TGTTTGATCAGAGACGCTCTCGGCATTGTGTG
CCACGCCGATGGCGCCGGCTAATAAAAGAGACAATGGAATGGGGGCACGAAACCACGCCGAGTGCCCC

AGTGGAATATAAATAATGTGTGGGCACCGGGT
GTTTAAACGACTATAACGAGTGTATCCTATTTTATTATCTTCGGGCAAGACATGCGCACCGATTTGGA
GGCCACGCAGCAGCGTGCAGTTTTTTCTTGTT
TTTTTGATTGAATGCATAATGGAAGACGGATTTATCTTTTCTTACTTTCCAg
>scaffold1085.size6980
ATCCATTCTCACCCAACACTCCCGACGCCATGCAGCCACCCAGTGCGTTGGTGTCAACGTCCCTCGCA
TAAACACCGCCGGCAACGCACCACAGCACGCAG
AGGGCACACACCAGCAGCACACGGCCGGTCATCGTCATAGCCATCACTCCTGTCACTTCCGTGTAGCC
TTCAACACAAACTAACAACACGTCAACGCAGA
GTGAAAGCAACAAAAGACACGACACAGTAAAAAACAGCAGCAGCAGCAGCAGCACGCATTTCATGAGA
AATATTGAACCAATGAAAGTGAAAAAAGACAC
AATGCGCTGCAAGTAAAGAAAAGCACAGAAAACAGGGAAAAACAAAAGTGAACAGGCAGCAATAAAGT
AAAACAGTAAAAACACACACAAAAAAGAGCTG
CACAGTATGGCTCCAGCGCGCACGCCTCTATCCTCAACAGTGTGTCCACGCAAACAGTTGTGTGTGTG
TCGTGCGTATGCGTATGTTTGGGATGCGTTCA
ACGTGACGGATGTGGCGCACAGCACGGAGCACACAGCAGAGCAGCGGAAGCAAAGAAAGCAAAAACAC
AATCATCATAATTGCTCTTACTTTATACATCG
GACCACAGTGGACGCACACATTTCCCCGTGGCTGAGTGCTGGCACGCACAGCCCGCCCACACACCTCT
TCAGCCCAGAGCGGTGTACGCCAGCGCGGATG
CGGCGAGCAGCAGCGGGGCACACACCCACACAGAGCTGCTGAGGCTGCCGTCCATTTCGCGAAGACGT
GACGGTGCGGTGGTGGTCGTGGTTGCCGCCTC
TGTATTCATCGCCGTATTACTTGGTGCCTCTGGCGCGGTGGTTGTTGTGGTAGTCCTACTTGCAGTTG
GTGCGTTTGTCGTAGTCGTCGTTTTTTGCACT
GTGGAATCACAACTTTTGAAATCGTTGCGGACTTATCCGCAGCACTTGTTTTCGCTGACTCAACAGAC
GCAACTGGTGCATCTTGACTGTTGGCATTCTC
ATTGGCTTTCGGAACCGTTTTTTCTTCTACTTTGCTATTGTCTTGATCCGTTGGTGTAACGACCCCTG
GACCTGGGCCCTCGTTTTTACCTTCTGCCTGC
CGTCCGTCCCACTTATTCCCGGTGGTGGATCGGAATTCTGAACGGATTGAGCTGGTTTATCGCTACCC
TCTGTCGCGCACACGGACGGACAGCAGCACAG
GGCGAGCACCAACAGGGCGCACAGCAGACGGCACGTCATCACCGTGAGTGTGGTCGTTCAGTGGTGTG
GAGCTGATCTGCACAGTGAGGGGCATGGAGAG
CAGCAGGGAGACGAGAGGTGCGGCAGACACACACAGAGGGCACACCACATGAGGAGAGGGCGGCAGCC
AGGCATGCACCGTACGCCACACACTCCAATCC
ACGGCAGCAGGCACACACACACTCCTGCCGCAACAACAACACACAGATGGAGGAGGGAGAAGCAAACA
GGAGCGGCACACAAGGCTCCACCGCACGCACA
TACGGCGAGAGCGGTCCGTCGCGTCGGCACCCCTCACGGAGGGTGCGCACACACACGAGAATAGGTGC
AGCGCACACAGCAACACAAAAGCAAAGTAACG
GTAAAAAGGAGGAGATCCCTGCACACCACGTGGATTGTTGGTCCACGGGAAGTGATGCCAATATTCAC
CACAAAAACAATTCATTTTTCTTTGGCCTTCG
TTCATATTCAGCGCACACCACTTCATGCAGTCACCGCCACCACCACTGCAGCAGCAGGCATACGAGAC
AATAAGACGCAAAGGATGGGAGGTGGTGTGGA
AGACCGTGGTGGGGCTGTCGCTGTCGGCGGTGGTCGTTTTTCCTTTTTCATTGTTGGTGAATGATGTT
GTCGTGAATTCTTCCGGTTGCTTGACTAGGAG
TGATGTTAAAAGGCAATTGGTTCCCTGGCAGTCATTTGATTCCTCTCCTGTCCTGTCGATTTCTGTGC
TCGCCGCTGTGTTAGGCGAGACGCTGACAGGG
CTGCCAGCACCATCTGACTGAAATTATTCTGCCGTGTTCTCGTCCCCCAGTCCCACTGCACCAGCTGC
TGGGGGCAGTGCAGCGTTCATGCCAGAAGCAC
CAGCAATGGGCTGGGGTGTCGGCCGCATCAATCAGTGGGTGTGTCCGCAATCGACAGGAACTTGCATC
GACGAGGGAGTTTGCACcgacgctcgggaatg
ggNNNNNNNNNNNNNNNNNNNNNNNNNNNNNNNNNNNNNNNNNNNNNNNNNNNNNNNNNNNNNNNNNN
NNNNNNNNNNNNNNNNNNNNNNNNNNNNNNNN
NNNNNNNNNNNNNNNNNNNNNNNNNNNNNNNNNNNNNNNNNNNNNNNNNNNNNNNNNNNNNNNNNNNN
NNNNNNNNNNNNNNNNNNNNNNNNNNNNNNNN
NNNNNNNNNNNNNNNNNNNNNNNNNNNNNNNNNNNNNNNNNNNNNNNNNNNNNNNNNNNNNNNNNNNN

NNNNNNNNNNNNNNNNNNNNNNNNNNNNNNNNN
NNCAAACAGAACAGTGCGTGTCACCCCGGAAAATCCGCAAGGCGTATTGGCGCGGGCTGCGTTGTGGT
CTCGCGGCGACGGAAATGAATGGCGGTGCTGG
TGGCGTCCTCGTCGTGCGTCCCCTCCTGCCACTCCTCGCAGTAAACTCATTGCCTGAGTACATTGGAT
TCCGCCTGAGAGACCGCTTTAAAATGTTCTTC
CCTCTAAATGTGTTGTACTCGCATGTGGCCACGAACGCCCGCAATGCCTCCAGCGGATACACCTGCGA
TGCACGGTCAACACATTCCACATCCACTCGTC
GTGCACCATCCAACACCATCTTTTCCTTGGAGCTATGAACAACACCGGGCACACACTCCTTTGCGGCC
GTCCACGGCATGCGCCTGCGGTGAGAATCCGG
AGAAAGTTCCATTCCTTCTGTGCACAAATTCATGGAGCTGCCGCCGTTGCGTGCGTCCCCGCGAAGGA
AACAACAGCCCGCCGCAGCACTCACGAATGCC
CACCCGTTGCCTCCTCTGCAAAAAAGAAATAAACGTTGGAGGGAAAAGCGTGCACAAATGCCCCGGCG
AAAAAATTCAGCATCAGTGGATGAACACTGAA
GCCGTGGGTGCATGCTCCACAAATGTCCTTCCTCTTTTAACCGCATTCATCGTAGCAAAGAAATGGCC
CGCAAACGCACACGCAAGAATACAAAGGTAAA
GGCAAGCAGCGCAGCCGTTGAATCACACATACGCACATCGTATGAAACGCTTCTTGAAAAGGAACTGA
ATGCAGGAAGAAGCGCACACAATTTCCACAGA
CACCCGCAATCGTGTGTGGTTGCTTCTGTTCCATTATTTGGCCCTCGATGCGTTCCTGTGCGTGTCTC
GCCAGTGAGTCACAGTGCCGTAGGATGCCAAG
TCCCTGCACACACACACCTCAGGACGCGGCAGCGAGGCTCCCCAATGAACACCTCACGGCCGTACCTT
TTGGATACCTCATGGACCGAGCGCCTCTCGCT
GCAGGCCACACCACCCAAAACAAGCAGCACTATGCGCTGAGGAGTTGTGAGGGCAATCGTCCCACCAC
CGCCACCCAGATCAGGATGATTCTCCCATTTG
CCACCACACCCTTGAAATGGCCCGGCGGTTTTTTCATGCTTCTCCACTGTTTTGGCCACCACTCGCGC
CATTTGGCCCCATCGCACGATCGCGCTGCACC
GATGCCAACGACAGCCCGACCCGCAGCGTCCTATTCCTGCCCGTCTCCATCATCATACAGGCCTGTTT
CAGAAGCGGATCCTCGTCCGTCGCGGCGTTGC
TCGCAGAGGCACTCGTGCTCCGTCCGCCCTGCGCCGACGCCGTGCTGTATCGTTGCACGCTCCCGGCC
CTCCTCGCCACATGCTCAAAGTTCGCAAACAA
ATGAGCGTCGGGCGTCAGTCCACCGTCTCGGATCAGCTGCTGCTTCTTGGCCTCCGTCAACTCGTCGG
TGCTGCTCGCAGTCACAAGCAACAGGAGAAGA
CGCAGACGCACTGGCCGGGGCATCGCAGTATCCCTCGTCAGGCGCCGCACACCATCCAGGTTTTTCCT
GAGGGGTTTGTGTCCAACGGCGATGTCTTGTT
CCACCTCCCGCACCTCCGCCAGTCGCTTCTCCCGGTACTGTGCCACAAGCCTCGCGCAAATGTCGATG
TGCAGCGACAGTCTGGTTTGCCTCTCCCGAAA
CTCCGGCGGAGACCGCACCGCACTGCCCAACTCCGCAAGATTGGTCTTCGGCTCCACGTCCTGCACCA
ATCCCGGGTGATACGCGTGGGGTTTTTTCAGC
ACCGCCGAAAGCTCCTCCACGCACTGCGCAAGAAAACAATGACGGTACGAGCACCAGTACACATCTTC
CTCACCAAGTACTGCCGCTTCTGGTCCTCACC
CAGACGATTCCTACAGGTCTGCTCATACAATTTATTCTCCAGCGGCATCAGGTCATCAAGCAGACACT
GACATGTGCGTTGGTGCATCAGCGGCGTCACC
GTGACACAGCCCCAATCAACGATGATCCGCGCCGGGTTGCCGCGGCTGTCGGCGCCCCTAAGTGAAAT
CCGGCTCGTCGTGGTATTGCCTGGCAAGCCCT
CAAAAAAGGTTGTGACAAGCCCATGGCAGATGTTGCCTCTGTTTTGGTGGCGAATGACAGGCACACTA
TTGTTCATGGCGTGAATAGCCGCCACAGGACG
TGGCGCCGCAACAACCACAACGGACCATCAATTCGGAGGCGACAGCAGCTGAGGGAACCCATTCTGCA
TGCTGAGGTGGAACACCAGCACCTCAGGGGCG
GCGAAGTCTAACATAATGTCCTTGGAACGCATCGCCCTCGGCGCTATCCGAGCCCGCGCGAGTTGCTC
AGGACTGCGATGCGGGGCGCACCCTAGCGCAA
AGACGTGTAAATCCCTGTAAGGAACCTTTGCCATCCAATCCTCAACCACGCGGCTCACAGACGCATCC
TCCACGGCAAAAAAGTAGCGCGCAGAACTACT
CATAATCGGCTGCCGCGGCGTCATGAGGTCCTCGAGCAGCGTCACGCCGTGCTCCATGAGATCATGAA
GACGCAGTGAGCAGTTTAGCACAGCCGCAGCA
TGGGCATCGCAGTAAAAAACTTTGGACAGTCCATGAACGACGTCAAGCATGTGATGGAATATATAATC

CCGCACACAACCTCAAACGCCATTGCTGGGCT
CACACAGCTGCCCGCTTCCGTCGGCCTTGCTCGCACCAACCCGCCCCGCATACCATGTCAAGTAGAAG
TCACACAGACAATGTGCTTGGATATGCGTGTG
TGTGCCTTGTGTGCGCACATTTGACTCGCATCGTTCTGTGTGTATGTGCGTCTCAGAAAATCTAACAA
GCACACAAGCAATGCCATACGACCATACGGTG
GCCCCACATCGGCAACCAACAAAAGAAGAGCAGTGCTCATCGGAAGTAACACCAANNNNNNNNNNNNN
NNNNNNNNNNNNNNNNNNNNNNNNNNNNNNNN
NNNNNNNNNNNNNNNNNNNNNNNNNNNNNNNNNNNNNNNNNNNNNNNNNNNNNNNNNNNNNNNNNNNN
NNNNNNNNNNNNNNNNNNNNNNNNNNNNNNNN
NNNNNNNNNNNNNNNNNNNNNNNNNNNNNNNNNNNNNNNNNNNNNNNNNNNNNNNNNNNNNNNNNNNN
NNNNNNNNNNNNNNNNNNNNNNNNNNNNNNNN
NNNNNNNNNNNNNNNNNNNNNNNNNNNNNNNNNNNNNNNNNNNNNNNNNNNNNNNCGGAGTGGGCACA
GCACATGCATATGGAGAACGACATCCATTGAA
ATGGATGTTTTTCTATTGGGCTTCCAGTTCTGGGTGCAGGCCGCGCAGGAGGAAGCGCAGTCGCCTCG
CAGCGCAACGCCGACCACACCGCAAATCGATA
GAGTGTTGCCACGGCGGCGGCAGAGGCAAACAGAAGAAGCAGCGGACAGGGACAAGCAGCAGCAGCAG
TGTCATCACCGGGAGTCGGGGACGGGCCTGCT
CGCCGTCCGTCTCGCTGCTTCGATCCTTGCCGCCGAGAGAAGATTCTCGCGGTCTGTCGGAGCATCCT
GGCCGGATCCACTTCTTCTCTGGCGACGGATG
CTTCTGGGGAACAGCCGTGTCGACATCCTTTGGTGGTGTGCCGCGACCGGTCTCAATGCTCTCCGCAA
CGGGGACGGATGCCCGTAGCAAAGCTTGTTCA
GTAGATGCCGGTGAAACCGTCCTGCTCCACACCCTCTCTCCTCGTCGTCCCACCGAGCCACGGGAAGT
AGGCTGCCGCTTGTGGCAGATATCGTGCACAC
CTCCGCGTGGTCACGGCAAATGACGTTTCCCTCATCGTACGGTCGAGTGGCTCACGCAATCGTCGCCC
CGTCNNNNNNNNNNNNNNNNNNNNNNNNNNNN
NNNNNNNNNNNNNNNNNNNNNNNNNNNNNNNNNNNNNNNNNNNNNNNNNNNNNNNNNNNNNNNNNNNN
NNNNNNNNNNNNNNNNNNNNNNNNNNNNNNNN
NNNNNNNNNNNNNNNNNNNNNNNNNNNNNNNNNNNNNNNNNNNNNNNNNNNNNNNNNNNNNNNNNNNN
NNNNNNNNNNNNNNNNNNNNNNNNNNNNNNNN
NNNNNNNNNNNNNNNNNNNNNNNNNNNNNNNNNNNNNNNNNNNNNNNNNNNNNNNNNNNNNNNNNNNN
NNNNCTCTTTTCAGTCACTCTCAGACGCACCC
GCATGCCGAGGCACCTCAATGCCGCATGCATGGCAGCACCGAGATGTGTCTCCTCCGGGTCCCTCGCA
TCCGTCGTGTTGGTGGGAAGCGTGTCCAGACA
CCACGAAGAGGGCTTGTCCCCAGGCACCGCAGACAAATATTCTCCCCTCCAAATAACAGCGGCAGCAT
CCCTCACATCCAGTGCGGGATCAGTGATGGGG
CAACTGTCCATCGGGCGTTTTGCTGACCTTTCATGGATAATCCGTCTGCACGGCAACCACTGGAATAC
CGATCCGACGCGC
>scaffold1086.size6565
CATGTCCCGGCGTGTGTCTACTTCCGCGGTGCTTCCTCTCTGCTTTGTGCGGATGTGCTGCGGCCCTG
TGGAGCTGTTGTGGAGAGTAATTCAAATTGTGT
AATTATCTTTCACTGGAGAGGAAGCAATTTATGATTCAATGGATCCCACGAGGGTGGTCAGTTGTGAT
TTGTTACGTGTAACTTTTCTTTTTGGAGTGAG
TGGTGTTGTTGTGGCCGTTGCTGAAGCGCATTTCAAAAACAACAAGGATGGCAAATCGCGTGTTGGCA
TTGCTGCAAAGTCGATGGAAGTCAATGGAGGG
ACGTGGGCGAATGGAACTGGCATGATGTTTGATTATTAAGAAATTAAAGTTGACCGCCTTTTGAGCCC
AACGACTATTATGGCTGATGGGGAAACATGTG
CTCTTGTCGGGGGTTATGGCGAGTCAGGGGCTCCATGGACGAAGGTGGCTGTTGATGGCTATTGGTCG
TCCAGGTATGCGAGGAGCACCGTTTTGTCTGA
AGATGATGATAAGGATAAGAGGGAGTTTGAGTGGAATCAATAAACAACTTTGAGAGTTTTTCTTGACA
GCTTTTTGAAGGATTtCAAACCCAATGCCGAA
CCCCACAAACAATTTCTGGGTGGTGGCGGAGCACGCATTCGGCTGGAGGACGATGATTCTTGTGTGCT
GCCCATTCAAGCACTTACTAATGGTGGAGAGG
TCGTTTCGTTGATCATACTCGCCAACGGTTTAGTGTATGATAAGGAGTTTTCAAATGATAGGAGCCAT
GAGGGCTGCATCCAGCCTGCGACGCTTGAATG

GGAGGGCAAAGGTCTCCGCATGATGACGTCGTGTGAGGACGGCAGCCGCAGGGCTTGCAGGGCGGACG
GAAAGGGGGATTCGGTGGACGGAGGCATACGA
CACTATTTCAGGCGTGTGGGGCAACTCACGGACACGAACAGGGCACGGTGTGCCGGGTGGCTTCGTCA
GCGCGACGATCGATGGACAGAAAGTCATCCTC
GTCAGCCGGCCGGTATAATCTGGGAAGGAGGGAGAAGAAGAAACGGGTCGGCTTCACTTGTGGCTGAC
GGACATGAGCTGAATTTATGATGTTGCGCCGA
TATCCGCTGAGAATGAGAACGTCGCCGCCAGCACTCTGCTGTACACCGCAGACAAAGCGTCATTGCGT
AAGGAAGCAAAGAAGGAAAAGTGAAAACTGCG
CTGCTCGTACGAGTTCGCTGCTGCGGAGGGAGGTAGGCAGTACAGCATTGCTTTTTTGGACTTGACGG
GGAATTTGAAGGAAATGAAGAAGGTGTTGGCT
GCCTGGAAGGAAAGGGACGCGTGCATTGCGAGGGAATACGGCTGCGGGAATGAAAAGAATGACAAGAA
GCGCAGTGGTTGTGGCGGTGACTTTGGCAAAG
GGCTGGTTGTCTTTTTATCCAGCACGTCAACTGTGAATACATGGAGTGGCGAGTGCCCCTGCGTGAAT
GCAACCGTTCACTGGGCAGTGACAAGTACTCC
TGATGGTGGACTGACGTTCCAAGGGTCTAGAGCGTGGGCGGAGTGGCCTGTTGGCGAGCAGGGGCAGA
ATCAGCTGTACCAATTCGCGAACTACAAATTC
ACTCTTGTGGCGACGGTGTCGGTCCCGGAGGTGATGGAGGGCATCGGCAGCTCCTTTCCTTTGATGGG
TGTGAGGATGAATGACCACAAAAGCACGCTGC
CCTTGATCTGTTGTACGCGAAGAAAAATAAGTGGGAGATAATCTTCAACGGCAGTCGTTTGTCGCTGC
CGGCGGATGCCAAGTGGGAACTGAACACAACA
CATAGGATGGGACTGGAAATGGATTACTTTAAGGAGTTGCTTGTGTACGTGGATGGATTGACGGTATA
CGACGGTGGTGGTGAGGATTACAGCTTCTTTG
AAGAGACAAAAACAAATTTGGGGTTTCACAGCGTCTCACACTTTTACATTGGCGGCGACGGCAAAAGG
GACTCAGGAAACATTCATGTGACGGTGAGCAA
CGTCCTGCTGTACAACCGCGCGTTGTACGATGACAAGCTTAAAGCACTAATGAAGACGAAAGCAGTCG
CTGCTTCAGAGGCGAGGGTGCCGGCCCCGAAA
GGTGCGCCTGAAAGCAGCCACGCAAGTGAATCTTCTGATGAGCCTGCCAATGGGTCCGTCGCCATGGA
TGAAGCGCGGCAGTACGCCACATCATCATCAC
CACAAAGACAAGACTCACCGGCGCAACCATTAGGAAATAAGAAGGGCCCAACAGTTTCCAGGCAAACT
TTTTCTTCTGACGTAATTGGCCTTGCCACCCC
AGCTGCCAAGGGGGAAATGGAAGAAGAGGCGGCGAGCAGTGGTGGAATGGCGCCTGCGTTGTCTCCGA
CACCGAGCGCGGGCAGTCGCCAGGAGGTACTT
GAAGCCAGGAAGCCTTTCGGTGGGGAGCGCCCTGAGGGTGGCCAGGAGCCCTTGCCTCCCAACTTGGC
TGCATCGACGATGGGGCAGGCGGGCAAGGCGG
ATGAAGGCTCTCTGCGGAACGGAACCGAAGACGATTGAACGCAGCACAGAACTTCACTGTATGTTTTG
AGGAGCGTGCACAACGAGCCGGCCAATGCCGA
CACGCTCACGAGCGAAGAACAAGACGCTGACCGTGAAGAGGACACGCATGTCCGCACTGCCGTGGGCA
CGAACAGCGGCCCCGATTTACTCAACGGCACT
TACGCGACGCCCGCGGATGCCACCACAGCCACGCCTGCGTTCAACAGAGACCAGACAATTGTTCACCA
CAAGGATAACGTGTCGGATGACGTTGGTGCTG
CACTGACTCCCACGAGCACAGCGCCTGGGGGGACAGAGACCCCATTGGAGTCCAACGCAGCGCCGCCT
TTGGGCAACGACATTTTTGATTCGGAGAATAT
CACCGAGTTGATGTCCATTGGTTTAGTTGGCGACAGCACCGTCCATGGATGTGTGTCTCGGGCGTTTC
TGCTTCTGCTTTTGGGACTGTGTGGAGTCGTG
GCAGGCTCATGAGCCAAAGGGTGTGGATGTGCGGCTCCTCACGGAGAATATTTACCGCTTTTCCCGCG
GTCCTTCTTTTGTTTTGTTTTCTATAATATAT
ATATTTTTTGATGTTACATTATCATTGCCTGCTGCTGGTTTTCTACTGGGACAATCATCACTTCTGCT
CTTTTCTTCTCTTTAATGGTCGGTTGCTGCAT
GCCGTCATGAGTGACGTGCTTCCCCTCTTCTTTGATTTTTTTGCCCACACGCGGTGCAGACCTCACTG
CTGTGCCGTCCCACCGACCGCACAAAAGGAGG
AGTGGAGGGGACAGGCCGGAGGCCGCATGAAATGTGAGGGATTTTGTGTGGACTCGTGGAACAGTGTG
CTCTTTTTAAAATATATTTCGTTAATTTCCTG
TTTGTGTGGGTCATACGTAAGTTCTGCCGGAGCCTCACCATTTTTTGTTTTGTTTTtgcccCtttggt
gaatcccagTggctcgtttcTGTCTGTGCCGT

GCCTAGTGTCACTGGGGTTTTCCGTGCCGACGAGTGTGTGCGGAGGGAGCAACGCTGACGGTGACAGT
GGGAGTGTGGGGAAGGGGCGTGGGCGATGAGC
GCTCATTGTTGTTTGTGTTGTTGTCGTTTTACTGCGCCCTCCTTCTTGTTGTTGTTGTGGTGCGTGAC
TCTCATCGCTGGATGGGACGGTGCGTGGTTGT
TTGACTTTTTTCTCTCCTTTGTTTTTTGCTCTGCGTTGACGGGGAGTTAGTTTGTGCTGAGGGCTGCA
CGCAAGTGACAGGAGTGATGGCGATGATGATG
ACAGGCCGTGTGCTGCTGGTGTGTGCCCCCTGCGGCCCTGATTGGTTTATCGCCCGCCGCTGGCATCC
GTCCACTTCACGGAGGAATGGTTGTCCGCGGT
GCGACATTCAGTCTTGATGAGGCTCAAACGGAGCATTGGAGACGCAGCTGCGAGTGGTGGTTTGTGGC
GAGGGTGGCCGCCCTGACGTGCTGCCAGCGTG
CGGTGCCTCGCGAGGAGGGAGGGCGGGAGCCCGCAATTTGTCTCGTCGACTTGTTGCAGGCGTGACGG
TGGCGGCGTGCAGGCCAGTGAGTGGTGTGGTG
CTGAGGGTGCCTGGGAAGAGTGCGGATGGATTCCTGCCCAGCAGCTGCACGTGCCGGCGTGTCTCTCC
CGCTGTGGATTTGACgGCGGGGGACTATCTGG
CAGCGTGGGGTGATGCGTGCCGGCGGAGAAGTCGCCGGGAGTTAGCTGGAGGGTGTGGGGAGGTGCAG
CAGTGAAAGACGGGCTGCAGCGCGTAGGCACC
GCGAAGGGCGGTTGTGCTCCTTTGCATCCCGCTGGTTGATGTGTGTGTGTGTGTGTGTGTACTTCGCT
TGGTGTGTGCTGACGCGTGACGAACAACACAC
CCGCCGTGGCTCCTGCTGGTGGTGGCCGTCGCTGTGTGTACCGGTGCAATGTTCCTACTCCTTGTTCC
GTGTGTCTGTGTGCGTGAGTTGTTGTGGTTGG
GAGTGACCGCGGCGGGGATGATGGTGCCANNNNNNNNNNNNNNNNNNNNNNNNNNNNNNNNNNNNNNN
NNNNNNNNNNNNNNNNNNNNNNNNNNNNNNNN
NNNNNNNNNNNNNNNNNNNNNNNNNNNNNNNNNNNNNNNNNNNNNNNNNNNNNNNNNNNNNNNNNNNN
NNNNNNNNNNNNNNNNNNNNNNNNNNNNNNNN
NNNNNNNNNNNNNNNNNNNNNNNNNNNNNNNNNNNNNNNNNNNNNNNNNNNNNNNNNNNNNNNNNNNN
NNNNNNNNNNNNNNNNNNNNNNNNNNNNNNNN
NNNNNNNNNNNNNNNNNNNNNNNNNNNNNNGCGCAGTGCGGGGAAAAGAAGGGAGCCGGCTGCTGTGCT
GGGATTGTGTCAAAACACCTGGATATAAGTGG
TTACTCAATGGATATTTCTACGTCGGATGAAAGTTTGTTTTGCACACAACTTGTGGACACCGCCGCGA
ATAATTTTGGGATAACAGAAGTGTTGCGACCA
ACGACGGTTGTAATTGGGGACAGTGTTTATATGCTCCTGGGAAACTACAGCCATACAAAGAAGCAGAT
TCAAGGTAAGAATGAGCCAACCCTTTTACTCG
TGAAGGGAACTGTCACGGTGGATGGTGAAAAGAAGAAACTTGTGTGGAATGAGACCCATGTGGTGAAG
CCTCAACCCAAAGGAAAATCTGGTTCCTTGAC
CGAGTTAATTGGCGGTGGAGGATCGGGTGCTGTGATGCGTGACGGCTCGCTTGTGTTTCCCATGCAGG
CCAAAGACAAGGATGGAACGAGCTTTTTACTG
TCCATGAGTTTTGACCCGTCAGATAAAAAATGGGAGCTTTCATCTGAGACGCCTGGTAAGGACTGCAG
GGATCCAACCCTGGTGAAGTGGGaNNNNNNNN
NNNNNNNNNNNNNNNNNNNNNNNNNNNNNNNNNNNNNNNNNNNNNNNNNNNNNNNNNNNNNNNNNNNN
NNNNNNNNNNNNNNNNNNNNNNNNNNNNNNNN
NNNNNNNNNNNNNNNNNNNNNNNNNNNNNNNNNNNNNNNNNNNNNNNNNNNNNNNNNNNNNNNNNNNN
NNNNNNNNNNNNNNNNNNNNNNNNNNNNNNNN
NNNNNNNNNNNNNNNNNNNNNNNNNNNNNNNNNNNNNNNNNNNNNNNNNNNNNNNNNNNNNNNNNNNN
NNNNNNNNNNNNNNNNNNNNNNNNGAGACGCC
TGGTAACGGCTGCAGGGATCCAACCCTGGTGAAGTGGGAAAAATACGAATACGGCGAAAGACTCTTCA
TGATGGCTCATTGTGCTGGAGGCTACTATGAC
GTTTACATGTCCACTCCGCATGGAGTCAATTGGATTGATCACTTCCAACCAATTACCCGCGTGTGGGG
CAACTCACACAATCGAAAGGGGCACGGTGTCC
AGAGTGGATCCACCACCGCAATCATTGAGGGAAGGGAGGTGATGCTCATCACCGCGCCGGTGTATGCG
AAGGAGGAAAATGGAAAAGGTCGGCTCCATCT
TTGGGTGACGGAAAATGCACGTGTGCATGATGTTGGGCCGGTATCTCGTGAAAATGATGACGCCGCCG
CCAGCTCGCTGTTGATGAGGGATAATAATAAG
GAGTTGATTTCACTGTACGAAAACAAGAGCGACGGAGCATACAATCTTGTTGCTGTGCGTCTGACCGA
GAAACTGGAGCGGATAAAGGAAGTTGTGAAGA

AATGGAAGGATTTGGACAGCGCCTTGCAATCATGCCGTTCCGGTTCCAGCGCCACTGTTGACCCGAGA
AAAAAAGGTATGTGCAATGGTCGTGTTCCCAC
TGATGGGCTTGTTGGCTTCTTGTCTGGTAACTTCTCTGAGAACACATGGAGAGACGAGTACCTCGGGG
TGAATGCAACAGTGACGAATGGGGAGCCAAGG
GTTCGCAATGGATGGACGTTCAAAGGACCCGGGGCAGGGGCGGTGTGGCCTGTTGGCGACATGGGGCA
GACTGTGCCGTACTACTTTGCGAACACCGAGT
TCACTCTTGTGGCGACGGTGTCCATCCACGAGGCGCCGAAGGAAGCTAGCAGCCCCATCCCC
>scaffold1087.size6355
tGGGCAAGCACCAACAGGGCGCACAGCAGACGGCACGTCATCATCATCGTGAGTGTGGTCGTTCAGTG
GTGTGGAGCTGATCTGCACAGTGAGGGAAATGG
AGAGCAACAGGGGAGACGAGAGGTGCGGCAGACACACACAGAGGGCACACCACATGAGGAGAGGGCAG
CAGCCAGGCATGCACCGTACGCCGCACACCAC
GCACGACACACACTCCAATCCACGGCAGCAGGCACACACACACACAGCCCTGCCGCAACACCAACACA
CAGATGGAGGAGGGAGAAGCAAACAGGAGCGG
CACACAAGGCTCCACCGCATGCACATGCGGCGAGAGCGGTCCGTCGTGTCGGCACCCCTCACGGAGGG
TGCGCACACACACAAGAAGGTGCAGCGCACAC
AGCAACACAAAAGCAAAGTAACAATACAAAAAGAAGAGATCCCCGCACACCACGCGGATTGTTGGTCC
ACGGGAAGTGCAGCCAATATTCACCACAAGCA
GTTCATTTGCAGTGGGCCTTCGTCCACATTCATCGCACTCCACTTCATGCAGGCACAGTCACCACCGC
AACAGGAGCTGCTGCACACGCAACAAGAAACA
AGAAAGGGAGGTGGTGTGGGAGACCACGGTGCTGCTGTCACTGCCGCCATGCGAAGCTGTGTTTATGT
TGTTAGTGGCGGTGGCGATGTCCCTCGTGAAC
CCCTCGCTGTGGGCTGCGGGGTAACTGTGCCGGCTGATGTGCCTTCGGTTTTGTTGTTGCATTCTTCT
TCTCCACTTCTTCTGTGGAGGGTCCGGCGTCC
TCTCCGTTCAGGTGGCTCGCATTATGATTGGTCGTGGTGCTGCTGGCGGCGGCGTTAAACTCTGCAGC
CGGAAGAAATGCAGTGCTGTTGTCAGCCTCGG
CAGCAGGCGCTTTGTCTCCACGCCGGTACGTTTCTGTTTTCTCTGCAGCGACAGAAAGTGGAGGCTGT
TTGAAAATCTCCTGCACGATGCATTCCTCTTT
CCAATCTAATGCAGCAAAGCCGCCATTTGAAAAAGACGATGACTGCGATGGCCTCTCGTGAATGGGAG
ATGGTGCATGCTGCACGGTGTCTTGGCGTGCT
GCAACTTCCTTCCTTTCTGCCTCATCTCCACCAGCTGCCTCTACTGCAACTTCTCATCCCGATTCTCC
TTGTGGTGCATCATCCGGTACTTGTGCAAAAG
TTGTTTTTGAGCCCACATCGTGTTTTGGCGCTACCTTCGCCATTCCCTGGCCATGAAACTTCATGTCG
GCTGTCAGACCCTTCTGCCCCTTGCGGCGGTT
CCTGCCCCTGCTGTTTGTCTTATTTGTTCGCCCGGTACTGCGGCTGGTTTTGTTGGTAGTGATCCTTT
TCGCCCCCCAATGGCAGGCTGTGTTGGGGGCT
GGGCTCCTCCCTCTGTCCCACCGCCCCCGATGACTCAGCGACTTTCAACTTTTCATCCTGAGCGGATT
CGTTTTCCCGACTGCCGGTGGCGCGACTGCCA
TCCGAAGAGGGCAATGCTCGCTCCCTTTGGGGCACGAGTTCCAGGCGCCCCACAGTGACAGGAGCCAT
CAGCATCTGAGCCTTGCCTTTTGAACCATTCA
CAGTTGACTCCTCCCGCTGTCCAACTCCACTCAATGCGTCGTGTGACTCCGACACATTCGTTTTCATT
TCGGGCGGCAAATAACGTCATCCCGCTGATCC
TCCACATACTGCCGGTGCCGCTGGTGGCGCGCCGCCTTGTCTTCTCCTCGTTCTAACGGCAGGCGATC
TACGCGGCTGGTGACGCATCTCGTTGAGTGTT
CATTGGATTGCAGCATACCCCCTTATCCTCGGCGGTGTCAGCAGCAACGGCAATCCCTTGGCGCCTCC
AGGGACGACGGCCATCCAATTACGCACGCATT
CCTCCACGGCGGACGCATTCGCCCTGCCTCCTGTCCTTCTGCTGCCATCCATCGCGCACTCCCTCCGC
AGCTGAGCACGCCACCGCAAGAGCTAATACTC
ACCCGTACCGCTATCGTCACCACAGCACGCAGAGGACACACACCAGCAGCACACGGCCAGTCATCATC
GCCACCACTCCTGTCACTTGCGTGCAGCCTTC
AGCAAAAGTAACTCCCCGTCAACAGAGAAGGAAAGCAAAAATAGACACGACCGCTACACCACGCACAG
CAACAAACGACAGCAACAAATGGCAGCACCCC
AGCAGCAGCGCACACACCACGAGACAGATGGAATCAGTACGATTGCAGAGCTCGCACACCGCACCGCA
AACCACAAGTTAAAAAGCAATAAAAAACAATA

ATTAAAAAGCAATcttATTAAAAGAAAAGAAAAACAAACCCAGGGCTCCAGTGCGCATGCATATGAGT
GTGTCCGCGATGCGTTCAACGTGGCGGATGCG
GGGCACAACGCGGAGCACACAGCACGGCAGCAGAAGCAAGTAAAGCAAAACACAATCATTATTTCCTC
TTATTTTATACAGTGAACCACAACACACGCGC
ACAAATCCCCGCGGTTGAGTGCTGGCACGCACAGCCCGCACACACCTCTTCAGCCCACAGCGGTGTAC
GCCAGCGCGGATGCGGCGAGCACCAGCGGGGC
ACACACCCACGCAGAGCTGCTGAGGCTGCCGTCGATTTCGCGAAGACGTGACGGTGCGCGGGTGGTCG
TCGTTGTTGGCGCCTCCGTAGTCGTAGTGCTG
GGTGCCCGTGTTGTAGTGGTGGTCGTCGTGGTCGTTGTGGTTGGCGCCTTTGTTGTGGTCGTCGTTGT
CGTTGTCTGNNNNNNNNNNNNNNNNNNNNNNN
NNNNNNNNNNNNNNNNNNNNNNNNNNNNNNNNNNNNNNNNNNNNNNNNNNNNNNNNNNNNNNNNNNNN
NNNNNNNNNNNNNNNNNNNNNNNNNNNNNNNN
NNNNNNNNNNNNNNNNNNNNNNNNNNNNNNNNNNNNNNNNNNNNNNNNNNNNNNNNNNNNNNNNNNNN
NNNNNNNNNNNNNNNNNNNNNNNNNNNNNNNN
NNNNNNNNNNNNNNNNNNNNNNNNNNNNNNNNNNNNNNNNNNNNNNNNNNNNNNNNNNNNNNNNNNNN
NNNNNNNNNGGTGGCTGTGGTGGAGGAGGCGG
TGGAGCGGGAGCGGCTATAGGCCCATTCTCAGGATCTGCCCCGCACACGGACGGGCAGCAGCAGCACA
GGGCAAGCACCAACAGGGCGCACAGCAGACGG
CACGTCGTCATCGTGAGTGTGGTCGTTCAGTGGTGTGGAGCTGATCTGCACAGTGAGGGACATGGAGA
GCAACAGGGGAGACGAGAGGTGCGGCAGACAC
ACACAGAGGGCACACCCACATGAGGAGAGGGCAGCAGCCAGGCATGCACCGTACGCCGCACACCACGCA
CAACACACACTCCAATCCACGGCAGCAGGCAC
ACACACACACACACAGCCCTGCCGCAACACCGACACACACAGATGGAGGAGGGAGAAGCAAACAGGAG
CGGCACACAAGGCTCCACCGCATGCACATACG
GCGAGAGCGGTCCGTCGTGTCTGCACCCCTCACGGAGGGTGCGCACACACACAAGAAGGTGCAGCGCA
CACAGCAACACAAAAGCAAAGTAACAATAAAA
AAGAAGATCCCCGCACACCACGCGGATTGTTGTTCCACGGGAAGTGCAGCCAATATTCATCACAAAGC
AACACTTCAGTTGCCTTCGGCCTTCGTCCACA
TTCATCGCAGACCACCTCATGCAGTCCCGGCCACCACCGCAGCAGCCGCACACGTAAAAACAAGCGCA
AGGAATGCAATAATGCAAGCATTCATTTATTT
GTATTTCCTTTTGGATGTTGGCGGATGTGTGCACATGTTGGTTTCTTTTTTGTGAGTGTGCAAACCTT
CATGCCTGGGTCGTGTCTTGGGATTCCTGCAG
GTCGCACAATTATTTGTTTTCTCTCGTGAATTTTTCTTGTGTTGGTGCAGCCAACAATTCGGTTTATG
TTGTTCCTGCCTTTCGCCGGTAAAGTGCTGCA
ATAGGTGCTTTTATTTCCTCCGTTCGTTGCTGTGGCACCCGATTCCTCACCGTTTATTGCGGTGGAAA
TGAAAATATGTGGGTGGACTCCCACACACCCG
AACGCACCTTCGCTCCTATTTATTGGTGCACGGAAGGGCATGTGTGTTTGGTGGTGCGTCGTCCATAT
ATTTTGTGGGCGTACATTCCCCTGCAGGCTGA
GGGCGGGATGACGCTCCGTCGCGGTGCACTTCCCTCGCATGCCGCCCACTGTGATTCATCATGCATCA
CCCCCTCACATACTCCGTGGTGCCACACAGCT
GATATTATTGCAGCCGCTGTGATTCCGCGTATTTCACGCATTTGATTGTGCCACAATACATCCGCTCG
TGTGGCCGCCGCATCCAAACAGCGGTGCCCAA
CACCGTGAGCGGCAGTCCACATCCTTGCATGTCCCAACGCAGTGGTGTTTGACTGTCAGCCAGGCGTC
AGACAACAAGAGAGTCTTGTGCTGGCAGTACA
GCACACGGAGCTGCAGTGAATGACTCGGGCGGCGACGCATCCATTTGTCGCAAGGCAGTGAAAAGGAG
TCGGGCAAAATAATGCCGGCTGCATTCACGAC
ACCACATCCACAGCCAAAGCATGCGACGTGATGTGTGTTTCACTGGTGCTGTGAAATGCCTGCAGACG
ACGTCACTAGTGGAGGCATGCCACCCATGATC
AGCGGACAGACAATAATCGGCATGGGAGGCCACAAGAGGCAGCAACGACGTCAGCAGCTCTCTCTCTC
TGTTGGCTCCGAAAAGTAAATATTCCACCAGC
CACAGCTGACTGTGCCATCGTGACACTCCATGCACGTCGTGGCTGCGTCAACACATCCTGTTCCCTGC
AGCGCCTCAAAAGATGCCTCACTCCGTCCACT
AAGTGCGTCGATGTGGGCCGTGCCTGCGCTGTTCGTGAGGACCACAACGATGTGAGGGTCACCGAGTC
GCAGCATGTTTGCTGTGAAGTTGTTGCATGGC

ACCTGCACACACACACGCAGCACCTGCATCCCTCCCTGTTGTGTCCGCCCTCCGCCGGAGTGGCCACA
CACACGGGCAGCAGCAAAATCACTTCACCCTC
GACAATCCAAAATCAACACCGACATCCCTCGCGACGCGAAGATCTCAGTTGCCAGCACGGGCCTCATG
CCGCTCTGCAATCGACGCTGAAACACGGCACA
GTGCACGCACTTATTTTCCTCGTGTGCAGTCAGCCGTTTGCTTCACACATTCTTCGACGGACCGACGT
TCACAAAAGCACGCCACAAAAGCCACTGTGAT
CGCACAAGCCCTTGCGATGAGCGTCGGTGTGTGCGTGCATCCGCTGCCCACAAGTGCTGGCAGGCAGC
CTGCAGCCGGTGCACTCGTGCCAGTTCATCGG
CTCCAGCACGGAAAAGAGAAAGTGGCCTATTCCCGATGCACTTATTCCCACCGGCAGCACCAACACGC
TGGTCGCCAGCCGTCATTCCTCAGCACATCCA
GGAATGCCGAAAAGTGACGTGGAACTGTCAGCCAGCAATGCCACAATCCCGCACACATGTCGGCTCCA
CCAGAAAAGAAACACAAATCACAAACAACACC
CTCCACATGTCTTCCAAACATCATTCCGACACACCGCGACACGCCAAACAGAAGTATTCGTCACTCCA
ATGCACCTCCCCACACAATCACATAGTCCATG
CACCACGCACGGCAGCGGCTCACACGCAGTCAAACAAATGTTCCTCGGATCAGTGGCCTCCACGTGCT
GCGGTGGGAAGTAAACAAAAGAGACGAGCCGC
CAAACCCCAAAAGAGTGCCACGACTGGCCGCAACTCAACGGAACGCAGCCACACAAATGAATTCAGTC
CTAATGCCTTCAGCCTCTCACCTTCCCCACAA
GAACTGCAAAGATTGAAAACAAACGTACCGTCCAATAAACTCTTTGTGCAAAGCAAAAAAGAAGCCGC
AGTGCCTCCTGCTGCCCTCACAGTCCGTTGAT
GACGCCTGGATGCGGATGGTCACCCATCGGTACTTCCAAACGACGCCGCACACCCGCGACGCATCCTG
CGCTCCAAGAAATGCGGCCCTCATTATCACCA
GAGACGCCCACGCACCGTCAGTACGCAACAAATCGTCAAGGAACGAAGGATAAGTCAGGCTGACAGCA
AGACACCCGCCGACAAATCACAGCACAAACAT
CATCACAAGCAGAACCAAAAGCAATGCAGGCCGTAGAGAAGCGTGAATCAACTGACGCATGTCAAAGA
AAAGAAAAAAACTCAGTGGCGTTACAAGAATA
CAAGAAGCTGGCACTCTCTCAACAATAAAGGAACAGTATAATAATACACAAAAA
>scaffold1088.size7460
ACTCGGCGCTCTCTCCGTCGTGCTTTTGAGGCCCTCTCCGTACGCGTTACTGTTCCCATGCTACGGTT
TATTGATGTTTGCGCCCTGTCGCCCCTCCCCAC
GCCAGCGGTGACGACGCGGCGATTCGGCTCTCTCTTTGTGCGAAGAAGGGGGGACCCGTCGCATGAGC
GGCGTGCTGTGCGGGGTGTTGCCCCTCTTCCT
GCCCCCGCCAAAAAAATGGGCGCTGCTTTTTTTTCTCCTCTTTTTTTGCCGGCAAGGTAGGAGTGAAC
GTCACACAAACACCTCACGCAACACACGATGA
GCTTAGAGCTTCGAGTGGGTAACCGCTTCCGCCTCGGCCAGAAGATTGGGGCGGGGTCGTTCGGCGAG
ATCTTCCGCGGCACAAACATGCAGACGGGCGA
GACCGTGGCCATCAAGCTGGAGCAGGCGAAGACGCGCCATCCACAGCTTGCCTTTGAGGCCCGATTCT
ACCGCATTCTCAACGCAGGCGGCGGGGTCGTG
GGCATCCCCAACATTCTTTTTTATGGCGTGGAGGGCGAGTTCAACGTGATGGTGATGGACCTGCTGGG
ACCCTCGCTCGAGGACCTTTTCAGCTTCTGTG
ATAGGAAGCTGTCGCTGAAGACNNNNNNNNNNNNNNNNNNNNNNNNNNNNNNNNNNNNNNNNNNNNNN
NNNNNNNNNNNNNNNNNNNNNNNNNNNNNNNN
NNNNNNNNNNNNNNNNNNNNNNNNNNNNNNNNNNNNNNNNNNNNNNNNNNNNNNNNNNNNNNNNNNNN
NNNNNNNNNNNNNNNNNNNNNNNNNNNNNNNN
NNNNNNNNNNNNNNNNNNNNNNNNNNNNNNNNNNNNNNNNNNNNNNNNNNNNNNNNNNNNNNNNNNNN
NNNNNNNNNNNNNNNNNNNNNNNNNNNNNNNN
NNNNNNNNNNNNNNNNNNNNNNCACCGGGAAGAAGGGGCACCACGTCTACGTCGTTGATTTTGGTCTC
GCCAAGAAGTACCGCGACCCCCGCACGCACCA
GCACATCCCGTACAAGGAGGGGAAAAGCCTGACGGGAACGGCACGGTACTGCTCGATCAACACGCATC
TTGGCATTGAGCAGAGCCGTCGCGACGACCTG
GAGGGCATCGGCTACATCCTGATGTATTTCTTGCGTGGCTCACTGCCGTGGCAGGGCCTGAAAGCGCA
CACGAAGCAGGAAAAGTACTCTCGCATATCCG
AGCGCAAGCAGACAACGCCCGTGGAGACGCTGTGCAAGGGCTTTCCCGCGGAGTTTGCCGCGTACCTC
AACTACACCCGAGGCCTGCGCTTCGAGGACAA

GCCGGACTACAGCTACCTGAAACGGCTCTTCCGAGAGCTCTTCATTAGGGAGGGGTACCACGTGGACT
ACGTGTTTGACTGGACGCTGAAGCGCATCCAC
GAGAACCTGAAGGCGGAGGGGTCCGGCCAGCAGGAACAAAAACAGCAGCAGCAACAACAACGAGAGCG
GGGGGATGTAGAGCAGGCGTAGCCCACCTGCA
CAGTCCGGGACCCTTGTTCCGCGCACTGTTTGTACGACGTCTTTGGACGTCCCACACGCTCCATTGTT
GAAAATATCACTAATTTTAGGAAATGAACATT
CTTCTCTTGCTTTGAGCACGAGGAGGTGCGTGCACGGTGATGTGTGGTGGCAGGTTAGGTGAATAGAA
AACACCCGCTGTCTCCCTTTTTGCCCTGTGTG
CCTTACTCGGCGCTCTCTCCGTCGTGCTTTTGAGGCCCTCTCCGTACGCGTTACTGTTCCCATGCTAC
GGTTTATTGATGTTTGCGCCCTGTCGCCCCTC
CCCACGCCAGCGGTGACGACGCGGCGATTCGGCTCTCTCTTTGTGCGAAGAAGGGGGGACCCGTCGCA
TGAGCGGCGTGCTGTGCGGGGTGTTGCCCCTC
TCCCTGCCCCCGCCAAAAAAATGGGCGCTGCTTTTTTTCTCCTCTTTTTTTGCCGGCAAGGTAGGAGT
GAACGTCACACAAACACCTCACGCAACACACG
ATGAGCTTAGAGTTTCGAGTGGGTAACCGCTTCCGCCTCGGCCAGAAGATTGGGGCGGGGTCGTTCGG
CGAGATCTTCCGCGGCACAAACATACAGACGG
GCGAGACCGTGGCCATCAAGCTGGAGCAGGCGAAGACGCGCCATCCACAGCTTGCCTTTGAGGTCCGA
TTCTACCGCATTCTCAACGCAGGCGGCGGGGT
CGTGGGCATCCCCAACATTCTTTTTTTTGGCGTGGAGGGCGAGTTCAACGTGATGGTGATGGACCTGC
TGGGACCCTCGCTCGAGGACCTTTTCAGCTTC
TGTGATAGGAAGCTGTCGCTGAAGACAACGCTGATGCTGGCGGAGCAGATGATTGCGCGCATCGAGTT
TGTCCACAGCAAGAGCGTCATCCACCGTGACA
TGAAGCCGGACAATTTCTTGATGGGCACCGGGAAGAAGGGGCACCACGTCTACGTCGTTGATTTTGGT
CTCGCCAAGAAGTACCGCGACCCCCGCACGCA
CCAGCACATCCCGTACAAGGAGGGGAAAAGCCTGACGGGAACGGCACGGTACTGCTCGATCAACACGC
ATCTTGGCATTGAGCAGAGCCGTCGCGACGAC
CTGGAGGGCATCGGCTACATCCTGATGTATTTCTTGCGTGGCTCACTGCCGTGGCAGGGCCTGAAAGC
GCACACGAAGCAGGAAAAGTACTCTCGCATAT
CCGAGCGCAAGCAGACAACGCCCGTGGAGATGNNNNNNNNNNNNNNNNNNNNNNNNNNNNNNNNNNNN
NNNNNNNNNNNNNNNNNNNNNNNNNNNNNNNN
NNNNNNNNNNNNNNNNNNNNNNNNNNNNNNNNNNNNNNNNNNNNNNNNNNNNNNNNNNNNNNNNNNNN
NNNNNNNNNNNNNNNNNNNNNNNNNNNNNNNN
NNNNNNNNNNNNNNNNNNNNNNNNNNNNNNNNNNNNNNNNNNNNNNNNNNNNNNNNNNNNNNNNNNNN
NNNNNNNNNNNNNNNNNNNNNNNNNNNNNNNN
NNNNNNNNNNNNNNNNNNNNNNNNNNNNNNNNGGCTTTCCCGCGGAGTTTGCCGCGTACCTCAACTAC
ACCCGAGGCCTGCGCTTCGAGGACAAGCCGGA
CTACAGCTACCTGAAACGGCTCTTCCGAGAGCTCTTCATTAGGGAGGGGTACCACGTGGACTACGTGT
TTGACTGGACGCTGAAGCGCATCCACGAGAAC
CTGAAGGCGGAGGGGTCCGGCCAGCAGGAACAAAAACAGCAGCAGCAACAACAACGAGAGCGGGGGGA
TGTAGAGCAGGCGTAGCCCACCTGCACAGTCC
GGGACCCTTGTTCCGCGCACTGTTTGTACGACGTCTTTGGACGTCCCACACGCTCCATTGTTGAAAAT
ATCACTAATTTTAGGAAATGAACATTCTTCTC
TTGCTTTGAGCACGAGGAGGTGCGTGCACGGTGATGTGTGGTGGCAGGTTAGGTGAATAGAAAACACC
CGCTGTCTCCCTTTTTGCCCTGTGTGCCTTAC
TCGGCGCTCTCTCCGTCGTGCTTTTGAGGCCCTCTCCGTACGCGTTACTGTTCCCATGCTACGGTTTA
TTGATGTTTGCGCCCTGTCGCCCCTCCCCACG
CCAGCGGTGACGACGCGGCGATTCGGCTCTCTCTTTGTGCGAAGAAGGGGGGACCCGTCGCATGAGCG
GCGTGCTGTGCGGGGTGTTGCCCCTCTCCCTG
CCCCCGCCAAAAAAATGGGCGCTGCTTTTTTTTCTCCTCTTTTTTTGCCGGCAAGGTAGGAGTGAACG
TCACACAAACACCTCACGCAACACACGATGAG
CTTAGAGCTTCGAGTGGGTAACCGCTTCCGCCTCGGCCAGAAGATTGGGGCGGGGTCGTTCGGCGAGA
TCTTCCGCGGCACAAACATGCAGACGGGCGAG
ACCGTGGCCATCAAGCTGGAGCAGGCGAAGACGCGCCATCCACAGCTTGCCTTTGAGGCCCGATTCTA
CCGCATTCTCAACGCAGGCGGCGGGGTCGTGG

GCATCCCCAACATTCTTTTTTATGGCGTGGAGGGCGAGTTCAACGTGATGGTGATGGACCTGCTGGGA
CCCTCGCTCGAGGACCTTTTCAGCTTCTGTGA
TAGGAAGCTGTCGCTGAAGACAACGCTGATGCTGGCGGAGCAGATGATTGCGCGCATCGAGTTTGTCC
ACAGCAAGAGCGTCATCCACCGTGACATGAAG
CCGGACAATTTCTTGATGGGCACCGGGAAGAAGGGGCACCACGTCTACGTCGTTGATTTTGGTCTCGC
CAAGAAGTACCGCGACCCCCGCACGCACCAGC
ACATCCCGTACAAGGAGGGGAAAAGCCTGACGGGAACGGCACGGTACTGCTCGATCAACACGCATCTT
GGCATTGAGCAGAGCCGTCGCGACGACCTGGA
GGGCATCGGCTACATCCTGATGTATTTCTTGCGTGGCTCACTGCCGTGGCAGGGCCTGAAAGCGCACA
CGAAGCAGGAAAAGTACTCTCGCATATCCGAG
CGCAAGCAGACAACGCCCGTGGAGANNNNNNNNNNNNNNNNNNNNNNNNNNNNNNNNNNNNNNNNNNN
NNNNNNNNNNNNNNNNNNNNNNNNNNNNNNNN
NNNNNNNNNNNNNNNNNNNNNNNNNNNNNNNNNNNNNNNNNNNNNNNNNNNNNNNNNNNNNNNNNNNN
NNNNNNNNNNNNNNNNNNNNNNNNNNNNNNNN
NNNNNNNNNNNNNNNNNNNNNNNNNNNNNNNNNNNNNNNNNNNNNNNNNNNNNNNNNNNNNNNNNNNN
NNNNNNNNNNNNNNNNNNNNNNNNNNNNNNNN
NNNNNNNNNNNNNNNNNNNNNNNNNGCTGTGCAAGGGCTTTCCCGCGGAGTTTGCCGCGTACCTCAAC
TACACCCGAGGCCTGCGCTTCGAGGACAAGCC
GGACTACAGCTACCTGAAACGGCTCTTCCGAGAGCTCTTCATTAGGGAGGGGTACCACATGGACTACG
TGTTTGACTGGACGCTGAAGCGCATCCACGAG
AACCTGAAGGCGGAGGGGTCCGGCCAGCAGGAACAAAAACAGCAGCAGCAGCAACAACGAGAGCGGGG
GGATGTAGAGCAGGCGTAGCCCACCTGCACAG
TCCGGGACCCTTGTTCCGCGCACTGTTTGTACGACGTCTTTGGACGTCCCACACGCTCCATTGTTGAA
AATGTCACTAATTTTAGGAAATGAACATTCTT
CTCTTGCTTTGAGCACGAGGAGGTGCGTGCACGGTGATGTGTGGTGGCAGGTTAGGTGAATAGAAAAC
ACCCGCTGTCTCCCTTTTTGCCCTGTGTGCCT
TACTCGGCGCTCTCTCCGTCGTGCTTTTGAGGCCCTCTCCGTACGCGTTACTGTTCCCATGCTACGGT
TTATTGATGTTTGCGCCCTGTCGCCCCTCCCC
ACGCCAGCGGTGACGACGCGGCGATTCGGCTCTCTCTTTGTGCGAAGAAGGGAGGACCCGTCGCATGA
GCGGCGTGCCGTACCGTGTGTCTCCCATGACC
GGACCACTCGTGGTTCTTTGTAGTGCGCTATTATTCAGTTTTACTCTTTGGGGATGCTGTTGTCGTAT
TTATTATTTTTAGCAGAATTGTTGTCAATGTG
TGTGTTTTTTGTTGTGGTATATGTCTGTTCCGTTGTTGTTTCATTTTGTGAGGCGGATTGTTCTGATG
TATCGTTTTCTTTTGTGTCTGTCATGAATAGT
TCGCTGTGTTGTGTGTTTTTTTTTTTTGGgNNNNNNNNNNNNNNNNNNNNNNNNNNNNNNNNNNNNNN
NNNNNNNNNNNNNNNNNNNNNNNNNNNNNNNN
NNNNNNNNNNNNNNNNNNNNNNNNNNNNNNNNNNNNNNNNNNNNNNNNNNNNNNNNNNNNNNNNNNNN
NNNNNNNNNNNNNNNNNNNNNNNNNNNNNNNN
NNNNNNNNNNNNNNNNNNNNNNNNNNNNNNNNNNNNNNNNNNNNNNNNNNNNNNNNNNNNNNNNNNNN
NNNNNNNNNNNNNNNNNNNNNNNNNNNNNNNN
NNNNNNNNNNNNNNNNNNNNNNNNNNNNNNGTTTTTTTTTTTTTTTTTTTGGGTATAAGCGTGTCTGTT
TGTTTTTTTTTTTCTTAATTTATTTGTGTGTT
TTTCTTGTTGTTGCTTTTGTTTTTATGGTTTGGTGGTTTTTGGCTTTCGCTGTATCCCTTTTGCTTTT
GTTTTTCGCATGCCTTTTGTGTGTGTCTGCTG
TTCAAATGTGTTATTCCTTTTTGTCTTTTTTTTTCTTTTTTACTTTTTTTCTATTTCTGTAATGCGTGC
ATGCATGTACTTCTGCGTATTATGCGTGGCAA
CGGTTATTATTATAATTATTATTTTAATACAACGCGTAGGGTTCTATCTTGTGTTGGTTTACTGTTTC
TGCTGCCGCCGCTGCTGCTGCTGCTTCTGTAA
AGAGAACAACAAAGGAGATTTAGGAGATCCAATACAGAAGGAATTAAAGAAGAAAAAGTAGAAGGAAT
ATTTCATTTTTTCTTCTTTTTTTTTTTTTTTC
CTTTTTTATTTACCAATTTGTTGTTGTTATTGCTTTGTCTGCGATAGAGAAGGATGACGAGCATGCAT
TGCACACTGGTACCACCTCCTCCACCACCGTT
TTATGGCATTTGCATGGAAGACACCACGTCGCTTGGGTACGCGTGCGGTTCCGGTGTAGAAAGCGACA
TGGCCGCAAAACCTATCTTGGCAGAGCGCTAC

AAGACAAAGTTTTGTCGCAACTACGTGCTAACCGGCATATGCCCCTATCAGAGACGTTGCATGTTTGC
ACACGGTGATCACGAGCTTCGGACACCGGAAA
TGAACATAAATGATGGGCTCGTGACGGAAGAAGCTATTCGTCTCTTTCGGCGTCAATTTTTATCCGAT
ATATGTGCATTTGACAGGCCTCGCCGACGGGG
TTTTCCGGCACCGCCGTATTTCTACCATGCCATGGGTGTTTGCGTGTCGGACCGTGGGGTACCAGTGA
GGATGGCGCCGGCAAAAATTTATACGCACAAT
CCCTATAAGTCCGACATCATTCCGTTGCAAAGTCGTCTGTATAGTCAGCCGCCCGCTTTGGCGATGAT
GACAGCCGCGACGGTGATGCCACAATATTATG
AAGAAGGGACGATGTGCCGCAGCAGCTGTGATCCCAGTGAAAATCAAGATGACTTTGAGCCCTGTGTT
GAAATTATAAGTTCACCCCGCTGTTACGCATG
CAATGAAAGTGATGACGCTGAACCCTCCGCGTGCAGCAGCATTGGCAGCGACTCTGAGGACAACCGAC
AGTCAACACCCTGCAAGGAATACATCAATGAG
TCAATGCCTTCTGAGGAGGTGATGAGCGGGGAGGCGGCATTGCAGTAAATCTTTGATTTTTTGGGGCG
GGTCGTGTTTTTTTTTTTTTTAATTATTCCTC
TCCTTTTTTCTTTTCTTTTTTTTTTTTTTTTCCTGCTCTTCTTTTTTTTGTGTTTTTTTC
>scaffold1089.size5474
GGAGGAGACATATTTCAGTGCTGCCTTGCATGCGGCATTGCGGTGCCTCGGCATGCGGGTGCGTCTGA
GAGTGACTGAAGAGGGTAACGTCCGTTGTGTCC
CATGCACGGCCGAGGCGACGATTACGTGGGCCACTCCGCCCTACGACGAGGGAAACGGTATTTTCCGT
GACCACGCGCAGGTGTGCACGATATCTTTCAC
CAGTGGTAGCCTATTTCCAGTGATTCCGTGGGACAGCGAGGAGAGAGCATGTGGAGCAGGACGGTTTC
ACCGGCATCTACTGAACAAGCTTTACCACGGT
CATCCGTCCCCGATCCAGAGNNNNNNNNNNNNNNNNNNNNNNNNNNNNNNNNNNNNNNNNNNNNNNNN
NNNNNNNNNNNNNNNNNNNNNNNNNNNNNNNN
NNNNNNNNNNNNNNNNNNNNNNNNNNNNNNNNNNNNNNNNNNNNNNNNNNNNNNNNNNNNNNNNNNNN
NNNNNNNNNNNNNNNNNNNNNNNNNNNNNNNN
NNNNNNNNNNNNNNNNNNNNNNNNNNNNNNNNNNNNNNNNNNNNNNNNNNNNNNNNNNNNNNNNNNNN
NNNNNNNNNNNNNNNNNNNNNNNNNNNNNNNN
NNNNNNNNNNNNNNNNNNNNGCAGCACACCACCGAAGGATGTGGACACGGCTGCTCCCCAGAAGCATC
CGTCGCCAGAGGAGAGGGAAGTTGCCTCAGGT
GCCACGACGGCAACGGTGTGCTCTTGCCCAGCGACACCGCGGGTGCGCCGCAGGAGGCGGAGAAGAAG
CGCGATCTTCCGGACATCGACGATGTGGCCGC
CGATGGTGTGACGTCTGTTGTGGGACAATCATTAGGAGATGAGGCCGTGAGCAACAAGGCCGGTGGCG
GTGCCACAGCCACGGGACCCAAGGGCCCTGAT
GCCGCTGATGACGCGCAGGGCGCCGTGGACCCTCCGCCGAAGGAGGATGATGCGGCGGTGGTGTGCGG
GGGTTGGGAGGCAGACATGAAGGACGCCCCGC
CTCCGATCCACGGCAATAACAGTGCAGCTGCTGCTTGTCCCTTTCCGCTGCGGCTGCTGCTTGTCCCC
GTCCGCTGCCGCCGCGGTGCCAACACTCAGCA
GTGGGTGCAAGAGAGAAAGGCCTATATCAACTGGCATGTTCCTGTCAGTATATACATACACTCTGCTT
GTCCGTATACTTTGTACCAGGGACATGCTGTA
TGCACTCTCCCTCTATTGTCGGTTGCTAATTTGTCGGCACCACGCCTGTATTATGGGTTGGGATAAGA
AATTGTTTGACTTCTTCTGAGGGATACTGTGC
TGTGTTTGTGTGCGTGTACGAGGGATCTGTGAGGTTTTGATTTCCCTATTTTTATTTACCTGCTTTGG
CATTGTGTAATGATACTTCCCTTCTCCATTTT
GTGTGTTCTGAAGTGTTGCTAGGGCACCGGGAGTGGATGCCTGTGGACGCACGCCGTGGATTATGCGC
TACATTGGTGGGAGGGGAGACTGACATAACTG
GCGGGATTCAGTAAGGCGCATTTTTGCCCACGGACCCTGCAGTTGCAGCAGCCTGAGTGGCGCTTTGC
ATATGGACGTGTGGATTGGTTTTCTTTTGCGA
GACCTTTCTTACTGCTTGTCATTCTCCGCTCGGGTACCGTGCCCGTGGCAGCAGCGGCGGCATCAAAA
GGGCTGTGTGCGTGGAGCGTCCTTGATTGAGT
GCCTGTCTTACTTCTCTCTGTTCTGTCGATTGATTGTACCGTGTGCCGGCCAAACGGTTGACATGACG
TTCTTCTGACGGCGGGGTCCACGTGACACGAT
GTTGCGTCGCGTGCTTTGTTGCGGGTGTGTGATTTCAGCAACGGTTTTTGGGCGGTTGACTGTTGGGT
CGTTGAGGGCGGCGTGGCGACGGGTGTGGATG

GACCCGATTGTGTGCCGTGGCTGTTCGTGCGGGGGAGCCGCGGGAAGGAGGAAGGCAGCGGAGCGATG
GACTCACGAGGAGTGCGGTAGCGGGCAGCCAT
GAAGATTGGCGACAGGGAACTCACTGCCGCTGTGAGTTGCCGTGTGTGGTGTTTGTTTCTGTTATTAT
TACTTGCGTTTTTGGATGTGGCCGCTGTGAGA
GAAGACTGTTTTTTGTTTCTTTTCACGCCGTTTTNNNNNNNNNNNNNNNNNNNNNNNNNNNNNNNNNN
NNNNNNNNNNNNNNNNNNNNNNNNNNNNNNNN
NNNNNNNNNNNNNNNNNNNNNNNNNNNNNNNNNNNNNNNNNNNNNNNNNNNNNNNNNNNNNNNNNNNN
NNNNNNNNNNNNNNNNNNNNNNNNNNNNNNNN
NNNNNNNNNNNNNNNNNNNNNNNNNNNNNNNNNNNNNNNNNNNNNNNNNNNNNNNNNNNNNNNNNNNN
NNNNNNNNNNNNNNNNNNNNNNNNNNNNNNNN
NNNNNNNNNNNNNNNNNNNNNNNNNNNNNNNNNNGGACGCCACATTTAGCGTCAGTCGTGGATCGAGA
TCCATGCCGAGAAGCTTCGTAGTTCGCGATGC
CCCCATGGTCATGACTGGGATTTGACATCCAGCCACTACCAGGGGCCACTTTCCTTCACGATCATAAT
CTGACTCCGTGTGCGTGGATAGTGTGAACCAA
GCAGCTTCACACTTTACGGGTTGATGGCCAGGCCATTTTCCTGACTCCACGCATGCAGCTTTTGAAGA
ACCATGTTGGCAGTGGGAATGGCGTCTTCCCG
GGACTGCCCCTTAACGATAATAGAGAGGTCGTCCGCATACATGTCTGCTGTGACACCCGCAAAGCTGT
TCAGCAGACTGAGAAGTGGGTGTACGTAAAGG
GAGAACAAATACGGTCCCAGCACGGACCCCTGAGGAACTCCTCGCAGCATGGGACGCCGATCGCTGTG
TCTGTGGCCAACTCGTGTCTTCGCGTGGCGAC
CACATCGAAAGTTTCTAAGCCACCTTCTACAATTCACCCCCGGAATATTAGCCAGCAAGCGAATGAGG
ACTTGATGATCTATGGAGTCAAAGGCCTTGCT
GAAATCAACCAGCACTAGCAGGGTCCGGTGGGGTCTGTAGTGGAGTTGATTGCCAATACCGCTCTTCT
TGGGAAGGTTCACTTGGAAATAGTGATTTCTA
TTATGCTCCACTTCGTGTGTCAGGTGTGCCAGCTGCATCGTCGTGGTACGCACACTACGATATGCATA
CTGGTGGGGGTGCGGCGTCCACACCCAAAGCA
ATCTCTTCAGTACCATTTTCTCAGCAACCTTACTCACAATGGAGGTAAGGCTGATGGGTCGATATGAC
TCAAGCAGTTCCGGCGACTTACCGGGTTTTAA
GAGTGGAACGATGGTAGCGCGTCTCCACTCAACCGGGATCGTACCGGTCGTCCAACTGTGGTTAATGC
AACGAAGAAGGAATCGGAGGCCCTTTGACGAA
ACATGTCGCAGTGCCTCACTGTAGACTTCATCCGGTCCGGGGCCTTGCCATACGGCGTATTACGTGGC
GCAGCAAGCAGTCCCGTACGATTAAAAGGTGC
ATCAAGAGGGGAGTGAAGATGAGCAACTAAACCAGATGTGGTGGCAGCTTCGTTTTTATCCTGTGGCT
CAGAACTAGATGAGAGTGAAGANNNNNNNNNN
NNNNNNNNNNNNNNNNNNNNNNNNNNNNNNNNNNNNNNNNNNNNNNNNNNNNNNNNNNNNNNNNNNNN
NNNNNNNNNNNNNNNNNNNNNNNNNNNNNNNN
NNNNNNNNNNNNNNNNNNNNNNNNNNNNNNNNNNNNNNNNNNNNNNNNNNNNNNNNNNNNNNNNNNNN
NNNNNNNNNNNNNNNNNNNNNNNNNNNNNNNN
NNNNNNNNNNNNNNNNNNNNNNNNNNNNNNNNNNNNNNNNNNNNNNNNNNNNNNNNNNNNNNNNNNNN
NNNNNNNNNNNNNNNNNNNNNNAAGGGACATG
GATGGCACCGGGAAGTATTGCAGCTCAGAGCGGTATTGCGCAATGAATTCATTGGTGGCAATTGCGAG
TGTGAAGGTGACGTTTTTCTATGCTGAGGAAA
GGGAAATATTTGTTTCGTTATACTTTGCAGCCACTGTCCGTTAATGGCCTGCGGGCTGTCCGTAGCGA
TGTGGCCAGTTTTTCGTCTTGAACGGTGCTGT
CGTGCGAGAGGGTGTGTTTCCAAATAGTCGGATGCTCAGTTGTCGAGGACGGGGGCATTCCCACTGCG
TGTGCAGCAGGGCCTCCCGTGGATTGTCGGGG
CTCAGCGGCTGATGGGGGTTGCAGCAGAGGAGTGGCTGAAGGGCTACGGCTGTCATTGGAGCTTGCAG
AGGTGCGTTGTTCATTTTTGCTCGTTGATACG
CTTGACATTTTCGGTAGTGACCTGCGCGTGAGGCACTTGTTGGGAGGACCGTGGAGCTGTCGCAGAAT
CCACATGTGACGCCTTCCGCAACGATTGGAGT
GCCGGGGCTAGTGCGTCGGGCGTGTGTCTTCATATTGTGGACGGACGAGAAGACGCTGCCGCAGCTTG
GGCAGGGCGACGTTGATCCGCTGCTCGTCGTT
CTACGAGGAGGTGCCGTGGACACGCCAGGTGGAGGTTCCGGAGGAGGTCGGGGCGGTCCTGCCCCTTC
AGTGTGAGGAGGAGCAGCACCACCGACACAAA

GGGTTGCTTGTGATCCGCAGGGCGTGTGCCGGTCTGCCGAGGAGTGGGCAGGAGGACGTCAATCAGCC
GCTCGCTGGTCGGCGAGGAGTGGTGGTGCTCG
CGTCGGGAAGAGGTTCCGGCGGAGGCAGAGCACAGACGCGGTCCCAACAGCACCGCAGCACAGCCGAT
CCCAGAGGGCAGGGACGTGGCGCCGCCATGCC
TCGGGGCATCACAGGCCTGGGATCGAGAGGGCACCAAGCTGTTTCGCACGGACCTCTGGCAGCATGGA
GAAGCCACCGCGGAGGGCATGATTGCGGTCCT
CCCGTTTGCGGAGCACTTCAGCTCGTGGCAGGCTGCCGCTGCAACACACTCGGCCTGCTCCATTCCGT
CCGTACAGATTCCTGTGGGAATGTCACGGCTG
CCACTCGTACGGTGCCTCGGTGCATCTCCATCCATGTGATTACAGTGGCCAGAGGTGTTCCACACGCC
GAGGTGGAAGCATCACAGTCAGCTTCACGCAG
GACAACGGCGACATGTTTCCGTCGTGGCAGCATGCCTGCCTGTCGCGGGATGCCGTCCTCCGTCCCTG
CAGTGTGGGATGGCAGATGGTGGTCCGAGTCC
ATGTAAACGGGTGGACGTCCAGTGGGAGACACGCAGTACGCGTTGCGCAGTCACGTCGGGAAACGAGA
CGCTGCGGCCGCTGATCCACGTTGCCTGTGCC
GGGTCGTCGGAAAGCTCAATCTCCATGCCGCTTGGGGTGGCGGCGAGGTCCTCTCCTGCCGTCGTGGA
TGGCTAGCGACCGTCACGCAGTTCATGGTGTG
AATTCAGGTCGCAAATAACCGCTGCAGGGCCAAGGCGACGAATGGTGCTGCGCCAGTCGAGCAGCGTG
GGCAG
>scaffold109.size81049
GCACATGGCTTGTCGACCCCGCACTCGCGAGACGCAGCACAGCAGTAATGATAGCACCGTGCAACTGT
TGCGCTACTCCTCTCTTCGCTTCTGAAAAACGG
ACAATGAGTGTATTTGTTTCCTTTTGCCTGCGATGCGTGGCTTAGCGGACATTTTTGTGCTGGAGGAT
TGAATTTCTGCGCAGCTGCGTGGTGGCGAAGT
GGCCTGGAGGGAGGAGACGAGTCCCAGCACCGCATGCGCCCACGTGCGTCTTTTAATGTGGGCAAGGC
AATGGGAAGTCGCCGTTATCCAATGAGTGCGG
GGGTGTCCGTGACTGCTGTGCTGAGGGCGGGGACGAGGACCCTCGCCGCGGCGTTCCACGCAGACAGG
CCTCTCCTGTGTTTTATTATTATTCATTTTTG
CTGCGGGGGAATGGCAGGCGCTGAGAGTGGGCGGCAAAACTGCCCAGCCGTGACCTCTTTGCCCAGCT
TATTGGTGTGGGAGCCCGCCCGACGCGATCCT
CCTCGCCCCACGCAGGAGGCGAGCGAAAGACGCACTCGACACCTCCTTTTCGTCAGTGATGAATGGTT
GTGGGAGTGGGAAGTCACGCCGGGCTTGCCGT
GGGCCACGGAATGCATCCGTCGCAGCCATGGCGACAGCATGACGAGGGCACGAGGTGTTTACATTTGT
TTTTCCTTGACTTGTGCTGGAGGAAATCAAGT
CGTCGACTATTTTTTACTTCTCTCTCCTGTGCCCTCTCCTCACTCCGTGTGCGTGTCGCTGCGTAGAT
GGTGTGATGGTACTTTTGAGTGCCTGTGAGAA
GAATGTTATCGAGAGGGCTGGCTGCCCAATTTAATATAAGTTTTGGTAAAAAGACGAAAATATTTCTG
TGTGATCCCTTTCCCATCTTATGGTCCTTTGA
AAGGCCCCGATGGAAGGAGTTAAGGAACCGATAGCGGCAAGACGAATTTTATTTTTATTTTTGCCATC
CACCCTCTTTGATTCCGACCTTGCGGCGGAGT
CTTGTGGCTGGAGGGCCCAAAGTGTTggtaaagcatccaAATAAATGTCTCCAACCCGAATACACCGC
ACCGTGGCACGAGGGTGCCTATCCGAATATAT
AGATGTAGTTGGGACAAACATTGAGGATGTGGCGATGCCGCTGCCTCTCCATGGCCCCCTTTTTTCCC
GTCCTATTTTATTTGTGCGTATCAGTTCGGGA
TTCTGCCAGCATCCCTGCAGAACTTTTTTCTGTGCTTCGGCTTTATTTGGAGGCTTTGTTTATTTTTT
TTGTCTGTGTGTGCGCTGCGGGAATGATGTTT
ATTATTTTTTTTATTTCTTTTTTTGCCGTTGTGAAAGTGGTGTAAGTGGAATTTTTTTTTTTGTGATA
TATAGGTTTTTTTTTTGACCAAATGTCGCGCGTGC
CAATCAGCGAGGAGCAGGCGCAGCAGATGCTTATGAAGCAGCAGGCGCAGCAGGATCGAGTGGATGCG
ATGAATGAGCAGAGGGAGGGTCTTTTGCGTGC
GTTTGTGACGGCGGAGGGGCGCGAGCGGCTGAAGCGGATTGAACAGGTGAAGgNNNNNNNNNNNNNNN
NNNNNNNNNNNNNNNNNNNNNNNNNNNNNNNN
NNNNNNNNNNNNNNNNNNNNNNNNNNNNNNNNNNNNNNNNNNNNNNNNNNNNNNNNNNNNNNNNNNNN
NNNNNNNNNNNNNNNNNNNNNNNNNNNNNNNN
NNNNNNNNNNNNNNNNNNNNNNNNNNNNNNNNNNNNNNNNNNNNNNNNNNNNNNNNNNNNNNNNNNNN

NNNNNNNNNNNNNNNNNNNNNNNNNNNNNNNNN
NNNNNNNNNNNNNNNNNNNNNNNNNNNNNNNNNNNNNNNNNNNNNNNNNNNNNCGTGCGTTTGTGACG
GCGGAGGGGCGCGAGCGGCTGAAGCGGATTGA
ACAGGTGAAGGTGGAGCGGGCGCAGGCCGTTGAGAGTTACATCATCCAGTCGGTGCGCCAGGGGAAGC
TGCAGCCACCCGTAACGGATGAGACGGTACGT
GAGATTCTTTCGCAGTTGGCATCGCAGGGGGTGGAGGCGAAGAGCAGCATCACATTTGCGAGGAAGCG
AATGGATGACGACTGGTAGTTTCCGACGTGCG
AGTGTTGTGCTGAGATGCGAGGGAGGCGGCAAGCAGAACGAGGGAGCGGAGGAAGAAGTAAAGAACGA
TGAATAATTAAGAGGAAGGAAAAAAACAAAAA
AACAGAGATGTAGAGGGGTGTTGGAGGGAAATCTCAAAAGTGAGCGCGGATGCAAAACAGGAATGAAG
CGAGGGATACGGGGAGAACCGAGAAGTAACAT
GAGGGCAAAGAACCGTTTTTTTTTTTTTTTNNNNNNNNNNNNNNNNNNNNNNNNNNNNNNNNNNNNNN
NNNNNNNNNNNNNNNNNNNNNNNNNNNNNNNN
NNNNNNNNNNNNNNNNNNNNNNNNNNNNNNNNNNNNNNNNNNNNNNNNNNNNNNNNNNNNNNNNNNNN
NNNNNNNNNNNNNNNNNNNNNNNNNNNNNNNN
NNNNNNNNNNNNNNNNNNNNNNNNNNNNNNNNNNNNNNNNNNNNNNNNNNNNNNNNNNNNNNNNNNNN
NNNNNNNNNNNNNNNNNNNNNNNNNNNNNNNN
NNNNNNNNNNNNNNNNNNNNNNNNNNNNNNNNTTCGCGAGGGACATATTCACCACCTTTATTCTCAGCTT
GTTTTCTCCTTTGCAGGCGCATTTTTACATGC
GATTTGGTGATTATTGACGGTGGTTGTGTGTTGCGTGGGCTTATTTGTTATTTTCTGTTTTGTTTTTC
CTTGAGTAAACATCCTGCAGAACCGTCGGTGG
CCTCAGTAAGTGGGAGTGGGGCAGAGATGGGGGCTTTTTTCCCCCCCTTCTGTTGACTACTACGATGG
AATGTGCCTGTTTGTCTATGAGAGTTAGAAAG
AAAGGGTGAGAGGAGGAAAACATTTTTTCAGTTTGCATTAAAGCTTGTGTGCACTTCCCTTTTACTGT
TCTTTTATTATTATTATTATTATTATTTTTCA
GCGTTTTCAGTTTACATACACTGTTGCCTCCCGCTCTTATTTTTTCTCCCTTTACTTCTCTTGCTGTG
TGTGTNNNNNNNNNNNNNNNNNNNNNNNNNNN
NNNNNNNNNNNNNNNNNNNNNNNNNNNNNNNNNNNNNNNNNNNNNNNNNNNNNNNNNNNNNNNNNNNN
NNNNNNNNNNNNNNNNNNNNNNNNNNNNNNNN
NNNNNNNNNNNNNNNNNNNNNNNNNNNNNNNNNNNNNNNNNNNNNNNNNNNNNNNNNNNNNNNNNNNN
NNNNNNNNNNNNNNNNNNNNNNNNNNNNNNNN
NNNNNNNNNNNNNNNNNNNNNNNNNNNNNNNNNNNNNNNNNNNNNNNNNNNNNNNNNNNNNNNNNNNN
NNNNNGCCGGTGTTTGCTACGCCTTGTCAATC
ATATCGCAGGGGAAAAAAAGAAAGAAGGGTGACGCCGGGCGGGGGAAGTGCCGCCTTTTTTTTTTTTT
TAGGGCGATTAAGTGATGCCAAAGCGTCACCG
TGAGCCGGAGAATTTGTTGCCGTTTGCCGTTGAGGACTACGGCGACGGGGTGGCGGAGCAGGAGAGTT
GCGGCAGCGATGAGTTTCACAGCAGTGAGATG
GGGAACGATGGGAATGTCTCCTCCCCCGCCGGCACGCCGAAGGACGATGCGGACGACAGCTCTTCTGT
CATGATGGGCAGCAGCAACGACGACGGTGATG
GAGAAAACGCCAGTCTCATTAACGTGGAATTTGGCGTGTTTGAGATGCGGCGGGATGTGAATGCTGTC
ATGCACCTCATTGACCAACTATGCCCGGATAA
AATGAATGAGGTGGACCGTGACGAATTGGGTGCGGCGCTGCACGAAAGCCCCTTCACGTGTGTTGTGA
AGATCGGCGACGACGACGACGACGACGAAGGT
GAAGAGGAGGGGGAGGAGGAAATTTATGGCATGGCGAGTGTTTTGGACGCGGTGCACGACACCCGGCT
GGAATCGCTGCTTCATACGCTGCGAGAAAACG
TTTGGCGCACCGTCGCGCCGGGTATTCTCCCAACCGAAATTTTGAAGTCTATCGACGACGCGACAGGG
AGGGCGAAGTGTGTGCTGCTCGTTGGCGAGTA
CGTCCGAAATGTTCCGCTGGAGTTGACGTCTCAAATCCTCCATGACATGATGGAACGCGTTGAGACCG
CGTTTAACGCGCGGAGCTCCATGAGCAACAAG
GCCTGTGAAGACGCACCGTCGACGGTTCAGGCCATATTCCCGTGCATGTTTGCGATGCTGTCTAAGAT
ACAGCGGGCGGTGGATGCTCCTTTAAAGACAC
CAACACCAATGAACGCAAGACAGGATCCCCTGCCCAAGAAGAGGCGTGGACAGAACGCAGCGCAGCCG
GAAGAGTTTGACTTGTGCCGATATCTGTTTTG
GCGTGAGGAGGACTCAATTATTTACGAATACCGAGACAAGCGCATCGCCACTTTGGCCTACCGCTGTC

GCCCGCAGTACGACGGCCAGCCGGAGAACGAT
ATTCCAATTTCAATCTTGTATGTTGTGCCGTACGGCGGATTGATGCAAGCAGTTGCGGAGATCCGGAA
GCGCGAGTCGACACAAGTAAATGTTGTGCGGT
ACTGAATGCACCATGGTCCCTTTATACTTAGAAACACAGGGACAGGCAACAAGAAGAGGAAGACGAGG
AAAAAAAAAACAACAGCCGGATATAACGTGGA
ATGAAAGAGGAGGTGCGAAATAGCATCGCATCCTTGTCTGCATATTGACGGGGTTGGGTGCTGCATGC
CTTTGCTGCTTGGTGCGGTCAATTGGGGCTGC
TTTGTTTGCCCGATTAAAATAATGACGAAATGAGCATCATAGAGGAATGGAAAGCAATGGTCACATAA
AATGCAACAACGAAAGATGCCTTTGAAGGGAA
GGGTGTGAGTTTGGCGTGATTGACGTTACAATTCCACTTCTGAGGAGAAATGACGGGAAGGCGCGCGG
GTCGTTGGTGGTCGCATTTGTTTTTTTTCTTT
TCTTGTATTGATTGTTTTCCTCTGACCTTTTTTTGATTCTTTTTTCTTTTTCCTTTGTTTTGCCAAAG
AAATCCAAAGGGGTTTTAAGTGTAGTGTTCGA
CAGCAGCGGCGCTGTGGGGACGGTGAAGGCACACGCACAAATATTTCCTCCCACCTCCGCGCGGCCAC
GCTGTCGCTGCGGTTTAAATGACAGCTCCGAG
TGAGTTGTCCGGCGAGGCACTCGTGTTTTATGTGATGCGCACGAACGGCAATCCGGTGCGGCTTTTTG
GCGCAGACGGTGTGACGGCAGAGTCGGCTTTG
AAGCGGGTGTACAAGGCGCTGGCGCTGCGTCTTCACCCTGACAAGAACGGACACCCCCGTGCACATGA
GGCTTTTCAGCTCCTGCAGCAAAGCTTCGAGG
CCACGCTGGAGTCGCTCCAATCAAAGGCACCAGAAGAGAAAAAAACGCGACCAAATTTCCCACCACCG
TGCCCTTCTTCTTCTCCACCGCCACCGCCACC
GCCACCGCCACCGCCATCTTTTGCTTTTGCCAAGTCTACACTCCCTGGAGTTCGTAAGGAAGAACAAA
AACAACGGCAACATGCCTTTGCAGGAAATAGT
TTGTTTGAGATACCGTTACCCCCTGATGTTTTTGGCGCATTTGCGGCGCCGGATAGCTCTGAGCCTCC
CGTTGCGTCGGTGTCGACGGGTTGCCCCGGGA
CGAAGCCGCCGGCAAAAAGAACGAGGCCACTCCCTGCTCTGGATCTTTCTAGTGACGATGAGGAACAC
GTGGACGAGGCCGGAGGTCTTCAGGCATCACG
GCGAGATGATGAACAGGAGCATGCAAGCCAAAAGCGAAACGCTGAACGGAAGGCGGTGCAGAAGGAAA
AACGGAGTACGGGCCGGCGAACTCTAATGCAG
CTTTTTGCAGAGTTCAATATCTCAGACGACGAGGAGGAGATGGCGCCTGTTAACCACCCGACAGGAAA
AGAGACAGGCCATCATTGGGAACCAACTTCAT
GCCACGAGACAAATCGGCGGCAGCAATCTGGCGCTGGGAAACCCGCTACCACTCTGGCATCCTCGAGT
GTAGCTTGCCCTTGCTGTGGTGCAGGCGAGTT
CTCTGCTCATATTATGCGGGGTGTGGTGTGCCAGTCCTGTCGCGCACAGTTTGTGCCGGCCGCTATAG
GTATGATGGCTGCTGAGCGTCATGCAGCGGGG
CGTAAGAAGGACCGCGCATCACGCGGCGAGTTGTGTGCTTGTGGGAAGGCGAAAAAAGGGTTATGCTT
TCTCTGTGACTGATGAGCACGCATTGCAGTTG
TGTCTGCTCCGCGGCAGATGCCGTGGGTCCTTTTTTTTTTTTGTGTGTGTGTGTGTGTGTTGAGTCGA
ATGTGGGGGAGGAAGAGAGGGGGCGATGCTAG
TTGAAATTTGGCACCCTTCTGAGTGATCCAGTTTTCTTTCTTGTTGTATATATATATGTATATATATA
TATACGTGTAGCATTACGTGTTTTCTTTTGCT
TGTTTCCAATTGGTGATAGTTGTTCCTCTTATAACCGCGAGAAGTGCATTTTTTTTCTTTCGATTTCC
CCTATCCTTTTTTTTTTGCTTCTTTTTTCGAG
AAGCGCTCAAGGTGTCAGGAGTGTAGTGAAGGAGCGGGTGTCGGTGGATGGGGCCCATACGCGTTGAA
CGGGCCCCTGTTGTGGGTCGTGAGTATAGCGT
GATTTGCCCGGTATGCAGCAACAATGTCACCTTTGACTGTCTCAGCAGGTCCGTGTACGGCATTCAGT
GCCATTTCTGCCGCACTTTGATTGACTTGCGT
AACCCGTCTGTGCCTCTCCCTCCAAACGACACGGGTGTACTCGCCGAGGACGTCAACGGTGGCTTTTT
TTTTTCACCCGAGGTGGAGGAGTCGGGTAGAG
GCTCCGCCACGACGCAGCTCTCCATGGTGCTTGCAGATTTTGCAGAGCAGCAACAGAAAGAAATGCCA
ACGACCATGGGCGGGGAAGCAAGACAGGCATT
TTCAGCACGCCGCATACAAGCTCTTTCCGAGAGGTTTTCGTTTCTTTACCCTTTGGTGTACGTGATTG
AGTACATTGTGTTTGGATCCAAGCCGGTTGCA
AGAATAGGCCACGTGCGGATATTTCGTAAGCGGCTACTGTTTTTTTGGCCGGTGAATATTCCCGTAAC

AAAAACCTCCGTCTCATATTCTGCCCCACTCA
CCGTTGCCATTCTTCTTTACTTGATGACCACATTTTACACCAAATCACTTGATGACGAATGGATGTGG
TGCATGCACCGCTTCGTGACTTTTTTTTTGGT
GTTATTCATGGTTCTGTTGGTGTGTTGTGTGTACACGGACCCCGGCTTTGTGCGTCCTGCGTACCTGG
AGACCAACGGCAGTCATATGGGTGAGTTAACT
CTGAAAGAAATTGAGGCAAAACAACGGGAGAGTCGGTGGGAGATGGTGAACGGACAACCAGAGGAGCG
CAAGTGGTGCTCGACGTGTGAAATTTATCGGC
CTGTGAGGGCGGCGCACTGTTACCTCTGCGGCTTGTGTGTCCATGACCACGATCATCACTGCTCCGTG
ATCGGTGTATGCGTGGGGCGTCGGAACGTACG
CGTCTTTTTTTTCTTTGTTATTACTACTGCTCTCTTGGCGCTGCTTCCCGGATTGTCTCTGCTGGTGA
AGCTTTTTCTCGATGCAAACTCCCTCACCGCG
CGGCAGCTTGGCATTGGTTTGCTGTTGATCGCGGTGTTGTTGGGTTTTGGTCTTGTGGTGGGATTTCT
CGGCCTAACGATGTTGTATTCCCTCGCGGTGG
AGACGACAACACGTGAGCGCCTGCAAAATACCTACGCGAACAAAAAGAACCCTTTTAATCGTGGTTTA
CTGCGGAATATCTTCTGGCATATGTGTCATCG
CAGTCTACCTTCTCTCTTTGATGATGACTTTGTGCAGTTGTGTGCCACGCAGGTGTAGGGGGCCACAG
GTATCGGCCATGTGTAAGGACCACACAAAGTT
ATCTGACTCTATTTTCCTCTTTTTTTTTTCCTTTTTGTTGTTTGTTTTTTCCGGATCTCTCTGTGCGT
GTATTTCTATCTTTTTCCTTGAGAAGACGAAT
GAATGGCCCATAAATATACACGACGAGTGATGTTTTTTTGAAAGCCTTTCTTTTTCCTTCTCTTTTTC
TTTCTAATTTTTTTTTTTGTGTGTCTTTTATTA
GAAACCGTAGGACCAAAACCCCTCGTGGTGGTGAGGTTTTGGGGGAAATTGCACGGGAATCCAGCCAA
CAACTCAGAGGGGAGAAAGAAAAAAGAAGAAA
GAGGAGGAGGAGAAGCAAAGCCGTAGGTGCACGTGGGCTGTGCGATGGACGGTGACAAGGAGCAGAGG
AACAAGAGCCACAAGGGCCGGCAGGCTGGTAG
CAAGGCTGCCAAAAAGGAGGAATTTCGCAAGAAGAAGGCAGGCATCGAGCTTGAGCCCAACCGTGGCA
AAAACAGGAAGGCATTTCAGGGTCCAACGGCA
GGAAGTCGCAAGGGACAAAAACTTCTTCGAAGTCTTGAAAAACGCGAGACGGCGCTGCATGTACCCAC
AGTGGATAAGACTCTTCGACACATTGTGGCGG
AGCCCCCACTGCTCGTTGCTGTGGTGGGACCACCAGGTGTTGGCAAGTCAACATTGATCCGCTCCATG
GTGAAGTTCTACAGCAACCGCAATGTGCAGAC
AGTGCGGGGACCCATCACGGTGGTGGCGGGTCGTAGTCGTCGTGTGACATTCATCGAGTGTCCGAACA
CACTGACGGCGATGTGCGATATCGCCAAGGTG
GCGGATCTCATTTTGCTTATGGTTGATGGCAGTTTTGGCTTTGAGATGGAGACGTTTGAGTTTCTCAA
TATTTCTCAGGTCCACGGGTTTCCCCGTATGT
TTGGTGTTGTTTCTCATCTTGATCAGCTTAGGACTGGCAAGGCACTGAAGAAGCGAAAGAAGTTTCTT
CGACATCGTTTTTGGCATGAAGTGGCAGCTGG
GGCAAAGCTGATGTGTTTAGCACCCATGGTGCGTGGCATGTATCGTTCCACTGACGTTCTCAAGCTGC
ATCGACTCCTCATTTGCGTGGAGCCTAAGTTA
CAGAGCTGGCGGAATACCCACAGTTGTGTGCTGATTGATCGCTATGAGGACATTACGGCCCCACAGAA
CATTGTGGAGGATGAGAACTGCAACCGCACCA
TTGCCTTCTACGGGTACACACGTGGAAAGCCGTTGAAACCGCAACAAGTTGTGCACATTCCTGGATTG
GCAGATTTTCCTATTGCTCACATTTCTCAACA
AGAGGACCCCTGTGCCATTGATGATCACCAAGGCAGTGGCAATTCACAGGGGCACAAGATGCGGCATC
TCAGCATGAAACAAAAACGTATTTATGCCCCT
CACTGCAATGTGAGCGGTGTGATGTACGATGACGACGCTATATACATCCAGGAGGACGCAGAGAAGGG
GATGATTGAGCGCTCCGGTGAGGGACTGCAGT
ACATAAGAGAACTGCAGCGTGCGAAGCCTATGGACGCCGCCGCCACGGATTTGGACGTCGTCCGTCGT
CCTGTAGTATTTCGCGACGAGGATGCGATGGA
GCTTGATATGGATGAGGTGGAGGCGCCTTCAGATGAGGAAGACGAAGGCAGCAGCAGCAGTAGCAGCA
GCAGAAACGATGACTTGGAGGATCGTATGCCT
AGAAATGACTTGTCTCTTTACGGTCCACAAGAAGTCGACGAATGGGAGGATCAGGAGGCCGCGGCGGG
GGATGAAGGTCTCGCACCACGTGGTCGCAATG
CGCCTGACGCCATCCGGGAGAATGTCGTGCGTCATGACTGGTCTGAGACCGCATTGCTTCGTCGGCTA

AAGAACCTCTGCGTCACGGGAGACTGGAATTG
TTCGGCCGATGCAGCAGAGACCAGCGGAGAGGACCGTCTGGGGAGTGAGGCGAGCAAAGGGTATGAGA
GCGAGGACGAGGAGTTCGACGCCATAGTCCGC
GGCGAGAGGCCACTTACCACCACCTCCGCCCGTGCTCAGTCGGATGATGACCACGAAGACCAATTCAA
TGGCGACACGGAGGGGGAGGAAAGGAGGGAAA
AGGAGAGGCATGGACGACAGCGAGTAGACGCACTGGCTGCAGTGGGCAATGAGTTCTCATCGTCGTCA
ACATGGGGTGCATTCCGGCCTGCGGATACAAG
ACATCATCGTGGCGACTGGGGGGAGAAGAAAAGGGGGCACGATGCGTATGATGATGGCATTGACGGCA
GTGACTACGACATTCTCGCCGAGGATGACGCG
GCGGTGTGCAAATCCGCCTTTGCAGACGCCGAACTGGACGATCTCGTGTCGACTTTTTTAGCGCCGCG
ACAGGCAACAACAAAAGGAACCACCACAACAA
TGGCGGCTGCTTCTGTTTCCGGAGCGGTCTCTTCTCTGCGCAACGACGGCGACGAAGATGCCATCCAG
TTTTACGACCCACACCATAGTCGCGTTGGCGA
GGATAATGGACGGAATAACGGCGACGGCGATAACAATGATAACAGTAATAATAACGACGATTTGGAGG
AGGCAGCGGCTATTGTGAGTGGTAAAACACTG
ACAGAAGAACAGGAGCGTCGTCTGCAGAAGAAGATGGCGAAGAAGAAGGCATTTGATGAGTCCTACGA
CATGGGCGACAGGAGCAACACATACGCGCACT
ACCACCAACTTACCCGTGTGGTTGAGGAGAAGAAACAAAGACTTGATGCGGCTCTGCAGGAAATGGGC
GAGGACGTGGCGAAAAAAAATTCAACTCGTCGG
GTACTTCAGTGGATTGTACGTTCGGTTTGTGCTTGAGAACGTCCCAGTGGAGTTTGTGCGGCTCTTCG
ACCCGACGATACCGCTGATCGCCGGCGGCGTC
AATGCAGGGGAGGACCAGTTTCAGATTGTACACGCAAGGCTGAAGCGTCACCGCTGGTACCCAAAGAT
TCTTAAGGCCCAAGACCCGCTGTTGTTATCCA
TGGGATGGCGCCGCTTTCAGACGCAGCCGATCTTCGCTACAGAGGACCCGAATGGTCGCGTGCGGTAC
TTGAAGTACACACCGCAGCATATGCACTGCAT
CGCTGCCTTCTATGCACCGGTGGTACCGACAAACACGGGATTTATTGCCATTCCTGTCAAGGAGCAAC
GATCTCCCAATTTCCGTGTTTCTTGCACAGGA
TACACGCTTGGAAACGATCATGCTACAAACATTGTGAAGAAGCTCAAACTGACTGGAACACCGCATAA
AATAATGAAAACCACAGTGTTTGTTAGGGGGA
TGTTTAACAGCGACATGGAGGCAACCAAGTTTGTGGGCGCGAAACTGAAGTCGGTCTCTGGCATTCGT
GGCATCGTGAAGGCGGCTATCAAGGGGAAAGA
CGGATTGATACGCGCCACCTTTGAGGACAAACTGCTCCCCTCCGACATTGTCTTCTGTAGAGCATGGA
AAACGGTACATCCGCCGAAGTATTGTTCCATG
CAGCGCAACCTTGTGGACGCGAACTGGATGGGAATGCGCAATATGCGGGAGCTGCGCTGGGAGCACAA
CGCCCCACTTGTCACAAAAGGTGATTCGGAGT
ACAAGGAGATCAAACGCCGCCAGAGGGACGACGAGGACTATGCGGCAGCGGATGCCACCAAGGTGCTT
CTCTCCCGCAATCAAAAACTGCAACTTCCGTT
TAATATGAAGGAGGAGTTTATTCCGCTtGAGCGAACCACGGCGCTGCAGCAGCGCCTTGCCGGTGCCG
TTACTGTTGCGCCGGAGCCGCGAGACATGCGC
CGCACCGCACTGCTGGATGTCTTTGCGAGTAAAGCGGACGCGATGCTGAAAAAGAAGGCGGAAGCAAA
GAAGAGAACGCGGCTGCGGCACCAGCGCGAGT
CAACCGCCGAGGAGGAGGAATACTTGCGGCAACTCAAGAAGGCGAAGAAGGAGACCGCACGCCTTCGT
GAGTTCCGCTCGCAGCATAAGACACGCAAGTA
AAAGGGAAAGCAACGAAGACAATGAGAGAAGGGGAAGAAAAGCAAAAACGACAGGGAAGAAGGAAAAA
GAAAGAAATGTGGAGGGGAAGGCCATTAATGA
CGTGTTTTCCTTTACTCTCGGTGTCGGCAGTCAAGTTAACCGTTTAAATCATCAGGGGAAAAGGGGGG
CAAAAGACAGGGAGGCTGTTGAAGTGGTGAGT
CTACACTATGCATGGCGAACCAACGTTTTTCCCCCTTCCCCCCCAACTAACTCCTTTAGGGCTTAAGG
AGACCGTAGCATGCTGGAGAGGAATATTGCCT
CCCAGGATCTTTTTTAAATATCCCTTTTCCATACTCACACACACACACACATGCATGTAACATATGTC
GATTAATTTTTTAGATGTTTTCTTTTTTTTTT
TTTTTCCTCCTTTATGTTCCTATGAATGAGGCGTAGACTATTCAAGCAACGTTTCACGAAACTGGGTG
TAGGCATGGGGGTTGTTCAAGAGATAAAAAGA
AAAAACAAAAACAAAAACAGAACGGAACAGGAAAGGGAAGAGAGGATAACATTATCGTGCAATTAAGT

TGCCAAGTATTTAATTATTTGATTCACTCATT
GTTTTACTGGCTAAATGTATGGTAGGTCGGTGGCCCGCGGCTTCCGGCACGCCTATTGGCTGCTATAC
CGCATGAATTATTTTTTACGGAAATTTCTATT
TGCTCTTGTTGCGGTTTTACTTCTTGTGATTGTTATTATCCTCCCACCTGCAAGCAGCACGTTACGTG
GGCGCTTTCTCTTTTATCATATGGAAGAATTG
CGTCAAAAAGCAGGGGACAAAATGCCGGAAAAACTTTTTCTTGACGTGACGACTGGAACATGGGATGA
TGTCACTTGCGGCTTGGTGAAGGCTCCAGCTG
GAGAGAATTATGTGGAGTTGAATGTGTTTGTTGTTTATGACAGAAAACTGCGTAAGACGTTATATGGG
GGGGTTAATTGGGGTCTTCATTGCTACCGTCC
TCTCCAAACTATTTGTCACCTTTTGAGAAAACAACGAGGGAAGTGATGAACGACCATAGGACGTACCG
CAACGCAGGGACTGAAGGCAGGTACAATGGGA
ACCGTATTTACTTTCATGCCACGAAGGAGCCTCTGGTGAAGTCTTACCCGCCAAATAAGGTGGACGGC
CGGCTGTTGCGTGAATGGGAAATGCCTGGATT
TGTTGCCAGTAGGAGTTTCCTGCAACCCTACAGCGCTATTATGACCGTCTACAGGGGGAGAGTGCTCG
GCCATTCTCTTGACAGCGGCACTCTCACCGGT
GTGGCCGCAGGGTTTTCCTCAACATCTGCAACGGAGAAAAGCCGAGAATGGGTTGACTTTTTGGAACA
TGCTGTGCAAATTGACCAGCCGCTGTTGTGTC
GTATACGTCAGCGTATTCTTCGGCATTCGTTTGGCTTGCGCCGCTGTTGCGTCTTTTACGGAAATTTG
CACAGCACGTCGCCTTCGCTCAATAATACCAT
GGGCCGTCGACTAATGTATGAGTAAAACGCATTCTTTCAAACATCGTTTACCCTGCGGGATCTACCAC
CCGTGTTTGTGACGCGGGCTATTTTGGTGCCA
TCGGTGCTGCTACACCACACTGTGCCATTTCTGGAGCGAGTTGCGGAGCTACTGACGGTCAATCGCAC
AGTTCTTCAAAGCGGCGGCTAAACCGCCGCTG
TCATGACGGGCCAGGAGAGCGGTTGGGGCATCAACAACACTCCGGAGTGGTCGTTGAAAGGGATGGAA
GACGCCTTGGCTTTCAGCTTTGGGCTAGAACG
AAACTACGTGTATGTAGAGATGCCCATCCGCCGTTTCACAGAGCCTGAGTTGGGGGAGTTGGGCTGAA
AATAAAAGAAAAAAGGGGGAATGGGGGAGTTT
ATTTTTTCCCCTTCTCTTTCTCCCTCCTTATGCCTGCAATGAGTTTGTCCGTCCGTGCGTCATTTTTA
TTCCTTAAATTTTTTATTTTATCTTTGCTTTG
GCTTTGATGTGCCGAAAAGGAAAACAAGAACAAAAATGAAGGGTTGTTTTCCTTTTTGCATTTGCCTT
TCCTCTCTCCCCACCCTAACCCTTTTTTTTTC
TTTTGTTTCCTTATGTCCAACTGACGGACGTTGGGGGGAGTCCATCGTTTTTCTGTCCACTGCACGTT
TTTCTCTGAATCACCTCTGCGCATGATTATCA
ACTACACAAAGGGAGAAGCACGCGTTCAACGGGGAGAAGGGTCAGCTGCGGTGCAGAGAAGAGTGGAG
TTGGATGCAGCACGCCTCTCTCGGGCCACACC
CGCCCACATCCCTGCTGTTATTTCTGCAGTCTGCTCCACCGCAGGAGACATTTGAGGCATTCAAAGAT
GACTGTAAGTTGTTTGGCATGCCGTGGTACTG
TCTCCAGGCGCAGCAATTATGTGTTGTGTTGTTGGTGCGGCATCCGCTTGTGCAAAAATACCCTCCAC
GCAAGTCGAATGTCCGTGCATTCCTGAAACAT
TTTATCACTCAGCTGGAGCTAACATGGGCCACACCACATGTGAAGGAGGCGCTAAACGATGAGGTCAT
CGATTCTCAGCTCATTGAGGCATATATATGTT
ACTCTCTGAGAGCCGATAGCGATTCTCAGACCAGCATGTGCTACAAGACATTTTACGCTCCTGGAGCA
CGGGAAACATTTCTTTCCGTTCGTTTAGCCGC
CGAGCAATTTGCAAATGTGGGCCTTGCGCTTTGGCCAGCGGCGTTTGTACTGGTGCAACTTCTCTTGG
CGGAATGCTCCAGTCCCACACCTGTTATTTTG
CCACATGAAGGTGCTTTGCGAATTTTGGAACTTGGCGCTGGCGTTGGATTAACGCCAGTGATGCTTCA
TCAATACTCATGCTACAAGAAGAGGGTCCAAG
GAAGCGTTCTCACCGATTATCAGCCGGAGTTGGTTGAAAACATTCTCTTTAACCTCCGTTCCCATATC
ATGAGTGGAGAGCGGCCACATGAGTCTCTGTC
CGACACGGAAGAGACGGGTGCGCCACATGTTGCTGAACTCCTTGACTGGACGGAGCACGAGCGAAACC
GGGAAAAGTTGCGGGAATGGGGATGCAATGTC
ATTCTGGCTGCAGACTGCATCTATGAAGTGGATTTAATTGATTCTTTTGTGACGACACTTCACCAGGC
TTTAAGCGTCTCTGAGGCGGCTGTCGCCATCG
TCGTACAGACACATCGTCAGAATAAAACGATGAAATGTTTCTTTGACGCCGTTCGAAAAGCGGCGATG

TGTGTTTCCTCCTACCGGCTTGCCAAGAGCAG
TAGCCAGCCGTCGGAGAACCTCATGCTTCTTCACAGTTCGCTCTGCCAGGATGATGCCATCGCTGGCT
CTGCCGTGGTTTGTGATGAGATTGACGCCGAT
GGCAGTTTTAAAGGGTGTCAGGCGGGGAGCTGCGTACGTGGATGGGTGGGTCCTTTTTTTCTGCACTC
TGAGGCGGCGGTGGGCATTCACGTGCTTCGAC
TCGGTTGACAATCAACACGCGTCATCTTTCGTGTGCCCCGCCGTTGCTTCTGGTGGTTGTTCTCCAAG
ACTTTCTGGCCCTGACTTCATTGGGGAAAGCC
GAACACACACGCACACACACATACATACGCATGCGGCTTTATTGGAGAGGGATCTTCTTAAGACATTT
CTCCTGACGCTCCAGCAGAGTTCTACCTCTAC
CAATGGGAGGGATGAAGGATTCGTATCCCTTCCTCTTTCTCTTTCTCTTTCTTTTTTTTTTTTTTTTG
TGTTTTTCTGTGTTTGCAAACTGCGTATTGTA
TAATAAACTCACAAAATTTCTTTATTCTCTGTCTGGCTGTGGGTTCTGCAGGCACCACTAATAGAGTG
TGGCGGGCATAACAGACACTAGAATTAAAGCA
GGCCACAGAGAAATGGTGTTTTCCATCCCCTCCTTCCTGGGCCTTGGTGACCGGTACAAGCATGCGGT
GGATGAGCGGGCGCAGGGGGTCAATTTTGTTA
CCTGCTACCCAAAGACAGGCCACAACCCCGACGCGCTCTTTGACAAGGAGTTCAAGTACATGTACAAC
GGCGACCGCCGCTCGGACCCGGAGACTATGGC
TCGTCGGGAACAAATGGAAAAACGAAAGAAAGATATGACAGCCACTGGCTTCGCATACGTTGGCCCAC
CGCAGAAAGGGGAAGGCCTTGGGTCGTACTAT
GGCTTGATTCAGCGGGAACCGTACCCCCATATTCCCGACAATCCCATGCCGCGTGGACCGCAGCCCTT
TCCCAAGTCAATGCCGCGTCAAATATTGACAT
CTCCTGGCAAATTGGGCAGTTACGGGACACCGGGATTGGCCCTGAGCGAGATTGGCAACGAGTACATC
GCGACCATCTACGATCAGCCTCGTATTAACGC
AAAGAAGGAACGCGATGCGTGGCGCCGTCGTATGCCTGCGGTTCCATTCATACCTGTTGGACGTCGTG
GCTACACTTTCGATGAATCCCCGTTCGCTGGG
GTCTCCACGTGTTACGTGATGACCAAACCGTTCATGCCCAAACTGGTGCAGCCCTCATTCAAGCATTT
TGTAGTAGACAAGCCGTGGCGGCCTGCCGGAT
ACGTAGAGGACAAACCCACCGCCATGGATTACCGGGAGGATCCGTATAACGCATTTGATCCCAGGGAG
GATCCAAAGTCGCGTGTGAAGAAGCCAAGCGA
CGCCGTTTTTAGGACGGGCTATCGCTGTGACAACTTTTGTTACACACAGAGTATCGTCTTTAGGCGTC
TTTAAGCACTAGGCAGTGTGTGTGTGTGTGTG
TGCGTGTGTTTGTATGAGAGCAACTCCTATATATGTATACATGTACACTGTATTGGGGAGAGTAAAAA
GAAAACTGGATTGTGACCAGCGCCAACAACAG
CAACCAAGGAAAAAAAAAAAAAGAGAAAAGGCTTTTACTTAAAATTGACGGACGGTTGAATTTTGCTT
TGTGGGGAATTTTAATCATACTTTAGAGGTGT
TAGCCGTCACCGATGCCGACATTTTTATTTTTATTTTTATTTGCAGAGGGATGGGTGTGACATCCCAT
AACACACGCAGGCACACCGAGAAGGAGAAACA
CGGCATAAATCATTGTCCATCTATGTAGAGAGATCACTCTTTTTGTTCCGTTTCCCTTTTCAAAACTT
CCATGTAACTATCTGTAAGTAGAACCTTTCCA
ACTGCTGCTTGCATGTTAGTCCCACGGTGACTGGGGATGGACTTGCCGATGCACGCTTGTTGCTTTGA
CGGTATTTGTGTTTACTTTTTGTGAAAATGTC
TTATTTTCCTTTTCTTTTTCTTTTTTTTTTTTTGCTTTTCTCGTGAACGGATGGGAGAAGTTGTTTGA
GGGGCGAGCACACCCCTCCAGAGACAGAAGAT
AGAAAGAGGGCGCCACTCGGTGAAATTTTATTTGCTAGAGCGGGAGTGAGGAGAGGTGTCGTCACGGC
GTAGATATAAAGAGAAGAAGCGAATCTCCTCC
CGTCGTTTTTTTTTTGTTGTTGCCGTTGTAGGCGGGGCTCGTGTGTGTATTTTCGCCTTGACGCATCG
CACATGCAGCAACGTGCCAAGAAGCGGCAACG
GCAGAGTGATGAGAGCAATACCCGACCTGCACACAACCGCAACGATGGCCAACCTCCTGGGCGTCGTT
TACCACTGACCAGCAGTGTTGCCTCGGTCACT
CCTGGCGGGGATGCCTTCCCCTCCTCCTCTTTGCATGCACCGCTGACGGCGATAGATTTGGTTTGGCA
ACGCAAAACGGCGGCGGTGCAGAAGCAACTTC
TTCAACTTGCCAATCTGTACGCACAGAAGGCACAGGAGAACGAGGCGGTGCTGGATGCCATTGAAAGG
ATGATAGCCTCACATGAGTTGGAGCTTGCGGC
GGTGGCGGCAGAGAGTGAGTCCCACACATGTCAACATCTTGAATTAATTGCGGAGCTGCATCGTCGGG

AAGATGCAACGCTTGCAGAAAAACAAAGTGCC
ATGGATGAGATAAAAACGTTACAGGAGCGACACGTGCAATTAGAGCAGGCACGAGAACGTTTGCAGGA
GGACATTGTTATCCAATCGGACGCCTTGGAGC
AGTTGCAGAAGACGCTGGAGGCAGAGCGCCAAGTGACAGACGAAGAGCAGAGAAAACTGAATGATGTG
CTTGAGAGAGAGCGGGTTATGCAAGATGCCAC
CTACGGTCTCTCCGGGGAGGTGAGGGAAATGGTAGCAGAGATGGAGCAGATACGCAAGGCGAAGGAGG
CTGCTGAACGTGCATCACGGGAAACCGAGATA
GTCCGACGACAGTTGTACTCGCAGTGTGAGGAGCTGAAGGGGACCATTCGTGTGTACTGTCGGGTAAA
GGGCAGTGGCAACAACACCAACGGTGAGGTTT
TGCTCGTTCCTGAGAGCTCTCGTGAGGCGTCCATTTCTTCGCACAGTACAATTAGCCTCTCCACGGAG
GGGCTTCACAACCTCGCCGCAGCAGGAGGAAG
AGGTGCAGAGGGCAAAAGACAAAAAATACACGGAGGGCAACAAAAGACACTGCATGGTCGATTTGAGT
TTCCCCAGGCCGAGGATGGGGTTCAGAGGACG
TTGTGTGTGTGCCAATCGCGGAACAACGCGACATCCACAGGTACGCAGGAGTCCAAGGAGACATTCAC
GTACGATTGTGTCTTTGATGGCAACGCTACAC
AAGAGGCTGTTTATGCCAACGTGGAGCCGCTTGTCAACTGTGCGGTTGATGGCTATCGTGTCTGCATT
TTTGCGTACGGCCAGACGGGATCGGGTAAAAC
ATACTCCATGGAAGGAGAAGTGTTGGATGAACAGAAATGTGGCATCACTCCTCGCGCCCTTCGTACCA
TATTCCGTCGCCAGGAAGAGTTAAGCTCCGAC
GGCTGGAAGTACAGCCTCAGTTGTTACTTCATTGAAATATACAACGATGTCATTCGGGACCTGCAGCA
GGAGCCGTCCGTGTATGAACCCGGTGGAGCCG
CCGCCTCTCAGCCGAACTATCACGTTATCAAGCATCAGAACGAGTCGGGGTCAACGAGTATCACCAAC
GTTGTAGAGAGGCGTATTCGCTCCTTTGAGGA
CTTTCAGCGCCACTACAAGACGGCAATGAAGAATCGTAGCACCGCTAAAACGCTGCTGAACGACCATT
CATCTCGGTCGCATTGTGTCTTTGTCCTGCGT
ATTGAGGGAGAAAATGCACTCATACGGCAGCGAAGTGAGGGGACGCTCTGTCTGGTGGATTTGGCGGG
TAGCGAGCGAATGAGCGAGTCGGGGACACGGC
AAGGGCAGCATCTCAAGGAGACCATCAACATCAACCGCAGTCTGTTGGATCTTGGGAAGTGCATCAGC
GCCCTTAACAACTCCGGCAGCGTCGCACCGTG
GCGCAACGGCAAGCTCACATACCTGCTGCAGAATTACCTCGGGGCAAAGGGCGGGAAGATGCTCATGT
TGGTCACTGTCTCAGATAAGGAGGAACACATG
GCGGAAAGCCTGAACTCACTCCGCTTTGCGAGTCGCGTCAACCAAACCGTTGTTGGCCCCTCCGTGAG
GCGCGTAACCGACTACTGAGCGACTCACAGAA
AATTGGGGAATTAAAATCCAGAAGTGCCTGCAGTCAACACCCACACCTTTTCCCTTTCTTCTTATGGC
TTTTTTGTACTTTTATGTCGTCAGAAAGAAGA
GGGGGAAAGAAGGAAATTCAAGGCTAGAATAGATTGCGTGAAAATCTCCCTCTTTGCGTCCTTATGAA
CGCATTTTGTCATTTTTGTTTGCTCATGTCTG
TGTCTTTCTGTGTGTATATATTTGACGGGTGCCACGGTCTTCCAGAGCAAACTTTGAGGCTTACTCTC
TTCTTTCTTTCTTTCTTTTTTTCTTCTCCCAC
TATTTTAAAGCAATCGACGTCACACAGACTTGACACACATTGTGAGATATACACATATATATATATAT
ATATATATATAACAGAGGCATAAAGAAAAAGA
GAGGGAGCGAGAAGGCACGGCGTGAGGAACACGTCATGCAACACCCGAGCAATCAGCAGATTCAGCCC
ATTGACTCATTGACGCCATTCTTGGGCGGTAA
GTGGTGGATACGGGCCCGAGTAACGGACAAGTCGGATGTGCGCACGTGGAATAAACCGACGTCGCAGG
GAAAACTGTTTTCCTTTACGCTCATCGACGAA
TCTGCATCTATCCGCGCGACGGTCTTTAACGACGCCGTTGACCTGTTCAACCCGCTCATTGTGAACGG
CCAGGTGTATTACCTCAGCGGTGGCCAAGTGA
AAAACGCAAATCGCAAGTTCAGCAACGTGAACAACGACTATGAGCTGACGTTTGATAGCTCGAGTCAG
ATTTCTTTGGCGCGGGATCCCAGCAGCGCGAC
AATTCCACTGCAGCGGTACAACTTTGTGCCTATCGCCATTCTCAAGCAGCGGGAGGTGGGGTCGCTTG
TGGATGTATTGGCGGTGGTGCTGTCGGTGGAG
GAACTGAGCTCCATCACGCAGAAGTCCACTGGAAGGGAGTTGATCAAACGGAACGTGAAGGTGGGAGA
CATCACAGCCGCCGTTGAGGTGACGCTATGGA
ACGACCAGGCGAAGGAGTGGGCATATTCTCCGGGTACTGTGTTGGCAATGCGCCAGCTGAAGGTGGGC

AGCTTTGACGGCGTAACGCTCTCTGCAACATT
TCAATCCGCATTCGACGTGAATCCAAACATTCCTGACGTAAAAAAACTTGCTGAGTGGTACATCTCCA
CCGGCGGACGCGATGTTTCTTCGCTCTCTTCC
TTGGGTGTGGGGGCTGGCAACAATGCAGGCGGGGAGAACTACCGTGGTCGGAAGTATTTTGACGACAT
TTCCGCGGAGGGACTCGGCCGTGGCCCTAAGG
CAGACTTCATTGATGTCCGTTGTGTGCCGGTGTATCTGAAGCAGGACGCGCAGTGGTACGACGCCTGC
CCACAATGCAACAAGAAGGTGACATTGGAAGG
AGCTCAGGGTGAACGCTACCGCTGTGAAAAGTGTGACCGGCAGGTGGTTCCCACGCAGCGTTATCTTG
TCTCCATTCAGGCATCCGATAATGTTTCCCAG
AAGTGGCTTACCCTCTTTAATGAGGCGGGCGTGGAGTTCTTTGGCATGCCAGCCACCGAGCTCAAGAA
GCAGCAGGAGGCGGACCCCATGTTCGTCACAA
AAATTGCGCAGTTGCGGATGAATCGCCCGGTGCAAATGCGACTGCGGGTAAAGGAGGAGAGCTCTTCC
AGCGCAGCTGGTGGCGAAGAGTCTGACCGTGT
GCGTCTTACGGTCATGCGTCTGAACGAATTTATGCCGCTTGATACCGTTTCCGAGGAAAAGCGCCAGG
CGATGGCAGCCCAACTGAAGACAGAGTGTGAA
GAAATGGTCCGGTGCATAGAGGCCTATTTGTAACGTCCCTGTGGGTTTTGTAACGGGGGGATTGAGAG
AGTGAGAGAGGGAAACCAAATATTTATGTGAG
CGGAACGGTGGGACAAGAGCCAAAATTGCCGTACAGAGGTAGGAGTGAGAGTGGAGGTGGGAGGGAGG
GAGTGTAAGTGAAGCAACATAGAGACATATCC
ATACCCCTCCAGCCAGAGAGGAGAGAGAGGGAGGGAAATAAAAGAGGAGCGAGGTGATTAAAAGAACG
AGGCAACGACATACAGAGAAATGCAAAGTTAC
GTCTGTGCATAGGAACTCAAATGTGCGACATGCATCCTATTATTATTAATATATTATATATATATATA
TATATATATATTTCTGGTGTTGCCATGGTTGT
GGCTTTCTGGCTGGTTGACTGCTATTGGGTGGTGCGGGCCCCGATCACCTTCTGCTCCACACATCAAC
ACCCAAACACAGAAGGATATACATCTACATNN
NNNNNNNNNNNNNNNNNNNNNNNNNNNNNNNNNNNNNNNNNNNNNNNNNNNNNNNNNNNNNNNNNNNN
NNNNNNNNNNNNNNNNNNNNNNNNNNNNNNNN
NNNNNNNNNNNNNNNNNNNNNNNNNNNNNNNNNNNNNNNNNNNNNNNNNNNNNNNNNNNNNNNNNNNN
NNNNNNNNNNNNNNNNNNNNNNNNNNNNNNNN
NNNNNNNNNNNNNNNNNNNNNNNNNNNNNNNNNNNNNNNNNNNNNNNNNNNNNNNNNNNNNNNNNNNN
NNNNNNNNNNNNNNNNNNNNNNNNNNNNNNAT
ATATATATCGTACATGTTATGTGTGCAATGGTTGCCTATCCGTAGAAGGACGGGGGGAGGAAAAAAAA
TGCGCATGTGCACGCATTCATGTGGGTGAAAA
AAAAAAAAACAATCACGAGTGAATCATGACTTTGTTTTTCCCATATTTTTTTTTCTTTTTGTTTTCC
ACAATATACCCACGTCTTCTGCACGACGTGGT
GGGACATGTGCCATTCACGGGAAGTGAGCGGTATCGCATAGAAGGAAGGGTAAAGAGGGAGGGGGGAA
GGGGTACGTGCGTATATTACATACATACACAC
ATACACTAGTGAGGGAAGAAAGAAAAGAGCCGAGAGGAGGGGGAAAAAAAACAAAAAGGAAAGGAAAG
GAAAAGAAAAGAAAACAATAAACACCCAAAAA
AGAAAAAAAAAGAGAAGCAAAAGAAGGAATAAAAATTAAAAGAAGGCAGGAAATGCGAAGGGGTCGAAC
GGTGGTGGCGGCACTTTTTGACGTTTGTCGTC
GCCCCGCGTGCGGCGTACAACGGCAACTTTCATGCTGCGCATTTGCCACAGCCACAATGATGCCGATG
TTAAGAGGCACAACAGCAGTGGCTGCTCTTTA
CACGACTGTGAATCATCAAACTCGCCGTCTGCACGTGTCGAGTCTTCTTCGCACCACCGCAGAGAGCC
CGGCGGTGCCGGGGACGAACAGCAGCAGCGCG
GGCAACGCATCTGCCGACAGCGGTGTGACAGCGCTTGATGTAGCCATGCGTGTCAACAAGCTGAAGCG
GCTGCACCAGACAGGCGGTGGCCCGGACGGGA
AAAAACGCATTGAGTTAGACGCATGGAGCGAACTGAACAGCCTCACGGAGGAACAAATTAACTCCGCC
GAGGGAAAGGCCGTCTCGCTGCTGCTCAACTC
ATGGGCGTACTTTGCGAAGTACTGGGACAAGGGCAAGGACGGGCCGCTGGGCATGGCGACCCCTTCCG
CCGCTGGGGACTCGTCTTCGTCAGAAGCCGCT
GCCTCCAGGGAGACACCACGAGACGGTTAGAGAGAATTTGTGTGATGGAGCCCTCGAGCACATGCATA
TGTATGTGGCTGTGGCTGTGTGTGACACGATT
AGACGCTTGCATTACATGTAATGCAAAGGGAGGGGGTTGTGTGACTGACACGCCACAGAATTGGGGTT

TTTTGTTCAATGCAATGAGGAATAACGTGTGG
GCGAGAGAAAGAGGAAGAATTGCGGTCCTTTTATATACATGCGTGTGTACTGACCAGCGGCAACGTGT
AATCCAAACGAAAGGAAAAGCAAAAAAAAATA
ATTTACAGACAAAGTCATATAAACGGAACGAGAGAGAAGTGAAGGCAGAGACAGAGAGAAGGAGAGAA
GGGAACGAAATCAGCGCAGATACATACATGCT
GCGTTCATTTGCGGATTTTTTTGCTTTTGCTGCTTCCTTTAATTGAATGGTCGCAGTAGAAAAGAAAA
GAAAAGAAAAAACAACAACAACATGACATCAG
TAAGACTGCCATTAAATTATGAGGGTCTTTCTATTATTATTTTTTTATTTTTATTTTTTGCTGTTTT
GGTTTCTCCCTGATACTCTCCGATATATATAT
GTATATATGTATATATATACATGCATAAATGATCTTCACATTTTGGTGAAGGACGTAAACGAAGGGCC
CGCGTTTTCTTTTATGGGAGTGTGTGCGTGTG
TGTGTGTGTCTATTGGGCACCCAAGAATGCCGCCTTAGCCCTCTCACACACAGTGCGTACATACACAC
AAAGGCATATATATATATATTTCAGCGTGTGC
GTGTTGGGTTGGTTCAGGGAAGAAGATGTTGCGGGAGGGAAATAAACATCATTACAGCGAAAACAACA
ACAGCAGCAACAAAAAAGAAAAAGAAAGAaAG
AGAGAGAGAGAGAGAGATAGTGAGGGGAATTGGAGGGGGCATGCGTGTGTTCGCCACCGTATTTTTTT
ATTTGCCGCCACGTTGATGACACCTTGACGCC
AGTTGAGGACGTTTGCCCACGCGAGTTTTGTTCCAGTGTATTTTCTTTATTATTTATTTATTTTTTAT
TTTTCTGCATTCCTCTATGTCCGCTGCGTGTT
GTGCGGGTGTAGGCAGAGGAAGGTGGCGAGGAGGGGGGGAATAAGAAGAAAGAAAAAAAAAAGAGGGA
GCATGCGGCGGGTGATTGGGGCCTCGGGATGG
CGTTTCATGGTAACAGCGGGAGCTCTCGCGAAGGGTGAATTCGGCTGCTTCTCATCGTTCTACTCCTC
CACCGCTACCTCCTCTTTTGTCATGATGCAAC
GGGCCCACCGAAGCCAGCGGCGGGAGCGGCCACTGCTGCGGCACCANNNNNNNNNNNNNNNNNNNNNN
NNNNNNNNNNNNNNNNNNNNNNNNNNNNNNNN
NNNNNNNNNNNNNNNNNNNNNNNNNNNNNNNNNNNNNNNNNNNNNNNNNNNNNNNNNNNNNNNNNNNN
NNNNNNNNNNNNNNNNNNNNNNNNNNNNNNNN
NNNNNNNNNNNNNNNNNNNNNNNNNNNNNNNNNNNNNNNNNNNNNNNNNNNNNNNNNNNNNNNNNNNN
NNNNNNNNNNNNNNNNNNNNNNNNNNNNNNNN
NNNNNNNNNNNNNNNNNNNNNNNNNNNNNNNNNNNNNNNNNNNNNNGGGAGCGGCCACCGCTGCGGCA
CCAAAAGCGACCACGACAAGACGAGAGGCATC
AACGTCGCAGCCATCACGAGGATGGAAGGAAAGCCGCGAAACCTCTCGGTCGTCAAAAGGGGGGATTG
CCGGGTCAACAGACAGCCGACGGCGGGGCGAG
AGGCAGTGACGCGGGACGGCGGCACAACGAGGCGGTGACACCAGAAACACAGTTGACTGAAAAGGTCT
CGGCGACAACCGCCGGCCGCTTTTCACACCGT
CATGGCGGGTTCGTACAAAAAGTATTGGAACGTGGTGCCGATCGAGTTGTCACGGGCGGGGCGCGTCC
ACGAGGGAAACAGGAGGAGGTTGAGCCGCGTG
TGTCATTGGCCGCATTTGAGCGTCTTCTCAAAGTACATGTGGCGATGAGGAACAACTACGAATGCCTT
CAACGGAATCATCAAGTTGTGAAGAGTTTTTC
AATGGCTTGTGCGGAAGCTTTGCTGCATTTAGGGTTGGCACGATGTCGTGCAGGGAAGAATGAAGGGA
AGAGGGAGGGGACCACTTCTGATATAATAGTG
AATATTTGTAACGAGTCCTCGGCCAAGGGCGAAGAGGTGTCCTCAGCGCCACCGCCCCTTCTCTTGTC
CGCGGACACGCCGCTTTACACGACGCTGATCA
GCTTCCCACCACCGCCCGTAGAGGCCCTACTGCACATGACCGGGTCATCCAAATGCAGAAAGGACGGT
GACAGCGGGGAGTTGTTGCTGGCCGCCATGGA
GGTTGTAGACGGCACGAACGTTGAGGGATTAGTTGAGGATTCTGAAACGGTTACGAAAGGTCGCGCTC
ATGACACAGATCCCACAGTCTCATGGGGCGCC
AATCCGCCAGGTGTAGAGAGTCTTCTTGCATGCTTGCAAGGTGTACCGATGACGGACATTATAAAGCT
CATGGAAACCGTCGCCTACTTTAAATGGATAA
AGGATGAAGAGGTGCTGGAGTGGAAGAAGGGGTCTGCGGTGGAACAACTGCGTCGTGAGATCTCCGCG
CTCTATGCCGCTACTACGAAGAAGAAGGAGAC
GACATCAACACCTGACGTTTCTGATGAGGCAAACGAGGCACTTTGTGTAGTTGAACACACGTCAAAAA
CGTTACTGAACCGCATTTCCCGCGTTGATTGC
GTAGCAACAGCGGCGGCGTCTTTTGCAACAACAGATTCTTATGTGCATGTTATTGACCTCATGTTAAA

TGTGTTAATGCGTGTGTGCCTGTGGAAATGGC
CTCGAATGTCATGTGTGGATCAGATGTCTGTTGTGCTCATAGCCACATTTCCCTGGTTTTCTCGTGAT
TTAAGTGTCGAGGCTGCATCATGGATGACGTA
TTATCATTTGCTTGCCATTTCAGACAAGGACGTGAACATGAATCATTATCGACTATTTTTATATATGC
AGCGTATCCGTCGACTTTGCTGGCCGTCTGTG
CAGGATTTAAGCTCAAACATTGTCCGAAAGTTGTTTTTTTTAAGGGAGACGTCGGATTCAACAGGAAA
CGCGGAAAAGGACTCAAATAATGACACGAGAG
AAATGCAACATGAAGAGCCCGAGGAAGCAGAAACGGATGCAAAGCTTATTCCCTCACTTAAAGATGTT
GCATGTATTCTGTACGGCTCACAAGAGAAGCA
GCGTGCGGCATACGAGTGTCTGTTGGTATGGTTAAAGCAATCCGATCAGTTTGACCTTCGCCTTGCCT
CGCCAAATGGACGTTACGTCGGTGCCATTGGT
ATCGCTATTGTCGATATTGATCGCTTTGCGATGGAGCGATTTGGCAGTGAGATTCTGAAGGGTGAGTG
GGTTCTGCTACGCGTGTCGGGGAAAAACGTAC
CGGCATTGAAACGAGTTACACAATGCACCCAAACGCCATATGCCTTGACACGACTTTTTTTCTGCGGC
TACCACAATCCGCTCTCACACATATGGCATGG
CATCCATGCGGCTCGGCGGGATTTGCTGTGGAGTGCGTGGCTCCGCAGTGTTGCTCGACCGATGGTGG
AGGCCATCCTTCCTGGCTCTGCTAGCAGGGAG
GACAAGGGAGGAGAGGTATGTGCATCCCAGTTTATAGAACCCTTGCTTGCCAGTCTAATTTCTGATGT
GGTGTTTCAGCGATTAATCACACAACTTGTTA
TTCTTTCGTTGAACAAAATGAGTGCTGCAGTCAATATGCAGCCGTTTGATATTATGTGCGTGCGGCTT
ATTGAGACAGTCTACACCGGCGCTCTTGTCCT
TTACTGCGTCTCTCTCAATAAATCTGCTGTCGATGCTCCTCCGGAGGCGACACCTGGCGCGACTAAGA
ATAAAAGCCCGGTGGAGGGAAATGCACCGGAA
CAGCAGCAAACACCTGCGTCTGTTGCGATCCGGCGCGCATTTAGAGAGGTCATCTCTGCCATGGTGCG
AATACTGAGTGCGGAACACGTCCCGCTGGTAC
ACCGGCTTCTTGACGCGTTGTTTGGGGTTTGGATCAATGACACCGCCAGGGAAACGCCCCTGGACGGC
ATCCGAAAGGAAATTTCAACCATCTGCGACGA
AAAGACTTATAACATGCTGAATGAGTGGCTGAACCGACGAGCAAATTTGCTTTTGGTGAAGGTGGCAC
TGCTGGAGGATCCGCTTCTTCGCAGCTGGCAA
CAAGCCGTGCAGGAAGTGGATGCAGGTCTGCCAACGACACTCCCCACTCTGATGGAGTGTTTTGTGGG
ACTGCTGGGGCTGCTGGAAAAACGGGGCCATC
CAATCCTGAACACCGAGCAGAAGAAGCAGCAGCAGTCTGCTGTCGATGAGGCCATGGCTGTTGGAAAT
GCCGCGGGTTCCTGTGCAACGGTGTTTACTTT
GCCTGAGCTTGAGGTGATTCTGACTGTGTTTTCCCGAGCCATTCTTTACCGCGTTTCAACACTAAAAA
CTCTAGGGCTGACCTCAATCTCACAAGAATTT
GCTGCCCTCATGACGTCATTGAATGGCGATGGGGGAGACAACAGGGACGGTGGGAGCAGCAGTAATAA
TAATACCAATAATAATACCAATAATAATAATA
ATATGAGTAGTAGTAGTGGTAGTGGAAACACCAACAGGAACAACATGGAAGGAAGAAATACCGCTACT
TCTGCTTCTACTGCTGTTACTGCCTGCAC
CACCACCGCCACTCCCATGTTAGCTGCTGATCACGTTATGCTCCTGGAGAAAATCCTTTCGGCTGAAG
TTGAATCCAAAGCAGACGGGCTGACGTGGGGC
TCTCTCCTCGAAGCCATCCTGTTGCACCTTGCGCCGTACGGCATACTAGAAAGCTCATACAATGCTTC
TTCTAATAGCGAAGCGGCGGGCACTAGTGGAA
ATGTTGCCCCAGTGAAAAAAATTGGAGGAGGACATGCAAGTGTGCCACCACAAGCGCCTTGTGAGCAGT
GTTGTGCTTTTCCGGTGTTACGTCTTTGCGAC
GACACATGATGGGGGGTGGGCGGATGCCACATTACTGCCTCAAATTTCTGAACAGGAGACGGATGTGA
GCCATGCGAGTGACAGCGGGGATGTAGCCGTA
TCCCACGGTCAAAGTGCTCAGTTGCGTGAAATAACATGGTTGCTTTTAGAGGTCTTTGTGAAGAATTG
GGCGAGAAACTCAAAAAATACGAATACGAAGA
GGAAAACTGCAGCAGGAGATGCGGGCACTACGGAGAAGGCGGAAGGAACTATGCGGCAGGGGCAGTTT
GAATACATTCTTGCTTGTCATGCTCTGGTGGT
CTACATGCGTCTTGCACCTTTATTCTCGACACGCTCACTCTCTCTGCAAGAGGCATACGAGCAGTTTT
TGCATTCCGCTGAGCACACGACGACTACCTTT
GGCGGGAACCAGTACAATGGATGCGACTTGTTGTTTTTGTGGTTGGCTGCAAGCGCCAACGCCTTGGG

TGTGCACTTCTCCCCTGCGCAGAATTACAACT
GGTCGACGGTGGGAGGGGTGACGAAACTGGGATCAGGCAGCATGAAAACAACTGCCTCGCTGGGAGAG
GGGACGATGCGCCTCATTCAAATCCTCGCACC
GTATGTGCAGAGCGAAACGCTTCGAATGAACCCTCGCGTGAAGTTTCTTGTTAACATAATTGTGGCTA
TCCACACGTTTGGGAAGTTGGGCATTCACATG
GATACGGGGGGTCTAAACATGGATCAGCTACTGCTACGTACTAGCTCAGATAGACGGCTGCTGCATCG
CCACCTCAATCTCTTTCTTGTTGGATGCTCCG
CATTACCCAGTACGCGACACGCGCTGCTAAACGTCGCCTCTTTTCTCCGCGAGGCGCGGTGCCTTCTT
TCAGCGGCGGAAATAGATCGGGCCCTTGTGGC
AGTTGCTCTCTCTGCTAACGCTTTTGCAAAAAATCATGAGGCGCAAGCTGATCTCCAGCAGAACGGTG
ACCTTATGGATGTCACCCACAGCACCGCTCCT
ACGATGCGTGTGCCGCCCACGCAACTTCGACACGCGTGGAACGCCTTGACACGTTCTGTTTTGGAGCG
CGCGGAGGGGATGCCGACGAGTGTTTTTGTGC
GGGGTGTACAGTGCGCTGGCGCTGTGGCGTGCGTCGATACAATTTTGTGCCAACAGCTTCTCTCTTTC
CTGGTGGAGTTTCGGCGGGACGAACTCTCGTT
ATTAGACTGGACGGCGATTCTCCACACGGCGCGGCAGTCCTTTGAGAGCCGAAGAATATTTGAGGAAT
ATTTGCAGGAGCCACTGACCTTGTTGCTTTTG
TCCATGTCGAAGGAAAATGGCAGGGGAAACGCATTGGAAAGTCAGCATGTAATATCGAGTGGAAAACG
ATTAGTGGATGATCTTTCCCTCTTTGTAGAGG
CACTGCCGGAGTTGTTCACAGGTGATGCGGAGTTTTGGGGGCTGGTGCGCACCGCTCTTAAGGTCCAG
TGGATCGCACAATTCAATGCCGCGAGTAGCAG
TGAACAGCACCAGCAGCAGTTGACCTTGTGCCTACAGGATCTTGCAAGGAGCTATGAGTGGGCCGTGC
GCACTGCCGGGCATCACGAATTGGGTGCGATT
CCTATTGTGTAGAGAGGCAACGGAGTGGACAGACGCACGAAAAAGACAAAAAACAAAAACAGAACAAA
GATAGCAAAGTTAAGAAACACACGTACGCACA
CAAAAGAAAACACGGTCCGATGATATGGTGCTGACTGGATGGAGGGCGGTGCTGTTGTCCTTCTTCTT
TTTTTTTTTTGGCTGTGGCATTGTTGTTGGAT
TATTTTTCTTTCCTTGTGCCCTCTTTATGGCAGGAAGTCGACATGGGCGAGAGAAAGGCAAATGGTGT
TGTTTTTGTTTTTTTGAACCTTCACGAATGTT
TTGCCTTGGGGAGAGAAATTCTCTTTTTTTTTTTTTTTTGTGTGTATTTGCTCGGTTTCAGTTTGTTT
CTTTTCTTTTCTTGTTTTTTTTTTCTTTTTCA
TTGTGGCTTTCCATGCGNNNNNNNNNNNNNNNNNNNNNNNNNNNNNNNNNNNNNNNNNNNNNNNNNNN
NNNNNNNNNNNNNNNNNNNNNNNNNNNNNNNN
NNNNNNNNNNNNNNNNNNNNNNNNNNNNNNNNNNNNNNNNNNNNNNNNNNNNNNNNNNNNNNNNNNNN
NNNNNNNNNNNNNNNNNNNNNNNNNNNNNNNN
NNNNNNNNNNNNNNNNNNNNNNNNNNNNNNNNNNNNNNNNNNNNNNNNNNNNNNNNNNNNNNNNNNNN
NNNNNNNNNNNNNNNNNNNNNNNNNNNNNNNN
NNNNNNNNNNNNNNNNGAGTAGGCCAGTGGCAAGGAATACATTACTCTCACGGAAAGGTACACACAG
AGGGAAGGAGGGAGGGAGCCATTGTTGCTGTT
TTATTATTTATTATTATTTTGGTTTTGTTGGATTTACAAGAAACACACACGACTTTTTTTTTTTTGCG
GTATTTGTGTTTTATTTTATTTTATTTTTTTAT
TTCTTTTTGCATCACTTCCCTGTCGTTCGACGCCAACGGGTTCATGGAAGAAGTTTCTGTCGTGCCAG
TGGAGGTACGATGCCGCCCGCCACGTGTCAAC
AGGACGGAGCTGTCAAAAGTCATCACAAGTTTTATACGTCAGCGCCACGCTTTTATTCACCCGGGAGA
AATCCTCTCGCTTGACACCGCCTCAGACCTCA
TAGCCTCGACGGTGGAGCTGCTTCGGGTGTGCGATGTGTACTGCCCGCAGCTCGGCATTCCCGCGGAA
GAAGCGGACTACGCCATGGTGATGTACACATT
GTACACCGATGAGAGTTGTGGGGATTCGTTGAACTCTGTGGGGCCCGATGAGGATGCTGGCGCGGGGC
TGGCGCCCTGCAGTGTGTTGTCGCTACCAAGC
GTCTCCCTGGAAGGACTATGGGAGACCTTGTTTTATGGTGAAACACGAAGCGATTCCGTCCGTCTTAA
ACGTGATCTGCTGCAGTACATGCGTACGGCGA
TGGTGTTCTCCAGGGCTGGTGTGGACCCACACATTGTTGCGTGGAATCGGCTGATTTTGCTTTACGGT
CCACCTGGCACAGGCAAGACATCGCTCTGCAA
GGCCCTTGCGCAGAAGCTGTCCATTCGCCTTAATGACATGTTTCCTTTTGCCCAGTTGGTGGAGATCA

ATGCACACAGTCTCTTTAGCCGCTGGTTCAGT
GAAAGCGGAAAGCAAGTCATGCTTCTTTTCAAGCAAATACATGAAATGGCTGATAAACCGAACAGCCT
TTTGTGTGTATTGTTGGATGAGGTAGAGACTT
TGGCGGCGACACGACAATCTGCATTGAGGGGAAATGAACCATCCGATGCCATTCGTGTCGTCAACGCT
CTCCTCACACAGCTGGACAGTCTACAACGTCG
GCACAATGTGGTGGTATTTGCCACCAGCAACATCACGGGGGCCATTGACGTGGCATTTATTGACCGCG
CCGATAAAAAAATCTTTATTGGTGCACCGGGC
TTTCAAGCACGACTCGAGCTTCTAAAGTCAAGTACGCAAGAGATGATTCGACGGGGGTTGGTTATATT
GGAAGCCTCCTCTTGTGGGGAGTTTATCAATG
AGGAGACGTGGCCGCCAGTGGTATCACGTGACATGGAGTTGGGCCAGAGTGAGTTGCTGCAGCTGGAG
TCCATTGCCAGGCAGTGTGAGGCATTTAGCGG
CAGAACCCTGAAGAAGCTGCCTTTTTTGGCGTACAGCGAATACGCGATGGGCGGCTGTGGCGTTGCAT
CGCACCTCATTTCATTCTCGACATTTATGAAC
GGCCTGCAGATGGCTGTTCGAGCCGAGGTGTCGGCGCGGGAGACAATGATGTCCTCTTAGTCTCCAAG
CAGAGCCTTGCGTTTCCGTATTCGTTTGCACA
CACACACACACACACAATTATTCCTTTTTGTGTGTGTTTTCATACACACATACATATATTTTTTACTC
TTTGGCTTGTTTGGCCGTTCTTTTCTTTTTTT
TTTTTCTTGGGAGGTTTTTTTGGGCGTCTTCAGGCACGTGCACTCACACTCGCATTCATTTGCCTGAG
ATGCACCAGAGAAAAACTAAAAATATGACAGA
AATTTTCTTTTTACCGTGGAGGAAGTTACTTCGGGTGCACTTCCCCCTGTGGTGCATCTATCCCCATG
CATGTATGCGTGGACCCCCTGCTGCCGCTGTT
GCTGCTGCTGCTGCTGCCGGGTGGTGGATGTCATGGAAGGAAGTTCTCTTGCTTGCTGCTTCTTTTAA
AACTTCGTTTGCTTAGATACAACAGGCAAATT
TTGCACGCACAAATTAGAAATACACGCACTTACGGTGGACGTGCAATATTTTTCTTCTTTTCTTATTA
TTCCCTTTTGTGCGTCCATACCGGGGCGTTGTG
TGAGAGTGGGTAGCTGATAGGAGGGAAGGGAAATGGGCCGCACCGTACTGATGGTGGCGGAGAAACCC
TCCCTTGCGGAGAGTATCGCGTTTCATCTTTC
CAACGGTCAGGCGGCGAAGCGGTCACGTGCGTTGCCAGTGTATGAGTATTCGGGTTATTTCTTCAATA
CTCCTGCCTATTTCAAGGTCACCAGCACGACC
GGTCATGTCTTCTCCTGTGACTTCACGCCGCAGCACCAGAGCTGGGATCGCACGGAGGAAGAGGACCT
CTTCAGCGCTCCTGTGGTTTGGAAGGACGAGG
CGGGCAAAGTCAGCCGACATCTCGCACATGAAGCAGAGGGATGCGACACGCTTGTGCTTTGGCTTGAC
TGCGACCGTGAGGGGGAAAATATCTGCTTTGA
GGTTATGCAGGTGGTTCGGCGAAGCATTCACGACATGCGGGATATTTGGCGTGCCAAATTCTCTGCCA
TCACCCGTGAGGAAATCACGCATGCCTTTTTA
CATTTGGGAAAGCCTAATAAAAATGTTTCTGACGCTGTCACTTGCAGGCAGGAGTTGGACCTGAAGGT
GGGTGTTGCCTTCACGCGCTACCAAACAAAGT
ACTTCCAAGGCAAATATGGCGATCTGGACTCCAGCGTGGTCAGTTACGGTCCCTGTCAGACACCAACA
CTGGCGTTTTGTGTCCAGAGACATGATGAGAT
TTTGAACTTTAAGCCAGAGAACTATTGGAAATTGGTGCCTGTTTCCAATCGATTTGGGTCCTTAATGA
CATTTGACTGGGCTCGCGGTCGTGTGTTTGAT
GAGTTAATGGCACGTTTGTTGCATCAAAAGGTGTCCCACCATAGAGCTGCGAAGGTCACGGATGTCTC
CGTGGGAGTTGACACTCGGGCGAGGCCTACTG
CTCTCAACACCGTCGAGTTGATGAAAGTTGCGAGCAAAGCCCTCGGTATGGGCCCACATCACGCCATG
CAGATAGCGGAGAATCTGTACATTGGTGGCTA
CATTTCTTACCCACGCACCGAATCCACTGCGTATCCGTCGTCATTCAACTTTATGGCTGCCCTCACCG
CGCAGGAGCGAAGCCCAATTTGGGGGAATTAT
GTTCAAGAGCTGCTCGCGCATGGACACACGCGACCAAAGGCAGGCAAGGATGCCGGTGATCATCCTCC
CATCACACCGATGCGTTTAGCAACTCCTCAGG
AGTTATCCGGTGATTCGTGGCGGCTGTACGAATACATCGCACGTCATTTTATTGCAAGCCTCTCACCG
GATTGTAAATTGACTTGGACGAAACTTTTGAT
TGAAATTGGTGGGGAGTTGTTTAGCTTCACTGGGAAGGTGGTGGAGGATCCGGGATTTACGACAGTAC
TTCCCCAGTTTGCCGTAAAAGACGATAAAGTA
CCAACAAACATTCAGGTTGGGAGTGATTTTCCCATTTCGGATGTTCGACTGCAGGCGGGCCAAACACA

GCCGCCAGGATACCTGACCGAGGCTGACCTCA
TTGGTCTTATGGAGAAACATGGCATCGGCACGGATGCGTCCATATCGCAGCATGTGAACAATATTGTA
GAGCGTGGCTACTGTGCAGTAAAACCAGGTCG
TGTCATGGAGCCCACGAAACTTGGGGTGGTTTTGATTCATGGCATCAAGAGCATTGATCCAGAGCTGG
TTTTACCATTGGTTCGAAGCAAGGTGGAAGAA
TATGTGACTTATATTGCCGACGGTCGTGCCTCACTGGATGAGGTGCTTTCCTATTCACTAGACCTTTT
TTTTGGGAAGTTTCGCTTTTTTAAACAAAACA
TTGAACGATTTGATGCCCTTATGGGGGCCTCGTTTTCATCGCTCTCTGCTTCAGGGAAACCTATCACT
CGGTGCGGAAACTGTATGCGGTATCTGAAGCA
CCTAGAGGCCAGACCGCAACGTTTGTATTGTGCCTACTGCGAGGTTACCTACGAGTTACCCCAGGGAG
GGACCATTAAACAATACTCTAGCTTTAAATGT
CCATTAGACAACTTTGAGCTTGTCATTTGTCATGTGGATGGGGGGAAGTCTTTCCCCATATGCCCCAA
TTGCTATAACAACCCACCATTTGAGGACATTC
AAAAACAGAGAGGAGGACGACAGCATATGGCATGCGATGAGTGTCGTCACCCTGTATGCTACCATTCC
CTGGCTACGAACTATGTGGCCGATTGTGTGGA
TGAGCGGTGTGACGGATGCATGGCATTTGTTCCCCGCACCTCAGGGAAATGGAAGATCTGCTGCAACC
ATTGTACCATGATGATTCTTCTTCCACCCACC
GCTCAGCGTGTATACGTAAGCCCAGAAGAGTGTAGCGAGTGTGGTGCCATGATGATGGATTTACAGTT
TCCCGAAGGAAAATCGCCACTTCCAAACCGCA
AAGATCGCATTGTGGCATGTGTGTTCTGCGACCCCACCCTAAACTCAAACGTGACTGAGGTCCGAGGG
CGCTTGGGCAACTTCAGCCGACGCGGGGGAAG
AGGTGGAGGAGGCGGTGGCGGTGCTCGCGGTCGCGGTCGGGGACGGGGACGCGGCGGTAGGGGCAGAG
GAGGGAGCAGGGGCCGGTATTAACACCGAGTA
ATAGATAGAGAGGGAGAGAGGGAAGGAGACATTGGGAGATACTCTATTGGTGAGGTGCTGCAAGAGGA
ATATGAAAGAAGGGGAGGAAGCCGATGATGTT
GTGCTTTATGGGTTCTGGCCTCATTACCGATGTTGTTTGGTGGGAGGGGGGAAGAATGATAACAAAAA
GAGGTCCTTCTGGAAGGCCTTTCAAAGGGCTA
GAAGATGAAAAGACCAAACCATTAAAACGTTGTCTTTTCCTTCTTTGCATGTTTTTTTTTTTTTTTTA
GAGTTTTATCGAGTCGTATTTGTTTGTTTTTC
TGTTGTTTGCCTTTTGGTGCTTTACTTCTTTTTTTTTTTAATTCCACACACATGTTCGATGCGTGCAC
TTTGTTAAATACATGCGCGTATTTTGTTAGCA
TTGCTAGTAGGGAAGGTTTTAGTACACGCATTTGCACACGCATGCAGCCTCGTGCTGTGCCCACGGGC
CGCAAGCCTATGACCCAAAAGCTACTATTAGA
GAAACTTCTCCATTTGCCGGAAAACCGCGAGTGTTTTGAGTGCTCGGCGAAACAACCCCGCTGGGCCA
GTACCAATCTTGGGATCTTTTTGTGCCTTCGC
TGCGCTGGCATTCATCGTGCTATGGGGACACATGTCTCCAAGGTGCGGTCGACAAACATGGACACGTG
GGAAGATCCTATGATTGAGTGCTGTGAGTGCA
TTGGCAACAAGCGAGGGCGAGTGCTCTATGAGCACGGCATGGATCCTCAATTGCGGCCAACCGCTGCT
ACCGACAACATTTCGGTGGATCGCTTCATACG
TGACAAATACGAACGTAAGATGTACTATAACCCGCAGTACGAGACGCTTCTGAAGCAGTTTTTTGATG
CCGGTACCTCCGTTAATGAGGAAAAGTGCTCG
GGTCTTGTGTCGTCCAAGCCGTCGGAGCAGACAGGAGGAACATCGGCGTCGATGCCAATGTTGTGGGG
TGGGAGCCCTGCCACGGCTATGGAGGCTCCTG
CTTCCTTGGAAGGCAACACAAATACCAAGAATGGAATCGATATCAATGAACTCTTCACCACCACAACG
AGTACCGTTACCAGTGCACCGATGCAACATCA
ACAGCAACAAATATTCTGGACACAAATGGCGGGGAATGGAATGGTGGGTTACCACCAATACTATCAGC
AGCAGTCGCAGTGTCCCTCCTCCGCACCCTCA
CAGGCGGGCCACCAGGTAGACGTCAAGACGGAGATTATGTCATTGTTTAGCACTCCACCCAATTGTTC
TCCCAATCATGTATACAGTGCGTGGCAGCCAC
AGTAAATTTCCGGCTTTTGGCCATTGCGGTGGGCTACAAGGCGCAAAAAAAGGAAATAAAATAAGTGG
ACAACAAGGGGGAAAAAAAGGGTGAGGGGGAA
AGGACGGCAGTGAAAAGCAGGAAAAAAAAAAAACAAACAAAAAAACAAGAAAAACCAAAGGAAAACGA
AAATAAGAGACATGGGAGAGGTCTGCAGATCC
GCACTTTTGGCTTCTCAACGTTTGCTTGGGCTTGTATTTATTTGCCCTTGTTCTTGTTGCTGCTGTCT

CTAGTTGACGCCGTTTCTTTTTTTTTTGTTGC
TGTTCTGAGAATTGCTTTTGAATGAAAGGTTTTTTTTTTTTTTGTTATGCGCTTAATTATTCTTTCT
CGAGTCTAATGACATCGTCCTTTTTTTTCTTT
TTTCTTATTTGTGTGTGTGTGTGTGTGTTTGGTTGCATGCATCCGTGGGCGTCTCACATTTTTTTTT
TTTTTCTTTGCTGTGATGCTGCCGGTTTTTGG
ATGTGTGCGTGTAAATAAACGACGGAACGAAGAACACGCGTCTGTCTTTACCTCTCTCTCTTTCTTTT
TCTTTTTTTCCTTCTTTCCCTCTCTCTCTCTA
TATATATATATATTTATGTTTTTGTGTGTGTGTGTGTGTGCACTTTTTTGGGGCGTTGAGGATTCAAT
TTCTCTGTGACTTTTCCGGCCGCGGGTTTAAT
TATGCCGTTATTGAAGTGAGTAATTAAAAAGGGGGTCTTTTACTGTAGTAGTAGAGCAGCGTGCATAC
CGTACAGCATCCACACGGGTGGATATCGCATA
TATATATATATTGTTGGGTTATGGTATTATGGGGAATCAGCTGGCAGCAGCATCCTCCGTCAACGTCG
CGGATGTCTCGTCAACATTGGCGCTCGTGGCA
CCTCTTGGGAGGGAGCATCGTTATTACTTCCGCTCATTCCACTGTGCGAAGCTGGCAGGTGCGGCGAC
CAGTACCACAGAGACGCTCCGCTCGCGTCATA
TTTTTACCTCTACGAATGACGGCCGTGCGAAGGACATGTCTTTGCGCCACCCGTTTCAGCGAGCGGGT
AGCACGGATGTCGTTGTGAAAGTTTTTGTACG
TACTTTTGGAGACAACCAACAGAAGCAGTTGGTGGAGTTCTGCTACGAGAAACTTCTAGAGATTGATG
GGCGAATGCGAAACACCCAAAGCGCCGCATCC
AACAGCACGTTTATGAATGGCGGCAATAATACGGCCGTGGGGAATCATCATCACTCTCCTGCTGCCCC
ACCAGCACCATGTAACGTGTTGTTTTACTCGG
GCATGGTGGTGCATAATGATCGATTTTGTATGCTGCAACGATCTTATGTCGCCTACTCACTTTCGGAA
CGACTTGCGACGCGGCCGTATTGGAAAATGGG
CGAGCGTCTCTTTGCCGGGTATCAACTCCTTCAAGGGCTGGCGCAGTTACATGATAAATGGGAAACCG
TTCATGGGGACTTGAAGACAGATAACGTTTTG
GTAGACAAGGCTGGGTGGATTTACGTGACGGATGTTTGCCCTTTTAAGCCCGGCCTTCTTCCGGCAAA
CAATCCGGCCCTGTTTGACTACTACTACGACA
CAAACGAGACGCGTATTTGTTTTTTGGCCCCAGAGAAGTTTTTGGACGAGACGGTTTTGTTTAACCCA
TCGAACGTGAACGCAAACGCCCATACAAAAGC
GATGGATATTTTCTCGGCGGCATGTGTATTGGCGTACATCTTCACGGAGGAGCCACTCTTTTCGCTCT
CGGACACTCTCAGTCTCCGCCTAATGCAGACA
CAGGAGGAACGTGAGTCCAGTGTGCAAAAGATATTAATCGAACGTGGCGTTCCCCCAAATGTGCGCCG
CATGCTTCTTGACATGTTGTGCAACCCCCCAG
CGACTCGTCCCACGGCACGACAGTTGTTGGAGACATTTACGCCGTCGGTGTTTCCATCGTACTTTGGC
TTTATTTACGGTGAAATTCTTCCACCACTTCT
GGCCCGCCCACCAGACCTCAAAGTACAGCTACTGCATTCCCGACTGACGGATATTATGGATGAAGTGA
CACAACGCTTTGGCAAAAATGAGGATGCAACC
AGTCATCTGGCGGTTATTCAGGATGCCAAACAGTTGAGTGTGCAGCTGCTTCTTCCCGTTATTTTAAA
CGCCGGCCTCCACCTCATTACAGATGACGCTT
TCTGTCGTCTTATTTCCATTCTTCAGAAGATTCTCCCCTATTGCTCATCGGAGGTGCAGAAAGATATA
CTTCTCCCTTACGTTCTCCACTATTTGAAGTT
GGAATGCCACCCGGTTATCGCGCGCATAATTGCACTGCGGACCTTGTCTATGATATGCCACTCTATCC
CTTTTTCTGCCAGCGATGCAACCATTTTTGAT
GATCTTGTCCTGCCGTGTATCGAGCAACTCTTCTTGAAAACGGACACCACCGATGTTGCCCTTCTTGC
GGAGGTTGCGGATCAGTTACCCAGGATCTTAT
TGCGGGCCCGCGCCTTTTTGGAGCACTGTCAGGCGCTTGCCTCTGCTAATGCGTGGCAAAGCGGCTTT
GGGGAGCAGCTGAACAGTCTTCTGGATCGTGG
CTGGGATGCCTTTCGATTACTATACAAGCATCATCATACAACGGTTGCTGTTGTGACGCTTCGCCGCA
CGGCGGATGTGGTGGCGTTTCTTGGAGAAGAA
AGAGCACAGGATGATCTTATTCCCTTTCTTACAACAGCCATTGCCTCCGCCATCGATATTCAACGAGA
ATTATATCCACAGGCGATCATGTGTCATTTGC
ACTTGCAGACACCGAAACTAAAGACACTGCGTTTTTTTGTGGAAGAGGGACTGAAACAGACGGATGTG
ACGTGTCTGGAACGAACCATAGAGAGCATCAC
AGTCATTGTTGAAAAGGAATGTTTTGCCGTGTGGGACATTATGCCGTTGGTGCATCAGGCATTACCGT

ATATTATGAGTTCCAATAAATGGGTAAGCTCG
GCAGCATCTCGCATGGTGGAAAAAGTGGCCTGTACGCATCGCGTGAGTGATGTATGGATGTATTTAGA
GTGTGCTGTTCGACCCTTGTTGCGCCACCCAG
TACCGCTTTTCCGCATGGCTTCATTTCCTTCAGCTGTGAAGTTGGAATTATCGAATAACATCTACGTC
GTGGAGCAGCTGATGAGGAACATGAATTTAGA
GACACGCTTCCTGGGCGGCGATGCAGCTTTTTATGAGCGCATATTGCGCAGCAGCACAATTTATGATT
CGCCTTATTTTAATTCATTTGGGATTGCAAAA
CGTTCCTTGCAGGGTATTGTCTCACGTGCAACATCCGTGGACGTTACTTTCGATTCAAGCATGTTGAA
GCCAGAGGTATCCTCTACAGAAAAGGCGGAAG
GGACGCTGAGTGACGGTTCTCGGAATAATAAGTGGGAATTATTTTTTAGGCCCACAAAGCGTGAGCAG
AAGCGTTTGCTTTTACGGCAAAAACCACTACG
AGATGATGGGGGCTTCAACGCGATACCGTCCCTCTTTCAAGAGCGACAACAGTCTGTCGGCAGTTTGC
ATACCACACGAGGAGGAAGTGGAGGAAGCCGC
CGCAGTGGGAATGAGGTGGCAGGATCCCCGACCCTTTCGATGACACAAGTCACTTGGGAAGAGAAGGA
GTTAAAGCCGATTGCCGCGCCGTGGTTTACGC
GGACTTCTCACCTTGGTGGGATCTACTGCTCCTCCGCGGCAAAAGGTGGGACGTTGGTGACAGCGGGC
GGTCGAGGTGAGGCGATTCTTTGGTCGCTAGG
CTCAGAAGGGGTGGACTACTCTCATCGCATCCAAACCAACGCCTCTCTCATGAGCACCTTTCTGTTTG
CAGGCTTTATGCGGGAGGGGCTTAGCCCACTT
CTCTGCATGGGCGGCACTGACGGGGATTTTTGTGTTTTTGATGTTGCGTGCAACAACATTGTCTTGCG
GCGTTTGTTGGACGGCAGTACACTGACATCCT
TTTGCTTGATTGGTAATTTTACCACACTTGTGTCCACTGCGTTGGGTGGTGTGTTTGCTATTGATGTT
AGGGTGGGGAAAGACGTCTGGCAGACAAAACT
GCCGCCTTCGGTTGGCCCACCGAGTGGTCTTTTACCTCTTTTTTTTGATACCCAGGCGTACGGGGCGT
CGGTGGTAACATTGACGGGGGGAGTAGCCCTG
TTTGACCTGCGCTTTCAGCTGATGGTGCAGGAGTACCGCCTTGGCAAGGATGATGGCAACAGTCATGA
AAGCGGAAGGGTCAGTTGGCCAGGTACCCACG
CTATGGATAATCCAAATGCCATACTGTGCGTTTCCCCGGACACTACATGCACTACTCTAGACAGGACC
GACGGTCCGGGCCTGTTTTTAGGAACACAGGC
AGGGACGGTGCACTGGATGGATCTCTCCACTGGTGAGAATCGGGTTGTACTAGAGCCTGCCACAATCG
GAAAGCCGACGAGGGCATTACTGGTGCAGCCA
AAGCACTCAGTGGTCATGACGGCAGGAAATGACATGTGGATCAGAAAGTGGTGTTTGACCGCTCCTAA
TCAATCAAAAACCCTTGTGTGCCCGCCGTACA
CGTCCCCCGCGTACCACGGAACCACCCGTGGGGGAATTGCAGAGGCTCAGTCACAGAACCAGTTTGTG
AGTGCACGGCCTTTCAATTCTAATGAGCCGTG
CCTGACAACTAATGGTTTTGTATGTGATGGTGCTGTGCCGCGGCACCATGAGGACGCCATTCTCACTT
TGTGTGCTGTATCAAACGGAAATGCGTCGTAC
CTGGCGTCCGGGTCTCGTGATGGCACACTGACGGTGTGGCTTAATTCAGAGTAAAAAACCGAAGGACG
GAAATTGTGACATTTGCTCCATCTGTTGGGTT
TTCCACTGATATGCCTATTTTTTTTTTTTTTCGAGCGGCCTTGCAAGACGGGCGGAGAACGGACAATAC
AACACACTTCAGAGTGCAATGCATTCATATGG
ATGAATTTTTTTTTTTATTGCAGTTTTTTATTTTATTTTTATTAAACCCGCATTTGCCGTTGTCGCTG
TTCTTTCCCCCCCCTCCGCTCTCTCCTCTTTT
TTTTCTTTTTTTTTGTATTTGTGTGTGTATTTGCGCGACTTTTCCATTTATTTTCGATTGCGGAGGAC
GACAGCACCAAAAAAAAAAAAAAAAAAAAGGA
AAAGGGGGAATGACAATACAAAGGGATGCGGCGGATGAAGGCTGCGGTGATCTTTTGCGGGCGGTGGA
GGAGCGTATTCCATGTGAGATTCGGTGCCATT
TCACCGGCATGGCAAGTTACCGTCAGCTTAGGGATGGCAACGAGGGGGAATCGACGTTTGTTTCTCAT
CCAATGAAACGGTTTAGAGAATTCACGTGTGA
AAAGGAAGTCTGCGTGGAGAGCTGCATAAGCCATGTGCTCTCGTGTACGGCGTGTCAGATACTAAAAG
GGTATTGTGTGCTCTGGGCCGAGTCTTTGGTG
AACGTTGTAATGTACGAACCATTTGTGTCACGCACGGAGAGACGAAGAAGGGGAGATGCAAAGACCCA
GACAACAACGACGAATAAAGAAATACGCGGGC
GTCACCAGCTGATTTTTCACACCCTTCACATTGTTGACAAGGCGGCATACAAGCACCCATTGCAGCAT

TCCTTTGCCTTGCAGTCAGTCTGCCAGCCGGG
AAGGGTCCTGCAGCTGGAATGTTATCCCTACCACATGATCCACTTTGGATTGCTGATTTATGCTCTCT
GGCGAAGCCCGGATGAGGTTTTCAGTGCCATA
CGAAATATGCCACGTCAAAAAAAGGTGAATACGCCCCTTGAAAAAGGGGCTTGCATGACGGAGACCGC
AACTATGTCTTTGACACCATTGCATTTACCAC
TTTTGGCGACAACCGAGGATGCGTCGGTGCTTATCCTTGGTCTCGGCGGGAATGTGATTGGGAATTGT
TTGGATTCCGCCCTTCCAGTTGATGTGTCCAT
CGACGTTGTGGAAGTGGAGCCGGCTGTGTTTGAAACTTGCCAAAAGCACGGCCAGGTTCCACCATGTT
CCGAGGTTCGGACCAAGAAGGAAAATGGGACA
ACACGTGTGTGGGTTGTTCGAAAAGGGAGGCAGCGGCATCCAAATTACCGCTTTATCATTGGGGATGC
CCGTGTGGTGCTGCAGGAGGACAAAACACACC
AAAAGGCTGACAGTGAGGGGCCGCTCCATGATCGTTGCTATAGTCTTGTGTTTCTAGATTGCTACGAC
CCCGAGAAGGAGCGCATGATGCATGATGGCAG
TCTCATTGATGTGTGCCAGTCTCGTCTTTGTCCAGGTGGTGCGCTGATTGTAAATGCGCATATTTTGC
CAACGAGAGAGGCGCTGGAGGAGCAGTTTCTC
TCGCGTGGCTTTGCCTCCGTGCAGGTGTTGCGTGTGGCGGGGTGTGTTCAGAGCATTGTGGTTTGTTT
GGCGAGGGACAAGGCATCGGCGAAAAGTCCCA
ACGAAGAGAAGAGTAACAAAAGGGAAGGGGGCCAGCAACAAATAGGAAGACTGGATCGATTTTGTGTA
AGACACGCCCGTCATTTGGCGTCTTATATTCA
ATGTGCCTCGAGAGCGGACCCCGGTATGTGCCGTTTATTCCGCAGTGGTTTTTCTTTGGATGCAAACT
GGTTGAGGTCCTCACGGTCAGTGGAAGGAGCC
TCTTGCAACACCCGTGTGTGGGAACACTACGATTAGGTGGTGTGGCGCGAGAAAGCGGATGGACAAAC
GTATTTACACGCACACGTACCGTGTTGTATGC
TTCTTTTTTTTTTTGTCAGTGTGCACGTATGTGTGTGTATGCGGTTTAAAGTGTTTCTTTATTTTTTC
CCGTTTCATTTTTTTGGACCATTTGCCGTTGA
TGAAAACGAGGAGGAGGTGTTGTTGGTGGTGAGAAATAAAAGGGAAGATGGACGTCTACCGGCAGGCT
AGTGAAATTGTGTGCGCCGTGCGTGAAGGAAA
TGGCACGGCGAAGGCTCTCTGCCTCCGGAAGGAGATGCAGAAGAAGAAGCAGACATATGCGGTGGTCT
GCGAGACGCTGCGGCACTACGACCTCTTGCAG
GACGTGCTTGACCAGGCAGAGTTCTTCAAGTACTACCCGCGTGCCAACAAGGGGTTTGCCATCTGCAT
GGCCTACGACGTTGTGCTGGGAAAGGGACTGA
ACACCAACCGCGACACGACGGCACAGGCGATCAAAAAGTCCGCGCCGTACTTGCGGGAGGCGTATTGG
CACGTGAAGAAGCATCACGTTATCCCCTCACG
TGCACATGAGCCCTTCACGGAGGACAGTAACGGTGGGAGCAGGAATGATGAGGTTGATAATAACGAGG
ACAATTACAATCGCCGCTGCACGCTCACTCAG
CAATGCTTTTCTGGCGATTCGGACAGGGCGGCTGCGGTGCCAGAGCTGCAGATTCCACGGTATGCACG
AGTGAATACGTTGAAGATTGACGTGGTTACTT
TAGTGGAGCGTCTCCACCGCGCTGCTGAGAAGCGTGGCCGTGAAGATGACGGAAGCCAAGGGAAGGAA
CAACAACGAAAAGAGAAAAAAACCCATCATAT
GAAAGGGTTTCGTCAGTTGTCGGCCTTCACTATGGATCCTGTGGTGCCGTCGCTGCTTGTATTCCCAG
CAGGCACCGACCTGCACGCTCACCCGGCGGTG
CGCAGCGGTCAACTCATCCTGCAGGATCGTGCATCGTGTCTTCCCGCATGTATTCTGCTGGACGCGGT
AGAGGTGGTTAGGCCGTCATCCGCTGGGGCCG
GTGCACCGCTGGAGTACATCGTTGACGCCTGTGCCGCCCCTGGCAACAAGACGACACATCTCGCAGCG
TTGGGCGCCCCACACGTGAAAATAATGGCGGT
CGAGCGCGATGAGAAGCGTGCGGACCTTCTTTTCCGCCGTGTGCAGAGCCTCGGAGCCAGCGACTATG
TGAATGTTGTCAACTTGGACTTTTTCCAGATG
TCCTCAGCGGATCGTGAGGCAACGGAGGGGATTCTGCTAGACCCAAGCTGCAGCGCTAGTGGGGTGTT
GACGCGCGTGGATGTGTCACTTATGCGGCAGC
GCCAGGCCCGTGTGGCCGAAGTCGGTGAAAACAACAGGGGTGAGGATGGAGCTGGCAACAATGAGACG
TGGGCCATCAGCTCAGAAGAACATGGTACCGA
ATTTGCCAGCAACGCCGATCGGGTCATGGGCCTTGCGCGTCTGCAGCGGAAGCTTCTGGCGCATGCCC
TGCTCTCATTTGACAATTGTCGTACGGTCGTG
TACTCAACATGCTCCGTTCACGTGGAGGAAAACGAGCTTGTGGTGCGGCAGGTGCTGGAGGACACGCG

GGTGCAGGCACGTGGCTGGGCGTTGAGCAACA
TCATGCCAACAACATGGAAAACACGTGGGCAGAAAACGGAAGGCGACACATTCCCACTGGACCACACC
ATCCGTTGCGACCCCGTCGCGGACGCCACTAA
CGGATTTTACGTGGCTCGCTTTGACCGCGCCGTTTAAAAAAAAAAAAAAGAAAAAAAAATGAAAGTGT
TGACATTGACATCGATTAAACTCCAAATTGTA
AGGAATTATTTTATTTCTCTCCCCGCTCGTGTCCACGCCGAGTAGTATTGTTTGAATTGACGGCGCGG
GCGAGGACGGCAACGATGCCTGGGTGCTGTGA
ACTTCTCTTTTTCTTTTTTTCTCTCTCCGTATATGCATGTATGCGTATTCTTTCCGCGTCCTTTAATG
TGGTCAGGGAGAAAACTCACGGTAAAAAGGAA
TAACAACACCACAGACCAGAAGAGAGGGAGAGAGAGTCGCACCGGCATTCGGATCTCGACAGTCGCAG
AGAAGTAAAAGGGAAATAGAGTGAGAAAAAGA
GAGAGAGGGCATAACAAAAAAAAAAAAAAAAAAAAAAAAGAAAGAGGGAACAACAACAACAACAACAAC
AACAACAACAGGAAGCATACAAACACACACAC
ACAAAAAATGGTTGTTGGTCTCTCATCCTCCAAAGGCATCTTGGCTTTATTAAATGAGACCGATGCCG
ATGTAATTCAGTTTGCCCTCAAACGTCTGCTG
GAGCTGGTGGACACCTACTGGTATGAGATCAGTCCCGATCTCCCGTTGATTGAAGAGCTTCACGAGTC
GTGTGATCTCTCTGAGGAGACTCGAATGCTTG
CCGCTCTTGTTGCCTCTAATGTTCATTTTCATCTTGGTGCCTACAATGACTCCGTACACTTTGCGCTT
GAGGCCGGGACCGCATTCAACTACATGGAGCG
CTCACTCTTTACCGACACCATTTTAAGCTGCTGCATTGATCAGTTTGTGCAGTATCAAGAGCAGCCGG
AGGAGACTCGCGGCAAGCTCGATCCACGCCTT
GACAGCCTTTTTACTGCCCTGACCAATAAATGGATCTTGCAGGAGGGGACACCCGTACGGGAACTTGT
CGGTTTCACCATTCGTGCTCGCTGCCTTGATT
TTTTGGAGAAGGTCCTCCGTCAGGACATCGCCCTCACGAAGTCTGCTGCCATTCTGAACTACACCTTT
GACTTGGCCAGTGTTATGCTTCGTGATATTGG
CTTTCGAGGGAAAATATTGCGTCTTCTGGCGGATCTGTATGCCCAGGGAGGTCTGAGCACCATCGACT
ACTTTTCCCGTGCACAGTGTCTCTTATTCCTT
GGTGACTCGGAGTCCACGACACGACTTATAACCGACTTGATGCAGGCCAATGACAAGGCGACAGCCTA
CCAACTTGCATTTGACCTGTACGAATATGGAA
ACCAGGAGTTTCTTTCTGCGGTTGTGGCGGCACTTGAAAGGGAGGCGGCAGAGATTCCCCACCCAGGC
TTGACGTCATCTCCACCTGAGAATGAAGGAGA
AGAGACCTCGACAAAAGAAGTTGATACCAGCAATCTCTCCGATGTACACCAAAAACTCCTTGCCGTTC
TTACAGGGGAAACTTCCGCCTCTTTTTACCTG
AAGTTTCTTTATGCCCAATGCAATGCAGACATCCACATTCTCAATCACATCAAACAATCGATTGATGC
TAAAAAATCTGTGCCTCACAACGCCACTGTAA
TTGCTCACGCTCTTATGTACAGTGGAACCACCATTGATGCATTTCTACGAGGCAACATGGAATGGTTA
GCTCGAGCAAATTATTGGGCCAAATTTGTTGT
TACAGCCTCCATTGGCGCCATACACCGAGGACACATTGATGAAGGACTTGCTGTGGTGGACCCTTATC
TTCCAAAGGAAGGTGTGGGACTGCTTCCGTAT
CAAGAAGCAGGCGCCCTTTATGCACTAGGCCTCATTCACGCTCCTGTTGGTGTAACTAGAAACCGAAA
AACGATTGATTACCTTAAAAATGTGTTAAGGA
AATACAGTACGAGTGAGCAAATGATTCATGGTGCCTCGTTAGGCATCGGTCTTGCAGCAATGGGACTG
AAAGATGAGGAGCTTTTTGATTCTCTTTTTGC
TTGCGTCAGCGGCTGTGACGCCGTCGCAAGTGAGGGCGCCGCTCTTGGCATTGGACTACTCATGATGG
GAAGCGGGAACCGGGGCGCCATTCAGAGTATG
CGAGCACTGGCGTCTGAGGATAATCAGAAAGAAAAGACCATCCGAGGTATTTCCATGGCCATGGCGCT
TATGATGCTTGGTCGAGAAGATGAGTGCTGGG
CCGTGGCGAATGAGCTTCTGGATGACTCTGATCCATGGGTGCGGTTAGGGGGTTGCTTCATGCTTGGT
TTGGGGTATGCGGGGGCAGAGAATACGAAAGT
ACTGGAACGTTTGCTGAGTGTGGCAGTCAAGGACACCTCCGATGACGTTAGACGCAACGCCACCACCA
TGATTGGGTTCCTTACTATAAAAGATCCAGCG
CTTTGTTTAGAGCTTACCCGTGTCCTGGTTGACAGTTACAATCCACATATACGCTACGGTGTGGGAAT
GGCATTGGCAGTGGCGGCTGCGGGAACAGGTG
ATTCCAGCGTAATTGAAGTTCTCTGGACTTTAAAGGACGATTTGGTGGATTTTGTCCGTCAAGGGGCA

TACATTGCGCTCGCCATCGTCATGGTGCAGCT
GACGGAGGCGGAAGAGCCGAGGGTGAAGGAACTTCGTGAGATTTTCAGCAAAAAAATTGCCGGTCGCA
AAGAAGACATGTGCTCAAAATTTGGAAGCATC
ATTGCCACTGGCCTTTTGGATGCTGGAGGTCGCAATTGCACCTTTTCCCTGCACCGTCAACGCCATCG
CTTGGATAAGGCCGTGGCTGGCATGTTTGTAT
TCCTGCAACACTGGTACTGGTTCCCATACCTTCTGATGATCACACTGGCAATGCGACCTACCTGCTTC
ATTGGCCTGAATGACAACTTGGACGTTCCGGA
GTATACTTTTAAATCAAATGCACCAGCGAGCCGCTTTGCACTTCCCAAGAGCGTTCTTGCAGAGAAGA
AGGAAGCCAAAGCGGCTTCCGTGAAGGCGGTT
GTTCTGTCCACAACGAAGAAGGAGGAGCAATTTCATCGGCTCAAGCGGATGAGCGGCAGCGGCAGGGC
ACATGATGCAGCGGAGGCTGTAGCTGCTTCAA
AAAACGCAGCCGCCGCTGAACCAGCACTCACACCGTTAGGGGCGGAGACGGAGAAGAAAGAGCCAGAA
CCATCTTTTGAGCTCCTGAAAAACCCGACCCG
TGTAACGTCCCGACAGTTCGCTGTGATCTCTCACGATGTGGATGGACGCTACGTGCCACTTAAACCTT
ACCCAACGGGTATCTGTCTGCTGCGCGATACA
AAGCCCTCAGACGGCAAGCAGAAGATTGTGAGTGAAGTGGGCTGGAACAGCGCCGACGATGCACCGCC
ACCGGAGCCGTTTACATGGCCATGAGCTACAC
ACAGAAGATAAAGAGCGAGAGAGGGAGAAAGAGAGAGAGAGAGAGGAGGAAAACAAAAAAGAAAGACG
AAAAAGAAGCAGAAGAAAAGAAAATGAAATGT
GAGCTCAAGCAAAATATCAATATCAATATCAATATATATTTTGCTGAGGGAGAAGAAAAAAAAAAAGA
GGAGGCAGAACTAGGGAAGATATGCGGAAGGA
TTGATGCGTTATTTATGTTTATTGATTTACTCTTTTTTTTTTGTTGTTGCTGTGGAAAGGCGTAGACT
TTACTGGCTGCCAGCAAGGAGGTTCGTGTGGA
TTGGGAGAGGATTTTTTTAATTGCTTTTGTGCACTTCGCTTTTTTTGTCCACGCTGTGTTTGTTTGTG
TGTGCGAGGAATGAAGATTGCTGCCGGGGGGT
ACACGTGAGGGAAGAGGGGAAAGACGTGAGAATAAAGCCAATGACGCAAATATTATCTGTACGACCAG
GCATGCACGGGCACATTGACACAGAGGGGAAG
TACACAGGAGGCCTGAGAGGAATAACACAACCCCCGCTGCATAGGAGTATCTTCTCATGCACTCCCAC
TCTTTCTTTCTTTCGTGCGCCAAGAAAACGGA
GATGCATGTGCCTGCACGTGATTTGGGGAGGGGGCTCGTGGTATTGTGTCATTACGGNNNNNNNNNNN
NNNNNNNNNNNNNNNNNNNNNNNNNNNNNNNN
NNNNNNNNNNNNNNNNNNNNNNNNNNNNNNNNNNNNNNNNNNNNNNNNNNNNNNNNNNNNNNNNNNNN
NNNNNNNNNNNNNNNNNNNNNNNNNNNNNNNN
NNNNNNNNNNNNNNNNNNNNNNNNNNNNNNNNNNNNNNNNNNNNNNNNNNNNNNNNNNNNNNNNNNNN
NNNNNNNNNNNNNNNNNNNNNNNNNNNNNNNN
NNNNNNNNNNNNNNNNNNNNNNNNNNNNNNNNNNNNNNNNNNNNNNNNNNNNNNNNNTTTTTTTTTTT
TTTTTTTACCGGAACCACAAGAAACCAACAAC
AACAAGAAAGTTTATAGTGAATGATTTTGCAGTTCAACTGGGACCGCCTGGACGTCATGGCGGCCGAG
GCGATTCGAGCGCTGGTGAATGCCAAATTGGA
GGAGGACCTTCGGCGTCGTACCGCCTTCTCAGGGGGTGTTGACGCACCTGCTGTCGGTGGCTCCACTC
GGGCAGAATCTCTTTGCGTCACGGGTATTGAG
TGGGGCATTGCACCGCCATTCATTGAGATTCTGGAGCTTGACGACGCGCAGGACCTCAGTAGGGCGAA
CTTCACCCCGCATGACACCATTCATAGTCGCC
AGGAAGCGGCGTCGCACTGCAATCTATTCCGTCTCTCGTCCTCTGTTTCCTCCCAGTACGGAGGTGAA
CACGGAAGTGAGGTTGACTCCTCGCCGGGTGG
ATGTCCCTCGGCGTCCGTAACGAAGCGAATGCCTGGAGCATGGATGGGTAGCAGCATTCCACAGTCCG
GCTCGCCCGTCGTTCCGTCCAAAGATACTCTT
GCCCCTTTTGTTGGTTTGGGAGGACTTTATATTTTGTTGCATGTCACATATGGTGGACAAATGCGGTT
GTCCGTGTCATGTGTGTTGCGTCACGACATAC
CCCTTGGCTCTATTAGTCTTCCTGTACGCATGCCACTAACTCTCCTTGTGTCAAAGATGGATATGGAC
TTTTATTTATCTATCAACCTTCATCAGAACAA
GTGCCGTGTCTGGATGGAGCCAGGGAAGCTATCAACCTCCCCTATAACTAGAATGAACATTAAGGCCG
TGTTTGGCGAGCGGCGAACATACTCGTCACAA
TTACTGGACAACTGGACTGGAATTCCACCTGTCTGCGAGGATGCAGACACATCCGACGCGTGGACGCA

TGTGGCAAGTAGTCACCTTGGCTCAGAGGAGG
AAGATGATGCAGTTTTCACGGAGGAAAATGTCATCTCGCGATTTGTTTTGAGTGAAATTCGTGCGATT
CTTCAAGAAAAGATTATTTATCCCCATTTTGT
AGAGGTTCCGCTGTTTCTATGACGACTTTTTGAAAGTTTATTTGCGGTTCTTCTTCTGCTGTCATTTG
ATGGGAAACGTGGCAATTTGTGATTGGAGTCG
TGTGGAATCTCAAAATATCAGCATTGGCCATTGTTGCGCTGTACCTGTTGGCAACCTCTTCCTTTTTT
TTTTTTTTTTTTTTTTCTCCCCTTTTTTCTTT
TTANNNNNNNNNNNNNNNNNNNNNNNNNNNNNNNNNNNNNNNNNNNNNNNNNNNNNNNNNNNNNNNNN
NNNNNNNNNNNNNNNNNNNNNNNNNNNNNNNN
NNNNNNNNNNNNNNNNNNNNNNNNNNNNNNNNNNNNNNNNNNNNNNNNNNNNNNNNNNNNNNNNNNNN
NNNNNNNNNNNNNNNNNNNNNNNNNNNNNNNN
NNNNNNNNNNNNNNNNNNNNNNNNNNNNNNNNNNNNNNNNNNNNNNNNNNNNNNNNNNNNNNNNNNNN
NNNNNNNNNNNNNNNNNNNNNNNNNNNNNNNN
NNNTTCTTTCCTTTTTTTTTTTTTTTTCTTTTTGCTTGCCTCTTTGAAGAAAAGAAGGAAATACTCTT
TGACAAGGAAGAAAATTCAACGCAAAACTTAA
ATCACAAAAGGGATAAAAAGACCGCGTCCCTGTCTGTGTGTGTGGCTGTCTCTGCCCCACGAACGGTA
TTGTCGGAAATTGCAGACAATACGAAGAATAC
ATCCACACAAGACAGAGAGACGCAGAAAGAAGGGGGAATCATTACCACACCTAATGTCTGCGTCATCA
GAAAGCTTGGCTGTGAACTCCACACAAACGAC
ACCCTCAAGGCTACTCCAACGAGGCATGAGGCGGCGAGAAGTCTCCGTGTCCATTCTCTCTTCAGGCA
TTGATCCCACGCCCAGTCAACAGGGAAGCGGC
GGGAAACCTGAGTCGTATGCAAATACGGTTACAATTCCACTTTTTATGACCCAAAGCAACGAGGACCT
TCTCTTTCTGCCAGTGTGCAGCCGAAACAAAA
GAGTCTTATCTCAAGCCACAACACGCAGCAGTTCCGCAAGTAGCTACACAGAATCTCCGTTTCAGCGT
CAGTTGAACTCTGTGAGAATGAAGCTTGGGCT
GGCCGAGGCCATTGTGCAGAACGCATCCATCACCGCCAAGGTGCCGATGGCACCGGAGAAGCGTCGCA
GGCAAATGTTGCAGCTGGAAGTGGCAAAGCTT
ATCCTTTTTGTGGCGCGACAACATAACATCCGCGAGGAGCTGCAGCAAGTTGCCGGGAACTCCTTGGA
TGTGTCGGATGAAATTATGTACCATCGCCGTT
CCATTGAGAGCAAGATTTCTTTTTTGGCGAGGGAGCCGCCGCCCGATTTGTCGCTGAAAGAACTCAAG
CGAGCCACGTTTGAGATGGAGAAACATCTGGA
GAAGATGAAGGATAAAAAATCGAACGCGCCGTCCGTTTCCGCCCACGGGGCCACCTCCAACCTTTCGG
AGTTTCTTTGGCTCATTGAATCGATCGAATTG
GAGACGTTGGATCCACAGGACGTTTATTTGGGTGAGGAAGCGGCCAGTTTCATTGCCCCTTCCACTTC
CGCCAACACCTCCACACCTCTGGAAGCATTAC
AACGCCAACATGAGCTGCCGCAAAAAAAGCGCCCGGAATTGTTTCCCCCCTTGTTACATTCTACACCC
AGTTTGCAAAATGAAGGGGAAAGCTTTTCGAC
CCTGTTAAGGTCACCAGGCTTGTATTTGTTGGAGTACCCACCACACCTCAGCACCGACAAGACAACAA
CAAAAGCGGAGAATGAGACGGAAGAGGAAGAA
CTCGTTGGATTCTCTGTCGATGTGGAGTCTCCCGGGTGTCGTGCCGTAGAGGCGGCGGGGTTGAAACT
GCTTCAGTTCATGTATGATGCTGTCGACGCCA
AGGACAAGGGACTGAAGCTGACGAATGGAAGTGAGGAGGAAATTGCAGCCATGTTTAGAGCAGAGAGG
ACAATGCAGTATGGAAATGAGATTATGCGCTC
TCTGAATACCGAAGAAGCAGACGCCGCTGAGCCAAAAGAATGTCTTTTAGGATCTGATGGGACGATGC
GGAATTTTGATGCCACCCGACAGACCCAATCC
GCAAGGAACAGAGCCACTGGCACTCTCTCCAACGCCCAAGAGGAGGAACTGAGCAATCAGTCAAAAAC
AAGCAGCATATCAGCTCTGGGAGAGGATTCCT
TTTTTTTTTCAGGTCACCCCGCGGGTTTTGAACCGTCATTGGTTAATTCTTGGATAAGGCCAGTCCAG
TCTACGGTGGAGGTGCATCCTCTTCGGACAGG
TATTATAGATATCCTAAAGAGGCTGTACGGGGACTTTAATTACAAGCATTACTTTGAACACTGTAAGC
CGCCCAAGGCGGATAAAGTGCCCAAAACAAAG
GAGGACTACTATCAGGAACTAGAGCGACGGGTAGCTCGGAGGAAAAGTGAAAAACAACAACGCATAGT
CAGCGATCAGAATGAAGAGGATTCAATGCTCT
TGAGGTGGAAAGGGAGAAGCAGTTCGTCCAAAAGTTTAACCGTTGGGAAGCGAGACTCGAACATCAAA

ATACAAGAAGAAATCACAGATGATCAGCTGGA
GAGCATGGCGACTGGGACTGCAGAGACGCCTCCGCTGGAGACACCCTCTGTTGTTACCGCGCCGCCCA
CTGAAACAACAGTTGATACGCATGGTGAAGGA
ATTGTGCTTGTAGTGGAGCTGGAAGAAGGTATTCACTTTTTGCTGAAGGGGCGGAGAGACGAGCGGGC
GGGCCAGCAGAGTGCCTCTGGATCAATGAATT
CCTTTAAAAAAGGGAAGGACTTAACGAAGGGAGGAAGCATTGAAAATAAGAAGGCTGTCAGTGAAAGA
GGTAACGCGGCACAGAGCGAAAGTCTAGAACC
GGATTTTGACAGCTACACCTTGTACGCTGTAAACCAGTCTGACATGCCACGGCAGGCGACAGTGGAGA
CGAATCGAACAAAACTCAAAAACTTGAGACTA
AAGGCACTGCTCAAGTACTTTGGGAAGAAAAGAAATGCAATTGCTGTGGATTTGCCTCCACGCGGCAC
CCAGTTGGTGTTGGTAATGAAACCCATTAAAA
AAGGACGAAAAGTTGTCTTTGATGAGGCGTTGAAATTTTACGAAGGACCTCCAAAAGAAGAGGCACCC
AAAACGACATATGCAATCGCCAAGCAAAGTCC
ACCACAAGTCCAATCTACCACCACGCCACCGAGAAGTGCCCCGTTGGCCGAGCCCGAGGTGTTACGGG
ATTCCGGAGGTACCGGCGCGGAGGCCCAGGCG
AAGGGGGAATCTGTTTCTCAGGCTGAATACAGTGAAAATAAAACCTCAACCAAAGCTGGTAAAACTTC
TTTGGAGGTGGCTGACGCAACAGAGCCAAGCG
TGCACGGAAACGAGGAGGAGGATCAGGGGAAAGCTGCGGCACTCAGTGGTGCCCGTGTCCCGTCCATG
CAGACGTTTAAGCCTTTGTTGTTAAGGAACAC
CGGTGGCTATACAGAGGATTTTTCATCCGAAGCGTATTCTTTTGACGGGAGTTCATTATCTGGAAATG
ATGTTTCGGCAAAAGGGGTCAATGACTGGGGC
GTCAGTGTTCTTCCTTTAGGCGAAGACCTTGAGGATCAGCAAGAAGAGGGCTGGCGGCAGAAAGGCGA
CATGGGTGTTCCACGGAAAAGGCCCAAGGAAT
CAGACAACAAAGCCGCGAGAACAAGATTCATGGGTGAAAAAGGAACCAAAAGAGGAAGTGAAACGAGG
ATTGGATCCAGTAAGGGTTCTCAGTTTGAAGC
AGACAGGGCTTCAGGGGAAGCAACGCGGGTGCATCAATGGGGAGTTCCCCTAACAAATGACGATGATG
AGGCATTTGAAAATAATTGTGTGGTCTTGTCT
GTGTTGCAGGCATCTGAGTGGCAAAGCGGCTCGAAAGTTCCGAGACGTTCCTTGGGAAGTAAATCTGC
CGTTCATAGTGTGGAGGGTGGCGAACCCAAAG
AGATGAATGTGAAGGATGGGGGAATGGATGACGCGACGGGGAAGGCCACGGAGGGTGGGATTTTGATG
GAATCTTCGTTGCGTACGACGTCTGAGCGGTA
CCCAAGCGCGCGTTTCCGAGCGACGAGCATTGATACAAAGGAGGAGGATATTGTCACGGCCGCGCTTT
ACCCCGCTGGGGACGGAGATGCTTTAAAGACG
GAGGAGATAGTATCTAGTTTTCTTCAACGTATCGCCGAGGCGGAGCAGTTAAATTTGGAGGAACAAAT
CGATAACGAGGATATTTCTTGCCCGAAAGGAG
GTGAGGAGACACCTCTGCCTGCTGGGTCAGCGGCACTATGGCGACGTCAACTTGTATCTCAATCGCAA
CAAGATCCCCATTCTATTGGATCGCAGGAGGA
ATCAAAGCAGCTTCTCTCCTCGTTAACGCCAGGGGTCGAGACAGAGTTCCCGCTTGGATCAGACAGAG
AAATATGTAAGGTGCAGGGTAGCACCTCTTTG
GCGGCTTTACGGGCCGAAAGTGAGGAACACGAAAAGGGATCCACTCTTAGAGCATCTTCTTCTCACAT
GTTTTCTTCAGAGCAGTTAACTGCAAATGCAA
CTGTGGTGCAGCGACCAAGTGTCACCTTGGAAGGAGGGTCTTCGGCCCAACGCATGAAGATATACGGA
CTTCCCCAATCTTCCGTAACAATGAATGAGTC
TGTGACTCCTGTGCACTTGGATGAAAATTACACCAGCACCTCTTATCCTCAGCAACCTTCTGTTGCCA
CGAAGGTGGACGGCGGCGAGATTGGGGATGAT
GTAAGTACTCCTGTGGAAACTACTTCCGGGCTGGGAACGACGAGCCGAACGGCGGACAAAAGATCCGT
TTCAATTGAGCCTTTGCAAGGGAACAGATATG
CCTCACCAACCATGGAGCGGCCAGCATTCAACTTGGATCTTTTGTTAAAGAGTGTCAAGAAGGCGGAA
AAGAAAGAGGCTCCTTTGCCAGGACTCTCCAC
TCGTATAGTAAAAGATGAATACGCCCTTCCAGAGCAAGTGATGCCGGCGAGTCCCAGTCGCCAGCATG
CATACGCAGAGGCCCGACCAGATGGACGATTT
GCCATTTTTGCTCCTGAAGAAATGAACGTTAAGGGAGCAGCCCATACTTCTGACCGCATGGACAAAAA
GAAGATGTCGACGCGGCTCTCAGGAGTAGTGG
AAAACGCGGCGGTGTTCGCCGAAGAAATCCGCACAACTTTTCATGCCTTTGCGCGTCAAGCCACGTCT

CAGAGGAGGCAGACTTTTGCCCGCTCAGAAAT
ACACGGGAAAGCAACCGTCGGAAGAAATGTGAGTGTTGATGTTATGCGCCATGCAACACTTCCGGCAC
TTTCAATGGTTTATTCCCCTGAGACGGCTCAG
GATGTGGAAGACATGGAAGCTGCGGAGGAAGAGGCAAAGGCACTTTTTCTACAGGATCCTGCTCGTTA
TGACTGTGTTATTGACGAACTTACAAAACGGG
CCATAGCAAGAGTTTACGAGGAGGAAGAACGAGAGAAGGAGCGACGGGATTCCGCGGCACACGGAACG
GCTGGAAGGAAACTGAAAGAGGGATTGATTGT
AGTGCCAGGTCGAATGCAAAATCGTTCTGTAGCCTTTCGTCAGTTTTCTGTGGTGGGGTCACAAATGG
GTGGTTTCCAGGCAATGAATATTAGTAGTACT
GGTAGCATGGTCCAACTTAACGGAGACCCTGTGCACTTTACTCACGGGGGGAGTTTTTTAAAGGGCCG
TCACCCGTCACTCTTACTGGGGAAAAGCCACC
GTGATAAAGTGCGTCATGACACGAAGTCTGAGTTGGATCAACTCCTTGATGAGCAGCGTCGTGCAAAG
AAGATTGTGGCATATATTCTTATGGAGATGCG
TCGCATGTGGCGGCGGCGGTGTCTGGAGTCCAATCTTGCTCTCCGCAGTGCCGTGGATCGCTTCATTA
AACGATCCGTTCTTTTTTCATGGCATTTTGAG
AGGCGACGTGTGCTTAATCTACACAAGCTCATTCACCTCCTGGACCGCAAAACCGGCCGCGTGGGTGG
TTGGTTGCCGTTTTACATGAAAGATCGTGTCA
TGGAGGACCGATTTGTCTGGGAGCCCGCCCCACTGCACCTCTCACGGCCGCTGCGTGTTGTTCACCGG
GCAATGTGTCTTGCCAGACAGCAACGGATGTT
TCCTATTCGTCTCGTGCGGCAGCAAGCTTTCCGAAGGAAAAATTCGCAGCATATATTGTACGGTTACA
AGCGTACCATAATGGAGCCGTTTAGGAGCCCT
GCCGTGCGCCTTCGTCATGGGCGACGCTCTCGCTACGCCGCGCCGTTTCAGAATCCCACAATGCGTTT
TTCTCCGCAGGTTGAGTGGATGGACGGAACAC
CGAAAAAGTAGGTCTGATGGAGGCCCTGGTATTGCCAGTAGTAGTAGTGTTTTAAGTAGCACTACTAA
AAGTGGTAGTAATAGTCGTATTGATAGCTATT
TCTCAGGCTCATTCTTGCGTAAAAAGGAGAGATTTACATCAACCAACCATACAAAAAAAGTGGGATTG
TTGGATGTGGCATTCTGTGTGAATGCGTCATG
TAAAATGGAATGGGGTGACCTTTGGTAACCCAGCAGGTAAAACAAACAAACAAACAAAAAGAGGGATT
GATGTTGAAGGAACAACAAAATGTCTGATACT
TGTGTCTGCAAATGTTTTAATAAATCAATGCGGCCTTTTTTATTTTTAATTTTTTGCTTTTGCTTTGC
GTTAGCCTCTGTTGGCACCTTTTGTTTCTTTT
CCGATAGAGTCTGTCTGTCTGCGTGTGTGTGTGTCGCGGTGGTGGTGGGGCGTGTTCACATTTCCCTT
TTCAATGTGAAGACACTTGGAGTTGTTGTCTG
CTCTCTCTCTCTCTCTCTTTTTTTTTTTTTTTTTCTTTAAATTTCTTCTTCTTCTCTGCAGCTAGTTTG
GTTGATTGGTTGCGTGTGTGTGTGTGAGTGCG
TGCGAATTCTGCGCCGACCGAGTAACTGAGACCGAGGGAGAAGAGGATAAAGAGAGAGAGAAAGAGAG
AGCGATGACTGCACAGTGCCCATCGTCGTTTG
CCTTTAGTTCCTTACTGTTGTCATCGTTTCTTTTGTTGCTGACGGGCGTTTTTTTTACCGAAGGCACG
GAGGAAAACGGTGATAACATGCCGGAACCGTC
GATAGGGAATGCAGCCGCGCTCTCAATGATGCAAGATGTCTCTGTGGGGAAGAAAAATTCCTCCACGA
CGTCACCTACTCCACCGGAACCCCCCGATGAT
AATCCCTCTTCTGATTTGGCGAACCCCCCGCCAAACAGCACATTCCTCATCGCAGCTAATAATTCCGT
CACGTCAACGACGGGAATGACGCATTCACCAA
CGACTTCTCTCACCGTCTCACCCACCCCGTCGCTGGAGGATGTGCCGGACAACATGTATACACGGGCG
GAAAGTTGTGATATTCCCATAGTAAATAAATG
TGTGGAGCGATTTTGCAATTGCATCTCTCGGGGACAGTTACAGAATGCCAACCGGTCGTGGTATATCA
AGAACGGGGTTTATCAATGCCACGAATTTGCA
AATGCGAATTGCTACTATAACCTTTATTGTGCGCGCGAGCGGGTGTCATGCCTATGGAGTGCCTCTTT
TTTGTACAAGGCGTACCGTCCACAAGGGAACA
CTTTGGCGACATCATATGAATTGAGCCAGAAAAATTCCCAGGGCAGCATGCCATGTGTTGGTCTAGAG
GATATACAGGGCAATTTTTCCCAGCTTACAAA
GAACACAAATTACTACGACTCTCTGTTTTATAGTGAGTGTGTGGACTATGTGACATTTATTTTGCAGC
GTACGGGTGATGGTATTTGCGCTGCAAGCATT
GTTCCCATGTATGTTTGTGGGGACTCTCTGTTTCCCGTTCCACCCACACGTCAAATCCACTATACCCC

ATTAAGTGAAGGGGCTCGGCGACTTGTTATTT
CCATTGTGGCACGAATTGTAGGTAACTTTACCCCCATATTTAATTTGGAACGTGCCAAGTATAATGAA
AAATATCAAATGAATATTGAAAATCAATTAAG
TAATGCTATGTACAGTTGTTTTTTTGACGCGATCGGAATCAAAGGAGTTTTTCTGTGAAACACGAGG
ACGAATGTCTTGTTGTGAGATATGAAGTGGGT
GTGGGAGAGGCTGATCCATGGGTGGGCGACGTTGTGACGGCAAATGCACTAAGTCTGATGGTGCGCAG
TGCCTCCTGGATGGACCCCGCGCAACGGGTGA
TTGGAAACATTACGGACTCTGTGAAGCTTAGTGCACCCATTGTTGTATATTATGTGAGCTTTGAGTCT
GGAACAGCCCAACCGGAGGCCAAACTGTGTGA
TGCCGGGTGTTCGGTAGCTATTTCATTGGTCCTGTTTTTGACACTTATTGCATTTGCTTTTTCGTGCG
TTTTTTGTTGTAGACTGTCTCGAAGGCCGTAT
TTTATGTTCGATGACGACCACGATTTTGAGGAGATGGAAATTGTTAAGGGTCGAAGAGCCATCGTACC
CAATAGAAAATAAAAGCAATGTTTTTCTTTTT
TTAGCACCGTGTGTGTACGTGTGTGTGTGTGTGTGTGTTTTATGAATTCGCACGTCTACCTTTTGTTC
ATTCTTTTTCTTTCAAAAAAATTTCTTTTTTT
TTTATTTATTTATTTGTTTGCATTTGCGCATTAGGCCTGATGTAACATGACTCAATTGTAGGTGTATT
CCCTTGAGATCTGCCACTCGGGCTGTCCTTTG
CTACACATGTGCAGATGTATATACATTTCTTTTTATCTAAGAACCCCCCAGTATATATATATATATAT
ATATATATATATATATCTGCTTTTTTTCCCCT
TTTTTTATTATTTGTGATTCAAGAAGCGTTGAGACTATTACTGTTATTATTATTATTTTCTGTTTTGT
TTCGGGCGGAGGCAGAGGTTTAAAGTGCTGCC
ATTTGAGTGTTGTGTTGAATATACTTTTTTTTTTTTTTTTACTTTCCATCTTTTTATTTATTTATTTA
TTTATTTTTGTGCGTCCTGTGCCATTTGGTGC
CTGCAAGGTAACCCATTTACTCACGGCAGCACAAAGTTGGTGGGAGGATGGGCTGCAGACATAGTGGT
CCCCGGATGCCCCCACTGGAACAGCAGCAGGG
GCACTCATTGGACCGGCGGAAGGAACGCGATGTAGTGGACATGATGCCGGGGGATGGTGTAGACGCGA
AAAGCGCACATGTGGCGGGTTTAAGTGGTGCA
ACGAGTGTGGATGGCGCACAAGGCAAGCCGTCGCGCAGAGATGACGTCTATTCCCCAATGCCGCCACA
GGTGCCGTCAAACTCCTGGTACGCACGGGAGT
TCACGGAGGTGACCAGGATGTCTCTTACGGAGTTTCACATGAAAAAAAGGGGCAACGTTGTCTTTTCC
AACGCATACATTCCGGTCGGAGCCTCGGAGTT
GGAGAAACTTCCCGGACGCGCCCCGCCGATGCCAGCGACGCTGGAGATTGGGAGGGACCTCGTGTACG
GCATGGCGTACCTTCACAACACACCGCTCTTT
ACGGAACACCAGTGGCGTCTGCGGGTGGCCCATCACGCGAAGTGGCGGCAGGATGAAGGAAAAAAGCA
CGAAGGGAGCGAGGAGCGTCGGTCCATTTGCC
GAAGAGAGTGGGCGAAAACGATTTACGATCGGCCGGGCATTGACACGTTAGTGGGCATACGCGTTTTT
GTTGAAAACAAACTCGTGCCCGACGAATACCC
CACAACCAGGAGTATTCCAAACTGCACTGCCACGATAGACGCCAGCGAGGCATTCAACAATTGGATTT
GTATTCCTATCGTACTTGTGGGGTATTTGAAC
AACTTTCTTCTGGGTACTATCACGTTGTACCGGTTTCTTTCACTCAACTTTGCAGGGTACATGCTGCG
TCAACCCATCAACCTCAGCGGAGTTGAGGCAG
CTGCAGCCGGCTATGATGGTGACGGTGAAGGGGCAGATGACTGCAGTGATGATCATGCCAGTCGAAAG
TTTGAAATTTATGTTGAGGTGGTGTACGGATG
TTCGTCGGAGTTTAACTTCTGCACCGATTACATATCAAAAGGTAGCGTCGAAGTTATTATGACAAGGC
GATCAAAGTTGGCTTTGAAAACTTACAAAGAG
AGGCTGCATAAGTTGATTCAAGGTAGTCTTCTTTCGCGCGAGCAGGTGGTTCCTATGGACCGATTGGA
GATGGTGGCACAGCAACAGTCATCGCGTAATT
GTTTGTTTTGTGGATCCCCGCTGCGTTACACATGCACCATATGCGGCGCCCAGCTCTGTGGTACGCTT
ATGTGCGTCTGGCGCCCCTTTGCTGGGTATCC
TAGCGGATGTTCCGTCCATAAAGCACAGGTAATAATGTGAAGCTTTTTATTGACACCTTTTTTTCCCG
TTGGGCTCTTTGTCTCTCTCTCTCTTTTTTTT
TTTTTCTTTTGCCCGGATTTGTCACAAAGAATGGGAAGGGGGCAACACACGTTCTTTTTTTTTGGTGC
AGGCAAAGTGTGTGTGTGTGTGTGTGCGTACC
GTCAGCAACGGCGGGGAAGGAAGAATAGAAGGAGGGAGTTGGTGCGGAAGAGGGACGAATACATGTGG

CATGAATGATTTATTTATTTCTAATCCTCCCC
CCGATTTCACTATCATGTCCCTCTTCCATCATTCGCATGTATGGATGTAGTCTGCTGAAGAGAGAGGC
TGTAGGGGCCCTTTTCTTTTTGGATTATTGCA
GGGACCGTGGTCTCTTGTGGAGTGCAGAGCGAAAGGCTAAAAAAGGGGAAGAAACCAAACAAATAAAA
ATACATTTAGCGGTATGATTTCAAAGCTCTTT
CGGTTGGACCCGCAGCGGCGTGAGAAACATGAAATCAACCCTCACGATATCAAAGCATTGCGTGGGAT
GGCTGCCACGCACAACATTACCTCTGTGGCAG
TTCCGCAGTATAGGGTTCGAGAAAACTACGTAGAGTATGTGATTGAATGCTCCAAGAGAAGCGTAAAA
TGGCAGGTGTTTCGACGATACCAACAGTTTAG
GGCGCTTGATCAGAAGCTGAAACAGTTGTGTTCAATGGGTTCCCAGTACCACTGTGAATACGGCGTCC
TCCCAGTGCTTACTGGCAGCCACTGGGCAGAA
GTGACAAATCAATCCATCGACCTTGTGGAGAAGCGTCGTCGGTATCTTGAGATTTATCTCGAGCAGCT
CCTTGTACCAAAAAATCTGTTTTATGTGGCCA
AGACAGCACTCTATAAGTTCTTGCACGAGGGGGAGGTTCCAGTACGGTGCAGGACGAATGTTTTGCGT
CCTCTCATTGGCTTTGCGGCAGTTGAGCCGTC
GCGGCGGCCGGAGAAGAGTGAAGACGACGCCACAACTGTCGAAGAGTCACCGAATCCGCTCGATATTT
CCGCTGCCGGCGGCAACCTCCCCGTGACGACG
ACATGTCACACCACGCCTGAGAGAGGGGTCTCCAACTCTGTAGAGTCGTCTTCTACGAACAGGTCGAG
TGGGATGGAGGAATACGACGACGTTCCATCAA
GGGAAGACACCGATAACGAAGACATCCACATGCCACCAGCTTCACCATTGTGTTTGCAGTGCAATGCC
GAATTCTCATCCATGCTCTACCCACATCGTTG
CTTTTTGTGTAAACAACGTTTCTGCAGGAACTGTTTGCGCAATGTGGAACTTGAAGAGGAGGTGGTTA
GAGTGTGTCTACAGTGCTACGAGAACCATGAG
CGGCACAGTAAGAAGAAGCAGACGGCGAAGGATGCCACTCCTGAAACGCCTCTTTCTCAATTTAAAGG
GGTCACGGCTGCACCGGAAATTCTACAGCTCC
ACGAGGGGAACCAACTGCGGACAGATGTCTCTTTGTCTGACTTTGAGCTCGTCACAACGCTGGGCCGT
GGTACATTTGGGAAGGTCCTCAAAGTCATATT
CCGTGAAAACGGCCAAGTATATGCTATGAAGATTTTGAATAAATGCATCATCCACAAGCGGCGTATGG
TTGAATACATTAAGGAAGAAAAAGATATCATG
GCGTCGCTACCTTTTCATCCGTACGTTGTGACATGCCATTTTGCCTTTCAGACCGATTATCATTTATT
TTTTGTGTTAGACTTCCTCCCGGGCGGGGAAC
TGTACTCCCATATCTATCCAAAGTGTACCCTTTCCCCAGTGGACGTGCGGTTAATTATCGCGGAGGTT
GTTTTGGCTTTGGAGCACCTGCATCGTTACGA
CGTTGTGCATCGAGATCTTAAGCCGGAGAACATTGTGTTTGCAGCGGACGGTCATTTGAAGTTAACAG
ACTTTGGACTAGCAAGAATGAACTTTTCCCGT
CATCGTCGGTACAGCTTTGTTGGCAGCCCAGAGTACCTTGCCCCGGAGACAATTCGAGGGGAGTGTCA
GTCAAGGGCGCTGGACTGGTGGAGTGTGGGGG
TAATGATGTACGAGATGCTGATGGGATCAACGCCGTTCCACGCTGCCAACAACAACGATGTCTGCAAT
AACGTGCTGAACCGTACTCTCGACCTCACCGC
ACCGTGCTTCACCCCTGAGGCTGCCTCACTGATTCGGCAGCTCCTGCAACGTCAGCCAAAGCAACGGC
TCCGAGACGCAGAGCAGATTAAGGCGCATCCG
TACTTTGCTTCTCTCAATTGGTCGGCGCTGGAGAATAAATCTCTTCCCCCACCGATACGGCTCAACCT
CGAGGGAAACGACACAAAGTACTTTAAACGGG
AGTTTACAGCGGAGTGGGCAGTGATTGCTAGGCCGCAGGAGGTGAGTCGTGCCACGTTGGATATGTTA
AAAAAACAATTTAGCAATTTTGTGCATGTGCC
CGATGTGGGTGCGGGTGTGTCTTTGCCGTCACCATCTTCCGGAAAGCGGGCATCACTCCCACAATCCG
TAAAGGCGGATGCAGATGAGGTGTTGACCGTT
CAGAGGCTGGTAGGCATCTGGCGTCTAGTGAGGGTTGAGATGACGACAGATGACGGAAAAATTGCCTA
TCCGTGGGGAAGCGAAGTCTGCGGTCTTCTCG
CATACTTCCCTAATGGTATTTTCACCATGCAGTTTACTCTGTCTCGTAGGCCGCACACGGGTTCCCAT
TTTCCGGAACAGGCTACCGTGGATGAACTCGT
GGAGATGTGCAACTCTTACGTCGCAAGTTTTGGGCGGTACCAGTTGAAGGCCGAGAGTAACGCTATTG
TTCACTGCCCCAATGGTAGTCTTTTTCCAAAC
CCATCATGGAACCAACAGAAGTTCTTTGTTGAGGTATCTAGTGAAAAATCAGTAGGTGGTGTTGCTGC

ACTGAAACTTTGTACACCACAATACAATCTTC
AGGAGGAACAGTTGTTAGCAAGAACTGTGCTGACGTGGGAACGAGCCGATGGTTTTAAAAAAATTTT
TTTTTTTTTTTTTTCGTTGCTGTTGTTGTTGT
TATTATTATTTCCCTAATTAATTTGGAGCTGCGGTTGTCTTTTTTTTTTTTATAAATATATATGTTG
TGTCTTTTTGCATTTGCATTTGGTGTGCTGTG
AGGTTGTTTTTCCCCATTCTTGATCCGTGCATTCATCCCCATTTCTGTAGATATTTATTTACATTGAT
ATATCGATAGAGGTGTGCCTATCACCTTTTAA
CACCTTTTCAACAGTCTCTGCGTCTCAATAAACCAAGCAGCGTGAAATACCGTGACGATATACATTAA
CATGTGTTTATGACTGTGGTACCGATGCTCTT
GAGGAGCCTATTTTAGGCTATTTTATGTAAATTTTATCACTGCTGCTTTAGTTGTTGTTTTGCTTGTG
ATGCTGGCTGCTACTGAATGCCCTTTTTATAA
TTTATTTCATTTTTTTTTTTCTTTTTTTTTGTGCCCTTTGTGAGGGTTCCTTCTCTCTTGGTGTTGTG
TGTTTCCTTGCTGTTTATTGGACGCACGGAAG
CAGTGAGAGCGCCGGGTCACGGGGAATCAGGGAATACAAAAAGGAAAGGGAGGGACAACTGTTTATTG
TGTGATGCGCTGTAGGAGGATGCTGTGCTTCT
TGCCGCTGCGGCGACCCGCCTCGCTGCCGGCAGTCACAGGCGGTATTCATGGCTGCCCCGTCGTGGTG
GGTCTTCGGTGGCACTTTGACGAGGTGCCATC
CTCCTCAACTCCCGCATCGTTTGCGTCGAGGCCGGTGATATCGAAGGAGGACCGAAAAGTCATGCCGT
GGGTGGAGGGCGGCAGCCCGGCGGATGCAGCG
GCCGCCTCAAAGACTTACAGGACCGAGCTGACACACGAAGCTTCTACCGCTGCTGCTGCTGCGGATGG
TGGTGACGGCTTTGAGAAGACACCGCCAAGGG
GCCAGCCCTCTGCAGAATTGTCGCCCACCGCGCGGTACGCCCCGTGGGAGATCCGGGCGTTGCTGTTG
CCGCTTATTCCTCTTGGAGGCGCCACTGTTGC
TCTTCGCGAAGTCATTCATCTGCTTCCTACCGCAGCCCGAGAGGAAATGCAGCGGGATGGTGGCAGTC
CGCTGGCGTACGTGCGGCAGCACTTCTCTGAC
GATGTTGTTGTGCGGGGTGTTGAGCTGTCGCGCCGCACCGTTGAATTGGACGGCGTCGTACCACACGG
AGGAGCGAGGCAGGCAGCGCCACCACCACCAC
CCCCGCCGTTGCCATCTGCCAATTCGAGTCGGGCAGCTGCACAACGACCTTCGCAAGCTGCCGGGGGT
GCGAAGACGGTTTTGGAGGTTCTAGACACACT
GGTGGAGTTCATTCCGACCTTCTTCGTGCAGCAGCAAATGGTGTCCGACCAGCTGCCATCTGAGGTCA
AAAAACAGTACGTCGATAGTAGTTTCTTGTTT
TACATCAAGCGCTTTCGACATTATATTGATATTAGGGCACATCATGGGAGTACCGAAATCCGTCTGCG
TCCTGACTTTGCCCACCCAAAGCGCGGCGCGG
CGGACACCCGCTACGCAACTGGCCTTAGCAACAGTGACATGTTGAGCCAGTTGGCACGGGGACGCAGG
CCACCACGAAATTCTGAGGCAAACTTAATTCA
CTTTATTATACCGAGAATTCCAAGCACTTACACACCACTTGCAACAGTACTGCAGGATGTGTCTGATA
TCGTCTCGCGGCACCCTTCGTTTGATCCACGT
CTTGGAGTCACAGGTCTGTTTGAGAAGTACCCGGAGTACTTTCAAATTGTTGATTCGAAGCTGCGTGT
GCGGCCGTTCCACAGTGCACCAAACTCGTTAG
ATGACTTGAATGCAACGACCTCGCCCCTTCCTACAATTTATGCCAAGATAAAGCAACTTGTGGATGAG
TATGCGGAGGGAAAAGACTACACCGTGGAGGC
TGAAAAGGCAGCAGCGGTGGTTCCAACAGGGAAATTGTACGCGTTACTTAATAACACAGAGAAGAAGG
AAGTAAAGACACGCTGTCGTTCTTTTCCACGT
TTCCTGCGGTTACATGGAGAAGAGATTGTGGTGTCTGTAGATAATATGAAGGTTTATAAATTCCGCCC
CACGTACGAGAGGTGCGCCGAGACGATTATGG
ACCAGCGTCTCAGTATGGGCTCCCTTTCTCCGGATGATCCCGTGTTGAAGATTCCTGCCGCAATCGAC
GACAACAGTTCAGCTGACTGGGCGGTGCGGGA
GTTGTACGACGCGCTTCCACTCATGCAATGTGCGGAACTTGACGAGGTCATGTCTCTGGTCCCACCCT
CCGTCAAGAGCGCCCTTCCACAAGAGCAGGAG
AAACTGGTTGAACATCTTGCACTGTACCCGGATTACTTTTTGACATGGCCGTACCCGGATGATCCACG
CGTCATTGTGGTGCAGCGAGCGAAATTGGAGC
CATTGCCGATGGAGGGGGAGGACATTGCACGTGTAGTAATGCCGATGATTCCACAAGGGGGTATTGAG
GTGTCAAGGCTTCTGCGTCGTGTCCCACTCCC
GCTGCAACGCCACTTCTACCGCCATGGTCTGAAAAAGGTGCTAGGCGAGATGAAGAATTACTTCCTGA

TTGTGGGAGAAAAAGTGATGCGGGTTGGGTGA
TAAGAGAAGCAATACGATTCCTTTGTGTACTGGCCCTTTCTGTGAGGGAAATGACTTTTTGCTCATTG
CCATTGCCTTGAGACACCAGCGGGGAGGAGGG
TCATACACGGAAGAGTATGGCGGAGCCCTTTGTTTTTTTTTTTTTTTAATCTTGGCGTGAGGGCAACAG
AAGTGGTATTAATATTTAAATGCATATATATA
TATATCAACAAACGNNNNNNNNNNNNNNNNNNNNNNNNNNNNNNNNNNNNNNNNNNNNNNNNNNNNNNNN
NNNNNNNNNNNNNNNNNNNNNNNNNNNNNNNN
NNNNNNNNNNNNNNNNNNNNNNNNNNNNNNNNNNNNNNNNNNNNNNNNNNNNNNNNNNNNNNNNNNNNNNNN
NNNNNNNNNNNNNNNNNNNNNNNNNNNNNNNN
NNNNNNNNNNNNNNNNNNNNNNNNNNNNNNNNNNNNNNNNNNNNNNNNNNNNNNNNNNNNNNNNNNNNNNNN
NNNNNNNNNNNNNNNNNNNNNNNNNNNNNNNN
NNNNNNNNNNNNNNTTCTTTGTGTTTGTTGGAGTCGTTTATTTACACATCTGGTCGACATCGATTGTG
GCCGGACATCTCCTTTTTGGCGAGTCTCTTGT
ATCTTTCCTCCTCTTGACGATTGCTTTTTTTTTTTTTTTTTTTTACTTTCTTCCTCTTGTTGTTATTA
TTATTTGGTTGAGCGCAGGAAATAATTGAAAG
TAGCTTTTTACAATCTCAATAGAACGACATTGGGCGCCCACATCCAGAGGATCAGTTCCAGTAGATTG
TGTGGTTACACCACAGCCATATTTCAGGTAGC
AATGATACGCAAAAGGGTACTTGGTGTCGGTGCATCATCTGCCACCATGCTTGTGCAGGACACGGAAG
CAAATGGTGTGTTGCGTGTGCTAAAGCGAATT
AATGTGTCGTCGTGGAATGCGGCGGATGTGACGGAAACGCTCGAGATGTACAAGACAATTGCGAGGGC
GAAAATTTCATACGTGGCGGAGGTGCACATGA
CGATGCTGCAGGGATATTTTTTGAGCCTTGTCATCACGTACTACGCTGGAGGTGACCTTGAGAGTTAT
TTAGAGGATGGGTCCGGGAGCCCGTTTGAGGA
GTCCAAGGTGGTGCAGTGGCTGTTAGTCATGGCGAGGGTTGTGGAGCAGGTCCAGGGTCTCACGGGGG
GCTATTTTTATGGTCTTTCACCCGATCGCGTC
TTTTTTGCGGATGGCGAACCGGGGATCTGCGTGGGGCTGCCGATGCCACGTTATTTGTACTTCAAATG
GCTGATGGATCGCGAGGCTCTTGGTGTGGCGG
TGGAGCGTGAGTACCCGCCGGAGGTGGTGAATGAGCGGAAGTACAAGCCAATGGTGAGCGATGTGTGG
CACCTCGGCCTTCTTGGAATGAAGATGCTGAG
TGCGCACACAAGTTACTCCTCCCGATCCTCAGAGATCCGCTCCATTATTTCTGCCATGGTGGAGCAGA
GTGTCGAGTACCGCCTCACCATCGCCGGGGTG
GTGCGGAGGCTGACGGAAATCGTCGATGGGCAAAACGCGCCAAGGCCGTTTGCGACGCAACGGAGCCC
AACCCAATCTCTGGGCCCTGAAAAGCTGACGT
CGTACGGCGCAGGGCTCTACGCCACAGCACCCTCAACAGAAGGCTCCACTGGAGATGAGAACAATGGA
AGCAACGGTAACAGCATCAATTGTAACATCCC
CGCAAAAAATGAAGTTGCCCCTGCACTGAACACATCGCAACGTGTGTCACGGATGAGATTGCAGGAGA
GCTGGCACCGCCGCGCAATGGAGCAGTTTGAG
GAGCTACAGCGGCTAAATGCGAGCTCTCCTGTTGGTAGATCCAGAGGTTGCTCACCAACAGCGCGGTC
GAGACGATCGCGGTCAGCAGGAGTGGGGCTGT
CGCAGTCAACGAGCGCGATGGATATGGCTTCAACAGCCCCATCTCCGCAAGATATACGGACATCCGGT
CAATTTCAGGCACGAAGCCCGAGGAGCTTCTC
CGCCAAAGTGACAAAGGGGCAGACAAATCTCACAAGTGTAGTCCAAAATGCGCTCAAGCAACATATGG
AGGAGCAGCGACGACGGGATGCAATTTGGCAA
GGTCAAGAGACAAGACGATTGAGGAAGCAAGAAAAGGAGCGAGCGTTGCAAAATTTTCACCAGAACCA
TGTAGAAAAGTTAAGGAAAATCCGATCCAAAC
GAGATGAAGAAACGAAAAATGAAATCCGCATGCACATTAAGCAGTGGAGGGACCAACCTAGGGTTTTG
GCCGATGAGAATGTCATTGTGAACAGAGAGGG
CGACGTCTCCGTCTTTGTTCCGAAGAACGGCCGACCGCCACCAGCGGCAGCCACTACAGCACCACCAC
CACCCATATCATCAGTAGGAACTTTAGGGAGA
AAAGACAGGGAGAAGGACCCTTTGGCAGTAAGAGCAGGGGAATCGAAAGAAGTAAAAGAGGTGGAGGG
AATCCCCGTCCTTGCATTGCCGCATCGAGCAA
CAAGTGTCCCAGTTGGCTCAAGGACGACACCTCCTAGTGTGGGTCCCCCACGTTTTGCTGCCCTTTAC
CGTGAATCTGGCGTCAGCCCACGTGTTCCCCA
GACGCGCGCCAAGGTGGTTTCCACACCGCCTCCGGTGGGCACCATTAGGGGGACAAAAGGGATTCTTT

CCATCTCGTCCCCAAGATCGGCCAATGGGAGG
TCTTTAAGCTTTTTCGCGGGGGTTGAGGCCATTGAGATGCCTCCACCGCTACTGGACCCGGAGAGCAT
TTTGGATACATCCGTAGCACGAGAAACCGAGG
AGGTTGATCCGTTTTCTTCCTCGAACAGTGGACCCCGCGATGAGACAAGTCTTTTTTTTTCGCTCAAC
GGCTCCATCAAGGCCCTTAATGAATCACTTAG
CAGTTTACTGGCAAACCATCACAAGCACCGGGAAGTCATTGTTGCCATGGACAAATTTTTGATGCGGT
CTGAAGCCGAACGTTGCAATCCACGCCTCAAT
GCTGTTTTTATGAGTACCCTCCGTGGGATTTTGAATGACGAGCAACTTTTTCTTGCGGCGGCACCGTT
ATTTGTACAGTTGATGGCATTGCGGGGCCTAT
TAAAAAAAAACAGTCTTGCAAAGAAGAGAGATTTGAAAATGGAATAAACTTTTATGGAATGAAATGCT
GGGTGTCTTTTTTCATTTCATTTGTAAAGAAT
AAAAAAAGATTAGAGAGGAAATAATGTATAAAACGTAAAGGCCAAACAAAATGCGAAAAAAAAAACAA
GACGTTTTTTTTTCTTTTTTTTTTCGTGTGTG
TGTGTGTGGAATGGGCTAATGGGAGTGAAAAACAAAAACAAAAACAAAAAACAAAGACAAGAAAACAG
GGGAGGGGAGAAGTAATATACACGCATGACCAT
TTCCCCCTTGTTAACAGTATAATTATGTGGTTTAATGTGTTTTGGAGAGGGGAGGGGAGGGGGTTTTC
TCTTGTTTTTTTAGTGGGAAGGACATGCAAAC
TGCTGTCTTTTCCTTCCCAATGTGTGCGTACGTGCCAGGAGCCAATTCTTCTTTTTCAATGTTTTCTA
CTTGCCGCATACACACACACACACACACAAAT
ACACACGGAGCTATATGCTTTGTGAGTGAGTGCGTAAGTGCATTGTGAGCCAGAAGAGAGTTGAGTGG
TGAAGAGCTAACCTCTTCTTGCTTTGCTTAAA
TTTTCCAGTTTTTTTTTTTCATTTGGTGTTGTTTTTCTTCTTCTCCGGCTTTGGAGAAAGACAGGCAC
ATCCTTTTCCTTTTGTGAATACAGTCTTTAAA
TAGAGGAGTAGAGGTGTATGAGATCTTGCTTCGTGTGGACCTGCCATTTCTTTGAGGAAAAACGTATT
GGGCGCACGTCATTTCTACCGTACATGCTGCT
TCATATATGCATCTTATCTTTTTTGTCGTTTTTTCCTTTTTTTTTTTTTTTTTTTGTGCGCCTACGTG
TGAAGGTGCGTGTGAATTTGTGGTGCCCACAG
AAGTGTACGGGGGGGTCTTTGTAGAAATTTGCCGTGAACACCTTTCACGGTCACCAGACTCAAAGGTG
TTTTTTTTTTTTTTAATTTTTTAAAAAATTCT
TTTGTCTGTTTATGGAAGGTTTGGTGCTTGTACCATAGCCATATTACATATATTAAATATATATATAT
TCAAAACCACTTATTACCTGTATTGTACTTAT
TGGGTGCGTTTATAATTACACGTGACGATGGGAGCTGTCCCAAGCAGGGAGTCACTTTTGCGTGGTCT
TATCCGTGTTACTCCCAATAGAGAGTCGACCG
ACCACACCGCTGCAACGTCAAGGAGAAGTGCTTGGGGGAGCGCGCAGGAGATGGTCTTAGTACCGCTT
ATACCGGATTACTTTCCATCTGCAGAAGAGCC
TTCTTTCCTGCTTCTTTTTAATGTATACAAGGAGGCTTCACTGTGTTGCGATTGTGTGCATGAAGAGG
ATTACTTAATGAACATGAAGCATGTAGGTACA
GAGAGGGAACTGTACGAGCATTACATGCATATTAGCAAACAGCGTCTTTTTGAGACGGACAGTGAGTT
AATAGCTGTGCATCGTGCAGGGAGAGTGTTTA
GTGCCGTGCCATGTGCTGTAGCGTTTGTTCAGGAATGTGGCTCATTAATATCATCATCATCATCATCA
CCCGCACCATGCAGGCGCTCTCCTTTGGGTCA
AACGATACCAACGGTTCAGTTACACGGTTTACACTTGTACATTGTTGGTTTTATTGGGGATACAAGCC
TCTGTCCACCCTCCATTCCACAGAACAAGGCA
AAGGATAATCCCTTCAGGATGTTATTGTTACTCTCAAAGAGTGTGGGTGGAAATCAGCGGATGTTGGT
AACAAGCCTTGCGGCGTACACGCTTATCACAC
AATTGCGGCGGATTGGTATTGTAGACGATCCCATGATGTTTGCATATCTTCATAGTGCCTACAACGGG
ATAGGGGAACAACAGCAGTTGGGGTTAAATAG
CCTTCAGCTCAAAGAAGGAACTGCATCTGAGGGTCAAGAAGTGGATAAGAAATGGAGTTGGAAATCGA
TGAATATTAGTGGGCATGTCATATTCCGACCT
CTGGAATTTACTGTGGCGTTATCTGACATCCCGTGGATGTGTTACGTCCGTGAGTTTCGTGCACGACT
TTCTGAGAGTACTTTGGATGTTCCACCCACAA
TCGGAAATGAAAAGGAGTTGCTGTTGAGTGCAGACACGCTGACAAAACATTCTTTAAAAACTTTTGTG
GAAAGCAATTCCCTCTGCGGGACGATGGATGC
AGGTCCGTTCAAGAAGTGCGTCGAGTGGGCCTGTTGGCATCATGAGACACGGTTTCAGAGTCTTGTTG

GGGCCCAGGCAACGGAATTTCGTGCGGCATTG
GCCAAGGAAAACTTAAAATGCGTCTTAGAGGGGAACGTCTTTGTTGaCTCTGGCGAGCCCACTGCTGC
TCAAAGGGGTGATGTGTGGATTGTCgGGTCCG
ACAGCTACACACGTGACAGGATCCATGCTGCCTACACTCCTGTCACACGTGGATCCGTCATGTCTCAC
GACGGTAGTCAATGGTGCCTGCAACCGGATCG
GTTTGCGGAGGAAATTTTGTTGTCTGTTTTTTGCGATGAGTGCAAACGAGCGCGAACTGCCGATCCCG
ACGACCCCCATTGGATGCGTGGCACAGACGGC
TCGCTGCTCTGCAGTGAGACGGGTCTCTATTGTTGGAAGTTTGTGAGGCACGGACTGACGCTAGTGTA
CAGCACGGTGCCGGGGTTTGCCAAGCAGCGAT
CGCGGAAAAAGATGCATGCGACTTTGACTCACGGGAATGGCGGTATTGTTTCCGATAAGCCCGTAGAC
TCTGTGGGGGCAAAGAAGAAGGAGTGTGGGAG
ATCACCGTCCCTGAACCACAGCGGGCCGTTCTTGGCTGTGGGCCGCTCATCTGTCGTGGGATTGTCGA
GGAATAGCCTCAGCTTTGAAAATGCCTTTGTT
GGAAGCCACTATGAGTCCCCATCGCAGTGGGGGGCCGCCAGCTACGGAGAAGGTGGAGACGTCTCTTG
CGCACATCTCTCATCGGAACAGGACTTAAAAA
ACCCCATACGTTCTTTTAATTCATGCGAATTGAGAAACGCCGTGCTCTCCCCGCCTGGACCTGTATCT
CACCCGGTCGACGTCCGGGTCCCAACCATTTT
TTCCTCACAATCAACGCAATTGTCTGTGACAGCGCCTTCCATTGCATCACGGCAGCAAAAATCGTGTT
TGGGGTCTTTCAGCCAAGCTGCCTCGTTCCAA
GCGGATGCCCCGAAGATTATCAAGCAAACTGAACACTGCTTGGAAGCGCACGGCAAGGGGACAGACGG
TAGCACTTCTGGGGGCGGTCTGCGCGCGTACG
ACAGGGAGGGAAAGTACCGCTGGGAGTGGGGACGGCGCTTTAAGTGATTTTTATGGCTGCGATTCGTA
CGGAAACCCACACAAATACATAACTAAAAGAT
AACGTGGAGGATAAGTAACTTCGACGGTACTTTTTTAGTCTTATTTATTTATTTATTTATTTTTCTTT
TGCGTCCGCATTTTTTTTTTTTTTTTTCTTACC
CCTTCTCTCTCCCCCCTCAATTCTTGTGGTTTTGCCATTTTCGCTAGTGGGAATGTTGACATAGTCCA
CCGTTGTATTTATAATCCTTTTTTTTTTTCTTT
TTTTTACTTTTCTTACTTAGAGTCTTTGAGAATAGTAGTGGGGCTCTACATGTGCCAATTTTCTTTTT
TTTTCTTTTTCCTTTTGTGCTTAATGGCTATG
GATAATGCGAGGTCTTCTTGGGTGTTTGCTAGGCAGTGATTTTATTTTATTTGTATAGATATCAGGGC
ATTATGGAGGGAAATATCTTTTGTTCTTTTTT
TGCTTTCCATCTTTTTGAAGGGAGGGGAATTTTTGTAATGTTCTTTATTAGCGATGTGGTGATGTGTT
TTGTCTGAAACAACAAAGAAACCAATAGAAAC
TCACGGAGGGAATGTGCGATCATATGGGGGAAGGGAGGCTAAGGAGGAGGTTGGCGTGGTTTTCTCAT
CCCCCCACCATTATTATTATTTTTTCTTTTTA
CATGTTGGTGCGCTGCAGGACATCGAGTGCATTTCTTTGTGTTTTCTGTCCTTGCACTCGCCCACTTT
CTTAAGACTAATACCGCTGCAGCAACTTGCCA
CGCGGAGGGAATTCTGGCGNNNNNNNNNNNNNNNNNNNNNNNNNNNNNNNNNNNNNNNNNNNNNNNNNN
NNNNNNNNNNNNNNNNNNNNNNNNNNNNNNNN
NNNNNNNNNNNNNNNNNNNNNNNNNNNNNNNNNNNNNNNNNNNNNNNNNNNNNNNNNNNNNNNNNNNNNNNN
NNNNNNNNNNNNNNNNNNNNNNNNNNNNNNNN
NNNNNNNNNNNNNNNNNNNNNNNNNNNNNNNNNNNNNNNNNNNNNNNNNNNNNNNNNNNNNNNNNNNNNNNN
NNNNNNNNNNNNNNNNNNNNNNNNNNNNNNNN
NNNNNNNNNNNNNNNNNNNTTGCGGTGGCCCTTTTGAAGACTGCCGCCTCAGCGTGGTGCGCCAGGGT
CAATCGCCCCTTATTGTCCGGGGAGGCCAGGA
GCCAGCCATGTTTCCCTCACGGGCCTGTTGGTGCAGGGCTGACGGCATGTATTTTGTGAATATCTTTT
TTTGTGGGTGCGTGCGTGTTGGATAAATTGTT
TCTGAATTGTTTGCGGGATGCTGGACGCATTGAGCCAAAATGTCAATTGTTATTTTATTCTTCTTTCT
TTCTCTGGCAATAGGGAGCTAGGTCTCAGCCG
GGCAAGCTTACTGCTTATTGCCGCTATTGTTACTGTTTTTGGTTTTGTTCTGGATGGTACCATTATTT
ATAGTTCTTGTATGTATGCCTGTGTGTGTGTG
TGTGTGTGTGTGCTGGTGTGTAAATACCTTTTAATTTTATTTTTTTAATTATTTGTTTTGCTGCGTTT
TCTCCCATTCACAATTGTTGCCGTGTGACGTG
GTTTTTTCCCCTTTCCCTGTTTGTGTTAACTTTGAAAGTTGAAAAAGCAAGCCATCAAATGCAGATTG

TCGACGGCCAGCACTTCAGCGGTGGCGTTCAA
CGGAGTGAGGCGCTGGAGGATTCCGCCACGGCACTGAAGCATTCCATGCAGATGGGCCGTTTTTTTGC
GTACTACACGCATCCACACCGTCTGCCGCTTA
GTCAGAGCGCGGAGGAGCCGATGTGTGAGAAGCTGCCACGCGATGAGGCGATGCTTGCGCCAGGGGAC
TTTGCTTTTCTCCCCAACCCGACGTACTGTGG
CCGGACAACGGTTGACAAAGTGCTCTACAAGCATTGCCTGAGCCTTGATATTCTCTTGTAGCTTCCTT
CTCTGCATTGGAGCTGCTGAATTGCGAGAATA
AAAAAAATTGTGTGCATGTGTGTGATGCCAATAAGGCTGACAGATAAGAGAGAAATAATTAGAGCAGC
AGCAACAGTGATGGCGGTTGTATTTGTTTTTA
CTTTCGTCTTACTCTCCTTTTTTCTCCTTCTTCTAACATTCCCTTCTTCGAAGGGGGAGGTAAGGCTG
TAGTAAAGGTGTCTGTCTGTCTGGGGGTGGGA
AGAGGAGGAGGAGGAGAGACACGGGCGAGAAGGGCAACTTCACACGGTAGGAGGGAGAAGGCGAAGGA
AGTGTTCTTCTTCTTGTGTTGGTCTTCTTACG
CATGGCACGGAAAGCGGAAAATGGGCGAATGACGGCCAGTGGCGTTGGCGGTGGCACAACACCCAGCG
CCACGCCGCCACCTCGTGAAGTAGAAGTCACG
GTCTATGACAAGTTGCTTAACGAGGTGCGTACATTCCGCTGTCATCAACACCTTTTGGAGACATCGAT
GGCGTACTTTGAACCTATAATACGGCGGTATC
TGGAGGAAGAGGCGGAAGAAGAAAAGAAGAGAATTCGTGCGGAAATTGAAAAGGGTAGGGACAGCATT
TCTGGTGCTAGTGCCGCCACGAATCTAGAGAC
GAGTATTGGCGTAACGGATGGGTCTGCTGACAGGAAGGCGATTATTGGAACGAGGGGGCAAGGAGACG
TGGGGCCGTCCTCAAGAATCAGTGGGAACACT
CCACTACCACCCATTCCTATTCGTGTAAACTGCGATTTGAGTATCTTTTCGTGGCTGATGGCATATAT
CCAGGGAAAGGATCGGCCATTCACTCCTGAGA
ACGCTGTGTCAATTGCCTTGTCTTCAAACTTTTTACTTATGACGCAACTTGTTGATTTACCCCTTCAA
TACATCAAGGAGAACCTCGTGCAGGTAATGCT
CTCTGGGGTAAACATGGAGTGCCTTACAGGTGAACTTTTGGCGCGGCTGTGCACCATTTTATCGGAGG
GCGACCTCGCACGCGCCTTTTTAAGATTGTAT
GACTGGCGAACTGAGTACTCTTCGAATAGACGTTTCTTGACAGCGCTTCTTCGTCATGTTGTTTGTGA
TCGCCTTGGGGAGCGACGGGCCGGTGGGTTGC
GTTGGTGTCAATACTGCGGAGTTCTGTTTGACCAACATGAGCTTCTTCGTTTGGAACGGAGTGGGAAA
AATGTGAAACCAGCACTTTGTCCGAGGCTGAC
AAAGTCCTCCATTGGGACTCGTGGTGAGATTCTCATCACTCATGTTGCTTCAAAGCAACCTGCCAACC
TGGCCTTCCCCTCAATTTCTTGGGGTGATGAA
CATGTGGAACGGTGGGCGTGGCGCATTATTGGAGCAACGTACTTTTTTTTATGTCGGCAATGCAACCA
CCTTGTGTCTCTGACAGATACCACGACTCACG
GGTGTTCTGGAGGAGTGGCGGCTTTTTCCTGCGTTGAGGGCGGAAATCGAGCCGATAGCGAAGCACTT
CTTGCGTGGTATGATTACTCCAAATATCTTTA
CGTTGGTGGTCTCAATCCCATTTACTCCCGTCAGACGGACGTGCCACGCGAGTTCGTCCTGACGTTAT
CCCCCATGGGTGATTGGACCTGCGAGGACGGG
GGTGACTGTGCTGTGCCAGGGCTGTGGTGCACTCACCCCATCGCCGTGGAGAGATCGGTTGATACAAA
GGGAAGGGTGAGCCTCGATACACAGAATGGAT
TTGAGCGATGGTTAATGTTGGAGCTGCAGCAAATGATGGAGGACTTTGGTGCAGGGGGTAAAGCAAAC
GCCTCCGTGAGCGATGCACAGACGCCAATACC
ACCGTCCAAAGCACCGACCACCAATTTTGCCTTGCAATTGTCACTCAAGACAAGCAACCGGGGGACAG
AATACATGGCTGGACCCGGGGCCAGGCGGACA
TCGTATCGCGGCAACAGAGGAGAGAGTCGTAGTGGGTCTGGCTTTGGAAACGATGTGGTAAACAAGCG
GAAGGAGACAACAAATAATAACGGCATTCAGC
GTGTGAAGGATCCATCCGGTGGTGCACATGTGGCCTCCAATTCAAATAAGAACAACGTTGTTTCCTCT
TTCTCTAACAGCGCTCGCGTCAGGTCAGCGCA
ATCGTCGAGGCCCTCTGCGAGGAAACTAATTAACTTTGACCCAGAGTCGAGTGGAGGCAACTCCTTTA
TTTCACAAGCTCAACGGCAGTGAGTATAATCA
CTATCATAACGCCGGTTATAATTCCTCCGTAATGTATATTCATTCAATTCTACAGGTTCTTTTATTTG
GGTTATTCCGACTTTATTATGTGAGGCGGTGG
CACCACTTCACCGATGGATGCGTCTTCTTTTACAAATCCATTTTTTTTTTTTTTTTGTGTGCGTTTG

TGTGTTTTATGCCGTTTCCTCCCATTTACACC
TTTTTGCATCCCAGTGTCACGCTACGGGGGATGAGCTCCCTGGCACAGAGTCGCTTGTGGCGCAATAA
GAGCGCATGGATTTGGACTGTTGATAAGGCAA
CGCTGCACGCCATTGACTTTCTTGCATACCGACTGGGCCTCTTTCACCCACCCCCAGGAGGAACTGTC
TGTGGCGCGGCAACTCTGGCGAGGTCGAAGTG
TGATCGATTAAGTATCCTCTCAGAGCGCTCTTCTATTCATTCCTCCTTCTTCCCCGTTGTGAGGAAGC
AACAGACATCTTGTGGGGAGATCCATTTTTTT
TCGTCCCTTGACACGATGAAGCGCATGATGCGGGAGTGGGAGAGACGACAGCGGAAACGGGCGCGGGA
TGAAGAGGAGGTGGCGATCAAGGAGGAGGCAG
ACGTTGCGGCCAAGTTGCAGCACCCGGAATTTCTCTTTTCTGATGAGGACTCAGTGTTGTTACGCTTT
GGCATGGGGAGTTTTGCGTCTCCCACCGCCCC
CGGCTTCTCCGGCCTCTTCCGGCAACATTGGAAGGATTTCATTGTGACGGAGATGGTGACAGATGCGG
CCGGATTGGCGACGGCCATCCCTCGAGAACAT
GATTGGACTATACCTGCCTTGCCACCCTCGCTGCTCAACGCCACTGAAACACGTGAAAAATGGAGGGG
TGACAGTGATGCTGATATGAACAAAAACGATG
CCGAGGACGAAGGTCGGGGTGACGTGGGTCCGTCTTCTTCCTTCTTTGCGGTTGATGTCGAGGAACGA
GTGGCGGAACTGCTCAAAGAGGGTAGCAGAGA
AGAAAATCCCTTGGCTTCGGGAGAACCGGAAGAGGGAGAGAAGGAGAAGAAGGAGGATGGAAATAATG
ACTGCGTGGCGGGCAAGAGGGATTTTTATCTT
CAGTGTTACCTCCACAAGCGCCACATCGCCCACAGTGTGGCACTCGCCAACATTGCGCAGACACTCCG
CATGCATCCTTCTGGTATTTCCGTCGCTGGTA
TTAAGGATTACATTGGTGACACTATACAGCGGGTACGGTTGAAGAATATTACGCCGGCGGCTGCTTTA
GAGGCAAACAGTTTTTTCCGCATGAAAAAATG
GTCAATGACTTTGTCTGACTTCTCGTACCAGAATGAGCCACTGGTGCCGGGGCGACTGTTTGGCAACC
ACTTCAGGATCGTTTTGAGAGATGTGACTGCC
GCACGGGAAGACGTGGAGCGCGCATTGCATGCGTTTGAACAAAACGGTTTCCCAAACTATTACGGATG
TCAACGATTCTCGTGGTTTGGGGGGAAAAATG
ACGCCGCCTTTGCGCTGCTCCATCACAATTGGCTTGTGTTTGCCTTTCGCTTTCTTGGATACACAAGT
TGTGAGTTTACATTACGGGAACTGTTGCAGCG
TGAGAAGAAGTACCCAAATCCACTGCAGGATGAGTACCGGCGCAATGTGGTCCGTCGTTTACGGCGTA
TAGCGACGGAACCAACGGAATTGGATGTGGCG
CCTTTCCTTTCGTGTCCTCCGCTCGGTGCCCCACCGACATCCGCGGATGGCGGCCCTATTAGTGACAA
GCAAGAGCTCATTATTTTACAGTTGCGCGAAG
CATTTTTGGATCTGAACGTGCGAGACCGTCGTTTAACAGCGCAGCGGTTGAGTAGCTATCTGTGGAAC
CAGGTGCTAACCCTTCGACTCCATCACTTCGG
ATTGAAAGTATTGGTGGGAGATGTCGTTTTGCCAGAGAGCTTTCGACGTGTGGCGGCGTTGCCGGTAG
ACCGCACGGATTGTTACCGTGAAGGCATACGC
TTTATTGCAGACGAAGAAGAGAAGTCGGCCTACACCATTACAGACGTTATGCACCCTGGGTTCTCCTT
TAATGGTATTCAACGACCCATGAATACGGTTG
GGGAATATTATCTTCAAGTCTGTGGCAAGTACCACTTGGATTGGCATGCACATCATGCACAAACGGGG
ATTCACGATTTTCTTGAGCCACCGCGACCCAT
CATTCGAAAACCCATAAATTTGCGTCATGAGTATAATATAGCGGAGTCAAAGTTGACATTGGAGTTTG
TCTTGGAACGTGGAAGCTACGCCAATGTTGCC
ATCAGTGAACTTATGAAACTGCTTCGTTGTGCCGGATCAGAAAGTATTCTTCTCCTTCCTGCCCCGGA
GGGCATCTGGGACATTGGAAGCCGTGACCCTG
GGTATGTGAAGTCCATGCAGGACATCTACAGTGGGTTTGAGGATGGCCTTGGCTTTGTGACGGATAAA
CACGATTTGCCGTTGGTCGGGGAGGGGAAGGT
GTGGGATCATGACGGGCCGCTTTTTTTACCAGATAGCGCCGATCCTGTCAAGAAGGCGTATCGATGGG
GCGAACAGCACCTTCTGCGAAACATGGAGCGC
CGTGAGCGAGATGAGGAGGCGATGAAGATGCGTCTTTTTGAGAAGCCTCTGGCAAGGACACTCAaGGA
CGGTGAGGTGGCCCGGTACGCCGGACACACTG
TCCCCATGCTACCCAACAGCAAAGCAAAGAGGGTGTTTTTCACCGTTTTGCGTCGACAGCAAAGATTC
CCTTGTGCTCCAAAGACGGTGCCGCGTATCCG
GCGTGGAGCTGAGGTGCGCAACCGGACCCCCGTAAATGTGCCGTTCAAGACCATCAACCGAAATACTT

GGAATGTTACATGGTGAGAGAAGATCACACTG
TGCCAGCGGCGCACAGGATGGAAGTAGGCAACGTGGGGGGGGAGAGGGGTGGATACACGAGCCGAAGA
TGCACCGAAGTAAATTAATTAAGTGAAGAGTG
GATGAACCCTTCTTTTTTTTTTTTTTCTTATGTGTAAGTGTGTATGTGTGTATGTATGTTGGCAGGAG
GGAGAAGGAGAAGGAGAGAAATGTGGGATGGA
TCTATATATTTTTTATTTGCGTCATGATATTTTTTTTTTTTTTTTTATTCTGAACATGTTTGCTGATC
AATAATTCATTTCCACGCACACGCACACAAAC
AGTCAAGGAGGGAAAGAGAGACGAAACAAGGTCAACTTACAGATTCTTTTGCAGCATCTGTCCTTCAT
GTCCCGTACTCAAGCTGAAAACGCAGGGAGAT
CGCCCAAACTTGATGCTCCGCCGTATGACCCCTCCTTGTGGGAGGGAAAGGACTGTGCCTTTATTACT
ACCTTGAAGGATGTCTATCATAGACAGGAGCG
GATGGAGCGTCAGCTGCGATTGGAGACGTCTGCAGTCGGTGGGACTGCTATTCAACTGCCTGCTGCTT
CCCATACCCAGATGGTCACAAAGCCCTCTACA
TGGTGGGGAAAGCTTTTGGGTAGCCTCTTTCCGCAGTACTACGCACGGCAGGCATTAGCCAATCTTGG
CCTTGGCCAAATGAGCAATGATGAGCTTGTCA
ACCGCATGCATTTTGCTCTTCAGGCCGGGGATACGGATCTCTCTGCTCTCATTGCAAAGGAGTTGGCC
CGACGTCGGGCGTGTTTGTATGAAGAAGAGGT
TGGTAGAACGAGCATAGGAAGAGATGCACTCCATTCGGGAGGTCGTCTTAGCACGGAGCCCAGGATGA
GGACGAGGCTTAGTACTGGCTTTGGTGGGCGC
GTAAGTGGTGTGGCACCAGACAGCAACACTCGTCCCTCTACCGGTTC
>scaffold1090.size6942
TGTGTCTGCCGCACCTCTCGTCTCCCCTGTTGCTCTCCATGTCCCTCACTGTGCAGATCAGCTCCACA
CCACCGAACGACCACACTCACGATGACGACGTG
CCGTCTGCTGTGCGCCCTGTTGGTGCTTGCCCTGTGCTGCTGCCCGTCCGTGTGCGTGACAGCGACTG
GCGAAGGTTCCGATAAGGACAACTCCAGTTTA
TCGCCCGCTCAATCAGAGGGAGCAGGTGGTTCTGGGGCAAAAAATACTTCCACGACGTCAAGTTCAAC
AGGTCCGACCGATGTGTTACCGAGACCCCAAA
AACAGGACAGTGAGGGACAAGCATCAGACGAGACGACCGATACCGGAAAAGGGACAGGTGGAGTGGAC
GTTACGTCAGGGATTACGGGGGACAGTGCGGA
ACAGCCACGAATTGATAACCACACACAACAAGGAACAAGTTCCGAGAAAGATACGGTAAAAAAAAAAG
GTGAGGAAGAGACATCAGGTACGACGCGCGAT
ACCGTACAAGAGACAGGCAAGGGACCCGCTCCGCCAGAAACTCCGGACAAAGCAACTCCGGAGCATAT
TCCAAAGGAGGGAGCCGGTGCGTCCGATGGGT
CAGGGTCGTCAGGTGGGTCCGTTAGCCCAGCTGCAGCATCAAGTTCCGTGACCGCTCCTGTACCGGCA
CAAATTCAGAAGGAAAGTGCGCCAACAACAAC
AACCACGACTACGACAAAAGCACCAACTACCACCACCACTACGACTACGGAGGCGCCAACTACAACGA
CCACCACTCGCGCACCGTCACTTCTTCGCGAA
AGCGACGGCAGCCCCAGCAGCTCTGCGTGGGTGTGTGCCCCGCTGCTGCTCGCCGTATCCGCGCTGGC
GTACACCACTCTGGGCTGAAGAATTGTGTGCG
GGCTGTGCgTGCCGGCATTCAACCGCGGGGACTTGTGTGCGTCCACTGTGGTCCGATGTGCTAAACAG
GATAAAATAATGATTGTGTTTTGCTTCACTTG
CCTCCGCTGCCCTGCTGTGTGCTCCTTGCCGCTCCCCGCATCCGTCACGTTGAACGCACCCCGGACAC
ACTCATGTGCATGGCACGCACACAACTGATTG
CGTGGAGTCGCATTTGAGGATGGGTGCGTGCGCGCTGGAGCCATACTGTGCAGCCCTTTGTTTTGTGT
GTGTTTACATTTTTACTTCATTGCTGTTTGTT
TACCTGTGTTTTGTTTCTAATTGTTTTGTGTGTTTTTCTGTTTGCTTGCTGCGGGGCGTTGTGTGTTT
TTGCCCTTTCACTGCTTTCAACTGCCTCATGG
TGTGTGTGCGCTGCCGCTGCTGGGCTGCTGCCATTTGTGACTGTGCGGTGGCTGTGCGTGGTGTAGCG
GCCGTGTCTCTCCTTTTGCTTCCGCTCTGCGT
TGACGGGGAGTTAGTTTGTGCTGAAGGCTGCAGGCAAGTGACAGGAGTGATGGCGATGATGATGACTG
GCCGTGTGCTGCTGGTGTGTGCCCTCTGCGTG
CTGTGGTGCGGTGCCGGCGGCGGTGGCTGTGATGAGACAACGCAGGCTTCGCCTTCTGGCTCTGGTGG
CGGCACGGTTGTTGGTACTGGTGGTGGCCCAA
GTGGTGGAGCTGACAATGGTTTGGTTGCTTCTGAGCCTAATGGGGAGAGCGAGGGGGATCGTTTGGAG

GGGACGGATTTGGGGCAAATAAACTCATCCCC
TCGCGGTGGAACTGTGGGGCTAGACAACAAGTTGGCAGTGATGCCTGTGGGATCTCCGGTAACACGCA
ATCAAGTGTTGTCGAATGAAGCGCTGTCTTTG
CAGTCACCACCATCACCAAAACCATCAGCACCAGAATTACAGGATTTACCAGAAGGCCCACCAGTATC
CGGTGGCAGTCAAGGTGGTGAAGCTGACAATG
CCGTTGGTGGTGTTGGCCCGAGTGGAAGTTCTTCTGGAAGTGGTGTTAAATCCATGGTATCCGCTTCT
TTTTCCGCCTGTACGCTTTCACCCCCCAATCC
CGTTGACTGGGATGTTTTGCAGGGCGGCAAGGGTACGACAGCCACCCTGAGCATGGATTCCTCAGAAA
AAAGTGTTAAGTCACAATCAGCAGAACCTCCA
GCACCCGCACAACCCAACTTGGATAGACAAGAGGCGTCAACATCTAATGCAGAAGACCACTCCCCTGT
TAAGCCACACACCGCAGGAAATCAAGTTGTTA
ATGGCGGTGCAACTGGAAAGGAGAACACGCAGAGGGCCGCAGTGACGGGAGGACCTTCCAGCGACACA
CGATCAACAACACTGCAAGCAGCTCCGCAAGA
ACAGCCAAAAACAGAACTAATAATAGTGCCCGCAGGAAAAGAACGCGCAACAGACCCACGTACTCGAG
AAAAAGAAGAAAGAGACTCCAAGAAAGCACCA
CCGCCACCACCGGCAAGTTCAGCAGGAGGAACCCCAGCAGCAAAAGAATTACAAATTTCAGTGGAGGA
AATGCACCAAACGTCACCGTCAGAAGCCCAAA
AGCTCCTTTTGGGACAAGAAACCCCTTCAAAGGATGACGCGGAAGATCAAAACACTGAGGGCACAGCA
ACATCGGATTCGGTGAAGGACGAAGCTGCCAG
CAGCTTGGTGGAAACAACAGCTCCACCCATCTCTACAAGTGGCGGTGCTGATACAAAGAGTGTAGCGA
GTGAGGATGCTGATAATGCTCAGCAGCCCAAC
CATAAAGAGGCACACAAAGACCCCGAGCCTCAAAATACGAGTCCTGCTTTTACAGCCACTGAGGCACT
GCCACAAACAGCAATACCACTCACCACCGCTC
CAACAAATGATACGACGACACCTGGCGACAGTGACGGCAGCACCGCGGTCTCCCACACCACCTCCCCT
CCTTTGCTTCTTCTTGTTGTTGCGTGTGCGGC
TGCTGCTGCGGTGGTGGCTGCGTGAGAGTGAGGGAGAGAGAGCCGTGCACCGCCCGCACACACACTCG
TCCTTCACTCTCTGTGTGTGTCCCCTTGCATG
GACTCTCGCCCTCACCTCACTCCACGAAGCACACACAATAATGTGCCCATTGTATATATGCATGCACA
CCCATGCAGGCCCGTTGGTGCTTTCTTTTGCA
CGACACGCACCGCTGCCGTCCTGCCTGGCTGCATGGGCGGAGCACCGTAACTGTTTCTGTTTTGTTTT
TATTGCATTGAGTGGGCGACCTCTCTCTCTCC
CTATTGGGCTTTACGGGAGCAATGGGAGTGTGTGCGCTGGTCGTCCCACCTGTTGCTGCGCGTCCCCA
CTGCTGGGTGGACGCCCCCACGCTTCGGCTTT
TTGTTGTTGGTATTTTTTATTCTCTGCCTGTGTTGTGGTTGAGTGCACCCATCTTACTTTTTTTGATT
GCTGGTTTGCTTTGACTGCGGACGCCTGATTC
CTCTTTGTGTGTTCTCACATTGTTTTCTTTCTCCTTCTAAAAGTGTCAGCCACTCGTATCCTTGTAAA
GCCACAACGTCCTTTCTTTTCCACATACGCCA
TGCGTCAGTTGATCCACGTCACCCCACGGTCTGCAGCGCTGCTGGAGCTGCTTGTGATGATGCCTGCG
ACGGGAATTGCCAGCGGGTGCTTTGCTGCCAC
CCCGACGTATCCTTTGTTTCCTTGACGATGTAGGGCGTACCGACGGCGCGCGGGCGTCTGTGGTGATG
GTGAGGCACGTGCGTCTCAAACCGTATGATGC
GTCGCAGGTGTGCAGCGTCGTTTCGAAGCACCAATGGGCGTTTCTACTCATCATGGCGTCCACAACGG
ATCCGGATGGCAGCAGGAGGCACTGCGATTCC
TCCTGGTGTTGCACAGCGACTTTGTTGGACGGCGCGCCTGATTGGCGTCTCTTGGGGCTATTGTGGGA
GAAGGTGGGAGGCTGAGGGCATTAGGACTGAA
TTGTTTTGAGTGGCTGCGTTCCGTTGACTTGCGGTCGGTCGTTGCACTCTTTTGGGGTTTGGCGGCCC
GTCTCTTTTGTTTACTTCCCACCGCAGCACGT
GGATAACACTGGTCCGAGGAACACTTGTTTGACGGGTGTGAGCTGCTGCCGTGCGTGGTGCATGGACT
ATTTGATTGTGTGGGGAGGTGCATGGAGTGAC
GAATACTTCTGTTTTGCGTGTCGCGGTGTGTCGGAATGATGTTCGGAAAACATGTGAGGGGTGTTGTG
TGTGGTTTGTGTGTCTTCTCTGGTGGAGCCGA
CATGTGTGCGGGGATTGTGGCATTGCTGGCTGACAGTTCCACGTCACTTTTCGGCATTCCTGGATGTG
CTGAGGAATGACGGCTGGCGACCAGCGTGTTG
GTGCTGCTGGTGGGAATAAGTGCATCGGGAATAGGCCATTTTCTCTTTCCCATGCTGGAGCCGATGAA

CTGGCACGAGGGCAGCGGCTGCAGGCTGCCTG
CCAGCACGTGTGAGCAGCGGATCCATGCACACACAGACGCTCATCGCAAGAGCCTGTGCGATCACAGT
GGCTTTTGTGGCGTGCTTTTGTGGGCGTCAGC
CCGTCGGAGAATGTGTGAAGCAAACGGCTGACTGCACGCAAGGAGAATAAGTGCGTGCACTGTGCCGT
GTTTCGGCGTCGATGGCAGAGCGGCAGGAGGC
CCGTGCTGGCAACTGAGATCTTTGCGTGGCGAGGGATGTCGGTGTTGATTTTGGATCGTCGAGGGTGA
AATGAATTTGCTGCTGCCCGTGTGTGTGGCCA
CTCCGGCGGAGGGCGAACACAAAAGGGAAGGATGCAGGCGCTGCGTGTGTGTGTGCAGGTGCCATGCA
ACAACGTCACAACAAACATGCTGCGACTCGGT
GACCCTCACATCGCTGTGGTCCTCATGAACAGCCGAGGCACGGCCCGCATCGATGCACTTAGTGGACG
GAGTGAGGCATCTTTTGAGGCGCTGCAGGGAG
CAGGATGTGTTGACGCAGCCACGACGTGCATGGAGTGTCACGATGGCACAGCCGGCTGTGGCTGGTGG
AATAATTGCTTCTCGGAGGCAACAGATAGAGA
GAGCAGCTGCTGCTGCTGACGTCGTTGCTGCCTTTTGTGGCCTCCCATGCCGATTATTGTCTGTCCGC
TGATCATGGGTGGTGTGCCTCCACTAGTGACG
TCGTCTGCAGGCATTTCACAGCACCAGCGAAACACACATCACGTCGCATGCTTTGGCTGTGGGTATGG
TGTCGTGAATGCAGCCGGCATTATTTTGCCCG
ACTCCTTTTCACTGCCTTGCGACAAATAGATGCGTCGCCGCCCGAGTCATTCGCTGCAGCTCCGTGTG
TTGTACTGCCGGCACAATACTCTCGTGTTGTC
TGGCGCCTGGCTGAAAGTCAAGCACCACTGCGCTGGGACATGCAGGGATGTGGACTGCCGCTCACGGT
GTTGGGCACCGCTGTTTGGATGCGGCAGCCAC
ACGAGCGGATGTATTGTGGCACAATGAAATGCGTGAAATACGCGGAACCACAGCGGCTGCAATAATAT
CGACGGTGTGGCACCACGGAGTATGTGAGGGG
TGATACATGATGAATCAGAGTGGGCGGCATGCGAGGGAAGTGCACCGCGACGGAGCGTCGCCCCGCCC
TCAGCCTGCAGGGGAATGTACGCCCACAAAAT
ATATGGTCGAGGCACCACCAAACACGCATGCCCTTCTGTGCAGCAATAAATAGGAGCGAAGGTGCGTT
CTGGTGCGTGGGAGTCCACTCACATATTCTCA
TTTCCACCGCAATAAACGGCGAGGAATCAGGTGCCACAGCAACGAAGGAGGAAATAAAAACATCTATT
GCAGCACTTTACCAGCGAAAAGCAGGAACAAC
GCAAATTGAATTATTGGCTGCACCAACACAATAAAAATTCACAAGGGAAGGAAACGGTTGTGCACACC
GCAGGAACCCCAACACACGATGCAGGCATGAG
GGTTTACCCTCCGCAAAAACAATGGCAACATATGCAGACATCTTCCACCCTCCAGAAAGAAACACAAA
TAAATGGCTGCTTTATTCCATTCCTTGCGCTT
GACTTTGCGTGTGCTGCTGCTGCTGCGGTGGTGGCTGCGCTTCCCGTGGACCAACAATCCACGTGGTG
TGCGGGGATCTATTCTTTTTCATGGTTACTTT
GCTTTTGTGTTGCTGTGTGCGCTGCACCTTCTTGTGTGTGTGCGTTCCCTCCGTGAGGGGTGCCGACA
CGACGGACCGCTCTCGCCGTATGTACATGCGG
TGGAGCCTTTTGTGCCGCTCCTGTTTGCTTCCCCCTCTTCCATCTGTGTGTTGGTGTTGCGGCAGGGC
TGTGTGTGTGTGTGCCTGTTGCCGTGGATTGG
AGTGTGTGCTGTGCGTGGTGTGCGGCGTACGGTGCATGCCTGGCTGCTGCCCTCTCCTCTTGTGTGTG
TTTGCCCTCTGTGTGTGTCTGCCGCACCTCTC
GTCTCCCCTGTTGCTCTCCATGTCCCTCACTGTGCAGATCAGCTCCACACCACTGAACGACCACACTC
ACGATGATGACGTGCCGTCTGCTGTGCGCCCT
GTTGGTGCTTGCCCTGTGCTGCTGCCCGTCCGTGTGTGCGACGGAGGGTAGCGGAGAACAAGTTGGGG
ACAGTGGCTCAACGCTCCCTCAGCCACCGGGA
GAAGTTGGTGCAGGGCCAAAAAATGCTTCCACGGCGTCAGGTTCAACAGTTCCGACCGTCACGTTACC
GGAAACCGGAAATCCGGAAAGTCAGCAACCCG
CTCTGCCAGGGAGTCCAGGTACAGAAGGTTCGGAGTCTATTCCAAAAGCGAAACCAGTTGGGTCCGAT
GGGTCAGGGTCGCCAGGTGGGTCAGTTAGTCC
AGCTGCGGCATCAATTTCCGTGACCGCTTCTGTACCGGCACAGAGTCAGAAGGAAAATGCGCCAACAA
CAACAACCACTACTACGACAAAAGCACCAACT
ACCACCACCACTACGACTACGGAGGCGCCAACTACAACG
>scaffold1091.size6704
AGAGAGTCCCCAACGTTGCCAATGCGGGATTGGCCGTCCAGTGCGGTGAATACAACGCGCCGGATCTG

TCGGAGGATCGTCGTGCACACTTCACTCCGCCC
GTGGCAGATACAATCCTCGGGCAGCCACACCTGTGCAGAACAGAACACCCTTTTGGAGAACGCCGAGC
AGCTCCACCGACGTGTCGGAGAACCTCACAAA
AAGAGGGCCGGTCAACCTGCGGCACATTACCGTGCAACTTTAATACCTTGACAGCGAGAAGAGCATTT
CTACCTGAGAGTGTTCGCCCCATCGTCATCAT
CATCACTGCTCAAATCATCTCAACAGGCAACAATGGCATCGTTCTCGTATTGCTCATGCCCGTTGGCC
TCCTCCACACGCCGCCTCACGTCCGTGTGAAT
AAAGTCTCGTCATGCACAGCGATGCGCCCTCTCGTTTCAGACAAATACCAATATTTTATAGTCACCAA
GGTAGCAGTTCGGTTCTCCCTCCACAGGCACA
CCTCATAGAGGATGTCCTTCTGTTCCCTTTGCCGAATCAACGCACCGCACCCAGTGGATAAGCCATGG
TAGTTCCACCAAAAAAATAAGGGTTCGACTCCC
ACAGTAGCGAATACTCTGTGTGATATGAAACGCACAGTTTGTTGTGCTAGTGCACCGCCTCGACAGCC
GCCTCGCAATGGTGCAAAGGTGTCATTGCCCC
CGCCTGGGTGAGGCCTAAGCCCCACATTACACGCACACAGAGAAGCACACGACGCATTTGAAAATATA
TTAATAATTTTTCACGGCACATTTATTGGAAG
GAGCACCACCCCCGTATGCATGTTGGCAAAGCAACGGCCACACAACGGCGGTGCTAAAAAAAAAAACG
CATCCAGAAACAGAGGAAAAAAGAAGAAGGGT
GTGGAACACCCACAAGAAATAATAGATAGCCCACTTCAAACTGGCAGCAAAAAAAAAAGGGAGGCAGC
AGTAGTACTATCAGTATTAGTAGTGACCATCA
TCTCCCCTTTTTTTCCTTCGCCTTATTCACTAATAACCCAACCACGTGAGACACAAAGGTCCGTTTTT
CCATACAAAATCGTGCCACAGTTATCCACCAT
CTAGCCAGCTGCCTCAATACACCTAAAAGTGCACACAAGAAAACCACAAATAGCCATGGGAAAAAAGA
GAGAAAAACAATAAAAGATCCCACGGCATTAG
TACGGAAGCGGAACGACACGCTTTTCGCACAAGTAGTCGTAAAGTTGGCCGGAGCGGAACAGATCCAG
GCCCGAGCAAACTCGGACGTCATAGGCGGTTA
TTGTGCTCCGTTGAGACTTTTCCGCCaGCTCCTGTTCCAGCCGAAGGAAGAGGGCTGGCGGAATGTGG
TTCTCGCCGCAAAGAGCTGCTGCATCGGGGGC
CAAAGAAACAAATCCTCGCATATTGCGAATGTCAGGTGGGCCACACCACCGCATTTCCACGTCATTAT
CGCTCGGCTGCGCACGCGCGTCTCTTCACGTA
CCCCGTCGTTCCTCAAGAAGCCTTTCCGCAGGAGTCCTCAAATGCAGTAGACGCTTGAGAGCAACAAG
CCAGGCCTCGCAGTCGCTGTCAGTCTCACAAA
CCACTTCCAACGATTTCCCACCGCGAAGTAAAAGTGCAAATGCGCGGTAGTTGTTTATTCCAAGGTTG
GATGGAGTGATGTTTTGCGTGGGATCATGGCG
GTACGTCCCATTGTCAGAAACTGCCTCCTGGCGTGTTTTTGCCAGCTGTGCCTCGCCGCTGTAGTTCA
CAAATGAGGTTGTGTACAGGCCGATGAGAACT
TTTCTGATGTCACGTATCCAAATGGTGACTGGAGGCCGTTTGCGATTTAACGGGAATCCCTGATGGTC
CAGCTCACAGGAATGAATACGTTTGCAGTCCT
CCGAGAGAAAATATAGGCGTCTGCGAGCATTGCCCTTGCTTGATATCTTTGTCATGCGGACCCCGTTG
CGTAAGTAATTCAGCACCTGATCCACAAGAGA
ACTGAGGTGGTGTTCCTTTGTCTCGGCGTCATGCAGTTTTTCGTTTTTCTTTGCCACGTAGTCATCAA
GCATACGCTGTCGATACACCTCGAGTCCTCGT
TCCAGCCTCTCCTTGTGGACCCGTTCCTCTTCAGCGCTGGCCAAGTATTTCTGTAATTATTGCGTGAA
GACCTCGTCAAACTGGCGTCGAATGATGCCCG
TCTCCTCCGGATTCACAGGAATGTCCTCAAGGGAAATTAGCTTCGGCAAATACTCCTCGATCATTTTG
ATGTCGGCAGCAAGAGCCCTCTCGGCGGCGTC
CTCCTTGGCTTTCGCCTGCTCACGCTTCTTCTTTCGCTCCTCGGCCAAGGGACGCACCGGTTCCGGGA
ATTTTGCATTCATTTCCTTAATCTGATGAGTC
CACTGCCTCCGCTGGCGACACCGCTCCTCCCGGACGGTCCTTCGCACCGCCTCAATAGAATGCCGCTC
CCGCATGACGGCCTCTCGCCCCTTCGCCCCGT
CAAGTTGAANNNNNNNNNNNNNNNNNNNNNNNNNNNNNNNNNNNNNNNNNNNNNNNNNNNNNNNNNNN
NNNNNNNNNNNNNNNNNNNNNNNNNNNNNNNN
NNNNNNNNNNNNNNNNNNNNNNNNNNNNNNNNNNNNNNNNNNNNNNNNNNNNNNNNNNNNNNNNNNNN
NNNNNNNNNNNNNNNNNNNNNNNNNNNNNNNN
NNNNNNNNNNNNNNNNNNNNNNNNNNNNNNNNNNNNNNNNNNNNNNNNNNNNNNNNNNNNNNNNNNNN

NNNNNNNNNNNNNNNNNNNNNNNNNNNNNNNNN
NNNNNNNNNCATCTCGAGTTGCTTCTTCTTCGTCTCAATGGCGCTCTGCCGCAGGCTGAGTTCTTGCT
CCAGCTGCTGCATCTCCTGCCGCAACCTCTCC
GAGGCACTCTCCTCGTCCTGTTTCTTCTTTCGGATCTTCTCCAACGTCTGCTCGTAGTCCCGAATGCG
GCTTTCGCGGTCGGCCTGCAGAGCACTGTTCT
CCCGCTTTTTGGCCTCCAGAGTCCTCTCAAGTTCATCCAACGTGGTCCCACTTCTCTGCTCAAGCTCT
TTGATCATCTTCTCCGCCTCATGCTTGGCACG
CTCGCGAACCACACTCAGCTGCCGTTCCCGCGCCTCCTCACGCTTGCTCTGAATGGCGTTGATGCGCT
TCATCTCTTCCTCCTTTATGTTTGAAATGGGC
CTGTTGGACTCATAGATGCAAGGCCTGTAGTCGTACACGGTCCGGGCAATCATGAGTTCATCCCGCAA
CTCCATGATGTCCTGATCAGGACGCCACTTGT
CAACGGCAAGATCCCTTGTGGTGGATCCAATTCCGGTCGAGCCAAGAATATTGCCAACACGACGAGTC
GTGGAGAGACAATGGAACGATTCCTCCGCCCA
CACGGCATCCGAAAAGGCGTGAGCCAATAAAGCCGTCTCGGTAGAACCAGTGAGGCTATTGTTGGGGT
TGCCTTTTTCCGCATTGTACGCTCGGCGCAAC
AACTGACTGAGTTTTGACTTGCCGAAGGGAATGCGTAGACGACTGCAGCGAATGCTGGAGACAATTCC
GGCCGCCGACACCAGTGTTCGTTGGGCCCTTT
CGTAGTGTAGCTGCGCGGTGGCGTCAATGCCACATAAAACGGGCCGTTCGGCATCTCCCAAGGCGACA
AATGTCAGGGAGTTTGCATCCATATAATCTGC
AAACTCCGCCTCACCATCATAGCGGTAAAGCTGCAGAACGACGGTGTCGGGCAAATACGAAGGAATTG
CCTCCAGCTCAGGCGTTGGCTGCTTTTCACAC
AGCACGGAGGCAAAGTATTTCTCATAGTTTTTGACAATAGTGTCCATCGCCGCTCGAACGTCGCCGGC
CGATGAGACACGAATTCGCGCCACCATTCGCA
CACGCGGTCCCTCCTTCACGGAGTCGACAATAACGCCGAGCTCGTTGTCGACGTTAAGGAGGTCTGCA
AGGTGCTCTGTTGATCCAATGACGTAGCACGA
GAGGGCAAAAATGCCCTTTTTGGCGGCCGACTGAATAACCTCAGTGATGACCCGGGCAGCATATCCTT
CCCCGGCTGCAGAGCCGTACATGAGCTGTCGC
TTTGGCGACGAACGCACGCCGTAGCTGAAGACCACCATGTTCTTGCGTTCCTGAGTAAAGTTGGGGGC
GGCAGTGGCAAACAGTTTTCGGAAGAGTGTAC
TTGCGTCCGGCCTGTCCAACGCCACATCGACGTCAAACGAGGCCTCAGCAACCGGAAATCCGGCATCC
CTCAGGGGCTGGGGGCAATTGAACTGCACCGT
GTTGCCATCCACTCGTATTGTCGCGGGGAGCGGCGAGGACGGCGTCGTGGTTTGGTGGGAGCAAAAAA
CGACCTTCGTCTCCATTGGAAGGATATCACTT
TGAATGCTGCGCTGTGTGTTTGTTGCATGCACAGACAAGAAGGTCAAAGACAGGCAGAAAGCAAAGAG
GAGAGAGAGAGGGAAAGGGAAACAACACTGTA
GATGCAGTGTAATGGATGCACACTAATTTAATAATAATAAATATATTTTTTTTAACCCACAAATAAAA
AAAAATGATAATAATGATTAAACACCACACAC
ACACACAGACGGAAGATCAAAATATGACTTGTGGGCTCCATGCTGTGAGGTGACGGAAAAGAGCCCGC
GAGAGCACAGAAACAATCAATGCGGCAATGGC
CAGGAACCGGCTGGGATGACTGCTGGCCGTGATGCTGAAGCCCCGCACCATCCAAAGAAGAAAGAGAA
AATCCACCACAACAACCAGCGGAATGGGGATG
CGCCACACCAGGAACACGAGGTAGCCGACCTTCGCTGTGTAGGCAAGGCTCACAGATGTTGCCCACGT
GGTCATCGCATCATCCTGCCCAGAGGCAACGC
CGCGCAGTGACTGAACGCCGATCCAAACGGCAGCAATCAAACAGAGAAAGTACTCCATAGAGGCGTAC
AGGAACAGCGTCGGCCACGTGTCAACGTACAT
CATCAGCGGCAGCATATCTGAACGGAGATTTCTCACAAGCTGCAAACTATCTTCCCCACTCAAGTTGC
CGCCCGGCGTTGCTGGAAAAAAAGCTTTCCTG
TTCCGTATGTCGGCATAGACCGATGTAGTTCGCACAACGTAGGCCTCAATCAGGCAGCAAACAAGTGA
CGCAAGAAACAACGATGAAAAAATCCCTCTCT
GATTAAACAACACATGCATCCAGGCACGTCTTTCCAACAAAACAACATCAATCCACTTTTGCACATCG
GCGAATTCATAGTACTTGTCGCAGCATCTACC
GCACGACACGCACTTCTCGACCATTTCCGTCTCGGGATGAACAATTCTCTGAACAAAAGCGCTGCATT
CGATACACCGGGGCATGCGCCCATTCCACACC
CAGCACTACACACTGACAATAATAAAGTGAAGAGGGAAGGGCACACGCACACAAACACACAGAACCGC

CACCCCAACGTCCGTGGGACCAAAAAGAAAGA
AAAGAAAGAAAAGAAAAGACATTTCATCCATTTTGGCTTTTCGTCGGCCAGTCAAGTTTTACGGGACC
CACACAAATATTCATCTCCCGGCAGGATGAAT
GGAAGAAATAAAAGACTGGATAATCTTCTGTGCCGCTGGCCGAATCCTGAAATTTCTTTTAGAAACCA
AAAAAAGGTAGGGGAAAAAAGTGGGGTTGCCG
CAGAGGTCGTTAATTGCCTCAATGTTTTCTTTTTGTCCAATCAATAAATTCGATTGACCGTTCGCATG
GTCTTTTGTCCTCAGTTAGAAGCTGAAGGATA
ATTACGTACGACGCGGCAGACTTTTGGGGTCCTCCGAGCACAAGACCCCGCCGCGTGGTGGGAATCAA
AAGGGGTTGGGTGGGATGGCAAAATAAAAATA
AAAATAATTTGCCTATTCGCTTTCTGCTCTCTGTTCCCCCTAAAGTTGTGTTCTTCCCACGTTCATAA
AAAAGAAAAAAATTATTGTAAAGAAGTGAATTC
GTTATTATCAGAATTTAAAATTAATTGAGGCAGTCAGCTTTCCTCTGTGATCATTCTCCTCACAAGCT
CCTAAAAAAAGGAAAAAAGTGGAGGGGTATC
ACACTTCCACAGATGACGTGCTCTCCTGCGTGGCACGCAGTGGAATGTGTTGCCGCACCACACGCGCG
ACGGTTATGGCAGGGCTTGAAGGCTTCGTGGT
ATTGAAGGTGCCCTCTCGGACGACAACGCCGCCAAACTCCCCTCCCACATTCTGTTTTTCCTCGTGTT
TTATAAAATAAATCCGATGGTCGGCATTGCGG
TAACACGTGCTCTTCAGACCTCATGGTGTTACGACTGTCTCCCAGCAGGACGCGGCCGGTCAGGGCGC
TGGTCGATGCCGTCAGCCCCGTGGCCACGCCA
ATAACTCCGTGAGGGAACCATCGCAGCGCTGTCAGTCCGCGGGTGAGAGGATTCGGCCCTCCACACAA
CAAACGCGCACACCGCCAGCAGTCACACACAA
TGGACGAGGACGAGAAGGGACACACCGCCACCGCCTCCAGTTCAGTCCCACGGCCAACGGGATACAGC
CACAGCCCCGCAGCCAACAAACACAACGCACA
CGGAGCAAGCAATCACCCAAAGAGCCACACGCAGAAGCGATTCCTCATCCACCCACGACGCGGCGCCT
CGCTCCATGCGGTCACAACGCAGCGCACTGCT
TCCC
>scaffold1092.size12021
AGGTGGAAGGCGCATTGACAAAGCGACGCAGACGCATCAGTGCCATCCGTGTCATGTTTTCTCGCTCC
TCCATTTCACTGAGCCGCTGTGCTTCCGCCACA
ACATCATGCGGAAGTCCCGCCATGCGTGCTACCCGCACCCCAAAACTAGACGGTGTTACACCCGGTGT
AGGCTTGTAGGTAAACACCAGTTTGGGCTCCC
CATCTCCTCCGTCTTCATCTTGCTCCTCAGAAAACCCCATATAATAGCACGATACTTGTTTTCCCTCG
GAGGTATTATGAAGGTACGGCTCACACAGATG
TGAGTAATGCGTCACAAACAGCATCGTCGTGCCTCGTTGCAAAAGATATTCAAGCGTTGCCGCAGCAA
CGGCAATACCGTCAAAGCTGCTTGTACCCCTC
CCAAGTTCATCCAACAATACAAGTGAGGAGGATACACGTGGCGAGCGCAGAATTCGGCTTGTCTCTTG
CATCTCCTTCAAAAACGTAGAGGTTCCCTCCA
GTAGAGAGTCACTTGCCCCCATACGACAATATACGGCTGTGAACAATGGCAACTGCGCCGCAGTCGCA
GGAACGTAGCAACCCAGCTGGGCCATGATAAC
AAATGCGCCAACCATGCGCATAAGTGCAGACTTTCCACCCATATTGGGTCCTGTTAGGATCCATGCCC
CACCTTTTGCCAGGCAAAAATCACAGCTCACG
TAGCCTCCCCGTAAAATGTTGTCCAGCACTGGATGCCTACCACCCACAATCTTCAGACACGTTgCACC
ATCTGAAAGCTCCGGTGCAACGTAGCCACGGG
AAGAAGATGTCACAGTAAGGCACCGAATCGCATCCAGCACGGCCACAGCATCGATGACAGCCTGAAAG
GCATTCATGGCTAAGGAAGAGTTAGACAACTC
ATCCTGCTTTTGTTGCCATGAATCTTTTGCCGCAATAAGAAGACGCTCTCGGGCAGAACAAAGTAAAA
CATTACGATCAACAATGTTTGGTGTGTGAAAT
CGAACATGACTTTTTGTCCGTGTCAACACTAACCAGTCTTTTGGTACACGATTACACTTTGCTTGTGG
CACGTCCACAATAAATGGCGTCCCAGCGATGG
TGCGATACTCCAAATCAGGCATATGCAGTGTCTGGCGAATGTACTCCAGCTCCTCATCAAGGGCATGT
AATGCCTCATCACGAGCCGTTAGGTGGGTCTG
TATGGCAGAAGGAACAGAAACACCATGCGATGTAAAGTACTCTAATGGGGATGTCGCGTGTGATTCCA
TTTCACATTTGCACAACTGAATAAAATTGGCC
GTTTCCGCGGAAGTGACCCTCTGCAGAGTCTCGAGAATTAAATTATCTATGCGCCCGTCATTGCGGGA

TAATACGGCATGAGCCAAAGTGTGGACTGTTT
CCAGAGCACGCAGAAGTTGAATGTACTCTCCAACTGAGCAGCGTTGCGCCCGCATCTTTCCCAGTGTT
GCTTCAATATCGCCAAACCGGGCACACTCCCG
AAGCAAATGCGTCACGGTGTTATCATCCTTTCCGTGCAACAAGAAAGAAACGGCTTTTCGGCGTGCTG
TGATTGTGTTCATTTCACAAGGCGGTTCCGAC
AGCCACAGACGAAGGCGTCGTACACCAGGCGTAGTGAGACTGTGGTCAAGCAGCGCCATCAAAGAGCC
TTTGGCCCCGATGCTGCTGTGAAAAATATCCA
GCGCGCTCAATGTGCTACCCGGCAACTCCATTTTTCCCGTCACACTGTCGATGGCAGCATTCCGTGGC
AACGTCTGACCAGCCTCCAAGCACATCTTCTC
ATACACTCTATTCAACCGATAAGGGGCAAGATAACTTGCAATCGTCTCGTCTATGGAACCGACCCCCT
CGAACACACTGAGGGAGATCGACCGGTCGTCC
TCCTCTCCGTTAATGGTGGGGCCAAAGTGCAGCGGCAGCACTTCATTCAAACAAGAGGCGTACACCTC
CGGCAAGCACCGCAGCGGGGAGGATTTGGGCG
ACAATAATTTGTCATTCGCATTCTCCCCTTGGGGGGGCTCGCAGAGAAGAATCACCTCCACAATGTCG
TAGCGGTGCAACACGTCCAAGAGTTCCACCTC
CGATGCAATGCGGTGGCGTGTCACGGCGTAAGTAACAAAGCTGACAAGTGTGACATTCATCGCGGGAG
CTCCTGCTGCTGCCGGCAATGATGATGGCGAT
GGTGCCTTTATAAAACACATGAACAACTCGGATGATTCTTCCTTCATGGGAAAATTCACTCCTTCTGG
TTCCGCTTCGTCATCCTCCGCCCCGGCTTCTG
CGACATCTGCCGTATCCACAGACGAAACACCATTTGAATTTGTCGTCTTCGCCGGGTTAGCGACACCA
ATCACCTCGGACGGGAGTAGTGTTCCTCGGCT
GCACACACGTGTGATTTCTCGGGTAAAAATACCCTTCGCGGTCCCACTGTTTGCGCGAATGGAAGCGT
TTTCTTGGTCAGCAAACGCCACCCGGTAACCC
ATGGCCACAAGGCGGTGAACGTACAGGTTTACTCGCACATACGGTACACTGCTCGACTCGAACGGGGT
GGTACCAACGCACATGATACCAAAACGACGGC
TCGCCACgCGGCTATCGCGGCCGTAGAACTTTACACGATAACCGCACGCAACCATCAGCACCACATCG
GGCGGTAGAGATTCCTTGAGAGAAACCACCTG
CTTCTCAAGGGGCGTCAGTGTGACCGCTTTCACCTCCGTATCATTCACGGGGCGTTGGCATATCAACT
CAATGGCATGCCGAACTGCCGCCGCTTCCGCC
TCGGCATCCCGCTGTCGCTTGCCCATCACAAACTCCTCACCGGATCCTCTTTTGTATGGCTCCCCCCA
CCCACCCCAAAATAAACCACAGAAAATAAAAT
GAAATAAACCGCCGCGATGGGGAGAAAAAAAAAAAAAAAAAAAGAGAGAGAGGATCACAGGAGAAGCAG
CAAAGAAATGTAATGCACGCATCCATGACGAT
ACGGGCAGAANNNNNNNNNNNNNNNNNNNNNNNNNNNNNNNNNNNNNNNNNNNNNNNNNNNNNNNNNN
NNNNNNNNNNNNNNNNNNNNNNNNNNNNNNNN
NNNNNNNNNNNNNNNNNNNNNNNNNNNNNNNNNNNNNNNNNNNNNNNNNNNNNNNNNNNNNNNNNNNN
NNNNNNNNNNNNNNNNNNNNNNNNNNNNNNNN
NNNNNNNNNNNNNNNNNNNNNNNNNNNNNNNNNNNNNNNNNNNNNNNNNNNNNNNNNNNNNNNNNNNN
NNNNNNNNNNNNNNNNNNNNNNNNNNNNNNNN
NNNNNNNNNNCAATCCACTGAAACACCACCATCACAAAAAAAAAAAAAAAAGGAAAACAACAACAACAA
AAAGTAGAATTTTTTTTTTTTTTGGACGCCCAT
TATTCAAAAAATCAAATGCTGCCTCCTTGTGCAAAAAAACAAAAAAACAAAGTCGAGTAACGAAAGAA
AAGCCCAACGTATCAAACGCAGAAATTTATGC
TTGTATATGTATATTAACCCATATGTACATGCGAAGACTAAAACTCTACCGCTAATGACGCAAAATGG
CACTGTGTGCCAGTAGAGCAAGCATGTAAAAC
TTTTCAATTTTATCCGTCTGCAGATGGTGCTTGGTGTTTTTTCGACGATCATCTTCAAGAAGTTGGGA
GAGCTCTTCGTTCAAGTACCGGCCTGTATTGA
GTTCGTAGTGAGTGTAGGGTTTGTCATACATGGGGTCGTAATTATTAATGAATCGCGCCTGTACCGCC
CCCGTAACGGAGTATCCTTCCTTTATCAAGGC
GTCTAGTACTTCCAAATCACGTTTGATAAAATCGTCCCCTTCTGTAGACGCAAGAGTGGAGTGCATCG
TCGAGGACACTCCTCCTCCTTCCACTTCGTCC
TCAGCTTCCACGTGCATCCCAATATTGGAGCTTCACACAGACCGAAATGGGCGTGTATCCCCAACCTA
AACCACGACAGGGCACGAAAATAAAGAAAAGA
AGAAGACAGCATTAAACATCATGGAGAGACACACGCCATTACACACAAAGGAAATGAGAGATGTAAAA

AAAGAAAAAAAGGAAAGAGCCCAAAAGAGACAG
TAAAAAGTAGAGGATTCAATGGAAGTCAATATTGTTACATACACCACACATGTGTGAGAAGGACACTC
CCCTCCCCTCCAAAAAAACTGGAGTCTCACAA
AAAAAGAAAACCACAGCACCGCACGCCAATTCTTTTTACCCTTTTTAAATTTTTTTTCCACTTCTCTC
TCTCCCTCTTTTTTTTTTTCTTTGTATCCTTT
TACTTGTGTTTTTATTCCGCTTTTCCTTCTCTCTGCTGTTTTTTTTTTTTTTTTCCTCTCCCCCCTT
CTTATTCCTTGGGGAGGAGGTTCCTTGCCGCC
CTACTTTTGAGGACAGGGGGCCATGGCACCCTTCTCCTTGTCCTCCTTGAGCGTCAGCACCCGGTTCA
TGACGAGGTGGCCGGGACGCGTGTCTGGGTCC
ACAACAAAATACCCAAAACGCTCGGCCTGCACGGATTCAAACACCTTCGCGTTTTGTATTTCCGGCTC
GGCGTAACCCTGCTGGACCTGCTCGCTGTCGG
AATTGATGTACTTTAGGAACTCTGGGTCAATGGCGGCACGATCGTCCTTCAGAAGATCTCCGTAAAGC
CGGAATTCTACGGGAATTGCGTACTTCTCACT
GACCCAAGTGATGTTCGTCTTGGGTTTTGTCGTCCGCCCAAAGTCCACCTCGACATGAATGAGAGAAG
GTCGTCCAGCCGCATCGCACTCGTATCCGCGG
CAGATGATATTCCCGCTGTACTTCAGCCCCACAGGACGCGGTCCAGGCGCCAGGCCGTAGAATTTGCT
GTTGTTATCCTCTGTACGGAAGTCACTACGGT
CAATATAAAAGGTTTTGGTAAACATTACTTTTCGCGACTGAAGATCGGGCTTTCGTGGGTGATTTGGG
CACATCACCTCCATTTCGCCGTCCCAGTTGTC
AATGACGACCTTGACAGGGTCCACAATCATAAGACGACGCTCGGTGCGTTCGTCAAGGTCGTCACGAA
GAATGTGTTCAAGCATAGAACTCTGAATGACG
TTCATGGAGCGTGTAATGCCGACATGGTCACAGAATCGGTTGATGGCCTCCGGTGTGTAGCCGCGTCG
TCGCATACCGGCCAGCGTCAGTAGACGAGGAT
CATCGAATCCACGGACAATACCCTTCTTCACCAGCACGTTAATTTTTCGCTTTGACAGCAGCGAGCCT
GTCACATTGAGACGACTAAACTCCCAAACAAA
GGGTCTCCAGAGGTTCAGTTGTTCCAACAGCCAAAAATAACTTTCGCGCCGTGTTTCAAACTCCAATG
TGCACAAGCTGTAGTCGATATCTTCCAGACTG
TCAACGAGGCAATGTGTATAGTCGTAGCTAGGGTAAATACACCACTTGTCCTTGGCGTGCGGGTGCGC
AGAAAACTTGACCCGGTACGCAATAAAATCAC
GCATATTTGGGTTATCACTCTTCATATCAATTTTTACCCGCAGTGTTGCTTCCCCTTCTGCATATCGC
CCCTGTCGCATGTACTCAAAATGAAGAAGATT
TTCTTCGATGGAGCGATTGCGCCACGGGCTTTCCTCGCGACTCTCGCGCTGCTTCTTAATTTCATCCG
CGGTGGAGTGGTCCACATACGCCTTGCCAGCC
TTAATAAGTTGAATCGCAAATTCGTAAAGTTGCTGGAAATAATCCGAAGAAAATGTGATCCAGTCCGG
CTTCCACCCCATCCAGCGCACCATTTCCTGGA
TGGCGTCGATGTACACCTGCTCCTCCGTCTCGGGGTTTGTGTCATCGTAGCGCAGATAACACTTGCCA
CCATGCGCCACGGCACAGCCAAAGTTCAGGTT
CATACTCTTCGCATGGCCAATGTGGAGAAAGCCGTTGGGCTCCGGCGGGAAGCGGAAGTAGGGCTTCC
CTCCCGTCAAGGCATGATGCTTTTCCAGCAGC
TCCGGTGTGTTGGAGTACCCCGCCACCGGCCGCCCTGTCTGCAGCAGGCGAAGATCCTTTTTTACCTC
CAAGAAGGGCACAGCCGTGGAGGCAGCAGTCG
TGGATGGGGCTGTCATTTTCCTCCACAGCAACAACGTGAGGGGGCGAAACGTTCTTCGGGGTGTCGAG
TTATTGTTGAATAGACGCTaCGTGTCAGGGAA
CCAATGTGGGAAAGAAGAAAGAAAGAAAAGAAAGGAAATTCAAACAACAGACACAGACACAGAAAAAC
CACACACCTGAGTTCTCTGTCTCCCTCCTTGT
TAGGTGGAAAACAAAATAAAAGCACCGTGAAGCAGACATTTAAAGCTCCATAAATTTCCCGCTGTGTT
CCCCTCCCATAAATGCTTGTCCCACACCCTTG
ACGGATATCGAGATAATATCAATGCCCCATGGTGTCCACAAACGTTAGAGCAAATTCAGACGCACCAC
CTCCATCATCTGTATTTTGTAGCCTTTATTGG
CAATGTTGTTGTTTTAGCTGCTGCTGCTGCTCCTGCCAATGCAATTACTCTTCTTGAATTCTATGGAT
TTTTATAAAATAGTCATAAATAATAACAATAT
ATAAATAAATAAATATATCAAGCCCCCCTGGCCTTCATAAATAGCCCTCTTTATTATACGTGTTTTTT
TTTTCTTTTTTCGTTATCGTCCGCCCTCCTCT
TATTTCACCACCGCACCGAATCCCTCCTTCGTTTTTTCTCCCTTCCCATCCCGCCCTTCGATCGTCGT

GTGCGTCTCGTGGCGGTCAAGAAAAAAAAAAA
AAAAAGGCTCCTCACATCACGTCATCCATCTTTGGGGCAAGGAAGTAGCGCAGGTAGCCCACTTGATC
GATATTGAACTCCACCATGCACGGGCTGTCTG
GAGCAAACTTCAGCGACACACGGTCGCTCAGCGTAGATCCCTTCGCAAAGACGTTCATGAAACGCAGC
GCAAAGGAGAGAGTAACAGGCTCGTCCGTCCG
CACCTCGACCCCAATGGCCGTGTGGCTATTCTTCGTGCCTGATGCGTTGGCGCGGCTGATGGGTGTAT
CGTCGTCCTCTTCCTTCTTCACCTCCGGCGCG
GTCCTCACCGTGCGCCCCGCTCCTCCAGCGGCGCGCAGGAAGGCGTAGCCCTCGCCCACATCGCCGGA
GGAGCTGAATTTCACACCCTCCTTTCGAATCT
CAATTGTGACTGTCTCGCCAAAGACGCTCATGTCACGAACGATTTTGGCAAACTCCTGGGAACTGAGA
GTCACCGTTGAGCCGTACTCCATCTCCGGAAT
TCCCATAGCCTCCCCCTCAATCTCCAGCAGTTTCAGTTGGTACTCACACTTGCGGGACTTCTCAACAT
TTTCCGAGGTCAACACAACGACATCAGAATCA
TCCTCGTGGTGCAGCGTCAAACTGTCTGAGCCCTCAACAATCTTCAGCACCTTTGAAAGAGAGGCGAG
ATTGAGGCCAAGAATACTGTTCCGCTCACACT
GGTACTTGGTGAAGCAGTCGTCACGGAGGAGGAGATGGACAAGGGCGACGTGACTGGTGTCCATGGCT
TGAATGGAGAGGCCACCAGGATTGCAGTCAAA
GTTCGCCTCATTCACAAGCCCACTGATGCATTCAATCAGCCGCTTCCACAAGTTTGCCTGTTGAACTT
GAGCCTCCAGCATGATCAAATGATAATCTTCT
TCGCGGGTTCTGTCGTTTCACTTTATTATATCCCTTCTTGTGTTTCCTCTCGTTTTCTATTCTTAGTT
ATGTTTAACCGCCTGCTGCGTTTACCGCGTAC
TGGGCGAATGGAAAGAAAGAAGGCATGGTAAGAGTAAAAGGAGAATTGGAAGAAAAAACATGCGTAAA
AACAACAACAATAACAACAGAGGACACAACAA
CAACAACGCGAATATTCACCTGAATGAGGCCCAGCTCCCCTCCTATTCCTACAAAGAGACAAAACACA
AAGAAGACCACAAGTTGGTTCAATGCACCCGA
CATCCCCCACAACTCGGAGGGGATTCAGACAACACTCAATACGCCCATCAACAAATAGATGCAAAACA
CACCTGCCGCGATAACCGGGCATGCCGCAATA
TCACAAATTGGCATCCTTTCGCGCCCCACAACCGCCGGACTCCCCTGTGCAGAGGGCAATTGCAGTAT
TATTGTTATTATTATTATTATTATTGGTAATG
GGATGGACAAAGAACTCACACCCCCTACTTCTCTTCAGAAAAAAGAAGTCCAGCGGCATCCTCGCGCA
CCAACAATGGGCAAACACGCATAGCCGTATAT
TCTTCCCAAAACGACTCGACAGATGTGCAGGTCTCGTCGCCGAACACCAGGGCAAATACGGCATCGTA
ACCCCCCGCCCCGGGACATCCCACGGCAAAAA
CACCAGGAATTGCGCAGGTGGCATCAAGCAAGGCGGTCAACTTATTCGGCTCAATCTCCACAGAAGCA
GCCTGGCCCATTTCACGCAAATAACGTCGACT
CTTCGAGGCGTAGGTCGCCGCCTCCACCCAAAGTCGTTCGGCATCGTTTTGGGGAATATGCTGAGCAA
GCACAACATGCTTCAAGACATCGACTGAGTTC
ACATGTTCCACTGTCTTTTCTTCAGCCTGTAGAATCAGCGCCTTTAGTGATGCCACGTACTTCTCATT
GTTTTCTCGGAGTTGTTCCCATAGATTATATG
GATTGTCGGCAACAGAATTCCGCCATGCCATTACCTTGGAAACCATTCCTGGCGTGCCTGAACCACCC
TGGTGCACATCTCCCAACAGCAGCTTCAGCCC
AAGGGGAAGATGAAAAGGAACGCGCTTCACCCACTCCTTCTCCATATTTACACAGTCCGCAAGCGTTG
ACACTACCACGTTCGAGGGAGACTCGTTGGTA
CTCATAACACTCTCAACAAATTTTGCCGGAAAGCGGCGGTACGCACAGGTTCCGTATGCCGCTGTGTA
AACGTCGAAACCACTTCCAATTTTTCCCTGAG
CCACACTATGAGCTACTTGAGCAACGCGGTGCACAATCTCCTTCTCCGCTGAAGTGTCTGTTTTTGCC
GCGTTATTATTTTTATTGTTATCACTGGTGCT
TTGGGCGGTAAGCGAACGGTATAGACATGCCACCATGCTGGTTGTCATTGCAGCCGAGGAACCCAGTC
CGGTCTTGGATACATCACCAACGAGAGGCAGG
TGTGGGGGAAGCAAACGTAAGTTGGCCGCGGTAACTTCCTTTCCCTGTGACTCCAAGTAGTTTCTTTG
ACTGTAAAAGTCGTTATCAGCAAGAAGCTCCA
ATGTTAACTCCTTGAAAACATTTCCACCCTGCGAAATTGCCGCAGCCACGGTATACAAGACGCTGTAG
CGTAAGAAGGGGGAATGAGTTCCCTCTATCTG
AGCCACACAGACGGCGGGCTCTGGCGTGCTCTCGACCGTGCACTCAAACGCAAACTCCTTGCCAAACT

GGGGCGAATGGATGCGCACACAACACTTCCCC
TTGGCTGCATCCCGCGTCTTCAGTAGCCGTGTTTCAAACCGCGCGGTTGTGCCGACGGAGATCCCAAC
ATTAGGGGCCTCAACGATGAGGTAGCCACCAA
GGATGAGAACTTTGCCAGAAGAAGACGCCCTCGTAGTCGCAATAGCAGTATCCATTGACGGCGGCAAA
AACAAATGACAGTTGCAACGTCGAGAAACAAA
ACAGAAGGATTTTGCCTCCAACACGGATGGGGAATGTAAAATAAAGCCTCCGACAAAGCCACAGAAAC
CGATAATGAGATGTAAACGCGCAAATACGGAT
GTTTACTAACACGCGAGCACAAATGGCTGTTGGCGTCGCACAAACGCCGCAGTGGAAAAAACGGGGAA
GGGGGTGAGAAAGGGAGAAGAAAAAAAAGAAA
ATTCAAAAGAAAGAAAAAGCAAATTTGAGAGGAAAAAAAAAATGCAACAGCCATCCGTGACGCGTACC
GCAAGTTTATTCGTGAAAAATCGCCCAGAAAA
ACAAACAAACAAGCAGAAGAGGTGTTGACTTCCAGAGAGAAAAAGTCCATATCGTTCAATTTGCACAC
CTCGCACTAACGAAATCCCCCAACGCCCGATC
TCTCCTCCCCTGGTGGCGGGAGCCCAATGGCCCTAAAAAGCTCCGGGTCGACTGTGACTTGTCGCTTT
TTGCGCAGTTTCTCTGCCGTTGCGTGGGCGTC
TGACGCCTGGGCGGCCTCAATCTTCTCGCCCATCTCTGTGACGGCTCCCGCGGCGACGTTGGCCCGGT
GAAGGCGCCACAGGTAGTACATGCTGAGCAGT
GCGCATGTTCCCGCAGAAAAGAAGAAGACAATGAAGAACACATACCCCCCTCTGCTCACACAACACCA
TTCGGCACAGTCCGTGATGCCTGCGTCCAGCA
GCTTACACATGCCCTCCGCCAAGCACGGATCACGTGCGCAGTCAGCACGCAAAGAACGATATCCAGTG
CATTCAGCGACACACTGAGTTGCCATCAACTA
AATGGGCACAATCACCGTTACTATTTTTTTTTTCCTTTCTTTTGGGCTTCAAATTCTCCTCTGTTTGC
TTGCCGCCGCGCAAGAAAAATGAGGGGGAAAA
AATAAGTCCTTTTCAACAAAAAATCAAAAAGAAAAAAAAAAAGAAAAAAGGAAAGAAAGGCACAGATA
TGCCGCCGCGGGTAGAAAAAAAGAAAGCATCG
ACAGTTAAGTACGACTGCAGCACGTGAACGCAGCGCAACAACAAAAAGCACCAGCAAAAAGTTTCGTA
TAAAAACGAGTAATTTATTTAATTCGTTTAGC
AGCGACGCCCATTTCTCGAGGGGCCTTCATTCTTGTGCCGCTCCGTCAATGAGGGCCTTTTTCTTTTC
CTTTTTTTTTTTCTTCTTCCTCTTCATGTCTT
TTCTTCTCCATCTGTCCATTTTTTCTTTTATTTTCTTNNNNNNNNNNNNNNNNNNNNNNNNNNNNNNN
NNNNNNNNNNNNNNNNNNNNNNNNNNNNNNNN
NNNNNNNNNNNNNNNNNNNNNNNNNNNNNNNNNNNNNNNNNNNNNNNNNNNNNNNNNNNNNNNNNNNN
NNNNNNNNNNNNNNNNNNNNNNNNNNNNNNNN
NNNNNNNNNNNNNNNNNNNNNNNNNNNNNNNNNNNNNNNNNNNNNNNNNNNNNNNNNNNNNNNNNNNN
NNNNNNNNNNNNNNNNNNNNNNNNNNNNNNNN
NNNNNNNNNNNNNNNNNNNNNNNNNNNNNNNNNNNNNNCGTTTCTCGCTGTCCTCCCATCGGGTGGGGA
GGAAAAAAACAAAAGACAACAACAACAATAAA
AAAAAAAGAAAAAAACTTTTTTTTTTTAAATTTTCCATTTTTATTCATTTTTCCTTTTTCAACTCTTC
GTGCGTGTCTTCTTGCCGTTTGGCTTCTCTCT
CTTTTCTTTTTTTCTTTCTTCTTCTTCTTTGAATGGGTTGAGGCTTCTCTTCGCCCTTCATTCATGTT
CCTCCCCCATTGAGGGCTTACAAAAGACGCTC
AATCTCTAGCTTGACAGCCTGCACCTTGGCGGAGACGGGGCTGTTCTCTGACTGCAGGATTGCCAGGG
CCACCGCGTTGCGTGAGACCTGAGCTGCCCGT
CCTAGTGCCGTCTTGGAAGGCACAAAAACATACGGCACGTTCTTGTCCTCACAAAGCAGCGGAAGGTG
CAGCAAAATCTCAATCGGGTTCGTGTCGCCCG
CCAGTACGATCAAGTCAGCAATGCCACGGTTCAGTGCTTTGGTGGCCTCGTTGGCCCCTTTCTTGATC
ATCTTCGCGTTGCTGGCCTCTTGCACTAGGTC
CAGGATGGTCTGCGTCAGGCGATCCCCGGCGAGAGGAAATGCCTTCTCACTGATCTCAGCCGTCATTC
GCAAATTTTCCTCGTAGACACGTCTATGCGGG
GTGGTGGTGGTAGTAGGGACCGGGGGCGGCGGCGGCAGCACCGTTGGGTGGGATTCGTTTTTAATGCA
CGGCAATAACGCATGCAAAGAGACCGAGAGGG
AGGGAGAGAAGCCCAAAAAA
>scaffold1093.size14801
CCTCCTTGTCCCTGAACGGGCATGCCACCACGACCGCCAAACGATGGTTGACGCTGCATACCTCCTTG

TCCCTGAACGGGCATGCCACCACGACCGCCAAA
CGATGGCTGACGCTGCATGCCTCCTTGTCCCTGAACGGGCATGCCACCACGACCGCCAAACGATGGTT
GACGCTGCATGCCTCCTTGTCCCTGAACGGGC
ATGCCACCACGGCCGCCAAACGATGGCTGACGCTGCATGCCTCCTTGTCCCTGAACGGGCATGCCACC
ACGGCCGCCAAACGATGGCTGACGCTGCATGC
CATTTGTTATTTGCCCGCTTCCGTCCATATTGAGGGACTGCGTAGCTTGATTATTAAAAGCCAAGGAA
TTCTTAATTGGGGATAACGGGTATTGAGGTGA
TCCATTTTGTCGCAAATTTCCTCCTGAAGGCTTTTTCCGGCGCAAAAAGTACACGAGAGTGATGATAA
TTCCTATAAGAGCCAAAACCCCCAGAACAATA
ACTGCAATCGTCCACGTAGGCAACCCATGCGATTTGTCAGTATTCGTCCCACTGGGTGTAGGTGCTTC
AGTGCTCATTATTCCCGCTTCTTTTTGATCAA
GTAGACCCTTGCTGTCCTCTTATCGATACTCCTCCCTACCCCTACCCCCAAAAAAGAAGGTTGTGTAA
AAAATAATGTCTCCTGTTTTCAGCTAGCATTG
TATATGCAAAAAATAAAAATAAAGAAATCAGATGACACACAGACAAAAAAGGTTCAAAACAACAACAG
CAACAAAAGAATCATTCAAAAAAAAAAAAAAA
AAAGACTTCCCCACACAACAGCCACGCCAGATCAAACAAACAAATGTGCTACACATGAATATCCCAAC
GCTTGGTGTTCCAGGGGAAAGAGCATGCGGTT
CTGTAATAGAACATCTTCCCTCCCTTTACAATGATATGAAGAAGAGACGGCGCAAATATAGTTGCCAA
AGAACAATCCGCCAAAAAAGATAGCCTCGTCC
TTTCTTTCCTCCGGCAGGCTTCGCTCACTCGTAGCAGTACCTATCCATGGGGGTAAGCTCTGTAATGA
CACCCCGGACAAACAAGTTAGGGTCTTTAAAG
CGTTCTGCAGCCTTGGGCACACCCATCACGCGCTGACGCCAGTGTGGCTCCAGTAGCAAATCCATCTC
GGACCTGTAGTGCGCATTTTTTTCCACCATCT
GTGATGTCATACCTCGAGCGGCCAGTGCCCTTCGCATTTGACTGCTTCGGTACACCTTCTGAATGTCC
GGCTCCACGTCCAGCCATTTGTACCCGGGATT
AATACGGCTCTCCCGTATTGTCATCACACCCTTTGTCGTGGCATACAGAGTAAAGGTGTTTGGGTCCA
AGCGGACATTTTTTCCCGGACGATACTCCGGC
AACCGCGAGTGACGATCTTGGCCGTATCGACGTCGTTGCTCCTTCAACTTCTCCATGGGCGTTACCGG
GGTGCCGGGTATGACGTGGAATAGTTTTGCTT
TGCGACGGGGGTGTCCCGCCTGATTTTTCGCATTTCCCGCAGTGACTTTGCTCGCGAGTCGAAGAGGC
AAAAGACGCAACATTTGCGGTCCACCCCTTCT
CTACAGTGCCCAAAATTGCGACATCAAAGACACCCACACCGCGAAGTAAAGAAAAGAGCAGAAAAAAA
AAAGAAAAGAAAAAAAATATATATATATATAG
ACAATTCCAAAACTGTGTGGCCTGAACCACAACAGGATTGACAGAAGCGCTCATATGCACACATGCGG
GGGGACATGTTGCACTTCCTTTTTTTACATTT
TCTTTTTTCTTTTTGCCTTCCTATTCTCTTTATTCCGTCTATTGGCAGACCCAACCCCTTTCTTCTTG
CACCCTCCGTCCTGTCGCTCGTACTCACTTGC
GGCACCGCACCGCAGCAAAAAACAACAACACACGCCACATATTTAAGTCCCATTTCTCTCTCTCTCTC
TCTCTCTCTCTCTCATTCTCTCCTTTTATTTT
GATTGCAATGGCCCTCCGTTCAACATAACTTCACGTAAAATCAACGCTCTGTTCGAGACACGGCTATC
ATGTGCTCCTCGAACAAACAAACAAACAAACA
AACAAAAAACACATAAAATAAATCCACAAAGCAACAGAAAAAAAAAAAAAAAACCATAAAATAAATCC
ACAAAACAACAGCAAAAGAAAAACAAGAAAAA
AATATTATTATTATTTTTTTTTTTACACTAATTTTTCTTTTTTCTTTTTTTTTCCATTTCCTATTTTT
TTTTTTCTGTTATTTTTATTATTTTTCGGGTA
TTTTCTCAGGAAAAGGGGAATAATTCTGTTTTTTATTATTGTTTGCCCTTCGCAATCAATGCATTTGT
ATGGATGTATGCGGAAATGAATCCGTATTTTT
GTATCCACGTTTCCTCATTCTCAATTACAAAGAAATTAAATATTGATATATAAATAAATATATAAATA
TATCAATATAAATCGTTTTATTATTTATATAT
TTTATAGATAAATATAAATAAAGAAATGCTTGGGGCAAATGTGGGCTGAAAGAAAAAGTGTCTGCTGT
TTTTTTCCTTTATATCCCTTTGCCCTCCACAT
TATGTCTTTATTGCACGCAAATACATGCAAAATGGGCTTCCCCCCCCCCTTCGGCTCTCCGCTTTTC
CTTCGCTTTTTCCCCCTTGCATTACATACAAA
CACCTCCGCCCCACTTACACTCCCTGAGAAACATTGTGTAAGAGGGATGGATGGATGTATCTTCATTA

CCAGCCATACCATCTTAGGGAAGGAACAAGAC
CACAACGATGGCAATAAGTGAGGCAAAGGATGCACCACCCAGCAGCGCAATTCCCATAAGCTGCCCAG
CCATTACCCTCTCCCCATCCGTCTTCAGCTCC
GGTGTGCCGGGACCGTACACCATGGTCAGTGCCCCAAGGTAGTTTGTCAGACCAATAGTGAACATGAA
AATGCACGGCGCCACGTGGCCAGGGATTTGGT
GAAAGACGCAGAGAAAGATTAATGGAATGTACAGAAAGCGGACGAATGATAAATATAGCGTTTTGCGG
CGAGAAATCCAGAGCTTTCGGAAGGAAGTCAA
AAAACGGCCCAAGGCGTCACCACCGTTGTAGCAAAGAATGGCGATGGTGGCAAACCACTCATCGTCAC
GGTCAATGGGAATCACCAGGCTGGGGAAGATA
ATAAGTGACAAGAAGACGCAAAAACATGCAATCTGCATGGGGTAAATTTTCTTGATCACTGGAAAAAC
GGGTGTTGTCAGCAGTTGCTCTGAAGTTGTCA
TTTCGGACTGAACAATTACCGGTGCGCCATTTTCATTCCTGCAGGAAAGGTTTTCACCGGCTGCCTTA
CCGCTGTCCGCATCCTTTTCAACCGCCTTGTC
GATGGGTTCAACATTGCCGTACGCCGTGGCTTCTGCACTTGGAGTTTCTCCATCCGTCGCGCGTTTCA
GCATGCGGTACTCAGCAACGTGTTTCTGCGCA
AAGGAGTTGTACCGCAGCGACAATGCCATAAGAAGCGCAGCGAACATGATTCCCAGCGCAAGTGAGAA
GTAAATGTACGCCTGTGCAAGTACCGAATCAT
ACGTGTTTTCCATCGTTGCCTTAATGATGCACTGCATCGTCGACGCAACCAGTCCCGAGAAGCCGCAA
CCAAACATAATAGCCGACATAAACTTTGGGGG
CATTGTTCCAGCCAGCGCGTAGCACGTAGCCTCCAAGTACGACTTGCCCGCACCTGCCACAATGCTGA
CAATAATAAACGCGACGATGGCAGAATTCTGT
GAGACCTTTCCCGTGGGCAGCAAAAGAACCACAATAACCTCCAGCATCATACATGTGAGCGCAAAAAT
AAAGCGATTGCTAAGGGAAAATTTTCTCGCCA
AATGCGTCAACACGGTGGGTCCAAATATAATTTGCGTCACCAACGACACAACGTTGTAGAAGGTGAAA
ATGTTGGCCCAAAATATTGGCACGTTTGCCTT
CGCATGGGGGTCTCCTGAGACGTAGCGGTAGTAATCCACCATGTATCGAGGTGCAGATGTCAGGGCGT
TCATTGGCATCAACATGGAGACGCCCAAAGTA
ATGCATGTCGTATAAACGTAGAACTCCGCCCAAGAAGAAAATCCAAGTGCCATCGTGAAGTCGGTCAC
AAACAGGGCAATCAAATAAGGATTACAGAAGG
AAAAAAAAAAAACAAAAAACAAGAGGTATTATGCGCTTCTTGGCGTCTAGCTGTACAGTAACAAAGGA
AGCCTTACCTGTTCACTCCCGTAAAGTATATT
CCCTCGTGTGTGTGTGTGTGTGCGTATATATATATATATATTTCAGTGTAGTGTGTTGATTACAAACG
AAAAGGGGAAACAAAAACAAAAAAAAAAGGTG
ATTGCAAAAATTAAAAACACAAATACTGCAACTTCATTGTGTGCGCGGCGACACGGACGTACAAACCG
TCAAGAAGAGGAGCATCTTGAAAACATGCACA
GAAAAGTTCACGAATGCAAAAAAAGAAAAATAATCAAAATAAAATTAAAAACAAAGTTGCTCTGCACC
TATTTTCGTGCCTTGCGTACGCCGCGAGTCTG
CATGTTCTTGCGTGCCTCAGCAGCGATAAGCTTCTCCTCACGCTCCTGAATCACGTTGATGCGGAGAC
CCTGTTGCTTCGGCAGCGTCACAGGAACGCTC
TGCATGTCCTTGCCAATCACAAAAATGTTGGACGCTCGAGTAGCAAACTCGTGTCCCGCCGCATCCTT
CAGACGTGCAATGTCGAACGCACCAGGGTGGC
GCTCAATCGACATGATCTCGCCAATACGGCCGCGGTTCGCGCCGCCGGTGACCATCACCACCTTGCCG
TTGCTGGACTTGATGAGGTCCACCACCTTCTT
CTCCTTCAGGTTGTATACCAGGGTGTCGCCACGGTGGGTGCGGGGGTCGGGGTAACGAATGCGGTGAC
CGTCGTGTGTCATGGCAACAGGAATGCGGCCA
GTGCTCGTGTAGACGTTCTCCACCTTCAGGAGCTTGATGTTCCCCTCAGCCTCGCCAACCTTCACGAG
GGCAAAGCGGCCCTTCACGTCGTACAGAATGC
GGAAACGATCCCCGGTCCGTGGGATCTCCACAACGTCCATGAAGCCAACCGGATACTTGGTGTCCTTG
CGGGGCTTACCGTCCACGCACACAAGGCCCTG
GCGGAGAATCATCTGAGCCTCGGCGGCGTTCAGCGCATACTTCAGACGATTGCGGATGATGATCATAA
GAGTCATGCACTCACGCAGCTTGTGGGGTCCA
GCACGGGGACGTGGAGCGAACACGCCCGTGAGCTTGCTCAGCATCCAGTCCTTGGGGGCATAAAGGCG
CTTCAGGTGCTTCTTGGTCATGTTTTAGGTTC
CTTTTACACGTATGTACAAGAGTACGGGTAGATGCAAAGGGTGGAATGGGAAAAAAAAAGGCGATAGAA

ACTAAATGTATAATAAAAAAGAATATTGCCTC
GTTATTCTTCCCACTTCGCACGGGGTCACAAAAAATTCAGCAAATGAAATCTGACAATAAATCATTTT
TTCCAGAAAGCACGTTTGACCACCGAATCCCA
CGAGTGCCAGTGGCTGCCATCGCATAAAATGTTACCCGGCAAGAAAAAAAGGAAGGAAAAGAACGCTA
AACCCACCCGTCACCTCATTTTTTTTTATTCA
TCAGCTTGGAAAAAAAGAAAAAAATAGTAATGCACAATCAAAAGTAGGAAAAACAAAGGATTCGTAAT
GTCGCCTGGGGTATCAAAAACACATTCGCAAG
CGCCGCCTTTTTCGTCAAGACCCTGAGACGCCATCAGTTCACCTCGTGAGATTTCACCCGTGGCACTC
CCTCGCCAAAAGCGTCTAAAAGAAAGAAAGAA
AAAAAAAGCCTTTATGCTGCAATACAGCCGCCCCACGGCCCACACGGCATTGTCTGTCCTTTTCAGGC
GCTAAAATTTTCAACGAAATTTTCCATTTCCA
GTGAGCCTCGCGCAAACACAAGCAGTAACTTTTGCCAACATTTGACTGACGTTGGACTTGTGTCTGTG
AAACTGTTTTGCGGAAACCTTGTTTGGGGTTA
GCTGCTTTTGTCCTCCATAAACGCATACACGCTTCCCGTGTGCCCCAATCTACAGCTACCCAGCCGAA
GCGGCCGCGAGAGTGCAGCCATATTTTTCATG
CACTGCTGCACTGCCCCGTTGCAGTTGGGTCGCATTATAATACGCATCTGAATGAGGCTCTCGTTCCC
CTATTCCACACACACACACACACAAAAAAAAAAA
AAAAGACACCACAATGGGAGGTNNNNNNNNNNNNNNNNNNNNNNNNNNNNNNNNNNNNNNNNNNNNNNN
NNNNNNNNNNNNNNNNNNNNNNNNNNNNNNNN
NNNNNNNNNNNNNNNNNNNNNNNNNNNNNNNNNNNNNNNNNNNNNNNNNNNNNNNNNNNNNNNNNNNN
NNNNNNNNNNNNNNNNNNNNNNNNNNNNNNNN
NNNNNNNNNNNNNNNNNNNNNNNNNNNNNNNNNNNNNNNNNNNNNNNNNNNNNNNNNNNNNNNNNNNN
NNNNNNNNNNNNNNNNNNNNNNNNNNNNNNNN
NNNNNNNNNNNNNNNNNNNNNNAAAAAAAAAAGACACCACAATGAGAGGTGGCAGTTTGGCTCACCGC
ACCACCCGACATCCCCCACACTTCAGACAAGA
TTGACCCAACACCCACACACAAGTAAACAAACAAACAGGAAAATACACATAAATCATACTTTGTCCTA
ACCGGACTCGTAGTGAAATGAGGAAGCGGCAG
TCTTTTTGGGCTTCACATCCGCCGGACAACTCCGCATGAAGAGCGGCAGCGACAGCGGGTGGGAAAAA
ATAAATAAAAAAGCGCCCACCACAATTCAACA
CACGCTCTCTGACAAAAAAATAATCCAACAATCGTAAAGGAAACGGGGCAGTTAATTAATCCACTTTA
TCATCTGCTCAGCCCCAACCAGTCCCAATTCT
ACCTACAGACATCAAAGTCAAATATAAATCCGTAAAAAAAAATTCAACAATTCTTTGCTCCTATAAAT
TGGGGTATGAAAAGAAGGAAAGGTAGCAAACA
AAAGATTAGCACGAGTGGCCAAATTTGTTTTCATCCCCATCGCACCTTTCAATAGAAAATATGGTTGG
CGGGTTGTGGTCCTCATCCACTTCATCGTCAC
GCTCATTTGCCGGCGCTTGTTTCGTGTTGGACCCCCCTTGGCTCCGTGTCGAGAGCAGTTGACGGCGC
AGGTCGATCGTGACAACCTCGCGACGAATCGA
ATTGCAAGCCGAGATCAGCTTCCCAAATCCCAAAACCAAATCCTTCGATTCGCTTGGTTTTCCCCGTC
CTACCGCAATGTTCCACTGAAGCCCACTGGTC
TCGCATTCCTTCATGGTACCGTAGTTAATAAGACCACATGTCACGCCCTCACAACGTGCATTCCGTAC
CAACTGTGTCCAACCGTTGAACTGACAGGCAA
ATATGGTGTTGTTGGAGTTTATTAAAACAACATGCGCGCGTGTGCTGGCGGATAACATAACCGAGATT
GAGGCCATTTCGTGGTCAAATCGGCCACCAAG
CGCACCCATACAAACATACGTGGGTAGTAACACAGCCTCCTCCTTGCATGCCTCTGCGCATAATTCGC
GCTCTCGCGCAGGATCAAGGTCAAGGCCAATG
CTCCCAGCATTTGGGATAATCGCGCCTCCAGTTTCCAATAGCCGCTTACCACTCTCCGTTCGTAGGAC
ATGTGGAGTAATAGGAACAATGTCTTCTTTAT
GTTTCTCTCGCAGTCGATTTAGGAGTAATAATGCTTTCATAAAGTCCGTTGTCGATTGACAACGGACG
TACAGCCAAAATGGAAGCGATAATAATTCATC
CCTTCCCTTTGCATCCAGCTTATCCCACTCTGCCAAAGACGCCCCTTCCATACTTGTACTCATTCGGG
TAAATTCCGCAACGGTAAAAGTGTACCTGTCA
TGAATTTGATCCAAAACAGAGGTAGGAATATCGTCCACCGTCGCATACCCTTCAGTGGGCGCCGACAA
TGAGAACCCAGCGCCATCAACACTCCCATGGG
TACCGTAGTTGTGGGGAGGGTTCTTGCAGGAGTCCATATCGCCAATGACAGCGTCACAGAAAGGCACA

TGAGAAAATGGACGTAATTCCGGACGATGTTT
TCGCTCTTCTTCAATATATGCCTGCAGCCGAGAAAAGGCACCATCTGCGCAAAGGAAGTAACATCGAC
CAACAGAAGACTCCGGCTGCTGATTATTAAAC
CGCCGCACACGCAAGAGTCGTAGATACTCCTCATATTCCCATCGGCCGTTTGACGGACTGTTCATTAA
GACAACAGCAGCGACCTGCCCTGCCGCGTCGT
AGAAATCATGCTGCACCACCTTGCGCTTCCATACACCAGCCATACGCGACATCGACCACGAGCTTTCT
TTTGCCATCCATGAAGTAAACGCGTGGGTGAA
TAAAAACGATAACACCACCACCAAAAGACGAAGAGGGAGAAAAAAAGAAAAAAACAAACAAAAAAAAA
ATACTGATGATGTATTACCAAGGAAAAAGAAA
ACCACAGATGTAAGTAATGATACTATTATATCGTAACGGTTTTCTGAATCAGATCCTCGGCTGTGTAT
CCTCCTCTGTCCTAATTATCCTCAGTTCTTTT
TCCTCCTCATGCCAAGGCAAACAAAAACAACAACAACAAGCAATATGAATCTACTCAAAGACTCAAGA
GAGAGAAACACACACACACACAAAAAAAAAAA
AATGCATATATATGACAGGTTTTTTTCTTTCTTTATCTGACTTTCCACTTCTCCCCCCCCCTTAAATG
CCAATACTCATTCTAGCCATCCAATGGCCGTG
GCTTCATTCCCCTTAACAAAAAAAGTGCTCACAAATAGAACCGATACAAAAATAAAACAATAATCAAA
AACTGAAAGAAAATGCGTCACATTTAACCTTA
TTTAACTCCTTTTTATTCAGCTTAAAAAAGAGGAAAACAACAGATATTGCGAGAATAATTTTCAAAAA
CTCCTTCTGCTTTATTGTTACCAATATGATTT
TATTTCATTTTTCCTAAATATGCATATATATATATATTTTTCTCCCTTTCCCTCCCCTTCAACCCATA
AAATTAAAAAGTAAACATGATTATAGCAATGC
TTATATAAAAAAACTGATATAGATTGATAAATAACCTAACTGCAACACTCCCGACAGCGAGGATCAGA
AAGAACCGGAAAGAGCAAAATCTACCTAAACC
CACGCTTTGGAGTAGAACTCTGTCACATCCCTGGCGTCACGGGAAGCCCAAGCTGCATTGTTACGCAT
CCCCTCAACGCCCTGCCGAAAGCTGCGCGACA
TGCCCATCTGTTCCTTTTCATTAAGTTCATTCCAGAAGACCTCCATTTCATTTGCCACTGTTTCCTCT
GCCCCATATTCAAGTAACTTTAGTGCACGACC
AAGGATAAGACCCGGCAGCCTCATTGCGAGAATTGGCCACATGCGCCTGAGCTCCATTGCATATGCTT
TAAACGTCTTTGTATTACAAGCCAATGAGCTG
AGGACGTAGAAAGTGTCTTGGCTGCGGACCTTCCCAGAGACAGCATATTCAAAGAGTTCAGCCACCAA
ACCATCAACCCCTGAAAAAGCAAGTGCACGAA
GATAGTGTGTTCTTTCCATCGCATCCGAAGCCGTCTCGGCAAGCGCCTTTACCTCATCAAAGGCAGCT
CTCCCATTTCTTTTCACAAAGGCGGTAAAAAC
AGCAGCCCGCAGATCTGAAGGTATTGGCGTTTTTTGGCGTTCCGCGTATAATTTAAGGGCATACGCAA
TAGTTTCTTCATCTTCGGCTGCCACAAGACGT
GCGAAAAGTGCAGCCCGCAGCTGAATCACATTATGGTCATCAACGGATTCAAAGGCATAACCCAGTCT
TTTTATGGCGTTTGAGTACAATGTTCGAAAAT
AAGCGTTATGCGCATTTATGGCCTCTTCACCTTGAGATGCAACAATTACTTTCAATTTGGTCTCAAAA
TCCACAATAGAGAGCCATACCGTGAGGTCGTC
TTCATCCTTATAGTATGAAAGAAGCTTCAACACCTTAACCGCACTGCAATATCCAGCGCGAGTAAAGG
CATGATAATCAGAAAAGATGCTAAATCGATCA
ATATTAGACAGTTTCTTCGTCGACAAAGCAGAAAGTAAATTTTGGAGTAATTCTTCATCCTCATATAA
GACACGACAAAATGCAGACTGATCATTATTCA
CCTTCACCCATGAAGGATGTGGAACAGGTACTGAATTTTTTCGTTCTTCCAAAACTTCGCGTTGAACA
CCATTTTCTGGTGTGGTAATAAGTAAAGGAAT
CTTCCAGACAGTTTCGTCCTCTCCCTCACCGGCGTCACCCGTAGCAAGAAAGCGATGTTGGGTTATTA
GTAGGCTCTGTTTGTCGCGCAAAGAAGAAACC
GTGAGAAATGGATAACCTTGCTTACCTGTCCAAAACTCCAAGATGGGCGCCAACGGTTTCCCTGCCGC
TTTGCCCAAAAATTTCCACAAATCTTCTGTTG
TCGCATTGCCATATGCGAAATGCTTTAAATAAGAAGCCATGCCCTTCTGGAAGACATCTTCTCCAATA
AAATTAACGACCATACGAACGATACCTCCACC
TTTGCTGTAACTGATCGCATCAAAAATATCATCAATCTCCTTCGCATTTTGAACATCCACTTCCACAG
GATGCGAACTTCGTAAGGAATCGAGTTCAAAG
GCTCGCGCGATTTCATCGTGTACAAACTGCGTAAATACATGCCAGCCAGGAAACAATTTGTTTACAGA

CCAATATTCCATATAGGTGGCAAATGATTCAT
TGAGCCAAAGTTCTTTCCACCACTGCATCGTCACAAGATTACCAAACCACTGATGTGCCAACTCGTGT
GCCACCACCAAAGCAACATGCTGACGATGAGA
GGCAGCTGAGTTCTCATCACATAAAAGGGCCGTTTCGCGAAATGTAATAAGACCCCAATTTTCCATTG
CGCCTGCAGCAAAGTCGGGTATAGCAAGAAGA
TCGACCTTAGGTAAGATATAGCTGGATTCAAAGAACTCTTCATAAAGTGGCAATACTTTGCAGGCAAC
ATCCAAAGCGAATGAAGCCTTGGATTTATTCC
CTTCAGTTGTAAAAACACGAACAAGTGTGTCTTCAGGCTGACCATTCCGTACTCCATGCGTTTTTTTA
ATACTTTGCTCAATACATTCAAATTCACCAAT
CGTCCAGGCAAGAAGATACGTCGACATTTTGGGTGTGGGAGCAAATGCCCAAGAGGTCTGCCCATTAA
CCTCTTCTTTGTAAAGATGTGGCATATTAGAA
AGCACCATCATATTTGATGGGGCAGTGATAACCATTTCGAATACCGCTTTTACTGCAGGTTCGTCCCA
GCAAGGAATTGCACGACGTGCATCCACAGCCT
CAAACTGCGTGGTGCCCATGTAGCACTCTTTTCCCTTGACAATGTATTTGCTTCGGTAAAACCCGGCA
AGTTTATCATTTATTTCTCCCTTATAGTCGAT
TGAAAGAACAGCTTCTCCAATAAAAGGTTTCTGTAAAGGAAATGTCGCTTTCATTTCGACCGCATCAA
GAATTATAGATTCAATAGGGATCGTTTCCACG
ACAGAAGAATTCCCTGCGAGAGCAAGGGTCACCTTGACAAAGGATAACTCATTATAGTTGAGGGTAAT
GCTGTTTTGAAGATTTTTTGCAGTTATCTGTA
TATCAACATGACCCGTGAACAAAAAAGCATCGAAGTCCGGCAATATGATTATTTTATAATGACGCGGC
GTAGGATCATCAGGAAGAACATTTCTGATTTT
CGCCATATTTTTCCTTTTTCTTACAGTTTTATTTATGTATTTTTATTTTTTATTTTTATTTTCTTTTC
CAAAGTAGTGGGAAAGGTCAAAAGACAAAAAA
GGGTTTGCACAGAAATGTCTTTTCCAGAAAAATACTGAAAAGGAAGAACGATATACTGAGGAAAAATA
AAATAAAACAAAATAAAAAACATAATAATAAT
AAAATTATTATTATCATTATTACTATTATTAAGTAAaTAGAACAAAAAAGTGAGCAAAAAAACAATCT
ACATAACACTCATATGTATATATTATACAGCA
ATAAAGAATGACATGAAGTTTCTCTTCAGTCTGTGGTGATAAACTGCACCCCAAACAGAATTCTTCCG
ATAAAGTAAACTAAAAGAGATAAATAAATAAA
TCATTCTAAGGAAACATCACAAATAAAATTTATATACATTTCTGATGAAATAAATACAATGTAACACT
CTTTTTCTATTGATGTGAAATTGCTCATGTGG
GGAAAAAGCACAATTTGCAACGACAATAGTAATTTAACAAAAAAAAAAAGTCACATAACCCAAGACAA
CTGATTTTNNNNNNNNNNNNNNNNNNNNNNNN
NNNNNNNNNNNNNNNNNNNNNNNNNNNNNNNNNNNNNNNNNNNNNNNNNNNNNNNNNNNNNNNNNNNN
NNNNNNNNNNNNNNNNNNNNNNNNNNNNNNNN
NNNNNNNNNNNNNNNNNNNNNNNNNNNNNNNNNNNNNNNNNNNNNNNNNNNNNNNNNNNNNNNNNNNN
NNNNNNNNNNNNNNNNNNNNNNNNNNNNNNNN
NNNNNNNNNNNNNNNNNNNNNNNNNNNNNNNNNNNNNNNNNNNNNNNNNNNNNNNNNNNNNNNNNNNN
NNNNNNNNGATGATAAAATGTTTTAAAGAGTC
AAATACAAAATGGATTCATAGGGCATGGTTCTTCCCATTTTCCATGATGCATTTCGTTTCCAAAGTAG
CCCCATTTCCTGTGCCTATATATATATATATA
TATGTGAAAAAAAAAAAAAAACAATGAAATAGTAATAACGCATAATTGGGATAAGGTTATAATATCAA
AATAAAAATATTCTAAAGTTTTTTTCTAAATG
ATAATAAATTCCAATGATACTTTTTATTCATTACATTTATGTAAACAAATAATTTTCTCGCGAAAGAT
AGTTGACTTATGCTCGCGCGGTAAAACCCCCC
AAAATGCTCGAGTAAAAGATACCTTACAAGCACGTCTTCTCTTTTGAAGAGGAAAAAGCTCTACAGGC
AGCGTTTGATACTCTAGAATGCATTCATGTCT
CTTCGAATGCTTTGTATTATTGATGGTTCCCGTTCCGCGCACAGCAAGAGATGTAGAAAGAACCTTTT
CCGCAATAACACTCTCCGACAAAGCTATGCGA
TCCTCAAGCTCTTTTAGCGTTACGCGAAAGCGATTTATGTCAGAAAGTTCAATTTTTTCAAAAGGATT
TTGAATACGCTTTATCATCGGAGCCAACTTTT
GAATTTTGGCTTTACTCATCGAAATAAATTGCCGACTTTTTTTCCCTTCATTCAAATGCTTTGTTCTA
ACCGATTTTACAGCGGCAGTTTCATCATTGGG
TATAATAAAGGCGATATGTTGATGAGAATGTACATCCCATAAGGCCATATACAATTCATCTGGAATAG

GAACAATTGATTTGTTTCCCTGAAAAACGCAA
TCCAAGGAATTTTTTTCCTTTCTAAATAGAACACCTGTTGATACGGCCAGACATCTAGAAAAAACCCA
ACCTTTCCTTCTTTTCAATGGACTTAAATTTC
CATCATATTGATTCGCAACAAATAGCTCGTCCAAAGTTGAGAACCCATCGCCTTCTACATAACATCGT
GAAATTCGTTCTAATCTTTTCCATATACCTCG
GTTAAGAGACTTGTGTTGTAAAACAAAGTTGGAAGCCAAAGAAGCCTCAAAACTAGTAGTAACGCACG
TTGCCACAGATGCAAGTGGGGCCATATGCCCT
TTATCATATGCATGCCGTTGTAGTTGCATTGGTTTTACCTGAATTTCCCTCGGTAATGTCCTGTCAGT
ATAAAAGGCATGTGCTTTATGTTTGTGACGCG
CCACTTTAACCATTCCCGAAGTCAAATAGAAGCCACACCATAAAGCCATTTTTAGATTTTTGTCGTAC
AACAGTGCATAGTTACTACGTGATATTAACTG
AAGCGTGTCCTCTCTTTTCAAAACAGCCATGTCGTTTATGGCATTTGTAGTTTTGAAAGATATTCTGG
ACCCACGAAAATTGTTTGGTTCCAACCACGCT
GGATTGTGGAACCTATGATTCAAACAACGTTGCCCAGCCCCCATCACGCGTCTTACGCGGGTTCGAAC
CCACAAGCCGCTAAAAACCCCAATAAAATAAG
AAGCACCCACCAGAAAACAGATTGGCATACTGAGCACCATCCAATTTGGGGTATATACGGTCTCCAAT
TGCCTTTTATTTATTGAAGAATTATTATTCCA
CCACATGCAGCGTCACTTAAACAACCTGGTAAAATGTCTTATTAGTAAATATAGATAGATACAAAAAC
AAAAGTAAGCTAAAAACAACAACAACGAGGGT
ATAACCCAAGCCTTGGTGTAGTTGGTTAAATACATTCCTCCAGAAATCACCCACAAAAGTAGGACAAC
AACAAAAAAGTATGAGTAAGTTTAATCATTTT
ATGTTGCTGCAAATAGAGATAAATATAAATTCCCTGATATATATATATATATGTTTCATACTGGTTTG
TTGGAAATCTGCGTTCGTCCCAACAATACAAG
TAAGCAAAAACAGTGGCAGCCACACAACCCGCCCCGATCCTTGCTCAAAAAAAAAAAAAAAAAGACAA
AAACAGCCTGTCCCACCAAAAAGAAAAATCAC
GC
>scaffold1094.size6319
CTCTTCAGCGGGGAAATATTTTGTTTCGAGAGATGTGAAGGATGGAGAAAAGTGAGTCCTCTCCATGC
TCCCAGTCTTGTTGGGATGAATGGTGAGGTGTT
TGCCGTTGCCGAGGCGTGGTGCGAGAAGGATAGCGTCATTTTTCCTTGCATTGCTTCACAATTCATGA
AGTTGGACGACAAAACCCCCAAAGCCCTGGAA
GCAACTGAGGTGAGGACGGAAGTGCTGGAGGAATGTCCAACCGATGGAGGGAATTGCGCCTCCAAAAC
AGAGGATAAGGATGATTCCCAAGGCGGGACGA
GAGTACATGTGAGCCGTCCAACAACAGTTGTGAAGGGAAATGATATTTACATGCTTACAGGAGTCTGC
ATGAGTAAAGGTCTCTCTAATTGTCAGGTAGG
TGTGGCTGGCGTGGCTCAGTGGGGACTTTTGCTGGTGAAAGGGAACGTCAGTGGTGTGGATGAAAGTA
CCAAAAGAATTTATTGGAATGATACCATCAGT
ATTTTGTGCAATGCTATCGGTGACAACGATGACACTTTGACAGGACACATTGGCGGTGGTGGATCGGG
TGCTAAGATGGAGGACGGTACGCTTGTGTTCG
CAACGGAAGCCGTGAAGGAGAATAGGGTTAATGGCACTGCGGAGGATGGAGGGAATAACAATGTTTCG
CTGCTCATTTCCTCCTCGGATGCTGCAAATTG
GACGCTGTCGAAGGGGATGTCTGACGGCGGCTGCAGTGATCCCTCCGTCGTGGGGTGGAAGGACAAAA
CACTCATCATGATGACTGCGTGTGATGATGGC
CGCCGCAGGGTGTACGAGATCGGTGACAACGGGAGGCCGTGGACGGAGGCACTCGGGACACTCTCGCG
TGTGTGGGGCAGCAACCGTAAGCCACATGAGA
AGGGCGTTGGAAGCGGGTTCATCACAGCGACGATTGAGAACCGGGACGTGATGCTTGTTGCTCTGCCG
GTGTATTCCGAGAAGCTTGAGAAGGGAGGCGC
TCAAAAGGGTGAACTCCATCTTTGGCTGACGGACAATACGCACATTGTTGATATTGGGCCGGTATCCG
GGGAAGGGGACGATGCTGCCGCCAGCTCCCTG
CTGTACAAAGGTGGCAAAAGTGGTGAAGCTAATAATAATGAAGAGTTGATTGCACTGTACGAGAAGAA
GAAGGGCGCTGGGGGCAAACCATCACCTGGTG
TGGTCTCTGTGCTCCTGACTGAGCAGCTGCAGCGGGTGAAGGAGGTGGTGGCGACCTGGAATAAAGTG
GACCAAAGCGTTTCCCAGCTGTGCCCCCCAAG
TGCTGAGAAGGACCGTCCAACTGAAAATGACTGCAGCGGTGTTAAGATCACGGATGGGCTGGTTGGCT

TTTTGTCCGGCAACTTCTCTGAGAACACATGG
AGGGACGAGTACCTCGGGGTGAACGCCACAGTAAGTAATAATGAAGGGGCTGCGAAGGCAACATTGCA
TGCCGGCGGAGTGACGTTCAAAGGAGCGTGGG
TGGAGTGGCCTGTTGGCAGTCAGGGGGAGAATCAGCTGTACCACTTTGCGAATCACAACCTCACTCTT
GTGGCGACGGTGTCCATCGACAAGGTGCCGAC
GACGGAGGGTCCTATTCCTTTGATGGGTGTGAAGATGAGTGACGCAGAGAATCCAGTACTTCTGGGAC
TGTCGTACAACAACGAAATGAAGTGGAAATTG
TTGCAGAGTGGCGGAAAGACCGAGGAGCTCAGCGGTACTTGGGAGGCAGATGCAAAGTACCAGGTGGC
GATTGTGCTACAGAACGGCACCCAGGGCTCGA
TCTACGTTAATGGCAAACTTGTGTGGGGATGCGCAATGTGAATTGAAAAATACGGATATTAATGTGGT
CTCCCACTTCTGCATTGGAGGGGATGGAGACA
AAACAGGGAGCAACGAAGGCGTGTCTGTGACTGTGACGAACGTCCTGCTGTACAACCGCCCGTTGGAT
GGCAATGGGATTACTGCGCTTAACGCAAACAA
AGTTTCTATTCCGAAGAGTGTGGCTGCGCGTACGTCTTCGGCCGTGGTGTCTAGCCCGAAGTGCTCTG
CTGATCATTTGGCGGCGGGAGCAACGGGTGGC
GTTGCCGCTCGTGAGAGTGGGGCACATGGTGGTGGCGGCACTGTGTTTGGCAACGAGGTGCTGCCATC
GCTTGTTCTTCTGTTGGGACTGTGGGGGTTTG
CGGCTCTGTGAGGAGTGACTGGCTGCCGCGTGGGACACTGCAACCCTCCCTTCACAGAACTACTGTCC
TCCCGTAAACGAAATATTCCCCGGATAACATA
TTTTTTGTTCTTAATTTTTAAACTTTTTTTCTGTGCCTTTTTTTAAATTTGAATTTTGTGTGCTGCCA
CCGTGCGTGATTGTTGATGTCGGGGCCGCACC
GCATGTACCGCTGCCATCCAGCTGTGCCTCTTGCTGTGTGTCCCCTTGCATGGACTCCCGCCCTCACC
TCACCCCACGAGGCACACACATAATGTGTCCA
TTGTATATATGCATGCACACCCATGCAGGCCCGTTGGTGCTCTTTTTTGCACGACACGCACCGCTGCC
GTCCTGTCTGGCTGCATGGGCGGAGCACCGTA
ACTGTTTCTGTTTTGTTTTTATTGCATTGAGTGGGCGACCTCTCTCTCTCTCCCTGTGTGTGTGTTTT
GCTTTACGGGAGCAATGAGAGTGTGTGTGCTG
GTCGTCTCACGCTTCGGCATTTTTCTTTTTTTTTTGTTTTTAATCTTTGCTTGTGTTGTGGTTGGGTG
CACCCATTTTACTTTCATTGATTGGTTTTCTT
CCTAAAGTGCGGATGGCTGAATTTTCCCTTTTGTATTCTCCTGTTCTTTGTTTCTTCCCACGAAATCG
GTCCTTGTTGCTTCAGTGAGCCACGAAATATT
TGCCTAACACCCACATCAATGGCATACTTGCTCTACACCCCACATTAGTTTGAGACGCTGGTGCTGCT
GCATGTGATGGTGTTTATGGTGTGATTGCCAG
CGGGTGCTTTGCTGTCACTTTGACGTACCCTCGATTTGTTGAATATGTGATGCGTGCCGACAGCACGA
ATTGGGTCCGCGTTGTTGTGAGGGACGCACCC
TTTTTGAGCAGTTGGTGCGCTGCGGTCATAGACCGTCATTGGGAGGGATGGATGCCCATCCGCATCAA
CGTATCCACAAGGGACCTGGATGATACCGGGA
AGTATTGGATCTCAGAGAGGTATCACGCAATGAATTCATTGAATGACAATTGCGAGTGTGAAGGTGAC
GTTGTATCCTCTGCGCACGGGATCATTCTCGA
GGATGAAGTTGTCCCTGCTGCTTTCAAACCGCATGCGGATCGTCTGCTCGTGCAACCACTGGAGGGTC
CTTTGATTGTGCCCCCATTTGCCACGGGCAGC
GTTTGCATTCGGTTCACGGTGCGTGCTGGGCATTGTTCCACTGGTGTTGCCAATGCTGACATGGTGCT
GTGCGTGGCAGAGCGCTGTCGGTGGTGGTGCA
CTCGACGACTGCCGTGGTGGCTGCGAGGAGCCCCGCGACAGCGACGACATTGATGGCATGGAGCTGCA
GGATGGCGATGGTGACGGGAGGACGCTGGAGT
CGCACTGGTCGCAGCGCCACGCGAGGGATGAGTGGATGGCACCGATTGGGAGTGCCGGCTGCCGCACG
GAGCTGACGCTGGCCGCACCGGCTGACCTTGG
CTGCATGAGGGTGAAGTGGGAGATGGCGGAGCCGATGCGCTGGTGAAGGAATTCGAACTGTGTGCTGC
TGCAGAGGAAGTGCTCCGCACCGAACATGAGT
GAATATCCCCAGATGTTCTGCGAAGCGGGAGTTCCTGCCAGACGCTGCACGTTGGATCGGTATTCCAG
TGGTTGGTGTGTAGACGAATTATCCATGAAGG
GCCAGCAAAACGCCCGATGGACAGTTGCCCCATCATTGGTCCCGCACTGGATGTGAGGGATGCTGCCG
CTGTTATTTGGAGGGGAGAATGTTTGTCTGCG
GTGCATGGGGACAAGCCCTCTTCATGGTGTCTGGACACGCTTCCCACCAACACGACGGATGCGAGGGA

CCCGGAAGAAACATACATCTCGGTGCTGTCAT
GCATGCGGCATTGCGGTGCCTCGGCATGCGGGTGCGTCTGAGAGTGACTGAAGAGGGTAACGCCCGTT
GTGTCCCATGCACGGCCGAGGCGACGATTACG
TGGGTTGCTCCGCCCTACGACGAGGGAAAAGCTATTTTCCGTGACCACGCGCAGGTGTGCACGATATC
CGCCACCAGCGGCAGGCTGCTTCCAGTGGTTC
CGTGTGGTGGCGATGTGAGAGGATTTATGGAGCAGGACGGTTTCACTGGCATCTACTGAACAAGCTTT
GCTACGGGCATCCGTCCCCGATCCAGAGAGCA
TTGGGACCGGCCGTGGCACACCACCGAAGGATGTGGACACGGCTGCTCCCCAGAAGCATCCGTCGCCA
GAGGAGAAGGAAGTCGCCTCAGGTGCCACGAC
GGCAACGGAGTGCTCTTGCCCAGCGACACCGCGGGTGCGCCGCAGGAGGCGGAGAAGGAGAGCGATCC
TCCGGACATCGACGATGTGGCCGCCGATGGTG
TGACGTCTGTTGTGGGACAATCATTATGAGATGAGGCCGTCAGCGACAAGGCCGGTGGCGGTGCCACA
GCCACGGGACCCAGGAGCCCTGATGCCGCTGA
TGACGCGCAGGGCGCCGTGGACCCTCCGCCGAGGGAGGATGAAGCGGCGGTGGGGCGCGGGGGTTGGG
AGGCAGACATGAAGGACGCCCCGCCTCCGATC
CACGGCAATAACAGTGCGGCTGCTGCTCGTCCCTTTCCGCTGATTATTCTGCTTGCCGCTGCCGCCGC
GGTGCCAACACTCAGCAGTGGGTGCAAGAGAG
AAAGGCCCATATCAACTGGCATGTTCCTGTCAGTGTATACATACACTCTGCTTGTCTGTATACTTTGT
ACCAGGGACATGCTGTATGCACTCTCCCTCTT
TTGTGTTGTTCGCTGATGTGGCGGCACTACGCGTGTATTATGGGTTGGTATAAGAAATTGTTTGACTT
CTTCTGGAGGATACTGTGAGGTGTTTGTGTGC
GTGTGCGAGGGATCTGTGTGGTTTTGATTTCCCTATTTTTATTTAACTGCTTTGGCATTGTGTAATGA
TACTTCCCTTCTCCATTATGTGTGTTCTGAAG
TGTTGCTAGGGCACCGGGAGTGGATGCCTGTGGACGCACGCCGTGCATTATGCACTACATTGGTGGGA
GGGGAGACTGACATAACTGGCGGGATTCAGTA
AGGCGCATTTTTGCCCACGGACCCTGCGGCCGCAGCAGCCTGAGTGGCGCTTTGCATATGGACGTGTG
GATTGTTTCTCTCTTGCGAGGCCTTTCTTACT
GCTTGTCATTCTCCGTTCGGGTACCGTGCCCGTGGCAGCAGCGGCGGCATCAAAAGGGCTGTGTGCGT
GGAGAGTCCTTGATTGAGTGCCTGTCTTACTT
CTCTGTTCTGTCGTTTGATTGTACCGTGCGCCGGCCAAACGGTTGGCATGACGTTCTTCTGACGGCGG
GGTCCGCGTGACATGATGTTGCGTCGCGTGCT
TTGTTGTGGGTGTGTGATTTCAGCAACGGTTTTTGGGCGGTTGACTGTTGGGTCGTTGAGGACGGCGT
GGCGACGGGTGTGGATGGACCCGATTGTGTGC
CGTGGCTGTTCGTGCGGGGGAGCCGCGGGAAGGAGGAGGGCAGCGGAGCGATGGACTCACGAGGAGTG
CGGTGGCGTGCAGCCATGAAGATTGGCGACAG
GGAACTCACTGCCGCTGTGAGTTGCCGTGTGTGGTGTTTGTTTCTGTTATTATTGCTTGCGTTGTTGG
ATGTGACCGCTGTGGGGGAAGACTGTTTTTTG
TTTCTTTTTCACGCCGTTTTGGGATCCTTTTCTGCACCGGCATCACTGTGTTCCTGCCGTACTGTTGC
GCCCTCTTTCCCTTCCATTTTGGGGGCAGCTG
CGGGTGGGTTTTACATGCCGTGGAGTTGTGTGCCGTGCTGTCCGGTGTTTCATTGTTGCCTGTTCTTC
CTGCGTGCACGCGTCCCCCATGCTCATGACAG
CAGCCCTCATGTCAGCACCTTCCCTTTTTTTCCATGCTGCTGATGTGTGTGTGCTCTTCTCATCCTCC
TCTCTTTGTTCCCTAGACTGTGTGTGCAGCCG
TGATGATGACGATGACTGGCCGTGTGCTGCTGGTGTGTGCCCTCTGCGTGCTGTGGTGCGGTGCCTGC
GGTGGTGAACGAGCTGAAACAACTCCAGGTTC
TTCGGGTAATCCGTCCGA
>scaffold1095.size6467
TGTGTGTACATCATTATACTTGTTCCTGTATCTGCAGACACGCTACATTTGGGTGATGGTGAATGACT
GGAATAACTGCTGCAGTGGCAACGATGATTGCA
TGACTCAGTGTAGAATGCTACGCTGCTTATTGTTTTGCAATGCCCTGCTTTATTTAGTTTCACTTCAT
TTCTCGACATATTTCCTCTTCTCTCCACTTCT
TTAACACACACACGAGGAAAGAAGGAAGACAAGCGATAAACGCTACTGTGAAGTTGGAAGGCTGTGAA
AGGACCAACAACAGCACACACACCGCCCACAC
ACACATATTTATATGCTCTCACGTGTTGCTGCAGTGAAGGCACCCCGCACACACAACCGTTGCCGCGT

GACCGGATCCAGCGGAAGGAGGAGGAAAGGAA
GAGAGAGTGAGCGGCAGAGGCCCAACATGTCCCGGCATGTGTTTACTTCCGCGGTACTGTTCCTCATC
GTGATGTTGTGCTGTGGATTTGGATCTGCTAA
CGCGGAAAGGCAGCAGTCAGGGGACGTGCGAATGCCACAACCGGTTGACATCTATGTGCCGATGAAGA
CGCAAGTGGTACCAAAAAAGTGGTTCTGGCAA
AAGTGGAATGAGGGATTCATTTGCTGCGCCTTCTCTTGTCCGTGCTGGTGGAGTGACGATTGCATTTG
CTGAAGGTCACGCCGAGTATAAAGGTTCACAC
TATAGATTGAATGTGCGTCCCTCTGATATTGTGGCGGGGTACATCAACGCCGCGGAGACGTGGCCGCT
AATTGCCGCCGAGAACAACAAGGATAAATGGA
GGGTACGCACGCTGTTTAGTACAGTGAGTGAAGAGGATCTTTTTGGTGTTGCGTGCAACCCCACAGCG
ATTGCAAAGGACAATAAAGTGCTTCTACTTGT
GGGGAGCTACAATATGTGAAAAAAATGATGGTCGCTAGAATACGGTTGGCTGGGATATTCAACTTGTT
GAGGGTGATGCCACGCACTCCAATGACGGCGT
GCAAAGTACATTGATCGACTGGGCTGAACCCAAGTCACCTTCACGGCAGATTCCCCAAAGGATTAAAA
GCAACCTGTCGGATTTTGGGGTGGTATTAGTT
CAGGCATTGTGATGGAGGGTGGCACACTTGTGTTTACCCTGAGGGCGAGGAATGGGGAAAATCGATCC
TTCTCCAGAATCACTTATTCCACGGACAACGG
CAATACCTGGGTGTTCCCAGAGGGCATTTCTCCTGCGAACTGCACTGACCCCCGCATCACCGAGTGGG
AGGGATCACTTCTCATGATTGTTGACTGCGAG
AACGACCAGAGGGTGTACGAGTCGCGTGACATGGGGACAACGTGGACGACGGCAGCCGGGAAACTTCC
AGGCGTGTGGACCAAGTCAAAATCGGAAGACT
ATCCGGAAGGAGTCTTGCGTGTGGAAGCCCCTATCACCGCGAACATTGAGGGAGTGAAGGTGATGCTG
TACACTTGGAAAGGGTACTTCTCGGGGGAGAA
AAAGGGAAAATGTGTTCCATCTTTGGGTCACGGACAACAACCGCTCGTTTTCTGTTGGACCGGTTGCC
ATGGATAATAACTTGGATCTTTTCCACGCCCT
GCTGTACTCGGATGGTGCGTTGCACATTTTGCAGGATGGGATTATTAAAGCAAGCAACACCATTTCAC
TTGTATTACTGAGAGGGGCTACAGATGATCAA
TTACGTCTTGAAGAGTTGGGCACAGCTGGATTCCTCCTTCTCTGAGTCGTTCACACCCACGGCGGGTC
TGGTTGGTTTCCTTTCCAACGCATCTTCCGGT
GGTGACACGTGGATCGACGATTACCTTTGCGTGAATGCAATTGTGACGAGGAATGCAATGAAGGCCAA
AGATGGGCTCAAATTCACGGGCTTGGGTCCGG
GGCAGTATGGCCCGTGAGCAGCCGGGAGCATAATGGGCCGCATACCTTTGTAAGCTGTGATTTCACTA
TTGTGGCGAGGGTGATGATTCAAAATTTTCCG
AAGTTGGGCACTCCTCTACCGGGCGCGGGTCTGGATGCACCCGTCAGCACGAGCTTAATTGGGCTGTC
GCACAACGCGGACAGTGGGTGGGAGACAGTGT
TTTACGGAATAAAAACAGCATCGGGAAGCACTTGGGAGCCGGGGAAAAAATACCAAGTGGTGCTCATG
CTGCCGGACGGCCACAGGGGATGTGTGTACGT
GAATGGCGTGATTGTGGGGAGCCCAGAGACGATACGAACACCTGAGACGCGGGGGCTGAAGTCTCATT
CTTCTACATTGGGGGCGACGAGGGAGGCATCA
ACAGCAGCGATGTGACAGTGACGAAAGTCTTTCTGTACAACCGCCCACTGAGTGTCGGTGAACTAAAA
ATGGTCAAGAAAAGCGGTGACGGATCCATGCT
TGGGGGCGTGTTGCGGGTACTGCTGCTGGGCTGTGCGGCTTTGCGGCCCTCTACGGGGCGTAACGCCG
CGGGACCCCTGCCTCTAGTGGGTGCAATATGT
GCCTCTCCACGCACCCTCCTAAGTAATTGACGTTTTGCTTGGACGTACCTTTTATTTTATATATTTTA
TTTTTGGTTTGAATATCCCTTTTCCGCCTGTG
CAATTCTTTATTTAAAAGCGTGCTCTCCCTCCCGGGACTGCACCGCGGGGTATTTGTTTTTGCTTTAT
TTTATATGCTTGTTTTTTTTATAGTGGAATAT
TTTGAGATTGGCGGGATGTCTTTAATGTTTCCGTCGGCCGATGACACGTGCAGTCGGCATTGAGCTCC
ACGACGATGGTGCGGTGAAGAAGGGATTTAAG
ATGTGCAGCAACACACTGAATGTTTCAATGAAATGTGGGTGTTGTGTGGACACCAGGAGGTTGAACGG
CTGACGCAAATATATCTTTACTTCTGCAGGAA
GAATATACACGTGTTTTTTGTTTTTCCGCTTCACCGTGTTATTATTATTATTATTTTTTGTGAGCGTG
AAAAACCTCTTCAAGGGGACTGTTTTTTGCGT
TTTATTTTTTCTTTTAAAAGTTTTTATTTTTTGTTTCTGCAAGCGTATTTTGGGTTGCTGTAGGTTGG

GCTGCCGTGCCATCTTGAGCTTTGGCTGTCTT
CTTTCTTTTCTCTTTGTCTGTCCTGTAACTCCGCTGCGGTTTTTTTTTGTTTCTTCACGTGCGTGGCT
CTTTTTATTTGCGTGTCTGCAGACACACAACC
GCACGACGTACATGTTGCAGTGCAGCTTGATGCTTCTCGTACACTCGGTGCTGGCAACTGCTGCATTT
TGCGGAAATGAAAAAGAAAGATGCGCCGAGAG
TGCGCCGTGGCTCTCATCTTCTGTGGAAATGCTGCACGGCGTGTGGCGGTATTGCTCTGAAATGCCGC
ACCATCACGCACGATGACGTGGCACGGAGTGT
CAGATCTCTTTTTGTGTCTGCGTGGTGTGGGTGCGTTTGCGCCGGACCTGTCGGCACGATGGCCGCAG
CGTGTGATCGTCACTCTGTTCTCTTGGGAAAT
ATGCTGCCACGCTTTTACGTGTGTGTTGTTTCGGGGACAATGTGCTGGCGGCTGTGCGATTGCCGGAT
GGAGGGAGAGGAGTGGAAGGAAGTGCGTGAGC
GCAGCGGTCAAGGGTCGCGCTGGGTTTCTCGCCGCGGAGGGGATTGCCCACCCAGCCCAGCACCGCTC
CTGTCGGGCACCCTCTCACTATGCAGCGTCGT
AGTCGACGCCAGCAGCACGCAGCGAGGCAGCGGATGCCGCCACCGCCACGAGTGCGACCGGAGGGGAC
ACTTCAGCTGTTGCGTTCCGAGGGGTCACTCC
GTGTGGCACAGCGGCGTGCCCACAACCCCCTCGCGGACTCCGCGCACTACACCGGGCACTGGCCGCGG
AGTGTTTGCGTCGTGGAGGACACTGCGGCTGA
GTCCCTCTTCCTTTCAGGTGGAAGGACGCCTGTTTTTACTCTTTTTCCCACCCAGGTGGATGGCTGCG
CGATGGCACCACAACAAAATGAGGCAGCCGTT
TTCACCATGGAGGGGCAATATCTGTGGTGCTACGCATTGGGGATGTAAACGGCGAATGTAATATTGAA
GGCAGTGTCATGGTTCTTTTGTACTGTGCGAA
TTGCAGAAATTTTTGATAAAAAATATATACATATATTTTCCCTCTGCGAAGAGTTTACCATGATGCAT
CGCTGTTGTGGCGTGTGTGATTTTGGGGTTCT
TGCGTGTGGCTGCTTGTGCACCGAATACTCCTCCCACTTTTGTGTGTGCGTTTTCGTGTGTCCGTGGC
GGTATGCCCGCACGTCCTCTTGCAGAGTGTGT
GCGGTGACGGCGGTGCCACACGGGTGTGGGACCGTAGCGTGAGGGATGCGTGGACGAACCCTCCGTGC
GCACCACGTTGTTATTGTTGGCAGCCACCAAC
ACTGTTCTGGGTGGATGGCACCCACTCGCCGTCGTTGTGGTGGTACCTGTGCATCGAGTACAGTGCCG
TCACACCGTGTGGATGACTCTTGTTTTGGCTG
CGAGTCATTTATGTGAATATTTTCTTTTTTATTTCTTTCTTGTTTGTTGGTTATTATTCAGCTTTAAC
TCAATCCAACCAAGAAATCAAAGGGAAGCAAT
GCCTCCAAAGCGCAATAGAGTGCAAGGTGGCAATGCCAAGAGTCGAGCGTCTGCCGTGCCGCAGGGAG
ATCGTCAGAGGAGAGCGAGGCCGGAGTCTGAG
GGTGTCACGGATCAACCCGCTGCGACACAAATAAGAGTGGAGGAGGCGCGGCAATTAATGTGGACCAT
GAGAAGCACCGTAGAGGACATCCTGCTGGAGG
GAAGCACTAACAGGAGCAACATGAAGCTGAATGAATTCATTCGGAGCTACTTGGGCAAGGAGTGGGTT
GTGGAAAGAAATGGGAACGTCGCTATGGAGGC
GTTTGTTCAGAATCCCGAGACGTTTATCAAAAAGAAAAGGTTATTACACACAATAAAGGCATTGCCGT
CATACAGAGAGCTGGAGAAAAGGAAAATTCTA
TTGGAGGCTATTTATAAGCTTAATCACGAGGGTGTGCACTTTCTCGAGCAATGGAGGGACTATGAAGG
AAAGGATACGCTCACTCTACGTGTATGTGAAA
GACTAAACGGAGTTCTCACTCAGGTACTGAGAGAAGAAAGGCGGGAGGCAGGAGAAAGGGCAAGGCGG
GAGGAACAAGTAGAACTTACCCTAACTACCAG
GATCAAAGATGTACTGTTTAAAGGAAGAGTTCGCTTCATGGACATAAAGCTGAATGATTTTCTTACGA
TGGAAATGGAAGGCAGAGGCATTTTGCACGCC
AATCGGAATGTCTTACTGAGGGATTTTTTCAGTGATCCCACGAGGTATATCCCTGATGCGAGAGTATT
GGTCGAAGTACAGAAAAAAGGTTATTATCTGA
GTTTGGAGAGCACTGTACGGGAGGAAATGGGTTTGGAAGAAGCTGTACGCAGGCTTCACGAGAATGGT
GTGGACAGTCTACTTGGATGGTCATTAGCTAC
TGCGAGGGCAAAAACAAGTGTTAGCAACAGCACTAAACAATCACTGGATGCAGCCCTTCATGTGGCGA
GGAACCCAACGACGACGATTGCACCGATATAC
CTGGAGGGATTCTACGAGTCTGTGTACAATGCGAGGTGGCATCATGTCGTGGAAGTTCCTGGTGAAGA
GGGGACGGTAATGAAGGTAAAGGAGGGGGAAC
CACCGCAGTCATGGACGTACAGGGCAGTTGGCGGCACTTTCGAAAAGAAAGACGGTGTGTTGCAACCA

GGTGTAGAGCGTCCCAGACTGATAGTGCTCAC
CTCGGACAGGGGATGGCCGTACTCGTGGAAACGGGGGGAGAGTGGAATTCTTCCTGACTGCTATTTGA
ACTCTGAGGTGGAGCGAGTGTGGCGGATCGTG
AAAAAGGATCTAACCAAATTGTTCAGCAGACACGGTAAGAACAAACCTTCGTGTCATCCGCGTGTGTT
GATTGGGACGCCCGGGATTGGGAAGTCGATGA
ATGTCGGCTCTTACCTCCTTTACCAGCTGCTGCACTACGATGTGGAGCAACTCCAAATGGTTGCGTAC
GTTATTGCGGATCGAAAATTTCTTTTTGACAA
GACCGCCCAAACCGTGAAAAAATACGGTGAGGCGTATAGTATcGTGGATATTTTGGACGGGTTGTCTG
GGCGTGGTGTGAAAGGGTATATTATCTACGAC
GTGGCAATGAAGGGTCGTCAACCGTCTACTGGTTTGCCTTGCGAGGGATGGGGCATGATTGTTGTGAC
ATCACCGAACATAAAAAACTACGAATGGTGGG
CAATGCAAATGGGAGCGGAACGAATCATAATTGATTGCCCTGACGAGAGCGATGTGAGGGCAATGTGC
ATTTGGAAGGAGCACAATGGGCAGGTTGAGGA
AGAAGAGGAAGAAGAAGCGGAAGAAGCGGAGGAAGAAGCGGAGGAAGAAGCGGAGGAAGAA
>scaffold1096.size9562
CTGTGGGCATCGCAGCGGAGATCTGAAGGAATAAATAAAAGGGAGTGTGTCTGTGAGGAGGAGGAGGA
GGAGGGGATGAGCACTCGGACAATTCTCACGGC
GGGGGAGGCAGCGGAGGTTTTGGCTTCTGTGCGGTACGTTCTTCTTGACGTGGACGGTGTGTTGTGGG
CCGGGAAGCATGTGTTGCCCAATATTCCAGAG
ACACTGTCGTACCTTCGCTTACGTGAGAAGCAAATTCGTTTTCTTACGAATAACGCCAGCATTTCTCG
TGCTGGTCTGGTGCGGGAGTTTCAGCGGCGAG
GCATTCAAGGGGTACGGGAGGGCGAGGTGTACAACAGCGGCTTTGCGGCGGCGCTGCGGCTTCAAAGT
TTGTTCACTGCGAATAAGAGCACAGGGTCGGG
CAGGCCACTGGTGGAGAGGAATATATTTGTTATCGGTGAGGAGGGGCTTCACGAGGAGCTGCGGCGTG
TATTGGCACCTGGATACATTGCCTACGGGATG
GAGCTGCATGACGCGGAAAAGTGTGGCGGCTACGACGCACACGTTGTGGCGTCTGCGTGGAAGCAACG
CGTCCTTCCAGCGCCACTCCAATCTTCCGCCA
CTTCCTGTGGGATAGCAGCAACGGGCCATGCAGCGGGCGGTATCTCAATTTCCGATTTATCGCCTGCT
GCTGTGGTGGTGGGGCTTGATATGCATTTTAA
TATGCTCAAGCTCGCGTACGCTTCCCTCTGTTTACAGGAGCGGCCTGCAGCACAGACAGCGGGCACGT
CGTCATCTACACCTACGTACTTTATTGCCACA
AACGAGGATCCTCAAATACCAGTTGGTGAAGACTTCCTGCTACTCCCGGGTGCTGGTGGGATGGTCAG
CGCCCTGCGCACTGTTTCTGGGCGTTCTCCTG
ACTTTGTATGCGGCAAACCGCATGTGGACATGGCGAAGGTTCTCTTTGAGGCTGAGGGAATCACGGAT
CCCCAGCAGTGTCTGATGGTGGGGGACAGGCT
GACAACGGATATCGCGTTTGGAAATGCCGCTGGATGTAAGACGATGCTTGTGCTGAGTGGAGCAGAGA
AGATGGATCGCGTCAGGCAGGCGGAACGGGAT
GGTCACGTGTCCCTTTTACCCGACTTTATAGCACCGTCTCTCGCCATCTTTTTACCACCCGCATAGGC
GTAGAAATGATACGAATTTTTCTTTTTTATTA
TTATTTTTTTTTGTTCTAAACGGAATAAATTGGAGGTGCTGGACTTTGCATGTACAACCGTAAGCGTG
TGTGTCTTTCTGTTTTCCTTCACTGCTGCTCA
TGTTGAGAAAACACTCACACTCTTCCGACTCACTTTTTTTTTTCCTTTTCTCTCTCTTATATTTCATT
TACTTTCCTTTTGGATGGTGCGTAGATACATA
CATGCACATGTATACATGTATGCTGCATTCAGGAGGGGCGTTTGTGGGGTGACTGGGCTTCAGTCCGC
AGTGCAAGCAGGAGGGAAGGCTAATCGTGTCG
GGGGAGGGGGAGGATTTGTGTTGGACTTCTGTTCTTCGTGGTGTCTTGATGCCTCATGTGATGGAGTG
ACGCGTTGCTCCTCATGCTGACGATGGCCATC
GCAACGCAGGGCACCCTTTTTTTTTTTTATATTCTCGTCACACTGTGACTGAGTTGTTTTGCAGGCGG
TTCTGGCGAGGCCCTTCCTGCCGTGTGGCAGC
GCTCTTTTCCACCACTGCCAACAGCCGCACGCACACACGCTTATTTTTACACGACGGCTTCAATTTTT
TAGTATGGCACTTGTGACATTGGCATTTCCTT
TACCCTCGTGTTACTGGCACAATATTTTTTTTTTGTATGTGTGTGTTTGTGTCTGGGAGGTGAGTGCT
TTGGCATCCGCACGAGAGGGCGAGTGACTTCT
TTTCCATTTATTCACTTCAATCACCCCATGCGTTTGGGTGCGACGATGAAACTACTGGGAGGCCTTTC

TCTTTTCTTTTTTCTTTTTTTTTGCGTGCAAC
TGGAGGGGCCGATGTGGTGCTTGTGGCGTAAACCGTCGTTTCTGCATACGACGTGGCTGCTGATTACT
TTGAGACCGTCATTGCAGTGGAGACGATGTGT
GGGGATGGCCACGCACCTCCCACAGTATACATGCATGCACATTGATGTATAAAGATTGACTGTGGTTT
TTATTGAATCTCACTTCCTTCCTTTCTCCTTT
TATTATATATATTATATATATATTTTGCGTGACTGTGGGTGTGTGGGTCATATCCGTAGCAGTGGGCC
TCCATGCAATATTGGCACCCAACACACTGCAC
CCTGCAGCTGAATTTTGTGCAGTCCGCTTCTCAGCGTGATGACGGCCGCGCCCTTTTGTTGGCGGGTG
ATGTATACAGTGGGAGATCTACTATTCGATAC
GTGCGTCGTTCATTTTCCTCCGCGACAATGCACGAGGAGTTTCCTGCACTTCGGGTGGAAAATAAAGA
CGGACTACACTTTCATGCCATTACGGCCTCCA
CGCACGCATTTCCAAAGGCCAGGCCTCTCGTTCTGCACTCCATTAAGGTGCATCAGCAGTACAACGAT
GCAGAAACCTTAAAGGTGCTGCAAGTTGTGGA
GAGTGAATTTAATAAGGATGACAACGGTGGGGCGGAAAGTGGTGAGCAACACAGCGTCACATCAAAGG
GGGGAAATCCACGCGGAAAGAAGGCGAAGAGT
AAAGTTCACGATGCCAATGAGACCGAGCGCCAAGGGTCTCATGATTCGTACGATAAGCAGCAAGGTCG
TTCAGATCCGGGGTCGAAACATGTCTTCTTTG
AGGGAGCTGAAAGCATGCCTGCCGGCTCCATCGTCACGTTGGAGATTGCCTTTACTGGTAGCATCCAG
GGGTGGGATCAAGGAGGCATTTACGTGAATGA
CAACCCGGAAATTGTGAAGGCGGCGGCGGCAAATTACGGTGTACTCCTTACCCACTTTGAAGTTGCCT
TGGCCCGTCTTGCCTTTCCATGCCCGGACGAT
TCTCAGTGCTACCGCCTCATCTGGCATCTGCAGTCACTGCAATTGCCCTCAAGCTACAGTGTGGTGGT
GAGTAACACCGCAAAGCAGTCAGAGAAGGCGA
TGGGTCAGAAGGGGACGCAACACACCTTTTTTCCTGTGGGTCCTTTACCCGCCTACGTGTTGGCCTTT
GCCGCCTTTAGTGGTTCCGTGCAAGTGGTGGA
GGAAACTCTTTTTCTCCGTGGGCCGCTCACACCCGGTGCGGAAGGCCGAAACTCGAGACTCAACACTG
TAGAAAAAATGGTACCGTTGCGAGTTTTGGCA
GCCACATTGAGTGGGGTGACATTGGATACACTCAGGCAAATTGCCGACACCGTTCGGGAGGCTGTAAA
CCTCCTAGAAGATTTTTTTGCCTCACCCCTTC
CTTTGCAGCAGATGCCCTTCTCAAATGAGTTTGAGTGGCAGGAGGATGTCCTTACGATTGTTGTCGCT
CCTACGATGCCGTACATATCAGGCATGGAGCA
TCACTGCTGCATTTTTCTCAATGAGGCCATTTACCGATCTTTTTCCCGCGATGGCACTGTGAGAGGGA
CAGTCCGGCGTCCCATGACGGAGGCAAACGGA
ACACATGAGGTCAGTAGAGTGATGCTTATTGTGCATGAACTTGCACACCACTGGGTGGGGAATACTCT
TGGTATGCCTTTTGTGCTGAAGGAGGGTATTT
GTCTTTTGCTGGAGCAGTGTTTTGGTGACGTCATTGTCGGGAAACCAATGCGGAGTATAAAACCTTCG
GGTGACAAGATGATGTCCGACACCGCCGTACC
GTCCCGCAGGGTAGTGTCGAGGATAAAAAAGGCGTCAACACCGCCAGTGATTGTGGTGGACACAGAGA
AGGGCAAGGAGTTTACGGAGTACTCGTACCAG
AAAGCACTAAACACACTGCGTGATGTGGTGAGTGGCATGGGATTTAACGACTTTAAGGAACGCATGCG
ACGCATCTACCAAACAGAGGTGATGGGATGCA
AAGAAAACATCGGCATGATAGACGGAAGGGATTGTGGGGATTTATTCCTAGCGCCGTACATCACCACG
AAACAATTTTTAAAATATATGGAGGCATCGCG
TGTAACTGTTTGAGTAGGTGCACAGAAAACAGGTTGTTGGGAAATAATACTTCTTTACCGGAGCGGAA
ATGAGGCGGAAAAGGGGGGAACAAGAAATCTC
ACTTGTTTCTGCTGTACAATGTCCTTTGGCAGTCCTATAAGTTTTTCTGCTTCCCTTCCCTATGGTTG
CGACATGACGTGAGCGATTAGGTGGTGGAGAA
GATTATAATATACATGCCAGCCTCATTATACACACCGTAAAAGGCAGTGGTATACTATGGACGTCTTG
TGCCTTAAGTATTTGATGGGCTTCTTTTATTT
TATTGCATTTTTGCTTATCCCTTCTCTTTCTTTCTTCCTTTCTTTCTTTTTTTTTTTTCAAGTCATAC
TCATACGAGGATACACCCAACCATGCGCCGCG
TCATTGCCTCATGTCTTTGTGCCCAACGTCTTGCCCAGTGTGCCACAACGTCAACTGCTGTACTGGCA
TTCAGCGCCTCGATTCGCCGTCAAGGCACACG
GCAAGGGGCTCCCACCGGCGTTCCACCGGAGTTACGCGCCCCGCCGCAGGGGTCTCGTCCGTCACACC

ACGAACACCAACAGACGGAAATGAACGCTGCG
CCGGAGGAAAACTTTGAGCCGCCGCAGAAACCCGCAGAGCCCCCGAAGATGCTGAAAATAGACACAAA
TGAGAAGGGCATTACCACCGTTCAGCTTAGCC
GGGCCCCGGTGAACTCCCTTAGCCTGGAGCTCTTTGAGGAACTTAATAAATGGATGCTGTGGCTTGGA
AGCGATGAAGAATGCAAGTCCGTCATTCTCAC
GTCTTCTATCCCTACCGTGTTTTCAGCAGGGCTAGACATCAACGAGATGTTTAATCCACAGCCCGAAC
GCCTCCGTCGCTTCTGGCAGGCGTTTCAGGAG
ACCTGGCTGATTCTCAACAGCTTCCCCAAGCCCATTATTGCCGCAATCAACGGCAATTCCCCTGCCGC
TGGATGTATTTTGTCTCTTGGCAGTGACTTCC
GCGTCATGGCCCGTCATCCTTCCGGGAAGCCAGATCGTTTATATCGCATTGGGATGAATGAAACAAAG
CTGGGCATTACGGCCCCCGCATGGGTGATCCC
TGCGTACGCATATGTTGTTGGCTCCCGCAACGCCGAGCGAATGCTGCAGTTGGGAGAGACACCAACTG
CCGACGAGGCACTCAGAATTGGGCTTGTGGAT
GCCGTGGTTGAGGAGGAACAACTACGCGAGGCTGCGGTGAGGGAAGCGGAGCGCTTCATGGCGATCCC
ACAGCAATCACGATGGATGTCACGAGACATGA
TGCGACGTGAGTTCCTGCAGCACATCGCAACAGACGAaGATAGAGAGTATGACACGCAGTTTGTGGTG
GAACTTATGATGAACCCAGAGGTGCAGAAGGG
ACTGGGTGCCTACTTGGCTCGTCTCAAGGGGCAGCACGTGAAGAAGTAACTTATTTTTTTCTTGTTTT
TCCCTTAGAACCATGCATGCCCACGCAATATT
TATACATTTATACACCTATGCATGCATGTATGATGACGGCTGATGGTACAAGCCGGAAACGGCGGGAA
GACTGACTGAATGCGTGGGAACGCAGCGGAAA
GAACGTTAAAAGAAGCAAAAAATAAAAAAGAAAAAAGAAAAAAAGANNNNNNNNNNNNNN
NNNNNNNNNNNNNNNNNNNNNNNNNNNNNNNN
NNNNNNNNNNNNNNNNNNNNNNNNNNNNNNNNNNNNNNNNNNNNNNNNNNNNNNNNNNNN
NNNNNNNNNNNNNNNNNNNNNNNNNNNNNNNN
NNNNNNNNNNNNNNNNNNNNNNNNNNNNNNNNNNNNNNNNNNNNNNNNNNNNNNNNNNNN
NNNNNNNNNNNNNNNNNNNNNNNNNNNNNNNN
NNNNNNNNNNNNNNNNNNNNNNNNNNNNNNNNNNNNNNCAAAAAAAGAAAAGAAAAGAAG
GCAACATTTGTATTTTTTATTTTTTTTTTAAT
TTTTTATTTTACACAACAGAGAGGGAGAGAGAGGGGGAGGGGAAAGTGGGGAACCAACGAAGAAAAAT
AAACAAAATATGACAATTGCCCTTACAAATAA
TCCCGCCTGCAGAAAACACTTCGTCATTGCCAATACAACAACAAAAAACTATAAAAAACAAGAAAACT
GAGGCAGTTGCATAAAAGATCCGGAAATAAAT
AATGAAAAAGAAGAAAAAACAAAAAAAAAAGCAACACTTTCCGAAACATTTTTTTTTTTTTTTCTTTT
TTATGCCACACAACATAACACACGTTTCCCAG
TATATATACATATATATATATGGACGACTTTAATTTGTGCCACATCAATAAATACCACGCGGAGAATA
CAGAAACACATCGGCAAATATAAATATAAATA
TATACGTGCGTAACCACATGCCAAATACAAAAACCCTCCATACAACAACCGTCTTGACAAACACTTTA
CATGCATTTTCACGAATTTGAATATGTCTTAC
TTTATTTTTATTTTATTCACAAAGAGTGGGACAAAGACGAAAAGAAAAGAAAAAAAAAACATTTGATG
GGGATAACAATTCATTATTTATATAATAAAAT
AACAAATAAAGACACATGTACCATACAACAAATGTGAATTAAAACAATAAAAAGCAGAAGATGTGGTT
TGACCTTGCGCACCGCCTGTTCCTGACTGCGA
GGTACATTGCGAAGGGCGATGACGACATGTTCCTCCGTGTGCCGCTGTTTGTTGCTCACCTGCGCCTA
CTGCCGCGCCGAGGGATCTACATGGGATACCA
CATGCTTCCTCCCTCTGGTCCGGAAAGATATATAAGAGGTTACACGTTTATGGTTGGCTGGTGCTACA
CCCTGTCGAGGGATGTGGCGGAGGCGTTGGTG
TCGTACAAGCCGCTGCGCCGCCTGGCCTACCTGCCGTACAGCAAGGAACGCGACCACGAGTTCGCACC
CCTTCTTTTTTATGAAGAAGACGTCATGGTCG
GTTGGGTTCTTGGGAGTGTATTAAAGTACAAGCCAATGGTATACGTCGAGATGCAGGATTGTCATTTC
CATGACGTTCGCAATGGCACGGGGCACTCGCA
GGTGGTGCCGACCTCGATGTGTGTCCATCACGTGCGGGAGGACGACTACGCTGCGCTGATGGCGCGCT
TCGGTAACGACACATCGCCTGTTGCCCGTGTG
GAATGGGTCTCCGAGGACACGATTTCTTCTCAGTGCGATTGACAAATGGACGAAATGATCTGTTTTCT

GTGCGTGTTGAATTCTAATTAATTGCTTGATT
GGTTGCTGCATTCCGGTTTGTGCCCTGTGTGCTCTCTGGCCGAACGAAGTGGAATGTGTCATTGACCG
CCCCGTGCCCCCGTGGAGCGTGTCACTCCCTG
CCCTCGACCCGCGTGCGGGAGGTGGTTGTGGAGTCAGTGGGCGCCCGTTGCGTTGTGCTGCGTGTGTG
CTGGGCCGCCAATGAACGATGTATGTGAGTCT
GTGTTTCTGATACATTTTATTCTGTTAATTGTGCTGTTGGGAAATGGAGTGACTCAGCATTTTTTTTT
GTGTGTGTGCGTTGCTTGCTTCGCCCTTCCCG
CGCCTGAGCCCTCACCTCGCGGTTGGGAGCCGCCGGGGATGCATTCTTGTGTTTTCTTGTGATTTATT
TGTGAGCGGAGCGTCCCACGGTGCGGCTGCTG
TCAGTGCCGATGGTGTGGCTCTCGGTGTGTGGAGAGTGCGTGCCGTGACGCTCACCCCGTCGCCGATG
CGCCGAGCGGGCGGGACAACGCCAGTGGCACA
CAGTTCCCTGCTTGTTGGCGACGCAGCTGTCCTTTTCTTTTCACTGCTTGTGTTCACTTGTAGAAGGC
TGCACAGTGTAGGGGCGTCGCGGACGGACGGG
AAGCAGACGACGGGTGGACTCGCTGGTGGGCGGCGGGCTTGCGGCGGCTTTCTGCGTTGCGGTGGAAC
GCCGCCGGCCTCACTCTGGCGAAACGTCTGGC
GCNNNNNNNNNNNNNNNNNNNNNNNNNNNNNNNNNNNNNNNNNNNNNNNNNNNNNNNNNNNNNNNNNN
NNNNNNNNNNNNNNNNNNNNNNNNNNNNNNNN
NNNNNNNNNNNNNNNNNNNNNNNNNNNNNNNNNNNNNNNNNNNNNNNNNNNNNNNNNNNNNNNNNNNN
NNNNNNNNNNNNNNNNNNNNNNNNNNNNNNNN
NNNNNNNNNNNNNNNNNNNNNNNNNNNNNNNNNNNNNNNNNNNNNNNNNNNNNNNNNNNNNNNNNNNN
NNNNNNNNNNNNNNNNNNNNNNNNNNNNNNNN
NNAGCGTTGGCTGCGGATCGTCGAGCCACCGCGGCGGCGTGCTGTCGGCTTCTTTGCCGCCGTGACCC
CTTCACCCGCACCGTCCCGGATTCGGGTGGGG
CGCAGTGGGTTGGCTGACGGGCATTCGTGTACTCGCGTGGCTGCGTGGCACCGCGTGGCCGTGTTGTT
TCTGGTTGTCGCCCGTGCTGTTTGTGTCTGCT
GATCGGTGTTACTACACACTCTCTCCGCCCTTTTTTCTTTTGCCTAGATTCTCACACCAAAAGCGACC
ATGGCGAAAACAAAACTGCCGACGATGAAGCG
TAAGCTGAAGCGGATTGCCGTGTTGCTGATTCTCTTCATTGCGGTCACGAATTTTCTGTACCTCAGCT
TTGTCCCCGCTGACGATGATGGTGCACCGGCA
CCCAACAACCTCCGCCACCCGCGAGCCTCCGCAGTGGACGACGCAGCGCTTGCTTACATCCCACGCCG
TGTTGTGGATACGTGGAGCCGGCGGGAGTATC
TGGTTGTGCTGGGCATCCCCTCCACCGACGACGAGGCGAGGCGAACACGACGCAACCTGCAGCGGTCG
ACGTGCTGGCGGTTCCCCGGAGTGGCGACGAG
GGCGAACGACTTCACCGGTGCGATGCTTGTGCTGTACGTCCTTGGGCGGCACCCGTCGCACGGCTACG
ACTACTCTGCCGCGCTGCTGGAGGAGGCGTCG
CAGTGGAACGACGTGGTTGCGCTGCCAATGAACGAGGGCCGTGTGACGACTAACAAAACAATTGGTTC
CTATGGTTCTTGGGGCGACGAGGCAAATGTCG
GTTTCAGTCGCAAGACGTACATGTGGCTTGACCTTGCGCACCGCCTGTTCCTGACTGCGAGGTACATT
GCGAAGGGCGATGACGACATGTTCCTCCGTGT
GCCGCTGTTTGTTGCTCACCTGCGCCTTCTGCCACGCCGAGGGATCTACATGGGATACCACATGCTTC
CTCCCTCTGGTCCGGAAAGATATATAAGAGGT
TACACGTTTATGGTTGGCTGGTGCTACACCCTGTCGAGGGATGTGGCGGAGGCGTTGGTGTCGTACGA
ACCGCTGCGCCGCCTGGCATACTTGCCGTACA
GCAAGGAACGTGATGACGAATTTGCACTGCTGGGGTTTTTGTACGAGGACGCCATGg
>scaffold1097.size6932
TATGATCTCTCGCAGCACAAGGGACATCCACTGCTGATCTACAATGTGGCGAGTAGGTGTGGCTACAC
GAAGGGTGGCTACGAGACGGCCACGACGCTCTA
CAACAAGTACAAGGACCAGGGCTTTACTGTTCTGGCGTTCCCGTGTAACCAATTTGCCGGGCAGGAAC
CGGGGACGGCGCTGGAGGTGAAGGAATTTGCC
TGCACGCAATTTAAGGCGGACTTCCCCATCATGGCAAAGATTGACGTGAATGGCGACAAGGCACATCC
GCTGTATAGCTATTTGAAGCGCTGCCTTACTG
GATCAGCGGGTGTGCAAGCCATCAAGTGGAACTTTACTTCGTTTCTTATTGACAGGCATGGTGTTCCC
GTGGCGCGTTTTTCGCCTGGTGCATCTGTGGA
AGAGATTGAGATGCAACTACTTCCACTTTTTGAGGGCGAGGTTGTTTCCGGGTCCTCGTAGGTTTTTA

TTACGATACGTTGACGCTTACTACTTTGTGCC
CATTTATTTATTTTGTTCTGTTATATGTTTCCCTCTCTTTTTTTTTGGGGGGGGGGGGGGGGTGGGGG
NNNNNNNNNNNNNNNNNNNNNNNNNNNNNNNN
NNNNNNNNNNNNNNNNNNNNNNNNNNNNNNNNNNNNNNNNNNNNNNNNNNNNNNNNNNNN
NNNNNNNNNNNNNNNNNNNNNNNNNNNNNNNN
NNNNNNNNNNNNNNNNNNNNNNNNNNNNNNNNNNNNNNNNNNNNNNNNNNNNNNNNNNNN
NNNNNNNNNNNNNNNNNNNNNNNNNNNNNNNN
NNNNNNNNNNNNNNNNNNNNNNNNNNNNNNNNNNNNNNNNNNNNNNNNNNNNNNNNNNNN
TCTTTTTTTTTTTTTTTTTATTGACTCCCCTCA
CGTTCTTTCGTTGTTGTTCTTTTCTGTCGGCCGAGATGAATTAGCAATTGCCATCCGTCTTCACTGTT
GTTTCCATAATGGAATTAAACGATAGGAATAT
TGGGGATGGGGTAGCTGTTTGTTTTGGTTTTTATTATTATTATTATTATTATTTCCCTCCATTAT
TATTATTATTATTGTTATTATTTCATTTTACG
ACAGGAATTGCGGAAAGGGAGGAGAGGAGGAAAAGAAAAGAAAAAAAATAAAAATCAAGAGGAACTAT
GGTTTATCATTTTATTTTTGTCTCTCACTTTC
ACTCTCTCTCACACACACATTCTCTCTTTATATAATATATATAATATATATACATGTGTGTATGCGAA
GAGTTCCTTGTTGCCATCTAATTTCCTACCAA
GACGAAATACAAGCCTGGCGGTGGTGGGAAGGACACGAGTATCACGGCGCCATAAAAGAAGAGAGAGA
GGAGTAAATGAAAATTAAAATGAGGCGAGTTA
AAATGATAGGAGTAGAGAGAGGGCTATTTGATGGGTGCAGTAGTGGCAAATCTTTTTTTTTTTTTTTT
CTTAATTTGGAAGAGGGGAGGGAAGAGGAAAA
ACCCCTCTTTTCCCCTCTTTCTTCCTACACCCTCTATGTAATGCGTGAGGTGGGCTGTCTGGGGCACG
TTACCCGATGCCAAAGCGCCATTTTGACTGGA
TGCACATGTTTAGAGATTTGTATGTTGTACGTTTTCTTCTTTTTTTTCTTTTTTTTGCCTTTTTTTGT
TTTCTTTCGTTTGTTTGTTTCTTGTCGTACTT
CGTTTAGGCTGCAGAGAGCTGAAGGAAGGGAGTCTGAGGAGGCTATTTCCCGTATACACGTGTTTTAC
GTTCTCAGGACCTTTTCGTGCTTTCTTTTGCC
TACTTTCGATACTCCGTGCAGTTTGTGTGCGTGCGTGTTTCCGCGGTGCTACATCAGTATGCTTGGAT
ACATTATCAACTCTGAGTTGAATGCCGACTGG
GAATGAGACGTCCATTTTGTCTTTTTTGCGTGCAGGAACATTAGCAGGTTGTCCATATGTAGAGCAGG
TAGCTTCTACTGTGAGAAAAGTCACTGTACCT
CCTGCTGAATCAGCCACCTCTTTGTCATCTCCATTATCGCCTTCACTCTCTTCCTCTTCCTGCCTGTG
GCCTCTTGCATTTTGTAATTTTGAGAATTGTC
GTCAAGAGAAAACGCTTTTGTGGGCCACTGCTCTCCGCCACAAAGCCATCACGGCAGAGGCCAAGAAA
TTAGGTTTGGATGCGCTGCAGCGAGGTGAGGT
GCGGAGACGCGCTAAACTTTTCTTCCATGTGGAGGGGCGGCATAAATGCGGCCCGAATGACACCGCTT
CGGATGCGGAAAAACATACATTGGCGGACATT
CTGAGGGAGGAGGTGGACGGGTGCCGTGCCTGGACAGATGTGGCCAATGGCACCAGATTTCAGATGAG
TTTTCTCTTAGGCGGCGGTGATATGGTGGGAG
TCGCAAAGGAGATGTCTGCCCTTGAGGCCACAATGTCCGGTCATGGTGACACAAGCCAAGCTAATCGT
ATTCGACTGTTGGTATGCGCCGTCGCCGTGGT
GTCGGCGATGTTGAATCATTTTTGCCCACTTCGCTGGGTCATAACTGCTTCTACAGGAGTTCCGCATG
TTTGGGATCAGGCAATTGCTCTTGTTGAGCAG
CTTCTTAACGACGAGGCTATCGTCTCTTTTTTCATGGCCGAAGCGCTTGCATCGGCGATTATGTCTAT
TTCTTCTTCAGACGTGACGATGCGACGCTACG
ATGTCCTTTTGAAGGAAAAACGGAAAAGGATGTTGGCATGCATCCTTCCCCTCCAACACGTTTTGGGT
GGGGGAGAGGCCGCACCCCTCTCCATTGCGGG
TGTCGCAAAGAATACGCTTCACTTGCGTACAGAGACGCAGCTGCTTGACTTGAAACGAGGTTTGCAGA
AGGAGCTGCGTGAGCGATTGGCAATTGCTTCA
AATCTGGATCAGCGTAGCGTGGATCGGGCGCGGCAATTCAAGGAAGCATGTGAATTGTGCCGAGCGAC
AGTGAAGGAAAACCAGACGGACGGACGCCACC
CGATACTTCAAAAAAAAGGCCTTACCGCTCTCTCCCGTACTGACATTTTGCCTGGGCGGTGTGTCCAG
TTAAGCGATGTACCCGGAGAGAACAAGTTGAG
AACGGAAACAAAAAGAAAATTGAGCTGCGAAACCGACACCAGCACCCATGAAGCAAAGGTTACATCGT

CCTGCTGGGAGCGCACAGTTTTAGAGGTTTGG
AGGGCACGTCGCGAGGCCGTCCTTGGAGTGCTTCGGGAATACTGGATTGTTGATAATGGCTCTTTGAG
GAGGAAAGGCATAGAGAAAAAAACGGAGACGA
AGGAGAAGGCTGGACCTTTGTGGATGACCGATGCGTTGCTAGGGACTCTTTCACGTCAACTACTTGCA
TTGATCGGTGTACAGGGTCAGAATTTGATTTC
TTTGTTGGATACTCTCTGGAGGTGGATTGGATGTCAGCGTGACGCACTCGATGAGGGAATGTACACGG
CACTTGCAGTTGAGTTGGTCGTTTTTCTTTTG
GTTGCTAAATATTACTCTTCTCGTGCCGATAATTTGCTTTCGGAATTGATGGCTATTTTGAAGCATCT
GGCGGGAATTCGTGTCCGCGATGATTTCTCTT
CTCCATTACTCCAAAAGGTACCGGACGCGTTGGCTGTGGTTCTCTTTGATGCGGTGTTTTTTTCCATT
TCAACAGGGATTTCCTGTTTCTCAGAAGGGAA
TTCTGACGGTTCACGCGACCGTCGACTGTCATCGCCTGTCTCAACCTTGTTGTGGGAAATTCAATCCT
CGGTCACGTTGAGGCATGCGGTGTCGATTCTT
CAGGGCGGCAGTTCGCGCCAACTTCCAACAGGAAGTGGTGGTGTGAACCCGTTGTCATTAACGCCCTC
TTTGGGGGACACTGAAGAAATGACGCTCTTAA
CCGACGTAGCAAGACATGGAAGGTGCGGCGGATTTGCACTGAATGTGTCGCCCAGGCATGATGTGTGT
TTTCTTGTGACGCTCACGGAGCTTCTTGGGAC
GAGTGTCTTTGCTCATTTGAAGGAAATTCCGATTCGTACAAAGAGGCCGCCACCGCGGTTTCTCAGTG
AGGCACTATGGAATGCACTTCTACCACTGACC
TCAGGGCTACTTCATCACATCCAACGCATGCATATCGAAAATGGGTCGAAGGCAGTACGCCGTTTCTT
GGAGTTTAGTCGTGCCCTTGTTGAGGAATTGA
CACCGCGGTGGGAGTACAACATTGTGCCTACGCGGCGTCTACAAAAGAAGGGGGTGGCTATTTTCAAC
TATACCGCCGTGGAGCAAGAGTACGAGGCGAA
CCATGTTGTAGAGGATCGTGTGGTCAAGTTTACTTCTCTTCTTGAGTGGTTACTAATGGGGGCTTCTG
AGTTCTTTCCGGAACTTCTCACGGATGGTCAC
AGCTTTTCCCGTATGTGCAATGCTGTTGGGCTTATTTTATACGATGTGGATGGTCGTACGGGACATAA
GATTATGACGCACCTCCTCCGTCTTGTACCGC
CTAAGGAAAAGATGGACATTGAGGCTTATATTGCAGCCATCCGTTGCTTGTCTTCTATGCGGCATGGT
TGTACACTTTGGGAAGAGGCTGTAAAATACTA
CCGAGAAGCAGTTCATTTTATGGAGGATGATGCCAACGCAAAGGACGCCATGAACTCCCATCGTCGTC
CACACGTGCCCGCAGATCTCTCCACTGCCCGT
GTGTTGCGTTGTATTGGAAGTAGTCCCCTGCCTCACGTGCCTCGGTGTACGTTGACGTTACAAATCGT
TGCGTTTGCTCGGGCATCCGGTGTTCCTTTAA
GTCTTCAGTCGTATTCTTCTTGCTTACTTAACTTTACACTACGGAAGAATCCGCCCACGAAGGCGTTT
TTTGTTGCACCGTGGTGTGATGCATTAGAGTG
GTATGACGATGTTATGTCCCGTAGTGAGCAGGTGCCTTTGCCGAAACAAGATCCCTTATGTTTTCAGG
TAGCATGCTTACAGAGTTTACTTGCACAGGGA
GATTGGCCAGAGGCACTTTTTTCACTTCCATTGGCAGATATTGTCCGTCAACATGATGACGACGCGGC
TGAATTGAATTTAAGTCAATTTTCAGGGAAGG
TGTCTTTCCTGCTGAGGCTATGTGCGTCCGATCGAACACGGAGCTGCGCCCATGCCGCGAAGTCGTTG
CTCTACGTGTTTCAAACCCTACCCTCTCTTAA
ACCTGGTACGCAGGCGGCAAACCTTGAAGATATGCAGAAGTTGTGTCATGTTGCTAATACATACCTTC
CTGTGGTTTCTGAACCGAAATTAACGAACGGC
GCACCGCTGTTTCGTCACGAGACACCACTAATTCCCAGAATTTCATTGGAGCTGGAATTTGAATGGGC
GCGCTCTTTGGAACCGTTTGGTTTTTCTTTTT
GCCATTTCTACCACATCCAGGATTCACTGATGGAAAAATTGGAGGCGGCCATTTTATCCCGCCTGCAT
AGGAGAAAGAGTGATACCGATGGGCGTGATGG
AAAAGATTCAGATCAGCCTTCTGCAATTATACGAGTTCGTGAGCGTGTGTCTTGTACATTTCCTCTCC
TTCAAACGGTGTGGTCAGCAGAAGAGTTTTTG
AAGAAAACAAAAGAGGCAGATGGTGTGGTAAGGGGAGGTGATGATGTCCGCTCACGCATGGACACGTG
CTATGCCAGTGTTATGCTGCCCCTGCGACGAC
TGGAAAGAATTCGTGCAGAGAAAAGACGAGGTATGCGGAGACGTGATCCGTCTTTGCGGCAAGATAGA
GGGGGGAGGAGAGAAACGGGACGGAATGAGTG
AATAAAAAAAAAAAAAGGAGAGAGAGTGAGAGGGACAATGAACGAAGGAAGGTGACTGGAGGTTGAGG

CTACATGGAATGTAGTGGAAGTAGAAGTTCTG
CTTTTAGGTTTCATTTCTACTTTTTTTTGTCGAATGCTTTTTTTTTTTTTTTGTGTGTGTGTCNNNNN
NNNNNNNNNNNNNNNNNNNNNNNNNNNNNNNN
NNNNNNNNNNNNNNNNNNNNNNNNNNNNNNNNNNNNNNNNNNNNNNNNNNNNNNNNNNNNNNNNNNNN
NNNNNNNNNNNNNNNNNNNNNNNNNNNNNNNN
NNNNNNNNNNNNNNNNNNNNNNNNNNNNNNNNNNNNNNNNNNNNNNNNNNNNNNNNNNNNNNNNNNNN
NNNNNNNNNNNNNNNNNNNNNNNNNNNNNNNN
NNNNNNNNNNNNNNNNNNNNNNNNNNNNNNNNNNNNNNNNNNNNNNNNNNNNNNNNNNNNNNNTTCTG
GGATTTATATAATAATAATAATAATAATAATA
ATTGCAGCTTATCGTTATTGCAAGCTCAAAAGAGAAGAAGAAGAAGAAGAAAAACAAACAAACAACAA
AAATTGTCCGTCACTGAAATAGATGATGCGTT
TTAGTCGGTGTTTAGGGGCTCCTGTCTTTTGCCGTATTATTGATGTTATGCGGCAAAACGAGCCGTAT
GCCCGACCAGGGATTTTGTATCCCATTCGTGT
GGAACTGGGAAATAATGTGGAGAAGTGCACATCTCGTGAGCTGCAGGAAATAATTGACAATATGCTTT
CTCGTTCCCAACGCTTTGCAACTGTGAAGACG
GGAATGGTTTACAACTCAGATCAATCCGTGGCGACAGAAATTAAAGCATATGGGCGCTATGTAGTCAC
GGGCAACATGCGTGGTGGATATATTAAAGGTG
ATCACCGTTTTGGGCCGCGTTTGACTGGTTACGGGGGTGCCACGACGAAGCCGGGACTGCGATGGAAT
GGAGATGACCTATGAATAGCTGTCAGGAGACT
GTTTTGTTTTCTGTAGGCATTTTATATTGTACTTTGCCACAGGGATGATGTTTTTCCCACAGGGATGG
GAATAAAAGTTAGTTGAAATCCAGTTGTTTTT
TTTTTAGTTGTTGTTCTTTTTTTTTTTTTTt
>scaffold1098.size7402
CTTCTGCTCGTCCACACACTTCATTTACTGCCAATCGCAAAGCACACATCATAGAACCAAAGAACCAT
CACACAAAGCCGAGGCAAATAAATAACAATGGA
GGAAATTACAACCTACACAGGCGCCTCGCACACACGCACACACAGACCCTCTCTCCATTCACTCCACC
ACTCACAAGGGTGAAGAAATGGGCAGGAAAAT
CCCGCCGCACAACGCATGCACGAGGGCGCACGCAGAGGCACCGCCAGCCACGTCGCACCGCAACAAAG
GGCCATGGGCACAGCACACCCACGGCCGCAAC
AAGAGGGACGGGACGCATCCAAGCACACGCCGCAGTCCTTTACTTCTCGCTTCCCCGACGGATAGGCA
TCACGCCTCAGAGCAGACCGTGGCATCCCACA
CTCGCAGTGAGGCACGGCGCACACAATCCGCTCCCCGAGTGCACTCAGCaggccggcgcccaGTCACC
CCTGTGCCCTCGCAATGGGGAACAGAAACAAT
CGTCGTCGCGGACCATCGGTGCCCCTAGCAGGAAAGAAATTAGAAGAAATGGAGGCATGCCAGCAGCA
CAGTCAGCAGAGAGACAACAAAAGGGACACGG
AGACACACAATGACATGGGTCACGGCACAGACGGAAAGAGACGCAACGATTTACCAAAAAAAAAAGAA
AAAAAAAAAAAGAAAGAAAAAAAAACGATTTG
GCGGGAATTTCATTTCGTTTCCTCTGGAGTACAATACGACCGTGTTTTTGAGTTCATACATACTGTCC
ACACACAAAAAACAACAAAAAAAAAAACACAT
TGTTCCACGAGCCCACACAACATTCCTCACATTCCATGCGGCATCCGGCCTGCTCCTCCACTCCTCCT
CTCGTGCAGTCCGTGGGACGCACGGCAATGAG
GCCGGCACCGCGTGTGGGTAAAAAAAGCAAAAGCAAACAAGAGGGGGAAACACATCGCTCATGACGGC
GTGCAGCGAGCGACCATTTGGGGGAAAACACA
CAGGGGTGCTGATTATTTCAACGGAGCACCAGCAAAAAAAAAAAGAAATGAAATGAAATGAAAAAGTA
AAAAAAAAACAAAAGAAATTAAAAAAAACAAA
AGAAATTAAAAAAAAAAAATGAAATGAAAAAGAAAAAAAAAAAGAAGTATTCTTGACAAATTTGAGTGC
GTAGTGCTGGGAAGCTGCACATTTGTTGTTCC
ACGAAAAGCCGCAATTACACCCTCCCTCACACCTCAGCAGAGAGCCGCAGTGCCCCACAGCCCCAGAA
GCAACAACAACACCCGAGACACACACACATGC
ACGGTGCTGTCACCAATTAGAGCCATGGCCGCCAACTCGCGGAACTCAAGCAAAATGTCGTGGTCCGA
GGGTATTGTTGCGTTGGACTCCGATGGAATCT
TGGTTTCCCCAGGCGCTGTGCTCGGAGGAGCCGGTGCGGCATCAGCGCCACCCGACACGTTAGTGCTG
CTGAATGAATCGGGGCCGCTGTTCGTGCCCAC
GCTCTCCGAAACATCAGGTGAAACGCTGTGCGGCGCCTGATCATCGGTGTTGCCGATCCGCGGAGAGT

CTTCATCTGCCGTGTCCACCTGCCCCATCAGC
GGTAAATCTATATTGGGAGGAAAGTGCTCCCCACTGACAGGCATCTCGGTTCCAATTAACTCATGACT
GCCCGCGTTCTGCGTTTGTGACGATGCAGACG
CCAAGGCACCGCTGTCGGGTGCCTCTTCTTCCACCTTTCCCACACCAGCTGAGGTGGAGGGGACAATG
ATATCAGGAAATGTTTGCTTGAAATCTGCTGG
GCCACTTTCTCATTCTGATGTTTGCGCTGGTGAGTGTTGAATTCGTGGTGATGATGTGGCGTCTTGCT
GCGCTTCAGGCGTGATGACGGGCACATTGGCG
GGATGACGAGAAGGTTCACTCACATTGGTAGTTTGAGCCGCAACTTCCTGGGCCGGCACCTTCGCCGC
TGCAGCAGCGAATGCGTCCTTCTTCATTAACG
CATTGAGCTTTGCTTCGTTCAATGCGCGGTTGTACAGCAGGACGTTGCTCACCGTCACATTAATGTCT
TCCGAGTTGCTCTTGTTGCCCCCGCCGATGTA
AAAGTGCGAGATGCCGTGGGACCCCAACAGCGTTCGTAACTTTTGGGTAAAGTTGTATATATATTTTT
TTCTTATCTTACCAGTCGTAGTAACTACACCT
CAATTGTGCAGCTCCGGTAAGCGTACGAAGGAATTCAGCAGCAAGGATAACCTTACTCCGCGGGCGGC
CATTATAGAATGGCTCCGACATCTGCCCTTCC
GGCCCGTAAAACTAAGTAACAACTACTCATCGGGTTTGAAGCATGAACAGTAGGCGcttagcttcctc
tctagcaaagagtNNNNNNNNNNNNNNNNNNN
NNNNNNNNNNNNNNNNNNNNNNNNNNNNNNNNNNNNNNNNNNNNNNNNNNNNNNNNNNNNNNNNNNNN
NNNNNNNNNNNNNNNNNNNNNNNNNNNNNNNN
NNNNNNNNNNNNNNNNNNNNNNNNNNNNNNNNNNNNNNNNNNNNNNNNNNNNNNNNNNNNNNNNNNNN
NNNNNNNNNNNNNNNNNNNNNNNNNNNNNNNN
NNNNNNNNNNNNNNNNNNNNNNNNNNNNNNNNNNNNNNNNNNNNNNNNNNNNNNNNNNNNNNNNNNNN
NNNNNNNNNNNNNGCTTAGCTTCCTCTCTGGC
AAAGAGTGCTGGACCCTGGAACCACGTTGAGGTGGCCGGCTGAGCCAGGGGATCCACGCAAGTTTTTT
TTTTTTTTTTTCTACTCTTACCAGCGCGTCTA
GCTATACAATGCCATCTGAGTGCCGAAGAGGTGGTCGAGCAACGACAAAAGTTTCCTGCTATGAAGCA
ACTGCCATTGAGAGAACTTTCCGAGGCGAAGG
TCCTGTTCAATGCCCAGCCGTTGACGCACAGCCAAGGTGCCTGGACATTCTAGAAGCAGATGGTGCAA
GGTTTCCTGCGGGCCGATTGGCGTGCTTGTGG
AAGCTGGCTGATCCTGCGGCAGCAGAGATCGCCGCCTGGCACTGCTGTTTCGATGCCGTGTGGCGTCT
GGGTTGTGTGTGCAGCGACTTCTGTGCTGTGC
GGTGCTTCTCCGTGTGGGGAAAACCATGTTGCAGAAATCACATTTGAGCTCGGGAAGCGGTGGAGGAT
GTTCCGGATGAAATGACCGACAGTGACTGACG
AGACCCGAGAATCCCGTCAATGTGGAATCACAGTGCGGACATTTCTCCTTCCGACGGCGTGTGAGGGT
CCGTCTCCTTACTGGAGAGACGTCTTCATCCC
TTGCCGTCGGGGGAACAGTGTCCGTCCCCGGTGCGGTTGGATCTTTGTTAGAGCGCATATATGCAGAC
TGTTCCGGTGAGATGTTGCAGAATCGGCATTG
GTTCGTGCAATCTCTCACGGCCCAGCATAGTCGGCCCCAGAGCTCAGATTCCCCGCACCTTGCCCTTG
CGAGGTGAACCAGTTCTTGACGGTGTAGAACT
TCCTGAGTGATCAGGTCCTTACCGCTGAGATCTGATGGCCTTGTGCCGCAAAGCAAGTAGCGATGAGT
GTCTTGACGAATGGTACTACGCCACTTGTTGG
TGAGCTGGGTAGTAAAACATGTCAGCAGATCCGTATGCCATAAAGGCGCGATTCCTTGCTCCGTGTAT
TGTCCACTTTCCATAGTCTGCGTCGCATGCTG
ATCTGCAATCTCGGTAGCATGTACTCCGCAATGTCCGTAACAAAATTGCAGGTGCACGGACCAGCCAG
CACGCGTCAGTGTCAAGAGACGCTGCCAGATC
TGATCCTCCGTCCAGTCTGTCTGACTGAGCGGGCCCTTGTTTAGAGCCGCTAGAAGAGACTGACTGTC
CGTAACCACAAGCAGGGTTTTACGCCTGTGGC
GGATACGAGGAATCATCAGCTTCTCTAGGGCTAGAAGCAGCGCACGGGATTCCGTCCTGTAGCTGCAT
GGGCGTGCACCGCAGTTTACGCTGGCCTTTTC
TATTATCTCCGATGAGTTGAGGGATGAAAGCAGTATTCCAACCCCTGCTGACTTGGGAACGTCGACAC
CGCCATCGGTCGCAAGTATCCACGAACGATGG
CTGTGCAGCGCCAGAGCCTCTTCGCTGACTCCGCGCTGTTCCCGTATCAGCTCTTCTACGTCAGTTAT
TGAGAAGTCTATCGGAAGTTCCACACCGATGG
TGACCCGGTGAGCGGAGGCCGCAAACCAGGGAGGAATGGAGCTGCGAAAGAACCGCTCTTCGCGTGGT

GAGCTCTCGGTGATGCCATAGTCCTTTGTGTA
TGCGTCTACGAAGGCATATAGCTCGTCCCGAGATAATGGCGAGATGCGGAAATCTGCGCGAGGCGGTG
GCTCAGGCGGGGGTCTCCGCATCCAGTCCGTC
TGACATGCGCGTGTGTTCTCAAATATTGAGAGCCGCGCGCGCAGGCAAAGGACATGGAGCGGCGGCAT
ATTAGCCTCCAGCAGCGCGGATTCCGGGTCGA
CAGTGCTCGGAACTCCGCTGACAATGCGTGCTAGAGTTGCGTACGTCTTCTGCATCTCATTCTTCGCT
GAATCCGTCGCTACTCCCCATATGAGCTCGCT
GCCATAGCGTAATTTGGAGCACCGTATCCAATGACGAAAGTGCGTAGGTCATGTGGAGATGGTCCCGC
CTCTTTGTGCGCTATGCAGCGTGGCTGCGATA
TCCGTTGCGAAGTGGCAGCGCATTGCTTGGAGGCCGCCACATTTAGCGTCAGTCGCGGATCGAGATCC
ATGCCGAGAAGCTTCGTAGTTCGCGACGCCCC
CATGGTCATGACTGGGATTTGACATCCAGCCACTACCAGGGGCCACCTTCCTTCACGATCATAATCTG
ACTCCGTGTGCGTGGAGAGTGTGGACCAAGCG
GCTTCACGCATTGACGGATTGTTGGCCAGGCCATTTTCCTGACTCCACGCGTGCAGTCTTTGAAGAAC
CATGTTGGCAGTGGGAATGGCGTCTTCCCGGG
ACTGCCCCTTAACGATAATAGAGAGGTCGTCCGCATACATGTCTGCTGTGACACCCGCAAAGCTGTTC
AGCAGGTTGAGAAGTGGGTGTACGTAAAGGGA
GAACAAATATGGTCCCAGTACGGACCCCTGAGGAACTCCTCGCAGCATGGGACGCCGATCGCTGTGTC
TGTGGCCAACTCGTGTCTTCGCGTAGCGACCA
CATAGAAAGTTTCTAAGCCACCTTCTACAATTCACCCCCGGAATATTAGCCAGCAAGCGACTGAGGAC
TCGATGATCTATGGAGTCAAAAGCCTTGCTGA
AATCAACCAGCACCAGCAGAGTCCGATGGGGTCTGTAGTGGAGTTGATTGCCAATACCGCTCTTCTTG
GGAAGGCTCACTTGGAAATAGTGATTTCTATT
ATGCTCCACTTCGTGTATCAGGTGTGCCAGCTGCATCGTCGTAGTACGCATACTACGATATGCACACT
GGTGGGGGTGCGGCGTCCACACCCAAAGCAAT
CTCTTCAGTACCATTTTCTCAGCAACCTTACTCACAATGGAGGTAAGGCTGATGGGTCGATATGACTC
AAGCAGTTCCGGCGACTTACCGGGTTTTAAGG
GCGGGACGATGGTAGCGCTTCTCCACTCAACCGGAATCGTACCGGTCGTCCAACTGTGGTTAATGCAA
CGAAGAAGGAATCGGAGGCCCTTTGAAGAAAT
ATGTCGCAGTGCCTCACTGTAGACTTCATCCGGTCCGGGGGCCTTGCCATACGGCGTATTACGTAGCG
CAGCAAGCAGTTCCGTAGAATTAAAAGGTGCA
TCAAGAGGGGTGTGAAGATGAGCAACTAAACCAGATGTGGTGGCAGCTTCGTTTTTATCCTGTGGCTC
AGAACTAGATGAGAGTGAAGAACCTGAAGTAG
ATGTAGCGGCAGACTCGTTGTTGTTCCGCTGCCCAAAAAGAAAAGAAGCATTAATGTTCAAAGATGCA
CTCTGTCTGTTGATGCCTTTGGAAAACCTAAC
TGCCTTGTGAGAGGGGTGAAATGGAAGAAAGATGCGATTGAGAGCATTCGCTGCTCTCCTTGCTGTTT
TATAAGTGTGTTGGCCACCTCCTAATAAAATT
GATGTGGGAGATGGAAGTGATGCCTTTTTTCGTCCGGAAATGTATTTCCATGCGAGTGGCTCGCCTGG
ATTTAACTTGCTNNNNNNNNNNNNNNNNNNNN
NNNNNNNNNNNNNNNNNNNNNNNNNNNNNNNNNNNNNNNNNNNNNNNNNNNNNNNNNNNNNNNNNNNN
NNNNNNNNNNNNNNNNNNNNNNNNNNNNNNNN
NNNNNNNNNNNNNNNNNNNNNNNNNNNNNNNNNNNNNNNNNNNNNNNNNNNNNNNNNNNNNNNNNNNN
NNNNNNNNNNNNNNNNNNNNNNNNNNNNNNNN
NNNNNNNNNNNNNNNNNNNNNNNNNNNNNNNNNNNNNNNNNNNNNNNNNNNNNNNNNNNNNNNNNNNN
NNNNNNNNNNNNACTTGTCCGTTGCATTTCTG
CCTCACGGTCGGGCGTCGGTAACTGTAGATAGCGGGCCTTGCTGTCCGTAGCAATCCGCTCTGCCTCC
ATGAGAGTGTCGTTCCAAAGCCTGGGACCGTC
CTTGTGCATGCCGCGGGGGCAATGTCGTCGATACGAAGTTATAAGCGCTCTCGTGAAAGCTTTAATGC
CGGTGGACTGCTTTTTATAGTCGTATGCCGGA
AGTTGTGCGTCGAAGTCCTTGACGAATAAATCCCACTTGCACTTCCCCCAACTGTAGAAAAACTTAGG
TGCCGAAGGGGGTGCCGAAGGTATGGTATCCT
TAAATGCCTGATGCAGCGTAAATGTCAGCATATAATGATCACTAAGCGGCGACACAGAAGCTGACCAA
TCCGACACAGCCAAATGCTTTGACCACGTCAG
ATCgagagcctcgtGGAGGAAGAATTGACTCCTTGTCCGTTGCATTTCTGCCTCACGGTCGGGCGTCG

GTAACTGTAGATAGCGGGCCTTGCTGTCCGTA
GCCATCCGCTCTGCCTCCATGAGAGTGTCGTCCCAAAGCCTGGGACCGTCCTTGTGCATGCCGCGGGG
GCAATGCCGTCGATACGAAGTTATAAGCGCTC
TCGTGAAGGCTTTAATGCCGGTGGACTGCTTTTTATAGTCGTATGCCGGAAGTTGTGCGTCGAAGTCC
TTGACGAATAAATCCCACTTGCACTTCCCCCA
ACTGTAGAAAAACTTAGGTGCCGAAGGGGGCGCCGAAGGTATGGTATCCTTAAATGCCTGATGCAGCG
TAAATGTCAGCATATAATGATCACTAAGCGGC
GACACAGAAGCTGACCAATCCGACACAGTCAAATGCTTTGACCATGTCAGATCGATACAGGATCGTTC
TCTTGTGCCACGGGCATACGTGATCTCACCAG
G
>scaffold1099.size6276
CCCCATGAAATATAAACGATGTGCGGACACACAGAGGGTCGGACGGTTGAAACACATTTAAATATCTT
TTAATTTTATTTGCGGGCAAAATATGCAGACAG
TTTGGTGTGGCCACACGGCAGCCGGCAGCTTTTTAAATTTGTGTGGAACAGTAATGGACGATTTCTT
TTTTATTTATAATTATTTTGTGTGATGGGGTT
TGTATAAGACGAGGAGGAAGCTTTTTTGTTTTGGATAAATCTTGTTTGAATTGCCGCTGCTTGGGCTG
CGGTGCAGTCGTGAGTTTGAGCAGTCTTTTTC
TCTTTATTTCTGCCATGCACCTCCGCTGTACATTTTTGTGCATCCTTCCACATACTCTGCTTCCTTTT
CTACTTCTTTGCTTTGTGTTTCTGCAGGAACA
CACACACAAGATGCACATTGCAGTGGATTTGAATGCTTCTTTTGTAGCGTGTGCTTGCGGTTGCGGGT
GGCTGCTGCTTCCTCCGTGGGCATGAATGGAT
GGAGGAGAGAGGGAGCGCGCACCGCGAGTCACTCTTCCTGCGGGTGCGATGCGCGCGGTGGGCTGTGA
CTGCTGTGGAGGGCCGCATCCTTTTGGTGTGC
GCTTCTCCGCCGTTGTGTCGTGCGTTGGCGCATGTCATTTGTTGTGCGCGTGCGTGCTGGGCATGTTT
TTCCGGAGCGTTGGGCGGCCGCCATCTGTCTA
TGGCGGCTTGTGTGAGTGGGCACGTGCGGATGAGGGTCAGGGCGTATGGAATGAAGGAATGGCTCGAG
GAAAGGGGAATTTGATTTGGAGGGTATTTGGA
CCAGATTCATTTGACTGCGGTGGACACATGCACGACGGATGTGTTTCCGCATAATGGGGTGGATGCGA
CAGGATTTTCCGAAGTGGCCCGTCAATGCAGT
TCGGGTTTCATAGCACCGAGGCATAGTGTTCTTCCTGCAATGGCTGGTGAGGGCCTCTAGTGGACCGG
TGTATTTATGCTGCTGCTGCTCGAGGAGGCGT
AGAAGTGCGATTTGAGGTGAGTGGCGGAAGGGCCACACATCCCGCAGTGACGGTCGTCGTTGGCCCAC
CGGGTGCCTCTGAGGGTTTTGTTGGATGCAAC
GTCCTTATGCCCGTCTCCGGATGGCGGCATCCCTGATGCAGCCAACGAACGGGGCTGGGAACGACGGT
GAGACGCAAGCACGTGTGGATGTTTGGAGCGG
CCGGCGCACCGCACGCTGAGAGCATGGCGCGGTGAGGGCCGGACCCAGAAAAGGGACACCGCGTGGGA
CGTCAATCTTCACTGCGGCGACCGCCACCGAA
TTCGGATTTGCTCGCACCTTCTGCGGTCCCTCTTCCTCCCCTCACTTCTCTGCCGAGGACTGTGCCGC
GGGATCCAGCAGCGCGATGTTTGGCCGCAGGA
ACATGGCCCGAACAAATGATTTGTGGGAGGGGGATGTGCACTGCGACGAGAAGGAAAAATGTTGCGAG
ACGACCACCACCACAAGACCGGAGCGAAGTTG
TGTTTTTTCCACTTGCGCGAGGGCGGCCTGCCACCTGGTCGGTTAGGAGGATTATTTCTTTTTTTTTG
CGTCGTTGCGCTCCCTTTTTATTCCGCTGCGT
TTTCTTGCGGACCTTCGAAGGGAGATTTGCAAGAAGTTCCTGCGAGGCGTCCGTGCCGTCGTCGGAAA
AGCGGATTCCCCCTTGAAAAGCGCTACTTTTT
AAATTTGTTTTGGGCGGGGAGTTCCTGTCGATTCACTGCGACTTTGTTTTGCGGATTTGATGCGTGTG
TTTCCGAGCGGTTCGATCTGTGCTCCCGGTCC
ACTCGGATGCGGTGTGCGCGTCGACGTGATGGTGCCTGCCAGCCGTTTTATTGCCTCCCCGCTGTGGC
TGCCGCCGCTGCCTTCTTCCCCTCCTGAGGTC
CTGCGGCTTCTTGACACGATGCTTTGGAGAAGGCGTTGCCGCTCTTATCGTCCATGATATTTTGATTC
TGGTGTCGCGGTCAGGAGACATTGAACGAGAG
GGTTTGCTGCCCTCCTGTTTTATTTGTCCTCTCTGCCTGACGGCCAGTTCTACACAAACGTGCTCCTC
GGCACCTCAGTTGTCGCTGGCAATCGCATGCG
CCAACTCTTCTCTGCTCCCTTTCAGTGTCGCATTTTTTTATTGATTGACGGACGCCATTCTGTCCGGC

GTGCCACTTTGATTGGATTGTGTGGAACTGCT
TGCCAACACCATCGGAGAAGGCAGGTCAACTTTTTCACTTATTTAAACGTCATCTTGCGTGCAAGTAT
TTTTTTCACATTTTCCGTTGGGCACTCAAACA
GCAAGGTGTCCGGAACTATGCGTGTGTGGGATTATTCGTGCCATGCGAACCGCGGGCAACACCGAGGG
GATGCCATTTTGACTCCGCGGAGTTGTTGTTT
CTTTTCACAGGGCTGGCAGGGAAATTTCGTACAGCCTGCTACTGCTGGTGTGTCCTCAAATGTTGTCA
CTTCATGCGAATAATGTGCTTCAGCGAGTGAG
GGGTTGATTGGGTTTGTGTGCACTCTGATTCTGTTTGCTACTTTGTGCGGGCACGCAACACTTGAGTG
GTGGGAAAATGGAGCGGGGCGATGCCTGGCCG
CGTCTCGCCCATTGTTCACTGCACGATGGTGTGGTGCATATTGTGGTGTTGACTCTGCGATGTGACTG
CGGTCTCCTGTGCGGCAATGGTGGTGGGTGGA
GGAGAGTGTTGACCCGCTGCGTGACGGTGGGATCGTGTCTCCTTTTCTTTTTACTCCTTCCCTTTTGT
GAAGGTGTGCACTTATTTTTTGTGTCGGTGAT
GGGGCAAACAGACCGACAACGTGAATAAAATAAAACAAAATAGAAAAAGGGGCGATACTGTGGCGGTG
TTGTTTCGGCCGATTGCACGTTGTGTCGCTGC
GGGGGCGGTGGGCCTCGACTGTTTTGCCGACAGGCGTTGCAGTGCGGCTGCATGGCGCACCGACCACC
GAGCCGTGCGAGTGCCACGCCCAACGTCATTG
GGACTGCGGGACGAAACAGCCGCGACTGTCCTTTGGAGCCAGCGGCACCTGCTGGCCGCAGTTGGGCG
GTGTGAGTGGGATGCTTCACCGCACGGGTGTT
GTGATGACACCTCGCCGTGGCTCTTGTGATGGTTCCGACGCTGCAGCTCGACATGTGGTGGGGAGTAA
AGTGTGGCCGCAGTGGACCATGAGCAGCACCA
ACATGAGGCTGAACGATTTCCTTTGGAATTATGTTGGCGGCAGGGCGGCCGTTGACGAAAGACTACAA
TGTGACGGAGATGTTTGCTCGGGAGCCGGATG
GTTACGCACAAGACCAGCGGCTTCTTGAAATTCTTAACTTAAATGCGTACCAAGCGCTGGAGGCCATG
AATAAGCCTCTTCACGAAGGTGTGCTCTCTCT
CGGGCAATGTGGGAACTATGAACGAAAAGGATACGATCACTCCTCCTGCAAGGGGAAAACCAAACGGA
GTACTCACGCAGGTACCGACAGAAGCGCGGCG
GGAGGCAGAGGAAAGGCTAAGACAAACGCAGGAAATGAAATTTAGCATTTCCACTAATATCGAAGAGG
TACTGTTTAAAGGAAGAGTTCGCGTCAATGAA
ATGAAGCTGAACGACTTTCTTACGAAGGAACCGGACGGCAGGGGCGTTGTGGACACCAATCGGGATGT
CTTGCTGGAGGAGTTTTTCAAGGACCCCACGA
AGTATGTCCGTGATAAAGGAGCGTTGAACGAAATGCAGGCATCCGGTCATTATTTGGGTATGAAGCGA
GCTGTGAAGGGTGAAGTGATCTTCGACGAGGA
TATACGCAGGCTATGCGATAGGGTGTCAACAATCTACCGGGGTGGTCATTGGCTGCTGCGGAGGTAAA
AGCAACTGTTCATAACTCCACTAAACATTTCT
TGGATGCAGCTGCCGAGGAGGCGAGGAACCCAACGACGACGAGCACACCGGAGAAACTGCAGGGATTG
TACGAGTCTGTGCACAATGCGAGGTGGAGCCA
TGCGGTGGAACTTCCGGACGGCGTAGAGCGAAAGAAAACGGGGACGGGAATGGGGGTGCATGAGGGGA
GGCCAGAACAGTCATGGTCGTACAAGAAAGCT
GACGACGCTATCGAAAAGAATGACGCTGTGCAGCAATTCGGTGCAGCACCTCCCGTGCTGATGTTGCT
CGCCTCGTACAATGGATGGCCGTGCTCGTGGC
ATACGATACAGGATCTTCCTAAGGACGTTTTGTAAACTGTGCGGTGGATCGAGCGTGGCAGATCGTCA
AGGGCGATGTAACCGCGTGGTTCAGTCCTCAC
GGTGGGACCGACTTTGAGCCCAAGCGACGTGTGCTGATGGGGACGCCCGGGATCGGGAAATCGGTGGC
TGCCGGCTCGTACCTCCTCTACCAGCTGCTGC
ACTGCGGTGTCGAGAAACTCCCTGTGGTTGTCCACTGTTTTGGTGGAGGAGCTGCGTACGTCTTTGAC
AAGACCACCAGAGCGGTGACAAGATGCAGCGA
TGAGGACATGTGCATCTCTGAGTTGAGAAGTTTGAGGGGGCATGGGAGGGATGTGTATATTATCTACG
ATGTGGCAAAGGAGGGAACGCCGCCACCGGGA
CATTTTGCGCCTACTTCCGGATGGGGCATGGTTGCGGTGTCATTCCCAAATGTAACCAACTATGATGA
GTGGGAGACGCAACTAAAAGCCAGTTGAATCA
TCATGAATTGCCCCGACGGGGTGGATGTGAAGGCGATGTGCGCCTGGATGACGCGTGATGAGACCAAA
GAGAAACAAGCGGAGTGCTGGAAAGAGGTCAA
GAATACATTTATCTTTTGGGATCGATTCCACGTCACTTCTTTGATGAGGAGGCGTTTACGGAACGCTG

TGGAGCGGTGAAGTTTGCATTGCAGTCGATCA
ACGAGGTAACTGTGAAGGAGCACTTTTCACGGGGAGGTGAGTCGCCATGGTACTCTGAGGATCCGTCC
CACAGGCTTGTGAAAGTTGTTCGGGAAACATA
CGCAGGCGGTGTGATCCTTTTCAATGCACCGATATCTGATTGCCTCGAGGAACGGACATTGGAACGCC
TCCCAAGTGTAGCAGAATAAATTAGCTTTTGA
GGCTGATGTTGGCGTCGAGGGATAATATTTTGCCGATGTTTTTGGATGGCTTGCTTTGAAATCGTTC
ACGGAGAGTGCCTTTGCCAAGGCAATGGCGAC
GTAACCCACGGAGTTTGAGCCGCCAACAGGACGTCAAGCGAAGCCCTGCGTCTTGAAGTTGGCCCCTG
AATTTCATCCCAAGGGAGATGAAGGTTTGCTG
CCGAGTGAATATGAAAAAACGATCCGTAAAATGAAGCACGGGGTGCTGCACACACCGTGGGCTACAAA
CTTTCCGCTGGTGGACGCCTTTTTTCTTCTCG
GAGTTCAATCCAATGACGGTGGTTGGGCTGCGGATGGCTACGGCGGGTGGGCGCCACACCACCGCCAG
CACTGTGCGGCGGTTCACTGAGTGCCTGGCGG
CATATTTTAATGGTTGGGAGGAATTGTCCCGAGACATGTCGTGGGAGATTATTTACGTGCAGCAGACA
GACAGCACGCCGAGGAATGGCTGGCAGAGATG
TGATGTCGAGAATTCCAATAATGTGAGCGACGAGAGCCGTGAGATTCGGCGTTTTGGAAGGAAGAGGT
GCGCCAGTACCAAGTGTCAATATCGTACAGAG
ACTTTTGAAGAGACGAAGCTTTCCGAAGTGTGCAAAAAATAATAACAATAATAATAGGAAAATAGATT
AGTAAATTGAAAGTGAACGAGTCATTTTGATT
TGTGGTAAAAAATATTGTGGGATTTATTTATTTTTGGATGCTTGTGCCTGAGCATGCGGTGCGGGTGC
ACCATCTCCATGCGGCAGCACCGCACACAAAT
GCCAAAATGTTCAACGCTATCGAACATAACAGTGGCAGAAGAAATTGGGGAGGGAAAGGGGTTGCTTG
GCTTCTGGTGGAAAAACATCGGGTGATTTTCA
TATGTTATTTTGATTGTGTGCGCATACGAGGCATAATTGGAGAATTGCCCCAGTATGAGAGAATCATT
GTGGCTGGCGGTGTCACGGTGTTACTGCATTG
TCCATTTCCTTCTATTGTGTTCGAAGGCCCATCACTAACAGAAGCATCTTTAAGGGATGGACGACGAA
TAAGTTCTATTGTGGAAACAGTGCGCTCCGTG
TGACCCCGTGGAGTGAGGCGCCGCGTCATGGCTGGATGAGGATCCGCTTCCGCGTGTGGCTCCTTGGG
TGATTGT
>scaffold11.size67638
CCGGCATCCAGTTTGTGTTGTATAGTCAGCTGAAGTTTTTCTTTCAAGACATCTGTGAGCGGTACGTG
CGACCACGCAAAGGTTCCGCTGGGGATGCTCAT
CACCGCGGCGAGACTCTTGAGTTGTTTCTTATGAAGTGTGGCGCTGGTGGTATCTCTGCCACTATTGC
GGGAGTTGCAGTTTACCCTGGCGAGGTGGTGC
GACTCCGCCTCATGTCTGGCGAACGGCAGTTTAACGGCATATTTAACACCATAAGATGCATCTACGCG
GAGACCCACTCGTTGCGAAATTTTTACCGTGG
ACTGGGGGCATCCCTCATGCAGCGGGTGCCGGATATCCTTGTGAGCTTTGCGGTGTACGAAACGATGA
AGTACCGTCTCATTGAGAAGGACTTTCTCAGT
TCACACCCCTCATACAAGAACATAGTTTCCACCGTCGCTGGCGGTGGGGCAGCGGCCCTGGCATCCAT
CCTTGTTGCATTTCCACTTGATGTGGCCAAGC
GACGAATTGGCATGTCGGGAAAAGGGAAGAGTGGGGTGGTGTACGCGGGTGTGCGGGACTGTCTGCGT
CAGGTGTATGCGCAGGAAGGTATCCGTGGCTT
GTACGCGGGTGCGCGTGTTGAGGCAATACGATGTGTGCCGCAAGTAGTGCTCATGTGGTTCTTTATTG
AGGGCTTTCAGAGCTTCTTAACGGGTCATTGT
GTTCATTAAATGTTGCACCCGTGACGGATTTTTTTTTCTTTTTTTCTTTTGTTTTTTGTTTTGTTGAA
TATAAAAGGACCGTTCGTATGATAAGGTGGAC
TGAAAAAGAAGAGAAAGAAATTAAACATGGTTTACTGGCCTGGGTGTTTTTTTGCATGCGCGCATATA
AATATATACATGAATACCGGTAAAGGTTTTGT
GTTCCATAGAAGAAGAAAAAAAAGAAAAAGAAAACAGAGAGAAAAAGGGGAAACGTGTTTCTGAGTGG
TGATCTCTCAGTAGTTCCCTCTAGCATAACGT
TCCTTTTTTCATTTGGATGAATAGAAGAGAGTGTGGGAAAGAAATGGATATATATATATATATCANNN
NNNNNNNNNNNNNNNNNNNNNNNNNNNNNNNN
NNNNNNNNNNNNNNNNNNNNNNNNNNNNNNNNNNNNNNNNNNNNNNNNNNNNNNNNNNNNNNNNNNNN
NNNNNNNNNNNNNNNNNNNNNNNNNNNNNNNN

NNNNNNNNNNNNNNNNNNNNNNNNNNNNNNNNNNNNNNNNNNNNNNNNNNNNNNNNNNNNNNNNNNNNNNNNNNNNNNNNNNNN
NNNNNNNNNNNNNNNNNNNNNNNNNNNNNNNNNNNNNNNN
NNNNNNNNNNNNNNNNNNNNNNNNNNNNNNNNNNNNNNNNNNNNNNNNNNNNNNNNNNNNNNNNNNNNNNNNNNNNNNNNNNNNNTGG
ATGAATAGAAGAGAGTGTGGGAAAGAAATGGA
TATATATATATATATATATATCAGTGTGTGTGTGTGTGTGTGTGTATTTTCTCCTAACTAAAGCGGTGTG
TTCCTGTTGGTGGGGAAGCAGAGAAGGAGTGG
ATTTCCATATCCTCGCTGCCCTTCACCCCACTCATGCGGTTAACCACTCCGTTGGTTTGTTTTTATGT
GCGTGTGTGTCTATTCAGTGCCGCATGGTAGA
CAGGTGAGAGATGCAGCCTTTCCTCATAGTTGGCGGAAAGTCACACTGAAAACACATTACTGCTTGAA
AATTAAATGCCCGTTCCTTAACTACAATGGGA
AAGGGGTTTCACGCGGCGTGTAAGCATGCGCGTGGGTCTGTCTGCAGTGGGCCTCTTTTTTTCCCGTC
ATACGTGTGATTTCTTTTTTTTCTTTCTCTCT
CTCTCTTCCTTTTTCTCTTTCTTTTTTTCTTCTGCGGCTTCCGTTGAGGAATTCTCCATTGTATTGGG
GCGAGGGAAAAAAAAAAGTAGAAACAATATTG
GAAGCAGCGGCAAGCCCGGTATTTCTGGCCACACTAGGAGGAAACAGACACACGCGCACACAGAGGGA
GAGAGAGAAAGAAGGAAAGGAAAGAACGCAGA
AGAAGAGAACAGAAAAGAAAGAGAAAAACAGACAAAAGTAAACTATGGAGGCCAAAGCCCAGGCGTAT
TTGGAAAAACTCAGCAACTCCCTACAGGCCGC
TGGTCGCACGGCAACGGCGGATGTGTTAAAGCGGGCCAAGGAGGAATTTGAGCGTTCCTTATGGTGCG
AGATGACCGACGACTTGCTGTTGGCCGTGCGT
GATCCTTTGGTGCTGGAGTCTGCCTATGAACTGCATGAAAACGTCTTGAAGGTGACGCGCATGGACAT
GTCGCCTACGGCCTACGTCAAACTCCTGCACT
ACATCTGCTTCTGCCCAAATAGCACTCTCCAAAAGGCACTTGATCTTCTCAATGGCGCCGTCGTCGCT
CTCTCTACAAGCTCAGAGCAGGGCGAGAACAT
GGCCAATTGCATCCGGGCCCTGTTGCTGCTGGACAGTTACAACGACAAGTCCTCGCCCGCCGGAACGG
CTGCCTCAGAGGCGAGCCGGATTCTTGACAAG
GTAGAGAGCTTCATCCACTCCAAGCAGATGCACGAGGTGGAGCCCGTTTTAGCCGCACTGCATTGCCA
GGCCCGTGGAAAGGACTACGAGATACGCCAGC
AGTACACAAAATACTACTCGAACGCTTTTGAAACCGTGAAGAGTGCGGAGCGTGCCGAGATGCCGATT
ATTGAGAGTGACCTCTTGTCTCTTGCCTACAA
AGCTGCTCTTTCGGCGCTCCTTTCGGAGGAAATTTATAACTTTGGTAAGTTTCTGAACGAACGTCACT
TCACAGAGCGTCTTGCGGATTCTGTGCAACAC
GCGTGGATTCTCGAATGGCTGCGTCTCTGCAATGATGGCAAGGTGGAGGAGTTTGAACAGTACGCCGT
TGCCCACAAGGCGGAAATGGCGGCGAGCCCCA
TCCTTTCCGAATCCTTTCCCTCCATCACGAAGAAGGTGCGACTGATGGCTCTTCTTCACCTTGTTTTC
TACACACCGTTTAACGAGCGCACGTTCACCTT
TGATGTGATTGCGCGGCGTTGTGCGGTTGACGAGGAACGGGTGGAACCACTGCTGCTCGCGGCACTGG
CGCAGGGTATCATCATGGGGAAGATCGACGGC
CTCACACAGGAGGTGCACATCACATGGGTGGAACCACGTGTATTGAGCCTGCAGGAGGTGAAGGAACT
TGcCGTTCACGTTTCGCGCTGGAAGGAAAAAA
CTCTGGGGCTCATGCAGTGCATGAAGGAGGCTGCTCAGGAGACACACAATTGAAGGAAGAAGGCACAG
GGTGACGCAACCGAGGAAAAAGGATGTATGCA
AACAGGCTCAAGGAAAGAACGGGAGGGGAAGAGAAGAAAACAACTGGCACAGACGGACAAAAAAAAAA
AAAAGAGAGAGAGACCTTAGAATGCGCCAGCA
CAAGGAAAGGCTCCGCTTTGATGAGTGCGTTCCCTGCTGGTGCCGGGAACGAGAGGGAAGGAAGAAGA
CAGGGCGTGGCCGGGGAGTTGGTCTGGAGGGT
CAAAGCCAAAAAGAAAAAAAAAAGGAGAAAAGGCGTTAACTCGTTTATTCAGGAAAAATGAAGCGGAA
AGACTGCATGTCATGTAATTAAAAAAAGGCTA
TCACTTTTTTTTTTCTTTTTCTCTCTCTCTCTTTCTACCTGTGTAGTTTTTGTTTTTTGTTTTTTCTG
TGTGCATGTTTGTTGTTTTGCTTCACGTCTCT
CTGTGTTGGCGCGCAATTCTCTCGCTTGTGCACTGTGTGCATCTATCGCTGTGGATAGTTTTGTGAAG
GGGGGAGCCTGTGGAGTCCGAGAGTGAGAGAG
AGAGAAAAGCAGAGGGGTTTTTTGAGTTTATGGCACGCGAGGCAAAAGAAAAACAAGAAAGAGAGAAA
ATAAAATAAAATGTAGACCGGATGCGACGTAT

GCCCGTTTCCTCTTTCTTCCCTCTTGTGGTTGTTTTTGTCTTGGCAAACCACAGTCCTCTGTGTAGGT
ATTTTTGAAATGTAAATGACTCTTCTCAGGTT
GTTGTTTTTTTCAATTCTCACGCATGCATCTTTTCCTCCGGCCTTTCTTTTTATTTTATTTATATTTT
CTTTTTTGTGCTTGCATGTATGATTCTGAGGG
TGGGTTCCTGCAGCTGTTGTGCTGGAGGGAGTGACAGAAGAGCTATCAGATTGTGCGAGTTTATTCCC
ACACACACAAACACACACAAAAAAAAAAAAGAG
GAACACAGCACGTCGCAATCATATAATTCACGTGCGTGCAGCACAGCTGCGTGGATTGGCATACGTGT
CTGTGACGGGCGCCTCCCATTTTTGCTCTAAA
TAGCAGTTACGGCAGACCTTTCTGCTTTTCCTCCCGGATTTGCATGGCAGCGGAACCAGGCTCTCAGA
TTCCACGCTTTCGTGTGGGCAACGCGACCATT
TCAAATAAAAGGTGGCTCGAGAACTTTGCCGCCCTGAAAGAGGAGACATTTACGACGACTCTGCCCAC
GACAACGAATGATGACCCAAACTTTTTTTTGG
AGACGCTGGATGTCTTTTCTTGCACCGAGGAGGAGTTTCTGAACGGACTCGCCATAATAAAGGAATTT
GCGCTGCGTTCCAAGATGAAAATAATGAAGAG
ACAGCTGAAAAAGGGTTACGCAAGGCGAAAAATGGAGGCCATGCAAGAGGAACTTCTCTTCTACCGAA
TTGCGGTGGTGAGAGCCTGCACGACACTGAGG
GAAGTAATAGACCGTGTGAAACGCCGACTCTGTGAATTACGGCGTGACGAGGCGCTACTCCATGAGTA
TTACCAGGCTCTGGATACGGATATTGAGCGCG
GAGAAAGAGAGGTGCTGCCCAAAGAGGGCAGTTATCATCTCCGACAGCGAGAGTTGAGGCGCGTCGCG
CCGCGTCAGAGGTACCGAGAAGACCGAACATT
TGTGGAAGCACGGCACGAAAAGTTGAATGCACTCCTGCATGAACTGGAGGAGGAAGAAATTGCAATCC
AACGCGTGAATGAAGAGAATGGTGCCACTGAC
TTAGACCGGGAGTACGGCATTTGTGACCCGAAGCGAGGGAAATTGGAAGATATTTACGACACCACCGC
TGGTACTGAGGCTGAGGCAGGTAGCAGCAATT
TGGAGTTGCGCTTGCGGATACGACCGTTACTGGAGAAAGTACGGGAACTACGCATTGTGCCCCCTCTG
TCGCCCCGCAGTGTCCAATCAAGATTGCGAAA
AGAACTATTACTCCCTTTCTCTGCGCGTGTTCTTGCATTGAAAATTGATGACGACAACAACGCTGGGG
AAAGATTTGTGGAATGGTATGTTAATGCTGCC
ATTGATCCCTTGGCAATGGCACAGCTTGTAGAACTGGTACCGCGTGAGTATCTTGTAACAACGACCGT
ACGGCTTGTAAACGCTTGCGATACATATTTGC
CCCTGCGACAGACGCAAGCCCTCCCAGATGTCCTGCATGTGTTGTTGCAAGCTATTGCAGAAGGACGT
TTTGATGACCTTGCGTCTCTTGTACTTTCACC
TTCAGACTCCTACTTGCATGCATTGGAAAGCAGGGAGCATGCGGGAATCATATTTTATGCAGCACTGA
TAACTGGGCAAATGGAAGTACTGCGGCATCTT
ATGTCAGTTGGGTTTTACAATACCAACGCACTCATTGTGTGGCTCTTTTTGAATCCCAACCGTCATGA
TCCCAATTACATGGCGTACGTCACAGAAATGA
GGTCCAATATTAAAAAATATATGCGGAGCGTGGCCCATCAAGCCCAGGATAACACAAACGCAGAATCT
CTACGCGGCGAGCTGGAGGAAGACTATATGGA
TCCAACATTAGTCACGGATTCTATGCAAAACAATGAAAACTTTCTGTCAGTGAACAGGGACTTGTGTG
AGACGCTGCGACGCTTCTTGCAGGAGCGAATG
AGGTGGAAGAAGCAAGTGGTTTCCCGGACCGATAAAGTGACCATGGATACCGACAAGAATGAGAAGGG
AAAATTATCTGCAGAGAACCCTCCCGTGACGA
AGTTGGAATTGGAAGAGGACGAAGATGCGCAGGCAGCGACGCGAGTCGGTTCCTCTTCTGCGGCTTCT
GGCGTGTCCTTTTCGCTCATTCTTGGGAAGAA
GCGTTTAGACCGCCTTCAAATCTCGTCGGTCAAGTGTGCCGTATTACCAGCCGTTACTACATCATTTA
TGCGGCGGGTGGACAGGTGGGGGTACACACCT
CTAAAGGCAACGGACTCGCATTTCCTTCTGGTTGGAGACACGTCTACCGCAAATGCACATGTCATACA
ACTCCCACCACGGCACGCAGACTTATGGCCCG
ACGATGACCCCACAGTATCACCACACGTAAGATGGCAACGAGAGAGGAAAGAAGCAGCAGCAAAAGCG
CACGGCCTGACTTTCTACTATGAGGTGCATGT
CTCCTTGAATTTTCTACTCAGCGGTGTTCCTGTGAGCACGAGCGTCTTCAACGGCTTCTCCGCATCTT
TGGAACGGACTCCGGAAACCATGCCAGGACTG
CAAACTTCTACTGTTTTTGTTGGGTGGTGCTCAGAGGAATGTACGTCCGCTGCCGATGGACGTTCGAC
GCATCCTCCcCTTGGAAGTGACCGCAATTCCA

TAGGAATTTCCTTTGATATCTTGCATCGATCCAAGGTCAACCAACTTGTGGAAGAAGTCATCATACGC
CCCATGAAACATGAAAGGGGAAGGAACGCCGT
TTACCAGGTCTTGGATGAGGACGCGCAAGAAAAAAAGGAGGTGGAGGACAAGGAGAGGGACTCGGTGG
ATTTACCTTTTCCCTTATTGAGGATTCTGTTA
GAAAACAATAATGAAGGCAATGATGTTGGATTTGTAAGTCCCACCAATGGACCTTTCTCATCACCAAT
ATACGAAGCTTCAGCCTCCGCACAAACTAACG
AAACATTGGAAGAAGCCCACATGCTGTCGGCAGCGTATGCTGCAAAGGAAAGCATTGTTCCCACCGTT
GAAGTTTTGAGAAAGAAAGGGAATGGTTCCAC
GTCAGACTCAGAAGAATCAAACTCCATTCATTGGAACTTGGCGTTCAGCGGCAATTCAGTCGTCCTGA
GAGGGGTGCCAGTCTATAATTCTCAGTCCCTG
GTTGTTGGTTGCCACGTGGATTTTGGTGCCCACAGTGCATCGTTTACTGTCAACGGAAAGTCTCTTGG
GATTGCCTTCACGACGTTACCATCGCCGGTTG
GTCTCCGACCTGCTGTGAGCCTGCAGGCCTCAAACCGAATTACGGAGGTCCTCCAGCGCGTAAAACCT
TCCGCTGGGGTCATTGTACGTTTTGTCTTTGA
TGAGTCACATCTGCTGGCTAGCCAAACGGTGACAACAGAGATGAGAAACACTTTTTTGCTGAATTCTT
CTCCTGCAACGCGGGTGGGAACGCCAGATGAG
TCGGTTGAAACGTGCCGTGAGTTTATTCCTGGAGTATTTCCCCTAAAATTACCCCTGCTCTTTGACGA
AAACATGATGCACTGTGCGCTTATTCGGCTTG
TCACCTGCGGCGAAAGCGCGCTCCTCAGCGGCCATGATAGTCACGAGCTCATTCAAGCTGAACGAGAG
ACCCTTTCACTGGGGCACACTCTGCGTGTCAC
GGCCTCGGGTGTTCGTCAGCTGTTAAATGCAAAGTCCGATAAGGAGGATCTGTTATTTTTTCCTCCGG
TGCTTCTTAAGCGACGCAGTGACGATACGAAT
AAAGGTGCCATTGGAGTCGCTATCATTCCAGAGGATGTTTCCTTGGTGCCTTTATGCATCGCACTCAC
ACAGCGCCAGCGTGTTGCGGCGTATCGCATTG
CCGTACACCCCTTTACAGATTTTGTAAGTCAGGACGATGCAAGTAAACGTCAGAGACGCATGGCGCTA
CTCGGTTCCGCAACACTAGGCTATACGGAGGT
TTTGTACGCCCTTCTTGAGCGTATGTCTATTTCAGAGACCCTCAATTCATTTACACTGAAAATGGTGG
GGAATGAGCCGGAGTATTTTTTAACAGCAAAG
CAGATGCGAGTTTTTTCTCCCGGAAACTCTCGACAGTGCACTTCTGCAACCATGCTACATCGCGTTGA
GTACACCCCGCTCCATTGTGCTCTTCTTGAAA
AACACCAGGAGTGTGTGCATCTGCTGCTGTATTATCTGAGTCGATTACTTCCAGCGGAGCATAAGCGA
CACGCGGTGAACGTACTAACGCGTAGTGGTGA
GACGGCACTTCTTATGGCATGCCGATTTGGATACACAAAAATTGCAATGCGACTTCTTGCAATGGGAG
CTTCACCGTCGAGTTTTGATCGTGTGACGAGA
ACGAATTGTCTGGAACTTGCCTGTGCCAGTCGCAGCGAGGAAATTGCGGCGGCCCTCTTGCAGACGTC
ACATTACCGCTCGCAGGTGGCAGTAAATTTGG
CGGGGGTGGCACCCCCAATATGTTGGTGCGCGCTAAACAATATCGGATCCCTCATACCACCGCTCCTT
AAAAATGGTGCGAATCTCAACGTGACGCTGGA
CGGTCTAACGCCGCTGCTTCTGGCCGTCACGTTTGGTAGTCAAGAGGCCGCTCTTAAACTGCTGGAAT
GCTGCGATGCCGTCTCCAATGTGTCCAACTAC
AAGAACAGTTCAACTCGCACCATCCAACATCGTTCACCTAAAAGAGAAACCTTTCATGAAGACCCTGC
CGAAGAGGGCGAATCTCGATTTCTTGCCGTGT
TAGACGTTGATGCATTGGACCCACTCACACAGAGTAGTGCGCTGCATCTGGCGTGCGAGCTTGGGCAA
TTGGAAGTGACGCGAGGACTCATTGCGAAGGG
GGCCTCATTGAACCTGCAGAACAAAACAAAGTATGCAACCCCGCTTCAGGTCGCCATTATGAACAGAA
ATGAACACCTGGCACTTGAACTCTTGGAGTAC
GCGAAGGACAAACTTCGGCGTGGTTCCAACGTCCTTGATATTTCAGCGATTGAAAAGAATGGAAACAC
CGCCTTGCATCTTGCCGCACGAGAGGGCATGC
TACAAGTGATTGAATACATCATTTTTCAGTTTAGCGACGAGGAAATTACACGAATTTCCTGCATTCAC
AAATTTTTGAAACGGCCCGATGCGGTAAATAT
TGTGGAAAAGAATCGCCAGGGCATGACACCGCTTCTTGTTGCAATTCACGCGCATCAGGAGGCCGCAG
CACAACTTATTGCGTCACTCATGCCAGAGTCG
TTGCCGAACCCAGGTGGGCCAGTGATTGATGGCACCTGCGTGGCGATCCTGTCGAGCGACGCGGAGAG
TCTTGACAATTTAACACTTTACTTCCTTTCAC

AGCCACGTTACTTTGCCAATGACGCGTTCCGTGAGGAGTTTTTCACCCGCTACAACGCGAAGCGGCGA
GAGATGGACCTTTGCGAGGATGGTGCGCCCGC
CAAGGTGGAGGGAATAAAATCCGTGCTTTATCATGACGGGGAGAAGAGAAGACAGCAGAAGAGGAAGG
CACGTGAAATTCAGGAATCACGGAGGCGGACA
CGATATCAGAAAAAAACGAGCGTCTCTTCAGCACTCCTGTGGCGGACGAGTCTCACGCCAGAATCGTA
TGCAGGGAAGTTGGGCGACCAAACCGGTACAC
AAAAAGCACCGCCGTCACGCCGTCGCAGCACGCTACACACCCGGATATCACGGACAGGAAGGCATCGA
CGAAGAAGTACATTTGTGCAGATGTTGCTTGC
CAAAGGGGGAAGTGGTTTCACCACATTGGCATCTATACGTATTTTGGAACAAGGATTTCCGCTTGAGG
AAGTGTACGTCATGGCGGACATCATCTCCACC
GAGACCACGCACTCGTCACAAACTGTGAGTGCCATGCAGCATGTGGAAACACTCATATCTTTTTTGCG
TGAATATGGTGGAGTACGCATTGCCACGGGCG
ATGCTGTTGTATTTTCTCGACAACTTCTTGCCACTCCGTCCTTGGAGAGAATGATGGACTCGGACCAG
TTAGAGAATGCCAAAAAGCGCTTGGCGGAGAT
GCGTCCGCTTTGCAGAGACTTGATGTCTATTATACGCTCCTATGGGCAGCGCGCAGACTGTGTGGAAG
ACTTGCGCCACATGGTTGAACAACACGGCGTT
ATTGGTACCGCTTTTGTGGAAGAAGTTACAAGCCCTTTTGGGTTCACGGTACTTCAGCTGGCTGCCAC
CCTGGGTCTATCGCATGTATGTGAATTTTTTC
TGAATGAATATGAGATGAGTCCATTGTATGTACCGGCAACAGGCAGCATTCCTTGCAGCGAAAGCAAG
TGGTTTCTGTCGCCGTTTCGTCTCGCATTGCG
CTCTGCCGATGTGGACACGGTTTCGGTATTTCTCTCTCTTGGCCTTCGATTGGGTTCCATACAGGAGA
TACTGGAGCACAAGGAGCTTCCAAAGGCGGAG
GAACTGCAACAGACGGCAATTCAGGAACTCATTTGCCAGCCAGTTGATACGCTCCCCGCTGGAACTAC
CATTGACACACTGCACATTTTGCGTCTGCTTC
TTCAGAGCGGGGCCGTTTTGCAGGGCAATTTTGATGCCGAGGGAAACGATGCATGGATGCTTGCTGTG
CGTGCGTGTCCTGGAAAAGGCACGAAGCTTCA
GGTCTTTTTGGATGAGCAGGTACCGCTCGGTGAAGAGGAAGAAGACGATGTAGTGGAGGAGGAAAGCT
CGCAGGAAGTCACAAGACAAAGTTCGCTATTC
ATGAAACGTGACTCAGTGCAACGAGGAGAAAGCAACAGCGGCATGTGGACAACGATTTCGCCGGATGC
CAAGGAAGGCCTGAAGTTGCCACTTGCGGTCC
CATTTGTTGAAGCAATTGCGACATCTTCTTGGATGGACTCCCCCTTTAGATTGGAGGGTGCTCTCGAA
TTCCCACGCATGAGCCCAAAGAAAAGGGTATA
CTACACCCAATTGGTCTTTCTGTGTGCTGAATATAACCCGCAGAGTCTCATCGCACTGCTGGAACAGT
ACTCATATGTTCTTTCTCCCTCCGTCATTCAC
CCTGTGACGGGTGATACTCTGCCGATTTTCCTCCTGCGCAGGGCGGTCCACATCTACTCAGTGGAGTG
CGGCAGTCATGCCTTTTTTCAGGGAAACGCAA
GCTTGGGGGAGCTTTTTTTTTCCACGCGTTGTTTTTCGTTGGATGTGGATGTGAGTACACGGGTTTCC
GAAAAAGATCCCGCGCCGTACCGTGACCCAAT
CACCATCCCTGCTGTTCGTCGCCTATTATTGGTCGTGGAACGATTGCTGCGAAAATTTCGCTTTGATA
ATTTGTATTATGAGGACAGTGATGGGCAAACC
GCGTTGTCATTGGCCGCACGGTTAAGCTACACCAATATTGTCTCACTAATAGTGCAGCAGCGGGTTTC
ACGGACCGCGGGTGGTGCGGGTGCGCGGAATG
GCGGGGAGGAGGAATTCAGCTCTATCAGTCGTTTAAATACGGCCAATGCAAGCTCCGACAGGTGTGCA
ATAAATGAAGATCTCGTAAGAAATTTGGAGGC
CTCCTCCTGTTGGGCTATACTCGCTACACGTCTCTACTACGCGGAAGAAGAAATGGATTTGCTCCTTG
CCATTCTCAGACATCTTCCAGCGGCGTCCACA
AAGTTTTCATTCCTTTCGATGGTTTACTCCAACCTACATCCCATCGTGGCTCTACGAGTTGCAGTGCT
TTACGCAAATGACGTGAAGCATATTCTCATGC
AGCCAGAAGCCATCAATGCTTTCTGGTCCAATGTTCTTTATGCCCAGTTTGTGGGCCGACTTGAGGAG
GTGACTGTGACGCCAGCAGAGTGGACTTCGCT
TTTGAGTGTGCTGCTTCCCGCCGCCTCGGCCATTCCAATCTATTTGATTAAATCCACCCCATTGCTGC
CAATGAGTTCAGCGTCCGATATTTCGTCCCCA
ACAACGCAATCCCCTCTTAGTTTTACCGATATTGGAATGTCTTCACCGGTGAAAAATGAAAAGACAGA
GACATCTCCCAGCTACAACTGGAGATTTATTG

TAAAAAAGACAAGACAGTACGTTCGTCAGCTTGCAGCAGCAGCGGTTTGTGTGGTGTCACCACCATCT
GCCATTGCGGCGGCTTATGGTGGTGGGGCAAT
TCCAAGTTCTCAGCGTCACGTGCTTACAATCATGTCGAGTCATTTTCATGAGTTAATTGAATTGGCCG
TACGCTTCGATAACTCTGCTATGCTGATGGAT
CTCATGAGCTTGTCTCCAGCAGAGCTACAAGCCTGTGTCAAACATGAATGGCGTTACTTAATGGAAAA
GTATCACATTGGTGTAATTGCTATTGCCGCAG
GCAGCATTAATGTATTGAAAGCTATTTCTGATGTTCCCGACGCGGCTGCACTCTTAAGTGCTGAGCAA
TATGAACGCATTGACCCCAATACTGGGCTTTC
GGAAGATGCGGGAGTGGCGGACGAGGCGGCCCCAGAAAACCAAAACACATATACAGGGACCTTTTCTA
TTCGTGGAACACCACCGCCAATACGTGGGGAT
CGAAAGAATAGTACATTTGGCATTCACGCAGCACGTAAAACCCCCAATAAAATAGCTGGTTTGAACGA
AAATAAAGGGAATACCAAGAAGGCAAGGGAAA
ACAAGAGTGCAATGGTGAATATCCTTGCACTCGATGCAAAGACGCCTGATCCGGGTGTGGAAGGGGAA
AAGGGCGATGTTGACAAGACTGAAACCAACCT
AAGGTACATGGATGTGATGACGGCGGCTCGCGAACGACAAACGGCTTCGGCGGTAACATGGGGCATTG
GTCCAGCCGCACGACCCGTATCATTGCAAGAA
AGAACAAAATCAATTGATCGTACGGCGTCCCACACACAGGCCTCTGCTTCATTTTCTACGCGAAAGAA
AATGAGTGACGGCACAACGGACATCGGGGCTC
CTTTACGATCACACGCGCAGCGAGGCACAGAAAGTACGCTCTCTACGGCGTCCGTGCAACGAAAGACA
CTCAGAAAATCTGTCTCGCTGGAAGGGACAAA
AAGCCTCAACTTGTCGCTGCGCCAAGGGGGAACGCCGTTTGAGACAACACCCCCGCCTCTGCCACCAC
GGTACTTTGCGTATTATTTGTGTGACTGGGCG
CTTCACACGACCCTTCTCCTGCACGCACCGCGGCCGTCGAGTCGGACGATTGATACCCTGCTTTACCT
TATGGATCAAAGGGCCCCCCTCACAACTTCGG
CAGTACAACTCTTCTTGGCAGCCTCCCGACCTCTGAATTGCTGGGAATCAAGTGACGGCGAAATGATC
ACACTCAACTACGTGACACGCAAAAATAAAGA
CACAATACTACACATTCTGGTACAGAATGGCCAGCTTCGACTTACCCGATATTTTTAGCGGCCGCCT
TGTTTTACTTCACATACTATCAATATGATCCA
CCGAGTACGATGCCACCAAATTTTGCATTGATAGATATGCCAAAACTACCGCAGAAAACTCTTTCCCC
AGAGTTGCGCGGAGCAATGAAAACTGGAGACG
TGGATGCATTGGATGATGATGAATCACACCCGGCCGTTTTCTTACGCAGCATGTTGCGAATGAATAAG
CATGGCCTTACACCTTTTGACTACGCCCGCGG
GCCAATGGTGTCACTACTCATGGAATACGGTTGCGTCCCGCCTACATACCGCCCGAATCCCCGTGGAT
TTTGCCGCGCAATCCGATTGCCCTTTCCCGCT
TCATCTTTCTACTCCGTCCCTCGAATCCTATTGGTTTCTATGAACTTTATTGAGCTTCATGACGAGGG
AATACCAAAACCACGAAACCACATTGCAGAAC
TTTTGGCAAAAGAAGTCGCCGATGCCACGTTGGTCAGAAATCAGGCGGCTAGTCAACGTACGACGAGA
ACGGCTTTCCTACCGCCGCTTCTTTCAGGCAA
CGTGAGTCTCCTGCATCTGGGATTGTGTTCTGCTGATGACGAACTTGTGTTGCAGCACATCAATGAAA
AGCGTAGGCAACAATTGCGAATTCTTCTTTTG
TCGCAAATGCCGCGGACTTCACCAACCCCGTACATGATGACGCAGATGTCGACTTCTACTTTGGGATA
CCAGCGACAGGATAAACTGGTACATTCTTCCG
TCTCTGGAACGAAAGATTTAAACGGGCAAAAATCCTCCACAACACGTGGAAAGGACGAAAAGGAACTT
TCAAAAGCGTTGAAGCTTGTGGATACTCTTTG
CGATTGTGGCTTTGTTTTATTTCCCCTTGTTCTTCCTTTGGACGTTGAGGCAAGTCAAATAATTGAAG
AGGACGCGGTAAATGAAGGCACGGCAATTCTT
CTCACCATGACCCCTATGGCACTTGCAGTGAGTGCAGGCGTCGTGAAGAAGGATGAACATTCCCAGAG
TAGTTCTCCCATGATGAAACGGTCGCTGAAAT
CCCCCGATATTTTTCGAAGAGCTTCTAAGGATGGGACTGCAGCCGTTCCGCCACGTTTCACACCAGCT
CCAGGGAAGAACCGTCTGCCTGCAGACACTCT
CGATGCGTGGGTAGCCTCCCGCCGTTTGGCAAGGTCAAACGCATCTGGAAAGGACCCGCACATGCATC
TGCTGGTTAGGGCGATTGGAAGTGGTCTTGTC
GGGAGGACGATTCACCACCTCGGTCTTTCCACAGCTCCGTCTTACGAGTCGCACAAGTAGCACTTGCA
TTGCAACGCAGGGACATTTTGCTTGGATGTAT

TGGTCACATATTTTTCCTTCTCTTTTGGAAAGGGGAAATTGTGAACGAAGAGGACAAATAAACAAACC
ATGTATACTACGCCTGTTCCAATTTATGTTTA
TTATCTTTTACCATCGGTTACAACTTTTTTATCTCCGAGGACCATGGCACCATTACACTTAAATGTTT
TCATTGTTGTTGTTGTTTTGACTCCCACGGGC
CTAATCATCTTATTTGAAGCAAAGCATGGGTATTTATGCATGCGTATTCAATTTGTCCCTGTCCTTTT
GTTTTTCCTTCTTGTTTGTTTTTCTCTATGAG
CCATGTAACAGAAGTGTCACATTGAAATAGCGGCGTGTGGGAGCTCTTTCTATTTTCCGTGCCTAATG
AGTTGCCCGACTGGCGATGAAGCGGGTAACTA
AATGAAGAACATCCTTCAAGGTGGATAATACACGCAAATCCCTTATTGACTATTCATGGCGTGGGAAA
TGTGTCTGACGGGAAACAGGCGCGCCTGTGCA
TTCGCACCTGCTTGGTAGTTTCAGAGGGGCGGGAAGTGTGGCGTGCGTCATTTGTGTTGGACATCTTG
TCTCCGTCTTTTTTTTTTTTTGAGGAATTAAGA
AGGGAATGTGTGTGTGTGTGTGTGTGTGTGTGGAAGGAGGTTAAAATCTTCCAATTGAGTTGCAAATGTT
AAAAAGAAACGCAAATTGTTTTTGGCTCTTTT
TTGCGTGTTTTTGCGATGGTGTTTTTTTCTTCTGTATATGTGTCTATGTGTGCTTTTGTGCATGTGGT
AGTTGTCATTCTTCCTATGTGGTTTGTTTCGC
TTGTTCTCTGTTTGTCTTTTGTTTGTTTTTGTCTCTCTGAACACATGTGTGGGCAGCACGGAGCAGGG
AATGTCAGGAGAAGCGGAGGGGAAAAATGGGG
CAAGTGAGGAGACGGCAACGCCGCATCACACCAACACGGTGGATCACAGCAACGCAGTTACTCGGAAG
CGGCCACGTGTGGCGTGTCTGCGGCAGAATTT
TTCCCGTCAATTGTACCAAATGATGATCCGGCAGTTACACCATGATGGGTACTTGGGGGCAGCCGCGG
CGGTGGCCGATGCGACAGGAGTTGTGGTCCCA
CGGTTAGAAAAAGACAGCCAGCGATTGTCAAAACTTGTCTCGATGGGCCTAGCGCACGAAGCAAGCAA
CATAACTGAAATGGAGAACTTCATGACGTATG
AGGTGGTGGAGCGCTACCTCGGTGCCTCCAAGTTGTATGTGCCGCTGCACCTCACGGAGAGTTCGACA
GTGGGCCAAATGGCATACCGAATGCGGGAACG
GTTTACGACCTCTTCACTAGGCGGGGTTGTGCGTCGTGTCAATATATCGCCGGATGGTTCTCTGGCGG
CCTGTGGTGGAAACAACGGGCTGTGTGTTGTG
TTTTCACTCAAGACGATGGAAGACCTGATGGCGCTAGAAGAGGTGCGACAGGAAAATCAGTTCCGTGG
TTTGGACGGCAGTGGTGGGAGTGGAAACGTTG
GGGTTCGTAACGCGAGCAATAAAATCACTGAACTGGCGGAGGCACGAAGATTCCACGAACACACTCAG
TCTGTTGAAGCGATGCACTTCCATCCGTCACG
ACCGCTCCTGTTGACAGGCGGACGTGAGGGGGATTTGTACCTGCGTGACTACAGTCAACCCAATAATA
AGGTGTTACATAAACTGCATGATACATTCCCC
ATCCGATCGGCCGTTTTTCATCCCTCGGGTGAATACATACTTTACGCCACGGACCACACAGCTCCGAG
ACTTTTGAATTTACGCACATGGAAGTTGATGA
CACCCAATACAGTTTCCGGTAATGAGAATAATGCGGCGGCTACCAATATAATTACAGGCAGGAGTGGA
ATGTCGAATAGAGGAGGGTTCTATAGAAGAGG
GACAGATGATAGCCACACAGCTGCTCTGTGTGATGTGGACTTCTCTTTGGATGGACGGTTGCTTGCTT
CGTGTAGTCTGGATGGTTCGCTGATTGTGTAC
GATGGCGTCAGCAGCCGTCCGGTGGCCAAGGTAAACAATGCCCATTCGAGTGTACCTGTGACAAGTGT
GAAGTTCAGTCGCACAGGCAATTTTATTCTTT
CTGCAGGAATGGACTCCGTTGCCAGACTCTGGGACTTGCGTCGTTCGGATGGTGGGTGCGGCACGGCA
GAGGTGATGTCATTTGGTGAGCCTGGAAAGTG
CAACCATCGCAGCATCCACGCCGCGTTTAGTTGTAACGAGAGCCACGTGCTATTGCAGGACACGTCCC
TCTTTGCTATCCATTCTTACTGTGTTTACTCT
AGTGATAAATCGTACTCGTTGGTGGTACCGAATCATATGCAGCGTGGATTTGCGGTAGCACCGTTTTG
TAACGTCGTGGTGAGCGGTGGCGATGATTGTC
GTGTTCGGTTGTGGACACCCGCGTGGGCAGCCGCATGAGATCTCCTGTCAGTTGTCTTTTTCCTTTTT
TAGAGACGGTGGTGCGTTTTTGTTTTTGTTTG
TTCTTGGAAGGTGGCGTTGATGAATGATGGTAACTGATGTGTTTAGACGGAATTAAAGGGTGGCTGGA
GGGGAAGAGGAAAAAGAGAAACAAAAAAAAAA
AAAAGAACAGCGGATGGCGCCGCTCTGTGCATCGAGCAGAAGAAAGTTTTTCTCCTTTACCTTTCCCC
ACGGCTTGTGTGTCCTTTATTATTATTATTAG

TATTTGTTCCTGCTGTGCAGAAGCATCACTCCGCACCGCAATTTACTCCGTTTTTCTTTTTTTCTGCT
TCACCACACACGGAGGGAGAAAAAAAAAAAAA
AAAGGGGGCCCGAAGGAGGGAGAACACGGTAGAAGTCAATCAACGGGCGGGGGAAACAAAATACGTCA
CACAAAAAAAGAAAAGACAGCAACCCTAAAAA
GAAAGAAAAAGGGAAATCAAACCTTTGTTTGGCTCATCCCCGTTTTTTGTTTGTTTGCTTGTTTGAAT
TTGATCTTGGCGTGCCGTGTGGTGACGGTGAG
CTGCCATTTATTTTTTTTCTTTCAGGCTCACTTCCGCGCGCCTGAGCAATGGCTGGCCTTACATCGGA
CAACATTTCAATAGCGTCCGCACGTCAATACG
GCTTTTCAAGGGAGCGTGACTTGTGCGTCTATTGCGGGAAGAATTCAAAGCCGCCGCCTACAGACCCG
TTTGCGCCAGTCTTTGTCTTCTTCTCCCTCGT
CGACTGCCGCCACTACGTGTGCCAGCCGTGCGCCCTCGTCAACTGCGACAACGCTGGTCGCTACATTC
GCTGCCCAACGTGTCACTCCATCTCGCGTCTT
GCGCAGACGGGTAAAAAGCGTACTGGACCCGACGAGTTGCGTGTGACGATTGACGATGGCGTATCCTC
TGCCGCCTCACACGCATCACGCCGCTCGGTAC
GTGTTTCGGCAGACAACGTGCCGGCACGAAGCGCCCTTGCGAAGGCTGACCGCAGCAAAAAACGTTCT
GCCTCTGTGCAGTTTTCGGCGAACCCGACGAC
GTCAGTTGTGCCGCCACCTGAGGACAGTGAGAACAAAAAGCGTAGAGATGCTTCTGTAACTTCACCGC
TGACGCGCGATGCTGTTGGAAACCTGCCAAAG
GATCCAAGCTATAGGCAGCGCGAGCGTATGAAGGAGGTGGTGACGAGGTCGAACACCAGAGAAACTGT
TGCAGTGAACCTGTACACCATCAAGAGCTCCT
CTGTGACGCGGCGTCGATCTTCTGGCAGTCGTGCGAGCAGTGTGCCGTTGCCGCCGACTGAGCACCGT
TATCTCGCACCACCGATACCTTTTGCGCCCCC
GCCACCACTGCGTATTCGTACAGTTGAAGAAGAAGAAGAAGAGGTAGGGGAAAAAGATGTGCAACAGG
ATGTGCATGTGTCTCTTAGCGCTATTGCAGCC
GAGATTGAAGAAAGCCTTCTTGCTGCCGTGGCGAAAGAAGCCCAGGAGCGCTCCACCATTTCAATGGC
GGAGGAATATCGTCGCAATGCCATGAGTAAGC
AGCGTGAGATTCGGGAAAAGGATATGCTTGCAGCACAAGTAGATATCACGAGCGTGTTAGATCTTGGT
GTCAGTCCCCGTACCCCTCTTGACGTCTCGGA
GGGACAAGTCAGCGCGACGTCGGATGATGAGACTCCAGCAAGCCTTCTCAAGAGACATCCTGTTGCTC
CACTGCTGTACGATGAAGAAGAAAAGCAACCA
GTTTCAGTAATTGCATCGGATACAACAATACAACCTGAGGGGACGCAGACAACTGAAGTGGTTTTGCA
GAAACTTGAGGACACTGAAAACAGTATTGCAC
ATGAGAAATTGGAGCCCAAAGAAGAAGTTGAGGAATTGAAACAACGTGATACTCAACTTTTGCAGCAT
CAATTGGAAAGTCTGCAGTCTGAAATAATTTC
AGCAATGGAAATTTCATTTGGGATTGGTGAAATTGAGGTACGAGAACGACAAATGATTGAAGGTGTGG
AAGGTATGGAGCGCCGTCTGTTGCATAGTTCC
CGTGTACAAGCCGAGGTGGAGATAAGTGCGTTTGAAGCTCAAAGACTGTCTGCCAACCGTGCAGCGCA
GGCGGCAACAAACTATATTGCATACATCTCCA
AGGAATTTGACGAATATGCATCAAGTGAGATGGAGCATCGTACTTTCATTGAAGATATGCAGCGCTCC
GATTGGTCAACGCTGGAGAGGGATTTCCATGC
GTCTTTGAAGAGATTTTTTGAGGTGGAACTTCTTCGTTTGACCGAAAAGCATCAGATGGAGCTCACGC
GGGCGTCCATCACACTGACAACAGAAGAGACA
AAGGCTCGTATGTGGCTGGAGCAAAACGCATTGGATGATCTCACGCGCCTCCACATGAATTCTATAGA
GGCGCTTGCTGAATGTCGTCGTCAGCTTCAAA
TGAAAGAAATGCAGCGTGGGGTCTCGCGTATTGTGGCGGTGCAGCAGCTGTACATGCAGCACTTGTAT
GATCTTGCGGTGGAGGAGGACAACAAGCGGTG
GAAAATCCTTGAGGAAGAACAACGCGACCGTCATGCGGAGTGGACGTCTTTTATTACGACGGTACCCA
CTCTTAGGACGGCAACGGCGTCCCCAAGTTCC
GTCACATCTGCATCCGTACGCGGGGCTTTGAGCTCTTCTCATGCTCCCGAATATTCTCGCACTGCAGC
AACGGCGTCCCTCTCTGTGGCGTCTCAGTACT
TTGCCGCGTTGGTTGAAGAGGCGGAATTAGAAGCACTGCAGCTGCAACATATGGAGCGACACGAACGT
CACGCTCTCATTAATCTTTTTTTGGCTCAGTC
TCAACGCCTGAAGGTCTGTGAAGCACCCATGGACACAACATATTCCGATACACCACTCTCACAGCGTG
CAATTGCTAATAGTTGGCGACGTCTGGAGCTT

CGAGAAGAGGAGAGGCGGATTGAGCTGCAACAGCGATCGTGGGCCTCATTTCGTTCCATTGTCAAAAA
GGCAACAGAGGAGCATGAACAAATCGATAGAA
TGGAAAGAATGCTGCGATCCATCGCGGAACGATCACGGAGTGTATTAAATCGTTGCTTTGGTGAAGTG
GAATTGGCTCTGATGGATGAAGAAAAGCATGA
GCGTCAGAACATAGAGGTCCAGGAGCGCCGTGCACGCATGAATCACTCCCGCGAGGCTGTGAGACACG
AAGAGGTGCTTCTTGCTGGAGAGCGCTTGCTT
CATGACGCCTCTGGAACGCGTTTTTTGGATGCAATGGACGTTGCGGTACGGCAATTCTCTTCGGAGGC
AGTGCGTATCGCACAGGAGGAACATCTAGAAC
GCCTTTCTCTCGTGCAGGCAGAACGTTTTGAATGCTTGTCACTTACAGCGCCTCTCGAAACACAGGCC
CGGCGAAGCAAAAAAGATGCAGTGGAAAAACT
TTCAGAACTTCACTCTACAATAGAAAAAACAACGGCGGAGGGTGCCTGGCGTGCACGTCTTGCTCTTG
TGGAAACAGAGGAGCTTCGTGTCCGGGACGGT
ATTTATAACGATGAGGCGGGTAACCGGTCATGGTGGGTGACGGACGAGCGCCGCCAAAGGATGGAATT
AACATCTCTAGAAACAGAACGGCTCAGGCGAG
CTAAAGAGGTTGCGCGGCTGCGTGACAAAATGGGAGAGTGCGAAAGTGTTGAGGAGCAACGCCGTATT
GGAGTACAAAGGGAGGAGGCAAGTGCATGGGC
AACTCTCGTGAGGGAACAGATAAGAGATGCGGCCAACGCATACAAGAAGGAGGGACAACGACGCATAG
AAGAAACACGTCGTGAGCGGTACGCCGATCGG
TCACAACGTTGTAGACGCGCCTTGGAAGAATTGTTACAGGATGAAGATGATGATCGGCATTACATTTT
TGAGGATGAGCGGGAGGCACGTAGGCGGCTAC
GGCGTCAAGCTCTTCAGCTCTCGTGTGACATTTTGGAAAAGAAGAAAATTGAAGAAAACATCCCGAGT
CAGCCAGTGTTGGCATCTCAAGGCATTGGACA
TGGCCCTGTAAATGACCCCTCAGCCGTCACGGTTGACGAGGAGAAGATAAGGCAAAAACGGTTAAAGG
AACTCGCACGTGAGGAGGAGTTGATTGAGGCG
CAGACGCGTCTAAGGGAAGCGGAGCTCCGTATTGCGGAAGAAGCAGAACTTCGCGCCAGGTCGGAGGA
AGAAAAACGCGCGGCGCTGGCGGAGTCTGTGC
GGTTGCTGCGTGAAGCCGAACAGCGAGCAGAAGAACGCATCCGCCGGGCACGTCAAGAGGCAGAGCGA
TATGTTGAGCAGGAAGTGAGTCGATTACGGGA
TGCAAATGAGCGGCAGGCAACACATGCGGCGGCGCTGCGGGCGATCGAGGAGGAAGAAAAAAGTGCGG
TGGTTGAGGCGAAACTGCGGGCAGCGGAACAT
GCATTGAGGGAAGCGGAAAGACGCGCTGCAGATGATGTCCGTCGTGCCAAGGCAGAGGCAGAGGCATT
TGCGGCGGCAGAGGCGCAGCGTGTTGCCCTCG
AGGCGAAGCGGCAGTTGCAGGAATACCGAGAACAGCAGGAACAGCAGCTGCGGCAGAAGGACCTCGAG
GTTCAGCAGAGACTGCGTGGTATTGAGCAACA
GGCGCAGGAAGCCATTTTGGCGGCGAAAGTGGAGGCAAGTCGCCTGGCGGAAGAGGCGAAACGTCGGC
TAGAGGAGTTGGAGGCCCGTCGCGTTGAGCGG
GCGGCGGCTGTTGACGCCGCCAAAGTGAGTGTGCGTGCGGCTCAGCAGCGCATGCGGGAGTTTGAGGA
ATCTCGGGAATCAACGCCTCAGCAGCCGCCAT
TGGCGTGGCCCGTGGAAACAGCAGCCTCTACCGCCGAAGCTGCCACGGCGGCGGTGTTAAAAGGACCA
AAGGAGGAGGGGAAGGATGGGTATACCCTCAC
TGCGGTGCTTTGCACCAATGTCATTCGGGCCGCCATTCGTGGAGCAGTGGAAGAAGTTGTGAAGGCGA
CAAAGGAAGCCTGGTATCTATCCGAAGAGGCG
GAGCACAGACTACGCACGGAGCGACGACGAATGCGGAGACGGCAAGCAGAACGCGATCGTGCGGAGAA
GGAGTTGATTAATGTCGAGAAAAAAAGCATTC
TGTGGGGAGAGGAGGAAGAGCAGGAGGAGGAAGAGGAAAAGGAATCCAAACATCGGGTTAACACCGGG
GATGACAAGGAGGCTTTCAGTACACCGCTGAA
GCAATCGACAAGGCGTCAGTATCATAAAAAACGAGAAGAGAAACAATCAGTTCGTTTGCAACCTCCTG
CTGATGACGGTTGCGGTGTTTGCTACCGAAAA
GATACGGTAAGCCCCTGTGTGAAATGTGATTCGCAGGTGTGTCTTTACTGTGGCCCCGCTGCTGTCCA
CAGCGCATGTTGTGAAGAACACCACATCAATG
TTATCAACGCCCACCGCCGTCATTTGTCAGGGGAGCCACAAAAAGAGAAGCATGAAACCAAAGAAGTG
GAAAGAGAGAATGGAGATGACTCATCTTTGTG
GGAAGTGGAAGAAGCGGTGGCCGTGCCACGAAAAGATGAAGTTGGGAGGTCACCGCTTGGTGTTCGTC
GGAAGTTGTTGCCTGACGACTATGATGAAAAA

GAAGAAAAGGAGGATCAGGAAAGCGAAGCGAAAGAGGTCAAGTCACCTGGAAGCGTGGAAGAACCCAG
GACAGTGCGAAAGCGGTACGAGGCCTTTTTTG
TTCCGTTATCCCGCGATGCGGAGCCGAGGCGACCGAAGCCGCCGACGCCAAGAAATTACGTGCCGGGT
GTCTTTGCCGCAAAGTCCAAACAAAAAAAGGC
GGAGAGGAGGTGCAGCCCGCCCGTACGGCGTGCACGCGCCAACAGACACATGAGCGCCGCTGCTGCTG
AGGAGTTGCAGCAGTTGCAGCGACGATTCCCC
AGGAATGGCCTCAAGGAGCATCTGGCAGCAGCCCACAACAACCAAAAACAACAGCGCCAGGAGTACCC
ACGCCAACCCTTCGCTTCGTTGAATCCACAAT
TTAGGGAGCCGCCCCCTGTCCACGCTGACTCGCGGCTGCGCAGACGCGAGGCCGAGCGGGTCGTCATG
GAGGATCCGCTTTCTGCCTTTGGAAGGGCACC
CTCTCCGAGTCATTTGGGAGTTCATTTCCCCGCCAAAACAAGACGCCCATCGCGACGCATGATGGGGC
GCATTTCTCCCTTTGAAGCATCAATTGAGCTC
GCTAAGGGCTTTGACGACTTTGCCCACCGCGACCCTACCGGCCTTGAGCGCACTTACTATGATGGAAA
GCCTGTACGACCTATTAAACGGGATTATATGT
CTGATGATCTGGCAGACTTAAGCAACCGCTCCAGTGTACGAAGGCAGCATCAAGAACAGTACAGATCA
CGGGCAATGGAGTTTAGAGAGGAAGAGGGACA
TGTAAACGTCAGTCGCGAACGCGTGTTCGTTACGAGTTACCATGAAGTGCCTCTTTACATGGGAACAC
AACGCCATCACGAATCGTTGCCGTATAACTCA
CATCAACGTGAAGAGTGCAATGTGCGCACCACGCCAACACTACATGAGTTAGATCAGCGTCTTCGGCA
CCTTCGACAGCACCGCCTCTTTGATCGTGTTT
CTCAACGCTCCCCTGACCGTCACTGTTCACGTCATCACTTCAATCAGGAGGGTGAAATTTACTGCGGC
TGCCATAATGCAGGATCAAGTAAGATACGACG
GCCCACACCATGCTCCCATGATCCTTGTATCACCGTTCACCTCTTTCCTGACACCCGTATTCCCACAA
CAGCAGCCGGACCAATGAAAATGACACGCGGA
GGAAGCAGCCGGCGAAATACCAGCCGTACTGATAGACGTTCAACGCTTCGGGTTGTATCACCGCCATG
GCGAACAGAACTCAATTCTGGACCACTGCGAC
CGTCGTGGAGTTTTGAGCAGCCGCACTCATTTATAACGAAGTCATACAAACGAACTTTGTTGCCGGTC
GAACCGTGAGTGAGGGAGAAAAAGGCATCTCT
CCACCTTTTCCTCTCTTTCTCTTTCTTGCTTTCTCTCTCTCTCCCTCTGTGCGGCTGTGTGTAGAT
GTACGCACACGGCGTGTGGTGGTTTTTTGTTT
GTGTGTGTATAGGATGTGTGTGTGGTACGCGAGAGAAAGCAAGAGAGGAACAAAGATATAGAAAAAGA
AAGCCGGTGTTCTCAGAGAAGTGTGGGACTGA
CACATGTGAAACAAGGAGAAATTTATATTTATATATATATTGTATTCATCCCATACATTCCCTCCCCT
CTTGTTGTTGTTTTTGCATTTTTAGAAATATG
GGAAAACAGTGTTTTTGGCTCTGCCCATGCTTTTTACTCCCTTTCTTACCTAATAGCATTCCCTGTTG
CTTCTCCTGGTCAACGCCACGACCACGACGAA
GTTGCTGTGGCTGTGCGAGTTGTTCTGGTGCATGCACACCCAGCACGCATGCACTCCACTCTAGAGAC
AAATAAATAAATAAAGTGAGGGATGATGAAAT
GGTGGGGAAAAAGAGAGAGGTGGAGCAATTTTATTATGGGGTAAGAATCATAACTTTTCACAACGTGC
GTGTTGTGACCTCCGCTTGAATAAGCAGATGC
ACACGCTCACGCACTGAAGGAGAGTGGCATTTTTTTTTTTTTTTGGAATGTATTTACCTATTGACACC
AGAACTGAAAGGGAAAATGCTCTCCGTCTGTG
TTTTACGCCTACTGAANNNNNNNNNNNNNNNNNNNNNNNNNNNNNNNNNNNNNNNNNNNNNNNNNNNN
NNNNNNNNNNNNNNNNNNNNNNNNNNNNNNNN
NNNNNNNNNNNNNNNNNNNNNNNNNNNNNNNNNNNNNNNNNNNNNNNNNNNNNNNNNNNNNNNNNNNN
NNNNNNNNNNNNNNNNNNNNNNNNNNNNNNNN
NNNNNNNNNNNNNNNNNNNNNNNNNNNNNNNNNNNNNNNNNNNNNNNNNNNNNNNNNNNNNNNNNNNN
NNNNNNNNNNNNNNNNNNNNNNNNNNNNNNNN
NNNNNNNNNNNNNNNNTTCCTGAAACCTTTTTTTTTATATCTTTTGGTTTCTGAATTTTTTGGTGAAG
TGCATGATTCTATGTGTGCGTTATATATATAT
TCATATATATATATGAATGTACATACATGTGTATCTGCTCGGGCTGTACGTTGCTGGTGTTCTTCTGT
TGTTGTTGTTGTTTTTTTTGTACTGCGGCCCT
GTGACGAGTCGCTCTGTCTCTCATTATTCATGTGTGCCCTTCTTCTTCTTTGGCTATTCCCATATAAA
CACACCTATGATGATGTGTCCTGCGTACATGT

GTGCCCCTGTTGAAGTATACCCAAACAAAGCTATTCTTCTTATAGGCCATTCAAGAAACATATAAGAA
AGACATCAATACAACAGTAACAAAAAAAAAAG
AAAAAACAGTGTGAAAAAATACGTGATTGCTTTGACACACAAACAATCAAAGAAAACAAAGAGGAGAA
ACAGAAAAAAAGGAAAAAAGGTTAACAGACAT
CATGGCGGTTGTTCAGACACATTGCTTCAACTGGATGGACCATGATGGTACCAGGTCCGAGACGCTCA
ACTTGGCCCTGTTGCGCCACAGCGACACGGGC
AATCTGCGATACAGCGATTACCGGCAGCGGCAGGAGGTAGTGGCTGATATCAGCATGCGTCTGAAGAT
TATCAAGCGTGAGCTCGGCCTTCTGCCTCCGG
AGGAAGAAGTGGAATGGAACGAAAACAAGGGGCAACCGGAGGATCTCTATGTGAGATTGGACGACGAA
GAGAGCGAAGTTTTCTTGCAGATCCAGGAACG
ACGAAAGAGGCTTTTCGCACTCATGGAGCGTCCAATTGAAGGCACTGACGAAAGCGATGTGTACTACT
ACCCTGGCACGCCGGCAGACGAGATGCAAAGG
GAGGCCGCGGCTCCGTTTGCCCTTTGTGTGCAATCGCTGCTTGTCCAATTTGGCGAGACGTTTGTTTC
GTACCTTGACGGCAGTGAGTTTTTGACCCTGT
CAAAGCTTGTACAGCACAACGAGGCTCGTCGTGGTGAAGTGGAGGCCGCTGCCGAGGAGGCAGGCGTA
TTGCTCCCGTCGGAAAGCGAATTCGATGTGAC
TTCTCTCCACGCCGCACTCGAAAATCGTGAGCAGGCGCGGATGAAGCTCGACGCCGTGCGGGCAGCAA
GCGACCCGAGTTACGCAGCCAAGTTTGTTCGC
GCGGAGGAGCGTGACAACAGCGGTCAGCCCATCACGAACGTGTCGCCGGAAGACGGCACGGAGGCGCA
GCTCTCCGCGAAGTTGACAGCGGCGAAGGCAC
AGTTGGAATCCGTGCGAATGGCGTACAACAGACGAGCGCATGCCTCCGCGCGGCGCGTTCAGAAGGCC
CTCGCCAACAGGGTTCCCCCGGTGGAGCTGTA
GGGAGCGGGGAAGGGCGTGAGCCGAGAGGAAACGCACCAAATTACCGCAAAAAAAATATATATAAAAA
GAATGGAAAAAAAAAAAAGTGAAGATATGACGC
GTCTGTAGGGGAAAAGAAGAAGGGAGCTGATAGACGTGGGGACGGAGAGACAGATTTCCCGCTCTCTC
CGTCCTCCCACACCCCGACAGGATTTAAAAAC
AAAAGGGAAACTCGGGAATTTTTTTCAGGGAGAAGCCACGCAAGTGAAAATGAAAAGTTGTTAAATTT
GGGAAGGAAAAAAAAAAGATGAGGAGAGAAGTA
GAAGGAAAAAAAAAAAGAAGAATAAAGGGGAGGAAAGGGAGTTGTTTTTGTCGGGTCATGTCTGGATTT
CTTCGCCATGCGTCTAGTGAAATGGAGAAATC
TCTGCTGTGGGAACACAAGGGAGTGGATGAGGGACCTCCGTTTTTTAAGACTCTGTCTGCCTTCTTCT
CCTCTCCAGCAAACCACATGATGCATAAATAG
AAGCAGAGACCCCAAATACAAAAAAAAAAAGAAAAAGAGGGATGAATTAAAGAAATATGCTACAAATAT
ATGCGTATACTGTTTATATCTTGTTGTCCACT
CTTGTTTGTTTGTTTGTTTGTTTCCTATGTACTTCTGCGCAATTTACACAAGTGTGCACTTTTTTGTT
TTCTTTATTTTTCCTGATTTTTTTATTGCTTT
CTTCTGGATGGCTGCTGCGGAGGGAAGATACGCCCTCTCATGTCGTATTCGTTTCTCCACCACATTTA
CTGGCTCTTTCCCCTCCAATTGCATCTGCACT
GGTGTGAAAACAGATAGGTACAGTGTGTGGGTAGAGAAGGTGGTGTAAGTGATGCTTGGACTCTCAGG
TGTTCAGCAACAGCAACCCTATCATCAGCATC
CTTCACAACAGCAGTTTCTGCGTGCTAAAGGGCGACGATGTCTTTACTGCCAGCAGCAGGGATGTCCG
GGCTTACAGAACTGCTGGTGGCCGCGCCTTCG
GTCTTTAGGGTCCAGACCGCAACGCAAGGTGGAATCCGATTTTCCACAGCATTTTTGGTGGGATGGAG
ATGTGAGGAGCGAAGAGAAGAACGTGAATGCC
ATGGAAGAAAACTTGAGGGCGCAAATACGTGTCTTGGAGCATGAGGTGCAGCTACTCCGCGCTGGCCT
TCACAACATTGCCTCCGTCAATCGCCGCGGTA
AGAAGGACGTTGCCACCATGGCCACCCCCGTTGTGCATCTTTCCACTGACTATCCCTCCGATCTCTTG
TTCAGTCACAGTGTTAAGAGTAGCGGCAGCGG
TCTTTCTTCGCCGAGCCACCCCGTCGTTTCCTCTCTGCCTGGTGGAATTTTGCAACAACAACAACACC
TCCGGGAAGAGCATCAGCAGCAGAAGCCTGTG
AAAGGGGTTGGTTGTGTTGACTTTTCCCACCCAGAAGGGAAAGTAGCAACCGGGGTGGCGGACGTGTC
TGCATCGCCACCGCTCCAAGGAGACCCGGAAT
CGAAAGACGTGGAGGGACTGGATCACCTTTTGAGGGAGCTGACCGCACAAAACGTGGCACTTTGCAGA
AGACTTGCAGAGATGAAAATTGTTCTCGAGCA

GCGTGACACACTTCTCCAGTCTATTCTGGCGGAAAAATTGAAATTGAATGCCACGCACGCTTCATCGT
CGGTGCAGGCGTTCGCCTCCGCTCAATTAACC
GGGCAACCGAAAGAATTATTTTCGCACCTTCAGGAGGCGCAGGAGGAGGTGGCACATCTTCGGGAGGA
GACACGACAATTGAAGAAGGCACTAAAAGGTT
GCAAAGCGAATCTGGAAGAAAACAACGCCGAAACAGAGGAGCTTCGGCGTCAGATACGGCCGCCCACA
CTTTTTTCGAAGGATTCGAACACCATTGCCGC
TACGCCACCCATTTTGTCGACCGATGGAAATGCTCCTTCACTGCCAACGGCAGCCGCAGCCGCGGCGG
AGTTTATGACCAAGGAGCCCACCGTCATTGTT
GTGAATGATTCCACCCCCGTGCCACACGAAAATACGGCAACAGACGCTGCGTCAATGAAGGCAGAGAT
GCGTGACCTCCACGAGCGCTTGGCATTGGCGG
AACGAAAGGTTCTCGCCTGGGAATCGTGGTATAGAGAACAGACGCAGCAACCATCGTCCAAAGAAAAG
GACTCACGGAAAATAACGCAAGAGGGAACTGA
TGTTTTTAACGAAGAAAACGTCTTTCCGATGATCCAGAAAAGTCCACAACGTGCATCCAAAAAGGTGC
TGCGAGCTTTGCCTGTCACAGCTCCCGCAAGT
CATGTAAACTGGCGTAATGTTTCCTCATGGCAAAAAGAAATGGTGTCGGACGCACTGACAATTGACAC
ATATGCTCTCATGGCACGGGAGGCCGCCCTGC
GTCAAGTAGCAGAGCAGGAGCGAGCGCATGTTTTGGCTGCGCTCATGACGGATAGTGCAAGTGATGAT
GACCAGCAGGAGTGAGTAGACGGACGGTGAGG
CCAAATCATAGAACAGAGATGGTTGAGGAGTTCCCTACATTTTTTTTCCCTTTTTTTTTCTTTACGTG
TATACATTTATATATTTGAGGAAATGCTTCTC
TTTTTTATTCTTGACTTTTCCCTTCTCTGTATTCCGTACCCCACAAACAATATGCAGATGCATTTAGT
GTGTGGTGAAACTCTACTGCTGCCGCACCAGG
AAGAAGAAGAATAAGGGGGAATTGAGGTGAGGTGGAGAACATTCGTGTTGCCTCCTCACATTCAATGT
CCACAGCCTCGCCGGTGTCCAGCCGAAGCACA
TACGATGAGGAAATGGAATTTCTGATGAACCCTGTGGCAGCTGCGTCTCAGGCTGAAGAAACACCTCG
ACAGAAATCAACAGAGTTGATGTCGCAGGAAC
AGTCACAGACTCACTACGCCGTGAACAGAGCGGTCACAGAAGCCATGACAATGGAGCACGCCGTTCGC
GTCCCTCATGCTTTGCAGGCGGCCACACGGCG
CACGATGCTTGACGTTCTCACACAAATGTGGCGCCAAAAACAGAACGATTTGGCTAACTGCTCTGGAG
AATACCTTGCCGAGAAGCCATTGTGCAATGGT
CTAACAGAAGAAAAACCACATGATAACCTGACTGTCGCAGATGAAGCGGCCTCATTTCTTCTCGCTGA
CACAACCAACATGGGGGCAAACAATGCAAGCA
CTCTTAAATCAGTCCTTAGTCGGAGTCTACGGCATCTTATGCCTTCCCACCGCGCTTTGCCAGGTGAA
TTGGAACCGCAACAATACACACACGAGCAAAG
TATGAGGGAAGACAGTATCTCTGACTTCGATGATGACAACAACGTTGGTGGATGTCTAAGACAGGTCC
CACAAAGGGTTTAATGGAAAGACACTCTGAA
GACGGCCGTTTTCTGCAACGCCACTCGTGGAATTCCGCCGTCTCCGAGGAGGCCACAAGACGGCATAC
AGAGCACAAACGTACATGGGCAAAACTATTTG
ATTGTGATGATCTACACCAAGAGGAACTGACAGGACATGAAGATCGAACCACAATGTTGCCAGCAGAG
AAGTGCACACGTGCGTCACGCACGTCCACGAA
ATCATCTCGTCCAAGCGCGAGTTCATTTAAAGACCTTTGGACAGAAAGTGAAGGTGGGCCGCGACAGA
CGACGGTTAGTCGTACATCGCTTGCAACCCTA
CTAAACGAGGCTGAAGCCAAAGATGATGAAACATATGCTCAGTCTATGGCAGCTGCCTTTTCTGCCTC
AAATAGTGATGGAAAGTCGTGCCGTTTCACAG
ATGTATTGGCCAACGAGGAAGCCGAAGAAGGTCGTATGCCTTCCTGCCATTTGGTTGGGAAGCCCATG
TTTTCCACCAGGCTTCCCACACATGGCAATAG
CCCATCGGTGCCAACAGCATTTGCTGTGTTGCACGGTTCCACGAGGCGAGTTGGGAGCCGCGGAGGAA
CGAGCGCGGTGCTAAAACAGTTGTCGGAGAAT
CCTATATCAGCACGCGTTGTTGCGTACCTGACGCGTTCCTTTGAGGATTGGAAACTAGATGCCGCGCT
CATACGTCGGGCGAATGAGGAAGACGAGCCAC
ATAATTTATCCTTTTCTGGTCCCAGTTTGCTTGAAACAAGTGGCGTGCAGCTTAAATTACTTGTGAAG
GTGGTGGAAAGTGCCTTTTCTCTCTACTGTAT
CCGATGCGAGGTGGTCTGGGCAGATGCGCAGGCATCAAAGGAACTTTTTTTTGATGCGCATACGGCTG
ATAGTATGATATCGATGCAGAAGGAGCATGAA

GGAGAAGGAGGAGGAAATGAAGCAGGAGTGGGGCATAAAATGAAAAAGCCACCTGTAACATGGACGGT
TGTTCTGACGGCGGCTACATTCCGTGCCATCC
CCATTGTTGTGGGGAGCCATCTTTACTTGGCACGGCCGTTTTTCTGCTATCCTTCCATCCGCGTCATT
GTTGCCTCATTAAATATTACTTCCGATGCGGC
TGTGCAAAGGCTGCAACCCACAGCCCGCCGCCAGCGTCGTCCACGAGATGCGGATTCGTCGGAGCCAA
AAACCGGAGGCCGCGCCCAAAGTGGCGAGTTT
TGCGGTGAGGAGCTGTCGTTTGGTGCGACAACACCCGAGCGAAGGAACAGGCCGTCTGCGTCTCTGAC
GGGCGTCTCGCCGTTCCGGGCGTATGATCCGT
TTGTCAACCCACCGACAAATCTGTTTTGCAAGAACAGTGTTGCTTTGGGTCCATCAAATCCAGTTTCG
GAGAGAATTCTGCGGCGGTCGGCCTCTATGCC
GCGTAAGACTACAATGAACCTGGAGAGTGATGCATGTGGACAATTATTGGCGTCAAGGTCACAAATGG
TAGCACATGACGTCATTGGTGCACAATTCCCC
CGTGGCCCCGGTGAGGAGTGGGATGTGCCACTGGAAGTACTGGTGGCCTTGTGCCCACCTCGTAAACG
GACAAGCTCCGGCGAGCCAGAAGCGCATCGTC
GCAGAACGAACAACCTGTCCCTAACGCCTCAACGAACCTGCAACGAAAAATATTCCCCCCTCTCCTCC
CTCTCATCTATTGACGTGGAAGAGCCTTCCCC
CCTCGCTGAGACTCAAGCCAGAGGTGGGGTGCCGCTATGAAAGAGCCCTGTTGAGTGATTATTGAGAA
TCAGCAGAGGAAGCGTTGTTGTTGTTTTTGTG
TGTGTATTTTTTTGTTGTTGTTTTTCTTTAATGCTGTAAATGCACCCTGCCTTTTCAGTCTTTGTAGC
TTGTAATGTTTGCGTGTGGGTGGCCCACGCTT
CTCCATTGCGATGCTTCTCTCACTTCTTGACCACTATTCTGAGTAAGAGAAATTCTTGGGGAAAAACA
AAAATTGAAAGCAAAAAAAAAAAACAGAAGTG
GCACAGCGGTATGAGCGTACATGAAATGGAGGATCTGCTGCTTGGGAAAAAAGTAGTGCGACTCCCAC
AGCAGTATGACGCGTCCAGCTTTTTGCAAATG
TGTTCACTCAATGAACAGTACCGAATTCTTGCAAGCATATGCCGTATTGAAAGTCGTAATCTTTCGCG
GCGGCCAGGCGATGACTGCAGTACACACAACG
GCGGACTTTTCGCGAATGCCGCAGAGTCATCCGAACACCACCACGGTGGCATGCCCGAAATTTATTAC
TCTGCCCGTCTCTTGGCCTTTATGAGTGACTG
TGGGCCAGAAGCAGGAGTATTGAATATACTCAGGAAGGAGCACACCCGTAAGCAGCAACAAGGGGAGA
AGGAAGAAGAAGAGAAAAATAGGTTGTCACGG
GAACTTCAAGAGCAGGAGGGGAAGGCGGTGGCAGAGGGTGGAGGATGTGAAGGGGGAGAACAAGAAGG
AAAAGAAGCGTCCAATGGGGAAGTTTGGTACG
ACGAAGAGGGTGGTTACTACTATGACAGCTACGGCTACTACGATGCAGACGGGTATTACTATTACTTT
GAAACAGCGGACGCTGGAAACCAGGGAGAAGA
GGAGTTGGGACAAAAAGAGGAGGAACAAAATGAAGAAGCAACTGCTGGAGAAAATGAAGGAAAAAAAA
AAGCCATGCCGTATAACTCACAAAGTGATGAG
GAATTGCATGGCGTGGCGGAATCGCATAACTCGAAGAACGATGGCATGGAGGAAAATTCGGTACGTTA
TTGTCGTGTGGTGCCAGATGAAAGTCAATTAG
CCTTCTCGCCCTTTCTTCGTGGCGTTTGCGCTTTGAACCGTGCTGTCAAATTGATAGAGCAATCCACC
GCTCCTGTGGACGTTTATATGAAAGAAAATGA
TTTGCTCCCCGCATTTTCAGTCTTGGTTGTCTCCGACTCAGAAGTCGCTGGGGGAAGAGGTGTTGATG
TGCGTCCCGAATGTATTCACGTTCTCGCCAGT
AGCCAACACCTACACATTGTGTATGTACCGACCTCCCTTCCTAATGGACAGGAAAAGCAGCTAGGGGG
TGAACAGGAGGAGAAAATGGATGGCTTGGCGC
CATTGCGTGATCTCACAACGCCAGGTGGTGTGCTAATGTCTATTCCCCTAAATGACTGGGGTGGTAAC
TGCACCACCCACTACGGAAGCTTAGGAAATGG
CCGCGTGTTTTTTGAAATTCATATGTCAATGGAAAATAATAATGGAGTGACGCTATCAATGGTTTTTG
GTATTCCTCCTGATGCGTCATTTTGGTCTGCG
CCGCTGGCATCCCGCCTTGTGGAATGTTACGAGCAGCGTGTGTTGCACCACCTGGCAAAAAGTGAATG
CGTATTGGACGAATTACTCCTCGAGGAATTGG
GGAACGCAAGTATTGGTTTCTGCTGCGCATTGGATATATCGCGGCCACTCTTCAGTTATTCCGCCCTG
CTTCTGAGTGAGGATGCTGACAGCACCCATAG
TAATAATGACGGTAACAACACGTTGCACGAGCTTGTGTACGATGAAGCAAGCAACTCGGTATTGTTTG
GTAAGGAGCGCGAGGAAGTTGAGCAAAAGGAA

CAGAGATTGGAGTTTCTTTACCTTGCCCGAGATATGGAGCCACCCCAGCGGCGTTTTTTGCGTGAAGC
ACTCAGCAAGGTGCAGCAGGACGAGTGTAAAA
ATCGTGATAAATTGATTGCCGAAGCATGGAAGCCCTATCAGGCAAGCCAACAACTCTCCTTTCACACC
ACGGCCTTTGAGATACTGCCGCTTCTGCGGAT
GCAGGAGGCTTCTGTTAAGAAACTTATTGAAACCGAACAAAGTCAGTGGGAGGGTATTATTCAACGTT
TTTCGAAGCGTCACCAATGGCTTTTTGCCGAG
GAAACACGGCGCCGACTGGAACAGCAGTTTGCATTTGAGCGAAGAATACGATTGCTACAGTGCTCCGT
CGATCTTGACATTCTCATAGAACGAGAAATGG
CAATGCGTGCGATGGCAGAGCGCGATGAGGTGCTGGAGCGGCACAGTGGTGAACTCATGGAGTGCTGG
ATCAATTTGTTGACACGCCGGCGTGCAGAAGA
AATTTGCCGGACGACACCTGTGAGGGAGGACGAAGGCGTGATATCTGCCGCCAGAAACGCGGAGGAGA
CGGATGAAGAAATGGAAGGACGTCTCGGGCGA
ACGGCGAGCCGCTCGCGCGAATCGCAGCAGCAGCTACGTGCCTCAACCCACTCCGTCCATTCACCGAC
AACTGAAGAATTCATGCGGATGGTTTCGGTTG
GCCACCGCGGCAACCTTGCTCCGTGTCGCACACAGCCTTTGCGGCAAGTTTGTCTCCCCCCGGCATAA
TGATGAAATTGAGAGAGGGGAAAAGGAAGAAA
TAAATAAAAGATGGAAGAGGGATCGATATAACGAAAAGAATGAACAAGAAGGGAAAAAGGAGCGAGAG
GGGAGTGGAGGGGAGCGTTGTGTAAAGGGCAC
ATGAAATTCTGCCCACTTATCGTAGTTTTATGTAACCGGCACATCTCGTGTCTTATTATTTTCCGCGT
ATTTTGTTGTCGTTGTTGTTTTTGTATTCTTG
TACTTGTGTGTATGTGTGTGTTTTATTCATATCACTTGTTTTTTAATTTGATGGGTGTCTTTGCGCAT
TTAATCTGAGGAGAAGCAGGCACAGCCGTGTG
AAAGTGTGAGAGCACTCTGCATGTCTCCTGGCAGGAAAGGAAGAGAGAGTGTGTGTGTGGCATGCATT
TTGTTTCACCATCGTTGTCTTCTTTGCAGACA
ATTTCCCTTTTTTTCCTTTTTTCCTCACACGGGTGCACGTAACTGTCGTTGACACCCTGTAAAAGAAA
ACATACGGCTCAGACACTTCTCTGCACGGCAA
GCGAAAAGAGGAACCAAATAACAAGGAAACGGAAACGTCTAATTCCACAGCAACAACAGCGGTGATAT
TTTTTCCAAGAGAAGTGTAGGCGGGAAAACGG
AGGATTTGGATCAAAAGAAAATAACAAGGCAACAAAATGGCAAACGGTGTAACGGCGAACTCCATGCG
TGTTAATGTAAAGGCGCCTGACTTGGGTGCCT
TTCCACTCGATCATTATAGCGAGTGTAAGAGCCAAGTTGAGGCATACTACAGATGTCTGAAAAGCAAT
GAGTACGTCACACCCTTGTGTCGCGATCAGAT
GCGGGAATATCTACAGTGCCGCATGGACCGTGGCTTGATGAAGCCGGCGGACCTCGACGGTTTTGGTA
TTCCCAAGACGGAGTTTGTACCAACCAGGCAG
CATCGGATAGATTTAAAGCAGCAGTGGCTACGGCAGAAGATGAACCAGGTGACAGTGGTGTGGGAAGA
AAACTATAGGAGGGATGATCTGCAAGTGCCTG
ACGGCTACGAGCGAGAGAAGGGTGGTGGGGATGGTGGTGGTGGGAATCCGAAGCCAGTGGGCGAAAAG
GTAAATACGTAGGGGATAGAGGATATATATTG
TGGTATTTGTGCTGCCATTGTTTGTTCTATTGGTGCTGTATGTGGGGGAGAGAGGAAGAGAAAGCATT
GTTGGGTGGGAATGGAGATTGAGTTTTCTAAC
GGCTCATTGTTCCAGCAAAGTGGAGTTTACATGATGTGTAAGTTGAAACGTGAAAAATCTCAGGGAAA
CAAAAAAAAAAAAGGAAAAGAGAAATGGGACA
TGTAGGGAGGTTTTTGTGCATCTCTTGTGCCTCCAAAATTATTCTCGACGTGTTTCAGCTTCTGCGTT
TCTTAGTTTTGTGGGGAGGAAGAAGGTGCGTA
CACGCCGCTTCAAAAGAAACAAAGTAGGCGGGAGGATTACCGTTCATTTAGTACTAGTTTATTTATTA
TTTTTTTTTGTTGTGCGTGTGAGCAGCATGAG
CACCGAAAAGCGGCCCGTGGTGCTTGACAATGGCAGTGGTTTTATAAAGTGTGGCTACGCCGGCGCCA
ACTTTCCCACGGCCGTTTTTCAGACTGCTCTT
GGTCGCCCCGTGTTGCGGTCAACAAAGAAACGCAGCCATGGCAGTGGCGGTGGGGAAGAGGGGCATAC
GCATGTGGACCCGATGATGCGAGATATTGTCT
TTGGGGACGAATGTAGTGGCGTGCGGCATCTTCTTGACATGACGTACCCGATACGCAATGGCATCATT
CAGAACATGGATGAAATGTGTCATCTATGGGA
TTACACCTTTGGGGAAATGCTTCAGATAAACCCTTCGGAGCATCGCCTGCTTCTCTCAGAGGTACCAC
TATTTAGTAGCAAACACCGCTGCCATTTGTAC

GAGGTGATGTTTGAGAAGTACAATTTTATGGCGATTCAATCGGCTGTTCAGGGCACCCTTTCACTGTT
CTCCAACGGCCTACAAACCGGTGTGGCGGTGG
AAAGTGGGGAGGGCATCTCCCACTGTACACCCATCTTTGAGGGGTATGCACTGCCCAAGGCGAACCGT
CGCGTTGATCTGGGCGGACGCAACATCACAGA
GTTTCTCATTCGACTGATGCAGCGGCGCGGATATAGTTTTAATCAGAGTAGTGACTATGAGACGGTGC
GGCGAATGAAAGAACGCTTCTGTTATGCGGCG
GTGGATAGTAAGCTCGAGAAGCAGCTTGCCCTTGAGACAACAGTGCTGGAGCAGGCCTTTCTACTACC
AGATGGCTCATCCTGTAGTATTGGACAAGAGC
GTTTTGAGGCTACTGAGGCGTTGTTTCAGCCTCACTTGATAGACGTTGAGTGTGATGGGCTATCGACA
CAGCTGTGGAACTGCATACAGGCCGCTGAAAT
TGACGTGCGGGCACAGTTATACAGTCATGTTGTGCTGTCTGGTGGCTCAACGATGTTCCCGGGCCTTC
CGAGTCGTATTGAGAAGGACATGCGTGCAATG
TTCCTTGAGAAAGCACTTAAAGGTGACCGCTCACGTCTTGGTCACTTTAAGCTGCAAATTGAGGACCC
GCCACGGCGGAAGTATATGGTCTTTCTTGGTG
GGGCGACATTGGCAGCATTGACAGTGGACCAGCCAGAAATGTGGCTCACACAAGCGGAATGGCAGGAA
GGAGGTGTGTCAGCGGTGCGGGCCCGTTTTGG
TGCTTAGGGGCTATGGAAGCGGAGACAAAAGCTTATATATTTAAATATATTCATTGAGAAAGGGAACG
AGCGAGGAAATACCGTGAAATAGACTTTGAAT
GCGTTGTGCGATGTCGTACATCTTGTCCAGACCCACGAACTACGTGGGAAGGCTGCAGTACGAAGCCT
AGTGAGAAAAAGAAGGCTCGGAGGGAAAATAG
GGTGTATTTTTCTGTGAAATCCACTTTTGTTTTGTTGTCTTTTCCTGCCACAGTACATATGTAGATGG
CTTATTTATCCTTCTTGTACACATACATGTAT
ATATATATTATTTATTTATTTATTTTACTTGTTGTTGCAAGCAAGAACTTATGTTGTGTCTCTCTTAC
TGTTTCTTTTTTTTTCTTATTTGTAAAAAAGG
GAGTGGTGCGAAAATACGCAGGAAAGGAACGGGGAGAAGTGTATCATTGTGTGCAATGGGAAGAAAGA
AGCGTGTGGCCAGTCATGCCATCAAGAGTATC
CTCATGAAGGAGAAAAGTCTAGCACAGAAGAAAAAACGGGAAATGGATGGTTATCAAGACACACCAGA
ACCGGTGCTTTCGCAGGTCAAGGATGAGGCTG
CTGTGCGATTTCAACGGAATCAACTTACTGTCGCCGCGCCAATGCAGACAAACATGTTTCTTTCATTT
AATAAGAGTCTTGGACCACCGTTTCACATCTG
GCTGGATACCAACTTTATCAATTTCTCGATGCAGAATAAGATTGAGATTGTAGAGGGACTCATGGACT
GCATGCTTGCGAAGGTCATTCCGTGCGTGTGT
GATTGCGTCATGGCAGAGTTGGAGAAACTTGGCAAGAAGTTTCGAATTGCGTTGAAAATTGCGCGTGA
TAAACGATTCCGGCGGTTGACATGTGATGGCA
AGTATGCGGATGACTGCGTTGTGCGGACGGTGACGAGGCATCCCATCTATATTGTGGCAACATGCGAC
CAGGAACTGAAAAGGCGACTGCGTAAGATTCC
TGGGGTGCCCATTATGTACATCTCAAAACATCGCTACACCATTGAGCGGCTGCCGGAAGTGTACGGTG
CTCCTGGATGAGGTAAAAAGAGACATTTGGCG
ATGCGTATAAGCCATATGCGGACTGCTTAAAAGCATGCGAATAAAAGGCACTGCTGAGGCTGCTTGGG
TACAAATAAAAAATAGTTATTGGGGAAGTGAG
AGAAGGAGCACTTCTGGCTATTGTCTCTGCCCTATATATTTGTTCTCGTTGTTGTTATTTTCTTTGTA
TGAGCCTTGGAGGCCATCAGGGGAAGACGCTC
TAAAAAAGGTGTTTGTAATGTCCCGAGTAAAAAACATTGACGCGGATGTGTGTCTATTTGAAACCTGT
TTATTTCTTTTTTTGTATGTGTGTGTGTGTGT
GTGTTTTGGCTCTCGCTAGGCCTTTGGCGGCGGGGGGTGGGATTTGCTCCTCTTTGCAGCCTGCATAT
GTGTGTGTATTCTGTCTTGTTGACCTCCGTCT
GAGGCGAAGGGAATCATGCTGATAAAAACAAGGAGGGTGCGCTGTCCATTGGGTCCGGCCACAAATAT
TGGTACAGTACGTCGGTGCCTCACGACAATTG
CTGCTGGGGCTTCTGTCCTGGGCGCGCCGTCGTTGAAATCCACATCCGTTGCGTTATCGCCGTCGCAA
TGGATGCGGTCTTCACTGCAGGAGCAGCGGCG
ACATCGTAGCTTTTCTTTTTATCTCAACCTGGACGATCCCGCCGAAACGCGACGGCGTGTCGAGGAGG
ACATTGCCCGCATCGACCAGGAACACCAGCGA
GGCACGATGATGCATCTACACGCACTACTCAACCTTGCCCTTAGTTGCTATCAGTCAGGGGAGTACAT
TGCCGCCCTCGAGTTTGCGGAGCATGTCCACA

ACAAGACGCGAGAATACAACAAGAATGCAACACTTTTGTTTTTTACTGCCAAAACATGCTCTCGCTGC
GCCCTGGCTGTCGCAGATGAGTACGAATCCCA
CCTGCGGGAAGCAGCGGAAAGGGCAAGTGTGTCTTCAACGTTGGCACCACGTCCTTCCGTCGTGTTTT
CAGCGGAAAGGACCATTGCGAAGCTACGCGAG
GATGCGGCGCGTTATGATGGCATCGCACAGAGAATATATAACCGCCCCGACAAAGCCTTCATGCGAAA
CTGGAGGCCTACGTCTACAGGATGGTCCGAGG
ATGCGTGTGGGATGGACGAGGAGCCGGAGGAATTTTTTGGAGGGCGATGGAAGGAACGCCGCAAGCGA
CCAGAACACGCGGCAATACGCCAATACTATCA
ACAGCAGCAACAAAGTAATGGGGGCGGGGTCGGCGTGCCAAAGTGAGCGGCGGAGGGTGGTTCCGGAG
GAAAGGGAGTAAAACCCGTTGCTGTTGTTGTC
TTTTCCTCGATGGCCACCATGGGGAGAGGGGGCAGGGAGGAGTTATTGTTTGGGGATTCACTCGCCAG
CAAAGACTTTCCTTTTCCTTGTCGTCTTGTTA
CTTTTATCTTTTCCGTTCCTCCCCTTTTCCTCTGCCTTTTTGCCAACAGGTAGCCTCCCTTGTGTTGT
CGGAGGGGCACACGCTTTGCCGCCGTGGCGGG
GAGGCCATCAACGTGCTTTTGAAATAAGTTTTTGTTTTTGTCCTGGGGATAAAACTTGGAAAAAAGGA
GAAAATCAAAAAAAAGAGTGGGGAATTGTGGG
AGGAAAAGAGAAATGTCCCACAGACCTGATTCTACGGGCGGTGGCGAAGACAGGGGAGGGGCTGCCGC
TCCCAGCCGGACCGGTGTGGGGACACGGCAGA
GAGAAACAGCCGGGTTGGATCATACGCCGATTTCCAACGTCGTATCTCTGCAGTCTGCCATCCAGAAG
GAGGGACTGACGGAAAAGATATTTTTTGTTGT
AATCGATGTGACGGATGGTCGCCGATACAAAGTCTTCCTGCGTCCCGACGATATATCGAGGCTGAAGG
TCTCCAAGATACGTAAGACACTCAGCAGCACC
TCAGGGAAACCACCCTTTTCATTTGTTCTGTGGCTGGGGGACGAGCCGCTGATTGACAAGGAGGATGT
CAACTGCGCTGCGCTCGGCATCACGCCGCGGA
CTGTGATGCGCATGTGCCCCACTAAAGAGGAAATGCCGCATCCCATAGGAGGATCTGAAGGGAATGGT
GGCGTGGACAGGAAGAGAGAGGCGAGGGACGC
GTTAGATAATAGAAAAAGCTCGGTGTCAATTGCAGACAAAATTATGGAACAAATTTTGGATGATCCTT
TGGAAACCCTGTGTTTGGTTGAGCTCAACCAA
CGACAGCGAGAACTGCAATTCCAACTGTACAAAGAGGCAAAACGATGTCATATTTTCTCGAAAGATGA
CGCGGTGGCTGGAATTGGTAGGCATAGAGATG
ACGAGAACGACGATGCCTTGGATGCCCTTTTATCCCCCCCTCAGACAGCAAAGAACCCACAAATGTGC
AGCGTGTCATCTGCGAGGATTCCCGGGGAGCC
AATTTGTGAAGATGATAGCCTTATGGGTTTTATTGAGGAGATTTTAAACGAGGGGCCGAAGCGGCAAG
ACGGCCCACGTCCGACACCACCGTCTGTGGAA
TGCTGCCCAGCCATGCCTGATGAACGGCAGTCCTGTGCTGCTGCAGCAGCAGCAGCAAAACAACCATC
ACTGACTCCAATTGGCTCCAACAATAACATAT
CAGCATCAGAAATAATCTCGCATGGCATGCGGCTTAACGAAATGTATTCTCTGGAGGATACCGGTGAG
TTTGTGGAACCTCCACGTTTCCTCAATCGTGA
TGCATCATCGGAAAGGAAAAATAGTGGTGAAAATGACAAGGGGAAAAACGATGCGTTGCCAATTAAGT
TTGGGGCGGCCATAGCTGTTCCCATCGCGACG
CAGGGGGAAGAGAGAGAGGTCACAAGGTTGGTGGAAGATCTGAGGGATGACGTTAATAACCTCCTTGC
ACTCCACGTGCGAAACGAAACCGCAAAACGCG
CTCTTCAGCGGGAGCTTTCGTCCTCACAGCAGCTTGTAAGGCGGTTGGAGAAAAAGGAGGCGGTGACG
CGCCAGCAACTTGATGCACTTTTGCTACGCCT
TCGTCACGCTCAAGAGGCTAACGGCATTCAAGGGTTCTCTGAAGAAGAGGTTCGTGTGGCACTGCGCA
ATATGGCGGCTTTGCGTCAGAACGAACTCCTC
CAGCGAGAAAATGCTGCACTTGAGCAACGACTTGCCGAGGAGGTACGCAGGCGTCGTGAAATGGTCCT
TGCGTTGGAGGACATGAAGGGACATATTCGCG
TTTTGGTACGAGTGCGGCCGCCATTTCGTCTTTTGCATGAGCAATACGTTGGAATGGAAGGAAACAAC
CCAATGTCTGTCCGTGACACTGTGGACGAGTC
ACGCATGACGGCAGACGAAAGGCGCAACACGATTACTATCACTGACTCTCGTGGGGAATCCAAGCAGT
TCAACTTTTACCGCGTCTTTTCGGAAAGCTCC
ACGCAGGCGGAAGTGTTTGTTGAAGTTTTGCCGCTTGTGCAACTTGCTTGCGATGGGGTGAATGTCTC
GGTGTTGGCGTATGGACAGACTGGGAGTGGCA

AGACGTACACCATATTGGGCGGCGACGGGAATCAAAGCAACACGGAGGCAAAGGGAGACGAAGGAAAT
AAAGAAAACGATGATGATGGAATTGTACCGCG
CTCGTTACGCATGTTATTTCAACACCTACAAGTTGAGGCAGCGACAGAGGCAGTCGTCATGCCGCGTG
AAGATGACAATTGTAATGACGACGAACGCGCT
TCCTCTACTTATGAGGTTTCTTGTAGTCTTCTGGAATTGTATAATGACAAGGTAAGAGATTTACTTGC
GCCCCCACAAGTGATCATCCCCAATTTTCAAT
TGCGCAAAAGTAACGTTTCAGAGCAACGTGGAAAAACACAGGTAACACCACAGTGTAATTTGAAGATT
GGATCCGATGGCCTCACGCATGTAGTGGGCCT
TACACAACACGTGGTGTGTGATGCAGGGGAAGCGCTGAGGACTCTTCGTGAAGGTGCCTCTCGACGTC
AGGTCCATGCTACACAACAGAACTGTCATTCA
AGTCGATCCCATCTCATCTTCACTGTTTACCTCACACAGAAACGCCGCATTCCCTTTCGAAACGACGC
ATCCACCACACGTGCATCGTCTACTTTCCTCA
GGCAAATGGAGAGCAAGTTGGTTTTTGTGGATTTGGCGGGTTCGGAGAGGATTTCAAAGTCACAGAGC
GTGGGAGACCGCTTGTTGGAGGCACGACACAT
AAATAAGAGTCTGGCCGCATTGGGTGACGTCGTGGCAACCCTCTCCAATGCGACAATATCTAACACCG
CAGCAGTTCATGTTCCTTACCGGAACAGTACC
CTCACTGGGCTTCTGCAGGATGTGATTGGGAACCGTAGCAAGACGGTGCTTCTCGCTTGTGTGGGTCC
AAGTGATCCGCCATACGAGAATAACACAGCCG
AGACGGTGACCACGCTAAAGTTTGCCAGTCGAGTGAGGTGTGTACGAAACTTTGCGCCGCTATTCCCA
ACAACAGGAGCAACTGGAGACGGTGATACCAT
GAAGGGCCCCGGGACGGATAATTTGGAGCCGGATGTGGATCCCTCACACATCTCCTCTTCCTTGGGAC
GGGGCGCCAAGAAACTGTGGATGCGGCCGACA
AAATTGTCAAAACAAAGAACCAATATTGCAGCTCGTTTCAGACTTGTTTCGGCGAACGAGGCTGAGAA
AACGGAGGACGGCGGACGGACGCGAACACCCG
CACCGTCCTGGCCACCGAAGGGCAATAACCCTCATGCCGAACAGCGACCTAGAATGCGTTTTTAGGTC
TCTGCGGCGCTATCGTGGGAGAGAGGCAAAGA
AGTGAGGCGGGTGGAAGACATGAATCAGTTCATCTTTTATCGAATCAGCAATAAGTATGCGTGTGTTT
ATTTATCTTGTTTTATTCGTGATGGCGGTATG
GTAAGAAATGTTGCTGCCCAAAGGGTTGCAAAAAGAAAAAAACGTGAGTTTTTTTTTTCCTTCCCTC
TTATTTGAAAGATAATTATCGCCATGCGGGCG
TCCTTCCTATGTGGTTGAAAGTTTCCGAAATTTTTGTGTCTCTTGCTCTTCTTTTTTTTGGGAGCTGT
TTTCAAGGGCCAAAATGTGTTTGCGGCGGAGT
AGTATATGGAACGGTTGGAAGGATCACAAATTTGCCGCTTGAGCAAGAGCTGGCGCAATGGGTGCTTT
TTTTAGAAACTGATTTTTAGGTGAAGAGTGCT
GCAGCCTTCACGTACAATTGTGTGTGGGGGGAGGGGGGAGGGGGGAAATGGCAGAATGGGGGCAATTT
GAATCCCCCGGACACTAGCACCTGGATGTGTT
TGTCAAGGGAAATCAATTCCTATTTTGCCTCCGTGCTTTTTCTCTGGGGAAACTCCGTGCTAGAAGCT
GATGAGCAGGCTTTGGGTGATTTTCCATATAA
TTGCCTGTGGGTGAATTTGTTTTTGTATTGAAGAATACTTTGGCATTTGAAACTCTTATTGCTGCTGT
TGTTGGGTGGGGGATGCATTGTATGGCTTTTG
GGTGAATGAGGGAGGAGGAACTGGGTGGAAGGTGGAGACTGTGACAGTTGTTCATGCTTCCTTTGAAT
GGATGTGTTGCCAGGACTGGAAAGTAGCATGG
GTATTCTTTGTATTTAGCCGCTGCCGCATCTATGCCTTTCCTTTTATTTATTTATACTCCGTTGTTTG
TGGGGGCAACTCTGAGGATTTTGTCTTGTTTT
TTTTTTCTCCCTTATTCTCCTTGAATGCGGGGCAGTTTGGGTGTGCAGGCAGATAGCTGCAAGGGAGG
ACGGGGGAGGGGGAGATGCTGCGGTGTGCGTC
GCGCGTCTGTCGGGCCGCCTCCCTGTTGGGTTACCCGCTGCGCCGGGCAGCCGCGATGGAAATGTTGT
ACGGCGGTGTGCGTGTGCAGCACCTTGCACAG
TCGCCGTTCTCCCTCCGGAAGACGCGGTCAGAGGCGCTTCCGTCTCCGTCGCTGCAGCGCGAGCGGGA
CGATCTGGAGCTGGAGGTGAAGGACTCCGCCA
ACAATGTGATGGGGCTCCGCCTTTTCCCCATTAACATTGGGATGCGGGCGAGGAACGAGGCGGTGCGG
CTGCGAAGCGAAGACTGTTACAAGCGACTACT
GGCACAGCGGCAGTGTGCAGAGGTGGGCGAGGCGCTGAAGTTTCCCGTTCCGTCGAGCCTTTCCGCCG
GTGAGCACAAAGACGCCACCGCGTCTAGGCAG

CTTTACGCAACGCATTTTCACCCACCATCGTCTAGGCTTTCCATCCTTACACGGCCCGTTGACTGTAG
CGACTGCAAGGTGAAAACAACACCATCCGATG
TTGCGCTATATCACCCTCGTGCATGGAGACCCTTGCAGATGTTAAAGCCGATGCCGCACAACTGGAGC
CCCACACTGCGTTCCTCTGGAGTGCGCGGGCC
GCACATGCAGCTTATGCAAGAGCGACTGGATGGAAAGGGATTTGGATGGAAACGCAAATCCAGATTTC
TGTGGCAACAAGATATTGATACCGCTGGATTT
AGGCCGAAGCGCTTCTTTTAGAAGTTGGAAGAGAGCGCTGCGTGAAATAACGTGTGGTGTTCGAATTA
TGGGGAGATGGATTTGCCAGAGGAGATGCATG
ATGCGTGGATTGCAGTGCCTTTGGCCGGGTTTTTTCTCCCGTCTCTGGTGCTTTCCCCGTTCTTCCCA
ACTTTTTGGTGTTTGTTGAATGAGTACGGACG
TGTTCATTCCTGTTTGTTTGTTTTTTGGCGAGAGGGCGACTTTGGACCGCACTCTTCTTCGTCTGCTG
CTGCTGGTGGTGGTGGTGGTGTTTTAATTATC
CGATTTTTTTCCTTCTTTACTTTTCTTCTTTTTCCCTTGTAGTTTCTTTGTCTTTTGACGCCGTGTTC
ACTCACGTCATTGGTGGCACGGTTTTTAGCTG
TCCGATCCATATTTTTACAAGGAAAAGCACACAAAGGACCTGCCACTGTAAGCATAGGCCCGTAAGTA
ACACAAAAAGTCCTTTAAAGGAAACAAGAAGC
AAAAGGGTAGAAGACACATAAGTATCGGAAGATTATTTTGTTCTTTATTTCATCCTCTTTTCTTTTTT
GTTGTTTTGTTTTTTTGTTTTTGTTGATTTTT
TTTTCTACATTTTCCGTCTGTCTCTCATTTATTTCACCATCTCTGTTGTGCTTTTGTGTCTGTGTGTG
CGCCTGTGTTTGTCTTGAACCTTCTGCGTCTT
CGTCGTTTTACTTCATTCGTCGATCTGCCTTTGCCGCCCTTTACCGGTGCCGTTCGGTTGGGCTGCTT
GTTGGATTGTCTTCCTTTCATTGACCCAATCA
TTTTCCACGGGAGGGATTGTGGAGTACTGCGAGCAGCACACCCTAACCCATCACCGTCACAAGCAACA
ACAACAAAAAAAAAAAAACGAAAACAGCAGCG
CCGCCGACTTTTTTTTATTTTTATTTTTCGCCAGTGCACATTACAGCCGAGCAGAAGGAGGGAAAAGA
GGGAAGTCACCAACTACGTGCATCGGAGGGGG
CGTCTTTGAATTTGACTCAACCTTTTTTTTTTTTTGANNNNNNNNNNNNNNNNNNNNNNNNNNNNNNN
NNNNNNNNNNNNNNNNNNNNNNNNNNNNNNNN
NNNNNNNNNNNNNNNNNNNNNNNNNNNNNNNNNNNNNNNNNNNNNNNNNNNNNNNNNNNNNNNNNNNN
NNNNNNNNNNNNNNNNNNNNNNNNNNNNNNNN
NNNNNNNNNNNNNNNNNNNNNNNNNNNNNNNNNNNNNNNNNNNNNNNNNNNNNNNNNNNNNNNNNNNN
NNNNNNNNNNNNNNNNNNNNNNNNNNNNNNNN
NNNNNNNNNNNNNNNNNNNNNNNNNNNNNNNNNNNNNNNGTGCAGCGTGAGTTGAGGAAGCGGGGGGGAA
ACACATCGGTTTAAGGATCAAGTCGGTTGCTG
AGGGATAAAATTTAGCTGAGCTATTGTGTGTGTGTGTGTGTGTGGGAAAGAAAGAAAGAGCGCAGAGT
ACTTTTTCCTTTTCCCAGCGGGCAGGACCTGT
TAAAATTATGAACGCCAATCTATTCTACACGCAGCAACGGCCTGAGCATCAACATCACGGCCCGCAGC
AGCAGCAGCAACAACCCGCGGGTTCGGGGTTT
TTACTTCCCGCGGCTCCGTTTACTGCGGCGGGGCCCGGAGCCACGAATGTTTTTGCCGGCTTTTGTAT
GCCGCCAAGTGCCCCATGTCCACAACAACAAC
AACAGCAACGGCAAGAACAGGGACAGACGCTTGAAAGCAACGTGGCAGCAGCAAATGGCTTGTTGCTT
GCCACACAACTTGAAGCAAAGGATGGAAGACG
TGGCACCGCCTCCTTTTCAGGGGCAATACCTGACGTGTCCAATTCAATCTGGGCAACGCAGGAGCCAA
ATTCCTCGACGACAACAGCACTTCAGAGTTTG
AACGCTAGTAACTCGATAAACTACCCTCCTTTTGGCATTAAGTACATGGTTTCACCAACCAATGCGTC
TGCATCCACTCAGGAGACACCCAATCAAACGT
ACCTGTATCCAGTTTCTATGCAGTCCGAGGAAGCCGCCCCAGCTCTTATGCTTCTTCCAACTGGAAGC
AAAGATGGATCTAGCTCTATGAGTCCTGCGTA
CTATGTTCTGGTAAACTCTCCTCAAGCCTCTGGAGCTATTCATTACACAAACGGTACTATGCAGCCCC
CTCATCCAACTTCGTTTATTTTGGGGGCCAAT
TCATCAACAAAGACGACAACGACCAATTCATTCAATGCGGCACAGCAGCCCTCTCCATTTCGGATGCC
CGTGGGACAACAAACTCAAGGTGTTTCCAGCA
ACTTGGGCATTTCTTGGCCGCCTCAGTTCGCATCCAATATGGCCACAACGACAACAGGGGTGTTCCCT
GGGCTGATTGAAAACCCAAGACCTCCGCAGGT

GGATAGTGCGGCCTTTCCGTCTCATGGCCAACGAGCCTACTACACGAGCGCTGTCTTCACTCAGTCAC
TAAACTCCACGCCATCCCCCACGTTGTGTGGT
CATCGACGTCCTCCGAGTGGGAGTGGACCTGCCGAAGGTGGCACAATGCGGAGTCGGAGCAGCAGAAA
CAGTGGCACCAGCATAAACAGCATCGGTGCTG
GTGGCAGTGGCGGCCAAGAAATGGAGCGTCGTGGAGGCCTGCCGTTTGAAGAAGATCCCATCCCTATT
GACACCACCAAAACCCAGCTGATAGTCAACTT
TTTGCCGCAGTTTTTAACCGACGATGGGTTCCGTGAGCTCTTCACCCCGTTTGGTGAGATCCACACGA
AACCAACAAAAATCATTTACGACAGGGGGGCC
CGGCGCGGCAAAGGCTACGGATTTGTATACTACAAGGATGGCCGCAGCACGGAGGCTGCCATCAAGAC
AATAAACGGGCTCTCACTGGGTGGCCGCAGGC
TCAAGGTTCGCTATGCCGATCAGCAGCGGGGCAAAATTGATTGCGACAGCTGCGTGGACGAAGAAGAG
AAGGAATAAACCGTCGAGTAGACACCAGGGGA
TGTCGGGGCACAAGGAAGCCCGCCCCTCCAGCAAGTATGGCGGTACTCAAATGGATTCAAAAAGAGGC
TGCGGCAATTGGGTGTAGCCAAAGCAAAAAGA
AAGAAGGAAAAGAAATTTTGAAGGAAGTAAGAGGACGAAAAAAAAAATTTTTAAGCTTTTTTTTTTTA
TAAAGTCATGACAAAATTTAAAGCAAAAGCAA
AAAAGAAGAAAAAAAAAGGCACAAAAACACAAGAAGGAAAAAAAGGATAAATAAATATAAATATATACG
CGAAAAAAAAAAAAAGGAAAGGAAAAAAGGAAA
AAAGAGAAGAAAAAAACCGAACAGAATTGAAAAAAAAAAAAAAAAAAAAAGAAGAAGAAAAGAAAACATA
GCGATGACGGATATATTTATATATTTATATAT
TTATAGAGAGAGACGGTGTTTTTGTTTGTTTTTTCTGTTCGTTGCTGTTATTGTTGTTGTTGTGATTT
TTGTTTTTTGGCCATTTTCATTTGGCGTCATC
ATGGATGACACGTGGTTGTGCACAGACATTGTTGTCGGACCTTTTTTTTTTTTATTGTCCTTTCTCCG
CATTCCTTCCTTACTTTTTTGTTTTTTGTTTT
TGTTTCTTTGGGATTTCACTTCCTTTCATATTGTCCCGCCACTCGCATAGGCAGAACAATGTGTGTTG
TTTTTGTTTGTTTGTTTTTTTGGACCGATGCG
CGGACTCGTCGCTTTGTATACGAGTCGGACACGTTTTCTCTTCCTGAGCAAANNNNNNNNNNNNNNNN
NNNNNNNNNNNNNNNNNNNNNNNNNNNNNNNN
NNNNNNNNNNNNNNNNNNNNNNNNNNNNNNNNNNNNNNNNNNNNNNNNNNNNNNNNNNNNNNNNNNNN
NNNNNNNNNNNNNNNNNNNNNNNNNNNNNNNN
NNNNNNNNNNNNNNNNNNNNNNNNNNNNNNNNNNNNNNNNNNNNNNNNNNNNNNNNNNNNNNNNNNNN
NNNNNNNNNNNNNNNNNNNNNNNNNNNNNNNN
NNNNNNNNNNNNNNNNNNNNNNNNNNNNNNNNNNNNNNNNNNNNNNNNNNNNGGGGGGGACGGGGAGG
GACGGGAGGGAAAATTTGTGGGGAATTTGGGG
TGTTTGAAACGAGGGCGAAGAGAACACACACGCAAAGGAGCAAGAGGACGGAAAATATTCGTACGACA
GACCGAGAGGGAAGGGGGAGAGAAAGAGAGTC
AGTGGGGTGACTTCTCTCTTCTCCTCCCCCCTTTCCCTTTCTCTCTGTCTCTGTCTGTCTGTCTTTGG
GTTTCTTTCGTTTGATGCGGTAATGTTGTTGT
CAGCGTCAGTCATGACGGCGGTGATTGCAGTGATGTCCTCCGCAAAATGAATAGCCAGACGTGTATTC
CCTGTTCTTCTCCCTCAGGGTTTTTTTGTGTG
TGTGTGTTTGTTTTTTTTTTGTTTTCTTTGCCTCCGTGTAATATTTGTGTGCATGTGACTTGTCGTTTG
CAATCATTCCTCTACGTCCAGCGCCACCGTTG
CGTCCATACTCTTTTTTTTTTCCCCTCCCGAATCCCCCACACTCCGCACGCACACACGCAGGCGGAGG
GGGAAAGAGAGAGAGAGAGAGAGAGAGGGAGAGT
GGGTGACTGAAAAAAAAGAAAGAAAGGAGAGACTCATAGGTCGTCTTTTGATGTTTTTGTTTGTTTGT
TTTGTGTGTGTGTTTGGTCGTTGATTTTTTGT
TTTGTTTTGCTTTTATCTCTGAACGTTCCTGTGCGACATTTTCCCTGGTTTTGTTGTTGTTGTTTCTT
TTTCTGCTTCTTCGTGTGAAACTTCTGTCTTT
TTTGACCCCTTCTGAATATTCTGTGCGTGTGAGACCACACGTCCGTGTATATATGCATGCATACATAT
ATATATGCATGTGTGTATATATGGATGGTCTT
AGGTTTATTATTATTATTATTATTTGTTGATGCTTTTTTCTTGTTTTGGGGGGGTTTCCTTTTCCTTC
TTAACCCACCACCCCTTTCCCCTCCTTCCTTC
CCCCCCCCCCCCCCCCCTCTTGATTCATTCATTCATGCCGCGGCTCATGTGCCCGGGACTGTGCTCTGG
AAACGAGGGGGAGGGGGAAGGATGGAAGGGGA

ATGAGAGAAATGAGGGAAAAAATAATGGGATGCGTCTTGTAGAGGAAGCGAGAGCATTTGGATGGAAG
GGAAACAAAAAAAGTAAGGAAGGAAGGGAAGA
TGTGTTGTTGTTTCTTTTGTTAAAATGTATATATTTATATACGGTTTGGAGCTTGACAAGGTGGTTGC
GTAGCGGGAATGGTGTCAATGAGAGAATGTAC
AACGAAATGATGATGTGTGGAAAAGAGAAAAGAGAGAAAAGCGATGATGTCTGAAGTACAGGCGTTTC
TTGTGCGAAGTCCAGCCCTTTTTTTTGTTTTT
TCTTTTTTCTTTTTTCTTTTTTTCTTTTTTCTTTGGTAATGTCGACACCACCGCAAGAATATACCCTT
TCCGTTTATTTCTTCCACTTATCTATTTTAAC
ACACACACACACACACACACACGCACGCACACATTACATATGACATGACATTGCATTATATATACATGCA
TAAATATAAGGATATATATATATATATTTATTTA
TTTATTTATTTATATTGTAAAATGTAATATGCCTCTCTTTTGACTTATATATATGCGTACATGTATAA
TTGCAGAATTCTTCAACGCTCACTTCATTTTC
TCTTTTACTTCTTTCTTTCTTTTTTTTTTTTTTTTTTGTGCGTGTGTGTGGTGTTTGTTGTTTTGCAG
TTATTTTTCCGTGCGTTTTTCTTTCACTTTCT
ANNNNNNNNNNNNNNNNNNNNNNNNNNNNNNNNNNNNNNNNNNNNNNNNNNNNNNNNNNNNNNNNNNN
NNNNNNNNNNNNNNNNNNNNNNNNNNNNNNNN
NNNNNNNNNNNNNNNNNNNNNNNNNNNNNNNNNNNNNNNNNNNNNNNNNNNNNNNNNNNNNNNNNNNN
NNNNNNNNNNNNNNNNNNNNNNNNNNNNNNNN
NNNNNNNNNNNNNNNNNNNNNNNNNNNNNNNNNNNNNNNNNNNNNNNNNNNNNNNNNNNNNNNNNNNN
NNNNNNNNNNNNNNNNNNNNNNNNNNNNNNNN
NaTTTTTTTGATTTCTTTGGTTTTCCTTCCAGCTGTGGAGGTAACAAAGAGGAATTTGGGTCTGTGGT
GTATTCTTTTTCTCTGGCCACTTAGTCTCTTT
ATCCCTCCCTCTCTGTTTATTTTTTGTTTTTGCTTTCTTCTTTCTTCGGCCGTTTCTTTCTTTTTTTT
TTTTCTTTTTTCCTTCCTTCCTTTGTTTGTTT
GTTTGTTTGTTTTTCTATCTGAGATGATACACGCACGGCCACATGTTTGTGTGTGCATATGATTTGTT
GTTGTTTTTTGACCTTTTTTTTTCCCCCTTCT
TTTTGTTTTCTTTTTTTTTTTTTTTTACATGAAGTGATATCTCCGCGTCAATACGCAAAGCACCTTTTT
TCTTTTTTCTTTTTCGCCGTGGCGTTTTCTTC
TTGTTGTTTTTTCTTCTGTTTTGTGGAAGAGGAGGAGGAGGAGGAGGAGGAGGGTCCCGTCTTATTTA
ATCGCTGCTTGCTGCATTGTGGGCGTGTGTTG
TTTGTTTGTTTGTTTTTCTTTGTTCACCAGCCTCACGCCCCCCATCCTTTCCTCCCCTATTAAAGATG
ATTAAGCCGCACACACACACACACGGAGAGAGAG
ACACAAACACGCACAAAAAGAGGGAGAGGGGGCGCAGTGTGAAGGCAAATTTGAGGCCTTTGTTTCCT
CCCCTGTTTCGCACCGCCCCAATCACCCACCA
CCACTCCGCGTCTACATGTGTGTGTGTACGCCAGTAGTCGCATGTAGGAAGGAGAAGCGACGCGTTTG
GCCCTCGACGGCTGTGCCTCATGTGAGTTGCC
AACGGCCGTCGTGGTGCGGAAGGAAAGGAAGGAAGAAAGGGGGAGACAAAAGGAGGAAAATCAAAAGG
GGAGTTGGGTGTGGAGGGGAAATGGAACGCAA
GGAGAGAAAAAAAGGAAATGATCCAAAATAAAAACGCAAGGAAATAAGTCACGTGGCTTTACCCGTGT
CAAACCTCCCACTCCAACGCATTTATCTATCT
ATTTCTCTATCTATCTATCTACATGTGTGTGTGTGTTCTTTATGTGTATTTATCTAGCATCTTTTATG
TTATTGTTGTTGTTTTTTCTTGGTGTTGTTGT
TGTTGTTGTTACATCCTTTGAAATGCAGCCGTCATAACCCTCCTTTCCTCTTCCTCTCTCCCTATCTA
TATCTATATCTATATCTATCTATATATATATA
TATCTGTCTGCATCTACCGATAAATATCTTTTTTTTTTTTTTCCTTTTTTTTTTTTTTTTGGTTTCTTT
CTTTCTTATTTGTATGCGTTGTGAGCCTCTTT
TGATTTTTGTTTGTGTTTTGAATGCACCGTAAGCACTTTTTTTTTTCTTTTTGCTGTACTCTCAGTTT
CGTACACGCGCACACAAGCAACGCAGCGTCTG
TAATATCAACCCCAGATGTTTGCGAAAATATATATAAACTTACAGGCACGCTTTTTTGGTTTCCTGAA
TTCAATTCCTTGTCTCCACCTTTTTTTTTTTT
TTCTTTTCTCCTTCTTGTCGTTGTTGTTGTTGTTNNNNNNNNNNNNNNNNNNNNNNNNNNNNNNNNNN
NNNNNNNNNNNNNNNNNNNNNNNNNNNNNNNN
NNNNNNNNNNNNNNNNNNNNNNNNNNNNNNNNNNNNNNNNNNNNNNNNNNNNNNNNNNNNNNNNNNNN
NNNNNNNNNNNNNNNNNNNNNNNNNNNNNNNN

NNNNNNNNNNNNNNNNNNNNNNNNNNNNNNNNNNNNNNNNNNNNNNNNNNNNNNNNNNNNNNNNNNNNNNNN
NNNNNNNNNNNNNNNNNNNNNNNNNNNNNNNNNNNN
NNNNNNNNNNNNNNNNNNNNNNNNNNNNNNNNNNNNNTTTTTTTACTTATCTTTCCTACTTATTTTGGTTT
TTTGGGAGCTTTTTATTTGGTGACTCATAAGG
TGTCTTGAATGTCCGTTCATATGCCTCTGTATATATAAAAATATATGTATTTTTTTTTTTCCTTTGTA
ACTACTGTGCACATCCGTTTGATATATATATA
TATTTATTTATTTATTTATTTATTTATTTAGTTTTATATATATATATATATAAATCCATATCTGCATC
GTACTTTAGATCTCTCTTTCTCTCTATTTATT
TATTTTTACCTACTTTATATATACGTATATATATGTACACCTTTTTGTTTGTTTTTGCATGGGACGAC
ATGGAGGCAGGGGAGCGGTTGAGACTGCACGG
GGAAGGGAGGGGGGGGGGGGGGGGGGGGGGGAGAAAGAAACACACATGTAAATACATACATACATATAT
ATATATATATATATATGTGTGTGTGTGGAAAG
GAGACAAGGCTCCTCTCACTTTTTTTTATCGTTCTTTGGTCTTGGCACCTGTTGATGTACGAGGAGGG
AGATGAGGAATATACCCTCCATGCTTTTATGT
ACGTATACAATTGCACGGCAAGATGCACTTAAANNNNNNNNNNNNNNNNNNNNNNNNNNNNNNNNNNNNNNN
NNNNNNNNNNNNNNNNNNNNNNNNNNNNNNNNNNNN
NNNNNNNNNNNNNNNNNNNNNNNNNNNNNNNNNNNNNNNNNNNNNNNNNNNNNNNNNNNNNNNNNNNNNNNN
NNNNNNNNNNNNNNNNNNNNNNNNNNNNNNNNNNNN
NNNNNNNNNNNNNNNNNNNNNNNNNNNNNNNNNNNNNNNNNNNNNNNNNNNNNNNNNNNNNNNNNNNNNNNN
NNNNNNNNNNNNNNNNNNNNNNNNNNNNNNNNNNNN
NNNNNNNNNNNNNNNNNNNNNNNNNNNNNNNNNNNNNCTTGAGAATTGAATGTACTTAGCGTTTTATTTTAT
TTTATTGTATTTTTTCCTTCTACGTACCCTGT
TTTTTTTTTGTTGTTGTTGTTTTTCTCTTTTTTTGCTTGTGCATTTTTGTTTTTATTATTATCATTAT
TATTATTGGTTGTTGTTGGTTTTATTACCCCG
CCCTTTTCTTTCTTTCTTCTTTTCCTGGGGGTTCACAGTGTTGGTGCATGACGACTTATTGCGGCACA
CGATTGCACCATGATCTTTTTGTTTTTGTTTT
TTTTATTCCCTCCCCTTCTCTCCCCGCCCCCTTTTTTCTTCTCCTTTTTTTTTTTTCTTTCTTTTTTC
CTTTCTTTCTTTTCTGTGTGTTCCTCCCCTCG
CAATCACTTACATGCGCAACGAGGCGGCGGGGTACGACGTACCGCAACGGCATCATATGAATATTGCT
GGAAGGAGAAGGGGAAAAGCACGAGTTGCGTG
GGATGAGGAGTGGAGAGAGTGCGGTGCAGTCACCCAGTCCCATTCCCCCACTGCAAGTGTGCCTTTTT
TTTTTTTTTTGGAACTCACATTGGATGCTCGC
CACAAATGTTACGGCATCTGTCTCTCCGTTTTTCTTTTTTTTTTGTTTTTCTTACGGTTTCCTTCTAT
TTTTTTTTGTGTGTGTGGCTTCATGTCACCGA
GAAGAAACCCGCCGCAAACGACAAAAGAAAGAGGAGCCGAAAAAAAAAAAAAAAAAAAATTTGGAGAG
AAAAAGGGCGAGGGGAGGAGCGTGTGTGTTTG
CCATTTATGAGAAAATAACCGATAAAAATTGTCCCTTTTTTTTTGTTTGTTTTGTTGTTTTTATCTTC
TCGTTGTCGTTGTTCTTTTTCCTGTTTTTGGC
CCTCTCTCTTTCATTCTTTCTTTCTTTTTTTTCTCTCTCTCTTTTGTTGTTTTGTTTTGGGGAGGAGG
GCGCCTCTTGTGTGCACGCGGGCATGTGTGCA
GGAGGAACGCCTGCCATCCCCCGCAGTGAGCGAAGTTGAATGTGTGGGTGTGGAAGGCGAGAGAAAAT
ATTTTAAGAAAAAAAAAAAAAAGAAGAAATGGA
AGAATGGAGGACGGGAGGACTGTTTGGATGCGTGTGTACACGTGCACTTGATGTGGGGGAGAAAAAGA
AAAAGAAAAAAAACTTTTCACTGGCTCTTTCGT
ATCGTTCCTCTACCTTCTTTCTCATTTGTATATGTACATTAGAATATTTATGTATACATGTGTGACAC
ATCCCTCCTTTTTTGTGTTTTGTTTTGTGCTCC
GCCCTTCACACTCGTACTGAGTTTGTTCGTGTATGTGTCTTGCTGTGGCGTTTCCTCCGCAGCGATTT
GTTTGTTTGTGTGAAGGATGGAGGGATGGAGG
GATGGAGGGATGGAAGGTGGTGGTGGCGATGGGAAGCAACTCAACTGAAGATGGACGAAAGGGGGTTT
TTTTGTTTCTTTTGGTTTTTGTTTGTTTGTTT
TTTACGAAACAAAAGTGAGTCATTGAAGAGGTACTGTTGTGCAACGCTGTGATTGCTTTGCCGCCGCC
CTGTAATTTGACTTTTTGTCTCTCTCTCTCTC
TCTGTGTATGTAACATGGGGCTGCAAATGTTCTGTGATTCGTCTCTCTGTTAACTTCTTCTATTTCTG
TGGCTGATCTTTTTGTTTTCCGACTATCTTGT

TGTGTACATGCCTTTCATACATGTATACATTTAATTTTGTTTTTTGTTTCTTTTGATTTTGAGAATTT
ACGGGAGGAACGCCTTTTTTGTTTTTTGTTTT
TTGTTTTTTGTATGCGTTGAACGCCGATATTGACGAGTACGGCACATGAGCCTTTGTTGGCCTGTGTG
TCGGCGAAGTTTGTTTTGTTTTTATGGTGACT
GATCGTTCTTTTTTCCCTCTACCGCCTTCAGTGCCTCTGCTTGGGCATACGATTCATTCATCCACCTT
CAGCTTGCGGGCGGCGCATGGACGCTTGCGGT
TGTTGTTGTTTGTTGTTGTTTTTTTTGGCTGATGAAATCCCTCTTACTGCCGCTTTCTGCCTGCGTGA
ATGCGTGTGTTCTCGCGCTGCGTGTGTGTCTG
TGGTGGGAATTTGGGTGGGGGACGAATGGCAGTTGGCGGAACCGAATACCGAGGACAGTGGAGAAGTC
ATTTGAAAaAAAAAAAGAAAAAGAAAAAAAAA
GAGGAGATATAAGAGAGGGAGGGAAGCAATAAAAGTAACTCATGAACGGTACCGAATTGCGGCAGGAG
GTGGGGCGGTGGTGGTTGCCGTCGTGTTGAAC
TGCGGCCTTAAACGCCTCTGAAAACCGCCCACACGCACATACCGCACGATTTGTATCATGTCTCACGC
TGTAGTCAAAAGAAACACAGAGAAAAAAAAGC
TTTATGCACACAAACAGACACACACACACACACATATATATATATATACTTATATACACTTATATACA
TGTGTACATGTGCGGGGGAGAGAAAGAGTGGG
AGTCTCAGTGTGAGGGGAGGAAGGGGAGAAGTGATGATTGTGGAAGGACACGGAGATGTGTGTAAGAA
TACTGCTGTTGTGTTGGCGCTGCATCTCTTTT
ATTTCTTTATTATTTTTCTCTCCTTTTTACGGAGCACTGCTGGACGTCTTCGTTTCTTTTTATCCACT
TTTTTTTCTTTCTTTTTTTTGTTCTTGTTTTT
TTTTTTTTTGTATGTGTGCGGATGTGTGCTTTCTTTTCTCGGTTTTTTTTTCTTTTATTTTGTTTTTT
TCCGTATCGTTCCTTTCATGTTCTTCAATTTG
TTTTTGCGCTGGCGATGATTGTCTCATAACGTCGCCACGGCATATTTTGTCTATTTTCCTTTTTTTTT
TCCTTTTTTTTTCCCCCTCACATTGTTTAGCC
ACAGCATCACGACTCCCGCCTTTGTTTTTGTTTTTGTTTTTGTGTGTATGTGCGTCTGCGTGCTTTTT
TGTTGTTGTTGTTGTTGTTGTTTGGTGAGTTC
CTTATGATTTCTCTTTCTTTTCTTTTCTTTTTGATTTCTCGTCAGTGTCGGTGTCTTAATAAAACAAG
CAGTGAATTTCAATACGAACCGTGTGTTTGTT
TTACTTCTTTTTTTTTTTTTGTTTCTTTTTTTCTATAAATATTTATATTTGTATGTGTGTGTGTGTGGC
TTGCCGCGCATTCATCTGCCACTCCATCGCTG
CTGCTTTATCGTTCCAATAAATGATGGATAACAGTGCCCACATCAAAATACATATTACATGCATACAT
ACATACATATTATATTATATTATTATTATTAT
TATTTATTTGTGTGTGTGTCTCGGTAGGTAATGAGGGTTTATTCCTGCATGTCCATTGGCCGCACATT
GCTGATGGCGCATGTGTTGTTTTTGTTTGTTT
GTTTTTTTGTACGCATGTTTTTTTTTGTTCCGCGCATTTATTATCTGTGTGTGTGTGTGTGTGTTCCT
TTTTTGTTTTTGTTTTTGCTTTTCTTTTTTGC
GTGATGTTGTACGCACACGCNNNNNNNNNNNNNNNNNNNNNNNNNNNNNNNNNNNNNNNNNNNNNNNN
NNNNNNNNNNNNNNNNNNNNNNNNNNNNNNNN
NNNNNNNNNNNNNNNNNNNNNNNNNNNNNNNNNNNNNNNNNNNNNNNNNNNNNNNNNNNNNNNNNNNN
NNNNNNNNNNNNNNNNNNNNNNNNNNNNNNNN
NNNNNNNNNNNNNNNNNNNNNNNNNNNNNNNNNNNNNNNNNNNNNNNNNNNNNNNNNNNNNNNNNNNN
NNNNNNNNNNNNNNNNNNNNNNNNNNNNNNNN
NNNNNNNNNNNNNNNNNNNNCAAAAAAAAAAAAAAGGGCGGGTATTTTTCCCACTGAAGAAAAAGGGA
ATTGGCGCACGCAAGGATGGACCTCCCCACAA
TACGCCGCTACTTTGATGTGGCGGAGGAGTTGTACGCAGCGGGTCATCGCTTGCATGCATTTGCCACG
TTGCATGCCCTTTTCGCTGTCCCGACGACCAT
CCAGCTGTACTACCGTCGGGAGGAAATTCTCTGTCGTGTACGTGCAGCCGAGATGCTGCTGTACAACT
CCTCACCGCCGCAGCAAGGCAAAAACAGTACC
GGGAGTGGCAGTTCTGCGCCATCTGCAACGGTGGCACGACTGGATTTGGCGTACTCCGTCTTGTCGCC
GATGATATCTTCTCGATGGTACTTGGAGGGCG
TCATCGTCTGCCCCCTGCTGTGGGATGACGGGAGCAGCGACGGTGCGCCAGCCGCAGATGGGGAACGA
GAAGGAGAGAAAGCGGGAGAGGACGGTGAGGG
CAGATTTGTTCCTGTTGCGTTAATTGTGAAGGCGTACGTGTTGAGTGCGATTGTGCATCGTCGTCGTC
TCCGACACACGCAGGCGGTGAAGTGTATCGAG

GCGGCTGGGCAGTGGTGCAGCACCGTCTTTTGCGCGCCGCTGACCGGTCTGGCCACGAGCCTCCCAGA
CGACGCGGAGGCGGAGGCCCAGCAGAGGGAAG
AAAAACGCTGCTGTGAAGCCTTTCTTTCAGTGGAGTTTTGCCGGGTGTATTATTCGCAGCTTGTGAGC
GCCGGAACGGCTGTTGTTGCGGAAAATCGCAC
ACCGAACACGGCGGTCCTTCATGCTGCACGCACGCACGCATTAGATCGGCTGCTCTATCATGCAAAGG
AATACGCGAATTACTTCACATCTGACGGCCTG
GAGCCACTCCTTCTGGAGGATAAAAGGCGCAGACAGTGTAGTTCACTGTGTTTTTCTTCTCCAAAGAA
CTTGTTTTCCATCCGAGCGGCGGCGCGCGTGC
TTTCTCACTACTACTGTGCCGCGTTAATCCTTGACCACCGCTTGGTAGAGGCGGAGCGGCACATGCAG
CGGTGCGGAGTGTTGTTTGGTTTCTCTCCCGA
GATGCTGTGCATGATGCAGCTCCTTGCGGTGGGACTAAGGGGACCATTAGCATGGAAGGGGCCGCGTG
GAGTGAAACGACAGCGCAGTGAACAGGTACCC
GATGAATCTCAGGTTTCACAGAACGAGGGAAGCATGTCACCGAAGGAGGGACTTAGATGGATACAGGA
TGGGTTGTTGGTGGCATTGAGGTGTTATTTGA
CCATGCATGCAGCTTCTATGGATTGTGCGACCGCGGAGACAACGTTATCTTTTAACTCGGTTTCAGAA
AGCATCCCTGTTGATGCAAACACAGTGGCCGT
CAATAACACTCAAAATGGGAATAATCCGGTACGTATTCTGTTTCAAAGAACACTGCGTTGTATTGATG
AGCAGTTGACGCTCCTGACGACAGAGGTAAGT
CAAAATGGTTGCGAGGTAAATAATGCCCGAAAGGATCTTCTCTACAGTTCCCCCTCAGCTGATATTCG
TTTTTTGGTGCTGCTGAAATGTGCTGCACTTG
TGACGATGACTACATATGCTCTTTCGCAATTGTGGCTTGTGGAAGCAGTGAGACACCTCCGTCAGTTG
CGTCTCTACATGAANNNNNNNNNNNNNNNNNN
NNNNNNNNNNNNNNNNNNNNNNNNNNNNNNNNNNNNNNNNNNNNNNNNNNNNNNNNNNNNNNNNNNNN
NNNNNNNNNNNNNNNNNNNNNNNNNNNNNNNN
NNNNNNNNNNNNNNNNNNNNNNNNNNNNNNNNNNNNNNNNNNNNNNNNNNNNNNNNNNNNNNNNNNNN
NNNNNNNNNNNNNNNNNNNNNNNNNNNNNNNN
NNNNNNNNNNNNNNNNNNNNNNNNNNNNNNNNNNNNNNNNNNNNNNNNNNNNNNNNNNNNNNNNNNNN
NNNNNNNNNNNNNNGTATTCTACAAGCACACG
ACAAGTGTGCGAGTGCATTATCATCTGATGGTATCCCAACTAACCTCGATGCTTTCATTGCGTCATCT
CTCAGGCAAAAGTAATGTGGCAAATTGCACGG
AAACGCGTGAACCCAAGCGACTCTCGGAAAGGGAAGAGGAAGAGCGGACTGCGGGGTTTGATGTGGGA
TTGCCATATGCACACGTCCTTGCTGCGGAGAG
GGCGACATTAAGTGCTGCTTCTGTATGTCTCTGTGGCCCATCCACACGGCTGCTTGTGTCGCTTATGA
AGGGAGCCGCGATTTCACACGCAGCCCGTGTG
GGGTCGGCAATTCACATCACGGATGCCGGAGTCACACTTACTCATCTCACTGTACACCAACGGCACCG
TTGTGCGATGGCCCTCACCAAAGTGTTGGAAG
TTCTCAAGGCAAGCTTTGTTGCAGTATCTGCGCAGGAAGAAGAAAAGAGGAAGGAGGATGATGAAAGC
AGTCTTCGACAAATAAGGTCCGCGTGGAATAA
GTCCAACCAAGTTTTTTTTCTTCTCCTTTGCGGTGCGGCTGCCATGTCAGAAGAAAAGGACGAGTTAA
CAGCTATCAGCATGTTTAAAAACGCCCTACAG
CAGGTGAGACGATACTTTGGCCCCACGAGCGGCGTTGCCTCGGAACCCTTGTACCTTCTCGCAATGGC
ATACAATGAGTCTGAACGGATGGAGCTGCATC
CATCCGAGGTGTCCACGCCAGTCTCGGAAGGTGGCAATGAATATCTTCTTGCTAGTGCCAATGGGGCA
ACAGACAGTGCACAGCAGACCTTTTCAGTGGC
CTGGCAAGTGGCTTCACAAGCGAGGCGGTTGGCACTGTTGCACTGCGTCTTGGCTCTGCAGGAGGGTG
GCAGTAAAGAAAATCACCGACATATTGTGGGC
GAATGGAATATGGAGGTACACCGTGTGTGTTTGCTGATGCGACGGGAATTAATGGAGGTCGTGTCTTT
GTAGTTACTATGCGGACTCTTCTTCTTTTCTT
GCATCTAAGACCGCACCATCGCCGCCACCTCCGTTCTCCTTTTTTTAAAGGACGGGAATGTTCTTGGG
CGATGCCGCACCAACAGAAACAGAAGGTGAACACG
TGTCTTTCTTTATTTTAATTCTCTTTTTCTTGTCTTTTTTTAAAAGACGTGAGGAGGGGGATGGCCAC
CCACGCATTGGGTGGTGTTTCGTGTGCGTGTC
TGTTTGTGTGGTACTCTTTCTCTTCCCCCACGTCTTGTTTATCTTTTTTTTCTGCGTGTTTGTGTGTA
TGTGTTGTTCCTGTGGGATGCGTGTATTTTTT

TTTTTTTTTTTTGGGTGGGGTTCATCCACGGAAANNNNNNNNNNNNNNNNNNNNNNNNNNNNNNNNNN
NNNNNNNNNNNNNNNNNNNNNNNNNNNNNNNNN
NNNNNNNNNNNNNNNNNNNNNNNNNNNNNNNNNNNNNNNNNNNNNNNNNNNNNNNNNNNNNNNNNNNN
NNNNNNNNNNNNNNNNNNNNNNNNNNNNNNNNN
NNNNNNNNNNNNNNNNNNNNNNNNNNNNNNNNNNNNNNNNNNNNNNNNNNNNNNNNNNNNNNNNNNNN
NNNNNNNNNNNNNNNNNNNNNNNNNNNNNNNNN
NNNNNNNNNNNNNNNNNNNNNNNNNNNNNNNNNNNCGAAGAGAAATTCATATACCTCCTCCCCGCGTCT
ATCAGGTGTAAGGAGCAAGCGGTGAAGAAAAA
GTGAAAGAAAAAAGAAAGCAGCAACAAGAATAGGAAACAAAAAAAAAAAAAAAACCGAGGAGTCGGTA
AACGTGTTTAGTACGTGGTAAGTGTGACGGTT
TTGTCGTGCACTGGTATTATTTCTTGGATTGACAGTGGTAGACAGTCATGTTTGAAAGCAACCTGCTC
TCGCTGATCAACACCGCATCGCCGCTTCCCAC
GTTTGTGGAGGTGGACTTTGTGAACTCCCTTAACAACTCACTATCAAAGGCCTTCCAGTTTGCCCACG
TGTTACTCGCTGAAAAAAGCGACATAGCGGCC
ATGTGTCTGCCGTGGGATGCTGAGATTTGGCTCGTTCTGCACTCTCTTCTCGAGCACCGACTTCTCTT
TCATGCGAATACAACGTTCTCGGAGATGTTGT
TTGGACTTCGTCGTGGCTGCATTGCATCGCCATCGAAGCCGTCTGGGTCTCAAGGGAGGCTGTCGTGG
TGGGTTCGCGGCCCCCTCCCCGCTCCCTCATT
GGAGGAAAAGGCGTTGCAAGCACCGGACGCTGCTGCCTCCCCTGCCGCATTGGGAGCTATCTCCGAGT
CTCCATTGCCTCAACGAAATGGTGAACTCACG
GCAGCGGAGATGCAGGCGGCACAGGGTGCCTCTTATGGCCACCTCCGTTTTATTCCCATCACCAACAG
ACAGCGGTACATTACACTGTTTCTCATCACGG
TAATGCCCTACCTGAGAGAGCGTGCTGCTAAATGGTACACGCATCGGATGGATTCTTCTCCCGAAGCC
AACTCTATGCGCATTGCTTATGCCACTCGTTA
CCCCACACGGGCGCGAATAAAAGAGTTATTGACTCGACTGTATCCTCTTGTTTACACGGCATTAAGGA
GCTTGAGGTTTTTTTACCAGATACTTTTTCTT
CTGGAGTTGACGCCATATACCACACCGCTTCATCGTTTGTTTGGTATCGTTTTGCGGCGTCTTACGAG
AGCTGATCAAATTGCTGCGTCGAATCCACGAG
CTCAAAGGGCTCTTATGTTGGCACGTGTATTGGTTCTCCTTATTTTTCTTGGATTCAGGCTGCTAGAA
CTCACTGGTGCAGGTAATGCTGCTGGAGGAAG
TACGGCACTCACATTGACGGGTTCAGGTGGAGAAGACCTTGCAACACCACCACCACCGGTGTGGGGCG
TTGATGTGGTGATTCCTCCAGGGACGAAAGTG
CCGCAGCCTGGCATCTGCCCGGTGTGTGAGCGACGCGTAACAAACGCTGCAGTGTGCACGGCAAGCGG
TGTGGTTGGGTGTTACCCGTGCCTACTGGGTT
ACGCCAGGACAAATGGTGCATGCCCCTTGACAAAGTGTAGGACCACGGTAGATTGTATACGACGCATA
TATGAGTGCTGATATGTAATGAGCCAGAGACG
ATAAGGACAGCAAAAAGGGCGGGACCTCAGTAGTATGGCCAGGAAAGTGGATTGTGTGTGATTTCTTT
TGTGGCTTCTGAAGCGCAACGTTTTCCTGTTG
GAATAACAAATCCAAAAAAGGTAGCATGGAGAATGGTCTGTGAACTACATTCAGGCGGTTCATGGAAA
AATACAGTTTGCCTCATGGGGCGAAGAATAAA
TGGTTATACTTACTCTGCACTTTTTTTTTGAATTTTTTCTTGTGGTGGTGAGGTGGTGCATGGAGCTT
TGCTGGTATGGGGCTTCTGTGAGTTACTCTTT
TTTTTTTTTTGATGGCAGTGCCTGTGTGGGCAGGAGGGAAGGAACTGGCAGTTGGAGGAAGAGGGTGC
AGGGTGGAGGGCCGTGGAGAAAAACAATAAAA
AAACTTTTTTTTTTTTTAAAGGGTTCTCATGGCGAGCATACGCGGCGGCGTTGGTGGGTTTCTTATTC
GCCGCGCGGCCGTAAAGAGCGTTCGGCAAAAG
TACCAGACGGGCCCACAGTTCAATAAGCGCAAATTCTTTCAGTTCCCAAAGGGCTACCACCGTCTCCA
CCTTCGTATCGGTGGCGTTCAGTTGGGCTCAC
CGACGCAACAGCGGGAACACACGCGCTTCTCGCATCTTCCAGGTGACACTCGAACGCGGCCGCAGTAC
GACTTCACTTTTGGTGAGAGACGAGCAGATGG
CGCCCTGTACGCCTGGCGGAAGCGCGGTAACCTGCAACTGTACCAAATGGGCGGAAAGCCTGAAACAT
TTGTCTGTTACCGTTGTGGCTACCCGGTGCGC
AGCCAACTCGTGGCGATAAAAGGGGACAACTGGGATTACCGAATGTGCTACAAGTGTTACACGACAAC
TGTACACCACGGGATGGAGAACGATACATGAA

GGTCTGCGTGTGGGTCTGTGTTTGTGAAACGGAAAAGAGAGGAGTTTTGCGTCCAGCAATATATGCGT
CTTCTCTTTGGGAGCGGCGGGTGGTCCCTGTA
GTGTTGTTGTTGTTGGTGGTGGTGGTGGTGAGGTTGCTGATTCCATTTTTCTTGCTGTTGTGGATCCA
TTCTGTCGCGGTATATTGAAAAAAAGAAATAT
TTAAAAAAACGGGCATGAAGGGGATGTCTTTTGTTTTGCAATAAAAGGAGTGCGGCTGCACTCAGAAA
CAACTTCTGGAGACGCTGTGAGCCCCGCCGTT
GGTGGTGTCGACGTCTTACTTTTCTTTCGGTCTTTTTTCTTTTTTTTTGCCCTCCATCGTAGTGAACT
TTCGACACGGCGGAAACGAGGGAAGGGACGGA
GGGAGAAGAAAAAAAAAAAGGTGGGATTGGCGGTGATGAGCTACGCGGGAGGCACGTACTACGCCAATC
AAGAGGCCGATTCCTACTACTATGACCAGCAG
CAGCAAATGGCTGAGGAAGAAAACACCATTGATTTTCGTCCACCCGACATCACAGAACATCAGGACAA
GATCATCCAGCTTGTGGCCAAATATGTGGTGT
TTTCCTGCGATGGGGCGCGATATCAGCACCTACTTATTAGGAGGACGAAGTTTAACTCCTACTTTGCC
TTTGTTGCCTCACCAGAGCACAAATATCATGA
ATATTACCAATATCTGATTAGAAGCTACATCGTGTGGCGCGACATGGCTGCTGCTACCTCTGCGGGAG
AAGGGAACACAGATGAAGGGTACGCCTTCTAC
ACGGAGCAACTGCGACAGCAAGAACAACAACAACAACAACAACAGTACCAGGCTAATATTTATTACAC
TGCATCAACGGATTATTCAACGCAGTACACGG
ATCCAACGGCAGCGGCAGCGATGACGACAACTGGGGGTTACTACGATGCCAACACTGCCTATTCTCTG
CAACAAAAACAAAATCAGAATACATCCGTCCT
TGGGGAGTCTTCGGCCGTTGTAGAGACCTCCCGTGGAGATGATGTCAGCAGAAAACGCGCCAGGTCAG
CAACGCCGCAAGAGAGTAGTGATGAGGAGGAC
GATGCTGGACGGGAATTGGTGTTTGTGATGGAAAACGGGGTTGCGAGAGCCCTTCCCAAATAAAAAGA
AAATAAAAGAAATGCAGGCGTCAAAACCGCAG
CAAAAGAAGATTCTTTTGTGCGAGGTTATAGTCGCTGCTGCTGCTGTTGGTGTTGATGTTAGTTGTGT
TTAAACTAGCCGGTGGGTGTGGCTTGTTCTTA
AAGGTGGAAAGACAAGGTGGCTGGGATAAACACGAGACGTTTTTTATTATTATTGTCGTTTGTATGGC
AGCGACAACTGCGTTTTCGCCGAGTGGATAGT
TTTTCATGTCCTTGCGTGTTGCCACGTCGTAATGGCCTTTATCCTTTATCCTTTTTCTTTTTTTTTTT
TTTTTTTTGCTCTTTGTTTTTTTGTTTTTAGT
TTGTGGTGGGGATATCCAAAAAGGGGATGCTGCGTGCTCCGGCTTCCAATGATCCGTTTGGCGACCGT
CTTCTCCCTGCCCCAGTGCAGGAAACGGTTTA
TTCCGTTGATTGGTCTCACATCACTTCTCCGCATGGCATAATTTACATTGGCTCTGGGGCATTTAGTA
CCGTGCGTCTAGCATGGTTGCAGGAGCGGAGG
AAGTGCTACAAACGCATTTTGACATCTAATGGTGAGAAGACGAGGACGACAGTTTTTCTTGGGGCGTG
TGCGGCAGTGGCAGTGAAGCGGGTGATGATAC
CGAGGGCGAATGATGGCTCTTGTAAGTACCGCCGACTGCTGTTGCTACGCGAACTCCAGGTGATGGAA
CGTATTCGAATGTATCCGCATCCGAACATCGT
GCAGTGTTGGGGTTTTCTTTTTGGAAAGGGGGAAGAGGAACAGACATTTGAAAGCTGGAACCCCTCAG
GCGATGGCACAGAGAACAGCACGGACGGGAAG
GTTCTTACAAGCCTGAATAAGGAAGGACACCGGATGGCGTTCAACGAGGAAAACAATGACGGAGATGA
ACGGACAGATCAACAGATACCGGAAGGAACCT
CGTACGTTGATTTGTGTCTCTCACTCTGCACGGGTGGCACACTGCGGAATTATGTGGCGCTCAAGGCC
AGCGAGGTCCCCCACGCGGCGACAGCTGGAAC
AGAGTGTTCAGCGTCCGAATCACAGATGGTGAATGTCGCAATGGCGTTTGAAAAGGTGAGTTTTGATC
ATTTCAGTGGCGCTCAGGGAATCACTGGAAGC
ACCAATGAAGACACCTCTCCATACGGCTGTGAAGATTATGATGCGTATCATTCTTACCCGAAACAAAT
TTTGGGTGAAGGGAAGAAAATTGCCAATGCGT
CCTCAAATCTACCACATGCGTCCGTTTCCATCTCCGCTCACCTTGCGGTTCCTGAGCGTGACATTGTC
GCCATTGTGTATGCACTGGTTCATGCACTTCG
TCACACGCATGAGACTTTAAAGACGTTGCATCGTGACATTAAACCCTCAAATGTGCTGATATTCAGTG
GGGTAGGAAAGGCACCAGAATACGCGTTGCAG
CCCTCTTCTCTCCCGATTCCGGCGTCCGGTGGAGAGCCGTCGCAAGAGCGCGACAAAACGTGTTCGAG
CCAGGCCAAACCCTTAAGCCTAAGAGTTGACT

CGGAGATCAGGAGTACTTTGGGTGACAGTGATGAAACGGTGGAATTTGCTCTTTTTCCTCAATGTCCA
GCAACCACGCATGGGTCCACGGGGGCACTGCG
GCAGAGCGCAGGGGGGAACCTTGCGACACCGACAGCAACAGCAGGGGGAATGTCAAAACTCAACGATG
AACCTCCCACAACGTTGGTGGTGCCTCTGGGA
TTGGGGTACTATCGTTTGCCATGTACTGCTACGGATTCTTCACGGAGAGTTTATATTGATTGCTTGCC
GCAGGCCGAGGGATGGCGAATACAGTTGGCGG
ACTTTGGGGTTGCTACTGGGGTTGACCGGCTTGCTGGCTCAGGAAGGTGTGGCACCCCTCCGTTCATG
GCCCCAGAGGTCGCTCGACATGCCGACCAGGG
TGTTCCCGAGCAATACACGACGAAAGCAGACATTTACAGTCTGGGGGTAACTTTGCAGCATACAATTC
TTTACCTTGTTATTGAAGGTGAAGAGGGAAGT
CCGAGAACGTGCAGGTATGCGGACGCATTGGGGGCGCAGTGGATTGATGAGGACGACACCGCGACGCA
ATCACGGCTGGCGTTTGGCACAGGCCTTGAAA
GTAACAGCACTGAAACAGATTTAAAGCCGAACTTTATTGTACCTAACGCCTGGCGCTGTGACAAGGAG
CTCGTTGAAGGGGAATACATCCGCTGCGGTTC
ACTGGAAACGGAGTTTCCCAAACGTGGTGTGGATGATGAAAAGGAGCATGAAAGTAAACCAAAAGGAT
TTGTTGTGCCCCGCGCCTGGCGATGCCGCGAT
GAGCTGTTGGAACGCCAATATATTCGCCGCGAGTTGTCCCCAAGAAGGAATGATACTGACATCCATGC
TCACCTTCTCACCTCTTTGCCCAATGAGCTCA
ACGACTTCAAGGAAAATTCCGCGGCGAGGGAACGTCGTGGGTCACGATGCAGCTCGTGTGGAAAGATT
CATCGAAACCTGATCGAACTGCTAAACGCCAT
GACGGTAAAGAAACCATCACAACGGCCAAATTTGTCTACTGTTCTTCAGAGTACTGCGATTATTGAAC
GAGGCACATTTCTGGAAAAGGGAAGGCAATTC
TCTTACCCCAATAATGCTGCTTGGGATGTTGGCGCTGAGGACCACGGAAACCAGCCATCCATGGACAC
GAATTCCTTGGAGAGAGGAGCGGCGGCCCAAC
GCTTTTCCCGTGCACGACGGTGGGCAGAGGAAAGAAATCGTGTCCGGTCTCCTCCTACGGGATTTCGG
TTTGACATGACAGAACCGCGTCAGAAGAGCAG
CCAGGGGAAAAACCGTGGCGGTGAAGATGACACAACAGTGTTGCTCTGGCGTCCACCTTCCCTCAGCT
TCCTTCGGGCTTCCCGCCACAAGATGGAGGCA
CACAACAGATAGTATACAGATAGTAAGGACAGATTTGTTTGTTTGTTTTTAAAGCGTGGAGGAGTGAG
ACCTTTTTTTATTTTTCTTTGGTTGTTTTTGA
AAACCTGCGGGGGGAATGGTCTGTCCCAGTGGTTATTTGGCAAATAGGCATCTTTACATCTTGTTTTG
GAAGTCCGCCCCACTCATCCCTCACTGCCCTG
CGTGGGAGgggtgtcaaagacgcttgcag
>scaffold110.size29389
ACTGAATTATGCGGACCCGCTGGATGCGAGATATGTTTCCATGGGAACAAGTCCCCACCCATCGCAGA
GCATGTCGCCGGTGAACATGCGAGAGTACCAGC
AGCAACAGTTTTTGATGCAAAGAAACCAGCAATACCGTGAACAGCAAAAACAAATGATGGCGGCGCAC
AGTTCGCATGATGCGACTCCAACGATGGGAAC
GAACGACAATTCGCCCATGGGGCTCAGGGAGCCAAAACCAAGGTTGTTGGCTCAGTCACCATCCTCTG
CCCTGTCACGGAGGGCTCAATGGACGAATGAT
GACCCAATTCAACAACAACAGCCAATGGGAGCTCAGCAGTTTCCTCACCACTCTCATGCGTTGTCCTC
CCCGGGGGTAGTACGGCGTGCCATGGAAAATA
TAGAGGACCCTCCAGCGCAGTCCTCGGATCGTCTTCCGGCTACACAGCAGGCAAAACGTCCAGACGCT
CCTGAGAGTTCAAAGGTGACCGAAATTTATGG
AGAGCAGGGAATATCCATGAAATTTACCCGTGGATCAGGAGATTTTTCCCAAACCCCTACTCCGTCGG
ACTCATACGTCTCGGAAGACAATGAAGAAGCA
AAGAAGGATGAGCAATCCATGCAATACGTCCCTGCACCCTCCAGTGATATCGAGAGTGAAATTGACAA
CAATAAAATGGCTCGTTTGGCACTGGACTGAA
GCTGATTTTGCGGGGTCAGCCCGAGTGAAAGGTGCGAAAATTACTCGGTTTTGGATTTTATGATGAGT
GGACGCATGAATTTGAGATGGAACGTGATTTT
TTTTTTGCCTCTTTTTGTTTCCCATATGCTTATTTTGGATGTTAGATTGCTTCTGTGAATTTTCGTGT
CTATTGGGTTCTTTTATTTCGTTGTTGTTGTT
GTTGTTGTTGCTATTTTTAGTTTTTTGTGGGTGTTTCTCCCGACAGAAAAAAAGATAGCCATGATATA
TATATTTCGTTTGCTGTTGTTGCCATTGGTTT

TGTTCTTTTTCCTTATTGGTTGTAATGTTTTTTTTTTTTTGCATTGTTACTTTTCTTTCCAGGCATAT
ATACTTTTGTAGGGAAAAAGGGGGAAAAAAGG
CGCTTTGGCAAAGATGTCCTCCAACGTCGTTAGTGCTCCGGGTGCCGGGAAAGAGAAGAGTGCCAGCG
CGTTTGTTCGTGAGACGCGTAGCGTCAAGGTT
CCCCACCACCGTTTTCCTCCCCTAAAGAAGGTGTGGTTAGAGGTCTACACCCCCATTGTCGAGCAGTG
CAAACTGGAAATCCGCATGAATTTAAAATCTC
GGTGCGTTGAGCTGCGGACCTGCCCAGCCACCATGGAGGCTGCAGGACACGGTGGAGACAGCAGCAAA
GTGCTCCTTCAAAAGGCAGAAGATTTTGTCAA
GGCGGTCGTTGCAGGATTTGAAGTGAAGGATGCCATTGCCTTGTTGCGTATGGACGATGTGTACATTG
AGGGCTTTGAAGTGAAGGATGTGCGAGCCAAC
TTGCATGGCGATAACCTCTCCCGTTGCATTGGGCGGATGAGCGGATCCCATGGAAAAACGAAGTACAC
CATTGAAAATGTGACACGGACGCGGATCGTCA
TTGCCGACACGCACATTTGGTTGATGGGAACAACGCAGAACATTCGGGTGGCAAGGGATGCCCTTTGC
GATTTGATCCGTGGAAGCCCAGCCGCAAAGGT
GTACACCCGCTTGCGCGCCGTAATGAATCGCGTGAATGACACGTTTTGATGCTTTGTGAGACAAAAAA
AGAAGGGAAGGAAGGAAGGAAGCATTCTGGAG
GAGAAAAGGAAAGCAATGAAGGAGAATGAAGGAACATCACGCCCAGATGTTTGCGTTTTTCTTTTTTT
TCCTCCCCTTTTTGTTTTTTTTTCCTCAAAAT
ATATATTTGTATTTATTTATAAATACATGTGTGGCGGTCACGGCAGACAGTGCCTGCGTATTGTTAAG
GAGATTATTGGCACAGATGCCGTGCTGCGTGT
AATCGAATAACAAGGTCAAGAACAAATGGGAGCCCCTTGTCTAAGTTGTTCATTAAAGTAATTGTGAC
ATTTATGGGTGCGCACGCACCCCCTCTCTTTC
TCGGTTGTCGGGTTTCATATATTTTTTTTTCTTTTTTGCATTTTTGGGAATATTAACTTTTTATCTCA
TTTTTTTTTTGTTTCGTGCTGTCTCATCCTTGT
TAGGAAAGAAAGGGAAGGAAAAGGGAATATCAACTAAATTGCGTGTCGTTCCGTGTCGGATGCTCTCG
CATATTGACCGCGTGGAGTTGTACAACTTCAA
GAGTTACGCCGGCAATGTGACAATCGGGCCCCTGAAGGACTTCACGTGCATCGTCGGGCCTAATGGCG
CCGGCAAATCAAACCTCATGGATGCGCTGTGC
TTCGTGCTGAGTCCCAGTGCCACGACAACGCTGCGCGGGAAGGACGCAACGGACCTAATCCACCGCGG
CGCGCAGCGAAAGGAGTGTGCTGTCACCGCCG
TTTTCTGCCACACCACACCGATATCGCCAGCAGCAACAGCAACGACAACAGCTGCGGGGCAAGGTCGG
GATACTGAGATCTCCTTCACACGAGCGGTGGA
CCAACGAGGGAGGATTACGCATAAGATTAACGGCGAGCCCGTGGACGACCGGAAGTATTTGGCGGCAA
TGTCGAAGTTTAATGTGGGGACTCGGGTGAAT
AACTTTTTGGTGTTTCAACACGAAGTGGAAGCGATCGCTCAGAAAAAGGCACGGGAGCTCACGGATCT
GCTGGAACAAGTCAGTGGGAGTGCAGAACTGA
GGGAGGAGTACAATCGCTGCAAGAAGGCTCATGAATTGGCAAACCAGGAGTTGACGACGGCCTCTGCT
GAAAAACGTGATGCGGTGGTGGCTCTTAATCA
AATGCGACTTCACAAGAAGGAGGCGGAAAAGTACGAAGAAGTTCTTAGGCGCATTGGTGAGGAGCGGC
GTGATGAGGCGTTGGTGCAGCTGTTTTACGTG
GAGTCTAACCTGGAGCGGCAGAAACAGGAGCTTCATACCTTCACTGAAAAACTCACAGCCCTTGAGAA
GAGTATCGCCTCGGATGAAGACATACGTAAAA
TGAAGCGTGAGTATGCCGAAAAGCACAAGACGTACTTGGAGGAACTTAAAAAGATCCGCAAAGAGGCG
GATACGCTGCGGGAGAAACATAACACACTGGA
ACGCATTAAGGTCTCGCTGAACCATTTGAAACGCCAACATGAAGTGAAACGGCACGGATTGGACAACT
TGATGAAGACAGAGAAAGTTCAGTCACGTGAG
GTTCAACGCATTGAGGAACAGTTGCAGCAGCAGAAGGCAATTCTAATGGCATTTGATGAGCGGTGCAA
GAAGGAAGACGAGGAGCACATGACACTAAGTG
GGTCGCTAACTGCGGAGCAGCTGTCAGAGTATCGTCAGCTTCGCAAAGAGGCGGAGTGCGAAACCGTG
GTGCTAAGACAACAAATGGAGAGAATCAAGCG
GCAACAGCATTCATTGGTCGAGGGTCAGAAACAATGCACAATAGCCATTGAGAATGTCAGGTCTCAAA
AACAGGAACTTCAACAAGGAGTGCAACGAAGC
AACGAACGTGTGGCTGAGCTAAAAAACCGCATGAACGATCTTCAGGACACGGTTCAGGAGCTTACAAG
CAACATAAGTCAAAAACGGGCGGACCTTTCCC

AGAAAGAGAAAAGGAATAGAGAACGGGAGGTGGAGCTGGCGAGGATTCAGGAGCAGCTGCATGAACTT
CGTTTTATGAAGGAAAACGACAAACATGGGTC
GAGAATGGCCGGGGCATTGCAGGCGTTACGTGCTCTTTATGGCGTTCGTGGACGCCTCGTGGATCTTT
GCACTATTCCCAATGATAAATATCGACATGCG
GTGACGGTGGCTTTGGGCAAGAACCTTGAAGCGGTTGTTGTGGATACCATGGAAACGGCCATTTCCTG
TGTGCGGTATCTAAAGGAGCAGCGCCTGCCAC
CCATGACGTTTCTTCCTCTGGATTCGGTGAAGGGTAAGGAGGTGAATGACCGCCTGAGGACGTTTGGT
GGGACCTGTAAGCCTGTAGTGGATGTGATTCG
TTATGACACGGCAATTGAGACAGCAGTGCAGTATGCATTGGGACAGACACTTGTGTGCAATGGAATGG
CGGAGGCGAAACATGTTGCATACGGCAGCGAG
GATGGGGAGCGCTTTAAGGTGGTGACTGTGGACGGTTCTGTTCTTATGCGGAATGGTGCGGTCCAGGG
AGGTCTTGCGTCGATCCAGTCCCGTGCACGAA
AGTGGGACGAGAAAAAGTATGAGGATCTGCGTGCGGCACGGGATCGGTTGCTTAATGATGCGGCCGGG
GGATCTGAGGCAGAAATGGCACGGACGCAATG
CGAACTCCGCGACATGGAGGCACGATTGGAGTTTACACATGGTCGCATCAAAGTGATTGCGGCAGAGT
TGCAGGCAACAGAGCAGAAAGTTTCCAACATG
AACCGAGAGATGAAAAATCAGGAAAATGAGGAGCGTACGATTGAAAAACGTCACTCCACTTATGAATC
CGAGCTTAGACGATGTCTGCATGAATTGCAAG
AGAAGCACGGGTCTATCATGCAGGTAGAAGAACGCATCTTTTCTGAATTTCAGCGAAGGGTAAACATT
CCAAATATATTGGAGCTTGAGAGTCATGAGGC
CCAAATATTGAGGGAAAGGGCAGAAAAACGGCAGCAGATGCAGCTGCTGGTTCACAAGTTGGAAATTT
CTTTGGAGGCAGAGCATAAGCGAATCGGCATG
CAGTCCATCGACGATCTCCGAGGGGCTTGTGTGCGTCTTGAAGAAGAAATTCAGCGATGCGAACAGGA
CCTGGCAGCATACAGCGAAATTGTAAAGACCG
CAGAAAAGAAGCAGAGTCAGTCACGAGATAGCGTCAGTGAGGCAAAAGTGCAGCTCGACTCGTTAGAG
GCCGATATTCGTCAACAAAGTCGTACCTCCGA
ACAAGAACTTGGCAAGTTGGCACAGGCGCGAAGAGGGGTTACGGCTCTTCAAGCCGCTTGCGATACAC
TTCGCCTGCAGCGCATGAACATTTTGCGGCGA
TGTCAGATGGAAGAAATTATGTTGCCGCTCAAACCCGTTGAGGCCCGCGGTGTCAAACGGTCACGGGC
GGCAGAGAGCGGCGCTTTGACTCAGAGTGAGC
CATTTGTCTTGCCGGAGGACGCTCCAGCTGCTGCGCCAACCAAACAATCTCAACCCTCTCAATCTCAG
CGGTCCGCGGCTACAAGGGAGGCACAGGTCGT
GGTGGACTTCTCAGACCTTCCAGAGCCTCTAAAGGAGGCGGCATCTGACAGGACACACCTTGCGGCTT
ACAAGCAACGCACAGAAACCCTACTGGAAAAC
TTACAGCGCGCCGCCGAGTCTCTTGCTCCCAACCTGAAGGCGGCATCGCGGTTTGCTGGATCAGAGGA
TAGGTTGGGCTCTTCCTCCGCTCATCTTGAGG
AAGCACGTGAAAAGGTCAGCAAGGCGTACTCGGAGTTTTCGAAGGTCAAGGAGCTCCGCACACAGCGT
TTCATGGAAACGTTTGAAAAAATTGCGGAAAA
TGTGGACCGTGTATACCGCGAGCTCACCCTGAGTACTCGCGCGCACGCTGTACACGGTTCCGCGTACT
TGAGTCTGGAGGATGTGGAGGAGCCGTACCTG
GCGGGGACGAGGTACCACGCGACACCACCACTAAAGCGATACATGCCAATGGAGCTCCTTTCCGGTGG
GGAACGCACCATGGCAGCTCTCGCACTCTTGT
TTGCCGTTCATGCAGTTTCACCAACACCATTTTTTGTTTTGGATGAAGTTGATGCCGCTTTGGATGCT
GGAAATGTCGAGAAGTTGGCCAACTACATGCG
GAAGAATTGCAACACGACACAGTTTATCGTCATCTCGCTGAAGGACCAGCTCTATCACGTTGCAGATT
TGCTTGTTGGGGTGCTGAAGAACAAACAGAAG
GAAACCTCTTCCATCCTGACAATGGACCTTCGCGGATACCCTTTTTGACGCCTCCTTAAAGATATATG
TCTTGTCTTCTTTTTAATTATTTACCCACCCC
CCTACAAAGAGCCTCTCAAGAAAGTAAAATTGAGAAAGAAAGAAAAGGCGTCGACCTCTTGTGTCTTC
ACAAATAGTGATTTTATTAGCCACCAAGAGAG
TGAACAGAAATACCATAGAGTCTTAAAATATGAAGTGCCTATATAACGAGGAAGAGGAGGGTATGAAG
AACAGAAATGGGGGGCAGTTGATAAATGTGCT
TCCAGAAAGAGTTAAGTGGATATGCACACGCCTACCCATTTGTTTATTTTGAGACTATTTAATTTTAT
TATTTTTTTGATTTTGTTGGATACAACTTCTC

TTTTTCTTCTCTCTTTCCTTTTTTTTTTTCTGCTTGTGTTTTGTTTTCTTCACCGTCGTCACGTTATG
CATTGCAATATGTTTGGTAGTTTGTTTTTGTT
TTTTAGGAACATTCCCCTGTGATGTCTTGGGATGCATTGCCCTCAGTGCCCGTGCGACCTGTGGCTCT
TCACCACGCGCTTACACACGATGGCTATTTCT
TGGTTGATGAAAGTGGGTTGCAAGAGGACAAGGAGAGGACTGGCCGCAGTGGTGACCGCAACTCAACC
TTACCGATTCCTGCAAGTCTAAATGCGATAAG
CGATATGTTGTTACCCGTCACGCTGGAAGAGTTTGAGTTGGCCGAAGAGGTGCTGAATGCATGTGAAT
CCGGGCATGCACCGTCTCTTTGAGGTGCTTAT
TTCATTCGGGCGCTTGATTAGCGTTGTTGATGTCATTATTCTATTATTATTATTCTATTATTATTATT
CTATTATTTTTGCTGGTGGGTGCTGAACAGCA
ATGTTTTGATGGCCCACGTGTCTGTGTAGAACAGTCTTAATTTATTCTTGTGGAAAATGCGCAAAAAC
ACATGCATCGGCGTCAAATGAGTGTATTTTTT
CCGTGTTTCTTAATATTTATTTATTTATTTTTAATATTACTAAATGCTCCTAATTGCTTATGTCCGCT
GCAGGGGAGACAAGGCGGATGAGGTCATGGAC
GCGAAGGAAGCCTTTGATGAGATTCGTGCGCATTTTGCCATGCGGTCACAGTTTTCAGAGGCGGGAAA
CAGGTTGAGTGCGATGGTCCGCCGTTTTCAAG
AGTCGCTTCAAAGACAGGACGGATCAGACATCCAAGGCGTGAAAGGGGCGAGACATGTTCAGCAGCGT
CCTTCAAAGTCGTCGATGCAAAAACTCCCTCC
CCCGATTTTTCGTCCGGCTGCCGTGGAGGCTCTGCTGCACTACGCATCTCCCCTGCCAGTGCGAACAG
AGGAAGGAAAAAACGTAAGGGGTGGTGCCTTT
ATTCTGTACAATGACAAGGAGAATGACATGAGAAGTAATCGTATTGGGGATGATTATTTGAGCCATAA
ATGTGTGGCGCAGCCTTGTGCATCAGGTGAGG
GGGCTTTTCAAGGTAAAAGGGACTCCTTCAGTTCCACGCCAGTGGCATTCCTTTCATCTGGAAGAAAT
CAGGACCAGCCAGTCGATGTTGGGGCGAAGTT
TTCCCACAACGATGGGTTCAGGAGTCGCTACACGCATGAGGCTAGTGCCGCGCGATCGTCGCAATGCA
TTTTGAGAAAAGAACAGGACATGTTTCCATTT
GTGAACGGCGACAGCGATGGCGGTACGCAGAAACCGAATGGGTGTTGCGAGGAGCGGGACGACGCTCT
TCACTCGCTAGGGGAATTTGACGGGGAAACGA
TGGTTGATTGTGTGTGCCACGCCATGCAGAAGAATAAATGTCAGAAATCAGAAATTGAAGAAGAAAAG
GAGAAAAGTGAAGAGGAAAACAAGAAAAAACGA
CGAATTGAGGGCAAAAGGTATGGAAGAGGCGCCAGCTGACACAGCGAGGAAAACAACGCGGTCTATTC
TTGCAGACCTTGACGAGTTGGAGAAAACTCAC
CACGACGCCGTTGCTGTGATGCTGATGAGAACCCCCTTGCGCCCACGACCGCAAAAGGAGCAGAAATT
AGTGACGTCTGACGGTGGGCAACGCTTAGACC
AGCATGGCGCTCCTCATGGAATTCAGCACCATGTGCATCCCGAGCAGCGGGCAACCGCGGTTAACTTG
GACTTGGATGTATTATCAGGGTCTCAAGCGCT
GCGGTGCCGCCCGCCGAACGGGCCGATAGATCTCTCGGTCAGTAACAGTGGAACTCGTGTACTGTCAA
ATTTAGGCCAGGGGAATGTAAAAAATGTGCCC
TATTTTGTGAAGGGGTCTTCCTCAGTTTTAAATTTTTCAAGCACAAGCAGCTCTGATGCGTATGATGC
AAAGGATAAGTGGTTAAATGTGTCGTTCCTGT
AACTATTTTGAGGAAAAAGCAAGATTATATCTTTTGCATGAATGCATGCATGCATATGCAAGTGGTTT
TTTTTTGTTGTTGTTATTTTTGAAGATGGACG
GTCACGCTGTCTTGTGTTGTATGCATATGTGGCTGCGGTTTCTTTTTTGTTCCTTTTGTTGTTTACCG
AGGAATCAAGCGGGACAGAATTCCTTTAGGAG
AACGCTGAGCAAGAAGCAAGGATGGGAAACAATGGCTCCAAGGACGAACGCTGGCAGAGAGATTCTGA
GGCTCCATCATGCCATTCGTGTGCTGTCAATT
TCTCCATTAGCACACGGCGTCATCACTGTCGTAATTGCGGCTATGTGTTTTGTGGAAACTGTTCAAAT
TTTAGCTGCAGTATTCCGATGCGTGGAATTCA
TGTGCCCGTGCGGGTGTGTGCCGATTGCCTCCACGCATTACGCAGGGATTTTGATGGAGCTTTAGTCG
GTGGAAGCTTTAGACAGTGTGGTGGGCCTGTG
CCCCCCGTCACGGGTGACGGCAACGAGGCAGGTGCCCTGGAGCAGGAATCGCACCTGAACATGAACAA
GGCGTTGAATGGGAACTTTGATTACGAAAGCA
GCTACTTTATGGGGTCTGGTGGCGGTGGTGGTGTGACAAGCAACGCATACGGGAATTTGAGGGAGTCC
GATGGATATTACGGCAACGATGCCAGATCCGC

GGAGCACTTGATACATAACCATTTAGAGACTGATAAACGGGCTTCTCAATTAGAGAGGGAACTTCTTC
TAAAACGTTGGGGAGAAGTGCGTCAGCGTGCT
GTATTCACGGATATCTTGCTACAGCAGGTGGAAATAGTAGAGGAGAATACCGAGGTGGAGTATTTCAG
GGAAATTGGTGACATCAAAGTGCAGCCGGAGC
ACCCTGATCTACTCGCCGCCATGTTTCCATTTCCGGAGGCCGCGGAGGGGAACGCGGATTTGTTGATG
GAAGCCATTCACCTACGTATACCAGATCCACC
GGAAGGATACGAATTAGTCAAACAGGCTCTGGGGAAACGTGCAGCTCGGTTGTTGTCACCACCTGCTG
CGTAAAACAAAAATAGCAATTACATTTTGCTA
CAAAAGTCCTAATATTTTACATTTTTTTTTTTTGTCCCATTCTGAATTTGTTGTTATTACCATATATA
TATATATATACATACATACATCTTGTTGTAAT
TTTTTTTCTTGGTCAGACTGCGGCTGGTGGTGTGGAAGGTGTTTGAAATGTTTCTAAGACTTGTCTGT
GCGGGATTGTACGTGGATGCAGTAAAATCTAC
TCGTGGAATAAGAGGGAAACAAGTAAGCGCATTTTGGGGGGCGAATTTTCTTTTTCTGTGTGTGTGCG
TATATGTGCACTTCTTGTTTTTACGACTATTT
TTCTTGTTTTTTTCCTTCTTGTTGTTGTTGTTTTTTGTTTTAGTAATCTTGGGCCATGATGCGCTGCT
CGCGGGGGTGTCGGCGGCAGCTACGGCCGTAC
TACAATCTCCCCTCCAAATCGGATCACGGGCGAAAGATGACGGGGTTCCTTACGCCCTACCGCCATTG
GATGTGGAAACAAAATGAGTTGTGGCGCAATG
TCCACGAGGCGCAGTTTGAGCATCTACGAAAAGTATACAAGCGCCAGTGGCTTGAGTCCTTCCGTGTC
AATGCAGATGAGTACATATACAAGTACAACAT
CACAAAGGCAGCCCAACTTGCTCAGTGGGAGTACGAGATGCAGGCACAGGAAAAGAAGCGGCTGGAGG
CGAAGCAAATGACGGAAGGACGTCAAGCCCTC
AAGAAGAAACATTTGGACCTACTGCGCGAATTCCACGAGAGACAATTCTTCTTTTgGTACGAGCGCGC
CAGTGAGCGGCTGCAGAGCATGAATCTTATTC
AGTACATTCCACAGTCAGGGGTCCAGGAGCATATTGAGAGGGAGTTGGACAAGTACGTCGCTGGAAAG
ATTGAGGCGTACCCGTTGAACTTTGTTGGACA
AATGCCTTTGGTTGAGGATCGCGAGGGAAATATTGTGGAGGTTCCCGAGAGTCTCCTCACGAACCACG
TCTCAGAGCACCCAGATTCTACGGCCAAACCC
CATCAGCCTCACGAGAGCACTTCTGTCAGCGTGGAGGAGCAGTTGCTGCGCACCATGGCAAGTGCGAG
GGAGGAAAGTCTAGAAGAATGGCTTGAGGATG
ACTCACGAGCGCTCTCTGAAACGATTGATGACATTTCCCGTGAAGAGGAGCAGCGGGATGAGGACACA
CGGGTGGCACGCAGCATGGAGGAAACAGACAA
CGAGCGTGAAATTAGTCGCCGCATGTATATTGACCGCGGTAAAACGGGCTCAAAGGCCATATCCCGTC
GGCCGGCTTTAAGCGAGACCGACGGCGGCTCC
GCTTCTGGCTCTGTTGGTGGTGTTACCGCACCACCTGCGGACACGAGTGCGCCGATGAGGCGTCGCAA
GAAAGGCAAGCTGGACAAGGCACATGCTTTGC
AGGAGCAACAGGATGCAATGATTGCCAGAATGTCTGCGAAGAGCCTTAAGGACGGTGAAAGCTCAATC
TCAATTGTCAAGCGTGGGGAGATTGCGACAAG
CAGAGGTCGCATTCGTGACAAGGCTGCAATACCGACCCAGGAAGTATTGATGCAAAAACCTGAATTGG
CAGCCGGGAGTGTACCAAACGCACGCATTAGC
TTCAAGGACAAGGTAGACCAACTGTACCACCGCGGTAAGTACAAGCAGAAGAAGGAGGACGACAACAA
CCCAAATGAAGACCTTTAGCCGTCCAGTACTG
AGCCACATTGAGGCTGAACCCACCATGGCTTGTATGAATTGCTCTCAGGGATGGCTAATTATATATTT
GTGGGCACGCATTCTTCATTTTCGTCTGGTAT
TTAAATATTTTTTCTCCTTTTTGGAGCCTACTTCAAGTTGCAATCGTTTAATTTGTGTGGCTGTTTTT
TTTTCCAGTTTATTTTTGCTCTCTTTTATTGG
TTTTCTCTTACTTCTTTTCTATGGTTTGTTTTTTTTTTCTTCTATCAGAGATTGAAAACAAATTTCAA
GGGGAAGGCAGTGGGCACTCCGGGTCCTTGGC
GTTTTATTTTATTTTAGGTGTTTTTGTTGTTGTTGTTGTTTTGTTTTGTTTTTGAGAATTTTTCTGGA
GGCGCAGCTGACTGTGTGGAATAAACCGTCAG
TGGACATAGTTGGGAGCCCAACAAGAAGGGAATAGATCGTCGCTGTCTGCTGCTTTCGGTGGTGAAAA
AGGATGTCGTCACAGAGGCGAAGATACCGCTT
AGAGATAGCCGTTAAGAGGCTTCATGGTCTTGTGAACATGAGTTCCTCGGCGAGGATTATTTTTGTTG
TGCGTTTCTCAAGCTTTCCGGAGATTTTTATT

CGGCCACATATCAGCGGTGGTGGCGGTGTCGTTGTGCGATCCTCCACGGGCGATGGCATTCTCTCGCT
AACGTACAACGTGTGCCACAAGGCCGAATTTT
GCCTGAATGACGATGAATGTAGAGAAATGTTTCCTGCTCAGTGCGTCTGCCGCATGTACGACGAGGGG
CAGCGGCAGGCATCGGTGTCATGGGTATGCCC
ATGCCCGGTGTTGACGGCGCAAGCAGCTGAGGGCCGTCGTCACGGCAACTACGCCATGTGTGACAGTG
CCGGTAAGACGATGGGTTTTGCGGAGATGTCC
TGTCGTGTTGTTCACATTGACGAAAGGATGTTCTCTTCTTCGGTGGATCCAGGAGCCATCGCTGCTTC
CGTCGTCTCTGGCCCGGCGCCACCGTTCACAC
CGAGTGCAAAATTGACGGTCAACGGAAAGCCTTTTGTTATTCGCGTGGTTCTGGACGGCACCGACGTC
AGGGAGGGGAATAAACGTCGTCACCGTAGGAA
GCAGTATGGCCATCAGGATTCCCCACCCGAAAAATCACCGTCGTCAGGAGCTGTCATTGCGGTTGACA
GCACACCGACACAAAAAAATGAAGAGAAAAGA
ACAGGTGATTGTCGACTAGAGAATGCGGATGGATTTGACCAAACAAAAAGGGGTTCAACTGCGAAGAA
GAAAACGCTTCTTTATATTCTGGATTATGACG
TGGCGTGTCAGATTCAGTCAAATTGCGAGATGCTTTACGCCACACTGAGGCGGGAATTTACGCCCCTA
GACGCAGTTAACGTGAATGCGAAGACAAAACC
TATGACGGAGAAGGCCGATTGTGTGGTGCAGAGAATCTTACAATCCGCAAATGTTGTGATCCAGCTGG
CCAATCAGCTTGCCGAGTCATCTGGACAGGAA
CACACAAGCACACCCGTGGAAGCTCACGGACGCAGACAACCTGTAAATGAGTTGCCCCTCCCACGCGA
GGGAAGTGTGGCCCATTTTCTCACGTATCAGG
TGCTCTTTCAATTGCAGTGTCTTGGAATGAATCTCCATCATATTACAACCCTTTACTGTCGGGCACTG
GATGTGCGGGTATCTACCCTACATAGTCAGCA
TGTCAAGTTCATCAAAAAGCTATGCGCAGATGTTCAAGAATTGACAAAATGTATCAACATTATTGTGC
AGTCAACGATAGATAGAAACCATAGTGATGAG
AAAGCTGATTCGAAGTCGTCAAAGACAACGTATTCCTCTCACTTTAACAGCTGTAGCAGTAGCAGCGA
ATCTTCCTCCTCACTATTGTCATCCGGTGGAC
GATCCACACGGCGAAGAAGCTCTACAGTTGAAAGTGCCTTCTCTATATCTACGGCAGGATCCAAGTTG
TTGGCCACGCCCTTTTCTACTTTGACGGCATC
GACATCAAAAGCCGTTCCGAACCCTAATGTTTTTGATCCAAAGCCATCAGTGCCTTCCCCACAAGGGT
ATTCCTTTGATACTCCCTTGCCATCATCACCG
TCTAAGAGTGATGGACAAACAGTGTCAGCCGCTACTGAATATGGGTACAATCCCTCACCAATGTTTAA
CCTTCGAGCTGTTTCTTCATCTCCACCAGTCC
ACACAATAAATTCTTTGGTGGGCCAAGGGGCCGCAAATAGGGTAACTGTCTACGACAATCTAACGGAC
TCCTCTTATATCACAGCTAGTCTTGCCTCCAA
GTCACCATGGCCGCCACACTCAGTTATAATTAATTCAGGGACGGACGTCCAGCAGAAATCGTCTCTTG
GTGGCAGCAGCGGGGTGCCGGTGCCTGTTCCT
CAGGCGACCCAGCCGGTAGTGGGAGGCACATCGACGGCGGGGACGGACGTCCAGCAGAAGTCGTCTCT
TGGTGGCAGCAGTGGGGTGCCGGTGCCTGTTC
CTCAGGCGACCCAGCCGGTAGTGGGAGGCACATCGACGGCGGGGACGGACGTCCAGCAGAAGTCGTCT
CTTGGTGGCAGCAGTGGGGTGCCGGTGCCTGT
TCCTCAGGCGACCCAGCCGGTAGTGGGAGGCACATCGACGGCGGGGACGGACGTCCAGCAGAAATCGT
CTCTTGGTGGCAGCAGTGGGGTGCCGGTGCCT
GTTCCTCAGGCNNNNNNNNNNNNNNNNNNNNNNNNNNNNNNNNNNNNNNNNNNNNNNNNNNNNNNNNN
NNNNNNNNNNNNNNNNNNNNNNNNNNNNNNNN
NNNNNNNNNNNNNNNNNNNNNNNNNNNNNNNNNNNNNNNNNNNNNNNNNNNNNNNNNNNNNNNNNNNN
NNNNNNNNNNNNNNNNNNNNNNNNNNNNNNNN
NNNNNNNNNNNNNNNNNNNNNNNNNNNNNNNNNNNNNNNNNNNNNNNNNNNNNNNNNNNNNNNNNNNN
NNNNNNNNNNNNNNNNNNNNNNNNNNNNNNNN
NNNNNNNNNNNCCAGCCGGTAGTGGGAGGCACATCGACGGCGGGGACGGACGTCCAGCAGAAATCGTC
TCTTGGTGGCAGCAGCGGGGTGCCGGTGCCTG
TTCCTCAGGCGACCCAGCCGGTAGTGGGAGGCACATCGACGGCGGGGACGGACGTCCAGCAGAAGTCG
TCTCTTGGTGGCAGCAGTGGGGTGCCGGTGCC
TGTTCCTCAGGCGACCCAGCCGGTAGTGGGAGGCACATCGACGGCGGGGACGGACGTCCAGCAGAAGT
CGTCTCTTGGTGGCAGCAGCGGGGTGCCGGTG

CCCGTTCCTGTACGTTCTTTTAACGTGCGAAGTTGATTTATTTTTTGTTGTCTTTGCTGTGGGTTTAT
GAGGCTGATAACTAGCCATTTTTCTTTAGCAG
TAGCAATAAAAACATCATTTTGCTGCTGATGTTTTGTCGTAGATGGCGACGCATGTTTTTTTCTTTTT
TTTCTCTCTCATTGTTGTTGCTATTTTTTTTT
TGCAAGCCAATAAATTTACTTTTTGCGTCACGGTCTGCCCTGTGATTTGATGTTTTATGGGCGGTTTA
GCACCTGGGTCCATCGATGATGTCACAGATGG
TGATTTCCCTTTGGGACGCCTTCACATCGATGGTGGAGGTACGACAGGTTCTTCTTGCTATTCTACTG
CTTTTGGGGAAGAGACTGCGAGTATTTATGTT
GATGAGGAACCCCCCTTTCCTTTACGCTCCTACAGTTGTGATGGGGGTGACTTTCTTCACATATTCCG
GCCACCCAATCAGCCAGAAACAGGGTCGATTC
ACTGCTACTCCCCTTGGACTGTTCGCGGAGATGACGAGTTTTCTCTTGTGGCCCATGTTGAGGTTGAG
GATGAGGAAAAGGGATTTACGTTGGGGATCTG
TGGTGCTACAGAAAGCGTTGTAGGCACATTTCTACAGTATTTTTCGGCCGGGCAAACGATGCATCCAA
TGGGAACACAGGAAGTGCGGAGACTCTGCATA
TGTCCGTGGCATCGACGGGCAGTTCTTTTGGCTTTGTTGGACGATCGAATTATCTCTTGTGTTTATCT
CCCATCTGCCGAGTTTTGTCTTTCTAGAAGTG
GGAGAGGAAGAGAAGAGGCAGGATTAACGCAAGAATCACCGACTGCGTTGTTTGAGCCAACACCCATC
GATGTGGACGCGTGGCGTGTTGTAGTGGAACA
CAAGGGTCGGGGATTTGTGCGCTTCTATACTGTTGAGCGACGAAAAGGTTCATGCTACTGTGATGCCC
CCCTATCTCAACTAAGTTGAAGGAGGGCCTGT
ATAAAGTTTTTTCTATCCATCTATCTATCCCCTCGCATGATATTTAAAAAGAACCTTGCAAATATCTT
GGCATTTTCACATAATGCTCTATTACTGGTAC
TTCAGTATGTTTTTTGCTGCGTTTTGCCCTACCTGCTTTCTACATTGTGATAATAGTACAGACTCACA
TTTTACCAAAGGTATCGTCGCATTCGATTTGC
GCGCGTGTACGTGTAAGGAAATAGGCCAACAAGAAAAAAGAGAATTGCTGAAGAAACGAGAATGAAAC
GCGCGTTAGTCTTTTCTGGCCAGGGAGCCCAT
CGCCAGGGAATGTCTATGGAATACCTGCGCGTTCCCGCGGTTGCTCGACTCTGGGAAAAGATGAAGGA
CCAGATGATGCAGAAATATGGCATTTCTCTAC
AGGAGGTCATGACAGTGAACCCACAAAAGATACATGTGCGCGATGACGCACTGGACGTAAGTGTAATT
TGCAAAAGAGGTTCTTTGGAGGATCGAAACGT
GGCTGAGTCAGCTCCGCGGAAAAAGTTTTTTTCATCCCAAGAAGGGGTCATGTCGCTGACGTTTTTAA
CACAACCATGCATGTTGGCAGCGCATATGTTA
GCACTGGAGTATCTCCGTTCGACACGGGGGTATTCGGTCGAATCTGCCCTTGTCATTGCTGGTCACAG
TCTTGGAGAATTTTCAGCCCTGTGTGCATTAG
GTATTTTTTCACCTGAAGTCGCGGTTGATCTTGTATACAAGCGTGGAGTCTTAATGGATTACGCTCTT
GAGCAGTTTTCAGCAAATCGTGATGATTACGT
CATGTACGCCTGTCATCCCGGAAGGGCGAAACTCTGCGAAGAGGACTTGGATGTTGCGGTCGATCAGT
TTCACGTACTTGTGGAGCTCGTGGCTCGAGCT
CTTTCCGCTACAACCTCATTCGTGGAGGTTGTAAATTATAACATTGATCACGAGCAGTATGTTGTTGC
GGGAGATCGAGTGGGGCTGTCCGCTCTGGGGA
AATGTCTGGATCCGCAGTTTCGAGCGAGTGTTTGTGGCCCATCCTCTTCTTTGGATCACATTGCTCGC
ACAGCCGTGTCGTCCGTCTGGCAGGATAAAAA
GGATGGTGTCACGATGCACCCCAATAAGGGGCCTTTACCGGATTTCGTAACGTCATCCGTGAAAAAAT
ACGGTGTACGCTCGACGTTTCGTCGCTTTTTG
CGGGGTCCAGACGATGGGTACACGCCATCACTGGAGGAACTAACGCATCTGACCCTTCAGGAGGATGG
ACGAAGTGGGCTAAAAAAAAAAGTCATGGTTTG
TTCCTCTTCCTGTAAATGTCCCGTTTCACAGCAGCCGGCTTCGCCGGGCGATGGATCTTTTCTTGCCT
GTAGTTCAAGATGCGATGCCGGAGGAAGATGT
GCTGCGCTCCATGCTGGGTGTGGCTACAAAGCCCCTTGAGGAGTCCAATTCAGCTTCTGCAACTTCAG
CCATAGAGAAGCGTCCTGTGTGGCTGACGAAC
CTCACGGGCAGTGCATTTCGACCCTTGGATCCCGAATTTCAGCGCCAGGCGCTGGAGATGATGCAGTC
AATGAATGTGGGCGAGGTTCGGCACCACGGCC
GGTACCAAACGGACTTGGTGGAAAAAGCCTTTAACGACGGTGTGAAGCATAACAGTGTACGTGAGATG
TGTGCGGCCGTTCTTGCCGCTCAGTTGGCGCA

TCCTGTGCAGTGGATTGACCTTATGGATGCTGCCGTCATTCACGCAGGTATTCGCGAGGTACACGAGA
TTTCACCTGTGCGAACTACCGCCGATATGTTT
AAGCGTACAGCGTTTTGCGAGGCTGGTGAGGCGGGAGTTGCACTGGAGATGGTTATCCGCTGCCTTCC
CGCAGAGGAGCGATTTTTGTAATAAAAAAAGA
AGGGTTGGGAAATGTAGACGATTGCTGCTGCGGATTGCCCAGCTTTTTTTCCTTCTGGAGTTTGTTCC
CGTTGGGATTGGCGCCCCATCCATGAATAATG
CAAACATATAAATTTTCTTTTGTTGGGAAGTCCGTTTGAAGAAGTTTTCTCATTTTCACATTTTTATT
ATTTCCCTTTTTTTTTTTTAGAGAATGGCTGC
TTGATTCTTTTTTCTTCTGTTTGCCGTGCGGCATTTTCTTTTTCAAGAAGGACTGGGTTTCTGGGGAT
TGTGTGGAGTGTACTTTGCTCTTCTTTCTGTT
TTTCTTTTGTGTGTGTGTGTGTGTGTGTGTGTTTTCTGTCGTTGCTGCCATGTATAGTAAATGTTCAGAC
TGCGGACTGTTGGATTTGGTTGCGCGGGACCA
CTTTGTGTGAGTGGAGCGCGTCTATTGTGGTGGGGGTACAAGGAACATGACCTCCACCGACTTGCTCG
TCAGCGTCCGTGGTGGGAGAACGCTGCAATTT
TTGCCGGGCTTCTCTGCTTTATTCAGGTTACGAGTATGTGCTATTACTTTGGGAACCGCATGTACCAA
GCTTGGCGGCCATCAGGTAACACTCTCTATGT
GAATAAGGCAATGGACACTAAAGTTTTCGCGCGTCCTGATTTGTCCGTGGCAGCGGTACGTATGGTTA
TTCATTACGTGCTTTCAGGCACCCAAACACCA
TTTGGAGCCCCCATGGTGTATCCGGAGTACATGCGTCCAGAAGCGGAGGAACTGATGAAATTTATTTT
TGATTTACACAGTGAGCAGCCCATGTTCTCTA
TTCCTGATCTTTGGGCGATGCTGTCGGCAAAGGCGCTCGCTCGCCTGGGAGGGCCGAATATTTCTGTA
CGTCGCGGGAGACCAGACCCCCCGGGTCATTT
GGCAGTTGAGCAGCTTCTCGTTGAGACTCCTTCCCTTGTTCCGGAAGGGAAACGCGATGTACTCAAGA
TGAAGCGATTGCTTGCGGGGCAGGgATTTTCC
GTGGAGGAGATTGTTGGCATGATCGGAGGTATTCGAACAATTGGCTTTCATGATAGTTCGGGGTTTCA
CACAAAAGAAGAAGTAAAGCCACAAATGCGTC
GTCACCCGAGTCTGATGGGACCCCAGGAGGACGTGTTTCATCTTCCTGAGACGATGGAAAAATGCACT
TTAGATCCCTACGTGTTTGGTGGGGAATATTT
TGATCTTCTTTTGGATTATAACTGGAGGCAGCGAAGTCTTATCGGTTGGCGGAGTGGACCCTTTCGCT
GCGATGAGAAGGATCGTAAACGTGAGATCACA
CTTCTGGATCCATTTTCAGAGCAGGCCGTAATGAGGGAGCGGAAGCGTCTGCAGGCGCAAGAACAGGC
ACGGGAGGCGGTTGAGCGGGCGGCCCAAAAAC
GAGCACAGATCACTGTACCTGAGGAAGAAAGGGCGGAATCCGAAATGGGACGTGTCGGCGAGGCGCAG
TTTATTCCTGGTGTGGAAATGCAGGAAAGTGT
GGATGATGACACGACGGAAGCTGTTGGTTCGTTGGTGGTGCCGGAGTTCAAGAGCCCCTGCTCAAGTG
TTTCGATGCGGGAGATGGACATGATGTTGCTG
GACGATGCGCTCTTGACTGGGTGGCTGCACCGCTTTAGCACAAACGAGATTCGTTTCTACACGGTCTT
TGGCGAAGTCATGGAGAAGATCCAGAACCGAG
GATATAACCTCAACTCCCTTTACGTTCCGACGTGAGCGTCTTTTTTGTTGTTGTCTCGCTCCTCTTTT
GTTTGTTTTTTTCTTACATGTTCTGAGTCTAC
TTCCCTTATCCTGGAGTCCAGAAGGACTCCCGACTTGGGGGAGTCCGTCTCCACATATTTCCTGATTA
TTATTAATTTTTTTTTTGCTTTTTATTTATT
TATTTTTTTTGTTGTTGTTGTTAGGAGTTTCTTTTGTTTCTCTTTTGTCATTTTTCACATGTTTTGCT
CGTCTCTTCCCCGATGCATTTTTTTTTGTTTT
TTGCCGCTGCACTGTAATAATAGCAACGGTGCCGAACGGCGTGGATGGTGGTGCTTGCAACTAGCATT
CCGAGGGGGTATCATGAATGAATCAAAACAAG
AAGGAAAGACAAGAAACGTGGCGGCAGCGGCAGCGGCCAACGTGGACGGGTATTTACGCAATGCGTCA
AGGCGGGTTCGAATCCCCAGTGCGTCGGGCGG
GAGGGGTACACGGCGCAAGTTGGCGATGGTGACAGCGGCGAGTGTGGAACAGGCTCCGCGGTCTGTGG
TCAACACGAACGCATCCGATGCCGTCGACTTG
GATGACGAGTGGGACGAGGTGGTGCTGCCGAACGCCAACGCTGCGGCGGTAAAGAAGGATGAGGGAAA
ACTCAAGGAGGAGAGCTCTCAAAAGCCCAAAA
TGGATGTCCCCGTGGCGAATTCGTGTGCCGGTAGCATCAAAGCAGAGGCATTGGAGGTGGAAGACGGC
GAAGTGGTGAGGGGCGATGAGGACCCGAGCGC

ACAGTCGTTGATGGAGGGCACACGGCCTCCACTGACAGGCACATCGATCCAACGGTGGAGGCGAAATG
AACATTTCCAAGAGCGCATGCCTGAGCAGCAG
CAAATTCTCGCTGCTCAGCGACGTTCAGAACGAATTCGGCGAGCCGCAGAAGGAATGTCTGTTTTAAT
TTTTGCCTTACAGAGGTGCAAAACACTCGTTA
GAGAATCCCGTCACCCGCAACTACTGCGGAAACTGCTACGCGTATGCACTGTCGAAGAGAATGGTTCT
AAATCGTTCCCTTTCATACAGGCTGTACAGCG
CGCAAGAGAAGAATATCAACGAGCTCTTGACCCTACAGTGAAAAATCCCGCCTTGTCACCCTGTTGGG
TTACCGTGGGCAAGGACACCGTCCGGAATTAT
ACCTCCAATTCCATAATGGCTTTGCTTAGTGGCATCACCACGGTATTTACCCTGGAGGATAAAGTTGA
TGCCTCAATCCTCTCTAATTGGGCTGCGCCTA
TTTCCTTTGGGCATCTCTCCAACCTGTTGTCAAGATCGCATCAGTTTCTTATCTCCCCCGATGCCAAG
TTGCGCCTGCCACATTCTTTGTACTTCAGCAT
CATATTTCTTTCGGTTGCCAACATTTTGGACATGTCATGTCGACTGGTGATGGCGATAAAAAAAGAGC
GGGAGCGACAGAATCCTGTGGCGAATGAAAAT
GGGAATGTTTCCGAAAAGCCGGCGCAGATGACAATTTTTTTGCCGCGTGTAAAGCGTCAACGAAAAGA
GGGTACTAATGAGGAAGATAAATTGCCACAGA
AGCTGCCATCTTCTTGCTTCTGGGTAGAAGTCTGGTGTCCGCAACGTGAGAGTTTCATCTCTGTTAAC
CCGTGTAAAGGTTGCACCGCACTCTGGGGAGC
CCCGTACACATTTTCGGTTGGTGGTGATGTTGCCATGGATGTGACGCCGCGGTACACTACAAAATACA
GCAGCGCATTTACTTATCGGTGGGGCCGCTGT
GATTCATATCGCTtTTTCTGGAAACACCTGGACTGGAACGATACCAGAGAAGCCTCTGAGGTCATACT
CGATGCCTTCCGTAAGGATATGACCCGAAACA
CATGCATTCAGCTGGCTCGAGAACGTCGGCAGCTCCATTCGCTTACATATGCGGAGGAAATACCAAAA
ACGCTTACAGCGTTGCATAAACATCCGTTGTT
TGTTCTTGAGAATGAGCTCGCACGACATGAAGGAGTGTATCCGAAGGACAATACGACGATTGTTGGAA
GCGTGAAGGGTCACACAGTGTATAAACGATCT
GCTGTTGTCAATTTACGCAGCCGAGATGGCTGGCTTCGTGTAGGACGCTGTGTAGTAAGCGAATATGA
GGCCCCATATAAGGTAGTACCCCCGCCGGCCT
CACGTCCTTTTGGATCCTCTTCTGGCTTCTTTGGAGTGTGGCAGACGAAACCGTTTGAGCCCAGTCCC
CTGCGCAGTGATGGGTCGCTGCCTTTGCATGG
CAAAACGCGCTGGTACGTTCTTCTTGGTAAGCCCGTACCTGAGGGATTGGCGTATATTCAACGGCCCA
ATATCGCCCGAGTAGCGCGTCTAGTGGATGTT
GAATTTGGACATGCTGTCATGGGATTTCAACGGCGCCGCTTGGATGAGCGTCGATTTTCACACTGGGA
GGCTGTCCTTGATGGTATCGTTGTAAGGGAAG
CGGACGCGGCGAACCTCTTGCATGCATACGATGAATGGAGACAGCTGACAGAGGAGCAAGAGGCCACG
AAGCGACGACAGCGAGCCAATCGATGGTGGCT
ACATTTTGTCCAGCGAATGCTGGCTATGCAGCGTGTTCGTCAACAATATTTGGATGGTGCATCTCACG
GATATCTTCCGTTGCATTAACCTCCTCCTTTA
TTTTGCTATTATTATTTACTATTATTTCCCTCCTCTCTCTCTTTATTTGTTGCTGGGGATGGGGATGA
GGAAATTCTTTATCCTGTGCCGCACCGAAGTG
GCGCCTATCGGAATGCTTTCAAGAGTTGGGAGACTTGGCTCATTGTTTTTCTTTTTCTTTTTGGTGTT
TTTTATGACAAAGGAGGGTCGACAAGGAAAAA
CAGTGAGAGCAGTGCCGAAATAGCAATGCGCTCTACTCCGTATCTTGGCGGCGGCGCACGTGCCACTA
CAAATAATCTCCCACCGTTTACGGGCGTTTCG
CCCCGTGTTTCCTCACGCAGCGTAGCGATGCACCGCGGCGAGTATGTCTCCATTGTGCTCAAGCCTAC
AGCGCTGAATACACAACGTGGTTCGCTGGAGG
CGCTCTGTGTCGCGACGGATGAGCCCGTCGAGGTAATGGGACTCCCCAGTGGAGCGGAAGTGGCGCAG
GTGAATGAGTTTGTGTGCTACGTGAACCCCAG
CAGCGGAGAGCTGGAGTACTTCCAACACGGCACGTACAACGACGAATACGACGTGGAGACGTATCGCA
AACTGTTGGATCTTTGCCCAAAGTTTCCACAG
CTTTTTTAAAAGCTGACAAAATGACCGCTGCCCGAAGGCAAGGCAATGGGTTGCGTGTTGCCAGCACA
GAGGACATAGAAGGAATGAAAGAAGAAGTTAA
TAAAAATAAAAATAAAAAGAGGTGGGGAAGAGCATAAATGCAGAGGCCTCTACCGCTGCAATTATCGC
CACGACGTCATATGCGTGTTTCTGATATTCAG

CAACAAACATGAGCGGAAGGAGGGTGAAGGAAAAGAAAGAATAAGCGAGAGAGAGAGAGAGAGCGATA
GAAAGAGAGAAGTGGTTGCTTCACGTGGAA
TTTACACAAAATACAATAACACAGAAAAGAGGATGGTCTGCTTTGTGCTACTTGTGTTTGTGTGTGTG
TGTGTGTGTGTTTATTAACATCGCATGGGTGA
TGTTTTTCCCAACCGTCGTTGACACCACTCGCTTTTTTTTATATATCTTGGGATTTTTGAAGTATTAA
AGAGCCACTTTCTGTGTGTGTCTGTTCATGTA
TGCTGACGAGGGTGCAACTTCTTAATAACAAGAAATATATGGATATATATATATTAGGTGGTTTACTG
TTGCTTTGTAGCGAGAGGGGTTTTACAAATAA
GTGAAAGTTTATAACCTTTCAAACTATGGAGTCCACATTGCAGACATTTCTAGAGGCATATCCTTTTC
GACATCCTGAGAACCCGTACGAACAGACGAGG
GCCATCTGGTCCAAACTGGTGGGTTGGGAAGGGGAGTCCGCCGCGGGTCCATCCCCCTCGCTTTGCGT
GCAACCCATTCCCCTAGCGCTTCTTCAGAAGT
GGAAGCGTTACTTTGCCTTCTGTTCTCGCGTCATTTTCTATGAAATGGATTGGAGGGACGTGTTTGTG
TACATGGGTGATGTGCCAAAAGCAGCAACGCT
CTCAGTGCCACGTGATCATGTAGGATCTGACAGAAAAGCAGAGGTGGGAGTTGATGCGGATCGGGGAG
AAGGCAATGGGGAAAAACAGTTGGAAACGACG
GCGGGTGCATATCATCGCTCGACGACGGCTTTAATTCATACACTGGCGAATGGTTGCGAAAGGCGTTT
TGGAGCATATCCATTGCGCTTTCATTATCAAT
GGCTCGTGCGATGGACCAAAATGCTGGCGCAGTGCATCGAATGGCGCTTGTTGGATATATCCAAGAGG
GGTGGATCTGTGCCGCTGGAGGAGCTGCAGCG
GTATCGTCTTGTTTTTGCTTCCAGGGCACGGGAACTTGTCGGTGCGGCTGCCTACATACGCACCGTAC
TTGAACTTTTGGAAATAGAGGAAGAAAAAGAA
CTCTGCAATACAGATATTTCTTTAAAGGGTTTGCCCAGTCTCTCTCTTAGCCAAAATTTTCCTTTGAC
CTTGACACTTTCCATGAGGTTTACGCTTTCTC
GCGCCACATCTGTTTCTATAGAGCAGTGTTTTTCTGCGCACTCAGCACGTGTTCTGGACTCGCTCTGG
GCAGACGTTCTGTGCATTCCATGGGATGTGTT
GGACCCAAACAGTATGCCTGAGAAGGTACGTAGAAAATTGTTAGCCTCCTTGAATAATGCGGAAGGTG
ACACAGACGAAATATATGTGTTGTGTGGAAAT
GGCGGCTGCCGAATGCGTTCCGCGACATTGAAATTTATTTTAGAGAATGAAACCCTGGAGAGGGCAGG
AATTGCAAATAACCCCCTGTTAGGGATTGTCA
GGCTTACAACAGCGCCGCTTGAATTTATAGAGCCCCACCAGTTGCCTAATGTGAAACATTTGCTAGAA
ACGGTTGGATATGTCATGCTAACAAAAATTCG
CTGTCACTTCGTAGAGAAGTGCTATTACACGCTTCCTTTTTTAGCAGATTTTGTGGCTGCAGGAGTGC
TACTTAAAAAAGCATTGGTTTCCTTCTCACGG
GTCTCGGCAGACCCAACATTATCCAAAGCAGAGGAGTCCAAAGTGGTATTGGGTATGGGACTGACGCC
AATGCTGCTTCGAGGTCTCTTGGAAGCTACGG
AAGATTTTTACGCCAGTTGCGTGGATAGCCTTCTACATGATTATTCAAAAAGGGCAATGAAGCACTTT
GTGGAATTTTGTGGTGGCAGCGCGACCGTCGT
CGTGTCTCAGGTGACGCTGAAACTATTGGAGGTTGTCGTGGAACCGACCCTAGAGGTGTTGTTTATGC
TACTGAGCCACGCTTCCTCTTCCAAGGAAGAG
TGTATTTTTATTTATTTTATCGCCGACATGCTTAAGTTTGTGCTGAATGAAAAGATGAGGGAAGCCAG
AAGGGAATGTAAGATTGGCTCTCGTGTTTTGC
TGCAGTGCCGTATGGATCTGTTATTGCTGATGAGCTACATTGATGAACATCCCCTGATGCGACGTCGT
GCCTCCTTAATGTAACTCTCCTTCTTTTTTCC
TTGCCTGTTTTTTTGTTTCTTTGGAAAATGAAACCTGCGAACCACCAGCAACAGCAACGAGAAGGTAT
GATTTTACTCGTGTTGATTGCTTTTATTCTTC
TCTTCTATGAGGCACCCGATGTTGACAATTGGTTTTATTTTTTTCCTCGTGAATTCTTTTTTTTTTCT
TTTTTGTTGGCCTTTTATTCGGGAAGCAATGC
TGCGAATTTGCTTGCGGCCATTGTGCCGCCCCCCTGTCTTTCAGGCGGGCGAGTCTGTCGGTGGATGC
CTGACGAACTCTTTGCATTGCGATGGGAAAAC
GACGCCTTTGGAGCGGCTGCTTGCACCGTTTATCCCAGTCACATTCACCGCCCTCACGCAGATAAGCC
GTAGTGTCGATGCGGAGCTGATGGAGAGAGTT
GCGGAGGCGGGAGGCCTACGGAGCTTCCTTGGCCGCCACTCGGACTTGTTTGCTGTCTCAAAGGTGGG
TGGCGTTTACGTGGCCCGCCGCCTCCGACGTC

GTTGTGTGGGGTCCATCCCCTCTGAAGGTGCGGGTCTTCGTCTGTCTTGCGAGGCACACGGCCCCGAA
CCGGAGCAGCAGGTGGAGGATGCGGGTGTATT
TCCACGGATCCAAAGCGTCCCCTTGGAGGTGTTTCGCCTATTTCCTACTTTCTTTGTGCCCATGGAGG
CGTTATTGGCACACTTGTGTAAACTGCCGTCG
ACAACGTTATCCAACTCGAGAGGTGAAGAGGAATTGGTTTCCGAGGACCACTCCGGCAGGAATCCTGA
GGCCGTAGCAGAATCTCTCGTCGAGAAGTACA
ATAAATTTATCGAAGTTGTTTCATTGAATGACAACAATGGCAACGGGGAGAAGGAACAACTTAAGAAA
TTTGTGCGTCTTCAATCGAAATTTGCTACACG
TGCTGCCTCGTATGAGGAAAAGGAAGTGGAAGTTACAGGAGGCCGTGAAGGATGCATCAGTCAAATTA
TGGAGCCTTACAAAGTGGAAGAATATGAACAA
TACCGTGTAGCTCGACTGATTCCAGTTGCAGAGGAATTTTGTCCCATTTCTGCAGAAATGCGACAGGC
GGCGGCTGTGTTGCTTCCTGAAGGACGGAGCT
TAATTCATGTGCTTTTGTCGGCACCAGATCTCTTTGAAGTTCGGGATACGCCAGAGCTGTCTGTTCGC
TTTATTCTGGACTCGCGCTTCAGACCGACCAC
CACGTGCACGAAAGAAGAAATTGAAAGGAAGCTGACCGAACTGAAGGAATCGCGAGTGAAAAATGGCC
TAAAAATGCCGATTGATAGAAAAAAACGACGA
ACTTTATCGAGACAACTTCAGTTTTTTGTTAAACCCACGCCATACTTGGACGAACGTGTTTGGGCCCA
CGCATTGCTGGATACGTTGCCGCTGGACGGTC
CCGTCGCATTTGCTGTCGCCATGTCAAATCTTCCAAGTGAATGTCTGGCCTGCACTCCAGTGAACTTA
CAACGTCTTATTTCTCAATATTCTTATCTGTT
TAGCCTAGTTGAGGGGGAGAGGGAGAAGTTGCTGCAGCGTGCAGATATTCCCGCAGTTGAGCAGCGTT
CAGTGTCAAGTATCGATACGGAGGAAATACTG
TTGACGATCTACAATTACTATCCACGCCGTAGGCATCCTAACTGTGGGACATGTCTTGAGCGTTGTAT
TTATTCTATGCCGCTGCTGCTCCGCACTAGGT
TGCGTCAGTTGGACTTTGTAGAGGATGTACTTCGTAAGCATCCCGACAAGGTTGAGGTGCTCGGTATG
CTTGACGAGGAACTGATGATGCATGATAGAAA
CCTAGCTGAAAAGTCGCAGATGTGTCAAGTATTTCGTTTCGTCGGGGAGTATCAGGCGGAGTTGATCA
AGCGCTATGAGGCACTGTGTGTCAAACTGGGT
AAAGACCCCAACACAAAAAGGTTGGTGTAGAAGATGAGAGTTTTTGCAATGAAGAGGAATCTTGTGAG
TCTTTGCTTGTTTGTTTGTTTACCTTTATGTA
TATGTATGTATATATATATACATACACATGCGTGTTGGCGGTTGTGGTTTTTATTACGACGGTGCCAT
TTTATTTTGAGATACTTCTAATGTGACTCGTT
TTTTTCTGTTGTTGTTGTTGTCGTTTTTTTGGTGTGGTGGCTGCTGTGAATTTTGTGTTCAGTGGGTT
GCGTTGCGCAATGAAGCACCGACCATCTTTTC
CTTGTTTTCTTTCTCTTTTTTCCTTTGCGATGATGAATGACATGTACGTTACAAAAGTTGACAAGAAC
GTTCTCCCTCCATTCTCCCAACGCACGGCGTT
GATGTGGAGGAAAGCTTCAAGCACCAAAGCAGAAAATTAAAATACAAAATAAAAAAGATGATCACAAC
ACACATGAAATGCATAATGATGAAAACTCGGA
TTAGTTTTTAGCTACACGAGAGGAATGACCACATGACTCTATCGAGTTCGAGAAACGTGCGAGCGATT
ACTGACGCACACCCAACACCCGTCTTACCCGT
TTATTCCTCCCCTATCCTTCCTTCCCTATTTCATTCCGTTAGCCCTCGAAAGAGTCATTCCTCGTTAT
TTACCACATGGCGGGGCAATGTAGTTGCGGCG
GCGAGATGACGTGTTTTGGCAGCCAGCTGCTCCTTTATTGCTCTTTTCTCTTTTTTTTTTTTTTTTGG
TTCTTGCGTGTGAGTGATGATCTAAACAATTT
GCACGTCAGTTTGATTCCGTTAATGAGATGTCACAATTGTTTTANNNNNNNNNNNNNNNNNNNNNNNN
NNNNNNNNNNNNNNNNNNNNNNNNNNNNNNNN
NNNNNNNNNNNNNNNNNNNNNNNNNNNNNNNNNNNNNNNNNNNNNNNNNNNNNNNNNNNNNNNNNNNN
NNNNNNNNNNNNNNNNNNNNNNNNNNNNNNNN
NNNNNNNNNNNNNNNNNNNNNNNNNNNNNNNNNNNNNNNNNNNNNNNNNNNNNNNNNNNNNNNNNNNN
NNNNNNNNNNNNNNNNNNNNNNNNNNNNNNNN
NNNNNNNNNNNNNNNNNNNNNNNNNNNNNNNNNNNNNNNNNNNNNNGCCACACTGTGGCTTCACTGACTG
GAAGAAGGCCCTTTGCTTTCGTGCACACACTG
CGAGACGCATTCCCTCACAATATTTTTCTCAGTTATGGTCTGTTGTGTGTGTGTGTGTGTATTCGCGC
TTTGGGCTGTTGCTTGGGCTTGTTGTTTCTGC

GTTTATTGCTTTTTTGTTGTTTTCTTTTTTTTTTTTTTTTTTTTTTTTACTTGGCGTGTGAGTTTTTT
GGTCGGCGCTGAACGTTGGTAAAGCAACATTT
CTCTTGAAGAGCAAAAGTATACATGCCACAAGGTGGACGGGGTCCCATGGTGCTGCTAAAGCAGCAGA
GGTTGCCCGCATGGCAGCCCATCCTTACACCT
CCACATGTTGCGCTAGCTTTTTTCCTGCTGAGCATATTGTTTATCCCTTTAGGGGTTTTCGTGACGCT
AATGAACAAACAGGCGAAGGAGGTCACCGTTC
GTTATGATCATATTCATCGCTGCACAATTACACATAACACAGGCGCCTTTATGTATGAAGGCAACAAT
ATGACGTTTAAAACTGGATGTATGACGGAAGT
TTCCTTTGATATCACGGAGAAACTTAAGGCCCCCGTATATCTTTATTATGAATTAACAAGATTTTATC
AAAATCACAGGCGGTATTCAATCTCGCGAAAC
GATGAACAATTGGCCGGTAAAGCCGTGAGATATTTGCCAGACACGTCACCTCTTACCATACCAGGGGA
TATTTATGGAATCTCTGGGACTCCCATCAAAT
ACGTGGATGGTTCGGATTTGCGTTACAAGGATTTTTTGTACGTTCCAGCCGGCCTCATTGCCTGGTCC
ATTTTTAATGACACGTTTACTCTTTACACAGA
GGCGACGGATGGTGGAACTCCAAGAAAACTCATATGCAACGCGACGGATTTTTCGAAAGGGAACAATC
TTCCTCTTAATGGCTCGGAGTCAGAAAATATG
TGCGTCAAGAAGGGGATTGCTTGGGATACGGACGTGGAATATAAGTTTAAGGCCCCGGATTTAGAGGC
GAAAAATCGTTTCTGGACGGCGGCAAAAGAAT
TGTACACGGGGAAGGTGCCTACTCCAGAACTATCAAATGATGACTTTTTCAACAAGGGGTGGTACGCG
GGTGAACTGGGTCACGCCATACCCGTGACAAC
CGATGAGGATTTTATGGTTTGGATGCGTCCTGCATCGCTTCCGAGTTTCCGCAAGCTGCATCGTGTCA
TCAATGTTGACCTTCCGCCGGGGAAGTACGTA
ATGGTGATTGGGGAGCACTTTGACGTCTCATCATTTGGTGGTACAAAATCCTTTGCGCTGGCCACGCT
CTCTTTCTTGGGAGGGAAGAATGTGTGGCTTG
AGGCATTATATTTCTCACTTGGTGGATTTTCTGTGGTGTTTGCTGTTGTGTTGCTTTTGGTTCATCGT
TTTTCtGGAGACCGTGCCTCGAAGGCCATTGA
TGAATTGATGCAAAAGTGATCCGTCAATCTTCTTTTGTAGGCGAAGAAGAAGAAGATGATGATGAAAA
GTGTTTCCTTTTTTTTTTTTTTTTTT
>scaffold1100.size6428
ACAAACAAAAATAAAAACGAAACAAGAGTTCTAGTAATGGAAAAACGGGCATGACGTCGTCTCTACCG
GTGACAAGTTCATATCGTTATCTACCAGGTGCG
CCAGCGACACGGATCGCGAGAACGCACGGCTGAGCCAAAATAAATCCCTACTTCTTCGGCGTTCTTTT
TGCTTGTGGCGTTTTCGCATTTTCCAGGGTTA
CTCTGTTCTTGGGAGACTTCATTCTGCCGTTCCTGCTAACAGCGCCACTCAATGCGCTTTTGTCGTCC
TTCGTGGGGATCAACTTTTGGGCAGCTTCCTT
TTTTTGTGCGATTTTTTTGTTTTTGTGGTGGCTTTGTGCATGCGGCATGTCTATCGTGTTTAGTGATT
TTGTTGGTGGCAGAGGTGTTGCTTTTCCAATG
GCCCTATTTTATGGGGGTGCCAATGAGAAGATTGTGGGGGCTGTCATTGGCACGCTACTTCTTCCAAG
ACCTTTAGAGAAAGAAAGATCACGCGTAAGGA
AAAAGAAGATCAATAAAGGTTTCCGTAAACACATAATAATAATAATAATAATAATAATAATAATAATA
ATAATAATAATAATAATGTTTCTTGTGGCTTC
GACGTGTATTTCCGGTGGCGTATATTAATATGGAAAGGAAAAATAAATAAACAAAATAACATTTTTCT
TTTTTTCTTTTTGTGTGTGTATTTGTGTTCTC
TCCTGGTCTCTCACGAATATTTCATTGCCATCAAATCCAAGTCTCCTTCTAAATGCAATTGGAGGCGA
TAGGGGTACATTTCTTTTCTTCTTTTTTTTTG
GCTGTTGTGGTTGTTGTCTAATACTTGCCCCCCGCGTGAGCACCCCCGCAAGAACTAATAAAATTCGT
TACATAAATAATATATATATATGTATATATATGT
ACCTTTATACATGTGTAATATTTTTTTCATCTCTGTTGTATGCGCCTTGTCCAATGGAAAACTCATAC
CCCCTCATCCACACACATGCAGACACACACAC
GGTCTCCTGATGAGCGGCAGCCCCTGCAAAATCAACAATAATAACAAAAGAAAAAACAAAACAACAAC
AACAACAAAAAAAAACAATGGTTGCTGCACAT
GTTATTTTAACTGTGTGACTGCTTCAAGTGTACCCCTCTGACACAATTTTTTTTTTTTTTATATGATT
TCTTTGGTTCCCCCTTCCTGCAGCATTGTTGA
TTCTTCCCGCAGCAGGAACGGCTGTGTTGCTCAGGCGATTTCCCCCACCACCACGGCCTCTTCCTGCT

GCATGATGACTCCCTTCTGTCAGACAATACAA
CCCCTATTCAATGAAAGAAACACCGAAAAAAAATAATTATTATTGCATATGATGGAATCAAGCGCGTT
GTGCCTTTTGTTTACGGCGAGAATTAATGCGG
CGAAGCATTTCTATTTCGCGTTCAATATCGCCAATTTCTTGTTCCGCCGCCATCTGCTCCATTTCGAG
TTCCTTCTTTTGAGCGTCTGTAAACTTTTGGT
GGGCCTTTTCAATGCGTTGCATCTCCTTCCTCATGTTTGCAGCTGTACGTATGAGTCCAGTGGATCGG
CGATCCAGATCCGCGATATCCGCATCAAGTAA
CTCCTGGCGAGCCTCACATTTTTGATGAATACGGCACAATGAAAGAATTTCTTTGCGTAACTGCTCCA
CAAGTTCAGCGTCAACGCGTTCTTCGTCGGCA
TTTTCCTCGCTGCCAAGTGCATCACCTATCATTTCAATACGCCTCTCAAGTTTGGCAATCTGCGTGGC
CCGGTGACGAATAGAAATCTCGTTCTGCCGGA
GAGTCTCCTCTTCCCTTTCCAGCTTGTGAACGACGGTGCGTCGCTCGTTTTCTGCTCGCTGAGCAAGC
AGTAGTTCGTCGACTGGCTTTTCCTTTTCAAG
CTGTTTCTCCTTTCGCTCTAAAAGGCGCCGCATGGAGTACACCTCCTCACGAATGCCTTCCAATTCAT
TTCTCTTTTCCTCAAGCTGCTGTGCCGCCCTC
TCGCGTTGCTCATCGAGTTCCTTGGATTTAACCAAATCAGCCAGAAGCCCTCGCATATGCTGTGTTTG
CCGTTTCAGGGTCGTTTGAATGGTTTCACGAT
CACGGAGCGACTGTAGAATGTCGTGCTTCAGTCCGTTCTTGTAAGCCACAGTGTCCGACGCGTCCTTC
AAATGTAGCAAAGTCTCCCCATCGGTGTACCC
AATGCGTTGCTTTAAATCGGTGTTGAGTCCATTGGCCTCTTCTGTAGCCGCCGCAGAACTCCTCAATA
TTTCTTCAAGTTTTTTCTTTTCCTTCAACAGC
TTTTTCTGTTCTCTTAACAGCAATGTGACATCACCGGCCTTGGAATTCAGCTCACCAAACACCTCAAT
GGGAAGAGTGATTGTGCGACACTTGATTTTTT
TCGAAGCTCCGTTGCCATCTGAAGGAGTTGCGCCGATCGCCCGGACATCCCGCTCTCTTACGCGACAC
GTCTTCTCAAGTGTCGCAACTTTATCTCGAAG
CTCACGGCATTCCTCATCGGCCTTGAGAAGAGTCTTGACTAAATCCGTCGCCTCCGCTGTTTCTCCCT
TCATTTCTTACACACACACACACACAATCACT
TTCTCTTTCTCTCACTTTCTTTTTTGCCTTTGGCTACCGGTTTGATGGATAATTTTCCTTTTTTTTTG
TTTGGATCTTTTTTCATTGGGAAATAGAGGGG
GGTTTGTGAGGAACATGTGTGTTCATGGGTTGCAAGTGGTAAGGATGCAAGAGAAACAATAAAGATAA
TTAATAATAACAATAGTGGCAACCATGCAGAC
ATAATAAAAATTGGCAACCCAATATGTTGACCAATAGGGCAGAGAAAGCGTGCCTGATAACACCGCAC
TTTTTTTAATATTTCTTTTTGTTGTGTTTGCT
CTCCTGCATCATGCCCCCTCTCCTTCTTTATTGGGTATTCTTTCCGATTTAGGCACACACACACACAA
ACAAACACACACACACTCAAAGGTGCACTCAT
GCGAGGGAACAAGCACATGCACAAACACAACCCCGCGGACACACGCAGTCCAATGTACAAAGATGCGA
CTCACACATAGAAAAATAAAAATTGATGACCT
CCAGAACCAAATCTCCACTGCTACGACGTATTCTCGCTAGTTGCCCTTCGAAGACGATGGATCACTGT
CCGGTGATGTAATGTCACCCATATTTTCGTTA
ACAGGCTTCTCAATCTCTTCTTTGCCCAGGCCATATTGTCTAACTACTTCCTCAGCAGCCCTCACACC
TTGTTGAGCTGCTTGGGCGGCCTCTTCGACAC
TATGTAGCCTGTTCACGTCGGTACAAACTTTTTCAGGCTTTTCGCCCTTGTCGGTGAATCTCCTTGGA
GGGGGTTTGGGGCCATGGCCCTTCGTGTGAGG
GAATCGGAAAATGTCATTCTTCAAAACGTCATCTTCATGCACTTCAACACGACTGTCGTCGTTTAAGT
CTTTGTTCTTCCGAAGCACAGAGACAAACTCG
TCGTAGTGGCCACGAGGATCCGTAAATCCCTTTGCCCCCTGGGCCTGAGAAAGAGCAAAGTTGAACTC
AGCAGCCTCACGCATTTGTTTACGTGTTTGCT
CCTCATTTGCCGAAAGACGCTCATTATAAACTGGGACGCCGTGGATGGTGTCCACAGCGCGTTTGATT
CGACGTGTTCTGTCAAGCCACCACTGGAACTT
GGCTTGATTGCGATCATCTTTCCCGAAATCAGCAGCATACACGTGTTTGTAAACTTGCCGTCTTGTCC
CATAAGGCTTTGGTTTAATGCCCAACACCTCA
GGTTTCTCAAGAAGTTCTTCTTCTGTGAATACGGCCTCACCGGCTTCCAACTCTTGAGTTCGACGCTC
CTCCAGGAGGTCTTCCATTTCATTTGTTTGCG
CCGCACGCTCTCTCCTTTTTTTCTCGCGTAAGCTGGCTCCCAAATCATCAAGCGACTTCACGCCACGA

CTCCGGTTGGTGAGGTACTGCTCCTCCTGCAA
CACTTCTTGTTCGGTGTACCCAATCTTCCGCAATATTTCGGCCTCGCGCGCCCTTTCAATCTGCTCTT
TTCGCGCTGCGATGTGTGTGTTTGCATCTTCG
ATAGTTTTATGAAGTTTCCCCTTGTAGTTTCGGTACTGCAAACGGGGAAGTTCCACGGTATCCAGAAT
ATCCTGTAGCACATTTCTTTCCAGTTTTGTCG
AGGGTAGAAGCTCATCTACCCCCTTCTTGCGCTGCTCCGCGGCAAGCACCCACTTTAAATCCTCGTCA
TCAATGCGTCCACGGACCAATGCGTAGGGCTC
AAACTTGTCACGCTCCCACGAAAGTGGGAGATTACCGCTGATGATAACTTGATCTGTGCGTTGGTTGT
CTACCATCTCCATGTAAGTGTTAAGGTACCAG
CGGGGGGCCGTGTGGTCTGTCAGCTCCGTGCTGCTCGCATCCTTTGAAGTTTCCACGTACATGAGAGG
CTCCGTGTTGACGTCTTCGAGGCGTCTGTTGA
TGCCTTGGGCGGTAAACTCCGGTTTCTTGAACTGCTGTTCAACCGGAATCCTGTCGGCACGCGGAATC
TGTTTTGCTACATACCGCACAGGCGCCTCGCG
TGCCGGTCTGCTTCTCGCAGCTCCCATACCCAACAGAAAAAGTGGACCACCTTTTTTTTCCTGCGGAT
TACAACATTAATTTACACGAAAAAAAAAAACGC
CCTCACACAGAGAGTACCAGCATGCACACAGATAAAAAAAAAGCCCACACACAACACAGAAAGGAGCT
CAATTTAAACTCAGGAAAAAAAAAGAAAAAAAG
CATTAATAATCCACTTAAGGCAACTCTTAGGATCACGCACGAAAGCTGCAGGAAATGCGGTAAAGCAC
AATATATATATATATGGAAAACGCAATGCAGT
GCATGATACCAGCAACGCTCACCCCGTGTCATTGTCTCGGCCATTATTGTCATTCACATCACACTCCC
AGAGGTTTTTTGCGTCAAAAGAGACACCTCTT
TTCCAGAAACGATAAGATGCATCTTCCAAAAAAGTGTGCCAGTAGAAAAAGCAGAAATAAAGCAACAA
CGACAATCACAGAAAACACACTGGACAGATAA
ATTCGATTATTGTTCACTTTGTCATGAGCATCCGCCCAACGAATCCATTCCAAATAAAACTTCTCCTT
CGCAAAACGCATGGCGTTGGATAACTTTTCCA
TACCTGGGGGAAATCTCTGAATCACCTTTTCTCTACGAAGCAGCACATAGGCGCTCCAAATGTGTTGC
CGTACCAGCATCATGCATGGAGGATCCAAGTG
AACACCATCCGACAGCGTACAAAATGTATAATTTCCATTGAATAAATGTTGTGTTGGAATCACAGGAA
TATTTAAATGCCAGGCCATCGCCATAAGCACG
CGTCGATAAAAAGGCACCACTACATGTGCAATAAAGTCCCCAAATCTTTTAAACATGTTGGCTCGTCC
CGTAAAAAGACTCGCAGCACATTAAGAGGCAA
CTCCTCAAGAAACATAATAAGGGGACGACGATGCACGGGTTTCTGCTGTAACACATGCTGAATGTTCT
CCATCAGTTTTTGAACGTGTTTTGTCCAATGC
TTGATTGCGATAGTAAATTTACGCGCGGTTCCCCCCAAATGTGGAACTTTGTTCCGATGGTTGATTTG
CCAATTAAAGTCCCACGTGCCCATGTAGAGCA
AAATCACACATGTCCCATACCCTCGGCCAATGGTGCGACTGATCAAAGGCAGTGGCAGACTGATATAA
CGAAGCTCAAGGAAATGTGCCGTGGAATTCCT
AAAATCACCAATAGGAAATATATTAGAAGTCGCATTTGAATCCACACGCGTATAACTTGCCGTATTCA
GCGATGCAACTTCACTCCCTCAAAGCGAGACA
TCCGCCACCACGTGATCATCCACATCCGCAATCCTGCCATATCCAAAAATTCGCATGAACGGAAAAAA
AGAATTCACGGCAAACACAAAATTCCTGTAGG
CAATGCTATCAACAACAACAAAACTGACTCTTAGGCTCTCTAAAAAAGACTTTCTGCTGCAGCTACTC
AGCATCCATGACGCGAAACACACATGGATTTT
TACGCCAACGAAAGTCACAAAAGTTTACGAGGTCCACGTATTGGTGCATGTGACGAACACACCACCAC
GTCGGCTGCACATCCTCCACGGGAGAGCCAAC
ATCACCCGGCAACGTGGAAAGAACAATCAAAAACAGAAAACACGGGCAAAGAAGACAGCAAAAGAAAC
TCCCCATTTTGCTTCAAAATAACAAACATACA
ACATTCAACCTCAACCACATAAAAAGAAAAAGAAAAAAACATCAAAATAATAACAACCAAACATCAAA
TAATAACAATACTAATAATAAAAGAACCGATA
AAAACCAAAAAAAAAAAAAAAAAAAAA
>scaffold1101.size9673
TGGAAGAATGTTCTTCACCCACTTTTCGAAGTCAGGGGCGGTGCAGTCAAAGCGGGGGCAGATGGCGC
CGCACTTGTTGAGGCGGCCGTTGAGATTCACCA
CAATTTTACCCGCGCGGTGATCGTCGATAATCTCAAACTCGCCAATGTAACCGTGCTTCTGCATCACC

TGCAAAAACTTCACGACGACCTTGGAGGATGG
GCGAATAAGAACCTGACGCTTGCCACGGCGCTCCGCGCTCGCAATACAGCGAAGCGCGTTGGCCAACA
CGCTCATCATCGTCATTTTCTCTTTTTTCCGG
GCCACTATCAAATGCACAACAATCAATTCATTAATAATAATAATAATAATAATAATAATAATAATAAA
AGAAACATGAATATAGAGTAAAAATACACAAA
TACATACAAAAAGCAAAAGCTCGGCGCACCATGAAGGAAATTACGGGCTGATACGAATCGTAAGAGAG
TAGAGGCAGAGGAGGAAACAAACAAACAAACA
AAGACGGTATAAGAGGGGTACAAACCGAAACTCCGCCTTAGCGGCACAATGCGCTCAAAGAAGAAAGG
GTTTCATAACGCCCGATTTTTCGGCGCGCAAC
CACGCGAAGATCATGTGATGCCTGATCAAAACAAAGGGAGACGTTAGCCTCATGTGTAGGTGAGGAGA
ACAATGAGGCCCCCAGAAAGATAAATCCATCA
CCGAAAATTGTTTCTAACCACGGCCCTCGGCCCATTGGTATCCGTGTATCCGCGAATCCTAACAAGAG
GCAGCTCCAGCGAAGAATGTCAGTCGATCGAG
ACATCAAAGAAAACTCAGAATCTGCGTCCGCACACATATCATAAACCTTTTGTGCCTCCCTTACACCA
TGATATGGTTCAACCAAAGGCTCTGATCCACG
AGCAGCATAATATGCAGCTTCTTCGGCCAAAATAGCGGATGGAACACCGGCTTTCGCCCAGAGATGCA
ACACCTCAGATGGATCCGCACAAGGCAATTTT
ATTGGTTTCAATGTACAAAGCTCTTCACCCTCTTCCACATCGTCGTTACAGATCCATCTGTGATCCAC
CAAAACAGCTCTGGCCATCGTTCCAATACGGT
CCCCAAGGGATTCCCAGGCCGCATTGGTGCCAAACCGTTTCACACTTTCTTTAACACAGCAAGCAGCT
GCGGTGCGCGCGCCAAGCAAATATAAAGCAGA
AGCTTTTCGATAATATGCTTTGACGTTCATTGGGTCAAGAGAAATCGCCCTTTTTGCATCGGACAAGG
CATCATCACCCCTGTCAAGTTGAATCATCGCA
GCCGCCCTATTGGACCATAGGGTACTGGACGAGGGTTCATATTTTAGTGCTTCACTATAGCAGCGGAC
AGCTTTCCGTAAATTACCTTGCTTCATTGCAT
CATTACCCCGCGCTCTGTATTCCTCTGCCTTTTCAGTTTTCTGAGGGACACTGGCTGCAGTGCCACTT
CTCACCACATGTAGAGATTTGTGCTGCAAGGG
TGTATGGGATACGTCAGAACGAGAGCCGCTCCGCATCCAATTGTTGTATCGCTCCAGTTCTTTTGGAA
GAGCATCTCGGGCTATACGATACACCTCTTTT
ATTAGTTCTTCTGTCTCGGCCCGAGTGCGGAACACGCCGGTTTGGGGGTCTACGTCCAGTTCAGTGGA
AGCATCTACGTACACCTCTTGACTTACAAGTT
CCCCATCCACAGGAACGGTCACCAATCGCTTCTCCACAGTGTGCAACATAAAATGCCAACTCACTACA
AAAAAGAGAAGAAGGAGAGGCGGAACAGCCGT
TTCTATCATCCACCAAGAAAAGGTAAATGCACAAACAAAAAACAACGACAATGGCAAGGAAAAAATCA
ACAACAACAACAACAAAAACAAGTACCGCCAC
CATTAATGATCCTTTCGCAACACCCGCGGAATAAAGCGCACCAATAGCGGAGGGTAAGGTAAATTACA
TGCTGCAGAGAAACATCATGTTTTACCGGTGG
AAAACAAATATTTCCACTTTCGACTTGGTCTTAAGAGTAATTTGCTATTTAGCACATAAAAAAAGGAC
TCTTTAAACGAGCGCAATCCGGAATAATAACA
GGAAAAATTTTACCTTAACAAAGAGAAATAAACAACAAGGTTATAAAACAAGAGGGTCAATGCTCATT
CGCGTAAAAATCTATACACGCAAGGAGCAAAA
AAGACAAAACAAAGTAATCGTGTTTACAGTCTCTGCTTCATTCTTTTTCTTGTGAGTCGCAAAGTTGG
AAAGGGCAAATGAATCAAAAAGCAACAACAAA
AACAAAATTAAAACAAAAGTGACATCACAATTTTAAAAGAATGAAACACCCGCATAACCCCATTCGCA
GCACAATACAGAAATGTGCTGGCGTCGACACA
GAATTTTCCACGCATAAATGCAAACGGGTCCTTTCAAGGTTGTTCATTTCTCCTGTCAAACATGTTTG
TGTTGGTTCTGCCCCGACCTATTTGGGGGCAA
AATCACAGGTGCTTTGCGCCTCTGAGCATTCGGGCGTAGGCCAAGAAAGCGAAAATGGGTGGGTGCGT
GCGGGTGCATCGGGCTTACGAATCGCCTTTTA
GCACAAAAAACGATGGTCCACATAAAAAAGGTAGGAAATAGCGAAAGGAACAAGTGATAAATAAATAA
GGAAACAAAAAAAGATGTATGGGCCATAAAAA
CAACCCAGGGTAGTTGTTAAAGGGGGAATTCTAGTGATAACAAAGTAAAAATAAGTATTACATAACAC
CGCTAACGCTTCCAAGAAAAAGGAGCACATTG
TGGTCAGAACTTGTCGTTACCCAAGCATTCAAATAAATCCTTCCTAGAGAATTCAAAAACAAAGAAGT

ATCACCTTTTGAAATACGTCTGCGCTCCGAAG
AAGAAGAAGAGGCCGATGGCTCCAAATACCAACGTCGCAACGAGGAGCCGCCCACAAATTGACGAACT
GAACGACTGAGAGTATTAAGTAAAGAATCATT
ATTAACGTTCATATTCCCTATCCCTAACAACCCTTGATTACACCGTATTTCATGTTCTCTAAACAATC
TGCTATATACTTGGCCCCGCATAACATACTCA
TTCACTAGGCATTCAATCTGAAATTGAAGTAGTCTAAATGTCCGCAAATTAGTTTCTGAAGAAAGTTC
CAGGTAAAATAATTCCACGATCCTCGTGACAA
TACTGGGAATTGGAACTTCTTCCTTATTCTGAGATGAATAACGCCTTGTACCTGCAAAAGCGTGCACC
CGTTCATCATATTCATGAACGGAGAGTATATA
GCCCTCAGAACTAACATTTACACAAACCGTATCCGTTACTGAAAGTTCATTTTCAATCTCATTTTTAT
GGTCAAGCTGTTGCCTCTGACAGAGACGTTCG
CTAAAGCTGATGATTCTATTCTCATATGGCATCAAGTACTCTTCTGCAAAAACTGCAAGGCGTCTTAA
TGGTCGTGGCAAGATCTGAGATAATGAAAGTG
CTAAATTGGCGCACTGCACACTATCACATCCATATACAAGAATTTGGATTCCGCTTAAAAGTGCACTC
AAAAGCATTTCTGTCTCCTGCAATCCACCAAT
GTGTTTCTGCATAAATTCCAAAAGCCAAACAGCAAGAGGTTTTACTACAAATTTCTCACCTACGGCCA
GTATTCTTCCATTACCCACAGCAATACAGCCT
TTTTCCCCAGGGGGAGAGTCCAAATCAATTCCAACGATATTTACTAGAGGAAGTTCGACTTCAAGGCG
TTGTTTTATATCATTTACAGACGTTGGCTCCA
ATACCGAATCAGTGACCCTTAATGGCTCAACGCCAAACAAAGCAGGGAAAAAACGCTCAAAAAAACTA
TGAACACGTTTAAAAGCTTCTTCTTCAGATTC
CGTGTTTTGAGATATCAAGCACATCAATGAACGCATTGAGCGTTTACGCGCCTCTTCACGACACCTTT
CTAAaTCTGGAAAGATGGAATACTCTAATGCT
AGGCGCTTTTTTGTTGCAGTAGACCATTCTTCCAATAGAATATTTAGAAATAAAGAGACCAGTGGCCA
CTGACGCACAAGTTCAGCACTTTGAGGATGTG
TCAGTATAATACAGAAGCGACGCTTTTCACCGCGGGCTAAAATATCTTCCAAACAAAAAGACATACCG
GCAATTCCTCCGGCCAGTGGATCCAGCATTAG
CACGGATTCTTCATTAGATTCATTTGAAGCAAGAACACGTGCGACAACGGCCTTCATCCGCTCCTTAT
ATTCTGGACACAAGGGTCCACTAATCACTCGA
GCACTAAGAAAAGTCTTTCCACAGACAGCAGAAAGGCGTAACAAGTGAGGTCCAAAAATATCCACTTC
CTGATATGCATAATACTCTGATACACAGGTTG
CCTTAGCCCCCTCGAGGGCGTCAAATTCCCCCAAAGTTACCATGGGAACACTCCAAAATGCTCGCGGC
ATTGGCTCTATATAAATGTTGGTGAATAATAA
TAATAATAATAATAATTAAAAAATAAATAAAAAAAAAACTAGTATGGGAAAGAGCAAAATAAAAGCCA
TTTTTTAAAAAAAAGAAAATGTATATGCTTATT
TATTAGTCATCAAAATATTAAACAAAATATTTAATTCTTCTCTCTGATTTACTCGCATATTTGCGTTA
CCAAAATAATAAATAAATAAGTAAATATTATA
CATATAAAAAATAAATTCCAAAAGGGTTTCTCTCTTTTTTTTTGTTTTATTTTTTTTCATCCTTCTCG
TTTACAATGAAAAGGCTGAAGACCACGTTAAT
CCAAATTTGACGTGTTCAAACAGATTTTTATTGCCATTTCTCCAGGTAACATTTCCGCTCCTATCAAT
ACCTACAGGAATTGAGAAATTCAATTCAAAGC
GTCCGCTAGGGGCAACACCAAACAATGGAATTCTGGTTACCACCACACCGAACCCTACTGAGGCAGCA
GAAGAGCGAAGCCACTGGTAAATGTCCAAAAA
CTCTGATTGATATCGTATCATTTTTAAATTACCCAAATTCGCAAAAAGATGCATAGCCGCTAATCCAT
TATGAGGAAAGAAGATAAATGGAAAGTTTACA
CTAATTGATGTCGCCCAAAGTGCATTACCGCCTGTTGCGGCAAATCGACCCCCTTCTTTTTGAATAAT
ATCAAGAGTTGAAGGCCCCACACTTTTAAACC
CACGAACATGGCAGTTTGAAAGAAAAAGCCGGTCGTTTAACGGTATTCTAGCCTGTCGATAAGACCAT
ATACCCGCTAATTTAGTATTCCATTGAAAACT
ACAAAAAGGTCCAAGCGGCCAATATTTTGCTGCCTGTATTTCTGTTTTCAAAAACGTAAATTGACCTC
CAAAGGGACCTCCAGCGAGCTCATTTTTTATA
CTTATTTGGTTTCCTTGTATTGGTAAAGGATACATGTTAGATAAAAAAGGATCCGCATGATATCCTGT
ATTCGATAAAGCCAATTCATGTCTCATATATC
CTTTAAACGTAGTAGAAAAGTCATTTAAAAGATCTGAATTTAAATGTTCTTTTCCCTTTGCAACCAAA

AGTCTTCGCTGAAAGCCAACTGAAAACTCTGA
AAAAAATCCCCGTTTTTCATTCTTTGCAGTAGCTCGAATTTCTTCAATTCTTTCACTACTAGCGGGGT
GATATATCTTATTTTCAGTCCGTTTTCCAATG
TAATACTCTCCCACTTGTCCCAAAAATGGAACATTGGAAAGAATCGAAAAGGATGCGGCATGAGTTCG
TGATGTTGGAGGGATATAGTTCCCAGACAATG
TGTACCTACCTCCAAGAATATTTTCTAAAGAAATAGTCAACTCCGGAACTACAGTTTCAGTTGTAAAG
ATGCCAAATGATTTTTTAGGTTTGGACTCTTC
AACATCTATGTGGACACAAACATCATTGGCTTCGCCATCGTAGGTGGGCTCTAAATTAAAGTTTACTG
AGTTAAAAACACCCATTTGTACCCATCTACTC
TTTGCTTCACTCATGTTGTTCGTGATTTCAGTTATTGTCCTTGAGCGTTTAATAGCCTCAACATCACG
ACTTATTACGCGAGGATGAATTCTTTGAATTC
CTCGGATACGCACATGGGTACGTATCGGCAAATCCATGACTTTCTCAATATCTTTTTCCGTTATGTTT
ACTTCCTGCGAAAATACGGATGCATTCTCCTG
ATTATTCATTTTTTTCTTATTATCTGCCCTCACTCCTTTGAAAAATTTTTCTGGGGTAAAGAATATGA
ATATCAGACTAAAAAGAGAGAAGAAACAGAAA
GAAAAACCTTGGCACATTATAATTCAATTACTATTTTTTTACCGAGCAATTAATAATAATAAAAAGCG
TTGGCAAAAAAATATTCAATTAAATTCCATAC
ATATATAATGGCGAAAAACAATACATGGCAAAAGATAAACCTAAAAAAAAAAAAAAAAAAAAAAAAAA
AAAAAAAAAATTAAATAATTAAAAAAAAAAAT
AATAATAATAATTTATATTCTATTTTCCTTGTGCCAACAGAAACCGTAATTCAGATAACATTCTTTTT
CCAAAATTTTTTTCTTCACTCTTTTAAAGCCA
TCTTTGTATAACTACAAAGAATATACACATATATCTATATATATCCAAATTTGTCCTATATTCTATCT
CCTTGATATGCTCTACCACGATCAAAATTTGG
AGGAGACCACTCTTGAAGAACGAGTTGTGGAAAAACATTTGGTTTTAACAAAGAAGTTCCAAGAAGCA
TATTTACGCATTCTTGAAATCCTTGAGTAACA
GTTGGATAAGCAGTGCGAAGCTCGCTCAAATTGTAAAGTGTTTCTTTATTATGAATTGCCAATGAGGC
CAATTCTATAACACTACTTGCATGTGGTCCTA
TGGCTCGCACACCAAGAACTTGCATCTCGCGATCATTTGTAACAATTAATTTAACAAATCCCCTGGTA
TTTCCCATAGCCACAGCTCGGGCCACAAATTC
ATATCCATAACGAGCCATTTTGTATGCTATATTTTGTTTCCGGCATTGCTGCTCATTCAATCCAACTG
CAGCAACTTCTTGATCCAAAAACATAATTGTA
GACAAATTATCTAATCGTTGAACAAATTCACCCTCAGGATGAGGATGATAAATGTGGTCAATGCAAGA
GCGTCCTTCCAATTCTCCAACGTTTACAAGAG
CAAATTTGGTAGCCACATCCCCACATGCATAAATGTGCTTGTGGGGTTTTACACGATTAAACTGATCT
CTTTCAAGTTTCCCTCGTTGAATCCCTACTCC
GATATTTTCCAACCCCAGACCACTTAAATTGGGTTCTCTTCCAATGGAAACTAAAGCATTGTCCACAA
TATGCGTCGTCAATTTGTTGTCTATTAAATTC
CTTAATGTGTAATGAAATTGTCCATTTTTGACTTTATTACTTTCCAGGGCTGAATGATGATGAAAAGT
AACCCCTTTATTTTCAAGTAGTTTTTGGACAA
ACTCTGCCACATCATCATCTTCCATGGGTAAAATACGATTGCTCTTTTCGATGACGTTAACTTTTGTC
ATACCAAAGTTTGCAAATATAGATGCGAATTC
ACAACCAATCACACCCGCACCAATTATAACAATACTCTTCGGAAAAGCCCTATTCATGATGTCATCTG
AGGTAAAGACCACTTTACCGTCCCAAGTCACA
GAAACATGTTTCCGTGGTTGAGCTCCACAGGCAATAACAAAATAATCCGCCTCCACCCTCTCGGTGCC
CCCAGAGGCAAGTTGAACATCTACAGATTGAG
GTGACATGAATGACCCATATCCCTCAATAACATCAATATCAACAATTTTCAGTTGATGAACTATTTGA
GCATATCGAAGCTCAGAAGCATTTTTAATGGC
CTTTTGTGCATTTGAAGAGTCGATTGGGGGAAGAGGCATAAGGGGTTTCATAAACCGGCTTGCCGTTT
GACCAGTCATTAATCGGACAAATTTAGACATT
TCCCACAAAGTTTTGGACTGCAATGCGCCATTCCAAAGGTCCGCTCCTCCAATTCGATTCGCTTCAAC
AATGCATGCCTTTTTTTTATAGTCAACCGCAC
GTAAAGCCGTGGCAATACCTGCGGGACCGCCTCCAATCACACAAACATCGACTTTTTTTAGTCCCCAA
AAGTGGCTACGATGTAACATTATCTCTGATTA
TGAACTGAAAAATAAATAAATAAATAAAATAAAAAACACGAATGAATGCCAAAAAAATTAAAAATAGA

ATTGCAAGAATAATCAATTCCGCAAAAAATAA
AATCAGACACCGGCCCTTTTCTCCTTTCCGCCGCCACAACCCTAAGAACAACACGCGCTCCTGATTCC
GGCCGCCTTCGCCCTGCTCTTCCGTTCAGCCC
CCGCGAGCTCCACAACAATTCGGAATTCGCACTTCCAATAAGAAAAGGCCTTTTTTTTATTATCGCGC
GCGGCGGGCATCCCAGTAAACATCCCGATCCC
ACGGCGCCACAGAACAACACAACAAAACAAAATGCGACGCAAATTCTCACAAAAAAGAACTTGACGAA
ACTTCCGCGGTCTTCTTTTTTAACATCACAC
TTAGCCGCAAGCGTCGGAGTCACCATACCCAAGTGGTTACGGGGACTGACTAGAAATCAGTTGCGATC
TCGCGCGCAGGTTCGAATCCTGCTGGTGACGA
TTTTTTCCCTTCCAGCCATTGAGCCTTTATCAGAAGCTTGTGAAGAGAATGATCGCGGGGGAGCTGGC
TAAGTTAATTAATGTATGTACATCGTGATAAA
TGAATGCATTCTTTATGATACTTTCTACCGTGTGAATCCTTTGGGAAGAACGCGACTTTAAGGGCATC
AGGGAACCGATAGAGGCCGGATAAATTTTTAAT
TTTATTTTGTCATCCCACCCACCCCCTTTTGATTCCCACCACGCGGCGGGGTCTTGTGGTTGGAGGAG
CCCAAAGTCTGCCACTTCGTAAGTAATAATAT
GTTAATCGCAACTGAGGACAAAGGACCATGCTGGTGGTCCACAGAATTCTATATATTGAATGAAAAGA
AAACATTGAAGCAATTAACTAACGAACCTTTT
TTCCTAACTTGTTTGGTTTTTATAAACAATTTCAGGATCCGGCCAGCTGCCCGGAAGATTATTCTTTT
ATTTTTACCATCCCACACTTAATTCCCACCAC
GCTGCTCCGTCTTTCGGCTGGATGGGCCCAAAGTCTTCCACTTTGTAGGTTGTGAATACATACATACA
CACACACTTATATATGTATATGTACATGCACG
CCCAATCCGAATGGAGCGATCAAGTTACGACAGTAGCCAACACAATGTGCATATATAAATATGCGCTC
ACTTGGTGTAAATACTCATTGGGGATTTGGCG
AACCCCCATCCCCATTCACCCCTTCTCATTCCCGTCCTGTGTGAATCCTATTGACAGCCGCAGGATCC
GTTCGGCGGCCCAAGTGAATATTCTAGGAGCT
CTTTTTGTGTGTGTGTGTGTTTTTTGCTTTTGTCGTCACGGCAATTTGGCTTTTTTTTTCTGTGATTT
ATTTTTTAAGATTTGGGGCGCTCTTGCCTGTG
ATGCTTGTGTTTTTTCCTACCGTTGTTGTGCCTCCTCCTTTTGAGGGAGGGGAGGGGAAGGAATGGGG
GTGt
>scaffold1102.size13227
TGTTTTTTTTTTTTTTTTTGCTGTTGTCCTCTTTTTGAGTAGCGAGGGATGACGCATGCTCTTCCTTC
ACTGGAGACTGTTCGAAGGGACGTGGGTAGTGG
GGATAAGAGCACCCGCTCACGTTTGGAGGATGTATTCAATCGTCTCACGCGCGATAGGGTGCCATACG
CCTCACACTACTGCGAGGAAAACACATATAAA
CTTATTGAGATCTTGTATGCCGAGTATGCCGTTCCTGATGACGCTGTTTTTGCTGTTTTTGTGAGCAA
TGAGCGCAAACAGACGCCTGTGTGGATGCAGC
GTCTTGCCGAAGCAGATGGGCGGGATCCGGTTTTGTGGGACTATCATGTCATTGCTCTCATGACGGAC
ACATCGGGTAATTGGTGGGTGTGGGACCATGA
CACGGTTCTGTCGTATCCGTGTTCAGCAAAGCGATACATAATGGAGTCCTTCCGACCAGAGATGCAGA
TGCGTGAGGAGTACAGGCAATGGTTTCGCGTC
ATTTCTGGACGCAAGTACTTGGCGAGTTTTTCATCGGATCGTTCACACATGAAGACATCGGGCGTTCC
TCCCCCGCCATGGCCCATTATTCAGGGTTCCT
TAGCCGCTACAACTATGCAACTACCTTTGTATTGGAATATGGCCAAGCAGGATGACAAGAAGGCGAAC
ATGGATGGGTCAATGGGAGACGCTTATGGGAA
CGTGGTGAACATTGAGGGAATGATGGCTCTTGTTTCTAATGATTAAAAAAGATTTTTAAAAATTGTGG
GAGGGATCATATCACACCTGTATGTCCGACAC
ATTTTTTGTTTTGTGTGCTGTGAAGGCAGGTTCCTCTGGCGGTGGCGGAAGGAACCGTGAAAATGGTG
TTGGTTCCATCGCAATTGTCGTGTCTCGAAAT
AGAGATGACCAAAATGCCTGCATTGACATTGAATGCGTGGGTGATGATGATGATGATGAAATTGATGC
GGATGGCTTTTGATGTGTATTTCCTTTTTTTT
GCTGTTGCTGTTGCTGTTGCGGTCATGTGGTGGCGTTCCGCTTTGCATATGCCGTTTTTATGCCCATT
CTTTTGCGTTTCTGTTTTTGCATCTGTGCCGG
GAGGGAACCCAAATGAACGTGTCTTTCTTCTCCCACTGGGTTATTGGTCTCTGCCGTTGTTTTTGTCT
CTTTTTTTCCAATTTTTTTTTTTTTTTTNNNN

NNNNNNNNNNNNNNNNNNNNNNNNNNNNNNNNNNNNNNNNNNNNNNNNNNNNNNNNNNNNNNNNNNNNNNNNN
NNNNNNNNNNNNNNNNNNNNNNNNNNNNNNNNNNN
NNNNNNNNNNNNNNNNNNNNNNNNNNNNNNNNNNNNNNNNNNNNNNNNNNNNNNNNNNNNNNNNNNNNNNNNN
NNNNNNNNNNNNNNNNNNNNNNNNNNNNNNNNNNN
NNNNNNNNNNNNNNNNNNNNNNNNNNNNNNNNNNNNNNNNNNNNNNNNNNNNNNNNNNNNNNNNNNNNNNNNN
NNNNNNNNNNNNNNNNNNNNNNNNNNNNNNNTCTT
TTTTTCCAATTTTTTTTTTTTTTTTTTGTCTTGTGTGTGCGTGTGTGCTCGGGAGAATTCTTACGGTCAA
GGGTGATTCATGTCGGAGGAGACTGGCGTGGG
ATCGGCGCCGGCCACCAGGAAAAAACGTGTGCGGCATCACCGCCCGAAGAGGAAAGGCTCCACCGCCG
TGGAAGCGGTCGATGATTCCCACAGGTCGCTG
GAGGAAGACGCCGAGGATGTGGCGGTGGAGAAAACAACTGAAGTCCCCGCAGCAGCAACAAAAGAAGA
AGGGGCGGGGCGGCGCGTGAAAAAGGCCCGCC
CGGAGTTGCCTTCCCAACAGGAGGAGAATGCGGAAGAGGAAAAACAGGAGCGGTCCGTAAACCCGGAT
GACGCAACGAGGGCTCCGAAGCAGCGGGAAAG
GGCCCCATGTGTATCCGATTACAAGGCACTGAATTTGAATCCACACATTGTCACGGCCCTTGAGAACG
ACTTCAAATTTGGGGAGTTGACGGGCATTCAG
GGACGATGTGTTCCCGCCGCCCTCAGCGGTCGGGATCTTCTAGCCGAGGCAAAGACCGGGTCCGGTAA
GACACTTGCATTCCTCATTCCAATCGTGGAGA
TCATATCGCGGGCAGGGTTTCGACCAAAAAATGGTACCGCCGCTGTCATCATTGGCCCCACACGTGAA
CTCTGCCTGCAGATTGAGGGCGTGTTGCTGAA
GCTGCTCAAGCATTTCAATGGATCTATCACCTTTCTCTGCTGCATTGGAGGCCAAAGCCGCAACCAAG
AGGCCTTTAAACTTGTAAATGGTATGATGATA
GTCATTGCTTCTCCCGGACGTCTTTTAGACCATTTGAAACTCACCACCGATTGGCTCACGAAGAATCT
TCTTGTTTTAGCCGTGGATGAGGCAGATCGTG
TCTTGGACAATGGCTTTGAGGAAGATATGCGCGAGATTGTCTCTCTACTGCCCAAAAAGCGTCAGACT
TTTTTGTTTTCTGCCACTCAGACAACGCGGGT
GGAGCAACTGGCACGAATATCGTTCTACCGACCTCCACTCTTGATCTCAATGAAGCGTCGGGAAGAAA
AGGCAACGGTCGACACACTTGAACAGGGATAT
GTCATGTGTCCATCTGAGCAGCGACTGCTAGTCCTGTATCATTTTGTGAAGCGTAATTTAAAGAAGAA
GATGATTGTCTTTTTTAGTAGTCGAAACGCCG
TTAGCTTCCACTGTGAGTTGTTTAATTACATTGACGTTCCGTGTGTGGCTTTTCATGGAAAGCAGAAA
CAGCATCAACGTTCGGCAACATACATGCAGTT
CTGCAATGCGCCTAGTGGAGTGCTCTTCACAACAGATGTGGCCGCTCGTGGCTTGGACATCCCTCATG
TAGATTGGATTGTGCAGTTTGATCCCCCAGAT
GATCCCGTGAAGTACGTGCATCGTGTGGGTCGTACAGCACGCGCTGGACGTTGCGGAAATGCACTCAT
GTTTCTTCTTCCTCAGGAGAAACTATTCCTTA
AATATCTATACGATGACGCACATGTCACGGTGAGCGAGTACACCTTTGACCTCAGCAAGGTGAAGCAG
GGTGTGTGTGAGCAGCTGGAGCAGCTTGTAAG
CACCAACTATTACCTTCGCACTTCTGCCCGCCAGGCCTACGAGGGATATCTCCTCAGTTACAGCAGCT
CGCAGCTCAAGAATGTATTTGACATCCACAAT
CTTGACTTGGCAGCTGTGGCGAAAGGGTTCGCGTTGAGTGAGCCCCCACCGCTGAAGTTGGACCTCTC
GCAATCCGCGGCGCATATGAGCAAAAAGTCAC
GTCGTGAATTCCGCCACATGAAGGAGACCAAAGACAAGAGGCGTCGGGCAGTGAATGAGGAGACCCTG
AAGAAGCGCAAATCGAACATTAGTGGAGAGTG
GTGAGGTGTGGGGTGTTTGCCTGAAAAGAGAGAAGAGATGTGTTGTTCTTCACCTTGACGTCTTATTA
ATATTCACACTCAACGATTAAAAAAAGGAAAA
AAAAAGGCCATAAAAAAAAAGTATTTAATTACTTGTGTGAAGAGAGATGGAGAAGAAAATAAAACGGT
GTGGGTCCGAAAGGAGAGAAAAAAAAAAAGAG
GGATTTTTTGTATGATTTTTGGTGTTGAATTTTTTTGTTATTTTGGCTTGTGCGTGTAGCGTCTTGTG
GGTGTCTGTGGGGTGCTTATTTCTCTGACATG
CATGTCCGCTGTCTTTCCTATGCTTGTTTGTACTTTTTACACGCATACTTGTATGTGTGTGTGTATTT
ATGTATGGATGGATGGTTGCTGTCTACATGTG
CGCGGAGGGTTGGATGCATTTATGGCTTTTTTTCGTTTGGGTCTTGTTTTTCCCGGCGATGATTTTTT
TCTTTTTTTTTTTTTTCCTTTTTNNNNNNNNN

NNNNNNNNNNNNNNNNNNNNNNNNNNNNNNNNNNNNNNNNNNNNNNNNNNNNNNNNNNNNNNNNNNNNNNNNN
NNNNNNNNNNNNNNNNNNNNNNNNNNNNNNNNNNN
NNNNNNNNNNNNNNNNNNNNNNNNNNNNNNNNNNNNNNNNNNNNNNNNNNNNNNNNNNNNNNNNNNNNNNNNN
NNNNNNNNNNNNNNNNNNNNNNNNNNNNNNNNNNN
NNNNNNNNNNNNNNNNNNNNNNNNNNNNNNNNNNNNNNNNNNNNNNNNNNNNNNNNNNNNNNNNNNNNNNNNN
NNNNNNNNNNNNNNNNNNNNNNNNNTTTTTTCCT
TTTTACTCGAGCGCATCCCTTCTTTTTGTGTTTCTGTGTGCCTTTCGCGACGGAAGGACACAGAAGTG
GAAGGAGCAGATAACCGTGGCGTCCTCTTGTC
AGTTATACCCGCAGTGTCCACCTGCACACCACAGCGGGGGAAAGAGGAGGCAGATCGAAGAATAAAC
GTCTCCCTCCGTGCGTGCAAGAGAGAGAAGTC
GCTGGCGTAGCCCTTTCTAATTTATTATTATTCTGTGTTTGTGTGTGTGTGGCTTCTACTTGTATTCC
CACCGCCTGCATACTCTCTCGTGTAGCCGCGG
TGCAATGAAGCGGGCACGAAGCAGAAGAGGCGAGGCGGATCAAAGTGTTAGTTCTCCACGACGACGGG
TTGAAGCACATGCTTCCGAGGATGTGTCGGAG
GGGCAGTCAAAACATCGACAAAGGGACGACGACACGGGGCTGTTGACAGCAGCTCAGCGGGAATCGCG
CGGTTTTGCGTACGTGCGGGACGCCAGTGAAA
ACACATCGTTGCCTATGGAGACACCAGGCGCCATGGAGGGTGAGCCCACCGCACCGTTGCCCTCTGTG
AAGGGAGCCGTCCCAAGTAATGCTGATGAAGA
GGGCGAGGAGTTTGTGTCGCCATTTGGGGCGCGGTCTTTAACGCGTGGGTTTTCCCATTTACGAGAGT
CCATTTCCTTTGCATTGCCACGACTCCGTGGG
AGTGAGCATAGGGTTAGCATTTCACGCGGCGGAAGTGTTCTTTTGCCTCTTTCTACGCCCCCCCATGA
ATCTGCGTCGGGTAGCAGCATTTCATCGTCAC
ACTCACTGCGTGTGAGCCGAGGAAACAACCAACATGCTCAACAACGACAACAACAACAACAACAACAA
CACCATCAGCAGCAGCAGCAGCGGCGGCGGAA
GTATCATAATCACAGGGTTAATGGGAGTGATCAGAGTTTTCTAAGCACGCGCAGTGCTATCGAATACC
TCAACAGCACCGGTTCCTTGGGCGGAGTGCTT
TCTTCGAGGGAAAGCAGGCGGTCAAGTCAGGCCACGGGCTCTCACCACTCCCATTCAAAATCAACTTT
AGGACCAATCGATCTCTCACCCATATTGAAGG
AGAGAAAACGTCAAAGAAGGCGTGCCGCTCCTCGTGGCGGCGTTGAAGCCAATGCTCTTAATGGCGGT
AATAATAATAGTGGCGGTGGCGGTGACTTCTC
CACGCGTCGACCGCCTCTGAATGGGTTGCCGATCGAAGTACCGGCGGCGATGATGAATCCAGCTGATA
TAGAAGTAAGAAATTCACGGAGTTTGGCGTTA
GAGGCGTTTCATCACTCGATGGCGCGCTACTCACGTGCGACAAATGTTTTTCTCATAACTTGTTTATT
TGTTTTTCTTTGGGGACTGCTGGCCGCGCCGC
TTTTAATACAGCAAACTGGTCCTGAAGACGTCTTTGTGCGACACTACGTGGACTCCGTATCCGAGTTG
CATTTTATCTATGAGGTGCCTTACATGTCTCT
CAGCTTTCACGAGGCGAGGCGTTTGTACCTTCGCGTCTTGAGCGAAACATTGGAGCGTCTCGAGCGAG
CCACCTCACACATGAGGCCCCCCGATACAGAT
GTCAAGGCGCAGATCCTGTACTACAAGCAGATGATAAGGGCATATTTGGCTCTCAGACATCGTGCACG
ACTTTATGCACGTAGTCGTGACCGAAGTCGTT
TAAATCAGTTTGTGTTTAACCCTCTACGGGATGTCTGGTATTATGGGATTTTAAGAAACGGTTTTAAG
ATGACGTTGTACGAGTTAATTTTAGAGCCTTC
ATTTTCCCTTTTTTTGGCGCGAGTGAAGGATATTGCTCTGTGCCTTCGTCAGGACGAGAAGTTGCCGT
GCCCAACACTAGCGTACCTGGAGGAACGGGAG
AAAAAACAGCGGCAGTGGGAGGGGGTTGGGGAAACTCAGCCTGACATTTTGACGGACGATGCGGTGAA
GGATTCGAGTTTATTCCATGAGGAAAAGGCAG
TGGAGGAGTTGCGACGTCTAAACCGGACGTTGAACTATGTGAAGGAGAGTTGTCGCCAAAGTAACCGC
GAGTACAATCATCTGTACACTATAGACAACAC
TCAGTCGATGTTATGGTGATATACCGTGGCGATATATTTCTGTTTTCCTTTGAACCGTACCACATTGG
TTGAGAATTCGTTTGCTGCGTGTCGTTGTGAA
GGAAATTGAAGAAGAAAGAAAAAACACTCACACGAAATCGGATTTTGTTGTTATTTTTTGTCTGTGCT
TTTTTGTTTGTTTGTTGAGGCCCGTGGTTTAG
TCGGGTGACGGGTTGTGGTGAGAGGGGCGAATTTTCTGCCCACGTCTGCTGATATGTATGCATGTTTA
CTGGGGTGTCTTTTTAAATTTCTTGTTGTTGT

TTCCTTTTCTCGTGGTAATTGTGGCCGTTTTGCGACTTCTGCCGCAGGGGAGCGTTGCGAAAAAAAAA
AAAAANNNNNNNNNNNNNNNNNNNNNNNNNNNNN
NNNNNNNNNNNNNNNNNNNNNNNNNNNNNNNNNNNNNNNNNNNNNNNNNNNNNNNNNNNNNNNNNNNN
NNNNNNNNNNNNNNNNNNNNNNNNNNNNNNNNNN
NNNNNNNNNNNNNNNNNNNNNNNNNNNNNNNNNNNNNNNNNNNNNNNNNNNNNNNNNNNNNNNNNNNN
NNNNNNNNNNNNNNNNNNNNNNNNNNNNNNNNNN
NNNNNNNNNNNNNNNNNNNNNNNNNNNNNNNNNNNNNNNNNNNNNNNNNNNNNNNNNNNNNNNNNNNN
NNNNNAAAAAAAAGAGAGAAAGGAAGGAAGGAA
GGAAGGAGTACCAGGGAGGCGGGAAGGGGCGAGGAGGGCCCTTAAGAGTGGGGTACGGGGCGGTTTTT
TTGTTGTAGAAGATTGCATGCTTTGGCGGTCA
CGGCAATCTTGTGATGGAGGTCACGCCATGCAATTTAGTTCTCTTGGGCTCCGAGGGGGTGGGGAAGA
GCGCAATATTGCAGCAGTTTTTGAAGAAGACA
TTTCGTCCGGATTACGTCCCGACTACATTAGAGACATGTGTGCACGTTGTCAGCGTGGATGGAAATAC
GTATAGACTGCATTTTTGCGATTGCTCCGGCA
GTCGTGGATTTAGCGTTCACCGTGCGCCATATATCGTGGAGGCGCACGGTGTCATGCTTGTTTACTCT
ACCACCAGTAGGAACTCGCTTGTGCATCTTCT
GGGGTGCGCAAATGAGGTTTTTTGTACACGAAGGCAACATGGCGTTAAAGGGAAGATTCCCTTTATTC
TTGTAGGCACAAGAGCGGATATCGTGGGGCGT
CGTGTCGTGACTATAAATGATGCAAATCGCGTGGCGCAGGGGTGTCTGCTGGCGTTGGGTTTGAAGAT
TTTTAAAGAGAAGAAAAAAAATAGGATACAAGG
GTGGGTGCAACAACGGAGGTATGGAGGTTGATCCCATCAACGACCTACCTGTGTTGGAAATATCAGGC
GCAAATACACATGAGGTATCTCAGGTAGTGTA
CACAATGATTCGCATGGTGCGTTACTTCCGTGCAACGTCTCGGCTGCCCAGGTTTAGTTTGATTTGCC
CAGGCGTGGTGCCGCGCCGATATGTCGTAGTA
GAAACAAACTTAGACGCTCATTCGGGCGGTGCCAGTAGCAGTACTTCCCCGGACGGTCGTTTCACACC
GGCGTTGTCTGATAATTCACAGGCAACCTCGC
ATCGCCACGCTTTTCACATGTCGCCAGTTGGTTCGGATGAATGGAGTGCCTCATATTCTCCGAGGAGC
GTTGGTGTAACACAAAGCGTGCGTTCAAGTGG
CGTGGAAGAGGACCGGGCGATTGTTCATCCATACCTGCAACAGGTAGAAGATCGTGAGGTCATCTTCA
GCTTACCGCCGCGAATTTCCTCTGATGCATCC
GTCAATGTATCTGCGAGTACTAAGACGCAATTGTCATCCTTTGCATTGGACGGACCGTCGGAACGACG
GGCACCAAAAGTTGGACCAACTGACAGGCAGT
GTCCCCGCTGTCACTTCATGTAGCGTACATTAGGCGCAGTTTTTTGAGACCTTAGGATTTACGATGCC
TGCAGGATATATATATATATATATATATGTAATA
TCACTACTATTATTCCCCCTCAATAACTTGTTTTATTTGCATTTGTTATTTTCAGTGATGGATACCTA
TTTGGTTTGAATTATCAATAATATTTTTCCCT
TTGTTTTGGTTTGTTTCTGCTTGTTTTTAGATTTTGCCTGAGTGCAGCTCTTTTTTTTGTGTGTGTGT
TTATTTATGCGTGTTCTTTATCATGTTTCACT
GCACTTCTCCATGCGAGTGTAAGAGGCAGTGGCTCTCATGAATGGAATCTTTGTTTGATATTTTGTTG
GCGGCTCTGGTTGCGCTTATTCTCTCTGTGCC
GCCACAGGTGTTTTTGTTGTGTGTGTGTGTGTGTGTGTATGTGTGAAGACTGCATTGGTAATTGGAGA
AACCATGCGTCCAGGTGTGAGTTGGAAGGGTA
GAGAGTGTGTGAGATACATATGACGGCGATAGGTAACAGGTGGAGGGAGTGATGCGGCGAGTTCATGA
ACGCTTATCCTCATCAATTAACCCCCTCTTTT
TTTTTAGTCTTTCTGGTGCTTACCAGGATGTATTTGCGACCGGCGTTTGGTATCGCGGTAAAGAATTG
AACTCGTGGCTGCCAGCTACATGCTTTGCTGT
AATGTGCGGGTGTGTTTGTTGCGGCCCAAGAGGTGGAGAGTTGTGTGTGTGTGTGCATCTTGATTTCT
TTGCACGGCTTTTTTCTTTTTTCATTAGGCCC
GTCACACGCAGAGACGGAGTGAGGGGAAGGGAGGCAAATCAAAGGACAGATAATAAAAGGAAACGAAG
GAAAATTAACACCCGGGGGAACCGGCAAAAAA
GACATGTTGACTGTCACGGAGGACAAAATTCCCGTTCCCCTTATTCCCGATACAGAGTTATGTGATGT
TCGGATCGGGGCGACGGATCCAGCGCTTTTTT
TTTGCATCAACTTGGACTCGCCATGTCAGGTGAAGATTGAGTTTCAGGAGTTTAACACTGCCGCGCGG
TGTGTGCTGCTACTCAGTTCCACAGATCCTCG

GCCAAGCATCCATACCGCCGTCTGGAAGTGCCTTTCCCCCGAGCAAAAAAAGACAGTTACACTATTGC
CTGCTGATCCCTCTTACCGTGTGGGACCGTTG
TACCTTGCAGTACGCTATGTAGAAAAGAGCGGAAATACTTTTGTTCGTCTCAACTTGACACTGCAGGA
ACAGTATGAGGCGGAGTGGTATTCGCAGTCGA
ATGGTGCCTTGTACTACGGCATGTGGTCCGGCTTCTGTTATCATGGACGTGGTCGATGCATTTACGGC
GTGGAGCCGGAACTGATGGAGAAGGGGACTCT
CAAGTGGACCGGAAGGAGTTTGAGGCAGCAGAGTAATTCCTTGTCTTGGCAATTTGACTCCACACCAC
CGATAGAGGACGGGAAACTAGCCATCAATCGA
AAGGATTTGGGATGGTCCATTCTTCCCCTACCCTCTCCGAAACTGGAATTGTACGATGGGGAATGGGT
GCATGGGAAAAAGGAGGGTCTTGGGGCCTATC
AGTGGGCCGACAGGGCGTATTGGGGAATGTGGAAGGGTGGTGGGCGAGAAGGGTTGGGTGTACTATGT
ACACGTGATGGATATCGTTACGAGGGTGAGTG
GTTGAACGATAAAAGAGATGGAGTTGGGAATGCCTTTTACCCGGATGGGACGCAGTATATGGGTGGGT
GGAAGAAAGATAAGCGATCGGGTGAGGGTTTA
TTTAATTACACAAATGGAATTGTTATTAGAGGTACCTGGGAAGAGGACGTATTGTGCCCAACGGTAAC
TGCAACCTACGCCGATGGATCTTCTTATGTTG
GCGAATGGGCCCACGACTGTCGTCATGGTAAGGGACGTCATATTGATGCAATTGGAAACGTTTTTGAT
GGAATGTGGGATATGGATAAGCGTACAGGAAA
CGGAACCTTGCAGTTTATCAATAATGTGATATGCGTCGCTCTTTGGGAGGAGGATGTAAGAAAAGATG
GTACGTTTAATTTTCCTAATGGAGAAGCATAT
GTTGGGGATTGGAATGATGAAAAATACATTCGTGAGGGACAAGGAAAATGTACCTACCCCAATGGTGA
TTTTTATGTAGGGAGCTGGAAAAATGATAAAA
GGCATGGTTTTGGTAAGTTTGTTTATTCTGGTGAGAAATGTGTTTACGAAGGTGTACTATGTACACGT
GATGGATATCGTTACGAGGGTGAGTGGTTGAA
CGATAAAAGAGATGGAGTTGGGAATGCCTTTTACCCGGATGGGACGCAGTATATGGGTGGGTGGAAGA
AAGATAAGCGATCGGGTGAGGGTTTATTTAAT
TACACAAATGGAATTGTTATTAGAGGTACCTGGGAAGAGGACGTATTGTGCCCAACGGTAACTGCAAC
CTACGCCGATGGATCTTCTTATGTTGGCGAAT
GGGCCCACGACTGTCGTCATGGTAAGGGACGTCATATTGATGCAATTGGAAACGTTTTTGATGGAATG
TGGGATATGGATAAGCGTACAGGAAACGGAAC
CTTGCAGTTTATCAATAATGTGATATGCGTCGCTCTTTGGGAGGAGGATGTAAGAAAAGATGGTACGT
TTAATTTTCCTAATGGAGAAGCATATGTTGGG
GATTGGAATGATGAAAAATACATTCGTGAGGGACAAGGAAAATGTACCTACCCCAATGGCGATGTTTA
TGAAGGGAGCTGGAAAAATGATAAAAGGCATG
GTTTTGGTAAGTTTGTTTATTCTGGTGAGAAATGTGTTTACGAAGGTGAATGGAAAGAGGGGAAGCGA
CATGGTATTGGGACGCAAGAGACTGAGGAAGG
AACGTACCAAGGGGAATTTCAAGACGATATGCGTTATGGACATGGGCTACACCTGGGTCGTGCCGGCT
CCATGCACAGAGGCATTTGGAGAGATAACGGT
TGCATTGGTCCTGGTGTCAGGTTTGATTCCAAAAAGGGAATCATGTATGATGGTCTTTTCCTTCTTGG
AAAGTTACAGAGTGTTGGCACATCGCGAACAG
AGAATGATATTTATGAAGGAACATGGTGCGATGGTGAGCGGCATGGCGTAGGTTTGGTGTCGCTCCCT
AACGGTGATGTTGTGCGCCATTTGTGGCACCG
CGGGGTCCCTCAGGACGGACATGTTATCTACATGTACCGTGATGGTGATAAATATGAAGGAGAGTGGA
AGGATGGGCGTCGTTGTGGGAAGGGGACACAG
TGGTACGCGGACGGATCTGTTTACGTGGGTGAATGGCTTGATGACAAGCGGCATGGCAGTGGATCATA
CACCGACGTCCGTGGTGAGACTTTGGTGGGAG
AGTGGTGTGGAGGGGAGCGCATGGACGTGAAGGGGAGTCTGCGCTTTTTGGATGGCACCGTCTATGAG
GGCGAAATGCGTTTGGGCAAGCCGCATGGTAA
CGGACGTCTGAAGTATCCCGATGGTACCGTATTTGAAGGCCGATTTAGTAACGGCGTTTATCTCCTGT
AAACTATGGAGGGGGGTTGGATGGGATCTTTT
ATCTCTTTTTCTCCATCTTTCCCTTTTTATGTATTTTATTGAAGAGTTTGTGAGTTACAAGCAACAAA
CGAAACCACAATAATAATAAAATATGGCAGTG
TTGGAAACAAATGGCTTTAAGGCCTATGAATTATATCTCAAATGTGTCATGGGGCCTTCTTTTCTACC
AGTGCTACTTTTTTTTTTTGTTTTTTGTTTTA

AGGAAGCAGATACAAGGAGGAAGGGGAGAGGAGTAGAGAAATATACGCCTAGAAATTAGTTTTTGCAG
AATCACTTTGGGGACGAGACGAGATGACAGCT
TCTCCCACTGCCTTAATGGCTGCCCCAACTCACCCGGACGAGACGCTTCCCATCTTCATGGATGACTC
CGCGTTGATGGATGCGAAGACGGCAAAACGTC
GCCAGGAGGACGATGAAATCCGAAATTATCTGATACAGCTTGATGCACTGCGTCACGAGTGCGAGCAG
CGCGGTCAATTTCGCAAGGCGCAGAAGTGTTT
GGATCGTATGCGTGAAGTGAACCTCCGGTACGCCAAGAAAATTGAAATGGAGGCCAGCAAGGTGAATG
TGGGGTGGAGGAAGCAGATGTTGGAGGAGCAG
CGGCTGGAGCTTCTCACCTTTCACGACATGTGGGAAAACAAGTTGAAGGAGTATGATGAAAAGGCGGG
ACAGCTGACACAGGAGATGAATCGTCAGCATG
CGATGGAGTTGCAGTTGCAGGAGGAGGTGATTCGCGTAGAGCTTATGAAAAGACGTCCACGGCCGTCC
AAGACGTTGGTGGAGCTTCGTGATAATCTGAG
GACATACGTACAACGACGCATGTACATGGACGCGGATCGTGCGAAGAAGGCAATTCACCATCGGGAAT
CGGAGGAGCTACGAGAGTTCGAGGCGGATATT
GAAAAGAAATTTCAGGACCGTCTGCAGGTCGCTTCAGAGCGACTGCGGTTGGGGAGGGCGGCTGTGGA
GCAACGTCTTGTCGTAAATCGTGAGGAATTGT
TGGCCCAGCGTCGAGACGAGTTTAGCAAATTGGTGAAGAAGCATCATGCGGCTTTGCACGCGCAGGAG
CAGGCCAATGCTCTTGTTGTCTCCAGAGCGCG
GGATTGTATTCGTCGCCAGGCGAAAGCGTACATCAAAGACACGTTCAAGACAGGACTGGAACTGGTGC
ATCTAACGGAGACGGCGATGGTTGGAGGAAAG
ATGGATTGGAGTGCGGATGGGCGGCAAGGGACATCTTTCAAGAAATGTGACCACCGCAGTGTCGACAG
AGGAGCTTCCGTGCGGCGGCATAGAACGCCTC
TGAAGAGGACGACGACGGCGTTGCCGCGGCCTCCATGGCAAGATTGAATCCCTGTAGAGGTGTGAGTT
GAGCTAACGTCAAAGGAGCACACACACACACA
AAAAAAAAATACAAACGTATGTGCAC
>scaffold1103.size6729
CTTCGGCGTTATTAACTTCTTTGTCCGCGGGTGCACCGGTGCCAATTTCAGATCCTTTATTACCCTTC
GCATTCTCTGTCAGTGATGTATTTGACACGTTA
TTTACTTTTGGTCCCGGAACGACCAAACCTGGTGCCTCTGGCTCAATTTTGTCCGCAGCCGGCTCATT
GGCTGAATCATCAGCAGCCTGACCGTCCACTG
GTAGTCCTCCTGTTTGAGAGGGCGCTGGACCGGCGACGTTTCCATTAGGTGCAGTCGCCCTAGATTCA
GCTGAGGTAGGCGACAAATTGGGGTCTTTTGC
AGTACCTTGGCCTTCAGAGGGCTCCTGAGTGTCTCCCGCGTGCACGGACGGGCAGCAGCACAGGGCGA
GCACCAACAGGGCGCACAGCACACGGCACGTC
ATCATCGTGAGTGTGGTCGTTCAGTGGTGTGGAGCTGATCTGCACAGTGAGGGGCATGGAGAGCAACA
GGGGAAACGAGAGGTGCGGCAGACACACACAG
AGGGCACACCACATGAGGAGAGGGCAGCAGCCAGGCATGCACCGTACGCCGCACACCACGCACGACAC
ACACTCCAATCCACGGCAGCAGGCACACACAC
ACAGCCCTGCCGAAACACCAACACACAGATGGAGGAGGGAGAAGCAAACAGGAGCGGCACACAAGGCT
CCACCGCATGCACGTGCGGCGAGAGCGGTCCG
TCGTGTCGGCACCCCTCACGGAGGTTACGCACACACACAAGAAGGTGCAGCGCACACAGCAACACAAC
AGCAAAGTAACAATACAAAAAGAAGATCCCCG
CACACCACGCGGATTGTTGGTCCACGGGAAGTGCAGCCAATATTCATCACAAAAACAGTTCAGTTGCC
TTCGGCCTTCGTCCACATTCATCGTAGACCAC
TTCATGCAGGCCGGCCACCACCGCAACAGGAGCTGCTGAACACGCAACAAGAAACAAGAAAGGGAGGT
GGTGTGGGAAACAGCATCGATGCTGTCACTGT
CGCCAGGCGCCGCTGGGTTATTCGTTTTGTCTGTGGCTGTAGAAGATGTTGTTTCCTTCTCGCTGCCA
TCCTCTCGTTCATGTTGGTGTTCTCCCTCCGG
CACAGCGGCATGAGATGCGGAACCACCGTTCAAAATCTCCGCATTGCCCGCGTCAGCGTCATTGGCGG
CAGCAGCAGCATCCGCAGAAGAAGATGGTGAA
GCTGTCTCAGCCTGACCCTCAGTTATTGCATTCTGCCTTGCATCCGCTGTTGGTTCAGTGCCTTTTCT
GCAGATCTTCTGTTGGCTTCATGGTCGCTGGG
ACTTCCGTTGAGGATGGCAGTGCGCTGTGTGGTGGTGATTTCGGTGGCGGTTCTGATGGGCCTGCTGG
CGCCTCTTATTCTGCTGCTGTTGGCGGCAATT

CCTCCCATGCTTCTTTTTTCTTCCGGTGCCGCCTCCTCCTCTGCACCTCCTTGTGCCGGTGGTTTTGG
CTGTCGCAGGGGCATTGGCCGTGCTTCTCCCT
GTTCTGCTGCACCGGTCGTAAACGATCGATAATTTCCAGAGAGCGACTTTGAAGCAGACTCCGTGCCC
GGATGTCCAGGGGAACGACTGGTGCTTGAAGC
AGACAGTCATTCGGGTGCTTCAGCCGCAGGTGCATGCTCTGCATTGTGCGCGCTGCCAGTCAAACATT
ACGGATTTCTTTTTGCTCCTTTCACCACATCC
CCTGGTCCTCCTGAGCCCATTCTGGCACTCCCTGTACCACGGACACACTCTGTTCAGCGTCAGTCGCT
GTTTGAAGCTGCGGTGTTTGCTCTTGACTGTC
GCCGTTCGATGCGCCCCGTGTGAGACCCGAGCTGGGTGCAGCCGCATGCTCTGCTGAGTCCTCGGACG
GCTGCGTCGCATGTGCTGTTTGTCCTTCTGCT
GGCGCTGGTGCTGGAGTTCTCCGTGCGCTCTCCCCCGTCCTGAGCACGTGTGGCCCTTCTCGTTCCTG
TTGTGTCCACTCGCTCTGAATATTTGTAACTG
ACGCGCTGCCTTTCTTTCCATCCCTCTCTTCCACGGTGAGAGTTGCCGACCCCGCAGAAAATTGTGGC
GGTTGGGAGCACACACTCGACATTGACGGCTC
TTCGCCGCAGCATGCTGAGCCTGCCTCCTCCCTTTCTCCTGCCACACTTCCACCAGCGGGAAGATCGT
CTTGGACGCCGGAGACAAATGCGGGCTGCTGC
TTGGGCCAACACCACCACCAGCACCCGAGACAGGAGTACTGCTGGCAGCGTCTGTATCGGCAGCAGAA
GATGAAGCCATTGGTGCAGCCCCACTGCCGCT
GAGCTTCAGTCCCGCTCCCTGCATCACCGACCGGTGGTTGTCCTGCATCACTGTTGAGCGTTCCGCTT
GCACCAATGCCGCCGTCCTCACCGGCCGGTAC
CGCCGCAGCTACTCCCCGTAAGGCAGCACTCGACACGCAGTCCCTCCTCGCAAACACCACATCGCCAT
CCATTCCCCTCGCAAGCCCCGCGTCGTCCATT
CCACCATCAGCACAGTAGCACACAGGCCACGCATCACCACCGACGACACCGCATCACAGCACGCAGAG
GACACACACCAGCAGCACACGGCCAGTCATCA
TCATCGCCATCACTCCTGTCACTTGCCTGCAGCCTTCAGCACAAACTAGCTCCCCGTCAACGCAGAGC
AAAAGCAAAAGGAGACAATTCACAAAGCCACG
CACCGTCCCATCCAGCAATGAGAGTCACGCACCACCACAACAATTAGGTGAGCGCGGTTCAACAACAA
TCAAAAAAAGAGGTGCTCCTCGCCAGTGCCCT
GTTCCTCACACCCCCACAGTCACTGTTACTGTTATTCCCTCTCCTCAGTCCCTCACAGGCACGCCACG
CCCACTTCAACACGCGCCCCTCCAGCACACGC
AGTCCGTCATGCGCTCCCACTGCGCCGCAAAGTCCGTCACCTCCTCCCAGTCGAACAGCTCCGTGTCC
TCAACGTCCCTCACAATCGTCGGCATGCCACG
GCCGCGCAACACCCACAGCGCCACAATGTCGTGCCGCCTCTCCTCCCCAATCATGAACGCAACGCCCA
ACGCGTACTGGCGCACGTGCTCCATGCGCTGC
AACCACAGGCCGATCAGATTGGCCGTCATGAACTGCATCTTGTCGTCCTCCGTGTACTTGGAGCAGAC
CCTCACATCCTCTCAATCCACGGTCTGGTGCT
GCGAGAGGCTGCCGAGAGGCGCTACGTGTGTGGAAGAAGCAAATGTGCGGAAACAACGGCCACCCTCA
CTCCCTCACACATCTCCACTTCCCTCCACTGT
CCCACACAAAAGGGCGGTGTCATGGGGAAGAGATGCCCGCCGCACAGCACATGCACGAGGGCACACGC
GGAAGCAATGCCATCCACTTCGCACTGCAACA
AAGAGCCATGCGCTCAGCACACACACGGCGGCAACGAGAGCGACGGGGTGCATCCAATTACATACCGC
ACAGCCATTTACTTCTCTCCGCACGGCTAGTG
GCGTCCCACACTCACAGCGAGGCACGGCGCACACCATCTTCTCCCTCCGAGGGCACTCAGGAGGCCGG
CGCCCAGTCACCCTCGTGCCATCGCAATGGGT
AGCACAAACAACCGTCGTCGCAAACCATCGGCGCCTCTAGCAGAAAAGAAGAAAAATATATGAGGGCA
GTTCAGAAACACAGCCAGCAGAGGGACACCCA
ACGACACACGGAGGCACACAGTGACAGTGGACACGGCACGGACAAAAAGAAGTCCGCAGGGATTTTTT
AAAAAAGAAACTAAAATGAATCAGTAGGACTT
CCATTTCGTGTGCCATGCAGCACAAGACGATCACGTGCGTATAAATATTACCTGCACAAGTAAAAAAT
GTAATGAAAAAAGAAAAAAATCTTATTACTCC
CAGCCCACACAACATCCCTCACATTTCACGCGGCATCCGGCCTCTTCTTCCAATCCCCGTTCCTGCAG
TCCGTGGGAAGCACAGCAGCGGAGCCGGCACC
GCGTGTGGGCAACAGCAAAAAACAAACACACAAGAGAGAAAACACGTCGCCCATTCCAGTGCACATCA
ACCAAACATTAATGAGAAGAAAAAAGGGCACT

TATTGCTCCAGTGGGAAAGCATCAAAAAGAACATTACAAAGAAGAAAAAAAGATTTAAAAAGCGATCC
GTAAATCATTAAAAAAAGGAGAAGAAAAAGTG
ACATAAAAACCGCAAAGTACGCGGCAAATATTCCCCGTGGGGAGTCACAATTCCACCCTCTTTCGCTC
GAAGGACTACCACAACNNNNNNNNNNNNNNNN
NNNNNNNNNNNNNNNNNNNNNNNNNNNNNNNNNNNNNNNNNNNNNNNNNNNNNNNNNNNNNNNNNNNN
NNNNNNNNNNNNNNNNNNNNNNNNNNNNNNNN
NNNNNNNNNNNNNNNNNNNNNNNNNNNNNNNNNNNNNNNNNNNNNNNNNNNNNNNNNNNNNNNNNNNN
NNNNNNNNNNNNNNNNNNNNNNNNNNNNNNNN
NNNNNNNNNNNNNNNNNNNNNNNNNNNNNNNNNNNNNNNNNNNNNNNNNNNNNNNNNNNNNNNNNNNN
NNNNNNNNNNNNNNNNCACAACCCCAGCAGCA
GCAGCAACGCCAGAGACGCGCACACACGCGCAGTGCGGCCGCCAGTTGAATCGCGTGGTATCAACTCC
ACGTCGTCAAACAGGGCGAGGCGGCCGTCTGG
ACTCACGCCGCTCCAGATCGCGAAAGGTCACATTTCTTGATGCCGTTTTGGTGTTCCGGGGACGTTGT
GACCATTCATTCCTCCCGCAATTTTCCCAGGA
ACGTGCTCGCCGCTGCCGTCAACGGTCTCACTCTTACGGGCATCCTTCGAGCCCACTGCCTTCCTCAA
CGCGGAGGCGGGCGCAGTCTTCGTCACGAATG
CATTCAGATCCATTTCTATGTTCCGACTCATGGCAATGCTAGGCTTACCCTTGGGTGGTGTTGGAGCC
ACGTTTTCTTTCGGATCGGTTGCAATGCGAAG
CTTTTTCATAGACGTGTTTGTCGCCGTGTTGGATGCGGGACTCCGATCGACGGTGTTGGGTCCGTCAG
CCGCCGCAGACTGTCGCCAATCCTCGTTCTGA
CGTGACGGCTCAGCATTAACTTGCGGCGCAGTTGTCTTGCCAGATTTTGTGTCATCCTTCCCTTCACG
GGGTGGCGGCAACTGACAGATGCTCGTCCGGT
CAGCATCGCTGCTCCGTGCAGGCGGATAGATGGTTCCATCGGGGAAAATTGACGCCGCCACAATTCAT
AAGGGCCGGCATATTTATTTGTCGTCACATTC
ATCGTGAAGGCAACGGTGGCCCCTTCCTCTTCCGTTGCACAGCTGACAACACAGTTGCTACATGTGTG
CACCGCATTGACCACATGGCACACAGCACCCA
GCTCACCATTCCTGCTATGCATGCGCACATCAAAGCCACTGAAAGTGTAATTCTCATGCTGAGCCTCA
GCAGCGGCAGGAGAACACTTCGTCCACGCCAC
ACACACACGCGATAGCTCAGGCTGCCGTCACTCTGAGCACACGACACATCCACCGGCACAACCACCGC
ATCCGCATCACCAGATACATCTCCAGCACTCC
CAGCAGCCGTCACGCACACGGAGCGAGCACCCACCACACCAGGCAGAAGCGGAAGCAAACCCTCATCC
ATCCATGACGCGGCACAAAAACCTACAACCAT
CCCGCCCTCAATGGGACACGGAGCCCAACTCAACGCAGTCACACGCGGTGCACCACACCCGCAGAAAA
GGAGACTCGGGCTGCGCACTCCCTCTCCTCCC
TTCATTCTCATGGCCACGGAAGAAGACAACAGCCGCCACACGCAACTGCCAGCATGCACTGCAAAAGG
AGCATCCAAATCCACTGCAATGTGCATCTTGT
GTGTGTGTGCCTGCAGAAACACAAAGCAAAGAAGAAAAGAGGGAGCNNNNNNNNNNNNNNNNNNNNNN
NNNNNNNNNNNNNNNNNNNNNNNNNNNNNNNN
NNNNNNNNNNNNNNNNNNNNNNNNNNNNNNNNNNNNNNNNNNNNNNNNNNNNNNNNNNNNNNNNNNNN
NNNNNNNNNNNNNNNNNNNNNNNNNNNNNNNN
NNNNNNNNNNNNNNNNNNNNNNNNNNNNNNNNNNNNNNNNNNNNNNNNNNNNNNNNNNNNNNNNNNNN
NNNNNNNNNNNNNNNNNNNNNNNNNNNNNNNN
NNNNNNNNNNNNNNNNNNNNNNNNNNNNNNNNNNNNNNNNNNNNNNGGCAACCCACACAAGATTTATC
CAAAATAAAAAAACACGTCCCCCTTCGTCTTA
TACAAACCCCATCACACAAAATAATTATAAATAAAAGAACAAGAAATCGTCCATTACTGTTCCACACA
AATTTAAAAGACAGCCGGCTGCCGTGTGGCCA
CACCAAACTGTCTGCGTACATTTTGTCCGAAAATAAAATTAAAAGATATTTAAATGTCTTTCAACCGT
CCGACCCTCCGTGTGTCCGCACATCGTTTATA
TTTCATGGGGTCATCTGCGTGTTGCTGCTCCCCTCAGCCCTCCCATCGGCGCGTGAGTGCGGCCCGCA
GAGCACAGCCACTCGTGTGTGTGGGGACTGAG
AGAAGCACGCAGACACGACGGCAGTCGCGTCACTCCATCGGGAGAGTGAGCGCAGCAAAAAAAAAAAT
CATCAAAGAAAAGAAATGCTCACGAGGCCGAC
CCCGAAGGGTGCGCCACCGCATGAAAta
>scaffold1104.size6841

AAAAAAAAACGGCACCAGCAATGACAGTAGGACTTTTGAGAAGTATCAATAAACATTAACTGCCGTGT
CACTGCTTTCCGTGTGCCTGCCAAAGTGCATTT
TCCCCCGTTTCACCTTTCATACATTTTTTTTTTCTTTCTCCTCGTCTCCTCCATCTCGGGCAAACGCA
CCGGCGTGGAAGTAAAGCATTTAACAGGTTCG
CTATACATCACAAACTCAAAACACTAACTTTTATTTGTTTTTCTGTCCTGTTTCTTTTTCCTTTTAT
TTATTTTTTTTTTTTTCCTTTTACCATTTCTC
TTTCCTCTTTCCTCTTTCTTCCTTCTCTTTTCTCCTTTGCATTCGATGCTCCTTCACTTCCGCTCCTC
ACGCGGTTGTCAGGCGGCGATTAGATTTCATC
GAGGATGCTCATCTCGGTTTTCCCATTCCCAGTCGATGATTGAATATCTTGCACGGTGGGCACGGGCA
TGTTGCGTATCTGATCCAGCGTGAACACCTTG
TCCACACTCACCTCAACGTAGTACTCGTTCAGGCAGTTGACAATCTCCTGCAGAGTAATCGTGGAGAC
GTCGATGGGGATGCTGTACTGCAGGAATTTAT
CCGCCAGCAGCCGGGGGTGAATAGTCTCCAAGTCAGTCACGAACTTCGCGCCAAGAAAAGCCGTCACG
GGGATGGCCGTCTTTTGCATCGCGTTGTCGCG
TTGGGTGCGAGGCGAGATGACAAGCTTGGACTGCCGCCTCTTTGGCATTTTTGCAACCTGCTTGTTAA
AAAACTTCGTCGCGAGCCGCACGACGGCGGGC
GGTAGTCCCCACCAGCGGGTCTGGGTTTCATCCGGGTTGATCCCCAAACTCTCATCCTGCGACACGAA
ACTGCCGCCGCCAGAGGAGCGGAAGCTGTCGA
GAAAAGACAACTTTGAGATACTGATGAGCCGTGAGCCAGCGCGCATCATGGGCGGGATGTAGTCCTCC
TCGTGCTCCTGCAGAAAGCGGAAGCGCcTTGG
CAGGGGCTCCGGCTGCAAGACGGAGCGGTACGCCCGACGCAGGCACGGGATGGAAAGTCGCACCACCC
GCTGCTCCCCGCTTGCCGCATCCTCGGTGTTC
TCCTTCTTGTCGTCGCTACCCGCAGCCCTCGCAGCCTCAGCCTCCTCCTCCTCCGCCTCCGCCAGGCA
TCGGGCATCGGCGTCAATCTGCAGCTTCATGG
CCGCCAAAACAATCTCGCGGGCCTCCGATGGGCGAAGCTCGTGAGAGCGGATGCGCAGACGAAACAGA
TCCTCCGGCGGCAGAACGGGCAGCTTCCCCGG
CAACGGAACATCCGTCATGGATTCCTCTTCGTCTTCCTCATCGTCGTCCTCCTCGTTTCTTCTCTGCT
TCTTCAGCTCATTCATCTCCTTCTTACCGTCG
CCCTCCATGGCATCCAAGAGACGCTTCCAGTTGACAATAATGTTGTAGTATTCGGTGTCCGCCTCCTT
CGCCCGCTTCGCAATCTCCACATCTGTCTTGT
AGTTGTAGTACACAAGCTCAAGAAAGGTATTGACGGATATGGTGCCATCGGAGCGTTTTCGTGGCCGT
CGAAGAGCTACGCGCTCAATGAGCTGCTCCTG
CAAAACCGGGAAGGGTGCGTTGAGCACAAGTCCGCGCAGTGAAACCCAGCCGGAACCGTCCCCGTCAA
GATAGTTGAAGATATTCTTCATGCGAAAGTAG
TCGTCCGCAAAGTAGTTCGCAACCTCCTCGTGGTTCTTGTCGCAGAGATCGTTGATGAGGTTGGCCAC
GTTGGTGCCGATGTGCGTCCCCTCAAGAGAAA
ATCGCACGATGTTTTTATTCACTCGCAGCAGCTTGATGATAGGGTCCGCGCTGTACACTGTCACAAGT
GGATTGTCGCTCAGGTCCACGCTGCGGATGCA
CTGATGGTCCTGCAGCGTCTCCGCCAGCTGGGCGACAAACTCGTCAGTGACATGGCAACCAGGCAGGC
TCACACGGCGCAGGGAACGACTGCAGAGCAGC
ACGGGCACCAGCGCCATGCAGCCCTTCGGACCAAGAATGAAATTTTTGCATTCGAGCACGCGGTGTTT
ATCCCAGTCACGCGGGGCGTCACTGAGCAGTA
GACTCAAGCTCTTCTGCGGGTGAATGCCCATCTCCTCGCAAACCTGTGCGTACGCCGCACGCGGAGAC
TTCCGGTCAAACCGCGCCAGCGTGGCGTCCAC
CTTCTGCATCGTCGTCATTTTTGCTTAATTTTCGACTTGTCTGTTTTTGCTTTTGTGCGTGCTCGTGC
AACTCAACTGCACGTGGAAGAGCAGGAAAAAG
AATATTTGCAAAAATAAAATAAAAAGGTGCAATAACTCCCTCCCCACCGACACCCACACGCAATTGTG
CCTCTTATTATTACCCGACACCTTCAACCCTC
GCACCTACCATGAATGAAAAACAACAACACGCGTCTCAGCGTCGATTCATGTAGAGAGTCATCATGGC
ATCCATGGCGCCGTCCTTGTCGGGCACGCTGC
CCAACGCGAGCTCCAGCCACCGCCGCACCGTGCTCGGCCGCACAAGTGCCGCAGCGCCCTTCCGTTGG
CTCTCACACTCAGCGGGACAAAAGACGTATTC
CAGCAGATATCGCAGCAGGCGGGGACCAACGGGTGCGTTGTCAGCACCGCCAATGCAGCTTTCCTGCC
GCATCCACCGTCCCCACTCGCCCTTCCAACCG

CCACCATCATCATCATCATCATCGACAACAATGGCAGTACCACTCGTGGTGGCAGCCGCACGTCGCTC
GTTCTGTTCATCCCGCAGAATTGCTGCGCCAA
TGGTGTACGCAATGCAAAAAAAAAAGTTGCTCCGTCGGCGGTAGAACAAGCGCCATGACGGGCACCGCA
AGGGGCGCAGCCGCAGTCACTGTCGTCACGCC
GTCCAAAAGCATCCCATCTTCGCCCGCCGCAAGCGACTCCACGGCATGCCTGCACGCAGTGACTCTCG
CCGCGACCTCCAGGGACACCCGCAGCCGTACC
TCCTCCAACGCGGACTCTGCCGCCTCCTCATCCTCCCCGCTGCTACTGCAATCGCCTGCTCGCACACC
AAGACGGCCAAGGTGTGCTGCCGTGGCATCTA
GAACCATCTGCGTAACCCTGCCGTCGCTCGAGACGCCCCTCTCCGCCTCCGCCATCTCATCGTGCATC
GCATCGTCTTCCTCCCCACTCGCGCCGGGATT
CACCGGCTGGCTGCCGCTGTTGCCTTTCGCCGCTGCCGCCACCACGGGCACCTGTTCGTGGCGACAGA
AATGTGTCACGGTGCAGAAACACGGGAGCGCG
ATCCACGGAATGGCAGGCGCTGCCGCTGCCGCTGCTGACGTCTCCTCCTCCTCTCCCGACCCAACGTT
CGTGCAGACCGGAAAAAGCAGATGCTCCAGCG
CCGCCTCCTGCCGCTCCTTCCGCCGGTGTTGTTGCTGCTCGGTGGCCGCTGGAAGCTGTAAGGGTGCA
TCCGCGCGGTAGCCGCCTTCACGCACAGCAGT
GCACACTGCACACCACGCCTCCTCGCAGGGCAGCGGCTGCCGCCAGTACGCCAGCTCCTCCTCCACCC
GTAGCGCCTCCTGCGCAACGCGAAAAACCACC
ACCACATTGGAAAGGCCGCTCGATTCGCAAAGATCACCGGATGGAAGCACACGTGCAAACCGCTTTAG
TGCCTGCATCAGGCGGGCGCGGTTCTTCTGAA
TGGCCCTGCCACGCCGCAAGAGATCGGCACGGAACAATGGCGTGAGGGCCTCCGCACGCGCAAGCGCC
TGCTGCTGGCGGATGCGGTCACACGCAGCGGC
CACACGTCGCCGCAGGCCTCCTCGGTGGCTCGCAGCCTCCCGTATCGCCGTGTCAACTTCTCTCTGAA
GGCTCTCAGCGAAAGCGGCGGCCATGTTCCCT
CCCTGCTCCTCCGCACCCTTGCCTTGCTCCCGTCCCACACCCCTGTCCCAACTGCCACACCCCTCTAG
TTTTCACGTCCCCCTACTGCTGCACAATCCCT
GTCCGATGCCACTGCCACCTGCGGGCATCAAAATGAAGGAAAAAAGCGCAAAGAAAAAAAAGGTAGTA
AGACGCTAGAACCGGCGGAGCCACCCTCGATC
CGGCGCTGCAGCCTAAAAGAAAAGGGCTAAACCGTGGAGCAACAAGGGGACACACAAACACACACACA
CACACATGCACACAACCGCACGGCCCCCTCCA
CACAGACAGAGGGAAGCAAAGAGAACACAGCAGCAAATGAAGGGCGTCCGATCGCTAGTGGTAGCCGG
TCACTGCGGCCGCTCCAGTGCCTGCCTTGGAG
CTTCCGATCCGCTGCATCTCGGTGTTTGCCCGAAGTATCTCCTCACGCGATGTCTCCAGAACCATGCC
GCCCATCACCATTTCCATGAGCAACAGCTGTA
CCCTGTCCGAGTGGAAAATCAGATCTAGTTCACACACGTTTTCGAAGGTGCGGTCAAGCGCCTCCACT
AACACCTGGATAAGATCGAGTATCGCCAGCTG
GGACTCGCTGCTGTCCACAATGAATACAAAGCAAAGCGTGGCGTAGTGGCGGTACACCACACGGGTAT
CGGGGGTGGGCCAGTCGCGGAAGTTGTCCACA
AAGTTGCAAAGATCGGCGCTGCGCCGCGAGATGAGGCGATGAATGCGGCGCACTAGCTCCTGCTGGAC
GCCCAGTGGAATGTCCTTTTCATACACGTTCA
GCAGACGGATCTTCCCCGCCGTGTTGATGATGATGACGGCCTTGATCATGCTTCGCGCCACCTTAACG
ACCAGATGATCCCTGCTCCTGCCGTCCTTTTC
CTCGCTGGCTTCGCGCTCCAAACAAAGAAGCTGCGAGGCCAGCAGAATCAATGAAAAGAACAGAAGAA
AAGAAAGAAAAGATGCCCAAAAGTGACAAGCA
ACGACGGCTCTCGTTAACAACCTTTTCTCCCCTCCGGCGCCCTCACAACACCCCTGTCGGTTCTGTTC
GGCCCTCGCTTCCCTCGCCAGGCAAGGCGGCA
CCCATCACCTCCCCTCGTCGTCGTCCCCCGAGTCCACTGTGATAATCTCGGCGCCGCCGGCACGGTTG
ACGTGGTCGTGGACAGCCGCATTTGAATTGGC
GCCAACGGACGGGCGCAGCGGCGTGCGGGCGTTAGACTCTTCGCACATCCGCAGCAAATAGTCGACGC
CCATCAAGTCCACCGCTGGGTGCTGTGCAAGG
AGAACAGGCTCCACCTGAGAGGGCATCGGAGTTGCCCCCAGCACTTCCTGGTCAATGGCTGGATGCAC
CACGCGCTGCTGCTGCTGGGGCGCAGGGAACA
GCACCAAGGAATCGGCGGTTTGTTGCAGCACCGGCCTCAAGAAAGTAAGAACGGTTGGTGGGTGCAAT
ACCGTCGGATCGTGCGTGGTGCAGGCACTGCA

TGGCGGCATCGGGCTCCCAGGCACAGTGAATATTTGCGAAAAACATTCTGCCACGCCGTGTTCCTATC
GGTCCACGCGCAACGACCCGGAAATAAACGGG
ATGAAGGCGTTAACCCACAAAGATGCTCCCACCAAGAGATTCGCCGGCATCCGCAAGAAAAGAGGTTG
TCAACCTTGCGTTCACTNNNNNNNNNNNNNNN
NNNNNNNNNNNNNNNNNNNNNNNNNNNNNNNNNNNNNNNNNNNNNNNNNNNNNNNNNNNNNNNNNNNN
NNNNNNNNNNNNNNNNNNNNNNNNNNNNNNNN
NNNNNNNNNNNNNNNNNNNNNNNNNNNNNNNNNNNNNNNNNNNNNNNNNNNNNNNNNNNNNNNNNNNN
NNNNNNNNNNNNNNNNNNNNNNNNNNNNNNNN
NNNNNNNNNNNNNNNNNNNNNNNNNNNNNNNNNNNNNNNNNNNNNNNNNNNNNNNNNNNNNNNNNNNN
NNNNNNNNNNNNNNNNNCGGCAAGAAGAGGGT
AATGAAAAGGCAGCGGCCCCGCCACATCCTCAATGTTTTTCCCAAATATATTCTTATATTCTGACAGG
CACTCTCGTGCCACGGTGCAGTGTATTCGGAT
TGGAGATATTTATTACTTGCTAAGTGGAAGAATTTGGGCCCCTCCAGCCACAACACCCCGCCGCAGGG
TCCGAATCAAAGAAGGTGGATGGCAAAAACAA
AAATAAAAATAGAATTCGTCCTGCCGCTATCGGTTCCTCATCTCCTCCCATCGGGGCCTTTCAAAGGA
CCATTAGGTAGGAAAAGGATCACAGAGAAATA
CTTTTGTCCTTTTACCAGGACATACATTAAATTGGGCAGCCAGCCCTCTCGATAACATGCTTCTCACA
GGCACTGAAAAACACCAACACCCCATCTACGC
AGTGGCACACACACGGAGCGAGAAAGGAGCACAGGAGAGAAAAGCAAAGAAAAGTCTGCGACTTAATT
TCCTCCAGCACAAGATCAAAAAAAAAAAAATAC
AAACACCTCGCACCCTCGCCATGCTGTCGCCATGGCCGCGACGGATGCATTCCGTGGCCCACGGCGAG
CCCAGCGTGACTCCCCACCCCCACGATCATTC
ATGACTGACGAAAAGAAGGTGCCGAGTGCGCCTTTCTCTCGCCTCCCGCGTGGGGCGGGAGGATCGCG
TCGGGCGGGTCCCCACAGCTCACCCTGCGCGC
GACCTCCACACCAACAAGCTGAGCAAAGAGGACACCGCTGGGAAGTTTTGCCGCCCACTCGTAGGGCG
CAGCCATTCCCCCGCAGCAAAAATGAACAATA
ATAAAATACGGGAGAGCCCCGTCTGTGCGGAACGCCGCGGCGAGTGCGCTCGCCCCTGCCCTCAGCAC
AGCAGACACGGACACCCCCGCACTCATTGGGT
CACAGCGACTTCCCATCGCCTGCCCGCACCAAAAGGCGCA
>scaffold1105.size7575
TGCTGCTTCTTGCTTTTCCCCCTCTCATCCTTTTCTCCTCTTGTGTTCCTGCGTGGAGTGGCCGCATG
CTCACGGGCCATGTTGATTGTGAGTGCCTCTTG
ACGGCATCCATTCATCTCCCTCCGCCTGTCTGTCTGTCATTCCCTGTTGGGTGTGGCGTGTTGCACCG
CGTGAGGGCTGGCCCGCACACACCGAGGGCCC
GCCGTGACTTGCCGTGTTGCTGCTCTTTTCTGTGGGTCATGGTCCTCCGTGCTGTTCCCTCCGGATTG
TCTCGTGCCCCGCTCTCTGCGTGGGTGCTCTG
CTCTGCTCCTCACTGACGTGTCTGGTGTATTGCCGCTGTTGCTGCTGTACTCTCCAATTGAACACAGA
CACACTAGGAAAGAAAACAAGCGCTAATCGCT
ACTGTGAAATTCGAAGGCTCTGAAAGGACCAACAACAGCACACACACACCGCCCACACACACATATTT
ATATGCTCTCACGTGTTGCTGCAGTCAAGGCA
CCCCGCACACACAACCGTCGCCGCGTGACCGGATCCAGCGGAAGGAGGAGGGAAGGAGGAGATAGTGA
GCAGCAGAGGCCCAACATGTTCCGGCATCACT
TCTACTTTTGCGGTGCCGCTACTCCTCCTCGTGATGTGCTGCGCCACCTGTGGAGCTGCGGCCGCATC
GACGGGGGAGAATTAAGTGATTGCGCGGCTGC
CGTGGGTGGCTTTTATTTTTGTTCCGGGACGAACGCATTTGGAACCGAAGGGTGGGACTGGTGCAAGT
GGAAGGAGGGATTCGTTTGGATCGCTCGCTCT
TGTGGGTGTCGGTGGAGTGACGGTTTTCTTTGATGGGGGTTGCACGATGTTTAATGATAGCGATGGGG
CACCGAGTGGGCTTTCTCATGCGGATATTGTG
GCGGGTTCCATCTTTTTTGCGTGGAATTTGTCGTCTTTTTTTGGTGAGTTTAATAATGGCCGGAATAC
ACACATCGTGTTGAGCACAGCGAATATAAAGG
GTGGTGTAGGCTCCGCGTCCAATCCCACGACAATTGAGAGCGGCAATAGGGCGTTTCTTCGTGCGGGA
AGCTAGCAAAAGTTTGGTACGAATGGTGGGTT
CAATTGGGCGTTGAGAGACCGTGATGTTCAACTGGTTGTGGGTGAATTCACGCAGCCCAATGACGGCA
GACGGAGCGAGCTGATCCACTGGTGTGAACCC

AAATTGCTGTGATCACAGATCATTCCCGAGACTCACGAACGCGGCGCGAATGGATTGAAGGTTTTTGG
CGGCTCAGGTGTTTTGAATGGCACGCTTGTGT
CCCCGTGGTTGCGTATAATGAAGAAAAAGACGATTTCTCCATGATCATCTGTTCGACGGACGGCGGCA
GCAGCTGAGTGCTTTCAAAGGGCATTCTTCCT
GTGGACTGCATTGACCTTCTCATTGCCGAGTGGGAGCAGGGACANNNNNNNNNNNNNNNNNNNNNNNN
NNNNNNNNNNNNNNNNNNNNNNNNNNNNNNNN
NNNNNNNNNNNNNNNNNNNNNNNNNNNNNNNNNNNNNNNNNNNNNNNNNNNNNNNNNNNNNNNNNNNN
NNNNNNNNNNNNNNNNNNNNNNNNNNNNNNNN
NNNNNNNNNNNNNNNNNNNNNNNNNNNNNNNNNNNNNNNNNNNNNNNNNNNNNNNNNNNNNNNNNNNN
NNNNNNNNNNNNNNNNNNNNNNNNNNNNNNNN
NNNNNNNNNNNNNNNNNNNNNNNNNNNNNNNNNNNNNNNNNNNNCTCCCCGTGGTGGGAGAGTGAGGC
CCATGTGCTCTACGTTTGGGTGACTGACAGCA
ACCGCGTGTTTTGTGTTGGACCGGCTTCTATGGACGCTGCTGTGAATAAGCCGATTGCCAACGTCCTG
CTGTACTCGGATGGTGCGTTGTACCTTGTGGG
AGCGAGGCACAATGAAGTGAAAAGCAGCCTGTTTCCTGTCCCCCTGACGGAGGAGCTGCAGATGATCA
CTTCCATCCTCCGTAATTGGGCGGATGTGGAC
GCTTCCTTCTCCGCGTCGCCCGTAAGTACGGTGGGTCTGGTTGGATACCTTTCCAACGCAGGCGCTGC
CAACACGTGGATCGACGAGTGCCGTTGCATGA
TTGCAACACTGACGAATGACAACAATGTCACAAATGGGCCTCAATTCGCAGGGCAACATTGCCCCGTG
AACCGTTGGGAGGATAATAATCACTGTGGCTT
TGTGAATTACGACTTCGCTCTTGTGGCGACGGTGGCTATCCGCGAGACACATGCAGGCGTCAAGAGTA
TCCCTTTGATTGGCGCGAAGGAAGCGGGCCGC
ACTGTGCACTTTTGGCTGTCGCACAGCCATGAAAGGAGTTTGGGGGTGATGCTCAACGCAAACAGACA
CACCGGCTTTTGGGAGTTGAACAAACAGTAAC
GAGTGGAGCTCACGCTGCAGGACCGCCAACAAGGCCTTCGTATAGATCAATGGTGAGTGTGTGTGGGA
TGCTGAAATGACGTCATCCTCAGGAAAGGGGC
CACGCGCGAATTCACACTTTTACATTGGAGGGGATGGGAGCAGTGCGAGCGACACCGGGAGCCAAAAC
AAGTATGTGACAGTGACGAGTGTCTTTTTGTG
CAACCGCATATTGACCCGGGAAGAAGCCGCAAAAGTTGGTGCCAGCAGCCTTGCTCTTCCAGAGTCTC
TTTCTGGGAAAATGCTTGAAGGGCCGGATGAT
CCAGCGGGAACTGGCAGTGGCTCCAAAGATGGAAACGCCTCTCAGCCCACGAACAACACTTTTTTGGA
GAAGGGCTCTAGTGGCTACGGCACGGTGCGTG
TGTGTGTGTGTGCCTCGGATCTTGTTGCTCCTCTTTCTGGGGCTGTGGGCATTGCAGCTCTTAGCTGT
GTGAGNNNNNNNNNNNNNNNNNNNNNNNNNNN
NNNNNNNNNNNNNNNNNNNNNNNNNNNNNNNNNNNNNNNNNNNNNNNNNNNNNNNNNNNNNNNNNNNN
NNNNNNNNNNNNNNNNNNNNNNNNNNNNNNNN
NNNNNNNNNNNNNNNNNNNNNNNNNNNNNNNNNNNNNNNNNNNNNNNNNNNNNNNNNNNNNNNNNNNN
NNNNNNNNNNNNNNNNNNNNNNNNNNNNNNNN
NNNNNNNNNNNNNNNNNNNNNNNNNNNNNNNNNNNNNNNNNNNNNNNNNNNNNNNNNNNNNNNNNNNN
NNNNNATTATTTATTTATTTCTGCTGAACCTA
CACCTTTTTTCTTGTTTTTCTGTATTTGGCAATTCGCTGCGGCTTGTTTTATTATTTGCTGTGTTGAA
TGCCACTATGTGTCTTTGTGTCCCATTGGGTG
TCCCGCTGCTAGCTGTGCAGTTGACACTCCCTCATCACTTTTTCTTTTCTGCTAGAGGCATCGATAGT
CCGCGACGACGATTGTTTGTGTTCCCCCATTG
CGATGGTACGAGGGTGACTGGGCGCCCTGATGAGTGCGTTCGGAGGGGAGCGGATGGTGTGCGCCGTG
CCTCACTGTGAGTGTGGGACGCCACCGCCACG
ACCTGCTGTGAGGCGTAACGCTATTCCTTCGGGGCAGGGGTAAGTAAAGGACTGTGGTGGAATTGGAT
GCGCCTCGTTCCTCTTGTAGCGGCCGTGTGTG
TGCTGTTCGCATTGCTGTTTTGCGGTACGATGTGGATGGCGGTGCTTCTGCGTGTGCCCTCTTGAATG
CACTGTGCGGCGGGATTCTCTTCTCCATGTCC
TTGCCTTTGCCCTTTTGTGTGTGGGGGTGAGTGTNNNNNNNNNNNNNNNNNNNNNNNNNNNNNNNNNN
NNNNNNNNNNNNNNNNNNNNNNNNNNNNNNNN
NNNNNNNNNNNNNNNNNNNNNNNNNNNNNNNNNNNNNNNNNNNNNNNNNNNNNNNNNNNNNNNNNNNN
NNNNNNNNNNNNNNNNNNNNNNNNNNNNNNNN

NNNNNNNNNNNNNNNNNNNNNNNNNNNNNNNNNNNNNNNNNNNNNNNNNNNNNNNNNNNNNNNNNNNNNNNNNNNNNNNNNNNNNNNNNNNNNNN
NNNNNNNNNNNNNNNNNNNNNNNNNNNNNNNNNNNNNNNNNCACGTGTGGCTCATCTTGGCAGCCTCTTGCAGCGTCCATGCCTCGTGACAGGGAGCGAGGGAAGAG
TGGCGTGATTCCTCCTGCTATAGACTCATCAATTCCGAGCAGTCGGAGTGAAACTAAAGGGGACAAGCGTGTGTGTCTTCATCTTCTTGCGGGAGGGCGC
GCAGACGTTGGTTTTCTTTTGTTACTTTGTTCGGTCACTGTGTCTGTGTTGACGTTATTTGTTTTTTTTTTTTTTTCCAGAGGCATTCATTGTGAGATTTG
TGCGTGTTTGGTGTCCAACGTTTTCAGCACCACCGAATGGGGTGTTGCGCAGGATCGGAATACATGGATGTGCGAGAGCATCGTATCGCTCGCCCGTAAT
AACACACACACACAGTTTCATCGCGTCTGATACGTCACGTTCTTCAGAACGGTCGAAATCACTTGTGGGACGTCACTTCATTTGCGGCGTCGGGAAAGTG
AAGTAAATGGAGAGATATGCCGAGAACGCAATGCCCCTCCATGATGCCGCAGGCTGGTGGCGACAAGGATCAGAGAGCCTCTACCCAACATTGAGAAGAC
GGCAGCACTCTGATGATCAGGTTTTGCGCTTTAGGAAATTCTAATGTAGTGTACACCGTGTGGGCCTTTTTGCTGCGGATATGCAGAGGGGTGATGTGGG
GTGGAATTATTGTTGAAGGCAGCACGGTCGCCGCCTGACCTTTATTGGAGGGGTGNNNNNNNNNNNNNNNNNNNNNNNNNNNNNNNNNNNNNNNNNNNNNN
NNNNNNNNNNNNNNNNNNNNNNNNNNNNNNNNNNNNNNNNNNNNNNNNNNNNNNNNNNNNNNNNNNNNNNNNNNNNNNNNNNNNNNNNNNNNNNNNNNNNNN
NNNNNNNNNNNNNNNNNNNNNNNNNNNNNNNNNNNNNNNNNNNNNNNNNNNNNNNNNNNNNNNNNNNNNNNNNNNNNNNNNNNNNNNNNNNNNNNNNNNNNN
NNNNNNNNNNNNNNNNNNNNNNNNNNNNNNNNNNNNNNNNNNNNNNNNNNNNNNNNNNNNNNNNNNNTTGCTGCGGATATGCAGAAGGGTGATGTGGGGTGGAATTATTGTT
GAAGGCAGCACGGACGCCGCCTGACCTTTATGGGAGGGATGCGTGGTGTTATTCCGTGCTTCCTGGCCTGCGTTGTCGCACTCATGCTGTGTGTGCGGAT
GGCCACAGTAGTGGAGCTGAATACGGTTCAATGTGTGTCGCTCACGGGGACTGCGCGACACATGTATTTTTTGTTGGGCGGCCCAAAAGGAGGGGCGCAT
CGCCGTTGCGTGATTCCcgcggcagtCCATTACTCTGCGACTGTGCATGCCGCCGTCCGCTGCGCCACAGCAAGCCTTCACCTTTGTGAGAAATGGGGAG
CGTGACTGCTGTGGCGGTGGTCGAATGTTCTCGAATTGGTGCTTTTGGTGATCCCACTTGGAGCGCCGAGCAGCGAGGGGCCGCACACGCCCAGGAGGTG
GGAGTGTGTGCTGCAACCGCACTTGCGATTTGCAGTTGGGATGTGACATGTTTCTTTGGTATTTTTGTTTTTGGTGATATGCGGGGCAGCGGTGTGTTGG
CGTGTGCGGGCTGCTCATGGCTGCCGGTGTTGCCTTTCCTGCTGTTCTCCCGCTCCACTCCTTTGCGTGTGACGGACCAAACGTTCTTACGGGCGTGTGG
TGGGGCCGCGTTCTTGTTTGGAAAAAAAGCCTTCCGCAGGCATAATGAGACTGCCGTCCGTGCCACACGAGGCAGTGGGGTATTTCCTGGGATTTATTGG
CGGGGTGGCACCGCACCCATGCGGTGTGGTTGATTTGCGTTCCGCAATGCACGGTGATGGGGATGTGCGGTGACAACCTACTCCAGTGAAGCGACACGAG
CAGCACGGGGAGCGAAACGGAGGAGTGGTGGCTGCGTGCTTCCAAGTGATACGTGAATGGCGAATGCAATATGAAAGGCAGCGTCACGGCACTCTTTTGC
CATGCACACCGCAGATATTTTTAATAANNNNNNNNNNNNNNNNNNNNNNNNNNNNNNNNNNNNNNNNNNNNNNNNNNNNNNNNNNNNNNNNNNNNNNNNNNN
NNNNNNNNNNNNNNNNNNNNNNNNNNNNNNNNNNNNNNNNNNNNNNNNNNNNNNNNNNNNNNNNNNNNNNNNNNNNNNNNNNNNNNNNNNNNNNNNNNNNNN
NNNNNNNNNNNNNNNNNNNNNNNNNNNNNNNNNNNNNNNNNNNNNNNNNNNNNNNNNNNNNNNNNNNNNNNNNNNNNNNNNNNNNNNNNNNNNNNNNNNNNN
NNNNNNNNNNNNNNNNNNNNNNNNNNNTTTTTATTTTGTGTGCGTGTCAGGGTTTATGTGCCCACGTCCTCCTGCCGAGTGTGTGCGGCGACGGCGGTGT
CACAGGGGTGCGGGACCGTGGCGAGCGCCTATTGTATTTCTTTTTCTCATTCTCCGCTTTGGGTGGTTATTGTTTAGATCTAACTCAAACTAACTAAGAA
AGCAAGGAGAAACAATGTCTGGAAGGCCGGAGAGTGTACAAGGCGGCAATGTGGAGAGTCAATCGTCCAATGTCTCGCAGGGAGGTCGGAGAAGAACGAG

GTCGGAATTTGAGGGTGACACAGATTACTCCTCTACGACTCGCATAAGATTGGAGGGAATGTATCGAC
CGCAGTGGACCATGAGCAGCAGCGTAAAGGAC
ATTCTGCTGGAGGGAAGCACTCTCAGGACTGACATGAAGCTGAATGAATTCATTCGGAGCAACTTGGG
CGAGGAGTGGGTTGTGGAAAGAAATGGGAATG
TTATTATGGAGGCGTTTGTTCGGAGACCCAATGCTTACGTACAAGACCAGCAGCTTCTTGAAGAGATT
CTTAACTTAACTGCGTACCAAGCGCTGGAGGC
CATTACtaagctNNNNNNNNNNNNNNNNNNNNNNNNNNNNNNNNNNNNNNNNNNNNNNNNNNNNNNNN
NNNNNNNNNNNNNNNNNNNNNNNNNNNNNNNN
NNNNNNNNNNNNNNNNNNNNNNNNNNNNNNNNNNNNNNNNNNNNNNNNNNNNNNNNNNNNNNNNNNNN
NNNNNNNNNNNNNNNNNNNNNNNNNNNNNNNN
NNNNNNNNNNNNNNNNNNNNNNNNNNNNNNNNNNNNNNNNNNNNNNNNNNNNNNNNNNNNNNNNNNNN
NNNNNNNNNNNNNNNNNNNNNNNNNNNNNNNN
NNNNNNNNNNNNAAGCGTGAGGCAGAGGAAAGGCTAGGGCGTGAGGAAGAAGAAAGGCAAAGACGACT
GCAAGAAATGAAATATACCATTTCCACCACGA
TTGAAGAGGTACTGTTTAAAGGAGGAGTCCGCGTCAGGGAAATAAAGATGAATGATTTTCTTCTGATG
AGATTTGGCGGCAGGGGCGTTGTGGCCACAAA
GGATAATGTCGTGCTGAAGGAGTTTTTCAAGGAGCCGGCGAGGTATATCCATGATGCGGGAGTATTGA
ACGAAATACAGATAACCGACGCTTATTTGAGG
ATTGAGGGGGTTGTAAGGGAGGAAATGGATAAGGGGGAAGATGTACGCAAGCTTAAACAGGCTGGTGT
GTACAA
>scaffold1106.size5795
CAACAAAAACAAAACAATAGAAAAATAAAAACAAAAATTCAAGTGTCCGCAGTCAAAGCAACCCAATA
ATCCACAAAAAAATATTAATTCGTGCACTCAAC
CACAACACAAGTAAAACAAAACAAAAAGCCGAAGCGTGGGACGACCAGCGCACACACTCTCATTGCTC
CCGTAAAGCAAAACACACACACAGAGGGAGAG
AGAGAGAGGTCGCCCACTCAATGCAATAAAAACAAAACAGAAACAGTTACGGTGCTCCGCCCATGCAG
CCAGGCAGGACGGCAGCGGTGCGTGTCGTGCA
AAAGAGAGCACCAACGGGCCTGCATGGGTGTGCATGCATATATACAATGGGCACATTATGTGTGTGCC
TCGTGGGGTGAGGTGAGGGCGAGAGTCCATGC
AAGGGGACACACACACACAGAGTGTGAAGGACGAGTGTGTGTGCGGGCGGTGCACGGCTCTCTCTCCC
TCACTCTCACGCGGCCACCACCGCAGCCGCAC
ACGCAACAACAAGAAGAAGCAAAAGAGGGGGGGTGGCGTGGGAGACCGCGGTGCTGCTGTCAATGTCG
CCAGGTGTGGTCGTATGATTTGTGTTGGCGGT
CCCTGTTGATTGAGGTGATGCCTCACTCATTTTGACGGTGCTGCTCGCATTTTCTTCGGGCGGCTGCA
TGGAAATTGTTTGAGTGGTAATGGCCGGTGCG
GCTGTAGAAGTTTCCCGAGTTTGTTCCAAAGAAGGAGTGACATTTTCAGGCTCCTGCCTCTCTTCTGA
ACGTGCGGCATCAATAATTTCAGTTCTCCTCG
GAGTATCTTCTGTGAGCTCCTCACTCTTGCGGCTGACAACATCGTTGTTAATTGCAGGAGATGGCGGA
TCTTTTCTCTGTTGATCTCCAATTGATTCATT
CTTCCTGTCCTCTTGACTGTGAGAAGCCGCCAGTCTCCCTAAAGAACCATCGGGTGATCCACCTTGCC
GCTCTTCTTGCAATGATTGTGAATGTGACAGT
GAATCCGTAGGCTCGGTGTCGAAGAGTGTCTGCTGCACTGTTGATTCAGGCTGTAAAACTGCTTGCAA
TTGCGGAGCCACGAGTGGCTGACCCCCAACCT
TTTGCTTCTGTGACTGCTCTCCTGAAACGACTCCTTCTCCACCATGCACCGTATGATCGGTGTCTTTG
TCAGGAGCCACAGAACCACTCTCAGAATTAGC
AGGTGAATCGGCAACACGTACCACCTCCACGTCACATCTACCGCTGGCACCGCACCACAGCACGCAGA
GGGCACACAGCAGCAGCACACGGCCAGTCATC
ATCATCGCCATCACTCCTGTCACCTGCGTGTAGCCTTCAGCACAAACTAACAACACGTCAACAGAGAG
TGAAAGCAAAAAGAAGAGACAAATCACAAAGT
AACACGCGCCGTAAACAACGGCAGCAGCCCTGCAGAGGAACGCACACTGACCTGCACACCAGAAGTAA
AAAAGCAATACCAAAACAAAAAGAGTAGTTGG
GTGCCCAACAGGAGTCTCCTCCTCCAAAAAAAAAATACACTTCAGCAGAAACATCATTTCGTACGCCA
TGCAGCAAAGCAGGACTGTGTTTATATGTACA
TGCGTACAGTACCTGCACAAGGAAAAAATTATTACTGTTCCACAAGCCCACACAACATCCCACACATT

TCATTGCGGCATCCGGCCTGTCTCTCCACTCC
TCCTGTTGTGTCGTCCGTGGAGCGTACAGCCGTGGGGCCTGTACCGCGTGTGGGCAACAGAAGCAATC
AAGAGGGAAAACAATTCACTCATGACGGTGCG
CAGCAACCGACAATTAAGTAAAACAAAAGAAAAAGGTACTCCCATTGTTCCATGCAAACAACATTAAA
AAGAACATCAAAAAAAGAAGGCCCGCAGGGAA
CGCGGCAAATATTCTCTGTGAGAAGTCGCAACGCCACACTCCGTGGCTCAGAACACTGCCACAACCAC
ACTCAGTCCCAAAAGCAGAAGCAGCAGTGGTG
TTTCCACACACGAGGTGATACATTAGCCACTGCTCACCGTCGGCAAGGACAAGTCACCTTCGAACCAG
ACTTTTCCACTCTCTTTGATTTTTTTTGTTTA
AAGAAGATATGACCTGTGTGGCTCTGCTGCACGCACCGCTGTTTGTGGGATCCTTAGTGGCGGGGCGT
GTGCTCGAGGACCATCGATACAGTCCCATCGC
CCGTACACATCGGTGGTATTGGACGTCATGAGGTCATTATATTTTTTTTCATGATCTTACCAGCGCGT
CTAGCTATACAATGCCATCTGAGTGCCGAAGA
GATGGTCGAGCAGCAAAATGAGTTTCCTGCTATGAAGCAACTGCCATTGAGAGAACTTTCCGAGGCGA
AGGTCCTGTTCAATGCCCAGCCGTCGGCGCAC
AGCCAAGGCGCTTGGACATTCTAGAAGCAGATGGTGCAAGGTTTCCTGCGGGCCAATTGGCGTGCTTG
TGGAAGCTGGCTGATCCTGCGGCAGCAGAGAT
CGCCGCCTGGCACTGCTGTTCCGATGCCGTGTGGCGTCTGGGTTGTGTGCGCAGCGATTTCTGTGCGG
TGCTTCTCCGTGTGGGGAAGACCATGTCACAG
AAATCACATTTGAGCTCGGGAAGCGGTGGAGGATGTTCCGGACGAAATGACCGACAGTGACTGACGAG
ACCCGAGAATCCCGTCAATGTAGAATCACAGT
GCGGACATTTCTCCTTCCGACGGCGTGTGACGGTCCGTCTCCTTACTGGAGAGACGTCTTCATCCCTT
GCCGGCAGGGGAACAGTGTCCGTCCCCGGTGC
AGTTGGATCTTTGTTAGAGCGCATATATGCAGACTGTTCCGGTGAGATGTTGCAGAAACGGCATTGGT
TCGTGCAATCTTCTCAGCCCAGTATAGTCGGC
CCCAGAGCTCAGATTCCCCGCACCTTGCCCTTGCGAGGTGAACCAGTTCCTGACGATGTAGAACTTCC
TGAGTGATCAGGTCCTTACCGCTGAGATCGGA
TGGCCTTGTGCCGCAAAGCAAGTAGCGATGAGTGTCTTGACGAATGGTACTACGCCACTTGTTGGTGA
GCTGGGTATTAAAACATGTCAGCAGATCCGTA
TGCCATAAAGGTGCGATTCCGTGCTCCGTGTATTGTCCACTTTCCATAGTCTGCGTCGCATGCTGATC
TGCAGGCTCGTTGGCATGTACTCCGCAATGTC
CGTAACAAAACTGCAGGTGCACGGACCAGCCAGCACGCGTCAGTGTTAAGAGACGCTGCCAGATCTGA
TCCTCCGTCCAGTCTGTCTGACTGAGCGGGCC
CTTGTTTAGAGCCGCTAGAAGAGACTGACTGTCCGTAACCACAAGCAGGGTTTTACGCCTGTAGCGGA
TACGAGNNNNNNNNNNNNNNNNNNNNNNNNNN
NNNNNNNNNNNNNNNNNNNNNNNNNNNNNNNNNNNNNNNNNNNNNNNNNNNNNNNNNNNNNNNNNNNN
NNNNNNNNNNNNNNNNNNNNNNNNNNNNNNNN
NNNNNNNNNNNNNNNNNNNNNNNNNNNNNNNNNNNNNNNNNNNNNNNNNNNNNNNNNNNNNNNNNNNN
NNNNNNNNNNNNNNNNNNNNNNNNNNNNNNNN
NNNNNNNNNNNNNNNNNNNNNNNNNNNNNNNNNNNNNNNNNNNNNNNNNNNNNNNNNNNNNNNNNNNN
NNNNNNAGGGCTAGAAGCAGCGCACGGGATTC
CGTCCTGTAGCTGCATGGGCGTGCACCGCAGTTTATACTGGCCTTTTCTATTATCTCCGATGTGTTGA
GGGATGAAAGCAGTATTCCAACCCCTGCTGAC
TTGGGAACGTCGACACCGCCACCGGTCGCAAGTATCCACGAACGATGGCTGTGCAGCGCTAGAGCCTC
TTCGCTGACTCTGCGCTTTTCCCTTATCAGCT
CTTCCTCGTCAGTTATTGAGAGGTCTATCGGAAGTTCCACACCGATGGTGACCCGGTGAGCGGAGGCC
GCAAACCAGGGAGGAATGGAGCTGCGAAAGAA
CCGCTCTTCGCGTGGTGAGCTCTCGGTGATGCCGTAGTCCTTTGTGTATACGTCTACGAAGGCATATA
GCTCGTCCCGAGATAATGGCGAGATGCGGAAA
CCAGCGCGAGGCGGTGGCTCAGGCGGGGGTCTCCGCATCCAGTCCATCTGAAATGCGCGTGTGTTCTC
AAATATGGAGAGCCGCGCGCGCAGGCAGAGGA
CGTGGAGCGGCGGCATATTAGCCTCCAGTAGTGCGGATTCCGGGTCGGCAGTGCTCGGAACTCCGCTG
ACAATGCGTGCCAGAGTTGCGTACGTCTTCTG
CATCTCATTCTTCGCTGAATCCGTCGCTACTGCCCATATGAGCTCGCTGCCATAGCGTAATTTGGAAG

CACCGTATCCAATGACGAACGTGCGTAGGTCA
TGTGGAGATGGCCCCGCCTCTTTGTGCGCTATGCAGCGTGGCTGCGATATCCGTTGCGAAGTGGCAGC
GCATTGCTTGGAGGCCGCCACATTTAGCGTCA
GTCGCGGATCGAGATCCATGCCGAGAGGCTTCGTAGTTCGCGATGCCCCCATGGTCATGACTGGGATT
TGACATCCAGCCACTACCAGGGGCCACCTTCC
TTCACGATCATAATCTGACTCCGTGTGCGTGGAGAGTGTGAACCAAGCAGCTTCACGCATTGACGGGT
TAATGGCCAGGCCATTTTCCTGACTCCACGCA
TGCAGTTTTCCAAGAACCATGTTGGCAGTGGGAATGGCGTCTTCCCGGGACTGCCCCCTAACGATAAT
AGAGAGGTCGCCCGCATACATGTCTGCTGTGA
CACCCGCAAAGCTGTTCAGCAGACTGAGAAGTGGGTGTGCGTAAAGGGAGAACAAATACGGTCCCAGC
ACGGACCCCTGAGGAACTCCTCGCAGCATGGG
ACGCCGATCGCTGTGTCTGTGGCCAACTCGTGTCTTCGCGTAGCGACCACATAGAAAGTTTCTAAGCC
ACCTCTACAATTCACCCCCGGAATATGAGCCA
GCAGGCGACTGAGGACTCGATGATCTATGGAGTCAAAGGCCCTGCTGAAATCAACCAACACCAGCAGG
GTCCGATGGGGTCTGTAGTGGAGTTGATTGCC
AATACCGCTCTTCTTGGGAAGGCTCACTTGGAAATAGTGATTTCTATTATGCTCCACTTCGTGTATCA
GGTGTGCCAGCTGCATCGTCGTGGTACGCACA
CTACGATATGCATACTGGTGGGGGTGCGGCGTCCACACCCAAAGCAATCTCTTCAGTACCATTTTCTC
GGCAACCTTACTCACAATGGAAGTAAGGCTGA
TGGGTCGATATGACTCAAGCAGTTCCGGCGACTTACCGGGTTTTAAGAGTGGAACGATGGTGGCGCGT
CCCCACTCAGCCGGAATCGTACCGGTCGTCCA
GCTGTGGTTAATGCAACGAAGAAGGAATCGGAGGTCCTTTGACGAAATATGTCGCAGTGCCTCACTGT
AGACTTCATCCGGTCCGGGGCCTTGCCATACG
GCGCATTACGTAGCGCGGCAAGCAGTTCCGTACGATTAAAAGGTGCATCAATAGGGGAGTAAGAAAAA
CCCCTGATGTAAATGTAGAGATCAACCCGTTG
CTGTTCTGTGGCTCAGAAATAGAAGAAGAAACAATTTTCAAGGATGTGTTCTGTCTGTTGATGCCTTT
GGAAAACCCGACCGCTCTTTGAGAGGGGTGAA
ATGGAAGAAAGATGCGATTGAGAGCATTCGCTGCTCTCCTTGCTGTTTTATAAGTGTGTTGACCATCT
CCCAATAAAATTGATGTTGGAGATg
>scaffold1107.size6519
GCACCGCATGCTTTGATGGTATTCTGCGGGAATGTGGACACCTCGCCGACCACGGACCCCGTGAGACA
CTGAAGTTCTACGCCGTTACCTGCCGTGGTGCG
ACCCCGCATGAAGGCCTCTGTGCCGATCCCGCGAGCAGCATGGCGGCACTGCAGTCCTTCGGTCTTGA
CGTTGTGACGCCGCAACCGGCAGTAGAGCTGG
GGACAGACGAATACGCAGTCCTGCGAGACGGCATGGCGCGTCGTCTTAACCGTGAGGGTGCGGTGGTG
AACGGCTGCAACGAGGCGGGGGTTGTTGTGCG
GATGTGGAGGCAGCGGTCCCACGCATACGCAATGGAGCGTGCGGCGCAGGAGGCCATCGTCACGCACC
GTTTGTGCGGTGTGGCGCTCCGCTCGAGGCTC
GCAGGCAAGCTCGCCGGGCTGCCAGAGGAGGTGCGCCGGTGCCTCGGCGACTGGGAAGCGGAACGCCT
CGAATATCTTGTGCAGTTTGCAGCGTGGCTGC
ATGTGACGGGGCGGCAAACGGCGCAAACGGACCTCGGCGGGCTGCAGGACCTGCGCCGCCGATGGATC
ACGCTGCAAAACCAGTTCGCGCAGTGTGTCGC
CGTCGATGCGCACGTGCGGTCCCAAGTGATGCACTACGAGCCGTCCGGTGACGACGCCGTCACCAGCG
ACCCCGACGCTGTCGTCTGCGTTGGTCCGCAG
GGCTGCGGCAAGTCCACATTCTCCCGCACCCTGTACGCTCTGCTGCGCCAGGCAGGGCTTTCGCCATG
TTGGATCAACCAAGACGAGGCCGGCGGAGGGC
GGCAGTTTCTTGACGCCATTCGGCGCGCGCAGCGAGGGGCCACACGCACCTNNNNNNNNNNNNNNNNN
NNNNNNNNNNNNNNNNNNNNNNNNNNNNNNNN
NNNNNNNNNNNNNNNNNNNNNNNNNNNNNNNNNNNNNNNNNNNNNNNNNNNNNNNNNNNNNNNNNNNN
NNNNNNNNNNNNNNNNNNNNNNNNNNNNNNNN
NNNNNNNNNNNNNNNNNNNNNNNNNNNNNNNNNNNNNNNNNNNNNNNNNNNNNNNNNNNNNNNNNNNN
NNNNNNNNNNNNNNNNNNNNNNNNNNNNNNNN
NNNNNNNNNNNNNNNNNNNNNNNNNNNNNNNNNNNNNNNNNNNNNNNNNNTGAGGGACTGCGTGTGC
GACGGCGGTGCGCAGATATACGTGCGGGGCTA

CAGCGGCGAGCCGGCGAGTGACCGGAGCCTGGAGGTGAGCGTGAGCGGGCTGTCCGGGAGCTACTGCT
CGCTCGTGTTTGTGCACAACCTGCCGGCACAC
ACGAACGTGACGGTCCGTGACTCGACGATTGTGACGCCGGGGCCGATGCGCTACTCCCAGCTGAGCGG
GCTGACGGACGCGGTGGCGTCGCCGCTTGTGC
TGCACGCGACGTCGCTGTTGCAGACGCAGCTGCGTGTGAGCAGCACCGTGCTGAGGTCGTCGCAGGCG
GGCGGGTCGGCGGTGTACGTTGGCGGCGGTGT
GGACCTGCTGTCGAGCGCTGTGGTGCTTGACGGCGTGTCGCTGGAGGCGTCGGGCGGTCCGACCGCGT
CCGCGATGCATGTGTCGTCCTCGTCGCGTCTC
AGTCTGCGTAACCACTCGGTGTTCTCCGTGACGAACGTGTCGGTTGTGAGCAGCGGCGGCGGCATTGT
GCTTGGAGAGCGCCTTGCGGTGTCCGACTCGG
TGCTGCGCTTCGTGGGCGTTGAGGGGTCTGCTGCGTCGCCGTTGGTGCACTGCGGCGGTGGGACGGTC
GGTGCCGGCGGGTGGCTGGACCTGCACGACGT
GTGGGCGGTGGGCGAGGCGTCGTCGGTGGCGTCGCTGTCAGGGGTGACGCTGAGCGGCGGCGCCGTGT
CGATTGCTCGCTGCGCTGCCACCGGCGCGACG
CTTGCCGCCGGGCCGACGATCACGAGCGGCGCTGTGTCCGTGCAGTGCAACCGTGCCGGCGGCCGCGT
GCTGCAGAGCAGCGGCGACTACCGCTTGGCCG
GCCTGCCCTCCGTGAGCGTGGTGCCGTGCGACGGCTGTGCGGCTGCGCTGGCGTGCTTCGATGCGCTG
ACGGCGTCGTTCTCCGACTGCGTGTGCAGCTG
CCGTGCCGGCGGCGTGGGCGAGGCGTGCCTGCCGTTCGACGTGCCGCCTGCGAGTTCCGGTGGTGGCG
GTGGGCAGGACTGCGTGAGTGGCGTGACGCTG
ACGGAGTCCGTGACGGTTGGCGGCGGGCAGGCGACGGCGTGCTTCGACTCGGTGGTGTTCAGCGGACC
GATCACTGTGGCGGTGGACCTGCGCTCGATGG
ATGCGTTCGCTGACGCGCTGAACGTGACGCTTCGCCACTGCGTGCTTGTGGGCGGCGCGCAGCTGCGG
ATTGGTGGCCTCAGCGAGAGCACAGCGCACCT
GGTGCCGCACGTCCTCGTCAACATGACGAATGTGACGTCGCTGGAGGGCACGATCGTGCTGCATGGCA
CGATGCCGCTGAACTCGAGTGTGCTGCTGGCG
AACTCGACCCTGCGCGCGACGGTTGGCGGGTCGCAGTACGTGCCGACGACTCCTGGCCACGAGAAATC
CCGGCACGGCTCCGCGCTTGTCCTGGACGGCG
TCTGTCTTCTGTCGACGCGGTTTGTCATGACGCGGTCGACTCTGGTGTGTGGCGGGGGTTCGTGCGCT
GCGATACTTGTGGAGCGCGGCCTTGTCGTGAA
CCTGTCCAGCGTCTTCTACATGGACAACTGCGCTGTCATGTCGCAGATGCACGTGATGTATGCTATTG
CGTCGGACCTGCGTGTTAGCGGCGGCTCCGTG
TTCTCCATACAGAACAGCTCGTGGAGTGCGCCGAGTACCGAATACTACAAAGGCGCGTGTGTGTTTGG
GGACGTTGTGGTGGATGGCGGCAGCGTGCTGC
AGGTTGTGTCCAGCGAGTTCCGTCTCGGCTTCGCCATGCTCATGGCAACCACGCTGACTGTGACTGGC
GGCAGCTGGCTGGTGCACCGCGACAACGCGTT
CCGCACTGCCTACGTTTTGTACGTGGCTAGATACGACGGCGTGGCGTTCCGCGATCGGAGTGTGTGGT
CGATACTTTACAACGACTTTGGGTACGGCTCC
TATTCGTCGATCACTGCATACATGACAAGCTTTTGGCTTGTACGATACGATGCACGCCCGATCATCTA
CGGCACGTGCAACGAGATAAAGGGGTCTCCCG
TGAAGAATTACAGAAGTGAGCTCAATATTGTGGCGCCCGTGACGGTGTTGGACTGTGGAAATTGCACC
GTGGACGCCGTGTGCTTTGCAGCGAGGACGAG
CAGCGTCAGCGGCTGTGGGTGTGTGTGCGCTGCTGGCGGATACGGTGACACGTGTCTGCCAGCTGCGG
TTCCGGACGGCCTTGGAGCGTTCCCGCTCCCC
GATGCAAACGATACGGAGGTCCGCTGCGTGCACGGTGGCAGCATCAGCTCCGTGGACTATCCTGATCC
CGGTGTGCGTGGTCTGTGCTTTGTCAACGTGA
CCTTCACCGCCGCGATTTTGCTGGAACTGTCGTATTTTGACGCTCCACAGCATACACTCAACATCACG
CTACTGCAGTGCGTCCTCATGGGCTTGTCGAT
CAAGGGGAGTGGTGCGCGCGTGCACGTGAGCGTGACATCCTCGATGCTGGATTCTGGTGCATTGGAGT
TTGAGGGAGATTTTGGCGCGAGCTCCCAGATA
CTGGTGGCAGGCAGTAAGCTCTTGTCAACGTCGAGCCATGCCATTCAATTTCCAAGGTTTACTTTGGG
TGCGAACTCGACGCTGCTCCTGCTCGACAACA
ACATGGAGGGGGAGAGTTACGCAGTTCATTTCCCCGATGCTGTTGTTGTTGATGGTAGTGGGATCATT
ATTAAGGGAAATATGCTGAAGTCAACAAAGAG

GGGTTACAGCTCGGAGTCTGCCGTTTATTATAACAATGTTGATTTGAAGAACGGCGGCTATATTGACG
TCGAGAACAACACGATTAGCGCAGCCATCGGC
ATCTTTTTTCTCGGGGATACTACTGTGAGCTCCGCGGGGCTGCTGAGGGTGGCGGACTGCACCTTCAC
TGGCAGCACGGAGGCAATCAATTCTGCTCTGG
TGCAGTTGTACGACTCGGTTGCTCTTCAGGGTGGTGCGCAGTGGCGTGTGGAGGGCAACAACGTGAGC
GCATCCTCAGTATTGAGTATGCCGTTTTCCTG
GTACAGTATTAGACTGTCGGGCAGCGGCACCACCGTGTCGCTTGCACACAACCGTCAGGCGGACTCGA
GTAAAGCCTTTGCGAAAATTACTTCATCGAAC
TCGATTGTGACGTCACCCGCGCAGTTTGTGGTGGGGTGCAACATGCAGGACGAGAGGGAGGTGTCGTA
CGACGGCGTGTTTCCGGAGAAAGTGGTGGTGT
TCGGGTGCGGCACATGCAACGACGACGCGGCGTGCTACATGCCCGGGACGGAGTCGGTGGACCGCAGC
TCATGCTCGTGCAGTTGCAAGAGCGGATGGCA
TGGCGCGTCGTGTCTTCCCTTCGGGGTGCCCGACACGGTTGTGCCGCCCTTGCCGGAGAGAGCCGTGG
ACGGCGACACGAGCTGCGTGGTGAACCAGACG
CTGACGAGCCTCGCGCTGAACATGTGGAAGACGCACCACTGCTACGTGGGTGTGACATTCAGCGGCGT
GGGTGCTGCGCTCACGTTCCTCCTCAATCGTA
TGCCGCTGCATCTCCCCATCAACATCACGCTCACTGGGTGCACATTCCGTGACGGAGCCGCGCTGCAG
TTTGTTGGGGGCACTGAGGCGGCCGAGTCAGC
CGGCGTCCTGATCCGCGTGAGCCAGACGGTGATGCGGTCCTCCGTTGTTCTATTTAGACGTGCCCTCC
CGCAGCACTGCGACATCGCCGTCACTGAAGTC
GACGCGGTGCAGTTATCCGCGGTGCAGTTGCCGAATTCTGTGAACTACATGTTGAGCGTCGTGAAGCT
GGACGACGTCGTGTTGAGTGCGTCCTCGCTGT
TGGTCAGCAACGTCAAGGCGCGTGCTTTGGGTTACAGTGGTTATGGTTTGTACTCGACCGGGACGCTG
ACGCTGGTGGGCGGCAGCTCGCTGTACGCGCG
GTACTGCAGCTTTGATAAATACACACACATGTTGTACATGTATAGGCTCATTGCCAGTGATCGCTCCG
TTTTTGCGCTGCTCAACAATACGATGTCCTCC
GGGACAAGTTTCCTGTACCAGTACCACGACCTCACCGTGTCGAATCACAGCGTGGTGCGCGTGGTGGG
GAACAGCGGCTCCTTGATTTTTGCCATTCTAT
TATATGATTCTTGGACCGTCCGGAATTCAAGCTGGCTGGATTGGCGCGACAACGACGTGGGAGTGGGT
GCGATGTTCTACTACTTTTCCTTTGGCGGTTC
TGTGAACATCGATGGCAGCAGTGTGGTGACGCTGACGGGCTGCAGGATGGGCTCAACGGGGTTGTATA
AGTCTCCGCTGTCGCGGATTGACGCCGGCTAC
GGGTTCGTTGCCGGATGTCTGACGGTTGCGGGCCGGGTGCTGACGACGGCGGCTGAACTGGAGCTGAA
TGGCATCACCAACGTGACGACGGTTTTCGCGT
GCGGGGAGTGCACGAAGGAGGGCGACTGCTTTGCTCCGCTGACGACGGCGGTCAGCGACTGCAAGTGC
CAGTGCGCTGCTGGAGGGCACGGCGATGTGTG
CGTCCCGGCGCCTGTGCCTGCTGGTCCGCCACCACCGCCACCGCCGCCCGCACCACCGTCACCGCCGC
CGATTGGTGAGTGCATCAGCGACATGGTGTAC
CCCGAGGTTGCGCAATCTGTGGGCAGCGGGCTGTCGTGGCTGTGCTACCGCAACGTGACGTTCAGCGG
GGGTGGCATGATCCTGACGGTGCTGATTGGGG
GGATGACGGGCGACGTGGCGAACATCACGTTTGATGGATGCACGTGGAGGGACGGCGCTGTACTGTTG
CTGGCGGGCGACGCGCACGCCGCTGTGGAATC
GCTGAACGTTTTTGTCACCGGCAACACGTTCGATGACGCGCTTCTTAGCCCGGAGGGTGATTTTCCAC
CGCACACGAACATCACCATCAGCGGGAACCGC
TTCAAGGTCACGAGGCTGATCTCTCGGTCGGGCTTGGACATTACAAGGCCGTCGTGTGTAGCGATGAA
TGGGCTGGTGGTCAGCAATGACTCCGCGGTGG
TGCTCAGTGGCAATGTGTTTCAGAGCGTGACGGCATCGTCAAGTGCCATTTACGTTGTCAGATCTTCG
CTGAGGGTGTCGTGGCACTCCCTGTTTGCGGT
GGTGGGCAACACGTTTCATATGGACGACGGTGGCGCTACGGTGATATATCTTGGAGGGTCTTTCCAAT
CCTCGTCGCTGGATGTACTGAACAACTCCGCC
GTGGTGATCCGAGACAACATTGTGTCGAGGCCGGTGAGGTACTTCATGTTATTTTATTGGGCATTACG
TGTGGAGTCCTGGTCAGCGGTTGTGTTTCAGG
GCAATGACATGCAGGGAAGT
>scaffold1108.size7380

TCTTTCGCGGATTCTTCGCCATTTTCCGCGGGGTGTTCATGCTGTTGGTGTTGTTGGACTTGTGCTTC
ATCACTTTGCTGCTGTTCATTTTCCTCTTCCTC
TTCTTTTTCTTGTCCTTTGTGTCCAGTGCCGGATGTCGTTCCTGCCGTTCCTTCGGGTGGCGAGGAGT
CGTTAGCCTCTGCAGCAGAAGACTGATCTCCT
GTTGAAGAGTCCCCTGTTGTGTTATATGAATTACTGCCTGAGGACCCCGCTGTACCACCACTGCTGCC
AGCTGAAGTATCAACAACGGTAGCATCAGGAG
CAGGAGAAACAGCAGAAGCGGGAGAAGAAGCAGGAGGAACAGCAGGAGCAGGAGGACCTGAAGGAGGA
AGAGCAGAAGGAGAAGCATATGAACCAGAAGA
AACGTCACCGGTGCCATGACCGCTAGTGGAAGATTCACTGCTTCCACCACCAGCAGCAGCAGGAGCCC
CGGCAGCGCCAGCACCACCAGCAGAGCCAGAA
CCAGAAGCCCCGACACCAGCAGGACCAGAAGGAGGAGCAGAAGCATCAGAACCACCAGGTCCACCAGC
AGCAGAACCAGTGCCACCAGTGTTGGAATTCT
CCCTTGAATGCGAATTGTTCTTTGTTGCAACAGCATCACTTTCGCCAGTCGAGGCTTCAACGGCAGTA
ATGGGCAGTACTGACCGCAAAGGCACCGTAAT
CGCAGTTTTATTACATCCGTCAGGCATGTGGGATCCATTCTCACCCAACACTCCCGACGCCATGCAGC
CACCCAGTGCGTTGGTGTCAACGTCCCTCGCA
TAAACACCGCCGGCAACGCACCACAGCACGCAGAGGGCACACACCAGCAGCACACGGCCAGCCATCAT
CATCGCCATCACTCCTGTCACTTGCGTGCGGC
CTTCAGCACAAACTAACCCCCGTCAACGCAGAGTGAAAGCAACAAAAGACACGGCACAGTAAAATACA
GCAGCAGCAGCAGCACGCATTTCATGAGAAAT
ATTGAACCAATGAAAGTGAAAAAAGACACAATGCGCTGCAAGTAAAGAAAAGCACAGAAAACAAGGAA
AAACAAAAGTAAACAGGCAGCAATAAAGTAAA
ACAGTAAAAACACACACAAAAAGAGCCGCACAGTATGGCTCCAGCGCGCACGCCTCTATTCTCAACAG
TGTGTCCACGCAAACAGTTGTGTGCGTGTCGT
GCGTATGCGTATGTTTGGGATGCGTTCAACGTGACGGATGTGGCGCACAGCACGGAGCACACAGCAGG
GCAGCGGAAGCAAAGAAGCAAAAACACAATCA
TCATAATTGCTCTTACTTTATACATCGGACCACAGTAGACGTGCACAAATCCCCGTGATTGAGTGCTG
GCACGCACAGCCCGCACACACCTCTTCAGCCC
ACAGCGGTGTACGCCAGCGCGGATGCGGCGAGCACCAGCGGGGCACACACCCACGCAGAGCTGCTGAG
GCTGCCGTCAATTTCGCGAAGACGTGACGGTG
CGCGGGTGGTCGTCGTGGTTGGCGCCTCTGTATTCATCGCCGTGTTAAGTGGTGCCTCTGGCGCGGTG
GTTGTTGTAGTAGTCGTTGTAGTGGTTGACGC
AGTTGCATCGCTACCTGATACCTTCATTACGGCAGTCACATTTGTTGGCTGCTGCGCATCGACTTGAC
CTGTCACTTCCAAACTAAGGGGCCTCTCTTTC
GCTTTGTTTGCTGACTCAGTGCCCCCAGTAGGTGGCTGCTCCGGATTGGTTGTAATTTGTCCCACCGC
AGTAACGGGTGCACCATCGTGTGTCTGTGGCG
CGCTGTGAAATTCTAGTGTCTGTTCCTTATCATTCGTCGGATTGGACGTCTGACCTGCATATTTCTGG
CTTCCTCCCTTCAGATTGTCAGGCGTCCCTGA
TGCCTGTTCCGTCTCGACTGTTGCCTTTGGCGCAAGGGTGTCTGAACCTGCAACATCCTGATCCTCAT
CAACCGTTGTCCCGCACACACGGAAGGGCGGC
AGAACAGGGCGAGCACCAACAGGGCGCACAGCAGACGGCACGTCATCATCGTGAGTGTGGTCGTTCAG
TGGTGTGGAGCTGATCTGCACAGTGAGGGACG
TGGAGAGCAACAGGGAGACAAGAGGTGCGGCAGACACACACAGAGGGCAAACACACACATGAGGAGAG
GGCAGCAGCCAGGCATGCACCGTACGCCGCAC
ACCACGCACGACACACACTCCAATCCACGGCGGCAGGCACACACACACACACAGCCCTGCCGCAACAC
CAACACACAGATGGAGGAGGGAGAACCAAACA
GGAGCGGCACACAAGGCTCCACCGCACGCACATACGGCGAGAGCGGTCCGTCGTGTCAGCACCCCGCA
CGGAGGGTACGCACACACACAAGAGGAATAGG
CGCAGCGCACACAGCAACGCAAAAGCAAAGTAACAGTAAAAAGGGAATGATCCCTGCACACCACGTGG
ATTGTTGTTCCGCGGGAAGCGCAGCCAACAAT
TCGGTTTGTGTTCTTCCTGCTTTTCGCTGGTAAAGTGCTGCAATAGATGTTTGTTTTTTCCTTTCGTT
GCAGTGGCACCTGATTCCTCACCGTTTACTGC
GGTGGAAATGAAAATATGTGAGTGGACTCCCACACACCAGAACGCACCTTCGCTCCTATTTATTGGTG
CACAGAAGGGCATGTGTGTTTGGTGGTGCCTC

GACCATATTCCTCTGGGTGCACATTCCCCTGCAGGCTGAGGGCGGGGCGACGCTCCGTCGCGGTGCAC
TCCCCTCGCATGCCGCCCGCTGTGATTCATCA
TGCATCACCCTCTCACACATTCCGTGGTGCCACACCGCTGATATTATTGCAGCCGCTGTGATTCCGCG
TATTTCACGCATTTGAGTGTGCCACAATACAT
CCGCTCGTGTGGCCGCCGCATCCAAAAAGCGGTGCCCAACACCGTGAGCGGCAGTCCACATCCTTGCA
TGTCCCAACGCAGTGGTGCTTGACTGTCAGCC
AGGCGACACACAACAGGAGAGTCTTGTGCTGGCAGTACAACACACGGAGCTGCAGTGAATGACTCGGG
CGGCGACGCATCCATTTGTCGCAAGGCAGTGA
AAAGGAGTCGGGCAAAATAACGCCGGCTGCATTCACGACACCACATCCACAGCCAAGCATGCGAAGTG
ATGTGTGTTTCCCTGGTGCTGTGAAATGCCTG
CAGACGACGTCACTAGTGGAGGCATGCCACCCATGATCAGCGGACAGACAATAATCGGCATGGGAGGC
CACAAAAGGCAGGAACGACGCCAGCAGTAGGA
GCAGCTCTCTCTCTCTCTCTGTTGCCTCAGAGAAGTAAATATTCCACCAGCCACAGCTGACTGTGCCA
TCGTGACGCTCCATGAACGTCGTGGCTGCGCC
AACACATCCTGCTCCCTTCAGCACCTCAAAAGACGCCTCACTCCGTCCACTAAGTGCATAGATGTGGG
CCGTGCCTTGGGCTGTTCGTGAGGACCACAAC
GATGTGAGGGTCACCGAGTCGCAGCATGTTTGTTGTGACGTTGTTGCATGGCACCTGCACACACACGC
AGCGCCTGCATCCCTCCCTTTTGTGTCCGCCC
TCTGCCGGAGTGGTCACACACACGGGCAGCAGCAAAATCACTTTACCCTCGACGATCCAAAATCAACA
CTGACATCCCTCGCGACGCAAAGATCTCAGTT
GCCAGCACGGGCCTCCTGCCGCTCTGCCATCGACGCTGAAACACGACACAGTGCACGCACTCATTTCC
CTCGTGCGCAGTCAGCCGTTTGCTTCACACAT
TCTTCNNNNNNNNNNNNNNNNNNNNNNNNNNNNNNNNNNNNNNNNNNNNNNNNNNNNNNNNNNNNNNNNNN
NNNNNNNNNNNNNNNNNNNNNNNNNNNNNNNN
NNNNNNNNNNNNNNNNNNNNNNNNNNNNNNNNNNNNNNNNNNNNNNNNNNNNNNNNNNNNNNNNNNNNNNNN
NNNNNNNNNNNNNNNNNNNNNNNNNNNNNNNN
NNNNNNNNNNNNNNNNNNNNNNNNNNNNNNNNNNNNNNNNNNNNNNNNNNNNNNNNNNNNNNNNNNNNNNNN
NNNNNNNNNNNNNNNNNNNNNNNNNNNNNNNN
NNNNNcgaagtgtgtaagaagttTGGGCTGACGTCCACAAAGGCATGCCACAAATGCCACCGTGATCG
CACAATCTCTTGCGATGAGCGTCGGTGTGTGC
ATGGATCCGCTGCTCACACGTGCTGGCAGGCAGCCTGCAGCCGGTGCCCTCGTGCCAGCTCATCGGCT
CCAGCACGGAAAAGAGAAAATGGCCCATTCCC
GATGCACTTATTCCCGCCAGCAGCACCAACACGCTGGTTGCCAGCCGTCATTCCTCAGCACATCCAGG
AATGCTGAAAAGTGACGTGGAGCTACCAGCAA
CGCCACAATCCCCGCACACATGTCGGCTCCACCAGAGAGGACACACAAACCACAAACAACACCCTTCA
CATGTCATCCGAACATCATTCCGACACACCGC
GACACGCCAAACAGAAGTATTCGTCACTCCATGCACCTCCTCACGCAATCAAATAGTCCACGCACCAC
GCACGGCAGCAGCCCACACGCGGTCAAACAAA
TGTTCTTCACATCAGTGTTGTCCACGTGCTGCAGTGGGAAGTAAACAAAAGAGACGAGCCGCCAAACC
CCAAAAGAGTGCCACGACTGGCCGAAAGTCAA
CGGAACGCAGCCACTCAAATGAATTCAGTCCTAATGCCCTCAACCTCCCACCTTCTCCAACTAAGGCC
CCAAGAGACGCAAATCAAACACCCCGTCAAAT
AAAGTCGCTGTGCAACACCAAGTGAAGCCGCAGTGCCTCCCGCTGTCATCCGGGACCGTTGTGGACGC
CTTCACGGGGACAGACACCCACCGGATATTAC
GAACGGCGCTGTACACCTGCGACGCATCATACGGTTTGGGAGGCACGTGCCTCACTATCACCAGAGAC
GCCCACGCGCCGTCGGACGCCCCACACAACCA
ATGACGCAAGTATACGTCAGAGTGACAACGAGACATCCGCTAGCAATCACACCGCAGGCATCATCGCA
AGCAGCACCAGCAGCGCTGCAGTCAACTGTGG
GGTGCCATGGACCAACTGACGCATGACGTATGTAGAAGAAAGAGCGTGACGGCTTTAGAAGGATACGA
GTAGCTAACACTTCCTTAACAGGATGAAAACA
GTATGGAAACAATAAGAAAAATTCAGGCGTCCACAGTCAAAGCAAGCCGCCGATCAAAAAAGCGTAAA
AGGTGCACTCAACCACAAAACACAAGTAAAAC
AATAAAAGAAGAAGAAAACAAAAAGCCGAAGCGTGGGACGACCAGCACACACACTCTCATTGCTCCCG
TAAAGCAAAACACAAACACAGAGGGAGAGAGA

GAGAGGTCGCCCACTCAATGCAATGGAAACAAAACAGAAACAGTTACGGTGCTCCGCCCATGCAGCCA
GGCAGGACGGCAGCGGTGCGTGTCGTGCAAAA
GAGAGCACCAACGGGCCTGCATGGGTGTGCATGCATATATACAATGGGCACATTATTGTGTGCCTCGT
GGGGTGAGGTGAGGGCGAGAGTCCATGCAAGG
GGACACAGACAAACAGAGAGAGAAGGAAGAGTGTGTGTGCGGGCGGTGCACGGCTCTCTCACACTCAC
TCTCACGCGGCCACCACCGCAGCAGCAGCCGC
ACACGCAACAACAAGAAGAAGCAAAAGAGGGGAGGTGGCGTGGGAGACCGCGGTGCTGCCGTCACTGT
CGCCTGTATTTGCGGTGGCATTTGTACGAAGG
GCGTTTTTATCTTTCGCGGATTCTTCGCCACTTTCCGCGGGGTGTTCATGCTGTTGATGTTGTTGGAC
TTGTGCTTCATCTCTTTGCTGCCGCATTACAT
GATCTTCCTCTTCTTCCTCTTCTTGTCGTGTGTGTCCAGTGCCGGATGTCGTCCCTGCCGGTCTTTCG
GCTGGCGAGGTGTCGTTAGCCGCTGCAGCAGA
CGACTGATTTCCTGTTGAAGAGTCCCCTGTTGTGTTAGATGAATTAGTGCCCGAGGACCCCGCTGTAC
CATCACTGCCGCCAGCTGAAGAATCAACACCA
GGAGCAGCAGGACCAGCAGTAGAAAAAGGAACAGGAGGAGCAGCAGGAGGAGCACCAGAAGTACCACC
GGTGCCTTGACCTCCAGTAGGAGAAACGCCAC
CGCCGCCATCAGAATCTCCCTCTCCAGAAGCACCTGGAACAGCACCAGAACTGCCTCCACCAGGAGAA
GCACCAGCAGGTCCACCAACAGCAGAACCAGA
ACCAGAAGCACCTGGACCACCAACAGAAGGAGGAGCAGAAGCACCAGGACCAGGTCCAACAGCAGAAA
CAGAACCATCAGGAGAAGAAGCAGCGTTGGAA
TTCTCCCTTGAATTCGAATTATTCGTTGTTGCAACAGCATCACCTGTTCCAGTCGAGGCTTCAACGGC
AGTAATGAACAGTACCGACCGCAAAGGCACCG
TAATCGCAGTTTTATTACATCCGTCAGGCATGTGGGATCCATTCTCACCCAACACTCCCGACGCCATG
CAGCCACCCAGTGCGTTGGTGTCAACGTCCCT
CGCATAAACACCGCCGGCAACGCACCACAGCACGCAGAGGGCACACACCAGCAGCACACGGCCGGTCA
TCATCATCGCCATCACTCCTGTCACTTGCGTG
TAGCCTTCAGCACAAACTAACGCCCCGTCAACGCAAAAAGCAACAAAAGACATGGCACAGTAAAATAC
ACCAGCAGCAGCACGCATTTCATGAGAAATAT
TGAACCAATGAAAGTGAAAAAAGACACAATGCGCTGCAAGTAAAGAAAAGCACAGAAAACAAGGAAAA
ACAAAAGTGAACAGGCAGCAATAAAGTAAAAC
AGTAAAAACACACACAAAAAAGAGCTGCACAGTATGGCTCCAGCGCGCACGCCTCTATCCTCAACAGT
GTGTCCACGCAAACAGTTGTGTGCGTGTCGTG
CGTATGCGTATGTTTGGGATGCGTCCAACGTGACGGATGTGGCGCACAGAACGGAGCACACAGCAGGG
CAGCGAAAGCAAAGGAAGCAAAAAACACAATC
ATCATAATTGCTCTTACTTTATACATCGGACCACAGTGGACACACACATGTCACCGCCGTTGAGTGCT
GGCACGCACAGCCCGCATGCACCTCTTTACCC
CACAGTGGTGCACGCCAGCGCGGATACGGCGAGCAGCAGCGGGGCACACACCCACGCAGAGCTGCTGA
GGCTGCCGTCGCTTTCGCGAAGACGTGACGGT
GCGGTGGTGGTCGTCGTGGTTGGCGCCTCTGTATTCATGGCCGTGTTAAGTGGTGCCTCTGGCGCGGT
GGTT
>scaffold1109.size9287
ACCGGCGGTTCCGCTTTGGGCCAGTTATATGAGGAAGGCAATTGAGGTCGTTCGAGAGATGTGCGCGC
CGTCAACGTGGGAGCGTGGAATGTCCCTCGCGG
GGCAATTCACAACCTTTTGCCGCACGAACGAACAGCCGATGGACGAGGAGAGTTGTGCGGTCTTTTTG
ATGGCAATCGAGGTGGCGCCATCGACCCGACT
GCAGTACGACAGGATGCTGCGGTCCATGTTGCAAGTGAATGGAACCCCGCTGGATATGGCGGCTTTGA
GGTTACAAAAAGTTGCTGTGCGATCGGAGACG
AGGCAGACGAGGCAAGCGCGCCCCTTGACAGAGGAAGAAATGAATCAAGTTATCTGCAGCCGGACCGA
TTGGAAGGAACGTGTTGTCGTACGACTCGCGT
GGATAACGGCGAGTCGTTTGTTTGAGATTGCGGTCTTGACTCCCAACAATTTCGCACtGGAGGCGGAC
GGGGCCTTAATTTTGGACTGGTCCCTGGCGCC
GAAGACGGCGAGGGCGGATCCCCACCGCGCCTTTCGGTTCGTCAGGGCACGAGGGCAGGATGCGTTAG
ACACTATAAAGCTATGCAGGACACTTCAAGAA
AACGAAAAGCTCACGAATATTACTAATGCCAAAGTCGAACGAGCTTTGGCTCCTTGGGATGCCAAGGC

GCATTCCATAGAACGCGGTGCGTTGAGGCACG
CTGCTCAAATCGTGGAGACATACAATTTGAACCCACACGTGATCTCGCAGTTGGCGAAGCACGTCGAC
CCGTTCGACCTTCCCCAGAATACTGTTCGATA
TTTGTGGAGGTACACCACACTGCTGACCCAGGTGTCGTCGCTGGTCGCATTGATGTGAAGGAAGGAAC
GGCGGAAGGGCAAGAACCCTGGCTGAGAGCAG
AGGAGCGTTTTTTCCATGATTTTTATCACAATCATGCTGCTACAATAACGAGACGCGCGACAAGGACG
CGTCCGGCCACTCCACAAGATGAACACGCTAT
CCCGTTACAGCAAGTCAGCGTGCCCGTATTGAAATTAGAGTGGATTAAAAGTCGGTTGAAACCGGCCA
CCTTGGAGAGTCTCATGCAATTTTGGGGACTC
GTCGGGCGGTCCCCTCTCCCTCCGTCTTCCTCCGGTAAAGCGCGCGAAGGCTCACGGAGAATTCCAAC
CGCTGACGCGAGATTGTTACGAAAAGCGGGAA
TAATCGAGGAAGGTTCGTCCAAAATAACAGGCGGATGGATAATACCTTTCTCGGTTGTGGAGGAGAAA
ACCACCGGTTTACGACGACGATGGATCGCGTG
GCCACGCGACAAGAACAGAGACGACCCTTACGAGGCTAATGTTCCTCTTTTACACATTTCCCATTATT
TGCCGCCTGTGATGGCTGAGGCCGCTTCCTGC
CTGGATGTAAAGTCGTCCTTTTTTCATAGTCTCTTTACCGCGGGAGACTCGGCATCTCTTTCGATGCC
GCGTGGAGGACGGCACGCTGGTGGAGCTGACA
CGGCTCCCTATGGGATACAAGGCCGGCCCAGAAATTCTCCAGATTATTACTTCGGCAATTGCGGGGGT
GACGACGGTGGTTCACCCCCTCTGGGCCGCAC
CTCCATCGGTGCGTTTCGACGTATGGATCGATAATATACGCATTGCAGGGTCGAAAAGCAACGTGACA
TTGTGGGAGGCCCAAGTGCTTCGTAATGCGGA
CAGGCGTCACGCCACGATAGGGGAAGGCCGCGAATCGGGCGCCACGCAGTGCACCTTTCTGGGGGTGC
ACTTTGATCACACTCAGCGTGCGGTGTCCCTG
AGTGACGAGTTTGTCCGGTCTGTACGCGCCATGCCGGCGCTCAATTCTTTAACCATCGCGGAAATGGA
GGTTATGGCGTCACGCTTTTTGTACGCGGCGG
CCATTTTGTGCACGCGTTAATGCGACTACTATCTTTTCATCAAGGCAGTGCGACGACGATTGTCCGCA
CTTAACCGGGGAATTGTGCCGGAGACATCCCC
GGCGAACCTACCGCCGTCAGCGGTTGGTTTGGGCGAGAGATTGCGACACACCATCGAGAACAATCGCA
AGCGAATCATCAAGCCCACGGAAAAGGCATCG
GCTGCCATCATCACGGACGCATCGCTCCATGGATGGGGAGCCGTTTTTATTCCAGACTTCGGCGACGT
TAAAATTGCCGGAGGGAAATGGGAGAAGAAAC
CTTTTCTATCATGCAGGCCGAGGCGCGTGCGGTACGCTTGGCCTTATCGGCCTTTCCGCCATCTTGCC
ATCCACCATGGACGTTTGGGTGGACAACACTT
CGCTGCAAGGAGCGGCGAATAAAGGCGGCTCAAAATCGCACGCCATGACGTGGGAGCTGCAACGGATA
TTTGAGTTTTTAGACTCTCGCGGAATACAAGC
AACTTTTGCCTACGTGCGGTCTGCAGAAAACCCCGCAGACGGCATATCACGCGGTCGTGTGTTTACAC
TTCAGGACTTGGCGAAGGGGAGCAGCGGAGTC
TTGTGGTTGGAGGACCCCAAAGTCTGCCACTTCGTAAGTAATAATATTTCAAATCCTAACTGAGGACA
AAGAACCATGCTAATGGTCCACAGAATTCTAT
AAATTGAATGAGAATAAAACATTGAAGCAATTAACTAAAGAACCTTTTTCCCTAACTTGTTTGGTTTA
TATAGTTTATTTCAGGATCCGGCCAGCTGCCT
GGAAGATTATTCTGCGGGCAATATCTATACAAAAACACATGCTCTTATTGTGCCGCTTGGGAAATAAA
ATAAATGTCCTGCCGAATGAATTTATTTATTC
TTTCCGAGAGTCCTGCCGGACACTCAACTTTTGTTTTCCTTGCTTAACCTCTGGTTTGCAGTGCGGTG
TGCGCTGCTGCTGAAGTGTTGCCGTTTTTCAC
CGTGCGGTGGCTGTCCGTGGTGTGGCGGCCGTGTTTCTCATTTCTTTTGCTCTGCTTTGACGGGGAGT
TTGTTTGTGCTGAAGGCTACACGCAAGTGACA
GGAGTGATGGCGATGATGATGGCTGGCCGTGTGCTGCTGGTGTGTGCCCTCTGCGTGCTGTGGTGCGG
TGCCGGCAGATGTCAAGAAACACTCTTAGTTT
CTCAGTCTGAAGTGCCCGGTGCAATTGAGGGGGACAGAGGACAGCCACAGGGTTCATCAGGCTTTAAA
ACGCCAGAATCACACGCACCAGGCTCAGAAGC
CTCAGTACGAGAGGTTCAACAAGAACAAGATCCGTCCCCACCTCGTGAAGAAACTCAGCTAGGAACCA
AAGGTGGTAATAAGGTAGAAAAGGCAAAAGAA
AAAGGTGCTGCTAAAGACGCTGGAAAAGAAGGGGAAGTGGATGGAAAAACTATTGAGCTAAAAAAGCC

TGAAGACAAACTGACGTCTGTGGAGGGCCAGC
AGTTGAATAACAATGAAGGCTTGCAGCCGACAACGTTGAATGGACCTCATGGAGTTGAAACGACAACA
CCATCAAGAGAAATTTTGTCAGTTCGTGAAGA
TCAACAAGATTTGTCGAGTTCTCTGCTGCCtCCGTTACACAACACCTCACCGGGTGGAACTCCACCAT
CCACTCCTGCaGCTGGCCGTGCTGACACAAAA
AGCCACGAAAACGACATTGGCAAGAAGAATTCTCAACAGTCTGTGGTAGGAGAAATTGGACAGAACAA
TGACACCGGCGAAAAAGCTGTCGATTCAACGG
GTGAAGAAGAAGGTTCACGATTGTCAGTCGCGACAGCTGAAGAAATGAGTGAAACAACAACGAAAAGC
GACAGCGGCGGCACCGCGACAACTACAGCTGT
GGGGACTGACATTGGCACAGAAGAAACTCCGCCCACCAATCATCCGAGCAAACCCTCCACTGAGGGCG
CCACACCACCCGGAACGACTTCCGATGGTGAA
GCCGCATCAACCAACAAATACGATACAGTCTCTCAGAATGCAGGTTCCACAACAGCAGCAACCACAAA
CGCCACAACAGGTGACACGGCGAAGAGAGGCG
ACAGCGACAGCAGCACCGCGGTCTCCCACACCACCTCCCCTCTTTTGCTTCTTGTTGTTGTTGCGTGT
GCTGCTGCGGCTGCTACAGTGGTGGCCGCGTG
AGAGTGAGTGTGGGAGAGCCATGCACCGCCCGCACACACACTCTTCCTTCTCTCTCTGTGTGTTTCCC
CTTGCATGGACTCTCGCCCTCACCTCACCCCA
CGAGGCACACACAATAATGTGCCCATTGTATATATGCATGCACACCCATGCAGGCCCGTTGGTGCTCT
CTTTTGCACGACACGCACCGCTGCCGTCCTGC
CTGGCTGCATGGGCGGAGCACCGTAACTGTTTCTGTTTTGTTTTTATTGCATTGAGTGGGCGACCTCT
CTCTCCCTCTGTGTGTGTGTTTTGCTTTACGG
GAGTAATGAGAGTGTGTGTGCGCTGGTCGTCCCACGCTTCGTTTCCTTGTTCTTGTTTTTTCTTTTTT
ACTTGTGTTGTGGTTGAGTGCACACATTGCAC
TTTCTGATCGGCTGCATGCTTTGGATGCGGACACTGGAGTTTTGTTTTTATTTTTCTATGTTTTTGTT
TTTGTTGAGGAAGCGCCAGTTTCTTGTATTCT
TGTAAAGTCATTAAGATTGTCTTTGGCTTATGAAATGAGTCCTTTGATTCACGTCACCATGCGGTCTG
CATCGCCTCTGGTGCTGCTATTGATGATCTGT
GCGATGTGATTTGTCGGCGGATGTCCTGCTTTCATCCTGAAGTATTCCTTATTTCGAGACGATGTGGT
GCGCTCCCACAGCGAGGTTTATCTCGTTTACG
GTGAGGAAAATGCGTCTCAGGGCGCTGGACGCTTTGCGGATGTGCAGCGGGGTCCGGAGGGACCGNNN
NNNNNNNNNNNNNNNNNNNNNNNNNNNNNNNN
NNNNNNNNNNNNNNNNNNNNNNNNNNNNNNNNNNNNNNNNNNNNNNNNNNNNNNNNNNNNNNNNNNNN
NNNNNNNNNNNNNNNNNNNNNNNNNNNNNNNN
NNNNNNNNNNNNNNNNNNNNNNNNNNNNNNNNNNNNNNNNNNNNNNNNNNNNNNNNNNNNNNNNNNNN
NNNNNNNNNNNNNNNNNNNNNNNNNNNNNNNN
NNNNNNNNNNNNNNNNNNNNNNNNNNNNNNNNNNNNNNNNNNNNNNNNNNNNNNNNNNNNNNNNNGAA
GGTAGACCATTCCAGCAACCAAACACCACCGC
CAGCACCGCCATCGGCGGTGACATCACCAGTTCTATCAAAAGAACCGGCACCTCCAGGAAAAGAACCA
TCACCAAGCAATGTAAGCCTACCAGCACCAAA
TGTCCCACAGACCACAAAAGGTCAAGAGCAAAAACCGTCAATTTTAACAACTAAAAAGGAGTCCAAGA
CACCGGAAATTCCTTCAGAGGATAATGTGTTA
CAGCAACAAGGTCAAAACACAACCACAGAGGGATTGATGGAAAATTCATCTCCAGGAAGTCAAGCGAG
AAATACAGAACCATCCACTTCTACAAGTGGCA
GTGGTGAGGCCCAGATTACGGCGGATGCGGATAATGCTCAACGGCCCAACCCCAGTGAATCACAGGAC
GATCTTGAAGGTACTGACATCAACAATTCTCC
TACAGCAGATGAGGCAGCACCTCAATCAGCAATAACACTCACCACCGCTCAAAAAAATGAAACGGCAA
CGGTTGGCGACAGCGACGGCAGCACCGCGGTC
CCCCACACCACCTCCCCTCTTTTGCTTCTTCTTCTTCTTGTCGCGTGTGCGGCTGCGGTGGTGGCCGC
GTGAGAGTGAGTGTGAGAGAGCCGTGCACCGC
CCGCACACACACTCGTCCTTCACTCTCTCTCTGTGTGTGTCCCCTTGCATGGACTCTCGCCCTCACCT
CACCCCACGAGGCACACACATAATGTGCCCAT
TGTATATATGCATGCACACCCATGCAGGCCCGTTGGTGCTCTCTTTTGCACGACACGCACCGCTGCCG
TCCTGCCTGGCTGCATGGGCGGAGCACCGTAA
CTGTTTCTGTTTTGTTTTTATTGCATTGAGTGGGCGACCTCTCTCTCTCTCCCTCTGTGTGTGTGTTT

TGCTTTACGGGAGCAATGAGAGTGTGCGCTGG
TCGTCCCACGCTTCGGCTTTTTTTTGTTTTACTTGTGTTGTGGTTGGGTTTACTCATTCTTCGGGCTG
CGTGTGTGTGTGTGTGATGATATGAGTAATTT
GCGTGTGTGCTATCTGCCTCTCTCTGCCGTTGCTTTCTTCAGACTCTCTTGTGGCGCACAGCTGCTCT
GCAAGTGTAGGCGCTGCCAAACACACAAGCAC
ATGCACACGCAAATGCCGTGTTTAAAACGCTGAGAATGCATGCACGCACGGGATTGTGGCTGCGGCGG
TTGTTCCTTGCGAGAGGGAGTACGGGAGTATG
CGATTTGCTTCGTTGACATGTTGCTGTTGTTATGTTTGCGGCGCGCAGCCAGTGAGTGATGTGGCAGT
GGGGGTGCCTGGGATGAGTGCGGATTGATTCC
CGCTTTGCAGCTGTAACTGCGGACGTTTCTTTCCCACTGTGGATTTGTTGATGAGGTATTCTCCGGCT
GTGCGGGTGACGTGCGAGTGCTCTGGAGGAGA
CAGCTGTAAAAGGGCTGGTGGCAGCACGTCGGTGCTGCGAAGGGCGGCTGTTTTTTTTTATCCTGCTG
GTTTTTCTTTTTGTAAGGGCGTTGTTCAGTGT
GTGCTGACGCGTGACGAATAACGACGTGCCAGGCGTGGCCCCTGCTGCTGGTGGCGGCAGTCCCTGTG
TGTTCCCGGTGCACTGTTCCTTCTTCTCCTGT
TCCGTGTGTCTGTGTGCGTGAGTTGTTGTTGTTGGGAATGACTGCGGTGCGGATGATGATGCCACTCA
CTGTGGATGAGTGACGGTATTCATTGTTGTAC
CTCTTTTTATTTTTTGGCTTATTTTCTTTATTTCTTTCTCACACACTCAGGCTAGGAAAGCAAACAAG
CGCTAATCGCTACTGTGAAATTCGAAGGCTCT
GAAAGGACCAACAGCACACACACCGCCCACACACACATATTTATATGCTCTCACGTGTTGCTGCTGTG
AAGGCACCCCGCACAGACTACCGTCGCCGCGT
GGCCGGATCCAGCGGAAGGAGGAGGGAGGGAAGAGAGAGTGAGTGGCAGAGGCCCAACATGTCCCGGC
GTGCGTTTACTTCCGCGGTGCTGCTGCTCCTC
GTCGTGATGATGTGCTGCGGCACCGGTGGAGCCGCATCCAGTCAGGACGTGCCGTCAGGCAAGGGATC
TTCACCGAAAACACAGTTTGTTTGGGAGGATG
GGAAAAAGGGAGAAACAGTGAGTTCGCTTCGTGTTCCCGGTCTTGTTGAGATAGATGGCGAAGTATTT
GCCGTTGCCGAGGCGCAGTTGAAGGGTGAAAG
CGATTTTAATGGGATTGCCTCGGAGCTTTTGGCGTTGACTGATAAAGCATCAAAAGAATTGGTTAAAG
CTCAAATGAGGACACAAGTGCTGGTGGAATGT
TCTTCCGACAAGGAGAATTGTGCTTCCCGTGAAGCTGCTTTGGCTGTTTCTCAGAGTGAGAAGAAAGT
ACGTGTGAGGCAGCCAACAACAGTTGTGAAGG
AAAGTGATATTTACATGCTTGTTGGAAAACACAGCCGTACCGATGCCACTGGTGATCAGGCAGCCGAC
TGGAGACTTTTGCTGGCGAAGGGGAATGTCAG
TAGTGAAGcCGGCAACAAAAAAATCTATTGGAAGGAAACTGACGGTGTACCGTGCAATCTAGGCGACC
AACAAAACTCATTGAAACGTCTGATTGGCGGC
GGTGGATCGGGTGTTAAGATGAAGGACGGTACGCTTGTATTCCCAGTGGAAGGCACgAAAAAGGATGG
CACTGGAAGTGATGGAAAGACCGTTTCGCTGA
TCATATACTCCTCGGATACTCCGAGCTGCACACTGTCGAAGGATGTGCCTGACGGTGGCTGCACTGAT
CCCTCCGTCGTGGAGTGGAAGGACGGAAAACT
CATGATGATGACTGCGTGTGATGACGGCCGCCGCAGGGTGTACGAGTCCGGTGACAAGGGGGATTCGT
GGACGGAGGCGCTCGGGACACTCTCGCGCGTG
TGGGGCAACAAACAAAAGGGAAATGTGAAACTCGTTAGGAGCGGGTTCATCACAGCGAGGATTGAGGA
AAGGGAAGTGATGCTCGTCACTCTGCCGGTGT
ATGCCAAGACTGAGAGCGAAGAAAATTGAAAGGAAAAGGAAAAGGGTGAACTCCATCTTTGGCTGACG
GACAATACGCACATTGTTGATATTGGGCCGGT
ATCTGTGGAAGGAGATGATGTTGCCGCCAGCTCCCTGTTGTACAAAAGTGAAACGAATGGGAAGGATG
AGTTGATTGCACTGTACGAGAAGAAGGGCGGT
GAGGAGGAATCACTCGGCATGGTCTCTGTTCTTCTGACTGAGCAGCTGGAGCGGGTGAAGGAGGTGTT
GAAGACCTGGAAGGAAGTGGACGAACGTGTCT
CCAAGCTGTGCATCAATTCACTTGCTCAGAAGGATCCCTCAGCTGACACTGCCTGCAGCGCTGCCATG
CCGACGGCTGGGCTGGTTGGCTTTTTATCCGG
CAGCTTCTCTGATAACACATGGAGGGATGAGTACCTCGGGGTGGACGCCACAGTAACTGGTGCAGCAG
CAAAGGCAGAGCCTTCCGATGGTGTGAAGTTC
ACAGGGCGTGGCGCAGGGGCGGAGTGGCCTGTTGGCAAGCAGGGGGAGAATCAGCTGTACCACTTTGC

GAACTACAACTTCACTCTTGTGGCGACGGTGT
CCATCGACGGTGAGCCGGAGGAGGGTTCTCACGTTCCCGTGATGGGTGTGCGTGCAGGCAGTGACGAT
GAGAATACAGAGAATTCCGTACTTTTTGGACT
GTCGTACAACAATGAAAAGAAGTGGAAGCTACTGTGCGGTGGCGCAACGACAAAGGAGCTCAGCAGCA
TTGGGGAACCGGGGACAACACACCACGTGGTA
ATTTTGCTACGGAACGGCAACCAGGGCTCCGCGTACGTCGATGGTCAGCGTGTGGGTGTGGGTGCGCA
ATGTGAATTGGGAAGCACGGAATCCCAAGAGA
TTTCACACTTCTACATTGGAGGCGATGGAGTCAATGCAGAGAACACAGCGGGTGACGAAGACGTGTCT
GTGACGGTGTCGAACGTCCTGCTTTACAACCG
TCCGTTGGCATCCGAAGAGATTGACGCCCTCAACCCGAATAAAGCCTCCATTTCACCTCCTGAAGATC
TTACCGCAGCGGTGGTGG
>scaffold111.size49133
TGCCATGCGTGCTGTCAGTGCCGCTTCAAGAGCAAATAACCACGAAAACGGAGAGAGACGTCGGACTC
CTCGCCGCAATCGACGAACGTCAAGGAGGCTTT
CAACGATTCGGCCGAACGGCCACCAGGGTCCATTTGCACGTAAGGCACAGTCCAAGTCGTCATCCAGA
GTGAAATCCACAACATCCACAGCATCCTCCTC
ACGGATACACCCAAAAAGAATGACGGTAGGATCTGATGGCACCACCCATGGCTTTCGCACATAACGTG
GTGGTTGTTTTTCCAACCGTTGCCGCCGCGAT
ATGGGCTGCAGACGCGAGAATGGATCGTCCGCGTCAAGACTATTATTATTCACATTATTGATATTGCC
ACTTCTGTTGTCATTACTGCCGCTGCCGCTGC
CGTCCAGTGTTCGACCCAAACGTGCCTCAAGAGCATGGAGCTCTCTAATGACCTCCTTTATGACCTGA
CAGAGCAGCCCGTCGATAGCGGTGATGCTGGA
CGACACGCCTACAGCAACACATTCCACGTTCAATGGCCGCCCCTTTGCAAGCTGCTCAAAATGCTGCA
TCACACTGAGACGAAAACGTGGAAACAGTTGT
ATTTCGTCCACGTGCACCTTTGTCAGAAACGGCTCCTGACCAATGCGCCTTCGCTGTATAAGATACCG
TACAAAACACGGGTTATCTGAAATAAGAATAA
AAGGTGGATGACGCCTACACGATAAGAGTTGCGTCGCACCACCACCTCGCAGAAGAATTTCCGCACAG
AATGCGATGCTTGGCACAACCCTATCTGAGCG
CATAGGGGTTCCGTACGGCAGCAACACAATGGCGATACCCACAAGTTCACGTGACAGACGTCTGTGTA
GCAGATCCGCACACAACATGCGGCTGCTCAAC
ACCACCACACCACCGGTTTGGTAATAAGTGCAGCGAGTGCGGGTGGAGGTGCCCTCCTCGGCCACGTG
CACCTGCAGACGTTTTTCGTCTATATTCTTGA
GGGAAGACGTGAGGGCTGCGGCCACCCGCGACAGCGTCCCGCTTTTTGTGCCATCGGGTAACACAACA
CAAGAGACGCAGGCATGGAGTGATGACAACCC
CACAAGGGCGCGGCGGATGAGATGTGAAACGATGCCCTCAACGTCAAAGCCGCAACTTACGACGCATA
ATTGCGCGTTGATGTCCTCAATGCCCTGCCCC
GTGCACCATCCTGCAATCTGACTCGTTATGGTGGGCAACATTCCCCTCATAATCCACCGAATGTCATA
AAGCAAGCACCAAGACAAAAAAAAAAAGTAAA
ATGAAAACATTAGAGGGTCACACACACAGACAGGGAGATAGAGAGAGAGGGAGAAAAAAAAAAAGAAA
AGGAAAAAGACAAAAATCATTGATACATGAGT
CCGCCATACACAGACAACTCTGTTGACAAATGCAAAATGGCGTTACGGCAAGCAAGCATGTACTGATA
CATAGTTTCATCCAATACATCCAAACAACAGC
CCCACGGCCGCACGCCACTGTGGCTCACATAATGTAGGTGCAAAAACAACAACAACAACAAACAATTA
TCATCCTGTTCCTTTTATAGCAGGTTTCTCGC
ACGCAGGATTACATATTCAAACTCGGCATAGTTCAAAAGTAAGTCTTCCTCCTGAACAGGTACTTTAC
GCAGCAGTTGTGGCTCCATACGGCCAACATGA
ATACACAAATCCCGCTGTACTTCACCGAGCTTCTGAAACGACAGGTTTTTTCCATAAGATTCCCAACT
AAGAATTAAATATCGAACTTCTTTCCACGGCA
CAACACCGGAACACGATGGGTTAAGATCTCTGAACCACTGCAGTATAAGAGGATCGGCGTAAAGGGCC
TCCTTTAACAAGGGCGCAATAAATACAATCTG
TCGCCGTGTGATGGTAAGGGATCGATTAGGGTTTTGAGCCAAAATAATACGGTACTCTTCATCACCAA
GACAGACGATAATGACGGCTGGTAACTGTTTG
TGAAGTGCGACAGTCACCTTATCACGAACGCTACATTGCTCGGCATCCGACGCATCCAGGAGATGGAT
ATCATTATGTAAAACACGCTCATCGATACCTC

CATCGGATTCATACTCCAAGGTGTATGTGACATCATAATTTACAGCAAGAACACGGGCGATATGCAAT
GAAACATCCGAACAGAAACGTCGGGCCAATAC
CCGTGAACCAACACAGAGAGGAGAAATGGGATCATGTAGGGCCAGTTGTGGTGGCAATTGTAGGTCCT
TCAAAGACCACAACTCCATTCCCTCTAGTAAA
TTAACAACATCTTCATTACTACTGCTACTATCACCTGCCTCTGCATGACCCTGTCGGAAGGCATCCTT
TTCTCCATCAAGGGGAAATGCCTTTTTAACCG
CCTCTTTCCAACGTTTCGCAATGTCCATGAACGCTGCAATGCCTTCGGTTACGACTGGCGAATTGCTC
ACTTTGGCAATCAACTCAACGTCCTCTTTACG
GACTCTCTGCCTATCTGGGCCGTACTGAATGAGTTGCAGCGGTGCACCACTACAATATGGACGTACCA
CAAACAATCCACTTGAAGCAACTCCAACAACC
AATCCTCGGCTTGTATGTCGCCCTGTCACACCAAAGACCGAAGATGGGCTTTCCATTCCAAACGGCAA
CACGTCAAACATGTGGAAATATCGTTGCTGCA
AAGGCCGTTTCGTGGCGTGACATTCGTAGTGCACATTGTCGTTTAGAACAGACTGAAACAGCGCATAC
GATCTGGGGCTGTTCTCGCCTATTGGCGTCAC
AGGAACACTGACGCCAGAGGCAATGGAGAAGCGTCGCGGTGTTGGATAGCTGGAATTTGTTTGTGAAG
ACGAATTTTGTTGACTATTGCCGTATGCAAAG
TAGGACTTGGGAGCGGCCGGATCACACAACAGCTCTTTTTCATCCACGCACCGTTCCACAATAACGGC
ATCCACAACCAGCCCTACATTTGTGACCACAG
CCGCGTGCATGAGGGATCGTTTGTCTCTCGAGCAAGCAACTACTAAGAGGCGAATTAAAAGGAAAAAG
TCTACACACAGAGACTCCGATGATGCGACACA
ACAAATGAGTGATCCTACATTTTTAAATTTGCGACAAAGTACAAACAAACAAAAAACAGTAAGAGGGC
GTAAAAAAAAAACAACAACAGAAAAAACAGGT
ATAAGTACTTTTGGGCAAGAAAAGGAATAAAATACGATAAAGGTGGAAACAAGAATGAGTACAATCAT
TCATGTGAATAACAACACCAAATTACAGAAAA
GAAGAACCGATATTTCTCGTTGACATTGTACGTAACGCGTCTCCTCCCTGTTTTATTTATTTATTTTT
GAAACTTCTTCCACAAACGCGCTTGTTACTCC
TTCCTCGGTTTTTCAGGCTGGGCGTGGCACTGCTGTTCCGGCTGTTCCTTGCCCATTGGCTGTGGAAT
GCATGCGGGCCGTGGTGTCTTCTCTTCCGTTA
AATTCGGTACCACAGTCCCACGCTGCCGTGTAAGGTGAATCATCAGAACTGGAGGCGATTTCATCTGA
AGCTCTCTACCGCAGGCATCGGCCTGTGTCGG
TAAATAACGACGCGCAGCTTCACCGGATGGTAAGAGACGACTCGACGCAATTCGCTGTAAAGGCGTAC
GGGCATGAGAACCCCGTGGCGTCACATTCCGC
GCGTTTGGTGGGGAAGTTTTCCGCATTAATGGAGTCTGTGAACATGACGGAGGGTTGTTTCCTCGAAT
CTTTGATTTCTTTTGCTCAGGGGGACGTGCTG
TGCGTGGAGAAGTTATCATCGACCTTGTTCCCACCTGAGAGGACGGAGGTGCACGAGCGCTAATAGGG
ACGCCACCGCTTGTCGTTGGAGTATGTACCGT
TGGTTTCCGCGCAAAACGTGGACTGGTAATATTCGAAGTCATCCGCTGTAATCTAAAAGTGTCTGTTT
TGAAGTTTATAGACATCCTCGTCCTCGGGTAA
TGTGCTGTGGTAGAATGATTACCGAAGGATGCAGCACCTACATGCCTTTCTGTGAGCGGTACTATTCC
TCCAAAGGTCGATGAGACAGATGCAAACGGTC
GCGGGGTGGTCACTCGTGCAGGAATACTCGATCCTGTCGTCGGACCCCGAGAAGCATGGAGCTTAAAA
TGCATCGGCGTATTGGAAATCATAATTTTGTC
GGTGGTCGCTTTCGTCCGACGGTGTGAAGCTATCGGATACAAGGAATGAATACCATGATTGACAGGAG
TGATAGAGTGATTCGCCATTATTGTTCTTGCT
GTTGTGGCGTTGTTAACGGCTGTGGTTGCCACAGGTGTAGCAGCAGAGGGCTTCCCACCATTTGTTAA
GTGTCGTCTTTGCGACATGGAGAGCGATGCGT
TCAACGATATCATGCCTAAATGAGGCGGAGGAGAACAGATGCCCTTCTTTTTGGTGCTCGACCCCCCT
GTTACGGTCGTCCCGCCCATCTTTGAGGGACG
CGGTGTGATAGGGTTTGGGGAGACAGTCCATGTGGAGATACGGGATTCTCGACAGGCGCCTAACTCCA
CAACCTCCACTTCCTTTTCCCTCATCTCAAAG
ACTTTGGACGTGTTATGCGGCCTCCCTCCAGTTTTTCGTTCGACGCCACTTGAATCTTGTTGCTGGGG
CTGCGGAGAATGCTGTTCTTCTAGCAACAGCG
CCGAGCCCCGGTCACCTAGAAATTGGGAAAAGGATGGAAACCGCGGTGGTGGACTGTCCGACCGCGAA
CCAGACACACTGGGCGTTGGTGATGGCTGCGT

ACCGTCGATACGAGGAATATCAACTCGGCCTCCAACTTTTTTGGTGGCGTCAATACGTAAAAATCTGT
TTCCACTGCCCTCATCCATCGGCCTTCTCACT
TCTGCTGATAATTCGTGTCTTTCGACGTGCGCCGTCACCGCGAATTTTGTTGGCGCTTGAAACTCCGC
AAAGAAGGCTCCATCTTTTTTGTTGTTGCTGG
TGGCGGAGGGAGTGGTGGTGGTGCTCAGTTTGTAATCCGACAGGGAGAAACGTTTGGGTGGAGAGTAA
GAAGAAATGCCGTTGGGTGTCCGTGCACGATA
TCCTCGCACTTGCTGTTGTTGTTGTTCTTGCAGCGGAGAATAACGTTTGATGGCTGTCTTTGCACGTG
CCATCCGTTGCGTCGGGTGCTGCCGCAGGGGC
AGGCGACGAGGTGAGTGCTCGACTAACACGCGGCGCTGCGCTCCCGACGATCCCGTTGCAGCTCCCGC
GCCATCAGACGCCTTCACAAGGAATAGCCGCC
GCTTCGGCGTCTCGCACCGACTCGCGGTTTGCTTTGCAGGGGTCTCGCGTCGTGCCATGCACTGCAGT
GCCGCGGCAGGAAAGAGAAACTTCACAATACG
CGTTGAACGCGTGTCGCAAAACTCAAGAACACGCAGCAGCAGACCGACGCCGTGGTACGTCGTCTGGA
AGAGCCAAATAAAACTTATAGGACGCACGCAT
GAAGTACACGCCCACATATCGGCGGCATGTGGCCGCGGCGTGCGACAATTCTGGCACACAGGGCACCC
ACACCGCCAGCATGTATACTTGAAGATCACGG
CAATTAGCTTGACTTGAAAAACCCGTTTGCATCGGTGGCACCGGCGCGAACAAAACACATCCCCCGTC
GCACCGGCGTCTTCTTGGGGAAAAAAAGCTGC
GGAAACCGGATTTTGTCCCGTGACCGGCACATCATTTTGCCTTGCATACGATTCACCAAATGTGTTCT
TATTTTTGGGCCGCTGCATCACGTTAACACAA
ACGTATAAAACTAATCCTTGTGTCGGTTTGGGAAATCTCTCTCACTCTCCCGATATATATGTAATATA
TATATATATATATCTATCTATGTGTCTCCTTG
ACCTTCTAATAGAAATGTCAGCGGGGGGCCCTCAACACCCCAAATCTTTGCGCCTTTTCTATTTAACG
TTGCGTGTGTATTAAACCGCAAGCACTTACCC
AATACCAACAATACGGACAAACACACCAGAAATATGCCAAAAGAACAAAGAAGACAATAATAATAACA
ATAAAAGAAATTACATGTATGCATACGTATGA
AAAGTACACGTTTCTTGTCTCACCCAATATGCCCTCTCAAGCCATGGAGGCATTTGCTCATTCCGCCA
ATTAATTCGTTCCTGGCTAAAACCAGTGACCC
CACATCAGCGAGCGGCGACCAAAAGAACTACCAGGCACCACCGCAAGACGCTCCGCCAGACCCATCAA
ATAACTTTGAGGAGGCCTGTACGCGGGGAGTG
GCGTCACCCGCTTGCTGCGCTTCGGTGCCTGCAATTTTGGTTCCCAACTTGTGTTGCTTGTGGAATTA
AACTCCGCTAACGCCATGGCACGTGGAGCGTG
GATGTAATATTTCTCCAGCTGCAACTCACTCGGTGGCCGGTTGGGTCTTACAAAGTGAAATAACGTAA
AGGACGCACTCGCCTTATTTGTAACGGCCTCC
AATGACGCCAATGACGCATATGACGCAACAGGCAATCGTAGAGGTCCTTGAAGTGTGACGCTGGTGCA
TACAATTCGAAGATCATCCGATATCAACATCC
GCACCGCCTGCACAGTAGCAACTAAACTTTCCAGGCGGCCGTAGTACTGCAGTGCCGTTGGGTCTAAA
ACGCGATTGGCGGTAAACCCCTCTGGAATCGA
ATCGCCATCCAACATGTAAAACATCCCAGGAAGCAGCCGTAGCCGATGCTCCGGTGGCATCTCCAAGT
GTTTCTTACCAACAATGTGTGGCACGTGTGTG
GAGGCAGCAAGCGTCATAATCTGCTCATAGTGTGGTGCAACATGCATGTGAAACTCCGGGGTTTCATG
GTTGAGTATTTCCCACGGTAGTTTGTTGTACG
ATACCAAAGAAGGGTACTTTGTGTTGTAGCGAAAACGCTTCGCCAGGCAGAGTGCAGACCGACCAAAC
ATGCTGCTTCCTTCTTATCGTCCGTCGCGTAT
GTGCACACACACACACACACACACACCAGTACAGCTCGCCTTCAGCTGCGCTTTGGAAGCAAGAGGGCAA
ACCACAGACAGAGAGAGAGAGATAGAGAGGAAAA
AAAAAAAAAGGGTGGAGAGAGAAATAAATAAATACATATTGGCAGAAAACCAAAGAAAGTGAGAAAAA
AAAAAGAATGTTTTGAAAGAGTCCACGTATAC
CTGTTTACATTCTATTACATACACAGACATACAGACGGATAGAAAAATGAGACAGATATCTGCACCAA
TAAAATGAGAGAAGTACGAAAAAACAACAACA
ACAAAAACAAAAACAAACAAACTCGAAAGATGAAGCGCTTCACTTTGCCACGGTTGTGTGTACTCGAC
CACTGCCGCTGCCTTCGCCTCCACTACTGTCA
CTTCTCACACCCCACTCATGCCGCGAAATAAAGTGAAGAACATGTTCATGCCGCTTCAGTAACTCTCT
TTGCTGAACCAAATTGAGGTGCATTGCATTCT

TTTCGTCCTCCAGAGCCCTCACCTTCTGCTCGAGCTCATCGATATGGACGGCCTGCCGGTTCCGCTCC
GCCAACAATATGGCATTCTGCGCCTCCAACTG
CTCCACACGTGCTGACGGTGACGGTGTGCGAGGTGCGGCAATTTCTCGTGACCTCAATTCACTCATTA
GTTGCAGATTTTCCTCTGCGATGGCAACTGCC
TCCTGCTTGAGAGTATCTCGCTCTACCTTCAGCTCCGCAACAAGTCGCCGAAGAAACTCTTCCTGATC
ATGGGAGAAATGCCGTTCCGTGGAGGTTTGCA
GATCCGATACATCAGGGTCTAATAAACGCCGCCCATTGGCGCCCCTTAAAACGACATCTACTAGACGG
TTAGACACTTGTGTGCACAACGACCGCATCGC
GTGGGCCTCCCGGCGCGGCTCAACAGTATACTCTTCCAATTCATTCAGAAGATTCATAAGTTGCGTTA
ATTTTCCATTCGCCCCTTCGTCGATGAAGAAG
CAACGTTTCAATAGGAACTTAAGCCACCTCAGCGATCTCTGCGCAATGTACTTCGTCGTCTTCGTTGA
GGTTTCTTTTCTTGTGGCGGCATCGGTGGCTG
CGCTTGGGACTCCGTCGCCGCCATCCCGGTGGACGACGGCATCGCGGAGACTGTCGCTGCCACTCACC
GCGCGGGTGGTTTGCGTCTCCGTCTGCCGCGT
GCGGTGAAGTGCCTCCAGCTGCGTTCTCAGAGCCTCCACAGCACCGCGGAGATGCAAGAGCTCCTCGT
CCCTCTGCCGCAGCTCCCTTTCACGGGCACGA
AGAGAAATTTCCATCTGCGTCAACTTTCCCTGCAGTTCCCCCTGCACGTCGACTCGCTCCTCGACGGT
CACCTCCAGGAGTCTGTTAAGGTGCTCTACCT
CATCTAATATCGCCCGGTACTTTTCATGCGCTGAGACGCGTGATACGCACACGCCGCCAATTGCTCTC
TCTGACGGGGATGCCGCCAGCGTATCCTCACG
GATTAATCGAATCACCTCGTCGCACTCATCGTGAAACGTCCGCCTCGTTGCCGTAGACGAGGTTCTCA
CGGAATTCCGTGAGGGTAACGGGGGCGGTGGC
GTGTACGTTGGGTAGCTTTTTGCCGACGATGGCGTGGCATGCAGAAAGTTGTCCGTCTCTGTCGTCAT
CCTCACCGTCACACACACCACCAACCCTTCTC
CAGCGGCAGGATTGTAGATATATAAATATGTGTGTATAAGTGGGTGTATTGTGTGCAAAGAATGGTCC
TATTTTCAGTACGCCCCTCTCCACCCCCTCCC
CTCCTCCTCCTCTCGGTGGAGGTCCCGCTACAACACAGCCAAAAGCAAAAAAAAAAAAAAAAAAAAAA
AAAAAAAGAAGGCTGCATACATAAAGGCAGGA
TNNNNNNNNNNNNNNNNNNNNNNNNNNNNNNNNNNNNNNNNNNNNNNNNNNNNNNNNNNNNNNNNNNN
NNNNNNNNNNNNNNNNNNNNNNNNNNNNNNNN
NNNNNNNNNNNNNNNNNNNNNNNNNNNNNNNNNNNNNNNNNNNNNNNNNNNNNNNNNNNNNNNNNNNN
NNNNNNNNNNNNNNNNNNNNNNNNNNNNNNNN
NNNNNNNNNNNNNNNNNNNNNNNNNNNNNNNNNNNNNNNNNNNNNNNNNNNNNNNNNNNNNNNNNNNN
NNNNNNNNNNNNNNNNNNNNNNNNNNNNNNNN
NTAAACAGATGGCATGCAGACATGCATCCACACACGCACACACATATAAGAATGCTCACGAACAATAA
AGAAAACAACAACGACAAAATAATAATAATAA
TAATAATCAAAAGCAACAATAAAACGGAATAAACCAATAAAAAACTAACAAAATAAAGAGTGAAGGTG
AAAAAACAAAAACAAACCAACCAAAAAAAAAA
AAAAAAAAAAAAACGGCGTATGTGTTTTCGTTCATCAGCCACAGGAATTATTATTGTGCGGCAAGAAGA
AAAAGAAGAAAAAGAAATAAACGGGAGCGTTA
AGGGTAAAACAAAAAAGAAAAAATATATATTTTTTCTTGAAATTTTACCCTTAGCCCTTATCTTTTGC
ATTCACTTCAGTTAAGAGTTTTTTTTTTCTTT
TTTCCTTTTTTCTACTCCTCCTCCTTTTGTTTTGTTTTGTTTCCCACCCACCCGTTCACAGTCTTCCT
TCCGTCTCCTTTTGAATAAATAATAGCGGCAA
CTTCCTTTCTTTCGTACTCCCCCCCTTCCCTCCCCCCCATAATTATGCCTCCAACACACACACACAGA
GAGAGAGAGCGAGAGGGCGAAAGACGGATATG
TGAAACATCGTCCTTTTTTTCCTTCTTCGTCTTTCCTTTTTTTTTTTTTTTTATTATTTATTNNNNNN
NNNNNNNNNNNNNNNNNNNNNNNNNNNNNNNN
NNNNNNNNNNNNNNNNNNNNNNNNNNNNNNNNNNNNNNNNNNNNNNNNNNNNNNNNNNNNNNNNNNNN
NNNNNNNNNNNNNNNNNNNNNNNNNNNNNNNN
NNNNNNNNNNNNNNNNNNNNNNNNNNNNNNNNNNNNNNNNNNNNNNNNNNNNNNNNNNNNNNNNNNNN
NNNNNNNNNNNNNNNNNNNNNNNNNNNNNNNN
NNNNNNNNNNNNNNNNNNNNNNNNNNNNNNNNNNNNNNNNNNNNNNNNNNNNNNNNNNNNNNNTTAAAT
CATCATCGTTTTCCCTTTTTTAAAAAATTTCC

GCAGTTGAGTTCATTTGTCTCTTCCAGTCAAGATTGCACCCAATTCACCCCCACACATGCACATGCAC
ATGTATATATATATATATATATTACAAGGGGG
GCAGCAGAAGAGAGAGAGGGAGGGAGGGAGAGAGAGAGAAAAATATGTTGAGTAAATCAAAATATTTA
TAATAATAATAATAATAATCCCATCACTTTTC
ACACGCTCACAGATGCATGGTGGATAAAAGTAAAGCCCTACATGCAGGGGATATCTTCCATTCGAAGA
GGATACATACATGAATGGATATATATATGTAT
TATTATCTCACACAAATCCTCTTTCTCCGTATTATTTTACCCATTCACCCCCTCCACCCCCCAAAAAG
TACTCGCAGTAAGGGAGAGCGAGAGAGACAGG
AAGGCAAAGAAACGGTCACACATACACACACACAAAAAAAAAAAAAATGGGTACAAAACAACAACAA
CAACAACAACGGTGATGGTGATGATAGCAGCG
GCACATCTTCTTCACGTTCCCTCTACCGTGGGTGCGCCCCACCCCTCGGTTGAAGGAGGCGACAGCCA
CAACCCCCCACGCAGAGGGACAGAAGTGGGGA
CGGGAAGAGGAGACAGACAGAGAGACATACAGACAGGGGAGAGGCGCCATNNNNNNNNNNNNNNNNNN
NNNNNNNNNNNNNNNNNNNNNNNNNNNNNNNN
NNNNNNNNNNNNNNNNNNNNNNNNNNNNNNNNNNNNNNNNNNNNNNNNNNNNNNNNNNNNNNNNNNNN
NNNNNNNNNNNNNNNNNNNNNNNNNNNNNNNN
NNNNNNNNNNNNNNNNNNNNNNNNNNNNNNNNNNNNNNNNNNNNNNNNNNNNNNNNNNNNNNNNNNNN
NNNNNNNNNNNNNNNNNNNNNNNNNNNNNNNN
NNNNNNNNNNNNNNNNNNNNNNNNNNNNNNNNNNNNNNNNNNNNNNNNNNGAATAGTTGCACGCAACA
GAAATAAAAAAGGACAAAGAAAAAAAAAAAAA
AAAAAAGAAGAGGACGGCACAAACATAATATAAATATATATATAATATAACCAACAGACCAGTAAAAA
TAAAAATAACAATAGAAACAAAGGGAAAAACA
AAAACAAAAAAAGAGGAATGGGGCCCATCGTTCACAGTGACGGCAATAGGAAAAGGAAATGAAAACAA
CATGTCAACCACCATTTGATAGGAGAAAAACG
TACATAAATAAATACAGAGAGAGATATAAAAAAAAAAAAAAAAAAAAAAAAAGAAAGATGGATAAAAC
AGAGAGAAAGAGACATGCACATCCTCAAAATA
AAAATAAAAATAAAAATAAAATAAAAATAAAATAAAACACACGTAATTCAACCCAAAATAACGGCATG
ATCACAAAAGTGACAGACAACAAGAAGCAGAA
AAAGTCCAATCGATAACCCAAATCAAATCAAATCAAAAAAAAAAACAACAACAACAAAATTCGGTGCA
AGGGATACACGCATACATCCACGTACACAACA
AGAGACAAAATAAATAACACACACACACACAAAAACATTAAAAANNNNNNNNNNNNNNNNNNNNNNNN
NNNNNNNNNNNNNNNNNNNNNNNNNNNNNNNN
NNNNNNNNNNNNNNNNNNNNNNNNNNNNNNNNNNNNNNNNNNNNNNNNNNNNNNNNNNNNNNNNNNNN
NNNNNNNNNNNNNNNNNNNNNNNNNNNNNNNN
NNNNNNNNNNNNNNNNNNNNNNNNNNNNNNNNNNNNNNNNNNNNNNNNNNNNNNNNNNNNNNNNNNNN
NNNNNNNNNNNNNNNNNNNNNNNNNNNNNNNN
NNNNNNNNNNNNNNNNNNNNNNNNNNNNNNNNNNNNNNNNNNNNACAGAGAGACAAGCATTTTTCCGA
CTCATTAACACACACGCAGTCTTTGAGTAAAT
AAACCACAACCCCCCCCCCCCCCCCCCCCTGACTACCCCCACACATTCACACACACACACACGCACAT
CCCCCACTCCAACAGCACCACGACGACAAATA
CACGTGACCATAAAAACAAAAGAAAAAAAAACAAAAACAAAAAACAAAAACAAAATGGACGAACTAAA
ATGGAAAACGGGTAAAAACAAAAAACAAAACA
ACAAGCAGCTATGAAACAAACAACAACAAAAAAAAAAAAAAACGTGAAAAAAAAAAAATGGAATCAAA
AAGAAAAAAAAAAGAATATCATAACGAATGCT
TCAATGTAAATAATATTCAAGGAATAAATCGCCAAGAATTCACTGTGCTTGTGTGTGTATACGTGTAT
GCTTAGAAGTGTGTGTGTGGGGGGGCAATATA
TATATATATATATATATAAGAAGGGGCAAGGGGTTTGTTATTATTTTCCTTTAATACTCTCCCTGCGT
GTTTCTCCCCGTATTTTTTAATCTATAACATT
TATAATGTAAATGAGCATATTATATTTGCCAATACATATGTACTTTACGTATTGAAAGGGGTGTAGAT
GCTTCTAAAAATAAACACGTAATTACACACAC
AAACACACACACACACACATACTTATACATATATATAATATAAGTATGTGACTAAGTACCTACAAAAG
TCAAAATTTATCCATCGATTTGTTTTCATTCC
CAATTCGCCGCCGTCCCTCATTGTTTTTACTATTATTCTTCTTAAAAACTACATATATATATATATAT
AGTTTATTTCCTTTGGTGTATTTTTTTTTGCC

TCTTCTCCACCCCCTTTTTTTCCCCTTTAATTTTCTGCGAGTTGCGCTCCGCTCCCTTTTTTTTGTTT
TTCTTTTTTCGGCTTTTGTTGTTTCCTTTGCT
TTTTGCCGGTCGTTGCTTTTTGAACTCAATAGTTTATGTCCCTGCGTCGTTTTTTTCCGCGTCTCCCT
TCGGCGTGTCCTCCTCCCCCTGAGAAGACTTC
TTGCTTCGGGTGTTGTCACCATCATCGCAATGGGCGTGTGCTGTTCTCGACAGTCTTATCAGTTTGCT
GGGGATCGTGAGCCGCAACTCTTTACCATCCA
CGACACCGAGCACATTGAAGAGCTCAGGCCGTCGCATGTATAAAAGTTTGGCCAGCCGCACACACTCC
GCCGCGGAGAAGAAAACGAGGCACAACGTATT
GCCATCGGCATCGTTACCGCAAGTTTTGACAGAGATGCTGCTGCTGCTGTTGCCGTCCCTCCTGCCTG
AAAGCCCGAAGGAGGCGGGAGGGTCATTGTCG
TCGTTGCCGCGCTTGCCCTGGTCAACGCCGTTGGTATTGGGAACGGCGATGTTACGGTTTTGGTTCGG
TCGGGAGGAATGCATCATCACCTCTTGCACAC
AACCAACATGATCCTCAAAGAACTTGTGAACAAACGACGACGCCGTATCTCGTGGAAGGTTACGCACA
GCAAGACCGAGTGAACTACAGCGTCGCGTTCG
CTCCTCCACGTCCATCGACTCCTGATACGCCCGAAGCGCGTCAATAAAACCCAAATTTGGTTCCACGC
GAGGCCGCCGGCGTGTGACGTATTGCAGCGCC
TCTGCTACTGTCCAGCCGTTGCGGTAGATCAGATAGGCGAGCACAATGGTGACGGAGCGGCTCTTCCC
GCGTTGGCAGTGTACCAGAGCGCGGGGACAAA
CACTCGCGCCACGTTGCTTCGCATCATAGTACGCTTTACGGACTTGCTCAAGGAGTACGTGGGTTGGG
CGAAAAAAACTGCTGAATGTTTGCCTCGGTGGT
GTCATCCACCGCAAATGGATGAATAACAATGCTGCGATCAACAGACCAATATTCCTCACGCGATACAT
TAAGAATAGTTACAATATTCTCTCGACGCAAA
AATTCCGCATTTGTGGCATCACGCACACCCCCAATGTAAAGAAAGTCAAGAATTTTTGTCGGTGGCTC
ACGGCCACACGTCGAAAATGAAGAGATTTTTC
CCAAAGAAGAATAAGGTGGTACAGTAGTGCAACTACTCAACAAACTGGATGAGCTACCGTGCGTGGAT
GGGCATTGTTGTTGTTCTTTTTGTGCTGGTGT
TGGTGGTTGACTACTGTGCGGTGCACGGATACACCCTCTCGGGGTGCCGTCACCGCCATTCTTCCTAT
TACTGTTCCTACTAATACTATGACTGTTGCTA
TTCCTATTACTCCTGGTGTTGTTGTCGTTGGTGCTGCTGCTGCTTCTGTCAAGGCGGCAGAGATCGAC
GACGTTCTTGTCGGAGAAGCGGTGATGTCGGC
GGTTGTGTTTCAACTTCCCGCTGCCACGTACGCCGTTCGAATGATACCCACCGCGCGCAAAGGCAGGT
GGTGGTGGTAACGGCGATGGCGGCGGCGGCGG
CGGCAAATCGGAGGCCGCAGTGAAGGGTTGTGCGTTCATTCCTCCTCGCGGCGTTCTCTCTCTGTTTT
TCCTTTTTTTTTCCCCTTTTCCTTCTTCTACC
TCTTTCCACCTCCTTTCCCTTTTTTTGTTTTTTAAAATTTATTTATTTTTCTTGTTTCGCTTTTCCTT
GCCTCTTGTCCGGCCGCCTATCCCGCACTCTG
TCGTTCGCCGGCGTCGGCCGGTGATGGGGGAGAAGGGGATCGGTGATTGCAGGCTGAAAAAGAAAAAA
AAAAAGAAAGACAGCGTGCATACATTTTTATT
TATTGCAATCATACACTTGTTTTTCTCGCTATCGTGTGTACAGAGGTACGCACAGCACAGAGGTGAGT
CGTATCAACAACGCCGATAGCAACAGGGCGGT
GGCCCTTCATGTACCCCAGTGCATTCGTAATTACAGCTGTGAGGCGTGAAAAGTCAAGTAAAAATGGC
AGACAAACAGACAAAAAAAAAAAGACAAAAAA
AAAAAGATTTCACGGTCCTTGTACTAACGCCACACAAAAAACAACAAGATAATAATAATAATAGTAAT
AATAATAATAATAATAAAGCCACACACGTCAA
AAAGCCTCCTGTATCATGAGTGAGGCGATTTCTTGTACCAGCGATTCCGCCAAACAGCTCTGATGGGC
CTTCACCATGATAGTTATACCCTTCTTGTCCC
CCTCCACCGTGTCCTTGCGCAGAAGTACAGCAACGTGTGCCTTCTCTGCCGTCGACTTCCGCGCATGC
AACACACCGTAAAATGATGCGACATCCTTAAA
TATTTCAACCGGTTTCAGCCGTAGTTTCTGTTGTATCAGCGGCATTGCAAGTGGCACATGCGTCAAAT
TGACGTCAACAAATCCAGATACCATTGGTGCC
GCCAACAGAAACTTCCCAAGGATGGTCTGACTGAATTCTGCAGGCGGAACGTCCTTCGGTATCTTCGA
GAAATAAGGATAAAAGAGAGGCAACTGTAACG
TTGTGGCTGCCGGTTTCTTGCCTCGCGTGTAAGCAAGAATAAACGTGAGGGGCTTGACAAGCGAGGAT
GGAAGCTGCGAAATAAATCGAATACCAATTTT

CCTGCACACAGACTTTGAGCCCTTGATACGTTCAGCGAGACACACACCTGTGACGCCGCTCCCACCAT
TCCCCTTTTCTTCTTCTTCCTTCTCCTCCCCC
TTTTTTTTCTGCTGCTGCTGTTGATTTGAATCATCTATATTTTCCCTCTTTTTTTTTTTATCATCATT
CTTATGAACATTATTTGAATTGCTGTTGTTTG
TGTCACTGAAGTCAAGTGACTCCAACTGTACACTTTGCTCTGTAAAATTGTCTTCATCGTATTTAATG
CAAAGCTGGACGTCATATGCGCTGCTGCTACT
TGCCAGATTTGACACTTCCACTTCGAGAGTTAGCAATGTGCACTCCTTATGCACATGGCATGATACCA
CATTCGCCGTAACACGAAGGTGCCGTTCTTCT
AGTAGCACAACGGGGTCAAACACGGCCGCTTCTGCTGCTGCTTCCGCTGCCCGTGCTTCCTCCTTTCC
ACTTCCCACAGAAGCACGGAGCTGCAACGATT
GTGTGTATGGGATTGGCTCTGGGAGCTTCTCATCCGCTGTAAGGGCCCGCGTGACGTCAACATTGCGG
AACTTTTTCGTAGCCTCATCTTCCTCCACTTC
TTCGTTGCGTCGTCGTGCGGGGTTCGCCGCGACATAATTAGACGGCCGTAAAAGTTCACGGTTAATGG
AATGTGTCTTGCGTGGCACATGGAGACCGGCG
CCATGAAGGTGCAGGGAGGCCGCTGTAACCTTCTCCAGTCGGACATCAACGGGCAACTCCTCTCGCGT
GAGCGTCGTGTCTTTGATGTAAAATGCTGCCA
CCTCTCCACGCCGCTCCTCTTCCTTCCGTCGCTGAAGCTCCACCGCGTCGCCACCCAAAAAGTCAAAC
TCGTCTTCGTCACCGTCCATGTTCTCATCATC
GTCGCTATCGGATGGCTGCAGTAACGGGGGGAGGTGCGTGCAGAATGGTTCGCCGAGATCAAGATCAT
CCGGCGGTTTCACCGCATCCTGCGCTCCCACG
GCGACAGACAACAACTCCTCCACGTAGAAGGTTGGGCCAATATCTGGGTTCGTGTCGACATGGTAAAA
GACCAAATGGGCCCGTTCCTGCACGTCTGGGT
AAATGCTGTGACGAAAGAGCTCTAGGCCAGAGTACTTCTCCTTCCCCTTGTCATCATCTCCGACGTCC
TTTTCCTTTTCAGAATCCTGTTGCGGGAGAAT
GACCGCCCGTAATTCGGCATCTGTCACTGGATCGTCTGGTAGTTTTAACTCCTCCTCACCATGTGACA
AAGTTACATGTCGTGGACAAGGCTGATGCACA
AACGCTGCTATTTTCCCCACTGCAGCAACACACGCCACCTGCGCCTCGGGGTGCATCGCGGCAATGCG
ATCACTCAAAAGTCGTTCGCATGTGAGACGTT
TATCCAGCAGCCAGTAGGGATACTCACCGCAGAGAAAAGCCGCCGCTTTAATTATCTGCCACTGAGTT
GAGCGTGTAGCGTCACAGTTGAGCAGCGTTGA
ATTACACAGCAGTTCACTTACTGCTTGAACACCAAACTGCCGCACCGCATTCACACGCGTCAGTACCG
TTACCAACTCTCGCTGCACAAGAGCACCATGT
TGATAGGCGGTGAGATTCACGAGACTGATGTCCAGTAGTATACTAAAGTACCACTCAAAGTCTTGCAA
CAGGGTATAGTCATCGGTCTGGGCAACTTCAA
TGACTGTGGCGATAACACGGTTTGACCACTCCTCATCCGGGGGAGACCGCACACGTCGCTCCATCATA
CTGTTTATGGTGGATACAATATTCTTTTTTGT
TACAATTCCTTGCAGAATCTCCAGCGCTTTACGTCGGATGGTGGCATCCATGTCATTCAGACATGCAA
GAATGACTTCCCGTTGATCAATAATAAGTTTC
GGATTTTCTCGCATCATGCGAGACATGGCATCAAGCCCCAAATACTTTAAATTTTGATCGGCATCCTC
CACAAATAGTTTTATTTTCTCTGCCGCCAACT
TCGTTAATGATGGCACTTTACTCATGCCATTGGCTACAGAGAGTATGCATTCATATTGAACAGATTTT
GCTCCTGTTGCACAAATGAGGTTCGCTATCGG
CTCCACAAGTTTCTTCCCCAGGCGAGGTTCATGTGGGGCAAAATAACCAAACACCTTCACAATTTTAA
TAAGCGTCCAATTGCTGTGTACGGTGGAAAGC
ATCGAGAAAAAGGGCACAGCAAGACCAAGAAAATTCGCTGGATTGCGGCGAGCAAGTTCACACAGGAT
ACACACCACCGCCCCACGTACGGCAGGATCAT
TGTCGCAACGTTCGCTGTGATCATCCAGCTTTTCCTTTAGCCGTGGGTACGTTGGCCGCAGGGCGTCA
GGATACTCAAAAAATATGCGATACAGACTAAG
CACCGCCTTCTTGCGGACGTAATTGCGTGGATGATTCAGCAGATTCACAACATCCACAACAAGGTCCC
TCGCAATGTCCTTTGTGCAGATAGAGGAAATA
CAGTAAAGCGCAAACCCCACTTCGTACTGATTCGCACTTGCGAGATCGCGCTTCAGCAATGCCGTCAG
AAGCGGCAAGACGTCTGTCTTCGGGGTGAAGG
TGATGCATGCCGCCATGTATCCAATACGCTTGTGCGCAAATGATCTATCAGCCATGACTTCAATAATG
TTAAACGCCCCGTATGCTGCGGAGTAACCGAG

CATAGAGAAGTACACCGCCTTCAGCACCGCTGTGACCTTCACACTCTGCACCGTGGATGTAATCTCCT
TCCGTGTCTCCGCGATGCATTGCTCAATCACC
TCCGCCTCGCTCTTCTTGGCGGAGCGCAACTTCCGCACAACTTCCGCAAGGGAGTTTTGGAAAAGAAT
CTGACTCGCCACCCGCGTTGCCTCCGCCATCA
CAAAGCCGCaGTGGCAGTACTAACCGGGGGGGGGGGGGGGGGGAAACCGACCAGTCGGTGCAGCAGGT
ACCGCAAACAATTCTAAAACAAACCAACCAAA
CAAAAACAAAAAGAAATTAATCAGTTCACAGCCAAAAGAGCAACTGACTTGTGTACAAACAAAAAGGA
AAAAAACAACAACAAAGGAATAAGAAGCAAGA
AAGAGAAAACAAGTGAACAAACAAAATGTAATGGCACACACACACATCAATAAAAACAACAACTAATG
AACAGACAGGAGCAGACGCGCGACTTCTTTAT
TATTTTTTTTTTTTTTAAAGGAAGGGAAGTTCCGCAGCAAGAGAAAAAACAATGAAATGAAGACGCAG
CAGCAGCAGCAGCCTAAAGAAAGCAAAACAAA
ACAAAGAATAATAATAATAATAATAAAGAAAAAGAGAAACGTTTATTCGTTTTGCATCCAAAAGGAAA
TGGGCGGTAATGACAATATCAAACGTAGAAGC
CATCACATCTCTTGAAGAAACTGTCCAACCGCCCAGCACCCTTCTTCTTTTCGTTACTCTCCTGTACT
TCCCTTCCCACCACATGTTGCATCCTCTTTTT
CTTTTTTTTTTTTCTTTTTTTTTTTTTTTTCCTTTTCATTGTGTGTGTAAAGGGGCATTATTCCCATC
CCTCTCTTTCCCTCTGTCCCCTTCCTTACAGT
TCGGCCCCAAGAGCCGACACTTTATTCTGAATTCGGCTCTGCGCCTCTTCCTTGATTTCGTTCAGCTT
CTTGACCGCTGCACGCACAGCCTCCACGGTCT
TTCCAATAAGTAGCAGGTACAGCGGCCGCACGCCCTCACGCAGGCGGTGGGAGTGTTTGTACTTTCGG
TCAAAAAAGACACCCTTTCGGATTATTGTAGT
TCCCGTTTCCTCCTGCACAGAACGCAACAAATTCGCGCTCTGCATCCGCAACCGAATGCGCTCGGGAA
GATCGTTGATGGGATACTCGGCCTCAAAACGA
GCACATGTACCGGAAGGATCAACAATGGAGTCAGTCGTCTTCCGCGCGTACGCAAGTGCAGATGCGAG
ACGTTCCTGTTGTTCTTGTTGCTGCTGCGAGG
AGAGTGTAAGGGCGCTAGTGTCCCTGTACAACATGAGGGCTGTAGAATCCTCCTCATTGCGACTGCCA
CCACCTCGGGTTCCATCTTCGTGCACTGCAAC
AATCTCAATACCGTCCTCCTTCCCATCTTCATCACCTTCAGACAAATCCGCTGTACAACATGAGGGCT
GTAGAATCCTCCTCATTGCGACTGCCACCACC
TCGGGTTCCATCTTCGTGCACTGCAACAATCTCAATACCGTCCTCCTTCCCATCTTCATCACCTTCAG
ACAAATCCGAGTCCGTGCAGGAGCTGGCGGAT
AAATACTCTTCAAGCCCTGCTGCCCGCGCTGCCGCCTTAAATTGTTCTTCTTGACCGCGGTCTGTAAA
GCGTAGCTTCTTTGCCTTCATGTAACCCCTGT
AAAACCCCGTGTTGTGCCGTACAATGCCCTCCTTACGTTTCTCTCGTAACTTTGCAGCCCGCTCATAT
AGTTCCTCTGGTACCTGCTGCTGTGCACGTTC
AAGGCCCTCACAGAGTTCCACGGATAAGTCGTCATCCTTCCCACGCATAAAAAAGCTTACCGCCTTGC
CTTTTTTACCAGCGCGACCGGTGCGACCTATG
CGATGCACGTAAGCTTCGTAGTGATCTGGAAGTGTGTAGTTAATGACCAACTCCAGATACGGGATGTC
AAGACCCCGCTCCGCAACACCTGTTGCAATAA
GAATAAACTGATTGCCAGGAGCAAAATGCTCCAAGGCAAACTCACGATCCAATGGGTCCATGCCACTA
TACAAAACGGCAATACGCTTCTGGTAACCACA
GGCAGAAAGACGCGCAAAGAGTTCGTCACACTCTTCCTTGCGTTGGGCAAATATGATAATGAGATGCT
GTCCAGTACCACCCTCATCACTGAGAATTTGC
AGCAGTTTTAAAAACTTTTTATCTTCTGTGCGATTCGCCTCCTCCACTTCATACACTTCTTCATCAAA
AAAGAAAAAGCGCTGCTCGACATTCGAGGCGG
GAGTAGGTTTGCCCCCCACTGTAACCTCCACAGGATCTTGCATATGACGCATGATGACTTTCTTCAGT
TCTTTTGGCATGGTTGCACTAATGAGCGCCAA
TTGTCTGTCAGGACGAATATTTTTTAAAAAGGCCTCCACGTGTTCCATGAAGCCACTGTCAAACATGC
GGTCTGCCTCATCAATCACGACAAAAGATGTG
CGCCGCAACGAAATGACGGCTCCACCATTCACGGTGAGAAGATCCAAAAGTCGACCTGGGGTGCACAC
CATGACTTCACATCCTGCTCTGCAGTGTCTGA
TGTTGTCTGCAAGCGGTGTTAGGCCGTACGAGGAAACAAATCTCAGCTTGGCTGCCACACACAGCTTT
TCTGCcAGTTGGGCAATTTGCGCAGCCAGTTC

ACGTGTGGGAACAAAAATAAGGCCAATAGGCCCTTCACCCTTTCCACATGGTTCTTGCCCAACACAAT
GACGAATAAGCGGCAGGAGATAGGCGAGTGTC
TTTCCAGAACCTGTTTTGGCTGTAATAAGGAGGTCGCGACCGCTCATCAACGCTGGAGAACCAAGAGA
CTGCACGGCAAATGGTTGTTCAAACCCTTCAC
GCGCTAGCAAATCAAGAACTGAATCTGGTAGACCTGAACCGTTCCAAGTACGCATGGGTCGAGGTGGA
TCGCGTCCACGCAATTTTGCTCCATCCAGCTC
CGCTATGAGCCGCTTTAACTCATCCGCATCGAGGTCCTTGACGTCTGGAGGGGCAACGTAAAACTCCT
TTCGAATATGCGGATATTGTATGGTGCTGTGG
TCTACATATTGCAACTTACGCAGCCGTTTCACATCCTCAGGGGGAGGAGGCGGTAGTTTGGCCGACAC
AATTTCTGCCATAAGATCGTGCGACTCATCTG
TTGCACTCACAACCGTGAGCGCAGTTGACATGACGGGCTTCAGCAACTCCTCCTTAAGATGCTGCAGC
GCATGCGCGGGTGTTACACCGGCGACGGTTGA
GTCTACAGTACCAACTCGCTTTGCAGCGCAGGTGAGTTGAACGTGGGCAAGTAGTCTCTCCCGAAAGT
CCTCGATGTCGCCATCGTTTTCGCCCGTATCA
GCCTCACCTTCGCTTGGTGTGGACGCGGCAGCTCCAAAGGGATCATCGCCGGAGTCCTCATCGCTCAG
CACAACATTTTCATCCACACGAAGGGTACTAT
TCAGAATACTATTTTTATGATGATTGCCTTTTATTTCCCCACTGTTGCCCGTGTGATGCTGCCGGGCA
TTTTCATCATCATCGTAGGAGTCACTGCTATT
CCAGGCCCCACGGCGTTGCTCCAGCAACGATATCTCCTCGTCCCGCGTCTCAAGCAGAGCTTTGCGCT
CCGCGAGTTTTTCCCATGTGGCAGCGGCTGAC
TCCGGCTTATCGTTGTTGCTTTCACTATCCTTATTTTTACAACGTGGGTGTTTCCGCTTTCCCCGTGT
GTCATTCCTATAATCGCTGGAGGAAGAGAAAA
GCGAATCTGAGGAGCTTTTGCTGCTCCCGCTGCTCTCCCTCTCACGCTTACGCATACCCCCCCCTCCT
CCCCCGCCTCTCGTTCGTTCGTTTTCCCAAAA
GAATAAAATCACCTCAACCCCCACACTTTCTTTCTTTCTCTCTCCCTCTCTTTATTTATTTATTTATT
GTACGGTTTCGTGTGGATAAGTAAACAGATGC
ACACATAAATACACACACAACCACACACATACACGCAGGTGCCGCAGAGAACAGAACGCCACGAACAC
ACGCACGCACAAGAAAAGCAGAGGAACACAAC
ACAAGGGAGAAAAGAAACAGGAGCAGCATTTGTGGAAAAGAAAAGAGGCATTCACATCACACAACGGA
TGTAACGTCAACAGAAACAACACAACGCTTAG
TATCAACCGACCAAAAAAGTGACCAAAAGAAACACGCAAACAGAATGAAAACAAAAAAAAGAAGATCT
ACCGTTTACAATGGTCCCAATTACATAATCTC
CAAAAGTTGCTTTCGCGCGGCAGTGTGCACTTTGTGATCCATACGAAATAACTTATGATCACCGTTCA
TGGGCAATGGAAAAGGCAGCGCCTTGGCTTCC
GCACAGATGAACGTAAACGGTGAGTCTACTTGTATTTCTTCTTCAGTGAACATCTCACGGTACGTGTC
ACGGTCCAGACACTCCACCAGGTAAACATATC
CCAGCAACACGCGTGTAGGATAATGTTTTGGAAATTGTTGAGTCGGTTTCATAAACTTTGAGTAGTGT
TCCTCTACTTCTTTAATATCGTGTGGCTGTGT
CGAGGCTGCGTGGATCCAAAGACGACCCCTATAATCTGTATGCCACACACGTCCCTCGTGGAACTTGA
TGCCACTCACAAGTAGCCCTGCCCATGGCTGA
TGCATTGATAGGCAGATACCGTCATCATTTATGCGCATCGTGAGTGAAGGTGTAAAACGGGTGGACAC
ACCAGTTGAAATGTCTGCCACCACGGCAGTCT
CCCCTGTTGGCGCCACATTCGTCTCGTCTATGGTTGATTCCTCCGCATCCGCACCGTCATGCGCCGCA
TCAAGACCATCCGATTCAACCTGAGAATGCTC
CTCCTGTCGTTCTTTCTGCGCCTCCAGAAATAAATGCGCGTCGTCTTCGTAGTAATTGACCTGAACGC
GACGTGACAGTTCTACCACACGACGTCCTGTG
GGTTTTTTCACCGGTGACTCCTCTCTGTTTGTGACGCCGGTGCTACTCGCTGTGGAGGGTTTCTCAAC
ACTGCCATCCAGACTATACCATATTTTTTGCA
TGAGAGAGGGGAGTGGTAATGCCCGTGCTTCCTCAACGTTGTATTCGCCGTCGTCTCCTTCTTCAGCG
CAACAGCTAACATACGACTCAGAGGAGGCAAG
ACTATTCGACTCCACCGCTGGACCTTCTGGGCAGCCGAGTGATACGTTTTGGTTCATGATATCCAAGT
GAACCCTATACGCCCCCTTGGCGCGGTGCATT
GCGGCCACTTTGCGACGCCGTTCTGCAAGCGCTTCGTCCTCATCTGCCTTTTGGCGTTCCTTTGGTGA
CATCCACGCATTCTTCGGTGATAAAAAGACGG

CTGTCTGATCGTCAATCACCTTTGTACGCTTAGCACGTGTCTCTGCGTAGTGCAAGAGCTCATCCCGC
TTCCTCACGGATGCCTCGTATTCTTCTTGATT
TTTCGCTACCGCGGCTGCACCTATGAGGCCCTCCTGTACCTTGATTTCATACGTCACACACGTTGATG
GGTCCAACCCACAGAAGTAACATGTCTCATTC
GACTCCTGTTCACATATGATGCGTCCACAGTTGGCACAGTTCCCGCGAAAAGCGTGTGTTGTGGCAAA
ACAACCGCATTCAACAAATCCTGGTTTCAACG
CAGCGGCGCCACTTTTATCGGCAGCTTTTGATAACAACGTGCCTTTCCGTTTCCCAGCCTTCCTCCCT
GTTGCCGCCAGCTGTCGTCCCTTACCTCTATT
TCCATCCGCACTCCTCCCCTCTGGGGCAGACATTGTGGGCGCAGCAGGGGTTTCATCAAAGGGAGGCC
CTTGATCGACACGTCGCTTTGTCACCTCTGCC
GCAACGGCCTCCCCGATCATAAGTGTCTCACACCAGTCAAACACCTCCCGCTTAGAGCCACATGTCGC
CAGCATGTTCGCCATGTTGTCCAACTCCTCAC
CGACAAGACCAAGAAAGTTGTTGTCCTCAATCTCCTTCTTGATGTAACGGACAAGAGCCTCCTTGATC
GTCTCCGCAGGGCTCATCATGGAAGGAGCATT
CCATCGTTTGGGTGGCATCCGTCCTTGCAAAGCACACACTTTCAACAAAAAGCAACGTCCGAAATATA
TATATTACCAATAGACCAAAAAAAAAAAAAAA
AAAAACAAACAAACGGAGAGAGACAAATAAAAGAGACAGCAGCATCAACAACAGAAGAAAAAGGATGG
TAATAGTGGGGGAAAAAAATAAACACACACAC
ACACACACACACACACACACACAAATACACACAAGAAAGAGAAGCATCCCCCAGTCCGTCCCATATAT
ATATTTATATACCGGATAGAGGTAGACACTTC
AGAAATGAGGCCTGTTTAAGAGTTCACATTTGTTTTATGTCTCCAGAACATCAGTATTGGCAAAATAC
GGCAGCATAATGCCTTCGCGCCACGTTATTTT
TCTCCTTACTGCAAGCAACATCTTGCCAATATCCCCGTGACAGACAAGCAGTACACGTTTTCCAGCAA
AGTTGGCGTCCACACGCTGTAAAACACGGGCA
GCACGATCATAGCATTCGTCAAACGTTTCCGCCCCATCGACAGAAAGAAAATACAAGACCCTGTCCCC
TTGTACGACATTTTCCCCGGCGTGCGTTCTGA
TCTGTTCCATAGGCTTTCCCGTCAGCACACCAAATTCACGCTCCACTAGTTCATTCTCCGTCTCCACA
TTAACAGAAAGAGCCTCACCAATGATGCGAGC
GGTCTCATTCGCCCGCTGCAATGGTGATGTTAATATGATGTCATACGTCATTCCTCTCTCCTTCAATT
TCTGCGATAGCGCAGTCGCCTGCTCCCGCCCC
AACTGTGTGAGTGGGCGATCACGTCGACCGTTCAAAAGCCCTTCAAAATTGTCTTCGTCTTGACCATG
TCTGCAGATGTGTACAACAGCCATGAACCAAA
GCGGAACGATTACGACGAGGGCTCCCTTTCACTGGAATGAACGCAGAACAATTAAAAAGCAATAATAA
AAGGAAAAGAAAAGAAAATAAAAAAAAAAAAA
ATGAGACTCCACAATTACCCTTTTATGCACCCCTTTGGTATACATGCGGTATATGGAGTAGGATCAAG
GACAAAATATCAACAGGCCTACAGACTCTGTC
GCGACATCTCCTTCTCGCTCACTCATGAGGGAGAAAACCAATGGCATCTTGGGTCGTTCTATACGAGA
AACGTCACGGTAAACACAAAGTTCGCTTCTCG
GCGAAAACAACCCTAAAACTGTGACTCAGACCACATCCCCACAGAACGCATCACCCGGAAAAAAAAAT
TTCTACGGATAACGACAGGCATTCACGCAAAT
AACACTTCCCTCCCAGTTTTCCCTTTCCATTTTCTTCTTTATAATATATATTATAAATTCCTTCTCCT
TTTTGCATTTTCATCTCCATTTTTTTCTTCTA
TTTTTTTTTTTTTTTTTTTTGGCCCCCCTCTGTCCTTCGCTATGTTACATCTCCTTCTTGCCTCCCGC
CCCACAAAAAGTGCAAGGAAAAACACAAGAGA
GAAAGAAAGAAAAAAAAAAAAAAAAAAACCATGAGGACATACACACACACACAGACAGAGGGCAATAAT
ACATATATATGTTGCTGCATTTATGTAATTCA
TCAAAGAGGCGGCATAAAAGACTGCACGATGTTGGCGAACTGCAGAAATAATATCAACGCCGTGGGAA
TGTTTGTGAGGGTAGGAAGGGGGGAAAAGTCT
TTTTTGTTTCTCCCGTTCCATTGTCTTTTTACACATTACGGGGGTAGGGAAACGGAGAAGGAAAAGCA
CGCGCATCGTCATAAACAAAAACAAAACAAAC
ACACACACACACACACACAGAGAGAAAAAAAACGGGCACAAATGCATTTTAAGAATTCCGTCCTTTTC
CTTTTTTCCTTCTCCTTCTCCTTCTTCTTCTT
CCTTTTTTTTTCTCCATCATCCCTCGTTTAATTTGCGAGTTCCAGAGGGAGGGGTGTAGAGAACGGTG
TGCGGCCTACGGTCTTTCCATGCCCACTAGAA

AAGGAAACTGCCTCCCGCCCTCCCCCCCTTCCTTCGCCTTCGCCATCGCCTGCCACTCCGACCACGAA
AGAAGTACATGAAGTGCGCTCGGCTCGTGGTC
GTCCAGAAGCGACTCATTCACTTCACGGGCAATTCTGCACGGGGATGCCTCCGAACACCCCGTCGCGT
CCGTCCCCGTGATGGTTGCCAAGGTTTCAACA
TAAATTTGATGTGCCATCGCTGCATCAGACATGTCGGCAATCAGTGGACTCACCTTTTCACATAGAAA
GAGTAACGCCGCTGATTGATCACCCCTTGCAC
GCTTCATGGCTTCCATGGCCAACAGTTTTACTATGAACACACTTCCCTGAGGAAGGGATGCGGCCGCG
TCCTCGTACTGCTCATTTAGACACAATAACTG
CAGACGCTTGCGCTTCTTCATGGACTCCAGTACAGTTTCAACACCTTTACAATCAACTGAATTATTAT
TATTAGTATTATTATTTTTATTATCATTGGAG
ACATGAATTATACCACTGTTTTTTCCGCTTCCATTGCTATTCTTCCTTCCATCATCATGTGACACGAC
ATCGTTGCGTAATTCCTCTTGGTTGACAAAAA
TGCCTTTGCCATTACGCCGTACACGACGCCCGGTCACCTCCTTCGTTTCTGTTGTTGCCGATGCTTCC
GCCATCGGTGTCGTCCCTATCCTTGTCATTGT
CTGCAGTTCTCGTAGCTCCTCATTAAGTGCGGTGAGAGATTCCACAAATCCCTGTCGGATGAAATACG
ACTGCAGGATGGAGGCAAGAACAATTTCCTCA
TCGTAGCCTTTGTCACGTATCACCGCCTCCTCCACAAATTTCCAACCAACGTTCTCGTACGACCCCAT
TTATTCCCTCCTTGTCCTTTTTTTTTTTTTTT
CCCTTCTTCTTCTTCTTCGTCCTCNNNNNNNNNNNNNNNNNNNNNNNNNNNNNNNNNNNNNNNNNNNN
NNNNNNNNNNNNNNNNNNNNNNNNNNNNNNNN
NNNNNNNNNNNNNNNNNNNNNNNNNNNNNNNNNNNNNNNNNNNNNNNNNNNNNNNNNNNNNNNNNNNN
NNNNNNNNNNNNNNNNNNNNNNNNNNNNNNNN
NNNNNNNNNNNNNNNNNNNNNNNNNNNNNNNNNNNNNNNNNNNNNNNNNNNNNNNNNNNNNNNNNNNN
NNNNNNNNNNNNNNNNNNNNNNNNNNNNNNNN
NNNNNNNNNNNNNNNNNNNNNNNNCCTTCCTTCACTCACACTCTCACACCCGCTCGTTCACAGGGTAA
AAACTAAACACACACACAAAAAAAAAAAAAAA
AAAGAAAAAAAGGAAAAACAACACACACAAAACCGACCAAACAAACTTCTCGTTGCTGTAGAATCACAG
AATGGACTGTTTGTGCTCGTGTGTGTGTTGCC
GCCTTGACCTACCTACCCCGTTGTGATGCGCACGGGGAGAATGCAACCTAAAACAAACCATAAGAAAA
GGAAAAGAGGCATGTTACGGAAACACACACAG
AATGCACAGATAGAAAAGAAAAGAAAAGAAAAGAAAGAAAAAAAAAAAGGAGGAGGTGAATAAAAACCA
AATAAGACAAAACACCGCATTCACTCCTTCAG
TTTTATGTTCCCTCTTTTTGACGCATAAGTCCATAAACTGCAAGGGATAAGAGAGTTTTACACTCAGT
TCACAGGGGGATACCTCTAAATTTTAATATTA
TTTCCGAATAATTCAAATTGCATTTTTTTTTTCCTCTTCTTTTTTTTTTTTTTTTGACATTCCTTCCT
ATTTCCTTTTTCCCCTCATTGCAGTTTTTTTT
TTTTTTTTCCCCACTCCCTCCCAGTTCCCCCCNNNNNNNNNNNNNNNNNNNNNNNNNNNNNNNNNNNN
NNNNNNNNNNNNNNNNNNNNNNNNNNNNNNNN
NNNNNNNNNNNNNNNNNNNNNNNNNNNNNNNNNNNNNNNNNNNNNNNNNNNNNNNNNNNNNNNNNNNN
NNNNNNNNNNNNNNNNNNNNNNNNNNNNNNNN
NNNNNNNNNNNNNNNNNNNNNNNNNNNNNNNNNNNNNNNNNNNNNNNNNNNNNNNNNNNNNNNNNNNN
NNNNNNNNNNNNNNNNNNNNNNNNNNNNNNNN
NNNNNNNNNNNNNNNNNNNNNNNNNNNNNNNNNCCCCCTCTATTTGAAAAAGGAAGGAGAAATCAGTTT
GTTTTTTTTTCCTCTTTACTTTGGATTCATTT
TCTTCATAACGAGGGGTGTATGCCCAAAGGCTCCGGCCTCCATGAGTTCTTATATACGCACATCTCCC
CTCATACAGACCCATATATATATATATATATA
GAGAGAGAGAGAGAGGAAGAGGAAGAGGAGGCAATAGACATGTGGAAGTACATAAATTACGGTTACAC
ATGCATATGCATAGGAGTAAAGATATGTGTAA
ATCAGGAGCACACACACACACACACATATATATGTATATATATACCCCCAATGAAATTGTCAACTCCC
ACTGTTTCACTTCTCTCATATTCCAATGAGAG
GGGCGATTAAACCGGCAAAAAGAAGACATAAGTATATATACCTTCTGGAAGTGGGAACATCAGGCCAT
TTCCCTCTCAACTGTATGCGTGCACGCAAAAA
AAAGGCTGCAAAAAAAGAAAAGAAAAAGTAAAGGTTGGATTACCATTAAAGAAATAAACAGGCAAAGC
AAAAAGAAAAAGGAAAAAAAAAAAAAACCGCA

AAATTCACATTGGCACACAGAATGGAACGCACACAAAAATAACGACATCCCATTTTTTTTCCATCCAT
CGTACTTTTCCTTCTACCAATCTCCTTTTCTC
CCTTACTTTCCTTCTCTCTCTCCCTCTGCCCCTGTGGGATTTTTCCTTTCGCTCTATTCTTCCATTCC
CCACCCACAACGTTCTCTCCCGTTCTCTCTCA
TCCCCCCTCAAAGCAATCGCCACACACACAGACAAAAAAAATATTTCTCACTTGCAATTTGAAAAATT
TGGCTTGCACGCAATGACCACAATAATGAGAA
TGACGACGCCAACTGTTCCAATAATCATAAGTGTAAGTTTAATATTTTTCAGGCACATACTCCGCTTT
AGTTGTGTTGATCGCTGCTTAAATTGTTCCGC
CTGGTCGGCAAGTGTGTTGGATTTCTCGTGCAGCGTGTCGACACGGTCGCCGCGGGCGAGGACCTTAT
CCATGTTGTCCATCATGACGTCTACTACTTGA
TTGATGTCATCATTCAGCGCCGTGATCTTGTCGTTCTGAGGATTATTGTAGAAATCCATCTTCTGCTG
CAGGAGCTTTTTGCCGTTGCGGAGCTCCGTGC
CGGGGGGGCCAACGTTTCCCCGCACAAGCGGCTCGACCGCTTCCAGGAACGCAAACACCGTTCGCATA
CGCATTTCGTTGGTCGTCACGCATGCCACGAT
GCGGCCCTCGCCGTTGCTGATGTAGTGGTAATTGACACCCCCCTCCTTGTCTTCAATCGTCTTGCGGC
GGTACTCCTTCCCAGGGACGGTGGCGATCACC
TTCTGCATCGTCTCCGCCAGCTTCGGCTGCTCGCCAGGCGGGTACTCCGCCAGCAAGACGGTGCCGTC
GTTGATGCAGCTATATTTGATTGGCATCCGTC
ACGACGGGTTAAAAAACAAAAACAAAAACAAAAGCAAAACAAAAAAAACTCCCTTTTTTTTCTTTTGG
ACCCTTCCAACACACACACACACAAAAAAAAA
AAAAAAAGGAAGGGGAGTAAGAAGATGACATCTTAGAAAATAGGACACATGGCGCGTATCCGCAACTC
CTAAAATATAATTCCACAGGTTCATCACTCTT
TTCTTCCCCCGTTCTTTCCTTTTTTTTTTTTTTAAATTATAAAAGGGACTGACAGGAAGGAAGTACCG
CCTTGGGCATCACTCCTTTCTCTCCCCAGTTT
CATTCCGATAGAAACTATTCGCTTTTCCACACCAGGAACACACAAAAACACGTCAGATCCCATCGGCA
GTTTCAACTCACGCACCCCCTCTCACAGACAT
GCATAAACACAAAAGGAAACAGTGTCCTTATAAATATCTCTGTAACAATTGTCGACCCGTGATGGTTT
GCTCGTTTGAGCTACCAAGCGCTTCAAATATA
CCGAGAATGACTTGTCTTGCAGGCGTCTTGGCGTGTGGACCAAGTACAAGGTCCTGCGGCACCACCCC
CGCTTCCTTTAGCGCAGGCAGTAACTTTGGCT
CAGCGCGAATGAGTCGCAAACACTCTTCGATGCCCTTGTGGTGGCGTTTCTCTACAAAATGTGCCACG
GCCAACTTGAGATGCTGGCGATACAACGCAGC
CGGGTCGTGTGTAAGGTCATCGTATGTGAGCAGCGACACATAATTGTCCTTTTCCACGTCAAAGTCAG
CAATGATCAGTAACTCAATGCGATTCACCGCC
TCTGCCACATCACGCAGATCCCGTGTGGCAAAAGGAAACTCCTCGCGTACCCGCTGCACGATGCTTTG
CGCCTCCGCGTTGTCTTTCGTGGCCATCGCAC
ACATGGCAAGACCGCAAAGTGCCTGTGGACTCACGTTATAGCACCCCTCACGCTTGAGACGCCCCCAC
ATCTCTTGTGTCACCTTCCTTTCCCTTGAAAA
CCCCAGCCTTTCACGCGCCACCTGCGTCTCCGCCATGGCCATATCATATGCCTTTTTGAAGAGCTCAC
GAGCCTTTGGGATCTCTTTATTTTTCAATCGA
GTGTGAGCGACGTTCAACAAAGTCATGGCATTCTCATCGTCCTCGTCGCTACGCTTCACCTTTTGAAA
AACGGACGTGCCGCTCATCTCATTTTTGCTCT
CCTCCACGGCGTAGTCAATAAACGCATCAATCGCCTCCTTTACCTGTGACTCGGGGACAATCCCCACC
ATCTTGTCAGCGTACACCTTGTTGCGGACAAA
GTACACAATGGGAAACATGTGCGGGTCGACACTAAATTTTTCCGTCAATCCCACTTCCCGGAGACAGT
CGACAAGCCCTAATTTTATAGCAAGGCCTCGA
TCTTTGCCAAACTCCTGGTACTTGTCAGAAATGCCGCTACTTTGCAGGCGGCGATTCGCTGCATCAAC
CTGCACGCTGAGCCGCTCTGTATATGCCACAA
CATCCGGATTGTTTGGGACGTGAAAGTAAAGGAATAAAGGAACTTTTGACGACATTTCGTCGTTGATG
TTTCGCGATGTGAATTGGATAACCCCCAGCCG
TGCCGGGGGTGCACCTGACGGGGGTGGTGTTGTCGCCCCTGTTGAGAAGGGAAAGAACTGGGCGGCTG
AAACCCCTACGGCCCCCATGTGAGCACGCCAG
ACGCGGTACATCCCACGGCTACTGCTCCCGTCACGCTTCTGTCCAATTTTTCTTGCAGCAACGACGAC
GGAGGCGACGGTGGTTATTTATACACACACAC

ACACACTCACAGAGAGAGAGAACTAAAGGAAGAAGAGAAAGAGAGTTTGTGGAAATGTACATACAGAG
ATGCATAAATATTTCGCTTTTTTGTTTCCCCC
GGAATGTGTTTGCGCGCATTCCTTCTCCCTTTTTCTTTCTTCTCCGTAATAGATGTCGTCTTATATGA
AGAACCGATCGTCTATGAGAGTCTTTGAACTG
ATGTTCGCCACGCCTTCACTCATGCGGTACCACCGTATGAATAACTCCAACGCAAATGTAATGACAAG
CGCCACCCACACATTATGCATAGAGGCATCCA
CAGAGGCATACAACATGGTGTAAAGTACAAACAAATCAACATCTAGGCCGTACAGAAATGCATTCCCG
TAGGCAAGTACATCATCCACAAAAAAAAGATC
TTTACCGGACTTATCACCGGACTGAGGCCCATTCATGTACAGAATATTTGGTGCCAGATCAAACCAAC
GATGAAAAATAAACTTCATAAGCAGTGTTCCC
TCAGCACTTCGAACACTCTGCTTTAGAACACTATTGATACGCGTTTTTATCGCTAATATCTCTGTGTT
ATACACACGTGACTTACGGGAAAAACCAAGAA
CACACTCTAATAAATGCCATTGACGGCCCGGTCGAATGGGTGCCAATGCCTTCCCTGAGGATCGCTGG
TACTCTGCCTCAATCTCTGCGTAGCACATGTA
GAGATATTGACGCATGTATATACCCATAAAGACCTCAAATGTTTGGCACTTGCTTTGTCCACCAAGAC
CACGCACCGGCAGGTTGCCTTGAGACTCCAGG
TACAAATTTTGCTGAAACTCCTCCATGCTGACATCGGAGTGTGCATGAATCGACTCGCCGTGAATGTA
ATAACCCCACTGGGTCTCGGGAAGAATCATGA
TGGAGATATTCGACACGGAGCAAAGGTCCACAAATGCCTGCAATGGATGCACACAAAAAAAGCGGTAG
TAAATCTGAAACTCAAGGATGTTTAACACAAG
AGAAACAGAAACCCAAAAGAACGTCCCCAACGCTATTCGCATTACTGTGAGGGAGTAGGTTCCCGCCT
CGTCTGCCCCTGACGTCACAGGAACACTCTCG
GTGTATTTCAGATAGTTCAGACCAACGAGAAAAAACAAAACGATCGTCATGCTAAGAAGCGGCCGCCA
CTGACGAAGAACTTGAAGTTCATTCAATTCAT
TTGCAACAAATGTCGAGCGCCACATGCTGACAGGTAAAATGCGATTTTCACGTAGTAATTGCCCTTTA
CTTCGTTCCCAATCAATGACAAAATAATCCGC
ATTGCACTGCTCCACAATTTTATAGACAACTGCAATGCCCTTTGCCGCAGTGGCTGCGTATAACATCG
CCTCTAGAAACACGTAATTATCACTCAAGAAC
ATACTCAGATTGGGCTTTTGAGCTCTGTACAAAACAAATACATACCATGATGCCAATGACACAACGAG
AGCAAACAAATTCCCAATATGGTTGCAAAAGT
ACACCATAAACCTAATGACGGCCCCAATGCTAAGCATCACGTTCTGTCGACGTCGCATCCATCCATAC
GTGCGTATCCAGGCAGAACAAATACACAAAAG
ACAAAAAATAATCATAGTAATCATCATCCCTTTATCCACTGAACTGGAAGCTATAAAAAGCACAGATA
CACCACCCTCTACTATGTTATTTGCAGTGCGT
GCCGTCAAATCTCCATTATGAGCCTTCTTCAGGCCTGATGCATACTGAAGATAAACGACAGGTTTCAA
AGAATGATGATCTCTATAAACATCCACCACTT
CCAAAGCAAATACCACATGACGCAGTGCTGTGACGTAACAGGGTGTCGTCTCTGTGACTTGTGGGACG
TCCAGCTTACAATCCGTAAGAGTGTCATACAG
GTAGAATCGTCGTCGGTAACCAGTTGCCATGGCACGCCGTAACGTCCCATCGCGTAGGCTTTGGGGAT
GGATGTTGCCAGTGGTGTAGTCCATCACAACA
GGGACCGGCACAAGGCGGATGTGTTCGTGAAATCCCTCATAAAGAGCTCAAAAAAGTATGTGGGACC
GGCGTCCTGTAAAAGTTTGTACCAATCAAAAA
AACACCGAAGCCTGCGATTGGATCCGACTTTAAAGAAACTTGACATATCGGCATCTGAAATGCAGCAT
AGATTCAACACCTCGGATACATGGCGAAGTCC
CTTCAGATTTCCACGCCAATCATACACTGCAACAAGAAACGTCATTGGATCACCAGCGGCCGTGATAT
TTGTGGTTGCCAGCAGGACGCCATCGTCGCTG
GTGTAGGTGTCGGTATTATCGCCGTCAAACAATGGCGGCAGTCGGCGGTAGGAAGTGCGCTTATCGCG
GACCGAAAGATACAACTTGCAAGGCAATGAAT
CCACATCATACATCATCAACACACACATATTGAACAAGAGACTACAACCCACTGCCATCCCACGCATA
CACTGCACGGCGGAATCCATGGCGTAACTTCG
CACCACCACTGATGTTACTGACGGACCCAAAATCCCTTTATTGTTAAGATTCAAAGGTTGAAACGTCG
TTGTGGCACCTACGGCCGCCACGACCACCCCA
TTATACACCTCCGTAGGAACACAGGACGCATCATACAATGTTGTGTAGCCGCTTGCACACACACACTC
TCCCGTTTCTTTCAACGAGTATGGAAATACAC

ACCCAGAGCAGCCATCAGGGCCACAGGGAACACACATTTGAGACTCCAAGAGCCGTTTGCCTACCTGC
TCCACAAGTTCATAGTCCGCCGGGCAAACGCA
CTTTCTTGTCATCTCATCGTGTGTGGGGTCCACGAGCTCGGAAAAAGTCCTATCACCTCCACACTGCA
CACAGCGCTCAGACTGATTTAAATATGGAATC
ATTCCAACAACTCCTCCTGTGAGGTTGCAATCCACGCACTCCGTGTCTCCTGTTTCCACTCGCACAAT
CCGCGAAAAACCCGGCAAGCAATTCACCTCCA
TATCCAAAAATAAAACGCCAGCAAGAGCCATACTTTGCTCAAGTGATTTCCAGTGTCGCAATGCATTC
GTGTCTCCCCGCACAATGCCGTCCGCTTTGGA
CGCACAGTAAGCCATACAAAGCGCCTGAACAGGCAAACAAACGCAGAACAGCAGGCATGAGAGGCAGG
TGAGAAGTGACAAACGCATTGTAAGAGTACAG
AAGCAAAGAAGCCACCGGCCTAAAAACGGCGGGACAAAATACCTGCCACCCCCCACCTACAGTCAACA
TCCAACAGAAAAAAGAAAAAACGAAAAAAAAA
ACACGCATTCGCCATTGAAGCACACGCAGCCCATGCAGAGCATTAAAGAAGAAGAAGAAGAAAAAGAC
AGCTAAAATGAAACATTAAGTTATCCACACCT
CAATATAATTTATTTATTTATTTTTTGTTTCTCCTATCCCACTTCCCGTCCCAGACAAAAGGAATGAA
CAAAACAAAACAAAACATCAAATTCTCCACAT
GCCCGACGACTCGTTCTCAAATAAAACAAAACAAAAACAAAAAACAAAAAAACAACAAAAAAACGTGA
CGTTCTCAAAAACAGGGAAGTTCCCCCTTTTT
CTCCCCTCACTGTGGAGTGCGATGCGACTGAGATGTGACTGGACTGCCGAAACTAAAAGAAGAAAACA
ACAACAACGGCCAGAAGAAGAAGAAAAAAAAA
AAAAGGGAAAAAAAGGAGGCAAAATTCGTTTTCCATTTAATTTTTGTTTTTAAATTATTTCTTGCCTT
TCGTCTTCTGTCTTTTCTTTTTTTTTTTTTTT
TTTTTTTTTTTGCGGATGAATCACAGAGAAGCATTCGCCATTGAAGCACACGCAGCCCATGCGGAGTA
TTAAAGAAGAAGACCGCTAAAATGGAAAACAA
AATAATTTGCCAATGCCATACAAAGAAATATAAAAAACAACAACAATCCAACAAGCAAGAAGAACGTG
TGCCATGTCGCTCACAGGTCCATTTCCCTATA
TATATATGTATGTATGTGCCTACCTTTCGTACATATAACCGAGTTGTCCTCAACACGTTATCAAAGGG
CAAAGAAACCAAATAATTTCTTAATACCTGCA
TGCACACACACACACACACACACACACACACACACACACACACACACACAAATACCTGCATGCACACA
CACACAAACACGGAAAAGAAATATTTTTGCAC
GTTTTCAGTTTTATCGCATGAAAAACAAAACAACAACAACAAGAATGAAAACAAAAAAAAACAAAAAA
AAGGAAAAAAAAGAAAATGGGCGATGACAGAA
GCAAAAAAAGAATTCGGCGATTTACGCATGTATCGTTTGACAAAGCACAGATTTCCTCCCCACTCTCA
TCCCCTCCCAAAAAAGAAGAGCAACAACAAAA
ATAAATTAAATAAAAAAAAAAATGGGTTTTTGAGGAAACAGAAAACAATCAAAGACAAGTAGGCATAG
ATTATTTGTCCGTAGCTGACCCTATTCCTACC
AATATATGCAGGATTTTCATATAAATAAATAAATATTGAATATTGACCGCACAATGGAGCATGATTAC
TTGACAACACCATATATGGTCGATATCGTTCC
AAAGCAGATTACAACAATACCGGCAAAGAGCAGTAAATATGTGAAGGTGTAAAGAAAAAAACCGGTTT
CTGCATAAGTGAAGCCGCCGCTGTAAATGAAA
AAGAACGCAGGAAATACAAAGGCGATAAATCCACCCGTCAGTGAGCCAAGCAGGCCAAATACAATGTT
CACGTTCGGAATAAAAAGACCGGCAATAAGTG
CAACAAGGCACACACCTGAACAAAGTACCGCATTCTTCCACCATGCAACGGTGTCCAGTTTCCAGCCC
AAAAGATGATGAAGCGAATCACGGCACGGAAG
AATGTGGAGAGAAAAGGCCACACAAAGCTTTATGACAATGCCAATATACGCGACAAACATCATCGCAT
CCCTCATTGGCTTGAACATCTTCAAAATAGAT
GAGGTAACATCCGAGCCAAAGTCCGCGTAACCAAAGAAACCGGTAAGCCAATAAAGAACGGTGCAAAG
AAACATACTGATTGCTGTCTCCAAAGTCATTC
GAAATGCAGTACTCTTCGGCTTCATCTCGTTCCATACCTCAAACATGTTGCTTTGGCATAGGTATGCA
AACATGAGCTTCCCAAGTCCTCGAATCCCCTC
ATTTCCACCCCTGAAAAGAACAAGATCGTCACGAAGCCCATGTTTTAAACCATTTCGCACACTGTGGA
GAATGCCGACAATGACAAAGTAGATAATAAAC
GAAACCGCAAAAAATGACACATATCGTACTGAGTTGATACGCTTCGGAAGCGACAATGGAAGCATAAA
CAAGAAAAAAACAATGCAATTGACAACGCGAA

CAAAACTTTTTGTTCGCCAGTACCCAGTCACAGAATCATCGCTAAGAAATGCTTCAATAACATCGCCA
ACTGAGATAACATACGCGACACATGCACCAAA
GCACTTTACAAACATGACTACCGCAGTGAAAATATCACCGCCACGCCCAAAAAGCTGACGAGCCATTC
CTTCATAACTGCGAATGCCGGTCTTTAGCGAC
GTCACTGCCAAAAGATACATGGAAAAAACGGTGAGTAGGTAGATGGCAATTAAATAGATGGTACCCGC
CACAATACCCGAGGAGTTAAATGCGGAGGCAA
GGGCGAGAATTCCAGCACCAAGACTGCTTCCAGCAAGATTACAAACACCAGAGAGAATACCGCCATCA
GGAATCAAGCGCCTTGTCGTCTTTACAATGCA
GTTCGGTTCCTTTGGATTCTCACTGGTCTCTTGGATCCTGGAATTCAAATCTCCATCTTTTTCGTACG
CCCCGTGATTAGCTTGTGGAAGATATTCAACT
TTGTCGGCCATTTTTGGAATATCCTAAACCCGGCAGGGAAATCGGCTTCACGTAGAGTACAAAAAAAA
AAAAAAAAAAAAAAAAAAAAAGTAAACCATAA
GACACGATTAATAGCAAAATGCATGCGCATACACAGAAACAGAATATAACAACAATAATGGCCCTCCC
CATGGCACGCCGACACCCACTTCGCCAGTGAA
TTACACAGCCCGAAGAGAAAACTAAAAAAAGAAAAAAAGGTGAAATTCCGACATGTATTATTTAAAGG
GAAAATACCAAACGACAATCCGCATCGCATGT
GTTTCCTGGACACCAAAACACCATGACGCAATCTCTCTAAAGAGAATAGCAATTGAAACAACAGACTC
CTCCGTTTCTCGCATTCTCTTCATTATCGACA
ACACACGCCTTACAAGCATTTTTCCCACAGAGGCAGCATAGAACGTCACATTCCTTGCACACCCAACA
CCTGCAAACGCTACAGTTCCCATTTGTTGTCA
ACCCACAAAGTACACATTTTGTACCCCCATTTGTTACCTGATCCCGGTCGCTAAATGCAGCCGCTATC
GCTCGGGTTATTGAACGCTCGTCGTCCAACAC
ATGCAATGAAATCGAACCATACTCACTTTTTGGCAAAGGAAGGCCACTTAGGTAAACAGTGGCGGTAC
GATTTAATGTGTCGATGTGGATAAACGGCGCA
TTTTCATGCTTTTCTGCATTATTCCTTAACACAGTGAGTTTCCTGGGGAAAAACAACAGTTCTCGCTC
CAACTGAAGCACCCCACGTGCAATTTTCTCCA
GTGTATGGCAAGAGCATCGAAAAGGGCCATGGTACTTGCAGGGAAGCCATAGTCGCTGTCCAATAAGG
TGATCACACGGTCGGTTCACTTCGCTAAGGGA
GAGAATATGGCATTCATTTATCACACCAACCACACGTGGTGCAAAGAAGAATGCAAGCCCAGGAAACA
TGTCCTCATCCAGCGTATCAAAGGCTATCGGC
CTGTATAATCGCACACAGGTGTTCCAGCCACCGCAAGAAAGGTACACGCGACAGGTTTCATCTCTTTC
ATCGCCCTCTTCCCTTTCTCGCTTTCTGCTCA
TTCATTCATACGCCAAAAAAGGAAAGGCGATTTACTCTCCCAAGGCAAATTAAAACGCATATGAAAAG
GGAGAGAAACTCACAAAAGCAACAAAAACATA
AACAACAACAGTAAGAGATTTTATCGTGAGACAATCAAACACGTTGCAAGGGCACACACACACACACA
CAGAGAGAGAGAGAGGGAGGGACACTCCATCC
TCCACGAGTAACAAATACCCCAAAATGATTCAACATATATATATATATATAGATCTAAAAGCACCCAT
TTACAAATTCGTTAGCACTCACATACAAAAGT
AACAACAACAAAAACAACAAAAATGCAGCGTTAAAACTGTGCAAAAAGCCAATTGAGCAAAGCCTCGC
GATCTTCTGCATCCAAGATTCGATACAACCGC
ACCAGTTGAATCAACCTTGTCAAGTTATGTATTACAAGAAGTGTTTTACTATTCATCTCTTGCACTGT
AAGAAGATGATGGATATATGCACGCATATGCC
GCTTACACGTGTAACAATTGCAGTCTGGTGAAAGAGGTCGAATATCTTTTGTGAAGCAGTCATCATTC
AAGTCTATTTGTGTTTCATAACGTGCAGGAGA
CACACCACTTACTGGATTGAAGTCAAACACCAGGCCTATTCCTTTCTCCGCCAATATCCATGGAATTG
CACTCTCAATAAAACATACATTGTATTTTAAA
CAAATCAGTAAAGATGGTAAGGAAATGGCAGTACTCATAACCTGCCCTTTATTGATAGCCTCCTTTAC
ATGGTATGTGTATTGTTGTAAAGTCTCATTTT
GACAAAGTTCCTCCAAAAAAACAAACTCGCCATTATTCTCCGCAGCACATAAGGGTTTCATTAACTTA
CAATTGCCATTGCCTTGAAGGGAGTCCGCAGA
TGTTGCCCACTTTGCCGAACGGGTCATTGCTGTCCGTCGGCGCTTTTTTTGAGGCTCATTTATTGAAA
CAGAGTCATAGGGAGGAATGGCTATTTCCGGG
CGAAGTGGCAAAACCATTTCCTTCCATTTATCCATACCCAATGAAATTCGACCCTTTTCATGATTCGC
ACACAATCCTGCGTCTGTTGCAGGAGCGCTAG

CGTGCATGCCAATATGAGGACTTCGCACAGTGAGGATGGTCCTAAATTCCCCCAGCCCACAGAATTCG
GCAAAACCCTGATTGGCTTTTTTGCAGGGATC
CACAAATTCGCTTGCGTCAAAGATGGACACACTGATCACCCGTTCTTCCTCGTTAAGAATTGCTCGTG
CCTGATCGGGCGTCAGACAAGGAATGCCGCCC
CGTTTGCAAGGAACCACCAAAATGGGGTATTCTTTGGCCATTAACCGCTTTAATCACTTGCCCCACAG
AATGAGATGAAGACACGCATCAAACACCCCAT
ACACGCACAAAAATAATAATAATAATAATAATAATAATAATAATAAAATAAATAAAATAAAATCA
AAAGAAATCCAAAGAAAAGTGAAAAAAAGCCT
TTTTTTTTTACTCGGGGCTTCCACCATCCTACATGATGTAAGAGGAGATCATTTCAGGCACCTCGCCA
TTCGCGTCTACTGGCACAGCGCCGCCAGCCAA
CATAACCATTATCGTGCACCTGCGTAAAACTCAAAAAGAAACCCAACGACGAATAATTAGCCTCCTCA
CAGATCTGCTTAAGCCGCCGTCCTCACCCACA
TCACTTCTATTCGCTTAAAAACAGATATATAGGTGCCATTACATAAACAAGTGCACAAATAAACTGAG
TGATAACGATAAAAGTGTAGAAATAATAATAA
TAAAAACGATAATAACAAGGAGAAAAAGAGAACAACGCACAATATCTATCTATCCGTCTCTCTCCTTA
CCTATATAGTCATATCAAAAAAAAAAAAAAAA
AACGTTATTGCTCTGATCCAAAGACGCTATCCCAATCTACGGGAATCTTTTTCTTATTCATCCTCACC
TCAGTTTCATATGAACTTCGCGAGGGATCCAT
TTTACTCCTTCTGTCAAACCCACTCATTGAAGGCAGTGTAGAATGTGAATTCCCGTTGTTTGAAGGCG
AAAAGGAATGCAACGACGCCTCTATCGTTCGA
TAAGGGCCACTGTATTGAGTGTCTTCTGCACTTTGTGCTACGGATAACCTCGAATAATTCTCCGAAGC
TTGACCCACATCCCCAATCCTCATCCATTCCT
GCTGTCGAAGCCAATTTCTTTGCACGTGACTAAAAATGGAACTTTCCTTCGATGCGTTGGATGAGAAA
CGATCTGGTGCGACACCAGGGCTTTGACTTTC
CCGTTTTTCCTCGTATGATCGAGCTCGGGAAAGCGCAAAATCGGTGATTGCCCTATTAATGCGTGCGT
CCGCAGCTGCACTTCTATTAGCCCTGGCTTTC
ATTCCGGCAGACTGCGGCCGCAATACTACCGGGGTCTTTAATTGCAACTTCTCCATTCTTCGCCGTCG
TTCAGCCGCAACTTTCATGTAATGTGAGCTAA
TATACTGGTGCTGAGAAAATAACTCAAAAAACGCGTCAGTTTGTTCCACCTCAATGTTTAGCCGCATG
CGGTCCTCTTTTCGCACTAATTCACGAATCTG
AATACGTGTTTTCTGTAATTTCTTCTCCAACAACCGTTGCTCCTCCTGCCTCTTCTTGCCCCTAAGAT
AATCCATGGAGCTTAGCCATTGCGTCCATGAC
TTTTCATCTGTGATCCGTTGAGCATCCAGAGCCCGCGCCTCTCTTCGCTCTGCCCCACAGGCAAGTTC
ACGCTCATATTCCAAAGAAGCGCGCCGCTCCT
CCAATAGTTGTCGATACAAGCGAATGCGTACAAGCTCACCTCGGACGTGACGTTGAATACAAATAGCG
GCTCTACGGCGCAATTCTTCTAGACGCCTCGC
CTCTTCAACTCGTCGACGCTCATCTTCAATTGCTTTCCGACGACGCTCTCGCTCTGCAGTTTCAATTG
TCTCTTGGTAAACTCGACTTTCGGCTTCTAAT
TCTTTTATGAGTTTCCACGACGTGTCTTCCTCGAGCTCGTATGAATGTCGAAGGTCCGCTTCCTGCAA
ATGCATATCTTTTGACGCCTTATCCAAGTTTT
TCCGCGCGATTTCGACCTTGTGGAACAGTAAAAAGATGTCATATACCCCGCGCCGCGTTAAGTATCCC
CGAGCAATTCGCTGAATAATCAGTGATGTACA
AAGCAAATGCCGGTCCCTCAACCTTTCTAGCAATTTAAAACCCTCTGCCTTAAGAAAAACCTTAGTTT
TGCCTATTTGAGCCAGAGAAGGCATGCGTATT
TGAGCCATTTGGAGTACATGCTGTATAAATTCCTTTTCGTCTTTCGTAACCACAGATGTACACATTCG
ATAATTCCGGCAGAACAAAGCTCGTGGAATAC
GTACTGGATATCCGGCTTTTCGAAGCTTCACTGTGGCAAGAACACCACTTGACTCCAGTTGTCCCATG
ACTTCCCTACCGTAAAACATTCTGGGTTTTTT
ATCAGGATGGGGCTTAATACACCGAATCCAATGTGGATTCGTCTCGCCAATTAAATCCATAAGAACTC
TCAGCTGATTACAAAAAAAACCACCCACAGTA
AAAGCTTTACCTCCCTTTTTCCGCTCCGATAAGGGGATGGGTGCTGGAAGGCAGCTTGCAATTACGCG
ATCACCGCTAGCTCGTACAATGGACTTAATGG
AATCTTTAAGAGTATCTCTATTTTTTTCAACCCAACCAAATACGTTGTACGTGACATCCGCTGCATAG
TGACGCACACAAAAAGTTTCCCCAGAGACACG

TTCCTTCTTAAAAAAGGAGTTGGATTCATGAGTATGCACCACTTTTTCTAAAAATCCAAGGTCCGTTC
CTCCTCCAAACCAACAACATTCATCAAGAAGA
GGAAAAATGCCGCCCTGTGCGGTAAGCAGTTTTAAACAGCCTGTATTATCAGTAAACTTAATATCTGT
CACATCCACCCCCTCCATTCTACATTCGTCCA
TATCACGCTTAAATATGTGCAGGTTATAATGATACTGAAGCTGCTCATTCGTCATATTGATACAAAGC
TGTTCAAAGGAGTTCTTATTAAAGTTCTCAAA
TCCAAAAATATCCAGCAAACCAATCCAATTACCATCGTTTTCCACATTACACAAGTCATTGCAGCGGT
CAACAATCCAGCCAAAAACACCATCATACAAA
TGTTTACACATGGCATCACGGATGTCGACAGCTGCCACAACGTCGTTTTTCTTTAAAGCAAACTCATT
GTCGGGAAGTGCACGAGTACTAGTCAGAAGTT
CATGTACAAGATCATCTTCACATACATCAAGCAATTGGACAGCCTTTTTGGTATATTTCATGGTTTTT
GGATCAATAGAACAGCCATCTTCTTCTGGAAG
AAACCGAACATTAAGTAATGTCATTACAGCTGCTGTCACACGCCAGACAGAGATAAGCTCGACATCTG
TCATCCCAACAATAGACATGGCCCGACAAACG
TCATTAAAGTCTTTTTCAGAGTTATACTCACTATTATTCATTGTCCCCCCTGAAGAAAGGGACGTATA
TTGTTTCTCATGCTCAAGACCAAGGACACGTG
CCATACTCCCTTGAGCACGGGCCAATAGATAAAATGAGTGGAATACGCGCTCGCCCTGAGCAGCAGAT
ACAACACGACTTTTTTCAAGAAGATACTTTAT
GTTTTCAGCGCCAACAACAACGTGTTTGGCGTTAAATTTTACTTTCATGAATTTGCCAAACCGAGAGC
TATTGTCGTTCTTCACAGTTTTCGCATTTCCA
AAGCACTCCAAAATAGGCGAACATGCAACCAACTTTTCACCAACACCCCGGGCAGCTTCTTGTTCTAA
TTTGCTTCCATTCAGTGATGAAACAACGCCCA
AATAATTTAGAACTATTTTTGTTGCTTCTGTTTTCCCAGCTCCAGATTCACCGGAAACGATAATGCAT
TGGTTTTCCTGACTGTCCACCATCTCATAGTA
AGTGACATGTGCTTGAGCCCATGTGTGTGGCAATAAATTTGAGGGCATACCAGAAACACTCAAACTAC
GACCCGCATTTAAATATTTTTGCATTTCACTG
TCCTTATACCATGGGATAGCAAAAGTAAATGGATTTAGCGCCACCACTATTGCACCGATATTGGTATA
GACCACGTCATTCATGTAACGCATATAAAGGC
ATCTCAATAAAGTTGTTTCGTGAAGAACGGTAAGATCAAGTAAGTCGTCTGGGGTGTCATTGAGGGAT
TCTCCATAAAGTGGTTCTACGTCTTCAGGATT
TTTTATTGTGATTATTTCGTTTTGAGTGGCTTCTCTTCTCAATGAATCCTTTGTGACGCAAACAATTC
CTCCTTTAACGATACTTTCAACTACTCCACAA
AGCCAGGAGTGTTCTGGATGGCGAAAAAAAACATGGTCTCCAGCCTTGAGCGCGTTCATTTGCTTAAA
AAAAAAAAATAGCGACACTAATAATAATAATG
GTAACAATATTAATAATAATAATAATTATTATTATTATTATTATAAAACACAAAACAAAAAAGACACA
TGTAAAGGGAAGAACAAATATTTATTCGCCTT
GCAAACAAAAGAAAGTAATAAATAAATAAATAAATAAATAAGAGGATTTAATAAACAAAAAGATGAGG
AGTCGCATCAATCACCCAACCAAAAAAATAAA
ATAAAATAAAATAAAATAAAAAAAATAAAAAAATAAATAGTAATAATAATATTATTATTATTATCATT
ATTATTATTGATAACGATAAATAAATAAATAA
AAAGGCAGTATTTTTATTTCTTTCCTTCTCGCTCTCTCTCCCTCTCTCTTTCGCATTTATGTAGAAGA
GTTCCCACTTTTACCCAAAAGAAATAACAGGC
ATGTCAACCCAATTGGACTTCTACCATTAAACTCTGTTCTATGAACAACTCACACTTCTGTTTATTCT
CTTTTCTTCTCTGTCTCTATGTCCATAATA
ATAATTACTTTTACTCCTCAATCTCTCGTGTCCTTTTCCTTTCTTCAAAGGCTGCCTGCTTCAATAGT
TTCACTCCCTCCTCAAGAGAAATGGCCTGAAT
CCGACTTGTCTTGTTATGAAAGAACACGCCAAGAACGCGATCTGCGGCTTCAAGCATTTCCGTCTTGA
AGGTTGAAGTGATAAATTGGCAATTTTCAGAG
TCTTTCAAGATAAGTTTTGCGACTGAAGAACGGTATTCTGCATCTAGTGCAGCATCAATTTCATCAAA
TAGATAAAACGGTGCCGGATCACAGCGCTGAA
TGGCAAAGATTAATGCAAGGGCAACAAGTGATTTTTGTCCACCACTTAACTGCCCAAGGTCTGTTATG
GCGTTACCAAGTCCAAAAGAAACGCAAATTCT
AGCACCAATATATGGATCTTCTCCTGCATTTTTCTTGGCTGCTGATCGAACAAGTTGTAATTCCCCAT
GGCAATCCTCAGTAGCTACAATTTCTTTAAAA

ACCACCTCAAATTGATACTGCATCTGTTTAAATGTTCGCTCAACCGCCTCATCTTTTTTCTTGTCAAG
ATGTTCCATTAGGTCGTGAATACTTTTTAATT
CATTTTGAAGAATTTCCTTTTGTCCAACCAATTCATTTTTGGTCTCCATAAGAGATGAATATTGATCC
ACTGCTTTCCGGTTCACATGTGAATACTTCTT
AAGCTTCTCATTATTTTCTTTTAGACGATACATTAACATTCCGAGAGACTGTCCAGAATATTTACTTG
CATCTTTGGGAACAATACCCAACTTCCGTATC
TTTTCCATGGCATCATCGCGGCGCTGTACAAGAAGTGTCCGCTGAATCTGCTTTTTTTCATCATTGTC
TCTTCGTTCCTGAACAGCCCTGGCTGAAGCAA
ATTGTTTGTTTTTCAAGACTTCCAGCTTCTCTTCACTTGCCATCTTCTCGTTTGTCGTTTCAGCAATT
GATTGTCGAACAGTCTCAATTCGCGCAGAAAC
TAAAGAAATTTCCGCATCCACATTTCCTTGTTCCCTTGTCAAAGTCTGATTGTTTGTATTTACCCAAA
GAAGTTCACGAATACGATCACGCGCAATATTC
AACCGACGCGTTATATTTTGAAGTGTATCTTCCAATAACTGAACCTCGGTGGTTATCTGAACACGTTG
TAGCTGAAGCGCCGAGAGATCCTCTCGAGTTT
TATCCACTTCCGTAATGACAGACTCTAGATGACTCTCTTCCTTTTCCCCCCATGATGACACAAAATCT
TCTCTTGCCTCCTGTTGCAAAAGTTCCAGAGA
ACTGTTTGCATCAGCCATACCCTTTTCCAAAGATTTTCTTGTCTCCAATAATTGTTCCCGATGCTTCT
CCAATCTTACTTTCCGCTCGTCATGAAGACGA
GCATCTCGAAGATCTGCATCTGCGTCATTCTCTGCCCTGGAGGCCTCTCCTCTTAATGATTCAATCTC
GTTCATGGCGTCGGTTATCTTCTGTTCAACAA
CAGCCACTTCTTGACAAAGCTTTTCAAGTAGAGTCTTCTCATTTGCCAGTCTTTCAGACAGTTTCTTT
TCATTATTGAAGGATAATAGTCTCGAATTGCG
AGATTCCAAGTATCCACCTGTGATGCCGCCCTTGCGGCTGATTTGATCCCCTTCCATTGTCACAGCAT
CACAATTATATTCTTTCACAAACTTGGAACCT
TCCTCCATTGACGATACAATCGCCGTTTTTCCAAATACCTCAGCAACGACTCCCTTAAATCTCGGATT
ACAAGAAATATGCTCCATGAGAGAACTACAGC
CTTCTTTTCCATCAAATCGGATTGGTTCACTTTTGCATGTATCCAGTGGGAAAAAAGAAATACGTCCT
GGTTTCTTCTTTAAATTGATTTGATCGAGAAT
AAGGGCGCCCACATCAAAGGAATCAATAACAACATTAAAAAGTGCATTACCCGCAGTCACCTCAACGG
CCGTTTCGTATCCTTTTCCAACATCAATTAGC
TCAATTAATTGGCCATGAACAGCGTTGGTGAGTTTTTCATCGGCAAGTTCGTGTAACACTTCCCTAAG
TGATTGTATTCCCTGACGAGTATCATGTCGAA
CGGCACGCTCAAGTTGATGTCGTGAACGACTCCATTCGTCCTGTAATCTTTGAACAATAATTTCTTGT
TCATTGACCTTTTGCCACAAACTTCTTCGCTC
CATATTTAGAGTATTTCGCACAGTTATTGCTCTACCCCTGCGAGTTTCATGATCGGCCAATTTTGTTT
CCACCTTCTTTGTCGCAGCCTCTTTTTCCTTC
TGATTCTTATCTTCTTCGTGGATACGATTATCAATATCCTCTATGCTCTTCTTTATGCGAGTAATCTC
CTTTCTGTGAGTCTCTATGGTATTTCTGTTTC
GCTCTATTTCTCCAGCTAACCATACGTCACGTTCCTTTTTATTCTTAAAAAGCTTCCTCCTCCCTCTT
TTAGCAAGGAGAGCTTCCAGCTTCTTTTCCAA
ACTGGCCAATTCATTACTTTTGTGATCTGTTGTTCTCTGGTGTTGATTCAGGTTGTTTCTGCTTAACT
CCAAATCCTTTTTCACTTTTTCTGCATTTTTT
TCCAAATTCTCGACTTCTTTTTGAAGAGCTAGTCTTTCCCTTTCATTACGGCTTATGGAGTTCTTAGC
ATCGACAACATCCAATTGGGCAATGGCTTTCT
TACTATTCAGTTTCGTTGCCTCTCTTTCCAGCGTCTGCATTTCACCTTCCAAATGAGCAATGCGTTGC
GCGCAATTACGAATATTTTTCTCTTCTATTTC
AATAGAACTATTCGTATTGTTTTCAGCATCTCGTTGTTCATTTAAAGAAGAAACGTATTTACTTCGTT
CTTCATCCAACTTCTGTAGGCACTCTTTGGCA
TTCGCCAATTCCAAGAAAAAGATGGAGTATTCGACACATTTTCTCTCCCTCTCAATTTCTTGAAGAGA
CTTTAATTCAGCGGTTTCAGCCTCAAGTTCTG
TCAATCTTTTTTGTAACTGGTCAATAGAATCTTCTATTTTCCTATATTTGCCTGAGGTTTCTTCCAAA
ATTTCCTCACTTTCTTTTCGTCTAGACTCATA
GACTCTTGTGCCAGCAACATCTTTGATAAGTTGGCATCTCTCTTCATCACTCATATTGGCCATGTTGA
CAATCTTACCCTGCTCCACAATATAGTATGGA

TTGCTTGAAGAAAATCCTGCACTTTCCAGCAGTTGCCTTACCTCTGTTGCTGTAAACCGACGATCATT
TACTCGAAATTCGTCCTGTTTCAATCCCAAAG
TACGTCGTATTCGTACTTCTTTCTCTTCAGCTCGACCAGGGATGATAAGACGGCCATCTGAGTTATCA
AAAATAATTTCAACAAAAATGGACAAGGCCGG
TCTTCCAGAACCCGCATGAAAAAGTTCCTTTCTTTCAGCAGCCGTTAAAGTAGTGTACTTTTCACTCA
AAACAAACTGAACGGCGGCAAAAAAGTTTGAT
TTGCCGGAGCCATTCTTCCCCACAATAACGTTATTCTTTGGTGAAAGGTCCACTTGAAACGATTGATC
CCGGTAGGAACGAAATCCCGAAATCAAAATGT
TTTTTATGTGCATTTTCACCAGCCTTCGAGAGAGAAAAAAATACAAGCAAAAGGGAAAGTAGAGAGAA
AATTTAAAGGAAACAAGCTTCACAGACAGGGA
TTTACTTTATGGGACGAAATAAAAACTTGGTAATAAAAAATAACCTCCCTCTTGAACGGCGGCGTCTC
TTTTTGGAGCGATGACCATGTCAGAACCGTTC
TGCTTAGGACATTTTCAAAAGCTCCCGTTAAAAAGAGTCTTGACAGCCTAAATGCGCCCCGAAAATCG
CAAAAAGGCCCGCAAACCAAAATGCAAATCCC
TGCTCGCTTTCCCGGCTCTAAATTTCCCTGCGAAAACGAAAGCATAAACAACACACTGGTGGCCCGGC
ATGGGCGCCAAAAACCCAATAGGAATGGAAAA
AAGAAAAGAGAAGCATGCGCTCGCCGCTTGCCGCCGAACGCGGCGGAGGCGAGGGGTGGGGCGACGCC
TTCAGGCGGAGACGGCGATCGCGCGAGTGCTT
ATCGTCTCCGACTTCCCCTTGGGTTGGCGTTCTGGGGCGTCTTCGCTCTCAAAAAAGCAAAGCTTTCC
GTCGCGCGATTGCCTTTTATCAGGGCTTCCAC
TTTATACTTCGTTTCGTTCCCTTGATTACTCTGTGTGTCGACCTGTCCGGATTCGTTTTGCTTGTTCA
TCTCAAAAAGTGCTATTGACCCAGAGATTTAT
CCCTTGAAAGGACGAAATTTCACGTTGGTCTGC
>scaffold1110.size6753
CGGGCCGAAGAGGTGGCATTTTTTTCTTTTTTTTTTGTTTTGTTCTTCTGAATGGGATATTCCCGCTG
CTGGGCGTAGTGCGTCTCCCTCCCTTTTGGTTT
TTGTTTTCTTCTTCTTCTTTTCCCCACCCCCCTACCCGCGGCGGCAGCAAAAAAAAAAAATGAAATGA
AATGAAATGGAGAGAAAACACGTTTTTGACGT
GAGTGCGCAGAACGCCAGGCGCACACGCCGGTGTTGTCCACATTTGTTTTTTCTCTTCTCTTCTCTTT
TTTTGTGTTTGTTTGTTTTCTGCTGCTCCGCC
GGCACCAAAAGGAGGAGGAATAAACACAAACAATCGAGAGAAAAAAAAAGAAAAGAAAAGAGGGAGCC
ACGCGGAGGTGCACGGCACAGTTGTGTTGTTC
TGCGGCCAAAAGAAGAAAAAAAAAAAAAGGACGCAGAGGGAGTCAATCAACAGGGAAAGAGAGAGTGTG
TGTGTCGTAAGCAGTCTGGGCGATCACTTCCC
CCCTCCCCCCTCCCCAGTGCGGCGGTGCTCTCTCCCACACACTACACAAACACTACACATTTACCGCA
GGCGATTCCTCTCAACAGATGCCAGAATCATA
TATTGTTCGTTTTAAGGTATTGAAGGTGGAGTCTGCCGCGTTGGTGGATGGCAGACAGTACACCATTT
CGTACCGCCGCGGCGAGACATCCCGTTCCACG
CCCTGTTACATCGCGGAGGGAGGTGCCGTCGACTTCAGTACAATGCCTGAGGGGGCGGCCATTGTTCA
TTTCAAGAGCGGTGGAGCGAGGTTCCTTCCAA
AGTGGATCACCTTCCGCCTGGAGGAGTATATACGTGGTCGGCCCCGAAAAATGATTGGGGAGACACAG
CTTGACTGCGCAGAATTGCTTGGGCTGCACAG
CATCACCGCATCTGCACGTCGGCGTGTGATTTTCAGCGTGTATGGAGCATCCGCCCAGATGGTGGTTG
CGCTCCTCGTGTACCCCGCAAATCACCCGGCG
CTCTCATTCGGCGACATTGCCTCCACGACCGGGAGCGACGGCCAAAGGAGTAAGACGAAAAATGGGGA
GTTCAAGCGGATGACCCGCGGGGAGGCGATGA
CGCTGCTGATATCTCTCGAAGCCATGCTTGAGCGCCGCCGCAAGGAGGAACCGAAGGGAAAAGAACCG
TCCAATTTGGAGCGGTGCCTGGCGGAGCTGGA
GGAACGGCGCCGTTCACTCACCGGCGTAGATGGGATGGCTACCGCAGTGGTGAAGAAGCGCGCGGAGG
ATGTGGTGGCGGCGCAATTTCTTGCCCTCTCC
CGTAAGTATCGCGCCAACTACGCGGGGGAAGTTGCCGCCTATCTCCGTCAACTAGCCGTCACGAGTGG
ACTCCCGCTCACCGCCGACATCTCTGAGGAGG
CGTCACCTGACGCAGAAGGGGCACGAGAACGGCTGCAACGCATCAACGCCCGCATCGACACTTTGTCA
GGCCAAGTGCGGAAGCTGGAGGAGGAGCAGCT

GTCCCTTGGACGACAGCAGAATAGGACTGATATGAAGGACGAATTGTGCGGCATCACAAATAAAATGG
AAGCCCTTCAGTCGCAAATTCAGTTGCTGCTG
AAGACGCGCACGGCTCTGGAGGAAACGATCCGCGGCAAAGGCCATGACGGCACGCCGATTGGTCGCGA
GGTGCACGACATCCGCCAACGACTTCGCGTCC
TTACGGAAGAAGAGGCAAAACTTCGGGAGCAGGTACGCCGCATGATGAACGTCGCCAGCATGCACGTG
TTGAAGTGGGCACGCAGCAAAAACCCGCCGTT
GGAAGACATTCAAACTGACCTCGCGGCTCTTTTTGCGGCGGATGCGCCATCACAAGAAGCGAAGCAGG
AGAAAAGCACTCCGAAAGTCCTGAGTTCCGAG
GAGCGCGGCCAACTCGTGGAGGCGTTGTCAAATGTGGCCAAGGTTTCCGCCCCTCCCGTGCGATCGGA
GGTTTCGAGTGAGATGGAGGGGCCACCGAGTC
GGCAGCCGCAGCAGCAGGTGGATTTATTTGGGGCCACGGGACTCCCATCGGTGGGGGACTTTGGCGGC
TCTGCGACCAAAAAAGGGCGGAATGAATCCAC
CACCTTCTCGAAGACCGACAAAGACGCTGAAAAACACGTGGATCCGTTCATCTCCGATCTTAAAAGCG
ATATGTTTGCCCCGTCGGCAGTGTCGGAGCAT
AAAAAGGACACGTCTAAAGTCCATGAGTTTAACTTTGGCGCCACAGCGGAGGAAGACACCGGGGCGGT
GGGGGTGGGCGGCAGTAGTGAGTACGCCACGT
TCTTCAAGGACGTGCATGACGCAGCAATGGATTTGGGCACACCGCCAGTTGTGGATTTGGTTTCTATT
GAAAACGACCCCATGTATGATTTTCATCCGCC
CCCCGAGGCCGTCAGCACAAAACCCTTCACCATTGAGTTCGAAGAATCGCCGCTTTATCACGGCCTCG
ATGGGGATGCCGCAACAAATCCCCCTGCCTTT
ACATTTGAGCCGGAGGGTCATCAATCAACCAATTCCAACACACACGCTTTGCCGACATATAGCTTTGG
TCCCGATAGTGCAACAAAGAACTCTCGCACCG
CAAATGCCCCGCGTCTACCGACGTATGACTTTGGCAGTTGAGGTTATGGAAAAAATATATATATATAG
AAAAGAATAAGTTGATATGAAGAGTAATGCAA
GATATAAAAAAGAAAAAAAAAAAGGAAAAAGAGTACCGCTTACAATGTACGCAAGTGAATTCGAATAT
ATATTCATTTATATAAGTGTTAATGGCCTTCC
CCAATGAAAAAAAAAAAAAGAAAAAAACCGAAAAACCGGCGAAAAATGAGGGAATGAGGAGAAACCAG
AGAGTTTTTGTTTTTTTTTCTCACAATGGGGA
TGGAAAGGGAGGTAGGATTGATGAGCGGTTCATTTGTGAGAGATATGATGGTGTCGGCTCATATTGAT
TGACGCCATCTACCTCTTTTCTTTTTTTTTTT
TCTTCCTTGTTTGTGCGTGTGTTTATCAGACGCACTGCGTGAATGTGCATTCCCTGGAATCTCCTTCT
TGCATCTCACCCAGTCTGTGTGTGCGTGTGTG
TATGTGTGGGTGGGAGGGGAGGGGTTTTTNNNNNNNNNNNNNNNNNNNNNNNNNNNNNNNNNNNNNNN
NNNNNNNNNNNNNNNNNNNNNNNNNNNNNNNN
NNNNNNNNNNNNNNNNNNNNNNNNNNNNNNNNNNNNNNNNNNNNNNNNNNNNNNNNNNNNNNNNNNNN
NNNNNNNNNNNNNNNNNNNNNNNNNNNNNNNN
NNNNNNNNNNNNNNNNNNNNNNNNNNNNNNNNNNNNNNNNNNNNNNNNNNNNNNNNNNNNNNNNNNNN
NNNNNNNNNNNNNNNNNNNNNNNNNNNNNNNN
NNNNNNNNNNNNNNNNNNNNNNNNNNNNNTCCGTTAGCAGGCGTCTTGTTTTGTTTTTGTTTTTTTTC
CTTTCCTTTTCGCGTGTGACCCTGATGAGAGA
GCCGCGGTGTTGGGCAAGACGGCAGGTGCGGCGCGTCTGTTGGGTCAGGTGAGAAGCAAACCAAAAAA
AAAAAAAAAGAGGGGGGTGGAAGCATACATAT
ACAAGGAGGGAAAAAGGAACATGCGTAAACCGATCCTTTTTTCTTTCCGCCGTGCCTCTGGCTGCGCC
ACTGCCCGCATGTTGTATCTTCGGTCCGCGTG
TCTCCAGCAACTCCGTTTTGGGCACACGCTGCCACCAGGTTTGAATGACGCCGCCCGTAGCGCTGCTT
TGGCATCTGAAACAAAGGCGACAAGGGGAGAG
GCGAGTCGAAGCCCCTCGCTTGTGGCCAAAAGCGTTGGAAGGGCACCTGGTGTTGCATGTTCTGCAGC
CCACGAAGACGGAACGTTTGGCAGCCTCCGAC
TTAGCAGCAGCGGCCCTTCTGGTCTTATCGAGGAGGCTACGATGGAAGGTCCACGTGATGAAGTGATG
GCGCCGAGTCACGCGGGCGATTGTGCCGGAGA
CTGTGCGGAGGCCAAAAAATTGGTTCTGGAGGAGGGTGTGGAGCAGGCAGGTGGGAATAACGTGAATG
GCACATCTCACGCCGATGAAAAGGAGAGTGAG
GTGCGTGTTTTGGTACGGGATCACATGCGCAAAATTGTCGATGTGAAACGTCTTCATTCCTTTGCGCA
GCTGAGGAGCAAGCTGCCGCGGTGGTTGTTCT

ATGGTATCCAGCAGTCGGGGTACATAAAACCAACTCTTGTACAGTCGGTGGCCATTCCGCTTTTTATG
GAAAGGCGAGATGTCGTGGGTATTGCCCCAAC
GGGGAGTGGCAAGACGGTGGCATTCGCACTGCCAGCCCTTGCTGCCCTGCCACCATCTGATAGCCGTG
GAAATCACGAGAGCAATCGTGGGAATGCGGAC
AATCCCGTGGTTGTGAGCCCCAACGTGTTGGTTCTATGCCCCACGCGGGAACTGGTGCAGCAAACGCG
GAATGTTATCTCCCAACTGGCAGGGAATGCTG
TGCGTGTGAAGGGAGCCTTCGGCGGACAGGGTCGGGAGCAGCAGATGGAGCAACTGCGGCGATGGGGT
GGCTGCGATGTTCTTGTCTCTACTCCAGGGCG
CCTCTGCGACTTTGTGGAGGCCGATGTTGTGAGTCTAGAGCATGTCGGCTTTCTTATCCTGGACGAGG
CAGACCGCATGTTGGAGCTTGGCTTCGCCCCA
CAGTTGGAATTCATCATGTCAACTATCCGAAAATTCAATCGGCCACGGCAAACGACAATGTGGACTGC
AACGTGGAACGCCACGGTTGGTAGTTTGGCCT
CGCGCTTTTTAAAGCCAGAGCGCGTGTTTTTTGAGGTGGATCGTGAGCATAAAATGAACACCGACATC
ACACAGAACTTGTATCCTCTTAAAGATCCCTC
GCAGCGCATCCACGCCATTGTGAAACTGTACGAGAATGCCATCATCTCGAAGAGGCAACAGGTCCTCA
TTTTTGTGAATCGCAAAGAAGATGTTGAGCAA
CTTTCGGAGGACCTAAAAACGGCACTGCGTGCGCCACCAGATTTAATTCGGTGCTTACATGGAGGGAT
GAAGCAAAACAAGCGAGAGCGCATCCTGCATG
GTTTCAGGGAGGGCAACATTCGGGTTCTTTGCGCCACGGACGTTGTGGCCCGTGGTCTTGATGTGCCC
GAGCTTGACCACGTCATCAACTATGATCTTCC
AGGCGACTCCGACGCGTATGTGCATCGTGTCGGCCGCACGGGACGCGCCGGCCGTCGAGGAACGGCGC
ACACGTTTATTCTCGCCGGCGACAATCGTGCC
CCGCTGATTGCCCGCTTCATCGCAAAACAGCAGGGTACCGCGTTGCCGGAAGATGTGCTTGCAATTAT
TCATGACATTGAGCTCCATGGCGGAGCGGCGA
CGGAGCGTCCAAAGTACAAAGACCACGCACGTCTCGCGGGCAAAGACTGGCGCGTGTATGGCAGCGCA
AAGTCAATAAAAACAGAAGAGCGTCGATATGT
CCGGGGATACGGCCATGTCTTGACACTCAAGCGGCCCACCCCACGCAAGGAGAAATAGTCGGTTTCAA
GTTATGCTCATTCCTGCGTCACGCCATGATCA
CCCACATCTTTATGCATAAACCCGTATATATACATATATATATATATTGTATGTATGTGTGTGNNNNN
NNNNNNNNNNNNNNNNNNNNNNNNNNNNNNNN
NNNNNNNNNNNNNNNNNNNNNNNNNNNNNNNNNNNNNNNNNNNNNNNNNNNNNNNNNNNNNNNNNNNN
NNNNNNNNNNNNNNNNNNNNNNNNNNNNNNNN
NNNNNNNNNNNNNNNNNNNNNNNNNNNNNNNNNNNNNNNNNNNNNNNNNNNNNNNNNNNNNNNNNNNN
NNNNNNNNNNNNNNNNNNNNNNNNNNNNNNNN
NNNNNNNNNNNNNNNNNNNNNNNNNNNNNNNNNNNNNNNNNNNNNNNNNNNNNNNNNNNNNNNTGTGT
GGCGGGGAGAATGTGGGGGGAAGTTCCTTTTA
ATCCACATCCATACATACATGTGTACATGTGCATCTATACATATATATATATATATATACCGACACTT
CACTTGCTTGTGCTTCCGTTCCCTGCTACGTG
ATGGTCAGCACGCATCTCTTTTCTTCTTCTTCTTCTTCTTCTCTCCCTCTCTCTCTGTTGCTGCC
GTGATGAATGCAACCGAAGGAACATTAAAAGA
ACAGAAGCCAAACAAAGAAAAAAAAAAGCCAATACATATACATACATACATTTATATATATTACTAAT
AAAATAAGGCAGAGGGCATTGAGTGTAGCCAA
AAAGTCAAAACAAAAGGAAAAGAAAAACAAAATATATAAATATTTATATATAAAGTAACCAAAAACTT
GTAATTGAGAAGGAGGAGGAGGAAGACAGAAA
AACATCTCCTCCTTTTTTTGGTTTGTTTGTTTTGTTTGTTTCTTTATTCTATCAGATAAAATACATAA
AATAAATATACACATACGTTGCTGACAACATA
AAAAGTATATTTATTTATTTATTTATTTATTTATTTGTTTATTTTATTTTACGCTACACATTTAATCA
TTTATTCACGACACAATAGGAAGTGAGAGG
TGGTGGTAGGACGGCAATATATATATATACATACATATATATATAGGAAACGTACACGCAAAGCAAGA
GCGAGCATAAGAAAAATAATTTTAAAGGAAAA
AAAATAAATAAATAAATAAATAAATAAATAAATAAGAGCGGACGAAAAATTTATAAAA
>scaffold1111.size6203
CGGTGGTGGAGAAGCAGTGGAGGCCGCCTTCGTGGCTTGGCCTGATGTCTCACTGGGGGCGGCGGAAC
GCGAAGGATGAGCTGATGGCGAGCTTCGTTGTT

TCCGGCTACTACACCGCGATCACGATGGCGCTGTTCACCGACCTCGGCTACTACAAGGCAAACTTCGA
GATGGCGGAGCCGATGAGCTGGGGCAGCGGAG
CCGGCTGCGCGCTGCTGACCGACAAGTGCGTGACGGACGGTGCCACGCTGTACCCTGATATGTTCTGC
ACCGACTCGAGCGACGCCACTCTGCGGTGCAC
GTCTGACCGCCAGTCGCTGGGGACCTGCGCAATTTACCAGGACGATAACATCCCACCGGAGTTCCAGT
ACTTCGCCACCAAGCAGGGCTCCAGGCGAGGG
GCCATGATGGAGTACTGCCCCTTCATCGCGCCAATGAATGGCACTGGGTGCATGGATGGCGACGAGGC
CATCATGCCTGGGAGCGTTGTTGGCCCGTCCT
CGCGCTGCTTGACTGGGGAGAACCTCCGGATGGGCAGTACGGTTGTTGGTGACGTGTGCGTGCAGTTG
AGGTGTGGTGAGGGCACGCTAAGTGTGCGGTA
CAGGGGCAGCGAGGAGTGGCATCCCTGTGCACCGGGTCAGACGCTGACGCCCCCGGCGCCATTCACGG
CGGGCAGCATAAGATGCCCCTCGTTCAGCGAT
GTCTGCCTCACCATGTTGAAGCACAGTGATGCGAGCAGCACCGACGTGGAAAGCCACCCGAAACACCT
TGCGATGGTCAGAAATAACGATGGCAGCGCTC
TGGCGGTCACTTCTGCGCCGCTTCTCCTCCTCCTCATTGTGATCGGCACGGTGATTGTCGCACCCTGA
CCCGTACCTGTTGCCGACACCGCACCTCCCCT
GCTCCATGGGCCTCTCTTGGAGTCCTTTTGCTATAGGAGCTCGGACATTTTCTTTCCTTCTTTTTTCT
TTTTTTTTTCTTGTTTTTCCTTTTTTTTTTTT
TGCTTTGGAAATGACCGGTGCTGAAGTCGTGCATCCCCTCTGGCTGTGGTGCATCTGTGTGTTCGGAG
GCGTGTTGGCGGAGCGACAGCGCAGGGAGCTG
ACTTCTTCCCCATTTCCCAATATTTTTCTTTTACTATTATTATTGCGCACTGCGACATCCCGTGAAAA
CGCGCCGAGGGTTTTGAAAGAAGTCTTTTTTC
GGTGATGGCGGAGGCGCCTGATAATAGGTTTTTCTTTTTTTCTCTTTTGTGGACGGGCACCGAGTGGA
TGTGCGGGTGCTTTACTTCCTTCTTGTTGGGC
TCACTGGGGATGTTTCAGCGCTCTGGGGCGGTAGGTGTGTTGTGTTTTTGGAGTGGAGGCGGGTCTAG
CCCGATTGCCTGTTTCTTCTTTTTGTTGCCTC
TCTTGTTGCTTAGGATGTTTTGCTCGGCTGCCCTCTCGCGGGATGGACCGGCCGTTCGTTTTTTATTA
CCCCACTGCCACCACCACCTCTGCGGTGTGAG
TGCTGCCCCAGATTCCACGGCCATTGCGCGGAGGGGAAGGAGCGCTGTGCGTGCGTGAGTGGAGCGTG
CTCCGTGGGTGAGGCGGCGGCCGTATATCATC
ATTTCTGTGCCGTGACTGTCTCAATCGGGCCCGCAAGAGCAGCACCGACGGCGTGGAACGCGCAGGGC
GACTGACGACTTCATGATGCGATCAATCGAGG
CATTTGTATGACCGTGACGATGAGTTACACCTCCGTGCACCCAGCTACCTACAACAGGCACGGGACTG
ACGCGCCCCCTCTCTCCACGGCCCTCTCCGCC
GCCTTCTCCCTCTCTGTCTCTCTGTGCCACGGTGCACCACCTCGCCTCCTCTGTCGTCCCGGAGTGTG
CTTCCCGGGCTGTGTGTCGCATTCTCCGTTTA
ATTTCTAATCCTGTTGTGCTGACAGTGTTCTTGGTATAAGGCTTTGTTGTGCCTGTTATTTTTGTGTG
ATTTTGATTATACACCCGATTGTTCGTTTGAT
GAGACAAAACCCACTGAGCAGTAGGTGTGGGGTGCGCGTATGCGCAATAAAAGAAAATTGTGGGTTTG
CACCTTTGTGGAGCACGATGTTTCATTGCACC
AACGACGGAATTGCCCGTGGGAGAGCGCACTGAAACCCTCGCACGCCTCAAAGTTACAGGTCCCTTGG
TGGCTGTATCTATCGATGCCGCTTCACAGGAT
ACCCATTGAGGCTAGAAATGAGGATGACCTCCTCATTATTTTTTTTGTTTTTGTTTTGGTGGACGGCC
GGAAATAACAAGTTTGGTTTGAGTGATTGTTA
TGGAGTTTGACATGAGGCGCTGCTGCTGCTCTTCGAATTGGGCACTACATTTTATTCATCGAAAGCTA
TGGAGTCGTTGTGTGTTTACGTCCACTCCTAT
CTTTTGAGGTATATGACTTTTGTTCTTGTAGGGTTTTTGTTCTGATCCTGGCAAGTACAATTTAATTG
ATGTTGCCACGTATGTTTTACCGGTTTTGCGG
TAAATACATTTTGTTTTTTGTTTGTTTTTTTATTTCACGAAAGGAAGATGTCACTTGATGTAGGCTCT
CTTTGTTTCTTCAAGCACCCCCTGGAGTCGTG
GGTTGTTTCCCGCGTCATAGCGAGGGATGCGAGGGGGTACGTGGTGAAGACAAGTGACAGTGAGCGGA
AGTGTGTGGGCGAGGTGTTTAGTGATGTGACG
GGGGACCGCGTCGCCGCCTGCCGGGACGACCTGCTGGACGAAGAACCGGATGATCTTCTTGCGCTCAC
CGTGCTGCACGATGCCCCGTTACTACGCTGCC

TGTACCTTCGTTATTTTCGTAACGTCATCTATACGAACATTGGAGCCATTGTGGTGGCGATAAATCCC
TTTAACTATACGATCCCGTGGTACCAAGACAG
CCAAATGGTGAATTACCTGCAAGAGGGCCCCGTGATTGAGAAAAACCTGCCACACTCTTGGGCGCAGG
CACACAACACTTATTACGATATGATCTCCGAT
CGGGTGAATCAATGCATTATTGTCTCTGGGGAATCTGGAGCAGGAAAAACGGAGACGACAAAGATTGT
GATGAAGTATCTCGCGCAGGTGTCATGCAAGG
AAGGCACGGAAGATGAAAAGGCGAGGAGTCTCGAGGTGGGTGCGAAGTTGGATGCCTGCTCACCCATC
TTGGAGTGTTTTGGGAATGCCCGAACTGTTCG
TAATGACAATAGCTCCCGCTTTGGCAAGTTCATGCGGGTGAAATTTAACGAGAAGGGCCAACTCGTCG
GGGCGGAGACAACAAAATATTTGCTAGAAAAA
TCTCGCATTGTTACTGCCGCAGAGAAAGAGCGTGTCTACCACAGTTTTTACCTTCTGGTCCGAGGAAC
AATGGGCAAGACGCTGTGGCTTGAGGCCGATA
CCGCGTACAAGAGCCTGAATGCGGGGAGGTGTCTACAAAACTCGGAGTACAACACTGCTAAGGAATAC
AACGAGGTCATTGGAGCCATGACGAAGATTGG
CATAAGTGACGAGGAAGTCCATTCGTTGTGGAGGTGCGTCGGCGGTATTCTTTCATTGCTGAACGTCA
CTTTTGACGCAGATGGGGAAGGAGCGCAGGTG
CAACAAGCAACCGAAAAATACCTAAAGGATGCTGTGCGTCTCTGGCGTATAGAAGAAGCGACACTACG
GAAGGAGATGGTAACCACAACACTGGTTGTAC
AAAAGAACGAAACCATCAAGTTGTTGCGGCCAACCCTTGCACTGGACGCACGCGATGCCCTTGTGAAG
GCCCTGTACGACGGTCTCTTTGGCTGGCTTGT
TGACAAGTGCAATCGGATGTGTGATGTGGCGGTGTCGGGAAACTGGATTGGTCTTTTGGACATCTTTG
GATTTGAGGACTTCACAAAAAACAGCTTTGAG
CAGCTCTGCATCAACCTGACGAACGAGACACTTCAAAACCACTACAACAAGTACATATTTGAGAGGGA
CATAAATGAATGTCGCGAGGAGGGCATTGATG
TCACCGAGGTGACATGCCCCGACAACTCCCCATGTCTGCAGCTTATCGTGGGCAAGAGCGGTATTTTT
GCCTTGCTTAACGAGGAATGTACACTCGGGAA
GGGCAGCGAGCTTGCGTTCCTTGAGAAATTGGATCAGGCACATACGGGGAAGAATTCCTTCTTTGAAA
AGAAGAAGGTGAGTCGTGACACGTTCATTATT
CATCACTACGCTGCGAGTGTCACTTATGACGTGAATGGCTGGCTTGAGAAGAACCGTGACACCCTCAA
AGACGGCGTGAAACGAATGATGCGGAATTCAC
AAGACCCACTCATTCGCGAGTTTCTGGAGGCTCCGTTACCTCCTGAAACACGCGGCAAACGACTGACC
GTTGGCGCTGTCTTTCGAGGGCAACTCGATGC
TCTCATGGGGGTCATAAACACCACGAATCCTCACTGGATCCGATGCATTAAGCCACACCCGGCGAAGA
AACCATTAATGTTTGACGGATTGCAGACGATG
CGACAACTTGAATCCAGCGGAGTGTTGGGAACTGTGAAAATACGCAAAGCAGGATATCCTGTGCGAAA
CATCTTTGAGAAGTTTAACAAGCGCTATAACA
TTATACTTGGGGCCAAGGCTCAGGGAAAGAGTGGTCGTGAATTGGCGCAAATGATCCTTATCGCGTGC
GGTATAAATAGCCGTGCCATTGCACAACTGGG
GAAGACGAAGGTGTTTATGAAGGCGGAGGCTTTCCCCATCGTGGAACGCCTGAGAAACGAATCGCTTT
TAAAGTTATGTCTTCGGCTGCAGAGCTGTGGA
AGGGCATACCTTGTGAGGGTTTACGCGAATCGCGAACACTGCGAACAGAAGTGCAGGCGACTTGCCAT
GCTGCTCACACAAGAGTTTCGTGCGTACATGA
AGCGTTCCGTCGAGTTGAGGGAGGCAAAGGCGCGTTGGCGCAAGGAACAGCTGGCACTGTTTTCCCAG
CGGGTTGCGGCGTTGTATGAGGAGTGCGAGGA
AGAGAAGGTGCAGATCCATTGTGAAGCAATTCGGTTGGTGCAGGGACTGCAAATAGCTTTAAAGAAAC
ATGTTGAACTAATTAAATCCTGGGAGGTGCAG
AAGTCAACAGAACGCCGGCGTTTGGTGGATGCCGATTTGGCGGCACGAGAGCGTATGCTGCAAGAGGC
CACGTTGTACCTTGAGGGGCTTCGCTGTGTCT
TCCGAGAGGAACAGCGGCTGTTCTTCACAATGCTTTTGGGGGAACTGCTCGAAGTGGAGTTTGGCCGG
CGTGCACAGATTCGGAGTGTGGAGAGTGAGGA
ACGGAGCCAGTTGTGGCGTGCCTATGGGTTACGGCGTGCGATGCAGCAGCTGGAGGTTCTCTACAGGG
AGGCTTTGATGTCACACAGGTGTATGGAGGCC
ATGGAAGTTATTCAACGCGACGAAATAAAGGAAAGGCGAGCGTTGGAGCAGAAGAACCGCTCTTATTG
GAAGTCCTTCAAGCGACAGTTTCGGGATAAGG

AGGGTATGGCAACCCGAATGTCCGGATGTCGGGAGCAACGTGAGAGGGCGCGGGTAAGTCAATTAATG
GCGCTGCACGACGTGGTGGCAAAGGAATCCAG
ACAACAGCAGGGTCGCTCGCATTCGTCACACATGAACCCCACAAATTTCTCTACGACAACGCACGACA
TTGGGCCGGACCTGCAGAATCATCCACGCTCA
CCCTCAGGAATTGACACCCAGTGCTCAACTACCCCGCGTCTTTTGCTGCTTTCGGGAGAATCGCGGGC
GCAGTCCGAGACGCCTTTTCCTGCGGCATTTA
TAAATGGTTTTGTTTCTACGAATCGGCCTATGCAGTTTTGGGGGAAAAAACCTAAGCAGGGCGCGAAA
ACCCCTGTGCACGTTTCTGTAGTGCCGACGTA
TGACTTTTCTACCTCTCCCATGCCATCTGCCGTTCTTGATGAGAAATGGGGGAGGAAGAAGTTGATGA
ATAACCACGACAGGGCGCGGCCATCATCGCCT
TTTTGTGAGAAAGCGTCCAACTTGGTGTCGTGGCCATACGCAGTCGCCACACACACCTCAGCAGACCG
CCGACTTTTCCCGAAGCTCTCGGCAGTAATTT
CACCGTCTGACGTCCCCCGTGGAGCGCCTCCGGGAAAACTCCACCCATCAGCTGCCGGGGCGAATTTG
GACGGTACGGTTCAAGAGACCGTGGCATCGGG
GT
>scaffold1112.size6847
cggTGTGCGCAGTGCCGCCACTCGGGGACCGATGTCCGGGTCAACACCAGGAATAGAGCCCTCGCCAC
ACCGACAGGCGCCTGTCACGCGGTGTGCGCGCC
TTCCTTTTCCTCCTGGGCATCCTTCCATTATAACCTCTTGACGGCGGTGAGGAGGTCCACGTGCCAGG
CATTCACGCAGTCCTGCGAGAGGGATCGTGCA
ATCGTGACTTCGTCGTCAGCGGCCTCACTGCGTGGAATACCGCAGTGTGATGGGAAAATATAAAATCC
ACAGGGTGACCTCGCTTCACCAAGGAAAGCAG
CATAATCCAAATTTACTCAGTAACGCGGTCTCGCACGGTCGGTGGGCAACGCGGGGTGCCTCCAATGC
CGAGAGGGAGTCCGTTTCCTCCAGCATTCGTG
TGGGCGATTGGGATGTTCCCGGACAAGGAGGTATGAGATGTATAAAAACATTTTCGATCGCAGGGCAG
TCCGCCCTATATGAGCATGCGAGGCTGCCAGC
TGCTGCACGATGTACCTCCACAGGGAGATCATTTGTCGTGGTGGAGTCATATAATAGCGCAGCGGATC
CAGATGCGAGTGCAGCGAGAGAAGAAGGACCG
TCCGTCCACAATTCAAAATCACAATCCCCCAAAGAGGAGTGAGTAGAATAAAAAGCCGTGCGCCCGAC
ATCGTCAGAGGCGTCTTCGGCAAACAGTGGCT
GATAAAATCTCACCTTGTTGCAGCTCGCAGTGTCCCAGGGGAAAAAATCGATGTTGGGCAAGAGGCGC
GCAAATGTGGTTGTGGGGTGTCCCAATAATGT
CACGGACGCGTAGCAATCCATTAATAACAGCATCTCTCGGAAGCGGGATCGATGTCGCCGCTTTCCCA
TGCATGGACGGCGGCATAGGTTGCGAGTAGAT
AAGGGAACGCAAATCCTCGTGATCATGGAGGCGTATATAGCGTTCGTATTGTGTGAGTGCACGACGCC
AGAGTATCTTCCGGAGCGGAAGAATATTGGCC
TCCAAATAGACAGATGTATCTTCCGTAGAGGTGCTCAGGCCAAGAATGTTTTGCTACCGTCGCGGTAC
CGTACCTCCATATTATGGAGGTGAGTGGGGGC
GAGTCACGGGACAATTGCCTCGCCATTATGAAATAGCACACTATGAACGTATCCAATCAAAAAGGATC
GCatggtgtgatgggacgcNNNNNNNNNNNNN
NNNNNNNNNNNNNNNNNNNNNNNNNNNNNNNNNNNNNNNNNNNNNNNNNNNNNNNNNNNNNNNNNNNN
NNNNNNNNNNNNNNNNNNNNNNNNNNNNNNNN
NNNNNNNNNNNNNNNNNNNNNNNNNNNNNNNNNNNNNNNNNNNNNNNNNNNNNNNNNNNNNNNNNNNN
NNNNNNNNNNNNNNNNNNNNNNNNNNNNNNNN
NNNNNNNNNNNNNNNNNNNNNNNNNNNNNNNNNNNNNNNNNNNNNNNNNNNNNNNNNNNNNNNNNNNN
NNNNNNNNNNNNNNNNNNNTGATGGGACGTGC
CCGCCTCTGCGACCAGACGCAGCTGATAGCGGCGTAGCATGGCCCTCTTTGAAGCGGTGGAAGCATGC
GTAACAAAATTGAGAAGTCGATCGAAAGTGTT
GCCCAGTAAGCGCACTGGGCATGACTGAATACGTAGGTTCCCGCTAACAAGATGGAGATCGACCATGT
CCTCGTCAGACCGTGTGTGTGTGAAGAGATGT
AGAATAGAGCAGCTTCGCTCTTATCAACGTTGATTTTTAGGCTGTGCTCGGCAGCCCATGTGGCCACA
AGAGACAAGAGCGGTCGCATTGCCGCAGCACA
GGCATGGATGTCCGCACCTGAGGCAACAAGTGTGTGTTCGTCAGCGTACATGAATGCGGAGGCAGAGT
AGATGTTGCCCAGGCGGCGCAGCAGACCATCC

ACACAGTAGAGGAAGAGTTGGGGTCCCGGGACGCCGCCTTGCAGTACTCCAGCTGAGGCGAGTTGTTT
CCGGGAGTGCTGTTCGCGAGTGCATACCCTAA
CATAGCGGCCCCGCAGGTAGTTGTGCAGCCAGTGTTTGGTTCGAGGGCCAAGGCACGGAAGTCTGTCA
AGCATGCAGAATAACTTCCCATGGTCAGTGGT
GTCAAACGCAGTCGGAAAATCAATCAAAACTACGNNNNNNNNNNNNNNNNNNNNNNNNNNNNNNNNNN
NNNNNNNNNNNNNNNNNNNNNNNNNNNNNNNN
NNNNNNNNNNNNNNNNNNNNNNNNNNNNNNNNNNNNNNNNNNNNNNNNNNNNNNNNNNNNNNNNNNNN
NNNNNNNNNNNNNNNNNNNNNNNNNNNNNNNN
NNNNNNNNNNNNNNNNNNNNNNNNNNNNNNNNNNNNNNNNNNNNNNNNNNNNNNNNNNNNNNNNNNNN
NNNNNNNNNNNNNNNNNNNNNNNNNNNNNNNN
NNNNNNNNNNNNNNNNNNNNNNNNNNNNNNNNNNTGTGGAGGCGTCGTCTCCAGGGCGCTCATACTCC
ACAATGCCAAACTCATTCAATCCACGAGTAAT
TTGTCGATAACAAGTCCCATCACGTCAGATGCAGAACGCGAAGGTGTAAATCCGAAATTGACGTGGGT
GGTGCGGCAATAACGCTGACAGACGGCGGTGA
ATCACTCCTTCGGTTAACTTTGGTAAAACAGAGAGTGGCGTGATAGGTCTGTGACTCTCTGGGCGGCA
TGGATCCTTCCCAGGCTTGGGGATAGGAATAG
CGTCTCCCATTTTCCAGGAGCCACGCAGGCTGCCATTTGCGAAGGAGTTGTGGATCATCGTTCTAAGT
GTGCCACACGCGACGGGCACGAGACGGTGCAG
GAATTCATTCAACATGTCGTCGGGGCCGGGGGCTGAGCCAAGCAAAGAGTCACGGATGGTCACGTCAA
GTTCATACGGCGGGAAAGGCCGATCAATTTCC
CAGCCACCAGGAACCAAAGGAGTGCAGCGAGCAGCAGCAGCAGTATAAGGCGGTACGGCACGTGNNNN
NNNNNNNNNNNNNNNNNNNNNNNNNNNNNNNN
NNNNNNNNNNNNNNNNNNNNNNNNNNNNNNNNNNNNNNNNNNNNNNNNNNNNNNNNNNNNNNNNNNNN
NNNNNNNNNNNNNNNNNNNNNNNNNNNNNNNN
NNNNNNNNNNNNNNNNNNNNNNNNNNNNNNNNNNNNNNNNNNNNNNNNNNNNNNNNNNNNNNNNNNNN
NNNNNNNNNNNNNNNNNNNNNNNNNNNNNNNN
NNNNNNNNNNNNNNNNNNNNNNNNNNNNNNNNNNNNNNNNNNNNNNNNNNNNNNNNNNNNNNNNAACC
GCACAAAATGACGGACAAGCAGATTTGCCTGT
TGCCGAGGCAGGGCGCATACGCGGTCAAGTTCAGTGCGGAGCACAACCGATTCCAGAGGATGAGGGTG
CGGCGCCGCCATGCCGTGAAGATATCGCCAGC
TCGGGCTGGGTGAGGCTCCACGTTTTTTCGCGCGGGCTTCCAAAAGCACAGCGGAGCAACGGCGTAAG
GCTTGATTTCGTTCCTCCTTTTTTCGGGTACA
TTCGGCTCAAAGTAGGCTGTCTCCAGGTGAGGCAATGTGTGTGCTTCGTGTGCTGCTTCGGCGGCGGA
CTCGGCCTGTTCCATTTCGTGTGTCCATGTCT
TTTTTGGNNNNNNNNNNNNNNNNNNNNNNNNNNNNNNNNNNNNNNNNNNNNNNNNNNNNNNNNNNNNN
NNNNNNNNNNNNNNNNNNNNNNNNNNNNNNNN
NNNNNNNNNNNNNNNNNNNNNNNNNNNNNNNNNNNNNNNNNNNNNNNNNNNNNNNNNNNNNNNNNNNN
NNNNNNNNNNNNNNNNNNNNNNNNNNNNNNNN
NNNNNNNNNNNNNNNNNNNNNNNNNNNNNNNNNNNNNNNNNNNNNNNNNNNNNNNNNNNNNNNNNNNN
NNNNNNNNNNNNNNNNNNNNNNNNNNNNNNNN
NNNNNNNATGTTGGGACAGCCTGGCGGAAATACACGGACAGCAATGGTCTCCAGGAGGTCAGTGCCAG
GGAGCGACAGGCGCTCGCACGGTGGAGAGTGG
CGCACCAGAACGGCAACACCACCACCGCGCCCAGTGCGGGCCTGAAGGTAGTCATTATGGCCAGATAA
TTTCGTCGCAACGTCACCACGGTTACTTATTT
CCTGCAGAAGAACGATGTCAGGTGGACGCTGCTCCAATCGCGCAGCAAATGCAATATTTTTCACGGAC
GAGAGGCCTGCAATGTTAAGCTGCCTAATATT
GATGAAGCCATCCACCTCGGGCCATGGTTCTTGTGGACGTCACCGCCACGGGTGATATCAACGGAGTG
ACCGCTGCGAAACGGTCGTGCAGTTCTGTCCG
ACAGTCTGCAGGCAGACCGCCTCACCTCCCCACACCGGGCATGGCGNNNNNNNNNNNNNNNNNNNNNN
NNNNNNNNNNNNNNNNNNNNNNNNNNNNNNNN
NNNNNNNNNNNNNNNNNNNNNNNNNNNNNNNNNNNNNNNNNNNNNNNNNNNNNNNNNNNNNNNNNNNN
NNNNNNNNNNNNNNNNNNNNNNNNNNNNNNNN
NNNNNNNNNNNNNNNNNNNNNNNNNNNNNNNNNNNNNNNNNNNNNNNNNNNNNNNNNNNNNNNNNNNN
NNNNNNNNNNNNNNNNNNNNNNNNNNNNNNNN

NNNNNNNNNNNNNNNNNNNNNNNNNNNNNNNNNNNNNNNNNNNNTCCCTCGTATCCAACACAGGCA
AAAACCCTGCTTGTGGTTACAGACAGTCATTC
CCTTCTAGCCGCCGTCACCAAGGGCCCGCGCAGCCACACCGACTGGACGGAGGATCGGATCTGGCAGC
GTCTCTTGTCACTGACGCGTGCTGGCTGGTCC
GTGCAGCTGCAGTTTTGTTACAAACATCGCGGAGTACATGTTAACGAGCCTGCAGATCATTAGACGCA
GACCACGGCAACCGGGCAATACACGGAACATG
GAATAGCACCCATATGGCGCAGGGACCTGCTGACATGCATTAACACCCAGCTCACCAACAAGTGGCGT
AGTACCATGCATCAGGACACCCACCACTACCT
GCTTGGCGGCGCCAGGCCATCAGATCCAGGCGGCAAGCACCTGATTACTCAGGNNNNNNNNNNNNNNN
NNNNNNNNNNNNNNNNNNNNNNNNNNNNNNNN
NNNNNNNNNNNNNNNNNNNNNNNNNNNNNNNNNNNNNNNNNNNNNNNNNNNNNNNNNNNNNNNNNNNN
NNNNNNNNNNNNNNNNNNNNNNNNNNNNNNNN
NNNNNNNNNNNNNNNNNNNNNNNNNNNNNNNNNNNNNNNNNNNNNNNNNNNNNNNNNNNNNNNNNNNN
NNNNNNNNNNNNNNNNNNNNNNNNNNNNNNNN
NNNNNNNNNNNNNNNNNNNNNNNNNNNNNNNNNNNNNNNNNNNNNNNNNNNNNTCACCACAGTCATTT
TTGTGGTTTGGTCGGCTTCGTGTTGTGCTCCG
GGTTCTCCTCCTTCATCGCCCTGCGGTGCTTCCTTCGCCGTCCCTGGCACCTGTGACTGTCCCGTGAC
GGTTTGTTCCGAAGCCTGACTGCCCTGGGAGG
TCCTCCCGCCACCAGCATGCACTCCACCCGCTGCCTGCTGAGATGCGTCGGAGCGTGGGGATGTTGTG
CCTGCGGCGTGCCCTCCGCCAAAAGACTCCGG
TCCATTTGCACGCATCTGAGCGGCAGAGGCGCTGCCAGTGCCGGACGGATTGCAATTGTTACTTCGCA
GTGGGTCGCTGCAGGATTGATCCTGCCTCGTC
GGAGCAGTGAGTGGTCCTGCGGGGGTGGGATTAGGGCCCTCACCCACGGTGCTTCCTCCACCCTCCTC
GTCTTCCTGATTGCTATTGTCCTCAGAGTTCA
CATTCCCCTTTCGTCCAGAGCCACCGGCCACGGGTCCACCTTCCGCAGCCATCACAGCTTCCGCNNNN
NNNNNNNNNNNNNNNNNNNNNNNNNNNNNNNN
NNNNNNNNNNNNNNNNNNNNNNNNNNNNNNNNNNNNNNNNNNNNNNNNNNNNNNNNNNNNNNNNNNNN
NNNNNNNNNNNNNNNNNNNNNNNNNNNNNNNN
NNNNNNNNNNNNNNNNNNNNNNNNNNNNNNNNNNNNNNNNNNNNNNNNNNNNNNNNNNNNNNNNNNNN
NNNNNNNNNNNNNNNNNNNNNNNNNNNNNNNN
NNNNNNNNNNNNNNNNNNNNNNNNNNNNNNNNNNNNNNNNNNNNNNNNNNNNNNNNNNNNNNNNTGCC
CAGCAGCCACCGCGCCATCACCCGCGGCCGCA
CACGCACCCCGTCGCAGCAGCCGTCACTCACACGCACGAGCAGCTGCGCATCACTGCAGGAGCCAACA
GGACGCAGAGCACACGGCCAGTCATCATCACG
AACAGTGGATTCGCCTAACGCTGCGGAGCCAACCTGCGATAGCATGTGAAAAACAATAGGAGAGACAC
AGCCGCTAAACCACGCACAGCCACCGCACAGC
AACAAATGGCAGCACCCCAGCAGCAGCAGCGCACACACCACGAGACAGATGGAATCAGTACGATTGCA
GAGCTCGCACACCGCACCGCAAACCAGAAGTT
TAAAAAGCAATAAAAAACAATAATTAAAAAACAATAATTAAAAAGAAAAAAAAAACAAACCCAGGGCT
CCAGTGCGCATGCTTATGAGTGCGTCCGCGAT
GCGTTCAACGTGACGGATGCGGGGCACAACGCGGAGCACACAGCACGGCAGCAGAAGCAAGTGAAGCA
AAACACAATCATTATTTCCTCTTATTTTATAC
AGTGGACCACAACACACGCGCACAAATCCCCGCGGTTGGGTGCTGGCACGCACAGCCCGCACACACCT
CTTCAGCCCAGAGCGGTGTACGCCAGCGCGGA
TGCGGCGAGCACCAGCGGGGCACACACCCACGCAGAGCTGCTGAGG
>scaffold1113.size6697
TGTGTGCGTGTGCGTGTGCGTGTTTTTGTCTCCCGTTTGTCTGTCTGTCTTTCCGTGGGCCAAAATGA
GATGAAATATGCGTTGCGGTTTGTGATGTTTGT
AGACGTGTGGACGGGGGTGCCGCGTGTGGGCGCCCTCATGTGTCTGTACGATACGACGTCGATTTTGT
TGTTGTTGTGAAATGCAAGCAGACATGAGTAT
TGCATCTCTTCTTAATCCTACCGGTAACGCATGCACATGCGTGTACATGCGTGTGGATCAATGGCAAG
AGAGAAGTGCGAGAAGAGGAGGTCAAGAGAGA
GAGAAAAAGAAGAAGAAGAAAAAAAAAAAAAAAAAGAGGGCGTAAATGGGGAGGAAAGATAAAAAAGG
AGGAAATGAAGGGAAGGGAGAAGACAGCGTAA

TCGATCATAAAAAATAGTGATATGCATTTGCTATTAATAATAATAATAATAATATAATTATATATATT
TACGGCATGAATAACTGAAAAGGCAAAAGGCA
AAAGAAAAAGAANNNNNNNNNNNNNNNNNNNNNNNNNNNNNNNNNNNNNNNNNNNNNNNNNNNNN
NNNNNNNNNNNNNNNNNNNNNNNNNNNNNNNN
NNNNNNNNNNNNNNNNNNNNNNNNNNNNNNNNNNNNNNNNNNNNNNNNNNNNNNNNNNNNNNNNNNNN
NNNNNNNNNNNNNNNNNNNNNNNNNNNNNNNN
NNNNNNNNNNNNNNNNNNNNNNNNNNNNNNNNNNNNNNNNNNNNNNNNNNNNNNNNNNNNNNNNNNNN
NNNNNNNNNNNNNNNNNNNNNNNNNNNNNNNN
NNNNNNNNNNNNACAGCAGCCGCAGCAAAAAAAGAAAAAAAAAAGAAAAAAGGAAAAAAAAAAAAATG
TGACACGCAATTATCGCAACGGAAAGAATAAG
TGAAAACACACGCAAACACAAACACGCACATATTTATAAAAAAAAAAAAAAGAAGAAGAAGAAGGCAT
GGTTACAGCGTGAAATTGAATTTAATTTGCAC
GATTTTGTTCTTCTTGTTTCGGTTTGTGTATTTATTTATATTTACATGCATTTTTGTGCGCACATCTA
CCGATGACGAATGAAAAAGAAAAAAAAAAAGG
TGGTGGTGGTGGTGGTGGGTGGGTGGGGGGAGAACGGATGAACATCGGCAGGCGACCACCGGCTTAGC
GGCGGTCAGTAAGACATGTGGGCGTGGAATGA
AGAAGGGAAAGAAGGGAGGCGCGATTCCCTCCACACGTAACGGCACGTAGAGCAGGAAAGAANNNNNN
NNNNNNNNNNNNNNNNNNNNNNNNNNNNNNNN
NNNNNNNNNNNNNNNNNNNNNNNNNNNNNNNNNNNNNNNNNNNNNNNNNNNNNNNNNNNNNNNNNNNN
NNNNNNNNNNNNNNNNNNNNNNNNNNNNNNNN
NNNNNNNNNNNNNNNNNNNNNNNNNNNNNNNNNNNNNNNNNNNNNNNNNNNNNNNNNNNNNNNNNNNN
NNNNNNNNNNNNNNNNNNNNNNNNNNNNNNNN
NNNNNNNNNNNNNNNNNNNNNNNNNNNNNNNNNNNNNNNNNNNNNNNNNNNNNNNNNNNNNNNCTTCCC
TTCTTTCTGACATGTAGCCGCTCACTGCCCCT
CTTTTTTCCCCCTTTCGATCAGTGTGCCTTGCGGGTGGTTGTAGAGAAGGGGATGCGGGCATGTACTC
TTGACAATGAAGATGGGAAAAGAGAAGGAAAG
GAGGAGGAAAAGTAATATTTCTTTGCATACATGTCTTTCCTTTTCTTTTCTCTGTTTGGTTTTTGCGT
GTATATAAATATAAATACGCCTGACCTTTCAT
TATTATTATTATTATTCTTGCGTATCGTGTTGTGTGTTTGTGTGGCGGCCTCGTTTTTTTCTGTTTTT
TATTTATTTATTTTCCCCTTCATTTTTATTTT
ATTTTATTATATTTTTTTTTTTGTGTTATTTATTTTNNNNNNNNNNNNNNNNNNNNNNNNNNNNNNNN
NNNNNNNNNNNNNNNNNNNNNNNNNNNNNNNN
NNNNNNNNNNNNNNNNNNNNNNNNNNNNNNNNNNNNNNNNNNNNNNNNNNNNNNNNNNNNNNNNNNNN
NNNNNNNNNNNNNNNNNNNNNNNNNNNNNNNN
NNNNNNNNNNNNNNNNNNNNNNNNNNNNNNNNNNNNNNNNNNNNNNNNNNNNNNNNNNNNNNNNNNNN
NNNNNNNNNNNNNNNNNNNNNNNNNNNNNNNN
NNNNNNNNNNNNNNNNNNNNNNNNNNNNNNNNNNNNNGTTTGTTTGCATTTTTACCCAAATTGTTTCTG
CTTAGCCGTCGTCGTCGTCGTCGTCGTCGTTG
TTGTTTTGTTGTTTATTATTCTTCCACTTCCTGCTGCCTTTTATTTTTAATTATTTTTATTTTTATTT
ATTTAAACAAAAAATAAAAAAATAAATAAATA
AAAATAAAAATAAACGAAAAAAAAAAAAAGAAAAAACGATCTTCCCCCTCATGTGTCTGTTGTATGCAT
GCATGCATGTATGTGTTGTTGCCAACGGCAGC
GCTCTTTCCCGTGCAGGGATGAAAAATTGTGATGGGATGAGTGGATGAATAACAATAAGAAGGGGGTA
GAAAAAAGAGAGGGAGGGGAGGGGAGGGGAAA
GAGGGAGGGACGTGCCTCTCCACGGAAGCACGCATTCACCGCCAGCTTGCATGGGTGTGCACCGTCGT
AATATTGCCTGACTGCATGTCTTTTTTCTTTT
TTTTCTTTCTTTGTGTGTTTGTGTTTGTTTGGCCGGCCTTTTGGCTCAGTCCATCTCACACAAATATG
CGGCCGCCACAATGGCTCCTCCTTTTCTCTCT
TCTCTTCTGTGTGTGCGTCCGTGTTGTGTTGACTTCTTTGAGTCGAGTGTATCGCCTGAAAGGGACTT
CGCCTTTTTTGTTGTTTGTTTTTTTTGTTTTT
TTGTTTTTGTTTTTTACTGGGCCCATCTCTCTTTTCTTTTCTTTTCTTTTTTCTTTTCTCTCACGTTA
TTTATTTTAATTGTATGATTGTGTGCTTGAGA
GGGATTTTCTTTTCTTTTTTTTTTTTTTTTCTTTTTTTCTCTACTTCTTTTTTACACGTTTTTACACTT
GTCTAGTTGAGCTGTGTACATGAGTGCATGCA

TGCACGTCTCTCTCTCTCTCTCTCTCTGTGTGTGTGTGTGTGTGTGTGTGCCTTTTTTTTTTTTTT
TTCCCTCCCGCTTGCTTTTCTTTTTTTCTGTT
TTTTGGTGTTTTTTCTGTTTTTGTCTTTTCACTCACTCATTCACTCACTCCCAAAGAAAAGGAAGTAA
AAAACAAAAACAAAAAAACAAAAAACTACACA
TTCTTCCTGACTGTATTCCATTAAATATTTATGTTTATTTCTCTCTCTCTCTTTCTCTCTCTTTTT
GTGTGTGTGCGTGTGGGTGTGGGTGTTTATTG
CGCCCCCACTTCCCTTTGGGGAGACGGAGGCGGCTGCGTAGTCCTTTTCTTTTTTGCTGCAGCGGCTG
CTGCTGTTGTTGTTGTTGTTGTTGTTTTTGTT
GTTGCTGTTTTGCTTTTTTTGTGTGGATATTTTCTTTTCTTTTCTTTTATTCTGTTTGGCTCCCTTCT
TTAAAAAAAAAAGTAAGTTTGGTTCTGTTTTT
GTCCTTTTGTGTGCGTTTGTGTGCGCACCCGTTCGCATCAGGCGCAGGGGGACAGAAGAGAAGGCAAC
ACAAAAGCGAGAAGATATTGAAGGAGAGGAGG
GGAGGGAGCGGCTTTTTTTTTTTTTTTTTTGGCAGGCAGGCAGGCAGGCAAGAAACGGAAAGGGGGA
CGCTTCATTTGCAACCAAAAGGGTGTGTTTGT
GTGGGGAGGGAGGGAGGGGGGGGGGGGAAAATTTGATAGAGAAGGAAAGGAATAAAGAAACCGGGGTG
TGGCAGCAAAAATAAAAATAAAATAAAGAAGG
AAAAAAAGGGGGAGCACGTCAGTTTGATGCATTCAACCGAGAAGCGGGGACGAGAGGAGACGGAAGTT
GCGGCGGTCCCAACCGTACACGAGACAGAAGG
GGTGGTGGTGGTGGGCAAAGGGGATGATGATGTCAGGAAGAAGAAGAAGAGGCCGCATCTGGAGAATG
ACGCCAATAATAATAATAATAACAGCACCACC
AACAACAACAACAACGACGTCACAGACACGTTGGACGCACATAAGAAGTTTGCAGGTCTTCGGGAGGA
GCGGCCGCAACAAAACGAACCTTTGCAACATC
CTCCCTCCGTTAACGAGGTGTCAGTAGACGGCCGGGGTGAAACGGATGGCAGTGAGGTGGGGGCGAAT
GATGTTGATGTGGAGGAGCCCACACTTTTCTT
CGGCCCCACACATGCGGCGCGTGACGAGAAGAGGAGTGATGGAAGCGGCGTCGATTTACGGTGTGTCG
CCTCCGGTGGTCAAAATCAACGACCGGACGCG
GCGCATGATGGCTGCCGTGCCCCTCACACCGTCGGTGCGGTCACGACTTTGTATTCATTGCGGCAACT
GGAGGAACTTATTGGCAGTGCACCTCTTCCCA
TTGCTCATGCGGGTGTGTTGAACGTCACGCCATCGTCATCGTCATCGGCCCCACCGTCGCTGCGTTTG
AGGGCGGGCGAAACGGTTCTCATTATGTTGCA
TCGGTGTAAGGGAGAAGCAGGAGTGTTGCCGCAGCACACGACGGAACAGCTCTCAAATTGCCGTATCT
TTTTACTGGATCTCGGCGAGCTGCCGCCGTGT
GAGTACGAGGAGAAAAGCAATGTGGCGTGTCCGAACGGCCATGATGAACGAGGAGTTGCTACCGGCTT
CCTGCAGTTGCCTCCGACAGACACCCTCCTGT
CGCTGGCGTCACAGGGGGCACACGACATGGAGCACGTCATGCGACGTGCGAGTTCGTTGCTGTGTGTA
GATGAAGTATTGAATGCAAACGATGCCGACTC
CTCCAAGTCCCAGACGTCCGAGAAGGGCGGTGGCGACGAGAACGCATCCGCAGACGCCACCCACTCGT
GTAAACGTGAGGAACTCGCTCTTCCAGCCTTA
GTCATGTGGCGAGCGGAAGGAGGGCTGGCCGATGAGTATTGTTACCCGAAGGAAAAGGGAAAAAATGA
TAGCCATGCCAACAACCACGACAATCCACAGC
CGCCGTTGGCCGGCAGATACCATCATGGACGACCGTTAGTGGTGAAGCAACTGACGTCGTTGGATCAA
CTGCACTCCTTTTCATTACTGACACCTGTTTA
TACGGTGACACATTTTCTTCGCACGGTATGTGAAAAGGCGCTTTTTCCGGATGACCCCGCTGCAGGTG
CCTCAGGCACCCGATCTCTTATTTACTTTGGT
GCGTCATGGTGCCCGCCGTGCATGCGGATTGTACACGCCCTTCCAACAATGATGAAGGAGGATTTTCC
CAGCAATATGACGTGCTCCGTGAAGGCTGACA
TGGACCTTGCCACCCCGTTGTTTGAGTTCTTCGGGGTGGAGATCATCCCCACCTTTGTCATCCTTGAC
AATGAATCGCTGCGAACAGCCGGGGACTGGAA
AGCACTTCGTAGTTGCTCCATTTCAGAGGAGGCCCTGTCGCAGATCTATGAGGGCCTACGCCGCAGCG
AGCTAGGACGTATCCAGAACTCACAACGTGTT
ACTGTGCGCACGTTTATTGAGAGACACTCGGGTATGCTGAAGTTTGATGAGGACTTTTAACAAAACAC
GAATGAGAGGGGCTTCCAAAAGTAACGAACGC
AACCAGACACACACACACGTACATACACAAAAAAACAATACATGCTCCCCCATGGTACTGGCGCGTCG
TGAAGATTTTATACGCATGAATCGTGTGCATA

AAACGACGCGGAAATTATACTGGAGATATGTACAATTATATACATGCATATTTGTGTGTGTGTGTG
TTTGTGTGTGTGTGTGCCTGTTTTACTGCCCG
TTTTATTTTTATTTTTATTTCTCTTTCTTTCTTTCTCCTCATGCTTCCTATTATTATCACCGTTATTT
AACTGCTTTTTGTTGTTGTTGTTGTTTTTTCT
TGCGTGTCCTTCCTTCAATTGTCGTAACCCTCGTGTTTTCCCCGTGCAGGAAAAGAGAGTGAGAGAGA
AGGAGAGGCAGATGGAAGGGGGGAGGACCTTT
TTAATAGAATCCATAGAGACTGCGGTGGGTGGTTTTTTTTCTTCTGCCATTTGCAAGGAACCGTGTGT
GTGTGTGTGTGTGTGGTGGGGCAGTACACATG
ATGTGTTGCATGTACATGGCCACTGTGAGGTGTCTCTCTGTCTGTAACGCACCGCGTGTGAGAAGAGT
ACAGAAGGTGGGGGACTGGGATTGGCATGCAT
CCATCAAAATGTTGCGGCTCCACCCTCGGCAATATGCACGCATGCATGTCTATTATTACCATTATTAT
CATTATGACGTTACGATTACATCTTCCCTCCG
ATACGACTGGAGCAAAAGTGGAGTTTGAGGGGGGGCGTAAAAGGAGGTGGACCCCTTCTTTTTGTTGT
TGTTGTTGTTTTGTTTTTTTACGCCTTTCCTC
ACAAACTTCAAACAAACGAAGAAGAAAACAACTAAAACAAAAAGAAAGAATAAGAATGGGGAGGGGGG
GGAGATGGAGTAACGACAAGAGCAAAACAAGA
AGGAAACAACAACAAGAAGTGTATACACACATATATATATCGGGCGTGAATGAATGAGGATGCAAGTT
GAAATGGATGAAAAAAAAAAAAAAAAAG
>scaffold1114.size6406
GGTGCATCGCGTGCGCCACGACGTAAGTCATGCTCGGGAAGCGCATCGCAGCCCACGCACTCGCCGCG
TCCACGCCGCGCCACCGCTGGAAGGCCGCCGTC
ACACCGCAGTGCACGCACCCAAACACAGCGGCGAGCAGCGCGTTGCCCACCACCATCCACAGCGGGTC
AAGGGCAGCAAACACCGACAGGAAGTACGGCA
GCGCGACAGTGCTCGCACGCTCCTGCGCCGAGGCGCATGGCATCCTCGCGAACACGCCGAGCACCTGC
ATCTCCAGGATGGAGCCCAGCCCGCCGGTCAC
CACCACGGCAACAGCCGCCGCACCCGCAACGACGCCGGTGATCGCCGAGAGGGTGCTGGCAGCGGCGG
GCAGCGTGTTCGACCTGATCGTGAAGGACCCA
AGCAGCACGCCCTTGCAGCCGCCGGACACAGCCACAGCGTCGCAGCGAATGACTAGCGTCTCGTCGGC
AGCAATGAAGTAACCGCGGTGCACGGGCACAG
CCAGCTCCAGGACGGTGGACGGATTCGTCGCGTTGCGCACCCACGTCGCGTTGCTCAGCATCGCTCCC
CACGGACCGCCAAACCCGCTCGGCTGCNNNNN
NNNNNNNNNNNNNNNNNNNNNNNNNNNNNNNNNNNNNNNNNNNNNNNNNNNNNNNNNNNNNNNNNNNN
NNNNNNNNNNNNNNNNNNNNNNNNNNNNNNNN
NNNNNNNNNNNNNNNNNNNNNNNNNNNNNNNNNNNNNNNNNNNNNNNNNNNNNNNNNNNNNNNNNNNN
NNNNNNNNNNNNNNNNNNNNNNNNNNNNNNNN
NNNNNNNNNNNNNNNNNNNNNNNNNNNNNNNNNNNNNNNNNNNNNNNNNNNNNNNNNNNNNNNNNNNN
NNNNNNNNNNNNNNNNNNNNNNNNNNNAATTC
ACCGTGGGGTTAAAAACAGCAAAAGCAACCACTGGGGACACGCGGCAAAGGTTGCCGCTTAACTGCAG
CCAGGAGTTGTAGTAAATGTAGGAAGAGTGGC
TTGAAAAGAAAACAGCCGAGCTTCCTTGCATGTCATTGCCCTGAAACACAACCGCTGACTGAGACTCC
ACACAAAATACCCAAAGAAACAGCATGAAGTA
CTGCACCGGCCTCGTCACAGCGTTGCCTCGAATCACCACGGCAGAGTTGTTCAGTACACACAGCGACA
AGAATTCGGTATAACCTTCAAGATGTATCAGC
GTACCCTCGCCGCCAGTCATATGAAACACGTTGCCCAACACAGCAAACACGGAGTGCCACGACACCCT
CAGCGCAGATCTGACAACGTGAACGGCGCTTG
ACGATGCCCTCACGCTCCTCGCCGTTCACAGTGTTGCACGCAGCCACAATCGCTCCGTTTGTGAGGTC
GCTAGAAAATAAGGGTATCTGCAGCATTGTCG
GCGTGAACGTGCTGGAAATGAATTGGTTGCCGGACACAGACACCGTGGAGTCACGGAGATTCACCGTG
GGGTTAAAAACAGCAAAAGCAACCACTGGGGA
CACGCGGCAAAGGTTGCCGCTTAACTGCAGCCAGGAGTTGTAGTAAATGTAGGAAGAGTGGCTTGAAA
AGAAAACAGCCGAGCTTCCTTGCATGTCATTG
TCCCGAAAAACAACCGCTGACCGGGACTCCACACAAAATACCCAAAGAAACAGCATGAAGTACTGCAC
CGGCCTCGTCACAGCGTTGCCTCGAATCACCA
CGGCAGAGTTGTTCAGTACACACAGCGAGAAGAATTCGGTATAACCTTCAAGATGTATCAGCGTACCC

TCGCCGCCAGTCATATGAAACACGTTGCCCAA
CACAGCAAACACGGAGTGCCACGACACCCTCAGCGCAGATCTGACAACGTGAACGGCGCTTGACGATG
CCCTCACGCTCTGAAACACATTGCCGCTGAGC
ACCACCGCGGAGTCGTTGCTGATCGCCAGCTCATTCATCGCAACACACGACGGTCTGTCAATGGCCAA
ACCCGAACGAAAAATCAGCCTCGTGACCGTGA
AGCGGTTCCCGCTGATCGTAATGTTCGTGCGCGGTGGAAAAACACCCTCCGGGCTAAGcaacgcgtca
NNNNNNNNNNNNNNNNNNNNNNNNNNNNNNNN
NNNNNNNNNNNNNNNNNNNNNNNNNNNNNNNNNNNNNNNNNNNNNNNNNNNNNNNNNNNNNNNNNNNN
NNNNNNNNNNNNNNNNNNNNNNNNNNNNNNNN
NNNNNNNNNNNNNNNNNNNNNNNNNNNNNNNNNNNNNNNNNNNNNNNNNNNNNNNNNNNNNNNNNNNN
NNNNNNNNNNNNNNNNNNNNNNNNNNNNNNNN
NNNNNNNNNNNNNNNNNNNNNNNNNNNNNNNNNNNNNNNNNNNNNNNNNNNNNNNNNNNNNNNNNNNN
ATGTCTGTGTGCGGTGGGAACCCACCCTCCGG
GCTAAGCAACGCGTCATGGAACGTGTTGCCGGTAACGACGATTTTCAGCGACCCCACAGCGGCGTACG
CGTTGCCCAACAGCAACAGCACGGCGCCGTCC
CTCCACGTGCATCCATCGAACGTGACGCTCGCCACGTCGCCCGTCATCCCCCCAATCAGCACCGTCAG
GAACATCCCACCCCCGCTGAACGTCACGTTGC
GGTAGCACAGCCACGACAGCCCGCTGCCCACAGTCTGCGCCACCTCGGGGTACTCCATGTCGCTGATG
CACTCACCAACCGGCGGCGGTGGCGGTGTGGG
CGGCACTGGTGGCGGCGGTGGCGGTGGCGGGCCAGCAGGCACAGGCGCCGGGACGCACACATCGCCGT
GCCCTCCAGCAGCGCACTGGCACTTGCAGTCG
CTGACCGCCGTCGTCAGCGGAGCAAAGCAGTCGCCGTCCTTCGTGCACTCCCCGCACGCGGCAACCGT
CGTCACGTTGGTGATGCCGTTCAGCTCCAGTC
CTGCCGCCGTCGTCACCACCCGTCCAAGAACAGTCAGACACCCAGCAACNNNNNNNNNNNNNNNNNNN
NNNNNNNNNNNNNNNNNNNNNNNNNNNNNNNN
NNNNNNNNNNNNNNNNNNNNNNNNNNNNNNNNNNNNNNNNNNNNNNNNNNNNNNNNNNNNNNNNNNNN
NNNNNNNNNNNNNNNNNNNNNNNNNNNNNNNN
NNNNNNNNNNNNNNNNNNNNNNNNNNNNNNNNNNNNNNNNNNNNNNNNNNNNNNNNNNNNNNNNNNNN
NNNNNNNNNNNNNNNNNNNNNNNNNNNNNNNN
NNNNNNNNNNNNNNNNNNNNNNNNNNNNNNNNNNNNNNNNNNNNNNNNNNNTGCATGTACCCATCAAAGC
TGCAGTACCGCGCGTACAGCGAGCTGCCACCC
ACCAGCGTCAGCGTACCGAACGAGTACAAACCAAAACCACCGTACCTCAAGGCATGCGCCCTGACGTT
GCTGACCAGCAGCGAAGAAGCAGTCAACACAA
CGTCGTGCAGCAACAATACGCTACACATGTTGTTCACGGTGTCCGGCAAATGGACCGCGGAAGACTGC
ACCACGTCAACCTCCGTGACGGCGATGTCGCA
GTGCTGCGGGAAGGCAAGTGCAAAAGCAACAACGGAGGACCGCATCACCGTCTGGCTCACGCGGATCA
GGACACCGGCTGAATCGGCCGCCTCATCTCCC
CCAACAAACTGCAGCGCGGCACCCCCACGGAATGTGCACTCATTGAGCGTGATGTTGATGGGGAGATG
CAGCGGCATACTGTCAAACGAAAACGTCAGCA
CAGCACCCACGCCGCTGAATGTCACGCCCTCGTAGCAGTGGTGCGTCTTCCACATGTTCAGCGTAACG
TTCGTCAGCGTCTGGTTCACCACGCAGCTCGT
GTCGCCGTCAACGGCTCTCTCCGCCACGGGCGGCACAACCGTGTCGGGCACCTCGAAGGGAAGGCACG
ACGCGCCATGCCATCCGTCCTTGCAGCTGCAC
GAGCACGAGCCGCGGTCCACCGACTCCGTCCCGGGCATGTAGCACGCCGCGTCGTCGTTGCAAGTGCC
GCACCCGAATACCTCCACCACTTCCCCAGGAA
ACACACCGTCATACAACACCTCCTCACCGCCCTGCAGGTTGCATCCAACCACAAACCGCGCAGGCGGT
TTCACGACAATGCTCGATGCAAGAAGTTTGCA
AAAGACAATGCTCCCGTCCACCTGACGGTTGTGTGCGAGCGTCACGGTGGTGCCGCTGCCCGACAATA
GAATTTTTTGCAAGGCATCTTCCATAGTTATT
AGGGAGGCTGCGCTCACGACATTGCCCTCCACACGCCACTGCGCACCACCCTGAAGTGTCACAGAGCC
TTCCAAATACAACAGCGTGGGAAAAAAAGAGC
CCACGCTGCCAAAAAGAGTGCAGTCCGCCACTCTCAGCAGCCCCGCGGAGCCCACATTTGTATCCCAA
AAAAAATAAATGCCATTAGCCGCGCTCATCGT
GTTGTTCTCAACGTCAAGGTAGCCGCCCTTCATCACATCAATAGTTTCAACAAAAACGGCCGTCGGCA

CTTCGTCCTCATTTGCCGTTGATAACAGCGTG
TTTCCCTTCACAATGATTCCGCCACCATCAACAACAGTGTCGGATATATAGAATGCGTAACTACTTCC
CTCAATGCGGTTGTCAAGCAGCAGCAGCGTCG
TGTTCGCACCTAGAAAAAAATCCAAAAAGGCAATAGCGTAGCTCAACGTCGACACAATCGCACTGCCA
ACCACCAGTATCTGGGAGCTCGTGCCAAAATA
GCCCCTGAACTCCAACTCACCAGAATCCAACATCGAAGATGTCACGTTCACGTGCACACGCGCACCAC
TCCCCCTGACCGACAGGCCCACAAGGACGCAC
TGCAGCAGCGTGATGTTGAGTGTCTGTTGTGGTGCGTCGAAACGCGAGAGGTCCAGCACGATCGCGGC
GGTAAAAGTCACGTTGACGAAGCACAGACCAC
GCACACCGGAATCAGGATCGTCCACGAAGCTGATGCTGCCACCGTGCACGCAGCGGACCTCAGTGTCC
TTCGCATCTGAGAGCGGGAGCGGCCCAAGGCC
GTCCGGAACCGCAGCTGGCAGACACGTGTCACCGTATCCGCCAGCAGCGCACACGCACTCACAGCCGC
TGATGCTGCTCGTCCTCGCTGCAAAGCACACG
GCCTCCACGGTGCACGCGCCACAGTCCAACACCGTCACGGGCGTCCCAATATTGAGGTCTTCTCCGTA
ATCCGTCACAGGAGAGCCTTTTGCCTCGTTGC
ACACACCGTAGATGGTCGGGCGCGAGTCGGATGGTTGTGaccaatcgtttgtcatgtgtgcatgagtc
gacgagtacgagccgtacgtgaagttgttgtg
aaNNNNNNNNNNNNNNNNNNNNNNNNNNNNNNNNNNNNNNNNNNNNNNNNNNNNNNNNNNNNNNNNNN
NNNNNNNNNNNNNNNNNNNNNNNNNNNNNNNN
NNNNNNNNNNNNNNNNNNNNNNNNNNNNNNNNNNNNNNNNNNNNNNNNNNNNNNNNNNNNNNNNNNNN
NNNNNNNNNNNNNNNNNNNNNNNNNNNNNNNN
NNNNNNNNNNNNNNNNNNNNNNNNNNNNNNNNNNNNNNNNNNNNNNNNNNNNNNNNNNNNNNNNNNNN
NNNNNNNNNNNNNNNNNNNNNNNNNNNNNNNN
NNCCGCGCTCGACAGCAGTTCCACGCCGCCGCCAACGTACACCGCCGACCCGCCCGCCTGCAACGACC
TCAGCACAGTGCTGCTCACACGCAGCTGCGAC
TGCAACAGCGACGTCGCGTGCAGCACAAGCGGCGACGCCACCGCGTTCGTCAGCCCGCTCAGCTGCGA
GTAGCGCATCGGCCCCGGCGTCACAATCGTCG
AGTCACGGACCGTCACGTTCGTGTGTGCCGGCAGGTTGTGCACAAACACAAGCGAGCAGTAGCCGCCG
GACAGCCCGCTCACGCTCACCTCCAGGCTCCG
GTCACTCGCCGGCTCGCCGCTGTAGCCCCGCACGTAGATCTGCGCACCGCCGTCGCACACGCAGTTCC
TCAGTTCGATGCGCAGCGCGCCGGGAAGCATC
GCCGCAACGTCGAACACCACAGCCACGCCGTTCGTTATGTACACGCCGTCCATCAGCACCGTGTCCAC
GGCTCGGCCAACCGTGATGGCTCTCTCCACCG
TACCGCCCCGCAGTCGCAACACCACCGCATGCACAGCAGGCATCCACGCCGCCGCCGCAACCAGCGCC
AACAGAACAAGAAGCGCCACACGCACAGCCGC
AATGGCCCTGCGCCGCACACGGTGCCGGCTGCGCGGACACGCACCCACCGCAGACCACATCCACCGAC
CCAAACAGGTACCAAAAGGGCTGTCGTGGCGT
GTCGCACTCGCCCCGTCGACCGTAACTCCTACTTAGCAAATCTCTTCCTACAGATTGAAAATCCAGAG
ACACAATTCCGATAAATCGTTATCCCACTACT
ACTAA
>scaffold1115.size6672
GCACGGCGCACAGGCGCGATGCGACTCCTGCCCACTCTTCGACAGGCCTGCACGCGCCCTGCGGATCA
CAAGGAACCCTCTGTGTCGGTGTGCCGTCCCTC
CCTCACTGCGGTGACCGGCCCGCCTCTGCCTCCGCCGGAACCTCTTCCCGACGTGAGCGCCGCAACTC
CTTGTCGACCAGCGAGCGGCGGATTGACGTCC
TCCCGCCCAACCTGCGGCAGAGTGCGCTTGTCCGTCCACAGTATGAGGGCACATGCCGGACGTGCTCA
GCCTGGCGTTACAATAGTTCGCGAAGGCCTCC
CGTGCGGATGCCGCAATGGCGCCACCGTCTTCCTGACAGGCGCCTCGCGCGCCGGTCTCTATCGAAAA
TGCCAAGCGTGCCAGCAGGCAAGGCACGCAGG
AAGCACAAACGCGGGTACTCGAGATGGACCAGGCTCTTCCTCCACTCTGCCGGAACCACCACCAGCCG
CCTGATACCCCAGAAAGTCCAGGGGAAGCCCT
GCCGCACATCCTGTGGGAATTCTCCTGTCCTTGAAAACCTAGCGTTGGATTATTTGGGGGCACCACCC
TCGCATGACAACGCCGTTCGGGACAAAAAAAA
ACAGCCACATTTATACGCTCAGTTCTCCGACTACTAGCGAACGGTGGCGGCAACATCGAAAAAAAATT

ATCGCGTGCGACGCCGACAGTTGCCACACACG
ACGGGAATGAAAAGGGGAGAGCGGAATTGGGAATGAATTTCCAGTTCCCCCACGAGTGTTTACACCAA
ATGGGTGCGTATTTATGTATGCGCGTTATGGT
GGCCACTTTCTCGGCGTCACCCTTACGTTTGGATTGGGCAAGCGTTTGTGCATGTGTTCATTCAAAAC
CTTCTATGTGGTCGCGACGCGAAGACACGAGT
TGTTCACAGACACAGCGATCGGCGTCCCATGCTGCGAGGAGTTCCTCAGGGGTCCGTGCTGGGACCGT
ATTTGTTCTCCCTTTACGTACACCCACTTCTC
AATCTGCTGAACAGCTTTGCGGGTGCCACGGCAGACATGTATGCGGACGACCTCTCTATTATCGTTAA
GGGGCAGTCCCGGGAAGACGCCATTCCCGCAG
CCAACATGGTTCTTCAAAAGCTGCATACGTGGAGTCAGGAAAATGGCCTGGCCATTAACCCGTCAAAG
TGTGAAGCTGCTTGGTTCACACTATCCACGCA
CACGGAGTCAGATTATGATCGTGAAGGAAGGTGGCCCCTGGTAGTGGCTGGATGTCAAATCCCAGTCA
TGACCATGGGGGCATCGCGAACTACGAAGCTT
CTCGGCATGGGTCTCGATCCACGACTGACGCTAAATGTGGCGACCACCAAGCAATGCGCTGCCACTTC
GCAACGGATATCGCAGCTACGCTGCATAGCGC
ACAAGGAGGCGGGACAATCTCCAAATGACCTACGCACGTTCGTCATTGGATACGGTGCTTCCAAATTA
CGCTATGGCAGCGAGCTCATACGGGCAGTAGC
GACGGATTCAGCGAAGAATGAGATGCAGAAGACGTACGCAACTCTAGCACGCATTGTCAGCGGAGTTC
CGAGCACTGTTGACCCGGAATCCGCGCTACTG
GAGGCTAATATGCCGCCGCTCCACGTCCTTTGCCTGCGCGCGCGGCTCTCAATATTTGAGAACACACG
CGCATGTCAGATGGACTGGATGCGGAGACCCC
CGCCTGAGCCACCGCCCCGCGCCGGTTTCCGCATCTCGCCATTATCTCGGGACGAGCTATATGCCTTC
GTAGACGCATACACAAAGGACTATGGCATCAC
CGAGAGCTCACCACGCGAGGAGCGGTTCTTTCGCAGTTCCATTCCTCCCTGGTTTGCGGCCTCCGCTC
ACCGGGTCACCATCGGTGTGGAACTTCCGATG
GACCACTCAATAACTGACGAAGAAGAGCTGATAAGGGAAAAGCGCAGAGTCAGCNNNNNNNNNNNNNN
NNNNNNNNNNNNNNNNNNNNNNNNNNNNNNNN
NNNNNNNNNNNNNNNNNNNNNNNNNNNNNNNNNNNNNNNNNNNNNNNNNNNNNNNNNNNNNNNNNNNN
NNNNNNNNNNNNNNNNNNNNNNNNNNNNNNNN
NNNNNNNNNNNNNNNNNNNNNNNNNNNNNNNNNNNNNNNNNNNNNNNNNNNNNNNNNNNNNNNNNNNN
NNNNNNNNNNNNNNNNNNNNNNNNNNNNNNNN
NNNNNNNNNNNNNNNNNNNNNNNNNNNNNNNNNNNNNNNNNNNNNNNNNNNNNTAGCCCTAGAGAAG
CTGACTATCCCTCGTATCCGCCACAGGCGTAA
AACCCTGCTTGTGGTTACGGACAGTCAGTCTCTTCTAGCGGCTCTAAACAAGGGCCCGCTCAGTCAGA
CAGACTGGACGGAGCATCAGATCTGGCAGCGT
CTCCTGACACTGGCGTGTGCTGGCTGGTCCGTGCGCCTGTAGTTTTGTTACGGACATTGTGGAGCACA
TGCTAACGAGCTTGCGGATCAGTATGCAACGC
AGACTATGGAAAGTGGACAATACACGGAGCAAGGAATCGCACATTTATGGCATACNNNNNNNNNNNNN
NNNNNNNNNNNNNNNNNNNNNNNNNNNNNNNN
NNNNNNNNNNNNNNNNNNNNNNNNNNNNNNNNNNNNNNNNNNNNNNNNNNNNNNNNNNNNNNNNNNNN
NNNNNNNNNNNNNNNNNNNNNNNNNNNNNNNN
NNNNNNNNNNNNNNNNNNNNNNNNNNNNNNNNNNNNNNNNNNNNNNNNNNNNNNNNNNNNNNNNNNNN
NNNNNNNNNNNNNNNNNNNNNNNNNNNNNNNN
NNNNNNNNNNNNNNNNNNNNNNNNNNNNNNNNNNNNNNNNNNNNNNNNNNNNNNGCCAGGCCATCCG
ATCTCAGCGGTAAGGACCTGATCACTCAGGAA
GTTCTACACCGTCAGGAACTGGTTCACCTCGCAAGGGCAAGGTGCGGGGAATCTGAGCTCTGGGGCCG
ACTATACTGGGCCGTGAGAGATTGCACGAACC
AATGCCGATTCTGCAACATCTCACCGGAACAGTCTGCATATATGCGCTCTAACAACGATCCAACCGCA
CCGGGGACGGACACTGTTCCCCCGTCGGCGAG
GGAGGAAGACGTCTCTCCGGCAAGGAGACGGACCCTCACACGCCGTCGGAAGGAGAAATGTCCGCACT
GTGATTCCACATTGACGGGATTCTCGGGTCTC
GTCAGTCACTGTCGGTCATTTCATCCGGAACATCCTCCACCGCTTCCCGAGCTCAAATGTGATTTCTG
TGACATGGTTTTCCCCACACGGAGAAGCACCG
CACAGAACAGAAGTCGCTGCGCACACAACCCAGACGCCACACGGCATCGAAACAGCAGTGCCAGGCGG

CGATCTCTGCTGCCGCAGGATCAGCCAGCTTC
CACAAGCACGCCAATTGGCCCGCAGGAAACCTTGCACCATCTACTTCTAGAATGTCCAGGCACCTTGG
CTGTGCGCCAACGGCTGGGCATTGAACAGGAC
CTTCGCCTCGGAAAATCCTCTCAATGGCAGTTGCTTCATCGCAGGAAGCTTTTGTCGTTCCTCGAACA
CCTCTTCGGGAGTCAGACGGCATTGTAGCAGG
AAGCGCTGTCAAGAGCAGAAAAAACACAAGGAACTTCACCCCGAAAAAATCCGAACAAAAGTCGCCAC
ACCGCCCCGCCAACCCCCGTTCCAAAGTTCAA
ATTGCCCCGCGCAATCGGCGAGGAGGTCTACCGAGCTCCCCCGCCAAAGACGCTCGACTGCGGTGCAA
AGAGTCACCGGCATCCCCGCGCTGGGCCAGGC
ATATGGGGGGCGAACGATGTGGCTCGAGAGACCCGGGCGCCGCCGACGTGGGAGCGGAGAACGACGCT
GGCGGGGCAACTCGCAACCCTTTGCCGCACAC
ACGAAAAGCCGACCAGCGAGGAGAGCTGCGTGGCCTTTTCGATGGCGGCGGCGGAGGCGGTGCCTTAT
ACCCGACGGCGGCGCTCCGGGATGCTGCGGCC
AATGTCGCAAACGGATCGAACCCCGCTGGACATGGTGATTCTGGGGTTACAAAAAACCGCGGCCCGAT
CGGAGACGAAGCAGGCGCGCCCCTTTACAAAG
GAAGGGATGGATCAATTTATCCACGGCCGGACCGATTGGAAGGAACGCGTTGTTTTCCGACTTGCGCG
GACAACGGCGAGTCGTTGGTCCGAGATTGCGG
CCTTGACTTTCAGAATTTCACACTGCGGCCGGGCGGGGCCATAACTTTGGATTTTCCGTGGCGCCAAA
GACGGCGAAAGCGGATCACCACCGCGCCTCGC
GTTTCTCAAGGGCATGAGGGCTGAGCGCTTTCGGCGTTTAAAAAGGCCCTCTGCTCTCATCCAGGGTT
GCCGCCCTTCCGCGGTCGCTTCCTTCACACCA
ACGCGGCCAGCGACGACACCTGGGTCAGCATTGTTCCGCACCTCTCCGAATATCGCGCTTTTTTCTGG
GGAGGGTCGAACGGGTCGGCGTGCCCCGTCAC
CTGCGGGATCGCTTGTGGGTCCAAATTTTGCGTCTCCGCGATTGAAGCGGCGCGCCGCAACGCGCCCC
GTTTTTCGGAACGCGCCGTGGCATTTCAATGG
GCCATGGCTCGCTCCACGCGGGCCGTCGCGAGGTTCGTGAGCTTTTTGTTGCGTCAAAGTGCGCCGCA
TAGCTTTATCATGATTTTTATCATCGCACAGC
CGCAATCACAGGGCGCGCGGCAAGAACGCGTCCGGCCACTCAACAAGGTGAACGAACCATCCCGCTAC
GGCAGGCCAACGTGTCCGTATTGAATCCGGAG
TGGACCAAGAGTCGGTTGAACCCGGCTACCTTGGAGAGTCTCATGCGAGTTTGGGGACTCGTCGGGCG
GTCCCCTTCTCCTCCGTCCTCCCTCGGTAAGG
CGCGCGGAGGCCCACGGAGGATTCCAACCGCTGACGCGAGATTGTTACGAAAAGCAGGAATTATCGAG
GACGCTTCGTCTAAAACTGGTGGCGGATAATA
CCCTTTTTCGGTTGTGGAGGAGAAAACTGCCGGTTTACGACGACGATGGATCGCGTAGCCACGCGACA
AGAACAGAGACGACCCTTATGAGGCCAACGCT
CCTCTTTCGCACACTTCCCATTATTTGCCGCCTGTGATGGCTGAGGTCGCTTCCTGCCTGGACTTAAA
GGCGTCCTTTTTTGAAGTCTTTACCGCGGGAG
GCTCGGCGTCTCTTTCGATGCCGCGTGGAGGACGGCACGCTGGAGGAGCTGGCACGGCTCCCTATGGG
ATACAAGGCCGGCCCGGAAATTCTCCAGATTA
TTATCACTTCGGCAATTGCGGTGGTGACGACGGTGTCTCGCCGCCTCTGGGCTGCACCTCCATTGGTT
CGTATCGACGTTTGGATCGACGATATACGCAT
TGCAGGGGTCGATGGGCGATGTGGCATTGTGGGAGGCCCAAGTGCTTCGTAATGCGGACGGCCGTCGC
GCCACGATGGGAAGGAACGCGAATCGGGCGCC
ACGCAGTACACCTTTCTGGGGACGCAATTTGATCACACTCACCGGGCGGTATCTCTGAGCGACAAGTT
TGTCCGGTCTGCATGCGCCATGCCGGCGCTCA
ATTCTTTGACCATCGCGGAAATGGAGGTTGTGGCGTCACGCTTTTTGTATGCGGCCGCCATTTTGTAC
ACGTTTTTGTGCCTACGACTTTCTCATCAAGG
CAGTGCGACGACGATTATCCGCACTTAGCCGGGGATTGTGCTGGAGACATCCCCGGCGAACCTTCCGC
CGGCAGCGGTTGTTTTGGGCGAGGGATTGCGA
CACATGATTGGGAATAATCATAGGCGAATCATCAAGCCCGCCGAAAGGAACGCGCCGCCATCACGGTC
GCGTCGCTCCATGGATGGGGAGCCGTTTTCAT
TCCGGACTCCGCCGACGTCAAAACTGCCGAAGGGAAAACGGAAGAGGAAACCTTTTCTTATCATGCAG
GCCGCGGTGCGCGTGGCACACTCGGCCTTGTC
GGCCTTTCCCGCCATCTTGACACCCACCATGGGATTTTGGGTGGGTAACGCTTCGCCGCTGCAAGGGG

CGGCGGATTGAGGCAGCCCCAAGCTACGCACC
CAGGCGCAGGAGCCGCGGAGGATGTCCAAACTTTTTGGCTCTCACCGAATGCGAGAAAGCCTCGATCG
CGTGTGGTCTGCAGAAAATCCCGCACACGACA
TATCACGCGGCCGTGTTTTCACACGCCAGGACCTGGCGAAGGGGGGCGGCGGGGTCTTGTGGCTGGAG
GACCCAAAGTCCGCCACATCGTGAGCAGTCAT
GTCGCAGATCCTAATCGAAAACAAAGACTAAGCAAACGGTCATATATATATATATATATGTGTGTGTG
TATTTGATGAGAGGGAAGTGTTTTGGCAATTG
GCCGCCTCTGCTTTGGTTCTCTTTTCCCTCCTATCTTGTTTGTTTTCTTGAGATAATTTCAGGATCCG
GCCAGCGACGGCTCATTTCAATATTCCGGGAG
CTTGGGAAGACAATGTTCTTGGGCAAGCGGTTTATCGAATTTGATATTTAACCTGGTAAAGAGAACTC
TCTACGATCTTTTTTCCCTAGTTTATGATTGT
TTGGTTGACCGCGATTAAAGGAATCAGGGGAATAATAACGGCCAAACGAATTTGGTTATTTTATTTTT
ATA
>scaffold1116.size6235
TACGTTGATGCGGATGGGCGTCCATCCCTCCCAATGACGGTCTATGACCGCAGCGCACCAACTGCTCA
AAAAGGGTGCGTCCCTCACAACGACGCGGATCC
AATTCGCGCTGTCGGCACGCATCACATATTCAACAAATCGAGGGTACGTCAAAGTGACAGCAAAGCAC
CCGCTGGCAATCACACCACAAACATCATCACA
TGCAGCACCAGCGTCTCAAACTAATGTGGGGTGTAGAGCAAGTAAGCCATTGATGTGGGTGTTAAGCA
AATATTTCTTGGCTCACTGAAGCAACAAGGAC
CGATTGCGTGGGAAGAAACAAAGAACAGGAGAATACAAAAGGGAAAATTCAGCCATCCGCACTTTAAG
AAGAAAACCAATCAATGAAAGTAAAATGGGTG
CTCTCAACCACAACAGAAGCAAAGATTCAAAACAAAAAAAAAAACAAAAAAAAAGCCGAAGCGTGGGA
CGACAAGCGCACACACACTCTCATTACTCCCG
TAAAGCAAAACACACACACAGAGGGAGAGAGAGAGGTCGCCCACTCAATGCAATAAAAACAAAACAGA
AACAGTTACGGTGCTCCGCCCATGCAGCCAGG
CAGGACGGCAGCGGTGCGTGTCGTGCAAAAGAGAGCACCAACGGGCCTGCATGGGTGTGCATGCATAT
ATACAATGGGCACATTATGTGTGTGCTTCGTG
GGGTGAGGTGAGGGCGAGAGTCCATGCAAGGGGACACACACACAGAGAGAGAGAAGAAAGAGTGTGTG
TGCGGGCGGTGCATGGCTCTCTCACACTCACT
CTCACGCGGCCACCACCGCAGCAGCAGCCGCACACGCAACAACAAGAAGAAGAAAAAGAGGGGAGGTG
GTGTGAGAGACCGCGGTGCTGCCGTCACTGCC
TTCAGACTTCAGTGTTTCATTTCTTTCGGCGGTGGCCGTATTCACTGTTTCTGGTGCGGTCTCAGTGG
GTGCAGAAGCAAGGTTGGTATTATCAACTTTG
TGGTCGTTTGTTTCTGTGGGGTTGGGCCGTGGATCATTGTCATTATTCTCAGCCCCCGTACCCTGGGC
ATCGTCACTGTCATTTATAGAGATGGATGATG
ATGTCGTTTCCGTATGTTTGCCGTTATCGGCAATCTCCACCAGATTTGATGTGTCGCTCTCTTGTTCT
TGTTGCTCTGCCTCAGCATCCCCTGGATGCCG
TTTCAGTGCTTCGGACAGCGCTTTGGTTTGTGACGCTGTGTTTTCTTTATTTTTTTCTCTTGACAACT
GGGAGAGATTTCGTGCCGCTGAGCCCTTTCCT
GTTTCTGGAACAGTCACTGCTGGTGATGCTTTTGGCTGTAGTACTGCCGAGATTGGTAGTGGCGGTGT
CTTTATCGTAGGGGTCGTATTCGTTGCGTTAT
GTGCCTTTTCCGCAACCTCTCCATTAGGTACTGGCACTGCCGGATCATGCTTTGTTGTTGTTTTTACC
ACCAATTCTTCGGAATTACCCGTTGAACCTGG
GGAATCGGCGTCACCAACACCTGGTCCAGGCAGACCAGACGTAATTGCTCCATCCTGTGTGTTGGACA
ACCCGCCTGATCCCCCTCCCGCAACGGCCGAA
ACTGGCTTATGTGACGCACCGGCACTATCCGGTTTCTTGATTGTGTCAGGATTTAAACCTGAAGAACT
GTCACCCGTACTTTGCACCTGTGATTGTGTGA
GCGGTTCATTGGGGACACCTGTTTCTGCAACAACCGCTTTCGTGCAGAAAAACGGGCAGCAGCACAGG
GCAAGCACCAACAGGGCGCACAGCAGACGGCA
CGTCGTCATAATTGTGAGTGTGGTCGTTCAGTGGTGTGGAGCTGATCCGCACAGTGAGGAAAATGGAG
AGCAACAGGGGAGACGAGAGGTGCGGCAGACA
CACACAGAGGGCACACCACATGAGGAGAAGTCAGCAGCCAGGCATGCACCGTACGACACACACTCCAA
TCCACGGCGGCAGGCACACACACACACACAGC

CCTGCCGCAACACCAACACACAGATGGAGGAGGGAGAAGCAAACAGGAGCGGCACACAAGGCTCCACC
GCATGCACACACGGCGAGAGCGGTCCGTCGTG
TCGGCACCCCTCACGGAGGGTGCGCACACACACAAGAAGGTGCAGCGCACACAGCAACACAAAAGCAA
AGTAACAATAAAAAAAAGGAATAATCCCCGCA
CACCACGCGGATTGTTGGTCCACGGGAAGTGCAGCCAATATTCACCACAAAGCAGCTTGTTATCTTGT
TTTGGTTGTATGCTGCTACAATAGCGAATGAG
TAGCGCAAGGACCGTCCGTACAAAAGTGGCAATTATTCCGTCGTGCGGGCTGGGGCAGTCCCACACAG
TCGCAGTCCATATTCTCAATGACAGGGCATTT
CTGCAGTGGATGGGGCGGAGTCTCCCGTGGTCCGTCGGGGATCAGCAGCCCGTGGCGTCTGTGATGAG
GCGGTGGCGGAAGAGCCTGGTCCATCTCGAGT
GCCCGCATTTGTGCTTCCTGCGTGTCTTGCCTGCCGGCATGATTGGCAACTTCGGTGGCGACCGGCGC
TTAAGGCGCCTGTTAATAAGACGCTGGCACCA
TTGTGGCATCCACATGTGAGGCCCTCGCAAACTATCGGAACGCCAGGCTGAGCACGTCCGGCGTGTGC
CCTCATACTGTGGACGGAAAAGCGCACTCTGC
CGCAGGTTGGGCAGGAGGAAATCAATCCGCCGCTCGCTGGTCGGCAAGGAGTTGCGGTGCTCACGTCG
GGAAGAGGTTCCGGCGGAGGCAGAGGCGGGCC
GGTCACCGCAGTGAGGGAGGAACGGCACCACCGACACAGGGGGTGCCTTGTGATCCGCAGGGCGCGTG
CAGGCCTGTCGAAGAGTGGGCGGGTGTCGCAT
CGCGCCTGTGCGCAGTGCCGCCACTCGGGGCACGATGTCCGGGCCAACACCAGGAATAGAGTCCTCGC
CACACCGACGGGCGCCTGTCACGCGGTGTGCG
CGCCTTCCTTTTCCTCCTGGGCATCCTTCCATTATAACCTCTTGACGGCGGTAAGGAAGTCCACGTGC
CAGGCATTGACGCAGTCCTGCGAGAGGAATCG
TGCAATCGTGGCTTGTTTGTCAGCGGCCTCACTGCGTGGAATACCGCAGTGTGATGGGAAAATATAAA
ATCCACAGGGTGACCTCGCTTCACCAAGGAAA
GCAGCATAATCCAAATTTACTCAGTAACGCGGTCTCGCACGGTCGGTGGGCAACGCGGGGTGCCTCAA
TTGCCGAGAGGGAGTCCGTTTCCTCCAGCATT
CGTGTGGGCGATTGGGGTGTTTCCAGACAAAAGGTATGAGATGTCTAAAAACATTTTCGATCGCAAGG
CAGTCTGCCCTATATGAGCATGCGAGGCTGCC
AGCTGCTGCACGATGGACCTCCACAGGGAGATCATTTGTCGTGGTGGAGTCATATAATAGCGCAGCGG
ATCCAGATGCGAGGGCAGCGAGATATGAAGGA
CCGTCCGTCCACAATTCAAAATCACAATCCCCCAAAGAGGAGTGAGTAGAATAAAAAGCCGTGCGCTT
GACGTCGTCAGAGACGTCTTGGGCAAAGCGTG
GCTGATTAAATTCCACTTTATTGCAGCTCACAGTGTCCCAGGGAAAATACGATGTTGAATAGGAGGAG
CACGATTGTGGTTGTGGGGTGTCCCAATAATG
TTACAGACGCGTAGCAATCCATTAATAACAGCATCTCTCGGGAGCAGGATTGATGTCGCCGCTTTTCA
TGCATGGACGGCGGCACAGTTTCCGTGTAGAT
AAGGGAACGCAAATCCTCAATACATCGAAGAAGTGTGAGGTGGTCGTGTTGTGTGGGTGAGCGGAACC
CAATGGTCCTCCGCAGCGGTGCTGGATTGGCG
GCCAGGTGGACAAAAGTATCCTTTGATGCGCGTGGGCCGAGCGATGCAGTGCAGCTGCCGCCGTGCCG
CACTTCCAGGCCGTGGAGGTGAATGGGAGCCG
GACACGGAGCGATTGCCTCGCCGCTGTGGTGCAAAACGCTATGTGGACGCACCCAACCAGAAAGTATC
GCGTGGTGTGCCGGGACGCCCCAGCCTCTGCA
CCCAGCCGCAGCTGATGGCGGCGTGGCACGGTCCGCATCGCAGCAACGGTGACGTGCACACCAAAATT
GAGGTGTCGATCAATCGCATTGCCGAGCAGAC
GCACCGGGTGGGACTTGACGCGTTGGTTCCCATCGCCCAGACGATGGTCGGCTGTGTCCTCGTCAGAC
TGTCGGTGTGAGGCGATGCAGAACAGAGCGGC
CCCGCTCCTGTCCGCGTTGATATTCAGGCTGTGCTCGGCAGCAAATGTGGCCACAAGGGAAACGACAG
TTGGCATTGCCGCAGCACGGACACGGATGTCT
GCACACAAGACAACAAGCGTGCGGCCGTCAGCGTGAATGAACGCCGACGCAAAGTGAATGCTGTACAG
GCGGCGCCACAGACCATCCACGTGGTGGAGTA
GGAGCTGCGGTCCAAGGAAGCTGCATTGGGCTGCCCCGGCTGAGGCGAGCCGCTGTCGGGAATATTCT
TGGTGAGCACACACCGAAACGCAGCCACCTCG
CAGGTGATCGCGCTACCAGCGTTGGGAGCGACGGCCCAGGCGCGGTAGCCCGTCGAGCACGGCCAGCA
CTCGCCCATCGTCAACTGTGTCGATCGCAGTC

GAGATGCCGATCGGGACGGCGAGAGAGCGGTGACGGCGACGGTGCAGTGCGGGACGCCGCCCCTAGCA
GGGCAGAGAGCAGCGGGATGGGCCGGCAACTC
TCCCTGTGCGGCATGTGTCCTTCCCGTGTCTGTGGATGCGAGTGGTGTCCCTCTTTTCCCGGGAACTT
CGCTGGGCTGCCGTCAGCGAGGGAGGTGTTGA
TCATCGTTCTGTGCGGGCAGAGGGTGCAAGATGGCGCAGAAACACATTCAATACGCCGACGGGTCCAG
GGGCCATGCCAGGCAAACAGTCGCGGATGGCC
GAGTCAGGCACACACGGCGTGAAGGGCCAATTCATACCTCAGTCATCAGAGGGCACGCAAGCACAAGG
TGTGCGGACAACAGTGAGGATCGCAGCAAGTG
CGACGCGGACAGTGTGACGGACGAGCGGGTTGCCCGCTGCAGAGGCAGAGCACAGACGCGGTCCCAAG
AGCACCGCAGCCCAGCCGATCCCGGAGGGCGG
GGACGTGGTGCCGCCATGCCTCGGAGCACCACAGGCTTGGGCTCGAGAGAGTACCAGGCTATTTCGCG
CGGACCTCTGGCAGCATGGAGAAAACACGGCG
GAGGGCATGATTGCGGTCCTCCCGTTTGCGGAGAGCTTCAGCACGGGGCAGGCTGCCTCCCGGTAAGA
AAGTACGCTCGCTGCATGCTGTCGCCGCAACA
GACTCGGCCTGATACATTCCGTCCGTCGTGATTTTTGTGGGCTGTCACGACGGCCGCGCGGTATGGTA
CATTTGCGCATCTCCAACCATGTGTTCACAGT
GGCCAGAGGTGTTACCACACGCCTAGATGGAAGCATCACAGTCGGCTTCACGCAGGAAAAGGGCGACA
TGTTTCCGTTGTGGCAGCATGCCTGCCTGTCG
CGAGAGGCCGTCCTCCGTCCCTGCAGTTTGGGATGGCAGATGGTGGTCCGAGTCCATGTAAACGGGTG
GACGTCCAGTGGGAGAAATGCAGCACGCGTTG
CGCATTCACATTGGGAAACGAGACGCTGCGGCTGCTAATCCGAGTTGCCTGTGCCGGGTCGTCGGACA
GCTCACACTCCATGCCGCTTGAGGAGGTGGCG
AGGTCCTCTCCTGACGTCGTGGATGATGAGCAACCGTCCCGCAGCTCATGGTGTGAATTCAGGTCGTC
CGCAATAGGCGCTGCAGGAACAAGACAAAGAA
TGGTGCTCGCAGAGTGAGCAGCGTGAGCAATTACTGTAGGAAGGCTGCAGAGGTTCACCACGTACATG
CTGGGGCTGGCCGGTGGGCACACGCAGAAGGC
GGTGGTCTCCAGGCAGTTGGTTCCAGCAGCGACAGGCGCTCGCACGGAAAGGTGTGGCGCACAACGAA
GGCCACGCCACCGGCACACCCACTGCAGGCCT
GGTGGAAGTCAATGCAGACAATGAGCCTCGTCGCTGGCACTCTGGTGCCTGCACTCCTGCAGAAGAGC
CATGCCGGGCCGGTGCTGCGCCAGTCAAGCAG
CAAGGGCAATCCTGAGGGGAGAACGGCCTAAAGt
>scaffold1117.size7130
ACACACCCACGCAGAGCTGCTGAGGCTGCCGTCGCTTTCGCGAAGAAGTGACGGTGCGCGAGTGGTGG
TCGTTGTAGTTGGCGCCTCCGTAGTCGTAGTGG
TGGTGGTACTTGGTGCTTTTGTCGTAGTCGTGGTTGTTGTTGTTGGCGCATTTTCCTTCTGACTTTGT
GCCGGTACCGTACCGGTCACGGAAATTGACGA
TGCTGCAGCTGGACTAACTGACCCACCTGGCGACCCTGACCCATCGGTGCCCGGACCTGCTCCCTCTT
TTGGAATAGACTCCGGACCTTCTGTATTTGGA
CTCCCTGCCGGAGCGGGTTGCTGACTGTCCGGATCTTCGAGTTTCGGTAACGTAACGGTCGGACCTGT
CGAATTTGGCGCCGTGGAAGTTTTTGTTGCCT
CTGAATCATCTGCTCCCGGTTGCTGAGCAGCCGTTGAATTGGGGCTGTCCCCAACTTGATCTCCACCA
GTCGCTGTCACGCACACGGACGGGCAGCAGCA
CAGGGCAAGCACCAACAGGGCGCACAGCAGACGCCACGTCATCATCGTGAGTGTGGTCGTTCAGTGGT
GTGGAGCTGATCTGCACAGTGAGGGACATGGA
GAGCAACAGGGGAGACGAGAGGTGCGGCAGACACACACAGAGGGCAAACACACACAAGAAGAGAGGGC
AGCAGCCAGGCATGCACCGTACGCCGCACACC
ACGCACAGCACACACTCCAATCCACGGCAACAGGCACACACACACACAGCCCTGCCGCAACACCAACA
CACAGATGGAGGAGGGAGAAGCAAACAGGAGC
GGCACACAAGGCTCCACCGCATGCACATACGGCGAGAGCGGTCCGTCGTGTCTGCACCCCTCACGGAG
GGCACGCACACACGAGAATAGGTGCAGCGCAC
ACAGCAACACAACAGCAAAGTAACCATCGAAAAAGGATAGATCCCCGCACACCAAGTGGATTGTTGGT
CCACGGGAAGCGCAGCCACCACCGCAGCAGCA
GCAGCACACGCAAAGTCAAGCGCGAGGAATGGAATAAAGCAGCCATTTATTTGTGTTTCTTTCTGGAG
GGTGGAGGATGTCTGCATATGTTGCTATTGTT

TTTGCGGAGGGCAAACCTTCATGCCTGCATCGTGTGTTGGGGTTCCCGCAGTGTGCACAACTGTTTCC
TTCTCTTGTGAATTTTTATTGTGTTGGTGCAG
CCAATAATTCAATTTGTGTTGTTCCCGCTTTTCGCCGGTAAAGTGCTGCAATAGATGTTTTTATTTCC
TCCTTCGTTGCTGTGGCACCTGATTCCTCGCC
GTTTATTGCTGTGGAAATGGGAATATGTGAGTGGGCTCCCACGCACCAGAACGCACCTTCGCTCCTAT
TTATTGCTGCACAGAAGGGCATGCGTGTTTGG
TGGTGCCTCGACCATATATTTTGTGGGCGTACATTCCCCTGCAGGCTGAGGGCGGGGCGACGCTCCGT
CGCGGTGCACTTCCCTCGCATGCCGCCCACTC
TGATTCATCATGCATCACCCCTCACATACTCCGTGGCGCCACACCGTCGATATTATTGCAACCGCTGT
GGTTCCGCGTATTTCACGCATTTCATTGTGCC
ACAATACATCCGCTCGTGTGGCCGCCGCATCCAAACAGCGGTGCCCAACACCGTGAGCGGCAGTCCAC
ATCCTTGCGTGTCCCAGCGCAGTGGTGCTTGA
CTGTCAGCCAGGCGCCAGACAACACGAGAGTCTTGTGCTGGCAGTACAACACACGGAGCTGCAGCGAA
TGACTCGGGCGGCGACGCATCTATTTGTCGCA
AGGCAGTGAAAAGGAGTCGGGCAAAATAATGCCGGCTGCATTCACGACACCATACCCACAGCCAAAGC
ATGCGACGTGACGTGTGTTTCGCTGGTGCTGT
GAAATGCCTGCAGACGACGTCACTAGTGGAGGCACACCACCCATGATCAGCGGACAGACAATAATCGG
CATGGGAGGCCACAAAAGGCAGCAACGTCAGG
AGCAGCAGCAGCAGCTCTCTCTGTTGCCTCCGAGAAGTAATTATTCCACCAGCCACAGCCGGCCGTGC
CATCGTGACACTCCATGCACGTCGTGGCTGCG
TCAACACATCCTGCTCCCTGCAGCGCCTCAAAAGATGCCTCACTCCGTCCACTAAGTGCATCGATGTG
GACCGTGCCTGGGCTGTTCGTCAGGACCACAG
CGATGTGAGGGTCACCGAGTCGCAGCGTATTTGTTGTGACGTTGTTGCATGGCACCTGCACACACACA
CGCAGCGCCTGCATCCCTCCCTTTTGTGTTCG
CCCTCCGCCGGAGTGGCCACACACACGGGCAGCAGCAAATTCATTTCACCCTCGACGATCCAAAATCA
ACACCGACATCCCTCGCCACGCAAAGATCTCA
GTTGCCAGCACGGGCCTCCTGCCGCTCTGCCATCGACGCCGAAACACGGCACAGTGCACGCACTTATT
TTCCTCGTGTGCAGTCAGCCGTTTGCTTCACA
CATTCTCCGACGGGCTGACGCCCACAAAAGCACGCCACAAAAGCCACTGTGACCACACAAGCTCTTGC
GATGAGCGTCTGTGTGTGCATGGATCCGCTGC
TCACACGTGCTGGCAGGCAGCCTGCAGCCGGTGCCCTCGTGCCAGTTCATCGGCTCCAGCATGGGAAA
GAGAAAATGGCCAATTTCTAGCGCACTTATTC
CCACCAGCAGCACCAACACGCTGGTCGCCAGCCGTCATTCCTCAGCACATCCAGGAATGCCGAAAAGT
GACGTGGAACCGTCAGCCAGCAATGCCACAAT
CCCGCACACATGTCGGCTCCACCAGAGAAGACGCACAAACTACACACAACACCCCTCACATGTTTTCC
GAACATCATTCCAACACACCGCGACACGCCAA
ACAGAAGTATTCGTCACTCCATGCACCTCCCCACACAATCAAATAGTCCATGCACCACGCACGGCAGC
AGCTCACACCCATCAAACAAGTGTTCCTCGGA
CCAGTGTTATCCACGTGCTGGAGTGGGAAGTAAACAAAAGAGACGGGCCGCCAAACCTCAAAAGAGTG
CAACGACCGGCCGCAAGTCAACGGAACGCAGC
CACTCAAAACAATTCAGTCCTAATGCCCTCAACCTCCCACCTTCCCCCCACAAAAGCCCCAGTAGACG
CCAATCAAGCGCGCCGTCCAACAAAGTCGCTG
TGCAACACCAGGAGGAACCGCAGTGCCTCCTGCTGCCATCCGGATCCGTTGTGGACGCCATGATGAGT
AGAAACGCCCATTGGTGCTTCGAAACGACGCT
GCACACCTGCGACGCATCATACGGTTTGAGACGCACGTGCCTCACCAGCACCACAGACGCCCGCGCGC
AGTCGGTACGCCCTACATCGTCAAGGAAGCAA
AGGATACGTCAGGGTGGCAGCAAAGCACTCGCTGGCAATTCCCGTCGCAGGCATCATCACAAGCAGCT
CCAGCAGCGCTGCAGACCGTGGGGTGACGTGG
ATCAACTGACGCATGGCGTATGTGGAAAAGAAAGGACGTGGTGGCTTTACAAGGATACGAGTGGCCGA
CACCTTTAGAAGGAGAAAGAAAACAATGTGAG
AACACACAAAGAGGAATCAGGCGTCCGCAGTCAAAGCAAACCAGCAATCAAAAAAAGTAAGATGGGTG
CACTCAACCACAACACAGGCAGAGGATAAAAA
ATACCCACAACAAAAAGCCGAAGCGTGGGGGCGTCCACCCAGCAGTGGGGACGCGCAGCAACAGGTGG
GACGACCAGCGCACACACACTCCCATTGCTCC

CGTAAAGCAAAACACACACACAGAGGGAGAGAGAGAGGTCGCCCACTCAATGCAATGAAAACAAAACA
GAAACAGTTACGGTGCTCCGCCCATGCAGCCA
GGCAGGACGGCAGCGGTGCGTGTCGTGCAAAAGAGAGCACCAACGGGCCTGCATGGGTGTGCATGCAT
ATATACAATGGGCACATTATTGTGTGTGCCTC
GTGGGGTGAGGTGAGGGCGAGAGTCCATGCAAGGGGACACACACACAGAGAGAGAAGGACGAGTGTGT
GTGCGGGCGGTGCACGGCTCTCTCACACTCAC
TCTCACGCGGCCACCACCGCAGCAGCCGCACACGCAACAACAAGAAGAAGCAAAAGAGGGGAGGTGGT
GTGGGAGACCGCGGTGCAGCTGTCACCGTCGG
TGAGAGTCACCGTGTTTATCTTATCAGTGGCGTTGTCTGGTGCTTTTGTGGAATTGTTGCTGTCAGAC
GCTGTGTTGTTTGTGCCATCCGGCGTTGTTTC
AACTTCTGTATCCACAGCGTTTTGTGCCACGCTTTCATTGGAAGTGCTGCTTGCACCCTTGGAGATTT
GAGTGCTGCTGCTACTGTCGCGCGGTGCCGCG
TTATTTTCGGTTTTGGCAAAGGTGGCTTCAATTTGTGTTGTTTGTATGGGACGATCCGTCGTGTGCCG
AAGTGTGGGATTTTCTTCTGTCACTGCGGCAC
CAGAAAGAGGAATAGCGTCCAGAATCGTCTTCGCAGCACCCTCGTCGGTGTGATGTGTGGCAGAAATG
TTCTCATTGGGAGGTGACGGTAGAGTTGTTTC
CGCTTGAAGTTTAGTTGGTGCATTGTCATTGGAATTTGTGGCAGATGGACCACTTCCTGTTGGTACGT
TTTTGTCCGAAAGGTTTTTTTCTTTTTCCGCT
TCCCCTTTCTTACCATTTTGGGGATTCTCTGCCGCAGTGAGTTCCGCCCCCTGGTCGCTGGCAGGAGA
ATTCCCTCCTAGAGAAGAAGATGACTGGAGTG
CCTGCTGTTCACCGCTAACTAATATTTCCTGCGTTTTATCATCCTTACCTGCGGTATCTTCTTCCACT
TCTTTACTGTCTGGTGAACTTACAGGTGAAAT
ACTTTCGGGGATGACCTGCCTTATTTCTTTTGTGGGCAACGCCTCCGTGCGAGAATTGGAGTTCGCAT
TCAATTCCTCCGATGTTGCACCTTCTGGTCGT
ACAACACCGTCACGTGATTCAGAAAGTAAATCCCCCCTCGTACGTGCGCATCTTCAGACCCCGACCCA
TTACCTCCCGACAAAACGTTCCTTTCACTGGA
GGTGTTCTCCGTGCCAAGCTCATCGGCGTTCTCAGCAGCAGCAGCAACGGCAGGACGTTGACGCCTCC
AACGACGACGGCCATCCACAACGGCAAGAACA
CCGTCCATCCTCTGACGCATGCATTCCTCAACGGCGGACGCATTCGCACGGCCTCCTGTCCTCCTGCT
GAACTCCTCCGCGCATTCCCTCCGCAGCTGAG
CGCGCCAACGAGAAAACAAACACTCACCCGCACTGCCATCAGGAACACCGCCAGCACCGTCGGCCGCA
ATTCCAGAAAAACCGCACCACAGCACGCAGAG
GGCACACACCAGCAGCACACGGCCAGTCATCATCATCGCCATCACTCCTGTCACCTGCGTGCAGCCTT
CAGCACAAACTAACTCCCCGTCAACGCAGAGC
GGAAGCAAAAGGAGAGACACGGCCGCTACACCACGCACAGCCACCGCACAGTCACAAATGGCAGCAGC
CCAGCAGCGGCAGCGCACACACACCATGAGGC
AGTTGAAAGCAGTGAAAGGGCAAAAACACACAACGCCCCGCAGCAAGCAAACAGAAAAACACACAAAA
CAATTAGAAACAAAACACAGGTAAAAAAACAG
CAATGAGTTAAAAATGTAAACACACACAAAACAAAGGGCTGCACAGCATGGCTCCAGCGCGCACGCAC
CCATCCTCAAATGCGACTCCACGCAATCAGTT
GTGTGCGTGCCATGCACATGAGTGTGTCTGGGGTGCGTTCAACGTGACGGATGCGGGGAGCGGCAAGG
AGCACACAGCAGGGCAGCGGAAGCAAGTGAAG
CAAAACACAATCATTATTTTATCCTGTTTAGCACATCGGACCACAGTGGACGCACACAAGTCCCCGCG
GTCGGGTGCTGGCACGCACAGCCCGCACACAA
TTCTTCAGCCCACAGTGGTGTACGCCAGCGCGGATACGGCGAGCAGCAGCGGGGCACACACCCACGCA
GAGCTGCTGAGGCTGCCGTCGCTTTCGCGAAG
AAGTGACGGtgcgcgggtcgtcgtcgtgcttggcgcctctNNNNNNNNNNNNNNNNNNNNNNNNNNNN
NNNNNNNNNNNNNNNNNNNNNNNNNNNNNNNN
NNNNNNNNNNNNNNNNNNNNNNNNNNNNNNNNNNNNNNNNNNNNNNNNNNNNNNNNNNNNNNNNNNNN
NNNNNNNNNNNNNNNNNNNNNNNNNNNNNNNN
NNNNNNNNNNNNNNNNNNNNNNNNNNNNNNNNNNNNNNNNNNNNNNNNNNNNNNNNNNNNNNNNNNNN
NNNNNNNNNNNNNNNNNNNNNNNNNNNNNNNN
NNNNNNNNNNNNNNNNNNNNNNNNNNNNNNNNNNNNNNNNNNNNNCGCGGGTGGTGGTCGTTGTAGTTGGCGC
CTCCGTAGTCGTAGTGGTGGTGGTAGTTGGTG

CCTGTGTTGTAGTTGTCGTCGTGCTTGTCGTAGTAGTGGTGGTTGTTGTTGGCGCATTTTCCTTCTGA
CTTTGTGCCGGTACAGAAGCGGTCACGGAACT
TGACGATGTATTTGGGTTAACGGACCCACCAGACGACCCTGACGCATCGGTGCGCGGACCTTGTCCTT
CTTTTGGAATATGCTCCGGACCTCCTGCATCA
GGATTCCTTGCCGGAGCGGGTTCCTGACTGCCCTGATTTTCGGGTTTCGGTAACGTAACGGTCGGATC
TGTTGAAGCTGAAGTCGTGGATGTATTTGCTT
GCCCTGCACCACCTGTTTCCGGTTGCTGAGGGAGCGTTGAACCGTTTGCCCCTTTTTGTTCTCCACCA
GTCGCTGTCGCGCACACGGACGGGCAGCAGCA
CAGGGCAAGCACCAACAGGGCGCACAGCAGACGGCACGTCATCATCGTGAGTGTGGTCGTTCGGTGGT
GTGGAGCTGATCTGCACAGTGAGGGACATGGA
GAGCAACAGGGGAGACGAGAGGTGCGGCAGACACACACAGAGGGCAAACACACACATGAGGAGAGGGC
AGCGGCCAGGCATGCACCGTACGCCGCACACC
ACGCACAACACACACTCCAATCCAcggca
>scaffold1118.size6260
CAATGAGCACCACCGCGGAGTCGTTGCTGATCGCCAGTCCATTCATCGCAACAAACGACGGCCTCACA
ACTTCCAAACCCGACCGAGGGATCAGCCTCGTG
ACCGTGAAGCGGTTGCCGCTGATCGTGATGTTCGTGTGCGGTGGAAACCCACCCTCCGGGCTGATCAA
CGCATCATCAAACGTGTTGCCGGTGACGACAA
TGTTCAGCGACCCCACAGCGGCGTACGCGTTGCCCAACAGCAACAGCACGGCACCGTCCCTCCACGTG
CATCCATCGAACGTGACATTCGCCACCTCGCC
CGTCATCGCCCCAGTCAGCACCGTCAGGCTCATGCCACCCCCGCTGAACGTCACGTTGCTGTAGCACA
GCCACGACAGCCCGCTGCCCACAGCCTGCGCC
ACCTCGGGGTACACCATGTCGCTGATGCACTCACCAATCGGCGGTGGCGGCGTGGGCGGCACTGGTGG
TGGTGTTGGTGGTGGCGGTGGCGGTGGGCCAG
CAGGCACAGGCGCCGGGACGCACACATCGCCGTGCCCTCCAGCAGCGCACTGGCACTTGCAATCAATG
ACCGCCGTCGTCAGCGGGGCAAAGCAGTTGCC
CTCTCTCGTGCACTGCCCGCACGCGGCAACCGTCGTCACGTTGGTGATGCCGTGGAGCGCCAGTTCAG
CCGCcgtcgtcaccaatcgtcccgcgaccgtc
aggcacccagcaacgaacccgtagccggTCCCCACCCGCCTCAGCAGGGTTCCCGACAACCCAGTCCA
ACCCATCCTGCAGCCCGTCAGCGTCAAAGCAC
TGCTGCTGTCGATGCTCACAAAAGCGAAGCCACTGTCGTAGAACATCGCACCCACACCCACGTCGTTG
TCACGCCAATCCAGCCAGCTCGACTCCCGGAC
GCTCCAAAAATTATACGCACAGATGGCGCAGGAAACAGAACCGCTGTTCCCCACCACACGGAACACGC
TGTGATCCGACACGCTGAATCCGCGGTCCTGA
TACAGAAGGCTTGTTCCAAAGGACATTGTATTGTTCAGCAGCTCAAAAACGGAGTGATTGCGGACACT
GAAGCCCTCCACGTAGAACAGATGGATGTAGC
CTTCACAGCTGCAGTACCGCATGTACAGCGAGCTGCCACCCACCAGCGTCAGCGTTCCTTTCGAGTAC
AAACCATACGCAACACGCTTCGTTGCATGTGC
CTTGACGTTGCTGACCAACAGCGAGGACGCGGTCAACACGACTTTCTGCAGCATCACGACGCTCAACG
CGTTACTTCCAGTTTCCGGCAAATGGACTGTG
GAAGACTGCACCGCATCAACCTCCGTGACGGCGATGTCGCAGTGCAGCGGGAGGGCACTCATAAACGC
AACAACAGAGGACCGCATCACCGTCTGGCTCA
CGCGGATCAGGACGCCGGATGACTCGGCCGCACCAGCACCCCCAACAAACTGCAGCACGGCACCGCCA
CGGAACGTGCACCCAGTGAACGTGATGTTGAT
GGGGAGATGCAGCGGCATACTGTCGAGGAAAAACGTCAGCACTGCATCCACGCCGCTGAATGTCACAC
CCACGTAGCAGTGGTGCGACTTCCACATGTTC
AGCGTGAGGTTCGTCAGCGTCTGGTTCACCACGCAGCTCGTGTCGCCGTCGACGGCTCTCTCCGGCAC
GGGCAGCACAACCGTGTCGGGCACCTCGAAGG
GAAGACACGACGCGCCATGCCATCCGTCCTTGCAGCTGCACGAGCACGAGACGCGGTCCACCGACTCC
GTCCCGGGCATGTAGCACGCCGCGTCGTCGTT
GCATGTGTCACACCTGAATACCACCACGCCCTCCGGAAACACACCGTCGTACGACACCTCCTCACCGC
CCTGCAGGTTGCACCCAACCAAAAACCGCGCG
GGTGACGTCACGACAATGCTCGACGGAAAAATTTGGCGAAGAAAGTCCCCGATTCCACCTGATGGTT
TCTTGCAAGCGCCACGGTGGTGCCGCTGTCCG

ACAGCTGAATATTGTGCTTGGAAAGCGATATACTTAGTACTGAGGCTGCACCCACGCTGTTGCCCTCC
ACACGCCACTGCGCACCACCCTGGAGAGTCAC
AGAGCCGTCAAGATACACCAGCGCGGAATCAAAACCCTCCGCGCTGCCAGCAAAGGTGCAGTCCGCCA
CTCGCAGCAGCCCCGCGGAGCTCACGGTGGTA
ACCCCCAAAAGATAAACTCCACTAATGGATCTCATCGTGTTGTCCTCAACGTCAAAGTAGCCGCCGTT
CCTCACTCCAACAGCGTAAGCGCAAACAGATG
ATTCCATACCGTTATTCTCCTCCGTTGTCCTCAGCGTATTTCCCTTCACAATGATACCACCACCATCA
ATAACCATACCACTGGAGAAATAGATTGCGTA
ACTATTCCCCTCGATGCGGTTGTCAATTAGTAGCAGCGTCGAGTTCGCACCGAAAGTAAACCCCAAAA
AGGCAATGGCGTGGCTCGAAGTCGTCACAAGC
GCAATGCCAACCACCAGTATCTGGGAGCTCGCGCCAAAATCACCCTCAAACTCCAAATCACCGGAATC
CAGCATCGAGAATGCCACGCTCACGTGTACGC
GCGCACCACTCCCCTTGACCGACAGGTCCATGAGGACGCACTGCAGCAGCGTGATGTTGAGTGTCTGT
TCTGGTGCGTCAAAATGCGACAAGTCCAAGTC
GATCGCGGCGGTGAATGTCACGTTGACAAAGCACAGACCACGCACACCGGGATCAGGATAGTCCACGG
AGCTGATGCTGCCACCGTGCACGCAGCGGACC
TCCGTGTCCCTCGCATCAGAGAGCGGGAGCGGCCCAAGGCAGTCCGGAACCGCAGCTGGTAGACACGT
ATCGCCGTAGCCGCCAGCGGCACACACGCACT
CACAGCCGCTGATGCTGCTCGTCCTCGCTGCAAAGCACACGGCGTCCACGGTGCACATGCCGCAGTCC
AACACCATCACAGGTGCTCCAATATTGAGCTC
TTCTCCGTAATCCGTCACAGGTGAGCCCCTTAGCTCGTTGCACATGCCGTAGATGATCGGGCGCGTGT
CGGATGNNNNNNNNNNNNNNNNNNNNNNNNNN
NNNNNNNNNNNNNNNNNNNNNNNNNNNNNNNNNNNNNNNNNNNNNNNNNNNNNNNNNNNNNNNNNNNN
NNNNNNNNNNNNNNNNNNNNNNNNNNNNNNNN
NNNNNNNNNNNNNNNNNNNNNNNNNNNNNNNNNNNNNNNNNNNNNNNNNNNNNNNNNNNNNNNNNNNN
NNNNNNNNNNNNNNNNNNNNNNNNNNNNNNNN
NNNNNNNNNNNNNNNNNNNNNNNNNNNNNNNNNNNNNNNNNNNNNNNNNNNNNNNNNNNNNNNNNNNN
NNNNNNCCAGTCACGGTCAGCCTGTTTGCAAT
GAGCATCGCGAAGCCGAGACGAAACGTGCTGGACACCATCTGCAGCACGCTGCCGCCAGCCACCGCAA
CGTCTCCAAACACACACGCGCCCTTGTAGTAT
TCGATGCTCGGCGCACTCCACAAGCTGTTCTGTATGGAGAACACGGAGCCGCCGCTCACACGCAGGTC
CGACGCAAGAGCGTACATCACGTGCGTCCGCG
ACAGGACAGCACAGTTGTCCATGTAGAAGACGCTGGACAGGTTCACGCCAAGATCACGCTCCACGAGG
ATCGCAGCGCACGAACCCcCGCCGCACACCAG
CTTCGACCGCGTCATGACAAACCGCGTCGACAGAAGCCGGACACCGTCCAGGACAAGCACGGGGCCGT
ACCGGGATCCCGCATGGCCAAGGGTAGTCTGC
ACGTACTGCGACCCGCCAACTGTCGCACGCAGCGTTGAGTTCGCCAGCAGCACAGTCGAGTGCAGCGG
CATCGCGCCATGCAGCACAATCGTGCCCTCAA
GCGACGTCACATTCGCCATGTTGACGAGGGCGTACGGCATCAGGCGCGCCGTGTTGTCGCTGAGGCCG
CCAATCCGCAGCTGCGCGCCGCCCGCAAGCAC
GCAGTGGCGCAGCGTCACGTTCAGCGCGTCAGCGAACGCGTCCATCGAGCGCAGGTCCACCGCCACAG
TGATCGGCCCGCTAAACANNNNNNNNNNNNNN
NNNNNNNNNNNNNNNNNNNNNNNNNNNNNNNNNNNNNNNNNNNNNNNNNNNNNNNNNNNNNNNNNNNN
NNNNNNNNNNNNNNNNNNNNNNNNNNNNNNNN
NNNNNNNNNNNNNNNNNNNNNNNNNNNNNNNNNNNNNNNNNNNNNNNNNNNNNNNNNNNNNNNNNNNN
NNNNNNNNNNNNNNNNNNNNNNNNNNNNNNNN
NNNNNNNNNNNNNNNNNNNNNNNNNNNNNNNNNNNNNNNNNNNNNNNNNNNNNNNNNNNNNNNNNNNN
NNNNNNNNNNNNNNNNNNNCGCGGCAGCTGCAC
ACGCACTCGGAGAACGACGCAGTCAGCGCATCAAAGCACGCCAGCGCTGCCGCACAGCCGTCGCACGG
CACCACGCTCACGGAGGGCAGGCCGGCCATGC
GGTAGTCGCCGCTGCTCCGCAGCACCCGGCCGCCGGCACGGTTGCACTGCAGCGACACGGCGCCGCTC
GTGATCGTCAGCCCGGAGACAAGCGTCGCGCC
GGCGGCAACGCAGCGCGCAATCGACACGGCACCGCCGCTCAGCGTCACACCCGACAGCGACGCCACAG
ACGACGCCTCGCCCACCGCCCACACGTCGTGC

AGGTCCAGCCACCCGCCGGCACTGACCGTTCCACCGTCGCAGCGCACCAACGACGACGCAACAGACCC
CTCGACGCCCACGAAGCGCAGCACCGAATCAA
ACACCGCGAGGCGCTCGCCAAGCACAATGCCGCCGCCGCTGCTCACAACAGAGACGTTCGTCACAGAG
AACACCGAGTGGCTCCGCAGACTGAGACGCGA
CGAGAACGCCACATGCATCGCGGACGCGGTCGGACCGCCCGACGCCTCCAGCAACACGCCGTCAAGCA
CCACAGCGCTCGACAGCAGGTCCACGCCGCCG
CCAACGTGCACCGCCGACCCGCCCGCCTGCAACGACCTCAGCACAGTGTTGCTCACACGCAGCTGCGT
CTGCAACAGCGACGTCGCGTACAGCACAAGCG
GCGACGCCACCGCGTCCGTCAACATTTTCAGCTGGGAGTAGCGCATCGGCCCCGCCGTCACAATCGTC
GAGTCACGGACCGTCACGTTAGTGTGTGCCGG
CAGGTTGTGCACAAACACAAGCGAGCAGTAGCTCCCGGACAGCCCGCTCACGCTCACCTCCAAGCTCC
GGTCACTCGCCGGCTCGCCGCTGTAGCCCCGC
ACGTAGATCTGCGCACCGCCGTCGCACACGCAGTTCCTCAGTTCGATGCGCAGCACGCCGGGAAGCAT
CGCCGGCACGTCGAACACAACAGCCACGCTGT
TCGTGATGTACACGCCGTCCATCAGCACCGTGTCCACGGCGCGGCCAACCGTGATGGTTCTGTCCACC
GTGCCGCCCCGCAGTCGCAACACCACCGCATG
CACTGCGGGCATCCACGCCGTCGCCGCAACCAGCGCCAACAAAACGAGCAGCGCCGCACGCACAGCCG
CAATGGCCCTGCGCCGCACACGGTGCCGGCTG
CGCGGACACGCTCCCACCGCAGACCACATCCTCCAACCCACACAGGTACCAACAGAGCTGTCGCGTTG
TGTCGCACTCGCCCCGTCGACCATAACTCCTG
CCTAGCAAATCCCTTCCTACAGATGGCGAATCCAGCGACACAATTCCGCCAGATCGTTATCTCACTAC
TAATAATCCTACTGCTGCAAGTAAAACCTAAC
ACCTTGAAACGATAATTGTGCAGGTCAATCCAAATTATCTCTATCAGCTCGGTCAGAGTAGGAAGAGG
CAGCAACCACAGTAACCAAACAGAGTGGCACG
CAAAAAATCCGGCCACAGACAATCCCCATCACAGTACAACCACCGACAAAAAGAAGCGCAGACACAAT
CACGCCTGTTGACAACAACGCCCTCGCACCGC
CCACGCAGCCCCACACAAAAACCATGCACAGTGGCAGGATCCCGGCAAAAAGATACATGACAATTGGC
ACCGCTTCTGCAACCAAATGTTTCTACCACAG
AGCAGCCCTGATGCCGCGAAAGACACACAAGGACCGAGACAGACCCCTATGGCGGCGTTGCTACAGCC
AGCGATCGAGCGTGCAAAAGAATGCACTGGTG
CTGACGCAGAAGAGGGGTAGAACGTTTGCCGATCGGCAACAAGAAAATGGTCATTTCTC
>scaffold1119.size7542
GAGTGCACCCATTCCGCGGCTGCGTGTGTGTGTGTGTGTGTGCGATGATATGAGTAATTTGCGTGTGT
GCTATCTGCCTCTCTCTGCCGTTGCTTTCTTCA
GACTCTCTTGTGGCGCACAGCTGCTCCGCAAGTATAGGCGCTGCCAAACACACAAGCACATGCACACA
CACATGCCGTGTTTAAAACGCTGAGAATGCAC
GCACGCACAGGATTGTGGCTGCGGAGGCCCTCGCGAGGGGGGAAAAAGCCTGCATTTTGTTTTTTTTT
GACATGTTGCTCGGTGTGTGCTGACGCGTGAC
GAATAACAACACGCCCCAATAAGCAGAAAAGAAGTAAAACAAAATGGCGCTGAGCGGCACACCGGTAA
GCCAAAAATACGCAATCACCAATAAAAAAGCC
GGCGATCACCCAGCAGGCACATCAAGCCTCCTAAAAACCAACCTGGAACAGACAAAAAAAAGAGACAA
TCGTAGAAAACAAAGATCCCCGCCCCCAAACA
GGAAAAACATTAAAGAAAACGCGATTCTTTTTTTTGTCCTTACTGCCGGGGTTGGCCGCTGCAAGAAA
TAAAAAATGGTAGGGCGGTGGCCCCGGACGTT
GGATACAGGACGACGTGTCGAATTATCCAAGTTTGCAGCGGCTGCCCCGGACGTTAGATACAGGACGA
CGTGTCGAATTATCCAAGTTGCAGCGGCTGCC
CCGGACGTTGGATACAACACGACTTATCGTAGTAACCACGCTGCGGCTGCGGCCCCGTACGAGAGATA
TATGACGAACTTATCGTAGTAACCACGCTGCG
GCGGCGCACCCTAAACATGTCGTTTTCGTTTTCCAGGGGACAACGCAAATGTGCTCGATTGTTCGAAG
ATCGAAATTAATACTTTACGTGAAAAGGGGTC
GCGCGACCGCATTTTTATAGCTCAGTGGCAAACACGCTCACAATGTCATCCGGGGACGCGGGTTCGAT
TCCCGCAGAAGCCTAAGTTTTTTCCATTTTTT
TTTTTTTGAATTCTTGTTTCATATTGCTTTTTAAACTTCTGGTTTGCAGGACGGTGTGCGAGTTCTGC
AATCGCACTGATTCCATCTGTTTCGTGGTTTG

TGCGGCTGCGGCTGCGGTGGTGGCCGCGTGAGAGTGAGTGTGAGAGAGCCACGCACCGCCCGCACACA
CACTCTTCCTTCTCTCTGTGTGTGTGTCCCCT
TGCATGGACTCTCGCCCTCACCTCACCCCACGAGGCACACACAATAATGTGCCCATTGTATATATGCA
TGCACACCCATGCAGGCCCGTTGGTGCTCTCT
TTTGCACGACACGCACCGCTGCCGTCCTGCCTGGCTGCATGGGCGGAGCACCGTAACTGTTTCTGTTT
TGTTTTTATTGCATTGAGTGGGCGACCTCTCT
CTCTCTCCCTCTGTGTGTGTGTTTTGCTTTACGGGAGCAACGAGAGTGTGTGCGCTGGTCGTCCCACG
CTTCGGCTTTTTGTTTTGTTTTCCTTTTTGAT
TTTGTGGTTGAGTGCACCCATTCCGCGGCTGCGTGTGTGTGTGTNNNNNNNNNNNNNNNNNNNNNNNN
NNNNNNNNNNNNNNNNNNNNNNNNNNNNNNNN
NNNNNNNNNNNNNNNNNNNNNNNNNNNNNNNNNNNNNNNNNNNNNNNNNNNNNNNNNNNNNNNNNNNN
NNNNNNNNNNNNNNNNNNNNNNNNNNNNNNNN
NNNNNNNNNNNNNNNNNNNNNNNNNNNNNNNNNNNNNNNNNNNNNNNNNNNNNNNNNNNNNNNNNNNN
NNNNNNNNNNNNNNNNNNNNNNNNNNNNNNNN
NNNNNNNNNNNNNNNNNNNNNNNNNNNNNNNNNNNNNNNNNNNNCCGCTCCTGTTTGCTTCTCCTCTT
CCATCTGTGTGTTGGTGTTGCGGCAGCTCTGT
GTGTGTGTGTGTGCCTGCTGCCGTGGATTGGAGTGTGTGTCGTGCGTGGTGTGCGGCGTACGGTGCAT
GCCTGGCTGCTGCACTCTCCTCATGTGGTGTG
CCCTCTGTGTGTGTGTCTGCCGCACCTCTCGTCTCCCCTGTTGCTCTCCATGTCCCTCACTGTGCAGA
TCAGCTCCACACCACTGAACGACCACACTCAC
GATGATGACGTGCCATCTGCTGTGCGCCCTGTTGGTGCTCGCCCTGTGCTGCTGCCCGTGTGTCTTCA
CGACAGCGGTTGTTTCGGAAAAAGATGCCCCC
GATGTACAGCCCACACAANNNNNNNNNNNNNNNNNNNNNNNNNNNNNNNNNNNNNNNNNNNNNNNNNN
NNNNNNNNNNNNNNNNNNNNNNNNNNNNNNNN
NNNNNNNNNNNNNNNNNNNNNNNNNNNNNNNNNNNNNNNNNNNNNNNNNNNNNNNNNNNNNNNNNNNN
NNNNNNNNNNNNNNNNNNNNNNNNNNNNNNNN
NNNNNNNNNNNNNNNNNNNNNNNNNNNNNNNNNNNNNNNNNNNNNNNNNNNNNNNNNNNNNNNNNNNN
NNNNNNNNNNNNNNNNNNNNNNNNNNNNNNNN
NNNNNNNNNNNNNNNNNNTTACGGAGAGTTACTTTTGCACTAGGCTGCGCGCAAGAGACAGGAGTGAT
GGCGATGATGACTGGCCGTGTGCTGCTGGTGT
GTGCCCTCTGCGTGCTGTGGTGCGGTGTCTGCGGTGGTGGACTTGCTGAAACAACTCAGGGTCTTTTG
GATACTGCGTCCGAAGAGAGCGAGAGTCCTAA
AAAAAACAAAGATACTGGTGGTAGTGCTGGAACAGATGGCCCAACCGCTGAAACGAAAGCTCCACAAG
CAGCAGACCGAGGGTCGAAGGAGCAGGAGGGC
ACAGTGGAACAAGCAGCAGCATCGGTGCCAGGCGTAACGGGAGCGGGAGCGGGGATGGAGACGGCACC
ACAACCCGGAAGAGAAGCAACAGAATCGGCAA
AAGGAACGAGGGGAAACAAGGAGGAAACGAAAAAAGAAGAGGAGAGTGAAAAGGAATTGGAGGAGGGA
GAAGAGGAAGAGGAAGAGAAAAAAGAGGAAGA
AAGGGAGAAGGAAAAGAAAGAAGAAGACGTTACCGACGCCAAAAAGGGGATTTCAGCAGAAAGTCAAG
AGCAGCGAAGTTTACCCTCTGGTGCGGAGGGA
GCATCGAATCAAACAAAGCCTAAAAGCGCTCAAACAACTGACGTCAATGACCCCGCAGCTGAAGGTGC
AGGGAAAAGAAAAGAAAAACAAAATGAAAATA
AAGAAGCCAATCCGAAGAAAACACCAGTCGAAGCCACAGCAATGAAAACGACAACGGCTACCCCTGGC
GACAGTGACAGCAGCACCGCGGTCTCCCACAC
CACCACCCCTCTTTTGCTTCTTCTTGTTTTTGCGTGTGCGGCTGCGGCTGCGGTGGTGGCCGCGTGAG
AGTGAGTGAGAGAGAGCCATGCACCGCCCGCA
CACACACTCGTCTTTCACCCACTCTGTGTGTGTGTCCCCTTGCATGGACTCTCGCCCTCACCTCATCC
CACGAAGCACACACATAATGTGCCCATTGTAT
ATATTGCATGCACACCCATGCAGGCCCGTTGGTGCTCTCTTTTGCACCACACGCACCGCTGCCGTCCT
GCCTGGCTGCATGGGNNNNNNNNNNNNNNNNN
NNNNNNNNNNNNNNNNNNNNNNNNNNNNNNNNNNNNNNNNNNNNNNNNNNNNNNNNNNNNNNNNNNNN
NNNNNNNNNNNNNNNNNNNNNNNNNNNNNNNN
NNNNNNNNNNNNNNNNNNNNNNNNNNNNNNNNNNNNNNNNNNNNNNNNNNNNNNNNNNNNNNNNNNNN
NNNNNNNNNNNNNNNNNNNNNNNNNNNNNNNN

NNNNNNNNNNNNNNNNNNNNNNNNNNNNNNNNNNNNNNNNNNNNNNNNNNNNNNNNNNNNNNNNNNNNNNNN
NNNNNNNNNNNNNNNNTGGCTTGCTTTTTGGAC
GCCTGATTTTTTTTATGCTTTCTTATACGGTTTCTCTCTTGTTATTGGTGTGTCAGCCCCTTGTGGCT
TTGGCAAGCCACCAATTGCTCTCTTCCACATA
CACCTTGCGTCAGTGGGTCCACGTCACCCCGCGGCCTGCAGCACTTCTGGTGCTGCTGTTGATGTTGC
CTGCGATGTGATTTGCCGGCGGTTGCCTCGTT
GTCATCCTGACGTATCCCTGTTTTCTTGTCGATGTGGCGCCTGCCGACAACGCGCGGCAACTCCGGTT
GTGGTGAGGGTCGTGCGTCTCAAAGCGCAGGA
TGCGTCGCGGGTGTGCAGCCTCGCTTGTAAGCACCGATGGGTGTCTGTCCCCGTGAAGACGTCANNNN
NNNNNNNNNNNNNNNNNNNNNNNNNNNNNNNN
NNNNNNNNNNNNNNNNNNNNNNNNNNNNNNNNNNNNNNNNNNNNNNNNNNNNNNNNNNNNNNNNNNNNNNNN
NNNNNNNNNNNNNNNNNNNNNNNNNNNNNNNN
NNNNNNNNNNNNNNNNNNNNNNNNNNNNNNNNNNNNNNNNNNNNNNNNNNNNNNNNNNNNNNNNNNNNNNNN
NNNNNNNNNNNNNNNNNNNNNNNNNNNNNNNN
NNNNNNNNNNNNNNNNNNNNNNNNNNNNNNNNNNNNNNNNNNNNNNNNNNNNNNNNNNNNNNNNNNNNATTG
GACGGTACTTTTGCTTTGCAGCTCTTGGGGCC
TTTGTGGGAGAAGGTGGGAGGCTGAGGGCATGAGGACTGAATTCATTTGGGTGGCTGCGTTCCGTTGA
CTTGCGGCCAGTCGTGGCACTCTTTTGTGGTT
TGGCAGCCCGTCTCCTTTTTTGCTTTCCACCGCAGCACGTGGAGGCCACTGATCCGAAGAACATTTGT
TTGACCGAGTGTGAGCCGCTGACGTGCGTGGT
GTATGGAGTGACGACTACTTCTGTTTGGCGTGTCGCGGTGTGTCGGAATGATGTTCGGATGACATGTG
AGGGGTGTTGCTTTATTGGACGGTACTTTTGC
TTTGCAGCTCTTGGGGCCTTTGTGGGAGAAGGTGGGAGGCTGAGGGCATGAGGACTGAATTCATTTGG
GTGGCTGCGTTCCGTTGACTTGCGGCCAGTCG
TGGCACTCTTTTGTGGTTTGGCAGCCCGTCTCCTTTTTTGCTTTCCACCGCAGCACGTGGAGGCCACT
GATCCAAGGAAAATTTGTTTGACTGCGTGTGA
GCTGCTGCCGTGCGTGGTGCATGGACTATGTGATTGTGTGGGGAGGTGCATGGAGTGACGAATACTTC
TGTTTGGCGTATCGCGGTGTGTCGGAATGATG
TTCGGATGACATGTGGAGGGTGTTGTTTGTGATTTGCGTGTCTTCTCTGGTGGAGCCGACATGTGTGC
GGGATTGTGGCATTGCTGGCTGACAGTTCCAC
GTCACTNNNNNNNNNNNNNNNNNNNNNNNNNNNNNNNNNNNNNNNNNNNNNNNNNNNNNNNNNNNNNNNNNN
NNNNNNNNNNNNNNNNNNNNNNNNNNNNNNNN
NNNNNNNNNNNNNNNNNNNNNNNNNNNNNNNNNNNNNNNNNNNNNNNNNNNNNNNNNNNNNNNNNNNNNNNN
NNNNNNNNNNNNNNNNNNNNNNNNNNNNNNNN
NNNNNNNNNNNNNNNNNNNNNNNNNNNNNNNNNNNNNNNNNNNNNNNNNNNNNNNNNNNNNNNNNNNNNNNN
NNNNNNNNNNNNNNNNNNNNNNNNNNNNNNNN
NNNNNNTTTGTGTCTTCTCTGGTGGAGCCGACATGTGTGCGGGGATTGTGGCATTGCTGGCTGACAGC
TCCACGTCACTTTTCAGCATTTCTGGATGTGC
TGAGGAATGACGGCTGGCGACCAGCGTGTTGTTGCTGCCGGTGGGAATAAGTGCATCGGGAATAGGCC
GTTTTCTCTTTTCCGTGCTGGAGCCGACGAAC
TGGCACGAGGGCACCGGCTGCAGGCTGCTTGCCAGCACGTGTGAGCAGCGGATGCATGCACACACAGA
CGCTCATCGCAAGAGCGGGTGCAATCACAGTG
GCCTTTAGTGGCGTGCTTTTGTGAACGTCAGCCCATCGAAGAATGTGTGAAGCAAACGGCTGACTGCA
CAAGAGGAAAATAAGTGCGTGCACTGTGCCGT
GTTTCAGCGTCGATGGCAGAGCGGCAGGAAGCCCGTGCTGGCAACTGAGATCTTTGCGTCGCGAGGGA
TGTCGGTGTTGATTTTGGATCGCCGAGGGTGA
AGTGATTTTGCTGCTGCCCGTGTGTGTGGCCACTCCGGCAGAGGGCGGACACAAAAGGGAGGGATGCA
GGCGCTGCGTGTGTGTGTGCAGGTGCCATGCA
ACAACGTCACAACAAACATGCTGCGACTCGGTGACCCTCACATCGTTGTGGTCCTCACGAGCAGCCGA
GGCACGGCTCACACCGATGCACTTAGTGGACG
GAGTGAGGCATCTTTTGAGGCGCTGCAGGGAACAGGATGTGTTGACGCAGCCACGACGTGCATGGAGT
GTCACGATGGCACAGTCAGCTGTGGCTGGTGG
AATATTTACTTCTCGGAGGCAACAGAGAGAGAGAGAGAGAGAGCTGCTCCTGCTGCTGGCGTCGTTGCTG
CCTTTTGTGGCCTCCCATGCCGATTATTGTCT

GTCCGCTGATCATGGGTGGCATGCCTCCACTAGTGACGTCGTCTGCAGGCATTTCACAGCACCAGTGA
AACACACATCACGTCGCATGCTTTGGCTGTGG
ATGTGGTGTCGTGAATGCAGCCGGCATTATTTTGCCCGACTCTTTTTCACTGCTTTGCGACAAATAGA
TGCGTCGCcgcccgactcattcgctgcagctc
cgtNNNNNNNNNNNNNNNNNNNNNNNNNNNNNNNNNNNNNNNNNNNNNNNNNNNNNNNNNNNNNNNNN
NNNNNNNNNNNNNNNNNNNNNNNNNNNNNNNN
NNNNNNNNNNNNNNNNNNNNNNNNNNNNNNNNNNNNNNNNNNNNNNNNNNNNNNNNNNNNNNNNNNNN
NNNNNNNNNNNNNNNNNNNNNNNNNNNNNNNN
NNNNNNNNNNNNNNNNNNNNNNNNNNNNNNNNNNNNNNNNNNNNNNNNNNNNNNNNNNNNNNNNNNNN
NNNNNNNNNNNNNNNNNNNNNNNNNNNNNNNN
NNNCCGCCCGAGTCATTCACTGCAGCTCCGTGTGCTGTGCTGCCGGCACAAGACTCTCGTGTTGTCTG
ACGCCTGGCTGACAGTCCAACACCACTGCGCT
GGGGCATGCAAGGATGTGGACTGCCGCTCACGGTGTTGGGCACCGCTGTTTGGATGCGGCGGCCACAC
GAGCGGATGTATTGTGGCACAATCAAATGCGT
GAAATACGCGGAATCACAGCGGCTGCAATAATATCAGCGGTGTGGCACAACGGAGTATTTGAGAGGGT
GATGCATGATGAATCACAGTGGGCGGCATGCG
AGGGAAGTGCACCGCGACAGAGCGTCATCCCGCCCTCAGCCCGCAGGGGAATGTACACCCAGAAGAAT
ATATGGACGAGACACCACCAAACAGACATGCC
CTTCCATGCACCAATAAATAGGAGCGATCGTGCGTTCTGGTGTGTGGGAGTCCACTCACATATTATTT
GTTTCCACCACAATAAACGGTGAGGAATCAGG
TGCCACAGCAACGAAAGGAAAAACAAACATCTATTGCAACA
>scaffold112.size29762
AAAAAAACAATGCAACAAGGCGCATTCACAAAAGACAAAAAGAACTTGAGAAATGTAACAGTTTTCGC
TACTGTTTTTGCTTAACAGACACCATAGCTAAA
AGCTGAAAAAGATAGAACTACAACCTGTGCATACATCAATCAATCTACATAGATAGATAGCACGAATA
TTTGCTTTGAAACCTCTTTTTTCTTTTTTCTT
TTAATTTTTTTTTTTTTTCATATTCGCAAAACAATAATAAAAATAAAATAATGATAATGAAATTCGTC
ACGATTTAACTCGCGTTACTTGCATAAAGCCA
TGCATCTGTTATCCTTCACTACATGATCTGTCCGCTCCAGCTGCGCTACCACGGGCTGCTTTCCTCAT
CCGGGTCCTTCTGCAACGGCTCCAACTTCCGA
CACCAGTACTCGTCAAAGGCAATTGTGACGCGTTTTAGTCCCTCCTCCAAGGAATCGTTCGTCGTGTC
AAATACCAACACCTCACCCTTGAACTTTATGT
TCTTGAGAAAATCCAATTTCTTTTCGACACTACCCTGACTTTCAACGCGGGCCTCCGCCTGCGCCTCC
GTCAAGTCATTACGATCCATCACACGCCGTAC
CGCAACGCCCTTGGCACAAGTCATGATCCACACATCGTCACAGAGTGAAAGCAGACTGCTTTCCGTGA
GAAGTGCGGCTTCCAGTATAATTAAAAGCACA
GATTTGTTTTTGGAAATTGCATCCCTCGACATAAATTCAATTTCCTTTTCAACTGCCTCATGGATGTG
CGGCCAACACATATTGTCGAGTTTCCGTAATT
CTTCTGAATGCGGCTCTGAGAACACGCGACCGCCGAGTTTCTTTCGGTCAATGAATGGCTCGCTGTGG
GAGGAATGTGAAGTGCCTGACATCTCCTCCGA
CTTCGCATCGTGCAGAATTCCTTTTCCGAATGCGTCAATCACATCGTAGTAGCACGGCTTTCCTGGAG
AATAAATATCATGACCTATTTTGTCGGCACTG
ATGGTGTGAGCCGTAAAAGAATATATGGGCTGCCGTGCACGTGACTGCGCCAACCTCAGCAAGTGAGT
GCATCGCACGGACTTTCCGCTTCCGATGCCAC
CCGTCACGCCGAGGATGACCACGCGTGGAAGAAACGCCACTAAAAAAAGCCTCCGGGAGGAATTCAGC
ATTCACGCATGCAGGCCCAGTATTTGCCCCGC
AGACTTCCCCGGGATATACGTAAGTGTATAGATGTAACTTCCAGAGCCCTCCTAACGACACACAAATA
AAAAAATAAAAATAAAAAATAAAAAATAAAAA
ATAAAACAAAAAAATAAAATAAAATAAAATACTTCAATTGCTGCAATCCATAAATGCAAGAATACAAA
AATGAATAAAGACGGGAAAAAACGGACACACG
CACACACATACACACACACACAAAAAAAAAACAAGGGTTAAACAGGTGGGTTTGTTTGCCAACCGCAA
TGCTCCAGCTCAGACACATCCTTCGGGGGAGG
CACAAAATAGTCATTTANNNNNNNNNNNNNNNNNNNNNNNNNNNNNNNNNNNNNNNNNNNNNNNNNNN
NNNNNNNNNNNNNNNNNNNNNNNNNNNNNNNN

NNNNNNNNNNNNNNNNNNNNNNNNNNNNNNNNNNNNNNNNNNNNNNNNNNNNNNNNNNNNNNNNNNNNNNNNNNNNNNNNNNNNNNNNNNNNNNNNNNNN
NNNNNNNNNNNNNNNNNNNNNNNNNNNNNNNNNNNNNNNNNNNNNNNNNNNNNNNNNNNNNNNNNNNNNNNNNNNNNNNNNNNNNNNNNNNNNNNNNNNN
NNNNNNNNNNNNNNNNACTTGAGATGCATCCTTTCTTTTTTTTTTTTTTCTAAAGAGTCCTTTACGTCTTTCCATTGATTTTTAATTTAATTTTTCTTA
AAAACCCTTCTTTTCTTTCTCAGTGTTGCGGGTGTGTGCCGCCCTCCCCGCCCTTTTTTCCCCGCGTCTTCCACCTTACTGAATTCCTGCACACGGAAAC
ACGCACACACGCACAAACATGCAAATACGCGTACACATGTATGCATACTTGCACGCTCTTTCCAGCTCCTCGTTTTTTCCCCCTTTATGCATGGTGCCCT
GCTCTTAGAGTGCTCTGATTAGAAACAACAACAACAACAATTACAGTCATAATGAATTTGATTATGATAATGATGATAATAATAACAGTGCCCGCGCCCC
GTTTCCCAACACAGGCCCCACACTACGTGTTCGTGTGTGTCTGGTTTGTCCCCACCCCTTCACTCATCCCCACTTGCGTCCCCCTTTCACTGAACGAAGG
GGGGCAAGAAGATCGAAAAACAAATATATACCGAAAAGTAACACCCGCCAGTATCCTCAACTGCGTGAAGAGACGGGAATAAACAGCAGAGGGGAAGCTA
ACAGAAAAGAAATGAAAGGGACCATGAAAAAAAATAATAATAATAAAATAAAAAAATAAATAATAATAATAATAATAAAATAAACCAGCACCAACTCGAA
CACTCACCGGGCAGGCTCCTAATTTACTCCATCATCCCCTCAAGTCTGAAAATTCCCACATCCACTCTCTCTGCCACGTGGGTCTCTGCACCTTCGCGTT
TCATCTATCCATCTTTTCTCTGGCCTTCACATCACCAGTCGCACGTCCGCAGAGATAAGAAGAAGAACAACGACAACAACAGAAGAAAAAGTCCCGTAGA
ACACCACATTTTCTTTCATTGCTCCTCTCCTCTTCCCCGTTTCCTTCCCAACTCCCGTTAATAACCCCCCTCTTTTTATTTTTATTTTTCGTTTCATTAT
TATTATTTATTATTTTTTTTCTGTTCCTTACGGAATTTGCCCGTTTTCTTTTGTTTTGTTTTGTTTTGTTTTTCCTTCCTTCTCCTTATCGACGTTTCTA
TTGTTATTTTTACCGATCCTTTCGCTTCTCTTTTCTTTTTTTTTCCCCCTTTTTTCTGCCGCAGTTGTGGGTGAGTCTCTTGATTGATGTCTAATGTTTT
CTGCCGCACTGTCTCATCATCCACACATTCGTAAAGAGTGAGAGGGGGCGCATGACACACACACACACACGCAGACGCAGACGCAGACGCAGACGCAGAG
ACATAAATATGTATTATATCGTTGCGAATGGTGGGCCTCCTCCCCTCCAAAAACACAGATTGAGGAAAAAAAAAAAGGCGTAGAAAAGAGGTTGAAATGT
CTTTGAAGCACTGCCAGCGGATACGCTCTAATTTCCTCAAACATTGCCGCAAATATCCGCCTCTTCCTCACACAATTTAACTTTTTTCCCCCCTTACATG
TATTTATAACATAGCCGTCGTTTCCTTTTATAAATTATTATTATTATTATTATTAATATTAATATTATTAAATAATACATTTAGTTTTACGCCTAAATAG
TCTCAAAGACACACAGATATATAAATATATATATTCCCCCCGCTCTACCCTACACAGGCGGACAGATTGCCCTTCTTGTCAGTTCACTCCCCTCCCCTTC
ATATCCTCCGCATTGGCATCAGACCACCCCTCTGTGGCAATGAGGGCGTCTCACCTCAAGAAAGTCACCAACTTCACACAAAAACAGTAGGACAGGTGGG
CTCGGTAAAAAAAAAAAGGAAACCGTTCTTTCCGCAGACAAAAATTCAAACGATATGGACAAATATGTTGCACGGAAAGAAAACCAACAACAATGACAAC
AACAAAACAGAAAACAAAAAAAACAAAAAAAAAGAACAGCAAAGGATTCTAAATAGGAAAAAAAGGAAACCCAAAAACGCCACGTACAACATCATACATA
TATGTTTGCAGGCAGCAAAAATGAAGCTCTTCCATTTATTACATGGTGCAATGTGAATGTGTAATTGTGTGTAATTTTGTGAAAAACAAAGAGCGTTTTA
CCCTTCCTCTTGGGTGTAATGTGTTTGTGTGTATTATTCGTTTTTCTCTCAAAATTCACATATCGCACAAAGACATTTTCACGCAGATACATCCACTGCC
ACATCATTAGGCGCGGCCGTATACTTCTCGCGAACCTCGTCAATCTGAATACGATGACCAGCCACACTCTTTTTTCCCTTATGAGCTGCCAAAACCTTGG
CTACTGCCGCCTTGACGTCATCACCAGCTTCCTTAATGGCGTTGTGGTCCAGTTGAACAAATGCAAAGGTGCGCATGACATTTGGGTCTTTTTTATCGGT
TCGTGAAAGTACCTTGATAAGCTTAGTTGGTGCAGTGGGATCCACAATATCACGGACTGTCTGTTCTGTTGCGTCCGTGGGAAGACCCTTTACAATGATA

TCGTAAAATACCACAAAACGGCTCAATGCACGGCCATCTGCGTCTTTCCGTTTATTATTGACTTCGCT
GCTGTTGTTGCTTTTCCTGTTGCTGCCTGTAG
CCAATGTCTTCCCGTTCTTCTTTCCATCGTTAGCTGGCACTGATGTCCCAGCCGTCCTCTTATTCACA
CCGTTCTTGGGAACAGTTTTCGTGCCGTTTTT
TTCCACTGGACGAGTCACCGCCTTTTTTTTCTCCTTCGCTGGAGTCAAAGCTGTTGGTTCCCTGCTCT
CCCTTGCCAGCACCGTAAGCAGTGTCGTTTGT
CCCTGTGAAGGTCCAACACGTTTGGACATCATGATAGCCTCTGCAAAGCTTTTGGGCTTCTTCACCTC
CGTAGTTGTGGCGGGGACAGATGGATCCGCCT
TGTCTGTTGTCTTCTCTACATCTTTGGATGCCTGAGACTGACTCGTCGTAGAAACAGTTGTACCGGCA
GCAGGAACTGGAGATGCAACCTTTGCGGAGGC
AAGGGACTCCGCCCTCTCCAGTTTCTCCAAATCCTTCTTTGTTGTGGCATTCTCTATTTTCGCCAGTT
CCGTCGTTGAGACCGGTTGTTCAATCTGCTCC
ATGATGCCATGTGCAGGTTCCTTCGCAAACATTGGGGGTGTTTCCAAGGCCCACCTTCTCGGCGCCTC
TTCGCTGAAAGTGTTTTCGCGCTTGTCAGAAA
CTATCCCAAAGCTGCCCGGATGCGACTCATGCTCCATGAGTTCCACCAAATGGATAAATTCCTGGCTC
ATGCCTGGTGGAGTTGTGATTGAGCCTTTGAT
CATCACATGAATGTGCCCGTCCCCATCTGTTTGCGTGGTCAGACTTTGAATCTTCACCGATGCGTCAG
CCTGCATGGGATACAGCTTCGATACGGCATCT
TCGATCTCCATGATGTCAGTTGCCTTGTGTCCCATGTGATTAAAGGACGCATTTTGGGTGTAGAAGGA
AGGCAATCTTTCCGGCATCTGCACCATCAGTG
TGTAATACTGGCGGGCAAAACTCGCAGCAACACGTTGCTGCCGTTCATTTCCGACGGTCATCTTATAC
CCTTCTTTCTCCACACGTTGTGACGGGGGCAG
CGATAAGAGGGGGGAGAGGAAACAAGAATGATGAtAGAGAAGTGAAACAGAAAACAGCTACATAAAAA
AAGCAAACCACCTCACCCCCCCCCCCCAAAAA
AAAAGAAGTACAAAAAAGAGAAAAAATAAAATAAAATAAAATTCAGTGCCACAAAAAACGACAACAAC
AAATACAACCACAACAACAGCGATATACCGAG
AACACGCATTAAGTACCCGCCCATCGTTTTCATCGGCACACCTACTTCAACATTTTTGTTGCGGAGGA
AGTTCCTGCTAGGGGCCACAGGAGTGCCTTCC
GTTTCTCGGGGCTCATCGAGCCCTTCGCGTCAAGAAGCTCCTGAAAGTCTACTACCTCCCACGATGAA
TGGATGCGGTGGCATTTCTCGCAACGGCACGT
CACGACATACCCTGACATCCCGGTGCACTCGCGCTGCTCACGGTGGTAACTACGGCAATGCGCATCCG
CGTCAAAGAGGATGGGATTCGGAATCGGCCTG
TATCCTTTTGCGTGCCGTTGAATGGACTGGCAGTGACCACAGCGCAGAAAGTACTTCTGAAAAAATCG
GCTTGACATCGTCACAAACTGTCTCTTCAATG
AACAGCCTTGAGTTTTGGCAAGCCAAATTTGAGATGCAACTCAAAAGAAAACACACACAAGAAAACGA
AGAAAAAAGGTAAAAAAAAAGCAAAAAAAAAG
AACAGGAATTCGTTCCTTCTTTTTAAAGAAGGACACAAAGAATGAGCTGAGAAAAAGGCCATGGGACC
ACCACAAAACACCAAGAGAGCAATAGCAGTGG
GAAGATGCAAATAACTGCGGTAATTGTGGTAAGATCCGTACCCTCTCAAGTAGGGGAGAGAAGAAGAC
AACGACTGGCTCAGGAGACCAACTTCTTGCGG
CGTGGGATACGCGCTGGCGGTGCCGACGCATTTTTCCTCGATTCTGCTTCTAATCCAAGGAACTGGCC
GGTGCGCTGCACCTCCTCCGCAATAGCCTCCA
TATTTGGCTTCCATGAACATGACGGACAGTACGCCGACCAATCGACAATGTTTGGATGCAGCGGGCAT
TGGCCACTCTCCACCATACCTGTGCGACTGCG
TACGCTCGCGCGGTACGAACCGCCACAGCCCTGACATTTCCACTCTGCGAGAAATTTGCTCGTGCGGG
CTAGCGACCCTAAATTATCCTTTTTATCCGTC
TCCTTCAACTGTTTTATCAGCTCCGGTGCAGACGCTGCAAGCGACAAACTCTGATGCTGTTCGCGTAA
GACTTCGGAAGGGTACCTGCCCTTACAAAGGT
TGCATCCCGCATGAAACTTCGTGCGCACACTGACACTCTTGCGGTAGGAGGAACCGCAAAAGGAGCAT
TTCCAGTAAATGCGGCGGTGATCATTCCAACG
GATATCTTGCAGAGTGATGCCAAGTTCCTCTTCAGTTTTCTTCACGTCAATGTCGTTAATAACGGCTT
GGTATCGTTTTGAGAAGTTCGTCAAACGGTTG
GACTCCCGCGTGACCCAATTCACCCCCTTCATGGGAAGCTTTCGGCCAATGCTGTAACTAAGGTACAT
GGCACCGTAATGCATGTTGGGCCAAACGCTTG

ACCAGCGGGACTTTTGCTTGAACTCCGCCGCCAAAAAAAGTCGTGTTCGATCCATCATTTTTTTTTCT
TTTTTTAATTGCCGTTTGCCTCTGTTTCTATA
CTTCCGACTATTGTGCTAATAAAAATTTCAAATTCCTTTTTGATAACTTCGCGGCTGGCCGGGGCGTT
GAAACAGCGGCGATTTATGCTCCACACAGCGG
CAATAACGATTGACGACGGATGGTGTTGTGCACTGACACGCCCCGTGCACGGAGAGTCTTCTTCCAAA
GAGAATACAAAAACGACAAATGCCCACTGAAG
AGCGAAGTGCTGGACAATCCGTCACCTCTCAACGTGCCTCAAATTTCAACCAGCCGACAGGGCAAAAG
CAAACTCTCTTTAAAAAAAAGGAAAAGACACA
AAAGACTTACGGGCACGTTCACATCCAAAAAAAAAAAAAAAAGACACGCCAGCAGCATGACACACGCGT
ACGACAGGAAAAAGATAAACACAAAGAGAGGA
AAAGCATATTATAGCCCACTGCGTAACGCGGCCCAGCTGTTTTGCGGCTTTCATACTTTGGGACCAAA
GAAGAACCAGAGATAAAAAATAATAATATGAA
AACGTACAACTCGGGAGTTCTGCAAGTCTCAGCAATAATTAAGAACGTGCTATATTTGATTGCGTGAA
TAGAAAGGCCATGTCATAATGTCTACTTCTCC
TGCCCTGCCTCCGTTAACGCCGTGAACACTCTGGGGGTCTTTGGCCACACAAATATAAATTTACAACG
TCCCTAATTTCGCTAAAGAAGGGGGCTTTTTA
TTATTAGCATATATATTTTCCTCGAACAGTTTATTTTTGCTTCGTTTTCTTGTTTTCGGTTCATGTCC
TCACGCCACCAATGAATTATTCATGAGCGGTT
TGGGCGGTGGCGGCTTCCTCGGACGCTCCGGCATGGGCGCCCTCCGCACGCTCTCGTACTTGGGTGGG
AATGTCAAAAAGAGCGTCGTACGCAGTGAGAC
AAACACGACGGACCAAAGAATGATGACGCGAAACCACGCTTGGACCAAAGCTTCATTCCCTGCAAATG
GGTTACGACGGGGATCCTTCGATGCCGCCTCT
TCTGCATCGTCATCATCCTCAAAGGGTCTTTTCTTAACCTTCCCTGTGTGCATCTCCTCGTAAAACTC
GTCGTAACTAACTTTGCTCTTGTGCTGACGTG
TTGTAAAACGGAAATTGCTGTAACGATTGCCACCATCAGAACCTCCACGGGCATCCGTGGAGCCTCCA
TCGGCATCCCGCCTAATCCAAATACCGCTCTT
TACTTGATCGTAGGCCTCCTGAATCTCCTGAAACTTCTCATGTGAGCCGCCCGGTGCGTCCGGGTGGT
GTTTGCGGGCAAGCACGCGGTAAGCTTTTTTG
ATTTCCTCCTTCGTTGCGCCTTGCTTGATGCCAAGTACTGCGTATGGGTTGGTGCTAGCGCTGGCAAA
CCGTGCCTGGAAAAGCATGGCCGTTGCGCCAG
CTGCCGCCTGCGTGGAGAAAGACGACGCCAATGTACGATGGGCCCCGCCTACAAGGAGAGCACGTGAA
CCATGCCACATTGTTGGTAATAAAAAAACAAA
CCAACTCCGTTAAAACACACTTTGTATCTGTGTATTCCTTTGATCCTCCTTCGTTACCTCTTCCTCAA
TGAATGTTTTACTTTCCCTCTCTTCGGTTAGA
TGTCCACTCGGCGTGAGTGTGCGTGTAGCCTAACGACGTATGTATGTGTAGTGATGGTTGTGTCACAC
ACACACACACCGGAGCGGAAAAATACACACCT
CAAATGCCAGAGAGAGAAATATATATATATATATAAATACGATTTAAAGTATAAAAGAAGGAAAACAA
AACACACTCACACCAAAGAATATAATCACAGT
GAGAATGAATTGATTGATTGACTTCGAAGTTTCCTGTAAACCTCTTTTTTCCCTTTCCTTCTCTTGTC
TGTGTGCGGGATAGAAATATCACAACTAATAC
AAATAAATACAGGAAAGAAGAATACAAAAACAGAAGGAAACATAAACTGTAACGCCAATAGACATGCG
CACTCTCACTCACAGTGCCGGAAAATATTTCC
ATGCTCACCAAAATAATAATAATAATAATAAAAGACCAATATAAAACCCGAGACTTAATTCCCCCCTG
CTTCCAAGGCATATATATGTATGTGTATATTT
GTGTGTATATTTTTCAAATTTTCCTCCTTGTATCCCTTTCGTACGTAACACGTGCGCGCATGTACCTG
CAGCACCGCGTAGATGTGTACGATGATATTTA
CGGCTCCAATTCCAATTTTTTTCTGGACAAATGGTGCTGAGCATAAGTAGCAGCCTCATTCACCCATT
TTCACATAATACCGCATCGTCACTGAAAAGAA
CTTCTTTTTGTTTTTTTAGAAACGGCATCCGTCTTTTTATTTTACATGCAAAGCAACAAAGCAAAAAA
ACAACGAAAAAGAGGCAGAAGAACGAAACAGT
AAAATGCGCACGATCACGCAGAATAAAAAAAAAAAAAAAAAAGTGCTTTCCAACATCCGCAAGTAAAT
AAAAATATATATGAACGAGATGCCAGTGATAA
AAAGATATTCTTACGCATGCATGCATGCATGCAAAAAAAAAAAAAATCGTCACTGTGCATTTTGCTGAC
GATGTTTTCCACACTTGGAACAGAACTTCACT

TGCGCAGAACGAAATATCGCCCCGCAGCCCGTGCAGTGTTGTGGCAGCAGCGGCGTTGGCTCTGATGT
GGGGCGCAATTTTGGTAGTGGGATCCGCTGCA
GTGCATTGCGCTTGTCACAAGAAGACGGTTCGTTTGTCTCCAGTGCGGGGTTGCGAGGCGGGGCTGTG
AGTGCCCCGCCGCCGGTTGGGTCGTCAAACAC
CAATTCCCGCGCAGACCAGTCGAGTGCCATCAGGGGAAAAAATGCTTCACGTACAGGCAAACCCTTCT
TGAGACGGTCCTGCTCCAACTCAAAAACCGTC
GAATGGGTCTCGCCATCGCGGTCACACACGGCACACAAAAAATGACTCGTAAACCCACCACAACCGCA
CTCCTTACAGTGACGCTTTTGTGGATCATGCT
GCTTATGCCCATGACCGCAACGACACTTGGCGGACCACTTTAATGGGTCCCAACTACGTCTTCGCGAC
AGCCATCCCTCCCCAATCTCCTCTGGTTCGTT
TGGCACGTAGTTAAAACCGCCACATGCGCATGCTTGACATCGGGGTGCCGGCACGCAACCCCGTGACG
GTTTCAGCGTAGCGTGCTCTTCAAGTGAATGC
CCACAAAAGCAACGATGCTGGTACCCAATCCGTGCACAGAAGTCCGTATTAGCCCCTGACAATTTGGG
CTCCTCACATCGCCAGACAGCATATACACCAG
TCTTTAAGGCCAGCATTGCAGCCTCATTCTCCCGGTTAAGAATCTTCTTGGATTGTGCGGATGGGCCA
AAGCGACGGATGCCCTGAAGAACTTGGCCTTC
TGAAGTGGCATTTTTCTGAAAATTTCCCTCAATAACCCCTTTTACCCGTTTAGAGAGATCACGTATCT
CTTCATGTACCTCGTGACTATCAACTACCATG
CCGTACGACTCAGGCGCACTCCGTACGACGGTTCCGTGTGAAGGCGTTGCCGTCAGCTGCTGCTGGGG
ACGACTGCGACATGGTCCCACCACCTCAGCGG
CGCTGCCACGCGCAGCGGACCTCCGCATCGGAATAAAAACAACAATAGGGACAACAAAACAATGGATG
GCTCCCGAGCAAAAAAAAAAAAAAAAAAAAGAA
AAGGATAAAAAAGGACTACACACAAAATCAATTTAAAAGAAAACTTTTAACTAATTGTGCTACCGAAT
CCACGAGAGAACGTCCAAAAAGCCCCCCACAA
CCCCGCAAAAAAAAAAAAAAAAAAAAAGCGGCAAATAAGAGTAAACGAATCTCAGATACATTTGCAGA
AGCACACACCATGTATCCACTCGCCCATCTTC
TCCCCTCCCACAACTCCCCAGTGAGAAACAGATATTTATATTTATATAACTTAGACTCCAAGAAAACA
CATTCATTCATTCATCCTCTTTTCCTTCTCTC
TCCCTCTCTCTCATGGCAAGATAATTTTAGCGTCACAACGGCAACCATTGACGCCCTGTCGCATCAAA
GAGCCTCATTCTCAACGTTAATGCGCGCCACC
TCCAGACACTTTGAAAGTGAAATTCCTTTCATCAACTGCCAGGTAACAGCGCATGCGCACAGCTTTGT
GACAACATCCGCGTCTCTGATGTCGTGATATC
CTTGAATGGCTCCCTCAGTAGAGGTGTTATGCCTCTCCTCCGAAGAATTGATTGACAACGCCGTTGGA
AGATCAACAACAGAATTAAACATTGCAAGAGG
AAGGAGTAGACTGCTGGTGAATTGAGCCGCAACACAGAGCGCAACAATCTGATATGGATTCATCGACA
TGGCCAGGAGCGCATCTCGCACCTTAAGTGTG
GCGCCTATGGGAATGTGTGGATGACCAATGCCCACAATTCGTGGCACTTCCACATTAAAGGCACGTGA
AAACCAATCCGCAATGGGTCCAATGACACGAT
CCTGCTCTTCCGCAATGGGGGCAGCACGGAAGTAAATGTTATCATTCTGATAAAAAGACATCAAATAG
TCCACACGCGGCGCAATCATCTGAGGGGAAAC
AGTTAGTGCTCCACTGCACATGTCCGTCATAGGCATCAAGAGCTTGTTCAAATACCCAGACTGCTCCG
CATACTCTTGTGCACAACAGTATGCCATTGCT
TCATTCGGAATTGCAAGAACATGCGTGGACTCAAATGCCTTTACTTTACGTCCATCTACCAGGACAGT
ATACCACCCCTCGTGACCATCGAGAGGTCCTA
CATTCACATCCTTCCAAAAGACACGCACAGCGGCTCGAGAGCGGTCTCGCATTGACACATCCATTTGA
TACAATGAGTCCTCCGTGAGTACGCGGCTCTC
CTCCTCTTCCTGCTTCTCTAGGCGATCATAAAGCTCTTGAAGTTGCCGTGGGTTCATATTCGACCACT
CTTCTAATTTGCGTTCCAGCTCGGCTGTTGAG
AGCTCTGCATGAGCCCCAGGATCTCGAGGGACACGATCAAGAAGGGAAGAAGCGTCTATATTACAGGA
TATACGTCGCTTTGTTTTTTTCGGCGCGATGG
TGGGTCCACTTGCAGTCCCATTGTTGCTGCTGCAACCGGAGTTTCCAGATGAGGAAAGAGACGAACAG
CAGCGTGTCCCGCTCAAAAGTACAGACAAAAA
ATACGGTAAAGAAGAAAAACGTGGCACCACAAACTTGCGCATGGTGCACTATTTTTTCCCCCCTCTAC
TTTCCTTGTTGCTTTGCTCTTATCTTGCCTCT

CGCTATGGCTGGAGTGCGTGCCGACGTATGCGTGTGTCGTCTACTCCCCGTGTGTGCAGCGCCCAGGA
TAGAGGGCAGTCGATGTGAATCAACTGCAGGG
TTGTGACCACGCCACCCAAAACAAGAAGAAACAGCAACAAAAAGGATATGTAAAAAAAAAAAAAACATG
GGAGTTTCATCTACACAACAAAATGTTATAAA
CAATAACAGAAATAAACTGGAAAACAACACCAAGGGAACACAGATAAGGAAAAGCCAACACTCAAAAA
CCGAGTCGCCCACAGATATTATGGCAATTCAC
TTAGTTCATGAAGAATCTCAATGTTTTGTTTCTTTAGCGCTGCATGCTGCCAAGCCCACGACGACTCT
TCGTCCTACAGCAGGTCATATTGTACATCCAC
TGGAACGGGATCCTGTTTGCGATCACAAAAAAACTCCTCCAATTTCCATTTATGAATAGCCTTCGGAT
CATTCACTGCATCAAAGTGTGACGGCACCGAC
CTCGGAGGAGGAAGCCTCGCACGTTCTTCACCATAATTGAAGTTGTACCACGACATCTGCAGAGCTGT
CGTCTCGTTGGGTCCGACTTCACACTTTGCAT
GCTCCAGAAGTTTCAAAGATTCAAGTGACCAGCGTGAGATAACCGTCAACCCTGTCAATGGATCCAAA
TGATGCGAATAACTCTCCAATGTCGCATCCGA
TGAGGTGAGGCAGATCACATTGGGTGCTTTAATTTCAAGGCACATAGACAATGCGCGGCATGTGTCCA
GCATAAAAACCACCTTACGATACCGTCGTTGC
TCCCACATCATCATGAGTGTATCCGCGATATCCATGGAGCTCAAAAATTCCGAGTCTTGAAATTTAAA
AAAACTCTCTGCGGCGTGACCGGCCGCGTAAA
TCAAGATATGAGAGTTTTCATCCGTGTTGAGGCGCCGCGAAGGCGGAGTATAGGCATCATAGCGTCCC
TGCAGAACACCAAGAAAGCGACGCACATCCAC
ATCGTATCCGGCATAGTCTATTTCAACGCTGCAGCCATACAAGTTTATTCGATCGGGGCCTGAAAGAG
TGCTGTAAATGGCAGCCGGGTAGGGATTGCGT
GGGTCACAGGCATAGCTGTCACCAACTAAGAGAATGATATGGTCGTCGTCTATTCCATGCTTGCGGCA
GAGATGATAAATTGTCAGCGCATTGGAGGTGT
GGCGTATATTAAAGAAGTAGCGTGAGGAAGACAAAATGACAGCCCACAAGTTTGTCTTTGTTTTGTTG
CTGCTGGCGATGACGGTGTCGACTGTTGTCAG
CAGAAAAAAAAGGATGCAACAGCACCACAAAACCCCCATCTGACGCTTCATCTTAAAATGAGGCTAGA
AACTCTCCTATGATGTCGTCTTTGTGCGAGTG
CGTGCCTCTGAAAAACTTTGCCTTCTGTTACTCGTCCTTTCCACGGCATAAAAAAACTCAAAAGAAAC
AAAAAAAAGAAAAAAAAAAAAACGGAAAGAGGG
ACACATCCACACACACACACACACACACACACACACGCACATACACTTACGCATGGGTGAAAAATAGC
AGGTGACAAGCACAAAAAAACCAAACAACAGG
AGAATTGGGCTTTCCCACACCGCTAGAAGAAGCGGATTTCGCAACAGGCAAGCAGTGAAAAAAAAAAA
CATTACTGATTTCAATGGTAAATGAAAATACA
TTAAGGCAGAAATGGCTCATACGACGGAAACAACTAACGTCACCCGTTTAGAATACATCCGCAGCATC
CTTTATGGAAGACTCCAAAAAAGTCAGTAAAG
GGCTTCACCTCCTCGTTATCCCTCGCAGCCACCCTCACTCCCCATTCCTCTCGTCATGTCAACAATCT
CTCACTGCTGTCCATCTGCTGCAGTCGTGGTG
GCATCCGCATATGGGAAATGGGAATTTCAAGCTCATTCGCACAGCCTAAAAACGATTGTGTTACGCCG
CCACCTTTAGCATCTCCGCGACACGCAGGGCC
CACCGGGCCATTTTTCCAAATCAAGTCAAAGCCACCCACGCGGATTTCATCTCCCGTACGGCGTTTCT
CAATATCAATAACGCTGAACATGTCCTCTAGC
ATGTTAAACTTCAAGTGGTAATCTGCGGGTGTCTCAGCAGAAAGCGAAGGAGAAGCGTTTGTTTCAAT
AAGCCAGGGATGCAAATCATTATCCACCATAA
TATCATATCCGTAGAGCTCAAAGCAATGTTTATCTTGCACAATAATTTTTTGCACAGCATGCAATGAA
CGCAAAATCATGTTCTGTATATCGTCAAACAG
CTTCTGTGCCGTCTCTACCCCACGCGACGCGGTAATGTACTGTCGAAGGTTGCGAAGTCCATACTTGC
AGCCCGATGACGGTGTATATTTGGGATTTGTT
TTTTGAACGGCAACATTCGTGACATGAATAAATGTGTTTTCGATATCCCTTAAAGAAAACCGTTGATG
ACAAAAGCGTGCAAATCCTGTGCGATGCAGCC
ACACCGTGAGGGGAGAGTAGCACGTCACAAGAACATACACACGCAGGTCATACTTTTTCCCACCCACC
AGGTGCGGGTTTTCGATATAGCGCTGCGCTAA
ATAGGGTTCCACCTGCTCGGCCCCGTACGGACCGCCCGCCTTTTCCCCCACTGCTCCGACCTGTCGTT
GTTTGTAGTCCTTCCGCCACTCCGCAATCTGT

GAGATCTTTGAAAAGAGAAATATGCCTTTTCCCTGCGCCTTCGCCGGCGGCTTCATAATCCAAATCGC
ATTTGGTTGACGGCGAAATTCCGTCTCAAACA
TGCCGTAGTCCTGCGGCAGCGAGAAGGTGACGGGAAAAAAGTCAAACTCCGTCGCCTCCTCCACACGC
CCCTCCCGTTCGAGGCTTTTTCGCATGCGCTT
CAAGTTTTTCACCATAAAATCCTTCCGTGTCAGTTCAAAGTGATTGCGGAAGTGGTTGATTCGCTGCA
CGTCGTCCAGCCGCACATGATCGTGCACCTCG
CGGATCCACCCCACGTCAGCCCAAAACAAATCCCAGCCAAGGTCGCTGTCGGTCTCGAGCCACCCGCG
ACTGCGCATCACATCAAGAACGGTGTTGCGCA
GAAATGTTCTAAAGCGTACGGGCCCCACACGACCGCGTCGCTCCCCTGAAGGCATTGCAGCACGCTGT
TGCCGCAGAAATGGGAATAAAAAAAAACGTGT
GAAAAGTGACCACCCGCTCCCTGTTGTCGTTTTTGTGTTGCCGCCGCAAAGGACACACACACACAAAA
AAAAAAAAAAGAATGACAGAAAGTACGGAGGA
AACAGAAATAACAATAATAATAATAATAATAATAATAATAATAATAATAATAATAATAATAATAATAA
TAATAAATGGCAATGCCTGCAAAGGCCCACAA
GATAACAGACAAAAAAAAACTACTACAAAAAAAGGAAGGATGCTTCTTTTTTTTTTTTTTCGACACGC
ATGTGGGCTATTCCAAAGTGTTCCTTTTACTC
GTCCTCCATTCTCGTTATTGTTGTTGTCGTCGTTGTGGCCTTTGCCGCTGTCCTTTCTTTTTTCTTCT
TCTTCTTNNNNNNNNNNNNNNNNNNNNNNNNN
NNNNNNNNNNNNNNNNNNNNNNNNNNNNNNNNNNNNNNNNNNNNNNNNNNNNNNNNNNNNNNNNNNNN
NNNNNNNNNNNNNNNNNNNNNNNNNNNNNNNN
NNNNNNNNNNNNNNNNNNNNNNNNNNNNNNNNNNNNNNNNNNNNNNNNNNNNNNNNNNNNNNNNNNNN
NNNNNNNNNNNNNNNNNNNNNNNNNNNNNNNN
NNNNNNNNNNNNNNNNNNNNNNNNNNNNNNNNNNNNNNNNNNNNNNNNNNNNNNNNNNNNNNNNNNNN
NNNNNNNTCTCTCCCTCCTCTTTGTCGCGCCA
CTTCCCCCCACACTCCACCCCCTTCTCTTAGCCTCCAAAGCCGAAAGGACGGACTGAGGGGAACGTCG
CAGTGAGTGAAATGGATCCGCGACACCACCAC
CAAAAAAAACAAAAAAACAAAACAAATCTTTTTCCTTGCTTGACGAGCGGTTTCTTTTAACGGCACTC
ACCACGCGGAGAGGGTTTGAACTTTCGATTCC
CTGCCGAACACCGTGGTGTAAGACGCGGGCGCATCTTCTTTACCAGAACAAATGACTGTAAATAAATG
GCGAAGACGGACACGGTGGTACGCAGTGCAGA
AGTCCAGCAGTTTTTCCATCCTCTCAGTGCACACAATACCACAAAATTGCATGCAATTCGTCAGCAAC
TTAAAACATAACTCAACTTCATTTCGTTCCAT
ATGTGGAGCAATTGTAATATGAACCGCCAATTCCTCAAAAAGGTCACCAATGGTCCTATCTTCTAAAA
ACGAGCTGCCATGCACAGAAGTATTTCCCAAC
GAAGTAAAAACCGCATCCACCGCAAGCATGATAACTGGCAAAAATGAGTAGCGCAAACAATTCAAAAA
GTACTTCAACAACCGTTTCCACACAAAGTGGG
TGTCCAAAAATTCTTTTAACTCAATCCCAAGATTTCCACTTGACAAAAAAGACTTCATAAGCGCATCA
AACACCCGTTCACGAGAATTCTTGTGCGCCAT
CACCAATTCGGCAGATACGGAAAAAAGAAGCGAGAGAAAACCTCCGTATAAAAAAATAAAATCTTTAC
TGTAAAACAATGCGACACCCAAGTTGCACCAT
TCACCACTAATCATACTGTAAATAATGTCTGCAACATCATACACATAAATCTCACTCACCACACCCGG
CGGGATTTCTTCTATTGGTCCCTTCCAGTTTG
AGCCTGACAAACCCAGTGTGTGCATGACACCATGTACGGCACGGAAAGCAAAGCCAACCAACCCCCAA
CGTGTTTCACATTTGTTATCTTCCACAAATAA
ACGTGTTGCCAAGTTACAGAGTTTGGCACATAACCGCAGCAATAAGGGTGCGTTTGCAGGTAAAGCCC
GAAGCAGATCCTCTATTTCCTGACTCCGTTTG
AGTAGAGCATCCAGCAAGCAATAAAGGCTTTCCACCTGATTGATGCCATCCGCAATAGCACACAAGTC
ATAAAAAACTGAATGAGGATCCGCCGTTAAAC
GATCCACATCCAATGATCGGGTGACGATACAAGAGATAAGGTCCAACGTTGGGAATCCCGCATAATGA
CGGCACTCAGTAAAGGATGTCAGGGTGCTAAG
CATTCTAGAGCGGGCCTTTCTCCCCTCATCAGTAAGTTTATCGCTAGTGATACGGAGGAGCTCAATTC
GTTCCTCCTTCATCGCCATAAGAATGGAGAAC
ATCTTTTCCGATGACAAAAAAAAACCGCATACACCCTGCATTAGCACCCTATTAAGTAGGAGGTTTGC
ATCATGGGTAAGACATGCCTTTTCGCTGTGGT

GTAAGTAAACGTAACATAACAAGCTGATCATGATTTCATAAAATTCACTAGAGGGCTCAGAAAAAGAC
TCATATAGACACACACAGAAAAAAAAGAGGGC
ACGTAATATGCCCAAGTGAACTTTACCTCCTCCTGTCGTTGAAGAAAGTTGAGAATCATCATCCTCTA
CATCTACCAGAGAAAGACTCGTTAAAAGAGCG
CTGGTCAATGTACCATCCACAGAGAGCAACTCACGCGCAAACCGAGCTAGAATTCCAACCAATTCATC
GCTTTGATGTGCTGCGTTATTTAACACGGCAA
TCGCCACGGTAGAAATAATGACGGAAAGCCGCGATAAGCAGACATGCATAAGAAAAAGGGGTGCATCC
AATGCAGTGAGGGTAGGAAATACGGCCTGAAC
ATTCACTGCAAGAGACAACAAGAGATCACCCATCCCATCCGAATCACCGGTCTCCCGGATACGCACGC
ACAACTCATACTGCTCAATAGTACTTCGCAAC
CGCTGTGCAACAATGCCGTAGATGTCAATTTTCTCGCAAAACAGCATTTCGGCAATAGGCAAAAGGGT
CTTTTCGTTGTGTAACATAATACCCAAATCGG
AGCGTGCTTCGTAGGAATCCTCAGCAAGGTGTGCTTTGGCAACAACATTGTCAAAGTAACACCGGGAT
ACTTGCATAATGCTTCTCATGATCGGCATTTC
CCGTGTATTCTCACCTTCTCCCGCCAAACAAGGAATGACACGATCACCAAGGAGGAAAAACAAATGCA
TAAGTTCCTGGCGAAGCTCTTCTTCACCATCC
TCCCATCTTTCCAGCACACCAATGACCGCCTCTTCCCACTGCTCAATAAGATATGGGCGATTCAACAA
ATAATTTGAAACCCTGCCTTCGTCGCGGTCAG
ATACGTTTACAAGAACTGCACAGCCTAATTGTAACAGTTGCGGCAATCCACTGGACTTATACATAGAA
GGTAATCTGGAAAACAGTTCAAGAAACGAGTC
ACTTAACTTGTTTTGCGACTGAAGCCACTCAGGGTCGACACAAATTATCGAAGAGGCCATTCGTAGAA
ACCGGACACACTTTCCTCTCAAATCCAGGGAC
TGATAAGTCAAAAGAATTTGAATGCAACCATCAACAAGTAGTGAAAGCGCAGGAAACCATTTTTCACC
CTTCTCCAAAAAGATACTTCCATCCATCTTGT
TTTTCACTGTGATAGGTGCTGGAGAACGCGTTGACATAATCTCCATGATAATGCAGCACAACTCGAGT
GCACTTTTTAGACAGTAAGAAAATTGGAACAC
AACAAAAGTAAGAACCCTCGGAATAAATCCCTCTACAAAATTTACTCGCATTCTATCCAAACCATCAT
TTGCTTCCTTTTGCGCAAAGTATGTAAGCACC
TCCAGTATGCAGTCTAGCACATCGCTTAAAGGAAAACGAGAAGTAAGTTCTGAAATGTATATTCCAAT
CGGTCCAGGGACCTGATCCGTGGATAAGATTT
GCAGCCTCCAATCCACGTAGAGAGCACATGCGTAAATAGAATACACCTCATTTTTAATATATAATGGA
AGCTCTTCACCGCGCTCACGCATCATAGTCAG
GTAGTCCAGAACATACTGTTGAATTTTTTTCCGTTCATTGGGTCCTAATTCATTACTTGCCATAAACT
GCATCGCTTTGCATACAATGAAACGCAAGTAA
TTACTCTGGCACAGCGGCAGCAAACGCTTTACTTCTTCCCAATGTTCATAACGGTTCATAAAACGGAG
TCTAGCGGCAGCCCTATCGCGTACAGCCTTGT
CTGGAGATGTCAATGCCTCCTCTGCAAGCTTTGCGAGTACATCAATGTCCATCACCTCCTTTGCTTTT
CTTCTTCTTCCCTCAAAACCTTATCTATCAAT
TCACTAACTAAAATAAGTGGTCCTCATGCATGCCGATAAAAATAAAGAAAAGAAGCAAACAACAAGGA
GAAAAAAAAATCGCGTCACGAAGTTGGCGCAG
TATCAGCGTTGCCGTTGCTTCCTCGTGTGGAGGAAAGCATCACAAAAAGAAAATAGAACAAGCAATAA
AATAATAAATAAATAAAAGAAAATAAAATAAA
AACAAAAATAGAATCAATGGGCGCCACCTATACAAGTAGCTGACAAACTGCAATGTCGGGAAAAAGAC
CCGTTCGAATCCGCTACAGGCCGCACACCAAA
CCGATGACTATGCACATTTTGCAACACACCATCGCCTCCGAAAAATTTATTGCCACCAACGTGGCCGA
TTTCAATGACATCCCCATTGGTATCATAACTA
TCACAGGAAAGAGGCACTGCGAAACGTCCAGAAGCGGTCCGCAGGCTCTTGCCTCGTTCGCGAGCCCC
AGATGCGGCACCTGCACTCGACACGCTTTTTC
CTTTAAGCGCGGGGGGGAAAGAAAGAGCTGAAAACGGTGGGATCTTATGGTGTTTTGTCGGTGGCGAT
ACGAAAAAGTCACTACGGTGCTTCAGCCACAG
CCGTGCCGCGAGCATCCACAGTGCACGTCCGTACGATAAGGGTGAAGTCTGCCGCAACTGCATTGTCG
CGTCAGTAACCGCAATGAGGAACGCAATCCAG
TCCCGCCGGCAACGAAACCGCAGCACCAAGACAGCTGCGCCATTGGGATCAATTGGACGTCGTCGATG
CGACAGTGGCAAACCGAGAGCTCCGTTCGTGT

GGTGCGGCGGGCTCTGAGATATGAAAAAGATACAGTAAGAGGAAGCGTCATCATGAGCAAAAAGCGTA
CAAGAAAATTCCATCAGCAATTGCCGTGCGTG
AGCACCACACAAGATACAGCTTATATTCCCGGTGGAGCCACTACCAATTGTCCCAAGAGGATACAACA
AAAGACCATCCATAAGCGAAAAATGATGTATT
TCCCGTACACGACGCGTCGAGCCTCTGTCGACTTCTCTGTTTTCCAGTTGTTGTTGTTGTTGTTGTTG
CTTCTTTTGTGTCGGTCCCATCTCCGTCTGAA
ATTGCTGTACCCCCTGCTTATCGTGTGATGGCATCGACGTTACGGATGCTTGGAATTCACGTCCAGAG
GATTTCCTAGCCAAACCGTTTAAATCCCGTAT
AATTCGCTCCACCAAAAGAAGGACTTCCCGTCGGTCCTTCGAAAGTTGCACCAGGGCGGGTATCTTCC
TCCCTTCACTTGGGAGAACGTCTGCCAAAATG
CCTCTCTTGTGCAGTACAGCCAAAGCCTGTGAAGGTAAAAGTGAATTTACCGGAGGGTCTAAAGAGAG
CTGCTTTGTCGGCGACAAAGTAACGCTCGGGG
AAAGAAGAGGACGTGCCCGTGATAATCTGGTAAGAGCACAAGTGCGTGGCAATTCCTTCTTCCCATTG
TTAAATGTCAGAGGGGAAGAAATCTCTGAATG
TGAAACCGGGAACAAAGAAGAAGATATTCGGTTCTCTGGTATTCCTTCCGCAGCAACTGACAAATTGC
CAGAAATGTCAATTTTGCTGGACGAAGCAACC
GTGTTGCGAGAAGTAAATGGAGGTGGGGGAGAAGTTCCCACAGGCTCTACAGGTGATGCTAACAAATT
TGCCAGCAAAGGTGTTGAAGACAAAGTAGCAA
AAGAATCTCTATCCACAGATGGAGAACGAATCTTTTTGGGCGACGCGCCCCCGTTCGATGGCGATGAA
ACATCACTTGAGGCCAATAAGGCAACAACTGG
AGGGGGAAGAGGAGGAGGGGGACGAACGGGCGACTTGTTCTCGGCTTTAATGCCCGTGCATGCGCGGA
GTACCGCAAGCGCTTCCTCGTGCACTTCAATT
GCTCTAACTTCCTCCTCAGTGAGGACGCAGAGTTGTTGAGTCTCCGCCACAGCGCGTTTCATTTCACT
TTCAATTTGAATTAACTTGGCGACGCGCTCCG
ACTCCGTTCTCTTAATCTGCGCTTGCGCTTCGGCGAGTGCCTTTTGACGGGCGGCCATCGCCGAGCGG
CGCACCGTACGCAACTCCTTCAACCAGCTTTC
TTCCTCTGCACGACGCTCGCTAAGATGTGTACGAAGCGTCTCCTCCATCACCGCCACGTGCGTCTCCG
CTCCTCGAGGTGGCGAGCGCCTCATTTCCCCT
TCCCGTTCCCGGCGTTCTCGATTCACCCCAATGTCGAGTGCGGCCGGCGTGGACAACGACGCCGGCGT
CAACGAGGGTTGGGGTTGCGGCTGAGAATCCA
ACTCGCCAGAGTGCGAGGGGAGGGTCAATGGACCTGAGGTATCTAATCTATCGGATTTGACATCCAAA
CGCTTTCCCCGTCGTTCACGCGCACGCTTCAA
AATTGCTAAACGTCGCTGCGCATCCTCACGTTCTGCTCGTAATTCGCGCCGCTGCTGCTCCAAAAAAG
ACCCCGCCTCTACGGACGCATGAGGAACGGGA
GAGAAGGTGCCACTGGCCTCCATAGCGTCGTTTGCAGACCCACAGAGGAAGCTCAGGCTCGCGTAACG
GCCATGTAGCAAGAGCGTCTCATCAATGACAC
GCATAACGTTGCGCAGACGGCGACGAAGTGCACTCCGAGCATCATCCGAATCGAGGTGCATAAGGGGC
TTCCCTTGCAAATTCAATTCCTCTTCAGTCCA
GTGGCCAGATGGAAAATGAAGAGGAAAAAAAGAGCAAGAAAAAAAAAAAGAAAAAGAAAAAAGTGTTA
TATATGCAGACAAATGCCCCTGTTTTTCCATT
TGTGCTCACAGAAAGTGAACCCCTCATTTGAAAAGGACAAGAAAATAAATTAAAAAATGATCGCCCTA
ATTCATTATTCCAAAAGCGACTGTTGTGATCC
TCGCCACTACCACCCGCAGTGGCGCTTAAGATACATATACAATGAGAAAAGAAATGAGAAAATCATGC
AGCTTCAATCGATTCTCAGGCGATTGTTGAAT
AGGAAGTAAGGTATCAAATTTGATCTTCGCAAAATATTTCCCCTTTCCCTCTCCTCGTTCATGTCAAT
TCACCTCCCGTCTTTTTCCATCCCCGCTGCTC
TGCACCACACTCGTGTGGCCGGAAATAAATGCAGGTTCTGCCAAATCACACCTTTTTAAGATTAACTT
TTGCTTCAGAACCTTTCTCTTTTGTTATTACA
TTCGTCCTTTTTTTTTTTCCGCCTTTTCCTTCTCCTCCTTCAGTGGGTTGGCACGTGAAGGTCAAAAAA
ATGATACATGACGGTTGCTGCTGTAATAAGAA
CGCAACCCAAAACAAACATTACGGATGGCATATGCCCCCAAATAATAAAAGACATGAAGCCACTCACC
GCCACTGCCACCCCTGTTGCAAAGCCCTTCAC
AATATTGTCCGTGTACTTCACCACGATAGCAACCAAAAGTCCACCGAGTGCATGAATAAAAACAAGCC
CAATGGTTAGCCACTGGTCATACCCCCGCCAA

AAATGGAACGCTTGACCGTGTTTCCCCTCGGCGGGCAAGGTGGGAAGAAAATCGAGCAAGAACACAGA
GAGCGCAGCAAAGGGGATCCCAAAAAGCGAGA
GATGAATGTTCCGCACCGCCAGCGATGGCGCAGTGGTTTTGACGACCTTCTCAAAATATACACCAGCG
TAACTCGAAGAAAGCCCCGACAGCACGCAGGA
AGTAACTCCAAGCAGAGGCCTCTGAGGACGCTGTTCTTTGAGCAAAAGCTTATTTGGCCTCTTCCCCC
CACCATTCTGCAGCTGGGCAAGAAGAACACCA
AGTGTGAGCACAAGCAACGACAACCATTGCATCATGCTCAGCCGTCGCCCTAGTAGTATCAGAGAAAA
CACGCCGGCGGAGATCAGCTTGGTCTGTGACC
AAACTTGAAAAGACAATGCGTCAAGCTCAGAGAGACCGATAAAAGTAAGGTAATTCTGCATGGTAAAA
AGAGCCGCGGGTACCGCCAGCTTAAGAGTTTC
CTTTTGAAAAATCACACCCCACAAAAGTTGTGGAACGCCACTTTGGGCAGTGGAGGATTTACCGAGTT
CAAGCAGAGGGACTCCGCACAAGTAGTTTTCT
ACAGTAAAGATAATGATGCAAAACAGCATCTTAACAATTTCCTGGTTAAGCACAAGTGTGGAGGCGTG
GTATTGCCTCTCCGGCGGCACATTAGCACGGG
AGTAACGCGTCATGACAACCAACAGACTGTTCTGCAGCACCAGAACCAAAAGACTAACGGCGGCCGGA
GAAGCCCATTGTAAAGTTGGCATCTAAACAGA
ACAATGCGCACACTCAAATATCACAGTGAGACGAGCAAAACTAAATAAATAAATAAATATGAATATAT
GCAGAACGGAAAATAGGCAAATCGGTTCGTGT
ATCCCCTCTCCCTCCTCCCCCTCCTTCTCAAAAAAAAAAAAAAAAAAAAAACAAAAATAAGTAAANNN
NNNNNNNNNNNNNNNNNNNNNNNNNNNNNNNN
NNNNNNNNNNNNNNNNNNNNNNNNNNNNNNNNNNNNNNNNNNNNNNNNNNNNNNNNNNNNNNNNNNNN
NNNNNNNNNNNNNNNNNNNNNNNNNNNNNNNN
NNNNNNNNNNNNNNNNNNNNNNNNNNNNNNNNNNNNNNNNNNNNNNNNNNNNNNNNNNNNNNNNNNNN
NNNNNNNNNNNNNNNNNNNNNNNNNNNNNNNN
NNNNNNNNNNNNNNNNNNNNNNNNNNNNNNNNNNNNNNNNNNNNNNNNNNNNNNNNNNNNNNNNNTAT
ACCTGAGCCTATGCAACAATAGCCCAATCCAA
TCAAAATGAAAAAAAAAAAAAAAAATTGGTGCGTGCCAAGTATCGTTGTGCGAGTAATGACGTCTATGT
ATTATTCCCAGAGGGAAAACAAAGCCTGCCTC
GCACCCTGTTCTACACCTGTCTGGCAATAGACAACAAAGAAAAAAAAAAAAAGAAAAGAAGCGTTTCA
CAGGAAATTAAAACGGATGGGATGCAACAACA
AGGCCCAATGTACAGAGACACAGAAGCTCAGACATAGATACGCAGTATCCACAACTCATAAGAAGACA
AAAAAAATCCCCCATTCCCACACCCACTCCAA
CAGAGTGCAACACAGGCACATAGCTGTAATTACTAACTAAACTCTTATCTTTCATCACGGCATATTCC
AAGCTGACAAAACGCGAGCAAGAATATTGTGA
TCATGTCTAATGCCAATATTTCATCACCTGGTGGTTGTCTCCCCACTAGTTCTTCGACGTTGACGTTG
TTGGCGCTGTCTCCCATATCATTTTTTCCTTC
TCATATCCCTTGCGATTGTCATTATGGAGTTTGCGATGATTATGAATTCGTGTGTACATCCACTCTGA
AAGTTTTACCACGTCCGTCTTCGTCTCCCACC
CAGTTCGGAACCTCGCTGGTTGTTTATGCCCGGTGTAGTCTCTCGTTTTCATCCGAGATTCGCTGCGA
TTTTTCTCCGCAATATTTGCCATGGCAATATT
GTTCTCGGCTGTTTTTGGTTTGTGATTGAGGATGCCATTTACACCGGCTAAGGTTGCTGCCACAGAAC
GCCTTGACGGACGACGAATTGCGAGGTCTTGT
AAAGAACATGGCGCATCCATCTGCGTCAACGCCACTGCATGCACCTCCGGCTGTGTGGCCTGCCACTT
TGAGCGGACGGATGGTCGCGTGACATCAACGT
AACACTTTCCCCGGCCCGGTGGTGGCGGCACCTGACGCTCCATCATCCAGGATGGCTCCAGGTGGGCG
GCCTTCTTGGATGTTGGGAACAAATCCCCGCC
ACCAGCACTAAATCGACGCAACGTCCTCTGCAACATCTGCAGATGTCAACAAAAATATAAATTATCGT
CCCTCCCCTAAGAACCACCAAATATAAATATA
GATGTATGCATGCAAAGAGGGAGAAAGAGGCGTGATGCACCCGTTGTGTACTTCCTCTGCAAAAATCC
GGTACACCAGTGAATCGACAATACACGCAAAT
AGTAAAAAAGAGGGAAAAAAAAAACGTTGTTCGGGCACCAACAAATCGAAAAAGAAATCGCTCCATCA
GAAAAAAAAAAAAAGCCGAGAGAACCAAACAGG
TCTTTGCGAATTATTGCCGTCACCAGAAAAAAAAAAAAAACTCTTCATACGTCATCCCTTCCGCATTGC
GTGGTTCCCTGTCTCCACTGCCTCACTAAGGG

ATTCAACACTAATGTCATCCTCTGCCTGATGAATCTTGAGGCGGCGACGCATGCCATACTTACCCTTT
TCGTGGCTGTTGTGCGGCAAAGGCGAAGGAAT
GTGGGGGGTGCGAGCATAAAATTCACGCTCCGCCTCTCTCTCTGCGGCGTAACGCGCATCCATCTCTG
CTCGATGGGAATGAAGTTTTTTCATAGGCTCA
ATGCCTTCATGCAGCTTTGGGGGCGGCGGCCCACCCTGAACAAACAACCGACGAGCCACAATAAAAAA
ACCTGCAGCAACGTTCGATGAGAACTCTAAAC
TAGACAGCCATGTGCGCTGCATCTCGCCCTTCCCACCAATGAGCGCCAACTACCAACAAATGCAATTG
TCGTTTATATCTTGCCACAGCTGCTCACGCAG
ACACAAACAAAAACAGAGAGAGAGAGAGAAAAAAAAAAAAAAGAAAAACAGATAGCAAAGAAACACAA
AAATGACATCAGATCATAAAATAAATGTTGGA
AAGAGCACAATTCAAACCCGAAAAGAAAACAAAATCCGCACAACGCATTCAATAAAATTCGCTTAGGA
ATGTTCCAAACACACACACAAAAAAAAGAATA
GTAGAAGGGAAAATACCTAAAAAAACATCAAGCAGCAATATGTATATATATACATATATACATGGATA
TATAGTGCCCAAATAAATTCCAGTAAAAAACT
TTCACGCGGGAATGTGTGCGTATAGATATGCATACATTTGTATAGCGTGCCTTTAAAAATATATATAT
ATATCAATTAAAATAAGGTTTCCATGCGTTAT
TATTATCTCTGCACCTAACATTTATAATAGGTAAATTTCACTATTTAAAAACATTTCATATACTTTGT
GAGTTCAAAGATTATTCCGCGCCGTTCGATGA
ATTCACTGTACACATTATCCTCAACTCATTCTCCTCAATTGGATTTTGAGCCACCGATCCCACGGTAG
ACGCGGCAGAAGGGGTGCTAAACGATTCGCTC
CGTATCACCCAATCTCCTGTAGGACCCACCACAAACGAAAGAAAGACGGAAAACAAAGCAGAGAATGT
ACCAACAACCGCGCCCCCCACAATGTCGGAGA
AGTGGTGCTTGTTATCGCGTGTGCGGGAGATGGAAACGACGAGAGGGAAGATGAGGGGAAGCAAGGAG
GCGGCGGCTGTGTAGAATGCACCAGTACGAAG
GGTTCCCGTCCGGCAAAGTAAATATAATACCAGCGGAACAAACCCGGCGAATGACGACGAAGCGTGGC
CGGATGGAAATGACAATCGCCCCTCACGGGCC
GCGCCACATATTTGATCGTGTGTGAACTGATTAAAATTACTTTCCGTGATGCCTTCCTTCGCCAGACG
ATGAATGAAATCAGGCCGCAGTCTCCCCGCGT
ACAGTTTGCAAAGGTTCACAATGCAGTAGTCAAGGGAAACGCTGAAAGACTGCACAAGGATCCAGGCA
TTCAGATGCTTGGCCACCATGTGCTTCCCGTG
TTCCCGCCAGGTGAAGTACTCCCCCGTAGCGTAAATGAAAATGACAAGAATCACGGCGATGAGGACAG
AGTACGTCGGGGATGTCTCGGTGTGGCTTGGA
GGTTGGCTGATGCTGGTGTCGTCAAAGACAAACGGACGGCAGTGTGGCCGTACGAGGATGCCGACGAG
TGCCCCGACAAGTCCAAAGACGACACAAGTAA
CGTAATCAAACACCCTCCACCTCCATACAAAGTTCATTTTGCCTGACTTGAACATCATATTGCCAAAA
GTTCTCTTTTGGGTAATAAATAGCCACTAAAA
GAAAAGAAAGCGGAAAAAACCGCCGTTTTAAATAAAAGCAGCAACAAAACAAAAAAAAAAAGAAGGAA
AAAAGAAAAAAGAAAAACAACGAACATCGACA
GAGAAAAAAACCGTCAGTCAAAAAATAAAGTTCGCCTTTTATTTTCTTCATGACGCCACTCCCTGCAA
ATCCACACAATTTTCCACCGCTGTACTCAGGT
ACACTCACACTTCAATATCTCCAAAAATTCACCAAACCCCCCGCTACACTCATCGGTGTAGAATGCAC
CATAAATAAGTCATCAAAAAAAAAAAAATATTG
ATCGCTTAAGAAGCCCACATCACAATAAAGAAAATAATAAAAAAAAAGGACAACGGAATAAAAAAAAA
TCCTTCAGTGAAGGACAAAGCGCGTAATCACC
TCTTTGAGGCATAATCCAGTCGACGTGGTAAGAACATCCCATTACGTTTCACGACACGAAACAGGACA
GTCACGGAGAGAAGTGCACAGCCTGTTCCAAT
GATGCCTCCAGCCAAAACATCAGAGAAATGATGACGGTTGTCACGTGTCCGGGAAACAGCAACAACAA
TTGGTAAAATTAACGGCAGCATAGACAAAAAA
AGACGCAATGGGGACGCAAAATAAAATACTTGCAGATGCCCCACAAAAAACAGCACCAATGTAACAAT
TGAAGAAAACGAGATGGCGGCGTGACCCGATG
GAAAAGACAATCTCCCTTCTCGTGCAACTTTACACCAGTCCACCCCCGTTGATTTCTCGCTGTAACCT
TCATTTTCCAATCGCGCAAGGAAGTCAGGACG
CAGACGACCGGCGTATAATTTCGTGATGTCAACAATAAACATACTCAGCGTCACAGAAAACGCCTGAG
CGAGAAGCCAGTGATTCAAATTTTCCAAAAAG

AGCCACCACCGTGCATTGCCACCCGCCTCTTCCCCGTCGCTTGTATTGATGACAGTGCCACCATCAGC
TTCTTCGGTGTGACACCGTCGCATATTCATCC
GCTCACTGTTCCTTGTTTCCAGTGCATTGGTGACCAAGTCCAGGGAGGTGGAAGGCCCGTGAGAGGGT
CTCGTGTCGGGTCGCCACAGCAGCGCGCGGAT
GAGCTCAACGACAAAGTAGATTAATGCGGGCAAAATGGCGATGGGAGCCAAAGACCAAGAGGGAAATA
ATCCAGCCCCAGCGTAGGTGTGTCCGATTGTT
GCGTCTTGCCAAGAAAATTGACGACAATGCGGACGCACTTTTTTCCCAATTGTTAAAGCGGCAGTACA
TAGAAACACACAAAGCAAATAGTCCACTACCC
TCAATTTCTGCAAAACCTTCAGAACCTTTTGATACGGCAGCATCCCTGGATGGTGTGAAATTACTTTT
TTTTtNNNNNNNNNNNNNNNNNNNNNNNNNNN
NNNNNNNNNNNNNNNNNNNNNNNNNNNNNNNNNNNNNNNNNNNNNNNNNNNNNNNNNNNNNNNNNNNN
NNNNNNNNNNNNNNNNNNNNNNNNNNNNNNNN
NNNNNNNNNNNNNNNNNNNNNNNNNNNNNNNNNNNNNNNNNNNNNNNNNNNNNNNNNNNNNNNNNNNN
NNNNNNNNNNNNNNNNNNNNNNNNNNNNNNNN
NNNNNNNNNNNNNNNNNNNNNNNNNNNNNNNNNNNNNNNNNNNNNNNNNNNNNNNNNNNNNNNNNNNN
NNNNNTTTTTTTTTTTTTTTTACGTTCCAGTCT
TTTTTACTTTTCACTTTTATTTCGATTTATCCCGTTGGCTGAATTTTGTTTCGTTTTATTTTTTCTTT
CTTATTATATCCTTCTGTTTTATGCTTCGTTT
TGTTTCCCTTTTTCGCTGACAGTGTTTCGAGGCCTTCCTTCTCGCCAACCCCTCCGGTGGCCAATCAT
TCCACAAAGGAAAACAAGTGAAGAGGGGCACG
AAGAGGCAAATTGCAGAAAAGTGGGGAAAGGCAGGCGCAGTGCGGAGAGGAGAAAAAAAAAAGAACAT
TACATAAATAAATAAATAAATAAATAAGATGT
AAAACAAAAAACTAACAAGACGGAAAAGAAGATCAACCACGCAGAAAAAAGGGGGAAAATAAAAAAa
>scaffold1120.size8286
TGTTTGGTTGAGCACATCGCAGGGAAGAGAGCAACACGTGTCAGCAAGGCATAGTGCATCCTATTTAT
TTGTATACGCATATACATACTCAAATAAACATA
TAAACAAAGAGCGAGATGGGGAAGCTGCAGGTGTGTATTTGTGCAGCCCGTAACTTGCATGACAGCCA
AGTCTTTGGCTTGCCGGACCCCTACTGCCGAG
TACGTATGGGTGACCATGAATACAAGACGAAGGTCATCAATAACTCGCTGAATCCCGTGTGGAACGAG
ACATTTCGATTTCAGGTTGCCGATGAGTCGAC
AGCACAGTTGTGCGTTGAATTGTGGAATAAAAACATCATTTCCGACGATCTCATGGGGACATATAGTC
TGTCGCTGGGTCACATGACACGAGGCGTGGTG
AACGACGAGTGGTACCTGCTGGGACACTCCAAAACAAATTCGGAGCTGCACTTACGTGTCCTCGCGTG
TGACTTTGGTATTAACCCATCGCCCAGTGATG
TGTGGAAGGTCACCACGGACATTAACAATGACCCGGCACTCGGTGCGGGGAAGAAACCCCCGTTGGCC
GCCGCCCCTGTGACTATTTCGAGTAACGTGAA
CAATACGCCGATTAATCCGAACATTGGCCCTGCTGTTGTTCCCGCCGCAGTTTTAATGCAACCGCAGT
CATTTCCACAAGCAGCTGGCCCTTTTCCACAC
CAGCCTATGTATTTTTCACCGCCACCGGCCCCAGTTGGGTATTACCCGCAACAACCAATGTACTACCC
ACCANNNNNNNNNNNNNNNNNNNNNNNNNNNN
NNNNNNNNNNNNNNNNNNNNNNNNNNNNNNNNNNNNNNNNNNNNNNNNNNNNNNNNNNNNNNNNNNNN
NNNNNNNNNNNNNNNNNNNNNNNNNNNNNNNN
NNNNNNNNNNNNNNNNNNNNNNNNNNNNNNNNNNNNNNNNNNNNNNNNNNNNNNNNNNNNNNNNNNNN
NNNNNNNNNNNNNNNNNNNNNNNNNNNNNNNN
NNNNNNNNNNNNNNNNNNNNNNNNNNNNNNNNNNNNNNNNNNNNNNNNNNNNNNNNNNNNNNNNNNNN
NNNNATGAAAGAAGGAGAGTTAACATGAAGCA
AATGATGAAGAAAAAAAAAGATGTATGTTGGTGTGTTTGTATATATCCGTGGTTTTTATATATACACA
CATATTGACTTACCATGCATCGGTATACAGTG
AAGATGGAGTGAATCGGATATGTGTGTTTGGAAGGTTATTTTTACTTTGCGTTGTTTATTTTTATTTT
TCTTTTTTTCCCTTATTTATTTATTTTATCCA
TCTTACGTGCTCTCTTGCATGGTATGAGCCGTATGCGCTCATCGCTGAGTTTTTTGTACATGTATATT
TGGAAGTGCATCTATCAGACCTGCACGTATTC
TTTTTATGTGCATTCGAGTGTGTGTGTGTGTGTTTTGCATTGGGGGGCGTAAGTAAATGTATGAAGGC
AGAGTGAATTGTGGACGCTGTAGTTGAGGGCC

GCATTGTTTTTGCATGTGGAAAGGGGTGCCTTAGCACACACACAGAGAGAGAGGGAAAGAAGGGTAGA
GGATGGGGTCAAATGTGAGGTTGATATCCATG
CAATTTATTTCCTGCTGTTTTTATTCAATTTTTGTTGCCTCAGAAGGGCATTTTGTGCGGATTGCCCT
TTGCAGCGGTGTTCCCTCTTTTTGAAGGCACC
TTGACGGGAGGGGGGAGAGGGAGAGAGAAAGAAACAAATAAATAGTAATAATAAAAAAAAGACCACAG
GAAGTAATTAGAAGAACAACATCAAAAAAGGG
GGCGTTCATGAGTGACTCGGAGTCCATCGTATTCCGTGAGCAAACGGCAAACAACTCCCTGCAGAGTG
AAAATATGCAGGACGCTTTAATCCAATCCTCC
CAAATGCCGTCGGCGTCTCCGGCGCTGGACAAACCGCCGCTTCACCACACCAACCAACGGGGCTCCCG
TACGATCACTCAGCCGCGTCCAGAGGCAAAAC
ACACGAAGAAGTCTTTTGCCGCTGGCGAACGGTCGCACGACGATGCGGTCTCCGAACACAGCCAGAGC
ATCCACTTCTCCTTTGATGGTGCTGAGCATAA
CGCCTCCATCGCGAGTAGGGCCTCTGTGAGGCGGGCGAACGGTCTCCAGCCGCCCCCCAAACGTGGCG
GAGGTCATCTAGAATTGCGATCAACTCATCTA
CGCCGCTCGGGAAGTCAGAGGACGGATTCAGAGGATGAATCCATTCAGTTTGCTTTGGAGACCTCCAA
TAGCCACGCACGGCCAAAGACCACGGTGCATT
CCGAGGCGTCGCCAAACGACAGTATCTTCTTTCACGTCGTGGATGACACGCGGACGGATGGCGCCAAT
TCCAGGGCCAGCGGCAATCCGCAGCATGACGC
CAGCACGGATGGCATTGAATTTACCGTTTTGAATGAGACGGAAGGAGCGGCGGAGGGTTCTTTGGGGA
AATCGGTGCAGACACGCACCGACGCGGAAAAA
GGGCGAGGATTCGAATTGCCAGTTGAGAAGAAGCGTTTGAAGAAGACAGTTTTATCAAACAGGCTTAG
ACACCCACCCACGGTTTTATTGCGTAGCGGGA
GAGTCTCAAACCCACCGGCCACGAAGCGCTCTAATGGGAGAAAGTTGACGGAAAACATTCCTCCAGCT
ACATTGTCGCCTGCAGAAACTGGGCCTTTGGA
TGCCCCTGCGATACCGAAACCAAAACCACTGGAGGCCACTGAGGCGCGGCTTGAATCAAATCGTATGC
CCCCAACTGCCGTTTCTATGTCTTTTAAAGAT
GACCAATGCACGTCAAAGGCTACACCGAATGAATTGGGCAATATTTTATTTCCAACGCGACGTTCGCC
AAGCACCGGCTTCACGGATCCGATTGGTATAA
AAAATGCGTCAACTGCTGCGGCCAAAAAGTCGACGGAGGGTTCGCCGCCTCGCATTTCCATTGAAGTA
CCGTCGCCGTACCCCCGACCGCAATCGGAGGT
CATGCAGCCTTCTCATTCAGATACGCTGGACGAGTGGATTTTTCAGGCTAAACTCTGCACGGAACATT
GGCGTCTCTTCAATGACTTTCAGAGGGAAGAG
ATTTTGCGTTTGATTGAGCGCATTGCGAAGGCACGTCAACCGGACTCATTTCTTCAGTTCATTGACAC
GGGCGTAAAGGAGAGAAAGCCGACACAAGCTG
CACCGGGTGGATTTGCAAATCTTTACAAGCAAAGAGTTTTTGCCGCACAGAACGCTGCCTTTTGCCGA
GGCAAATCTAACACCGTGCCCTCGACAATAAA
ATTGGCTTTTAAAGTCCCACCGCGGGCACGTTCAGCGACGTCACACGGCATTTCGACGTGCAGATCAC
CGCTCTCTGGACGGACGCAGGACCATCGTCCT
CCCGTTGCCAGGCCACCAAAGACGGCTGAGGCAGGACAGCTTACCGCAGCCCCGCAGAGGCCCCCGCA
CGCACCGTCGCGGAGGTCGGCAGGCGGCAGAT
GCCTTTCTCTTTTTCAAGACGCGTCTTCGTCTGATGGCGTTGCGAAACTCGCCCAGACGCTTGGTGTG
GTGTGGCCTCCTTGGTCACCGGATGCACTTTT
TTTTGTGTCCGGCGCCCGCCTGACTCCGCAGCAGAGGGATGAGTTCTACAAGGCGGCTGAGCTTCAGA
AGGCAGCCTTTGCGAAGCTCCACGCGGAGTtA
CAACACTCTTCTGCGGCACAAAAGAGGGAGACAAAAGGTGCCCGCCAGAAAAAAGTGACGCTGTCGCG
TACTTCCATCCTCCGCAAGGCCTTTGATTTGA
TGGATGTGGACACACGCGGCATCATTCACACCGCAGATCTTCCGGCGTTGCAGCATTTACTTGAGGCG
GAGCGGCAGGAACTTGAGACAAAGGCGGCTGA
TGGAGTTGCGGCTTCTAGTTTTCTTACAAGGGCGAGGGGTGAGCAGCGCATTCTTAAAGGTACGATTG
ATTCCAAACGACCTGCTACAAAACCCGAAGAT
GATGCCACATCTATCGCCAAGAAGATTATGCTGCACGGTTTTGTTTTAGAGGTTATTTTTCCGCTTAT
TCGTGCCTCTAATTTAGTTGTGGTTGACTTTG
CCTCCCTTGGCCTTTTGGTGTTTGGATCCTTGTGTCTTTCTCAAAAGGGCGCGAGTGCGAGTTTTGTG
AAATGGTTCCAGGCCGCATTACACTACTTTGA

GGCTCTTGCAtAGACCGGTTCCTTTTTCTCGTGGGTTTTTTTTACCTGCATGACCCTTTTTTCAGTCA
TTGTGTGGATTCCATTTGGACGGAGTTGCCCC
CCCTTTTTTTTTTCCCTTCCTTTTTCTTTGTATTTGCGTCTGCCTGTTGCGGTGTGACTTTATCTGTA
GTTTTTTTCTTTCTCGTATTGTTTTTCTTTTC
CTCTGTGCGCCGCGCTCTTCATAAACACGTTTTGGCCTGTTCTGCAAGGATGAGTAACGGCGACGTTT
AATCACAGACGGAGGAAGCAGGGAGCCCCAAC
TCAAGCGTAGAGTTACGTGTTTATAGAGACGGATATAGAAAAAAAAACAAAAACAAAACAAAATAAAA
GCGACTGGACAATTGGGGGTGCGTGGGCGAGT
TGACACGTCGATGCGCACACCATATTCTTCTGCAACAAGTCTGGCGGATAGGGCCGTCTCGCCGTCGC
AGTGTTTATACCCTCGCACGACTGCCAGGCAA
ATCCGCCCACTGGTACTTTCTCCAATGACACCGGTGGACCCAAAAACGATGTCACCACATTGCCCCGA
GTCGCCTCAATCCCTGCAGAGCGGAAGTCCCG
AGTCAGAGGGAATTGGCAGGACGGAGCCGCAGTCATTGCTGCCGACGTTTTTGGCTGCGGATTTTGAT
GTCGTCGATGTTTCCGCACGATTGGAGAATGA
GTTTTTTAGTGCTGCTCAGTCCGCCGAGTTGAATGGTAGCAGAAGCCGTCATCTACAGGAGACGATAA
ATTCCCATGAGAACTGCTCGTCAGCAGAAAAA
TTACTTCATCGTAACGGTAGCTGCTGCCGTGTGGATGTCACTGAGGAGATGACGCAAGTGTTCCAGGA
ACCGATGACGCCGCTGCGCGAGGCATCTTCAC
TTATTGAGGAGAACCGCGAGGGCCAAGATGACGCCGAGGCCGATCGTGCTTCCAATTCCCCTTTCTCC
GTGCATACTCCGCCTGAGAACATCATTCGTTC
TTCGTTGATGCTGACGCCGCCGTGGACGCCCGTTCTTGCAACTCCACGGAAATCTGCCAAACGACCCT
CACCGCAAACCTTCACGCCGTCAGCGCACCGG
CGCGGTGTGTCGTCACATAATTTAAGTATGGAATCGCGGGAAGTCTTTGGTGTGCTGCCGCACATAAC
TCATGCAGATTACCTCCCACCCAATAGAAACC
CGTGTTCACCGTATCTGGAGTATGCGGATGAGAGACAGCTCCTGGTGCAGCTTCGCATGGAGGAAGAG
GAGGAAGAAGAGGAGATGGATGGTGGTTTGAT
TTCTATAACAGATGGGATGCTGCAACGTGCGGATGCATTTGCCATTCTGCTTGAACAAACTGTTCCTA
TCGCCAGTGCGGTGCGAACAGAAAGGTGTTGG
TCTCCGTTGTTGCATAAAGCAGGAAAAGGTGGTGGGGGCGCGAATACATTTCCCAATGAGGTGCGCGA
AGAAAATGATATCACAGGCCTTTCTCCTGTTT
TAGTGGAAATGGACTCGGCTATGCGAAGAAATGCGTCTACGGCAGTGGAGACAGGCGACGTTAGTTCA
GGGAATCATTTTTTGGACTTTTGTGGATTTTC
TGACGACGGGACGAGTTTAGGGACTATGCAGAATGAGACGCCGCATCGTTTGTCTCGTGAAGCCGGGG
ATGTGGCATGTGATGCGTCCACACGCAACCTT
ACAGATGGCCAGACATCTTTTGAAACGCTTCCAAATAAACTGCTATGTACACGTGTTTTTTATGACCG
GCGCGGCCAGGTGGGGGAGCAGACATCTCCTC
CTTCAAACTCCCCATGTATATTGCACAATGCGGCACAATGGGACCGTACTGTTGGTATGCATTTCTCA
AGGAATAGGATCCATGAAACGGGTCTTTCTTT
TCAACCGTCGCTCTTGTCCTCCTCTTTATCGATCCGCGGAGATTCTCAGACCTGCTTTTCTGCAGACA
ATTCAGGAGACCTTCCGAGTCAATTGCAGTTT
CGCAATTGTTTGTCCGTGCATTTCCCAGTTAAATTTGATTTCCCCACTGCGGGATACCCAGTGAGTGA
TGCGGAGGACGAGATACCGAGAGGACATGTCA
ATTCCAAAGAGGAGGAGGAAGAAGAAGAAGAAGAAGAGGACGAGGAATTCGAACCCGGTGATCGGTTT
TGTAATCATCATCGCTATCAATTTTTGCATTG
GCTGCATTGGCGAAAAGCATATGATTTTGTTCCCAACCATTTGGATCGCAACGGGGTTGTCTGGCTTG
TGACACACCGCGTGGATGGCCTTCCGTATGCC
GTCAAGGAAATACCGGGCACGCAGCAAAACACTCTGCAGACGGAGCTGGAGTGCCTCACACTCGGAAA
CGCCTCCGTGAACGCGTTGCAACAGCAGGCGG
CTGACTACATCACACGGTACTTTTCTGTTTCACCGTATAGTGTTGTGTCATGCGATTCACATGAAAGT
ATGGCGTTCCTGCTGCAGACAGAGTACTTTCC
CATGGGAAATGTGATGGAGTGGGCGTTGGACCCACACAGACTTCGAGGCAAAGAGTTCCGCGTTCCAC
CGGAAGATTTTTGGTCGTGTGTGCTTCAACAC
GGCCTTCTGGCGATGGAGAGTCTTCACGCAGCTCATATTATTCACGGAAACCCTCTTCCATTCAATCT
GTTTATTTACAGCCCAAGGCATTACAAACTCG

GTAACTTTGGTTCTGCAGTGAAGTTCCCGAGTACATACCCTGTGGGCTCTCCAAATGTATTTTTGCCG
TCAAACATGGCGGATGGCAGCCCATGGACACC
TTAtGAGATGGATATCTTTCTCTTTGCCCATAGTATGCTGCAGCTGTGGAATCACTGTATTGACAAGC
GGAAGCTGGTCTCGAATGCGGTCCAAACCTTT
GATCATGATAGCGAGGGCACACTTTGTGATATATTCATGAAAGATGCCATGTGTATATCTTTCAGTGG
GTTTCAAGAGGACCGGGTGAAGGGTTATCATG
AAATCTCCGATCATCTTTGGAATGTGTTGCAGGCCGCCCTCCGTCGGACCCCGATAAGTACTCTCCTG
AGAATGTTGGAAGCCCCCTCACGGCTCCAGTG
GACACTTTTCTCGCTCTTTGATACGGAAGAATTTTATCTGCTGCGGCGGAAGCGTCGGTTGAAGCGGA
TGCTCGAAGAACGTTTACAGAGGAAGGCACAG
GAGAAAGTGAAACGCCAATCTCTCCCTTCGACGAGTGTGAACTACGTGGACGATCCGGTGATTCTTCT
TCCTCCTGCGGAGTGGAGTTGCCAAGAACGCA
CCGCCCATTGTTGCTCAGATGTTCAAATCCGAGGGAGCATGTCAACGGCGGCACCACCACTTCCTTTT
CAATTGCCACTGGCCTCCAACACATTTGCCTC
TTTAGGAAATGGCGTTGCTTTATCCCCGGGACGGGGGAAAAATGAGTCTTGTAATCATAGAGATGCTG
CATGTAACCGTCATGGTCGCAGACTATTCAAT
GGACCGAAAAGAGTAAAGGAAGACACGCCGTTGAGTTCGTGTGACACCTCTGTGGCATCATATTCACC
TCATATCCTTCATGCAACGAAGAAGTTTAGTC
TTTTAAAAGACAAAAACTGCAGTTTTAATTATTTGCCTGAAGAAGATATGGACAATAAATCGATGTTG
AAGTGTGCACACGGTGGACGGCCACCCTATAT
ACGGAGACGTGTTGACGCCAACATCTCTTTTGCCAATCGAGACCGATTGGACGCAAATCTTTTATGGA
CAATTCGTCAAATTATGGCCGTTATGAATTGG
GAAGATGCTTTGAAAGGGATTGCTTTGCATTTTGACGAGGATGTCCCTGCACGGGGCTTCTTTTCACT
TTCACGCTACGCCGTGAGACCCCTGACATCAT
GGGAATAGAGAAAATTAGGTTTTTGTGATGGACAACAAGGGCGTAACAAAAGAATAAAGCAGTGGCGA
ATACACATGAAAGAAGCATGAAGCCCATTTTT
TTTTTTTCGCCCAAGGCGAAAGGGGGTTTATTGCGTGCGTGCGTGTGTGTGTGTGCTGCGATTCATCA
TAGAGATTTTGTTTTTA
>scaffold1121.size6153
ACACTGGACGCCATACAACCCTGAACTATCCGCTGCTTGGAGGAATTTCGCGAGCTCCTGCCCACGAG
CTGCAGTCGGCGGATCGTTTTCGAGCGGCTGCT
GCACCACACGCTGTGGTCTAAATTTCTGTTTCGAATTATGAATGGTGGTGGTCAGAGACACTCTCTGT
CAATGTCTGTTTGCGTGTTCACACACTGGACA
CCAAACAACCCGGAACTATCCGCTGCTTGGAGGAATTTCGCGAGATCTTGCCCACAAGGGTGCTGCAG
TCGGCGGATCGTTTTCGAGCGGCCGCTGCACC
ACACGCTGTGGTCTAAATTTTTGTTTCGAATTATGAATGGTGGTGGTCAGAGACACTCTCTTTCAATG
TCTGTTTGCGTGTTCACACACTGGACGCCAAA
CAACCCTGAACTATCCGCTGCTTGGAGGAATTTCGCGAGATCTTGCCCACAGGGGTGCTGCACTCGCC
GGATCGTTTTCGAGCGGCTGCTGCACCACACG
CTGTGGTCTAAATTTCTGTTTCGAATTATGAATGGTGGTGGTCAGAGACACTCTCTTTCAATGTCTGT
TTGCGTGTTCACACACTGGACGCCAAACAACC
CGGAACTATCCGCTGCTTGGAGGAATTTCGCGAGATCTTGCCCACAGGGGTGCTGCACTCGGCGGATC
GTTTTCGAGCGGCTGCTGCACCACACGCTGTG
GTCTAAATTTCTGTTTCGAATTATGAATGGTGGTGGTCAGAGACACTCTCTTTCAATGTCTGTTTGCG
TGTTCACACACTGGACGCCAAACAACCCGGAA
CTATCCGCTGCTTGGAGGAATTTCGCGAGCTCTTGCCCACAGGGGTGCTGCACTCGGCGGATCGTTTT
CGAGTGGCTGCTGCACCACACGCTGTGGTCTA
AATTTCTGTTTCGAATTATGAATGGTGGTGGTCAGAGACACTCTCTTTCAATGTCTGTTTGCGTGTTC
ACACACTGGACGCCAAACAACCCTGAACTATC
CGCTGCTTGGAGGAATTTCGCGAGCTCCTGCCCACAGGGGTGCTGCAGTCGGCGGATCGTTTTTGATC
TTCCGTGCGCATTTTAATGTTGGTAACAGGAG
GACGAGCAGGTCAAACTTGCCTTTGACATTTTGGTCGAGTGGATCGGTGTACTGGAAGTCCTGCAGGA
TCCCACCGATCGGATCATGAATTTGGGGAGGT
CTCTACTGAACACATTCCGGATGCAGCTCACAATCGCGTCCGATCCCGGCATCCGGCCTTCCAAACTG

CGCGGCCGTCTTTACACCGCTTTTCACCAGAC
CGACACCTATGCCAGGGCTGCCGAGCTTTTCGTCGATCGGCGAGAAACCCAACGCACTTTGCGATGCC
AACAATGTCATACCTATGGACACGATGCGTCT
ACATGCAATGTGCGCCCGAGAGGGAATTAATTACATTATAGGCGTCCATCAAAAAACGGCAGAGGGGA
TGCCGCACGCCGCTCATGAATACTGACGACGG
CAGCTCCATCCAATACGCCACCTCCTACCAATCCTCACAGGCAGCGCACCGCGCCGCCGATGCTCCAC
CCGATCTTTCAAATTGCCCGACCAAATCTTCG
GGAAGGGCGATGGAGCTTATCCGTAAACGACGCTCGACTGTGTTACGACGAAACACCGACAGTTCCGC
TTTGGGCCAGTTATATGAGGAAAGCAAACGAG
GTCGCTCGAGAGATGTGGGCGCCGTCAACGTGGGAGCGTGGAATGTCCCTAGCGGGGCAATTCACAAC
CTTTTGCCGCACGCACGAACAGCCGATGAACG
AGGAGAGTTGTGCGGTCTTTTTGATGGCAATCGAAGTGGCGCCATCGACCCGACTGCAGTACGCCAGG
ATTCTGCGGTCTATGTTGGAAATGGATCGAAC
CCCGCTGGAAATGATGATTCTGGGGTTACAAAAAAAGTTGCGGCGCGATCGGAGACGAAGAAAGAGAT
GAATCAACTAATCCGCAGCCGGACCGATTGGAAG
GAACGTGTTGTCGTACGACTTGCGTGGATAACGGTGAGTCGTTTGTTTGAGATTGCGGTCTTGACTCC
CAACAATTTCACACTGAAGGCGGACGGGGCCT
TAATTTTAGACTGGTCCGTCGCGCCGAAGACGGCGAGGGCGGATCCCCACCGCGCCTCGCGGTTCTTC
AGGATACGATGGCAGGACGCTTTCGACACTAT
AAAGCTATGCAGGACACTTCAAGAAAACGAAAAGCTCACGAATCTTACGACCGCCCAAGTCGAACGAG
CTTTGGCTCCTTGGAATGCCACGGTGCATTCC
ATAAAACGCGGTGTGTTGCGGCACGCTGCTCAAATCGTGGAGGCGCACGATTTGGATCCACACGTGAT
CTCGCAGTTGGCGAAGCACGTCGACCCGTTCG
ACCTTCCCCAGAATACTGTTTGATATTTGTGGAGGTACACCACAATGCTGACCCAGGTGTCGTCGCTG
GTCGCATTGATGTGAAGGGAGAAACGGCGGAA
GGGCAAGAACCCTGGCTGAGTGCGGGGGAGAGTTTTTTCCGTGATTTTTATCATCATGCCGCTGCAAT
GACGAGACGCGCGACAAGGACGCGTCCGGCCA
CTCCACAAGATGAACACGCTATCCCGTTACAGCAGCAAGTCAACGTGCCCGTATTGAATCTGGAGTGG
ATTAAAAGCCGGTTGAACCCGGCGACATTGGA
GAGTCTCATGCAGGTTTGGGGACTCGTCGGGCGGTCCCCTTTCCCTCCGTCTTCTTCCGGTAAAGCGC
GCGAAGGCTCACGGAGAATTCCAACCGCTGAC
GCGCGATTGTTACGAAAAGCAGGAATTATCGAGGACGGTTCGTCCACAATAACAGGCGGATGGATAAT
ACCCTTTTTCGGTTGTGGAGGAGAAAACCACC
GGTTTACGACGACGATGGATCGCGTGGCCACGCGACAAGAAAAGCGACAACCCTTACGAGGCGAATGC
GCCTCTTTTACACATTTCCCATTATTTGCCGC
CTGTGATGGCTGAGGCCGCTTTCTGCCTGAATGTAAAGTCGTCCTTCATAGTCTCTTTACCGCGGGAG
ACTCGGCATCTCTTTCGATGCCGCGTGGAGGA
CGGCACGCTGGTGGATCTCACACGGCTCCCAGTGGGATACAAGGCGGGCCCAGAAATTCTCCAGATTA
TTATTACTTCGGCTATGGCGGGGGTGACGACG
GTGGCTCATCCCTCTGGGCCGCACCTCCATCGGTGCGTGTCGACGTATGGATCGGCAATATACGCATT
GCAGGGCCGAAAAGCGATGCGACATTGTGGGA
AGCCCAAGCGCCTCGTAATGCGGACAGTTGTCACGCCACGATAGGGAGGATCGCGAATCCGGCGCTAC
GCAGTGCACCTTTCTGGGGGTGCAGTTTGACC
ACACTCACCGGGCGATGCCCCTGAGTGACAAGTTTTTCCGCTCTGTATGCGCCATGCCGGCGCTCAAT
TCTTCAACCATCGCAGAAATGGAGGTTGTGGC
GTCACGCTTTTTGTACGCGGCTGCCATTTTGTGCACGCGTTTATGCGACTACTACTTTTTCGTCAAGG
CAGTGCGACGGCGATTGTCCGCACGTAACAGG
GGATTGTGCTGGAGACATCCCCGGCGAACCTTCCGTCGGCAGCGGTTGGTTTCGGCGAGAGACTGCGA
CACACCATCGAAAATACAACCGTAAGTGCATC
ATCAAGCCCACGGAAAAGGCATCGGCTGCCATCATCACGGACGCATCGCTCCATGGATGGAGAGCCGT
TTTTATTCAATACTCCGGCGACGTTAAAATTG
CCGGGGGAAATGGAAGAGGAAGACTTTTCTTATCATGCAGGCCGAGGCACGCGCGGTACGCTTAGCCT
TATCGGCTTTTTCCGCCATCTTGCCATCCACC
ATGGACGTTTGGGTGGACAACACTTCGCCGCAAGGAGCGGCGAATAAAGGCAGCTTAAAATCACACGC

CTTGACGTGGGATCTGCGACGGATATTTGAGT
TTTTGGACTCTCGCGGAATACAGGCTTCCTTTGCCTACGTGCGATCAGCGGACAACCCCGCAGACGGC
ATATCACACGGTCGTGTTTTTAGAATTCAGCA
CTTGGCGAAGGGGAGCGGCGGGGTCTTGTGGCTGGGGGACCCCAAAGTCCGCCACTTCGTAACTAATG
TTATTTCAGATCCTAACTGAGTACAAAAGATC
AAGTGAGCGGTCAACAGAATATATATATATTTAATGAGAAAACAACATTGAGGCAATTAACTGCCTCT
GCTTTACCTTTCTTTCCCTTCCTATCTTGTTT
GGCCTTTTGAGATAATTTCAGGTTCCGGCCGGCGGCCCAAAAGATTTTTCCGTTTCTGTGCATCACGC
GCCCGCGCGTGGCGATTCCGATAAAACGAGGG
CGGTGCGTTTTACGCTTTCTGCCGTCGACTTTCTACTTTTGCGGCCGAATCCTTTGCTGCCGAAAATC
TTCTAAGGGCATACCGCTGCCTCGGCGCGAAG
CCGGCCAAGTAGCAACAGAGAATTTTTTTGTTTGTCGGAAGAAGAGGCGCGCGGCGTTTCGCGATTGT
CTGTCGGTGTCGCTGCTTTTTGTTTTCACTGG
TCTGATGGCGGGCGCGTGCTTGCTTACGCCCTGTCGCCCGAACTCTTCGAAAGGCTTTGCCGGAAGGT
CGCCGGCACAATCGGGAAAGACGAACCTCGCG
CGAGCCCGGGGGTTGTTTGACACCCGTTCCGGCGCCATCGCTGTAACAAATGCCGCCCCGCGTTTCGG
TGCGATGCCGCGGGCGAGGCTCGACCGGCGGT
CCCGGCTGTTTTGGACCCTCGCGCGCGCGCTCCCCGCCCGCAGGCGTGGCCCGGGCGCTCGGTACCGG
TCCCTGCCCCAGGACCTCCCAGGACGGCCTTC
CGCCGCACAGGGCGGTGCGTGGCGGCTGGCTGTTTCGCCGTGCGGCGCTCCTTCGGCTTCCCCTGTCC
CACCGCTCCGTCGAGAGCGGCCTGCCAATTGG
CGGATAAACGCTACCAAGGAAGCGCTGCCACCCGTCCATTGCGGCATATGTTCCTAACGGGAGGAATA
AAAAAAAAAAAAAAAAGAGAGCTGCGCGTTGG
TGGTGGCAGGCGGGCGTCCGCCTCCTCTTTCCCCCAAGATTGTCTCGGAGGCTCCTTTTTTGTACCAT
TACCCTGTGCGCCGCGGCGCTCGAGCAGGAAG
CCGGCATGCATTGAGGAGACAAAAAATTAGGTGAAGAGAAATTGCGACGGGAGAGGATCGAACATCCG
AGAGACCGCGTGTGAGGCCAATGTCCTGAGCA
CCACGCGGTCATCACCATGCACGCGCCTCAACGAAGCCCGCGCCGTTTGTCTGCACCGTGCCGGCTGC
TTCCACGCGACTCGTTTTTTTTCTTTTTTCTT
TGCCCTGGGTGGCATATCAGTTAGCCGCATATATTTCCCTCCGCCTGGGGTAGGAATGCGAGTATCGC
TTTTGGCCTCACCGAAACCACGCACACACACA
AAGAGAGAAAAACACGGAGAAGAGAGGAGGAAAAACAATTCCTTAGCATTACCGTTTTTCTTTTACG
CCTCCATTCTATATATATATATTTTTTTTTTC
CGACTTCTTTCCTTTTTCTTCCTTTTTCCTGTTTGTTTTATCCATGGAATGCTGCAGGCTTCAGGGT
TTCTGTGGAACACTCGGCCATTTTGATTGGTG
CGGGTGCTGCTTTGCGGCATCCCATCTCCTTGCAAAGGAATCGGTGCGTTTTCCAGTCATCTCGCTGA
CATGTGACGGAGCAGTAAAACGTGACGCGACA
TCCGCTGCATTTCAGCATTTCCTTCACCTTTCCGGGACAATCGGGGTTGCCGCAGCACAACTTTGCCC
CTTTACCAACCAACGGATCCTTGCTGTGCACG
GCAAGATGGAATTCTGGAGCGCTTACAAGAAAAAAAATCTTTGTTATGTAATCTTTTAATGTCCGCAA
TTCCTGTGATGCATTCTCCGTTCCTAGACGCG
AGTAGATGCTGTCCGCCGTTGCCAAGGCCTTGTTGACAAGTGGCAGATGTTCATTTAGGCCTTTCATC
CCCCTCATCTCCCGTCGAGCAATATACAGCGC
CAAAACGACATTGTCAATCTGCGGTGCAAGACTGGCAGGGGATGCCCGGAG
>scaffold1122.size7015
GCCTTCAAATTCCAGCGTAAGAAAATCATTCAGCTTTACGTCCATGACGCGAACTCTTCCTTTAAACA
GTACCTCTTCGATCTTAGCAGATAAGTTAAATA
TTATTTGTTGCTGTTCCCGCCTTGCCCTTTCTTCTGCCTCACGCCTTTCTTCCCTCAGTACCTGCGTG
AGTACTCTTTTTAGTTTTTCATTTGCAAGAGG
AGTGACCGTATCCTTTCTTTCATAGTCCCTCCATTGCTCAAGAAAGTCCACACCCTCGTGATGAAGTT
TATAAATAGCCTCCAATAGTATTTTCCTTTCC
TCCAGCTCTCTTTTCATCTCTCTTTCCAGCTCTTGGTACGACGGCGATGCCTTTATTGTGTGTAATAA
CCCTTTCTTTTTGATAAACGTCTCGGGATTCT
GAACAAAATTCCCCATCGTGACGTTCCCATTTCTTTCCACAACCCACTCCTCGCCCCAGTTGCTTCGG

AGGAAATCGTTCAGCTTCATGTTGGCCCTGTT
AGTGGTTCCCTCCAGGAGAATATCCTCTAAAGTGCTGCTCATGGTCCACTGCAGTCGATACATTCCCT
CCAATCTTCTGCGAGTCGTAGAGGAGTAATCT
GTGTCACCTTCAAATTCCGACCTTGTTCTCCTCCGACCTCCCTGCGAGACATTGGACGATTGACTCTC
CATATTGCCGTAAAGTCCTTCCTCGGGCCTTC
CAGACATTGTTTCTCCTTGCTTTCTTAGTTAGTTTGAGTTAGATCTAAACAATaGCCACCAAACAAAA
AAGGGGAATGAGAAAAAGAAAGACAATAGGCA
CAGTTATGAATCGAAGCCACAGCAAGAGACTCACCCACACAGTGTGACGGCACAGTATTCGATAAAAG
CCAAATGCACGAGGACCACCGCAACGACGGCG
AGTGAATGTCATCCACCCAGCACAGTGTTGGTGAGAGCAAACAACAATAATAACAACGTGGTGCGCAC
GGAGGGCTCCTCCACGCATACCGCTCGCCACG
GTCCCGCACCCGTGTGGCACCGCCGACACCGCACACACTCTGCAAGAGGACGTGGACACATAAGCCCT
GACAGAAGCACAAATCATACACACCGCAACAG
CGATGCATCACGGTAATCCCCCACAAAACAGAAAAGAGTGCAACTTGCATGCTTTATGGAAAATATCT
GCGGTGTGCATGGCAAAAGAGTGCCGTGACGC
TGCCTTTCATATTGCATTCGCCATTCACGTATCACTGGGAAGCACGCAGCCACCACTCCTCCGTTCCG
CTCCCCGTGCTGCTCGTGTCGCTCCACTGGAG
GAAGTTGTCACCGCACATCCCCATCCCCGTGCATTGCGGAATACAAACCTGCCACACGGCATGGGTGC
GGCNNNNNNNNNNNNNNNNNNNNNNNNNNNNN
NNNNNNNNNNNNNNNNNNNNNNNNNNNNNNNNNNNNNNNNNNNNNNNNNNNNNNNNNNNNNNNNNNNN
NNNNNNNNNNNNNNNNNNNNNNNNNNNNNNNN
NNNNNNNNNNNNNNNNNNNNNNNNNNNNNNNNNNNNNNNNNNNNNNNNNNNNNNNNNNNNNNNNNNNN
NNNNNNNNNNNNNNNNNNNNNNNNNNNNNNNN
NNNNNNNNNNNNNNNNNNNNNNNNNNNNNNNNNNNNNNNNNNNNNNNNNNNNNNNNNNNNNNNNNNNN
NNNCCGCACCAATAAATCCCAGGAAACTCCCC
ACTGCCTCGTGTGGCACGGGCGGCAGTCTCATTATGCCTGCGAAAGGCTTTTTTTCAAACAATAACGC
GGCCCCACCAAACGCCCGTAAGAACGTTTGGT
CCGTCGAACACAAAGGAGTTGAGCGGGAGAACAGCAAGAAAGACAACACCGGCAGCCATGAGCAGCCC
GCACACGCCAACACACCGCTGCCCCGCATATC
ACCAAAAATAAAAATAACAAAGAAACATGTCACATCCCAACTGCAAATCGCAAGTGCGGCTGCAGCAC
ACACTCCCACCTCCTGGGCGTGTGCGGCCCCT
CGCTGCTCGGCGCTCTAAGTGGGATCAACAAAAGCACCAATTCGGAAAAATTCTACCACCGCCACAGC
AGTCACGCTCCCCATTTCTCACAAAGGCGAAG
GCTTGCAGTGGCGCAGCGGACAGCGGCACGCACAGTCGTAGGGTAATGGACTGCCGCGGGAATCACGC
AACGGCGGTGCGCCCCTCCTTTGGGCCTCCCA
ACAAAAATACATGTATCGCGCAGTCCCCGTGAGCAACACACATTGAACCGTATGCAGCTCTACTACTG
TGGCCATCCGCACACACAGCATGAGTGCGACA
ACGCAGGCAAGGAAACACGGAATAACACCACGCATCCCTCCAATAAAGGTCAGGCGGCGTCCGTGCTG
CCTTCAACAATAATTCCACCCCACATCACCCT
TCTGCATATCCGCAGCAAAAGGGCCCACACGGTGCACACTACATTAGAATTTCCTAAAGAGCAAACCG
GATCATCAGAGTGCTGCACACTTCTCAATGTT
GGGTAGAGGCTCTCTGATCCTTGTCGCCGCCAGCCTGCGGTATTATGGAGGAGCATTACGTTCTCGGC
ATATCTCTCCATTTACTTCACTTTCACGACGC
CGCAAAGGAAGTGATGTCACACAAGTGAATTCGACCGTTCTGAAGAACGTGACGTATCAGACGCGCTG
AAACCGTGCGCGTGTGTGTTATTACGGGCGAG
CGACACAATGCTCTCGCACATCCATGTATTCCGATCCTGCGCGACACCATATTTGGCGGCGCTGAAAA
CGTTGGACACCAAACACGCACAAATCGCACAA
TGAATGCCCCTGGAAAACAAACCTCCAATAACGTCACCGCAGGCACCGTGACAACAAAGTAACAAAAG
AAATCCGACCTCTGAGCGTCCTCCCGCAAGAA
GGTGAAGACACACACGCTTGTTCCCTTTAGTTTCACTCCAACTGCTCGGAATTGATGGGTCTATAGTA
GGAGGAAACACGCCACTCTTCCCTCGCTCCCT
GTCACGAGGCATGGACGCTGCGAGAGGCTGCCAAGATGAGCCTCACGTGTGTGGAGGACCCCAATGCT
CGGGGCAGGTCGACAGCCACCCACACTCACCC
CCACTCACAAAAGGGCAAAGGCATGGAGAAGAGAAGCCCGCCGCACGGTGCATTCAAGAAGGCACACG

CAGAAGCACCGCCATCCACATCGTACCGCAAA
ACAGCAATGCGAACAGCACACCCACAGCCGCAACAAGAGGAACGAGGCGCATCCAAGTCCACCACAGT
CCTTTACTTACCCTTGCCCCGAAGGAAAAGCG
TTACGCCTCACAGCAGGTCGTGGCGGTGGCACCCCACACTCACAGTGAGGNNNNNNNNNN
NNNNNNNNNNNNNNNNNNNNNNNNNNNNNNNN
NNNNNNNNNNNNNNNNNNNNNNNNNNNNNNNNNNNNNNNNNNNNNNNNNNNNNNNNNNNN
NNNNNNNNNNNNNNNNNNNNNNNNNNNNNNNN
NNNNNNNNNNNNNNNNNNNNNNNNNNNNNNNNNNNNNNNNNNNNNNNNNNNNNNNNNNNN
NNNNNNNNNNNNNNNNNNNNNNNNNNNNNNNN
NNNNNNNNNNNNNNNNNNNNNNNNNNNNNNNNNNNNNNNNNNCCAGACGCCCTCGCACCA
TCGCAATGGGGGAACACAAACAATCAACGTCG
CGGACTATCGATGCTTCTAGCAGAAAAGAAAAAGTGATGAGAGCGTGTCAACTGCACAGCTAGCAGTG
GGACACCCAATGGGACAAAAAGAAACATAGTG
GCATTCAGCACAGCATATAATAAAACAAGCCGCAGCAAATTGCCAAATACAGAAAAACAAGAAAAAAG
GCGTAGGTTCAGCAGAAATAAATAAATAAATA
AATAATAAAGAAAAAAAATCGCATCAGTGGACTTTCATTTCGTGTATCCCTCAGCGCAGCAGGAATTG
TGCATCTCCATATGTTTCTTACAGAGAGAAAA
AAAGATCGCAGTGTTCCACGTGTCCACACGAAATCCCTCACATTTCATGAGGCACTCGGCCTCTTCCT
CCACCCCTCGTTTTGTACAGTCCGTGGGACGC
ACAGCAATGAGGCCGGCACCGCGTGCGGCGCAACAAAAGCAAATAAAACAGGGGGAAGCACGTCACTC
ATGAGAGTGTGCAGCTAGCGGCAAACAAGAAA
TAAATTAAAAATAGTAAAAAGGCAGGGGTTCTGACCCCTCCAACAAGGCACCAGCAGCACATTATATC
CAGAGGTGAAAACAAAAGGGATAAATAAAAAG
TCCCCAacggagtgcattgagtcttcctgaggNNNNNNNNNNNNNNNNNNNNNNNNNNNN
NNNNNNNNNNNNNNNNNNNNNNNNNNNNNNNN
NNNNNNNNNNNNNNNNNNNNNNNNNNNNNNNNNNNNNNNNNNNNNNNNNNNNNNNNNNNN
NNNNNNNNNNNNNNNNNNNNNNNNNNNNNNNN
NNNNNNNNNNNNNNNNNNNNNNNNNNNNNNNNNNNNNNNNNNNNNNNNNNNNNNNNNNNN
NNNNNNNNNNNNNNNNNNNNNNNNNNNNNNNN
NNNNNNNNNNNNNNNNNNNNNNNNCATTGAGTCTTCCTGAAAAGCTGCACACCCTTTTCC
ACGGGGAGTCGCGGCTCCGCTCCCGCCGTTTC
ACACAGCTGAGAGCTGCAATGCCCAGAGCCCCAGAAAAAGAAGCAACAGGATCCGAGGCACACACACA
CGCACCGTGCCGTAGCCACTAAAGCCCTTCTC
CAAAAAAGTGTTGTTCGTGAGCTGAGAGGCGTTTCCATCTTTTGAGCCACTGTCAGTTCCCGCTGGAT
CATCCGGCCCTTCAAGCATTTTCCCAGAAAGA
GACTCTGGAAGAACAAGGTTGCTGGCACCAACTTTTGCGGCTTCTTCCCGGGTCAATATGCGGTTGCA
CAAAAAGACACTCGTCACTGTCAAATACTTGT
TTTGGCTCCCGGTGTCGCTTGCACTGCTTCCATTCCCTCCAATGTAAAAGTGTGAATTCGTGCGTGGC
CCCTTTCCCGATGATGATGTCGTTTTAGCATC
CCACGCACACTCACCATTGATCTATACGAAGGCCTTGTTGCCGGTCCTGCAGCGTGAGCACCACTCAT
TATTGTTTGTTCAACTCCCAAAGGCCGGTGTG
TTTGTTTGCGTTGAACATCACATCCCAACTCCCTTCATGGCTGTGCGACAGCCAAAAGTGCACAGTGC
GGCCTGCTTCCTTCGCGCCAATCAAAGGGATA
CTCTTGACGCCCGCGTGTGTCTCGCGAATAGCCACCGTCGCCACAAGAGAGAACTCGTAATTCACAAA
GCTACAGTGATTATTATCCTCCTAACTGTTCA
CGGGTAACATTGCCCTGCGAATTGAAGCCCATTTGTGACATTCTTGCCATTCGTCACCGTTGCAATCA
CGCAACGGCACTCGTCGATACACGTGTTGGCA
GTGCCTGCGTTGGAAAGGTATCCAACCAGACCCACCGTACTTACGGGCGACGCGGAGAAGGAAGCGTC
TACGTCCGCCCAATTACGGGGAATGGAAGTGA
TCATCTGCAGCTCCTCCGTCAGGGGGGCAGGAAACAGGCTGCTTTTCTCTTCATTCTCCATTGCTCCC
ACAAGGTGCAACACACCATCCGAGTACAGCAG
GGCGTTGGCAATCGGCTTATTCACAGCAGCGTCCATGAAAGCCGGTCCAACACAAAACACGCGGTTGC
TGTCAGTCACCCAAACGTAGAGCGCACGGGCC
TCACTCTCCCACCACGGGGAGCTCACTTCCCAAGCCTACAGCATGACGCTCTTTCCTCCAACGGTCGC

GGTGACAATGGCTCCCACACGCAAGCTCTTTT
CCCAAAGAACCGATTGTGAATTGGTCCAAACGCCCGCAATTGTCCCGGGTACTTCCACACACACTGCC
CCCCTGTCACGTGACTCGTGCACTCTGCAGTA
CCGGCTCCCATAGCAATGAGTAATCACGAGAAGTTGTCCCTGCTCCCACTCGGCAATGAGAAGGTCAA
TGCAGTCCGCAGGAAAAATGTCCCTGAAAGCA
CTCAGCTGCTGCCGCCGTCCGTCGAACAGATGATCATGGAGAAATCGTCTTTTTCGTCATTATACGCA
ATCACGGGGACACAAGCGTGCCATTCAAAACA
CCTGAGCCACAAAAAACCTTCAATCCATTCGCACCGCGCTCGTGGGTCTCGGGAATGATCTGTGATCA
CAGCAATTTGGGTTCACACCAGTGGATCAGCT
CGCTCCGTCTGCCGTCATTGGGCTGCGTGAATTCACCCACAACCAGTTGAACATCACGGTCCCTCAAC
GCCCAATTGAACCCACCATTCGTACCAAGCTT
TCGCTAACTTCCCGCACGAAGAAACGCCCTATTGCCGCTCTCAATTGCCGTGGGATTGAACGCGGAGC
CTACACCACCCTTTATATTCGCTGTGCTCAAC
ACGATGTGTGCATTCCGGCCATTATTAAACCCACCAAAAAAAGACGAATTCCACGCAAAAAAGATGGA
ACCCGCCAAAATATCCGCATGAGAAAGCTCAC
TCGGTGCCCCATCGCTATCATTAAGCGTCGTGTAACCCCCATCAAAGACAACCGTCAGTCTACAAACA
CCCACAAGAGCGAGCGATCCAAACGAATCCCT
CCTCCCACCTGCATCAGTCCCACCCTTCGGTTCCAAATGCGTTCATCCCGGAACAAAAATAAAGGCCA
CCCACGACAGCCGCGCAATCACTTAATTCTCC
CCCGTCGATGCGGCCGCAGCTCCACAGGTGGCTCAGCACATCACGACAAGGAGGAGGAGCAGCACCGC
GGAAGTAAACACACGCCGGGACATGTTGGGCC
TCGGCGGCTCACTCTCTCTTCCTTCCCTCCTCCTTCCGCTGGATCCGGTCACGCGGCGACGGTTGTGT
GTGCGGGGTGCCTTCACAGCAGCAACACGTGA
GAGCATATAAATA
>scaffold1123.size6023
GACTTCACCGGTGCGATGCTTGTGCTGTACGTCCTTGGGCGACACCCGTCGCACGGCTACGGCTACTC
TGCCGCGCTGCTGGAGGAGGCGGCGCAGTGGAA
CGACGTGGTTGCGCTGCCAATGAACGAGGGCCGTGTTTTACCAGAGAAAAAAGTAGGTGTTGGGGGGG
ATTCGGGCATCGAGGCTTCTATTGGTATGACC
CGCAAGACGTACATGTGGTTTGACCTTGCGCACCGCCTGTTTCCGAACGCGAGGTACATTGCGAAGGG
CGATGACGACATTTTCCTCCGTGTGCCGCTGT
TTGTTGCTCACCTGCGCCTACTGCCGCGCCGAGGGATCTACATGGGCGTCCATGCAGGAGATTCATTT
TTGGTTAAGAAATGTTTTCTACATGTGGCGTT
TATTGTAGGATATTGCTACACCTTGTCGAGGGATGTGGCGGAGGCGTTGGTGTCGTACGAACCGCTGC
GGCGCCTGGCCTACCTGCCGTACAGCAAGGAG
CGTGATGACGAATTTGCACTGCTGGGGTTTTTGTACGAGGACGTAATGGTTGGTTTGGTTCTTGAGAA
GGAAGTGAAGTACAAGCCGATGTTGTACGTCA
AGGTGCTGGGTTGTCATTTCCATGACGCTCGCAATGGCACGGGGCACTCGCAGGTGGTGCCGACCTCG
ATGTGTGTCCATCACGTGCGGGAGGACGACTA
CGCTGCACTGATGGCGCGCTTTGGCAACGACACATCGCCTGTTGCCCGTGTGGAACGTGTCTCCGACG
ACGTGGTATACCCGTCGTGCGATTGACAAATG
GACGAAATGATTTGTTTTCTGTGCGTGTTGAATCCTAATGTATTGCTTGATTGGTTGCTGCACTCCGG
TTCGTGCCCTGTGTGCTCCGTGGCCGAACAGC
GTGGAATGTGTCATTGACCGCCCCCTGCCCCCGTGGAGCGTGTCACTCCCTGCCCTCGACCCGCGTGC
GTGTGTGCTTCTGCTGGGGGCACGTGATGAGG
ATGTGCGTGTGTGTGCTGGGCCGTCAATGAACGATGTATGTGAGTCTGTGGTTGTGATACATTTTATT
CTATTTTAATTGTGCGATTGGGAGATGGAGGG
GATTCAGCGCGTGTTTTTTTTTGTGTGTGTGCGTTGCTTCGGCCTTCCCGCGCCAGAGCCCTCACCTC
GCGGTTGGGAGCCGCCGGGGATGCATTCTTGT
GTTTTCGTGTGGTTTATTTGTGGGCGGAGCGTCCCACGGTGCGGCGGCTGTCAGTGCCGATGGTGTGG
CTCCCGGTGTGTGGAGATGGCCCGCTTTTTTC
ATGTGTAGTTTGGTTCTGGCATTTTGGCATTTATTTTTCCTTTGTGTTTGGTTTTTCTGTGTGCTGCC
CGCACGTCCACATTTCTGCCTGTTTGTTTTGT
CCTTCTTTTCTTTTTTTTCCCGCCCCACTCCCGGGTGGGGAATTATTTGACCAGGCATCTCACCCGCG

CTGTTTGTCTTGTTTTGTACGTGGCGGGGCGC
CTTTATTTAGGCCGCTTGGGACGAGGCCGCACGGCTGGCAAAAGGCGGTAATGAATGTAAGTGAATAT
CAGGGGCTGGACAACGGTTAAAACGAAAAAGG
AAAATGGAGCGAAACGGCATGTGCGTGCGTGTGCGCGTGTATTTGACCGCGGTAGAAGCGAATGTGCG
TTTGTTGTTGCGCCGTGGTTTGTGGCGCTCTT
TTTGTCTTTTGCCGTGTACGTCCGTCCTCAGTTCTCTCTCGCCCGGCAACAGCGAGAGTACTGGAGTT
TGTGGTGCCGGTCTGCACGCCCAACGTGCGTG
GAGGACACACACTGAAACACAAACACGCGCGGAGGGGGAGGGCGGCGTGCGTGTTGAGGGGCTTTGGC
CCATTTTTTTTGCTGCGGACAGAAGAAAAAACA
GACGACGCTTCGTTGCCAGGTGCCAACAGCCGCCGTCTCTCGGCCACGGTGCGGGTCGGAATTTGTTC
TTGAGGGCGTGCCCGTTTTGGAGCCGCCGGAT
GTCCTTTCCAGCGACGTTTCCAGCGACGATGAGGACGGCCTCGGCCTCTTCCGCAGATGTGCAATGGT
CCATCAGCAGTCAAATAATAGACTCTCTGCGG
CAGACAAATGGGGACGATGAGAAGACAGGTATTTGCTGTTGCTCTCCTTGGCAGTAGCGAAGTCGACG
GAGACGAGGATGAGGAGGGGGACGCGTGGAAC
ATTTGTGGGAGCAGCATCTATTGAAACGGGAGCGACAGCACTCTGGCAAGTCAGATAaCTCTTTACAG
AATTGCTCTGGCAGCCTCTCCCCCAGTCTCTG
CCTGGAGAGACACAAGAACGCGACCGAGTGCACTGATACAACCAGCCTCCACCGCACAGAGGAGAACT
GCTTTGCGTGTAGCGAGGACGTGTCCGCCGAG
TCGAGTGAGGGGGTGGAGCGCCTGCCGCAAAACCGCCTTTTCAATAAAGTCAGGAGCCGCTCTTCGGC
ATTCGTCACACTGAGCGGGAGTGTCCACGGAA
TGTTCTGTGTGCAGGAGGGCTCACTGCATGGAAGCGTAAAGCCCCTGAGCGACCTCACGGATCGGATG
GACAGTGGCGCAAGCCCTTTCCGTTATTCGAG
CAGCACCGACGGGCACGCTGGAGTGGCGTCAACGGTGTCCCCCGTAGATGCTTTTCTGTGCTACCTCT
CAGTAAAGGACGGAAACTTTTATTTTTTACCT
CGATGGCGGGGGAAGTAGCATGGCCGCAGCCGAACAGACGCCTCGGCGCCGTCTCTGACCGCTCTGAG
GCTTGCGGCTGCTTCTTGTCGGCACAGGAGTG
CGGCCCATATACACTGGGCGAATGGATGTTCGCGTGGACCCCTGACGCGGAGTCTGTGGGGTGGAATG
ATTATCTTCAAGAAGACTATCTGGCACGGCAA
AAAAGTGCCTTGACCACGCAGGCGGGGAGCTGGCGGCAACAGCAGCTGCAGTGCGGGGACAGTTTGCA
TCGTGTGCCCGTGGGGGAAGGACGACATGGCG
CAGGATGGGCTTCTGGAGGCTGAGTTTTTTGCCCCGGTGGATGGGAGCTTCTAGTCCAGCAAGACATG
CTGAGCATGGAGAGAAGGAGCTGTGACCGCCC
CGTATTTTGGCCTCTGAGCCAGAGTGCACCCTGTCGAAGGCAAAAAGAGGCGAAGTCGACACAATGCG
ACTTGTCCACGAAGCGCTTCTTCCTCCCATGC
TCTGGCAGAGGCGTCCCGGGAGGATGCTGCGAACAGTTGCGCTTTTGTTGTTGGCTGTGGTGGCGGGG
AGGGCGGAGGGGCAGACTCAACTGACAGGCCT
TGGTCGCGGAAGGTTGTGAGAAATTACTGTGGCGCGGCGCACGTTGTACGTCTTCCCCATCGGAGTGT
CATTTGTATCGTTGACGTGTTATATGTTCTCC
TCTTTCTTTCTTGTTTTGCCCGCATGGCCCTGTTTTTTCTCTTCTCCATCCATCATCCTATAAGGCGT
GCCGCCAATCCTGACAGCGGGATGTGAGTATG
CAGCGTTTGTACGTGTATACATACATGGGAGTGCCCATGCCTGTGGGTTCTGTGTATTCTTGTGCAAA
TACGGCACGCATATATTTTTATACGTGTATAT
TTGTGCATGTGGGTTGACATCTATGTGAATTCCGTTGTTTTTGGGTTTTTTATTCTCATCATGATGTG
TCAGCCTATTTGTTTGTTGTCTTGTTGCTTTT
TCAAAATGGACTGCTCCTTTCTCCTTCTCTTCTCATGCTACTGACGGCCGGCCTCCCCGTGTTTGTGC
AAGTGCGCCATGTACCACTGCGATGACTATGG
CACGCTGGATACCACCGACCCACTTGACTGGATGGATGCCCTTCTGCTTTGACCCATCGATGTCGACG
GAAGTTCTCCCCGGCGGCCACTAGTCAGCAGC
GGTGACGGTGGGCCTGCCACTCACCCCGAACTCCACAGTAGAAGCGGCAGTGACGTTTCTAGCAGTGG
CGATGCCTCCTGCGACTCACTCGCCCGGGAGT
CCTGCCACTCATGCCGCTTCCTTTCCTTACAGCTGGAGGAAGACGAGGAGCAGTCGGCGCGCAGGGCG
CGTGGCGCATCCACCCTTGTGGACCGGTGGGG
GGAGTTGACGCACGGTGCTGATGGTGTACTGTTGGCGAGTTTTCTCTCCCGGGCAAATAACACGTCCC

CGACGCAGACGCGGCGAAACAGCCCGGGNNNN
NNNNNNNNNNNNNNNNNNNNNNNNNNNNNNNNNNNNNNNNNNNNNNNNNNNNNNNNNNNNNNNNNNNNNNNNNNNNNN
NNNNNNNNNNNNNNNNNNNNNNNNNNNNNNNN
NNNNNNNNNNNNNNNNNNNNNNNNNNNNNNNNNNNNNNNNNNNNNNNNNNNNNNNNNNNNNNNNNNNNNNNNNNNNNN
NNNNNNNNNNNNNNNNNNNNNNNNNNNNNNNN
NNNNNNNNNNNNNNNNNNNNNNNNNNNNNNNNNNNNNNNNNNNNNNNNNNNNNNNNNNNNNNNNNNNNNNNNNNNNNN
NNNNNNNNNNNNNNNNNNNNNNNNNNNNAGAG
TCATTTCCATCCACCGTTTCTTTTTGTTTGTTTTGTTCTGTATTTGCTGTTTGCAAAATACGCCCATT
TGTGTGGCGGTGTCCATTTTATATGTTTTTAT
GCGTGTGGTGCTTGTTTCTGCTGGGGTGGCGGGTCTTCCTTTTTTGATTGGTTTATTGTGCGCATATT
TTTGTGATTTTGTGTGCCTGCCGCTGCGTGTA
ATTTGTGCGGCTTTTGTGTCTGGAGGGGATATAAATGGGATGGAGAGAGGAGTGGCACGGTGTTATTT
TGTTGACAGCAAGCGGTGTTTTCCGTTCCACT
TGCACCATTTTACATGCAACGACATGCGTGGGAGCGTCGTGGTGGTGTGTGGGGCGTCACTCCGCGCC
GTCTCGCTCTTTCTTCCTTTTTTTGTTCTGTT
GCTGCTTTGGCCTTGTCGTACTCCTTTCATTGGTGATTCGTTATTTTTCTGTTTTTCCCCTCCCTCCC
TGTGTCTGCGTGTTTGCCCGTACCTGCGGCGG
CACGGGGCCCACGGATAAAAGCGCGAAAGGGACGAAACACGATCAAAGAGCCTCCGTGATGCCTTACC
CGGAACCGTACGGCGCAATGCGGCTGATGCGG
CACAACACGCAGCGCCGGGCCGTCCAACAAATGTTTGCCGAGACGCGGCGGCGGATGACTCAACAGGG
GTGCATGGCTCGAATGAAATGCCGCCGTCGCC
GGAGGGCTTCGGGAGCATGGGCAGCCAAAACTCCGGATGGGGCCCGTGCCGGCAACAGAGCAGCAGCC
ATGAGCCGACGCCGGCGGGCTATTTTGGCGGC
TTCTCCCCGGCACCACAGTTCCTCCCCAACGATCTTGAGAATATGCGTATCCCGGAGGAGCGCTACGG
AAACTACTGAGGAGCCGAGCGCCGAGACTCAC
AGGGGAGGAGGGGGGCGTGCCGCCGTCGAGGGTGACCCTGCATGCCGACACGTGGGAGGGGGCTGCAGC
CGTGGAGACCGAATATATATACCGAGGTGACA
ACGCACCGGCCGCCGATGGTGGGTGGTCATGCGTACAAGAAAAGGCGGGCCGCTTTTCTGCGAGGACT
CAACAGGCGCGCAGGACTCTTTTTGTCCCCTA
CTTTTTAGTTTTTTTGTGATCAGAAATGTTGAGTGAAACCAACGCAATGAAGTGAAAAACGTCTCGCA
AGATGGAAGAAAAACAAGAGCATTTTTGTGTG
TGTGTCGGAGAGAGTCTTTATGTGTCATTATATTTTTATGACTGTTTCTTCTTCTTTTGGTGTAACTT
TCTGGGAAACGTGGTCTACTCCTCATCGGGGA
GGAGGCGGCGCAGCGTCCCACAGGCGCCGGACGAGCCCTTGCGGTGTTTCCCGTGCCTCCTGTGCGCT
GGTGTCGCCGCCCACGCTGACGCCGCTCGCTC
CGCCGCCCTGACCCCTTCACCCGCACCGTCCCGGATTCGGGTGGGGCGCAGTGGGTTGGCTGACGGGC
ATTCGTGTACTCGCGTGGCCGCGtggcaccgc
gtggctgtgttgtttcttgttg
>scaffold1124.size6236
TCAGGGCGTCTGAGCGAATGTCACCGGCCACGGGCCGCGGCGGTGCGGGTGGTCGGTACGACGACGCA
ACGGTGGTGCCTGAGGCGTACGACGATGACGTC
ATTTCGTGGGGCTTCTGCGTCTCGTCGCTCTCCTCGTCATCGCGCAGCAGCGCCTCCAGCCCTCCACG
CCGCGGCTCGCGCAGCTCCTGCCAGTGGCGGT
CCTCCGCGTACCACACAACGACGCTGTACACAGTGACCGCAAGCAGCACAGTCGTCAGCAGCAGCACG
ATGGCCGCGTACGCCCTCGCACCGCCGTCGCT
CGGCACAAGATGGTTGGCCGCGCTGACCACGCACAGCGCCGCAAGCAGCACAAAGCTCGCAGTGCGCA
TCACCGTCAGGAAGGACGAGCGCATCATGTTC
GTGAAGGCAACCACGCCCGCACCCGCAAGCAGCACGGCCGCCATGCAGAAGTACTGGACGTGGCAGCC
TCCCACGGGCGGGTGCACAGCAGCAATCAGGC
ACACCACACACAGCACCGACAGCTGGAACACGCACCAGTACACGTGGCTGCCCCTCATGTTCGTCAGC
ATGCCGCCGTACATCCGCTGCTGCGCCGCGGG
GTGCCAATACCCGACGGGGTACAGCAGGCGCTCGTGCAGCGGCTTCCTCGAAAACTGCCAGTACCTCG
TGAAGCTCGCGCCCGTGTGGCGGGCAATGAAG
TAGCACACGCCAGCAGGAAACGCCACACCGTACAGCACGCCAACGACACCGATCACGCGGTGCTGCAC

GCGGGCGCCGGGCATCGCCAGTGCAAACACGG
AGCCAAAGAAGATGCCGAGGTGCATCGTGTGCGCCACAACGTACGTCAGGCTCGGGAAGCGCATCGCA
GCCCACGCACTCGCCGCGTCCACGCCACGCCA
CCGCTGGAAGGCCGCCGTCACACCGCAGTGCACGCAACCGAACACAGCGGCGAGCAGCGCGTTGCCCA
CCACCATCCACAGCGGGTCAAGGGCAGCAAAC
ACCGACAGGAAGTACGGCAGCGCAACGGTGCTCGCACGCTCCTGCGCCGAGGCGCATGACATCCTCGC
GAACACGCCGAGCGCCTGCATCTCCAGGACGG
AGCCCAGCCCGCCGGTCACCACCACGGCAACAGCCGCCGCACCCGCAACAACGCCGGTGATCGCCGAG
AGGGCGCTGGCAGCGGCGGGCAGCGTGTCAGA
CCTGATCGTGAAGGACCCAAGCAGCACACCCTTGCAGCCGCTGGACACCGCCGCAGCGTCGCAGCGAA
TGACTATCGTCTCGTCGGCACCAATGAAGTAA
CCGCGGTGCACGGGCACAGCCAGCTCCAGGACGGTGGACGGATTCGTCGCGTTGCGCACCCACGTCGC
GTTGCGCAGCATCGCTCCCCACGGACCACCAA
ACCCGCGCGGCTGCGCCGTCGACACCGGAAAGAAGCTCAGGTGCGTGCCGAGCTGCGGGTCGCGTGCC
CACCGAAACGGCGGCGGCAGCGCCACCATCAG
CAGCGTCGGGACGGCGCTCCCACCGCCGCGGATGTCGCTCTGCGTCAGGCTCCCACCAGCCGCCGTCG
TCGTCACGGCGAGCGTCGGGCACGCCACCTCG
CTCCACCACAGCGTGCTGCTCACCGACGCCGTCGGGGTCCGCGTGGGAAACAGCGCCACCGTCTCCGT
CACCTGCAGTGTCTCCGTCGGCCCGTACTGCG
TCCTGCTCAGACTCGGTGTCGGTGTCCACGTTGAAGTCAGGCTGCGCGTCTCCGTCAGCGTCGCCGTG
TGGCTCAGCAGTGGCATAGCGGGCGTGCGGTT
GCACCCGTCCACGTGCGGCAGGTACACCGGCAGGCAGTCGCGCCCGTACCCACCATCAGCACAGCGGC
AGACGCACGACCCCGACATCGACATCGTCGCA
GAAGCAAAGCAGCGCACGTCGGCGTCGCACACGCTGCACGCCACGGGGCGGAATTTCCCGATGATGCC
GGCGGACCTGTACTCCATCGGCTGCAGCGCCT
GCCCGTTGAGCGTCAGGCACCCCACGACGAACTTCCGGCCGGCGCCCACAAGCGCAACGGTGCCGTCC
ATCACGACAGGCCCACTGCCAGCGTTCCCATA
GAGCGTCAGCGTGCTCTGCACGAGATCCGCACTGCGCGGGTACGACGGCACAGACAGAAGCCTGCCGC
TGATGCTGTTGCCGCGCACCGACAGCCACGAC
CCGGACAAAAACGACGCCGACCCCCGCAGCTCCAGCAGCGCGCCATCCGTCAGCGCACCGGCGTTGTC
GTTCACTACGACGGCGGACGCGTTGGCCACCA
CTCCGCTGTCGAACACCAACCCATGCGTGGCATACACCTGGCTCCCCGACACGCGCAGCACAGCGCCC
CCAGACGCAACCACGCGCGCACTCGCGTACAA
CGCATATTCGCCACCGAGCACAGCCGCGTCCAGCGCAACACCGCCACCAACAAGCTCCAGCACAGCGC
CGTCCACCACCACCGTCCGCCCACCAGTCACC
ACGGTCACAAGGGCCACGCCAGACACAACCAGCACGGACCGCACGAGCCGCAGGCCCGACAGCACCAG
CACCGGTGCGTACGGCGAGGACTGCGAGCCGG
GAAGATACACAAGCGGCGTCGGCCTCGTGGCAACCAGCAGGGAGTCCGCCACCGTCAGCACGGACCCC
GCAGGGAACGTGCCGGTCAGCACAAGCGCTCC
GTTCAGCGCCTCCACGTCGCACACCGACAGCTCCACCGGCCCGCCCTCGTCGCTCCCCGCCGTCCCCG
CACCGCGCAGCGCTCCACCGCCCATCACGTAC
AGCACAGCACCGTTACGCAACGACACGTTCTGCACAACGATCCGCACGCCGTCCCGCGCCATCCACGG
CACGTAGAGCACCGGACTCACACCGTCCAGCA
CAACGTGGCGCAGCGTCACCTCGCTCGCGCCGGCAGGCACAACGAACTCCGACCGCACCGTCACGCCC
TCGCTGACAAACACACTCGACGCGGTGCCCAC
TGCGGGCGGCAGTGCGGGAGCGCCCACAGGCACGCACGCCTCGCCGTACCCGCCGCTGCCGCACGCGC
ACACGCACACACCGTCCGTCATGCTCGCAGAC
GCCGTCCCGGGCGCGTAGCAGTGCGTGTCCCCGTCGCAGTCGCCGCACCCAGCAACGTCGATGCTCCC
AGCGTCGCCAAAACCAGCCGCTGCGTACTCCT
CCGCCGTCGACAGGACACGGCCACCAGCGTCGTTGCACGCCACCCTCAAATGCGCCCCAGCCGCAAGC
GACAGGCTCACCGACAGAATTCGTGGCAGCAG
TGCTCGGTTGCGCACAAAGCTCAGCGTGCTCCCAGCAGCGGTCACGGCGCCAGCGGCGTAGAGCATCT
CCCTCGAGACGCCGTCGTGCACGTTGCCCTTC
GCCAGCAGCATCGACCCGCCATCCAGACTCACCGTCGAATAAAACGCCACCGCGTAGTCCTCCGCCGA

CAAAAAGTCGCTCCGTACAAGCGCCACAATGG
AGCTCGACACGCTCACAGCCCCACGCACAGACACCGCGTGCTTGCACAGCAGGAAGTCGCTGTCAACA
AACGCCAACACGGAGCCGCCACTGGCCACAAC
ACCGCCCTCCACCGACACGCACAGCCCCAGCGCCGTCTGCACCGACAGCCCGTCCACGTACAGCGCAC
CGCGCGACGACAGCGCCACGCCGCCAACCACC
AACACGCCAACAGCGTCGCGGAACACAGCCGCCACGTGCGTGCGCGCAATGGTCAGCACGCTCCCAGT
CAGATTCACGTTGTGCACCACGAGGCACGCGG
AGAGGTCGCCAAGGTCGTAGGCGCCGCGGTACGCAACACGATTCTCTGCAATCAGCACCGAGTCCACC
ACAGTGACGCGCGAGCCCGGCGGAAATCGGTT
CGCCACCACCACGCCGCCGCCGGAGCGCGACTCAAGCCCGCTGATCAACACATCAGCACACAGGCCAG
CAGGCGGGTCGGACCGCCACCCCAAAACGTAC
AGGCTCGCTCCGCCCACAAGCGTGCAGTTCGCCAGCGTCACGTTGCGCACGGTCCCTGTCATCGACTC
CACGTCCACCACGACGTCCACCGCGAAGGTCA
CACCAACGTAGCACACCACCGACGTCCCGAGACCGGCGTTCACCTCCACATCCACACGCACGTCCCGC
ACGCACAAGTCGGCGCCGTCGGTGCTCGGTGG
CTCGGGGACAGCAACGGGCAGACACGCATCGCCATGGCCGCCCTCAGCGCACCTGCAGGCGCAGCCAT
CACTCGAGACCGTCGTCGTGTACGCGGGGAAG
CACGACGTCGNNNNNNNNNNNNNNNNNNNNNNNNNNNNNNNNNNNNNNNNNNNNNNNNNN
NNNNNNNNNNNNNNNNNNNNNNNNNNNNNNNN
NNNNNNNNNNNNNNNNNNNNNNNNNNNNNNNNNNNNNNNNNNNNNNNNNNNNNNNNNNNN
NNNNNNNNNNNNNNNNNNNNNNNNNNNNNNNN
NNNNNNNNNNNNNNNNNNNNNNNNNNNNNNNNNNNNNNNNNNNNNNNNNNNNNNNNNNNN
NNNNNNNNNNNNNNNNNNNNNNNNNNNNNNNN
NNNNNNNNNNCGGAGATTCACTGTGAAGCTCAAAACAGCAAAGGTATCCGATGGGGATTCACGGCAGA
GGTTGCCGCTCAACTGCAGCCATGAGTTGTAG
TAAATGTAGGAGGAAAAAGTTGAATAAAAAACAGCCACACTTCCCTGCATGTCGTTACCCTGAAACAC
AACCGCCGACAGAGACTCCACAATTAGGGCCC
AACGCAAAAAAATGACGTACTGCACCGGCCTCGACACAAGATTGCCTCGGATCACCACGGCAGAGTTG
TTCAGTACATCCAGCGACGAGGATTGGCCAGA
CCCCTCAAGATATATAAGCGTGCTATCACCGCCGGCCATATGAAACGTGTTGCCCATCACCGCAAACA
GGGAGTGCCACAACACCCTCAGCGAAGATCCA
ACAAAGTGAATGGCGCTCGACGATGCCGTCACGCTCTGAAACACATTGCCGCTCAGCACCACCGCGGA
GTCATTGCTGATCGCCAGCTCATTCATCGCAA
CACACGACGGGCTGTCAACGTCCAAACCCTGCCGAGAGATCAGCCTCGTGACCGTGAAGCGGTTCCCG
CTGATCGTGATGTTCGTGTGCGGTGGAAACAC
ACCCTCCGGTCTGAGCAGCGCGTCACGGAACGTGTTGCCGGTGACGACAATGTTCAGCGGCCCCACAG
CGGCGTGCGCGTTGCCCAACAGCAACAGCACG
GCGCCGTCCCTCCACGTGCATCCATCAAACCTGACATTCGCCACGTCGCCCGTCATCGCTCCAACCAG
CACCGTCAGGCTCATGCCGCCCCCGCTGAACG
TCACGTTGCGGTAGCACAGCCACGACAGCCCGCCGCCCACAGCCTGCGTAACCTCGGGGTACACCATG
TCGCTGATGCACTCACCAACCGGCGGCGGCGG
TGGTGGGAGCAGCATTGGCGGTGGCGGCGGGCCAGCAGGCACAGGCGCCGGGACGCACACATCGCCGT
GCCCTCCAGCAGCGCACTGGCACCTGCAGTCA
ATAACCGCCGTCGTCAGCGGAGCAAAGCAGTCGCCCTCCTTCGTGCACTCAGCGCACGCGGCAACAGT
CGTCACGTTGGTGATGCCGTGAAGCTCCAGTT
CTGCCGCCGTAGCCAGCACACGTCCCGCGACCGTCAGGCACCCAGCAACGAAAAGGTAGCTGGCCTCT
ACCAGAGATCGCAAAGGCTCCGACAACCCCAT
CAAGCCCATCGTGCAGCCTGTCAGCGTCACCACACTGCCGCCGTCAATGATAAGAAAAGTGGATTCGG
GTTCGTGAAACATCGCACCCGCTCCCACGTTG
TTGTCACGCCAATCCAACCAGCTTGACCGCTGGACGGTCCACGAGCCCTCTGCATAGATGGCGTATGA
CACGGAACCGCTGTTCCCCACCACGCGCAACA
CGCTGTGATCCGACACGCTGAAGCCACGGTACTGATAAAGAAGGCTTGTCCCGAAGAACACTGTGTTG
TTGAGCAAAGCAAAAACAGAGCGATCGCTGAC
ACTGAGGATATCCAAGTAAAACAGGTGTGTGTAACCATCGAAGCCGCAGTACCGCGTGTACAGCGAGC

TGCCACCCACCAGCGTCAGTGTCCCAATAGAG
TAAAAACCATACCCGCCGCGCCTCAACGTATGCGC
>scaffold1125.size6607
ACGACGCCCTCCAGAGGCGCCTCAACTCGGCTCATACACTAACAAAAAAAGAGGTGCTGGAAACGCCC
AGCACCCCGCCCTCCCCGCGTGGGGCAAAGTGT
TTTGTGTGGGTCCTCCCGAGGCGCGCCTCATCATTACCAACAGACGGGGAGGAAAACAAAACAAAAAA
ATAAATGGGAAAAGAATGTTTTCAAACATGTT
GTTTAATCAAAAAATATTTGGCCTGACCCTCACACACACTTCCCCAAAGATGAAAACAATTAACACTC
GCAAATTAAAGACCAGTGCTTTTAGGGTATTC
GCCTGGGCCGCTGGCCGGATCCTGAAATTAAATGTAAACAAAATACGAGAATGAGAAGGGGGTAAATA
AAAAGGCAGTGGGATCGCCACTTCCTCAATGT
CTGCTCCAATTAAATAAATTTATGTATTCTGATGGCCACCCTCGTGGCACGTTACGCCTTGTTAGAAT
TAAAACATTTATTACTTAAGGCGTGCTGGATT
TTGGGGACTTCCAGCCAGAAGACCCCGCCGCGAGGTGGAAATCGAAAAAAGAGGGTGAGTGAAGTAAT
GAAAACTAAAAATAAAAATAAAAAAAATTCCGT
CTTGaAGTTCTCGGTTTCTTGACTCCTCAACCCGCGATCCTCCAACAATTAATAAGGAAGAATCGAAA
AATATATATATTCGTTTTATTAAAATGTTTAT
CAAGTTAGATAGCTAGCTTTCCTCGCGAACACCCTCTTCACAAGCTCCTACTAATCACCGCTTTCAAT
TATGTCAGCTTTGCCTCTTGAAAAAGAGCGCA
GCTTCCCTCAACTCCCCATATGTTGCACTTGCATGTCGTTCACACCTGTGGAGTACAGCCCCTCATCA
ACACGCTCCTCTCCTCCTATTCCAATAACGAC
TCCACGATGAGGTAGCAGAGCAATTCCTCCTCTCAGTGCCTTCCTTTCTTTGCCGCAATTCATACAAC
GCAGCTCCGCCCCTCTCCACATTTTGTCCCCT
TGCACTTGGTGAAAACGCCATCGCACAAGAAACAGATCCCTCGTCGGCTGCCGATGGAAAAGAGAGGC
AGAGAAGGAACACCGTGTATCCACCCAAGTAT
ACGTATATCTACGTAGCAAAACAAGCCCACACTAGAGAAATAAATNNNNNNNNNNNNNNNNNNNNNNN
NNNNNNNNNNNNNNNNNNNNNNNNNNNNNNNN
NNNNNNNNNNNNNNNNNNNNNNNNNNNNNNNNNNNNNNNNNNNNNNNNNNNNNNNNNNNNNNNNNNNN
NNNNNNNNNNNNNNNNNNNNNNNNNNNNNNNN
NNNNNNNNNNNNNNNNNNNNNNNNNNNNNNNNNNNNNNNNNNNNNNNNNNNNNNNNNNNNNNNNNNNN
NNNNNNNNNNNNNNNNNNNNNNNNNNNNNNNN
NNNNNNNNNNNNNNNNNNNNNNNNNNNNNNNNNNNNNNNNNNNNNNNNNGTTATTGAAGTACTGCAGCGGAC
CATCCAGCGTGTTGTCAATGATACTGGGGGCG
GACGTCGCCAGCAGCTTGGCGCCTAATATCACGAGAACAGCCATGGCAGCACTCCACAGCACGACGTA
AATGACCCACATCCAGAGAAAGCAGCGGGACG
CGTCACTGCTCCGCCCTGGCTTCGGCTGACAGCAGGAGTGACAGCAGGCAACGCTGAGAAAAACAAGC
GGGAAGCTCAGCAGAGTCACAACGAACAGCAG
CGCACAAACCCCCGGATAAACAAGGTAGCGCCACTGCCCCTTTATGCGGTCGGTGTTGGACAGGCATT
TTAAGACGTCGTTGTCGATGCTGGGGCCATCC
CACACCTTTTGGCAGTCGAGTGAGCGCTGGGCAGATGCAACGGCGGGCGCGACCACGAGAAGCGCCAG
CAGTGTCGCCGCTGCAAGCGCAGTTGCTTTGC
CAGAGGGCATTCGTCGGCCTTATTTGTGCTTTTGTGTGGCTTCGCTCTGGTGTGTCTTGGGCGGAGGA
CAAAGAGGCGCAAAGACAAAAACAATAAACAA
ATGGAGGAAAAAAGAGCAGGCAGTCAAGCGGCGCCACTCAGGCATGCACAAGGCGGCCACGCCCCAAC
CCAGACCGCAGCGCATCAACAATACTCCTCCG
CAAGGAGAAACTCCGTCAGCCGCACGTACACATCCCTCTGCGTGCTGCTCAGGCGGCGCACGTCCATG
TCCGCGTCGCATGCCTCGCGGCCACAACACCT
CAAGCGCCCTTTTGCCGCGCGCCGCAGCTGCCGGTGCCCCTAATTCACCCGTATAAAGAGAGCAGAGA
CAGCGGTGCAACAAAAGTAGTGTGGGAAAGGG
CTGTAAAACAGGGAAACTCCTCCATCTTACACATATCCGGGAGAAATAAGCATCGCGCATCCAATTCT
TATAATTCGTTTTTTTCGCATTCCTGAGCTCC
CTCGTTGGGGACCGTTCGAAACAAACCAGCACTCACATATTGACTAATATATATATATATATATATAC
ATCAAGGAGACACATAGATATACACAAAAAAA
GACAGTATATATTCAAATAAATCTGTATATTTATCCGTTTCAAAAATGCGCACCTTTTATCGTATGCC

ACGCGTATTTTCTACATATTTCATCCTCGGTC
TTAACATTACCAACAAATCACAAACATCAATCAGCCGGAAAAAAAAAAATCGTCGAGAAAATGAGGTCA
CGCACGCAAAAATAGGAAAAGAAAAAAGACAA
CGTCAAGAAAACATACACTCAACTACTCTGGGCAGCATTTACCGACCCTGACCCGTTTAGGCCACGAG
GGCTCCTACCCTGTTTGATTGACCGGGCGTCC
CAAATGCAGGAGCCGCGGAACATGATGTAGATGGCCAGACCAAACATCAGTCCCCCAACAAAGAAGCC
AGTGCCAAGCATCAGGGTCCCCGCCTTCAGTT
CGTTGCAGTCACTCATGGCTCCCAGAAGCTTGTCCACCACAAAATTGCAGTCGAGCAACGGCCTGGCG
TACGACAGCGCAATGCTCGCGTTGCTTGCCTG
CGCCGCAAGCTGCAGAATCACTGACGCCACTGCTTTGACGTCTGTGTTGGTGCAGTTGGCGGCGCACT
CAAACAGCGTGCACGATTCGCCCGCCACGGGA
CACGCCATTGTGAAGGCCTTGACCCGGGTAGCACTCAGCACGCTGGCCATGGTGCCAAAGTTGGGGCA
CTGATCCTCCGATGTGATGTTGTTGCCGCACA
GCAAGGGCTTAAATAAATTGGGCTTGTTATCGCAAGACTTGAGCAATTTCTTGCACGCGTCTTCGGAG
AACCGCCGCTCCGCATCATCCGCCTTCCTGTT
GATATCAGAAAAGTCAAATGTCTGCTCACAGTACGGCACAAGATACCACTGGAACACACCCGGCTGCC
GTTGTTGCTGCAGCTCCACCTCGCCACAGGCG
GCCCAAGAGAGATACGTCAGCACCGTCACCACAACCGCGAGCAGTGCAAACACAACCCCGAAGAGCCA
GTAGATGCAGGAGAATAAATATGTTGTGCACG
GGATGCCGCAGCGGCAACAGGCCAACAAAACCACAAGAAGCATTAGCACAACACCCACGCCACCGACA
CAGTACGACACAATCGGTACCCATCCAATGTA
CTTCGAAATCTTGTGCTTGGCATCCATGAGTAAATCGGTCACATTGGTGCTGATGTTGGTGAAGGCAG
TGATGTCCAGGTCGATGGAAGGTATGGGCTCA
CGCTTACCCGAGGACCAGTTGGACGTGAAATCAATGATCCTCTCAGCCGTGTTATTGAAGTACTGCAG
CGGACCATCCAGCGTGTTGTCAATGATACTGG
GGGCGGACGTCGCCAGCAGCTTGGCGCCTAATATCACGAGAACAGCCATGGCAGCACTCCACAGCACG
ACGTAAATGACCCACATCCAGAGAAAGCAGCG
GGACGCGTCACTGCTCCGCCCTGGCTTCGGCTGACAGCAGGAGTGACAGCAGGCAACGCTGAGAAAAA
CAAGCGGGAAGCTCAGCAGAGTCACAACGAAC
AGCAGCGCACAAACCCCCGGATAAACAAGGTAGCGCCACTGCCCCTTTATGCGGTCGGTGTTGGACAG
GCATTTTAAGACGTCGTTGTCGATGCTGGGGC
CATCCCACACCTTTTGGCAGTCGAGTGAGCGCTGGGCAGATGCAACGGCGGGCGCGACCACGAGAAGC
GCCAGCAGTGTCGCCGCTGCAAGCGCAGTTGC
TTTGCCAGAGGGCATTCGTCGGCCTTATTTGTGCTTTTGTGTGGCTTCGCTCTGGTGTGTCTTGGGCG
GAGGACAAAGAGGCGCAAAGACAAAAACAATA
AACAAATGGAGGAAAAAAGAGCAGGCAGTCAAGCGGCGCCACTCAGGCATGCACAAGGCGGCCACGCC
CCAACCCAGACCGCAGCGCATCAACAATACTC
CTCCGCAAGGAGAAACTCCGTCAGCCGCACGTACACATCCCTCTGCGTGCTGCTCAGGCGGCGCACGT
CCATGTCCGCGTCGCATGCCTCGCGGCCACAA
CACCTCAAGCGCCTCCACAACTGCAAGAAGGAACCATACTTGCACGCAAGCTGTGTCGCAAACCCGGA
CGAGCATCCACGAACACAAATAAGCATGCGGG
CAAAGGTAGTGCGCGCGCGGAGTGCTCGCTTTATCCTCGCGGTCACGTTGAGGCAGGTCGCACAGTCG
GCCAGCTGTGGTGGTCGTGCGCCACTCCGCGC
GGCATTTGCCAGCGCCGTGACGTGGGCAACTCCAAGGGAGCGCAACCAAGAGACGGTGTCCTTCAGGT
GGCGCGTGCGGATGACACGGAAGCGTGGCACC
GCGGCAAGCGAAGCGCAAGCCGACAAGACACGACGCCGTTCATCGGGGCGCAGCAGTTCGGTGGTTCC
CTCCACCACCCACACCACCGACCGAAACGGCG
AGACAGAAAGTAGCCGCCGTTGCTCGTAGTAGCGGGACGTCGCAATGCTTGCACAGAGGTCCTTTACT
GTCTTGCGTTCCACAACAACAGAAAAGAGTCT
TTGCGCAGGAGCATCCCCACACTCACCCGCAGCTGGGACGGAGTGGGTGGCACCAAGACTGTCCAGTT
CCCTCGCAGTTGGGGGACTGGCGTGGCTCCCA
CAGCTGTCACCGGGCATGTCGATCCCAATCATGAAGTCACCACATGGAAGCGTGCAGGATGCAGTGGG
AACACCAGCCGTCGTGAAGGCTTCTATCACGC
GCCCATGGGCACCGTGGATGCGCTCTCGGTTGTCAACAAGAATTCGCCATTGGTATCGCGAGACCTTT

TCCTCTGAAGCAACGTGTGCCAAGGATCCACC
AACGCGATCCTCGCGCTTCGCTTCCTGTCTTGGCGCAGCAACAAACTCTTGTGGGGTGCCCACGAGCC
TGCGTATCCGTGGCTGATCTCTTGGGCGCGGT
GACGACGGGCGGCTGGTAGATGGCGATCGGAACGGTGTTCGCAGCCGCTGCGAGTGTTCGAGCTGCTC
CTCGTGCGTCGACATGAATCGCCCGGGCAGAG
CACCGCCACATTCCCCACTCTCCACCGGCTGTTCTGCCTGCCCCTCGCCGTCCCGCTCCGACCATGGC
ACCGGTCCCGTCGGCTGCGACACACCGTGCTG
ACGCAGCCGGGTGAGGAGGACATTCTTTGTGCCGGTTGTCAGGAGGCCGCGTGAGGCGCACCTCGACC
GCAGCTCCCTCAAGCTGAGCTTCTCCGCCGTG
GGCTGGGCGTCGGCGTCGCGGCGAGTGATGGGCGGCAACGGACAACCCGCGCAAATTTCCCTGCGGGG
AGAATGCGACAAGTCCGGCGGCGTGAGAAGGA
AACACTCGCCGTCCTCGGCGCCGGTCccaaacagccacgggagcggggaggggtcgccggcgccgtGC
GACACACTGCTCTCCAGACGCCGCAGCTTAAA
GCAGCGGAACGGCGAGTGCATTGCCACCGCGTGGTCGTCAGCCCACGATGGGCTGGGCGGCGGCGACA
ACAAGTCCAACTCAGCGCCAGCGCACTCGGGT
GCTTGGCAGCGTTGCTGCACGTCGCAGCAGTACGCGCCGACCGCCCTCACGGCGTCTGTGGAGCAGCC
ATGCAGCACCGTGGCTGCCGCGCCGCTCGTGA
CCGGCAGCGGGTAGCGTCGCACGGCGGTGCAGAACTCCGAGAAGCGCCCTGGCCACGTACGCTCCATG
TGGCGAGCCAGCAGCTCGTTGTGCGTTACGCA
GTGGTGGCGCATTGCACACGCAGCTCCACCCCTAACAAGCTCCTCCCCTCCCGCCTTCCAGCAACCGC
TGCCGCCAGTCACATGCACAAAGCATATAAAA
GAGACAAAAAAAGGAAAGAAAGAAGACAGGAAGATGCACACAACACAACGAGCAACCGCACGTAGTGG
CAGAGCAGAGGAGGAAAAAAAGGGTGGGGACG
GGGGTCAAGTGAGAGCTGTCCACTTCTCTTGTCTTGCCGATTTACTCCTTCCTCCTCTTCCATTGAGA
CGCACACCCGTAGTGCCGCATATTTACATGCG
TGCGCCTCTTCGGTCCTCCAGTTTTCCTTCGTCACAAAAACACGCGCACAATGAGAGGCAAATGCGTA
AATTAGCCGCAACCCGTCCTTTCTTCGCCCAT
TACACC
>scaffold1126.size6048
CAACACCACTCACTGGATGCGCGCCGCCAACATCACACCTGCAACACGTCGAAAAGAAATACCGCAGT
CTCCCGATCTCCCTCCTCACGAGAGCCGCCGCA
GCCAGACCTTGTGCATGTGTGCAGCATAGCATGTCTGTGTGGGCTTGTGTGTTTGTCAGCCCCTACAC
TTGTAAAGCAGCTGTCCGACACAAGAGAGTCT
AAAGAAAAGCAATGACAGAGAGGGAAGCGAATCGCACACACGCAAATTACTCATATCATCACGGACGC
AGCCCGAAGAATGAGTAAACCCAACCACAACA
CAAGTAAAACAAAAGGAAAAAAAAGCCGAAGCGTGGGACGACCAGCGCACACACTCTCATTGCTCCCG
TAAAGCAAAACACACACACAGGGAGAGAGAGA
GGTCGCCCACTCAATGCAATGGAAAGAAAACAGAAACAGTTACGGTGCTCCGCCCATGCAGCCAGGCA
GGACGGCAGCGGTGCGTGTCGTGCAAAAGAGA
GCACCAACGGGCATGCATGGGTGTGCATGCATATATACAATGGGCACATTATGTGTGTGCCTCgTGGG
GTGAGGTGGGGGCGAGAGTCCATGcaagggGA
CACACACACAGAGAGAGAGAAGGACGAGTGTGTGTGCGGGCGGTGCACGGCTCTCTCTCCCTCACTCTCA
CGCGGCCACCACCGCAGCAGCCGCACACGCAA
CAAGAAGAAGAAGCAAAAGAGGGGAGGTGGTGTGGGAGACCGCGGTGCTGCCGTCACTGTCGCCCGTT
GAAGCTGTGGCACCCTTCATGGTCGTGGCTTC
GACTGGGGTTTCCTTCGTATTGCCATGCTTATTTTGACTGTTTTTTGTGTCGTTTGTTCCTGCACCAC
CAGCAGTTGGATCATCGCTTGTTATCCCTTCT
TCAGTGCCACTGTTAGCGTCGTTACTCTCAAAAATGCTTTTAGTTGAAGATGTTGTTTGTGCTGGTTC
ACCATGACCGTTAAATGTTGCATCCCGCTGGT
TGGCAGTGGCGGAGGCGCTGGCTGGGTTTCGTGGATTGTTGTTTGACTCCTTGCTCTCGTGAACGCCT
TGTGAATGTTGCACCAACACGACATCTTGAGG
ATCGGGCTGTTCGCTTTCTTGTGCCGGGTCAATATCTTCCGATTGTTTTTCATCCTTTTTCCGCGCCG
CTGCTGGTGTGCTTCCTTGTTCTGATGGTGTT
GCTGCCGGTGTTACTGTTGCTGCCGACGCTGCTGGGACTGGTGAATCTTCTGGTGATGGCGCTGCCTT

TGGCGGCTGTCCTTGCGTCGTTGGTAGTACTG
CCGTTGCTGGTGATTGTGCTGTTGGGATTCCTTCTGCTGCTGTTGGTGTTGGCCCTTTTTCGTGCTTT
GATTGTGGTGATGATGATGCTTCCTCTTTTGT
TGGACCATTGGGTATTTCTGTCTCTGCTGGATCTACTGTTTCCTCTTCTCGCTCTTCTTCCTCAAGAT
CATCAACAACAACAACGTTTTTTTCTACTCTT
ACCAGCGCGTCTAGCTATACAATGCCATCTGAGTGCCGAAGAGGTGGTCGAGCAGCGACAAAAGTTTC
CTGCTATGAAGCAACTGCCATTGAGAGAACTT
TCCGAGGCGAAGGTCCTGTTCAATGCCCAGCCGTTGACGCACAGCCAAGGTGCCTGGACATTCTAGAA
GCAGATGGTGCAAGGTTTCCTGCGGGCCAATT
GGCGTGCTTGTGGAGGCTGGCTGATCCTGCGGCAGCAGAGATCGCCTTCTGGCACTGCTGTTCCGATG
CCGTGTGGCGTCTGGGTTGTGTGCGCAGCGAC
TTCTGTGCTGTGCGGTGCTTCTCCGTGTGGGGAAAACCATGTCACAGAAATCACATTTGAGCTCGGGA
AGCGGTGGAGGATGTTCCGGATGAAATGACCG
ACAGTGACTGACGAGACCCGAGAATCCCGTCAATGTAGAATCACAGTGCGGACATTTCTCCTTCCGAC
GGCGTGTGACGGTCCGTCTCCTTACTGGAGAG
ACGTCTTCCTCCCTCGCCGACGGGGGAACAGTGTCCGTCCCCGGTGCAGTTGGATCGTTGTTAGAGCG
CATATATGCAGACTGTTCCGGTGAGATGTTGC
AGAAACGGCATTGGTTCGTGCAATCTCTCACGGCCCAGTATAGTCGGCCCCAGAGCTCAGATTCCCCG
CACCTTGCCCCTGCGAGGTGAACCAGTTCCTG
ACGGTGTAGAACTTCCTGAGTGATCAGGTCCTTACCGCTGAGATCGGATGGCCTTGTGCCGCAAAGCA
GGTAGCGATGAGTGTCTTGACGAATGGTACTA
CGCCACTTGTTGGTGAGCTGGGTAGTAAAACATGTCAGCAGATCCGTATGCCATAAAGGTGCGATTCC
TTGCTCCGTGTATTGTCCACTTTCCATAGTCT
GCGTCGCATACTGATCTGCAGGCTCGTTAGCATGTACTCCGCAATGTCCGTGACAAAACTGCAGGTGC
ACGGACCAGCCAGCACGCGTCAGTGACAAGAG
ACGCTGCCAGAGCCGATCCTCCGTCCAGTCTGTCTGACTGAGCGGGCCCTTGTTTAGAGCCGCTAGAA
GAGACTGACTGTCCGTAACCACAAGCAGGGTT
TTACGCCTGTGTCGGATACGAGGAATCATCAGCTTCTCTAGGGCTAGAAGCAGCGCACGGGATTCCGT
CCTGTAGCTGCATGGGCGTGCACCGCAGTTTA
TGCTGGCCTTCTCTATTATCTCCGATGAATTGAGGGATGAAAGCAGTATGCCAACCCCTGCTGACTTG
GGAACGTCGACACCGCCATCGGTCGCAAGTAT
CCACGATCGATGGCTGTGCAGCGCCAGAGCCTCTTCGCTGACTCTGCGCTTTTCCCTTATCAGCTCCT
CTTCGTCAGTTATTGAGTGGTCTATCGGAAGT
TCCACACCGATGGTGGCCCGGTGAGCGGAGGCCGCACACCAGGGAGGAATGGAGCTGCGAAAGAACCG
CTCTTCGCGTGGTGAGCTCTCGGTGATGCCAT
AGTCCTTTGTGTATGCGTCTACGAAGGCATATAGCTCGTCCCGAGATAATGGCGAGATGCGGAAACCG
GCGCGAGGTGGTGGCTCAGGCGGGGGTCTCCG
CATCCAGTCCATCTGACATGCGCGTGTGTTCTCAAATATTGAGAGCCGCGCGCGTAGGCAAAGGACGT
GGAGCGGCGGCATATTAGCCTCCAGCAGTGCG
GATTCCGGGTCGACAGTGCTCGGAACTCCGCTGACAATGCGTGCTAGGGTTGCGTACGTCTTCTGCGT
CTCATTCTTCGCTGAATCCGTCGCTACTGCCC
ATATGAGCTCGCTGCCATAGCGTAATTTGGAAGCACCGTATCCAATGACGAACGTGCGTAGGTCATGT
GGAGATGGTCCCGCCTCTTTGTGCGCTATGCA
GCGTAGCTGCGATATCCGTTGCGAAGTGGCCGCCACATTTAGCGTCAGTCGTGGATCGAGATCCATGC
CGAGAAGCTTCGTAGTTCGCGATGCCCCCATG
GTCATGACTGGGATTTGACATCCAGCCACTACCAGGGGCCACCTTCCTTCACGATCATAATCTGACTC
CGTGTGCGTGGAGAGTGTGAACCAGGCAGCTT
CACACTTTGACGGGTTAATGGCCAGGCCATTTTCCTGACTCCACGCATGCAGCTTTTTAAGAACCATG
TTGGCAGTGGGAATGGCGTCTTCCCGGGACTG
CCCCTTAACGATAATAGAGAGGTCGTCCGCATACATGTCTGCTGTGACACCCGCAAAGCTGTTCAGCA
GATTGAGAAGTGGGTGTACGTAAAGGGAGAAC
AAATACGGTCCCAGCACGGACCCCTGAGGAACTCCTCGCAGCATGGGACGCCGATCGCCGTGTCTGTG
GCCAACTCGTGTCTTCGCGTAGCGACCACATA
GAAAGTTTCTAAGCCACCTTCTACAATCCACCCCCGGAATATTAGCCAGCAAGCGACTGAGGACTCGA

TGATCTATGGAGTCAAAAGCCTTGCTGAAATC
AACCAACACCAGCAGGGCCCGATGGGGTCTGTAGTGGAGTTGATTGCCAATACCGCTCTTCTTGGGAA
GGTTCACTTGGAAATAGTGATTTCTATTATGC
TCCACTTCGTGTATCAGGTGTGCCAGCTGCATCGTCGTGGTACGCATACTACGATATGCATACTGGTG
GGGGTGCGGCGTCCACACCCAAAGCAATCTCT
TCAGTACCATTTTCTCAGCANNNNNNNNNNNNNNNNNNNNNNNNNNNNNNNNNNNNNNNNNNNNNNNNN
NNNNNNNNNNNNNNNNNNNNNNNNNNNNNNNN
NNNNNNNNNNNNNNNNNNNNNNNNNNNNNNNNNNNNNNNNNNNNNNNNNNNNNNNNNNNNNNNNNNNN
NNNNNNNNNNNNNNNNNNNNNNNNNNNNNNNN
NNNNNNNNNNNNNNNNNNNNNNNNNNNNNNNNNNNNNNNNNNNNNNNNNNNNNNNNNNNNNNNNNNNN
NNNNNNNNNNNNNNNNNNNNNNNNNNNNNNNN
NNNNNNNNNNNNNNNNNNNNTTGATGCCTCTGGAAAACCTAACTGCCTTGTGAGAGGGGTGAAATGGA
AGAAAGATGCGATTGAGAGCATTCGCTGCTCT
CCTTGCTGTCTTGTAAGTGTGTTGACCATCTCCTAATAAAATTGATGTGGGAGATGGAAGTGATGCCT
TTTTTCGTCCGGAAATGTATTTCCGTGCGAGT
GGCTCGCCTGGATTTAACTTGCTGATGCGGCGTAGATACGTGTTGCGCGAGCGCTCTCGGAGTAGGAG
GAAGAATTGACTCCTTGTCCGTTGCATTTCTG
CCTCACGGTCGGGCGTCGGTAACTGCAGATAGCGGGCCTTGCTGTCGGTAGCCATCCGCTCTGCCTCC
ATGAGAGTGTCGTCCCAAAGCCTGGGACCGTC
CTTGTGCATGCCGCGGGGGCAATGTCGTCGATACGAAGTTATAAGCGCTCTCGTGAAAGCTTTAATGC
CGGTGGACTGCTTTTTATAGTCGTATGCCGGA
AGTTGTGCGTCGAAGTCCTTGATGAATAAATCCCACTTGCACTTCCCCCAACTGTAGAATATCCTAGC
TGCCGAAGGTATGGTATCCTTAAATGCCTGAT
GCAGCGTAAATGTCAGCATATAATGATCACTAAGCGGCGACACGGAAGCTGACCAATCCGACACAGTC
AAATGCTTTGACCATGTCAGATCGATACAGGA
TCGTTCTCTTGTGCCACGGGCATACGTGATCTCACCAGGGGTGTTAACCAAGGTGAGTCCCGCATCCG
TGCACAGTTCTAAAAATTCTGCAGCAACCTCG
CTTGGAGAAGTCTCCAAGAATGGCTCCCACTGTGGGTGATGCATGTTGAAATCCCCACACAGCAGGAG
CGGCGACGAGGCTGGAAGGCAGTTTACTAACC
GCCTGAAGGATTGCGTTACTTGCGGTGGTGGTCTCATATAGGCACTCGCTACAACAAGATCACGGTTC
TGGTCCAGAGCAACCTGGACCACCACCACTTC
AAGGCTGGTGTCATGCTGGGGGATGGTGAGAGGTATACGCTTGGAGCGGAGAGTTTTCCGCACAAGCA
CCGCCACACCGCCTCCCTTGCCGATGCGCGAC
TGTGCATAGAGCACGTAATCTCCAGTGTGCATGCTGGCGATCTGCTCCGACGACTTCCAAGTCTCCTG
AATGGCTATTATGTCTGCTCCTTGCGCCATTA
ATGTTGCGAGTTTTGACGGCGTGAGGCAAGAAACGTTCATCTGGAGTACTGCTATGGGGCCTGGGTGC
TGCTCAATGTCTCCGCTTAGCAGTAGCTTTTG
TCGTTGCGCGTCACATACGGCACGCAAACGGTATGTATAGCGCTGATAAGCGCCGATGGAATTCAGCA
GCGGATGGAAATACCGCTCCGCGGGCAGCCAT
GTAAATGGCTCCATCTGGTGCCCTTCCGACCCGTTTGTGCGGCGCCTCCTGCCTCTCCGCGTGCAGCG
GATGGGCAGCAGGCGCTTAGCTTCCTCTCTAG
CAAAGAGTACTAGACCCTGGCACCACGTTGAGGTGGCCGGCTGAGCCAGG
>scaffold1127.size6610
gACTCTTCCTGTGAGGGGTGCCGTGTGTTTCCCGCCCAACTGCTGCACTCGCACACCCACCGACACGC
TCATGACGACGGCCCTGTGCGCCTCGCCTGCTG
CACGCATGAGGAGGGGCCGCGACGAACGGCGCGCTGAGGGAGCCGTGACGTGTGGGCGTGCGACGAGG
GCCTGTGGGGCGGCGCACACATTTCTTCCCTC
TTGTTTTTTTTTGCGGCCAGGGCTGGCAGACACCCGCAGCGCCACACACACACAGCCACACCCTGCCG
CCGTGTTTTTCTTTTGTTATTTTGTTTTTTGT
GTTTATTTCACGCGCTGGCAGTCCCGTCAGCATTTCTTTCCCTCCTGATGATTTCTTTTTTTTTGCTG
CGCTCACTCTCCCGATGGAGTGACGCGACTGC
CGTCGTGTCTGCGTGCTTCTCTCAGTCCCCACACACACGAGTGACTGTGCTCTGCGGGCCGCACTCGC
ACGCCGATGAGAGGGCTGAGGGGAGCAGCAAC
ACGCAGATGACCCCGTGAAATATAAACGATGTGCGGACACACGGAGGGTCAGACGGCTGAAAGACATT

TAAATACCTTTTAATTTTCTTTTGTGGGCAAA
ATATACAGACAGTTTGGTGTGGCCACACGGCAGCCGGCTGCTTTTTTAAATTTATTTGTGTGGAACAG
TAATGGACGTTTTCTTTTTACTTATGATTATT
TTGTGTGATGGGGTTTATATAAGACGAAGGGGAAAGCTTTTTTTTGTTTTGGATAAATCTTGTGTGAA
TTGCCACTGCTTGGGCTGCAGTGCAGTCATGA
GTTTGAGCGGTCGTCTTGTTGCTTCCGTTATGCGTCTCCGCTGTACGTTTTTGTGCGTCCCTCCACAT
ACTCTGCTCCCTTTTCTACTTCTTTGCTTTGT
GTTTCTGCAGGCACACAACACACACACAAGATGCACATTGCAGTGGATTTGAATGCTTCTTTTGCAGT
GCATGCTGGCAGCTGCGTGTGGCTGTTGTCTT
CTTCCGTGGCCATGAGGAGAAGGGAGGAGGGAGTGCGCAGCCCGAGTCTCTTTTCCTGCGGGTGTGAT
GCACCGCGTGTGACTGCGTTGAGCTGGGCTCC
GTGTCCCATTGAGGGCGGGATGGCGGGAAATTTGTTGCCGCGTCATGGATGGATGAGGGTGCACTGGC
GCGTGTGGCTGGTGTGGTGGGTGCTTGCTCCG
TGTGTGTGACGGCTGTTGGGAGTGCTGGAGATTTATCTGGTGATGCGGATGCGGTGGTTGTGCCGGTG
GATGTGTCGTGTGCTCCGAGTGACGGCAGCCT
GAGCTATCGCGTGTGTGTGTGGCGTGGACGAAGTGTTCTGCTGCCAGTGCTGAGGCTCAGTATGAGAA
TTATACTTTCAGTGGCTTTGATGTGCGCATGC
ATGGCAGGGATGGTGAGCTGGGTGCTGTGTGCCATGTGGCCCATGCGGTGCACACATGTAGCAACTGT
GCTGCCAGCTGTGCAAGGAAAGAGGAAGGGGC
CACCGTTGCCTTCACGATGAATGTGACGACACATAAATACGCCGGCCCTTATGAATTGTGGTGGCGTC
AATTTCCCCCGATGGAACCATCCATCCGCCTG
CACGGAGCAGCGATGCTGACCGGACGGGCATCTGTCAGTTGCTTCCACCCCGTGAAGGGAAGGATGAC
ACGAAATCTGGTGGGACAACTGCACCGCAAGT
TAATGCTGAGCCGTCACGTCAACACGAGGATTGGCGACGGTCTGCGGCGGCTGACGGACCCAACACCG
TCGATCCGAGTCCCGCATCCAACACGGCGACC
AACACGTCCGAGACGAGGCCTGAGTTTGCCAGCAATCCGAACGAAATGATGGTCCCGAATGCATCCAA
GAAGAGGCCTTCCTTTGCCAACGGTCCGAGAA
TATTAGTGGCGTCGAATGCATCCGGGATTATGTCTGTCACTTGTGGCGGCAGGGAACACGTTGCTGCG
GATAACACGGACGAGGTGAATGAGCGCAGTGC
CACTGGACACAAGACTCTCGTTTTAGAAAATACCGTTGCAGGCAGTGGATTTGTTGCTGAGGGTGATG
CGGACGGATTGACTCAACGCAATGCCTCTGGA
CACGATAGCAAACCCCAATTACGAGAATTCACCGCGCAGTAATCCGGAAACGGTGTGAGTCCCGACGA
CTGCTTTGCCCTGCACAACGACGCGAAATTGA
CACTGCGAGAGTTAACTGCTGACGGCACTGTGCGTGCGTGTGTGTGTTGGGAGTGGCTGCTTCTTTTG
GGATTGTGTGGATTTGCGACTCAGCACGGAGG
ACATGTGCCGCGTTTCCTGCGTGTTTTCTTTCATTATTTACATTTTACTGTTGGTTTTCGTTGTTCTC
CTGGAATAATGAGCGTCCTTTATTATTTCATG
GTCGGTTGCTGCACACCGTCATGAGTGACATGTTTCCCCCTCTTTTTGTTTGTTGCCCACACGCGGTG
CCGGCGCTGCGGCTGTGCGTCCCACGGACTGC
GCGAAACGATGAGTGGAATAACACGCCGGATGCCGCATGAAATGTGAGGGATGTTGTGTGGGCTCATG
GAACGATACGTTTTTTAAAGTTTTATTTCCTC
TTGTGGTGCGGGCAATATATGTATGCACAACCACACGATCCTCTTCTGTGCTGCATGGCACATGAAAT
AAAAGTATTGTCCGATTGATTTTTTTTGTTTT
AATTTTNNNNNNNNNNNNNNNNNNNNNNNNNNNNNNNNNNNNNNNNNNNNNNNNNNNNNNNNNNNNNN
NNNNNNNNNNNNNNNNNNNNNNNNNNNNNNNN
NNNNNNNNNNNNNNNNNNNNNNNNNNNNNNNNNNNNNNNNNNNNNNNNNNNNNNNNNNNNNNNNNNNN
NNNNNNNNNNNNNNNNNNNNNNNNNNNNNNNN
NNNNNNNNNNNNNNNNNNNNNNNNNNNNNNNNNNNNNNNNNNNNNNNNNNNNNNNNNNNNNNNNNNNN
NNNNNNNNNNNNNNNNNNNNNNNNNNNNNNNN
NNNNNNCGGGAGTTTTGCCGGACTCTCAACTCTTTTGTCTTCCGTTTTTTGCTGCTGTGTTTTTTCTT
GCTTGTTTAACTTCTGGTCTGAGGGACGGTGT
GCGAGTCCTGCAGTTGTAATGATTCCGTCTGTCTTTTTTGGTGCGCGCTTCTTCTGGCTGCTGCCATT
TATTACCATGCGGTGGCTGTCCGTGGTGTAGC
TGCTGTCTCTCTACTTCCTTTCTCATGCTCCTGCGGATCGAATCCAAGTTATTCAACAGCGATAGTTT

CGATGATGCGCCGTGTGTTCTGTGTCGTGTTG
GCGCTTACACTGCTGTGCTGCTGCTTGTGCGTGTTTGCCGCGGAGGAGGAAGTGGAGGAGGAGGTGCT
GCCGGTGGATGCGGTGTGTGCGGGTGACGGTG
AAAACGTTGGGCGCTGGCAACTCCGTGGGAGGCCACTGTGGTACAACTGCACGGCTGAAGCTAGCGGT
TTGGGAAAGATGATTTGCGGCATGGGCGCTGG
AAACTGTGCCCCGGAGGATGTTAAAAAACGTGTTGAAGGGAATGCGAAGGATGGCGTCTTCGAGATCA
AATTCCATACGCCCACTCCAGGCCGGGTGAAG
AGTTGGTGGGAGAGTAACGTGGAGCCGAAGCCGGCCACCGCTGCAAGTGGTCCGGCCAAACCTGCGGA
AGAAAGACCTCCGGTGGATCGAGAGGAGGAGG
CTCCTCGTGACACCACCACAATAAGTTCAGGATCTTCTCCCGCTGGCGTTGGGGGTTCCGCTGGTGCC
CTTTCTTTGCCACCTACAACTCCGCGTGACGC
TGGTGCTAACGCTGCTGCTGGCGCTGCGGGCAAGACGGATCCTTCTCCTCCTGCGACTTTGCAAGCGC
AGAATCCCGCTCCCTCTGGTGAACTTCAAGGC
GCTGCACATTCTGCTTCTGCTGCGTCAGAATCCCCACCAGATCCGGAGCCCACAGAGAGCGGGGAACA
CTCCTCCCATGACCCTCCAGCGGGACGACAGG
AAGAGACTGTTGATGCGCCACCGACTCGGTTGTCCCAGACCGGCGAGGACGGTGGCGCAGCGGAAGGC
GCCGAAGAGGACACCGCCAACACCACCGACGC
CGCCACCAGCGAAGGCAATGCTACGGCGGCAGGCATGCCTGGTCCCCTTTCCTCTGCGCCCACAACGA
GGGCGTTGGAGAACAGTGCGGGAAATGACGCC
TGCTTCCACGACCCCCGTCTGCATACCCGGCTGCTGCTTGTTTTGGCTGCGCTCACGTACGGGACACT
GGGCTGAAGAATTGAATGCGTGCTATAATTGC
CAGCACTCAATCACAGGAATCCGTGCGCTTCTTTTTGTTTTTACGTAATAAATAATAGAAAATAATGA
ACATTTGTTTTGCTTCATTTGCTTCAGCTGTG
CTGCTTTGTGCTTCATTTTGCTGCTCCCTTTACCCGTCACGTTGAAGGAATCCCAGACACACTCATAT
GCACGACACGTCCAGTACTGTTTGCGTGGACA
TATTTGTGTGTGTGGGTGCATGCACACTGGATCCATTTGTTGCGGCTTCTTTTTTTTTCGTGTTTTTA
TATTTTTAATTTCCTTCTTCTTGCTTGCTGTT
CTTTTTGCTTTTAGCTTGCCGCGGGACGTTGTGTGTGTTCTTTAATGATTTAAATTTCTTAATTTTTT
CTTTTGTTTTTTTCTTTCATCCCTCTTTTTTG
CTTTTGGGGGCAATCACGATGGAAGTTTTTTTTTCTACTCTTCCCTCTTTAATTGTTTGTTGCTGCG
CTCTTCCCGTGAGGGGTGCCGTGTGTTTGCCA
CCCAACTGCTCCACTCGCACACCCACCGACACGCTCATGACGACGGCCCTGTGCGCCTCGCCTGCTGC
ACGCATGAGGAGGGGCCGCGACGAACGGCGCG
CTGAGGGAGCCGTGACGTGTGGGCGTGCGACGAGGGCCTGTGGGGCGGCGCACACATTTCTTCCCTCT
TGTTTTGTTTTCTTGCGGCCAGGGCTGGCAGA
CACCCGCAGCACCACACACACAGCCACACCCTGCCGCCGTGTTTTTCTTTTTCTTTTTTGTTTCTTTT
TTTTTATTAGATGGACGTGTGCCGCCACCGTG
CGTGGTTGTTGATGTTGGGGCCGTGCCGCACGTATCGCTGCCATCCAGCTGTGCCTCTCGCTGTGCTG
ATGTGCCTCCCTGTGCTCCTCCTCTTCTCTGC
TCCTCCAGCACACACAGAGATAAGTGATTGCACAGTGCTCTGCAGGAATGCTCTTGGCGACGGTGGGA
CGTTGGCTTGCATCCCGTGCCGAGATGGCGAG
GCCACTGCGTGTGTGCCTGCGGTGGGAATCACGGTGCCATTGGATGGATGTGTGTGGGACACCTTTTG
CATTGTGCTGGAGGCCGGCTGACGCGGCTGCA
CGATGCCCGCCATGCGTGAAGGTGTTGCCTGCTCTTGGGGTCATTCTTCTCTCCTCGTTGTTTTTGCT
TTGCCGTGTGCGTTGCTCGGTGTGTGCTGACG
CGTGACGGACGGTGCCACGATGGCCTCCTCTGGCGATGACAGTCGCTGTGCTCCTGGCACACATCCAC
ACACAATTCCCTGTGTCTCTCTGCGGGAGCTG
GATGAGTTGAAGATCGATTACAGCCACACGGACACCCGCACAGTTGCTGCGCCGTACTTCTTCCAGCA
CTGCGATGCCGCCGGCTGCACGACGTTGTGGT
ACAACAAGTACAACAAGATGCAGTGCATGACGGCCAACCTGATCCGTGTGTGGTTGCAGCGCATGGAG
CACGTGGGCCAGTACGCGTTGGGCGCTGCACT
CATGTTTGTGGAGCAGAGGCGGCACGACATTGTGGCGCTGTGTGTGTTGGGCGGCCGGGCATGCCGGC
GATTTTGAGGGCCGTGGTGGACACGGAGCTGT
TTGACTGAGAGGAGGTGGAATTTGCAGCGCAGCGTGAGTGCATGACGGACTGCCTGTGCTGGGAGGGC

CCGACGATCCCGAGGCCTGTGCTGGAGGNNNN
NNNNNNNNNNNNNNNNNNNNNNNNNNNNNNNNNNNNNNNNNNNNNNNNNNNNNNNNNNNNNNNN
NNNNNNNNNNNNNNNNNNNNNNNNNNNNNNNN
NNNNNNNNNNNNNNNNNNNNNNNNNNNNNNNNNNNNNNNNNNNNNNNNNNNNNNNNNNNNNNNN
NNNNNNNNNNNNNNNNNNNNNNNNNNNNNNNN
NNNNNNNNNNNNNNNNNNNNNNNNNNNNNNNNNNNNNNNNNNNNNNNNNNNNNNNNNNNNNNNN
NNNNNNNNNNNNNNNNNNNNNNNNNNNNTCTG
AGGAAAGAGAGTAACAGTAATGGTGACTGTGGAGTGTGAGAAAAGGTGCACGGCGAGGAGCGCTCCTC
TTTTGCTTTGTTTTGATTGTTGTTGAGCGGCG
CCCACCTCATTTTTGTTGTGGTGCGTGACTCTCGTTGCTGGATTGGGTGGTGCGTGGTTGTGTGATTT
GTCTCTCCTTTTGCTTTGTGTTGGCGGAGTGT
TAGTTTGTGCTGAAGGCTGCACGCAAGTGACAGGAGTGATGGCGATGATGACTGGCCGTGTGCTGCTG
GTGTGTGCCCTCTGCGTGCTGTGGTGCGGCGT
TTCGGTGG
>scaffold1128.size6731
GGTCACAGAAATCAAAAGGGGACAGTGAGTGCGTGGACTGCTCACCACAAAAAAAAAACAACAACAAA
AAAAAGACAACGGCCACCCGTAAAGAAACAAAT
TTTTGTTAGCCGAACACCATAACCACATGAAAGGGAAAAAAAGCATGCAAGACTAAACCGCTCCATTT
CACAAGTGCTCAGTGTGATCACTCATGCGGGT
ATTCATCCCACACGGATGTTAAAGGGCAAAATACGTAATTACAACACACACACACACACACACAAAGA
AAGAAAGAAACGACAACTGAGGTTCAATGGAA
AGAAGAGAGTGACGATGCTGATAAAAATCACAACTTATGGCTCCCAACTATTCAAAAAGTTTTTTACC
TTTATTTTCCTTCCCTACAGGGAGTTGTAATC
ATCATCTTCATCCGTCAACACATCCGAGGGCAGTACACCACAGGCCAACAAAACCTCTTTAGCCATGC
GGACAAACGGAGGAGGGCCGCAAACCATAGCG
AGAACAGAAGATGACGGACCCAAGAAATCCCCCACAAAACTCTGAATGTCCTTCTCAGCGGGTCGCCG
ACCGCATGTAAAGAGCGACCACAATGACTCAT
CTCCGGCTGCTTTCGCTGAGAGCTCCTGCTGCTTCGTGAGGTAGCAATGGAACTGGTATTCGAGGTGC
TCTGTTTTGGAATGGTGCCACCGTACAAAATC
CTCTAATTTTGCAACGCAGGAGACCCGTCTTTCAACATGGAGGTGAATGATACGAAGTTTTGGTATCC
CCTGCATACGTTTGCCCTCAAATTCTTGACGA
AGCGAGGAGTAAGGCGCCGTCATAGCGATCCCAGCAGAAAGCATCACAACACGTCCCCCCACCTCACG
CAGACGGCTCATGTGATGCGTCAAGGTGAAGC
TCCCACCATAGTGAAGTAATGAAAATCGCTGACGTAACGCGTTGTTTGACATCCACGCCGTTCCACCG
TTGGAGTGTTCTGCAGTCTTGATGATGAGAGT
AAAATCACATCCCTCCTTATTCAGTTCAACCATGGTCCACCGTCGCACAAAATGCCTCCCGGGAGGGG
CCGCAATGGGCAGCTTCAAGTCAACAAACTGC
CCTGGTACCAATGAGGAGGTGTCAAGCTCTCCCTCAGATAGCCGAAAGTAGTACTCTTGTGTATCGTC
ACACACAGGCTCGACTGCTACACACGTCAGAA
TCGGGTGATTCAAATAACTGAATCGTTGCAGTCTATTCCCCGTTACCATCCCAGCAAACACATGCCCG
CTACGAACGCCACTATGAATCCCTGCACCAAG
ACCATTCATACTACGGTGGGTTCTTCTACTATCCTCCAAAGGAGAAATCATCGCTCCCTGCATGTTAG
CAATGACCATTTCCGCGTAATTATCACTTGCA
AGACGCGGCCCGGAATTATTTAACGTCACAAACACCTCACCGTCCTCTACGGTGACGATGTGGGTGCG
TTGTTTCATTCCCTTGGAGCATACCTTCTGTG
GTGCCTTCTTGTCCGGAACTCCCTCCGGCGTCATCTGCAGGGCCCAGTAAAGACCCTCACCGGTGTCA
AGAGTGATGCGGTACTCATGCCACGGGCACAC
AACGCACGGATGACTCCCCATTTCCTCAATATCGCCTTCTAGCAGCGGCCCGCCATGATGATAGCAGG
CGTTGTCTAGCGCGTAAAACCGATCCTTGTAG
CGATACACGGCGATATTCCTCTTATCGCACTTAACCAGCTGCCGACTTTTTTCCTTAAACGATGCCGC
CGGCCCAACGTAAAATCGCCCTGTTGTTGCCA
TTGCCACGTTTCCTTCAGTGTAATAATAATAATAATAATAATAATAATAACAAACGCACAAGAAA
CATATCCCATAACTTTTACCATCAGTGCAACG
CGAAACAAACAAACAAAACAAAAAATAACAAAAAGGACAACAAGGAAAGGAAAAAAAAAAATATGGAT

ATGAACACAGATCTGTTTACTTCTTTGCGTCT
CCAATTGTTTTTTAAACGCTTATTTTTTATTTCTTCTTTTTAGCACCAAAAGGGAAATACATAACTAG
CACCACCTCTGACCATTTTGTTACATCTCCTT
TTAATTTTCGTTCTCCTTATGTCTTCAAGCTCCCTTGAGAGAAAACTTCTGCTTCTCGGCATTTTCAA
CAACCCGTTCGCGGTGGAGTCCCTTGCGGCGG
CGAACACGCTCGAGTAACTGCCCCGCAATGTTTTCATCACCACCTCGTTGCCATATTCCTCAATTTC
TTCTTTTGTTGTCGGTGTAAAATACGGGTCCG
CATCCACAATGCTCCACAGACTCATTTGGAGTTGTGCCGTCGATGCCCCTTGCGTTATGACACGTAGC
TCATCCTGCAGCCCAAACGCCTCCACAGCAGG
CAGCAGGCAGCGGATGTAGAAGAGATCACTGCCTTCATTAGGCACTTCTTCAATAATCTCCGCGCGAC
GACGGCTGAGAGACCCATAAATCTTCCCCTGT
GTCGCACCAAACGAGTATACAGTGCACTCATATACGGGCTCCACGAGTCGCCGTGGGTGAAGCTCCAT
CGCCGCACGGCAGGCGTCCCGGACGCATGGCA
ACACCATTCCACCTGTCAGGCCGGAGGAGGTGTCAACTGAAATATCCGTAATGATAAACGCAATGTTC
AACAAAGGCTCCTGTGCCATGGGACCACTCTC
AGCTGCTGCCTGAAATCCCGCTATGATGGAGTCCTTCCAGTCCTCAAATAGTCGCAACTTTTCCAAAG
ATGTTTCGTCGTCACCAAACTGCAGCAACAAG
AGTGCTCCGAGAAACCTAAGCTTTGTTGGACCAGATGCAACAATCCCCTTTTGCATTTCCGCTGCCCA
TCGCTTGTTTGCCATTAGCGCAGTTTCCACCT
GTCGCTGCACCTGCATGCTGGAGCCATGGTTCATGCAGTCATCTTTAATTATATCGAACACATCTGGA
GGCATACAACGGGCGTGTAGAGTGATGGAGAA
GGCACCATCAGCAGTCGTGCCCGTATGCAACTTGGGCTTCATACTCGCACCTCCAGCAGCAAGAGTCT
CTCGAAAAGACACAATCGGTTCAGACGCCAAA
ACCTCCACCCGAGCAAAAGTATCCACAAGATCTTTTAAACATCGCTCCAAGTGTACTTCCCCAGCTGT
GCCAATCACATGTTCACCCGTGGGGAGAATGC
TCACCTCCACCTGCGGGTCAACCCGGAACAACAACTGTAACCCTTTCACCAATTGGAGGTGGTCTTTC
GGATCCTTTGGATAGACAGAGGCACGCAAGAT
GGATGTGCTCTGCAAGACGAGGGGTTTAAACGGCGGCACCCCCTCCATCGTTGACAATGTGGCGTACT
TTGTGATGTATGACGTGAGTCCACCAACGCCG
CAAAGTGTCCCGCTACAGGCTTCGGTCGCCTCATCCAAACCTGTGCCGCGGAAAAGAAAAACTTTCCC
CACATTTACCTCAACAACCTTTTCATCGCTGT
GAACATGCACCCTTTGACCTGGAATAAGGCGACCACTATAGACTCGGGCAAATCCAATAAATGCATCG
TCCCTATCTTTCTCTTGTCCAATCGTTTTACC
CACAAGAAACTGCGTATCGATAAGCCGACAGACATAAAGAATGCATGGGGCATCAGGACTGTTTGAGC
AATTGATCAACGCATCCTTTACTTGACTAGAG
GCCTCATCAAATCGCGGAATAAGGAGGGGCAGTCGACGGCGTTGCGCTACAACAGGAGAATCCAACTT
GTCGCAAATGACGCTTAGAACACAATTTGCAA
GCGGAAGCCATGAAGACAGCAACGCCTTTAATTTTCCTCGCTGGTCGCGTCGAGGGTTATTCCACAAG
CGCTCTGGAACACCCAGTTTAGCGGCCATACG
CAACTGCACCTCCACATTATCTTTCTCTCCAAGAAACGTTCGGTATAATTGCCAAATGGGTTCCAGCA
TTAACTGCACTGCTAGCGGTTGCTGTCCAGCT
CGCCGTGGAGTTATATCGATGGCCTTTGTTTTGGCATCAAGATAATACTCTCCCCAAAGAGATGCATG
CAAATTGGCAATATCCAATTTGCTCTCGTAGA
GAGAAGCAAAAAAATTGGTCGTGAAGGCCCATCCATCGTAGCAACTACAAAAGAGTACGTTGCCTTTC
GTCGGGCAGAACCATACATCATCACTAGGATC
ACCACGATCTTGATGCATGAAAGATTCCTCACCCTCCTGAATCTTGAGTTGATTGGCAAAAGCAGCAA
GTGCAGCGTTGCATGATTCCACAATGGAACGC
ATTCGGTAATATGCCTCCTCTGGGGTAATTTGCTGCACCATAACCAAAAGGTCTACCTTGTTAAGTAC
AAGACACATAGATAGACCCTCACGATACGCGT
GACGCAGCATGCTGCTTGTCTGCTGCGTTACGCCGTCTACCACATCGACAATGATGACTGCACCATCA
CACAACCGCATTGCTGTAGACACTTCACATGA
GAAATCAATGTGTCCAGGCGAATCCACAAGATTTAAAAGGTGCTCTACCCCTTTGTGGACGTGCCGCA
GCGCAATGGAACTAGCCTTCATTGTGATACAT
CGCTCCTGTTCGTCTGGGCGGGAGTCCAGCAGCCGAACCTCGCCCGCAAGTTGCGGAGAGAGAATGCC

GTTGCTCGCAACGAGGTAGTCGCTAAGTGTGG
TCTTGCCGTGATCCACATGAGCCACCATGCAGAAATTACGGGTTCGCTCCGGCTTCTCGCTAAGACGT
TGCAGGTAGGCGGTGGTCTCGAGACTCATGAA
ATTGTGAACCGGTGCCTGCACAAAAATATGAATAATAGTTTCTAAACTAAAAGAGAAAGCCTTTCACT
CCTCTCCCACCTGACGTGTATGTAATTATTTA
TGTATGTATGTGTATAATGAGAGGCGTGTTTCCTTCTCTTTCTTCTTCTGTGTTTACACGACCGCACT
TTATGTACCCTTACTCTTCCTTTTCGTCCTTC
TTCTGCAGACTCCCGTTTTTGCTTCCCCCCTCCTCCTTCCCTCACGCACACGCACGGCCTTTCTCTCT
CTCTTTTTCTTATTGTGCCTGTTTGACGCCAC
AGCTCCCGTCCCCCCTCCAAACGGCATGATGCCAGTAAAAATGCACACACACACACCAAAAAAAAAAA
AAAAAAAAAAGNNNNNNNNNNNNNNNNNNNNN
NNNNNNNNNNNNNNNNNNNNNNNNNNNNNNNNNNNNNNNNNNNNNNNNNNNNNNNNNNNNNNNNNNNN
NNNNNNNNNNNNNNNNNNNNNNNNNNNNNNNN
NNNNNNNNNNNNNNNNNNNNNNNNNNNNNNNNNNNNNNNNNNNNNNNNNNNNNNNNNNNNNNNNNNNN
NNNNNNNNNNNNNNNNNNNNNNNNNNNNNNNN
NNNNNNNNNNNNNNNNNNNNNNNNNNNNNNNNNNNNNNNNNNNNNNNNNNNNNNNNNNNNNNNNNNNN
NNNNNNNNNNNAAAAAAAAGGAAAAAAAAAAAGG
AAAAAAGGTACATAAATGAATTAATCGTCTTGTCGACAGGTTCAATTTGCCAGGCCATTCAAACGAAG
CAAAACCAAAAGACGCTTGACATCTCATTCAT
TCATTCATTCATTAGCTCATCTCACACCCTTCACTCTCACTGCTTTCGTTTCATCCTCTTCTTCTTCC
GCACAGACGGTTTATGCCGCTCGGTGCCAAAC
GAACCCACACTTCACCGTGGTGTCAAATAAGTTCGGTAGTCTGCATGCAACTTCTTTTTTTCCTTTTT
TTTTTTTTTTCGTCTTCTCCTTTTCCTTGTAA
ATTAAATACATATCTGAACCAGTTTTGTAAAAATATGTGGGTTTCGTGCACCAACTCCCTACTACTAC
GTGCTAATACGACTCCAAGAATACGGCGCCTA
AAACAACTCTGGTCTAGACTTTGGCAACATGATCCAGTGGCCATCAGGTCCATGCAGGCCACTGAACG
ACTTCTCTTTAGGCATCAGGAAGTACAGGTAG
ATAAAGGCAATCAGATAACCCCACACCTGAAAGTACTTCTTGCGCGATTGAATCTCGATCAATTCGGT
CGTGGAAATATCCCATGGCCTTTTTGTGTGCC
GCTGGTAGTACTTCGCCTCAGGGGCCGGAAGCTTTTCGATATTCACGTCTATCGGTTGAAAGTACGTC
CATGACATGTAGGTATCATAGATAGCGGTGTT
GGGCTCCTGGAACGGGGATGAGGAGCGGACACCACGCTCGTTCATGGGGAAGGCGTTCTGGATGTCAC
ATGTGTTCATGCACTCACCGCCTGTTGAGAAA
GTGGGGCCGTTGCCTACCAACGCCTTGCGTGTGGCGGCCACAGCAAACATCGGCGTCTTCCGCATCAT
TTTTGCTTAAAACCTGGCACTTAATTTTTCTT
TTTTTCTAAGTGTAAAAAAAGAAAAAATAAAAAACAACAACAACATTGAATAATCTTCTGGGCCACTG
GTCGGTCCCTCAGTTCCTCAATCATAGAAAAA
CAAACCAAATAGAAATAAGAAAGGGATGTATGGAATAAAAATGGAAATTCACCATATCCGCAAAGTGT
AACACCTGAGAAGTCCATATTTATAAAAATCT
GTCATCCACTAGAcGCAATCGTGGGCCGTTCACTGTATTCGGATGGGATACATATGATTGTATTTATT
TATTACCTACGAAGTGGCAGGCTTTGGGGTCA
CCCGAACACAAGATCCCGCCGCTCCGCTTC
>scaffold1129.size7652
GCCCTCTCGGTACTTCCGACCTGCCACAAGAATTTAAAAAAAAAAAAAAAGAGAGAGAAAAGAGTACA
ACTAAATATCATGTGCAGACAAACTATGGAAAG
AATTCTTTTTTTTTTTCCCCTCCCTTCTTCCTTCTCTCACTTAATATATATATACTCCCGCAGATACT
CTTGACGCAAGTCATTTCACTCGCCATGCCAC
TCCGCTGTCTTTTCTAGGCGTTCCTGAATCCCTCGTGCCATGCGTTTCGTTTTTGCCATGTTTTCATC
CAAGACCTTCATGTTATCTCGCATAACGTGAA
GTTTCTGTGTGTATGCTTGCAACTTCTCAAAGATTTCCCCCGCCTGCCGCATCTGCTCCTGCGTCTCG
TTGAGTTCCGCCTTCACCTCCTCGATGGTTTG
GAGTGTCGCATTCTGCTGGGTTTGCATGCCACGAAGCAGCTCAAAGATACTGCCAAAAACCCCATCGC
GAAGAGCCACAAGCAGCTGTTCGGGGACGAGA
CTCGTTTCTTGCGGAGGTGTCCATGCACCGTGCGACTCTTCGTTCTCCGCCAATGCCGTGGCTTCACT

TACGTGGGATCCTTCCACCCGCTGCTTCTCCA
TAGCGTCGTCCGGCTGCTCATCCATCACATCGGACCCCTGAGCAACAAAAAAAAAAAAAAAANNNNNN
NNNNNNNNNNNNNNNNNNNNNNNNNNNNNNNN
NNNNNNNNNNNNNNNNNNNNNNNNNNNNNNNNNNNNNNNNNNNNNNNNNNNNNNNNNNNNNNNNNNNN
NNNNNNNNNNNNNNNNNNNNNNNNNNNNNNNN
NNNNNNNNNNNNNNNNNNNNNNNNNNNNNNNNNNNNNNNNNNNNNNNNNNNNNNNNNNNNNNNNNNNN
NNNNNNNNNNNNNNNNNNNNNNNNNNNNNNNN
NNNNNNNNNNNNNNNNNNNNNNNNNNNNNNNNNNNNNNNNNNNNNNNNNNNNNNNNNNNNNNNAAAAAA
AAAAAAAAAAAAGAGAGAGAGAGAGAGAGAGGA
AAAAGAGGGAATTCAAACAATAAAAACAAAGGGGATATATGTAGCAATGACATGATACGTGCATTGTC
CATGAAACTCAATTGGAAACAACGGTAGGGTC
ATGATGGATGTGGTGCGGGTGCGGCGTCGGGGTTGCTGTTTTCCGTACGCTCCGCCCAAACGGCAGTT
GGGTTAACGACGTACTTCACCCCCTTGCGTCT
CTCCGTTACAACCCGACAAACGGCCTTCCCACAGACGGTACAACAGCCCTCCAGGACAGTCGGGTCGC
TTCGCTTATTCTCATCTCTTAGGCACTCCTCA
CGCCATGTACGATGCTGCAGCCTCACACAACGTCCACTACGCGTCTGCCGCAGCATGTCCCCCTCGAC
ACGCAACCCGGAGGACGCAAGAGTCACAGCTG
GCGAAGCCCGGCCGCCATCCTGCAGTACACACTTCTGAAGGAGACTTCGTAGCTGAAGCACACTAGTA
CACACTGGGGCTGTAAACGACGGCCACCCGCC
ACTGTGCCGACCCAAAGaACTAGACGCCGCATACTCTGAACTACAGAAAAGAGGCGATTTGCAATTAC
CACAGACGTACAGCAGAAACAGAGGTTCACCG
CTATTCGCCCGCTGTTTCAAAGCCTCCGACTCACCTGGAACCGGTGTAAATGGAGGATCGAGTAGAAG
TGGCGGAGAAGGAGAGAAGGAGGCTGAAGACG
GTCCGTTAACTGCGGGCTCCTTTGAAAGGGAATGCGATGCGGTGCATGCCGTTTCCTGTGAAGGAAAG
ACGTGATGTGCAACATTCTCCCTGTTACGCGA
AAACCAGAGAAGAGACTTTGACTGACGGCCCGAGTAGACATCGCGACGGCTTGTCCCACTCGATAATT
TCGATGGCAGCTTCGCCTCACGCAAACGCAGC
CTCAAGAACAGGAGCATTCCCCGAGGAAAAATCATCGACAACTTACAAAACGTGTTCCCCTCCCAATA
GATATATTTAAATATGTTTGCAGATGTAATGT
ATATACACTTCTCCTCCTCAAAACAATGCCGTTACCGCCGTCCCATCAAAAAGCAAAAAAGAAAAGAA
AAGAAAGAAGGACGTTCATGTAATTCACATGT
TCATGTGAGTGTGTTCATGTGGGTAAGAGAAAAAAAGAGAAGAAAAAAAAGAAAAAGAGAGACGGAAA
AAGGTGTGAGTGTGGGTGATACAAAAACAAAA
ACAAAAAAGGTGAGAGAAAAGAAGGAGAGGGAAAAAGTAGGTAAGTAAAATAGTTTTAAAAAAATGCT
TCTTATTTATGTCGCCTTTTAGAATTCCTCTT
CGACGAGCCAGCGGCGGCATGCTCTGCACCCGCGCTGTTGCTCACCGGCTGGTGTTGTTGCTTGCCCT
CCACAGCGTCTTCCCCGCGGGGAAGTTTAGAG
CCGCTAAGCTCCCTTCGAATGACCTGCATAAAGTTGGATTTAAAGTAATTCACAGACTTTGTGGAGGT
GAGAATAGCCGTATGCTTCTGTCTACCACTTC
CTCGAATGCGAACAATGCACTTGGTGGGTAGCACATCCCTTGCCTGCTCCCCACCGCGTGCATCGCCA
CTGGTTTCGCGGAGACCCATCCCCGTCACTGC
GGATGCCGCCTCAAGTGATGCGAGGGGTTCAGCAATGGGGAAGTAGTAAAGCGACGGCAACTGGTCCG
CCTCATGAAGCACCGACTCACCATGACCCTCC
GCTTCTTCTTTTTCTTCTTCTTGTTTGCTTGCCCGCGGGGCAAAGCGCTTCAAAATAACATTTTCTCG
TGCAATCACTCCCACCCACTCATCCCGTGAGA
GTTTTGTTGATGTCGCCGCGTTATCCACGGAGGAGAACGTTGGTGCTGGGGTCGTCAAAGACATTGGG
GCGCCACTAGGAATGGTTTGATCCACAATTTG
TTTCAAAAAAGCCCCAACCGGCTCACGGCGAAGCATTCCAGCCGCAACCTCGTACTGTTCGCAAAGGT
ACTGAAGGCCCTCGTCTCCGTTGGGTCCACTG
CCATCGTCAAGCAAAAGGCCCGCCTCCTGCGCCTGCTGCCACGCCGTTTGTATGTACCGTTCTGCCTC
CTCCAGTGCCTGCACACGACGACGTTCTGAAA
GACGTCCACGAAACACTGTGTTATGCGCAATCTTATTCGTCTTTTCTGACGTCGGCACATGTGCGAAT
CCGCCCTTCAGCACCACCCACACGGAACCGCC
ACGGGTGCTGGTCTTCCGAGATCTTTTGGAGCCCGTCCTGGGCGGCCGTCCCTCCTTGAGGTCTTTCT

TCGCACCTGTCGTCTTTGTCCCTCTCGTTGTT
GTTGGGGTCGTGGTGGTAACGGTGGCCTTTCGTCTCCTCCTCTCCGCTCGGTGTGTGTTTCGCTGCTC
TCGCGCGTTTGCCCGCGCCATGTGAAATAATT
TGCCTAGTTCCGCCAGGAGCACCTTTTCATCCTCCAGAACCCTCATCCCGCCCTTTCCCATCTCCGCG
TTGCTCCCCTCTAATCGACCAATCCTTCCCAC
GTCGCGTTGTGTTATGTAATGTGCGGAAGGGGGGGAGGAAAATAACAAAAACAGAGAAGTGTAAAAAG
AAGGGGGAGGAGCGCAGCGCCATACACACACA
CACACACACACACGTGCAGGAAGAGAGACATTCAGAAAGAAAAGAAACCCCCTCCCCCTCCCCAGTCC
AACAACAACAACAACAACAGAAAATAATACCG
GGGACAAACAAATAAAGATACACACAAACATATATATATATATGCAAAAAGAGAGAGAGAGAGAGAAA
GGGGGTAAAAAAAAAAAAAAGAAAAAAAAAAA
AGGATACACACAGACAGAAGCACACATGCAATCAGACCTCAATATATATTTATTTATTTATATTTACT
GTTCGCAACTAATTTTCTTTTCATTTCTTTTC
CACCCTCCTACACTCATCATCCTTCTCTCCTCCTCCTCCTCCTCCTACTCCTTTCCTCCTCCAAT
AAGGCACACACGCATGCACAACAGCCGCACAG
CGAGCAAGCAGCAAAGGAAAAATACGCATGGCAAATAAAGAAACAGCAAAGCAGAAAAAAAAAAAAANN
NNNNNNNNNNNNNNNNNNNNNNNNNNNNNNNN
NNNNNNNNNNNNNNNNNNNNNNNNNNNNNNNNNNNNNNNNNNNNNNNNNNNNNNNNNNNNNNNNNNNN
NNNNNNNNNNNNNNNNNNNNNNNNNNNNNNNN
NNNNNNNNNNNNNNNNNNNNNNNNNNNNNNNNNNNNNNNNNNNNNNNNNNNNNNNNNNNNNNNNNNNN
NNNNNNNNNNNNNNNNNNNNNNNNNNNNNNNN
NNNNNNNNNNNNNNNNNNNNNNNNNNNNNNNNNNNNNNNNNNNNNNNNNNNNNNNNNNNNNNNNNNTT
CCCTCCGTAATAATAATAATAAAGATGTAAAG
ATAAATATAACAATAATAATAATAATAATAAAAAAAAAAAAACAAGTATAACTCACGGATCTATTCGCG
TGATGTAGCGAAAGGCAAAAGAAAAAAAAAAA
AAAAGGAAAAAAAAAAAAAAAAAGCAAACAATGATCAAAAAGCAGAAAAAAACACGGCTGGCACGGTAC
CCCCCAAAGGGGAAATAAAGATATATATGGGC
ATAAATAAACAAACAAATACATACATGCATCCGAGAAAGTACACAAGGAAAATAAAAGAGAGAAAAAA
CAGAGATAAGGAAAATATATATATTTTTTTTC
CCCCCTCTTCCATGCATATTGATGTACTTAATTACATATAACAACAACAACAACAATCATAATAATCA
TAATAATAANNNNNNNNNNNNNNNNNNNNNNN
NNNNNNNNNNNNNNNNNNNNNNNNNNNNNNNNNNNNNNNNNNNNNNNNNNNNNNNNNNNNNNNNNNNN
NNNNNNNNNNNNNNNNNNNNNNNNNNNNNNNN
NNNNNNNNNNNNNNNNNNNNNNNNNNNNNNNNNNNNNNNNNNNNNNNNNNNNNNNNNNNNNNNNNNNN
NNNNNNNNNNNNNNNNNNNNNNNNNNNNNNNN
NNNNNNNNNNNNNNNNNNNNNNNNNNNNNNNNNNNNNNNNNNNNNNNNNNNNNNNNNNNNNNNNNNNN
NNNNNNNNNCTTCTTCTTCTTTTTTTTTTTTT
GTCGTCTTCTTCTTCTTTCTTCGTTCTTTAATTTCGGTTTTTGTTTTTGTTTTTTGTTTTTTTTTTTA
TTTTATTTTATTTTCCCTCTTCCATGAAGCGT
CAAACACATTTAAATATAAATATATTCATATATTTAAGACTCGCAAGGATCGCAATGGTTGCAGCGGC
AGTGGCAGCAGCACCAAAACCGGGTAAAGTGA
GCATCCCTCTGGATACGCGTACATGTAAATATATGCAGGCATTCTCTCTCTCTTTGGGCCAAAAGGCA
GGTTAAACAAAAAAGGAAAAAAAAAAAAAAAG
CATCATCATTCAATAATAATCATAGTAATGATTAAAACCAACATCGCCATAAAAGAAAAGAAAAGAAA
AAAAAAAAAAAAGCTGTCAAAAATATGGAAAT
TAATTTTGGTGCACGTACTTGTGCTGAATATATTTATCCATCCCCTTCCCCCCTCTCTCCCCCCTGCC
CTTCGCTCTCCTTCTATATATAATAAATAATA
AATAATAAATAATATATCCCCTTTCATTCACGCAATTTTTTTTTTTCCTTCTTCTTCTTCTTCAAATT
TTATTCGGCTACTTATATATAATACATGTACA
TATATGTATGGGATGTGATGTAATGCTTCCTGCTTCGGTAGCCCTTGGAAAAAGTATAAAGCATGTAA
AACAGAAAAAAAAAAAAAAAAAAAAAATTTTC
TATTCTTTTCTTCATGGTATCTTTATATATATATACCCTTTTGTTGTTGTTATTCTTTTTGTTGTTGT
TATTATTATTATTATTATTATTATTATTATTA
TTTTCACTTTCAAGGCATGTATTTTTTTAAATAAGCCTCAAGACATGCATTTAATACGTGTGCCGTAT

TTGTAAATCAAACACGGCCTAAATCCGAAAC
TCTCAATATATATATATATATATTAAATAAATATATAAAATAAGAAAGCACAGTGGAAAAAGCATATA
TATATATATATATATATATATATTTCGTTTTTAT
TTGCCGCGTCATAACAGTGGCTTCGCAATTCCATTGAAAGGCCTCATTTTCATTGGTAGTTTGCACCG
TACCGAACACCGTCCCATGTCGATGAAACGGG
AGATGACATGTACATTGGCAACGACATCATAGACATGTTGCTTTGGTACATGAGATGCTCACTTGGTT
GGGGAGATGGTATTAAGTGTGACACGGTCGTT
GGTGGCATGGCATGTTGCGGTTGCTGGTGCAGTTGTTTTTGTTGTTTTTGTTGTTTTTGTTGTTTTTG
TTGTTGTGGTTGCTGTTGTGTCTGAGGTGGTA
CATGGAACATTGAGGGCTGCATAAACTCCATTGGATATGAGGAGACAACTCCATTGTTGTTATTTCCA
TTAGAAAATCGACTGAAATGCGCCATAGAGGT
AGAAGGCATCAAAAGAGGACGTTGTGTCACTATTGAATTATAAAGTGGACCTTGTGAAGACATAGAAA
GTCGCTGCACATATGGAACCAAAGAATGAATA
AAATTGCATGATTCTCCCAAATTACACATTTTATTAAACTGAAAATGAGCACAGTGACGCGCCTTGAC
GTTAGTCAAGTTTCCACTCTCGCTACCAGATG
TGTCAGCAGTCTCAGTAGTAAGCATATCCCTTGCCTTACCGGTTCCAGGGTAAGCAAATACACTGTAT
ACATTCGACGCACCCGCGGTGCGAAGAATCAT
CTCCGATGGAATTAGTTGCGGTACACTTTTCCCTTGATTTGGTGGAAATACACGAAAAATAACCCCAG
ATGGCATCGTTGGATAACCGTGTTTATTATTT
CCTCCACGCAAAACTTCCGGTGGCATTTCAAGTCCTTCTACCACCGCGGCACTAATGACATTCTCATT
TACATGTACCGACGTTGGGGGAGGTACCAAAT
GGGTATGGATATACTGACAATTCGCCCCTCGTGTACAACGCTTATTTAAGTACCGTTTACATAATTGA
AACCGAAATCTCGATTGTGCTCCTCGAGCCTC
ATTCATTTTTAAATATTCCACCGACCCATGCGTCGCCACGATTTGTGTGCTATCAATGTTGTAATGAT
TTTGAATTTCCGCATCGTACACAGAGAAAGTA
CAAGAAGGAGGGCACATTACTTCTTTAAACGGATCCATCATCATCATTTGTTCCAATAGATTCCAATC
AATCGGTTGTTGTGCATTCGTCGCCTGATTTA
ACTCCTGTGGGTGCGTCGGATTTCCCTGGGATGTTTTTCGTGAAGCCATCGTGGTCGGATAATCGCAT
GTCCCTTCAGGGTCAAAAAAGCCCTCACTCGC
AGATCTTGGCGATACCCCTGGAGAAGCCTTGGAAGCAACCTGCTGTCCCTCCCTCATTTCTTCCATGC
TCAAGTCGTTAAACCCTACTTGGTCCATCGAT
GCATTCACCTCATTTGGACGCAGTGTGGTGTTGTACTTCTCAACCTCACGCTCATCATCGTCAAAGCG
CAAGTCCCTAGAGTCGGTGTTGGCGTCGTAGC
CTTCCATGCTCCCCTTTTCCTGGGAGCTGGAGGGCCGCACCTGCACTACACCCCA
>scaffold113.size32590
CAATCAGCCCGCGCAGCAGCTCCACGCGCTCCTTCACCATGCTGCTCTCCCCACCGCCATTCAGCAGC
ACAGTGTCGTCCTTCGTGATCGTCGCCTTCTTC
ACTGTGCCGAGGATTGCGGGGTCAAAGTTCTCCGCGTCCAGCTCCAGACCACTGCCCTCCTCGCCGAC
AAGCCGCGCGCCCGCAAAGATGGCAATGTCCT
GCATCATGGCCGTCTTGTTGTCGCCAAAGCCGGGTGCCTTCACGCACGCAATCTTCAGCTTGCCCTGA
AGCTTGTTGAAGATCATCGTCGTCAGCGCCTC
GCTCTCCACGTCGTCGGCAATGATCAGAAGCGGGCGGCCGGTGCGCACCACGTGATTCAGCGCCGGCA
GGATCGTGTGGACGCTGCTCACCTTCTTCGCC
GACACCAGCACAAACGCATCCTCCAGCTCGGCCTTCTGTGCCTTCGCGTCGGTCACAAAGTATGGGCT
GATGTAGCCGCGGTCAaTGCTCATGCCCTCCA
CCACCTCCAGCTCCGTCGTCATCGTCTTGCCGTCCTGCGTGGTGATCACTCCGTCCTTGCCCACCTTC
TCCATCGCCTGACCAATCAGCCTGCCCAGCTC
CTCGTCCCCGTTGGCGGAGATCGTGGCCACCTGCACAATGTTCTCGGTGCTCGTCACCTTGCGGCTCT
GCTCCGCCACGCTCTGCAGGATGACGCCCACC
GCACGGTCCATGCCACGCTTCATGTCAATTGGGTTCGTCCCGGTGGCAATGCACCTGAGGCTTTCACT
GAACACGCTTGCCACCAGCACCGCCGAGGTCG
TCGTGCCGTCGCCAGCAAGGTCGTTGGTCTTGTTGCACACCTGGCGCACAAGCTGTGCTCCCATGTTC
TCGAACGGGTCCTtGAACTCAATCGCCTTGGC
AACGGTCACGCCGTCCTTCGTGATCTTTGGCGCGCCGTACGACTGCTCAATGATCACATTGCGCCCCT

TTGGCCCAAGGGTCGTTGCAACGGCGCTCACG
GCACGCTGCACACCCTTCTGCATGGACTGGCGAGCCTCCGTGCCAAACCGGATTTCCTTGCCGGCAAA
ACGAGCAGCAGAGCGAAACATATCGTGTGTTG
CACTTGTATGTTAGATTTTTGTTTTTTGTTTTTGTTTGATAGATCTTGTTTTCAAAGAATTTTATACA
GTAGCGCGTTGTTTATTATTAAAAGCGTCTTT
TGTCTTATAACCTTGTTTGGGAATATATTTATTACGCGTCAAAATGTTTTCTCTTTTACTGTATATAA
GTTATTCTTGTGACTTGTCGTTTTTTTGTTGT
TGTTTTTTTCGCTTTATTGGCTCTATTTATGGTGCCTTGCGTTTGGATTTCTTTCTTTCCGAAGCTCG
TAGTTCAGTGAAAAAAAAAGGAGTATGCACGT
AGTAGAGGACCCTGTCGCCTGTTCCCACAGAATGAGAAGTACATGAGTGGGAAATGCCGGTTTGCTTC
ACCGCACCCAACACCCTCCATAGCTCGGAGAA
TATTCATCTACCACTCACACGCCAGTAAAGACACTCACTCCAATGGCGTTCTATAATAACTGGGCTCA
TTGTGAAGTCATGGCGCGGCATTCTTTGGCGT
CCCACAACCACCCGGCTCCTCCGCGTGAAAAGCAGCAGGAAGCGGACAAGATGGAAAAAGAAACCGCA
GTCGCCGCATTCCAGCGCCCGGTATCATTGAA
AAAAAGCATTATATAATCGATGAGAAAACAAGGGAGTCCACAAAGAGGCGTCATCATCTGCTGAGCCC
CTTTTCTCGTGGAACATTCCTCCGGCAATATG
GCAAGAAGACACACACACACACACACACAGAACGGCAGCTGCAAAAAACTTTTAAAAAATGAAATGCA
AAAAAGCTAAGCAAAAAAAAAGGGATAATAAG
CGAGTGTTAATAACGCGCCGCCCAAGCCCACCAGCAGAGTTTGGGCAAATAATTACGTATTTTACTCT
TTGAGGGACCATCCTCTATGGATAAACGACAA
AAGATAAAGAAGGGCCAAAGATGTGAAAAGGACTAGAAGCAACTATTAGGTCCTTTTTTTCCATGCCT
CAATGATATTCTTTTATTTGTGTGTGTGTGCC
TAATCCCAGTCCTTCCCCATTTGCTTTATTGACACCGTTGTCTGCCCGGATCTTTTTACTTCCATGCC
ATTTTTTTTTTTTTATTATTTTCCTTGCAACA
TGTTTGTATTGGAAAATGACAACAACAACAACAATAATAATAATAATAATATGTTACGCATGAGGGCA
AAGCGGCTCATACTCCTTTTGCTACAAACAAA
CAAATTTGGCACCGCCATTTTTTCCTTTTCCCCCGCCGCACCACCACACAACGATACCGCTGACGAGC
CAGGGAGAAACACAGTGGGAACCTCACCACTC
CACGATGAGACAACGTTTCTTTGGTAGAGCACGTTGAAAATTCTCTTCTTTTCCTTTTTTTTTTTTTC
CACCGAGAAAACACCGGTATCATCGCCACGTA
CATCCACGACATCACATGGAACACAGGTGCTTCTCTCTTGCCTTTTCCTCTTTGACACCCACAGACAC
ACACCACCAGAGTGGCAACTATACACCGGGTG
CATTTTCTCAAGATGCCTCATGTGTTGGAGATTCGAAAGACAGAGTTACACAGAAACCAATATATAAT
AATTAAAACAGACAAATGAGCAAAAAAATCTT
GCTTGTTTTCAAGGTTCTTCAGCCAAAGTTGGAATAAATCGTTGCCCCTGTGCCAAATACAACACATA
TCACACCCACAAACAAGGATGTGTACGTCATG
ATGTATTGGAACCAACCGACCCTCTTCAAAGTCCATCCACCAGAATACATATAAATCAACGCTGGGTA
AATAAAGCCTATGAATCCGCCACAAAGCGCAC
CCGCGAGGTCAAATGCGGTGCTAATTCGTGGAACAAATAAACCAGCAACAAGAACAATAGTGGAGACA
AGAGTTACGCATGCGATGTGCTTCCAGTACGA
TATGTTATTAATATCCCATCCAATGATGTAGTACAGTGTATTTCGGCACGGAATCATGTTCAACGCAT
AAGACACACACAACTTGATGATTAGTCCCACA
TACGCCACCATCATCAGAGGCTCACCAACAGGGTCAAACATGTACAGCACGGACTCCTTAACTTCCCT
GCCAAATTCCATGTAGCCAAAGACACCAACCA
GCCAATACAAGACAGAGCAGACCGTCATACTGAGAGTTGCACTGAGGAGTAGTTGCGTTGTACTACGA
GCCTTCATTTCAAAATACACCTGAAATGCGAC
AGCATGACACAAGTACGCAAAGACGAAAGTACCAAGTCCCTCCATGCCAATATTTCCCGTCGAAACAA
TTTGAACCTCCGATCGCTTTGGAAGCCCATTT
TGGCAGGCATGAATCACGACGAGAATGACAAAATACACCACAAAACCAACTCCAACCGCAGAAAAGTA
TCGCAATGTGTTCACTTCTTTCGGTATCACCA
GCGGAAGCATCCCCAAAAGCCAAACTAGACATACCAGCAGTCGTATACCTTTTTTAAGCTTAAAAAAC
TCCGGCGTTGCTGTAGAGTGTTGTGCAATTGG
AGTAATTAAGCTCCCCACCGCAATGACGTATGCAACTGCCGCACCAAAGCAACTCGTACCCATGACAG

CGGCCATAAGATAATCGGCACCCTTCCCCAAT
GTGCCTCTTGACATTTGTTCAAAGGCCCGATAACCCGTTTTCTCCATCACATAGCCCAAAATTGTAAT
AGTGTACACCGTGAATACCGTCATTAAAATAA
GATAAAGAGTGCCCATCAAAATTCCACAGGCGTGAAAAGCCCACGGCAACGTTATGACACCAGCGCCT
AGTGAAGCCGAGGCAAGACTAAAACTGTTGGA
AAGAAGTCCACCATGCGGTATCACTGCGCCAAGCAGCAGCGCCAAGCAGCCGGCAATGCTTTTCCCAC
ACTCTTGCTGGGGTGATAAAGCGCCGTTAACA
GCAGATGCCCTCTCCTTTTTTCTCTCGATAGAAACAACATACGCGGAGTCCGAACTCTGCTCTCCCTC
GCGTGAGATTTGGGGTAAACCCTCCATTGCGT
CTGTCTGCTCTTCATTTTACCAAAGGCGGAGAAATAGAAAGAAGCGAATAACGCACACACAGGTTTCA
TTTATGTCGGTTTTTAGAAGAGAATCAAAAAA
AAAAAAAAAAGTAAAATAATAAAGAGAGATCAAGATGTCGATTCTGGTGGCGGTGGTAGGGTCCTCTC
TCAATTGCCGTTGTTTTTCTACTGGCATGTAT
ATTCATCGCTCGGCGCGACGCAATGAATTTATGACGGACATTTTTTCTCCTGTATGTACATAAATACG
AGGAAACAAAAGGAAAAGGGGGTAAAAAAGCA
AAAAAAAAAAAAAAGAGAGAAATCAACTTCGATGAGTTTCTCAGCTGCCATTCACTCCCGCTCACTTC
GGCGGGAACCCATTGGCTCGTATGTATAGCGG
CTCAGTCGCGCGATATACTCTTCATTTTGTATTCTATCTGATTCCCACTTTTCAACGGAGTACGCCGG
CTTCGTTTTCTGTAACCGCTTCAGAATCCTTA
AGTTTTCCTCTTCAATGCGTTGCTGCTCCCTCCTCCGGCGGCGCTCCGCATCACGCGCCGCATTGGCG
CTGCGCCAGGGCTCCTTGTTATCAATTCCACC
GCCTCTCTTCATAATAATTGTCATGTTGTGGACAATTCTTCTGTTTTCCTCTTCGATCCGTTTGCATC
GTTCCTGCTCCATCAGCGCCCTTTTGTTGCTC
AGACTCAACGCGTGAGGCGGTTTGTTATCAATGCAAGACGTTTGTTCCTCCACTCGCCGGCGATGCTG
CATGTATTCCCTCTGGCGCTGTTGCTCAAGCC
ACAACGTTGTGGCTGTCGGCTTCGGCTGCACGGGCATCAGTGGACTGCTGTTGCTTTGCCTCTTCTTG
ATCCTCGTTCCTTTTTCCCCGCCCCCAGTAAT
TGGTGTCTGCGTTCTCCTTCCTTGGCCTCTTCTATTGCTCACCGCGCAAACACCGCCTCTCACCCTCC
CCTTTTCCAACGGAAAGAAAATAACAAAACCA
AAAGGAAGAAAGAAAAAAAAAGAAAAAAGGTTGCGTTGTTGTTGTTGGTTGTTTTTATTTTTCTTCTT
TTTTGGAAGGAAAGGCGAATCCCCTCAAGTAG
AAAAAGTGGAGACAAGAAGGAATGACGGGGGGCGAACACACCGCGTCATTCAGCAGAAAAAAAAAAGG
CCCTCACTCACTCACTCACAGTCACACAAAAA
CGCACACACAGAGACACGCTCTCTCTCCTACGTCTCAACCCCCACCGCCTCCATAAGAAAAGGAAAAG
GAAAAGGCAACAATGGATGCATCAAAAGGGGA
ACAGGTCAATTTATTGGTGGCAAAGATAAAAATAAACACTGATTTGCCGTGGGCACAGACCCACCTGC
ACCGAAGTGTTAGTGATTTGAAACCAAAGACA
AGATAAATGAAATGCAACTCGATATAATTTCTTATGAAACATACATAAATACGTTTGATGTGCAGCTT
GTATACCAAGTAATATGGGGTAATAGGCATGT
AGCACAGAAATAGTAGTCCAACCAGAGGGATGAGAGCAGAAAGTGAGACCGCAGCGGTACAAACAAAA
GAAATTTATTTACATGCGACATTCATAGACAA
ACAAATAAATAAACAAAAAAACAACGTTTGCCTCTTGTCGCCATTACATGTACGGTTTGTTTTCGTGT
GTATATTTGATGCACCACACCTTATTAAAGGC
GAGCCCGATAGGATTCAATGAGATCTTGACGATCGCCTGGTAGCAAGAGCGTGTAACCCTGAAGTAAG
ATTGACAAAGGCACTAGAACAAATCCTGTCGG
CTCAGGTCTGTTTTTATCCACCGGCTGAAGCGATCTGTCCTTCACGACATAAAAATCCGGCTGGCATA
CCATCTGCATGAAGAGCCTAACCTGCTGCATT
AGCGTATCCGAGGCGGTGATTGAGTTTTGCGCCTCGGCGAGCTCCCTGTTGACTCCTTGAAGTTGTGC
CTGTGTGCGCATGAGGTCCAACTCCAATTGCT
GTTTAACTTTTAGAAAACTAAATTCATCCTTATCTTTTTCTTCCTGCAGTCTTTTACACAATTCCTCC
AACCCTACCTTTGCTCTCTGCGCTTCCTCAAG
TTGCTGTCGAAGAACTTTGTTTTCTGCCTGAAGGATACTTACTCTTTGTTGCACCTCTTCTTGACCGA
GCCTCGCTTCTTGTTCCTCCCTCAGTTTTATA
ACAGTCTTATCGTGCTCCTCCGCAGCCTGTTGAAGAAGACGGCGAAGCTCGGCGTTGGTCCCACGCAT

GAAGTCCGTATGAATTTGCATTAATTCTAGGA
GATCATCTATCGTCGATGTTCGCCGCTCCACTTCCTCCCCCAGCTTAAGTTCTGCCACTGCCGCCTTC
CGTCGCAATTTCCTTTCCTCCTTGTTTTTCAC
CCGCAACTTGTCCAGCGCGCGCTGTAGCTCTAAACTAAGCTGCTGAACCCTTTTCTGCTCAGTCGCAA
CGTCCTCCTCCTGGCTCCTTCGTATTCCCCTA
GAATCCGACAACCGCACCTCACCCGTGCGATTACCCTCCACGACAGTGCTAAAAGGGACAAAATTGGG
CGCACGGCGGGGCGACCCAAATGTTATCACAG
AGTGTTGCAAGGGCAATAGAGTAAGTCTTTTCATTTTATTGCATGCAACACTATCCCTCCTCGTGGAG
TTGAGCAAAGGTACGAAATTAAGATCGGTACG
GTTCATTGACTATGCCCGCAATGATACCCCCTCAGCCTCTTCCAAGGAAGAGGTACAGTAAAGAAAAA
AAAAAAGAGAGGGAGAAAGAACATGAAAATCA
TGCAAAGAAAAAAAACAAAAAACGCTCCACTCCACTCAGCATACAAGAAATACATGGCAAAAAAAGAA
AAATAATAATAATAATCGTATGGTGTTTTAAT
TTTCTTCATTTAAACGACTGAAAATAAGGGCATTCACCAGCAGAGCCCTCTCAAGATGATGCCGTCAC
GGCGTCAAGCAAATCGGACAAATTCTTGAGTT
CCGACCGTGAGAAACAAACGGATGCGGCAGGACCGTAATTTGTGACCAGGGGATAAATCTTTTGTAGA
AGGTATCGCAATTCGTGCGACACCTCCCCTGT
AACAGCGTCCTGTTCCACCACCTTGTTCTCCTGATCCTCTTCCGGTTCGTTTTCGCCCTCCTCCTCGC
TTACTTCCATCTCCTCGTTTTCTTCTCCCTCT
TCGTAGTCCAAAGAGACCGGCGTGGCAAGATTAAGCAGACCTTTTCCAACAGCAACAAAAAGCGCCAC
ATCCAGAGGCAAGGTACCCCTTTTGCAATCCT
CCTCAATCCATGCCAGCAATTGGCCACAGAGTCTTTCATCCAATGGGCATTGTATCAAGAGATCTAGC
AAAGCATCACATGAGTGAAAAAGAGCCCCTTT
CACGGTGAATACACTCATCTTTGGCAACCATACGGAAAACATTTCAATCAGAATCCCCGATGACCACA
AAACATTAATAAAAGCATGCATATGGAGTTTC
ATTAATACAGCCAGAGGAAATACAAGTTCCTGCGTCACCGTATCGGTGCGAACCCTCATAAGACGACG
TACAATGGTTGTAATAAGTATTTCAATTTCAT
CATCCTGAAAAACACTCGTATTCTGTGCAATGGCAACCAAAACTCCTCCCACATTCATCAACAAGGCT
TCTTTTATTTCCTCCCCCAACATTTTCATCGC
AATCGTGACAAGCACGGAAGAAAGGGGATGCGGTTTGAGTTCATCCCTCTGCAGGGTAGGGATTTCAA
CAAGATCACTACAGGATAACTTTTCCCCATTC
AATACCGAAAATGGCTTCTCCATTGAGGGAATGGCTACAAGAACCTTCATTGCTCCCACTTGTGGAGA
ACGCTGCAAGAGAGCAGCAAGACAATTCGACG
CAGACGATATGATAGAAGACTCTTCAGTCCCAAGCAACAGTCTACAGAGGGCGCCAGTAAAAAACTCG
GCCCCCTTCCTTGCCACCTCATCAGATCCCTT
CTCAAAAATGCATGTCAAAATTTGCACAACAAAAGGTACAACACAATACTCTGCCGTGCCACAACAAC
TATTCAGTACATCAGCTAGCCATGAAAAATGA
CCCGTGAGAAACTCATCCCCACTGTACTCTGTGATTAATTCACCAAAAAGAGATACAACCAACTCTCC
CATATTTGGATCCCGAATATGATTCCTCCAGC
AAAGAAACAGGTTATTGTAAGATTCCGAAGAAGCAACATCTCTTCTCTTACTGTGACATCGAGCAATC
CACTCTGTGTGGACTTCAAGACACACGTACAG
CAATTCACCCGAAGTAGACTCGTTCAGCAGAAGCGAAAACAGGGCCTCTTGACCCTTAGAAAAAAAGG
TTAAAGCATCATCCACATGGGCCAAAGGAATA
AGGCGATGAAAAAGTTTGCATGCTGCAGCACGAAGTAATGGAACAGAACTTGACAACGAAACGACCGC
CTGAGGAATAAGCGAGGCACAAAGGGATTCTG
CACCTATCCGCTCCCGCTTCATCATTGACAATAATATACCCAACAATGAGAGTGCTCGAGAGGAAAGA
ATAGCCGGCCCTTTTATATCTTTTTCAAGAAG
TACTTGCAAAAGTTGAGAATGACAACCTACTCCTCTAAGAATGGTCTCCCTGGGACATCGGCGTAAAA
GAACTTCCAATACAAATAAGATACTTTCCCTC
TTCTTCCATGACTCTTCAGCAGAAATTAAAAACTCCAGAAGTGCCCGCACAATTTCTCCCCCGTCTCC
AAGAAGTGATACACAGATCCTACAAAAATCTG
CTGCAGTATCACGAACATTCCATAAGACTTCATCCTCACGTTCTTCTTCCTTACGAAGAAAGTCATTT
GCTTCAATAAGCCATTCTTCGGCTTCCTTATC
ACACAACATCGTGTGCGACAAAACGACATTTAAGAATGATGACAAATTATTAATAATGGCCTTCCCTA

AATCACTCCTCAGGGTCTTCAAATGTACCATA
AATCCCAATAGTTCCCAACGTTTCGCAGCAATCTGTTGAATAACAAGAATTGAATCATCATCATTCTC
ATCATTTTCTAAAGTTTCCATGTAGACATTGT
TATCCGCTTCAATGCGCGTCAGTAAACCACAAAAAATATTAGCCGTCATTCTTTTTCTTGTCCACTTG
TCAACTTGCAACATGTCATTGAAAAGCGCCGT
AATTCCCAAAGTCAACCTGTTTCGGACAAGGTATTCTGGTTCCCCTTTTGACGAAAAAAAGGGCGTCA
GTAGAGCACCCGATAAGGCCGGAAAAAAAATTC
TCCCATGTTTTTTTTATCAGTCCCGATAAAAAAAATTCTCGTATTGATATTGCAGGAAGAATGAGCTC
TCTCAGCAAATTAACAGCCAAATAACTAGCCT
CTCTCGGGGTATTAGAAACAACAATTTGAAATAAACGTGGTGTGAGGGAAAAAATCTCGTTGCGAGTG
ATGGAGCCGATAGGAAGATGGTTTATCAACTC
GGAAAGTAGTATCATAAAGTTCAATTCATGTCGATTTGAATTGAATTTAAAAAATTCATCAGACAGCC
ACTTTACAAAGAAATGAAGAGATCCAGATACC
ACCATGTTTTCTGTTTTAATGGATTCCATTCTTGACACAATAATACTTATAACGGCAGCAGAAAGATT
TGCTACAGGGCGATCCTCTTCCAAAGGAAACA
AATCCATAAGAATTTTACTCACCTCAAAACATTCTTCTATTGCGATAAGACTGACTTTCCGTCTTAGA
ACAGTCAAAACCAAAAGACGATGACTTCGAGG
AACAGTCTCCAAATTCAAATAAGCGTAGTGACTTAGCTGTGACAAAAATCCCTGAAAGAGAGTAAGGC
GATCAATTTCTTTTTCCGCCTGAAATCGTACG
GAATTTTCCGAACTAAGTGCACGTTCCAAAAGCGCAGGTAGCTCCTTCAAATCAGTCATGGAAAGAGC
TACAATGGTCATAAACCACAGTACAGGCCTTA
CTATTTACTTGGACAAGGCAATCAATCAAAAAAAAAGACTAGTGAAATACAGAATGGGTGCAGAAAAC
ACCAATACAATGCAATCGTAATCATATAATAG
AGCATAATCCATGTATTAACAACATCTTAAAGAAAATACGCCAATAACCCAAGATAAAAGTATACAAA
TATTTCCTTTTCTTTTTTTTTCCTGTCCTCTC
GTCTCCTTCGTTGCGTTTTTGACGTTGCTGTCTCCTTTGCGTATTCTTTGTTCCGCTCTCATTTTCAA
ATGTCCATAATATCATGGAGTTTAATCATTTG
CATCCCTTATTTAGGAGTGAGTCAAATACATCATGAAGACATTCCATCAAAAAAGGAAAAGACCAACG
AAATCCGTTTAGATGGTTTGGAAGAATATAGG
GATGGGGAGAAGAAAAAAACTGCGTCACATCCACCTTTTTACCTTCAATACGAAGCACTGCTCCTTTG
TTATCAAATGTATCGCAATAATTAGGGGCTGA
GAACACTGACATCATACATGGGAAGTTGGAAATACTATTAAATCGATAGAGTTTGTACCCTTCATCCT
GCAACTCATGGCTCCGAATGATGCAAAGAAGA
CTATTTGCCATAAGAAAGTGTTTTAGACTCGCAAAGTTAAACAAATAACTACACCCTCGTTGCTCATT
AACAAGAAAAACGGGTTTGGATCCATAGGATG
GACCAGCACTAGGGGTATAATAATCGCTTTTTGATCCTCCACTAACCGATGATGGATTTTCAACATCC
CAAAGTGGATCCGACCAAAGCAAATCACACAT
GGCACCTCGTGAAGGAGGCTCTCTAAAACGATGAATAAGTTCAATATGATTTAATTTGATTACATCCG
GTGAAAGGCCGCCGTGAACGCAGAAAAAAACGC
TTATTTACTAATGCTGCTAACGGCAAACAGTCAAACGCTTCCATGATCGCGCGGTAAACTTGTTCATT
ATACTTACAAATGCATTCCTCCTGAAATTCAA
AAAAATCCGTCATTAAACGCGATTCGTGATTTCCACGAAGCAAAAAGACAGATTTGGGGAAATTAAGT
TTAGCTGCAAGTAAAACCAATATGCATTCACA
GCCACAAAAGCTGCGATCGGTGTAGTTTCCAAGAAAAAGGTATTTTTTTTTATCGTCAAACATCCCTC
CTATTCGAAGTATTTTCGTTAGGTCATATAGT
TGCCCACGAATATCACCTACCACAGTAATTGAATCATCATCAATAGTTAACACATTTGGTTCTGCACG
TAAAATTCCCGACGCATTTTGAATTATGCTCA
AAGCATCATGTTTTCCAAGACGCCCCTGGCGAAGAAAGTGGGCAATCATCACATTTAGGTTAATTTTG
CCTTTTCTATCAATCAAAACCGCTCGTTTTAT
TTCATCCCAGCATGGTGGAGGAACAGATGTCTTTCCCCTATTCACTTTAAGAGAATCTGATGTAGACA
ACATTATTATTGCTATACCAAAATAAACTCAC
TACTGTGCGATAATATGTATATATAATTATAACTAATAAATAAAGTAAAGTAAAATGAAAAAAAAAAA
AATATTGAGTCCGACAATATCAATTTAAAAAA
AGCATATACACATATAAAAATCAAAAAATATAGTATTAGTAACGCATATAATAGAGATAAATTTGAAA

AAAATAAAAAAAAAATGTAGTCAATTTGTTACTT
TGTATTCGCAAGGCAAATGCTAGTTATTTACACAAATGTGGAGGTAGTTTTTATTACATTCTGAATAA
TTTCCCGATCGATATCCAAATCATTAGAAAAT
TCTCTAAACGTGAAAGATATGCGACGATGTTTAATATTTTCCAAACGTATACCATGCAACCAATCATA
GCGACTCCCATTTGATAGTAAAAAAAATGCGC
CACGAGGTAAAAAAACGTCAACAGCCGTCCCCTCATTATTCACAAAGGTCATCACTGTGTCCTCTAGC
AAATTTAGACCACCAACACGATTGCCCCAAAC
CCAAGTATCATCAATGTGAGGATCAATACTACTGCTATTTTCACTTTTATACTCAAGTGCTGAAACCT
CCACGATATGATATTCTTTTGAAGTTATATCT
CGGACAAATGATTTGACTTTTTTCATAACACTCTCCAGCTGCTTAGGCATACCGCGTATACCAGACGT
ATTAAGTTTCCTTTTCTTAAAATTGGCTCTAG
GACCAAAATCTTGTTTCCTCCTCCCACTCTGGGAATGCTTCCAACCAGAAAGTGACGACGGCTCATCA
AGGAAACTAACCAATTTTTCTTCATCCAGTAA
AGATAAAAATTCCGGAATAACAAAAAGACCTTCAATTCTAATATCGAGGTTGGGTGTTGCGCCTTCAT
GGTCTGCGCAGCTTTTGAAAGCCCCGTGATGC
ATTAAAAAAATTGTGTTGCATTGCCAACAGAGCCCATAATGGCTTAATTCCACACAGGTAAAGGAGCA
GGAAGAAGTTCTCCCCGCACCATATTGTTGGG
ATATGACGTCTGAAGACTTTACAAGAAGAACGTTCTGATGAATGATACCTTGAGCGCGCTCAGACTCT
ATACAACGCCCACAAAATCGTATTCCCGAGCA
ACAACAGCCCTCTTCTTTCTCCATTTGCGACATACAGGGCATTCCTTATCTCTCTATTAGAAGATGTA
GTAGAACTACTAATAACAAGGCAAAAGAAAAA
AAAAAAAGGAGTGGTAAGAATAAAAGAAAAAGCGTCAAAATTCGGAGCAGATGGAAAATAATAGGGAA
AAAGAACTATTTAAAAGCTGAGGAGAAATAAA
ACGCAAAGTGATCTTTAAAAACATTGTGTATGCAAACGAATTACCTAATTTCATTCAACCCGCTTTTT
GTTATTTAGAAGAAATCAATTTGTTGACTTCA
TCAACAGTGTCTTTGTATGACTTTACGGCGTGTCCAATGATTCTTTTGTCAGTGTAAATTTCATAATC
ATTGCCACCAGCATGTGTTTTATCACCAAAAA
AATGAATTTCGTCAAAATCATTTTCCACAAAACGCAGACAATAGCTTTTGTCCCATCCAACTGGAAAG
ACATCAAAGCTAATCTGGCCTCCAATTGAATA
CTTTAAGCCGTAATCAGGAAAAGAGTTCTGAAGTTCCTTGATGAGTTTTTCACGGACGTGATGCTCTT
TGTCATATACCTCAAATTCATCTCTTTCCGAT
TGCGTGCAGTTGCGACCAATTGGACAGACATTTATCATTCCATTCCTGTATTCAATGAATGTGCCCCT
TTGAACAGGAATATCAAGCTCAGAAAGGAGAC
GCAGGGCTCTTCTCACAAACTTCACGATGCGCTCGTTTCCAAGTTCTTTAAGGAGGTTTTGACGATGA
ATTTCTTTCCCATGCTTATATGCCAATAAACC
ATTTTCAGCAAACACGTAGTCAAACTGTTGAAAAACATCCTCTCCGAGTTGTTCAATCTGCTTAGCCA
AATCCGATCCACCCACTGTCCCAACTGTAAAT
CCCACACTTTTTGCACGCCGAATCACTTCCCGAACCTCATCTGGCTGAGAGAGCCTCGGCGGGGTCAG
CGTCCCGTCAATATCAAAGAGCAAAAGAACCT
TTTTCATACGTCTTGAAACAAAGGCCCAAAATCACCTTTAAAAAGGCAAGAAATTGATGAATTATTTT
TAAAAAGCAAGAGTGAAACAAATGCATAAGAA
AAAAACACAAAAAAAAACACCAAAAGCTAGAGAGAATTTCAAAAAAACACCAAAAGAAAAAAAGCACA
AAAAAAAGCGGAAGAGCGCGCGGAAAACCACT
GGCCCCGCCAAAAAGAAAAACACGAGACAACAACGCAGCCCTGCTAGAAGCCCCCTCCCTCGCCCGTT
CTCCTTTTTTGTGCCCCTTTTCCTCCGCCCG
AGACCGGCCCAAAAATTCCGGGCCCTGAAGCTCCGCGCGGCCAAAAGGGCGGACGTCCTTCCGAGACA
CGTCCTGGATTTCATTCGCGCTCGCGCCTGCC
GCGCCCTCCTTTAAATTTTTCTTGCCGCTGTTGTTCCGGTTCTTCTTTCTAGTAAAGCATGTTATTTG
AATATAGCTCTAATATTCTTGTTTTTCCTTTT
TTTGGCGATGAGAAGGGCGAGCGGGTTTTCCGCATTGCTGCCGCGTATTGGTTTATCCCCCTGTTTAG
GACGGCGCTCGTCTTGTGATGGAAAAAAAACG
GACGGAAACCCACAGGATCCTGAACCTCCTAGTGAATCGAAAGAACTCGATCCTTTCCGTATTGGATA
CAATATGGGAATGCAAAGTGTTCATTTAGAAG
CCATTCGAATGAAGCTAACAGATCATTTGGAGGTTTTTAAGTCGCATTTTCATGCACTCACCGATGTG

TTGAAATCGAACCATGATCAAGTCAAACAGAT
ACATGATCAGTGTAACAAGACGATTCTTCATGAGCTGAAAGAATCCAATGGCCAAATTTCTTCACAAC
GTTCTGAGCTTTTACGACAAATAAAGGAAGTG
GAGGAAAATTTTAAAAGCAGCATTGACGATAGGGTGACTGGGCTTCAGAGACACATTGATGATTGTTT
TGAGAATCTGAAGGATATGATAAAATTGGGGA
TTGCAGTACTTATTTTTCTAAATTTATGGATGTTTTACACAATTTCGCAAAACCATCGTCAGTACATG
AACGCCCAAACGACACACACGTTGAATGGTTT
GTCCTCTCGAAATAGAAAATAAAGAAAAACATTTCCCGAAGCGAAAAGTGAACTGGTATTATTATTAT
TATTATTATTATTATTATTATTATTATATTTA
TTTATTATATCATTACTAGTGCCCTTATTATTATTATTATTTGTTTGATTTACGCTGTTATATTTGTA
CTGGAGAATAATCTTCTACTTGTTTTTGCTTT
ATTCTGTTGTTGAATTGGATTATAATCGAATTTAAGTTTTTTTGCAGGTAAATTTAATGGTTTTGGAT
TGGTGGTTTTATTTTAGTTATTATTTATTTGT
CGCTGCTCTTTTTTTTTTTTTTTTTTAGTTTGTTTTGAGCTTTATTAGATGAATGAACGTTCCGATCGC
ATCATGCTTCCAGCAAAGGGTCGCGGGAATAC
AAAACATTTTGTGCTTATGCACAGTAGATTTTACTCGTGTATTGGTGTCCTGTTTGGGCACCACGGAT
CTATTTCTATTGGATGTTGGTAGCAACGCCAT
CGAAATGGATTATATTTATGTGGTCAATTTAGGAGCGCCAGCAACCAAATTAAGGCATGTTTTGTCAA
TGGATGTGACGTTTGTTTTAAGTAATGGTGTG
CTTTATTCGGCTACCTTGAGGAAAAAAGCAATGAATTCTCCTCAAGAAATATTTACATTGAGCGAGGG
AATAGTTACAGACTTTGACATTGTTATTTTGA
AGGAAGGTGCGTGTATTTCCATATTGGAAGCACCTGAAATATCTGTTTTTTTTACACGAGAAATGGC
AAATTTTTGCTTAGCCGATTTAACGCAGGAAA
TATTAGTTCATGTAACTGGGTAACAATGGATTGTTTGTGTTGCTCAGGAGAAGGTATTTTACATATTT
ACGGTGCTGATGGTTCATACATGCATCGTTTG
GGTTCACAGACAGCACCAGCACGCTGTGCGATTCCGCTATTTGAGTGTTTTTCTTCAAATGATAAGTC
ATCGTGCGCCAATAAAAAGAACGCTTTTGCTG
TTCTTTTTGAAAACAGTGAGTTGTACTTTGCGTATTATTTTACTTTGTTTTCTCAGGTTGGCGATGTC
TTTACGGGAACTTTTAGATTTCCAATTTTTAG
TAGCTCATTGCCCACGTGTATTTTTTCTGTTGGACCATGGATTTGTGTTAGCAGTTCATTTGGCTTGG
AGTTCTATGATCATAAAACTGGTTCTTATGCT
CAATCAGCATTGGAGAATGAATCTTCTGCTAGTTACTCTTTTGCAAATATTTCTTTTGATGCTACCCT
TGTTCCAAGCGATATTCTTTACTCAAGATCGC
GATTTGGTGTAGTTGTTACGAAAAATGCTCTTTATTTATTGTGTTTGCGATCAATTTTAGACATTGTT
GATGAATTGAAAGATGATGCCGCGTTTCACAC
AGTTTTGTTGGAAAGTTTAAAGAGTTTTGGTGAATCCGTTCGGGCGACAAACATTACGCGGTCCTACT
GTCGATGTGATACCATATCGAGTTTAAGTATT
TTGTTTTCGGATTACGGTTCAATTTTGGCAAAATTTCGTATTTCCCTATGGGAATTTAAATCTCAAAA
CAGTGATGTGGGTGAAAGTAAGAAACAGGGTT
CATCATGTGCCATTTGTGGTTTGGAAAGCTTTTTTAGTTTGAACTTTTATAACTGTGCGTTGTGTCAC
CTGGTTGTGTGCCGAAAATGCTCATTTTTGCG
AAATTTGCGTCTGTATGGCTATTTCACTATGAGCGGAAAAGGTGTTCTTTGCGCTCACTGCGATGGAG
GATGTGATCTTAACTTGTATCGATTGTTTATG
TCTCGTCGATATAATCAAATTCTTCATTACATGGAAGAGTATCCGGAAAGATGTTACAAACTATCTCT
TTCTGAAGTCATTCCTTCCGTTCTTCTTCAAT
GTTTTGGAGAGAGAAAATTCGATCTTTGTGCCGAATTGATTGAAAAATACGTTCCCAAAAATGATAGT
CTTTGGGACCAATGGGTAGTTCGATTCAGTGT
ACAGAACCAGATTGAGTGTCTAGCTCGTGTTCATAACCCTAACCGTACCGATCAAGCTATAAATACAG
GCCTTTTACTCCAACTTGTGAAAAGTGTCAGT
TATGAGTTGTATGTGCTTTTACAACAGTGGCCTGGTAATTCATATAGTGCGGAAATGGTTTTACATGG
TATTCTCTCGCGTCTTTCATACAAGAAGCAGG
AAGCTTATGATTTGCAGGAATCGTGCCAATGTGGTCCTTTAAATTCATTAGAATGGCAGAATGCCGTA
AGCGAGATTTATTTCGATGATGATATATATCA
ATTGCTCCGGGCTTATCTTTACATTTGTTTTCAAAATGAAAAACATTTGGATGCTGCAAAGGGTTATA

TGGAGTATTTTGTCATGATGCTTCCTTTGACA
AAAGAACAACGTCAGCGAAGTTATGAAGTTTTTGAAGACAGTATCTTGCGTGGAGAATTTTTAAGTGA
ATTTCCACCATCAGATCTCATTAATTTATGGG
ATTTATTGACAAGCCATGGTATTCTTGAAGAATTTATTTGGTTGAAAGATTCTGGTGGTAAGACTCGT
TTTTTGATTCCGCTTCTTACTCATTATCGTGA
AGAATTTGTTCGATATTTGATTGATTCTGTTCATATCGATCTTCTTGAAGATATATTACAGTCTACCG
TTTATGAGTTGAAAGATCAGCCTGTATTACTT
TTAAATGTACTGGATTCCATTACGAGAATTTCTCCTTTGGCAACACGTTCTTATCATTCGCTTATGAC
GGAGCTTTACTTGGAGCATGCCCCAGAGAAAT
TATTATCTTTTATTCAACAACCTCAGGTGGGTGGATTAAATTTGAAAAAACTTGCCAGAATTGTTGAA
AATCGAAAGATGTTTCGAGAACTTGTTTATCT
TGTTGGAAAAACTGGTAATGATATGGAGGCCATTCGCATGGCTATTCGATGTATGAAGAGTGTTCCAC
TTGCGATGGAATACATTAAGGATCACCCTGAT
GATACTCGGCTTTGGGACTTGGTTATGAAGAATGTGGTTCAATCTCCTGTTCTTATAGCTGATTTTTT
GGAAGCGGCTGGTGACCTTGTGGATATGAAGG
CATTTTTGCAGTCCATTCCCACTCCAAATCGTCTCAATATTTTTCATGGAGGGCCGCGTTTAAAGAAA
GTGCTTCAGGGTAAATCGTGCTTTGAACGCAT
TGCGAGGTCCTCTGTAGATTCTATCTCCGTAGATAATTACGTTTCACTTGTCCGATTGAGACAGCAAA
ATTGCAGGGGGGCGCGTTGTATACCAGGGGAT
TTATGCAGTTTATGTGGTCAATTAATTTTGGGGGAATACATGGTAACTAGCTCTGGAGCAGCGTTTCA
CTCTGGTTGTACTCCTCCTTTGTCTTTGCAAT
ACAAAAAACATGAAAACAAGATAAATCATCAGTATGGAAGGCCCAGCAAGAATCGTCGAGATGTCTCC
AAAATGGATTACATTGAGTATCATACTAATAG
GTCTCTATGCACCTCTAATCCAGTTAAATTATTACCAGAAAAGGAAGTTTATCGAATTCTTTCATTTT
TGTCTCCAAATGAACGAGAGGTTTTTCGGGAC
GTGAGTCGTGAATTCCGAAAGGTCGTTAAATCTTTTCATATCTTTGCCAAGAGAGAAGACGATAATCT
ATTTGGTCGTTATTATGCTCGATTGGAAACTG
TTGGGGAGAATAATTGCCGGTGTACTTTGCAGTTGGGAAGGGTGCCCATATTTAAGTCCCTAAGAGGC
GCACAACGTTATCGTGTCGATACTGTTAAATT
ATAGGAACCTTTATGTATGATTTCTATTTTAGTCTAAATTTGACTGCAAGCAGACTGAGAAAGTAAAT
CAAGACGAATAATATGTACACAGGAAGATGTG
GCGTTTTGTTATTCCTGTTTTCTTCTCTTTTACTCTCCACTTCTCCATGTATTTTTGATTTCGTTGTA
GAGGCTATTGCTTTTTCATCCAGTGGTCAAAA
AATATATATATATATATATAGGTGTCTTACTCACATCCAAGTCTGGTTTAAAATTATTTGTGAGGTTTTG
GTCCTAATGTTATTTTCCGGTGCGTTGTTTCA
TTTTGGGCAGATGAAGATGCTATTGTAGATGGAGCCACTGTGTGTGTGTGTGTGTGTGTGTGTGAGGG
GGAACTCCTGAGGACGGTAGTACCACAAGCTC
GTTTCTAAATTTTGCCCGCTCGGCTGGTGACTTTGCTAGTTTTTTTGTTTTTCATCTCTTCTCAAGTG
AGGGATAACGCTTTAGGGTCCACAAGGGTGCC
TTTTAGCCGGGAGCGTCATGTTTCTCTTGGCTCCCCCACAAGTATGATTTGGTTAGAAATCAGAAAAT
CATGGATTCTAAAATAAATCAAAAAAAAGCAC
GGAATAAGGTGCTTTCCTCTGTTGGGAAATGCAACTCGCTCATTATACGTGTGGCATATACAATTGAA
GTTTTGTCCTTTTTTTAGACGTTTCTTGCACT
TCAGGCTTTTGCTGTTTTGCGCAAGATTCCTTATGAAGAGTTAGAAATTGATATTGAGTGGGCATCGA
TGTGGCACTTTAAAGTTGACCACTATCATCAT
GGAGCGACATTGATGCCATCCGCATCGTTAAAAGTGATAAATCTCCTTATCAAAAGGGTGGATAACAA
AAAGAAATTTTTTGTTGAGTGGAAATCAATTA
AACGAATTACTGGCTCTGATTGTTCATCAAGCAGTGGCCGTGACGCGCCAATAGTCAGAATTTGCCGA
ACAATGACTCAATCTGTCGCAAATACATAAAG
AATGTGATGCAATTGTTAGTGGTCTCAATCAATCAAATGGCCTGAGGCAAACCCAGAAGAATGGGACC
ACGCATATATCACGAACAGCCAAAATAATGAT
GATTCAGAGGAGCTTGAAGGGCGATTCTCACTTGGACGAAAAGAAGTAGAAAATACCTCCAATGAGCC
AAAAACAAAACACTTTGCGCATAACATAGATG
TGAGTGTAATAACGGGACGGTTGGATCAAATTGAAGCGAGACCAGGAAATGTGGAGTCTCAAATTGAC

GAATACTTTCGTAACAACAAGGATTTTTTTGC
TTTGACTCAAGGAACACTATGAATAATTTTTCTGAGCTGGACACACAGTTGACTTCCCCTGAAAAGAC
TTTTCAAAAGGATCCTTGTTGGAGCACTTGTT
TCTCAATTATTGGAGGCGCCAGAAAAGATTCGGGGAGAATTGGAGGTTGTGACGGCGCAGCTCATGCG
ACTTTTTAAGGTTTTGGAAGGCATTCGACGTC
GTTTTTTTTTTTTTTTTTTGTTTGTTTGTTTTTTGTTTTTTTGTGTGATGTTTACGTCTGTTAGGAGGG
GGGGAAAGTCAGTTAATTTGAAGTTTTTCGAT
TGCAACTGCGCTTCTTTTATGGGTCAACGGCAACCTGTGGAAGGGCAGGGAGAAAAAGCTGAGGTTCA
CGGGGAGGAACATGAGGAAATGCCCACGATTA
TACATGCATTCTCCTCTTCAATTGTCTCTAATGACAGGTGTTCGCCCGTAAATAGCACGGCTTGTGTT
GCCCCGCGTATTTTAGGCTTTGCCCATGGCGA
GGAATTACATCGTATTTTACCAGAATCACTTAGTCTGATCATTTTTAATGCCCTTCCAACGAGCTGCT
GGCCTGTTTCCCCTCAACAACCCCAGTGGTAT
TTACTTTACAGTAGTAGGATTCATGGGCGCTCATTTCAAAAACTCGTTCAAGGCCTAGTGGACAAGGG
TCCAACGTTGATTGTGATCAAAGCGTGTGAGT
CATCGAATGTTTTTGGTGGATTTTGTGGGGATCCATGGCAGACTGTTGCCGCAAGAGAAAAACAAGAG
AGGAGTCGTTCTGCGGCTGCTAGACGCGCGAC
GCGGGAGGGACAGGGAGCCCAGGGTATCACTTGTCGACCTGCAAACCAAAATAGTGCCTTCTTTGGTG
GCGAAAACTGCTTCTTATTCACTAACAGAGAT
GGTGGCCTGATTTATCAGTCCAAACCCTCCATAAATTCAAATTTCATGTACTTGTTTGATTCGCATCC
ACTTGTTGACAAAAATGGGGTTGGCATGGGTG
GGCAACCCGGTCATTTTGGTTGGTTTATAGACCGTTGGCTAGAGAAGGGAAAATGCTGCGGTGTGTGC
TGTGCCACTTTCTGCAACCCCCGCCTCACTGA
CACGGAAGAATGGACAATTGACGGCGTGGAGGCGTACGCGTTGCATCCGGAAACTGTGGAGTCGCTTT
CCAAGGCACCCCGAAAAGGTACTGAGAGGAAT
TCGTGTTTGCAGAATCCCGCAAACGATGCAGACAAGCTATTACTTGGGCTTCATGGAATTTACGATTT
TGATAGTCAGGAGCAACCAGAATGCTGAAGAG
GCTTTATGTTTTCGTGTGTGGGATGTTGGGCACATTAAAATAAATTGTTATACTCCCTTGTTTTTTTT
TTTTTTTTTTTTTTTTTTTTTTTTTTTTGGGT
GGGGGAAGAGACAATTTCTCCTCCCACGTGCAGGATCATTTATTTATTTAATATCCATTTTGTTGTTT
TTGTTGTTTGATTGTTTTTCATTGTAGTTTGG
GCTGCTGCCATCCGCGCTTTGATTCGCTTTCTTATTTAATGGCTGCCTATCAAGTCATGCAGGAAGGT
TCACACATACACTTACACAAAAATCAAACATA
AAAAAAGCAGAAGATCACTTTATCACTTCAACTGGACGTCCATCTTCGGTGCCTGTTGTTGTATTTTT
TTTCCCTCCCTCTTTCGGTCTATTTTCCTCAT
CTAATATATATATATATATCATATAAGCACAGTGGGAACGACAGGACGAGGGAAAAACAAAAAAAAAG
GGGGAGGGGGGTAATTCTTTATTTTTATTTTT
TACTTGGATAAAAAGGAGAAGGAGGGAGGGCCAAGTTCGATTTCTGTTGTGTGCGTGTGTGTGTCTTG
TGGCTTCTTCCTTTTATATATTGTCTTTACTA
AAAGCTTCTGTACAATGAGTAAAAGTGTTTTGCAGGTGATTGCCCGTATTGCTTCATTGTGCAAGGGC
CGTCACGCATACCTTCGCGAGGTTTGTCTGTA
TACTCCGGGGGCGCTGGAGCAAACAGGCGGTGCGGGCGCCGCTGTAAACCTTCTTTTTTATCCCCTGC
TGATTGCGAGTTATGTGAGTAACGAAGAACAA
ATGTTTCTTCCCTTGGCGTTGGAAGGCATCCTGTGTCTCTTGCGGCTTCACATTGTTTCGGAACGTAC
AATGTACGGCCCGCCCGAGGTACCTCTGCCTG
AGAAGAAAAGGAAGGGTCTTTTTGCAACTACTGATGTCACTGCGCCCGGTGAGGGTTTATCGTTTAAC
AGCGCGTCTGCCACGAAATCAACTCCGCTTAC
CGATGTGATCCTTGACACGGTGAAGCGGCACAGGCATTCCGTGTTGGAGGAGGTTTCTTTTCTTGTCC
TTCAGTGTCTCACGGAACTCATGCTTAACTGT
GAATGGATTAAAGGGTCAAAACTAGTAGACTGTTTTCAGAGTGCGTTTGAAGCACAACTGGAGGTGAG
GCCAATGTCAGGATCAGGCGTTTCTAATTCTC
TTCTTCCGCATGTGTTGTCTCGCATATTAGCAGGACACAGAGGTGCCAACTCTGTGGAGCAAAAAAGA
CCATCAATGGATACCACATTGTCATGCAGTGT
TGACTCCACGATGGGAAGTAACGAGAAACAATCCACTTTGGATAAATGCAATTCTCTTATAAACTCAA

TGAATATTACAATAGCTGCCTCCTCAACAGAT
ATTGTAGCAAACCGGCAACACTCAGCGGGTGAAGAAGAGGATGAAGAGGATCAGCAACAAAAACAACA
ACTAACATTGAGTGATCATCAGGACTGTTATG
CGCTTCTTGTACGAATTTGTGATCTTTCCATCAAAGTGGTTGAGAGCCGTACCCCTGCGGATTCAATG
GAGTGGCGTCGTAAAAGACTGAGCACTAGACT
TCTTGCTCATTTTATCAATGGAGTGAACGATGAGTTTGCAAGGAGTGATTTTTTTCGATCCGCCTTGC
ATGAAAAGGTAACCCTCAGTTTGCTTCACAAC
TGTGCATTAGAGGATCCAGTGCTGCATGGTGTGGCACTCAAAACACTTTATCAGCTGGTGATGCGTTA
CCGCAACCTCCTCAAATCAAAAGTGGTGATAT
TCATCCTTGGATTGCTCCTTCCCATCGTAAATTCTAAAAACACATCCTACGAGCAAAAAGCCACAATC
TTAACGTTTTTTGAACACACCCTGCGTGATCC
CCAGCTGCTGATGGATTGGTTCACGAACTTTGACTGTGTTCAAGGCATGCCGAACCTTTGTGAGCAAC
TTGTGTCTGGGCTATCAAAAATGTCAAAGATG
TCACACATGTCATCTTGGGTGAACGCCAAACAGGATGCTTTACTTCGTCTTCAGTGCATAAAGGCACT
TGGAACCTTTGTTCGGTCTCTGGAGGGCATTG
CGAAGGAGTTTCCCATGGGTGGCGGCATCACCCCTCGCAGTCAAGAGAGGGAATTGGAGTCAAGGGAA
AATCAAGAGATGAAGTTGGTGGCAGCGGAGAA
CGAGAAGGGGGAGACGGGGGCGCATAGTAAAAACAATGTTAACTCTGGTTCGTTGTCAGAATGTGGTG
TGGAACAACTTCTTCGTGGGAAAAAGGCGTTT
GATGCGGTTGTAGATAAGTTTAACTCGGGTGATCATGCCGCTGCCATCGCGATGGCTCTCAATGTTCA
CCTTTTGAGTAGTGCAGCACCAGAAGCGGTGG
CCCGTTTTCTCCTGCAGAAAGAACTTGACCCTGTGGGAGTTGGGGAATATCTTGGGAAAGACAATGAG
GAACGGAAAGCTATTCTCCGCGCCTTTATAGG
GCTGAATGATTTTTCGGGTTTGCCTATTGATGACGCGATGCGTTTATTTTTGGGTAAATTTAAACTCC
CCGGTGAGGCGCAGGTTGTGGATCGCGCCATG
GAGTTGTTTGCAAGGGAATACTGCGCGCAGAACCCTTCCAGTTTCTTTGGTCCTGGTCCGGCCTTTAT
TCTGGCGTTTTCCATCATGCTTCTAAACACAG
ATGCCCATTCCTCTCATGTCACGGATAAAATGACTTTAGAGCAATTTGTTCGGAATAATAGTGGCATT
GATGATGGGAAGGATCTTCCACGCTCGCTCCT
CGAGGGGGTTTATCAGCGTATTACAGCGAGGGAGATTGTACTTGAGGCTCGTGGGGCGGTGCCATCGA
ATGGGTTAAGAAAGTGGTCCTATGGAACAAAG
GATATGCATCCTTTATCATCATCGTCATCATTATCATCGTTAGGGGCTCGCAGGAGGAATCATCGCTC
CTCTCGTCATATGGAGCAAGCGTATCTTCTGG
AGACATCCGTGGAGCAGATAACGCGAGATGTATCGTCAGAGCCGTATACATCCATAAATTCCTCAGAA
CTTGTGGGTGCACTGATGGAAAGTACATGGAC
CGCCCTGTTAGCAGCCTTCTCCATCCCTATGGAAGAAATGGAAAACATTGAACTTATCGACGCCTCTC
TTGAGGGTATTGAATCGGCCATTAAGGTGTGT
TGTAAATTTTCATGTCGAACACAACGAAAAGCGTTTATTTCTGCTCTTTTGGCTTTTACGCACCTGAC
GAACTTTCGGGAAATTGAGTACAAGAGTCTCA
AGTCCATTATCGCTCTTACTCGCGTTGCCCTTGAGGAGGGTGATCACCTCGAGACTTCCTGGTATGAA
GTTCTTCGTTGCATCTCCCTTCTTTCCAAGTT
GCATATATTGGCTGAGTCTCCATGGACGTCATTGCTCAATGATCGGAATGGTAACCATGCGGCCCCCA
AAGCCCCCAACACGTCCCTGGAAGGCCAGGGC
CGGTCCTCATTGCAACCACAATGGGAGCGAGCCAAGCTGGAACGGCAAAATGCCGAAATAATAGCGAA
ATATATAGATGAGGTTGAGGTGCACCGTCTTT
TCTCTCGCAGCAATTATCTGAAGGATGCAGCGGTAGTTAGTCTTGTTGAAGCCCTTTGTTTAGTGAGT
GCTGAGGAGCTGGCGGAAAATCCACCGCGGAT
ATTTTCTCTGCAGAAACTAGTGGAAGTAACGGATACAAATATAGGGCGTCTGCGGTATGTTTGGTCCA
AAATGTGGACCAACGTCTCTCGTCATTTTGTC
AAAGTTGCTCTGAGCTCCAATGAACTGGAGCCCATGTATGTGGTGGATCACCTGCGGCAGTTGGCAAC
GAAATTCCTTGCTCGAGGCGAACTTGGGGACT
TTAACTTTCAGAAGGGGGTGCTGCAACCATTTGAAGCGATTGCCTCACGGACGCAGTCGACAAAGTTA
AAGGAACTTCTTGTGGCCTCTTTGGGTCAAAT
GGTGGAGGCGCAGGCGCAGAATCTGAGGAGTGGTTGGGGTACTGTGATAGAGGCACTCGCACACTGTG

TGCAACACGACACAAATCCCGATGTGGTTTCT
TCTTCTGCCTTCGTGCTGCAAAACATCACTTTGTGTCATCTGCATCTCCTTACCACGTCTGATTTGGT
GAAAATTGTACGGGCATGGGCTGTTGTTGCCC
GTTCTGCTTTTAGTGATGATTTTGCCCATTCAGCCGTGTGGTTCGTGCGTTATGTGACTATTGCCTTG
GCACTTCAGGCAGATTCCAATAGTTCCTGCGA
GGGCGTGCAACACTCACATGCGTCACAATGTGGGACATCATCCAACGGAGAAAAAGAAATACATGTCG
ACGAATCACATAGCCATGCTATTAAGGAAAAT
AACAAAAGCCATTCTCTCCTTTTGAAAGAAGAAATTAAAGTTTTGGCTTTGCGTGCTGCATTTCCTGT
CGATGGCGATGATAACAACAAAGTTGAGAGAG
GGCGAGATAGAACAAAAGAAAATATGCAACTTTTTCCTGTGGCCGTAGGAGCCCTCGCTTCTCTTTTG
GTTCACCGCTCAATAAGTGGTGAGGCCATGGA
GGCTATGCTATTTGTCATGACGGACCACCGCGCAGTCTTCACACCGGAGGAGTGGTGGCACACAGTGG
GTAGTGGAGTTGCCCCCGCGTTGGATTTCCTG
TTGCAGCAATGCAGGTTTTCAAACGAAGCGGAACGTGACGTCTGGCTATCACTCTTTAAACGCGCCGT
AACGAATGTCGTACAGTTTGTTGGGTACAACT
TGGATGGAAATCCTCTACCGGTGGATCATGTGCTCTTTTTGTTTCGGGTCTTTGTCGGAAGGGCGTGT
TGTGGTTCTCACGACAAGGCGACAGAGCTTGG
ACTTCAGGGTGCTCGCCAGATGTTGGAACTTTTTAAAGAACACGCCGTCGTGGATGAATTACTCTGGA
ATGGCATGGTAGAATACATCCAGGAGATTGGG
GACATTCTTTTAGGTGCCTTGGACTCCATTTGGCGCTACCCAATGGACCCCACACAACAAGACACGTT
GACGCCCACGTTGTTGGCGTTACTGCAGTACG
TCAAGTGGAATCGTGACGCGGCGTTGACGCCACAGTTAACAACACTGTGCCGAACGAAGGCGGTAAAG
GTTCTACTGAAAACCCTTCAGCGATTCATGTG
TCTTATGCGAACCTCCATCTCCTTTCGAGACAGCGTCACGGAGAAAAAAGATGTTTTTGACTTAGCTC
TGTTCATTGCCTTGGAGTGTTCTTTGTGTAAC
GCAATCCTTGATGCGGAGAGGCGGTACGGTGAAATTTTGGAGAATGAGAGCAACACCCTTTGGATGCT
TATAATAGGGCAATTTTTGGCGGTTACAGGGA
AGTCTTCAAATGTTAGTCGTGAAGAGGCGAGCAGTTTAAGGGGGATTATTCGGGAGTTGTTAGATGCA
ATGCTTGAGGCTCCACAGCGCGTAATGATGGC
GCGTTGCAGGGTCTGCATGCCGTTTCTTTGCCAACTTATTGCGCTAAGCGACGATGTCTTTGCTGAAT
CACTTGCCAATTTGTTTTTAAGATACCATACA
GCCACTGTTCAAAACATTTGATGGACCCTTCTTTTTTTTTTGTGTGTGTGTGTCTGTGAATGTATTTT
CTGCCCGATAAATATCGCTATCGTTTTATAAT
TCAATTTGTGCTACAAGGTCCCGGGCATTTCATTGCAGAATTGAGGCAGATTCTGCTGTTAATCGTCA
GGATATATATATATATATATACTTATACGTGG
GGGTTCTTTTTTTTTTGTGTGTGTGTGTGTGTGTACTTCACCAGAATCATCATTCTTTTGGTTGTTGT
TGTTGTTTGTGTGGTTATTGCTGCTGTAGTTC
TCCTCAGCTCCTCACCCCACATGGCGGTGAAAAAAGGAAAAGGAGTGGCGAGGGCGTCGCGGTTCACC
AGGGCACTTTTGATTTGTATTGCCGCATTTCT
TTTGGTGACATTGTTTTTGCTGCGAAGCCTGGAGAGCTGGGATGGCCACGCATCCTCGTCATCAGCAG
CGGCAGCAGAAGGTGGCCTCTCACAACCGACG
CCACATGCGCGGCCACCAGGAAGTGTGACGAGCGGAGAAAATGCACCCGACCACGCGAAGATGTCAAG
GCCGGCCGGTACTGAGGCCATGGATGACGATG
GTACGCTGGTGCCGCTAAGAGACATCCCACCGTCGCCGTATTATGCGTTTGGCCCACGCACAATGCCT
CGAGCATTTCTGCGTATTTTACAAAATTCTTC
ACGACGCATTAACAAAAATTGGATTCGCTCCAAATTCAATCGGTATGACAAGGCCAACATCTCAGACT
GTGATGTCGTAACACCAGAGTGCGGCTTGCAC
AAATACCTCATTAAGCGTACTGGGGATGATCTCAATGCGCGACCCTATCGAAAGTGTTGTGTAGAGCA
CAAGGCTCTTCTTGAGACAAACGTATGGGTGG
TAAGGAAATTGAATTCGGCGAACATAACGTACTTTCTCTCAACAGGTACTGCCCTTGGGGTGCGGAGA
CACGCAGGCTCTATTATCCCGTGGGACACAGA
CGTCGACATCTCCGTTTATCCAGGCGATGAGAAAAAGGTGGAGCAGCTTCTTCAGAATAACGGAGAGC
ATTATTTTCGGAGGGATTGGAAGGGACGATTC
ATGTTTTGGGTTTATCATTCGAAAGATGGGAAGCCTCAAACTGGACCACATGTGGAGATTTTCTACGA

CCCCGTTTACACGCGTTTTCCACACCTGTTGC
TTCCACTGGAGGATTGTCTCTTTTACAGTGAGCCCGTGAAGTGCCCGAACATAAAACAATTCCAGGAG
TGGTTCCCGAGTGGCTGGACCGTTTACAGCGG
AGCTCATTATCACAGCCCCGAGAAGTGCATTATTTATGTGAATGGCATGAGAAGAGTAACTAAGCGGT
GTTGATGACGGGAATGGGGAAAAGGTTTGCTG
TATTTCCTGCTTTTAACGTCAACAACCAACTGCAGCAAATCAAGAGGAATGAAAGGGGAGAAGGGAAC
TTTATGGGCATCAAATTTGAGGTTTTTATTAT
TATTTATGCATGTGCGTGTATGATATTTTCTAGTTTTATCCTTTTTTTTTTTTTTATTGNNNNNNNNN
NNNNNNNNNNNNNNNNNNNNNNNNNNNNNNNN
NNNNNNNNNNNNNNNNNNNNNNNNNNNNNNNNNNNNNNNNNNNNNNNNNNNNNNNNNNNNNNNNNNNN
NNNNNNNNNNNNNNNNNNNNNNNNNNNNNNNN
NNNNNNNNNNNNNNNNNNNNNNNNNNNNNNNNNNNNNNNNNNNNNNNNNNNNNNNNNNNNNNNNNNNN
NNNNNNNNNNNNNNNNNNNNNNNNNNNNNNNN
NNNNNNNNNNNNNNNNNNNNNNNNNNNNNNNNNNNNNNNNNNNNNNNNNNNNNNNNNNNNGATATTTTC
TAGTTTTATCCTTTTTTTTTTTTGTTCTGTTT
TGTGGGAAGATGCCTCAGTGTGGACAGGCAAGGTGCGGCCGCCAGTTGTTTACTGAAGCAAAGGGGGG
AATGGATGTGTAAAAAATTCTTCTTTTGGTGT
ATGAGAGCTTTTTCCCCCTTTGCGGTGTCTGCAGGTGCAGAGTGTTTCGGATTGGTGATTGGTGGTAG
CGTTCTCTTTTACACCGCGCTCTCTCTACTCG
GCTGATGCTTCACGTGGAGAAAACTGCATTTATGCATATATGCATTCTATTTGCTGGTATGCATTTAA
TCATATGTTTGTTTCTATATGAGGCACCTCAC
TCTCAGGCTCTGTGTGTGTGTGCTTGTTTTCTCACGGTTGTAGATTTGTTTGTTTGTTTGTTTACCTT
TTCTCCATACACTCACTTAAACAGGAAACGCA
TTGCATTCTTACGTATACGCCGGTTTCCATAAACGACGAAGACACCTCTTATCTGAGAAGGCATTGAT
TTGGTTGGGCTCTTCCTACGGGAATGGATGAC
GCTCTAGTTGACTGTGCGGACGTTTACCGCATGGAATACACCTATTCTTTCTTGGCGGGAATAGATCC
TATGCGGCCGCCCTCGTCGATTACGATCCCCG
CCAAGGAACTGGACGCCATGGCGGGCATGGAGCGGCAGTACTGGGAGATCAAGGCAAAGTACTTTGAT
GTGTTAATtCTCTTTAAAAAGGGAAAGTTTTA
TGAGCTGTACGATCAAGACGCCGCCATCGCACACCGAGAATTTGGCCTCAAGCTTGTTTTCAGCGCCA
CCAACCGTGGCAAGATGCGTCTTGCGGGGGTT
CCAGAGCAAAGCTTCAGCGAATGGGCACGCCTTTTTGTCTTCCGCGGCTACAAGGTGGGCAGAGTAGA
ACAAATGAAGGAGGAAACGGATAACTCCCCTA
TCAAGACTGCACGCACGAAGGTGCTACAACGTGAGTTGGTTGAAGTGCTCACACCAGGCACATTGACA
GATCCTGCAATGCTTAGCGGTTCCGGTCCTGT
CTTTATTCTGGCCCTCTGTCCCTTGCAGGAAAATGTTGTGGACGGTTTGGCGGTGGATCTCTCACGTC
ATGTGGTTTATCACTGTCCATGTGGCGTCGGG
AGAAATGACGTGTTGCGTAAGGAGGAAACGCTGCTCATGGTGTGTGCCCTCTTGCAGCAGCTCCGCCC
GCGTGAAATCATATTTCCACTTTGTTTTGTTC
CTAATGCTGCGCAGGCCGATAGGAAGGCATCATTTGCCAAGCGTTTGTTGGATTGGATAGAGGGCGAG
GGGTTTCGGGTGGAGTTGGTGGATGTGTCTTC
TCTCTCGTTTTCAAAAGGCAACTCCGATGGGGTGGACAAAATTTTGGACGCGCACAAGTTCTTGGCAC
ACTACTTTCAGACGTTGAAACTTTACCATGCC
GTGCCCATACTCTCAGAGGCAGAACCCTACACCTTCCATCTTCCTTACGCATCCTCCAATACCACCAT
TGCTCGCAAGCATGAGGGTGAAGTTTCAAATT
CTTCATCGATATTATGGCATGAACGAAGAGTGGACCGTGGACTCGTGCTGGATGCCACAACCGTGAGC
AACTTGGAGCTAGTCAGCAACTTGCGGGATGG
CGGGGAACGGGGCTCACTGAATCAACTTTTCAATCGCTGCTGTACAAATGGAGGAAAGCGTCTCATGC
GATCATGGATTTTACGGCCTTCTGCTTCCTCC
CGTGTCATTCTGGCTCGACAGGAGGCGATTCGCTTCATTATTGAGCACAAGCTTGACGAGCTTTGGGG
CGAGGGTGGAGAGCCGGAGACGACGTTGGGGA
TTGCAGCGGCGTCCCCCACACTGAGAGCAGAAACCCCTCGTGAGCATGAACCAATTTGTGTTGTGAAA
CGAGAGCGGCCGGTGGGTAGTAAATTTGAAAC
ACGTTTCTCCAGTTTGGTGGATGTGGATTTTGAGCGTAATCTTTCTCGCTTGACGGACTTGAAGAACA

ACAGTGACGCACAAGTGGCGTTTGTTGATCCC
CTTGTTCAGTACAAGAAGCAACTTCAAATCATTGTTACAACGGTCCAGGCTCTTGAGGACATGGTGGC
ATGGTCACACGACATACAAAAGGGGATAACCT
CGTCACCGCCTCTTTTGAAGGAGCTCTGGGCGCAAATTGATGCTGCCGCTCCCGCGGTAACCTCCATT
GGAAACTGTTTTGACCGCCATGCTGCCCTTGC
GTCGGGCGTGATTGTTCCCTCTCAGGGAGCATCCTCCGTGTATGATGAGGCATCCGGTACTCTCGACG
CCATTGAGGGAAAATTACAGGGGGAACTACGC
CGATTACAAGAGGAGGTTTTTGATGGTACAGCGATCAACTACAGCGTCGTTGGTCACGAGCAATTTCT
TGTAGAAGTGCCAATTTCTGCTGTACCGAAAA
CGCCTTTGCGTGGATTTGTGGAAAGATCCCGCAGTGGCAAGAGTGTGAAGTATGTTGTGGCCTCCCTG
GAGCCACTGGTGGAGTCTCATAAGAAAGCAAA
GAAGGAAAAATCAAACGCTCTTCTTTTGGTGCTGCAAAATGTTGCATCGCACATGTGTCACTACTTCC
CTCTCCTGTATGGTGCCACGGAGGCACTCTCA
TACTTTGATTGCCTCTTGAGTCTTGCGTCACTGCGAAAATGTGGGGTCACAACCGCTTGGCCTATTGT
GAAGGATGACGCTGTGGGGGCGAGTCTGGAGG
CCCGGGAACTAACTCATCCTTTTCTAAATAGAGACTCTGTGCCGAACGATATTTCCTTGGACGCGGCG
CACGGGCGTATTTTGCTCTTGACGGGGCCGAA
CATGGCGGGGAAAAGTACACTTATGCGTACGGTTGCTGTGAACGTCATCATTGCGCAAATGGGAGGAC
CAATTTTTGGTGCATTTATGCGTCTTGCTACG
GTGTCCCGCATCTTCACACGTATCGGAGCCCGCGATGCTTCTCACAAGGGACAGAGTACACTGTATGT
TGAGCTGAGCGAAACAGCTGACATCCTTCGTC
ATGCAGACCCGTGGAGTTTATGTCTGGTAGATGAATTGGGACGTGGTACCTCTACACATGACGGTTAC
GCCATTGCACATGCCACATTACACTCGTTGAA
GGAACGCCTTCCTGTTTCCCCGCTTCTCCTGTTTTCTACCCACTATCACGCTCTTGCACAGGAACAGC
TTGGCGGGGATGCCGTGAACGTATCGTCTTTG
GCCCCAGATCATGGCTCGGTGGTGCAGTTGGGATACATGGACTTTGCTATATCGGATACAAAAAAAGA
TGGTGTTTCGGCAATTACATTTCTTTACCGTC
TCGTCTCTGGCATATGCACACGTAGCTATGGCGTTGAGGTGGCTTTACTGGCTGGCATTCCCTCTTCA
CTCGTGCACATGGCAGCCATCAAGTCACATGA
GCTTGCCTCATGGAATGACCGGCAAAAGGACATTCATACCATACGACAGTTCCTTCATGGGCCTAACG
CTGCCACACTTTTCAAGAAAAAGAAGTCGTAG
TGCACACAGCTTCGTTTAAATGACGGACAACAAAATTATAATTATGTGAAACACAAAACGCGTTGGGA
AACAGACTATAGAGAAAAAGATACAAGTAAAA
GAAAGAGAGTGATAAGGAGTTAGATATGCATTCCCATCGATCAGTAGTTAAAGGCGGATGAAATAATG
ACTGCAATGGCGGGTCCTTAAAGGAACCATGC
CCAGCAATTTAACGAGAAGAGCAGCATTGTGTCTGTTTTTATTTTTTTTTTTCTTTTTTTCCTTTACT
GTTTTTGCCCCATTTTGCAGTTTTCGCTGCTG
CGGTTGCCAGCTATTTTTCTGGCTTATCCAGCATCCCCTGGAAAGGAAGAGAAATCGCAAAGACACAA
AAAAAAAAAAAAGAAAAGG
>scaffold1130.size6135
ATACATCTCATCGGCAAATTGCATCCAACAACAGTTTCACTGGGATTAATGTACCTCCGAATAAAATC
ATTCAGCCCCATGCTGGTGATGCGATCCTTTCC
CTCCAGCAGTAAATCCTCCACCCAACTGTCAAGGGTCCACTTCGGTTGCTGCCTTTTCCCCACTCCTC
TGCGAGTCGCATCGGGTTGATCGGTGTCAGCA
TAGGACTCCGGCCTCGTTCTCTTCCGACAATCTCCCTGCGGCACAGTGGGCGCTTGACTCTCCAGGCA
TTGTTTCTCCTTGCTTTCTCAGTTAGTTTGAG
TTAAAACTAAACCAATGGCCAACAAACAAAAAAGGGAAATGAGAAAAAAATACAATAGGCACAGTCAT
GGCTCGCAGCCACAACAAGAGACTCACCCACA
CAGTGTGACGGCACAGTATTCGGTGCACACCAAATGCACGAGGACCACCACAACGATATGTGGATGTC
ATCCACCCAGCACAGTGTTGGTGGGTGCCAAC
AATAACAGCAACGTGGTGTACACGGAGGGTTCGCCCACGCATACCGCTCGCCACGGTCCCGCACCCCT
GTGCACATTAACCCTGACACACACACACAAAAGT
GGGAGGAGAATTCGGTGCAGCAACGGCCACATGCAGGGAGGACAAATCATACACACCGCAACAGCGAT
GCATCACGGTAATTCCCCACACAACAGAAAAG

AGTGCAACCTGCATGCTTTGTGGAAAATATCTGCGGTGTGCATGGCAAAAGATTGCCGTGACGCTGCC
TTTCATATTGCATTCGCCATTCACGTATCACT
TGGAAGCACGCAGCCACCACTCCTCCGTTCAGCTCCCCGTGCTGCTCGTGTCGCTTCACTGGAGGAAG
TTGTCACCGCACATCCCCATCACCGTGCATTG
CGGAATACAAATCAACCACACGGCAAGAGTGCGGTGCCACCCCGCAAAAAAAATCCCAGGATCTTCCC
CACCGCCTCGTGTGGCACGGGCGGCAGTCTCA
TTATGTCTGTGAAGGCTTTTTTTCCAATCAAGAACGCGGCCCCACCACACGCCCGTAAAAACGTTTTG
TCTGTCGCACGCAAAGGAGCTGAGCGGGAGAA
CAGCAGGAAAGACAACACCGGCAGCCATGAGCAGCACGCACACGCCAACGCACCGCTGCCCCGCATAT
CACCAAAAATAAAAATAGCAAAGAAACATGCC
ACATCCCAACTGCAAATCGCGGGTGCGGTTGCCACACGCACTCCCACCTCCTGGGCGTGTGCGGCCCC
TCGCTGCTCGGCGCTCCAAGCGGGATCAACAA
AAGCACCAATTCGGGAACATTCGACCACCGCCACAGCAGTCACGCTCCCCATTTCTCACAAAGGCAAA
GGCTTTGCAGTGGTGCAGCGGACGGCGGCATG
CACATTCGCAGAGTAATGGACTGCCGCGGAAATCACGCAACGGCGATGCGCCCCTCCTTTTTGGGAAA
CCCAACAAAAAACATGTGTCGCGCAGTCCCCG
TGAGCAACACACATTGAACCGTATTCAGCTCCACTACTGTGGCCATCCGCACTCACAGCATGAGTGCG
ACAACGCAGGCAAGGAAGCACGGAATAATAGC
ACGCATCCCTCCCATAAAGGTCAGGCGGAGTCCGTGCTGCCTTCAACAATAATTCCACCCCACATCGC
CCCTCTGCATATCCGCAGCAAAAAGGCCCACA
CGGTGCACACTACATTACAATTTCCTAAACAGCAAACCGGATCATCAGAGTGCTGCCCTCTTCTCAAT
GTTGGGTAGAGGCTCTCTGATCCTTGTCGCCG
CCAGCCTGCGGTATTATGGAGGAGAATCACGTTCTCGGCATATCTCTCCATTTTCCCTCGCTTTCCCG
ACGCCGCAAATGAAGTGACGTCCCACAAGTGA
TTTCGACCGTTCTGAAGAACGTGACGTGTCAGACTCACTGAAACTGTGTGCCTGTGTATTATTACGGG
CGAGCGACACAATGCTCTCGCACATCCGTGTA
TTCCGATCCTGCGCAACACCATATTTGGCGGTGCTGAAAACGTTAGACACCAAACACGCATAAATCGC
ACAATGAATGCCTCTGGAAAACAAACCCCCAT
TAACGTCAACACAGGCACAGTAACCGAACAAATTAACAAAAAAAATCCAACCTCTGCGCGTCCTCCCG
CAAGAAGGTGAAGACACACACACTTGTTCCCT
TTAGTTTCACTCCAACTGCTCGGAATTGATGGGTCTATAGTAGAAAACACGCCACTCTTCCCTCGCTC
TGTCACGAGGCATGGAAGCTGCGAGAGGCTGC
CAAGATGAGCCTCACGTGTGTGGACCCCAATGCTCGGGGCAGGTCGACAGCCACCCACACTCACCCCC
CACTCACAAAAGGGCAAGGACAAGGAGAAGAG
AAGCCCGCCGCACAGTGCATTCAAGAAGACACACGCAGAAGCACCGCCATCCACATCGTACCGCAAAA
CAGCAATGCGAACAGCACACACCCACAGCCGC
AACAAGAGGAACGAGGCGCATCCAAGTCCACCACAGTCCTTTACTTACCTCTGCCCCGAAGGAATAGC
GTTACGCCTCACAGCAGGTCGTGGCACCCCAC
ACTCACAGTGAGGCACGGCGCACACCATCCGCTCCCCTCAGAACGCACTCATCAGGGCACCCAGTCAC
CCTCGCACCATCGCAATGGGGAAACACAAACA
ATCGTCGTCGCGGACTATCGATGCCTCTAGCAGAAAAGAAAAAGCGATGAGGGAGTGTCAACTGCACA
GCTAGCAGTGGGACACCCAATGGGACAAAAAG
ACACATAGTGGCATTCAGCACAGCAAATAATAAAACAAGCCGCAGCGAATTGCCAAATACAGAAAAAC
AAGAAAAAAGGCGTAGGTTCAGCAGAAATAAA
TAAATAAATAATGATAAAAATTAAATCACCTCAGTAGAATTTCATTTCATGTATCCCTCAGCGCAGCA
GGAATTGTGCATCTCCATATGTTTCTTACAGA
GGAGAAAAAAAAGATCGCAGTGTTCCACGTGTCCACACGAAATCCCTCACATTTCATGAGGCACTCGG
CCTCTTCCTCCACCCCTCGTTTTGTACAGTCC
GTGGGACTGCACAGCAATGAGGCCGGCACCGCGTGCGGTGCAACAAAAGCAAATAAAACAGGGGGAAG
CACGTCACTCATGAGAGTGTGCAGCTAGCGGC
GAACAAAAAAATAAATTAAAAAATGGTAAAAAGACAGAGGTTCTGATCCCTCCAACAAGGCACCAGCAG
CACATTATATCCAGAGGTGAAAACAAAAGGGA
TAAATAAAAAGTCCTCAACGGAATGCATTGAGTCTTCCTGAGGAGCTGCACACCCTTTTCCACGGAGA
GTCGCGGCTCCGCTCCCACCTTCTCACACAGC

TGAGAGCTGCAATGCCCAGAGCCCCAGAAAAAAAAGCAACAAGATCCGAGGCACACACACACACACGC
ACCGTGCCGTAGCCACTAAAGCCCTTCTCCAA
AAAAGTGTTGTTCGTGAGCTGAGAGGCGTTTCCATCTTTGGAGCCACTGCCAGTTCCCGCTGGATCAT
CCGGCCCTTCAAGCATTTTCCCAGAAAGAGAC
TCTAGAAGAGGAAGGTTGCTGGCACCAACTTTTGCGGCTTCTTCCCGGGTCAATATGCGGTTGTACAA
AAAGACACTCGTCACTGTCAAATAATTGTTTT
GGCTCCCGGTGTCGCTTGCACTGCTCTCATCCCCTCCAATGTAAAATTGTGAATTTGCGCGTGGCCTC
TTTCCCGATGATGATGTCGTTTCAGCATCCCA
CGCACACTCACCATTGATCTATACGAAGGCCTTGTTGGCGGTCCTGCAGCGTGAGCATAACTCGTTAT
TGTTTGTTCAACTCCCAAAGGCCGGTGTGTTT
GTTTGCATTGAACGTCCCCAACTCCTTTCATGGCTGTGCGACAGTCAAAAGTGCACAGTGCGGCCTGT
TTCCTTCGCGCCAATCAAAGGGATACTCTTGA
CGCCTGCGTGTGTCTCGCAAATAGCCACCGTCGCCACAAGAGCGAAATTTTAATTCACAAAGCTACAG
TGATTATTATCCTCCCAACGGTTCACGGGTAA
CATTGCCCTGCGAATTGAGGCCCATTTGTGACATTGTTGTCATTCGTCACCGTTGCAATCACGCAACG
GCACTCGTCGATCCACGTGTTGGCAGTGCCTG
CGTTGGAAAGTTATCCAACCAGACCCACCGTACTTACGGGCGACGCGGAGAAGGAAGCGTACACGTCC
GCCCAATTACGGAGGATGGAAGTGATCATCTG
CAGCTCCTCCGTCAGGGGAGGCAGGAAACAGGCTGCTTTTCTCTTCATTGTGCCTCGCTCCCACAAGG
TGCAACGCACCATCCGAGTACAGCATGGCGTT
GGCAATCGGCTTATTCAGAACAGTGTCCATGTAAGCCGGTCCAACACAAAACACGCGGTTGCTGTCAG
TCACCCAAACGTAGAGCGCATGGGCCTCACTC
TCCCACCACGGGGAGCTCACTTCCCGAGCCTACAGCATGACGCTCTTTCCTCCAACGGTCGCAGTGAC
AATGGCTCCCACACGCAAGCTCTTGTTCCAAC
GACCCGATTGTGAATTGACCCAAACGCCTGCAATTGTCCCGGGTACTTCCACCCACATTGCCCCCTTG
TCACGTGGCTCGTGCACTCCGCAGTACCGGCT
CCCATAGCAATGAGTAATCACGAGAAGTTGTCCCTGCTCCCACTCGGCAATGAGAAGGTCAATGCAGT
CCGCAGGAAGAATGCCCCTTGAAAGCACTCAG
CTGCTGCCGCCGTCCGTCAAACAGATGACCATGGAGAAATCGTCTTTTTCGTCATTGTACGCAATCAC
GGGGACACAAGCGTGCCATTCAAAACACTTGA
GCCACAAAAAACCTTCAATCCATTCGCACCGCGTTCGTGAGTCTCGGGAATGATCTGTGATCACAGCA
ATTTGGGTTCACACCAGTGGATCAGCTCGCTC
CGTCTGCCATCATTGGGCTGCGTGAATTCACCCACAACCAGTTGAACATCACGGTCCCTCAACGCCCA
ATTGAACCCACCATTCGTACCAAACATTTGCT
AACTTCCCGCACGAAGAAACGCCCTATCGCCGCTCTCAATTGCCGTGGGACTGGACGCGGAGCCTACA
CCACCCTTTATATTCGCTGTGCTCAACACGAT
GTGTGCATTCCGGCCATTATTAAACCCACCAAAAAAAAGACGAATTCCACGCAAAAAAGATGGAGCCC
GCCAAAATATCCGCATGAGAAAGCCCACTCGG
TGCCCCATCCCTATCATTAAGCGTCGTGTAACCCCCATCAAAGACAACCGTCAGTCTACAAACACCCA
CAAGAGCGAGCGATCCAAACTAATCCCTCCTC
CCACCTGCACCAGTCCCACCCTTCGGTTCCAAATGCGTTCATCCCGGAACAAAAATAAAGGCCACCCA
CGACAGCCGCGCAATCACTTGATTCTCCCCCG
TCGATGCGGCCGCAGCTCCACAGGTGGCTCAGCACATCACGACAAGGAGGAGGAGCAGCACCGCGGAA
GTAAACACACGCCGGGACATGTTGGGCCTCGG
CGGCTCACTCTCTCTTCCTTCCCTCCTCCTTCCGCTGGATCCGGTCACGCGGCGACGGTTGTGTGTGC
GGGGTGCCTCCACAGCAGCAACACGTGAGAGC
ATATAAATATGTGTGTGTGGGCGGTGTGTGTGCTGTTGGTCCTTTCAGTGCCTTCGAATTTCACAGTA
GCGTTTAGCGCTTGGTAGTAAATTCCTAGCGT
GTCCTTGTCCAAGAAGTGGAGAAGGCAGCAACAGCGGTAATAAATCAGACACGTCAGCGAGGAGCAGA
GCACATTACTCACGCAAAGTGCGGGAAAAAGA
AAATCCGGAAGGAACAGCATGGAAAACAATTGCACACAGAAAACAGCAAAAACAGCAACACGGCAAGT
CACAGAAGAAGCCAACACACATACACATGGAC
GCGGAATTTTATTACTCGGCGACGCCATTCAAGGCCATGCCGAAACAGTCCCGCAAGTGATACAGTGA
TCAGTCACACGCTCCCGGTGGACCGCcgcggc

aaagacgcggacacaaacgaaaatatgtgcacaa
>scaffold1131.size6376
GTTGTTTTTGTCGATACTTTTTCGGTTCATGCGGACTGTGTCTTTTTTTTTCTTTTATTTCTATTTCC
TTCTCCGTGCACTCAGTGCATTCATGTCGGGAC
CACTCCTGACCCTGGCATGGGTCGACAACCACCTCAAGCGACGTGGTCTGCCAAGTAAATATGCATCG
GAGTCAAGGGCGTGCACGGGAGCGATCTCAGA
GGCACACTTCGACGGACCGCATGTGGCACCATCATCATCGACGGATTCTGTGCCCCCGGTGGAGGAGT
TACTTGCCGCGGTTGGTCACCGTCAGATGCCC
GGTGGCAAGCGCGAATCGGTTCAATCAATTGGGGACGGATTACAGAACACAGGGGTAGACGACAGCAG
GGACGCCAAGAGTGGTGACTCCCTTATTCAAT
CTCAGCGGTCAACTCCGCAACGCGTGGCTTCGGGTGTGGCATCGTTTGTGGAGCAACAGGCTGGACGC
ACGGGCACCTTAAATGGCCCCACGCAGGCATG
CCTTGTTTTGCACACAGTTCTTACGGCGTGTGTGCGACATGCACTTCGTTACAGTCGAATTGATCAGT
CTTATGACGCTGCTTCTTCTGTTTTTTTTCCA
TCTTCTCCCTCCGCCGCGACGACGGACGTTACCTCAGGCGCTTTGGGCGATGCTACTTTGGAACACGG
GGCAACACTTGAGGAGGTCGCAGCCTCCTCTC
AGATCAGATTTCCCTGGTCGGCACGTGTGGCGTCGGAACACTTGCTGGAGGTCATACAAGATGCCGCC
ATACAGAGGGGATTACGCTCCTTTCTCTGGAA
GGTTGTGGACAAGGAACACAAATGCATCATGCGGAACGCCGCGGCTCTGTGCTTGAACGAAGCCGCTC
TGTTGGCTGATATTGGTGTGGAGGAGGCGGAG
GTGCGGAGGCTATTTAGCATTCCATCGGATGTTTTGAGCTCAGCACGGGAGGCTCTTGCCTTTTCTTC
TTCCGCTGCGACTTCCTCGGTGCGCATGTCAT
GGTTAAACCAACAGCCACTGTCATTTTTGGGTGAACATCATTTTGTCTACACGACGTCGTCTCCGCGT
CTCGCAGCGTTGCTAAAGGAGGGGACGTCTGA
ATTGAAGCCAATAGAGCTGGAGAGAACCAATGCGAGGGTGAGAGTAGGCGCTGGTGTTTCGTCGCCTG
ACGGGTTTGCACGGCAGTTAAATTTGCTTTTT
TTGGAAATGACGCGCGTACGAATGGCGTTGGTGTTAATTCTGCAGTTTGAGATTCTCATGCGACGTTA
CGCGGCGTTTGGCAAACAACAGCCCGGTGGTG
TGACGACAGCGTTCCTACCCTCGTCATCACCATCGTCGCTGGCGCATGAATTGTGTGCGTTGGCCATA
GCTGCTGGGTCTCGTGCACTGGAGACGAGTCA
GCATCGCAAGCGATTTGTCACCCAGTTTCGTGTATTGGCCCGTAAGCCAGAGCAAAGGCAGCATACTA
ATTCAAATCTCGATGACGCACCGCATTGCAGC
CTCAAGTCCGTCTTTGCCGCACTCTCTCAGGCGGCTAAAGCCAACGCGGCGACGGAGAGGGCGGCAGT
CGATGGAGTCAGTGCTTCGTCTGTTCCTCTTT
CTACCGCGGCCTCAAGTTTGTCGGCACCGGCCACAGGGTCGGAGGATCCGCTGGAACACGATCCAGTG
TTGCATGCGGTGATGTCATGGAAGCCATTAAA
GCTGGAAAAGGCAACGGACCGCAACGGCGCTACGCATGCATGCGAATCGTTGAAGGCATCTTCAGCCG
AAGGATCAACCGATCCGCCTGTTCGGAAGAAG
AAGAAGCAAAAGCAGGCGCGTGCAGGAGCAGAGCCATTGCCTGAGGAACAGGAAAATCAGGCAGGGTT
AACGTGGCACTTCCGTTGTTGTGGAGGGGGAAT
TGGGGGCGGAATTCAACGCCGTTGACGCATTGCCATGTGGTGAGACGGATGCGAAGGTGCAGCTGCTG
CGCCCCTCGATACGTGTTCCATGGCCCTTCGT
CCTGGAGGTGGTTGCGGCCGGCAAGAACAAGAAGAAGGACGCCATGACAATTTCCCCTTCAAGATTGG
GCGCTTTGGAGAAGTTTACTCGACCCCTGCCG
AGTGCGGCGATGAGGATGCGTTACAATCCCGCGTGGATGACGGGTGATGAAAAGATCACCCCGCTTGA
CGCGCGGCACACCACGCAACTACAGCGTCTGC
TCAGACTTCGCAGTGACCGCCTGCTGATGATAAACGGATGCCCTGCGGAGAAACTGAAGTCGGTGATG
CGCATTGCAGAACGGAGTGTGCGCCTGGTGGT
GCGATTTCGGTAAGGCGGGAGTGGGGCGTCGGGGGGCACACGGTGCCGGATAGAAATGCGGTCGATTT
CACGGAGGAATTGACGAGGGGGCGAGCGTGAG
AGAAGAAGACGATGAAGAACGAGAGAGAAAAAAAAATTATTTAGGAGGGCGACTGGGAAAATCACGAG
AATACGCAAGAAGAGGAGCGTGTTTCCCACGC
ACGTTTTGTCTCTACCTTTTCTTTCATGTCTTGGTTGAATTACACGTATGCTTTTTTTTTTTTTTTCC
CTCCTCCTGGCTCTTGTATACACACACACACA

CACGAAGATTAGGGATTTTGCGTGAGCACGTGACGGCTGGGCAAGCTACAGGAAACACAAATACACAC
AAAAACTCTTTTTGTATCCTTGTCTTTTTTTT
TTTTCTTTTTTATTTGCGTGCGTTCTTTTTTTTTTTTTTTAATTGCCATTGTTGATTCTTGCTGGTGC
AGCTGCGGTGGCGAGTAAAAAAAAGAAAAAAG
AAAAAATTTTTTCGAGGGGAGAAGGGACCCGAACGAAAAGGAAAGAAAAAAAAAAAGAAAGGCAGAGT
GGCTGAGCTTTACGGGACGGAGGCATCATCGC
TACCACTCGAGCACAGGACGACGTAACACACTCACACTCACACTCTCTCTTACTCCCACGGAAGAAAC
AACACATCACACAGCAATAAAGTTATCAACCG
GCAAAAGAAGAAAAGAGAGAGAGAAAGCTCAAACAGTTCAGCCAATTATATATATATATATACAGACA
TTACCCCGAATTGGCAGAACAATAAAAAAGCT
AAAGTAGTAGCAGTTAAAGAAAGGAAACACACAAACAAAAACAATATAATCCCCAGGAACAACAAGAA
AAGGCAAAAAATGGGAAACGGCGCCTCAGTCG
GGGAGAACCGCCACTCACAGCTGTTCCAACAAGGCTACAAGTCCCTCCAGGAGGGACGCTACACGTAC
GCCGAGATTTACTACGACCAGGCCATTGAGAG
GCACGAGGGCCATTCCTTTTGGACCCGCCTTGATGATCTTATAGAGTGGGAAAGGAGGGACAAGGCTG
GCCAAAAAGGAACGGATGCAAAGCAGAAGACA
GCATATCGGGATGGAGGGCAGGAAGAAGCCGCTTTTCCAGAAGTAAGAAAAGATTCGTTGAAGGAAGA
AAATGAAGTGAATGTTGAAATGGGGGAGGCGG
CTGGCGTGGGTGGCTCTGGGAAGATTGAAATTCCGTTGGACAGCCGGCTGGCGGACGTCGTGGATTAT
CTTGTGCTGCGTGCCGATATTGCGGAATCTTA
CATGGCAGAAGGCCGATACGAGGAGGCGACGCCGCACATGGACTTTCTCTCTTCCCACACACGTGTAA
TGTTAAAATTTCTTCAGATCGCCCGCATGACA
AAAGCGGAGGAATCCGCCAATTCGTCCAACCCGCTGGAGGGGTCGACGGCTGACGCAGGCGCTGACAA
CGCTGGGGGAGGGAATCGTCGAGCACACCAAG
GCTTTATGATGGTGTCTGACGTTGAGCGGGATTCCATACTTGATTGTTTTGAACAGTTTCTACGTATG
CACTGGGTCTCTGCCATGGCAAACTCCGTCTT
CATCGTCTCCGAAAGGTTTAAAAAAGGAAAAAGTGAAAAGAAACACAAGAAGGAGCTTGACACTGCTG
TGGTGGCATGCGCAAAGATCCAGGAGGAGCTG
TACAATGTTGCCAAGCGCCGTGCGCGGAGCCTTTCAAACAGTGTTGTGCTTTCTTTTCTTGAGGGCAT
TATGGAGGAATTGGATGCCGAATCCTCCACGA
CGTCCATTACAAATGAACGTATGGGTACTGCCAATGGTAAGACTCGTACCAACACATTATTTGAAGAG
CAGAACAATAGGTCGACGGGCAATAGGAGTGC
CAATGAAATCGATATGTTGACAGATCGCGACGAATGCCCCATCAAGGTGCACCTCCAAATGGTTTATG
ACCGACGTTCACTATGCCGTGAAGTACCTGCG
CCACAACAGGCGCCTACACTGCGGTACATTACATGGATTGATGATGAGATCCGCCGTGTTGTGCCCGG
CACGAATTATAACCTCGAGATTCAATGCGGCA
TGGTGGACGAAAAACGGAAGAAACAGCTACAAAAACGTTCTTTGAAGTTTGCCGCCCAAAGGAATTGC
AACTACAACGATCGCGTCCTCTTGGGAAAGAG
CTTCGACGAAGAGAACGCGCTGCTTTTATTCCCTGTGTTTCTTATTCAAGCCGACGTGCAGTTTGAAC
TTGGCGCCGCCACAAAAGGTATTAATGCCCTC
GACATTGTTGACAAACTTTCCACGCAGCTCTACGGCGTGGGATCCCTGGAGCGGCAGAGCATCATGCG
GTCTGTGATGGAGTCCCGCAAGCGTGGCGGCT
CCCTCTTTATGATGTTTGAGGATTAGCCCGGTGACGATGATGATGATGATGATGTACGCAAATGATAC
ATGAGCCGAAAGAACTCGCCATGACGACAAAA
AAAAAATAAATACAAACAAAGAAAAAAAACAAAGACAGACACATACGAGGGGAGGGGGAGAAGAAGAA
GGAAAAGAAAATAAATAGTAGAGAAGAAAATG
ATGAAAAAGAAACAATACAAATTTGGATTTTTTATCCCCTTCCCCACCCTCCCTTTTTGTGTCGCCTT
CTCCACGAATTTTTATTTTTGTTTTTTATTGG
TTTTATTTTTATTTTTATTTTAATTTTATTTTTTGCGTGGCCATGCACTTCCGCAAGTATCTATCCGT
ATATAAATATAAATATAAATATATACGTATGC
GGATAGATAAGGTTGTATGTATGTATGTTTATGTATGTTTATGTAAGCAATGATGGGCTACAAGAGGA
ATGATACAATTGAAATATATATTTCAGTGCAT
GTGTAACTGCGTGTGTGTGTGTGTGTTTGTGTGGAGGGATATATTTTTAAATGTTCTCACAGCAGTTT
TATCTCTTTNNNNNNNNNNNNNNNNNNNNNNN

NNNNNNNNNNNNNNNNNNNNNNNNNNNNNNNNNNNNNNNNNNNNNNNNNNNNNNNNNNNNNNNNNNNN
NNNNNNNNNNNNNNNNNNNNNNNNNNNNNNNN
NNNNNNNNNNNNNNNNNNNNNNNNNNNNNNNNNNNNNNNNNNNNNNNNNNNNNNNNNNNNNNNNNNNN
NNNNNNNNNNNNNNNNNNNNNNNNNNNNNNNN
NNNNNNNNNNNNNNNNNNNNNNNNNNNNNNNNNNNNNNNNNNNNNNNNNNNNNNNNNNNNNNNNNNNN
NNNNNNNNNTTTCTTTCTTTCTTTCTTTTTTT
TTTTTTTTTTTTCTTTTGCGTAATGCTGTGCGGCTCTTCTTCTTTTTTTTTTTTTATTATTATGGAGA
AGTGATGTTGTAAGTCGTCTGTAAATGAGTGT
CTTTTTGGACGGACTCATTTGGATGCAGTTGACTGTCCACTTCTCTTTTTTTCAAACGTGTTTGCTTA
CGGCTTCATGCGGCTTCTCCCCCTTTTTTTTC
CCCCCCCTCCTGCTCTCTCTTTTTGTTTACATAAATAAGTTTTTGAGTGCATGCATCCTTTTTTTTTT
TTTTATTTTCTTTTTTTTTTTTTTTTTTTTGG
GACGCTCCTCCTCCTCCTCCTCCTCCACACACCGCCACCTTTTTTTTATATATTTATATTTATGTGCC
TGACGTATATGATTTGTCCTGGAAGAAGAGGG
ATATAATTAAAGTGAATATTTTTTTTTGTTTTTTGTTTTGTGTTGTTTTGTTGTTGTTTTTGCTCCCC
ATTTCTTTGGTCATTGCAGATATAAATAAAAA
TAAAAAAAAAATATAAATATATGTATGTGTACGTATGTATTTATTTGAGTACAGTCAGGCATCCATTT
TGAAAGATCCGTGTTTTTTTTTTTTTTTTGTT
GTTGTTGTATAAATAAATAAATAAATAAATGAATAAATTTATATGAATTTATTTAAATACAAAAGCAA
AGAGATATGCCTGTACACACACACATATGCCT
GCATTCATGTATTTTATAAATGAGTGCAATACAACCAATTATATATCTTTTTATATATCTCTCTAT
>scaffold1132.size10819
AAAGAGTAAAAACAGGCGTCCGTCCATGTGGAATGAGAAAGAACTCCACCGCGGTGTCCTATTTGACG
CAAACGCTCCGCGGCCAGTGCCCGGTGTCGTGT
GCGGACTCCACAAGGAGGTTTTGGGCACGCCACTGTGCCACACGGAGTGCCCCCTCGAAACGCAACAG
CAGGCGTGTCCCTTCCGGTCGCACTCGTGGC
GGTGGCGGCATCCGCTGCCTCGCCGCGTGCTGCTGGCGTCGACTAGGATGCTGCAGTGTGATAGAGTG
TGCCCGGCAGAAGCGGTGCTGGGTTTGGTGGG
AAATGCCCTCCGCGGCGAGACACCCAGCGCGACTCTTGACCGCGGCGCTCACGCACTTCCCTCCGCTC
CTCTCCATCCACCCGGCAATCGCACAGCTGCC
AGCACATTGTCTCCGAAGCAACACACAAGTGAAAACGTGGCAGCATATTTCCCAAGAGAACAGAGTGG
CAGCCACACGCTGCGGCCATCGTGCCGACGAG
TCCGGCGCAAACGCACCCACACCCACAGCAGCAGACACACACAAAAAAGAAGAAGTCTTCCGCTCCGT
GCCACGTCCTCGTGCGTGATGGTGCGGCGCCT
CAAAACAATACCGCCAATTGCCGTTCAGCATTTCCACAGAAGACGAGAGCCACGACGCACTCTCCCTG
TCACTCCCTTCCTTCTCTTCATTGCCACGGAG
TGATCCAGCAGCAGCCACACGCCGCTTCCACCACCGAGTGCAGGAGAAGCATACAACTGCACCGCAAC
ATGTACGCCGTGCGGTTGTGTGCCTGCTGACA
CGCAAAGAAANNNNNNNNNNNNNNNNNNNNNNNNNNNNNNNNNNNNNNNNNNNNNNNNNNNNNNNNNN
NNNNNNNNNNNNNNNNNNNNNNNNNNNNNNNN
NNNNNNNNNNNNNNNNNNNNNNNNNNNNNNNNNNNNNNNNNNNNNNNNNNNNNNNNNNNNNNNNNNNN
NNNNNNNNNNNNNNNNNNNNNNNNNNNNNNNN
NNNNNNNNNNNNNNNNNNNNNNNNNNNNNNNNNNNNNNNNNNNNNNNNNNNNNNNNNNNNNNNNNNNN
NNNNNNNNNNNNNNNNNNNNNNNNNNNNNNNN
NNNNNNNNNNGGCGCTCAATGCCGAGTGCACGTGTCATCAACATACGGAAACATTAAAGACATCCCAA
CAGTCCCAAAAAGTTACACTAGATATATATAT
ATAAACAAAAACAAATGCCTTGCGGTGCGGTCCCCGGAGGGAGAGCACATCCTTCGTAAAGACTTACA
CGGGCAGGATGAGGTAGTCAAACCAGAGATAG
AAACAGATAAAATAAAGGGTGCGTCCACGCAAAACGTCAATTATTTCGGAGGGTGCGTACAGTGGCAC
ATATTTTCCCACTGGTGGCAGGGACTCCTCGC
TCCGACCTCTCAGTAAAGGGCCGCAATGTCCCACAGCCCCAGCAGCAGAAGCACCCGAGACACGTCCC
CACGCATGGAGTCGTCACCATTACCTTTTGTG
GCGTCGCTCTTCTTGACCATTTTTAGTTCATCGGCACTCAGTGGGCGGTCGCACAGAAAGACCTTCGT
CATCGTCATACTGCTGTCGCTGTCGTCTTCGC

CTCCCCCAATGTAGAAGTGCGAGACTTCAGCCCCCCGCACCTCAGGTGTTGGTCTCGTTTCCAAGCTC
CCCACAAGCCGGCCATCCACGCACACGGAGCT
CCTTGTTGCCGCCCTGCACCATCAGCGCCGCCTGGTATTCTTTCCCCGGCTCCCAAGTTCTGCCATGT
GCAGTTTTATTGCCGTCAAACACTATCTCCCA
CTTACCCTCAGTGCTGTGCGACAGCCCAATGATAGTCGTGCTGACCGGTGAGGTCAGCACCGCACCCA
GCAGAGAAGTGCTCAACCTCGGAAAATTTTGT
ATGACCACCGTCGCCACAAGAGTGAAATCGCGGCTTACAGAAGTGTACAGCCCAGTTCTCTCCCGGCT
GTTTACGGGCCACGTTGCCCCGGACCCAGGCC
TCGTGAATTGAAACCCATTTTCAACCTTCACTGCATTCGTCACGCTTGCAGTCATGCAAAGGCAATCG
TCAATCCACGTCCCGTCACCACTGGCTGCATT
GGATAAGAGTCCAACCAGACCCGCTGTGGGTGTGAACGAATCTGAGAAGGAGGCATCCAGCTGTGCTC
AAGTACTGAGGGCGGACCTGATCGTCTGCAGA
TCCTCCATCAGGCGAGAAAGTGAAATGGTCCTGCTTGCTCCAATAACCGCATACTGCGAAGGGTGCAA
CGCAGCATCCAAGTACAGCGGGGTGTTAGCAA
ACGCTTGACCCACATCATCCTCCACAAAAAACAATCCAACATGAAACGTGTGGTTGTTGTTTGTAAAA
AAGAAGTTAGAGCGCATTGGCTCTTTTCTCCC
CCGAGAGCTACCCTCTCTGGGTGTACAGCATTAGTTTCCCTCCCTCAATTTTCGCGGCGGTAAGGGCT
TCCACACGCAGGCTCATGTCCCAAGAGACTCC
TGTTCGTGACTTGACCCACACGCCTGGGAGTATTCTGACAGCCCCCGTCCACGTGTTCCTCATGTCAT
GCAACTCGTACAACTTGCGGCCACTCTAAAAA
AGAGCAACCATGAAAGGTGATCCCTCCCATTCGGTGGTGTGAGAGTCAAGGCATTCCGCAAAGGAAAT
GCCTTCTAGGAGCACCCAGTTATTGTCGTTGT
CCGTCGAATAAATGTTAATGGAGACAAATTCACGCTTGTCATTCCTTGCCGCAAAAGGAAACACAAGC
ATGCCATCCTCCATGAAAACACCGGCGCCAGC
AGTGGGGAAACGCTCCTTCGCTTCTCTTTTAGTCCGTGGAATCCTCTTTTTAACAGCAATTTGGGTTC
ACCCCAGTTGATCGGTTCACTCGGCCCGCCGT
CCCCGGAACGAGTGGCCTCACCCACAACCAGTCGAATATCTGAGTCAATTACGTTCCAACTCTTCTTA
GCATCGTAATACTTTTATTCATAGATTCCCAC
AAGTGGAAACGCCCTCTTGTCATTCGCAGTGGCTGTGGGTTGGCGCGCAACATCCACACGTCCCGTTT
CATTCACTGTACTCAACACCGTGTGCGTCCTC
TACGCATCCTTAATGGCCTCAGCGACAAGAGAAGAAAAACCCCACGCAGGGTTGGCGTACCCCGCAAC
AAAGTCAGCTGGATAAACCTGTTTTGTTGGGC
AACGCGCCAAAGCACCCTCAGCAAGCACAGTCATTACCCCACCAGCACCGACGAGAGAGGGTGAAACA
AAACAATCCTTCACaTTACTTTCAGACCCATC
TTTTGGTTTCACCGGCGTCTCCTGCAGCACAAAAATTTCGGCACATTGCAGCAGCCGCACACACCATG
ACACACTCCCCACAGCGTGTGCAGCCCCACTG
CCGCAGCAAACCAACACAACAAGAAGCACCAGAACGGCAGAATAAAAAAGATGCCGGGACATGTTGAG
CCTCTGCGGCTCACTCTCTCCTCCTTCCCTCC
TCCTTCCGCTGGATCCGGTCACGCGGCGACGGTTGTGTGTGCGGGTTCCCATCACTGCAGCAACACGT
GAGAGCATACAAGTGTGTGTGTGGGCGGTGTG
TGTGCTGGAGTCGGTCCTTTCGGGTCCTCCGGGCCCCGAcGCTCGGCAAAACCGAGGCGCCGCCGCGA
AACGAGCGCGCGCGCCCGCGACGAGCGAAGGA
AACGGGGAGCCCGGCCCGACCCCAACGCGGCCCGCGTTCTCTCGCTTTTGGAGCGGTTGTTTGAGTGA
GGAGCCGGCGCTGAAGGAAATTTTGGAAGGGG
GAATATTCCGTGGCGGGTTGCGGCTTCGGGTGACAGCTTTTAGGGCGGTAGGATGGCTGATCGCGAAC
GTCTTTTGGTGGGATCTTGGGGCTTTTACCCG
TATTGGTGCTCCGCGTTTTTTGTTGTTTGTTTTGGCGCGTTTTTTTTTCTTTTCTCTGGAAAAAAACG
TTGAATTATTTCCGCTTATTGTATTGAAAATT
TTCGCTGTTCATATATAATTTGTATGTTCGTTTGTTTGTTTGTTTGCTATTGTTGTTGTTTTATTTGA
TCGCTATTTTTACTATTATTTCTTTAAGAAGC
AAAGATTTTAATTTAAAAACAAAATGGCTTCTTTTACATCTTGTGCACGTATTCTGTACAACACAACC
AATGTTGACCGTACCATGCACACGATCGTTCA
TTGCAGCGATGGAAAATACGTGGAAGTGCCAAAGGCGGTTGCGGATAAATGCCCCTTTATTGAGAAAG
TCAAAGAAGAAATGATTCCTGAATTTGAGTAC

CCCGCAGCCGTTCTTGAAAATCTTGTCTGCTGGACAGAACACTATGGTATTGACGGCTTTGCCGAAAG
TGAAATTGTAAGACCATGTATTTACAGAAATA
TTCTCCACGTCCTGAAAAACAAGTGGGACAATGGCTTTTTTGTTTCTCGCCTTACCAGTGAACTAAAC
ATTGGACACTATCTACAGACCGTAAACGCAGC
AGAAAAGTTTAATATGAAGGGTTTGCATTGCTTTCTTTGTGTTGGACTCAGCTGTAAGTTCCGCAGTG
AGGACGATGCAGAACTCATTCACAAGATAATG
GGGCTTCCATCTAATGTGCAACCGAACCAAGAGGATATTGAAAACGTGACCACACGGTATCCTTGGCT
GAATGAAGCCATTGAACCGGTTGTGAGGAAGT
AAAATAATTTAGAGATATGCTGAAAGCGCACCGCCTTATTATTATTTATCTATTTATTTATTTATTTA
TTTATTTGTTTATTTATTTATTTATGCGGCTG
CCCCCATACATGGGATTCACAGTGGACCACCTTCCTGAAGTTTTTTATGCCGGTTCACATTTAATCCT
CCTTTGATTGAAATACATACACGTTTACACAG
AAAAAGTTAAGTGATACGGAGAAAAGCCTAACAAGGGAAAGGAACAATATGGGCAAACCCGGGAAAAA
GGCTGGAAAGGGATTGCTCCCGCCTACCAATG
TCAATCGTCGTGTCGATGCCAACAAAACTAGTTTGCGAGACCAGAGGACAATTAAACGATTAAAAATG
TATAAGTCCAAGCTTTTACGTGATGAAAAAGG
TAATATTGTGAAGGGGAGTGTCTTGCAGGCATCCGATCGTGTTGAACAGCAAATGGTTCGTGTTGCAC
CTGATCGTCGGTGGTTTGGTAACACTAGAGTT
ATTGGTCAGGAAGCCTTGCAGAATTTTTTGAAGGAAATGGGTGCAAAATACAGAGATCCTTACAGTAT
TGTTATTAAGCAATCTAAGCTCCCACTGAGTC
TTCTTGAATCAAGCGGGATGAATGAGGGCAGTGTTCGTCAGCAGATGGAGTGGGAAAAAAACCTTTGGA
GCGAAGGCCAATCGAAAGCGCGTTCGTCTGGA
TACCATTGATATGGAGGGGTTTGCAAATAGGGCCGTATCGAAAGGCGAGGAGTATTTGACTGAGAAGA
AGGGAGTCGATCGAAATCTTATTAATAAAGAA
GAGAATTTACGTGATGATCGTTCCAAAAATAGGATTTTATTTAAAAAAGGTCAATCCAATCGTATTTG
GAATGAGCTTTACAAGGTAATTGATAGTTCTG
ATGTGGTCCTATATGTATTGGACGCAAGAGATCCATTGGGAACTAGAAGTTCTTTCCTTGAGGAATAT
ATGCGTAAAGAAAAAAAATACAAACACTTTAT
TTTTGTATTGAACAAGTGTGACCTTGTTCCTTTGTGGGCTACTGCTCGCTGGCTTCAAGTTTTGAGCA
AAGACTATCCAACCGTGGCTTTTCATGCAAGT
ATCAATCATCCGTTTGGTAAAGGTAGTCTGATTTCGCTTCTTCGACAATTTGCAAAATTGCAAAATGT
TACTCATCGAGGTAGTAAACGAACAAAAACTC
CTATTTCTGTTGGTGTGATTGGCTATCCAAACGTTGGTAAAAGTTCTCTGATTAATACTCTTCGGCGA
AAATCCGTTTGTAAGGTTGCACCTATACCAGG
AGAAACGAAAGTTTGGCAGTATGTTGCTCTCACAAAGACTATTTTTCTTATTGATTGTCCCGGTGTCA
TTTATGATCGCGAAACAAACAATGATGTTCAG
GCCGTTTTAAAGGGAGTTGTACGTGTAGAACGCTTGGGAAATGTAGACAAGACAGATGTTGTAAATAC
GGTTCTTGAGATTGTGAAGCCTAAAGACATAG
TTGCCACATATGGTGTTTCTGACTGGAGGGATGTGAATGACTTCCTTGAAAAACTGGCAAAGCTTAGG
GGTAAACTTGTTAGTGGCGGTCAACCCGATAC
AGAAGCCGCATCGCGTATGGTTCTCTACGACTGGCAAAGAGGACGAATCCCGTGGTTCAGCGCTCCAC
CTTTTGATTCTAATAAGCAATATCGTGATGCC
TTGGAGCTACCAGAGGCAAAACAAATGAAACTAATTGAACATTATGGCACGTTTAACATAGTTGAAGA
TGCTATGGATCACAATGAAAAATCTGATGAGG
AAACATTGCAGAATACTTGTGACTATTCTGGGGCTGAtTCTTCCAATGAAAAGCAAATTCAGAAGAGT
AAAAAAGAAACGAATATCCACGCGACAGTTGC
TAGTTATATGTCCGAGCAGATCATTTTGAAAAAGAAGGAACAACGAAAGAGGTCCATGTCCGAACCTG
CCATTAAAATTCCTCGAGCCAAGGAGACGAAT
AACAACTATAATGATGATGATTTATGGAATCGTTTCCTTGCTGCAGCCAAAAAATGAACAAGAAAAGT
GTAGGTTTTTGAAGCAGAGAGTAAACTAATAT
GAGTTAACATTGGTAATAAATAATAATAATAATAATAATAATAATAATAATAATAATAATAATAATAA
TAATAATAATAATAATAATAATAATAATAATA
ATAATAATAATAATAATAATAATAATAATAATAATATATCGTATTGTGTGTAGTAAGTGGTTTAACAG
CGCTCCCTAATAATGTTGTCATTATTGTTGCT

TTCTAATTTAACTTTTTTTTTGTTTTTGTTTTTCTTTTCCTAAGCAAGATAAAAATCATTATTTAAAA
TGATGTAATTGTACTCAAATAGGGAGGTTCAT
ACAGTATATAGTTGCTCGCGTGTATTTCTATGTATTTAATTTCCTCTATTTACTCTTTCTCAGAGTTG
AGCATTCTAGACACACCACCCTTCAGGTTGAC
GTTATTTATGTTTGTCCTTTTTTGTTGTTGTTGTTGGAAAACTTTTGTAGTTTTTTTTTTGAATACAA
AAAGAGACTTTGGTGTTGTTGTTCTATGAACG
GCTATCCTCTCTGCAGAAGGGAAAACTTGTCATAACGCCCATTTGTGTCCCAAAGGGACATTAGGTCT
GATTTCTATTCCAGTGTTCATTGTATGATTTA
TATCTCAGCTCCCTTACTGATTTAACTTCTGTATTGCCATTTATCTTCCTTTACCATTGGCCCTTTCC
GTGGACAAGGAGAAGACAGTATGTCATGATTT
TGCTTATATTGAATCAATTGCTGTTGGGCGTCTATTGGTCTGCTTTGTTATTGTCCAACCTGTTGGAT
CTATTTCTAGTGGATTTAATTAATTCTATTTC
GGATGTGTGGCTGGGTTTTTTTACTCTAGGCTGCAATTGCTTGAATCGGACTTTGTTTTTCTTGTTAT
TTTGACCGCTGATTTTAATCTATGCATTCCTG
TTGTACTATCGGTTTAGCCATTTCTGGTAATGTTATCAAAATTTTAGGTGCTTGTGAAGAGATTTTTC
ATAGGGGGAGGTAGCTAACTTAACGAATGTGT
ATTTATCCTGATAAATGAAGGCATTCTTTATGATACTTTTCTACCGTATGAATTTTAGAAAGGCCACG
ACTTTAGGGTGCCGATGGCGGCCGGAGAACCC
TGATTTTTATTTTACCTTCCCACCCACCCTTTTTTGATTCCCACCGCGCGGCGGGGTCTTGTGGCTGG
AGGACCCCAAAGTCTGCCACTTCGTAAGTAAT
AACATTCCAAATCCTAACTGAGGACAAAAGGCCACGTGAGCGGTCAACAGAATATAAATATTTTATTA
GTAGAAGACATTGAGGCAATTAACTACCTCTG
GGTTCCTCTTTTTCTCTATCTTGTTTGGTTTTTATAAATAATTTTAGGATCCAGTCAGCGGCCCAGAA
GATTATTCCCTTTTCTCTTGGCCAAATTTTAT
CATGGGACAGAAATTGAAGTCTCTATTTCTCTGGTTTTACTGACTGGATGCATAATTGGATTTGCGAC
GCCTCCTGGTCGTTCAGCGATAAAAAGCTTAG
CCAATAGCAACTATACATAGAGTTTTATCTTTGCTTTTGGGTATGCCATCAGCGTAAAATATTTAAGT
TACTTCTTTCATTGGAGACGCACTTAAGAGTT
AATGGAAGTAAAATAACTGTTGGTTTTCAATATTCTCTCTAGATGTATATAAAAGTTGCTGGAAAATG
TTTACGACATTTTGGTCAGCGATCAGAAAGGA
GAATTTTAAGAAAAAAGGGGGAAGGGGCAACTAAATGACGCTTAATGGAGCAATGCGCAATGTAGCCT
CCGTTTTATTAAATTTTGCTATTATTTTTTCT
TAATAATCTTGCTGCATTATATATATATATATATATATGTATAATGTTATTATTATTATTTGCATTTG
TATTTGTATTTGTTTCTGTATTTTGAGAAACT
TCTAATTAGATGTAACATCTGACAACCATACTGGGGAGTAGAACGAGGAGGAACGTTAAATTCCTTTT
GCCATTCATCATTTCCAAGAGAGAGGAAAGTC
TTTCTTTTTTTTACTCATTTGCTTGGCCCTTTTCAAGGCATGGATCAACCCGTAGAATAGATTGTAGG
AAGAGAAATATATCGGAATGGGGTTAAGAACG
GCTCTTACGAAGCCCTTTGTCGCGTTTGTAGCTTCTATGGACCGACGCATCGATCTCATGGATGAACC
TTTGAAACGTCATATCCAACACCATAACAATT
CAGGGGAGGACCTATGCGCCAGTGTTTGGCATGAATATTGGTCGTACCAAAAAGCGCTCTTACCGTAT
CGCAAAACAAGGCTTTTAAGCGAGTTGAGCTA
TATGGCAGGGGGGAAAATATCGCTGGCTAGCATGGGCGTAAAAGAAATTATTCTCTTCTCCCGTGTCT
TGACAAAATGTTTGTTTCTTTTTATCATCTTT
GTAATTATAGGTCGACGCTCTGTTTTTCCGTCCTTGGAGCCCGATTCTGTCTTTGTCCAAGAAATCAA
AACAAACTGGCAACCAAACCACAAACGCGGTC
TATTGATTAAGGAACTTTAGGAGGTGAATGAAGGACAGGAAGAACAAGGGAGAAGTAAAAAGTAAAAA
AAGGAGTGATTTCCTTAAAAGAGAACAATGAA
GCGACGCGAGGATACAGCCTTGTCACGGAAAAGCCTTTTTTCAATACTAGCATATACGCACTAAAATT
AAAAGTTTTGAGCATTTTCATCTTACATGTGA
TACGTTGGCCACTACATTTTGCAAAAAATGAAGCCACGAATCTAAAAAAGGCTTCTCCGATAAGAGCA
TTTCATTATTGGTTCCCCCTTTTTTCTTTCTG
TCAATCAGATCCACAAGACCTCTAAAAAAGAAATAGATATGCGCCGTTTCTTTTCCAAGTTTGCTGCG
GGCCTGCCGGCACGCTTCGCCTCCACAGCACC

ACCGGCGGGCAGCGCGAAGGCCGCTGCCTCGGCTGCCGCGGGACAGAAGTCGTTCTTTAAGGCGACGG
AGATGATTGGCTATGTGCACTCCATCGATGGC
ACCATTGCCACGCTCATACCTGCACCTGGCAACCCTGGCGTGGCTTACAACACCATCATCCAGATCCA
GGTGAACCCGACAACGTTTGCCGCCGGCTTGG
TATTCAACCTGGAGAAGGACGGCCGCATCGGCATTATTCTGATGGATAACATCACGGAGGTCCAGTCT
GGCCAGAAGGTGATGGCGACGGGCCAGCTGCT
CCACATTCCCGTGGGCGCGGGCGTGCTTGGCAAGGTGGTGAACCCACTTGGCCACGAGGTCCCCGTCG
GCCTGTTCACACGCAGCCGCAGTCTGCTGGAA
CAAACAGTCGGCAAGGTGGACACCGGCGCTCCGAACATCGTGTCACGCTCTCCAGTGAACTACAACCT
GCTCACCGGTTTCAAAGCTGTAGACACAATGA
TTCCCATTGGCCGTGGCCAGCGCGAGCTGATTGTGGGCGATCGTCAGACGGGAAAGACATCCATTGCC
GTGTCCACGATCATCAACCAGGTGCGCATCAA
CCAGCAGATCCTGTCGAAGAATGCGGTCATTTCCATCTACGTTTCCATTGGCCAGCGTTGCTCCAACG
TGGCGCGCATCCACCGCCTCCTCCAGGCGTAC
GGTGCGCTGAAGTACACGACTGTCATGGCCGCCACCGCTGCCGAGCCCGCTGGCCTCCAGTACCTCGC
ACCATACGCCGGCGTGACAATGGGTGAGTACT
TCATGAACCGTGGACGTCACTGCTTGTGTGTGTACGACGATCTTTCGAAGCAGGCCGTTGCATATCGT
CAGATTTCGCTGCTCTTGCGCCGCCCGCCCGG
GCGTGAGGCGTACCCTGGCGACGTCTTTTACCTGCACTCGCGTCTATTGGAACGTGCTGCCATGCTTT
CGCCCGGAAAGGGCGGTGGCTCCGTGACTGCT
CTCCCCATCGTGGAGACGCTCTCCAATGACGTGACGGCGTACATCGTGACGAACGTGATCTCCATCAC
TGACGGCCAGATCTATCTCGATACGAAGCTTT
TCACCGGTGg
>scaffold1133.size7010
GGGTGAGCCGTTCTTCACGCGTCAGCTCACAAAGAGCAACGCCCACACGAAACAACCAGCGAGGTGCA
AAAGAACAAAACCTCCCTTCGCAGCACCCACGC
GCTTCCACCAGTCGTTTTACCGTTGTCTCCTCCAAAGCACCCGCTCCGCAGCCGGCGCACACCACGTC
GCACAGCCGGATAAACCCCATCAACGGATCCA
CAGCATGGTAGAAACTTCGGCACATGAGGCTGCAGGACGGGAAATAATCCGCACTCTCCCAGGCACCC
TCAACATTCCCAATACCCACCTGCCTGCATGC
CGCCACATTCACGCCCGCAGCACGTCGAAAAAACACATTGCGGTCTCCCGTCCTCCCTCCTCAGGAGG
GCCTCCGCAGCCACAATCCTGTGCGTGCGTGC
ATTCTCAGCGTTTTAAACACGGCATTTGTGTGTGCATGTGCTTGTGTGTTTGGCAGCGCCTATACTTG
CAGAGCAGCTGTGCGCCACAAGAGAGTCTGAA
GAAAGCAACGGCAGAGAGAGGCAGATAGCACACACGCAAATTACTCATATCATCGCACACACACACAC
ACACACACGCAGCCGCGGAATGGGTGCACTCA
ACCACAAAATCAAAAAGGAAAACAAAACAAAAAGCCGAAGCGTGGGACGACCAGCGCACACACACTCT
CATTACTCCCGTAAAGCAAAACACACACACAG
AGGGAGAGAGAGAGAGGTCGCCCACTCAATGCAATAAAAACAAAACAGAAACAGTTACGGTGCTCCGC
CCATGCAGCCAGGCAGGACGGAAGCGGTGCGT
GTCGTGCAAAAGAGAGCACCAACGGGCCAGCATGGGTGTGCATGCATATATACAATGGGCGCATTATT
GTGTGTGCCTCGTGGGGTGAGGTGAGGGCGAG
AGTCCATGCAAGGGGACACACACACAGAGAGAGAGAAGGAAGAGTGTGTGTGCGGGCGGTGCATGGCT
CTCTCACACTCACTCTCACGCGGCCACCACCG
CAGCAGCAGCCGCACAACAACAAAAAGAAGCAAAGGAGGGGAGGTGGTGTGCGAGACCGCGGTGCTGC
TGTCGCTGTCGCCAGGCGTCGGCGTGCCACCC
GTTGTGGCGTTTGTGGCCAGCACTGATTCCTTCGGATTGGCTTCTTTATTTTCATTTTGTTTTTCTTC
TCGTGTCCCTGCACCATCAACTGCCGGATCAT
TGTTCGGGATGCCCTCTTCAGTGCTCTTGTCAGCTTCATTATTGGCGAAATTGCCGTTTTCCGTGGGT
GGTAGTGGCTTTGTTTCCGCATGGCCGTCAGA
TGTTTCATTCTGCAAATTGGTTGTGATGGGGGGCAACGCCCTCAGTTTGTGAATTGTTGTTTGACATC
ATGCCGCTTTGGGGTTGTTGTAGCTGTTGCAC
GGGCACGGTATCTTCAGAGCTCTACTGTCCATTCACTCGTGCTTGTGTTGAATCTCCCTCTGTTTTTC
CAATTGTTTTCAGTGCCACTTCTGCTGGTGCT

TCTATTAGTGATGGTGATGTTGCGTGTGGTATGTTTTCAGTTTCACTGCATTTTCAAGTGTCCCTGTT
GGTGAATTATCATTTTTTTTTCTTTGTGGTCT
TCCGCCTCTTCTTGTACTGCCTGTTTTGGTTTTTTCTTTTGTCTTCATCTTCGTTAACCTTTTCCAGT
GCTACCACCCCCAGTTCGGGAGTGTTAGGAAA
AATATTAACGCCACTGTCATCCGTCGGCGTGAAATTGGAGGATGCATTACTCTTGGAAGGATTATTCA
AATTGTGATCTTTGCCAGGTTGAGTGACAGCA
CTGCCAGATGAAGATTGACCAACGCTTTGGCGTTGGGACTCCGTTGCCTTACCACCTGAGGGATTCCT
GGTATCGCCAATTCCATTTGCATCAACCGTTT
TTAAGAGCTTCTCCTGTTCAACTTGATCTGATGCTTTAGCTGTCGGCAGTGGTACTGTTGGATCAGGC
GTTAAAACCTCATCATTACCTTTTGACGTGGT
ACCAGATAATAATTGCTTGGGCTTTTCAATGCGACTGCACCACCCAATTCTGCTCCCTCCTTGTCCCC
AAGCTTCAACCGCCCTTATTAATCCCATCTGA
GTGGATAACAAACTGCAGTCAGTTCTCGGAAATACCCCGTCACTTTTAGCATTATTCATGTGCGTCAA
TAAATTCCCGTTACCTTCACTGCAGCGGTCCG
CCATCGCGTACACAACCTCCGCCGGCACCGCACCACAGCACGCAGAGGGCACACACCAGCAGCACACG
GCAAGTCATCATCATCGCCATTAATCCTGTCG
NNNNNNNNNNNNNNNNNNNNNNNNNNNNNNNNNNNNNNNNNNNNNNNNNNNNNNNNNNNNNNNNNNNN
NNNNNNNNNNNNNNNNNNNNNNNNNNNNNNNN
NNNNNNNNNNNNNNNNNNNNNNNNNNNNNNNNNNNNNNNNNNNNNNNNNNNNNNNNNNNNNNNNNNNN
NNNNNNNNNNNNNNNNNNNNNNNNNNNNNNNN
NNNNNNNNNNNNNNNNNNNNNNNNNNNNNNNNNNNNNNNNNNNNNNNNNNNNNNNNNNNNNNNNNNNN
NNNNNNNNNNNNNNNNNNNNNNNNNNNNNNNN
TTGCGTGTAGCTTTAAGCACAAAATAACTTTCCGTCCACGCAGAGCAAAAGCAAAAAAAATACAAGTC
ACAGAACCATGCACAAGCAGCAGCTGCACACA
CAAATCAGTCGGTTGGAATAAGCACAATTGCAGGACTCCCACACCAAACTGCAAACCAAAACTTTAAT
TAAATTAATATAGATATATATAGATTCACCTG
CGCCGCTGGCCAAATCCAGATATTTTCAATAATAAAAACCAAACCAAGTAAAAATAAAAAAGGGGTGA
ATGGAACAAGAATGGGAGTTCACCATACCCGC
AAAGTTTAACACCAATTAAGTATATATACACCATCTGTTTGCGACAATCGTGACCAGCTTACGTGGCC
TTTTGAACTCATGTAGGATATAAAACATTTAT
TACTTACAAAGTGGCAGACTTTGGGGTCCTCCAACCACAAGACCCCGCCGCTGCCCTTCACAAGTTCC
ACCCCTTCGCCAAGTCCCGAAGTGTAAAAACA
CAAGACCGCGCGGTATGCCGTCTGCGGGGTTGTCTGCATACCGCACGTGGGCAAAGAATGCCTGCACT
CCGCGAAAGCCAAAAACTCATATATCCGTTGC
AGCTACAACATCATGGCATGTGATTTCGAGCTGCCTTTATTCGCCGCTCCTTGCGGCGAAGTGTTGTC
CACCCAAACGTCCATGGTGGATGGCAAGATGG
CGATAAAAAGGCCGATAAGGCCAAGCGGACCGCTCGTGCCTCGGACTGCATGATGAGAAAAGGTTTCC
TCTTCCATTTCCCCCGGCAGTTTTAACGTCGC
CGGAGTTTGGAATAAAAACGGCCCCTCATCCATGGAGCGACGCGTTCGTGATGATGGCAGCCGATGCT
TTCTCCGTGGGCTTGGCGATCCGCCCAAAATT
GCTTTCGATGATGTGTCGCAATCTCTCGCCCAAGCCAACCGCTGCCGGCGGAAGGTTCGCCGGGAATG
TCTCCTGCACAATCCCTTGGTGAAGTGCGGAT
ACTCTTCGCCGCACTGCCTGATGAAAGAGTGGTAGGCACATAAACGCGTGCCCAAAATGGCAGCCGCG
TACAAAAAGCGTGACGCCACAACCTCCATTCC
CGCGATGGTTAAGGAATTGAGCGGCGGCACTGCGCATACAGAGCAGACAAACCTTTCACTCAGGGATA
CCGCCCGNNNNNNNNNNNNNNNNNNNNNNNNN
NNNNNNNNNNNNNNNNNNNNNNNNNNNNNNNNNNNNNNNNNNNNNNNNNNNNNNNNNNNNNNNNNNNN
NNNNNNNNNNNNNNNNNNNNNNNNNNNNNNNN
NNNNNNNNNNNNNNNNNNNNNNNNNNNNNNNNNNNNNNNNNNNNNNNNNNNNNNNNNNNNNNNNNNNN
NNNNNNNNNNNNNNNNNNNNNNNNNNNNNNNN
NNNNNNNNNNNNNNNNNNNNNNNNNNNNNNNNNNNNNNNNNNNNNNNNNNNNNNNNNNNNNNNNNNNN
NNNNNNNAATTGAGCGGCGGCACTGCGCATAC
AGAGCGGACAAACTTGTCACTCAGGGATACCGCCCGGTGAGTATGATCAAATTGCGCCCACAGGAAGG
TGTACTGCGTGGCGCCCGATTCGCGGCCATCC

CATATCGTGGCGTGACAACTGTCCGCATTACGAAGCACTTGGGCTTCCCACAATGTCACATCGCTTTT
CGACCCTGCAATGCGTATATTATCGATCCATA
CGCCAACGCGCACCAATGGAGGTGCgGCCCAGAGGGGGTGAGCCACCGTCGTCATCCCCGCAATCGCC
GAAGTAATAATAATCTGGAGAATTTCTGGGCC
GGTCTTGTGTCCCATTGGGAGCCGTGTGAGCTCCACCAGCGTGCCATCCTCCACGCGGCATCGAAAGA
AATGCCGAGTCTCCCGCGGTAAAGGGACTTGA
AAAAGAGATGTCTTTAAGTCCAGGCAGGAAACGGCCTCAGCCATCACAGGTGGCGAATAATGGGAAAT
GTGTAAAAGAGGAACATTCGCCTCATAAGGGT
CGTCTCTGTTCCTGTCGCGTGGCCACGCGATCCATCGTCGTCGCAAACCGGTGGTTTTCTCCTCCACA
ACTGAGAAGGGTATTATCCATCCGCCGCCTGT
TATTGTGGACGAAGCTTCCTCGATAATTCCTGCTTTTCGTAACAATCTCGCGTCAGCGGTTGGAATCC
TCCGTGAGCTTTCGCGCGCTTTACCGGAAGAA
GACGGAGGGAAAGGGGACCGCCCGACGAGTCCCCAAACTTGCATGAGTCTCTCCAAGGTAGCCGGGTT
CAACCGGCTTTTAATCCACTCTAGATTCAATA
CGGGCACGTTGACTTGCTGTAGCGGGATGGTTCGTTCGTCGTGTGGAGTGGCCAGACGCGTCCTTGTC
GCGCGTCTCGTTATTTTAGCAGCAGAATGATT
GTGATAAAAATCATGGAAAAACGCTCCGCAGCTCTTAGCCAGGGTTCTTGCCCTTCCGCCGTTTCTCC
CTTCACATCAATGCGACCAGCGACAGCACCTG
GGTCAGCATTGTGGTACACATCCTCAAATATCGAACAGTGTTCTGGGGAAGGTCGAACGGATTGACGT
GCTTCGCCAACTGCGAGATCACGTGTGGATCC
AAATTGTATGTCTCCACGGTTTGAGCAGCGTGCCTCAATGCACCGCGTTTTATGGAATGCGCCGTGGC
ATCCCAAGGAACCAGGGCTCGTTCCACCTGGG
CCATTGTAATATTCGTGAGCTTTTCGTTTTCTTGAAGTGTCCTGCATAGCTTTATAGTGTGGAAGGCG
TCCTGCCCTCGTATCCTGACGAACCGCGAGGC
GCGGTGGGGATCCGCCCTCGCCGTCTTCGGCGCCACGGACCNNNNNNNNNNNNNNNNNNNNNNNNNNN
NNNNNNNNNNNNNNNNNNNNNNNNNNNNNNNN
NNNNNNNNNNNNNNNNNNNNNNNNNNNNNNNNNNNNNNNNNNNNNNNNNNNNNNNNNNNNNNNNNNNN
NNNNNNNNNNNNNNNNNNNNNNNNNNNNNNNN
NNNNNNNNNNNNNNNNNNNNNNNNNNNNNNNNNNNNNNNNNNNNNNNNNNNNNNNNNNNNNNNNNNNN
NNNNNNNNNNNNNNNNNNNNNNNNNNNNNNNN
NNNNNNNNNNNNNNNNNNNNNNNNNNNNNNNNNNNNNNNNNNNCTCGCCGTTATTCACGCGAGTCGTAAG
ACAACACGTTCCCTCCAATCGGTCCGGCTGCG
GATAAGTTGATTCATCTCTTCATTTGTCAAGGGGCGCGTCTGTTTCGTCTCCGATCGCACCGGAAGTT
TTTGTAGCCCCAGAATCGCCATGCCCAGCGGG
GTTCGATCCATTTCCAACATGGACCGCAGCATCCTGGCGTGCTGCTGTCGGGTGGATGGCGCCACATC
CAATATTGCCACCAAAAAGACCGCACAGCTCT
CCTCGCTCATCGGCTGTTCGTGCGTGCGGCAAAAAGTCGTGAATTGCCCCGCTAGGGACATCCTCTGC
TCCCACGTTGACGGCGCACACATTTCTCGAGT
CGCCTCGTTCGCTCTCCTCATGTAGCTTGTCCAAAGCGGGGATGTCGGTAATTCGTCGTACCACAGTC
GAGCGTCGTTGGCAGATAAGCTCCATCGCCCT
TCCCGACGATTGCGTCGGGCAACTTAAAATATCGGATGGAGCATCGGCGGTGCGGTGCGACGTCGTTG
CGGAATAGCGGGAGGTGGTGGAATGGATGGGG
CTGCCGTCGTCCGCCTTCGCAAGAAGCAGCAAGCAGCCCCTCTGCCGTTTCTTGACGGCCGTCCGCGC
TGCGAGTAACTTCCTCTGGTTTCATCTCGGGC
GTTGCTTTTAAATGTTACGTGTCCATGAATATGACATAACTGGCATCGCAACGATATTTGCGTCCCTC
GTCGTTCCAAGGGAATCTGGATTGCCCTCGCA
AAGGTGTCAGCCTCATGAACGGCGGTGCAAGGACGGGCGAGAATGTTGAAGAGCGTGATGCTGGGGTC
GGATGCCATCATGAGATGCATCCGGAATGTAT
TCAGTAAGATCCTGCCAAAATTCATGAAACGATCGGAGAGGTCTCGCTCTGCCTCCAGCACGCCGATC
CACGCAAACAAACTGTCAAATACAATTTGAGC
CTAATCCTCCGCCCGTTGCACATGATGGTATGCGCACCGGAGGTCGAACACGACTCCGGAAACGCCCT
CGTTCCATATACGTGTGGCCCACGTGGTGACG
TAGAATGCGCGGAAACTTCGTTCCTCTTCTTCTGCACCAGAATGGTCGTCCAAGAGAAGTGCGGGAAG
GCAAAGGCATCGAGGGATAGAAATGTGGTCAT

CAGAAATTGCCCTGAGAAGTCTGGAGAAACAACCTGCCGCCTCTCTCACCGGCCTTCGTTGGGTCGTG
CGTGACGAGGCGCCCACGGCCATCAAGCGATG
CGtctga
>scaffold1134.size5903
TCTCTTTCATGTTCAGTGGCAACAACGTCTTTTTCCGTTACTCTCGGGGATTCTCCCGCATGCGTGGG
TGTTGATACCTTCTGAATTCCCGTTGTTTCTGT
TTCTGAACTTGGGCTTCCTGATGCGGGTGGTTTATGTTCCGTGGATTCCGGCGGAACTCCCCTTCCAC
TCGCGGGATCACCGGGTTCACTCTGCAACAAA
CTCGGTGGATCTTCTTGTCGTTGTTGAACTCTTCCTTCTGGCGGAGTGTCGTCGGAGGGTATCGTTTT
TTCACCTCCTTTTGCTCCATTCGGTGACGGCC
GCCGTACGGTCTCATCGTCGGTTGAGTGCAATTCCGCCTCTGCTGGTGCATCGCCTTCAGACACCCCT
TCACCTCTTCCTTTTTGAACGGTTTTCACTTT
TTCACCATcaactccaaNNNNNNNNNNNNNNNNNNNNNNNNNNNNNNNNNNNNNNNNNNNNNNNNNNNN
NNNNNNNNNNNNNNNNNNNNNNNNNNNNNNNN
NNNNNNNNNNNNNNNNNNNNNNNNNNNNNNNNNNNNNNNNNNNNNNNNNNNNNNNNNNNNNNNNNNNN
NNNNNNNNNNNNNNNNNNNNNNNNNNNNNNNN
NNNNNNNNNNNNNNNNNNNNNNNNNNNNNNNNNNNNNNNNNNNNNNNNNNNNNNNNNNNNNNNNNNNN
NNNNNNNNNNNNNNNNNNNNNNNNNNNNNNNN
NNNNNNNNNNNNNNNNNCTCCTCCTTTTTGAACGGTTTTCACTTTTTCACCATCAACTCCAACTCCAC
GGCGATCCGCTTCATCTCGCTCAGCAACGACT
TCATCCTTTTCTTCCAAAACATCGTCTGAGCGTCGGGTCGTTGATTCTTCCTTAACTTCTGGCGCCTG
TGGCTCCACGCCCGGTATGACTGGGGATGACA
ATTCCGTTTCTTCCAATTTCTTGTTGTCCCCACCAGAAATAAAAACATCGCCACCGCCAGCATCATCG
GCCGCAATTCCAGACACCGCACCGCAGGACGC
AGAGGGCACACACCAGCAGCACACGGCCAGTCATCATCGCCACCACTCCTGTCACTTGCCTGCAGCCT
TCAGCAAAAACTAACTCCCCGTCAACAGAGTG
AAAAGCAAAAATAGACACGACCGCTACACCACGCACAGCAACAAATGGCAGCACCCCAGCAACAGCAG
CGCACACACCACGAGACACATGGAATCAGTAC
GACTGCAGAGCTCGCACACCACACCGCAAACCACAAGTTTAAAAAACAATAAAAAACAATAATTAAAA
AaAGAAAAACAAACCCAGGGCTCCAGTGCGCA
TGCTTATGAGTGTGTCCGCGATGCGTTCAACGTGACGGATGCGGGGCACAACGCGGAGCACACAGCAC
GGCAGCAGAAGCAAGTGAAGCAAAACAAGATC
ATTATTCCCTCTTATTTATTACAGTGAACCACAACACACACGCACAAATCCCCGCGGTTGAGTGCTGG
CACGCACAGCCCGCACACACCTCTTCAGCCCA
GAGCGGTGTACGCCAGCGCGGATGCGGCGAGCACCAGCGGGGCACACACCCACGCAGAGCTGCTGAGG
CTGCCGTCGATTCTGCGAAGACGTGACGGTGC
GCGGGTGGTCGTCGTNNNNNNNNNNNNNNNNNNNNNNNNNNNNNNNNNNNNNNNNNNNNNNNNNNNNN
NNNNNNNNNNNNNNNNNNNNNNNNNNNNNNNN
NNNNNNNNNNNNNNNNNNNNNNNNNNNNNNNNNNNNNNNNNNNNNNNNNNNNNNNNNNNNNNNNNNNN
NNNNNNNNNNNNNNNNNNNNNNNNNNNNNNNN
NNNNNNNNNNNNNNNNNNNNNNNNNNNNNNNNNNNNNNNNNNNNNNNNNNNNNNNNNNNNNNNNNNNN
NNNNNNNNNNNNNNNNNNNNNNNNNNNNNNNN
NNNNNNNNNNNNNNNNCCGGATCCACCTGAACTATCTTTTTTAATAGGCGCCGGACTTGTTGAACCTGG
ACTTCCTGCCAGAGCGGTCGCCCCACTTGGAT
TTTCTGGCAGAGCTTCTTCCGCACCTGGAGCCCCTTGCGCATCGGACTTTCCACTTTTTCCCGATGAC
GCAACGGCCGCAGCTGCTGAACTTGACGTCGT
GGAAGTATTTGTTACCCCTGAAACACCTGTTCCCTGTGACTGAGTAACCGAAGAAGTGGGGCCGTCCC
CACCTGAATCCTGACTACTCGCTGTCGCGCAC
ACGGACGGGCAGCAGCAGTACAGGGCAAGCACCAACAGGGCGCACAGCAGACGGCACGTCATCATCAT
CGTGAGTGTGGTCGTTCAGTGGTGTGGAGCTG
ATCTGCACAGTGAGGGACATGGAGAGCAACAGGGGAGACGAGAGGTGCGGCAGACACACACAGAGGGC
ACACCACATGAGGAGAGGGCAGCAGCCAGGCA
TGCACCGTACGCCGCACACCACGCACCACACACACTCCAATCCACGGCAGCAGGCACACACACACACA
GCCCTGCCACAACAACAACACACAGATGGAAG

AGGGAGAAGCAAACAGGAGCGGCACACAAGGCTCCACCGCATGTACATACGGCGAGAGCGGTCCGTCG
TGTCGGCACCCCTCACGGAGGGGTGCGCACAC
ACACAAGAAGGTGCAGCGCACACAGCAGCACAAAAGCATAGTAACAATAAAAAAAGAAGAGATCCCCG
CACACCACGCGGATTGTTGGTCCACGGGAAGT
GCGGCCAATATTCACCACAAAGAAGATTGTTATCTTGTTTTGGTTGTATGCTGCTACAATAGCGAGTG
AGTAGCGCAAGGGCCGTCCGTACAAAAGTGGC
AAGTATTCCGTCGTGCGGGCTGGGGCAGTCACAAACAGTCGCAGTCCAGTTCCCAACGACGAGGGCAT
TCCCGCAGTGGATGGGGCGGAGTCTCCCGTGG
TCCGTCGGGGATCAGCAGCCCGTGGCGTCCGTGATGAGGCGGTGGCGGAAGAGCCTGGTCCATCTCGA
GTACCCGCGGTGGTGCCTCCTGCGTGTCTTGC
CTGCCGGCATGCTTGGCAACTTCGGTGGCGACCTGCGCGCGAGGCGCCTGTTAAGAAGACGGTGGCGC
CATTGTGGCATCCGCATGGGAGGCCCTCGCAA
ACTATTATAACGCCAGGCTGAGCACGTCCGGCATGTGCCCTCATACTGTGGACGGACAAGCGCACTCT
GCCGCAGGTTGGGCAGGAGGACGTCAATCCGC
CGCTCGCTGGTCGACAAGGAGTTGCGGTGCTCACGTCGGGAAGAGGTTCCGGCGGAGGCAGAGGCGGG
CCGGCCACCGCAGTGAGGGAGGGACGGCGCCA
CCGACACAGAGGGTTCCTTGTGATCCGCAGGGCGCGTGCAGGCCTGTCGAAGAGTGGGCAGGCGTCGC
ATCGCGCCTGTGCGCAGTGCCGCCACTCGGGG
ACCGATGTCCGGGCCAACACCAGGAATAGAGTCCTCGCCACACCGACAGGCGCCTGTCACGCGGTGTG
TGCGCCCTCCTTTTCCTCCTGGGCATCCTTCC
ATTATAGCCTCTTGACGGCGGTAAGGGAGCCCACGTGCCAGGCATTGACGCAGTCCTGCGGGAGGGAT
CGTGCAATCGTGGCTTGTTTGTCAGCGGCCTC
ACTGCGTGGAATACCGCAGTGTGATGGGAAAATATAAAATCCACAGGGTAACCTCGCTTCACCAAGGA
AAGCAGCATAATCCAAATTTACTCAGTAACGC
GGTCTCGCACGGTCGGTCCAAGGCGGGGTGCCTCCATTGCCGAGAGGGAGTCCGTTTCCTCCAGCATT
CGCGTGGGCGATTGGGATGCTCCCAGACAAGG
AGGTATGAGATGTCTAAAAACATTTTCGATCGCAAGGCAGTCTGCCCTATATGAGCATGCGAGGCTGC
CAGCTGCTGCACGATGCACCTCCACAGGGAGA
TCATTTGTCGTGGTGGAGTCATATAATAGCGCAGCGGATCCAGACGCACGCACAGCGAGAGAAGACGA
GCCGTCCACCGCCAGCACAGTTTCCAGCGAAT
AACAACACGTTGACCATGAATAGGAGACTTATGCCTGCACTGCCGTGGGCACGGTCAGCGGCCCCGAT
TCACGCAGCAACAGTGATCTAATGCCCTTGGA
TGGTACAACAGCCACGCCTGAGCCCAGCACAGTCCTAGCAATTGTTGAACGAGTGGTAACGTGTCGGG
TGGCGGTGATGCCGCACAGGATTATTTGCGTA
CAACTTCTGGGAAACCAGAGATCCCATCGGATCACAACGCAGCATCACTTGTGAGCAACGGTTTTTGT
GAGCAGGGGCATCTCACGGATTTGTTGGCTAT
GGCTGAAATTGGTGACATCGCCGTGCATGGGTGTGTGTCTCGGGTGATGTTGCTCTTGATTCTGGGGC
TGTGGGGTACTGTGGCGCTCTGCTGAAGTGTG
AGGGAGGGTGGAATTACGGCTTTTTGTGGAACAATAACTGTACAGCTTGCCAGCATTGCCCACTGAGT
TTTGTCAATAATTTAAGATTTTTTATTTTCTT
TTGTTTATTTGTTTTTGCTGCTTTTGCCCTATTGGAATAATCGGTATCCCTGTGTTTTTCCCCAATGC
CCGTTTGCTGCACTTCGTCATGAGTGACGTGC
TTCTCCTCTTGTTTCCTTCCGTTGCCCACACGCGGTGCCGGCCTCATTGCTGTGCGTCCCACGGACTG
CACGAAAGGAGGATTGGAAGAAAAGACTGGGT
GACGCAATGAAGTGTGAGGAGTTGTGTGGGCTCACAGAAAAGCACGATATTTTTTGATTTTTTACTGC
AGACATTACGTATACAAGAACTAGTGGTCGTT
TACGTTGCGCTGCTTGGCACATAAAATAAAGGACGTCCCTGATCGATTTTTTATTTTTTACTTTTGGG
GAATTTTACCGGACTCTCAACTCTGCTGTTGT
GCCTTTTTTACTGCTTTTTTAACTTTTGGTTTGGGGAGCGATGTGCGAGCTCTGCAATTGTACTGATT
CCAACTGCCTCATGGTGTTTGCATTGCCATTT
GTGACTGTGCGTGATTGAGTGACGTGTCTCTCCTTTTGCTTTTTGCTCTGCATTGATGGGGAGTTGGT
TTGTGCTGAAGGCTAAACGCAAGTGACAGGAG
TGATGATGGCGATGATGACTGGCCGTGTGCTGCTGGTGTGTGCCCTCTGCGTGCTGTGGTGCGGTGCC
GGCGGTAGATGTGACGAGGAGGTGGTGGAGAT

TCCTGTCGGTGGTGGTAAAGTTGCTGTGGGGTCGACTTTAAATTCAACGCCGGGATCAGATAGCTCAG
ATCCAACAAAAGGTTTGCTCAAGGTTAATCAA
TCAACAGAAAAGTCTGAAGGAAAATCTTTGGAAGGAAAAGAAGTTAAAAATGATCAACTGTTTGTTGC
TCCTGGAGTGGAGGGTGAAGATAGAAGCTCTT
CAGAGCAATTAGAAGAACGTTTGGAAAATGCTCCAGAACAAGGAAAAACAAAGCCAGAAATACAAAAT
AAAGAACATGAAGTAGGGCAACCGCCAAAAGC
GCAATTAAATGTACTACAGCAACCGCAGCCACAACCGCCATCAGCACCTCAGCCACACACTCCTGCCT
CAGAAAGGGTGAAGGTGTTGGCGAGAACAGTA
AGGGTGGAGAAGGTCAGTCCTCATTGGGAGCACAAGATATTGGAAACGAGGATCCAGAGAACCCCAAA
GAAGAAGACTTATTAAAGAGTCCAGGTGAAGA
ATCAGAAAGCAGTGAACCGGTCCAAACGACAGTGCCAATACACAGTACCGCCGGAGCACAAAACCCGA
AAATGAAGTGTTAACTCCGGAACAAAAGACAA
ATGAAAGCCAAAGCACGGATACATCCACGAACCTACCCGAAACACAAAAGGAAAATAAAGAATATCCT
GCCTCCACGGAAGGTACGGCGCAGAGTACATC
AA
>scaffold1135.size6330
ACCGCAACGAAGAAGTAACTCCTCCTGACGCACGTCATCATTTACAGGTTACACCTCACTGAGGTCTC
AAATACTGAAAAATAGAAAATATATATGCGTAT
ATTAATCGGCACCCAAAGATACCGACGTTTGTTGTTACAAACGTATTTTTTTCTTTTTACTTCTAAGC
ACAGCACTGGAAGAAGCTGGAAACAAAAAAGA
AAACACAGGCGGCGCAAATGAGGTAGAAGGCCTCGGAAAACTTGTAGCCCTGATCCTTAAAGGGACCG
TACTGCGAGGATAGCACGGCGTCGCTCTGGCA
GTAACCCCTCATGTACCCATACACAATGAGCGCCAGACACACTACACACGCGACAAATGCAATTGAAA
TCAACAAGGACGTGGCACACCCCAAGCAGCAA
CAAATTCTCTGAAAAATCATCTGAATAATCACAAATAACACGACGACGAGGCCAATGCCTGCCGCAGC
CACAGCCGCACCCTCTGATACCTGAAAAAATA
GCTTGTACTGCCCGCACAAACTATCACTCACATCAACCTTGGTACCAACGCCACCTGGAACGGTCGTC
TGGTTGTACCACAGTGTCTGCTTTACCTTCGC
AGCGTCGCTCTCCTTCTTGAACACAGGAAAGATGTTCGCACACACAGCGCAGGCGGTGGCCAGCATGG
CGAGAACCAACACGATTACACGGCAGAAAGAA
CACATGTTTCCTCCTTGACGATTGTGAGAGAGCTTGCTTCTTGCCTTAGCCTTGCTTCGGGGAGAGGG
AGGGCGGAAGGAAAAGAGGAACATGGGGAAAT
GCACACCAGTTCGGCAAGGAGGGGGTCATCAGCGCGTGGAAACACTGACAGCTGAGCGCGTACCTCTG
TGCGCGACAGGACAACAAGGGAAAAGCACCCG
CTCCAGCACGCTCTTGCCCTTCAGATGTTTGTGTATCGCTCGTCAGCGGGACGGTCAAAGTCGATGTC
CAGTTCGCATATTCGCCAGTCCTCATACGTGT
TGTCCGGAAGCACACGCCGTACGATGAGCGGTATGACACCCTCGCGAAGCTCCTTTTGGGCGATGATG
AGCGGATCCGTCTCGCCCTCCAAGGCCACCAG
CACCGGCGCGTTCATGCTGATCTGCAGCGCCCGTGTGCCAAGGATGCGGGCCCGCTCGTATTTTGTCA
GCACCGGCGCAGTCACACGGTCCTTGCCGTCG
CGTCTTACGGCAGAGCCGCTCGCCGCACGCCCGGACAGGACAACGTCCGCGCCATCGCCCTCGCTCTG
TTCGTCATCACTGCGCAGGGTGCTCTCCTCCT
GTTCCTCGCCGAAGTTCAGACTCAGCCGGTCGTCATCCCCGTCACGGTCGGACATCTCCCTTCCTTCC
CCAGAAGCAACCCACAACAACACCCACAGAGC
AAAAAGAGGCAAATATCACACCAGCTACGTGCGCCCGTGCCCCTCGCACGCAGAGCACAGCACAACTG
GTGCAGCAGACCGTCACTTCCCCGCACACAGG
CACCATACACACGCGCAACAAATCACCGGGACGCACGTGCGCACGCCAAACAGCGGTGGGCAGCGACA
ACGGCAAACAGAGCAGCGCACTCGCACTAAAC
CAACCACTGGTTTGTCGTCGCACAAAAAGCAATAACATACGACAAACAATTACAGCGAATGGCTACTC
ACACAAACCACGCTGATGGTGGCATTCCGGCA
AAGGTATTGCCGAGCACACGCAAAGCAAAAATGCTCAGAGGAGAAGAAAACATAAAACTGAATATGCT
AACACCACTAAAAGCAACAGCAACAAAAATAA
AGACACTTCTTTTCCGTATGTTTCCAAAGAAAACCAGGTGGCAAATCAGTGCGCAAACAATACGTTCG
CCACACAGCACTTCCCTCCTGCGCTATCCATG

GGTCGCGTGCGGCGCTGCCGCTCTTTACTTCTACGCACAGCACTGAAAGAAGCTGGAAAGCAGAAAAA
GACCGCAGGCGGCGCAAATGAGGTAGAAGGCC
TCGGAAAACTTGTAGCCCTGATCCTTAAGGGGACCGTACTGCGAGGATAGCACGGCGTCGCTCTGGCA
GTAACCCTTCATGTACCCATACACAATAAGCG
CCACGCATGCTCCGCAAGCGGCGAACGCAAGGGAAATCAGTGAGGCTGCGCAGCAAGTGAAGCGGGAG
CTCTTTTCTTCCAGAATGAGCTGGGCGATAGC
AAGTCCCAGAACGACGAGGCCAATGCCTGCCGCAGCCACAGCCGCGCCCTCTGATACCTGAAAAAATA
GCTTGTACTGCCCACACAAACTATCACTCACA
TCAACCTTGGTACCAACGCCACCTGGAACGGTCGTCTGGCTGTACCACAGTGTCTGCTTTATCTTCTT
CGTGTTGCTCTCCTTCTTGAACACAGGAAAGA
TGTTCGCACACACAGCGCAGCCAATAGCCAAAATGGTGAAAATCAACACAGTCACGCGGCAGGAGAAA
TTGTTTCCTCCTTGACGATTGTGAGAGATCTT
GCTCATAGCCTTAGCCTTGCTTCGGGGAGACGGAGGGCGGAAGGAAAAGAGGAACATGGGGAAATGCA
CACCAGTTCGGCAAGGAGGGGGTCATCAGCGC
GTGGAAGCACAACCGCAACGAAGAAGTAACTCCTCCTGACGCACGTCATCATTTACAGGTTACACCTC
ACTGAGGTCTCAAATACTGAAAAATAGAAAAT
ATATATGCGTATATTAATCGGCACCCAAAGATACCGACGTTTGTTGTTACAAACGTATTTTTTTCTTT
TTACTTCTAAGCACAGCACTGGAAGAAGCTGG
AAACAAAAAAGAAAACACAGGCGGCGCAAATGAGGTAGAAGGCCTCGGAAAACTTGTAGCCCTGATCC
TTAAAGGGACCGTACTGCGAGGATAGCACGGC
GTCGCTCTGGCAGTAACCCCTCATGTACCCATACACAATGAGCGCCAGACACACTACACACGCGACAA
ATGCAATTGAAATCAACAAGGACGTGGCACAC
CCCAAGCAGCAACAAATTCTCTGAAAAATCATCTGAATAATCACAAATAACACGACGACGAGGCCAAT
GCCTGCCGCAGCCACAGCCGCACCCTCTGATA
CCTGAAAAAATAGCTTGTACTGCCCGCACAAACTATCACTCACATCAACCTTGGTACCAACGCCACCT
GGAACGGTCGTCTGGTTGTACCACAGTGTCTG
CTTTACCTTCGCAGCGTCGCTCTCCTTCTTGAACACAGGAAAGATGTTCGCACACACAGCGCAGGCGG
TGGCCAGCATGGCGAGAACCAACACGATTACA
CGGCAGAAAGAACACATGTTTCCTCCTTGACGATTGTGAGAGAGCTTGCTTCTTGCCTTAGCCTTGCT
TCGGGGAGAGGGAGGGCGGAAGGAAAAGAGGA
ACATGGGGAAATGCACACCAGTTCGGCAAGGAGGGGGTCATCAGCGCGTGGAAACACTGACAGCTGAG
CGCGTACCTCTGTGCGCGACAGGACAACAAGG
GAAAAGCACCCGCTCCAGCACGCTCTTGCCCTTCAGATGTTTGTGTATCGCTCGTCAGCGGGACGGTC
AAAGTCGATGTCCAGTTCGCATATTCGCCAGT
CCTCATACGTGTTGTCCGGAAGCACACGCCGTACGATGAGCGGTATGACACCCTCGCGAAGCTCCTTT
TGGGCGATGATGAGCGGATCCGTCTCGCCCTC
CAAGGCCACCAGCACCGGCGCGTTCATGCTGATCTGCAGCGCCCGTGTGCCAAGGATGCGGGCCCGCT
CGTATTTTGTCAGCACCGGCGCAGTCACACGG
TCCTTGCCGTCGCGTCTTACGGCAGAGCCGCTCGCCGCACGCCCGGACAGGACAACGTCCGCGCCATC
GCCCTCGCTCTGTTCGTCATCACTGCGCAGGG
TGCTCTCCTCCTGTTCCTCGCCGAAGTTCAGACTCAGCCGGTCGTCATCCCCGTCACGGTCGGACATC
TCCCTTCCTTCCCCAGAAGCAACCCACAACAA
CACCCACAGAGCAAAAAGAGGCAAATATCACACCAGCTACGTGCGCCCGTGCCCCTCGCACGCAGAGC
ACAGCACAANNNNNNNNNNNNNNNNNNNNNNN
NNNNNNNNNNNNNNNNNNNNNNNNNNNNNNNNNNNNNNNNNNNNNNNNNNNNNNNNNNNNNNNNNNNN
NNNNNNNNNNNNNNNNNNNNNNNNNNNNNNNN
NNNNNNNNNNNNNNNNNNNNNNNNNNNNNNNNNNNNNNNNNNNNNNNNNNNNNNNNNNNNNNNNNNNN
NNNNNNNNNNNNNNNNNNNNNNNNNNNNNNNN
NNNNNNNNNNNNNNNNNNNNNNNNNNNNNNNNNNNNNNNNNNNNNNNNNNNNNNNNNNNNNNNNNNNN
NNNNNNNNNGCAAGGAGGGGGTCATCAGCGCG
TGGAAACACTGACAGCTGAGCGCGTACCTCTGTGCGCGACAGGACAACAAGGGAAAAGCACCCGCTCC
AGCACGCTCTTGCCCTTCAGATGTTTGTGTAT
CGCTCGTCAGCGGGACGGTCAAAGTCGATGTCCAGTTCGCATATTCGCCAGTCCTCATACGTGTTGTC
CGGAAGCACACGCCGTACGATGAGCGGTATGA

CACCCTCGCGAAGCTCCTTTTGGGCGATGATGAGCGGATCCGTCTCGCCCTCCAAGGCCACCAGCACC
GGCGCGTTCATGCTGATCTGCAGCGCCCGTGT
GCCAAGGATGCGGGCCCGCTCGTATTTTGTCAGCACCGGCGCAGTCACACGGTCCTTGCCGTCGCGTC
TTACGGCAGAGCCGCTCGCCGCACGCCCGGAC
AGGACAACGTCCGCGCCATCGCCCTCGCTCTGTTCGTCATCACTGCGCAGGGTGCTCTCCTCCTGTTC
CTCGCCGAAGTTCAGACTCAGCCGGTCGTCAT
CCCCGTCACGGTCGGACATCTCCCTTCCTTCCCCAGAAGCAACCCACAACAACACCCACAGAGCAAAA
AGAGGCAAATATCACACCAGCTACGTGCGCCC
GTGCCCCTCGCACGCAGAGCACAGCACAACTGGTGCAGCAGACCGTCACTTCCCCGCACACAGGCACC
ATACACACGCGCAACAAATCACCGGGACGCAC
GTGCGCACGCCAAACAGCGGTGGGCAGCGACAACGGCAAACAGAGCAGCGCACTCGCACTAAACCAAC
CACTGGTTTGTCGTCGCACAAAAAGCAATAAC
ATACGACAAACAATTACAGCGAATGGCTACTCACACAAACCACGCTGATGGTGGCATTCCGGCAAAGG
TATTGCCGAGCACACGCAAAGCAAAAATGCTC
AGAGGAGAAGAAAACATAAAACTGAATATGCTAACACCACTAAAAGCAACAGCAACAAAAATAAAGAC
ACTTCTTTTCCGTATGTTTCCAAAGAAAACCA
GGTGGCAAATCAGTGCGCAAACAATACGTTCGCCACACAGCACTTCCCTCCTGCGCTATCCATGGGTC
GCGTGCGGCGCTGCCGCTCTTTACTTCTACGC
ACAGCACTGAAAGAAGCTGGAAAGCAGAAAAAGACCGCAGGCGGCGCAAATGAGGTAGAAGGCCTCGG
AAAACTTGTAGCCCTGATCCTTAAGGGGACCG
TACTGCGAGGATAGCACGGCGTCGCTCTGGCAGTAACCCTTCATGTACCCATACACAATAAGCGCCAC
GCATGCTCCGCAAGCGGCGAACGCAAGGGAAA
TCAGTGAGGCTGCGCAGCAAGTGAAGCGGGAGCTCTTTTCTTCCAGAATGAGCTGGGCGATAGCAAGT
CCCAGAACGACGAGGCCAATGCCTGCCGCAGC
CACAGCCGCACCCTCTGATACCTGAAAAAATAGCTTGTACTGCCCACACAAACTATCACTCACATCAA
CCTTGGTACCAACGCCACCTGGAACGGTCGTC
TGGCTGTACCACAGTGTCTGCTTTATCTTCTTCGTGTTGCTCTCCTTCTTGAACACAGGAAAGATGTT
CGCACACGCAGCGCAGCCAATAGCCAAAATGG
TGAAAATCAACACAGTCACGCGGCAGGAGAAATTGTTTCCTCCTTGACGATTGTGAGAGATCTTGCTC
ATAGCCTTAGCCTTGCTTCGGGGAGACGGAGG
GCGGAAGGAAAAGAGGAACATGGGGAAATGCACACCAGTTCGGCAAGGAGGGGGTCATCAGCGCGTGG
AAACACAACCGCAACGAAGAAGTAACTCCTCC
TGACGCACGTCATCATTTACAGGTTACACCTCACTGAGGTCTCAAATACTGAAAAATAGAAAATATGT
GGGTGGAATAATCGAAACTCTAGATATCGATC
GTTTGTTGTTACAAACGTATTTTTTTCTT
>scaffold1136.size6083
TTTTTTTTTTTTTTTTTTTGAAACCCAAACAACAAGAAACAACGACACACACACACACACTCTCTCTC
AATAATAATAATAAAGGAAGGAGGAAGAACCGG
CCGTAAAAAAAAAAAAATTATGCCCGAATTTATTTGGTTCTGCTTCGCTTTTTCTATAAATAAAAGTC
CTGTAAATAAATATATATGTTTGCTTATTAAG
CCTGTTGTTTGCATCTTCTTGACAACCTCGTCCCAGCCATTTCCAACTAAAAAACAAGGCAGACGCAC
ACACACACACAGACGCAGAGAAATAAATACAT
CGGTTTATACTATTCTTAGCCAGAAATGACTTGGAAAATAAGGATTTTTTTGCAGTAATGGGAAAATT
TAAACACGGGGGAATAACAAAACAACAACAT
AAATTTCCAAATTGCATGCATATGGGTAGAACAGAGTTTCAGAATATTAATAATGCCGGTAAACTACA
TACACATATATATATATATATATATGTAATCAGC
ACCAGCTTCAACATTCCCTCTGATTGCTGGGGAATGGAGACTTAATTACGGCGGCTCAGTTCAAATTG
ATTTATATAGAACATGGTCCGCATTGTCTTTA
CTAGTTCACTGCAGAAGAGATCAATACCCACAAAGATGGCGATGCTCATGTTCATATCATGGTTGATT
GTAAACTCTTCCGGACGAAGCATAAAACTCGC
ATTACGTGGTGCCGTGAGCTCCTCCCGTTCATCCCCTTCTTCCTCTTCGCCATAtGGACAAATGCTGG
CGTTGCCATGCGGCACACTCGAATTCTCTCTT
CGCCACGGCATCAATGATAAGAGGCTGCTGTTGTCAATTACCATCGACAGTTTCAAATTGTTCAAATG
GAGTTCGTTCGCCTTCCCTCGTATGGCAGACA

ACATTTTTCCATGCGCCATGGAGAACATCTTCTTGAGCTCCGCGTTCAAGCGCAGCAGGGTGGAGAAG
TGATCTACCGTTTTGGCACACGACCATGTCTT
TGGCCGCTGCACCGCATCAACAAAGTCTTGCATGGCGTAAACACTGTATAGGTTAATGTTCTTTAAAA
GCTGTCTGCTGCTTGTAAGCATTTCAACCAAT
TCATCCGCCACAGGGCCCAAGCGTGCACGATGGAGAATCGTCAGACCCAAAAGCATGATGTCCAGCGA
TGAGAGCAGGCGGCGCAACACCGGGTGAAACT
CATCTTGCTCATGGACAGGGTAAGGTTTACCACGGATGGTGGGCTCCAAAGAGGCTGTTTTGATTTTG
GGCGGCAACTGCCGTACGGCACGCCAGAGACC
ACTGCTGCATGCTTTCAGTGCGTGGATCTCTTGCATCACATCGGGCTCTTGGTCGTCGACGGGCGCCT
GCAGGAGACGGATCAGGCTGGAGAGGTAGTCA
GAGATGCCATCCATCACCCACACCTCCGAATTGTGTAACACTCGAATGGTGTCGACGGGAAAAACAAA
GACAACAATGGCATAATACGTCATGACGGTAA
GTGAATTCGAGATCACGCGGGAAAACATCATCAGCGGCATGGCTACCATGTGCATCATGGAAGGAATA
AGAAGTGCACAATAGAATGAACAGTTAAAATA
GGCCGGGAAAGAAGAAGTAAAAAGTGCTACTGCAATAAGCACAAGTGCAATGGGGTACGCTTCGAGAT
CAGACTTATGATGTCTCCGTATCACATAGGCC
AAAGCCGCAGCAAAGACAAGACCCGCCACACGCTGTAATCCCGCCACCAGTGAATCACCCGTGGCTTG
AAGTCCAACACCTATGAGTATCGTCATCCCAA
AGTAGTATACACTATCAAAATCAATGCAGAGCGTGTAAACACAGGCCAAGGTGTAGCCACACGCAAGT
TTGAACGCATCTTTTGCAAGCCGCACATCATT
CGGTGTGGCCAACGTAAGACGCTTCGGTAGCTCTGTCCAAAAATCCCTCCAGTGCTCAAGAAAAAAGA
AATCCAAAATGCGACGCGATGTGGACGTGTAC
CGCGAGCAATCAAAGTTGCGCATAAGTTCAGCGTACCGAAGCAGCTCCTCGCCAATGAGTACCATCAT
GAGGTGGGAAAACACAATTTGCATGTACCACA
CCGAGTCGACATTACCAAAGCGGCCTTTCATCATGTCGTAGTGGATCTTGTCAATGGTGGACTGCAGG
GATGAGGTGGCTGACTGAAGGTCATCAAACGG
CACGGTGGCCACCACCTCACGGGGATTCAAAACACAACCCAATTGCTTCAATGTAACTCTCAGGGCCT
GCAAATATGGCTCGAGTGCTTCTTCCAGGCGC
CCACGAATAACCCCCACACCGGGGCTGACGGCGTATTGATCGTTATCGAGTGGTGACCGCGACCGCTG
CGCAGCGTGGAGTGCACGCACTACACCCACCA
AGGCATGCAGCATAGGCATTATGCCACCAATCACCTTAATACGTTCGCTTCGTAGCTGCAATCGTAAC
GGCGACTCCCATGGCTCGTAATGGACAAAACT
GAGTTCAAAGGGCACGGTTGTAAATATTTTATGGAGTGGTTCCGATGACGTCTTTGAGAGCGCCACCG
CCGCGTCAATAGGGTTCTCAGACCAACATGCC
TTGACGGAATTATGCACCACCTCCCCTATGTAGACCCACGCCTCTGCCATCGCCTCATCCACGCGCTT
GCACATCGTAAATGGCGGTATAAAAGACTGAA
AACAAGCAAATCCTAAAGCAAGAAACGCGTCACGCCCCACAAGAAGAGGCAGAAGAGCCCCTTTTTCT
ACCATCAGCTGGGCCCGTTCAACCTCCATAAC
AATCAGCGTCATTAAGATCGCCAGTTTTCTACCCCGTACGTCCCCAATTAGACCTATGCCAAATGCTC
CAAAAAACACAGCACACCACCATGCAGTTTGA
TGTGCAAGCAAATTAAATAAATGCATCAACGAGCCCCACAGCATCATCCACAGCAACACTGGGATCAT
CGTGCTTATCATACATATTTGCTCCCCCACAG
TATCTTTGCTATATGCAACGCCAAACAAGGCCAAAAGAGTGTGCATCGGAAAAAATATCTGGATATCTA
TCTCCAATATACATCAACGGAAGAGCCGAGAA
GAGTGAGGTACGAAAAGCCACATACGCAGGGTGGCGAAACTTTTGCGAGAGAAGGGTACACCAATAAG
ATTCAAAGCTCACCGAGTCATCATGTATATTT
CCCCATATATTGAGTTTGGGTGGCTTTATTTCCTCCGCGTCTTTTTCCATCAGTTGCTTCATGAGGTT
CTCCGCCCACAAGCGGTTCTCTGATATGTGAA
TCCACTCTATGGATTCCATGGCGTATCTCCCCCCCCCCTCCCTTCCCTCCCCACCCCTTTTTTTTTTA
TTTATTATTTCCGTGTCGTCGTCCCTACACAA
TTAGAACTCCTTCTGTGCCCTGACACCTTCCCCCTTGGGTGGTGGATTCCAAATGCTACACGCCACGC
CTTTTCCTTTATTTTTTACTTGTGAGATGTGG
GGAACTTCCCCTCCCTATTCCGGCCAACAACAACAACAACAACAACAACAAAAACAAAAGAAAACAAC
GCACAATAAGCAATATTATATACACGGAACAA

AAAAAAAAAAAAAAAGAAAGCGACATCTTTGGAGAAATATCCCCAAACAGTGCGTGACTGCTTTCCCCC
ATCCTCACGGCAGGAGAAAAAACTTTATATCG
GACAGGCTAGACACGCATAATTGTTTATGACATGCACATATGTATATATATATATATTCTGAGAGAAA
TGCCAAATCATCATGGAACATACGCATACATA
CAAATAATGAGCAAAAGAAGAGTTGTAACTGGAAAGAGTTATGGAGTATGTTTTCTGTGTATGAGCTT
TAAAAAAAAAAAAAAAAaGAATTGTTTATTTA
TTTTTTTTTTTTTTTTTGGCATTATTGCCTTCACAATAATTCCCTTTACTTCTACCGTCAGTTGGTTA
TGCCATCACATTTTTGTTAAACCTTGCCGTTA
TTTCCCACCACGCACGTTGCTCCAACAGCGTGACTTGCTTCCGCTCCACTAATTCCACACTCATGCAA
ATATGAAGGGCAATTTCATTGTCATTACTATT
TCGTGTCCGCCCTGTCGTGTGCAGTGGCGGAGGTGACTGGGAGACATCGGCCACCAGATGCCCCATGG
CACAATTCAAGAGAGAGAATATATTCCCAACA
GTGTGCTCCTCTGAACCACTCAACGCCGTTCTGCTCTCCATGTCACAAGGACTGACATCATGGGAAGA
CGCCATGCAAATTTCTTGCGCTTTTGCAGGCG
AACTCGCAGGTGGGGTGCATACCGCTTGTGATCTCGCACTTCGACTGCGCGCCGTTTCCACTGCCCAC
GTTCGATGCACACCAATCGGCGTTTGACTCTT
GCTGGGCCGGCGTTCACTGGCAACAATTTCCCGCTCGGCAACAAGCAACGCAGAGGCTGAACGAGGGG
CGATGGGCGTGGCGGATGGGCATCCATTCAGA
TCGGCAGCCAACTCATAGATAAGACTTTCCTTGGCGGCAATAAGACGGCGGCTATTGGCATGGTTCAG
TACCATTTGCCATCGCGATAATAGAAGCTTCC
ACGAGAGATGAGCCTCCCCTTCAATATAATCTCGTTGCGACTTCTCAGCATCACACAATAAGGCATGC
GCGTAGGATGCCCAGCCCACATTTTCTACGGT
GGGCCCGTTGACAGACAACGGCTTTCCCTTCACATCACAAGAGAGTCTCGTCAACTCTTTCTTTCCAT
TTTCACAGGAATACATCGCGGCACCATGCAGT
TGCGCTCGGCATGCCTCTTCATATTCTTTCGCCCCTCTTTTCACAGTACTGATGGCGGATGCCAGAAT
ATCCTCCTGGGCATCTAGTTGCTGCTGCATCC
ACCGCATAGCCTCCCAGAGATGTTTGTCCCAAAGCACTGAGGGCTCCACGATACGTGCCCGTGTTTCT
GCATCCACCCCCGCAGCCAATTCCTCACGTGC
GGTAACGAGAAGTGTGACGCGTCCCAGTCGTTCCTCCATAAGGATGGTGTGCCGCCGAACCGCTTCCT
CGGCGTCACATCGGAGCGACCACAAACGCCTC
TTATCAAAGTGGGCCTGTAATGCTATTTGTCTGGCTTTCCACCTATCAAACCCCAAGTTACATACAAT
GTTCTCTTCCGGAGATTTATGTACAACGGGGA
TAAGTGAGGATTTCCCTACCGCCGGTTTTATTGCTATTGCGGTTGCTGATGGTAATGATAATGCGTTA
ATTTCCTCACATACACCGTGTGGGGCCAAATA
AGACGGTGACGAAAGTCTCGTATCGCCTGGACACGCACGGCTGCGACTTCGACTCCAAATGCCACCCT
CCGGGGTCTCGTGGTATTTCTGGGGAGACTCG
TGTAGATGCAACTCCTGCGACTCCTGCCGATTGGAGGAAACGGCATCATAAGCCAAAGAAAACGCATT
CGACACCATTTTGTCACCTTCCTCATTTGAGG
AGATATATCCACGTTGGGTGTGATTCCCCATCGCTGCTGGGGGGAACAGCTGCGACGTCACCACCGTC
TTTCCCTCACACCACGCAATGGCCACATCCAC
CTCGTTCAGCCGCCGCAGGGCGTGCACCATAGGAGCGTCATCGCGACCGTTTCTGCTACTGCGGTGGT
AGTTTTCGTGTCTCGTGCCTGCATCCACCTGC
GGAGCCGCCATGGCCGCGTCCACGGCAAAGCGTCGCTGCTCCTCTAGCCGCATCAATGTCCCGCTGCT
TGCCAGAGCCTCGTAGGCAACGATGATTCCAT
CCACAAGAAATGCCTGATCCTGAGCAAGCGAGTAGGCGACGCTGTCTTCAAGCGCCATCAGACGCCGT
ACGCAAGATAAACGTTGCGCCAAAGTCAAAGA
TGCGGCGGTCGTGCCCTCTCCCGTCGTCAACATCGTCAATGAAAAAAGAGTATTAATAACAATAATAA
TAATAATGAAACAACACAGGACCGACGGGGTC
CACCTGCCCCACAAAAAACAACAAAAACAACAGATATGTAATAAATAAATAAAAAAAAAGGGGTAGAA
GAGGAAAAAAAAAA
>scaffold1137.size6505
GACCTACGCACGTTCGCCATCGGATACGGTGCCTCCAGCTCACCTTATGGCAGTGAACTCATATGGGC
AGTAGCGACGGATTCAGCGAAAAATGAAATGCA
GAAGACGCACGCAACTCTGGCACGCACTGTGAGGGGATCTCCGAGCACAACTGACACAAAATCCGCAC

TGCTGCAGACCAATACACCGTCGATCCATGTC
CTTTGCCTGCGCACGCGTTTCAGATGGAGCGGATGCGGAAACCCCAGCCTGTGCCTCTGCCTCGCGCT
GGTTTCCGCACCTCGCCGTTATCTCTCGACAA
ACGTTGCGCCTTCGTGGATGCACGCACACATGACTACGGCGTCACCGAGAACTCACCACGCGAATAGC
AGCTCCATTCCTCCCTGGTCCGCGGCCTCCGC
TCACCGGGTCACCATCGGTGTGGAGCTTCCGGTGGACCACTCGATCACCGACGAAAAAGAGCTGATAA
GGGAAAAGCGCAGAGTCAGCGAAGAGGCTCTG
GCGCTGCACGGCCACCGATCGTGGATACTTGCAAAAAATAGCGGTGTCGGCATTCCCACGTCAGCCGG
GATTGGCATACTGCTCTAATCCCTGGAACTTT
CGAGGGTAATGGAGAAGGTCAGTACCAACTGCGGCACACGCCCATGCAGCCGCACGACGGAATCCAGT
GCACTGCTCCCGGCCATGGAAAGGCTGAGTGT
CCATCGTATCCAGCACAGGCAAAAACCCTGCTTGTGGTTACAGACAGTCAGTCCCTTCTAGCCGCCGT
CACCAAGGGCCCGCACAGTCATACCGACTGGA
CGGAGGATCAGATCTGGCAGCGTCTCTTGTCGCTGACGCGTGCCGGCTGGTCCGTGCAGCTGCAGTTT
TGCTACGAACATTGCGGAGTACACGTTAACGA
GCCTGCAGATCAGTATGCGACGCAGACCACGGCAACCGGGCAATACACGGAGCATGGAATAGCACCCA
TACGGCACACGGACCTGCTGTCATGCATTAAC
ACCCAGCTCACCAACAAGTGGCGTAGTACCATGCATCAGGACACCCACCACTACCTGCTTGGCGGCGC
CAGGCCATCAGATCCAGGCGGCAAGCACCTGA
TCACTCAGGAAGCCCTCGACTGACAGGAACGGACAAACATTGCATGAGCAGGGTGCGGGGACTCCGAG
CTATGGGGTCGCCTGTACTGGACTGTGAGAGA
CTGCACGAACCAACGCCGTTTCCGCAACATCTCACCGGAACAGTCTGCTTTCATGCGCTCCAACGACG
ATCCCACCGCACCGGGACGGACGCTGTTCCCC
CGACGACAAGGGAGGCCGACGCACCGCCTGTGGAGAGACGGCCCTCCAAACAGCGTCGAAAGAAGAAA
TGCCCGCACTGTGACGCCACACTGGTGGGACA
CACGAACCACATCGGTCACTGTCGGTGCTTTCATCCGGAACACCCTCCACCGCTTCCCGACCTCCACC
GCGATTGCTGCGACACGGTCCTCCCCACACAA
AAAAAGCTNNNNNNNNNNNNNNNNNNNNNNNNNNNNNNNNNNNNNNNNNNNNNNNNNNNNNNNNNNNN
NNNNNNNNNNNNNNNNNNNNNNNNNNNNNNNN
NNNNNNNNNNNNNNNNNNNNNNNNNNNNNNNNNNNNNNNNNNNNNNNNNNNNNNNNNNNNNNNNNNNN
NNNNNNNNNNNNNNNNNNNNNNNNNNNNNNNN
NNNNNNNNNNNNNNNNNNNNNNNNNNNNNNNNNNNNNNNNNNNNNNNNNNNNNNNNNNNNNNNNNNNN
NNNNNNNNNNNNNNNNNNNNNNNNNNNNNNNN
NNNNNNNNGAAATCGCTGCGCACAAAACCCAGACGCTGATCGGCACCGCAGCAGCAGTGCCAGAAGGC
AATCACTGCTACCGCAGGATCAGCCAGCCTCC
ACAAGCGCAACAATTAGCCCGCAGAAAACACTGCACCATCTGCTTCCAGAATGTACAGACGCTCTGGC
TGTGCGCCAACGGCTGGGCATTAAGAAGGACC
TTTGCACCGAAAGGTTCTCCCAATGGCAGCTGCCTCATAGCAGGAAACTCATGTCGCTGCTCGACCAC
CTCCTCGGCACCCTGGTGACATCGTGTGGCTA
GACATGTCGGTAAGAGCAGGGAAAAATAATAATAATGAGCATTCCCGCCACTATAGAGACGTGGTGCA
TCTTGCGATCCCGCTGGCGGCTCTCCTCTTCC
ACCGCTGTTTTTGCGCAAGAGGTCACCACAGTCATTTTTGTGGTTTGGTCGGCTTCGTGTTGCGCTCC
GGGTTCTCCTCATTCATCGCCCTGCGGTGCTT
CCTTCGCCGTCCCTGGCACCTGTGACTGTCCCATGACGGTTTGTTCCNNNNNNNNNNNNNNNNNNNNN
NNNNNNNNNNNNNNNNNNNNNNNNNNNNNNNN
NNNNNNNNNNNNNNNNNNNNNNNNNNNNNNNNNNNNNNNNNNNNNNNNNNNNNNNNNNNNNNNNNNNN
NNNNNNNNNNNNNNNNNNNNNNNNNNNNNNNN
NNNNNNNNNNNNNNNNNNNNNNNNNNNNNNNNNNNNNNNNNNNNNNNNNNNNNNNNNNNNNNNNNNNN
NNNNNNNNNNNNNNNNNNNNNNNNNNNNNNNN
NNNNNNNNNNNNNNNNNNNNNNNNNNNNNNNNNNNNNNNNNNNNNNNNGCTTGCCCTCCGCCCAAAGAC
TCCGGTCCACTTGCACGCATCCGAGCAGCAGA
CGCGCTGCCAGTGCCGGACGGATTGCAAGTGTTGCTTCGCAGTGGGTCGCTGCTGGATTGGTCCTGCC
TCGTCGGAGCAATGAGTGGTCCTGCCGAGGTG
GGATTGTGGCCCTCACCCGCGGTGCTTCCTCCACCCTCCTCGTCTTCCTGATTGCTATTGTCCTCAGA

GTTCACATTCCCCTTTCGTCCAGAGCCACCGA
CCACGGGTCCACCTTCCGCAGCCATCACAGCTTCCGCGTGCCCAGCAGTCACCGCGCCATCACCCGCG
GGCCGCACACGCACCCCGTCGCAGCAGCCGTC
ACTCACACGCACGAGCAGCTGCGCATCACTGCAGGAGCCAACAGGACGCAGAGCACACGGCCAGTCAT
CATCACAAACAGTGGAGTCGCCTAACGCTGCG
GAGCCAACCTGCGATAGCACGAGAAAAACAAAGGGAGACACAGCCGCTACACCACGCACAGCAACAAA
TGGCAGCACCCCAGCAGCAGCGCACACACCAC
GAGACAGATGGAATCAGTACGATTGCAGAGCTCGCACACCGCACCGCAAACCACAAGTTAAAAAGCAA
TAAAAAACAATAATTAAAAAACAATAATTAAA
AGAAGAAAACAAACCCAGGGCTCCAGTGCGCATGCTTATGAGTGTGTCCGCGATGCGTTCAACGTGGC
GGATGCGGGGCACAACGCGGAGCACACAGCAC
GGCAGCAGAAGCAAGTGAAGCAAAACAAGATCATTATTTTCTCTTATTTTATACAGTGAACCACAACA
CACACGCACAAATCCCCGCGGTTGAGTGCTGG
CACGCACAGCCCGCACACACCTCTTCAGCCCACAGCGGTGTACGCCAGCGCGGATGCGGCGAGCACCA
GCGGGGCACACACCCACGCAGAGCTGCTGAGG
CTGCCGTCGATTTCGCGAAGACGTGACGGTGCGCGGGTGGTCGACACAGCTGGCGCCTCTGTAGTCGT
AGTACTTGGTGCCTCTGTTGTAGTGGTGGTCG
TCGTGGTTGGTGGCTGTGTTGTGGTGGTCGTCGTCGTTGTCTCCTCGGTAGAATCTTCAGACTCACTA
TTTGCCGGTTCTTCGGATAGTCTCTTGTCACT
GGGTAACCCTGTCGTACCCTTATTACCACCTGCCTGTAACGTATTGGACACCTCATTTGGATTCGTTC
CCGTACTGCTAGATCCTGATCCCTCACCGCCA
CCATTTCCCGACTTTCCAACGGGCCTCGTTGAAGGATCTGAATGACCTTTCCCATGCGCTCTCTCTAA
TAGGAGGGGCGATTGACCGGAGTCATTTCCAA
CAGCTTGAATATTAGCCTTGTCATCGGCAACTGGTGGCTGTTGTGGAAGAGGCGGTATACCGCTGACT
TGTTCTTCATCTTCAACCTGAGTAGCAGCAAT
GGTTTCATCCGTAGAATCTGGCGCACACACGGACGGgNNNNNNNNNNNNNNNNNNNNNNNNNNNNNNN
NNNNNNNNNNNNNNNNNNNNNNNNNNNNNNNN
NNNNNNNNNNNNNNNNNNNNNNNNNNNNNNNNNNNNNNNNNNNNNNNNNNNNNNNNNNNNNNNNNNNN
NNNNNNNNNNNNNNNNNNNNNNNNNNNNNNNN
NNNNNNNNNNNNNNNNNNNNNNNNNNNNNNNNNNNNNNNNNNNNNNNNNNNNNNNNNNNNNNNNNNNN
NNNNNNNNNNNNNNNNNNNNNNNNNNNNNNNN
NNNNNNNNNNNNNNNNNNNNNNNNNNNNNNNNNNNNNNNCGCTCCCATTTATTGGTGCATGGAAGGGCAT
GTCTGTTTGGTGGTGTCTCGTCCATATATTTT
GTGGGTGTACATTCCCCTGCAGGCTGAGGGCGGGGTGACGCTCCGTCGCGGTGCACTTCCCTCGCATG
CCGCCCACTGTGATTCATCATGCATCACCCCC
TCACACATTCCCCGTGGTGCCACACCGTCGATAATATTGCAGCCGCTGTGATTCCGCGTATTTCACGC
ATTTGATTGTGCCACAATACATCCGCTCGTGT
GGCCGCCGCATCCAAACAGCGGTGCCCAACACCGTGAGCGGCAGTCCGCATCCCTGCATGTCCCAGCG
CAGTGGTGCTTGAATGTCAGCCAGGCGCCAGA
CAACGCGAGAGTCCTTGTGCTGCAGTACAACACACGGAGCTGCAGTGAATGACTCGGGCGGCGACGCA
TCTGTTTGCCGCAAGGCAGTGAAAAGGAGTCG
GGCAAAATAATGCCGGCTGCATCCACGACACCATACCCACAGCCAAAGCATGCGACGTGATGTGTGCT
TCGCTGGTGCTGTGAAATGCCTGCAGACGACG
TCACTAGTGGAGGCATGCCACCCATGATCAGCGGACAGACAATAATCGGCATGGGAAGCCACAAAAGG
CAGCAACGACGCCAGCAGCAGCAGCAGCTCTC
TCTCTCTGTTGCCTCCGAGAAGTAAATATTCCACCAGCCACAGCTGACTGTGCCATCGTGACACTCCA
TGCACGTCGTGGCTGCGCCAACACATCCTGCT
CCCTGCAGCGCCTCAAAAGATGCCTCACTCCGTCCACTAAGTGCATCGATGTGGGCCGTACCTCGGCT
GTTCGTGAGGACCACAACGATGTGAGGGTCAC
CGAGTCGCAGCATGTTTCTTGTGACGTTGTTGCATGGCACCTGCACACACACACGCAGCGCCTGCATC
CCTCCCGTTTGCGTTNNNNNNNNNNNNNNNNN
NNNNNNNNNNNNNNNNNNNNNNNNNNNNNNNNNNNNNNNNNNNNNNNNNNNNNNNNNNNNNNNNNNNN
NNNNNNNNNNNNNNNNNNNNNNNNNNNNNNNN
NNNNNNNNNNNNNNNNNNNNNNNNNNNNNNNNNNNNNNNNNNNNNNNNNNNNNNNNNNNNNNNNNNNN

NNNNNNNNNNNNNNNNNNNNNNNNNNNNNNNN
NNNNNNNNNNNNNNNNNNNNNNNNNNNNNNNNNNNNNNNNNNNNNNNNNNNNNNNNNNNNNNNNNNNN
NNNNNNNNNNNNNNNCAGGCATCATCACAAGC
AGCTCCAGCAGCGCTGCAGACCGTGGGGTGACGTGGATCAACTGACGCATGGCGTATGTGGAAAAGAA
AAAGACGTGGTGGCTTTACAAGGATACGAGTG
GCTGACACTTTTAGAAGGAGAAAGAAAACAATGTGAGAACACACAAAGAGGAATCAGGCGTCCGCAGT
CAAAGCAAACCAGCAATCAAAAAAAGTAGGAT
GGGTGCACTCAACCACAACACAGGCAGAGGATAAAAAATACCAACAACAAAAGCCGAGGCGTGGGGGC
GTCCACCCAGCAGTGGGGACGCGCAGCAACAG
GTGGGACGACCAGCGCACACTCTCTCATTGCTCCCGTAAAGCAAAACACACACACAGGGTGAGAGAGA
GAGAGGTCGCCCACTCAATGCAATAAAAACAA
AACAGAAACAGTTACGGTGCTCCGCCCATGCAGCCAGGCAGGACGGCAGCGGTGCGTGTCGTGCAAAA
GAGAGCACCAACGGGCCTGCATGGGTGTGCAT
GCAATATATACAATGGGCACATTATGTGTGTGCTTCGTGGGATGAGGTGAGGGCGAGAGTCCATGCAA
GGGGACACACACAGAGAGTGGGTGAAAGACGA
GTGTGTGTGCGGGCGGTGCACGGCTCTCTCTCCCTCACTCTCACGCGGCCACCACCGCAGCAGCAGCC
GCACACGCAACAAGAAGAAGAAGCAAAAGAGG
GGAGGTGGTGTGGGAGACCGCGGTGCTGCCGTCACTGTCGCCAGGCGTATTCTTATCATTTGTTGGAG
CGGTGGTGAGTGGTATTGCTGTTTGTGGTAGT
GCCT
>scaffold1138.size7289
CAGGGCGCTCATACTCCACAATACCAAACTCATTCAATCCATGAGTAATTTGTCGATAACAAGTGCCA
CCACGTCCGAGGTAGAACGCGAAGGTGCAAATC
CGAAATTGACGTGGGTGGTGCGGCAATAACGCTGACAGACGGCGGTGAATCACTCCTTCGGTTAACTT
CAGTAAAACAGAGAGTGGCGTGATAGGTCTTT
AACTCTCTGGGCGGCATGGATCCTTCCCAGGCTTGGGGATAGGAATAGCGTCTCCCATTTTCCAGGAG
CCAAGCAGGCTGCCATTTGCGAAGGAGTTGTG
GATCATCGTTCTAAGTGNNNNNNNNNNNNNNNNNNNNNNNNNNNNNNNNNNNNNNNNNNNNNNNNNNN
NNNNNNNNNNNNNNNNNNNNNNNNNNNNNNNN
NNNNNNNNNNNNNNNNNNNNNNNNNNNNNNNNNNNNNNNNNNNNNNNNNNNNNNNNNNNNNNNNNNNN
NNNNNNNNNNNNNNNNNNNNNNNNNNNNNNNN
NNNNNNNNNNNNNNNNNNNNNNNNNNNNNNNNNNNNNNNNNNNNNNNNNNNNNNNNNNNNNNNNNNNN
NNNNNNNNNNNNNNNNNNNNNNNNNNNNNNNN
NNNNNNNNNNNNNNNNNNGCGACGGGCACGAGACGGTGCGGGAATTCATTCAACATGTCGTCGGGGCC
AGGGGCTGAGCCAAGCAAAGAGTCACGGATGG
TCACGTCAAGTTCATACGGCGGGAAAGGCCGATCCATTTCCCAGCCACCAGAAACCAAAGGAGTGCAG
CGAGCAGCAGCAGCAGCATAAGGCGGTATGGC
ACGTGAAACCGCACAAAATGACGGACAAGCAGATTTGCCTGTTGCCGAGGCAGGGCGCATACGCGGCC
AAGATCAGTGCGGAGCACAACCGATTCCAGAG
GATGAGGGTGCGGCGCCGCCATGCCGTGAAGATATCGCCAGCTCGGGCTGGGTGAAGCTCCAAGTCCT
TTCGCGCGGGCTTCCAGAAGCACAGCGGAGCA
ATGGCGTAAGGCTTGATTTCGTTCCTCCTTTTTTTNNNNNNNNNNNNNNNNNNNNNNNNNNNNNNNNN
NNNNNNNNNNNNNNNNNNNNNNNNNNNNNNNN
NNNNNNNNNNNNNNNNNNNNNNNNNNNNNNNNNNNNNNNNNNNNNNNNNNNNNNNNNNNNNNNNNNNN
NNNNNNNNNNNNNNNNNNNNNNNNNNNNNNNN
NNNNNNNNNNNNNNNNNNNNNNNNNNNNNNNNNNNNNNNNNNNNNNNNNNNNNNNNNNNNNNNNNNNN
NNNNNNNNNNNNNNNNNNNNNNNNNNNNNNNN
NNNNNNNNNNNNNNNNNNNNNNNNNNNNNNNNNNNAGCTCAAAGTAGGCTGTCTCCAGGTGAGGCAAT
GTGTGTGCTTCGTGTGCTGCTTCTGCGGCGGA
CTCGGCTTGTTCCATTTCGTGTGTCCATATCTTTTTGGAGCTGTCACGGCTGCCGCGCGGGATATGGC
GCTCAGCTGCTCGTAAGATGCCTCCGCGAGGA
TCCAACCATGTGGACGCGGTGGCAAGGAGGGTTTCACACAACGATGTAAAAGCAGGCCAACCCGCTTT
ACGCAGGGCAAAGGTGGCTTCCTTCCGTCAAG
GCAGCGTGTTTGCTAGTCGTGGAGTGCCATCATCCATCTCTACCAAGTAAGATATTCAGCAGTGGTCC

GAGTTTATGTAATGGGCGAATGTCCAGTGGGA
GATGCGTAGGGCGCAATACGCAGTCACGTCAGGGGACGAGGCATTACGATGTGTGATCCGTGTTGCCT
GCGTCGGGTCATTTGCCAGCCCAAAATCCATA
TCGATTAGAACAGCGGCGAAATTATCGCCTCCAGTCGTTGATGTTATATATGACTGTCCCAGAGTTCA
CGATGTAAATTCGGGTCACCCGCGATAATCTC
TTCGGGCCCAAGCGACAAACGATATTGATAGATGCGGATTATTGACCAATTGTTGTGGGAGGGTTGTA
GAGATTCGCCACGCAAATGCTGGGACAGCCTG
GTGGAAATACACGGGCAGCAATGGTCTCCAGGAGGTCAGTGCCAGGGAGCGACAGGCGTCCACACGGT
GGAGAATGGCGCACCAGAACGGCAACACCACC
ACCGCGCCCAGTGCGGGCCTGAAGGTAGTCATTATGGCCAGATAATTTCGTCGCAACGTCACCACGGT
TACTTATTTCCTGCAGAAGAACGATGTCAGGT
GGACGCTGCTCCAATCGCGCAGCAAATGCAATATTTTTCACGGACGAGAGGCCTGCAATGTTAAGCTG
CCAAATATTGATGTAGCCATCCTCCTCGGGCC
AGGGTTCTTGTGAACGTCACCGCAACGGGTGATATCAACGGAGTGACCGCTGCGAAACGGTCGTGCAG
TTCTGTCCGACAGTCTGCAGGCAGACCGCCTC
ACCTCCCCACACCGGGCATGGCGTGGAACCTTCTCGAGGGGTGAAGTCACCTTGGTCTCCGTCGTGCT
CGCAGCAACGCCCTGCTGCGGAGTTCCTATAC
GCAACCAAAGCCTCAACACCGCGACAAGGCGCAACATCATCGCTGGGGACCGTGGATACTCGCAACGG
ATGGCGGTGTTGACGTCCCCAAGTCAGCCGGG
ATTGGCATACTGCTCTCATCCAGCAACTTTCGAAGATAATAGAGAAGGTCAGTACCAACTGCGGCACA
CGCCCATGCAGCTACAGGACGGAGTCCCGTGC
ACTGCTCCCGGCCATGGAAAGGCTGAGTGTCCATCGTATCCAGCACAGGCAAGAACCCTGCATGTGGT
GACAGACAGTCATTCCCTTCTAGCCGCCGTCA
CCAANNNNNNNNNNNNNNNNNNNNNNNNNNNNNNNNNNNNNNNNNNNNNNNNNNNNNNNNNNNNNNNN
NNNNNNNNNNNNNNNNNNNNNNNNNNNNNNNN
NNNNNNNNNNNNNNNNNNNNNNNNNNNNNNNNNNNNNNNNNNNNNNNNNNNNNNNNNNNNNNNNNNNN
NNNNNNNNNNNNNNNNNNNNNNNNNNNNNNNN
NNNNNNNNNNNNNNNNNNNNNNNNNNNNNNNNNNNNNNNNNNNNNNNNNNNNNNNNNNNNNNNNNNNN
NNNNNNNNNNNNNNNNNNNNNNNNNNNNNNNN
NNNNATACCGACTGGACGGAGAACCGGATCTGGCAGCATCTCTTGTCACTGACGCGTGCCGGCTGGTC
CGTGCAGCTGCAGTTTTGCTACGAACATCGCG
GAGTACATGTTAACGAGCCTGCAGATCATTAGACGCAGACCACGGCAACCGGGCAATACACGGAACAT
GGAATAGCACCCATACAGCGCACGGACCTGCT
GACATGCATTAACACCCAGCTCACCAACAAGTGGCGTAGCACCATGCATCAGGACACCCACCACTACC
TGCTTGGCGGCGCCAGGCCATCAGATCCAGGC
GGCAAGCACCTAATCACTCAGGAGGTTCTACACCGTCAGGAACTGGTTCACCTCGCAAGGGCAAGGTG
CGGGGAATCTGAGCTCTGGGGTCGCCTGTACT
GGACTGTGAGAGACTGCACGAACCAACGCCGTTTACGCAACATCTCACCGGAACACTCCGCTTATATG
CGCTCTAACAACGATCCAACTGCACCGGGGAC
GGACACTGTTCCCCCGACGACAAGGGAGGCCGACGCACCGCCTGTGGAGAGACGGCCCTCCACACGGC
GTCGGAAGAGGAAATGCCCGCACTGTGACGCC
ACACTGGTGGGACACACGAACCACATCGGTCACTGTCGGTGCTTTCATCCGGAACGCCCTCCACCGCG
ATTGCTGCGACACGGTCCTCCCCACACAAAAA
AAGGCACCGCACAGCACAGAAGTCGCCGCGCACACAACCCAGACGCCACTCGGCACCGCAGCAGCAGT
GCCAGGAGGCAATCACTGCTACCGCAGGATCA
GCCAGCCTCCACAAGCGCAACAATTAGCCCGCAGAAAACACTGCACCATCTGCTTCCGGAATGTGCAG
ACGCTCTGGCTGTGCGCCAACGGCTGGGCATC
AAGAAGGACCTTCGCACCGAAAGCTTCTCCCAATGGCAGcTGCCTCGTAGCAGGAAACTCATGTCGCT
GCTCGACCACCTCCTCGGCATCCTGGTGACAT
CGTGTGGCTAGACATGTCGGTAAGAGCAGGGAAAAATAATAATAATGAGCATTCCCGCCACTATAGAG
ACGTGGTGCATCTTGCGATCCCGCTGGCGGCT
CTCCTCTTCCACCGCTGTTTTGTGCAAGAGGTCNNNNNNNNNNNNNNNNNNNNNNNNNNNNNNNNNNN
NNNNNNNNNNNNNNNNNNNNNNNNNNNNNNNN
NNNNNNNNNNNNNNNNNNNNNNNNNNNNNNNNNNNNNNNNNNNNNNNNNNNNNNNNNNNNNNNNNNNN

NNNNNNNNNNNNNNNNNNNNNNNNNNNNNNNN
NNNNNNNNNNNNNNNNNNNNNNNNNNNNNNNNNNNNNNNNNNNNNNNNNNNNNNNNNNNNNNNNNNNNNNNN
NNNNNNNNNNNNNNNNNNNNNNNNNNNNNNNN
NNNNNNNNNNNNNNNNNNNNNNNNNNNNNNNNNNNNTCGTGTTGCGCTCCGGGTTCTTCTCCTTCATCGC
CCTGCTGTGCCTCCTTCGCCTTCCCTGGCACC
TGTGACTGTCCCGTGACGGTTTGTTCCGAGGCCTGACTGCCCTGGGAGGTCCTCCCGCCACCAGCATG
CACTCCACCTGCTGCCTGCTGAGATGCGTCGG
AGCGTGGGGATGCTGTGCCTGCAGCGTGCCCTCCGCCCAAAGAGTCCGGTCCACTTGCACGCATCCGA
GCAGCAGAAGCGCTGCCAGTGCCGGACGGATT
GCAAGTGTTACTTCGCAGTGGGTCGCTGCTGGATTGGTCCTGCCTCGTCGGAGCAGTGAGTGGTCCTG
CCGAGGTGGGATTGTGGCCCTCACCCGCGGTG
CTTCCTCTANNNNNNNNNNNNNNNNNNNNNNNNNNNNNNNNNNNNNNNNNNNNNNNNNNNNNNNNNNNNNNN
NNNNNNNNNNNNNNNNNNNNNNNNNNNNNNNN
NNNNNNNNNNNNNNNNNNNNNNNNNNNNNNNNNNNNNNNNNNNNNNNNNNNNNNNNNNNNNNNNNNNNNNNN
NNNNNNNNNNNNNNNNNNNNNNNNNNNNNNNN
NNNNNNNNNNNNNNNNNNNNNNNNNNNNNNNNNNNNNNNNNNNNNNNNNNNNNNNNNNNNNNNNNNNNNNNN
NNNNNNNNNNNNNNNNNNNNNNNNNNNNNNNN
NNNNNNNNNTTCCTGATTGCTATTGTCCTCAGAGTTCACATTTCCCTTTCGTCCAGAGCCACCGGCCA
CGGGTCCACCTTCCGCAGCCATCACAGCTTCC
GCGTGCCCAGCAGTCACCGCGCCATCACCCGCGGCCGCACACGCACCCCGTCGCAGCAGCCGTCACTC
ACACGCACGAGCAGCTGCGCATCACTGCAGGA
GCCAACAGGACGCAGAGCACACGGCCAGTCATCATCACAAACAGTGGAGTCGCCTAACGCTGCGGAGC
CAACCTTCGATAGCATGTGAAAACAAAGAGAG
AGACACAGCCGCTACACCACGCACAGCCACCGCACAGCAACAAATGGCAGCACCCCAGCAGCAGCAGC
GCACACACCACGAGACAGATGGAATCAGTACG
ATTGCAGAGCTCGCACACCGCCCCGCAAACCACAAGTTTGAAAAGCAATAAAAAACAATAATTAAAAA
ACAGAAATTAAAAAGAAAAGAAAAACAAACCC
AGGGCTCCAGTGCGCATGCTTATGAGTGTGTCCGCGATGCGCTCAACGTGACGGATGCGGGGCACAAC
GCGGAGCACACAGCACGGCAGCAGAAGCAAGT
GAAGCAAAACACAATCATTATTTCCTCTTATTTATACAGTGAACCACAACACACGCGCACAAATCCC
CGCGGTTGAGTGCTGGCATGCACAGCCCGCAC
ACACCTCTTCAGCCCAGAGCGGTGTACGCCAGCGCGGATGCGGCGAGCACCAGCGGGGCACACACCCA
CGCAGAGCTGCTGAGGCTGCCGTCGATTTCGC
GAAGACGTGACGGTGCGCGGGTGGTCGACACAGCTGGCGCCTCTGTAGTCGTAGTACTTGGTGCCTCT
GGCGCAGTGGTGGTCGTCGTAGTTGGTGCCTT
TGTTGTGGTGGTCGTCGTGGTCTCGGCAGGATCTTTGGCTTCCTGAGCTGCTTTATCTGCGGATGTAT
TAGTGCCATTTTGTCTTCCTGTCATACCGTCC
CTGCCTTCTGCCTGTAAATTATTGGTCACGACATTTGATTGCGGTCCCCCAGCGGTCACACCTGGTCN
NNNNNNNNNNNNNNNNNNNNNNNNNNNNNNNN
NNNNNNNNNNNNNNNNNNNNNNNNNNNNNNNNNNNNNNNNNNNNNNNNNNNNNNNNNNNNNNNNNNNNNNNN
NNNNNNNNNNNNNNNNNNNNNNNNNNNNNNNN
NNNNNNNNNNNNNNNNNNNNNNNNNNNNNNNNNNNNNNNNNNNNNNNNNNNNNNNNNNNNNNNNNNNNNNNN
NNNNNNNNNNNNNNNNNNNNNNNNNNNNNNNN
NNNNNNNNNNNNNNNNNNNNNNNNNNNNNNNNNNNNNNNNNNNNNNNNNNNNNNNNNNNNNNNNNNNNNNNG
TCGTCGTGGTCGTATTGGAAGAAGCGGCAGCT
TCAGGCTGAGCGTCTCCCGCGTACACGGACGGGCAGCAGCACAGGGCAAGCACCAACAGGGCGCACAG
CAGACGGCACGTCATCATCGTGAGTGTGGTCG
TTCAGTGGTGTGGAGCTGATCTGCACAGTGAGGGCCATGGAGAGCAACAGGGGAGACGAGAGGTGCGG
CAGACACACACACAGAGGGCACACCACATGAGGA
GAGGGCAGCAGCCAGGCATGCACCGTACGCCGCACACCACGCACGACACACACTCCAATCCACGGCAG
CAGGCACACACACACACACAGCCCTGCCGCAA
CACCAACACACAGATGGAAGAGGGAGAAGCAAACAGGAGCGGCACACAAGGCTCCACCGCATGGACAT
ACGGCGAGAGCGGTCCGTCGTGTCTGCACCCC
TCACGGAGGGTACGCACACACAAGAAGGTGCAGCGCACACAGCAACACAAAAGCAAAGTAACAATAAA

AAAAAGAGATCCTCGCACACCACGTGGATTGT
TGGTCCACGGGAAGTGCAGCCAATATTCATCACAAAAACAGTTCAGTTGCCGTATGCCTTCGTCCATA
TTCATCGCACTCCACTTCATGCAGGCACGGCC
ACCACCGCAGCAGCAGCCGCACACGCAACAACAAGAAGAAGCAAAAGAGGGGAGGTGGTGTGAGAGAC
CGCGGTGCTGTCACTGTCGT
>scaffold1139.size6952
TCTGTCCCACAACAACTGACGACACCATGTTGTTTTGCCTTCTCAAACAGTGTCGTAAAATCATGAAG
CATCCCCATGGCATCTGGAGTTTGTACCAAAAT
TCCACAAAGTGAGGAATCGCTCCAATCAATCAAATTTGGATCACCAACAACGACCTTTATTTTAAGGG
GCTCAGCCTTTGTTTTTACCATCTCTACGCAA
GATGGCAAGACATCCCTCGATAAAATAATAAAAGGTCATGCGCTCCTTTCGACCATGATGAAAGGCAA
GGTACATTGCCTCAGCACATGCAGTAGCTTGA
TCCAGAAGAGATGCATTTGCCAAATTCACAGCTGTCAAGTCTATAATCATGGACTGAATATTCAACAG
GGACTCTAGACGGCCTTGAGATATTTCAGCCT
GGAAAGGAGTGTATGGGGTGTACCATGCAGGATTCTCGATAACTTTACGAAGTATTACAGGTGGTGTG
CCCTGGCTCATAGTAGCCTTGCCCAATCATGC
TCCTTAATACCTTATTCCGTGAACCCAGTGAATGGAGAAGCGAAAGGGCATCCTGCTCGCTCATCGCC
GGAACCTCTTTTAAAGGACTTCGCAAAATATT
CTCTGGGATCACAGCAGTCATGCGGTCCGACAGACTTTCCTTGCCAATTATTCGAAGCATAGAAATTG
ATTCTGCCGCCGATGGTGCAATATGGCGATTG
ACGTAACTGTTTGTAGCAGTCCGTCGTACAAACGCCAGTGGCAACGTGGAGAAATGATGAAGGCAACG
ATACATTATTATGTTAGATATGTGCTCGTCAC
CCTCCTTCTCTCTCTCTCGCACACGCGTTAGTTTTCCTTTACTTTGGATAAAAATTGGGCTTCACAGT
TAAAAATCCACAAGAACAAATACAAAATATAA
TTAAAAAATAAAAATAAATTTAAACAATAATCAAAATAATGATAATAACCCATTAAATAACAAAAACA
ACAACCGAAAAGGATTGTCAAGGAAAATAAGC
ACGCATTTATATGAGCAATTCCAATTTTCATCCCCGCACACCACACGTAGCCCCAGCGTCCCTTTATC
CATGGACCTTCACGTCGGGTATTCACTAAAGT
AGGCGTGACGAAGGGCGCCAGCCGCAGTCAAACGCTTCTGAGGATCCAGGCAAAGTAATCTTCGCAAC
AAATCCAATGCACATTCAGAGGCATCCGGGAA
AAATTCACGGAGAGGAAGAGGACGCTTCGGCTCAAAGTGCACCTTTCCCCAATCGGGAAGCAGCACCG
CCGATGGCCACGATGTGGGCGTCGGTGTCCCT
AATACATCAAATATTCGTGACAACTGCCAAATATCTCCATCGCCTGCAAAAAAAGCACCACCGTACCC
CCGTAGAAGTTCCGCAAATACGCACCCTGATG
ACCACACATCAATTTCGGGTCCGTAGTGTCGCTGGCCCAGAAGTAACTCGGGAGCACGGTACCACCTC
GAAACGACTTCGTGAGTCATGGATGCTTCCGT
ATCCCATGCACGACTTAGTCCAAGGTCGCCGAGGCGTAGTTCCCCCTCCGCAGTCAAGAAACAATTTG
AAGGTTTCACATCTCGATGAAGAACACCCTCG
CTGTGTACGTACGCTAACGCCAAAAGAAGCATCTTAAATAAAGACTTTGCCTCTAACGCGGGCAGAGG
ATTGCTGGCAGTTCTCCCCGAGAGGACTGTTG
CCAAGTCAGTCTTGCAACGCTCCATCACTAACGCCAAGGAGCAGCCACACGGAACCACCTCATATGTC
CGAATAAAAAAAGGATGCACCAATCGCAATGC
AACGAGTGGTTCCCTCGCTGTGGGGTGCGGAATACCCTCATCGAGACGTGGAGTTGTGACGAATTTGA
TTGCGGCTGGCGCTCCAGATTCCTTAACAACT
GCAGCACGAACGACGCCAAAGCGGCCCTCACCAATTGGGGATCCAACAATAAAACGATCCATTGCCAA
GCGACCACCTCTCTCTTCTTTCTCTTCTCCAC
CCCTCCCTTCCATTCTACGGCATCGTGAAAATATAAATGGAACACAAAAAATTGACTCCGGTGATGCC
AAAATCCCTCCGCACCCAAAGACCATCCTCAA
TGCCCCTCCCTAATGAGCACATGCATGGAAACTGAAAGAACTCCAATTTATCTCAAAAGAATCCATTT
TTTGTTGTGGTGGCAGTTATTGTTTTCTCCAC
GTCAAAATCTAATTATTTCAAAAACTTTCTCGTATTTAAGACTTCTGACTCTCTATTCACAACAGGGG
CAGTCGCACCAGAGTACATCTTTGCTCGAGCG
AACAAACTAACTCTTTCATTCCTGGGTTTCTCATGGCACACTTCCACCTCAACAGGGGGAAGAGGAGG
AAGAGAAGAAGGCTGCTGCTCTTCCGTGGAAT

ATTTCGGTGTGGCTGTGGCCACATCACCCTCATTTTTGGCTTGCAGTTGTGCCGCACGATCCTCAACc
aaaaagggNNNNNNNNNNNNNNNNNNNNNNNN
NNNNNNNNNNNNNNNNNNNNNNNNNNNNNNNNNNNNNNNNNNNNNNNNNNNNNNNNNNNNNNNNNNNN
NNNNNNNNNNNNNNNNNNNNNNNNNNNNNNNN
NNNNNNNNNNNNNNNNNNNNNNNNNNNNNNNNNNNNNNNNNNNNNNNNNNNNNNNNNNNNNNNNNNNN
NNNNNNNNNNNNNNNNNNNNNNNNNNNNNNNN
NNNNNNNNNNNNNNNNNNNNNNNNNNNNNNNNNNNNNNNNNNNNNNNNNNNNNNNNNNNNNNNNNNNN
NNNNNNNNAGCCGTGTTCTCAGGGGCAATAAG
ATCATCTACACAGACAAGAAATTCCTCAGCGCCTGTGCTCAATGCACACTCCCGAGAATCATCCGCAA
AACAACTGCCTGTATGCTCCCTCACCACCAAT
GATTGACTGAGAGAGTTCCTTTTCCGCAAGAGCTCATCACGTAGTTTATTAACGGTTGACGCGCCACC
CCTCTTGGCTGCTACTCGTAAAGGAATGGAGT
GAGTTGCCCTCCGAACTGTCGCATTGGGGATGCTGCTGTCCGTCTTTGGACACCGATTCGTCCGGTAC
GTTTTTATCTCCGTAAAAGACTCGCGTGTACG
GCCCAAACCTCCCTCTTGCGTGCGCCGCCAAAATGCATCATACTGTTGCCTATTGCTTCTCAGAGATA
TGAAGTCGGCGCATCTTCGTCTCCACGATCGC
TGATGCTCTGCTCACTTTCCCCCGAGTCATCCGGAGATAAAATACGTCCAGTTGTTTTTGTTGCGTCT
TGTGAAAATGCGTTTGTTACACCTGCGGCACC
CGCGCTCCCTCGCCACCAAAAGGAACGCGGTCGCCAGATTCCTTCCCCGCCCTCTTAAAACTCCTTCA
GAAAAGCGGAAACGTCCATTTTCTTCAAACGT
AAAAGAAAATAAAAAAAAAAGAAATGAACATGTGCTTAGATGAGGTCGTTGACGTCTTATCCTCAGAA
ATGTGAGAGGAGGAAAACACAACAAAATGGGA
GAAGACAATTCAGCCACACATCGCTCCGCATCAACGCGCTCCAAAGAAAAATTATCCAGCACGCATGC
ACACGCAACAAAAGCCATCAGACAAATAAATC
GACAGCAGCCAAACTCGTCGTAAAGTAGCAGAGCCGCAGCCATAATTTAGTGCCCCTTTTGGCCGCCG
GACTCCCCCGCACCGCGTGAGGCGCAATGAAG
GAGGCGGGTGGGACGGCAGAAAGAAACTGCAGCCGCCACCTTCCCGCGCAGCGCCATCAAAATTCCCC
CACAACTCGCACATTCGTTCCAAAAAGTTTCT
AAAAAAACAACTAAAAACGCTGCAAATCAATAAATATGAGATGTTTTGAGCACTGCGGATTGCAAGGC
GGAGCATCCACGTATTCCACAGGAGGGCGGAC
TGGCACTTCAATTGGAAGGACAGGAATTCACAAGTTTTTCCTCCTCCCTTGTTTCCCCGTGAGTGCGG
GAACGCAGACGAGGGGAAACTCACAGTTTTTT
AACGCAAAAAAAAAAAGACGGCAGCCAAATGAGGGTCTTTTCTCTGCAAATTGTGCCGGAGACGAAAG
GAAAAAATAAATAAATAAATAAATAACACACG
CGGAAATCACAACAAAAACACAAACACGCGAAAGTAAAAATAAAAAGGCAACAACCAACATATCATAC
CGTCCTTATTTTCATTGCAGAGTTCGCTACAA
AAAAAAACCCTCCATAGGATCCCCATCGTGCGCATGCTGCAGCTCGGAGGCAGACAGAGTCGAGAGAA
CCAAGTGTTTACAATGAGATGCCACATCACTC
GCATTGGCCTCATTCTCTACAACAATGGATTCACTTGAAAACCTCATTTTTCGCAGTAAAATCTCTCT
CTCAGCCGTTCTAATCGACTTGAAAGATCCAC
CNNNNNNNNNNNNNNNNNNNNNNNNNNNNNNNNNNNNNNNNNNNNNNNNNNNNNNNNNNNNNNNNNNN
NNNNNNNNNNNNNNNNNNNNNNNNNNNNNNNN
NNNNNNNNNNNNNNNNNNNNNNNNNNNNNNNNNNNNNNNNNNNNNNNNNNNNNNNNNNNNNNNNNNNN
NNNNNNNNNNNNNNNNNNNNNNNNNNNNNNNN
NNNNNNNNNNNNNNNNNNNNNNNNNNNNNNNNNNNNNNNNNNNNNNNNNNNNNNNNNNNNNNNNNNNN
NNNNNNNNNNNNNNNNNNNNNNNNNNNNNNNN
NAAGTGCAAATCCACCAAACGCTTAGCGAGCATCAACACGGTTTCCCCACCACTCACTGCAACTAAAA
TCACCTGACACCTCGCAGCTGGTACATAAATA
AGAACATGAATTACGTCAGAGCCCTCTGAATACCGAAGGTTGTCCTGCGGTGCAGCAAGAAAGGCGAG
GCCACCGGTGTGTTGCCCATCACCCATAGAGG
AGCATACACCTGCCACAGCAGCAGAAACATCGTCGTCGCCATCATCCAAATCAGCATCGTCAGCATTC
TCCTCGTCGGGAAGTGTGCCTGCGGTATTTCG
ATCTTCTCCTCTTCGTGGCGTCCAAGAGACAACAGTTGCTGCCTGGGCGAATTCAGTAGCCAAAAGAT
CGGACTCGCAATTACAGGCATTCACACCGTTC

AACTCTTTATGGTGGCTCTGAANNNNNNNNNNNNNNNNNNNNNNNNNNNNNNNNNNNNNNNNNNNNNN
NNNNNNNNNNNNNNNNNNNNNNNNNNNNNNNN
NNNNNNNNNNNNNNNNNNNNNNNNNNNNNNNNNNNNNNNNNNNNNNNNNNNNNNNNNNNNNNNNNNNN
NNNNNNNNNNNNNNNNNNNNNNNNNNNNNNNN
NNNNNNNNNNNNNNNNNNNNNNNNNNNNNNNNNNNNNNNNNNNNNNNNNNNNNNNNNNNNNNNNNNNN
NNNNNNNNNNNNNNNNNNNNNNNNNNNNNNNN
NNNNNNNNNNNNNNNNNNNNNNAGCTAAGCCGCAGACACACACACTATATATAGGTAAAGAGAGACAG
AAAGAAAAAAAAAAAGGGAAAACATTGTTGTTG
CTTTTTGGTGTTGTTTCGTTTCACAAAAATTGCCTCTACCGCCATGTGCAACACCTTCAATCACGGCC
CCAGTGAGTCCTCACCATCACTCTTTTTTACC
ATGGAAGACAATGGCAACCAAGAAATATTCACCTGAGACGGTGAAGAAGAGTCCCCGTCGTCACTTCC
GCTCATGGAGTCTTCACGCAGGCTGCCGCTTG
CGAAGGAGTTGTGGATCATCGTTCTAAGAGTGCCGTGCGCAACGGGCCCGAGACGGTGAAGGAATTCA
TTCAACATGTCGTCGGGGCAATGGGCTGANNN
NNNNNNNNNNNNNNNNNNNNNNNNNNNNNNNNNNNNNNNNNNNNNNNNNNNNNNNNNNNNNNNNNNNN
NNNNNNNNNNNNNNNNNNNNNNNNNNNNNNNN
NNNNNNNNNNNNNNNNNNNNNNNNNNNNNNNNNNNNNNNNNNNNNNNNNNNNNNNNNNNNNNNNNNNN
NNNNNNNNNNNNNNNNNNNNNNNNNNNNNNNN
NNNNNNNNNNNNNNNNNNNNNNNNNNNNNNNNNNNNNNNNNNNNNNNNNNNNNNNNNNNNNNNNNNNN
NNNNNNNNNNNNNNNNNNNNNNNNNNNNNGGC
ACTTGAAACCGCACAAAATGACGAACAAGCAGATTTGCCTGTTGCCGAGGCAGGGCGCATACGCGGCC
AAGTTCAGTGCGGAGCACAACCGATTCCAGAG
GATGAGGGTGCGGCGCCGCCACGCCATGAAGATATCGCCAGCTCGGGCTGGGTGAAGCCCCAAGTCTT
TTCGCGCGGGCCTCCAGAAGCATAGCGGAGCA
ACGGCGTAAGGCTTGATTTTGTTCCTTCATTTTCGGATGAATTCGGCTCGAAGCAGGCTGTCTCCGGG
TGAGGCAATGTGTGTGCTTCGTGTGCTGCCTC
GGCGGCGGACTCGGCCTGTTCCATTTCGTGTGTCCATATCTTTTTGGAGCTGTCACGGCTGCCGCGCG
GGATGTGGCGCTCAGCTGCTCGTAAGATGCCT
CCGCGAGGATCCAACCATGTGGACACGGTGGCAGGGAGGGTTTCACACAACGATGTAAAAGCAGGGCA
ATCCGCTTTACGCAGGGCAAAGGTGGCTTTCT
TCCGTCAAGGCAGCGTGTTTGCTAGTCGTGGAGTGCCATCATCCATATCTACCAAGTAAGATATTAAG
CGGTGGTCCGAGTCCATGGAGGGAGTGGATGT
CCAGTGGGAGATGCGTGGGGCGCAATACGCAGCCACGTCAGGAGACGAGGCATTACGATGTGTGATCC
GTGTTGCCTGCGTCGGGTCATTCGCCAGCCCA
AAATACATATCGATTAGAACAGCGGCGAAGTTATCGTCTCCAGTCCTTGATGGTGTATATGACTGTCC
CAGAGTTCACGATGCAAATTCGGGTCACTCGC
GATAACCTCTTCGGGCCCAAGCGACAAACGATATTGATAAATGCAGATTA
>scaffold114.size32926
ATTTAATTAATTAATTAATTAAACTCACAAACCCCAAATCAACTTTACTCATCTCCCCATCCCCTCAC
GAAATAAATAAAAACTATCTATTTATTTATTTA
TTTATATATATATATGTATGTACGTATATAAGTTTACATGTATGAGAATGACCCCCAGAAAATAAGTT
CCTGACATGGTCTTTTTTTTCTTTTTTCTTCT
TGTTTTTTGTTTTGTTTTCTTCATGTTGTTTTGTTTTTCCTCCTCCTCAATTAATTGTGCGCACTCGC
AGGGAGGGAGGAGTGGTGGTGGTGGTAGTGGT
GGTAGTTTCACCCCCCGCTCCCTTCCCCCACCGTATTTGCCCTCCATCCCTGTCATCTCCTCCCTCCC
CTCTCCGCCCGCTGCCCCTGTGCCTTCTGCCC
ATAACCACTCTGTGACAAGTGGTTGGTTCCACTGCCGCAAGCGACAAAACCAAAAAAAAAAAAAAAAA
AAAAAAACTCAACTTATATACCTACAGACAAG
CATACATACATCCTTCTATTTCTTTCTTCTTTCTAGATGGGAACCCAAAACCGTTCCCGTTGTTGCAT
CTCATCACACAGTAAAACACGCACAAATAATT
TAAAAAAAAAAAAGAAAAAAAGAAAGATGGGGCAGGTCAACTCTCATAAAATAAAAATGAAGATAAAA
ATAAAAAGCGTAGATACATGTCACCTACAAAA
TGGCTACCCATTTCTCTCTCTTTCTTTTACATTCCCACTCATACATAACCCTTCAATTTATATCGTTA
TTGCATCTTCCTTGTTCCCTTTGTTTTTTTGT

TTGTTTGTTTTTTTTTTTTTTTTTCAAAGTTAACATTGCAAGCCCAACAAAACAAACAAATACACGTAG
AAATTTTTTTTCCTTCCAATCCAAAAGTAAAA
AATCAAAATCAAAATCAAAAAAAAAAAAAATTTTTTTTTCGGAGGAAGGGAGAGGTTCATAATATCCAT
ATTATTCAGGGTTGCTTACAGTGACGTTGCAC
ACAGCAGCGTGTATATTTGTTTGTATCCGGATATATATATATGAGAGAGAAAGAAAGAAAGAGCACAA
TCGGATACTTTACTTAAAGGGCACCACCCCTC
TTGAAACCACCTGAAAAACAAAAACAAAATATAAAACAGAAAAATAAAAATAAAAATAAAAATAAAAT
AATAAATAAATAAATAAATAAATATTAATCTG
CATATTTTTATACCTTTGTATACATTACATTATGGCATCATGTCATGTAACATCGTGTACCGTTAATA
ATCAATTGTTTTATTTTCTTTTTCTTGTTGGT
TTTTTTTTTGTTGTTGTTGTTGTTGTTTTTGTTTTATTGTTTGTCTTTGCCTTTCATTTTAAGTTAAT
GTTTCTCTTCACGAATCTATGCCTCTGTGGCT
TTGGGTGGAATGTTTGAATGCATATCAAAAAAATTGTACCCGTTCTTTACAGGAGCAGTCATGTGCCT
CTATACACGCACACACGCACACACGCTCCATT
TTGTTTGTTTGTTTGTTTGTTTTTTATTGTTCACTTGTTTTTGTTGTTTTTTCTCACGTGTCTTCGCT
CCCTCATCACCCCGCCTCCGTTCATTCCTTTA
AATTATCGCCTCGCCGTCGTTGTTTTTCCATTTTTCTGTTGTTTTTGTTGTTTCTTCCGCATTTCCCT
CCTCAAGGTCCATCGGAAGCTCATGGATTCCC
ACGGCCGAGGTGTCTCCCACTCACAGTTCCCAAACACGCAGGGCTGCTGCTCAAAAGAGAGCCGTCGT
CAGTCTTTTATTATTTCTTCTCGCTCGTGTAA
TTGCTTGACTTCGATGCATTTTGGCGTCGCAGCGGGACCAAATAAAAAAAAAAAAAAATGACACGTGAC
ACCGCAACGGACCTGCCATCGTATGATCGTCA
TTTCTGGTGATTCGTATTATTTCTGTCATTGAAAATGGTGCACTCGCTCATCAAGCGCGTATTACTGA
GCCGTTTTCCTTGTGTCTGAAGACTTAGAATC
ACCCAGTGTCAACTCCCCGGACTGTTGATCGTTCTCCGCCTCGGCCACCACATGGGCATCACGCTCGG
TAAGTGCATTGGTCGCGCTCTGTGCACCACCG
CGGCAGGAAAATTTTGCCTCGCCACTGTTCCCATGATTCAGCGGCGGCGCCGGAAAGGTGGGTGCGTC
ATAAATATCATCCTCATCGGTACAACAAAAGT
CATAATGTCTGTTGCTACTCTCAGTGTCCGTTTGGTAATCAACAACCGCAGCAAAGGGCAGAACAGTG
GTGTCTGGTAATGGCTCTGGCAGTGTGGCTCC
TGAGAAATTTTTCTGTAGGAGGACGTAACTGTTGAACTCCGCATCGACAATATCATGCCACTCCTCAT
CCGCCATGTTCGCAACACGCTGCCGCCGTACC
GTCTCGCCCTCAAAGAGATATCGACATTCGTCATAGAAGCGCTGCCTTGTTGTGTAACTCCCTGAACG
AAGGCTTTGGACGGCATCCGTACGATTATGTG
GCAGAAATGACGCAGCGACTGTCGAACCTTCTTCCGCTGCGACCCCCCCCTCACTCTCCATCTGAACC
TCTGTGGGATCCATCCATGCCTCCGCATCTTT
TTCCCTCGGCGCTGAAGCTCGACGAAAAGAAGCGGAAGCATTCTCATCACCCGCCTCCAATGGATTGC
CCCAATCACTACTCAATTCAAAGGGAACCGCC
TGCTCCTTCTCTTCCAGGCTCATGCGCTCTATTCCATCGGTGAATGCAAGACGATAATTGTATCGGTC
CCGTGCCTCCTGCAGAGCAACCTGATCCCGCT
CGTCCGACTCAATGCTTTCCCAAACATTCATCAAAGTCACTCCCCGCAAACCCACTCCCACTGGCGAC
TGGCCCATTGCGCGATCAACGGAGGTGCCAAC
AGAAATGGGGGTTTGGGATGAGCCGGTAAATCGGTCTCCATTTGCGGCGCCAAGCGATTGATCCTCAT
GCATCGGTAAAGGACTCACAGTTGTTGAACCG
TATATCGTCAGCGGGTTTAATTCTTGCTCGAGCATCTGCGGGTAGGCCAAGACAACCTCCCTCTTCTT
TTTCTCCTTCATCCGGCAGAACCGGACTGTCA
CGCAAATCAAAAATACAATCAGTAACACTCCAACAAGCACAGAGAGCACAATAATTAAAATAAGCAGC
TCTTCACTCATAGGAGAGCCAGATGTGCCAAT
GTCAGAAGTACATGAATCGGTACTCACCTTGACACGGCCCAAATTACTGCAATATGAGGCATCTTGTG
AGTCCCATGCACAAACACTGTATTCGGTTTGG
GGGGGATCATCGTCCAACATTTTATGATCCATTCTCAATGTGCATAAATTGCGACTTCCCCGTTCTAT
CACTCCAACAATGTTCAACCGATCCGCAAAAC
TGCAGCAATCACCCACAATGACCGTAAAAAACTTACCCGACCACGACGGATCCAGCGACGCACATTTG
CCCAACTGCAGAGGCTCACAACGCGGAACAAT

TGATGGAAGAGCAAAAATTGGTGTCAAAAACCGAACTGCGCCATGGCGAGGAAGAGAGACAATAGAAC
GGTTCACGGAAACAGCGGCACACAAGTACAAG
GCGGGAGTAGGTTCACCCGCGTAAACGATAATACGGCCTAATCCACGTTCAGTACTGGCTCGCAATGG
CTCCCAAAATCCATAGGACTCCACTTGCTCTT
TGCAACTCCTGTCCATGCTAAAACCACGACGTAAGTCGGTAACCTTTAGGTCATCAAGCAAATACAAA
ATACTTGAAACACCAACAATAATATCCGTACC
ACGCACATTGTAATACACGGGGTTAAAAGCCTCAATCGTGGTAAGAGCCTGTAAAGGCACTCCAGGAG
CAGCCATCGCCATTCCTTCTTGATCAAACGGA
GGTGCGCCATCATGCCCCGTGTTTTCCACGCAAAGCGTCCATCGACCTGCGGAGGGCAAACCCGCACC
TGAGGAGCGTCGAAATAACAACCTCCCGCGAC
CCTCACTATTCGTTGTGAAGGACGGTACAACTTCCTCACCCCGACAGTTGTCATCTTGTCTTAAGTGG
AATATTCCATTCGGGGTAAGTGGAATAAACAC
ATCCGCATCCGCACCAAGAACAAACTGTGTAGGGTAAATGACGGCGGTAGAAATTTGAATGTCCGCAA
CAACGGAGAGGTTACCTGCGGGAGTACCCGCA
CAGAGGGAGAGGGACTTTGTTCCCTCTGGTATATCCTTCGTTGGTAGGACAACGCGTCCAGTATCCCG
AACATACACCACATCGCTCCCATTGACAGTAC
TTCTGCATGCATCAATCGAGAAAAAAAATACCGCGGAAGAAAGTGGTATAAAAAGATGCAAACGCATAC
TGCGCGTCTTCCCCTGGAATCAAAGAGTACAA
CTGTGCTTGGCCACTCACTGTTGACACCTGTAACGTCGCGTGAAACGCAGCACCAAGAACGAGTATTG
GATCCAGCATCGATGGTATGGGAGGATTGACA
ACAAGGATTTCCGCCAGTGCCACTTTTCTCCTGATATCGCCTTGAGGCGTAACGCACACGTAGCTCAG
TCCTGACGCTCCCACGAGAAACAACAATACAC
GGGAAGGGTTCACCGCAAGACCACGTAAAGATACCAGAACATTACCGCAGCTCGCACTGTCACTCAAG
AACGTCATTTTCTCACCTGTACAATAATTTAA
ACTCAACTCCGCCCGCATCTTGGCACCAAATCCTGCCACCAAAATACCCGGAACAGATTTCGGTGAGA
CCTTGAAGTTGTCCACCTGAAGCGTCGGAGAG
TCGGTGGTGAACACGTAATCGGGATCGGTCCTCACACTCACGCAAACAAAAAAAATTAACTTCTCATC
ATACCTCGGTAAAAAAAAAGTTTTTACCAATTG
GCAACTTCTGGCAATCCGATTTCTCCGATAGCGCAAAAAAATTGAAGCGGGCCCGATCGATTCCGTAA
TTAAATATAAACCTTCCAGGTGGCACTGTGAT
GGGCGCAATCTCCGAGGGGTGATCAGGGTCACAGCTCCGGAGACGAACTAGCGCCATCGCCCCACGGT
AGTCAAAAACTCTGCCACCATCTAGGCTTTCA
CAGTAATGTACAGTTTTGAGATCCGCATTGCCACCCTCGCAGCCATCCACCCGAAGTTCTGAGATGTA
GTTCGTGATGAGCGCGGGTGGAACGCTCATGA
CACTCCCCGCACAGTCCGGTAGGGCAGTGAGTTTTGCCACCACATTTTCCTTCCACAATCCTGAGAGC
ACAGGTGTTGTGCGATCCGTCACCACCGTCGC
GGGGGTCATGTAAGATAGAAGATCAAACACTTCTGCAAGAAAGATGTCCCTTGGGGAGGCCTTTACAC
ACAACCGTACTTCTTCTGCAAAGGGGTTGATC
ATTGTCACGGTGATATTCCCAAAAAAAGCTCCTGCAGCAAAAATAAACTCTTCACTTCGTGCATCCGT
TTCATTACATTGTAATCCTGGTGGCATTGTAA
AAAGCGTAATGGGCTCGGAACGTGTTTCCACATCACCCCCAGACAATCCTATCCTGACTGCACCAAAT
TGCCGCACCATCGTATCATAAATGGTCACCAA
AAACGGATCCACAACAAATATAGGAACCGTTGAAGCCACAATCCCTTTTCCATTCGCTTCCTCTTGAC
ACAACACATAGCGGCCGACCGCCGCACCTTTT
AACGGAACCAATGAACTTGTATAGTAGCGTTTTGTCGCTTGTAAACAGACTTTCACCGAAATGGGGAC
GCCACACATGCTTTTAGAGGGACATGACACAA
GAAATAATGAACCGTCATCTAGCACTGGAAATGCCAAAATGCTATTCGTGCGTGCGCGAATGGCATAG
CGTGAAAAAGACCTAAATGGTACAATAGCTAC
GCTACCCCCATAGACAAAAAAACTTTGATTTTTATCTTTCCATCCAACACATACAGTCAGTGCACCAG
TCCGTGCCACCACGAACGGTTCGGCAACTCCA
TTGGTTTTATTAATAGAGCCCTCCCCCAATATTAAAGTTTCCTCGTCACTTGGTAATGCGCATGATTT
CACATTGTCCGTAATAAAAAATAGTATTGGCT
TAAGAGCGGACCATGATGACCCACCAAATTTCATTTTGACCGGCAATCCAGCGGCGCCTGATGGCGGA
TTCGCAGTCATTTCCAATGGTGCAACAGAAAA

AATGGTTTCGATGCTTCTGAAAGTGCTGAATTCATTTCCCAAACACACCTCCCATGCTCCAGCTTCGG
GAGGTGTAAAAAGAATAAATGCCCGTCCTTTA
TTGGGGACATTAACAGGCAACACAGACCCCGTGGGGGGTGATTGGCACGAGCCCTCCGCTCCATGAAC
AAATGCCTGGAATCCGGTGAGAGAAGTAGCAT
CAGGTACAATAAAGTTCACACGGTTTACCTTGGACACAATCACAGCCATTGGAGCGACATGAACCTCT
CTAATTGGCAACACTCCAACGGGTATTATATT
CCAATTTCCCTCATCCTTGCTCCCGCCATACCCTACACATACAGTCAAGTTTGTCTGTCTAGCGTAAA
GCACCTGAATTTTTAATTCCCCATTCATGTCT
GCTACCCCTTTAGCTACTGGTGCAAAACTGCAGTCTTTTGAGGTTTGAATTTTCACGGGAGCATAAGG
AGGAACCAACGACACCGACACATTTGTGAGGG
TACCCACAACGATGGAAGTGGGGGCCAATTCTACTGCACCATACACTACAACTGTAGCAATGTGAGCA
TACGCTGAGGCATATAGAAGCTTTACACAGAG
GGAATACTCCCCAATATCCGTAAACAGCACATTCTTCAATACAGTCTCATTCATCGAGAATTCGGCCC
CAACCATCGTTTCACATGTTGTGTCATTGGAA
AACATTCCTTTCGTGCCTGCTTCAAACTGCACATCTTTGTCCAATGTAACAGCTCCTTTCTTTCCCAC
AAACAAGGTATCGTAGCAAACAGTGTAGGATC
GAGTTGTAACTTCTTCGATAAAATAAATGTAGCCATAATCAATATTCTCGTCCAAATGCCCTCCCGTA
TGGAAAAGAGGGGTAGAGACGCAGAGATAAAT
CGTTTCAAGAGCATCTTCTGGAACGCGTAACGTGGCCTGATCTCCTGTCTCTGTCGTTGCAGACCTGG
AAACAAGTTGTAAACAGTTAGGCGTCAGTGAT
AAACCAACGGTAATGACTCCAGGAGCGGACGCCTCACGATTATTCAATTTCACTATAGTATCAATTCC
CCTGATAGCGGGAGATCGTGACACAACTGCTA
TTGGTGCATCCAATACCGTAATATTAGCTGCCAAGGCCATGGTGCTTCCGCTTGAGTCTGTAACCGCC
ACACATGCGTAGTACAAGCCTGGCAATGTCAC
AGTCCATGTTCTCGACGTACTCCAAGAGATAACTGGATCCTTTTTGCAACCTGGAGAAAAAAAAAGCG
CTGTAGCAACGGTAGCATTAGTTTCCAATGAC
GCTGCGTCCAATGTAAGTTCCATTGTGGTAGGCAAGCCGGCGTACATTGTGTGTGGCTGCAACCCGTA
CTCCTGAACCGTGATAAGGTTGCTCGAAGGCA
CGTACACTCGAAGGTTTTTGTAATAAGGTAATCCAGTACAGAAATAATAATCTCCTTTAGGGACGAGA
ATGGTAAGTATTATGTTTTGCAACTTAATACC
CCCCACCGGTTCCTGCAAGAGTTGAAGACAAATTGGAGAAAGAGAAAAACTGGCAAATGGAATTTGGC
TGAATGAGTCTAACTTAAAGAGTTGTTTGGTG
TATCGAAGAGCCTTCGTCTCACTCACTGAAAAAGGTGATACAGGCAATAGTGTCGCCGCAGGTATATA
AGAGGAGTAAAGTCCATCATCACCATACGGAA
TCCGGGCGCAGAGATGAATGACACCAACATCACCGGCAGTCAAGCTTAAAGTTCTGTGCATTCTCAAT
ACTTTTTCTGGCATATTTTCAATGACATCATT
GCAATCTTCGTCGCTGAAAAAGCCAAAAATACTTCCCACAGGAGTCGCATCCCCAACTGTCACAGTAG
ACAAACGCTCCAAGAACACAAAACGTGGAGTA
ACATCGATCACATGCAATTGCAGCGATTGTATGTACCCTTGAGAAAAGGCGCTAGAGCAAATATAGAG
CGGCGGAACGTACCTTTCGTAGTGTGGACTTA
CACCCATGCTCTTTGAGGTAACATTAAACTCGCAGGTTCGATTTGCCTGAAGGGTACAAAACGCGTCC
ACCAATTGTTCCACGTCACAGACTGAACTACT
TGAAAGAAACAAATTCTGTCCCTGTTGCAAAAATTCAGCAGTGACGTAAAAAGGAACATTTAGTAACG
GCAGGGAAGGGTCCACAGCAACGTACACCGCA
TGCGTCAAAAGACAAACAAACAAAGACAATATCATATACACTGCTGCGATCCATATTGCAATTCTTGT
CAAATGACTCCTGCCCTGCCCCCGTTTGCCAC
CATGATACACCATCATTGTGGTTTGTTTCTCTGCAAACTCCACACACACTCTCTCTCTCTCCTTTTGT
TGTTGTTGTTGTTTCCTATGCTTGCTTCTTGC
TGTTGTTTGTTTTCCTTCTCCGACTTCGTCGTAATTTTTTCGTAGTGTTTCGTTCTCCGTCCAACACC
TGGGTAGGTCCCCGACAGAAATCTACCTCAGC
CACTTTACACCAGGGAAACAAAAGTATTGCTTCTTCTTTTTTTTCTCTGTTGCTTTTTTGTCGGTGTT
GTTTGTTTGTTTGTTTGTTTGTTTTTGGTTTT
GTTTTGTTTTTGGTTTTTTTGGTTCCCGCTTTTTTTCGTTTGTTTGTTGTTTCGTGAAAATCAGTCCT
GAACAATGTTGCCGGCGTGTCTCTTGTGTGAT

GTCAAATGCTCCTATTGCCGCTGCTAACGGCTGCTCTGTTTTTTTTTTTTTTTTTTTTGGCGCACACA
GTTGCTTCTATTGAGGCAATAAATAAATAAAA
AAAGAAGAGCACACAAAAGCAAAGAAGCAGAAAAAAAAATGTTGGCAAACGTGCCGTTGATACCCCCCG
TCGCGTGTGAGAATGTCAACTCACCTGCGCGG
CTTGCACTCATCAAGCGGTCCCCCCTCGGCGACGTTATTCTTTTCACTGCCACCCACCGCCTCAACTA
GGCAGACCCCACATGCATACACATCAGTGTAC
ATATATATGTTCTCCTCCGTCTTTTTTTCCCCCTCCTCCTCATGTTTCTCTGCACACTTCGCACAAGT
TAAAAATACCGCACAAACACACACACAAAAAA
AAAAAAAAAGAGAGAGAGAGAGAAAAAGAAAAAGAGGGGAAGATGGCAGTTTGGTTCATTGCACCCAACA
TCCTCACTCACTCACACACACAAATGTGGCGA
TGGCCGGGGCCGCTGTGAACGCACAAAAGTGGCAGTCCTTTAAAAGAAGGCCCCGCAGCCGGCAAACC
CCTCGCCGCGTGGAGGGCAGTCGTCATCAGGG
GGGTGGGAACGAAACAAAAAAAAAAAAAGCAGAGGAGTTAACCACTAAGTGCTATTATCCTCCGACCCC
CTTCAAATTGGGCCGCACAATCCCTCCACACA
CCTCACGTAAATTAATTACTGTCTAAAATTAATTAATTGCATTTGGTTTGAATGCAGTGGTCTTTTAC
ACCTCCCAGCATCCACCCACGTCCATTTGTAC
ATAAGTAAAAATGTTGTATATTTTGCCAACAAAATTCTGCATACGTGAGTAATCATATGCCCAACGAT
GTGCCCGCATCCTACTCTACGCTACCCCCAAT
CGCTAAATTGATTTTTTGCGTGTCTTTCACTCTACGTGGGGCAAGGCATCCTTTTTCAGTGGATCACA
GTACCGTATTACAAGACCTTCCACCACCTCGC
ACAGGCTGTTGCACAGCGCCTCACTACCCGTCTCTGCGTACACGCGAACCAGTGGCTCCGTGCCGCTT
GGGCGCACAAACGCCCTCGCCACAGAGTTTGA
AGCCAAAGCCGTGGCAGCCACAGCCGCATCAATCGCCTCTTGCAACCCCGGCGGAGTCAACGCCCGTC
TTTCGTCAGGTGTGTTGGTGATCACTCGTGGA
TTGGGTACAGTAACTTTGGTTTGACGCGAGGGCAGATCCGTGTAAATATGATACCACGCATTAAAGTC
CATCTGCAACGCCAAAAGCGCAAATTCGCAGG
CAAACAAATCAGCAATTCCGTCACCACACACCTGTGAAAGAAGGACCGGAAGATGTTGTAAAATGACC
TGGGCGCGTGGGTCAGCCAATGAGGAAACCTT
GGATGCAATAGAGTCCTTGTTGAGTAAAACTGTGCCGTGACCGTTTGCCTCAAAGTACACACCAATGT
CACGCGCATGGGCAACGGGATGCAGATTTTTC
ACGCCCGTGGCGGTGGTGTACACGCGAAGCCCAAGCTGGCGCTGTAAGTAATCTGTTGAGGCCCCGTT
AGCGTATGCCGTCTGTACGACGCCAATGTCAA
GGACCTGAAGTGCATTCTGGCCAAGACACTGCCGGAGCAGCATGGCATACAGGATGGCAACACGATCG
CCATCTAACAGATGCCATGTGTCTCCTCCATC
GCAATCGTGAAAGAAGGCTACCACACGATCTGCATCGCCATCCAGCGAATAATAGTGGACTTCCTCTC
GTTCTTCTCGAGGTTCATTACAATGCTGCTTC
CCATAATCTGCAGCCCATTCCTTCATTTTCCCAGACGGTGAACGGGTCTTCTGTGTGAAGTCAGCACC
ACACAGATGATTAAGAGCGCCATCGTTTTTCA
CGTCGTCGTGGAGAACGGTAATGTCAAAAAGCTCCTCCAAAAAATTCTGTGAGGACATCTGCCGCGCA
GCCTCAAGCAGTGAATGAATGGCGATGGCACC
CACGCCATTAGACGCATCCAAAACGATTTTCCGGCGCCCTCGTCTATTCGGCGTTGGCTCCGTCAAAG
TGTTCAACAACGCCCCCAGAGACGACAGTATA
TCCTGCCTGTAAAGTGCAGTTCCGAACTCAAAAACGTTTGTTACTCCTGTCGCCATCGATGGTTGATT
GGCACGCTGCACCAAGTAATGAAGTTGCGGTG
TTGTGGTTATTCCATGGTCCCTAAATGGGAGTTGCAACGACTGTAGCGATGTCTGCACAGCGGAAAGA
ATTTCCATACCAGAAGGCCGTGTATCACGTGC
AAGATGCAACACTGAAAAGGGACAACGCGCCACAAGTTGATCCATGGAAAAAGAAGAAACAGAAATAT
TGTTCCTGACCACCCACTCCTCCAACTCAGAC
ATAAGCTCCTCCGCCGTGGTGGCGTTAGCAACGGCCGTGCAGACTTTCTCCCAGGAGACTTCCAGCAT
ACCGCCATCAACGTCTATCAGCTTCAAGCCAT
TGTCAGTGGCGGGGTTGTGCGAGGCCGTAATCATACATCCAACAGAACAGCCAGCAGGCTCCGAGCGA
CTAACAACGGCCCACCACACCCGTAATACCGC
CACAAACGCGACACGAGCCGCCACGGGGGGCAGCAAGACTCCTAATGTACGAAACCCCGACGTCCCGT
ACAATAATGGCGCTGTGCGCGCGTCATGTCGC

AACGGAAACTTCGAGACGCACTCTTGCACCGCCTGTGCAAATAGTAAAGGTAATGGCATGACTCTTCT
ATTCCTCTTTACACAACCATGTGATGTGAAAT
CAAAAAAAAAAAAGTGTCAACAAAAATATATATATATATATATAGAGAGAGAGAGAGAGAGAAAAAAAA
AAGAAAAGAGAAAAAGAGATGGAAGAGATAAT
ATTTAAAAAGTCTGCAATACACATGATCATAATGTAACCACCTTTCATGCATAAATCACCAGATGGTA
GGGATTATACGCTCGTCCTCCTCCTCTTCTTC
GCCCCAGCAGCAATTCTTCCATTTCTGTCCTATAAATCTTCATGGATTTGGCATTTATTATTATATTT
TTGTTGTTGTTGTTTTCGTTTTTGACAAAGTC
GCATGTTTTTCTTGGAAATACGCAGAGCGTGTCGTCCAGCTACAGAGTTCAGTTGGCATGAGGAAAAA
TTAAAGAGCGATGTAAGGAAGACAAACACACA
GGCGTACAGAGAATAATAGGACAACGCATTCAGTAAGAAAAAGTAGATGCCTCTACCATAAATTCCTC
TCATAAACCAAAAAAAAAAAAAAAAGAATGAAA
GAAAAGAAAGTAAACATCCCACGCACACGCACACAAAAGCACATATGCAAGCCCTTCTTGGCGCCAAG
CCGCATTGCAATGGAGGAAAAACTGAAATAAC
GTGGACTGCTCTGTGACTCTTCGTCTGAAATAGGAAAAGGTGGTGGCAGGTTTCATCCTAATTTCCAG
TCAAGGGTGTGGATGGGTTCTTCTCGTTGTGG
TAAGAAGGAAAAGAAGCGGCATGCGGAAATATGTGGATCCAGCTCGAATGCAGTCATCTCATTATAAA
ATTTATTCAACAAATTTTATCCAAATGTCTCT
GCTTCTTCGTTCCCGTTTTCCGCCCTCCCCCACTCTTTATGATTATTTATTTATTTACTTGCCTTCTG
TTTGTGGTTTTTTTTGTTTTGTTTTTTGTTTT
TCCTTTTGATGCGAATGTGCCCCTTTTCATTCACTCCTCCCCTCTCGCTCTTTTTTCTTTTTTCTTTA
ATTTTTTTTTTTCTTCTTTTTCTTCTTTTTGC
CGTCTAAACAGTTGCGTTCAATGAAAAATAAGGAGGCATTCCGCATCTACCGTGCCGGTAACGACATG
AAGGCACGTTACTGGATGTTCTACAGCATCGG
TGTTTTCCATTCCAATACGCGAGGCTCAAACGTCTAATCACGCATTTCTGACTATGTGCGGTCATTGT
CCTCCCTGCATGGGGGTAAGCCAAGATGAAAA
GTGGTGCAACACGAAGATGGCAATGACAATAAACAGGCTGATAAAAATAAACAACAACAAAAACTAAT
AATAAATTAAATAAATTAAATAAATAAATAAA
TGACGAGGGAACTCACAACACCAACAGGAATAACAACACACCTTCATAAAACATAAACAACAACAACA
AAAAAAAAAAAAAACGGGAATAGTGAAGCTAAG
GAATATCAATCATGACAAGAGTGCAAAAGGGAAAGATATTCCCTCCCACCCTATTCTTCGAAATCCTC
AACACACTTTTTATACCCAAAAATATTTCCGT
CGCTATTTTTCCTTCGTTTTTACTACTAATATTATTTTATTATTTTATTATTATTATTATTTTCCTCT
TTTTTTTTTCCTTGCATTCCTTTCCTTTTGTT
TCAAAGGAACCAAGGGACAGCGTAGGGTAATGCAATTCACAAACGAAGTGATCAATTCAATGCGTCAA
ACCACGCTGCATTGCAACACGCGTCGTTGAAG
ACGAAACGGATTGTGGCAAACCGGCATTACTTCTTGAGTCCCTTTCACCAATCAAACCATACTGACTG
AGATTTCTCGTAGAAGGCGGCGGTGGCGGTGG
CGGCGGTGGAACGGAAACGGATGCGGCATTTGTGGAAACGAGAGGTGTTGGGACAATGTTAAGGCTGC
CAACACGGTTCTGATCCAGTCTGGCACAGAAT
GTTCGAGGGTCACTGAGGTACTCCGATGGACACAACGTTATAGGAATACAAAGATACGTCTCAGTCAT
GGTGTTACCATCATCGCAGTTGTTGGTGGAAA
CAACATTCCCATGACTTGTTGTGGATGTTGACGATTGAGCGGGAGGTGCCGAAGAATACATCTGTGTC
GACAACGGTGGCAAGCGGGAATTTCCCACGGC
TATGGCAGGCAACCTTGAGGTGCTATTGACATTTTCTTGCCGTGATGAAGTAAAACGACGGTAGTTTC
CTCGTCGTGACGCCACGGAAGATGAGGAACTT
GAAGAGGGAGAGCGGGATCGGCGGCGGCGGAGGGACGCTCCTTCATTCACGTAGCTACGCTTCTCTGA
GCGGTTGCCTTCGTGAGGACCTCGACTACGTG
CCTGTTCCACAGTAAGCACAACACCCTTCAACACGTGTGGGTCCTGTCGCGCAATTCCCAGGGGTGGA
CTTCTGTACGATATCCAGCCGTACCCCCGCGA
ATTGCCTGCCGAATCCCGCTTCATTGTAAACCCCTCAAAATCCCCAAAGGCTGCAAAGTAGCGTTCAA
TGTCTTGTTGCGTGATGTGACGCTGAATACCT
CCAATGAAGTAACGATAATCCTTCACGGAACTGGAGGTATCCTCTACCGAAGCAGCCGTTGGATCCGA
AACTTGCATCGGTTATATTTATTATTACTGAG

AGTCCTCGATGGATAAATATTGAGCGTGTTCTTTTCTGTTGTTGTTGTTTTTGTATATATGTTCCTGA
GTGCTGCTTGATCCTGAAAATGATGGAGTTCG
CCCAGGCACACACACACACACACACACAAAGAGAAAAAGAGAAGGAATACTCGTGGCAATGGCGTTTGCG
TGTGCGTATGTGTCTTTACGTAAACTCTTATT
TTATTCGGTGTACAGTGAGAGTTTTACGCCAAACAGATGAGTTAATGGTGATTGTACGACGGTGTTCG
GTGGTTCGGCCAGAAGGAAATTATTACACTGT
ATATATATATAGATATATTGCCTTTAAAAAGGAGGGAGAAAAGGGAATAAAATAATAATAATAAGCCA
AGCGAAAACACACACACACACACACAAAAAAA
AAAAAAAAAAGGAGTTATTTCCCTCACCTCTCCCTTCCTTCTTCATGAGAAGGCGGCGACCACACATT
TGTCGCCACCACCTTTCACCCCTTCACAATCC
CCATCGCTTCCGTCAAATACACAATAACAGCAAATTGTGTCTATGTACGTATGTATGTATGTATCTAT
GTATGTTCTTTTTTTTTTTTTTTGCGGATATAC
TTGTACGCATACGAGACACTCTTCAATTTGTTTTCCTCCGCATTTCCTGCCGTCTCTTTTTCTCCTCC
TCCTCCATGAGTGCCAGTGCAATGGCACGGAC
AACACTCAAGTCATTGTCGGTAAAATTAAGCGCTGTATTACCTCTTTTGGTCTCAGAAATTGGCGCCG
TCTGTCGTGCGGGTGTCGTGTCGTCATCGTCG
TCTTTTTCCCGCAGTGGTACTGCGGCCTCTTCTCCTGCGCCCCAGCCGCTTTGATTCTCCATACCAAC
ACCACCATCACACTCATCCGAGCCGAGCCCAC
AAGCGAGTCGGAATGGCTTCATGGCAAAGGCATGACACAAACGGGCACTCATCTCGGATAAATGCAAG
CGTTTTCGTCGATCCTTCTCGCTCAACGACTT
CTTCAGCACCACAGGCTCAACATCAAAGTCCTCTCCGTCGTCTTCTGCATTGTGTTCGACAGAAGAGA
CCGGTGAGCTGCTTCTCTGCCTCCAGCCCATG
TCTTCACAGAAGTCCGCCTCGGCCGCTCCAACGAAAAGCGTCGGTTTTTCGCCACACCGGGGACCAGC
GGAACCAGCGGCGTATAAAGGTCTGTCATGTC
TACGCCCCAACGAAGAACGTCCTGTCTTTCGCCGTGGCCTCGCAAACGGGGGTTGGAAGGAAAAAGAA
ACCGATGTCAACTCGGAAACGTTCCGAGCGGG
CTGCGTCCCCATGTCCGCCTCCTCCCTCTCGCACACTGCGGACCAAGCCAACTGGTGATGAATCGTTC
GCTGCTCCTCCCCAGCGCTCTGCGACGGATGC
GTGAGAGCATCCCAGGGAAAGGCGTGGCATTCTTCATTGTCGCGGAGAAACACCTGCTTAAAGGACAT
CTCTACAGGCAAATCAAATATATATATATATA
CAACTCCCTTTTCCTACCAAAACAAAAGGGGATTTGTCCCGTCGTCAGAATGCCTGCCCAAAAATATT
TATATCGTAGATAGACTTCCCCGGCCCCGCAA
CGCCCACAAAGACACACAGACAGACAGACAGACAGACAAAAACGAGGAGTTGGCAAGTGGCCAAAAAA
ATGACTCAAACTTTTCTCTGCGTGAACTCGAT
CTCCGCCTTAAAAGTTAAAAGCAGTTATCAATATTATTATTTTGTTGTCGCTTGTTATCTGTACCCAA
AGGCCAACCACGGGCGGTGGGTAAGTGTGTGC
ATGTGAAAAGGCATTTAAGGGAAAAAAACAAAAAAAAAAAAAAAACAAAAGGAGTGAAGAACCAAATT
GTGTGGACACGCGCCTAAATTACCAACAAAGG
AAGAAGAAAAGCACCACTCACCAGACACGCAGAGAAAAAGAAACGCAATTTTTTTTTGTTTGTTTGTT
TTTTTTTTTGCATATTTGTTCGTAAATACAAG
ACAAAAGGGCACAAGAGTAAGAGAGTAAGAGAGGGAGAAAAAAAAAAAAAAAAAGAAAATCCAGGTA
AACTAATCTCTTCGCCTAAACAACAGGCGCCA
TTTCCCTTTTTTTTTCCCCCCCTCCCCTTCCATTCTCCTAAAATGCACACAACACCAACAACACACAC
AAATATATAATGGGCGCAGACACAAAAAAAAA
AAAAAAAATGAAAATGAAAAAAAAAGTTGAAACAAACAAATCACATAAAAAAAGGAGAGTGAGAAACG
GGCACCATCCCCTCTCCCCTACAATTGAACAA
ATGGCACTCTCATTCGTTTCGTGCGGCTTTCTGCTCATTGTGGAATACGTCACCAATGATTTTTTTAA
TCCTCGTAATCCCATCAACTTCCACAGAGGCA
GTGTTACCATTGCTAGTACCCGTATTCTTACTCGTACTAGCAGTAGCAGTATTAGTATTAGTATTATT
AGCAGCATTAGTCACGGTACTATCATTTGTTT
TACTCATGGTGGTTACCCCGGGTTGAGTAGTAGCAACAGGCGTAGCGAAAGCATGGCCTTCATTGGAG
GGTTCCTTGTCACTCATATTTTTCGCATTTTG
TTTCTTGTCGCTCCACTCACGGCCTGTTTGCATATTCCGCAGCTGTCTATACCGCAAGTCTTCTTTTC
GTGGCTCAAAGAAAACGACACCGGGTGGATCC

TTTGCACGGCGTATCAATCGACCCTTGTCTTCCGTAAACTGCGGCCACCCCTTCGCCTCCTCGCCCAA
TTCCATCTGTCGACGACGGGCGTACTCCAAAG
CACGGAGCTCTTTCTCTCGAGAAGAAACCACCGAGTTGTAGTCCCTGTTTTCATCATAGAGGGCTGCA
AAAACCCGTGCACTAGGATTCTGAGCGGCAGC
GGCCTCACGCGTGGAACGAATGCGTTCATTCATCTCTTGTCTCAAACGATGCAGCTCCGAAATCTGTT
CATATCGTGCCAAACTTTCAGACATTTTCCAT
CCGTCATCGTGTAGCCAATCACGGTATTGACGATAGATCAACAATAATAATATCGACACCCCAAGGTA
CATCACAAGACCACGTAAAAGAATAGACGAAA
CCGGCATACTCCATGCTCTTCGAAGTTGTGCGTGAAACCCAAACGGATGGGAAGATGAAAAGGGATTC
TCGCTATATTTTTGCTGTCGTTGCTGGTAGTT
GTAGAAGCCCTGGGACCTCATGGGACCCTGACGAAAATACTCACCAAATGGTGAGTCGTAAAAGCCCT
GCGTGCCGTTTGAAGCATGGGTGTTATACGCC
CCGTAACCAGCAGGCGACTGGTAGCGGTATCCACTGCTGCTGCTGCTGCTGCTGCTGGCGCCTGCCCT
TGTGGCATAATAACTTCCCCCGCGATAAGTAT
CCTTCTGTAATTTAAACTGTTCCCTCTCAATCTTTGACATGCCTCGTAGTCGATCGTACGCGACGGTA
ATACGACTCATCATTGCCTCATCACCTCCAGC
ATCGGGGTGATTTTTCCGCACCAAGTCACGATATACCCTTTTTAAATGCCGCTGATCGCAATCCACCT
CTACGCCTAAAATATGGCACGCCTCCGACACC
TCTGAGAAGGTGGGAGCGCGACGCGGACTACTTGCGTTGAAGCGTCGGGCGCACAGAAGAGTGCAGAA
ATGCAGGGAGCTGCGTTGATACATGGATAAAA
GAGTGGTGTGCAGGGCAACGTGACGGTGAACGGAGACAAAACGGCAGCCAGTGGCACATGCCGACACA
CTTCTGGGACGCACCTGAGAGAATTGAAATGA
AGACGACGAAGACGACGACGACGACGACAAAGAGCAACCGCCCCGCAATGGCGTTTGGGTTAACTCCG
GCAGACAAGTTTGAAGAAAATTACGCCGCAAA
TGCATCTCGCCCCTGTCCTCCTCCCTTCTTCCCCTTCGCTTGAAAGTCGCCAATGTCTCCGACAAATG
CCACCACCTGCTGCCTGCCCCGCACAGACACA
CACAAGTACACACGTACCCCCGGCCTCCTTTTCGTTTTTGTGTTCTCCGACTGTTGTGGTTCCCTGCT
TCCTTTTTATTCTGATTCTTCTTGCGAATTTA
AATCTCGCTGCTCTTACTGAATGGCTCGCGGTTCCCTCTAAGACAAAAACAAAAAGAAATAAAGGGCA
CAAAAAGACAAACACACAAACGAAAAAAAACA
CAAGCACACACAACGCAAGAGAGGCATGAAAGTATCGGGCGCAGAGAAAATAAATTCAACGTGGAGAC
AACTGCACACCCCTCCCCACGCATATCACACA
TCACGTCCACTGCAACAAATCGTTTTTTGTTTTTGTTTTTTCTTGAAGCACCGCTTTGCTCCATGTAT
ATACGCATCGGCACACAAAGAGCCATGCTCAA
ACAAACAGTCCAACACGCATAAAGAATATTTGGACAACGCGGAGAAAAGTACAAAGAAAATTAAATAA
AAAAGAAACAAGAAAGGAAATATATATATATA
TGTATATATACCTGCAAGCAAAAAGAAAAAAATGCATCCCACATGAATGCACCCTCCTTTCCGAACAC
CACTTTACACACATACTCATTGGTCCTCACGC
ACAGAACAAAATATATCTTGACAACACTCATTTCATTATCGGCCACTCTTCTTCTGATCATTTTGCTG
CCGGTTCCAAGTTCAAAGTCGCCTCACACACC
TGGTTCCCACCTCGCCTTCTCTCCCGTAGCATATATATATATAGATATATATATTTATGTGCATCTTT
GCAGAGAGAAAAGATACGCGTCGTTCTTCTGT
ACTTACATTTTCTTTTTCTGTTATTATTTAACATTTTTGTTTTCCATTTTCTTCTTTTTCTTCCTTTG
CTCACTGCAGCCCAAACTTCATCTCGGCCAGT
GAGGAAATCCCTTTGCTGTCATATGTGCAGCGCCAGATGGTGCGGTGCGGCAACTCCAGCCGTACACA
ACTCTCCGCGGAGGTGCTGCCAAAGTACGCTA
GCAGCGTTCGCAGTACAGCCTGATGGGCGACGACGACGACGATGCGGTCGGCGTTTTCAAGTTCCATG
ATCACCGGCTCCAACCGCAGAACTAGGTCCTG
GTAGCTCTCCCCTTCCGGATAACGAAAGGTGTACTTGTTGACTTTGCGGAAATGGTCAATCATCGGGT
GCTGCGTGCGCACCTCTTCAATTGTGAGATCC
TCACAAATACCAGCGTGAATTTCATTGAGACTACTCCAACGTACATTTTTAATCCCCAACAACGGTTC
ACTGTTCTCGACGGTCTGAATCGCACGGCGCA
GCTGACTCGTCCAAACCTCCACTTTCACCGGCTTCTCACACTGAGCACTGCAATCGCTACTGCAGAAC
CCTCCCGCTTTATCATCGTCGTTGTTCACATG

TCCTTCTCCGTCTGCCGTCTTTTTTGTATCTGCGCAGTAGTACCTCTTCAATGAGGCTAAAAAGCCTA
ATATTTCATTGGCTCCCTTTTCCCCATCGGCG
CTCAGGACAGAATCCCCACCAATGCGATTCTCAGCGTTGTATATCGACTCTCCGTGTCGAACAAAAAA
AATGGGATGTTGAATCTTGTCTTGGGTCAGAT
TCATTACATATGAGACAATGCGGCTTGCCGTATAACCCGTCACACCGTGCACTTCAATCTCCACCGAA
TTCCACAGTTTCATATACCGCCGCCCTCGTTG
CTTCCTTCGTGTCGTTTTCCCCGGTACTTTATCCTTCTCACGTGACACATCGCCCAATGTCACCTCAT
CCTCAGAAGGTTCTCCAGGACTCTGGCATGTA
GACGAGAGCGTGGAGTACACTTCAGTGAGGTATTCAATACGGCGTGCAAAGTCTCGTTGAGCCTCACT
TGCATTCACGTCATGTGGGTACTCTGACTTAT
CATGCAGCTTCAATTCATTAAACTTTTGATTTCTTTCATTACTTCGGATAACTTCCATAAAAAAGGTT
TGTGAAGCCAACGGAGAAAAAAGGGCCTCCGC
CTCATCCCGCAGCTCCTCCGTAATGAAATCATCGTTCACAACGAGTACATCTCCGCCACTAGCATAAA
ACGATTTTGCGTCGACAGCATACCGCTCGAGT
ACACGTCGATAAAGACGACGTTCGACACTATCAGGGTCACAAGTAAGTGCACGAAATTGTTCTGCGCA
CACGTAGTTCTTTTTCTGTGAATTCCCCATCA
TCGGCAAAGAATACTCAGGCAATGAAGAGACTACGGGATCGGATATATCCTTTTGCTTCGCAGTTATT
TCCGTGTCTCTCTCACTGTTGCTACAAGTTAC
TGTCGGTGATTTTTTCGACGTCCCCTCCGATTTGGCAGGCGTACATTTGGCTGAAGAGGAGGCGTCAT
CAACCGGTAACATGTTGGGAGCATCGAGTCCT
GAATGGCATCTTTTGTATTCAAGTAACAAACGCCGCCACGCCACCTGAATGTTCAATACTTTTGCGCG
ATAGCCGTGCCATCCCAAATTTCGGCATATCC
TTTGCGCCAGAAACGTTTTGCCCCGCGCAGGGAGTCCAACCATGATAATGCAGAGTGACGCCACACTC
TCTTGAGCATCACTGCTGAAGTGCTCGGGTCC
CTGACGCGTAATAGGAATTGTATAGTTGTCGAGCAAGGGAGTCAATACATTCAGGGTGGAGTCTATAG
CGGACGGCCGGCTGCTCTGGGAAGAAGATAAA
ACAGTCTGGACAGCTTCGCAGGAGAGAGCCGTGTGTATACGCCCGCGCGCCAGAGAACATTTAGCGGA
AATCGGTGGGTTACCGCAATTTTCTGGCGATT
GCACCGAAGTGTCTTCAACTGTCATTTCTGGCTTGGCATTCGTACGACACTCTTCTACATTTTCGGCT
TCACGCAGCTCTTTAAAATATGCAGATTCACG
CCAACTCCATTCTTTAAATGGGCGAATCGGACCTTTAATTAGTTCAAGTAGACGTGATTCACGCTCGT
CTTCATCTGCCGCACTCTTACGTGCCTCATCT
GCGGCTGCAGTGGCGAGGGGGTGGGTGTCGGCTGGAGCGATGGGGGTATCCGACGGCTCCACGGATGA
ATGAATCGCCCACTCATTCACCTCCGTGTGGT
CATTCGCATCAACGCTCATTTGGAGGCAAAAGATACCAAAGAAGCAAAGGTGCTCCCAGACAGTATGG
CCAAAAAAGGATGGAGAAAAGGGAGAAGGAAG
GAAGGGAGCTATGTGTGTGAACTATCGTGAATATGTTTTATACAGACAATATATATATATATATATAT
ATGCGTTCCCTTTTACTCGTTTCCTCCGCGTG
AAAGGAAGAATATACTTCCACAGAACAATTGCCACCACGCCTATCCTCTACCCAGTTCTCTTGTAGCC
CACAAACCAAAAGCGTTATGTATAATGAAAAT
ACAAATTTTCCCCCAACCAACCCCCGCTCAAACACACGTGCGGCAAAAGGCCAAAGATAAAAGAAAAA
AAAGAAAAAAAAAAAAGTGAGTGGGTCTCGCAG
TGCGGCCATTGCCGCGGTACAAGTGACATACGAGGAACCACTAATTCAGCGCCGCCAATATGTATTTC
CTTCTAGATGCCGAATCTGCCGCCATTTTCTT
TTTTTTTTTTTTTTTTGTCACAGGCGGCAATCCCAAAAAGGGGCGCGTAAAACAAACAAAATAAAAAT
AATAAAAAAATAACAAGAGGGTTCGATTTTCT
CGCAGCATCATATCTGCATGGTAACCCTCTCCTCCCTTCTGTAAACATTTGAAAATCCAACTCTCCTT
AAAGTCAACCCTGCCCTCATGCAAAAGACCCT
TTTGCGCCGTTTTTTCCTGTTCTGGCTTTCATTGTGCTGCAGGAATGTGCATGTCGTTATTTTTTCTG
CTCCTTCCTTTCCTCTCCATGCCCCTTTTCTC
CTACGCCTCCTTCACTTGCCATTATGGAAGTGGCTGATGCTTCCGTTGTGTAATAACCCCGTCGTGCC
ACTCGCTGCCGTGACACGGTTGCATACACCAT
GCACACAAAGGCAAAGAAGACAAGGGCAAAGATCCCAAACACAAGACGGTACCCTACGGCCGCATCCA
CAACAGCTGCCCTCTCCGCTTGCTTCCTCAGC

TGCATAAACTGCAATTCTACCTCTGGACTGAATGCGGCTGAAGTGGACGCAAATGAGTTTGGAGGGTA
CTGGGGACGGCCAGGGTCGCCGCCCAGGCCAT
CCCCCAAGCCGCCCGTGCGCTGATATTCACGGAAAAGACGATCCATCGCTGCCTGAGCGGAATGACCT
TTTGGTCCAAACATGAACGAGAGCCGATGTAA
AGCGCCCTCCCTCCGCCGCCGCCTGTCCGACACATTCCACGGTGGGGCCTCCTCCCGCATCCTCTCCA
TGCGCCGCTGAAAATTATTAAATTCCGCCGTT
GGGCGTCGTCCCCGGCCTGACGTCGTGCCATTACTCGCGTCCTGTGTGCCCTGACGTTGTTCGCGGCT
TTGACCCTCGTACGCGCGTTGCGCTCGCTGCT
GTGCCTCGCCATCCGCGCGAGATAAAGACAAAATCATACGTCTCTCCTCTCGGCTGTATTGTCTTAGT
AACCGATAGGCTTCCACACCTCGTCGCATTTT
GTCTGCAGCCGTTGTTTCACTCTTTAATGTCTCCGCGTTGACACCACTGTACTTCGTTTCATGGTCGC
CGTGATCGTGGGAATGGTCGCGGTGGAGTACA
TCGGGGTGACTGCTCTTGGCAAGCTTCTGAAAGCAAAATTTAATGTCGCCGTCCGTCGCATCGTCTGG
GAGGGAAAAAACACGCAGTGCGGCGCGAAGCT
CCTCGCTGGACACGGTGTGCTTCGTTGCGGTACACCGAGACGAGCCAAATATCACCGCACACTCTCTT
TCTGCCCCACGGTCTCGCACAACTCTTCGTAT
TACAGCCGATGTAAAACGGCCGCCGCCCATACCGACATGTTTGAAAGATCCATACATAAAGATTCCCC
CACAATGGACCACAACCACCATATATATATAT
ATATATATGTATGTATGTATGTATGTATGTATGTATAACTACTCCCTGTGTATTTGTGTGTATGTTCT
ATGCTGCTAGACCACACAAATAAAAATACAAT
GCAAGAATGTCATGTAAGATACACCCAAAAAAAAAAAAAAAAACGTAAACGGTACAGATGCCACTGCTG
ACACGTCACAGTTACCCAAAAAAAAAAAAAACT
TCATTCTTTTTTTTTTTGTTTGTTTGTTTATCATGCAAGAAGGAAGAGTGTTAATGCAAACGGTTACC
AAATTTCACAGCAATTGACATCTTCAATAAAA
ACATAAAATACGAGTTGCTGATAAAAACATGTACAAAAAAAATCACATTCATACGCGTCCTCGAAAGC
TGCGTCAATTCACTTGCAAAAGAAAAAAAAAA
AAAAAAAAAAAGAGGGGGGGGTGGTGAGTTTGGTGGAAAAGAACCCCACGCGACTCCATTCAAACACG
ATTCTTCCCTCAAATTAGCAACGCAAAAAAAA
AAAAAAAAAAGTGGAGAAATGAAACAATCAGACCAAAATACATACGCAAATTCTCAGGGTGCCGGCGT
ATAACTGCCACAGTCAACAGCCAAAGTAAAGT
AGTAAAAAAAAAAAAAGATGGAACGTGACGCATTCCACACGCAAAAATAAAGAAAAAAACTAACGTAA
AAGCTGGGATTTACAAAGGAAACAAAAGGAGA
GAATAGCTCACGAGAACGCCACGCAAAAAAAAAAAAAAAGAAAAAAAAAAGCAAAAAGAAAAAAAAAA
AAAAAAAAAAAAAACGTCACTTTGGAAACATT
GTTCCATAACCAATTTAAAGTAACATGCATCTTGCGCTAACCAAACAAATATACATCTTCTCTTGTCC
ATTAAATTCATTCATTCTAGTATTTGATGAGA
TGCACAGAAAGTAAACGATTGTAATGGGGTTTTGTTCCTTGCTCGATGTTTAGCCCTTGTACGTGTAA
TCCCTCACTCCCCTGCTCATCCACACTATTCA
TATTGATATTTACATGACGCCTTCTCTCATAAGGAGCGAGAAAGGCTCAACGTCCGCTAGACTACCAA
TGTCTTCCTCTGTCGTATCCGCCGGCGCCACA
AGACGGCAAACCCCCCGCAATTCCCTTGGATGCTGAAACGTCACAAGGGTAAAGTGCCTCATGTTGTA
AAAAAAACGCTTTGTGCTCTTTTGAATCACTG
GCTCCGACGGATTTCTTCGCCTTGCAAGCTCCAGAAAGGCAGATTTTGTAATTTTCACCCGTGTCAGG
TGTCGGCCTGGTCTGTGGCCCTTCAATTCGTG
TTCACAACCATCTGGCGGTAAAGTTGTCTGCTCGGGATTTTGATACTCTACTCGACGAAGATACAACG
GGTGGGAATCATTTGCACGGGAAAGGCGAATA
ATCACCTCGGTGCGGTAATTCGCTCGTAATGGAACCAATGTTATGGTGACAACATTTGCCACCACAGC
ATCCGCGGGAATTTGTGCAAGATCAACACGAT
TCACAAAAAAATGGCGCTGTAAGCCGCCACGTTCGGCATGCATGTCCATGGCAAATGCAAGACTCGGT
GAAGAGGGCGCCGATGTGCGCCCCAGTTCCCC
ACCGCCGCATTGCGTAACAAAATCAGGCGGTGTGAGCGGTGAACATATGGAATTATCATTAGTATTAT
TATTAGTATTGTTATTGCACAATGAATGTGCG
GAGACTGTTGCCATGGATGCGCTGTTTGCAAAGGCACGTGTTCCTGTCGCCAAAAAGGCCATGTGTGC
AACATCATCCTCCCCATCCCTCTCATCTCCCC

CTTTTTGGTCGTGTGGAGACAACACTGTTGTGAGCTGCTGAAGGAAAAAAGGTGTTGTTAATGATGGC
GATTTTGTCGTCTGGGCTGCGGATGATAGGGC
TTCCGGCGTCGCTGTTGTGATTGTGGTTTCCCCGGCAGACCTAGCAGTTTTCTTGGGGACCACAGACT
CTGTTGCATTTTCTTCCAGCGTTGAAACACCC
GTCACAAAACCGATAAATTCCGCCACGCGTAACATTTTCTTCTCAAAATCCGTGTCCTCGTTATGCAC
CACAGAGACGAGGTAGTGACCACGCCATGCAT
TTAGAGTCATGGCATCCAGGCGTCGTGCATCCTCCAGTCCCTCGGAACGAGCAGCGTTATTTTCAATC
GTGTAAAACTGCTCGGCACCAATGGCTGCCGT
TACCAAATGGACAACTGCATCATACCGTGCATCGCTTAACTCCTCATTCGTATACCCCGTTATCTTAA
GAATGGCGTCCCACTCCTCGTCCGAGCAATAC
GCACTACCGTCCATTGTGCCACGATCATAGAGAATCAAAGATGGTCGAGAGCGCATCGATGCCAACTC
GTAAAAGGCATCCTCCAACATCATCATCATCT
GAAGAATAACGCGCTGTTGTGTGATACGCTCCTCGCTGGTACTAAGTATTAAATTAACGCCCCCACTA
TGCAACAGGGTTGCAACTTCGGGTACACAGAA
TACATTAAAGTTAAGTTTCTCAAACACCCGACGAAGGTATGATAAACATGATGTCTTCCCGCTGCATG
GCCCGCCAGTGATAACTACACGATAGATGGGA
TCATCCATAAGGCTCCTGGTGGAGCGAATGGACTGTGTCAACTGCGACAGGTTGCCATCCTCCTCGGT
ACTTTCATCCTTGCGGGTTGAAGATGGTGGAT
GCAGAAGCTGCAATATTGAACGACATCGAACCGCTGCCACAGAGGGCAGTCGTGTGATGATCGCACCG
CTGTGGGTATGATAAAAATGGCGTTTAGAGAA
AACGGCGTCATTCTGTGATGCGCCAAGACGCTTACTTCCACCGGGAAAATCCAGACCGCTGAAGGCTG
TCCTATCCAAAATACAGTTCACCGAGTAGGAT
CGACCTGAACGTCCAAGACACCCTGTTGCGGTCTCCTCCTGCTTCGTCGTGCATGCATCTTCATTTTG
TTTGGGATTATTACTGTGCTGTTCTTCCTGCT
GCCCTTCTTCCTCGTTGTTGTCCTGCGATGACGCGTCGTCCTCAGAGAAAAGGCCCTCCAATGTGCTG
AGGCTGCTAAATTCCGCTTCGCCTGAGGTAAA
AAACACATTTTGTACATTTCCTCCCTTCTTCTTCCCAGAGCCCTTAGTACTCGGTAGAAACGCTCTCG
TGCCTTCCTCCTCCGACCCGACCGCGGAAATG
GGAAATTCGGAGTCGTTCTCCTCTTTCCTCTCCTCCGCGTCACCTTCATCTCGATCCTCCTGCGACTC
AGTGCCCAGACTTTCCGCGCCTAAGCATGACT
TCAGAATTAAACTAATGGAGCTGCTGCGAGAGTTTTGACCTATCCTCTGTGCTGCATACGACTTCATC
GAAAACAAACCGGGATGTAAATCGACCCCTGA
ATTCAAGCGCCGCGAAACTCGCCGCTTCTGCCCGCCAATAATTTCCTCCTGCTGCAAATGCCTTTTAC
TGGATGACGCGGCGCCATCCTTCCCATCATCA
TTGGAAAAAAAAGATGCGAACCCCGCATGCCGTAAAGACCGGCGCCGCGGTGAGAACTCCCTCCTGTT
GGTCCTATTGATGTACGTAAGGGCAGCCGTGG
CACCTATTGCTACACCGAGCACAAGGGATGCCGCGGCCAACAGAGGTGTGGGCACAGCGAAAAAAGCG
ACTCCCCCCATTCCTGCCTATCTTCCACTCTC
GCCTTTATAGCGGCAAAGGTTTTAAAATATATATTTGCTGTCAAATTAAGAGAAGATGGAAGGGATGG
CAGAATTACATTCCTCAAAGATCAAAGGAAAA
AAAAAGGGAATAAAGCATAAAATTGTCAAAAGGGCAAAAAACAGTAAAATTAAAAATAAAAGGACACG
ACCTCATCTCAGGCTTAAAGAAAGTGGGTGTG
TGTGTGTGGGTGTGTGGGGGTGTTTACGTTTGGGTTGGAAAGAGCCAAGATGATCTTGTCGTCACGTT
GTCACTTCATCGACCTTCCTTACTAGCCTCGT
CTGTGGTGCTGAGTCCCTCCAACTGAGAGTCACGCTGTGCCCTCCGTTCTCTGTCCGCCTCCTCTTCC
CAAGACCGTTGCTGCGGGGAGGAGATGGTAGC
GAGGTGGATAAAGAATTTCACCGACATCTCCAATTCACTCACCGATACATTGAAGCACACACACTCGC
TTTCTCTCTCTCACACTCACACGGTACCTCTA
CTTATTGTCTTGTACGCACTAGAGATTGCGCGGTCTACTTGGTAATAATTTACGATAATCTTGCCTGT
AATATTTTCAACTACTGTTGTTGTGTTCCAGC
AGACACGAGGAGTTCAACAGGGCATGCCGTTGTGCCAACTCTGCTGTCGTCCTCCAAGGTCTGATGGA
AAGTTCATCGGAATGGCGGTAGCGTAGACACA
AGGATGCCGTGTCACGATCGTTGCTTTCAGAAGCAACAACAGCTGTCTACACCCCGTCTGGTGATGTG
GAGTCAAAAAAATACACCTTTGTTCCATCAGA

CAATTCCAACGTCGCCATGGCAGCTTCAAGCTCCTTCTGAAACTGGTGGACAGCCCACCGCCGTCCAC
GAATGATCACCTTCACCCCATCCCTCGTAGCG
CTGGATCCCTTTTCCCTTTCAGTTCCTCTTTGTTCCTCTGGGGAATGAGGAGGATATGGATTGACCTC
CAGGTGCTGCTGGTACTCCAGGCTAAGGCCAA
GTGCCCGCGCACGGTGCCGCAACCCACGGCCTCCGCCCCCCGTAAGGCTCATTTCTCGATGACTCACG
TACTCATTATCATCAGCGCCCTCCTTCTCAGT
AGAGCGGTCTCCTTTGTCGCCCGACATGGCAAGTTGCAACGCGTACAAGACGCCTGGAGGAAAGGCAA
AAACACGTGACACAATGGCGTTCGGGTCGCCC
TCCTCTCTTCCTTCATTCCAATCCCTTCCCGTCCTGTCGGAAGAAACACCGCCCGCTTCCAACAACAG
TGGTGGCTTAACTGCATTTTGTCGCCCAACGC
AGCGCCGCTCCCAACAAAATCGATTGCATGTCGGAATAACAGATCCCGTCTTATCACCTCCTGCCCCA
AACACCGGAAGGTGCAACCCGCACGCCCTCAC
CTCCTCTCGCAAAGTATGAATTTTCTCCATTTGCTCCAAATACTTCTTCTCGGCGGCAGAAACAAACG
CCTCACGCCGTCGGTAATCCCACACACGCCGC
TGAGCCTCCTGCAGCTTCAGTCGCAGCGTATGACACACCGACTCCCCTGCCTGAATTTCCGTTTTACG
CGCCTCATTATTCTTTTCTTCGGCGAGAATAC
TGGCGTAAAGAGAGCGAAGTGCCTCCTCCTTTGCGGTGTTATTTAATCTGCACAGCGTTGCCTGCTCC
TCCAACTCCCGTACTTGACTGAGTAATTGGTT
TCGCAAAGATGCGAGCAGCTGGTCTACTTCCACACCcGCGCCACCAGCATCTTTATCTTCCAATCCAG
TTGGTGGTGGCGTCGACAAGTCGGAATAAAGC
CGCATTAACAATTCCTTCTTATTGGTGTGTATCTCAGCCTCTAGCTCTGCCACCGCGCGGCGCAGCGC
ACAAACCTCCTCCTGCGTCGTCCCAACAAGGC
GGCGCAGTTCCTCCACGTCGTGTAATAAATTTTCATCACCGTGTGTAGTTGGAGACTTTTCTGTCGTT
TTCATAAAGTCAGGATTTTGAATAACAAGCAT
GGGGCTGCGCATGAATTCTGTGATGCTGGAGTGAAGACTGCTGGCTGTCTCCAACTTCTTCGCTGGTG
AATCCATTTCATTCGGAGAATAATGCAACAGC
AGCTGTCGCGATGCTGACAATAACACTGGCGATGGACCCGACAGTCCTTGAGGAGACGCAGTGAGACT
CCCCTCCCTCAGCATCGTGAAGCACCAACTGC
CACAAAATCAAAACACAATCCTCCCAAATAAAAATATGGTTCTAATAGTTTTAAAAAAAGAAAAGAAA
AAAAAAGAAAAAAGAAAGAAAGAAGGAGTGAT
TGCGAAATAACCCGCCAAGGAAGAAAGAAAGACAAATATATATATATATATATATATTGGTTCAAAGT
CCGCAAGTTCTCAAAATAATTGAATAGATCCA
AACGCACACACAAACACACAACACAACGCAGGCAAATAGATATAGATAGATATAGATATAGATATTAG
TATATACACATTTCATGGATACACATTCAATG
TACCCTGGCCTCCCCATCGTTCTCTTCTCTTTGTTATAATTCCCAAGCATCAATATCAGTAATATTAA
CATCATTACTCCCAATCACCATCACCATCCCT
TCCTTACACTACACCCATTCCCTTAAAGACCCTCTTTCCTGTCTGTCTTGTTTTCTTCTCTTTATTTT
CCATGTTCGTTTTTTTTCTTCTTTTTCTTCTT
TTTCCGTCGCGTTACTCCACAGTGATGCTTAATGAAACCGCGTTCCGCAGTAACAATGCTCCTTTGTG
CAAGGTGGATGTTTATGGCGGTGGTGGCGATT
ATCACCGTGCTGGCAAGCAGCAGGATCGCCCTCATGGCTTCCATGACGGCCGTAATGGTGAGCATCAC
CTCCTCTCTGTTGGTCGTTGTCACCAATAACA
CCGCCGCTTTCGTCACCGCTGCTGCTGCTGCTACTACTACTACTTTCATCGCTGCTGCTCTCCCCAAA
AAGTTTTTTTTTATGAAAAACACAGCAACTTT
TACTGACGCGCTGGTTTCTATTTTCCCGTAcATTTCTTTCCCACGAAACACGACGCATGCGATCCTCC
TCGTTCTCCGCGGTTGCAGGACGCAGATGAAG
TGTCACCACCCTGCGTGGCACTAATTCCTCCTCAGTTTGCCTCACATCGTCCGACTGCTGAGCCGCCT
GGCGTGTCAGAGACAACGGCATCCTTCAGCCC
CCGCCTTCCCTCTCTCCCGCCGAGCCAACCGAATTTGTTTAATAAATCCCTTTCCCGCTCTTCTGCCG
TCGTCCGCTCTGCTTGCGGCCTCCACTTCTCT
CCGTTATGCGGAAGGCACCACACAAATATGTCTGTCGGTATGTATGCGTGTATGGATATTTATTTATT
TAATTTTCCTCCGTTTCACTCTTTCTTGGTGT
TGGCGGCTTACTTTTACTTCCGCAGGTCACTGCACAAAAAACAAAACTATTCCCCGTTCCTGTCCCAC
ACGCCTTGGAAGGGTGAATGTTATGTCAATCA

TAATAATAAAAAAATATATTTTTTTTTTTAAATAAATAAAAGGAAAAAGAAGAAGAAACAAAACAACA
AGATCCAGCCGGGGAATCATGTCGGCCACAAG
CCATGCCTACTCCCAGTTTTTCCAACTCTTTTGGCCAAAAACAATATAAACACGCCAAACAACTTGCC
CCAAATCTTTGTATCACCATCACTCCCACGAT
TATTTCCTTTTCATTGTATGTGTGTGGCGGGAAATGGAAAAGCACTCGTTTTCCTCTTTCATCCCCTC
CCCTCTCCATCTTTTCACCAATGAAACAATAA
ATAATAGTAATAAAAAAATATATATGAGTGTATACGTATGTATAATTAATGACATCTTTTATATTCCC
CCCCCCCCCTTAAGCGCTCATCGCCGCTCTCT
CACTCACTCACTCTCTCTTTCCCTCCAATGGCAGTGCGACAGAAAATAATAAATAATATGCACACATA
ATAATGGTGATTCCCTGCATGCACCCATGACC
ACATGCCGCTCTTCGTACACGCAACCAAACACACACAAACCCACTCACTCACACTCTTCTGCCGCGCA
TACACAAGGAGTGAAAGAGGGGTGTGCAGGCA
GGAGAAGGGGCGGGAGGGGGGGAGGCAACGGAATACATGTGGATGTACACGAAAAAAAAAAAAAAAAA
AGAGGTTTAAATCCCCATAAAAATTCAAAAAA
GAAAAAAGAAAAATAAAAAAACGAACAAATGAACAAATGAAAATTAAACCACAAACTCATAAATTTGT
ATAAAAGTAATATTAAAACACAGTCGTTTAAT
TATTATCGTCGATTACAGGTGTCCTTCCTCCCTTCTTGTTCTGTTCTTTTCTTCGGCTTTTCTTTCCG
TGCCTGAACGTAACTTCATGCATCCAAACCCG
ACCTCTCCGCACCGCCGCATGAATGACGTTCGTCTTCCCACCAGGACCCCAAAAGAAGTACCGGTGCA
TATTTTGACAGCAGCGGCAGCAGTCATTGCCG
TCAGCCACAGGCAATCGCAGACACGGAGATACAAACACACAGAGCACCGCAAATATATAAAATATATT
TATTTATTTAAACTCAAAAACAAGAGGCATCA
CTCACTCACACAAACGCAAAATAATAATAATAATAAGAGCGTAAAGATCAACAAGCAAAGTTTAACTT
CCCCACTTCTCTGCAACTCCCTTTCTCTCTAT
ATATTTTATCGACACCAGCTGCCTTCCTTTTATTATTATTATTTATTTATTTATTTATTATTTTCGGC
TTTTTTTTTTTGTTTCTTTCCGCCCCAATTTT
GTTTTGCCACCAAAAACAAATGCGTACATACACGTACGTACATATAATAAAAGAAAGGCAGCTGGATG
TGTCCTCCCCTCGTGTGATCCCATCACTTTTA
ACCCACACGTAACTACCGATATATATATCCCACAGAGATAATATACTGAGGCTTGTAAAAGTCCCAAA
AAATAAAAAAATAAAAAAATAAAAATAAAAAT
AATACAGGAAAGAATACCAAGAACAAAAACAAAAAATAAATAAATAAAATAACATAAAATAAAATAAT
ATAATATAATATAATATAAACGACTGTGCAGG
TGTGCATAAATGCATACAAAATATATATATATATATATTTATTTATATATCATAGAAAACAGAAATCA
TATAAGTGAATGTGTACGTATGTACTTATGTG
TCAACATGCATTTAAGCATGTCACAGGGAATACGAAACAAAATTTAACTGCGGAAACAACAAACAAAC
AAAGGCAAAAAAAAAAAAAGAAAGAAAGAAAC
AGTCCTGTGGTAACAGCAGTACTGATGTACACAAGTATCTACAGAGAAATACAAAATCCCTCGCACAT
AAGCGAAAAAGAAAGAAAGTCTCGTGCCTTTC
CCCCCTCCCACACACACACACACACCCTCCTCTTTATTTATTTTTGCCTGCACGTATGCATGTATGTA
TTTATGTGTTTTCCGCCAACTCCCCCACCGGC
GGCTCCCTTCTTTTGGGGTGTCTTCTGCGCCGCTCGCTGACGCGGAGGCGAATAGCCGTCCAAATTAA
CACTCGGACGGTGACGGATCGACGGTCCGAGG
GCGGCGGAACGACGCACACAAAAACAAACAAACAAACAAACAACCGCAAGGGCAGAACAGAAAGAGGA
GGAAAAAAAAAAAGACGCCTATCGGCTCCCTT
GTAAATAAAACTGGTCGGCATGCGTGTTTTGTTTGTTTTTGTTTTTTTTTTTGCCTGCTTGTTTTTGC
CTTTTTTATAAATAAATATTTTTTTCTTTTTT
CTTTAATATTCCGCCTCCCTTTCTTTTCCTCCTCTCTCTCTCTCTCCCCCCGTGTCCATAAAAAAAAA
AAAAAAAAAACAATAGTACAGAGAGCCGGTAT
ATAACAACTGTGGTA
>scaffold1140.size7548
AAAAAAAAAGGTAGGGGAAAAAAGGTGGGGTTGTCGCAGAGTTCGTTAATTGCCTCAATGTTTTCTTT
TTATCCAATCAATAAATTCGATTGACCGTTCGC
ATGCTCTTTTGTCCTCAGCTAGAAGCTGAAAGATAATTACTTACGACGCGGCAGACTTTTGGGTTCCA
CCGAGCACAAGACCCCGCCGCGTGGTGGGAAT

CAAAAGGGGTTGGGTGGGATGGCAAAAATAAAAATAATAATAATTTGCCTATTCGCTTTCTGCTCCCT
GTTCCCCATAAAGTTGTGTTATTCCCAAAAGA
TTCATAAAACAGAAAAAAATTATTGTAAAGAAGTGAATTCGTTATTATCAGAATTTAAAATTCATTAA
GGCAGTCGGCTTTCCTCTGTGATCATTCTCCT
CACAAGCCCCTTAAAAAAAAAAGAAGAAAAGTGGAGGGGTATCACACTTCCACAGATGACGTGCTCTC
CTGCGTGGCACGCAGTGGAATGTGTTGCCGCA
CCACACGCGCGACGGTTATGGCAAGGCTTGAAGGCTTCGTAGTATTGAAGGTGCCCTCTCGGACGACA
AAACCGCCAAACTCCCCTCCCACATTCTGTTT
TTCCTCGTGTTTTATAAAATAAATCCGATGGTCGGCATTGCGATAACACGtGCTCTTCAGACCTCATG
GTGTTACGACTGTGTCCCAGCAGGACGCTGGC
CGACGCCGTCAGCCCCGTGGCCACTCCAATAATTCCGTGAGGGAACCATCGCAGCGCTGTCAGTCCGC
GGGTGAGAGGATTCGGCCCTCCACACAACAAA
CGCACACAGAGCCACCGCCTCCAGTTCAATCCCACGGCCAACGGAACACGGCCACAACCCCGCTGCCG
GCACACAACGCACACCGAGCAAGCAATCTCCC
AAAGAGCCACGCGCAGAAGCGGATCCTCATCCACCCATGGCGCGGCGCCTCACTCCATGCGGTCACAC
GGAGCGCACCATTTCCTCAATAGATCTTATCC
TTCGTCCATGCCTTTAAGATGCTTCTATTCGTGATGAGCCTTAGAGCACAATAGAAGACTTCGTAGTA
TTGAAGGTGCCCTCTCGGACGACAAAACCGCC
AAACTCCCCTCCCACATTCTGTTTTTCCTCGTGTTTTATAAAATAAATCCGATGGTCGGCATTGCGAT
AACACGTGCTCTTCAGACCTCATGGTGTTACG
ACTGTGTCCCAGCAGGACGCTGGCCGACGCCGTCAGCCCCGTGGCCACTCCAATAATTCCGTGAGGGA
ACCATCGCAGCGCTGTCAGTCCGCGGGTGAGA
GGATTCGGCCCTCCACACAACAAACGCACACAGAGCCACCGCCTCCAGTTCAATCCCACGGCCAACGG
AACACGGCCACAACCCCGCTGCCGGCACACAA
CGCACACCGAGCAAGCAATCTCCCAAAGAGCCACGCGCAGAAGCGGATCCTCATCCACCCATGGCGCG
GCGCCTCACTCCATGCGGTCACACGGAGCGCA
CCATTTCCTCAATAGATCTTATCCTTCGTCCATGCCTTTAAGATGCTTCTATTCGTGATGAGCCTTAG
AGCACAATAGAAGAAAATGAACAATGCAGTAG
CACTGCGACACCGCTGGTGACAATGATTCCCTCATACCGGACAATTCTCCAATCATGCCTCGTATGCA
CACGCAATCAACATACTATATAAAAACCACCC
AACGTTTTCACCAGAAACAAACAACCCCACTCCCTCCCTAATTTTCTTCTGCCACTGTTATGTTCGAT
AGTGTTGGACACTTTTGGCGTTTGTGTGCGAT
ATACTGCCGTCTTGTGCTGGTGCGCCCGCACCGTACGCACACACACAAGTATCCAAAAATAAATAAAT
CTCACAATATCTCTTCACCAAAAGTCAAAACT
GCCCCTTTCCCTCCCCAATTTGTTTTCCTATTTCGGCGGCTATTCCTCGCTCCGGAGTGCTTCGTCTC
TTCAAGCGTCTCTGGATGGTATTGACACTTGG
TACTGGCGCACCTTCTCCTCCCAGAACGCCGCTATTTCACGGCTTTCATCGTCGCTCACATTATCGGA
ATTTACAACATCACATCTCTGCCAGTCATTCA
ACGGCGTGCTGTCTGCGTGCTGCACATAAATAATCTCCCACGACATGTCTCGGGATAATTCCTCCCAG
CCATTAAAATATGCCGCCAGGCACTCAGTGAA
CTGCCGCACAGTGCTGGCGGTGGTGTGGCGCCCACCCGCCGTAGCCATCCGCAGCCCAACCAGCGTCA
TTGGGTTTGAGTCCATGAAGAAAAAGCCGTCC
ACCAGCGGAAAATTCTCGACCTCCGGTATGTACAGCAGCCGGTGGTTTATACAAGTCTTCTTGCCAAT
GCGTTCCGCCGACGGCAAAAAGTAGTGGCTGG
TGGGGCCCTCCTGCGAGTGCACGTCCGGCGCACAGCGGTGTGACGGACGTCGTGTTGGGGGCTTCAGC
TCCTTCAGTTTACGTCTTATTGCAGCCATGAA
GGCCACATTCAAAAATGCAAACATGGCACACTTCCCAAAATTTGCGTGCATGAGACAATCCCTGATCC
TCGATACAAATAAGTTAAACTCAGCCTGCGTC
ATTAACTGTGCCAACTTGCACAGTGTTTTACTTGCGAGGTGGGCAGTTATCGGTGCATTGAGGGGCGA
CTCGCCATTGCGCTCTCCTCGTGCTCGGACAA
TCCTCGCAAGATATTCAAGCACCTTATTACCCTCCCACAGTTTGGAAGTTCCGAAAACAGAGTAACTC
TGGATTCCCCAAGAGGTCGTTCCATCCACAAG
TTTGTGGCAATCTCCAATCCAATATTTATAACGTCCCTCTTCAAAGATGTAGCGAAGAATAGGTCCCA
GTTTGTCCATGCGACCCTTCACCTCTCGCCAG

TATTCCGCTTGCTGCGGCGGAGGCTGATTGCGCTTCTTCCAAACGCACATTGCCTTCACGTCGCTCTC
ATCGGGGCAATTCATTATGATTCGTACGGCTC
TCTTTTCGTTCGCCCAGTGTATGTAACTTGTTGTCTCTGGCGTTGCCACCACAAGCATGCCCCAGCCT
CCGGGAGGCAAAAAAGGAGACGGAATATCTAA
ACTGTATACCACGTCGTAGATGATATACCCCTTCATCCCGCGCCGAGACAGTTCCTTCACAGCTTTTA
TCATCATGGACTTATCCATGTATCTTGTCACC
GTCTCGGCGGTCTTGTTAAACACGTATGCCTCACGTCCAGAAACAACGAACACAACCACAGGGAGTAG
CTCCGCATCGTAGTGCAGCAGCTGGTAGAGGA
GGTACGAACCGGCAGCCATCGATTTCCCGATACCGGGCGTCCCAGTCAACAAGCGCGGATGAGGCGTA
AAGTGGTCCTCAGGACGACTGCTGAACCACTC
GGTGAGATCGCCCTTGACGATCTGCCACACTCGATCCACCTCACAGTTCACATAGCAGTCGGTACTTG
CATTCCCGCCCAAGAAATCCCATTTCTTGCCC
AAGGTAAGCACCATCAGCCTGAGACGTGGTGCACCGGATGGCTCCACACCGTCATTCTTTTCGAGGGT
TCGGCCAACTGCCTTGTACGTCCATGACCGCG
GTGGTTCCCCTTCATGCACCTCCATTCCCGTCCCCTCGCCGCCAGGAACTTCCATGACATGATGCCAT
CTCGCATTGTACACAGACTCGTAGTCTTCCCA
CAGCGTGCGTCCTTCCACCCGACTGTCAAGAGCCCACATTAATCGCCGCACCTTCTCCACTCTTCCGC
GAGTCGCAGCGGGTTGATCTGTGTCACCCTCA
GACTCCGGCCTCGCTCTCCTCCGAATATCTCCCTGCGGCACAGCAGACGCTTGATTTCCAGGCATTGC
TTCCTTCTGTTCTTTTAGTTGGATTGAGTTAA
AGCTGAACACTAACCACCGAACAGGAGGAGAATAAGAGAGAGATGGGGTAACTAGGCACAGACATCAC
GGGCAGCCACAGCAAGAGAGTCATCCACACAG
TGTGCGACGCACACCACATGCATGAGGACCGCCGCAACGACGGCGCGCGGGTGCCAACCACCCAGCAC
AGTGTTGGTGAGAGCAAACAATAATAGCAACG
TGGTGTGCACGGAGGGTTCGCCCACGCATCCCGCTCGCTACGGTCCGCACCCGTGTGGCGCCGCCTTC
ACCGCAAACACTCTGCAAGAGGACGTGCGGGC
ATATCGTCACGGACACACGAAAACACACACAAAAGTGGGAAGGGAATTCGGTGCAGCCACGGCCACAC
GCAGACACCCAAAATCACACACGCCACAACAG
CGGCGCATCATGGTAAGTTCCAACACAACAGAAAGGAGTGCAATCACATGCTTTATGAAAATCCCCGC
AATTCGCGCGGCAAAAAAGAGCCATGACACTC
CCTTTCTTACTGCACTCGCCGTTCATATTCCCAATGCGCAACCCTGCAGATGTTGCCCCGCCATGGTG
AAAACAACTGCCTCGCTGTGGTGTCATCGCGC
AGCCATCCGCCTGAGTAAGAACAAAAGGAAACTCAGGCGTCCGTCCACCTGCAGGGAGGAGGGACTCC
ACCGCAGTGTCCTTCATGACGCAAACACCCCA
CATCCAGTGCCCGGTGTCGTGTGCAGGTTCCGCGAGGAGGTTTTGGGCACGTCACTGTGCCACACGGT
GCGCCCCCTCGAAACACAACAGCAGACGTGTC
CCCTTCCGGTCGCACTTGTGTCAATGGCGGCTTCCGCTGCCTCACTGCGTGCTGGTGGCGTCGATTAC
GATGCCGCAGAGAGTGAGAGAGAGAGAGGTCG
CCCACTCAATGCAACAAAACAGAAACAGTTACGGTGCTCCGCCCATGCAGCCAGGCAGGACGGCAGCG
GTGCGTGTCGTGCAAAAGAGAGCACCAACGGG
CCTGCATGGGTGTGCATGCATATATACAATGGGCACATTATTGTGTGTGCCTCGTGGGGTGAGGTGAG
GGCGAGAGTCCATGCAAGGGGACACACACACA
CAGAGAGAGAAGGACGAGTGTGTGTGCGGGCGGTGCACGGCTCTCTCTCCCTCACTCTCACGCGGCCA
CCACCGCAGCAGCAGCAGCACACGCAACAACA
AGAAGAAGCAAAAGAGGGGAGGCGGTGTGGGAGACCGCGGTGCTGCCGTCACTGTCGCCACTTGTCGC
CGTATCATTTTTTTGAGCGACGGTGAATGTTT
CTGTTTTTACGGTTTGTGGTGCTGTCTCACTGGGTGTAGAAGCGACTTTGGTATTGGCAGCTTTGTGG
TCACCTGTTTCTTTGTGTTTAGGCCGTGGATC
ATCGTCATTATTCTCATCCCCTGTACTCTGGGCATCGTCACTGTCATTTGTGGAGATGGATGGTGATT
TTCTTTCCGCTGAATTGCCGGTAGCTGCATTT
TTGACCAAAACCGGTGTGGTTGCGTCGTGACCGTGTCGGTCTGCCATGCCATCCTTTAAAGACGTTTT
AGGTGTGTCGGGATTTGTTTTGGTTGCTGAAG
TTGGCATTTCGTGTTTTTCTTCTGTTGCCGTGCGTGGGTCTTTTGGTGCTCTGTTTGGCTGCAGTTCT
TCTGCCGTTGGTGGCTTTACTGGTGGTGGTGG

TTGCACTTTCGACTTGCCGGCGGTGGACAACGTGTTGGAAGAGCCGTCCGTCTTCATGTCACTCTGCG
TCCCCTCAGCTTTAGTTGTGTCCTTACCAGCA
ACTGATGTTTCTGCTTCTTCTTGTGTATCGGAGGCGCGTGACTTTTCTTCGAGCTTTGACACCTCTCC
TGTCTGCCCGCTTGGCGACACGGGCCCTGGAG
ATTTTTTTCCAGACGGCTCACCTTTTTCCGAGGATTCCCTGTTCTGAATGGATGGCGTATTGCTGAGG
GTCTTTGAAGGATCATTCCCGTTGGGATCGGA
AGATGAACTTGATGGAACACCGGAAGAAGGAACTTCACTTAAGCCATCGATACCCCCAACACCATCCA
CAACACCGCCAGTGCCACCAGCACCACTGTCT
CTGTCAGAATTACCGCCACCAGTGTCGCTGTCACCGGGTACTGGTGGTCGGGATGAAAGCGGTTCCTT
GGATTCTTTTTCCGGTGGTGGTGGTGGCGGCA
CTTCCGATGATATTGTGGGTGCCAATGACTCTGATTGTTTTATTGGTGGCGTTACCGAAGATGGATTG
AAACCCGGACTATGTCTAATTGTAACAGTAGC
TTCTTCTCCGTCAGTCGAGGCTTCAGCGGCAGTGATAGACAATGCAGACCGCAACGGCAACGTAATCT
CAGTCTTCTTACATCCACTCGGCATGTGACAC
CAATTCGTACCCAACAATCCCGACGCCATGCAGCCACCAAATGCGCCGTTGTCGATGTCCCTCGCATA
AACACCGCCAGCACCGCACCACAGCACGCAGA
GGGCACACACCAGCAGCACACGGCCAGTCATCATCGCCATCACTCCTGTCACTTGCGTGTAGCCTTCA
GCACAAACTAACTCCCCGTCAACGCAGAGTCA
AAGCAAAGAGGAGAGACAAGTCACACAGCCACGCACCGTCCCATCCAGCGACGAGAGTCACGCACCAC
CACAACAATTGGGTGGGCACGGTTCAACAACA
ATCAAAATAAAAGAAGAGCGCTCCTCGCCGTGCACCGTTCCTCACGCTCCCACTGGCACTGTTACTGT
TGTTCCCTCTTCTCAGGCCCTCACAGGCACGCC
ACGCTCACTTCAACACGTGCCCCTCCCAGCAAGGGCAGTCCGTCATGCACTCCCGCTGCGCCGCAACG
TCCGCCACCTCCTCCCCAGTCAAACAGCTCCG
TGTACTCCACGTCCCACACAATCGCCGGCAAGCCACGGCCGCGCAAAACACACAGCGCAAGAACGTCG
TGCCGCCTCTCCTCCACAATCATGAGCGCAAC
GCCCAACAAGTACTGGCGCACGTGCTCCATGCGCTGCAAACACATGCCGATCAGGTTGGCCGTCATGC
ACTGCATCTTGCCGTCCTAACTGTGCTTGGAG
CAGGCCTTCCACATCCTCTCAATCCATAGCCTGGTGCTGTGAGAGGCTGCCGAAAGGTGCCACTTGTG
TGGAGGACCTCAATGTGCGGAAACAACGGCCA
CACTTCCACACACATCTCCGCTCCCTCCGCTGTCCCACACACACAAAAGGGCGGTGTCATGGCGAGAG
AGGCCCGCCGCACAGCGCATGCACGAAGGCGC
ACGCAAAAGCAACGCCATCCACGTCGCATCGCGAGATGGAGCCATGCGCACAGCACACACATGGCGGC
AAAAATAGGGACGGGACGGACACAAATCCACG
GCGCTGTCCTTTACTTCTCTCTTCCCCGACGGATTGGCACCCACACTCACAGTGAGGCACGGCGCACA
CCATCCGCTCTTCTCCGAGTGCGCTCAGCAGG
CCGGCGCCCAGTCGCCCTCGTGCCATCGCAATGGGGAACACAAACAATCGTCGTCGCGGACCATCGAC
GCCTCTAGGAGGAAAGAAGGCAGAAGAAATGG
AAAATGTCAGAAGCACAATCAGCAGAGGGACACACCCAACGAAACAA
>scaffold1141.size5978
CCGTTGTTGTTGCCGTTGCGGTGTTTTTATTTCTCCCGACTGGAGTGAGATGCGTTTTGTTTCTCACT
TGTACGCTTCGCTGACTCCCACTGTGGGCGCTG
AGAGCGCGCAGCGGAACGATGGCAGGCTTGCGGCAACTGCCTCGCATAGAAAGGGAGGGCTGTTTGTT
GTGCAACTAGGGGGAGATGGTTGTGGTTCTCT
CCTCCTTCTATTTTATGGTGGCTGTTCTGTCGTTGTGTGTGCGTGTTTCTTGTGTGGATGTCGTTTTG
CGGTGCGTGCAGTGTGCGAGTTGTGCCTGCCG
CGGGAGGTCGCATCATTACCGCGCACGCAACGGCGACGCTGCGACGATGTGTGTGTGTGTGTCTTTTT
GCTGCACCTCTTTTTATTTCTCTTTGCCCTTC
TATTCTCCACTTGGTGTGCAGATCTGCTCACCGAACTCAGCAACTCTACTGCTGCTGCTGCTCGCACG
TGTACGCGAAGAGAGATGGCGATGGTTGTACG
GTGCGTGTGGGAGCGAGGATTCCGGTGTGCACTGGCGCCTATTCGGCGAGAGCAACTGGACTGCGCGT
CGTACTGGTAAGAAGCCGGATGGTCAATATGT
AACGGAAGAGGAGGCTTTCAATGCGATATTCTGCGATTTTGCGGCGAGGAGCTGCTCGGATTACGGCG
GCACGAATAATTCCGTAGGAATGGAGGAATAT

ATGAGTCCTGACCTCACGACGCCCAACACCACCGATGCGTACAGCGATGGGACAGCATCCATGGTCCT
CGAGCACCCGCCCCCGCCGTCAAGGAGCCGAC
AAACAGCAGTGCGTGCAGCAGCGCCACACAGAGCACATCTCCTTTAAACAACGAGAAAGTCAGCGAGA
GCAGAGGAATCTCTTTTGAAGCTGACTTGTCC
TTGCCGACGGTGGACGGCAGCGAAATTATCGCCTCTTGTGGGTGGAGAAACACTGCTGCCTCCGCTTT
TGGGACTGTGTGGGGTTGTGGCAGTCTTCTCA
GCCACGGAGTGTCGATTTTCGACTTCTCACGGAGAATATGAGCTGCGTTCCCCGCAAGTTTTGTGTTT
TTATTTTTTTTGATTTTGCCTAACTGGAATAA
TACGCGCTCTTTTCTTTCTTTACTTAATGGTCGGCTGCTGCACACCGTCATGAGGGAAGTGTTTCCCT
CTTGCTTGCTTCTGTTGCCTGCACTCGGTGCC
GGCCCTGCGGCTGTGCGTTTCACGGACTGCACAAAAGGAGGAGTGGAGAGACAGGCCGGATGCCGCAT
GAAATGTGAGGGTTGTTGTGTGGGCTGGTGGA
ACAGTACGATTTTTTTTCTTGTGAGGGCATTGTACGCATGTATGTATGAACACAGACATGCTGTGCCG
CATGGCGTATGAAATGAAAATGTTGCTGAGGT
GTATTTTTTTCTGGGCGGAGGGACTCCTGTTGGACACTTAACTCCTTTTTTTATTGTTTTGGTATTGC
CTTTTTGCTTCTGATTTGCAGGTCGGTGTGCG
TGCTGCCGCAGGGCTGCTGCCGTTGTTTACTTTGCGCGTTATTGTGTGACTTGTCTCTTCTTTGCTTT
CACGCTGCGTTGACGGGGAGTTGGTTTGTGCT
GAAGGCTGCAGGCAAGTGACAGGAGTGATGGCGATGAAAATGTGACTGGCCGTGTGCTGCTGGTGTGT
GCCCTCTGCGTGCTGTGGTGCGGCTTATGACT
GGCCGTGTGCTGCTGGTGTGTGCCCTCTGCGTGCTGCTGGTGCGGTGTTAGTGTGGTGGCGGCCGATG
TTGCTGGCGGAGGTGACGGCAGTGCGGTTGAG
AGTTTGATCGCGGGACGGCGCGCTCAGCTGCGGAGGGAGTGCGCGGAGGCGTTCGGCAGGAGGACAGG
AGGCGGTGCGAATATTTCTGCCGTGGAGGAAT
GCGTGCGTCGGGGGATGGACGGTGTGCGTGCCGTTGTGGATGGCCGTAGCCGTTGGAGGCGTCAACAC
TCCGCTGTGGTCGCTGCTGTTGATTCTGATGA
CAAAAGTGGCGAGGGCAGCGATGGCACATTGAAGGGATTATCTTCTTCAGACCAGATGCCACAGGCGG
GACAAGGTGGAGAAGAAGGACAGGAACATACA
GGTTCACTGGAAGTGACGAATAAGTTGGAAGGAAACGAGGATTCACAAATAACAGGCGAGCGGAATGT
CAGTTCAGATGCCGATCGTCCCAAACAAACTG
GGAGTCAAGTGCCAGCAGGCGGCCAACCGGAAGATGAGGAAGGCACGAAAAGTCCCCAACCGCCACCA
CCACCACCTCCAGCAGAATCACTACCACAAAT
ACCACGACAACCACCACCAGGAACAAACACATTACAGTCAGAAGAAACACATCCACCAATTACAGCGG
AAAAACACTCAAAGCCACAAGACGGAGGTCAC
TCACACGAAACGAAGATGCCACAAACGAAAGGCTCAACAAAGCACGAGGAAGGAGCATCCTCCTTGCA
GTCACCGGAGAGCGTATCGCATTTGATTCGAG
AGAGTACAACACCATTATCTACCTCCACAGGCAGCAGTGGCGTTGCCGCAAGCACGGTGGAGAACAGT
GGTGCTAACAATCCCACACCTCCTGCCGCCTC
AGTGACACAACATGATTCCGAGGGAGATACTGGCCCCGCTGCCCTGCCAGCCAGCAAAAGTGAATTGC
AATCGGAGGAAATACCCAACGCAGGTATAATT
GCCACGACGTATGATGACGACAACGACAGCAGCCGTGCAGCTCCTACTGCTGAAAGGACTACCGCTGG
CACAAGAAGCACTCAAACCTCTGGTGATGCGA
GTGACCTTGAACGGGCCAATAACGACGACGTCAATGATGACGTGGCACACAACGGTAAGACCGCCGAA
TTTGAAGGCGCCTCGGGAGACAGGAACACTGA
GGCAGATAATGATGAGAGATACACAACAGTACCAGAGACGGCCACCAATAAAACAATCAATACAGAGA
TACCAGCTGACAGCGACGGCATCACCGCGGTG
TCCCACACCACCTCCCCTCTTTTGCTTCTTCTTGTTGCGTGTGCGGCTGCTGCTGCTGCGGTGGCCGC
GTGAGAGTGAGGGAGAGAGAGCCGTGCACCGC
CCGCACACACACTCTTCCTTCTCTCTCTGTGTGTGTGTCCCCTTGCATGGACTCTCGCCCTCACCTCA
CCCCACGAGGCACACACATAATGTGCCCATTG
TATATATGCATGCACACCCATGCAGGCCCGTTGGTGCTCTCTTTTGCACGACACGCACCGCTGCCGTC
CTGCCTGGCTGCATGGGCGGAGCACCGTAACT
GTTTCTGTTTTGTTTCCATTGCATTGAGTGGGCGACCTCTCTCTCTCTCCCTCTGTGTGTGTGTTTTG
CTTTACGGGAGCAGTGAGAGTGTGTGCGCTGG

TCGTCCCACGCTTCTTTTTGTTTTATTTTTGGTTGTGTTGTTGTTGAGTGCACCCATTCCGCCGGCTG
CGTGTGTGTGTGTTTGTGATGGTAAGAGTAAT
CTGCGTGTGTGCTATCCGCTTCTCCCTATCTTTGCTTTGTTGGGACTCTCTTGTGCCGCACAGCTGCT
CTGCAAGTGTACACGCTGACAAGCGCACTAGC
AGATGCACACGCCATGTTGCGCACGCTGTGGGGCGGCGTGAACGCTGAGGACGCACGCACGTACACGA
TTCTTGCTGCGGCTGCCCTTGCGAGGAGGGAT
GACGGATGACCGCGATTTGTTATGTTGCCGTGCTGCGAGCGTGATTTCCTAGGCTTGCTGCCCATGGG
CGGCGTGGCGTTTTGGTGTCTGGGATTATTGC
GGATGGGCTTCAGCCCAGCAGCTGCACGGTTTGACGTGTTCCTCCCGCTGTTCGTACGCCGACGGAGG
ATTCACCGGCTGTGCGGGGCGATGTGCGGGTG
CGTTGGAAGAGACAAGAGTAAAACGACTGGCGGCAGCGCGTGTGTGCCGTGAAGGGCGGCTGTGTTTT
TTTATCCTGCTGGTTTTTCTTTTTGTGAGGGC
GTTGCTCAGTGTGTGCTGACGCGTGACGAATAACGACGTGCCGGGCGTGGCTCCTGCTGCTGCTGGTG
GTGACCGTCGCTGTGTGTTCCCGCTGCATTGT
TCATCCTTCCCGTTTCATTTGTCTGCGTGCGTGAGTTGTTGTTGAGGGTGACTGCGGCAGGGATGACG
ATGATGATGCCACTCACTGTGGACGCTGGCGG
TGTTCACTGTTTCACTCCTTTTTCTTAACTTCTCTCTTACCCACACACAAAGGAAAGAAGTTAATAAC
AAGCGCTAATCGCCACTGTGAAATTGTAAGGC
ACTGAAAGGACCAACAGCACACACCGCCCACACACACACATATTTGTATGCACTCACGTGTTGCTGCA
GTGAAGGCACCCCGCACACACAACCGTCGCCG
CGTGACCGGATCCAGCGGGAGGAGGAGGGAAGGAGGAGAGAGTGAGCCGCAGAGGCCCAACATGTCCC
GGCATCTCCTCTACTCTGCGGTGCTCCTCCTC
GTCGTGATGGTGTGCTGCGGCAGTGGAGAGGCTGCGGGAGTTGCGGGGCAGCCATCTGGTCCTCAGTT
TGAATGGAGGGGCATCAATGAGAGTGATGATG
TAACTGTGGACTCGTTGGGTGCTCCCAGCCTTCTAAAAGTGGGGAATGACGTATTTGCCGTTGCCGAG
GCGCAGTGCAAGAACGGTGGTGTCGGTTTTAC
TGGCGTTGCATCTCAACTTCTCACGACAAAAACTGCTGACACACCGGAGGAATTGTTTAGTGATGCCT
TAAAGAAGACACAAGTACTGGAGGAAGGTGCT
TCCGAAGACCAAAAGAAGAAAGTGGATGTGAGCCGACCAACAACTGCTGTGAAGGGGAATGATATTTA
CATGCTTGTTGGACAGTACGGCAGTAAAGTTG
CCGTTCCTGGACAGGATGATGCGGCTCAATTGGGACTTTTGCTGGTGAAAGGGAGCGTCAGTGGTGGG
GATACAAGTAACAAGAAAATTGACTGGGAAGT
TATAGAAAGTTCCCCGCAAGGACTTTTTGCCACACAGCTCGATTCCTGGACAAGGCTGATTGGAAGCG
GTGGATCGGGTGTTGAGATGAAGGATGAGACT
CTTGTGTTCCCAGTGGAAGGCACGAAGAAGAAGGCGGTGGGGGGCACTGAAGAGGGTGTGAAGACTGT
CTCACTGATCATATACTCCTCCACGGATAATA
AGAATTGGACGCTGTCGAAGGGGATGTCTGACGGTGGCTGCAGTGATCCCTCCGTCGTGGAGTGGGAG
GAGGGAAAACTCATGATGATGACCGCGTGTGG
TGATGGCCCTCGCAGGGTGTACGAGTCCGGTGACATGGGGGAGTCGTGGACGGAGGCCCTCGGGACAC
TCTCGCGTGTGTGGGGCAACAAAAAGGACGGA
CAAGCGACGGCCGTTAGAAGCGGGTTCATCACAGCGACGATTGACGGTGTTGGTAATAAAAATGTGAT
GCTCGTTACTCTGCCGGTGTATGCCGAGGAAG
GCAACGAAAAGGGCAAACTCCATCTTTGGCTGACGGACAATACGCACATTGTTGATATTGGGCCGGTA
TCCGGAGAAGATGACGCTGCCGCCAGCTCCCT
GTTGTACAACAGTGGCGGGAACACTGATGAGAGGAAGGAGGAGTTGATTGCACTGTACGAGAAGAAGA
AGGATGTGAAACCATCACCCGGTATGGTCTCT
GTGCTTCTGACTGAGCAGCTGAAGCGTGTGAAGGAGGTGCTGACGACCTGGAAGGAGGTGGACGGCCG
TGTCTCCCAGCTGTGCACCTCATTAATTGCTG
AGAAGGATCGTGCCTCAACTGACGATGTCTGCGGCGCTGTAAAGATCACGGCTGGGCTGGTTGGCTTT
TTGTCCGGCAACCTCTCCGGTGACACATGGAG
GGACGAGTACCTCGGCGTGAACGCCACAGTAAATAATAAAGGGGCAGCAGCGTCAACATTGACCAGCA
CGGATAGGGGT
>scaffold1142.size11699
GGGGAACGGTGCTTTGAGAGGGTTGCTAACGATGCCGCTTCTTCATTTTTACTACCAACTGGACGGGT

CTATGACGGTGACGCGACGGATGCCGTCGGTAT
CGTCAATGCCTGCGCTGGAGGCGACGTCAGGAGGAGGCGAAGGAGGCGGAGGAAGAGGGGCGCAGCGG
CCCACGGAGCACGACGACATCCGCCACGTGTA
CATCCCGTCAGTCACGGTCACGACGACACCAATCATGAACGAATGGACGTCCGAGGCGGCCTGGTCCT
TCACAGGCAGCATGCATGCCCCTTACTCGTCG
CCGCAACGGACGTGAACGACAACGTTGTCGGTGCACGGAGTGTTGTGAGGCAAACTTTTCAGGAAAAA
TCTCACGTGGCATTTTGGTCAGAGACAGTACA
CGCACATGTTTTTATTTTATTTATTTTTATATTTTATATTATATATATATTTTTTTCTGTTTGCATGT
GGTGTTTACCACAGGCATCTTCTTTTCTTTTC
TTTTTTTTTTCTTTTTTTTTTCCCCTCCTTTCCTTTTGATGCAGTCGGATGTGTGCATGTGTCTTTGT
GTGGGTTTGTTTGTGCCTTCCACGCTATGGAC
ATGATCCTCCCTGTTTGTGTAAGCAGGGAAGCAGTTGTGAGGGGATTTGCAGATGTTGGTGTGAAGCC
AATTACATACATTGCATGAAAAGTACACAATA
TATATATATAAAAACAAAAAGCAAGAAAAACAAGAAAAACATATATATTATTTTTGGCTGCTGCCGTA
GTTTATTTAAAAGTTTCTTTAAAAGCCGCATC
CACATATTTACCACTCAGTGCACGGATGTGTATGTGGTGTGTATTGTTGTTTATTAATCTCGTTTTGG
GCCCTCCTTTTTTTATTTTGTTTTATTTATTT
ATTATTATTTTTTTTTGTTTTTGTTTTTGTTTTGGTTGTGTATTTTATGGTGTCAATTTTGTCATGGA
TGAGCACAGAGAGAGAGAGAGGGAGAAAAGAG
AAAAGAAATACACACAAAAAAAAGAGGGGGGGTGGGGGGGGGGAGAAATGGGAATCGAGAAATTAAAT
ACAAAATAATAAAAATGATTGTTTGGGTCATG
TTTTCTTGTCGCCTTCTTTTTTTTTCTCTCTCTCTCTGTGTGTGTGTGTGTGTTGCTCCTGCAGTCAA
ATCCTCTTTTTGTTGTTGTTTTCCCCACCACC
ACCACCAACAGCTTTTTTTTTTTTTTTTTTTTTTTTTTTGCACAAACACATGTGGGTAAATGTCCATGT
GTATAAAATGTACATAATTTGGGGTGACGTGT
GTGTCTGTGGTGGTGGCAGCAGCGAGTAGTAATAAAAAGAACAACAACCAAAAAGAATAATTGGGTGG
ATTCTGATGTTTTTTTGGGGAAGTTACAACTT
TCAGACGCCGAATCTCGTGGAACACGAGAAGAGGCCCGCGCTGCGATATCCGCTGTATGTGCCCCCCA
CCCTCGCCATGGTGGGCGACGGCGGCAGTCAT
GTCCACAACGTTTATGAAGCGGATGATGCGGAAGGCGAGAGGCTACGTCTTATTGATGCGCGGCGTAT
CGTAGGAAAATTACTGGCGTGCGGACAGAGGC
ATCTGGTGGCGGTGTTGGCGTCTGTGGAACACAGCTACGATGTGGCAGCGGGGGCTGCGATGCCTTCA
CCATTGGTGTCGTGTGCTCAAGACAACATGAA
CTGGTTTGTCTACGGCATGGGGAGCAACCACTGCGGGCAGTTGGGCCACCGGGTGCCGGAGTACACAA
CGACACTTACAAAGCTTCATTTTGAGGAACTT
TTGTCGGCGGGTAACACCGTCACTGGACTCGCCTGTGGCAATAAACATACAATTATTTGTACGTCTGC
GGGCACTGTGTTTGCTGCTGGTGATAATAGCT
TTGATCAACTTGGTGTCACCGCGAAGGTGACTGGTTTTGCGCCGGTGGCGGGCCTTGCACACATCACG
GCAGTGTACGCAGCAGGAAATGCAAGTTTCGC
TTTGAGCAATAAGGGCCAATTGTACTCATGGGGTGAGGCACAGTACGGCCATTTATGCCACGGCGATG
ACGGGGCACGCATGGATGCACGCACGCTTCAA
ACGGTGAAGACGAATGTGAAGATCCCCGCTCTGGTGCAGTGGTTCGTGCGACACCATGTCGTTATTCT
GGAAGTGGCAGCTGCTCGTGGACATGTTGTCT
GTCGCAGCTCGGACGAGGTCTACACCTGCGGTGAGGGCTCCTACGGCAAACTTGGCCTGGGTGGTTTA
GCGTCTTCCTTCATTCCGCAGCGTGTGCGTTT
CCCTGAACGCTCCCACCCTGAGCGGCTCTGTGGCATTGCGGCTGGCGACGACCACACACTTGTCTTGC
GTGAATCGTCTGCGGTGGGTGCGGTGGTGTAT
CACTTTGGTAAGATGGGCAACGGTGATGGGCAGCTCACGCCAATCATCATTGCGGCCCCGGCAACCAT
CAACCGCATCGGCGCAGGTCGCGGCACGATGT
CAACAGCGCTGACAAGCGATGGCCTCCTCTACGTGTGGGGAAAGCATTCCCACAATAAGGTGAGCAAC
GGTACGAACGCTGATGCCAAGCGCAACGACCC
ACAGATGGTAACGGCGTTACGTTCCTTTTTCGTCACAGACGTCATCATTGGTGGGACATTTGTGGTTG
CTCTCGCAAATTTCCAACGCGGATTCATTGAG
GGGACGCAGACGGCACCGGAAATGAATGCAATCAACGAGAGAAACAACTGCCATAGACACACCTGGGA

CGTTGTGGTGCCACAGGACGCACGTGTTCGTG
CACGTGACGCGGAAGTTGCAGATTACACATACGAAACCGCCGTGCAGGCATTTCTTGCGCAGTACTTG
GGACCAAAGCTGGGACCCATGTACGTTGCAGA
AATGCCCGAGGCGCCACCCCTCACTGACCACAACGCCAACGCGTTTGTCCGCATTGGGGCGCACGCGT
TAACGACAGGGCAGAAGGTACGCCTGTGGATG
ACGAACGTGTACGCACTGGGAACCGTCGTGGCAGTCCTCACGTCCCCACAGGCGGAAAGTAACATCGA
CCCCATCGCCATGAGAAACGAGGAGGAAGAGG
CAGGCAAAAGCAAGGAAAACTCGCCTGCCACGGATGTCAAAAGGGAAACAAAAAATAGCACGGAAGCC
CCAGTGGAAAAAAAGTACGGATGCCGACTCCG
CATTGAATGGCAACGAGATGACTGGCACGATGAAGTCATCACACTTTATTCGGATGACGAAACGCTTG
ACACGCACAACCCCAATCGATGGCAGCCGTTG
TGGTTTCTTCCCGACCCCGAAAACAGCGGCGACTTCCTTCTTGACAGGTAACAAGGAGGAAAAAAAAA
AAAAACAACAACCAAAAACAAGANNNNNNNNN
NNNNNNNNNNNNNNNNNNNNNNNNNNNNNNNNNNNNNNNNNNNNNNNNNNNNNNNNNNNNNNNNNNNN
NNNNNNNNNNNNNNNNNNNNNNNNNNNNNNNN
NNNNNNNNNNNNNNNNNNNNNNNNNNNNNNNNNNNNNNNNNNNNNNNNNNNNNNNNNNNNNNNNNNNN
NNNNNNNNNNNNNNNNNNNNNNNNNNNNNNNN
NNNNNNNNNNNNNNNNNNNNNNNNNNNNNNNNNNNNNNNNNNNNNNNNNNNNNNNNNNNNNNNNNNNN
NNNNNNNNNNNNNNNNNNNNNNNNAGAAATAGA
AGAAAAGAAATTCAAAAAAAAGAGAAAAAGAGATTGAAATTATATAAAAAAAAAATATAATAAAACTC
ACAAAACAACACAAAAATTGGGCCTATTGAAG
TCTTTCACGAACAAAAAACTGTTACTAATCTACGCTCATTTTAAAACCAACATTTCTAACTCTCCTGT
TCTTAAAAGAAAACAGTGCAATATTACAGTAC
ACCATCTTAGATAAACCAACAGACGCGGTGCACGAAACTGGAGAGCAAGAGATGTCCACGACCCCGCC
TCGGTGTATATATATATATACGCACATGGCTG
CAGCTTTGGGATTTCTGGAGACGGATCTGGATATGATGATTGACGTAACATCACAGACTTTGACGAGC
CCGTGAGCAAGAAACACTGTCGCCCCAGTCTG
ACCGCGACCCCGACGCCTCCTCCCTCCCTGCCCTGGCGCATGTGCATGGCGATGTGATCGACAACCAG
GCCGATGTAAAGCCGTCGCAGATGGACGTTGA
CACAGCAATGAAAACAGCGTTATTTCACGCCCCGTCTGATCGTCGCACCGACCCTCCCTCTCTTTCAC
CCTCTCCCCTTTGCCTTCATTACGCGATCCTG
TCACCTCGGGGGCGCTCTCTCAGTGGGCGTACGGCCTCGAGACGGCATGCGCGTGGCTGTCGCGGCCT
CGCTGTCCCTTTTGGGGGCGTGCGTTGGACGC
GTTCCGCTGCGTGTGTGTGCACGGGGGTCGGAGAGCAAGGGCGCGCGCGGTGTGACGCTCGGCGCCCT
GTGCGCCTGATGCTCCGCCGCGTGACTATCCC
GTTATGCATATCCTGTGTGCGCTCTAGCTCCATCGGGGCCCTGACGAGTCAGCAACAAACCATGCATT
ACACATTTTGCGCAGAGTGCCCCCTTCCCGTG
AAAGCTCTTTTTTGTAGTGCGCTCGTCGCTAAACCAACAGACGCGGTGCACGAAACTGGAGAGCAAGA
GATGTCCACGACCCCGCCTCGGTGTATATATA
TATATACGCACATGGCTGCAGCTTTGGGATTCTTGGAGGCGGATCTGGATATGATGATTGACGTAACA
TCACAGACTTTGACGAGCCCGTGAGCAAGAAA
CACTGTCGCCCCAGTCTGACCGCGACCCCGACGCCTCCTCCCTCCCTGCCCTGGCGCATGTGCATGGC
GATGTGATCGACAACCAGGCCGATGTAAAGCC
GTCGCAGATGGACGTTGACACAGCAATGAAAACAGCGTTATTTCACGCCCCGTCTGATCGTCGCACCG
ACCCTCCCTCTCTTTCACCCTCTCCCCTTTGC
CTTCATTACGCGATCCTGTCACCTCGGGGGCGCTCTCTCAGTGGGCGTACGGCCTCGAGACGGCATGC
GCGTGGCTGTCGCGGCCTCGCTGTCCCTTTTG
GGGGCGTGCGTTGGACGCGTTCCGCTGCGTGTGTGTGCACGGGGGTCGGAGAGCAAGGGCGCGCGCGG
TGTGACGCTCGGCGCCCTGTGCGCCTGATGCT
CCGCCGCGTGACTATCCCGTTATGCATATCCTGTGTGCGCTCTAGCTCCATCGGGGCCCTGACGAGTC
AGCAACAAACCATGCATTACACATTTTGCGCA
GAGTGCCCCCTTCCCGTGAAAGCTCTTTTTTGTAGTGCGCTCGTCGCTAAACCAACAGANNNNNNNNN
NNNNNNNNNNNNNNNNNNNNNNNNNNNNNNNN
NNNNNNNNNNNNNNNNNNNNNNNNNNNNNNNNNNNNNNNNNNNNNNNNNNNNNNNNNNNNNNNNNNNN

NNNNNNNNNNNNNNNNNNNNNNNNNNNNNNNNNN
NNNNNNNNNNNNNNNNNNNNNNNNNNNNNNNNNNNNNNNNNNNNNNNNNNNNNNNNNNNNNNNNNNNNNNNN
NNNNNNNNNNNNNNNNNNNNNNNNNNNNNNNNNN
NNNNNNNNNNNNNNNNNNNNNNNNNNNNNNNNNNNNNNNNNNNNNNNNNNNNNNNNNNNNNNNNTGTTTTTCT
TTTTCTTGTTTAAAATTTTTTACTGTAGTTGG
ATATGTTTGTTAAAGTATTCTTTGGTGGTGTTGTTGGTGTTTTTTTTTCTAGTTTTAGTTCCTGTTGG
TGTGAAGGACCGTCGCAGAGAGGTATTTCTCC
TGATGGCTTAAGGAATTATACTCTTGAGAGTTTGCCTCCGCCTACCAAGGAGGAATTGGAGTATGTAA
AGACCTGGGGTGTGCCGTGGGTGAGCGATTGG
GATCGTCCTGGCGTGCGTGGCATTCGTGCTGATCGTCATCGTTCGTTTCAGCGGCAGATTATTTTGAT
TCGTCATGGACAGTACCAGAACGAGAGTTCAA
ATGATGACCGTATCCGCACTTTGACGTTGCTTGGGGAGGTGCAGGCTAGACGCACTGGCGAATATTTG
TGGAAGGCGTTCGTGCAAAGCGGAAATAAAAT
[truncated: 416,687 more chars]
