# Supplementary material for: Assembly of highly repetitive genomes using short reads: the genome of discrete typing unit III Trypanosoma cruzi strain 231
Source: Microb Genom. 2018 Feb 14;4(4):e000156. doi: 10.1099/mgen.0.000156 (PMC5989580; doi:10.1099/mgen.0.000156)
Supplement: Supplementary File 4 [file mgen-4-156-s004.pdf]

>TCRU_0 Histone-like transcription factor (CBF/NF-Y) and archaeal histone, putative
MLQDLTRLAAAEAERQRRRTVTYADIARVVHHFDRFSFLSDIIPPVSVVAGGAKAVAVGS
ATSLAAESKTSRSAAREGAVRAQLKSTETGGGGASMRQTTLRF*
>TCRU_1 hypothetical protein, conserved
MDEPLQQWRDQRETGERHRKPTICFLVRFDAAPLEAADGVFRGAQAFRSPSEIVVPLKMA
LEEASLTALQSDFMSRTFRRFPQLSNRTVEAFFVSAEEHFMASSTGLSRPYLLRLDACMN
GDDTLDSFFTPVATNAPIVYEVIAHWAPEKLTDTVSSHITLEGEEEEENGDTLPSDTVLE
DTIPAPVVYPFAHRCTYARLIPIQPETPVTDGSTIDEVKAEAPPILNAMRRKEAARMARR
EMEFVEGTERRKLLEEERSARGTVAELEEESRIQTVTLLRLMYDEVPECGVNYIQSIAPR
QRYPVDTLPLAPFPSSVYMKGLSKCAEETARLSHVFATTAISGSAAAPTKHEDTLVAPVP
ADKLNNEKIQEETQEAIPCSDSGEELVRHYADLRRDLLLWERTTFRALTTEMRRILEKQK
KIDEEIVQKAFLEEVENASILDFDSYVHHYSHEHLLEGRSDTPDTVTNYIPGECKRSSHA
ASLVFRRPTESPKFPAAQKEVETTVGAEVGATPTTLSLPGAIRTPSPSLPRPRSPRSLLA
EEKQFREALERSKARAKAALEAIALVDRHWHEPVAFKLAKHRLLLDEKGSRNAILAEEME
SWENHVKEVKKVEIGWVMKAISARKHLFGPVIGEEEKFRADLDEQQERHFAMLLSLYYLG
MER*
>TCRU_2 asparaginyl-tRNA synthetase, putative
MAFRLIFERRLAPLSNSCRRCCRTMFSGPTPRITSVTRSGLRGYVLWQSPPHGSCICPAL
SSSRAFIHLPERTTMASSEELLESLQRIVGLDEKNSKDLSTKPERVQDLLGFFANQNMTP
ESPREQKMMLFNLWAKVKLPKHRDLLADYVKRKKLDSTQKVDAAIRFVDGMKEDALFDAA
KFEKECGVDVVVSKDDTLRLVKTALAEEDLKALKTSWVKNPNMLLGKMKRVPGLQWADFT
LVKTILEELVPPMIKDVVIDEAEPKTEEKTATPPRTKGAEVAATQKCPRNADWKCVVDLH
SLHRIGDLSSMQEGTKVRVVGWAHRVRHQSRLSFVVLRDGSGYIQAVFNGETEPFHRETS
LAIRGTLKYEPKAATELQPPYELHVDEYAIIGNSDGSIENAITPESSPDKLLDDRHLVLR
GTYGSTTIKVRHELIRAFREYCWSVNMFEVTPPTLVQAECEGGSTLFDVIYYGTTAHLTQ
SSQLYLETCTSSVGDVYCILPSYRAEKSKTRRHLSEYTHLEVEYSICDFELFLSKLEDLI
VGAFNRTIERAGDLIAFMNPEQLVDPKANPCDPNNWKFAPKKPFRRLKYADAIKLCNDNG
ILNPETNAPFQFGDDITDQPERAMVAILGEMVFMTHFPAVMKSFYMARDPEDPTLTESVD
VLAPGIGEIVGGSMRMWDHEELIEAYRRKDLDPSVYYWYTEQRKYGSTPHGGFGLGVERF
LVWLLNLDSVREACLFPRYMGRCKP*
>TCRU_3 Clathrin adaptor complex small chain, putative
MGKTRLAKFYVPCDDAEREKTKREVHALINGRDARMSNYAVCGSLKLVYRRYAGLFFCFG
IEKDDNELFYMEFIHLMVEVLDMFFRDVCELDLVFNFHKVYLIIDEMILAGEVEEVSRAV
IMDRLRTLDITNT*
>TCRU_4 hypothetical protein, conserved
MLSWDRFIFPAGLLLLAHSVYLALCIREQLQEHHHSGGGGSVTSAYFPMSSYPTGVMAHM
NASTLPIFVELLVGVVVAIVGFVKQSTFKRARLIDKNCHQRYDDTIFTGVGFMHFNHRGS
VMGNHSGGAVNGDASVSTSKKKV*
>TCRU_5 hypothetical protein, conserved
MDLIFAEGTRTPSAKPFAIPSEVKSRLRARNPAEREHVGGTEEAGKRKEGEFSGAVAARG
LPGSLVKYIQRCIEYGQNSTHLEEELQAANGLTDSTRRLAALQSALSILCHIAAMDKSYC
FISSRSTAPRKQPLARTSRNRSQTTKVISDPFFSLTDIYTTVRSLKLEYEDVLKQQKSGI
GKQEHMSVNSVAASVSTIEARVDASLALLEFTVLQMLLNFDRARDISVGYVADMLRTMPQ
IFATEKRPLTVLQGDDGKNRERKRFRDGGNVVDDVANAANSIVLKALQALKSKDCLRTLS
RANSGNTVRASDGSLPVSFDGMMTNALDGTYRGRGIYGALHDMKMLLYGLYATAPSEQEE
HIALARLQFEHSLREEYDLFLRKTGLRGEVEDPLLPERTMFDVNPLFADADSTSCTDTQL
KSLKLLDLIREVDNENWFEYPVFDMANIELSSIEKWVRGSIFGMKTNREAFLALRDVLEQ
MVDNCVLSYGPTSPFSQVITLMRQRLVDAAREVGLL*
>TCRU_6 twinfilin, putative
MLTVHFRLDSTLQEAFATFREANAPFRALLVTVEDEVMRLHGKMLESSANMDNDLAKVRE
NITTEKLEAAFIIVKISEGEFALVSFCLDSLKPRVRMLYASGAGHLADVSGLNYILKEHA
MRVEEITPSLFSRENSASRTELMTEKEKLYAAIEKMEIAPMQMTMPGVTMPITEEGVNLL
KELCRGDLNAISFAVDTDKIVVDKVLEASEDLLSSFSLSEFVETVKVLLPEDHPRFVLLR

FPSNETANIIMVYVSPSASKPRERMIYASSKSSFVLQAEQHGINFLRRSEVGSVDELQEV
VADAFGMEQKEGSEE*
>TCRU_7 hypothetical protein, conserved
MYFRRVIHPRRHLLFSAAVLSCMRCVSSGSASAKPLSPVNVDSFTLNEEVVKKDMEALQR
WNELSQMIERSRAEKKNESILAAVEKGLAMLSEMGAVNAPIQCETLLLLEASQAHYNLQQ
FEAALKSAQKAKNMLLETPSAVRDAALLAEVNQLMAYVLLESGKTEEATNAFTEILRWID
VDSKAAMPMQAVAAVNMRRSVVTGIGFCYQKRAEKEAATGGDGKELYGKALDLLIEALNA
HIDENDYDLVKKTLSGVLRCFEGVNDISQAISTGEKYVSWCRRHSDEAGVAQGTAWLKEL
QEKHGKGGDTIL*
>TCRU_8 hypothetical protein, conserved
MDEEDEKCVDLRHAWRTFMVPHSNKKEGPPCSRFDGKYTLLRSYRLVGTDTDDQPLFFLS
LISPNRELISTTAHLFVQFNGQFRDCVAENENRMMAMRALHERQLADLVEAENRDTRDMV
VGSELTRNFVLKHQRELEELQRECREREISVELEVRQSLLAFLKTSAPDALTALAREAGR
EKLRADTAQLTRITDMAHFVATPVPVRAVELPNPLCHEKVGAHSRDVSLRPVVVDVSPTT
SLQACLTSLYSAERETAPAVAEHLLRLSQQTHAVLLLIGSQAEAESLLSTPAAELFLKNS
FYSGGSPHFVPLKRHCHLKMMLSTRFWGANIIFLWTPPKQEGHPSGWPLVVEDALQLAFS
WNADMFSVAVMKSSSYWQGTSYRSSIEQAQDVSMAVLRQLQRGVARLAHEAAGVKLSTTW
GMPEAPDTHTLPQPQRWEDGYVVKSPASVGKPQVKFNTHIAIRAFFPFSVSQTEEEEGLS
LRDSAPVINSFSSATVAADNSTDRGRRTGRRRRSMSTASLSSGGSFSSELAVSHKRSSRL
QQPMSFKKLLVYTFGNAVVVH*
>TCRU_9 sarcoplasmic reticulum glycoprotein, putative
MSSKQIASPQVNLTPRSADESWDKHIDAVLSELRRIYFERVRPVETKFQYDVFRPSWFAE
SIVQKRPFVTFLGPFSSGKSTFINYLLQTNYLLTGPQPLTDKFTVIMHGDEFQQIPGRVL
MADSSQPFRGLSQFGETFAEVFQGLIVPHPLLQSVSLIDSPGVLEAAGDIHSRRYDYVKV
CRWFVEKSDLVFFLFDPTKLDAGTELRMVFKHALRGHESKIRIVLNKADSVGPQELLRVY
GALFWNLSGLISTTEPPRVYISSFWDQPYQKDTDHCLFTDEKSDLLYDLTELVPLQSLDQ
RVSSVMLRAKHVLIFALVCASFKSRMPWLFGKEKARAEFFAQLPQISEDLANTFRLGLTD
FPTREEMSDFLSRVQTDDFYGMDHLRKKGWPQLLKRTIEEDLPALLNPIKHTEVVDPRDR
KHAIMMQRNYIKQMSNQLAGQRGIQGGLGQQQAPGHRVQTMPNAASLNTGLNFPTQQTQQ
TFLSQMPADQMSMMMQMMQNMMLQSQQQQPQLLQLEQAPTQQLTPDQMSMMMQMMQNMAS
NNSS*
>TCRU_10 RNA helicase Prp43
MARLEGVNNANTVSVESEINPLTNKPFSKQYYEIRALRATLPVYEKAELLKRYVRDHQVV
LLVGETGSGKTTQVPQFIAEMGYAGIIACTQPRRIAATSVAARVATEMDVKLGEEVGYHV
RFQSMMGEKTRVLYMTDGILLREAFSDHNLSRFSVVIVDEAHERTIDTDVLLGTLRLLME
RRSEFRLIVMSATLEMQKFQAYFSQAPLLQVTGRKYEVQVKYTTQPVKDYVETSVQRVYD
IHMNEPPGDVLCFLTGEAEIEKAVTWTKMKLDALLVRDSEATHDSRQGPMWVQVLPLYGS
LSMEEQKRVFASPGPHTRKVIFATNIAETSLTIDGIVYVVDCGYHKQSLYNSEVRVDYLL
PALISKASAEQRKGRAGRTRPGKCFRLFSPQDFATFPDQTHPEVLRSSLVNTVLLLLKLE
VENPYQFSFIDPPSQQSIMDAYYQLSFFGAVDEDLHLTEFGRLMAEFPVDACLARMLIRS
AEHRCGADAAVIAAMLEAGNVYVRPSGRGNEADQQHTTFQHPDGDHLTLFNVFHAFWRNN
QSPQYCFDHFLRYHALLQATRVYAQLTKLMGKKSIPVVSTYDDKTRLFDSVALRKTVLEG
FFTQVAYKPPSAEHYKTVKDSQWVVLHNQSVLSKKKRPPWIVYDRLEVHGKEGTFIRFAS
VVEPEWLLVVSDFFTDPAELSDGEICHILQRLKNNDELNLSSAHP*
>TCRU_11 Kelch motif/Galactose oxidase, central domain containing
protein, putative
MELQQNGDGKIELEVVRKDGRPIVCRPIDVNELLSKQGHTIDLGDGVRVRLEEYSSDTFS
SSEEGTEETDSEASASDSRTSDRTNVPPGRRRLTGDGAAFDLHGGEEKAKTQKLSFHDPE
RNKGMATQHGTISGSNVSEKVLHSRRDIWASFAPPIDPERNHNWLQSTSIIPGATSRRFR
LPPLMLHATSSYLGPSDQGQRILVYGGITNFGKKVEKELYEFSVLTGHWRRVEGKHFFFP
GNYGHAAVVVERLQRLVVIGGMSPGGQPVQGERYSDEFSKLRSRILFPCQDKTKTKRPPS
VRMEFKESEKPTLWSLAMRAEEEPPIGFMPVLFEMNLKTLRWRVVQTSPEIPVALHTAVA
TSNTIYLFGGVTNRLLVSAQLIGIHAENNSISLIRSSAVSPKARYLHSAAIYGNWMIVYG
GFDAYNEPLADVWAFDLVNERWEQLECHGTPARGAHASCILGSSLIVIGGFEGAITTETK

PTSSIYELQLVPTSTGQYLWKKITVRPAIPPLAFSAACHCADDVSFIVYGGCTVTGIKAK
KRPPDSKRVGQLAFTKSRDARNISRHDSDGNVYNDNFWQGLVPLDDGFAFTFPLKKNDAT
GSHGDGSGASIHVNELGVVTDPEELTPEFKAFVRRQEEFILKKNASRSQAMKRTILDELE
NMEPNLYLTSEEIELLLEKSNLLCESLTGYNMEAMPHNIPEREQRLQHTEKCISLSRQIR
DVLMSMKGYAPGVTAVKSKTHRIKSGEKFEDHSVAKPFRRVIVMGLVREIKTELKRIHNL
NIVLKTVEWPEKKEYLDAVETMQERKTEFVQAVKDILEKYIERRVESLISAADRQKDNMR
RLAEIVAKIRHEKIFQTEGPDPQRERVRQMWSKGEGQETPQTDTKRSVSRSHHHHRRSRS
TGAGYYKDEAKAFISLQPKELQKLLKKIHLVHKVATSFSSYCQSKKSDVQKSSSEMPPPP
SPSTAPPALSNPESSSLAVPAALAIVETVPPSNVSPATHTADADFGTASPSELVIRQSDA
IREEAIEIATSVGQSVCAFAKELETSFEADTKPNSQTDVNKDEKKPTLLFLSSLRPLLSC
KMQLAQLREKISRIRVNRWAVDDVAGAPQDVEAQKRCEKLSKCLAALVQRLTASFLSKAG
ARPPRLTFRRAHSSGALKPPSSSLASPPRSSPSLFSSSSSPATDKHRRVLQAISTVDSSD
VAALEKGDDSSPQAQEETAEQEPEKQSTCVFGPLSTPLMLLDTPPLLKAAAMPEIPRTIC
LTSNEAQSLVPPFCTTSSICNEGAIIPSVRDTKVISNTHQEASTWSNKDSSIMVPHKEFF
TAVTCKDSSQSPPPRIFVSKGPQTTTAAAAMAIPAELPPFGESLSAIHIRSNTDGDKNTS
SCMVSLSPGCFHGSSTSGVAVDPINRVDPQQEALCLEEDYFLFGRRYAAEPVASPCVVED
ARNRQVLVAKDSPLVVRSMHAVKVSPPVGTPAAPTVTAPRKRVVNSIFRKHFTSASQPGA
PEDVSAKPLKKGLFSGKLTPGEMHIIQARERLRTSSSRR*
>TCRU_12 Glycosyl hydrolase family 65 central catalytic domain/
Haloacid dehalogenase-like hydrolase, putative
MPFWRRSPLAREYEVTKRPIFLLDGWNIVQGTDAAAYVSGSVTDGVMYGTNGYLGVRHRS
ECETVGFESNVFVNGVYHNVPVDRQIDSEALPYEMQMGATVCTVNLDCLIDGDCGTLRSV
PPRRLDLRNATYHSECRDVMSGDGGLMFDGDYRRVVSMKELDLWGADIVYSNFTFGSSPY
DLYLGESPNIFYANNMNSTNPDASPSYLLEFVVTITVDLREWAILRGDSVDDILLQSRTS
GVVESIVTQSTCAVDYENSSVKITPEHQLLCETGDHGLMEQNFSAAALSCPSDSESDPGF
GGRIISSFENQKKKTLSFKKRFILSITPDTLPTRVTVTYLARHHSGGIPSARLFPTISEI
MEDQRLILNEFWDHFFIDLQQQEELTPDRLRLSLLFNAFRLFCVSHNLHNGLPRGGCSAT
KTSLLYDLHQYLYHGIFYTLTSPPRALALLRGLYSLLPQARVNAHLISLKHGAIFPRSTI
KGTECEQHNTVSQARLHVNAEIGYIINFFFAAVETVDQKDRLWLLELMLETARVWPQLGE
WVESEGVFRLDNIAGPDEYNSTASGNFYIHLSAKMHMRYVLDLYEQQLALLGEEAMTNLL
KQIDMDKSELENFRATSDGIVIRRNDHLGVYLVHDYFHTLKEWKGERPKHPLSMNYHPLA
IFSHMLVDIPEVLLGMLLYQEEFEKKDLVRNLAHYEALCTYDSPESLAIVAAVDFQSNGS
FAHGLPLLRSLMSLDVDNVIYTADEGLHFGVMSSSWIAIVSGIGRLKLGKRTLYLDPCFP
AGLSIVKFTICWRGSTLSTTVDKDYVTYELIAGDSIRFVHGDGHRIHLHTGFHRYTAKRQ
VSVPRLIRSGEGEFDGAVFLCETLFDEITDIHYMAWYKTLESLFENYRALHLREIQPLTT
EEFFEKVIYQAEEREIAFSGIHNVLLDRGIDLELGTPDDAEIVETRYGLANAKVAEMAEI
LSRMTPRVNAGMHALLKDLSNSGIALAVVTYSRSLKTLMHYNPEVMPLFLAHIDGEEAYD
RHIKSRPHVDIFLRAAEKIHVNPARCVVFSMYLDRGFKASSLANFRMFFDVEGIFATKRM
SQQTQPYPTLSNDAAAAVGRDGVPLILRLSRENLPTTIDALEDMLYGRCNAVDEQHVASY
TK*
>TCRU_13 hypothetical protein, conserved
MSVTDTLGPLFTFLIIINVIGTALPLLRHRFVLYPLAFVLFGFAGLFLILAVIFLLGDAT
VSVYNVNAKGQVKVQANVPFDLPAFFRLNLTMTTWMLSSVLAMVTWLCYQSRKGWHVYIV
PMLQDLNTLETSCWSNTLDLRNRVAPNTM*
>TCRU_14 Endonuclease/Exonuclease/phosphatase family, putative
MARQPNTVIVLHPVNSAIITLDICLQLDDSLSSIWRKNMARSEDEVAMRTIERLQILLVQ
QKEGKNSRRMKREDLLKDSQPIHVFGLEIDEHEAEGQATAPTSNTLKPLDTSITNKLFWK
RAKEMLIGSTVVNIKYNVPTITSIVPPSALYVGLPALCCGITTLFTSEVGILYEWCLCST
DSREHTNISVISTDAVFTPLEEHLGKTLVLRVSPEGDSGLWTQVDLPPVRAALTPVDRWK
HTLAPAEAPAFRVVTYNVLHDEFCSTSAAKRRIYPFATDDILSLEYRQVRILQELLAYRA
DVICLQECGEKVYRQFFERILHHSGYDGRYTNKNGGVKEGCACFWKRTRFCMNETLVFPL
NWTTLQEDHPDLVARVSLYPEFREALEKVTSIGALVLLKDLQTKEELIVGNTHLFYHANA
CHIRLLQVYMLLYKLKIFAVSQPSVVLCGDFNFTPTTGGYRLVTRGQTEAEHHSWKKGEL
FYWGCDRMLGVGTEEMEGVEEAAEEGASSSVPVTQPDKRQPPFGAFRETLSAPLQLRDAY

SETGQELPWTNYAMTFREVIDYIFFSPTRLSVLRTVPIPPESELSENVALPNKQYPSDHL
ALIADLVYTSSSSSSSSSC*
>TCRU_15 SET domain containing protein, putative
MLSITRRRADFGKELYGKLSTLPGDFYEACRSHFGITLHQDTFIGVRSSQVRGLFLSTSS
TPLEANKPIATIPLTSLYTVSNIQSKPNTLRHVTLDGVRNAVREEEFKMMSPQFYLGLQF
SAIISSIPDITHARDAEEVERITQILRGGAMPWARLIDDEDFNEQFIFGMYGMALDMWQR
KSYEEMTKLFHHNITAIHEKFQPPFSVDTFRRIARLVLARVEHMPPPDYYDGSAVWRRVR
HYMRQWIKKPDPSEVCMVPLLDLVNHSNRPNCGLRVGPSSVVGGKGAITLYSIARINPGQ
EICRHYNFAINRPNALFRYGFLPFDLISIVEHDAIDEYLVKNQHMLREESEEVRMKQQKE
REEIQKLEKIFQHARSGR*
>TCRU_16 hypothetical protein, conserved
MQLLDVQPYETALQRLLRAGVCSEDQLHDYHLSQAFRSLAYAVNRASLSVAALLPSHGPP
ISNEAPLSPPSSGLTSSSTTNADATGSAVVLCRMCTCALASQASLRTLIYVSTRLLCDEA
SAFGEGNERPCCTTAWVEALCSPGVRRSFLHKQMGVHRRLTLGYLLLLSGTVRAVTHGCA
KGKRLHSHFVQFAQQLLADVVPYVALIADTREATTLADAAFVDGDDASLRDGTKKELLVP
SLMTVEANNTLLHLFHGIALDVLEVHQHDEKQTTTSRRAVLDRDAPSRIWSVVSILQRAL
LRALEWVQSEIIWEMSSINSADPSTKTQAVNETFAVVRIALEDGFAQLIKKQETLECSRR
AVEKQFGQNSKDYHAISGMMWTNTLRLFFFCRSLSNAWTYIYGGLGEMGVQHTDEQILAL
IASFVSSVPSFEGEEKEHDVFLATTMLLSGALAAVAAAPAPSMQAALWFSELNLTQQIHN
TIVQRGLRYRDENVQKLSALLSRLILYSSSNNSAFRIIADSPDQLLELLSNGRDGASQCV
IGVMIRAAVERPHFILPSLFRLLQHGDTNTRRNVLDVLSALPKASDDSAYGGAIAPERMR
PVLRLLSEELLLRIQDEELQIRLQSSKLFAKVWPEDVCRSLLKLATQRDRSVKRKSSAQQ
ALRLVVEAHTSDGSVVLLLIKESLTLLGENVTSICATPSTPADILAFATLHCGTLQDTVE
SQQVTNSSTEEEGNINERIMRLVALLGRHWAEGVINWTKACILPLLQRAMEARAPLEQEL
LVRVMSQIAYVCGATNEGLVALSLSCLHIMKPTEEGTSNSTVWLENLTGPSTALSSDASI
YQIIAPLLFLRGCPRRIFATFVTVTQMPPPVAEIWEFLWSVLLNPNYREELSLDAQRLIL
ELVCKYNASVMMEQLRHLGSRYMKEGFFSLVDGSIAFFICRVGFFCIGFLLSQNQALFCP
KDMKSNLGLNEHEAEEREMVGMVATAEKEREEEMEFTELVSGAQMVGRWAEDVVLPWLLS
EDDAGICDVPFPVDFQRLSRSAVECLAIVMIAALNRQAFSDTVTPFVMLPLTQLAQHCRE
RVNFWKENCSATDALVEWSPSPHLLGRFEFAIHIHQRALNLLRAHPSGGTTIGLYFKHYM
PPLIELANAICTAVLRLGKKDIAQVAVESCHALFLAVMASTGASPGLSGGETPKGRAEAS
KSVLMFLDVTDRNALVTFAVGSARYSDLPQVQSEGVKLLSALLGAAPEIFVTEANMDEAP
LGEALTVLGSVALMHPDAKTRVIAEQVLGILQAN*
>TCRU_17 hypothetical protein, conserved
MGEAFELLRFAALSSAVHADAVECLVDDTRSVLSRIAMDTERLEALEEQLAGLLEEQRRI
HAVRDVLSRSRSTRNTTEPTKRGKHPTEIRSKLDTRSPGASPPGTSRLGTENVLRFASRA
VPEEDVPTMSRAPRREHVATVEILTDDLREEKATEGEKCIDEMVERVKSEIRSLESMSVK
TKLRELEALKRRIKGLDANAIIERYVEPFRFGDEVERLWDAPDAILDNTNMDLFISRACQ
RFSGRYEDALGAISDFSSYEDGDAAQVFGSLMYLEALTSSSNYDAAAVRTLQGLSKESLL
EYYREKRRAATVSTTVRRSRVETVLGPEEFGFLPEDWWSSRALFIPAGIQLPPARHILTA
RFSFADAHELNTLQSVRVDFQRSVVDAFIRMPKALAELREQIEFQSKHDEERDVSASLLR
LLVENEKGGGGDIWTTIARCS*
>TCRU_18 eukaryotic translation initiation factor 2-alpha kinase,
putative
MPDASIRIGLLILCILTLGLSFGVGSTTLLSVSGDQAHAQDANLLKNVETESAGDEISAS
RPDNKELVPSYRLHPSNRLLLLHKDGKISSYGLDNGVPMWTVDTGGDMLRVEIEKHPTER
VIRQDPLALPFLLEGGMLFTRRCVAPFGCKKQMPERCFDRKGIEVETRPEARPGEAKTRS
GSGDSCEIPFSSTHPRCFMNLSTLIEKKHVQVGETDIFVTTSAHIMDVDSNDGKPVPETN
SLFSYLHVVRYDFTVHVRRPGEYSWHMSIAQYKLSEKSPLQSSEENLSDHDPLFVSRVLQ
SLLQKGDCGDNEYVPSTNEENTVDKVNIEPVVEEGSHNDIRNKQLMIREVDRNTYVLWSK
LKHAPVWSSVVNPLSTVMSAFVWFSDRGKVHSLPVYRLETPKSIADTVEILEPENTDSLY
LPSSLNSDQHRSKMSFNLHGFPNTWEFDIVADELELRELQNLYQQCSWIHGCDGSSGNIP
NHSPNQRNPGMSSQMSLVSRFPLSHTDTGNTLLLLFNVSCYVVSFLSAFLAVLFRWYLWN
TTGVGPDAFVDRISNSSRPGSIKMNPLDPKISASQSVKSIDSAYKELKASHATEGGRSFD

SITPLAEAEMLPPSTVNVVGTKSMHHVDSWWRYSGDDEFSISDKPSSECRIQPNSTVSSQ
LFQQQFQKPEKIGSGAEGSVFRVKHQFTGVSYAVKAIRIPDDNEIYIQEAVLHSTFDSVN
VVRFFNAWIERVPRNFAETLGILHRDDTMDNISAETLESFHETPFTESASSDACYTVLFI
QTEWFERGTLADHFVRRKGFTRLENLKHLLQISEGLQYLHSQCVVHCDLKPRNIFMSDSG
IMKIGDFGLSRKNRKRPHKLRGDADGFTSNSEAGDEGHAIAAFTPLYCSPEQKRGDAATT
ASDIYSLGLIALEFYCVFTTQHERFCTLGEARQGVFPKEFADTYPVERALFQKMLSEDES
CRPLMKDIVKALRQGIKEEEEVMDGLIMRSKTLSPIDEYESSNPASPLEKFKEKGKLTRV
APKFTRLSLSSLESTEFTQNT*
>TCRU_19 hypothetical protein
MHSGGVQKAAAGAPQLPPTAAFWEEMEMQKKESQELQAKIEAFCSLFCPEQMHLAPFLSV
EFCGREEELEGILSAMYGHSLSKMPRAKGDEEKEKGAHSTGGAVTDSDNDFMAQAVALSS
FFGLGRHFVPAVYAQTKRTRNRRRMSWGAWLPKDEEELGAVEGVGKEEERHIKQTVLFEV
IAALFPDFLCTVLEEGDQRTAFVAKRLAAFLSFHEPESDVKKYLVSHGGRTYLGRSFRNV
FMKHYFSFLKECSGTNLSLDDLDEVGSLSSSSVVTIESAGGGEDTTSRRRKTKAVLREWT
TAYGLRAHSTPMPSRSCPELHNVAVDTEELSAMERMRAIKLRLSRTEMGTMTQSQPPAFG
FDSTKPATRRFYGTGTPFWGGAHNEIHAAAARWKQKWLQNPEEHILPNPSIVLETNSCQK
CLFLEAQLAETKAAFQRASRKFVANEEQQYYNEASCYVKQKEILYPFDLSEANGSSSKKI
MPQHFVQPSHGCLMCQDLRQRIKCLEQRLRESYDNCRELIVKLRRSQLPLPPHMCVISSS
VNEAKKRDVSTMTELTGQQLDECIGYMILESL*
>TCRU_20 multidrug resistance-associated protein, putative
MSRKEQESVTTTVYAVRLVHAVVLWLAVVFFGISVVFLKRRLPRATPISFYFCCLLGIGG
VFVSALYQLAGPRSARGGGGGIFWVTWAGLVCWLLLLMRSVRHANLPDVLFVFLLLALVG
MECTGIVLQFISTRSRIADILMDAFIAVPHMLVFVTLVGWDWLMVNDIALVFRENTPIEG
GRYEGNNKDDDDDDDGGLGHVHDKNPHAFFLLPPSQDIFIQSSAASSLFGESLRFDGHTS
IIRTIVRHWGPTYFLLALVRLLYDFGGLLPAYFLRQLVDNLMQAKTSGKKGKDGGADIHM
SIISVLLIVIITLTCTFLRVHYNMKLQKMSLYNRNLLTIELFHSAVRCRRHQLGNQREGE
IMNYLSVDVQRVADAVQTINDLWALPLQLAFALYLLYVQVSFAFVAGVVVTIILIPVNMQ
LAKRIKHVQTSLMRENDERVLTITEIMNNILCVKMCGLSHIVQRWVEIPRSRYMKHLCWL
KFLDAFCVFFWAVTPTLVSLLTFITFIWMGGKLTPGKAVTALALFGSLILPLNAYPWVIN
GFVEAYVSWQRLKPFFSSSKDQGCFFDGTTSVIPGADDEVVLCDVRENIINTNLLEEEQK
SLHMPGENQPLLQATNQLMCGVHPVEQHQLNSPKQRQEISSDVLVDIQQGDLILAASSGD
DVLEQLFTLRISRFQALRGQLIAVVGHSGSGKSAFLSSLAGELIMTPTFSLTVARSSMAF
VEQAPFLMTGTIRENILFGLAFDSARYESVIRAVALDEDLRHNFAPDLDRTFVGDRGRKL
SGGQKVRVALARALYANKDLYLVDDFMGCLDTTVAHHIVQEVFVTAAQQGKCVIVVTHNE
ELIHYADAVYECAGGWLAITERSKATSASSPTWMPREMDHKGQTEEGIGKKTEIDSIEDA
KPSFRALEVSEHGVIAWSTMNCYLRRVGWGLTVFIVISVAAMQVARNASDQYVVVWSKDG
DGDTAAFIHVLSALAVINSLLAFIRGFSFAIGGLRAAHRIHNELLHHVMSATFHFFSNTP
PGRIINRLSSDIYTIDDSLPFIINILLAQTFLLMGSMMIIILNSTGIILLTLIPLSLLYY
RVQLPYRIVSRELRRLEAAARAPVLDTMREVLLGGVVIRTFGVSVARFYMHRVNEQVGVL
LRTQYNALMLNSWFALRLELVGILLLILVGVLAIYYHGNSHAPMLGLALAYVQPLTSYVN
GVIGAFSSTERDLISVERVLQYFYLEREDVMDRDVPLLFFDSWPTRGKIDFVGVSMQYDD
SGPKVLQRLTFHVEAGEKLAILGRTGAGKSSIFLALLRLVKLTEGYIAIDDIDICQLPLD
VIRTRLNVIPQNPVIFHGTLRKNVDPLGLHSDDEIRAALACVNLADLSLDSNIVDGGNVC
GGKQHQIAVARVLLKRSSLLLLDEPTSQLNSEAESLLWHVLGTHLKETTLLCITHKLSHI
DFFDRVIVIDKGRVVNSGSPSILRRDGAWPFA*
>TCRU_21 NAD dependent deacetylase, putative
MKPRRQMVTILTGAGISAESGLSTFRDKNGLWENHRVEEVACQYAFFRNPLLVQRFYNER
RRALFSDCVAPNPAHLALAKLQREYRGGRVVLVTQNVDNLHERAGSVSVLHMHGELLKVR
CTATEEVFDWTQDVVHGTSRCKCCNAVDTLRPHIVWFGEMPLHMDEINAAMDETDLFVAV
GTSGNVYPAAGFVTRAREKGARTLELNLDSGTNFSEFDESIYGKASVIVPAWVDRLLQDA
V*
>TCRU_22 ubiquitin, putative
MLVKVKTVSNKVIEVASLNEENTIAELKEKLEESEGIPGKMIRLVYQGKQLEDGKRIKEY
PIAAGATLHMVVALRAGT*

>TCRU_23 hypothetical protein, conserved
MSFWPFRTGNDDRFQVSSKEETIYDAIKGEMPPHRYETEVSNYLEWRWRRGLIQSVFPVT
CYSAVLGLSLGFRQARLEGRYIGRYRVLWRYSSTFAAVGLLTTAFHHLLVVRNKYNDRMH
YPMIAGASGAVVLTMASQMGTISQGMLAGSFIGVLYTAGCYGMTYYHKRRLKIFLSQQQL
QQVPIHRVSPELQPMYRAYLYDNRPLEEEDFLKRQAVVLSLGEEDVGLDAKRIVSNMVPE
IVDWVNFPDWWPLKFPTQTEEERMILERKRHEEVERRKRLILESEDGALLKRVNRAKNYR
DA*
>TCRU_24 cardiolipin synthetase, putative
MPTSGCKFRVALQRLNIERLLPPPLREKRGSNTVGRINNNSGGVELRESVNKDFKDVCSA
VGNGARGVYCNGLSFHVRWDRIQRNLAVLGRVSNGNTVVPYRDSAEAFRVMWKAVDEAKR
SVLWQTYICKDDLIGQMTVEKIAAAQRRGCQSELLYDCGGNITGRRRLIDKLKQSGANVI
EHRPVLHHVLPYLFGGMKWERSPAIRNHRKILIVDDKIGFCGGLNIGNEYCGRSQGGTGR
FRDTHCSVVGPAVAHLREAYEDTKAPKPWQWSWTRWRKIASTTMARRYKQGRNATDDLLG
PMTKSVQQRGKLYLQQQMERAEEKRVRFMERIRWKRQRERLELFMRWYPKKAAKLLPQNL
VHVDVEDGTKFPTERAVDTGASTVMKQRMEHYRMRALNRAKAALERRLAERPLSKAPLSD
ADPVPGAEEYGKIRSNATQIICCNPHTRDWSIPYAFWQVSQKAHRRLWVTTPYYLPHRKL
LRAILYAARRGVDVRILAGSNCTTDPWFMWHASNYITERLLAAGVRIYEYKGDQIMHAKT
VVVDSIWCSVGSYNWDMMSNKNMEVCLCHLGYEMAREMEGHFLKDLQTSIEVKYEEYQRR
SFWLRFTSWFFYVGLQIAEKLTFRTFWDADLSSKVDDLD*
>TCRU_25 hypothetical protein, conserved
MQEQDLLISIRDVFVGRLGLQIKQNAAFCSNNARTLRLISSANHIDVNVTVDRTEAVEAS
AVLQELLNDSKSAHPVIIFMKVILKQFNVHSSQLTPYVLTLMVVAFSRFCSYMSPLFSFN
YYPHTRETVEPGYLLFSFLSFFSPLPRGQFDPTQMCLVPIHPHGVVYETNEGDHLSQLGK
NVGGIYWRVMEPTRRGDNAAASCVLIDKCRDLFERILVVLLHHYTGVVFTCPVFVDDAHA
QILGAEEVPKTEKIPIDNHLISLLLRDWWLKKQSEFF*
>TCRU_26 hypothetical protein, conserved
MWKIRVWSAFSHRTLTLALQRLDVVSAASAHCFYPAWHVARRECTTGSEGHPLQSLKDAD
APPQTTTTTTKISTTASTKSNDIDWDDNTVVEEIRSILTLELLPRKGSCDEGGDNTVPLY
ALFRALGPTLRSQLQNSSFGALSKAVKSRPDWFIISADGARVGLTDAAYSCRATNHDAIE
SNKKGIDGLRQDKSCRASTAASGNVMQTPCLEKRAITRSKSYVLVRGCFGSTPLDVLKPP
LTSAEIEEWKLTRILLPVTLYFLMDKEPITLFPYGEKPRRLSPDLEEAESLYVSKGLFPP
SEVAAAFVHITPTFFVETRHVLAAMSPDVAQCFESHISKDRVVDLFFKKYPMIFKIKTGK
FQKTAVKLNLDFSFIQNYPGCGRADRLLRRHNQEYRATVGGVVSRQITSPSREQEKQGRS
GESHVDVKIFEILLRNLPRFPSDKSLSGAELEERRCAPIPLVKWIDGFPQEDLDVLNSVP
QRRVLTLLTRYSHIFQLVCRGEDSNLFAEHMYLNSSKNSNTPRRAQQDDGSKNSFVRPLD
STSVCELNEIPHADESTVEEEKADKLEADTPLPPETEMKRREANIAAALRDDLIGVDDIL
SSDAESVPTLTDEGEDTELGDKLNTDEDEVYSAEGEEGGEFFDANEVKEEEEDVDGGESQ
RNLEERGGDGVKKDPPLTDMSYADDESDGPYIPSQYDIIYVRRLPRRIAPRSLSDYNAAN
SPEPELLQHIASFLNPPPSLREAKQQIMQSNRKARKVPLFTSGSAVGPWRWVPIQRIYAS
LSKEQKRLLRPFKGLVHFMRLHGEIFEISMDFLHVIAHDPEGQIAPFVPTQLVFHSEDRV
LLPQKFDDDEKSKASLIADLERNKFKSILGASQIPTDRPQLLLLDPLNPVLNHEILCEEV
SLFMPDHPVPLHQLINRLPPILKAALSLRHKNNFKTSKHLKVWNEGNRTMLQKSQLAVPE
SALMGEETISVEDAIESVREVIPDEGVMIGHLHRMLPYAAKKTLNTHFGNVYNAILGYPQ
YFYIDKVEENRSNSTVFLVERLQEEIQC*
>TCRU_27 hypothetical protein
MDEVLDEEVGVPYLTPGQVPLQAAAVLPFLTPPAGAPTFPRLYAASLGEEYIDTAECVEY
ASSNDAGFMVKSCFVLVGYGGVLCGCLADSLEPNEPTAQRSFTELLEAKINETRNTDYSL
DTITFIPFFPDDTLDPALGSLLGIFSVVEDPTWCLFVSHAKREENGSDCILHVKILRIPI
VEEADRLHRALNSLVLHHCGGPNGNSICRSLMSVEELFATARKYSDCVVAGPAWDRLFSL
RLTNEEIFCIAKKMILSQPTFLMDVNGTKLEEIPPLAVAVRIEAFTNVPKSEGESGGVEK
AVALEEKGTAAQQREVLRELASVRAQYRAVLREGNDLMACIDEEEHRWNKIEMHREEEQR
KRKEHPAAPPSEHDEHIRKNVCALETLQQKREKEEGEEESAQATARAFVAEKKRKKTGKG
SRGGGGSDKKFASTSNFTSKEAEIRNVRAHNTALRRLLEKLAHELKEEEQTMENLSIDLR
GFMSDINKLEKAKLLLTVEHSMLMNTTARLQQSLRDQKEKLRQEQEAAAAEQREISRRLL

VQKERTKKSLHVKNGTTELLHRLKTNLSK*
>TCRU_28 hypothetical protein, conserved
MKRDLNPNAGEFHPTGGRLTPKPSLNEGGSRSSSLQGGSAPAAASSSQQNNANPNDRTPF
QVSEGLVEACYFVPATRFLMDCRAAFYEHPETTKCKHMEFERLLRRCFFGAHEANELSIS
LASASGQNRPFWVSYSAAYFRMAHDVVQEVELGTPCPSTQLLPPAAAGASLAMPMIPPNL
TSFTNNSTEEERQRESLREVYLSRLRPVVPYSIRKQLAFRAKRPGVDLQEDVTRAMTYAL
DIFFFFTRLQFLRFLDASSGLSYSACVQFLTRLRERLMKLEVADSFLSVALVLMLVSTAH
ITFAPRAVEVALNETNSSKLKAQWLFCRYFSALVVAFLSGSSLQSVEELRHSFVSTKQDD
SLPVLRACPGALAAIREVISYNNKTAMLWSITLSEVVKYALRHLGQTDRTKRPNRAVPFF
LSESTTLDDHLHVKFPTVLAALLGSTNTVAEEKESQHVTQQKGGVSSRSKEFHALATKAA
STSGGSEYETVYFSLLALTNMGCFSPTGLSWLFKALEDSYNSDSHPSALDAFLSSTVCAT
VSPTARELLDVLRTLMRVGHKPLQLQALIEFKGILERQQNALPSDGTPTKQPTKP*
>TCRU_29 Protein tyrosine kinase/Protein kinase domain containing
protein, putative
MPPKLSPLPRKPTVQPSFLSADDDIWLGNIHVGPGGVEFTGEGDLHVPCQDLSLDSLDCV
KCLGKGTQGKVSKCINQKDGAVYAVKRLLIPRTTDRMNKSTVAGELRNIFSQGTNAYTVE
LYNAFYRDGTLSLVMEYMDWGNLEELIAICPTIPEPACAYIASQILHALQILHTKANHPT
ETDQKGRRQIHRDIKPANVMLSKNGVVKLTDFGIAASTETIGVNSFVGTATYMSPERIQG
RTYSTPSDIWSVGVLTAQLLLGHFPFSAAEGGFMALLRQVTECCSLSLVDQCACSQKAEN
FINHCICRNPDDRSTATELLQHEWIVQNASNGKDVLTELLGTVGEPVASAEYGNS*
>TCRU_30 hypothetical protein, conserved
MHQKKRFFETFALEVEDKDVNAITSQLEKKGGCGTLSPDSGKHKKLNTTSSFTGGTHVSS
PFMQQNDSNVIEPLRMLHNCIRHFASHPETYGSNLHALVDREICHVMRVKASEAALLTPG
MVPVASYPLPAINNTKARNNKYQHPAMNKCASLMFCPRSIEDAVSAGTVAGVDGMFGFGA
TTFVEGTPGFFSLLDKASRLRTLHEEPNALEIADTAGDVSALASSATDLVTLGKDSSLQD
GTPQQESTRVRFVAFSDGFSGNTPSSSALRTGAGPKLLVDGTSYEVSFLRSQLQQVEAAY
NAKCISHHELQQENAQLKSELSKTEKKLQQYTTKNQEMRVQLESLKRELDAWKQRSSEFN
LAGHAGNSHTSSEGSRRVQAMQLGHAKRELSHMSRLWRETEKSLQETKRTLESAEKETQL
SHRYLDDAFFLIERLERRVLRRDHYIEIQQRRQSSLEEKYEKLMWCFEELRTMNGGSSYV
DFLLSQDNGWSLFLFVRLQRRSMGYAQMEEPFPANPGPLFFRRTPFGGRFCDMYSPNLVL
KLVTDYTQRKLGNTEGMTEMAWGVSIPRWRGNYIIDYTPPSVGGNGEGINTENVMTSWEG
LYRSFPVLSLASVLLPQPSTALTGNVPIEEDIPNSAQYDQATIRLIIYCFWSERLLQYKK
EMERRVRVFKSSRREPNSGQLQGEAKGEGDVTDRPIYPSTFLAALVDFVQRTFPLNTKVA
EENAEEETKRLVVRGVVGKNEKAVDSVRMLFSTIFYIMKDGKEVPVVLSSQARELLFSLY
YYAEEYKEVDADFRLFYLVAHQQVPELVAINFWASLEALRKDCEELLCHHIGPSSSFRSS
LSKMHDSDTAFCKKTPQEELIHDTPFNALKRAVEVIDTAPLVEQSTSIEPDSDGEGYGLI
LPGSNVSSSEKTSSHDRAAEPARSDFYTLKHNIHEYLFRFKEESAVSSAVPEKPEKGDTP
FKDVAKDKSAEKKEIPTAWRTLDERIGAVLGPHASFLEAFKANQGPDISKKQRIKRQSFN
KETTSFSASRGLLPLDEVLELMYKHCFATYAVSCCGAARFSLVETLTRTGDSSTVDGILN
SLEHLTKVGWLPPTETQMRRLRFAISMDQPSTLVRFTDLFDVDSRYGLPSHFHDEYLQLT
LDAYMEHQEAWMNTILNFVIPVDDGYGVRNSDEIDMLVPFPIIRRAVLNVFHQLLQKPEQ
GNTLAKHFLLYCNLLREDENVRLGQFTDAPFFINALPLSPAEAESKFAKIEKRRWPVEEE
SASCSLLHLSLAIRMTHIIWGSSSTPSARLAGLVLSSTASEQARGVELPGGHGTPSKGMR
DGAFLNNVKEEFSDALLRVKTGVMQHNVLRHLAAEFTGAMREMEETPSPLDLTVDSKSAL
EASFHIRRLYAPWDDDQRRALPSRGSVPRGGRRSYTPRRMTSAFSGLNLQPETTVDRHYT
PEVASLTRFLRLSRIGKSRGDKKRKSKSPRGFQETETIFLAPCASYETAYMSHQARLVRR
LVEAASTPQPLPAVSAEETSAGPQLRTDSETAIASSCFAPAADRFNSIDSNIVEAAVAGA
ANISSMSTTDDSNIALPDVILDENGSTQKGKETIGGRNAVYQRHVSVRQLHAGCATLSLM
*
>TCRU_31 FtsX-like permease family, putative
MAQTMDRGAVSDPNSILNVELEAKSESWMAFMRRLCAERKRIFPMTAVFMKNALMNAKAH
KVSYCISFFSVFLVVFLCVILMSTIFNLPIVFLRLGEVHNGKNDLAITTGGELHSASSLN
YSIVEEIFPLTDPLRGYHSPRIRFGGYILKYSTCSGITKPQDLWYDKKKELCLPACLSEY
CDGTETPTVIFAINEERETRMGFGISWKGRKLKKGEVILSIGVAVAANNTRVGDKVLLLA

SLGNPLHEFFKSYPSLIRNTRTIVPLKVVEIVVPDRSKFDSDDFVVLDYDTFVSMVSEGL
FPKHDQEDVKKFGESNPKSCATSIHFNLGPSERTNTYKTTDYATVRLRISSWVSSIAEPL
GFNQITVNTPILNFLYTVRFFSLFVELIVSIILLSLAFLSIMLIYSLLNLSIENQVYELG
VRRMIGFSRGTLVFMLFVNAYAFTIPAWILGLIAGQVVFLGLRSVLRDIIGVNLPYIVPG
QAFGWATLLGLLLPLFGALLPVINLLRQNLPEALNASRGRNVGVIYKIERGGYSRSVNYM
VFGIGTAFAVFGFLLHYFFPIGLMHMRLGLLFYIFFGILIGMLAGMVLLSLNFERTLQTV
SSYIFFFWESAAVFRAFQNCLVAHRKRNRKTTLMYAFSLGFIIFITIAFQVQLRSFQYGI
RRSMGAEVVVDFEGLDLFTFSRMSHFIKAALPQVSAVTYLSHTVTADFNIQNVSLYSPGR
YQGVFINSLVAISPNYLDALNKDFLLVKKYNKESRKYSISGAVYTEKRNQIIVSSSVFST
LQSSSLEDPEMLVTAIMTDAKERSVENVRHAVKPSAVLDAAPQVSMTKFREMDGVVLTSY
PSLTRFSGVEYLSVRSIKISTVSLRVPDSRYVNYVSRVIEKYLRRRRITKTSVRDDGRTA
QYLGIADRTLGFFFIFVQVVTLAICFFSLLSSMTANISESTKEIGIYRCIGMTRFQIYRI
FIWESFVVVIASGIVGIIVGLTIGYSMQLQNYLFTQLEIPFALPYTQLLIVLFMAIGAAL
LASYPPVAFLLHLPSITHILRRTT*
>TCRU_32 radial spoke protein RSP10, putative
MSKQKKEVVINLDEVDEQTGYKSAELFVWLKFDETYLQKPEDDREADVDAWVDAFCEQME
EEGMNYDRNFVRSVCVLGIYAGARDEFQQRMGSGKAVYPNGDRYDGEFFEGKKHGTGRYC
FVSRGKSECDRIVEKEMQKLGGAVPDEQFITNVAEKYKISRQIVSWIIEYGVYPCYHGDY
VRGVRTGQGLMKNKDGTVYKGDFLANKRHGQGMFYYLNGDIFSGTWRNGLKHGYGTYHFA
GGSEYRGSWVDGVFTQGQWIFPDGTYYEGLFNKKNRPCDDAASMHFPSIKMAQTGVFRRG
VWAPSSALQVCEETPVDGMSWID*
>TCRU_33 hypothetical protein, conserved
MVYTRWKCDRLPVFQLKLFSQEYPLHTTVGILCMMFLWKHMGHCSEETERKHGWWAGYPY
WRDPIARRNEAKYKQLINKNDVDITDPKWTGCSFEQLEKLKKLI*
>TCRU_34 hypothetical protein
MIQTESEQPLISRANEGGRFPCCITLEDISVDVCTNFTSALSSCVETPCWDEMRVVFFRD
GVTQTTRWLSPAVAMHQMMSASAKRIGIDKGSSERLPGITEVKWSPINAAAYTVYFRFLD
KDADHSLDPGYERPVFSTNLKYLDSYNTDDADRLRSKVSPKVIHPFYFSIECRNPNRAFV
YTGGEGERPTSAKVEAMSAYYSRCVSKISTVFRKASIKLSSGSTEGSRSVEGEAADRPQT
KGHGHCSNYSSSFYVCDKYGVDPSEAYNAPGGRRCISLPLIPSHSKLPQNKTGVPLTLHL
TMVAHLNDPLMRPVRDYHSMVVLVDQVILDVMNVEAETGGKDASSARRVEAYGFGLTSNT
SQTAELRFSSPFLAPPDIPLNQEGGVRLVDFSVLHPSQNERELTALDLAKNPTSICIVSS
GCKVGNKRRIGNRPVLVSRFKTGVDGLCKSSYKSRLVMGLIRRYNTGEVEVERCSHPVDL
ASILNQEEYSDKIGAMIFSRGEYMSFRLRRRCFAEPERYRTELLMPYDYDNSGVEISDVV
DDDDIVMFSAQGARILPEENASKSGGGDDVPNGDGGRPDESRMTPQAATPTGTTGQLVEK
PMPSVTDSVMTSYSHEYQSYVPAQKNEKEAATGSSNRFIIGKPQRDGDSPVVKTSRDIHA
EDSESLRGKGNAEGAESNADGRVSSRDVSLLRETGMAVETGQTNPFEKTTSNFLFDSDAP
KEGAAHESLSLVTFELNAMGGINGNDDTQFALQVEPYISETIDAPRATENPRVYWWES*
>TCRU_35 hypothetical protein, conserved
MFRGILGGAGRQWRSERRLATVMNATSRNPAQKSTYRISRTNLAKQSAVRMKEDAKRRQK
SAVAQSVSRGDLSHADAEFLRHQQDEALAEHDARVRAFTKDTGSAHHHRHAPNPPRHLEY
DSVDANSGHSIIQQRFDALVHDAVLKYGPPLPAEFVSENDIVSPFGSRGDFNASSPFQEE
NPNAHYFYDEVQIRPQELFGIIRKLDPSFSVRTHADGVPFSLLIKNCLYFRVWGGKVHFM
RCLKRVPLRTGEAGEDDEEMRGEVTTTLSKYKMRESPSFDGLRQGPQPWLYNYRMM*
>TCRU_36 hypothetical protein
MKEEGKGATCTLNVNARHVVEGISALLKPALGVEHISRSVLLQQQRLLREIESLNDELEN
NRTHFETLNEAQREKLDIISAKIARCRSSAALLFRRLSEIKERLLRVHDVILQRRLKKPL
EKVAPEEEPVTTPSEQGNVAVVLFATEEVEASRQLNRHPEDVDNQHEEAVLPAEGEAHDE
*
>TCRU_37 Got1/Sft2-like family, putative
MHQAWNLVRDEPPEEVEVMEMPRENGIMGDLQEMSSLTYHQRFVGFFATLGMGLCFIAIA
TVFAPSVAVFPKKFAFFLTAGNLFCLGSTTFLVGIQQQIRSIFDAKRMEAAVMYAVSVIL
TLVSVLHWKSSVLAIVFAVAQVCCLLWYALSYVPFARRTIGIVWSYAWYIIWPVFCAVSQ
ALRRCCGLLISSVK*

>TCRU_38 brix domain containing protein, putative
MGKSKVMPGTVDTAEADKATPKSIIIYRGEVGNNVRALMHDWRAVFLPWSSRNLHGKNSS
LKDFLAIASTFSVSHLQLFTAPLHGTSLRIMRFANGPTLYFRVLSFTLRHEIVATQRRPI
SLNQAVWEAAPIVVLNNFTHPDARRRPEVPLLEATFRGMFPTVNIQLVKNSDIQRVCLFH
YDHVEHLVEVRHYFINAKAVGVSKTVKKLLERRCPTKLGTLESIDDVLHREDVWSDTDGE
GEEVPLAQPFRQHREQCRIKLHEIGPRLTLQLVKVTNGFAGGEVLFHRFESKTPTEVAEN
AVKVRARRSEKAKRKAEQDENVRRKQNMKEERLRAKRLRREETMRQQAENPLEVVDSGYG
ADAE*
>TCRU_39 TFIIF-stimulated CTD phosphatase, putative
MWPTRGHMKRLVKSVVPCIVCFFQDAVLDCVVAWKNIHKTLYVLFCFFTGRSHLAYPHPL
VQLRKKKAELALHRSRSTNPTVSHASSSSCTTLERLGSSWDDASPRESSGKEGNSLLMGR
KPALSMAEGDTAENECWIQRHVRRGHKGELSERVLKTRASPLVSPAYHLCHSESLHRLVS
NIIGPIQQPTVSALPTRHTAPQWPPHSSGRSLARHDGRGDDFSITTDEYSSTTSSQCHPL
RSVGVQSNTTSGSVDARSNSSFRAVPSVVYRRESSKQVQFDRSKGSRMPLQPIASITSHH
VLSYQATRQKVLIMDLDETLCFVSTKSTSSCSPPTFSEVIPTASGAELFYVWERPHVRLF
IGTLAKLFNLALFTSATKPYADSILRRLDPERLIRRRYYRHHCRQIPRSACGVSPGGRSL
AESVGSSTKTAAQNVTSLTGQTERSCGTAANDVVAGRMGDTVPPSCVHEGARKSDGGEGV
SGTVLVKDLRILKVPPELMIMVDNTEESVLANRENALVVPPFFPPAVMGTGDNAASDDVL
LSLIPLLEALLVVPDVRSVLRHGRVYWPNLNEK*
>TCRU_40 hypothetical protein, conserved
MLFRLGRHVVSRVALVALVPGGSVRLLSNSALQLAPKRRTKTSEPGKAAALGASASPTQK
NDPPPRRRQRKTKGDGHDTEVLNPRGSLASLPRAKKTKGVQSVAKEVRGPTPTTSDETSE
SATPSFTVDVLRVVEEWGAACANVQPEDVESGETQVENKNAASHVAVAAGTKGKPQLGAT
ALAALLVRAVKLPEEYPTLHGVLQVYQQTPLSRSGVVVLHAIELVRNVDHLSTLVAFLRV
YRTSAFSSTPVYEAVMEELVRATTMWWHQDQNSTKRGERDEIVMRTTLMWILLEFRRNYF
EMNATSTRISDASKGNKKKVGDKRGASIEHSKWLEETTQKLLADAAVPAADHIAQEAGDR
KLQTLTEGVVNMALFLLSSAMATELQMYSSHKETWERGTSQVQTAIDVYLSHNSSEDSLN
ASMKHLRAHGFDLLDALRLLSMLDTALSPLLRERLKRTASECFLKLCGAVGARPTLFTEV
LCTPEMAQRVRALTSNDIGDYVKAALFTGAALFCERADDAAVNRIAQQLQAVKRVSAKKE
GDLCVDADEKEEDDAEVTSPRSRRATIAYGLFSAGFDPEAVLSSGLCVGHNAMEVLELLQ
AAALFSRSFALSPLPARLSMLLTTEVAALIEARIRAENRAGVTPTDCIEGPRPLAELLEN
FSPVEKNTSGKKDGLVPLEVIFHLAFGTAVELLVRGEEKKLSSQLVRLLTLLEWEGSYKY
LVDGLSLLIDGFVERFRTAGRCQHDRHQQKSSMTTSTEVEQSLSEVTAFFVPRVVEAVQR
RLAKTKRASAMSDAEEKEAAQLLQLVLHFTVYVAACGMNATTAKQLVGLHTMVVKGLTSG
GSDTHLASLSETVIRMNILVSYLTAKQREFGVARHPESPGLRHCSIFLNDVVAVHLKPIA
AVVRRGGTIVVAQERYQVLSLVSLLFRLVSEYTVEAEPAELRAFRTDVLDPTLTRVLQEQ
NNGDVQCNETLDLDALSEITVVMCVPHTIHHLSSQLLDQLAAAVVVAAPKQLQLARTEDQ
RRHSSKTATRLLVSAAAVCQLRQDKLTARYLSLVQALGPLLLPAEVSAVLCSFVAFNVPS
DTDAVVELRNRLTEEAVSTNSSLSVPETISSLQVLCELEDTTRLAHAAILRRIIYSGHRL
TPGEVGILVRITTRLQQLNGTLPEDDLKFLQDLPFSLAEEVLRVRQHCSASDLPLVLQGY
SQLEEDRYGELNDRVMKAYILRTIQVRPLLGPKEVVECLESYAAARVSHQYLFGVLLARV
ADVKLKFSLPLAVRLVRCGVNASWDKNIQTACITAAQPVFIGLVRHLLQNDPASLPSVSS
NAVVVLQCLTEAFPNDPTSDLLLQNMASHHTALSLSTMMAVLELIARRSSTEYNILRCMT
EHAASALFPQTSPSAFAELTRLFVRCGVRSRVLFTALSRRFKEISNKCDGNVLATLAEAF
AVAHVDLEEGIITTFIERIVALSEGKQLLLQLNHCISILKLFSHIDAEKVEQATNLLLES
ARKSLEAKEMVHDGVKIQQAQEINILLSASLRTAPLRHRALAKQCADALLGIVGAEGVGE
EKNCGAWQTDLMLIVQLASNIVRLGLRNHPVVPYLFDALYEKRSALMRRRLLLQITAETI
QIAGEHTHKQLYALLVQGKLVA*
>TCRU_41 leucine-rich repeat protein (LRRP), putative
MGREQSADFEKAFADLLLAGLRRQGEVHGDERVRSIMRRVLVERGGDGREETGTVASAVF
SLAGAGLGDKGAIAVAEALLKLGSPQMVKSHGVRRWTVLLNDNGLTDRFSAHVSLLVSRC
APPVVLLDARGNAMTPAAIQRIEEARELSPAPCALRLPPTAGTAGVAAAASLRESASSAS
TAGGWRRGLDAEALRKRRSALFASGAASSTAVGSELGGRGRPRSRQSEGEAPHDVAAVEV
AARAPSSASGPAPEERSRRMEASGRRHSAGAGKSEKREEEAPPRGGDCGEPLDVTANLSP

LLAVVLDLSNAVPTGRLQALYGTDDIWSVVASSGEAVSHAFPPKQTPFLRAGIKSMVSFH
SLTVLCIRKNRLESLKILPPSLLRLDVSENELRELTGVGQCRMLTLLNARRNRLQSIRGL
ERNLALSHLFLAYNEIAFVEGIAHLLLLETLDLAHNRLKTQASIRPLSLARGLRHLLLRG
NPVVKCIKGSYRPVLRNLCPSLLSIDGERLTFSRFAAKAQQECSDIRIPYAPGDSIGGIN
VSLISAEDGKATKVTQCANFMHLLTRGTAVGAGSGYSDAARAVRTAKALQARGAANDKKG
KEQRRPAYLADGEPLRKELLKRLAQESRKYLESMLGERLSNIQASHVYDNLPGSGESRDG
NNESEGLSNDTSAQRESSGQGSGPHLFSGDRNRKAPEFTQGLFPVEVASEENIEKHRTIP
LEKKMPAVHRDATLDHEQWLRLRECRIRRSAPFGDTPQRISPSPKTPDISYTKSEEKEVY
ISPIRKDHDECQAASINVTEILPDMEMLDSRFRADGKVSGEPNGRPRLVKSDLGRPMLLS
PSAECSPVKAHRVYSSLQSQKGNKPILSSSATPLHGVKAANEPVIFSPPPPLLSPCSLHS
NARSYAVKKWVNQLREDAEAVQEALETLVSILDSRWWSKAAITGKPSFIPPEVLEERKRC
VDILTESGMLLDTEIPLDVVEYYHFTPDELRHTGEEDYGEEGDCAANPLSVVTACDSCRE
KREILHCIRVIGDGKTCLRYLVLLIEEMKEEELQQYVKELQLLTTCE*
>TCRU_42 hypothetical protein, conserved
MKITDFSAKLQGPPETETLLNCLVGVLLGVTRRKATEGPTRKGISTSPDGNSEAGVISSL
RSVNPSSEDDSFAQDVYLNELCSYLSSGTSSRETGETPGASPPIPLPLPKIGSFQKARTL
FTRLAKSHYWRRRFLIPLLRRWWREEGHSVFGFLLLQSITDAVARELVCIQSDSGEAVMG
NEGQETCHDDGMGNNKKLLCPGAMQDLEELLKSTGSFRGLCIGVDAIIRNNLSNLLRCVR
KLLAGGACKDEKDALTGSARAEMECLVDGSTKSVLKDLFAFFFGTMNDVEKAFRESVWTF
LVALLSAYTGALILLKRDTNSDAQDNLFFLEAQRMFIGKMQEHLRGYLTVRLSSDSVAMG
NSPVTVHNVVCRQSFFWRASMEVEIAMESLPPAFISNSLVVSWARKLVAAAWGNAFCLVQ
SLVRAQEATEEVPGTVETTDSIISAIRRRHRELSEEHLERLVSAELRCFSGTKRPRMLET
DAVGDKTKGGSRTVPTTEETPAFGFSSYATEYFLRTYHPSCFAVLSLVEQDTLVLLLRQL
YAVIVNKEREAIRRSLLTVTVPDGSNVTDTQKSLLQWHLHHVHTQVVQALGEDALLLMCL
LHAVFIFLERIGKTNLNVCDVLSDTLLPILAMLCSERHGGFAEHEDGEQYYEIQQCIMML
LASGFLVLPWCLRGIRIRQVLYNYIWANLRDSFSAEEAFTEVSHIFVQYADVLNPAEDAA
AALCGDLSDDPFLRMLASEGEAATTTMEVMDDLLRLETPVQFPAPCGALLLISIFRTLSL
NFSRLFAEWEQQKQHVREGAMDYLGREATFCTIRDIGVFRVLIQLAHRMVLGFQLQPVGL
LRLWLSFLTHLFTHFVPHLTEDDEIVLMTLHTKDGSRSRAEEEPPRRAYKRSPNETSQGE
LRAILGELLADLALMPLVDAEAVMQCPVAVPHRPGSAQWIQKQSERSQDVLILQRVLPVL
VLEELCFALRIPIEDLDEREADQAFVRQVGKTVEQSFLGVLGVFAFCQRMELFVVPGTFT
DKRFAELLQTLWLAVEAGSMVMAPT*
>TCRU_43 hypothetical protein, conserved
MRTGSVQLGRLFSPSLIGRNRYCTPHLQQANYLLPFVAGACCSVEDARRLFARCCGCYHH
YRAEGEDESHGGDNSTDTESVMEESVMSLQRLGLISHTHEEQAVRGELQVRRIYREPIIT
INLHSINVTSLQRGEKETEALFQMLHGVIIPLMFSRLASSSATSPTNCSPHFEEEVSAQL
CFSLLQQSRGTCSKVNDTTCRFMVDLVNEALLLGVRERVLFPCGGGLFRLHREMTQRVSP
CLLTFQWWWWRRVDSFLRHVVASRAAGKDVASEAMIDAAQERVRYWIKRRDEYLPHFFLH
ASMDDTMALVADAVRCWGVPQSPVQGQKAFSVPKWRLQLSDEAKASLALETLNSIRSCLV
RRPAGISVDELSTLIAWSTISSLLQKKSILKTLNSFPSNFVVKTVQGVTVAQLQLCLGET
NTINATEARTWVLGRRNESSDTTVSFLQEVDFVIRSVLFLRKRHLLEKRVTFEDLRAVLL
PEKDMESTDAFLEVLRRYDVVTGFEAEKPVSWLSLQQERVFLSSRESVALKAIEASRKRK
GSKYQLYAEAISPFLSRCSEEATAAGISDASMPIALLERWLQSDRLLLNRADLFKMLREC
SLPFVMDEKAQSIYLGRGKMADLSQCAVPPSPVDPMPSTLPPPPPPLPTPVTPLQKLTFD
SQLSRALKGQHPDALFRFAEATIHAVFGLILPLVSVPSGMRLQNLIRRVRWGSVAATLGV
LPSFLEAFDGLFFDLFADTSGPMEDMVVTAYRGHVGAWLLYARLISRLFPTDVNIPLRLV
AEGLSWGVWFAPRFGDLPTLLRRVGRSCREGYLLAKNECTVVPSAEDDAALWEILENVRR
EEKDEQKPHKVLTEIRSYLLVKPTCLIRYMRGNKETDSSGDDMWMQYAETAIRRFPLFFQ
WNTALQGATPLLRIALKCAPAPDGLAALVEEYVCPLLRQRPQDGTSIAELDDRLGWSRGN
FDTHPASSRALGVVASGTSLYGILQRYVEAVDPPRILLQRGSTRATEHESVMVRPNTCIY
RSPEDMLLEVNGLEYMTNENATLRPSGRPVSLFELLAEEQELQGGENVPSLPRTEQWRVS
LQCNGEKAAEWEVELANGRVDVLVWCES*
>TCRU_44 hypothetical protein, conserved
MGGGDETIERIESIGDTCHTAVGVDEHATHGGEEAFLVENNSTSSHSSKAVHPTEKAVEL

QERRKGTSSTGTARREGDTFPEYSLSTMLENTAAASVDPYETDGRMVKENPALTAVEITT
VSSPSSCSHSVSATTGLNELSDGDANGDNCINDGEDCGGESYMSPIVVQANAFQETRRNT
VDAYYPPLNYMRPTMSWRRQHEGLEWPTRLRPSQGRSRSVTSNYSLRSGNERNWKENTLV
HLIRIPFEEGPGVWGDVEMPRRPFGYDALGNKAYEMTNAVCCRAPVTTTISSSARRRDAA
CRFMAEGSTSRSPSRARSSISCHANGTPTAEKENQEFFEDPTTLENLCGIVGRKFVAPQH
RITATNADRISTTRRLRSMTPSYVDAFYKLDREVAGRKRFLASLRRGATLRESSRRAATT
PCRQHSQHPYRRSDGGNISGRGLTLSPGTSCMNFSSSPWSPKPKPWR*
>TCRU_45 hypothetical protein, conserved
MSPGEKLCDGCGNPAFSFQCPLCQAEIATDRGFFCGQECFAKNWRNHRNTFHKKGVVRAK
QPPGPASVEAAGDCQKRLTGGTKEAGGSIKKRRKLEKEVGVKTKCPGAVPWLPPPRGSPA
TAEVTPPLPPETPRAVVGCPSADRRIAFWSAAVAASHHIAAAFSQGEGVAPFSVLVVTSS
PLAAHAMAWAARCAGLAGVMRLVVSAPSEAVGANLLKHTSDERRVIITTQEIVRCVADGS
VAVWLPRAESLLVTLPGVVDACDLQAVHLRAVFFVGRTAGSDSTEDNWCVEEEIVHHVSP
PLSPPAALLHQVDVFSIPTEDSSATGNKEATDTTPKLKLLRFDNDVSTSLSRGDLSGALQ
HLVRLYSASRGMFEEVFLSTFLSALGAASVTHAHHMLAYAARYLVDHSARFAGPPGGTLK
EMTLRAVTKVVRLFPPIKEAEEGKVATKKKGDAVTTDATGNGDLSEFLTQPSAIEKRKFL
NYYETLPNLQLQATICYLYEHAPPGTVDAFGAAWGVDKVVPGLEALKLADRKREDAVQRL
RNRYGSKLSSAFANYLVVLMHFIYDAVAGYSLAVEELERRTLWSLTLEPEVGSLRSFLSN
CFLLQKEDTSCAESKRGETARLSSRKVSNKPPSRTEILRSFPVRTSVFEEPLFLSWLTLP
LVGKRQRDMERLQRQAVEEILMAMPRNPRPMYIGDVGNLIGKWNHFNARFEGALGVSLME
FLMQHPEHFRVVGNLVTRRSAGVSEQVRMRFDNDDSGDDDDNGKESDQERKAKDRALLTG
VKGSGGRGLNAKDLPARARKKATVKAFNKARFNRNYKPMDPAAKVPGYVKHGPRKVKGRG
RKVNKRVTKRGG*
>TCRU_46 hypothetical protein, conserved
MRASPRLSASFSSSAISSTPTAVAAAASSSSLDGTLHEVERCFLRHVIPMRVQGAGSAAA
LYRHAFLHQESLIQRVLGALESVPLPALPRMLLAEGFQRMLDGETPQRDIRALFEEAENE
ARRVLIQAEFHSSLNSQHCDGHFYAAIKTNPLMRLLAYELRAACGYYAQIMSIRSQPEDG
AAMLLRTLIAADIRTDPFLVDVMLKFAALRRNVDTGERLEACPSPVEEFVKECLLLERDA
FGRFRFDARGDNRHLLHCIKLSDITRAPRDFAVLLDPVLRQYGNFCIQSTSVHQGRWMEH
RLSCAEESHRVDPHLPLLETVEVPSNKESELSVGDAVGVAHGVSGRPPLVFFVRYNDPMC
RRHKETTREKGAEKDHLEVFELAVEDKSRTFWEKWFMDR*
>TCRU_47 choline/ethanolamine phosphotransferase (CEPT)
MNNSQKPENVRGNASLRIDQVSFGSYLPENAEERMRAYVYRGTDKSLLYKYVWRPLCARI
VTYLPVWLSANMITFAALIFVCSTHVLLAFYIPKFSVASGGNGVGGFCEIGEEGNEFMKE
NFPPPPSFVFILCALSLMAYTFLDNLDGHQARRTRMASPLGLIIDHGCDSFNSIILSLSI
SASTLAGPTWKTWTFLLCMMTGFFMNTWEEYYLGELLLPLINGANEGVMAAVGVYLFTAW
MGGPQWWFMNGVELPERLIPTVLTLSPPVATVTIEASVLRTVCPCISVPYFYVPNSTLSL
LPLLFGLNCSDAYISAPPIPINAANQTSHAVYIGHNTLQNTILGLYDPSHPNLFWVRFNT
VVVLFFACSTLVTVLGNIIRVYRALHRHNDMSISTSGSIVCRIYFVHALTRLMPLVSIMF
FATLWLFTSPSNIFRRHPRIFCWTFGLLFAKSSIHLMIAHICNVKFHPLHRTFWPFFFLA
IHLTLTYCRNVVEWIQHYNHGDTLALLSARNVWGVALDEEQALWCLFVVCVWNYIHLVCT
VVRDTAKRLGIPVFTVPRSNQVTLRRQIEEERAANEKAGEKILGATHLLKDDGTPMDEGG
RGKKKGSVSGE*
>TCRU_48 hypothetical protein, conserved
MLQTARIRTEKPPVFRRRHRQGVSSTTTTPFPGFIPGASFDATAPSRREALQQFHRLEMM
LEVEERLRAEDGLVHSDTGGGSEDGRSEWGDKTGDKSRLESAAKAALHDAREELAGRTLH
ADPYCVENVWSEAQRECQVEEGHRMGEDEAMFSARKGIDFEPSNLLEADTHAAIMEDLEK
LHSRIQQARAQKSRHSSPLGGDAEGASSTSESSIHLSEEVEFDPERFVAPLLRHSDVIDG
AELWTPQFGIDGGGVVCFRTAHDIFSDTTTVQEALQLLQYSVHSNRPDVVACVQRCQEHQ
RQHQPITTIPKLPTGETVNPPRRKRKVVIPATNVGKQHEEEAKLYRPLPSESPPSAEELA
AILSKTLPAIPDELRNELIDQRRLLERLAAAQEASFRQLAREQQESARRRIMAPRADTTM
PLLAPSKLQYSEPFEIAETLQNLQADPKRPLPPVITISTVRPQPTKILPPSPPLLPPHYI
EEELRRLHRHYEKILRDERRAWTDIMQKQKATFEMEKNAAVQTLRSAAEQRQNENTEEIK
RAIALQEIKRRRWQYASESKQRIEFQKMLYKTFPLGDVGHGHGGGKIGVAARITPGIRQR

QTDIAEGAKFFSSNKQIHSPEAEREEEDEKKKKKKNEKKRDLKRPFVRGPFSVVLPEEPG
VEPTATAHDASLERVDEVVMLSDGEEKNGIQQARGEASVGSRALTDKLRIGFSSPAGKQR
RVLGPQRSPYAVSSKPSVLRGTAKTSSGGGVKSAAAAASGIKHARAPSHSRGARPQHRLL
SKGSQEKSPHAFAVHDARIGDERNFYNTTETVLTESVHSLDATLTPPPVVGGTSHLHENT
LDIGGSIAGRAVFPRSILLLSDPLTSEEMERQHSLNCAFNALLPPPPRATSKEGSGQIQR
PCDIYYFGEEHGALTPRRRAKLRAAEAQTRIEARRRLEHIKALSLTSKGQKLGEPVPHFP
ESQLFEARSEQRLADLLANETIHSIIGGAAEESLFADLFKELEDELVSAELRRLAFNNLK
QRDESGQEQKLMEEEYEGDTKPPDMVSMERAVASAKIFDAPINEEIFLEETVLRLLEESL
LHFLLQEKSDEKEEEKTIDNENIEGGEATMVSSQRGQTVLMSDGAYETETGPLWVREVVS
ATTDPLWHGAEEEVNPPRESSIQATTMVSSEEHAPVLSAPPLSAPPTSKKAPLTPASTAQ
MGVVITMEGARGNVVPDTAGTVRIVLDIAPVMEHLTAIAPLDAVQPTRPAQVDAILREDP
RLPCAKEEELISCEVVDAPTITGTQRLTVSTEVKRDEMQEVRPPLLVSDIQLGESHPPAL
TKEEKTNVPQPSPLPLQGYVTVTEVPKARQSQHIEIERQKECARLCDNELIQRQMLEEQE
DTLRASWHMMWDWQQMNVAMLIQHQYQKNPFDVASQPFSAAVPFTETQTVNKVEPSGNNV
LPPAPLDSTIRDPITRFIFEWMHDYDEKKKEETARKRLVELPHEASCVPEGKTLQEGSSG
TCTTGTEVRRRLLFDIDNAESSSWLSSTHLSSFAEDGFPRFANRPQPRVVRPQRKYRRSG
DENFSSETTETTRYTNSSSILWLAQEAWSRGVSPPPVFPTAAVGNATSTTVTTSSSSRTW
PSTSSSEKPHLRETMLRHLRRIQSSDPYQQGEQESQREEYPSVEELRKEVERVQASEQRA
ATHDASRLEGADQESRHTENFVALLPLLQRPQSW*
>TCRU_49 6-phosphofructo-2-kinase/fructose-2,6-bisphosphatase
MNSVTGARGSSETAPQAADDDSGDATASGNTNGNVASGNSAQAKATSEQAVETEITTTTT
TTKTPAAAAAVGVHHRGCPGNSRGGECGFCLFEEVVVEASPPSSSYLFPQNASASPGVDS
LQQCRPLDSFLDTACGKASGHLSSSTRICPSETESTATFVNGVVQSDRNLKESRRIWATS
FPSSEFFSAAETEKALQHQMQHETAASFPLSDKPAPERRHALPLLQVPAQDTNADSVIKK
QNIAALICVLERQKFALQRQLKEIRKELSAQNIFSLVRASNASQLQYLLENKICDVNKRD
YNGCTPLHVAALEGNEVIVRVLISFGADLMAVDNTGRTPLDWAAANRHSSVCHYLMNVIQ
RLMLTQEQKKPQIQRNNDAESVESTEKNVQRNTSFGVSSTNNSMMQSTHLTSALRESTCS
LPTPSLPLDSEIGNEQQNEHGLLSSFLTCLPPDMISLMRSRSALSANPIEAPLHEDESEF
EEQGTLSNFSSYTTISGTVPLVVCMVGLPGRGKSFIAQRISRYLNWKGVPCRVFNAGNYR
RRLLGVEGTSDAGFYDPNNANGKQMREKMAEFACEDLVEFISRHRIAVGVFDATNTTKAR
REHLVEFFNKAFKQRHMESRVMFIESICNDDTIITENILRAKCDNDDFKNVGDTSKVIAS
FRSRISEYEKVYEPLDRADDLSFIRIIDVKRHVVMKNIPCGLASSIAFFLMNLHPVAHPI
YISLPGETEGEKNHMYGGDEHLTPLGNKFALALKRFILERHFPHMIVLHGTNHSVLNTLK
PLAQSLQEDSGDGADKVEEEENSAFSGITCQEELLCPLPGLDSINYGLFSGRTVQWVRKR
YAKLSRLLYVASPVCGDESALNLYNSKKLYAVDVDDKKKGIIETHEYPKKQDQGEKDARK
QHFDKSARATSRFLHVPNGTDPRLSYCVQFPNGESCRQVNVRLEPALMAVMRVQGPVFVV
ATSVPAQGVLAFFTDTMPEVSPTLRLPQHAVVEIGVKGEITVHQLVEPVDHVPQPGSIRE
GR*
>TCRU_50 AGC/RSK family serine/threonine kinase, putative
MSETKMVNKSKNGIREKLHSAIAWLFTPGHKSIEYCKNQGEGDHQVSAAVDGSSSLAGDG
CPLTTPRSNLNVKINREGLQQAAVSAATSQHAGELSTRAAGNNLDLDLDATSIDHSEFFS
FLEDVIGGKHVEMLKELNQLHESNSNSNVEANAVDSPSSAPVESGEGFRESVLRASSFLA
PCVEAQGQSERISEVKQDTLANKKERDGASSAARSLKGYELIAYLGKGAFAEVTLARHKA
TQKLFALKKISKRKVREEGCVQRTFTERQLLASLKHPYLVKLYQAFQSLTHLYLVLEFAQ
GGDMYFFLESKPWLREMRRKVQKLRRLSFCSDTACPFPGGAQDESDPTSNHSCPPKHLIS
TVSSFSSTIQLAPDQSRAPIRLVAFYAIELALVLQYLHDHGFVYRDLKPENVLIARDGNL
MLTDFGVAKYYGGTKLKKSGAERTNSFTGTTPYMSPEMLLGEPQDARMDWWSFGCILFEM
ATGRRPFEGESQYAVVQSIVERDVEVCQDDFLLTSLEIERRVMQLQERYEAYLVRVVEQI
KKCKENGSMTEEERTQFSGSLEDAQLTFTVDAASALHDTSSCGSKNLLGDGTRSSLGEAF
CRTFGSETLNAKSSMTIENQSLFMNFAVGELDEACDLLKDLICSLLERRTDRRLSGPAVL
EHPFFSCPYVCSQIYYKHDARVDPGDISGFYVPTKWRNSFSSGGHFAEDEKKNENGEKND
GHDAEDDDGGTTELQKHLGAARFLAQSPTQRPDNWRELFLEGVVSSLYVPRLLSADDLRY
FPHAVTATGDSVVTQQRALREQIRRKHGALHPRGGEGDHQASPLSVTQKGGHPLHQENQM
SEEDEDVETLPQSASQRSSLVESLRGSKHAAFAHEEPLFMGETVSSLAFPHHDNFSVYSV

SSLEVSPAKYPSFLAKSYGRSYNKECCNEIGKETVTEPMEESSNRLKLSALDLSYSTSAE
AAKATAKTPEATLGGVLAEVAPSLISHSFPLQLGESQTAAHQELHPDLGRAYTAPSEIFT
STPTKSRDALPSFNLSTGNLGAREVLSSDTSYVTTEDEDSTGNAFDDIKGQTQGTLHGIA
YDVDDYFPSCTKQKRLLLKSKTGIPTQRPSKTNRSIFSVQRLRRSLLDKRATKFSRQLVA
SARASPTVPQQLPMCASYETFAFKSPKKNSHKTDSEYDNRRCSEDMEGRRLSLLSVYSPM
LYTATVSPRSPKKTEFLNSDSLVHETNILPGLCSTSRTGANSDSVEERPNSPTMESSSHP
GNGGAGTSVQHYLGFTFDSRAGNAFLLGNTSNGGSNDKWCF*
>TCRU_51 hypothetical protein, conserved
MHQPAATSHSQRVCHEHGEAETELAGHNLQHAAAIHRNVPKHTRTFVEANARRTPRAVLY
GEHGAAADTQVRRKGVHHVRLRQDSAVVHVEDAGQVRHEVALHKDRSARAPATHQRRPRH
DKPRRQKPDTVQDKRGAVPGPRMATGRRHVLRPANRRAQR*
>TCRU_52 hypothetical protein, conserved
MQHNRALQRRHIRHVDEGVRNQARRALAEAANPQLRAAHKHTVAQRHVQRVSGQVHRAQV
HRHSDRPAEHHRVEARRRTPAANRHGLRQRHATHAVLRTAAAGPRRRHVERQARVAHTAG
AAAAHAVGERRRQRIEARQRCRTAVARHHAHGGQAGHAVVAAALQHAAARTVALHGHSAA
RDRQPGDKRRAGGSAASNRHGAAAQRHTRQRRHRRRLAHRPHVAQLQPPADTDRPTVAAH
QRRRDRPLDAHEAQHRIKHREALAKHNAAAAAHYRHVRHGEHRVAPQTETRRGRHMHRGR
GRTARRLQQHAVKHHRARQQVHTAANVHRRPARLQRPQHSAAHTQLRLQQRRRVQHKRRR
HRVRRPAQLGVAHRSRRHNRRVTDRHVRACRQVVHKHERAVAPRLPAHAHLQAPVTRRLA
AVAPHVDLRTAVAHAVPHFDAQRAGKHRRNVEHHSHAVRDAHAVHQHRVHGAANRDGSVH
RAAPQSQHHRMRRGHPRRRRNQRQRHKQRHTHSRNGPAPHTVPAARTRTFRRPHPPTQTG
TNRAVVACRTRTVNRNSCLTNPLSSDGEYRYTIPIYRYPTNSAIPNTAINN*
>TCRU_of Trypanosoma cruzi region 4, putative Dispersed gene family protein.1
MDGTVALGGAGGKFVVGCLTLSGQALQPMDYRSAGIIGEFRPVACGVCDADVRCFAAATR
AMSGSCGCRCAEDGYGHDCLPVYLPHVDGCNRTLAPPLLSHTAAPTETLTPTPTWTPGMS
ATHYSPTETLQVTETVALSPTRTPTASVSSTLWWSDVACPTLAVTTRRLVGA*
>TCRU_54 hypothetical protein
MPARCCSRATEHRRRRLVRARRECGCGGERRFRDVGGVLRVCDLCGGRRGGRGLDVGERA
QRDAGELHACGRSEPVRCRVAVRPDCWPACRCVDQRARVALRRRRGCGEPLPAGLARHCG
GLGADCREARCVPRRLRPWRCVRVPCGAQRESDGERADHCAHAGGGGVRRRCWRVGCWRH
GAVVARCAVLGRAVGADGAGAVRVGGGRCRGQRRLRGGVC*
>TCRU_55 hypothetical protein, conserved
MWSAVGACPHSRHRVRHRAIATVRVALLVLLALVAAAAWMPAVHAVALRLRGGTVDRAIT
VGRAVDTVLMDGVCITNGVAVVFDVAAMLPGALRIELRNCVCDGGAQIYVRGYSGEPASD
RSLEVSVSGLSGSYCSLVFVHNLPARTNVTVRDSAIVTPGPMRYSQLSGPADAVASPLVL
HATSLLQTQLRVSSTVLRSSRAGGSAVHVGGGVDLLSSAMVLDGVLLEASGGPTASAMHV
SSSSRLSLRSHSVFSLTNVSVVSSGGGIVLGERLAVFESVLRFVGVEGSVASSLVRCDGG
TVGVGGWLELRDVWAVGEASSVASLSGVTLSGGAVSIARCAATGATLVSGLTITSGAVSV
QCNRAGGRVLQSSGDYRMAGLISVSVVPCDGCAAALACFDALTASFSECVCSCRAGGVGD
ACLPFDVPPARAGGGGDTAQDCVSGVTLTESVTVGGGRATACLDSVVFSGPITVAVDLRS
MDLSADALKVTLRHCVLAGGAQLRIGGLSESTARLVPHALVNMTNVTSLEGTIVLHGTMP
LHSSVLLANSTLRATVGGSRYVPTTPDHEESRYGPALVLDGVRLLSTRFVMTRSTLVCGG
GSCAAILVEHGLGVHLSSVFYMDNCAVNSQTHVMYALASDLRVSGGSVFSIQNSSWSAPS
TEYYKGALVFGDVAVDGGSVLQVVSSEFRLGFAMLMATTLTVTGGGWLVHRDNEFRTAYV
VHVDKYDGVAFRDQSVWSILYNNFTYGSYSSTTAYMTSFWSAQDDTSPTIYGTCNELRGS
PVTDYDEDLYIRTPVTALDCGTCTVGAVCFAARTSSIRGCECVCAAGGYGDTCLPAAVPD
GLGPFPLSDAKDTEVRCVYGDRISSVDYPDPGVRGLCFVNVTFTAAILLDLSYFNAPEQT
LNITLLQCVLMGLSIRGSGACVHVNVTSSMLDSGALEFEGDFGASSQMLVAGSKLVTNLG
YAIHFPRFTLGANSALLLLDNNMEGDIYAVYFPVAVVVDGGGIIMKGNTLRSKKKDYGSA
SAVYVHTVDLKNCGYFDVENNTMSAAIGIYFYGDTTVSSAGLLRVADCTFTGSTEALNSA
LLYLYGSVALQDGAQWRVEGNNVSAASVFIMPYSWHSIELSGSGTIVSLAHNRQADSSKA
FARIASSNTFLASPARFVVGCNLQDDEEVLYDGVFPGEVEVFRCGTCNDDAACYMPGTES
VDRGSCLCSCKDGWHGASCLPFEVPDTVVPPAPERAVDGDTSCVVNQTLTNLTLNMWKTH

HCYVGVTFSGVGAALTFFLNSMPLHLPINITLTGCTFRDGAALQFVGGTEVAESAGVLIR
VSQTVMRSSVVLFRRALPQHCDIAVTEVDAVQSSDIQLSGTASSRLSVVMLDDVVLTASS
LLVSNVKARLLDHGGYGLYSTGTLTLVGGSSLYTRYCSFDKYTYMLYMHRLIASDRSVFA
LLNNTMATGTSFLFQYHDFTVSNHSVLRVVGNSGSVSYAIYAYDSWTVRNSSWLDWRDND
AGAGAMFYYFSFYASVNIDGSSVVTLTGCKMGSTGLSESPLSRIDAGYGFVAGCLTVAGR
VLTTAAELELNGITDVTTVAACGECTRDGDCFAPLTTAVSDCKCQCAAGGHGDVCLPAPV
PAGPPPPPMPLPPPPPPVGGCISDVVYPEVTQAVGSGLSWLCYRNVTFSGGGMILTVLIG
GMTGDVANITFDGCTWRDGAVLLLLGNTYAAVESLNIVVTGNTFDDALLSPEGVFPPHTN
ITISGNRFTVTRLISRPGLDVDSPSCVAMNGLAISNDSAVVFSGNVFQTVTASSSAIHVV
GSPLRVSWRSLFAVMGNTFHMDGGDSTLMHLGGSFQSSSLDVLNNSAVVIRDNVVSRPVQ
YFMLFLRALRVESLSAVVFEGNDMQRSVAVFFTNYYTYVYYNSWLQLSGNLCRDSLSNAF
VHLSPRVNLRDSTVSVSGNQLMSNTGTCHIADLQRLMHPCGVVLPRVHDDGLE*
>TCRU_56 retrotransposon hot spot protein, RHS3, putative
MQASGHYLGMKRAVKGEVTFGEDIRGLCDRGFNNLPGWSLAAAGVKATVHNSSKHFLDAA
AEEARNPTTTSAPEKLEGVYESVHNARWSHAEELPDGVERKKTVTGMGVHEGRPEQSWSY
RKADDAIEENDAVQQSGAAPPVLMVLTSEKGWPYSWHTIQDLPKDVFVNCEVERVWQIVK
GDVTAWFSPHGGTDFTPKRRVLIGTPGIGKSMAAGSYLLYRLLHCDAEKIQVVVHCFGGG
GAYVSDKTTRAVTRCSDEDMCISELRSLRERGRNVYIIHGVAKEGTPPPRHFAPTFGWGM
IAVPFPKVTNYDEWAKQLQAARIIVNCPDETDVKAMCVDNA*
>TCRU_57 retrotransposon hot spot protein, RHS3, putative
MYLLGPIPRHVFDEEAFTERCGAVKFALQSINEVTAKEHFSRGGELPWYSEDPSHKLVKV
VREIYASGVILFNAPISACLEERTLERLPSVAE*
>TCRU_58 BNR repeat-like domain/Concanavalin A-like lectin/
glucanases superfamily, putative
MEDGTLVFPMMAKSEEEDVYSMIIYSKDNGSAWALSEDMSPAECLNPRITEWEGSLLMIV
DCEDGQRVYESRDMGTAWTEAVGTLSAVWVKSRSFLWDLSLHVEALITATIEGRKVMLYI
QRGNFSGKNKANALYLWVTDNNRTFFVGPVGMDNAANWMLASSLLYSDGSLHLLQRSING
EGSVISLSRLTEELKTIKSTLSTWSQNDVFFSSLSIPTAGLVGFLSNTSSGGDTWIDDYR
CVNASVTKAAKAHDGSKFTGPGSGAIWPVNSREDNNQHRFVNHRFTLVATVTIHQVAKGS
TPLLGAGLDAPVSTNFIGLSYSMNKTWETVFDGKKTTSNTTWELGREYQVALMLQDGNKG
SVYVDGKIVGSPEKIPTLETQGHEITHLYFGGEGDINSSVTVTNVFLYNRPLSVGEIKMV
KKSDAKKGKR*
>TCRU_59 hypothetical protein, conserved
MVCQCGETQVQMTFIDGNQLSDETLTVAPSATGLCLFHGPVSKAWQDSTLVGHIANLRAP
QIPNFETHVLLPAMGVARELGHQQGLITAERERLNFDCANAVADVRNRNAYLNSRAQEAH
HNLSVRESTIAGAGNQVLELQQQLEAAHAQLREREFNLGVLNAQLQEREARHNAQQPARD
AETAGRDESPATRHVANDGVTTLPHQTDILMAGGVPSSAARRSSAAYRRPHGNVGRPHFH
PSTPLPSRTSPLDAQLPMEEDRGFCAFDVTTWPSRIWNEGASGVVIDLRCAYQCWQQDDE
QVKLAFDNLVEWIGALED*
>TCRU_60 RNA triphosphatase
MLSDRQGISRHEVVSMGNAEQRAVAKALFDAVNKHLSNPFIEVEMRLGQFKVGEDALREN
TGDQNGAQTNGLRIVDANFTACVSTEDYERIKTYLMTEMGNSSMTRSVTHDVSLRGWRHT
YATDEDGNPTCCVSIVRKKRILVKNIVVPLGAYNLRFAVSTETPGDLRFSGAGPRAGHTR
LKDRLSITDGMFRYDMTQVTEKGVLMHEVEIEGVFSSHETQLTESWLEELLSRAMRLATL
RTNSTHGVALN*
>TCRU_61 syntaxin, putative
MQGIMKRLQLINGTSENTKGAPLLPLREELHMQPQKTMTPYEKAQLEVASRIRRIRELVT
QLDGLSADASRKFRVEVSQQIRKEELQAKVEMQNARRVAQSEGRMQDFELLSRHFNDTLD
IVRERYGGGLPVAKPGGSTFEREPLPFTDLEYAVNQSAFLASNSGNVVPKPGNAYNLHED
VEFLQFFYETQKNDEVIDKSLDCIYVGVQRLNQNATVITSELCTQEQMLDETEKKMDDVH
GRLGKLNVKLKKALERMDRPMMAVYLICCLILLSIIVIIYFMAKK*
>TCRU_62 kinetoplastid-specific phospho-protein phosphatase,
putative
MGRKYAQLETLHNVNGRVVIVGDIHGCLAQLEDILSVTDFVRGRDQLITAGDMVNKGPDS

FGVVRLLKSLGARGVIGNHDAKLLKLRKKIRKHGTLHGKNSQSSLAPLAMSLPQDVEEYL
LQLPHILHIPAHNILVVHAGLHVQHPLERQLVKEVTTMRNLILQDDGLYRASEDTTDGVP
WASLWQGPETVVFGHDARRGLQRYPHAIGLDTRCVYGGELTALVCPGEHLVSVPGWTSNR
SKV*
>TCRU_63 Domain of unknown function (DUF4498), putative
MGPQTRFSFEPLTHRRLEIVLTDPAYKEAQRRWGLHSQTRVCALRFEQENNSRIAQEGAS
HGGGVGNGAVVAGAMRFSGFHKGVLQEFLVSLFDSTAVRDALALSPALRLTRDAVEYTEL
HCSWTTLEPFTRLVKAGVVRHVEVQGDVCHDEAEAAIFDGCVRLPVVRRPDFYLPHDIAV
TDELRALFLLAHNRAICHEGDEYAEEEAWDAINYGSGAGLSLAALRRVFTEAERSEFLYH
ILWRLVAGGGSNNQWDDDLGVYLDVARAWYKGLVAIRAVQKNEAPSAGGGMDEPDHAAYV
DGTVLTPEVVSTVVSVQAVSGFSLFTRTDDAEPSNLNYCYVCIDPVEAEVVVWYHCL*
>TCRU_64 emp24/gp25L/p24 family/GOLD, putative
MPNLRERLRGQQSSPPQLSFLLFLLSTALLSLTVCLIGVEAATTTVGSGEKLCLREVVPP
QSRVTFQFQVVGGGNHDIRASVADQEGHILKEWGETSDGLYEVLAQSGTKAIVACLDNTY
AHYTPKLVVFHFRYHVDYTSVAKQSELDPVERKVEHISSLMRQVESLQMLLRTQQKEHRA
TVEESSERLLIWSVFQVLTLVIMSCFQLYFLKRFLERKSFV*
>TCRU_65 monoglyceride lipase, putative
MGSCCSCCDASHALKFSKPSRVPPDPELFPRYFQNKQGLWLRFAEWEPPREVPAVRAVLF
LVSGVAEHTARYDPVALTFAREGYHVFCMDNQGAGGSEGKRLHVEHFYDFVDDFLLFKKI
ILSRYPGYAVLPHFLLGHSMGGLIAAHVAFRDPGAWAAVVLSGPALELDPKLTTPLLRRI
APMVSRHFPKLAVRSLDIDLISGNRPVVELAKQDPFRVSVPLTARYGAEMMRAIDDVWKN
MERSTFPLLIVHGSKDLLCAVGGSRRFMELAVSTDKRLIEYEGLMHEVLTEVTWRRVLSD
IQGFLDGHCLSR*
>TCRU_66 hypothetical protein, conserved
MQRGSGRTARRLQQHAVKHHRARQQVHTAANVHRRPARLQRPQHSVAHTQLRLQQRRRMQ
HKRRRHRIRQPAQLGVAHRSRRHNRRVTDRHVRVCRQVVHKHKRAVAAGQPAHAHLQAPV
TRRLAAVAPHVDLRTAVAHAVPQFDAQRAGKHRRHVEHHSHAVRDVHAVHQHRIHSAANR
DGSVHRAAPQSQHHRMHRGHPRSSSNQRQHNEQRHTHSRNGPALHTVPAARTRTHRRPHP
PTQTSTNKAIVVCRTRPVDHNSCLANPFLQIANPVTRFRQIVILILLILILQVIPNALKR
*
>TCRU_67 hypothetical protein, conserved
MSMAKPRRKVLDTICSTLPPATTTSSNTHAALPYSVLGALHELFCMENTEPPPTRRSDAR
AYITCICELTAQLSMKKTLDRFTPRPRSTRIAAHEPPPHTSVDRVMTSRVDRSRTPSRAS
VGPYRDPAWPVVVGTYCDLPTVARSVEFASSTLECSGIAPRSTIVPSSEVTFVMLTRAWG
IRRAVLSLRPPIRSCAPPTSTQWRSVTFSASANASIERRSTATVIGPLNTTESKHAVARP
PPTVTDSVSITPLTQSSAPPPLPPGLATGTSNGRHASPTPPSRQPHTQSENDAVSASKHA
SAAAQPSHGTTLTEDRPAKR*
>TCRU_68 hypothetical protein, conserved
MALKNQLTRIQHRGDHVHVHACTTPLDRQAHEDALQQCDVECLLWCVKIRQVQHNRGSEG
HVDKAQTTHTGIRNVHGADAATVHAADLRVLRVGGRKRPKAVRNRSWQTRVTIAASSTYA
LTAADAARPRCEAHGAHGARAAVQHHHGCSNIEVDSVIRHRRAPCRVAHAVDDRVRVGWW
*
>TCRU_69 hypothetical protein, conserved
MQHNRALQRRHIRHVDEGVRHQARRALAEATNPQLRATRKHAVAQRHVQRVSERIHQAQV
HRHSDRPAEHHRVKARRRTPAANRHRLRQRHATHAVLRTTTAAGPRNRHVERQARVAHAA
GTAAAHAVGERRRQRIKARQRSRTAVARHHAHGGQAGHAVVAAALQHPAAGTVALHRHGT
ARDRRPGDKRRAGGRAVHNRHGAAAQRHPRQRCHRRRLAHRPHVAQLHPPADTDRPTVAA
HQRGRSRPLDAHEAQHRINHRETLAKHKATAAAHNRHARHGEHRVAPQTETRRGRHMHRG
RGRTARRLQRHAVKHHRAQQQVHAAANVHRRPARLQRPQHSVAHTQLRLQQRHRVQHKRR
RHRVRQPAQLGVAHRPRRHNRRVTDRHVRVCRQVVHKHERAVVAGQPAHAHLQVPVTRRL
AAVAPHVELRTAVAHAVPQFDAQRAGKLRRHVEHHSHAVRDGHAVHQHRVYGAANRDGSV
HRAAPQSQHHSMHRGHPHSSSSNQRQHNKQRHTHSRNGPVPHTVPAVRTRTHRRPHPPSQ
TRTNRAVVACRTRPVDHNSCLANPFLQLANPTTLFRQIVIPLLNLILQVTPNTLKR*
>TCRU_70 Trans-sialidase of Trypanosoma hydrophobic C-terminal,

putative
MPPAGKPSEAPGEQATLQQPPEERETQENTTVGESASTQQVPANRSQGSVGSAAALNSHA
VGGATGDGGTVRGSGLLPSLLPLLLGLWGFAAL*
>TCRU_71 hypothetical protein, conserved
MEIELSDDTAQATRISGRSVSFPDVTCKAYFVSPTVRPPVYMDSDHHLQSHTAGTEDGIP
RLAGMLPQRKHVAFVLRDADWNASTSACGTPLASVTTWLGMRKYTIPCGCRDRPEESRRT
EWSRPSVLQRQPAPS*
>TCRU_72 Trans-sialidase of Trypanosoma hydrophobic C-terminal,
putative
MGVRESTSLQEEVPPHPGTEVIPKADVERPIHEEEATSPEGATERQTQETTAPLVENEDS
EDVGTASGNTSTLPGETKIPSGSNATAPSGTGILLENGHFEELAGMALFAESTMHGCVSR
VLLLMLLGLWGTAALC*
>TCRU_73 Protein of unknown function (DUF1253), putative
MGKAGGFFKRLKAKQKRSASRRRRGTKGASKEERRVKHNERVDYYAAKREAEALAAISSS
SSDDDNDAVLSDSEMEARRQEHALLRLRRLIGVAGGSKRSRMDDSTNKPNGNGHNSRKYQ
DDKCTDATEMAEQLSEDDDWKAYLSEEDEEEQEEEEEDETDGDEEMSLDEMPGDDGDDDE
YEPEEEEEEEGEDAEKEDEYEDDESVVDYSDDDDDVEKEGGKQRQQRMEEAVQLLPQFRP
GTTGVTALRHVVTANDPWFVKYHQDKHGGIDKTPMQSREEHWRQQQAAGDGKMEDVSRVA
NIAVAASAHAVRHFLHPPFVFGKKQQQKPPYMHETLWAKWLEYCAAESRNPMTAEERGLL
DLLQGYPDVMDCCRSWENADARREIFILHALNHWFKARSVVLAHDALLQERRKNKKGRQK
KRKIQSSKKNRGEKEQEEGDDDADVDQDEEEYEFRDRGFGKTRVLIMLPMRNIAHRYVTT
LVKLLRATPDECAKLSTFNEDFTELAEAVDPTFKRRPRDYQRQFAGNIDDSFCVGVRLEP
SRVRVYAHPLNSDLIICSPLGLRRRLERSGDVLVSLASIEVCIVDEAHVLLMQNWQHMTE
VLRLLNKRPKDTTHGLSDLRRVYAWALAGHSGRHRQTIMSTNLTNATLLATFRSCVNNSG
RVLLQHREEAGVLAQVMVPVRQHFLRFDPGTSLEKCDDTRFDFFTREVYAKKISPLVERD
VRTIIFVPSYFDFVRLRNYMIREHRDSFAAICEYTSLQHQRKALGQFTDLERPLLLVTER
FYFFKRYFVKLAEVMVFYSPPLFPAFYTSLVGRLVATSPNAFALTLFCRFDTHELHRIIG
TRRTRQLLEREADAFSFVTN*
>TCRU_74 hypothetical protein, conserved
MSEERLDSLFDTALEAVAELERRRSLLHSLLQAAHFLYSDVQWEMDRAGLVLGLAAVPAL
EGAVCPQLGVAWRAKSTEEMDKEGGEGSHDRYELSVVKMAAGEGKDPARWFASVPSKRLR
ACQQQFREVLNACVQVVQAQERALAAAENYRVAAL*
>TCRU_75 hypothetical protein, conserved
MLSLTRSFRRRIVNPSVRESTIAGAGNQVLELQKQLEADRAQLREREFNMGRLTAQLQER
EARNNAQQPARDAETAGRDEASATQHAANDGVTTIPHQTDILMAGGEPSSAARRSSAAYR
RPHGNVGRPHFHPSTPLPSRTSPLDAQLPMEEDRSFCALDVTTWDPRAYGMRAPPESLST
SGAHTNAGNRTTSRLNLPLTIWSSGSARWKTGRIPLIES*
>TCRU_76 hypothetical protein, conserved
MQVWRLVERSSFPPSSSGKAREGSRRIPTADARLLNEAGIIEDASSTITGGWIIPFSVVE
EKTTGSRRRWIAWPRDKSRDDPYEANVPLLHISHYLPPVMAEAASCLDLKASFIVSLPRE
TRHPFRCRVEDGTLAELTRLPVGYKAGPEILQIIITSAIAVVTTVAHRLWAAPPLVRADV
WIDNIRIAGSKSDVTLWEAQVLRNADSCRASMGEDRESGATQYTFLGVHFDHTQRAVSLS
ERFVCSVRAMPALNSSTIAEMEATVSRFLYLAAILGTRLCDCYFFIKAVRRRLSALHRGI
VLETSPANLPPSAVGLDERLQHIIENIIVGESSSPRKGICRHHHERIAPWMGSRFHSRLR
RR*
>TCRU_77 hypothetical protein, conserved
MVAPLMLDLMDFRRMMCNISVPIRLLVLVQNGREAMLSLCLQELGRVYGWSGRLIVSRHP
ENIGYSAAVNIGLRLALSLPREEVPFVFVTNSDVKFLPDLLPNLLRDVHEMTRHGAARMD
ELAVEAANEPSEYSFVLRRGLRVLRSTVNDNRLSTSALLPDRIRYASAKEREKAFSKHYG
HFCAYCKSSCFVSVMLTRLAISTVGYFDENFYPAYVEDVDYSLRLRLLGFRGRNVLYGKF
VHRGSSNIRLSEQLELPDALWYRRVKSLMTNQPYAVMKWNGLKACCDGCKGPYDGMVPLD
VWVKDEARIQRIRVYGHDEEQGVPKVDYDRTLLYPVRTKGR*
>TCRU_78 BNR repeat-like domain containing protein, putative
MDILTSDISLLCMQHGDTAANNFGTTELLRPTTLVLGDSVYVLLGNYSHTGPQVGGTNER

GLLLLKGTLTDEGGRKKIKWNETHVVEPQGRGDSRSLTELVGGGGSGAVLRDGTLVFPMQ
AKDKDGTSVLLSMRLSNSENKWKLSSETPGNGCRDPTLVKWEEKKDDERLFMMAHCAGDY
YDVYMSTPHGVNWYTSGQPITRVWGNSHNRKGTASRADPPPQSLREGR*
>TCRU_79 Protein of unknown function (DUF3676)/Trans-sialidase of Trypanosoma hydrophobic C-terminal, putative
MSSGSYAEEMPEEEGEADGRSGGSTSSVGASSDMDTATETVDSERQLQQSTELSAENEGL
RSTGTGTTGAEQSLGLEAGDRNSERTMGSDSSLTPSGIDAEPTFAEDTDDVSRTEGTEVS
FEDGKEVPQTVDTKPENTNTTPGGEGIPSTKGAARHSDNDTFTGEIAELLSMGLSRDSTV
HVCVFRVLLLLLLPGLWGIVAL*
>TCRU_80 hypothetical protein, conserved
MQHDRALQRRHIRHVDESVRHQARRALAEAANPQLRAARKHAVAQRHVQRVSERVHQAQV
HRHDDRPAEHHRVEARRRTPAANRHRLRQRHATHAARRTATTATGPRRRHVERQTRLAHT
AGTAAAHAVGERRRQRIKARQRGRTAVARHHAHRGQAGHAVVAAALQHPAAGTVALHRHD
AARDRRHGDKRRASGSAARNRHGTAAQRHPRQRRHRRRLAHRPHAVQVQPPAGTDRPTVA
AHQRRRDRPLDAHEAQHRIKHRETLSKHNAAAAAHNRHTRHGEHRVAPQTENRRGRHTHR
GRGRTARRLQRHAVKHHRARQQVHVAANVHRRPARLQRPQHSVAHTQLRLQQRRRVQHKR
RRHRVRQSAQLRVAHRPRRHNRRVTDRHVRVCRQVVRKHKRAVAPGQPAHAHLQAPVTRR
LAAVAPHVDLRTAVAHAVPQLNAQRAGKHHLHVEHHSHAVRDVHAVQQHRVHGVANRDGS
VHRAAPQSQHHRMHRGHPRRRRNQRQHDEQRHTNSRNGTVPHTVPAARTRTHRTPHPPTQ
TRISRAVVVCRSRPVDHSSRLINPFLQMANPDTRFRQIVIPIILILILRVIPNTLKR*
>TCRU_of Trypanosoma cruzi region 4/Dispersed gene family protein.1 Dispersed gene family protein.1
MFHYSLITTVNVDGSSVVTLTGCTMGSTGLSVPLLSQAAAGYRFVAGCLTVAGREVTTAA
ELALNGITNVTTVAACGECTKEGDCFAPLTTAVIDCNCRCAAGGHGDVCVPAPVPAGPPP
PPLRPPPTPSPPPVGECISDMVYPEVAQAVGGGLSWLCYRNVTFSGGGMSLKVLVGAMTG
DVANVTFDECTWRDGAVLLLLGNAYAAVGSLNIFVTGNRFSDALLSPEGVFPPHTNITIS
GNRFTLTWLVPRSGLDIRRPSCVAMNGLVISNDSAVVLSGNVFQSVTTSSSAIRVSRSAL
RVSWHSVFAVVGNTFHMAGGDGTLIHLEGPRYSSSLSVLNNSAVVIRGNLVTRPMKCFVL
FIRTLDVESFSAVVFQGNDMQRSSVVFISGDSSNIYYNSWLQLSGNLCRESPSEAFAFLY
PKVNLRDSTVSVSGNQFVSSSVTPTVLRIPQSSRDLTNGAIVAACNTVNGEEGAYYAIPS
VYNATIMTCSDPCTLAASCFPAYTTTVSSEGSACRCAEGGHGDACLPVAVPEPPGTDGTD
LCVRDVRVEVEVNVSFGTSVACYVGVTFAADVVVDVESMSGSVRNVTLVNCLLVGGASLY
VVGWRSDPPAGERADVLISGLESCSGGGVLVANRFPPGSRVTVVDSVLIAEARVAYRGAY
GLGDASACLVLHNVNLTGSVLTIARTHVAAVFRDAVGVLFVGGVALSSRGALYVDELLVQ
TALGLCVSVEGGVAASGGSVVAFVDSDFLLCKHAVSVRGAVSVSGSAVAFVRSEFLSTED
YAVAFYSTVSLDGGSMLLAKGNVHDGASREMLYAAGAVTAAGSTLSFVRNRALLPRMLSL
SLSLAAGAHVRVACNDAGDRVLSTAEEYAAAGFGDAGSIDVAGCDACDRDTHCYAPGTAS
ASMRNGVCACLCGSGGHGEACVPVGAPALPPAVGTASSVFVREGVTVRSVFVVPAGASEV
TLRHVVLDGVSPVLYVPWMARDGVRIVVQNVSLLNGAVLYVMGGGALRGAVSAGSDEGGP
VELSVCNVEALNGALVLTGTFPAGSVLTVTDSLLVAARPTPLVYLPGSQSSPYAPVLVLS
GLRLVRSVLIVSGVALVTVMTGGRTVVVDGAVLELVGGGVALDAAVFGGEYALYASARVV
ASWGAVLRVSGSQVYAAHGLVFGSGVEANASAVVVNDNAGALTDGALLVLRGSASFASGS
WLSVRGNSISGRLLSVPPYPRRAELVQSTLTLHGNAGSGPVVMDGTVALGGAGRKFVVGC
LTLNGQALQPMDYRSAGIIGEFRPVACGVCDADVRCFAAATRTMSGSCGCRCADGGYGRD
CLPVYLPHVDGCNRTPAPPPLSHTATLTETRSPTTTWTATPTPSLSTAHYSPPWYGPTET
LQVTETVALPPTRTPTASVSSTLWWSDVACPTLAVTTTSAGGSLTQNDIRGGGSAVPTRL
MVALPPPFRWARDPQLGTHLSFVPVSTAQPSGFGGPWGAMLSNATWVRNATNPSTVLELA
VPVHRGYFIAADETIVIRCDAAAVSGGCKGVLLGSFTIRSDTLPAAASALSAITGVVACA
TAVAVVVTGGLGSVLEMQALGVFARMSCASAQERASTVALPYFLSVFAALDPLWMVVGNA
LLAAAFGCVHCGVTAAFQRWRGVDAASAWAAMRFPSLTYVVAHAMHLGIFFGSVLALAMP
GARVLHRVIGVVGVLYGVVFPAGVCYLIARHAGASFTRYWQFSRKPLHERLLYPVGYWHP
AAQQRMYGGMLTNVRGSRVYWCVFQLSVLCVVGLIAAVHPPVGGCHVQYFCMAAVLPAGA
GVVAFTNMMRSSFLTVMHAASFVLLAALCVVSAANHLAPSDGGARAYAAIVLLLATVVLA
VTVYSMVVWYAEDRHWQELREPRRGGLEALLRDGEESDEETQKPHEVTTFSYVSGATAAS

SYRPPAPP*
>TCRU_82 Mucin-like glycoprotein, putative
MPVQEGEKKGTTEDENDTTAPKSQSKGSTVEQGEADVGGGKSLTAPKRQSGDVIDTVHEE
DGSTTGPQSRTTKSAEQTDRETKDAAATTTTTTTTEAPSTTTTEAPAVSTTRAPSRLREI
DGSLSSSAWMCAPLVLAASALAYTTVG*
>TCRU_83 hypothetical protein, conserved
MLAFALSPAVISAPCIEADPFTVEFTQQLSSGCAAGPALALSHVFQRCLADDLMCVFRAA
ASSFLFVPNKKHFALRWVFICVAVDARDDAQRVCLLFSGHEGDFGVCQQHRGSLRRRIEA
KIREVCPLHLSRQLRGAPDAQSEWMDAAQLGRPVVFSALRCQGNRESTASLPAAFYCWGS
TVLKLPV*
>TCRU_84 BNR repeat-like domain/Concanavalin A-like lectin/
glucanases superfamily/Trans-sialidase of Trypanosoma hydrophobic C-
terminal, putative
MSRRVITSAVLLLLVMMCCGTGGAAQAAEPTIPEAPVPKSSFGWRDTTGDETVSSLRGPS
LVEVNGKVFAVAEAQCKNGETTVLTGIASRLLTMEKGNEPKDVLKEAKVKTQVLEEGGSG
KGKKKVDVSRPTTVVEGSDIHMLVGKHSHEDLANCQVTTETINSGILLVKGEVVEDGNKV
NWNDTDGVPCTLGPKHESLSQLIVGGGSGLKLKDGTLVFPVEATKKKNEEDKDVKAVSLI
IYSLKDATNWTLSKGMSDDGCSDPSVVEWEKDKLMMMTACDDGRRRVYESGDKGESWTEA
LGTLSRVWASKQKGKVEIVGSGFITAALGDGDDKRNVMLVTLPVYSKEEGNGKGVLHLWL
TDNTHIVDIGSVSGKEDDVAASSLLYKSGDNEKELIALYEKKGGEQPSPGMVSVRLTEQL
QRVKEVLTTWKKVDDRVSKLCPTENAEHDSSTDDACSTTDKITAGLVGFLSGNFSDGTWR
DEYLGVNATVNENVGAEPADNGVKFTGHGAGAEWPVGKQGDNQLYHFANYNFTLVATVSI
DEALKSGSHITLIGAKMDDNEKSVLVGLSYNNKEKKWILLCDGGENKEHSSHLETENSEH
VVILLRNGNQGSVYVDGQRVGDAQLDLKDIKDKKISHFYIGGDEGSAEGQEGQEGVSVTV
SNVLLYNRPWTSEEIAGLAKNKITISKPEGPKTSMDTQSSATSGSPVQGAVSLSNSARQL
PSEQETLKENIGADGVSGAASVTTPSADANTPASNGEGKVEPAVKKEMSVSSGEDAETVG
GTDVQEGIHPQAGEVNATALNSSLGHLSQGNNSDGGTVRGSGLLPSLLLLLGLWGFAAL*
>TCRU_85 hypothetical protein, conserved
MALPGWVRRFEVYAICMVWPIATIVGLVSTYKVFMWSYGGRDCFRYVAIQEPFKEQWYQA
NPKTAIGLDTPLAHVPKYLSTPGHSTGGEEHLHHRNY*
>TCRU_86 aminopeptidase, putative
MSTAYKRQRTKSAYEEHNVSAFVESCIHFRTNLTFHTAAEGMQLANEALQNHPQEPNIVL
LLGNQKQLQEKKALSTFFPYYSKAVESAINVAASGKNYLAEITPHARVLVGMVSSKASRH
NCPLRPDMISTLVKDAVHQARTGDCEKSVLRSVDIYCCESQNVLCVATAIARAANRSFSA
KRGRAEEGYWERGLPVRVVFAANTTKPGAAVDGDGFACGSRRALEVAVTVVQLCQRLVDA
PTNLLDTITFTEIAAGHVEKLKGEGRDVSIGIIAGEELREKGYGGMYGVGKAAEFPPHLV
TLSYQPSNGIKPQEKLAFVGKGIVYDTGGLSIKTLGGMCAMKHDMGGAAAVFCGFLGLAM
IEAPQQVSTVLCLADNAVGPRSQRNDDIIRMKSGHTVEINNTDAEGRLVLGDGVYHASAL
LPYTPDIIVDMGTLTGTQGVATGRFHAALYANNEEIENRLLKASRICGDLCFPILHCPEF
HSLEFNSSVADSRNSVTNRGNASSSCAAYFIGTHIDPKYQGSWAHIDMAAPVSRDEATGF
GVTLLLQTFAMSIYNPA*
>TCRU_87 Unc104-like kinesin, putative
MPQSAQSEEGRITVCVRARPLNEREKRLKSPSCLTFHEGKKVQIFRKGFAADGVEGLDES
FVGKVFAFDHAYDTDVPQSSLYEDLGLPVLDSSFKGFNTCIFAYGQTGSGKSYSMMGPSG
GRDVFVDPGIIPRLSKGLFAMLRERQAKNQSDREAARANAVEEHALPPEVNVIVLVSYLE
IYQERVNCLLNSKLDNLKVREHPVLGVYVEGLREVKVSSEEEIMQIMERGNQCRHTAATK
MNDRSSRSHAIFAISLIQERKSITKDGKVTSTMLRAKINLVDLAGSERAKSTGAEGDTLR
EGANINRSLTVLGQVISALAQASKQKSDAAGLKHVPYRDSTLTFILKESLGGNSKTFMLS
TLSPAAANYDETLSTLRYADRAKAIVTRAVINESAGDKKIRELEDEVRLLREKMRHYEEM
IARGEVMSQMSISGSPQLRDKIGKDGESKAIVEDDMVYANAKTPRQAGDAAVLERELHRA
ENLVQNLVANKGISLESFIMPAFHRNTSTIRVNGNDPFILNLDGAGDWVVEYIVARITYL
GVEASATGNDARYIVVSGSQTAGIAPQHCCFVRQDGGQVLLRPLGGNRTYIDDDPDPVDG
DTPMKSGTVLCLGDEYVQFKFIDPSAPQSAVRRRTVRNSESIPMQSLSIDAKSSSSNSNH
SLKSSPLLTGAGSGNAGHPPVPALKLKEALQRMQSNSARTKKAHRDPMTPTGTSLTSAPH

GISVVGITPRTTRTAGKVVISPSDELTVIYRHTFLFLGACNSGKSALRENLQKPDRWYSM
FANDKLHVRPTFGVDSSLIETFAGNQPVQMNLMELSGNRCFSFFESLLPTRHVSYVLCFS
LRELPTLETLQPLLEFILCRTFNRDATVILIGTHLDEISLDEIKLAHLFAIIETEINNYF
FLVQAIPEKRPSILGRFAVDNIGRVVVSPGFSKFKKFPELLSWFSDQALQRCRNDVEFPN
AQVPLRAVALSKKIREINREGQWYLTSANYKAIAKAVDARYGLSRHDLHRHTQLLVGWGV
LHHHFRHLTMKKYVVIDVQWVFRVVAVLACCTYSRGYGTENNSLLQPLPLLFCRKTMEDM
QNVIPFDVKTVMASDLCCVFNQGVVSMKTAMSLFRGVLGEKGLDSCHLTSILELLRGYDL
IIMGSRLKFSSFMGELRENKSHGMMDTSVLRGNDPVENDDDVDDEEEEEEEEEETAGASV
LSSEFFMLIPACFTCQPPSAFNLHLPSFLFGPFYRFTLNIVPHNFFARVMSRVAHFAEKI
YIGPVSARLVPVENLVSVDCSNITIRGSICHAFGDHDSRNVSVGTMEGRRAGTSQEKTSM
VGKSHFWGSTAWIISSARSRALIRMVHHSLLITFHDLDDDEEFYNGLRHVVRNLVYESPG
VKCEESILCSRDFMEEGEKASEVHGDKVYWHDVDENLNSLEKIRQREQFALMTARSSTSR
TLSGGAEGRSNIDALMEEEDDNMVIPFVRNRTECVDVEASVRRVCEDSLLTDDVLHGVRE
ALHAMNKARRRGSGAGQCRAMDRLVDVLAQA*
>TCRU_88 hypothetical protein, conserved
MNVAQFLTGRVEELAAFSALLFRKRPRHENERAFSRTARSVVHYRRPSSANVPLSDVMRL
ANFRSRRSKRFVRLLAAEEPPPATPKTVEEEKGRVQKKKRPLTYRAWCLLLRRRRRFLLR
RRATTRRRIRRRIIALRYGKTQLPLCSCGPPAAAPQPSLPQCLSTQAFSSGGGRTAVLWL
PTHWRFCRRFHYSVLKTRINGCSSSPDDDFSSRLVTVRIAVPTTSQRKQHRVLQRWAARL
PSCCALGKPPRRLKSKPMAVPPPLCFAAERSHMCVWAIQPRENGHPLTTEEVMNILMIQT
CKAPPVTLHTRDLATMAETPVNSLPYETCWRRTESGVFYGYMWSLGKAISKAKWLLRGGV
ELFDTDTTPIVVPVILLRNEDVDGGTYHLVAPTPVHIGARARRGLEMRLVSHWNPCGAPK
PMCSMFELWCCADAVSVKGADMSSTRDGSFILEWIRGRVHATVGRWCRVNKISSFAQQEG
YETNNNSSSLIKRTTVCVFPSIPPNLETIDHTSFPVHRCVMSILVFSKESFPQLDTTGSA
VRRDRMCHFQFARKLFASLTDATATAWRTMRHDDEKIAWAFRLCQSLGYRRNVRIIGEEE
RETLLSLIGCPAFTDVSGKNASFQISRMARLRCSPSNAHQRVLLKFAARGGREHQREGSA
LFVLKPGSPAGLFHVGAVTSAPFFSMRFGTRMAYAWVWDDAGWELVKYYTRFSLRNVFRE
GTEVFHEGPKRGGATEHSGAGSGSNICFFLAAAEKVSFLVPFLKNSPIRIPGCSASSCPV
EGEVSRQRWHHRHGTKRLRTGDPVVSAKMKSWRAMETMLCDGDTCLPVEVVLLGTAGARR
VGLSSRHSCDSDVTRTTTV*
>TCRU_89 hypothetical protein, conserved
MEAGHLLLWARCRRFVGDVCAGRDATHGLAHMETVTGNALLILHMEHGAASCPSLTISRV
ILVAMLHDVADHKYDAAGRLIQQVREFLGAEKGAITAAGMDGEEAFSHALTTIEAISFSK
EKKQCMRWFEAKLPSDWVFVRDVVSDADKLEAIGEEGLRRCLVYTMHTLLIREGRQGWDA
VLQQRCLQGVKEHFVEKLSRLSKEFIVTTAGKFLAAPRHAAMERALQEWESQGLPSFF*
>TCRU_90 SET domain containing protein, putative
MEDASCLVLRWAKRLEGACGDSGNSVNGGQTRGLGQQRQAQTQAAVAEAASVGMALLKLL
LQMEQKEDEMADDDKDAKEGTETALPHQRLTQLLVEVVLAILDSCIQWTGTKNAVQAVCC
ELMAASSREKGQEDLAEKLCDWWDHEEVAEQAWRWCEAHLTLLPADEAAWRYGASHGVAT
PTDWWWRPDAMTSANSNSLSETTAAAARSLFQEVYATVSQQCIAESPRTELATAKELLHN
LFTLPASFFFYRIAVSRGVLQRLFTMKATALLHQAHLLTCLAFLKNAAQPRYTEKSRKTS
GGGSSHELLWVSRECLSLAWKNLISSITAVQVATIHFGASCGGLRQQVRRALFGVKEVAS
ETDGTFSSCEDFFYVHPSVEPSENACRVTHGVGLVATGKISEGSVMLQERSLLFHSSPDQ
TAAKTEMQNNEMSTVTGAALELFRRGGMTFISDAVTSQVVESLQSGAFFGEPTQAGEMWN
ALHLIATMQPADRNSIGTEDGFARDIADLVRCWTERAIPFCPVEEFRIELEGAPGEGKGL
YPFAGFINHSCSPNALFVFGEEEGACCSSRGGVLRVVALCDIEPGEEITVTYLSSLLLSR
ELKRERNGFYCCCTFCLSYAALLEGVVCPTCQQLVYDDHNDKEEVSSKAVDVVSSVRANN
TANGRVKPYAHLPGCAYAGYSSYKDLAGPMKSGFKKIIDNIHCELLGKSITDAVHEHGNC
MQEGFGAGETEKYKEEEDDDDKKYAQRAQKAVRQLMDLDTLVSGLPSTHHYRLQARMECL
ATSLNAIMTPNDVTRLMQLCEKLLEDLEVLLPRNYPLLTGIRLHYALARSRYLIATTDIN
DPAADGYPEGCCGSVREQAEMMRLPFMRDAIIRECVVRSFQEHYVYTGWRFAEANDEEIL
QSFLRRYEAELFACGVEELSHFNMLSLMFDAAEGGSG*
>TCRU_91 hypothetical protein
MEVIGTLSAAVALSKTTNTLNPATESSGSYVLASISHLRSDFVRPNGLRVCVHSGEMLMR

THILSDPTPHLEKRQQEEEGSDHQTHQGEGSGAMPNGEVFGRLLSRVGSGSDMSNLGSRH
EVTSAVFSWRWLVERNDVAPFNHELKSTISSGKRTDDRILTPPGPIHTPRCAGSGRMDDE
ALIAIDMLRDWGEKEKEELFLRHPRLEAYATRELHTPLSAVLTGFPLAGTSMNDRNARPV
AMLAPLPNLPSQGMTTTNKRHALASHPSSTDLGLLLNPRVSTERNYTPQLGKLLSGFAQE
GRQVPLEVASVSHTDDIPEEDDLLSRGLCGRGDPILGDRGRVWPWRKGGRIDKRSLPLND
VLLECHFFGVTDILEVLRYDCEGDGSDDVLLTNFQDNSELSRSVIGRVVALADRSLRRHA
GEDEYGEISTNTDVHFSTAGMQERRNDALQASRILVDSYQSAEDELGYVVDLWRMAAECA
EPSSSLTPSTETEVIT*
>TCRU_92 SEP domain containing protein, putative
MPKNEDDEVATLIAFMPWLQREEALKLLKNYGGDAESVMRFLEEAASSQGEPQASPSPAD
QRKKTGAKKAEGKKMTEKYFVGGGSSSGQEVIGGDGGVDNVIESLFDKARQQLGEEGAEA
QFFFGRGRRLGHTTSESPFIESTLKPRREIVLTVYRDSYRVDDGPRMPKESEEGMAFFKA
LEAGVVPEGIAAIYPNTSISVRLVDCTQQDAPPTFLPFAGEGHRLDSNTSTTAPAASSVA
KTGADATPVNMSDNFELHEDEEVTKLAIVNLIGERKEFRVNPNRHTVGDVFFLAARHAQP
GALAFQLIARDVPPRPLTDRSLTIDVANLRNATVIMRRT*
>TCRU_93 carnitine O-palmitoyltransferase II, putative
MLRRSFVLCIATAPIAVPKDIHDWSRLRQYLEGFTVGACTDTGVLDASIIPTRHLQRSLF
RLPIPKLKSTCSRYLDAVKPLVTPKQFDTTSKIVKDFESGEGLSLHEELVRTDKANKHTS
YISADWFDLYLLDRKPLPVNTNPCLITRRDPDKGDMLTRATFWITSSVAFYRMYLENTLK
PDIFNFGSRDHYSRKDWFERTVALSPRSISAKVCVFGSNFHAFPLDMSQYANLMNSTRIP
GLIKDEVRGFGFAPHIIVQFRGHQYVVTVADSECRPLPEEQIYARLKAIIDLNPMPPRVD
IGVFTSTDRTTWHSVRTEMLRDSENQKSFELLDSAMFVLNLDDDVEVDFLTSRGAVETIR
VMLVRNVNRWWDKSLSVIVTKDGFLGINFEHSWGDGVAVLRYTQDVFNHSIMRSRRSMRH
DAAPTEGVRQLKWCLTPKLEQEGAKAKRRIDADIRRMDCCSCLVNEIGSSDPVFRKGPIK
VDPFLQLAMQLAWWRLYKSTVSTYESASTAAYLHGRTECIRSATIESQAFTILMDNPRAT
TNEKFEALLKAAKKHLQISKNAKMGEGLDRHLFALQNVALRRNPNSIPDLFLDPSYKTLG
SNMMSTSTLRSDALVGGGFGPVSPGYGIGYASERKCLCFNVSCWKEFGPRYSADEFTHAL
YSAVVDMYNIMLSVKQNE*
>TCRU_94 hypothetical protein, conserved
MAPKKKNASAAAAAKEETEECAPAEVTTTTPPTSPQTESLPSACNAVELYATLQAAVSED
PVKLNDFVRELLQALSDAECPDAPLLVRVRTLEFISKYGQHVRDGNALKKIVTSLVKILS
GSEDTPQLLVAAVQGLSSLGPVSVLDKKWEYLSREGADVLMQVMLDEEGFAESVRQAASK
ALDSLIQTAFRPVVTKLLHWISDNRETEDEDQLKKERRMALTRLRKLAHTPSLQCQWTEE
VQEHVLSLIVRVLPTVTVQEFVQLTSIAASLPIVRAEAGVPFLKAFLAQNAINTDRVLES
LSIIGEHIGATPYDIVPVLEDAGLLTTPVEKNTARGMWHAKVLLLAARLATPDNIDKLYN
AVLEQLAHVMHDGSALPENMTTLEVLLFAVMAAGQKKPVEMLKYLNDDAFVAKCMGLAEL
VAQMEPLLIYAVKKRMQKSSAGQKDAEVIGCCHNVRVILSAFSAKHMPTGTLTESWLHKN
KLPSVKHARDPAATAKATETTAGIAGAAAGQLGVLPPPPGTEEHAKKRSRGQESGYNKNV
NRNKGGWNGNGRYKRARRGGVY*
>TCRU_95 hypothetical protein
MVRHHKRKPIGVVAKCQTLLKGPPKMLQKAANAFIPQISWHVMRFSHIPTGVIDKMQAQL
ARGGVNLWLKLRTKSWKFNGHDIRQSRRILLVQALLVIFERWLLR*
>TCRU_96 cystathionine beta-synthase, putative
MAQNPSAVNNKKEVPDRRTCILDTVLDAIGSTPCIRLNHVPKKHGVCCDVVAKCEFLNPG
GSVKDRIARQMILDAEASGRLRPNQTIIEATSGNTGIGLSLVAAVKKYPMIITMPKKMSH
EKEVTLQALGAEVIRTETALPWDHPDSLIGMARRLEKEKGYVLLDQYRNPSNPKAHYEGT
AQEIYDQCGGKVDMAVFTTGTGGTMAGVARRLKELLPNIVIVGVDPYGSILADPSVPLDP
KPYLVEGIGYDFVPDVCERKYVDRWVKSADKESFALASEVHREEALLVGGSSGAAMWGVL
QAAKDLRPDQRCVVVFPDGIRNYMTKFPDKNWLVENGLEEGEVTRPTYGSLEAQLEEAKK
KLSEYEAKCGGSVEEGKRCCEKAK*
>TCRU_97 zinc finger protein family member, putative
MWRNAECPAADEGSVLLQKIFPPADGVATAKRKRKKKRRRVLYDQYLADSGSECSTGSSE
GGGEVLNAYEFEEFEPGMDINVLIQQTVQNVAQSVSLEDYDGLSQFGDYDLICFAAAVKH
NSSILSLQIRYLDVSDVSLVPLCRALEGHPCIRALDLSGTRGGNATIKAVFRLVCTNPNI

LFVRLDDTMVASHDAGEIELATRYNALGCPDPTNNPFHLGLLRKIGDMEEEKQKYKEQLS
AHSWLFSDHTGGAANQATKRTKKFLFAEKVAKSRIGADVCAQFMSGRCNYGSRCRYIHPD
KTAALQNAITLSQYKISKELEGDVNSLRTCAALLSSGSKMRLQSRLRPAKFALKEHACSR
IVAEPKDVDSCSEVDDFSTPNVVWAHAVWIFSVTVVVCSVVVAVNY*
>TCRU_98 BNR repeat-like domain/Concanavalin A-like lectin/
glucanases superfamily/Trans-sialidase of Trypanosoma hydrophobic C-
terminal, putative
MSRRVFTSAVLLLLVVMMCCGSGGATATDNGGSTPRTSPEKHFVWRDKNGEETVSSLHVP
SLVEVDGGVFTVAEAQLKEGEGDFFTGIASELLEWTDKESKELDATKLKTQVLEECPAGN
NSCASPKRAREASASQSRTKVRVRRPTTVVNGSRIYMVAGNYSFEVTDRAAGNTAAAAKW
GLLGAVGRVTNDGSSGKKKIYWKDAFVISLTDFEKHESLTRLIGGGGSGVKMKDGTIVFP
VEGTKKDGNAVSLIIYIATEDGKLSKGMTDGGCSDPSVVEWREGKLMMMTACDDGRRRVY
ESGDKGGSWTEALGTLSRVWGNKHKGNEKGVGSGFSTATIDNRDVMLVTLPVYSNRRGNG
KGELHLWLTDNTHIADIGPVSGNDDVTASSLLYKSAGSGDNNNEKLVALYEKKGDGKPSL
GMVSVRLTAQLKRVREVLATWKEVDDRVSKLCPSTAARNPSTGNDCSAGKITDGLVGFLS
KNFSGGTWRDEYLGVNATVKKGTNGVATGYADGVTFRGAWAEWPVGSQGENQLYHFANHN
FTLVATVSVHGGPKGDNPIPLMGATMNDKNTVLLGLSYNKEGKWQVLCGGKTTKELRSNS
EPGTTHQVAIVLRNGEQGSAYVDGQRVGDASCTLKNADSKGISHFYIGGDGGSTGRKEDV
PVTATNVLLYNRPLDDNEIRVLNANKISIPKLTDLRTVAAGATGVGTARHFGANGDGSAV
CGGGLLPLFLLLGLWGIAAS*
>TCRU_99 hypothetical protein, conserved
MNCGGVNFSPDGTIHPPARSGDADRTGIRQLPPPREGRDDTKSGGTTAPQVNAEPSHQNA
DWRQSAAADGPNTVDPSPASNTESNTSETKPELAYV*
>TCRU_100 hypothetical protein, conserved
MDTVLELCAVCAVEDGPAGTLIESVLSFIVQKTARVALPHMSAMERGALTLQLISAGGLQ
HDVCRQFLFKELSPPYSTTAIVRQSHELYIRPAERMARIDDNENTTMLSRRKKCSLPFLS
LLRKSGEMERNQPPVDFHGEDAEQLLAALLQSWNSSNAASLPHSLSLRELPPGLATYLTL
HYPARTLQLLRTTIVGPSVPQTWRDLWRQIFFFAADANAAADTMHMLLSQGACANFWPSA
LRALVHPLTGTVGDTEPEAKLLANFEGPSTFLTHALLCLWLSESRHDYDMGRMEALSGNA
ELRKETSSFLVAPLVSSKSSSFLSMTTDEVHPLWRRTLIHGLRLVEKDEAYGSHEEPQRG
RAAVVQAAYALLLSLYHGHSMASQYVRSALDHPDVTLFVPSFEDLNGELSEGAQWGGDEG
ICSISAFLAALALRIPAAQLYIAERVWRHLETMLAPGEVEARERQTAELDARLRILLNSF
AFSDVPLVVAHGGRGTSVPPVHTTVATLGEGSQKREDGPRLTLRLKRPRADGQIPQTADD
GRKDTVKSKSQTRMVPTSSDISLAASLLRNALIDFATEEIVQPSTLVHSSAPGSTFTATS
DGQLSRNKTRLMLLIPPHMKSFLGCFSAFSSSSPCSLMVCNVCCVIMSVFLYRQCYALQR
WLLHEAKSGLKAAVVGRFVWRVRRWLRFLAPLGVFCYAPVSDVLLPECLREMEKQKEKTV
DDGSGATATAILKELCGVLMSAI*
>TCRU_101 aminopeptidase, putative
MNRPPATDSGASEFVRSCVNFKTNVEFTDVDAYKKTHTAGLASTFVVILGTHAQLREDAL
KELPFYCPAVAEAIQRVKKGETYAVLAEGVKNEANERFVRVLVGEVPSEASRTNCSARPD
VVASLLSAALEEIKGPETKVDVFVRSTAALAIAVAAARSAKRNFTAKEGLAARGYCDNCV
RLTVVFPEAPNPSPSELAVVATSTQLCQRLVDAPTNLLNTATFAEVAQSYAKELGCAVDV
ICGEELRERGYGGIYSVGKCAVEAPRLVTLSYKPKDETRKKVALVGKGIVYDSGGLSLKP
TNFMTGMKRDMGGAAAVFCGFLTAVRLQMPIELSCTLCLAENAIGPDAYRNDDILTLKSG
KTVEVNNTDAEGRLVLGDGVFHATHEISFKPDVLVDMATLTGAQGIATGHRHAGIFVNDE
EEEFSFLKAGRVSGETCFPVLYCPEYHVTEFRSPVADMRNSVKQVNNASVSCAGHFVANH
LSPDFKGKHIHVDMAFPAFENDKATGFGPALLMEYLRNLR*
>TCRU_102 hypothetical protein, conserved
MDRHGFWYAENMEQYLRLKEYCESVRRLPQQIRHFQTDGLPCMPLVVELSRSVNAASVTS
LPAPPSFDEVAPIATMERNRMKLRHRTCLK*
>TCRU_103 conserved protein, unknown function
MGGVPSRDAYPTFFSLRYGSHEIALIPLLRRYFCNENKELRKQFVRMRNDPSVRYNFMRS
PKYSMKELPPASYTEKDLDTFLDVIEETLYDSLKEMVEDEDAPLRPVPCAVCFVYVNGRE
GTDCFLFYGRRINVIGQFGCHRYEDKKDDEPCLYLQKAILKAAGDRTLERVVTLAAMALK

EEFFRAVTTVNREANAKEMFLRRISRLPLVSSTFWNSYPVLKFLYDTAAWSWSVGNVQEH
NDAPSFRFYEVPESGESGLVMEMPGSLLLRFIFWACSSINPSAVRGMWEQYGMDDTAMHK
KEKMIANWLETSPDVNMFVGGVEFTSRVKKHLETLLSEVVRARETPTEDLSVWSAEQECS
HDGKIYRIFDSPLCGPFLTTVKVKRDKKNGTTSGAEGNRSNQTLDPLLNIAEEHKKRDDI
PPVSRVRVGGASQPHATRPPPEYEEAVNSMMPSPFLPSGPSTICPVSLPATVLPHGNVSI
TAPQNSSVMMLPQSLNPASSSFSLPASSASVGPTFIPLYSEPPKPAPLPMQSFGIPYVVL
TPSGPAVPQQNIVPLNSGASTTTTTTSPNLTTMESSKNDGVSGNAGNITTENGATSTLFP
SIPSTPFVPTGATFTGVLNGPMGLQSRSVQPIGNYVLLPDGNIGVLNSVPVTPINYWNT*
>TCRU_104 conserved protein, unknown function
MGAFPSHDTPELFYTTFVPRPSTETQVEVCLVPLLPRFFNDTHKKAKRKLNDILSRRGND
PPPAAATPSGTTTPGSATASANSRRTETTSGGPIVDCIIASNDPFFLVKVKDAIISSDAL
LGNKPHIKKKLTTPDLPSASAAGSATEMNTSHHHQQQSTSHSSPVPLLRPVPFAVCILDP
INPSNESSSATSKSHSHLNRAQQNIAANSGNVLIIKGHRLQIVGFISCCLGRPVHLINPA
TFTKTQALGGGGSSAYSSHTGIGDSKKPSIPVVLDVIFDDVEAHGYLTESVVLLASLAFR
KQFVNSSALYYPAEDRDVEASFDYYMERLQLRIILQHNNYRELRMFYSQYALLDMIKELR
MHETAPPHFSMYENHLGEYGLEVTFSNKFLGLWLRYVENKMRVDSVKGQHIRCNTIDFLQ
ERQRLMQERHDSGAFLEEEEEDERLYGGSLLASTLKQWLLKGSQSALQQQAQLAAAQQMI
MYPPTSGTATTAANFWSHMPTMFMGAPPGWPTNVMPFNAAATSIPLMPGQQQQFMFPGSR
PQLQYASPQTTLPGASPFSSPPGLHVLSPATSSYGVAAARGVPPQQILYVLPGGPIAQSS
AMPHAPTATAAYPPGAAPPMYFAQPPTYGVPYSSYMFSQPPATAPAPPE*
>TCRU_105 Overproducer of inositol protein 10 homolog, putative
MAVAPNVLPPFAAILPGLPLVTEFNCLDGVRWIAPLGNAPESIVVFLTSPAPLAPGICLG
IYLAREDDGAFLYVGHLSNMRPSTILRVPPVLLNVDFPVRLLIGISQEKEEDVANLGATQ
EQPLQQEQAATRLAMAERIVEDLFNFVMSYGRVLSADPTDAQSEETVYLPASFVSRWRER
VLTKMRKDASFWN*
>TCRU_106 serine/threonine protein phosphatase 2A regulatory subunit, putative
MTEHFDSVKSMINNLRSEDPEERLTSMRGIHLIASTLGPERTRDELLPYLTDYLDENEVV
LRVFANALGTMLQEVGGADHLQSILTPLELLSSLDEITVRDEAVTSLQTIGGQVFRETGE
AAAQAQRDFLDLVHRLGKATTQCRSSASYLIATAYPHVSTAIKGQLMSLFIELCNDKEIM
VRRAACISLGKHMVHVLGNRSTELINALTAFSQDKFDAVRLQTVEAAAALLKALPYELHA
NVFSSIKALMSDYSWRVRHMAADRLGKLAEAMSPAEVKNILPFFRSLSQDAEAEIRASAV
FSMASLLAVFRDPSAKRELLTAGCRLVNDENAHVRMCLASAVLRSVAHVAKELWATTIVP
TCTQLLKDQEADVRLALVSGFSSMGNTPEAREMAPKLVPVVVALAGDPNWRIREVVISQV
PFLITSLGKNAEDVVELCVQHLVDRVATIREAAVRSCCTLVAENGTAWSRASLFPRLSSM
ASTNNYLHRVALAHFYASLASVQSLDCGTASQHILPMLRLFAQDSVPNVRLNCAKALLAL
KKGRRLLDSDTEPLISRLRKDADVDVRFVASED*
>TCRU_107 rRNA biogenesis protein, putative
MLFRDYRVHHAAPDGIVVQLPGNGGFGRLTVETLGGDLLAEKLLKRLASREQVRAVARRT
RDAQQYRILSIQLRELWSDVATYPPPVATLLPNLFRKNVLIGLGTTTMARVVKASPERGA
VLSIAGGLTALAALEHVDAHAGAVEVRLLNYDVQTGVANVTARTEVVERTPSDVIAMQTI
FSGLQAGDVVPATVLLSLTDDHCAIVEVPCGASSLIGYYVYNWHGTAASGEPPVVGASMQ
LVIEFVPHGSLLQEVLPFLVLSSNRTVFTLPHWRRAPLPPGMKGLLGLFPWRDRKRQHSL
EGKQQQRQQRHPETESDENDSIDDNDGTTDKNGGREQKLRKRKIEEAIDAYERSMETTVP
SSPDEFRRLLLAAPNNSYLWVQWMAHHVALQQQEEARLVAEKALSTIGVRETQERLNVWV
AYMNLENIHGTVESLSAVFKRALQRAPDQLVVYERLADIFSATRKPNQLLALCRNMVSKF
RNEQRTWERLGKVLIDQKKRDQLRRMVKDMGGALRRDAYSLTVVRLAVHEYKSGSVENGR
ALFEGLVVRMPKKSDVWSAYLDQEMALLVRRDESAAVPLVRALLERAVATNFSAKVMQQF
LTRFMSFERAYGSPADVEKVKTRARSYVEAKIHASVGDGADGIVTAVHVSAKNNNGNKTK
KKDSGVTDETDE*
>TCRU_108 hypothetical protein
MNGDGVKTPVDQNTTGDGAVANSKKGTSGQQRKQHPPKRGAEKQRTEDDVRSPVGKGLNT
QDTARTVTSSGLRRLSHKKDVKRSCSPDPITEIQCEAGVATRNRTRRVNPPDSLCTLVSI
GGTKFNEGKPDEKKKAGHNFSNDAKSENWTAAAATNTKGFNGKSLPSKVESPPSLSPAWI

LHRGGIDPKQFQQQRRQQTGKSLLTEDELSVFAGLVILDGNDGCGDDNDDDGSVEYEDES
VVVCPFRLVGSCVKGQHTEREMLSSDTIHSHLMSLAGLVHELRTEQRITEKECQKLHVRL
ESQKITIERLERYILAQCPASGKLLQTEATSPHEIGPEAPRTDAGKNPKGNILPGSILKP
AQDRSVPMKPEMNSLSNSKLHFGGESFSERVRSKTSVETLAFEPCNAVATKKGEFVGYEG
DRHGSVKYMAEELGTPVDLSRAALDLSQVSATGGASRKGANRHASGKQRHQKQEMPDWRG
HKGKAQDDRYGGAVGVVDDSRGDVLSPIPTVVGLQDNDMSSNDDVVVAVASSVHPSGDAV
QLPDLGERCTASSKVLLGKNNGRQNGTREKAKKRTIQGNSHSFGSSNEKTKRQGKNLRCV
DSALEEAHLSILRPNPKTFVECEQLRLRYHKACCFILYLFFIVIIFFIWSYFFGVFA*
>TCRU_109 Quinonprotein alcohol dehydrogenase-like protein, putative
MSVGIYSLKAETNASSTGGSRGLNRMRRLPAIQLDSWEFRKHAREGSVMPNALVASDVDG
DGVEEIVVGTTEGQILVVKPDHRMPIFSRTLAATISIVLYSATYRRLVLVTLEGTCEVTE
GFLSGGCKVPPSASSNALQSKGQCDWGLPPSLEPSNHMNHGWGGHDEGKKSNPPSTSSHV
FCIVPNCTCGDITAEVGGTTIFLGAHNCIYVYSITTDGECLGSLLLQAPVTSMKCFTVPF
ATEGHLRIINAPLFPCRNAAATPQTTFDGSTPRNDAVFIGSSTQTVAGATLLLVSCGERL
VLLNASSQDVKEWGGSGNLRCVATQECPLGDFPLTGGPNIHTAYSSSFYTTDVLNPYEVT
GTRRTPLQPLWQCSMSRTSEMQQRQRWPLKSSMDEGHTQSGFFDLSTVSLPVSSPDTCNV
VPREELLLSPRRQQLQRGVCDTAAAGALLNYKEDSETALDLLENDLPVSFAVDVAVSKNK
LQFAIVSEDGRWLILEMSPACGEGSMGAVPRTLSTKQHENGNEYPPNKWFSATIVCLASG
CLKPQWFLSCVCNFCVRQDEFCTVFLSTEGNCYLVGSNSEISESRLGADASSFTILKGGF
MGNSVHPAWASSNCATTVMPTGVSSALVRDFTEFPQEQQQPSLLRASSGRGVCLVCVTLD
EIVISSVGSELFLSAQQSSVPLAVSRGLNFAGGDGIHGWNETRSSFGITAEGGAHFVDEM
DEREEELMLQLGTALLEAEGYADPTALFSPEERRFAARMLCAAGYTEREWELLWQMDAEL
SE*
>TCRU_110 Mitochondrial Editosome-like Complex TUTase
MALMVLQELGLLPVFSKPTGDFGELTLPDVQLQLESFKLPAIYDSLQGDDEKLGEAVFFC
LQRFAEYYSKYDFRSGTVSLIHPRRHRSVYEKVVKQYLERLGVKKRIEWEKHLAEHKEDG
AFDENDFSAAMQNEATQRPCTSPFVVEDFVNYVNCGRRVQATRVQHVQQEFNRLHEMLVD
KEEELDFEEVFRESDVVPRFHGFEGVGARDPRVKTFRAR*
>TCRU_111 SET domain containing protein, putative
MPVYENRHLLRLLLTPQEQARQFVEYCRAHQVITRGLCRIGTTKRGGLRGLIANKEIKKD
ENVVMLSVRASLHPGTALRDKQFFDLLPLREREMYFLRRPFIENSRLADRSIIRHNQFLL
ATYMSYLILRNDVNEGSQALKYVNFMPRNEGDFEQLMCNMSGALDSVEVCQVCQSALASF
FKVTQAEVRPLLLWSLSMIFSRMAPIDHRWTLRHAFSGTPFVDAFDITGPDDDENLVKEP
IVFLCPVIDMCNHSDHENVALMVPEKTPTAHEPVICLRSLRDIENGEELTMTYGGSPTEL
KVIWGMQHVL*
>TCRU_112 ribosomal RNA methyltransferase, putative
MLHGNWKYLRQGGRQWMEKQARDPFVLEARRRGYVARSAFKLLEMDDKFGLFARNHTRVV
VDIGCSPGGWLQVARERCGDRCALFGVDLLHVSPTVTGAVCIQGDFTDMAIQQQLLSRME
NVHANGLVDVVMSDMCLNRGSGGTDDRQKQTQLNMEALAFATSQLRTGGNFVCKVLGTPT
AFEELRGELQRWFVRSTLCKPFSSRRGSDEAFMVCLAKRETPRPRGCNIAEGETRDFGLD
DWPGFLRRPRQQR*
>TCRU_113 hypothetical protein, conserved
MWLIIFVILTAVLPGCAEASAHYMYDGEYDVLRVGEVRFNDTMAFPASMATEVRRRYVLT
GVELGTRYLVHLSHLGSPSIEYRIHVGRVHRSAIKGKIWPAKELAPGRRKLADVIMLHFK
THVDELLFEYIDGDGDVDNGDIEYGVKNDELVVLEIRGTRNAFPVQPDKWRKFSYNLRVE
AIGSGSGINRLIMWHAMLVITAVAVVVVLVPRLIGNTIACDAPMPTRPRYDFLIFLVNKR
TKTKA*
>TCRU_114 acetyl-CoA synthetase, putative
MENLTVEEARHRHDKDVVQPYKANSAKSYVGKSISDRQALYAESKKDPVLFWSKIAADNF
YWKNLWPSEKPAMEFNFHKSKGKVFVKWFDGARTNICYNALDRHLAQHKDRVCYYWEGNA
EGETSSVTYGNMHEAVLQLAAVLRHQYGVRKGQVVTLYLPMIPFTVQLMLAAARIGAVVS
VVFAGFSGKALASRIIDSASELVITADSSFRGEKPIPLKEVVDVAIAECESEGHHIQCLV
YERHGRENIPMKSGRDRWYGDVVAAMTASQLADCPIEWVDAEDPLFMLYTSGSTGKPKAI
LHTVGGYMVFAGTTFKYSFDYHEDDVYFCTADIGWVTGHSYVVVYGPMLNAATSVLFEGTP

TYPTPSRWWELVDRYGVTVFYTAPTAIRSLMQYDDEPVKRTSRATLRVLGSVGEPINVEA
WRWYYGVVGEGHVDISDTWWQTETGGHLITPLPGCTPLKPGSATLPFFGIEPALLDPMTN
AEIDGPGEGLLVIKSPWPGQARTIFGDSERYEQMYFPVDGYYMTGDGARRDEDGYYWITG
RVDDVLNVSGHRIGTSEVEDAVNTHPLVVESAVVGIPHNTKGEGIYVYVTFHQNTIIDKD
LLNAVRATVRKVIGPLATPDHIHPAQCGLPKTRSGKILRRILRKIAAGIFDGLGDISTLA
DQTIVAKLIEERNGVSI*
>TCRU_115 Domain of unknown function (DUF4200), putative
MAGTLESITAATQLRRAVMEVQKELDKKRELYMVRMARVREVEDVIAADRSRLQDKLVQY
YKFIQENEIRRGRAVRKAATEERIKREREEQIVELTAKLDSLNKRREELRQQYDAYAKYQ
QYLEGVLQRNDCDEYQSPRDIIQRWNTLQDNTKVLQRRKTQLEEELLRNKNSLNLKRQKK
NNESVELQNQLNELQATYETMQKSIKIKQDELERCINQRSSTSRTVSHVRMACKNLYDRC
IAWTAPYSGRGKFDVREADVLFQLHVIGDCLRDFRDVIAAHHNSQQQQQQQQQQIAASRA
EKEEEDE*
>TCRU_116 nucleoporin 48
MYQPLLELPSEAAAVFVAGDRLLGEAAGSLYGSSKRVSFEAQGGAVYPLWKLANRNVLGR
RAGREKPSSLSSSVFSLSWWKKQQTFGVVRWLLRPLVSVMPLLRFVFPFVVPASANCAPR
LLNAADCPECVTAVAFHPDRMVLAAAVDEGAGCSRVVVYDVAEGKETCVLTHAFQRHVRC
VAWKPLSRDVLAVGCDGGALVWSLSFGVSPVVTVYGRSNNGHSEKGLGAGEEDDVDRKAH
CLFYRCTTHVPTTCISFSCRDGRYLACGSTEHIALHFHDIRLPPSNSSLRKNVSVEGATE
DVLFADDDTFAVKLVCGASVVVMIAFPSCVESVVPTAAAVLKMTKAQALGPNHFFLQCAH
VEGVVIAHANPFIGVQVIALVSTGIAGGIGGNVRRMACSDRRLYIALETGHLLVCHYGRR
GGAFTLIPVGVAELDAAHLAVFDGFTRGSLLAAVEASDQSVIFMPSYHS*
>TCRU_117 N-acetyltransferase subunit ARD1
MQIRRATMEDMYQMQHCNLRCLPENYNLRYYFYHILSWPQLLYVQEDYNRNIVGYVLAKM
EDEEKPANIHGHITSIAVLRTHRRLGIASRVMRASMKEMEKEYDANYCSLHVRKTNDAAL
HLYQETLGFRCANVEKGYYVDEEDAFHMKKFFKGPNPGLYVAANRQLVRQQNSAAAAVTG
MAALQRNGGTAPPPPPPSSSSVGGQRGENDTADLEKEQLKLVAELLEESNKESGKGRQQK
QKQQQQSKGGNKRGKTKK*
>TCRU_118 hypothetical protein, conserved
MFREILECVLEFVGSHSEQYGCSGKELLSFVSKDALPYHGLVDHASLRQMVLDLLLSESS
EVSVKRDGNPVVPSELLNGTVPYEQCTFYPSMALQEKALGVSLRFKNTRIAVNYACDNLV
TGVRRFQNSAAGEEERRPSEAGLGGGIRRLLREQWLRVFYVCVDEGAPVVPHLFPHFATP
PPPNQPKQHLRPLEAYLARRRGELQPPLIVIDPLVARQIVLSSPERRLVFEDAIHAIFAA
HGHEVPVINGRRMYLALRRMLRAGGLRIAIAPIFIRKSKRNARVVVANSSPDASDADADD
DDDDNDNDNDNDGEDDDGNMSGASNSSDEDDSDADEGNDDSKEHGGGAAADLNANAKRNK
ESFGVDPSYPLSLQLVKEAERRPLVVESVLPRVTLYDLRATGNELLRFARQFEERWETIE
STNVLSAHSKAFTRLLRPKGWGDDRHGSADKMSSMEGKELDLVKGVSSWAVNKVMEALHV
APLQALLLNDLLRIVDRRTLGRVIPFLRDKGLVATTGLSTPKGRRLGVVTIAGVELTAEL
RAKLMHSHLDGASVRGQKRKRSTALIKHLPDPKKGDEDDSAAAAVATKPCSRSVRMVAKV
TMVRNGYSRIGLFRLSRLHLELWRLWLALRQTPSTPMTVERVLHEMSFSSFCIIVGVADV
DVGEYVGGRSMESCLWGTPICRLPPSLQTRCRRSGVQPLLHSLAGLQSKGLLVCERSLVA
YLDVPLAEVSIALVSTALDERRRRHIFCDGTDMTPQDTCRAVLNFWSESWTTAAVTHEPF
RAMELISEVVATEPNLSNAQLIALSRLLRIDPVILAEHVLQRQGVVRARKRTLRDVIQVE
ESNKRRGEGHRASCTRRRIEVNGVTMAEALGAILQSEQPYFGLANLQRLVRGYSKELCSS
RYDPQRKRVHGSIQTLIQVLMNVIERQQQSGMPPAVHSVMTSVLSPNFSLAPDDGEQRAS
EQEDGAPTGPAIQENSAMDASHGVPCEALQDVLRMILLTDEAHYDAIAAKALLAQFPEEE
QLRCIDWLFKFPSFRTRSGGSGRIPRIELSPIFTFVPATLATTVTHCQSRASNFLQEITT
SLLFTSPPNCQRWCVPPFAVTENTTGRDSLCQAPRLVEQPSMNLDSFAEEIREQKGLRVL
RLPRFAWPSHPAAAPKTTLPPGVAAANAAVIPEAVSAATLTRTKTEPYPARALLRPWRHL
NHLELRDIPVDSAPSVEQVIMARSASATPKNEGLPLPRFPSIFHHVDGSFHEFVWRSFLF
ALYSLVHHFPGITKEQLVQRLKVSGLVSDASCHVALEFLKASLVIVARHVLVPCDGEVQS
PFSSSGSACKYSECYFCTVSRDGPWRIMEL*
>TCRU_119 hypothetical protein, conserved
MSLSAATVLYNLAEKVNQIDNVQELTLILQQQFVELFRGGEMPSSPSKPKVSSLASTEEE

KLLVVRAVEAFHSVGLQRHGVFFQSDSYHTNAAFFIHELLASLSGSRRIWFQKLQKASLH
ACVAVVKAIGCRNTRLCLPGIVSACVHYIDRAHHGTDAVAVRTGAVELLRVSLKASLVSQ
GDDTQWLQSTVGHLGHSMTRLLDPSRLALGAKTLPFAQSLLHLTTDLLLLPSLGTFLDTP
FMREVIVGWFVLSNVNFILSGGVFGGATDSTTQIPSGFFQERAVVELLQAQLKKLRGVEL
LHFSSALLRCKDTRATAFPVEDHNQHLMQRVLQRCIQVVGTEMSVEDLYSAKVSRSYPCI
VGDTFIECAAYAVTATTTTTTTSEDPAGCVTVGEQVLKDFMAECEATLADWDLYMLHPPT
IYVLTRLVLWQFRTPAWVTEEEEKGRPAEEFILSDTFEQLWSIVAQPHLWNITEDENLCS
YQQLRHRQTVAATILCFLETAAGLLRDEPVIHRAKRKRAVQRLNLLVLYIVLEKATGPTF
VRDAAMGVMKALGEAGFYPDTLAFFLSQSGYIVDEAARAFTEEDLRVSAASVLRGGVSFL
QSKLLSVPGKEEFTAITSRQWVDVPRGRNPAAVVLVTSAVPKVSDFVSSAVKVASDGCRR
STLQEETTGALASVALLLDCFSVSAALNRSVPQLGIHEDQDCMTTAAEPRVQILQLAVLE
AVQMLLAYCTRNDAVAPLAISAVTRGLTVFLTTPRAETWINETESNGDQPPPVFEWEDNT
PAVLPRNHLRTVYQVYLSFLAILAEPVSGFTTTSNSKRLSAQERRVLEAVQPTPAVIAAM
EGLKALFALATDFLSRRVVEEVLPVVFTWHERGALPRIPTKTDERLQTAAKKFLQDACVI
DPSLEKQLHAECAFLLPPLASVSAAITEEA*
>TCRU_120 long-chain-fatty-acid-CoA ligase, putative
MQNVRLFLRILTFTGCVGGTNSRCGGFAFQLSYRFGTSVAGTYYSESDVASGGSMNNDMR
LGLVIKPQSTLVELWEQAVKVWPLRRFLGTKMWVRGQLGYVWATYESIEAEMAAMRTLLH
KMGIDKGSRVVVISENRYEWVVVHFATLQLGGHFVVLPTNVNPMEAQQVLKSTQARVLFV
ESTSSYATVKGWIGEVGELQHVICFEDQIGEGSYAVAINIASDVPEKIPPRNDITAEDTA
MIMFTAGTTGPPKGVMLSHRNLVANISSIYAHFGGALTHTDLFMSLCSWCVAGAITVELY
QAICKGACICIPPEQLEGFQDLPLVQPSVITSVALPFQRAYANMVDDIMNRGSLTKDITR
FTLGRITENRLMLQKPGQMLRVASHVLLGKFKEQFGKELRIAFIIGSQLTRDQMELLADL
DVFTVSTYGCLEAGGLIATDIDVPLRLKALPGIEVRVVNEKNEIVAPGDIGELLVEAPHA
MQGYFDIHIDPDEAKNSLVAYGGRTFVRTSDYGSVTGGWISIKGNKDVLITLANGAVVDP
LEVEGTLIKSPFIRQIFVHGENRPYLTALVVPNAKAIASHLRKVERRDGVPIVSEREKAD
CIRAELRRVSQGLPSRSHVRRFVFIEECTLANGFITCKYGFARRRIEKHYVHYFNAMYDE
TPKFFGHAVDDYDDLF*
>TCRU_121 radial spoke protein RSP4/6, putative
MSVRPLVEDVEEMFGRTKAYLMNANKDGVSVYDQLTRLMEQLLDENPHDIAADPSRFNEI
FTLLQQSSFVNGQSTPACNEPCSVPPSELARLADNERLFDRPPPETLTTIEQPDPYTTVT
TTRIKPHTAPPYDSVAQHNLYWAWAGCGVTEEEAFLLDRSITMLAMEKKLRDVRFFGKIF
GTKADYYVVSSQRYVQDGEKVYKEVNTMPRPARRKLEVPVQPEPGFVGVNRLSFWVTSHP
AAEWKLLPDVTPQQILAGRRIKRLFTGDLNAAVIASPPFEWNEAVYLRVQLSRIASGTYI
SPMGALEEPEEEVDEEEEAEDDEGNAAKKPREAKYRPLTQPVKGFASEEDSDVTQWAKLD
QWVHSEGYIYANGRQTKVPEKQEDEEDEEAEELQQQQQQEEEEEEEEGEEEEEEERELFTP
INLDFLYAVINIPEPPKVDEEDEEEEEEDDDPAEDAAEAEKDFDDMPPKPVTDADVPDDD
PMKVKIAAWTVRVVNNNSKKHRVVVVRSLRWPGAVAFAAEGGKKWGCVYFGNGLNKTDFS
FTPTLAPPVLPECGDITEVDDPTPATEKLVRRGEDPPEPDSEDEVEEEEEEAEGDM*
>TCRU_122 serine/threonine protein kinase, putative
MENYLIVELIGEGSFGKVYKARRKGTGQVVAMKFIVKKGKNDKELLNLRSEIEIMTKLDH
DNIITLFEAFETQQEFVVVMEYAQGELFEILEDDKKLPEDVVQRISKQLVQALYYLHSNR
IMHRDMKPQNILIGQNGSVKLADFGFARSMSYNTMVLTSIKGTPLYMAPELVQEQPYNHC
ADLWSLGCILYQLYYGKPPFCTNHLYKLINQIVNDPVKFEEPISPDFKSLLKGLLTKSFS
ARLNWPHLLNHPFVAFGEEDGKWQKKIKEHEMVMKEHMDRLECFRLCAQPSRFQPNSSGH
TPRSTGVSRQSSLGSKESCLFSASSIEIISSSKNSQEIIETFKGLAKAADTLTSSPSQST
ILLERIPQMGILGPTLRRINNGQTIDIVRYALEFIRILVFPENGSVFPFPSQYPSETIMS
ALQSRSELPPFDFLIRQQVALELMEKPQETLDFMMNEFIENRKNLREDCVKVIFQCVKWG
TGFGAALIKMRIFPIFWASLLDSVKESTVRNGSHFQFYAAVAFHTVSALVPHIKLSSPDQ
INAHKVSEFVSTGLTAVCYYDPSASGDIAALNCAAAAALLVAFVHREMKDTIVFEPDSIL
MDGLCAIVEGVRRISDQLMVSRALGTSYGFADYGFLDGVTHMLSVVFSDPKSIVYQQNSD
SLNRNFLDNDKWGSLRTVIGLLRDSDSKVELSPNGVLAALRCVQQALQNQQEGMQSMSLL
IESVEPYYGDGGGPISVIAIVCRQLRKNYLKQLRLWPEYRGGGTVGVNAHLTIVVQILLV
TFQPAKQGSRSDDDTIAAVQQILYKEGVMEMLIAALDYTEVAFWGSPFTVIIKLVMGSPL

FAKAFVEGDGLHPERIRKVLDSRKASIGLVSDGLNVLSQLARISRDFYPPIHSANLYESF
LDLMRNRDAGIRSKMCNLLGNLCKHSPYFYEPLAKHKLIEALVERCSDEDPTTQKFAAFA
AGNAAFHNDFLYEILRPSIPVIVKLLSSSDEKTRQNAAGAVSNFVRNGGSLTSALMGNSV
VEALLRILRKDKVGLRKIALITIGSFCAYEECKKKFLWLGLEDALTQLEQSDAIEAEPSI
AKYVTRIKQRIGSS*
>TCRU_123 ALBA-Domain Protein
MSAENAERPKNAVRVGFHGSKFLYVDITKHLLHDGEKEVSISALGKAINEAVSVVEMLKD
QQMVLVKKISTSRGGGEGARNNTVDKIEIVVTKAPGFNEKYEEQQKVREAKKLEKGNENV
AK*
>TCRU_124 ALBA-Domain Protein
MSCDAEEPSGPIQVRVGVDRRNFAYVDFTKHRLHEGKPEVIITGLGRAISDAVAVVEILK
NQGLVTVKKITTSRGNVKSSSTSVIDKIEILVVKSKEFDSIYEEQQKRKDESAEKGQ*
>TCRU_(Ubiquitin related modifier), putative Ur.1
MSSYRRITVQFAGGCELLFDKQTQLQLEGIVPHGTTINGLVQMLKVNHIKERPDLFVDQS
GTTLRPGILVLVNACDAEVVGGLDYVLTDGDTIEFISTLHGG*
>TCRU_126 selenocysteine-tRNA-specific elongation factor, putative
MTGEETLLANINVGILGHVDSGKTSLAKALSSTASTAAFDKSPESQRRGITLDLGFSACC
ISSAVVAPPLHAAGIEQIQCTFVDCPGHASLIRTILAGAQIIDIMILVIDACKGVQTQTA
ECIVIGEVLCKPLVVVINKIDAVQGSTPEEKRVTLDKLRKRLQRTFERTRWPSVQMVEVA
AAPRRDEATSGEVSHPINMEEVISAVLGLVDSTALKDQLTTPLRSEDFVMMFDHCFVLKG
QGTVFTGTVICGQVSVGDNILIPDSHVVKKVKGLQAFRKPLQHARRGDRVGLCVVQFDAE
GVERGILCSAQHQRGNAISNVPLLFESTSVIVAEVNRVRFHQLLCDAHTKFHITIGHSTV
MGTMKYFSRPTTIAPEEKEASFDIWQESMYVEELTEDAFLTFHSTEGQPQSGQPVAVKRG
PLTYYAIILLERPIMAAVGTPLIATRLDMERDNMCRIAISGSVRAFLSSGTSADASIISW
RELPIVRFKSRALVVERVVNEKTCIASGVVGFEAPETRGSSINQKSKEKDTLHAEVKKFI
GMRVHFRPTGEEVNDATILTGVKEGIIDSAFGKTGKVKLVFKDPIFAENEPSTTGRTKRG
GVTFLTDGVVELTLKKYPLAVHCQLQQ*
>TCRU_127 Bacterial transferase hexapeptide (six repeats), putative
MNRLEVIRKSISYVGLDPAMFKPYPFNVSSSALSELMDCMSYIVFPEFSEPPMNPVTVPE
QRLGGSALLQWTLTRMADIINSQIYSALMLQGHTKCSKDEAGIPHSLNAESGSNDDGFIP
QNETHAFGEVSSFHLEAKKKAEEITEAFLMRKLRNIRWLLRTDAESILNNDIASNSLSEV
VLCYPGLRCMKHQRTAHALHELGAPSNFTRLLTEMAHSTTGIDIHPATSIGHHFFIDHGT
GIVIGSTAIIGNHVSIYHGVTLGTRSFPVVAKTGERVRNLARHPIIEDRVTIYANAMVLG
RIRIGKDSVIGSYCLVLKDVPPKSMIRRTPNAKLPTPYITI*
>TCRU_128 hypothetical protein, conserved
MFSASALWLRGQYKTRLKKRMVGFIPKVIPRKIKNNMVALRSEANTGHMEGYMKTEAERL
DATGRKVQKVLWDPVLQRHCLFKETKIRGPFMTKANIVKKMDFPIGAAGTASLK*
>TCRU_129 hypothetical protein
MPLQKSRVAAVVDGEDDSDDGELPHTANAERVPFVEELKQNVTLLYQRAAVQSSSQQATP
PRNDPHVVELPPPPPAPSRELQVRESDGSLSALQRQLTALQSVLKSSQERFQVRDDATAR
RSLPIVTASQFKRPISEDFLHELRECRLTQSGGDENTAHLKEGFSCHCENNLRTAYNRWR
GILAEIHANALKRTSTIASKLDSEIKGQKQFLEEARQRIKGTILLTSTLSDAITCFTRYS
PEHYGALQENRKTPI*
>TCRU_130 ribosomal protein L3, putative
MSHCKFEHPRHGHLGFLPRKRSRQIRGRVRSFPKDDPSQKPHLTSFMVFKAGMTHIVRDV
DRPGSKVNKKEVVEPVTILEAPPMVVVGIVGYRQTPVGHKTIGTVWAHHTSVEFRRRFYK
NWKQSAQLAFTKRKQFARTTEGRLAEARTLKAFAKKADIIRVVAHTQLRKLRNNRVGVKK
AHVSEIQINGGTIAEKIELAKSLLEKEVRIDSIFQQSETCDVCAVTKGHGFTGVVKRWGV
ACLPRKTHRGLRKVACIGAWHPARVMYTVARAGQHGYHHRTHLNKKIYQLGRAVSMEPNQ
ATTTYDLTAKSITPMGGFVGYGTVRNDYIMLKGSVAGPRRRVITLRRPMAPQTSRKLTEQ
ITLKFIDTSSKIGHGRFQTKKEKSQWFGPLKKDRIRREERLRKERVARAAERKAKGVAVA
AAPKKAKK*
>TCRU_131 Patatin-like phospholipase, putative
MSRGSANDDSKVFDLTFGFAAGGWFGMYHFGACQAIVDSGIIDRLTAEGKRVRFAGSSAG

ALVALCMVLKNYRFKEMRDFLMGCAEHYRYSWLNLFRIDRYLDDALDQFGGHLRELEMKQ
ELFDTLNDGALEIFVTTLPKMKSKIFTEFHSHEDVVEAVKASCCAAPFVGVPFRLRGTGE
WVCDGGLSIVTPHNGEPRTITVSPFYCSTATVHPTIFVPFWWGLRPPGKVALRNLFALGY
NDMIEGLVVNGYITPEKGESLLRPEVQFLNGGGMFRRYGLYLLDFLAFIFVRPVIVLCIY
VELAFMSFFYFLRGLFCLDNRPIRRMYDNLRNIVSIRTLGRLIFGENVPHNVERLEKSSR
VYRVFNPILLGGKNTTERAVMSPTGVFSGVMPTLSSKTRKRATSAAQEKSEKIYRRL*
>TCRU_132 hypothetical protein, conserved
MGMASTVFQEPGRLPFAKGLWIIVPSVPRATGRDGAGIHSTCRRGQGLSQAKSRGWSRQV
HTAGKADPFPSHQEPKECSEQQQQYKAVWHVKPHKMTDKQICLLPRQGAYAAKFSAEHNR
FQRMRMRVRRRHAVKISPPRAG*
>TCRU_133 hypothetical protein, conserved
MHSRTALPETTRLNWSTTRQRPGTQLFLHCVDDLPRRMDNIYSASALMCADEHILVASGA
GIHACAAAMQPALSPITTWAAEHNLKINVDKSEAALFHISTHTLSDEEMVDLLLGNRNLR
IQSRPVRLLDTTFDRLLNSGPHASTAAKQTTPRRYQLRPVAQAGAFHHNTQSFSIGYGHG
VSLYSGEAIVPCLAPTYLHNTELLYRNSCKTHLGISAPTEDTSF*
>TCRU_134 hypothetical protein, conserved
MEQAEPAAEAAHKAHTSPRQETACFEPNSSKKWRTKIKHYAVASLCFWKPARKKLGPHPA
RAGDISAAWRRRTLIPWNRWCSALNFGLRSASATGKSAPPSFCAGLTCHITLYCCCCRRT
PLLSGDREMGRPLTPYELDVAIRDSSLGSAPGHDNMLNEFLHRLGPVARGTLRTMIHNSF
ADGGLPGSWNTVDTIPIPQPGKGPCRPESCRPTTLLSVLLNRKNDSPPSVSV*
>TCRU_135 hypothetical protein, conserved
MKSHLFAVLGLWLRIGTPLLCIAASTTETKTTPPLEKVPRHARCGEVNRSACRLSGKTAR
PFRCGHSVDVTRGGDVRKNSGTEEDGYITNWQLNIAGLSSAKKIASAARLEQRPPDIVLL
QETNTRIATAAKLTGHNSFLQAALGAVVAVSLWCATLCRASA*
>TCRU_136 retrotransposon hot spot protein, RHS3, putative
MLAAGRLLVKDYNVTMEVFVQDPDDYVHDQRPLEEILNLTAHQALEAITRPLHEGVLSLS
GNVGTMKEKHTITPLARRTPNGVLTQVLTEARREAEERLRRTQEMKFAISTNIEEVLFKG
RARVNEMRLNDFLTRELDGRGVAETNRDVMLEEFFRDPTRYIRDKGALNEMQASGRYLGM
KRAVKGEVVFDEDTRRLCDKGVNNLPGRSLAAADVKAAVHNSTKRFLDAAAEEARNPTTT
SAPEKLEGVCKSVHNARRSHAVELPDDDERIKTGTGMGVHEGKPEQSWSYREVGNAFEKD
DAVQQCGSAPPVLMVLASEKGWPYSWHTIQDLPKDVFVNCEVERVWQTVKGDVTAWSSSH
RGTDFKPKRRVLIGTPGIGKSVNAGSYLLYQLLHCDATKLQVVVHCFGEEMRTCLTRPAG
R*
>TCRU_137 hypothetical protein, conserved
MNSHIVAVLGLWLHIRTPQQGVAASTTETKTTSPLERVPRHARCGEVRRSACRLSGKSVR
PFRSGHSVDITRCGDVHKNSGTEEDGYITVRRLSIAGLSSVKRLLLLRDGSDIKLTPFFC
RK*
>TCRU_138 hypothetical protein, conserved
MAMMMTGRVLLVCALCVLWCGASVAVLSADGAGGGDGSAGEYLPLQWRTRFLTECAEEVG
RRTGGRANASSVEECVRQVMESLHAAVDGRSRWRRQLSAVAVAAAAEGGGKENPNTVEDE
KTSETLATSGKASTGKKTAVASEQTSVNPAPAIVPPARETLVQGKHNETNAKEEKEEEGG
EKDDSEEEEENHKEDDEQEDAEEGEKAEEREEDKKGEESEKNTKEEEAKYGVEEDEEDKE
KDDDNTSEGMPAVSQEKRNSTSGPEGAPNKTNTEGTQTPGDIDGSTAVSHTTSPLLLLVV
VSAAAAVVAA*
>TCRU_139 retrotransposon hot spot protein, RHS3, putative
MKGYIIYDVETQGTQPDPGFAPSTGWGMILVSSPEVSNYDGWEEQLKVSRIIMNCPDETD
VKAMCAWMKRDETKKKQAECWRMVEERMEKVGPIIRHIFDADEHGKRTKDVMRALEWINI
GDRGKYFTQGGENEWHSEDPSQKLLKVVRVREHGPIEDFANAPICEYLGVLTVSRLAKVL
SPHDILFLVLGMKNAIQSADLKKYALNAFLNEDFVTSMVKDLKELPPPSPSKPRPSVLTL
NPHGYPTEAAAITELNLIDGLQKLKRRLLYIPTSPTFPLVDCFFFMESPRRTLVGLRMTT
ASAHHTTASTVRQFTERLAAYFEGWEEFSQGLSWEMIYVQHADSTPMNDWQRCDCVNPNN
KIDAEKKIVAFWDGKVHQYRVMLKGKFPEEIKFSDDSLPEGDKKNRKI*
>TCRU_140 hypothetical protein, conserved
MGVARELGHQQWLITAERERLNFDYANAVADVQNRNAQLNSQVEEAHINPSVRESTIAGA

GNQVLELQKQLEADRAQLREREFNMGRLTAQLQEREARHNAQQPARDAETAGRDEASATQ
HAANDGVTTIPHQTDILMAGGEPSSAARRSSAAYRRPHGNVGRPHFHSSTPLPSRTSPLD
AQLPMEEDRSFCALDVTTWDPRAYGMRAPPESLSTSGAHTNAGNRTTSRLNLPLTIWSSG
PARWKSCRIPPLES*
>TCRU_141 hypothetical protein, conserved
MRGQPSLSWIGEKPNALCDANVVISLDTRHLHAMCAPEGINYNTGVLQKTAEGCRTPLMN
TDDGSSIHSTTSHQSSQAAHRTADAPPDLSNCPTQSSRRAMGLVCQRRSTVVRRNTDIPA
LGQLYEESERGRSRDLGAGNVGAENVPSGAIHNLLPHARTADERGELCGLFDGNRSRAID
PTAAHQDAVDHVGNESNPAGHGDSGVTKIAARSETRQVRSSTKEGMNQLIRSRTDWKERV
VLRLAWITANRWSEIAALTPKNFKLRTDGTLILDWPVAPKTARADPHRASRFVRIRGQDA
LDTTKSSRILRLPKSNEPWFLGMPRRIPQNAVR*
>TCRU_142 hypothetical protein, conserved
MQVWGLVGRSSFPPSSSGKAREGSRRIPTADARLLNEAGIIEDASSTITGGWIIPFSVVE
EKTTALRRRWIAWPRDKSRDDPYEANVPLLHISHYLPPVMAEAVSCLDLKASFFQVFLPR
ETRHLFRCRVEDGTLAELTRLPMGYKASPEILQIIITSAIAVVTTVAHRLWAAPPVVRAD
VWIDNIRIAGSKSDVTLWESQVLRNADSCRASMGEDRESGATQYTFLGVHFDHTQRAVSL
SDKFVCSVRAMPALNSSTIAEMEATVSRFLYLAAILVRRLCDCYFFIKAVRRRLSALHRG
IVLETSPANLPPSAVGLDERLQHIIENIIVGESSSPRKGICRHHHGRIAPWMGSRFYSRL
RRR*
>TCRU_143 hypothetical protein, conserved
MAAEGGPVAGGSGRRENVNSEDNSNQEDEGGGGNTVGEDPNPTSAGALTAPTRQDQSSSD
PLRSGTCNPSGTGSASAARMRASGPESLGGGQAAGTTSPRSDASQQAAGGVHAVGGRTSQ
GSQASEQTVTGQSQVPGTAKEAPRGDEGGEPGAQHEADQTTKMTVVTSCAKTAVEEESRQ
RDRKMHHVSIVAGMLIIIIFPCFYRHV*
>TCRU_144 RNase H, putative
MQPHDGVPCTAPGHGKAECPSYPAQAKTLLVVTDSHSLLAAVTKGPHSQTDWTEDQIWQR
VLSLMRAGWSVQLQFCYGHCGVHVNKPADQHATQNTAIGQHTEHGIAPIRHTDLLSCINT
QLTNKWRSTMHQDTHHYLLGGARPSDPGGKHIITQEALDQQERINTARQGAGNPSSGADY
TGP*
>TCRU_145 hypothetical protein, conserved
MLLQQRVSSARAVRTKVASIPSCGLGQSHTVAVPVPNDEGIPAVDEAESPVVRRGSAARG
VRDEAVAEEPGPSRVPAVVLPACLACWHAWQLRWRPAREAPVKKTVAPLRHPHGRPSQTI
ITPG*
>TCRU_146 hypothetical protein, conserved
MQHNRALQRRHIRHVDESVRHQARRALAEAANPQLRAARKHAVAQRHVQCVSERVHRAQV
HRHNDRSAEHHRVEARRRTPAANRHRLRQRHATHAILRTVATTTGPRRRHVERQARLAHT
AGTAAAHAFGERRRQRIKARQRSRTAVARHHAHGGQAGHAVVAAALQHPAAGTVALHRHD
AARDRQPGDKRRAGGSAARNRHGAAAQRHPRQRRHGRRLAHRPHVAQVQPPAGTDRPTVA
AHQRRRNRPLDAHEAQHRIKHREALSKHNAAAAAHNRHVRHGEHRVVTQTEARRGRHMHR
GRGRTARRLQRHAVKHHRARQQVHVAANVHRRPARLQRPQHSVAHTQLRLQQRRRVQHKR
RRHRVRQPAQLRVAHRPRRHNRRVTDRHVRVCRQVVHKHKRAVAPGQPAHAHHQAPVTRR
LAAVAPHVDLRTAVAHAVPQFDAQRAGKHRRHVEHHSHAVRDVHAVHQHRVHGAANRDGS
VHRAAPQSQHHRMHRGHPRSSCCQHQRHKQRHTHSQNGPAPHTLPAARTRTHSRPHPPTQ
TRISRAVALCRSRPVDHSSCLSNPFLQMANPVTRFRQIDIPVLPILILRVIPNTLKR*
>TCRU_147 Concanavalin A-like lectin/glucanases superfamily/Trans-
sialidase of Trypanosoma hydrophobic C-terminal, putative
MGAKVNDGDKNAVLLGLSYNREKRWELLHSGEKTLEHSSHWEPGTTYQVAIVLQNGIQGS
VYVDGQRVCESVQSNLENTKSKGISHFFIGGDGGNAENTGGEEGVSVAVTNVLLYNRPLS
SEEITGLAKNKINIPTPVAARTSSPAVSRPKRSSESLAGGAAGVGTARHFGANGDGSTVC
GSGLLPLILLLGLWGFAAL*
>TCRU_148 hypothetical protein, conserved
MGRNDVVQLLCEKMKLGPLAVQVTSSCLVNAILNAARRAKRRQILLYGIMASTDPIPKAK
LHSSCILLELTVATWGVGVRAVVMFTVPTDTPPQKKRIFFFWKRSDVTNLQRNVADSMMA
KRSGRREGATHPATTPRWDHHRSNFAVQHAAFGAAAVTDRPTTAQHATEVDLTL*

>TCRU_149 hypothetical protein, conserved
MGENCKFVHADVDYSTLEGQPIHVNYIWRDEKLCIYERLPPGDVLEVLLPNCKHPAVMIS
SEYVLATRGARSFSKGRPQTLSHCAHYYFNYLCSRGEDCSFIHAIYVDPTISEVCKRAAG
RSHRQRVPGALRSSVESADSDSQLQCSRQRKKCGVPPEIPVTIDARNSRGDGVMAPTVNA
EGLNSWAGLNGPSEASSEGPRHSSSGEEGTLEPLVGLKCREPNGVQMIGSACGRAAMRRA
RVYRHDPYGIF*
>TCRU_150 hypothetical protein
MCTPNGNGFYGDAMYQTHFCIRNKRENGLIGVTKSRFFGSYGDSASCNSIGNSVSLLPYG
YMGSTNNGGSPTSTANQDIVPATSFPLAVNTASVSIATPTAVIPPASHGSTNTVATSLSQ
RLQRQAVLLVKLADGQPISCMPVQQLVLARPGPLNPASLLTSEDFQGSKRCSSGVSSPAE
GGPRDAGEGVISPLKSTSFLDEQAINTSRATVFSVMSPVTHVSSRPLSFAYHPSATPLAQ
NGNNVLPPGPDSIRKSDFYRRNPTTTNNKGTISIKNQGDFAGDGLRVDVYSSDFSCIISL
SPSAIFHKPHPSLGVSRLVLCRKYFPGRPDSCWKGAMCKFVHADTTGAPRYSIHVNYSWR
SAECCTYPRLPAGEMIHVLAPNGRQPLETFPSERILVTRGSINWKNHIGPLSRCAHYYFN
CLCNRGEKCNFIHTVYVDLSVEGVFKRAPASRTIPPTKKRASRAKLFASSRSDVAANGCA
SVSKKKPVLMQPSRGHNNILSEDFDIDGDTVPTANQNLDLAIPKHVTDSFSHTLLSVRDG
SARSSTSGETRPIEWTFVDNSEDSILSDGVSALQLS*
>TCRU_151 hypothetical protein, conserved
MLTKSGGVEAKLEALQSKLEAMHADIKKIVPHSVSFLNEDNSVTRSTPVQSFTNGAAVME
MPTIPIGRGSRLNAASPAAEASPNTTPSSFFLPVISGGGSPKRVGFQEKGHGAETTNSLN
TRRKADAARQKLQDFVKPMILSPGEENVAELRSAPCAWLLSIALACSYVSGESITIEDIL
RQNRLGLHYVSFPSVTLAELFDVTNEFLTDHPKLREMRVHCEVATFDSETVDELGEVMGT
GERLPIMTLSQFRKDLSNCDPNSICVLNFDPYLIEQHEIRMRLNYMELNETDYPMETIVP
RWPAKNQGTFGLILNFSPALHTVTVGTPILLENGRIVIQEHTVPVQVLYKALCVKDNYCN
RSRGFVRVFISDQPVETVPSIFPLNVLDGSLTGGLLTTALDTSISPHILGLSIMHHLVTN
VLLDDTERQRQNATNFCDVMLRGIPVTLVCQQLGLGIATIVGGSNKASVPSAFSWYRVLL
KKLKIEDAIALGIVLVERRGGAEDGPVNISDDTFMRHIELAVESGSVMLMGFDVNVALNV
KVDNRPEPCHFAIVIGMDQERGIVRLADVNVKKYRKTWHLPVTRLYSAVIGYGYIVAAKS
RKTIEKLNATGFEQSVLSEARYALPPTQRLLRFEYPKKNYVVTILADAFARLGFKADVES
VVNFSGFHLSFMLSEHLPLENAATVARNYSHTHAEDGVSVATRHMDKGATGSTLQGLLQQ
IRFALQFPESRCLIVNFQTSIIHANKAVWNGSNGGSYAIVLHFDESSSLVTLSDSNHGSF
YRTWVCPLDVLFDAISAVDSIALRARGTLMLTTTSQRDMYLDCYGYDMRHSIVHHPFKPS
VWPAFHCLALVASEMSRGDSTTGQSVQFSAEDFLYSLSSFSVHNVLRNELESEHIAALAN
TAFERLEIPLEANAVDVTISGSFIKACCDETVNGKPVTMTLLGYDTRPIHRVAGFSVAAI
NRVRGTEKEGFVQLVEGNGCTFGSVWERPAQELQFAVTAMVRIRRR*
>TCRU_152 acetyltransferase, putative
MSGSEIVRLPPRGGVPIEIKLRWGIQKEDLTALGVLHDAAFPVKYESEYYDWMLTDSCLA
LLAFATENCILSLLKAAEHQQNTETERDEENSFGDVEDDGSSSLYSVMVGFCIGQIAYGQ
RRDGRIDASPTGYLGSFAVDSRMQRCGVGGALLDRFLSYMFFTVSVPPHLFLDYSPPYLG
CEGLWGLVSSAYPTLFDWMSRWWFGRDRKGTNHVPQETLKDKKLCDSVNPTVDSQSGRIQ
YGVGEVWLHCIAADTKLLLYYMKRGFSRVLVAPNFYFFGGRYHEGALLVCRKDGAVIDLH
DLNSARATGKKLEDGAGQETFIAKGLSHGFRLRSGNAVFPATVSEEDSGEGEIVDLLLMT
DLAGLRRKWRSSVGKTAFFSDLLAS*
>TCRU_153 mitochondrial carrier protein
MSTDKKIRRDAPLPKSWTIGIAGFSGMFGWVFTHPFEMWKNTVMMAPPGNSHLDVLRKVR
ERGAFKGLSAGLLRQVVYAPARLGCYPIFRDALIMLKGASPETAPSIADRALAGALSGAF
ASVLSSPVEVCLVLQTTSPMKVSILNAATQVYTASGITGYWRGVGALASRAALVGVCQVA
VHDQMLTFLRRHNKCRAEQKGVSPYGDNLVVNTASIFTALFYAFCTMPVEMARVRMSAEA
RKGKLPSGQAAELKYKNVLQAISRIAREEGVLTIYDSFIPYFCRCATHTVVCFFVIECLT
RNAKLWRSSRQTKENSFGSRRVDVPA*
>TCRU_154 ttaggg binding factor
MSYTQCPVSTSPPPMDFTVLHEIMRDHIRRGNHFAVLGILALTVVHEDPAFWQTCTYHDG
IASAVIANYVGNDRVKTHPPQESRCQTEEIAPNLNQEFLTENELYLMLRRVCGKDPLLCR
VIHQLEMDPVKENNASNGRSKELDAAVSVPSEAMRVNLDELFAVLHERFCSLDERRSQSQ

GTTIPSATIGPQALKRARESDGSEENLGVIATESAAVAPPKSRYLEPVDGVDGAGCSDAL
EVLAHIHALPGTTCALNSFDAVAGLVDDGERGGLESHSSRQQGLEPKGTSRSDIAPVGLR
PKQRRHKFTPEEDEAILQGVARFAKGPGRFESILYAYRGVWHRDRTATQLHDHWRGTLRQ
RAVLQQGGYRGKNSIATRGGKSEME*
>TCRU_155 pre-RNA processing PIH1/Nop17, putative
MPLETINLSGKDAQDGEKFTPTPEELRTIQEKMKDPKFVELFQSYMKSMEDPETRREEEA
YLKQVEHQAKAGGDYSFDFVFPKPGFVVELLEPSTSYQVTHPSLKAESKKKKHVPRVYVN
MCSSEKIDPFSEQSTGDREKSNWLVPVSVSKPRTELFSEEAATSGKTGEKQTHESGEGTN
PVVVYDAVFHPNTLQLTDRSDRFCCFLVNIAVEHINSGYGDCHGFEFRRLPSRIQSVGTP
QNQTISREKGKSPFAAAMNEQTLTHPTKVLPPLTSTSEKGRTTPCNKGKETSGVQAKKET
DAEAGDKVEKKEHIPHFTIAHQGHIDLTDTWGWKIVDRRVGVPEALVVKMDFTGVQSAAD
LNIEVESTYVIIAKSSSHPYYGSVTLPFSVESTPLEAKFERRKSRLTLVLRVVPPAPTGI
TAEDMRQELLSAGNPPDSVTEHAEDVVTERKNNDSAQDSATAGCVANDETSTVSDLLPAE
ARPTAVELPKKTPSVPQFSCVGDPDRVRIVMEKVQAARLEREKAAAAAEEQETSFAKTGS
DPQEKENDDDPAKEPAGNPEHEPQREVVTGSTALEGAASTSTFTLTEVSATAGADGAVHV
GDGTETAEDAAGLELLRQRQDAWQREIHRRTEARHDEDREKALIADREARREAERLRRRA
EAARLQEAAAVKLAERMAELPLRNRHIFTVD*
>TCRU_156 hypothetical protein, conserved
MGVTDNPACFLCFSRREMDSGEKETAADALQSTSFPLSELNKFMHNALSWPATDLTPDSS
LPALQKFSSGQPMRLCLSPGTYACKIIENTASCLVAGASSVRDTVVSLTDREGESGFVGK
DGFSSMAVFQQPMKIYSRASLVFHRVWFMNFVEVVGGGPVFFSQCFFGASTCGGNVVGLP
GSSQFASVHLAMDGDCFFFDCDFDYRCGVAGWEGNNEVPAVTASGLTKLHILNSSFSGPG
VVSTALALFDTARLEADYISVVRCGGHSLVLRDKSSAIVSHALFDRNDSGVWVKDNGKLD
IRESSLELVRHGRSGLVLSDGGHCLAHKCIFSAWAAGPQSIGRCIVVAGDNSYVSLSLCE
LFWRNGEDSINSQELIKGPDPVPDSDTCEKMKDFGLCHVLLGGRSEATISRCVFRLPLTV
ESNGQSSVIGVLLRSVPKRDSITFYAVENTIVFGWSMEGDDVRRARLPERGKSSHTWGLV
LPYVSGRSEEKRLKQLATMWMTDNCFLESNEEPLKDASLVYSSYEGYCRGTGVSLVFPDV
AKNKDEDDANAVCSSTVSFMTSDFVNSPPVQDLENHSVNVHWTPRKSVTSLLSGGAALTP
TSLCEDSPPWREHTCPEQGYLSLVEEESSDSTSLTSYSGSEKEGRGITVAKRASLSLPEV
GNSPYACAQNNVGILRSPSEFPSLNGVHFHTKRCRKISGVAAIGLPDRKQEIVLEPFPQM
TVASTEAPRPLSVSFATDEETPTSRPGLKEGGRQFCIFAGGRRRGVCQRRQLLMKQEVID
CQRKLRSLMSRRSKGTIVSSSHTGTAQAQKVRNSLATKKSNTRIFSELFPYQSELGPRRR
PLLAGRESPSERLVEKGNVSSKEVWPWEVPTLRRFLMEKLGMYSSIEAQKRAFCGRGKDE
EREEGEDLVWSPQRKRAPANRVNVGSLNGNTRRMCSTSPRRETRLRSPSLVSTPADSFCA
RRPSPGKAGYVPLRLYEKFWKKWCKTHFVRETSDQPQRDQREGECRSRGVMEHQAPRLHS
SGSTDKKWGPATRVAGSARNYAFQQRDATQPSKIRELTAEVRQPSSAVVVNQTLHDFSDT
EEEKAEENDAGHGATVIPPRMPLFRPPLTAARVEGRNRFAFKRRNPAYSSV*
>TCRU_157 SET domain containing protein, putative
MSHSVPSLLLRNLHCSLPAIAQLYKEAAGVGTGGVVEGDRGIPSCSSLPLTVSTTNDGEA
VLDFFCRELGMGASSLRHWRSGLTKAAHRRWWRGGKLPSQNAADASLFTDTDDVLFMQSA
IWDLKAVVSPFLTLTKPRRKSAVRGSSFMATRRLLPGVYLLSLPVEAIFFASPPPATDPI
TSFFMYVEELVGQLVVAGDDVSENDWRPSHAGYVNYLKKSVIPSNNLPFLEREEIARILQ
SDKTKPLLGAIGESHTVSNKSPALELWEYFHNDMQGLPLSAYLRQRLSKEEYAWWVSLVL
SRRAGAATLIPLVDKLNHHPEPNCYYTMATEETFCGIDVFDNLLAGVPSDLLYEPFLHTF
SIREIQEGEELTLCYASPTINPNDRDSRSAGGMSVTSQEGRAAWQLQWGFFPENDSPYSS
TDLKEVAAIVAERRVNLRRQYFPPDG*
>TCRU_158 hypothetical protein
MEKEVVFSKRIQQLCRRIDRARPVFEQHASGELRVLSEAQSRQVEMYPLWLEDLEHLRQG
RPPPLRPSKLPNASIKAPDGATSQEELPARDTAVVVGTESRNELELADDVAAQRSEEGSA
TVEKNGNEEEEVMDERGSSQQQSRCTETNKRGNAKKQVVANGFVGNFESEKERVVEHLRS
LPPAARETFICKYLRKINDADELRILKKRFESANNLRQKMISGDVSCPSKSQLEIIKGLP
ELRRTLSRLKRHAKMEGRRAMKFFLQLLEEIKEEEEWGKQPASIVLLENEDEKRDDENGA
TETSANGETAPDNSTVEEITETHVEMQLPIVNTLAAEQTDSAIKLEGNFALIRRGVVDSS
GNENSTSVQTEFKSEGEANIDPSLSGHTSLCGNNAVRGVVVSPRATHEVGPSSTGDRERD

RIYNPLVDCSNFNFSKNGSRPEIKALHFNQVNKKEEQEQQQQKQRENDGKKSEGLIRSDD
ESAFIGTDQKTREMAKQKVLEMPSHITREELGFIQLHIPIFYRCKASEGREQSAHMEELE
PRTAAVLRRVEKELQYILQHPIPNVTVINSQLRLEKGKCSHAKIEAESESITLARWNVYV
SPSDGPLAGQLLHIVVDFSQHYPFVPPLIYSTLFLPLTTMMVSESGQPNTIRLFSTCAPA
VAHDGKRLANKKCTIWCPESGMHEVFSRLPSFFDSGAFAASREALGKELYGSSVAEARLA
CGRSAVEFLMGNGKVKVPTPTTIEMVNGPFLVYAPAAGDAKGEKKKYGNSKIKGKKGAFG
SETVQYYEDGDRGVIQGPFRGCWGHLLQMQLPFARARRNQKRRSKKISRGLNGPVVTARV
WARAGEVFPELELVAISEQSAISKELHWAESVLGTPRTEGPNGEKNQQPSGSLAVIAHCK
VKLTALEDRKGAVVRFFVEEEEGVTRDNDENGKRNLPRCENGDQAGVTHVYLPRSCASLS
PEGEETSLSWAIVIYVGDGCSATVAFSVPALGSNVEDVRNAVEAGTHVSDVALDLVAGTV
SDSAERTFARRISSCYDPASLFCVASLAAGREAGILGVLCKVIYPSPSTHSFVPENVVVF
PRLILSENAFDAHFLCGMKKLFFTSSPFSPCTFFLPLTAPHETLPHAVQNRLKLSLTQVQ
RNPDASTAYAPPFKEKWALRFFVLALRSLGDQAKDVHDTGEAKDETRESFLHSVYKHTAY
SFTQLVRGEAGLQKLCHFIAAGDFPLDRPSATRTSHKRDAEELEALEWLLASWSIRTGLV
AFEAENNTNFIASSATTSKNTILQPTREDWHATTNKLMASLNESKKKDLLSQIQLHANFL
YDATSK*
>TCRU_159 bifunctional aminoacyl-tRNA synthetase, putative
MSASDCRGESEMLKILRALGMDLPTISHEEKHTVEEANKELGRFGVPCTGTKNIFLKSKK
EDLVLVTALHTTRTNMKVIQDALSLKDLRFAPEDVLRTHLGVVQGGVTPLALVNNESKNI
KLVLDKALVESTVPVVLHPCRNDKSCLMTPKQLQDFLRKLEYPFVVVDFSAEATGGTPSG
DNTRAKSSTSLKETGKKTSQPAAAATTGGANVTGETKLGILVKREENFPQWYVEVITKAE
MIEYYDVSGCYIIRPWAFFIWSRIQKFFGARIERMGIEDCYFPMFVSKSCLEREKDHVEG
FAPEVAWVTKAGETDLEVPVAIRPTSETVMYPYYAKWIRSHRDLPVRLNMWNNVIRWEFS
HPTPFIRTREFLWQEGHCAWQTEEECSQEVLEILDHYAAVYEELLAVPVVKGRKTEKEKF
AGGYYTTTVETYIAAIGRGCQGGTSHNLGQNFGKMFNISFQDPEKNDGSTLIPWQNSWGL
STRVIGVMTMVHGDDSGIVLPPRVASVQVVIIPVGITKNTTDKERKSLLEGCKTLEKVLV
SGGIRAKADLRDNYSPGWRFNHWEVKGVPVRVELGPRELASLKISLVLRHDGSRRSLLWD
KELPTAMTLLMDEIHNSMFAKAKRERQENTKKLTEWSDFTPALNRKCLVLAPWCGEMSCE
EQVKKDSAEESKAMQSEEMKDDARAPSMGAKTLCIPFDQPESVEGKTCICKSCSRPAKHW
VLFGRSY*
>TCRU_protein (LYR family), putative Complex.1
MVNLSFCLRHKLRDAAGNAHNFAFDIMTKVYREAVYAKDARYAGNPMYPERQTSATAAGI
ARSPPASASTVAELGKIQKGDQTGASTSASSEMPRSQRRSGVQVDILNLYRDMLRALQRL
EDPRTRKDLRKFIRSEFDSNRDIPRRCITRIEWHLHHGKNKLEELRTMRPDTKFFLVK*
>TCRU_161 hypothetical protein, conserved
MRRAAWWCLHLIGVVAPFSSTGGGRCVHLPADGDSADGSRCTESRPSGPSKTTARLTLNA
LSTDVRRLLENSATRFWETPGSVDTVLTILLGHQRGSHGGNSDILHPLLGVEILKGLGFA
VWKGGTIGGGGGWARRSGISKEDLGKVARCVLQPLLPHVIGHPRVRSLLPVIVEWLVCIS
DIPLSEWLPQLSLYLDDFAALKTNGRCFMGPSPGFFLQYMTPKLLHEAVDTQREVSLDLR
QVLDRMVQLLATGDGVALTPEKTLSDGSRSVSTSNDLIDCIFWQGEIPLILRNNFVREAY
VRLCHSTRCEANEYFPHAGFVGKESQTFLILPVVDDDVFRRLLLRLMPTSFCSAASETAA
ISLDYLVLVKPLLKLEDPSIIFRFITPLSEFVSVVLDGVFSSPLRLSFNGIRALIYSIIG
ELQQLQKRPPPSCFSLLPHKPRIHLLKALCDRLLCDAERKLKVSGADATFPGVPLQDQVN
ILRSFSHAARRLVRAVQTEKTASLLEEGPEGSALQAGGVVVKHNDVTTILRNTAFRVLET
SLFRYYDALEKWSSLSTGGDARNNDHVADALTEIEHHAGGRSTFFTARHLLQHPSRHDVT
AVIEALRIVAIQALLLRLPECREWLRANFATAIAVAFFDVWHRTMKKSLSCNMRGLNQEE
ESFSVFMESENLVSWRLLWTMLLLHQSCCSCDASMKSQLGINVGSASICAVFRSLLDLLT
LHGTCTEFSSFGSSLPLQDVEVLSTAEAEAMPEVKEEKRDETPKSLPKCVAYSAMGGFSI
GGSTTRWELQEELLSLGSGVGRGLWRWLGTPRAVWEVVSAGANNTTTVTGTMPAGSGGHY
AGYLLPWTTVAHSRHSLRLYDESGQSAAAASAFDANLSALFQLEDRPVVRVVGLTEEAAL
FREMAFTPELLLGSGVSVVHPEACRRSRGRDFLMEMEVDDDDGLD*
>TCRU_162 hypothetical protein
MEQQTEIFGNSLDALLMETLEAVKKAHENHLTQAEWRNTASPERRRGVRKERDLNQRAQD
ESLDALRSELKSVRVELAEKESAIRTLLRLQMRQGEKLHQELRNEPVPKTPGSLGNSPAS

FSRVRTSSTSLGGEKFGDDTSNGPVLDSPKTAPRSGGALCATEDGVQHFHSADRTGMIRG
TLSSFVPATVSGHGKPNQPRHHRSHSQHMPRTQFRSLLPSGEIVSSSSSSSGRFSNSLPR
EATDEHHSCSNRVQRGVMRKHQEVWMRQAEMMGILTDVSQYGASSYRTTSLQTEN*
>TCRU_163 hypothetical protein, conserved
MQHKRRRHRIRQPTQLGVAHRSRRHNRRVTDRHVRVCRQVVHKHKRAVAAGQPAHAHLQA
PVTRRLAAVAPHVDLRTAVAHAVPQFDAQRAGKHRRHVERHSHAVRDVHAVHQHRVHGAA
NRDGSVHRAAPQSQHHRMHRGHPRRSSKQRQHHKQRHTHSRNGPALHTVPAARTRTHRRP
HPPTQTSINRAVVVCRTRPVDHNSSFQNPFLQIANPVTQFRQIVILILQVIPNTAINP*
>TCRU_164 hypothetical protein, conserved
MDTVLNKLIANALLYSDGALHLVGARDNEEKSSLFPAPLTEELQMITSILRNWADVDASF
SASPVSTVGLVGYLSNAGTANTWIDECRCVIATVTNGKNVTNGPQFAGQHCPVNRWEDNN
HCGFVNYDFALVATVAICETHAGVKSIPLIGVK*
>TCRU_165 hypothetical protein, conserved
MAIAAMRVALLFLLALAAAAAWMPAVHAVVLRLRGGTVDRAITVGRAVDTVLIDGVSITN
GVAVVFDVPAMLPGALRIELRNCVCDGGAQIYVRGYSGEPASDRSLEVSVSGLSGDYCSL
AFVHNLPAHTNVTVRDSTIVTPGPMRYSQLSGLTDAVASPLVLHATSLLQSQLRVSNTVL
RSLHAGGSAVHVGGGVDLLSSAVVLDGVSLEASGGPTASAMRVASSSRLSLRSHSVFSVT
NVSVVSSGGGIVLGERLAVFDSVLRFVRVEGSVASSLVRCGGGTVGSGGWLELRDVWAVG
EASSVASLSGVTLGGGAVSIARCVATGATLVSGLTITSGAVSVQCNRAGGRVLQSSGDYR
MAGLPSVSVVPCDGCAAALTCFDALTASFSDCVCGCRAGGVGEACLPFDVPPARAGDGGG
GAQGCVSGVTLTESVTVGGGRATACFDSVVFSGPITVAVDLRSMDAFADALNVTLRHCVL
AGGAQLRIGGLSESTAYLMPHALVNMTNVTSLEGTIVLHGAMPLHSSVLLANSTLRATVG
GSQYVPTTPGYARFRRGPALVLDGVRLLSTRFVMTRSTLVCGGDLCAAILVERGLGVNLS
SVFYMDNCVVMSRAHVMYALASDLRVSGGSVFSIQNSSWIASSIEIYEGACVFRDVALDG
GSVLQVVSSNFRLGFAMLIAETLTVTGGSWLVHRDNEFRAAYVVYVANENGVVFHDRSVW
SILHNNSLTARTRQPL*
>TCRU_166 Metallopeptidase family M24, putative
MGRGYSNSRSKSARPTLRMKRRRSLSLQQLRGSVRPAHTTRQKVAAVPKPEAQTPTVDAG
HKTSAKRAPTPQRGGSARVRSASTIRQKLALMEKELEGELEPAERVAETIVKPDVRTKYK
SAGRALDEVMDILTAACVPGATTKQLCDRGDEELLQRVRAMFSKAKDADGNRILRGLSYP
TNVSVNYVLCNHAPLVEEEAVVLRGGDVVTIHMGCHIDGYPVTAARTVFVPHDVTAANEE
VRQLTQGASNAVEAARVALHGMIHLLQPGMLNADITDFIHRVGNYFEVQGLEGVLSNRTK
RWVPDGMECIITRRVSAEDPHQDVADCTIGANQVWTLDVAFTDHPSYKVSPADGVTTIYR
RNEVEFQSDARVRSVLASLQEITEKHQCFPFSLKHLENPLKARMAVAMLRRQGVIDPLSV
LRIKGDRHITARFSATVAVSERRVTVLCGLPPAEPLVVPDDVRPPPLPDDIAAVLSEPLV
FAKGGEKQQEKKADKEEKKKGLHAAADFNANEKEQQKE*
>TCRU_167 hypothetical protein, conserved
MRYSQLSGLTDAVASPLVLHAMSLLQTQLRVSNTVLRSLQAGGTAVHVGGGVDLLSSAVV
LDGVSLEALGGSTASAMHVASSSRLSLRSHSVFSVTNVSVVSSGGGLVLGEHLAVVDSVL
RFVGVEGSVASSLVRCDGGTVGTGGWLDLHDVWAVGEASSVASLSGVTLNGGAVSIARCA
ATGATLVSGLAITSGVVSVQCNRAGGRVLQSSGDYRLAGLPSVSVVPCDDCAAALACFDA
LTASFSDCVCSCRAGGVGEACLPFDVPPARAGGGGDGAEGCVSGVTLTESVTVGGGRATA
CFDSVVFSGPITVAVDLRLMDAFALVLNVTLRHCVLAGGAQLRIGGLSEITARLMPHAFV
NMTNVTSLEGTIVLNGAMPPNSRVLLANSTLRATVGGSHYVPATPGHAGSRYGPALVLDG
VRLLSTRFVMTRSTLVCGGGSCATILVERGLDVNLSSVFYMDNCAVMSRTHVMYALASDL
RVADGSVFSIQNSSWSAPSIGVYEGACVFGDVAVAGGSVLQVVSSTFRLGFAMLITNVLT
VTGGSWLLHRDNEFRTAYVVYVVHENGVAFRDRSVWSILYNNFTYGFYSSTIASMTSSWS
LPSDSRPTIYGVCNEARGSPVTDYQEDLNIGTPVTVLDCGACTMDAVCFATRTSSISGCE
CVCAAGGYGGTCLPAAVPDGLGPLPLPDARDTEVRCVHGGSISSVDDPDPGVRGLCFVNV
TFTAAIVLDLWSFDAPQQTLNITLLQCVLMGLSIKGSGARVHVSVTSSMLDSGALEFEGA
KPRSRVYDHHPLF*
>TCRU_168 hypothetical protein, conserved
MRAELRVACPTKRRRQRHHQQRRDNAPTAADVVLRQAPSSRRRRHGERRARHVAPPQRAA
HRRRRGPRGRQRHRLRHLQRLRRPVLRRTVVRRPRWRHRRAARLRQRRRATQQRHAGRAV

APVHVRQVHRQAVAPVPALSTAAAARPRHRPRRSSKAAHVGVAHATRHGAEFPDDAGGPV
VHRPQCLPIERQAPHNELPAGAPERNGAVHHDEPAAGVPVERQCALREIRTARVRRHRQE
PAADVVAAHRQPRPGHERRRPPQHQQRAIGQRTGVVVHYDGGRAGLHTAAKHQPVRGVHL
APRQAQHSAPRRNHARTSVQRIFATEHSRVQRNTATDKLQHGAVHHHRPPTSHHGHEGHA
GHNQHGTHKPQARQHKHRCVRRGLRAGKIHKRRRPRGDQQGVRHRQHGPRRERAGQHKRT
VQRLHIAHRQLHRPALVAPRRTRTAQRPAAHHAQHGTVQQRHVLHNDPHAVPRHPRHVEQ
RTHAVQHNVAQRHLACAGRHNEH*
>TCRU_169 hypothetical protein
MTMAVDSHDPSVRYLFFEEQRQQQQQVPVDGTSSGRLPLLYSARPACHPVNDVRSAVALR
RITEGFLNLDQQMKMWRRHLHRKLGNLESRLSRCESVLLLKKTPVPQPAVVEAPTTTYND
IYDGVEDEFKRLHLTVTALREKAAECCRDGQSFFPPNRQGKKGRGKPAGKHAMCDDEDVS
IEGGEVFDSHCEHVGVAKRMSPTADTAAVESGESVGLTPDGAEMPSACLFLGETVVDGDW
QHVEGMQPREASCCLYKLQNQSSFQPTPSFSTMTPVSEQKYFAMDSTCRHDWSPFIHEPC
RPEKGIIRGGKPATTDKTPPNVPDLRMGSTATTTQEKCCEINESPPLQASSLERRLIYSP
ETPKRMQGQLSPVPSDAARSASSSTKRGMEIPLSNADSVEHLHNLAEPALPSKPVTNGQK
GPDVTPSSQHGGAFVDAFASAVAGVPSSSPLYVLPQQIYSGRGDSCDRRSEWDNTEDEEK
ASENSNEEVLSAVALSMPTVGTVWQRVVSLAHAEQYYDQLSRLNHPSLSMLSSDILEISE
LTVQET*
>TCRU_170 Sperm-tail PG-rich repeat, putative
MVLIGGFTEWAHLRLRDHRPGPMSYIPVRNDVPERSILKKYPARPAEATVGPGEYQIPST
VGQCRSTVFGPASGPESAADAKAGSEKSSKENLRKKKGRKLSPLPKQYSLALSPDTPAYS
IYGRLRDRPLGSGVGPGEYVISSSFDSSGKGPHFGERTKLLTTRDIVPGPGAYQLPRFGD
DTSKRKEYITSVRRVPERENGPGPGNYDDPTTIAARLAPKKLRLYEGPLFGGRHSKSNCS
LSTGPGPAEYGDVSRIMRKSIRCTPKFHPPVQTVPPHKTVRGTMELSQGPYTYYLPSDFD
RDYNKGVSLGARVFYQPKTAASDAVGPGLYDLGKPPMTSNGFRFAAQPYDAIALAEMEEK
AAAAASNAASGPNMYYPNLDAVRPSRYRAVAGFGLGERFLVQNAGGPLCYDVKPPEATRA
TVFYRGDYRRSKNLHGLGDGGPSYVVENDTIADSTRKGKGFTFGIRYPARATHQVCKPYD
ATTNINCEYPDETTWMTKLK*
>TCRU_171 oxidoreductase, putative
MSKITSKGWCYARCGPISRVLKHVTFEVVPKKEEVIVDVLQAPLHRTDAAVVNGTALGRR
RFSSAGFPRVGGCEGVGKVVEAGASQNVKDGDTVWVAPLHGTWATRVAVDHKMVHKIDPK
HVSLAVNASNYVVAQHLLNGFASLRKGQVIVQNGGSSPTSLAVAALAKPLGVKVFTACTP
GERFKGAKERHAKYSSEVFEYNGKGARAMRQAIGNVGATLYLNGVGGRHFDTFLGLVGCG
GHVVSYGAQNGVGLMISGSNLIYNEVTIQGLFLPSYLAGISYSERQTQLDFVLQQLSSMG
FSYPTSVAASLEQLPNVWDDAFVHGGKKGVVIVGK*
>TCRU_172 hypothetical protein, conserved
MYVVNLCSLPRQLLTLSLCEHHSSLGPAAVICDLNSHHELRDGCSPSTTAGEDLAATSTG
MEIELSDDPAQATWISGRSVSFPDVTAQRVLRVSHWTSTRLHGLGPPSAIPHCRDGGRHP
ATGRHAATTENMTPLSCVKLNVMLPPRRVEHLWPL*
>TCRU_173 hypothetical protein, conserved
MLRSMLEMNRTPLGMAILGLQKIAARSETKQARPLTKEEMNQVIRSRTDWKERVVFRLAC
ITASRWSEIAALTPNNFTMEADGALILYWSVAPKTATTDPHRASRFVRIRGQDAFDTIKL
CRTLQEKEKLTNITNVKVERALAPWNATTHSIKRGALRHAAQIVEALNLNPHVISQLAKH
VDPFDLPQNTVRYLWRYTTLLTQVASLVALM*
>TCRU_174 hypothetical protein
MGGACLPASGRGRAGVVGGGHGGEGRGPTESLPPGLLFWNAVRGSAEKTAAAGRRPPYGD
AFLATMEAGGSRTSGEPAGRGAVVALLLPRAEEQHEARAVFASYFAAAEGQRTRRAGENA
LGRRRGARV*
>TCRU_175 Glycosyltransferase family 10 (fucosyltransferase), putative
MHTRSRHRRGIIFLFCTIAIFSLWNAGGGDVDSFAANVEGVLSPQSSDGHPRHVRTFNGV
NDVLVVPTRPGTYLCEYMSNATLLPEGCVQSCGNDGQENGKDFNCYVHSRHRVAEFRRAD
IAINHNGPVPSCPFGQRRPHLTLFYTGESNHTDKKRSTEAYQRLYDVVISFHHHRPYYFT
WAHRQQQQFLKILHGNNQPLANKDAFDWGKWQQRHSAVAVFVSRCKARRAEFIRRLSEHY

PVHSFGACARNRAIPSECARRTGRYPQKLCVFQKYKYAMALENSNETDYVTEKVYHALLS
GAIPLYWGAPNVEDFLPSGRGSIVELERLLPGQVGDDARATANAKDAFRGFGEKLRRLEE
DEASVRRLLRWRRAKRAAEWGNSFLGNLYHPDPFCAVCAEARLKRSKDGKEK*
>TCRU_176 CorA-like Mg2+ transporter protein, putative
MREGEKGAKVHGLAMDAQEVGSAAVGIVQPMQRCLVVHTATAVPFSTNVFCNPDRVVDDL
RKRMQDGEVSPDGPCVWVDMQGCTDEQNRNVLQLLFPDLQQSQIEWMIRCDTYDTVELQP
VKGEYLIGSLACPPNRGPSLSAPAASEVEKENTVVCTFACNDRFLVTMHAAPFVGLAELL
RHVEVEGSGGHRGGASAAPNTASPQMQRSIAPAPSLRGITSAAILSLLVCFTCEAFLPDP
TSLLAEVGNIDEMVLLISPGEQDQRDLLRRVALLRRRISNFRTGLYLKEKLLQELIAPTM
RLSFVAKEPDTVRDYKETLEKVRQVSERLDDARDMLNQANLNFVTGVSMRMAQASAGLDF
KMSLLNQLAAICLPIHLVISVFGMNCKVPFNRDEYPTLVPFWIICAAFVFWALLCASPLL
RIAIQGLRATPVTSYE*
>TCRU_177 Concanavalin A-like lectin/glucanases superfamily/Trans-
sialidase of Trypanosoma hydrophobic C-terminal, putative
MLQGNKASVYVDGKLLGEEETMLTGETPLGLVRFCFGACEIHNFPVTVKNVFLYSRPLNP
TEMRAIKERRHIPRIEGVRAKAPAGSGLLPLLLLLGLWVFAAL*
>TCRU_178 hypothetical protein, conserved
MAMMMTGRVLLVCALCVLWCGVGCGFADKVVEAAAGVVASNRTDEENLILNWYVLTQGEC
ANESTTGGKLNVTAERICMHKVMKEVCDAFYNKTSGVTHDRDVEFICTYYATIPEEPVEP
PTPQGPPSHSAVSNATSDEGTPKNAPESDVAGRGEGKEDDKEHGNTKQKAVETAAVESIT
RSTDSEVSTAVSHTTSPLLLLLVVACAAAAAVVAA*
>TCRU_179 DHH1
MSSEDDWKKNLLAPKKDTRKKTEDVESRRNVTFEEYGLRRELQMGIFEKGFERPSPVQEE
AIPVALQGKDVLARAKNGTGKTASFVIPVLEKVDTREPHIQALLMVPTRELALQTAQVTK
ELGKHISGLEVMVTTGGTTLRDDILRLQNPVHVLVATPGRVVDLASKRTAKLDRCRIIVL
DEADKLLSQEFTELIEDLYTYLPADRQSLLFSATFPVTVKDFADRYLRNPYEINLMEELT
LRGVTQYYVFVEERQKIHCLNTLFNRLQINQSIIFCNSVNRVELLAKKITQLGYSCYYIH
ARMQQQHRNRVFHDFREGHCRNLVCSDLITRGIDIQAVNVVINFDFPKYAETYLHRIGRS
GRFGHLGLAINFVTYDDRYNVYRIEKELDTVINAIPGEIDPELYAA*
>TCRU_180 methylglutaconyl-CoA hydratase, mitochondrial precursor,
putative
MRRTMWLHTTAAHLLLDGECRLTRMTDHVGVLTLNRPSRKNAIGQTLLRELSECIRMCRD
PANAIHCLLLESQVDGIFSAGADLKERRTMSLEASRAFVKDLRDTFTAFEDLPMPTIAAV
EGKALGGGCELALCADMRVAGAAAMFGLPETSLAIIPGAGGTYRAPMAMGLSRALYFILT
AEQLTAERALQFGLVNEVVPEGGAAAAALSLAERIARNGPVALAAAKAAVRHGFGRPREE
ALRGEEAQYDVVLQTEDRVEGLRAFAEKRPPKYMGK*
>TCRU_181 hypothetical protein, conserved
MSTAVLAARACARTILWQSLRLSAFQGAVPHVLLVVDRLPCTLQKVLLEGYMQELDAINM
GSSNPVEGPPRLDLDLLATTPSYSNGLDKDWKVTYGVLPFSAGSFLEDVQTLQAFLRQCG
APVDTEEGRIGGQWSEEKMKGVLDSLAPILSPYRADDNADGSVEWKDALLHTLRAGQPRE
LLGCILIQRNAFQNELEKYRLRLDLFKMGLRVAEHNHLECMSRTADTLASGKDGGSRERC
LEEEEIIYYMQSCSLQPHVAESMGRAIADSIDALSWSRRTVAADGSDGVKLDVVPPASFY
ESLSNADLLSVYNAGVLRWEGSCYEKNTVTGMDPSAASWNISGPGAPLRIICQDGQVLSF
EGGLEECLLNTGYYAAAHAPERNCVQGHRQRFPVRDAGEANKVEEGAEEKEEGDIASMRK
ASDAEKRGRLKKKEYLAMLKRTGKKNTAGRTSSPSDENDDGVRRRGNDCGACVGSSIGGT
FPVGEVISESFDLSKLNGSCSVFAYPSLFKEVTMSDLNPVTMRIENGIVTDIGPNAPEEL
VELVSLVRQAEGACYVRELGIGLSPHVGRGRIVSDVTAFERQFGVHVSLGQRHPLFVKQP
AKRNADGSVAVQVDGPVLKRKAGKYHIDVFLDAARLEMGAFVVDFTKGVCSFSSAAAAAV
SCA*
>TCRU_182 hypothetical protein, conserved
MPYFVNSLSGRACGLLAGAATAVLFAFGGFSDGEAGWLRRARRQDTVPLFDASAIERVEE
YARYPCQAFPGYSVVVSNYYTRVQKDDVHRNGDGGETLLKRLLSLFSNDTHTAADRGDSS
ADENLLHLVREVVGDSRAPLPDTDTSTTAVNSPFVASSTWPLGTAKYAAMTMEPSSSFSA
AGEPVDVMRRLADRTTNQSLIRCDPFASPSMNCVADASYLGAPYLRMMLSAFLLLPSPLP

RLRVAVLGVGGGSLPSFLQQHFSRDIMRLDLVDAEPQCFRAAVEDLGLRETMRGGAMSCH
VSDAAAFLKGAVTGLSEDAAAVFYSGASPPFVKEAPVVVKGRQRAQRYDVLFVDLFVGST
PPAFLASSSFLQLCHGVLSSVGVAAFNLPAPDPEFMVRCRRVFGRGNVFQIPVPASANVV
VIARRVVGSEMVDGVPISRRHFYRRAEALQKSHALPYDLAGHYPFWWRFW*
>TCRU_183 3-keto-dihydrosphingosine reductase
MLLSLLVTIGFALVVAVAVLAAWSVQHVPPFNIKSCRALVTGGSAGIGLETARQLVRQGA
KVVVISARRVDLLRAAAEELRREVGKTGTQVFYVEMDVSNEASVAHAVEEIETTMTGGAG
LDLVVCNAGFSIPARFVDTSSEDVRKMMEVNFFGCVHVVRVVLPAMLERHYGRIVLVSSL
ASRCAIAGYSSYAASKAAVRAFAHSLDMENSCLGVRCQVVCPPDIETPGLQSENLLKSPE
CKAISSFGGNVPYTAEAMAKCIVKNIKRYRFEVSLGMDAVLLGLGSAGIEPATSLLELTL
QFFFSGWLRLVMAAYSKVHYEIVRRVRGRESTTVHKKDT*
>TCRU_184 serine palmitoyltransferase, putative
MPSYVTTETLIDVKGEVEQITAKTDIWMGSPSETCNKESTTTPPLSPQETDDARQEEQEV
VKTCQQAEQPLQEPRKNGEEMESCSLLFKIGVYMSFGMLTLFSLFYEFYYRLFQKEKERD
GYAPLLKGFDRYWQKYFYQRIRDCFNYPIDSRPSRVIGVMERVSYDYNENFSLTGNILPC
VNLSSYNYLGFASDIPSITRQVLDSIDRYGIASCSPPQLAGQHQPVVQLERAIADFLSKD
DAIVCGMGFGTNFRGLPALFGRESLVISDSLNHSSLVNGIRMSGARTKVFKHDDFQQLET
ILREAVVLGQNPRGEYLPWTRIIIIIEGIYSMEGEIANLPRVVALKKKYGALLFVDEAHS
IGAIGRTGRGVTEHCGVDPKDVDILMGTFTKSFGAIGGYIAGEQRVIDYLRIHSSISLHC
DTIAPPCAQQVLAALEVISGRDGTDIGRKRIHQLKENSQFLRRGLIAQGLIVLGDDSSPV
IPVMLYRTGAMPVMARECMRRGLAIVIVGYPATPLLGGRIRFCVSAAHTRDDLQLVLDVI
KDMSKHVQLQLRPIPKSLQE*
>TCRU_185 hypothetical protein, conserved
MNMNDLLFQAATQTIDALSFTQSHVIDEDFRAHSKATINSVRSLQREQRSRIEQNYGPRA
VSSDAIAAQLRATYRGETTSLELMKEANERGVPPAFMANMSHSKCQVFKKEMKRRIHEWE
RAFREEHGVAVTAHDKASLHHVYELYKAAKNRLKETESPRKTVFEPKVEDTSELHQPQYY
EKKHEPYQPQQAVYEGGGNNGDAGRDERGGTVGVQPSQIRGSQMDIAGSKQFTAPASVTS
SINVPRQNSPARPIRKGKPASQMSDDDLATEKRYLKYILHRFESDFEQKNGRPPTKNDRR
VWTAEYTRYGELKNEILRRSANGNQGEASNVEGGAHTRVSAGPVTIPENF*
>TCRU_186 hypothetical protein, conserved
MQGSSLSRRVRVGGVEPEDAVLRAELFRTFASYGVIEEACFEEDRAFHSGAAIIQFQRIS
ASEKLQNDVEETGRRWFLSFLPPIVHVGAELLVTSPKAIDTSYLRSLVGEGVKIEEKLLS
FPGAKRIGSTTAEEGAVDYGSKNAGARDVFIKALSSVRAHAIGRFAVLMQFATVHDANEF
LTTHQMPFLTSNEMYVVHVGPDAALQHALKHILLTRRSTKDVARGSVLRGFVLPQHESGN
IGGSGGGGGGGNRMIKCEVDAGLIRKGRPVFLQTQTNFVKVSPWDIVEVRLTSADGEASE
NLEAELRGVVNAAADTAEAQKPTVLFQRLRQAGVSSSSSSSISTAASSKKALAGKLYQAL
HARKTEPDNAGEQLHSFPQGLHAFSVVSVCIQRVGDDGLYARTILPPHATSNTPSREWPV
FVPSIFVPCEGGMGWKDFAVPGERMTVTLLYATAVGGSEAALRLVASKREADMRRASAFF
VSAATPGAKDGAGGGFNDTKTLSVGTSFSGIRAVWLPRTPSTENPVYILLPSSSSPSVLF
TITVLKHATESPTDHQTPETMTMDAGLHPFVIASIVNSDRLGHYAVAVEEAVYRQMEAER
RAAEIQKESEQDAMLQRIMTEVFSNGASVPAGDGDNSAQGRKRPRYSQEE*
>TCRU_187 60S ribosomal protein L30
MAKKNKTKVDTINTKIQLVMKSGKYVLGTKQTLKTLRQGRSKLVVISSNCPPIRKAEIEY
YCTLSKTPMHHYAGNNLDLGTACGRHFRSCVFSITDVGDSDITSA*
>TCRU_188 60S ribosomal protein L30
MAKKNKTKVDTINTKIQLVMKSGKYVLGTKQTLKTLRQGRSKLVVISSNCPPIRKAEIEY
YCTLSKTPMHHYAGNNLDLGTACGRHFRSCVLSITDVGDSDITSA*
>TCRU_189 DnaJ domain containing protein, putative
MSLSFIESGFVPSDGMRRGVEAADTSAAAELLHLAVPPLMNAGGKTRVCVAFYEAAQCPF
DSRCEHAHHFSELNGYTQNKLLETVPVESIPKHFVAPLNSNSSSGNNKNDRTFYATDGNA
ANYTATAAVDGGVAHRSLGGEHVEKEKTSTNRRSKRTARLYDISGSNTNLCDNSLSSLAS
STDTLLLLGSVHDSKDVSPQKGTRRDEGMEAFRIRLPPRCRYPHRNTRGTYYDVLGIQRT
ATQEEIIASYRRWQKEYKSMKQVDPQGADAHDTIVVEARNVLGHPVLRSEYDRTVSAFWS
SNISCSKDLTRGTATLSSANSSRSINMEATTNTMRIANGPSSADMMSLTQSGLPEDSIW*

>TCRU_190 NDUFA5/B13 subunit, putative
MRGSHSLRAALRRTAALFTISDEYIPRAFPVKSTTGLAGVAVEPLWKPKLLAAAAELQAF
LTTSDIPPESTYYNIAMTLVKRINHGIKECQDDWCTLEKRYFWGWPVEYILQVTWRELET
AQKWNEWRFWELDPEQVKKVAREDQNIGREGLGYNSPWEQVVREDFDKRKKALTQEEMAE
LKRMDTERMARETAAYKERKDRIRDDLEKARGDMLKKFLNKRFAVDKDLQRMQPGKRYSG
KTSEDLIAELKSSVQNNPQKQDPKK*
>TCRU_191 tubulin gamma chain
MPREIITLQAGQCGNQVGSEFWRLLCAEHGIRHDGIVEPFASAGDDRKDVFFYQADDDHY
IPRALLIDMEPRVINAIQRGSMQRLFNPENVYIHSEGGGAGNNWAHGYEMGDSVQETLFD
MIEREAENSDSLEGFVLTHSIAGGTGSGMGSYLLEHLNDRFPKKLIQTYSVFPNQSRGGD
SDVIVQPYNSLLAIKRLTLHADCVVVLDNTALNRIATDNLHISSPTVEQMNGLVSTVMAA
STATLRYPGYMNNDLMSMLASLIPTPRCHFICTGYTPTTLDTSNIQSSVQKTSVHDVMRR
LLMPKNMMVSTSMKSGCYISLLNLIQGDVDPAQVHRSLERIRERSPNFIPWGPASIQVIL
SKKSPYLDTRHRVSGLVMANHTSINSLFQRTLKQFDLLFNRGVFLEQYKRYGPMKENLDE
FKHSREVLESLVSEYKACESSDYIRNF*
>TCRU_192 Ankyrin repeats (3 copies), putative
MASRVEEILLAVEHGNVEFLSKNLTPQNVNYVDTERRMSLLMWAVALRKHSIAEMLLNLG
ASMYPRDMFQFNVFHYAAWSSDVKMLEILYYSTSCSGCKSADLNCLCLAHLKNRQFRPGS
RSLVDLPHPYTGRTPLMLAAVRGDVEMVEFLIKKAGCSLSVRDFRGSTALDLAACCGHKA
VVILFLSNGDDSADFVFESTRRQAEANCENAMTLQQLEVKKDLNLLLCREWLPQSLVAIS
*
>TCRU_193 hypothetical protein, conserved
MLLLDTTAESLLRDPQYLLRLYHKVIQYLVKCDPSSFARSLSSSFNQIDTRYRVRSREQA
IEVWSLKGILRQILPVSVMSDRELSIILAMLPLEDYGGNGTGNGGDDVLVSPVVLLLCLR
KMCPVQASLVLEMLRRIDTRPKRPHPYESACGKALLVSARDGRGDACVFERAAILDYLTE
SYDMTLSEAFFLTDYCSMGLPPSSSTVAIDGSYLYAFLYQRPLPSDVKYPLLMSVFAEAI
CDPNIGAPLGTLALIEGLHRLSPKPNHGMHREEVFDVNIDTGGELEHYSLTRKSFEDLCR
YLRVGLLLEEVHQLFYYLRGESSEELLSAHTLLCEFKRHFVPVSESLFQIVEEAVRRYLV
KSGGMLALPRLHLALHGGPLSVARFIDVLRVAGVPEAVSDVELEWLRFKGWDRERLVSLL
SGRFPANREALVRQLFDQLKNVKGLTMKQDHVEVERVLALFHPEKVEGTLIGSSDDWRFV
MTQCFDGNISKTLTYDQFFYFWRAVSAACSDDSVFTMILWRSFNMHTSR*
>TCRU_194 kinesin, putative
MNGTNSFEETYTHLMKEVETLRRRVCTLQEEVNIEKQRSALVSKDDSLNKTPAKAANRCK
ADDNSVLVEENPEAARMRRHVLELERQCAVFQSILRDRENELNAFESRGDIAEKVRRELQ
QQAREREEAQRALEEAAPQLETSELLRKVTDTIVWMQEQLDGVCQQKKFMMKAMEGMRCE
QHRIMTDLLEARKNVLQLENAQEESFRKFNDVFAENTKLKGIIDELNLKLLREYAERAIR
EETMSWFKALTERDEQCERLRAAFEDQRNLCDVLQSRLNSTEQELDRSSKHQGALLQEMR
KKDEAFRTIWLFLTPQQKARFMSFGDQSEGANGTPSSSLHQGHENVQLRSQIRTVKRQLE
EEFLRNKELEYRIEDLEKENELLKKRLQNCEDEYKSLVSLEQDYREEREHMEQQIAYLFT
YIEEHNAKQQVSERQLCELKSEWERLNEEHRNAQREVVELTAKLEQAKGEIWAQGVDNNK
LKDRLKEVKKQDALLAGLHRQLTATQHIGDEASRRLGIADRMQQHVASEANIYNHHGDII
KRKAIRRNSDTLLKALMQRVDSAQRVRGAAVQPLPLNNRVQRLRNEVSQQFRLCGPSSLS
FNGTARTREESFGSSHTHHSGSRRRKRTMLHKPHDLRSKPLFRSSNFVVAEIPSFIIDRT
RRRNM*
>TCRU_195 electron transfer flavoprotein-ubiquinone oxidoreductase,
putative
MFRRSCRIFCAPVERMVEEFDVVIVGGGPAGLSAAIRLKQLAGDAKDDFRVGLVEKGSEI
GAHTLSGACIETHSLDELLPDWRKATDLPTMTAVKNDALYLLRDKERSMKSPLLPPTLHN
RHSYIVSLGSVCKWLGERATELGVEIYPGFAAAQPVYDGSGEALEGVQLNDVGINKKGEK
TPQYEPGMIFKAKQTIFAEGCRGSCTKQLEKKFGLRGEGNFQTYGLGIKEVWEVPAEAHR
PGTVMHTIGWPLTDKGHHNTYGGSFMYHYGDGLISLGFVIGLDYSNPHIRPYMEMQKWKT
HKLISSQLKDGKPLLYGARTLVEGGYVSLPKLHFPGGLLVGDCAGFLNLPKIKGTHTAMK
SGILAAEAVYDDAFASGKEKRDGVECRSYDDRFRNSWLHKELYAVRNVRQVFARNFFQGV
LYTGITTTITRGWEPWTLSHHKPDHEALKVAADCEPIEYPKPDGKLTFDLLTNHSRSGTA

HNADQPAHLKLKDSSVPEKVNLAVYEGPEGKYCPAKVYEFVDGKLVINAQNCLHCKACDI
KDPTQNINWSVPEGGGGPNYSAQM*
>TCRU_repeat, putative Se.1
MSGLFLPQLRLLRPQGSVTPVFLVLIISLFFLPSTALSSSSSPSSSSGPSSSVSTASGDS
EEDPRDAVKTATERAALPVKEYPELQRENEISAASEKLTQSKKDALGIKEADRSVVEPSD
SLALAHVMEEKQEKNPLQASEEKRHEKRRNDAMREFALVVQQEDDGVMPIVNDVDTLTTS
VSAAEYSVNDRDVDETNHHMNRDNAVDAAAEFGIEEIYSKEFPTTETETEGADAYSFVPS
QLSNVEPSELYQNVVRSIYNGNLSSTFELIKEGTLHGHGRLHWLLGVLHANGIGVPQSDA
KAVLHYTFAALEKVFEAHMALGRRYTDGLGVAKSCQDALEHYREAADVVVTNYEGMPNPT
ERFSRQFSADALKHGGHSNSKMVQMLMFRADEGSTDAIISLGYAYFKGIYGLRRNWHQAR
LYFLDALAKGDVAAYGALGRLYATGDSTANPAIGRDLATAATYFSQGAEKKDAVSLNGMG
YLHAIGFFNDGNSSAAKGRKPNFEKAAKFFAESVDRGSVEGTHNLGVLLLHGRGVPYDPA
AAIKQFGLAAMRGNVLSIWQLARHEQRKGNCEQAMQLYSRVAGFDSIFDHSNDVPYFALS
DGSSSSSSLYSSSFASSADNIFPQLLEMLAMAETGHAASRYQVAQLLDKLPIIEETDDEL
EDGVFAKSGRPPERALRMDGGVVWLQWYHNSMRPVPTGEASIIPLADARLLSQREALNML
RLRMYGLSALSGSAEANLRLGDFYYYGESQLVGVSMSRALLHYEAAAAKQDPKAYFNLGF
MYQLGLGVSGRDQVSSVSDALYRIVTNTLLEEETEGGGKSGSEANFSRTTSTKNIKDALL
AEEMRLYLAKRYYDRAMEFGSGGSGYAVKLALMTLNLQWWWLYFSHQQFGFSWMLRLPMP
MARGGGGGDGGSGAENETEKASMAPELHEQQQQQQQRRQEHQGPKQLLSTGSETLPDVVS
HEWRWDDYTFAASATTLFLLLLLRHHVA*
>TCRU_197 Molybdopterin guanine dinucleotide synthesis protein B, putative
MRRTRRLLFTGTTEVSAGAEETTTRGGGIPLVHPWAPSDAVTALPEGGNRDDMDTAPPVK
RLKLPDKVLTRLGKTPPASGMGNVEGDSAAKGVAEVIHSKKLAAAKHHASASDIEAGDDL
RALVSVRRIPRPQPRQGEGQQRSGELNPPAEHFANSNDEFIRFAATGRAENSDSSSLISS
WSDEKLQDDDGNADARTEKQRRVIDRHFAGYESENIESLSEEEEEEEEEEETSGSESSEN
DDGGEDDKDNNVPPKNEGDRAGFGLLERRRHQQREQQSQKGKMGAVKDEGGVDAATNHTS
DRRYRYTSMFFEMHIAREAMRPCHHTLICLSGPVTVQGPCGIIGFGIGEVAVGGFFLGKK
QINLYSEADRVVLTPVQRLKSKNAGLESLPVPVPFSFKAKNDPVTVVPPTIRESYEDAAD
DNDVEIFGAIDWVWVETTIGSWRGRFANKMPSIVLVVQPYDPYGLPPTRRHRLKGKGKKL
TDAFMEVPRFVARGNDVGIDAVLLPEVIPAIVKQGTGVVVVLGSQNIGKSTLARFIANAL
FSQHGVCYWLDLDLGQPEFSPPGVFSLYCVQQPLLRPRDTRKVKLVKGFFLGGSRPRCPV
ATAIAIEQLCAIVGPLQRRFPVVVNTHGWVLSTGRRMTVEAIRRLQPKHIVHLVKEGETR
WARDSELLMCPTKGLNSEVLHKRFFVRRAELENTKRKVNDVIGRLPYPHSVMNRPEYATT
AEWSGRVHTVNVTRSEGSLQQGIQPKASAIRSAIWQQHLAPIFCYYETLDEKRRKNGGNE
LQLTEADESTTLFRGQLRHFESIVLAGVDDARDMTDDMVCAALEHSVVALSFSLVPSSSP
LACVTQRQEASNGPLGNYRSLMDMPKGFSFSCFGFVESTEAEMRVSKEVKIRLPYHRDVV
RGMLSKEDDTKMIRVSLACTLAACTDTAALEAYFYGDDGGGK*
>TCRU_198 cleavage and polyadenylation specificity factor 30 kDa subunit, putative
MFTDSAAHTSLAFEDALPDEPHATVKRREICQPFQHGKCRHGTACPERHVISQFKTMRLE
VCKHWLRGACVNGENCLYLHEYDDRYVPLCAFYQRLGECTNPECPFQHVIQVEKQPECAA
YRRGFCPLGPKCHLRHVFRPSCPFYMAGFCPLGPKCTMGHPVQELYNRNSVSERLRQRML
IERVDDPTFNKNATCYRCFDPGHLSPNCPGVQSGLLRRMLMAVQEPGEQLYFQSDGRSAR
KCCFFCGDESHEVRDCPKKPKNHQWHTHRRA*
>TCRU_199 hypothetical protein, conserved
MRFSRVICGRLRRRTHGNVPVMQQQKQQCAWEREETLATLPLSNAERTASALAFQLLERH
RRRHASDLHGRAAVGIDGTAEVGSHPTQEEIQEGPLNNSRTRPSHLPAVDAFTGYGKTAV
TTNLAHMADPWVVDTRNLLESTDREWEALTARSKMSATLTDTGDRPTMRAWVRYSRGNAS
AHLPEKFGEKIITPFDAVMRRYNSVVTNHRFRVVTVDAFCSWRVNMPASLTQRGTMRCVL
ARKEQLDRQNEWRKLRKSQLPLGSDIGDGKFDKERRNVEKLSSSRDFSSMPFASPVFRRY
CLQFGSHTLRRVAFLTDPTLYFVLCNWRSTGNESLMEVRQQAECLSHRYMSMAYLP*
>TCRU_subunit, putative N-acetyltransferase complex AR.1
MTTYRDFKLTDIMEFNFVNLDQLTETYSTSFYGEYLTHWPEYQRICVHPTTGICMAYALG

KAEGKGEDFHGHVSAVTVAPIFRRLGLGAALMKELEATTSLVHNAYFVDLFVRQSNKVAQ
EMYHKLGYIVYRRVLGYYRGDGPKGTFKDDEDALDMRKAMLRDRERGRSSVIPLAKPIKP
EELEWN*
>TCRU_201 ATP-dependent DEAD/H RNA helicase, putative
MSLAEMDAQQHLAVLGLSKCSENDTLDRLLAGAERGPSVDLNDWGDGCDLCVDAPSQPSP
AARAAPVMEDKSEPKRLKRKRTSTLDAEEDEFRSKATAESRAFRGQFPAFADAPLKNKNI
PPSSSLSTVFEADKACVDAESLPPLNELVHSKLLRPLTECLHITSLTCIQKQSWTPMVDR
TRDVLLRSETGSGKTLAYALPLLHQLLCECDARPIQRQIGSIIIVLCPTRELVVQVTDVL
SVLTRCALFLTVGGIHGGENRHKEKARLRKGVPLLIATPGRLLDHLRATVSFCVASTQTI
VLDEADRLLDMGFERAIKEIMGLLLEKTENSACSCDEIFTETREKYTLKRVLVSATITAE
VERLSHFALRSNVVRVGETEDTFSIPSSLRQHYALVPIKHRLSTLIGFLRSQIDAGAQRI
IVFVSTADSAEFHYRLLSRLQSPFCDRRKEVLFKGASKQNARQYGVKRRVEEANRHVQNQ
SEAIVTFEDDSGSEDDNDEGQTLSGRNALLDVNILKLHGNMSQVDRASVFKAFKHVGGAA
RRSLKGVLFCTDVAARGLDMPRVDWIVHYDPPTDPACYVHRIGRTARIGNVGDSLLFLMP
HEAGYVPYLSKFIAKESGSSFTGNEAAAVVATMEKRNYESFLYYLAKLDPKSNHIWMQST
ATLERAISRLVMNREEPVNELPAGDASRNDDLTRLALFAYQSYIRAYAGHSRELKTRFFN
LDMLHLGHIAHSFGLDKRPSEVRAQLQNLIMEDRKIAREASSLNTQERVRGCDGSRVRRR
LQVEVRHDDRYRSMIVQKQRKVTRDWFQGKKDGVPKNKTLQFTEFDA*
>TCRU_202 ATP-dependent RNA and DNA helicase, mitochondrial, putative
MRRIQVAFLKKLRQRRNCGSFNERALVQRTCEHIYCSSSAREQLRQLIHESSVENALRHL
GEFSNDASVLKGSAWLGQLSKAPDVVGRIAVILPQFEKWYVARKAPEKVTMHEPWDWYPK
ARFMRRRFVFHYGPTNSGKTHAALETLVKAKSGVYCAPLKALAAQVWKRIDASVPCDLLI
GDERRFGGGAEHVSCTVEMTPIDYQIDVGVIDEVQMIADRDRGWAWTRALLGLPAREIHL
CGEERAIPLIRKVLYKTHELQRLEVVPHKRLVPLEVSPSLDGDLRRIENGDTLVCFSRRA
IFDMKKKLENISGMAPHCIYGSMPFSVREAQADAFNNGTRDVIEGKSGKKHVLISTDAIA
YGLNMSIERIIFTSMRKFDGKQMVTLPQATILQVAGRAGRFGVLRSHKFGRCTTLDSNDF
AVLCEAVNSRLPSLQKAGLLPTAEVLELYIRLREAEEGGRVTENNLASFYQRMREFSECC
QASDLFFPCDLSRSLLQVAKELEAVTDLSLSDRILFCYVPLNDRGKETYDLLRAFGHDHA
AGRHVVLRIDEEFERLVKQCDLVCSGDTRRAHQVLTRMEHLYRVAEMYCWLAWRFGKTFV
HLETATALKERAALKMEEILQHL*
>TCRU_203 haloacid dehalogenase-like hydrolase/Putative hydrolase of sodium-potassium ATPase alpha subunit, putative
MGFMGVLCFWRSKKRGKDDEIRIVHAGRPSMEYCDNTVKSSKYTLLTFLPANLFEQFHRP
INLYFLLLTALQFTPSVAPVSPFTSLVPLTIAFLITAIKEGIDDWYRHKLDDECNSRWYS
IIDMETLNVERVQCRDIRVGAVLLLLPMEVVPCDVVLLLSSGENGEVVISTEALDGETVC
KKRYAILSDMYAQRPTTSVATVDSMQEDETNIRVAAEFIRDTECVIESEGPKPFLNSFTG
VARVQVPHDTEEEVTIGVNAENVALNSSVLRNTRFALGVAVFTGNETRVSMTRTTPPTKW
SLIDQRFNQMVIVVFVIQFILVTVYVGLSDAALEKDQHALWYLGMYEEKIALKEYVILLP
LRFFLLVSPMVPLSFKVMVDASKAFISILIKWDEEMSDENIKTEVHNSSLMEDLGQVEYL
MTEKTGTLTRNTLTLKSVVATSGKIFNPGTPHEASVVRREMRQDESLLQLMRVLVYCNTV
ESTRGITLNVAQTSAPKSPPSSAWVSPSPDEVALVEGATFLGVKLLERKRRRAFVMFGNN
VESATVLKFLPFTPERRRSSILLQIDGDTQFLLLTKGSDESVLPRCIKTSYNRQRVLHRL
SCLSSEGLRTLVSAYRYLTDEEAEIWLADVRRKEAIVTAAARAVNVGEAYESLEKDLIYC
GITAVEDQLQDDVCRTITQLRSAGIRVWMLTGDKSQTAKQTAIASGLVGPDDRVIALTPE
DVESLSVQEHFEYVERRVNYVLRKRKFQEAEKKHNIHRSWYVRSGLRDFVGEFFHHESIL
SLLYHPSSKSFSQDCDEYFTREDVAENEQEDLLSNSVTIQSGGYVILFSGKTIEFLKECG
NKELFVNFKRVLLHASTVICSRMAPEQKSFVVRTVKKAGHVTLAIGDGGNDVSMIQSAHV
GIGMRGREGTQATAASDVVVSQFCFLSRLLLFHGHTAYQRSAIIVQQSFWKTVALAWMQL
LFNIYTKFSGVSFWDSFSLMMYNSLFTVPVTFLCALNIPLSASVLLNNPQLYILCQRGRY
LNMVTFYGYVFRGFLHGTIIFFLAFGFLNDSSSGVMRFGHPMDRACDFYVSCFALVTLHS
CIILLETHYITFFHWLALIWSVMALFLCLSLYGLLSENHSLLQLLRNASFYLQWNIVVAV
PFISLSVYSAIKLFFFPDVLQLQRLIIHKMSKVPFLSGLWPFWVDENPSRFWGSFSVRHW
CHDKSLTPCGSQSVGGITSLDMQAQINAEDHCGEPHIVKMALDRVRDSTALSEPFIKRV*

>TCRU_204 arginine N-methyltransferase
MTTTETPAPTKAPVAGSATGDYYFDSYSHYGIHMEMLKDYHRTTSYRDAIWRNAYLFKGK
VVLDVGCGTGILSMFAAKAGARKVIGVDCSSVTVQARQIVKDNGFEDVITIIQGKMEDLQ
LDEKVDIIISEWMGYFLLYESMLNTVLYARDRWGTPDVKLFPDRANMYACGISDQQYKEE
RFDVWDDVQGFDFSYFKRLSYIEPLIDTVDRNQILTNMSNFFSFNINTVQEAELAFTTEF
ELIAERSGCVDAISVHFDTPFAAGHEVVVLTTTPLEAPTHWRQTVLYLFNSIHMNKGEKA
KFRMSCCPNKGNPRDLDIALHVDFDGKLQSSHYDQDFRLR*
>TCRU_205 hypothetical protein, conserved
MEGQSELVERVVRMQLTSPDFAEYCVAHVTDPKHRFLFSRNEDEAERRLYVRLLEGRGHQ
KRAETPAVVAPAFAPERTSSLMTHGGTAVLWMSLLDYFSSTPGVRGPHMPHMFRTEADLT
AFVDMLDGAQREHVSLEDVVQFMLRRLPMTEVFLYVLCYMCDVRIHEVRRETRRRSSGAC
VTDSADGASDEKTLQAAGTLLFIANMLLLQHDALERPLRYLSVFEFYMQRMVEALAGTVP
KNSPVDAFLRETVMVWSEGHVFGEEALDCMKRAIGCSLG*
>TCRU_206 hypothetical protein
MRRGGSCLTVEGKALGPLGSRSPVFIMLAILFTLFTNSSEFGPSDEVGTIITSCPRPTLL
QQLVSFAAMRDVAEEEQLDWGVPATLGRQNTYRLQVKPAEQSEGDHAGENPHGNAASSAS
STGNDVNSANAPSQSRRRPHRRQRRKKKTESESLGEDVGTPVSLGTCGTKSLNGASSSGG
SNLYSSDVGEHAETVFTTSQRLWAQLHMVEVAFSCGDNNLLHITERFLRCGNMYPIIYRC
HLSQVQAAHEQALLDAPNTGLQKSCFWSCRSCGKAHDLVCESCMRCKRQSGPYKKLIFDQ
LLPETDYATSVLRLLHATHPEVTIHRIETQPHVDGGGRSSVAVYVSADAAPELVEKLNGN
VFFDMDDDDDDERTGGGVRVHYVYGSQRPWLWAFINMRKAQCKDPNKLPWGALVVVMETS
KEKISGGTATRAFRPTRTKRNLFLMSN*
>TCRU_207 hypothetical protein, conserved
MMASASAITLRGTPQCHGEHLKPTVRTRSTSESGTVQISSVTSSCAASEPRRSASIAERE
SVAAGTRVGDRDAAAMRGTENCLWPSSVAGHSARQEHARLPAAAR*
>TCRU_208 hypothetical protein, conserved
MSSDRVASVGEATACHVARIVSRLEGRSFDSTHRDEGGTGECGPFMQRLLPVVEETRVDR
RLDDVFRRAHSIFEPLSQATVAAGDDAEALMADVASRASRLVVEEVLQPCDSTKSSPSSF
SESPLRTQQAAWCRTGQLSLGIIRESTCGFYFAPSTGANSAPQRVPVLLQRRIPWRCKAN
DGSGSHDAVGRDCDADSGREEGDAPMLSGVTLQWGAVLGFLQLLSRAVGLTTSVDGEYTA
VSSLTEGEHSLLHIVADSIIVMMERNHALLQEARDMNQLGMAAEVLIIAANNFAWIRQRC
QRIPMANAREVEEILTQSLAGVLQDIIDGFVEEIQYILSMYMTSSGGGGGGRQRVVTYSF
LATRVTAICRLSNGLVHLLPRQEVMSIVLEQSLRPVVEQLLVGDKQNEVERGGITTGVLK
RVGDIFLPADAFLRHDAAHVESGVDENGSGTLAQRLSEVLQILESEEAFGAAICQRSPPF
RTLRRLLPTMTF*
>TCRU_209 hypothetical protein, conserved
MSRERRDAASSDAGDDVTPAKFNFSEGTGTPLAELPAVRAVGNRGAAWKQLETRLEPLHR
MVFGRPGRTGERRRALESFCGFPEAMGEVVLQSVQKLRKDKLRELIQALGMPVQFTITHA
RMADGVAAFLMQPHVAGTRVSSSGKTPQPQKKAVKRERATTVSESGGAAKTPKREVDPAS
TGAEKVKESLRRMKNEVPVAESSSAVQPPSDDTIRVAVYKRVLATSRAERSHLTTKALRL
ELERQFSDLEGRKVVIREAASECLAALIAAEEAAIISASLGNAAAVASLNGGDRTVEPSP
APHPPQFTEPPTVNASRSQAPPPVTPDGSVALNSSVPTFPAHPPTTLPTVSNPPQLPPGA
PMF*
>TCRU_210 hypothetical protein, conserved
MPGDEPSLWFLDTLSTNTTDARNPEETHLNAVAHAALRCLGMQVHLEVTEKGNVRWSHAR
TERRLRGPLDPALGETLFAVTTRRCARYPPPAAAYFRWFRGTARREHVEQDGFTGIY*
>TCRU_family, putative Se.1
MAKVPHRDAHIFALGCTPHRRLQQLVRARIAPKAMRLKDIMLDFVATEVLVFHPSMQNGF
PQLLSPLSPPTRESVLNVAESLIVAAIHTMNNGVPVIRHQNRGSICHGFARTFFEGFSGS
ATTSRISLRGADSRGNPVRIIVDWGCVTVAPLMHQRTCQCLLDELMPLEKKVYEQTCRNR
LGVGSKRQ*
>TCRU_212 hypothetical protein, conserved
MGLSPDPHRRRMPWTAAKECVPGVVRSSKEKMVLDGAQRVDVECVDRASQVYPLEALRAA
VATCEYNIFRGKNIFNWSLRWNPMCSGNALRQGVAGGDARRGRHSHRHLFPSPRDHNAVR

ANTPCGFSEAACAVLFVATVPPSPSVGAEVPVDATSYRLRTHPLIDAAGTAPSSWCSGTG
DENTADQVQSDGAGSPVSVSPTTAASTEIDGKGEESNDCQENNCLLTSLPVKQPEE*
>TCRU_213 Mucin-like glycoprotein, putative
MMTCRLLCALLVLALCCCPSVCAAETEEITVDLEAEDEVDGRDNRQSPHAPGIPMNQNQT
QAESPEAAPSLPPPPSVAQSQGTGQPGSSSNGAAAKHGNNAGDTGTLPGPHSQSSGAHNT
VQREGTEDKSGEKSGASTSTEQAIKKVDDSAETTMTTTTTTTKAPTTTTTTTTTEAPTTT
TTRAPSRLREVDGSLSSSAWVCAPLLLAASALAYTAVG*
>TCRU_214 RNase H, putative
MTMGASRNTKLLGMDLDPRLTLNVAATKKCAATSQRISQLRCIAHKEAGPSPHDLRTFVI
GYGASKLRYGSELIWAVATDSAKNETQKTYATLARIVSGVPSTVDPESALLEANMPPLHV
LCLRARLSIFENTRACQMDWMRRPPPEPPPRAGFRISPLSRDELYAFVDAYTKDYGITES
SPREERFFRSSIPPWFAASARRATIGVELPIDHSITDEEELIQEKHRVSEEALALHSHRS
WILATDGGVDVPKSAGVGILLSSLNSSEIIEKASINCGARPCSYRTESRALLLALEKLMI
PLIRHRRKTLLVVTDSQSLLAALNKGPLSQTDWTEDQIWQRLLTLTRAGWSVHLQFCYGH
CGVHVNELADQYATQTMESGQYTEQGIAPLWHTDLLTCFTTQLTNKWRSTIRQDTHRHLL
CGTRPSDLSGKDLITQEVLHRQELVHLARARCGESELWGRRYWAVRDCTNQCRFRNISPE
QSAYMCSNNDPSAPGTDTVPPSAREEDVSPVRRRTLTRRRKEKCPHCDSTLTGFSGLVSH
CRSFHPEHPPPLPELKCDFCDMVFPTRRSTAQHRSRCAHNPDATRHRNSSARR*
>TCRU_215 hypothetical protein, conserved
MAVELVVDAACERLYEVITIALRKMFLDAKCIHAREDHNIIPSTVFVNNDSTTSLLLLLI
TLDDWMLMVRHNCVCNYLKVVYDASNCPVYVVILGQLTGHRQSRFWEEVSCLCADDSFLS
ILVGFDIVSSVEQLAEVAVAHGLKAIKQQNLEGDPIVRSEGRRKCDPTDFHALYLDMLLE
ISSVSERRASTIAGTFPSMFHLLEFIDSGAHNRVQAEEYYEKRGTSVLDPYIAEVLSTDY
NTTEAQEMMNGLFDASAGIFS*
>TCRU_216 gamma-glutamylcysteine synthetase
MGLLTTGGKPMRWGTDENNRAIPHVSSHGIQQFLTMRAGKKEEKNFPFYWGEEVEHQLVR
VEGGKVKLSLNAADVIEQLTKSSQKDNAVWHHEYGSFMVESVPGVPYTLEVDSLCSVEGN
IRRRCELLDSVAGENTFSLTLVTFPLMGVGDFITSDSKEVPYSRSLFVPDVCINQSHPRF
TNLTRNIRLRRGHKVCIQVPIYIDTYTMEKTVDPRLNIDRTPHNIDIDCSSTEVTKAKGE
KFTINDSVISKEKENNSSNNNNNKDSNISSKYPSAEEMTHFYTPATHYYYAQYFKKSQME
LVKQRYDACPCPVPSVSHPCIYMDCMAFGMGCNCLQVTMQLENETQARHVYDQLGVLCPL
FLAMSSSTPFQKGILCDSDVRWLTIAASVDDRKREEVPHIIKSRYDSFSVFVSLTLPNLE
EFNDEEFVINDTYLEVLKSAGVDTRLAKHVAHLFIRDPLVVYDQMIDIDDTTHTEHFENI
QSTNWQSVRLKPPSLDGNTGWRVEFRIMDVMPTPFENAAFSVFVPLLARAIIKYNPLFYT
KMSIVDENMGYAHNRSPCRQKYVMRRDIFAKNISTDPSENSEFTVNEVFNGKDGEYYGLI
PLVRRYMEEENMLSSTLEGYLCFLSMRAAGEIPTAAEYLRNFVMQHPDYGHDSRLTERIA
YDLVLHVRKLASGEVKDDLFLPMNKFMPKRSRE*
>TCRU_217 chaperone protein DNAj, putative
MNGEDVRNYPARVLLAHDDRLVQFLDASVCQDWNAAVEEWQMKKQEEIAFQKKARIPTCV
DVPSTSKTGLFSDEEFFFLTKQLENITAALSAHSATKAGVHYGENDFVYSPNEVPDLFLL
DPAMHLLPLDGEKRRKCQEASEAYQRGSYGKAAEIFSSVIDSCLPNMLNAALISNRAACY
LRVENYKLSLRDAIRSYEMDNDYILGVCRALRSLICCGRVAEARQTIEKFRRNKCGYNFE
REMTDISLYEKYGAFLEGNEHSTALMHLNELLERVPCATFEVLKVQLLAIEEGNEVALAH
VNNTLRIYTDFPELLYWRAQLCFLESASMAELQAVLPFCSVTGSTDSTGRLRQVLQRVQY
CVDLCREMESLVSKSEWMRLIELCSKSVKTLFIGDRLRAGILAKRARGFYEIKHYYECMD
DIDAAIRNITSGNVRAELLLLKALSEEKLLRWSDAIRSAELAMREHRTIETVEAWKRLCD
RKKEYEWRKQQKQRFHSGYQDGEEGEEGKDSKDGSAPERNGFPRTHASGSGENERRKQHT
RGPDHHVVTQLYAQLSLPTGASAERVKKSYRALAMRWHPDKWCSASDQQRKEAEEKFKIL
KAAYEELMCIVGG*
>TCRU_218 hypothetical protein
MEKNLIINVTCNPPVISILGPVKESTIEKLNDVLPGACTTTNTGKVHFAFVRKEEPPHWY
GELQTHFASEDIGQSQLFVSLLDAVEEEGMWKLCGSNANVHDYDKATHKFFFVRA*
>TCRU_219 hypothetical protein, conserved
MEERARRPPSVLTSEVYNEAETIFTMLENKEKLVSFLDCMTLLRGMGMNPTQTDMDYLRE

RMADPVLRLEQWRREEELRREKERRREELREKRKSSSAYSQKKPRKSTVERAMQVGATEG
QVPQQNVKIVPVEEIKNIDWNIFISCAEEIYRDAATEQKEVLGALKVFDKEGAGEMTVDD
LVRIVTTNGETVLNPVEVQQLRSIIPPRCSLVELAARLQGTYIPPTKEELERKALEEIER
RRQQEVSEKRFSDDAMLLP*
>TCRU_220 membrane transporter protein, putative
MNPQHRWENDDDNACMLSGNADGRVSIHALVCRTIKVALPTDFSSVLWMASQMVTMIFVG
HCLGEDGLSQYSAGILVFNVTAMSIISGLNSAIDTISSQAFGRNPRSSVIGETLQRALII
NMVLWIIMSVAFLNSKPIMVYVFGEAVGDGAARFLRMCPLYLLTQILTGIISKTLYAQCL
SSLVVYSNAAAALTSPVANYFLIFMGIEGAALSQGLTGMMCAVTDVLLCLFHPEVVVREA
QWPSPALCHKSGWIHFLKVGIPSLIAVCSEWWAFEVQALFAIAISPLALAVFGVAMNIIS
VMFSIALGLSVSASTIIGNALGGQRPLFASQYARFILICDVMIGICTAVAMGYFGGHIAR
LYTNVPEMASAVESVMPFVILCHIGDSLQYCLQGVFRGAGRQEQAARGVVFTLWLVGLPA
SALYVFVFNWGVRGVLGGLLTGFCFEIPLLYYLMSQWDWRVLAQEAQVLSSLQDFVVLES
VFQNDESSEHDSDVSVKKEVELEAVVYNRTDSQYKVR*
>TCRU_221 hypothetical protein, conserved
MEIQLGEKVALLTTHKTYRDDLEGGVRAVASYLPFCKYVATCAEHGPVPSTFIIRSIRRV
AQRIVGLIMEVECVTRSGKIVQTVDICDSNPVIIFPVATVNGIRYAILVRKRQVSVGCNF
IVEALCGVENNDGSFTIPNDELLIPLGVDLQKTHALSSKRYTIGNEGTTPYKVHSVTLEM
TPESLQKLQNTPKDLENSLIALPLDEVLSTVGDVKASLAASLLLVS*
>TCRU_homologue predicted S. cerevisiae Go.1
MDSLYKWNDSTKIGVALTGLGALFTFLGVIMLLDSALLTMGNILFVAGVALVMGPKRFQS
FFFSRRRASGCFFLGMLLIISGFCFVGLLLQGFGALNLFGNFFPMIARVLESVPLLGPIM
LSKPVQRLLTLLGLQDHRSRNV*
>TCRU_223 Noc2p family, putative
MTTKDKKSFKKYAKKHLATVIENRRKSAKAKKEKLERVERKKMREVKEVQREEREHQEQL
DHLKEIDPEFYSYLEEEDPTLLQFGEEEMEVVDDGVGSDAETEMDGEDEDNNDDDEYKAR
DCYLEKSAKCFSSSRITRKEIEHLVKGRSIEACVDIFVSAVRELGYNVKENPKISSTRKF
DDPSLVKESVFRIARAIGSNLSHFITGKGSFKSYKTRFLMRRLLISLVTIVSEGGNVDAV
LTAGLLHALTPFVPILHYIKGITKSVLRASLSLCSASEESVRIAAYVVVRAVATRATGTR
SMYQSAAFKGIFLTLVKKAYHYNIHNASLIAFLINCVVDLYGTDMEAAYQHTFVYLRQLA
IYLRSALHQQTQSNVRAVVNWQYLNALRTWGAVVSKYSEPSQLGPLIHPIVQIALGLMDL
FSSPRMFPMHLQVIEVLNHISLRADGVFIPVAPYLLRMLTSPSIALNRPTQGEGEKFERV
DLQFTIRMKKAQSRSSSYHQNIFFECLYLLTEHLATHSHTLGFPEAFWVVETTLKRLRSE
VKIPKIHSQLTILLRHMQTTTQRIIARRDQAGNGPCDLAAVKQLEDELKAAGNPLSTYHQ
MLRQQRVEVFATKQKNANGGERTTLEAIMEGGRERKSRKRSVRAFEV*
>TCRU_224 kinesin, putative
MGRRVQLVIRLRPGEGGASCVSVGVNKKEITVVDLSGTHASSTPREFHVDQVVQEDCGNE
ELFRSLVLGRIRSSVDEPDTSCFLAYGHTSSGKTHSIAGTDKEPGLLTLSAEALLHTYGV
AEVAMLEVYGESVHDLLAQGERRMIRRRAGPDGTVIMVENLTSCSLTSMEEWKALSEFGM
LARRTAPTERNSRSSRSHAIFTIKTRGMRLCLVDLAGSERQTTYSPQLNKESIAINKSLS
RLSTVLEALSSARLKSDGSTSYVNFRDTTLTVLLQRYLSGASMTVFLACIHPDTAFYYET
MSTMRYTQRLKCINTKNAPKKPNEDMSLFQLGENQKLLEELMILRRIVGRQHEQREEYHY
YDGEFRKVSEETVIGRLPKDHCSDSLFQNHESALQESSPMISASIVGHRQRLLRYKDAMR
VAGWLLSRILSELPELSVGFDDYFDTYLPSQVQVVGYISLMTCMAPRDVNEAMGGLAFLD
VGDIAMGLSMLDAGIPACVGLHRLTCGESNTWEAHEYDGVNRVFVLAFFEINEHLVEIMG
DTHDAFECCGGLLTLEPLVPLAIVLCTPLDSSDELKENVLQHLVTLQGEQDGALESSIGG
SSSLSQLTPMHTGIDGAMENPKLNLLKAMTQWRDDYVKEVTSSSDTESFSAAPLAEEIER
QALNKLQVCSPFQESASVNSGEAFSNEKPTIIHLDHKLHVIYASSSSFSSESSSDSSVIL
GSKLSLLSPAVADEGQCQEISRTHMSSECPSETECDAEKGVGMPQSIDREVHGANQFGVS
VSPADVHVDMYNRPYLPHAPQTNCYSIEEFPAYAVIARERSKSDKKDDCADCSATASVSP
PEKKRIYEKNEKKKKKKNLHRKRKPVLQGCHNCVVL*
>TCRU_225 SNARE domain-containing protein, putative
MLFFTLIVRQRDALALAADTDTSSAELEQCKVAAKTLLRKLAAEHSNPSLCVPPLLTIVW
KNYVFHILSESAVSFLTMCDTAMPAGIAVAFLEDVAREFLQQYGSQVEAVTRPYCFIKFD

LYLQRTKKVFATANSSRSMNVSRVGRTAPVRRTFEDVMAGIDSKTGSSMSSRGGGVRDTE
KTRRFLIICFIAAVTLLLFCGIFYYAALA*
>TCRU_226 60S ribosomal protein L17, putative
MVHYSRKPQVSSKSAKAKVSDLRCHFKNTFETANVINGMPLRKAQQLYRQVLAKTRCIPF
KRYNGKIGRTAQAKEWGQTKGRWPRKSVVAMLSLLKNAEANAIEKGLDPNKMVIKHVQVD
QAARMRRRTYRAHGRITPYMCSPCHVQLFMTQPQERVPVPKSKPKK*
>TCRU_227 hypothetical protein, conserved
MRSLRLNRLITVAALARSQSGSGSGRYQQREQQQQQQQQQQRFGGPRDGRPLRGRPPQN
FQQGGYRNSLNEPQLVAKLLELYPSGKSWMPINKWASSIPEEIQEALVAFGGLAQFTASQ
SNFFIVRKENGTVVVSLSTMASELCAKREKNIKKREEKAAAFSARRRDDSRRQRGRD*
>TCRU_228 CutC family, putative
MILKEFCAEGYERIPEAIASGAQRVEICARLDVGGVTPSADTIQKTVEYCHSRNAMVMVI
IRCREGNFYYSDEEFQVMLKSVILARRLGADGVVFGAIDVLGNVDPRIGILLSAAKGMSL
TFHMAFDEIPTEKQFQAIDALTAYGFHRILTHGGSASTNIEENLDWLKELVLYAKNRISI
MPGGGVTSLNAEKIARDLGVTEVHGTRVVRFV*
>TCRU_229 clathrin coat assembly protein, putative
MNVKDSNELKRGAGYLKEKAIIGLARVTGDELDRAIMKVTSHMLKAPKEKHMQRLLATTY
GHYKNDTRDGKSICGHIVAELEKRMHTHNWIVVLKTLVTLHRLMTDGSNEFNACIKRNRS
IFCARNLKDLSESVEGAAQALFIRQYLSYLEERAISQEAIGVDNRLESVDFSSYLRSMDV
DSLTPFFGALLGQLAALVAVEYQEAIVDNFCTLEAYQRLVCDGKMLYQLLSNRVIFVLDG
FDDFSLPLKKVWLELYRQYSVVVEKLRLLFDSILRSSKVFMQPPPQLKPLPDSFLGQLED
DVRLSSIPREDVTETLGKLGICGSEIKSPPRETVEKKSPPLSPSVNAQPRSGKESPPIPP
VATEKKEAAFSMDELFVSAPAAESGPAVPSAPADPLVDNQHFPLFSPQSEENIGPENLGD
HYAALNTVIDDGWSTGAPVSWEGTPSVQYSTAAPVADGMSHTVWGGTYSSATDAIPKQGN
ENVFKELYNEEQKGWHANNI*
>TCRU_230 hypothetical protein
MSTTPVGGKIEFSSHREIAHVRQDLAVTKEILAIVKEENERLCSVAEERAVEVARLNKDL
EEAKRQFQNQKHAHEMEIARFQSGRTSLEGELAEALAERKRLSDLLHSLRGQVDGRLTKL
VEELQRKDETIHGLDYRNREYEREIERLNRMVKTDSLTASELEETQGKYSKALAEVLLLK
EQVAQYQLELSSTRERVQQLSDNSVDDSQKSFLREAVSLLMLYRQWLVEFNVSLWDIEDR
IETSLNSGIDALAQMTNPGVPYRPPTMERERLKEMKKFERERKNFDSMVYPDKNETCENA
FAFSVHVIRDTWDSSRHSLEKVKVISKQLLETLKSVVQVSEDLHTKTVLASPSEIVTNAK
VLRYECDQSRLVDTLKENFEMQLQLREEEQRKKEAEITNLKSMISTLEGEKDLLSNELLK
TKQDVINASLSSTSKLNELECKLRETVRSTEDMNAKTLSVLKLEQEKERKEMAEANSRLK
KTLEEEGANIESMKKMLLESKRRRLILKDENTALRKQLQKTEDLVNALRLELLGAKQTIE
LLYAELHFAENNHERQPNSSSSKAFLSFKKGIQKSSLEAKAVYLPRAL*
>TCRU_231 endosomal integral membrane protein, putative
MARWGHFALLSLLLLFAAPGVSCALLDHLSSPTIYQAGTEIPITVNSLTSKRGLLPYNFY
SVKTCQPDERRMRDERIHENLGEILLGNRILPSMYSVKVGENITCREVCFVAYSESEMKR
LQKLIEQQYCAHMFLDGVPLLERPLNASTEQHLRVGYQLGVPAASDESTKTTIHNYLHFK
VTYTNAEQGGFSITGFYVVPSSVNVLTGCPDPETTEGGSIQPATPESMDVKYFYSVSWEL
DTEKIEFVATRWDVYARAGHPASKRGHLMAIMNSLALLSFLGIIVMVILTRTVRKDLLSY
ADADLAEENSEESGWKLVRGDVFRAPPNALLFTSLVATGCQVVFMAGVVVIAAVLGVVHP
*
>TCRU_232 60S ribosomal protein L21e, putative
MVHSHGYKCGTRHLFAKKFRKHGVPSVSTILTNFKVGDYVDVVADSAVRAGMPHKYYHGR
TGIVWNVTPRGVGVIVNKPVRNRTVRKRICVRFEHVRKSRCQEAFKQKQKDFLTFQAAKK
AGKPLPPLKRSSRNGGIVRAKRVELLARRAAEYEAMIPY*
>TCRU_233 hypothetical protein, conserved
MLLPRTDSHDPDANDADPRSTSSAPSVSAPVLSFTTTADALAITPLSNTNPCAAYTWLPD
TRSTAPSDATTRALAYNATSPPSTAASSATPPPTSSSTAPSTTTVRPPVITVTRATPDTT
STDRTSRRPDSTSTGAYGEDREPRRYTSGVGLAETSRESVTVSTDPAGNLPVSTSAPFSA
STSHTDSSTGPLSSLPAATAPRNASPPITYSTAPFSSDTFCTTIRTPSRAIHGT*
>TCRU_234 hypothetical protein, conserved

MLPSRKRQAHRDHPRQQRSVAHKAQRAPSSGHGASRVEHLPRDAVVHVALRQQHRPASQA
HSRIKRHRVVLRRQKVAPHKRHSGARHAHSPTHRHRVLAQQEVAVNKRHHGAAAGRNTAL
HRHAQPQRRLHKKPVHVQRTARMQRHTVNQQHASSVTEHRRHLRARDGQHTPRQIHVVHH
EARGRVAKVVVAAVRNTRLCNQHRVHHCDARARRKSVRHQHAAAGARLKPADQHINALAS
RRVGPPPNNVQARSAQKRAVRQRHVAHTPRHRRHVHNDIRRKGHTNVARHRRPETGVHLH
THTHVPHAQVGVVGARWLGNSNGQARIAVAALRARAGAAITQGCRRVRGEARRRKGAWVA
AGQNGGIVHGRNGVFRLLLAVQSVARSHNRSVCEILGPCEYPKYLRHSCAGDESVAGHRH
RGVAEIHRRIKKEESFRWGFTAEVVAQLQPGVVVDVGEVA*
>TCRU_235 hypothetical protein, conserved
MMRRCLCYAARCKWGQRTWSPTASSGGAAPTNGVSAQEALQIVYRPMPLPQTVEYEEDFG
HNLMIHREFVSKRHRDQLSFELGALAYSEVELRRGRQHLAGVMNRERRGVAIGAAGAPSD
QLHMQTDVDPDTREVLTARYLFDEKRMQFCDRFQNFLQSRLEGHAAQSSDGGKGGEDKHY
LFSLMEACAVIYGCETHAAREVYFRMFLSLDLESLEAEQEALRHRIAEGKLVRQALEQSE
EKEGEGNAVLPVSRGNSSTTLDDDAAFIASIPELSLFEDTPEVDEGGSVKNETHPPALAD
KEFLPAGVVDDSGSLRNSSHSAIELPEEFEEYAPLYKAYLAHAKGESGIASYDVSTLGST
GITAERRRWRNLMDKIVREEYHTMTETEQMDALILNEQLHTVKFFDLKVGDTVREILQLL
QRETGCGSSAHRDEPLCISPHHPERRI*
>TCRU_236 Thioesterase-like superfamily/Thioesterase superfamily,
putative
MVFLTLLRTAAYFIYGAYQRRRMLSTNPQAVIEPMKKHYTCVYAGIRMADIFGHVNNSKY
LEICELARWHYGGYVGMMKILWELRASFVVASVNVQYVREILPCRRYLVTTEVLRFEDAG
SSSDKPISGKDESGVARNLPPAGRMVIMHEIWSMDKKTLYAGILLRAALVGDAKSPLAIP
SGGKRRGRYALLDCRAVYAAIRGADGANELANENMRNFYNRLGFQPNQEDFNGDPWIEAS
MRLEKEWRSKLQSVREP*
>TCRU_237 hypothetical protein
MAALAPKNGSFLHALQLENDALRRQLRRAHEDMERVLQQLQRESKGGRGTAEGGSHGGRH
EGGGDAWHVERLERRCELLATRLVGIVRDRQLSECAEIHFLLRTQEEERRFLHDALVTLL
ERQKGSSGGDEYGAQTEKEIHPGMLSALIMRVAENMQSSRSQLKLEQTERIMLLGQTIDL
ILDIQKAIEKAARWVYGYHCDFLHAGSLQELTLWREAQNSGIVGLRSLAEVTPDVPPRTS
FRKRSSNHSERRWREEVDCSIEFPSSSVHQTEAAANFSSRRGDLLEACDVLRACHQALTG
VRTLLGDAMSNVTSSCSSRKGKPTSVEELLKGFHRDLQEMVRSNERCRETLARRLEAEIE
AHRRTVQKFEARLKLAERELIDHLTSSHKFQKPLGSNDADAPSFSNYPSKVKAYHGETGA
TTPPTPTGTTATGNRRTVRVNNNTRFQFAPEKTVPLSPRRDLPFVLEGQNDKKQKRRGDT
RPGTPHKGSACDVVSFFPPSLPSFAALAGPLPETRGRQHEDLQSFSSRDNFEDGTRSNST
GEGSVAHSSPPLPASRRERHPSSGKIQEGREAVSSQRCVRWPRGAVLLSPSAF*
>TCRU_238 zinc finger protein, putative
MPPSHHGPSAISSPSFSQQNYSQSVSKTDYLECFPSCSGPRGGDPTSHSSDASREDGNSK
VSLASSMAEIMNSIPNEEGLEEVKVVAQNHLRATEEGGGKEEMLRPPSPFVVLGYGERGG
DLTTNGVADVSIVDLETELANLRKELLKLRREEEKRRQQLYEVWRQKNPKVTRGLSSPLC
TSRGRKNRNRKAASTVDDVTVVKSNGSDVTSSVICNTKAICNGILTPPAEDEGAHDGEKD
TGNAAVLDQPFHGHNNDNNFLFSGRRPRATRNGMVADPSDPFGKCTSLGNDGGSLASRFN
PPNLYDFPKAGKCWLGGRRARKSTYTVVAAADGHGNDDAPTGDNRGEEETSLPCVDKKSG
VNGWKTHDGELDFSYYSLSLVQTGSAGMLEFVFDEDDAVKRRAFRDAENQLMLAEQRILL
QMETLRHGRSQAGRHYWQSNESVTHCTRCGKLFSITVRKHHCRRCGILLCNDCCSQVGRD
MYALQHDKVSEQEEDEEDVVVVDDNGEIQATVAVESTNNQKKEYDWMFDTYELTDGTNWR
QEEGDEDKLFSQRSKVATSHTTRGSGRGRGGLKVRSAPWCRICNCCYLICLRARIERNYS
DVLSDGRRRFHVLRDDEQAFTNLNMAWEARVAQLDVLKHLIVERATSAAQGQMGHFAGLF
KEWMRSLGRRTEQNHIESNSDRSNCGSESQ*
>TCRU_239 Der1-like family, putative
MDFEFFRTIPLVTRSFLLLSVASVMLVSFGVVHPVEVVFSPLLVFQERQYWRLITNFFYF
GHLDLNSILELHWLCVVSSGIELQYFRRRKVDYCITLFAGMSLLLLFRCLRVVDTPYLSF
SLCNALAYLFSRLMPEQEANIFLLVTIPVRLLPLFFLAIAIIFDMQRSIRLIVVENLVGH
ILWYFLEIFPCITRVHPLRLQEMFMQ*
>TCRU_240 SAM domain (Sterile alpha motif), putative

MNTLSVWCERQTASFTKAFKKRYVTFDPESRKISYAENEQKPPKGVIIVTKIVRCCQVME
VKTSDLFTLMVDGSHEDGKEDQWTLRMPNRQVFEEWTEVIGNVCAAAGLMQPLNFGLPSI
DPRTNLPFAQVPLEYLFKFAVLEKAVIHFFGTVTLVTSTTGKDNHPARHNLVVGDKAIYI
FTQNATILYCSLISHIRRIYHGPEATFFAVHVKSPAPDIVVKDFSEGAQVAFILARLFHV
TTGKSLSVMPINVPTAEVLVESQQLRLNGDEGYKLHVVSPTTKLRLRQVIDTNKDGRSLS
GSVNMNANGGGQKQGVDKALAVEPTVVVPTDPLTTLLHKIGLPEYAPKLLSQHVDLDVLQ
CMDVSDLMTFGVSNKSHCQMILDAALSSDTADPENVDDDALDAFDPKTGYPSGKPVGGVT
LSDDEDDEIDLFVPPQRTVTLDEDSDEELLPSRTPAVNVSKPPAIVIDDDDL*
>TCRU_241 conserved protein
MTEYKKLCAILAQLREEVTSLVRAFEGGEGRDTVALHSLSTSIQTLVTNAQPRLLKILRK
ATETDPNRQIYNEAMCAAIKQLFDDFCELLGCLFGVPMKEMVLSEGKINFEDSPSISWTE
DVHNNYLLHLAQTEAWKKRIAANIADLVFFEEETRAVYFAEERKARETLLQTKRNEKTNI
LHMLKEREAAKWEAEVRRRNDEHKGLMNASSFYGVQNIATVLLRVPEPFRKLLAGNMAQL
VRALRTTPEDPNIRRIRCNNRRVMMDYSHIVFCVECETCRILVAAAEILWYIMGYRVEYS
TAPTSSLRTVIDNNPPILLPCGRYASEHAIAVIGFEEYSERFFTLHEPDPMRDSDEWMVW
YATLEALIARLEDCLV*
>TCRU_242 dual-specificity protein kinase, putative
MYGTSKIQGGGLRGSLHYCTQQRFLPSANRSPGFVNARGKVTASKAATSSEYHAAYLTEY
EKSEIRGYKDVYYVGQNCERKIQAPVEGGYNCGYDDERGDYTIMLHDHIAYRYEVLGTLG
SGSFGQVVKVADHQNGTTVALKIIRNKKRFMSQAKIEVQILSHLRRGDPNGIYGIVQMLD
SFTFRSHVCITYELLSINLYEHLKQRNFHPLSLSAVRKIGAGVLVSLSYIWRENIIHCDL
KPENILLKTPDRATVKVIDFGSSCFENARIYTYIQSRFYRAPEVILGCSYNKHIDLWSYG
CVLCELATGVPIFPGESEQDQLGCIMEYLGAPPHELILQSSRKQELFDVNNGYAPKLVPN
SRRKIRYPGTRSLAAFLGLPQDDGFVSFVRQFLCWIPEERVSPRRAMRHPWIADIFEGPP
PPLSQVSKTQTNLAVRAAISNAHRITGQALKVPHLPKIGEH*
>TCRU_243 hypothetical protein, conserved
MVGRADQAQRRKEHKARSVHHRQEGRAHHVREGNNARARNQHSRHAEVLDVAASHGRLHS
RNQAHHTQHRQLEHAPVHVAAPHVRQHAAVHPLLRRGVPIPDRVQQALVQRLPRKLPVPR
EARARVAGNQVAHASRKRRTVQHANDTNHAVPHAGTGHRQ*
>TCRU_244 hypothetical protein, conserved
MHRVRHDVRQAREAHRRPRSRRVHAAPPLEGRRHTAVHAPEHSGEQRVAHHHPQRVKGSE
HRQEVRQRNGARTLLRQGAWHPRKHAERLHLQDGAQPAGHHHGNSRRTRNNAGDRREGAG
GGGQRVGPDREGPKQHTPAAAGHCRSVAANEYRLVGSNEVTAVHGNSQLQDGGRIRRVAH
PRRVAQHRSPRTAKPARLRRRHRHEAQVRAELRVACPAERRRQRHHQPCRDGAPTAANVV
LRQAPTSRRRRHSERRARQVAPPQRAAHRRRRGPRGRQRHRLRHL*
>TCRU_245 hypothetical protein, conserved
MMPADLYSIGCSVFPLSVRHPTTNLRPAPPSTTVPSITTGPLPAFPWSVSVLCARSALRG
YGGTDRSLPRMLLPRTDSHDPDANDADPRSSSSAPSVSAPALSFTTTADALASTPRSNTN
PCAAYTWLPDTRSTAPPDATTRALAYNAYSPPSTAASSATPPPTSSSTAPSTTTVRPPVI
TVTRATPDTTSTDRTSRRPDSTSTGAYGDDCEPGRYTSGVGLVATSRESVTVSTDPAGNV
PVSTSAPFSASTSHTDSSTGPPSSLPAATAPRSPPPPITYSTAPFSSDTFCTTIRTPSRA
IHGT*
>TCRU_246 Mucin-like glycoprotein, putative
MMMTCRLLCALLVLALCFCSSVFASESETAQVEDIVSGIYSPSHVPGGPPGVSEPLPPIT
LSTARGAPMTVIGQSMSSGEVSGLVPRKGGGRRADVADALQKKDRIDMTAPETSDTSPAD
GLLGNQDREGLEPSTKEQTVLGTGEGGTTVSQSRTRQPAEETIESVGGLAKTTKTTKIKS
PEGASQGDLPAKATHLTPSPPVEGPLPNADKAPVPQPEEDSVSMKPNKDLAEEDIATTVD
TDQNETSSGRATSTTTTTLVASNNANNEADRSTGEDIPNNTPDSDVSGTEERRDENKYNN
TKKTPVQVADIKSVTVASGNSDSSTAVSHTSPLLLLLVVAAAAVVAA*
>TCRU_247 hypothetical protein, conserved
MTLHLVAVLGLWLRIGTPQQGVAASTTETKVTSPPEKVPRHARCGEVRRSACRLSVRTAR
PFRSGHSVDIARGGDFHKNPATEEDGYINVWQLNIAGLSSVKNIAFAARLEQRPPDTVLL
QEISNRGEAATKLCGHNDYLQASLGAVLPLWCATLYRASACRSLALTSWRPLLSVYFHRA
VAAFAWRISAILPQQLVNNPHLSISFVACARRDYRG*

>TCRU_248 hypothetical protein, conserved
MRNGTRRCRNSFNMSSGPGAEPSKESRMVTSSTYGGKGRSISQPPGTKGVQRAAAAVQGG
MARETAQNDGQADLPVAEAGRIRGQVQCGAQPIPEDEGAAPPCREDIATSGWVRLQVFSR
GLPEAQRSNGVRLDFVPPFFRVHSAQTRQSPGEAMCVLRVLLRRRTRPVPFRVSISFWSC
HGCRAGCGAQLLVRCLREDPTMWTRWQGGFHTTWWKQANPLYAG*
>TCRU_249 NADH dehydrogenase subunit NI2M, putative
MMRRSCFLLSAGMVEYHEWHLHEPLLQNYEGWRILDNPRYESKSEAYIYDFIGSVRHLDP
IIDDPRLTHKQRVCRLYRWALKELQMWLVQLNAHKFNLAYKVVRRRFEMYRYVTDPAMCD
MMVRETQKYLRENANFYFLRRNNTSPWSTHTLANPMFHPDGSQVYDHWTHPEVMWYDDAK
LHRWTGHHPMYAGAGEMSDRFGDMDVSPHLRIFTIAIFGCISLWSICSFLAVFDRDVDPH
FEKWCESFDDNLKGALYAAERNSRSRTSMLGWDWDRIMGKVQHQSGYHLYNVQRFQ*
>TCRU_250 superoxide dismutase, putative
MRRSAFSTGVVPAIAAILSPAALQASRRGGLVMHTPKSDIVLGRFSFMDQYNEFRNRSGR
APDYYVEKYRDTYWRVDEYIRRAEHYIQSQGFFYLPTLEFPWYRGCLPLFSSYQIRLHYG
RHHRGYVEKLNQLIEGTPLYGSNLDEIILRTHDDVMLTGVYNNAAQHYNHCFFWKCIQPY
GSNIPPDLKAAVEKQYGSVEEFERIFTDAALNLFGSGWVYWVYDTRLGQFDILSLSNAGC
PLTNTHVVPLLCVDVWEHTYYVDYENNRAAFLAKYFNVVDWHWAERHWKRATGQEYYEMK
FW*
>TCRU_251 WW domain containing protein, putative
MQDEAVRAALESGVWREKKDPKSGKTYYANVQTRVSVWNLAKELARLQQQQKSTEEMELR
KQKKLQSLAEERQAQAKKWQEEEETLMQRMAELEREKARLESEITILQGPTEVEAASLQE
EQSRLLDAKQSLELIVSEELRKRRESAAELQQLQARLEALRQERQEELGMFESLRKRLEQ
LRQEHAEALGDLQREEAVEESLREELQREERQLEDALHEEVRLKELLKMREDEVDHMKAE
LKAVCQRKAELQQQHQRLVEEVAESAMGAAEDTLQEPPSSSSLLLSHLQREVANRKKSLR
HLMQQQRLAKEAEWMEAESAELRRFTALAEREAQRLAQFSRELSDQVRAVTPLLEAVKHD
VQQLEEEMEELR*
>TCRU_252 variant surface glycoprotein (VSG), putative
MEAKLEAVVAALHGRFDKNGSFSHPSHAYGIAWELVNALAATERRCQLQRLLLLFEGNRD
DYTVGQIVSAGSLVRACGLLLEHDVWRWERAMHSILVSHPPFSRDVVSSVCFCVAQMAVC
GDSVALIGVAGASLQNTALGESLPEEVASELLAECTDVLVRRHREEEEEKEEGDTNEGKD
PRGKSSSRRAALRDALIGYALDALSCKRGAEALSSLLSCRVLIPLLLSGGHQSLESHHPR
VQSLLQSISKETARQGEVVVLVTALFEYLFRFTDGDKDRDYRCSPHLWNTLYAAFWDALT
CDADAGRRGNMQLRVEYLLKRIVDLTHGQETHEKWEKTVFFHEQFVWVPAASHNQWGLFF
LVLEATNEFGLHIVQPALLKLDVLVDSLTCDAVAWAAIKDGGNTEAAVSGPSLVGSLHPA
WVELLLLKVLLHPNTAVRKTGLRRLWSLSTSVLRLFSSVFLFTSAVEASLDSRLCADVDR
TPHATHLSMFKGFEGAEVRALEGMAGPMADQLERFYACIFRDVLCTAHKRKEAMCRLLHG
VVERPSVYGVTMLLCILHGVAETLSLDTEARSREATLEMITDPNVLQELLLLLRDAAHDG
VPFWLDVRLSAVAFSALLHFARIDPTLVHQCDGLWKLLCVCGPLGNSCGGNASAIDSVGH
IGALGTCIHPRFLNEQLIRMSIATPIGGSNFRFARILLRTDRLLEDVRQFLRFASFDEAR
VKQLFFLCGALDIAESEDRRLLADVAQEIGTTLLSFAKRPYSPSESFLAALAAFVEFHRS
VGAVACKRYYSLATVVDLREALHTVALSALRQVFRAMTQVNSFDDFSTDVWALQHTSEWD
VVVAAVLSAGQIAVAHDSTGGNTWFQTRQHVIDVLQLLLLDADGTFAAASVGDTTKVTRH
FAKMVVFRNASRLLQSVLSGVITLGTPGNTLEGLNAAEITCRLRIENADLLHWLRSLMRF
PSLRLLTCDVPCPLTSLSWATLLAQYYGGVYNAVFVLCSCVDPQQSPDLVLEVQDYALDH
VERCWGTNLASVYDILAWVANSGTFTENNAVREKCVDHRAIINAMFGHLKDVGARDHGRV
SVLAFNVLRCGAVEHEELLKSHISRALLDEADSERSVYLAAVTVATEVLRDPLANWPRLK
DLLLHVAVLYNSGRDEEASEMMVAATEPLILLFPEALRVQYPPTVRLSAVGRALAISTIL
WCCRLRNDWATEISLELLDWNTTHAAVKHEPCMPNSRPHRCRIRLWQLLCALLPMLDDAA
AQQSILGRVIKTCLPLTNMGSVRRLMELYALKILERQPALYTLLDAAMTDYNLRPQVCGS
YVLIMAHMMLQQLRGEVERVDGLFHVLLHRLFQQSTSHQHLLRVICHIGLHQIHETCTKQ
GVKFLPTEEMVFDYIARAPEHVKFREKHAEELFFNLAEACTPRQLFCVKRKEGNNILTES
IPAAAFDRMRFLDHEFRCLIGALSPVEMLRVRIISDRFESRRILEPFKSFPYIPHTESHT
TYCVDYTAEAMALLTADNNNNNTNNRMIDGDENIQRKVTPWWNGLVYNELHPRALKQEKR
QPLIVLSSLLQNPVNVAGLFRCGEIFAIEKVVVSDQAVFAHPHFVAAARSAELWMPWDAV

MAKNLPEYLGSLRQDGYTLIGIEQTAGSVSIASYRFPERAAILLGAEGHGVPAELLPVLD
VCVEIPQFGLIRSLNVHVTGALVMYEYTRQHLMENK*
>TCRU_253 hypothetical protein
MSLCYNSRGLLFKSVFVLVAVYFCFFVLWPGHNANKQGKRSTQIHQAAHGLGAQINGSQG
FQLGWSGRTCGPGARLIHKSLLSRCRTFANRRGSVSLAFCDRILRPGPPPCGFFVGGKFK
QPLRLLGLSELKQLRGDGRFEACASRRMYDGVQSNSTLHAVDVGNGSSFAFEVRPALCPS
YRYVSPLEALSILHHASIGRNGHGIIVTGDSMLRQLLLRLMFFLRGEEVFAEHEFHRDAL
YVVYEDRDELILSFTCVHAALIETYFPGYQGHMKNKTSCAHFLQSERVVAIFLYLWDPLV
NDFREDAFRIRSPPVHLASFMYWWQRGEERLGGLTRYFHRLRSRMVFDKKAFGVPATKYV
FFTTPRTEALPSFLFPDDLRLERNAFAWESLQSMEEELDTGGFGTAMMSLVDFAALADLR
RISRTDDLIHFMCTWRPKSPRKVSSMKTAHVTCQDPVNLAVVQWTLHLLWTGL*
>TCRU_254 hypothetical protein, conserved
MEELSAIDSVLSTWAPKDIFFSSLSIPTVGLVAVFFDAVSDDTWNDEYLCLNATVRNATK
DSDGLQLTGLESRAIWPVNTRSDKVRHVSLSHDSTLVASVTIEVTPSGNTPLLTAMLADT
ASNHTVGLSCSHNTKWETAFEGKTKARSSNREPKEEYQVGLMLQCNKTSVHIDGESLWEE
EVLLAGERPPEVLRFCFGACGGHESRLTVTDVFPYSRPLNSTAMRAPKDRVPAPTREAAP
TPPERIRVVDSVSEHVAGNIGEEK*
>TCRU_255 hypothetical protein, conserved
MLSRVAAVKAPRTQYRRSESRTITESNTSETRPEFAFVRNTKVAPTPPEKRSKPVTEPKE
DPVDDAMSGSINVELLQGVDLFVPQTTLVQSEDGIVPVTTRDSFASPSPVSAGGVIAAFA
EGHTDAEYQGGQLSEPFSSDVVAGCIDSAWNWSTLVGEVNKRYMEGTRCAWCSGGKGEFG
CCAPPHNNYEGPRSVSSCGRL*
>TCRU_256 hypothetical protein, conserved
MPFPTYEGNFPDDVLRHPHLFYLTTRRNAGLVLSDESPPSRLEPKRRAHEETCCSRPVDA
WPVRCLSATVEHLICLIAAPSTNCTVLVLLSNADKGGLHDGVLLLEGSVRRKNVFFLFLD
ETNLSFDSSTNALLQFLLAVGGFGVCSNQWLSLAIPRPNIDWCRRSWAHVAWPINYLCNW
TTKFSVGVDHVLQNMTTAAAERAGVCCVLHDSDDVLADFTLLRVSNVTSVPLRVAEA*
>TCRU_257 hypothetical protein, conserved
MLLPRTDIHDPDTNDADPRSTSSAPSVSTPALSFTTTADALASTPLSNTNPCAAYTWLPD
TRSTAPPDATTRALAYNATSPPNTAASSATPPPTSSSTAPSTTTVRPPVITVTRATPDTT
STDRTSRRPDSTSTGAYGEECEPERYTSGVGLVATSRESVTVSTDPAGNVPVSTSAPFSA
STSHTDSSTGLPSSPTAAIAPRNAPPPITYSTAPFSSDTFCTTIRTPSRAIHGT*
>TCRU_258 hypothetical protein, conserved
MEQPRDRKTVPADRDVCVRWKHTLRAEQRVNKCVAGDDDVQRPHSGVRVAQQQQHGAVPP
RASIERDIRHVARHPPNQHRQAHDTPAERHVAVAQPRQPAAHRLRNLGVHHVADALTNRR
RWRWWRWRWRASRHRRRDALIAVPSSSALALAAADRRRQRSKAVAVLRALPARGNRRHVG
DAVQRQFSRRRHLPSRDRQTPSNETVAGVSPR*
>TCRU_259 hypothetical protein
MLRLGVVQRTDHCGGGPALDGRVRRRAERDAAPLRACGRRAAADWRPQREHGAPDAARFR
QHDECDVAGGHDCAAWRDAAALECAAGELNAARDGWRVAVRADDSWLRRVSVRPRACPGR
RPASVDAVCHDAVDAGVWRGLVRCDPRGARPRREPVQRLLHGQLRY*
>TCRU_260 hypothetical protein, conserved
MAMMMAGRVLLVCALCVLWCGAGGRCDGDEVSSSALSGPRGGVDGPQSSSKSVAGVSEDA
RQLLYAQNGKNENQGRGSVIEPSLDDVYEEEKGEETETRETPPPAGGGIKAPSASAGPGV
IPTSGSSSLSGVDSSSRIGGGTGPTVSTSSNTVGKASKEDMALQEKESQHTQLGDGEAAG
THNNGSQQNSSAPPPTGGQNDNDAVTLSADGLRTLGVQIAVKGDLEASRSEEPSDGPSTN
LPESGRAPPESSQAAAAPEHTPQHKVMTSVKEETERMNMNASANLPGAPKRSNEDPASTA
TTMQSTSTGGQEGEATPSSNGIPKIQEEKLTGNNTTENVQPSDTAETEIRQSVNTSKVGD
SDGSTAVSHTTSPLLLLLLVVACAAAAVVVAA*
>TCRU_261 BNR repeat-like domain/Concanavalin A-like lectin/
glucanases superfamily/Trans-sialidase of Trypanosoma hydrophobic C-
terminal, putative
MSRHLFYSAVLLLFLVMICCGTGSASSKEKSPVQIPPPKTYFDWRDVKVEETVSSLRVPS
LLKVNGDVFAVAEALCTGKKDTGEGSFTGIASEVLELTGENPKEELGKNKLKTQVLEECT

FEGGNCPSQNAAEEGGSRSQTTVHVSRPTTVVEGSDIYMLAGSYSWTLTADDQAGGWGLM
LVKGNVSTVDSKKRIYWSDTYGLPWSYKVKQNEPLMRLVGSGGSGVQLEDGTLVFPLEGT
KKLNGAAEDGKTTTVSLIIHSVKDIASWKLSEGMSADGCGVPSVVEWEKDKLMMMTACDG
GRRRVYESGDKGESWTEALGTLSRVWGNKKREKVELVGSGFITATIDKKKVMLVTLPVYS
SKDGNGKGVLHLWLMDNTHIVDIGPVSEKDDDEVTASSLLYKSVEGKKDELIALYEKKKG
VEETSSGMASVLLTAQLEKVKEVLATWKEVDERVSQLCTSESVGRDTLPGTACSADIKIT
DGLVGFLSGNFSENTWRDEYLGVNATLDNEGVEQVETGVKFKGRGAGAEWPVGSQGENQL
YHFANYNFTLVATVSIDGEPTEGNIPLLGVLLDGEKKTTLMELSYDREKKWILLCGGETH
KKHSSTWDPETTKKHHVVILLRNGNQSSAYVDGKRVGEDVPCALGNTDSKEISHFYIGGD
GKSAVSTGNGEDVSVTVTNVLLYNRPLTSEEITALNTNQLSIPKLEDLKTEARDAPPPAG
TEPHTLGAVSSTIHGGHQDTEEKLLKISNDAGGGGASNSAKTTMTTHSVGGQSAEQLASG
GSHDVNKNVNVDSSSNGNPAVGTVGGDTKQGNGSPQIPVGISDTADANTPTTEGEGQYGP
TVNPEAGVSSGENGETVKGTNGQEEEEIHPQDGELNATALSSSLGNVSQGNNSDAGTVRE
RRLLPSLLLLGLWGFAAL*
>TCRU_262 hypothetical protein, conserved
MDLAEKRRQLDEIRAAREAKQRSVQQQLQQQQKKGSQHHVSSSSLARSGTPQSPMASSVG
GDPLNRVASTLSAVSVREEQQTGDSRTTSAAASRRPPVTLSTRISSGDLRSKVNRRGTGS
GTTEAVGGEGSAWKVEVGVGAFALKPTVEEGESDHSVRDGMMAASVVGALSALEVEDASA
CATERTQNCEASGQQRVVPPPDVAMQLAHSLRAESFCNCKDRCVLDVVMLDPKISGGNTG
VSDDCSGFLVAFALSEPHQADEQSDSAPKLHSGRGIDAQSLLPLPLSTSTATTAAAVAEV
LGELRGLVLLWCISRGKTGDASYSVIPLCFDSEIRVLTYTRHRQHLLFGGAANGCVVGWR
IDHALQGHSCAVIANGLERFSVAPSLMPLPAGGVAAVEPSERSFPSPHTHQSRVIGMAVH
GDVNYHHLYTISQDGRVCMWAPPQLGLPTSSRDGMVLGQPIGCVGSCAAFVDKAADAMSK
VVVGCLDGRVLEGRTKSSSLVEMSPINAMPAFSITNGVLPRSNSTQRTSVDGPAHHATVV
AVAPHPLHADPRISDTVLTAAADGSCILWLGSRRIAIDGFTSQVNCLRWSPTHPAVFVAG
ESNGRVAAWDLSRNSFSPVAVVYLRAVPSAAGWASTPSATSAVSAGSYRSAAVTNLAFSD
DGAWLSCGTAAGEIHLLHLRSDLTVENAATTAAKEVKAGPLSRARNEEVAFKDGCDDATV
TAAAVGRHATSSWIDARFGF*
>TCRU_263 'Cold-shock' DNA-binding domain containing protein, putative
MFRLAGWRLAAVASMTPLLQKASISKKTEENATAQPAKRRIGTVVSFMHRRGYGFIRELE
SSPGKEKKTHKAAAVAPADADAPGSSAASNFFFPRSSLDGGFYVAEGQTVAFDVRLTEQS
KNINAHLAEANISSSAASASSTPLRTDGTDEEVDGEQSKKLTFAHRIRLYDARGGKEKPV
TPIMLVGYVESWDQLAGKGVIAELDLSGRLNEDAPRFTVGLEDLDLAGHSELRRGRYVRF
CLESGDRTAARRVIIDRGAERKRAAQLSAAAEDQVDGNDGRQYGTVREIVEGRFGFILLD
ETGESVFFHMSNAEGGVVKQGDTVSFIMREVTQGKHAGKRTCLRVRRCAARNSTSSAGAA
AGNGQKSRAAQDFDDDDEFELLE*
>TCRU_264 BNR repeat-like domain containing protein, putative
MTCCGIGGGASAGGSNVRKAVDALMGIGWEKLDNWEDFSDAGGKYGSLRGPSLVDLQGHV
FAIAEAHCKDGGDCSDVGFTGIASKYLDLSGVADPTEILTADASIFGADLLKEGSEVISA
RNGITRPTTLVLGDSVYVLPGNYSHTGPQVGGTNERAFYF*
>TCRU_265 BNR repeat-like domain containing protein, putative
MQAKDKDGTSVLLSMRFPKSENKWELSFETPGNGCRDPTLVKWEEDEDDERLFMMAHCAG
DYYDVYMSTPHGVNWYTSGQPITRVWGNSHNRKGTASGVDPPPQSLGKEGDAHHRAGVSE
GGK*
>TCRU_266 Protein of unknown function (DUF3676)/Trans-sialidase of Trypanosoma hydrophobic C-terminal, putative
MSSGSYDEEMPEEEGEADGRSGGSTSSVGASSDMDTATETVDSERQLQQSTELSTENEGL
RSTGTGTTGAEQSLGLEAGDRNSERTMGSDSSLTPSGIDAEPTFAENTDDVSRTDGAQFS
FEDGKEVPQTVDTKPENTNATPGGEGIPSTKGAARHSDNDTFTGEIAELLSMGLSRDSTV
HVCVFRVLLLLLLPGLWGIVAL*
>TCRU_267 hypothetical protein, conserved
MQRRRGVLHARDGVGGPRLVLVQLQGRMAWRVVSSLRGARHGCAARGGESRRRRHELRGE
PDADEPHAEHVEDAPLLRRCDVQRRGCRADVFPRQYAAASPHQHHAH*

>TCRU_268 hypothetical protein, conserved
MAAGYFFILSLQTQPVWFKETALVPYTASEWCDIGARLWDLVRQRGDIEVQITFINGTEM
SDETLTVAPSSTGLCLILGNVTKPWEDSSLTGHIANLRAFQVPNFENQLILPIMGAAREL
GRVARARHC*
>TCRU_269 hypothetical protein, conserved
MRSGGVCLCLTALQLFALERRRTTTSYKYFEDTASIRAAFFRLTDAERELLEQSAMALRH
DVALTSVRGEEGCLEVATDDIAVQIDDPSGCAVDAAGDPAEEGGAGEICL*
>TCRU_270 hypothetical protein, conserved
MGSTVCITGATFSPENEAILWTHAMARGYAASVWVPQSLVERLQLTERGHTTAVPVGVNA
ANGRRFFYNVSQLECTEEEVRGRVSKMSVPATRRPAACESDFDASHPLNTNGEPLPPVFV
EEVHARFGRHHSAGHSRYWATVDEAELIFDSPFDPLFLNRENAVTLRASNGSSCVTYYNV
CGTRRPEVFNSDNCRRYQPVNFCGRRYSPGIATQMKASAISYGCMDSFIWLTTRRARKNG
IGVRRGVTPLTVCAEGIFHLVNIAMTNDPARLLSLALSRRLPGSMDDLSLTVSG*
>TCRU_271 hypothetical protein, conserved
MPGEPLGTYGGNYQAPAALDAMQHVASVCLSDGALYEGPLVEGKPEGRGVCVFPSGVICA
GRFSHGHLDGNAEVLLPTGALFSGSFKSSAAHGSGLYVQDGRLTRGRWHEGFMVEQAEDN
LGGGARAQLFTRIAFLLCDELQKLTAVTRAPNWQHLYVNSRISEPKVRLCRECSPEKNTM
LSNSEKPEYFIVNCSSTRIPSRAIISRSFVDRQASLSDTEESLTAATTQRQTAHAEIQMQ
EKPATKNALAMGASAYAIFACAAPASNEFFSFSHYVKYCLIFLFPFLSLPQFPFSPIRKA
RLEMEREFIVSGASLLRKYDPPFLSLHFVTLAMCCSFSAVVIAASKVNLGAVSDGRLTLA
EIVIPCVFWVLQSLFYAAYNSYMRVAHALERFERRLTPRLSAFTAGLVDTKAKVCIYTWD
EKGRGMVTNKHYRYRWLACSVVVGIVMGLAAPLTRVGFRHPFFGTGTYDMAAAVLVFISN
MLFSTILAYYVLKMTDMQRQILEQMRVISRLAYLEGRSLMRPSEYLNQRFNFDEPLNPHD
VFRGVAGWCVVRSLVFYASTCSNHAARSSAMSVYLAFALFGFLATILDIVHILLAGYLHS
GMHFSTGHSYAIVLCTLWGLMLLRYLYICVETVKEHKVHLYLLDVASLYHRMRQGPGGEG
SEVIARCREMIKEHEELPSVFSVQISPHILAAMAFIHVLALAVLVLNIYIAIHNRQSHRM
*
>TCRU_272 hypothetical protein
MHGRPEVQQLVGGGIARQEARWPQSQLGLDDVPDACGGGAFHELAEAAAQRYRSVGSRGW
MPFSPDWAGVAQPRVANSVGRPLHVGGYQTARGRHASPYDLDGAVPLNVFRRVRAPVPRA
TAVWCGRALPYLLGEVHRIRRDRWRRVGLWVPCCFRRLQLHEVADLQVSAEHWAIEIHGW
RAQGTRGLSTGVLPAACCRPCPRARSGVCASGFRMLRAAPPCDGGPAADRLLRGVRRQRR
GKISPAMEPRMAIAGGSSPGLLP*
>TCRU_273 Endonuclease/Exonuclease/phosphatase family/Endonuclease-reverse transcriptase, putative
MLEPFTWLPAERYFHPLLNSIGAYQRYTYRLRAVCDAQRQKPLLSGDIEQNPGPIAVLQM
NFSCLTPSKLATLMAQGADIIAIQETWKSSEQIASMHTGECVLYAQSRIGKGGGVAVLVR
KTLRSKRIPLTIPQHDTSHEVVVVQVALEQNRDIIVAGAYMRPTPQVTQSFRRLVNCLPA
SSPLLLCGDFNMHHPQWEPFLETSPSEVAAEFLELCTDAGLTLVNTPGEITYARGTRERS
CIDLTWSKHLTVSNWSASVSPLSDHHMLTFTLHQAFKDTIPSAPPSAPKFFYSWGKCKWD
LFIKDFDAQLPAYDYKKQSTGIKAFTRALITSYRRHCPAACTRTVPGFGKTLSWRRSGLP
RTARPAIYSYRRPTVRQKCNGQGVNSFSCSESAWATRIHTASAS*
>TCRU_274 hypothetical protein, conserved
MESGQYTEHGIAPLWHTDLLTCSTTQLTNKWRSTIRQDTHRYLLCGTRPSDLSGKDLITQ
EVLHRQELVHLARARCPESELWGRLYWAVRDCTNQCRFCNISPEQSAYMRSNNDPTAPGT
DTVPSSAREEDVSPVRRRTLTRRRKEKCPHCDSTLTGFSGLVSHCRLFRQEHPPPLPELK
CDFCDMVFPTRRSTAQHRSRCAHNPDATRHRNSSARRRSLLPQDQPASTSTPIGPQETLH
HLLPECYSTLAVRQRLGIEQDLRLGKFSQWQLLHSRKLLSLLDHLFGTQMTLYS*
>TCRU_275 BNR repeat-like domain/Concanavalin A-like lectin/glucanases superfamily/Protein of unknown function (DUF3676)/Trans-sialidase of Trypanosoma hydrophobic C-terminal, putative
MRDGTIVFPMQAKNSDEERILLSMSFTPSDKKWELSHTATGKGCRDPTLVKWKEDEYGAE
LFMMAHCAGGYYDVYSSTSDGYGWNTLGEPINRVWGNSHNRKGYGVQSGFTNAIIEDKEV
MLITAPVYAKDNEGGKGRLHLWLTDKARVYDVGPISREADDAAASSLLIKDENRELISLY

ENKKSDGSYNLVAVRLTGKLERIKEVVKKWRDLDSALRTCRSGSSGTVDLPTKGMCNGPV
PTDGLVGFLSGNFSENTWRDEYLGVNAIVHGPAEKRIVVPNGLTFKGSEAGAVWPVGDMG
QTVPYYFANNEFTLVATVSIHEVPQSGSPIPLIGVRMNDTSGTVLFGLSYTHDKKWLAIP
GDGEYFDDWEPNRAYQVVLRMDYYYWTVFVDGEEIHHTEYNTSLFDSHRISHFYIGGDSK
DQSATGGRVTVTNVMLYNEELSEDDLNELPDSKVTIPSLSVEEQPTGQAASTDDSVASGS
RSKESATAEKLTEGDRDKQDEESVLNLVPVAPPSTVVAGSSVPKPATAAESAGASLPDDN
IQLSGGENSPQTTMNEAKESMQRDSDVQTQDLQSAESTEFNDVEGSSESNDTEQTVEEGE
ANDKSGGTTSSVAVSSDIDTVAAPADGEHQVQQSVELSSENNDVRSTGTGTTGAEQSFSL
EARDGNSERTMSSDSSLTPSKSGAETTSAENADDVSRTEGTEFSSEKGKEVPQTVDIAPG
NTNTTPGETKIPSKSNATAPSDAGILLENGHLGELAGMALFAESTVHGFVSRVLLLMLLG
LWGTAALC*
>TCRU_276 retrotransposon hot spot protein, RHS3, putative
MGMEVYEGKPKQSWTYKAVGELFEKDDVLEQSGAPRPRMVMLTSDKGWPYSGIVGKYIHD
CHVNCEVERAWQIFKGDITEWLSSDRGTEFTPKRRVLIGTPGIGNSMAAGSYPLYQLLHH
DAEQLQVVAHCLGGTAYVFDRTISTVSVYMSEPRMIKFVKELSLSRVKAYIIYDVGCTWK
ILSPCLPPGEWGMLVVTAPETTSYIHWVGMKREPREP*
>TCRU_277 retrotransposon hot spot protein, RHS3, putative
MHKLGPILRYIFDERDYNYWIGNCHNLVDRTPSWGSESYFVFGTSKLWEGNNALEYIARI
VRVRGERNGESPFNAPITAHLASKTLCMLAKLMPQADFNLFVSRVRDYLISENFGKCAMF
AFLNGAFVGAIRRKLKELRPPARRPSHRCAPEVYSQEGSTRHDFLPSVEHRYQKTDVECG
VLYTSWATNFPLVDVFFFVKSNPKTLVGLRMATAGEHNTTAGTVR*
>TCRU_278 hypothetical protein, conserved
MVCVRCRCWAAAICDCAVAVRGVAAVSLLLLPLYVDVLLVCAEGCRQVTGVMAMMMTGRV
LLVCALCVLWCGAGGADASNLDNDAVGGCMASWVLNTNKSYTPSGCDKTALTLPLRSVLS
ITAVAASTDPLVTGTAENKSDLAPSSGADFGGAGSPPGRGGEGGGGRDDGAGGPGGVGGA
SGSQARGSKASGGGGGGGSNGGSKARVDGSLKSPASDVKSPSPVLGDGGSSPGAPTGSVGS
DGSKGFILGSFSSGGRSQPDVGSPGGDASLSAPVAGGNSPPSITDESDALEKTGAAASTK
GGKPQEAGEQSGANRARSELPELSAPAESTPEAQEDSPSKQEKPESEAAGRAATGDEKRR
SDAEAQGPSTVMSTASASNGRRRTTNSGGSSGRRKPNGTNRLANFDRRKDKNATVTKQKG
A*
>TCRU_279 hypothetical protein, conserved
MPVGSPVTRNQVLSNEALSLQSPPSPKPSAPELQDLPEGPPVSGGSQGGEADNAVGVGPS
GSSSGSGVKSMVSASFSAGTLSPPNPVDGDVLQGGKGTTATLSMDSSEKSVKSQSAEPPA
PAQPNLDRQEASTSNAENHSPIKPHTAGNQVVNGGATGKENTQRAAVTGGPSSDTRSTTL
QAAPQEQPKTELIIVPAGKERATDPRTREKEERDSKKAPPPPASSAGGTPAAKDKFQRRK
CTKRHRQKPKSSFWDKKPLQRMTRKIKTLRAQQHRIR*
>TCRU_280 hypothetical protein, conserved
MVSASFSACTLSPPNPVDGDVLQGGKGTTATLSMDSSEKSVKPQSAEPPAPAQPNLDRQE
ASTSNAQNHSSIKPHTAGNEVVNGGATGKENTQRAAVTGGPSSDTRSTTLQAAPQEQPKT
ELIIVPAGKELQISTEEMHQTSPSEAQKLLLGQETPSKDDAEDQNTEGTAASDSVKDEAA
SSLVETTAPPISTSGGADTKSVASEDADNAQQPNHKEAHKDPEPQNTSPAFTATEALPQT
AIPLTTAPTNDNTTPGDSDSSTAVSHTTSPPLLLLVVACAAAAVVAA*
>TCRU_281 oligopeptidase b
MKSGPIAAPKDHEVVFGYVEGENRGNNAMNPPRRRNDPLFWLRDDSRKNPEVIAHLKLEQ
AYFEERTADIKDFSETIFKEYISHIKETDISAPYRYDGYTYYTREVEGLSYKIHCRVPLG
MTPGKSEDEQVILDENKLAEGKSFCMVHEVKPAPPDHNLVAYSVDYVGNEMYTIRFTDNA
VTDVVEGTNGQILWGPNASCFFYITKDAAERNYKIWRHIIGRPQSEDVCLYTENDLLFST
AMAISGDGNTLIIGSGSFETTESHLLDLRKGNGHTTLEMVRPREKGVRYDVELHGTETLL
ILTNKDNCMNGKVVSATRNTPSDWENVIVPHSEDVFIGEIGVFAKFVVLSGRRGGLTRVW
AMPVGSDGLFRSGAFVQEVSFDEPVFTAFPVFSHMKMYDTETLRVSYTSMSTPTTWFDLH
VVNGTRTIVKVREVLGNFDAKNYTCRRLFATAPDRTKIPLSIVHDVSLDMSKPHPTVLYA
YGSYGACVEPEFSVKYLPYLDRGVIYVIAHVRGGGEMGRAWYEVGAKYLTKRNTFSDFIA
CAEYLIEIGLTTPSQLACEGRSAGGLLIGAVLNMRPDLFRVALAGVPFVDVMTTMCDPSI
PLTTGEWEEWGNPNEYKFFDYMNSYSPVDNVRAQDYPHLMIQAGLHDPRVAYWEPAKWAS

KLRALKTDSNEVLLKMDLESGHFSASDRYRYWREMSFQQAFVLKHLNARTLLRR*
>TCRU_282 cAMP response protein, putative
MSLAAQADNDLFNRNVLDFGSYEEYLDQQVTPEDLFYLGNQDAARQIVELGYKGKDFLSR
EEFEAACQLALEGPSRREETVDVICSAGKKLDDAPLLSALAKREELVRNGKLSTIIFLRD
IVRGQEVSAYIDYGHRLKTEDFTEYFTKRKKLTPKRTDLSYYNWKTHTLFYNNSFTFQVL
ADNEMGLLMKHKRDRKTINVDPKAPTPGDNSNRTVIQTSDYVQVTIYDHVTRRKN*
>TCRU_283 conserved protein
MPRKGSKRRGRGTEQTNPPQPPVKSNIPFNDPTGSLKEREPQLLSPQTLIASEFLENPIS
SSTHCSEIGTPSSSVPTLAMVKYLLQQVAEDEESDNTAGRMILIREESALRSIIENDASM
EHHAMMKAFYGRSPLSSVCGSVNSWSASSRRVHGVESETRAMKPCGLWRQQEDTEVPLAK
TAALPMSASNCGVQVTSPLQDLQEEEEEEKKKGSEQVLSRRFHLVPSYGTMEGLQGDANS
HLGHVNSREVSSNPLFFNFVTGEEDRTIAWVLYENELPVFKIVDDSDFDVTVEIEKEMKG
LLRVDVNFRDFFRAMARQVLFLILSMGIFFPSMAQSGLFASMDLLTNVEGSVTRRNMMKL
TLLFVDAAAIFILMPLIAFCFHGGNSIRFIILFTATITTVGSGICLMRGYTSGSFALMMI
SQLLHAVGFVLSVMMMLFINCAKMGAFLGTISLMFVTIYLWLMSIAAGIFIFLITLRLSE
GEVLEVLRGFVYLTCVGPIFALAICLLPPPPPTDCQGTTRISHSAIDAGTMFATLRRVEV
CFLLRCIACGCLLAVLFLIIEFESLFSSKLFAFTSPSFLAYFLLFTVIVALPLCLLPRFG
SFVRLCEFGLLTSLAAVSLFGLMFTNIPSEVATSIIPFSLPWMPAMTAILCASSFAAALA
GLMRSLASVKRALPLVVVPALSLTTLLSLCVALATGVAAVVLEFRLQSGNKKEGMTRSGA
NDTGETQAGQAVCVGRVAFGMCVGALFLQFITAARTFFAPNFQ*
>TCRU_284 hypothetical protein
MERQLQERDDALAALRDRLEEYGREKSALESRSSESVDALAAMERQLQERDDALAALRDR
LEEYGREKSALESRSSESVDALAAMERQLQERDDALAALRDRLEEYGREKSALESRSSES
VDALAAMERQLQERDDALAALRDRLEEYGREKSALESRSSESVDALAAMERQLQERGNAF
NIFDGPVNEICMEKPSLGVRLKTGTDMFVVFLRRLRGLGEGEYGVSFQDGLCTFHHAKDS
YFVSFLWGVFKSLRSLRNFVFGWNWNGNCRNEDWWDALGHVQLEFGCVEAELHEFSELLR
KLEEDCKDTEPTRRLQELIDIHAGYGENEDAALNLEENFEVLIRELRHRVCEMKTMLVGQ
SVRERLIQARIDALSSGIHRVANLSFVVNGEVDKDKRRRALEELHEKHDSLSEELSSLGR
GLLVLYALQQGREETFLRPLHEYSPFLAAKCLRNAGFLTDTIETGVFSLTSGVLMVAVIV
TLMYKRYSLI*
>TCRU_285 prefoldin subunit, putative
MSTEDGIYGPPSSFDAETGRMARLEHFLDAVLRQSLEKVLRERDTVYTMASECCQLRELF
QEMRALSSTHSFITDGDAPSLPATMAAGEGEKEDEEKRPQRNQIMVDIGNYFFVQCSVKD
GSQVWVNLGCGVVLPMSTAEADVFLQKKEKLLRERAARLSKEALRIKYRIRLVMEAISQL
YDRATGQRK*
>TCRU_286 hypothetical protein
MFLRLATVGAFVTVLVAFTLLHGDELWGFGPQRRRGNSPCECLTGVIGDSALLGALHRKS
LFLGLHWRATPAHVMAYLDPWQLKYANCISFHRLELSGIKNDTSAVDVLRERLEEDAAND
TSGSWMIRDRGLMASRQRHRQHGRLKTSAAEPTCHVWILPETARIPLTVWVALKGLLQEG
TIADVPVRLSARETKWSMYQPLGVLLWADPADRETLKHVVPPRTVMMFTTISR*
>TCRU_287 DEAD-box helicase, putative
MALWPSVGVLSAGMRCTVASGCRSFGGVVSNRNRTALLTPARGIIVPQQLKAYGGTFSRS
IGERSDHLVGNFVLQSLSPSRTAHSPLLAKNVKTKTVTSFEDMLLAPELRKALTQSLHVD
TPSPIQQTAIEVILQGKDTVVAAPHGEGKTLAYLLPLYQNMMKDRDVYKIPLRERRPRMI
LLAPTKELIAQLQHICATLDAATGLRSISFTSRKRAKYHLSRLLKNTLADVVIMDPKVVL
RLIRSRRLFLDDIRYVAVDEADVMLSSQHDHDAVHLLMKVQKRMMFKYLWPVQTQTVFAT
AYITRKLEFVIGKKFPNAVTCLQRQRMHRPPDTLSHRFFAIQREQEKMDVLQHLLKKHGN
HPRVIATDVDEVQAHTQTHYLSGTLEEFMERRRNTAPPSPQPNYQQRQQQQSDELPEFEE
EVVQEQEESHHYEGESNGSVNSLDCSSSPSYSLSLDVERDVNEEARRILEQVRPLRWEHL
TTVAAPFTCHIPRTVFGEGQRIIVFTRGIDSATAVYHHLRGRGFTCSLLHAALPWDVRRR
MFADFASGRTNILCATDLAARGLDVHVDVVVNFHMPTNALVYLSRAGRTARQGRLGRVYS
LYTKHQGVIVSAVRAFLKKNIPLEGVSNSKSHMMNPRYAEWRTHKMNAIARSYVSLITQK
TIPAHLERTYLHHNATWRPLFHPQTTGIHAGVPPRQQQRVMDAVTEQAVWFRRGQLARRK
GGRAKFGGRDAERGVWNSIGGIATSWVANAAQSANPGGPDFGPPQGPPK*

>TCRU_288 Uncharacterised protein family (UPF0160), putative
MPRRFVAEYVTKHLCGVRVLESFAETSTATGVIGTHNGSFHCDEAMACGLLRCSQNFGQS
NILRSRDPNALERCNIVVDVGSVYDEATLRFDHHQPSFHDTMKTPKAVYQTRLSSAGLVY
KHFGREIIQGYVESALASPYRVKLLDATKWGTDKKKLSEQELDTLFDIVYKNFVEHIDGI
DNGVNAYGPAGTEGEEGAVPVSSSSPSCVRKYNVSTTLSARIGNLMPWWNEEGNGKVENE
NAAFLQAMELATSEFFDSVHYHVFAWMPARGIVRAAFLEAMNVHPSGRIVVFKDCFCPWK
EHLLELEEEHGKVGHVLYVLFADKKGWRVQAVPKEASGFENRKSLPWRGLRDEELSQASG
IEGGIFVHVSGFIGGNKTYEGALQMAVKALTVVN*
>TCRU_289 ras-like small GTPase, putative
MSAKTNISFSASSSSLIKGTEPRIKIISIGSSGVGKSCVIKRYCEGRFVSKYIPTIGIDY
GVKRVSLRAPAHMSPAPPNFFVRVNFWDMAGRDEFLEIRNEFYYAVEGVLLFYDVTDASS
FAALDEWLKEMQTYVNAPRNTGGETLGVNPVKKPAIVVVCANKVDNEVDGGKKRVVSEAD
GRRWAEAHGYKYFETSACTGLHVTEMLETLFNDVVAAFM*
>TCRU_290 hypothetical protein, conserved
MNSYARFFCGDQSGLSWIGWNRLRDKQLFFGSLFFATSIYAAVHYSEEKERRRRHVSIEN
DIERERWRAKELGLEEPRDDGFADAYMKKLRGRQ*
>TCRU_291 hypothetical protein, conserved
MKTGSERNPSPRIRVHNAELYTDVMNASDWSRMIEREQQRERIAARRQHEAEEWQLQEQM
KLEEVRLQAARDTEAAKARQERRAREDTQEQELADFMRRFTAFVEEHENFNARCACQTRR
EERHLRRVVEKLHRAWKEDVFLPVQAFIDAQLQDVEGEEHRTLLRHNMLTAFLKECNRTD
RFGGRHAVYRDIIDEVRYDPLGQLQTCTIRYPAPPRRETAVDAQRRAAEEVQRKYAQLAI
DFHGLERESTTGVLTDPPLNDTSQIPATNLPITGVVSTGKEFHFASRTWSSDPREVATTA
TGIESNATLSTTGWTAKHCTSTAGRTTRRTVLPPLQHPPPPKDFRHLSYGINVNAVDSLA
KWTKERRMPRPREADLPVTMWGRFPETIHGWMATADGDVKPSSRRRVDAARKAQHSDVMF
DHYNFPRGLDGPLHTGKRVL*
>TCRU_292 hypothetical protein, conserved
MLTAFIRQTATLRAAYLPLAAAYTSSSSSTSNPLAVVATTGNATPITATGMTNATERVRS
LPGGGVGAGNDTMPLVGDPRVSIWRDEEALLRASLGRLEAERELARMRRRCEDELQSLRE
GLKVREGQVARIVRRVITRTPAKSRRGRHAHVVANNINSTHDTAISCVESGGEEVVAATD
ECEGHGATVMTQDEAHTEEICRPAHPGRANKKEKAHRLTLGNAGAMEVEVEELDGKGRVK
DDNNEVEAVNDAERSEGSNSFSGDDAADNADGDETGTTVPSGMELKMSLPGTRRGGLAGG
TVHVGSNATVTSTAGGKRQAATASTNGELTKDAVGRRGRRRAGTTKKGTGSSNNNNSSSS
STKNKNKKRETGRSPAKNKKAVMKTLTKQRINSKGRTVSHNAPSSKKKVQPKPRVKTGHP
TPKRKTSSSMRKQPKKQDHKNNKSKQSSLNKKGNDHTKKATNTRQRR*
>TCRU_293 hypothetical protein
MHSVGKRSGVNEDSGRVQDDTTCMVGQELNNGVGQREEESANVQLSLSFSEMRVGTHTEC
VDSLMRNPTPTLSVFGGGTTLGVSTDTENGAGNKDAAYQPLRLQIAWDGDICMSQVDGVC
VMLSAEEAQAEDVHSAAPHANSIDSELCPLGCDENSSATSWMLRQRRLQNRAEQWRRHEE
ELWRRRYEQSISQTCAATIVKFPPLTETTALQSHDDVHLGTDENGDVDGDAGGCDDSTEP
QTKRFSTANGRRLRRYRPEPIKSEDDDVAMPLWQKSTCRRNP*
>TCRU_294 Concanavalin A-like lectin/glucanases superfamily,
putative
MCTGPVPTKGLVAFLSGSSTETEWRDEYLGVGAIIKNTAAAVNGVTLKGAGAGAQWPVDM
NGPTVPHHFANREFTLAAAVSIHEVPESGNVPLVGVGVSDPNSTVLIGLSYTHEKNWSFM
TPSDANPDEPALFDWKPNTTYQVTFEMNTDGCYVFVDGRGLYMKKADEESFATLLEKHRI
SYLFIGWGSARGTESSRMTVTNVILYNHTVYGEGLEQLNASTVPIPQSGVAKQQRKHEGD
PSSGNSTVHLSVSPVLLLPALVLQLWGMTALC*
>TCRU_295 BNR repeat-like domain containing protein, putative
MSSCGDGQHQLCVSDGHGTSWYPNRVSINRVWGNSHDRKGYGVQSGSTTAVIAGEKVMLA
ALPAYSKDNEKGRLHLWVTSIVRVHGVGPISREGDDAAASSLLVKGNNDELLSLYENKER
SEDGSDGHVAVRVAAGLERVKDVAKKWTDLESALQ*
>TCRU_296 hypothetical protein, conserved
MWSAVDACPRSRHRVQRSAIAAMRAALLFALALVAAAAWMPAVHAVVLRLRGGTVDRAIT
VGRAVDTVLMDGVSITNGVAVVFDVAAMLPGALRIELRNCVCDGGAQIYVRGYSGEPASD

RSLEVSVIGLSGSYCSLVFVHNLPAQTNVTVRDSTIVTAGPMRYSQLSGLTDAVASPLVL
HATSLLQSQLRVSNTVLRSLHAGESAVYVGGGVDLLSSAVVLDGVSLEASGGLTASAMRV
ASSSRLSLRSHSVFSVTNVSVVSSGGGLVLGERLEVVDSVLRFVGVEGSVASSLVRCDGG
TIGGGGWLDLRDVWAVGETSSVASLSGVTLSGGTVSIARCAATGATLVAGPAITSGVVTV
QCPWLSRPPQRGARV*
>TCRU_297 Polyadenylate-binding protein 2 (Poly(A)-binding protein 2) (Poly(A)-binding protein II) (PABII) (Polyadenylate-binding nuclear protein 1) (Nuclear poly(A)-binding protein 1) (PABP2), putative
MSNFPAASPSIWVGGLDPNLNEQKLYDHFVRLGPVASVRVCVDSVTQKSLGYGYVNFQNP
ADAEKALDQAGVKLGSKHIRIAKIQRDPSKRRSGVTNIVVKKLPPSVDTYALKEMFSKYG
RLTAIGLATNENGESRGYARISYEKEESAIQAVQEVNGMVIDDCAITVERYQPHHREEQL
KQFTNLYVKNLDPSVNDEKLKEVFSAFGEVTSAKVRDLGANQTVGFAYVAYATHEAAAKA
VEELDEKESPLAKEGMKLSVCRFRSRDERQRERERLRRERQQQHSKYPNLYVKNFDDTVT
SERLKELFERCGETVSVSVMMDRATRVSRCFGFVSFKEQSAASRAIQELHGSTALGPRPL
FVTYALRKDARRQTLEDMRNKQPRMRQPPMGSLMGGMMGPQLGFMGPQAMFNGVPFVNPR
MSMMPTPMGMGGQLRPMGPTPMNQVRARPMPQRPPMQPIMAPPPQPQSLASQGQNLSTVL
ANLTPEQQKNVLGERLYNHIVAINPAAAAKVTGMLLEMDNGEILNLLDTPGLLDAKVQEA
LEVLNRHMNV*
>TCRU_298 retrotransposon hot spot protein, RHS3, putative
MRLPHEWGMAMVTTPNEGNFKEWYNQETAVRTVMNYPEEKDVKALCVWMKRDQPFGKQAE
HWREVKGCMDKVGLLLCYTFDKL*
>TCRU_299 retrotransposon hot spot protein, RHS3, putative
MEVKEGEPPQSWTYKAVGKTLEKDDGVQQSGAARPRLMVLTSDKGWPYSWEEDGSTRVCH
VNCEVERVWQIVKGDVTEWFNSRPEEHFTPHPRVLIAKLGIGRSVNAGPYLIYQPPHYDA
EQLQVVFHCLGDMT*
>TCRU_300 hypothetical protein, conserved
MESGQYTEQGIAPLWHTDLPTCFTTQLTNKWRSTIRQDTHRYLLCGTRPSDLSGKDLITQ
EVLHRQELVHLARARCGESELWGRRYWAMRDCTNQCRFCNISPEQSAYMRSNNDPTAPGT
DTVPPSAREEDVSPVRGQTVTRRRKEKCPHCDSTLTGFSGLVSHCRSFHPEHPPPLPELK
CDFCDMVFPTRRSTAQHRSRCAHNPDATRHRNSSARRRSLLPQDQPASTSTPIGPQETLH
HLLLECPGTLAVRQRLGIEQDLRLGKFSQWQLLHSRKLLSLLDHLFGTQMALYS*
>TCRU_301 Endonuclease-reverse transcriptase/Reverse transcriptase (RNA-dependent DNA polymerase), putative
MHHPQWEPFLESSPSEVAAEFLELCTDAGLTLVNTPGEITYARGTRERSCIDLTWSKHLT
VSDWSASVSPLSDHYMLTFTLHQAFKDTIPSAPPSAPKFFYSWGKCKWDLSIKDFDAQLP
AYDYKKQSTGIKAFTRALITSYRRHCPRGMHKDGPRLWDDTLMEAERIATDSKARYLQLP
TPDREAEMQRTRSQFFLLLRERLRNTYLRRISKLNPGEPLAWKYISGRKKASLPSPTSML
LGDGQHTYKTARRAANALNRIFLPFHPSHKAVRFSKGINRQSASLNINASFLFGQQNNSE
SAASFTSFSSTSSSSEPQNNNESAATFTSGSSLSSSSEPQDKNEAATTSGLVAHLHSPLD
APFNRTELLAALRNTPYGKAPGPDEVCSEALRHISLKGLRFLLRCINHSWTTGTIPVEWR
RATIVPLLKPGKSPEMLESYRPISLTSIVSKVAEKMVLKRLLWVWTPHPHQYAYRSMRTT
TMQLAHLIHEVEHNRNHYFQVSLPKKSGIGNQLHYRPHRTLLVLVDFSKAFDSIDHRVLS
RLLANIPGVNCRRWLRNFLCGRYAKTRVGHRHSDRRPMLRGVPQGSVLGPYLFSLYVHPL
LNLLNSFAGVTADMYADDLSIIVRGQSREDAIPTANMVLKKLHAWSQENGLAINPSKCEA
AWFTLSPHTESDYDREGRWPLVVAGCQIPVMTMGASRTTKLLGMDLDPRLTLNVAATKQC
AATSQRISQLRCIAHKEAGPSPHDLRTFVIGYGASKLRYGSELIWAVATDSAKNEMQKTY
ATLARIVSGVPSTVDPESALLEANMPPLHVLCLRARLSMFENTRACQMDWMRRPPPEPPP
RAGFRISPLSRDELYAFVDAYTKDYGITESSPREERFFRSSIPPWFAASAHRVTIGVELP
IDHSITDEEELIREKRRVSEEALALHSHRSWILATDGGVDVPKSAGVGILLSSLNSYGDN
RKGQYKLRHTPIQLQDGIPCAASSPREADDPSYPPQA*
>TCRU_302 hypothetical protein, conserved
MDTVLNKPIANALLYSDGALHLVGARHNEEKSSLFPAPLTEELQMITSILRNWADVDVSF
FASSISTVGLVGYLSNAGTANTWIDECRCVIATVTNEKNVTNRL*

>TCRU_303 electron transfer protein, putative
MLRKITTPYKISVRRTTSTAPEATSKKAILRLIRFDPTTNRQRIESYEYDKQHEYMVLDL
ITAVKAHQDPTLAFRASCCEGVCGSCAMNINGINSLACITFSQQVTTVGPLPNFPVIKDF
VVDLRHFFRQYAQIRPFVRNTNLDRSRVENIIERYDTLRKVIYGVSPHGIPTDSDVPRGG
NAETEVVGLLRLLDAISEAGNVTHLVSTLEELEAKGVQLDQAKVKELIETTLRKHGERTK
*
>TCRU_304 Ran-binding protein, putative
MTEGRVVELFGGAIRCLLPVTTVDVSEFRQVPDTQEVYTEAETGMCIIVELLARQSEVSD
RECGVFFFKDLAHANECRDGDYTLNAQEPLAPSDYPKVAQLPPGAAPGSGQECAYGCLIS
GTQRISKYKNERGKENEVFVAIGVLRFLPSISSDVLLSISAPQWIHPESSEARVVRQLRR
KEEVLAVLRRMLISFEVRDWSLFVPEG*
>TCRU_305 DNA polymerase theta (helicase domain only), putative
MRKTFVCRGGFHAPRKVTTAHDLPFAEVSQEKGAGDDISAVSLETAAIKRPRVESSPRDT
PPVTVARPLESSLPPNTTAPPPHGRSANGTAVETPPLENEEELQREVEEDQRLSTDLPFT
PSKTAAGLGTPPFVFSQPLLEEEQDDTKENANTAGVVAFHNNSDAVPVTSQPNNIDLSVQ
PRDIASSSSEVVLVDGGGVFYDLPQSAQNFFLNRRGIKKLYPWQHEVLMRDDVRGGGSLV
YSLPTSGGKTLVAEISLLRCLLNRKKSCFFVLPFVSLAEEKAVGLRPFGDALGFAVDGHY
GTQGRLPLPKRTAVFVCTIEKANSLLNHMLEEGLTEELGAVVVDELHMLGESHRGATLEL
FLSKLLCLPHQVQIIGMSATIPNLPNIAKWLRASCYLGQYRPIPLRQYAVVDGMVMEDGE
RNERNLVAAGYATETEQLLLLATEIQGASVLIFCASRQQCVDTARLIARNRKGMKDGFHS
ASTSTLGALQNELSSFGADVNVLSQLLPHGVAFHHGGLLGEERELIERAYRQREINILCC
TSTLAAGVNLPARRVIFKTPYVGRDFLTKSRYLQMCGRAGRAGLDEFGESFLLLSRRDCH
RGRELMRQEVEMCSSQMLSQASAFERALLECVAVGVIGSPSAAQAWMANLMCHHAAGPFD
PVYDVASRPLPVALEGILQSSMDQLIRSGLVQRVSLRRESDEGGDAATMQPAGNDDEQVS
LSATPFGACSVRSCFSIEEALLVRGELENLQRTGLILADDLHLCYFLTPLREIGECNWSA
YCEVLSQMSENRQRIANMVGVDEFFVHQRSMGLGDPYGGSGSRTFATRRFFVALMLADLL
SEVPMSVVERRYQCNRGQLQSLMRSASMFSSSITSFCRAMEWFSLEAVLSSYVKRLGFGV
KPDIVPLMEIRGLQPARARALWAAGFKDPAAIAASNPGELLQRVKNANAPENKSVKFFTL
RAAVALIREANLLLQHHIREKKGELIELTLRSRASPLP*
>TCRU_306 predicted WD40 repeat protein
MSILFDSNALLTASDDGVVKMTSLDTGVHLRTFFTSGSEQHSAVHDFAVNTQLRLLVTVG
PERYGIVWDFSHDAPLAILDAHNSPCRCCAVHIRQRQIFTVGIDGTIFIFDTHGYRLTQV
IHVHHLHPQCVVYDEKKLRLLCLASHPYYHGKQLSAAFACSNKYQGNMASMVGMIYNKTY
DLIVTIDLEGFVMTWKRSNGAPVFAFQLKDFSDSVVMNAARLSSFALDGLGRRLLTGFQN
GAVAVWNLVNGQTTNVITAATESFATTTLRPEVTSLESLTRDGTTFFFFAAAGHLFSTRE
SGTFTIASANKWEVPAVYGEILSMLPVSLQILVCGTSSGALIFYHFLAERQVGSALWVTE
PTESMHRSISFSSEKRRSDVQASVLTSRIIAIFPLHAVGPHILMSVHADGTVALWHTLRR
LLMDSVSLRSAFPAGRGETGLAHVAIDECNQCFVFADDRGDIHVCNLQLQAVNDPGSMRP
ARTPPLKGDAERPMAHWPGSPFRNYEFVGKSPRSKAKGQNDVAGQPNENQQQKGLEQEVD
VEKTLYVFLRFCQEYVFHCGFPSVSGVSIVKEPTRRDPRGVTTASTATETPLPIAKGAEV
GEQACGSLSEGEAEHFIGNYGPTSDAGFYLDLTSSTASAGIIVVSSGSDNFTRVFTLTGT
IIGECGMNIWTLGKESTYEFLGVKPTRRLPARGCSVQAVDFLQETPKGEKSHPVGSRGLR
LTRGTSKVGKILRDSFSGSELSTCHPMADTTELWTSPTTRFPPSAPPSGATPEINPLEYP
SMQPTGAEVREFGNRKKKDSHAKDRLLRRFLPKATTPFEEAGVLSTHIHSRRLKEENIQG
LVKSEFISRLRGLPVNGSDEDSAAHRTPLAFERNPRGGVYAEPSSESFLSASGVNNHNSI
NTNNHSGLTNVTAVVNSLPEETFTPLDTRNHVPPVFVVGGLSHSGRMAKAQAGAVAWKWD
LPAANDIEVTQTPVSCALRQNALSALTASPVEDEAQRQDLIQEHMEAQRRMLLALGRKDS
VLGTALSCRSSRHSSVSPAEKMGKTTGTQLLETLRIQSLGMMETRDRIARITARLHLAPV
SPIAPPSVIREREGDSPLKDGRKNTHSAALR*
>TCRU_307 nicotinate phosphoribosyltransferase, putative
MVDVARPIITSLLDTDAYKLHMQQAVFHLYPSVCTAFDFCCRDRDEHFGDAADAIREEVH
LMQNLALTDDEYKYLASKRYLRKDYLDWLRKYRFNPQQVTVRAVPSDDGNVDLAININGP
WVETILWEVPLLAIVSEVVHRRRTPNLGVAEALAHLEKKLEAFFAQTTEESIETFCVSEF
GTRRRYSFGVQEAVVKALHSHPKFGRYFCGTSNYLLAKNLGLPAVGTQAHEWFQAHQQLT

PILRDSQRVALTQWLKEYPHDLRIALTDCISMDSFLCDLTKDLADAYTGLRHDSGDPIEW
GQKAVKHYRKLGIDPKTKTLVFSDSLDLEKAAMLHRTFSNEINVMCGIGTQLSCSIPGAR
GINIVVKMTDCEGKPVAKLSDDPGKTICRDRDFVSELRRAFGLPMMAVALVTAVASVPNE
GICGLQA*
>TCRU_308 Mucin-like glycoprotein, putative
MMTMCRLLCALLVLALCCYPSLCASETQQNQAQADGNDSRTLPPPPEPSTTTTTGDPKPP
VSESARGDTGHSGSSSSGPVNEPETPTIGEKSSKSTVMQSVETKKTAKTDGTAETQSLTH
PSPHKAGQNAEASAQTTTSTTQAPTTTTTTTTAPEAPTTTTTEAPTTTTTRAPSRLREID
GSLSSSAWVCAPLLLAVSALAYTAVV*
>TCRU_309 hypothetical protein, conserved
MVDNEDDGTDKEKEERDKAQEGAPDGQTQEEEEVSPPPSPLSPAPLQSPPPPAASDGTQP
GGGPGADSSGGGSNGSGSSIGGGGSNLETLALNGGSQSAFGSLGYGGETGSFSGGAGKGG
VDIGSPRGDFSDSSLSADGTFLSPALDAPSSQDTQSSENVERSKTMPAGLPLPNVNALKG
PESKAGSLPPNTQEKSKFSDASGGAAGNKAKQSDVETDDPSSSFANTGDQTVEKTLLLPQ
TPAVLLPVPSAELSPEVPPVTPVQANPDKEV*
>TCRU_310 hypothetical protein, conserved
MTGRVLLVCALCVLWCGVSVVAADGAGGGDGGADEYFVLRWHAQLRKGCAEAFGSRSEGR
KDVFAVDDCVPLWMAVLSGDASVLR*
>TCRU_311 Zinc-finger double-stranded RNA-binding, putative
MTRRWDAPPGPAARVTGDSTRLISSDLLCPSEESAQRQRRYVAPGHAIPDGAGQAGSQNQ
QPHFRQSCPRPVTQNLVPPAPHVAPSTGRHQDLPHDHPSHSRGNYGVEENRRHVCIRMFL
PPSFPDTNARGEKKNRELRSWRDLPANPLNGLPGLATQVMPVDREECNFYCDACQKGFLS
QERYDGHMMDHVWCTMPGCRFTCLKGKEWKMEMHMETLHNRPDAPNLADVGAYLSQRRQR
FPTQDTVKSKVEELFYKASRGVIIPDERRRWLRQHGVLVKKNPRTEETYIDSDALRRPAN
AATNKAVEGHQGQKEKQQQQQQQQQNDEESGEKEENTEMNVPPVSIKNPHEEGITVKENP
AVAAPNPSAHGRPKRIIPLGPNGTLTRGQKVQLIRDRYRDAKTVPRFYVCHRCGEKGNHW
VDECPKRDDENFGRQTVWGEERLEPARQKHRTEGEKEEEKAKTKNINENEGQMEDIPSPP
GGGGRNTKGSTTAFCEGKDLVEDDGTLEIVEDDGNPPAESSARRDDAAGDSTNPTTEVSR
MLAPVAAATLTEKKGKAQQRVSSRSRTRPPPPPTLYERLVESRKANEQGLLLQAFRFFVM
RRFFEPL*
>TCRU_312 ABC transporter, putative
MASLWFIQFRAFLWKVFLQRWRAPVSTLAELLLPCVFTLLLALGYWFSTTTKVPVTDYSG
TQPMDMSSLLPAFFCQNMSSPRPFGRRLHVGPCGPAGPSTMCFPFVRDGALCVRDRGTFS
KVMFGVYYASGPIKVPTLDAYLALSAFVSHESRRTGSTFFSRSAQASLSHYGDLLVAGKD
GDTSFASGFVKYCSDVSALCAEVVHKDRVFPTLAEAEKFALKHDDRVWAIVELPTPLPGS
PAAAGVEGTIFSISMNYTATPWTFESKMQFFKGLGDNDYMLYVASGFMTLQNVVQQFYAR
YRLDKAPPPPPSGANATGIFEFVNLQGPPLIPMPAPPYVDNSFYSRWAYFMPLVAMLAAL
FPVTKLVSWIVEEKSLRIREAMQIMGLRWSCMALGWFISAFLMDFVASLLAAMVFRLSFF
SYVNFGVLFFLYFSFMQQNTALSLFLSSLFTNPRIAGAVGALCIFLCSMPYYSFPDGMSM
LRLVTMSFVPCVAYAKAFDELAKYASFGYKFTWKNTRVGDYNVAMAIGLMWASSGIMWIF
WIYLDQVLPSSIGRRRHPLFFLSWVRKLFPCCFCCCDREGSELSEKDLQNTRKFSNGLAS
ASVGTLPPHDGKPVDEEVNSHAVVFRKLHKVYETGGIIGWLYLFLTGLRRDGDRREAVRD
VSFNMDFGKINALLGPNGSGKTTLMGIATGMVTPTSGDVYICGHNAAYKLHKCREHIGYC
PQSDIVWNRLTVEEHLTFYARMKVNGGWDVREQVDAIVASMQLEEKRHCIAKSLSSGQRR
RLCVGIALIGKPDLLFLDEPTAGMDMRGRKAVYDALQKGRDTRAVMVSTHLLDEADRIGD
KILLMHEGALCGAGSSLFLKSKMDVGYVVTCVVESCSSEMEENICISRLTEFVREKSYPG
HSRNMSTELHSISTKCKLLGIERRGREISFRFPLSLLSASGSAIIRALEEEREALCLRSI
GLSLTTLQDVMEYLTKRQPSTMATAPSNEPLTDKATEHGISIQIEGMGSNQGSCFGTAVG
CEMNRDNSSMSLLNETRNLHDESEYYNPHRQSLARHFAVLFMKRVHCAKRDLRLVVFQIL
LPVVFLSLALLTDLMSPPKQPALTLDASLYPGYDTQPYSEVMWTVSSKFPDVFDVERSNM
STAFGPYYTPIRAKCDVENCSQPLSAELIPDIRTHPATRYIALALTSAQGKKGTPTSILM
HNVSARHSAPQSLNALYNVVNHQLFGQGSMTTARNVPMHVGPFEQKMVSAFRRVIFGIFI
LLPFTFIPSNTVSFMVRERQSGSRHLQWLAGANVVAFWLSSMLFDFCCYLVTEALAMIIF
VIFKRTEFIGEPKTVVASLTLFTVFGISSVPFSYVVSFFFSSPFVAQGVVLIANFVLGFL

WVMGEQIIGGIKELETFVTRTTHVLRVIPSVSFGEGIFTLSGVELANMMFPERKRPNLFD
KLEFVEGKFRGGIGTALIYMSCTLVVSLLLLALLEYARIQRVKWFFTRMSCSKRKNKKSS
NQNADAPPSAPCICHQREANMSIDASVAREEEEVCRNETGRIEDCITLQHITKKYGGTHR
PALDDLSLGVHKGEIMALLGLNGAGKTTAVGILAGEVVPTSGSAYINHLSVLRSASRSYV
GYCPQKDALIDHLSPGEHLRLYAGLRGATDKYIRKEIPKLLDALGLTSQKNTPAYSLSVG
NKRRLSLAVALVGGTTSVLLDEPTAGMDATARRQTCAVVRRLTRAKSVILTTHLLDETEA
LADRVAFISKGKLRCVGTPQELRTHYTNDSVYTVRVVFADGCGAIDATGDVMKRLRELFR
TSSSTEGEECVLEGVVGRTVTLLVQQSLSQICSVASEIREGGLEGLPPVVQVSATQATLE
DILLSI*
>TCRU_313 Golgi vesicular membrane trafficking protein, putative
MQPQSSVFRHRARLHEEAENSRANAGVISEATEEENNEIMRALLGDVRSVRHKFTAMGEE
VRRQNTLLESLDAVFARTRVRLGRTLRQLDVAGVTSVTHMWVLFVFVIVVFMLIYFMLKF
G*
>TCRU_314 Scavenger mRNA decapping enzyme C-term binding, putative
MMLLGVTAPRLAGAIRRTAGDAAAAGGPRTQLPFLRKVVRDALRSPNTHQSTTPFRPYPP
LGAGEEKQRRSGVIYKDTQCIVVNDAFPKSRLHCLVLPLDLSLHSLSALRIQHVPLLRHL
MDVADRYVQFLRKDASGSDFASLSFATGFHSLPSLPHLHMHLISLDFDSPYLKKKKHYNS
FATPFFLPADSVVEDLERNKCITLNQDVVGLKRMEEKELSCLWCGRVEAGMPQLKAHLRT
CPKSRAFLNSTDAK*
>TCRU_315 flagellar radial spoke protein-like, putative
MTGLTENEIRESPLWQHMKKVLLQVVQQQPSSALDAIIPSSFVLQTGSTVPPHATTIYAD
HRPRVINAVPSDALENLRWASSFGNVLAPPKPRRKPTEEDEEMPQEEAEENEEEVLLGDV
SNVVGEQAIFNSVGEGLQPEEAFRLVVGMKQLVKTEPLATARFWGKILGSGADYYIVETK
IDPERLPEDEEDIGMANDEEEEGIPIDNVADVLYSYGAKKQADTEVEPAGSGLNEWVYYA
AQTADPTSWTRLPDVTPNQVIVARHIRRGFTGDLESSVDTHPHFPGKEMNYLRAQIARIS
CACRVAPRDMYTSEGAVPEEEDEEGNVLPPPESVRAYTTLPPLTPQEVPDEEDTEAIEPI
KSWFYGYRDDELLQGKYWVHIAPTLLKTGRTVAREQEEEDTGFDHSEKIHPFLCEVSRDD
PLTYASHSRSQLPAWSFRKAFHNESSKTRTYVARSATWPGAFTYAVVELGQPGSQYQSVY
IGTGLKSLQGMNYAPKLPPRCLVEYPETELVLQRDATADDELEYAPPPPKADAADEEDEE
A*
>TCRU_316 hypothetical protein, conserved
MPPVTGRPIGKTPSPHPIIRRTRSWRGIMQVRFNDTRADRNKIVKNALFWAPMRIFGIGF
LTTTAMYFTIGHDRFMHTLFGYESEMHYEARVNPDASVVIGDTLLDKDRGWKSPLRELEM
PLHPKRQFDVMRDPNST*
>TCRU_317 Triple RNA binding domain protein 3
MNSRGKEPIGFRGGRGASGVSVAARGFGRSGGNGGRDFAHGRGRGRGGHLLRAGAERPES
SAVMSHEDSGSDDDEKGVMQGENVHLATGKCSTALHKQRHVKTMLTEEGTELPKKLNNKV
FIDGLPYEHKATPGSSTLEEELMQFVAAWKVGKPVRLIKREGQGFGFLVFHSPHSVDVAV
RVLNGRKFLGRKLRVEVPKPKDMTTNDVDIGLDAGKSSFARQVLLSDLAKITQPEIVREI
LRDVAPQLEKRLESIKMTSRNRKAFLTFLSADDVEPAVRFLDGFSMLGRRITAARAAAPG
SLPYTKSPGHVLSHKRSGASADEESHGEAMHAGILHGDADEEECGQPVLPLGVVNPPAAQ
QEARRAGRNVAKELKPSNITGVTEKYNLLEEGPAEVFVGNLGEEITEEQLRCHFSVCGRI
LSCDLLVNKSTSLPTGIARILFALPAYASYAQKHLHGSCLHGHVIRVDRGEEPSAPLASD
LAPHDGEDVVDEDGYMAHYGVKDKAAYFKGTSLEERRDGRNKSKRARETAVDTKKKRRKT
KDEAFKSSGFISAGGNDDDDEEEERFHDVDKVIVPVTASSGRREPKKRLIAVKRRAKRVK
KAFE*
>TCRU_318 NADH-ubiquinone oxidoreductase complex I subunit, putative
MLAFYARARGAPAAASAALLLQRRRVEYWGSSVTGRTQPFAQGDMLPRLDNTRNAINKSS
SAAASITGNHLGIAYDGNNIFSVEEYSRLEKEHYQPLRGVSRYKVEPINEGDGLTTQRLM
LELEEDTVVGIDAAWWGAVFKDFPSLKKPCTSEFKAEFADFYKQALGVAHDEAAVTALAV
PFVRRQMQTKFISFPMFWRIAEKLADALLTAAAGRRHEMRVSRIAVERVTKIALNTIEDE
PWAHQDTTSPLLFDVSNFRAWHEAGNW*
>TCRU_319 hypothetical protein, conserved
MDPLEVSQPRPPPPEEQQLISFLLHAPVHHATFFSLTLVEELLRHARRVDQEVSFLSLPL

PRSRESTRVLSRIVQENRYEEYWPNEHPALDEARQSAPSASYLLRIGVLRALGFNTTGVD
DFVDDVSLALGRRIVSAAPLPEDTASVPLRYREAAQEELRHVALGEWCEDLIHFTAYRGF
SAEQVRCVLLTAMHLMNVVADLSPDATDVEAEKRCTQLLEELLIEQACGLPRKVVETHKS
WRTVTSEIPDPEFVAEMETAISQEKNKKRRTALKEKLENAPRINYTRQELQKERFLTEIV
VGPYFTLQEVAQILDYFSSSVVRHWRLFRAFLAEPQPVETHDEVQVQWDIHSFCVPPLEE
FVRDDMYEIEQERRDLMSACEDAIESAFEEEFIQPLLALQREKDELLECLKERERQAEED
NIRNALDGPGYMQVTRGFILRLNKCIERNERVGKLELNEKPPDSTPPSPEPFSSQMSPSL
SLANKSRRSVTTRQGLGLAGVAGSHKLAAAAPPSSHSENPADAVFSLEEVEARLEKLENA
MRETRWSSHDRGRKKQTGG*
>TCRU_320 intergrin alpha chain protein, putative
MIAAPDIQLVRREVTTVASRSPRCMVVYPLGKKSRQRIGVADKNGIMSVFSLRKAMERVA
VFETPPQNRPVSCATIYEDQLFFVHGSTLEAYSRKGRRFFSFDTNVTDVIHTLFVSTPFI
ICCGNFMATGFREASELGFYMAPDRINAMAVFAVSSVANLEERNFDDYRCILGCNDRTVR
MLRSHKSIDDIRCEAPVTSLGVSDGMRRVYYGTSAGSLGCLDIGSGDSFLSRVFSFVPDD
HQAAVTALAVYDINMDEREELLVGRQDGSVQIFYVYAEEWADSGHPVCMWSGNTDESVLS
MAAGFITDSRRPGVLVHSFSGRITTFTLKEEEHAAPKEMEAVDAVLECTDAQINEAREEI
EGLKRSIEARTRELAAYSGVKKGDTPLLAVSSTFKTKVTLSQQKDSPALRLIVEVDTPLD
CVVLQSELHLHFLEESAEVIVQEEIPRNNPLTKTIAIVHPLETRNYSCSVAFWVEDGQWG
LLKVTALAAPAPRTAQVKMVQLRPLPLYARVGRVDKAVTEGNASPVLSTMEVRGNFSARD
MHSWIFRLLPGTPEMYQEKMSTLSYEDTFFQNPLEVKYGDGSALFTTELLQVLMVTERFI
RYCASERSLEVSFSVGIHSEAVRSNLSRIVPRIAACNKGLTSFRLLEALQELQGGQQTDM
VCFPKEYQRILSQADETKKEHAQAMVTRGYLQKAFVSLCSSVVEIVPKKPKMSLVTKEAL
AALTDCSDASEMMSRLDSLLLCDEGGEGLKSTVDEGGGDDTHNVRDRDGGRE*
>TCRU_321 Elongation factor Ts, mitochondrial, putative
MLCLTRLRANVNKVAFMEMVKDLRFRTEAPIAECGAALKETHGDVEKAMEVLRKKGAARA
MKKRSRVTEHGSVVACVGGLFGAAVITVCSETDFAARSAQFQNTCARVKDALQRKIIDSK
GDVLTNPMEAHRSLVEATAEDIRSSIAVLGENVTIKSVESLRLAPHVAEHISIGSYTHGS
LDVPDVGRIAGVVAVSRLDPTREVQASTLTDVARHFVASSGAEGNYAHQNFFGTEETVGQ
WLKHHGLCFSSSLVVDFGKEPITHTASQPRNAVKHPEG*
>TCRU_322 hypothetical protein, conserved
MPSTIRRKWLATALVFSVQDRRNRARALREVRERKAALLALSTNDVAQRLRAMRPPDDIM
DSRGDGIGEKTQKAGQNSKFTTSDDAATPFRPPMSYEQWQEEGFPVGCWRPLLLTELYHP
LEAEKNYLGKKYSLAPKDTERRGTAASRKRR*
>TCRU_323 peroxisomal targeting signal 2 receptor, putative
MPSFNFHPGFAGHGMRCNPWQPTQYILTAAEHFGVVGSGKVYIVNTAPGVPPGSPVSLLG
CWGTSDGAFDACFSEVDQNIVAVACGDGVKLYNVQQSLNLDGAPPLVHIMEHQGEVAGVT
WNRDNFLSCSWDGSVKIYQAANPNASCMTFQEHMKEVYEVACSTRNPASFLSCSGDGTWK
LWDARTPRSVLTQVGHNHQIVLSIDWNKYDGCLFASGGVDRTVRLWDLRRPTQPLASLPG
HENACRRVRFSPHSRVLLASAGYDCRVCIWDLNQPQRPLVGRYAHHREFVVGLDWSLAVP
NALASASWDGRVFFWVSGQQVTPSPPLPQLPAAVPPPRVRVPRNKVLPGLPPAPPAIPVQ
Q*
>TCRU_324 hypothetical protein
MAVRLVPQLPVRVLSPTEFATRLRRIRGDQSTSWVADSHAVVSRVLGSSNDSQFDAPEST
TAASPSPLRQNVEFVIGESPARDTSGTPWSLPDSDVQLLSPWSGNPCPSSASGDKKRSAS
PDLLPAGNTVTSKAKKAKKPTTAGNKRRRTIEEFFSRQR*
>TCRU_325 U3 small nucleolar ribonucleoprotein protein MPP10, putative
MATTKKDAALPRKLKGSVGTLQQALDNGLDHFFSSRKAGEETSRQCLSSLFVLASSLHTP
RLPYYKKSLETSAARHVTVEQIWGQLSMLLRPVLRRLQDNVRRLQKTVNASSASQKRGST
RVQTKRPRDDVNVDDSDALEEGKDVLSTRNSQLSESDLDEEIAQLLVEQQQQRRKLAKPA
STNGHNGDDLWRYALGKGDGDGNDENGDADVDGEGDDDEDSNSDLARIRRRHARAMAGAD
DDDDNEEEEEDERAVEELAAMKEMYGEDFVPDEDDDDGAEDDDDDPHFEEDLAWDDPTAV
FKESDGMYFGNEDILAGDGAQEEHQQHERGDAVGENDDDDAELNDPSLTMLQRERQRERR
LVERLEQERLYSTQWAMSGETSGNQRPRDALLDEELDFEHAMKAVPVITEAVTAKLEDRI

RRRILCANYDDVQRRTSLSAPGDLVTTRRDATIDSEKSKLSLMDLYEKEYLERARALEEA
SGDAAQSAEPLTEIERDELRAIQMWRRLAQHLDALSNFYYTPKPIQEELAARVRAVDGQA
PAIALETVGNFATTREAALAPQDLYRSADRKYADVGINELVPRERRALRRAKKEQAKAAS
DRKEKRAAQAKKKKQQLPEPQ*
>TCRU_326 heat shock protein 90, putative
MARHPIIQAILIALIVLGVAVTGVTVKDDGSVEKGRPISFQAEVSKMLDILINSLYTNRA
VFLRELISNGSDALDKIRMLYLTAPKEPKNKDGEVPALEMRVIIDNERKTLTLRDGGIGM
TKAELEEHLGSLGTSGTKRFMEKLKETKDDSLIGQFGVGFYSAFLVADRVRVASKSDDSD
VQWVWESAGDGQYYIYEDERGNTLGRGTEITLEMKPDALEFLSTDNVRDIVHQYSEFVHF
PIYMQKGEKWEVVNENKPIWTRKPSEVTEEEYHKFYKSLTHDYRNPMYYSHFNVEGEVEF
SSVLFIPQEASQDIFVNNEDTRDNIKLYVRRIFITDEFRQLLPRYLSFVRGIVDSNDLPL
NVSREVLQESRILRVIKKKLVRKALSMISEIAEKDARLKEGLEKEKSGEDGEAKDENTTG
KKSGDDKAKEPLYPKFWAQFGKHIRLGVLEDANNRGRLAKLLRYTSTKSNGTLVSLQEYT
DRMKPEQKHIYFLTGDSVKKMRQSPHIEEALERDVEVLFMTDAIDEYVVSQVQDFGNKRL
INLAKDNARLDEPTERDKAIEKERKKKYEPLTERLLTLFGKSQVRKVILTRRQSSEAFIL
STQENDMTPRMVNVMNQQAISSLHTMRYSRVLELNHRHPLVRDLLTRFEADANDQTAIDV
AWVLFGTANLQAGFPVSNQAMYAKRVNRLLRGRVGLAADDTILPPDDDEYDISDVKPDTT
GTDEGLLLPVDNDGDESSDKEDAEPTAAEMKPTKTEDDAGDL*
>TCRU_327 hypothetical protein, conserved
MSAVPGGGGRSGGHAEDGDGGFGGNPKDHVRAAAEMSSWMRNAVDEARRDPSKEVGISAE
ELQQRRKMASNPRAFVSEQTSRAFEVKSSARAQQIMNKGLLDRQRETRNKEVLKKMTILR
RIQVGTFFLGIGVAWWMGVEYLLPHYAAVQERNRLLQLRCEMAQRKREEVEAQRRQQQ*
>TCRU_328 U-box domain containing protein, putative
MATIQKLAERLESDVPLRVDEMTFWSRESEIGRAQVFYGPSLEEAKRCAQRISDKLVTKY
LNNPNTNEVPLDHKSRVCLILNNLALHKPIRGCIFDVLDKLEGIFEESIREDGTLPFNPD
LGRMTEHVAVLLMRVTGYKLKAVNVLEFTDGNAQFSVQLMLAILLKEPAYEFGLRGNCIT
IILGFTQPQAFFDSSKGVEEASCLLFTEKVDSLLALMLRLRAVQVISDVLTEQLDSTNVM
TPLLHVATCSVMRCVMNIFRFSSTKSTQWRQHILLSTTLLDHSVTLYLLMQCEALQKSLE
GASPDLSIEMLRGMSLGFKFASLCTFRMHRHARRVRLFCPYLHDMLQFSIRHVRQDNPAS
GVLMRLYVDMFHLMANIDALGGEKDLSIEEIPQQLLSEALLKTTEAFLQREIAPGGLAWA
RAWHTALKTVEADTLVDRSTHTFTTLEKLFGDLEARVAAHQPLPSPAPVAAGGRHRILGE
MPPLRSKTAEVKVPVESAAAPVDMKQYIEVVRKDVIEGVDPILLCALTGNVMKRPVVSPY
GHTFEQEAILAWLEQNGSVCPITGKTLTADSLRPNKEVAALIMKRVIQQSMSTYNQENEA
DLYDF*
>TCRU_329 stomatin-like protein, putative
MSCCFCVSTSSLGIVESCGKFQRIANPGCQCLIPCVETVRGRVTLKLQYASVNVETKTKD
NALVLITACLHYRVLPEEATNAFYRFANPEQQIGSFAANVIRGEVPKYTLDEVFVASRNI
KHAVEEELKERLSQYGFALEATLVTQIEPSTELQQAIAQTQLNAYRRTAAEHQAELEKIV
KIKDAEAEFEEKRLAGVGLAEERRAIMEGLQSSIESFVDGVPGVGARDVVQLLLMNQYFD
SLKEVGSTGRNKVVLLPPSGGQSVLTDLLAAQMNNKSMV*
>TCRU_330 hypothetical protein, conserved
MTMGRLAFSTSCLRRRATGGLTVKSLDRFESDVPIVLQRLPPPPPSSSAAEMPQGGTYKT
FRRSAYAVLRNRNTSPGVCAKNLLSPKEILSTPNRNRCCCAALLYRVTEQEPENSSFAVS
RSEEDRERDDFVFALKLADVEQPLRELVGEAKYEALVGPALAQAQQVGLCECLPQLEEAE
GVGKREGLWVARLLLTPEEDNRVKWLREDGELSSAAFARVPSFRSMYVAHHAGEKHRLEL
RQFESIEARMVENGVDWGMCDAAAVYSFAIYSRELRPYLFHVLRDVLVEYRKDSSNSEVE
KLTKVNLRHVLGAILLLQCLKQLKREADEVGGHVVTPPTYLFVAAEKLFSLATALPKTDD
LAHVLNFLAGICRQSVLSLAATYIVPADSPELTEFTLWLLKEVPNSSVCHDHFRRVVQHS
QADAIILLEKGGLLSAKFTDLRVVMTIILPLLDSSGHEEFFFLVIREVLMGMEERLRRGA
HSFSKVELGFVAALGARLFETQQQKNIPLHHGTGSCESMARGQLEELQVMDGSPWALVTF
SYRSLKEILRALVRDHRRLKRQVQAQMRNIVEAVTYDAPNDDNRGYSPREGDKAAPASAS
PPTTFSSAEEGRNAAATTATAAAVDRGNFNSDCGGGTMRPLRLSTPAPIRERFLAFVEEL
IKALVDETGANQQSCKVVSDELFAFVEKHVPLPNIHDEYRRKLFLLVNVFTEGKFGKEES
NAGNDSDGDTGENSDEIPAELRLLVTSLPLTLSPWASVMILREVLSESAGNNPRYTYVLN

AAIDLQLPISTTRSRIATTLNMWRFLSSQADKLTFKEQVRMKQRCFFNLPLSYILSSYWS
LLTWLSLLAIFGLNVVGMDFESQYVATRLFKEFAPPDEVLPPVTEAWNKEGAEELASMYL
DDSGPGGNGIHHQKRRRSSSELLLSSASYIFLGEDDQRRPMTIVSISSRGTQSAVERATE
ELACASAVLREMSLQLPFPFFLQVEKELPVELIISRVAERIRRVSFNNAWFVSRVIFRTF
PLGRYHTEYTLVSHTCAQANLTRRRMTFLLYVNQGVTLSPQLLFRLRESVLAQKGNLVVV
AHEDSLKKGGATPEVISELTDGGSVRFIPYNRQWATLAVHGAPPPRTIEKYFTEISSRWR
EMVSKCAADVFLLLRMLNRHVAQNGIREWRPTLHVGQAVFPHALQGEEEEEEERGQD*
>TCRU_331 Nucleoporin
MLKPGQASAGTGFAAAPAATAGKSASSGFGGFGANTATTGGFGANSTVGFSGFGAAKGFG
TAAAIGGQAQVPSYKGIKGPGFSTDWARSVNFSTFRDDVLFEELPPPLQQHLMELHNFIQ
AEHDAKRFVESFLTDSMGTSAGDYSSYMELQKKLVQLTAGDNAVDAIRVDCFEREVQGQH
LGMLLENCEEEVVQYSKHVWEPLAEQDFTQRLGGNRAELPSEPFQCCLSEVQTRMEEMSA
VLTELELAVLPNAQRHKRTTNNDKNTSNTTNAEPSASHFSRYTAEMKPLDTATGLRPTIA
SHPVVQMNTTLSNELATLLNLAAWTSRLHARADAARDLFAHLYGSSEAEVLLAQQQRQMP
VGPKRPFPLCSAEASSFSEPNHDTIGDIHRRSVGLDRLAALSNGERRMQYDVLLERQRQD
SAPASVGTNNTGFGAPVAPAAAIAATASAVPTVAATTAGTTTTTASTAAAGFGASAPSAT
GTGGTTAFGAPLGTASSTRTANKKGRG*
>TCRU_332 hypothetical protein, conserved
MFFDDDNEVNDGSEGARSPSSGASSVCDVPLPPLPPPAPPSSIYGMATDEIYSIIKELRR
QIDDPATHQDIKAMLKKEPSVLLAVARVLHESALLRRTVVNVDGRVEEQLVPLPEAPTPP
LPSGWSSWA*
>TCRU_333 hypothetical protein, conserved
MLAVLFGTFAAPQRLKALLPLCQRWSVPVPSGDGEGCDQDVCRLAMERLAAKVCGVIKKS
VSGDSIEGGLRTMSLSEGAVIWSDGALRLRRFSVERAGLLGELHRRSGFCQASLSTAEEV
WWMEEDPVATIQVRRRV*
>TCRU_334 hypothetical protein, conserved
MGCTNTKEKKDANSCGGKEQLAAGVEEFGKLAKENPVAAKLKEEWSAFVCSLSLPTPLEA
PREVWANTVDNPLTHRTVDKVGKLFLLYVKNDLTLREWGGNFDYTVVGLENQGFLKATAS
VDASSSTGRSKEAMWEVKVHYHSTAKTS*
>TCRU_335 hypothetical protein, conserved
MVVPLMLDLMDFRRMMCNISVPIRLLVLVQNGREAMLSLCLQELEKVYGWSGRLAVSRHP
ENIGYSADVNIGSRLALSLPREEVPFVFVANSDVMFSPDLLPNPLRDVHEMTRHDAARMD
ELSAEVANEPSEYSPVLRRGLRVLRSTVSDDRLSTSALLPDRVRYASVKEREKAFSKHYG
HFCAYCKSSCFASVMLTRLAIGTVGYFGENFYPDYVEDVEYSLRLRLLGFQERNVLCGKF
VHRGSSNIRLSEQLELPDALWYRRVKYLMTNQPYAMMKWNRLKACCNGYKEPYDGMVPLD
VWVKDEARIQRIRAYGHDEIRRVPSIDYDRRLLYPVRTKGR*
>TCRU_336 Nodulin-like/Major Facilitator Superfamily, putative
MVLGFNDRLEAPNSINESQRFGFMVCSMFCAIASGAVYSFSLISGKMTDDYGFTQNDITT
VSTVGIVLGYFTLPFGFILDYIGPKPLFAIGIFAYGLGAALFALTFSGRIGASVGSLAVI
NAIMNTGCAMFDMGPILSVISWFPVDRGLLVAAVKSMIGLASSVIATIYNTYFSGNHSTF
MFFLLAVFVAIGFWAFIFIQIPPYHMTGHRIKHYTEEEHAIARRVEHMYLIKKAPRRRFL
ILFVIVLSLLIVITVQSIVFVFVEGEVPFKTKNPPAIIMIVLCFSLFLVVLPFNCLDKPL
RGSRKSTSGSNEPLEESNKKNDSKVNTSAGDAKNEIMDGAFEGEERLVSNDDKNFPQYQT
GFFHNVLHSIPLWCFWLNAVIVSGGVHIVMLNSRQLFVAISEDPSSEQLPALYVALTSIG
NAISRLGVSFFEAWNASRPLEKRTPITITYCIPSLMMCLSCIFFLIVPARALIVPMLLGG
FANGSYAATLVLTVRTIFSIDVAKHYNSIFFFDLIGVIVFNRFMFGELMTRNSVRASDGR
VHCLGRSKCVRTSFTVLACLCALAFTASLLMHFVYMRFVRSRRAQEEAGRNVPLEMAGVT
SEEVQ*
>TCRU_337 Galactose oxidase, central domain/Domain of unknown function (DUF4110), putative
MGKGRDKRKKHEDPLKAVKRASRQAQKRAKGIYKGDAGDDEVKGFNNEEAPEVTLNRIRK
QEGKMRTTLVEENVPAPSPRANVVFTAHPERDHELMLFGGEYWDGEKTVAYNDLYFYNIK
RDFWSRLVTALNPPPRSSSQGVLYKHFLLICGGEFVSQSQSQFLHFKDVWRFDTKKFEWE
ELKGLKGGPSSRSGHRICIWRRNAVLFGGFYDNAQECHYFNDLWVLSHLDGSGKWTAVKT

APYAELPHPRSGHSMAVWNDTLFVYGGFSSEKFNRFKKSQATVHHDLWSLKLPQGIGAAD
NNNAECEFTLWTKIRLGGIPPPIRCGVGSAFKDNKMYLFGGVVDIESPGGKVVSSFCSDL
FVFHMDTKRFYPIVLRARRKTTVSESKGGDLQSELKALHLQNLKDASKFSSDEESDEEDE
EEAESHGNVEEDDKEEMKESFEVNKRGQIYPHRRMNASMLVLGNTLYIFGGQFESGLREI
TMSDLFSFNLHTCDTYRVLLSQDLSRLVWLGKAAEESDAGSWESGSTVVSAAFDMEYDED
DDEEAEGAEEAAVDGGVDFSRIPRSLECGDNDDEPPQVIPAELDTAVTPARDIVDGITTV
KGKKGLKVHKEQLLAQLGSGSAVPTPLREETPVLFFERTSAFWVSIATESFADAGGNMRQ
SEKRIYRRAMRFAFLRYHEAKKLLEQLRLVEEREEEETRFFKERRQQKEKEWEEHERKIG
EQEEDDDKSA*
>TCRU_338 peroxisome assembly protein, putative
MIRSMEADSVELAVQLELCILRAVTPKDYQCVLERNGKMDGNTVRLLQAIMQQDDKVHLG
QTILLSEHFPVALMVVDLAPATAGVLCDTTTLIVLPPCVSNGATDVCLAAHTTEENPKEE
EEEEEEGDIDEDQCTVVLDDSLSGSIAVSSERFFLRTLLMDGAVVCVGDAEYTTAFSICM
KAHGEDGADSVPTLHTSPGVFRNIRAAILLESLSSVEATRVQYSDQRLNTSLSVFRPALD
SACMPLTLGLSKNGFSELQMSCAVLVPAWPLAMLNAVLGDTYEKMLVLAINASKEMLSGR
MVSEGDVLIFHFTPQLFLERQLDYTDVTEAVLRHVAHLVQGDAAETTMRLRQNFDGTIAV
PLRVAEVTTSSGKPYGLLELDGQGTEVVLSIRSKLPCVDVTPLYPPLCSSGTVYLNLSFT
LAIREALGSFFLPLHSAGTHVFVVRAMPENFPYECITTALAMNGVAALIVDLDQWRTEDV
QRLVSEYMQGRGEVALVIKNAQILPDNPHLLHLFDTDDTISDTCLGVRIIFLVCEGMEAP
PPTVAARARNVEGVLNCANPSDTDRRIIAETLFQEAQRSFGVCKSLLLSFDSVASWTVGL
SAADVVAYMQECLSVLRCMRLPEGVRPVLSESLCGSVLQKYQKTHGYSLVSTKLHPVRWK
DVGGLEEAKRELRETIQLPLLHPELFLTGTKRRAGILFYGPPGCGKTLLAKAVATEMNMN
FMAVKGPELINQYVGESEKNIRLLFQRARDNSPCIIFFDELDALAPARGAKGDAGGAMDR
VVAQLLVEVDGVGHTRTDGSTTAQVFVIGATNRPDLLDPSLLRPGRFDRLCYLGLPATRE
EQLVALRALTRKFNLADDVDFDALLEPLSMDYTGADFFALCSDAMMFAVESMLQRTAAER
GDDTQSVRRHAEEAPSFFVQMRDFVRARDQLKPSVSKEELQRYESLRSKFTTW*
>TCRU_339 Glyceraldehyde 3-phosphate dehydrogenase, C-terminal domain containing protein, putative
MAWKIVPSVTGAAKAVGKVIPAQDGRLTGMAFRVPTPGVSVVDLTARLEKPATYEHSRAA
IKAASEGEPRGILCQTEDEVVPSKTQLVRALVLMPSAPRQQRRPREGNIAGSWLPPTIRS
N*
>TCRU_340 hypothetical protein, conserved
MRRLILYRRELCGREACVSLRRFLRRRGTPKKEEVESVDSVDVPSLRVGGMMAAPDTSLD
DIRTEILRMRSEHVRREEKLNENAASSVKEGSTMNDAWNDDIEKLRSNEVYVKGHDVLER
VKAKDRAVRKQLFLDAEEVLRKSNKEDTLRYLQLVYGRIGSYHREEVMESVKALVQLVEE
ETQKIKGRIQENLIDRDPLAELDAAISSDEVKQMAWVNALTNMSDEDLKKLWKWGILDFD
TIESLACMDMDNETRVVDMNLQENSETEKFFVSTTGQRQEGEDDSLLDPTIPQLSIAENT
EALQNVGEIPEFTATLYESLFLGFPNIDLAPVIQKVQEKRYESISESELRLLEHYEESKQ
SINLSAPSTITPSLNKTEKSEEKEVVKQGALGERFSKDTHFLDTKTLPCKMSLTEAMMDR
AFSPSNRFLHAVARCDPALRSSLEVMERIKRHALLDPVFQAAVEEAHAWEETSIQKHLQN
GGVSAKGETRKQRRLRRAEGSATPILGGPLAKPQHAADIPYYYGNIRSFVPPQGRYNMPA
PRLSREEAAQLRNQERKQKKSRMRFSQ*
>TCRU_341 hypothetical protein, conserved
MTSKKAAKEKDEYTPQERLTAAALEYKKLEGHNQSSIERMYGALSSPFRVSNKPCEVPRA
AVLRCFEEILGKTNSGENGSTDNLTETTVVGRSISGGNNADGRVLLSSDSRFPSVHRCYD
AVLQYESCVVEKTLAQHHSLLAEFEARQLQEVESTNEVPKAVASGA*
>TCRU_342 hypothetical protein
MAPRKKNAPKKELTAEDMRRLGMSEEDIARILAERGKSAEERRQEQEAAEARRRETEQRQ
KRMKELEKQKEELVRLEGGPRVAIRDEEDAEWEQLEETLTVEKKRRLRECAEKMVQQRFA
EARRKREAEAAAYQETLGHLTPEEREAFVAAQIAAEHARSEAEMQAKEQQQKREERRLAR
ERAARRAQRQRRRQDGEEDVDEEDADDIATVGGGAKASYERIKSIDELSADLHDKYD*
>TCRU_343 BNR repeat-like domain containing protein, putative
MSRRVFASAVLLLFVVLVCCGISGATPANEEEVKNELINIQNLQRVDLFVPQKTLVLPKG
GTGSVTTRDSFFAPSLVNAGGVIAAFAEGHMNAEYQGGQLTKPSSDVVAEYIDSAWEWST

LVGEVSESTWKAHTVLGATDGTNRVGVVLRPTTTMKGNKMFLLVGSSAVFYESGGNWS*
>TCRU_344 syntaxin binding protein 1, putative
MPRPVRLRLLLLLVTASSTDELTEAKKQQLIRDGGLTPDAHLFANFEHVARRAGSVQRYS
TASAQGGRSTSASASNAATDEDPLLKQACMMMETGRNRTLRVGLSLASVQRDRAMGPNGA
SGGQNSGEA*
>TCRU_family, putative Se.1
MLDVVHGLSKVFYCDAHAAAVLNCSLRLHDLMEHGVTLLEDLMTPRQPIMSSSARYFFAV
EDASVSRVVEDWMAKVPYRDLHVFALGCAPHRSPEQLARARIAPRAMRSKDIMLDFAAPE
VLVFHLSMQNGFPQLLSPPN*
>TCRU_346 hypothetical protein, conserved
MDVFLLGFQFWVQAAQEEAQSPRSATPTTPQIDRVLPRRRQRQTEEAADRDKQQQQCHHR
ESGTGLLAVRLAASILAAERRFSRSVGASWPDPLLLWRRMLLGNSRVDILWWCAATGLNA
LRNGDGCP*
>TCRU_347 hypothetical protein
MKTNVSESHDALSGVGQREESTVNGSKGKAQMLMAPVTVGRLELVPQRERALPSSDGSRA
TGSRENESAQDEKLKVAESSGAVGQREEPSPQHSLPLGGEKDHYQQNQPQYRANK*
>TCRU_348 hypothetical protein, conserved
MRVDRAVPCRSRQAFPLLVRDVLVRAGVAVLLGETKINDVDVVPLLPGAHQEIVRLHVTV
DDALAVDKLDARNHLLRQHQRCLQRQLPITEAEKVLERGSQQVHHHHVELALHAKKKNVG
DAHDPAACVENAVESDLKGKLWMARLRLLQLDGHGLARLYVCAAEDLAERPRPNLLAEAE
AVTHSKL*
>TCRU_349 hypothetical protein, conserved
MRVDRAVPCRSRQAFPLLVRDVLVRAGVAVLLGETKINDVDVVPLLPGAHQEIVRLHVTV
DDALAVDKLDARNHLLRQHQRCLQRQLPITEAEKVLERGSQQVHHHHVELALHAIKKNVG
DAHDPAACVENAVESGLKGKLWMARLRLLQLDGHGLARLHVCAAEDLAERPRPNLLAEAE
AVTHSKL*
>TCRU_350 Reverse transcriptase (RNA-dependent DNA polymerase), putative
MVLKRLLWVWTPHPHQYAYRSVRTTTMQLAHLTHEVEHNRNHYFQVNLPKKSGIGNQLHY
RPHRTLLVLVDFSKAFDSIDHQVLIRLLANIPGVNCRRWLRNFRCGRHAKTRVGHRHSDR
RPMLRGVPQGSVLGPYLFSLYVHPLLSLLNSFAGVTADMYADDLSIIVKGQSREDAIPTA
NMVLQKLHAWSQENGLAINP*
>TCRU_351 hypothetical protein, conserved
MDGDAPRHRTSGSRDIPTGICTDGMEQAECVAAAACHELKCSANGRTAIMPSAVASPCCQ
RSVRNSLVPSRSQACDAPRHGGATSLPSGIGCAAVLLGPRLCSASAGTSSRREHHHSSPT
SERLIDVLLPTPRQTGTRPADHKQPFVSVVLLLLTLKGQDRPDLLRNLHLACPRHLLVER
RAADQRRPAQAAAASSRPSTI*
>TCRU_352 p21-C-terminal region-binding protein, putative
MPKRHREPENLLPFAVEDYGDGVAEQESCGSDEFHSSEMGNDGNVSSPAGTPKDDADDSS
SVMMGSSNDDGDGENASLINVEFGVFEMRRDVNAVMHLIDQLCPDKMNEVDRDELGAALH
ESPFTCVVKIGDDDDDDEGEEEGEEEIYGMASVLDAVHDTRLESLLHTLRENVWRTVAPG
ILPTEILKSIDDATGRAKCVLLVGEYVRNVPLELTSQILHDMMERVETAFNARSSMSNKA
CEDAPSTVQAIFPCMFAMLSKIQRAVDAPLKTPTPMNARQDPLPKKRRGQNAAQPEEFDL
CRYLFWREEDSIIYEYRDKRIATLAYRCRPQYDGQPENDIPISILYVVPYGGLMQAVAEI
RKRESTQVNVVRY*
>TCRU_353 DnaJ domain containing protein, putative
MTAPSELSGEALVFYVMRTNGNPVRLFGADGVTAESALKRVYKALALRLHPDKNGHPRAH
EAFQLLQQSFEATLESLQSKAPEEKKTRPNFPPPCPSSSPPPPPPPPPPPSFAFAKSTLP
GVRKEEQKQRQHAFAGNSLFEIPLPPDVFGAFAAPDSSEPPVASVSTGCPGTKPPAKRTR
PLPALDLSSDDEEHVDEAGGLQASRRDDEQEHASQKRNAERKAVQKEKRSTGRRTLMQLF
AEFNISDDEEEMAPVNHPTGKETGHHWEPTSCHETNRRQQSGAGKPATTLASSSVACPCC
GAGEFSAHIMRGVVCQSCRAQFVPAAIGMMAAERHAAGRKKDRASRGELCACGKAKKGLC
FLCD*
>TCRU_354 palmitoyl acyltransferase 4, putative

MGPIRVERAPVVGREYSVICPVCSNNVTFDCLSRSVYGIQCHFCRTLIDLRNPSVPLPPN
DTGVLAEDVNGGFFFSPEVEESGRGSATTQLSMVLADFAEQQQKEMPTTMGGEARQAFSA
RRIQALSERFSFLYPLVYVIEYIVFGSKPVARIGHVRIFRKRLLFFWPVNIPVTKTSVSY
SAPLTVAILLYLMTTFYTKSLDDEWMWCMHRFVTFFLVLFMVLLVCCVYTDPGFVRPAYL
ETNGSHMGELTLKEIEAKQRESRWEMVNGQPEERKWCSTCEIYRPVRAAHCYLCGLCVHD
HDHHCSVIGVCVGRRNVRVFFFFVITTALLALLPGLSLLVKLFLDANSLTARQLGIGLLL
IAVLLGFGLVVGFLGLTMLYSLAVETTTRERLQNTYANKKNPFNRGLLRNIFWHMCHRSL
PSLFDDDFVQLCATQV*
>TCRU_355 AARP2CN (NUC121) domain/Protein of unknown function (DUF663), putative
MDGDKEQRNKSHKGRQAGSKAAKKEEFRKKKAGIELEPNRGKNRKAFQGPTAGSRKGQKL
LRSLEKRETALHVPTVDKTLRHIVAEPPLLVAVVGPPGVGKSTLIRSMVKFYSNRNVQTV
RGPITVVAGRSRRVTFIECPNTLTAMCDIAKVADLILLMVDGSFGFEMETFEFLNISQVH
GFPRMFGVVSHLDQLRTGKALKKRKKFLRHRFWHEVAAGAKLMCLAPMVRGMYRSTDVLK
LHRLLICVEPKLQSWRNTHSCVLIDRYEDITAPQNIVEDENCNRTIAFYGYTRGKPLKPQ
QVVHIPGLADFPIAHISQQEDPCAIDDHQGSGNSQGHKMRHLSMKQKRIYAPHCNVSGVM
YDDDAIYIQEDAEKGMIERSGEGLQYIRELQRAKPMDAAATDLDVVRRPVVFRDEDAMEL
DMDEVEAPSDEEDEGSSSSSSSRNDDLEDRMPRNDLSLYGPQEVDEWEDQEAAAGDEGLA
PRGRNAPDAIRENVVRHDWSETALLRRLKNLCVTGDWNCSADAAETSGEDRLGSEASKGY
ESEDEEFDAIVRGERPLTTTSARAQSDDDHEDQFNGDTEGEERREKERHGRQRVDALAAV
GNEFSSSSTWGAFRPADTRHHRGDWGEKKRGHDAYDDGIDGSDYDILAEDDAAVCKSAFA
DAELDDLVSTFLAPRQATTKGTTTTMAAASVSGAVSSLRNDGDEDAIQFYDPHHSRVGED
NGRNNGDGDNNDNSNNNDDLEEAAAIVSGKTLTEEQERRLQKKMAKKKAFDESYDMGDRS
NTYAHYHQLTRVVEEKKQRLDAALQEMGEDVAKKIQLVGYFSGLYVRFVLENVPVEFVRL
FDPTIPLIAGGVNAGEDQFQIVHARLKRHRWYPKILKAQDPLLLSMGWRRFQTQPIFATE
DPNGRVRYLKYTPQHMHCIAAFYAPVVPTNTGFIAIPVKEQRSPNFRVSCTGYTLGNDHA
TNIVKKLKLTGTPHKIMKTTVFVRGMFNSDMEATKFVGAKLKSVSGIRGIVKAAIKGKDG
LIRATFEDKLLPSDIVFCRAWKTVHPPKYCSMQRNLVDANWMGMRNMRELRWEHNAPLVT
KGDSEYKEIKRRQRDDEDYAAADATKVLLSRNQKLQLPFNMKEEFIPLERTTALQQRLAG
AVTVAPEPRDMRRTALLDVFASKADAMLKKKAEAKKRTRLRHQRESTAEEEEYLRQLKKA
KKETARLREFRSQHKTRK*
>TCRU_356 hypothetical protein
MNDHRTYRNAGTEGRYNGNRIYFHATKEPLVKSYPPNKVDGRLLREWEMPGFVASRSFLQ
PYSAIMTVYRGRVLGHSLDSGTLTGVAAGFSSTSATEKSREWVDFLEHAVQIDQPLLCRI
RQRILRHSFGLRRCCVFYGNLHSTSPSLNNTMGRRLMYE*
>TCRU_357 Putative methyltransferase, putative
MQHASLGPHPPTSLLLFLQSAPPQETFEAFKDDCKLFGMPWYCLQAQQLCVVLLVRHPLV
QKYPPRKSNVRAFLKHFITQLELTWATPHVKEALNDEVIDSQLIEAYICYSLRADSDSQT
SMCYKTFYAPGARETFLSVRLAAEQFANVGLALWPAAFVLVQLLLAECSSPTPVILPHEG
ALRILELGAGVGLTPVMLHQYSCYKKRVQGSVLTDYQPELVENILFNLRSHIMSGERPHE
SLSDTEETGAPHVAELLDWTEHERNREKLREWGCNVILAADCIYEVDLIDSFVTTLHQAL
SVSEAAVAIVVQTHRQNKTMKCFFDAVRKAAMCVSSYRLAKSSSQPSENLMLLHSSLCQD
DAIAGSAVVCDEIDADGSFKGCQAGSCVRGWVGPFFLHSEAAVGIHVLRLG*
>TCRU_358 Domain of unknown function (DUF4586), putative
MVFSIPSFLGLGDRYKHAVDERAQGVNFVTCYPKTGHNPDALFDKEFKYMYNGDRRSDPE
TMARREQMEKRKKDMTATGFAYVGPPQKGEGLGSYYGLIQREPYPHIPDNPMPRGPQPFP
KSMPRQILTSPGKLGSYGTPGLALSEIGNEYIATIYDQPRINAKKERDAWRRRMPAVPFI
PVGRRGYTFDESPFAGVSTCYVMTKPFMPKLVQPSFKHFVVDKPWRPAGYVEDKPTAMDY
REDPYNAFDPREDPKSRVKKPSDAVFRTGYRCDNFCYTQSIVFRRL*
>TCRU_359 C-terminal motor kinesin, putative
MQQRAKKRQRQSDESNTRPAHNRNDGQPPGRRLPLTSSVASVTPGGDAFPSSSLHAPLTA
IDLVWQRKTAAVQKQLLQLANLYAQKAQENEAVLDAIERMIASHELELAAVAAESESHTC
QHLELIAELHRREDATLAEKQSAMDEIKTLQERHVQLEQARERLQEDIVIQSDALEQLQK
TLEAERQVTDEEQRKLNDVLERERVMQDATYGLSGEVREMVAEMEQIRKAKEAAERASRE

TEIVRRQLYSQCEELKGTIRVYCRVKGSGNNTNGEVLLVPESSREASISSHSTISLSTEG
LHNLAAAGGRGAEGKRQKIHGGQQKTLHGRFEFPQAEDGVQRTLCVCQSRNNATSTGTQE
SKETFTYDCVFDGNATQEAVYANVEPLVNCAVDGYRVCIFAYGQTGSGKTYSMEGEVLDE
QKCGITPRALRTIFRRQEELSSDGWKYSLSCYFIEIYNDVIRDLQQEPSVYEPGGAAASQ
PNYHVIKHQNESGSTSITNVVERRIRSFEDFQRHYKTAMKNRSTAKTLLNDHSSRSHCVF
VLRIEGENALIRQRSEGTLCLVDLAGSERMSESGTRQGQHLKETININRSLLDLGKCISA
LNNSGSVAPWRNGKLTYLLQNYLGAKGGKMLMLVTVSDKEEHMAESLNSLRFASRVNQTV
VGPSVRRVTDY*
>TCRU_360 replication factor A, 51kDa subunit, putative
MQHPSNQQIQPIDSLTPFLGGKWWIRARVTDKSDVRTWNKPTSQGKLFSFTLIDESASIR
ATVFNDAVDLFNPLIVNGQVYYLSGGQVKNANRKFSNVNNDYELTFDSSSQISLARDPSS
ATIPLQRYNFVPIAILKQREVGSLVDVLAVVLSVEELSSITQKSTGRELIKRNVKVGDIT
AAVEVTLWNDQAKEWAYSPGTVLAMRQLKVGSFDGVTLSATFQSAFDVNPNIPDVKKLAE
WYISTGGRDVSSLSSLGVGAGNNAGGENYRGRKYFDDISAEGLGRGPKADFIDVRCVPVY
LKQDAQWYDACPQCNKKVTLEGAQGERYRCEKCDRQVVPTQRYLVSIQASDNVSQKWLTL
FNEAGVEFFGMPATELKKQQEADPMFVTKIAQLRMNRPVQMRLRVKEESSSSAAGGEESD
RVRLTVMRLNEFMPLDTVSEEKRQAMAAQLKTECEEMVRCIEAYL*
>TCRU_361 ATPase-like protein, putative
MEEVSVVPVEVRCRPPRVNRTELSKVITSFIRQRHAFIHPGEILSLDTASDLIASTVELL
RVCDVYCPQLGIPAEEADYAMVMYTLYTDESCGDSLNSVGPDEDAGAGLAPCSVLSLPSV
SLEGLWETLFYGETRSDSVRLKRDLLQYMRTAMVFSRAGVDPHIVAWNRLILLYGPPGTG
KTSLCKALAQKLSIRLNDMFPFAQLVEINAHSLFSRWFSESGKQVMLLFKQIHEMADKPN
SLLCVLLDEVETLAATRQSALRGNEPSDAIRVVNALLTQLDSLQRRHNVVVFATSNITGA
IDVAFIDRADKKIFIGAPGFQARLELLKSSTQEMIRRGLVILEASSCGEFINEETWPPVV
SRDMELGQSELLQLESIARQCEAFSGRTLKKLPFLAYSEYAMGGCGVASHLISFSTFMNG
LQMAVRAEVSARETMMSS*
>TCRU_362 DNA topoisomerase III, putative
MGRTVLMVAEKPSLAESIAFHLSNGQAAKRSRALPVYEYSGYFFNTPAYFKVTSTTGHVF
SCDFTPQHQSWDRTEEEDLFSAPVVWKDEAGKVSRHLAHEAEGCDTLVLWLDCDREGENI
CFEVMQVVRRSIHDMRDIWRAKFSAITREEITHAFLHLGKPNKNVSDAVTCRQELDLKVG
VAFTRYQTKYFQGKYGDLDSSVVSYGPCQTPTLAFCVQRHDEILNFKPENYWKLVPVSNR
FGSLMTFDWARGRVFDELMARLLHQKVSHHRAAKVTDVSVGVDTRARPTALNTVELMKVA
SKALGMGPHHAMQIAENLYIGGYISYPRTESTAYPSSFNFMAALTAQERSPIWGNYVQEL
LAHGHTRPKAGKDAGDHPPITPMRLATPQELSGDSWRLYEYIARHFIASLSPDCKLTWTK
LLIEIGGELFSFTGKVVEDPGFTTVLPQFAVKDDKVPTNIQVGSDFPISDVRLQAGQTQP
PGYLTEADLIGLMEKHGIGTDASISQHVNNIVERGYCAVKPGRVMEPTKLGVVLIHGIKS
IDPELVLPLVRSKVEEYVTYIADGRASLDEVLSYSLDLFFGKFRFFKQNIERFDALMGAS
FSSLSASGKPITRCGNCMRYLKHLEARPQRLYCAYCEVTYELPQGGTIKQYSSFKCPLDN
FELVICHVDGGKSFPICPNCYNNPPFEDIQKQRGGRQHMACDECRHPVCYHSLATNYVAD
CVDERCDGCMAFVPRTSGKWKICCNHCTMMILLPPTAQRVYVSPEECSECGAMMMDLQFP
EGKSPLPNRKDRIVACVFCDPTLNSNVTEVRGRLGNFSRRGGRGGGGGGGARGRGRGRGR
GGRGRGGSRGRY*
>TCRU_363 protein kinase, putative
MGNQLAAASSVNVADVSSTLALVAPLGREHRYYFRSFHCAKLAGAATSTTETLRSRHIFT
STNDGRAKDMSLRHPFQRAGSTDVVVKVFVRTFGDNQQKQLVEFCYEKLLEIDGRMRNTQ
SAASNSTFMNGGNNTAVGNHHHSPAAPPAPCNVLFYSGMVVHNDRFCMLQRSYVAYSLSE
RLATRPYWKMGERLFAGYQLLQGLAQLHDKWETVHGDLKTDNVLVDKAGWIYVTDVCPFK
PGLLPANNPALFDYYYDTNETRICFLAPEKFLDETVLFNPSNVNANAHTKAMDIFSAACV
LAYIFTEEPLFSLSDTLSLRLMQTQEERESSVQKILIERGVPPNVRRMLLDMLCNPPATR
PTARQLLETFTPSVFPSYFGFIYGEILPPLLARPPDLKVQLLHSRLTDIMDEVTQRFGKN
EDATSHLAVIQDAKQLSVQLLLPVILNAGLHLITDDAFCRLISILQKILPYCSSEVQKDI
LLPYVLHYLKLECHPVIARIIALRTLSMICHSIPFSASDATIFDDLVLPCIEQLFLKTDT
TDVALLAEVADQLPRILLRARAFLEHCQALASANAWQSGFGEQLNSLLDRGWDAFRLLYK
HHHTTVAVVTLRRTADVVAFLGEERAQDDLIPFLTTAIASAIDIQRELYPQAIMCHLHLQ

TPKLKTLRFFVEEGLKQTDVTCLERTIESITVIVEKECFAVWDIMPLVHQALPYIMSSNK
WVSSAASRMVEKVACTHRVSDVWMYLECAVRPLLRHPVPLFRMASFPSAVKLELSNNIYV
VEQLMRNMNLETRFLGGDAAFYERILRSSTIYDSPYFNSFGIAKRSLQGIVSRATSVDVT
FDSSMLKPEVSSTEKAEGTLSDGSRNNKWELFFRPTKREQKRLLLRQKPLRDDGGFNAIP
SLFQERQQSVGSLHTTRGGSGGSRRSGNEVAGSPTLSMTQVTWEEKELKPIAAPWFTRTS
HLGGIYCSSAAKGGTLVTAGGRGEAILWSLGSEGVDYSHRIQTNASLMSTFLFAGFMREG
LSPLLCMGGTDGDFCVFDVACNNIVLRRLLDGSTLTSFCLIGNFTTLVSTALGGVFAIDV
RVGKDVWQTKLPPSVGPPSGLLPLFFDTQAYGASVVTLTGGVALFDLRFQLMVQEYRLGK
DDGNSHESGRVSWPGTHAMDNPNAILCVSPDTTCTTLDRTDGPGLFLGTQAGTVHWMDLS
TGENRVVLEPATIGKPTRALLVQPKHSVVMTAGNDMWIRKWCLTAPNQSKTLVCPPYTSP
AYHGTTRGGIAEAQSQNQFVSARPFNSNEPCLTTNGFVCDGAVPRHHEDAILTLCAVSNG
NASYLASGSRDGTLTVWLNSE*
>TCRU_364 methyltransferase domain containing protein, putative
MTIQRDAADEGCGDLLRAVEERIPCEIRCHFTGMASYRQLRDGNEGESTFVSHPMKRFRE
FTCEKEVCVESCISHVLSCTACQILKGYCVLWAESLVNVVMYEPFVSRTERRRRGDAKTQ
TTTTNKEIRGRHQLIFHTLHIVDKAAYKHPLQHSFALQSVCQPGRVLQLECYPYHMIHFG
LLIYALWRSPDEVFSAIRNMPRQKKVNTPLEKGACMTETATMSLTPLHLPLLATTEDASV
LILGLGGNVIGNCLDSALPVDVSIDVVEVEPAVFETCQKHGQVPPCSEVRTKKENGTTRV
WVVRKGRQRHPNYRFIIGDARVVLQEDKTHQKADSEGPLHDRCYSLVFLDCYDPEKERMM
HDGSLIDVCQSRLCPGGALIVNAHILPTREALEEQFLSRGFASVQVLRVAGCVQSIVVCL
ARDKASAKSPNEEKSNKREGGQQQIGRLDRFCVRHARHLASYIQCASRADPGMCRLFRSG
FSLDANWLRSSRSVEGASCNTRVWEHYD*
>TCRU_365 proteasome regulatory non-ATP-ase subunit 2
MVVGLSSSKGILALLNETDADVIQFALKRLLELVDTYWYEISPDLPLIEELHESCDLSEE
TRMLAALVASNVHFHLGAYNDSVHFALEAGTAFNYMERSLFTDTILSCCIDQFVQYQEQP
EETRGKLDPRLDSLFTALTNKWILQEGTPVRELVGFTIRARCLDFLEKVLRQDIALTKSA
AILNYTFDLASVMLRDIGFRGKILRLLADLYAQGGLSTIDYFSRAQCLLFLGDSESTTRL
ITDLMQANDKATAYQLAFDLYEYGNQEFLSAVVAALEREAAEIPHPGLTSSPPENEGEET
STKEVDTSNLSDVHQKLLAVLTGETSASFYLKFLYAQCNADIHILNHIKQSIDAKKSVPH
NATVIAHALMYSGTTIDAFLRGNMEWLARANYWAKFVVTASIGAIHRGHIDEGLAVVDPY
LPKEGVGLLPYQEAGALYALGLIHAPVGVTRNRKTIDYLKNVLRKYSTSEQMIHGASLGI
GLAAMGLKDEELFDSLFACVSGCDAVASEGAALGIGLLMMGSGNRGAIQSMRALASEDNQ
KEKTIRGISMAMALMMLGREDECWAVANELLDDSDPWVRLGGCFMLGLGYAGAENTKVLE
RLLSVAVKDTSDDVRRNATTMIGFLTIKDPALCLELTRVLVDSYNPHIRYGVGMALAVAA
AGTGDSSVIEVLWTLKDDLVDFVRQGAYIALAIVMVQLTEAEEPRVKELREIFSKKIAGR
KEDMCSKFGSIIATGLLDAGGRNCTFSLHRQRHRLDKAVAGMFVFLQHWYWFPYLLMITL
AMRPTCFIGLNDNLDVPEYTFKSNAPASRFALPKSVLAEKKEAKAASVKAVVLSTTKKEE
QFHRLKRMSGSGRAHDAAEAVAASKNAAAAEPALTPLGAETEKKEPEPSFELLKNPTRVT
SRQFAVISHDVDGRYVPLKPYPTGICLLRDTKPSDGKQKIVSEVGWNSADDAPPPEPFTW
P*
>TCRU_366 hypothetical protein, conserved
MILQFNWDRLDVMAAEAIRALVNAKLEEDLRRRTAFSGGVDAPAVGGSTRAESLCVTGIE
WGIAPPFIEILELDDAQDLSRANFTPHDTIHSRQEAASHCNLFRLSSSVSSQYGGEHGSE
VDSSPGGCPSASVTKRMPGAWMGSSIPQSGSPVVPSKDTLAPFVGLGGLYILLHVTYGGQ
MRLSVSCVLRHDIPLGSISLPVRMPLTLLVSKMDMDFYLSINLHQNKCRVWMEPGKLSTS
PITRMNIKAVFGERRTYSSQLLDNWTGIPPVCEDADTSDAWTHVASSHLGSEEEDDAVFT
EENVISRFVLSEIRAILQEKIIYPHFVEVPLFL*
>TCRU_367 hypothetical protein, conserved
MSASSESLAVNSTQTTPSRLLQRGMRRREVSVSILSSGIDPTPSQQGSGGKPESYANTVT
IPLFMTQSNEDLLFLPVCSRNKRVLSQATTRSSSASSYTESPFQRQLNSVRMKLGLAEAI
VQNASITAKVPMAPEKRRRQMLQLEVAKLILFVARQHNIREELQQVAGNSLDVSDEIMYH
RRSIESKISFLAREPPPDLSLKELKRATFEMEKHLEKMKDKKSNAPSVSAHGATSNLSEF
LWLIESIELETLDPQDVYLGEEAASFIAPSTSANTSTPLEALQRQHELPQKKRPELFPPL
LHSTPSLQNEGESFSTLLRSPGLYLLEYPPHLSTDKTTTKAENETEEEELVGFSVDVESP

GCRAVEAAGLKLLQFMYDAVDAKDKGLKLTNGSEEEIAAMFRAERTMQYGNEIMRSLNTE
EADAAEPKECLLGSDGTMRNFDATRQTQSARNRATGTLSNAQEEELSNQSKTSSISALGE
DSFFFSGHPAGFEPSLVNSWIRPVQSTVEVHPLRTGIIDILKRLYGDFNYKHYFEHCKPP
KADKVPKTKEDYYQELERRVARRKSEKQQRIVSDQNEEDSMLLRWKGRSSSSKSLTVGKR
DSNIKIQEEITDDQLESMATGTAETPPLETPSVVTAPPTETTVDTHGEGIVLVVELEEGI
HFLLKGRRDERAGQQSASGSMNSFKKGKDLTKGGSIENKKAVSERGNAAQSESLEPDFDS
YTLYAVNQSDMPRQATVETNRTKLKNLRLKALLKYFGKKRNAIAVDLPPRGTQLVLVMKP
IKKGRKVVFDEALKFYEGPPKEEAPKTTYAIAKQSPPQVQSTTTPPRSAPLAEPEVLRDS
GGTGAEAQAKGESVSQAEYSENKTSTKAGKTSLEVADATEPSVHGNEEEDQGKAAALSGA
RVPSMQTFKPLLLRNTGGYTEDFSSEAYSFDGSSLSGNDVSAKGVNDWGVSVLPLGEDLE
DQQEEGWRQKGDMGVPRKRPKESDNKAARTRFMGEKGTKRGSETRIGSSKGSQFEADRAS
GEATRVHQWGVPLTNDDDEAFENNCVVLSVLQASEWQSGSKVPRRSLGSKSAVHSVEGGE
PKEMNVKDGGMDDATGKATEGGILMESSLRTTSERYPSARFRATSIDTKEEDIVTAALYP
AGDGDALKTEEIVSSFLQRIAEAEQLNLEEQIDNEDISCPKGGEETPLPAGSAALWRRQL
VSQSQQDPHSIGSQEESKQLLSSLTPGVETEFPLGSDREICKVQGSTSLAALRAESEEHE
KGSTLRASSSHMFSSEQLTANATVVQRPSVTLEGGSSAQRMKIYGLPQSSVTMNESVTPV
HLDENYTSTSYPQQPSVATKVDGGEIGDDVSTPVETTSGLGTTSRTADKRSVSIEPLQGN
RYASPTMERPAFNLDLLLKSVKKAEKKEAPLPGLSTRIVKDEYALPEQVMPASPSRQHAY
AEARPDGRFAIFAPEEMNVKGAAHTSDRMDKKKMSTRLSGVVENAAVFAEEIRTTFHAFA
RQATSQRRQTFARSEIHGKATVGRNVSVDVMRHATLPALSMVYSPETAQDVEDMEAAEEE
AKALFLQDPARYDCVIDELTKRAIARVYEEEEREKERRDSAAHGTAGRKLKEGLIVVPGR
MQNRSVAFRQFSVVGSQMGGFQAMNISSTGSMVQLNGDPVHFTHGGSFLKGRHPSLLLGK
SHRDKVRHDTKSELDQLLDEQRRAKKIVAYILMEMRRMWRRRCLESNLALRSAVDRFIKR
SVLFSWHFERRRVLNLHKLIHLLDRKTGRVGGWLPFYMKDRVMEDRFVWEPAPLHLSRPL
RVVHRAMCLARQQRMFPIRLVRQQAFRRKNSQHILYGYKRTIMEPFRSPAVRLRHGRRSR
YAAPFQNPTMRFSPQVEWMDGTPKK*
>TCRU_368 hypothetical protein, conserved
MPEPSIGNAAALSMMQDVSVGKKNSSTTSPTPPEPPDDNPSSDLANPPPNSTFLIAANNS
VTSTTGMTHSPTTSLTVSPTPSLEDVPDNMYTRAESCDIPIVNKCVERFCNCISRGQLQN
ANRSWYIKNGVYQCHEFANANCYYNLYCARERVSCLWSASFLYKAYRPQGNTLATSYELS
QKNSQGSMPCVGLEDIQGNFSQLTKNTNYYDSLFYSECVDYVTFILQRTGDGICAASIVP
MYVCGDSLFPVPPTRQIHYTPLSEGARRLVISIVARIVGNFTPIFNLERAKYNEKYQMNI
ENQLSNAMYSCFFDAIGIKGVFSVKHEDECLVVRYEVGVGEADPWVGDVVTANALSLMVR
SASWMDPAQRVIGNITDSVKLSAPIVVYYVSFESGTAQPEAKLCDAGCSVAISLVLFLTL
IAFAFSCVFCCRLSRRPYFMFDDDHDFEEMEIVKGRRAIVPNRK*
>TCRU_369 hypothetical protein, conserved
MGCRHSGPRMPPLEQQQGHSLDRRKERDVVDMMPGDGVDAKSAHVAGLSGATSVDGAQGK
PSRRDDVYSPMPPQVPSNSWYAREFTEVTRMSLTEFHMKKRGNVVFSNAYIPVGASELEK
LPGRAPPMPATLEIGRDLVYGMAYLHNTPLFTEHQWRLRVAHHAKWRQDEGKKHEGSEER
RSICRREWAKTIYDRPGIDTLVGIRVFVENKLVPDEYPTTRSIPNCTATIDASEAFNNWI
CIPIVLVGYLNNFLLGTITLYRFLSLNFAGYMLRQPINLSGVEAAAAGYDGDGEGADDCS
DDHASRKFEIYVEVVYGCSSEFNFCTDYISKGSVEVIMTRRSKLALKTYKERLHKLIQGS
LLSREQVVPMDRLEMVAQQQSSRNCLFCGSPLRYTCTICGAQLCGTLMCVWRPFAGYPSG
CSVHKAQVIM*
>TCRU_370 differentiation inhibitory kinase
MISKLFRLDPQRREKHEINPHDIKALRGMAATHNITSVAVPQYRVRENYVEYVIECSKRS
VKWQVFRRYQQFRALDQKLKQLCSMGSQYHCEYGVLPVLTGSHWAEVTNQSIDLVEKRRR
YLEIYLEQLLVPKNLFYVAKTALYKFLHEGEVPVRCRTNVLRPLIGFAAVEPSRRPEKSE
DDATTVEESPNPLDISAAGGNLPVTTTCHTTPERGVSNSVESSSTNRSSGMEEYDDVPSR
EDTDNEDIHMPPASPLCLQCNAEFSSMLYPHRCFLCKQRFCRNCLRNVELEEEVVRVCLQ
CYENHERHSKKKQTAKDATPETPLSQFKGVTAAPEILQLHEGNQLRTDVSLSDFELVTTL
GRGTFGKVLKVIFRENGQVYAMKILNKCIIHKRRMVEYIKEEKDIMASLPFHPYVVTCHF
AFQTDYHLFFVLDFLPGGELYSHIYPKCTLSPVDVRLIIAEVVLALEHLHRYDVVHRDLK
PENIVFAADGHLKLTDFGLARMNFSRHRRYSFVGSPEYLAPETIRGECQSRALDWWSVGV

MMYEMLMGSTPFHAANNNDVCNNVLNRTLDLTAPCFTPEAASLIRQLLQRQPKQRLRDAE
QIKAHPYFASLNWSALENKSLPPPIRLNLEGNDTKYFKREFTAEWAVIARPQEVSRATLD
MLKKQFSNFVHVPDVGAGVSLPSPSSGKRASLPQSVKADADEVLTVQRLVGIWRLVRVEM
TTDDGKIAYPWGSEVCGLLAYFPNGIFTMQFTLSRRPHTGSHFPEQATVDELVEMCNSYV
ASFGRYQLKAESNAIVHCPNGSLFPNPSWNQQKFFVEVSSEKSVGGVAALKLCTPQYNLQ
EEQLLARTVLTWERADGF*
>TCRU_371 protein tyrosine kinase, putative
MIRKRVLGVGASSATMLVQDTEANGVLRVLKRINVSSWNAADVTETLEMYKTIARAKISY
VAEVHMTMLQGYFLSLVITYYAGGDLESYLEDGSGSPFEESKVVQWLLVMARVVEQVQGL
TGGYFYGLSPDRVFFADGEPGICVGLPMPRYLYFKWLMDREALGVAVEREYPPEVVNERK
YKPMVSDVWHLGLLGMKMLSAHTSYSSRSSEIRSIISAMVEQSVEYRLTIAGVVRRLTEI
VDGQNAPRPFATQRSPTQSLGPEKLTSYGAGLYATAPSTEGSTGDENNGSNGNSINCNIP
AKNEVAPALNTSQRVSRMRLQESWHRRAMEQFEELQRLNASSPVGRSRGCSPTARSRRSR
SAGVGLSQSTSAMDMASTAPSPQDIRTSGQFQARSPRSFSAKVTKGQTNLTSVVQNALKQ
HMEEQRRRDAIWQGQETRRLRKQEKERALQNFHQNHVEKLRKIRSKRDEETKNEIRMHIK
QWRDQPRVLADENVIVNREGDVSVFVPKNGRPPPAAATTAPPPPISSVGTLGRKDREKDP
LAVRAGESKEVKEVEGIPVLALPHRATSVPVGSRTTPPSVGPPRFAALYRESGVSPRVPQ
TRAKVVSTPPPVGTIRGTKGILSISSPRSANGRSLSFFAGVEAIEMPPPLLDPESILDTS
VARETEEVDPFSSSNSGPRDETSLFFSLNGSIKALNESLSSLLANHHKHREVIVAMDKFL
MRSEAERCNPRLNAVFMSTLRGILNDEQLFLAAAPLFVQLMALRGLLKKNSLAKKRDLKM
E*
>TCRU_372 hypothetical protein
MGAVPSRESLLRGLIRVTPNRESTDHTAATSRRSAWGSAQEMVLVPLIPDYFPSAEEPSF
LLLFNVYKEASLCCDCVHEEDYLMNMKHVGTERELYEHYMHISKQRLFETDSELIAVHRA
GRVFSAVPCAVAFVQECGSLISSSSSSPAPCRRSPLGQTIPTVQLHGLHLYIVGFIGDTS
LCPPSIPQNKAKDNPFRMLLLLSKSVGGNQRMLVTSLAAYTLITQLRRIGIVDDPMMFAY
LHSAYNGIGEQQQLGLNSLQLKEGTASEGQEVDKKWSWKSMNISGHVIFRPLEFTVALSD
IPWMCYVREFRARLSESTLDVPPTIGNEKELLLSADTLTKHSLKTFVESNSLCGTMDAGP
FKKCVEWACWHHETRFQSLVGAQATEFRAALAKENLKCVLEGNVFVDSGEPTAAQRGDVW
IVGSDSYTRDRIHAAYTPVTRGSVMSHDGSQWCLQPDRFAEEILLSVFCDECKRARTADP
DDPHWMRGTDGSLLCSETGLYCWKFVRHGLTLVYSTVPGFAKQRSRKKMHATLTHGNGGI
VSDKPVDSVGAKKKECGRSPSLNHSGPFLAVGRSSVVGLSRNSLSFENAFVGSHYESPSQ
WGAASYGEGGDVSCAHLSSEQDLKNPIRSFNSCELRNAVLSPPGPVSHPVDVRVPTIFSS
QSTQLSVTAPSIASRQQKSCLGSFSQAASFQADAPKIIKQTEHCLEAHGKGTDGSTSGGG
LRAYDREGKYRWEWGRRFK*
>TCRU_373 hypothetical protein, conserved
MQIVDGQHFSGGVQRSEALEDSATALKHSMQMGRFFAYYTHPHRLPLSQSAEEPMCEKLP
RDEAMLAPGDFAFLPNPTYCGRTTVDKVLYKHCLSLDILL*
>TCRU_374 Mucin-like glycoprotein, putative
MTTCRLLCALLVLALCCCPSVCVTATGEGSDKDNSSLSPAQSEGAGGSGAKNTSTTSSST
GPTDVLPRPQKQDSEGQASDETTDTGKGTGGVDVTSGITGDSAEQPRIDNHTQQGTSSEK
DTVKKKGEEETSGTTRDTVQETGKGPAPPETPDKATPEHIPKEGAGASDGSGSSGGSVSP
AAASSSVTAPVPAQIQKESAPTTTTTTTTKAPTTTTTTTTEAPTTTTTTRAPSLLRESDG
SPSSSAWVCAPLLLAVSALAYTTLG*
>TCRU_375 hypothetical protein, conserved
MVCVRCRCWAAAICDCAVAVRGVAAVSLLLLPLCVDGELVCAEGCRQVTGVMAMMMTGRV
LLVCALCVLWCGAGGGGCDETTQASPSGSGGGTVVGTGGGPSGGADNGLVASEPNGESEG
DRLEGTDLGQINSSPRGGTVGLDNKLAVMPVGSPVTRNQVLSNEALSLQSPPSPKPSAPE
LQDLPEGPPVSGGSQGGEADNAVGGVGPSGSSSGSGVKSMVSASFSACTLSPPNPVDWDV
LQGGKGTTATLSMDSSEKSVKSQSAEPPAPAQPNLDRQEASTSNAEDHSPVKPHTAGNQV
VNGGATGKENTQRAAVTGGPSSDTRSTTLQAAPQEQPKTELIIVPAGKERATDPRTREKE
ERDSKKAPPPPPASSAGGTPAAKELQISVEEMHQTSPSEAQKLLLGQETPSKDDAEDQNT
EGTATSDSVKDEAASSLVETTAPPISTSGGADTKSVASEDADNAQQPNHKEAHKDPEPQN
TSPAFTATEALPQTAIPLTTAPTNDTTTPGDSDGSTAVSHTTSPPLLLLVVACAAAAAVV

AA*
>TCRU_376 hypothetical protein, conserved
MLDDYVAKKNEKLHDAETKEHHLSSLVDQVLNYLRNGVRMTKISSKGNARRRLYFLSEDC
KRIHSCELDHQGFPLNRKRPPVTIWIRDIRKVLIGLYTTSFVNYSGEAQLAKTRQEAVSD
NGTYRHDPTQNITPSNLGINNYRAFALLLRGGKSLEVVCETDSDCEAWLVALKRLLHLRT
PAERLLEERRGT*
>TCRU_377 hypothetical protein, conserved
MRERHSIEAVRRTVREERCRQRRQWTHQIKEMNAKFPEPVRPLAEERKKKREQAKAKEDA
AERALAADIKMIEEYLPKLISLEDIPVNPEETGIIRRQFDEVFTQ*
>TCRU_378 conserved protein
MHVEAEDEVEGGGVSSTMHSTLASTEGDDFIKRDLEVLDALIKEGYSVTGAVQARFINNY
DPMYDKPYTHYELNTGRYLNEELSQLLEDDRRKNTKHHLQTDKIEKFYMLALLAHSAILR
H*
>TCRU_379 glutaminyl-tRNA synthetase, putative
MTAPSTTAASTAVPFLEVKKDLRLLQTGRPVAGYSNTPELLEKHHALTGGKPYFRFPPEP
NGFLHIGHAKSMNLNFGCAVAHGGKCYLRYDDTNPETEEQVYIDAIQEMVRWMGWKPDWI
TFSSDYFQQLYEFAIQLIKAGKAYVDHSTADEIKKQRESREESPWRNRSIEENLLHFEYM
RQGRYAEGEATLRVKIDMKSDNPNMRDFIAYRVKFSAHPHAKDKWCIYPSYDYTHCLVDS
LEDIDYSLCTLEFETRRESYFWLLEQLNLWRPFVWEFSRLNVTGSLLSKRKINVLVKKGI
VRGFDDPRLLTLAGMRRRGYTPEAINRFCDHVGITRSMNVIQSSMLEHILRDDLDERTER
RLMIVDPVKVVIDNWDGEMEVMCPNHPRKPDLQSRKVMFTKTFYIDRSDFRTEDNNSKFY
GLAPGPRPVGLKYSGNIICRGYECDAAGRPSLIHVEVDFGRTTKPKTNITWVSEKYAIPV
EFRLYGDLLKDDRAAIDPEFLKYINSDSEQVQQGYAEPEIQNAKVFESVQAERFGYFVVD
PDTRPGHLVMNRVLTLKEDKEKGAMAPCPQK*
>TCRU_380 proliferative cell nuclear antigen (PCNA), putative
MLEAQVQQANLWKRLIECISGLVNEANFDCNPGGLSIQAMDTSHVALVHLLLRDDCFTKY
QCERNSILGLNLASLSKVLKIVEGSDSLTLHHEDDSDVVVLTSENVEKSRKCEYQLKLLE
IEGEAMGIPEMEYGSTVTLSSQEFAKIVRDMSVFGETVTIEIRKEGVKFSSSGDVGEGYA
FLRAAGGAGRTVRTAPEVKKEEDDDTPISRANASGTKNSHTAIGVEVRTDEPVTLSFALR
FMNVFAKGSTLSDRVSLKFAPDSPCMVEFNIDQVGYLRYFLAPKMDDVM*
>TCRU_381 phosphomevalonate kinase protein, putative
MDTAIATTRASSSGKVLILGGYLIVEAPNVGISVGTTARFETRLLKTRDAAKGKCCVRIH
SPQFGKEFAFECTVESTPEPAVCVAQIEGTHSPFLRYSVLYTVAAAISQGGNVFKELTLE
LLADNDFYSQRNYLESQGKEVTAANLRLLPPHLPLVGDVSKTGLGSSAAMTTSMVACLYR
SLTAQSTSDNNKNNNAAKTDTSAEKEIVHRVAQVAHSVAQGKIGSGFDVYTAAYGTCAYR
RFPAKFVESVMSTNESPSNVVVSTLADCVNMEKEWVKRVPFHLPLGLKLLLGDVHQGGSG
TPGMVSKVMAWRNSVADNPYNLWEQLRENNEKYVASLKALILQAEEKTVEHVNSVDVLKH
VVLAQHIPQNDAERLWVEAATYASKSRRYLREMGQAASVEIEPNKLTALLDATCAIPGVF
AVGCPGAGGYDAVFALVFGDETCTSVESFWEEYTAMRVCPLLVREDAAGLLFSEEK*
>TCRU_382 hypothetical protein
MATQCVAECTGYRSLRADCARDPCLAEGMCKLLDAGITDCAEWCCVSRGGYVFFIVFFFS
AGTCALLSMYYLWRLHRANVAAGAVTEMGEKIEAAQASDAHATAEKLRKKRQVTVDPELF
RAIGLPPPGEERSGVGGFR*
>TCRU_383 ribosomal protein S6, putative
MTAEISEKAFPLAGDRLTQTILDLVQEASNAKMIKKGANEATKALNRGIADLIVLAGDTN
PIEILLHLPLLCEDKNVPYVFVPSKTALGRAAQVSRNAVALAILQSENSPVSAKVQAVKL
EIERLL*
>TCRU_384 hypothetical protein, conserved
MLRLLPLRLASKVTAGNAKNQAGHPRRKAKLFHVIPGTPVTPMEKLKEQRRRYGQDRHSR
LPEYRPGKNVRLDPNTFTLYATTKGVMTIRESRINPGYKWLDVEPDIQKVYRSSQMRRAL
AARGMTSQMVEKNAHYRSEMDLLLEPHWRQRVMGVPKAAERFKDPNLFVRGVITELTPMD
RYCYE*
>TCRU_385 nucleobase/nucleoside transporter 8.1, putative
MALGFSSWAEFYVYTTCITLGVSMLMPMNALTSAPRYMVDYYRYVSGDPHAKANVPIFWA

NIFTFYNVVSLVTQIIFGPTVLTHLARKFSLSNRFIFALTCMMLEVIVVLLLPTGKVSQN
SAIVAFIIVSIVAGAGKSYLEATCYALAGTMPPKFMSAIMFGCGFSGLVASTMQCIIKAT
MENTYDSVLAQAYIYFSLALGIMFAALLMALSLRYNSFAQKHVAEYRMLKRATDGETPSA
EATAYGNVEPIDKAVEKDADSGKAAGENLSCRNENGAPVIVQSEMTTSEQLLTTPVFPVI
KKIYPMQIACFCVFLSLIIFPSLVIPIDRDDEWFATIAILCYNGGDALGRFLTSFRKLWI
SRRKTLYLSFVRFLYIPLIFLCVFHQIPGHVAPCIFMFTIGLTNYLGALTMVYGPGTPEL
KTDGERVMAGQLMGIALLGGASFASLIAIVVVLFLP*
>TCRU_386 40S ribosomal protein S4, putative
MTKKHLKRLYAPKDWMLSKLTGVFAPRPRAGPHKLRECMTLMIIIRNRLKYALNAAEAQM
ILRQGLVCVDGKPRKDTKYPVGFMDVVEIPRTGDRFRILYDVKGRFALVKVGEAEGNIKL
LKVENVYTSTGRIPVAMTHDGHRIRYPDPRTHRGDTLVYNLKEKKVVDLIKSSNGKVVMV
TGGANRGRIGEIMSIERHPGAFDIARLKDAAGHEFATRASNIFVIGKDMQSVPVTLPKQQ
GLRINVIQEREEKLIAAEARKNMQTRGVRKARK*
>TCRU_binding domain containing protein, putative Thiamin
pyrophosphokinase, vitamin .1
MSRMAGVWKRKVVQHDFYDAAGQVAAVVLMNSPSNGRWEYEEYLRLLRVRRFNNQQPESS
VGRCYFLCADGAFSRLQAYIEEERKHRPELRPFSHVPFCDAVIGDMDSCKNPPHNYGTHG
SVDGAGFSLSAPTEGYATVDDIPTSVLDQIHDRYTFTVAEFTRMSTSMEGASLAEWDKLD
AKGRDELLSLPFWLYVRCQSTTDFMKALLLLNRLREKHKEDIVPITPHVLRTESGKRLLE
TGGAIIPNAGSIGLDLDPARERELCAEACKEEAVLLPTYVCMGALGGRFDHEMASISVML
SASTRAHVVLINSNNTIFACQFNGWTQLVRNARCEGVTCGLINYGTMKECETSGLQWNIA
VGRGKPSESKDLVLGFGKLISACNSIRREVVTIDLRRQLLSTRSQGGSNTKQAPANERDD
EVDEDHNPPTIFSIERCDGDENKFGHSC*
>TCRU_388 aminopeptidase, putative
MAKIRNVLPDDPTPRHYKIIILPDFDAFLFTGHVDIQITAKNLQNSITLNYNELSFVKVT
LALAGNSSVVETIPIESIILDAVEMKATFPLQKPFIGEAVLSIDYKGEINDKLAGFYRSK
YIVKGKECYMGTTQFEAVDARRAIPCWDEPAVKAVFEMVITAPSNMMVLSNMPHLYKEEV
NGQTSWAFAPTPKMSTYLLAWTIGEFECIEQSIKKTHGVRNGQPEDTLVRVFTTEGNKSK
ASFALDVACKVLPLYEEFFESSYILPKVDLLAIPDFAAGAMENWGLITFRETALLCDENS
AASHRQHVALVVAHELAHQWFGNLVTMQWWKELWLNESFATYMEYWSVNKLFPGWHVFTQ
FVHDEIARAFELDSLRSSHPVEVDVQNAKEIDDIFDAISYSKGGGIVRMVVNFIGEDVFQ
KGMASYLKHFAYGNATTEDLWKFLGKAAGKPLAPILEFWTGKQGYPFLTVSSLRDKQSLL
ITQHRFLATGDAGEGEDETVWKIPLLITTPENGVQREVLEERKNSVPVPHPSWVKVNNDQ
SAFCRVLYEDEELLQNLLSALSTKKLSNIDRFSIFSDYHAFTRAGYCSAVKVLKLLSYYK
DEDDLTVWLSIVDFETKLKVIVASQGEEAINAHNAYFRTLYSNAIKRLGYAFESVDDHNV
IQLRAALFARLVAAEDEETIAYALKLYAERQKTPIPSDLRAAVFTAFVKRNGRAAFDEVK
ALAETASDAMERTHYLRALAFSGVDGLVAELFEYAVSGKVRSQDTFYVLSSLACNTKTFK
AYAMELRRMWPILAMRLPGLILGRALKLLEYGAEETVANEMEVFWNELNEKEQMGMSRSF
RQGVEGMRNNAAWASRDARDVTEFYSKAWV*
>TCRU_389 BNR repeat-like domain/Concanavalin A-like lectin/
glucanases superfamily, putative
MNGEVFAVAEAWCEKDSVIFPCIASQFMKLDDKTPKALEATEVRTEVLEECPTDGGNCAS
KTEDKDDSQGGTRVHVSRPTTVVKGNDIYMLTGVCMSKGLSNCQVGVAGVAQWGLLLVKG
NVSGVDESTKRIYWNDTISILCNAIGDNDDTLTGHIGGGGSGAKMEDGTLVFATEAVKEN
RVNGTAEDGGNNNVSLLISSSDAANWTLSKGMSDGGCSDPSVVGWKDKTLIMMTACDDGR
RRVYEIGDNGRPWTEALGTLSRVWGSNRKPHEKGVGSGFITATIENRDVMLVALPVYSEK
LEKGGAQKGELHLWLTDNTHIVDIGPVSGEGDDAAASSLLYKGGKSGEANNNEELIALYE
KKKGAGGKPSPGVVSVLLTEQLQRVKEVVATWNKVDQSVSQLCPPSAEKDRPTENDCSGV
KITDGLVGFLSGNFSENTWRDEYLGVNATVSNNEGAAKATLHAGGVTFKGAWVEWPVGSQ
GENQLYHFANHNLTLVATVSIDKVPTTEGPIPLMGVKMSDAENPVLLGLSYNNEMKWKLL
QSGGKTEELSGTWEADAKYQVAIVLQNGTQGSIYVNGKLVWGCAM*
>TCRU_390 Leishmanolysin, putative
MLTWCCAWQSAVGGGALDDCRGGCEEPRDSDDIDGMELQDGDGDGRTLESHWSQRHARDE
WMAPIGSAGCRTELTLAAPADLGCMRVKWEMAEPMRW*

>TCRU_391 hypothetical protein, conserved
MRDSFAAPSLVRAGGVTIAFAEGHAEYKGSHYRLNVRPSDIVAGYINAAETWPLIAAENN
KDKWRVRTLFSTVSEEDLFGVACNPTAIAKDNKVLLLVGSYNM*
>TCRU_392 BNR repeat-like domain containing protein, putative
MEGGTLVFTLRARNGENRSFSRITYSTDNGNTWVFPEGISPANCTDPRITEWEGSLLMIV
DCENDQRVYESRDMGTTWTTAAGKLPGVWTKSKSEDYPEGVLRVEAPITANIEGVKVMLY
TWKGYFSGEKKGKCVPSLGHGQQPLVFCWTGCHG*
>TCRU_393 hypothetical protein, conserved
MINYVLKSWAQLDSSFSESFTPTAGLVGFLSNASSGGDTWIDDYLCVNAIVTRNAMKAKD
GLKFTGLGPGQYGP*
>TCRU_394 hypothetical protein, conserved
MQRRSRRQQHAARQRMPPPPRVRPEGTLQLLRSEGSLRVAQRRAHNPLADSAHYTGHWPR
SVCVVEDTAAESLFLSGGRTPVFTLFPTQVDGCAMAPQQNEAAVFTMEGQYLWCYALGM*
>TCRU_395 p-nitrophenylphosphatase, putative
MSTRTILTAGEAAEVLASVRYVLLDVDGVLWAGKHVLPNIPETLSYLRLREKQIRFLTNN
ASISRAGLVREFQRRGIQGVREGEVYNSGFAAALRLQSLFTANKSTGSGRPLVERNIFVI
GEEGLHEELRRVLAPGYIAYGMELHDAEKCGGYDAHVVASAWKQRVLPAPLQSSATSCGI
AATGHAAGGISISDLSPAAVVVGLDMHFNMLKLAYASLCLQERPAAQTAGTSSSTPTYFI
ATNEDPQIPVGEDFLLLPGAGGMVSALRTVSGRSPDFVCGKPHVDMAKVLFEAEGITDPQ
QCLMVGDRLTTDIAFGNAAGCKTMLVLSGAEKMDRVRQAERDGHVSLLPDFIAPSLAIFL
PPA*
>TCRU_396 aminopeptidase, putative
MQYWHPTHCTLQLNFVQSASQRDDGRALLLAGDVYSGRSTIRYVRRSFSSATMHEEFPAL
RVENKDGLHFHAITASTHAFPKARPLVLHSIKVHQQYNDAETLKVLQVVESEFNKDDNGG
AESGEQHSVTSKGGNPRGKKAKSKVHDANETERQGSHDSYDKQQGRSDPGSKHVFFEGAE
SMPAGSIVTLEIAFTGSIQGWDQGGIYVNDNPEIVKAAAANYGVLLTHFEVALARLAFPC
PDDSQCYRLIWHLQSLQLPSSYSVVVSNTAKQSEKAMGQKGTQHTFFPVGPLPAYVLAFA
AFSGSVQVVEETLFLRGPLTPGAEGRNSRLNTVEKMVPLRVLAATLSGVTLDTLRQIADT
VREAVNLLEDFFASPLPLQQMPFSNEFEWQEDVLTIVVAPTMPYISGMEHHCCIFLNEAI
YRSFSRDGTVRGTVRRPMTEANGTHEVSRVMLIVHELAHHWVGNTLGMPFVLKEGICLLL
EQCFGDVIVGKPMRSIKPSGDKMMSDTAVPSRRVVSRIKKASTPPVIVVDTEKGKEFTEY
SYQKALNTLRDVVSGMGFNDFKERMRRIYQTEVMGCKENIGMIDGRDCGDLFLAPYITTK
QFLKYMEASRVTV*
>TCRU_397 3,2-trans-enoyl-CoA isomerase, mitochondrial precursor, putative
MRRVIASCLCAQRLAQCATTSTAVLAFSASIRRQGTRQGAPTGVPPELRAPPQGSRPSHH
EHQQTEMNAAPEENFEPPQKPAEPPKMLKIDTNEKGITTVQLSRAPVNSLSLELFEELNK
WMLWLGSDEECKSVILTSSIPTVFSAGLDINEMFNPQPERLRRFWQAFQETWLILNSFPK
PIIAAINGNSPAAGCILSLGSDFRVMARHPSGKPDRLYRIGMNETKLGITAPAWVIPAYA
YVVGSRNAERMLQLGETPTADEALRIGLVDAVVEEEQLREAAVREAERFMAIPQQSRWMS
RDMMRREFLQHIATDEDREYDTQFVVELMMNPEVQKGLGAYLARLKGQHVKK*
>TCRU_398 hypothetical protein, conserved
MPTGNETSILSFLRAGTLAGCPYVEQVASTVRKVTVPPAESATSLSSPLSPSLSSSSCLW
PLAFCNFENCRQEKTLLWATALRHKAITAEAKKLGLDALQRGEVRRRAKLFFHVEGRHKC
GPNDTASDAEKHTLADILREEVDGCRAWTDVANGTRFQMSFLLGGGDMVGVAKEMSALEA
TMSGHGDTSQANRIRLLVCAVAVVSAMLNHFCPLRWVITASTGVPHVWDQAIALVEQLLN
DEAIVSFFMAEALASAIMSISSSDVTMRRYDVLLKEKRKRMLACILPLQHVLGGGEAAPL
SIAGVAKNTLHLRTETQLLDLKRGLQKELRERLAIASNLDQRSVDRARQFKEACELCRAT
VKENQTDGRHPILQKKGLTALSRTDILPGRCVQLSDVPGENKLRTETKRKLSCETDTSTH
EAKVTSSCWERTVLEVWRARREAVLGVLREYWIVDNGSLRRKGIEKKTETKEKAGPLWMT
DALLGTLSRQLLALIGVQGQNLISLLDTLWRWIGCQRDALDEGMYTALAVELVVFLLVAK
YYSSRADNLLSELMAILKHLAGIRVRDDFSSPLLQKVPDALAVVLFDAVFFSISTGISCF
SEGNSDGSRDRRLSSPVSTLLWEIQSSVTLRHAVSILQGGSSRQLPTGSGGVNPLSLTPS
LGDTEEMTLLTDVARHGRCGGFALNVSPRHDVCFLVTLTELLGTSVFAHLKEIPIRTKRP

PPRFLSEALWNALLPLTSGLLHHIQRMHIENGSKAVRRFLEFSRALVEELTPRWEYNIVP
TRRLQKKGVAIFNYTAVEQEYEANHVVEDRVVKFTSLLEWLLMGASEFFPELLTDGHSFS
RMCNAVGLILYDVDGRTGHKIMTHLLRLVPPKEKMDIEAYIAAIRCLSSMRHGCTLWEEA
VKYYREAVHFMEDDANAKDAMNSHRRPHVPADLSTARVLRCIGSSPLPHVPRCTLTLQIV
AFARASGVPLSLQSYSSCLLNFTLRKNPPTKAFFVAPWCDALEWYDDVMSRSEQVPLPKQ
DPLCFQVACLQSLLAQGDWPEALFSLPLADIVRQHDDDAAELNLSQFSGKVSFLLRLCAS
DRTRSCAHAAKSLLYVFQTLPSLKPGTQAANLEDMQKLCHVANTYLPVVSEPKLTNGAPL
FRHETPLIPRISLELEFEWARSLEPFGFSFCHFYHIQDSLMEKLEAAILSRLHRRKSDTD
GRDGKDSDQPSAIIRVERVSCTFPLLQTVWSAEEFLKKTKEADGVVRGGDDVRSRMDTC
YASVMLPLRRLERIRAEKRRGMRRRDPSLRQDRGGRRETGRNE*
>TCRU_399 hypothetical protein, conserved
MMRFSRCLGAPVFCRIIDVMRQNEPYARPGILYPIRVELGNNVEKCTSRELQEIIDNMLS
RSQRFATVKTGMVYNSDQSVATEIKAYGRYVVTGNMRGGYIKGDHRFGPRLTGYGGATTK
PGLRWNGDDL*
>TCRU_400 Trans-sialidase of Trypanosoma hydrophobic C-terminal, putative
MPVSGEHFPPNIDLPLMGQVDTADEDSPRIGNTDDQAPHSVSPDVSESVGTNSGPDSFSS
TNVSGGADAAPAPPSTAPGETKIPSESNATIPSDHDILLEFRELAAMALIGDSTVHVCVS
RVLLLLLGLWGTAALC*
>TCRU_401 RNase H, putative
MQKTYATLARIVSGVPSTVDPESALLEANMPPLHVLCLRARLSIFENTRACQTDWMRRPP
PEPPPRADFRISPLSRDELYAFVDAYTKDYGITESSPREERFFRSSIPPWFAASAHRVTI
GVELPIDFSITDVEELIREQRGVSEEALALHSHRSWILATDGGVDVPKSAGVGILLSSLN
SSEIIEKASVNCGARPCSYRTESRALLLALEKLMIPRIRHRRKTLLVVTDSQSLLAALNK
GPLSQTDWTEDQIWQRLLTLTRAGWSVHLQFCYGHCGVHATEIADQHATQTMESGQYTEQ
GIAPLWHTDLLTCFTTQLTNKWRSTIRQDTHRYLLCGTRPSDLSGKDLITQEVLHRQELV
HLARARCGESELWGRLCWAVRDCTNQCRFCNISPEQSAYMRSNKDPTAPGTDTVPPTARD
EDVSPVRRRTLTRRRKEKCPHCDSTLTGFSGLVSHCRSFHPEHPPPLPELKCDFCNMVFP
TRRSTAQHRSRCTHNPDATRHRNSSARRRSLLPQDQPASTSTPIGPQETLHHLLLECPGT
LAVRQRLGIEQDLRLGKFSQWQLLHSRKLLSLLDHLFGTQMALYS*
>TCRU_402 retrotransposon hot spot protein, RHS3, putative
MNEKDTITPPARGKPNGVLTQVPTEARREAEERLRQTQEMKFSISTNIEEVLFKGRVRVN
EMKLNDFLTKEPDGRGVVDTNRDVLLEEFFKDPTKYVRDKGALNEMQASGHYLGMKRAVK
GEVIFDEDIRRLCDRVSTIYRGGHWLLRR*
>TCRU_403 retrotransposon hot spot protein, RHS3, putative
MTVVGLRMATAGGRHTTASTVRRFTECLAAYFNGWEELSRDMSWEIIYVQQTDSTPRNGW
QRCDVENSNNVSDESREIRRFGRKRCASTKCQYRTETFEETKLSEVCKK*
>TCRU_404 Mitochondrial carrier protein, putative
MKCGAGGISATIAGVAVYPGEVVRLRLMSGERQFNGIFNTIRCIYAETHSLRNFYRGLGA
SLMQRVPDILVSFAVYETMKYRLIEKDFLSSHPSYKNIVSTVAGGGAAALASILVAFPLD
VAKRRIGMSGKGKSGVVYAGVRDCLRQVYAQEGIRGLYAGARVEAIRCVPQVVLMWFFIE
GFQSFLTGHCVH*
>TCRU_405 proteasome regulatory non-ATP-ase subunit 9
MEAKAQAYLEKLSNSLQAAGRTATADVLKRAKEEFERSLWCEMTDDLLLAVRDPLVLESA
YELHENVLKVTRMDMSPTAYVKLLHYICFCPNSTLQKALDLLNGAVVALSTSSEQGENMA
NCIRALLLLDSYNDKSSPAGTAASEASRILDKVESFIHSKQMHEVEPVLAALHCQARGKD
YEIRQQYTKYYSNAFETVKSAERAEMPIIESDLLSLAYKAALSALLSEEIYNFGKFLNER
HFTERLADSVQHAWILEWLRLCNDGKVEEFEQYAVAHKAEMAASPILSESFPSITKKVRL
MALLHLVFYTPFNERTFTFDVIARRCAVDEERVEPLLLAALAQGIIMGKIDGLTQEVHIT
WVEPRVLSLQEVKELAVHVSRWKEKTLGLMQCMKEAAQETHN*
>TCRU_406 SPRY domain/Ankyrin repeats (3 copies), putative
MAAEPGSQIPRFRVGNATISNKRWLENFAALKEETFTTTLPTTTNDDPNFFLETLDVFSC
TEEEFLNGLAIIKEFALRSKMKIMKRQLKKGYARRKMEAMQEELLFYRIAVVRACTTLRE
VIDRVKRRLCELRRDEALLHEYYQALDTDIERGEREVLPKEGSYHLRQRELRRVAPRQRY

REDRTFVEARHEKLNALLHELEEEEIAIQRVNEENGATDLDREYGICDPKRGKLEDIYDT
TAGTEAEAGSSNLELRLRIRPLLEKVRELRIVPPLSPRSVQSRLRKELLLPFSARVLALK
IDDDNNAGERFVEWYVNAAIDPLAMAQLVELVPREYLVTTTVRLVNACDTYLPLRQTQAL
PDVLHVLLQAIAEGRFDDLASLVLSPSDSYLHALESREHAGIIFYAALITGQMEVLRHLM
SVGFYNTNALIVWLFLNPNRHDPNYMAYVTEMRSNIKKYMRSVAHQAQDNTNAESLRGEL
EEDYMDPTLVTDSMQNNENFLSVNRDLCETLRRFLQERMRWKKQVVSRTDKVTMDTDKNE
KGKLSAENPPVTKLELEEDEDAQAATRVGSSSAASGVSFSLILGKKRLDRLQISSVKCAV
LPAVTTSFMRRVDRWGYTPLKATDSHFLLVGDTSTANAHVIQLPPRHADLWPDDDPTVSP
HVRWQRERKEAAAKAHGLTFYYEVHVSLNFLLSGVPVSTSVFNGFSASLERTPETMPGLQ
TSTVFVGWCSEECTSAADGRSTHPPLGSDRNSIGISFDILHRSKVNQLVEEVIIRPMKHE
RGRNAVYQVLDEDAQEKKEVEDKERDSVDLPFPLLRILLENNNEGNDVGFVSPTNGPFSS
PIYEASASAQTNETLEEAHMLSAAYAAKESIVPTVEVLRKKGNGSTSDSEESNSIHWNLA
FSGNSVVLRGVPVYNSQSLVVGCHVDFGAHSASFTVNGKSLGIAFTTLPSPVGLRPAVSL
QASNRITEVLQRVKPSAGVIVRFVFDESHLLASQTVTTEMRNTFLLNSSPATRVGTPDES
VETCREFIPGVFPLKLPLLFDENMMHCALIRLVTCGESALLSGHDSHELIQAERETLSLG
HTLRVTASGVRQLLNAKSDKEDLLFFPPVLLKRRSDDTNKGAIGVAIIPEDVSLVPLCIA
LTQRQRVAAYRIAVHPFTDFVSQDDASKRQRRMALLGSATLGYTEVLYALLERMSISETL
NSFTLKMVGNEPEYFLTAKQMRVFSPGNSRQCTSATMLHRVEYTPLHCALLEKHQECVHL
LLYYLSRLLPAEHKRHAVNVLTRSGETALLMACRFGYTKIAMRLLAMGASPSSFDRVTRT
NCLELACASRSEEIAAALLQTSHYRSQVAVNLAGVAPPICWCALNNIGSLIPPLLKNGAN
LNVTLDGLTPLLLAVTFGSQEAALKLLECCDAVSNVSNYKNSSTRTIQHRSPKRETFHED
PAEEGESRFLAVLDVDALDPLTQSSALHLACELGQLEVTRGLIAKGASLNLQNKTKYATP
LQVAIMNRNEHLALELLEYAKDKLRRGSNVLDISAIEKNGNTALHLAAREGMLQVIEYII
FQFSDEEITRISCIHKFLKRPDAVNIVEKNRQGMTPLLVAIHAHQEAAAQLIASLMPESL
PNPGGPVIDGTCVAILSSDAESLDNLTLYFLSQPRYFANDAFREEFFTRYNAKRREMDLC
EDGAPAKVEGIKSVLYHDGEKRRQQKRKAREIQESRRRTRYQKKTSVSSALLWRTSLTPE
SYAGKLGDQTGTQKAPPSRRRSTLHTRISRTGRHRRRSTFVQMLLAKGGSGFTTLASIRI
LEQGFPLEEVYVMADIISTETTHSSQTVSAMQHVETLISFLREYGGVRIATGDAVVFSRQ
LLATPSLERMMDSDQLENAKKRLAEMRPLCRDLMSIIRSYGQRADCVEDLRHMVEQHGVI
GTAFVEEVTSPFGFTVLQLAATLGLSHVCEFFLNEYEMSPLYVPATGSIPCSESKWFLSP
FRLALRSADVDTVSVFLSLGLRLGSIQEILEHKELPKAEELQQTAIQELICQPVDTLPAG
TTIDTLHILRLLLQSGAVLQGNFDAEGNDAWMLAVRACPGKGTKLQVFLDEQVPLGEEEE
DDVVEEESSQEVTRQSSLFMKRDSVQRGESNSGMWTTISPDAKEGLKLPLAVPFVEAIAT
SSWMDSPFRLEGALEFPRMSPKKRVYYTQLVFLCAEYNPQSLIALLEQYSYVLSPSVIHP
VTGDTLPIFLLRRAVHIYSVECGSHAFFQGNASLGELFFSTRCFSLDVDVSTRVSEKDPA
PYRDPITIPAVRRLLLVVERLLRKFRFDNLYYEDSDGQTALSLAARLSYTNIVSLIVQQR
VSRTAGGAGARNGGEEEFSSISRLNTANASSDRCAINEDLVRNLEASSCWAILATRLYYA
EEEMDLLLAILRHLPAASTKFSFLSMVYSNLHPIVALRVAVLYANDVKHILMQPEAINAF
WSNVLYAQFVGRLEEVTVTPAEWTSLLSVLLPAASAIPIYLIKSTPLLPMSSASDISSPT
TQSPLSFTDIGMSSPVKNEKTETSPSYNWRFIVKKTRQYVRQLAAAAVCVVSPPSAIAAA
YGGGAIPSSQRHVLTIMSSHFHELIELAVRFDNSAMLMDLMSLSPAELQACVKHEWRYLM
EKYHIGVIAIAAGSINVLKAISDVPDAAALLSAEQYERIDPNTGLSEDAGVADEAAPENQ
NTYTGTFSIRGTPPPIRGDRKNSTFGIHAARKTPNKIAGLNENKGNTKKARENKSAMVNI
LALDAKTPDPGVEGEKGDVDKTETNLRYMDVMTAARERQTASAVTWGIGPAARPVSLQER
TKSIDRTASHTQASASFSTRKKMSDGTTDIGAPLRSHAQRGTESTLSTASVQRKTLRKSV
SLEGTKSLNLSLRQGGTPFETTPPPLPPRYFAYYLCDWALHTTLLLHAPRPSSRTIDTLL
YLMDQRAPLTTSAVQLFLAASRPLNCWESSDGEMITLNYVTRKNKDTILHILVQNGQLRL
TRYFLAAALFYFTYYQYDPPSTMPPNFALIDMPKLPQKTLSPELRGAMKTGDVDALDDDE
SHPAVFLRSMLRMNKHGLTPFDYARGPMVSLLMEYGCVPPTYRPNPRGFCRAIRLPFPAS
SFYSVPRILLVSMNFIELHDEGIPKPRNHIAELLAKEVADATLVRNQAASQRTTRTAFLP
PLLSGNVSLLHLGLCSADDELVLQHINEKRRQQLRILLLSQMPRTSPTPYMMTQMSTSTL
GYQRQDKLVHSSVSGTKDLNGQKSSTTRGKDEKELSKALKLVDTLCDCGFVLFPLVLPLD
VEASQIIEEDAVNEGTAILLTMTPMALAVSAGVVKKDEHSQSSSPMMKRSLKSPDIFRRA
SKDGTAAVPPRFTPAPGKNRLPADTLDAWVASRRLARSNASGKDPHMHLLVRAIGSGLVG

RTIHHLGLSTAPSYESHK*
>TCRU_407 WD domain, G-beta repeat, putative
MSGEAEGKNGASEETATPHHTNTVDHSNAVTRKRPRVACLRQNFSRQLYQMMIRQLHHDG
YLGAAAAVADATGVVVPRLEKDSQRLSKLVSMGLAHEASNITEMENFMTYEVVERYLGAS
KLYVPLHLTESSTVGQMAYRMRERFTTSSLGGVVRRVNISPDGSLAACGGNNGLCVVFSL
KTMEDLMALEEVRQENQFRGLDGSGGSGNVGVRNASNKITELAEARRFHEHTQSVEAMHF
HPSRPLLLTGGREGDLYLRDYSQPNNKVLHKLHDTFPIRSAVFHPSGEYILYATDHTAPR
LLNLRTWKLMTPNTVSGNENNAAATNIITGRSGMSNRGGFYRRGTDDSHTAALCDVDFSL
DGRLLASCSLDGSLIVYDGVSSRPVAKVNNAHSSVPVTSVKFSRTGNFILSAGMDSVARL
WDLRRSDGGCGTAEVMSFGEPGKCNHRSIHAAFSCNESHVLLQDTSLFAIHSYCVYSSDK
SYSLVVPNHMQRGFAVAPFCNVVVSGGDDCRVRLWTPAWAAA*
>TCRU_408 hypothetical protein, conserved
MAGLTSDNISIASARQYGFSRERDLCVYCGKNSKPPPTDPFAPVFVFFSLVDCRHYVCQP
CALVNCDNAGRYIRCPTCHSISRLAQTGKKRTGPDELRVTIDDGVSSAASHASRRSVRVS
ADNVPARSALAKADRSKKRSASVQFSANPTTSVVPPPEDSENKKRRDASVTSPLTRDAVG
NLPKDPSYRQRERMKEVVTRSNTRETVAVNLYTIKSSSVTRRRSSGSRASSVPLPPTEHR
YLAPPIPFAPPPPLRIRTVEEEEEEVGEKDVQQDVHVSLSAIAAEIEESLLAAVAKEAQE
RSTISMAEEYRRNAMSKQREIREKDMLAAQVDITSVLDLGVSPRTPLDVSEGQVSATSDD
ETPASLLKRHPVAPLLYDEEEKQPVSVIASDTTIQPEGTQTTEVVLQKLEDTENSIAHEK
LEPKEEVEELKQRDTQLLQHQLESLQSEIISAMEISFGIGEIEVRERQMIEGVEGMERRL
LHSSRVQAEVEISAFEAQRLSANRAAQAATNYIAYISKEFDEYASSEMEHRTFIEDMQRS
DWSTLERDFHASLKRFFEVELLRLTEKHQMELTRASITLTTEETKARMWLEQNALDDLTR
LHMNSIEALAECRRQLQMKEMQRGVSRIVAVQQLYMQHLYDLAVEEDNKRWKILEEEQRD
RHAEWTSFITTVPTLRTATASPSSVTSASVRGALSSSHAPEYSRTAATASLSVASQYFAA
LVEEAELEALQLQHMERHERHALINLFLAQSQRLKVCEAPMDTTYSDTPLSQRAIANSWR
RLELREEERRIELQQRSWASFRSIVKKATEEHEQIDRMERMLRSIAERSRSVLNRCFGEV
ELALMDEEKHERQNIEVQERRARMNHSREAVRHEEVLLAGERLLHDASGTRFLDAMDVAV
RQFSSEAVRIAQEEHLERLSLVQAERFECLSLTAPLETQARRSKKDAVEKLSELHSTIEK
TTAEGAWRARLALVETEELRVRDGIYNDEAGNRSWWVTDERRQRMELTSLETERLRRAKE
VARLRDKMGECESVEEQRRIGVQREEASAWATLVREQIRDAANAYKKEGQRRIEETRRER
YADRSQRCRRALEELLQDEDDDRHYIFEDEREARRRLRRQALQLSCDILEKKKIEENIPS
QPVLASQGIGHGPVNDPSAVTVDEEKIRQKRLKELAREEELIEAQTRLREAELRIAEEAE
LRARSEEEKRAALAESVRLLREAEQRAEERIRRARQEAERYVEQEVSRLRDANERQATHA
AALRAIEEEEKSAVVEAKLRAAEHALREAERRAADDVRRAKAEAEAFAAAEAQRVALEAK
RQLQEYREQQEQQLRQKDLEVQQRLRGIEQQAQEAILAAKVEASRLAEEAKRRLEELEAR
RVERAAAVDAAKVSVRAAQQRMREFEESRESTPQQPPLAWPVETAASTAEAATAAVLKGP
KEEGKDGYTLTAVLCTNVIRAAIRGAVEEVVKATKEAWYLSEEAEHRLRTERRRMRRRQA
ERDRAEKELINVEKKSILWGEEEEQEEEEEKESKHRVNTGDDKEAFSTPLKQSTRRQYHK
KREEKQSVRLQPPADDGCGVCYRKDTVSPCVKCDSQVCLYCGPAAVHSACCEEHHINVIN
AHRRHLSGEPQKEKHETKEVERENGDDSSLWEVEEAVAVPRKDEVGRSPLGVRRKLLPDD
YDEKEEKEDQESEAKEVKSPGSVEEPRTVRKRYEAFFVPLSRDAEPRRPKPPTPRNYVPG
VFAAKSKQKKAERRCSPPVRRARANRHMSAAAAEELQQLQRRFPRNGLKEHLAAAHNNQK
QQRQEYPRQPFASLNPQFREPPPVHADSRLRRREAERVVMEDPLSAFGRAPSPSHLGVHF
PAKTRRPSRRMMGRISPFEASIELAKGFDDFAHRDPTGLERTYYDGKPVRPIKRDYMSDD
LADLSNRSSVRRQHQEQYRSRAMEFREEEGHVNVSRERVFVTSYHEVPLYMGTQRHHESL
PYNSHQREECNVRTTPTLHELDQRLRHLRQHRLFDRVSQRSPDRHCSRHHFNQEGEIYCG
CHNAGSSKIRRPTPCSHDPCITVHLFPDTRIPTTAAGPMKMTRGGSSRRNTSRTDRRSTL
RVVSPPWRTELNSGPLRPSWSFEQPHSFITKSYKRTLLPVEP*
>TCRU_409 hypothetical protein, conserved
MLGLSGVQQQQPYHQHPSQQQFLRAKGRRCLYCQQQGCPGLQNCWWPRLRSLGSRPQRKV
ESDFPQHFWWDGDVRSEEKNVNAMEENLRAQIRVLEHEVQLLRAGLHNIASVNRRGKKDV
ATMATPVVHLSTDYPSDLLFSHSVKSSGSGLSSPSHPVVSSLPGGILQQQQHLREEHQQQ
KPVKGVGCVDFSHPEGKVATGVADVSASPPLQGDPESKDVEGLDHLLRELTAQNVALCRR
LAEMKIVLEQRDTLLQSILAEKLKLNATHASSSVQAFASAQLTGQPKELFSHLQEAQEEV

AHLREETRQLKKALKGCKANLEENNAETEELRRQIRPPTLFSKDSNTIAATPPILSTDGN
APSLPTAAAAAAEFMTKEPTVIVVNDSTPVPHENTATDAASMKAEMRDLHERLALAERKV
LAWESWYREQTQQPSSKEKDSRKITQEGTDVFNEENVFPMIQKSPQRASKKVLRALPVTA
PASHVNWRNVSSWQKEMVSDALTIDTYALMAREAALRQVAEQERAHVLAALMTDSASDDD
QQE*
>TCRU_410 hypothetical protein, conserved
MSTASPVSSRSTYDEEMEFLMNPVAAASQAEETPRQKSTELMSQEQSQTHYAVNRAVTEA
MTMEHAVRVPHALQAATRRTMLDVLTQMWRQKQNDLANCSGEYLAEKPLCNGLTEEKPHD
NLTVADEAASFLLADTTNMGANNASTLKSVLSRSLRHLMPSHRALPGELEPQQYTHEQSM
REDSISDFDDDNNVGGCLRQVPQRVLMERHSEDGRFLQRHSWNSAVSEEATRRHTEHKRT
WAKLFDCDDLHQEELTGHEDRTTMLPAEKCTRASRTSTKSSRPSASSFKDLWTESEGGPR
QTTVSRTSLATLLNEAEAKDDETYAQSMAAAFSASNSDGKSCRFTDVLANEEAEEGRMPS
CHLVGKPMFSTRLPTHGNSPSVPTAFAVLHGSTRRVGSRGGTSAVLKQLSENPISARVVA
YLTRSFEDWKLDAALIRRANEEDEPHNLSFSGPSLLETSGVQLKLLVKVVESAFSLYCIR
CEVVWADAQASKELFFDAHTADSMISMQKEHEGEGGGNEAGVGHKMKKPPVTWTVVLTAA
TFRAIPIVVGSHLYLARPFFCYPSIRVIVASLNITSDAAVQRLQPTARRQRRPRDADSSE
PKTGGRAQSGEFCGEELSFGATTPERRNRPSASLTGVSPFRAYDPFVNPPTNLFCKNSVA
LGPSNPVSERILRRSASMPRKTTMNLESDACGQLLASRSQMVAHDVIGAQFPRGPGEEWD
VPLEVLVALCPPRKRTSSGEPEAHRRRTNNLSLTPQRTCNEKYSPLSSLSSIDVEEPSPL
AETQARGGVPL*
>TCRU_411 CHCH domain containing protein, putative
MANGVTANSMRVNVKAPDLGAFPLDHYSECKSQVEAYYRCLKSNEYVTPLCRDQMREYLQ
CRMDRGLMKPADLDGFGIPKTEFVPTRQHRIDLKQQWLRQKMNQVTVVWEENYRRDDLQV
PDGYEREKGGGDGGGGNPKPVGEKVNT*
>TCRU_412 actin related protein 2, putative
MSTEKRPVVLDNGSGFIKCGYAGANFPTAVFQTALGRPVLRSTKKRSHGSGGGEEGHTHV
DPMMRDIVFGDECSGVRHLLDMTYPIRNGIIQNMDEMCHLWDYTFGEMLQINPSEHRLLL
SEVPLFSSKHRCHLYEVMFEKYNFMAIQSAVQGTLSLFSNGLQTGVAVESGEGISHCTPI
FEGYALPKANRRVDLGGRNITEFLIRLMQRRGYSFNQSSDYETVRRMKERFCYAAVDSKL
EKQLALETTVLEQAFLLPDGSSCSIGQERFEATEALFQPHLIDVECDGLSTQLWNCIQAA
EIDVRAQLYSHVVLSGGSTMFPGLPSRIEKDMRAMFLEKALKGDRSRLGHFKLQIEDPPR
RKYMVFLGGATLAALTVDQPEMWLTQAEWQEGGVSAVRARFGA*
>TCRU_413 Fcf1, putative
MGRKKRVASHAIKSILMKEKSLAQKKKREMDGYQDTPEPVLSQVKDEAAVRFQRNQLTVA
APMQTNMFLSFNKSLGPPFHIWLDTNFINFSMQNKIEIVEGLMDCMLAKVIPCVCDCVMA
ELEKLGKKFRIALKIARDKRFRRLTCDGKYADDCVVRTVTRHPIYIVATCDQELKRRLRK
IPGVPIMYISKHRYTIERLPEVYGAPG*
>TCRU_414 hypothetical protein, conserved
MLIKTRRVRCPLGPATNIGTVRRCLTTIAAGASVLGAPSLKSTSVALSPSQWMRSSLQEQ
RRHRSFSFYLNLDDPAETRRRVEEDIARIDQEHQRGTMMHLHALLNLALSCYQSGEYIAA
LEFAEHVHNKTREYNKNATLLFFTAKTCSRCALAVADEYESHLREAAERASVSSTLAPRP
SVVFSAERTIAKLREDAARYDGIAQRIYNRPDKAFMRNWRPTSTGWSEDACGMDEEPEEF
FGGRWKERRKRPEHAAIRQYYQQQQQSNGGGVGVPK*
>TCRU_415 Kinesin motor domain containing protein, putative
MSHRPDSTGGGEDRGGAAAPSRTGVGTRQRETAGLDHTPISNVVSLQSAIQKEGLTEKIF
FVVIDVTDGRRYKVFLRPDDISRLKVSKIRKTLSSTSGKPPFSFVLWLGDEPLIDKEDVN
CAALGITPRTVMRMCPTKEEMPHPIGGSEGNGGVDRKREARDALDNRKSSVSIADKIMEQ
ILDDPLETLCLVELNQRQRELQFQLYKEAKRCHIFSKDDAVAGIGRHRDDENDDALDALL
SPPQTAKNPQMCSVSSARIPGEPICEDDSLMGFIEEILNEGPKRQDGPRPTPPSVECCPA
MPDERQSCAAAAAAAKQPSLTPIGSNNNISASEIISHGMRLNEMYSLEDTGEFVEPPRFL
NRDASSERKNSGENDKGKNDALPIKFGAAIAVPIATQGEEREVTRLVEDLRDDVNNLLAL
HVRNETAKRALQRELSSSQQLVRRLEKKEAVTRQQLDALLLRLRHAQEANGIQGFSEEEV
RVALRNMAALRQNELLQRENAALEQRLAEEVRRRREMVLALEDMKGHIRVLVRVRPPFRL
LHEQYVGMEGNNPMSVRDTVDESRMTADERRNTITITDSRGESKQFNFYRVFSESSTQAE

VFVEVLPLVQLACDGVNVSVLAYGQTGSGKTYTILGGDGNQSNTEAKGDEGNKENDDDGI
VPRSLRMLFQHLQVEAATEAVVMPREDDNCNDDERASSTYEVSCSLLELYNDKVRDLLAP
PQVIIPNFQLRKSNVSEQRGKTQVTPQCNLKIGSDGLTHVVGLTQHVVCDAGEALRTLRE
GASRRQVHATQQNCHSSRSHLIFTVYLTQKRRIPFRNDASTTRASSTFLRQMESKLVFVD
LAGSERISKSQSVGDRLLEARHINKSLAALGDVVATLSNATISNTAAVHVPYRNSTLTGL
LQDVIGNRSKTVLLACVGPSDPPYENNTAETVTTLKFASRVRCVRNFAPLFPTTGATGDG
DTMKGPGTDNLEPDVDPSHISSSLGRGAKKLWMRPTKLSKQRTNIAARFRLVSANEAEKT
EDGGRTRTPAPSWPPKGNNPHAEQRPRMRF*
>TCRU_416 hypothetical protein, conserved
MLRCASRVCRAASLLGYPLRRAAAMEMLYGGVRVQHLAQSPFSLRKTRSEALPSPSLQRE
RDDLELEVKDSANNVMGLRLFPINIGMRARNEAVRLRSEDCYKRLLAQRQCAEVGEALKF
PVPSSLSAGEHKDATASRQLYATHFHPPSSRLSILTRPVDCSDCKVKTTPSDVALYHPRA
WRPLQMLKPMPHNWSPTLRSSGVRGPHMQLMQERLDGKGFGWKRKSRFLWQQDIDTAGFR
PKRFF*
>TCRU_417 RNA recognition motif. (a.k.a. RRM, RBD, or RNP domain)/
RNA recognition motif (a.k.a. RRM, RBD, or RNP domain), putative
MNANLFYTQQRPEHQHHGPQQQQQQPAGSGFLLPAAPFTAAGPGATNVFAGFCMPPSAPC
PQQQQQQRQEQGQTLESNVAAANGLLLATQLEAKDGRRGTASFSGAIPDVSNSIWATQEP
NSSTTTALQSLNASNSINYPPFGIKYMVSPTNASASTQETPNQTYLYPVSMQSEEAAPAL
MLLPTGSKDGSSSMSPAYYVLVNSPQASGAIHYTNGTMQPPHPTSFILGANSSTKTTTTN
SFNAAQQPSPFRMPVGQQTQGVSSNLGISWPPQFASNMATTTTGVFPGLIENPRPPQVDS
AAFPSHGQRAYYTSAVFTQSLNSTPSPTLCGHRRPPSGSGPAEGGTMRSRSSRNSGTSIN
SIGAGGSGGQEMERRGGLPFEEDPIPIDTTKTQLIVNFLPQFLTDDGFRELFTPFGEIHT
KPTKIIYDRGARRGKGYGFVYYKDGRSTEAAIKTINGLSLGGRRLKVRYADQQRGKIDCD
SCVDEEEKE*
>TCRU_418 hypothetical protein, conserved
MVSQLTSMLSLRHLSGKSNVANCTETREPKRLSEREEEERTAGFDVGLPYAHVLAAERAT
LSAASVCLCGPSTRLLVSLMKGAAISHAARVGSAIHITDAGVTLTHLTVHQRHRCAMALT
KVLEVLKASFVAVSAQEEEKRKEDDESSLRQIRSAWNKSNQVFFLLLCGAAAMSEEKDEL
TAISMFKNALQQVRRYFGPTSGVASEPLYLLAMAYNESERMELHPSEVSTPVSEGGNEYL
LASANGATDSAQQTFSVAWQVASQARRLALLHCVLALQEGGSKENHRHIVGEWNMEVHRV
CLLMRRELMEVVSL*
>TCRU_419 peroxisome assembly protein, putative
MFESNLLSLINTASPLPTFVEVDFVNSLNNSLSKAFQFAHVLLAEKSDIAAMCLPWDAEI
WLVLHSLLEHRLLFHANTTFSEMLFGLRRGCIASPSKPSGSQGRLSWWVRGPLPAPSLEE
KALQAPDAAASPAALGAISESPLPQRNGELTAAEMQAAQGASYGHLRFIPITNRQRYITL
FLITVMPYLRERAAKWYTHRMDSSPEANSMRIAYATRYPTRARIKELLTRLYPLVYTALR
SLRFFYQILFLLELTPYTTPLHRLFGIVLRRLTRADQIAASNPRAQRALMLARVLVLLIF
LGFRLLELTGAGNAAGGSTALTLTGSGGEDLATPPPPVWGVDVVIPPGTKVPQPGICPVC
ERRVTNAAVCTASGVVGCYPCLLGYARTNGACPLTKCRTTVDCIRRIYEC*
>TCRU_420 hypothetical protein, conserved
MASIRGGVGGFLIRRAAVKSVRQKYQTGPQFNKRKFFQFPKGYHRLHLRIGGVQLGSPTQ
QREHTRFSHLPGDTRTRPQYDFTFGERRADGALYAWRKRGNLQLYQMGGKPETFVCYRCG
YPVRSQLVAIKGDNWDYRMCYKCYTTTVHHGMENDT*
>TCRU_421 RNA binding protein, putative
MSYAGGTYYANQEADSYYYDQQQQMAEEENTIDFRPPDITEHQDKIIQLVAKYVVFSCDG
ARYQHLLIRRTKFNSYFAFVASPEHKYHEYYQYLIRSYIVWRDMAAATSAGEGNTDEGYA
FYTEQLRQQEQQQQQQQYQANIYYTASTDYSTQYTDPTAAAAMTTTGGYYDANTAYSLQQ
KQNQNTSVLGESSAVVETSRGDDVSRKRARSATPQESSDEEDDAGRELVFVMENGVARAL
PK*
>TCRU_422 protein kinase, putative
MLRAPASNDPFGDRLLPAPVQETVYSVDWSHITSPHGIIYIGSGAFSTVRLAWLQERRKC
YKRILTSNGEKTRTTVFLGACAAVAVKRVMIPRANDGSCKYRRLLLLRELQVMERIRMYP
HPNIVQCWGFLFGKGEEEQTFESWNPSGDGTENSTDGKVLTSLNKEGHRMAFNEENNDGD

ERTDQQIPEGTSYVDLCLSLCTGGTLRNYVALKASEVPHAATAGTECSASESQMVNVAMA
FEKVSFDHFSGAQGITGSTNEDTSPYGCEDYDAYHSYPKQILGEGKKIANASSNLPHASV
SISAHLAVPERDIVAIVYALVHALRHTHETLKTLHRDIKPSNVLIFSGVGKAPEYALQPS
SLPIPASGGEPSQERDKTCSSQAKPLSLRVDSEIRSTLGDSDETVEFALFPQCPATTHGS
TGALRQSAGGNLATPTATAGGMSKLNDEPPTTLVVPLGLGYYRLPCTATDSSRRVYIDCL
PQAEGWRIQLADFGVATGVDRLAGSGRCGTPPFMAPEVARHADQGVPEQYTTKADIYSLG
VTLQHTILYLVIEGEEGSPRTCRYADALGAQWIDEDDTATQSRLAFGTGLESNSTETDLK
PNFIVPNAWRCDKELVEGEYIRCGSLETEFPKRGVDDEKEHESKPKGFVVPRAWRCRDEL
LERQYIRRELSPRRNDTDIHAHLLTSLPNELNDFKENSAARERRGSRCSSCGKIHRNLIE
LLNAMTVKKPSQRPNLSTVLQSTAIIERGTFLEKGRQFSYPNNAAWDVGAEDHGNQPSMD
TNSLERGAAAQRFSRARRWAEERNRVRSPPTGFRFDMTEPRQKSSQGKNRGGEDDTTVLL
WRPPSLSFLRASRHKMEAHNR*
>TCRU_423 Pre-rRNA-processing protein PNO1, putative
MSSNVVSAPGAGKEKSASAFVRETRSVKVPHHRFPPLKKVWLEVYTPIVEQCKLEIRMNL
KSRCVELRTCPATMEAAGHGGDSSKVLLQKAEDFVKAVVAGFEVKDAIALLRMDDVYIEG
FEVKDVRANLHGDNLSRCIGRMSGSHGKTKYTIENVTRTRIVIADTHIWLMGTTQNIRVA
RDALCDLIRGSPAAKVYTRLRAVMNRVNDTF*
>TCRU_424 structural maintenance of chromosome 1, putative
MLSHIDRVELYNFKSYAGNVTIGPLKDFTCIVGPNGAGKSNLMDALCFVLSPSATTTLRG
KDATDLIHRGAQRKECAVTAVFCHTTPISPAATATTTAAGQGRDTEISFTRAVDQRGRIT
HKINGEPVDDRKYLAAMSKFNVGTRVNNFLVFQHEVEAIAQKKARELTDLLEQVSGSAEL
REEYNRCKKAHELANQELTTASAEKRDAVVALNQMRLHKKEAEKYEEVLRRIGEERRDEA
LVQLFYVESNLERQKQELHTFTEKLTALEKSIASDEDIRKMKREYAEKHKTYLEELKKIR
KEADTLREKHNTLERIKVSLNHLKRQHEVKRHGLDNLMKTEKVQSREVQRIEEQLQQQKA
ILMAFDERCKKEDEEHMTLSGSLTAEQLSEYRQLRKEAECETVVLRQQMERIKRQQHSLV
EGQKQCTIAIENVRSQKQELQQGVQRSNERVAELKNRMNDLQDTVQELTSNISQKRADLS
QKEKRNREREVELARIQEQLHELRFMKENDKHGSRMAGALQALRALYGVRGRLVDLCTIP
NDKYRHAVTVALGKNLEAVVVDTMETAISCVRYLKEQRLPPMTFLPLDSVKGKEVNDRLR
TFGGTCKPVVDVIRYDTAIETAVQYALGQTLVCNGMAEAKHVAYGSEDGERFKVVTVDGS
VLMRNGAVQGGLASIQSRARKWDEKKYEDLRAARDRLLNDAAGGSEAEMARTQCELRDME
ARLEFTHGRIKVIAAELQATEQKVSNMNREMKNQENEERTIEKRHSTYESELRRCLHELQ
EKHGSIMQVEERIFSEFQRRVNIPNILELESHEAQILRERAEKRQQMQLLVHKLEISLEA
EHKRIGMQSIDDLRGACVRLEEEIQRCEQDLAAYSEIVKTAEKKQSQSRDSVSEAKVQLD
SLEADIRQQSRTSEQELGKLAQARRGVTALQAACDTLRLQRMNILRRCQMEEIMLPLKPV
EARGVKRSRAAESGALTQSEPFVLPEDAPAAAPTKQSQPSQSQRSAATREAQVVVDFSDL
PEPLKEAASDRTHLAAYKQRTETLLENLQRAAESLAPNLKAASRFAGSEDRLGSSSAHLE
EAREKVSKAYSEFSKVKELRTQRFMETFEKIAENVDRVYRELTLSTRAHAVHGSAYLSLE
DVEEPYLAGTRYHATPPLKRYMPMELLSGGERTMAALALLFAVHAVSPTPFFVLDEVDAA
LDAGNVEKLANYMRKNCNTTQFIVISLKDQLYHVADLLVGVLKNKQKETSSILTMDLRGY
PF*
>TCRU_425 hypothetical protein
MSWDALPSVPVRPVALHHALTHDGYFLVDESGLQEDKERTGRSGDRNSTLPIPASLNAIS
DMLLPVTLEEFELAEEVLNACESGHAPSL*
>TCRU_426 hypothetical protein
MLLIAYVRCRGDKADEVMDAKEAFDEIRAHFAMRSQFSEAGNRLSAMVRRFQESLQRQDG
SDIQGVKGARHVQQRPSKSSMQKLPPPIFRPAAVEALLHYASPLPVRTEEGKNVRGGAFI
LYNDKENDMRSNRIGDDYLSHKCVAQPCASGEGAFQGKRDSFSSTPVAFLSSGRNQDQPV
DVGAKFSHNDGFRSRYTHEASAARSSQCILRKEQDMFPFVNGDSDGGTQKPNGCCEERDD
ALHSLGEFDGETMVDCVCHAMQKNKCQKSEIEEEKEKSEEENKKNDELRAKGMEEAPADT
ARKTTRSILADLDELEKTHHDAVAVMLMRTPLRPRPQKEQKLVTSDGGQRLDQHGAPHGI
QHHVHPEQRATAVNLDLDVLSGSQALRCRPPNGPIDLSVSNSGTRVLSNLGQGNVKNVPY
FVKGSSSVLNFSSTSSSDAYDAKDKWLNVSFL*
>TCRU_427 hypothetical protein, conserved
MMRCSRGCRRQLRPYYNLPSKSDHGRKMTGFLTPYRHWMWKQNELWRNVHEAQFEHLRKV

YKRQWLESFRVNADEYIYKYNITKAAQLAQWEYEMQAQEKKRLEAKQMTEGRQALKKKHL
DLLREFHERQFFFWYERASERLQSMNLIQYIPQSGVQEHIERELDKYVAGKIEAYPLNFV
GQMPLVEDREGNIVEVPESLLTNHVSEHPDSTAKPHQPHESTSVSVEEQLLRTMASAREE
SLEEWLEDDSRALSETIDDISREEEQRDEDTRVARSMEETDNEREISRRMYIDRGKTGSK
AISRRPALSETDGGSASGSVGGVTAPPADTSAPMRRRKKGKLDKAHALQEQQDAMIARMS
AKSLKDGESSISIVKRGEIATSRGRIRDKAAIPTQEVLMQKPELAAGSVPNARISFKDKV
DQLYHRGKYKQKKEDDNNPNEDL*
>TCRU_428 hypothetical protein
MGGLAPGSIDDVTDGDFPLGRLHIDGGGTTGSSCYSTAFGEETASIYVDEEPPFPLRSYS
CDGGDFLHIFRPPNQPETGSIHCYSPWTVRGDDEFSLVAHVEVEDEEKGFTLGICGATES
VVGTFLQYFSAGQTMHPMGTQEVRRLCICPWHRRAVLLALLDDRIISCVYLPSAEFCLSR
SGRGREEAGLTQESPTALFEPTPIDVDAWRVVVEHKGRGFVRFYTVERRKGSCYCDAPLS
QLS*
>TCRU_429 acyl transferase-like protein
MKRALVFSGQGAHRQGMSMEYLRVPAVARLWEKMKDQMMQKYGISLQEVMTVNPQKIHVR
DDALDVSVICKRGSLEDRNVAESAPRKKFFSSQEGVMSLTFLTQPCMLAAHMLALEYLRS
TRGYSVESALVIAGHSLGEFSALCALGIFSPEVAVDLVYKRGVLMDYALEQFSANRDDYV
MYACHPGRAKLCEEDLDVAVDQFHVLVELVARALSATTSFVEVVNYNIDHEQYVVAGDRV
GLSALGKCLDPQFRASVCGPSSSLDHIARTAVSSVWQDKKDGVTMHPNKGPLPDFVTSSV
KKYGVRSTFRRFLRGPDDGYTPSLEELTHLTLQEDGRSGLKKKSWFVPLPVNVPFHSSRL
RRAMDLFLPVVQDAMPEEDVLRSMLGVATKPLEESNSASATSAIEKRPVWLTNLTGSAFR
PLDPEFQRQALEMMQSMNVGEVRHHGRYQTDLVEKAFNDGVKHNSVREMCAAVLAAQLAH
PVQWIDLMDAAVIHAGIREVHEISPVRTTADMFKRTAFCEAGEAGVALEMVIRCLPAEER
FL*
>TCRU_430 peroxidase, putative
MFRLRTVGFGCAGPLCVSGARLLWWGYKEHDLHRLARQRPWWENAAIFAGLLCFIQVTSM
CYYFGNRMYQAWRPSGNTLYVNKAMDTKVFARPDLSVAAVRMVIHYVLSGTQTPFGAPMV
YPEYMRPEAEELMKFIFDLHSEQPMFSIPDLWAMLSAKALARLGGPNISVRRGRPDPPGH
LAVEQLLVETPSLVPEGKRDVLKMKRLLAGQGFSVEEIVGMIGGIRTIGFHDSSGFHTKE
EVKPQMRRHPSLMGPQEDVFHLPETMEKCTLDPYVFGGEYFDLLLDYNWRQRSLIGWRSG
PFRCDEKDRKREITLLDPFSEQAVMRERKRLQAQEQAREAVERAAQKRAQITVPEEERAE
SEMGRVGEAQFIPGVEMQESVDDDTTEAVGSLVVPEFKSPCSSVSMREMDMMLLDDALLT
GWLHRFSTNEIRFYTVFGEVMEKIQNRGYNLNSLYVPT*
>TCRU_431 DNA-repair protein, putative
MNESKQEGKTRNVAAAAAANVDGYLRNASRRVRIPSASGGRGTRRKLAMVTAASVEQAPR
SVVNTNASDAVDLDDEWDEVVLPNANAAAVKKDEGKLKEESSQKPKMDVPVANSCAGSIK
AEALEVEDGEVVRGDEDPSAQSLMEGTRPPLTGTSIQRWRRNEHFQERMPEQQQILAAQR
RSERIRRAAEGMSVLIFALQRCKTLVRESRHPQLLRKLLRVCTVEENGSKSFPFIQAVQR
AREEYQRALDPTVKNPALSPCWVTVGKDTVRNYTSNSIMALLSGITTVFTLEDKVDASIL
SNWAAPISFGHLSNLLSRSHQFLISPDAKLRLPHSLYFSIIFLSVANILDMSCRLVMAIK
KERERQNPVANENGNVSEKPAQMTIFLPRVKRQRKEGTNEEDKLPQKLPSSCFWVEVWCP
QRESFISVNPCKGCTALWGAPYTFSVGGDVAMDVTPRYTTKYSSAFTYRWGRCDSYRFFW
KHLDWNDTREASEVILDAFRKDMTRNTCIQLARERRQLHSLTYAEEIPKTLTALHKHPLF
VLENELARHEGVYPKDNTTIVGSVKGHTVYKRSAVVNLRSRDGWLRVGRCVVSEYEAPYK
VVPPPASRPFGSSSGFFGVWQTKPFEPSPLRSDGSLPLHGKTRWYVLLGKPVPEGLAYIQ
RPNIARVARLVDVEFGHAVMGFQRRRLDERRFSHWEAVLDGIVVREADAANLLHAYDEWR
QLTEEQEATKRRQRANRWWLHFVQRMLAMQRVRQQYLDGASHGYLPLH*
>TCRU_432 replication factor A protein 3, putative
MRSTPYLGGGARATTNNLPPFTGVSPRVSSRSVAMHRGEYVSIVLKPTALNTQRGSLEAL
CVATDEPVEVMGLPSGAEVAQVNEFVCYVNPSSGELEYFQHGTYNDEYDVETYRKLLDLC
PKFPQLF*
>TCRU_433 hypothetical protein, conserved
MESTLQTFLEAYPFRHPENPYEQTRAIWSKLVGWEGESAAGPSPSLCVQPIPLALLQKWK
RYFAFCSRVIFYEMDWRDVFVYMGDVPKAATLSVPRDHVGSDRKAEVGVDADRGEGNGEK

QLETTAGAYHRSTTALIHTLANGCERRFGAYPLRFHYQWLVRWTKMLAQCIEWRLLDISK
RGGSVPLEELQRYRLVFASRARELVGAAAYIRTVLELLEIEEEKELCNTDISLKGLPSLS
LSQNFPLTLTLSMRFTLSRATSVSIEQCFSAHSARVLDSLWADVLCIPWDVLDPNSMPEK
VRRKLLASLNNAEGDTDEIYVLCGNGGCRMRSATLKFILENETLERAGIANNPLLGIVRL
TTAPLEFIEPHQLPNVKHLLETVGYVMLTKIRCHFVEKCYYTLPFLADFVAAGVLLKKAL
VSFSRVSADPTLSKAEESKVVLGMGLTPMLLRGLLEATEDFYASCVDSLLHDYSKRAMKH
FVEFCGGSATVVVSQVTLKLLEVVVEPTLEVLFMLLSHASSSKEECIFIYFIADMLKFVL
NEKMREARRECKIGSRVLLQCRMDLLLLMSYIDEHPLMRRRASLM*
>TCRU_434 LEM3 (ligand-effect modulator 3) family / CDC50 family, putative
MPQGGRGPMVLLKQQRLPAWQPILTPPHVALAFFLLSILFIPLGVFVTLMNKQAKEVTVR
YDHIHRCTITHNTGAFMYEGNNMTFKTGCMTEVSFDITEKLKAPVYLYYELTRFYQNHRR
YSISRNDEQLAGKAVRYLPDTSPLTIPGDIYGISGTPIKYVDGSDLRYKDFLYVPAGLIA
WSIFNDTFTLYTEATDGGTPRKLICNATDFSKGNNLPLNGSESENMCVKKGIAWDTDVEY
KFKAPDLEAKNRFWTAAKELYTGKVPTPELSNDDFFNKGWYAGELGHAIPVTTDEDFMVW
MRPASLPSFRKLHRVINVDLPPGKYVMVIGEHFDVSSFGGTKSFALATLSFLGGKNVWLE
ALYFSLGGFSVVFAVVLLLVHRFSGDRASKAIDELMQK*
>TCRU_435 hypothetical protein, conserved
MKGETAEATDLVKTLLKADEECRELRDKVATLEKTCRVRERDVRAIGATPSDGNGASKKI
KCRTITLPIEVFGELNSKAGDVTLLLREQKKLLKEKKKLEEILRSSAAATEEANGLNTDL
KQRIGYTDGETLLHLKDASDTVAYKNGLKHDILQSLRDRETIQTTLKRQTQHMRGLLADL
VKSKELDEQRERAAQQLEEKRNELEGIREEVYSMRRLLERKEKQLEKEKPVDELLLAQRA
ENERRTVVHKLEREEETLRQNEISIRHRATQIAKLERRIEMIGDALGSEENADEERVDAE
LVEQLRKEILSLCRIHQKCEARQELLDADIADLDRRSTGLIRTAANMRKEMQRIEKAHQK
FTDAQKKELEMEQMAAEQEIGDIEREIEMLRRINSRRKQKAQRA*
>TCRU_436 hypothetical protein, conserved
MGAARSRPAREAPVRYVAKQIPRADRIPVEQQFKKPEFTAQGINRRLEDVNTEPLMYVET
SKDASSTELTDHTAPRWYLNTYMEMVDNQRTDQVIISGNLPLSWERDKFEPYALVRGRID
DEDLKWVLAAEQRKKGVDELLPSTKLERNVLQDILDTVELPRLQYRNYKGKLHKTIEDAN
THIAARKEQIERAREAEILRKIGYTEQEVLQEEQYLTNRSRGVKSLDDLGASLREKKRRE
RAAQTNEMEDLLEERRTQELEAGEAVFTEEELLEKPEVLGIKPKPYGTRRQVYKHVYAAD
FGKDDRNQAKFQWWLDRTRRIKRAVDTIHGVPVYNERLSANEEQTRKQMREAAEFNFALS
QAQGAKGFTDPRGHYDEFVSVLRKNKDLNDDSRVEVHEDDVLKNDIFRFPHTKGHGPKPP
PRRFTDKGEKPEKVCTDVNRLHSVEEAAQAAQQGVRAAEEVVRQYGLGKEEIEKPVNENM
GDITSPDSDPSSSKGN*
>TCRU_437 TPR repeat/Tetratricopeptide repeat, putative
MLHTVEKRLVTVPVDGELVSQEVYVDASTELDVDPQTGVFRTRAETEELIKEVYRIARDA
LPKELERYNNWMRSGSRSDVSHTPLQHKSLHVVRSGTAASVPQKTEKAEEYRARGNDAMK
QGNLRKAVRCYSEALKYEPSSSTLWSNRAAAMIQLDRGDDALSDAKRAISLDPMNVKAYY
RKASALYLLGARTAAACCVKESVKRFGTNAAWESLGDRIGTMARAVLVDHRWICNDDVEE
GEELCTLKPIKLPCADPSEVLHLWAKAGVPSAILAEEAAYYAARGSEPLVEPYHGVREAQ
KVYDMCADADSEFSLMSRSTDILRWSCLLLGFADTRIPMGRGPWLETIFGDGFIFLGASL
FSSPTHEANVSLCFDQASHDLRVVARRKIGRYETLSSLSALCR*
>TCRU_438 Vesicle coat protein involved in Golgi to plasma membrane transport, putative
MPRAFWSVPMVTLGEFDALEGAKATCVSEYYAYQEVDIFGPHLLRLSAVCGKTFLSARVI
SGPLCPEYKERMKAVVARVLASNESNEESVLMLDPLAGGIAGMSFCLEDILARGEKRRFC
IILTHPQSAELVRQWPLVSLFLNILLEEWSTATKKRLALEYSIFPDLERCREEARKRSMR
SLMCLISQNTESEEEAFKRVHSFFERFFPALFGVEPLRVTDSVLEPTSVNDIKQRLEVEL
PLVNIVGIDLDSPPGEKGCIAVGNGRILAVGEKFVVKPLAVWLLEFMQKHIGGLQETEML
LSALLSGIQILVYGCDSVQCANLALSLSQILPRPLRRLAVFAEEYLMPYENRIISFSERL
CQRQQLDHKNEIENELSVTDTVCVNVSSEGYILSVHEYDERVHAFAGTRRYSSQNKEEVP
IPSIVTRIVELFYLELSSETNLRTFRLLQFQIECLVNEYVMRGQVYSRLFREHEIRCNQG
LLGIGNMNVNNDSLLNTLSRSVRQFVGGSSLRRWYLEPSASSSSSERRRISKGDTSLFLN

SLGRIYLNAWVTTSSDHNVLLFLGSVSGVM*
>TCRU_439 Tob55, putative
MNNQENASVFSQEVNITEKDIEKVMDLPIRTHVRIRGIQRIHPRVISRDVEAIKRSRTIT
EITNNMSEAKSRWVQMGVFNSVNFNLEPTYDGEANDVCVHIDVEESKPKKSFGIFTTETV
VPELTISLENILGGRYTLSGNYIPPTSRTHAASFSILSNVPFLGQVGEYYIGKRTENKIY
HPASSERIEEIRATAKNEKRGFFSEFSVGFQRRLLVAKGKEHLNSDLLNDFSTTFKGYMR
HELALSNTGYHADPFLSNMYPLPIQGNQISIKNELAGGPFGGQFTFLKTEIQAAKYWPLG
PFCSFQWNTKLAGIWSYRQARIPLNDRLFLSNCHVRGFKSVGPSTLDIIQKEGGRFAATG
GNALWATSISVNFPFIFFPHNGLAAMHLFANLGNLKMIRYQSEFLDIYQWLRSSAASVGF
GVVVTRIPLFGVAPSGRFELNFSIPVGIDRSGNVTWRNGNKNLFEHVKFGLTWSSAFSL*
>TCRU_440 dihydrolipoamide dehydrogenase, putative
MLHRSHFWGLKKVDVCVIGGGPAGIATALRAVDYKKKACIVEANRIGGADLWNGALQSKT
LWEMSKFVRLMTGQTASRFMKPLMPLPPIDSSNAQKAIKNASELRYAQIVHQLKIVDIDV
IEGYGSFMSPQSVDVQLASGGTERVEADYFVIACGAQPRKHVSVTWDGKVVFTSDDIMNR
AFPKSIVIIGAGVIGCEFASIFANFGMTKVNVIEKSNRILPMEDDDVAEFVQKLLENKGV
TFHHHSALESNKVKNGQFHYTLRNLIDNKLTTHIVDNALVSIGREPNLSGLGLENIGVGI
QRGKLERDQFNRVKPHKHIYACGDVATKFALVNVGELEGRSCIDHIYHPHPEGEFVQRLD
NLSTIMFLDQEVAAVGLNEQQCRKQNIAYKMARYGYEFVARAVAMGNTRGFVKLIVTNDR
EMQVLGVRAIGPHASSVIELASLAIHNKETLYNLSELRTAYPTVTQGFQECVNMLLGTSL
LKPNVFPQLVLQEWSPPNFDRGRAYQGDRI*
>TCRU_441 N-terminal glutamine amidase, putative
MTHALPSLETVRRDVGSGDKSTRSRLEDVFNRLTRDRVPYASHYCEENTYKLIEILYAEY
AVPDDAVFAVFVSNERKQTPVWMQRLAEADGRDPVLWDYHVIALMTDTSGNWWVWDHDTV
LSYPCSAKRYIMESFRPEMQMREEYRQWFRVISGRKYLASFSSDRSHMKTSGVPPPPWPI
IQGSLAATTMQLPLYWNMAKQDDKKANMDGSMGDAYGNVVNIEGMMALVSND*
>TCRU_442 ATP-dependent DEAD/H RNA helicase, putative
MSEETGVGSAPATRKKRVRHHRPKRKGSTAVEAVDDSHRSLEEDAEDVAVEKTTEVPAAA
TKEEGAGRRVKKARPELPSQQEENAEEEKQERSVNPDDATRAPKQRERAPCVSDYKALNL
NPHIVTALENDFKFGELTGIQGRCVPAALSGRDLLAEAKTGSGKTLAFLIPIVEIISRAG
FRPKNGTAAVIIGPTRELCLQIEGVLLKLLKHFNGSITFLCCIGGQSRNQEAFKLVNGMM
IVIASPGRLLDHLKLTTDWLTKNLLVLAVDEADRVLDNGFEEDMREIVSLLPKKRQTFLF
SATQTTRVEQLARISFYRPPLLISMKRREEKATVDTLEQGYVMCPSEQRLLVLYHFVKRN
LKKKMIVFFSSRNAVSFHCELFNYIDVPCVAFHGKQKQHQRSATYMQFCNAPSGVLFTTD
VAARGLDIPHVDWIVQFDPPDDPVKYVHRVGRTARAGRCGNALMFLLPQEKLFLKYLYDD
AHVTVSEYTFDLSKVKQGVCEQLEQLVSTNYYLRTSARQAYEGYLLSYSSSQLKNVFDIH
NLDLAAVAKGFALSEPPPLKLDLSQSAAHMSKKSRREFRHMKETKDKRRRAVNEETLKKR
KSNISGEW*
>TCRU_443 hypothetical protein, conserved
MKRARSRRGEADQSVSSPRRRVEAHASEDVSEGQSKHRQRDDDTGLLTAAQRESRGFAYV
RDASENTSLPMETPGAMEGEPTAPLPSVKGAVPSNADEEGEEFVSPFGARSLTRGFSHLR
ESISFALPRLRGSEHRVSISRGGSVLLPLSTPPHESASGSSISSSHSLRVSRGNNQHAQQ
RQQQQQQQHHQQQQQRRRKYHNHRVNGSDQSFLSTRSAIEYLNSTGSLGGVLSSRESRRS
SQATGSHHSHSKSTLGPIDLSPILKERKRQRRRAAPRGGVEANALNGGNNNSGGGGDFST
RRPPLNGLPIEVPAAMMNPADIEVRNSRSLALEAFHHSMARYSRATNVFLITCLFVFLWG
LLAAPLLIQQTGPEDVFVRHYVDSVSELHFIYEVPYMSLSFHEARRLYLRVLSETLERLE
RATSHMRPPDTDVKAQILYYKQMIRAYLALRHRARLYARSRDRSRLNQFVFNPLRDVWYY
GILRNGFKMTLYELILEPSFSLFLARVKDIALCLRQDEKLPCPTLAYLEEREKKQRQWEG
VGETQPDILTDDAVKDSSLFHEEKAVEELRRLNRTLNYVKESCRQSNREYNHLYTIDNTQ
SMLW*
>TCRU_444 ras-like small GTPase, putative
MEVTPCNLVLLGSEGVGKSAILQQFLKKTFRPDYVPTTLETCVHVVSVDGNTYRLHFCDC
SGSRGFSVHRAPYIVEAHGVMLVYSTTSRNSLVHLLGCANEVFCTRRQHGVKGKIPFILV
GTRADIVGRRVVTINDANRVAQGCLLALGLKIFKEKKKIGYKGGCNNGGMEVDPINDLPV
LEISGANTHEVSQVVYTMIRMVRYFRATSRLPRFSLICPGVVPRRYVVVETNLDAHSGGA

SSSTSPDGRFTPALSDNSQATSHRHAFHMSPVGSDEWSASYSPRSVGVTQSVRSSGVEED
RAIVHPYLQQVEDREVIFSLPPRISSDASVNVSASTKTQLSSFALDGPSERRAPKVGPTD
RQCPRCHFM*
>TCRU_445 MORN repeat containing protein
MLTVTEDKIPVPLIPDTELCDVRIGATDPALFFCINLDSPCQVKIEFQEFNTAARCVLLL
SSTDPRPSIHTAVWKCLSPEQKKTVTLLPADPSYRVGPLYLAVRYVEKSGNTFVRLNLTL
QEQYEAEWYSQSNGALYYGMWSGFCYHGRGRCIYGVEPELMEKGTLKWTGRSLRQQSNSL
SWQFDSTPPIEDGKLAINRKDLGWSILPLPSPKLELYDGEWVHGKKEGLGAYQWADRAYW
GMWKGGGREGLGVLCTRDGYRYEGEWLNDKRDGVGNAFYPDGTQYMGGWKKDKRSGEGLF
NYTNGIVIRGTWEEDVLCPTVTATYADGSSYVGEWAHDCRHGKGRHIDAIGNVFDGMWDM
DKRTGNGTLQFINNVICVALWEEDVRKDGTFNFPNGEAYVGDWNDEKYIREGQGKCTYPN
GDFYVGSWKNDKRHGFGKFVYSGEKCVYEGVLCTRDGYRYEGEWLNDKRDGVGNAFYPDG
TQYMGGWKKDKRSGEGLFNYTNGIVIRGTWEEDVLCPTVTATYADGSSYVGEWAHDCRHG
KGRHIDAIGNVFDGMWDMDKRTGNGTLQFINNVICVALWEEDVRKDGTFNFPNGEAYVGD
WNDEKYIREGQGKCTYPNGDVYEGSWKNDKRHGFGKFVYSGEKCVYEGEWKEGKRHGIGT
QETEEGTYQGEFQDDMRYGHGLHLGRAGSMHRGIWRDNGCIGPGVRFDSKKGIMYDGLFL
LGKLQSVGTSRTENDIYEGTWCDGERHGVGLVSLPNGDVVRHLWHRGVPQDGHVIYMYRD
GDKYEGEWKDGRRCGKGTQWYADGSVYVGEWLDDKRHGSGSYTDVRGETLVGEWCGGERM
DVKGSLRFLDGTVYEGEMRLGKPHGNGRLKYPDGTVFEGRFSNGVYLL*
>TCRU_446 hypothetical protein
MDDAGLARGMDGDVVFARRDCVSSAALRGVAAAVPAGEDGGIGASGTLNSDAGQPPVGDA
GSGTEAQRQWGCTNGFIFCCRYRRCQQYSCLGCWWWCWPKQQPAFVSGVQDDLPAGGSVA
GEREEAGSACCGEEPSMSSVCSQPPQFSAGSATLTVEERDGKKGSASVTNIQSEWTQQER
EGPHVLRTGESARRTPAPAPAEGQTAHATQPSEDSAEHAAAPSSGLTRGASNGDSQEQTP
QLQTATDAEQSVSVVQGVPEWAQEDQGMW*
>TCRU_gamma, conserved domain containing protein, putative
Elongation factor.1
MQFMTANLIGLWLQRMEHVRQYALGVAFMIGEERRHDIVALWVLRGRGMPTIVRDVEDTE
LFDWEEVTDFAAQWERMTDCVCWRGAC*
>TCRU_448 hypothetical protein, conserved
MKKLRIATDPKENVAPTPPKGKPSIAMSRNIEMDLNAFVTKTAPASALRKAVGSKDARKS
ETVDGSGEHVPGKIAGGMNGHNVPGTPKRHQEM*
>TCRU_449 paraflagellar rod component, putative
MTTMQKVDATLARFDRKSPRAAYAQVCEEMGIHPQKSLSLLLSDAPRDWDKHRVLECKNF
ILGPKGCMALVPVLLCSRSLRRVSLPGCHVTDEFVAQLAETLQDHQCIRSVDLSDNPLVT
VYSADPIIKLLRVNKNIVRFSLEGTHIGTNVANLINDLCDKNHEEVANYFADDYFRMKNI
FNYLDGDGSGWVSLRGLVLNAPFPVLQEQLIERVALRRPRKRSDGTISVNTFLELVYYNY
KTDVEIAKRAKEADTEYYNIIVNWKRLLDAMEGDGKKEMNELKKQRRNEEDDDEEDEEES
MTDVPLPGKLPVLPPEDLFRLRIRSHELRPSEAREIVLAAMKLQIDADARCLAEAEEEEA
EAARAAGSDDKKENTEDAASGEQRVVRLSIPCLRRAYRSVLQPEPLPRRFRFLQEHEEDY
IPPMMRAGSRLISISKLSFLDSFRSSGGGSFVSQDESLGINPDETQTRWWGLPPAVVRLA
TKFFNKQVAKMPKRRQSKLVISPRTQRDNAMQKTAIPVTAFLGAKFVTDLETIHPRLLAD
KFLQYSIPIDVSTITLQEIVNCLNEYYVEVSVDKVFTLDQIRNMPVPTVQDIQSSTGNGK
TEMSILDEI*
>TCRU_450 hypothetical protein, conserved
MAAAFAESLQREVDTAIREAASHRGGLRRRVAAACDRIRQQQALARAEALTPLFRADLLR
RGRAIQKNRARLMQALKRFARVLPSGDLCESSGLSNVVVVFRVAQEALRVEEELAYWRQP
LPCEEAWCAVCTAVREGGYRADAPLQLPAATEQQQHRRKERQEAALEHLLFPVCTNVGSG
EEEETSAAAAAAPAIPWIALPCFCTVTHFCRHEQVPVVAAAAKGNSGSQPVNPGASGEED
DAMHDEMAEAERGVSSDGRVTQMVLDATAAHLGRLGVRAGDCSSSGEDEEAAESALEEVR
LRVSLEVAARVTACRHAVESLAAGEDGMLLDGVTTVTAAAPLAVPVMALVLPPTEQLFFC
IAYTIGAAILRDEQNERRAAATTSGTAIVVDDDDDDGGGWKGEWGRWMRQESCIGGADNA
PVGPRLLRYLLEYVFCPAECESQRKGAAALVRPSTVRRWLELALGSVPDKDGAMDAMMTL
YMNRR*

>TCRU_451 clathrin assembly sigma-adaptin protein 3, putative
MIKAVIIINTAGKIRLLNVYEKDIPLGVQQELVRRIHRLISRRSADLCNFVDNFRDWPTP
DTRVVYRHYATLCFVFIVDSSESQLAILDLIQVLVEALDRTFENVCELDLIFHSDRVQLL
LMEMVMGGMVLETSREEILRANTEMQRIGSSKAGTGAAAVTGYH*
>TCRU_452 hypothetical protein
MPPCSACTTHDPTVLHPPTVLTFLRPVLQQTADSLVLFPAPQQQQRVVHPAIDQEVLGAT
PMPSQVEPVLLAQHPAVDLMGVDYLLRMCEESNARTPLRPSVGANSNAAVHDHVNRAGGA
EIITVDSGDDDEGR*
>TCRU_453 hypothetical protein, conserved
MDAAVNKPIANVLLYSDGALYLVGARHNEVKSSLFPVPLTEELQMITSILRNWADVDASF
SASPVSTVGLVGYLSNAGAANTWIDECRCMIATLTNDNNVTNGPQFAGQHCPVNRWEDNN
HCGFVNYDFALVATVAIRETHAGVKSIPLIGAKEAGRTVHFWLSHSHERSLGVMLNANRH
TGFWELNKQ*
>TCRU_454 hypothetical protein, conserved
MAMMMTGRVLLLCALCVLWCGASGRCDVEVVRVADSPANSESGSVAPDKDTDHTVHGGEG
VVSGEQSQKQKVGGQPLVAPQLQAVLQPESTVQQTLFDTEPTDSLSHSQSLQEERQGGSP
DGSLGRLAASHSQEDRKNESIGDQQRKDPPSPAINNDVVSRKSEELTEDTPRRTEIIDAA
RSEERQEPENVTPSLEQTRETSTAAPAITTQTISMQPPEENASSTVKMSEASPQSTGTAN
TNHTTTPGDIDSSTAVSHATPPLLLLLVVACAAAVVAA*
>TCRU_455 hypothetical protein, conserved
MLWSVKIRQFQQNRGGEGHVDKAQTTHTGIRIVHGADAATVHAADLRIVCIGERERSKAV
RNRSWQTRVTVSASSAHTPTAADAARPRCKAHGVHGAISTVQHRHGRHNIELTSVILHGR
PLYLVARAVDDRACIVSYKPKACHVCSDRRIGAVPKVVVKYRPHTPIAERHAVVSSHVQN
VGSAERVVAVHQPAAASHSQRGCHEHGEAETELAGHNLQHAAAIHHNVPKHTRAFVVFGT
RRTPRAVLYGEHGAAANTQVRRNSIHHVHLRHDSAVVHVEDAGQVHDKAALHKYRSARTP
ATHQSRPRHDKPRRQKTDAVQDKRGAVPGFLVARSRRHVLRPANRRAQGRVRQQHTRVQR
HRAMQHDRALQRRHIRHVDEDVRHQVRCALAEATNPQLRAAHKHAVAKRHVQRVSERIHR
AQVHRHSDRSAEHHRVEARRRLPAANRHGLRQRHATHAVLPTATTGTRRRHVERQARLAH
AAGTAAAHAVGERRRQRIEARQRSRTAVARHHAHGGQAGQAVVAAALQHAAAGTVALHGH
SAARDRRPGGKRRAGGSAASNRHGAAAQRHP*
>TCRU_456 hypothetical protein, conserved
MQRRRGVLHARDGVGGPQLMLVQLQERMAWRVVSSLRGARHGCAALAGESRGRRHELRGE
PDADEPRAEHVEDAPLLRGCDIQRRGCCAHVPPQSYAAASPHQHHAHWVHIP*
>TCRU_457 hypothetical protein, conserved
MGYKAGPEILQIITSAIAGVTTVVHPLWAAPPSVRFDVWIDNIRIAGSKSNVTLWEAQVL
RNADRRHATIGEGRESGATQCTFLGVHFDHTQRAVSLSDEFVRSVRAMPALNSLTIAEME
VMASRFLYAAAILCTR*
>TCRU_458 hypothetical protein, conserved
MAMMMAGRVLLVCALCVLWCGAGRCQETLLVSQSEVPGAIEGDRGQPQGSSGFKTPESHA
PGSEASVREVQQEQDPSPPREETQLGTKGGNKVEKAKEKGAAKDAGKEGEVDGKTIELKK
PEDKLTSVEGQQLNNNEGLQPTTLNGPHGVETTTPSREILSVREDQQDLSSSLLPPLHNT
SPGGTPPSTPAAGRADTKSHENDIGKKNSQQSVVGEIGQNNDTGEKAVDSTGEEEGSRLS
VATAEEMSETTTKSDSGGTATTTAVGTDIGTEETPPTNHPSKPSTEGATPPGTTSDGEAA
STNKYDTVSQNAGSTTAATTNATTGDTAKRGDSDSSTAVSHTTSPLLLLVVVACAAAAAT
VVAA*
>TCRU_459 hypothetical protein, conserved
MHAAVVTNVGLVVDAVIVERCVDEKELLCDPAAPKSYFAYGNSQQNSSSQTNSSYPTPRR
FSIASGVSVPVTPIGENSPRSYALFQSVLNDNVHYECHATKRPLQQRYFHMFDVLPFGME
SPSSVFGVTGRHTSRGLVVGVASSGLFVVRPYCSGAPLQLIQYGPDRQRVRKEDVELIAK
VSNSPVVTEGIAAFMDIAKRWKEAVKKAFPLDGEKDAFRQGHAEAGDSSSSNEDVVNLLE
GMELWSLKDLQLPPQLALHDPISPLCVGSRVLARRFCSDVSLHIARVLAVNYDVTYTLEY
ESDGGIDERVLHNDIHLLDASDAEQCSVRDKVTVALHKQLPAVIIVCLGDEEYRIILAQN
PNRSLTITRRQIVFIAPLLKEALYADPLILQWFRDLNPSCSGVVPWKEVRYLILSWESYG
KNLSFQKLGEVQRDLCIHVGRMEPQLLRKVPVQEEDLLLNYAEFEYVILRARNLL*

>TCRU_460 hypothetical protein, conserved
MQRPKNKNTFGESYARQNDVPVTGQNPVSAAFFPQEDAGATGDVFCSRRCHRCKRVFQVK
LIAVIFKYTCWRCGCPVCQNCRTPRPHAADMWACTSCVRPISFIWLFQTTYHGVGLLLRV
LEFCDTRSTRIVKFLFPAAALQCMARRETPAKQTASRCETPKRRLFLVKASDGAGAATGS
SGAQRRVLVEHSPRRLPLRQHPTQRMARAKTAIKRYSPLQEQQQQQVRGYRARTPNGISS
YSPPKRFSLSDYKLSTTTTPSATSNNKKDGAFFAEFQAPTKFAVTAHVERHELSAEVRRP
MDEGSGNRFLRIDATKKVGGRVDIPRIDGTQPSPTPSVSGSRSDSPPPRFPSFSQFLGDR
GSALLLEEQHSPQPQQQDSSGVERKTGGRPHNTSKVFEMREKEVEVVELGACRESRISTW
TVSPNPITPRPSKMGGTTVTGGSSTKKKGICSPPPHLGMISLNASLSMSQRRHLTNGGKP
SAATPVATTAVNNATTARTIMANHSITPVNHGIHSLYPIASHRRTKATTDKIMISNTPMH
FKLHASRGPTTGSSIPARVTTPRPFASVSSTFGGIVPLTERHVGAASFGNHSTTAHYPRT
RMSINFKTDTFRLQRMTSNITSPRFARKPTVHTPTTSGGVPISARAPPSSQVGTRSMITS
PRTARPPEQKKSKIRGNNPPSCSQTPLMRKTSPPNARNVTPRGSHARTPLQRIASSRLLP
SGEAARRYLPTQADACGRELQMKSPPVLMIHLTRQRGTVVPNLTEEKTPRPACIPQPMGK
EQPEQQCHAQPEKPRKE*
>TCRU_461 hypothetical protein, conserved
MFGRSALCLAKRFRYNTKYPSLVSYNKLPWEILNHETPEFHMHVAPHYEQIMTLAASTHV
PHIVGKKHLEMPPEHRLRLLPGMFYMLDGDSIPEGFTANRVLDPTALQYYGRLESLVATV
QAVRMLISDDLRIVCTSVTLQGPLRLPVASYASLASLEAVTNKASASFTLFHFVRPNRPP
SELQLEKYYIHAPRAMALAEFNSTSNTSWEPKLQAPKRSKRVTPLPAYRPPQSYLMGLAE
RLAVVPGSSFGRRSLMWGHWF*
>TCRU_462 hypothetical protein, conserved
MTTETDNFLHATPSSAKSYPTYTPPPPLPSRNSVRTSSTATRRTFHDECDEVIRLIREDT
LAASPSERAIGGVCVSRVSAHEKYRAILDEVEHLNRLLEVTVEERVDVQGELQGKLTQME
ISLRARERELRQRDEELLHLRGAVEALRTQLEALHRTRQTETQTTRAVSGSDSLRDAVVH
RDGGDGVPSAATDAATRKETSTKTTKYIAQRSLRWLKFLLKRCFFIDEGANGKLTQLMNL
LNELEEYTVEPRREAHAMRSLCTQVSNRLVDVVLRGANGRRLLDPDVSDLQTSTERHFSH
DQEEFLRRLVAELKVERDTLKQEAVAIAEENLQLMSELRSREIAAPRTPSPSARVEQLEA
QNAILLAERNRQAVHIDELEQKVRALEDEKNAMHLNLVQQRELLKRHEHVLHFISRHEWG
VRSDSSGGEGSGSGRVHTTVAK*
>TCRU_463 kinetoplastid-specific dual specificity phosphatase,
putative
MNAQPFTAASDLPPPPPPSPLPPPPAFARGGYHSNGVRGSGKLKHNRRHHRFSDKNVVDL
CRLDRSSSSTNDNNTRSNRNSNSHSISRNSNRKNGGDGTPRGCIRAPHSSQPPTPAQKEQ
QQCPSTHGSSSSLLSSCTTVPPYSSLGKISSFSTCGREPPTKILDFLYIGGVRDATNAEF
LRRENIVTILNVSREEYWSVDRSIVIHPFAVDDTTEANIQQFFRPTHVLLEQVRKAYYDA
KQRGASVCPRALVHCQRGKSRSVTIVLAYLIYRNGWTVAEALQYVTRRRPRVEPNLGFID
ALRAYQESMDVEERTRRCSSLGLAVRNLPRDTASSFVHKFFEDHVGCVQEVMMHSSRPNQ
NRNIAVPNTNGVDQGKRGNDDNDPPASFGLSGRRDGNSSSSISVKTCGNDADGNTLCLVF
FSAAECVRLAKLLYMRRPELFNVLGVVDGKELRLTIPSKLIRLSRTAHAHCDDGDNTRSK
KSSQGEEDTPKGDAEKNDAGT*
>TCRU_464 adaptor complex protein (AP) 3 delta subunit 1, putative
MAEATRVASQILFQNSLAEVVRKLRSAKKSEAEVIEQCIAETRKEITSTVQSVKVTAVLK
AVYFSMLGYSAAYGAFNIIEVMADRSFAHKRIGYMAACITFTPKTDVLPLLTALLKRDLA
SANQYEVGFALYCISSICTKDIARDLVVDVVNLLNHPRNYVRKKAVLSLYRIFFEYPDAL
RPTYPRLKEKLDDHSERCDNDPAVRGAVVCILCELARRNPANFLGLAVPFFSMLSTVHSN
WTLIKIVKVFGYFAPHEPRLGKKLVEPIANLICATGAKSVQYECILSVANGMSKVPSLTK
LAAEKIKLFVEDADQNLKYLGLDAMSRMMRENPKLIIDQREVILACLNDMDATIRRKALE
ILQGIVTKKNIVSTINSMMERRVRSPPDEEWSNRVIATVIEVAQTDDYTLLQDFEWYFSI
LLDISLVNLTAYQHGALVQRELVTVLTRVNAVRQFGVQAVSELLCNSTLLNCDATRSTQW
QIIKAAAFLCGEYPYWLLDKRLTCERLLSDRIAAMHPEAQVACVAAVGKIAAFVHQPCPR
HVTLSHGEEELKLPDDPVTDAELRAVILPQQDSEKEKDVGDDDKGKEKYSGLELFRHSIY
PDVQERAHLVFYHVDTNPDIGPTFYVEELLSVAVGAQDAVKPPDDLDLGEPFCTHLPPLL
QPSDSDDDENMDGDEDEFDFLGGDAVELQRRKEEERRGEVAAFYIKDTTLTREELPVDVR

LEKVTAASLHLHGAGLHVPRKTHSINRELLRPSNYVAANPARRRNEEVEEDEATKKFRNV
DVTRALTADEKLPEPIPYTQSLQLRASVGSGKEEARAAEAAAEAAVFDPVVLLEERHLRV
TANVVSCHVHKECTLLTLEVEVSNLASSSSAYDVQLCIKYDEDNFTEQSVQLESLDFSDT
NNSNSNNVHKNDDKKKKRENIDDSNQQQQQKKKGEEKEEEEKGNGGSGVTGVCLAERIKG
SKSVCRKIGIRFISQLPSSLVKPLTFILAYTRGKKPAATTLQLPLFYPYFSKIPKDVPPA
EFSQTILGKFLLAAPMVSGFVDVNLTHVPLAMPLIQQKLRLKPVEIFKDVASFYGVLHAR
KSTAEKAHVAVLLRKDTVEGDKKGITIMVKAHQSCLAESLVQEIASLMIQEAF*
>TCRU_465 Putative zinc finger motif, C2HC5-type/ASCH domain containing protein, putative
MPPKRWNAPSMMSPAETIKEALVRYIKKEIEDNNFLGLVGEELDNMANMLATCGSKREVF
DWCETLMIGEAVAAEVTKRRVDQGPPFDETPAAPTMSAPEGRSADGNRGKGRQLAATGRK
AGKRKGTLLSKAADKSGAAALKPGFVECGCFATTHAFRGNCANCGRIICEQESNETCYFC
GLDPSTCVTYEIKVQEGLIGAAAVAKNQEEYEASVRKRDELLHYAETRAKRTKVIDDQTA
VFLSPKNAWMSPKERQKADEDEALAERRRKVAAMHRAKGAYRVHLDIMNQNVSLGCPEGP
AVESNSLASSESYVSCCAEEGDDGEYNVEEARALPLPSLMQKIWYSLDGSVEKPSTASST
GVTNREESPVKKPTGRRVVELSRRVQVNYYEDDAHLFLEAQKERQEEHSQVESDGLDAAH
DGADAEESTIDETNVAPTGETAVVADISTGVSTRFTPSLTMRINDDGICLSMHQPWAGLL
VSGIKFHEGRVWHTDYRGRLWIHAASTQPHDIKEVEEHYSKFMKPTQQFPKHYPTRVLLG
YVYLVECLDRDTYREMFTEEEIQVDSPFTFICAEAKALPFPLPMNGDHKLFRMDHKVHTA
ARKQLLEIM*
>TCRU_466 phosphoglycerate mutase protein, putative
MAVVHICRHGQDEDNFEGLLNGRRDRPLTQLGREQATALSQKLKERGMTYDIILTSPLQR
ANETARIIGEALSVNVETENELVEREFGVLTGKPMEQIRTHAGENVVQGDRVLYFLSVDG
AETFDECYDRAARVLQRVDANFAGKRVLLVCHGDIGKMLLAVRRKITWREGIMLPYFANT
DVLET*
>TCRU_467 hypothetical protein, conserved
MGSYENVGWKFVEEAVIRDKGYDEEIVLASILQSYFIRQGFVESLTALNEELRELQTMTR
IGTTPMAEASATTETKEVTGRRVRRNGKGIFVNQEELRNDVVSHDDGRKNSNGSGKNSGI
IHVSNDNKNNNTNNNNSVDCKGVETVLESMKKRKRLQLLCLNEQYEDAAASLPQGSVFIV
KLLAMEAMKRARGDQSAALLFLCEKVSPLIADMSDAAMAHQIYVETLATITGTDATGCSE
ASPCRIAREVNESLLDDHEPSALHVLLSWSEWQAMAKAKEGGEGGRQFPFLVGMERP*
>TCRU_468 vesicle-associated membrane protein, putative
MPIKYSCINDGTVLLAEYPPGEQPKLAETMQKVIATVPGKEYRRKTIEDKEGGVNYHYIS
NGEGRIVACVTTNEMRMRTVFAFLEAVEPLVRGNVGPPGTELRNGKKLLQQKMDFYNNPQ
NDKITALNDDINQVVDVMMDNMDKVLARGDRVDTLHEKSNTLADQAEQFKQRSTQLKRSM
CLKNIKLTLMIIGTVGVVILIIVVIACKPNFSNCK*
>TCRU_469 Transmembrane amino acid transporter protein/Tryptophan/tyrosine permease family, putative
MADKVEYLPQANHGAYEKDGDLNSRIQETSENPKEPNCIVKTTRRLIPDGGILSGVCNLA
GSSLGAGILALASAFNSSGIVAGTIYLIAIYLLTVFSMYLLAVTSLKTGIRSYEGMARQL
FGRGGDIFTAVVMFVKCFGACVAYVISVGDVIEAFLSDDSVTGYWRTKSFVRVVNCIVFF
LFMLPLSLPKRINSVRYVSFFAVSFIIYFVIVGILHSVRNGLKHGLRDDLVLFRGGNEGI
RGLGKLMFAYLCQSNMFEVWNEMKPKSTAFRMTLETAISMFLCTVLYWLTGFFGYADFGS
DVTSSILKMFKPMRDAMMFVAYIGIVIKLCVAFSLHILPCRDSLHHLLGWKLDTVAWWKN
AVLCSGVCLVALIAGLFIPNVNIVFGLLGSLTGGFIAFVFPAFFFIYSGGFTYAETGFFL
YTFTYLLLFAGIVVICFGTISTIYGVVK*
>TCRU_470 queuine tRNA-ribosyltransferase, putative
MAKEYPILVVPCKRGGIPCLTPDQARAILNEEERVISVSIFDASEFVDPCKKANQGFAEF
CGLGEFRTILTVRSPHIGMHASAPATDAGLCANHEKGRISLGMDKWKEMVLPLRPEIAIP
PYDSVSINEPQKKRRRTAMTRSAKWATSADSLQGNGNCKLMKPLCAAENNGEFVFLEELC
QNETLQQYTYHVKEAINKGQVMSTAISLPSLLICLKYNVCFIESAIPWILAEKGIGLVFD
FNPVSGVSPARYETQIDLNDDCFTKDIRPLSPDCNCYTCKRHMRAYIHHLLTVQEMNSKT
LLVIHNLTRLIQLVRLYRILDAEDREALLNWLFAQF*
>TCRU_471 structural maintenance of chromosome 3 , putative

MHIKNILISGFRSYRDQSFQVDLSPKNNVIVGKNGSGKSNFFAAVQFVLSEKYTTLTAAE
RKELFHAGSGRPALSIFVEIIFDNSDGRLIIPGRAEEKEVRIRRTLGLKQDEFRVNDRRF
TATEVRQLLESAGFSSSNPYYIVEQGKIVNMANMSDEERCQLIKDVAGTRVYESRRKESE
EILEETSGKYRKIEDSIDQLQKRLTELEAETAELKSLQEIERERKCVEYSIFFLELANAK
ECLQKLDEERSKYVSSLNEQRDAENNTNSSIEIEEKNIRNCAQRIAHLEGEMQTLEREAT
KLNSKKAIAQLDVVDAKNSISRNERERLALQKEVENLEKNAEKVKKDLELSRNNLNQHQR
TTDHKSNELASLEKKLEALLAKRGRRKLFKNKKERDVWLAGEIERNRNTIETHRKEITRI
KKSIEDIDNRIHEEDKNQKEKEAATKKVETKLADHETRRGRAITVRNTLNMERRSLWQKV
NEQEIIVQRLQDEWSRSRHQLERAVRHDTRQGIQSLREVLHELADEKLTNAVHGQLIELI
DVGKGYETAVEVTAGNALFNVVIDSFDVGALILDQINLKKKPGRISFFPLDTCKSEPIRF
DGKEGCSSLMEHISCNPRFKGVVAEVFGKTAIVSSMEEGSKFVKEYNCDAVTMEGDQISR
KGGITGGYLESRNSRLLSFNNEKKLSERLANEKTLLEKLCQEVAVVEQKITDAMNEIESL
RGEASRAENDADADLRDARLHDERKVRLEKHREQLLETRKSLEKGMADANSSLELLQQEA
REDFVSSWGEKEESHLESVITEVDKTREDLSALQLQRVQITTEVQLLEDTLQNITRRLNI
ARDRIRELLWVNTNNQTLTREQGNVDAEISLVSARIETVRQSIAETTNEKMASEEKLEVL
KNKQFASARAVQERRDNDEKKQIQRTLLVQRRDDAMEKIRKLGIVPKDASKYSGQSLGML
MYRLKENNEKLKKYSHVNRKAVDQYSSLMETKNELVGQKEILQNELKSIHDLMEHLDKKK
DEAVERTFKQMQYQFEVVFKEIVATEDCHGELQLVRSAAKKNAGEDPYIGARICVSFGLG
NAITDLGQLSGGQKSLVALALIFAIQRCDPAPFYLFDEIDAALDAEYRSSVAKLILKDSE
NCQFITSTFKTEMLEAADRVLGVFFHNKTSRIQAISLEEGVKLLKQAAFEERKRTREIEE
*
>TCRU_472 hypothetical protein, conserved
MPESYIVRFKVLKVESAALVDGRQYTISYRRGETSRSTPCYIAEGGAVDFSTMPEGAAIV
HFKSGGARFLPKWITFRLEEYIRGRPRKMIGETQLDCAELLGLHSITASARRRVIFSVYG
ASAQMVVALLVYPANHPALSFGDIASTTGSDGQRSKTKNGEFKRMTRGEAMTLLISLEAM
LERRRKEEPKGKEPSNLERCLAELEERRRSLTGVDGMATAVVKKRAEDVVAAQFLALSRK
YRANYAGEVAAYLRQLAVTSGLPLTADISEEASPDAEGARERLQRINARIDTLSGQVRKL
EEEQLSLGRQQNRTDMKDELCGITNKMEALQSQIQLLLKTRTALEETIRGKGHDGTPIGR
EVHDIRQRLRVLTEEEAKLREQVRRMMNVASMHVLKWARSKNPPLEDIQTDLAALFAADA
PSQEAKQEKSTPKVLSSEERGQLVEALSNVAKVSAPPVRSEVSSEMEGPPSRQPQQQVDL
FGATGLPSVGDFGGSATKKGRNESTTFSKTDKDAEKHVDPFISDLKSDMFAPSAVSEHKK
DTSKVHEFNFGATAEEDTGAVGVGGSSEYATFFKDVHDAAMDLGTPPVVDLVSIENDPMY
DFHPPPEAVSTKPFTIEFEESPLYHGLDGDAATNPPAFTFEPEGHQSTNSNTHALPTYSF
GPDSATKNSRTANAPRLPTYDFGS*
>TCRU_473 Leishmanolysin, putative
MSHWGRRNAKDELMASFVVSGYYTAITMALFTDLGYYKANFEMAEPMSWGSGAGCALLTD
KCVTDGATLYPDMFCTDSSDATLRCTSDRQSLGTCAIYQDDNIPPEFQYFATKQGSRRGA
MMEYCPFIAPMNGTGCMDGDEAIMPGSVVGPSSRCLTGENLRMGSTVVGDVCVQLRCGEG
TLSVRYRGSEEWHPCAPGQTLTPPAPFTAGSIRCPSFSDVCLTMLKHSDASSTDVESHPK
HLAMVRNNDGSALAVTSAPLLLLLIVIGTVIVAP*
>TCRU_474 Thioredoxin, putative
MHSTEKRGREETEVAAVPTVHETEGVVVVGKGDDDVRKKKKRPHLENDANNNNNNSTTNN
NNNDVTDTLDAHKKFAGLREERPQQNEPLQHPPSVNEVSVDGRGETDGSEVGANDVDVEE
PTLFFGPTHAARDEKRSDGSGVDLRCVASGGQNQRPDAAHDGCRAPHTVGAVTTLYSLRQ
LEELIGSAPLPIAHAGVLNVTPSSSSSAPPSLRLRAGETVLIMLHRCKGEAGVLPQHTTE
QLSNCRIFLLDLGELPPCEYEEKSNVACPNGHDERGVATGFLQLPPTDTLLSLASQGAHD
MEHVMRRASSLLCVDEVLNANDADSSKSQTSEKGGGDENASADATHSCKREELALPALVM
WRAEGGLADEYCYPKEKGKNDSHANNHDNPQPPLAGRYHHGRPLVVKQLTSLDQLHSFSL
LTPVYTVTHFLRTVCEKALFPDDPAAGASGTRSLIYFGASWCPPCMRIVHALPTMMKEDF
PSNMTCSVKADMDLATPLFEFFGVEIIPTFVILDNESLRTAGDWKALRSCSISEEALSQI
YEGLRRSELGRIQNSQRVTVRTFIERHSGMLKFDEDF*
>TCRU_475 hypothetical protein, conserved
MLGKRIAAHALAASTPRHRWKAAVTPQCTHPNTAASSALPTTIHSGSRAANTDRKYGSAT
VLARSCAEAHGILANTPSTCISRMEPSPPVTTTATAAAPATTPVIAERVLAAAGSVFDLI

VKDPSSTPLQPPDTATASQRMTSVSSAAMK*
>TCRU_476 hypothetical protein, conserved
MNELAISNDSAVVLSGNVFQSVRASSSAVHVVRSALRVSWHSVFAVLGNVFHMTGGEGTL
IHLEGYTEFFSLCVLNNSAVVIRGNAVTRPVQYFMLFLWVFCVESRSAVVFRDNDMQGSS
AVFFSSHSSYIYYNSWLQLSGNLCRVSPVVAFAVFNPTVNLRDSTVSVSGNQFISSTFTP
TMLQIPLFSSDLTNGAIVAACNTVNGEEREGIVKRRSRCQICAEGVVALRVCCVGQRVSY
DWRRGYADTS*
>TCRU_477 hypothetical protein, conserved
MYPSKLQYRAYSELPPTSVSVPNEYKPKPPYLKACALTLLTSSEEAVNTTSCSNNTLHML
FTVSGKWTAEDCTTSTSVTAMSQCCGKASAKATTEDRITVWLTRIRTPAESAASSPPTNC
SAAPPRNVHSLSVMLMGRCSGILSNENVSTAPTPLNVTPS*
>TCRU_478 hypothetical protein, conserved
MTTCRLLCALLVLALCCCPFFCTKAVVAETGVPNEPLTQSQVQSTGDSSSGLNPDTIKKP
DSAGASHKPVSAVAGGGSGGLSNTQDGAITSGLPGPGVGDADSPGSTGNSEELVVKTTTK
HDPAVPVPNGEVAEKAHNATNTTPTIKTPPLPISAVLQPKASPAVTVPETGKGSAARNLS
QLSREKNKENTASQTKALSEALKRHPGDAEAEQQEQESDTSNLVEIADNGKHTETTSSSI
SINDSDDAQGTGAENNDNDPRPNPTETNDHKVDNTNLASAPTETAPETVNTATAERNETL
KSEGSDGSTAVSHTTSPLFLLLVVACAAAAAVVAA*
>TCRU_479 hypothetical protein, conserved
MEGCPGGKGRRAHRVTGARRCGEDSIPGVGPDIVPRVAALRTGAMRHPPTLRQACTRPAD
HKAPPVSVVPFLPHCGDRPASASAGTSSRREHRNSLPTSERRIDFLLPNLRQSALFRPQY
EGTRRTCSAWRSDSLRGPHMWMPQWCQRLINRRLKRRSPPKLPIMPAGKTRRKHKCGHSR
WTRLFRHRLITDATGC*
>TCRU_480 hypothetical protein, conserved
MRVGRAMQCSCRRAALPGRGGEWEPDTERLPRRCGAKRYVDAPNQKVSRGVPGRPSLCTQ
PQLMAAWHGPHRSNGDVHTKIEVSINRIAEQTHRVGLDALVPIAQTMVGCVLVRLSV*
>TCRU_481 hypothetical protein, conserved
MVCVRCRCWAAAICDCAVAVRGVAAVSLLLLPLCVDGELVCAEGCTQVTGVMAMMMTGRV
LLVCALCVLWCGFSGIAADGAGGVPDGSAGECLFSRWRAQLRRECAEEFSRRTGGRANAS
AVEECMRQRMDGVLAVVDGRRRWRRQRPAVAAAAENADELGTENTSSERNVLSGGNGSGS
EDAHVRGGIYFLNHVTVLYDQKVQHRRN*
>TCRU_482 hypothetical protein, conserved
MQRRRGVLHARDGVGGPRLVLVQLQGRMAWRVVSSLRGARHGCAARAGESRRRRHELRGE
PDADEPHAEHVEVAPLLRGCDIQRRGCSADVFPRQYAAASPHQHHVHWVHVPWRCRAAVC
WGCWCGRVIRRPDPREPDGDAVLCCCVYECPPAALRHRRHGG*
>TCRU_483 hypothetical protein, conserved
MHRGRGRTARRLQQHAVKHHSARQQVHAAANVHRRPARLQRPQHSVAHTQLRLQQRRRVQ
HKRRRHRVRQHFQLGVAHRPRRHNRRVTDRHVSVCRQVVHKHKRAVAPGQPAHAHLQAPV
TRRLAAVAPHVDLRTAVAHAVPQFDAQHAGKHRRHVEHNSHAVRDVHAVHQHRVHGAANR
DGSVHRAAPQSQHHRMHCGHPRRRRNQRQQNEQRRTHSRNGPAPHTVPAARTRSHRRPHP
PTHTGTNRAVALCRTRPVDHNSCLANPFLQMANPATQFRQIVISLLIILLLQVKPNTLKR
*
>TCRU_484 hypothetical protein, conserved
MAMMTGRVLLVCALCVLWCGVCGGGLAETTQGLLDTASEESESPKKNKDTGGSAGTDGPT
AETKAPQAADRGSKEQEGTVEQAAASVPGVTGAGAGMETAPQPGREATESAKGTRGNKEE
TKKEEESEKELEEGEEEEEEKKEEEREKEKKEEDVTDAKKGISAESQEQRSLPSGAEGAS
NQTKPKSAQTTDVNDPAAEGAGKRKEKQNENKEANPKKTPVEATAMKTTTATPGDSDSST
AVSHTTTPLLLLLVFACAAAAAVVAA*
>TCRU_485 Dephospho-CoA kinase, putative
MLNSSRRLFLVAFLPRVVILGVTGGIGSGKSVRCTHLLRLAQSRARQPIYSFTAHTISAD
KIGHDIYSPGKPCYYDVIDAFGKGILHDAKSEEMSGTSHSSHSEPFIDRKKLGGRVFSEP
HSEELRKLDNMCWPHIHEAVEKEIEFMSRDAISKNKSVLLIILEAALLTESSLLSLCDDV
WIMTCAKGVAVRRVMDRNDLTEAQAEARVESQGSVEKKLDFLKNIKFKGEVLVFDTTNDS
LEEGLKRVTIAFDEYWCRKLEPLQKDPDEESSPW*

>TCRU_486 Nuclear transport factor 2 (NTF2) domain containing protein, putative
MTVGNERQQRVAASFARQYYTLMVQMPERLPSFYTQNASFNHMGHKATDIMEIEDAVSKL
YPMQADASVKIQSLTTQTDGDGHIHVMIKGSITTPPGMSQEFIHLVELMEHESHPGSFGI
VSDKRENTFSEEAPRRWALETPPMFAKEPAHGIMEQIEQPVSTTELAKIENATTKKDLEK
LERAESLASAKVASPVPAAGTTVSTTSQSQASKDVEKTTDKADPSVPATTTEVKKPKSFA
EAIMMSKRVGPSQGQTTLLTVLARESREPTALTPAKEKKKAVTRPVEKNGTKTVPKNGVN
KRTAGTSVPANDGKKNGKTLATGSNRKSNNSSEVNNKRKDADGRALSRFVVFYDIIVKGL
PTDATEQTVRDIVDPTAPTKLIKVLSRTDKKDPNVMRTFAFVQLDHNAIKEAGDDVKAAV
AKVLAAHKGKKSVAGHRIQIDEVREKYTAAPNDVAVDVSA*
>TCRU_487 hypothetical protein, conserved
MSSRFFQKYFLRCGHCQSIQRHAKGYRPIPNPILFDADAHCRSYHREQRECTGMSGYVVT
CRCEKCHRIHSSWEVVDFQELLDAKGSMSPEKRKALLWPLAGTSSATKMLK*
>TCRU_488 chaperone protein DNAj, putative
MWHGSRALLVGGAHRTLASSFSTQAAAGATAMLFQARFASASTNPYAVLGIKQGATKEEI
KKAYRVLARKHHPDAPGGSHEKFQEIQEAYDQVKSGIWIRRDADGGSTDARGGSDGGNRY
SNFRFTTRQHKSKVSYDEFYEEMHTGKVKKRPFEDDDDAEEAASKDPRRNPFAGNEALVQ
AWFRVIILWSVVFVSLRTTLFLTFPPKYESVRRAPMPERPRKPPPPKPLMNNSLVA*
>TCRU_489 Domain of unknown function (DUF4475), putative
MRRSAARGSAAEVVGPCRSRPQQQLTATPSHGTVVRSAPESYGMVVDSHEVHEEIRDLSK
RVKGVIEGNFQKNATSEGQVLQGIRRFGPSAQSKKILNRENEAAMLALKTGVYAVWRCEE
PKLSGANTDFCARIGYQHRCFCGHSLEEHATLKPSRGCVPAPRCQACACGGFNYVPNEPE
EIGEGWLSRRRSWDPLKWSAKCRCGHGHKQHDPQKRHCKECGCGGFTSHFLCAVCDRDGE
THSTVFELEQDRLKKGLPVREAFFPLMALDWSARELVFDDPTGGGALTAPPRNPALETNE
PSSCDKRNALQRIPLPKLRPTSEPTPLLPQHCTGCGAIFRSAQVKFCSKCGKHRQQNAQ*
>TCRU_490 ATP12 chaperone protein, putative
MRKFVVPRFSSLPYFLSVLLSGTRCCSSLSSSGNSGCSSNNGTASGPTIAPKKTKRRISC
NIDASSLLDRVPRDPGAHAELSTAELERKLEEWSNMNPRQLQELYDRLEKQEEEESRVLT
EDSLYQMDVSMRDRSRAAVRVFWKDVNVGPLDGHEGWYTVLVDGRKVKAFESTHVLAIPN
EAMAYCCAQEYAEQSGYLNKLLMPMTDMCSGALTVSPQMIAPRVDYLMSFYQNDNIYFRA
APIAEEQDRVIGPIADWFSRAFNVEVPRIVGIGHPHIPIGATLKVRDALLAMSMNPYQIV
ALCVAAQFTSSLLLPLAMFNSVVDLPTALSINSSEERHNTSTEGAIQGYHDIRDADVVTK
LCACAVTWQLMKGISLSKCLEVARINVENEAL*
>TCRU_491 GPI-anchor transamidase subunit 8 (GPI8)
MKRQMGVLWCCCILFFLLTTVDTVIASSNKTKTNLWAVILSSSRYFFNIRHTSNALTIYH
LCRKHGIDDDHIILLVGDSYACDPRNPYPAAIYSTLSGPDRINLYGCSVEIDYAGYDVDV
RRFLGVLQGRYDAYTPPSRRLNTDENSHILIYAAGHAAESFFKFQDSEFLSSMDIADTLM
MMWEQRRYRKVVFMLDTCRALSMCLEIKAPNVICLTSSDATLESYSHHLDPLTGLTVISR
WSLESLKLLEHAKCEVGPNETTALQMSWYNFNYGEERARLPPPRSVPSHFDAVNDPKAIH
KWKLEEFFCDRKQDPVPVDVQYDLL*
>TCRU_492 tubulin tyrosine ligase protein, putative
MPSGERRGRVGPVRFRTFLRNTVLDVMRSRGWLETDSDLGWDLFWADVGWIREVHDHVRL
DDVQRINHFRNHFELTRKDFMVKNLKRMRKSLEREGRVEEATEFDFFPVTFSLPQDYGMF
ETEFRRQPNAIWIMKPPAKAQGKGIFLFSKISQIAEWRKDYKQRQVGAVGEKAGGPYGAE
QVEPYLAQRYIENPHLVGGKKYDLRVYVLVTCYSPLTVWLHRTGFARFCHQRFSLRDIEN
TFIHVTNVAVQKTNPKYTPSSGCKYGLRNLRQYITASRGVETAQKLFDDIQNMILRSLHA
VQKIIVQDKHCFELYGYDIMVDNDLHPWLIETNASPSLSAETPADYHLKFNMLEDMFSVI
DIEKRRTGDEIRVGGFDLIWKNGPVGPACRGDAKGGGVTQSFLGCANELEIPISHMRMPP
RLQQMDSSERLLT*
>TCRU_493 hypothetical protein, conserved
MDIDVLAKLAEEALTSPDKAVRDRAAARLRFMNRYEHWEEVKRLLPLCQSNYLRFIVCKA
MQFMASNELGPNERKKIQQYVLDYLTMMRERGEELPLYIKNEVYSIYACALYVDWRLQIL
STDQVPGPIGIYISELTSRFPLSDVLDCILEVLTYFAQKEANDGLDRMRVNFVEGFIPRV
LTFVVFQFSYCLKSALELCCIIMEIMSTRSPAPITVKNKMDGSIFLEKGEKWFPALSLLV

DGCIQILLTYQSLDLRGKCVRFLRMASSIICVDPEWLQSQNKLSDSFLELFSRLPSMYKS
SGLPQLLQLGCAVLVNVSDRDEGRVSNYLLNRPYLIEQWEEAVIGVLERWEDGEEELRQE
LMHLFFLLGDRVIPCLAGEGENTREMPIMRSIMQVSRCYFDNVVAKAHLAEDSYEARSDL
GIMLHNEKTLLPIAEMLFCEKIDIYGIVAQRLRSTIEQYELCVRIRETGDSDGMGDLLLS
LAVNVQAVFPTLTALDAPLFLMHVCLSRLSVIISTVAIAVLNNAAHQSDELVGILARFAR
ELLSVDGTLTSALLTSLSLVDVEDDDSQLSSTTGGGKVHLGILRALFFFCVCLYESFSEP
SSEFYEIMISLLCYVYLHHSEKACLTHDANLLLNRVLMQGVCGFFLSSEKMFSILMAMKE
ERIELLRITSDKLTDEGRKARSRMLSTLTSFTECRHYAGFPTLDLISCIVTRSLDVDRLT
ADPHSVFYDLCAIADGINQVESLYCLLDALLKRSQEIEDLLRALPANAPLLLRLCAKLCN
LATRLFVEDNKCETRWGLVGFAFRAVHGVMHTLGLSGSNWKGPIEEIPPGVVSEIYVYDV
ADIIYSMISGEWCNLGVALFYSKDFIFLYGGFLSLLFSVSAELVMAHKNSRERVFDALMK
SFLSSGNLGIELKEFLDTHFVWKRLLKYFLNCLRYSFLPVIMLAVDAVFTSLGNTSVHGS
SFLEDRTIGDLFEELAVHITIAPHMERNEVELCFKLLTNCMQFCGIVCTERMEKLLDFCT
AYHRVRLRHLFTVICSGKEDAPASYTTVFGRESKVQTLSAW*
>TCRU_494 hypothetical protein, conserved
MHLDSDDARSALRRRLRNVMRVIDETLLLHGRYASLSFLCGSANDAMEASGTFSPVPHAS
VEAGSFLEQQRRELRAEREDAQRRLAILKRARERRGKRLDVKSDRLDTSGPLTLPSHSGE
LDSQPQPQPSLTPASLSTPAALDIGVNRERREREGEMRRSPPRGAETHVAVMEETLRTHL
SERRAEEESWLKELRTVRRSAMAARQKALAEAQAQIKRTESERVAKLIQIESEMKRAVAE
TQQLCVLTEEEVRAIEVHEEALAVLRACTGIKAENKSPVRPPPPLPPPVVALLASSDVSS
PSNGGASPKKIRSPSVDRDSFATLSSTPLLANLLASPVEPVGTSPPPPFTSRNTVASSSK
IDISGNLSVAAEGIPENRISSSLFPVSHSEISSPLTFNNGKKELPRTCALTRLSRARPLL
SPSVTLSPTKQLSLDPPVNSLLPSQALAVLHKRGILADVLPSEGRKIPALVQLSKDRREV
LLLVERIIRDLNGLARKSSGREFQASVTSMPSHDKQGVQQFQTEMGPTQKKQQQQQQQLE
NREVDRGSTRRVREIHHFSLMDGLLLYPLGTIGSGSTGNISCILCGAHARQLLMEFSCTL
FAHDDASSYCIFFISQSPPHHTNGALGLPLSHRRRPIDPNGAAVLVLRFRCRRDWIAFLI
AVTDATMQLRQTSPLSYGRALWMLAARLWLKHRSDFFVSPPTKHHKIPPFSALSFPPALK
GKSVSSAGAASGARERGKSLRTASGRFAVPLSCDSYDTNGDVIEIGHVGGNKFFGGDGVL
QNVHSHRFGVRPVADSNGSFSRHCSLSATCIGGAH*
>TCRU_495 UDP-galactose transporter
MPTLQWASPAAVSLLVLVLQNSLLVVMTRYSRANVPPERQYHASTLVLNQEIVKMLFCII
IFTVENYLCGVPLLELGKSSTAQSGVPQLLWGVIFQKETLKLAVPAALFTMQNYLTFIGL
SELDALSFQVWSQTKLISAGVFSLILLGRRLSMMQWLSLLVLTLGVLLAQLQNGGGKRPN
KLLLKEQRPQRPLLGVTSCVLSGLSSSYAGVYFEKVVKTTAPSLAVRNIHLSLFGIPFAA
LSVFLLDFLPTLPAEGKHGQAFHFWRGYDQWLTIGLVFIHALGGLLVAIVVKYTDNIVKG
FATGVAVAVSGFMSFIIWGHMPSVMFVLGCVLITAATVMYHFFDLHVPTH*
>TCRU_496 hypothetical protein, conserved
MQRTWLSSLEFSSNVAAGFFIVARRLFVQGGPPPPKLHEGIEPMKKLHSHRAEMDARYAA
EREAEREFYARTPHIPSPLPHNSHEKGKYGMRRRLKIHQAEDDISVESLSEAVETGNHAM
RKG*
>TCRU_497 hypothetical protein
MNEIRPQSPRVQFAKVHNAVVKGNAERLHKDPGIQMLGHHVLPVFPPGEVLPRSVNENDK
NHGDEDRVRRGCLGVAWRLADAGVVKDKRTAVWPYEDADECPDKSKDDTSNVIKHPPPPY
KVHFA*
>TCRU_498 hypothetical protein, conserved
MSDSESIVFREQTANNSLQSENMQDALIQSSQMPSASPALDKPPLHHTNQRGSRTITQPR
PEAKHTKKSFAAGERSHDDAVSEHSQSIHFSFDGAEHNASIASRASVRRANGLQPPPKRG
GGHLELRSTHLRRSGSQRTDSEDESIQFALETSNSHARPKTTVHSEASPNDSIFFHVVDD
TRTDGANSRASGNPQHDASTDGIEFTVLNETEGAAEGSLGKSVQTRTDAEKGRGFELPVE
KKRLKKTVLSNRLRHPPTVLLRSGRVSNPPATKRSNGRKLTENIPPATLSPAETGPLDAP
AIPKPKPLEATEARLESNRMPPTAVSMSFKDDQCTSKATPNELGNILFPTRRSPSTGFTD
PIGIKNASTAAAKKSTEGSPPRISIEVPSPYPRPQSEVMQPSHSDTLDEWIFQAKLCTEH
WRLFNDFQREEILRLIERIAKARQPDSFLQFIDTGVKERKPTQAAPGGFANLYKQRVFAA
QNAAFCRGKSNTVPSTIKLAFKVPPRARSATSHGISTCRSPLSGRTQDHRPPVARPPKTA

EAGQLTAAPQRPPHAPSRRSAGGRCLSLFQDASSSDGVAKLAQTLGVVWPPWSPDALFFV
SGARLTPQQRDEFYKAAELQKAAFAKLHAELQHSSAAQKRETKGARQKKVTLSRTSILRK
AFDLMDVDTRGIIHTADLPALQHLLEAERQELETKAADGVAASSFLTRARGEQRILKGTI
DSKRPATKPEDDATSIAKKIMLHGFVLEVIFPLIRASNLVVVDFASLGLLVFGSLCLSQK
GASASFVKWFQAALHYFEALA*
>TCRU_499 Protein kinase domain containing protein, putative
MRTPYSSATSLADRAVSPSQCLYPRTTARQIRPLVLSPMTPVDPKTMSPHCPESPQSLQS
GSPESEGIGRTEPQSLLPTFLAADFDVVDVSARLENEFFSAAQSAELNGSRSRHLQETIN
SHENCSSAEKLLHRNGSCCRVDVTEEMTQVFQEPMTPLREASSLIEENREGQDDAEADRA
SNSPFSVHTPPENIIRSSLMLTPPWTPVLATPRKSAKRPSPQTFTPSAHRRGVSSHNLSM
ESREVFGVLPHITHADYLPPNRNPCSPYLEYADERQLLVQLRMEEEEEEEEMDGGLISIT
DGMLQRADAFAILLEQTVPIASAVRTERCWSPLLHKAGKGGGGANTFPNEVREENDITGL
SPVLVEMDSAMRRNASTAVETGDVSSGNHFLDFCGFSDDGTSLGTMQNETPHRLSREAGD
VACDASTRNLTDGQTSFETLPNKLLCTRVFYDRRGQVGEQTSPPSNSPCILHNAAQWDRT
VGMHFSRNRIHETGLSFQPSLLSSSLSIRGDSQTCFSADNSGDLPSQLQFRNCLSVHFPV
KFDFPTAGYPVSDAEDEIPRGHVNSKEEEEEEEEEEDEEFEPGDRFCNHHRYQFLHWLHW
RKAYDFVPNHLDRNGVVWLVTHRVDGLPYAVKEIPGTQQNTLQTELECLTLGNASVNALQ
QQAADYITRYFSVSPYSVVSCDSHESMAFLLQTEYFPMGNVMEWALDPHRLRGKEFRVPP
EDFWSCVLQHGLLAMESLHAAHIIHGNPLPFNLFIYSPRHYKLGNFGSAVKFPSTYPVGS
PNVFLPSNMADGSPWTPYEMDIFLFAHSMLQLWNHCIDKRKLVSNAVQTFDHDSEGTLCD
IFMKDAMCISFSGFQEDRVKGYHEISDHLWNVLQAALRRTPISTLLRMLEAPSRLQWTLF
SLFDTEEFYLLRRKRRLKRMLEERLQRKAQEKVKRQSLPSTSVNYVDDPVILLPPAEWSC
QERTAHCCSDVQIRGSMSTAAPPLPFQLPLASNTFASLGNGVALSPGRGKNESCNHRDAA
CNRHGRRLFNGPKRVKEDTPLSSCDTSVASYSPHILHATKKFSLLKDKNCSFNYLPEEDM
DNKSMLKCAHGGRPPYIRRRVDANISFANRDRLDANLLWTIRQIMAVMNWEDALKGIALH
FDEDVPARGFFSLSRYAVRPLTSWE*
>TCRU_500 hypothetical protein, conserved
MNTDDGSSIQYATSYQSSQAAHRAADAPPDLSNCPTKSSGRAMELIRKKRRSTVLRRNTDS
SALGQLYEESKRGRSRDVGAVNVGAWNVPSGAIHNLLPHARTADERGELCGLFDGNRSGA
IDPTAVRQDSAVYVGNGSNPAGNDDSGVTKKVAARSETKKEMNQLIRSRTDWKERVVVRL
AWITVSRLFEIAVLTPNNFTLKADGALILDWSVAPKTARADPHRASRFFRIRWQDAFDTI
KLCRTLQENEKLTNLTTAQVERALAPWNATVHSIKRGVLRHAAQIVEAHDLDPHVISQLA
KHVDPFDLPQNTV*
>TCRU_501 hypothetical protein, conserved
MDAAVNKPIANALLYSDGVLHLVGAMENEEKSSLFPAPLTEELQMITSIPRNWADVDASF
SASPVSTVGLVGYLSNAGTANTCIDECRCVIATVTNGKNVTNGLQFAGQCYP*
>TCRU_502 UDP-Gal or UDP-GlcNAc-dependent glycosyltransferase, putative
MLVLYVLGRHPSHGYGYSAALLEEAAQWNDVVALPMNEGRVLPEKKVGVGGDSGIEASIG
MTRKTYMWFDLAHRLFPNARYIAKGDDDIFLRVPLFVAHLRLLPRRGIYMGVHAGDSFLV
KKCFLHVAFIVGYCYTLSRDVAEALVSYEPLRRLAYLPYSKERDDEFALLGFLYEDVMVG
LVLEKEVKYKPMLYVKVLGCHFHDARNGTGHSQVVPTSMCVHHVREDDYAALMARFGNDT
SPVARVERVSDDVVYPSCD*
>TCRU_503 hypothetical protein, conserved
MSPATGRGGAGGRYDDATVVPEAYDDDVISWGFCVSSLSSSSRSSASSPPRRGSRSSCQW
RSSAYHTTTLYTVTASSTVVSSSTMAAYALAPPSLGTRWLAALTTHSAASSTKLAVRITV
RKDERIMFVKATTPAPASSTAAMQKYWTWQPPTGGCTAAIRHTTHSTDSWNTHQYTWLPL
MFVSMPPYIRCCAAGCQYPTGYSRRSCSGFLENCQYLVKLAPVWRAMK*
>TCRU_504 hypothetical protein, conserved
MPRCIVCATTYVRLGKRIAAHALAASTPRHRWKAAVTPQCTQPNTAASSALPTTIHSGSR
AANTDRKYGSATVLARSCAEAHDILANTPSACISRTEPSPPVTTTATAAAPATTPVIAER
ALAAAGSVSDLIVKDPSSTPLQPLDTAAASQRMTIVSSAPMK*
>TCRU_505 hypothetical protein, conserved
MSLCVRLPPAAVVVTASVGHATSLHHSVLLTDAVGVRVGNSATVSVTCSVSVGPYCVLLR

LGVGVHVEVRLRVSVSVAVWLSSGIAGVRLHPSTCGRYTGRQSRPYPPSAQRQTHDPDID
IVAEAKQRTSASHTLHATGRNFPMMPADLYSIGCSACPLSVRHPTTNFRPAPTSATVPSI
TTGPLPAFP*
>TCRU_506 hypothetical protein, conserved
MLLPRTDSHDPDKNDADPRSSSSAPSVSAPALSFTTTADALATTPLSNTNPCVAYTWLPD
TRSTAPPDATTRALAYNAYSPPSTAASSATPPPTSSSTAPSTTTVRPPVTTVTRATPDTT
STDRTSRRPDSTSTGAYGEDCEPGRYTSGVGLVATSRESATVSTDPAGNVPVSTSAPFSA
STSHTDSSTGPPSSLPAVPAPRSAPPPITYSTAPLRNDTFCTTIRTPSRAIHGT*
>TCRU_507 hypothetical protein, conserved
MKRVAHHRKQGVPQHPQRRSNKVNGARRCRHALKHIAAQHHRGVIADRQLIHRNTRRAVN
VQTLPRDQPRDREAVPADRDVRVRWKHTLRSEQRVTERVAGDDNVQRPHSGVRVAQQQQH
GAVPPRASIKPDIRHVARHRSNQHRQAHAAPAERHVAVAQPRQPAAHSLRNLGVHHVADA
LTNRRRRWWEQHWRWRRASRHRRRDAHIAVPSSSALAPAVNNRRRQRSKAVALLRALSAR
GNSRHVGDAVKLQFCRRSQHTSRDRQAPSNEKVAGLYQRSQRLRQPHQAHRAACQRHHTA
AVNDKKSGFGFVKHRTRSHVVVTPIQPA*
>TCRU_508 hypothetical protein, conserved
MPSGKATALAAATLLALLVVAPAVASAQRSLDCQKVWDGPSIDNDVLKCLSNTDRIKGQW
RYLVYPGVCALLFVVTLLSFPLVFLSVACCHSCCQPKPGRSSDASRCFLWMWVIYVVLWS
AAMAVLVILGAKLLATSAPSIIDNTLDGPLQYFNNTAERIIDFTSNWSSGKREPIPSIDL
DITAFTNISTNVTDLLMDAKHKISKYIGWVPIVSYCVGGVGVVLMLLVVLLACCRCGIPC
TTYLFSCIYWLFGVVFALLAVVVTVLTYLSWAACGEVELQQQRQPGVFQWYLVPYCEQTF
DFSDINRKADDAERRFSEDACKKLLKSCDNKPNLFKPLLCGNNITSEDQCPNFGTMASVL
SATRVKAFTMACPVAGESCTLFECAANCTNTDVKAVASVILQLAAQASNASIALSYARPL
LDCNFVVDKLLGAMSDCNELKAGTLMLGTGFFVGGLMFGLAIYIMFRGSCIWDARSIKQG
RSPRGLNGSGSVNAAQSS*
>TCRU_509 DNA repair protein, putative
MRHHCVTHNELLARHMERTWPGRFSEFCTAVRRYPLPVTSGAAATVLHGCSTDAVRAVGA
YCCDVQQRCQAPECAGAELDLLSPPPSPSWADDHAVAMHSPFRCFKLRRLESSVSHGAGD
PSPLPWLFGTGAEDGECFLLTPPDLSHSPRREICAGCPLPPITRRDADAQPTAEKLSLRE
LRSRCASRGLLTTGTKNVLLTRLRQHGVSQPTGPVPWSERDGEGQAEQPVESGECGGALP
GRFMSTHEEQLEHSQRLRTPFRSPSTSRPSSPRPRDQPRIRRLVGTPQEFVAAPRQEAKR
EDRVGGSLAHVASEEKVSRYQWRILVDNRERIHGAHGRVIEAFTTAGVPTASCTLPCGDF
MIGIDMPGDSCGSHASPPTARELDSLGATHSVPAAGECGDAPAQRLFSVVVERKTVKDLC
ASIATSRYYEQRRLLSVSPFRSVVWVVEGTTELLRPDERRRVLSACASLAAVPRFRVIRT
RHLKDTVSWLRSLGVAHVTALANAARSGARPPQLADCATCLNVTARIKRALRARTTFARM
LICVRGCSSGFATQLACKYGSFLQLWRRLRCCGREACDADMDVRRLSSTQRDVYVRLTEF
LLAEEYC*
>TCRU_510 hypothetical protein, conserved
MMVPNASKKRPSFANGPRILVASNASGIMSVTCGGREHVAADNTDEVNERSATGHKTLVL
ENTVAGSGFVAEGDADGLTQRNASGHDSKPQLREFTAQ*
>TCRU_511 hypothetical protein, conserved
MMRRVFCVVLALTLLCCCLCVFAAEEEVEEEVLPVDAVCAGDGENVGRWQLRGRPLWYNC
TAEASGLGKMICGMGAGNCAPEDVKKRVEGNAKDGVFEIKFHTPTPGRVKSWWESNVEPK
PATAASGPAKPAEERPPVDREEEAPRDTTTISSGSSPAGVGGSAGALSLPPTTPRDAGAN
AAAGAAGKTDPSPPATLQAQNPAPSGELQGAAHSASAASESPPDPEPTESGEHSSHDPPA
GRQEETVDAPPTRLSQTGEDGGAAEGAEEDTANTTDAATSEGNATAAGMPGPLSSAPTTR
ALENSAGNDACFHDPRLHTRLLLVLAALTYGTLG*
>TCRU_gamma, conserved domain containing protein, putative
Elongation factor.1
MASSGDDSRCAPGTHPHTIPCVSLRELDELKIDYSHTDTRTVAAPYFFQHCDAAGCTTLW
YNKYNKMQCMTANLIRVWLQRMEHVGQYALGAALMFVEQRRHDIVALCVLGGRACRRF*
>TCRU_513 Rieske-like [2Fe-2S] domain containing protein, putative
MATTGRFYVGPAASFKEKSRQLVKCDKRNIAVYRYKDRFYALDNACYHHGGPLLEGDIEE
MGSHPCVVCPWHEYRITLDTGEGLYWALQMTPEGVPDKKAPQKVCSKGMKQRTHIVTVED

GEVFVTLNNSGPRLASDNYAEMVIANMQGAMISPLEDSRRTHRSMNGLGAGIHSGVRSGH
VFAGMVTGNRLQRFSYLNHPILTCVAVEPVCDDTQEYYFRLSEGELDTSSLVPGQFVDLK
LPIAAPPGRHFVRRWTMVELNKEGCDFTLIIKTAEHSNGGTAWMSNNALRQRFSLLHYGG
SFTLTHHMSRLREVGGRVVMLSAGIAMTAPYSSLRQEFEGKRMQGIPKLRIIHLHVERRV
SCVAKLEDFVRWHHSKTEHLEYQFHCYLTKQQELSAKAAGDESLWSLFTCGRRPAEKDIQ
SFVGDFLGPSSSVLAMVCGPPPFVRMAKEVLLACGVLPSDVLTDEDDDYNSL*
>TCRU_514 translation elongation factor EF-2, putative
MSLETTAYLQRLSEKPERTRNFCMVAHVDHGKTTLSDYLVASNGILSPQLAGEVRLLDSR
PDEQERCITMKASSIALRHVHKGVEHLLNLVDSPGHIDFSCEVSTAMRLCDGAVIIVDVV
DGVTQQTSSMLRHAYREGLSMCLVLNKVDLLVMVQQITPEEAYYRMRSIVESCNAALAAF
ANQLKIQEGEESFMHQDRGDPSDDVWFCPTKGNVLFCSCYDGWAFTTNFFASLYESKLDI
ANLHASLWGEYYLDAKTKAIDITPRRAGQQPLAVQLMLEPIWQLYRTFLGEKDNVEVQLR
MAAKLGVPERLWNNPRRDQRGKLKALLSSWLPLANCVLSVICDKLDSPVVAQRRRLPLLI
PRFDEASSQVKDALINCSNSPDAPCILYVCRLIDTQFLVGKTIGQEKDRDDAFIGFARVY
SGRLIPGQRVHVHSDEKVVEVNVGKVFLFRGTGLDEATEACSGTLCGVGGLTSYITKYAT
LSTMEGVPPFKPLVLQSTSILRASVYPKDPKDHLQLVKGLQLLFRVDPQVEVSILPTGEH
VIGTAGEVHLERCLKDLVDTFARVEVLASEPIVSFRETLAAGGASMKPKLHTGTTADGAF
SITLHARCMPPDVFDIIKDDCMNHGSSMQVQRQVETALMANKRWAAEMQKGIVASGPTKL
RFLGALLLLQFGDDETSLEKLRLFEDWKDSIIAGFQAAAESGPMAQEPLLNIAFIITDIS
VDTSSGLTGGMVLPCVRDACRAAMELHPRRLVEPVYECTVYSFGATQGKIYGSLSRRRAE
IIEEVPNEGSDLFYIRCLLPAVEAFGLQDELRVITQGASTAQLQMSLWSIVDADPYFTPT
TKEEIEEYGNEVVMKNIAGQLLERVRRRKGLHRERVVENAEKQKFSLKGA*
>TCRU_515 hypothetical protein, conserved
MMRKTPMFAVAATRKALVGNGPTFSTGGECMNTCDIQNAFPMNERGVRSSSPFQEPNTAI
YDTYMSWTYFQPIDVNIEKLPAPEAKYYQRHTKRPWDISTTELIEIQSRKKYFQVWGYLI
AFIYLYFLMPKEKSFSGLHGPDGHWIMLPKSRPELF*
>TCRU_516 Snapin/Pallidin, putative
MDEQPDDAMEKQRVEGSHVSEATALAENEESHGAWTPPQETSLVPEQLLVALRDGVFGSI
FELLRGMQTQQNATLQTIEEVKAELNETQEQMRQAGEIFEKLQAYTQKLHVMRDNMKVLD
ENMAKTKRMARGIQERLEKTAEWHGE*
>TCRU_517 hypothetical protein, conserved
MGKGGMRVLEDEKVLLAELGKLFHMARANAREQRNTHRAERRRRKATVTTTTPTTTRGTK
TTGAKKDLKEGRPPRTGSKRSRKTSTRGGSVWVVLKGGFAHVPTSEKTNKIAHNTVFRGR
LSERRRVQALEEAERYIQTAWQQAQEAGLLLDDGSGPNGDEGLQYLCEQYEVAAGMLRRE
PVGAFLKQIVDQTIPSGAPMSLTTPAPTFSSVDNAATSTKLSRDEWVGVIARENVILKRF
APRASKQEEEKEEAEGHGESVLHEADQLPSLYYFPIAEPLASLEAASAVTGMGLRETSGD
ARGGEQARDVLPTKCIVRIRGSGRQKHTAILTSTKSVNYFKSNFMQVIRRELSGSKLPRG
EDAVEGKQQHQPVSNSAGAEHAAAGSSKRNSKRRHK*
>TCRU_518 hypothetical protein
MEGYDANTDSRDLRFDDDEREVEKYNTTLRPNEVNASMDQVGFNDLSMEEMREGQQVASK
ASPGVSPRSASEGFFDPEGTCDYPTTMASRKTSQGNPTHPQELNQATNAQQPIDWNLLEQ
MMMMDPFKEVMCPPSCTFSVYDAEIQNHYNIDSTQIVATHGSVEYLKMNEARGAQSRFRF
QLCKRYLNKRCTRGANCQYIHTHLVPPPTSVHVNENVISAAVVEGLEMPPEVLRGGNNKH
GYPTMPSGVIFRVFPPNQGKSVPQLIPSEMILRTAGASNVYSVFAYPGTGKARDMLTTET
ADTSGSESGNLTNVKARHCAHFQFNKMCNLGESCNFIHSLVPYVQRLSMSSQGPLYNSIV
TQRPLLMPSTSMAHFSRFSNGNNNNGVVSSYPMEFMQPSMFHVPPQTQQQPQQQKQQKQQ
KQQKQLHQQPQHAMPPTTVSHLIPSPQPSEHLMYQSNMSMMSLPMYMSSPVSSTWDGVRY
GANYQ*
>TCRU_519 amino acid permease, putative
MEGLPQISREGEQSSDSAYVVSIERKKERASAVNGALSPQQECGKSIAGCLALLLGAVIP
HGGLLSNSFSLASASLGAGVITLPWAFHACGILMGTLYLILMTVFTVYTITILGYVMEKT
GYRAFEQMSRGTLGKGADYLMAAVMGTSCFGAAVAYVIAVGSLITPIAQHSTATPEFFKL
KKGIRLLVCLVWLLGMLPLVIPKEVNTLRYFSAVGVGFVVYFVILVVIHACQNGLPKRSE
VQIVSTGNIGMEGLGTFVFAYLCHAVAFQVYFEMKARSTTQLLLSATLSMTVCSVLYWLV

GVFGYMEFGREVKESVLYMFDPVGEPLMMVAYVGLIIKLCVSYALNMIPCRNTLYYIIGW
DINNISYWKHIACVTLVSTIVLVAGLFVPRISTAFDLAGALCGGFIGFIYPALIYMYSGG
WTLKRVGWFQYIMTYTSLFVGVICVVFGTGATIYSNFG*
>TCRU_520 KIAA1430 homologue, putative
MPVQPKPTATTLWLEQQRQREYMQHRRRVEEQTSCIDNKPPHALSLSNKRALMEQERCKR
IEEENRRIVHNMTIIMKRGGGIDNKEPWRSANAARDAERRRRREQQRIEEENLRILKRLQ
KTKPAYSVEKWESDRIQNEEYIARLSRYTYEPMGSRRSERE*
>TCRU_521 hypothetical protein, conserved
MNRTDLNFVPLLNSTRRDSVACNKMKRLTLLPLQHSVITFGSPRRAPNFVPFSTVVEGNR
TGEVRLSDSRGIRRSQEEDVATEQKRVQQLSLELQRALDKLRVKNKEERKLRRKAAVAEL
KLGEEVERRTSTIDDLLELMQIHTDFMRGTNAELRRLLQQAAEEHDKTVIKLREEQEARL
GQEEVQQRVSILQAENKVLRQQLEEAQRAKVGLEELCKRLQEEKDKDEFSFLKVKQQLEL
DLMRTQAQLQGVNRELAEAQNSITASDTLMQQVRLFMQMVCQPDFYVVKDRSLQPVDKNR
PEPTGFVLVPLSILLQGYTLLLPGDRQDLIESYRARL*
>TCRU_522 hypothetical protein
MTIVALSMTDLKELPALLERALSSENSVRFQAEKEIDRLTLFQGFLSQLSHYAYLNLETV
PRSHRLLVLTVLRRKVSLIAIEECFEVSKILMDLFPLEEDRPVANLSAAVISIIVSRMES
IKTENMVVSGSLHFFVKWLSDEFFKFNSNRHELNFMILLSELINHLPIGSITRNEIFSLT
PRLFQIVVSNTPREASYLAVNLLRELILPAISIREFFLSGLIKKTWENFFPALSGALLTP
FFSSKGEPEYLVRNRLTLGITALFNDMLQVDKWTRKRMTANIFCGLLTRIEADNNVYMET
LENDENDDDSILVIQQIAAKRWELLGFMVHLKTLRSDLGKAIINNLSSFLNVVLSHTMLC
DKEAEEWLIEANDFLRKEEEREDEVLWNVRDTAADFCRICVSLLGDGGEIVRALLEFLIS
AEESWKKRESILFVLEVLLRRCPRETILRGVGCHSQLLQVLLEKDIKGPAILSSRALSLL
GILLSMMKRERIGAESLCASLIPQAVVSLSSSVPLLRAAACKLFHRLIPLAHVDDALTFF
SKGQEALFSLLLNESTSGELLYVCLEVHTEWIARCHSKRRDVASSESYNNLFLCWRNHIR
DPNMGELVVSLFGELITEYSGDEFLTGHFSWLADVLNSCCGTAEYCVVPFVVQILTCIFE
KGSDEVARKGAEFFTGALCRLLLGTEESSIISSASNCLAALLQRSPQVGAMKVLVAIPSM
EKPFSVLNGEKLSCSDLVEIPTLQRDELKPHPLSSVLVTIAMKMLGEEIKEALLMNVGGV
LVAIAQNTSVFQDDEIEILITTIVRRLMRVRTDTVTQELVFPLAVLMKLHMHAFINVLWS
SGILIEMFSVWLPKMSVFTVKGALFHSCDALLDLLIQCPLDERLCGQLLAWIEEDCKRGT
LPLDVALFVAVGKGLLNLATPVSLDYEEGEENEEMEVSEEEGENEPEEDQENKVVEQDAV
TGEVSHELRYLLQKIYPLVTNYGPAASVCFSRSELKNLSDLLDAVTASS*
>TCRU_523 phosphomannomutase, putative
MKKVLLLFDIDGTLTPPRLSQPDEVREVIRRAKSVGFTVGTVGGSDLAKQIEQLGEDVFQ
QFDYVFAENGLLAYKHGKEIHRQNLLKELGNERIVKFVRRALRLLSELDIPVQRGTFIEY
RNGMINVCPIGRNCTQSERDEFEVYDKEHHVREKLIKELQNSFPDYGLKYSIGGQISFDV
FPVGWDKSYCLRFVENDFDEIHFFGDKTHAGGNDYEIYTDKRIIGHAVKSYKDTVDEVNK
LISSK*
>TCRU_524 TLD, putative
MGQRQPVEGQGEKAEVHGEEHEEMPTIIHAFSSSIVSNDRCSPVNSTACVAPRILGFAHG
EELHRILPESLSLIIFNALPTSCWPVSPQQPQWYLLYSSRIHGRSFQKLVQGLVDKGPTL
IVIKACESSNVFGGFCGDPWQTVAAREKQERSRSAAARRATREGQGAQGITCRPANQNSA
FFGGENCFLFTNRDGGLIYQSKPSINSNFMYLFDSHPLVDKNGVGMGGQPGHFGWFIDRW
LEKGKCCGVCCATFCNPRLTDTEEWTIDGVEAYALHPETVESLSKAPRKGTERNSCLQNP
ANDADKLLLGLHGIYDFDSQEQPEC*
>TCRU_525 Guanine nucleotide exchange factor in Golgi transport N-terminal/Sec7 domain/Domain of unknown function (DUF1981), putative
MSKSVLQVIARIASLCKGRHAYLREVCLYTPGALEQTGGAGAAVNLLFYPLLIASYVSNE
EQMFLPLALEGILCLLRLHIVSERTMYGPPEVPLPEKKRKGLFATTDVTAPGEGLSFNSA
SATKSTPLTDVILDTVKRHRHSVLEEVSFLVLQCLTELMLNCEWIKGSKLVDCFQSAFEA
QLEVRPMSGSGVSNSLLPHVLSRILAGHRGANSVEQKRPSMDTTLSCSVDSTMGSNEKQS
TLDKCNSLINSMNITIAASSTDIVANRQHSAGEEEDEEDQQQKQQLTLSDHQDCYALLVR
ICDLSIKVVESRTPADSMEWRRKRLSTRLLAHFINGVNDEFARSDFFRSALHEKVTLSLL
HNCALEDPVLHGVALKTLYQLVMRYRNLLKSKVVIFILGLLLPIVNSKNTSYEQKATILT

FFEHTLRDPQLLMDWFTNFDCVQGMPNLCEQLVSGLSKMSKMSHMSSWVNAKQDALLRLQ
CIKALGTFVRSLEGIAKEFPMGGGITPRSQERELESRENQEMKLVAAENEKGETGAHSKN
NVNSGSLSECGVEQLLRGKKAFDAVVDKFNSGDHAAAIAMALNVHLLSSAAPEAVARFLL
QKELDPVGVGEYLGKDNEERKAILRAFIGLNDFSGLPIDDAMRLFLGKFKLPGEAQVVDR
AMELFAREYCAQNPSSFFGPGPAFILAFSIMLLNTDAHSSHVTDKMTLEQFVRNNSGIDD
GKDLPRSLLEGVYQRITAREIVLEARGAVPSNGLRKWSYGTKDMHPLSSSSSLSSLGARR
RNHRSSRHMEQAYLLETSVEQITRDVSSEPYTSINSSELVGALMESTWTALLAAFSIPME
EMENIELIDASLEGIESAIKVCCKFSCRTQRKAFISALLAFTHLTNFREIEYKSLKSIIA
LTRVALEEGDHLETSWYEVLRCISLLSKLHILAESPWTSLLNDRNGNHAAPKAPNTSLEG
QGRSSLQPQWERAKLERQNAEIIAKYIDEVEVHRLFSRSNYLKDAAVVSLVEALCLVSAE
ELAENPPRIFSLQKLVEVTDTNIGRLRYVWSKMWTNVSRHFVKVALSSNELEPMYVVDHL
RQLATKFLARGELGDFNFQKGVLQPFEAIASRTQSTKLKELLVASLGQMVEAQAQNLRSG
WGTVIEALAHCVQHDTNPDVVSSSAFVLQNITLCHLHLLTTSDLVKIVRAWAVVARSAFS
DDFAHSAVWFVRYVTIALALQADSNSSCEGVQHSHASQCGTSSNGEKEIHVDESHSHAIK
ENNKSHSLLLKEEIKVLALRAAFPVDGDDNNKVERGRDRTKENMQLFPVAVGALASLLVH
RSISGEAMEAMLFVMTDHRAVFTPEEWWHTVGSGVAPALDFLLQQCRFSNEAERDVWLSL
FKRAVTNVVQFVGYNLDGNPLPVDHVLFLFRVFVGRACCGSHDKATELGLQGARQMLELF
KEHAVVDELLWNGMVEYIQEIGDILLGALDSIWRYPMDPTQQDTLTPTLLALLQYVKWNR
DAALTPQLTTLCRTKAVKVLLKTLQRFMCLMRTSISFRDSVTEKKDVFDLALFIALECSL
CNAILDAERRYGEILENESNTLWMLIIGQFLAVTGKSSNVSREEASSLRGIIRELLDAML
EAPQRVMMARCRVCMPFLCQLIALSDDVFAESLANLFLRYHTATVQNI*
>TCRU_526 LicD family, putative
MAVKKGKGVARASRFTRALLICIAAFLLVTLFLLRSLESWDGHASSSSAAAAEGGLSQPT
PHARPPGSVTSGENAPDHAKMSRPAGTEAMDDDGTLVPLRDIPPSPYYAFGPRTMPRAFL
RILQNSSRRINKNWIRSKFNRYDKANISDCDVVTPECGLHKYLIKRTGDDLNARPYRKCC
VEHKALLETNVWVVRKLNSANITYFLSTGTALGVRRHAGSIIPWDTDVDISVYPGDEKKV
EQLLQNNGEHYFRRDWKGRFMFWVYHSKDGKPQTGPHVEIFYDPVYTRFPHLLLPLEDCL
FYSEPVKCPNIKQFQEWFPSGWTVYSGAHYHSPEKCIIYVNGMRRVTKRC*
>TCRU_527 DNA mismatch repair protein MSH6, putative
MDDALVDCADVYRMEYTYSFLAGIDPMRPPSSITIPAKELDAMAGMERQYWEIKAKYFDV
LILFKKGKFYELYDQDAAIAHREFGLKLVFSATNRGKMRLAGVPEQSFSEWARLFVFRGY
KVGRVEQMKEETDNSPIKTARTKVLQRELVEVLTPGTLTDPAMLSGSGPVFILALCPLQE
NVVDGLAVDLSRHVVYHCPCGVGRNDVLRKEETLLMVCALLQQLRPREIIFPLCFVPNAA
QADRKASFAKRLLDWIEGEGFRVELVDVSSLSFSKGNSDGVDKILDAHKFLAHYFQTLKL
YHAVPILSEAEPYTFHLPYASSNTTIARKHEGEVSNSSSILWHERRVDRGLVLDATTVSN
LELVSNLRDGGERGSLNQLFNRCCTNGGKRLMRSWILRPSASSRVILARQEAIRFIIEHK
LDELWGEGGEPETTLGIAAASPTLRAETPREHEPICVVKRERPVGSKFETRFSSLVDVDF
ERNLSRLTDLKNNSDAQVAFVDPLVQYKKQLQIIVTTVQALEDMVAWSHDIQKGITSSPP
LLKELWAQIDAAAPAVTSIGNCFDRHAALASGVIVPSQGASSVYDEASGTLDAIEGKLQG
ELRRLQEEVFDGTAINYSVVGHEQFLVEVPISAVPKTPLRGFVERSRSGKSVKYVVASLE
PLVESHKKAKKEKSNALLLVLQNVASHMCHYFPLLYGATEALSYFDCLLSLASLRKCGVT
TAWPIVKDDAVGASLEARELTHPFLNRDSVPNDISLDAAHGRILLLTGPNMAGKSTLMRT
VAVNVIIAQMGGPIFGAFMRLATVSRIFTRIGARDASHKGQSTLYVELSETADILRHADP
WSLCLVDELGRGTSTHDGYAIAHATLHSLKERLPVSPLLLFSTHYHALAQEQLGGDAVNV
SSLAPDHGSVVQLGYMDFAISDTKKDGVSAITFLYRLVSGICTRSYGVEVALLAGIPSSL
VHMAAIKSHELASWNDRQKDIHTIRQFLHGPNAATLFKKKKS*
>TCRU_528 hypothetical protein, conserved
MSGPLLTLAWVDNHLKRRGLPSKYASESRACTGAISEAHFDGPHVAPSSSTDSVPPVEEL
LAAVGHRQMPGGKRESVQSIGDGLQNTGVDDSRDAKSGDSLIQSQRSTPQRVASGVASFV
EQQAGRTGTLNGPTQACLVLHTVLTACVRHALRYSRIDQSYDAASSVFFPSSPSAATTDV
TSGALGDATLEHGATLEEVAASSQIRFPWSARVASEHLLEVIQDAAIQRGLRSFLWKVVD
KEHKCIMRNAAALCLNEAALLADIGVEEAEVRRLFSIPSDVLSSAREALAFSSSAATSSV
RMSWLNQQPLSFLGEHHFVYTTSSPRLAALLKEGTSELKPIELERTNARVRVGAGVSSPD
GFARQLNLLFLEMTRVRMALVLILQFEILMRRYAAFGKQQPGGVTTAFLPSSSPSSLAHE

LCALAIAAGSRALETSQHRKRFVTQFRVLARKPEQRQHTNSNLDDAPHCSLKSVFAALSQ
AAKANAATERAAVDGVSASSVPLSTAASSLSAPATGSEDPLEHDPVLHAVMSWKPLKLEK
ATDRNGATHACESLKASSAEGSTDPPVRKKKKQKQARAGAEPLPEEQENQAGLTWHFRCC
GGELGAEFNAVDALPCGETDAKVQLLRPSIRVPWPFVLEVVAAGKNKKKDAMTISPSRLG
ALEKFTRPLPSAAMRMRYNPAWMTGDEKITPLDARHTTQLQRLLRLRSDRLLMINGCPAE
KLKSVMRIAERSVRLVVRFR*
>TCRU_529 hypothetical protein, conserved
MGNGASVGENRHSQLFQQGYKSLQEGRYTYAEIYYDQAIERHEGHSFWTRLDDLIEWERR
DKAGQKGTDAKQKTAYRDGGQEEAAFPEVRKDSLKEENEVNVEMGEAAGVGGSGKIEIPL
DSRLADVVDYLVLRADIAESYMAEGRYEEATPHMDFLSSHTRVMLKFLQIARMTKAEESA
NSSNPLEGSTADAGADNAGGGNRRAHQGFMMVSDVERDSILDCFEQFLRMHWVSAMANSV
FIVSERFKKGKSEKKHKKELDTAVVACAKIQEELYNVAKRRARSLSNSVVLSFLEGIMEE
LDAESSTTSITNERMGTANGKTRTNTLFEEQNNRSTGNRSANEIDMLTDRDECPIKVHLQ
MVYDRRSLCREVPAPQQAPTLRYITWIDDEIRRVVPGTNYNLEIQCGMVDEKRKKQLQKR
SLKFAAQRNCNYNDRVLLGKSFDEENALLLFPVFLIQADVQFELGAATKGINALDIVDKL
STQLYGVGSLERQSIMRSVMESRKRGGSLFMMFED*
>TCRU_530 hypothetical protein, conserved
MTASVTNAVKVENGFQFTRPGSGATWPVNSRERTGLYTSVSRDFTLVATVVIQNFPRLST
SLLGAVLTSPVSTTIIGLSHSTEGKWEIVFDGNKTAHGRTWEPGKEYQAALMVQGGNKEL
RVRGWPACGELGNETNT*
>TCRU_531 hypothetical protein, conserved
MSRHLFYSAVLVLLVVLVCCGSGAAHAVGSVSWCVRLLQCAEIFVLQETPVKPKDGSESN
VKDCFVSPSLVGAGGVMTVLAEGALARCPTKQVYPADFVAGYANPAWGFSSLVAEAIKDA
*
>TCRU_532 hypothetical protein, conserved
MASFTSCARILYNTTNVDRTMHTIVHCSDGKYVEVPKAVADKCPFIEKVKEEMIPEFEYP
AAVLENLVCWTEHYGIDGFAESEIVRPCIYRNILHVLKNKWDNGFFVSRLTSELNIGHYL
QTVNAAEKFNMKGLHCFLCVGLSCKFRSEDDAELIHKIMGLPSNVQPNQEDIENVTTRYP
WLNEAIEPVVRK*
>TCRU_533 GTP-binding protein, putative
MGKPGKKAGKGLLPPTNVNRRVDANKTSLRDQRTIKRLKMYKSKLLRDEKGNIVKGSVLQ
ASDRVEQQMVRVAPDRRWFGNTRVIGQEALQNFLKEMGAKYRDPYSIVIKQSKLPLSLLE
SSGMNEGSVRQQMEWEKTFGAKANRKRVRLDTIDMEGFANRAVSKGEEYLTEKKGVDRNL
INKEENLRDDRSKNRILFKKGQSNRIWNELYKVIDSSDVVLYVLDARDPLGTRSSFLEEY
MRKEKKYKHFIFVLNKCDLVPLWATARWLQVLSKDYPTVAFHASINHPFGKGSLISLLRQ
FAKLQNVTHRGSKRTKTPISVGVIGYPNVGKSSLINTLRRKSVCKVAPIPGETKVWQYVA
LTKTIFLIDCPGVIYDRETNNDVQAVLKGVVRVERLGNVDKTDVVNTVLEIVKPKDIVAT
YGVSDWRDVNDFLEKLAKLRGKLVSGGQPDTEAASRMVLYDWQRGRIPWFSAPPFDSNKQ
YRDALELPEAKQMKLIEHYGTFNIVEDAMDHNEKSDEETLQNTCDYSGADSSNEKQIQKS
KKETNIHATVASYMSEQIILKKKEQRKRSMSEPAIKIPRAKETNNNYNDDDLWNRFLAAA
KK*
>TCRU_534 hypothetical protein, conserved
MQDTSRKRKAHEYYNGPGGTSPGSLGCHGAFHKTRCIEARCSNRGDIQFGSTRDLAVGEA
RQSVRPSPEHCSIFEDVYHNADPGAVAGRIDVKGETAEGQEPWLRAAERFSMIFITIILL
LK*
>TCRU_535 hypothetical protein, conserved
MELICQRRSTVVRRITDIPALDKLHEESERGDSRNVCAVNVGAEDVPSGAIHDFLPHART
ADERGELCGLFGGNIGCGAIHPTAARQDAAVHVGNGSNPAGHGDSGATKTSGAIGDETDA
PLDK*
>TCRU_536 hypothetical protein, conserved
MEGCPGGKGGRTHRVTGACRCGEDSIPGVGPDIGPRVAALRTGAMRRLPTLRQACTRPAD
HKEPSVSVAPSLPHCGGRPASASAGTSSRREHRNSLSTSERRIDVLLPNLRQSALVRPQY
EGTCRTCSAWRYNSLRGPPMRMPQWRHRLLNRRLARRSPPKLPSMPAGKTRRRHHRGYSR
WTRLFRHRLITDATGC*

>TCRU_537 DNA-directed RNA polymerase subunit, putative
MSDRDGDDDRLSLNFGEEQEESTLRSDDEQSEGDGADVVLSGRAASGSAVRRDGKDRVTA
PVLTKYERARILGTRALQISMNAPVLVALEGETDPLIIAQKELREGVIPLIVRRVLPDNT
YEDWRICELDIDFDRPADERYTNI*
>TCRU_538 Amastin surface glycoprotein, putative
MSKISHNRQGGNNFSCRVTVLIFTILAIGCAVCANIFPVFKKESNTKKIKQTLWYSQTTV
PGGVGTKVDVSDSLCGQYKLFFQVSEGAAVAAAGIGLVVLGLAIAQLILEEKSSRFTCCA
ASLISLAFAACGACVALIVYGYMKGYCQSDAVLSSQYGPLKDQGYKFSEAFYLICAACGL
FLLSSFFQCCA*
>TCRU_539 DNA-directed RNA polymerase subunit, putative
MSDRDGDDDRLSLNFGEEQEESTLRSDDEQSEGDGADVVLSGRAASGSAVRRDGKDRVTA
PVLTKYERARILGTRALQISMNAPVLVALEGETDPLIIAQKELREGVIPLIVRRVLPDNT
YEDWRICELDIDFDRPADERYTNI*
>TCRU_540 DNA-directed RNA polymerase subunit, putative
MSDRDGDDDRLSLNFGEEQEESTLRSDDEQSEGDGADVVLSGRAASGSAVRRDGKDRVTA
PVLTKYERARILGTRALQISMNAPVLVALEGETDPLIIAQKELREGVIPLIVRRVLPDNT
YEDWRICELDIDFDRPADERYTNI*
>TCRU_541 Amastin surface glycoprotein, putative
MSKISHNRQGGNNFSCRVTVLIFTILAIGCAACANIFPVFKKESNTKKIKQTLWYSQTTV
PGGVGTKVDVSDSLCGQYKLFFQVSEGAAVAAAGIGLVVLGLAIAQLILEEKSSRFTCCA
ASLISLAFAACGACVALIVYGYMKGYCQSDAVLSSQYGPLKDQGYKFSEAFYLICAACGL
FLLSSFFQCCA*
>TCRU_542 hypothetical protein, conserved
MESIEWIHISENRLWAENLMKQLMEKDAEEIKPPKLNIWGNIHDDSVSFESYWCTLLSQK
FRHPAYVAFRTSLFSALPLMYIGDRYPDIFPMHTLLALFGVAYSKDTVGEQICMISTMIP
VLLWMMLWGSLMHLFNLLAHQTAWWCAVFFGAFGIGLIGDVRGRKLAILMTLIVMEVERA
QLMVEKGALLPLLVGRDAFLALGFACFQSFIPPFTMCKRVDEAMAEAWVYIGEVVHNSVK
ACWSENPIDAAVALSKTSSEPLHKIFTTVPFELSFVHYEPWESPLRLQLRSERIKVIGGI
MPMLHALVGVVRALHAAQRSRSPLDNDQYAVSPGVGVIRGRLEEALEPYLQALRVTLKQL
GCVLNPREVVATVPFDDLQSATSSLQSTIDKIHYDMMKGRFGNVDSVWYMQIVFSHLMMV
LIGEELLRYAELMRNFDCSRYTSTSRRILDFFFLEHWRDFWTELPKRLTLATPNDVRLAK
DAFKLACGYTLACVYTLCIDFDSVYYFGMTILIGVGLQATGDSLVAGLQRVAGLVFAAAL
AYVIRRHHKSDLEAYPIALVLIAVALFTSSFPAYFNCSFYCALLIPSMMHMVAMPLMMFS
RVISNSLTVMTYYAIVVFVFPVDTIRVLHNSEVWVMDGISDYLSSLIRLLQAPVDDQEPD
VMQEIHALKACSSGLWRAVRQLPPKIKTASLEPTIRGKPYPVHEQDEFHPVLRRLLSSLD
IMLLGLTILHRARLGPVADELVEMLTSSRQLLKNINLYSVYAMQDFVDAVQRPKTWSCAK
TVDHFSTLLRLNAELKKMFSMAHGKMLSAIRGKANELHLNNLKLSMVIDNSSLLSLMPWR
RENSSVPHGNASICPYGEEEEGDEREELTAPRNASFMLRPEEFTINHDMNMSIAIFVGID
LFCSELVKTMRTMFYINQFELSRRN*
>TCRU_543 RNase H, putative
MQPHDGIQCTAPGHGKAECPSYPAQAKTLLVVTDSQSLLAAVTKGPHSHTDWTEDQIWQR
LLSLTRAGWSVQLQFCYEHCGVHVNEPADQYATQTTATGQYTEHGIAPIRHTDLLSCINT
QLTNKWRSTMHQDTHHYLLGGARPSDPGGKHLITQEALD*
>TCRU_544 hypothetical protein, conserved
MHQDTHHYLLGGARPSDPGGKHLITQEVLHRQELVHLARARCGESELWGRLYWTVRDCTN
QRRLRNISPEHSAYMRSNNDPTAPGTDTVPPTTREADAPPVERRPSTRRRKRKCPHCDAT
LVGHTNHIGHCRCFHPERPPPRLLRHGPPHTKKGTAQHRSRRAHNPDATRHRSSSARRQS
LLPQDQPASTSATISPQKTLHHLLPECADALAVRQRLGIKKDLRTESFSQWQLPRSRKLM
SLLDHLLGILVTSCG*
>TCRU_545 Glycine cleavage system P-protein, putative
MSQGTPPVILRKVIENPAWYTPYTPFQAEISQGRLESLLNIQSMIIDLTAVNLANASLLD
QATACAEAMYLAFHHGRKERMTFYYFIEGCLAILRRDGKNKG*
>TCRU_546 phosphoacetylglucosamine mutase, putative
MPLPLLFAQAVQECVSKFPLRHDARTAPLLYGTSGFRTLGVLLPPVAARVAFVAVLRVWW

AVVSRSEPAGCSVGCMITASHNPATDNGLKLIDVDGGMLEVSWEKVCTAVANATTAEELM
SELEEWVVRNNISVSSFSMDQLVARCPFSVLHLARDTRPSGMEILSAVQTSLQSLQLPFR
DHGITTTPQLHYLVQRANQPSMATGVTNVFEFGTALYRQDILSSLGALLNTLTEPTPNRR
GRRKIVLDASNGVGAIAIHSLLEAARQMSSQNFLEELFDITVLHDDVKNDGALNHLCGAD
FTQKTRSPSGKMKEWAADYGKQHCNEPREEREEVHYYSLDGDADRVVAFFHDCDGGDTWH
LLDGDRVAILYAMLLRQCLGQNALQVLDIGVVQTAYANGASTDYLQRQLGLRVYTTATGV
KNLHPVAHARDIGVYFEANGHGTVLLNKDSIASKVSSLADPRAQVILQHLPVLLSQVCGD
GIADLFACEFALLALQMDFNAWYHIYTDLPSRQTKVTVPNPRVITNTPDERRALTPPGLQ
EAIDAAVAATALASNSVARAFVRPSGTEPLVRVYAETGSEALCNSLCEVVEGLVIRYCDP
LKKDALPHVE*
>TCRU_547 RNA-binding protein 33 (RNA-binding motif protein 33)
MQVSDPTAASVEDTSSSVKDYRYFIGGIQRHITQQDIERYFAAFGDFEGFTMKRDSAGNS
RGYGWISYRSPPLGIARQDPHVLKGVVLTVEQARSRGPHEGNRSEKRSYVNEGASLRRRR
SRSPSSSSSSSVASRRGNYRRFTSSRQENVNSTSRLPAIAVGNSRLPPLSTQMYSSAPPA
QSSTSTTSHGNVVSTNNCDDGNTMTETYLCIPITLCPSEYLSDPRTFCARLDQNRVGSLN
IVPTPLVSTNAASVSVPPPPPPPPPSTRNLSQYGLIGERDSRSNAGLPQSVSSSTTRVAM
QRGLTH*
>TCRU_548 hypothetical protein, conserved
MSFKQVFLRDNEECHAFPWDALTHPSQSAGEEQRTIHHQLAWSAVCEREEADMGTQPARN
VSELTSVSFSFQPPFARPRRKTGRSSLGRRHDRPLYAAGSAGPRCGEKPTLFVGAAEADF
CEDMGWRQRSSSPVSSVEHNAEDDGEDFDVEPVVLKKSLSEKDRRKRLHLSEMSARLCHA
FAMKPFRLACGLGSDECDGGVGMENQSGWGAGEEAAVPLREKDDDDDTTPARQTAPISET
KRGNTALNFTDNDLSVVRAIALALMEEEEKKRRQEMRRKTN*
>TCRU_549 6-phosphofructo-2-kinase/fructose-2,6-biphosphatase,
putative
MSVDANDHTEVNEWAIHSSVEPSDTPIAPADTHPLATAAADEARKSAADEDERESRLLEL
IKGPIRPFKEWSWRESAYFKELREAENVEECRTNAKPEMTVEDTSVQSPENCGNPPISAK
CSLARGRIHTALSCEAVQTVLSSSQSSRPSAIDSTLNVLTPLLDNYTIPITRQGPEHFSS
DAQESVASLCIIMVGLPARGKTFLAQRICRNLGWHGYRAKVLNIQVAWRRLLLEYKRCHS
GLDAPNMLPVDDASSSAKCTPAKSEGTSKKSPTVTCSNSERDTEITAKQKDISDPVVSSL
PEYSLPMMGNSQKKNYVCAEQFRALTCDPDSVERRLYRRVLERYAVDAKSFYASGGDVLV
VNDDFITEELRDEAEALFSPLASQTFFMEVIRSNERNQKFNELKLHDKSEYPHDVNASEA
QRDFARRIEYLTEVYSTLSSTCQSPGEPSEDEVTLGDVSREKDKVPGKTTRRKQRGRRYM
KLWNSVEIEVHGVTGYTASRIVSYVMNLTQDKIQHPIFFVRHGESIYNAENRIGGDSVLS
ADGEKGANEILGFLASLKRYYCADTKKTADGEGHVNNDDDKAGGFCSSDCSAQCEKPVKV
EVWTSQLRRAIQTVENSEPLLGIKNVRWSSLNEIHAGICEDLTIEEVRTQHPMIDHFRKV
NKYTFRYPEGESYQDLVLRLEPVIMELENADRIVVVVAHQAVLRTLLAYFGSTSAESCVR
LELPHRTIWRCTYDSKGISSLAEMKFGLQ*
>TCRU_550 AAA domain containing protein, putative
MGGVAFFAVPTPLLAAASLVLGVAIGATAALTYINRTNRREFSPRRRSLRHAGFASFFSN
DDGKDGAASSSKRHLQQEEIIGGQKRRVSRRLNSGVDLHPGLFSMKSYAAQRIGQNSRSS
SISLILKSCLGAESLGTESQEDRDEGDAEERKEENDSEFPISAVGSEEEGTRAFLPSTKG
SGKKKGGNVQNVFFTSGEAEFSSLSTLEGLFSEDDASSQDNNEEEGQQEEQHSNNPKQNE
DACTTKQEETATGCLGRSGRSYSVNCILDRTAFSGLDFPGGSKRLGASQNDAVFSKRHFY
HTHSGAIITRLPSVAAVRCRSILQLLHPPSSTRKDESTEEDGNLSQLTQSIRSTRSLMDD
PIYRVVITGGPCSGKTSCLSYLRRVFEKLNFNVFCVPEVATLLHSGGVNLILSTSEERIT
QQRVILQMMMMLEDAFYELASMRSRPSLILYDRGTMDGSAYCSDEEWDAILKITGYTNEE
LSDARYDAVVHLVTAAIGAEQFYTIENNAARSEGLEDARRLDAMTLNAWRGHYLVSVVHN
EDTDFEKKMLRVAEFIGFVTGVSTLEENATESVVPKKTARSAGETTITTATPEALSSAAQ
TTKSPSLTTPFFLQQLTTVLSPHDQKGGDERDGEDDVAHMAFLATGTRAFANSASMATVS
AHSLCNNNTNNNTNDNSICSPLTPPDFVTQCGGGELGRTSAPSSPSLAFAMDMHAERGGL
QRHFFVNRVDLAQIPADAVVANVVTITLVPLRANYRTEVIIRLSRANDSHPLYLRRVEYQ
NPEQTTLPPDGCEHELKGHRPGRHLTRVKITKSAFLELARRRNPSEPVIQKSTKRFFYNM
RHFTLVTFQHPRELRGVCRLVAPADTTEEDIGSLADVEPFSLLMREGVM*

>TCRU_551 hypothetical protein
MLREGSLTASPQGLSGPSPVLLSASRQLLLHYSPNEMDSPAKKLETASSLHSSITEFMRS
PMLVIQNPDFMKTTEKSPTTHGDENLLHDVEELRRLVGTTQEEVCALRRAVAELEAEIHT
NKKELLMRLYSDLSTPPPTGLEDKDAGGAGVEVDQLLASLRNQLLSQVRELEEQATLCRL
NNTAKEEALRSLYASILAEEKNNEARKTEIQAGESVCHTLRLKLQEAQRRVWDYRRREAF
VSAAEKKYLEQMEKIHTLREEVRACGLHLPVFGAGGDKTGSVIPTCNRFCWERRCVGRQN
AVKPPLLLEAGGVSSDRTGRDWNEGREEGDPNAIVSRVFAFPPGVLYALQLAMSGDKGDR
STEKEGADDNEYVSHREMSLTGGGGRGLRHRARALGLSLEYQQHLEVNPYPPHSPEEQRG
TEREKGSSATRDGVKVIIRGRRWAVHQFQKELEAAMATLELSDGTKVYFFDSTSPDGV*
>TCRU_552 retrotransposon hot spot protein, RHS3, putative
MPGNQASAVPQGDIRRRARPESEGDTDQPAATRGRVEKVRRLMWALDSRVEGRTLWEDYE
SVYNARWHHVMEVPGGEGTGMEVHEGEPPRSWTYKAVGRTLEKNDGVEPSGAPRLRLMVL
TLGKKWDFLGGNASTDCYVNCEVDRVWQIVKGDLTEWFSSRPEDHFTPHPRLLTGTPGIG
KSMAAGSYLLYQLLHYDAELLPVVVFVVSGREAYVFNKTAETVTRYMDKSMMIKAVKELS
RRGMKGYIIYDVVYSLDIPSPFLPPGGWGMLVVATPETTSYIHWANEKRAVRIIMNCPDE
SDVKAMCVWKKRNQPPPQQAEYWREVKGRMDKLGPILRYIFEEGRYKYWIGDCHKLVDGT
TSWGIQSYSVFGTSKLWEGNKVLEYLARIVRARGERNGESPLNAPITAHLASKTLCKLAQ
LMTQAEFNLFVSRIRDCLMHANFGKCAMFAFLNVAFMAAIRRKLKELKPPTRRPSHRCAP
DVHSQEGPTSHYFLPSAERIGKKTCINHRLLYIPEVENFPLVDGFFFMDSNPMTLVGLRM
ATAGGRHTTASTVRQFTECLAAYFNGWEELSRDMSWEIIYVQHADSTPLNDWQRCDVVNS
DNVSDDESREIAAFWEEKVRQYQVSIPSRDA*
>TCRU_553 hypothetical protein, conserved
MAMMTGRVLLVCALCVLWCGAGGVYARDIDNGAFGGCMASGLLGTNWCHMPSGCKKTEIT
LPLRSALSITAAEASTDGEEATVTIRHSPGFNPSSVTPPIKQSESLAPTISSEVPPPPPP
EKESKEPLSSRPPVPGDSDTGGGNSDRDSGAGGTGGVVDGVGGIDGLSEVPSSGVPSSSS
SDPNGNDPSKTLSNTPSIQNRESSEKGEPSGKKSPGPVSPSGQTGEVSKLEEKSRASDTQ
EEAETSVAGKDTTKAEGTQSDMKTDGSSNTLSTAGKSKVQPPPPVKPPTAEELQPNRAPK
DPRTATEEKHEMPTSATKTNPDTPKTSLKDGMADRHGHDATTPVLVKNAATGNSAERKSP
SISTNDSDDAQSTGDENNDDDPRPKHKETGDHKAANTKVASTPSETAPQTVKTETFTVAQ
KNDTATSGDSDGSTAVSHTASPLLLLLVVACAAAAAVVAA*
>TCRU_gamma, conserved domain containing protein, putative
Elongation factor.1
MQCMTANLIGMCLQRMEHVRQYLLGVALMIVEERRHDVLALCVLRGRGLPAIVWDVEYTE
LFDWGGGGGRCGAAGVHDGLPLLGGARVEVSVACL*
>TCRU_555 hypothetical protein, conserved
MDGVRAVVDGRSRWRRQHSAVVAAVDSDDKSGEGSDGTLKGLSSSDQMPQAGQGGEEGQE
HTGSLEVTNKLEGNEDSQITGERNVSSDADRPKQTGSQVPAGGQPEDEEGTKSPQPPPPP
PPAESLPQIPRQPPPGTNTLQSEETHPPITAEKHSKPQDGGHSHETKMPQTKGSTKHEEG
ASSLQSPESVSHLIRESTTPLSTSTGSSGVAASTVENSGANNPTPPAASVTQHDSEGDTG
PAALPASKSELQSEEIPNAGIIATTYDDDNDSSRAAPTAERTTAGTRSTQTSGDASDLER
ANNDDVNDDVAHNGKTAEFEGASGDRNTEADNDERYTTVPETATNKTINTEIPADSDGIT
AVSHTTSPLLLLLVACAAAAAAVAA*
>TCRU_556 Regulator of chromosome condensation (RCC1) repeat, putative
MFFWGSYNFQTPNLVEHEKRPALRYPLYVPPTLAMVGDGGSHVHNVYEADDAEGERLRLI
DARRIVGKLLACGQRHLVAVLASVEHSYDVAAGAAMPSPLVSCAQDNMNWFVYGMGSNHC
GQLGHRVPEYTTTLTKLHFEELLSAGNTVTGLACGNKHTIICTSAGTVFAAGDNSFDQLG
VTAKVTGFAPVAGLAHITAVYAAGNASFALSNKGQLYSWGEAQYGHLCHGDDGARMDART
LQTVKTNVKIPALVQWFVRHHVVILEVAAARGHVVCRSSDEVYTCGEGSYGKLGLGGLAS
SFIPQRVRFPERSHPERLCGIAAGDDHTLVLRESSAVGAVVYHFGKMGNGDGQLTPIIIA
APATINRIGAGRGTMSTALTSDGLLYVWGKHSHNKVSNGTNADAKRNDPQMVTALRSFFV
TDVIIGGTFVVALANFQRGFIEGTQTAPEMNAINERNNCHRHTWDVVVPQDARVRARDAE
VADYTYETAVQAFLAQYLGPKLGPMYVAEMPEAPPLTDHNANAFVRIGAHALTTGQKVRL
WMTNVYALGTVVAVLTSPQAESNIDPIAMRNEEEEAGKSKENSPATDVKRETKNSTEAPV

EKKYGCRLRIEWQRDDWHDEVITLYSDDETLDTHNPNRWQPLWFLPDPENSGDFLLDR*
>TCRU_557 Serine/threonine-protein phosphatase PGAM5, mitochondrial, putative
MFVKVFFGGVVGVFFSSFSSCWCEGPSQRGISPDGLRNYTLESLPPPTKEELEYVKTWGV
PWVSDWDRPGVRGIRADRHRSFQRQIILIRHGQYQNESSNDDRIRTLTLLGEVQARRTGE
YLWKAFVQSGNKMVRAKDCAADYLASDASSSGGVAGAALGENHMGGFLIATEPKFIHVSD
MTRAQQTAKLILEAFPSHVRRRLATDAALRERFPCDPEPVFRNKYASYKDMRAVEGVFEK
YFHRPTADESSVEIIVGHANVIRYLVCRALQLPPEAWLRISLSHCSITSIIISGNGHVRL
SCLGSAGHLPVDQITTRNVS*
>TCRU_558 IQ calmodulin-binding motif containing protein, putative
MNTCAYLQPLRPQLVASLMEAMAHASSLQHVELSAAITIQSFYRMWRDRRSYRVLRSAVV
CLQRMYRGHMHRKRVAELREEAEASHERAVYEYYATRIQACFRGYYVRCHIDDFYARKFY
IEQTVRASSHVQETANKMLQDRLQEELERRQSKQKQNYKEATEKLHHLISTTCVSGIYRR
PVIPCSTVTVYGTSVENDIRINSGAALRSQHGRRMREKLKKTVNDGRKDGMVATTITDGD
FKKSHLNCAGDKNEEEKNVWKFSTLRGAWNRSGVEQVSLPPIKTPVGSGTKYLGKVVSHS
CIDEFEPMYDTDTAALKRFVDAKEIEALHGKKPFVSSFSSGM*
>TCRU_559 hypothetical protein, conserved
MSVPLAFLSKRVESIGLSESALPAALTSEQMQKIITNLEGLSSAENRPCGRNFFGRDSVC
AEASSFLVSQGCPSQSLVESSFVAAGEHSELGQMLQWFARAVVALFVDGVKSGTKFPQAW
EESMRRHPTLASHTFELQGVGALFSFLMCQLAAVESAMSKCGVSGEIGAMRKVELRSDQR
KAKKRNGFDTVSGQRDGTQEHVLRKNVPTQKNSNEAAVPKDTSITNTAAAGATTTTTTTA
TATAQGDNVDRQTVITSATKPRLSLRTLKMKSVQPLWNASGTTDVSAPLKPPIPCPPLTS
LSCNKSQEAHSGSFLSPMQPRQQVACSGALAPGVGGLDHLSHYSKWPDENIVPPLACTHA
LTIRSLSARNNREPVDSDGDACDGTSNFPKLRGHLSGTASTTEEPKVEKKQPFNSQFKHL
RLEDIEQQSRVQELRKQYGVKNIINYARQEVEDFKTEMQKMRDILDAEFNRTEDAA*
>TCRU_560 hypothetical protein, conserved
MDAAVNKLIANAMLYSDGVLHLVGARHNEEKSSLFPAPLTEELQMITSIPRNWADVDASF
SASPVSTVGLVGYLSNAGSANTWIDECRCVIATVKNGKNVTNGLQFAGQCYP*
>TCRU_561 hypothetical protein, conserved
MQHDRALKRRHIRHVDEGVGQQARRALAEAANPQVRAARKHAVAQRHVQRVSERVHRAQV
HRHSDRPAEHHRVEARRRTPAANRHRLRQRHATHAVLRIATTAGPRRRHVERQARLAHTA
SAAAAHAVGERRRQRIEARQSGRTAVARHHTHGGQAGHAVVAAAPQHPAAGTVALHRHDA
ARDCQPGDKRSASGSAARNRHGAAAQRHTRQRRHRRRLAHRPHVVQVQPPAATDRPTVAA
HQRRRNRPLDAHEAQHRVGHREALAKHKAAAAAQYRHVRHREHRVAPQTETRRGRHMQRG
RGRTTRRLQQHAVKHHRADAIKSVTPLLSHCPHSPTQPPCQVAWSRQNKHDHWRS*
>TCRU_562 retrotransposon hot spot protein, RHS3, putative
MIVVPSPNVGNYDEWMKQEGAARIIVNCPDEMDVKAMCAWMKRGMDPDKKAECWRMVKER
MEKVGPIPRHILDANEFIAHSAAIEDALDAINSLDGEKHFTHGE*
>TCRU_563 hypothetical protein, conserved
MTLVASCGGIYFPETPVENILTLTWQVKEGSEAAVPYGCVVFKVKSSSPKSFYVHPRYGA
LLVSDGLGLPRDPCSTQVTFGLRPAATEVLSEAAATTAPRSATSIGVGNGYHERIVIEYI
HFSGDRAIYDRLSQGLAANSRLSEVVRGVWDLMTAGTLHATRGTPITLKVYTECVTRESG
KKGDGVVVVVPPTAKLVTPALREQLLSRMSTAHAREISTSSNALSASEKHTRGAGTCELR
ALKLGIDALRNEPEVATTTAPVATDDTYNSSSKNVPRLQADHTETDILMRMPKLDSATTK
GSKDGVPLFMVLAAMLFTYVITMLLLRTI*
>TCRU_564 cdc2-related kinase 2
MNPAAGGVVYDTLSSQPQRIMPQPHYAALEANERYFRQEKVGEGSYGVVYKCLDRQTGRI
VAVKRISLKLKDEGVPATAVREVSLLRELNHPYVVQLLDVSLQDSKLLLIFEYMEKDLQG
LLKQRNTPLVGGKLQRIMFQLLLGLHACHSRRFVHRDIKPSNILISRDESVVKLADFGLG
RAFRVPLQTYTTEVMTLWYRAPEVLLGDNHYLPAIDVWSMGCVMAELAKGTPLFAADTAI
SQLFAIFQVLGTPSESTWRGVSSLSHHNVDFPQWRPTSLSSVIPTLEPEGIDLLQRMLLY
DPRQRITAYDALRHSWFDDVRHDMSEEAAL*
>TCRU_565 hypothetical protein, conserved
MVKEDELSCFQECVFNVCEALLIEWLVEAADLVNSEGGSAITTGTLDVTGRTCPPDIKAH

DSDRSDSLGVLGGKSSKASPLQARETSAVERVQYGESVENTAGDSLLVSGDPTPVLHKED
AQSVVTVPISAAVVKADIIRKQLLSGDQVNIALGPAVTGEVWSCPTPGVKKGGGSNAAMT
EAVTVYIVEQAGVAVEAIGDAGDLSYATVEATLWRGLIGATSAAVASTDEVTEEWRAGLR
ALAERIASDYVYFASKQKLRKLDEYLWHAEAFLTPEEITARSIAAVTPRRFFNGLQRSDV
RFLIHHHVELARKGTLNEDIYTEARLRENLFERQHDDVASPLLSQQTSGTTTKKNNASGR
TILAVNRRRAGLTQLVLGHALENLLEETVAETSKWIASLVAN*
>TCRU_566 hypothetical protein, conserved
MLWSDTNNPREDCVFLRVKCALRRGGGPKSVVEALRQFFELEEEQRARGEKGVTHKLFID
LLRDFGMRFREGETEYLCAAFDDDQDGYITSDRFVRHLTGLNTRRHKAVLRAWEVLPKNE
RGEVSRDELHERYALSGTRGDLSDTFASPPYTQGGVEGTAAASAEAHASGGVRLEEFVAF
FSAVSPEFPLDEQFELYMLREWDADAPARPTMNETQREWDSNGDPLAISKSMFVQDALNT
ALGMSSKSYNFSHMQRSHPHVEPLPPLERDYMSVMKRDFCYFPKDKILTANTLCYR*
>TCRU_567 actin, putative
MQIAEGTAKILSNIPLPDEVAVVHRQAAIVDIGSYTTRIGFAGDDKPRVDEPTCVVKGTG
KESSSLCLKKAYNCRATTEVSRVIENGEVDWEAMEQLLSYLDELLQLNSKEVQTPLLLTE
KVLVPRTQRQKLAEILMEKHGVTAVHFAQSPVLALYAAGICSGTSVEMGYNACHVVPVFQ
GYPLFHATHMLNVGGDLCTRQMMSTGPELPAMVHPNHRIDVWSHLKEKYCETCPEGAVFR
QAREEEAANKVAISSGNSDGITSKYAVRHRLPDGTIITLDSNRFVPAEMLIDPSLVSSDV
LSGDQFFIDNVERLRTSTEPHGLPRLILGSLNVCDRDISPLLERSIHLSGGCSMIRGFPE
RLDSELNAFIPHTFHVTARTERRHAAFVGGSILASLPTFQEFWVTKLDYEEFGPSAVLRR
CF*
>TCRU_568 Eukaryotic translation initiation factor 4 gamma type 2
MEPLRAASVEKPRRTSSVPGSLTRASHRRSIPQSLVDALCAARESFERYGVISLLETAVR
APVDGVREATLTVLKDDRGENPQQQQQQMELSTSLPGVSPAPPTTVSSGETKMEACAAST
ERVIGETVEGEMSTTTAVTNKPTSIVSDPPKEKLTASFTCLSYDATVTREAMKRLKHVVE
YCPGRTAAFLEQLREIVATTVPEIPAAPPIAVSRLKSNSSAYHTARQLEALSELWGNRRD
APVSVSSTLGRKRGLFGEVERKELHHLVISILNRVSNDTEKYREVKNELLRLPIPEANDA
MLAKIVDVFFLKAVREQHFSNLYADLVSALCKVPGGQRIVGDKQQSLEYRMRQQLLHRCQ
MEFRQLEEQSIERAERGGEDRASQGAEESFKERRDRVCGIVRFVGELFLRHIVTEKVIGN
ILVTACTGLYGREGLCVPPPYTPTESQMDETITLLGIVDATFFRTPLGSSMLVECAQFLQ
HWSLRHPVSRIRFLLMSAVERLQKLVEERNAAIAASQEQLQLQQQQQQTTQTADDPQQVQ
QSVQDGKRLTNGSVILPQPLSPSNEESYFNSTGSRSTSAMANDMSVKSKQLVFVGGKPIP
APRSAASRQCSVMSITSSNCFTNFSVQLTKQLATSENVAQHMQNVTLATRSLEEVVEEIM
KSFADYIAVVGVWTERCLTVVKAVKDRSLYGAFLSSLNRYHKGTSKQELRKVAMSTFANA
VCQRLHEDLGIFKYWAAMILSDTMREVLDEDLLNEALHYLLVNDPAAVKVYLRDVAKHMQ
AASSSPRTPSDETTNFTRYRPLQVVHRHNTHRKREYELNVVNFPDVRLASMEIHVFCALI
TGTPSKEALFDAVRSSSYLRCPLMAPEIFSAILHAELCSNTTAYLEDNLDLLSLVVDGQD
REIRELLLVAELYVVLKEGSTESLRPLSAGAHVMAKLGDSIISDETRLRAQKYFESRDDC
AHHYIGAQSAQLQARHVPSSGSSSVVNNSSSTNSNNNNMNTNSVVRRPAIQRR*
>TCRU_569 hypothetical protein
MSLSVSPPANGNGADVNESLPSHAPAMGETSADDASPRPREEKQQQQDESTAQDDARGTA
TPIHSAMKGKSGRDRSTDMKPRFNPEVSVIETPSYFLVMNNEESGDEAEELAVFNRLYEE
AKSRLERSKAAATAGKDGEVEDPEAKECTFSPVVSDYAKQLGRYTKFAEFLQTQEERRKV
SAMHFEKKKERVLGEDCVPLFEDMPRKHSLRIISYLEKERNYKGPIKGWRERFRAYMNKL
QEQDRELNPNKPKTNASTPSVHSGITRDDAVFDRLYDEARVRAVSQRVVAMTQLERESRE
LYRPKTNESEYRGSDGHSSQVSPSKGRSNSRCSSSSQRLSSRGGHLASVNSDSMSSVFEE
LYAMGEEYRRRREIRAMEAIENQEEFTFKPATNPESSKIIMERAIARAKGELPERAARKQ
RLDAADENDGNETNARERKRNIRFNPDIFCSRLQRKEFERLKRLAELRHQLRMDEASECT
FRPSISRNSQIMAMQKGYGQITFAPSLHSSPRHSRQHTAGSGEGGQEHHDARNGDGDDDN
AFQRDVYRHHQQRRQEMAGRGNSKGNADDSVRRSSSGSSGGGGVNEPAGRLFDAEEVTEG
YISALSSEIQGVLSQWTEAAERAKAAI*
>TCRU_570 hypothetical protein, conserved
MQPVHTVRTSDENDTYVLDGCVCNDGSSLCLSVSDHTIRCYDTHTATFLFTLTGHTCAIK
DVTTPASQATVLYSSQEDTGVMISDMRQVNPAHFLSEFCGTGATGGTVGVTPNGRFLGVA

VNGDTHIVDTRMWHTTHVIANMHLDEITRLRFLDDTVYCSAGEDQMINFIDSASEVAEND
MLLQAISCGEVVTKMTAFSHLGVLGLVGSCENAYLFPFDLQEKEVRYPRPDQATYLVEMC
VLAGQPYLVRGVRDDDGNVGPLSLMNWATRDVTQLAPVHRETCRMAIGVGDCLVTGGEDG
LVAFWHTNDTAGAVTATTLTDAAALGRGVMKSRHHVTRPLRSMPYAK*
>TCRU_571 hypothetical protein, conserved
MLRMCEAYQVNITVPQPPELFDSFLRALEEHQLVTSFLVEAISDWREPLSRPFPFVVSGE
NYLYRIMDDCSLLDASLLGSLIGLRLTEYPLGSRNDRVAESKRKRGGFVRGVGGLMCVEC
EEKPVVMQGWPSLWRRKKLQRWIHEIDATSKRRCPLGRKFKCSPADLQRGEAVIKGEGIL
QRNLMDELSMKAEQGLFVTLLATPDIVRGASDGLPLLEGCSALWYERAVGGKFIHRHYSD
NNNNNDNDNK*
>TCRU_572 hypothetical protein, conserved
MNDDEELSSMTSVSSRSLEDMTPMLNRFGRHAEEDVSKSRRELKPSGLSVVGRKLTAPWC
PADVVEAAPLQGGDDGGRATEPNCIVPPASHDAQALRAAADTLSGRSITFTAEDKAACIA
TVAAVLQGILVQTRRVLPCYYCGEGQPLSSYRLHLDFCKIRTEALYARYGLNPLRFMMSV
PVLPIPALTATDAEKSAFAKACYRSVKESILPCPGCDMSFRIHELPEHQNVCNNSSKSNR
IGGDLPLKEKK*
>TCRU_573 conserved protein
MSSIPDGLFGRWLLTKFNGLDLRNEVRRFRMRFSRAQNDVEVRALVVNVMSGFMEYRDGK
LTGGMASTLSLAPPFFMGMENFLRNGFAAGMDVRLEGDVLTLIHGEDVLVLTAE*
>TCRU_574 hypothetical protein, conserved
MAEAASCLDVKSSFIVSLPRETRHLFRCRVEDGTLEEPTQLPMGYKASPEILQMITLAIA
RVTTVAHRLWAAPPLVRVEVWIGNIRSAGSRSDATLWEAQVLRNADRRHATMGEDRESGA
TQYTFLAVLFDHTHRAVSLSDRFVWSVRAMPSLKYLTIAEMEVTASRFLPAAAILRTRLC
EYHFFIKAVRRRLSALNRGLC*
>TCRU_575 Solute carrier (proton/amino acid symporter), TRAMD3 or PAT1, putative
MYYAQKQRQPTSVLGAHGFTTEVAGRDRVRFFRRPVEARSIPTLYADIDNVRFAKSEPNT
PTPTGSQLAAALFPQRTASASGQKTRPHHLPAIKKRVHHAGAGTSRTFTTPMDGAGVSRD
VGMQTVYRENEAQTVPYTPEYYIPEGGDPNPEVLGIRQLKWNDGLPAGMEEVELIQRLRR
RRAVEASLPPETDAKSAEERYRTLYELEKQERKECEDHMAKIRQRRLDSIRDALMERERA
REEANLLRLENVKAQKLAELRRTIERTEVKRILKGQRLLEDEASKTAPSVACGMYAVDPI
DSHKPKKDLIEFHSKNGMFATTPQVTHGRSMSPVMKEPSDHFRHYDVRPTMLTLEEGVKE
LEETKVPRIQRIRADAFLAPENYAINSLPTLYQRREATRVVDALEYVNAKIHKQDSPEEK
MRIMELYRATPRLQRPDTPELELEDDEGEELEEACILLQRLLRGRAVQNDFFDGKERCRG
LIEELQAASNAKYAERSPEEKEMDERAKRFEAIAEGLGDAAQGDVIVETLDYLFHELERQ
RDLSRLEELRREAEAVRAEREAKEAELRAQQRILHDKEAVQYAAYIRAIEDVVECYSHQL
YTTSAEECAMEEAIAAECSRLERPPTPAREFLDADATENLVCDMLDSFVLPAVVDMVCLR
QEELERKAPAAAALEEANGTIPPKETDEKPT*
>TCRU_576 hypothetical protein, conserved
MIRIQRLWTQRSPFGRSNVALRFSVFHRVVSIASSCCSHSGTIFLAVRCVQYEQTSATLR
GINKKKRALSAPWITKSTKDYMSEADAKATAVNPIKEKLLQNSKERKERFVRIAEETDRV
FDSEMDRMRDYNVRRWRKGINFFKAQGLGFTLLYIFAYLGCLVALYVGFATGILKKETAY
EFMFFLLGGYVDKEWFYMRVEAWGTYVDFGFAFLINEMLELFRFPFMIYTFYTFRPYLTG
VNRRVKASIFRWNAAES*
>TCRU_577 Protein of unknown function (DUF3773), putative
MWRRVLFFSASLTVALRHTHRLTVTRTTTSTKNSSPGTATLWCDVAVAAQQVPLATVEEM
FGRMEGDDYDEKGDESAPHPATASLRDGRDAVEEEFFEWLDDENNTAAAGTGDLSLDAVM
NAALAEPDPGGERNASDEIGGNSDTKSSDSVRASHGNGAGGGEGRAVSRMDDILAEEAVD
DYDGVVAAGGPDVLFDDNGGNDEREDHVDDVEDDDASAIPAEEEEEDQVEQLIRATQTAE
SAAALFFKDKQRVVKATVATPSFDDAAAEGAELEIVGEVMPSPQRF*
>TCRU_578 hypothetical protein, conserved
MNLSVQKKSTSSLLPSTYSASTDGWANGLSADTSFANPKTVEDRFIGIVDYKEFLHNPHD
MCAHAAGSVAEARYRKTLESRMGLGGSGLMLFHAGDLASNFPRLDLNGGDYYPFSAGCAN
RGDGITTMDGQLQLPSFNADLMTEVAQEPNDGFCTPPSTPIKNRIEGAQISFPSSQRTAS

TFSSPKPTFTAHVQLNNGCHTPLAVSKNWGMGNQETRSSGMNGGFISGSPYSYSQRCDTH
RTTPALSTNGFPSPLRGSRSGDVVSTLATSPHPGRNATLHSCASPQLRTPVRHENDLLCT
PTQGGHGGNITVSQGASPVSRRSVLYSQHATPRRQGETCDRVLSAEGLSVCDDQGRPNFT
PLSWGHMGVVVALQRSVYLWQESGKTYFLFETPTAATLVSAVASSMRPTEMGDVFLAVGM
NNGFITVLKYYVGGGDSIGCDASQFTPTGILLEKTTQSSFMGTTSHVSTLYLVGHFLFSG
CMDGILTVRNLRDGSVIWHSSGAYAQIDLSSYASRLADYSVDVGAPIYKIEATPDGEHIA
VGTNDSLFVYQTNCMGPENRTRRRVVFSGAPRPVRAFCWWSFPFSSSNGRAVDAHHGSGS
GSRFDFLHTVLLYGGGSDGSVLSVYFVGSRCHKTTYRMSAPILGIVSSEASEEIFVSLDI
AGGEVENVSNAEEIGVPPVVDGNVHRVADNITVGGFGGPEDWASSGSSSEEEGLPQAPSV
GQHLPIGVSGRGGHNRMNGNGAGGMTLGLSPALARRFIHRIEATRARPPLPHMTLFREST
RGAGLLQLFQLKDGGARLERLSGFVGPHIASLYMALSPDNSLLATAGSEMKLRIWRAFKL
KSPVRGVRCELR*
>TCRU_579 hypothetical protein, conserved
MGKFSLCIESNLREVNSLLAFHNVHPQVRADVQRRLQRLTYRVYDHIGTLVPGRSEPAED
VCAAVVQELVENTIQKALPGVELAAEERRSAAFQQRTRDLELELSKSEQQCRELGERLDR
FRKAHNFMLRCYFREVLLLRYQLQDAMWRRRRSHNSQAQGGHFGENFKDSTSRGLLAVIP
ETKTPKSEYALPTIQASSNLSLSEAVQNNSTSSGSAEAIMDSEPQLSTTSELGKNNAPSS
PSTTGAVAKQSNAVVKVSSLTTMPTGEVPAKHVTPIPAVLAVAGVSSLSQPAEVPHSSPL
ISGVARPRPAPRKVETNFVTVNAASVKGVVQLRTASTQTIPLMGDDSVGAIFDYERYIRQ
LNRPRRGSALSVEAEISKSPQPKVLLEEQRRRKAKRLQLMKQKFAQYQENAANATLSPSG
KKSGMLQAFILGRKKSAAMEGRKTLLEEFNEIYLGVRGLREQHFKDMVRLKKEMDSIESS
VALLLGFFGTYAEEVSRLAAVLSCEEGAASSTMNVTMKKEDDPYRRRAVMDYFSAAASVG
AKPVLSSEKRENEEMKEFITLEQQEQRKRQILFWENNPVTLRAKAAFFELQAAAAKLREE
SSFRKGQKGAMDLLPTVVGYDAFSSALFKDLIRGKSEQEEEEGNRALLLLSREEKLKTLV
QLRMKRISHRARHRTQLRQLADGAKKGKKGEYLQKILLRASLHEKTVERLTRMINKLLRD
LGLSGEDLEAQRAFFTKNLASSPYLCESGGNLREDEESGVVYVPLPVSTRHAGDLCAALS
SNPAGYIIFNDEVIPIANVTYSVRTGLPDNVEQLRGQRSAAAAEAGGRGTRRVLPFHGTV
VDYYPGVQLGIPLGDNHGPMYVFWGSDEGSFFLVEDGLDAERTASQRAPPLTARQGHQQQ
LGQKRNVNLSTILLSPPLPEPERMRPFVTHSSGAFVTSPRDASGIETSPVVTYALERVTP
APSLTLQEERGKLPSCSPPAPPTIRPFRPMIVGPNREERDLMRIESLSARRGAGGANKVT
SLSYGADTALARLKGAQRERQQKTVASSKQTPTNGKTQGRTPNDHFFRMRLPYKRSTLPP
VFE*
>TCRU_580 magnesium-dependent phosphatase-1/HAD phosphatase, family IIIC, putative
MTEESVPLVIVFDLDGTLWAPEMYELWGGGGAPFRADPSDPNGAFDRAGTNVRLLGETRE
LLQKLSTDPKWSSTSLAISSTCDEPRWAMELLQLFQFTDSSGRRVPMLSLFGDLVEIYSA
NKASHHRTILRKLREKEIDVKEDFSQFVFFDNQTNNIESVSSIGVTSVYCPRGMVSGVFE
RGIQQWREKVKRSVL*
>TCRU_581 hypothetical protein, conserved
MSHGRGNLLEATADEFLRSISVEPDCQIKAYPNGAEAGIAQKLPFDARIVEVDGQAVTDR
GELREILLSGDASRQVSVVYEKTNEPSERVKIEESVRCDGCVSGVAQQPLLETVLNKIAD
EDLSLPVSQETPVQSSLSKLSSQTAFLLDTGSCASQSAAMKDGEEDKTSVASSDLTAETT
GSAPCHSMGTGYLNKVNSVPELLEFEKIDTPARTEDSFHVNLRRPSENSMRRRLMRAVEM
VAVGRCIVCVCYDVNPIAEIELVAKTLNMHIVTIDLGVKLRLRPKAEVFVEQFKAAMYDG
AWFVLVNAHKSIASCRILEELLKESESRNFQGFSSNARVIICLEPHPHFPRFLLKEAEIT
RIQSCLSASVSELSMATSMSCNRLVTAESWYSRRTSGWGVPTTSDTVVGTTGDKPRVRIN
ASVDVVDIAPREVVSAPRERPLNIHGAVVLRSAFTGVPGDKFLCVVSDAVEGRFAVGSSM
GNVYFVDELGNSLMQAHTHEAAIWDISFKERYQFATGCEDGNAVEWSFEMGGIDGAALSP
TSSTSLGNDVYCVSYVHGQTVPPLLIGGLSRSIVLCSNDRPVQHLTISSNAQVMDCIPTG
SVVLVGGSDGSITVVDVASNSILTSFKGHSRKLPALTVRDNNQFFTGSFDSTILSWDYRV
PYRPTGKPIIDGLPSANFTHTLQLKDYVTGLHVDDVHLAASVGENLYLWDVRKLTEVLGG
FPQAWKGLSRGVWVSSISRCVVTASQDGYVRFWTFV*
>TCRU_582 kinesin, putative
MSIEVHVRLRPHVPNGVWSSAETVLYSTHNPNTRYVYNKVHHIGTSNQSIFQGMEALVHA

GFDGKNVTVMAYGQTGSGKTYSMNGCEADPGIVPRTAKLLLDLRTKFPGTQIVIYFTEIY
NESVKDLLEPQRGELGLHDAPDGGVYFDKKSVPIETIDDFVRLQNLAERNRKYGVTNLND
HSSRSHIILTFEIHRTYRQVSTINLVDLAGSESASRANTDGLSLREGGFINKSLLTLGNV
VDAIVEKRSYVPYRDAKLTRILRNCLGGSGMTFILCCINPSHENFEQTVASLRFTQRAMK
IKNDPVMVLNMPPLFTHQYEAGAMGLVQGFRELCDAYYQRGIRDAFMYTSSTVSSIVTHF
DGQVSDSLHAMANAQRLLIAHDHAIAVDQVGRLYNQLHEVTRQSLQSKEIEEHERKRQRE
TEEEVEIRRAKIARLEADVKEKDADVDTELAGWEYQLYEARQRRVTPLELLLSSERSKRC
RLQYEWAVCMERIAARCVPTIISLGPVTPPVADKNTALQQLRQQLQQSQRELADLTVAHD
MIKGDLPELRRKAEENSCVSTISPAASLSPQPRPHGVSIATEATDEEIDQRIRELEGEEK
ALMSRAVHVARCESTRRLRETLKPPRSRTPPTMRSAVRHDNFSPPKEQQTPMADMYSGNT
RGDCGRHVSPVQQRSPTPRTARTIFPIAEESSGVETFLVSEKSASPKNEQLTDGVRRALG
LLRDVRSKLMNHTRGRQRRAEAADRPTASPPVDAAENYANFPASVNARSSSRRCRRVGSH
APQDENMTLAEMYMQRDEGKGHEAVVGIAARRRIEKAPLRPNAERALWRMSISPTSPFVE
MKSRSRSNRG*
>TCRU_583 hypothetical protein, conserved
MMTMTGRVLLVCALCVLWCGTAGAAADGSDERADVSGMGGGSLPELKGIGNSPEGPQGKK
DEAGDVKGKKHPASSEDKEEDDADGDEEDYDVVVDDVEQEEREEETLDPAETEIPNDPTK
EEASSPPQSKHKKGPAPAEGIPTTQSPATPVQPTPQGQPPTAPPSPEDSPVPAASTATVT
PAVTPSEQGNIPAPARKKDEKQSEDIDPAQESEQPDPHDVVLVQHSQGVHESKESNNNPR
NPVSASATANQRDATFNGRGEPAQTTSSTESVKQSNDDNSGTEEGISSDDPTADGAGTKD
TKNSQNKHGNTKETPVEATAVKATTATTGDSDGSTAVSHTTSPLLLLVLVACAAAAAVVA
A*
>TCRU_584 hypothetical protein, conserved
MEGSSKTPRQPQENSQKSTGQPPKLQVPPAKSPSQASDLGEADAGPVGIEGTGRNVIGGS
LDKNKDPKGREGHISGPPSGGASSSPISNNGDASQMNGGALPTQDTTSLKNNEQSGPTTS
SGNAPLNRETAERSTPDAKQHSSDTQENETSQVADGDANYSAAGQNAMGMKGSSGVSTTA
SNAPTTPQPQLPAPPVPPTATVTGAPAEKPTAERSPPPADSTTGGNLAATTTAPTNDTTT
KPGDSDSNTAVSHTTSPLLLLLVVVACAAAAAVVAA*
>TCRU_585 hypothetical protein, conserved
MNQVIRSRTDWKERVVLRLAWITASRWSEIAALTAKNFTLEPDGTLILDWPVAPKTARAD
PHRAFRFFRTRGQDALDTIKLCRTLQENEKLTNLTAAHVERALAPWDATAHSIKRGALRH
AAQIVETYNLNPHVISQLAKHFDPFDLPQNTVRYLGRYTTMLTQVSSLIALM*
>TCRU_586 retrotransposon hot spot protein, RHS3, putative
MNAGSYLLYQLLHCDAEKIKVVVHCFGEGEAYVFDKTAKKVTKYVGSKASRSVFSSFPQC
GMKGYIIYDAPTNGSQLPIIFTPPIGWGTIVLASPKVRDIYEFLRQRDSRRITMNCPDEM
DVKAMCAWMKRDGTPQEQEKYWWMVCRQMAFLGPILRYIFDAKDFSKRYNELDRVLRSVK
SRDDAKRITHGGMTAWFTGNPFCKLMCVKREREKKGAEDFKTELLSRHLAGRAASFVIKI
IPLNEICSLQ*
>TCRU_587 Trans-sialidase of Trypanosoma hydrophobic C-terminal, putative
MPDTMLQSSAKPQPSEQETLTTNIGGVGGVLSRAASTATTPSSDFAQEAATRSGDTMLGN
GSPQTPEVGVSSGANEETTEGRDGQKEGIHAQHGEVNATALSRSLGNVSQGNNSDAGTVR
GSGLLPSLLLLLLGLWGFAAL*
>TCRU_588 hypothetical protein, conserved
MLTSEDPAEHAGQAGQAIRPPPSPESDGTRPSDSSRGDSSVSPLSTAGISSSSALDAPLV
QSTQPSEKGLRSETMSLGTALPNDQPKERSESKAKQGSSTSEEAAESPDDSGDAVEKKKK
KGDVGLKATKSLSSTTSNPPVTQTTPRSSAEPSPTETELQAGEETSTENVTITMRNATAN
TGDSDGSTAVSHATSPLLLLLLVVACAAAVVAA*
>TCRU_589 hypothetical protein, conserved
MFYHGSHPEHPLRTLKNPKAGVLCSICKACAHPSMAEWCFCMQAGCGYILCCKCSEIEEQ
LFDIPQVITRNPVLPHEDMSVCLRTRPRSNAHVLCPLYPGRLMIAVSSAKGTLNANETYY
RLRKGGWVNAAHVVRVLPSQQLIEELAKTHLRAEEHHSHEFMALEEILLCIDESFRLMDE
RSPYGTVMASLTFFPLLQYSSSLHLISSEERTVVVRASLGFIHYLFHFVLEEVSQWMNNV
DHRVKTTALTLYVELLGKSLLVARDVAESQIVALPSELGPLITRTAAVVLEILPIVECGT

APFGGATVSLRAPSIATRQVDLAEEQARLFVCCCNMVETTGWLLLHTPTFTYDLKCLRPL
RVAVSIVPNSESLERMLCQVVNNALEKMPSLRRRVHDLDVIEVAISLAGRATCAESQMAV
FRLISAIAHNSPRNISQSASLVIAPLIQLAMKAWNTVVANAAFDCFAELAYNDPRVDGFR
SDSQSEELMLPVCSAALHPQEAPYQMANVYEVGDHLQALCEYCSHSHPLINGVLVREPQF
AYFRCQCESCAHVQLARPLPPPNIPTTPAIQEMLKENIARVVLFWLKCSESAVANAVGRL
SLKISLPVEVTLTLYRALLKQNLVGYADAALAIAAQSHLPAVQEMRREYPCAAISQYLSC
QPAFRAAAFSKETPGKDSQNEDPDGLSPSPDVTTKKTEMFASLPCGVSPPLVDLFTGPQK
MYLDEVL*
>TCRU_590 Sperm tail/Sperm tail C-terminal domain containing protein, putative
MVAFADDERDERIAERRIRVRRRLKELCDSERSTQTVVGAENNVSDGNGASGAIGLGEES
VRQARCEMAEIVSTAHSAVTSWEVNVGKNENERREREERLVDERRQKRREELKANEIKQG
AIELRFESIYKLNAPHELKKSLEEQEQQCKELIAAKDRLIELLREQLEEREEEFVAILQR
NAADVNNLVAEMRGQTEKYLDAYTSQLREVEATYENERKEYLTRCNEEIQQLMKLRRTRE
TEYRKKREDEIMEAQRKMDDKHRGNCEEYNEIKREHLQEIHALMEELERCKAEFLLNGER
LSYNLQILRERVKENKSAQALHKRKLGRLQDTLSYLVARYAESEKKYQRTNKDLTAQLHR
VANQYRDLQKKFQKFERSDKEKYRQLWDMHEEKNIQLVHKSLQADRVLFEEILGVPWNPP
QLKYWLDDEAADAVEDNEVDVSSSDDEIELSEEALMLLAILHKQAPFIADENVYAAIRQV
QDATEDRAMVESILATLQIRKNEEMDDLLQYFIVDGPEETTALIRPQDAIRALSTFLTEK
QREKKQKEEGQKESAKRNKEMAKMKLEERRRAAEKEYWLRMARSVPKEHQRVWGALEKGL
KLYIAQLQQRKNLIVDTDSLRAQNAELRALLTQYLQSDVNYQLYIPPKLLLQSRAAS*
>TCRU_591 hypothetical protein, conserved
MPGGAQGHSTILQGLGPTSFNWHGSIPTNQADTHFEPNFRQFDFFLDTQAMPDESLAERV
YKVPEGESHWNLRTFWGQNLRCMPVVFTSLAGAAFVNFFYTRFLNRTGASIRWSFLKCYA
AIFAWNNFTKFVARERYFQRDFQRNQLYSFDEMRDQRDRQRVREALYEKQFVKNPIAEYR
VKAWQVAERFA*
>TCRU_592 protein-L-isoaspartate, putative
MAWTCSALSNASMIQNLERAALLSTPAIIASLQRVDRGWFVPDGMRSSAYRDEPLSIGYG
ATISAPHMHAIMLELLSPFLLGTPGSLPKAVLDIGSGSGYLTAILADICGDGSRVVGVEH
VEELQERSLKVVLQHFPSWVNEGRITFINGDGRDISKLFHPHTTMFDVIHVGAAAASVPK
DYLEAIKPGGCLVIPVGNENEVQHLHLYTKDEKGTITKKTHGGVSFVPLTSLRHQKTRS*
>TCRU_593 hypothetical protein, conserved
MDRPFASYELEVAVRDSSLGPTPGPDDMLNEFLHRLRYVARGTLRTMIHNSFANGSLPGS
WKMGDAISIPKPGKDPCRPESYRPITLLSVLLQLTGGMIHRRLSALLPHHPRQFRICTSA
FYVGRGGTCYRQITRGLNEFGIVEHERPGDDAPHDALVAIARSFD*
>TCRU_594 hypothetical protein, conserved
MKSSWSPSCSQKRPAAKFLKATRAPAPRQGPTSSLGTVDTGNGWRCRHSQFSRDWHTALI
PAFLGNNREGQYQRRHTPMQPHDGVPCTAPGHGKAECPSYPAQTQTLLVVTESHSLLAAL
NKGPHSHTDWTEDRLWQRLLTLTCAGWSVQLQLATNIAEYTLTSLQISMRRRPRQPGNTR
SME*
>TCRU_595 retrotransposon hot spot protein, RHS3, putative
MKGYVIYDVTEKGMPASWFSPFNEWGMIVVSSPEVSNYDKWERQKGAARIIMNCPAEMDV
KAMCAWMKRDETAEKKAECWKEVKERMDNVGPIPRYIFDANEFVAHSAAVEDALDGINSR
DGEKHFIPRGVRLWYSENASQKLVRVARARGEVGAEVFLNAPISVCLGRRIPHYFGERDE
*
>TCRU_596 retrotransposon hot spot protein, RHS3, putative
MTTAGGHHTITSTVRQFTECLAEYFNGWEELSRDMSWEIIYVQHADSTPMNGWRRCDVVN
SDNVSEEESQKIAAFWEEEVRQYQVSISSRDFRSDEALRSVEEPSK*
>TCRU_597 hypothetical protein
MHVCLPRRFGVLDPYFTLFGAAEVDGEVVLSRAARAWWTPLRDPVDDPSKSKCTMRFGST
RKEETLGGLGELQCTRLQKMTCIIEFTLTTTWGPFLEVVNPS*
>TCRU_598 retrotransposon hot spot protein, RHS3, putative
MKFTISTNIEDVLFKGRVRVNEMKLNDFLTREPDGRGVVYTNRDLLLEEFFKDPAKYIRD
KGALNEIQASGHYLGMKRAVKGEVIFDEDVRRLCDKGVNNLPGRSLAAAEVKATVHNSSK

HFLDAAAEEARNPTTTSTPEKLEGLYESAHNARRSHAVELPDGVERKKTVTGMGVHEGRP
EQSWSYRKADDAIEENDAVQQFGAAPPVLMLLASEKGWPYSWHTIQDLPKDVL*
>TCRU_599 retrotransposon hot spot protein, RHS3, putative
MYLLGPIPRHVFDEEAFNERCGAVKFALQSINEVTMKEYFSRGGESPWYSEDPSHRPVEV
VREIYAGDVILFNAPISACLEERTLERLPSVAE*
>TCRU_600 Leishmanolysin, putative
MAVPSLAPCTLTVSFISRTPRHPHRCAPVGPCYRVHLPAHGKPQHGEECCWCVWQGAVGG
GRLDDCRDFCEEPRDSDDINGMELQDGDGDGRTLESHWSQRHARDEWMAPIGCAGCCTEL
TLAAPADLGCMRVRWEMAEPMRW*
>TCRU_601 Thioredoxin-like/Protein of unknown function (DUF3638)/
Protein of unknown function (DUF3645), putative
MSAVQEALMTSTEELQKCIAALSTLSAADAADVRELTESVLHLANDIFAPIKKEGDNEIT
HTGHTANEVDDFVETRVQEYRLQRMQGCRSAVPLEWLLGALLSSDMASDLRAVNPFHGDI
ERLKAELVLLMLLANRSYMAEQAKQSVRQIVLFLELCARIRGASSDDGDDCSRRDFWSPR
PNPESSRSRLQELLQCFSIGVDVDALERGGRQPTSGGDANKSSEGSSKGSQGLRVLSDDM
VAVLEGRMLQLENESIELLNAKRHYATVGGGGDGERVSLDPRYLLFEFIHNILLRARQVE
MVRWFVDNTRAGVSRVQQMIMGQGKTTVVGPLLALILADGSQLVTQVMPTALLEQTRGIL
RRCFSVVVPKQIYTLQFDRAFDDTDTNNIALLEQKIAAAAKTRAILISAPECIKSVFLKA
IEQMHLLETTPTEELDAVQNSADERVSAQARGILRRTQQRSAMADAITPIIQLWQRGVLI
MDEVDVLLHPLRSELNFPIGLKHPIDLSGPRWTLPIHLLDAVFFYQRGRISCGAVSGTKL
FDVTPTSEVRQDDVGQFSPEEDGTSEERQTLQMTLLNTIRDAIGKGYEMQALQREPHLVL
LDHTYYKHLLLPALLPWVQLWLFHEIQCVSRHEFHVTTASFAASLNWQSFIESTTDFLLH
PERPSRESAVGSAINANFSPFAIQLLCLAHDWIHRLLPHVLAKIDRVSFGLLQPQDMEAV
TVEERERMPMSRRMMAIPFVAKDVPSRSSEFAHPDVVIGLTILAFRYEGMRFTDVKALLQ
QLKQDFARQSGPKEHRPAARLYHHWLRLSMSFDAQGEPHASCNMLTLPSPSAAAGVGAKL
PLHNMTGGVKTHDRSGIPLSQLQVTDEVAVHALHYRLRYLPEVVHYYLCSHVFPRTMNFQ
GMKISACGHELGSSMLFTSRIGFSGTPSNLLPLDLGDCFYEPGSDGRVLSVLTNPSVVTT
EVLPLDWTPLRVLDRIATSHPPYHALIDAGALITNMENEDVARYLLGKLSPALFDGVVFL
DSKDRQMILQRSNGLKVPVAQSGIALTRRFTFFDQVHTTGTDVKQAASVTAVVTLGKDLV
FRDYAQGAYRMRGVGKGQRLRLYLIPEVLSRIHATLGSATTGNSLLDVPAWLLLNSMQME
SLQFIKLSVQELANVWRKKALQYLLRDSIHFHEHSDLYTDMARCRRFHMQRPFVIEMEGE
PPLPDVSLLRSAIQEFREPVAYPVDSVIDTPLPFLQRLMDHMRERPQELIVGDAASQQLL
HDLMHRLASSLAIRGPELTTATNTDGSMNLDSEIVHEQEAEEEQEQEAEQEEQRISVASR
DDELHIPWRVETLQTHGKDHPTEGSCFYPLRRLRVRPQQPFICVDPVVQVSDNYYREFWH
GAGERRLKNVFLSLEWIPRQSNENAAHVIHSGLITLAEGESLRWMMHHQTPLTRLIGMAL
RVVSSGRYMDFTEPFVAAVPRVRAERVLTDPLTQRVSLISPLVSSVERDGALLLYRFFNN
DMFFLPEELAVLEVVMRDVPHTDRLQFFTDCLRARRRHRNHWDDAPVASLFVPVERPEAM
RPLAILTALRGRFENLYERALRAPPTAKLMTQLRFFHERFAMACKEQRSEVHPMGTSIVF
LPIDMMGRILHDVFPSVIKPFTPHDLEHALRYLSQQDAAGLDTPTPADMSNFYRVPLHRV
MEAFPVLQEEFFHTHLETMQASEKSQQEPWYCEVCTLLNNSTVRRCVACDSARPSAAADG
ECAATSMDMLDEPWPCPQCTFINGSRRQLVCSICLAANPTPLQEPKNGAGGVDADTSVVW
GCPAGCWVCSVEHGGCSKFNPNHFFYCQVCDKARPDLATLRF*
>TCRU_602 Leishmanolysin, putative
MADTAWSVRVAAHEIAHALGFSQECMEEKSLVKKRERSVRGTHRRMVAGKHVRDKAKAHF
GCDSLEGMVLEDEDGPPEKKSLTGRDATRGTS*
>TCRU_603 hypothetical protein, conserved
MFCGAKDAETPRCTSDRRHVGTCTATIVEDKESLVDKDVCPVVSLEFHEISSGTTYRTCS
DENVDYLRGSLTGGGLWCLDAELLETTDGDGHKSVRGVCAQVSCEGGTVRVRHLGSSGFQ
P*
>TCRU_604 hypothetical protein, conserved
MENIRKGNQVDSTGNTRNKEHDSATETLELLTASINTRHSGAPVNESNTTLLSGTLRSST
AGTEEQAKAGSMPLRQPKPPEEKLISEELPPTATEVEAPGGPSAPPPKSLPQSALPSSKK
VPDTTQSTEDPQSKGIEPTTDARQNAVTEGHAETTSPSSPVDDAAANDADKSNVEIANDG
SAVNAGVPEEEPHQEQVDGSEKQTSFTSTAKKHNATIGDSDSSTAVSHTTSPLLLLLLVV

ACATAAAVVAA*
>TCRU_605 BNR repeat-like domain/Concanavalin A-like lectin/glucanases superfamily, putative
MQLVAGGGSGVQTDDGTLVLPVEGTTKGESEKGGKSVSLFIYSLDDSCWTLSKGMSDCGC
GDPSVVEWEKDKLMMMTACDGGRRRVYESGDRGDSWTEALGTLSRVWGNKYEEQTKRVAS
GCTTATFENGDANKKVMLVTLPVHSKEKRSEKKEKSELHLWLTDNTRIVDIGPVSEKDED
EVAASSLPYKSAGGEDNNNEDRLIALYEKKKKGLDETPHSLLSVRLTEQLQRVKEVPATW
KKVDEHVSQPCPTSGAAKSALTDNACSAVKIADGLVGILSGSFSSDTWRDEYLGVNATVM
KGTKGVATGYAEGATFQGARAEWPVGEQGENQPYHFANHKFTLVATVSTDKAPKEEDNST
PFLGVRLGTEGGSKLMELSYNSEKKWKLFCAAETNREHSSTWEPEAAHQVAIVLQNGNQG
SVYVDGQRVGGE*
>TCRU_606 queuine tRNA-ribosyltransferase, putative
MTVDTTLFCFETNRGPRTRHDHDGDHCHHARRQQARSGLFHLPHGPLRTPIFMPVATQAA
LKGVTVEQLEELDVEIILGNTYHLGLRPGEDVLNEVTRLKNVQESAAKGNEANEPQPLRI
RNNMDGIHFLQGWRRNILTDSGGFQMVSLLRLAKITEEGVRFQSTHGGNGGGVQMVQNNN
NNNTNNSGNNNNNVAFNRTEECMEYTLMLRPEDSIRIQNAIGGDIMMQLDDVVHVLTTGP
RVEEASKRTVRWLDRCLSANMNPDKQGIFGIVQGGLDATLRAECIAEMVKRTACKGYAIG
GLSGGEAKDDFWRVVAQCCKGLPYDKPRYCMGVGYPEDIIVCIALGVDMFDCVYACRTAR
FGSALTSTGKIQLTKKAFANDMRPLDPACNCRTCNSFTRAYLHLIATKESLGSTLLSYHN
LAYLINLTRRARAAIEDGSFTEYVQNFFVSYYPKRNYPQWAVDALDSVGIELL*
>TCRU_607 Hydin
MYPSQTAASGVGGDGDGSTVIAAHGRLPKILTFEVNPQEICFREFEPHKTYESPLHLKNM
TKETQFVRVRQPQSRVLKLRPPRRNVTSVKVAPGLTVTYKVLFTPEESRNYSCDLVVTTE
REEFTVPLHAIASRGQLTLPESFTVAPCPVKSTGTTSLFIRNTGKLECQWRAEASLPFTV
TPSSGVLSPGGGVFPVSVGFAPTRLQQYESTICFLLGPEGDVVQKLPVVGNAVEVEVVLE
QNTLEFPVTFVTLESQALVRVRNDSGYTVHFSWKSDRSFAEERRAIEQSLLGQTEAVTHA
VLTSSKNEATRRRLREERRETLAGGGRVFDDDVFTLEPVSGTIYAKGSQEFIVTFNPQLA
MDYVATAYLDIGGRRQRLPLQVKGTGIGPQCELEYTRLDIGEVFVGAVHEYQVDIINKGC
IEARYAVLPQNTIMGRKFVFQPSEGVLAPGTQETLFIQLQTDLVGLISETFHIHLHGSLD
ELTIRIRGSVTGPALRFEVEELDFGNVSYNCLHSRVTQMVNVGQVPITFRLRVPESSLLH
GLITVSPSEGIILPKQQEEIHVSLVSDIVGNCETMLLVDVEGVGDGVDSIPIKAACLVPT
LCLSTERLQYGTCFVGHEYVMNLEVVNKTALLGKYNLVLLNEEGVRAANAIVVIDSPEGS
QAPKVIEPHSRSQVPVKLTPLAVGGLQMTLYVRVLGSNEPPLPVLLTATARGPVVTAEPK
SLAFGTLVLLQPSEREFVLTNTSPIPALFFANFPETSKHNSISTAFCIEPSKGEIPPHRS
TTVKVTARLDDARLFSEQIQIIVRHAEEDPLFVSVNASGKGYALVPIGPIEKIDFGDVFT
STMAEKNILLRNEGRRDVEVLWSGARARAGGLSPVFRIFPETVVIGAGSSCTFTVQGLVD
EAGVFTEEFLLKDKKEFRLIFTTCVTGNFVLPVIAYSKKRIVFDYVYGAEGETGNTVIMK
TFTMKNTTTQSLDVTLKCVEKSGEAASPFTLVSPKSFTLMSGETQIVAVNCDACYRHDNV
AHTAKGRILVAFANHPFSEYVSLLANIAFPSIRIEPEDRQVKFGSILTNTEGRSELTLTN
LSPVVPAEFCWSIEYRSEVPSEQPQRDQKRFDFVPFRGVIPPGGKRVVEAVFYGTRGRHD
ATATCTLKGGPSYNVSLIGWSDVTVRFESTSLDFGVMHYLERANKTISIFSPSRVAVPFT
VNTIALKQPGSIVVKPMKGVVSNKVVLTVSFCPMVPDEVVETFFVQVGHLDPQLITVRGF
GQAHTIMLSTSDKNVKLNRIEDSQYLRCMEELKDKEWVPYLEKSGFPCAAPNVLPVVLEA
ERQAYCRNLLLAAKNLTAGASVTEHIEEFRADTSKRERGVLARYVIDFGHMTRLDVRTIN
VSLVNTSIYPVGVILDKQVMESGPLCVEPRKIPTISPFSGTNIALTLFFGDTGNVTNGAN
QLEFFLDIHKGPRILVEVRCFVATPALTPCETVLDFGDMLLGRVKNLPLHFYNKEDIPCT
WRMTLTEPKQKPGAKNETDTANPVNPPLVVASRRQFKVHKERGTLPPQCGMSIMVSFMSF
TLGPAKTKLRLRYASNPEEQYVVLKGNVVGVNVELSPSQLVFPTVFPCQTVKHCFTIKNN
EDRSVEVFSKNFDEKHRVCVGILQRALATQKLNSGGEGEIHLPIREAGDSLPEPFLDFFF
TELQREERALEYRDENGLEIDFQRELRTGSCLSSRRRRASQARSELQTPLARTPEIPAGD
VPPTQPISKAKPQIVVITGPPFSGKTTQIRRLVEKLNVSQLDLDAMVRAEAEFDTAEGEL
IRTILSEREENRTPRVVEDVARSPSPVVAVDRIVKSNEVKILHALLLRHLSTLSSVTDVV
IDDFRSILTEDREAVLIAIERAASVTGRTLQVLSLGVSEATTGLRRSLDVQKKCREIAEA
AITLPLTEEEYETLDEEGRDAYNRRLLHYNACKRALTEASNEVSRYEAELENNPQLTVQE

HAEQNIIALEEQEALIRSASRSKKNVSEPRRRPQWEEISELEQYKELYTQLRRIGGVTHI
VVSGEAEEKDVNKAIVEQFFSGDNVMHSSPPSPIPFSLMEDKNESLSFLPNVGRSGMWEL
ITEETMGRTNTRVEQFCFFTVTRREALVKKTAKGSPAQTTLAMEETARWIVPPKSSVEVT
VVFCSDGLGQINIPFSFGITGTTQEITLPVFACTAYPEICRDYKKIFPFTKPRAIAGKRP
IRVFVMSRKTYEFGPLIVPGGVVSAQSSSFKKNRQLNESLSTFTRSTVPSNKESGAQVTM
TFTNTGIFPAEVSLSFGKEKEPNFSVTPNKFTLIVGETANVVLRATPETIGEVFSTLVVT
VRDNPTPLFVNVSCVGTRPAVTLNGKKELLVQYGRCLIKREVGKVINIANDSLMPVCWRI
AGVEKVPMEFKLSSTGGVLDVNGMYPLEIVFKPTRPGVHNISLRVEVTDAEETLFESIPL
TVKAEAHDVVIEWSNHVDFKLMHVGETKKENVLILNKSPYDVGYLLRLPKCLQNLISLNP
SEGVMRGLVGFKDAFLLNVEVTIRFDKEGEIPASLGVIEASFFDPVKNELLFPVQSIPVS
GEAWYNRFTIKPACIDFGFCVVNQSKKCSFELQNTGHFPINFRLFNYKNGPALLQQEDEE
GVGKKQRKSAKPDTGFQLGVFTVSPSSGTVAVGETFSFQVLTVQKTRSNNRETLGIYVDH
CDPETEAKGIPFELVASTTTPGIVSDLTSLVDVETIFEEQQVVSSYSQRNKTLRAYARDT
RTFYFGTTLVGSRVEERFRIANSSPLMCTVKLQLLPQYPREGKEKERDKKEREPLLEVDG
FQLSIDGEPEKINTLQLPSFESRFVTVSFAPSTLQSSRARFEAVVVGIPDPKASALEFEL
AGEGVLPSVEYILPPRLQTPFAVSLTTTMKKGSRVRGKEPRKIAAHQMAEFATENIVSDT
IQLPVTLVGNASSRTFTVRNTGELDAHIRVSAPDEFTSDISVSKLREVLSLPPGCAESFT
VNFAPTSIEKHRVRLRVAVTENPYEDRELWILAESYFNAISFENIDPGSEDQLTLGQCYV
SQPVEHVMLLRNNSPHAIRFEWSCSSSILQCTPQLGHMSPGSTKQLTLRLYTDTVSGETI
SSVFRVKSITTMEGDDWDNTQTKSRWAVLSPAEDSDAVENSTSSESNTRKNLRKVLEAVP
EPSYEVTGDISLQKTLYVSYATEFASYKITLPMSGDMEISDNLTFPLTKIFQRRIATLRV
KNTGLNVLPFSFEIHDPQRQSGREGGMPGVFTVEPASGKIDPRAYQDVSVIFAPKSIEST
SQVLVGSFPHSLEPKLYISLQGTAECPLVHFNVPASDYLVSRVDGEAGTNLDPNTIPLMF
YSCGLNSKCSVRFKVINPSTNTYRFEWISDPLSRRISPFRCLTPSGSIAAGKQYEMAFDY
LSNSIGIREAKWSFFISGHMSVSFLLVGKTDEPNVFLHVSRVHFGGVTVGNKDERIIELE
NRENTSFPFEFDRTMLNSEDCCVGVKPLSGTIPPKSIMPLTVSFAPKDEVSVNVRLLCRV
KRMSDPLTINVKGEGLRIHPSLTIEDDDDDDLAEPVFVPQLQMFHCFLGRVQPNTVVKKR
FVLTNDGQCSMDYVMLVPEHRFLKLSPLSGTVGPKQRAVMSLVYFPTEEEILRNFKIQCR
IDKVPVYNIRLGAVAYVPKVQLSFMNHDFGPCFITEFSSDSVVTKTLSMMNTEAKESVAL
DCILSKNGIFELDGTSFVLSPGEMRNLVISFAPPEVGDFTSELQITFNGSYSIYIPLKGE
GIIPRIEVPIRFIKLGGARIGERRVADLRLDCRSRAVTPISFVNCVDEALREKGISIFPN
EPFLMRPREVRTVTVSFKPTARMPEFQRELRMLVCGREMPLVVVSAFCEDAEVHLDVQNI
LFNDVVVGTTAFRRIVIMNSGDISQRFRWDPTLSTESEMRIVPPTGLVRAHSEQVCEFVY
TPKDAGNVFRRNVRVEFDDAPAATVNVEAHATERPKAEAVLTFSCCAKATTTKTVPVDNP
TDGTWTIKPVIDSRLWTAPKSFQINPRSTALLPVTYAPMFVSGEKDTATLFIPFHNGAAR
VIELEGVASHPMEEGKVAVKTIEAGTPHLENFEVYNGTTQPLRFQVNAQWTPELEKGMVS
VKAPASIDVPAKQMKICAITFVLLKEGDFRGTVQFCCPEREDHTQLFDLALRVVPKKVSS
RTELVAKVRNAAIHLMPVKNPLGKNAHANIRVENGSDVIFVDSSINIPPKGTVDVPIRFF
PLVHKEYPVATVTATSVEIGTVSCQLLLRSKPPEPEKVTRVSCPLGQTTVFPLRFTHYCK
SNCEFTVRFGSRVTPFSKVGSSSNVKMPGTTRPEGQEVSMEIQYEPTVVGEIKESIEVFS
PQAGLYIFPVLASCLPPQRQGPFLLRLGQVTSIPFRNVFLEPLTLTLSTDSPSFLVLKTT
ETVAAKKTINIQVTCKPEEGGSVSQTGKLTVSGVNNGETLQWVYYLKCGVVDAGPSVAKA
SFRGRE*
>TCRU_608 splicing factor 3a, putative
MFGGVLEKIRLFEADIEHHIDNIVQQKLLADVSNRRHQILRDHFILQEAEAIAPIADKLL
DLYLDQDDIVAVQEAPSEGEDVLSAALKEFEAQIADIREYHRTFRDLPPVKTDLDHPDAA
AMEQLFSVGERYGSCLDLEPHYHRYSNFMLTTSKRAQEEGDAVGSAWEGRVGYFAFVPAI
SSIILHDIDAHRKLYGFLQYKEFVDELLAYLIGFHDRAKVMEKDVLLSSLARCDTDAEEY
WSKLMEHKKAVVKSGSGNSDVSSADKTASSLIVPPPLRKYTKAFALWPITHLESMIVEGM
KAVSPSLAEVKTVIHAEGKIVMLLQTLLFDTLKETEKVLLRDYSKTAEELEWERKHLQEE
FLQSVEEARKKSATTVEGSVAQAAQYDVMATLNSKDVENDPKENTAVADDTSGQLLDRDG
KPVARWLIHLQQLHKKFYCEVCGGTVYVGPKAFKDHFGAERHAEGLRRLGVTVHLREYAG
ISSIRKVIEMREKVISNTASIRKRLHEDQEGEEMQDAQGNVITAGAYKRFQISRKGI*
>TCRU_609 hypothetical protein, conserved

MGSRRRAKRPSSAATEVAEKKVDEEANTKEAETTTLSPKVSEHPASESNVEQPPALPLPA
AEEPEAPAPKPSRRALQRLRGWEPVIKPPEGMAALPASPVVPTADVVWLDNSRRVTLIPN
SGGGVHTRRGAANLAAVSPPTSYFVCTRDPLIKLVRAGTQSVHVITSLPPGGDPCSILDN
VPSVGSFTQRSGEAVTRRSNVTSSFSSSSCFSRPREGLNGDSEGEGGEETGMGDEEELEE
ANGTNADAHAASCRKQPPNKVDTSTNSVEYGGTEADVGKGKKNATLKKSGNATPAATTAA
TAPPTRIGVLSPAQAMLPPPYRRMRRLKRKLHITEETPAFAIHSELRDKMPQLFPKAPFS
TEEDVLRSTIDWCDDMTLMYTRLLWEFAPDEFLKRALQNQQQHDNEDTKKPKLTENLLPL
HSLEEEEA*
>TCRU_610 hypothetical protein, conserved
MPLGEPQFATVSFLLGDAVEGTFRVPITDETTVRRLAKDAMHRLIARSNTGRHVIPRDKI
SVSEVFVKSGTDKAEIFAQDLVNQVVLVKEEVLYMRLRIRETVTAGMREEFLTATTSTVG
ATTSNIDKNGRMNSTTVQTVDVHSSTGEPPQEKASKGKKNSVATEKPVEHTSSSFLSSGA
AAGEKCEAQNGKRAKQEGGEETEVKKEDARTVAGGGKGKGTGRGLGWGPEAHKCFADNYI
TSPNRLMQKSRAKRKATAKKATTPVTKLKGKRELPEKEPSPEVDAVTQPKRRLGWGPEAE
KYFPDNYVASPDRIARLKRQGKRMQEKKKKEEEEAARQEREKKEKEEGEEEEAKMRKKNA
ASSAPTFSSIVQSLEKRLNENQHRDTSPDCTSTVTTRNIKGGTSEGGREPRTAPTALAVA
STPHPNHGNACRRSTGSNLSRKRRLGEIGEDGSALFPIVVERQLSFEAAEETSTTHTPKE
QSTRQLLEGGTAAGEPQQKAPPEGWGKEATRYFDVETYFDDPSKARLSAEILARPIRARR
DVVLPYYFSAIAK*
>TCRU_611 hypothetical protein, conserved
MLSRKGYIGRQLRPSQLASELLPADQTWCATCHLAVLKKNWPDHRVSRSHRLASAKMQKM
KRLSLDMWARHRAAPLEEESAHEESIEAEFERFRTEQRRRERMVQLSSWKR*
>TCRU_612 AAA domain containing protein, putative
MLQSPKGINILITGTPGTGKTSLAELLAQELGDFKHVEVGKIVKENHFYSEYDNALDTHI
VEEDDEDRLLDFMEPMMVNEGNHVVDYHSSELFPRRWFHLVIVLRASTEVLFERLTARRY
SGQKRDENMEAEIQGLCEEEARGAYDDSIVIVRENNTLEEMAATVDLIRLRVESLRGNAA
*
>TCRU_613 RNA pseudouridylate synthase, putative
MGRRIRYSRPKAALPEKLLEFLCSRFSYLSRDEWIKHLAAGHLSVVSASDSNAVTTSGEY
VLRQNDEIYFNPPRELEPDVDEYIHVVHDDDDVIVCVKNGNLPVAEGGRYSGNTLVGLLE
RRDAVSFVSEKYSAARQKRDRTREEDGDAGVSVHKMPASYYPIHRLDKETTGLVVLAKQL
AAARALSTQFEKQSERITAQVSKRTDPAGDGLSAAVFEKLLAEEKVVTKVYVAVLAGAAP
EGADFVMCDRIGLLFDSLWNEGDTEDAKKHNKLKKLKMACFPVNLKENPKRHGRPAASRV
RILSSDKKLGVSVARVDILTGRTHQIRIHCAQLGYPVLGDKLYETTTPGVAGGCCAVPDD
TYIARAKGNEGLVSAAAPRLRVKRHLLHAAVLSFLHPVSGERLMYFADPREWFLRDVMEE
EGAEGDAVRTTAGGSRSALEALRQLLDRAVGALGL*
>TCRU_614 hypothetical protein
MASGPTEPLIDEERFLLTVEVLLANIPSLVSDLSSAKYALLSFSDKFRNEVRQKSSLIPA
VSDAYREFLETRGSLQEEQRKLNELETQLVRSSPPNFLSVEEVHFEDTGGALLVDEPRRS
DGADAQEESLIEENLVKPHAASAGSTVSTAEGLVNIYETHKNSTLSEDASQTALVLSADD
IKRVRESESLLEEEREKLACLARETNVLEDQLELDRSQCEEMSEHVRSIRRNYQAILAEL
TAVESERRQYATLREEWEGRKGIYSEARRDMEEIFVLCRNEELAIISVVLEGVNEHVISI
ERELNEKKRSQEVELAVYQIAYKEQQAVVDELRDAVSYLQRHQWELELMAKRQEIEKYKR
KSIRRLLMASLSASLNTGEALKQELSEEYIKEAKEDHRAVSLVSARIGALEARRGVLAAE
LLRISSTEKDTTRMNCALRRMREILMDEITIDESMLQNEDELTPWYNRELLPPYS*
>TCRU_615 hypothetical protein, conserved
MQYSICVATDCDGEKANLRFLFDSYGPSVAQLLNRSIVAFNNLFRLRSVPRTFAVSAAVV
FNDVHSTWDRLERSTQLLHNSQVYLFQPDILDLPAEIPEPFEGHPLLGDEGSWPQCDASN
SPRAVGPSHYDPRPALTIESPRGLPMWSGHPKKSLLYGYGSRSRHGNHDYDVRGDGTNDR
YRRAAYATPKRGDNLDSIEKGQTSLLRRTLRYSSLSPPIRKPREPIFRSYSDGRRSILRE
EREKIEYQMRLPIDEMRRHLREETRQLERSISPMQRQLMNGSSQCSNSGEGRNLEMTKTY
KANSEGGYVY*
>TCRU_616 Endonuclease/Exonuclease/phosphatase family/Endonuclease-
reverse transcriptase, putative

MEPFTWLPAERYFYPLLNSIGAYQRYTYRLRAVRDVQRQKLLLSGDIEQNPGPIAVLQMN
VSCLTPSKLATLMAQGADIVAIQETWKSSEQIASMHTGDYVLYAQSRIGKGGGVAVLVRK
TLRSKRIPLTIPQHDTSLEVVVVQVALDQNRDLIVASAYVRPPPQVTQSFRRLVNCLPAS
SPLLLCGDFNMHHPQWEPFLETSPSEVAAEFLELCTDAGLTLVNTPGEITYARGTRERSC
IDLTWSKHLTVSDWSASVSPLSDHYMLTFTLHQAFKDTIPSAPKFFYSWGKCKWDLFIKD
FDAQLPAYDYKKQSTGIKAFTRALITSYRRHCPRGMHKDGPRLWDDTLMEAERIATDSKA
RHLQSPTPDREAEMQRTRSQLFLLLRERLRNTYLRRISKLNPGEPLAWKYISGRKKASLP
SPTSILLGGGQHTYKTARRAANALNRIFLPFHPSHKAVRISKGINRQSASLNINASFLFG
QQNNNESAATFTSGSSLSSSSEPQDKNEAATTSGLVAHLHSPLDAPFNRTELLAAPRNTP
YGKAPDRMKSTVRHCDMFRQRASDSFFVALTTVGRPVRSRLSGDALPSFHS*
>TCRU_617 hypothetical protein, conserved
MPGISVSSSAATTAVRSEASTEGSRTISDLSPSSSEDDARPKNTPDGDDAPHIAEDVVSP
SAGTPMPQRTAAQTDATATPGDSDGSITVSHTTFPLLLLLVVAFAAAGTAAVVAA*
>TCRU_618 hypothetical protein, conserved
MAMMMTGRVLLVCALCVLWCGSGGGFAEDESPAVGASADVGGRQPKPPQLETTPPNVQGP
KIPSADVGLPMLEVADTSLEGTDGDDVEEDDGEEEEQSAGKGNEGIRAQEQHGEHDRQND
QHVGKRNEMPMIQNQEKKEKRLEKTQRQREACSQLPRTAHRHIYRMMLEEAMRRSPEMRV
RQEMMIVDKIH*
>TCRU_gamma, conserved domain containing protein, putative
Elongation factor.1
MTANLIRVWLQRMERVRQYALGVAPIIGKDRRHDIVALCVLRGWGMLAIVEDVEDTELFD
WGRRWWRTLWRGGSA*
>TCRU_620 Trans-sialidase of Trypanosoma hydrophobic C-terminal,
putative
MLNASKVSIPNPGEEFLTEKEEETPSSPEEGKAEQEVGEALPRPQSSVEAPEGSDAAAVS
SGIRGSHGKREEETETDGSGELASPPALSSVGTSAHSGDDGMGVREGTTLQEEVPPPLGT
EDIPKADVERPIYEEEATSPEWATERQTQETTAPLVENGDSEDVGTAPGNASTTPGETKI
PSESNATSLSDHGILPEHGHYSELAGMALFAESTVHGCVSRVLLLLLLGLWGIVALC*
>TCRU_621 hypothetical protein, conserved
MPRGGRHAGGAHTAPNGIQGRPRNSPDYYLGNCGGDDGGSLLWAALPSVRVDVWIDNIRI
AGSKSGATLWEAQVLRNADSCHASMGEGRESGATQYTFLGARFDHTHRAVSLSEKFVRSV
CAVPSLNSSTIAEMEVVASRFLYAASILGTRLCDYYFFMAVRRRLSALYRGLCGRHPR*
>TCRU_622 hypothetical protein, conserved
MVSMRLNDLDRARHGAAGDVFSLHAALIPVYFLLGSFLFSTLMIFCFVCFFLLTEDGEAS
NSPHGDAATEGNDEQQGSEAHSGGEKGVEGGHDDDGKPSRKHSFFQESRRNRDVRDMPQG
APDTPTGSNGEMFLPQRPRQRLSDID*
>TCRU_623 DnaJ domain containing protein, putative
MTDYYQSLELPRDATQEQIRRNYRQLALRFHPDRAGPEGAERFKEVQSAYEVLLNPQKRK
IYDRFGASFVDNPVSDVLILQLGGRSILCIMACIPFVAASLVVIFTAFLVAYVAGRLVAR
DAHSVNDHSQAYWNYVKVFSPLFIVDIIIGIPTLVVFLCRCARYV*
>TCRU_624 Leishmanolysin, putative
MLTWCCAWQSAVGGGALDDCRDGCEEHHDCDDIDGMELQDGDGDGRTLESHWSQRQAKDE
WMAPMGSAGCRTELTLAALADLGCMRVRWEMAEPMRW*
>TCRU_625 ADP-ribosylglycohydrolase, putative
MVATLNHNSVPRTTNFYENSLKQLSDIQQKQTGLLIGAVVADAAARGLDGCSADEIAEFA
RAQAKFNSGGGEEEAEEELLVFVRAGPQRRSSGPSLTGALQHHSYTCLLLRQLLRAMASA
RGEFPVSYVKDHWVSIARAHPQWFAAEHASLLNVLGVVLPLPVIYPWADDTTLRSYATQF
IDFLTEPPVGQAAAEKTRSAVKSYTFSALGVALRCLQSNPDPSRNAAIMAVPGTADLFPE
DLALLCPARTHKELLSQLIDANSTGCAAGGAAATPISMCGGSSSCGAGSPSLPVSARPFA
RDVCVAREALVVARHARSFADGVRAAIRLGGPVCQRSLIVGGLLGARMGVRCISPSWLSA
THDHSPLATLAIEVAQWSWNPPHH*
>TCRU_626 hypothetical protein
MRAGNPGLELGLMENTASSLGGVAARSFLNDSITAVVLMEISDFSPDFGWLLLPELDMTL
LCMGERSRDVGVRFLPGVGTRFVPGERCRPFLIGGRKPSLQEASGPRRDINWDM*

>TCRU_627 hypothetical protein, conserved
MDAEPEHGSLQSDAVQADGDPAGDGDGGAAAHADPDGVGERHAVVERGGVPDTRRDDDGG
WWEPDAERHPRRWERRPDAADGGAAAAVSVGTRPAARHAPELCAGVDGAAARVWRSVGSD
AAQRDVGAQRDESVHRPGAGCARAPRLLHCCRRDDSHSLRRCGGVRRLQGCAAWVLHDQV
RHAARRRQRPLGDHRRCCGCGGCCGGGDRRAGLRPGDAGARRVCEDAMRLGAGACEHRCA
AVLPVGVRCP*
>TCRU_628 hypothetical protein, conserved
MVGRADHTQRRKQHKARRVHHRQEGRAHHVREGNHARARKQHSRHAEVLDVAASHGRVHS
RNQAHHTQHRQLEHAPAHVAAPHVRQQSAVHPLLRRGVPIPDGVQQALVQRLPRKLPVPR
EARAHVAGNQVAHTSRERHTVQHANNTNHAVMPAGARHRQCKHGAEEYA*
>TCRU_629 retrotransposon hot spot protein, RHS3, putative
MVPTLQLDMWRGVEETQRTEWTMSSTVEDILLEGSTLRTDMKLNDFLRNYVGGRGVEEFN
ENVYMEDFLISSNEFIQDELFLRTIEASPPYQEQKKEREEFYMLLEASNKLSHERIFTLR
QCRDYERKGYDHSSCKGKTKRSTHAGTDRSEAGGRGKARTNAGNEIYHLH*
>TCRU_630 hypothetical protein, conserved
MHSWVTHALEERYIEALQGLDGTEGVYCMAWEHEWYEDGIVMDWSALHRGLVASVCFPPV
AAQMDDLTRFGCCVRDDTLLVTSIDIRAPWRMIPLSDQAQRLSAFLLPPAANACGNGARV
AVEELRGYRFVKGPPGQRSVRDDVYALLQTVNRQLTRAGVKTYVFFSNVIVASKNFAQLM
HHTAAVFRALREAGFAINGNGSSFEPRRVFTVVPGRYWNTLTLDSLRREEDILAFLVDMF
YRWLEDCYVWLCETPPRSLLLSAGDDDKKLQLFSDFYTRLVYPRHDYIHFLAVKGMTLAD
VLRLAGPRLAKKMPCVGPQKWSTLELRLCLSALDAYNNFPCK*
>TCRU_631 translationally controlled tumor protein (TCTP), putative
MRIFKDILTNAEVVCDNDRPMDVEDEIVYVVKGSYIEVGGEDYGIAANVDEDAGEGAKGE
VDDSRQRVVDVVHNNRYTETSYDKNSYMAHIRGYMKQLLERIEDEEEKKKFQANAAAFVK
RVIKEIDEYQFFIPEGNEEDPDNGMIVLCKWDGEVPYFYYWKDGVKGERV*
>TCRU_632 START domain containing protein, putative
MAYLVRKTEFSSSPLGFASHKRGVPDNDDGLYREAVNFIDVERLLLADAPATGFKLIAQD
EDCGISLHSRPVEDCPMHLMRAEVELPCRPAEVLAYLNVSTRRRWDDYIAELRSVRILPR
PGGVLGESNVVDVDSVAELQLRPGQRRVALYYMAIHTPILLVQNRDFEMVVAEEVRHDGT
VWVKAFSTPLGYVEPLDPRQSKYVRALMLFSGILARPVINAKGNMESHVSYVALVHPMGL
VPSVLVNVVLGAQLNALKRLQRFIQQNPLSTLATEMAGKNSEHRSGAEYSGKAVIADRVR
QAAGSQSTDQSAANVAVSKKGKNRNDGGNMKNKEAARGGINPQIRRQFASLMGRVMGFMR
SKL*
>TCRU_633 hypothetical protein, conserved
MEEPKNEVTLGVRLATRYVPNQVLPSTLSWGFFMGVEDFMRLERCFAERSITHDGDPAIT
KEWLVHYMMQFLGDHLNLPASGVGTEEATVCGVQLLDEEKMQFYKEEVLRWASSFSIGVS
VHDPLWDACEVSNAELARAEHSMNSSTYASSFNKGASSSNKSGGYSATTTASSHEGAPRG
GRFGRTRSVVRMLLRYNRPAGHAQTQRKEPARHEIVRWSDLTTNLVRDYERFHQVDEKEL
SFTRVRTELTSGNSSLLRSVAGKEGLDADWSRVTEGRGDTETDVFTGSELHAQSADHILV
STVMDCVVTASKDGVVKLWNGKTGIFMRNLYNAGNAWVIGMFLLHDEEYILIATTKSQLT
VLDFPEGNVLQKYVGCISLQTALYEVVQLSANNIQRYGMKPGECGRHRVELRKDETAEHY
HARRRESQNCVFSKVLPAKPVVGFDAPTASWFDKYSGIFFFGTATGAVGAFDISADVRPS
ALLAGANSKPVCLLFLTEVHAGAVVGVFYTSYVTSVFTAGADGCIYRTPLGPTGKPSGES
RLVAQQARAIRSMQWWPPSKFYVTIHVDRRVVLWVIGRYGDPLHQFPSETQEVVSAALHP
RRQRLAVLLSDKTIKVYETHGNKSLATIAQPTPDSNFTATDATRQMMERSAVDEDGVVAW
HPYNSSIICCLRATVVYEPSKNQLQTESVGETEINFPEDERGAFSNRDVRDIAEDVKPKQ
RNLSAVGVVESRRVESHRSGVVAMAVQRQALRLHTFDEEGWRMWDLDTGVIIRQVNVPRV
ARMEHMPLKRATVASCSWTTSLQVRLVTGGQDASILTWDPVTCLPLAAEMLVHDVSEQLD
SDVNLMSHRNKLIAWSARLCRITTFNTGSILPSGIEESKSVLLRVPSLASITACCVVRDA
YLCVGTGDARIFFFSLRGGAPTHDCVLREKTEEGKGAVVHLIFLNEQNQNLLLVILDIGI
LFVYSYVKQAVVYRICLGRRFECRIRKALYIPEDGTVVYGDSLGRIRVVDVRGCTSLSVE
FRQAFILKSTFQGATDEITSLESFYSSGRRYLLVGSLDRMARLFYFDDTDWLHAPFSNVA
MQQNSVAAVKFVGAFGRDTWRLSDPTTFSEEPPTALRVPANNTAEEEMLLETILHPETRP
ESVRSLMDSLLDDDFSVSRARSRRGTKAITPLSASGSPRGMLTRPFFLTDISHPQEEVTQ

VPSLSFLSSTESARQPPVAQATQSTHEKQGPSIPSGDEEGGAPSTSQGIDRVSSMAPATG
RRPQFRPRPGEKFSNRCSVSLFTEPRPQLPSLDRQTEGPSEGGNPYQGFLAGSLSPPKKR
MALLTMTRRLEAVLPRKFANGEFFKYANELLMADAQLGLRRTKSGRARQEKVDLQLALEN
RRQLAERVEEYKKKQEKRRCDKRNTLMPFAARHELIPVVFPQPSVLSNEGNISFAWMLNA
SSMGLPQKPFAHASNTM*
>TCRU_634 hypothetical protein, conserved
MAWRPALRAAAGGGATTASFNSPSPISLLGRGVAVQLCRLTLFCPQRFKSTSTTATTGGW
SYEQVDTRTGRTPLDTLYEERLQRIRAVFESPLPPMPRDGNVSPDFLVGFAHYEYRLRDQ
YRAVLARALDLPVAAVRLSVAWSGRFDVARFNRVQKVCGVCVASGNTPHMAELQERIRRV
VRAINERQSESFLASHLVQASGAITEVEFAATERDAIVAERVHRWLCEHILRPHLVVVVY
GANNDARDGPNSNGVQKNGEFARQRAFIEEKWLKAFYHRRVVPHVLSFSRLKELFQDAVR
RGEVKGVALLQPKANPDEEARHAKKSLEAEAKSGPTASATAAPAEESGTPVKLLALDDEA
AWYTLLRSAVFRRCDNRVAFPLLFIHGDSVGGVEEMRYLLHNREALDAVLLHPNDVAFKK
KFMSRFRSSHSRLHMAEEMSV*
>TCRU_635 radial spoke protein RSP9, putative
MSSVAGDADGLLFVGRALSTAERAALQSSIPLLSARYETPVRFWGKILGYKGDYLVTQAV
PEGVFGKRFSFFSVDGGTSWRLLDPLTEDEVAFCDQLRGVYMGDPTFMYKVRRDIPPEPE
PTAAMPDADELLTAAREKYGAEKTGQEGEEEEEEQEQEEAEEEEEEEEDEDEEEHEEQPK
KKRPKFMIIAVPETVRLAHFIQLHDAACTLVVRGQYVFTPSGSGVSKNTMFAGQPVRHAM
KPSCYLRAFHPGNEERNRILYGPTYSSVTDRLSPITDDEPQGVWVVKYDPTASIVTVQNL
FYNGSLFWYRPGTNDCGQVYFGNGERDLETCFLL*
>TCRU_636 Domain of unknown function (DUF3437), putative
MLQGTRKGEGEAMMEPRDGNETIRTTTNSRTTRVFYEDPLLPYSLQCIHPRLRDAVEVKG
RAELLRGAQLLIEHGTPEDVARHISLKVRERESIVDQSSSPEEEDNLCAGIPLQPAFTSP
NSEYNSFYEADEELQHRCHYFLSTFTDEHESYNVPYDVQRRVVPLLYAWIVDWDFLRRNT
DCWVCVTQCFCRVVQSRRRRFSVRPDFSLPWRPLVEILISLFFQSDGVMDLNVFQGMRKA
AGRHLERLCELAALHFGADAWPGLWETFAPYFSADREEAPIMLCLLSHLFPIHHLSDDAT
GEPLPITERIVKFLLEDSLYWQQLSQNWLVYSLKILFKMSLHHVGVVDFDRYAEPLFSMV
LYELHLPIAEDMAVPKGPVGFGVLSKVSLFRIGENEMSAAEGFIGSMLPRRTDSPLWNHL
QRFVNATSVFLRPNATDKVVLRRVFAFYSSLVAVIHRRIKKQRSYQFMRATKKTKFEGRI
IREAYLWDRDTIDWFVELVAPVLRLLLHHRLEDAPRIVGLLASLSPQIVQRLFLPYVDAG
LRGHLGSPTQRGLALRLLAKCLFPLSECPATREECWLFLAEVLPLLLQWVNPGELSLSTL
VMNLILIACSMTKLRDLLGGQDAECSFALTLVERIFDCSAHVKFGKGCAFDLLMHVMNAL
SLNLSDETFSCCVSRVLKESEVQGGISKAHAVSCLLEPFARRAPERMMRWAIQTFAPPLK
NVSTPLNELQWCAHLLAGCIRGAGLAGFSYRHELLHCVQCQVSFITREEQILAAAALYNA
LFTAFTEFICTEAKAEPFSHSKVGDMAFCDTDELVYDKKQDEFEVSMCFCRSSAVQLTWR
EASEFHIAYVVDVYKRFVEDSVDIIRNIEQVLPPAEGERGEGLRSLLHIPLDKSADTKSD
DGVGKLGDAASATASVEADVDAFTPKNVLRGVLLWLRLVIEINQEFHADVFCAAQTFNMV
PWWAQDPKSTLQLLPQSLGSPLLSAVPEKIHNVLIEYAFRRVSGMTAAESITAKAFYPDR
SLVMQGRPRLHSTQVDEMDPRGLHLVLTILAELCNITPMTPLQYDHCYIYSQGPDVFANN
VVETPKGRLYLPAMYWRMKAESFTQKRRSLLTRVVSPKRLSELLRIAHTLLFSPYQLIQD
NCSHILTGCVPMMSRKATSQFLSEHLSVLEEISNLWSQENDVTEQHSHDHDPGNTTSPSF
RGDDDCETDYPADGPDGVKATTLKGSGRRRKHDDCKKKIAVDDESGYGDGDAPAPAPAAD
AAVVVCCCGGDGDGDGDDDKYEDMKGAMPHFSSQLRRIISSALSLMSAGFNSSYFYYDDD
LIYRTCELLVQFPDQLVVRRIISRVLTQSSYDLGCLSAIEGARAVRLCDKLLLLAMKFAH
TAPDRTLGCLLKIVALYRPSQIALLSPQSVPLLFRLAVNCHAKVRETSFHILQTLVLSLR
EPHPKAYVLTRRGPLEASENAAFFRSRYELLKRECPSFYGLRDLGLAFLPKAIRVDAEHV
SPDMFHDGEVAAVPAETVKLCKNYTEEKRDYFIDAAQGEAKQQEQRLAQEELKRIIGCLS
GILDDELNPSTDGEANMDDAARAKLRDGWIWKVMQLRHDQKTFSESRMRVWKSMGKLVGV
EPSVRFFTRVVRLWVNDFVELARSGSATAKESQQSLFSCLFDVLVAAIRISKRHPVAREE
AMRCYMDALRLVCTSALVPHEVLDSFLRSISALHDTLKVNEVWLIYEFLFAELKPFLRAP
LATDASTVKNETESGMTCCTSWCSGRTQEIVRVLNVMVHLICVFAQEINVELLPQLCKQV
LQNKELFLCSTSSSIQGCSAFMISEIMRLSLYQSEYIASNSAVLNEILSFVDCIEQLVEL
PPPPAPEPQPTLAPWSETPALGAVRAPCLSTADSMAVGSSPELPMEPCPPLIEGFSLDGG

RCTKPFGNLATVKTFVMCWCRPLPPLLNARVKSVLNVLVRALDIALPEMDALNLRVEAAL
QRIAYVRLPRNTIHDMLQMLCGILKEEQPYGKSRPAKVAVSRMLSRVLLTNLHRIGKFAT
MQNVASAALASIGHGDGRVRSEGRLLLAVLTRVASVEQIERIIRDCFMELRGLSSVAASM
TGGHLQSPHTLHENVRKRRRIALLLALCSILCATPGVVPPYVPRLMERLAAHAHDPAPEV
QRAVKRTFEEWWRSHREGWELEHKPRFVAANIQIDAMLPLLTAPAYLV*
>TCRU_637 hypothetical protein, conserved
MVSTSVNPEVKAETEDSATVRAAAAPSPLKGKRMPPEMNKVTAVRAPSVTPLLSVLFMEV
EQLANAVREPSTLLADTTDKLIQMRALLKTMMDSLRNCRHCIRRGQALSRLPRLVVTGPS
QSGKSTLIDFILQRRHAQTKLDSGATAVSKAEECDKSEHSLRKILSPRPMNSSSTSLESH
SVNRISDCSTSFSKLGGLPCQKPNGMNGMDEHEELEGILCSSTSNASIFVPSERGNKPDT
NHNVEDAAQHEVYNLPFLNERSRWRYGRGNDGARIRQPLRVVKLASISGRRMSKSAGSDV
VTGMDKNSPHGSMVVISGRFDITIVDRIRRKKGDAAHLLREGEKKLCDQANGIHSPTTNS
GVSGSRQTNAPSASAGSVGAGVRLFNPCRAPFWLLQENVGGGVRWEGEGTTAVQLSDSLC
AEAFFFTLPCEVVGTHRMVAIMNEFFEPFATLTEGDRCKDGGGEHVTSPSNQQQRHCSQE
KKGCAHDEKGDDYQSICQLISDITFYVLTFSDAIVLRSEGVQTTDKQLVNRSPPMANSLP
ELVAMFQREMLRLFDIHVDSWQVIPFSGPMSRVTRDVLTEIYEARLARMDLLDADDGPPP
TFSALEPLFSLNVSTLFPITSGVVNTEGKLQVAITEFCDVMYGQRFKESRGSFSPGQMEE
LVLQHALRDVWKDSGACQLLHIVRCFDWNSPHHTVSQIALSLVIWCRQMHLVLLQAQKIL
WQRLKSLQSDLQRINRDDDRAKSAVRQLKEESIPRQMTQSIVFKVQKQFEELTIRFWWTL
VTLLDEENVRYGVYRSGSAQMLTSTRSCPLLLPFSSLSMKARKRLCRQYHRFLVVYVTQR
YQTQMSQLQFIIEEKNQNLGGNTLGHAKERSMDQEEIRDSLDADASTLPPNLGSLMEDMR
HHIPFVAQNARELKLTMRKELSLLLARLNTEVINYFMAELANAFPALVKMCEVQRKIAKG
KLLQLVTTVNSRETYDSAERRLLRSMLSEIPRLRLLEMRPDQELEAFVTGLRSSLCQDQL
VLYVKHLDDGWNGQYAGCAAKTLAYLLKAEWESDARQLRSVDYIQPQNATLLQQLREYQD
VFWAPMEMTEMTPLPSDRGGNLACIGLGDQPSINGATGKAKEAAGTHVDNGSSVSKELPQ
EGEKGSLMGSNDILVSKRNPIGFFLAVWQEVFSSMSFRPWLLLHNVRYASLLNNITLIFL
RGQARLESVLKERSCETEAVLRSVRHEMHLLGVELPSSLKRLSSRAIATAHNMKKLNAET
IISLSS*
>TCRU_638 hypothetical protein, conserved
MADTVTAPTFRDYTATFSAAMPPVLSSQSASFSALLKPTPTALTKVPSIGIDCFPQRFGE
TGVPGAAMGRTFLGISGPMLHTRQMEAGLVRREIVLRSEFDRLRHADEERMRWDRQRERE
AMLAEERQRMLDLAKEIEMERAKRTEEQQKRLLAQERELEEWKHERAKEMEEQRRRRVEE
EDRLRARQHEREMLLMREEAELRLQAQKSVDDHKKSIETSVNSAAEAVMARLREEFRAIE
GQWTSRLERLQLEHINETASLRQEIERERQLVAHGESQLSETRNQLAKMTSQLEELCRQR
EGDAHSRGTETFMGEIHQKAIERLQRAFEEDKQSTKQWFVEEQQRILNSHERTLHEIQDS
HRAQVRRLEETLDASNRQLREMEIEVQQYKTKLAGAALKPSSASADELRLREELRTIRKQ
LNEAVDAKQRLERRSREIEQECDDGRKAQQALQLEMEALTVRHNAELRSLREENQQLMDQ
LTSAREAHAEELQQVRKNQRKSESAVTRDAAKEFETTLRRMNERHEETRRALELEKGQLV
RQLREAEQRCEDKQRRLEIELEKSEAARRDGERIQQKHDEKERALAELQESVEELKRKDC
SVADLSSRNRKLQQELEQLRHNHETATRAKDSELQELRHDLAETRRRVVGLQDELTAARR
MQEEVEVRMKSYVSDAELKEKQLRRSLEEATQSVQSWKDSYTQLQSQSASAAPKMQGLLS
EKETELSRLQETLDALKRDKNSLEKRTQELQTISATREKEMLDQASVVKGLNAEIVSLRE
QLSTAMRPDKEMSTQYLSRALDYSQSPRTPTVGGATDARPAQPKSTVEASKSSNLSLTAT
APMCSPQVVHPASDFTGVFSSSNPSMASRPLSVRALSHALTTDASPPSRSGNLSVPVQIS
EGVPLPTGRQSIPTVRPSLPGVFPPGVGTFNSGTHLPVGFSPLASAQANTSASASRSPSV
TVPAPSTSPTAPGHFSAQSVVPVPVPVHVPTPDPAVGHPVSSSTFPQTSQQINPSSGLLG
VSSGVSISQQLVPPPAPAAAATSNTASLVPSGVSSTISVPVPLSIPNPAAGNPRPQSVPL
TSAPVPVPIPLPIAPMATAPFSRK*
>TCRU_639 nucleolar RNA-binding protein, putative
MQEFHAFEVQPGKEHALTLPPLHGFHLSVVSIPHAGKGRSTLYVTVDGKSYAIATLDASQ
GILQNATDLIFNEAQKVIFRVKGNAPLHCCGYRQELDVDFHDANDRDGSVDDGPNAVPMN
GGKDKEEEEEDEEEDEEEDEEDYEEEDEEEDKEDDIVLHDPTQAPQNGYQGDKSEDYDTD
HEEEEDEDEEEMDDDEVQPMKRAKPEENSQSQKKPQQAQSPQNRPSNQQKPQQAQSPQGR
PSNQQKPQQAQSPQGRPSNQQKPQQAQSPQGRPSNQQKPQQAQSPQNRPSNQQKPQQAQS

PQNRPSNQQKPQQAQSPQNRPSNQQKPQQAQSPQNRPSNQQKPQQAQSPQGRPSNQQKRG
RE*
>TCRU_640 Domain of unknown function DUF59, putative
MTELVNPNPTVFRDALQHQPQRSAEELLQEQDESFRDPIDSLEVFHHIRSIRDPEHPNTL
EELKVVEPELIRVDEVKQTVRVQFTPTVPHCSMTTLIGLCISLKLQRSLPRGTKVDVYVT
PGSHEQEEQVNKQLNDKERVAAALENKNLLNVVESCLNEFE*
>TCRU_641 RNA-binding protein, putative
MKEAGGFFTVSCTDRGGVGSSQASSSVGKADNSPTSGQFSTPTDSSKGSEKQMHPGSVLP
AAGTSGGVFIAEKNTGFSVVAGNVPPSNWQTGSNAHWPQSSGKSTLFKSTLNPDAKEFQL
PSGQGNDALALTHASMFNPSLTHGVKSSTFMPLFAMPSTTTPWKTGSAVSSEAIFVSGIR
ADAMKWPQVSVHTRPFASRDAAGPPPSLLTPRTITRQVMEEYAEVCCSKGYHIVTVFNLE
PRTTQEELLAIFFPMCAQSAELLPIHCSPRSNRRSGVVFFLSKAIALMAVEKLDGFVPNK
QHQPLTVLYSGPDTSETGTRESSILSANSPQSVKSAHQQAQLLPQPAPETVTSGSPQMWR
HPTAVQMDQLHKEMWQFVKSTASSSSSGVMKHVVAVHGLTPENAQSVLLWNFINKGAERY
ELWPMNASGLSSATMPFKGISALVWFSQEAAAKEVVAWFEQQRREGGQALQIDAVYLNKL
DFAHENSSVGNAQLRNGVHDANNNGQLTAVASPSHMAIIENIDAFFFREYDDPKLYLVAV
HNLSPNTDKKALFSLFSPHGALDAEVYPGIVEFAGELRRSGVAFFDNEFMARKAAEKLNS
FVPFKQPNPLVARYLKKVWNSRMMTPLDCASSSSGMAESVAVAASGIKLNDMVVSVKKML
YAPVLDGERLMEKMLLVITHPDTTREVVEQLASVIVEALTAMSHHAGALSTPLVNALMSL
HEKLNIPQNVVKDSNVNEAVKNSELKKYMAYMQLIAHTVINLFMDKTKPHESRKVAAVLS
AYLFQFSYLVKTPYELSVLLMKKHEAALNRAREYMMKRHANIKPMAFLGDGEEEEHRPWL
TLMECLEQLTSLWRQNNKSRAKKDPFRKEYEQRVNEFSQGEISLKGGGSSLNTSTRPTPR
RVVAKVTPGTGSGGGGGSSSSPGLTHGSSNSSRIDPRTLGENNIMSHEISRESNASPVVV
LPVFCANDPNPSGAMRTDENVSMIASKLSQYQSQPQQTLPPQISRRDMPVRSGLSEGLCM
SEATKPTLGGSRISGGPVQSVYTHAELMERTVYITKLPSFLRRAQFRRILLHFGEFNKVR
LCRDDNQIQAKNEAFISASTSAVSAASSSVQGAEPSVILYFSFVEFAEQSSAKAMIEYFR
NIVTNPQPFSFLLDNSVVWPESSYFTPQNIQALLSVRTSPARNPIHDQLPMDAVLVPTAN
TPRHVLRNRPCVFGIDGGEKTLDATADANSSSSFSAGVLANPSAVCGSSGVGASGRKVAS
VVATASESLPLRPSSTSALQTERFHGDAAGEGLDLNAIVSAVAPAVMTNTWVSETVELPG
HNDHVFNPIQGSWLTDVTASTLGGDPLAGVTFSEENAHPTTAPAPDACTAEADGKDIWPP
CVPSCFHQFFDSESDADDDDDGKNN*
>TCRU_642 Integral peroxisomal membrane peroxin/Rab-GTPase-TBC
domain containing protein, putative
MSDPLRVTVECWENQRYHPLRGWGFHRLPMDRSTWSNKKGDAVICRDSFLTPVGWVWSSD
WEVAPNGNESDGWFYAADFSIPTRFCSAIPNAMSYVRRRRWVRTRMQVAADTGVDSVLLD
ALSLSSSFQADPRLVWLNTSFYGFVGASVKEQRENQRLQHISCYHLHRLLDISCSETSVD
NNDDDDDLCGEEKNGRGSPSPCRHRSVTTEVLRCVYRYGLPQCLRSALWLEWSGGSTLLR
ESPRMYDELLERSDRVHGQPLQDILQDVVRTAPRHPFFAKKGGGAEDLRNVLLALELACV
LPSYHQAFSYIVATVLLNLPAREAFWVVTALFSGFLPSGYHEHYTLQYDLQVYNEIVAHK
FPDVHVHFASKSIDVSVFASGWLQGLFCAHFPFCTAARVLDVLLAEGDSSTLVRFLVAFT
GKFRSEILTMDSSGSLGYFANEWARNCFNIEDVVRAMEKDGLRSVVLSRREELTGATVAS
*
>TCRU_643 DNA repair and recombination helicase protein PIF7
MWTGALKAHQHLRTMRRWNEARVGVGQDGMVCNERRCEGGEDSDRTTHATSHAHISIECD
RGVMKRSRDVATESDGNDSGSQLTPLLVGFTGSASVTEERGMMEEGGDDGAAHNAVEMVS
HSTEVAVCEVIVLDDDSQGAEKSNVLSSKNSTPASYSGKLEQLLNSEKSALVSSTPSGES
PPSMKLSAEQHHVFDLVVKYGRSVFLTGGAGTGKSHLLRAIIEALPRSSTFVTATTGIAA
LNLGGSTLHSFAGCGIVDFQTHSAEDVCDTVRGKSRAKRNWRFCKVLVVDEVSMMDAWFF
DVLEYVARKIRGDNSPFGGIQLVLAGDFLQLPPVVKHSGQDPRFCFESEAWCRVNPRVCI
LSERFRQNDEGFFRMLNEIRCGVLTAASLALLASLSSTTTVRFVQEATRDVRAEGVIEGT
LGTSPPSLLTARDVVDSRGRTRAERYDGFSILRARRAEVDAVNSERFNELKTEIFSYQGF
HWGEGKYPADLPAVVSVRIGCRVMLLKNLDVFVGLVNGSVGTVESFIDIRKVRDFSNKTT
PVDLRNMAKHTFLPVVRFDTCKDNGSKDSACGRLVMVEPHRWSVLQGDREVSYSVQIPLQ
LAYAITIHKSQGMSLSRVNVDFRGIFEEGQAYVALSRCTDLNSLVIENFDAHRVNPNAKA

LAYYKALADAVEAEEKAEKKMLHARLRHPWGYNDMDDNDNDDDDGDQEDNQSHSRIHSEA
ARKRRNMADVVEEVRQRITPSYILWSSLRQRVLSTAELASSVHGALLVMDATSLLALGSS
HNSTALYEKVFVKNQNMMRVPRLVKEELLRTASVSGVEVRVTPTVCLSFSSLRSNSPGGN
FVHEAAELAAGTLCLMEDAKSDFVLDEQRAGESRPLPPSVPEWEGFFSRCDWSDCELNNT
CVGPYSHPHQQHIELLEFSCYLLEQYSSHQAVLVCTESIELAARALAVGLRVCSMMYLCP
VDWARILDEGGKSK*
>TCRU_644 PPIase, putative
MVKGDCIRAAHLLIKFDGSRNCVSHRTGKSTADLTYDAALAELKQWAKRIADGEITFEDA
ARQRSDCGSYNSGGDLGFFGPGVMMKPFEDAARSLNVGEVSGVVRTESGLHIIKRLA*
>TCRU_645 hypothetical protein
MAHAGLAEEEIAAIRRELDTLKLQISTTSPDSSPSSLDGATSTPKRGGDDASPQRSVDSL
FERLTQEQQRTRQLEKELQALRMSQKQMNTESLDEISAKKEELDKLMRERDELRDWLQRG
VNECRRVAEEASRLKRDLEERTRRCQSLETVKEELQGKINKVQQLLIESQSQIERLQEKQ
RLLEASAGEHVIAEKKLMEVNEEMQKQYEALVAEAESHRLHAEKLEDAEVCVQLLTERLR
QLLTHVERLGQAVRKGCVEDVAVDEFFSRENVAEPQHPLDELVARAKSLPHTIAIGIQQL
QENIVAFVAEQRRLRAEQEATQRDHLEALHLEKQTVLHRYEKERETMTQEIAQLRGELLR
VLSKNAVDDDESPDRQRARIIEIEELLRSNKQQAEENERLRNENEELRQKLRRMKIDWRK
VHESQLRFQELQVEVNMIARTNEKLERENENLKMLIETRFGQLPPDKKIVEELHDDRRYS
GVNSKEKGVSLMRPSFSSVMMNSTTDGRHGSSGSPSVSYSHSRRSAEREVFQEWKRMVLS
GYIE*
>TCRU_646 KREPA5
MFTSTWRSLAPRPLNRVTLLGAMHDVQRGFLGQSSVFQFTLTCLLFEPPKRDQAEKKPVA
TPPSPTAKGGASQFVKEQYTVRCMGDEAYTEALNNTLEAGCIVRVIGRLKTFEVAEGGKK
QLFPTILVEQGRWSHVALLHSLRRQRRDWQLQNALTETRNLKQP*
>TCRU_repeat, putative Se.1
MNPSTASTRPNAVACTTHMFHVAKPSVPPKGRGVRRTKEAIPTRAAAVVAPLRRSELEER
LQYLLQAEEELASAVGPNDVMQVLEELRRRRQDRKAVALPRKRLSESLHFICPHVATLVR
ACESTSDEAVVQILREFYQSHYAAGGNEKGRSLVSTALAEVLLKRRANENDISEALSMLD
DAVANGHTGAMLLVGLCLRDGIGVPKDLEAALVWVERSADAGYAPAMFELGVMYEDGVED
CGESTLPADWGEAAEWYKGAADRGHTMAQLNLGKLLWKAAALARDAKPDNCDESTIVNLQ
TKSRAWLEMAASSGSEEAVRLLRRR*
>TCRU_648 cation-transporting ATPase, putative
MIPKNNIISEVRLLQRRHWSTWVTVWPFVVLYAVSFALYMDPELVWDRMTYLVHSTYIDF
FHAICTPVLLFLHGFLSLFTIWSVRFRSMILFKTLTREHMESATHILVCTHEHKGESEIV
PLMQASEDHPIHFVFQERKWKLDAKKGGFVKPHFPVKEPLSHYLDWQGLSGSAMCAEQLD
NFGPNKMEVVIPDFQTLLVDHALAPFFVFQMFCVLLWCLDEYWYYSIFTGVMMVGMECTV
VMQRIRNMRTLRDMAEVPVREVTVIRQGREVRIKTNELLPMDLMVVESNAACPVDSILVR
GTCIVNEATLTGESTPQLKEAPDRMELSLVMKKHGRHLLYSGTELLLSNGPHGKSDTERG
RALAVVLKTGFETKQGKLLRTILHSQGRVSENSGESFAFIGVLVVFALAAAGYLLKRGLE
DPNRSRWKLFLSCVQIITSVVPPELPMELSLAVNTSLLALVKLQVFCTEPFRIPFAGKVD
TCCFDKTGTLTTDEMLFGGVDMADGNGLLNQLKKIPKMTELVLVTCHSLLQLEGTDTVAG
DAMEKAALGALGYRVNLDDTVVYDPPAPKDTEAAGENNKNDGHGKSNKHKKHAEDGNRRY
KIISRFPFSANLRRMSCLVSTSEGKYVVAKGSPEAIAHLCTSVPPDYQQVADTHASRGYR
VIALAMRPLQEEERLKASVHNLQREDCEKNLKFVGLAVYECPLKKDAKSTIVMLQNGSHR
CVIITGDSVRTAISVGQDVGILQCRRQLVASGVGDGSGAVEWCDALTGERISLDQRAILE
KTFVRTRKHMSPLDDEWDLCVSAENLSPSSMTALIAAYNEQIAVWARCAPTQKEDIVTDL
KKKDHTVLMAGDGTNDVGALKQAHAGIAVLNAAAVAPQKTDGKVVNNSPQSHNEPDVPAE
HKLPPGFKLTVLPPKPPADAPFMVQMRWKMSEARRKAEIMQIARWNKQLDEVKKKKIAEK
AAVPLPPPESTSDFLMQSLFNEDDMDLGGPPQIKLGDASIAAPFTCRSKALMSVCDIVRL
GRSTLVTTLQMYKILALNCLTSAYSMSVLHTDGVRLGEKQMILSGVILSVCFLCMSRSQP
MPTLCPQRPITRVFHPYMICTIFMQFALHLYSMMRTVKLVEEVDSMEVASMREIGVEGEF
KPTLLNSAMFLLTTLIGGVTFAVNYRGEPFMQSMRKNKPMFYALIVLTLVVIYFASETDP
EGNALFEIVAFPSQEFRHRFIQLLLTDAGGCFAIEYACLFFLTDYM*
>TCRU_649 Dpy-30 motif containing protein, putative

MLGGDEGSATVAPPSEEPVRLLSVPEYLESTVMAVLTEGLEDLCRQRPANPIDHLALFLL
RRSKSNTVTEVPAKDAIPAEPPKKYE*
>TCRU_650 lipoate-protein ligase, putative
MMRRMGWCLCPTTTLFSFLTRHATATHSERQLTEARASVALVSNSRCIFENLAVEEALLR
GVILPPGQQLLFSYVNRPCVVIGRNQNYLQEVAVSAARRDGVPIARRSSGGGAVYHDTGN
VCFSFFTHRSAYHPERTIEIIRLFLCCAFDICPERLTTTFRHDLFLDRKKITGSAMRVQR
DIACHHCTLLVKSCSERLSAYLQPEGQYVFFTTSSIGSVRSSVTTLQMAGVLPEGYCSDE
NINGEDVVHCTQRSLADFFVRHVGVITAHSAPWEIDPSIFLSKRNEETKGSEEKTLHPSN
ESVFLLDVVGALRENTRIVDGEGRRPAAGSSKTVLEEVDRLRSADWMFSMPKFTTCVAVT
AKDLLAWENDTIVRSCVPPGVVTYLMQGRTRFDITTVVECRHITKLTACWAENAAGDVDE
MWCAAILRVLLEGVRVDDPTVDVSEENAVLVAALQLECRSLMDGMPLSAREENIACDENG
SGSLDEQSALLLFMRAVLSVWRVKNVFIPVISQ*
>TCRU_651 KREPA3
MRRIASHLSQRTYRALFPRGGGFYAPPFNMSTRFYRETSLLQAPKTETPAYQCGECGKTF
RLLNALNHHIMTKHAGHAKAMVLKAGKLEEVKPEEAKNKSSHATDATHAASTTATGATMS
ATQIPTGFPGMTYSPLGGTAAFGSPLGVASMPLTSTAAAGAPTNAGKNASGTESSVADED
ADKKLFVCTICQKTFRLEAALQHHYQAKHNMEMPTNSTSASSTRAASQSSTNLGAANAIF
GGATDDAVKSTTGVHYVHSQESVLPQAPQYHLDVAPNAPEEGDVAAHWRCVNYCVVLGPV
QNIQEGYVFEEKVVQFTLITDFEGPSPGDPDKDFHTVRIFDNSFCEQMKKELKEGDRFLV
TGRLRMIPQYDSAMKKYYHYPVIQVHPGCGSVVKV*
>TCRU_652 hypothetical protein, conserved
MTSVVRSGESSKRPSGMEPVESFKCEARCCCVAGNDVWVTELKGGIAVCDAQRGNVLERI
AVGGTDTRVQFKCMVLVHGDEVWAGSINGAIHIFDVPTKRWKQSMTILGVEARAPILSLF
FDGFSVLGGSETGRIVQWNPFTKEQVKVFVALAPVTAITVTGGFVVNGGRDGVVQFWDSR
SGEVICEHTQDKSAVTSILVDSTTSTLWVGRTNGTVSVYTLSVPMSFNCRLSVGNGAVTS
MLAVSGKVLATSYDRSVVVFAAKTREEVSRSRTAHNAFIHYAARVYAAEMARIWTIGNDG
VIYVWDVAGYFLPCRSSPTISPYDQAAVVHDARGALTVENAQLSSRVAESLSRVVDVKEE
LRKVRDEAHEMRLRLISFENVLKEKEMEIASRTARMKELEEDVNRLQKSVTDSNARVDAA
QRETNVLRADHNTAVQDASQTKAQLSAKISENANLKQQLSATCTEKSHLEIRLRDRDAEL
TQLRAENARLHGAMGQISSSQSAEREMYKKMLANTEGAMRQELNELRRRSQILGSLVVSM
EYTIRRKEEEERDMTALMNAFRRRVAERVTDPHLAALLNFTMLRNPDRFDMDCDEHTKEL
LCDRNGPFIQFLHLLRDRDPDSYGQLLEYLQHPDAGSQFSQLFDKILSLAQKEGNASGDD
DIVTFKKSLPALLDSLNAMKAASNRTEDAGGSVGNISKPGASVANANYLLPPNTAASSNQ
TGGVAGNVGLGSGASGVYSNGGNAATFSGTATLQPQTSSQQQAGAGAVEAGGALSMNVDD
FKTFLRSMNISIEQPDLVGQAPLGLTEDTYVKQLKSTFEFILQTRRDLVQQMGILFKRIV
KGRQVVDALTRNAREGLSPTHRSSGSLGGGSGRQNLGSIATSILEELHRFVLNLLAVYFT
AAEKQRLGLGA*
>TCRU_653 Bucentaur or craniofacial development, putative
MAHLRSLSISHDDENNNSSSGSSGESEELSSGRGELSSSSCSLTSDSSHVRGNPGKGCCD
ALAKERMERQQRLRRLLSQRNLGHAVALDDGKHVEPFISRKGALDADDSVILSIFSNSLL
FSRLLAVLNSSQNPAHLQNFDEVLSAASPHFYRSFKAPLEDSTRQPARVGALTRRECAAL
ERSGIVVRRRKRDRPASKVGSTFFAGKVRRGRAADNGQMTHGSSATSGIIANSLRNWEQH
LTEELKAEGVTIETFRQQRHGNSYLEERRFLQESQWADYQRELRLEEKRKEQEAKRAIRR
GEENKN*
>TCRU_654 hypothetical protein, conserved
MGRSAKITRGGDKTRVNQGRQEAKLRAQGAKSHATSVKEAVEARRVQKKRSQAIATELRS
KTQAQAAPIVKMAPKIKKPQE*
>TCRU_655 proteasome regulatory non-ATP-ase subunit 10
MPEACFLCLDSTEFMRNGDQFPSRFLGVQEAASLLSNAKIQMNAENTVGFLTLGGNACTV
FETLTLDVERVMCSLANISIRGKRCHFSQGLQIASLALSHRTNPRAEKRIVAFIGTPLSE
TDEELEKLARKLRKDDVAVDIVGICVEANVPRLSAFVEKLNKNGNSRFLNIPAGANLTDS
LMSSSILLGADSGGGANETQDTYQGFGVDPSNDPDLAMAIRMSLEEEQQRQAAAATIAAS
QSTAPVSTATRTDVPPPSAEPENLENMSEEEMLARALKMSLEEAQKAANTAVKSEEESSL
HEAEKEPERPKPTEKSFQSAEEDELHENEEKSSGEKQPRNKK*

>TCRU_656 hypothetical protein, conserved
MVLATIRVLVCGAEFHRVESALGSSVENTNFAWNRRYEISCTSSTGVSEADGRLLSMCHL
VVLTRGASPPLEVKSYCGKPTVSLESAPEGVVGMLRNCDFYYGVHALEKLRSLVVDIGLS
PPHVIWDGVLRTFTRNGELSIRRAFWLLDKDGDGVLNEEELLAWQRSVTSACFSKSDLAE
LCTHCSLNNFPPSINGETFMKMQENFLMNGDAKKVWATLHLTGVCPDGLPYSWRDIHAVR
VSKESNTYLSHTAIQFFRNLYKLRRFSDLNNMWDVTPGCPWKHVHGFLTTEIPLDRFIEY
WKYMALTNRSVVVQYARYWGYKGDTILLFLLRHARPYREVGEAVPNTIQVLVVGSAGCGR
RSLMFTLTALDEEVYKEDTSASQEMYVRTTTFPVRHGEGEALQTVVYVTVPIENLTKVLE
DDTLEKQLDVVILCYDGSHIEETTLPIMRAYLEAKALPLRCRNLPFVVVMTKAEASASTE
APTNESQVMERFCREHQLLWPPVVTSVETPEESDIAALNEYMYAVAKEPEIAVGNAPLTL
QRILRRVAIVTFFALAVGSVTRYFFRRMRGRRQ*
>TCRU_657 peptide methionine sulfoxide reductase, putative
MASGAPATFAAGCFWGTERFFVKKFGDALLSHEVGYMGGAESDEVVTYSAVKKGTTGHAE
VLHVKYNPEKVTYQQLLDFFFRIHNPTTVNRQGVDVGTQYRSAIFYHDDQQLKEAKDYIS
RLNGADPALREGFVKAFGDAQVATSLEKASRLHVAEDYHQMYLKKNPDRVCSHRIHW*
>TCRU_658 N-terminal region of Chorein, a TM vesicle-mediated
sorter, putative
MLQQAVSYVISSLAKIGDIDACNVESSLRERTLRIRDMSLRQDTLNTFLPFPIQCGKVGD
LRVKIPWPASNEPLLLCCEGMAFTLNGETSQSCTKEMTREEIRGGITEESILGTVVTPSV
PVNSHEEQRSAAPLASEENSEERETSWSAALEDDDYMSCQSVSNTSFASCDSQEDLTSLP
QNGKVFFCFLSIPPVVLCRGS*
>TCRU_659 N-terminal region of Chorein, a TM vesicle-mediated
sorter, putative
MVKTEPTLEAGAELMKVIVITFSGVAVCANAAKEQTQKIVEIDHLSIRITSVYSSAGILC
RKNVAIVFDGVSSLFFDEDALRSLTRCAVSHGIMKEVPEYCRPFYKLRSLSSRWSHAKSC
VFAFIRDRRRRYNFNSSHLHFYAEARTSYLALLDYCHRTRDIAEKREQLADIEGDIRYRD
VVLFLRRKVQERYSTTVLAGSTGAGVTPPPTQPSDARQFVVVSSLHVDAKITCVRLPAET
TVWLRDVAFVKRHGESNFSVGNVTLVAGGKSQLLRAPDSGEPFFSLQQTESNGMTFVRTT
LEALRFITTSNWMLQLVQPIMVAMPHVLRAIPETNCNAPETKLSNRCEKTTSFVFPLIDF
HVDDLRFHLKQLSLTHIERTSGDSRVGLLMKELSVRFGEETDVLSPMKVEMTEEEDILVS
AVTLNVSNALWEYLYHVRTEWNDALLRVSNMPGFVSRVQSTTGNTQIKKSFAEVCCEAEL
KEEFNSALKLQRTYLLGVVITFEPLNLSMQLGTMNMWRGAQTCGFPSVHANISSFEFSTD
HVEGGVLTVTCPSGILYSKTSSTSDASRVNVSIGVLESVWKASSTRNLLHIDAMQFHVYG
DATHCSAETLRSWML*
>TCRU_660 BNR repeat-like domain containing protein, putative
MSRRVFTSAVLLLLVVMMCSSGGASNAATSNSGNVQLPHTVDLFLPNQTLVVSKDGRSQG
TKRDAFVSPSLVSAGGVIAAFAEGRMETEYQGGPSIKPSSDVVAEYIDAAWNWSTLVEKV
NKGTWKAHTVVCAAEEEESLGVVLRPTTITKGNKVFHPAGGSDVSYEGVCWREGGLELKL
FVGDVTNSTPGDPGERIKWGDPRPLFDSKYSLNHKSKLNTLFPSGGSGVLMEDGTLVFSL
MARKENPNEAFYTIVYSKNGGKK*
>TCRU_661 BNR repeat-like domain containing protein, putative
MSPPNCYNPRVTEWEGSLLMIVDCENDQRVYDSRDMGTTWTGTIGTLPGVWVNSQSEDYP
DGGLHVDALITSTIGGRKVMLYTQRGNYWKGQTERTNALCLWVTDNIRSFYVGPVGMGNA
GEKELASNLLYSDGYLHLLQRRGNGEDSAISLSRLTEELSAINSVLSNSAQKDVFFSNLT
IPTAGLVAVLSNAASDDTWNDEYLCLNATVTTNAKKLRDGFQLTEPDSRVSLPVNTRANN
VRHVSLSHNFTVVASVTIEEAPSGNTPLLTAALGDAGPEYFMRLSYTADNK*
>TCRU_662 hypothetical protein, conserved
MAKGFLRVNWRACGGWLILQWQRPLPGTTERVAVSVLANGTTPLVAGLGSKACQRVRVPY
GRDSMFRVLSGCHGLRRMLSRRLISMHSTMSVTKLPVHGPAGTHQCQETLPDPVLVEGR*
>TCRU_663 hypothetical protein, conserved
MTMGASRTAKLLDMDLDPQLTLNMAAASQRISQPRRIAHKEAGPSPHDPRTFIIGYGASK
LRYGSELIWAVATDSAKNEMQETYAGLARIVSGVPSTVDQ*
>TCRU_664 hypothetical protein, conserved
MCSQYGPLRNSSQRGRPIAVSVANSCLRVAATQKFSKPPSAVHPRNIGRQATEDSMSNEL

KGLAGVYQHQRSPMGSAVELASSTALLGKVRLEVIGSVMLHFVYQVCQLHRRGTHTTARM
LVGTRRPQPKQSPQCHFLSSLTHDGRRADGSI*
>TCRU_665 hypothetical protein, conserved
MPHGELSAPPAALCPARGERRTGPPPPVSAAGTPPTVSAAGTPRRQPRLTPRRTVSDVVT
PVRDKTARGLSERIAVECGRCRGGVPRCTLTVAALSQSRPASRLRSAPLTMGECWLCPAG
SRACAVEPNTGCAATWEAAALG*
>TCRU_666 Trans-sialidase of Trypanosoma hydrophobic C-terminal, putative
MGSSGVNGGGASPSAVPTVSTPSAGKDSLQQVASGKSSDGTQTVDGGSFSDGEPTVETGE
GGADGREKEEIHAQNGDVNAAALSNSLGNVSQGNNTDAGTMRENGLLPSLLLLLGLWGFA
AL*
>TCRU_667 Concanavalin A-like lectin/glucanases superfamily/Protein of unknown function (DUF3676)/Trans-sialidase of Trypanosoma hydrophobic C-terminal, putative
MEGRVPWRECNCDEWGEKGSQWRNIQGPGAGAEWPVGDMGQTVPYYFANNKFTLSATVTI
HEVPQSGSSPVPLIGVRMNDTSSTVLFGLSYTHEKKWLAIAGNRATSKLVDGWEPNKTYQ
VLLQMDYDEWTVFVDGKEIHNKRYDKKLFNSHRISHFYIGGDSKHQGATGGHVTVTNVML
YNEELWGNDLHKLHASKVTIPSLGVEEQPTEQVASTGALVAPESRSEESTASHELTEGDT
DKQGENSVDDLLLAAPPSTVVAGSSVPEPATATESAENSRSDDNAQSSGDETFQQFTLNK
SNESMQRDSDVQPQELLSAESTGVTDVEGSTETNDIEQPEEEGEANDRSGESTPSVAVSS
DMDTVAAPADSEHQVQQSVELSAENNEVRSTGTGITGAKRSLILEARGSNSERTMNSGSS
LTPSKSDAETKSAENTDDVSRTEGDEVSSENGEEVPQTVDTAPENTNTTPGETAIPSDSN
ATTPSDTEILLEKEHLGELAGMYLIGDSTVHGCVSRVLLLLLLGLWGAAALC*
>TCRU_668 RNA-binding protein
MPAKKKGGRGKTINLVDFNDEYIPEDNLDWAVEVPREKSRDELAGEKIAKRDYSRTANNA
LMEKGGFRDSPSEPVNRTKGINDLQPPFVAHFGNLRNGTTEEEFLRNFHEDCIVTSRLIS
QDGKSFAFVEFKTAQALAIALAMDQTMVKGRKLYVDLATQKQVERLHGHNGISRQNPGQS
QQGAGLQQLNLTRDAFGSTQMTEREMGHRSGFCSRNDLHMLADLSRDTLGTATRSSESAA
SPVGQPDFSSWRNEGPVAQPEFHTGLPENRRSDAELDRRGGMGGGRNSKRQSPVSAGCLA
NWRDEPMTQPPPPPPLPPPGSGSGGDNSKNNGEDGRKGGWASDGAGRGNHRGPPKSQAAP
RDDSKWEALRR*
>TCRU_669 hypothetical protein, conserved
MMEIRQCESVSGANSSVLVPPLVPKSRGPSLPDGRRTPGGPPRTTPTRTASRYYCRRWHS
VGERLPSSEEPQVVRLIPKAANGGSTAWGEKSSIADSPSSFNIETVSEPATLHGDRASIV
FELEPGMDSGSKRLHMSAPTGSEDHLRGTPDAMKATPLPGVLTPRTIQRSAMPNPATATT
RSSPSRSPPSLRFHLVDHPVMYGDDDDADSLPSALPPPKPYPIVEVAAASQYGVNGTKLR
LYGNVMNNTLVSPRGRSNMRGTPNMYTEGTVEKGVKPYTTSIEVGAPSQTLVSAPSPPPY
MLNTPVQSLLNEVNAWVDVIHSVEDKNCYKIYLGSTEKALHDTQGEMKALATKHKLVNKE
LYAAKARHVERLDGNQARKKPVAQVKDAENKANTAWKRQNEEEEEVGEEKEMTREEEEWR
LEKGLLLAEKALLLQKRKELCYHLRRGTNIKNVAALALSGRSSWSRSDGSANDTVSENTA
PSVSDLETIQLRLELKEAQVQLMKLVDSHRILQGAQRTRTKEFEDEFVVLYRSANLAKQE
TEGHRGTLEATLRKAEQLLEKCQQKNEESLLLLLEQRVEEARAALRPRDDKLLTNDGFRS
RMPSIGRCSSTGSTARRVSGASVSPAFVTPKTTAKAEQNSLRAKMQ*
>TCRU_670 hypothetical protein, conserved
MGTARKLGHQQGLITAERELLNAEHANAIADVQNRNAELNWQVEEAHHNVSIHESNIAGA
SNQVREPQQQLEATRTQLQERKFQLGELSAQL*
>TCRU_671 hypothetical protein, conserved
MARTRRAPPSHSRTSAAPPPARPTSPSHNQQQHHTHQTASTPQQQPQVTNVYLQRGGGGT
GILGTMASVAAGSVLGHGISNMLFNREQPPTQPAQAQEMAQRVGDGACAPQIKSYSKCLE
ANAEHPENCKWAWDYFIQCQDEQQQGQ*
>TCRU_672 ubiquitin-conjugating enzyme E2, putative
MALTTRIIKETEKLQREHQEGIEARPSPSNPRYFHVKIRGPPQSSYEGGIFHLELFLPEE
YPMVPPKVRFLTRIYHPNVDKLGRICLDIIKDKWSPALQIIKVLLSIQLLMASPNPDDPL
DNEVAESWKRDEVAAMRTAREWTQKYASPQA*

>TCRU_673 hypothetical protein, conserved
MWRLSLLRRAAAAGGRVNSSGSASLPYRRVLKMLQAEGEGLPAAGGVFEQFRAFYRQVMA
DKAASERAVKPIEEFCGASEDVEPFALVMMKVLCDLQKMEDLRFLFAQCLLELHSPSIEL
FNVYLLAISLTDSFNQYEIENVVEVMRARAIHPDVVTRLSLFILYIRLGQDYSVWWPEIR
DEVLKIAKSGQRGQYPLLEVRLQHCFQTILRLHHDVTMIHECFDLLKLVDQKRISSRLLL
PYMLLSANNAAAPPATTVQIVELLEATRKLEDNTSEGASLRAVSEDAETERTNVGSTDPV
LNNELTALKLLAKCAKWGDVASAVSVREYLRKHPGIISPTSQPAVSLLFLEAASRGAMLK
EALTVLEDEVPETHCQPGPRPKFFLESRKVTLLNADPIMTLARLVAFGEASVEKGLAILE
ERREAGGEVSHKSLNVLMEAAALLKDEGKAAMVFAAFESLGAVRTSRTFLLLLLSMPDAA
SSTARIPEVLAEMSDLGIEVSSDFLRGALELAIDAQDVFTAMQMVGYHEKRSISIESKLS
IRLMRLLCLVVDVDSVRRLIAALKATESPVDSRTVSLCVATFKKWAIPCDDLKAV*
>TCRU_674 S-adenosylmethionine decarboxylase proenzyme, putative
MLSNKDPLSLMAMWGSVKGYDPNQGASFEGPEKRLEVIMRITDETHSEGLHALGDGVWKG
VVGSLNAQIVSKESNEYIRSYVLTESSLFVMRDRIILITCGTTTLLNAVPFVLEAVSDVR
GEVEWVSFMHKNYSFPWEQKGPHLSMAEEFNTLRTYFPSGKPFIFGPVDSDHYFLFVYDD
VIRPCETENDTQLSMTMYGLDRTQTKHWFSDRFISTGTETAAIRKATKLNKVADDSWKLH
DLQFEPCGYSINTIRGAEYQTIHITPEDHCSFASYETNTPAVNYSERINTVLGVFAPIRF
SVIVFIDPDSDVGRLYQKGQNVGVEAEYYPKYELQNRTVNEFAPGYVVMKMNYAMRAEVA
EKDSTDTVEE*
>TCRU_675 prozyme
MLESTWAAAREEVPESVHALMAMWGGFDGPRNANDCGIEKRLELDFRGVIVPSEVSVLDL
ESVMSHAGELLERHSAEKGLISLVFGNSLMQLTQRHVIVTSSSSVLLHEILGPLLDLLRS
HGIDIEWASFMRMNTTSPWSSLCEMSEIMAQEYAQLKSVFPSGHPYLTGPLDSDHFFYFV
YDAIERNVLGAPPEDDVQINVYMYNVKTDGSGDDDDALKSVQQVVPLSETEYEMLRVSTD
NMAHPFAAFETNSMIAATKKRELVRGLLEKFCPDRFTMVVLQDRCSPLAQKGSIFDEMEG
YTIMNRATNHFGQGYAFHQTSYIRAE*
>TCRU_676 valyl-tRNA synthetase, putative
MKRLTPQMAAAYTPKDVESEWYEWWEKSGFFRPASDLGRKHNGNTFVIVSPPPNVTGYLH
LGHSLTGAVQDTLIRFHRMKGDNTLYVPGTDHAGIATQVVVEKRLLRESGKTRHDLGREE
FLKRVWDFKENHAHVITRQLRQIGLSLDWTRERFTMDDHCAGAVLEAFVKLHEDGLVRRS
TRLVNWCCALQSAISDLEVEFADVPKNAKIAIPGYDKKVDMGVLTHVAYRFEDSDDEIVI
ATTRPETILGDTAVAVHPDDERYKKYHGKRLKCPFRDETIPLILDPVLVDVGFGTGAVKI
TPSHDPNDFEAGVRHNLPQLTMMDLNGRITIDGPFKGMHRFDCRREIVKELEKMGLLRDV
VPYEYRVGRCSRTGDIVEPLLMPQWFVDCSEMARKSVEAVRNKELRLYPSSHEAVWFHWL
ENIKPWCVSRQLWWGHRIPAYKCSGVVPSNHEDPWVVARNIAEARSKARAKFNLTDAEVE
QLVLDQDPDVLDTWFSSAMWPYSTLGWPGDTDDMHKFFPGNLMETGHDILFFWVARMVMT
SLHFTGKLPFSEVFLHAMVRDKNGEKMSKSKGNVIDPLFIINGITLEGLHSTVTNGNLDE
KEVPRALKLQKETFPNGITECGSDALRFGLLSYTQSGRSVNLDIDRIVAYRQFCNKLWNA
VRYVLYHALGTEYKPGLTVIDVLRAESYPLECRWILSRLDVAVEECTRGFSEGSYDFALT
TNAAYRFWLYELCDVFLELTKFSIHAGGDRKQLVQDVLLHVVEVALRLLHPMMPFLTEEL
WHRLPNYNTFGVESIMLAPYPAVTGWKNETVEEKMRIMMDTVRNVRSTKASYSLTNKHKP
DVWITAQTAEVKQLLMDHSYMVISLGVVGNVSVISPEEVEANVPKGCGFSVVNKEVGVNM
MLLGFIDVQKEVAKLDKQLVGLQKQIEGLKKKMSIPNYDMKVPADVRTANSEKLDTLTAQ
EKQILEGLEKMKSIL*
>TCRU_677 UNC119
MAANIQVTPEQVLQFSAPTSEFLCPFTANTYNIEFYRFTIRDMETGKVMFDVERNPEDYP
SIPTIAQLPEEVHRTMRTIYYNFPPSMLRRRAIGAKLVFGINSDFPVRNFRMIERHYFRD
TLIKSFDFTFGFCIPHSTNTWEAIYDMPALSEDWIRAIIDNPNETVSDSFYFVENKLVMH
NKAFYAYDAPEENAVI*
>TCRU_678 hypothetical protein
MRSRGSAPSSAPGEQHCTLRIRNPAADVVYHLVFEGNIRKLTGAQLRLHLEAICLIPAEQ
QVLTFNGVPVENTSTGASLGLHNKAVLELHTAGEASRIPSSRRGDWGDRRRGGRQTHPAE
EHLNQSDRIEFSHGLNTSATTASQCTPVPRPRQQRADRERAGERQRYPCDKHDCGGPRAA
TGTCAVAPLERTPSAQTLSPVDVIIEEDAIDGLGTAMIPFYDADVEDARLEQREYVWMME

QMRFETERMNRERELLRQREELEHEAEILEREKCIVKRRTLQERRKLVELHQWICEEMAM
EAQILNLNGHENDYNNCANDDGDGGDFIERGK*
>TCRU_679 mitochondrial carrier protein
MSTPQTHPATLVDARYNGRRFFFYHLVFGPTGAATLAGLCEILIFHPFDTTAKRLMSHQH
IVFDPTSLSNTWRVFLRVVFAGIEHDKPLGARITSLDRVRHMYPGSTYAMLYKVLQRVIK
FAGQSYMRDFLESRFGHIFLGDAQEQSKQQKQHTHRRNNHGAMALEATAGCLVGVFEVIL
LPIDRMKVLNQTNKGAIKNRSFFRILRQEGVMRMYAGIGTTAVRNAPGSFLLFGGVAWTK
EYVFHLEDYSDATVVQNLVSSTVGGFLGVWFTNPIDVVKTRIQNKRFEDGRVTGWSVMTE
TFRREGVTAFYKGMLPKLLTSAPRLVFSYTMTQFFIRCFRGEKPKPLRESK*
>TCRU_680 hypothetical protein, conserved
MSTKAIGSCNPSGTARHTDSEGPGQMSQSSPGRSEDDTVAHRGGPCPEKVTVNTQLADAA
TAEEERRRENTDATRKTFMEVSGQHRRPPPEQMLRQLLATVTAAPRSPSPLPHFASCLCC
CCRDGDCACMKWRVLRAREGQRKLHCPFDEYCLQLPRNMNSRGVRGLLFSCFIAFLLLWA
PRLHFCVKAVCERCGHDRRSRHVQYMWWGLVCRPCWSLPPPSVCCSRCMTVCVCVCLLPR
IGVCVVRGVRRTVHAWLLLSRLVWYALCVRLPHLSSLLLSIFLTVQISPTPLNDHTHDDD
DVPSAMRPVGARRVLLPVRERDSEW*
>TCRU_681 hypothetical protein
MMAMSKGESDGEFLKADWRLIAPLRLLGTVVSAEAKNDEERDGCGGKEGAFRDICGKPLS
SASLDVLGVGVRGDADSHLPPRALSAGGSSPPTGWVMLTTVVTHPQVSLGTQRILLMAAG
TRRGKLGA*
>TCRU_682 metallo-peptidase, Clan ME, Family M16
MLRCCLPRRGIVQTVKKDNYVLSRLSNGLRVLTCDDGNGITGMGLFMLNGTKFEDEKNTG
AAAVFESLPLRSNQIFTGREISEALGSLGNAFKVTNNKEAMSVMLMMPRYHQKDGLELLN
AMCLHPTRDEMEFQIAKEKTGERAGLHHRDATSVCLELVHEAGWNGRGLGQPLDPKKEDL
DKLTLEKFTAFHRTYTRPERTVLAATGVADHKQFAAEAELILSFDSETAPLGAPRKHPYT
GGSRLVQRTEAPESMNKFQEKNLSHVALFCQGVPMNHPDYYNISVIQTLLGGGTSFSSGG
PGKGMQTKIFREVLNREGFLHGLECITAWYSDGGLIGLYGSAPHEYVYALLKVMIYQAAS
ICQRVSPLHLEMAKNQLRSQLILLGEGREQLLSDMGFNMVVHDHIITASETTEGTKHITI
DELKRVCDEMVQRPLTFVVYGETTKMPSHEKLEETVRKTYERLN*
>TCRU_683 enoyl-CoA hydratase/Enoyl-CoA isomerase/3-hydroxyacyl-CoA dehydrogenase, putative
MRRVDTILSHVGHRRYCHWERSGDAAILILHHEPSNNLTPKMRAAIQHYFREAEADPAVR
RIILTGEGRAFSCGVDLADFSASASQMEYDGVLIPSLATLTNMVENSVKIVVAAINGLTF
SGGLELAVAAHYRVASPSSEFSFPEVKLGIVPCGGGTQRLPRLIGVRAAVDMITTSKKLT
AREALRMGLVDHLASCTKKHDKDRNDGDNAVSNKDGDHLRGNNDHLVAVAIQLSTYWKTP
RHLSCDNRRLGNSIVNRALFRWMQREIDKNAPKGVKAPMRCLEAIRASTTVASFSEGLQV
EMKLFFESLESPEAHAMQYLLRAANAAFCGNLASLPRGASDGLNIRHFAVIGCGTIGTGV
VLLMLRSQIPVTVVELNEEQCLRARLFVQEELDREVENGRLLPELCEQRLSMLSLVPYHA
DLVQALKEVDMVLECVGENPALKKQVFTRLSEVCPPHCILATSASTTSIGELAKVTRRPE
KVIGLHFIPPSDVTPLLEIVQGSCTDQFTILQALRVGHLFHKAVILTRDVGACLTTRLFS
AGLYQAFSMLEEGAFPVEVDRALRRFGFRLGVFALEDLAGLDFTAEIRTRLQKNNTTFSS
QADVYTIPRQLVEEGRLGQKRGLGWYNYEKGTFFNSIVTKLRGAKRAENEGVAALGVWRK
GGASLKRVPRPDRAVELLILDVCRKKDILRRDISEREIVERIIFAIINEAAKLLGEGAVL
SSADVDVAMTFGYGFPAWKGGVCYYADKFGIANIVHRMRIYNRAFGDAVFPLPCDVLASM
VTSQQTFRSMWQ*
>TCRU_684 hypothetical protein, conserved
MRKRERSASASPVMGATSQVEPPGQRVWLRQQVLFDVLRRTSLAEQKDREIVEWLTDAAE
WLQDSEYEKLGAGSQAELRFLVLGVLHTMPWWRVVEAVDPKRVHVASRGAQSATRAITRW
HEKIQLAFERVCMLLIEQSPHAVRGVLEVAMKPFQMRPPVADGARRLIPADSIMRIMNAV
STKYPSALVLDVLEQTMRSLVPKRRWAEERHHRACIALFLHLALEAHVPLFPTHTYGQIE
PFGNGASDPFCSTSHVISVQGSEDSATDAAASSSSNRYHQLHPLEMTCPVMSASDADFSD
ASMNISSTASGHFFPTRLTDFTMQNNKMLAYRNELIRFVLQQLLDMELSLQNSDNESLQE
LTSPSSSFYSIGPTDATRVPWRPLIGRFKERSADKPFVDILRGCTSIVFHRLAEDLVRQR
AAGVCGNAEWWRELLAFHLNFVIKMECPIFLLYLAPSLALLGGEEEAIDMMHKLVSLVTK

GYLPVQQSARRVTAAGPARVRMAPPCVGPLMIPMTHRVRAAVYIYPLFRFLRDRLGSGEG
TRKRLQKWVVQELKGTAIVSASLIVQVLFAQCAAMSQLLEADFVEEMNSQLPGSIALRSL
WEQYLFPEGMTSRACNGDNEGMSSVSTNNSYNGTNTTTNDTVDVGAQKRRMDLLKMEAQG
FLHPKLMDDCPWDWRRCQTERALE*
>TCRU_685 hypothetical protein, conserved
MKSVASQFLDTPYKEEGTVGTRQNGMTMGVRSDDAARCVNHPPLFSSVLAPRPVLIGPLL
RSPRISETVVLLDGVPCGAVCFSGRVVRCEGPQRHGGVSASRAYTALLISDNTGLISVMQ
PHTARVNVDVGAAWEGVTPCLTGGVAELAGNATDEMVHENDYVFVVGWLAFADTSREVRL
LLQGVVDFVTGGDGTKPDENCFCVRGTVRQIHDMNELHFWALAALETNVRLLAKAGDFPS
VFPTRRKNV*
>TCRU_686 hypothetical protein, conserved
MAGCPADELREIYQELTQPCKHDNTYVTVQPSWLRSAALRSTSGRTHPTAVGFSSRNGRP
VAAGYTSLPVNCREACEGASGSSEHTSTSGQNAMRKSPVSLGLVDTPSGSAGKGNRPTRV
NQSHRTGLESNICLPEAMTSKVPDAGGMNTRHSGLFLSTPTEHRWSCWLCETPCARPGWL
NAPELYLGEEEGADMDDYYQHHKHHLPVTLLTLCSRCSAACSLPADASHASVLRKAGATR
VLVQRTEVFVQFLRGFKRLLESLAAKIEEKMVSDARVYAGGTRPSLWDFHPDFQSSVMHQ
LIDMLASTLEADLLQSRRQQVDVLSAHERLLLEGERVWLQRQLSTIEENGPSPMWLLRTA
VVPLSTAENGIMSHDVSHAPQRGLQDERRKLHANLRPCFLSAKTEPGGWWHDQRNRVVQG
GEGSNEALQNMMERALTARWHQLGDESASSANVTDTPATKDDKRDNQQLHSPTFVSLPKD
AGGGGARLIHEDPDNILLHYFEGRLEDLPKFKLLSVDAARGGGNNECLPQPFRHVPGTHR
SSCGDLSNVSSDVVLVAADDAAAVSMDTEGLLKHLREEQKRREESEAALRESHQKIAALE
KATDHISGLALELEYVLGEQLARMWRFLRNATQGCVDVMMQKSLLFSDEMAALMRQAQRQ
AYGVSGMFPPNHAGRAVEARRDLPSPAPMKSPNGALAESNFERLRSCANSPASCAARIGP
THAL*
>TCRU_687 protein kinase, putative
MSLSKGEHLKLYEGDLIDSRFRVLRELGAGNFSKVYCCHDITRQRDVKRALVAVKIVKRE
YMEDAYFERDMLDVFRRKRAEGPMVCRMMEFFEWRKCPVIVMSIHGPSLRTRRLGFKNGV
VTRAKLVQLAYSLLTTLRYVHFDCQMVHTDMKPDNILLADLDAPKHSLGSQWVVCDFGSA
SLWRMDQLDADLITTRPYRAPEVVLGNQWFYAADMWSMGCILFEVATGQRLFDVRDDVTH
LQLMEKRLGPLPDLFTRNAKHSAKYFTSSGSFIAESESIRAGKVSMRKLKDVFSDDPELY
ELISAMLIYDPMKRLTAKEGLQHSIFDGIHLLSKKTDAIDNNGHNLNGNSIAFSPSVLHS
HIEGKLDTGSKQLEILSGVGGKIVTRRGSSVPAISIDNNTSLNPHPPKRLGLKFVGKAPR
VGGTTSRAFIFQKARPQDSSVVNVEPPRCLTPERGEKTSPDNSHPSGSFASSHGNDQTHK
MRRFPVVYASPIDMSTENETLVEGISKLCEKSVFASSSNTSEGDSLVRNPPGPFPIVIPT
PEKVLSSADCFSEYTNPHRERDQPPLKVSDISQSEKSLGVSQVDDTAGAGSPLGPLTHGI
NMNGLVLPTECESEVNVGNVLRPFPSHPLSCFSATLNSLDLNAAKGGSDWKDSTRKSGLH
ALATREMKGGCNSLTLHPAHTNVSELSWLSNSILRPSTSSLPPSHAPTTGKQSLPFNLAD
VISSPKTAQTRNLLKEGNVRVRVFPNGAQGAVMPRVIIKKSFEGTATNSTQHQLPSPTVR
NAGQQGLSGAMNSLAGLPRAGSSQSMSLRASTIKVRRRSDTHVHVLKTVNTVRSKTNHPN
SQEISFQKSNYPLKNGSTVAGRGVLMDMGTVSSETSSMFAPKLTASTGVVSTKISSFNSV
NPTVLGVPQKRRPSVDIRPTHSQLIQRNVVSSGKPCVCVNNPPGQEYLNQSDVSRAMQSN
GGADDDTFVSGVTAGMDGNATQHVGALMSTVEDASCEMRAPHMTRHFPTVQAPE*
>TCRU_688 glycosomal phosphoenolpyruvate carboxykinase
MPPTIHRNLLSPELVQWALKIEKDSRLTARGALAVMSYAKTGRSPLDKRIVDTDDVRENV
DWGKVNMKLSEESFARVKKIAKEFLDTREHLFVVDCFAGHDERYRLKVRVLTTRPYHALF
MRDMLIVPTPEELATFGEPDYVIYNAGECKADPSIPGLTSTTCVALNFKTREQVILGTEY
AGEMKKGILTVMFELMPQMNHLCMHASANVGKQGDVTVFFGLSGTGKTTLSADPHRNLIG
DDEHVWTDRGVFNIEGGCYAKAIGLNPKTEKDIYDAVRFGAVAENCVLDKRTGEIDFYDE
SICKNTRVAYPLSHIEGALSKAIAGHPKNVIFLTNDAFGVMPPVARLTSAQAMFWFVMGY
TANVPGVEAGGTRTARPIFSSCFGGPFLVRHATFYGEQLAEKMQKHNSRVWLLNTGYAGG
RADRGAKRMPLRVTRAIIDAIHDGTLDRTEYEEYPGWGLHIPKYVAKVPEHLLNPRKAWK
DVRQFNETSKELVAMFQESFSARFAAKASQEMKSAVPRYVEFARL*
>TCRU_protein, putative NUP.1
MRKVEEVNAELMKAQRDYRALERLLRSSGRLSISRVENNDGEEHEREMSFTPTFLRNTEG

AELAQFLQISSLQADLMLSRSTCHRLEARQLELQTALEQCEWRLASLPPGVEESHAELLR
VRGQLENHQLEYHRLEGRYKALKRQRAEEVEQLQRQNERLVQQLREKRDKIGSLSRQMRD
SEVAAKQQAEELAQAFNLLETQMQALREEVEGGQPRGRQARRQGVEETTEAEGKMLRGRI
TFLEHTLRQKETEMHRLQEELGRNEAQLDQFEDQVTVATQRLENTARRSTHLEQKVHELK
RTNQELREELGAVRACVVVQAERRSPQRAPSTDVLREALHGGRASSLASTPTQGEREGRA
ASALPRAATKRPRAIDASS*
>TCRU_690 Concanavalin A-like lectin/glucanases superfamily/Trans-
sialidase of Trypanosoma hydrophobic C-terminal, putative
MGAILKDAENPVLLGLSYNNKEKKWTLLCGDGQKKEHSSTWETQTDRTRYQVAIVLRNGT
QGSAYVDGQRVGEPCKVENKNSKGISHFYIGGDGSNKGSQEGVSVTVKNVLLYNRPLDGN
EITALNAIKPPITPPKDPNAQEVVLPSPGRTPQAGQEPLNGGEGADGLGVPKAASIASTS
IPVGQTVQQLASETSPDGNADVDVSSSSSGNPTVGEGSADTIQGDGPHTPSVGNTAAAAD
TNVLTAEGEGHDGPALTRDVSVSSGADGETAGGTDGQEGIHPQDRDVKSAALSSSLGNLS
QGNNTDAGTVCERRALPLLLLLLGLWGFAAQ*
>TCRU_691 hypothetical protein, conserved
MAVRQSRVRSAACFLRANQRDTLDARVCLANSHRRHKREFVIHKATVIIILPTVHGAKLP
CELKPICDILAIRHRCNHATALVDPCVGSACVGKISNQTHRTYGRREEGSVHVRPITGDG
SDHPQLLRQGGRKQAAFLFIVPRSHKVQHTIRVQQGVGNRLIQSSVHESRSNTKHAVAVS
HPNVERMGLTLPPRGAHFRAYSMTLLPPTVAVTMAPTRKLFFQRPDCELVQTPAIAPGTS
THIAPLSRDSCTLQYRLP*
>TCRU_692 retrotransposon hot spot protein, RHS3, putative
MPPKRNRVQGGNARSRASAVPQGGQRRARPESEDVTDQPAATQIRVEARRPEWTMSSTVE
DILLEGRTLSTNMKLNDFLRNYFGGRGVEEFNENVYMKDFLSSPNDFIQDEVLLSTIKAL
PSYQELKKGREEFYALSGASNKLGDERLLTLSHWRDFERKDTVIPLARAQISTAYSHVVR
VEAEGRGKARTTRTGN*
>TCRU_693 retrotransposon hot spot protein, RHS3, putative
MDNLLRWLVAAAEVKASVHEITKSLLDAAAEEARNPAKTSAPRYLEGYYESVYNARWSHV
VEVSDGEGMGMEAKEGNHRSHGITRKLTTLSKRMMVWSNPVHRVPGCWCSPRTRDGRTRG
I*
>TCRU_694 hypothetical protein, conserved
MDAQVQLVFDGLVLCRRALEIDRDPTDQLMDFGRTLLRTFRMRIMMASDPSITLSKLRDR
LHMAVHVADTFAKAALPLV*
>TCRU_695 hypothetical protein, conserved
MESTHPVEYCRLVVSGSKDPVEYFVRAKGDGRAVMPAQTETRTGWLMCPVTLRARLRHWM
RAEQVALRCLLASRPTRWPLLKGIQEHLSSEGVSVVWPAHCSTRSIWLFLCWEDGLLHFL
DPCGGRVMGQDAHRFGVGAPRACSRVTQEA*
>TCRU_696 hypothetical protein, conserved
MAMMMTGRELLVCALCVLWCVAADGDVVGPVTSGDPGGGGQSPLADGSHGGSGCSKADIS
HESKSEPLVAASGEESPAIHLDPNGVSSLSEEHLRPEKREPPKENGKSHNHRLKMHRRLK
*
>TCRU_697 hypothetical protein, conserved
MLAWLTDRRGRTFRTGCYSVWALLPCGAPQGSVLGPWPFEVFLDDLGHSFHPTHLPAAIV
TPIVHFPIIDHTITADIVAYACDVIVRATVPGITHLDVAMQPLFNAILEWTRAKALPLSD
RTYFMAIRIAGHHNSPVPDFFGINGRRCPCKR*
>TCRU_698 retrotransposon hot spot protein, RHS3, putative
MGAGSHLLYQLLHCGAEQLPVVAYFIAEQTFLFDNTTKTMSEDVVEPDTLNIVDGLLDRG
FKGCLIYDVAMKGCQPSTALSCKGWGMIFVTSPHAS*
>TCRU_699 SET domain containing protein, putative
MPLLGGNHFLFHMPITCGYRSAFQRMTLRFNLEKAMVLSTIETGARYFARVSPVQGKIDP
PKGGFVTDDVKIDMNANIGGAYARGVFACREIGYGREIMNLPAYAMYIGDSEEQTLREQV
LILTKEVFTKLVLGSPEEKLYIKHRVMTLMSGGFSYFTRERDVFEFVEDVRAPGAEGVLK
NGANYLLSGEFSSYDLQKLPLIIEFNRYDVEYNGRRGICLFPEAQYFNHQCEPNVEVTIT
YNSLKGRFFLSARTVRPVREGEELFINYMPGNTLPLSRLALAMKKRWGFECTCVRCKSRG
IGAVTMLFVVVLLPMTAYLRSVNVRRTQEKQRGV*

>TCRU_700 hypothetical protein, conserved
MSGNMFAIDDWRRELTAEDHVQRQHLAKTSLQRNFRGDAQRRVTSSTASSNMAVIDCCWA
RGHRTGGASSSFSTVLSNYLFCALSGGVVCILNTTNFMKMYLFEHDSSRRSRRDERAQKK
DVSRQHDSRREGVAAIDTVLFPRRGKRESSVSITTSGTALEGEGFLSLVCASAEGSLCLF
TVESEASSHTRSVSIKTGAVHKAVELPSDDAVPAKGRWEKAYIRFSPKGGRVVAVFSSTV
PCGATTVETNSFFLTGEIVEYVTLFCVLKWKEGESIETFTVETPWTRIRSPSNAASAELQ
YVGWWDDDESVFLTAWSDGAVSLMGVHLETLAHTSIFCTPAERMAGRVSMVTAVPPIPQK
KGSGRAFTYYQSGLVAMVLDGNIVTVFTVHDSQQHYGTASEDGPPLKFAKLETTKGHTEP
VREFTLRPGHHTYCSEDIPVSDIALFDNFLPYTLAVLLRSGALIALDTVTMEILYHRPLQ
RIFPSKGGKAASSINEKREAAIVGDGGNSSGNGGCVSEGMSYFMRPKERPMTMCVVEHNT
VLLLRPS*
>TCRU_701 hypothetical protein, conserved
MALRGVPIRLGVHRVGYTHPSTLPVPCAQRWDLRLARARIFQEYIEEKAPGAWQLEDERS
MSPEFKTFTGYPMREMRPGYGQNLPDFIMKKRLPNNTHYELFARRDIPNEDNAMYGKYLY
DMTVHGTSLPSTYRMHKDINKAQRNDRKLSGNRFRVLCSSGAKKPPSGWEPIPDATEEEE
*
>TCRU_702 proteasome alpha 7 subunit
MSGTEHDQSTDIFSADGRVFQVEYACKAVDNGSTAVAACCTDGVVVAVEKILTSRMLEER
SNDRIHAVDRQAGVCICGMLPDGRAVVSRARAEAENSRDVFATPIPGSVLASRIGEFMHV
YTTHYAYRPFGCSVIIASYADDGPQLFVSDPSGTVAGYYGIALGKGKTVAKTELEKLNFK
SLTCDEAVVKLTKILHDVHDKSKDKLYELEVAWVCNKSNCVFQHVPNDMIPKPPASQ*
>TCRU_703 zinc finger CCCH domain-containing protein 6, putative
MAERRQPRVLGAKRTVSDALGANALEGGGLAAVVRCVVAGTVDDSVTLGQLENVVAFYAN
HNSEYTRPGEDDIHVLMSEILNNFIRSVMLFSDFSSAFTDVEVYSISVAVQYNLSLEAIT
MNGVNVSDEAISMLCEALTKSRVSFIDLSNTPLEDEAGRSLAALAHVNPYLRTVVLDDTL
IAEEVLDEIDVACQFNQSNFEANDGAVEIGNPAELARLRHRLQHIIRAKHRKTYYCVAHL
LGMCPNGDLCLYSHSLMTTGAPDANANLHERLANLFASGGNWQDKLPSPPQAGASWKDPN
EADGQTLRLNLERCKAGASKVAAKGRGRNGVQHPTDGWRGSYRVVGATVALAAVVLVAGL
WSSRRK*
>TCRU_704 hypothetical protein
MKILLQEVSVGLIDEFCILCNVWLAGDVSQHTHGEKHLRWSEAAQAVFRQTAPAACVLAA
TLLRRVVEGEVRDHYCGRCSQHVGGKFRQAIHHLDSRRHHRKEEKQISLVSTVIKEASLH
SDGFVQGVFCSMCLCVVSVQQEKHLLSKRHQRNLRAAMSSASQTEAAEARQEMWVELLQP
LHGNICCSCAAYVPAEDIIQHFTSERHKRAHSFAQGIKDKDGDKGLRDFLENAEEHAADR
YCSVCDVTLRASMRTQHAQGKRHNMELLKAYRDPEGHIKLWRERFIANLAKERGQFPNVD
NTMPTVEVPSAPPDSLTSVAPTSATPEKCPQEPSGTCLLADMPLLFFFINGLCILCDCVV
APQSTGLVATPVGKEHCRTPKHKEALDAFVFQQHEMGQVDVATAWAARWYKDAFGALREE
CASWRECAERLSLYTSDDFMKLLQDDLRVDAFVEAVSLLRTAS*
>TCRU_705 alpha-tubulin N-acetyltransferase, putative
MSSTSQVALLPKLSLPDGVTVWDGTALEYERRCNNVDEHAVHLMQTINILGIRSKEAQCL
NTVLTSVARLRENRQARVYLLCQDGYGVGILKMGVKKLFVTHPSYSSLVEIDPLCVLDFF
VDTSFQRKGFGKTLFDAMLLNEGLNPGEVAIDRPSVKFLAFLQKYYGLVEYTPQSNNFVV
FHRYFDKWQPQRGKGHWGGNAVPTRSLVRPQNCLRVYPKYQSTTGPNNNFEEDATHRTPP
PPPLPPPLVPQGSVTSPGVGKKTAYELQYEEYLREQAYRRRQGGDPRLQPVPNPVSSSEI
VAASCGARRRMSPTRSGVQYNIISGTPEH*
>TCRU_706 ATP synthase subunit beta, mitochondrial
MLTRLRSAALRGAAGTRAASGMPTADHKGRVGYVSQVIGAVVDVHFAEGVPPVLTALDVV
EKLGRDEPLTLEIVQHLDAHTGRCIAMQTTDLLKLKSKVVSSGGNISVPVGRETLGRIFN
VLGDAIDQRGPVGEKQRMPIHAVAPKLADQAAEDTILTTGIKVIDLILPYCKGGKIGLFG
GAGVGKTVIIMELINNVAKGHGGFSVFAGVGERTREGTDLYLEMMQSKVIDLKGDSKCVL
VYGQMNEPPGARARVAQSALTMAEYFRDVEGQDVLLFIDNIFRFTQANSEVSALLGRIPA
AVGYQPTLAEDLGQLQERITSTTKGSITSVQAVYVPADDITDPAPATTFSHLDATTVLDR
AVAESGIYPAVNPLECASRIMDPDVISVDHYNVAQDVVQMLTKYKELQDIIAVLGIDELS
EEDKLIVDRARKVTKFLSQPFQVAEVFTGMTGHYVQLEETIESFSGLLMGTYDQVPEMAF

YMVGGITSVLEKGKKMAEEAAELEKLRRARAAQAGQKE*
>TCRU_707 BNR repeat-like domain/Concanavalin A-like lectin/glucanases superfamily/Trans-sialidase of Trypanosoma hydrophobic C-terminal, putative
MPNMSRHLFTSAVLLLLVTTMCCGSGAASTVEGNSEGTIIFKGEESFSDSENKSLVQAFD
SFRAPSLVYVNGVVVATVEAHYTNSTDSKSCVSIAAKSMEIDGRTWTKGSAIVFDHYDVR
IDRLLRPTAIVKKGGYEVSALLGGYHTSGAPLKEVGDGYWTPRMAEGEVSGEEDGKKEFE
WNQVASTTNLQGVLNDYNTNIKRFKQFLGGGGAGIMLEDNSYVLPIQALKDDGKEVSLVI
LAKGTHYGWEFSNDTSRDGCIQPAVLEWEDRELVMMTSCEDGSRRVYRSSTMGKMWTVGY
GTLSRVWGNSRNRTGHGVQGGFVSATIDGQKVILVSRPVYTEKNGKETGRLHLWLTDMQR
IYDVGPISAENENVAASTLLYATVEAQSLEEGEPNKEKEKLYCSYEVAAEGDKYSIAFLD
LTEKLEDVRRVLATWKENDAYIEKEYSCGNEKNNWSSDCDDKDLNKWLVGFLSNTSTGNT
WSDEYLCVNATVKNGVPGADGGLTFQGSGAGAEWPVGKMGQNVPYYFANEEFTLMATVSI
HKVPTEGSSSPIPLMGVKLSETDSTLIFGLSYTHDKKWRVAFNGSFRDPTHDGDLSWEPN
TKYHVALHMDSDDGLIVYVDGLVIYDSEEDYEEDNGFAEAFRSLMSSHSISHFYIGGDSE
RDSDNINVTVSNVLLYSRAFNKAQLKPLMKTNTVGTPDAEVPAPNVATQTIVASQSSGQP
ADEPVVTNEAQPSGNANGPAVSRETSFPSVLGVSTSAAAGEVKGEAPSSSVELLSALSPT
PSAGVRREVLETEESVSGVHMEGDQEYSPTNGTASTMEQAGRADEVSSQNVSTNDSMQHS
IDREEDTDVLTAEGTNSSTDPATVHRNDNVSGGASAAPTHLSTAHGEAKIPSEFNATIPS
GHELLLEHGQFRDLSAMALIGDSTVHGCASRVLLLLLGLWGTAALC*
>TCRU_708 hypothetical protein, conserved
MPSAGFSADRTYAKDACVRRESNNLYIRCSSHVIAFDFELPLFAAPRSEVLSTQTSMMDG
KMAEKADKAKRTARASACMIRKGFLPHSPPGSFNVDKVWDENWSPSMERCVRDDGDRRLF
RGLDDSLTIIPDDVSQSLAQTNRCRRTVRRGCLQHNPLVKCG*
>TCRU_709 hypothetical protein, conserved
MVPNASKKAPSFANGPRILVVLNASGIMSVTCGGREHVAADNTDEVSEPDATGHKSLVLE
NGVAVSGFVGEGDAGGLSQRNAFGHENPNYENSPRSIPGAV*
>TCRU_710 hypothetical protein, conserved
MAMMAGRVLLVCAFCVLWCGAGGGCDEEVVEAAAGGLLGTAGKGTVETSGTSTPGPDGSD
LPKGLPKVNQSTETSTGKSLEVNKVNVDQQDVAPGVGDEEDGSPSDHLEEPLKDDPEKGK
TKPELKNKGQEGREPPQLQVTVPQQPQPPLQQSQPPSAPQPQPSASEKGEGAGKNNKGGE
GQLSLGVEHKGNAAPEAPRKEDLLEGSGKESENSEQVQTTVPNKVTPEHKTQNGMLTPEQ
KTNESQSTDTSMNLPELQKENKEYSASTEGTAQSTSTGSQEQETEPSTSEEPSPFEEEHS
TGTKTSEDARTPDAAATEKRQTGDNEKVSDSDSSTAVSHTTSPLLLLLLVACAAAAAVVA
A*
>TCRU_711 retrotransposon hot spot protein, RHS3, putative
MDEVGPTPRYIFDERKYNNWVQRCHKTVDEATFSVIAQYSGLGCGASWDRTKVLYWLARV
VRERGEEFGYEFFSNLPVSAHLGNKTLFKSAKLMQQHYFNLLISWLRDYLISENFGRCTV
FAFLNGSFVSAIERRLRELRPSPQRRSHRCALAVYSQERSTRHHVLPPLEHFSERIDVEC
GVLYVTEVENFPLVDAFFFSESNPMTVVGLRMTTASEHHDNQHCEAVH*
>TCRU_712 MSP-B, putative
MRHTMLLLVLLLCCVSGSVAVAEHHCISDEIEKKVGSRTTAVVLELPTRESGMMRALTAS
DPDWAPVRFQLFTEDLNDPSRYCTAEGQIRPDFTGGTVECKERDIFTEEKKSIILNSLIP
RALKMHTDRLLVEPLMGRVIVPEFLSGACAQFTIPSSHHTEGVFGADMYLYVSAAPIKGS
TLAWAVACSALPDGRPVVGVVNYGPRSVTDSEYGVRALVHEIAHALGFTLEIMEERNMLK
EFLNVRGKASVLQVSSPKTVEKTREHFNCVTATGMELEDEGGERTASSHWKRRNAKDELM
AGLSGIGYYTALTMAAFEDTGFYRANWGKEEPMSWGNNSGCALLTEKCVMNGVTKYPEMF
CTAESRLLSCTSDRLALGYCALKLYDAPLPPQFQYFSNPKLGGSPDLLMDFCPYIEEYAN
AGCSDGNATDMRGSRVGPRSKCLKGDGLADFMGFIGDVCAEVSCDKGEVSVRYLGDDAWH
KCPEGSSITPTGLFMKGRILCPKYDDVCIVIDTINGTGDVSSLLSAFPPIPLIMLVLIFI
SMF*
>TCRU_713 Reverse transcriptase (RNA-dependent DNA polymerase), putative
MLRGVPQGSVLGPYLFSLYVHPLLNLLNSFAGVTADMYADDLSIIVKGQSREDAIPTANM

VLQRLHAWSQENGLAINPSMREAAWSTSSPHTESDYDREGKWPLVVAGCQIPVMTMGASR
TTKLLGMDLDPRLTLNVAATKQCAATSQRISQLRCIAHKEAGPSPHDLRTFVIGYGASKL
RYGSELIWAVATDSAKNEMQKTYATLARIVSGVPSTVDPESALLEANMPPLHVLCLRARL
SIFENTRACQMDWMRRPPPEPPPRAGLRISPLSRDELYAFVDAYTKDYGITESSPREERF
FRSSIPPWFAASAHRVTIGVGLPMDLSITDEEELIREKR*
>TCRU_714 Endonuclease/Exonuclease/phosphatase family/Endonuclease-reverse transcriptase, putative
MEPFTWLPAERYFYRLLNSIGAYQRYTYRLRAVCDAQRQKLLLSGDIEQNPGPIAVLQMN
VSCLTPSKLATLMAQGVDIIAIQETWKSSKQIASMHTGDYVLYAQSRIGKGGGVAVLVRK
TLRSKRIPLTIPQHDTSLEVVVVQVALEQNRDLIVASAYMRAPPQVTQSFRRLVNCLPAS
SPLLLCGDFNMHHSQWEPFLESSPSEVAAEFLELCTDAGLTLVNTPGGITYARGTRERSC
IDLTWSKHLTVSDWSASVSPLSDHYMLTFTLHQAFKDNIPSAPPSAPKFFYSWGKCKWDL
FIKDFDAQLPAYDYKKQSTGIKAFTRALITSYRRHCPRGMHKDGPRLWDDTLMEAERIAT
DSKARYLQLPTPDREAEMQRTRSQFFLLLRERLRNTYLRRISKLNPGEPLAWKYISGRKK
ASLPSPTSILLGGGQHTYKTARRAANALNRIFFHFTPLTRQLGFPKASTDRVHL*
>TCRU_715 NADPH--cytochrome P450 reductase, putative
MLRRWIAVAAVVAGISGVVLVARVWRSRHVAYVYDASFVSKYTALLGKQTELPSEQSDED
ARILVFYGTTSGNAEAYAKGLVADLRERGVAVALIDPSCWDYLGQYKSHQDHGQHPFRLG
TSGESPILVFVVSTTGEGEVPGNFLCLYSEMQSVLEGALKNGTKPFDGLMYAVFSLGDSS
YKYYCRGGLEVKTLLRRGGGTEVVPAGFGDARNPLRDDVFEEWEEKLMEAFERRCGVVFT
ADSNLPPKPQLVFRFAPEKHVGASPYPPPPSLLEPSMQFPAEFRVQAKTPRTERREDGSF
ILHLVLNIEGYTMSYQAGDHLAIYPANPPNVVEAYRSILKITEEEWLTPVELCASTNARA
RASLKNSLPACVALRTVFERYLDLCGRPRKSLLRVLSKYCSDPAEKSLFTELLHRGVKEN
GKDGENTMPTSLLMPSQTYFSSSHCHTLLDYLKKFPSCCCIPVGHFVEMMPRMQPRLYSI
ASDMMTHPTTVEAFIRIIPEGLASQYLGRIAVGEKVTAFIRKSKFHLPAKCGGRPILMIG
SGTGVAPMVGFCYRREALMKRHPGAAHGTMILFFGAQRRATEYFVRKEMECWCLHEEGKI
QTPDALPSSLLCLLDLAFSRDQSEKYYVTHLIEKHKDHLVQLLTSTEKGGCLLYLSGDAS
RMARSVEQALLKVLQCAGMTRSAASEFLRKMEQDRRYMKDVY*
>TCRU_716 Double RNA binding domain protein 9
MSLTEELFGLPGPSSAAGIKNERDVPEGMPLSFAPAKKAAFDLFALAPQVKKPTRKEKQR
LRLQKTFADKDGTQGSEGSDRQEAGGDPFKTVLLTEDLGVGKAAEKKGIKSNKHLRHALH
ENKEEESRTVFVGNLVNDVKRRIVEKVFKNCGAIECVRIRAQALEGGGEEGRRNENRHKS
VGRAIRVLRGEIKKGDQYSAVAYVLFKDEGSVTKALEMNGVVVDGRHIVVTTMDAKSREY
APETSVFIGNIAYDTNEEMVWNFFLEKGVTDVRRVRLVRDRDTGSCKGFGYVEFRSPASV
SRAIAVRGSHLNGREMRIVHVQKSRVVSATKTSRREKRKREAQAAAGRGQNDEKKTKKLR
AEEKGTKRHANAEGEFSWMGVVTSPRKKIPKDLRPLVEGKRGRPASGLRASVRRKMRNPE
K*
>TCRU_717 hypothetical protein
MSADRVFLEGSGAPSLELHVGGAPLAEVLADMMRLIRSQQKELESVRRDAEERIYRNECG
LKKHDGVLESFAEDLQIHVRPINYNGQPPMPTMADAVANVECRLWRVEREKKFYAAARLS
EVTRKKLMAAFYSQWWLLRKLRAKSHFLLKETQREIWRRYMRKWSRSLALRRRQAAWRRH
LMGLTAAHGRRIMMCYWCKWVQVVAFKSQARANRRISAYDISDTMLHITCRGIARRYLYA
WAQFVAAARDARCRAREALWLEQKTARILARRYFERWLDAHRWCRLPLMRLKALDALRVK
LSRGLARSFLRAWQDFVRNRCERRRVLRLIPHLHHANLAALARRYFSRLYAFRFVCRDAR
VVETLEQRLKTLSERADGLQRQLDMGLHTLAHTNSVLARVVDTVMVAGEPRDALQAFLQG
DTMISVPTVPSVEEKRSQQGASAPSLSPNSRRHEESASSTGRERQPTDEHRCVSASEMLQ
NLRVRLEKTYKMAGGEP*
>TCRU_718 hypothetical protein, conserved
MTLRQTKQRSDMMSGIDYSKWEKFCDSDESDGSDETPGNEPRITRLRYPSRVTVGPNGVH
MENTPPIATTSPFKEGIDATINKNTAAAATTTTNTASVMGPHSKQNTRKDVRGVEEAEED
EMMYHNLTRNGGREGQSHLWSQTRDTASVSFILPSMNTRAKEIMNFRLYSEEQPDRTSVC
VLTFGVKGVEGDRRCVFRYPIKVDNDVVEGCWQLHSLHSKSLRLMVVQLVKESVAVGMAL
WWDRCFVTDQTTVDTRTLVDRSSSEALRGEQFQRAWNEAHEEFKKRVNKGRGRQPATGEE
EEEFIE*

>TCRU_719 Yos1-like, putative
MKDSGIMGFSVASILQALLLCLNAMAILSERRFLSKYGLATTPLTEHGGDAGYVPQSFGT
GDEFVRARQPSPWKAQLASVLSGVRTLLRLPLIIVNTAVIIFTLVFG*
>TCRU_720 hypothetical protein, conserved
MDGSRFGTESLTPLVGDCELVLQRSTSYEPPMYLFPELLPKVPEDTAPFSVSDAVSGAVD
VEASSLNIAELFHLPQMEQRDDTGGSQGGDADGTTPATNPASAAPMTTLASLLAADRLQV
AGLSAEMLQRGKFSSGSIIEELIKLKDRAELNDNEAKAIMSNHFINVLAWRTLIEANNKA
LSDVKGENTLHGCRSFARPREAETAEVDIGALRPSDDPWGTHVDLQPGVFVSAAEQNVEK
RIELIETLSALYRRPWQTLQSRGIQTEKFTPGSINETAPHVDPIKLPAAMLDPEEDLGWK
QSAVEDASNNHWELRVTSSSTAASELIPVRGRFMHFGRSAGVTSAAHGTVTNVHVGLSAF
TNHPEFLSPHHFSLAVLSAKPPWSASDAGAAVKHEVDGNGENGDEGQFIWLMNYGRNGVR
VIGKQWALGDMVRLDPGDVLQPTDDIRIVIGGCAQKTEGDSSNAKSEVLMED*
>TCRU_721 hypothetical protein, conserved
MSFLVKALLGSNVVHPVEHEGSESPSLQQGDAEEEKQRVPTRTLILRVQSCTVAQDRRDA
LKELQACPDLPYVMAAKEVSYLCNVLRIFPDDNDVVESSLAVLSGITELTSYPSSSSNYA
VSEKEKRRIRDSFLHELVPEVPLFLNHVQAGSFWCRFHAVQILQRLQEHDPSAVHKLLLS
SHGIGMLLDILNDNRHCGALRSEGLLLITSITATDTELQTILAFDNAFEMLFAVIKEEGG
LDGGVIVCDCLTIAHNMLRNNKATQKLFCEMGCARLVCNLLEAVPERLRAAMHRSASLKN
QGGDTGENMEAIFTPLSDMQNDVIFKAVSILSCVLRGAEAHQEGPAAQEAFLQSGALGPL
SSLALGGVIADDASRVEALRTLAVLLQDRRVGIERFIQLQVTTLVSCEFSLWVEEWSALR
GILHVLLEIEDRVMQSAAAQVFHALLSVSVCEGDVAEKLLEGFAPSLSSLAPAAPAATAS
RGVLPARCGAALANTLFRPIPCSRSSQKYYSALLLDRLLCVSGVMRQLLGTNQREMASSL
SLGKPITPSETNGVFLAYVQYVIKCLRGQGEMDLNTLSACFRSLLRWVIGCEEAVQVFLS
DGKYYKSLLQRAGQDDGPVHVRFWSAVLCAALCVAAPSGLPKTAQEPPRAGGNGRMRECE
GGADGVPSSPSLNRRQLLELFLNYLGGPSIFDSFLFDVKASSPMWSEPPKNAFLRPTPAL
YDEKMKHTLLGVIKEFNEICPSRGKMQPTAMEESDLSVAPASTAAATRVTNTAFATVVDD
VDFFSEGPLLLREDLNENLGAVGTTAPKQQNLSQVEGCLKNAEDEACNALRNVYELRVKG
LLEKNAALERELQVMRGQLHADAESREVEKKPEKRHLEEVKGLRENVRLLEEALSAEEEE
HRLLAQSLNMLEEQLREARANRSNTAEVLQLRREIEKLTEERDRLLVLVAELDEENLPMN
YTGLGKTYPSPSPPPLPPPSAPYSFFSSTKAPQAICGEVNLLPATNYAGFAEGEQITPAE
PSHSLFEATGPNGFQTSPSPSVRPPQEQQQQSDLQPARRPFERTLGITNGLQEPVEKLAS
MERNLFSDEHPDGCVRGNELEARHMESASPAGAARRSAPLKSNPFANMVGPHDELSGGPW
*
>TCRU_722 Nop53 (60S ribosomal biogenesis), putative
MGKLRDLWHGDSERAVNAPQPVRVAPGWNVQPEALPAKPLAPRRRKYVHVRPNNRDAVAL
PHPGQSYNPLHDDHQDALRVAVRKLERKKTADEKFVAMMTNGRDVPITGNFSADKTWEEE
VQERPQKTQKKEKSTEKEECKKKKKKDKKKKAKKTSVQAARRALPHRRHPKHEIALKEA
DALDDIIVEHQQREKRREAARERCRAAKREKMQVKSFGRYHHTPLALDVAPTDKLVGSLR
HLNGGYIHPLTERMKSLEERNLVPARMRHTYNKRKVLKPKGEVRVKRETFGVMPETTF*
>TCRU_723 hypothetical protein, conserved
MAKKKSGTNALLPELTVNRRYIKKMKFIVKRREAAAAAARRGKAGEKKKKGGARNKAGDA
AKGSGRGKLSHHSAAKKSPGVSAKKKKTAANGGPAKNRRRNINDAKSGAASQQQKQLSRE
EIEQQMIARAIRGDEGTRNASLSAGALDGVDVDDLVQYPYLPFLEDYAEQKRSEVVSAET
LLSNAHAYFKDLGRKESAMRRAAARRLKELKNINRRGGDKDGFRYVVPRNVKEVVRQMMQ
NAEGADASGVDKTHLTSTFGGDEAQDTFERPMHRKRRKNSCYSDFYQFQVSKRWTCNAES
FLNRGRAYKSMVEASRPQRSIKKF*
>TCRU_724 ubiquitin-protein ligase, putative
MSVRSEVQTENSKQQLKAVQSQRNDPDKVLEPLEKLCISLVMGVEPYLRSFDAATAIPIL
LCMTEKEFFSFCGDTMTMLLRALALIMEYVPSAYESVHLYHKRLMKSVCQVLSRTLLAKM
KYNNTDNVMLLEESLRLIQFITKDEGNISVLRFGVTDVIRIAASENTLLARQAFDVLVTI
CSKIILPSDVEKLNIIGLSSFLICGRKKKSKKGCLESLDSHGKIRVYAVENVIVPFLHST
LQQYSASLETFPEAWTFLELTIHCLGMLMRRSMLSHRPSTTRKIVTRDMCRTLFRLLLFN
DTNVAIDQSVRSERILLCETVLDAAMAADSLVASEFLLAEEAVSFYKLLLGEQESGIITQ
LDDPFPVSGASSRSTQRRDKNNIVSIAAIRLFVLACPRVLSEAFGPKPKVLLPVHQWYWE

DELRNSNSISEEGCVALETSFCRLCKETPFRMHMKSLRANFQSMKLSGGFGSGERNISRK
IVPFVFHFVDEADLRLVKSAKEAIMLCNPTPKCRRELGVINASEAAAISKGLGELFIINR
DSVVPPLYMSIAEMFLESLCSFVVSVSGQMAMQLGTSACASLLQAAICTEENQRLAGLLS
KIIRPLCEMLREALVSGDKATKSVSLTMMVWLLCHPQASAWNFSETALRCGVVNQLEILL
SGGAVVSSKKTKPHKGLTKVGPEVKQTRTAYLAASALAVLRAINKQLSKTQSPRSERSLS
TMDVMVVRKAVNELKICAATPGLHQGVVARILELMEKSANTVTAYEFSTLGIADAVLKYL
LVEHYKDYCRDAVTMVGTATPSVATGSTEVKEESFVGGFGQELTEGDLLAFAGTRTANGL
HQRCQKERLQCFLESAMQRPRGVKALIENLVSALPLHSTFPLVESLLGPSRATCRPPTKA
VLVGSHISPIVSLCSKPDAESDAAKRWEFERKNSKTLIKRSKSCPVSMPMQPDAGLFSGG
GVEVVHSRDNCPRGHRLLAISEGHSLGLCDMCEEEIHTGFCCHRCEFYICKDCYTDPKVQ
YSNANSGASPSSGAAAGGILGFVAQSQQQQQQTSQERQRQQYSRPIIRAHLFSSIGDIE
RYFRTGSYSTRNTDVAPAPQSVLQNIDYFVERAKSFLARKRLNCSDEILRKEVHRILSSF
LLNGPTIDPEATAEDVTDVHPLGPKELQELRRLLRDAMEERYVLYTAPGGRCAYQETLIG
NFLRRAIHEGNLGVVEQLETCLLSIEGYCESSNGVQEIHRNESLLLRQDEVGLPPSWTDV
PERSSNARTYMFHHFEKDEDTRCLCGKASRAELNSMYPPKLVRKPSRLSQNPDTLLLILL
QKLLSPLIKQGAMDIDAGLFVSAPFTTLVVKSLAASALRVALLPPRLAVPRWADFVITEA
NFLLPLNVRERVARFFAYGARRALHGHLKASPLSRSNRSINVYPAEWGRWANHKYRVSRE
SLLHDAYTVLQKSADSRLPISIEFDGDVGVGQGPTAHFYTLIAEEVKKAKLRLWRTGAPA
TKTKDGSTHGDMSSGDARVVPPAEGLYPFVSAMKDQCRLFQVSSWQQPGGATMLTDRFAF
VGDYAEFDRERARCYYLIGSALGRAFTDELVFPLDLSPALSIFLSRGAPAPCVFFSGDAI
ANKGIERPIDFMGLSLDDVDLMDRSVATSLRSLLSLSGATLKSLAVPFTLPGHDNFEMIT
GGSEKLLTKDSVAQYIRRVSSGLLYESAVLPIQFLIRGFRDIVPCEALASLGEQEMMSLL
CGCRANDKKPLWTLAEIKSILVAEHGYQTDSPQMTMLQNILANRLTPAEQRSFLLFCTGC
PRLPVGGVGALGAITVVKRSDFTMFHGSSGRNSGGSDGIGGADRKEQNPKQPGEWNSLPN
ETDWPLPSVNTCFRYLKLPPYPTEDLMYKKLRLSIMQGGETFQLS*
>TCRU_725 Fumarylacetoacetate (FAA) hydrolase family, putative
MKSSLAFSVRHAVVPVMGCNLLFPVRRVYCVGQNYADHAKEMGGQVSKSNPFFFTKPPDA
VVSGETNHTAAMDVNIRYPPQTNNYQYEVELVVAIGESHGGREKYRAISPLEAHTVIYGY
AVGLDMTRRDLQLAAKKNGKPWDLAKGADESAPLSAIVPVKLIREKASALFYATDANYRE
AMNAKAQGGPTVLRRGDIELRVNGERRQKGNLSSFVTPIDELLSILSHSIELAPGDLIFT
GTPAGVSAVKPGDEVRATIEGVGEITARLH*
>TCRU_726 hypothetical protein
MRVYGLLLLMCTLLGTTTATGRLGTKSPEFTSVAPESRGIESFRVVIYLNATACSASEEI
PQTFFVAFEEDLLGGILPEISSMCGASSASPSLSCLAVCSIHSPSQELAFTEHFGGAGAS
FFQHPTIVCEWKADGSVQMPMGFYPTCRKRVQDLYAMENALEVIKAKKSHKKRNRYTFGR
SSEVYYKMTGDARHLVAVGGDCYLTDFSAESARKMCGRVTCLEASMIVLTAVIALMVVVA
VMYLCRRQLFVHFFNFGLRDGESSTSSQLIVHLPDKNESHSSTAYEAWKKSTEPHTPPAS
RLPVSFGDSNASPSRSPGSNEVDFTKMDKVLANASPRTEQSGGFSDRRQCLTISRTSSQS
HFLTPWRYRQPRVGSNDVAVSEGKKIDGLAASHQEKALPEACEVDISKEEREQLQRQRSW
LREHVIGLDAI*
>TCRU_727 Autophagy-related protein 24
MGETVFEFKVLDPEQRRGTGALDLAYWTYRIRASTTLSTYAQKDLEVVRRYSDFEWFRAQ
LCEAYPYCIVPPIPEKDVQGTLDKIVGSGSQSASRLRDYRQRALRKFLVRLGAHPRLHTS
HLLKDFLEMNEDEWERKMKAFAKNSEGFFTSSLGEGVNHAFTRQWNTSGAVAEAGAAYTR
VVTNTNTTPQVWEETRLYIQQLEDSIKMLRERLQLLVDRRRNTSNALHEFGVAFEKVGEI
ERGIESTPLSNALIAVGQHSEQLFFIYSDYANEEMKQVVETLNYYCGMCAAVRETLRRVH
NRSHYVESLRQHAAEVVAQKERALEKGGQAGRVARLESELAGLEDRLSTAKRELEAGETI
LKDELRRFHQEKQYDVKAILKNFAELQLKYAARMKETWESLRPTVEAVQFEGEGGGQGNA
LR*
>TCRU_728 cysteine peptidase, Clan CA, family C19, putative
MSAPAIRHGGPSGRLPTRQGHHFMLTAEPPGRSFATSATTPNVPKTTLPSYPGMHRLYSR
PFRGEPQFLGVSSSSHILPSTLHGQEDRRWNSMRSHSHRPPLLSLRGRPPQLQPLLAPSA
PAIGFARQASSTSEMQMPRTSEHQSPIPLRNFGNTCYMNATIQCILHSPWLFSSLAEANA
WSKRRLATSALLELGAARVDAPGQLLLTVKGEAAKFNAEFQDNGQSDAHEFLRTFLFVVH

SEINTSGGHNTPYEELKEVENESEEEAMRRWQEHYLRVDNSIIYDLFGGVMRSRCVCSSC
GKVSLTFDPFLDLSLPMIPGARKAATIESMLKISFDEAEEKLRGGNQLLCARCRRLRNGT
RSVKIIRWPKILVLHLKRFDDTGKKNSEPVVFPESFMTHDNSPMQYQLYGVVCHSGSENW
GHYTSYVRTLSGRWYHCNDAVISLSTVTEAMQALTTAYILFYSVK*
>TCRU_729 Flagellar Member 5
MASARSPVLPHLDFRPQSAEGRAPGVTDQAAYNELVANYTTLRAKVNAMLAKNVDIIRHS
PVVDLTRNYVGAAGLQAMTSLLAKNDNLQELRLPGSGLNNDAVVFFCRAMVKHPRLTSLD
FSCNNISLAAGLALISLLQQNKNVVHIDMSETQVPGAVMKKIQLGLQRNKLLAARAAAAS
PDAADDTTSVTRSTEGGFCSTIRETVRKVEWERREEEALHRLREITHSAAAETGNDRRFV
PLPDRESGWRLIEVAILSPPFLFESELSLLIKKVFPRLNEELRSSKVHLLPLLDPPDGPA
GTYLKHLRFAIAADVLRNVEKSRMVTIELIGDRAGEYQQLPSNEIMKRPQLLQSMRRQDG
AAKLIPAESKWAPPLSPVLQSAHELATALSNWIIVATRKETRRTGVPPSLAPLLTEEPNV
EHPDYLQSQIRRTVTVGSDVVCGTQTIECDASLKAKKWHEHQIFRENLLETAPVQELVIR
NYNATFDFCSEDGQVHFKNLDEFVEAIYKRILLIMIAHFPPCHDSHPAHYQGYNVEKFAA
KKLGLYQKRLHDLLRACVEHSFVKKSITDRLNLYIITPPSRNVFVLHGSEPVPVTALIGS
FTSRLLAKEEQKFRIAFHTTHATSLTEEPNELHEIICHVILQLFPEQEIYRRAIAEVDIV
RLQQMFLDLLTDKIKSTETFKRGGGGGGENQITVVILDGMDSVLPTVCPCNALRLSEDTG
KDIWGTPAPPAQQSARYGYIPVCLSRNLRLVLGCATNSDMHHQLGSRGPDSAELLSMGPI
TPNDFDEMLHPDMLSKIGATLSDDDFIVIRKKEEALCPEYIMLVLDTLRSLNEVPGIETH
SVVTSLPGTVKGMIQLKYNNLQQTFGTALVRKCTRLLLSSRWGIYEPQLRLMLQLPQRRF
NQLVRMMRPLLGYGTLFSLGIKEGNALLSRICIRSREFRALLEQEDLQEEGNEEDQKLWH
EMLARFYLNVVHDVIGREKTPPIFGLSSTNPYERISVKELPYHAVKAHAWHILFRFILSM
SFLMIVYRNRLGYHLVRELIVAFNTLYEMYELGEFPEENGATHGEKPSALLRLREYIYFL
REYNARLTEFPHLVIQTAVESAARYNFVGSDAKEYVTRKMSHHAPIVQRRTVFFEIIAGA
KTVIHHGAITTCLFSPKGNRITTGSVDRSVCRVSPVTGAVAAQLRQAPTRVEEILYCETG
AYHAVICHDRTLLIYDSAQMKLLSRCDGGIFGAPLSSVAFSARGRFYLVATEDLHLRVFE
TEKGRLLLHVDQRQFLHGYCDGVDINLLRGYSCVIPHRIEDDVFHSTCHNVLIKWQFSPT
RDEFASETRLGVSFTVSSGKAVLIGTHLLLHPIPFPAPTSGRRAPPRFVHLHSLLTGREM
AVLTSPCSQLFYQLSANERLLASALDDGSVAIHQLCWTSLQKEDGDMIAIPPVMELAAYR
ESPVATVRALRFRGDCKALFALGNSFEMKFWALPQRLLDGGGDNVSDADVSYPVEVDDAS
REEEKELVEGVADGEFILEPADVASWDVVQVPNTKDAEVIFGDSSGRLTMLRMWSPLM*
>TCRU_730 Spt4/RpoE2 zinc finger, putative
MVEEPPPQLPIGDRGYLACRQCRLILTETQFIRDGCSVCGTGPVSRDELHDVTTADFSNF
VGLIAPEKSWVARMIGRTGCPNGVFAAMLNDEDEEEIDSNDDGDDGAVEEDDKEEEVEVG
DEHTDGGGGHRDDTPPSSRPASPVMTDEEFLATEK*
>TCRU_731 Eukaryotic protein of unknown function (DUF1764), putative
MMRRAKKQQFDGTNSKAATVVGVASATAGCRGGRVVVEPKGESEIADIFAAMREKLHRDG
CRSRSGGDSIQRRKDVKTASQCVSDGLYHAPEKTLELSDPAFFDVASHRQQKKKKANAST
ATKFPEEVLRREGVDRIVTLEELRGITSSNPKAGTTANCPFDCDCCF*
>TCRU_732 aminopeptidase P, putative
MHSEECYVVEQNTPTKTTMASNNKQNGHKDNACTAYPFKVKREMYREQRQRLAAALLSSK
DATHAAFLQGGSEVPVNSSDINYLFWQESYFAYLFGCDIPDSFGAVLADGRGLLFIPRYP
VSYAVWMGELPTPESVKLATGLEEVYYTDEIEAALTSKGVQTVEVLDGVNSDSGLHVLTA
KLPEGSKVKISNKWLFGVLTQQRCHKTDLEAELLQYVCRVSSEAHIHVMQHCKPGMSQHH
LESTFLHYVYYHGGCRKVAYTCICGTGHHGAVLHYPNNDAPVEDGSMALLDMGGHYMGYA
SDITCSFPVNGKFTEDQRIIYNAVLDAHDSVMRQLKPGVNWVDMHKLALRVMCEHLVRAG
ILLGDVDTIMRKRVMGLFQPHGLGHLMGMDVHDVGGYLEGCPPRPEESDCCKLRMARVLE
KGFCLTVEPGCYINRTLLTDAFQNPEFKPHLNEAKLRSLWNFGGVRIESDVLITETGAIN
MTLVPRTVEEVEKTMAGAPFSREPEVFTH*
>TCRU_733 Poly(A)-specific ribonuclease PARN-3
MTSDITFITRQNFTEIFPHFVRDLSEANFTAVDLEFTGIDRESNSDFLRLPEEAFLQKMA
AARLFSPLQIGFSMFRGDTDLEGNSGGNSPGAGVNSQSYRDAMGWEAKSFCPCGLMGPML
KQIIESWVSKGQVDPNELAELEKRRREAENICGSVAEFNSVYERLRAHQTLNEINTVIAM
AQRWKGYAERKKRHVAVRNYSCYLFPAPEKDEGDRTVTLSSETAVFLLKNAMDLNKWVTE

GLFFVSFDEYAKAQQGALCIPFAADRLIEQGGRVAALYRWINALPPAELRGQLSAEFKNI
LSFLKQAEVGDSVELNIPFMKAPWFAEFENHMRVLGLHFSRRTITRVPSSGDEGVPIEKD
PRYFGTRLLETLVTATRCWKKILVIHNGLSDLAFLCAAMHQPPPETLGEFKRTIREMFPF
FFDTRTLTCAPSLQNIGILTATLKKTYFTLLKRNKRIQLHCPSSFEVAEQSEMKEHDAVI
DAFMTGSLFLFAQEELAQVGSDFRRLRGITPIYGCVFSVNFEDEVADCIVHPPSAPIYLV
HRPSHMSLNMETLRNKLSAVAEGVAFIFNGDYCLVKICDKTVKKGVKEHIEVVIRQWCES
LSMGFKPLDVASCVRAHNISYVSEIEGPLLVPH*
>TCRU_734 Importin 1
MSDVRQAILDALGAAADANPDIRHHGEAQLQRLKESPDIFFSSCAELFTDARVPTRGRQL
VGYLLKNHLAHPSCLQNKTLQVAVMESAVVDPECGIRAVACAIISAAVREAYWPVQPVVS
SLTGVFSTRSGELHAVHGAMRALSQIVDDTVQLLDMRQLTGVVVSAVLPYLRAQFGGREG
LEVQLKALDTVSVVLEQAGFDCSSFSYSSMRPYVLNVIESCFMNLQNPSSAQLSTKSVKC
LVLSLGYHDLISDELFHKITNLMSMATASPDGTDEDLRIEATEFWRGVLCFPRFASLVES
TLENIIPILIRSMVYSEMEISMLQASAEDWNVPDKIDDIRPRHYQARVNDTGANDADDDS
DDDDGEVEEWNLRRVSALTLDSIAEYYGERIIFTVLTVIEGMMQPNNSWKELEAAILALG
AIMDGCFDSMTPYLPEISTRLLQLLGDPSVHFLVWNISLWTMTQIGKHIVSVPEKLKGFI
TCILQKMESPSKLVQEGATAALQKTIVLCDEGQLDNEIPFIIDCMARCLRGYQLKNRVLL
LETLETFCEAMGEQLRVRPDSVELLMAPLGEIWGSTPNDSPLLFSLFKCISGVCRALGAA
VQPELAKNLFERSYCLLVMHVQARTEARRTNQDPPEYEFLVTAADLLSGLFDALGTGLGP
LVMQCHPPLVYTVLEALRDEDAEVRQSGFSLLGDLSKVCPVTVQGELERVVKAALENLAV
LDENTYGVISNIGWCMCNLLENQMDIDNLSTLDAANGMPQLFAAMARILGTVSHAADMRN
MAENLCICLGLMLYADPGVESQSGCQVSLFADAFCSYMRNVKDVPHKEQAVSGFLMAVRQ
QLPFVVLRLHLFFDLALSLASCRADVKRAMGELLQAARAHAGARWTQQMAEYSEQLRTRL
YHVYGIQ*
>TCRU_735 hypothetical protein, conserved
MRRAFALFRPTAVVRCVQPAAFRPTRVLHIPPQLVRLIVAGGLLVFQAFVVAHNREAQRL
REEADGTATGEGCSTALMSSSEALHILGMDANLRVPLEEKKDREEARRRFDHLFAIAKNN
NNAYLQGKFSAAYRLCVDANWDADENSCSNRGIDSSGSGGEQG*
>TCRU_736 hypothetical protein, conserved
MRRLAAVSCGGCLPLLLFYCPNLSAGTLFSSVQGKPGHSDDDALGAEDSFKAVEAALKDK
QTSKDQALPDEIDPVASAVNQRRNRRKRSVDSIFGDVLHDALRLRDMGHKPDADFVYKRA
QERMYEEEYGHKPPSAKDHAAKYVPFPPDLGLNRLIPLEHIIGDDPWPARSITAQNPVWT
RQQVEEDRVKHGGAQLKEWEKKMFGPREGFDPCGEFIDAMEELAYWEERFVKFIREAPLP
QRRTLPLLHEVYRYITHRLVRAERRYLGTRDAALKKARASSSVYKMTEANIERIRSLYSE
THRSLSSPNYDPVRMKKSLTGPLLQMSESEFEEWKNVRKHQRNRLVAEIV*
>TCRU_737 hypothetical protein, conserved
MLVFVTQALAESLTVTSDACCNMSINGVRGRRRKTRDPSLCPFEERLFKAFQRGFSSCPR
KPHALLHTFSLSPSAAKACAYLQRQTSPSMLERGYYVVDLDHIYMEAPTFFDEAHRCWKY
STRGLRMQKKTSISRHPELLMIVLSPPETAAVRGPGTMFPTLVCRCADNADGTEEGIYAR
LCGFPPSAKFSVLSEQLQQLSLSYPNKALITEGDGWNWLMAHLQSRDVSCDWCRASLISS
DFVKQSLFFLEWCVPEDFAWLPCPKTTVGWGSDVLGKLLRGISEQLVFAGGADSEPASKR
CRAEHEEAFLDMFIPTGLDAVASSMEALEEAKREVDMRFVEKIHSAVFRPRQGLLLKTLL
GQLEMVEEFFSGYNLYPCDESLVNAADMNFSPLPAGAPTGRVAQLLNEWERIQKSHRCSS
FPSSDTPHSRTRHGDAEVELPGEDAAAPFLEMMRRHFINHGSQGGVPFRLTGDGETLLEA
PCVAETTLREPPEEMVELADVAELLTALSASADIDFRLAVGDCGLDNQLPRGPLKSIMEA
SVAAYISLAFGAKTLAVGTAACGHDVVDSSREGDGDCRSTPSTAPLEVLGDVFFKFMNEM
DGGRNERSSELHDVLNEVMKLMCHVKADEAEAAGGYIILSPKGSGENNMDSDAAFPITSI
ATRLVDAARTAMRQAASGLTEVEAKSRGIRWLRHIAFLFSHASVPVGASSPIFLPAPMMS
KETEGDQVRRVVRGLEAHLFFLDAALCAVDEAIVRRWEDAHEALGKGIA*
>TCRU_738 hypothetical protein
MLYLEDCYNNFLEEGLRKLQGERGGSNSRGLGSLFQTKLLQTLLSPGLCVQPFAPQKKSS
NTNSHGEEYVNPLAMASRERSVVSTTPSVGRKLWTWVVDRIIVGVYGMPTRNKMERGTCW
KYGDSA*
>TCRU_739 hypothetical protein, conserved

MSSGAGRGGGFGTPTQLGVQYSSEEERSIYKKVATIDGYTFEVPPVNRFHSKAAESKFVQ
LTGSMNPKFDAERFDALRERLQQADKKGKDSHLQAIAVLYAESLGNNFLRAKNVDLAELE
RCFYPSHIPSYAAALRTVGLKKPLWWRQGPFVRLEDISRLPLSRCDVNKQTVMSFEEWYC
LFWNYYTPFNTLAYLLMVTAQSTFPTRELIDGTAEYLAYRHSLIEDPRARKAPILFLGSR
TGKLGGLLNQTGKIPVPVIHVHEKPNMNPYLLVIPPNKQDEFKPHPIIKMKDQVALEKYE
PAIVLISDMIMNNDPTSMIRRIGSVREYIYFGIPNSYIEGHPWDTWGNPRYREKGTDHIP
SYIRENWLKVSLPHLSRWMIYKTDSEMQMGNGAVTTWMRRPLKPSAKAILSWRLARYKPF
Y*
>TCRU_740 DNA excision repair protein, putative
MADDLSQLGVSWVEESALRDEVVAEVDAQVVAEAAMDEAELRRRWEALGEMEHVVAELQS
LAAQSQKGSASSRGVAEVLGGKNDKVGRREEENTAAVLQFRLAKAQREVENRAKLLRRWQ
VEMEGRQRKRLEEAKKQRQLAGKEEEEAAAERGKSNVSGNTAVASAGLHGVMDTTSITAV
MNVSGLQVASSFSSLISAASPLKAADDTRREPKIPLKTRVVSNDAATAVHSRTATQPQRC
HRSGSHPSVAMTASEYAGGAILPSLYRVTLTRSDLRSKSIKYADDGDLDVFHARQQKRRR
LEEVAATMAASHLALSHSGTVKLEAAGNTGSMEVKQENDEKGESGNTRKEAIDVDALMEK
VDDDDDKNVMRQLLMNSGGGCDNNSTVHSMNRREHETSVSIVPGISLLANIYNRLLEHQR
DGIKWLLRLHTQRMGGILGDEMGLGKTIQVASMLNALHHSQQLRDPCLIVSPLTVLRQWV
AEMHRWAPYIRTCVMHESSATNMSREKLIQSIRSTPAVLLTTYAAVRQHCHLLHTARFQY
VILDEGHKISNPEATVTLAAKSFPTPHRIILSGTPIQNTLKELWCLFDFVRPGLLGTMTK
FVEEFEEPINASKDVRASPLALATAVECAQALRERIAPYLLRRLKRQVMSDTLPQKYERV
IRCPLSDDQLEAYVELLTSSRVQRLLSSTLTSSQLMGGLNCDGRDANGCLHIAGRRFQLF
QRQYHGGIRLEAFRVLNELRQICNHVDIFRLRRAAKENGAEYFDDNEEDETDGVDGKTKR
RRGSHMSLRSNRLVNYSGSGKLQTLQKLLTVWQRGGQRVLVFSQTRMALDIIENMCEQEG
FKYIRMDGSTSGHHRQELMDRFNEDDSIVAALLTTRVGGVGVNLVGANRVVLFDPDWNPV
TDEQARERAWRIGQTRDVGVYRLIASGTLEEAILRRQLAKTYVTEKVLHNPALQRFFHQQ
ESLTETFYLGAEYDSRVPVGKKHILATQEISPLLEAEEDDDGREQDDGAKVMQEKGAAAV
YRVRATTMTNKLNASSTPSTSTAASASRAETALLQDLVDGQEVRVSGGDAIARRLARLSA
AQTMQRVTNTTRTIEQQQAEMRMRALREVKGENGN*
>TCRU_741 calpain-like cysteine peptidase, putative
MTEIKYENGQPTFEGPTVVKCFKDNGNGLLFRIVNEEKKQWAFYNDTKEYNMNVKAAFGK
DSKVEPLGSTKMEKDEATGEFKCEVQVPPLATVLFVEGEPNGYKLNFEANPVAK*
>TCRU_742 calpain-like cysteine peptidase, putative
MTEIKYENGQPTFEGPTVVKCFKDNGNGLLFRIVNEEKRQWAFYNDTKEYNMNVKAAFGK
DSKVEPLGSTKMEKDEATGEFKCEVQVPPLATVLFVEGEPNGYKLNFEANPVAK*
>TCRU_743 hypothetical protein, conserved
MLLPRTDSHDPETNDADPRSTSSAPSVSAPALSFTTTADALATTPRSNTSPCAAYTWLPD
TRSTAPPDATTRALAYNATSPPSTAASSATPPPTSSSTAPSTTTVRPPVITVTRATPDTT
STDRTSRRPDSTSTGAYGEDCEPGRYGSGVGLAATSRESVTVSTDPAGNVPVSTSAPFSA
STSHADSSTGPPSSLPAATAPRNAPPPITYSTAPFSSDTFCTTIRTPSRAIHGT*
>TCRU_744 hypothetical protein, conserved
MPRCIACATTYVRLGKRIAAHALAASTPRHRWKAAVTPQCTHPNTAASSTLPTTIHSGSR
AANTDRKYGSAAVLARSCAEAHDILANTPSACISRTEPSPPVTTTATAAAPATTPVIAER
ALAAAGSVSNLIVKKPSSTPLQPPDTAAASQRMTIVSSAAMK*
>TCRU_745 hypothetical protein
MSSISVLFPRLRLARHRGNNLLLGNCGGVLLPGDEVAPSRGAGALPEYKNIAAGGRCERD
GDHDDVIFADSRRISKTILSWFTVSKAWAWSTKTASVRCCGALQCMAALRCSNWSREELL
GRRPDGCGDNWDSMMSRLRAARIRYMSDPRQLLSATGRCEWVF*
>TCRU_746 hypothetical protein, conserved
MTEGCAGGKGRRAHRVTGACRCGEDSIPGVDPDIGPRVAALRTGAMRLLPTLRQACTRPA
DHKEPSMSVVPSLPHCGDRPASASAGTSSRREHRNFLPTSERRIDVLLPNRRQSALVRPQ
YEGTCPTCSAWRYNSLRGPPMRMPQWRHRLLNRCLARRSPPKLPSMPAGKTRRKHKRGYS
RWTRLFRHRLITDATGC*
>TCRU_747 hypothetical protein, conserved
MTEAASCLDLKASFFQVSLPRETRHLFRCRVEDGTLVELTRLRMGYKASSDILQIIITSA

IARMTTVAHRLWAAPPSVRDDVWIDNIRIAGSKSDATLWKAQVLRNAEGFHATIGRIANR
VLRSTPSWGCSLITLTPGGITE*
>TCRU_748 hypothetical protein, conserved
MNQVIRSRTDWKESFVFRLAWITASRWFEIAALTPNNFTMEPDGTVILDWSVAPKTARAD
PHRALRFVRIRGQHAFDIIKLCRTLQRNEKLTNITSAKVERTLAPWNATAHSIERGALRH
AAQIVETYNLDPHVIPELAKHVDPFDLPQNTV*
>TCRU_749 hypothetical protein, conserved
MAMAPCTATEWSFSGSRMLDLVRQQGNIQAQLAYMDEHRPLDVVVTVAATMAVRRWISGD
IAKYWGDPTLMGHFANLRAPQLPIFETQLLPQFVADVRNRNAQLYLQVEEAHNYLLACES
HIAVAGGLFAGRGDAVSTGSVHLSERAAR*
>TCRU_750 Concanavalin A-like lectin/glucanases superfamily,
putative
MKVLLYTQKVGYPPRERNDKALYLWVADSNHTVYFGPLSVETGAKRAFANTLLYYGDALY
LLQGMGNGKTDGVFLFRIIEELGKIKSIVRNWIEVDAFFSGLSTPTNGLVGVLSDSSDAG
KWNDMYRCVDATVLGAIRIEGGFKFTGHGAGGIWPVNSRGDNKQYTFVDFTFTLVATVVI
EKMPSGGSSVPLLGAMLEDGMGTKFLGLACAENRMWETVFDGQTGQGDFWVPKKEYQVAL
MLNGNEGYFYLDAELLGTSDTMRRREERAYDVLGFYFGGDESGRDSSASVKNVFLYNRKL
TPDELKMVKKTGSPKHESSSSEASGSDSSAHECVYFVPLVSLGLWALAVFYGV*
>TCRU_of Trypanosoma cruzi region 4/Dispersed gene family protein.1
Dispersed gene family protein.1
MTGSVRNVTLANCTFLGGASLYVLGWRSDPPAGERAEVLISGLESRSGGGVLVAKRYPPG
SRVTVVDSVLIAEKRVAYRGDHGLGDASACLVVHSVNLTGSVLTIARTHVAAVFRDAVGV
LVVGGVALSSRGALYVDGLSAQTALGLCVSVEGGVAASGGSVVAFVDSDFLLCKHAVSVR
GAVSVSSSIVAFLWSDFFSTEDYAVAFYSTVSLGGGSMLLAKDNVHDGASREMLYAAGAV
TAAGSTLSFVRNRAVLPRMLSLSLSLSAGAHLRVACNDAGGRVLSTAEEYAAAGFGDAGS
IDVFGCDACDRNTHCYAPGTASASMRDGVCVCACGSGGYGEACVPVGAPALPPAVGTASS
VFVREGVTVQSVFVVPAGASEVTLRHVVLDGVSPVLYVPWMARDGVRIVVQNVSLLNGAV
LYVMGGGALRGAGAAGSDEGGPVELSVCDVEALNGALVLTGTFPAGSVLTVADSLLVAAR
PTPLVYLPGSQSSPYAPVLVLSGLRLVRSVLVVSGVALVTVMTGGRTVMVDGAVLELVGG
GVALDAAVLGGEYALYASARVVASGGAVLRVSGSQVYAAHGLVFDSGVEANASAVVVNDN
VGALTDGALLVLRGSASFLSGSWLSVRGNSIRGRLLSVPSYPRSVDLVQSTLTLYGNAGS
GPVVMDGTVALGGTGRRFVVGCLTLNGQALQPMEYRSAGIIGKFHPVACSVCDADVSCFA
ASTRAMSGSCGCRCGEGGYGRDCLPVYLPRVDGCNRTSGMPLLSHTATLTETRSPTPTWT
STWTPSLGRTQYGPTETLQVKETVELSPTRTPTASVSSTLWWSEVACPTLAVTTTTAGGG
LTQNDIRGGGSAVPTRLMVALPPPFRWARDPQLGTHLSFVPVSTAQPRGFGGPWGAMLSN
ATWVRNATNPSTVLELAVPVHRGYFIAADETIVIRCDAAAVSGGCKGVLLGSFTIRSDTL
PAAANALSAITGVVAGAAAVAVVVTGGLGSVLEMQALGVFARMPCASAQERASTVALPYF
LSVFAARDSLWMVVGNALLAAVFGCVHCGVTVVFQRWRGVDAATAWAAMRFPSLTYVVAH
AMHLGIFFGSVLALAMPGARVQHRVIGVVGVLYGAAFPAGVCYFIARHVGASFTRYWQFS
RKPLHERLLYPVGYWHPAAQQWMYGGMLANMRGSHVYWCVFQLSVLCVVGLIAAVHPPVG
GCHVQYFCMAAVLLAGAGVVAFTNMLRSAFLTVMHTASFVLLAVLCVVSAANHLAPSDGG
ARAYAAIVLLLTTVLLAVAVYGVVVWYAEDCHWQELREPQRCGLEALLRDDEESDDESQK
PHDVTSSSYASGTTVALSYRPPAPPRLVAGDIRSDALSLFDRASSASCSINYVPLDR*
>TCRU_752 hypothetical protein, conserved
MDAPHLPLAIESSEIPITSSIYHKKPVGDAFKGKSAVYKEKIQQIESFLSVNCARHVAEP
QRGNNIRKALDDLSRLEPSISGILLSVRRYLNEWLTTTESTQESLRSEIRHVEERTFKKC
EEIFENRFLQLLKDKRKAEATIKSLRDEVLKLRIERDVTLMKAKEDVMIRLNDCERKEDE
FRSFRKLISSVFKTNEGLVNRVEDLEALLRKHRIEIPRVNDDLYAYNKPRGEAKSSKRDS
DGVKKIGDQVSLEFMYASREEMNLARLSLQRELLNSAFDDRTAYRLQVNGLRHENSDLQT
QIDALQNQIIGLERYIHEKRFLFSTTDGDEAPLTPRPRDVPFTIQTDLGIDLRNSTARIV
VELAALGTNLKHQLNSAVMTLRQLSSAVDWMEDETMLKIADEANVRGALPTFPTSAWANI
PHFLRTHVEPDVPNLRWSETDIAFLLFGLFSNYSSLKAACRTTRDSKMLAPRQYKLFERC
ENPMVRLDASLNELQRDENSVPFGYVVSYFVSNVLLVNVADSPYSSLIQPGTLNPSFSID
GKNASVEMELTKYSYNIWYAAQRYKEQQPLCRLFADVVDGRLPVEIFDVMKHVLRIVEAR

IRKFDTDGSGSFTYNKLVSSVLKLVADMDVQVGRCAILAVMETFKANQTPLVGGRIFLPS
IFADESAPKKKDAPRNTSNRSAVSTRSTRSYIDREPQGASVFTRFWRRLVIQRYERVYSL
MERILGPLVLESKVVIGLFLLPIPSAFQAVRDFDTQCGNMTEEEFNLKYPLLSEKTTSVG
GKSLLTSGVKGDVSRCTDEYEKLLTRNKTTLRYIISLEEVLNTTLGSVSLLSRSMHFPAH
EEERSEPEEKSTDVPVLEKPINSPVNTAGLKKEFSPKRKRGKTKVNAKSKAKVGTPYGKK
EPVQEKSVLEEPVRDIIPSEMTVRSDKEMAEWYGFCATLRQSVLKFPEAIFSDGIFQGER
VHFSGRSTDVSGSFDEELSADKSS*
>TCRU_753 40S ribosomal protein S15, putative
MTSNITQERYEQLKKERTFHKFTYRGLEIDPLLALSEEEFKTLVHARARRHMNRHANRRA
PVLLKRLREAKKDVKAGEKPRAVKTHLRDVVITPEMVGSVVGIYNGRQFNAVEIKGEMIG
HYLGEFSMTYKPVIHGRPGVGATHSSRFIPLK*
>TCRU_754 hypothetical protein, conserved
MMMTGRVLLVCALCVLWCCAGGGGCSEAAPVVPGTGVTGNGNNPESKNNTTDGVGGDGQT
GQKAVTQPAAGSVQKVPGTEAESAPKPGAQVSGTAEETIEKKDTTKKEDEVDKEMKGEEE
EDEDEEEGKDEDKEEGEEDDGGEEKKEKEEDDTSTTEGKSAIGQEEPISPSGTVKASNKT
QPQSTQTTGDKDQAADGAGTQEEKKNENKEANPKETTVEAAAMKTTTATTGDSDGSTAVS
HTKSPLLLLLVVACAAAAAVVAA*
>TCRU_755 hypothetical protein
MCHDGNGVLLPSDTAGAPQEAERESDLPDIDYVAADGVASVVGQSLGDEAVSDKAGGGAT
TTGPKDPDAADDAQGAVDPPPREDDAAVVRGVGRQT*
>TCRU_756 hypothetical protein, conserved
MKRIRVSLNLSSTLGSREFGMKASLEFYLKSGAHAISGAEGMLAPKSPLAFLVLWMRALR
LRGEPGKQFMDLGGIVPLAFRMRIVIKFDPSIPIQKLPARLHTVFHEDDTFARVAQSLMD
RRELDRNNNDRCYANCALFMDTRHLLATCVQKGVSRTTSERQKTAKGQPAAY*
>TCRU_757 WW domain containing protein, putative
MSVVLDCVPDASYEPSEAELLEYGKWLGMKFPEDKPFLWIAREGLKAPLPENWKACRSEK
GDLYYFNFKTGESNWDHPLDKQFKELLQSEKENPSPQSIAKGAKRLTIEIDNAAETVTEQ
TEAKESCISSSRGKSRTQKLKTLKLKKELPDNLRLKPIARVNSGYDTSERGSEPVGVGKR
TGSKACSILKQNIETLSTERSEGSLSSEKVTFEADLNDELRRFQSEKLREHQAAKDEYEM
KLKDSWKRYQKLQTEEYEKKMNSYKDELEKKLFEEKNNLEENYSTSVQKFGKEVAARVEL
EAKRMRDYLSEKYEEEKSDIEKGWQEELENFRRSKDLELDRLRASNFQSVAELKQVTDEL
WENALYFVETYKKAQDAFVEELTNTKIIFSKFGEHALSKLIENARELYNSELNEIKDSFH
SELEKIRCQFLEEIVAFQRKQKLLLEEKEKYDEENETLLNRVNKISDCTNLKPCMTHITQ
RQEKDPQENTRIQGVIEKRGDFASGIKGDCAQFDEDTMGVRSEKELFESVVTYMKEVQRK
ELEAVKRDLKQRAEEELKATLDEVVRDRLHLGFEPLPEEKDEIFSFHGSEKALPEIKTEE
KESEKERLSTALSDTIKKDDLTDFLTEALRRVFAGSPFIISSPKSGDAATVSTTAIFGAV
NGANGKQTYNAEGISVPCRTEVNTLDGFPVSFQEQKMLLAGEHQRVAEGKRFVENQRFGL
EERRQQLKMARHQWKQDVRTAKEEGVRASSKRGKLLNKLRIALENQARGLERDESILRDS
ERWLLMKEQSVYQMEKHIKELEEGKGRDTSINSIDTVGLVTDFFKPTMPSSGFYSPNHDA
MQGVVRKRDLDPIYTKTLDKIARRLEEVTSMMNIQKQKRGSSLPYENLPRRRSAQKPTKG
A*
>TCRU_758 BNR repeat-like domain containing protein, putative
MRPTTVASGDSVYMLLRNQGVTPSVDKLANARDWGLLLVKGSVSGGNEITWKETHAVTFG
NDEYVAKFSTQLVSGGGSGIKTKDGTLMFPMQATEKGTTSLLSMRFDNSENKWKLSRKTV
GDGCRDPTIVEWGEENRLLMMASCEQGYRDVYASTGNGVDWYTYGETLTRVWGNSHDRKG
HGVQNAFIKVTIEDKDVMLVTLPVYPKDDNKQKGRLHLWLTDNTRVYDVGPVSRETDDAA
ASSLLMKSGNEKLISVYENKKGDGSYSLVALSLREQLERIKSMVKTWKDLDSALRTCRSG
SSATVDPRKKGMCNDTVPTDGLVGFLSGNFSDNTWKDEYLCVNATVNNGERRVPNGLTFK
GSGAVAVWPVGDMGQTVPYYFANNKFTLVATVSIHEVPKESSSPIPLMGVRMNDAQGTVL
FGLSYTHEKKVGVHTSQRRL*
>TCRU_759 Concanavalin A-like lectin/glucanases superfamily/Trans-sialidase of Trypanosoma hydrophobic C-terminal, putative
MLYTHKRYRTLEKKEKALYLWITDNSRSFYLGPIYVESLLSETVSNTLLHSDGALYITRL
TYTRAGRVISLARLTEELKTIRSTLSTWKKLDASFSESSTPTAGLVGFLSDEASGDTWID

GYRCVNANVTRATKVHNGFKFTGPGSKATWPVNTWEENTYYGFVNHDFTAVATVVIHQVP
KGSTPLLGAGLGDGAGTRFIGLSYDANGKWETVFNGTTTASGSTWELGREYQVALMLQGG
NNGSVYVDGELVGSSETLPTPETRGAEITHFYIGGDEGDSDSSVTVTNFFLYNRPLSDDE
LKMVKKIDGSVRGDVSRVLMLLLLGLCGIAALY*
>TCRU_760 mitochondrial DNA polymerase beta
MFRRTFWRFNPDSRENIIRIFKEMADLNNALGEKYKVQSYIRALNSLKTNLDLPLRTVED
LERFPGFGSKLLKKAEEIIMTGKLEELEKKTKPKLKAIQELTQVHGFGPRAAAALFDREG
IFTVEELIEKADRIQLTDQQRVGVKYFHDINEKIPMHESVLHENFLRESVQARLTSDYEI
QVCGSYRRRHPFSGDVDAILARTLSAPPLDYPVTNTGVLGTLVDYLQERNYLEATMAQGP
LKYMGMGRLPPRTTNGTTKHYKARRVDIRLIEARSVPTALLTFTGSKNFNVIMRQAAISK
GYLLNEYGLFKLGSPEEVKALQERVRANKIAGTKNSPTDAASDPTVEEGVDPVVSVISGH
SAKMETLGLTKEELEVKRVHVTCEKDVFDVLGMPFAKPENRDP*
>TCRU_761 mitochondrial DNA polymerase beta-PAK
MMLLRATRLLRVSKRTVASAGAQGAVKRATTTAAAPVAESTTLREARAAAATKAAGQRKP
LHHSRGGSGDSTRPVTSLQASAQLKGVRTISKASSFVPDHREKVLQIFQRLAEINNALQE
RYKAQSYQIAVDNLKRDDFIFVHLPPNILPPGVDDAKRTEQLNAVSATPVVGEKLKAKIV
EILSMGDLAELHSLQAKPIIRAIQELTQVHGVGPRTAVTFYKKYGIKSVAELQQRVKDHE
AEKGIGDGDRVGSNCGNSGGAKKGSKKTSAGDDDPVLHLTEAQRLGLKYHRDIMQRIPYE
EVRLHEAFLKLRLRKYLGKGYELSICGSYRRRQPTSGDIDVLITRKMNGKGPAIRRTKSK
KGGHHHSDEQVLAPQEVLAAFVEALKRERYIEATLAQGATKFMGVSRLRSYNYNAGATSP
KLYPARRLDIRFVEPECFPAALLYFTGSKHFNVVMRAEAIKQNFVLNEYGLFHNDAATTV
AAAAEDNAVDDSSSCSSGSSGGNSKNKPNNNNSNNGGGGNTARGRWNAEAFQKLVRAQYY
SAKSPAFMGEDDGHGEEDDDGEDGADGAGEDTRSKKHGKKVSAARQRQQQQELAALWREV
ESRRVKVKHERDIFDALGMTYVQPEQREM*
>TCRU_762 Beta-lactamase superfamily domain containing protein, putative
MAAEYIQLVGTDVPGSYGSCCFSGQVQHEALRCPTQPLALSSPSPTPSTGVATAANGDGQ
LLFFNCPEGTQRFSSEANMRLVRTRGFFFTRWAVSAVMGMPGLLFTINDAGVRHANFFGP
SSHSLTVEGGDGAQTQQPPFSAGLEGMLGALRLHYFWYRTMSFQQLRVGAVLRQLPTALP
WNYVCEVPLHPLEEGEGGTSADPHDMSSSSSSASSFFVLALLLSSTSALLGFRVSRKAST
PSEETYGYAILHAPKACFDPKRAKAFGVPPGPMYGQLKQGNGVWIDDEQSPPCVAHSAEK
SRRFIHPRSVKQATVGSRCMYVSLVLDGDDSGEVAEALESLFGRDASYRGDQQEEGVNAD
AESERLRGGELRRFLVQWQPFLLFCDDDHAASGEATVTAHPPMERTLVLRHVVHVQCSDF
FSSFAAGATGSVEAHYVTSIFTRVQREVREQAVESFRHDRYDGDSVVATGAKTGTMHHFC
AAVQQQFSAFPTALAHRYHLNCIANAQFPITLPSAGEKEHERMKEDTGRIWPYAVKHLLI
PPEAAGHIPHNTYNLAMKGPAFHVATFSSLVPGSKQQKRKGGNSKKEDVSGLLRYPTARS
AVDMLSKEFRSIASRAHEQMQGVLLSPSSFSSVFHHSHGDTHDNFHGICGHKDSGNTWHL
DGGGALTFLGTGSAMPSKYRNVSGTYLEVMYQARSSTTLSRAVVVLDFGEGNAGQLAIFW
GNGFAQALFQFFNDLVFVFISHAHADHHLGLMTLLELRHRVRLQVEKQDSIDSGNLNEPL
LVVCPQEVHEFLQDTWGRCTAYRQWLEEEVVYDVFPAERHRVGGAPATSVLLPRLNAVCA
RLDEKTAATNHSERHGASPTVWGAEVFPVHHPANAHALLLRFPAPMGATTASRVFLFSGD
TRPSPLLIERCRRFTRWTCRLHEQREEEPKEEGDDGPGVFLCLHEATFGEDCGEEAVEKC
HSTLPEALQVAAAIRAEHVVLNHFSQRYPKLPGLMKSQLNGRDVDLHCKRRKPRPPDALT
ALTESSPFEEAVKSLEDVQRGDTPKRKPLDTPNSGELKMCFGFDFMRLSFASLESQQTAQ
LTPLFVQLLEEYESWGVGTTKRLRG*
>TCRU_763 protein kinase, putative
MNALDELNLRLGIGPTRPHLPITLQRSGYSAVRKLGKGSFGNAYLVYHAGRKQQYVVKHV
NMASMTARQRRDAHQEIVVLQQLEYPNIIRYVEFCEEHPHLYIVMEYADGGDVYTHLKNL
KSSVWALGGGSGGVGDGVSSTGGLTEEQVISLFVQTTMAVKYMHDRRLLHRDIKSQNVFL
TQNHVVKLGDFGISTVLMSTVAMAQTMCGTPCYFSPELCQGKPYNNKSDVWALGVLLYEL
CTTGRLPFEATTMNRLMGEICKKEPRRIPSRFSDELWNLILWMLKKDPRQRPDAGQILRS
PVLLRYIPNIIKKLSATDGHPEDEFRRVLTGDATPPTPIRPAMDLQLKQLGKETARDGPA
PSTGAIPVASAAAAAAAAAAAAGRQGFAQKRHDQYPFKRVNDDNAIKDRVGGILGMLAQQ
RQQIQKMKEEEEAMKRAPPANQPPLAEPLNYVPVLLQRNQKLTKNGEKVDVNSKFNKNDN

CNRNDNDAGGNTPKKEKNIFARTPLRPPKNVDGGNLKRENGVPHVDASKEQEQRKKAINH
LPLQDLFLLYDESKKRILNEKEQRLGPARQPMNYQPAVAAPARMRPAVVELQVGGETKKR
PLSPVLPNHPIHGSTKAFPYGTPSMAEEKSIPIPPIALPTGAKSSALTSLEMLDHDKNTK
DDGNRASGKIPAKNEPTGDLALVLKEMTNHLESMRSSAARAESNTEAEGPKGTPGTNSLS
AVSAFFGVDAVGNSPFPASEMNVDQVGEEFSDAMGTTLNVQELQRQRSASSFSHEEVNDG
AAGEFLPLCISRYNGVWSSRGSRVAPAGMTATPGTAKETSEGRETQPFNDIQPSKPGISF
PALNTGPNAVSPQLVEAVAAAQENEGGGGASLFTGGCLCDGVHFTGLASAIFGSFVCNCF
VCSRCSGALMGVEWLHLPDVPFEDLFADGFPSEAASPSTLPKMPIASPVDETSAGSGVSA
EQSALKADPSSALRDGARKASNAEIVEEEEERRDKEEFVRLPELLKKFVVQVPVVNEDGV
ETLGEYTVYFCGRCGSTVGMNHDDVAGGLVAKAMLSEASLGMLELFQKTMPLHNSEALTP
RKIR*
>TCRU_764 hypothetical protein, conserved
MRFSPLTACFYSGARRQTPLQSWCRYHGQDACRQQRFYTLGEYLQRLPRGIRDLLLWSPA
IAAAYFMLYKQAVYFLPSMGRQLLRRPITPATEPSNRSTDRAQGSGELRAVKVRMDDACG
SVVDGLLLPLVDREEEAAVASSSLSPPIPPFSTGAMMDAMPLRAVRDVDTGKVSGYTRL*
>TCRU_765 hypothetical protein, conserved
MLRRWLLMPLTAGLPATLLSSSDSVSRGSCVRCVGSWQQSAKQSRRFSKEAMMEGRKRHH
WQKEVAALRQHINEEHGLQQRQRAQLRQRCDRAATALIHGASRGSSASAANALATARHLL
TLQEATRKAMKSEKSRRKAMFVSGKKWRG*
>TCRU_766 radial spoke protein RSP2, putative
MTESAYLKSSVGPVLAKAVAETVLAQPSNPQEYLALYLLHVLQEEERRGADAERKKKAEI
QRQMWASERALREKRAADTIQRFYRQCRRKLQLRREEEEALWSKYEEAEGEAEELLDEEE
TAAAAGKSTDEVDKMDDKDGAAAAGGVDGNNEEEMIEDAASAVEEARRDFYKAQRFMLHI
RKALIGALKKELTDQREQIRVGQDRMYDLLDLAEVERREKEEAEFVAAVTAGAIPSSAAI
TELVKKSEIPRHERISTSMILYRVMRCWCYLFFDSTPKQTDTPAKVAALVKPFTFMQLLR
AFNPVASYQRNRPLRIGLEGKHEEGEGDADDMLQDGGDEPLPQPKPRQARRVSRVLRVLM
HDAEYVCAVNPADYLDEEEEEGAAKNSTAPERGAALATASERGAALATLVETAAKKHSVM
LYALLRMLRTASAYRDARDRWVSLLQQNGQDAPPNEEMPEDDDADSKNEEGLRDDDGEVD
EAAVRRLLLVIGTDTDEALAKLWADQDEMQRKKWQAMAAARRGEEEEEVATNDEGDDDAG
DD*
>TCRU_767 Protein of unknown function (DUF2453), putative
MTAGFLTVVCPVTFITSKKQLTQVAQSPSAKTGTSHCSDGDICAIGDDGQSMTKHSVECR
AISGMDHTVVDVSSGKCSDAFGSQMVDSNEALLEAVNRVRDVLQRCQGTHNSPDTISSLQ
SPPLTPFLAHQEKEMEGREEMLTAIAALLEKDTAFGPRFLSSLSGDALRSLLVIGTTQEY
FGQDAVEAQLRAVDKDQDDNISSQDYDAWVNTAVRERVAMRFELPRATAHVQDTTTSKAS
ISPNTTVAAAGYIPWGLWHRIALSSAVPFMAFGMLDNTIFITAGDAIDKRFAEAFGMSTM
AAAALGGVVSGTAGIQMHGLAERMAQRSRLARPPTLTPSQSASASNGSATRVGSTLGMIL
GLLFGMTPLLLLSVPVSRSEREQPAKG*
>TCRU_768 hypothetical protein
MHVPGGNKATSPPLHQQQLQQQWPAASLRRRAVQQRQQQSPGIRRAANSTTAPSSSESSP
TPLIMTYELENSSEDDSEEYSETTYTPSNVPHFLSMPFGPTGDGTASLIWGMSSTSSYCA
DVFSSQGTEIGVGSKTLSPYRILRQISLCDTHSNKSGIPQMPVIVSERNSPVHGEENQEL
RPSNLTRTNLMNAAISTQLPQIVGSEARIQHTQKPMVNLEPPHNESTSRVATLPPAPGKI
CTRPSKECVSPITARPSTPAAPAASCRGARSAVGGSGVSNVSRDSRTLLDDDAKRGVKAL
REEVEGEATPRLILNVAPPAHSSAETPVVAVVPAEEKVGTSRGNFATNLHEIQSSVTATT
ASPASQCVCVNATNGTTQLTRQAMTAATRRVKPKEVPCGSERRRIVSGSDAYYKLPSLFS
STNSVLPRVEKRQKFFAESLQNHPQQNQQQQQLSELAIVPRRLGDRGPLVQERIQRQRIR
RWETAERVAILNKIMEMKQIPNIPHAFRSKDVASCAQSPPQLLSQLKGCEANESDEVTGK
ILRSVNPIENWGSQHNATPQGLGTGLGSAKATDDPTRHFPENTKTGVKGLCPPRCQRMES
ARRRVRNQQMDMLPSIGGSRIKLTESSQILPGHEGNMT*
>TCRU_769 chaperone protein DNAj, putative
MGGEEEQAEEIRRLLHCAASKADAFAILGLDAQSCALSDINRNFRKIVLKVHPDKCTLAK
AADAFHVAENAHKLLSNEAVLVRLKIAHKKKQEREEEEAARRQATASAVGGGGTVGTVDE
ANLSKEERVRLRRREQLVEQQLEAARLAEEAARKKQRVERETRERAELAAELERQRKEWK

DLNLF*
>TCRU_770 AAA domain (dynein-related subfamily)/von Willebrand factor type A domain containing protein, putative
MLRYRAQPLFAMLTRYTEAKAKTPFFNTQLCAQGRHGSTDGLLLTIGDITVPVPENLSHP
ENVKWLLPGIVGCGADTDKPDNSDDNSTGNMVEKGFQLHGGNAFFQLNDSTTRSYLRWMC
QKEQLRQDMCLVGEPGPAMRWLVQMYSYLVRREMQCVSLNRDTTESDLGQRREIRNGTLV
YDDQCVVVAAREGQLLLLEGLEKVERNVLPVINNLLENREMHLENGMMLIHPERYDRLLQ
ELLSAKKRRYEHGSSDIKNGEKMSSHDAMTHAAAHEELRRMGFIRVSEHFRVVGITVPVP
PYDGNPLDPPLRSRFQCLYVSIPLPGLAAAKNMTAPASVREGYATGAAESLLKLRTIIDE
VNASTKDFIMGGKQAGNSGEPSNHHHNDNSSGIGGVTSTVRGLQTIGSYELAVVSRQRQL
FPDISLGEILPRAFPWMLYAQQTATSGVAHIEEAARCRREYERLLGACHIGVPLYECRHS
SIDEDGDVIGNNHEDGVYAGEEDATNSRAPVGILSTRGHARMSQTLREKLAAQRRRSSRG
GSEKSTEEPEVSRRPEDFNDVLSIENIRVAETNCGAVVGDACIASREKGGIYRLPICMGE
TAMHTANGETTNSFLVAARCPTPCNVFTTAHHHVLQSMLQLHIAGQHILLLGPTGCGKTT
LLREFAQLIGYRMETMYLYADMTNRDLFQRRTTDSSTGDTTWENSALVEAALRGRIAVLD
GIDKLPHGMLASLQHLLVDGNSSLYDGTILKSQREYEILKQKLMATDLEMKARRIFPVHP
AFRVVATARSTSSAASSGGGGKANQSRWRVTHEVTSLFGLVMMPATTQTILHAVLSRAAS
TELETWRNEMSSLTESDGGAMAERSLETIKRDVDRLLGLRDALERLQETEPKVPQLSLRQ
LLHLIRFRVRHPDDFTPGVESTLLLPLMNTLTAEQVRQAMHSCGFHGVSDHEEKQKQQQR
HYGTRKGTETVADLSEGRRGKRHDVLTEVSALKNTNAITECQGCGFMRVFAVHGRPVFCS
SRVYSEEERAMVPYVPYFHSNPVHESIIAWLAKQYAVGNNILLIGNQGVGKNKVVDAFLA
RIGVPRQYIQLHRDTTVGTLTINPTVEGGRVIWSDSPLVKAVQRGHCLVVDEIDKAPVEV
VQVLKGLVEDHEMRLRDGRQIRGPRARVVAADSDPKVEANVIALHPDFRIIVLANPPGFP
FHGNDFYRECGDLFASYVMGNPDALSQLRVLKAYGPNLPDTLLVRLISAFQTLQKSFEEG
HLTYPFSLRELIAVVKHMAAFPHDGIYDALNNVFNFDARNENTLHLVRQVMHLHLHPLVS
AGGMRRLLPLRAGQSLPLHFSPQSLQVPATASLKLSTTPSVSDATLLPVRFVTDADCDKP
YIAQSVPTWCEQYKESEFAAVVPNVESEGFTEWLAAPNIPFLRSGDVVVGIAPVEADVDM
GALITLAVLFLREEATNDTSNNNSSRIKRLLLAMVAPPSVASFGMVTMRRSHLSLGQIQF
FDLTRFFPDADFSSSSSALAPSLHAFNAEPHSSFLVTETQAGWLNDAFGAPIPLVAVVDA
QSGLVLLINTAAAAVQSCDVSPRQQGEEEECTRGAVLPVRIAPPSCEPSLCLCSLRPLSG
IVAMTRPRSPELFVWMNGQRIVVTLPAASIHKLQFVTDNLAMVSLRVGEMVSNCLLKMAV
SADGQAKASLLHLPDGISGDIVGFAGNQATALPSDHALLQPLKLEELKGAVVVPSFSETN
RGDVVYMTLDEYSPEKRMWSVRLFTDADAAAAVGTGSLRVSHPNHQLLATEWSSAEHRGR
IVAVDFQQHMVRRFAPPPTASSSSSSQDPGMPLFALNEGTGVALWYDNTAQRLLVADVVE
ERVQKRYRNWSTLLEGSVKQIGNVASTTRQPLSMTYSKPRAKPSGTLKHGKEDDEEHHGG
NTYAGGTGGTDTAGLGGIVGPYRLDKGWPVHQVSPEEKQRVPPHIIEEAKRMGKAALEER
LREIGMSAKEYEQYRALQERVQAPVALLRSVLSGLKAAEGERTWLRGQTDGIWDDSKIVE
GIVGERNIYKRREESTETNPFQSKHKKRLLFVLDVSASMYRFEGMDHRLSKLLESTVMVM
EAFAGFEEKIDYAMVGHNGDSACIELVSFGHSPANQKARMEVCQRMVAHAQYCWSGDNTV
EAMRQSIDLVTAEKGDAYLVFVISDANLPRYGIQPSELSAIMRSHKEVQMFCIFIATLGE
QASNLQAEMPPGHGFECLDTQALPHILKQIFFSVNLL*
>TCRU_771 hypothetical protein
MSGRGIVPILERHGDVYITGSTVVRHKVGSNGDELDLFPSGYDLQLVLSAKNLYKLRQRH
PPPRLLSRKPGDNRKEGKEEDELQQKKQWSLGEKSHAPLPPPPPPPEMMWRESLGLIRAL
VPLHNHPSRMWAAGRHELGANCYGFAIEYFSTKKGPKPSAQRTVLQRYMKEIQCRCVNDV
FTDVKLVALDGTPGGLRVREFHVGLMRPPQSGSEPARTLYASSEGSPFLEQEWCCVFREA
QMNYWHERLEDKLIEDPELFQYQIYAMEFPNTTTASTAAGRATQKHEKQPLLCQLVLSTE
RLYVVPRHDAKLLAVELPLHHSEMHLRWFTVAALQSISFDPYTGVIVCVGRQQHEGKQPK
SSLARQSLTHAGSAADSSKEVRFTLGFFSVAEAEEAVLEIQRVWELTHRHEDFPLA*
>TCRU_772 histone deacetylase 4
MKKRGPQRMGGFGAKLDGDVTVRRFLAPQLRTTTAIRRGEMLEPIGEVAMRVGVDAATVE
GLVDAQGLALFGKLQHGAEVFLAGAVAERSDKHNSNCDDSGGMLSLQPPDASVIWAYDSR
MLLHVPPIDRIPETPYRLQRAIELLRSAPRAGELLPEELLCPDKGKVDTMTGATRGDVND
TSLSGVIPPSYWLPPRLATLEEIALCHNVSRYREFVEQGTALPPPLKSDVYCNDMTSSVA

TRLAVGAVIDAARRALSGAPSFAFCLVRPPGHHCTSDTPGGFCLANNVAIAARQLLKDWR
DNNNGSGGSDGGPPPRIAIVDLDVHHGEGTQSFVEEEPPPHGGTSVSPLLYLSLHRYDHG
NFYPYDPRGDTAYIGRYRNVCNVAVHTAANDPACCEEVVSDAVFERVVDDIFVPRLKRFS
PDVVLLSLGFDAAHGDPLGRMAVEGGFTYAVRALKRFCRTQQQSSRGLHAGLVVVLEGGY
SPEAVAQGVVAVAHALRYPANDAEVVRYAARQLPKTWQELRRRMARQQYEMERQQQEVEG
DEGNAEASYEPVPVSPTQGTGAIISLPEDDVLLERHERWCDRLISRVLAIHEESNKRQ*
>TCRU_773 hypothetical protein
MGGGISNTNQRENAQDDNVNEKRQCRVDEGKRKFITSTVNSANKEYTEGMSDPLVARGIP
DPVESTTPRSKLTFVKGNSENPTSGAASAGNIGKGAGNSSSMNTMRNMRNASLAVQEDSE
NPTSGAAASSILVKGAGNSSSMNTMRNMRNASLAVQEDSENPTSGAASAGNIGKGAGNSS
SMNTMRNMRNASLAVQEDSENPTSGAAASSILVKGAGNSSSMNTMRNMRNASLAVQEDSE
NPTSGAAASSILVKGAGNSSSMNTMRNMRNASLAVQEDSENPTSGAAASSILVKGAGNSS
SMNTMRKMRNAFVAIVGDGASETVESEKSNLEVEPGATTSQGSVMNVAGPFICQSYNNAV
EKTKGLGRAPAPGFFRSVVGPSPVVSNLTRKKVTSFLRPCVATGSEEKISLDVAFDPPAM
PFVTPSTGSGSARGPLRMRLRIKQGNEGNPVARADTPEKGSLQKSGAKNKTVPPSAPYNL
DFDW*
>TCRU_774 Sperm-tail PG-rich repeat, putative
MEQTGSSHAVLVADDFQTQVDVLYAQILASVDSSIDNNLESACGAKSVTQRPVRKRGASV
ACSAPVVKDVAVGEKVCCFCADDVVDVSTTTASTILMSDVNRVCGCSCHSPCAARGLHPE
QQQQQQNSVNTSSECLGAMPRSVSSPQKTAGGAYGAYGRSKTGGLVPPRQRQPPSTFGSG
RSGMCDSIKKTSAPGPGSYFRGDDEAMRYSMATRSQSKNPPVGGGGGAVGNCGPLGTRHA
RRSAEDAARALQRQFTLLCQCYWKQFPELGISTNAAAHARNCPCHSIHTASPGGTDSHSS
SSSRSSCNDASFRVGAKKTSGRRPVVPLTLSPFRVVSPSTKRGAVFGTSPCRVPPTEKQQ
QQQRSTHTESVENGPRKGRGPLVRDNTPSRGRPMELASTPGPGAYNVASAFDAASARGKT
RSVSIGKAARRLHTPTESPGPGTYDTESKGQTGAQLKNNYFLTTSSRQLQWMAKHDGECS
PGPGEYSTENATRTDARHKNSPAFSFGRAKLPVDAKRLVDPSVMGKPDEVPGPGTYDVPM
MLPDGATVIGGRSAVITTARRDDNMWRGVYYPPDDVPGPGHYVILKETTGPQWTFSTRGL
PSILEEPSPGPGDYSIPIFDVRRGRSATSFFPTAPRLLHEEANRAANLPGPGTYDVEDVA
GVTRSAFIGTTQRFFKPESQEEGVPGPGAYVVKHTARERRMAVPILASTAPRFYGFDSVD
STNLIPGPGHYDVVDNMRTLHERGAIIGTASRALTLDTAVATAATDAGPGAYDITLPKHG
PSFSMRQRLMDRTVNGDPTPGPGKYDPRELSQGPSAVVGIAARFQSADEEMLCNSVPGPG
AYEAALPEKLSTTTFFGTSPRFVNNGAVMDEKMGGGLGPGVYDPREARSNIAGAAWGTSP
RFFKEEYRDIPGPGAYEVASPSPMRGAVFGNADDKSDWIIPADAANLPGPGSYLPQDAPD
RMASAVVFGSSPKNALSPEEREAFMQRHIGPGSYDAVLQVPHAFHATFGGADTKRLGEGK
PSDTTPGPGQYATTAESQAFVPRGVVFGTASRVPPLQDAAGTPGPGAYVPTMQPNDGSVT
GPVSFPTAPRFHGGSEGFGDGNTYDTPGPGSYVLDNAGNQLGGRGAVVFGTEPRIPDYEQ
RERALFPGPGQYECVQDATTTNGPAYSFGKNPRISMHGNNDDGGFVGGGQAGDVPGPGSY
DVVIPTLTAPTTRFGTSNRTVGPLLLSEVPGPGTYDVTKTTTVAAASAPAYRFSTAPRES
HVDASDTPGPGQYGDLAADGSMWRRFTGVSFPTESRFTTGKETDVILPGPGQYSPQHPQW
NASQVYSFPSGKKEFFVGNDELPGPGTYNPTISPAGNGIRFGTAERGGEQSGQDGDVPGP
GTYDVTLNRQEGSAFSMHRTAPRGPTLVEVEKSYIPGPGTYTLPPAFPATEGAGTGVSFL
KAERFKAFDTVVDNVDCVGSLTAPGPGYYDPQLPQSTTGVFIARAPRLAEVGDATAGVTD
LPGPGHYEVRRGDPLLTTPLDGGVAMRFTAARFPLRDEATASEMPGPASYSPVAPQNTTS
GASFPHAPRFVLNETQERAEQPGPGEYSLPSAWCPSQDVGYSFGTSKKIENAYTTQDIPG
PGSYDTNRYATRYLTGPAFTVQGKSTYKDGHAGAEYPGPGAYSPQPVETARTTTFAPPST
VDPDVVFAARVNATPGPATYAIPATLSSQTVGFGTAPRTLEKTDDLPGPGAYLTSGPIVI
PSQPAFSFGHAPRPDIVPQTQKNAPGPGEYHHPIDPGPPVPAFTFAGAERFVEAQSVSTT
VPGQYYHPVPASQPRGPSFGFPNTISTMTTTAAAAAAGVRAGAGSGLGPGVYFHPSHWDL
RERGPSFPVTLGHYPPALGADTPGPGAYHVEPRQNTPLAAMTAQIGVLNTK*
>TCRU_775 CYC2-like cyclin, putative
MYTSSALAAGVAAASHFAPSRPRDGDGGGQGYSTGGHHATNFAGVGTATTVAVENVEGGM
VDTGAMGTEDNNGEADDEGMKRTSSSNDEPTPETCTDTTPRTVIDEVFAQPHEGQQQNHH
HHQEEEEQQQQQQHQEGDALQGQQQGCNFSQDAPPLSCNSREDACKSEVSNEDGKDVDVT
GATEEAEACEMCAFSPVVVSARKPLQSQIDLVTARVAVTLFLENLSEDNKAEPVLTSDFH

SHRLPQMPIEAYLDRVVRHSGVSGETLIASLMLLLKYSHFINHPVSVYNVHRLTITSLLL
GAKLRDDQYYSNEYYSRIGGISNAEINKLELRFCGCLEWDMWLDEAEYETLENLLLQLVT
VFASNMEEIDEVTRRRFQRQFWVEELRPWKERFDVSSSQRRERIRRCAAEDFSSEQAKIQ
CLSPSGSLFPQNPLVYPGTGMPGVSLDQIILPAVYRQTSGGTVSNAHTTALVTVIAQNAA
GSTTSKPTHYYHSYHPIYQPPPRQQMQYGCGVYDANINNNTNSNVGNIGSGRSTNAGAGF
GILERAVSLSPTTPTTRTTTTTGLHHSGISIHAKPYHYKSRGSSTKKFFNKPHRYHYASS
QQMDSCHAGDIAMMDATTATYCSSSSRQNSIHNNNYNNNNDMEFASITSTVWRPSGTNNN
NNNNSSGRRPATSDSVTMERHSGGGGGSAVRGSGPLVFPSQPQPCTDAANEEVAERRNRR
KRPKPDHYRDYR*
>TCRU_776 kinetoplastid-specific dual specificity phosphatase, putative
MSLMPADGGEGVGPPGDGNQQQQQQQPPRPQQQRCCNKQKNREEEGENCEEEETCPLEAF
GSAPASNTSTDVSGAQGLTACKEEGSGDMTATTKTTTTTKTAAVVKVVDTNAGRSQQNGI
VAPARSGVTEYTCSGRSSSSSVGSSAHVSPSRFVVGSTRHISLTTKPPAPGNGARVSSGS
LSNSQMQAVIKFVSCRASGDKKQNVTQTSVPVGQSPLQTPGNDTSANTARASVDVTPMPT
ISLPLQGQSSKGQMASLSAIDLPLPHVSLRQCTSTPLVPNTVGTQPHSCYEKEEERDYFL
QKVTPQLHRMLPLWRKNFGNEMVTEEGMTVTTTTTTTEAETEAECQQEQWQEKILSENDV
SDRRLRQRLPEASSRLHIRLPASPMCNAANSAYNPGSSDFDVSVSSSSSSSRTLSTLQAS
SHHFVRSVCHVLTGTPTFSSCNVGPEENETEKTFGTSKAQTTTTTTTTTTTTTKATTMTI
DGGGGSNSSNSRSDGFVLTSHEQNDCICGVQAIGIDAPVEFVQENSTRQFLTKNGNQSGV
FTPSPLPPLPPLSHGACNSYSLDAAMVLPGLFLGSYSDALKIDALAAHGISLVINVAEEC
VMMETMIYNDYDIKFIKFPLKDHSDENITRYFGPITRIIHRQLHRRERALRSSRKAATGN
IRSYDDEEVMSISNKSVGGGVLVHCRMGVSRSAAIILAYLMIYGETLWVDDNCSIQNQHI
EEESFTPIFTPPNVSPISTLLLDGSDVEKENHSTNGKCCECCCCLAIQNPHITCNTDGVY
KPSVVKSYRDAFEFLKKRKKDINPNIGFVLALRELDDREGK*
>TCRU_777 hypothetical protein, conserved
MNVVCERRIKRLEQQLRCYRHAYDAQRKVLFELNAKLQAAETVEGAALNSGETTAPPAPT
RSEENGMQMSAVVPTVEWDEAFRQRLLQVLRDPLEQQQAVTFPTVASHDTCSDDANPLHG
DREIRMRSLGPPRHDASIRNGDGGSNTEDDGDSPPATRLPSARHGSKAMESEQLTASLVI
ASTDAATRFLEMMRRLWSAYFAHMHSQVQGVLRRMDDNFARFDASPSEETLVEFLEAHQR
LRPHLSGIFGMPEGEEKEEKEGQPRGAALPSGNPLEQEGVFVQQLSLCIAEAVSPLFALA
LRSGNNGTQVVEESHDEKKTRSSRQRRHDGHRTRTAAAAAASPVCSAPFSPSRSHSMSTV
FTDAEESVTESLKPNRDASQEELLDSHNGNRTRSSGESTTDSSEGRNSRALDVTGMGPKG
THPPSPVCPNPVRNDHKTDAGTPRLNEEDAENYPPQRRWRRDNDSLQVEAVLYSRGSSAS
MRSPLNGPAATSTAHRAIGEEEEVEVGGATTWQNPHTRLRRLRMQEERLSFTLIDIVAQR
HDYSSEAKCYAVNKRLARVRSAIIRTEREVNELRRIQEEKEAIRKERAARQRRLLYI*
>TCRU_like, putative Vt.1
MTSSSSLTVNVPDHWVATLRPYLQRADEFDKVQPAISYFLRTHVAHLAMKQRKKDDPVGT
QYLRQLLQVLEAEKQRLGQEVTEVDGRTVLTKTALTLFSKADDAERSGAPAEMGLVRLFF
TASILLDATAQFSENGELDPIAAKRRDYARYIAVRMKKAIESGTPYESPNAPETSGGLDE
MFSEDVNDDKRTQEQQPGFPASMYHPMMTPEQPPPPPPPPYQPPNPSPPPQPPAPAVPMM
VPNTGSVADGPSMDAMISAQKCAKQAVSALQFYDHATARKQLLTALKFLDGK*
>TCRU_779 Flavodoxin/Radical SAM superfamily/Wyosine base formation, putative
MTESVSEALWWVLLGALIPLVTLTLTTLLDRWVSRRRKRQMEVARAQAAKEGKTTWMEPN
VHNSTGKTNGNGSTIHDAAKGSKGGVDVKKILIAYATQSNTACKLAHKLFSTLSTALHES
RDEMQGGSSSCVCSEGPSSCCATSHTARHLPKLKVLQLTEEGRQCGVEHLLESQAYGLVI
LIASTYTDGTAPESSQVFANLLQDAYTDFRVPRDTLAKKKFAIFGLGDIAYGEKHFNRFA
KNLYMWCKGLGATFVVPPVYASEANTIPLFNVFAASLVKWVGRVTFTADGVTILKRQQKQ
LKEKKEKGQLKLCNDVELQGHATCNSEEDEEDEEREEEDEENGRKKRNHNSEGDVEDVVT
DDEDEGEDVNGQSGSVDHAVQALSSEPPELLYPRLRQNLQRQGYHLVGSHSGVKLCRWTK
AMLRGRGGCYKHTFYNIVSHQCMEMTPSLACANKCVFCWRHHTNPVSRSFRWKVDPPQLL
IAEGIAGHQKMMKQMRGVPGVTPERLEEGMHVRHCALSLVGEPIMYPEINTFVDLLHERG
ISSFMVTNAQFPEQLHALKPVTQLYLSIDAPTPEELKRVDRPLFEDYWERCLACVRELRR

KPQRTVFRLTLVNQYNTENVAAYADLVRLGWPDFIEVKGVTYCGTSAASTLTMKENVPRH
EEVVRFCAALCEALASDTAPRDKRTWQGTEPETGPLQRDLNCDEKRMSVNNMENNNRRCN
RKDEEEGVGINFSGPYGIACEHEHSCCVLIALRRFLVNGVWHTWIDYEKFNALARSGRTD
FTAAEYAAPTPSWAVFRSNEKGFDPTQVRVYRKSGNTTVVTSGC*
>TCRU_780 ubiquitin-protein ligase, putative
MNSAEVSVRLASRDDSAALVGELKSSLLVFKRGKSEDAFQRLLMALNRICVAFCFGDEEF
IRRLDVERYASVLVSTLRSVSRNEFSRLRSVTVQALALLADLVPRGGLAIAAAKGIRPLF
CIMKDVKLQGNETFLEELLKCISHVSAEAKASLVHEYAVSFITVLETRVETHHLRMLCLK
CLCHLFEAARLKDWNKYFLVPCNTLVVRFSQQVKRVFGESKDQPLERLSKENEAYMLRLT
ECVALIYDRILRTRELILKNGSILENSLPAFVNMLIRTASMGTQGSTFRDAVCSPLLTLF
FADPPISLRMFLKYHVLQSICGVLDTVLERTTKRISYSGVAFAEAFLSNDVSAIPFMAEE
KQIIHLLEFFLVIFPTALVARESDYYVTLPHFVWQCEDDFHNRTDCTDSLSKQMEGEYKR
QRRLSAFKPYEVCNASRTLKIDFDNMQYMSNELSSYPHSLGRHAFLSGYFHSRVMYCNCG
DKLLLDATDEKFISKQHGEYSSESPKRTYRHQYNPLMANSTEGSGAVNEDIFSTITARST
DAAYSHRIAHAGGLRGAAINRRLVDGNECGSWCAYIAGLVRQGERQTGRQRSGNKTVQRK
DLLLCEFFRNSREGILNYSIIRLHFLHDSMIQLILSWCLPVLTTMVRKTVSPIIARHCSI
LILRCVDLSVEFFRDDGNRFRPKVMEELRQRFNIVCSQLGPVLSYLATANTNHTITAFVQ
PFKEFQNDHSFLQALMAVGLKPNSRLPAMTLRCEIQMSALSSVLILQHACRSTMSIFNGN
QKLILCRMLENLKVRVSRHQGVLRSSIYGGTGIILCPFHVTAEKIEEVHAKLIEQAILVI
SLDLGKVYKGHGGTFLSTSFKLTSTSSSSNRDHANSSLARTAILVEPKKFNIPESCIISD
IMVRANMHSDLPTGVQKVFAIHEYMLECETNLSSLVYGHPEFLEELAGELQNSTIEPALN
KELAEAFSHVRKGLLSVINKNIGLVNAEDAPPIERQSVRPAHRLRRNKITLAMANHIWTP
LRVKLESLDFTETDEHSPSSVGIIIIADERYTVLDLSLERETVSILPTMPLSHLAKHIVL
QLNQKYMDAGKCKAGVRLGRRHTAPDAMEVQSLYSTNSPFLPKLERVEGDLGQSFLSRSY
SILDPHVSNEPESAESSFSNGSLVSWENNYESERRHSAASVRVQSTYDREESAEGFSIER
QTLPDPNDDASWNILPSNIVFFYNGKPVVDLSVSLLELFWDSATVLASGRAATTEKEKAG
EQLAATGMQRMLAMWLTTQTIHYLVVKEPFMQSLDGVTEDRAYGTRISSPKKVFTHLLSC
SETQSSLKVAAKLLNEMRRVLLSVKATLGSSEGRLCSALIYGFHNHIGIFMLPSVVWLPL
LCSSLNEQQDQHLASYILWNYPQIFSLDVRRALIQLIFSVRRVPIVMLAKLKESARLTGY
VAPVHGLEWVTPDVDMRSTKKVIVQRDNLLESGSNVLRTHAMCPLPLSVEFENENGVGAG
PAVEFYNLFSAQLQEQRLNMWRTEEFTDSSGALTKRVQLPLFPAVCQTAKSLSYFELLGL
LVGRVLLEGRVVDLPLHHCFIQGILGNLEKNRLRDIDPELDYHLSRLSSLSEEEFIACDL
TFVLPRPDAATAGEEIELTPNGSMKRVTGVNVSEYVTAVRQYYCRAVLTGPLMHFRQGLR
YAIIPSYLELFSVEELQLLISGPDGKIWERPEDLERIIILSHGYDKDSPVVNFLLEVVSS
WDAPLQRAFLRFVTGSSRLPLGGMRPPITVVRRTLGVTGEEGGNASFSAAPSFGEESRKM
QLLMDASLPTVNTCAHYLKLPSYSSKALLEEKLRTAVTEGQESFLLT*
>TCRU_781 Regulatory particle triple-A ATPase subunit 6
MKTAVNTSRSTHDPVSTYFLQKIQELRAQEKRLLDNYLRLESQRNELNRRVRQLKEEVQM
LQESGSTVADVVQVLGKNKVLVNVSSNQGKLIVDVEKTIDVAELTRNTRVALRNATYAIH
YILPTKVDPLVSLMKVEKAGSDSTYDEIGGLSRQVKEIKEVIELPVKHPELFEALGIAQP
KGVLLYGPPGTGKTLLARAVAHHTDCTFIRVSGAELVQKYIGEGARLVRELFVMAREHSP
SIIFMDEIDSIGSSRLENTGRGGDSEVQRTMLELLNQLDGFESTQSIKVIMATNRIDILD
EALLRPGRIDRKIEFPAPDEAARLEILKIHSRKMNLTRGINLKSVAEKTNHCSGAELKAV
CTEAGMFALRERRVHITQEDFELAVAKIMHKDQDKNVSLKMMWK*
>TCRU_782 hypothetical protein, conserved
MHGRNGELGAVCHVAHAVHTCGNCAASCAAEEEGATVASTMNVTTHKYAGPYELWWRQFS
PDGTIHPPARSGDADRTGICQLPPPREGRDDTKSGGTTAPQVNAEPSRQHEDWRRSAAAD
GPNTVDRSPASNTATNTSETRPEFASNPNEVMFPNASKKRPSFANGPRILVASNASGIMS
VTYGGRENVAADNTDEVSERSATGHKSLVL*
>TCRU_783 Glutathione S-transferase, N-terminal domain/Glutathione
S-transferase, C-terminal domain containing protein, putative
MSLTLWSGVNAGNTRTHRLLAAATLANVAVTPKTCEYGRENETAECCRNCSPCGRYPVLQ
TEEGCVFESNAILRHIARLDRSGGFLYGRTPLEGSQVDMWLDFSATELDAAPAPFVQHAF
RGGQRLANAMDCVHEVLRALEAWLETRTFLVDERVAVAFAPQWHYRPSGAEGEALTRKYR

NAYRLYNTVVQQPKTVEVLPPSRLCWTR*
>TCRU_784 hypothetical protein, conserved
MKVAPTPPKRKPSIAMSRNIEMDPNAFVTKTALANALRKAVGSRDARKTEAVDGSGEHVP
GKIAGGMNGQNVPGKPKRHQEM*
>TCRU_785 hypothetical protein, conserved
MARGRRHVLRPANRRAQRRVRQQHTRVQWHRAMQHDRALQRRHIRHVDEGVGHQARRDLA
EAANPQLRTARKHAVAQRHVQRVSGQVHRAQVHRQSDWSAEHHRVEARRRPPAANRHRLR
QRHATHAARRTATTVAGPRGRHVERQARVAQAAGTAAAHAVGERRSQRIKARQHSRTAVA
RHHAHGGQAGHAVVAAAPQHPAAGTVALHRHDAARDRQPGDKRRAGGSAARNRHGAAAQR
HTRQQRHRRRLAHRPHVVQVQPPAGTDRPTVAAHH*
>TCRU_786 hypothetical protein, conserved
MRLDVAHQQRRRTQQNVFQQHQYAKLSLPSVKQLDLEKLHRGNLRNGDVAVLREGAHKCN
KRGPHHRLAYTDQDACRLGRLSTPKKLQRGTATECAPGKRDVDGEMQRHTVEKERQHSTH
AAKCHAHVAVVRLPHVQREARQRLVHHTARVAVHGSLRQGRQKSVGHLEGKTRRAMPSVL
AAARARAAVHRLRPGHVARRAVVARATPEHLHFLRKHAAVRHLLTALQVAPNHKPRG*
>TCRU_787 hypothetical protein, conserved
MAMMTGRVLLVCALCVLWCGAGGRCDEVGTAGHGVQPGVSASGGSGAPDNASGQTASGVA
GKPSAEQTQLEKVEDSAPGSPQLQADLQNQLPTIFTLR*
>TCRU_788 BNR repeat-like domain containing protein, putative
MIICSTDGGRSWVLSRGILPADCIDLLIAEWEQGQLLVITHCYGSRYCRVHESRDEGTMW
VEVPGAIAGVWANSQSVRWDMSLRVGAIVTATVGGKSVML*
>TCRU_789 retrotransposon hot spot protein, RHS3, putative
MQASGHYLSMKRAVKGEVTFDEDIRRLCDRGFNNLPGWSLAAAGVKATVHNSTKHFLDAA
AEEARNPTTTSAPEKLEGVYESVHNARWSHAEELPDGVERKKTVTGMGVHEGRPEQSWSY
RKADDAIEENDAVQQSGAAPPVLMVLTSEKGWPYSWNTIQDLPKDVFVNCAVERVWQIVK
GDVTAWFSPHGGTDFTPKRRVLIGTPGIGKSMAAGSYLLYRLLHCDAEKIQVVVHCFGGG
GACVSDKTTRAVTRCSDEDMCISELRSLRERGGNVYIIHGVAKEGTPPPRHFAPTFGWGM
IAVSSPKVTNYDEWAKQLQAARIIVNCPNETDVKAMCAWMTRDETKEKQAECWKEVKKHM
YLLGPIPRHVFDEEAFNERCGAVKFALQSINEGTVKEYFSRGGESPRYSEDPSHKPVEVV
REIYAGGVIIFNAPISACLEERTLERLPSVTE*
>TCRU_790 hypothetical protein, conserved
MHFLALEVTIWAPRVWNEGISEVFVNPRSAYSYKCLRQADAQVQPAFDGLVSCRRALEIE
RDPADRFIDLFRALLQTFQLQMMTTESSVPISELRAGPRTQQQRSLRCRLCRIYGRDAST
CNVGDATRGSYSQRRWPSKNGKRADRRL*
>TCRU_791 retrotransposon hot spot protein, RHS3, putative
MDHWVLYKPWATNFPLVDAFFFVDSNPKTLVGLRMTTAGGYHTTTSTVRQFNECLAAYFE
GWEELSRDMSWEMIYVQHADSTPMNGWRRCDVVDSNNMSRAENREIAAFWEEEVHQYIAA
VSSADARRGEALRGEE*
>TCRU_792 Mucin-like glycoprotein, putative
MMTTCRLLCALLVLALCCCPSVCVTASDEDQGGSKEVKIPTPSPRVPEEVKVVETEQKLG
TSGEPGERSQNIVDQTPGTTDTEEPLNQVITPHTEEGAPATAGERLTTNSEQATTGETET
AQKTEERTLKPEVTDHSDVQELTGRIPQMKVSDIENTAKNKITTTTTTTKAPTTTATTTT
EAPTTTTTTTRAPSLLREVDGSLSSSAWVCAPLVLAVSALAYTTLG*
>TCRU_793 hypothetical protein, conserved
MAAPSLAGSECDNANDGKESDEYYGDDLHREADYLRVLAHESQEGLDALPTIPGLAEPLA
FALSPAVISAPCVEADPFTVEFTQQLSSVCAAGPSLALSHVSQRCLACDLLCVVQAAASR
FLLVPNKKHFALRWVFICVAVDARDDAQRVCLLFSGHEGDFGVCQQHRGSLRRRIDAKIR
EVCPLHLSRQLRGTPDAQSEWMDAAQLGRPVVFSALRCQGNR*
>TCRU_794 hypothetical protein
MRVIVRNSTALLCGAVVHIFFFFIFILLSGSGTAHVHAEVTGAVTWKSDESPAGNTNGLS
IPVLSADGKITGFAEVLPIQGVFLLRDTKGKIVSKITDNSLLDCTDPTGTSLINDGPAVM
IFCGGKSSFLYVMLSGGMGSMRVVEVSTETTFYPSSSLPPTPLVRIRGSSPDSRKDAVFA
VSKQGELDCIGQYAFEESVSLCDKDSILDFYFQKWVPITSDESNPEGFIITERKSAEGLK
IDSYYLPDFGFSAAPNASVTLEFVGSSKAVHTNVNDISQSEPLIAYVQGTAAETFKLAVV

VLDANSLVRKHSGSIDAGIAVSESTLVTGAYMVSTARGALLMVQTSALLMAVNYTILSEV
NAPLWTAKASSTAGVLFLPFRAMENSFKEYYRRMLSPSFTDYNPYHIIKATEVQFLESAK
NVTKLNILDGSLKSSFEVPWTGASFCSEADRLAAITVFEEPMVYLGCEKSGHYCLVDPIA
SKPHYSITFPSMGPAEDVTFGYLLDDPSVVLCNGSRSAGLALHPLLLYSESVLIPQRDAP
VGAYDRGRMSIVTNNFTDPGEMLFYFYVHDTAGNSGGRDPLPPSNGPVSLIKNASSAKPF
TTFFGDGNNYVAVATTPGIVSIFSAANGEPVMDLLLVGCVPSGGNPDSGVITFMKSVQHF
GNKEDYYVLLGGTDTCLFEVNDSLVVRVTSLQGYQSGVVVGTTRYSGYKFVPATRTLQAF
TSFFGPSSMPVYTEAGIDYYVVGSNVFIYVKDDEIICRSVRSDITVWKYKFSEKFQGPVV
HMTYYNNTVFVVSQREFFRFDVQYYEDEDRYSRLLFQNGLTEEDEKYLGAAVNQGIVFLY
SGQKVYCHDAVNGKLIWKTEPRIQYEAKDAKVEAVIYSPSDTGGVSTDDIGTAYIEVRMT
VSQGNNKARFTHADMYAQASGELLKSVLDAPNDLTGPVIWGNKYVTFSTLTKGLVTFRAL
NISQVDIPEQAKRPEDPMTDVYVPGEKPIEPPPPPLPRGDNFMAASPPMKFSVAGRAALI
STDRKRAVVGKVEHKNGMYEYKIRCFNVDDPQGNPIREWAFPPAPVPLETKFMEFNKDHF
AIFLPSNVQFYSWSNCKEVGKSISLTGIVEGLPSYISLGYQNQWLFLNSDQVTCLEPSTG
NEKWRRNVRGCITIRRVDNYIICDGGSNLVVLNAGSGAILFEDAIEGGITTMGLDFGGVV
RIPRGYEAAFYTTSGKVFQSSIAIPWSAGKVMGAIPGTLDKNVVVFFENLAASIMFDTSA
KKASLLWTSDLNGERARPGAAAYAGARGVNIVVGTENGIIMLNSSSGKGFLNISLPKRYE
EDTDEVQVLKMVAVKENTLYGISWNGYAVLLRVTPGMEEALFAFGLGRVRASQISFEDDF
LVISGGGSAGTVPIDATYWGAPLSCVGMSFSASTNFMAGWDQTNDTAVNLVVVTAGRTWS
SSHIPHERAILSSMSAASAVVFFGKRTISTIGGLEGIGVEFTIPRECEETKLKEVSVGGS
TTGASLVWYTGEVRDCIFVLGAGGAPPSFLAGLSGKYAAVVNVRYGTSFGLFTSTHLSLL
GYDGTRIDSIDVNNPIYVSSLSSPDTGDGIFLVVSADNQLICLQINGLFHVWNYTLSSPS
RAPATYYNEGVFISTEAGPYFFTLAEISYPCHDRAIFRVPRVPTEGRSNFHTEVVVNEEY
AIGFVTTFETFYAFDALTGEVLWQRPDLFAVSHATVLREGMLLVVSTKSSGVVFLNPFTG
VSLLYFANIVEESSEIFLSPDGTTVGFGGSMATAINVIEYAHYFDVVKANRPKQQTPPAF
RPYNYGPTDNPNDCSEPSGDGEPIHVQLSPEWVSGSKYGPSNPKQTTVAMSHGEKSGWAA
SLEKQKLYIARAVDGAFEETALFQPKESGCDLLNLYSLSAKKAYVGVRCGKKTHFYNASG
VEQGTLGRAAKNAICAVIKSNGYFVNTDGYFDAVNLNKIKPLPSSPSKARCKAKFDIVPA
NRLERLLFVCAGTGIRILNHATLAQIGSTITGGVSRPDFTMYEADDNLRVGIACGKNGTN
VYMVAFSLETAKTILMHNESVQGTPLCGIRYPDYPSRNGPLFVFAYPNGYRTWEVQGGKP
KTTQEAANTFPAAITSHGVIYHNTGNDYYFRSYGFPTSSLIVSSKSPATGSMMVKGPDGV
QVSPLVIIYGNAFTLAVDTSTVNLRIFWSGNGLITFTPEARFVSNLPFVYTTVGNSLVAF
MLAGDSFALEEVLRINTALIDQEQRVFLSQRGTIGCVDKDGKVLWNIGFSNSGTPQILFA
PIVLDNLDFALVSGGVRVAAIINKRTGVVARSVQFERCSISPDSTEADVVVNGTIVYVAI
RGWSCVHVVDLNLKNKSGKITGPEVKALEIENTSTGILGGYPMVAMVEFANHLVFALQPE
NGDMRRPTFVVYRVDDMSKSLWTQLWKKRLRADLGKISTYGYFLYTAVDSVLNVYDLLTG
RYVWGYIFEKSITGPVLGADRTLYVTTVNGIQSLRSTLSLSWSLRLMGNITLPNRMVTPS
DVTIGPVRTPYGHIIVSNSVGTFAVEHEREGSVSKLVWYTEKSISRFGVYDVDSKVKVGL
FDASDGTGIMDLTSGKMLWLGGVYPTDGKTNGVASPSFPFFVCFFVLYVPTCRLVPEKYL
MPSRNLPLYKYDTEPTEKTPDEWDPIYPGGGGSVPLHPSPRCQSMMHAYIPPFTECINKA
LRLIYPRGRGTRLVCPASLYIIGICQDSLKELADACPDVGVLLVESWKAQTMDKLSPLGL
CEAEEASARCQTKNGTVLNATCSVVSMTFVLNDYENPMGYELGYELPIFDFPSIRSAKSV
GAILGGVMAVLVVIAIGAAVGVHFYRKKKKVRDSRFFDDDVNASLLGSEGQDLPMQIIKN
ESHKLMSGEGSLANMDAVSSRVQENPPPELENGFSPSQGETNGVTHTAAAPAAIAGPKKM
PPKMPMKMPGKMPPGKGLGPKMPPPSRPVRKPANENPILDDDL*
>TCRU_795 hypothetical protein, conserved
MSLAASTFPPTRARVDRRRKLGLANAVAQGRPLYRVGIISESEIADIRRLANADEGAFDA
YRREEDARRREASKARTDLWPDTMQAKQEAFLRRRKDDKAEEERRANIILELDAQQQEEE
RRQRQAQLALKYLKEDPRGRNVRSLLMLDEAIKDREAQIAFKKELKRQEAENEKEKQELL
QREQGENRLTEEQEKLARRSRNVEEKNSHLQQMLFQIAERKRMKAEKKEMLAEVNRGAEE
EKQENQEELMMQKEKMVAVHAYNRSIAKPPLSKHDRLRRRIQQEEGDDAARELQEKKIEA
MKRVPMERLKKKQELFENRKEQSWRMFEEENKAMRHKERTQDVFEQCGTSIIDSMAAREE
RRKQEALEERRRRQEEFLRVTNGEVEEPPEGVRRAATHRAGFMDDEEARQYQKEMRTHPA
RVLAEQQAEAAKRREEAELLQHIHRLQAAEKKENERREAEELVEARRRQREAMETDDEHY

RTYVKSQLPSDMDPYLYAKAMKLHC*
>TCRU_796 RNA polymerases N / 8 kDa subunit, putative
MIIPVRCFTCGNVIADKYLLYLDLVAEGVSEEAALDAFNLQRFCCRRMFLTHVDFSDLLL
KFNPADTNAAIYPTGVH*
>TCRU_797 Uncharacterised ACR, YagE family COG1723, putative
MTDSESDVGAIQLRNRLLELVYTSGAVFTISQLALILAVDKARIVHELRRSDFRGIVYDQ
DEETVHMRPQHRMSPSDTILSGKMPTSRGIRRSPGIVETRYANYGSDGYHHYRRSTLGGG
RVVSTKSTYRKTKYRLPFDADVVSGDNTESALPKGRVGYVCVADEFHLRELEAHYSAQGY
DTKFDFDVLHIRFSDREMSAKGTRTSVLAAKSLTNFTNPTSAGCGTHGTNTTEGSNNENN
SNNNNNNTGGEKPSDRSSLPSAKNLCKTGFDLFVFEYGAIVWWGFDQRFFKIVENDFMLP
NSAISRYMENRYMTQLISENYPVWCTYTLERKDTLEPDEHFRERLRFDHFIIPFGRGDMD
TSNVSMLCASHALAQSAKIDYLELKVQELAENCSPLPRELREKGQVSITERRLLQLRGEV
LSYRLMLKSGSNLMDEPDFFWENAYLKPVFQATKECFEISERVEALDNKLDAANEILSML
AEEFSQRHGARLEWIVIWLVLAEVLIGILELLIDLKPWFYRGL*
>TCRU_798 Alpha/beta hydrolase family, putative
MKNLAYKTVRGGAWGKTSSRVVIAHGLLGNSSNWATVSRRLAEHDLLRSKLHEIDMLDMR
NHGNSPHASPHTNAVLASDLEVFTLQRQAVASPPDDGGVVLIGHSMGGLALMATLLRRAN
ESSLLLPSMEELQMRLARGDYYGWEDEQGFAQEMRSVNESMGFPAQQPLDALFGCKEAVS
PASSRHSPQKEEKRGPRVNAAIVVDIAPTSRVGSQSAREQSVAVTLDAMARADLSRMTSI
RDATEELERVGVREKAMRDFLLTNLILDSKTRKSKWRCNLPVLCTDYNRMELGLLDWFLN
APVEGGCSDGGMVAPTRCAIPTLFVFGSESPYNRPEDRLLIDRFFSNVTQAEVVGAGHFV
HYEKMQEFVETVAPFLARYLLNTEKGGN*
>TCRU_799 calmodulin, putative
MDHSISQKELDEFREMFDLVDTNHSGRITRGELRKLLKTLQLRPTEQELEQMFSDAEANE
SANEINFDEFVALMSRRVQSDYTLEQLRNAFKLFETEDMPPGYVSREVLEHALVSYGTEK
LTHDEATRLLAAVDPEGTGRINYIEFVSLVGGGDP*
>TCRU_800 hypothetical protein, conserved
MIVRRLRCASILLPGNDTLRRRRRAGLRHGSSVMDAFALTAADREHGSLHDNRGGLSPHD
TQPADDIYGSRHEESSETMLPFTDGAYCGGDEAEGGQHCVVEGKRSVGEKDETQSLRLSA
RTLRDAAEREIKAEIERDSRDGVAAIPPLPPSGWKVQHPAGSNYFVMTRTLRGGAKSAEQ
QTRRYQSVHDVFLKALKENQRQKVLRDTKRREEKEEEQQQGIQGQMQEQARNVMDDIASS
SSLQSRGSFKKMVVDYTGQDQHRAVLREMTASNDAEHVDRMDVCLTVFAPFRVYDPSFHD
PTVNICEWSSFDVLVRKKMAAEKQGSRGRTNNALNSLAHLVHDRALCMFVRLASVNSEMR
IRFIQFLSINEAQALEEHACFGRGEPGFLELMRRRRQMRARTRPFRSLRRYDEPLTLLHE
APDTQDASSTSPLAIGSQQGSRGSAYELLTECFDSTGEYSRTLCYSGPYLSELSKEMMGA
LQDYLQGDLALSQELCEYVCQMQFFLEQEEYMAWLGRLRHVATVASERSHK*
>TCRU_801 adenylosuccinate synthetase, putative
MDKQAERGQSAGPVKTPQGTQPPAHNYTYHTNAAQRAVYDYLKNVKPIPELAEPKTYKTY
EEASVEAVLYPIIEKHQVIMVAGAFFGDEGKGKTVNAVANHPGCTCIARVNSGENAGHTV
YDDAGRKFVFNLAPSGLLSKGKRNYVGPECVMDPISFMENEVKQLIEANVPYKEQLFIGN
VSIVTPYHKLLDLLSSAPNSSTLKGMAPIHASKVTKRGIRLDHIFNDQSVLRSRLTKDID
TYFGFLKVKGLSDADVLRRCEEENGDGVVRVPHYVMEFVQAEDKVEYLVKLYVDRVRNNK
NFPARCDVAHELRSALSRGEKVMLEGPQSYWLSNAREKFWESTTSADTTASGLLATAQYN
FQRYSSVVINVHKAPGSSRVGVGANPSSFVAQDYFSATGVKTLRDLPENMCVDFDSIQKL
FFTKAFHPETKEYNGIWEPLEFEDSTGKYNIGVAMAVASSRQHGECGAVTKKPRVCGFFD
CVLQYEVNAVQGPYLSISALDRGDDYDKLGVTIAYVYYNGKNDEVLNINGREYKNGDIIK
AGEAVPGEVALYYCHPIVKLINGWKQTPIAASKRKPGDPLPRGVCEFLSTVEYFTKAKII
SIGNGPRGKDIIYIKQ*
>TCRU_802 hypothetical protein
MPVQGQGGMQRQPSFGGYDGMPVQGQGGMQRRPSFGGRGGMPVQGQGGMQRQPSFGGYDG
MPVQGQGGMQRRPSFGGRGGMPVQGQGGMQRQPSFGGYDGMPVQGQGGMQRQPSFGGMPQ
SSAQRRVMQGRQM*
>TCRU_803 D-tyrosyl-tRNA deacylase, putative
MKVVVQRVLSGSVTVGDEVVGAVGRGIVALVGMHRDDTVDDVNYVARKLLGLRIWPSEDG

KKTWERCVKQIDGGVLLISQFTLMHVLKGNKPDFHLAMDPEHALEMFDCLRDTLKREYME
ERIATGQFRSYMNINLVNDGPVTILLDSHSRQ*
>TCRU_804 Glu/Leu/Phe/Val dehydrogenase, dimerisation domain/
Glutamate/Leucine/Phenylalanine/Valine dehydrogenase, putative
MKYTSVDDFINKCVKKRDPNQPEFEQAVHEVMTSLWPFLEKHPEYCQDSLLERLVEPERV
VQFRVSWVDDKGEVQVNRAWRVQFNSSIGPYKGGMRFHPSVNLSILKFLGFEQTFKNSLT
TLPMGGGKGGSDFDPKGKSDREVMRFCQALVTELYRHIGPDTDVPAGDIGVGAREVGYMA
GMTQKLTNNKACRFTGKGISFQGSQMRPEATGYGVVYFLDALLKKHNQELKGKTVVVSGS
GNVAQYAVEKCLQLGAKVLSVSDSRGCVHDAEGFTPEKLKVLMDIKNVKHGTVEEYAKGL
GLQYYAGKRPWHIKADIALPCATQNELELEDAKALIKNGVTVVAEGANMPTTTDATMEFI
NAGVLFAPGKAANAGGVAISGLEMSQNAARLSWTAEEVEQKLRDIMNSIHDACVKYGTVG
GKTNYVNGANIAGFVKVAEAMKGLGVV*
>TCRU_805 Centrosomal spindle body, CEP44, putative
MLSATFSLLHRRLSSLGFDGWDAVTEEDVYSGAPHCYAELMRAILFSFPHDTAALMRKYP
WLCIEGEDSALAHSVLRLLSLEGSRRIVIKATQFGEKKYAAAKMNVCIELFDLLSRLSWL
RENTQGTRAAARRAALARAIPFYPAACDASAFFLKARLGELNGRRKALDHHLDRE*
>TCRU_806 Concanavalin A-like lectin/glucanases superfamily/Trans-
sialidase of Trypanosoma hydrophobic C-terminal, putative
MLKDDKKPTTESGTWELKKEHQVALMLQGNKASVYVDGKSLGEEEALLTSGTPPELFAFC
FGACDFGDDGVGDDDEESSPKKIGKKPRVTVTNVSLYNRPLNSTEMRAIKDRIPVSTRAP
EPQVKIAPKPVAPAAPAVPGPRELPAAPGRTTVGRTANTQHAPAGRLTSAGNEGTAREKG
DGGANGDAGSAYGRELLPLLLLLGLWGLATA*
>TCRU_807 hypothetical protein, conserved
MWPQRCPEGIERPQGTAFAFIRGSVTRKMRAFRLLASRCLLTPYSVSLWVLSDVPAFYWM
IGAAEMWRTFAAPSFGMFVARRVRDGCRLPHCMGCCKAMHYATRLCCGCHAVEIFDVVEV
PLDGWSGDPPARVITDGFTRPFEFSVSSLYVHELTADIAEYI*
>TCRU_808 BNR repeat-like domain containing protein, putative
MEVVGKAVDPFQGTTPVPYKRQEMTGSEAAAGSLRVSSLAEVVGGVFAVAEAQCSERDGA
CGHAAIASTHIETGGGGSKAISAMDAGVSLIEPVDAASGTIRAREKMQPTTIVSGDSVYM
ALGDYEKKTSGGQVADADGWRLLLTRGTLTEDGGQRKIMWGDIRAVDPVAIGLTQFLKRV
IGGGGSGVVTKNGYPVLPMQAVEKDGRSVVLSMRFNTRMEAWELSSGTTGINCKEPSIAN
LEGNLILITSCAAGYYEVFRSIDSGIRWDMSGRPITRVWGNSYGRKGYGVRCGLTTVTIE
GREVLLVTTPVYLEEKKGRGRLHLWVREGARVHDAGPISDAADDAAASSLLYSSGGDLIS
LYENRSEGSCGLVAVHVATQLERIKTVVKRWQELDEALRTCRPTATIDPVKRGMCIRPIL
TDGLVGYLSGLSAGSEWMDEYLCVNATFHGTVRGFSNGVTFEGPGAGAGWPVARSGQNQP
YHFVHKRFTLVVMAVIHDEPKKRTPIPLIRVVMDDGDRTVLFGVFSPMMGGG*
>TCRU_809 Peptidase family M20/M25/M40/Peptidase dimerisation domain
containing protein, putative
MPLDSVEWLRRLIAFDTTSRNSNLELIACIRAYLVDECGLEKVLVQRAADDVHANLWATL
PGEGGVTEGGIILSGHTDVVPVDGQKWDSDPFTLTERDGKLYGRGTCDMKGFIAVCMSMT
PELLKMKRAKPIHFAWTYNEETDFAGIRQLVADAGIPVGAAAGCIIGEPTDFNFVVGHKG
IRSSIVHLRGKAMHSSLVPYACNAIEHGAEIVRFLRDLGREFRDSGPFEREYDVPYTTVC
PAMVEGGNARNTVPADCYITYEFRNIPGHSGKEIQKRIDDFVAATAQKMKEEDGSCGTES
SCLDEQEAFKGRSDSRVCEALKAASEESCKVVLAPFCTEAPNYQMCGVDTIVWGPGGLCA
HQPNEFVEAADLKKTERILLRALAQLTGSAVGSRL*
>TCRU_810 lysyl-tRNA synthetase, putative
MSSTNETRAQIDDLAATIARVKKEKGAASEECKALVAKMTELRKQLPAKKVEKAPELSYF
DTRLAMVKELGLLGAAYPHKFDRQYTIPAFKARFAPQLSEKGQRVEEVVAIAGRIVNKRS
SGSKLNFLTLQGDADTVQVISAISDYVDDTFAAVHGRIRRGDIIGVKGVASLSKTGEFSM
NAFEITLLSTCYHMLPDGWYGLSSIEQRFRQRYLDFIVNRENIQTFVTRSKVIRYIRNFF
EDLDFLEVETPVLNQIAGGAAARPFITHHNELNQRMYLRIAPELYLKELVVGGMDRVYEL
GKQFRNEGIDLTHNPEFTSVEAYWAYADYNDWMRTTEDLFYGLAMHIHGTPFVKYAPKDS
EGNQLPEVVFNFNKPFKRLYIIPELEKRTNVKFPTEFESDSSNAFLQELCSKHGVECIPP
LTTARLLDALISHYLEPECQNPTFVCDHPRVMSPLAKWHRNDPQLTERFELFLNKKELCN

AYTELNNPIVQREEFMKQLRNKEKGDDEAMDIDEGFVQALEHALPPTGGWGLGIDRLVMF
LTSQANIKEVLLFPAMKPETSSSLTYPPGTLLNGQGVPLL*
>TCRU_811 hypothetical protein, conserved
MAMMVTGRELLVCALCVLWCVAADGDVVGPVTSGDPGGGGQSPLADGSHGGSECSKADIS
HESKSEPLVAASGEESPAIHLDPNGVSSLSEEHLRPEKREPPKENGKSHNHRLKMHRRLK
*
>TCRU_812 hypothetical protein, conserved
MESTHPVEYCRLVVSGSKDPVEYFVRAKGDGRAVVPAQTETRTGWLMCPATLRARLRHWM
RAEQVSLRCLLASRPTRWPLLKGIQEHLSSEGVSAVWSAHCSVRSIGPFLCWEDGLLHFL
DPCGGRVMGQHAHRFGVGAPRTCGRVTQEA*
>TCRU_813 dihydrolipoamide dehydrogenase, point mutation
MLRRALLLWGGKPPLVRWRPVELSHFDVCVIGAGPAGIAAALRAVDYKKRVCLVEAKRVG
GCDLWNGALQSKVMWEMSNFFAKANGSGALRVYSTVISSFMEPDEEKMRESLVQASEVRE
KQIIEALRAANVSLMYGRAFFASPHQVEVRNRETKEYHAVTADYFIIATGSTPKEHPYVK
TDQKRVVTSDELMMMPLPKSMVIVGGGVLGSEFATIYARLGKTKVYLIDKEKCIMPKEDD
DIVERIQQSMENHGVVIHQDSVLYDMQSWEEDDAEMQAHPEDPTQRSGVRYSIMNRRTRE
IETYEVERALIAIGRKPNYIGLGLYNAGCKVREGALVLNPFGKCVDRDHIYAIGDAANDS
KLVSVGEAKGRLVVDHIFGVQQTSPLRHDFTRLIFLTIAVASVGMNEKECQRQRISYVMA
KYGFELCSRAVAADNTDGFVKILATNDSKKTLLGVRALGMNASTIIELASAAIRRNQSAY
QLSELLTAYPSVSQAFLECLRVILGSSTLKPGTFPGLVCQTWTPPDYERGRAYTSSSAAQ
NPPRGDATSP*
>TCRU_814 dihydrolipoamide dehydrogenase, putative
MLRRSWAWRKMPVEVTRNTSKLPHFDVCVIGGGPAGVAAALRAVDYRKRVCLVEASRVGG
VDLWDGTLQSKTLWEMAKYASRLSGPAAQRLFEEDAVAGVKNKISDARVLQALQDVSGVR
EQQLLDALRTAGVEIVFGKGMFSSPNELDVHSTGSGVYNVVRADYFIIATGGVPRTQFHM
EADGRRIVTTETIMRLPIPSSLVIIGAGAIGCEFASIYANLGRTKVSLVDRTPRILQVED
EDISQFVQDQLVRLGVTIHSNCTLFDLESWGDNEEEGGCIYSVRKNDIDVVSPVETYEAE
RALISVGRVPNVSGLGLENTSCKVTDGRLVVDAFNRCLPHKHIYAIGDVCARRALVNLGE
AQGRGAIDHIYSEKPEKSINAEALTNLSTILFLDEELACVGLNEQQCRARSLGYIVARYG
YGHLSRALAMGNTTGFCKVIVTSDSEKRLLGVRAFGVHAGSIIEVASLSIRRLESAYELL
KLTPSYPSAVQGLVECIRMILGRSALKPNTTPDATLKVWHPPHFRRGREYIDETGYEESV
MNSDDVKIART*
>TCRU_815 protein phosphatase 1
MAKGQEMAINLDDIIERLLLHPSHNGAAALASASIENPVGRQCKYSRQSRLTAFKNAQAM
DSYRQLNEMSSLKELSEVEVRYLVIQARMIFMSQPMLVEVTAPVRICGDVHGQYHDLLRL
FDLGGYPPEANYLFLGDYVDRGDQSIETICLLLAYKLRFPESFFLLRGNHESSSINRIYG
FFDECKRRYSVRLWKQFTDTFNCMPVAGLVEDRILCMHGGLSPELKQLDQIRCILRPTDV
PDSGLICDLLWADPGECSGWGENDRGVSWTFGEDILQATLKQFDLDLICRAHQVVDAGYE
FFSARRLVTVFSAPNYCGEFDNAGAFMIVDENLTCSFAQIEPTNIMLQYSF*
>TCRU_816 hypothetical protein, conserved
MLESGREGRLGKLWQRLLSSVCSEEALAEATRGPRVAWNPYRPARGEHLLQGILNSEVYE
WRSAVAVNSLRLHGTALSTDGLPQSPLNTVHNIKFFHLEANVSGITTNGLSSDRTAAQCS
ERTGPSCEIESETIEGMCNENNRENILRSTEPQDVIDDIFEVLWRTFAVPWIIERHASGH
QLRREKREPETHDNSTAILSFYCADPVRRFAGKPNRNNDLVPNRRRPTTGPAMRRGGTER
RAAPALPDEKKTPLLLLHTEASLKKEFVDVAREEGHPVAVARQGVQFLNEVGHSPKQKRV
SSFRLSVLASSGNAEAERRRSLLLGKPTIGRPTLHCKGDHPFSSVLHPRDNSSPDRRHSP
KGKKNNVFAKKLDRGAQNVRALSGCRGCKLAPLPRKARVCSPTIIKHQNEEV*
>TCRU_817 TIGR00269 family protein, putative
MPPRRLCIHCCEEKAAVKRPRNGQLLCQKCFFHLFEEEVHETIMAEKLFTSGDVVACGAS
GGKDSTVLMHLMKLLNERHGYDVQILLLSIDEGITGYRDDSLKTVHRNSAEYELPLRVIS
YKELYGWTMDEVVRLSGLRNSCTFCGVFRRQALDRGAALLGATKIVTGHNADDLAETILM
NILRADLPRLSRCTSAITAGDGLIPRVKPLKYAYEKEIVLYAHLKKLDYFTTECSYSREA
FRSEARTLLKEVEKLQPRCILDTIRSGERLRVKERESVPENPQGFCVRCGYVTSQKLCRA
CVLLQALGSRDPMTAVRGQHQKTQSEMDV*

>TCRU_818 centrin, putative
MGNNDFLRSTAQRLPPDLSDSQKDEIAEAFRIMDVDGTETITPNDLKVVLRALGYEPQKD
VIKKLVAEMDKSGVSSNLILPEFEAIMKGKFFAEDSEEVLLSFRHFTKGNSDYITLEDLK
RVVEEVGEEMPEDVLKAMIEEADVLDHDNRVSKEEFLRMLLPHKEK*
>TCRU_819 CCR4-NOT transcription complex subunit 10, putative
MADDAIKEQLENARKAFEAQNYQACEVILQKMPQEDLKAQHNLAIVRLLQNHADMEQTIA
ALADNSTEGLPMLSEREGEAKNSVSLLYEGHETAHFNRALLLARSGLLNEAAGILRGLLG
THNSITPAVLGRALCLLQTITRIPTKAHHRQRSDDELIQKVITENMSKFSKDPALLSMVK
GAFADSSTIHEMPHNSDADWAVYFNNLGVLTMGDSKMNVASLCFAKAEKAAAQCNTPLMR
HPIVYNAGLCALLRGEYDSAITCFLSVQEFMKKSPLFWVRFAEAAVGKLQTETRKRDMEE
YERMQNHFSEQLRNGKMLPNYEYLILPGAVICQGPLADPKDSSVSTALETLVCSTIHNAL
ILLLPQGHTAASAMEAFPHNVQLIQFALFYWCALEIFRNNYTVVVNVGYDLLSLHERRPL
SPNLHAALLSYTVEALIHLNEPERALKVLRRSSLSSLVVGSSLEPLDSHQQGRVEALFIN
LAITHIANGLWNQASSVVESLLAKVYEGRAAKEKEGAGSISSGSSTASNQGVILAYHMLV
IFLELAQGNQEKAQEALLRVSWVA*
>TCRU_820 hypothetical protein, conserved
MPHRESCRPLPLLSVLLGVRKGLVRRRLSALLAHHRRQPGSTPRRTASELVTPVSDETAR
GLSGCIAVECERRRGVIPRCTVAVAALSPSRLASRLRSTPLAMGECWLCSAGSRAYAVAP
NAGSAITCEAAALG*
>TCRU_821 hypothetical protein, conserved
MDSDHHLPSHTARTEDGIPRQAGLLPRRKHAALALRAADWNDSATACATPLAAVTTWLEM
HRGTVRAARRNIPHVSGRTEWSSPSVLQRQHGTRTQLHPEAACPELKLSANGRTVIMPSA
VVSPCCWRSVQSSLVPSRAQACDTPRHGGTTFLLSGIGCAVVLLGLCLCSASAAGKFARP
SHCPSLTCCDPHSCLHSLCLRAPC*
>TCRU_822 Concanavalin A-like lectin/glucanases superfamily/Trans-sialidase of Trypanosoma hydrophobic C-terminal, putative
MVSVLLTAQLQRVKEVLATWKKADKLVSELCPSSAEKDASPGDACSPTVKITDGLVGFLS
GNFSGNTWMDEYLGVNATVKGNDDGGKKATLHAGGVKFTGAWAEWPVGSQGENQLYHFAN
YNFTLVATVSIDGEPKEGSPISLMGVKMNDGDNPVLLGLSYNGGGKWELPCGGGPIKEHS
SAWEKDTTHQVVLMLRNGTQGSAYVDGQPVGGDEACALGNKDSRAVSHFYTHFYIGGGER
SAGDAGSEEDVSVTVTNVLLYNRPLSTAEIAALNPNKDSMPPAVPDNAQETLSQSSSGGK
SPSGSKLLNGNEGAGGGITSPSAPSTVTNSREESVKQLALRVSPGGSKHVDVASSSDGDP
AVGAEAGGAVQGDTPPQTPVDTPDTADANTLTATNVAQVDPAATTGVAASSGANEETAER
RDGQEEDDIHAQHSEVKAAALSSSLGNVSQGNNTDAGTVRGSGLLPSLLLLGLWVFAAL*
>TCRU_823 hypothetical protein
MLRKGKYLFRYTLQPLFVNGLRAVRSDVASFSLLNGAVVREGVPKESDAQLSRTGAFPQR
VRQGLPCIVGGSAAGGGCSRGVAEGLRLSLELAEVRCSSFAR*
>TCRU_824 hypothetical protein, conserved
MLGYALVNSTPGNNSPQTEHQKAASRDPTLLHCVDDVLHRLGNSYPAAAFMYTDNLKIVA
SGADIHACAAAMQPVLSLTTTWAAEHNLKINVDKSEAALFYNSTHTRSDKEMVDLHPGNR
NLRIQSRPVRLLDTTFDRLLNSGPHASTAAKQTTPRRYQLRPVARAGAFHHNTQSFSIGY
VHGV*
>TCRU_825 hypothetical protein, conserved
MVLVNITATGDINGVAAAKRPCSFARQSAGRPFHLPAPGMAWNLLEEWSRLGLRCARRNA
PQRSSYMQPEPQHRDKVRLHRWGLPNVVKDLI*
>TCRU_826 hypothetical protein, conserved
MLDDYVAKKNGKLHDAEATERHLSSVVDQVLNYLRNGVRVTKIPSKGSARRRLCFLSEDC
KRIHSCDLDRQGFPLNRKRPPVTMWIRDIEKVLIGLSTTSFGNYSGEAQLAKTSQEAVSD
NGTRRHDPTQNITPSNLGTNNHRAFALLLRDGKSLEVFVRLTATARLGLFLSSVLCI*
>TCRU_827 hypothetical protein, conserved
MRVDGNTVQFNCPQPLRDAGFAVAGASFDVDVALDRPDASTLFRKLCATAAPNFTQERQN
MVAFSYGVRSSPKRQLMYGSAAGEGYAARVITEVIRLEAKKGIFALSCRFIGPTEHRADL
LDADNELGVIVGSVKEGPRVRMVARIRVPLAGDVRGAMDTIVKDYEKYFSSVLCEKQPTP
ELEAMPSYLPDTVVLQLYRDDGEAESAYYMDANSLTFVALGDAERPALCGIDAAAQLHHE

GVQRALVSAAGIVSGIRRRRLRIPFGNSELSQLLRRAYNTEKGNRNNSLTGSTETALLAH
AFLDAVCAEESFHCLSTTRRVGNILVSTGIGSATRDPAVDKWRPDQDIMELRDELMIART
AYDYRPCIYESNRPISNIKEEEMKRINAIESNREKARERQLSVVRERAKHEAEKMTKELE
RRGGATLDEIERTLEAKKRESGALQADRESRIWEYEHTLEKIRTRKQTEESASERLRQAM
QQPEQGLSLRQSAIETKEQQLEIFQLDGAKGREAIMRERRSIEAA*
>TCRU_828 retrotransposon hot spot protein, RHS3, putative
MTGMGVHEGRPEQSWSYKKADDAIEKNDAVQQFGAPPPVLMVLASDKGWPYSWEEDESTH
DCHVNCEVDRAWQIVERDLTEWFSTHGGTDFKPRRRVLIGTPGIGKSLAAGSYLLYQLLH
CDVEKIQVLVHCFVGGDAYVSDKTTRAVTRCGDEDMCISELRSLRGHGRNVYIIYDVAKE
GTPPPRHFAPTSGWGMIAVSFPKVTKYDEWGKQLQAARIFVNCPDEMDVKAMCACMLRDE
TAEKQAECWKGVKKQMYLLRPIPRHVFDEEAFKERCGAVKFALRSINEVTVREHFSRGGE
SPWYSEGPSHKPVEVVREIYAGGVILFNAPISACLEERTLERLPSVAE*
>TCRU_829 retrotransposon hot spot protein, RHS3, putative
MLAAGRPLMKDYNVTMEMFVQEPDDYVQDQRLLEEILNLTAHQKRWRPLLSLFTKVCSLS
GNVETMKGKDTITPIARRNLNGVLIGRGKAERNAVTEARREAEERLRRTQEMKFTISTNI
EDVLFKGRARVNEMRLSDFLTRELDGRDIVDTNRDVLPEEFFKDPTKYIRNKGALNEMQA
SLAQPAASTWCQGLVLLLERKLSACCSCFKPDE*
>TCRU_of Trypanosoma cruzi region 5/Dispersed gene family protein.1
Dispersed gene family protein.1
MTGVNGTLIYLEGSMHSSSLSVLNNSAVVIRGNVVTRPVQYAIFFRWGLIVESFSAVVFQ
GNDMQGSLVVFYSSYSSNIYYNSWLQLSGNLCRVSPVDAFAFLLPKLNLRDSTVSVSGNR
FISSTVTLDVLLISIGSSDLTNGVILFECNTVNGEEGVQYIIPSVYNVTILTCSDPCALA
ASCFPAYTTTASSDGCACRCAEGGHGDACLPVAVPEPPSTDGADLCVRDVRVDVELNAGL
GTSVACFVGVTFAADFVVDVESMSGSVRNVTLANCTFVGGASLYIVGWRSDPPAGERADV
LISGLESRSGGGVVVANRFPPGSRVTVVDSVLIAEKRVAYCDAYGLGDASACLVVHNVNL
TGSVLTIARTHVAAVFRDAVGVLVVGGVALSSRGALYVDGLLAQAALGLCVSVEGGVAAS
GGSVVAFVDSDFLLCKHAVSVRGAVSVSGSAVALVRSEFLSTEDHAVAFYSTVSLAGGSM
LLAKGNVHDGASREMLHAAGAVTAAGSTLSFVRNRALLPRMLSVSLSLSAGAHLRVACND
AGGRVLSTAEEYAAAGFGDAGSIDVAGCDVCDRDTYCYAPGTASASMKDGVCVCVCGSGR
YGEACLPVGAPALPPAVGTASRVFVREGVAVRSVFVVPAGASEVTLRHFVLDGVSPVLYL
PWMARDGVRIVVQNVSLLNGAVLYVMGGRSLRGAGSAGSDEGGPVELSVCDVEALNGALV
LTGTFPAGSVLTVTDSLLVAARSTPLVYLPGSQSSPYAPVLVLLGLRLVRSVLVVSGVAL
VTVVTGGRTVVVDGAVLELIGGGVALDAAVFGGDVALYASARVAASGGAVLRVSGSQVYA
AHGLVFDSGVEANASAVVVNDNAGALTDGALLELRGLASFVSGSWLSVRGNSISGRLLSL
PSYPRRAEVMQSTLTLHGNAGSGPVVMDGTVALVGAGRKFVVGCLTLNGRALRPMDYRSA
GIIGEFRPVACGVCDADVRCFAAATRAMSGSCCCRCAAGGYGRDCLPVYLPRVDGCNRTP
ATTASVSSTLWWSDVACPTLAVTTTAAGGSLTQNDIRGGGSAVPTWLMVALPPPFRWARD
PQIGTHLSFVPVSTAQPRGFGGPWGAMLSNATWVRNATNPSTVLELAVPLHRGYFIAADE
TLVIRCDAVAVFGGCKGVLLGSFTIRSDTLPAAANALSAITGVVAGAAAVAVLVTGGLGS
ILEMQALGVFARMPCASAHERASTVALPYFLSVFAALDPLWMVVGNALLAAVFGCVHCGV
TASFQRWRGVDAASAWAAMRFPSLTYVVAHAMHLGIFFGSVLALAMPGARVQHRVVGVVG
VLYGVAFPAGVCYLIARHVDASFTKYWQFSRKPLHERLLYPVGYWHPVAQQRMYGGMLTN
MRGSHVYWCVFQLSVLCVVCLIAAVQSPVGGCHVQYFCMAAVLLAGAGVVAFTNMMRSSF
LTVMHTASFVLLAALCLVSAANHLAPSDGGARAYAAIVLLLTTLLLAVTVYSMVVWYAED
RHWQELREPQRGGLEALLRDDEESDEDTQKLHDMTSSSYASGTTVASSYRPPAQLQFMTG
DTRSDVLSLLDRVSSASCSINYALLDR*
>TCRU_831 hypothetical protein
MPPPLLTTETFVTENTEWLRRLRRDEDATRIADAVGPPDASSDTPSNTTALDSRSTPPPT
YTADPPACEDLSTVLLTRSRVCNSDVACSTSGDATASVSPLSWE*
>TCRU_832 BNR repeat-like domain containing protein, putative
MGRRGRSPWGHSIARLWANWHDGQGKGVLSGFITAAIGEGNVLRVTLPVCHKASEKEKGR
LSLWLADNVHIVGIWLASRNVYAAADSSLFYEGGEGEDDDELILLCGKRDDGEWARSHLS
VCLTAKLKRVKNLVRALKELYICVSQFCPYSNVKKNLLSVEGCDTFMPPAGLVGCLSGKL
FENKWKEEGLGAERYGDGWGSCGRRAFQWIGFQGRWFLGVAVRWQSGEESDVVPCNERVH

LLWRRFLFTSCHRLAAASF*
>TCRU_833 phospholipid-transporting ATPase, putative
MVSGADREEMEGNCSTTTSDSSVSASFVEINLLREDLNATRDYPDNSIRTAKYTWLSCFP
LSLLYQFQKVANIYFLIVTVLSLVPGASPVNPFSTILPLCIVLGAAIIKDLWEDGKRRQS
DDCANNTLAFVLRGEKFVSVPACSVHVGDVMLCRLGEEVLADVVMLNSSHPGGVAYIETV
NLDGETNAKTRRAKPQTIEALGTIEDIIESCLPDPAIQAYFMERGTTASWNAAAARDTFD
VVTPVNSSLVQKNRSGMRDERTWGANGQSGAREFPGRRREPFMPYPLGRRASLDSRLGKF
THVRSHTYGSWGRKPPETPVYHKRHLTFDERFAVGREMPVPSKILEMSRRYDEKHMGHHR
QRRRSCPFFADNEMMHLPNGEDDPDNFDGAEENSNQNCKSTGGIVILGAPPCSELYSWIG
QMRMRDGEIVPLGIEQLLPRGCVVRNTEWILTAVTYTGRNTKMLLNQRPKSVKFSVVARR
INILNCFLLLVHQSFLFFLCGLAVRWRNERLEAPEGTRSGYSTWYIQWNLSRYTAKEFFG
WRYLTNFVLLSYLIPLSLYVTMEFNKAIQMVLISRDRRMANFDEFIGELRYTRPKTSELN
CQLAHVRYIFTDKTGTLTENIMTYVGGCTATKTHNEQERPGGLGEEFIQLIQNYPNRSGT
RSVIQGNCFFRGRRRKFDFDESMMESEPLFQYLRALALCHSVVCFDRSSVDQAAIISSLS
CTHVSYGHEAENAHFRMQTGEQQELQALDPAITGTFNSSGAQVSGFHTSTASGVGKFLQG
HNGSSSWYVTRYNDMQTDQRSATMPRIVNELKDESKIYEAQSLDEIALVTASRDNLFALQ
RRSATHMFIKVVNKIMCYEIIAELEFTPQRKLMSILLCRSPTHDDEAFEQPETTISYHRL
NTATAFIPVPRELECEENGIVDIIPPSEDPSATRKREERAIDNVDMNVNATHGNTERSLD
NAEERPYLLLVKGADSSMMTILNKNNPRNAELWPTFQRELEAMAQQGLRTLVMGQRYLSE
REVREWLPSFHEAQCSMHNRMERLHRVYAMLEKDVDLVGTTAVEDRLQDGVPETIKFFLE
AEIVVWMLTGDKRETAVTIAGTSGLIDPQFEDCVIHLDVAEASLQFTSVTEAAADATLWE
SLQEQIREAVRKCNTSDEIYGSTRTVVLVVDGRTLDIIFTDVACTREFFEIGIRCRSAVC
CRMTPLQKAKVVRMFQKNTSSVALAVGDGANDVSMIQESHIGVGIMGLEGSQAELASDYA
IPKFRFLKRLLVVHGRFALYRDAHCITFSLYKNVVLCTGLATYAFFCGFSGMILVESWLL
AMFNLFFCSLQPLALGIFDKDVDDELAESTPSLYPALAREHMFFSWSYILKWLCDGVLDG
LALFFVIFYTVAKWDELYPYRSASLEDYGTLFFMMVLLLLNLRIAAVMTCYTAISVAVIA
FGFIAIPTLTMVYSAVPHILGSNWGVNIAMELMGTNKFWMLMFFSVGVFVVYGMAVNTYI
ALFWPWANGGAAMRAAWRSPYRLEYKERMKQRG*
>TCRU_834 Ubiquitin-2 like Rad60 SUMO-like/Ubiquitin family/
Ubiquitin-like domain/Ribosomal L40e family, putative
MQIFVKTLTGKTIALEVESSDTIENVKAKIQDKEGIPPDQQRLIFAGKQLEDGRTLADYN
IQKESTLHLVLRLRGGVMEPTLEALAKKYNWEKKVCRRCYARLPVRASNCRKKACGHCSN
LRMKKKLR*
>TCRU_835 DnaJ domain containing protein, putative
MRRTLVANRGALSLLGLDERLHYTEEEVKSAYRRQVKRFHPDAGGSAERFRELHDAYVFL
LGDRKATDTPAESCDSSSFYNNAHSRWSAGRYEGAGCGAAFARDASSGYARGDYEYDLGV
EWDGERRYANRSTRDFYRPYTSNPFAHGFTDEEVLETERLNRLRVLWAVLKHGLIFGGLV
YLLFVCWRDNRIQRAIEARNNGYVDASYWEKLKEDQQRGLTPTPRRHWADLQNEEFMQAW
NKRLEEQRRRGSPCGGGGGYACRPVAVTFQGRPFTATGVRGARSGVPKNTATYEGDEHCE
LDDAEGD*
>TCRU_836 hypothetical protein, conserved
MRGEGVERDEWLVGLQLFMDVRLRTVPFHVASRNRPGVSVPLPIPNTQYIMDGIMWSVRD
YIAWAPNAWLTIQNHLQSCSQRYTQLAERLSESHAHNNFQDGAEVERERLYSSHAYVQDL
YVAFCAVDAFLRHVAEASQSTAREAVEKELPRLVADHMPIWQLARSQYMHDAVEDYRLLF
LDVLRSWTVTKLLRPDTHRRIEEYVRYKLKTLRDNSTTHEGDQNVGRRSSEAISAGLRVL
LAGAGPSQSSTSSLSSSSPSAPATIDVVRRNMREFVVAIFPSHRSDSGRKRCAHCGAIFR
TLAAKIAHYRYHFYNRSYLTGEKIVRLPYPTLEDYISHEVTSRVTGDFVRVTENLLDVLR
VPEKRTVHVRKEKQEKNPS*
>TCRU_837 Kinesin-13 4b, putative
MLGNDQEKGLYLLAAAELFAQLTPEHEVYVSLYEIYCNSLFDLLMNRTPVVVREDHNHRV
NICGLSWHNVSSAEELFLLISSGTDQRRTGSTSANERSSRSHVVLTIRVFCRKDTSFCGT
LNFVDLAGSERASETSTNDKQTRQEGAEINKSLLALKECIRALDEKKKHVPFRGSKLTEI
LRDSFTGNSRTVMIANISASSCSYDHTVNTLRYAFRVKGLSIANIVPSKARNAPRPMRQA
VSEVPPLLNFSNIAAADAPAGKAKTPPTGTNGRRALSTQRGRSYTPKTGNLNVQRGNMNP

IHQSTIGDTHADKFSTYLRAVTHEKLLDLLPPAKLREMLNKDTSNRDGGASTSESSTVSL
EEKQLLAEMVSHVVEKLKVELGSDLTQNIKKRDRLIRKLRDENGALRKAIVTLEKRVGEC
PRCRQRARSNSIS*
>TCRU_838 hypothetical protein
MKCAVLRGLSVKEWELACLFPHGLPEDLKSVSERLDNLSNSALLPNNTLIDSLWSHCYAS
TDNSICSESLDVVTCDTIDMDCFCAARLDGEKCVGAELPPVGRSLSVGKRDFIDDYVEQF
VLLESFFRGSIAETAEREWLSILQREVLARSTLSDAEQNMGKNTALFRRLPVPLRLIARG
CLWLALMVMSFYFLIGSVFGTLFLSCFMNFLFFSMFIEPYKAFPYGCALLVTGCHCALFC
SRGIELARRTYNGELVDAFRFIGPRALLMQALACVAALIAFFVVFCVMASQYDVVSGIQY
SMATGWIVAVCIILLYHFLYAFGVTGYYKSLRKESAAVATLYFFLDVDMDANQTTMRGTA
TIEVTMASIVAMAAFAFGLSYVLDGSIFFLCATVTTTFLIGLFVVLFLNTGRDSSTDGLL
TVVAFYSGLTWLTIVFTTAAHGWQGRWGTSLGALLALALVTDTVVLLYTFLPVRNRVARR
WMFRALWMWIIAQFIAFLALAFISDYRLGVITIILGVHLMLHGMRVGGGCSNYGLSIVVF
IFLSALLFCIVGGWSTAQSVYKTPASTWLLPNYTWHGGNTPVTGVSLPQSLSFLPVCLVR
FSSTVDIVGMALFAKLSDAGSLDVTLADLHRWFPNFRYVYNDTFLPLSFLQTKIFTSSLK
GHNTTIVTLGTTDQMYPVMESMTIWINVVALSLLALITPSGWFVQLVSLISAADELSALL
WNSSIDRARDILSAYVKSIVSPTHDVYFVGHGLAGAAAAFFVSDLPENVHSVTFASPLSA
VAWRRGHNWMSDELSSRVLSVVSSSSLFSLRWMWGGLSYMIPCNLPEGECHNIDSMARLL
DALCGLNTKSDGN*
>TCRU_839 hypothetical protein, conserved
MFRGFWHDVTRRGRRFISIGGRHTRGGFSQPLGKHPQVKQGIAEGVPRRIPGTTKVTYTN
KKGRTFNFSIPVAEITHPQVELTNSSGNWREIDTSFSDLGDMEDDMPSAVDECLQMSPDA
TSLRSLYEAFVSFCKEYVLMDTTGMKNMPFYGELNVGPGYEHYDRRLKRKRHWLAIRHRF
EDVRYIIWPDDEAEVEELSPDAQLSVRQMLDALRWLEAASTFCVRKMHPADMTNEEEFLP
LDLHREVAVVARRARNDATLFSSLSTESDLFVACATLCLNHKVPFHLFFAAGDRNSTKGV
TAGDGSCILVNMPFVHTTLGAIRIMSVIGGDSTAYAFSNLAGAVERWDVLEGLSRLMSVE
AFGEKDALENVGEDEICIIMRFCVEVREKNAAFFSCDSTENVEDVSFRAKYRQVSHLALS
RCKYLLYRLDGPRTRVMSEGGYVPLVDLQKHAERTNKDTLVHYNLGIRSAQGLRRVTLGA
QSSAQLAELVDRLEAPNARVNGNTLLVDLVQHLSCKAAASKISLTLKEVNTLLPLLARMR
AETATGELDSRFDRLLNVMETAIGTAMQQNRSVEEIVDLLEGLAACNFVPSSFKQVEMVL
MRLMMARSCRMSHVTRVLASLSSLFNSEVSQVLLQTAASHAMFCTKKGTISGREVDELVE
LLEALASCRYAALPGLIAHCREECFSDEVLFSLKQRCGYASLLASAASGMRHQDESFSQQ
LANDSRHHFIEYIRNREEEKDLACFVDCITGLTTVGFGDAAENEEYYSTLVSFLRSPCLS
LERCATMSTMALATLLEGVLLWCTTLRVPPEGVVAPIEGTLLARICSFNDPGSTLDSSEE
LAESVCRLVGLQRASEDLRRAAAQALGKIILHAEAVAEAWIPTAQQNLSFEVVALANAER
ESKHMNTVLRYCAALQQSGMLHLVEGLKMQ*
>TCRU_840 retrotransposon hot spot protein, RHS3, putative
MKGHEPSTGLPCEGWGMIVVTSPDKNNYESWAEQMGAERIIINCPDESDVKAMCVWKKHN
RRLAEERAEEEAEEEAEEEEADYWKKVKGRIDKVGPIIRFIFNEQKSDARFNECKDAVND
ITPPMLQYYAGIGTGESCNGNHVSRKLVGVVRVRRGNNIEWPLNVPISPHFERETLSKI*
>TCRU_WW domain containing protein, putative Phosphorylated CTD
interacting factor.1
MQVRTRREGVDDCFGADESLADIARVCLVPSGAGRKAAYVLSCDSIQYNIRLGSRSAFLL
RGLTPRLGRSLVTGQTPEDAADARNDTKNTDGGVRRKGGVDTISCAEEEVLAARISADDE
LDRHDAFLDLSRALEEAFCWHGAFTAIDDTAGRGRLLMAPQAARELPARWVMQSLNETTG
VRANRDTVTLLDRGETVRLRHVHDATLYTVVDVANMAGGQYWVEIAPTEPSTMDSRRVDR
EDIILEPPRPVYFDPLLPADEFNNIRGTSTCLFVAATLALRGIRLPREKTMVTANKMKEF
SKNVTVRMGHYVRGLRKKLSERCLARRSCCDEPGMTLSGLVEVRMTQDCVTLRYGGRAVE
VLQRTVERLSLLWDARLQREREGTAVNGRGVESARNVWQCAVTQQRTSVSDGLHVFMTSE
PFFLRVFTMLLRYRSLFGEMGYNQGPHAAVPPLVMRQLCEAFDVQCEAFASPINAQLSHF
GSLFPDTDYYFGSLGSFFDLSLVAGHYEVNPPFVTAVLQRLQTCLLQDALACHDGEDDPS
LLFVIVLPSHDLDDAEVALHDSPTPPQHRHTVREKVGNIDNNRDGGGGDGHDGISRKRGR
PDSAGETMSVERALRESSFCLAHALCAAAESAYVDGHQHLLRAPLFCIGTPTRLIVLGNR
AARRRYDNAAERLAAVKDTWCRYTLEFKLEKRRA*

>TCRU_842 3'5'-cyclic nucleotide phosphodiesterase, putative
MKAPAAHVPVKRIADSRPNERLMALFIAQLRKRLAESTSLDEFNNWMNEQYQALEASLLE
PQSLRPLVDAVGEAVLTPTSQETLTECGDEYTEHGVYWAPETQGNPCTTFSSTTESFRVN
TPASIGKLNRASPRAANAAETNTQNLLQHEMNRLLLSPRSTGPGEDVKTEKEALSVLQLS
SVWAPSIFHRGIAKPLGRGKPYRGSETNPLGFGDSHGSENQSYSILPDSIHGASTVSHRT
GAPPLRNGVSILDIGSNGDFGEGMPAFSLDGRLTNQGPIFSRSSSRHSPVFRPIDQVQEA
LRSVGVPFLGDVNSSNVDILHYRQCYGPKAYVYTCLNVLLRYNFSVRFGFNVRRLYCFYN
TAVNYIFPGNPFHNAYHASDIVLAANQFLSEPHVAENFSDEELFSFLFASTVMKLGHVGM
TNAFLIKIHHPYAALYGFFSPQQSVSLALGLTLLDTPECRFPTVASSQESDHDSTPSVWT
HEKGVELYEMTAQLIISTDERRHQVLLNQLQQILREGAGNVVRESHLMIILQNVLHAADF
CYVFRLSSVYRIAAACAVAEVYQQNQTRIWLTERGLRQWPWNGPHAATAGEKRAKESNNN
ILGGFFSCEGSFYGMGECREPEKVPSMYSDLTEGLTPSNPSLTPSVGHIGEASGFSPLHH
AKVLQTAVLETVFLPFIFTLSPFVPDYWLTNAQSNLVWLGEFSADAFEADVMGLLRHPET
GGLRLRSIGANGFERRVLRRIVDPLFQQHTEETK*
>TCRU_843 hypothetical protein, conserved
MLLSQRIAALQRVELQLRRILKEEAIQRSPSLFHPGVDVRQNGEAQRRAAVKDKLLFRHV
PVAVSLLERSFLSLTSGKKTAAQKRPKEHETQPPRERSTKLVPPPSQSHSGVGPASPENF
KTDLQQGTILTASTALKSQGHFMRQPEYALRLLSSLHFMEQERRKLYRHHMSRLESRLQN
TEHALHTVRKRREDTVALNAARDAVTEAKADVTLLQGSLLSRPYISARLWRLLLREEIWV
TAVRDFRKQNGVNGVGTASSVIVMLLSLTRLGFGALQDSLKGHALNTRAPRFVASLPSRR
MRRRKLARLGLKGEKGLTVPASVLVPVNSNGDSKDPAMLMMMEMMKTTSETAKEEPPPSS
ASKATVEVSISETNSTGDSMKRIPPLRWLPPSSCEVSSLVLETLAMCSHVLPLLDVTAII
TCCGEAQELFDLLLVLQASASSLLVLYANEGNGEATMHQTLRRLEDAMMKCGENAKGALV
TQMNEMSLVLPPVKAARLLFGLESMKLIQTEEHVSQKLLMQLVKCMFPQLSTGTQRALLE
KSRQNSLFAFATRSQRAKLRREHGKQGTESAFLNSVRARVRLQQRQMQAALRMKHLEEVN
SQSVGQLAEELSPHSLLILFDLLVRAARDAPRRGMEVQSCFITAALFVVEAVCVARARTP
SSNSGTTLQPDDLPSLLFTLSVLWPLVTERYQMFRNAPNDRQYNQNHGYIPRRYRGQQGS
GVGDSVAKSELMIVTYVTDMVFSAVNAAMAERVQRAESVTEPNSERELGHTLSLKDVMRI
VDALFEWGKNSLTNKNSMSLETTARLIKRLLVTDLQLWDELRQLNETRREQFFCRLSDVL
LRLNMMDTTVTQVLFSVC*
>TCRU_844 L-2-hydroxyglutarate dehydrogenase, mitochondrial, putative
MTSKISTWAVRLGGVGFVGGVGAAVNYKYKYEYITTDAAATKHAVSIFSKDARREFYDVA
VVGGGIVGLAVVREIRKQFPNKRVILLEKEADVAQHQSSHNSGVVHAGMYYPSNSLMARL
CPRGHDLMMEYCKRNNLPYDRVGKIIVAANELEIPAVQRLYKQGIENGVRDLKILANEEE
IKKVEPNVCGVYALYSPDTGIADFSAVARHMLKELYEGSKGLFNAQFRFEAMDFVGLSFE
EEEKKAPSEMVLIRGREEGQLGPEKTIAAKNVITCTGLSNDITAKRSGPILSRIGKKVTQ
TFSFRGRYYQLKPEARDLVKMNVYPAPDETRGLSVGVHFTPTVDERRGRQVIVGPGSAVA
THRYGYSPYWFDAEYCWHSVTSRGGWVNFVKHFDTVIRTFCVDASRTVFLHEARKLVPSL
KDSDVEESFCGVMGLAIAENGALESDLCIELSRPRTVLSATLKPKKVIEVVENANTDGSS
TKGQNNPLILNLRNAPSPSATACMSIAESIVAVVKESFNW*
>TCRU_845 hypothetical protein, conserved
MPQLVGRYTHVSIRHRYLARVKVHGSSKWTTIIQQDAQSRPRPYTRSRKNIGVENHVGTN
HTHRVVLVWDGEVIAHPQTGSRNEDGARHGPHGETLRVQLNGGGESLPQNNAPPLLRQHV
VRPACYCAAVVVNAQIPRRSAVPQRVVHVLRGHLDADRSPAIVPILAGGGVRLHGALSPA
RHLTHHRSRQPAVFLHHRVKPTAVLHGGIRGNANTGATHHHHEPRQQWQWRGVAWLAHGF
GTVCLFLRCGQQQWY*
>TCRU_846 Leishmanolysin, putative
MRQPRHTALLPLLPWLMMVVCCAGVCVAADRSVKHRCGFDAMMKKYGRLPTAVVREVPRR
GQGAVQAYTAASEDGDDGWAPIRIKVSAEDMHDPLRHCTAAGDLRIDHDGRAITCGADDV
LTEERRSIILRQTLPAAIQLHAERLSVRPVTRPVLIPRTGLGMCDNFTIPDKHHTVGVVG
TDMILYANIFPTSGVRSWAALCVLLDDGRPFAAAVNFDPSQIAVSIGDVRAAAHELGHAI
GFDVSYFVTLHMISEVPNVRGMSKVLVISTPKTKAMARQYHNCPTLEGIELEDEGDYGTA
LSHWRKRNMRDELMTSDMGVGLYSALTLAAFEDMGVYVANYSAAELLWWGNNSGCGLLEK

KCLTDGISEYPDLFCNQFDKNVKFFCTYDRLSLGSCDLKRHEEALPEEYRYFADPRVGGD
DPYMSRCPYVEAYSNGGCTNGNPSLMPGSVVGPNSRCVKGQELQFDDEYIGDVCVDTRCG
DGTLSVRFLRDDAWHECQEGETVTPPSGPWRGSIVCPQYADVCTAFPNISSHPIPVVDPP
LADDPTSAEDAEGDEGEVPRKRPRRL*
>TCRU_847 syntaxin binding protein 1, putative
MPRPVRLRLLLLLVTASSTDELTEANKQQLIRDSGLTPEAHLFANLEHLTRRAGSVQRYS
TASAQGGRSTSSSASNAATDEDPFLKQNCMMMETAGRNRTLRVELSLASVQRDHAMRPNG
ASGGQNSGAAGTTPRHFKGVVAKRKIIQICVAVMLGRLPSQLLSA*
>TCRU_848 hypothetical protein, conserved
MGLSPDPHRRRMPWTAEKECVPGVVRSSKEKMVLDGAQRVDVECVDRASQVYPLEALRAA
VATCEYNTFRGKNIFNWSLRWNPMCSGNALLQGVAGGDARRGRHSHRHLFPSPRDHNAAR
ANTPCGFSEAACAVLFAATVPPSPSVGAEVPVDASSCRLRTHPLIDAAGTAPSSWCSGTG
DEIAAQQFQSDGAGSPVSVSPTTAASTEIDGTGKESNDCQGNNCLLTSLPVKQPEE*
>TCRU_849 Mucin-like glycoprotein, putative
MMCRLLCALLVLALCCCPFVCAAESTHTEVQVSDVTLAPTGLSSSSTEEGQVKGQSGPPA
PPVTEAAEKPGENAGAGQSLTGTPGLQIDTNKSAGETDQDAEASAQTTTTTTTRAPTTTT
TTTTTTTAPEAQRTTSTETPTTTTTHAPSRLREIDGSLSSSAWVCAPLLLAVSALAYTAV
G*
>TCRU_850 BNR repeat-like domain containing protein, putative
MSRRVFNSAVLLLVAMMYCGTGGAASSQDTSGKGSSPKTQFVWEDGKKGETVSSLRVPGL
VEMNGEVFAVAEAQLKDESGFNGISSELLKLNDEKSKELVTAQVKTQVLVECPSNKTNCA
SQADVSAGSQSEKKVRVSRPTTVVQGSDIYMLAGSYSFEVTHQTSSEAQWGLLLARGNVS
TDASRGNRICWSEAYVIPWIHFEKQHESFTGLIGSGGSGVRMKDGTLVLPVEGTKKEVPS
GRHEKGWKGRFADHTLLGC*
>TCRU_851 hypothetical protein
MSLVEELRRLQREAESNDGRIQRLLNELRLLDVKLEAKRNAKKRYAAEAQEEISRLEKDI
ASFKELWRSVTTAAAKHLVSSPSASLSTFHYSPTMSRDRSENTGNDLERQCEQLAELRAQ
RQGLEELLRRATVCYPHYRITDLFDVNNDLERVALARLTNTT*
>TCRU_852 kinetoplastid kinetochore protein 4
MARHHHQQKQQQQQQRDATNNVESKFVAQYGAAVLPPAPSLPSPPPLPLPTTTKTSLATA
SRTPRQRRPPHAPLQQQHSRSGQRSGTSSVVGGPTRSPSPVNPKRGAEQPRCFVVTSLTE
EERARVKAAVAAIGQHGVVLESDYEDPPPFTATHIVVRGPPRSMKALCGVVGSKWLVQPE
YVYASRDAGFWLDEYEEGGLRCFPPPLKCQRFLLTMPEGIVKEKLEQVIEYGGGEVVRPA
RDGRSYDQGVVVIASGDDLLHFATRIE*
>TCRU_853 THT2A - glucose transporter, putative
MSTDCSMYKKEVACNRVLNAECSWNGTRGECGWNGFTCFWGHGKDKTPCLDDSRCKWVYS
DEECKNPTGYSSSYNGIFAGAMIVGAMIGSIYAGQFAARFGHKVSFLIVGIVGVVSSVMY
HVSSATNEFWVLCVGRLLIGVVLGLVNVACPMYVDQNAHPKFLHVDGVLFQVFTTFGIMF
AAAMGLAIGQSVNFDKDTKMDARMQGYCAFSTLLSVLMVALGIFLGESKTKFTSGKHEDD
GTALDPNEYSYLQMLGPLAMGLVTSGTLQLTGINAVMNYAPKIMGNLGMVPLVGNFVVMA
WNFVTTLVSIPLARVLTMRQLFLGASLVASVSCLLLCGVPVYPGVADKNVKNGVAITGIA
IFIAAFEIGLGPCFFVLAQELFPRSFRPRGSSFVLLTNFIFNVIINVCYPIATEGISGGP
SGNQDKGQAVAFIFFGCIGLVCFVLQVFFLYPWEESTPQNHGDTNEESALPEQQSPIEVA
TPGNRQAA*
>TCRU_854 40S ribosomal protein S12, putative
MADETRVEKQGEEPTVTQQFDVEPENLRDAIRIVVRKSLEVNGLVRGLSEVARALDRRTA
HMCLLAADCEDEEYKKLIKALASQANIDFVEVESREELAEWAGLQKQKSDGSVKTFKCSC
VAIRDFGERTRALNMLLGQLAG*
>TCRU_855 target of rapamycin (TOR) kinase 1
MESCVHPLIGQLQPIFNELHSSTAATLPSVILKLEYIIGDEGDTFFQGSAGGEFRASHSG
LSTWVNTQIKDLFSKSETQLAGILCTKALLGTDYIGSNSLLQFFLQLLEGSVRSSDVVVS
EKSAEVWGLLLNNSGSFAEGTAQTRLKTCIKDLRMTGDAVDKVAASLFLCQLLKHTPSTV
VPYLHLVFEGIRVALRDLSERVRVNAACVLQYALSVMYTSMGSSTVIKLLDSFLGDSLNG
LESKKKDLQHGAILCFNAVLFSITNGISSYGAAVILSSASISYLHEIWQCVNAKIVNPSL

LPEIRRELLNSLILLASYDTVKFRERSIPVVLAAASTVFRAAACREEHSMMFLAMGKLAA
ILRDHVTLFLDRMMPHIQAALGQKSKDDRCLEAVTCFATLAEVSPKAARPYLRQILALLF
SFPPTVGFTRDIARICEAFPELRSTCLSKILEATKQQLLNAHHRLSISTGENDADAVLSL
ECLSALSSLDFSGYSTLPFLCDAVICYVSFPHEELRRAAINLCFKLLLSGCTGSQLPCRR
TPEGVVVHHGRDHTRLINTIVSKLVNAAVSDPESDIRLHTLESFTEEYDHALALQHVVSS
LFPALHDKHQNRLAVVRLLGRVSRRNPAHIYPMLRKIMVQCLTEIQFFEHAKKQEQAFSL
LGAIVESTPDFSKPYVPSLLNSCVKRLREPSQTAAVLTALLSCIGKLVRYAEGDDVRVAA
EIRPVVVQHILDSSHLPKKREALRALGDIIRTTRDVNIFEVHPELLSVLLQALHGGFKET
WPVRNDVLQLMGIIGAVDPIRVKEILRNSHVNDSNTEMVPSFGLRGEDSIAQTVVRSVLQ
ILSLPSLTDEQSVGAVNVIVKIISLREVSSSGLIPYHAEIISLILKHARLQVRKREDILI
CLTSFVSHIKEYVRPYLDELTLAVASFISASDISVLNQTLALVKQLRRCFREEFRPYLSS
LLIPILNLVEESPHTAGENVFGFLSEMGSLLDDHLDNVLPVVCDVIVNASFSNTSRVAAV
ETLRSFAQQLPSLRFHASRCVHCLCRVLKEQGGVKATARGSLGSAAITALLAIAQNLGKT
FENFTPIVFPVVAGHYGEMDGEYKRFCGILRDTINTRKNPEMQRCQEENNSGGTIASRSG
TNSDKLPAERPSDPFETLRHVLRNWVRSNDEDWNHWLTQLAVILLRSSPSPSHGCALTLA
ELHDPFARQMLYSAFATCYSEMNESTRKDVVGLLSEVLRGPRVPSEVLQELLNLSEYMER
LEIRQNAHGNGCGNASQDFRLFDLPTLMESSERCNLYSKALHYVEIEFFETIREYERSVF
RGIPRPLPSGVWQNLLQLCEKSIYLCNLLGQRESANAVLKFIQQNYSMLTGQEVTELFQM
MDARLFDKLQWWSQSYRAYERRLRQENNKISNMVGLMKALDALGSYPEVLNLWKQYSVQV
SKKEVVELAPMGAHAAWLLRCWDDMENITSFMSEDSYIGTTAVFYRAVLATRKRQFREAD
RLIKLCRKRLDSNLSALVAESYDRAYELFVGIQQLSELEELAMAARDVRNVGRWQELWEK
RLGSMAYEGWPGTLANHTLLVPPAQELDMWLRFVSLSRANGRERVSNDVLHELLGNQSIE
SAVANQMLQPAVALGAFQHLYETNRKKKAIEMLEQYVEKVEQMGTQHANRTREELAICHA
KLAEWLTHRKKSHSFAETLRSALHHLRCATDLDNKNGTIWHTWARLHHEAAMKNVSGKVS
PSVEDHIVCAIDGYLRSVCLSQELEDTLGFLSLWFMHASLPSIQGSSTIKAGIHQVSPTV
WLKVIPQIIARIYSRDAVIAEYAYHLMVIIAKSHPQAILYSLNVANTSHQMKVSSECVER
QKGTQRVLARIAEFHHNGKTMVNDAALACRELVRCAVLWPELWFDELERVWFQWGKDKNA
NNVLSALNPLLEQLKMPETMAESHFAMEFGQLLTEAFGYVEKSALLDSEGCMEEAWSRFK
TAVKRMDDQINGMSSLALQLVSPRLLQNGKNLSLVVPGQYKENGSYPRIASFRGALKVMN
SKQRPRRIYINGSDGNLYKFLLKGHEDLRLDERVMQLLGFANKILEKHLVTKRRGCLIQT
YSVTPLSDNAGLVGWVDHCDTLHQIIKEYRIHPKYLSTELNLMRSMSDELHHLTVIQHVE
PFELALERTEGDDLVRSLWMKAPSAETWLERRTTYVCSLATMSMVGHILGLGDRHPSNLM
IHAFSGRVVHIDFGDCFEVAQQRSNYPEKVPFRLTRMLVKAMEMGGIEGLFRHGCIMVMG
VLREEGSSLLALLEAFVHDPLVSWWRDEGGNDRCTNNKDNAKQSTSMTGSLRASHEEYDV
GSVHGTQRSLHRRGAISRVCIQGEEIALQQTRKAQSVVNRIKEKLEGFDFFHDRPSRGDD
GLTVEEQVSRLIQEATSNENLCVHFSGWCPFW*
>TCRU_856 hypothetical protein, conserved
MMGEEGIIVPFCASVDAYIKKGAGIDLIMKSVAGLSRLVSLYSQNEETSRNYAKFGQAII
NCRMLANHFRYAESLNFAIVKFKKRDGRPLLSWLCLVGSFFFRSLEQVFGDLNYYQSVFM
HHWSRTFLSLGYWFFKSLSLTCGFFYELMSLRATIASPEWRTKSAQGRSKILRAGCISLL
RYIFDMVVYYQWVPWYNPYKTVQYASAAAAGILGIYVSWKETQAARVAGAKKAPELRTAM
NGKEGNEIAELKTVVPAE*
>TCRU_857 legume-like lectin, putative
MNAFLLSTILLLPLALVREVSGGDQIQVHSVFPPILQNYWENGMRFWSFGLSTVVTDKYI
RLTGGSPNAKGYLWNKHANRMEAFELNVTLQIRGRQITGRDDVRDGGMGIWYTTSAMPAT
NTDVGFFGFHKRFYGVGVILGHSDEISLVSNDNTFELEPSSLLSRRHGYCSVPSLGELHI
TITILYKEGNISVLYVYHEGDEPRLEDYDRAVFCTTAPSTSFLGIHHFGVTAVNSATFQA
THEIHSVIMTPLSDITHHKEEENKAFQFRLFEEEENETRKELLDDLNEKQ*
>TCRU_858 Concanavalin A-like lectin/glucanases superfamily/SPRY domain/HECT-domain (ubiquitin-transferase), putative
MEEDLSDWGLLSIADILDFDFLRKNEALSSIVITLKDCEIPLLPCEPSYVVKYVQMLIKN
SKYNAENRHSNFEEMDRFRSIKLKRSLAASELIRERLLGAIESFALTVQKETHKPSFLQS
VVHLGSIVCPAIGSFCDALIEGAEAMALRGVSSKKDIGSCVEVLQLFRSRIDISVLTEEQ
KRRLFFAEFSLALRSERLGVVVNCLLQKSTKDALRGKYCPPLLEHRVSFVKAKTDGFLTR

TCELPFPIHSAQVNCFPSDEKLVVCSGNRGCVFDQLGMMGTRFKLREEFEVSIEKNTRII
LCNEKELFLANRIPSALGNICYSIQKLDCVSGRLLKKTKILFLFDDCLVASRDVVFDIYG
SILSCLVCSESSRISNSKCHLIQKDLDIIFSVDNHQFHVTSQCVLTRGQGLWHSTKKCYY
FSKGNALSIGRVTLKENFHPFTLEMWVYPCNALENQSVLSLGDKSLDEVLIEIEPTPEGV
LWRGGSRTPHLGASFVTFSVPGKLAFCQRWWHVALLFKGNSWELWVNDAIVSRAPALVSP
EALIDVECVLGKSFVGCLAEVRIWNCCRTPVQLARDSRRSLTTIEPTLFGYFPLDESEGD
VIADYASNGSHALLHHGQATWLLVDHFPIFPLNIVRCIDDFAPIAWRESSGILFLSSCPG
YIALAVPTEGPSLTICEYTTYDFRLVFQARVELPIRTPLKGMAYNALRHSLVCYASTAEH
PKRLLIWELHQQHYFPFLRKDAYDNILECERDLLTQCAMYAKRLVGAERCLVDQLSWSVQ
VPGLVVDTSDGLISSLLRFIHIVWMDQKDHEYTRQLYALLHANLLCRVENQREGLMKEIG
DLFPDAQALINAAQECGEAPNARAELLRLSFQESFFDSGLLSMTCRCLLSEKSRLAFLKN
QAGRGRLTEMKDMLFRSLLEYYGSLQASSLLLNNAENAQLFCSSLMAEEVFQVDRWLGGN
GKALEATKRTSHSLEVFQEILLARVVEKSQGEFCTFLTTYAKLLLRTSERIMEMFSKALR
KNPELYLGNLITCIEFSAVGALLPSFTVALPLLPTIIQAACLPFLKSCREALFTLTDTIP
RSEKLPYELGVALTSALCRIGCSLLVSSDVVTSEVELKYLHLLWGGIRKSGSERDAIIKN
LQQGVGSISRVVDELHKDDPGALIVLRDDRLVKLERLLMAAFCALLIPTESLRHATKQTL
APMFRYIHNMRALVLSKRQEDQESLEVLEERAKLLARFEPICGVRTEEPPQIKNSTRDTS
PQRKWKRFFQTWKALRVLKTKMPQQRETESGEPGAVILQFLWDKNLERESVERLVVQQTQ
RAHYRLSGMLLLRQLIEETRASDTLARIVLPVLAKTFTGWHYSDDVQCCSKGDFLRLHGA
FFQLLELVVFNVKSGVRDVWADVFLSLLTSELRVIDFRRVRSDVTTTLCFLWKFENRKTT
GIPHLNGSIPESMRNTLLSPSGAAPTLVIGRSGFTMKGLGGRGTCVVSSEWSLGKKNVPY
YFEVLIVDMYPGGSCCVGLGPSDYSVSRLPGWDSESYALQSEEGTLHESNTLGRPIGCTF
CIGDVMGCGWNTETREIYWTKNGKDIVASVEVLQHQLHPLIGMCGKGLIKVNFGAERFLF
DKVPGIKSIRRPLGENAWDVFRVFSLRASLGLVESCSGLRTHSADSIEGLRAVVEQCIEC
SCAEMERTLFLSMSESFVISLSSHLITLAKVLVSAKDDELTPCVQRVVMVLQRITHHTLQ
GAAWSSKLRCVLLKTWFTFMNLVNPNTITEDDKLPGFLDTLLAVGREIEWAVLQCPGASS
ESRPAVEAWTALAFLQYMNSDDPPHRWREELHKWIMQNLEGTKDKVNTSLALRILFGAPR
LLVPGDRVVVQKSRHTKLTAILIDYSLDDETCEIIENEERRQTISLRQTDITTEDDAVTF
PQTGNKFHERQMHQVLCLAERIWSDTTVDNGLTGTTLRCKILSILWRCVRKGVLVAPSEI
LPILAKFCDNGENFSEEHTVLAQERLVIEHCLLGRALSGSTGGGNAVDNVVVGGNDNRES
RTRLAQELSMRGYNLDLCFLALDETNNDIQLALQLLGDHHGDFMSARRRTASSSSEDEDM
TSDDAEMDAAEYDLHVLFPAESEDGMDEDVSVDHCDPSLSEGIRFLGGYICVTGERPLGH
KFTLDMHLYLFESSAMQVVLHQKAITGGWRLVAHIQNSFFYCGWMRVGDSVSSLCKFPIQ
YTDTLRWIQLTMVQDGTTFSLYKGGVFQSEMIFTHRCALLENELFFGGSSESDDTHFNGG
MKSVQMFATALTKEEVNNLVSMKTADIVSHHFLILECASDNIKAISRGTAYNANVTVMGA
VTWFEHSEVGGRPNDFCQHEIDFTTQNGEDGIFTVDTANESSSFTTLWRRRKDQRKDHLL
ASIIKLDRQLACYYAVAILTQMMCLTPNVAIFFTREQVRNMVRLTLNSLDADLLESLTAT
LKKLSGSKDGNLVNYAIEELVEVVQREQKMLVFESSHPFKSTSETAHEVYVPGRRAYELY
FDSRCSSSSVLVTLYSDHTLSSIIAQTPGYALNTLLIRAPRFFFDVRMDATTPQWGYKVA
VLYDASVPRSASRLLRSTLSAVISRGISNVEFLKTSVCLEGLANASRMNIGTTRRIILSC
LTDLFIHGAEHIEPMPHVARMHDLRRMAERLFRRTIGSQHLQSRFVQSVAEFYVALKDAE
HCWRVIGDERVRVFDEVYDAAVDTESFMQKRLEQRNLYRRREGMRVTIEKVLHPESLRIG
VNPNKTILAWSERSGSSVVADVPLGSGKWYFEVRMMATGDVFIGVLPSRLHDWADAPALE
TYLAFNGKTGVFHGVNDPTVAPRRIWKAKDYVGVVFDGVEKKCSFFVNGYETGLSFCFAE
NGAEAVSAAGAHLQDELDMSYYPFFVLEEEEGITINFGGAHFEFESPRDCFPLDPANLTL
GTLIPYNQLRAFHDLATYIASAGRTSLPTFFHEEANPFDGSTERFGPPHVSLLSSVGVQI
NLLLVRNTGTRFATVRANCRVSGGKWYYEVTLRSQGLIQIGWASSDEPQTGGVGDTSTSW
SIDLFRRLKWHDGKSEPLTASRRWVVGDIIGCALDLVERKMLFLCNGRPLCDNTLNDSTF
AGLPTTLTYEPAVSLRAGNEVLFNFGSSSFKYKPEGFCALGVPDSWNERMDAFYSTLRPS
TTLLRLRVLRDMWLSADDLAIRDKLKPYFFVVDAIQQYCRREGKSFAQITEEVCTEIFRD
LPISAQEAWKAYMVLVAFSRVSQAVIPFLHLDTRHPNISTKLFLACRSLLFASLRNEMVD
NILRVTNVRSENLRVSINRLKARANKNSWMSSVFGQTFSLLADQHPRIFRTNKRFWGVVF
LGEGSEDVGGPFREHIGEMCRELMSTALPLFVPTANNVHNTGTYRDAFVPAASAIGTAEL
AAFVFIGQLMGGAMRSNEPLSLFFPPIIWKFLCFYPIEESDVDDVDRICLQCIREFRGLR

NRAGSGDMFDEVFDAETFTTRLSDGSVKELIPGGSNTRVTLERCEEYADALSSARIGEYT
RQLEKIRDGLMNVIPETVLCLLTPSELEFRICGKPDYTVGELREGAVYEGLTSDDRRVQF
LWQALEEATPLQRRLFLRFVSGRDRLPVKLRVLPLSSPGDADSVLPRAATCFFALELPDY
SSVEVLKAKLYYSIENCADIDTDFNPREVDESESPQLMVGLEDTRQEEVDSTTLSD*
>TCRU_859 hypothetical protein, conserved
MEISSISETDKNAITRLLSSDLSRTVARHAIIVLHYFRTISDEDLPIDVLLGGCVLYAVK
QRQASNANYFLRECLERVKESDIVGFELLLVQVVRHNVLLIETCLRSVFHEVLLENPVAG
LDRERTIKVCLHLISFLYRTSWCLFPESAARGAFLVASEKCEVKLGKLSSAFDGPMVKHI
AKYLRDQFWN*
>TCRU_860 hypothetical protein, conserved
MRKKLVLPLSIGRGFSSWDDGSDGHEWKSRALAEKRLLALTYLGDVNKRVQIHDAIRLRG
EVNKQAIRACELPTFFQTKETNEAENLQEIDYHSLLSMIEGEFEVDTLAHIAPSDANFLK
TEFLQCDDSAEVKLLGQWSNLRRDTADYNNYASIPDGERNAWSAWYLRNVRPGKTEND*
>TCRU_861 hypothetical protein, conserved
MDFDLSTSSVTEALGDLQMKLQSVQEDAELYSNLEKKARSSFERHRAELTLLLQKERAIH
AAAEDRLRDELQRLLMENERAKEELTSMRLQREKQCRNEMRARQHKREGREKALQRELLE
LQEQVKATRTEADLAQEEKTWHEMQLREVLGRQRGLNDELQRLRQDVEGQHTREAAEAQR
QDRCSRRQGVYCGLEKVFLPSGPPDRCHTTSPSSHHIAAIVSTVERQNYYKPRLYYRRRG
ITSPMDRRLENSPRYDISPLGETKPKPLLGKCEPSGSFLGKETAVASASSSPPRHVVDSS
AKTRTPSRAVTKSGRHLEAVCRSLLREILSLRREYQDCSVALNDPVADSVEVSRRMRSIM
NELDRKVHQLRSLRQQQAKVEDKLRLHDMMMEIAAENNYCENIYSDLLELIRS*
>TCRU_862 DNA topoisomerase II beta, putative
MANRTVEEIYQKKTQHEHILSRPDMYIGTIEPVTEDMWLYDEAENIMKLRKCTWTPGLYK
IFDEILVNAADNKVRDPLGQTAIKVWIDAERGMVRVYNNGEGIPVQRHREHNLWVPEMIF
GYLLTSSNYDDTEAKVTGGRNGFGAKLTNVFSTRFEVETVHSRSRKKFFMRWRNNMLESE
EPVITPSEGPDYTVVTFYPDFAKFNLQGFGEDMVHMMRRRVYDVAGCTDKSLRCYLNDTK
IACSSFLEYVDLYPMMGEERKAASYARVNGRWEVCVRVSNIGFQQVSFVNSIATTRGGTH
VRYITDQVIAKVTEQAKRKSKTEVKPHMIRPHLFVFINCLVENPGFDSQTKETLNTPKNR
FGSTCDLPPSMIDCILKSSIVERAVEMANSKLTREIASKLRNADRKQILGIPKLDDANEA
GGKYSHRCTLILTEGDSAKALCTAGLAVKDRDYFGVFPLRGKPLNVRDATLKKVMACAEF
QAVSKIMGLDIRQKYSGVERLRYGHLMIMSDQDHDGSHIKGLIINMIHHYWPDLIKTPGF
LQQFITPIVKARKKGRSDGDDRAISFFSMPDYFEWKNAIGDGIRNYEIRYYKGLGTSGAK
EGREYFENIDRHRLDFVHEDATDDARIVMAFAKDKVEERKHWITQFKANTNVNESMNYNV
RTVRYSEFVDKELILFSVADCERSIPSVIDGLKPGQRKIIFSSFKRRLTRSIKVVQLAGY
VSEHAAYHHGEQSLVQTIVGLAQNFVGSNNVPLLQQDGQFGTRLQGGKDHAAGRYIFTRL
TNIARYIYHPSDDFVVDYKDDDGLSVEPFYYVPVIPMVLVNGTSGIGTGFATNIPNYSPL
EVIDNLMRLLRGEEVQPMKPWYFGFTGTIEEKEKGKFVSTGCANVRPDGVVQITELPIGT
WTQGYKKFLEELREKEVVVQYREHNTDVTVDFEVFLHPEVLHHWVAQGCVEERLQLREYI
HATNIIAFDREGQITKYRDAEAVLKEFYLVRLEYYAKRRDFLIGDLRSVASKLENMVRFV
TEVVDGRLIVTRRRKKELLEELRQRGYAPFPLQQKKKVSSTTIQQGEEEEGAADATHATA
EDVFLVLQPAVDEGGDEDNQETPEMRRAARDYDYLLGMRLWNLTAEMIARLQSQLQKARD
ELAALEKRTPKDLWAEDLNQLRPRIENLFEDRAKEIASIQRKKMEKKRSFDPRRLRVPLL
SEKARQALAKEFIKTEKKGGRAARVDGDAGGDRKNPSVGRKPAAKRRRKKNSDDDSDDID
ADDDLFMSDASGGGGGGGWRPGDEEGDGGSDGVVLDDDGPGNNAGGGAKKPQPKRPTAKR
APRKPKEEGLKRVKKEAAKKVVVTDDFDIDEFGIEALTRTAPKFNNKNTTTTAKTTTAAF
TPPPQSTRVASVIDTDTDDDLALLGISSVDKKTLAPVPPPKRSPQRPKSQPKKSQGSRKR
RRSSSSDEDSFFDDDDDDDEDDDDEASSGSSSYDFSD*
>TCRU_863 retrotransposon hot spot protein, RHS3, putative
MKFTISTNIEDVLFKGRVRVNEMRLNDFVTREADGRGVADTNRDVLLEEFFKDPTRYIRD
EGALNEIQASDRYLSMKRAVKGEVIFDEDIRRLCDKGVNNLPGWSLAAAEVKATVHKFTK
HFLDAAAEEARNPTTTSAPEKLEGCYESLHNARWSHAVEIPDGVERKKTGTGMGVHEGKP
EQSWSYKKADDDIEENDAVQQFGAAPPVLMVLASEKGWPYSWNTIQDLPKDVFVNCEVDR
VWQIVKGDVTAWFSPHGGTDFTPERRVLIGTPGIGKSVAAGSYLLYQLPHCDAKKLQVVV
HCFSGGDAYMFDKTTRAVTRCSDEDMCISELRGLRGHGRNVYIIYDVAKEGTPPPRHFAP

TSGWGMIAVSSPKVTNYDEWAKQLQAARIIVNCPDEVDVKAMCAWRTCDETKEKQAECWK
EVKNTCIFWDRFHFTSLMRRRLRNAVER*
>TCRU_864 protein kinase, putative
MRRQGKNTTKRSMLLRVGSFSLDAERTDGSTVMAGLSPTSGGIITPRRIVRSLNVSPAVT
QLVPLDAVAQRPLSTETSATVLSEEVLDDLISESVSATYVPEGSLPVLGVHNGPTEGMTA
SFDPGNQKSMSYSLTRMTDKDSLCSSSLSGGSSTHSFSHEYTLAFDVESVYSCSLSCVGD
AKHGTRSVSRQMASPAATAAAVSAAVAAATPNNSVPDCGDTITRDPEAGIVQLIATGDGS
NAVEVGRGRRIVNVRREERIGRGGYGDIFRAVDLDTGLPLAIKEILVTADISKDVEKQLR
ALEREIRVMRKLNHKHIVSYYSARRDESCSALLIYMEYVGGGTIAQKLRTNGPFSEDETR
HYTRQLLEGLDYLHQRRIVHRDLKGDNLFLTEDGVLKVGDFGTSKELQTTLVTDSVAGTP
NFMAPEVIACSGHTCMADIWSVGCCVLEMLTAHPPFWNLDNHMAVMFAIMKGRLEEQVPH
QISGDAKDFIRACLRNDPKERPTAAQLKQHRWLLCKEENEDLGSRVQSCLSLSSLGFSNG
DGKHREGQCATQEGSLTARKDAFKSSTAPVVAIIPDGNDTSGGSPVNSSKGGNKKTGKRK
RILDPPAPSKAAKSPSGSAPNLMPPLATPLSNGQLTNHTKQTTTRSVPHANGVKHGKRST
KVGIGSTRNGGVSGGHGEPKRGGLTSEGCVVTKTTKGGRLTTPRLEATTCSTR*
>TCRU_865 hypothetical protein, conserved
MRPSRKQQAQRQHPRQQRSVAHKAQRAPSSGHGASGVEHLPRDAVVHVAPREQHRPAGQT
HRRIKCHRVVLRRRKFAPHKRHSGARHAHSPTHRHRVLAQQEAAVNKRHHGAAAGSNTAL
HRHAQPQRRLHRQPVHVQRTARRQRHAANHQHANSVAEHRRHMRARNGQHAPRQIHAVQH
EARGGGAKVVGVMVRNALLCDQHRVHHSDARARRVSVRHQRTAARTRLKPADQHIGTLAS
RRVGTPPDNVQARSAHKRAVRQRHVAHAP*
>TCRU_866 hypothetical protein, conserved
MAMMMTGRVLLVCALCVLWCSAYCRCTEEESASLSRSGIGNSRTGSGVSGENRVKTDKTR
TMRTASEGDSHPSERDVSDGGSFDQAPPDKSMCKEKNDGSTVKPLTTDGEVLVDSQETVA
THLKGTDTDLADPNINQNHKNPKAIDGIDTQLPDAPAAPKAQPSPPTVPTANKTVNSKEE
LTAEETPEHSRATRNPNEGSETKNPTDQALKPDAGGIDTLASEDASSPSIDDAATSEATA
NTARVGSPIASLNNTDGKNEGSGDGESPTPSPSAAEPETITTAKPTTHDVSHPNKNGTVT
FTGEKNAPTTNPKSATEGKETADVESDSSTAVSHTTSPLLLLLLVVACAAAAAVVAA*
>TCRU_867 C-terminal, D2-small domain, of ClpB protein, putative
MTSNLGSEHLVHVGNSPKGLEATREKVWQVVRSYFRPEFLNRLDDIVLFRRLGFGELHEI
IDLIVAEVNGRLRSQDILLEVTDEAKNFVLENAFDAEMGARPLRRWVEKYITTEVSRMIL
AQQLPPNSTVRVLLDSSQGKLAFSVKRSFVSE*
>TCRU_of Trypanosoma cruzi region 4/Dispersed gene family protein.1
Dispersed gene family protein.1
MSVSGNQFMFGTGTPTLIKIPKRSSDLTNGAIVAACNTGSDEEEANYVIPSVYNATILTC
SDPCTLAASCFPAYTTTASSDGCACACAEGGHGDACLPVAVPEPPSTDGADLCVREVRVG
VEVIAGLGTSVVCYVGVTFAADVVVDVESMSGSVRNVTLVNCTFVGGASLYVVGWRSDPT
AGERADALISGLESRSGGGVLVANRFPPGSRVTLVDSVLIAEARVAYRGAYDLGDLSACL
VVHSVNLTGSVLTIARTHVAAVFRDAVGVLFFGGVALSSRGALYVDGLLVQTVLGLCVSV
EDGVAASGGSVVAFVDSDFLLCKHAVSVRGAVGVSGSAVAFMRSEFLSTEDYAVAFYSTL
SLADGSMLLAKGNVHDGVSREMIYAAGAVTAAGSTLSFVRNRALLPRMLSLSLSLAAGAH
LRVACNDAGGRVLSTAEEYAAAGFGDAGRIDVAGCDDCDRDTHCYAPGTASATMRNGVCV
CACGSGGYGEACVPVGAPALPPAVGTASSLFFREGVTVRSVFVVPAGASEVTLRHVVLDG
VSPVLYVPWMARDGVRIVVQSVSLRNGAVLYVMGGGALRGAVAAVSDESGPVELSVCDVE
ALNGALVLSGTFPPGSVLTVTDTLLVAARPTPLVCLPDSQFSPYAPVLVLSGLRLVRSVL
VVSGVALVTVMTGGRTVVVDGAVLELVGGGVALDAAVLGGEYALYASARVVALEGAVLRV
SGSQVYAAHGLVFDSGVMANASTVVVNDNAGVLTDGALLELRGSASFVSGSWLSVRGNSI
SGRLLSVPSYPRSADLVQSTLTFHGNAGSGTVVMDGTVALGGAGRRFVVGCLTLNGQALR
PIDYRSAGIIGEFRPVACGVCDAEVRCFAAATRAMTVSCRCRCAEGGYGRDCLPVYLPHV
DGCNCTPGMPLLSHTATLTETRSPTPTWTPSPTPSMSATHYSPTQYGPTKTLQMTETVAL
SPTRTPTASVSSTLWWSDVACPTLTVTTTAAGGSLTQNDIRGGGSAVPTRLMVALPPPFR
WASDPQLGTHLSFVPVSTAQPSGFGGPWGAMLRNATWVRNATNPFTVVELAVPVHRGYFI
VADETIVIRCGAAAVSGGCKGALLGSFTIRSNTPSALSAITGVVAGAAAVAVVVTGGLGS
ILEMQALGVFARMSCASAQERASTAALPYFLSVFAALDPLWMVVGNALLAAVFGCVHCGV

TAAFQRWRGVDAASAWAAMRFPSLTYVVAHAMHLGVFFGSVLALAVPGARVQHRVIGVVG
VLYGVAFPAGVCYLIARHMGASFERYWQFSRKPLHERLLYPVGCWHPAAQQRMYGGMLTT
MRGSRVYWCVFQLSVLCVVGLIAAVHPPVGGCHVQYFCMATLLLAGAGVVAFTNMMRSAF
LTVMHAAGFVLLAALCLVSAANHLAPSDGVARAYAAMVLLLTIVLLAITVYSVVVWYAED
RHWQDLREPQRCGLEALLRDDEDSDEETQKLHDMTSLSYASETTGASSYRPPAPP*
>TCRU_869 succinyl-coA:3-ketoacid-coenzyme A transferase, mitochondrial precursor, putative
MLQGRHTDVTPLDALQVTATGEIARLYGVEKVLFAETVTMQTLCGGGRGLVLLEHVDRDG
KPRIFEQRDMPVTWRGRVGTIILDLAVFGVVYMGDSACRLLLGELAGYFTVEELQYKTGG
PISFNCRIE*
>TCRU_870 hypothetical protein, conserved
MCSASSVADARCEESLPSRLAGASPATDAVTHATAESETIAYLLRHCRFPVECWWISLNL
LERWRLLQSETMSLGSFRKRTSFFPADMSPASCAHLMRNLSYTCVAWVDVLRLYELATRD
VAAPEAARRQRETTDRAGDEPRASRRVSLRWMRHTALTSLLQANQWKESLQFYRHMLYQG
DTPSHVSAGHLVQRLGKVGAWEAVFRIFELHLKLIAATPTYSTARQGNDGVEHLSARVAS
PTAAPTRVGEWGTMFSMSLDVVGRVCRRPLIAMQMFNEACNKNIDGSLFRWDGNFLSAVQ
SFPSELDRVNVLRRAHASGQLDHFKIIRGLVHHGKWLEAIAVFADGLATQQLHRRDIGRS
RLNILHASSGDSVQAVLYCLQRLTRRPENSLRVNDAEVECVLSKVPSMLPTSSGASFASM
AHWRFCLQVLSENYASFATSGGRGVMPGIEGRRPTQRMVSLLLRHSMPWNVALRIFELSC
RLNMAATPYDPAGASSTPSNVLIINRVAHILHAHGQRQRMIDFLDYVLCASPLSPSSELL
EFVPLEFFSSNRGVPERKPLMVEDKVLYHFVETTMNWERALTLLHLVEDQRAGAVTGNVK
PLQVISASVHCATLKMLRRCCAAHGELWLHSLQYFQHAVSGLHLLDCHTHPRRLAQQHGD
TDVNTILYCVLYETLLNLLEEPSGASIVLREKNCFSLMECVTARCGGRIPTHMLLPNQMD
RLLPRLGVGEPTVPNVEERTRVSMKLIMCVLVGLLGSTGKEGLKSPHMTPPDTVMFYELQ
KLVCRVAEYRHYLMRLHETGQSLSNSASPRKTAATEKRVVPQPFQMGLLWQTSLELLGCQ
CRACGVDSMMPGTLKLVYRSCATSGGQWEAALHATQYLLQHPGAVEGEHCAMYCSLFGWE
RALAVWCRHFPQQMLREVSGHAKGLEYCMQSLECALE*
>TCRU_871 hypothetical protein
MIHDAVRICQNLLRAAGRRTDKHARKIATTATETVVPEEGKVVRSNKATSTYVYVCGVAL
ARAQADASTPYAPRGLRCPVALVNVDMPAHSWSVEASWRQSMLPHTKSYEWTTFAMVPPK
DPVEPLIRLVTWQRGSYVTVAATTGNSPTAASISKEL*
>TCRU_872 potassium voltage-gated channel, putative
MRRRAVINAGGRVFETQFSTLLRYPNTPFAKMFPLPAGGRKKCEYFVDVTPEVFESVLGF
LRTSQLHLPKENEQLRAEIVHCLNIWGLLEHVFPAAPDDTSGSDIVELPGICVVQICDHM
QHDQGVKRHALTITFGADGFHLRRLTQRIRRDLEGLISSTYWQCCQTNERAVFFVTTKVA
NGTADLLTTSVNQQVIEHTEAMGYSLASSYVTLSPDVVHTSVRMLVHNFTFRRVRRTALE
VGDELALSGEGDGKEEEVIETHENILSMRVGPQKKSLYEERSLPPNSTRASDIWRAH*
>TCRU_873 protein kinase, putative
MDEEDAERTEAPTVRVLCDNNWEERVLVRRGKHSRFYRVHWAKGGVWLGLKEEECVDPLA
TVRNVLPIQRLHSKFILHHYDICVDANRDNLLLVLTELMDYSLSDVMHRHCSRGHLTECQ
ARAIAFLTLHALVDLHDRIGMVHGDLSPSNILLRATGELKLGDLSSAMPVNAAVDCFSGT
VLYTAVEVLRDRQWASSPSSDVWALGVTLHECVNGSHPFSGNSDENFWEFLSLMEAAMRA
QHQPLCPPCFSRDFHDILSAMLCWDPARRPTVRSLLEHAWFSQYSVAAAQRVLCLIAQ*
>TCRU_874 ATP-dependent zinc metallopeptidase, putative
MDPLSREEDASKSTSFYRGFYDAQQRRPTKNVNPTVSQAVGGAIFTALPFVILGYFFMRR
RVNVASSAFGAKRGNFLSNAMDMLQKQMNPLGDKNFRVEVRDTKFRDVVGVPEALEEVQQ
YVNFLKTPERFTRLGGRLPKGCILTGEPGTGKTLLAKAVAGEASVPFFTCSGADFIEVYA
GSGPKRVRELFAAAKREAPSVIFVDEIDAVGSRSNGQGALGLSSEENRTVNQLLAELDGL
QANEAVVVFAATNFPDNLDKALLREGRFDRKVEIPMPDQRAREELFAHYLNRIVVSDATT
NAQRLARLTPGVSPATIATIVNEAALAAAVQGDDKVTERTLLPAIDDVLVGKKHRSRMSE
ATARRVALHESGHTLIAWLLPRQSDVIKVSITPRGPAAGFTQQVGKETFDMPTDVSLFTD
ICVMLAGRLAEATRHAELTTGAQDDYRRATKTAIHAFLAFGMSPHVGFLACEPQRLDEGR
IYQKHSEKIQAVAEEEAARLVGTAQQYTNTLIAEHKELLHRLADALFTRKELLKEDLEAI
LGPRGAARLSLEAEKALTAFVQKSEKHGTTVRSTSG*

>TCRU_875 inosine-5'-monophosphate dehydrogenase
MVNVDLRTKTIRDGVTAEELFQSDGLTYSDFVILPGFIDFGASDVQVSGQFTKKIRLHIP
IVSSPMDTVTESEMARTMALMGGIGVLHNNCTVQHQVQMVRSVKMFRNGFIMKPKSVGPD
TPISVIHEINADKGISGILVTENGRHDGKLLGIVCSKDIDFVKDVSLPVSQFMTKRESMT
VERYPIRLEEAMDVLNRSRHGYLPVLNDKGEVMCLCSRRDAVRARVYPNSSLDRNGHLLC
AAATSTREEDKARVAALAGAGVDVLLLDSSQGNTIYQVSFIKWAKKTFPHLEVVAGNVVT
QDQAKNLIDAGADAIRIGMGSGSICITQEVLACGRPQATAVYKVCRYAASRGVPCIADGG
LRSVGDICKALAIGANTAMLGSMLAGTSETPGRYFFKEGLRLKTYRGMGSLEAMSQGKES
GKRYLSEKETVQVAQGVSGTVLDKGSVTKLLAYIHKGLQQSAQDIGEISFDAVREKMYGG
QVLFNRRSPIAQMEGGVHSLHSFEKNLFTSKL*
>TCRU_876 WD domain, G-beta repeat, putative
MSYLTEEEEKERKREEQRRFSRQQIIEHVKKNLPYTAFDVKWVPRGASFAVVGQYPNNKG
AISVLQLNKGELSVQCEIKGKVPIKCSTFGHNTSNWQSNITLCTGDFAGGLSLWDIERLD
ADPLFLVPHAHENIINAVDGAQHSGPPEIATCGRDGCVKVWDVRQSNKPVVALNPANPAH
ARDCWTVRFGNSFDPDERVVAAGYDNGDVKLFDLRIQKMLHEFNVNNGVCDLEFDRPDIN
MNKLIVSSLEGRVRVYDMRTLHPNLGYAYVEERVSNGTVWCTRALPQNREVFMSGGGGEL
TLCRYRYPPERMLRDPEGVAKGVAGSVEELNRAKLGDQPIHALDWNRAKEGLLVCASFDQ
SIRVVLVTKLSLLQ*
>TCRU_877 Protein kinase domain/Protein tyrosine kinase, putative
MQPESGTRGATGHILVPGFLGCSGSTDSKYTYVRYLAHGSSWETWKVLDVSDGKPYALKI
TSLKNYREPKLLRVRTEVRCLESIDHFACIRLHEKPETESHFFFVMEYCDAGDLRYQMRD
GGKLLEHQIVHILLKLVLALHYLHTKKKILHRDLKPANVLLKTTGLLKLGDFGFSNAYDT
ISGDVGLTICGTPAYVAPELWRNERYGAAADIWSLGVILYEAMTGRRPFEGTKYEELRHK
VLHEEVLPIKTSHSVDLKRTVYALLRKSSVERPDTTQLLREPLLVAALSNFPEVLRVSDV
EDSLLQRVLHDISPEQLLPRPRLDHSVLFSGMVWKLKPTQDYQERYMELADGQLSLLLRP
GGRVGFRGCIPLLRVEQVACIDPNHFALKIEGADWCFFQTPQAAVWEEQLRAALGI*
>TCRU_878 Choline/Carnitine o-acyltransferase, putative
MEPKSLEEESMMYLCGNGHNRWCDKSFNVIVTEGGHCGVHAEHSWGDALTLANVVEYSHA
SDEFKELYTEDGHVKKLPEDEVALTEGKFKVFVPNRIRFTTDAVMKRAVQGAQERCIREI
KDVDIRVCRFADYGKGFPKKQRCSPDAWVQMAMQLAYFRDQGHFDQSYEAASMRMYRKGR
TETIRTVSEESCAFVRAMEQPSLSAEAKLKLLRVACERHQKTSPDAIAGCGIDRHLFGLY
CASTGFEVASEFLQAAMQSKWKLSTSQLLTRQLPAKFYPPSDEPFETPNGGFGPVQDDGY
GVCYCLYGESLFYFTITSKHSCKSTSSTRLAEHIKRALRDMAALTGWN*
>TCRU_879 Choline/Carnitine o-acyltransferase, putative
MAEARFAVRPTAALHHVGFEVDTSRKQTQLYVSRRGLVLYVPHPYFIIKNMRRSFWHGVD
KVQFALYPIPLSVVTAFSFGVFLWVLNSPADAWIRVNCVSDILWRLDERNFISARIPSRY
RMPALCANVAFGAVTLFTALQRFVLRKLLSYNRWIYEGQGKLTRKTMLWGFILKTFFMHN
LKRTGAYGSCLPSQPLPDLKITVQRFMKSMVPFYEGKTAEWEHLKKLSEDFLRNEGPQLQ
RYLWLKYLLADNYMTDWWIKYVYLAQRESLCINSNWFGVAFAKYLPTPLQASSAAALVHN
LVKVKKSLDRRTFSPQFSGLVPLDMNQYRYVFNTTRIPGREMDVLVQHEGIKHIVVIHKG
RFYQLEVLHPLTNHQLTPYQLEMALESILHSEDETDPVEALIPAFTTAPRA*
>TCRU_880 hypothetical protein, conserved
MRRATNTARIHWAEQLRMYRTPLGGTYFEQPLPSSLGASRAKGKEISTAHITMRRRGPRK
EKKPLSVPLTAEGLRGKMRQIGEAMAVFKQRRADGGEVRIAGDSHHEMRQRAFHNTEENE
FTPMRIATELSPVILVDQDDMVLAETGEEEVEGKNTILPGSSVLGSDLDRLEREMMRDYH
QRGKELPHFDRVYAAFGSGRRSKKPYHVLAEEYRQQMAVRINTTVSGAAGRGVNFCRGFE
QRSEDKDEGEEDAMLPPEANPHFPVAMEFGAVSAVAATDMQLTENAASFEPSSPSSPPHD
ASSSARLAHGRLPELRGAEFWDDAVNRERLQQRLDHAAEGHTHEMLAEGAVDTGEIGYSA
NRVRRETLLYFQAHPINEMIQEPFVRIRSILPSDGQAEVHFPAGDADTDVDLPTSQARRM
ARELGLDLIRVGAIHTDRSDRRVVALCKIADHREQMRDMLRFKLQKLGVQPPPTKECIEV
PFRGGTHPHAIRFKSIGIAKHLLRRHVVRINLTEFGTPKEGFPVFRTILDEVKKQTLQLR
AYHTAGTVRANYNEVYCYLFPSTGRSPKTTVTHPTQAQLDAVRDQRILEDEREIYFDDLN
NKKTARERLAYIKKLEKGTAWADKDEGLSLQRQREIKVMLGYLPKGNRELYAARGDVDIP
PPFRASHPTSVERWTHPPETNLEQASRGAAVLGKRLAMTVSEMHDRQETADNPTLLDRFY

YRLQGPALEAGELKEALGLKRNRKSVPRHASGWGTLGMPKNAPGEPGYAAK*
>TCRU_881 UAA transporter family/Triose-phosphate Transporter family/EamA-like transporter family, putative
MFVTPHRFLIPFFLALNAFSSIAIVFCNKLIFEDHDFRASTTLTLIHFVMTFLGLVFCLA
GGIFKFKRLSLMKVMPLSVSFCGFVVLTNMSLMYNSVGFYQLMKVLTTPLLVLMETVIYD
KQFSKKIKLSLLLICFGVSVATVTDSEVNLVGTLVALSALFVTCQYQIWVGTKQKDLGCD
SFQLLLYQAPLSSVLLLPIAYFTELRRLHYPCNDTLSVILLSGFVAFIVNLSIFLVIGKT
SPVTYNVLGHFKLCVILLIGHVFFDGPMGSKRFLGVLLTLVGVFWYTHLKTAKHSGVEVI
ISTEEFKGKDEDSDGV*
>TCRU_882 eukaryotic translation initiation factor 4E, putative
MQTRLKSRPDEPKLAVHPTSCITPLWGVWEMWCVLPDPVGGAVGNNTGVRGSAVKKGCAV
HKEGNRATWLDQVRSIGLFDNAEGFWGIATCTLPPSQLPPGITYYMLRRNIAPMWEHEAN
RRGGRWVVRFQDQQHRQQRKGENEEEITEDRSSRVDEAWESLCIALIGEQLPCPETEVCG
VALRRAERRREWKLSLWTRTAADRETQLCIGRFMKTLLNLEDDVLQYLSHRELMQASKEG
CWKVPPKYEL*
>TCRU_883 hypothetical protein, conserved
MSGDTYFRRAIDWMYGDTAYLQRMPSPEIGEKHINELLGRHPFHPFDPEMKPDHPCYNAN
AIFYNCMTADHVEGYELHMKHVSCYFPYKTDLMKCLSGEKRRARAAARQGVTE*
>TCRU_884 oxidoreductase-protein, putative
MNGRAPKRPREPGPSDCCGSGCSRCVWDVYFDDLAKYEEFVKDLSGQGKETGGDEDDEFG
SSSHDDDDDDDDGEAHDFVGSVVIKYVDAPTNEERVITPLVAVSKKLERFLPIHDVQLVS
SSSSHEFDGNTLKEGQGQAVQIVDVLLENSCELKRPMNFITPSPGDVVEVFIPNDYDMHN
SGYSGEVEKLCDRLNLNPSQWCEIHRSPFVPPTHFPPWLPFQSQIQIRTLLAYFVDISSC
GYLLRPTFFQTLWRLATLSKTTQLPTVAEKGVGTMELLETCASKEMAPFIYGIVMNGGSS
LCYPRLIDMLNVFPFVKIPIERLLEVSGPLRPRRFSVSDYTLDDSSAEKRLRSIQLCLRR
VEVVGVTSDKVGNDEVNAARILAEKLRAAALHRGTMNGGNCNGTTPFLFKGHVSHPLCTF
GSRPHPLPMFVGTKMFGTTLFARGLKEAIMPFMTQTAASTTLPLLILVGAGTGIAPLVCA
VHELVRYRQGGVGCKTQIPNCWVVYGARNFAELVYHRKLQEALELNAISRYDVSISRSSS
EGYLKYVTDVLDAHSEELRGALLERGARLFACGPAALLKSLRLRLANNILRSNDDDESVR
EQRVLLLEKRGQLMFDVWSAVNIFE*
>TCRU_885 mitogen activated protein kinase 4, putative
MDKPQEVATLAEGRRLYLVQGQRFEVAKSYKLIKLVGCGAYGSVCSAIDEGSGDRVAIKK
VSRVFADIKEGKRILREMDILTTLHHTNLIRLRNFIRPYGKDDFDDIYMVMDLYDTDLHR
IIRSRQKLTNEHYQYFMIQAFRGLCYLHGAKVMHRDLKPSNLLVNADCALAICDFGLARD
DQWTVSDALTEYVVTRWYRPPEVLGMGSHQYTNAVDVWSLGLIFAELMVGKTLLPGPDYI
RQLIMILHLLGTPTVEDMEFLSDEARNFLCAQPPQKTRPFTEIFPMATPEAADLLSKLLV
FHPAKRLTAKEVVEHPYFAKFRNSTEEPAPNEPYVWRHNEELTPESLRDEMWRIIVTHSP
EP*
>TCRU_886 hypothetical protein, conserved
MAEAVSCLDFKASFFQVSLLRETRHFFRCRVEDGTLVELTRLPMGCKAGPEILQIIITSA
IAVVTTVVRPLWAAPPSVRVDVWIDNIRIAGSKSDVTLREAQVLRNADSCRASLGRTANR
ALRSAPFWGCSLIALTVRCP*
>TCRU_887 Protein of unknown function (DUF3682), putative
MAMMMAGRVLLVCALCVLWCGAGGVYARDVDTNALGGCMASGVLGENGSHMPDGCNKTAI
TVLLRSILPITVVEASDGKAGALATKNDSNSSEASDAGVNGSGSAAVPGAGDSDVGERAP
AAADGGGSGTSTGNHGTGSVSSGSSEGPSDPPAAPGVDSSAGSSDGKAGSPGTNPSNTTG
DSPTGNQTPAAAEANDTSPPERLAGKTSGTEQKGKEKEEEEENEKQQQSDETQVQQHQQH
EHPAENGEESAKDKTALRTNATANTGDSDGSTAVSHTTSPLLPLLLVVACAAAAAVVAA*
>TCRU_888 retrotransposon hot spot protein, RHS3, putative
MVEHTGLKYQFFTFVACVDWDARDRESMAAGLCPLYQLLHCDVEKLQFVVYCFGEGEAYM
FEKAIQTVTKYVDEEKCIMAVRSLRQRETKGHIIYDVAKKETQPAQFFFLLSYAWGMIAV
SFPKVTNYDKWEKQKGAARIIMNCPAEMDVKAMCAWMKRGLDPDEQTKYWKMVETHMEKV
GPIPRYIFDANKFLAHFAATEDALDGINPRDGESHFTHGGVRLWYSEDPSHKLVRVVRTR
GEVGAEVFLNAPISVCLGRRIPLYFWKRDE*

>TCRU_889 retrotransposon hot spot protein, RHS3, putative
MPGGQNGLQGGNAESLVSAVPQGDGLGRARQGFESETDEPAATRGREEERQRPQWTISST
VKEILLEGNTGMAKMWLSEFLRSNLDEEWVVDTNENVTMQEFVLRIPRVISAMREYWAKY
RHQIIT*
>TCRU_890 DNA-directed RNA polymerase II subunit 2, putative
MPLPTRGSDDAAYNTRKLALLPALIGMKGLLNHHIASFDHLINVELKRILLNESNREIKS
SIDPDFSIRYTNIRVCQPREIVGKGQLPKVVSPHECRTRDLTYRGDMIVDVEYTSRDRNK
RPCTETDIKIGTIPIMLKSSSCNLFGKTREELVAMRECPLDPGGYFIIKGVEKVCLVQEQ
QSKNRVIIEADDNGNIVAHVQSKTHYSISKCSVTFKKGRIVMVHRSFKEDIPIVVMLKAL
GMESDQQIAQHIGTTPAFESVLFACFEQAAALGVKTQDDALHFIGERRKETSWEVDDAQQ
RHIQKSKADNAAEFLGNVLLCHIREGQVQRDWNFRHKALYVCFMVRRMIEASVDSSLLDE
RDFYGNKRFETTGTLMGLLFEDLLKQFNRVVKTAMDQQLSKKDTTKPFNVKQLMESKLEV
IQSGMRVALSSGRWELKRFNMSRQGITQVLSRLSYIACIGMMTRLASSFEKSRKISGPRS
LQPSQWGMVCPCDTPEGESCGLVKNFGILSQVTLDMDDSFVRAAAHNLGVEEVDSISSRE
FLNYYTVFLNGTLIGIHRYPNRLCRGVRALRRSGRLHPHVSIAINDRQKSVQIGCDGGRI
VRLYIVVNHAKPAVTSAHLDQLSAGQCSINDFLAEGLVEFIDVNEANDCLIAVYPRDIET
YTTHLEVEPLSLLGVVAGIIPFPHHNQSARNTFQSAMGKQALGTIAYNQYIRTDTVLMLG
AYPQRPLCRTKAMDLTHYEKLGAGINAMVCVMSYSGYDIEDAQVYNRCSLDRGYGRCVVL
RKHEVDLERLPNGEYEIILPPDSNAGKCKALNSDGIASKGAIVRQFDILVNKYTPVASLE
PRPNPLVYKYPQPAVVDHVVILPPGDSERSMDVEQKIKVITREVRVPEPGDKFSSRHGQK
GVVGLITDGVNMPFNEKGISPDMIMNPHGFPSRMTVGKLLELVSSKVSALTGTFGDGTAF
GGDNAEDLGMHLLRNGFNYHGKDVFYSGITGEIMQAYVFFGPIYYQRLKHMVTDKMHARA
TGPRSMLTRQPTEGRSRSGGLRVGEMERDCMVGYGASNLLNERLLLSSDLFTVDTCRSCG
FLGYCGYCPYCNTKNTVSHVNVPYAFKLLLQELQGMGISTRLSLDFIGQH*
>TCRU_891 hypothetical protein, conserved
MHKDGPRLWDDTLMEAERMATDSKARYLQLPTPDREAEMQRTRSQFFLLLRERLRNTYLR
RISKLNPGEPLAWKYISGRKKASLPSLTSMLLGGGQHTYKTARRAANALNRIFFPFHPSH
KAVRFSKGINRQSASLNVGSSCISEPQNNNESAATSTSGSSLSSSSEPQDKNEAATTSGL
VAHLHSPLDAPFNRTELLAALRNTPYGKAPGPDEVYSEALRHISSKGLRFLLRCINHSWT
TGTIPVEWRRATIVPLLKPGKSPELLESYRPISLTSIVSKVAEKMVLKRLLWVWTPHPHQ
YASS*
>TCRU_892 Reverse transcriptase (RNA-dependent DNA polymerase), putative
MQLAHLIHEVEHNRNHYFQVNLPKKSGIGNQLHYRPHRTLLVLVDFSKAFDSIDHRVLSR
LLANIPGVDCRRWLRNFLCGRYAKTRVGHRHSDRRPMLRGAPQGSVLGPYLFSLYVHPLL
NLLNSFAGVTADMYADDLSIIVKGQSREDAIPTANMVLQKLHAWSQENGLAINPSKCEAA
WFTLSTHTESDYDREGKRPLVVAGCQIPVMAIGVSRTTKLLGMDLVPRLTLNVAATKQCA
ATSQRISQLRCIAHKEAGPSPHDLRTFVIGYGASKLRYGSELIWAVATDSARNEMQKTYA
TLARIVSGVPSTVDPESALLEANMPPLHVLCLRARLSIFENTRACQMDWMRRPPPEPPPR
AGFRISSLSRDEPYAFVDAYTKDYGITESSPREERFFRSSIPPWFAASAHRATIGVELPI
DHSIADEEELIREKRRVSEEALALHSHRS*
>TCRU_893 hypothetical protein, conserved
MATGQYTEQGIAPLWHTDLLTCFTTQLTNKWRGTIRQDTHRYLLCGTRPSDLSGKDLITQ
EVLHRQELVHLARARCGESELWGRLYWAVRDCTNQCRFCNIPPEQSAYMRSNNDPNAPGT
DTVPPSAREEDVSPVSRRTLTRRRKEKCPHCDSTLTGFSGLVSHCRSFHPEHPPPPPELK
CDFCDMVFPTRRSTAPHRSRCAHNPDATRHRNSRARRRSLLPQDQPASTSTPIGPQETLH
HLLLECPGTLAVCQRLGIEQDLRLGKFSQWQLLHSRKLLSLLDHLDE*
>TCRU_894 hypothetical protein
MDSIPVEASSTPLLDRLLLKQGSTAKQPFTVSSAPADILRDTDTYGISLIASLGTLARAR
QNPSVLLWLDGVARRQFRWRKKDATAQRLFRWASRQIERREALKSHLDPAKGLSFQPTIN
GNFVIKRHGPHVAHEDLVQWEQRRFKQLEEKNCSQPRQMSPGPQKILQLRGTVEDGEEGS
RRKGEHNDDVSTPQNTKSNSRRITKDYPPLLERMQDDIAVRRQRAAEREDYARRESAALR
LSPRQVATQISRLSQHADTKRELFFDEDVTFTTKTNGEIFSPPDKPPGVSVAIASSFQLR
RQGDTGPSSAPRRCPPPEMPTARAATSPIQIKAFCERQRLWETRRRDRLRCIGEEINARR

EEKEMAECTFHPTVNVSPLIHTPSFEETRSLSMRIPFGALEYKSPTHRRPLDYEDINELR
LRQPRLRKTPASANKSNAHAQGRRNASLYGRAASCTPSRRAGTRTYVELHTPRREDSKRT
DRSNVMEKSSVTRRASQSQKDIGSGALDLDEQTLLDLSIWWKDLLMQELQVSCGTCKKRE
DDALDLRLQDWALETPFTCTLAPKTVIQAIQELIYLSAGESNAMALERIFGNESSWIQTI
LQQGGRVTFAQFIEIYKKLLHTK*
>TCRU_895 hypothetical protein, conserved
MHQPAAAGHGQPFYHEHGEAETESAGHNLQHAAAIHHNVPKHTRTFVVFDARRTPRAVLY
GEHGAANNMQIRRKGVHHVRLRNDSAVVHVEDAGQVHAKAALHEDRSARAPATNQHRPRH
DKPRRQKPDTVQDKRGAVPKPFVATGRRHVLRPANRRAQRRVRQQHTRVRRHRAMQHNHA
LQRRHIRHVDESVGHQARRALAEAANPQLRAARKHAVAQRHVQRVSERVHRAQVHRHSDR
SAEHHRVEARRRTPAANRHGLRQRHATHAAL*
>TCRU_896 hypothetical protein, conserved
MHRGRGRTARRLQQQAVKHHRARQQVHVAAKVHRRPARLQRPQHSAAHTQLRPQQRRRVQ
HKRRRHRVRQPAKLREAHRPRRHNRRVTDSHARVCRQVVHKHERAVAPGQPAHAHLQAPV
TRRLAAVAPHVDLRTAVAHAVPQFDAQRAGKHRRHVEHHGHAVRDGHAVHKHRVHGAANR
DGSVHRTAPQSQHHRMHSRHPRRRRNQRQHKKKRHTHSRNGTAPHTVPAARTRIHRRPHP
PTQTDINRAVVVCRTRPVDHNSCLANPFLQMGSPEIQSR*
>TCRU_897 hypothetical protein
MGRHQAEKVERRTAIEDGMRTQGMAEPHGATGGRLGAQHLMAHNSGHPQDNATNPTRPRE
QQEGIHGDAVSSPRRRTLLIRRSTLPSSSKTKRSRTRCVTIRRAMEGRASQLATSTWGPF
S*
>TCRU_898 hypothetical protein, conserved
MRPSRKRQAQRQHPWQQRSVAHEAQRAPSSSHSTSGVEHLPRDTVVHVALRQQHRPVIQA
HRRIKRHRVVLRRHKFAPHKRHSRARHAHSSTHRHRVLAQQEAVVNKRHHGAATGRNTAL
HRHAQPQRRLHRQPVHVQRAARRQRHAANQQHANSVAKHRRHVRARNGQHTPSQIHAVHH
EARGGVAKAVGAAVRNTPLRNQHRVHHGDARARRKSVRHHHAAARARLKPTDQHIGALAS
RRVGTPPNNVQARSAQKRAVRQRHVAHAP*
>TCRU_899 hypothetical protein, conserved
MDRCPITDPALDVGDIDAVILRGEYLSAMPGDEPSSWCLDTLPTNTTDARDPEETHFSAV
MHAALGCLGM*
>TCRU_900 hypothetical protein, conserved
MAMMMTGRVLLVCALCVLWCGTAGAAADGSEERADLSGIGGGSLPELKEIGKSPEGPQGK
KDEAGDVKEKKSPASSEDEEDDDADGDEKDYGVDDDLEEEEQEETVDPAETEIPNDPTKE
EASSPTQSKHEKGPTAAVPAEGVPTAQSPATAVQTTTQGQPPKAAPSPEDSPVPAASAAT
VTPAVASSEQGNIAAPARKKDEKQSEDIDPAQENEQPGPQDFVLVQHSQGIHESKESNSN
PRNPASASATANQRDATFNGHGEPAQATSSTKSGFESNDANSGTEEGISSDDPTADGAGT
NDTKNSQNKHGNTKETPVEATAMKGATATTGDSDSSTAVSHTTSPLLLLLVVACPAAAVV
AA*
>TCRU_901 5'-3' exoribonuclease D
MGIAGFYLWLQRWYGDCVEDVPEEVVKAALDGQPLPASSDTTQRFDNFYLDMNGLIHPCC
HDTAPLPEPESEEEMFERIFAQVDLLYKIVRPRKCLVLCIDGVAPQSKMNQQRSRRFRAA
EERIESEVFSAQCAEDVVKKGLPLPQTRSRWDHNVITPSTPFMERVGLAIEWFIMKKINE
DPAWRHIAVIFSDAHVPGEGEHKIMHYIRELRAQLGYNPQTSHVIHGMDADLICLGLSTH
EEHVTILRNQLTETFQPDHNRFCYFSLYKYRLRLREDFGGIGQMDFERVLDDFIFLCFFV
GNDFLPHVPLISIKTKGIELLLDHYVRGFKDHSYLTHGGEVNYGNLAKFLRFFVVGYMEK
LKQEYGGVLRAKQRAKAKVEERVKELERELEEQLNSLQKDRKNAQQVSNAAHKLLLSIMR
ERARLVVDKEPLGFSYVNDDHRDKYYEKKFGWDPADREAFEKKIQLCCAEYLRGTQWVMR
YYTTGCPSWDWYYPFYYAPLLQDLAHFPGKVKVKMTLGAPRHPVLQLLAVLPRLSVEALP
EELHDAVKDPESVLGVFYPEKVDVDFSEAHFSYQGVLRLPFVNNSHLQSACQKLVRLEPD
FGFTLLLCHESSRLSRALESLLADKDGEKVMKPIPKVLAAKVPIGGKVGPHEMEWPRHAR
LHCPDDGIASETSYGAPIKSNAACCYRYELNTQSLYRPVLLRELRKTMRGAPAPASTADN
LVKNPAVRKSSAPADSLRSSEKKLIRGAKHGREEPQEQNTEVEVVVNKKDTSTAKRRRVE
NTVREPTLKAEPPKKAVMRKSKTRRNN*
>TCRU_902 RNA polymerase IIA largest subunit, putative

MSGGAPLPPSQMDLQKVVEVQFEMFKEKQIKDYAVCLVEHAKSYERGQPVRGGINDLRMG
TTDFEFACETCHRKHPECPGHFGYIDLAEPVFNIGVFDIVLQVLKCVCKSCGALLMDTND
PQVHKKLQHLTGVNRLRQVVKMCGMKCRMSTDATTPEEAVVARGCGATQPRIGRYLGIYP
TLIIKATLEEQDMVWHADTARQVLDRVSDEDARVMGFDALRCHPRDLVLTVLPVPPPQVR
PAISFGSLKSDDELTHQIMSIVKRNNQLRKDKESDVQVAVDRSRALLQEHVAAYFNNASM
YYKPTKVNDTKKLKSLTERLKGKYGRLRGNLMGKRVDFSARTVITGDPNIDVDEVGVPFS
IAMTLTFPERVSAVNKKRLTEFVRRPVYPSANYIHHPNGTTTKLALRRDRSKVTLNIGDV
VERHVINGDVVLFNRQPTLHRMSMMGHRVRVLNYNTFRLNLSCTTPYNADFDGDEMNLHV
PQSLLTKAELIEMMMVPKNFVSPNKSAPCMGIVQDSLLGSYRLTDKDTFLDKYFVQSVAL
WLDLWQLPVPAVLKPRPLWTGKQVFSLILPEVNHPATPQERPPFPHNDSSIMIRRGQLLC
GPITKGIVGAAPGSLIHVIFNERGSDEVARFINGVQRITTFFLINFGFSVGVQDTVADAD
TLRQMNEVLVKTRENVEKIGAAANNRTLNRKAGMTLLQSFEADVNSALNKCREEAAKKAL
SNVRRTNSFKVMIEAGSKGTDLNICQIAVFVGQQNVAGSRIPFGFRRRTLPHFMLDDYGE
TSRGMANRGYVEGLKPHEFFFHTMAGREGLIDTAVKTSDTGYLQRKLIKALEDVHAAYDG
TVRNANQELIQFMYGEDGLDGARIEGGQLFPLPFRDNKEMESTYKYEYDETGAFNEKVGG
AYMDPHVKKMLRADPENVRKLQSEFDQLMADRDWARQMMDLEEREKLKLNLPVNIGRLIQ
NARSTMGKRSQVSNLSPVTIIDHVRKLQEDMLKLFPCYHKGVDGRIHNLLSRQRIENALT
LFSVHLRQLLASKRVLREYKLNDKAFEYLLKEIRTKYLQSLVSPGENIGAIAAQSCGEPA
TQMTLNTFHNAGISSKNVTLGVPRLLELLNVSRHQKHASMNVSLYPPWDKKRTAQQAQHL
IEYCTLESVTKRIQFIYDPDPRETVVEADRAILELEWSVMDESDERARLQEVENGSPWIA
RLELDADMFVDKALNMKDVKQAILRVDDGYVIETGMANDTKERTIRMRPKKNDGVDSIPR
LKHAIPELLARVHLRGIPGVRKALLKDRTTFTVDPATGKMKGEKTWLIDTDGTALRRAFI
GIVDEKGNNIINATRTSSNKVPEVCSLLGIEAARYKMLHELREAYLAYGLNINYRHYTIL
VDTICQRGYLMAVSRVGINRSEMSGPLMRCSFEETVKVLMAAAAFGERDPVRGVSANLVL
GNQARIGTGLFDLVLNMAALQQAVPQEDAVAPGKEVNVYHGLASTQNLPSSMPYRAKDHE
ATPFVNDASLFFREGVGGSSSAAPLTASTLYGGMHEASAVHFSQAYPAMGMLDQRVRASS
VSQPYSVMSSASVYNPASMQVPSALPSSVDYSEASSFHIQSSMAMLSVPSSPQFEAPQAF
GGGRFGGLSPAYVPSVMSSVATQPYQCVGDSESQQSASYVPTLSPAYSAYTARRSVVASR
ASASRVDQQSQQQKEDDSASYLPKSPGLHGLPPPVGEFSLTEDPEEEEERHQ*
>TCRU_903 hypothetical protein
MSRDEDVASSDAILTVLQRLRGNPVLRQYLHGHEGSCGCDGMTLSALRAKGAVTAEGTLP
RREDRESLVAFQARWSREEEDRLQNSIQAQRAFQQALQSLATCREVPGAVTEGRKRTVVP
SSFSAASVGRVVFSFEPQDATRAEEKTSVDDIQDAALTMQEKLAFRDHTIQRLQKELEET
RARYEADIASMRAAHMQSKQETGREMAELKMRLAQLALAGNSATNACENSGEFLEALEFK
ERLLQEMHDAFEHASEEWKSDTMCTLASLRLAAEDEVRGIVSRAEQLVGDLQRALDALTT
PRNDTAASFTAGVARRLLQDEEGLLLSMETKLREHVRMTVKNAVRSRVKDILRPLVERQL
LSHSELHDPRRLSPLHIVFSVRTARGALSPTPLALSTLPSPIPAEGRSGEAPAIFQPMTS
RQEQRAPSQRRELSAGGAAAPAVLDELSRGVKAHDINRPSDGISRPSWLNRFCH*
>TCRU_904 calreticulin, putative
MRAAIFFCALLGLATLSAVHGTVYFHEEFKSMEHWTTSKHRDDFGKVEISAGKFYADAEK
SKGLRLTEDARFYALSTAFPTPINNEKKSLVVSFSVKHEQDLKCGGGYIKLLPSMDPEKF
HGETKYWLMFGPDRCGSQNRVHIILHYNGENREWSKRIRFPEDKLTHVYTLHIAADNSYE
FFLDGESKAKGQLEEDWSLLLPREIVDETDKKPEDWVDEETMDDPEDKKPEDWDNEPAMI
PDADAKKPDDWDDAEDGPWEAPMIPNPKSKGTWKPRQIPNPAYKGVWEPRKIPNPDFVED
SELHKVPEPLTHVGIDVWQVESGSIFKDIVIGDDLKEVLDLVEKTYGGLKKAEADALKVM
EDMEKEKRKKEEEEEREKEKAEEEKDEEELEEKGDGDKEDL*
>TCRU_905 hypothetical protein, conserved
MSLAGQFTTFCRTHEQPINEESWAVFLVAILDVAPSTRQQHARMLRSMFEMNRTPLDMMI
LGLQKIAARPETKQARPLTKEAETKQARPLTEEERDEFIRGRVDWKQRVVF*
>TCRU_906 RNase H, putative
MIPRIRHRRKTLLVVTDGQSLLAALSKGPLSQTDWTEDRIWQRLLTLTRAGWSVHLQFCY
GHCGVHANELADQHATQTMESGQYTEQGIAPLWHTDLLTCFTTQLTNKWRSTIRQGTHRY
LLCGTRPSDLSGKDLITQEVLHRQELVHLARARCGGSELWGRLYWAVRDCTNQCRFCNIS
PEQSAYMRSNNDPTAPGTDTVPPSAREEDVSPVRRRTVTRRRKEKCPHCDSTLTGFSGLV

SHCRLFHPEHPPPLPELKCDFCNMVFPTRRSTAQHRSRCAHNPDATRHRNSSARRRSLLP
QDQPASTSTPIGPQETLHHLLLECPGTLAVRQRLGIEQDLRLGKFSQWQLLHSRKLLSLL
DHLFGTQMALYSQTRW*
>TCRU_907 dephospho-CoA kinase, putative
MLLIGLTGGIACGKSTVSTMLEKQHHLTVIDADRVVRELQRPSMPCTRKIARRWPGCVNS
QTGEIDRAALGEIIFRDPQARRELARIMNFPIFSKVMLLLVRFWWESMKQRMRGEGPLLV
VLDAPLLYESNIYTWFIDRVVVVGCKEEEQLARLEKRNGFTREQAMQRVRAQMPIEEKCR
RADYVIHNSGTLTELFFSVKDSVEWMRQQSGFKMNTIVFVSAAGGTVCLVAVVHLLCHLP
WWQ*
>TCRU_908 alanyl-tRNA synthetase, putative
MTEWPVNRVRQEFLKFFEQRGHKFVPSSPVVPNNDPTLLFINAGMNQFKNLFLGTADPNT
EFGRLTRAANSQLCIRAGGKHNDLDDVGRDTYHHTFFEMLGSWSFGDYFKRDAIRWAWEL
LTEVYKLPKDRLYVTYFEGDPENGIAADEEAKQLWLELVKPEHVISGNAKDNFWEMGDIG
PCGPCSEVHFDRLGGRNAAELVNKEDPMVIEIWNLVFMQFERHEDGVLVPLPKMHVDTGM
GLERLTSIMQDVHSNYDTDAWTPLFEAIQAVTGYEKSYAEIRDQNDSDATVAYRVIADHI
RCLSVSLADGAMPDSVGRGFVLRRIIRRAVRYGLQFLGAKVGFFSELVDAVVASLGPFFT
HMQDPRTVQRIKTVLADEEASFAKTWDVGLKHFNKAVADAVDNTISGDNAFILHDRYGFP
VDLTCLLAEKAGLRVDMNGFHAAMKANQLSAGRVAAAKTFLDVHQLEELKQMGIAPTEDA
AKYIWKNRTAEVKAIFNKKTGTFVDMLMPGEEGEEDIGLIMDVTNFYAESGGQIYDTGRI
VAAADAVFEVKKVYNLGGYVVHLGRMMTTNSTAVPIPVTSSVDLQVDYKRRIAIAANHSM
THVLNWCLRRVLEDEAPENYMEVQQKGSLVTEEMLRFDFSYNSRVSQEDLAKIEKALNEK
IQQGLVVYRKEVELEKAKHIVGLRQMFGEKYPDPVSVISVGVPVDDLLAKPESAEWRAYA
VEFCGGTHLSNIKEAEVAVILSEEALMKGVRRIVVATRDAAREARARGELLEKEYRDIVA
GGMTDSTAKSLSVLNKKVGDSLIPLTVKNSLREEIDGSVRAAIANVKAMAAQLREKATVA
GRAEAEAYDAVENPFFVKRLSDFGAEREALQAYADGFTNAVQGDVGVFLFGTDANKALAL
VTLPKAFTAKKLSAVDWAKAAIGKGGGKPHAAQSGLTAADVDVAMEKALAEAAKMKTAM*
>TCRU_909 hypothetical protein
MSADEAKDGKSPPPVEKEEAAMERDENDIGLQPAPIALVLRHQWAAEALTAMKEEESVDR
LSLVRSYYSGFLAIVSQSIHTLHLDSIALSRVQAQQQLITGLRCMPVPMNSKKRVSDSAA
SRARRRRAVDLSDGDTEQGNDKESSKGFLQRLRLNSFRRMNQELIERQRQVEAFAAGELR
RSAATRKQEELLSARRCSNEYKYSMAKSMAVRDMRARQRTEKYEDQQNTVQARQSSLQES
RNRKPCDSAAEHKEMQLILNKRRILFEKAMKELQKVVDWINERHHGGCVGDTAFMASVAS
SIESSFPTAFYQAPKSRCKKRNTTPIREEEYARLCGLKGAAIRSVVAFSQELSPTAAAPN
NSIARELQHLCISPILWH*
>TCRU_910 hypothetical protein, conserved
MRWLTRGVDLQRATAHVGGLHFFSTWNVALAPKRAVGGRGRTHTAAVHGKTSSLESPPPP
AALGSARSRNSAALSVGDNRFGTNRSGGKSPKEAAAANKNSEDVDVEEEERLLAEGDTRL
AMRLLMQHHFLYQHNVRRPEERRLEEEREMLREKHRARMERIEEKTRNAMQLGPDVSVDG
ILPLYGRGNSAAAALASKPSASWMRLNPVEAETGDNDGDDGESRSITGALLTKPDMSVYS
VEHDHRIRSSEVLDEEQRRRREELKSGDATKKLWSQEELEMGGGLTPQRVMESQMLEDED
QDFAVEAYSNDYDE*
>TCRU_911 ribosomal RNA processing protein 45
MMAHRCAFQTIASPALTQREVEFTKSAWIAGLRPDQRGAQQLREVNVDFPLLSRDVSCVR
CGGTIVTAAIACELVEPFPCRPKHGIIDFSVRQPYTERDSSAKTAELKKLTAFLERLIKG
GGVVDTEGLCVLPGRRVWSISVDVTVLNDEGNVTDVAVWATVAVLLHYRRPELTIRGDSV
ILHPTHERDPVPLSIHHTPLSFSFAITMAPGDRNKLARGRRDSQEALEVVVDPTFAEATA
ASSSVVVAVNAEGQVCSVSKSEGSCIHLNEVNHCMETASLLAPHILSLIRDAMAAHDKRR
QEVLKRMFLWAQKRSGVTREVTEPESAKKLKTET*
>TCRU_912 hypothetical protein, conserved
MDASHIQDEELGASEVLWSPDLDARWHLLRRHITPFEKDRMAYHSAKYQKPVFVHMNPII
WRRLVEKQGAYRAVDLMLMLGFAPAPEGAANLVEWIEREAAVAARGTAGMSVATTASSSS
SPVSNGGGLLPGRRSLALPTVVGDEPRTNGGIRFWVLNHEDSRPSSFGSPVNQYISPHST
APGACTSSPAHGLTVAPVSLPASASAPAQATAVVGPQTEAEASASRIRNLEAEVRELTRQ
IRLLKSRDSDRDGSVLSDVTSQNEMPQPHIIVSPDRPFIASTNCHALVCLPVNYLDPDCA

PYVVSLGVSTGRTLLACSIVEHRYVIFLAPPHRRGSMAVVLLCTRDGTLRPYTRAVWLEY
RAEDPPNTLTHHAVNQLLQEIPLPSSATVVDPNPSEVEQTKSESAFEDDNTFCDTDTAVS
TSDQGLESEPRLPLTKEMLQLIPNGIITGPRVSNAAKSGQANTNGTNLSVVGSESSLCGG
DREGRARAGRLWNDNDEMKPGSASMSTGQPTSYQRGPQSGFSDEETTQLAFNCMTNRTEM
SSSRATSVISIESAVPNGGGGFAGIMASRGWVRRYLQGSPRSSQCFGTNGNAGSLSATAT
ILDEQLQCAESQPSWPRGGSVTSQ*
>TCRU_913 kinetoplastid-specific phospho-protein phosphatase, putative
MDTQSAVLSQLRLLKLSNGARVVDSVQRPESTPTTSVAAALKDVMGGRGLFNAVPQQADT
IKAGNSGRGSNTTSPMTLVTGSAGSSSHPSTSELKLVRGNQGSATATATATGGGGGGKTT
RNMRPTPRSLQQPMLNQEKEKEAVTPAQSPNNALKQLLQAVSSTTISTSRDAGSSSFPVA
IALTNVEPSAVRMSTLVAGTAADPRLLEPIDNFNAQKPIIRTAPPCEHRYIVVGDVHGCP
EQLEQLMLKVNYQKGKDCLILAGDLVNKGPDSIGAVRVAQKLGAIGVLGNHDSTLLNCIA
RCRKPKLTVQEELDPVMQLALSFPQGCQDYLRSLPHILRIPKYNVIVVHAGLNLNNSIEN
QDVFEIMHMRRLEKIGGSEDKKARTITKAKWKAVVKGTRGEPWAEHWKGPEFVVFGHDAR
SGLQEHPFAYGIDTGCVYGSELTCVVYGPDSPKGTLFSVPGLPKFTNEERGLPPPAAPVY
EQYIEDMERQILRPTSRATPSVVTNDYKPTFLSVPPTTSSPATSSTFLGASGGCANGKTI
SKQCGVERATLLSLSQAGELTAVNTLMSLPIYEAAWMSMLNNEAEDCTITFWIPFVQGIV
TKLLNVSAEFPMDCVDDVLQLALEVCDELETVRKIVAPQLHVLLTREGSGAISLSKATMK
LLRLITMTQQ*
>TCRU_914 hypothetical protein, conserved
MAAPSITDSECDNTNDGKNNDEDDDDLHRRADYLRVFVHESREGLNAVLTILGLAEILGF
ALLPAVISAPCVEADPFTVDFTQQLSKGCAAIPALALSHVFQRCLACDLLCVLSRPPPPV
FFWCPIRSTLRCGGYLIAWPSTHVTMLNGCVFCLAGTRETSVCADNSAKVFVDGSGRK*
>TCRU_915 Mucin-like glycoprotein, putative
MKTCRLLCALLVLALCCCPSVCVAQDTEKVEESAKTTTTTTTTMQPPDPKTNPKAGAQAN
LGVTISSSSSTEGIEGKDQPSPHAPPVNKPAEKPGEDAVAGKSLTGTTELQSDTNKSADH
TDKEDEEPAKTTTTTTAPVAPSTTTTEAPTTTTTRAPSRLRETDGSLSSSAWLCAPLLLA
ACALAYTAVG*
>TCRU_916 Concanavalin A-like lectin/glucanases superfamily, putative
MINYVLKSWAQLDSSFSESFTPTAGLVVFLSNALSGGDTWIDDYLCVNAIVTKNAMRAKD
GFEFTGLGSGAIWPMSIREHNGPHTFVSCDFTIVARVMIQNFSKLSTPLPGACLDAPVST
NFIGLLINADSRWETVFYGIKTASGSTWEPGKEYQVALMLQDGHRGCVYVNGELVGSYAT
IRTPETRGLESHSSKLGEAREAAAAVRR*
>TCRU_917 hypothetical protein, conserved
MRDSFAAPSLVRAGGVTIAFAEGHTEYKGSHYRLNVRPSDIVAGYINAAETWPLIAAENN
KDKWMVRTLFSTVNEEDLFGAACNPTAIEKENKVLLLVGSYNM*
>TCRU_918 hypothetical protein, conserved
MTKHVDRSRTPSRTSAGPYRNLSRPRVVGTYCDPPTVARSVEFASSTLERGGIAPCSTIT
PSTDVTFVMLTKACGIRRAVLSLRPPIRSCAPPASTQWRSVTFSASANASIERRSTATVI
GPLNTTESKHAVARPPPAVTDSVSVTPLTQPSAVSPPPLALAGGTSNGRHASPTPPARQL
HTQSENDAVSASKHASAAAQPSHGTTLTEGRPAVR*
>TCRU_919 hypothetical protein, conserved
MEQPRDRKTVPADRDVCVRWKHTLRAEQRVNKRVSGDDDVQRPHSGVHVAQQQQHGAVPP
RAAIERDIRHVARHPPNQHRQAHAAPAERHVAVAQPRQAAAHCLRNLGVHHVADALTNRR
RWWRWRWRWRWRWRWWRASRHRRRDAHIAVPSSSALALAVNDRRRQRSKAVALLRALPAR
GNRRHVGDAVERQFSRRRRLPSRDRQAPSKKPVAGVSPR*
>TCRU_920 hypothetical protein
MGRHASPWPPSAHVQAQPSLEAVVVYAGKHDAAIVHGSLQVKMAALYTDGMMYSTPSSPF
TVLHAAKIAPFVISRDPLGIHSTVGVPVLEINWLPDTDTVESRRFTVE*
>TCRU_921 tRNA synthetases class I (W and Y), putative
MGDMYPTVVQVQRMLTLSAVKNTFGLKDSDNVGKAAFPAVHAAPCFSSAFPRVLRRLAGD
PPLKCIFPCAIDQDAFFLLARNVAPRMKCCAPALLHTKFLPALKGAQFKMSSSAEGNGVI

TLHDTEQEVRKKVRKAFSGGGGTLEDMRAEGVNLHADVAYQLIRFFCPDDNLLAEVTSKY
SRGEMNSAEVKELAADVVVRHVLSGWQARRAGVTDADVEHFSGIRSILL*
>TCRU_922 Fungal tRNA ligase phosphodiesterase domain containing protein, putative
MLDGKKVQKAFHVTTLYLRRDACKDPVLLRQLVGLLGESIELTLTSVASDPKGTAIAVRN
EGEVPCENVHPHITIANAPGVPPAHSNELLDDSHADDPCRTVDSLPAGTRVTGTFVFRWP
*
>TCRU_923 retrotransposon hot spot protein, RHS3, putative
MLASKDNILPIFFGWFALKSFTESAFVRAMATKLTEFEPPTGRQAKPCVLKLAPELHPKG
DEGLLPSEYEKTIRKIKYGVLCKPWATNLPLVDGFFFVESNPMTLVGLRMATAGGHHTTA
STVRPFTECLAAYFNGWEELSRDMSWDIMYTTLRQHADG*
>TCRU_924 UDP-Gal or UDP-GlcNAc-dependent glycosyltransferase, putative
MAKTKLLTLRQKLKRVTVLLILFIVATTVLTLRFDTAADDEVPGPNNLRHSHATAAEDAA
LAYIPRSIVDKWSRREYLIVLGVPSIDEEARRTQRNLQRTTCWRFPGVATRANDFTGAML
VLYVLGRHPSHGYDYSAALLEEVAQWNDVVALPMNEGLVTTNKTVGHYGFFGAEANVGLS
RKVYMWFDLALRLFPTTRYIAKGDDDIFLRVPLFVAHLRLLPRRGIYMGVHIGVAQFANK
RFPGNTFMFGWCYTLSRDVAEALVSYKPLRHLAYLPYSEERDHEFALLQFHNDDAMVGWV
LEKELNYKPMVYVKMLPCHFHDARNETGHWQVVPTSMCVHHVRGDDYAALMARFGNDTSP
VGRVERVSEDVIYPCCD*
>TCRU_925 hypothetical protein, conserved
MGDDNLMGDDNLMGDDNHLMGDDNLMGDDNLMGDDNLMGDDNLMGDDNLMGDDNLMGDDN
LMGDDNLMGDDNLMGDDNLMGDDNLMGDDNLMGDDNLMGDDNLMGDDNLMGDDNLMGDDN
LMGDDNHLMGDDNLMGDDNLMGDDNLMGDDNLMGDDNLMGDDNLMGDDNLMGDDNLMGDD
NLMGDDNLMGDDNLMGDDNLMGDDNLMGDDNLMGDDNLMGDDNLMRDDNLMGDDNLMRDD
NLMGDDNLMGDDNLMGDDNLMGDDNLMGDDNLMGDDNVHSEMRMTHDRFSRKLSSPLVTL
LLVGVLLLVRLKMYYREPKCH*
>TCRU_926 hypothetical protein, conserved
MLAHGPLSKAWKDSTLVGRIANLRAPRIPNFKTQVLLPAMGAARELGHQQGLITAERERL
NIDCANAVADVRNRNAQLNSQVEEAHINLSVRESTIAGAGNQVLELQQQLENTRAQLRER
EFNLGRLTGQLQEREALHNAQQPARDAETAGRDDAPATRYAANDGVSTLPHQTDLLMAGG
VPSSAARHSSAAYRRPHGNAARPHFHPSTPLPYRASLLDAQLPMEEDRSFCAFDVTTWPS
RIWNEGASGVIIDLRCAYQCWQQDNEKVELAFDNLVEWIGVLDGGQDPTDRLMNLGRSLL
NTFRMQLMMASNPGIRLSKVRARPYTAVHQMDAYARAAQLLVDRRETQRTVRCQRCHTYG
HEASTCNVRPRGNYLQHRRPSTNGRGAAARRS*
>TCRU_927 hypothetical protein, conserved
MQDTLRKQKAYGSYDCPSLTHSGSLECHGAFHKTGCVKTRCSSREDVQFGSTRDLAVGET
RRTGRPSPGHSSIFVEVHHTADPGVVAGRIDVKEETAAGQELWLRAEERFFHDFCHNHAA
TITRLATRTRLATPQDEHAIPLQQVNVPVLNLEWIKSRLRPAALERLMQVWGLVGRSPLP
PSSSGKPREGSRRIPTTDAHLLQTAGIIEDDSSTITGGWMIPFSVVEEKTTGSRRRWIAW
PRDKNRDDPYEANVPLLHISHYLPPVMAEAASCLDVKASFFQVSLPRETRHLFRCRVEDG
TLVELTRLPVGYKAGPEILQIISSAIAVVTAVVHRLWAASSLVRIDVWIGNIRIAGSKSD
VTLWEAQVLRNADSCHASLGEDRESGATQYTFLEVQFNHTHRAVSLSDKFVLFVCAMPAP
NYLTIAEMEVVASRFLYAAANLCTRLCDYYSFIKAVRRRLSELNQGTVQ*
>TCRU_928 hypothetical protein, conserved
MISMTFANGLRLCGFQCGMEPRLTEASWRRPDILIVGLDTYAITDVTVTYAGRVTAYVSE
ESMEEADALRAARDRLTQKRQKYRYWALANGLDFEPFVMLTNGAIHPASRRWLRRILGAR
VLAYAGAQWVAERQ*
>TCRU_929 Endonuclease-reverse transcriptase/Reverse transcriptase (RNA-dependent DNA polymerase), putative
MHHPQWEPFLETSPSEVAAEFLELCTDAGLTLVNTPGEITYARGTRERSCIDLTWSKHLT
VSDWSASVSPLSDHYMLTFTPHQAFKDTIPSAPPSAPKFFYSWGKCKWDLFIKDFDAQLP
AYDYKKQSTGIKAFTRALITSYRRHCPRGMHKDGPRLWDDTLMEAERIATDSKARHLQLP
TPDREAEMQRTRSQFFLLRERLRYTYLRRISKLNPGEPLAWKYISGRNKASLPSPTSMLL

GDGQHTYKTARRAANALNRIFLPFHPSHKAVRFSKGINRQSASLNINASFLFGQQNNNES
AATSTSGSSLSSSSKPQDKNEAATTSGLVAHLHSPLDAPFNRTELLAALRNAPYGKAPGP
DEVYSEALRHISLKGLRFLLRCINHSWTTGTIPAEWRRATIVPLLNPGKSPELLESYRPI
SPISIVSKVAEKMALKRLLWVWTPHPHQYAYRSMRTTTMQLAHLIHEVEHNRNHYFQVSL
PKKSGIGNQLHYRPHRTLLVLVDFSKAFDSIDHRVLSRLLANIPGVDCRRWLRNFRCVRY
AKTRVGHRHSDRRPMLRGVPQGSVLGPYLFSLYAHPLLNLLNSFAGVTADMYADDLSIIV
KGQSREDAIPTANMVLQKLHTWSQENGLAINPSKCEAAWFTLSTHTESDYDREGKWPLVV
AGCQIPVMTMGASRTTKFLDMDLDPRLTLNVATTKQCAATSQRISQLRCIAHKEAGPSPH
DLRTFVIGYGASKLRYGSELIWAVATDSAKNEMQKTYATLARIVSGVPSTVDPESALLEA
NMPPLHVLCLRARLSIFENTRMSDGLDAETPA*
>TCRU_930 hypothetical protein, conserved
MLKSFQDTYIEYLKSTLHEIEVDVSIVGETLASLGLDERSISDILGGSLHACATGASSVS
SVSSDRDWDRRLRKGPEECSTISSTSKKSLLTPQREGMLRVDIGKFDREQMGGAALPASS
SSSLSVIESPRAGRGNFLHRGHDASCNVEEEDEEALTSPATAWDDPIVTPDRRDSYKEHL
EALEKLLGVVWRPVAGGGDDSVPLEVRSFLCRERVQNLLLSQFKARGIALPASPPKRNTP
HQRFKVRHEEKKQPEGSRNNSRRGRVKHSQCIRHFGSLLETKKSPQVGFRWQCEFCARLD
DKPILPSVSDHPRQPQVTPAAMGNACGVKMRPQAVNTVGSGRICTKLLATTLLYREDRVK
KALAYRDGWNASGCAER*
>TCRU_931 hypothetical protein
MSGVGDSGGGGGVKVVTHLDAAVSCYLSSPGVTPLEVYLTKVEDAAISLTQEFGSLVFSY
EEHASRAGRATLECSTALLQGTVFLEERLREAMALANGVVEGMTGIAAAVKGMEDLLSEA
RKVAQDVSLVEDAIKAAEAARGRDANS*
>TCRU_932 hypothetical protein, conserved
MFAKVSQDPGGRLGIIYFASVALSSAFVAWIAQKWWKRQHGTTGDEGIFDDVEESPLFDP
NFVFPAKLGVKRASDAGRDLECVGPLSMAEREGALRMARGLMRQLGGGAYNEEGENNNKN
EEEEAESSSSGDETNVSLNYHIATELEKEARDSLVEAVIHSELLKMEAQGKRIDPSECAN
ILAKLVEYFDISNEREKLRERRIAMRPNETTNNGFLWANSDGYNDRIAQQMAKINEAYAG
FFSRETEMDQRLRAFNKDGAEERFEMDWDGADDDDEEADLMNYLDQDDAAGYGSRMELRM
KAMGRDSLRMMDVDGEMNYRDDDDDDDDDDGECEEEEESDDIYEKTGFERMLLDKLHQLA
ASLGEKTTSSLGTGAAELPRSNGTVAACDAEREKEKEEGSSQNEWETESDDDDDEDEETK
EK*
>TCRU_933 high mobility group protein, putative
MSTELKSGPLPADVEEVIANIMREEGVSFLTSKILRLRLEARYRMEFTSHKAAIEGIITK
LMQLPEFKKQLENAVKEEKAASSIGGKKKKRSASAAADERNAKVNKKEKKPDDYPKAALS
PYILFGNDHRDKVKEQNPGMKNTEILQSLGKMWAEASDAVKEKYKKLAEDDKKRFDRELS
EYKKSGGTEYKRGGGKVKAKDENAPKRSMSAYFFFVSDFRKKHPDLSVTETSKAAGAAWK
ALSDDMKKPYEAMAQKDKERYQREMAARAS*
>TCRU_934 hypothetical protein
MGDGDRDGENFIDFFLDDFCCLVSRFLKISCRTRVGLVPAPPPASPPSSSPSFIKPVTDA
THVEEQLDAADSMLRLMSDAKPRVKTSEKKADFPTESAVFLLRLRSLLERQLNQDQQSDV
LPSTSKTTRSTRGESPSGGESKFSLTCSDAPCRRVRKHVHRFPNAMVEDIRGTSTLNRRI
ATEDAQCQTEFSEMLSMGREPSSLGQGVAVHLRRRVSSLSDAHGRLALILQQHTISSILA
LGHVLAEANRNPTKMQECDLRIQTRYVEGIQGVLEREQVRLNKECSNWNGIKEHEQLHFS
EIVQVLERLEMAVASVDFRAFVVEEVDEGIKGCDVHDKPVSIEGSSVTHLVDFFGKSCAG
FVSKLVVYCLQCFSSALTSASGSPLRWSYLEKVLFLTDSESPVPTVPMEMSRTPSRGSGD
MDVDKTPTQERSESVRACDVDELPKRRNTDTPWQEGENQLLRLMQKFRPCHEYEEVRQLE
IPV*
>TCRU_935 U2 small nuclear ribonucleoprotein B, putative
MGEPKQTLYIRGLPDKVSEKEMRRALYLYCTQFGPVLEVSYRKSRDMYGQAFVVFSDVAT
ATSARRELNERQFYGRLVQAFYAKRQSFSADPGERRRRDLRRERESAAKRLREF*
>TCRU_936 guanine deaminase, putative
MTVFCGTAFHVPERHKLEVLQDVLIVVSDTEGIIERILRPSDCDYHKVKEEAREAGNLVT
LRPRQYLIPGLIDLHVHAPQWPQAGKALHLPLEEWLQENTFPLEAKYKDVKFAEKVYNSL
VETLLANGTTTAAYYATIHVESSLELARICLQKGQRAVIGCVAMDIPGACPDFYRDRSAV

DSIDKTAVFVEAVKALKGNERALVLPSVCPRFIPTCSTESLRGLGELVKKYNCHVQTHCS
ESDWEHNHVIERFKKHDAFALDGFGLVTRHTILAHSNFLSTEDMDLVLSRGAAVAHCPLS
NFYFSNAVFPLKKALDKNLHVGLGTDVSGGNSPSILDNCRYAVAASRALEDGVDPKKDAK
ERSGWKGARINFIEAFWLATTQGGVSLDLNIGKFEPGYAFDSLVIDVDARSSNVKVFDSE
DSLEHIFQKIVYQSTSANITQTWVGGRLVHQL*
>TCRU_937 cytochrome b5, putative
MLKGLLQIIGLKKEWPVFTLDEVRQHNDRSSIWIVAGNSVYDVTHILDTHPGGLNALLRR
GGGVKDCTEDFYFHSRAARRTWNSLKIGELDLGEAMGIGTLAVSLTDSRDGSSAPEASPV
SHACASWECDGGKNCCCTSTVETHGSTSEPVPERLCSECLHRHSAWR*
>TCRU_938 hypothetical protein, conserved
MFYADTIFRAALASHRPVLASSISARSVVRRLLGDRGYRVPNVGAMDSVVTKTKGRGLPG
TACVVGRAALYVHHGNPEAAIAEINRVLPEIQEADLKRHAIGIRLRAKNDLLDIKEAALK
VDGGTTELDVCTLRSAIREDYEALKSASSGCWLVEMAAAEYALYEAKAEEAYKVFCDVAE
KTESFIASLEGLHISSEVASAENTQSFSLLSFLLKRAHDAEGVNPSASKIVEEGIKNILN
DDSVKGSLATFKKELGVSLTDEEAVEVACVLDAAHVRHHFHDFFPLEGEYNDWRSEGAKE
AKNEIVQVFTPQAVVISNELLENVKKSVPYKPFYAPEDQLRKFLSSGHKNYLAELRATFG
PSPACVLAKSDEAYFEAMNILKQGPPSADTAGDLSYPQKQNEICRKLARQLLYRTKVNTA
VALTQLDHLNEAVEILDKVIAANEFVYMWRAFLARGEAKKRLGFISNSDKDFRQLSEQKK
TFRRKM*
>TCRU_939 ADP-ribosylation factor-like protein 3, putative
MGLLTLLRKLKRNDTEPRILILGLDNAGKTSILRKLSDEDPTTTQATQGFNIKSINCEGF
KLNMWDIGGQKAIRVYWPNYFDEVDCLIYVVDSADRRRLDETASELENLLQEEKLREVPF
LVFANKCDLATALSPEDVSTALNLQSLRDRTWSIQKCSAKTGEGLHAGLEWAVKNLKK*
>TCRU_940 hypothetical protein, conserved
MTVARDASSMPIVAHFELHTSKVGHDDACMHWLKGRYFREGNIGVTQQKKTVEDTDILLS
RMENDLAACATHRWAHFSQRSPVYEATSPRTDNTQLNKVEVRIEECNATGKDGEFIVSVQ
DPHSRRVLGIREDKMLAEPSRHIFTEAVRKRNQTRLAMEIEAAGRLHDVTNVRRPYFGGY
FLPPHSVGPTRFLLFPQWMHDVHLDATQPQELPYFLRGTIDRKSGCTSDEGAEMSEDKRG
PLHKFLKSIIFNASFARKFPETMPRWGIFNGVDALRLELYANNENSERLFSSCANAEASK
NTQVKHKSRPLTLNFCDSGARLAFFFRDPRYSLTVSNAYRVAQHSSKSSAKQRAIASLTV
SDNYDSAVQRFSTPLLHRWFPSARYQLQWSAGPGVEYVSVGKPWTVFGKLGTEWWVELPF
FKRALFYIRNQSALVQPLSLRRTKEVDERNDDAHNSAASPEDNRSSAHSYFWATQGPRWN
PSLVRGFKDDYPSLHYASRWYTLISAELASDRRQRDKGSKTWQVPTWMIYANACFTDSFA
TPPRASVGFAFSGTPRGTTDAFNVFTPSSFECSFNWWLKFYQGRPSLVSGIVSGQRAEES
TLRLSPFETFRHFRCGLTWKA*
>TCRU_subunit mitochondrial RNA binding complex.1
MYRFCCRGAVWRPINRSHVLSGLAWCASQGVPPADDGKPRKAARHVAPSDEDVAEMLTAD
REVPTSRTGSKLSDAAMEFTRVDSVDDTGGRKHAEHFRDATDFDGEEDDDDDEDIDDVRR
RPSHVHGHGERRTAEDVLTDENELPHESDATASSPPIDILLGAEDEAAEPSVLHPIAERQ
AVARTMSAVELVEMLVLYLRATENPRLVSAEEEHVLFPVVMERLNELHLGQMLDIVECHW
ARSTLVRYGITFKDMVRDRIAMLASVAGKDAAQKPKRASRVDKGFGEDDDATNDVDDDVD
DDADAGEVYAHEVKTPTLDPILLQARDEMNAEGVLRCIIVMGMSAGRRKRDLQFFQAMGM
FLVHHVNHYKDPHDLVRVLTAFARAKIVPPKGFLALLGRRFAVLNKRKPLGALPSYRAFV
NLYKMGHDQMNNFRFLANCIYEFMDANVKSEKKRQRLLELQKKSGTTDAASADATKTTAQ
SESTESNEDGSTMERMMMAGDLDPQLLQFIKARERFKRLTELKPSMFTKLLLVLARFGAP
HQQYLRPTIVPLILPTLRCFPPPSFSRLLRALYLFRTTDLELIEPIIDYLADDLGVQSVI
PEDVLEMIRIVSPAEAPVPRNLSKILLLCESVYAGVDAAPVRLILPTDMCSVAAALLRIQ
MKEDVPLDALDPLTRLMEVFADRLYSLLQLHVVSLTHVDIFADLCRQQQHPDVSGKIARL
IQERRRLSSTEGDDDYYSRLDIDVRETFHKIMVVNDFKTYGQYRPTPGVLQVDFKQALTE
VSAFDVLEATHLFAQAFPNTLRPTVVRHLSRSIIAKLGDSGEEVITEDNTMVLRPPRELL
LTKEDLLKFVELVQATPLKRVKESPVVWRFIEEKARRLRMSELLKTVEQQAVVATA*
>TCRU_942 mitochondrial RNA binding protein 1, putative
MRRFALATRRVTAVTSPVRLCFRAASVPASNPVAAPETTTVREPQPRQKENVLKVPSMTL
AQLLTSVVGEMQKKSLTPTAHENVSQNKTEGSSEEAAKDASKEAKAGLLPYKLSPEKFIM

AVSTYIRREEEMALMSRSNFVAMRRQYLMLRAKQPRLSSSQRDILLHEASCIVEKREVPL
NVAARLLRPRWPNYFMDVVGVRHHAFLAQSLRGWTVELIRDGKLTAKEARRFLMQCPRLF
FGQVSLMGSVVECALSDLNTIDDPELLIKLMWSVNNAKTHAPDHFWRRIVDKLAMLNRSM
RDQVGDAIRIEKGKDEKKSEEEVTEANSVMVGHVFSGLTTRQLFRILRVLRKERWCGDVP
VIFDFMDKALKNIVFEVEATQVADTNSSEGPPSRRTVVQRVKKASDLSPVELLSLLSIAG
ELGADFHVSLARLSEYLLSPMTRYLDRDHLLLLTTCVRRTRCDSAQLVQTIVDTIVRRGV
TYSSSLALTKAVLRTILQKPALLSHLTLAPFMDHIFYLCDTHKRHLRAPQILAWVELLYA
LSRLYAPSSSVGVRVRAAVESFADPLIAMLAVEVVPTSIVSRMLEYTVILGMRKQPQYPL
TAKLWEDRNTLVNGRRAHADSLGSGDENDTAPPDHGPEEDVTSDTMEMEEVPKGEVTKSV
KRVYDDLISVYERQTVMQMPLLKGEKERVCETFQRVGLYSIFMGADIMKQLHLRDAASTS
SASTLRHRAPSSRPLSTWIERNVASIVDTRIRSAKISPESTDNDVLRLFGHRQCDAAKVR
RFQVLLMSSPLLLARESRSLWEYTSELARRFGGAQEKSLAEEMLRKALH*
>TCRU_943 rRNA methyltransferase, putative
MYRCRSAHKLLELDAAYHIFAWPCRTVVDLAAAPGGFAQVALQRMACTSLPTSTEDVRPV
VIAFDQRPIEPMEGLFSVTCDVNDHPRVMAQTARLLRTLRGENETQRKDVHVVLHDGVSV
VKGQSLFSVTYGQNQMALGALRLAGSFFALHTHHTFARRLACGDVKHHDDNNRSTSKFIT
KAMHSSHFNTLLEACQLYFYDVHVHKPSECKPESLETYIVARGFSARRWLLEEKRAKDLH
PHLSLPPRREDVVSGRQMMWRCWGCREVRLGTSPCPLCSAGH*
>TCRU_944 hypothetical protein, conserved
MEGCPGGKGRRAHRVTGACRCGEDSVPGVDPDIGPRVAALRTGAMRLLPTLRQACTRPAD
HKEPSVSVVPSPPHCGDRPASASAGTSSRREHRDSLSTSERRIDVLLPNLRLSALVRPQY
EGTCRTCSAWRYNSLRGPPMRMPQWRHRLLNRCLARRSPPKLPSMPAGETLRKHKRGYSR
WTRLFRHRLITDATGC*
>TCRU_945 hypothetical protein, conserved
MSVHVSCSRTDGRLGWRLPNETGWRKCANKPAEGGGGEDECALVCIAAGYVYKDAENPRL
CAPGGRPSVVWTGIGMFLSENVKNCSSAAAPTAAVVGQAQSGVGSPPGEAAEAPQKKSPE
HTDEGGSYPSLSENFAVSLLTSTDTNEHVASAPEASSQTPSSPSGGTATGMRDPGGADSS
DAATVWVRTSLLLVTALICAAVR*
>TCRU_946 hypothetical protein, conserved
MFKGKTTTRSSTWEPRKEHQVTLMLQGNKASVHTDGRSLGEEEVPLAGEKPPEVLRFFFG
ACGGHESRLTVTNVFLCSRPLNTTAMRALRERVPAPTREAAPTPPEKIRVVDSVREHVAG
NITEEK*
>TCRU_947 hypothetical protein, conserved
MDAEYQGGQLSKLFSSDVVAGCIDSAWNWSTLVGEVKKDTWRAHTVPGAAEGEEGLGVVL
RPTTITKGHGVFHPAGGSDVSYEGVRWREGGLEPKLVVGDVTNPTGGRQSGSNGATPNHS
RN*
>TCRU_948 N-terminal conserved domain of Nudc./CS domain containing protein, putative
MSADERFDTMLLAIAQQHDGIDSILDTFFSFLGRKTDFFTQPQKARESVQRALERHLVRA
EERLQKKQTEEALRKQKDAASRVEVLEDEEEAASKAAAKAAAEAAKRKAEMEKAANAVAN
AKEDEDGMKPKGLPPTAGNGFDYEHYMFSQTLQEVEVRVPLPIANAKGRDLDIVLQQRHM
RVGMKGKTPIVNGDLYAAVKAEECMWTIEDGSTVVITLTKMNQMEWWKTVIAGDAEIDLQ
KVVPENSKLDDLDSETRQTVEKMMYDQRQKAMGLPTSEEQQKRDMLAKFMAAHPEMDFSQ
AKIC*
>TCRU_949 F-box domain containing protein, putative
MPHLFSDSSVSFLKRFQFIQRGRHGEVARQGTIPTDTLSLMDVLLHTNSTFTLFFHLKHA
LDAAQRSENANDGTSSARRTDDLANASHHLQEDMTEFDGGLAEEILGPILLSFLNFYLDR
SIDMMSIRRTLCTLFETTPYLREMHDDEDKNQPLSSNIALLLLNLSRALLHCQAKGEMSL
RKKNRVVQQHALPLLPWLRSCARNATQKPTWYWWASQNSRTLSILTNGAFVYRSTTCEAA
ADEDAILVSYAAQPAPRKRGRGVDDDDVMEAAVEKNRRISSTSQTHLNGGKFSLGVVPIP
YAVQGIILEYLSPRALSTACLVCKAWWSLIDQSLTLRFYLFASQAVTRVFIQFFEDDWGK
AWVRLNELTTFRRLSLQRMYICLVQNHLHRQRASAASIGDFVLRLYNEFGKKSPQWEMLE
GLWNAMEVEMKIMPFLALRDVTFSSPLQNGTTPCCCNDISRAAALVQMKQVLRLLQSSDY
PFFSLFNELLRRWSVLLSAVHSPPAAMS*

>TCRU_950 pyridine nucleotide-disulphide oxidoreductase, putative
MLWRLFLGSVVGGVVSACRSNIRTTLAATAPTLELILDPSKDVVPVRCVIVGGGYTGSKL
AYMFDSMFDVTLIDEKNYFELTNDIIPIVANPWSELNEEACRRLFILHRYYLKRSNVLTG
TVDGVDEEAVTLRDGRRVSYDLLFIAPGERKPFPFATKQRTIAGRVQELRHFNQFLGTCK
KVAVLGGGPVGTSLAMDLARNRPEMQVHLYHSKSELIPALPTTSRKYAVNALQKCKNLSL
HLCTRVTDVDGCDSNGKRIDLNSSSLLTRLLGFGRPPAEPARFMVRYDKLHFEPAPQQSI
VSQAYFGRREPHLTGDKVESSGEEEFDYVFSTIGDVPRCIQAGKGKCNILQEHEAPDGHY
RVSTLMQLYKRPNIWAPGRCNNLPWVRSYGSSDVQVRTIFRGLNSVVYNPTERFLSSRDG
VQPQRMAIPRLLVRLGNNDAVGATPWSGGMVGLSAFHEFMQDRNHLVKEFQQPIFYKQQD
PAKVKQRISNWASHETTDIVDFSHL*
>TCRU_951 mRNA capping enzyme, beta chain, putative
MLHRIPAYRDPLLQVLSDALKRALDVYRREMSAGMSIEFECRLGTFSASKHTYAHPFPTH
TLTPALLDGQSTTPFFFFAGVAQPSFIAAHETLKTIGASMPPRSSTVLSVRTVGKDRLEY
AMNEACNSGHLLAIVEKERIFSHDVRCPGWGADFRVSVSREKTIDRETWNQAKWESFTPN
ISRLRQRKSVRIDPFLSVDMAMVRSFTDHSRFVRPWSPELQVPFISAPHISFDVELEVNM
PALHRVVKQTRLVESILGRTALDLLTVLQFLAAKRE*
>TCRU_952 hypothetical protein, conserved
MCDFDYEKLFSLRQDDDLELLRITEDLQRQMMVIDDDDDEDMNKKSLAAANSPILGSAVG
NATDDNNKSSANELRSGQGAKGLSNATITPPRNPPRTSRVMEPIEGQSFSSSHSRGHPQL
TSSGPQTPTQTSNENSNPRLPVLTVAPSPPSELSVLKMAPALVQDAIPKPLPPYSHQEEF
PPPYGWVGGVPAKQPPGVTSMPQNHQTVPAQNLRPGPTAHSIPQLPYATYGSNAQILPPN
GFPAGLQSRPMVLQPQSQPQPQQQMQPQLQLQPQLQPQPQPQPQPQQQPQLQLQTQPVAN
GAMVQPLNVNQQPPVLPQSLSHSLESSVQQDQHQSQSQTQHVTGTLPPGYAQVLVPVRTA
DGTTGYQLMMLSTQHISQGASSFSPTLVQAQMEQQQLQQQPRPHPQPQPQQSQQAQQQQQ
QQQQQQQQQQQQLQAMMQYMWQSPSQAQSQHQYVSQQPQPQPQPQQQQQQQQPGSIPQQF
LTPQYANIASAPQRYALVSANGQQQYLMALTLNPLQQVHQQQQNQQQQHLQQQAQQQAQQ
QQIQQQSHQFQQQLHSARPVYLTSQQPQQVFTTSMISSGMPPGYRFN*
>TCRU_953 ubiquitin-conjugating enzyme E2, putative
MSTALKRLSKELREASLNPDSDMDLHLLHAGSLFTWEAVLRGPPDTPFEGGEYRLLLRIP
QDYPMLPPTANFVTKVFHPNVNFDTGAVCLDILKSRWSPAWTLNSVCRAILNLLAEPEPD
SPYNCDAGNLLRAGDMEGYASLVRMYAIMDAGAPPFPELA*
>TCRU_954 Protein of unknown function (DUF3608), putative
MKAVGLRWRPRNSAKQSAKGQNDTPVTVVFRAHTDTVAKVPFLYSPKNYNRPMPTEEEGF
TEAIIELCVPNGMNIIGSSTAAGAGGGECSGNKNESKIGSTSPGCWQRHSGMGGCALSPG
AAAISPSLMEMGHRGAASPASEGAGLMGAARPAIFAGDAVLNSDALRRRRQQQQEQQQK
HTDPSTSSAGVSFFKYDSWVCRAYCFGEKDAIYANGAISVHQSLANKSQFINNIAKGKAL
ARVVPTYDEVRERFGLSHLELSFENQFVTRADMFLISEALRDRILYEGEEVTLCGFTMKV
NEMLRALQPVAAAESAAGGALQQGDGVKTNNTAALDVDGEHRGAAENSWTSLKTLGSQRV
PVQPHFSHPSLTLPSPQHQQDGKEESRYRRVQSGLVTKETLVNFRSLSPVHYILIEISKE
MWNPTVDGRITLCWVMKNFLREYLMQQVHKHKASPLIHILMVGRLYPKYAINGNVDVLHV
MKIPHDYRSTSLVEEVELQCEEFLRRVLQEVKKTFKKEKAPVSTEAAGETTCTPGEEVPF
VSQADVADTLTEQSLFVHAKNTCTVETINLVLEECIKGRWDTNTGYIISVVSAGKGLYQV
TNELSQTTCTRLFSIGIEKINIICMGRPPLHITPLFEYFSEDETLRSQYHLHVGQRSQRF
YERPEWARCFFYHPTVDASSCGLLAFDLLTREEWMQRHRAVPGSKSQFFSGLGLPMAPVG
EVLLPVMETRRKEQALSNSVRVADADSLVSPMPSSALSAVPEDSGTQFYPTLFRHSFEVR
EKAQPPLRLTGTRNKHEGITRGERFGMPGRTSGTTQPDGDAKSSPTPGHSRPLVFASWYC
HRGCAFSSQQTFSREYLYDFIIHQSHEHSGDGHTRNVAMEPEANGIVSCGLNILDSDLQS
GYVFLRLRIDSSALPEACWSPKILTDVDMWAVLMGAFSGEDNRLSEEEKKHFVSRFRALD
IFCRTFSAILEDGLTLILASHSSKLTFSTPEEIVFTFRQPLIVASSEVTPFSTTIAHLQI
RNSILFAIKPISPYRPTTESEIASSASRNITADVILRRRWQFAYPELPSSGAQKSPWATL
CHCKILPIYGTKSPYQRDCFFGVPTHQYAVSIRDNMQLLEYVLQRLQQQYQIVVVGSSPA
GIQWPREEPTRNARVELSIGHQIHELKIGEAGQSISVTRILHGGMYSNASVTHIISHSYL
LLNYLKPGFDLRTVHLETQIGEKLAFQWESLDSYLRDRHRANIFIPRGPDLSLREGKLCV
ALLPEAFEAPSVSYAEFSAFINHRFSVHFKMTDAAESEEEKVGMTTNFSETLNGSDGITA

RTVTLIHDNPLTLEPYCLMDHKTGRTLCLEAPTRNRMLSMEVSIPATYSPNCHCLLEISW
LVCITPIIADWIRSIFAGAARFRLRAVPLPCFSPSTKSGFFAVSYTVRARKEEEEPLFRS
RLLSLLTNSTYRYFPDVTVMSRICRLLHFSGLCYVTLHWGGTALARWFENSMLRTGQPEH
QQLLREFTEAVQIVRDEIEVS*
>TCRU_955 NAD+ synthase, putative
MSPVIALHEDLQKRLEEYRSTRGFSPVKWIEQKCAALNEYMASHGLKVCVTSVSGGIDSA
VVLALCSRAMRMPNSPIVRNVGICQPICSSAWALARGRENIQSCGALEVIVDQTELYKQL
SRIVETAVGIDGQNFARGQLRSYMRTPVAYYVAQLYSQEGNAAIVMGTGNMDEDGYLAYF
CKAGDGVVDVQLISDLHKSEVFRVARELGVPANTLQASPSADLWEGQTDEEELGFPYDFV
ELYTGWCLQLSDEERQSFLHSLSSDARQQYEKYKDACENTHRRNAHKLRGPINL*
>TCRU_956 Paraflagellar Rod Proteome Component 9, putative
MTDGTAAAAGALSELFIDEDKVHRSLIPDDVVIRDYLSCINDDTWVRHAELVQLSDLKET
PHEELVKKEVLRTFIEERRRSGVEMPRDLIMEGILWQERFMEEYKREQRSQDKKEASNYK
QSLMSKFRTKRTGVSDVTDSASRGTRAIEYKESQASAPEYPTENTDARDTYHELPNGNAS
AAQQDPNNDVPKKEEEEKIEPAAANEFDHEIDIAAEPCPVEDHTEPEQQADEALQQGHSG
ESKNEYPTEDEFPAGQQSAVAEPAGQAAVPGDVTQAAAEYPSEEGISAEKPSHEGEATAP
DTEAPDGTEYPVEDAKTPPNEPAADLADQPATDANADTDAEEM*
>TCRU_957 coatomer subunit epsilon
MADPFYDARNALAIGNYHQALAEASGAKTTLRKPDDLAIFNTEREALLSLAQIGLGQGES
VVTQLASASHPTLLAVKNWAQFSLAIKNKSPDGALNQTAASALTQLTEAAEEVDPARIQG
AVLAASALLASGDNAGAISLAKRWIGELPTPEGASNIRQQMELRAVVVEALLRMRRSEMA
RNEVKGMEQLDDESVLTVLCSGIVSLHEGIKSRDAYERAIQRFKELSMRCGQSVLVHNLI
ALAQMELGDFTSAERSLLDALAMRSNDVDTTANLAVVSSYLGKAADSTNRYTQQAVAIAG
TWSHQYAAMNARLEEAALQFSKTA*
>TCRU_958 Mitochondrial carrier protein, putative
MTDFLAGWMGGIAVLLVGHPFDTIKVWQQSRHAVDVALSIPAPRGSSFTAAMELYRSNGV
RAFYKGLCAPMCAVGFANSALFGTYGVIVNFFIRNESSQYYYDDDHHVTVMYTSRQINGN
HASGNTYQKQDLSEHFRTGGGLSAFENFIASTGGGVAHATIMNPFELVKIRLQTEHLFPH
RRYLGTVHCVRRLYENGGIKYFFKGFSATLIRDIPGAGVYLFSYSSLIQLLGSEKNYSIP
HILIAGGCAGLAQWIVIFPLDTIKTRMQVAKQGEMLGWTRCARQLYKIHGFTGLYRGMAP
ALVRAFAANGAAFAGVEATLQFFRTTGLEDIEVCDS*
>TCRU_959 hypothetical protein, conserved
MGVGSHKRSQGGAAFTQTSHAPPLPALLRPQFQVFGEDNLNAIDRRLRRGEAERVEEKKI
QERCARVRERRNAELQRNIEMIQKRTARHASAAGLVEERRLRAEFRQLCRW*
>TCRU_960 hypothetical protein, conserved
MSEHKNPARVDQYDSGTYTRKGQTTRVVVREKPFIKSRSQRFFEFLRPSTRTGFNVVQGF
ELAKVMVCIVFPIFMLLYWKRMQKRLPDQWESQFAGLQHRKLKEDVLQEQETDYFTIIET
FKERREKALQKKQKEAREANQQFRQ*
>TCRU_961 Ring finger domain containing protein, putative
MEDNNGPKRARSEAMEDTSSRSTTPLQVDQSTTPEATPARDPLLEEQMDQLGVDEDFKAS
LRCMSPNTRRDVMNDIIRGQQQERTTMATPFGLFSILGETSILPLFSGVDVRLSVEEDSE
DEQRGSDGSRAHSSSRRMSPSSGADDRSENSRMETPALREQVLRSQMLYLLHIMRGSHIA
NTISEAIAQRSLGITQDIDDMSYEQLLELQERIGFVSKGITREQMQQCMQDVPWPKEGSC
VVCQSEWQDGTEGNERSVELKVCHHIFHQRCIEQWLGSNKTCPVCKKDVV*
>TCRU_962 Vesicle transport v-SNARE protein N-terminus, putative
MSSGLFALYEDDFNESSRQVRAAAAALQESLKQDSSTYEPPPTTGPQSRGHHCEVMQQGL
AHMRELVTSMLYESNDVESGELRNEVRRRVEDYKQMVTVLEGELFRLRQDSKNADRMDLI
SGGNDTLDEANQEARAFMLENTQRLRQGTTTLERAERALHGASDLGTSTLNTLRTQTETV
RHFHATVHDVDSQVMESRRLVNQMQRTAMKHKLWLIGIIVGLFVCIILGIYVRR*
>TCRU_963 Vesicle transport v-SNARE protein N-terminus/Snare region anchored in the vesicle membrane C-terminus/Sec20, putative
MSTLFQSYEEEYRDVVKSVREGLDGLRATLNREADGYNAPPATGPASRLQRGAQLVKLTT
QLKELLNNMEYECNDVPVVHRPALRERLAECRRGARELEEEIAKTRGECATADRLDLMGA
PEKKGSAPVDARGLGLDDETARHRLQMMHNTEKVKQASGTLNRAERLLNETEETGAAVMK

NLRTQTETIQRINATTTGVNEEISEVSKILTKMHRTMVKHKSMLIGIILVLVVLIFVALY
VSFLKHKKETPVQPSQTPTDAPELPKWGPDSGGGLPVD*
>TCRU_964 BNR repeat-like domain/Concanavalin A-like lectin/
glucanases superfamily/Trans-sialidase of Trypanosoma hydrophobic C-
terminal, putative
MSRRVFTSAVLLLLVVMMCCSGGASNAVTSNSGNAQLPQGVDLFVPQMTLVVPKGGTSQE
TIRNSFISPSLVSAGGVIAAFAEGQVYTVNGAGQNEKTSSDVVAEYIDSSWDWSALVGKV
KKEEWKASTVLGTTNGTDNRADFFYHPTTTTKGNKVFLLAGSLYEYRQTVGKQTYSGFKS
RIELVVGDVSEPTSSEPTERIKWGEIRSLLNESTITAHKGKLTWFLAAGGSGVLMEDGTL
VFPLMAESGNGGYYSMIIYSKDNGSTWALSTGVSPANCTDPRITEWDGSLLMIVNCNYSR
RVYESRDMGKTWTEAIGTLPGVWVNSRLYFWDLSLRVEALITATIEERKVMLYTQRGYAL
GENANTLYLWVTDNNRSFYVGPVATDAAGNWEFASSLMYSDGNLHLLQQRGSSEHSVISL
SRLTEELSAIESVLSTWAQKDIFFSSFSIPTAGLVAALSDAASDDTWNDEYLCVNATVTN
AKKVKNGFQLTGHSSGVLWSVNTRGDGIRHVSLSHSFTLVASVTIEEAPSEKTPLLDAML
GDTNSNHTMGLSYTADNKWETMFGGKVTTESRPWEPKKEHQVALMLQSQKVSVYIDGQSL
GEEELPLKGESTLELVRFCFGACGEDAGQKTKVTVTNVFLYNRPLNSTEMRAIKDRIPVP
TRGPETQVEGMATTEHLAAVHDPREVPAALGRTTVERATATEHAAAVSFTSAGSGLLPLL
FLLGLWGFVAA*
>TCRU_965 root hair defective 3 GTP-binding protein (RHD3), putative
MVAFAGGARTEDAHLIDDEGQLLPVNGIEEYLFTALGASRGGDALHRVGITYHVVGVLGG
QSSGKSTLLNCLFGTKFQTMDETKRRGQTTKGAFISRANFEVLCGDDGEMDAGASALMES
YAGKSLPLFVVDFEGTDGFERGEDQSFERQLSLFALSVADVLLINMWAVDVGRFNAANMS
LLRTIFEVNLQLFSHDSYTKEEKPTLLVVLRDFTEVETRTHFETVRKSFDKIWDNIVKPE
AFKNSTIDTLFDLRYHVLPHFKLQRAAFDKETAKFRQWFYLSTCDEYLFHTRGMFRGVPL
DGIPSYLSSCWEMIRKSKDLDIPTQREMLARHRCLEVKKQILQLFTEFCSNYTERLQRGE
LVTHLTSLLEHDVDDKLRDFHQQTRLYRIDIVRKTEAELEEELLKVELKLVGEYAKFISS
KVLEELETAVSGTVDGALRWLLHQAQSIPFLSAEAGDAGGGTEYMSHLKSVERASSNDVY
ITDNEACNVLVHSFWKRLCRALQAEIELLYCDYSQQHQRQRESQTLNLYDRYASLVAEDP
ALQEAIAHFVSDAVFQKVSRRFASMAENAAETIHQAFEGVLNRNQDGTVRFFHTTKALQR
IEPQARQAGLVLLGCLLYYRVKVVADRVVYKLEDTDGLSRAAVHLLGERRKLIVRENSEE
QKFFLHYATISEAPRYPIGAPVAETDSADTSDNVVDRDCVLLSQQAVQRAFDLYTQKCEF
TMQLQLRSIESEKQNLPAWVLPVLLLLGWNEIWYVLSSPVLLVVVVIIAAVFLRGFLLTQ
WAIFEETGPTCVVVGVRVVVQQIRNIYKALVPMMPDDVKSNVARHRDPGSFSDVTASAVG
TSWPYAAAEPTVLPPSTTSATLTRRLKKEEEVPTQKE*
>TCRU_966 hypothetical protein, conserved
MSVKVPYGSVEYQSHHHCQCDFCDSRRKVGAKKFALSSAIAKQKAQQNQHNCCCPGHDAS
AKQPLSGNGAPSFELDDPEHHVNQTELNAKMGRTGLPDGNQYTTAVGGTRGLRGAGESGT
AKAKKREEMEEEKMEELDRLERLLILEHKARAKATADAEILSLRQSTGRGPCPKPLEAVP
PLPVEDGMDQTMQGTGAYQAGTARETHENAHESFVASPPHGETSQLPQQTVRDGHELARE
SMNQQQNNAAFGRSSQIGGVGGTTTRQVNSLQADASAAKGAGNSTVKRPWDYFGPRPEGA
GVVWRKIQPSIPEDTTTGVGGLGNVDNRHLWKKTLLKGSSAKENTANANSKKTAVAAPDP
SIMSPGALAALSLQKQQENDHQARDAVGSPVPTVCNADDTPI*
>TCRU_967 hypothetical protein, conserved
MPIKAKPIDPGRARLAALGQWNRQRRTTSTGPNSAKRRGSGSWANSTGSSTSTSSSEGSR
TGRRGRRRLCNQNGKREGNGNGGTPAFLTSSRTVDSDSGTSNSDPELYRAARERTERFLY
PNGRSGINVSSSNHGAVMEARGEAASNAENISLDASLPMAPVAPVFSGLQSQNQKKESDN
KGSDANLESSRKFSPPSPAVVPLVVQEALRGLQGTKDSASEREFARLMDLFAADVQKERE
KEKELQQEKELEVDTMDTISSMMAKNQNQKPAQGPWDETYKLYTSPPHTFDELQERALLQ
LYAANEENKDSGYFIATDHMAEAFTLATLEATRNEFPGESDAASGASGALNDNNEKFRPI
GAPPSLMEIVAIERARRNARAAFQKRREGEEGVTIDRNTEALLSMDTEVLAATAEENLNR
FLLAVRCIENAVRNRRVTDALGRFLFAHHKPFLRTTRGAGSSADAIREFPHEAFAVYERY
GHYVGKFITELLNRNVPKFNMEEFVLSLFDVNMNGEENNNASDAEDVSMDVLSYPAWRLL
QSISSFNEFCTFMDDFIAEEYGVEKVVAHENESTNGDAGVKASRYDGDGVVVVAGARGVR
ALLTKSKAKLPSARASSTCDASQQMRVPNEGAHESNDDFLFLPSEGGATETPLSSPPRES

QAHGDCSAAEFSTRTDSAPTVLSNSHPALSGSSRPPFGNSLTPPPAASLQGPRPSKNSLS
TANSIPQLPHGSRRIEAVRGRNLPPLSAAGGKEAETMFFTAPLKLSLGSEANVVPPGGFK
AEPVTTERTVRGTDSSGKIDKVSGRQAPSSSIGISYGHKKKSKDSRSRSGARTGAETTTT
TTKPRGKKLKSPQDA*
>TCRU_968 hypothetical protein
MSAAEKPSSPSSPESSAGKDVAASAWSRFVAPIDSIFRFPKKMWACTADGLSRLPYVFSL
WQQFGVGDVLLLSGPRQALFFAFTAARAVSNYVQRFLTEELLPFYCDVSAWLVLRPGETA
HTVGIKSVEGLRWVYIYFGFLSRNTTSPSRGRIICTGITAVTTAPPSSSSLTTSAVNSAA
PTNRSRRWKIKSGIPNWLRASFTFLFSPKRKASASPPPPSRSVEGGSGLCGMSRGAAPPA
KGSRVSQEPKPSAKEASQRETMPNTQRGLLEKAFLFLKWLTCGFSFVVSKPSGKSFGQKI
EMADKKSWIPKKAGVEIHSSSLEPLDLPGRSSFFSFKASEHAAEATLKSSKEKSISLTRA
ASPDDNNDKAIVHTVTPRIVTVHHFGFAVYVTGKPPHKFEEKIEGEFSLQVVQGTFIRDV
AHLFSSAFSIPIDNVNVRRHGSLYHVGFDVLQLPGGNFWKKREIDTHENEELPKSTSETK
SQSEHEEVLESEVEQLREYHAQRGMRVSGDFAPDLEHIIVSGENFQDHRAAFCEKTGSAG
SSVVACVVSLNSFLSAFYVHRHFCLKLVVLDLKGRCIFATGDKLVVFDCRWPIVSMRSIS
EPAEADGISEEYLKLNEVHDTIVMDDSPGLSERKLLAAAHVLSSDQHCAEIRTVVPSSAH
VMNWMDTSVVISGIVCTAVSIGFCVLAVQRSLLHRRGDGEWLMKRWQLARSELLLKALNE
RFSVLLEALPAHFGVPREEMFFLRRVVQAYFISCESHYIDLVRSIGMREQMVLQRREAAA
KYIQHVFDLKWLFQLYEVMLLEEQHRLRLCVEEGRNANRLLEQMMAVSNVQLEYFMGREG
ARAAAAVRVTCLADTEIVVASSQSYDKSDNTQDTHVASSSCSFSFAETPNERIHARGDAN
GLEELLFEMLLSENREKHALRLELCLLRRQLAEYTEMNFDRRSSPCNVTECNGND*
>TCRU_969 intraflagellar transport protein 140
MSLYFVNGVVSQGVVDAELVFAHPILPIVASVWKGPARLLVTDAEGEFIADSGAQLALKD
AGSITSMAWHPTHSALAIGCSSGRLMMWSLPPVKKTAISMIPLEPPVDTEKALPQHDEGS
VTACVWSGGGTYLLTASKKLRVVMWMVESTTVNTGENDKDGATTMMRFRLLPLWSVPTEV
AILQALHVTSSIALSLQQSAQRLQRYNTEGTSSPTSASHRGIDDEGDECAFFLASTGDKH
IFALTEDRKLFPLCSLDEPLMAVLYDSAERQLVALSAANVISIYHVSEEFKVKMVLRRKL
STPNVKTERFTLTMRWATPGVLAFGCGDNRVRFFDIRDDRVYVLSHPALAAAHITHIATL
LKKGLLAMATAEGPLAVFQRNAEAYFNGNSATTAAVNSGAAKMDNRPMGGNISKVENDPA
EQWDLLTVVDMEGCVDSLSFTTASHIVVALATGKMQVLRETVRKRAWDGVVAATQISMEM
VVVESVTGCQCLLKSGSKIRGMAAAFPIIGLWNGHQIDLYTVSEAKSTATLTNFIPTTSP
AFAVHPEGLFYVKDANRVLFTNFQLVTIGQIAFTETEGTPIVIDVMGDVVVTISSTNAMR
IACVSGRELRQLGPPRQLTLPDDSIVVTDAKVNAQGRRVALMTRRITDDLPDSRIWVYDC
DRDLLNFYDFAARGEIPDAVYWNTPEPNSSTIGELGYLLLACETHQIKNNTVPHSTDSTD
EGGECLTRNDSSKGPKLHSESSVIATSRATEVLGMESMPDLENFAEKKQRAEEESYAGGF
SGVNPHANRTHSVVTLFSTNKGLIVHNAVLLKKYQICLVGLTVPDFLLASVRINGNPSNP
EDYMIEQKRLRDFEGLKTEKDVAVLEALMKFSYYSTIGNMDEAYRCVKTIKNSTVWQSLA
RMCISSGRLDVAEVCLAQMQDGVAASALREARTKYPEEKDVHLATLACGLGLVKECEDLL
RKAKRFDLITDLLLACGKFEQAQRHAKQYDRIHIHPVAYKYAQFMESFSNFDSSIMWYCN
AGCLSTDVPRVFFQSNCLNELRDLIMSPSEKAANDASPKTGNSDNAAGEKQSNGNGNGNN
GPPGTASSVEDGKLDENSAGDGSVAKQQPNFRHLLAYNRDLLIWWAQHSERKCQFAEAIK
FYMLAKDFFNTVRLLYAENPPRIEEAIELVDKEIEKACMRANNAAGVAIARRESPTTETE
PVGAAFFVGLHYEHSNDVPNALKYYKHAGAWRAASKLAKAQRRYVDLLALSLASDDTQLM
LDSAAFLERNSVFDKAVELYHRIGDVQKAIDVCIKGGLYDTMHRISSTLDAQSDPEVFMQ
MAEHFVGSGHYNKAAEMYIFAKVFPRALELCTSHGVTLTDEMAESMSSDANCSNLSGEER
NALLRQIAGIAKDQGNWNLACKKYTEIGERLKAMKMLMRGGDVKKVIFFASHSRNTEIYT
LAGNFLQSQNWHTDSNIYKHIVLFYTKAKAFSNLISFIDAFAQLQIDENRNYYEAWCALN
ECVQVLERNRDAVYGGSSIMAKEEGLRTRRDIVQQVVMALKLLVDSASDDKKAKELIAVC
SDLIKRSRPNHQDSANVLAAIRIGDVFALLVRYYYENARSAKDAMRVMESMLKHAVQPRF
FVERDLLEAVCAANGRNVAEFLVEDAAAASAKGKGGNHESIEEEVAGL*
>TCRU_970 hypothetical protein, conserved
MRRRRPRSTSTSTRSGGPGGCRRLGGVCRQEKAEKRQSPLASSLAERPANTNRLSQHDCRNH
ERERRSQPALWNDLSHPSASCPSLRQLKEQPVFCLPHYRWSPLHTSYAHDVVREHEAVIR
WYEEKLRPCDVKIDDEFAVAWLATARSRRDALRNFVQEHRSLYGSLDAEKLQEMEVCLLR

LSLCVKRAEQNWNLGSFISSLGTPLGSSRMAPQQAVAREKHISFARPLSHFFSSPPLTEN
EDPLCGLACPFSAEGNTADGDGNSTDAPYTGRTSGVYYGNISSSPTVVNAVSSLEYIHED
EANTSSWQKEKHGQWLSGVIATENGAENVKTAVSFPYGSLPSFFFASGPGGWRRRELQGL
WIFLLYIFLLTTCCLLFFLAACWRRS*
>TCRU_971 hypothetical protein, conserved
MEQFPSADEIMRNPTRRRGFVEKGKFTKILPRGVFYARLLFLFLAFSLPFAGSTAATPQC
AWIFSVDEGETSTSVLQREWEPDACVGKYFTLKALPPILPGLVIPSVYHLVVQFTPTVTG
LTKGDVRYVSQELSVMCDVFTPVCEKASVYYVVHGTAKGEVTNDTTTTTTTTTTTTTTTS
KAPAPVAPSCPPGGVLPVVKTEVMVPTRIEPMSYVGNCAAPRRIVIRNVSAEQTKYFSVE
NGTTLWFDRRSENQKEGVFSVNFTVYCNEVASCFNVKATFFVVNPPSPTNCSHEYGFHLN
FLQSVLFTITPHAEDNCPAGQKAVFTPISLPKYNAEKFRLDRTTGQFNYTAPNGPNVTSD
AFVFVIGCTTDAGEAGRPLCTGHARILITSAPSPPPPPSPKEEVICPGNFVFSYGIDGEK
SDVPGTYNLFLESSRGENASFCPSNNYTNAELRAPTRNGTINNFNSTTGSFQYEPFGKSI
DHFEFALTCETENVCHGTATMQPCNKSAPTPEYPSTVYEIGKIVCKGTCDVSAWRSSPRY
PGLFDVTDGPQGNRSQLTPRHDAKSPDDMDFEVSVEGNSLLLRAYTIIGNMAARFVTFIP
VENPKSAFLTSAQVPKGAHPSFNATCLDQQAQSGVASSIWTWDDDSEPDGGTLLHGHLNT
VSGEYKSGENYFQKFGGKHQDCDVYRLDPCKYAPMLTPASVMDASDPKNKDAVSAIDWKL
YVNNCDATWVGNVSLNRLRMLRNSATNEPLFKVVNKSLLVGTIYTQVVKPASWEPPYNGI
LSMSRVYTVRISIDYSIGTSVEVSHVNYSSRTQKGAKPETKKDKTRRIKSGTKSSMDFSV
QSDGFAQDSFNVSVDVQFATGEDPRTGERLYGYSFLIYPYQLNYSQIIYSVPTLVHVNAT
KLRVAALELLNASWRTPNPQDCAQCTGKAVACEGTGSGLDLDCDSIIRFEPVGNVSLFPQ
FVLNNKGGGQFVNISLVARVIGSGTAQSATGYPEGSFAIRLKFASSETIIINVRQVTYIT
SVNSIAHRVLCRSSAYWPVPDVFGGSVPAKPFNSTQLLNDILSEPLDQKGKAKGKPYYGP
PKSLHTDAFCFSHVEPHRPGRVVLNSIATTVNEDGDTSGSSTVKLSLCAASNEQLYGVTD
WVYVTLHDKARPLDSSTIAKMSLEYLVLSVNAEDVLGVFQSEEMNSRNGQSKLYFLLDGA
SSPDIMSGGELGEEGERRRWMGETPVGNSTVYLRWEKYAAFLHYRRIYLQPCQSDNQTKE
CVTEPFHFAFIPGSLLHRGSKVRVTFYLHAGVRYTSTDDVSDSWVEEHIAIIPVSAQISS
ASRFDRDVYTPRVVPQRHIRHSNKMARTLFLVFALAIVAVLAASFCIETHYRRALHFAKR
RLRQQSANTKENAPRERPLGQAFAPPQEQNGTTHKNDLGVGGGMSGGHFENRLAPSGILT
RYGVVQLEPTRVEGSEEDSGCAVAPASTPV*
>TCRU_972 Serine/threonine-protein kinase Nek1-related, putative
MEKYTKIRVLGKGSFGSAILIKRRSDNALLVIKEVFLGKMSKKEREEARHECRLLQQLSH
PNIVRYVEHFENRNNLYIVMEYCDGGDLHTKVKLGPMKESTILYYYSQVCLAMEYLHSRH
ILHRDIKAMNVFLMKNGSVKLGDFGISTVLRNTMGMANTVCGTPYYFSPEICRNKPYNNK
SDVWALGVLLYELATGKHPFDGNSMQQLMQRIVKGSYAPLPSHFSPDFRKMVDWCLQKDP
SRRPSIRQTLSLPIIRHSLEQLEENLMLATQCKVRLKDIIDFEVGGENNNANNNNAAWEQ
VKQGRDPQRYPSPSSKPLEMSPGKAAALAMANQQNEHQQAPQRSPQRLSPGAEAAVEALA
QRPGGNFALLGPKPGAAPLAVDHNRIENYRRYLLQHYQKEANDNKDVAPAPGGNGNGVAG
GVPPAYAPQPVSALPFLRQQPQQQLQPQQRNLAPAAVEHRNQNPNDSPRQKVAGNGAGAE
GFKERMQRIDAIMQRYAQNVDPKSRETIQAYMKRKQEEYLQRQKLKQERIQQREEMRRKE
IAKVIEHQNIIARGDLLHQQRQQQQQQQQKVAHQNPLAVQVPFGAANCSPYSPRKQQQQ
QHAVFGRDPRFVHRPVKEMESPTPLQRVEPIRNNNNNANRRRHEAAVPGKKKVFAANSIP
VVSSPRSRQHPSFDPSPREDEARSPQPAAGAAGVKGGDKNVNMNRRLSPPQLVGGGAGLV
PSTKGGRVADTGAPRPYPSAARGPLPMFQQARPQGMLRLVSAPVLPSPVAGSHANREDAE
ENDEINKGKKAAAPTRCPANRLVRRSSVPLHSHIDDPNATNAKPFSSPVVGNKQMIRPTA
AAGFMPQGVSPPQRHLQGFLSPVNSPRKGLMVLGSKPSLISKEDLPAGVEDRRQYEQRRR
QLLQNADMKLFQEERPERADGALLLQAPNTGLEAPANNNRRKSSNSQLVDAKAPLRLDLA
SMLAKENRVDEKPRKNEVPPPIALAPLQIRQMQPSFPQCSSPNFSSSPFKANGALPPNTA
RAAGAKQCVPQSTGAAVAQLQLGIGQMPAVLGVKMPPQSREPPVAGAVRRESFSHNNSQG
EGLPILLDIGYGFSSPREDGTKQSQVPGTGLKAALPSITDLGAEQPDVLQQWRNVAENNL
GYKAAHRIAFTQMQTAATQETQVFSGDDDVLIAFQKANGMPLSIPSTLEVLRTFRDGEAD
DLDVPVEKCPGVSLALLKTKQKKKNETNSSVSQPSATPVLGLSLPIDTEDMENQREDGNP
SPKLPSSPATNSNANTPPNLFGLDRRDSVETEERAAMLAIVDGINDARANNDPDGANNSV
TSTDRPNEPSLEGYLAMLDHLRGLLYRRRPSSKMAFPAEKEERMENEQKRDDDDDDEKGK

IIKVPPPFVGKRNSDLEPLRPGGIPCSTAEDLSKSQGLQHTKAWNELCEQQANQILQSLG
KDEGKQPLTPGFKPSVEEYEDNAEEEIDEEDNDDDDDDDDDFEDALPLLPPCCGVVSDTY
RRLLDNAGYLSGVLAGQEPHDQADVNAGDVLLSAEKVEGTGEVGTSEIIDNMTFLAPAAL
EEEEEEDSETEDGFTVTVFPPTTESRTVAAERRDGAVK*
>TCRU_973 hypothetical protein
MIAGEEWRRTVGMRFSEFHIFDGNTPDSLDRAAVQNLNSPLEGLAPSACSASSTSNVWAL
EKSGLLEENPEPSAAPSSRSLSFRVLSRATVFSAGHSAVPSASCAAALASSVAPVDDTMK
PYVDSTARKDGSDAPLLPERDGRPMEPNVSTRRNRKVSESPTASDVYMKRLTTSSGGMSI
KEGKYDSYVEIPITRPPIVIPKRQPPTRGSVKSTVAPDGRRSWVLFAPRRRNDQSLDSVC
QESVPEWDEKVVAPPPGHLPRRVLDSVATGRLSARVVGKTIEEVFNRDNLLAVAGDHDSN
RIPEAKRRRSLEEKERRETQADKRNYKNDDNDDNDGAWSILRRWLLRRDRGVDGKLPYTE
SEAAAALQEGQGKLKTAAETLLSLLQPVCMDSEGGPQWFPLPREWIFPSELTAPSLDLRN
TESSRYMAESFMYMRNQLKSITDCGEDTMIFREPSEVSHSTLLRCDGPRGNFITKSLHEE
VRDDVNEEDYVKAFFNLDPLPIHEADDVIAYTSLLHKNGILAAAPGAYLPHKLPGGAAME
EEEEGEYDGNEKEEATEVGGATGKMRGSVQSTRIQSRQQGRETAGRYGLWCTMEPLMKEL
SGSGTATRQVSRLSANVRGSGKGRRDVQLPRWCIQVCGGALPGRHDVFSPFLENSAGSTS
LDGTDAPTVVPAHFEALFFPQPPDDPLNMFGPHITESTAISASSPICSIPLTPQLLRTTE
VSQYRRVIMRQEKRVLLRHEKEDGFVFGRGDDRDELFLLLTNVAVDISLQYALDLSVYLM
PCRRIFNDSSDSKTSSLAVLVEVPLFHSHNAMAATRCVHGAKLTFTTKSAFVKAYRALLW
ASEVNIIDMHPTPGIPSYLSANNGESVYVTIRCFASFSRKAEKFVYNSYREQCVRRVASV
FVRERDVFGQSPYRLEDFVLEATSSRGKRKPLSSAATPSLTPVTLNSAVAFDEYGVVYRA
LHTPSAQLFDVRVLPRNRTKLLWADAAAAGEYWGSEGDLVSRSMMEAALVAEYLSRMPYQ
NPIAAVLCDAYNCYIVCVPIFSVLSEIKHNAAFPSPLTPQHRQRFGVISLRSFIDKVLRP
PHASFATRWSLTRIVAAQLLLVLVSLHGKGVVLGPRTPEGLFVRWNPAAVSGISLSDNGD
KGVGLTTRDFHLVVPTLGVSSDAWVYERQQSGVLEYIPPRYLFHLAHGTVLGESGASEWT
IGDDYWLYLCLLFDLFSVTGESLVAVAPLPSMMGDREQRPFTSTQPGGKKNIWQLWKRIF
KATHHSLSENVAHHRQTAVALALQRFVHSRVEASILPNVEAWVMMEERRRRSEGSSLSGT
TGTGRDATHSTLRAVSLADAYAVALRSHPPSHLISKGGGDGADGLATVDPAAAAAHFAQE
IQSSRAFMLTIQHFFKILVDTSLQSTASCVDKTAWHQTWALLSHPFFIGIDFAMLFDGKL
RVPHAEFLCNKLRSKGLVSGATNTVSMKSHTDRRGLPSQSNAELQQLLSPLGASVANRFF
VSPFSLAMEEDEEGGGSASNNDDTFAPSSSRSPHSSMHYGTYKAGEIEEIKKVQEEVRRA
LNNFSLADTALPSFLAEERKQSKRYGYSHDLLTVDSVTGAQRKSPGGNKGETSSCISDWR
RLQERAKTAANNSDTYCDENTSDLLLPTVSDPTESRVDHASLMLPKFSTLHAISSADPLR
RIDRDKRQGLQHERISRVVNGYDESDEQRIGLRGSEKLLRVARSRRNSLQGVRRPQSGFP
SGETCFFARGDEVAVATEQMHRGDLGDSAGVEAMVALSGSKEEYWRNMQNLCGVGETPTF
SGKCGTKRPASVSGPLREKKMTTAMDGDFLFRVHGSSLTTVFPLTAVSEPATNKSGMGNE
FTLVPSGDEDEDEDDEYNDKAYARKEEKDEGRRMTVASASWVRNSHSVLTTFETLCRRES
GGVSYSSSTFRSSTRVASAHSLETSEIQKHERQQELQRPDDAVWELVRENDEEENEKETY
VFSWPTL*
>TCRU_974 leucine-rich repeat protein (LRRP), putative
MEVDLSFRSISELRFDRAGASEEEVLVAGHVRLLNVCNNRIERIVGLGALFNRLAQLDLA
HNSLGSRSGPDACVWLDALPSSLCVLNLAHNELCTFMTNEAVESGGTNRTEGCGSAVLAA
ETASDGMKMSLGRLRRACPITVAFFFNRRRFPHLRKLDLSYNALSLTLDESEIVEGMWQE
VVERPVALHAFNQEEKAVWLQCTTSAVTVLQLDGNTHLSALNGVLCGMESLTDLSAASCG
VSDLTAVSAAATFCPKLHSLDMHGNPVVEAFMHAPQTTIATFLELLLLPLILSDSGVDDQ
GCDGNEEEFEKRSALQCLVREVAQQHNEKIEALLLRQERSGCSTLAEVLYAALLQQVIPQ
VAELDGGVDVNAAREGLMNAAREVFEDVIKNIDRSITKRGDAKASRRSMNSQINSSPIGD
QKGHREKQSKPIVSDGSVNTSQNSSQAHMRGSSSVGWRGREVFSASEDSTDTLRRRERSR
SAQSMMKRGATVAAQLRPEVRVTGSASKNTPIIPSRRIASIGAFARDATDAENRASAYLS
KDYESATATTSMNASNGSNSGANCNHSSILQHSSPFLHREGNGDTPTVFRSSTVKAVGTQ
NGGGSFCEESGGGSRFQSAIAALTPILMQRLRREGLPDHSAIASDPSRFSLDDRDTSTFS
TETSNSNTLTVKEKLLQQARFLEEALLASQCRQRGMQEAVSLLKEQLKQDRRLIVDQRKE
AARLRRELDLLVESNKRTAGRVKKRQKEIAYGAAALRRREEAAQRKAAMDYIEREEKSLK
QAERRLREKLALSGVCSPTHLTDSSGIVQATTATSGIEKRPPRKTHADFLRENAAKERMA

AMRRADPLSYSPRKFRPSDYGLNGGDESSNPWTNSTSVNTQAERRQPLQETKRYSRSVDA
GREEKSNYGNTPWRDGESNSSSSSMQSTKSTRRVGQEEKGLQSMYDKGFSPYGAVEKSDG
EHDNKGNKYRHKSLQWLSLQQLFEEAAAIQRRQRSLNQRMNGVVNDMGNDADTRNGELEE
REMAFKEEVHHDGDGDKITFEEHTLSRCMGAIQGESIASTPYATTNGPSWSGINSKTQNN
GLNTDNNDDDDDSVDVVVPLAEGQLNGDFFVKTMTPHSPSSFSPVTKKKFTADTNWNTTE
PQEHYNGEDNSFLPFPGAAGEHFSMDACRIYAEILRQRQEQEKKQESSHHQQHEKEMKTG
GVNLEEKKQVKREELSSASNGPKEPSSTKNGSAAYKEGAFEESSVCGLHMEKEDNPGSAT
RRALFT*
>TCRU_975 hypothetical protein, conserved
MAGDEAPSTPSSTPQKQRVASVRHTCTYQGLQGTLTADAFGCYFTGDLLRAEFAWENVKS
ISVTTKYIKDTPTSVLAIKMTRGKSLSSRFNSSGKHYFYHLMDIVEASKELQALKDAASA
ANKTSGPECPVTSAGANESSNSISGGDGGRTMPLATSTDGSLPNSNQINQTLPVGGNTVP
RPNNPLNDGAKSSGMPCTTSPAASPHDDDLVSSSSTGRARKAANNRDDCEEENNGRSSAS
VGVNMPFIIGVPRHSEQKEGSFIVPQAQRFGGALLVNRRRRGRQRGADTTFWSRCFERIF
SNFSESPLRVAVTLGVVLLGIIGFLLVFRLLLGVVVGDRWYETLPEETVARVTRGLEQLR
QWRSEIHGLSMGKGAHRGGTSATVMLMTMRSPPPVRVTLKDMITVVKELLARYVTVQQEM
IAIRLQRMRRELVDAETIFDGSSRLVDGFSLDSLLRDKEDEENMHIKSDGDAIWDKNAAF
SQKEGYPQSTSRFSTARIKADLNRLVHYLREAITMVKWIFSRRISSDNHNNRIRSSFLRW
RVLKKSSGDSSIYGETGEKQKLKRYTEVLANGQLVTQDVTPEDWYERRNCLRLTRELLHL
SNLIEGVLMEYQDVVMGPLYEAYLHQMFSKFAGMNKMSTSVGAAVTEVTVGDEHGENGLL
SGQNGHILRSVLLRRQTLALLQFDPLIQPGRNRQKEAIRRPQKPRFFSNSENDSSEKVDQ
APSMMTPTREHVEAILREFVTSTSDPFNGYDSISAENFGLMMRNILAEVKYWYEHEEEWQ
TIVLWRLNLSSQNQSATQKSFNTTNEEKFKEQGHNDSFRKAIDFLPLFRGLWCFLEQPVR
LTPEYEGIKEGNASLASDSVPSQPPPNRDEVPEAVNNQRDELEGKEETTEETLAGDDGDN
VVDGELSTVEKSVTLAKSMDVQLSSHEFLDTDVVEKPSGGGETSMKEEMGTAEEEVEEET
DAGIRAVELISQEAQIRWLNAFDLFIRSHEDVLEQRSEDDKEWDWEKSHEQEMYEDNDES
NAAYFRQRFTRLIGWVKLHYGKYLSNDAITSFGTSGTLSEFMRECSAPQKWWLWWTFLWP
PSWFFEGTSVRPMSCWISRLAALDPAVRQIYKDMLRVPLGEVEDLPLMLLYTLSTPSELL
KVQYKIHTFSCFLSWFLLFGIAATIAMAFFSC*
>TCRU_976 Histone chaperone Rttp106-like, putative
MSSLTGVAVATNTLPVENWGALRWTTRQQQRKKSNANGDSGVVLANPKKDGKPDGTNGAN
SDGDEEALLQLVSLPKPQHHSSGTPASVVEKPIVSIPVSLIASASATTKNDLSLTLRPQS
SVTTTQSGGGVAVELTSIRFAIPNVCVDHEQTEEAGREILAETQEAIKKYQSRLLGEVGS
AANEGTDNQLLMMMAGRNREDERIVAVMDDITFSYPSGRYKLILSNYSLVLEEKKRGAAG
SGGVAPFAPLSDILQLYLCDIPDSGSGEQENEELAQYVVIILRNPLKVRTTTYRHIVISC
PAGFALDEAHPWRCQLETQEEIDSFLATLPHNQSNSSAAPTATVKSEEGSKKIKNTFAPM
MSGRVSEILIRVLKALTGVTALGGFNKEYQTSRGYSVLRCLHQGAHGLLFVLHSALLFLH
RPVTRALFSQVSHVEVDESEGGSATFQMVVYGQFGKHTGTTTENKLVFSALDKREKAALL
AYLEKKVTVRRIGLSMEDEEEEAEDDDDEEEEDESDDDDDDDDDDDEEDESDREKRSHKR
SRHKKEEKKQHHHHKHKKHKKERE*
>TCRU_977 hypothetical protein
MRPGKRFTSPNGPMPFYSQSSIAVERGRQRMAGGVAASSSYAQQRRLTKSRAASSNARRA
GTVGAMSEQGVEQRRAASTDGRQRGNYNTIANSNPATGISSEARRVSFVRGMKLSPDGSS
YVTPSTRNHKSHHGSGESGHQVRQLLLELVEISHTLAEYLQGEPSAFVLQQQASPINSDA
GFPTRPHSQKQATLKAQEIERIVKRVESHFSEKIASMEKKEKEQTAVILELRKELQRLQQ
KDRPFSPPLSSSREGDFRKSSRSGQYPTGASNGTYTVTTTTNANNNTAAANKNANGNGPS
EHTAEVETLRRMLQEEKRQRLFVEEQTQSLSEQHGRVVMTLERRLQKQEDQLCDLIASLE
HQSQLASSSPSAQSPPASRPLPSTALSSPRRLLRQQLTQHEQNKRVLEAYRRNLRGTQGL
TTITRDNLSADKSQTTSNHGNDDALDENDILVELGLADPKPVLCAEFGTRNVAQAPIHSS
LLKHSSPLTQPRQQQQAINQQPSPASRQQQQRPYDVEKVANAIDASVVNGVVEVDDIAAF
LDNITQELESIDAMENGREGGI*
>TCRU_978 Hsp70 protein, putative
MYAVSISLGSSSAVVAVAAATIATAAGEEKSAPIKLTANSAGHRSTPVMAAFAGNEEVLF
GEDARALYARAPEVVVPYLFAFAAVAECHQEQQRRCKMGGDPEEGNNDDAEKAALALKRE

IEEAAGKQFKGSCPLLTGEGDEAQKPGFCLPNRGDEVGEKNVFISAEDMLVGFFNHIKKH
TVDGACGLTATAENFSSQEETPQQAKQPEIILTIVVPRYLFQGAVMLNNSQQEEEISGSQ
DAGRRDAIQWLRNAILKSHLGAVAMHVSVLFSDEAALLAMDAVSCRPHQSHPMANRYFLL
PSSASASPMAAINGAVASSWPHSRVLVVKWGSLGVSFTRLCLEGGCLVDEPRTLFLRASQ
KAVNSPGAEFGFAATSCGGGDAVNLALRESLATTFAQKHRRVLGYQSLSDFPARAQRRLL
LATEEKKVALSKAAQVPVEVEALAEGIDMRDTVTFSRVRVDAALRGEWGFIESFERVLLE
YLHLLEPKEDGAIDAVVLCGGMLNMPCIAQSLRNIFTQHANMPREGRRLFARNLVIMEAG
KSTTLVVDGSKDSGTSSSQSVSNKIIFGAEELPCVGGCLHSYHLALGALQEQEHGYRDDT
KSRKNGKRRMCMGSKKQERAEGSRRVWLALTFGDAGKHSAGSQRCNEVVMLQNSIFLYTE
SNLNALRSAVALQKEKNAPLRLPKAALSLLFAAHTLLPARVFLPVTGERRDPFVLYLFMG
FQGTTQQQQQQKQERGASDEENALFHVAPLSDAAVTLPAVEEEEEQEQGGKWRNCIVFTL
REREKSDTESSGCEGKTVPLQLVLQLARVATHAFEPPVLGPDNVQTSVTLDLGEQ*
>TCRU_979 hypothetical protein, conserved
MTADGKSSSGMSGGGSGRGNSAAKPPGVANAVPPPKKNPHLFRLAAFYNTRIPLGIREFV
FLVPLLLITFGIAYNIPVLVPIEKFTQGLTPNTTPMTNYVLEPMYDEHGKLKGYRRILRH
RGEKGENT*
>TCRU_980 U2 small nuclear ribonucleoprotein 40K
MRLTLDIIRRAPQFTNTLLEREIDLRGLGLTVLDEQLLLMLNDSFDVLNLSQNPLVRLEY
FPIGGGGSAGTAAMEGTTGFVDPAKKMMLRLKSLIVHRNRLSHVSEATCAAVLPNVRAFL
ADHNEFSELRDLLFLSHWQKLEILSIEHNPVTLHESEARLRAFVVFLCPKLKLVNYQRVT
QDDRRNVEKMREEFEGLVREWRRLGEKSLGGRVNDGEKTGINLGCVVNGKKTRKRGREAR
AAAAANSIHSGDGLDEMASNAGGASTTNTTTTTTTDVAALDATHKAFDSTQARLEALEAK
MAAAETEEELLEVQQEILELEAAAKQQQQQQGAKSKRARTS*
>TCRU_family, putative AB.1
MLFRRFLGRRALASFVGVPVLSLGGLGIYVEYRKSIRPSISVSYPVFDEHGSMFIDGKLV
ERPGLLPIAGRMVELFLLFFPLALLYVVMRIRKEWYLRWLELLLRAVKRAGPAFVKAGQW
SCTRHDIFSPEFRSVFRRLYNEVDVHPYSVSLQVLREEFQKEPLELFSHIEATPVGSGSI
GQVHLATLRETGEKVVVKVMHPNIVETILKDFCIAMQVARILDKCFPSLEMYKLPALAHA
WRNHLSAQLDFRLEAKHLSIFRENFRHEPFVDFPRPLFSTRRVLVETFCKGELASPEFLS
AQEEHVRDILAHKGLNTWCKMLLHDNFVHGDMHPGNILVDISDPHDPRVSLIDVGLCQHI
TPEEAVVTHDLMESFVRWKADFCCSSLLRMSKTQKFVNKKKFEKEVEWLFEHWRPKKSSD
FAVSNILQSIFECIRENRVQMDPPFVSLLFSVLVLESFIMNLNPEFNMVRHAAPWLVGEG
HISYGLAKNILLTRLDVLKQDLGVLRSRVRGGVLGNLAQKESSRMMTSK*
>TCRU_982 hypothetical protein
MNEVTDLHAHADSFFLLQGEKERKSVTACGVHVLDGENRVCADDGRNTEDYGNSSQSSTD
APVEECWRRNKVLLEGILSDFFFSTLPSEASMSGRCNVSLEALQARRALRKWGSYVVDPV
HMKLSAVDALLSRAAQGGSGTESSESKKHFLPQSLPQKLNVPLTLRCAFCQRFDSLCSNC
HRNWKEILRKRQRVVALQSYCRLPEDRRCGLLFMTAAYGVGEFAAMQLEFDGEKCFKWRS
VFFPLKNGTTERQHSLVKVGEVHALHVAFAYDRVDVAEFLLAHPRCLMLKSEHRVDILNF
LPKPLTPQWEAVLNNSDVYVNHLLVEQAKRLRRAEDWEGAEALYAELLQRKPDSEHAHSG
WAKMRYDQGYVVECIHLCDSMISGETLVDWNEFSLESIKSLRGASLERYHEYCHQVETEG
VLKACGCVILDKVRVPIRKLPFSIIVDSIFLFCDGVSLWNIFKSTRMPLLRAVCEKLTAL
LPKWCLQILLMREPGFKGMSDKLLCKLPSSVGKLAVSPVRPLGLSVVAMADFNMFLVRAH
AACFSAKTVKNGFSALSLFGFGRKESKDGRQAPNEATEIVISKQYRIYRPSVEEPWCIKE
TGEWEVDELSVEVFESHLLQVKNLCDDK*
>TCRU_983 Trans-sialidase of Trypanosoma hydrophobic C-terminal, putative
MVPKASKKRPSFANGPRILVASNASGIVSVTYGGREHVAADNTDEVSERSATGHKTLLSE
NGVAGSGFFGEGDAGGLTQRNASGNEKTQLREFTAQYSGNGVSLHDCFALLNDAKLTLRE
LTVGSTVRVCVYWVLLLLLGLCGVVAVL*
>TCRU_984 RNase H, putative
MTMGASRTTKLLGMDLDPRLTLNAAATKQCAATSQRISQLRCIAHKEAGPSPHDLRTFII
GYGASKLRYGSELIWAVATDSAKNEMQKTYATLARIVSGVPSTVDPESALLEANMPPLHV
LCLRVRLSIFENTRAFQMDWMRRPPPEPPPRAVLRISPLSRDELYAFVDAYTKEYGITES

SPREERFFRSSIPPWFAASAHRVTIAVELPIDLSITDEEELIREKRRVSEEALAPHRHRS
WILATDGGVDVPKSAGVGKLLSSLNSSEIIEKASINCGARPCSYRTESRALLLALEKLMI
PRIQYRRKTLLVVTDSQSLLAALNKGPLSQTDWTEDQIWQRLLTLACAGWSVHLQFCYGH
CGVHANEPADQYATQTMESGHYTEQGTAPLWHTDLLTCFTTQLTNKWRGTIRQDTHRYLL
CGTRPSDLSGKDLITQEVLHRQELVHLARARCGESELWGRLYWAVRDCTNQCRFCNISPE
QSAYMRSNNDPTAPGTDTVPPSAREEDVSPVRRRTLTRRRKEKCPHCDSTLTGFSNLVSH
CRSFRPEHPPPLPELKCDFCDMVFPTRRSTAQHRSRCAHNPDATRHRNSSARRRSLLPQD
QPASTSMPIGPQETLHHLLLGCPGTLAVRRRLGIEQDLRLGKFSQWQLLYSRKLILLLDH
LFGTQMALYS*
>TCRU_985 Reverse transcriptase (RNA-dependent DNA polymerase), putative
MHKDGPRLWDDTLMEAERIAADSKARYLQLPTPDREAEMQRTRSQFFLLLRERLRNTYPR
RISKLNPGEPLAWKYISGRKKAPLPSPTSILLGDGQHTYETARRAANALNRIFLPLHPSH
KAVRPSKGINRRSASLSINASFIFGPQNSNESISTFTSGFCHCPLDAPFNSTELLAALRN
TPYGKAPGPDEVHSEALRHISSKGLRFLLRRINHSWTTGTIPAEWRSATIVPLLKTGKSP
ELLESYRPISLTSIVSKVAEKMVLKRLLWVWTPHPHQYAYRSVRTTTMQLAHLIHEVEHN
RNHYFQVSLPEKSGIGNQLHYRPHRTLLVLVDFSKAFDPIDHRVLSRLLANIPGVNCRRW
LRKFLCGRYARTRVGHRHSDRRPMLRGVPQGSVLGPYLFSLYAHPLLNLLNSFAGATADM
YADDLSIIVRGSPGNTPFPLPTWFLKSCMRGVRKMAWPSTRQSVKLLGSHSPRTRSQIMI
VKEGGPW*
>TCRU_986 conserved protein
MSSIPDELFGNWLLTTFNGLDLSDQGGPFLMTLEPAGGLVYLDIQITNILSGLLEYREGR
LYGHLVSTRMLGPPLHMQIEQAFGAGFEAGMNVLMEEAGMIFSQGGNTFFFVAQTDSSQQ
*
>TCRU_987 hypothetical protein
MDEEDAYWRSYRHYFLARCVEELLVALKKLHAYHPGLGAGRRAEGQSCGVEQSGAGSAGA
SGEAGQTVAAHRHLREACGTVPGEATGGCVGSGAKHYRRAQALYDKPQQHAAPGEGCRNA
PAGATASSPVAFGCEQQRRVDGGQEAAADPRRRTDARRPFVCDPRACDEEGRERATIQHG
VGAQRTENECFRRQRRDGRGSVSESGLHDYGDGEQEEDAAGWAFVGVDSARSCDEAKWRE
LCPKQWSSRK*
>TCRU_988 hypothetical protein, conserved
MAMMMTGRVLLVCALCVLWCVAGGVYARDVDNSAVGGCMASGVLGTRRLYMPRCNKTAVT
LPLRHVFPVSAVGASTDGQVSGTIDGGLLPIPAPPLSPAPNIPKAPEAPTSHGVSQPAVG
VHSGDGTPSLHIPNVDSSVGGGSGQSGNSSGSVLSVVEPPSTVVVISPSSISPGVGGLQN
KDGPPSNPKLGSPAVIVESGKILVGHNGETEKDSTPDDGSESSRIQQETESRATTEEARG
SEAGQSGVGTNGPPNVLSTESQQQHRETTVTETKPPPPSPGKQIPAVSLPPGVDAPAALS
PGPQASSPEPSRAAEDPEEEKRILPATTASHNSKGETHKASSLRTETVSAAPKTPSPDVV
LEQSSEGTVTKDAMKTDAYTDGLAATSESSISTSDSGVAQDKTEEEDDNEQRREAKGPHN
DPYAGNTNDAPTASDTAAETVKTITTPKSATSKSGDSDGSTAVSHTTSPLLPLLLVACAA
AVVAA*
>TCRU_989 hypothetical protein, conserved
MYARFRTRSKFYKRPERVLRAYNVSPNILRRPNVKPGLIRGVYSDEKVDLRDRERLELLE
SIRHPRERDFYQDHTYHNQWIRRDLEKHQKMQLSSRYRYFAPDYVITPWIWYPGDVVEVV
SGEGIGQRGAIIAVVKYKNEIIVQNINVQDVVIPASETRPEQVLQREHPISVTRVRHVDP
STNELCNLDLVKVRNKETGALEEKRMSLETGVLLPIPPLDSGMEVGDPLKDTPIQDADEA
TYDREAEMAVLVQRRLHAMEEYFVRSLRKSYEFHEPLRTKNAEDLKAFQSDVVDAASTAL
AEKLLPVDGTTPSTWWKDALAPYVESIEAEMRARAEEEEAEAAVAEGETLATQVAEEDEF
LDEEEDEINMEKDASSL*
>TCRU_990 MSP-B, putative
MRHTLLFQVLLLCCVSGSVAVAEHHCISDEVENKVGPRTTAVVLELPTRGGGMMRALTAS
APEWAPIRFQVFTEDLNDPSKHCTAEKQIRPDFIGRNLECNKRDILTKEKRSIILNSLLP
RAFRMHTDRLLVRPLTGRVIVPRYSSGVCAQFTIPSSHQTEGVSGADMYLYVSAGPTQGS
TLAWATTCLKLPDGRPVVGVVNYGPRSVTDSENSVRVSAHEVAHALGFAVWLLQERNMLK
EVLNVRGKAKVLQVSSPKTVEKTREHFNCVNATGMELEDEGGEGTASSHWERRNTKDELM

AGISGIGYYTSLTMAALEDTGFYKANWGMEEPMSWGNNSGCALLTEKCLMNGVNKYSEMF
CTAETTLISCTSDRLALGYCTIYLYTAELPQQYQYFSNLKLGGSNSSLMDLCPYVQPYSN
TRCSNGEASIMHGSRVGPRSKCLKGDGLVDFMGRIGDVCAEVSCEKGEVSVRYLGDDTWR
QCPEGSSITPTGLFTGGKILCPKYDDVCIIFDPLRGGGDVSSLLSVFPSISVILLVFIFI
SIY*
>TCRU_991 hypothetical protein, conserved
MQKLLIAINGTTTLRPSAPSKARHKSPPPLPREFGVRPMQQRGLGDPAWKGEPHDAPGEV
ELLLGLRGTQGLMREIRGWQGFARRIAVAPFGGVLECMLDATPGHSSWMRLGSLFRRRLR
QTPACRQRWEERGTLGVRQSRGIDVHGNSPVDRATAYTQSHGSSGQQKRILER*
>TCRU_992 hypothetical protein, conserved
MDRRAPFAAVLDKSSGRRWRSIAWPKPRNAQEDAEATFRHFTFHSVLARCETKPPLYWIC
MRLFTGGSLGGYARTSFRCRVGFLDLVESTLLHTGHECIPEVPRAAARVLAEDPEMVRPQ
SVAPRAVSTPVWICGIRTSGPRRSVVRRYQCAVKKWCQQENRWSLPRGVNLLAYPSSARR
RAPP*
>TCRU_993 Reverse transcriptase (RNA-dependent DNA polymerase), putative
MLRGVPQGVVLGTCLFFLYVHPLITLLNGFSGVTADMYADGLFIVIKGRSWEDAIPTAKM
VPKKLHAWSQENGLAINPSKCEAAWFTLSTHTESDHDREGKRPLVLFGCTIPVMAMEASQ
TTNLLGIGLDPRVTLDLAATKQCAATSQRVSQLRCIAHKEAEPSPHDPRTLIIGYGASNL
LHGSKLI*
>TCRU_994 hypothetical protein, conserved
MCRVFCVVLVLALLCCCSCVFAAEEGEEQEQEVLQVNAECAGKDKLGRWQLYGNSLWYNC
TVADGGVGKEICGMGDGNCTSEDVESRAKGVTKDGIFTINVTSPTPSAVKSWWESNVKSK
AVTVAIVPAKPPAAEPLGSREHKASGGTSTTNSESSDVEDSADVSPPPQTATPGSAVANS
AAGAAASQASDSAPAGGFQGPSHSTSAASAAPESLPDPKPTEGGGHSSHDHPLELQEETA
AAPPTQLSQTGEDGGAAGGAEEDTATTTDTASGEGNSTAASMPAPLSSAPKTNAPEKSVG
TDACFYDTRPLNRLLLFLAALTYGTLG*
>TCRU_995 hypothetical protein, conserved
MAMMVTGRVLLVCALCVLWCGAGGRCDEGEAAGHGVQPDVSASGTEGGLGGTKNAGPAPD
SQIQSSGEEQQHKSREGRPALPEGSLEEEEEEETVENKNLDEVTERGEEGRREDGQDANE
PDGSQTSGVLTKQKETLPPSTPAAPAGEGNQNASPQAPSVDSHGGGRTDRIEDSILGAPS
SGVALQHAGNASTNQKNNSLETGKHAENTITGGPVPSQAKTPKEPINDAEIHSVTGEDTA
SNQVVGRGTAEYEAQQRALATQDRSSVSFTESETKWQPTPHETPGTQVPPSATEPSSREG
QPPTAKTDSQTPNAQEHKKLLLPKKEYIAPERPSEDIVAEQQGQATATENLTENAPATNQ
AETTASSNSTSGSGEARIMADENTANAQRPNPNESHDDLEGSDTHPLPQTLRQHHKQQ*
>TCRU_996 X-Pro dipeptidyl-peptidase (S15 family)/Alpha/beta hydrolase family/Prolyl oligopeptidase family/X-Pro dipeptidyl-peptidase C-terminal non-catalytic domain containing protein, putative
MQKVVVSTLPNKVKTIENVFIELRDATRLSCRLWLPADDVPRPAILEYIPYRKRDGTRGR
DEPMHGYFAGHGYAAVRVDMRGSGESDGLMHDEYLQQEQDDAMEVIEWISRQKWCNGNVG
MMGKSWGGFNSLQVAVRRPPALKAIITVGFTDNRFTDGIHWQGGCLLNDNFWWGSIVLAY
QARPIDPEIVGDRWKEMWLQRLENMPVNIADWAEHQKYDDYWKHGSVCENYDSIQIPVMA
VDGWADGYTNAVFNLLSNLKVPRKGFVGPWAHVYPQDGVPQPAMGFLQEAVKWWDHWLKG
TENNVMDGPMIQVWMEHEMKPSVHRPVSEGHWVGVEAWPSKDVTEETMRLECGRIVDFAH
AVRQEPASVLLRTPLNHGLLAGEWMGAGVSGETPADQRMDDGMAMVFDAAELQHELEILG
RPILEVELTCDKPKAMLFAQLSDVSPDGAVSRISFGVKNVTQAAGDQRIEYLQEGKRVRV
RVLLNYCAHRFTKGHRIRLSLANSQWPMFWVSPEINTLSLDLSTATLRLPTYRGPAIAGP
NPQPETAAPTPLTVLRAGFVDRSITYDIVKDEWTCITDGVGGVFGDGVYRFDEIGTAVEH
NLRRELRLSNKDPLSARYDIKQKMKMGRPGWEADIDIDTRQTCDANYLYVTAEIQAKMND
EIVFEKNWSRRVYREGM*
>TCRU_997 cation transporter, putative
MQSTSHGDHHEDHHHVGHHHGGCSATTGPYSLGLHVGAIFILLFASFLGTVIPLAGKYVP
GLRLSPFLFVLGKCAATGVVLAVSLLTMIHHSMHSFAEDCIPKGLHADTYDAFALLFAMI

SAMLMQLLDVLLDGMLQSWSACDAGAQTSTTVGEPGNEQKQDGRCAGSCGMEGCGDQPGP
SCEMGGCCQNRGALSAAHLNSARRVAAAILMEFGLASHSVFLGLSVGIASDKDMRTLLVA
LSFHQLLEGIALGSRLVEASMSIMLEVVMTMIFSVSVPLGIAIGVITMKGTHTSMTGPAF
VALQGVVNAVGGGMLLYIAFSLIFNDFPADMRSVAGPTVAHCGWRRCAMFAAFWGGTGAM
AVLANWH*
>TCRU_998 ZIP Zinc transporter, putative
MNNAESSDAHLPSRVLQQAGAESQQSQASTAQMYARPELACTETKGEYSVALHIVAIFVL
LVASLIGTMLPLAGKYVSFLQLQPFLVVIGKCISSGVVMAVAMVHMMNHGVLGFMKDCVP
ESLQQSFDAFSLLFAMIAAMLMHALDVLMDLLLESWAKNNASEATSQIEQAQLPEMETTT
TGQEMPGAGCHNHGGIYTARLDSAKRIIAAVFMEFGLALHSVFLGLSVGVANDSQTRSLL
VALTFHQLFEGLALGSRLSEASINFRLELLMTFIYAVSVPLGTVAGLVTLKTSDISMTGT
GFVTTQAVLDSVCGGILLYLGFTLILNDFMSDLRQYAGVNAAHRGWKRLAMFVALWGGGA
VMTLLGKWA*
>TCRU_999 hypothetical protein
MTALKCGVHLESHVHKSFVELKLILLVGNVGNKLHRLLHAFHVVHNDSKALLLLSHPRKQ
ENHLLQELVFFAQCIYLLQLGKVCGILMQERKQTRRLVLNKKRRKELRMRLRQRGVHCLV
SGLQRLQVCPQITTLRPFQSLHDGRGCEKRSQQQRLCCFFLIHNSASHVNVFQEELDGPV
LVPGEQRRQGLQCFRKEFLFPYKTEELITLIHP*
>TCRU_1000 hypothetical protein
MADAAKKAAVLLLVRHLLAREGVDTGVETKPLPEKYEDLRDGAILYRILARISPQTFPEA
DEMTKQAPSSRQNWVVRKGNLVALVRHMNDYAHQFLGAPETLNLTLLISPTEIANLPELS
EESETPLELEKGMECLMELTDIFVVMVVLSGDPAVLQAVKSLPHAEQVVLSNAAKACITK
HALRPRRRHGTLALEDSLKSGLHRSVTAGCAESSSHAAHLSYSLVGEEALNHQILQLRRG
LDDANAKVVELDSKLHLALTERQEWESKYKNLLAEAELRRMTAAGEDHLRQLLAKKEEIV
QKLTATIEDQKKRISAFKEATAAQETALGSFRRKIKHTEEELMKKNVERREALDKLSVAE
RSLAAQMNIRAELERQLEDLRTELALANAQTHNGMENSEACLNRSFGSVNSVDRVVQLEN
ELDEARSQKEAAERLLQVLQRQVAALPSEGFGSSAAMGALKAQLRQAEKEKEELRNQLAA
TIARLEDAQGYWRRRGSTSFVDNETESPLLEGKIGGENADSPSGREETDASGQQHRPCEK
QDGVSRERTLLGSTLLLLGYRNLMLQQQLTMTSGGKFTASGDGEKENNGGFAGNTGSFLT
RHRYEVEQGLLRSVLSRGG*
>TCRU_1001 hypothetical protein, conserved
MPVPLKSFTKSKWLAFFAELTAKLAHHNVMVVMEGCEVIKRLLVSRVSVAAFLEEGELLT
HLVDIIAGEMLSSERRSGRPPLFMAEKLLFTQLETLSLFIDMMLALHADKNPYYCLLVLA
RAGAILMTLVKLLQRQHPRFFGIIGTMVKLLLSLPLGREVRGLPLNESQSVAFTCHLQQL
QAAVAQHEEILQETFETLQTLCFATDGPMRYCSHSLLNMAVHRLRRLKKRSKQGSSAGEA
SSSRHEVGLNRTEYIPADNFMESSLRSHDATCLAADETKISINTAMDGEDAILEEVADVS
IPEEEYTDCFDRLSKAGSHEEFLFVMNRLWCITVAAGDQLQRRFTSLNFSRAFKRFLETM
PSNQQDQVVFTSVLLWLSHMISGFHLRSETRDSIASVAGSSLIKIVLQDLNARDMLLPQR
DASDLSTIDTSTHAASAVSTSRVNMSASSPMRCERPGGLVERPSISLVVLSFLLIAERAC
GPQEMGQWIRDAGLFALIESLIRNVERTKSALIDTPTTVDGALSDEVYLACAMASTRTEH
TTLAALACKLLSSVIWNHYMVLNKLLAPETLQMLRSTIPLLMTIGIHNPTLTSAIESVSG
SAIHHLPSCQRASRHTSLGECSLVALDACFWLMEFSRMDAVCLRDVIPTLPAVSRATRHS
IHPPLRASVYRCLSRLCTSSAELMTILHEMPSVLSAAVNSVLEYTKTAPQWEAAAAAEWL
AHVIRIASSDAKVEQNFDFIQTALPVKLLELASSNGITYATAAMLSLMTSLFGQQGRLAE
FHQNTFSNVLLPYGKRSLECWLKLLLVATQTNEKLEHVSETPIPAATTTKDALSRFLSGI
EGISVQFSFVCSLAQGLLFLFASNTVVREQISDDVICDFIISVFALPLPDDIFDMLSSRF
GVRVEQHRTLEDSYEVMLQWVMEFFSLWFTPASRAEKNPLSLSSTFFTHVCKVIRNNRLS
QRIRAHVCSFVSGIVLVRCDALYEVLAASSGDLFVASLLVHPASSVCGMQCRLLSLFALA
NDKAHEVGWVAAKVTELDGQVQALKKNGGMVGGDEMNSLVMALSFVSSALAFSECRALSR
DVRQSFCSSFRILLGYETTQRAAFQASAALARSNEGRRCLLHEVCSTGDPLSRSCFGRIL
GVTLGVLQSGEHLQLQKENNDAMKHVPLGEREALRAAVVISGFDICATACGKGGVPFTHA
VMKLKGVEHIESLFLKHERQRVPTPLGLFRLLAAISFQLEAQLAIVRRAELMTILIETAG
STNHTGTLSLLTLRNLCFNSTLKTQICQDSRILLTFKAALMAIPSPQTGTHENSSPKPKD
CMKIVLSSPNEVGEKRASLQTFLEQRMSWQSRNSECVSSIDDSLGDLDIFRRQELAVTAF

WSLMYDNQRGKSYVRGVFNQSPTVNINKLLTTCSGLTVCAPDFAKYSEKMSEAIGNIKFL
GKGAI*
>TCRU_1002 hypothetical protein, conserved
MTADLVFCEVKKEQDGGRHSAVVEALEKWCFLPYPESTRSRLEQVNIFFVASCRTPATDT
TEGAEDVSSAMVGVVGVVWVPLAPGAQVEGYIQLVLVLPTHRRRHIAQQLLRRALRLVEG
GDPSRKIVRWRLHTMTPSQQTTAYLRRVLPDGDDSASREQLLEEMERLVAAVPRVYETLG
FDIRRNMYAYYDNSADAVEMVKVVQGARKRF*
>TCRU_1003 pyridoxal kinase
MSEEKTILSIQSHVTHGYVGNKAASFPLQLHGFDVDGVNTVSLSNHSGYPIIRGHRMDLQ
EYETLMEGMRANNFLPSYRYIITGYINNADIVRSIRDTVKEIRTLREKEGKKLTFFCDPV
MGDDGIMYCKQEVLEAYRELIQCADVATPNYYEASLLSGVNVTDMTTALQAADWFHAQGT
QNVIIKSFRMKEDPQNLHFLFSTMATGGKTPQRFTGVVPYHEGRYTGTGDLFAASLLAFS
HQHPMEVAVGIAMGVLQDVILATRNEGGDGTSSLMNRELRVTKSVQSLLNPATVAEVKPM
*
>TCRU_1004 calcium-binding protein, putative
MDTTLYSEVNRLERGDFLLFHCVQLSQHERDVQRYFFGCYFPRWRGFYLEEVRDMPGPLG
YKVQRHFPAYPFDVYLKDNGEHFLTDDFQEGSIFTLGASQNQRDGDSKRYKVVHCDDSRL
RTRTGTTLADIGNDITTKLNQTHRVPGEVIDLLREIRDAYVVYAGNGIPEIGIKAMGRHF
RHVSEDGKRWMSLENIGKLVRDSRAFSTTLSFEDTQKTNSTISNNARSIHEAFPQNEEGC
IDYDLFMDYVRGPMSQKRKDAVWEIFRKLDFDGDGYLNILDIQARYNAQQHPVVAVERLF
SADKLLKGFLTVWDENKQYGLIPYAEFIDYYNGVSAVIADDYIFFDILRNQWKVMRDWGG
TVGTRRGKCEVPTM*
>TCRU_1005 Zinc finger, C3HC4 type (RING finger) containing protein,
putative
MENGAEGLVPIECLICAATCEALAVFKCGHYVCYVCGLHIHSIDHGVCPVCREKGETVIV
TCTPPGENDDEDKFSAELIRTMEDVSGFDSHLQCFVHGKALANELRKMYEYVCPVPKCWC
RGVQEPFCEMYPLHEHLKTDHGLGYCTICLQHRSVFLCEHQVYSKENLRLHMDGVCPHDS
PSFLGHPFCRFCPKTRFYDEDHLLEHMKQHHFTCDICNRGEFLFRYYENRKKLLQHFERE
HELCDHPACASLDPMMRVFVSDLELALHKQRVHGVRSRLALFTTDASSTNPSSSPPTSTT
PATGFNANVISITFDYVHKQETVKLMQACAQRGSGGGFKGKRKGGGNNHMDWVRGDAPGL
PVHFRTRGVLKPLMRKVRERDVALQATSHPRAFGLSESAETYNTKNRVPLNPTELRGALD
AVLQEELPDLHRMEGFRECTADFMSGKMLTMRYYNCLRTEFFPSESAFQKVFPLVVATVP
LPERRDALVQIEKMRNSPEVQHAQRMQEEEEKKKKDSEKWKKQTEYLERIQQGSKRQKGN
NSGGRNVWLERGSAALINQAHGKQSETQEKEQPRQQQGSQRTLWGKDNKHASQKSGNERS
LSGDATSARPTAWGAAQERQQPRLSLLPIGAAGYYPNPEDFPALPTRNPSRALGLPQAKR
TSRPNAWYRK*
>TCRU_1006 AAA domain containing protein, putative
MGCGVSRQPFSTELRPLRATRECAPGGFDCAAGCGGHPKQITGESHENGISSERPSPDGG
SATKSDSPPPRFSAAETVVGGNSHRDDSRDEVKFSVEWNRSVSPVPGLSEYATLPLRHVH
KKLKHNHRVFRICLTGGPCGGKSTLLTQLQAKMPQRTGYKVFCVPEAATLLVTGGMVWNS
DLIEEQQLALLRVQIGLEDQFYNVAMASGAPSIVISDRGTMDGRAFCSEEIFERLLRRLN
VTLETLRDRYDAVIHLVTAASGAKEYYNLDNPSRYENVEEAVESDKKLRQVYVGHPMLRI
IDNSTTFEEKMERGLSLVGQVIGQRFSMQNTSYYLVRHCPKELPVQSVTSTLTITLLCNS
QINDVRLLIRREHPSGECMYFFNSIRCAGKGNSFGHKDGSEGSVTRDASPVRHAEIQAVQ
SAFMGTMESGQRVKTEQRISSREYATLICNRDAQRHDVVLNAVQFIHDDARYEVTTIVEP
PWAAGKQALTLECDDQENATFPSFVKVDREVPIDSLSTAFLISHTEMGPLYTSLVYAPVF
TRGILTRGTRADADGSCATSDSGHRTPRASKRDVEDEADEKKKLMKRKKKKRVKHTAVTD
EDESLPLQKAEKKADFTATVVEAVDAREIPPNLPSQSNLGPTDDTPNSPPVPSASTTPPL
PSGPPTANPRGAMTGMH*
>TCRU_1007 small nuclear ribonucleoprotein Sm-E
MSATTKQMVKPTSIVHRYMTEGQRVCVWLVHDTKMKIEGVILGYDEFMNVVLGDASEVHH
KTKEVVHVGKILLRSDNIGVIHPIGV*
>TCRU_1008 ubiquitin carboxyl-terminal hydrolase, putative
MLCSCTERVDPQRLRVKFANAWLKEGVLTSEPLTMYCTLCEQRGLMSGKTKSKTKLHGFN

IFRKTRDEADGSGENALDDLFLCFTCTMCFCAAHAKIHEEEQRRAAMAGNNNRGKRHFLF
FAVPFWAKTMGNYTPSGKNKTLFPTFHVDDLEHIRESGPLTLEKEAAIVFRPSSDSHGWV
YNVWCTHCSEKPAPLRFSEFQESDPIHVQIKLLGDIIARLRFLFYEGIRIELPAEWSALN
QGNFHTGESSSYPRACGDIYANRNTLHDLDVHASGTSSMGTSSFALRGSKGDAEALVLAH
VAGIENHRNTCYFNSVLQCVLKCRFFTRPLLSLEVGALPGPLSYRIYAMARHLSEQTLVD
VQTRAVYPFARAVLRCLCELAPLFAEDDQQDSQELFLCMINGVADEFDKGKSEEEKKKGP
RLSFEGVMRTEVVCTQCESRFPREELFMALSVPVEDSIESGLRKLFRPMHLREKDQYACE
RCFKGLTKAEQERHNAAIRAENERKEKAKKKASAEEKRSLNCVYSDAVVSTSISRLGGTL
ALHLLRFQSDGRDFQKVTRSVAFPMSLDLAPFVSKDVLREYEMARGLSALQARFPHVQRD
RLLRFFEHANGDLQRAERMLIGMEEKGALNGEDDALLEFKAGQGTSDGSIPGITTGRSSG
HTSCDVSPAGFSPVSSVIKTNGILGEGQEPLPALKRELVGIVAHRGSLHGGHYIAYVRDD
SRPNSWFRCDDEDVERVDKEYVLRCQSEVYMLFYE*
>TCRU_1009 RNA decapping enzyme
MTLTTHDAFSAVAGRHQTQIPVLECTSDDEVVVVAFDGLRYRRSVCVVIMNDQGQFLGCR
RCDNRQILQFVQGGAKSHETVQQTAEREVFEEIGLPAKHLRFVAEILPKTVGRELRAAFR
YRSKTWRKKDIIGQELYPLLYLAEAGVVDLLHFKAVPGVRQEFCEAKWMTLEELMQNCPP
SKAAVMANICIAVASFARGGLGNGSELNICGGNAPQLVGIVQGTDVRNEGTASGRGNTGG
GGGHRNRRGRKPRVQQE*
>TCRU_1010 hypothetical protein, conserved
MPLAEQRGLLPRGVAELNAHFLSSRGDIFTRGTLTVEFAPMMSSGLSGNGMTRDEGINNR
HAAILLTLSPLHEIGEVSVRLERRGEDAGDSMGECHQLPLNNLKLCETKRRVVRINDGWR
PAVASPDILAVDSASSVAVEHKLGLVEAEEKNEPKIDEDNGAEEVLELVFGSSDSENDTK
AGEYDDADDYANENCESVAEEDPLHSAFLEESGNDNTVQQFVLKLWPFPANGKDTMEDGA
WFISRCCIEVRLLPPSQLASLSYPRLTVSSAAGKTHSKQEEVWEFGSENYLLLVGQRFYD
YFICPRVAYAESGPDAAIFEAVDVYVTALRPFSDAIATSWEILPMCSFPVLPEDLVCAFV
RPQQGEDASLVAEETHAGVRWRVQHHSAYTVLSSIDEEDAIDGKDLYFFNCDHVLSDSLF
PLIRLAEEAWEEKNSRAAVRVDASFCRRKTTWVVTGCSNTLLSLRRGTLCTRGAVVLVAD
VLRTVRKTMTLSTPLFQVNLLRAKAAVLSSALEMLWRMSAGNGVAPEFEFTPSSSALTRG
WALCLLCNEFGWTELLCQERRIALTHISASSQLHPWWLTQRYVQETHETLMFLSSAFVVA
SIRQRRLFPWQFFEKHRKYKPSVEMLGESNVMVSSGMHLLLVQQMLTQRRHEGGGGGIPS
QPVVFHGSLYYEGIKQHATCCDINLRLNCVKANATNRLRMHGMRLSQSIWGIDVPFTVVF
FAVDASEASQMAPEGVRKEKRLQCRVTHVLPCCWRVSGLKWARGVDQQASFSFLGTVAEN
FERDFVRKNETMEKEESEEGNDEPLFMKIPMLVLNLKIVPAALSISVQHDWFWLNLISPL
DALARQAILQRIMEESVREGENKVEETDLIFTPLMNQLLEGVVGDHCCPSAKSEGNLPAT
LFLSALQTVDLDFTAIGSRIRPGLYTRFTRLVLKLNLTASVATDPAGALRRSCGDDERFR
LPGVVMGVEEKMLSNLRTVEAYDASLPRPNPRRRQRKEEENEGQGKQLGPRLVLSLRRRS
VVQQAEEEKGHQEMRAKNKLEMTPLDNPADLAAMVGRIITKGAESNVADDTFLAARSAAL
SLVLPLWLLLHSRPDMSEAQQAALCDGAEDIYESLRDSAVANDLDPVLLKGYECLAQKAE
SRMGQRDCSGHP*
>TCRU_1011 MA3 domain containing protein, putative
MQASSRRHGRDEGNDEEFDKRRDRKQRRRGDREARKEARIMAHEKWVEQRRIMREEMRKA
REMKRQNRKETHRKRLATSDASAFPPRRATNVAKSMKTVSGHGKTLADTLDAGDARSPLR
AFASQKGPSPPHEEEHSPMALAKTTTSASAAEVTTIASATETNSAKYIPPTLRGKRTREK
NATLDEITRRIINKLTVHNVVDLTRETSELFGGAVKGSSRASVLRSLSEHINCMCMLDTG
PLTPIASLSFAGLIRGLQLLHGNVVGSELIESLCFALQEHLKKYNETSASNGAMVLAHLY
VLNAVDSLLISSFIRSALQMKGHQGVCAAACTLTLLRACGEKLLKEAPGQMEQALTEAKS
YKRQQECNVRYSTLLSYIADILSGHTRSAKRTVDEEEVPVELLLNDMTTLLPSGGKPSNK
RVLYRIMSTTSVLTGVTWARMMRTDKPSRWFVLGAWDDVDGNENSSVLPKTNAEAIFFAD
SGSENTDDEEDEVEVKAERIRQLRQQEKAISGQRFNTENKREVFQALTNASDDLEAFTLL
IHRDPSFSRLHDTFAVLLQCAYQEKVYNPYYAQVIQRFCSAKESCKNTLQFALWDMFKLV
RVEPVDVTGYFNLSCLLAHLIEAGVFTLAVLRGLDLDCTNRTIGLFTRILLLRLIFQLPP
ARLAEVFFGGDGFRAHDVKIDTSVLRSNLSKVVERYFVDEREAGKWIPHFYDVVSRGTFF
DVHRRDGAEGIGGDEGVMDDETRLQLFMKRIQVVFKALRGGIS*
>TCRU_1012 hypothetical protein, conserved

MQRPQHSAAHTQLRLQQRRRVQHKRRRHRVRQSAQLRVAHRPRRHNRRVTDRHVRVCRQV
VHKHKRAVAPGQPAHAHLQAPVTRRLAAVAPHVDLRTAVAHAVPQFDAQRAGKHRRNVEH
HSHAVRDGHAVHQHRVHGAAHRDGSVHRTAPQSHHHRMHSRQPRRRRNQRQHNKQRRTYS
RNGPAPHTVPAARTRTHRRPHPQTQTSTNKAVVVCRTRPVDHNSCPTNPFL*
>TCRU_1013 hypothetical protein, conserved
MPSRTSSLDAHSGTEDQVLHFIAFGVATWALRIWNEGVSVVIAYLLSAYGYYWHQADAQA
QLAFGSPLSWTRALDMDRDFADRFKDFGMALLQAFRTQTMITSGPASRFLNFAPAFARNN
SDCCDANCAIFVDETRPHATCAA*
>TCRU_1014 hypothetical protein, conserved
MGDAVVRAGLSCSATSFPEGCSTALDSVSVSDGNFSFFSSFEFCATFGALRLPSPAEKLG
AVDFFSDGAGADRPGDGTAGDGANSGVGCCTGGLLAADNDLEGIPVSTVFCTFFFPVVTS
SVSGLSSVSCSVEDCSSAFGCVIAGACDLGSEGARGVVPECTPVSEKLFF*
>TCRU_1015 hypothetical protein, conserved
MGTVCVDGKTLGSSATTVTRGPQLLDVSCFYVGGDGCSGSYCSQVTLTHVCLCSRPLTRQ
ELQMPLLRGKQTEADCREGKATPRPGNFAAKLPQDLGVDAAAREVLPVPAKGELGLGNST
TDVNGSTVLLFPKGIGDGAVHGCVPWAFSGFTSGSAWGRGIFMSRRVRLCVF*
>TCRU_1016 hypothetical protein, conserved
MPRRTASDVVTPVSDEAARGLSERIAVDCGRGRGGVPRCAVAVGAFSWSRSASRLRSTPL
AMGECWLCSAGSRAWAVAPNAGSAIICEAAVFG*
>TCRU_subunit mitochondrial RNA binding complex.1
MRRILQPPTYGVSGLFAMAIKNATGLRSGTGACLASRPMTLHVSVRAQGGFGGGRPARGQ
RGGGGGGNEGRGRGGGGGGGGSGGGAAQSKGGAARRTVINKTGDGELSDFTRIVMRDDSHI
LKNIVKHIPQNGAISVKSLHAQLSPDVQEAIAEKYDGLKNFLEQRKQLFVVKTNPADGVL
YAAATPLAMQKLVAREKQRETMQEMLGINSPAGSGKRGGRGGNRGGGGGGNRGGGGNRNN
RGDGGGGGGGRGGFGGGGGRGGFGGGDGGGGGERFHRGRGGGGGGGRGGFDGDGGGGGGG
GRGGFGGGGGRGGFDGGGGGGGGRGGFRGRGNGGRIGGESRSFGTQGRR*
>TCRU_1018 hypothetical protein, conserved
MFVVGRGALRWKAVLQLLTHAGRGGTTTRGVTAFVRGTWPPSFVCHVSGAVVTQRRLHAT
SSGDGESTEKGASTSGSSDAPAASSTVSHQEAMERLLEEIQVVLSRPVPIGSLFRALSPT
SRKTLVKNKEPLEQLLMRYPEHFALYQQGNAKNKIIYCAPPHLVPSTARRMVFEEPPVVA
AVSSSSAQSKDGKGINERILQHDPVAERQKRINTVLQYIPNEWAAFTDLNIPEEVRVKCM
GKPSLRAFHYFEKYPQYFEVRQQGITDHSFYVRRSLALQRKTEKPP*
>TCRU_1019 hypothetical protein, conserved
MSRRVFASAVLLLFVVLVCCGISGATPANEEEVKNELINILNLERVDLFVPQKTQVLAKK
GTDSSGRDAFVSPSLVSACGVIAAFAEGHMNAQYTADGKVIKPISCDVVAEYIDSAWEWP
TLVGEVNESTWKAHTVLGKADGTNRVGVVLRPTTIMNGNKMFLLVGSFAVFYESGGNWS*
>TCRU_1020 CLN3 protein, putative
MIPVSVKKLEESVHVKHRIRNAFSLWWLGMINNFHYTLVLAGSDGIAEGYGMKKYVALIT
FANVFFGIISNFLNAFVIQRLSYNIRVTAASLMTTVAILLVSFAWGIGGHNNVAAFIVML
IGVTFVGVSSNYGSSVFLGYMERMPSWQISSWSSGTGLSGVSASLIFLGLSSAGMTSPQI
FLTSTVFVVVYWAMYFFGLKLPYCVPVKSSSTTMRNNGRSEVFRGEEEEVEYVCKAMVNW
KGTPWTSIVPAKPEFKPECLREMERARRASDFADSEHEEVANECEACGDAHLSGWARWKR
DVWPLIKVMHRATLWNNFNLALVYVAEYAVQFMAPFSFPRQVKESKNFWLKNSFVITQFC
YQFGVLISRSSLLCVRIRYVWIMSVIQVINAIAWFLQAKVKYMSDPNNMDRELKFAFVLF
AWMIFVGLMGGASYVNVFHNILEETRLMQEKELNEAMVFLASRQKNGGDAGGAHAERGEQ
ERNVVDGESKELCEAAHYITTVWKEKRDLAMNIGSIYSTIGITLGSLLDLLFTLVVLKGE
AEA*
>TCRU_1021 GPR1/FUN34/yaaH family, putative
MSNSNFDELQGEQFDGRHSNSRKTNDPLANRLLAALTADEEKSELMLTVLQNILNSNDLA
LVKKVALPKKRVNLSSLGMLGFGISTILLQSRNAGIFELGSVIPAMGLVMGGGMQLFVGL
LEYFDGNAFSAISFLSYGAFWLSLVGTWLLPHTEENALVVKTEGSLMGLYSLLWGFHTVC
MGFVAFLRSHRVMQFLFTSMAFLFFLLAAGDFSGKKEVTQAAGYVGIACGSSGLYIAFAE
ALEESLGYPVLPLFHVKRN*
>TCRU_1022 hypothetical protein, conserved

MRCCCVLQDKSMFAAKRRVIVPIHPTPNYPAHFIKASFTTDPLKEKQKARFSSGGEAMRE
VQMIPKNLEGERSRRELMSRGDTEFEALVEFIQGASYDQLISGRRFKKVYDKLSENDDTF
VWLCHTAMSVLNPGDVRSRLVYNHLRTLAEAVANGEMTLRTAFRFYESAVRSPAYREIAK
RQMEGGAATRLAGISAAADVMRRMGLTRRPMASYFELYQRIVERSEAMTPWGFPPLFQFE
ERLSLEPRLKFFSRASQQALERRRRGHIMSAYTTLQGRRIFWIPPTWNRAGRFLGPHVTL
YPGMTPD*
>TCRU_1023 protein kinase, putative
MNSCAPLIFDVPPVFKVESIIGQGSYGAVCKAVYGTDQIAIKKIPNYVKSEETLRRVLRE
IEILQNLQFCEQVVGCRLLFRPTSREKDVYVVMDYIPSDLSLVVKSKSIALSENIIRYIT
CQLLLALRAMHRCNVLHRDFSTRNILINYNSQVFVCDFGLSRFFDPDEQLSFGVVTQWYR
APEIILDAEYDAANDLWSVGVIVCELLLRRHLFPGKCNDSADQLNLIFHLVGTPPATVFN
EGQPFARASVNAKNYALSVIEKRPRKSMLRELLLRTPILQGKDAEKSLIIDLAENLLSFN
PRDRPTADKALQHAWFEPCRTFINEMIAAQDEQCTSPQFSSISSLSMEELMEKIEELVPM
FSEELLASEDAS*
>TCRU_1024 hypothetical protein
MSRRKDNKSHLKEFLRRLRDQGRQGTPQASAGRGRARTGEGSGRYALSFSGRSVVATAPT
RGRSYLPRSRKDNEDDSMPLTVANAALSSTDDARDQGREHQRRHRDEETRTSEESSRRRP
SRSTESDPHEVVLSESRASVLPSTSTGSRASGSTDPVSEHAARRADAGRGIGRRRGGDSE
KSLKRPRTGGP*
>TCRU_1025 delta-1-pyrroline-5-carboxylate dehydrogenase, putative
MLRRTLQFAFAYAKCPTIVNEPMDTFEAGSVSANGTLEACRRMRESVTDCPIVIGGKEYR
TDNVIRRTIPSDHKKHLATSYNATPELAQKAIDTALQASREWSQTSFRDRAAIFLHAAHL
ISTKYRHELRAATMLGQSKSPFQAEIDVIAESCDFLRFSVHYAENLYRDQPLSPSSGAVW
NSLDYRPLEGFVSTIAPFNFAAIAANLVACPALMGNVVLWKPSPHAVLSNYLLYKVFEEA
GLPAGVVNFLPCEPDVMTNFVNSHRDLAGVAFTGSTKVFMSINKQIYARLEEYRNIPRIS
GETGGKDFHLVHPSADLKLAAALTVRGAFEFQGQKCSATSRLYAPKSRWEELKNYMLGVH
EQLKMGQPDDFKSFMCAVIDETAFERNKKYIDIAKSSPSTYSVIAGGGCDKTEGWFVQPT
IIESKDSQAQLMHEEIFGPILTVHVYDDSKPGFWSDVCDVVNRSTKYALTGSIFAQDRQA
IRDATTKHLRYAAGNYYINDKCTGAVVGQQPFGGARASGSNDKPGSPLFLTRWVSARTIK
ENFDHSTQVSYPHQLPDRLFV*
>TCRU_1026 U2 splicing auxiliary factor, putative
MYQDRCIFFSKMGACRHGDHCTKVHVRPTSSPTVLFPMMYPNPMAIEHIQDRQWDFHFDR
KYLKRHFEHFYKETWRTFMELGRIAELRVVSNLGDHLLGNVYIRFEEAADASHIARELKA
KKLNEIILLPELSPVTNFADACCKEDLEGKCGRGAQCNYLHIIKVSRKLMEKLEKEQAKY
WKKKEKHSRGSDRKRERSKDRGRERSRSPRPHPSDLCHICGKSGHISRDCPLK*
>TCRU_1027 zinc-finger of acetyl-transferase ESCO, putative
MKRQRSLLEYFSKGREEALDVASASPTADPWAHGATKGVNDGLPLALETSAAASSSLAAT
TSKMPLRHCASDVAFHAAAPQIDDKENVGDGAPSRPPTFLRCAEKTRRMRNGARSPQAAV
QTSLDFGQNNAGGVLKCGLCGMLFNFTVKEDIRIHERCCSALSSGSVSATMIGRQFADGL
DMWIASEQLGKALEGVAQPKRRSQAVRDSTRKCTPEEFCTRVQETSDSELVLYVFDCSKK
DCVEDAVFRRLVESLQFSDVLSCASAYCLVSVVHRHAGCLLCAVAGRPFTRQQDPVLLLR
DSGDSTVTHCYTRSCMTFCDVPYVWCQSVAVLESTARDWWLTKTVTAIQSTRLAIQDFFY
PTRRNDNTQQRLHALLESALRCAVTTLGRHVSYGHTLCPRSEFSYDCSALSIDVLSRVDS
VTACIDSEGPLYTHNNGCDALVDSESSLSVVSYGD*
>TCRU_1028 ABC transporter, putative
MDLLQVKAVLDARHPDVVRVFQDNVPEVGLRQLDECVLEYLLKMLEGQMNPASYLPEETI
RHTLKLYLAEFAVCPSEDALNKAVDAICSEMETRGLAQKPKEDVSRLVNAVSIGRQYEEN
LKKATMINNVGKVTMINTNADWTWETKRNAAKETRRKRKEEEKKSMMAEEYEEFLRKRGI
ANATAIVKLHHKNEGGSYSSDIRCENIHIHMGKNVLLDNTDLNILTGHKYGLVGRNGTGK
TTLLRALTERELEGVSPFVQILHVEQEVVAGNETPLQVILAADVEREQLLREEQDLLKCN
DDEASTRLKEVYERLDAIEAHSAEARASSILNGLSFTREMMSSPTKNLSGGWRMRVALAR
ALYVEPDVLLLDEPTNHLDLFAVLWLEQFLKDWRRTLVVVSHSRSFLNNVCQEIIHLDDK
QLRYYTGNYEQFEVTRVEQLRQQQKNHEAQERQRAHIQKFIDRFRYNANRAKMAQSRIKM
LERMEVVAAIKYDPQFTFKFPEPEPVPGAYLQMVECEFGYKAGQTLFRDVNFGLDENSRV

GLLGANGVGKSTFMNLCYGKLEPRQGHVVRNKKIRVAHFAQHHLEFLSPQLSSIEFMRSK
FPHVEDQMLRAHLGSLGLTGDKALQPIYTLSGGQKSRVVLAWITFTRPHLLLLDEPTNHL
DIDSLDALIEALLEFKGGLLVISHDEHFITSLCDEMYVCANNTIKRFDGDFSEYREIVLK
QLR*
>TCRU_1029 hypothetical protein, conserved
MRYYPSGKDRDSHISGVRPGGKFGALHDVNKRRDILPEHVFATIRDQVIQEERLRLLDEE
ARAHVKLLLASGKNGKRMAGGAAGDERRELERVEKVEAIHARHRALLELYSLEKEQWRAE
LEHRGLSMDT*
>TCRU_1030 N-acetyltransferase, putative
MVAANNAAVNQSRYVGPELRPVEGLQIRTLDDPRVVERIRILDQYCLAVKYSDSYYDTYL
RPCGHQYNQIALYYDMLVGSCTCRLESTTNENEYRLYVMTIAVLAPYRRLGIGSHLLDTI
LRNVAKETKLCIREVTLHVQVGSGAMEFYKSFGFEVVEKVPKYYTGLEECDAFKLRKVVP
QIGTASTQSQRAKSGGVKNGKKK*
>TCRU_1031 leucine-rich repeat protein (LRRP), putative
MYSVIQPAASATEMHVSGQSRNIKEIDLAAVFLTRQDREILALITSFDLSHNNIEQLHQL
DALTALTRLNVSYNKIARIGFLPVTITELDLSHNSLPSLEGIGSLPHLRDLNVSYNCLKN
LMGLSRSQPLQVLRAGGNRIVSTIGLESMAQLRLLSLDHNLIDNANELHFLSSTTCLEML
SLRENPVANMNGYRTLVARLQLSVLSLDGVPLLRAETACPSTPKEVQKLTLAPVSLDTSR
KSPPPMSDAVTHATFPPATPMEGTLAGNNCLQEQAARGTEVPAPSKKVRPKCYSTRGEAA
PETAAGDMPALSECGQKNHIHDHNLPKYGSLQSQRQKIPEKTNQKFSQELLLASFNVEKS
GREPQTKKTTAVEKSKNLAILPSHTVERKLHEAELLLEDMRKENEYLRTRNKTIGDQLKE
ARRVITSQLDEINRLRLQLNSAEENEKKLKGRLDKMKRGARVVDMHTHNTVDAMAIEQER
LKAVYGAQIADLRQQLRCAHRQLATAVTAKITQPSGMIKEKSPPLSSRSVQGEADANDAS
VEIQAVIVDDTRAAPTSGDHNDTSLFTTNGETEEQTPGRIARLRAADIGKLAGELKNWLY
SEMAEDARRAEEERVLDKIRQSLDQDTTLKKE*
>TCRU_1032 myotubularin-associated protein, putative
MSVLIGRRELFAFAERYSSCRLKDYTDLADGVVLCNLFNLVFPEMRIRTASPQTHTLSQR
TQINWAALRSALDEVGIPQNLLDQGAIHEGDAKEGFSALVLFYFLYHLTKRTDFSAEFAA
DVSDVLTEFLQSIDSIAALFAGGALPLDAVPLQLQEQLSKATMARKRRTVSKHVQKGEGS
MAAASLFTSVEDNGARFERQLSFDSEVTSVGQFVLDDEAVRAKHVSNVPMMDTAHSGQPH
YHDKRRKSNKEKTASGKVPERNRSVSISSRSSQMTRGEKNLMDTDAVKREAELQQALIAK
SEECEKLKMQKMELPSKMEKMGEEKSVNVFSAMPDGKDATKQKLEEEQRRVAILEEELMY
LRRILQKTREERCNQIPLTGSFAEELLKDVVDTETGEVVNVHETATSLHGVILDALRDSP
RRRREAQRWLWLIVSAYHVIETRLVTACDVAAMEWQRLEKKIVDGNHHSHSGGADIGSNC
NSASEATAELPSTPGTIAKKLRGFQRVSPSSFSPGVAASTACVDLSKLETEMERRQQQFE
KQLADLHTNEKKLRQRVHQLEMRLRRLAKKTIERELNWKSLCASIHEAERTSFAIAEAES
AEEVEQLLTRRQLWYARADEESAVLVQQGADDTFDIESEPTGLHDVCEAMQTLVLSVTQD
RDRLQREIVELRKEMQTSVQLPETAAHTSALKDEIQNRLMEQKEYEQDEVWFVRAESRPP
YTLSCPSTKRASVTPQTTSRSVVQDGATPFARNLSVLLASVTPA*
>TCRU_1033 cytochrome c oxidase assembly protein, putative
MLCRSWALRCAAKGAGGKPRRIRIESGYEREGKKHYEERGQFFSAFASMSIMSLGCTFLF
VPLYRIYCAPTGRGADPKFYTPQAQREREEMDKSYPIAKKLLKVRFLSDVGNTMPIAFVP
LQKEVEVLIGEPALAFYSAYNRSNRTLLGVSSYTIAPPEATNYLNKIQCFCFEEQRFKPQ
ELVEMPVFFYIDRDFLNDPLVNWLDEVIVNYTFFNLEKTRDIIFRSNLA*
>TCRU_1034 DNA primase large subunit, putative
MQAIITTAKSFAGEEKRPRDVKAAAAAPVLDWATMYTRRPAGTSTLFTLEAMVAKRMELL
AWIDQLMNSPNVRSFSVVLDEIGARLPIERRKHVTQHAADHVVVLGRDDSPAADDADSFR
TPRLSVSRSSGSAALNAFVFEGDEDLISHLLARFAFCMSEQWRKWFVKTEEALLRARLKL
QMAKYETDFLPSLMRDNGLPCDPLSDEQWQDPLLQEYIVYFAVFQGKQQSRSREDYFSVP
MTLATRLIKTRSVLCLKGHAILHRDQAQEVFVTVFRSKLNKGLHEAYLARMRLTSNEDDE
ERETVMRMLDAFLEYFVSDPTSAIQEAVSGAVSAGEVRRIAQTHFPLCMRQVDEHLRREG
HLKHHGRFTYGLFLKAIGLSLQDSMTLFSSLMTVKGVAGNSEAFAKTAYGYNIRHNYGME
GKKTSYSSMSCATLLALPPVVDRFDCHGCPFRFKDEGAFRSMLLKEQLNPLGKEHPPLRP
SASDIEEIVQDCKGQHYTRACYKYFVATHPGVKRDTLFRSPFEYYTLSREVGERPEEILS

EQTTPARRLMTGDSLKRSMFTPTLKEDVIRERKSPP*
>TCRU_1035 glycerol-3-phosphate acyltransferase, putative
MRESAEKNVSSSKLEGVIDNGTHMKPHELHETQAVPTGDKDAEKAGKKQRKDQVSASVKT
GAGDAEMGEAARSSSLQRRRNSVKDRIEVTKHNKFVRCTVCGDDIPIGEWPDHRDRLRSV
RLLSLRVSKFHEFVVSLFIDFFMWVLMNIYFREVTVVNKEMVPKTGAVVFYGNHQNQFID
AMIIRANCGRPVRFIMAEKSFHRPIVGQFARMMHTVPVVRPQDVPIMPGEGFLLRMNGAI
VHGEGTKFHTSLANGDVIMWGKGTNNCTAQVHCIISDTELELTMPVLASNVVTQPTAFRV
SRRIDHSTMYASVYQTLQKNQCIGIFPEGGSHDRTSLLPLKAGVALFSLGAAERGISVKV
VPCGLTYFYGHKFRSRAHIEFGEPITPSEDLVELFSRDKRKATGIFLEHLNAELRGFTIN
VPNWSALNFLHGFRHLYQPPNCTLATRDYLRLARRLNAIMEAQKNNPEFIDFRNRVENYQ
DYCNALLVRDSQAATLGKLGTTEAQQLQLLFRRSFTLFLMGVILVPFFVVGLPIGFLASH
LSQRHRKKALSESSVKVVGADVEGSYKLLVGFFSVPVVFTFVSLVMFFYTDLRTALTVFF
SLPMAMYVSLLILQEAVIELRAALPLFMSVVSKHKQFRKLYERRQALVALTNELVAKWDP
ELAEEVQTYVRESKDDMKRREPSLFSLRHSGLRRLADRMS*
>TCRU_1036 hypothetical protein, conserved
MKSSEVQKDSIRAAILMECVKNKSLDVVYQRSVLYQRLTSQETRNLVYECCLELVEDPQR
RRDVGQLILNCISSFPPSQATLQAIANKCKPYATSRTPYSGIYQELKTVIDTYLHHKPLS
YMLGRTLPRGKEGLIFRQSELSRNPDYIQKCTLQPPIAQHTEKGKRIVPLSSWSNEELAV
TAGEKNRARSGLWTVKYHVVGCKDYVNQLPLECTEAVIDQQVRVLNSFWEKYTTNPALLR
ATSFLIKLMCDAYVSKIPLQSNYSFFRSVSICYLPTLFDLMGSTYACVRNHVYDILLTLG
IHFQLVDTQGIFPGCSKALEEELIWMLLTVLRKQSLHESSDEMVWTAAAKCALAIIPPHK
LYLIDVRALYQFLSLPVIVAYHPHLYTTFAEAFAKRILRWDPAGDATEEMLVLDEEEFKK
LGHQPVAKLLYLYRQSMTLGARLAFFQVLFTHAVTVLQSRSSLSRLYPRVLRRCYIELVK
VGFHWHLHSLLFYQSHQVFRELPGHITYDMDVKEMREYKTEAVAIVECILQIVEEDARLP
DVIMNAFRNTLGSRTEERADLATVLTDCLSMIPAMLKEDTDGDLYNIGWRMAFYCMRLSK
ENLGNERSTAIIETLATNLVSFSGSTDRLELAKIRSVCPDVLASLMFLGRKHANQAVEPF
STLILRFLLDTEVVDIQTVMRLYYRLIGSMSIQNGPLNKSAEADISQMLCSEQLVTAQSI
VDDIGARVLWALYRSLYPVQMFSVCRARHVLASFLDELEPVGGSSQAHSWKAVMADPYPP
VALIGAQKILQMYGRSAYDRFDEAMSIPQKSEKGDSVYALAVKVAQLAESRKE*
>TCRU_1037 methionine biosynthetic protein, putative
MEKVRLVPRKVDLFVQKAQEQGAAATEMADIVRLFPRRFELLGHVVVVKLNPGVTQEEFV
CYAKAFAESFSPRVVDVVLLDTEGITGELRLPKLVTLWAKEATLITMPRESLKYALKQFK
EQSYILSEDLKQFEAYADSPTFTTHVENGIRYSFDVCKVMFCSGNGTERMHFATIEARDE
VVVDMFAGIGYFALPLAVFGFVKVIHALEKNATSAGFLKYNTLQNRVGHLIRTHCGDNRE
VASELCGQCDRVLMGYIPSCTAFLPRAISFLKQSSHGDPVGTIHYHFLSDKKTAEETAKQ
HIRSELGEHVTSLMHIVALRTVKSYAPKIFHHVADIRFEAS*
>TCRU_1038 Dual specificity phosphatase, catalytic domain/Tyrosine phosphatase family, putative
MGLCCIAARRAVSLKKRRIKYKNAPGGTVDLDLVQIHRRIVCMGFPALGIESFYRNRYKD
VLRYLDHMYGTDYMVYNLCAEPKYRYDLTFFHGRVREFPFPDHWACPLIMIPRFVEDAVR
YITSVDHAREAVVVVHCKAGKGRTGLLTCCLLMEIEPSIGGLAKNAISYYGIQRTFDGRG
LTLPSQLRYVEYYERLRNEYGGEVPAEVPVVDIHQFHIRGIQAKVTITSCKWYTCERVCV
ISLNSSRCCNAQVIKSSPHFTTVNVANNDFFKKLSQDIRVEFLNGCGIVGVLTFHTLFIE
REYVVYRGLDKICVLKLPESAGISFDFTSSLEK*
>TCRU_1039 Myosin head (motor domain)/IQ calmodulin-binding motif containing protein, putative
MPAGRIGTLAVYFTPAHHWVVGKITNYDPRRDTYSFSATQPEFINVDNLPRDSSKLWFPK
EDALNDDFDDLLNLVELHEASVLFCLKKRYLRDVVYTNIGPILVALNPFNYFIPAYVEDK
MEDYLSEGSVIEKNLPHPWATAHNAYRELRESGRNQTILISGESGAGKTEAAKIVLKYLA
ALSTKNGTPEDRELSIQINKRVLASSPILESFGNAKTIRNDNSSRFGKFLRVQFDKHGIL
VGCHATKYLLEKSRIIGAATGERVYHSFYQLVQGPLAKEFSLRNPKVYRILNAGECINID
GVDDAVEYASTRQAMGDVGITEEEQMSIWSVVAGVVFLQNTDFVESEERAGLIARMDSNL
LPVLARACQLWGINEEEFMKELISTCIVTQKEQTVKPLNKVKALDMRDSVCKSLYVWLFD
WLVLKINQTTNREAVTTHWIGLLDIFGFENFQKNSFEQLCINLANEALQGHYNQHIFTLD

MQECQNEGIDTTHVTFADNNECVELLMGKVGVFTLLDEECAVSGNETSYLHKLMEKFAPT
KGKSGNAHFLRATGRAVDNHFIIRHYAADVQYNVEGFLDKNRDTLKERMKQIFMQSKVAL
IPEIIPKEEVKTNLKGTVGGLFIKQLLQLLAVINQTNPHWIRCVKPNAEKQCRQFSDGLV
LQQLRSAGVLETVRIRKAGYPIRFKYDDFLKRYRVLFKNGVHGKVASREIVQNCDIDPAQ
VQFGETKIFMKHEAYFKLNHRREQAICRFSFLFQAVGRGSLARGQLFRDYALLHRRRIME
ERRIREEALRMQREEEEKRRVREQAERERRESLLKERRHKAAVSIQKVVRGFLSRHKFLM
VYLSTLRAREEHSRDDEQQKYRLYEANRHRKMCVLEARRDGQHGPTNTHRRELLRERRER
TMRLLEEKRLLHASKLEKEVAQEKAQRAKERVQRRKQMLEMWKGTKTSQKESMDLSGASQ
IVEKRFFGEMIRKLQGHAKDSNESPTEFSQNVGGDSRYQDSTFDIVKKQWEEKERWKDER
ERILGPRHEAHGSALTPRQRHLEGQYRIRTVSNVLWQLESKAAALSFQKQQR*
>TCRU_1040 hypothetical protein, conserved
MAQELLKEGTDGGGASTTALSTVTTSPAGKESVDLLASGTSPDGTQAVDGVSSSDGNQTV
DTADGGKTQEDEPHKTPADNANAADAKAPNSEGGLQDGFAVGPERTSGEDGETAGGTDGQ
EEEGIQPRIREVNATALSSSLGNVCRRGITAMPAPCVSADCCHRFFCWDCGGLPLCEE*
>TCRU_1041 hypothetical protein, conserved
MYVVNLCSLTLAISHVAHFASTILRLCPAAVICDLNSHHELRDGRSPSTTAGEDLAATPS
GMDSELSDDSARATRISGRSVSFPEVTAQLVLRVFHWTSTRLHGLGPPSGIADCGDGGRH
PATGRHAATTETCCLFPA*
>TCRU_1042 hypothetical protein, conserved
MRCASSAVVLTPSVSWCVRCCWGAAICCCAWCSGCVSLCFLVLSQVGSAALGDSTVCDDD
WPCALRPVSSCSDAQLLVRVSEGCCDGVRLRPAGDGAVTAGHARGVMAAEGGPVAGGSGR
KGNVNSEDNSNQEDEEGGGSIAGEDHNPTSGGSLTAPTRQEQSSSDPLRSGTCNPSGTGS
VSAARMRASGPESLGGGHVAGTTSPRSDASQQAAGGVHAGGGSTSQGSQASEQTVTGQSQ
VPGTAKEAPQGDEGGEPGAQHEADQTTKMTVVTSCAKTAVEEESRQRDRRMHRVSIVAGM
LIIIILSCSYRHV*
>TCRU_1043 hypothetical protein, conserved
MLAGLFYTRAAKRQATETKEKGGDLEGDSATNANKREVPFFLPQHSVVANDFPPASDLGT
PDPRQRISDEFISSADIFGIGEPSATLAANENYIRQPILDEQASLEKIKPVLLGWLVRKR
LRSLLGRALVAQIVGMKNSNGVENKLGSLIEHKESQRSQFISRLVSFLENGVITNKEEYW
QKKIDKTSKNVPKESGRRAKKESGEAGINRQLIIQLYSSDESKKTEEELLGSLQEAVGMK
NIHRDKLLRDITPVFLFERRSAFLRTIFALEDFEERFPQVPRLTVGNGQNMLSSDRSFIS
TFGGEYFQLIDNVNHICGNLR*
>TCRU_1044 kinetoplast poly(A) polymerase 1
MRRFLKIYSAREGARRWIALPWKSSIPPINDRCWEGIGSFVVQQALTSDTGRLAFEECSV
ARQQLENIVQAAGYDANVFVFGGLVVLGLLEVGGDVDFVGVMDVEPGVGEAGEIVSRLSR
EMRRLGMKSSALSKARVPVIKVDRVSKLLPGTPLHHLSACGLFQFTRQLNDNETQSFKKR
MQENYAATRVDWNNNQQFATIEFNSTTALVSALTQVRRHEGIDIPFRLPVDPRNGPELYR
LPFDFCFSSIGLRNSYLLSNTLSHYEFSRHLLLLIKKWGRRSGVINSMDGLLASYALTVM
CAHFLIKVGKLPKVSTLRSTDEPQLLPFFPEYRPLNDGKGLDVAELGFLTAAFFEYYGHI
FDYEKNVVCTTNMNLLKKTMRWEKSPGLETGRPPFFEFAIKDPYGLDNIGRNLDREATEY
VKDAHIVALKYILDERNDPEFTINNITQSPPRPQWKDRTLASRGIASSNCSPDQLEAHHM
LKRMEFHERRKAMERFGQRTVRSTEQQRVVSSVANDVLGWIRSDDSQQ*
>TCRU_1045 kinetoplastid-specific dual specificity phosphatase, putative
MCDVLKKIQWSPQQRIVSSVETTSTETDISWLKLSNIFEGLFVTCEETICNRFFAQENGI
SLILTLNGSDYVAPYRVYEYSSDLQRCVYNKLSSFDAFVTQIDHYISKEIPEEIQLRKVF
IRSVSAEDSPAYDISRHFSELCTLIELVMLSRTKFKNCPSSLHAVVVHCLVGVSRSVSVV
MAYIMKRTGCSKNEALLLVKNARPMANPNPGFHSQLLYWEEGGFYRVVDSLTASLTANEL
RQDGALETYMEKYFPLLIRVNRFVCDREFFALVIAEAGLGEDSIRYVLEKVYSYIAFAIA
DELYVDIPAFFDNICETNASFARFLPTLFVHTEERTGYYFDDLLYYEFCKNLASVGDTGK
SFIIVKAFCSFFEAIGSKHFLNNPGGRPQTLDSFGKVVGTPYEELSLTFPFLPFLAPFAA
GFVPQQQLEALGKEKWTTQVMERNLSDLLSLFQENFLLTTFKTGISPDTFSAVGRWHANS
RLLFLESDMEVKVLESVERGSTLSDAVSPYLHLDHLDQNSCLTWMRKVTGAVIGTRFFYD
AVDRYFNAQFRDSISDDTAYLMADKFAPWEEIFKIIQSAGELYAKKCGGESPALLAWIRE

ELRPLVSIGVVSIPSP*
>TCRU_1046 calmodulin, putative
MASAFDEITRQFLKRNYDVFDRDKIGRVPLSDLITLLQVCGATPSELDVESWKKEADPDG
RGSMNFEGFCRVMMIAFQNMKTSRDLKAAFEGLDPEGKGLLSQTELRYYLTTYGDRLSTE
EMNEFIEEMRSEMDMEGNFVVTDLVYKMTPEMFR*
>TCRU_1047 RNA polymerase B subunit RPB8, putative
MVAVPVILEDTFTVTALNPDGVVYIKVSRIQCKNERGNITITSDINTEEFPVSLDDRLTI
MLANSIELSGQSGAKHYDQSVYHRETRLQDCDYAMHGRVYGHEVDESSLDVKVHISCGGL
LTQVIGTPQSLRDIHYNSDVYILIKRVGK*
>TCRU_1048 hypothetical protein, conserved
MRRSRNAVFISLDGVFLHHQRCSNRDAREPYRRRSSDPKTIREKGNFQRDMKFSKQRPLP
TAPWLQDKKRTYMDDAERLYGRKETWCNVKDEALREKLASLLQDYLKDMIDATRLDIRDA
KKYMRLCEQMDAEYRSYVAAATFSSPHLLRSWALTVCQKDEKESRMLSALGDSQEGSLSR
EDDVMDPLREEEEGDGANEEEHQETRLTRKRFLESRRRQGEEATLENTPATMDLLSGERQ
SAPYSAFPFLRQRAMENETTLDPSLVDWTAKYFPEDDDRTFSSPPKLHGDAEEGEPVLGS
SSTQVSSAGNNDFSSPKPSARRRLQRSLRLHQDMHRPTFDAEGVFYRVRRQGNDDDRASK
PREVRGSDACIDRDSEMVKVPWETVVKAKEQGYSVDILRAKERLMRLSRGEDPKTIP*
>TCRU_1049 Leishmanolysin, putative
MPAREHHDCDDIDGMELQDGDGDGRTLESHWSQRHARDEWMAPIGCAGCCTELTLAAPAD
LGCMRVKWVMAEPMRW*
>TCRU_1050 hypothetical protein
MTGNRLHSFGLLRGGFPLSLRGGGRGSATCVRMRRHAAWDFGFTWTLRSAKMPYSHEFHG
GHIRNPYVEQRLCAWRQLTSKVTESATSSCGVGVAGPTNFRPLFHLLTAKTRSALEESAE
NFIQGERRRLYAIYRPSPSGDDSLMPSPYDYVCVLQRLSACGMGVTSKIVYLILVELLKK
PLHPSQAGTEWDLPEEATVNVQARALEAVMSGLEGRDALEFAQSCLMPPLSSTTGTDGSR
HSGEMVLIHAIHRQLKGSGVNPTPVGVNGRIIDGRLIVTPFVVAAVAAALIRCNTNTKKQ
QAFELLQLASIARLRGLNLFDENVAERCAEAAALIGDAETVTRIIFILYRARGVLGQSVS
SENAAGENADDGVINSKGKGGGSLWDRVFLGLGLPASAEKRTEGDGDAAFRRATVRLLKS
VIHAALRRPNATDSREESLTAIREAVRLFGSLRMGCDWDGTASMAVELLTQMGVETRRYL
DHEPHDGDMEPLFYLHREALRVVIVLWDALPSRPSAYQHSVYAACAALAMKCYLVEVSIS
TENGMTVPVLAVPFFSSRIKQLDYLFAAITATLDLFDSMSPECQVQQMHLAVTFSAAAVA
ISSVRPNDGSVIEHIFSRLKELFFRTGLLTTNPTDMMSRFSSLGDQGTVCGALWLYMVLS
AGLLPVLLGEGEKMHAGEVLGEGMDKWVASALPSQRRLLWELLSSPVFHAYFSLMPSLLT
VMVSAVELALDTARTSPERRSEAVAMLRFLYSQRWCDSVADATARAIADRLLSSLLRFGV
TTASATDIAEKCSSDLETNFASEEVILVIGASGIQNARGTKRDLLELLREMASSCETAGV
SLHVGSSGAFTRRLILTLDAVILLVELSLRIDDGKCINEGALEVLSWLQQCPPLDPLFHQ
VLIMTPNSRVASLSVEMTRERRCSVWSQRFRDGVGESCAIALALADRITNPAQASRVAVF
LWDNDDEDDADDGAGRHTGAKTRYALLSRHGVRPLSRRRAQVQRAKADVMEILRASFPPE
SFTARHRRDQKKLCVKSSFERRGVVSPSSHIP*
>TCRU_1051 hypothetical protein
MNIWPSQSKAAERRKAAGHSPVDEMQRFRMLVCCMFCGICTSLIYAFDLFTTDFSNRFNL
SAGDQSTISTVGLVFCYFTIPYGFLYDYGGPFPLLIVCVLTAGVGALCLGLTFDGVITGN
LASISVFYALMNISSGVIDVAYIVTLAETFPRNLGPIIALAKVMVGLGSSVLASISVNLF
RGNISGFIYFIMVYSVVVCSVAAFVVVLPPYFINGWRRRGKTEEQIAALKSLEPAYRRQS
VPIRRLAVGYAVVALLLVFLSVQSPVVSYTRVSNGVSTAFGAITIVLVLSFFLMLLPVRW
LGGMDDRAGDESMRAIVSEEAVDRSEEISFTRADAAVTNAPDKEQCPLPEMTSDTADAAS
EIPQDPRYGGTLWDNLKRPDLWLIFLMFICQSALGVIVVYNASTISVALTGRKRSQQTSA
LYTAFFGVANSVGRVCMGMFEAFVQHQSPNKRRYLVTLALPLSPFLAAVAGTLLLTIPGE
AILLPYIIIYFEEGVFAAVTALIFPSLFASHHGVYYNVGFLTTVISVIGFNRFLFGFVVD
AKHDSLGFGPKEECSVAECVRLPLIVATCVETVGTVMAVIVHIRYSRFVREALRGRFAAG
AGRTVPLRVRCEDE*
>TCRU_1052 RNase H, putative
MTMGASRTTKLLGMDIDPRLTLNVAASRQCAATSQRISQLRGIAHKEAGPSPHDPRTFVI
GYGASKLRYGSELIWGVATDSAKNEMQKTYATLARIVSGVPSTVDPESALLEANMPPLHV

LCLRARLSIFENTRACQMDWMRRPPPEPPPRAGFRISPLSRDELYAFVDAYTKGYGITES
SPREERFFRSSIPPWFAASAHRVTIGVELPIDHSITDEEELIREKRRVSEEALALHSHRS
WILATDGGVDVPKSAGVGILLSSLNSSEIIEKASIYCGARPCSYRTESRALLLALEKLMI
PRIRHRRKTLLVVTDSQSLLAALNKGPLSQTDWTEDQIWQRLLTLTCAGWSVRLQFCYGH
CGVHANEPADQYATQTMESGQHTEQGIAPLWHTDLPTCFTTQLTNKWRSTSRQDTHRYLL
CGTRPSDLSGKDLITQEVLHRQELVHLARARCGESELWGRIYWAVRDCTNQCRFCNISPE
QSAYMRSNNDPTAPGTDTVPPSAREEDVSPVRRRTVTRCRKEKCPHCDSTLTGFSGLVSH
CRLFHPEHPPPLPELKCDFCDMVFPTRRSTAPHKIRCAHNPDATRHRNSSARRRSLLPQD
QPAFTSTPIGPQETLHHLLLECPGTLAVRQRLGIEQDLRLGKFSQWQSLHSRKLLSLLDH
LFGTQMASYS*
>TCRU_1053 hypothetical protein, conserved
MLLGTCIRWSKRTPGYVFWRQNTLVELEKTKRNYLLTERPVPIADIARRMVRVKSRYLDT
KDFHEQNLLRGEYKYFSGKLCDVRHARTEDMVAYVECGSFFGFWDTAQMNRVMDELLLKL
NELGSVELVHLFTSLPSLRKQSSNLYEYVARALVAVVPELSLDECIRVAVACDDGVPEEL
IYNLMRAIEPEVSRGGLSAVRAVELIDAFGTCLPSVQREFDSLFDNMKRTAVAGMMELSV
MDIAVLCTSLKLREELDASTETNAVRVFLSRLEETCARSTSMMFTAVSGTLAFAQAMEER
VVFLATDFTPSELLSVFSVYMDNYTSMYIVTSGDDGVVAPATKKSSSQSCIGELHFAQHR
QTTFLLVLRELMDQMILQMESATAYFLPAVQLQYLEVFSKAVENIGSNVSHLTSAVPVLG
RCLELLSSRVIASLQRYTYMELVALLQLCAPLGKLVADAAVGAAVQELIQREVGTSKEES
MELYSMLQRVSGLRAEHQRHIENFLLPKLRERSGWAG*
>TCRU_1054 hypothetical protein, conserved
MLRRVRLLHSPIDELTITGSCWSRLLARALPKTPPAPSPIKSTPFITLIPTLHVASVEFY
DKVLDYMVRAVQERENVVILLEGICDTEESEAQQMGEYFSIARNEELKAAMLQKADQNTL
YDDETVKTICEELAVNYKVLLKQQATVRLQECYLKPKMAALVGSYLRNGADLNMSEVKAI
LAEEGNGSGGTFFPFSHLGANPAVRQSRERKVARAAQLRCVEWIQRGCEGEVILPWGFYH
AEGIKHNIFATNAHDETVVFLESDDKLCRLPFGITKELLA*
>TCRU_1055 hypothetical protein, conserved
MGLCCGRSALPVVDDTGRQHRTLRPKVFVTPVPTLEFDIVAPEGSPSLALNMTRVSGGGG
IGSIIGGAACMDQSLHTVSEYSVSSPDPQGQSLSGTLRDEANRSFVTNGLNEVLSGNIDQ
FVTGVLEMTTSEPVNILEESLYAASTSTLSRTRNSRGIRQGLVTHSLSREQVRYLDAACD
MTERMARLVELEQMSRQEIFGAWRMNSALMILAMKRIMPTIRRFRGTPRTRRQKSAQEVL
IIHARDIELQMDEEKVKENGVARPGKLNRRHESSEQGPLFSPREEQDMSKRGQPVAEHYQ
MLALTAREISMTASVQAQVVNDTWDFLSLPEAANHLNTGNVLFRFLCAPVMKLGRDSLFL
IDTFINLSSFADEVQENNVPKIDIFSLEEQEKYVTILVKAMYLLPGVRLSNLTVDLLASL
DYQRNCQPGINKFEVSDVPNTFRTIKTVIRFVFSVTIQMKVSEINDPEVLNSFKDVDGMV
PAKVLLLERTKKNIHKIDSTVKVRSVLLYYPVNDGVLVTNQTILLNSSLPVVVSSLMQTF
GSQGATEAAQTAKLTRRYLIRRFGDCRS*
>TCRU_1056 hypothetical protein, conserved
MSVFVRKGAAGRVNADRLYAFNKTLKKDVLRERSYWEVSKDSHPVNRWSYRRWKRRKEEL
SIFEERLDKVPVDQKFKIKKLLTVANISVVGPIVLFFYFLFCYWRHRLWGITPIDGASAI
ARGVQNLPRPPGY*
>TCRU_1057 helicase-like protein
MTNRTRIFNCAYGFYIAMHPWLKGSGYVKVGYTANLSRRLEMASFTTCFTPEWSYFAVFE
LESIKEAFLLEQAVLFLFKDRRVSNRELIQGSATEVLEGARRVCLALNLNALVNMQPNYS
STESPVRGRSGRELFPDNVSSTHNALRPNTLGRYMEALEKLYDSVRKPALCDKITVDRGE
KFAENEEEEEYDLDVLETYKEESFSLTTLRPYQQEAVTRCREELESHGATICQMACRCGK
TPVAFKIIEYYLKNKRDTRVLYLVPGLTLLRQTVRKLYSYGLREIKYLLIGSYPTSVSLG
DGNSLMMTTDEGVIENVFKGSSSQMVVISTYQSSHIVSKLSVFDLTIFDECHRVCGSASI
TNFNSVLFQPRSGNRLFLTATPAYDTPIKMDNTHLFGGIAYRYYLREGIDAGYVNAFSLR
IVLGEEMDDMNPYFYEAMRIVDKMIVYCRSIEHAIQLTESLKCPFPDDTVPFNVMLAHSR
MGCRVVTDVLRDFSASKRCILFNVRLFQEGVEIPDLNAVFFAAPRYSSRDIVQSICRPLN
KMENKPKSYVFLPATIDKNLPETHPVNLQKFSTLIPFTDALMDEDPILFEYLIDPQKVSY
DIDVVGLRSLKLSSERLRKFVLPAIRRGVRYTSQNTDRLHRAARIPWKSAFGELRRIVLE
CNRYPKTNDAWVIGKKSVSMALFYRYARKGYRQYMNHEPSYLKVYQIRDLESLPLWQSYG

MQGPYPWEECLRTLRNYLSMHAHVPPLDVHKGGYIGLDATPFERLSGALMHINQCDAHDK
LRLDLSKQKDLDDLCGAYGLKWRKRRDASGKVIPGETTFINESYERFKKLYGSCHRNQFQ
KYIDQHFPGYPEKHERMEDPRNLKKGRVPPRHVSRDERNKMHSAGRVMCRVCRRHIAVKA
WERHLKSHPHLANLQQITL*
>TCRU_1058 methyltransferase domain containing protein, putative
MEVIRKSTRNCGGGKFSDPEAIQLHANDFSWEEKMEELSDDERKLVQNYLYNCEQKLLEL
NMKSITVHLPTPARKADAWDSHYRTNKRHFPLKNYIVLAFPLIKEMCSSSTQSKCLYVLE
CGCGTGSTLLPLMRQFGENVHFIGFDVSDNAVSVLLDHPLSKEFIEGQRLTVFTHDILCA
RERPVDGPEKSRVRKESGGLKSVILKRVSDCTHGVDLVLLVFVISSLPSLESMVYALKEI
AEVLKPNGFLLFRDYAVPDHNLFRFTRQGNEVVNDLAFRKGDGTLQMFFEANFIRKLFAL
AGFKEVDGHGLQYHCNRIVNRKNSKKMDKVFINGAFCLPPT*
>TCRU_1059 RING-H2 zinc finger, putative
MQEGCCEAVSPPEGNEGQRRFELKRWNAVALWSWDIQVETCAICRNHVMDLCIECQANQS
LSPTDCNVSWGACNHAFHTHCISRWLKTRNVCPLDNKEWSYQKLGA*
>TCRU_1060 hypothetical protein
MDRLKLSTKALLGMATRTVDQDHGMRHTCVKSLFQAVVQYEFAISKLVAALSTLSDSLEG
VEKAYKNVISNEKQVSVFDGLNEVTNHLEKCRTTVISGSIETFRANVAPSLSALKEHCEL
CERLHNERAKAVDLYDYHRDVVEKKEVQYASKGKLLDDSKRYKEEISKRDAAHDAYLAKH
SEYNKAYDEFMCKKSELTTLSGRLFFHELLDIAEKLKNEVSSMCDVVDQIPLNPVGYKET
SAPLGGGVDAETNVIEYTINRDSSEAKDADAAVLHESTDEQSPAEICGESKNNAENTTGP
AEE*
>TCRU_1061 syntaxin, putative
MPMSRDPFDESVSDVSELVAKAKSVQESFRAKGIVDRVLIEEVRRTLDAADEELELLKNV
LQVIEQRNGRVGDQVFSVNEVLRRQGVVRNLETELKGLRTFSDAVEVRVVEAEKRLIMNS
NEIQVNDSFLLGQEQIQRKEHMEQDIILDRLSHGLQELKETGVNVNDELQQQEILLVEAQ
QDMEGVQARLRVLNTKIDKLLSSMSNRKKICTILILLVTLVFLASIVFG*
>TCRU_1062 hypothetical protein, conserved
MEDAVTLSATLRRASPLTRRRALVILFALRGVAKKSDMTLSLTSITTALEFSMAGRPARF
LFATGGHASVLRSHDRGGTWRIAYRVKGTSNDHMEEGVPPSDKMVTMEDIAKFLPSSESA
KGQKWYLASECSATELKTQISHVTALDSMLAVCGSRAFLAVSVDRGMSFFCVKEEDIHSF
MKDEPSASCESWQIDGVAFLSDQLLMFFSKNSLFSIELIHHHHKQQQQQFGVVAFGRVRL
VKKFAVPVGCLKTFSQSGKFMCELFVSTKGFLHYSSDGGQSFLTFPHKIGIIRAIEPLDI
VLRRREVPQLPNLLLTHMQSAATEETKSIKTGKYIYRAISRRDSVEEKDDGKQNVVDSGN
WVTTGAFPYCSENVHYRVFLVGGIGTSILPYDYTALLFIAATLNDSNGRIGLLGSLAESV
DYIPYVRSGAAEPISIALCRRPGSKGCVFARSNASGVSLSSDMGRSWTTPTQAYTVAVIA
MDGGEFVCCNRRNVVSNIHGDSREVHSIPADLRVPYLTDAAVML*
>TCRU_1063 WD domain, G-beta repeat, putative
MSSVPYLKPTQFQSFAGLFGSGTAALTLRSCEDSNMPHHLIVGGEDGNLVLVDYELGTTT
RRWYGAHGRDVNSLTRPLSSGVFASASRDKIAKVWNLHSGEALCELHGHTANVTSVTMGA
EHNFLVSGSRDNTVRLWDLERAEEVQCCEIKQNIVQFTQWVPQLHCVAQGGEDLTVRLWD
VRMNANGKKALGLSLATTLACIEYHPICCELIPGEETCGLLTGHNGFNGHGAYVVEWDLR
MPKCLRIFHGHAGTVRSIRTLPRQDPANHGEFVSAGDDGAMIFFSMGPSSHETDVAYDPR
SIFKLNEGRVTCLDVCANGDIFSSMWDGAVSVLRCTSPKKGVKGTFKRFRCFGAEELPK*
>TCRU_1064 hypothetical protein, conserved
MVVHEIAAQPASKVADVARSLAVKGDRVEAVYIAHEILEENLAKLPVMIAETRRPASSYL
DPWKSISETMESLCQDVELCLQLFSMGANIQRFKGLRTVASLYDQKKPAADRLRGFFLIV
SLSFTVDDAVTPMPARILRKFAQAICIFDAIQAAEWLSVGVQNMLDAGRLSQLSDSFLVS
RSLICISVFGGEKTAPGCGKAKGTKASLMTAILTLLIRCRADAGVTRMEVHEREHELRGF
LEDDKLKSLLHFESVCATAMAEKKQRIQEKMARRTADKRATAATKKDGADSKEMNADAHG
DKTSYRRYLNSSLEYFLGSEWRQRPSRIVLVVAGVILLLLMIRQVASIVARGLRKMKSLP
HGARKTLTL*
>TCRU_1065 cysteine peptidase, Clan CA, family C2, putative
MTTLEDLYASYCRRRGCRPNSFLSRYLQEEFTRHSQQRVLESVDLSDNYVGAKGIIPVLD
LVKNVKTVRRLDVRKNMLEHEQLEHLVYCLALHPSIEEVDVSGNALDDGSVDIILKLLQA

NESITVFRVDGNHFASSSLSLINDHLERNKERKAERQKEMPPVYTGKWFRAQVRGSLTEE
TSGGHVHFSTWWKNQQYIMRVSRRAQVRVVMDVDDPKAARQAGFFVFYSDGTRKVIVADA
DHIAAESNVDHSHCFVTLLVEEHAAYSIMPFTFHPERSMSFTLMVELCQESLKSSEGWVT
LEPVDSALDWLVYVMHGEWTSASAGGSPTQHLWCRNPMIHVQYAGSIQSYRMASPATVFV
QLSKSIDADENDDRHIGFDVVTLDTTGNNKPPICCKEESRKCTCPHEHRTTVTASFVIMC
AALDLFIVPSTAIPGELGSYTVTVFSSVPLRLTTSAFPHGWNYRAVKGVWDEDNCGGSRE
RSMSWKCNPSLALHFDAHQSPPDLTLFLEESLPRPKSPVRRGSADSCGNLMDSLSAVEET
QKKKELKEFLHRHQLSHLEGCISLVEACPPMYRLLYSSEYSRGSVVSLVVPAVSEEFFLL
AATRHAGQLGNFILHIFSSRPFVTDELKTLALREREYQLLQCAQESQARQAANSVLAKKR
SGPKDSEDAVLAQNEILLRCMVTGEKYVDRDFPRGGSSLWIDPAKKTPAWCGKEIYWKRP
TELVEEVTFLREWKCDCPFPFSRREWFASVTYAIATKPLWLQNLTAGYNVTEGLAQFRFF
KSGQWTLVTIDDYLPFDSTMELCMGRPSRDNTDFFFPLLEKAYAKHHRCYEALELKVTPE
LSIVDVMCHGLMDLSGCAPVHFPLRGSVEMSAEQQNILWMKLKNAIQQDVLFTFLLRGDS
AEAAERISLGILSDHLYPALDARFVEGQRLVKLRHWGQVGEVRWGGKWRAMSTRWTTILR
DLLKFDEDDRETFWMSLDEVFFYFTDLIMTAGTKHTSWVSADFADCPKECGTPVMEGAQF
TLRLGDFPPDLNKTQISLGLHQPDARARVIRQKNALAAYRTAIGLAVVATEDNTVWLKEV
READVVKCLEPCKCRDVMCSLNIDMDNVKGSKRLTLIAFREDQKAANVPFLLSAWSDNCE
VALTPITRDIKTTVSGEWPIGYPVGSPSSSFWRDCPQYFVFPSESTEFLFVLRQDLPVGE
LPKPIGFTVHREMTCRSYLEYNPDTVVLYVQAVASACVEGTVRLLGMKERRGMPYIIVPF
CTEATPGGKFWIDAIANRSSRFCCIDPRLDWYRDRKSVSFTLADGSFGGSPRFSSWRSSP
QLALNFPVGGQGRLFVVVRNDDLSDNRTELGMMLLRGDNQWENGLRRKLFISSGDIVARS
EEKIGETVIDCNVDVQPECTLILVVYASMPYREAAVTVALYSASAVEVEPVKEWAQVAVA
EGSWELGYTAGGGSDQFGSWINNPFVALNTYRRTQIVALLLQYPQGPDKPLVKRAGKKKA
FLPPIIINPNNRMMIALDLNVQNSELTPIASTPYTYNSEVTLVAHVPAADSLPFLFIPHT
KLPEGNGEFKLFVYADSPIELYTLEKKRLPYV*
>TCRU_1066 DNA topoisomerase IA, putative
MMRRTLAALEKLVIVESPNKVIKVEGLLSDPNVVPDWSFKQEHLRRLGVGAEKAVAMATT
GHFMAMKEIQWTPRAISFPEVRGAGEEPFPPNGTVAEYTLEWQLLPGRRIQETLERYIQE
KLNNVTEIILATDPDREGELIAVHALQTIKRLYPKLKVPYSRAYMHSITEDGIRKAMRER
LVDKCDYDLANAAETRHAMDRIFGFLGSSVVRAANSQMRSIGRVQTPALILIHEREEKIA
AFLEKNKTNYIVDVMCQFPGPHGTTFSQVVTINPDVKGEASHWATEAEARRCLHGWKLNN
CSGFTVPLEPLIVPSEVPPPQPFTMATAIAKANRHLKMSSETVSGCLQDLFQLGHITYPR
TDSTRIDESALTDIYATIKKEFGKEFLYRLEDRTVSAPEGKKSKRTGKKNSTKQSKGADQ
PVGNVEDAHEAIRPTNIYADGESLSLPPMTRAVYDLVRRNTLASFMIPMKSEKIAATVKF
TSGSGDKLQVVLEGKRVAEPGWTRAFQKTDFTAAAAGAASSTNNTTDSSVNDKNTTGVAE
EVTEDITEKEGAPVIRSLSQEEFRAIANLRSALASLKQSRFFELRSPSVRENRPVPPLLH
SEGTLIEELKNNGVGRPSTYPMIVKTLLTRGYIAVNPKGRCETTPIGRLLVSTAKSTFPS
IVDIGFTATFEKKLDLIAKPNPAKRPTILRGTNVSQADYFLSMFLSTFLNYVAEATRAQR
ASIVERSMRLKHEQEEGSGKDDAEFEESVANAKKKVALATGDLMALPKTCHNFTALQRGL
HDYLRRHFPPSRLGSFATAPANADGNNMMMEGTSPHMISTKHKLEKSSEV*
>TCRU_1067 protein kinase, putative
MPWSARGQQNSATGAGVQLTTLPALGTVGASTAASNLYSGDLGKQMAAAEVSNKISSRAL
LNTTALRTARSISCMEVMNNESSPDLSSKEMGSIHAMLLDASPRQGRKDFKAADAGTSGK
ETTPILVKRCSDSSPSTPLGLNGLRIEETGEQNTVTLEEMLREASEVHGISLKWILYGVM
LVTVLIFLMLLGEVHLFVKDNIIIPEEELVGISSATAMNQLEWLQYGVTNMLHNFELIRG
DPHDTSTNYTLGILCRSIGGAPLVFARYGPLGTPLLESYACPSATTKPVVPPTSIGTQHK
LPTGFPRMAVYRSDFIIAMMTLRAGSEVLTVILHKEPVGRLLLANSIPTYSSLALKHSAT
SFFLPDFSSLKIVVALHSLDAEYTRYDVAQPTPLGHVLKNVFNGICAAGKFHTWHTVVRP
DTNPLPMRENDMNASHNALPGAYVAYRGLWGSVSRVVICGVLCSESEKNECSSDNPTRVW
FIVDDEPLRRETEIALTAVSIISSIAIFLVLVALVIAYLSISAPVQHLKSLVFNTVGDKW
KKTKQQQWVFHHTRNCWPGDLQALVRSFQVLSFCFRFNKKYVPQHVLEQQVVALRKRKDY
LWRTIVEEIESDDDEEYDGEEEDMVPGVNDRMDMEAFVKTVAIPDGVAAMGRFNGPEGMF
QRELSWCGLISPDVEILEASGNNFLGHSNIDLAGGGMTLVEGATVLVLVVRLCAVELAYFTN
YRAALKPHRRIMNLLLGRIRRYRGELFERSGDCIAAAWNAFESRTDHAQRAAACALSIAK

MLSCYREAGFRVGIVLHQGPFVCGVVEDNKEAFTTVFGTVPRQAIALAELASSLPYFRVL
VSEPVKQSLASLYECIMVDVIKYHEEDNPIVMFELYEERRLPLVKGALLSSSTFAEEHAR
VFFDFRNHDFARAMMGIEQLRLRFPGIGRRLLTRIEILCKYYMYHENELPMPYFRPFPIW
PNFEAIANTECSSDIGVQSTKEDSFLDPPRYLRPTKSIYEDEAVNFRQELHDKVLASKRT
QNQSPTLKAANDAPSKDAEANNAQSPHSLIMLSQTQNMLRSTPSLEPETGEGDDAASKEH
QSKPLLRVAVVSPHEEHAEQCVEADKKRCSGASAGKEEVNREEFAPSIREPTANACATAA
VSATGSDEKGDPGDEENDGAGRQRFSLTNRMGLAFPDCRSCSFFCGTDSLDSINSETVVT
APTFSLCNIDGSLTKVASEIPTEIVAKNGITYQRSSRILGKGSFGCVYLGMDILSGRMVA
IKFLPLPSEEEEIANVEREVVTMQKVKSNQVVEFISYAFKSNLIIIVMECMMAGSLQGMI
SAFGSIPGTTARLFIRDVLRGLHKLHSLGVIHRDVKPQNVLLTLAGNCKISDFGASAFLQ
ELVRRRMEGKGLQIQGTPVYLAPESARGTPEEKSDIWSCGIMFIQLVTGKLPYAKPFLEM
SPQILVYQIGSGAAKPIIPESLDEFDAEFVCNCLKDDPNERLSAQRLLELPLFAL*
>TCRU_1068 hypothetical protein, conserved
MYLRLQRLLTSSTEGRIFARGRSGFLSYPMNGRGLHLSGPRLSIGSGSGSFFAETGFEEE
QLQQQWASDTVNTASEISGDGVTRHTPTSGGEKGALNAQEVCGLTAASILREAVPLTSSE
DIKDLLKGCKNAKELRHVLDTVLYRNPLCHDVLNGHGYRVAVLQALTAVAPRTQCLHEWF
DCVDNFRRLGFVLTRTFAAEGFTVIREWLSHHFNYEGRSPLLVTEGISHIRELVQFCKED
RLVFDHVLYTRIVFLLTMIVGFFDRQNIYRSAFPENFTKRDGVVVEWVISCERCVDFDEC
VLQCDAVLEEVLEMLRQDIPSRPNFSIMYRLIDYYFATDNVEKMIAVMEDTESYGIDIAE
SSTAKLMQLACALNYPNVPELFMRWRVSLPQCVLATPDMSRLMFYYSRSGGGRPCPLCNE
PYNHRNVSVYVWQKTPEHQRNCQALMDARRRKGELDENTTLPQNQDWSEMAFGLWQLSQS
RAIEWGAVEWRGFLLCCMFSPRALEAKGLLDKFFDHTKMDDFLRATYVRLLRHHAPELVF
SALSDWREKGQRLSPIVLQESLMGSVMINEANERQRAVKHIWKALLEKDSYVMPFTKRLL
QRRLEELRARTTGGITSEETALFLEIIGMQPRHLSLLDMKDSTSDFVVGISKKNYYAPHT
SSAGAKIESGRQRRRA*
>TCRU_1069 serine/threonine-protein kinase, putative
MRELALPGYTHVQHICSGASHETLVAQSDDTKELVLIRVYALDYLRKHPSLRRRIERRAL
ASRVAHHPNIVKGLPAFVSDVDLFLVEEHCVGGELLIALDEYYRSTAAPSANDTGRQPAQ
QPFRCLSLSFIRRTMQDLMSGLHYLHSVCGLVHRNIKLESIFLDSSNRAKLGRLEMCAAI
PSFEVKDGMLQLCCASKHYAAPELVMGCPYRGELIDVWSAGVVLFVMLTGRFPFEEEKEV
VDGGDSLLFQRICTADEVLQIHPALGMVPDPLAVGLVRNMLRVNPECRLTVEEALQHPFL
LTS*
>TCRU_1070 leucine-rich repeat protein (LRRP), putative
MRRVAKHANAAISTMRWLWTLHDVAACPSIDVLRLHMCQRAQQETEWDLDFALLSSLNKS
HAPNEVLRCCCAAMSSAAHSVERIAVAVTSAAVGGALARVLCSAQVESLRTLHLTLHEAA
FLRDDLSLATLLRSTAHFTSNLNSLCITMVACDMQHGLQWNQKSLRALAANIPSLALRVL
SLEYINLKGANENDLMVLCEAIAHCHLLTHLSLRGTRGLLPRVPLLRFAVAQMRKLEVLD
LSSTLLGDVVMEQLVDTLRMSIGGWYRLRQLNLLETNVSVWGLRRLLRDVGETSHGETAK
IELLNFSSNSMDDEGAFVLASVCMHCGMLRELHLRHNRLTKKGAADIGSALIAAASLRRL
NLHSNPLSDEGLFALLQYANYWPELRSLDFTRCRLTARCLPALCAALPLFDRLEEISLDR
NDLRVVDAGDASTTEDKRETPEGDLPLFAYDPKFMQGGSRGDWKVSTSFELDRRDAIEGR
RRFKGTESFQTPLSSRESIGGVVSFEQLGLALSGCRELGRLSLSSCSLTDDCFLAMAGAL
VVRRLTHLDLSANPLFTRMESLEALAKLLRRASASMQKLDLSCTGLGSVGASLLVDGSPT
DDTNGDADEAVSALRSLTALTELRFSSCRIGAEGFEAIADAFPAMTLLERVFLDGNAVKK
LDTILYLLSALEALPSLRFVGLYGSVPPHLAATVGASREYAALQSKGVVVHL*
>TCRU_1071 hypothetical protein, conserved
MKGPKKRYRGDGDDHQSYHHHHQQQQQQPRVRYADLAPEVKLQRRREERQVKKEMERTL
SYFKSVQRALEEQASGAGDASTLASIVDGFFVELIKLLKADSTLHLLRNGLVCRVIESAL
ANSLLLHSKSLLYVLLGHVFDTVTSPTASYTMETLLASLAQALSALADDEVESFEAEMTD
GGPGVHAGSGVPSAATLITYVVEELSERAEEIIVHEVAARAVRSIVLVLGGFTIRGAPAL
EHVVKFTQPLGALGVALMRGLEATFRDHNSRHAADVWLNIANTPTASFILQSLLRVCEAG
TEVDVAVRQRLESINDDDGVALLRHLLMDPMGCHIFQAYIKVPVPLELLEACDVLSNRRA
RAAAELTSAKGRRLQEILRETADNVTDEEERVVALRAQEKTCWDKALDVVMEVLEDIFDP
AAEQMNPVHYVLQDLVLFAPTAVHLECVWERLLKPRIRMFVSLPAPMHVLAAFARKCAFS

GSLGPRDGGNNGNNYNDIAVAGAVESSSDALPKDIDQLLRTDMARGVRYFTVSVNFQETV
IKTLCSAINEECKKGAAQFLLVDGNAHEKGAELARYILHFQPLASAMFVHAMDKLSLKDM
EFLIRHGKGSLVVQQYLRAAAVVQTLPHAQGNKGEEKKLDKTGPMRFLRRIASLLHDLVG
DTYAAYVVEVLYEVGSLEVKEELVKLLVPIYSSLQHGHEKASKVEDRDESREVEEDGNAT
AANRPQMRDFIARKVMTKCCVELYMHRPDDWTKLARRQCQVMRLLQRMSAVSS*
>TCRU_1072 hypothetical protein
MANSRKELRAVQDRLEYVERREKEREQDLYGQLKSMQLELSQMRGNFTSAENECRQEMSR
LQREWQKATAAAKEAQKKAENIWRDLILEERERLATEHQQRERERFDSLHHVVESLANEL
ARLSDIVAATRTWNSQLSSHVEQELERHRSWLKRHKREESEFLKLFEETCTQLQDALLHE
RRAREESMNRIEKLLTRRGVDSAASPVSNLHRF*
>TCRU_1073 CHAT domain containing protein, putative
MGGKTNLCELYRTYCAEEGCRVNSAVLQYIESRGAMCPLEKLELGSNYLGARGLRPIIRL
VEYSQTLTSLVLDGNGADNETVDRLCEVLEKHLSIQYLSLRKNPITVTGGKRLLALVKQN
PRIVGVDLQDTDVFDALIYKITKVTESNHGRGMRERPQLTATELDGTQMSRGHIGAAATR
PSAERPTLSTLPAISRDAAGKEALSKVIGPLPHRSSVAPVITKIRTRPLEPALASKECGR
LPDLQRQELQRRYKEKAILSREINSSQASRAADRAREELMLLEKTAKSSSSRKKCGAKTT
TAVSLPENTKNRKSPLSHHAQTTDFTEMASPKINVDASLLPQQQKDKDNGMQGPEELDAA
ASISNSTGELIVKGKSGDNNEAGADATPLPDADAHVVAPAEQTDAEDAIVKKKTESELVA
TNNSDDIPLDILRDTPQWMMLDSNEQFQRLFDGGCRAYVRHDFDEAYTTWNEAMNIAASK
KNREWMAVLSSNLKRLSYEILVREGVEELKQDHLEEADLIFQRAQEVAQEARNAKWEADM
HKARKDVQHAVFQRCHEAALKVFERAQQVHEHKLTEDDYFVVPGTDVMVQHTETYVNVWP
RMLLVKEAVEMWAASRRVTQRIGGTESRVLQNVVNEALDTVACFLAKRCFDTEDTQSLSW
MRTSLYSYHECVMLSSLWTDMASCDVFAEHHKLFAALAAAQIGNFYLATNQLSEAQAQFD
TLEWLAGELDDPLLRAAGHTFSAIVNWQRSRYAAAEPLLRAAVLEWGALRDASSSQRKKE
NGGDDTADSNEDKTCGSKLLASVPTDYFSIMESVSFKYLVSSIASTYRYSEALEVLEHSL
VCKYRDLLFDKMRVNFGAQPTLERIVATSSLIRSPFIYQVATHRYDWSVDERCYVVDEKL
LMWVVLQRNEMRFVEVGVTKDFKVPSINGLIETLRRGLLLDPLSSSGNTETEITLSLPKR
AWVEPLQTLYAIFVDPVVEFLRALDPLFLSKNGVITMIPTEQLWLVPFNALIARNGNFLV
EDFAVQLAFCATQCAFSVLSAKRVLQRDLQRDVVLVQREIDAPALHLLSHVAFPFDSLRS
RKEGDLIVRTLAENKRSVLQSTRTTDSVCVTTSSETLVEDLDAFRELLPKSRTVFIATST
TSATRNDDNSAGAICMAVSHEEIDLLRSSEISRMRLFAELVVISNTNMSNARVAGTHDDV
QGLIRGFFSSGVPCVIVGQWCTPDMTPSELFSKFFQLLSEANNKPRRFSCSHEVATTAIA
TAKTTTSDAELKHPVRANDEDEVGGHDAACFYRHKALLLAWAIRALLEDNFFRFAPRTWA
GYCCVGYGSHQ*
>TCRU_1074 hypothetical protein, conserved
MADHQVLFFGCVAVTLTEVRVKDAVFPTKMQNNNGVNFFVRMAILPKEGNETEERMGEYV
DSSMHPLKSAEMAEELLIPVMLERALPVSAVAGGLSLSYETPKQNNEKDHIADDNGTAVR
GDNGHGMSVELHLEVAVARGTLQATIAHRKVIIESLLNIAHEKCGVVAAHVSADALRQCA
VWGKGFSLPTTDKSCVLEETHEDDENDDDGVCSGMEVEFIVRALDMSEPLRFMLAAECEA
RNLLHMPKREIQTTFLLPALRLPYGALPLFLLHQHYRRFYEHFFNKTLNPSDQVAKLLSV
DDGNIGACRWCALSVVDMRRNILGEEALAPLLATLYHCPSLSSLVLDQNSLGDFTCYWIA
ALFHKHRLLREVSLYRNNIHECGAEQLLRLTRRNKILTQLNVIGNPCSPQILQRIQRVTE
VNQAALKKDPFNVISSLYDYAVSPNSFSPDIVKKALILWAMLSSSSLKAADSTLESKQPS
LIPQCALAPLLNEVMRTVALEMSFLIQDHFIRMVFVDIETHWRATPSLQDEDGKPALEED
DGGRETGHGELRVWEGNDDEGEHVFPPVDVEELYSYSFLRVIVVTMRSMLREGDWAESKT
VLQAIGRSQQAIGVTPEDYRVALKVFIRALTIVCGGEHADAENSAAFLQLLALGVRTTLG
VQCHDCNK*
>TCRU_1075 major vault protein, putative
MDPRVVRLEPYEYVHILDNNTCKVTLLEGPCCITLLDHLVNLHKNAQHHIVIPPNHYCEV
QNPVVLSPDGGEPTYRMGHREVRLSQPPFPLYPGEVASDLKPMRILTSKEAIIVRALEDH
TTTEELTGKTVQRIAGEQWLVKGPGAYVPRVDEEVLRGVAPLLLSANEYIQLCAMADFKD
PDGTARRVGEKWNLLTQGVFFPGPYTKQEPVKKGITLSPTLALHVRAVHSFYDTRFGIQR
CLGDRWLVTHDEVALFLPTEDEDPETTVPLTIVGQQQYCILLRTVQDGAVHDGKRKLLKG
PCSFFLKPGESLQDNEVKDAYLIGDHEALLVEAVSSFAEEDDTWREVSSRWLVSGPCSYI

PPLDVCVLERRERLLLTGSQGVYVRNVRTGMVRAVHGQAIMLAADEILWEKPISPLVHRL
LQADTFSVYDAGALKSASEKEPYASHKVVFYKVPHNALVRLYDSSTNTSRVEEGPAALFL
APNEEFTPISLSGGRPKASNRIHSLCLFLGPDFMADVIEVETLDHARLMLHLAYNWEFDT
TDMERIKKIAFELPDFVGKACNTLANRIRAAIASESFEEFHRNSSSLIRQAIFRSHLGTT
EMREDGLYFPVNGLLITNVDVQSVEPVEPNTRDALKKSVQLAVEIITQSQENEASHQAML
LEQEARGTLELQIMKDKASEEAERVTLLQVTAENTAIELSGSSRAQTLAESEARLVELQA
ELDVTPNRCRALEFMTDAELDLQRERMEIEISHRRAMNDLAVQKAKALADFEATKVEKIF
SALGRGTVTAIARAGPELKAKLLQALGLKGFLVTDGTSPINLLGMANCMVHDPRGKRA*
>TCRU_1076 hypothetical protein
MAAPLREVVIGTHDSANSHNDNGASLTMPPWDDHLPELPELAEVEAHVASLRALVTNGGA
PIEPTHLQLIDTIVDRLDQVYYWAVEEGTTLLSEPSQLAQCKVPQLTVLCIRTKRPRIVL
AALRIMRHLLRHGQTGPQLVAAVAEKKEEKNISTVNADDEELFGMLASVMEQHAGLSDLQ
AEAAAVAAEAALIDIEGLLESPVISHVLAALERHGLSTAMQREGVLLFSTLVDIHRGGTP
RENAANDVRPTPVAILLSHVGVVVRFVVSVAKAHVGSVDVKRNAVHFFLRCASYPENLDV
LLREGVYPVLVGALPSAVRVPELFAELTEAIAHFIPLLDPLQQRSLVLVVRRVLLETTSP
ILVSLCAALLVRMLQVEPRRGDPMPCQSMQPGLSQEAALNLCVRGGERTLSDTIHLGEGD
IRVFMSSNFIPQLLCCAADTIGEDDAILRRVVAEAIELLSPYRWR*
>TCRU_1077 EF hand/EF-hand domain/EF-hand domain pair, putative
MPLSETQRERFSLQFLIFDKDSDGRITTEQLGPLLRIWGFCPSEAEVQAAVRLLDPDNTG
VLTKAQAFDAAESMPNQMITEVDVREALRVLDADGDGFLTTAELRHVLLNLGVRLSLEEA
DEVIADAYADEDQQVNTDDFARMLFSDASEGKMDI*
>TCRU_1078 Acyl-CoA dehydrogenase, N-terminal domain/Acyl-CoA
dehydrogenase, middle domain/Acyl-CoA dehydrogenase, C-terminal
domain containing protein, putative
MLCRFFCARRTAIAVRCHGGVLDCARRCGSFNYAEYNDGDPLLLQEQLTDSEVEIRRVVR
EFCKKTLLPRVTDAYRNEREDRKIFRELGALGVLGPTIEGYGCAGISSVAAGLISREIEA
IDSGYRSAWSVQSSLVMHPIYAFGSDAQKQRFLPKLATGELIGCFGLTEPNAGSDPMSMT
TRARKVAGGYSLSGCKTWITSAPLADIAVVWAKLHDTNKIAGFIVERDFKGFATERIEGK
LSLRTSCTGMIVLDNCIVPEENVLPHATGLKAPFNCLSSARYGIAWGVVGAAETCFNIAR
DYSLERKQFGKSIASMQLVQAKLADAVTEITLAMQSCLRAGRLKDEGKLSHQVISMLKRN
NTRKAIEIARAMRDILGGNGISDEYHVMRHVCNLETVLTYEGTYDIHGLILGRSITGMSA
F*
>TCRU_1079 actin-like protein, putative
MSGDRPVLIIDNGGYTLKALYIPDPSNTALQPPRQAVVPNCVGAASFAGRGIVGEQLFKM
PHFHGFMLRRPVDRGFVVDATLQSYIWEYLLQYFAIADESDVELVLTVPFAAPKQVGELL
HYLVAQRFKFRALTVVSATFLALVADASRDWLHRNVHESCREDDEAGGGCGMVVDFGFSA
TTVVPYVDFLPIRESAVRVDVGGKLLSNRLKELISFTQVNMTEDGWLVNHIMERCCHVAL
QPKASLRAAQRQKQRGKSSSSSSISDGGGGICYGNGNAAGLRYYLPTIPPLMPLGCREEE
LHAVLGGDGSGVERHELQHLLFHHEAFLIPELLFHPVDVGIQQMGVVDAIARGTGSRGML
RDAPTLHAALLHHIVVFGGTANFPQLRERLQEELRKEFTACSTSASLQPPVERLRMAPHA
IDVDDTAIGYSAHDSELQPLYGALALFTCPSRQPQLRLLRSRCHVDLTTLRKNKTGVGRA
QASVALKTQKTLLDAMQHLP*
>TCRU_1080 hexokinase, putative
MSARLNNLLQHIAVKDKDSDTMRHLKQRMALASLANQFTVGKDHLKQLMLYMVHEMIEGL
EGRESTLRMLPSYVYKTDPSKATGVFYALDLGGTNFRVLRVTCKEGRVADRVDAKFVIPQ
QALQGTAEDLFGFIAQSVKKMMEQKAPEDLNRTVPLGFTFSFPTEQKGVDHGFLIKWTKG
FSTRGVEGKDVVELLQKALKRMEVKVKVVALCNDTVGTLITNYFFDPDTQVGVIIGTGSN
ACYFEDAYAVTKEPSVAARGTTQTPINMECGNFDSKYKFVLPVTAYDEAMDAVTPNRNYQ
TQEKMVSGMYLGEISRRMIAHLAELHCLPSALASKMAKPWSFETKFMGMISADRMPGLQF
TRQVFQELFHVDVTDVADLHVIRDVCCLVRGRAAQISAMFCSAPLVKTRKEGRATVAIDG
SVFEKTPSFRRLLQQNMNAILGPGCDVTTALARDGSGIGAAFISALVVNDK*
>TCRU_1081 Pentatricopeptide repeat domain containing protein,
putative
MRRFLRSVLCRVTPWRLSPVLCAPVHQVHHTSSVVGNSSPLAHVMELMEAKNYDGVVQFY

EASYRADRMSLQEFSGGAFQARDMAVCLMRTFTALGRMDRVREVFAAGLRDLAGNGQGKD
SGRTVSLLNANFFNAYLEVLTQRKNFATEEVLYILKEMKAANVVPDTLTYHYLIELHIRM
GVDPVGLWNDMRRGSELQPLPCTVQALLLQVVPSSPDASFVVDVTREALRHGGNVLDKRL
LAGLMEQWLHNAQEYPPEYVLWLMFELEIRCVLDKASFVQFVQKQHMAELLQRCAKCADA
VTAGKALALMDRHVMTKTADVLSLVVWCWAQALELEKAFDLLELMARKGHLELTDPFKKY
TVDCLRQVMDRHFMMALADAISTTALLDRVLRHLRERRRRGQIVSAHSLDLVVLAAAKLG
EDRRAMLLVSEYIADWGIQPRTNTYNCLLVGLSSRRGASLQRTIYDAMLENGVMPNAFTF
RVLIRQAVLADNIDEAIEFLQRVTSHVGLRVEVEMVLPILERAARAGDAETVNRVSKFAL
ECDIGIDSVVLNGVIKMLTEAGQDVEVIKGHQPLHDALRSRSKTGRQRPRSEIAA*
>TCRU_1082 hypothetical protein, conserved
MENGVVFASPRAAGFRALRAARQTFAHLFLREMKERQSTSAAAEPIAVLDVSEADCAFSR
VAVHVGVEGGLELERDFHVSYVPLSEVAAAAPEEKDANITSLLRLVAHPGGRLEKEPLRA
LQSRYFVLGCYTAHSGRQRRAKGESVGIELAHVISKLHRGSTLLVNLLGGQQQQQEQEVV
GERRDISLLKREVTAMVSEALSRSGRWGVISDEVLPASDCYDAAVSVVVSLD*
>TCRU_1083 hypothetical protein, conserved
MMRRWILWEHTLLAVFFLLLLSGWGVDAWNVGEIIPVTLHMRLKRVAPTVTVGRNDMEEL
EDMQESGQGEQFLRPLPPDFCPRFGINRRVHQPAKRLFEHAHKQTKASGRSLSDVAVRFQ
LERGLSKSTAWLPLLKTTVRPSMSNRGQNASVRDENMLYLAAVTFSFGYQESTFNKITTF
YAEPVYAPVHHEEIELQYHWEERRAYNPHKALSACAFISVFATIAVLYMIIFSSRSLSKE
IKNKTFTLRSHRE*
>TCRU_1084 elongation factor 1-alpha, putative
MGKDKVHMNLVVVGHVDAGKSTATGHLIYKCGGIDKRTIEKFEKEAAEIGKSSFKYAWVL
DKLKAERERGITIDIALWKFESPKSVFTIIDAPGHRDFIKNMITGTSQADAAVLVIASSQ
GEFEAGISKDGQTREHALLAFTLGVKQMVVCCNKMDDKSVNFAQERYDEIVKEVSAYLKK
VGYNVEKVRFIPISGWQGDNMIDKSENMPWYKGPTLLEALDMLEPPVRPSDKPLRLPLQD
VYKIGGIGTVPVGRVETGTMKPGDVVTFAPANVTTEVKSIEMHHEQLAEATPGDNVGFNV
KNVSVKDIRRGNVCGNSKNDPPKEAADFTAQVIILNHPGQIGNGYAPVLDCHTCHIACKF
AEIESKIDRRSGKELEKNPKSIKSGDAAMVRMVPQKPMCVEVFNDYAPLGRFAVRDMRQT
VAVGIIKAVTKKEGGAGKVTKAAAKASKK*
>TCRU_1085 elongation factor 1-alpha, putative
MGKDKVHMNLVVVGHVDAGKSTATGHLIYKCGGIDKRTIEKFEKEAAEIGKSSFKYAWVL
DKLKAERERGITIDIALWKFESPKSVFTIIDAPGHRDFIKNMITGTSQADAAVLVIASSQ
GEFEAGISKDGQTREHALLAFTLGVKQMVVCCNKMDDKSVNFAQERYDEIVKEVSAYLKK
VGYNVEKVRFIPISGWQGDNMIDKSENMPWYKGPTLLEALDMLEPPVRPSDKPLRLPLQD
VYKIGGIGTVPVGRVETGTMKPGDVVTFAPANVTTEVKSIEMHHEQLAEATPGDNVGFNV
KNVSVKDIRRGNVCGNSKNDPPKEAADFTAQVIILNHPGQIGNGYAPVLDCHTCHIACKF
AEIESKIDRRSGKELEKNPKSIKSGDAAMVRMVPQKPMCVEVFNDYAPLGRFAVRDMRQT
VAVGIIKAVTKKEGGAGKVTKAAAKASKK*
>TCRU_small nuclear ribonucleoprotein C, putative .1
MEGSSELSQERRRRQRRRRLAAKSPETRRRRLQYSRRHNEKDGRLLYCDYCDLFVSSAQR
TWMQHLRGVRHMEAMEAYYAMVEARDPAWVKAFREDVVRAHVEERIRQQQLALGKGASVP
MPAMTLGYVGHQGIVVGGTQQPGGTHTRVGGGNNNSTSPQCGSPSVRVGGLLVAAATPPV
VKIDGKALPMPRSNQTTHQP*
>TCRU_1087 Vps51/Vps67, putative
MSAGNTERRRRIREQLREFYGDPTASRERHPVDSGKSAIPPEMDLDSEFFNVSKYVTDLL
RRESLKELVETDTQLLRNVRQLDGELQELVYRNYAKFISATDTIREMRDNITEMDAKLQA
LSSNVENIDKVSKSITDKLQSHRSRIEEMVLNNRMLRKVQFLVELADTMRRLIERQEYSA
CVRYWIVGDNFLAKHMNLSRITKIRADCMVLAEQLYKKLQETVVTTSLDDPEAMDVIRCV
VEDMRLMRATSLFANGAVKGTSTTAVADCSSPASFETEILNTLMKNVSGGFADGIQSVNG
AIRAALAVPDFASMDRTRWAEVMENARLQEALAQLKSLCALFHSNSERVYTLFHREGDAT
VSLRIVEEVQPVLIDIMSPITAQFSELLIAVIDAFCSDGEGALANPSIVKSGLSAMFTTL
AKRLKYYSGTLKTLGVNYLDNAHSQQSRQYAALVDQSVLEIFQRFLGALEAKLTEGINVP
DTIRSNVTAKDPAYQKPLQFCMTRFLFAHAANAADLDGITSLIDTETLKQTDVAGLQRQC
SCVMRAFLHRGIVLLGQLHLQWVAAASCPADAVDPTSTAAVNFGVSRSLYELVGQLGNIY

TLLRRGILPVNAAAGEAVKERRSVDGATGSSRLYMSTGSGRSSGMAASRKLVVTFARQTD
NALQLSVDRIFSKESQRNAELRTFPREIRPSSIVAAVTVYVLKGMIEYVRKQTFTRTGFQ
CVQITCTFLLHALTPFVPPASPGATTAGSDKCASPLSGEWLCECKDDSMMKSVPQLLNEC
CTCAYERCREKAPLTSVVVDKLVKSAIENMRGPSPPP*
>TCRU_1088 Protein of unknown function, DUF607, putative
MFASFLEVRVRFQCRFVCPRRYLRGVAAMELVRRECSSRSVDKKRRVECGGGLEEMETCI
NAVSTVPGNSLFTLEGLCNYRESHLRPLLIQKRHALQQMTEIKTRCEAVALRYANYYAAG
LFVLLTAQAVTLFYWVYFLFDWNLVEPMTYLLGYGGVWVAIACYATTQRDFTYEACMEMI
AAYYISRLYRQNNFDLQLWQRLTAEVKLLEERLRGLEGF*
>TCRU_1089 hypothetical protein, conserved
MNVEEYWHMKACEAVEEHRSLIQQQRDEIERLQRRCNDLLHERSMMRAATAEDVRQFCVE
YQRNQLQQHFAATAASSTISAGRLSAVSSVDSIPIYLLLQFLHQYSAGMVPMTENIRRKR
SRETMNSVCGPKTVGQQRLVQRVVARRRLRMREDDTIEELQQEHGQQYDQCPCADVAR*
>TCRU_1090 Rtr1/RPAP2 family, putative
MTSSGGAWEVVVDKAVLELSKYLAVTTDPFLPDSRTAQWMLTLIDKSTLRDVLVERNYGN
HCGMIGCSGCCRTARRRQGEISNERVSSSAGGGGNNNNANEEHNREEEEDEEGEEWEKDA
ADAFRQYERYREQRTQNAVGEPSIELGATMADRFCSPACAEEFLALMTKVPSSLVYERSD
VIHAVGGLFPNMSLAALHRLAGAESTSLGNISERNIQQEGGERQQPPSYLPNSIPVHSTA
VQEALSCIQAEGNTWSRDMRCAQVTESQVGQRRHMPLPLMVYDWCMTISTARTKVIFAVL
CHKSSLHSHVWKDSEGLAGRNRSVYVKCVLPIRMACVQRAEQELLSLGGEELGGEDPPCV
DPQLQQQRLALFATHVFSADTTAALSRLLLYDEGTLRQAWGVWQSSALLTSLQFPFALPG
AFVAGGVAPHVLFLALVLLAATGLCEPTVWAEWLRQDEDGSNALDELMEALGVTAEDLAA
CVRALVLE*
>TCRU_1091 Protein of unknown function (DUF667), putative
MFHNSYQSGFLSILYSIGSRPLQIWDTKVRNGHVKRITDEDIQSSVLEVVGSNVSTCYIT
CPADPKKTLGIKLPFLVMIIKNLKRYFSFEVTILDDKGIRRRFRASNYQSTTRVKPFTCT
MPMRLDEGWNQIQFNLADFTRRAYGTNYLETLRVQLHANCRVRRLYFSDRLYSEEELPPE
FKLFLPISQQQQQQQQQQQQQQQQQQHAQQQQQQAPQQQSQPQPQEAPASAQQQPQQQSV
EAV*
>TCRU_1092 NLI interacting factor-like phosphatase, putative
METAPHNIQTERFEADQYVRVQLSTGRSEIGVILSIDESGGTAVVATRHGYDITVRLSSV
RRAFLLVLDLNGVLVSRGRGSFFDRPGVCEFINFVMNNFVVAVWTSGLERTSNPIIDKVF
DGFQDRLLFKLYRDSCTVWPTPEKPYRTIKNLQRIFDSYPKSFNAVNTIIVDDSPDKCSH
PDIALCPVPFTDPVKQMDDNGLVLAMEVLKEVLRTESHAPLIRASEERLVALAAQEEQGK
KEEEDALAGGPSAGDANDVKSVELPVTFLWAARLCCDYISGGCAREDCRFVHSFDDGKRP
CSRKGACRRGHARRWIDEPLAEAEAPSTTTSLKTPGTSGPFNVPSVFSLFSTNPDRQKEH
QQHDYAWSGNAHSKQEWTKDTKHQLQQKQQQKGQQQTKQQQNKWQKEAKFVGRQKDPKDV
FFGQKDINRGVSNIAQFLGSHHGAPRNAHSLDSVAPGPKSMRFTGDRKDGNALLRQLQES
LSIAKEGRPANTRPIRGKDRGC*
>TCRU_1093 hypothetical protein, conserved
MMPRKCVSVFFVLLEILLVLCFVALPVAGAYCVTPTEIPANVCPSFTLTDQLSALFGRDT
AVASCYCGRVCQTSWDARQELSLRWEMERWIRANRTAGMFPEEYEFVMDELMRLTTPLRG
DEPWHVRQEKEVYAKRANATLRDMPWLLVSNGTFKRAQDFYHRNVRQRHIVRSFHLSREK
SRPGIIRFYLQEVAGWWAEAEKHVSRDLRVAAPLLPWISLEQLIGPPMTPAEIPQPEMAG
RPAAAATKVPAPMQRRSFRFAPARLRAPTAMVYYYSDDCVLCEALGVAFDVLPLIYRRLC
EYGHRSIVSCSPLFLFFRASAELSGFRTPAIALHGVELGVEPTWMPPMSILYDIDELVNP
ADAFGFLTLSVPVNTSLQETLVSLIRPLVALHVINLRYLSSDEITELGRLAYKIMKQKKR
QQQQNASAENSSDTKDAILSEDDEGKDEASEERNRDLAWQQGRVITREDYLHEVVVFHMR
ELWGNSTAEVESNEFPLWSQLEPPWIMVVSSVLSVLWLLILICVPKGFRF*
>TCRU_1094 hypothetical protein, conserved
MDVTRGDTGRHYRFTFFMPAPDPFCGGEKDKATGAVKYRNAVVRELLNQVSLEAALTADR
EWDVVFHGKNVLFESGAESLMSALLSDSCQRIPLALFYGLETE*
>TCRU_1095 rieske iron-sulfur protein, mitochondrial precursor
MFRRSCISAVRRTRLNHVSLVFKQLEGSNPLTVKDKPVNSWSDEFLKPPVSKEMMNKHGK

YAKYSDPALCTVDTSSEVVLNTYPDGAPQGRIETTAGVALKDYDASMWDEEFFRKFILKP
KLPEELEDRARVTDYALNSALLGFVILMARYAVLPLWYVGQPAMSMVGQMNIEAEVGELE
DRQCKTIVWRGRPVFVYKRSARQMKELQETPLSALKHPEADEARFPDHREKAVVIAICTH
LGCIPIPNEGLYNGFFCPCHGSHYDASGRTRQGPAPLNLEVPPYKWIDDNTIYIGKL*
>TCRU_1096 hypothetical protein, conserved
MDGRVKDLLRKSCSAGAPWEVAVQVVSSLFQSKNGSHHDVCLIYAVVGECMRLCIMHQKT
ADALRIYELFSRSGYVLGQSRHARLRSLPVLLSVESLRGLSADELALVALRQHPHRVHQS
RIVAQMIAAEDADLDDVRNTPVMTERPLASKAPPFRDGVQTFVNMDGGFAETFNVPFLGV
TEVTEAEQFRILLQQNMQLLRETRYFQQRLGLHYLLSQMRHVVDNKRLSSEVEMVPLVHV
GRAVLEHPFIFAKPSTETARQQLLFEEFGSLPVHKGLSEVQQTKLMALETTVEPLRPLCR
EPLKFMDSLASSWDALFVRRAALTLVLPREAHDVLPRSRTRGRGGGSINVSGGGGDCGRG
ERHLENSLTASTWTDASSELFGGVEARDAASRVELLINRRLHHNIVVPDTTFLLQNTNQL
LQLARHREVVIPHSVFLDLVNSASDDQGRRRFHSRRVLLLLMHATTTKKTVRDTHGSLDS
YSVHMKRSQGGVTLLGLQDEVVLLENSHERFFLADALAGQEQEGPSIGRSSLASVLVAKQ
LGKMVSSTPGYVTCRDASTNASEVDHLVAGVIASMKDESETQGDHGALVCIRGKPAPLFK
SRSRSFWARTPVVLATTSDVTRRVAFMVGLPMFPPVSAV*
>TCRU_1097 chaperone protein DNAj, putative
MESSRIDWIIANERNYYGILGIPRDADERDVRRSYLTLALQLHPDKNINNKRAGEAFRAV
GKAYAVLKDKRMRMIYDEGGVDRVHGLDAEDLSIKNLMAALSKLVAAKVLCIVARRAHAV
EILRQRHGWVDAFTSWGPADDFACLQKSPREAARRIVRKVFISVFMFFLCVSVGHTFLQF
IRRTPNEGSSEEPYRRLSRYNSNDTGATFTIRVLRGGKMTDTVIWRPSSISENDARALVL
QWIHEMCPTEKLLSWASRERYNRTATLRVGRKLRAAPSPNRALRRKWAPKGWKVKFQEFF
PITSAEDLYVPLPLCEGVG*
>TCRU_1098 hypothetical protein, conserved
MQECYNPQHRILPVGSGESAGAFVPRWTLCVTCLLNHFERLPTNAAVQALQDTEFLLQQS
FSPLFPNQGVGIGGEKPSVGSATPSVSPESLWESFMSFQDVLAQLAEMLCASSSLHLQAQ
LYRASLTLLSLLFTLLREAERQISKMNGTAYILEGDNLELGDRGTGKGGASICLVTLPVL
ARRMCDAVRMGLSKEGDSPAGLLLLTDLAEMSPTFLRVLSEHCVLSVCALLTSLSNAYRV
ELTLYLLHQVFETCAIHPTWLESLAVVLSGNMICDALGDSFLDFLAGTLDTFRDDEPVVV
NALSLARFLVTYETTRTALLFSKLTNTTRAGKGDFAQIVPLRLLRHLTLLLLDLDADIIV
AASETMREIIVATQPPGGECTNIADYVIESLRTASVYSAVVLVSLLNALPTEVVPAGPVL
RVIFELAGNNEIAFEEAVRGPHFLASVKGHLSNINYVAQSVAHAAECGGRTSLHCFLLQI
RALEAACGGINAGTCTIIPETVSVIANTLLQLVTKERGPALTEDELTDGQWLFNNRFFTL
TNEPLWDLIFGVAFLLPAVQGAFLPLATEIIAEALRRFSEFDFVSFDGRSLLFDGPTGNV
LLVLSAAVLEHMSADGDEGNTADRLVKRQVRQLFEKDLLTFLLRVSGDSFSHRGRILYHF
MRLVESNRNGRSADLPAATVLDGSSSPRALLEKSFRHHPHWAASLLLALAMTGCPQPVEV
QELEEFLFKQISILPWFLRDAKRALTADGAEDLCSTPMGNEDCAIAALTALKASAAYHKW
RGELPHREFFQVDSVLATGRVCDVPEELSFIFFQLFRSEERSWLEALKSCSWGTGLLAVL
VSSSIMHMKRDFPKESVKEAYDYLFSDGASQNSDVEMALCRLVVTLAPTCHLFLSQLFGI
VRRSVADNLSLGCCVICFLCQCVSLAVGEEVPNSTAFIRTLLEQHVTPCLLFRENPALTG
YVARLVVLTLARIPVAMASCCDHTLFRWSMQHVTHPKAQVYTWHVVFLLLERVHGNEFAL
RYESELEMKLRADGGEKLPPFFAAALATVKWMVCGAMRRRGACRMNPVVTMPLKEWSQDL
SQHVLLRAFIISTVERETRGNFPDASLLNVSPMLLSWANEMFARRYATVATARVFYTLVK
ISPKLGASLEALGLFLQMTNETETGASPTAESSQPSLIEQRAWVFAALVLSWPPWGVSAG
LREAVDAFLTTLEMPPRACKGKKRPREQEVDIFHTQEAGVRRAMEALVEACECRSEDHGT
FPLNRPKDFFLLRHAAQPDANVTHEVMFKAVHEGCELFIPS*
>TCRU_1099 methyltransferase domain containing protein, putative
MWLRRLPPTHSGAACSVTVLARWCSTQSGRGPEGKESSSKETTTTEGKSEDLSLTNTNSS
VEKKGGFLQPSDDVVLEYARHALEREGRDKKPEAVIWQWDTTYSPILSKGKQNFYDYTDD
IPKHVKPFWHHEYYQQREYFRLQREKRPLREQLKTWGIVVACLSVAGAALTLIRVWVEQP
REIRELREELLQQTYGKVLELAAGHGQNIGAYPYAVHEVIMCDSNAQQLQALRYRLPRTS
YPKYDVRRVRSENLDIFADGEFDCVVDMFGLCHLHDPVKALRQMQRVVKPSGLILLLEHG
SSPYPPVNWFLSYFEQRHKVNTHGCKWNLPIREFLRESRLEVKELRNMHYGTTYYVVAYP
EVLEAFKDRGAASASALATA*

>TCRU_1100 metacaspase 5, putative
MDLLLGVLSSGILQNALPFVAGVGRVKRPKRVKLEEAFREAHLCRPVIPYRAPTPYTGGR
VKALFVGINYTGTRNKLSGCVNDVRQMLGTLQRIQFPISECCILVDDMRFPNFTALPTRE
NIIKHMAWLVHDVRPGDVLFFHYSGHGTETKAERDSEELYDQCLVPLDFQVQGAILDDDL
FELLVKGLPAGVRMTAVFDCCHSASLLDLPFAFVGNNNFYSGGRHEMRKVRANNFSMGDV
VVFSGCDDSGTSADVSNVSSFGSGLVASGGAATQALTWALVNTSQLSYADIFIRTREILR
QKGYKQVPQLSSSKPVDLYRPFSLFGPITVNTSLIHYVPQQYLQPWGPPQPYYPPPQPQQ
PYYPPPQPQQPYYPSSQLPTQYNNLAPTAGIPLMTSSSELPPGQYPQALSGDQNGGVPPQ
YPSDQSTYYSSAQYLSGVGKPL*
>TCRU_1101 hypothetical protein, conserved
MKERSAELRFHPVMLAMLMVMVMIGFALGYHHGVKAWEKDFVNKYEQSHGRELHAGSMLG
VCGDELFLLSNNVSSKNQTIHAVQNENAGIEQEMKKLSQEFKSVTDLVDKCRAEITIIGE
EGSRRDGDPHLELKAIEDELRVLRELLSNLTEGEGMHRVHIHGLLGSMREKHKHFCGRLP
GCTLLSDESILRRWKEVLYNSSQRRMLKEADRMRRMKWRYDKQTPQERESIYFSEREEGG
KVPTYFGRTGNSSLLLAQGQMSQVVVSTALERVLLLQRFALCAYRTNNPNITFPSIFQWS
ASSENTVADLRDLVETPLLTFCMDCAEVEITSDIAQLCLKAPEGENGYGKRDFWAMRSML
LPHSGVVHDAKNFYTAHALTNRKVLAVVAHNSVRDCLAHFDDPRGDHFLYLLANFPEGRT
RFENHVSNNTLHQCSPTAAQLAKRITQVLEEGKLDGVNSSFDAVYMSVSSEIWKELRGIE
KKLPWWGMALFRQGEIASAHDELVDLTIIGRAHGILVSPFLAPSQYVLESFLLSHGLQPM
GRVWFF*
>TCRU_1102 Nucleoporin
MRSTASEFPPTLVAAVRSGFDAFVQLQRVRLGKCTDELPRSLAEGFGSCEPVATPAPITN
TAATAAVCTVNGSHNHHNDKSTTAFPQTLCGWSDLLLSRLKAINDVSVVDPTAEVVWELV
SVVIAILENGGDDGVIASGPYLEWYRSRFVSMDAIYGLRSTLNAVPPRATLSSAAEEDLF
EGLLGPYMISIQRAFLAGLFPVAAELISSLVDYLRSCEIDPFDAEDESALADVIRLFLET
PRNSTEHREWTSTANLLIVDCKRVFTRAGDESNLIVQAAQQLKSLCLDLLLMVSGDAALI
LDTCHLLELDVMDYVAAVCAVCKPYIALRQLHHLYQSALDTWGGSVKGPWYVGVVERLLA
SRCMADVVCAMELVGRAVRCKEYLPKPPSEDSNVVSSPPANSVEEEDDVSGVEITSEAPA
DPKLLRRFALLFMAAHLADICAPPLGAPVEQIEVTFVRNELVEDFVALFRHHPLLWRTAA
MYVVVYSPLMNPAILYRIVTERAGNAVSDRYTFLACHTFIYSSWEETSAFQVALRRLLREK
YPLSETLDKWIASVDYYRRKAIEVLHEKIINERWACGETAPAVWLAVECQQSKSLQIRFE
RILRSPGALESVEVYVVGQAVNNNFISVDTSSNLRMLNVLRVCAAVSEYRRAIGSLQGLD
GQKDPVRSPDTIHWVLRAAEKALCCTASFRMHPSTVLALVEQTVDVALRLPEEQRPRKFI
PLLSHALELVLAYHSEKRSQHAAHASAIREKIMRLL*
>TCRU_1103 Concanavalin A-like lectin/glucanases superfamily, putative
MKHTLSKEQLADFAQQAVISIRRFMSSSQEIMTLTQQDLISTSKSIEESWRKRRIVPDIT
VVELWAMSFQEELLSQLEVYSSNRDSNSNSQLLTEKDGAPNVSLAGPSSGRKSATRKFIG
ESSLLACTEVLKILLRTNGEKSAEVFTRFHLATHLTKYLMRAPMSKKMRDAFASVLLEIA
GYNSLLLEEFLSVSHELLVFPEGSGSGNVVTPQQVQLAWLLSSVFRRVDVNNVSADSARI
QCGRDGFLSGIIRTCSLLSGGTAEGGNEGMRFTAFLVYFGLVNCMVRGCAANKALCRGEL
RDELVRLWVIAASNVSKVELSLPIPTNVPGKDASLAVEGTQAWASELIEFLVEVATSDSF
SLRRQALHDDSMNNGGGRNMLNNTGFPPLSPSPLSSPGISRASGGYELLPAHWDTLLTGG
SAYAWHLALLLFNCVLSREGCDFIAEALLRIDTLNAVSEFIYRGVIATLMLLCVATPRNA
ELLAESPIIEALIALITCSQASVFAHEVIILEKLCTPCRALNAYAIQTTMAFMSLLSSLC
VAERTLPNLLHAVEEMCLQQTCVADADVIDSLLHVFTCSELPRGVLFFSGCGSVVCMVDR
FPTTRFNGYGFAAWIHPKCVWLEGCHLFSFTDGSSGTSVALIVVANGRSCSLMMRTRHST
DVTLSSIPDTSFPADAWSHVAFTHNMTGFLLFINGRKTENSLSISFPKTPTKKDRLQFAF
GGYRDEPSFFGFIASIELFDGSLSEKDVQRMYQAGPRSSREVPISCHPLLSVESQMVSGT
TQLCVSVNGGLAGKNKGVSVHSVMACSPLNVQEMFLHEDVVNWALRTLVKAKNSSTNFVM
IAKLCVQFVCTAMKLTTTEEELNRMVGEHVIGQLREDMLKWENIPIEVPAILIASTIPRG
GGKVMRAHSTTQDILSLLLDVIHEQGVHPTVILCILRELSDALLLSENVAIFRAVPGRFE
RILSLSLELPPECVESVITLVERLFKESREMEQTLRFLLSASNSKMADFVKTEMLRMMFD
IALTNTAMCDLIGGAFGNSGLSFLIGLVGGINHSSEAIRVFALRIISLMHHTNKRFREHF

MKYHGYEMLGAVMTDTSSSVPIRLPTFNCLFQMAFDAFQPTENEETTLLHLQQSSSSKMK
RRTTRHSEHSHISMASVGKSGPLQGYMPGLLSPELRVTRREYSFDFSHDLQCVSDIRPLL
DDTHRCPRSRANIHLVLQVPQAIQAVLFLLERLLRSVGVGTPPVLSVTGDSRLSSEVASD
ANVAVHEDRGANNAADSVSRVSVISPTQEDSKEIITLRVLGHIEKIVDRPENAKILLPFP
WLIWLWGAVGPVLDPASNSSTEPLSKMSKKHMVAIQQQFRNIVRRLAILDVICNTKAGIV
RTLRQTEQPPFLIRLVLEEIVAHFTKFRCEISSRSDATNIIKNLDFLFHGIEDLLRPFPL
PLGLEIVNAISAIAVNNNSWVRTKMKNSSRLFETRRNLSLALLISTKRFSKLESPALTQL
LGANGYEPNTIRVLLRRLISATSICDVDEVEQLLALTRQFLSGDPNHCQAILSIIGMEYR
SFAELLCGNAGNRKVNSLTCPERSLPGSTASSIRTEEQVTVDELPAAEVIRWSLEDKVRW
ETVRQRVYAATNSLGTVHGGGGSEDVTVSPLPRSKRIEERQQSIQHKVAAEVCRIKREVN
YYLNMNGSSVASHLPNTSFMKTSTPVDSAMTEIQRNISF*
>TCRU_1104 hypothetical protein, conserved
MNLVALPSAAEEMAEDVFFTFRLLSPGGEAYIPPCFIMTAAREMGYYPTSQCLRNCLSEV
IGRGKRLTFPLFLKLCSSLEATRTLNTGAIEECKRVFDVRNSGTLNRGEFRTILTSSAAA
EMTSGEVEAIVELLDPAHTDAIQLCAVQMILMRCLSKDARAIDDFFYTPRSARKEVSQPV
AGGDDHHLQLLPLNFVEGDRKDLSRDSKNLRANNKEAVRPLKAIEVNGRCASRPSRGCVN
TVQRKRQKSPVPTAPDDASDDTAEESLIYLHPPKTTAPSPCTSGPVTRATSLQQVPPLRE
EPLSRLETPTPPSEVAAPASLPIGDVEKSFMRGSHCSMPPSQTSHTVVPSAAERGKTTRE
QDGRTTLVASKSRFLGSGTRRTLPEPPLEELREVSLGDSCVLGNTSDVVHLHEDVAAADA
TVEGSRVLQSTHGSQGAANENASGRNQVRKKKAWVHNCCIQS*
>TCRU_1105 hypothetical protein
MAQRVAIPDDCVYEGYGNLYRGDAEVTPPQPIVGARGGNGVLYGTIDSEEALRQLRTALR
VDVHPQLKALVQEKKLDEVASSPASLPWLVCRPNAPQLRTLPSSFSSGTRYEIERMKSQC
RFLRDWLMCHETPEERVEAREFLRTAQRLLQKESVEELLGRTENLLQQLENWPAVVEKAR
PHIAEWNDDKSLTELREFVGDVLGTDEVCARLSKLTCRFSLLAPLLEEAAFRSAALEEME
QWSSEATVSSLRSLRASIHDWEVGVAKDQQNVLDSSQLLLERIAAVRNKRRESAGNLT*
>TCRU_1106 Thiopurine S-methyltransferase (TPMT), putative
MNMHLGTHNVKEFWTTVWRTQNTGWKLGERSAPFTRILFAFLKGAGILSAEELKKDPYDA
VLCFLAGKTVFAPLCGDSAIISYMVGCRVKHVVGVDLSEQALSLQCQVNFPNVSFQTTRL
DPVEGTAGDFVVHEATVGDSRVTLFQGDLMQLPCFKAYSELKVEFMYDRASMMAIPPDIR
GAYVQAVTRVFRANTTVAYERMVRVLPEERHWGPPFMVDMDEVLQMYRESTGREYRATRV
LADEMESTLCPEWYAIVPHNWMSSVHLIL*
>TCRU_1107 hypothetical protein, conserved
METAPRATGGAASSSVCSDLRELNEAKVANILSYLDEAVATRPQLAAPFAQNDEMLPESY
VSEGIPLSSSAAPNRLLLNKPPLAAPPPQLTQRQQQQQQMSSSASASCLTSYVIGGTLD
PYVGVYHGIKSKIEALRFSNDELRAENEELKARLSTARQRETENVEKLQAAARSEVDSLR
KGFQESEKEHNRVTQLLQREKAQLTKAVESLTTQLRQEMSRREEEVAKLESANAAAIAQL
KTRWQAQEKAAREKWKISEAKRIKESTLQSLEPDIVLLLNRHKAEKARLREEFENELRQR
DEVIALKEAEVEEVKAKLARDAEAMMTREQKEFRDRLREEIERVNRQLEDERRVARQKTE
NTEAFFEEQKSVMQREISRLQKELFALKEAAATEKATFHDAVAREVAGITANSNRIVSDL
KEQLMWEFSQREKETRAQNAQYLAAKEEELRRKYEAERDAAVAAATQRLEQEHIKSLTES
YNNDGVLRDRFAQMSRENERLRVEAELLQEQLKSALEAMNRKEEEIVRLREAAELTEQRV
KAIEDRIRGECETRLHVLDTEWQRKLRQFEIRHVEEVGGMQHELEKATIELEAAKNAAAL
EQRNIEQKHNAELTSINERVLVALATKDNTLKAQTEQISLLQEALRMRDEHIARHRELL*
>TCRU_1108 mismatch repair protein PMS1, putative
MIKRLDAASARKLSAGQVITDLSSVLKELVENSLDAGAHTVTVRIENYGLDKIVVNDDGS
GLALGDLLTCEGTVREDASLPLLACRATTKNLLEEADASVAQFRDSLGFRGEALHCLANL
SEVSIQTMSAESQPATLCITYDVKTHRTSVNVTQERKHPGTTIVVEGLFRSLPVRHREFN
KNRKKQILAATLLMKQYALSHPHVRLLMTHCLSPQSAPATLVSLTGTNDVHRALAEAYGG
RYLAEMNRVEWEFSFGTITGFVSKVTSGRLSADMQVLALDGRLVDLPIISKAVTDAYAEC
QPNAAQRAYPVFFLHLSCGNHVPYDVNLAPNKRKVLFTEESKYAEEVHSRALMEFRASTD
GIDVDRHIHIERTRRVDWKATQDFKLTRTPVSATSFTQFTYRRTDPLLTPSSTDDVLASG
LVEMSKIRSSIYYTQTEATILSTGTTVDIESKRISSGSSSASSRCSSTAERSCNEDAPST
RILKRESPVDSPCTPTQKMGGDESNSPVFLTDEANHPRPAGPRTGMRFPPLTELSMQPLS

HGPNECKSIPFKRTRKERQTFTSLTEQTENQLATYLDKSSFKEMIIHGQFNHGFILASLG
DDMFVIDQHAADEKFNYECLLSQYTARPQPLLAAVSVSMDPHDVDLAVLHSEELRQHGFI
VKRGEDANKLLVYSVPVLQYEAVGPHDIVELVQQIALYGNITKPLRSLWHSMATKACRSS
IMIGTALSEKTMRSVVSRLGELEQPWNCPHGRPTLRHVACVSSLMRSMKEASAD*
>TCRU_1109 retrotransposon hot spot protein, RHS3, putative
MDATAIVTNCPEENDVRAMRIWMKRNWPLQEQAEYWREVRGRMNNVGPILRSIFGKQACD
DRIKACQQAVDGSTASELERNLGIGCCYSSNDSDLSRKLVRVVRVRRGNSIESPLNLLVS
SHLERETLSRLEDEMKQSDFIFFVLRFWDYVPPYLIEKCAVSAFLNEDFLRAIRLKIKEL
WPPGRREPRSCALKEHSDTSFTRKEVLPPQERLSNPVAMDHWVLYEPKVQNFPLVDAFFF
VDSNPKTLVGLRMATARGHHITASTVRQFTECLAAYFNGWEELSRDMSWEIIYVQHADE*
>TCRU_1110 hypothetical protein, conserved
MRKGRVQAVREFVVKYRPHTPIAERHAVLLGHVHNVGGAELVVAVRQPAAASHGQRFCNE
HGEAETEITGHNLQHATAIQNNIPEHTRTFVDFNARRNPRAVLYGEHGAAADTQVRRKSV
HHVCPRHNSAVVHVEDAGQVHDEAALHKDRSAQAPATKQRRPRHDKPRRQQPDAVQDKRG
AVPGSCVARSHRHVLRPANLRAQRRVRQQHTRVQRHRAMQHNRALQRRHIRHVD*
>TCRU_1111 hypothetical protein, conserved
MDGVRAVVDGRSRWRRQHSAVVAAVDSDDKIGEGSDGTLKGLSSSDQMPQAGQGGEEGQE
HTGSLEVTNKLGGNEDSQITGERNVSSDADRPKQTGSQVPAGGQPEDEEGTKSPQPPPPP
PAEPLPQLPRQPPPGTNTLQSEETHPAITAEKHPKPQDGGHSHETKMPQTKGSTKHGEGA
SSLQSPESVSHLSRESTTPLSTSTDSSGDAGSTVENSGANNPTPPAASVTQHDSEGDTGP
AALPASNTELQSEEIPNAGIIATTHDDVNDSSRAAPTAERTTAGTRSTQTSGDAGDLERA
NNDDVNDDVARNGKTAEFEAASGDRNTEADNDERYTTVPENATNKTINTEITADSDGITA
VSHTTSPLFLLLVACAAAAAVVAA*
>TCRU_1112 hypothetical protein, conserved
MISSLETPPSVGGIPPNSLSEEGDGSQSLGDTLNSEDEVSSEVIGPSVQTKTPGQSDSPI
VKAPEKSGPETKTHISDKQGEAGSTEANEDATENEKGQSEVVKRDASSAPQSPPTPPVPA
FPTKETPLAPAELRNSVEEKLNTPPSQTKTDLEAPEPSLEDAEAEKHGEGATTSDSMKDA
AKGRSGDSTPSSISTSDSVDAQRKAEEEDGNEMEALNPNEDSILPLVNKNGQGKVIPSFP
AEQHEQKGQPLGKSSAGGASALSTVTSSSAGKESVKHLASGTSPDGHQNVDGGSTADGYQ
TATTGYGDKTQEDGALKTPTGNLANAEDANGPTTMGVGQDGTTVTPEVGASSAEDEETAG
ENKQEEIHPQNREVNATTLNSSLGNLSQGNNSDGGTMRGSGLLPSLLFCWDCGGLRLCEE
*
>TCRU_1113 hypothetical protein, conserved
MTVVVFSAHTSTHSPPCLSVGVIAAGAWSTAAAVGAAERDEAPRNKRANLLDEITMADES
CFVAFASCIAKNATCRRLTGCLHSAPFEALFCFIQCNATIIMAAPSLTGSECDNTNDGKE
SDEDYGDDLHRGADYLRVFVHNSQEGLDAVPTIPGLAEPLAFALSPAVISAPCVGADPFT
VEFTQQLSSVCAAGPALALSHVSQRCLADDLMSVLSRPLPPVFFWCPMRNTLRCGGYLLA
WLSTHVTMLNGCVFYLAGTRETSVCADASAKVFVDGWRLK*
>TCRU_1114 hypothetical protein, conserved
MLRRCTSPVFHGDELSRCRGNFRRSKPNLHHLLLTQSPLLREHDAEAGTPAEHRVVARIS
IIGGVTAAPSNQLDSDILLRLMGHFIAMAERPSDSLDDIMAWRVGVSLPLYPTEPALPRC
CCVDDDAVTEGTAVVYSR*
>TCRU_1115 Mucin-like glycoprotein, putative
MPLTVQISSTPLNDHTHIDDVPSAVRPVGARPVLPPFRVCGTTVDGEDQDVAGSDTLAPK
ATVETEQTSGTPDNLKGGSQKYAGQTSNPTNDKEQTLEFHSAPQTHDGAPVTAVGQITTN
PEQPPTGGTESANKAKERPLSLEVTGQVDAQQPTNVTAVMKVSGSDATASTTTTTTTTTT
APEALLNTAINTEAPTTTTTRAPSRLRESDGSLSSSAWVCAPLLLAASALAYTAVG*
>TCRU_1116 hypothetical protein, conserved
MMRSLPWSVGDQQPVASVMRRWRKSLFHIGFPRLCFLHVRSAGMLGNIGGDLCARRLLGG
RWRHCGVRMRGVLRTSEHKAENALILWTDEKTLPQIGQENVNPPLVGQQRFAVLTRGGGS
GGRGGPISSVREGRRYRHKGLPVICRACAILPKSGQESHRSCARSCGSWHNIWPDTSNGV
FAIPTIAGSGVVCAFLFLLDFLPA*
>TCRU_1117 Reverse transcriptase (RNA-dependent DNA polymerase), putative

MVCAREQHSRKQLISAGGPQGSVLPPQLFLHCVYDLLRRLEDMYSASALMYADDLALVAS
GAGTHACAAAMRLALSLVSTWTAEHSLKINVGKSEATLFRTSSDTRSDEDMANLHLGNGR
LRIQSCPVRFPGTTMDQLLNFGTHASTAAKRTMPRRYKLRLIAQIGAPHHTMQSFLFGYL
HSVSLYCGDAIVPCLVPTYLHNMEVRYRKGCKTSLGISAPTEDASVYLKANPPRSGRHSG
LLRSHNTNAIYASMITRICVLLSTRNPCLRPCIMKRRHQSRSRETPLLMNYDAYVTLLGH
PTTTIVLLLPNIELFPGTLCVAIKRDFISHLLSKDASDDFRRTAFDSIYSSLVL*
>TCRU_1118 polyubiquitin, putative
MQIFVKTLTGKTIALEVESSDTIENVKAKIQDKEGIPPDQQRLIFAGKQLEDGRTLADYN
IQKESTLHLVLRLRGGMQIFVKTLTGKTIALEVESSDTIENVKAKIQDKEGIPPDQQRLI
FAGKQLEDGRTLADYNIQKESTLHLVLRLRGGMQIFVKTLTGKTIALEVESSDTIENVKA
KIQDKEGIPPDQQRLIFAGKQLEDGRTLADYNIQKESTLHLVLRLRGGMQIFVKTLTGKT
IALEVESSDTIENVKAKIQDKEGIPPDQQRLIFAGKQLEDGRTLADYNIQKESTLHLVLR
LRGGMQIFVKTLTGKTIALEVESSDTIENVKAKIQDKEGIPPDQQRLIFAGKQLEDGRTL
ADYNIQKESTLHLVLRLRGGMQIFVKTLTGKTIALEVESSDTIENVKAKIQDKEGIPPDQ
QRLIFAGKQLEDGRTLADYNIQKESTLHLVLRLRGGMQIFVKTLTGKTIALEVESSDTIE
NVKAKIQDKEGIPPDQQRLIFAGKQLEDGRTLADYNIQKESTLHLVLRLRGGMQIFVKTL
TGKTIALEVESSDTIENVKAKIQDKEGIPPDQQRLIFAGKQLEDGRTLADYNIQKESTLH
LVLRLRGGVMEPTLEALAKKYNWEKKVCRRCYARLPVRASNCRKKACGHCSNLRMKKKLR
*
>TCRU_1119 Ubiquitin-2 like Rad60 SUMO-like/Ubiquitin family/
Ubiquitin-like domain/Ribosomal L40e family, putative
MQIFVKTLTGKTIALEVESSDTIENVKAKIQDKEGIPPDQQRLIFAGKQLEDGRTLADYN
IQKESTLHLVLRLRGGVMEPTLEALAKKYNWEKKVCRRCYARLPVRASNCRKKACGHCSN
LRMKKKLR*
>TCRU_1120 hypothetical protein, conserved
MQARGTVKVQGDANVDGKMSTGQHPHHQHLNSTQANATTTALEYRAMNRPLYRGPISHNI
ISEMAEGFYVLSGGYKKLFIPSKDVYALMQNVGMHLTEEEFHDALRVIGQSEPQNADELS
FSDFLLLMTREVDDTMADELRSAFFHYDKHKTGYVTRKQFTELFATLGERSTPEELEELL
AVAEVDETDDKIDYNRFVNELTSRVNCM*
>TCRU_1121 hypothetical protein, conserved
MMIVWLQKTPARSETQQARPLTKEEMNQYIRSRTDWRERVVFRVAWITASRWSEIAALTA
KNFTMEADGNLILYWPVASRTAKADPHRALRFVRIRGQDAFEIIKLCRTLQHNEKPTNLT
TAQVERALANWNATAHSIERGALRHAAQVVETHDLDPHVILQLAKHVDPFDLPQNTVRYL
SRYNTMLTQVLPLVALM*
>TCRU_1122 hypothetical protein, conserved
MPALNSSTIAGMEVTASRFLYATAILGTRLCGYYSSIKAVRRLSALNRGIALETSLANLP
PAAVGLGERLRHTIENNRKRIIKPTEKASTAIITDASLHGWEPFLFQTPATLKLPEGKWE
RKPFLIMQAEARAVRLALSAFSAILPSAMDVWVDNTLLQGAANKGSSKSHAIRVPMAVGQ
NYLFIINFAFTANPF*
>TCRU_1123 Leishmanolysin, putative
MNGALSSAHGNAVAEEVVPAAVKPHADRLLVQPLEGSLVVPSLATGSVCSRFTVPAEHCS
AGAANSDTVQYVAAAPGGVWALPCATLEDGRPVAGAMNIARPPYFTDASPPASPRT*
>TCRU_1124 Leishmanolysin, putative
MAGRGMVRNAACVRGGALLVVVHSTTAAMAAREHHDCDDVDGMELQDGDGDGRTLESHRS
RCHARDEWMAAIGVPATARS*
>TCRU_1125 Pyridoxal-phosphate dependent enzyme, putative
MARRLEKEKGYVFLDQYRNPSNPKAHYEGTAQEIYDQCGGKVDMAVVPTGTGGTMTGVAK
RLKELLPNIVIVGVDPYGSILADPSVPLDPKPYLVEGIGYDFVPDVCERKYVDRWVKSAD
KESFALASEVHREEALLVGGSSGAAMWGVLQAAKDLRPDQRCVVVFPDGIRNYMTKFPDK
NWLVENGLEEGEVTRPTYGSLEAQLEEAKKKLSEYEAKCGGSVEEGKRCCEKGK*
>TCRU_1126 hypothetical protein, conserved
MSCDIRRGLFCRLSRRWAARRSGIGRLFGCVACHRVPSWRVCFFPPGGLHPTAVLLRPQT
IKPNPAHTPSGKFDRSFEKLSGEIENRMPFRQQQILFGSKYDHNFGEENQDAMRQAAKDD
VYGREPERAAARQARSYFTGDDLSPTALHVKSPRLKVSQQQHRQKYDRNILKTSQESAEY

YYHRESTSERNVRRGMMTACGAVGLAGTWWGLRLLCQYMLE*
>TCRU_1127 kinesin, putative
MASETSAVCVAVRVRPLNARESPLECTVTVLNDHTITLTDVSNATMGASSSNTGQQHVFA
FDKIFWSVPPHVLPLFPSAVAPPTFTPRSSIGETATSTNVSRSASLFGAPSACSRTNSYI
GSLHEPISATTSRVPFSGLPCFANVPAYDDQASVYAFIGPRMYESVMTGYNSCLFAYGQT
GSGKTHSLLGPPEYVSLRSEERGIMPRLCEDLFELMRKEREEDESISYNVECSFLEIYCE
RVHDLLFHGGGGGRDTAAAAGGAGAAPLQSPSASSQSSLRIRQHPSRGPYVEGLSLVKVR
DAEGVMKQLMSGLRERATAETRMNEYSSRSHAILQLHITRVAVVREEAAVVTKTRVCKVN
MVDLAGSERVSQSGVTGDRFEEARNINLSLTTLGRVILQLSEKQSGKHVIPAYRDSVLTW
LLSDSLGGNSKTIMLATIAPSAYCYQQTINTLRFAGVTKKVINVATVNEDHHFQKLIAAM
RQQIVKLTLQLEKGKAAEVHREEIQAFRRERDELEAQIDSMRATMSTMVPATEVATLQRR
ITDLEEDNAQLQKEKNQVQRQLVSTTTALREELAQKRGEIMKLHEALLRKDGELQESLRR
YREETVRRETSSQQLEQQQHVASSSVVDTREGVGPSQSPPAAWKENVGSSGFEAPPPLVI
AGRSLRHEEELERRVGVLTKEVKAQQERAEEAEKAKSSLQEKMKMLQQEQDAMAQLLEET
QQRFAEKAHALDMAQSDLAATRTMLRAERGANSAAEGKNGLFAQLENVRAEYLSEKKTNI
DLLMRVSRVEQQLSKLKKDSATKTRDINELEQLLLEETETSERYYIWLRYSQDVLSILSS
FLRRTLSARGKRITALKGKMNSSVGSHAGDSVDLAGILWEQAVWDCARGEAYSRALLEQE
RLNAMIEMLGQKKVLYEISSGAAKDELMTLRALAAEAAEEPQRLRRKVKSLELLIEKASE
ERLNMQVTIQNSESARERLSRQVEELTNQLEQLQQNNNSLIEKIVEEHSSRNMLGTCSVV
SGGGSTSQEDELQEAEDTGKRQFQQLQRRLEKQVAQYQEELGLLHAQLEKERHDKAEKRH
SMQRERDEFTQELRRVRNECKASISESAQIVRDYEIRIKELQEMITTLRSALEEECNNAD
KAKDEMLRAGMEKRETLMELRRVIASRDALEMRRNDDEKRFNELRTQVRELQLAYYKLEQ
QLAGVKTHTPDVYITIDRSFGTDDGENSWMLKAQREKEVLERQKRSVQRINNELLATVRQ
RNESLRAVHRQITKIQRGGVSPDMAEMEIGDDNSPLGAQLKFE*
>TCRU_1128 Nitrogen permease regulator 2, putative
MFSLHSVLLIALDVVKGPYLHCHAPLNPFLPVAQEPTSSTPANTTTHSRDNNKNSHEKEN
MICPGDSGSKRKMRHDSSCKCDASSYRTTSADRLGGFSDVFVPRSEFCRRVMYLMEAESG
LLYLFYPEEIAGLHYQRKTLRYTLCFVFRVDVELVTPSLSLTERLIQPYSVVLTGIMEEL
RKTEVAYGYISMGLARVTSHHPSVVLPRHFSREREPSTTMAMTMTTDDSETQPPRVDVHA
ASGENVAAAASVNTTGVPATVAQVASSSVTNEDSVHTSSKGTSRVNVFLTPLQDPASLQW
TPLEEMIATLYACLSREERQETPRIRRTPRKGETPGAHENGLHTTIKDEEEQQQQQQQQE
EELCPPVEERKVTVRLSQTHSYHVCRMIQPYVLRYHTLDEVPIPIVEYTAEMLEWADLAI
QDVYKAVDGYRTIASIVQLLAVGHEPPGEETQKESRVLSPLSSPHPPASSLAAAAWALSP
RVKNAAGCSFASPNLRGTSAALPSVIGILSSTHGTETRVPSPLSQESRSGMSPVVTVSKG
PTSSVTFLVPHERSADEVGIGSHEGGQHWYGELEFIVLEALQHLELHNYVRIVRPCQLNS
KYNTTRAFYSVVSNWKHASRRIIGQRMLLVEEEFYRLRKKQPHTQQERQLMHRRRQHESK
SGVSSGFGDSPSETYALTLPAVLLAKAALDWQEKKRDEDEKEEKEEGDTGPAHLAAVEQS
PNTFSWSGVVPTPNDTLKETTIELEDAPAIGMAHSESDINAAAAAALCAIGKFTNNTVAN
VQWEMRRIPGWSTAFASWEEMCCKALVEIALLNDWLVEVGRPPTSYS*
>TCRU_1129 hypothetical protein, conserved
MSTSAPAAHYTIDTLRGVGLLPMQLALSRQPRLRPHVRHLKGLVYPLPYYAMWRGNHNKY
MYNQSTVSRWGEGETRHMYHQHYSHAKCPTDYGRGGREFEYLSVKRGRLVKKPLPQVQYV
SKGSKPTWLFKSWHTPLSSPTMWEREVQYAEHVPEHLGAKRPLAVVAPRTMHRYLFLMHM
EKITITISPFLFGYGHTLQKAVMDFYRRAISARAPFPKDKVFLFYAIDHITPRIEVTWLN
GKTYVPPLLEGTSSHDLIQMVMEEAWLAADRMGAEGRVLNPLAIDDYKWEQLIVFKKVRD
KEAAKGGGKKK*
>TCRU_1130 WD domain, G-beta repeat, putative
MDVFRSGLSGAFNGGGIEHLLQQQQQQQQQLTSATTTGTTVAGTAPSRMVGSGNVSAHTT
VHVTTSTEVDPFDEKRIDEIRRTLRMLEDIGHGRHIINWTELVDPSQPVESAKKISAALA
RPTTVPGAISSVAECITTPAHLSIVALLDPPFLAKVRDAVLQDLLNALTMVHESNPMPVC
SEMLVEMVKLNLVVLRGVASTLEALLSDPNSRRAAIAALGKLADQKRGNEVFLGAMQNLE
PLVRMIDEPEYEYDCLAISRLLGWGGAHKTASLVPVKRMDHSSPVTAMTYFRQRDELASA
TFDGTVTIWGTPHPSTGDVKAAVALDLPQYCVPVAMDGPPRGNYLLIAGMPFPAVNVQTM
ELERGSINSNVMNNSFNSSGKKRSSFATVPRGPIVRLLTCNESTGAWTSGETVTRKETVT

LTTAAALASFVVCTAESGPSDKLSESGLQHDLVLLNGHTSQFLRRFERVHDDYTTIIRAA
EDGDRILFTGSRDSVVKVWDIRSGNNRAGLTTAATMSALATPLHRLKGNHTDTITAILPH
RKALLTASLDGSFLVWDARRMTAPMHEVRLKDPILDLALADSGYVVISTARGLKLFSLES
LKAHDIIPNVAYTQLKANNDGSIIFAAGNSGVSIYALHR*
>TCRU_1131 hypothetical protein, conserved
MMRARRVVVALSPHAQLCVHVQWRLYTPIWQPDPAVDHVAPLRESDENRTLWASSAPIAN
VSDAIAAWIRFGNDPVLHTALPVIHVGQNERTRADGSSASLSLSSLPSPSSTSPFATVED
YMGTNMVFGSPEHVKDSAAVWASYFERRYLSQLRHSRRTAANHVGLVNAPDVFTDEADRP
ETKWSQDTRFRERAYMAEKFLKEKVVNLQQLEQALKQAKPAEYIAFHDALQQQTLTLIPL
PSPSVWHYGGARRTQWAERFLPLSHEAQQFFTTVLAEDLKRAGGAPEKVLQKVAAVFAEV
GKILLQRHRRCLGGREWSALAPHEKDEFCMKEVERWKQQVEVGEFDPPLDGDDDPTSTEW
QSEHDAIMQLMTATIDGLSFSALEFWTHTIRCEEMETEHIHTEKRVRAISAAARRAMYDT
TSYEAVLQGIVDAVAKGQLDMKAAGFKPHMNDIWCQLNYAKFGASTVTQHTTTARRQLNY
FHAGLLKEVAATAALYYATKPLSSSLDYASPYKFRRSLVGLFSTYGVEMVYAVQRPLLFS
AANLAKAEDLIRGVVKNVARPFGERRRAKLKQLRAKHRRLATPVQGVVVSAVVSDLLESG
ADVSEAKKAEKTQESVTFWPLGARRVVSYDWPTPHFDALKRRVAAAGSAVTAQSTKEIQE
IKRNAFVEVSLWRRVTAEETKQRHDAVEEETRRVADVVRTIPPLAQVQQYATSLYQRIED
AVPFPAATDTNAKSEQEDDESSWEFVVMLDDRVVLNANQAAELYLPYTDASGVPIPQGEC
RVRVRGFDVDVNPTLNPAFCSEAFSTPFQVFDAIPQLVQQFFGTAKPSVAEVSDISSSKF
IQFCAFLREAGLDVPVQCEFEAGQALNAEGDVFMEYFLNLLRSDRFHRSCAQAGLTGMQR
VIESSCRAHWEVHHPGANEAEWAEARRRVLDRAMEKEREWWFPNEMLDVMNMSPGSNHGL
RLPMYPATVRYGRELCTLLAAEGQFDNNSGLSATCAVNGTGAAESITFSTGDHISSTFSM
EEALAVAKGALRNAHDRQNTLAAFRLGPLSKHSQVLLFCGINATEFGGKYARTYTYAFEK
AKKELAETFVSGRVVPGVDEDELLRVSDKEGVDRFASSTHPEQRKTQFVPRVGPGGTPIE
DPTADQKTQWGR*
>TCRU_1132 hypothetical protein, conserved
MLPFLQRHWEAGLEGPLIEEMISSAFSDFAPYAGAEQQQLRFVRRQMDGTTSSFMYDDEG
DWAVNVENRNIGAVAFSTRREVLCDLRARVPLRLGSVENDNHNDKPGGNADRTEISHAVS
SQTRDSQRRFISSAVLPSRGRPGTIGVLEVLFSPAVERYGIGLRAGPFSLRTTTGPARSE
EKESPNSTQTDTARRTPIFIDGSWCVPVGPMLPDFLATALPSSLHLSVALQHPMGSAEGD
TLASLCFHSKPHERYEDTFHFLVVQTLISSNRLSHSANEIARSRSTLFSGDVSWRDLGTL
IGVSKSFGNGLFRLSASSMMHDGEMDYEAAMMLDATPAFANQTQFKVGINKVGCIGIGIA
TKIFEGLQLTLGVHHLRRSGTRFGLEIAI*
>TCRU_1133 UAA transporter family, putative
MKSTNGASSNKPGADMQQKNTPEKPKREASGSRKGASPPLSVIFVMLQIVFCVSGIYVCF
GLWSIKQERVVTKPYRAVRADGEHAEMAKLSTVFVLGLVQAIAGTLVGALLLLVEKMYSM
YVAHSAGTGSVGKMSKAGRLAAFRMTAVMGFTNAFGSSLSYAAMRRLPYPVVLATKMSKM
VPVMLVGFFWHGTRYSFSKCAACAITTGGVFCFYLLGEKHSRHEETQQHDGAALSSWLGF
LLLFFSLLTDGFTNSTQDVLVKRLGWGGNRLMFYTNLATGTWLLLVLLALEALHPLAVVI
LEAEELTSTALSSTSAIASALLSLDTGLRWYLREVAPLKDLSRTWHFFQRYPEALKDVLE
MSFLNATGQVFIFRVISFFGSLTLTAITLLRKSGSVLLSVVVHGHQVAIGQWIALLAAFA
GVVWEALMHVRKAPSARRSSPGAGNKKPAGSHGKEESDMPAPASATQGNTTRRRGREKNA
KAEA*
>TCRU_1134 hypothetical protein, conserved
MVGAPSSLTFAEKMQRLLQHRAERQPLPEGYMRCPLNPVHQVPIAAIVTHLERAHQQDAL
ALSPSAMYGSGTHRRRREFLEKQFHRHNRADHRQPQSGRHHHKRGRHRRSRRSSRDSSSS
SASSQNSLPQVNSHRSRRRYRSENQESHVRNSGPPAANYNGVGATSLPPHLSAASAAQCG
SYHPAVAAVPSPAPPTRALMGRYSIGFSIDKRSLMEYLLQFGSVLRCDVQPRSATFTVLY
DTLEAASRCFTQSYPSVIIDGVAVELCPTAGEAEVAAAPDTGMAVSGISPVAPMYSRECH
VTPPLSTESTSQHPCTLSWAQDAPPVITVAGKPVGVAMLSNVDDCTKINTIRGRNSKIEP
PKCVFIATLKKSEDGDKKKADERHIWEEFARFGDVSNILLVGPRVVVEYASHDGVEKITQ
ALQKGQEVFTCCTPLQ*
>TCRU_1135 Galactose oxidase, central domain containing protein,
putative

MRDSVDASEIKALLQELEGVVGTPTTNKGENERPFSPKKRGSSNVDVAVSRYAGHLGQSK
FSTYANTTNDSRRAVVPLWERLPPMNTVDITGDASCPVKSPVTASQKTFLRPKLVGGVVG
ASLQRMPLEGKVNNASPLKNDEQGLQQTKLTSQKQIQEEEKEPPTTSFSKEPVAQGEGED
REREVNFLPIMCARYINPAGITPRARWGHTLTPMQDQKLLLFGGMEIEEGETASLFEYDT
HHISWDPVCVSGGVTPTPRTNHAACASGGRWLYISGGTTDRGTRVLGDLYCYDIFTQEWT
CLWEYGGSSTRSKHEPTPRFGHSMVSWEDRLYIFGGKTPKRDQGDSQSTQHVTVASTDVY
VFSLTSRKWKRRIHAGKTMGVNGDGKNILNNRKTEETPLPAASSVTQLTNVFSERPANRF
FHAACALGDCMYINGGTGDGGVILSDTWFLDLRKQHWVLLHNGRTADSFPREKHHLFACG
EALLVVGGCSASSHSTRITLKYHNFVAVLPFEGNDSPCWIPVAMGNVSIVAPNRKSFGAA
FAGGFVHVFGGVSGSEPASNSMVRFLAADGYFETDSRSWAKSNADALTSMMRHIREASIS
TMFDTYAIPRSWDGETGIDTLSNRPYGVHRGFLQQRAPKFWEDLKRCRMEHVKPAGTGIA
MPSLDLTDPLKEAFTPSPVFPTTGGVATTSTAAGETHPSHAVAYFTEGNSRIQGLSVSLS
AEQLQCLLDYIYWGDVRHRILLEQEEEEYTMDANVSGQGDAMQEEAKVLQALRHAADVYE
LHPLMDICDALLARSRKKLKQAHERSTRQLQADLLSLLESSNSATATVLFVDPHTKQKSV
YSLHPFILMAASSFFNDLLRPLVSGEKTTFQVGPVAAKMRSAGRGSSQTRRDILVGPVSV
PYLAVKPLLRFLYTQMLTVPREAVFATLLGSHLLDVAPLQAYCESILAREEVNYDTAGHF
YSLGRKYHAPLLQETALLTAVLGYAEVCRSPAYKALSEKEAREIDAVAHELGATSWIAPP
QTVSEVKPAAVYQLRWNASSPLSK*
>TCRU_1136 hypothetical protein, conserved
MQRVSRPLWLSSGINSGRLRLAEQFATMQGWQAGEDVSFDAYVKEKRLKERYEAFDQRVE
RGYAAAAKLHRAEIQNAVKRRLKSTGTKFTAATLREMKVALEERMAWLRDVWTQIDADYR
SGDTARQETAAREISAALRGEPSDYMRWVYDKKRELRFAGPIKRGEMQGELQSAELPDVS
DEEVNRYHALRLNMMEVEYNVKSKYGLAGQQHWAELQATKDDEYVKKLDDAAEVYKQLLD
QNARLDESRRTELQRSHVERVHQAQVRFRAAMEMEKERERLIDAHREMKKERDLREKEQR
RALLQEAAEMRAKGKTSAEVLAALKERQIEADAQRQAEYELKEREDILRRKSQLINMIAQ
FKHDVEEREGRELLQRQKRTANRPTNVFGFSDDDAIDGSELSVDSTGNTESAPFAHSTKG
SAITATDGDSSRSSHSVRRPLAHPNKNELWKAINEDTYEDPFRTVHQARLDAAKTYDPTY
ARTFPLNLVLGRKYSRQGAGEMAAGNETDRQILQKGNAVALSFQWGIGNGTVHDLDAEGS
TDYFMDGNFHVRDKKTGDIDWRYEKKKGGVILKGPKFYRLGAQREAMDPGEHPMDPMPFT
KLPREHRWRSS*
>TCRU_1137 small nuclear ribonucleoprotein SmD1
MPAAESSTLIGFLQQLRGTLVEIELLNSSIVSGEITFVEPSMNTHMRHVKITAKGKNPVQ
AETYVVRGNSIRFVILPEAMNTDDVLNKATAAQKKTKK*
>TCRU_1138 hypothetical protein
MRHVRPVLLWSIANARRSAFGWGNSKGNSSSSSSGGDNSNNNTTNNTTNYNGSNTVGGGY
MQLTEFVGKAAALKRRHRGRGQYEFARREAEKFLQEEVPRTGTNVWMENGTNALALSQLL
SFFEIEPEPSLLALITNKLLRLLDDPNIDSHTRLQVAIALSSRSELYMERIFATVDDVLK
QATRDVAYNDNSGDFGDITHKWNVIELSAKVLQRNGKHAPSVLNESLTQMVIAQLERTPT
NELPILQSSLLRVYSWMVRTGKVAEANHLLVALVEHTGEFHSYDFATLMSSCLRHHQLSP
LPLPLLHRMARVGLMYSTLANGRDVAAILASIARMLGSLEAGKNGVTQRDVGELGGRMSM
LLEEYEPRVLRFLDSNDDLYWKHCDDITSIAFAYEMGGRLRYRHVFLAYQSYVAQNVTCF
EPQQLAIASGILRRSHLLSRELAMKLGERIETVLGEFSLSEISHICSTFAPISPSWMPEA
KAVATRLLVTNCSSYTKLTLSMAFPDDEALQSQINYKEISGRQLVDAMTLAKGIKFEGII
VAELTARLNAAQERFSPDDLRMVQASGRQELLAACMMYLSSRFAEAEWTADTLYSLPLAS
GIPHARDPVKALAAAKAVSISPEQFVSLVEILTSVFGEDSDEAMVNFVVTGGEELVSEEK
VQLRTVLRYLEAVRPIRKLHPRSEWLAIFNEKMLRFMRSLGTREMRSLLWSLQEVYGNVA
QQPLLQQTLTEIVTHAYGRITQPDEEAAKFTLLIAHIQYGMPNPPLTASTPLAVSVKSNA
ATYPAEVRDAVMNMSLPQEAQTKQVGRFTLRAVRALATPMPTPPTEPQSHDVNAALDADP
FATPLRATASDATTTGTTTLSPSQQQQQQQQSQPLSQCNVGTMPAFGAKDVSPAQMTKTT
GSQPAATTEAYENATRNHTNGKQEQTNDSASAPPSATHSGNTASASSGMRRWNLFGTAPD
TSMKPREPVMSAPPVAPQQQPPEESRTADSHTRSSYMKLFDPSRLSNAWHEVSQQELHGG
DMINQSNSNTIDEQLRPRRTGLPRVDKHGSSTGIHTPPAAKSSFGYDSPQFSYDGWNTSP
NAARTNAPPTSGWASPWSVPRPSMASAQGNHMGGQNATWLSPQMQPPARAFGGNGNSFGV
NTQHQQPMQQQQQQQQQQQQQQTQQKMPQPPPMNTSMMQQHSQQHQQQHEQEPQQQQQQ

QPQQHQQQQGAPSAYRMAPRGGVASPGNHLFIDPASSSRPRPVITKKNSSSKAQQSVPSS
SLDEDINQMYAKNAEGPNEGVSNFMKKAGLTKITGYNMGSTDGETTRGDQKGFWADLINQ
SVRRTKGESRRTNKKPNRLSEWLDAPPTRAVTAARKGRVATPSAPAAKGKSKPKEKRGNG
KEHTPLATAPTLSAKPTTAARGKGANAKASLPKAARGTSAKAPAAKVASKVAAKSTSKAA
VAKPKKQPENNNHNNININNNKKVVKKKNEKKPVMKKAAPVPSSVAANAKNKTPKKTKSS
SNRKK*
>TCRU_1139 hypothetical protein, conserved
MLTRLAISTVGYFDENFYPACVEDVDYSLRLRLLGFQERNVFYGKFVHRGSSSIRLSNEV
EPPDALWYRRVKSLSANDAYAMMKWNRPRACSGGYKEPYDGMVPADVWVKDEGRIQRIRA
YGHDEEQGVPRVEYDRTLLYPVRTKGR*
>TCRU_1140 hypothetical protein, conserved
MDRTPPDMMIVRLQKIAARSETKQARPSTRKAVDKCIRSRTDWKERVVLRLAWITASRWS
EIAALTAKNSTLEADGNIILDWSVAPKTARADPHRASRFVRIRGQDAFDIIKLCRTLQEN
KKLTNITTAQVERALAPWNATTHSIKRGALRCAAPIVETYNLDPHVISQLAKHVDPFDLP
QNTVRYLWKYTTMLTQVSSLVVLMRGEAAEGQEPWLKAEERFFMIFILTPPQLQDARQGR
VWPLHKRSEPSR*
>TCRU_1141 Endonuclease/Exonuclease/phosphatase family/Endonuclease-reverse transcriptase, putative
MCIWLPAERYFYPLLNSIGAYQRYTYRLRAVCDAQRQKLLLSGDIEQNPGPIAVLQMNVS
CLTPSKLATLMAQGADIIAIQETWKSSEQIASMHTGDYLLYAQSRIGKGGGVAVLVRTTL
RSKRIPHTIPQHDTSLEVVVVQVALDQNRDLIVASAYVRPPPQVTQSFRRLVNCLPASTP
LLLCGDFNMHHPQWEPFLETSPSEVAAEFLELCTDAGLVLVNIPGAITYARGTRERSCID
LTWSKHLTVSDWSASVSPLSDRYMLTFTLHQAFMDTIPSAPKFFYSWGKCKWDLFIKDFD
AQLPAYDYKKQSTGIKAFTRALITSYRRHCPRGMHKDGPRLWDETLMEAERIATDSKARY
LQLPTPDREAEMQRTRSQFFLLLRERLRNTYPRRISKLNPGEPLAWKYISGRKKASLPYP
TSMLLGDGQHTYKTARRAANALNRIFFPFHPSHKAVRFSKGINRPSASLNINASFLFGQQ
NNSESAASFTSFSSTSSSSEPQNNNESAATSTSGSSLSSSSEPQDKNEAATTSGLVAHLH
SPLDAPFNRTELLAALRNTPYGKAPDRMKSTVRHCDIFRQRLFDSFFVALTTVGRPVRSR
LSGDALPSFHS*
>TCRU_1142 hypothetical protein, conserved
MGTTTVSLLEKVPRSVGCRGARRFACSLTARTAQSVHCGRSVGITRCGGVRKSRSAHEDG
LVVVWQLRTLEFLPFRIALAARLAQQPPGMVLLHECWQQSAPATRLIGCGDFQQACSERA
AGVAILLLHTLP*
>TCRU_1143 hypothetical protein, conserved
MCALHAAAAVDSACSIPSVVIPLEPSRLPHGIARLAARTVPQCTSNHVARNVACAEVEAF
QSASRREKTACFHSGSMPACRGMPSSVPAVCDGRWWSDCMQRLDVKWETRSARCAVTSGN
ETLRPLIRAACAGSSESSDSMPVGVVARSSPAVVDAERPSRSSWCEFR*
>TCRU_1144 hypothetical protein, conserved
MLSDRTLRSAGACKATSGCAVRLHRQRGCPGSQAAARIQMHAERETTSRPSSGLRHTAQG
GAGRFKGRSSDTVQGVVGVPWLEDARPAAAHSVADENSVLSMRGADVVREDPHVAADASQ
FLEDGISRQRNGRRCLPKY*
>TCRU_1145 hypothetical protein, conserved
MAPRSPTQRESGRGHRHGTIPPAGLRQCLARGLSHCSQATLLEGCAGGTGRRAHRVTGAF
RCGEDSIPGVDLDIGPRVTASRTGAMRHLPTLRQACTRPADHKEPFMSVVPPLPHCGERP
ATASARTSSRREHRNSLSTGERRIDVLLPNLQQSALVRPQYEGTCPTCSAWRSDSLRGPH
MRMPQWRHRLLNRRLARRSPPKLPSMPAGKTRRKHKCGYSIWNRLFRHRFITDATGCWSH
GGPREGLPRPLRECPRRWELGLRLFVTAPARTTARMRRNTCLLSADSPFATHTFFPQDAT
KKLVNCFFDEYWPHLPWNNNPRGVRGSLIFECYFAFVFLWALDPLACVCAHPP*
>TCRU_1146 RNA-binding protein, putative
MDPLTVLAFCPTGEFARIERVIREWSGVEAVGSLRIKERKKDITNVFIKFNSEDIARSAS
LQIDEVPGLYLTDEKKGEKQKGAKRNATKGRSKTVEIELMNFGTYNQEKVALEQQRKKGF
ERNRQDRESRGRRTQRGFRGFINNRGSKLRPFIFPSAQVNVAFVDNIPFNMTNDQLTEVF
SPFGRVLDINRYELMAMIFYDSPESVLRCIQQLNGKTVRGNIISVSSGSVRIPGPVALQM
GI*

>TCRU_1147 hypothetical protein, conserved
MHRWDAFKPLVSSLCKKKPSWETAIAHYQRSLVEVGSSLELEHAQRIALLFLKQNGVPGQ
LSSWARLFSFEDIPTCSLMLKALVRHDLWRSAINLWVWNPNDATGEALIDELAFCNMWKQ
SIYVLQQLIMNEKFYSDSKKTITGDIIEEKHIDSDVALALNDAFRLANDPTFIPPEERRR
RGTIPHLVNTAFPLKSQWQRAISLLVRLSERDISSDTRKELLDVAAARLAYQAGKMEDTF
FWIKETEYFRGSRSLQRTFFRSAISIQRYEEAISILEILSNHGIEEIPSGSLQDLCKCFV
REYNAGKYVELLDRFSTVVLKCSSRIYSKETRDELRHFFQCNAVDTTALDFVGSLERTRG
STFIFGDRSHLEGIVTSATDIDKAASALLHKGYWKASLELLKRLLEVKPLDENEEVLIEA
ARVSLGSWKTAILFIA*
>TCRU_1148 paraflagellar rod component, putative
MVQNLVINLLFNSPEIRIMGPVKEQTIDKLNEVIPNATSTARSTRVAPSRFQYISNPNHW
YMKLDGQFCDEDGISYLMVLLLDALEEEGLWKLVSSTALRTPVGQSSKDYTETHVLFMNK
LVGDEI*
>TCRU_1149 intraflagellar transport protein 57/55
MSQEEILQQTTGPSVPGKGVIVDDGTMEDLIDKLRILRYEKDFCPRVKPPFKPLSKYYFS
GPSSIDNPNAQFHYFTSLCSWLMGLAGHNFEPPGQFDDPNATSTNILGELRAMNISAPNL
APNRIRQGNGEAVLTILSVLSDHALLKKGFTVRSVDYKNVEKYDELDGVTDADDDNGIGE
EVEDNVMIESDDEEELYVRATGERLVKEDAGVPLESEINAEEWNLEVERVGPLLQVRSDA
LQDWRSRIESAGVLLRAVEKMYPEVRQMLQHVADDLEKSRDRIQKREQTLAQQFSDQVED
YRVKLRELNTSQDSANMASQSVEQLSAELNQLSGLLDQVKRGIEDREAKISDTTPLMQVK
DAVTKVRAEIKQMSLRIGVLQHAVLHYVMKQTKAKREGLENGLGLDDWEEIESM*
>TCRU_1150 hypothetical protein
MLSNLFGSSLDGIGKLCSSSSTVEEIIAREDLSSYIQSQRKNGKGMLPLVEFIEHSIPEI
VKAALQPICEEECVGSVGKEGDADGHGPKKKTPGKKQVLFNENATELITFYLDVSQEAEV
FIRRIVAAAFLVLGIAAPLTPLVAQCFRRIILGAFDVSSVTTTEAIAAVLDEEMVLAIIR
HMSTSFIISDTLAFLFEPNLPTTEIVFFQPRPLIFTEACIRLRFAKHLASHMAAALRTVS
CQPYFNVWKELINHGCSQAAGPIVDELLKYDLLSVYFMDILLGCEEARKNPSAVPLAAQG
VELYHDIIALVRTSLIPPDVREMYFMNLMLIAPIKLLKMYTERFCDLIFSSETVITSCVD
SMRIELCAMFVEILRFHLRDTDSIIVESFFLEKLVAICAKYPDCHRLCLLLQQAVLSIFS
CEIKEKDNVVLQHLTVEDTSGKPGFLLECLGFCLDEGIRHTALSVIMVETWRTLATKTPV
VDLCGALRHTMELFLTNPNVQSRIQNMERRITGQGFVEVGSGCQEKVQHRPTINYITQQS
LGSDNSSFSSSEPTPSSAGEGSPNRGVNHTSAYIMEPLSEPEENLEAISPDGETTRDALF
TAPEALLYDAEDTVTMDLEWAEEAMLAEASPQARAYPKFADVRISFEVREEFPSDNPTEY
GADLFLRRRRSTDEHEDEDAWINGRRPRPRP*
>TCRU_1151 hypothetical protein
MNRNSATGNDEDATTRRVFLEVQIHGEEPHRFLQLEPHVNFKNVLKSIRRAGDLVWNGLL
LRPMETPKSVGMPRGLANVVFLRLFTRGPVTFSQKEGGRDNTPQALPLFVVPEPAGKARW
NGAPAEASQTHVFAGDHTATDVAGSDETRAPERSREVIGALEIRLRELRLELEEERGKRE
ALEREFCRLKWERRQPLHRSTAEEIHRWQMEELRRQRGVTWRTQSPRVVTSN*
>TCRU_1152 hypothetical protein, conserved
MRNSFFPVLCERQPTPELEAMPSYLPDTVVLQLHRHDGEAEFADYMDANSLTFVALGDAE
RPALCGIDATAQLHYEGAQRALVSAAGIVSSIRCSRLRIPFGMSELSELLRRAYNTEKGN
RNNTLNGCTETVLLVHAFSDAVWAEESFHSLSLHESSCWQCCLDRDWIRHKGSCR*
>TCRU_1153 hypothetical protein, conserved
MRTRCRRRIFHVCVFLARTAVGADAVVRLASNEGTGCCGVSTVAVEVAVSLSAPSLFRLR
HCALLRRERRLHDAVSRYLGPGVYLDSRWGSPELSDRVWRAMARNGFATMRCCRCVTGVR
RPNCSGPRNGTAEYFLLGDLVRSRRHVRSTLFGGNKCCGYQRETGA*
>TCRU_1154 casein kinase I, putative
MSSVKITLFQGKFRLDRLLGKGGFGEVYAGVQVSTGEPVAVKLERNKGRCFLFHEARVMQ
DIQNSMPGRTTPGIPELKYFGQEGDFRILIMSLLGPSLEDLHAKLGRFSLKTTVMLADEM
LSRLEFIHSVGYIHRDLKPDNYLLGVGRNVRHLYLIDLGLSVRYRSTNGEHREMATGKSF
VGTSRYASLRTHKGFSQSRRDDMEQLSYILIYLYRGHLPWSGLRMKDREAKEKAIGEIKQ
KLTTAQICSKCPSQFEDLLNYSRKLEFAESPQYEMCRILIASVLDTIGPNVKHDFQYDWV
NVRKRDNSPPPPPPCIVTHERRATENKINLKKGNENGEKDAATIGSKIFCFFPGLSSSAS

QEMVLVNEK*
>TCRU_1155 hypothetical protein
MQAELVAHREMRNARINERLSHPELCDSASPQMPPPNTEVPVAPEIVRSEPLYSVTIDEL
NKTTTLNEQLTRALAQREADNEKLAEDLAQREADNEKLAEDLAQREADNEKLAEDLAQRE
ADNEKLAEDLAQREADNEKLAEELAQREADIEKLTDELAQREADNEKLAEDLAQREADNE
KLAEDLAQREADNEKLAEDLAQREADNEKLAEDLAQREADNEKLAEDLAQREADNEKLTD
ELAQREADNEKLAEDLAQREADNEKLAEELAQREADNEKLTDELAQREADNEKLAEDLAQ
READNEKLTEDLAQREADNEKLAEDLAQREADNEKLTDELAQREADNEKLAEDLAQREAD
NEKLTEDLAQREADNEKLAEELAQREADNEKLAEDLAQREADNEKLVEELAQREADNEKL
AEELAQREADNEKLAEDLAQREADNEKLAEELAQREAVIEGAEADASKTIEGVYSRCREL
EELAARRELAATGDVDILERELADALVQLKALEGENAALAALLAAKKAEMVEASDAAGRS
VRELESRLSAAVSEASHERAMAEKLISTTRGALTAMVEENQKQVEQLRADHDQARATMQA
ELVAHREMRNARINERLSHPELCDSASPQMPPPNTEVPVAPEIVRSEPLYSVTIDELNKT
TTLNEQLTRALAQREADNEKLAEDLAQREADNEKLAEDLAQREADNEKLAEDLAQREADN
EKLAEELAQREADIEKLTDELAQREADNEKLAEDLAQREADNEKLVEELAQREADIEKLT
DELAQREADNEKLAEELAQREADIEKLTDELAQREADNEKLAEDLAQREADNEKLAEDLA
QREADNEKLAEDLAQREADNEKLAEDLAQREADNEKLAEDLAQREADNEKLAEDLAQREA
DNEKLTDELAQREADNEKLAEDLAQREADNEKLAEELAQREAVIEGAEADASKTIEGVYS
RCRELEELAARRELAATGDVDILERELADALVQLKALEGENAALAALLAAKKAEMVEASD
AAGRSVRELESRLSAAVSEASHERAMAEKLISTTRGALTAMVEENQKQVEQLRADHDQAR
ATMQAELVAHREMRNARINERLSHPELCDSASPQMPPPNTEVPVAPEIVRSEPLYSVTID
ELNKTTTLNEQLTRALAQREADNEKLAEDLAQREADIEKLTDELAQREADIEKLTDELAQ
READNEKLTDELAQREADNEKLAEDLAQREADNEKLAEDLAQREADNEKLAEDLAQREAD
NEKLAEDLAQREADNEKLAEELAQREADNEKLTEELAQREADNEKLAEDLAQREADNEKL
AEDLAQREADNEKLTEELAQREADNEKLAEDLAQREADNEKLTEELAQREADIEKLAEDL
AQREADNEKLAEELAQREADNEKLAEELAQREADNEKLAEELAQREADNEKLAEELAQRE
ADNEKLAEDLAQREADNEKLTEDLAQREADNEKLAEELAQREADNEKLAEELVQREADNE
KLAEDLAQREADNEKLAEDLAQREADNEKLAEELAQREADNEKLTEELAQREADNEKLTE
ELAQREADNEKLAEDLAQREADNEKLAEELAQREAVIEGAEADASKTIEGVYSRCRELEE
LAARRELAATGDVDILERELADALVQLKALEGENAALAALLAAKKAEMVEASDAAGRSVR
ELESRLSAAVSEASHERAMAEKLISTTRGALTAMVEENQKQVEQLRADHDQARATMQAEL
VAHREMRNARINERLSHPELCDSASPQMPPPNTEVPVAPEIVRSEPLYSVTIDELNKTTT
LNEQLTRALAQREADNEKLAEDLAQREADNEKLAEDLAQREADNEKLAEDLAQREADNEK
LAEDLAQREADNEKLAEDLAQREADNEKLAEDLAQREADNEKLAEDLAQREADNEKLVEE
LAQREADNEKLAEDLAQREADNEKLVEELAQREADNEKLTDELAQREADNEKLAEDLAQR
EADNEKLAEDLAQREADNEKLAEDLAQREADNEKLAEDLAQREADNEKLAEDLAQREADN
EKLAENLAQREADNEKLAEDLAQREADNEKLAENLAQREADIEKLTDELAQREADNEKLA
EDLAQREADNEKLAEDLAQREADNEKLVEELAQREADNEKLTDELAQREADNEKLAEDLA
QREADNEKLAEDLAQREADNEKLAEELAQREAVIEGAEADASKTIEGVYSRCRELEELAA
RRELAATGDVDILERELADALVQLKALEGENAALAALLAAKKAEMVEASDAAGRSVRELE
SRLSAAVSEASHERAMAEKLISTTRGALTAMVEENQKQVEQLRADHDQARATMQAELVAH
REMRNARINERLSHPELCDSASPQMPPPNTEVPVAPEIVRSEPLYSVTIDELNKTTTLNE
QLTRALAQREADNEKLAEDLAQREADIEKLAEDLAQREADNEKLAEDLAQREADNEKLAE
ELAQREADNEKLAEDLAQREADNEKLAEDLAQREADNEKLAEDLAQREADNEKLAEDLAQ
READIEKLAEDLAQREADNEKLAEDLAQREADNEKLAEELAQREADIEKLAEDLAQREAD
NEKLAEELAQREADIEKLAEDLAQREADIEKLTDELAQREADNEKLAEDLAQREADNEKL
AEDLAQREADNEKLVEELAQREADIEKLTDELAQREADNEKLAEDLAQREADNEKLAEDL
AQREADIEKLTDELAQREADNEKLVEELAQREADIEKLTDELAQREADNEKLAEDLAQRE
ADNEKLTEDLAQREADNEKLAEDLAQREADIEKLAEDLAQREADNEKLAEDLAQREADNE
KLAEELAQREADIEKLAEDLAQREADNEKLAEELAQREADNEKLAEDLAQREADNEKLAE
DLAQREADNEKLAEELAQREADIEKLAEDLAQREADNEKLAEDLAQREADIEKLTDELAQ
READNEKLAEDLAQREADNEKLAEELAQREADIEKLAEDLAQREADIEKLTDELAQREAD
NEKLAEDLAQREADNEKLTDDLAQREADNEKLVEELAQREADIEKLTDELAQREADNEKL
AEDLAQREADNEKLVEELAQREADNEKLAEDLVRREADCHKLVAELDVIESKLNSAMSGL

HVFSSGDGGVIEMLEGYMERASLVSGAEKDALERLSLSNKELAMLRSDFLAERDLMSREI
ELEKAEKARLLGELEVTQRNLETLKNAPVVASGENELSVLRQKICDLEEVVDVKNAHLES
LNERLEGLVEELHDKREELFHVIGQLDDFVVKFEKAREGEKVAVEKLAARDQELFELERM
HRELQTEHEQIVSHLQKQVEELRAQQSDDAIALEDLKSDIKGLRGSLQEVQGALREKNDE
INRLKEEILAQQEEAQALIDELAEERDQKSLEVSDSLKKLEEFVELMEDARKCEEEALQR
SADAERSLCRTQEELERLRTRQVEGRDNPKASELDTLRAALDEACDVKEHAEEENKKLKL
CVRLLTDALSKNQSRFTASSGVLEEACDALSNMQEG*
>TCRU_1156 hypothetical protein
MRRLGHRRGQTGLQLPHAPPCCIASFHHLGLLCSEQRSQGSVLPLQRLQLDESVRQFALQ
DIHITRSSKFPSRSKLLKFTTAAIHTLNCLTRVRLCTLNYRLPLCKILCELLDVSLPLRK
ILCELLIVSLPLRKILCELLIVSLPLCEVLCELLIVSLPLRNFLCELLIVSLPLRKFLCE
LLIVSLPLCKFLCELLIVSLPLRKFLCELLIVSLPLRKFICQLLDVSLPLRKILCELLIV
SLPLRKGARQLFVECCCLVELVDGDAVERFAAHYFRGHWHFSIWRWHLR*
>TCRU_1157 hypothetical protein
MSRETHNTWGHGGRLLQTVKLITETFGPGTTVIHLMDADRHLPRGADTTLHPGVGTDLVV
LVYTRVAFIIGAGLGAPLADDAPFHIKETALRVLQPDIHTI*
>TCRU_1158 hypothetical protein
MRRVERSSRGEGEDALATGEANVRKDTTVDVLRQASRHAQGAIETSWMTTRRLQVQGEQL
EEIDRALDKMEHDLGVGDDALRRMTWTGRVKRWFTSRRKQPSDGVESSTSSSVPSPSLPI
KPQGLREKNQQREGTRGSKREDVIGCGSSTSHLAMEEEKLLDALLNDVHAMREQATLQGA
MLTEHNCRLDVIATKTDAVHNHVNRTKQKVEKML*
>TCRU_1159 hypothetical protein, conserved
MLTRAWGIRRAVLSLRPPIRSCAPPASTQWRSVTFSASPNASIERRSTATVIGPLNTTES
RHAVARPPPTVTDPVSVTPLTQPGAPPLPPDLAGGTSNGRHASPTPPARQPHTQSEKDAV
SASKHASAAAQPSHGTTLTEGRPTMR*
>TCRU_1160 hypothetical protein, conserved
MTADPKRAREGTGKTIEEELATLLSGGHGGIMTAEEERDTTVLSPPCSDLVGKLLRNVAS
LPFSFFHVHVHLPSSALFLTPSTTKKNTATHEARVLFDKREEDRTEKSVVARLAGGLEPA
VPFAVGSPLVVRCGVTCGSSAGYEGVVADGMPFGHIQCLLRVCTRGGRSDGSEEPEEAAT
IPKACSEPPESLMHESKAFPVATNDRESNKRENVNDCHGTPNGILESWKLGVSLDPKIPF
YVRTMTEKRPAAACTGSTFDDVLSPSRSCGYTLLGRRDCETYILPQRELLFSFYAPAEVV
TMCAQQNEERMRRQAALGYSGPTHAFAEGPRTASRLLQGAHCNHVAAEEAAVAAASKYGT
NCGATPQNGPVVYEVRALPGDVVFVPRGWRYSVERIVGTAIMNAAATSTPTEGVVTRKKT
GSDVLPYGEMRTAFMHKKPSTLASLLGKVRKVSIKTESQLRCMEAAEICGVEVEAFLLCY
KPYPVLSAEQAAAYVPSNYVRRGVEEFYAKGGNDVFHKYV*
>TCRU_1161 hypothetical protein, conserved
MRVDRAVPCRSRQAFPLLVRDVLVRAGVAVLLGETKINDVDVVPLLPGAHQEIVRLHVTV
DDALAVDKLDARNHLLRQHQRRLQRQLSTTEAEKVLERGSQQVHHHHVELALHAIKKNVG
DAHDPAACVENAVESGLKGKLWMARLRLLQLDGHGLARLHVCAAEDLAERPRPNLLTNAI
PVCNH*
>TCRU_1162 hypothetical protein, conserved
MLSPRTDSHDPDTNDTDPRSTSSAPSVSAPALSFTTTADALATTPLPNTNPCAAYTWLPD
TRSTAPSDATTRALAYNAYSPPNTAASSATPPPTSSSTIAAVKATLTKHRPRIPGSGWST
ELMLPPCTQRTSVPFASESGSGPRPSGTAVGKHVSPWPPAAHTHTWPLMLLVLAAKRAAS
TVHAPQSNAVTGAPILRSTR*
>TCRU_1163 hypothetical protein, conserved
MRVWGLVRRSPFPQSSSGKAREVSRRIPTADALKVTLRHCVLVGGARLRIGGLSESTARL
MPHALVNMTNVTSLEGTIVLHGAMPLHSSVLLANSTLRATVGGSQYTPTTRGHEGFRCGP
ALVLDGVRLLSTWFVMTRSTLVCGGGSCAAILVERGLGANLSSVFYMDNCVVVSRTHVMY
ALASDLRVGGGSVFSIQNSSRSAPSTEYGEGACVFGDVVLAGGSVLQIVSSTFRLGFAML
MANTLTVTDGSWLVHRDNEFCTAYVVHVDKENGVAFRDRSVWSIIDNNFTYGSFSSFACM
TSK*
>TCRU_1164 hypothetical protein, conserved
MLRSMSQMDRTPLEIVILGLQKIAAQSETKQARPSTKEEMNQVIRSRTDWKERVVFRLAW

ITASRWPEIAALTPNNFTLEPDGALILDWSVAQKTARADPHRASRFVEIRGQDAFDIIKL
CRTLRHNEKLTNLTTALVERALANWNATAHSIEWGALRHAAQIVETYNLDPHVVSQLAEH
VDPFDLPQNTVRYLWRYTTILTQVSSLVALM*
>TCRU_1165 ribonucleoprotein p18, mitochondrial precursor, putative
MRRVSRQLIHATTVAAATRFAATSGAKKYDLFGYEVDTNTAPWIEKIKKCKYYDEAGEVL
VNMNVSNCPPDIATYNAALQRIYEAPSKQAQPVENESKFCAMLDLLEEMNHRNKMKPNEE
SWMWVMRECVKSGQFRLGYCIQKVMEAEFKSCPADLVKQNEANAERAKAEGKEHPSYLSL
QVGLFDVKIE*
>TCRU_1166 replication Factor A 28 kDa subunit, putative
MNLNAYGFSGQTASNAGNGAVGAAAGSNQPQRRMHPIRPVTIKQLLEAQRVGEGVMVVDG
REVTQATVVGRVIGYEDDTTNRVTGALTAKHYGYRITDGTGLVVVRQWMDADHQEEPLPV
QCYVRASGTVKVWQNAPIVTGTVRLVSDCNELNYHYLDVILTHLRLTRGDKHAKEEGAVP
AVSNTASTLGVQNMLPGGGGKVFATDVLLNAIKQNARGDAGLSMDELAAAARQYGFSVND
VRVALRTLAAEGSVYQTHDNRFNI*
>TCRU_1167 hypothetical protein, conserved
MESYKDVILSQAPAMYDKMGNSAGISMENYKGILLCARPANLPHGPSVQLRGENASAGPL
LTRGGEPAPAFVPGGATETQAGLGPSVEDRATMERNHRKQIENAIARRNRAFTVLTRHRR
WLRSFAEQVRKMKEEEIQREIQLARRAEQNRQRQAQKRAEAVSGGFSPVADPTHTAREQQ
QEQQERAATEAERAEAEKKKKKAASKKKKKPKWALTEDEALEDELADADELLDFAKKLDY
EKFINDYEVAGALAIMRERVEEIARENNWSKEVVERAASESVAEDLEGEGDADAGEGNTA
AKVGGPRGDDQTPQQTVCTAAARKAAPCNIALHKEGWNKSTSVGGVLRRAIFRDTLLLAE
RILASSMSMQKIHTKYSLARLLQRCVLNGDNALEAMQKPSMGGSKGLDVEPRVVKVNPEA
TKQESGGGDTDLGSQRILVAMQRSKERTQGLPYLYRCPAI*
>TCRU_1168 hypothetical protein, conserved
MAMMMTGRVLLVCALCVLWCGAGGGGCTEKLAASAEITVDSASGGVVPQLRRNCAEEVSR
RTGGRANASAVEECMRQRMDGVLAVVDGRRRWRRQRPAVAAAAAENADELGKENTSSERK
VLSGGNGSGSEDAHVRGGDLHSESRDGVVRPEGATSEELNVNSNSHTEALPTKEIRQVIP
ESISPVSSPDSKEVEEDTAGKDDKTQEILVSGEQQALQSSSSLGGNSPASDQGAELTAAE
NPQNDKKGEAEKEKNLSDKNVPTGINPSATNSNDNAPTKLQAETLYRHLPMRIFLPHITP
TRVLRRRFWTPLLFLLPQ*
>TCRU_1169 hypothetical protein, conserved
MKDVEGREFAEALQSGNTLLVEGHSGHRLRLYYDSLSSVAAKAGSGGERAPVPLLISSLK
CLSNYLRKLDFSRRYVFFVNRDLCVPCEVRHVLCQHYGAAARLQTMSLRQLHHSCGETLK
LLEALEQKCVAKLAAVNFRYSLADVSRHPLIIPEEYGMSGFASRSQNSTDATLSTGLVDV
HSYCSKWMPDLDKNLWTDTFVFERTWPSDPCGDFLPIFRGVVEALINREVVLIDVSDPRE
SVERVALCVALSSREKHRAKEFVERIVTGTRDMGWVLVAQIATPSSFDMPQRRIYLWCGA
RVVFRDGRMGTVKGFDKTSLCRSGKADWPFVMLDSGSASTVVLPSITFLQRPLNLRVSEL
PLRYADTMTVEELFLTPRVSLGKRFFVLRPSLFVGRLSHLRCAFASLPSLSCLALTGPLP
WKLLVTAENS*
>TCRU_1170 Repressor of differentiation kinase 1
MEEKVSEKTPEAAEVVQMAASKASESRHSAEYSMQNPLGSIFGKSAPIHPMPSFSLYNEG
HGLEEPEGEEARNATEDSVETDYEVKKSGFVISTVLAVALVISFGVIAVGLIGFLPGHMI
GFRAVSGTAEIIQVEAVRGVIIIMRSTILQLPDFAHTITANYMRNQTSAYTVNAFPKNSQ
SLLYSLLSVLSTFKDTISFFNFMDRRGLYGFASEILWPNESGSMLLLGAHNSTTGISPVM
TYNATILDVVSPDPLAYWNISDELSQEQGRFELIVDPWVKNKSITQRWFPSALNRTGLYF
DFIIPFEIDGFAGYCEVGMPTHRLLRESRPLIAALRKHGRVMLFDDKRDIVILTSWGENS
TWWAGDSYLSFGYLTLDGIKDPLAAAVARQVKSNRKQMSDVVNGTTELVFPFENSAVRAS
FARVTDKNGLNVMLSVAVLRSDFFSGIVQARTIIIVVVVAVVTMSAITAFVGACYLVKPL
THLVNALKKASNLELPGSDSKVMKPSRILEVGEIQADYVKLFRQLLMLRKFVPEAILARI
NLGDMSTLTASVDVNENADDRDSSFSSNTSRSQVVGNEIGVKRDVRLNSDVFREQLNLFS
PRHCTVVAVEFHASQLWDDSKKYMTLFIATATELSGCVEVLGPDKIVVSFGAHTTIPLHS
IEGCRFAFEFLSKLSPYEQGAVTIVVECNEFLVGTCGAHLRNARVLLGTDYLFDLTRAMS
GAPYHIAATSGVASQIQGYQAFPVDCVLIPSTSLPVVLYELRLLTTGEEQTQNVTKLFRL
GFSAMRQGNYKQAISHFQRVEKSDPQAQRLIRLCAQRRLAKDTTPYVRCVNNSYDIANNT

FQYSSGEIWNEVPEKRELINKTPVSLKRENSNLSREESMALFELCQSSSSSSESFQALED
GCNSADDLPLLLTDMNHRVWTRSLKKISEGAFSAVFLGMSEDGGQVAIKCIPRRRRDIMQ
ESLEAEMNVASKLRHPNIVQYVSCSVVQSHLAIIMEYVPGGSLHAVIKNFGRMSSLVARR
FTVDILNGLSYLHGLGIVHCDVKPHNMLLGMDGVCKLSDFGSTISEAADMARTMADEVML
RGTALYMAPEVAAGGRCTSQSDIFSLGISLLEMLLGRLPWKWSSKAPEGSDATSLQALFN
RDLLFVQSLARDYLEPEIPDFLDFEVAHFVRSCCHPNPAMRPSASALLSYSFVL*
>TCRU_1171 hypothetical protein, conserved
MADAEWEISALSFPTEVENEEGIQHLTVISPQLNTAAKCDANSGDSSRKQPDDFGVPPLC
PIPSGGGSSANAEVAQERHLVELLHKASQVIKEERARSATLQQELNELRQSSSAQLVTQQ
LQNENRELKRELLSLHNQMDKAQAYDLQPRRQVGKKKDQRSVRSGSLPSMERGTDSVTSR
PSASRTPFTPLTANTDAGGSYSSAQHRKRQAVSLQRRQLLAPSESSRERGSSGSQKIQEP
PSSPKKQQEEERNQKTKQQYVAKKRASSSSSNLPFVPQKQLPNAKEEEVLVLPQSLLKSP
AADEIWQANASYNRETAYSLGMITRLRRAMAPPPTKEQLGEVIHAMVQEIVRDARRRGIG
LKLTRQAPCVYLFELRGAASKKGKGDTARVVHLSIDSGRLSVKVGGGHENLLEYIERYHS
LSSV*
>TCRU_1172 hypothetical protein, conserved
MPDYTQFVKYFNEYPNIPASETSAYSRLADTTWRLLTASEKENAGGKGSSANGATCPSIV
IGRLLVSTEDRWRIQAHMRREEVKAGAGRKEYNKKVTRAAKNTKGPAQGEGFPPGRGRPV
*
>TCRU_1173 exosome complex exonuclease RRP40
MDQVSPTTAMIKGVRELAPLRSHVCLPGDPVLLLDPTAVVTLGNGLRPLPHAPPHGNTTS
GSAGGGNTVVVAELCGPVECSVESNHVRRYTLDAPAARRYMYSPRDPVVAIVVRKTSSYY
YCYTGAAMLAVLDVLGFDGATKSSRPRLQEGDVVYAFVKPKPSGAQPASGDACVESAGDE
VELSCMAGDVGLVAKDWTSAEAVFGPLTDGTVVRVPISYARSLLASDGASRLLSLLGERV
PFEVCVGVNGFVWVRGQTSDVDAAAAARRTVAVASCILESQDDATPEEMESRVKSYFPSE
*
>TCRU_1174 hypothetical protein, conserved
MPDASACARAAAAELQGWVYVTNQLLLESARNTVAQVEEPSRRASLNAEYVEGLTKLFYS
HAAAVTSLLFLSAAKSTSSAVVLDEEHEKKWEIERERVVAEVQELTRQKQALEQEVERLQ
KEEQRVGKAVSVARADADALRREVEREQKALETLRREMQEESVRQREEHEHQMFNARQEL
RQTQEEFAAEGRRLQKVRQQLRSLADVLAELRDGDGDGDERVLHEEPSESLRQLLEEFVA
YAEVTQRTQQELQEELVALREQMAAEEPVASRSRAQTLYQRILPPPAVNPVALTLQSVRE
QVRELRPQAAGTPCGVVDTPRIQNRQWQQHSCRSHIKGETSFTASPVSQCQTPQTWLPNG
VSSNLIAWREKMALLKAELHDLRREIEN*
>TCRU_1175 hypothetical protein, conserved
MDLVFVRVVLCLWLLFLPTCKGAWSLWVKSSSRSDPVKEGGAAATSVSGHSDDRVQIPDL
FTLRQKKDAGQDRQAAHLLNELEKKGRISSCWKRAVDALKEGCSSLQSDDGARSRLALTM
ASCDGEADGGRRAWSYCAFDTDVRKCVDHLDDAQYLMYVQYRLHADVLCLYIQEEAFQER
TEMAVQALYASALAASETLQALRSSSSELHSSVRKTVEQQASNLVETSKLNDQLRDLRSG
QSLAFESLRSSTEGMARSLTDARLSLNELHTALYESTAQAAAAVREVAREATAFQKRTED
HANTMVRVLERIEVFQQGLLEKTIGLSEMIRAAVILVILLLLTIPTRTAAARRPCIGVVA
LAYALRPLFLPYPRSGLGNNFFFATVILISGIILVFFAYTHRSPEYTLRMMLRSELRHVV
EYARPLFLESVRQVVIEGVTFVLHSLDYEKEAAQPRTPKDTALLLASEEARAEETCTPPT
VAKPQRKATHRRTLSRLR*
>TCRU_1176 hypothetical protein
MPTAQPQYCEFILPSRFAAERPRTWHLVMDDAGRSLAARYGGDVPLRNLPDEAALRSRDR
RMRDEVSRRRMGLASGARNSGTLDTHKFSRADANLLSLQHHRREEGLRTAAASFLSGRIA
AVQRNAPPRLQRTTPDDVIPREVPVSLSQ*
>TCRU_1177 hypothetical protein, conserved
MLPSAREVEELVVATLRGGNWARAVMLFAGALKLPCVPTSRTYAEVVAAVATRSTWEVTV
QVLDRLQTSPLSARAVYHTAADAILARNDGVLPEKLLAVILSARRRRVILDARHYMQCSR
LLLDSGDWHGALLVCSHERLIQHAPFLAQPTMLAAAAGHHWVAGLKIMSSLLALGNIPSA
AITESFIRCFSPTSPWWRALAVASVLTCRDAPEVKQRAHDLREEVTHHAKSPEINAKEGR
RRVLLRVTNGGPMPEDLTMGEAKEVLAMMKDRQWERALDLFCAFPALLELTPRPPALLLP

MTVLGNVARRLRYWEALWQAVSRTLIAAPMDNVISAASLFLAYMRIEEQGADAQEKAKEV
ARAKDAVIAFVNDRRMHEGRALEPAALHSFVHSTCTRGCWEDALSFTERPAMVFAEVVER
SNWQAAIAIYGTMLPSAQERAGPQLEHLFISMGLWIPALRYAQSYLPDDTFALRSLAIPL
CAALGQWRRALHMLFSHVPLPTAAEALLKRYCFLRHTEETIYEASESGDWMKALQAWNAV
RQIPYDRTILARRGGEGKTDEDAHPCISPHAVKCTAKLLLEHQRYDDCCLVLSSHNPTAS
DPLLLRFLLMFYYIRLSPLSYGRVLQKYREAHAPYRVVLDGYAVLALSVSGNYSGAFWIL
RSLSSTREAELENAIARGFDEVPHLKQMFSSLLLKGTKSSQVVDLEAVMGMLIPCVRSFE
EVNLFNEPLTAFWGSDSDALRECALSISTRLAQHMMAKELPVPWVVLRIVAGGIDRPAEC
STAHLLYEAVRQQGVLGTTEEVERVGDEKSGFWRPSKEERDIFFRRCAQAFIRMGHWSRA
LEAMQDCETTDEMTLFRTAVLLKEEKDCRMRMAHRVILEATSNDDKNTSEDNSSLVLGRQ
AEEALFALTQLHAWKAALVVFNGVVAGGRHWSRGSGGSLSSGVVNALLSSVASSGHWELA
LQILKTVQKEFSNVIAFIDKGITDVLRSVRRHGGAIMCSKLLLFFVEEMGGVPTPLQYEV
LLGSVLGSQLTAVERDVCAGNLKRLSQSLNEKQVIDLILAITISGRSWEEEDSDGAIVLP
FAGTERRQRMASSNHPRPTCWIPVERPPLSVEEFHQLCHLAPRIVLATSRGLETLSQEAF
SGRFRPFELKLLLQHYRMEVLARVRDTGDGALTLDTQLVEYCRESKLLHILLLPHERVWR
RTLPPVGTESAAAFIERLTSAVFHGFASPQTVAAVWEMCHPFVTQLAEKRWPWLCEYELL
RAVRRRGGKPPASLLTRPDASRVEITSYWATYGRAIWPELFFSGPELCQLAVYVPLVEPL
LGNEEELSELLAGIQLTHFSVEAIPQGEELLRQPRSVRAIRAVFKLAFKTLKRLHTQLDT
DARGGRFYYHHHPDASLWKRYSSEANMKERLPRWALALRIIHHYRRFTANTLPPRMLAAL
FSACASDAAEATCVSKKKAKQPKKLSFWELAIQCAQAVEPKFTPTLWYHAMQLACLQMPF
KGAHARASDEYCLEDMHDTSLQATSRVLLSHAKKRDGKIPFAESLLLIDQTKELMDEMHS
REYAQDECTQGEDVLTLWALLQNHGCLERMEPPHAVLLRRLFLEGNPSSEVL*
>TCRU_1178 methyltransferase domain containing protein, putative
MEPDDVAEYSLQRYWEKRYSKEEQYNWFPSIHQTSVQALCEELLNVFVQRKAQRAFCPGG
CHDASPVLRVLHLGTGNSSLCMDLHNAVQAHRLPFVLEQVAMDYASNVIENMRAKYPPEV
LPNMKWMVGDVRRLEEFCSLGPFDVVIDKGTMDALEADKNSPNMEKDIWAMLYGVNELLK
HAKGYGAFMQITWVVPYIRLYYTKRDAFAWGDQVRHALLGESDMYRLFVYTVKQ*
>TCRU_1179 hypothetical protein, conserved
MQHHVAESDTVLSESSSWNDSENSVIDDMFDRLAVAVQGLLATMRDEQRPYTGSLCTRRL
QDRIQRAFPVEYRAVVDGIYQGSWEQYLKEKAVVFCFTQKRDIKRPNIEWMIGLGAMRCY
FLSEDIELVNDNDELLGCLVRDHCRGSLENNCRTILMELFSALIGTEPVEEEDDKTSSVA
EYELGSLVLWRPVLRKLWLHPIKGRRVPLRDLERPCQQLGFHIRLRCRWATYVGNNLLLK
YIHICVLRVLLNVVGPPLQPSKGNQQEDTEGTLMVGFSESLGNYIVSLEKTDMWGRIPPI
PRNSLRRGRIFS*
>TCRU_1180 retrotransposon hot spot protein, RHS3, putative
MNCPDESDVKAMCVWEQRSKPPQQQAEYWREVKGRMDKVGPILRHILDEQSYDDRIEKCH
ETVEETISPETQYYTGLGNSTMWGGNSVFHWLAKVVRIREEACKGEFSLNLPISDHLCNK
TLCMLAKLMQQDDFNSLIETQA*
>TCRU_1181 retrotransposon hot spot protein, RHS3, putative
MERGDAWLRSRLMVLASDKAWPYSWGACEFNRDCYVNCEVKRAWQIVKGGLTEWFSGHGE
TEFSPHRHLLIGTPGIGKSMAAGSYLLYQLLHYDAERLPAVACFICN*
>TCRU_1182 hypothetical protein, conserved
MSPSRWSYDAVQRLHGSGLGLDGAVCGGGAIGVPSLALVLDKLVGGYSGSLGKLTFHSAR
ERAMPRELKPCVTTLTRQLQCLPHTGRYAGASDRGGVGSTGRLRSFRAKFFACLRWGIRD
AATACARDGELQWPQQPSVFVRNPAAEADQCGLFPLVRWSPCAWLPSRLSRWCGEVRA*
>TCRU_of Trypanosoma cruzi region 4/Dispersed gene family protein.1
Dispersed gene family protein.1
MSGSVRNVTLSNCTLVGGASLYVVGWLSDPPAGERADVLISGLESRSGGGVVVANRYPPG
SRVTVVDSVLIAEKRVAYRGAYGLGDASACLVLHSVNLTGSVLTIARTHVAAVFRDAVGV
LLVGGVALSSRGALYVDGLLVQTALGLCVSVEGGVAASGGSVVAFVDSDFLLCKHALSVR
GAVSVSGSAVAFLRSEFVLTADYAVAFYSTMRLADGSMLLAKGNVHDGVSREMLYTAGAV
TAAGSTLSFVRNRALLPRMLSLSLSLSAGAHLRVACNDAGGRVLSTAEEYAAAGFGDAGS
IDVVGCDACDRDTHCYAPGTASASMRNGACVCDCGGGYGEACVPVGAPALPSAAGTASSV
FLREGVTVRSVFVVPAGASEVALRHVVLDGVSPVLYVPWMARDGVRIVVQNVSLLNGAVL

YVMGGGALRGAVAAGSDESGPVELSVCDVEALDGALVLTGTYPAGSVLTVADSLLVAARP
TPLVYLPGSQSSPYAPVLVLSGLRLVRSVLVVSGVALVTVVTGGRTVVMDGAVLELVGGG
VALDAAVLGGEYALYASARVVASGGAVLRVSGSQVYAAHGLVFESGVEANASAVVLNDNA
GALTDGALLVLRGSASFASGSWLSVRGNSISGRLLSVPSYPRSADFVQSTLTLHGNAGSG
PVVMDGTVALGGAGRRFVVGCLTLNGRALQPMDYRSAGIIGEFRPVTCGVCNADVRCFGA
ATRAMSGSCGCRCAEGGYGRDCLPVYLPHVDGCNRTPAPPPLSHTATLTETRSPTPTWTP
SLSVTRYSPTQYGPTETLQMTETVALPPTRTSTASVSGRLWWSDVACPTLAVTTTAAGGS
LTQNDIRGGGSAVPTRLMVALPPPFRWERDPQLGTHLSFVPVSTAQPRGFGGPWGAMLSN
ATWVRNATNPSTVLELAVPVHRGYFIVADETIFIRCDAAAVFGGCRGVLFGSFTIRSDTL
PAAASALSAITGVVAGATAVAVVVTGGLGSILEMQALGVLARMPCASAQERASTVALPYF
LSVFAALDPLWMVVGNALLAAVFGCVHYGVTAAFQRWRGVDAASAWAAMRFPSLTYVVAH
AMHLGIFFGSVFALAMPDARVQHYVIGVVGVLYGVAFPAGVCYLIARHVGASFTRYWQFS
RKPLHERLLYPVGYWHPAAQQRMYGGMLTNMKGSHVYWCVFQLSVLCVVGLIAAVHPPVG
GCHVQYFCMATVLLAGAGVVAFTNMMRSAFLTVMHTAGFVLLAVLCLVSAANHLAPSDGG
ARAYAAIVLLLTTVLLAVIVYSVVVWYMEDRHWQELREPRRGVLEALLRDDEDGDEDAQK
PHDRTSSSYASGTTVASSYRPPAMP*
>TCRU_1184 hypothetical protein, conserved
MWSAVGACPRSRHRVRRRAIAAVRVALLVALLLVAAAAWMTAVHAVVLRLRGGTVDRAIT
VGHAVDTVLMDGVYVTNGVAVVFDVPAMLPGALRIELRNCVCDGGAQIYVRGYSGEPASD
RSLEVSVNRLSGGYCSLVFVHNLPARTNVTVRDSTIVTAGPMRYSQLSGLMDAVASPLVL
HATSLLQTQLRVSNTVLRSLQAGGSAVHVGGGVDLLSSAVVLDGVSLEASGDSTALALHV
PSSSRLSLRSHSVFSVTNMSVVSSGGGFVLGERLAVSDSVLRFVGVEGSVASSLVRCDGG
TVGAGGWLELHDVWAVGEASSVASLSGVTLSGGTVSIARCAATGATLVSGLTITSGVVSV
QCNRAGGRVLQSSGDYRMAGLPSVSVVPCDGCAAALACFDALTASFSDCVCGCRAGGVGE
ACLPFDVPPARSGGGGGAARDCVSGVTLTESVTVGGGRATACFDSVVFRGPITVAVDLRL
MDAFADALNVTLRHCVLAGGAQLRIGGLSEITAHLMPHALVNMTNVTSLEGTIVLHGAMP
PNSSVLLANSTLRATVGGSRYVPTTPGHERFRYGPALVLDGVRLLSTRFVMTRSTLVCGG
SSCAAILVEHSLCAKLSSVFYMDNCAVKSREHVIYSLSSDLRVGGGSVFSIQNSSWSAPS
TEYYKGACVFRGVVVAGGSVLQIVSSTFRLGFAMLMAATLTVTGGSWLVHRNNEFHSAYV
VYVANENGVAFCDRSVWSILDNNFTYGSYSSTIAHMTTNWSPPSDTHPIIYGMCNEARGS
PVTDYGEELNIVAPVTVLDCGACTMNAVCFAARTSSISGCECVCAAGGYGDTCLPAAVPD
GLGPLPLPDAKDTEVRCVHGGSISSVDYPDPGVRGLCLVNVTFTAPIVLDLSSFDVPQQT
LNVTLLQCVLMGLSIKGSGARVHVSVTSSLLDSGALDFEGDFGASSQILVVGSTIVATSG
HAIYFVKFTLSANMTLLLLDNYIEANRYAVYFFNGVVVDGGGIIVKGNTLSTTKDDDGVE
SSVCVNGVAVKNGGYFDVENNTMSSVNGVIFFGDTTVSSAGLLRVADCTFGGSEVFDSAL
LHLSGSVTLEGGAQWRVEGNSVVAASVLIISYSWHKIQLSGSGTTVVLAHNRQVNDSYPF
ADLALPSMVKKPPARLVVGCNLQGDEEASYDYVFPEGVVVFRCGTCNDDAACYMPGTELV
DRGSCSCSCKDGWHGASCLPFEVPNTVVPPLAERAVDGDASCVVNQTLTNLTLNMWKIHH
CYVDVTFSGVGAVLTFSLNSMPLHLPINITFTGCTFRDGAVLKFVGGAEAAESTGVLIRV
SQTVMRSSVVVFALAFPQHCDIAVTEVDAVQSSEVHLPDARRNKLSVLLLMNVVLSASSL
LVSNVKAHALRYDAFGLYSTGTLTLVGGSSLYVRYCSFEGYEYLFHVYRLSVSDHSVFAL
LNNTMFSGVSLLYQHHGVSVSEHSVLRLVGNSGSARYAICNDGLWTVQRSSWLDWRDNDV
EVGAMFYDAGSAFVSIDGSSVVTLTGCKMGSTGLSVSLLKRADAGYRFVAGCLTVAGREV
TTAAELVLHGITNVTTVAVCGECTKEGDCFAPLTTAVIDCRCQCAAGGHGDVCVPAPVPA
GPPPPPTPPVPPTPPPPPVGECISDMVYPEVAQAVGGGLSWLCYRNVTFSGGRMILTVLI
GAMTGEVVNVTFDGCTWRDGAVLVLLGNVNAAVGSLNIVVTGNTFRDALLSPEGVFPPHT
NITISRNRFTVTRLVLRSGLDIWIPSCVVMNGLAISNDSAVVLSGNVFQSVTASSSAIYV
VGSALRVSWHSLFAVVGNTFHMAGGDSTLINLEGSGQSSSLSVLNNSAVVIRGNLVSRPV
RYFLLVILALRVESLSAVVFQGNDMQGSLAVFFPRYSSNIYYNSWLQLSGNLCRESPSEA
FLFFNPTVNLRDSTVSVSGNRFMSSTVTPKTVLIPKRSRDLTNGAIVAACNTVNGGGERF
MS*
>TCRU_1185 WD domain, G-beta repeat, putative
MSKAKLKLSIEGKTVVYEGRPESRKKDLLDILVKMKVDPSVAVKAFRENIEDAARYTDEN
IQQQFLDAQRAYAEKKKRKMGEVISEEQQKEKPERITLSFKGKQFSYEGLPSSKQYAVIE

LLIKQDATRELVVETFRVNLPESSASDEEIMSMFTAAMDAKNARKAAKKKDTAGELAAQS
SSRSIEQPPPPPPPPPQQQQQIMNTSSTSGVDAIEEITGMSVEERDSISNFIKQVLEQPE
MDLTARMREESNTFSTVGAMTRNTGPGAGAEDDALTMTLRSTRGKIAIYCQEENSMANKV
LYISPNTTFEEFCAMVEKKFGRKMVMSFNEGEDLIDMDDDDVFCMFLEMSQNHTQEGKRM
KLICAPPETRKKVSDDKLTDTKGGDVFKIKAFSNGKLDVREERSYTGHASAVYCCSFSPK
GERFCTASRDRSVRLWNTVTGSSSVMKGGHNGFVLSCDFSPRGNRIVSSSDDRTIKVWNT
TTCAKVYTLKGHDDKVYCVQYNSTGDYIVSASCDHTVRIWNADSGTKMLTLRSHSLAVFS
CCFSNTDCGKYVVSGGDDRLIKVWDWAKDDEYCSMAGHTDTVWSCKFSHDDARIVTASMN
HELRVWDWKNRNCILSWKGHQVPIHHAAFSTNNKYIYSCARDWTVMVWDAATGELFETIT
GHHSTVYHLDVVGNKLLTSSLDDTLKLWTINEK*
>TCRU_1186 PH domain containing protein, putative
MRKYNPGKLGTIDGILKAYKGREEQMFEALVRKYGPEPSRDEPHLLEQKKPPPSGVKDVK
RLHSEGISTRNVIEKVLRVAEVSYEEVLLALWCQKRYLFDAISELPTTASKELRAESFSP
SSQSTASRERLSRFFTMYAPEKLSGMDALIEAAGAEGMNKLIDSLVAMYGPEPAGDYETE
DSRSAGNENTKKRFVSDGGDNNDCEERQLFANWLVGTGRGAENIFYTLMPEEQLLDFVGA
TFGDYENRLRRLFVRYSPARLQEVNSLVADHLGHEEELIERLTRELGPEPARSAPTALTR
LFIRRRIDADRSHIEEEKWSDNGDTNGGDNDRVNAAEHLPMTLVNGRALIDEYASNESAE
FSGDEAAAYPRDEEYWYRLFVHSCEDATNNDDDTDDEYEDNSLRTHCDSAKNDGLSTDCA
KTATLSSTTITGEHRSLDSPTTAELRKIRGGKQRIRVMLGNKFGQAIGTLCTACGESIVQ
SFEALWQPVGPQYLQLFSETVVKDGYLEKLSPDTRRMGVKWQKRYFRVNDKGMHYYETSA
TSEKPKGYKVFTRNSVIISGVDASTHPNCIDRRYHYFAVTVNESNDVFYLRTPSDEEKRQ
WVSFLQLALARMRLTTVGHDQNPSRWRARMTGTREANIALIELAQASANVLQSLEQKHNE
LHERIRSERAQRELMETQMSEMQKQQDFLAQRINEAKAQARLAEEDAEVKCLELAERAAS
IKQRRLDMKDNLEEARSSIDGITHSVLKLQRDIVDMENECRSREAQLRQVYNKWQRCEER
RAYEKGGEYRKFSS*
>TCRU_1187 39S mitochondrial ribosomal protein L46, putative
MRLMTLQPRLSGRSFHSQHRTPIIPQIPGGDTVPGSNTGVLVGYLLHRHPVLKHTPHPLE
MEMGFLLEREHQRYCRHEASESATHFMGQRGFSIDVMNRTDPRETKGNFFGLDLYQDAMR
VVLQRYKPERRVTPQDLWNPSELSATTPPPRHSIHRKMDDYLYLIIQDAASSKWTVPYTA
LKDRETLRMGVDRAIAKHNSDTLDCYVWSNAPQATVSNNVDNTCLFMYAATYLMGRPRFS
EFEPTLKDHAWVTRHEMLQYKQEFQSAELLEALLDISADGIFES*
>TCRU_1188 hypothetical protein, conserved
MDGTVKCSWRVHRHDCPPPNLMFCEDAQSARMHAEAVAASRADGNANTTAVVLDDENEAA
AAPWMTLQLQNPLRHLVVLSNARVLEVHVGESAIYTHKGVAVAAGLFLHDTECNAVAGQT
LKLKFFARKAKHVIDLVALSLVFDFVGPDDKSGGHDGHVADAASTVESRLRQLEMLLHTS
MKSVVLRLKELEARIGALESRGCDEQQC*
>TCRU_1189 pumilio/PUF RNA binding protein 5
MVMAASNDPNDHVAFSGPYLKQVLPPRTSTDINCSATTQGSAITAGRIRRYTHNPYASQS
ATTSCSSTNTSRQLNSSNNTSCDSSIQVYNNCFAREASGAELVLLSSNLLEQFFAVVGNI
PRTACTVTGRHLLVSVLRLQHTEKVQIIIDELLPQLNIVALDQQGCHVVRALIELISTEL
LETILPHFAPKTIIELAVSSQHTRRVVQSIFERHKSEALTPIVTTIAQSGQQLAVSQQGC
IAVIRTIENSLPHQRRSVISMLLPVLPSLTMNCYGNYVVQCLLQHMDHASLVSVVCHNFA
GHWLTLSCNKFASNVIEKVVWQLDGVARKEVVDELVMDASNLRCLMQDGFGNFVLQAIID
SSNDLEEFSEVSSRVRPLLHTSPYGHKIGNRLHGGLVRSLQVTPAPQVGGSAAPLMDE*
>TCRU_1190 hypothetical protein, conserved
MNDGVENAGPSTDTGAELSIKTIMEQQRVIDTLREMVTAMKSRLSTLEAAASNEVANPSA
TATVNCDRDASLHQKENLRLSRLVEQLASERDAAKAKCAELEAAVEKGDCRMAEALKCCR
QLKQMEGELRTTRCLLNFTESRYRETELDCRRLKEEMEALSFERSRWKALAMTITNRLDT
VTRERALMHISSLEREQSMRTAERRAVSPNSNPNTAAAAIASDNVTSLPTFDAMKLISAW
GTQQTAPLPPLPRGVANGGTRHSSPALPSRSITPRRKGGRYLVPL*
>TCRU_1191 hypothetical protein, conserved
MCNSPVPVYVLGRHMLDAYFFEMEGTADLDFVICDVAKNSTSDRERIVDYSWLEFAKKPC
FSPADSISSRVRALVRWIERSYTEKCTRELPEALRAHSKTMGFEYDVYLSSIVSHDGRRV
EGVVQAVDGCVYITLAIPLLLEERARVRRNLSNVVELYSKVLQLRLDTVPQFSRVEISLI

ISCCANSRDAPVDVLSERISMDLGEPNNSTEVSFMSFITSCVKFRRMPRYSSTLQRGASF
MDYIPLLERALFVSLREPLHFLELVCMMSSVFGRPISVNVATNYPGECGNAGDVSVRCAT
FCVVDDATQFGFCICVRYHNGWTLPSVGIIANQYADGTSQGSPLQGKLQYSEDVRQLEKR
CSNEEFLDVVALCEAIERGSFEVMAFLIAVCR*
>TCRU_1192 hypothetical protein, conserved
MRLSYSDQLIGLDQQRRAKAYGREAKRFHLKLVIFASALSYLAARVAAWIYPKSVSHTWV
DDVLVEVVEDDGLERVCSIFGCVFTASLFFFLFLTIRADVRRNFFPPSRGSRAAGERGRR
VKGWSESFSSLAVPASTIFGALPKADGPTATAVGSAQHLRLNQYHLPQQQQSIRTEAQLN
QFLSSKESSSENGGGAPLATVTAAAAASFPTATPSAPAVTSSAPCAGSVFNGPLVGGGIA
AGPSDGIRVQYLGGAEKQSAAQPVDTEWAGLGILSAERSILKARRWLSDLCQELMEETAA
CDRWFAERQISSFDTTHCLHETIPGPRPSNSAGVGVGLPTLLSSTTPAVLRKLDALLNER
QKLAGQAQNVQNFDVILHLDQRLSLEAKLDPSGTFPTASPLSVAEQQAQRQYVIKRLRAF
ASQKTLASYRHNHGDADVWREGFPTDAHLLIHIVRVCVDGFANYVKFLHQPMNPQQDLAI
FVGDTGEPYFYVRYRSGPDDKTYATHQGVNSLFEALLIFAAIVRAYHNDSYGGIWGVMDL
SQTGLLNLL*
>TCRU_1193 cyclophilin-type peptidyl-prolyl cis-trans isomerase,
putative
MRYLLLSKVTQLLLLLLLMLSAAVSARADPVVTDKVYFDITIGDEPVGRVVIGLFGNDVP
KTVENFKQLASGENGFGYKGSIFHRVIRNFMIQGGDFTNFDGTGGKSIYGTRFDDENLKI
KHFVGAVSMANAGPNSNGSQFFVTTAPTPWLDGRHVVFGKVVEGMDVVKKVENTKTGLND
KPKKAVKINDCGVL*
>TCRU_1194 Domain of unknown function (DUF1768), putative
MNKGVVRFHDVHEVPYGVFSPLSPHPIVIRHKQYPSVHHYFLTERFRDAPVTAALQSATS
VWELDRLVKEAERTGSQREDWNRVRVDVMLLGNYMKFKQNEAVRSLLLETGDKLIIDHTT
SDDFWGDRGDGSGKNLLGVILMAVRDRLRREMATKAREK*
>TCRU_1195 proteasome beta 6 subunit
MIEDHMEYGHHFPRKLADSTLSLPRQGVKEQQWSPYADNGGTIAAIAGKNYVILGGDTRL
NGDFCIHARDDRTKLFHLTEHTFLASTGMQADRLQLQQMLKYRIQWYQYNNGGKLPSTKA
IAKLTSTMLYQRRFFPYYTFNMVVGLDEKGAGVCYSYDPVGSTEPFRYGTSGSASSFVEP
LMDCLLTRQHMVQQAPAELSMTETLEMLKNAFTGAAERDIFTGDAVCFHIITADGIRSEL
FELRKD*
>TCRU_1196 hypothetical protein, conserved
MWVELPGAIAGVWANSQSDLWEKVLACGSLCHFDRWRKERHAVGSEVSSPWWESEAHALY
VWVTDSNRVFCVGPAYMDAALNKSIANALLCSDGALYLVGARDNEEKSSLFPASPDGGAA
DDHFHPP*
>TCRU_1197 hypothetical protein, conserved
MLYTQRGHASVKKRATALCLWVTDSNRTLSAGPVAVDNAANWTLASNLLYSDGSLHLLQR
RGNGECRVISLSRLMEELSAVRSVLSNWAQKDIFFSSLSIPTAGLVAVLSGAA*
>TCRU_1198 BNR repeat-like domain containing protein, putative
MWLRGASTLRGIGPLLLARSIKDTWRAHTVVGAAEGKESLGVVLRRTTITKGHRVFSLAG
GSDVSYEGGCWREGGLEPKLFVGDVTDPTGGKRSGRIEWGDPKPFPKLISSASHEGEWTE
FIASVGSGVVMEDGTLVFPLTAKDEAEDVYSMIISSTDNGSIWTLSEDISPAQCLNPCVT
GWEGSLLMIVDCENGRRVYESRDMGTAWTEAIGAL*
>TCRU_1199 Trans-sialidase of Trypanosoma hydrophobic C-terminal,
putative
MTALNTESVTIPKLDHAKPEPRGTPSTPAGQQPSELGHLVGSSKVASGGVEGADSQIRGV
NATALSSNLVNVSQENNSDAGTMRGSGLLPSLLLLLLGLWWFAAL*
>TCRU_gamma, conserved domain containing protein, putative
Elongation factor.1
MASSGDDSRCAPGTHPHTIPCVSLRELDELKIDYSHTDTRTVAAPYFFQHCDAAGYTTLW
YNKYNKYNKMQCMTANLIRVWLQRMEHVGQYALGAALMFVEQRRHDIVALCVLGGRACRR
F*
>TCRU_1201 hypothetical protein, conserved
MQLICQRRSTVAQRNTDIPALGQLYEESERVGSTDVGAVNVGAWNVPSGAIHNLLPHART

ADERGELCGLFDVNRSGAIDPTAALQDAAVHVGNESNPAGHDDSGVAKNCGAIGGEAGAP
LDKERDRGMYPQPDRLEGTCCRTTCVYNGESLVRNCGLDTQQFHNGAGRGHNFGLFRGSK
SVGAGLRRAPLPDRIRGQDAFDIINLRGTPRRSARLTNITNAKVERALAPWDATAHSIKR
GALRHAAQIVETLNLNPHH*
>TCRU_1202 hypothetical protein, conserved
MEEANDYFSTRASSAAVADGLARRESAFAGRERHLETTPETRGTPQCLGRVDKSGQLEVP
SSTRGTPDAPAHEAEDGVATTLRHERLAEGCAPVSAISDPFPRESDGG*
>TCRU_1203 hypothetical protein, conserved
MGTTTASPLEKAPRSVGCGAARRFACSLTDRTAQSFQCGHSVGITRCGGVRKSRSPDEDG
LVVVWWLRTSGPVLLFFSAARLAQHHPGMVLLRESRHESAPATRLVGCGDFHQACGGRAG
GVAFLVRHTLPCERL*
>TCRU_1204 hypothetical protein, conserved
MCQLIHVTPRSAVLLELLVVMMLVMCCASGCFAASLTCSCPSDKAMRADRAKSIPAVVRD
VPFGSEGASQKCSVIRRNGWAPTRIDVSTRDVDGTRKYCSSER*
>TCRU_1205 hypothetical protein, conserved
MAMMTGRVLLVCALCVLWCGAGGGGAEMNDETLVDSQHLSGVCEKAPKGLPESGERGPDS
AGHSLDKNNGINSNLQGGTGTGTTTERVGKKKEEDVGDDDGLEEEEEEAETDDDDDEEEE
KEKSKEKEDTPQAGKSESSSKEVAATIPTASGLSGVGGGSPSGVDDGGSNGSPKGIEDLN
LEVPGPVVNSPSPLPNAAAGGLQNPDGALPSQKNNFSETGVHSGKTLPTPSLPKPQAPAI
TQPKAEEQSSTEQGTEDSPETEEVTTGKENVQNTVETVRPSSSLSAASKPPVQQPTPLLT
QSPAAPSPERLAAATSEEKSIAPSFSAGEGSRRAPNVARNSKEEKNEKLPSENETESKAV
EQPSGNDEAEQDSPARTTASPIPASGGADDQRNEDADNSNARVLKSEGTHKNSETGYTNL
ASTAGDAETQTEKALADAKTNDTTRGDGDGSTAVSHTTSPLLLLLLVACAAAAAVVAA*
>TCRU_1206 hypothetical protein, conserved
MMVPNASKKRPSFANGPRILVASNASGIMSVTYGGREHVAADNTDELSEHDATGHKTLVL
ENGVAGSGPAAEGDAGGLSQRNASGHENPNYENSPRGIPGVAGVYTTALPCSTTRN*
>TCRU_1207 BNR repeat-like domain containing protein, putative
MSRRVFNSALLLLLLLVIMCCNTGGVEADAPAQGPRSLPENHFVWRDKNGGEKVSSLYAP
SLLEVNGKVFAVAEAQCTENDKVFTGIASGLLTLDKKQTKGELDASKVKTQVLEECPSDK
AECASQPEEKDVSQSETKVHVNRPATVVKENEIYMLVGKYSPTAVVVPPKNDAEDCGLLL
VKGKVGGGSSDSIIWGDTAGVPCVSTVEKEKIGSTRLIGGGGSGVKMHDDTLVFPVEGTK
KEGEAQKAVSLLIYTLKDTNTWKLSKEVPADGCSDPSVVEWGEKEKKLMMMTACDDDRRR
VYESGDKGESWTEALGTLSRVGQQKGRRSEGR*
>TCRU_1208 BNR repeat-like domain containing protein, putative
MLVTLPVYSSKNEVNRKGKLHLWLTDNTHIVDIGPVSGNDDDAAASSLLYKSGKDGDNKK
GEELIALYEKMKGDGKQSHSLWSVPLTAQLQRVKDVLATWKKADNLVSRLCPSESSVEST
SPKDACSPTVKITDGLVGFLSGNFSENTWSDEYLGVNATVKKENGAEPAENGVTFKGRGA
WAEWPVGSQGENQLYHFANYNFTLVATVSIDGEPTSGSVPVMGVRLSREGEKKTFELSYD
KEKKWQVLCDVESPEDQSRTLATDTTHHVVILLRNGTQGSA*
>TCRU_1209 Trans-sialidase of Trypanosoma hydrophobic C-terminal,
putative
MSHDGTAVTAELGAHSGASGETVGGTDGQKREDIHARDGEVKAAALSSSLGNLSQGNNSD
AGTVCWSGLLPLLLLLGLWVLAAL*
>TCRU_1210 LisH, putative
MNQSRSGMSDENFDPSLIDDNRTNEAAGSYDNDQHDTEEISDDEEEMLMESPSTDLVKDI
MKRLLKVQKQIKSLTHKIENVLNVNSLDLHPLGVNNENDIEGTILLLIKYDELHLFLWGR
MAKLLHEEHQQCADVYTDTLNLHDRVPFESLTYTIHHNTVFCEYMGELINITIDHTANHS
DVEGEHLRQNWAKHIRSMEKSQILATYTALLVVEGSRESFLDPFSTYDLCKIIVEEKIRR
KRLLALSLLFSMLEQDDQAMGETLNQQLPQILLQRVRHLVHRIFGYPEKQNVMFSREHRG
SPFRDYSTLIHSNSNSPFSSRRASPEIVNRNLVNDTRGTPLLSSALASLHNGTSSSHAAD
NQGITSAEAQMRELPIINMEFEKPLFQRVKHTRDNQESELTSRPAKAIKLEINERGPECQ
ESNENWEPQKGEKVGRGPAWSFENQAEACDIGEVLGVTTTEEILVSWVRNVDSAVDNDAE
SRVFRYKYKKPHEQVVSWQRFLSIKSMKSEDIDFKMEEFMLNCAVIGAICHQREAVLPAL
QFQVIESMIHVLNSIDLTDAATMAKNAEKSTEMRERTLLRMEEEDRAVDEDKLEIRLRWD

AKLYITKEHYRRVREIVARVGWVLNGLLSHKKLAFLFLELGGLRQIMTLINANLEVSTTY
GCCMVLSQLARSAVLEKLLRNHGDYFEPIMKFVLHHWKNAPSHDVQSSAGGFLFQALSFP
CVISFFDIHEGPQSTMATIERLLNNSEERFDVIYPGLTLAALKCFYVYLVSHLMLYTRVV
FRKHRFLSTLVTNSSQGVSLPRDPATVEWILSFLSSTSASIPDVTIETVHSLLTRERLVV
FRSLIENDFHRLLLRCTQFYFNQGRWELLAASLNVLCVLSVVPFVRPLIADAQYPESGIV
HLIMILSDLPSASRNGNTSRESHLIPCVATALQILLHLAVPPVDKSDEASVATFNQVCGM
IRANDGVRTLLEVLKIRKDAAMSAKLHFFPVVARALQLMVTLRRYADTRLLFDALDVNSV
ARELLKQYDDVQKEYISMMGSRYTAGDVHATGRFMENVKCFLFDKENKENLTVSVDPVEL
ERRQAIIAHSYINYSRESLLELIYRYLDSEGLQNAASVLRRDANLSSDFSQLQQSTHVNT
DVTAFSTIGAPTLDGIIRSYLRQQHEKCTNPISTLPQFDLRKSHVYYPLPAPVEYTRNTF
NRGLAQKMGIDFSLRMRNNENHFLYRYPGHLFGITGVGDEMQGDTIAFCDNGDTLIVGTS
DGTIALFDTFPDDSSVEKLLEQHLVFDDDGVSGVFVSDDGDMFAVVSTDCKVAVMRRDSL
PVVKYSVEGSRAARFSNSNKFLLTTCDERHTCRLYDISAQQEVRHFSDPSWVGENLDNVA
TFDAFSQLILSDAVLWDIRCGDKPIYRFDRLNESFCNAFHPSNLLVLIDEKIWDLRTLTM
LQTVPAFKKTSSFHINLFGQVIYSFREASSLGNSASSVLSVVESHTFETVFSTEIRPPFR
AFAIDPSDRYCAAIIDQDMGAVVRVFSTSSGPLPGQQTFPFPQSNGDNASETDLGENEDA
DEDELDHDSSSSSSDEDSDDDRSSDTVDEEGSEWDHTEVVSGADSEEVSDAFENGTLTTT
GDETSGGDESGETAEEMEEVSDESMSST*
>TCRU_1211 retrotransposon hot spot protein, RHS3, putative
MWRCWGWLDIALLRGRWASAASPTGVAVRLRGAPTTEPCECHAQRHWDCGTKQPRLSFGA
SGTCWPQLGGASGMLHHTGVVMAHRRGSCDGSGAAARHVAGSKVWPQWTMSSTNMRLSDF
LWNYVGGRAAVDERLQCDDGDVFSGAG*
>TCRU_1212 kinesin, putative
MCGSVRVVVRCRPLLSGEEGDACSRVHFEAEGGNVLVDGKAQKGTRCFTFDRALPPNSTQ
EDVMQEVSPMIEHVLKGIHATVFAYGQTGSGKTYTVEGIDYLRHGSGNPRPSLKTDPRQH
GIIPRAIQLVFDRAREMQLEDGNLRFQFCCSFYQIYNERVMDLLNPSTTHSPNTGLKVGW
CRGDVFAVENLFICNCDEPEKMRRCFFSGTKEKFMGSHAMNQQSSRSHCVFTIYVARCDA
DSPETPIRAELSIVDLAGSERVAMLSRSPSSSMLKDSPDINASLLTLGRVITALTANGTR
AKKGKPADRSHIPYRESKLTMLLKHALGGNSLTTMIACISPSDRYVDETLSTLLYAGRAR
NITNIPRVNEDAKSALIRQLRAEVASMKRELKYYTELLARLGVPHREQLNWGPPSLNVG*
>TCRU_1213 ATP dependent DEAD-box helicase
MRRVHQLSGTPWRDRHLFSVFVVIRCVIQSSQSNGLRSPLDQYEDFRLVQQLRKNRQGNY
YRYQQRLWPSTEALERSMTERLYSPTERSPLALLPQPKRTSPNRTLVLEGNGATALLEAA
ESTLAQNGQTTANEATTPSAPLSDMEEMLAEHLRVELQGPIGSKIITEADLEGTILESAR
ERARHVIEEDLLVNDLVSSSSSSASSAVLSLRRKVPSAKDEGGVAWISLGLDPVLSERAL
AKYGPAPTRLQCRLLPALLHEDHNDVLFNGVTGSGKTSVFLLALLQSVRSEAAGLNVFVA
KDGLTALHAYSQLRALCGPLGGTVVDRPRDDWSWLYLGSYREGYEKYYRALRRSLHSDHG
PVRIFITTADTMCELLFEKKMEFESFGYLRRVYIDDIGVQIPMIPPNAPVAEMRKRLRNP
LACELLLGTLHQLPGPHIRSILQLGLVSADMDERLKDHLKALCVKLERHTIVLSSVRVPS
TIHCLFSFHLFHEDLYEYLVKLIWNARDTIPGRAVIFIRQEDNLLQVRTKLRGLGMNAKL
FSEVCHDGEFCGKWKFLLLRESEAFGIEIPLVSHVFITFCPRTACSFQHMCGRTGRLGNL
GWVYTVTDKRDARSVREIATQLDVDFCNHVINVQLAQAPPKDMDHQTKEYELYGLDPQYA
VRQHYIVQTENPSMAYRSREFFSKPAKKQFQMEQYTPISVLHRRFVNSKKLAADINRDPS
VAVSLQKQGLLDQSLKPTKRLKFLLNKKSQKHGVSLYGGKNGIHDGR*
>TCRU_1214 ubiquitin-conjugating enzyme E2, putative
MKNISNKRIIKDLKLLLEEVDANNEANSSGSPHSAAIFSVHTDTIYNWILKVKAPADSVY
GGAGNTYQLSVLFSEDYPHEPPTVRFVTPVYSPLVTEEGGICDRMVNDVWTPDQRASDVI
KLVLDRVFSQYKNHSDDDVNPEARHHLEKFPQEFAARVRRGG*
>TCRU_1215 Hydroxymethylglutaryl-CoA lyase, mitochondrial, putative
MFSSRFFLAPIRLVECPRDAMQGLSHFIPTAQKILYLNALLKCGFYSIDCASFVSPRAVP
QMRDSAEVLAGCNHALLTGEDAPKLSVVVASLSGLKRALETPIVSTIGFPLSCSERFQQL
NTKKSIAEALNELTEMQKAVVDTNAALSSSNGMSLELNKKKELLVYLSMSFGNPYGEPCG
PEVAEQLVSNLVSMGVRTISLADTIGVSEPQKIHDLFTRLQKKFPDVFFGAHFHSNTATS
KEKIIAALKAGCQMIDSALGGMGGCPFAKENDLVGNVATETVLQAIDELNLLPSSIDRKQ

INECVLLKQHIFGVTVKDMLLSQSLRDEKRFFQLCQEHFKLYDVNDTGMLDYEGFRSSML
RVFEELGCDPPSEEKICSSFHKVDVQKLGCITLDAYTMGARRLLMKRLGFFANVKSGETG
GSRRIATA*
>TCRU_1216 hypothetical protein, conserved
MPTYYERLDNLFAVYAPEKRPLVAGILQKYSGREEEVLQSLVRKFGPEPSEVPNHTKGGN
SYRERLVCFYTKYAPGKLVTVDATLARCAGREEELFSALVEKYGPEPAISCGDEDSRSVE
KISDTQSDLSRTTSSMSLVPYNVHDFRWRLARILMEHAPGKLAILDQMMEKYAGREEEML
AGCVARYGPEPLPPDIQSPRSRLLRFIEHYVPRKASSVDVMLKKYDGRTEALFAALVHKL
GPEPPAPSPGNELSKPTYRQRLIRFYEFYARERLADVDTILRRYNGDEESMFRILVERYG
PEPDPAFDSDAPIEPATDYHTRLTRLFTKYAPERVDSVPYLLAKYKGSESNIIKALIKKL
GPEPPFDASGTQSAMVHLGNVNEGPGNGVPPGTSEFRDDDGKEGSEFHSKAEKPEEWLRH
LSLPLSAYRRYAVNRLGEARVSALEILFGVPRLLFNENGGDEKDEKRWVIVKAGMASGKK
FDAAQESEELKEPMIAECISANFACLVLILETVKFPLPAVYDEGKVFSLYPFCTLLGDRD
RFTLRICAAIMDLRKRVEAEVASLLHEEEVCRGLIQRMDGWKQQHIIRLKAKEAYKERLV
QKSIELLPEFEEKERQDIITVEQIEIKGKAFWFYEGLQTPMNTCEDLQVPYGRQRLGNEK
KMAVGGKHVGSPPPPAGCRGFSQLIFSHLLRGQNRENYGLRSLQSYKSSAEDTMDVSLVG
GQKMGSPATVTTPVSWGPKHSTQEENSPCNLLEASPPPRKPRGAVEPTVRVLRFSGERIR
VNGQVTHAGNKVDITGRDKWGPSKDGRISQIAAVPCRDTREAILLGEDPGKFCKSGNNWL
V*
>TCRU_1217 Regulator of chromosome condensation (RCC1) repeat, putative
MIRDAEFWMEARRKHSGGDAIFTAMLPMLHARQIVLDSGIEVDENEIGREDSAIFSFPSK
FVRKEPFEDNKVIHLPKSNASGKKVSSLVLRVLQQIMMFAVPLYSLQYNIPAMSMSWRTS
CVVDRTTKQLITFGAGDGVKVPKQLLCRGCVATDEFFAILTDQGEVWVSGGLSVLSLENG
KWTAGPLGKAESLSTVAEKALLIVGHGTRLACVTRAFVVRPLSILSNPVKSVIPSRHVRF
LDLGYGEDYYMVGIDSVLYKTLASKRTLGTPRRVMALSRTPVSRVSCGMGFCLIIDQNGH
LYTLGRNKKGQLGNGQQQNARRKPYLQKSLCHHFFVMVAAGDAHSLALTSSGVVYAAGSN
ENGQLGLGKSVDTMLYFTPISLPSKCVGIAAGPAGSMFACEDGRVYVCGLNDQKQLGLDT
SARIVYTPSFIPVIATGVEAYTMDFGAYQKPLITTRNSVQSSFQPNDSSAAVTNPEETIP
QLIPSELLQTSVVSDTNRHKQFEQTPLPRKEKNGKCVKCCATM*
>TCRU_1218 IQ calmodulin-binding motif containing protein, putative
MQGVTYPRFEEKRLQKPVVGSVQLPPVKMSRQPPTEVQKRQGISSSDVSPSPKMKKNHSG
NKKRRNTVRNVTGSATSVETVMEMNKKAMALMKDGKMKASYDVLQTALTAAEAGVRRFPS
LPMGSNSENQEKRDAWLLAFAATLSNLGCLQRRGNQPYEAIRYLQDARNVELQVFGRPSC
STMVNLSAVLLELGSTEEAFFIARDCALASEESDPMLHIIALHNYGVALSQHDSEEMRQL
AAPVLMKALREAEIHLGEEHPTTILIRQHCGMDLKKTTDGTDTCSSNNSGAITQLRETPE
NSIQPLPPGGRLPPLAPVPAHLMKAKEALQVLNYGGMPSMAQNYDSPVPDSIKALNRPES
ASNDGEALLSPTVAPVLRAVEIMIATSKRRCTSNEPAAPSVEEGVEATLARQGSSLVGTL
TAPLVPLQEPPNEEEEEMDEKKESEEMCEDDYGNKSNTLGAAVETLEEGRAESLIDCCVT
DPGKEGQEDTGVNKDAPGGALYDDDDESPMRTEEKRIDPAAVSNTFEETKRPAKNTIPGE
YFLIGAQGNRSEPSFYRFAPTDVSVTMNMYGGEQNVAAMKAPETTIEKETPLIDFDYEEE
EEEDDYDYGHGYHELRPFEQKRRQEEEKKRLDTAEKVIEKKREVFFKIIGANPTLRRQAR
IQQERLEEEVLCARIRKEAEEKAKKEYFESTIEAIIFRTRDRAARKIQTTWCMWWYSVGK
RRRELLVKRAEEKARRERSRQALFEHERRVEQERRRRKSNKGGFEVGTIPSVTSCGKKWL
EKTLCVRYLARCGISHKDQKEDFFVSKVSKIQAIWRSYRTRGKMREAASYRAQTWVLKRQ
MEREVYAAMVIQMFTRRCFARRAREKKFLERYRPPTIKIQRWFRSVMPCRRALGVDRVSK
WRRQYSARLIQRAWRAYLSRLAYFMERLRYKLDEDRRHERVASSLLQRVGRGFLSRSYVA
RRWAKSSNLRRTMYALEWGEKGMDANKGGMLTVAAPVGNYVPTPIEEVNAVAELEREKYH
IGLFVDVVAERHRAEWKEALRLRPFEVLRRRAHEDHLCELELNASRRDRAAVKIQREFRR
WLRIRDAPFSDKTLLLIARGNYQVLEYGRKIDFMRHACEQDAGRVIFGEQVALMREARAA
AQAELQAVRPLVRTQVPHHEVRTREERYEVEKELQRDEHLLKKQLLTDTLRERRESFMRR
AAEAGIVGSAIGRTKKQLTQ*
>TCRU_1219 ATP-dependent DEAD/H RNA helicase, putative
MLTGLGTWESAVDSVEAQDQQPKKKRGGGFQSFGLDKPVLDGVLRLGYNVPTPIQRKAIP

PMTQGSDVVAMARTGSGKTAAFLIPMLNLLKSHSKTVGIRGLVLAPTRELSLQILRFGIQ
LNKFLDLRFAALVGGNSLEQQFELLGSNPDIVVATPGRLLHIMEEASLKLSMVKCLVLDE
ADRLFELGLQPQIVAVMQKVPDACQRALFSATMPSVLAEFTSAGLHNPVVIRLDAEMKLS
DKLKQSAFVVRNDEKVAALIVLLKKVIGVAAGDTNDGTAQQALIFVESKFHVDYLQMIME
AYLISCSAVHGQMDQEARRNAVQAFSRRETHVMIVTDVAARGLDLPLLDNVVNFSFPSTP
KLFVHRVGRVARAGRSGAAYSFLTFEDFPYYVDLMAFIDRPLQCRRTEGDLLFTADDGCF
GRMPEEDLQLELDFLRRLALEDVEVRNMAKVVDNAHKKFTRTKKKATHDGIQEARQEKYQ
FDNTPLHPIFVERLEAKRIAADEARFDLKRFKAKESVLEMIHGEKMFQIMKPVTIQTLWK
EQREQAEEDDAVTGKGAAEEEPQKSDRAKITKRDVTVPEKKLSLGEKLLLKAQERKKRER
QEMDGATNEEDAVTVCPTRSMHRFESEYGGGSGKYRDQDYFMDTTKRSTMEDAHYSVKDA
TFDVGAESAQEAQQQRQVYAWSKKKNRFVRMNVNDAKAMLRGVKNEAGKAVNFKSKLEAY
SRWMKKSNMRIQDVGEEEDLGPLHRARAAQEQGTGGYNHDGDDDDDTVDISDPNQGKKLR
VGRKQRRLPKDGHIRTFEEMAHIKRKAEKERARLSRKKQKRKSK*
>TCRU_1220 Glycosyl transferase family 8, putative
MHRSKRFRPGLRHRRCWRCSWTSLLLTLFFLVLNSLVWLYGFRRIPSLTPFPFPTDREEP
AVVGPGAKEPRRAGHNSSQAPNFVLSGSDNERHENIILPKGFGHEIECVTSQRENSSLLY
VRFTDRKRVLTLKDIIKKVHHNLNKNDSNNHYHDVNVSICPPLPEDVKIPINDSFEIGMI
REFVGGMYHPLYCKQWKRHNSNRTENGNIEKDEVGGEYAASICSAETQHKLTEAINHPPK
YAYVMMVSNHRYLDGAIVLADSIREHSLLSRNHEADIVLIINENIKKEMFPLLQVVFDRV
QIFRGMQKWAPKSIFKATFDKMYLYMLEDYTGGILFLDADSLITDNPDFLLQMQEKIAAI
VKSEDPGYWTAARRAALSYPLKESEWKRLMSTQKMPFLFAVGDEEYFQTGLMILRPSLNM
FLKLYLEFRLGSYGYNRVGARDGVLFRTCFTTIGELVRRPHGVYHFTGTPKPWFNLERLD
HTLRPIQVKMRKQLPLSWKDCKLRYDWWTRYERLHLEHFQRIELETRACCGPAQDAELIR
WRNKYGDKIRQIPSDLHITKYGGAMLLRKRTRDLRLPLGINSLKEAIHASPKSYMWLMRF
SKDVEYLHPVREHMAKLRTYDAPLASMHELNSLLSAFNSFSTTMHYPKRIAYAFAAHPHG
KEGNTQDGLWSDCDFQCARLGLRCNASLLIDTRIADCQYSEPPSSMANGLIRCKRCLSYY
KAGAPFVIFKKNERNTLWIREAEVCFFNAWTRFAVPSCNATEPQIYEYELQHGEFLAPVC
ACQ*
>TCRU_1221 hypothetical protein, conserved
MQAVVHYGEQFVEVPDASVTLRDICGSFGMTGDLAGVCIKHRATGRVVATLPFECLPAAN
DAQGEEEKIYDVCAPSEVGVEESTPTTDDMTDILRQLVRLGATSLLSTESEQNRSLDPYD
PRMSPGLLMRTIYPPSLYSPYRHRGDPLNMQGRFANSPITGQMTGPPYVSAVEPYTVEAP
TELTLQLRPDLVPQSEEEARDILQGLVAAATRVKAMVAMADEEEKRHAPSDVKPNVALMQ
MN*
>TCRU_1222 Trans-sialidase of Trypanosoma hydrophobic C-terminal, putative
MAMMMTGRVLLVCALCVLWCGAVFVSAAGDGDGDGRGVSEKAPMGLSEPGERGANNAGHS
SNKNNEINSNLQGGTGTGTTTDRVVKEEEEEEEEAETDDDDDDDDDDEKDKSNEKEDTP
QAGKSESSSKEVAATIPTASGLSGAGGGSPSGDVGSSGSPDGSEDLNLEASGPVVNSSPP
LSNAAAGGLQNADGALSTQKNNFSETGVHSGTTPPTPSLPKSQAPAITQPKAEEQSPTEQ
DTEDSPDTEEVHPQDGEVNGTALNSSLGHLSQMNNSDAGTMRGSGSGLLPLLLLLGLWVF
AAL*
>TCRU_1223 Concanavalin A-like lectin/glucanases superfamily/Trans-sialidase of Trypanosoma hydrophobic C-terminal, putative
MNGDGNNVLLGVSYDSGKKWHVLCGDGKTEELSSTWETETQYQVAIVLKNGTQGSVYVDG
QRVGNEECALRNDESNEISHFYIGGDGENAANKEVSVTVTNVLLYNRPWDEAEITALNPN
KDPTPPVTPNAQETGASSALDGSQLTERGQSMGSSGAGSGGASPSAVPTVSTPSAGKDSL
QQVASGKSSDGTQTVGGGSFSDGEPTVETREGGTDRQKEEGIHAQNGDVKAAALNGSLGN
VSQGNNTDAGTMRESGPLPSLLLLGLWGLTAL*
>TCRU_1224 40S ribosomal protein SA, putative
MTSVESGAKVLRMKEGDVQKLVAMHCHLGTKNRSNAMKKYIHSRTKEGTNIIDLHMTWEK
LILAARVIAAVENPQDVTVCSTRLFGQRAIFKFSQLVGTSFLAGRFIPGTFTNQIQKKFM
QPRVLLVTDPRTDHQALREASLVNIPVIAFCDTDAPLEFVDIAIPCNNRGRYSISMMYWL
LAREVLRLRGTIPRSVPWDVKVDLFFYRDPEEALKHEEVNQAAAPVAEVDEGFGWVERDN

NAWEQ*
>TCRU_1225 Domain of unknown function (DUF4496), putative
MVLSSAPKSGGEKVGMMQGGLEEKRDPLINSAGCYACYGEIASIVASTQGLYYHQTRGNG
GTDLLNRIWRSMGELLEEQLLLKRSCIVTDFFHASVKVQNIQYYDGKHVFYKLQLVLLPD
FTSKFHLQNVLTIADAQYHASVPHIISYSKIADRSGTDRFVVEAAIIDSTREIGKYLMRN
AKSVLRIDIGIAFLEFRGREYRIKWTEEFLQRFRKAVGPRSVVSPYDPPLMSPAATGCRF
QTACLSEDSLHKSLINTVQREGRPAVVEMNSSGGGSWF*
>TCRU_1226 ARM-like helical domain-containing protein
MKSAAPKTSSKALKGKTATPNKAVEKAPKKLSKCEKFLHELEDLNQSQRWKKVVEMGRAA
HAGGEASSELALLLQLSQSGVPYERVLGAMGLCQTNCSSEIGRLLRDPCDRVATLAMMPA
AVHLSDNDLCELFKELPRRRLSFLCERLDHKKRLPAVADKYIATLPPLVRSSSAHALLCW
ASEAHLSRQNPLALRHFSRSEWYTVVKRLPDFALAFAEKGLKDAATLESASERIHVILNA
FRRFYPSVGLKLLTSAMNHFCVPDDCIVAYSNIFPEAVAELVVKHKRLCVFDVWRPPKNL
RHETFLALIDSGGMQHTHVTDFFLLAPPKLRSFLYNSRNDMFRNANGSMNVDVLFYVTDP
AERRREGMRALTEIAELRTDPIEWLKYLFVLPFPEALELAGRYLQVSCVNTRAAACASLV
KTGSFHPEHLHDILDFCVKRGKENDIVRHNFLSEISGIPPHLWKEDHYPKLVKIIQGAFD
SRDASTATFSSIMSLIRGTVRFNTVLADKFLLNILNRGFQFLLSNTKQIPRAVMEHLWKF
MLPWLKERVDKKAFSTVNYILEEVGPSLHFVAESSVPFMKTLLKSSESCAAINAFKVLFR
FFRPTAMELLPGLLEESLEWIGVDEVRHVVSLHLQGHWLDRYLVPIQTNGRLGTRKGQWL
VVFRNGHCWTAAKQEKYAKSISRNLSCANYSREFILGNLAALRKLYSANVTEALAPFIKE
EHTDPFLWSNAIELLGKLDDAASLKVLKDAFNDERVRVAVYAIRRRICNLPTAQIIEELD
VPLHSKLVAVQKVALRHVCDAGDDVAYAYLSQLRHGEKMHPNLEAVWLSAMFQFLEKREV
WDIFMDVAKGKEYILAKTLVDVPDDTLVEDWQLVNLDKLLAVLLSCEELTVVLAVLERLK
KRSLRRDEEVVRTMLHILKTTTINEIMNLTVGALCISSAEKQEVASAFLSLRPESRLWRV
ADTLIQLRHTACDRFKEIAVAYMKLLLENCCQTAVACQLICYLPVKDLLRHLNDVLQRGL
LHAGAVNSMLRSLKCTAEIVNEFEAVENQLRQHEHPHMQRIGLAMLESAASTGKWDEKRR
KALEVYRNASNGWVSDDAKMVDLPRE*
>TCRU_1227 hypothetical protein, conserved
MDSLSRTEALRVVPLAVRGYVTEESWIMLLSLIQRGHSVEFMFSPSHCGIPRNEAADDEA
MIARSLPQEGGPIWHVDFLTAVRRRNWRDAQKKGRARTPHERRLSVWQGLYSWHDQKIVP
RVAALRKGTVRFLPTPRHACACPSYQK*
>TCRU_1228 hypothetical protein, conserved
MAEAVSCLDLKASFFQVSLPRETRHLFRCRVEDGTLVELTRLPMGYKASPEILQIITSAI
AGMTTVVHPLWAAPPSVRVDVWIDNIRIAGSKSDVTLWEAQVLRNADGRHATMGEDRESG
ATQYTFLGVQFDHTHRAVSLSDKFVRSVRAMPALNSLTIAEMEVMASLFLYAAAILGTRL
CQYYFFIKAVRRRLSALNRGIVLETSPANVPPSAVGLGERLRHTIENNRKRIIKPTERAS
AAIITDASLHGWGAVFIPDSGEVKIAGGKWERKPFLIMQAEARAVRLALSAFSAILPSTM
DVWVDNTSLQGAANKGSSKSHAMTWELQRIYEFLDSRGIQATFAYVRSAENPADGISRGR
VFTLRKLRRGSAGSCGWRTPKSATS*
>TCRU_1229 hypothetical protein, conserved
MGYKASPEILQIITSAIAGMTTVVHPLWAAPPSVRVDVWIDNIRIAGSKSDVTLWEAQVL
RNADGRHATMEEERESGATQYTFLGVQFDHTHRAVSLSDKFVRSVRAMPALNSLTIAEME
VMASRFLYAAAILCTRLCDHYFFIKAVRRRLSALNRGIVLETSPANLPPAAVGLGERLRH
IIGNNRKRIIKPTERHRPPSSRMHRSMVGEPFLFSRLRRS*
>TCRU_1230 hypothetical protein, conserved
MGEEAFSYHAAEVRAVHLALSAFSAILPSTMDVWVDNTSLQGAENKGSSKSHAMTWELQR
IYEFLDSRGIQATFAYVRSADSPADGISRGRVFTLQDLAKRSGGVLWLEDPKVCHFVSNN
I*
>TCRU_1231 hypothetical protein, conserved
MIAVLPFAVHFSSGQAAAATHSACYIPSVQIHVGMSRLPLVRCLGASPTMRSQRPETFHT
PRWKHHGQLHAGQWRHFSVVAACLPVAGCRPPSLQCGMADGGPSPCERVDVQWETRSTRC
AVTSGNETLRPLIHVACAGSSDSSISMSIGVAAKSSHAVVDGERPSRSS*
>TCRU_1232 oligomeric golgi complex component 8, putative
MEVRMSDEEGVRRSIVEATVEHYSLLAETQEVNAKVASASGNALSCVRRLNDSLGRLQTL

SQEMIRVGKQWRREKQIALGTVSQHQKLLAFLEAPAVLEDCIRNEMYHEALMVLEHINRS
AQTMATVALFRRVEMEARCNLERALSEIVLPRLAEQLTVASAVKVTTFFRRLGVGEGQVR
QLFLWKRAEYVDGLLREAEESGVPYSRIFRYVTAFKVHVTETILQYAACFSPTIENGSCS
ELAAWCHERAYVFLEGFRASLEKITNGSELASVIEECNSCCATTTLLQMDVSGLINESLI
SRARALFAEQIVLAMQSYSASMLAFSWRPSNGLQCASSQKRVEAAPSSSNGIMSPPLSLV
QWLPLAYALNGVLTAFNTIRKCVVPGVELFCAAKLEGLLQTIAKDLSRDKDLLMAMEGGE
KQFYLLFVEAFVNDFYPHVLACMRELLGEDAQRLLINGVRESIESLRSLLFMESSTKQSG
APQQQMTDAAGTPADIQGDRITPNVVGA*
>TCRU_1233 BNR repeat-like domain/Concanavalin A-like lectin/
glucanases superfamily/Trans-sialidase of Trypanosoma hydrophobic C-
terminal, putative
MSRRVFASAVLLLLFALTCAAVGPVQAQSFGTRVCDSYGGHSSEQSPGEYRAANTAAAGH
IFRNPHLVNVDGKLLVIAGAQFNESAGSGSASMQLMARISNDDGVNWSPYSRPGDLDAFA
ARLHQMLFTTPFRLFGPLFAFVEGYDLRKGVRPHYANWWGGSGVESVIHFLGTGPGRSGG
FSMSSVSMSIRLPYPYKSDGLIGFLNDASTPITKMTDGTLVFPVQFLTMGGSTASTIMYL
NPRQQHWTFANSATHAGCTNPIILEWEAGKIIMITSCEYGHRRVYESTDKGNTWTEALGT
LSRVWSNSLAGLGLHIQSGFITATIGGKKVILLTQLEYSRHDSKGEIRLWLTDANRIYNV
GLLATGYGATSSSLLYADDRLYCLYEASGGSDSGAFFLDLTSELQRIRHALDTWAAKDNA
LGKCSSIASDAALSRGDCSVPFPTAGLVGHLADRFSGHKWEDEYLGVNAVVRGATKKVPS
GLTFEGQGAGAEWPVGKQWPTRPLHFANYGFTLAATVSIHEVPKGITPLMGLKKMGTTTL
LGLSYDKNMEWRVVNDLFPKHFTAWEVDKTYHVVLKMHDGMGSVYYVDGTLLWNMRLKNSF
NEGLDTVSHFYFGAYDEQLSSGKIHATVANVFLYNRPLNETEIGALNASKVTIPPPEREP
VPAAAATSPSVESASDTVATNTQPTVPSPAAAGPQPTDQKSLSASSVPSGGALSEPAASR
PEEPEPAESRPEEPEPAREGTADQPASVTSSDAASTDVGASSSDDAQTVGTEGGDMMQAD
QPAQFSVGTPDAANAATHNAEGKGQDGLHPQVNEAEAATLSSSLLNSSQWDNSVAGTMRE
SGLLPLLLLLGLWGFAAA*
>TCRU_1234 Complex1 LYR-like, putative
MSMNVYKRQEKLRLYRALLKGANRFPLRSRREIVAEEVRGAFRHPLNDSLTTKEVDYKLL
LGWERAAAIQTYAENMYWFHSRDEVSREMMHHSLERDRQRIREMERCNAVGNAEIKTPEV
TEFKSSMYHVHPDYHGKIGQMPLTHSRDIWRARGQYGSDVGGPRQKFYVRRFKALFPQGW
*
>TCRU_1235 hypothetical protein, conserved
MAEAASCLDVKSSFIVSLPRETRHLFRCRVEDGTLEELTQLPMGYKAGPEILQMITLAIA
RVTTVVHSLWAAPPLVRVDVWIGNIRSAGSRSDATLWEAQVLRNADSCHATMGEDRESGA
TQHTFLAVLFDHTHRAVSLSDKFVWSVRAMPSLKYLTIAEMEVTASRFLPAAAILRTRLC
EYYFFIKAVRRRLSALNRGLC*
>TCRU_1236 Elongation factor G 2, mitochondrial, putative
MLRQNVGCHWRQVTPLWQCNSALRVRRITQCAPLKNARAVTMSSGSKADALESSIARMRN
IGVVAHIDAGKTTTTERMLFYAGALKRIGDVDSGTTTTDFMKEEMERGITIQSAAVSFSW
RNHSIHLIDTPGHVDFIVEVERAMRVVDGVVALFDASAGVQAQSYTVLRQSRKFGIPVIG
FLNKMDKYNADFKKSVESIREKLEVEPLLLQIPLQSEDGAFEGVIDIVEMTACRFGGTHG
LDVEVKDLRLLDSEPVHIVQAALIARHELISTLTKLDDPLSDAVIALLDQTDGDEKRAED
LIPSDLLRAAIRRRTLRQGVPRPAVPLLCGASRRDKGVQPLLDAITYYLPSPRDRPLTGY
ARDGKIVPLPPATSAPSVPVVALAFKVTHATSPKGRRQPLVFFRVYSGKITPNMTLVNNN
CNRHENLEKLYVIHANRQLEVPHLVAGEIGAAFMQSTKTGATLFRAPQQVHLDADHTKNE
IFTLEGIQPPPPVISFSVEAATKQQIPLLEAALQELSFEDPSLCVHKNDFGQMVLSGMGE
LHLEIVMSRLEHDYKLKCRLLRAIVEYREVVLEAVELLHGIGMYNELPYVECSLRLQPLL
EEDGFCDPGQCCSFVLDETLTEKYLAGAAGSRNDRRRRMEDHHRNVKEELRIITDVFSRA
VTDCSHMGPLAGLPLHGVRVALLEFKKLGGTQLSEGPLQQAARSLVLELFRSVSKANLAT
MEPMMEVEVHLSEAAYIGGVVSSLSERKAIAVDIQDDGRSVKAIVPMRNIVRYTMELRKA
VKGHASLYARLHHYRVIEEKAVLSRIMKNLGIYEGH*
>TCRU_1237 ribose 5-phosphate isomerase, putative
MTRRVAIGTDHPAFAIHENLILYVKEAGDEFVPVYCGPKTAESVDYPDFASRVAEMVARK
EVEFGVLACGSGIGMSIAANKVPGVRAALCYDHYTAAISRIHNDANIVCVGERTTGVEVI

REIIITFLQTPFSGEERHARRIEKIRAIEASHAGNKGVK*
>TCRU_1238 leucine rich repeat
MNRVQSDAAAVYVEACNREHAECALELVQAFENNEREANLSDRSVQLNDVDLSCIAEALC
STAHSIRVLNLEGNAFGMAGLQALLEAVEKNPGILRELRLGRNKLKDQAAVVIGHALSRD
GCGLKVLDLSENDITKLGVIPIAAAIGDGSCDVVELSFHNNKIEADAATYLGQAIRHAGK
LRHLHLGYNAIRDAGAAQLAQCIPVTVSLSTLDLTANRIGPSGGKELARALMTNSCNMQR
LNLRHNLFDSETIEMYAEVIKTNTSLIQLFLGFMNPTPHSAALVLSALCANRSLLLLDIY
GWKLHVEDAWKIIDSIQRTNGTIAAIVTDACQSIAKRIDEGNDEREDHGQHPIYVGPDDR
DAYIATKSLRRFSRAQSRRQSRASSRGAMSSRGAMSSRTGSPSHRRAVERSHSYKDSSVH
TPHSTRAHQEILYQSTQGMSVAAKEELQHYRRTASGINDPVTIPSRENTLSQLEYQSGDA
MRQTVEQLRRELFEQKQIQRALEFRVAALESRLEGMCCNKTFDSVDRTHVSRAAGGSSHG
RGTLNRFNDGYKKNGSSYRSSSHNDAYRFSENIPYSHMAASVGVSHRKESIERSKSAEAI
SFELRQNGIAASGAAGGGGMAAAHSPSSGASGGPAEFPHISVARTERSEPPAEPLELPKE
RRPAEAMLQTSTRVVGNHQPQLAPSPSKDKDPPRRKGSIRSA*
>TCRU_1239 protein lipid droplet kinase (LDK)
MSMGKILGDRRYRIGCPIAKGAFSTVYRCVDLTTGKAYAVKVVSRALVEANKMQCAVVCE
INAMEVMPRSKYLVNLVDKLVSTRNYYLIMDLVEGGTLSDLIRSSPLMLSTQWCRRIFRQ
LLKGLSALHGSNIVHCDIKPENLLLNEEHTQLIISDFGFACYAPPGRLLHRACGTLKYCA
PELLQCDPRYDGREVDVWAAGVTLYVMLFNTYPFQGDEKCPDTLVQAVMAGKYTFPSPVS
PSLEHLFSVMLQPVPAKRWSVERLLRHTWVVGVDPPVAKASSRTAISLGDIVPNNHCCGH
ASEEFLVQSSLAKALEDDDLCGLSRRSRSCPLSSRKLSGDEVLGHTGLKGEVNGGAPKYI
SGIGNKRGRKGNANISTRASDASGEGEKCTELGGGGGGLSTDLDVVSNDSYNDNNSHIFR
TMSFVINSSGGSPTSLSTSSKVSRDGSPVSYTRDFILTLKAIVNFFLFCTALVVIGGMRL
LLDVDFNDLPIPSFLRAMVERLIKPPEDVISQHVEPQRNRNQTRGERLRQMFASAERAIS
ASNPRRRVVPT*
>TCRU_1240 D123, putative
MDLRVPLSYVLDTPLAQRHAIPHRTLFLDATHPFIQYLNSDDTRLPQHPTLTVEARPVDE
YETYVGEDDWRREKAVDSDNTDPAPRFDDVVQWVSETLNEIGGNGDGVVLCGRRVVADDC
GWAVFSRTPTLLSSRDVFIAMRNSSKFLRDLHHQLQGVALAGAEGPVKVEVTLAKALSGN
NAREFRAFLPYRLHTSAGDGCVEAWAGHMYAGISQRATDVCFPSLMAWDEATHDENYAIV
MRHIEHAKLLERALENDALLRTALFRQANKPSVADPAPKKKSPVVLLAVDVLFESPSLPV
YLLSAAVRCFHHDDVEDPACAFPFILDDDAKLGRGNDGNTTTTTTNIANNNNNNNNNNSK
ADGWSGESEDDDDDLLSDSVSFFRLFRDAANWNRHVEVWRRRGSTAGVGERSDTTRTQKE
RRFFVVASERGDLASTKEYMAKRGLPLEFFRPDLFKGDGNFLREWRERLMQRISMAEQSR
GD*
>TCRU_1241 hypothetical protein, conserved
MEPDADFGSVTAAFRGTEEYAAAFDLELWKAQQQARLRSELNEEKKRLKKEVAAEVRKKE
EQRLGDLDTVRQDLEQVARRLQIREETLQKRISQFEAREAVFEARRVKVAEQHEQHLLSV
EGRTRRQLEEAVVRQGVLQAQLREREDTITRLEEKLRAAESEHDALQRCMARHVAEDEDK
ARITRLEEELASANAAVTTLQLKLKERESELVVARKERDQFQEASKLSRDQLGQLARRYS
ELQKQWQQRERMLLDEEKRRLEEDKRRHQLVTLQAGSETKGVYRHDVLGHQFAEGIVSRE
VRDRVLKRGNAGDDSSTFLRELMAEVAQGLRELRVKEKKKGKKVKVRIADSSEPRLVYAE
VPPRHQDENSLHVRGGWRAVSSEEFVEQKRHQPRDFTQTTTACEDVANSTLSSLTQRESE
GRRELTEPYYSPLAEKPPAVKEEEEEADVDVSSSYCYADVESWIAEREDRRVLPREEAVG
KDVMESHLPTFPSAADAPPRPTPPSPEDFLQASRAAPPSASRESTREEMVEFVKKLRMNK
KRLLESGVYTEDDTLLKEMTDKIALYEDFLMRHF*
>TCRU_1242 NADH dehydrogenase subunit NB6M, putative
MLQRSIPRLVRDPVPTDTKAFYSWFSGQAYRQERVIPGGYPAVRVYPVYGKRWFTGRTVM
AMIAGFSLFGAWFRPEKERYNMEMMIEWSERQAAHLPYQKAEVNLRCLITSYKRHRYEQE
NLIDKGFVGLTSEFRKFFYHDDVWRPPLHDVLMHPYAKFGGPLFSYNWTIGYF*
>TCRU_1243 DPCD protein family, putative
MAERLAEPKSSVIVNGRKRITSKFVDGSEMVEEYDVITDELLLRKRRVKNTLGNFGDWQV
EVGSEVKTRNLEKELLIEANGSPEIVKQDTPEYHVFRIRNLPYAKDVFSVTVEHREPGDV
GEIVVRTSNKKYFKRLSIPELQRHHIQLDPGHLLYDVQHNTLIIRYKKHLAVLAADAAAK

KERAALPARRIDGADGNASDGCAQQ*
>TCRU_1244 DNA-dependent RNA polymerases, putative
MAFHPRDAFILHQEQIQRYNEGDEVDTSAEQKVQIELQRLTDSESTVTIAFSHEDHTLGN
PLRHVLMQNREVTSAGYAIPHPLEAKMLLHVQSTDYAVEAVAEGLERLASICDETLQSFE
DCMHRMRCEGAKQE*
>TCRU_1245 tRNA threonylcarbamoyl adenosine modification protein,
Sua5/YciO/YrdC/YwlC family, putative
MARIISASELEKAADALARGNLVAIPTETVYGLGGNALNKEAVEKIFIVKGRPLTDPLIC
HVSDVEKAMDLWDVHADPEAVELARVLGTAIWPGPLTIVLRANPLLPNSVTGGSGFVGAR
IPRHEITLRLLQLVNFPVAAPSANTFGHVSPTTADHVLKDLGPKDSSLLIVDGGPCDIGI
ESTVVKVNNVESVEILRRGGVTLTEVLRALGDKFHPLITIRDTRLRHLSPEMAMDGPGQL
LTHYSPNVPSRLLTPSSFTTNLPMDPEGALVEFSSNSSVAAPTEVLLRHTVIIDFGGKLQ
CLKPGCLAYRDLSVNAQVREACFAVFDALRWTETIPGAEVVLFPLVSGWPHASVDTDLLA
AVEDRLFRAASGKVARIRMQSRSV*
>TCRU_1246 CEP76 C2 domain containing protein, putative
MPGDPSHVNAERLKELRVAVHKFLQDSNVYSKIRDIVDSYVAENPGDSSNPASSADVMKI
IREKGLVQDLLNRLRGEKSPGIVQTPNKALSWKLLPGEYYLHLRLAGGKAFVDNLDLTPS
FAKDYQMYVAVHFGSQRYRSNLEDVSLDPPFRDEFLIDLDYKSFGSCAADLIEVSTPLHI
AVFREERLLTFSKMVGENIVDWRKVLRTGYLGLTVELCGDNPGIPAGILELQLELLPNKA
PRFSEEDVASRIDLQRSAITSADREFLLYARRWWMEFQDIRPTHKQRKIKVFASTSNGRM
VPTTHFISVVRPDKGIDTPLEAARFVSLIAVTDEENLTQEFLLGGLGNGKSANCWSSMFT
FLSQRRGEPCNHAALLCSLLLGFGLDAYCAVGTNKKGKTAMFVVSRKRLSAGVFDVMLWE
PTTGGRFALTDAHPWETLGSVFNHRSFYANIQPSDIITTISFDLDNEGLWKPLNPLKLRM
VPKYPNPSLLWISSDTKSLEEELLTLLIRQLTEYRDIRGITTRFNENLAYVLRQALSAYE
SHRRNGAPEDLSFFENCVKGVIGEGKTFKGVPVNVTHLNSRKILATFEGSASGKEILELT
ADSLTLAVQVKIYSFPEGVLSVWVMLAAAYAFSSV*
>TCRU_1247 glycerophosphoryl diester phosphodiesterase, putative
MVSCGVCAILFLGGYIMGAGIMVRVFPLRPAGERELLKERFPYPVTKIAHRGGSLIGPEN
TIYTFGRAISVGNADMLELDVRQSRDGEIVVSHDAALLRTCGSFHEEKNVFDIVVDGNPN
KTLPQSLREIPIHFESPQRGGCYKATDSVPVDETTRLCLLSEVFDAFPDVPLHIDIKSEG
AEITKKVLKLIERYGRETKTLVGTSNWRNRKHIYDYFVSGHLSESERLLKKRKRFRIFAG
PMDCFVVYVAYFLGVLPLVPLAFDVFSIPVFTKCKLKALGSCFTRFIAYFVNSPVLWKHL
QRRGILVLGWVLNDDEEFEEASNWPINGIISDDPIALDAFFKSREMPTAMNIL*
>TCRU_1248 RNase H, putative
MNPRIRHRRKTLLVVTDSQSLLAALNKGPLSQTDWTEDQIWQRLLTLTRAGWSVHLQFCY
GHCGVHANELADQYATQTMATGQYTEQGIAPLWHTDLLTCFTTQLTNKWRSTIRQDTHRY
LLCGTRPPDLSGKDLITQEVLHRQELVHLERARCGESELWGRPYWAVRDCTNQCRFCNIS
PEQSAYMRSNNDPTAPGTDTVPPSAREEDVSPVRRRTLTRRRKEKCPHCDSTLTGFSGLV
SHCRSFHPEHPPPLPELKCDFCDMVFPTRRSTAQHRSRCAHNPDATRHLNSSARRRSLLP
QDQPASTSATISPQETLHHLLLECPGTLTVRRRLGIEQGLRIGKFSQWQLLHSRKLLSLL
DHLFGTQMALYS*
>TCRU_1249 Protein-tyrosine phosphatase/Dual specificity
phosphatase, catalytic domain containing protein, putative
MEANGTLVECKRGESDAVVFRFLIIDAPSPSSVPAYMKLLQRHNVRHIVRACGPTYNAEV
FERQGIVVHGWNFDDGAPPTQTVIDNWLNLLEQEKNKSPPETIAVHCVSGLGRAPILVAL
ALVEYGGMPPLDAVGYVRGRRKGAINQVQLNWLMRYKPRHQEGNEGSLICARCTLM*
>TCRU_1250 phosphatase of regenerating liver-type phosphatase,
putative
MDVNCTLIECKDRRDVSRTLFNLLILDAPSPSNFPTYIKALQRRHVRHLVRVCGLTYDAT
QLEKNGIDVHSWPFDDGAAPPKSVVESWFQLLDKEKEKLLSGASTQPASIAIHCVAGLGR
APILVALALVEYGGMEPLDAITLVREKRRGAINQTQMQWLAKYKGRSAAGGGSSGGCIVM
*
>TCRU_1251 mitochondrial carrier protein
MAEEYFHTFVAGTFSGVVGVLLEYPLDTIKVRLQAYGSLYRGYWDCAAMLLKNEGMLSFY

HGVSTRFVGSGFEHAVVFSFYKWTLRRVGADEYHPLAWQIFLGGVGGGVASTVFLTPLEL
VKCHLQVANMLPAGQREYHGVTDCTVKILRRGGVTALYKGGVAMLAREVPGTAAYCGTYD
KMKEFLTPLGGSTAELSPWRLMFAGGCSGVAFWTVFFPADVVKTRMQVDPVFSRWSFGKA
LRVLYAEGGMRALYCGWSLTAVRSFPSNAAIFATYDLTMRAFTNQQRNTNSAAGAV*
>TCRU_1252 Las1-like, putative
MPKNTARKRSRAWQNSPNEKRKRGTCIKIGLGELGDLDAVAPTGTSGRIRADVSNTKAVG
SASAEGEFAFSTAPFLSFSRLAPSVFGHGWDEWKRVKQMLFDPQDGTGLTEARRKALNRI
QLVWKARERKNRQLPAYAEATALLMEAFSLDEGDQLSSSGAVSFYGAAISRAVHLMTGSF
ARGAADTYRKRARLIGFPEEAVEVRQRVAHGALPLLSELRWVCGLVLQFLFHHYWVEQER
HVHLMEEETEGEEDNTTSGHGVQQSAPSTTVEEMRQILEDLSDVGEDVTDYSNDRVEFLQ
DGWQLC*
>TCRU_1253 SMP-30/Gluconolaconase/LRE-like region, putative
MELVAELPGRLLTGLALDANDDVFAVCACSGELLRLNKTNGTMIPIMATETSPHALAIDP
RNGDVYLTDRSENAILKLEAVPNAKGHAEKDEKCDNDRGEVDYTVVRYLNSFENRQFVGP
TALAVAPDGELFFTDAGAEGDSSFADPVGAVFRTLQGHSQVVPLCRRGLIRPTGIAVTSD
KSVYVCEQGTNRVLRFVLRGTYYIGSVFAQLQGGMGPSAIAVRKSDGSIFVTQYDMKGSS
SSSSPNEGAGGIITVIGRDGEVSGIVRTTCPSLCAIALDSREETIYAVEADEASGGSRLL
RFRLSPAAQQVTE*
>TCRU_1254 SRP40, C-terminal domain containing protein, putative
MRPAPKKAAIPDSSSDDDDAPLRKPAQKAKASPAMRPAPKKTPRSECCGEQEPVKKSVRT
EVVSAGPGGKEWEDNNVNNTRKSNGNGGTALRRFQRIDPTKVVFVKDELRDNRPGGEHMA
FRQNQEMMRVKGKNFNKLKQKNKAKFYAAGVDTSVRSFQFDDSD*
>TCRU_1255 WD-repeat containing protein
MPLFPASASGNGNNTGVAEEKPLNLPFDPPPLLRVDPVAVLEGHEDRVWCVSWCPTANVL
ASSSADKTVKLWSCLQHAKAVDEQHQQYPQKGLSWECIHTLAGEHSRTVRHISWSPSGEY
IACASFDRTATVWKRASDDVNRFDFELEAILDGHESEVKCVAWLTDSTLATCSRDRTVWV
WDRVDVGEFECAGVLAGHAQDVKACAWIPPIDGGAKPLLLSCSYDNTVKVWAESHIRDDW
HCFQTLTRHDETVWSVAVQPVEQSIETLRAEEEEEGGEAQPACQPIICCCSDDKSVTFWK
RNAEGKFHVIVTATGFAERSIYTVGWAPSNCGAIVACGSGDNKITLLGLYQSRGYNEVHV
TVLAELPSAHEADVNSVAFCSIVNGALDDSKEVVNDNNNNSSSSSALLLASGGDDNMVRV
WRVARA*
>TCRU_C-terminal conserved region, putative PS.1
MLIAPLSDCTAGVIQEANKANNVACSELRKAIRRRHTHNPYLQAILTPVVTSCNFNDKKR
HEPNPNAPAFVPAEIPWNTYAPCVYNENSCVNETAYDYAVYGSSYYYSQRISPSFAKTTQ
MTYQLPAVPTESINTSSVADKTMNLSNNSTSCGNTGTSVVSTPVKRSNNLYKMYVDGRPK
VVRGVMYAEVKVKLRLGSEVFIVNDVPQVFGLIVNPEELVGRYVIVEGDRGEDMGIISAI
ERSEQEPVKQVEKAEEGDNSHPRVLREALAEEVESFHGLDRLEEEALRFCKAAINSLKIR
TPLQVKRAVFQFDRKKLTFTYSSDSYVEFKVLLRALNRQYQCRIWMHQLNWEANCKQRRQ
SSDTEGSRKSRRDPRKCGGTKKDGKRSQEADE*
>TCRU_1257 dihydroorotase, putative
MARVELPTLVDCHVHFREPGLEQKGDMRSESEAAYAGGIGVVCDMPNTKPPTQTITAFAE
KVRRAKAVEKLCDIRLFFGATAHEHLQELETLWTQPQHAALKARCAGLKLYLDNSTGNLK
SSADVTEAAFELCGKLDIPLVAHCEHATHNDEACSANPYTGPPSHSLRRPVHSESASIVY
GIELARRHGTRLHVAHLSTGEGLELVRQARAEGLHVTTEVTPHHLFLTTGDYGCCGARVK
VNPPVRPPEHHERLWEGVLDGTVDCIGTDHAPHTLEEKSDATNPPSGMPSIEVVVPLLLT
VCAGRWPHPTAAMPKALEARRLTIGDIVRLMHTNPNRIFGLGLSEKSQRFFDLSMEWVVK
ESQLKSKCGWSPYANWHLVGKAV*
>TCRU_1258 glutamine hydrolysing (not ammonia-dependent) carbomoyl
phosphate synthase, putative
MFEEKVKASLVLHGGECFEGYSFGYEESVAGEVVFATGMVGYPEAMTDPSYQGQILVLTS
PMIGNYGIPPIETDHFGLTKYFESMGGEIHVSAVVVSEYCDEPAHWQMWETLGQWLRRNN
IPGIMMVDTRHIVLKLREMGTALGKVVVNDKDVPFVDPNMRHLVAEVSTKTRSTYGHGTL
VILVIDMGVKLNSLRCLLKYDVTLIVVPHDWDITKETYDGLFISNGPGNPQMCTKTIEHV
RWAITQDKPIFGICMGNQILALAAGGSTYKMKYGHRGQNQPSTCRSDGHVFITTQNHGFA

VDFKSVSQDEWEECFYNPNDDSNEGLRHRTKPFFSVQFHPEGRCGPQDTEYLFGEFIAHV
KESKVKEASKYKPRKVLVLGAGGIVIAQAGEFDYSGSQCLKALSEEGIETVLVNPNIATV
QTDDEMADQIYFVPITAEAVERVIEKERPDGIMLAWGGQTALNCGLEMDRLGILKKYNVQ
VLGTPISTITVTEDRDLFRNALLQINEHVAKSLAVTSIEEAVGASKEIGFPLMLRAAYCL
GGQGSGIVYNEEELRHKVGVALAVSPQVLLEESVAGWKEVEYEVVRDIYDNCITVCNMEN
FDPMGIHTGESIVVAPSQTLTNDEYHMLRSASIKIIRHLGIVGECNIQYGLDPSSHRYVV
IEVNARLSRSSALASKATGYPLALVAAKIALGKGLFEIANGVTKTTMACFEPSLDYIVVK
VPRWDLSKFNMVSQNIGSMMKSVGEVMAIGRTFEEALQKALRMVDPSHTGFDVPPRLEAK
KNWDYMQDLKVPTPDRIFAICRALHEGVSVETIHEMTRINLFFLNKLHKLILLQNHMLQQ
YKGKMNTMPRDYLLKMKANGFSDAQIAKYFLCTADDVRERRMELKITPKVKQIDTVAGEI
PASQCGFLYTSYNAYHDDVEFTERMYAVLGCGVYRIGNSVEFDYGGVLVARELRRLGKKV
ILINYNPETVSTDYDECDRLYFEEVSEETVIDILLKERIQGVVISLGGQIVQNMALRLKQ
HGLPILGTDPVNVDKAENRHKFSKMCDELGVLQPEWILSTSVEQVHEFCKQVGFPTLVRP
SYVLSGSAMAVIASAADINRYLEEAALVSGEHPVVVSKYYEGAMEYDVDIVAHHGRVLCY
AICEHVENAGVHSGDATMFLPPQNTEKEVMKRIYNTTALIAEELDVVGPMNIQFLLTKDK
QLRVIEANIRSSRSVPFVSKTLGISFPAVMVSAFLSQHDSNLVPIKRARMTHIGCKASVF
SFNRLAGADPILGVEMASTGEIGVFGRDKKEVFLKAMLCQNFRYPQRGVFISCDVDAMAE
DLCPYFECIAHRFPVFTSKQTSRVLADYGIPHTVLTQRHEDSEPTFDTAVAVKEKFDLVI
QLRDKRQDFMLRRCTQENATPDYWIRRLAVDYNHSLLTEPNVVRMFCETLDMDVKEIEIE
PFRHYVPRVYNKMENDNYTMLHRHKVGLCITSTNDSKVLAISLREEKIALTCFHACLGGI
KNNSEEIAEQFRSIGVPVELIDLRTEMAELGFDMVMAMVDDDTNNWHLQKLSLHVMGLYL
LKAMRMRHMTVVAQSSSRGKKDLNFERYVRTFFPQMGVYNPWRDPSLLDQFPSEAHKIAF
LRMHGVEARIAQAESHSSVCGITYSFSDTRSMPTPQMVRPLRDCLSVPEFCSLTFRSARC
TNINRKEVTPLQALQMANEIAGRNGIGLVRTREGVMYETPGMTMLSKALRFLYDVCFDRG
AADMFRLYSGHLASQLATQGLMERHTQSALEAIRYLTSEVDGVVEVELNQGEVIFLKVSH
VRKPAKKRVAKLMTEEELEDVFQPGNGSFSDVQW*
>TCRU_1259 hypothetical protein
MGYISVSSPGSFSVAAGASGELHGFTVQLLENSRGRLRARSITLGKATVVILRVGELDDM
LRFMEESRREGETSTAPSPFSAQQHSHVYRYPLHEQENNVHEESLNALFVAVGSDAGYSA
VTLLRIAALVSELCLFLNIDPHRGGPNAPVLKQILLYMNSRLMQHDVSLLCQTIERLVRP
AIIATGFASDGASSLLDDLARQSLQQDATGCRLPNYIAVLLGHKLVLETSPRWSEETAKA
GGVEHVKLHDSDRYLAHLVTCAFFSTPLLHGCYEVSPQEQRLQALERRLRRFGIHRHNVS
RLAARRAIQTYAQALKKGDVNESRRAFFFENAYNELLATMNRQKPTCGTNKSPTARSPSV
AFNEYHQKDMDFNAVEGRTFELDNICFRNSDFVGFMQLRWIPAAGPHQQDSGDLEDENPL
MEKCPHGYGVFLLYLPPQSAREQESWRRSPTTADRPVFAQFTRDVLGSMETSFNCFQWLL
SPPVDAYEIPGLIHFVVVERRRNGGVVASFHRMMHQRPANVQDAVVTLRRLVALNISRAH
SFLQAGYSEGLWGHLGMQFFYSVCNFHDSPGKVVNSPLQTPQAPRLVKRRPFFFFGGESS
TSTSSYVPTRATSTVSSQQAAMSFDDSTASVKGADSLWGSLDGRLKLPAATLPHLALRPM
LIPDFTCDEPAGVCVMEVYAVFVGAMSPADVCREVEKLLRSRVVPL*
>TCRU_1260 CrcB-like protein, putative
MGEESHLQEEMDDGGLQEETAAEQGEMAFAGGQPPQPNKSILDDVWLTHVGAIIGVACWS
LAGTALRLAIEQTFVRNAVLTTYTTYGPNCIGCYLMGFIASLASSKSQNAIFPWIFRSLL
VGFCGSLTTFSNWMLDTVRQERVALCFGELVSGLTMPFVFFLWGRDTAFLLRAFIMPSLG
CIIGTSASRYELGGRQMRLVNVIFLFVSVVAAVAVPVAIEASVHRHLLNVSFINRRSVVI
SPLGAVPRYLLAQLLNGSSRFWNFPIGTFAANMIAVLLMGLLDHFYRTGGNVWCKIAMDG
LCGSLSTVSSFVNEMIGFYSSDNRRLMYIYAIISVAVGAALMSVCRHVF*
>TCRU_1261 BNR repeat-like domain/Concanavalin A-like lectin/
glucanases superfamily, putative
MLVTLPVYSKEKGSENKEKSELHLWLTDNTRIADIGLVSEKDEDEVAASSLLYESAEGEG
NNKEEDNLIGLYEKKKKGDEKPSHSLWSVRLTEQLKRVREVLATWKKVDEAVSKLCPTAS
AAKSALTENDCSAGEITAGLVGFLSDNFSGNTWRDEYLGVNATVNHNGGAEKAKDGVNFR
GAWAEWPVGRQGENQLYHFANCNFTLVATVSIDKAPEGDNPIPLMGVRAGSKGETKLMEL
SYNSGKKWKLFCGGDTTNELSSHWEAGTAHQVAIVLQNGTQGSAYVDGKRVGAAPCELEN
ADSEEISHFYIGGDGDSAEVREVVSVTVKNVFLYNRPLTFSGGDADLEEAPHLPPANPQP

TEAVPQSVEGDQKAMPPAGKPSEAPGEQATLQQPPEERETQKKIRRWGNLPPHSKCRRID
RREVWGVRRL*
>TCRU_1262 hypothetical protein, conserved
MRSRLPIGGSLVHIPHNCRVPALAVSLHHQQRRHLYQRQQQDEHQKEEHLCASESNGGDT
PVGNPRCPASTYAGVRVGSYILSPVFEEYHSLWQWRTVMWREKLHAVPFLLYCAPGVPLR
HPFLHGTLVESITTPAAKGRLREAATASKTTEFGVKQRYMPLAVPSPIGMLYPTTSGTSA
CHPAAKWVDELFHFLEQGAAQTAGVPNSFSSTYTDVFKSGDATISSLRGQTSWKKQHDQS
RNTATATDLKETSGSDNNVHHSTQRVLHSNWSVPLKNRNVIGCGLAEVVLAIDRYAAMLA
RREITYSKACWAVLVDSKADVQPYLRSSAVAIEKELIAATAAHEAVTAAVGDPARRPLLK
LADYPALARW*
>TCRU_1263 RNA recognition motif (a.k.a. RRM, RBD, or RNP domain)/
RNA recognition motif. (a.k.a. RRM, RBD, or RNP domain), putative
MPQSKPRARLFVGQLNFGATEDDIRAIFDFYGHVLSVNLLHDKSKNRSKGSAFVEYGSTG
EADCAIRSLHNRYYMERDKPLQVSYCERSELISPFGWQQALQLHQENSANPPPPDRSLPT
PAKRLL*
>TCRU_1264 RNA-binding protein, putative
MVLGQARLFVGQLNFDATEEDVCALFDFYGKVLHVNVLRDRTTNRSKGSAFVEYGSTEEA
DCAIMALHNRYNMEREKPLQVSYCGKSEVISEYGREHALRLHRENSANPAPPERTVPQ*
>TCRU_1265 RNA-binding protein, putative
MVLGQARLFVGQLNFDATEEDVCALFDFYGKVLHVNVLRDRTTNRSKGSAFVEYGSTEEA
DCAIMALHNRYNMEREKPLQVSYCGKSEVISEYGREHALRLHRENSANPAPPERTVPQ*
>TCRU_1266 trypanothione reductase
MMSKIFDLVVIGAGSGGLEAAWNAATLYKKRVAVIDVQMVHGPPFFSALGGTCVNVGCVP
KKLMVTGAQYMEHLRESAGFGWEFDRTTLRAEWKKLIAVKDEAVLNINKSYEEMFRDTEG
LEFFLGWGSLESKNVVNVRESADPASAVKERLETEHILLASGSWPHMPNIPGIEHCISSN
EAFYLPEPPRRVLTVGGGFISVEFAGIFNAYKPKDGQVTLCYRGEMILRGFDHTLREELT
KQLTANGIQILTKENPAKVELNADGSKSVTFESGKKMDFDLVMMAIGRSPRTKDLQLQNA
GVMIKNGGVQVDEYSRTNVSNIYAIGDVTNRVMLTPVAINEAAALVDTIFGTTPRKTDHT
RVASAVFSIPPIGTCGLIEEVASKRYEVVAVYLSSFTPLMHKVSGSKYKTFVAKIITNHS
DGTVLGVHLLGDNAPEIIQGVGICLKLNAKISDFYNTIGVHPTSAEELCSMRTPSYYYVK
GEKMEKPSEASL*
>TCRU_1267 ATP-dependent DEAD/H RNA helicase, putative
MSEFEQLGIHQWLSKQCAYMALQHPTPIQKLCIPAILAGKCVVGGAATGSGKTAAFALPV
LQILAEDLYGVFALVLTPSRELAYQIVDQFVALGAPLHIRTAIIIGGLPHEQQLDALKAR
PHVVVATPGRLKFMLEKFPEARKAFLHLRFLVLDEADRLTSDDIETDVEEVVRLLPPARP
ERRVLLFTATLEQRLTRVEEGGWLVRLGVTPAAQHLEVFATNTVTTVADDNGATTESYAV
ADSLKQRYLFIPNMVKLAYLVASIRAAGTEQSTIVFTNSCMRCEVVRLTLQLLGFPVCSL
NSVISQKHRVDNLATFKLGIARILVATDIASRGLDIPAVGLVLHYDLPKQVSTYLHRVGR
TARAGREGLSVAFVTEYDVNLVRRLERKMDCTMTLWKAKGVNEKEVLKLLDEVSAAKVQA
KIQVEDQFGRRVNTLKVHAAEKRARQREEQQQQQQETTARVCRVASKEDNTSKAALLHP
MPDQDPVKLQSEKKREKSLKKRRRADA*
>TCRU_1268 hypothetical protein, conserved
MVRHVNNDRSPPFFDNHYGNGVLIENWQEGRIYDACGRKFTLAMDNSVGPKRSTYTSDYT
NPGRTLLQPMMRRRGLGKALMFGEGNSTVFKSEPTSTTYGETQRHLIDSRYGTSHRAKDR
LNNTMGGLSKQNFKGTTMPKTLKPTADEELYGNFSSTKAAMDLPIECFHFQRQMAYNKVP
C*
>TCRU_1269 hypothetical protein, conserved
MVTGELPKLTNGIYPDRSNALLYDAFLPYEFLERHNFKRSGDRQRPDKFLFSFIQGHSRV
GLYEETTLVSNTTSEWSDIRARLLDLSRQRCDNRAQLVIMDGNETRNAKLTVADPSTDPL
SISGSNAEP*
>TCRU_1270 hypothetical protein, conserved
MGYKASPEILQIFITSAIAVVTTVVRPLWAAPPLVRVEVWIDNIRIAGSIGDVTRWEAQV
LRNADGRRATMGEDRESGAAQYTFLGCSLITLTVRYP*
>TCRU_1271 PRP3, putative

MASKKPRHRPISIDDDADIDFSAVTKKAAPARPAVPPTLLQCTVTDTAQKQKETAVVLQA
VDPRLNSQTHRVAARRRVGFGGAPAIPPVFNSTAPLAATGMTASGVTSNNSGGTDDAKRS
KELLDAFWREDAARKEEESCQTRRETYEQRLRALWHGEVITSEETLNAGRVDAAPLQKYF
LLDQFFEDGVPDVEPWDLWALSMPRYGAANLAAGPLDRVHHPVLPGTHYSQHYQHRAEER
PVEVKLMKTREELRAERRERLRKEKEVRDYAKKVAHQEQRNEAQFGGELTAASLGSTAKD
RLTNNNLRLNLFSDSVMNPLSTENKVFSQYQERFLEHQRRNHERHVAAIPQQVEKRKRDL
KRHSEERPVFRVYRIYPIFTPAHLGKLRNFANDGLLRGFILWVCRCDALVVLTGGEVAVR
HLERWILEKMRWESSETVAVRLVTCPLQDATTFSFVSEKSRKRGSHPARTREKEEEGKEG
DKKRMNRDEKKREDGTEHVYMNFVDSVQEGESFMRSMPTEGPWRDLSHIWRAALAPGVGR
NTNLS*
>TCRU_1272 hypothetical protein, conserved
MSRRCFMVYPLPSVADESFLNVIFDGGVCRSVFAGFGPRRFALVELRDDACAEAVTSALG
PTPSPHVNTHTSASSLLKDKDDDCGLEKLGSAPVAAASNSRRKGNAAGRKKDGDDPTGGA
DSDGCTGTSGIAYIGRLRFRNTPIQVLPSPITLDEIYRCGGQLPQRCSGRKKESDKPQGR
VGRGPLERKRQRPPATEETTTESENAAMAGGGKTTASNGDAYRRCKSAEHFTRQCSAELS
TEDADGKRCGAAGAATGSGPRGTSPRKRTAKHVFPADCCQKCGSSLHFTRHCDADVHSAG
ESARPQDHTGLDNAAAAHAVVPKKQDKAGHHEPPNKKKEKPQQKQEQRHVALCTSKDHCK
HCGSDAHFSRHCPSK*
>TCRU_1273 hypothetical protein, conserved
MAPRSENGAAKPACESARRLDFYLPPPDDRAYRRGRIQRLRDQITAWRRQPRRGGAQAYT
RAASAGTEQRGLVVDIPLWKASTVSPTRRNVSRHEKVFMLSQVSRSPPNAKHIFSSATQG
GELREKTPLRSSNYTNTKNSFNHKAQTTGRVMEVPYFHDQTIFCLPRDGRVPMTAATVDA
AAALSDAMDDGEGQQTDSNAGFCYGVTSNSEEMTRHSEHLRHERKADRDGKPLPEKTTFG
SDRFEGAPSVFLSLQSKGGRAASVPKSTESKGLTIDSTFAAMTRSVELATAVHAECTHRR
TAMQRSRSDSFRQRVLEQTMEIPVDWREFERVTPCGNASCLTTFPDEQCSAASMGRTHCT
ANFGSCSVGRGPMRLYSIRLRKRLLNSEGSMEKASSFPRLGTRVILSKSWV*
>TCRU_1274 hypothetical protein, conserved
MTDPTAAALLHNTATRVTLGNTPLHAVPVTVAKQLKPLLESSTSLTVLTVHTQFTPPESL
RLILLGASRCRSLQHMRLVGGISVLQSKMIMSAFMQFTFARRTSRTTSAVRPFSDPAGRC
PHTPLSKRPSPAQRIRRNVVVSDAKMTTGGCQTGGYGRLNSPALRNMEVSKSPSPRFSWL
NGVQMVLDLHHLDDVVAGFLLKGLERSERIIGLDLHIAPTSQAATRAAVRMLSHANVIVA
RNARRVRYLLSQNPPVDAAFFRGADKRDKTDFSLCSPLPEDVSRAEQREQVREGGEKEKE
EWGREATTMRQGLSTFDLNTLPPRRYSPPRLLSGAPPDPSLRPMQPHVVSASPRIFDNGE
LEMGHMSRRGEQKQMEEEASHENECFVCSGGSLYTPEKIQASKIPPNTINRCAGERREDG
SCAWPAPKDPLETTQPSPSFSRHTPCSPPSPRGQFVQHIRYLHGCMREINGVVVRYQMES
RDSIQRLVAEMHVLEEQLTERVTSKLTDVLLSLSELEKQVE*
>TCRU_1275 poly(A) polymerase, putative
MPPTALVVGQSVMKAFQHLSTQDLHTRRVEEAYRVVVECITAVDPAMRLYMFGSTAVYGV
HEKGSDVDFVALSKTDVEDGKGEDAGTQVAKGLQAEFLGKLARVVRQKHLSWNVEEVRRT
RVPVIRVKGGSSVDFDITVNRRNGVRNSALLRAYFEQDPPCRWLSMGVKRWSKRAGLNAS
VVGGSITSYGFNLMVVYYLLQRGALRFIEPHAIDVANVAPVPPYLPLEPPRNDGAELGEQ
VLDFLAFYMHEFDAARHVVSLSRPGITTKEQLNWTKNAEDLARMNGEKINYQWCIEDPYE
LNLNVGRNVTPLKRDFLRKHLEKARETALLTSTE*
>TCRU_subunit mitochondrial RNA binding complex.1
MRRWSGLPLAPLWGLIGRRRVSGDDRSGDSSAFLSDDEALRGELASALETEGEAAPLDIM
QHHPLEAATMSSSTSTIYLEKIARPPARFDLLTNSFVYKWQTKAALARKVSGPMREWAAE
LKYRTGVHMELEPTFPERIPDEESKTGEGDTYETADDVDITVYLFGSERGIFNCHKLMET
AIQQDPVYVRLGVFRRLHGSNEVEWLMLRRINREMRPPDIPPISLKLPGKWTLLYERYKE
AAIRTLWEETGITVDPTSVFPTAQLYQTSPKYYWRVPVRYFVAEVPHDVRVEGPQVVPTQ
YMRNWDVRLLRQSPDPIDRAWAQLADPITGCAWMKAPMIDELQKPLRGEKYMATRYTPPP
YSNLQEVVGLGDGDQDTQNKETEAADPNA*
>TCRU_1277 NOL1/NOP2/sun family, putative
MPPKRRQGARGGALGAKRSAGFEEFYAGVYGTRWPALRKALTAASPKKVLLWNRFCQLPF
AKVMANMKRMDEESLLQVFRCQDGCEVAPPASDAFNVKAYYPLDYASALVVEQLEVGAFD

RVLDVCAAFGGNSIGIAQFLSADGALTVNEPNHERSARLRRNIREYIPSNYVPVTITQRN
AETWYAPSMYHRVLVDAPCSAERYFLQRDGGAPVSPKMWTEQTSLELSRRGCVLLLRALE
TCRPGGRVVYSTRSLSPLENDGVVEDALRRTRCQVEVHPTTLCNMGEKTRYGHIVLPDTA
GGFGPLFCCVIHKVSDRREEVDSNEEGEECEYDDENGDS*
>TCRU_1278 hypothetical protein, conserved
MRHWSPWRCVGAAAATGALIHRRQFIADGLYDCRAGAAGKDDTSKYRLPLRRALHFVEKG
RQLAGAVVEREGGGGEALPSNREGSLLHEDAARYAAYHCHHMLTSFRAERRQWTKLKEFS
FDEFTRVMEYLFFAALLLHLTALYETADKYCEALIDVAMSCTIFLSKDADGLHHADMLRQ
GRSKSTDAKVAYLSNGEEFEARDEPWNSLHWAIVHFSRGVFAWGLLDHTELLFDMKRTMK
PYSGMVRTFAKIICERLAGMSLSAEDLAFLSCLSTEEGAGKWLGTANISAVSSTDVFARA
DKNIVEATLHFAQQRRLQILPLMWMALLTRHSAIIGAPRCFQPARHALLNAIPLFQEEAE
ADTALKLLAQCCYFPSSSLLKASSVEVGVLLSQMERHGVFSSSEFMLVELLQAWMRVEAP
EELSPSVKSAYGAYMAFRNIPRCALRVVGKPYTAKSDGEAFVHELLFHALVQWSSCLSVR
APFHREVEELLISARYVASLFDSIRQHGKDRDSVGAAVLVVEATFRAFAVRVADICTRGR
LVESMSPSDLAEFDRLVDHVLREGTEGSVGGCVPQLYEENRQLWIYSTRRNGS*
>TCRU_1279 hypothetical protein, conserved
MYARAQRASSVRESAGVGEHRLPRGFEKMPASPEARVACEREAELERLHQSVEAAAELVF
QQRLAIHRMKRSAFVSEIKRRPFYNSGSGGDAYSDALKQLVDLEDNLSQESSRCIRVSNV
TALHLLNEKQYSRTLQILLRVEALAQKGAGMWFPLFFSTECGMVGAGEFVHFAGDRRSGD
EEGVREIFVPFFTTRHEKMRQLVLAVIDNNIGLYHFKIAEYELAVRRMSRALHLEELLGV
ETIGVTYFNLAQAQYECGSLEDAFSAITLAEEAIEKRVHEYQSQNNHLRCTVDCSGSGVN
NIPGLERRRLDVYIGWREGVCLLSRVLEAHGRWLQSSNAYKPAINCYEQSDRWLSSVKRL
SAEENNWRQELRQRIQECRRFQRHSCCFPKSTVLFSSPAPGVPSGSPRSIVVTTTLPRKI
EITACATPFDNGQKGTGKEGSKGIDQRQTRQRKTKHKEHSGKSHYRDVYELSATTTSTSN
AAVTTGHHRSRTAPRRPEWDAGTQVVNPDTTRVRPATTATTSSVAHANSEARNSSRLTSV
AGTPLSRGSKHERGSSGKPDLSSETGLTSSDVRRTQASAPVAQCTTPFRAQASSNAMANR
AWNINISDKSLPLTRFTSSSTKLCPLSLMRCIHILQAFARSRVSLFEKEWRKLPCEAGFV
SPQSHSHSSFSVSLPSSWSGRHLYETDGASRLMCGDDSFLKNGGLFVGTPSAHATISLNS
CEILLAFLAARRSASLVCQRSIEQRYTKVMEGILRQHASCAGSPSTTVKMRVLRDSARSR
SLEETEPRRTISGGAAFSPHRTESFPNRNGELCHRISEGVSSGEGKKNGFYISEGRLHRR
YNSLLRMVPQGRETPDTCLDAVKANNRFNGDLASDTAGAMGSQLYSSMPVTPPLSEDSGS
RNSRADEIPTSTWRARRWSDFPYTRQSEKNPQKVKNHPQIVPQQRNGTMGAVNCEDFDAP
NGKVYGNGVQNGLPPRHPIIRRTPLLTCENHDDIAHTTAVIIRQRLLREEAAIIIQGAWH
KWRERRQLGAVRRVGYR*
>TCRU_1280 histone deacetylase 2
MAFTDAVAGKTSLPPPVAIIVGHNIDASAMPLTYERNRLVIDMLQHYACPVFSHKKSGDA
MNTVSSDVFEWVLEPLPVVGVEDMTAFHDRAYLNYLSIREALSEVDERASTVKRSRAEGI
QPPVSRRSSAQNQPPLRVLPDLVPIPTDEEYGLVNENMPFVGMWRTIQATVSGTLLAARL
LAQPGRFAAIHWFGGRHHAKQSTAGGFCFANDVVLGVLELKKLLSSDKKGILVVDVDAHH
GDGTQSAFLHDNSVLTLSMHAHGVGIFPGSGGIEEIGAGLGRGFTMNVPLPEGATDILAV
TLMYRSIHFAFKKLGEGLAAIVIVCGSDALSGDPLGALNLTVGGMQSIIRLLLKEAARRS
LKVLLLGGGGYVDTSCARLAGVVTKDVLSCAAAMRLGKTEYFGDSANLGDNLGVAVPEGC
EYFTRYGPSFLMHGLPPARVSKLYRLPYGSPLFMRMQRAATKEALRRRRNQEQDEEEDAE
DEEEEEEEEEEEEEEEEEVEEEEGEGEEEA*
>TCRU_1281 hypothetical protein, conserved
MTIRVGVSFDLLGTLVDVHRSSGYQYAIDFCRFVEKKGFDSPPINVDVLEENSKKSLKAE
IAKDRAEWTAKNLGVAEEMPFGGVTKESVVKFWCRVVNSTYDREGAFCGHDPKTISIMQQ
AQNSSEWDDFIVSVVQRFATPEPYSWLPETLPTLHSLKRWRELQLPLGIQCDPPRVLTNA
DYRLVSVIREMVQREGDEELLGPVFSADVVGVGKPSPCGIEVARKAACVPSASQWIHVGD
GGADRVAAERAGCHFLPCLQTKGVVWEELREKLEQVCRLAQEAAPCE*
>TCRU_1282 hypothetical protein, conserved
MSSILLLIPTVAEEGAVARARPLDAPVHTLRHERTGAAKTGQGISTSWYSGLDVRSVGFK
RSYIDGDEDADEGDAWSTFSDDALNNARQGGRKNREGKEEQRVVKRNKVETNEDTAANTN
GIEGTKGVKKYSDDVQQATNDGAPVSLLPTSAFSTLEGATQRVDKVDFSGINVEDLESLT

ASLLQQWASAGKAAMPFSEVTKVVLKSHPKYTEMKAKHQSPQGRQEALEWFRVSQKAVRS
YVLHLGYTIDSTNNVYLSRPSNT*
>TCRU_1283 Major intrinsic protein, putative
MPKSDSSPALEKPRSTTPQPAEPEASNKNKYTDPLDLEDNEIETVEMSTKTQAAVHLPPR
TLQQESPEACTAHGANTVHALAAGVPAYSGGEENDQNPFHKGKREISTTSQLTTSASAIQ
DSATEPDGKKFFISVEVTSDGVTRLEELDPRELARLYAQQQAALRSISGEFDILYVPDWV
RLHPLLGSYVAEAFGTFACVLTLSLVSVRNQSLFDRSNDTNMTALPIGFMFMCMVFTFGY
ISGGHFNPAVTMAVFLVRKLDFVRAVGYTICQTGASLGAGLVAMAIQGGTDIFVPYVDKA
YVSSGIFSELIFTFAIATVVLNVAYSRQSGNFFYGFAIGMTVAAGSASVGRISGGAFNPA
AATGLQLALCLTGNCEPLASFWIYWMAPLVGSALAALLYANLSQPENTRALTDAAVLRAD
SAANGVAPVQ*
>TCRU_1284 Major intrinsic protein, putative
MTEEVLAVEERSYLARKLPLVKLSAYQAKFICEGIGSFVFVMTVSLAEMNCGITSINGKD
RTRNLAPIAEGFMLCVLVFAFGYISGGHFNPAVTFSAMLIRAMRLEEAVAYWIAQVIGGV
LGALFGIVVNGFPRHLPAPQVYQNLPAYIFTGFLAEALFTGFLVTVVLHVAYSRQRNSHY
YGLAIGMCLLTARYSVGSVSGGAFNPAVATGLQLTKCIAAGYCIPLMHLWLYWAAPAAGA
LGASLLFKMTHPAPQETEEENQRKALQRSARHLYGS*
>TCRU_1285 hypothetical protein, conserved
MSEDDDESCGVTNKTNSSSIALFSSLPDVLSFTENSGTWISRLVCIFFARLASGEELLPL
IQYVRTELLLPTQAPLGARAIPVATLEASVANTTTDVPGTMLQGSNPRMEAMDGVSDQDK
LFLYRAAKVALLLSAEGEKAQDEATRLLCEPVVAQALRLAANQGDAVALPLPRKYDFFLS
SRVAVCCAKRWAREGRWDVGLRLLERAVATPMTKAAAVELLSNAGRWEMAVRCLGQLPPT
LWTEIEVSAAVRSLYRAVEGRRRNGMKVGDTPNAGLWRIALHILTLVRDNKVKLSAPSAV
NDALGLLGADGQQWVMACRLVKTMLLNLEKEGRTGESSGTVRPNMVSIYHMFRALRQHWH
LALQHASLMMREGDVCISDDREVTDRLLKACVVGYRWVEALRTVQKSLESNPSEEGRDFI
HTDVFLRVLGMLHERQKGFLATQLFQQPQLSRHFSKDTTGKAYNVMLRYCNTVSEARVWL
QTMGTKNILVENESYEHLMILYSREGVWQQALSLFNALLTDPQRQRLYIPCAKAHDAVQY
ALERVLPPGPSWEVSMTLFTRMCELNVPISEVAFQSVVKKCFSQGKADEAQSLFRFVVRH
GVRR*
>TCRU_1286 Endonuclease/Exonuclease/phosphatase family/Endonuclease-reverse transcriptase/Reverse transcriptase (RNA-dependent DNA polymerase), putative
MEPFTWLPAERYFYPLLNSIGAYQRYTYRLRAVCDAQRQKLLLSGDIEQNPGPIAVLQMN
VSCLTPSKLATLMAQGADIIAIQETWKSSEQIASMHTGDYVLYAQSRIGKGGGVAVLVRK
TLRSKRIPLTIPQHDTSLEVVVVRVALDQNRGLIVASAYMRPPPQVTQSFRRLVNCLPAS
SPLLLCGDFNMHHPQWEPFLETSPSEVAAEFLELCTDAGLTLVNTPGEITYARGTRERSC
IDLTWSKHLTVSDWSASVSPLSDHYMLTFTLHQAFKDTIPSAPRIFYSWGKCKWDLFIRD
FDAQLPAYDYKKQSTGIKAFTRALITSYRRHCPRGMHKDGPRLWDDTLMEAERMATDSKA
RYLQSPTPDREAEMQRTRSQFFLLLRERLRNTYLRRISKLNPGEPLAWKYISGRKKASLP
SPTSILLGDGQHTYKTARRAANALNRIFLPFHPSHKAVRFSRGINRQSTSLNINASFLFG
QQNNSESAASFTSGSSLSSSSEPQDKNEAATTSGLVAHLHSPLDAPFNRTELLAALRNTP
YGKAPGPDEVYSEALRHISSKGLRFLLRCINHSWTTGTIPVEWRRATIVPLLKPGKSPEL
LESYRPISLTSIVSKVAEKMVLKRLLWVWTPHPHQYAYRSMRTTTMRLAHLIHEVEHNRN
HYFQVSLPKKSGIGNQLHYRPHRTLLVLVDFSKAFDSIDHRVLSRLLANIPGVNCRRWLR
NFLCGRYAKTRVGHRHGDRRPMLRGVPQGSVLGPYLFSLYVHPLLNLLNSFAGATADMYA
DDLSIIVKGQSREDAIPTANMVLQKLHAWSQENGLAINPSKCEAAWFTLSTHTESDYDRE
GKWPLVVAGCQIPVMTMGASRTTKLLGMDLDPRLTLNVAASKQCAATSQRISLHSAQRGG
TIST*
>TCRU_1287 hypothetical protein, conserved
MLPPGGELGTVCGHARWQDAPLGRSMQVCQQLRGEAPQGARSSGESSGSAADPEAFSARD
NEPCVQRPASYGPREAQAASTADRLTPSRASLSVPWPEDAHRVAARSVQVTIRCSA*
>TCRU_1288 hypothetical protein, conserved
MVGLSVTADNKREVAFDGEAGEVQHGKSGLERNYRVAVVLQNGMGTVCVDGKTLGSSATT
VTRGPQLLDVSCFYVGGDGRSGSYCS*

>TCRU_1289 hypothetical protein, conserved
MGEDRELGATQYTFLWVLFDHTHQAVSLSDEFVRSVRAMPALNSLTITEMEVTASRFLYA
AAILGTRLCDYYFFIKAVRRRLSAVNRGIVQESSLKNVPPAAVGLGERLRHMIGNNHRRI
IKPTEKASAAIIADASLQGWGAVFIPDSGDVKIAGEKWERKPFLIMQAEVRAVRLALSAF
PAILPPTMDVWVDNTSLRIKAAQNHTP*
>TCRU_1290 hypothetical protein, conserved
MQEDYPVNLRVEMTPDGSSVVLPGSITITRTTVSVFKLCEASDEEPLFVGVCSVPLIRFA
RVPSTVLTSADSLVLTGASGHLFINAALSAGIAPAKDGSVVSARTASTSLFPGSSREEPT
GDELQRLDNPARWGNLRLDFPSDEDCDRCVEALLARHAEDITAEYMRSTRLVGNKTQTAD
KISKKILSVRAADESIASNRSTNQRSRNTQDYTVTKGDLSEFMQSCRSSPMDLESPRGDS
YLVQASKIPASRPSESFKELGTPQSELRTPHESQHGRILPNRSHPTASQRELPTPHASLN
GSNFMQGSQNAKGLPVRSNPTNSRRELGTPSELRTPHESQHGRILPNRSHPTASQREPPT
PHASLNGSNFMQGSQNAKGLPVRSNPTNSRRELGTPSELRTPQGSRIDSVRELPPDAVLM
PKSLNNNSVYAKTPHSTSSLRTGDDFGRTRHPGSVGRPTSLGPNAIPSTPGSSHAMHVAP
ASGQNDDPVGKFPYQSPSDLTVTQRSSRARTEHQRFLRNVMKFMHHRLHLMQYLHKELVH
EAYLVEEAKLFGGRGRGSRSASMSRSLSHSISRPTSVSPRSSRRAALSTIAEESSRLREI
EARLRSVEKLQREAEQDRQEALRLRNEAEKKLEAASQELANAPRHSFSSRNMLEPQKNDI
PVRSQSPILRGGQQHPLQKNDSTDVLHAPYPEKHRGSELSRMSRPSTVLTGSGQLAFASS
QQLHHFQPAGALTEVEYSNTILDRYIYGDEWHLLYPAREEDVRFCALIDTCLILKLPRRL
VTITNVSLDQPGMRITAEIHHSRDHLPRETIERRFTTQPFLFLKRLYEIRHCFPESPAAL
GQFYRGNRHGSRGTSRAVTPRGNHTNSLSYLRVADEEMDSVEDGEITTEGSMTGMNGRMR
ELERITQERVQQREQERIRERERKQKFFQQERLRQQLGQLERDEDSRRSRIVLSEAQERQ
DAGCKTDWNHKKPIAVSQNALEVEEQVQRAEVEAGYARQLQVLARAQNRNFTFVRMFTQE
RERRWRVADQEREDRLVLDLMMNPRVLVGVSSQTEVDERCCRQRLIEAEAKQRRQIYANM
RSFLIDVLQDRVEAIVGDERTARMILAADEMKEWLDIVCKGKTPTSGGVPRAAFFSPQAW
QALFAEEVAERTHLFSDEERRRRLFRREFIFTTEDAARNEIELKELDEHVFMLESIMHCN
RAMLERRRQERKDMTNLSNEEQYYRKGVLSEEQDEWEVLVELFDSGLNDVLVEEAARLAA
EAAMADLESPQRRRRVTGTPRQAKPFHYMTFVPEDMDHPPSANLAIEGILGCSINKNLEV
TSIERPLPRVEEDDLQFQAGDMILDVAGYSLHSLSHLREVLSNRTMQIQHEAREEFVDLP
EEELTVNPALQKYVDVLCEHHNFLVQVLRGCDIFQIIVKS*
>TCRU_1291 retrotransposon hot spot protein, RHS3, putative
MEYHTNNSIKHPLLCIPAVGNFPLVDGFFFVEPNPTTLVGLRMATAGGHHTTVRTVKQFT
ECLAAYFDGWEELSRDMSWEIIYVQHADSTPMNDWQRCDVVNSDSVSKREKQKIAAFWNE
KVRKCQVLISSGDLRGDEALRSVEEPRK*
>TCRU_1292 retrotransposon hot spot protein, RHS3, putative
MVDASKSRADQCEVLDEMDVKAMCAWMKRDGTTHGRAQYWKMVKERMDKVGPIPCYIFDA
NEFIAHSAAVEDALDGINSRDGGKHFTHGGVRL*
>TCRU_1293 hypothetical protein, conserved
MMTGRVLLVCALCVLWCGAGGSCEEDAGAPVSGGVPPPAAKELETAGQGTKNLKVGSPDI
DGKVIPAPSPPTEEAGDEDSDEEEETAEETKKDKKGEKEKNKLHPQTHESQNNEGAPPPP
PTIPSGGPAPSSTAGEGGPLPDQENLHNVTKAKKENTPSRSKTDSEAPEPPPGDATQGQH
NHDTDTKDSTKNAATGSPAETTTTSSTSTSGSGDHAQNKADEVDAQSSEEQHESLEIGNS
NVVPPLSETAPKTARTTTDAQTNGTVTKVGESDGSTAVSHTTSPLLLLLLVACAAAAVAA
*
>TCRU_1294 retrotransposon hot spot protein, RHS3, putative
MDHWVLYEPKVQNFPLVDAFFFVDSNPKTLVGLRMTTTRGHHITASTVRQFTECLAAYFN
GWEELSRDMSWEIIYVQHADRTPMNGWQRCDVVNSDSVSDDESREIAGFREEEVRQYQVS
ISSGALEETQLSEVRNSHGNGKRNWGGKGEVLISGKK*
>TCRU_1295 retrotransposon hot spot protein, RHS3, putative
MDATAIVTNCPEENDVRAMCIWMKRNWPLQEQAEYWREVSGRMNNVGPILRFIFGKQAYD
DRIKACQQAVDGSTASELERNLGIGCCYSSNDSDLSRKLVRVVRVRRGNNIESPLNLLIS
PHLERETLSRLENEMKQSDFIFFFY*
>TCRU_1296 Nucleoporin
MARSLKSGSRILRENFKPSSSAPEIRLVMQNDLKDDVSKTVKQLILNKMFAKRSDKVMGV

DQWIEKCPVYDEHLTAALQKFRAHYVQKVAEDSDVFQALYNECHESMEKELNRIVELRKA
VVQQQLRCIEGIMEQLKEGKMAEKKTFRLLKYYPANDVMRFRPFGKVSGISEMGESADVC
EPPAPVLVNPFNTAHSANSP*
>TCRU_1297 Golgi reassembly stacking protein (GRASP homologue), putative
MGQEGSKVKETLKGICGLQVARVLPRSPAHNVGLVPFFDIITAVDDCLLVDEDVAVMQFR
SFVSQRKNSQLLLKVYNLHVRAYRELVCFPSTSWGGAGLLGCSIEWCRAKDCLRRSWHVV
GILPGSPAAHCEELRAERDFIIGMQRPEEPVVTLLKDEDDFYGRVDLWRSLQRVAIQRLN
RNSMALPAGADGKAASSADIGRLLLLVYDALENEVKEVVLDVGPDVDAPLGLNLAAGLLH
SLIISSPMEERRSISGSNDSKDGNENDNDGGCATNRRLSLPVMTTFLTSLGVVERSLPAT
PLQTPFRKNVGMHSHDELPDPVPTMPPTPTPYRTLAPPDFCEITMPSSKPLIGREGVIQR
QECLATPGAVDMSRTLPAAGEVELGELRIPSVLHPDCKTIPAISQGSLTNTVGSGLEGLT
NPCTVSQEAASNALPPVITENGNKPLQPTAQRGEQHVGTSSSGMQRHGPVAHGASWNTFL
SANKNFNSPPQLTLLKIPPPLHFPIIRPTTPTSWK*
>TCRU_1298 heat shock protein 84, putative
MRRVYQRICRDALSHSTPSGRAAFAAAISTLSAAYDGSRGGNKMRAALATPMRFCSTSSD
AATKKPVDITDEDVVIDPTPAAKDGSTGADGASPSSSSSAKPNEDSERVVGESEEMGFKT
ETRQLLDIVACSLYTEKEVFIRELVSNSSDALEKRHLLEISKPEEYPREEDDEAPFIAIS
CNQSKSRFVIRDTGVGMTREELAENLGTIAGSGSKEFVRELQSAASGAQAAEKIIGQFGV
GFYASFMVAKHVKVYSRSAKKGSKGYLWESDGTGTFKITECEGVDKGTKIVLDVKDTELS
FCTPQVCERVLKKYSNFVSYEITLNGGKVNTVEALWMKDKNAVSNEEHIDFYKFISGAYD
SPMFRLHYVVDAPLSIRALLYVPQSHTEKYGGGRMESGVNLYCRRVLIQSKAKGVLPEWL
RFIKGAVDSESIPLNVSREHTQDGSMMRRLSTVLTKRIIRWLEEEAKQDRQKYERFIQEY
GPFLKEGVCTDQVHKMELAKLLRFETTKSDIDYPLVSLEEYRDRMLANQTHIYYINAPSK
EMALQSPYYEQYKEHEIEVLVCTEPIDDFVMQHLDTYAKHKLQNIEMFDASLDGSVQHKK
KLEGEKEDVKVEKQLTEAQVKGLSDFIAKRLVGRVGVVKSTSRLRDSPAVIADHESAQLR
KIYRVTGQMAGPPPKYNFHFNPKHPIVRKLYTLSISPTAEEVETAGLLVEQLFDNAVISA
GLLEDPRSIVSRLNNIMSRMVENVPEPTADK*
>TCRU_1299 hypothetical protein, conserved
MTRAFPQWMKRSLPWFVGGSAAHGVCDEAVAEVPGPSRVPAFVLPACLACWHAWRLRWRP
AREAPVRMTVKQSRHPHVRLSQTIGTPGRARRACAVILWTEESTLTKIRHVDFNPPLAGR
QGVAVLTSGRSSGRGGQAGHSPQRERAAPPTQRVPCDPQGACRPAEERAGVASRLGGDPP
LRDRCPGQHQEWSSATPRSACHAMCAPSFSSWNFFQYGRLTAVRRPT*
>TCRU_1300 Trans-sialidase of Trypanosoma hydrophobic C-terminal, putative
MLLPGGELGSVCGHAQWQDTPLGRSMQGCQQLRGEAPQGAPLSGESSGSAADPDDAQRDT
TSRPPPTQFPASNNTLATNRARMPALPWARIAAPIHSATVIIDNVSGGADAAQDYLRTTS
EKPEIPSEHDAASLVSNGFCEHGHFTDFLAMAEICDSTVHGCVSRVLLLLFLGL*
>TCRU_1301 hypothetical protein, conserved
MAMMMTGRVLLVCALCVLWCGAGGGFANDEKSGPGNRAELPLASQGPETAPQDSQGSQNR
APGEEENLTSVLGEEADGDDVDREKKAEEEEITERQGSEGGTAAVGSDSRETNLSDSEQE
TGQPIVPAGSISPSNSQESNANLTQTEVEGKKEPDKNTTTVEGALTTVNGEHTLPAGIAE
GNLPPPTEDSVDSREQDGEDATSEGKKNASSPETAATPQRDREKGSEGTGGDTKATTVTA
NTTDTTSTQNSDGSTGEFCNRTDSICFVVCAAAAAVVAA*
>TCRU_1302 protein phosphatase with EF-Hand domains (PPEF)
MGCTNTKESSERGGGNASELGKGGRVGLTGVLAANGLHGGGSANDQVHWPTCHFCGKRIP
EKEYNAHTVMCDEREVTCWNSWCRQIVKQGMLHNHLEECAKKQRALCYKCGADLLATELQ
AHRDGCQPQKCTSCGELCITRIMKWCPDNFITRVPIMHGPFATEVLLARYVKGGAQNKSG
GAAPVALVSYNVARMQLLWRWTKAKSYLEEAIFRLTSKEMNLKKEGFAIFKALDKVSDSH
VLEPRRSRSILQTPVVAPAISSHYFPTRMAEPITIEVVQRLINDLTDHVLLPYPAAWRVF
TDAMSHLNTMPNVVRLSPPVGARVSNGRINQGSKVVVVGDLHGQLADLLHILKECGMPNE
GTYYIFNGDFVDRGANGVEVLLILFSLMLACPKYVTLNRGNHECDYMNDEYGFDVEVSTK
YDRNVFRLVQRCFCALPLATIIGKKVFVVHGGLPRRKGVNIEDISRIQRFRQIPMPDYSQ
PEEDEIFQDLLWSDPVEDLQGWRESPRGAGVVFGADVTQEFLQNNGLELVIRSHEECLRG

YEEHHDGKLLTVFSASNYDGPETNFGSFAVFVGDNPEPSFHTYQVAEDDVEVQTLVDLGE
TFTPTLGRVSSFATLSQSKLLLRRRARDDVLRVLRERIYQRRHRLLAYFAKLDRTRKGSV
WKIEWVEAMRNVLNLDLPWFFLRGYLVADDENTRVWYSHFLVKFHNFFQPLWLNDWIQSA
CYHLTQQQRANHRSQYVAKAFNKEQVSYNEFCSVIRAIDYTMSEAQLFQLFVYLDEGGTG
HIDGPKFVNMLSEMAAYPLSDTLRWDLGAMEQLQSVVIQGRSQLPYLFKVTSKDRALPKE
KFMLGLAQLGRGMRKQLTQQQKEKIYEFLAERAPPSGVMFEHFLFMTAVFDVQAMPRSPA
VVLADVNLFGNYLKNSSFSSMCK*
>TCRU_1303 hypothetical protein, conserved
MSDDNNSNRSARGDEQPGGQADNSRRPKSGAVSMDAITSLTVREQVDALAQALLREFMHR
RGYTKTLQKFDCECPRDERTIASRQLMRQLLEIPSHAFPSRLSGEGGTHNTAVIIASGER
EANNEEKKSKKKKKKPAPTYMEELCSYRLQKREVKQRRQHEEEKKKETGEGTETTTIIDP
SDAEMEEWRAAAVEKERRIAEARERHEQLLRERRERKQRKREKRKKKQEKKEKKQKEGEG
SRHHTHEEDEGDGGDATVNFHNSVPHRYGKRRSHLDDVSDSDDEVLLRPMSGKAVAVPHK
SHRNGGGDSVDEGVEGAGARGGLAEPEFLLGRMEKRREQRPTGHTVGSHWIPCGGNVTEL
LGPGRAALFGPDTPMPSPSVTDSLSPSVNSERPPAFLLGEGMSFMSERMQAAKRLSALED
CRLPLSSPPPPAPSTSPLRKPASTIWGSRTMRGGAGFTSSPASNVWSRSSSREGSPGSAM
PIFHYTTAGQHMMSLSSSGRAHSPPLSLDGSVSVTLPPPPPTSSILKTSSLYSSGNRGFD
GKAQPASSAMRKDDTNTTDKNGDGASGEKKDSEREQSSGRKNRKVTILVD*
>TCRU_1304 primase 2
MLQLSRLLQCVRIASSSSSSSSLFASLRAFSNGRRVGKKALASATSGEAREVGGDEDTDD
SADMRHDTMSELGRTRPNIDFASFEADVNDGGMMPLPLRGRLQLPPSQHQKRQPRRLGLS
PPSSLSSLSALPEEVLQATNASDPIDAARRERRVRGAREEEEEEGEGEGEGDDDEEAGME
NEHDPVDAPAAFPNGYTNHHHNHNKHNNEYDVDIGKDTLSTTINSHNSNNNNNNYHTNIA
GLTNPFLGLHEHITRSYRLDQLLAVARPEDILMGRQEAKSGAFSFIRFESGPQFLASNAK
SSTTPRSEETPHNDCALVVAERLMAFPPHMRHFHSICGRENVPCDFFADVDLPNETPASG
EKLLLEMLNYLEVRLEGIGFHQPSFLVLANEVPSPDKVSYHVHARSMGAMDEKTTADDEE
GDDDANEHENEDEEADLAGTKGSPRGKKRNNNNNNNKKAAKAKKGQKQKKIIAFQDYRVV
RLLADEVNTTLGRTVVDEQCYRANGMLRCAFSSKIAPSPNGGVVRQKGKRLVPMLKAQDT
ALQRRLNEMSAYLNTLSEAEVLERTFGTRFAPITGSQKADTVKDSTRQFKLLRARNVLGP
KEAQAVEFDAYGNAVSLYLTEGAKWRRFKMVIEKLRRMPPRSAESYDIWVRVGLALHNFS
NEDHVFEEWVRFSLKCPQKYSRESCRKKWQQFERNPDALNWRRGFNYLNTTVWRSVYRE*
>TCRU_1305 alanine aminotransferase
MSGSRKEIRINPRVVAAEYAVRGMLPMRADEIRAALATPEGKAKYPFSSIVYCNIGNPQA
LEQKPLTFFRQVMSLIDAPFLLENEKVTSQFPADAVARAREYLRHIGDRTGAYTDSAGYA
FVRDIVARQINERDHEIKPLVDASSIFLTDGASSGVRLLLQVLVGDASDAVMVPIPQYPL
YTAQLTLLGGTPAMYYLCEKDNWALNVEELASVYDECVAKNNATPRVLVVINPGNPTGGV
LDRDVMEAVAKFCCDRGIVLMADEVYQENVYAAGKRFLSFREVVLGLPAPYNTDTVLASL
HSTSKGIIGECGRRGGYFCLTNFPAPVREQVVKMCSMVPCSSVNGQLMTALMCSPPRPGD
ASYESYWAEYNGIFASLKKRALLLAKELGTIRGFSCQPVEGAMYAFPTIELPEKYFQHNA
ELNAKEGRKLGPDTRWALELLESSGVVVVPGSGFGQRPNTLHFRTTILPPEQQMERMVKA
MRTFQEGIWAKYG*
>TCRU_1306 alanine aminotransferase
MLRKTLFGFRAIRINPRVVAAEYAVRGMLPMRADEIRAALATPEGKAKYPFSSIVYCNIG
NPQALEQKPLTFFRQVMSLIDAPFLLENEKVTSQFPADAVARAREYLRHIGDRTGAYTDS
AGYAFARDIVARQINERDHEIKPLVDASSIFLTDGASSGVRLLLQVLVGDASDAVMVPIP
QYPLYTAQLTLLGGTPAMYYLCEKDNWALNVEELASVYDECVAKNNATPRVLVVINPGNP
TGGVLDRDVMEAVAKFCCDRGIVLMADEVYQENVYAAGKRFLSFREVVLGLPAPYNTDTV
LASLHSTSKGIIGECGRRGGYFCLTNFPAPVREQVVKMCSMVPCSSVNGQLMTALMCSPP
RPGDASYESYWAEYNGIFASLKKRALLLAKELGTIRGFSCQPVEGAMYAFPTIELPEKYF
QHNAELNAKEGRKLGPDTRWALELLESSGVVVVPGSGFGQRPNTLHFRTTILPPEQQMER
MVKAMRTFQEGIWAKYG*
>TCRU_1307 hypothetical protein, conserved
MATPPVAEAAATTASLTACASCHRLCEVLDEVARKDSALQQFRALFEKMGRDYEVEQRNA
AQAKLELAALTTRHETQQQELIAVKAKSAMQESRIKEEDDAWERRTRQLRQELAAAVDER

DALAGEREVQWQQIRALRSRLEGQQRQLTVMEDAWKCRLEETRREAEEKVRTGMREREEL
LRLREVELQRSTSQLEGRLADMAAEVRRQHDASEALMLRIENLQRSLRRQEAMTQRAQDA
LSVEQKEKAQLGEYMALERQTYRQRQEQTETSYKAQLQEIERVCDQHVRERERLESEIAL
CQRESQKEAKRTREVWMQEKQQLEATIESLRGELIREHMRFDKAHESHQRVEALLVRTRR
ELEASQHQEVVLQETIASLQKAAAKRDATHLRLEGEITRLSASVAEREREAERWRSAMDR
MEWQSRLETARDARERERMHPEALPPPPLLPLYTSSAPRRDTTRRSAWVTPNKRWEQAES
IEHTPLTGSPSMKGAENPPRGGEDRDADMLRSIVQEVLQECVTRTEPSSSLTHASDSAAL
HPAAVATGDRHLRTPRRRKTSSRARRHAKNGDSHLGEDSLRNGMPCASPITRSTPPRDVP
TTKIRASMALAPTRISAPTPSMATSTNSSILQAAAPLFTSTSNNSYLDSSTVSQPQRNLP
LRDCDHGDVASHFSVGSPLAVPVERRSADADKAAATAALIQKYTLEREENEQRMRQTLRD
LEQRQQQLLYSVESGAEATNNTDAPPTRKEGRNGDGVKVHDISHASSAAASSSSLATGHI
SSHSSSARASTAAPAPPSRLLLLQERFATMPIIQ*
>TCRU_1308 TPR repeat, putative
MHRQESVSSGGGNATGRGSLTTAEVLDRAMNQCMQRGLFDTASWLGQLALNATDSVLRDS
ISATSPAVAALQDPPLTGRAHRYLVVALSLMQKGEYIRCHHELNIALKEFSAESKPEESE
KCARDNPPMSRGSGRSTPLPPPSSSSMLPPPQLQFLCLYSLYMAGECIKSTSSNPRKSSN
PHLRTLRGRLLTLLEQQRRSLSSSPASIKSSMRPAPLSSASMAVGAPAYGDPFLCWLHGV
VLRELGMKQESATYFLAALCNHPMLWCAWEDLCTLVSRENQIEEIEAIIASLEPRFMSEI
FLASVKAALNVAPMSLVPPSLSTAAAAAMAQRSTSPHCGSLPRQPTSALENQEQHYRPQH
HQRRGESGVSPRLVNSWEALLERFPNNLFLLANLAGYYYNVKKDLEKAQSLYKRLHEMNP
YRLESMDDYSIVLFLRGDRIGLSSLAQQVYQIDPFRAESNYVVGNYYVLMGAHDRGVLHF
RRAVAADPTFLAAWTLLGHAYLETKNSAAAVEAYRAAVDLDPRDYRGWYNLGQIYELLQF
YHHALYYYWHTTTLRPTDPRMWSAVANCLDREGRTGEAVLCLERAEAYESSSSDYYPPLV
HRLGLHYLGIRRLDRAVIYLEKLVFSEARRREDVLFAIPHVVPYYLQQARQLLDIPSRSP
SYEPQPHHSTTAGGGDGQLPQTMASAIGATNASTGGNVYRSSLADQWLTADAAARRNIET
RWEQATLCLTSSERHLENFASVLGIPVASAADNGARKSTEYGDTGVSGSGGVAGVTMDEG
RSQHTLQLACLYRELNKIRQYLTSQQEQVETAMRMRGEETHRK*
>TCRU_1309 hypothetical protein
MRFDPQQQQKHQGNKDAPLSNGHSQPFKASKLSLDFTGTNDSAPMKAATVQEIVDQQDAD
ADVVRAGRCCTVVFLPFIVVALLLGTILGVSFTNGRALDVEGVPRAVLFIIEGFKGTTFN
ELLEGQRLPHIRQMLLEQHGVYAACASLEDVRCARAVLVEDDVTGEAFISAGAGIATILT
GVSPRRNGVRNDTWISYAALSSTSVQFPSVALRVTLAKKRAAALGGPHLLNGLDARTGQC
NVPGVLDAECVTPPVSVNSVDANEFNRTQPAATLTDGVDHFDIPPMMTCLHLKSCNLFKR
ALTLSSGRDGREEREFTRGLIDLFGGLSREGQEAGNSYDNSLLIFNLNALALRAGEENLP
DFTYDRGSLEYAAQAFLIDSLVGQVLAFVQDRARSRKENWLVVGTSNHGGVGKSFGNFGD
EDEVVPFFLATYTSNRRGYRRLRPLTRPVTHMDAAPTILKWLGIAPYDEDDEEEEKQNYL
EGGDNTDRREETGWQHNDHDQSKPAASKTRNSPSNTGDQRTLLDGVPQGICGSGLRTKDC
AVVGEE*
>TCRU_1310 BNR repeat-like domain/BNR/Asp-box repeat/Concanavalin A-like lectin/glucanases superfamily, putative
MKGNKVFLLAGRLYKHKQIVGNWTYSGFKTQLELVVGDVTNPTGGEPSKRIKWGEIQSPL
NESAIAAHKGNLTRFLAAGGAGVVMEDGTIVFPLMAKSGNGGYYSMIIYSTDNGSTWSLS
EGISPANCTDPRITEWDGSLLMIVNCKSSQRVYESRDMGTTWTEAIGTLPGVWVNSRLYF
WDLSLRVEALITATIEGRKVMLCTQRGDFSGEKRERALYLWVTDNNRSFFVGPVGIDTSV
EEEFASALMYSDGRLHLLQQRDNGEDSALSLSRLTDEVSTINSVLKSWAQKDAFFSKLTI
PTAGLVAVLSNAASDDTWNDEYLCLNATVTNAKKVRYGFQLTEPDSGVMWPVNTRDNNVR
HISLSHNFTLVASATIEEAPSGNTPLLGAMLGDTNSNHTMGILYTADNKWETMFKGKITK
SGTWEPKKGYQVALMLQGNKASVYIDGESLGEEEVPLTGEAPLELLRFCFGTCGEDAGRK
TKVTVKNVFLYNRPLNSTEMAAIKDKAHTSNGPGEALAGWAALEGAAAREHAAVVGLTSA
GSGLLPLLLLLVLWGFAAA*
>TCRU_1311 syntaxin binding protein 1, putative
MPRPVRLCLLLLLLVTASNTDELTEAKKQQLIRDGGLTPDAHLFANLEHVTRRAGSVQRYS
TASAHSGRSTSASASNAATDEDPFLKHACMMMETADRNRTLRVGLSLTSVQRDHAMGPNG
ASGGQNSGAAGKNAGIFQGCGGKWGIILIWVAVVVGRLPSQLLSAFCCLFWVVWPAARGA

RSMRYPKGMAVRWSLGSLAAAS*
>TCRU_1312 Tryptophan/tyrosine permease family/Transmembrane amino acid transporter protein, putative
MTNAPIGEQIDDPLNDGVELVEDLQGQAGLPTGDRMDVAKIRIEKLNQERQERVARRRDP
QSCLKRGLHRIIPYGGIISSGCNLASSSLGAGILALPYAFNTSGLAMALVYLVVVGLLTI
YSFTLLGIAGKRTGLRNYEQVTRALLGKGADYFLAFLLWLLSFGAEVSYVISMGDVVEAF
VQNSPSSSEYLKSSSGRRLLTSMVWLVAMLPLCLPKEICI*
>TCRU_1313 amino acid transporter, putative
MSPSLPVVVSVADGVAESSGFYDEVFFTEQEPANTDETTCENLVKTTRVTSTLSPAEQRI
QELNAMRAERVARRRNPDNIFLKAMHVVMPYGGILSSAFNLASSTLGAGIVALPAAFKMS
GIAMSILYLFIVASMAVYSFVLLTVVGERTGLRSYEKVTRLLLGRGADYLLAVLMWILCF
GGDVTYVISMLGIIKGFVNNAESTPEFLKTLPGNRLLTSIVWLFFMLPLCLPKEINSLRI
VSTIAVFFIVFFAICIVVHAAQNGLKNGIRDDIVYIQSGNQAITGLSIYLFAYVSQVNCY
EVYEELYKPSVGRMTKSAALGTSLCTVLYFVAGVFGYLEFGPAVTDSVLLLYNPVQDKMM
GVAYAGIIVKLCVGYGLHMIPCRDALYHILHIDARLIAWWKNSLICATMAFLSLIVGLFV
PRITTVFGLVGSVCGGSIGYVFPALMFMYCGNFNARSVGWGHFIGAYVLLIVGVIAIVFG
TAAAIYSELQNG*
>TCRU_1314 hypothetical protein, conserved
MQLVLGHCGVPRNEACDAESEKASDLSQLTDTWSTGIVALAERIIRARQLRTEARRSTAA
GDWEATHTDFILTRGEEAALARLRS*
>TCRU_1315 MSP-B, putative
MRHTMLLLELLLCCVSGSVAVAEHHCISDEIKKKFGSRTTAVVLELPTRGGGMMRAMTAS
APDWAPIRFQFFTEDLNDPSRYCTAEGQIRPDFTGKTVECKSEDILTEEKKSIILNSLLP
RALKMHTDRLLVEPLTVNVTVPKFHLGLCAQFTIPSSHHTEGVFGADMYLYVSAGPIKDS
TLAWALACLELATGRPVVGVVNYGPSFVTDSEYSVRITAHEIAHALGFSFCMLIERKMLK
EVEGVRGKARVFQVSSPKTVEKTREHFNCMSATGMELEDEGGLSAALSHWKRRNAKDELM
AGHSGIGYYTALTMAAFEDTGFYKANWGKEEPMSWGNNSGCALLTEKCVINGVTNYPEMF
CTARSSLLSCTSDRLALGRCTIHLYGFSLPPQFQYFSNPELGGLPDRFMDFCPYIEKYVN
TWCSNGNATVMRGSRVGPTSKCLKGDGLADSEGLVGDVCAEVSCDKGEVSVRYLGDDAWH
KCPEGSSITPTGLFTGGRILCPKYDDVCIVIDTINGTGDVSSLLSAFSPIPLIMLVLIFI
SMC*
>TCRU_1316 NAD(P)-binding Rossmann-like domain/Flavin containing amine oxidoreductase, putative
MVELLTPKIVIIGAGPTGLGAAVRLTELGYKNWHLYECNDTPGGLSRSFLDENGFTWDLG
GHVIFSHYQYFDDVMDWAVQGWNVLQRESWVWVRGRWVPYPFQNNIHRLPEQDRKRCLDE
LVRSHARKYTEPPNNFEESFTRQFGEGIADIFMRPYNFKVWALPPCLMSTEWVGERVAPV
DLERIRQNIQENRDDLGWGPNATFRFPQRGGTGIIYHAIKAKLPSEKLTFNSGFQAIAID
ADAKTITFSNGEVVSYDYLISTVPFDDLLRMTKGTGFKGYDEWPAIADKMVYSSTNVIGI
GVKGTPPPHLKTACWLYFPEDTSPFYRATVFSNYSKYNAPEGHWSLMLEVSESKYKPVNH
SALIEDCIVGCLASNLLRPKDLLVSKWHYRIEKGYPTPFIGRNNLLEKAQPELMSRCIYS
RGRFGAWRYEVGNQDHSFMQGVEAIDHVLGLATEETTVANPGRVNGTRATTRFGLLQKDM
*
>TCRU_1317 hypothetical protein, conserved
MKASSKSGARAISGAEGTLAPESPLAFLVLWMRALQWHREPKKQFMDLGRIIPRAFRMRI
VIKFDPSIPIQKLPARLHTAFHGDDTFARVAQPFKDRRGTRSRQQRSLRCQLCLIYGHDA
STCKVRPKRVFPNYKRKLKNGRRAAARRSLMPATAAAPNLPPPAIPHRHRTAPLTPHPVV
RAADEIGERGGRDGLRNVGDVKLGAENAPGGANLKIWLHARTGNQQGELRGLFGGNRSGA
IDPTAVRQDAAVHVGNGSNSAGYGASGVAKVCGAIKGEASAPPDKGRGESSNPQPDRLEG
TCCLTICVDNGEPLVRDCGLDPNNFTMERDGTLILYWPVAPKTAKADPHRASRFVKIRGQ
DAFDTIKLCRTLQKNKGKLTNLTTAQEERALAPWKVTAHSIKQGALRHAAPIVETQNLDP
HVISQLAKHVNPFDLPQNTV*
>TCRU_1318 clathrin coat assembly protein ap19, putative
MIKYLLLISRQGKLRLAKWYVAYPKKEKAKLVREACQLALGRSARFSNVVEHRGSKYICR
RYASLYFVASIDKDDNELIVLEVIHHFVEVLDRYFGNVCELDLIFNFHRAYFVLDEVILG

GELEDSSKRTILKYIQVHDGASEEIEQAGSNGAAAAIAATVGALKIA*
>TCRU_1319 Degradation arginine-rich protein for mis-folding, putative
MPPRRYLRNGAAPLPWLLVLLWMAFMVTAAAAELTEADFKRMKIRELRDFLEDRGLTCPD
CQEKADFARYAYQNRDKKPTSEQGKREVPNASFWEVWRDIAKEVCEEAVKKRGLDVSAEP
QSEVCSALAYVTESFFLQHGKRTAGKLRKKPEALLKTSFKAVYYDAGRVLLGRLADHCLA
SAGNQNTCSSMSKLMALAEESKIADLAKWMTNVGIENTNPMYEFIDQRDDL*
>TCRU_1320 BNR repeat-like domain containing protein, putative
MSRRVFASAVLLLFVVLVCCGIGGATPANEEEVKNELINIQNLERVDLFVPQKTLVLPKG
GTGSVTTRDSFFAPSLVNAGGVIAAFAEGHMNAEYQGGQLTKPSSDVVAEYIDSAWEWST
LVGEVSESTWKAHTVLGTTDGTNRVGVVLRPTTTMKGNKVFLLVGSSAVFYESGGNWS*
>TCRU_family, putative ribosomal protein L4/.1
MRRVPLAVCLPSACARRAIIFSTRYDWRTSGVHDIAPRDEGDFVYEGAQQVLPGAHPLPL
YHPHNTVTRPLISPYLPSPQRSHPYFTEPLPELPHLNTTKPVVYTCGTMKERIIVPVFNL
KNEVTHTRELDPFVFGMYPETEELSKNLTYWLVRCQNYASKWDYETREIWRKAKKNWPNT
GMGMPRVSNRKNHQYPWGGRTKPSKPWNMLMPTMDVKTWSKSNRMMLTLKMLQGRLQVVE
RLTLSEPTQECYLGLCRTMSWDVRHTGGGVLFMDGGSRITPSIEFDRSFFFGSFFNGRNK
VVRPTLLCDEQYDYNKTASKQRMKGPKGPKNPIPINRFNVFDAMQHERLVITEGAIMQLE
EEMYEHKLHLLPPHIRNQLPERGYLDSETLGDCVPSLRTIQMEAAARTEEMESGMYQKFA
DNPYQLWTDEAHASYSVDAADGTIQQFIGGKKSSWSMLS*
>TCRU_1322 tRNA pseudouridine synthase D (TruD), putative
MKLASKGPISRWSPRNPMTGAFFSRNDWRSGRDLSREERDSGLLLRLRDVGGRLSDATLP
VAVVKAQSTDFQVNEIDANGRVAALEKAPKGKWRKISRQKTVLIEDNAEISFGDPTKTFS
PRVQSYGEYVRILGKLPREFPILRFVVYRDSYSLLSVEKRMGYVLSLQPGSTFLRESSGG
SFGCISQYGVCVGMTREFLPHASRQFNLHPLIFNPQTYHTQDDFQDLLRRPAGHYYRLVL
RCVDGDPDTINRRLKMLVEGGFVNYFGIEAFGVGSNRLFEVASFAAHGYFRQAMGALLQC
VAECDGVHHDYYIKYLNADPSTVLGVSQLWADAAKHMRSQKWLVDLLKGVAKYHEDGEKE
EHLRILWDSLPIRDRIQGSAAEFVWNAMASQRLLSKGLDVVEGDVVRVPIPDSHFAVGSC
SSYQYKLVTAEDTRLGIYAITDVVLPVPYGNDALNNCLFPQIAPLDRELYGEFAAKHGLS
FLFEEPIEPCAFPREYYRPLVVRPGDFQASVIKDPNSFTCLKSDLSMMQERKPIHSKDMD
YVSRVREPCVYNVSERFVEKMGPIKKTHTGCYSVVLFCRLPEGTSPFVMLRESFSLRYAA
FHDMYGVL*
>TCRU_1323 conserved protein
MRVCQATHDYINGGRHYLPLCREDVLFILEEHPSGWWTAQNVKGEKGIVPSTFLREFVIC
PPFEVLLHEHSLILTAKRFSVDLSSHAPAPLNKGEVLLQWETPCEKTALTELERGIGDLL
VQRETVRQVLRESLKDLDRNTREPFNDEGNCKKNAVFLETKLATLRQRMNLLEAHRKQLK
SALRSLRDNVLLTKCRAPVRLIGRVREFLGEEPVPTSRIPPYLNSLHDQLERLHGDVRAG
EERLFLLSKNIHELECSCQEAKELLSYRISLRNSKIRAFLKFWSEKAEVAKEKYLHRKLQ
SLHLESLHQEEELRLRQLMQKRRAKYMEAEDECRHWKEKTKQTQFFLEEKATLDQLNQAI
QQVTEEERVWRKKLLSTRINVQEPNTRGEEEGKD*
>TCRU_1324 phosphoinositide-specific phospholipase C, putative
MGLCTSKCNVEDKMCRYAPAAKRLVDDFFAQDNEVLGDAGAFFACMWGASRDILLCSGEE
ATAFLRRACFSNPKSEEALNLYLSSSNFGRALWDLNRAKFMMIWMKYDADNSGDISLREL
GKLVKGLNFPADLSKQLIAAVKAAGGIVNYAFLEKEYFLLTRLKELEYVFTGVAGPGRET
MTRDEFKSFLRNYQGEELNGERVKEILDYVCCMTTGEVYFTNFLKFLEDNRFCSIVDEEK
VGKVYQDMNQPICNYFINSSHNTYLTGDQLTSNSSADMYRKALLDGCRCVELDCWDGFAG
EPIVYHGHTRTSKISFLSCIKVIKEYAFKASEYPVILSIEVHTSSAQADRMAEIMCDTFG
DMLFQSPWAPAEPTTFLFSPENLKRKILVKSKRVATSSAISDAEITENEEEEENDEEEET
EGEEKELTTSGPNPLYRQFKEEKKKEKKKKIKVALEKLSRVVSIESAGFKGTSDTSYLEK
RQPYQCTSFVESKAKKIAVSHAMEFKLINQHCLSRIYPAGSRVGSSNYNPQLFWNCGCQI
VALNWQSTHTYAWRLNKGFFSDNGNCGYVLKPESVRSSAASRNGSQVRSLNLEIISGFCL
PKPKRSNKGEIVDPFITCFIEGPDGNSDRKTSGTIRNNGFHPVWRGKGLGSEFSWDVKDW
DLSTLVVQVYDEDKKTRDDFLAECILPLRVLKAGIRQIPLHDINGNSIFGSFLTCSLTFS
*

>TCRU_1325 hypothetical protein, conserved
MASTYALIVSAFLAVANAVAAEDNQTSTKENKKEFPLVWVCLGVTIVGIGMALYIAYRRP
DELHLPGSTVMVSTEMEEASGNNEPTMRTKLNDDLEKV*
>TCRU_1326 hypothetical protein, conserved
MLDLVRQHGDAQVQLTFIDEHGPLDAVVAAAATMTGLRLVSGDIAMQLEGSTVAGHLANR
RAFQVPNFDVQVLLPIVWGRPGSWGASKGSSLRRGRY*
>TCRU_1327 Protein of unknown function (DUF3682), putative
MAMMMTGRVLLVCALCVLWCVAGGVHARDVDTNALGGCMASGVLGENGSHMPDGCNKTAI
TVPLRSVLPITAVEASAGKGGSAGATGASGGPGPGDAGGSGGTQKNSISSETSDAGGIQN
NSHSMKTSDTGVPRGGDSDGGSAAGEKGGSGGGGSGTSTDGHGTGSVSSGPSAVPAPAPA
APPSAPGHSGGPSAPTDATGVDSSPSGSDGEAGSSGTNSSNTTGESPTGDQTPAAAEAHN
STPAEGLAGTTSGNGHKGQEKEEEEEKEEENENQQQSDEAQVQQQEHEHPAESGEESAKD
KNALRTNATANT*
>TCRU_1328 EF-hand domain containing protein, putative
MQRESVVLQDLVPDVRKQQLQLQPRNTDTFSKPGRPLTEDEVLRLAQRYFISPAVAKEAY
EVFGRYTAMEEQRRGRMNGSTMPYGRLTLTATKLPMMTLPEVTERCLSGNLEVPEEKDPA
VAKEKEKKDGGENDGVTLWEPDTVSMKSTLCGEGKGQQQQQHQQKKEEEDKREGAQNNES
EKESKEVMEERLGIEGLQQFFNDLGIRMSALEVSDMVHDLNDEPIDYVLRLRAMEAAAAE
SQAVAAGSSSAGQGQTRGSPLSAKAGSSRRRGNSKVDLSSGKETELSAPLFKDSSMEGVT
FSQFLFTLSHMRLEREESAEMKECEMRDLFHLLDTDGDGAITVRDIQTVVQRLIDEEVLA
DDRDIQQLSEMNTIELEAALMECDVDDDGYFTLEDMYGVLQP*
>TCRU_1329 exoribonuclease 2, putative
MGVPLLLTWLKRRFAPCFLSSSSCPAADCLYIDVNGLVYQAAALATASEASAVDIDAAIL
RKLFDIIDDIVLRLVRPRFLVYLAVDGISPMGKLAQQRSRRRRRSGGGSRGRNAVEWDSN
SISVGTPFMSRLTEALHFYCASRAERINGERLRDELQQVARGNTTGGDVPVTPITFIVDD
VWRPGEGESKIADAIRRFRSQSTYDPNTSHVICSSDTDVTVCSLILHEPRIHVLRYECSP
SHHSGGASRGGDSGSWRNQVGDAWASTFFSIHAFREELRNKLRLQTSRKDSAEEAERRNR
QFERALHDVVFVLLLFGNDFLPSVGCRIEEGFLDDLLELLATDFISRDRSIIDPVTNTIQ
FDSARYALDSLAEMRRERACGDGLCVDNGKEGATDWGFVDDTFRKQRQLKEETELAPKCY
AYWTMLQWSLQNSAGVVEHWGCYYPYGTAPPLHLLRKYCGMLSYDALMELAKKRSHSRHR
GATSDSNAVETTDKLWDDGPGDVLVQLLVLLPARSTALLPSAVRNAYGEIEHMVLAPVEQ
MNFAAISAWCEEKKAMFTEEERTRFRAYALAARPHMLAQEVHGAPIHGSEMVFVADWDAE
AFAMEVQQAKSARLSEITTAEGLKKSENGATAPAVSTSSLFVSSFFDTRPARKASSAAAA
ALIAASKKADASDRNNRNEKSACSNESHTNDVPLTPAVTVQETRPRLSEEVEMRQGRGFT
LVTAPTEVLGDVAMIKNVGSVVGDTFVCGQHTSPPLSRNPRTMRVRLRWRVASKPLLTSA
TFVPDLLPGYITPPSLPSAPSSSSHETNKAVVKRPASAMNVSDSNSGSSDNGDYVDKKGE
VEEGKGDFSTCNVISELEKKKEAVRRRLEALQSRVGGNAL*
>TCRU_1330 hypothetical protein, conserved
MLISRKGVEITSNPSVCSTVGEVEFSKAEEINSARSFQVSFDSPKDRRMIDFSVYDITNR
RHTSKLTGFEIPLAGMCSILAGESMCEKKSISFRIDGKPGRLEVVFRMHPASAPPPPLKT
ASAGGANDSAAASGKGGETQSSKPSGRLPVRAVGALTKAGIFPSETEVVEMDNDLAQEIA
ARTALLFPEERDLQEKVRTLEREIAQLEYDTQYAAKDKVISGSAALTALRDELKHWQNIS
DEIDAKSAHEAGIAPILPAVRTAPTIQTKALIAEVEDQLAAAQAKMRKLEALQIHRDVSN
DVIPLLDEIESLEAVLIDLKKNTEEGSLEKKQDSGTEKYVDQWEQLAGMLYDKESVVEQL
KHTVLALNRLQFEPYPAGIEAGFAEEAPKELPFIRDNKRRMVAEQSLDAALFGNDSPPRK
TPQLPAANSGRHVDLLDDLFGGPPPPQPPTAQPSEQAALTTTGAELLSVQPSQDPVETYH
PPVAATVVPVWAQPPQPPSEQQQQQEQREQQSAPEPLTLESAAASNVMNTERVPGSENKQ
SAPSSPSATGSTKQIVQPGGPQNGDTSASADISATPAATVSGEQWPIPQRSGEGDLPALA
ASPLESQPQQTASEPYSPIQQAQGQSAQQSPQNYASLQAQGQSAQQSPQNYASLQAQGQS
AQQSPQNYASLQAQGQSAQQSPQNYASRQVGQPQQQVAPSEAPVPPFVRRPATLEEIPYT
NFYNIPLMGRACPLDIYLDGKTPTRGTELSILNNSDYDFIIGGVELKQEDMLSPSPDSTR
TVPTRRWPQQLCVPGGGSRATCIIALHPSFPRASSLLMLVVVYIFSNGRYTPYAARFAV*
>TCRU_1331 Oxidoreductase family, NAD-binding Rossmann fold,
putative

MAPSPKTRIRVGVLGAAKVLRETWIAIHEAKHEITIVGCRDKLRGEKFVDELNAFIPFAK
KPLLGDYETVVASSNVDVVYIPIPVTARHKWVLECAKHRKHVVCEKPTAVSSLVLQEWLR
HFAAKRLLFVDGTMLSHGRRVQDVRLALREIGKLRRMEAHFAFLASEEFLQSDIRLNPAQ
EPMGALGDVGWYCVRYFLHMVDFVLPDAVMGRVAQCSGPKNGISAFSGELLFTVEGEPVV
ATFFCTYHAAYEQLVRIKGDDGIITVYGAIKPTDEDVPTFSIERNSFEGSEGTLSFHRNV
EKRRCDEDYTFQMVQLWRDVGLALMRIDPEEPSVVVPEKSEQWARMAFTTQYVCDKLMES
ARKSAELASSLSLVDNNNGNNTGRKETAPTQTPAASEEVHA*
>TCRU_1332 hypothetical protein, conserved
MAEEAMDASTKPSSGVALFLCDEDAHTPAFAPIQLHGDITLKELAEEYCITSVLECDSTG
CVQPRAVALDSPLDTLRGGKYYIARRDLPRVGCSPRVTFRGKITVEEFEADHVLGRQQQG
KEKENYGPVALSASTTTAANSIAPIKSATRKRLRHDEESNDDYDEDEYEKGTNMDGDDTD
DDGVNMSDVMDVARCEFVPFVGDLYGDGREAMIRLEDAKRLCENVESDELMFAARHAEAE
EILAEAKRVLLELTPCGNTGGSVGINLC*
>TCRU_1333 WW domain containing protein, putative
MDVAVSSPLLESLYSECVPLIDIMQRHLRRSNGDHRNGGDVMNRYDGDEEIAFLSSVQAP
SELCLALACVSWRREAARRSERGPLLVFVPFHEPKDEVFVEVPVAAFGDSRRQRGLQLLS
LNEYSSLFANIKDGLPPRKGFFVTFRTREAQQRFREAMNHLIRAYSRGGPLEDRNRLLRA
MGSSPNVRANHKEGKFSEGGGENVPEAIQPSHDAVFMPRSSREAIRAASIIDSLLAERDR
RREEYISSIHSSPPRNGQNHKDIRFSNNPSGQQQKVNRGVWSMIAGTPERQVAEAGPRVG
EHNGTMITQSEPWQQRNAYSFSHSLPSRVDQEVLLNELRSALETYDNMNSAQGSGEMSLM
DASRRFLRQHQDYLRQIQQGDCAPDQSSSAARQDYTENSLHLVAATTQRPITMDGSSDEA
PQPPRVVIPQQQQQSMQQTTPVTPNLVKEAAETSVQKLPVPSQLEKPTLEPTEAQSEPQS
RLRDELQRALEIESRILSSVHNMAPKPLADKRIFGRAPKASLLSSSSLGIDEEVKKEDGG
GGSDDKKSGNTTLAFETFNVQPSLAQVEVQGDDASRKQEEGAAKEVEVKAPTCGAGWKSV
RDPASGKSYFVHIATKKTTWKIEDTFKDVSQEASVKEIPACGAEWRAVTDPNSGRTYYVH
RKTKATTWKLEDTLKENGGSFSADLTDLQQKKEKNATEWAERKDPSTGKVYYYNKKTKKT
TWKRSETDLDPL*
>TCRU_1334 hypothetical protein, conserved
MNILHRVLVVEFSRLSEPLDEKGTPLLLSWQSLRFRLTIAFSNVNGELDREGAALMAAMG
ESDFHLMWRKLFHTVRIVPVILGSAASSSAGKTQNLPEPESRATTASGLQHGVGCFFSFG
VPQFWENRAPAEKNAPFLASSVMATAGKILTVAAEQCDEAVVAPAEVLKAEMPKPYLKGA
VPSHLHCTYTFSYALPELEDFHGPLTLGFVLLLSFPNGSGKGGTDREGDDDFNDDVLLRA
LVPVEDQRMAWYRPSYVVLGTCYYRAHVSKLLCCSSEARRVSRYRVLVNVNVVNIAGISV
RVQQVFFDIFSTWIGNSLGSLSSVPLWSEQRAGPRSGDLKYIELLRRVVTVTPVVVHEFC
APVMLEPEESMNFQFAIQLLPHLCYLFEPNSLESVYGKFLRGYMEDVAEEEIKTVLSSPS
GPMHISSQRITTSSTSVGSLKPVAVATGATDEISCEELLQLMSFTFTSHVYVYYEAISPT
SKACSEEANRSINGDGIYASLQLRHPVHWSMGLP*
>TCRU_1335 Poly(A)-specific ribonuclease PARN-1
MQVTRDKFPAVLPKFVELLKSCDFYAFDEEMTGISTAELPESITDTPEESYRAKRTVASR
YNIIQVGICLFHRENTAADSTLAHYVARPFNFVLFPHNSDEFNANERSRDVVLSPSSLAF
LRRHDMNFQSWVYQGMAYCDANQEEALRQRHEEKYRELDSIRNEGNQEVEGVSLLTEEEK
KWYDNAVTLAKTFSERIQATLEQAKVKNAGQTAGMAEVSAALDLIQSGGREVFLPPQRSK
SAREALERHVEQHFHNLLLTFRRQGAVHMGMLRAVFPEERNRLREKEQVFREGELMNMLG
FRLVFNALVESRKPCVGHNCLADLLFLLASLDGPLPDSLPKFKRRMNELFPIVFDTKYIA
SRQQFFPLERFGARYLSGLFGEYGFRSANAHVTLPLGFESYDPLAVGGGERHHSSGANAG
GPTHEAGYDALLTGTLLLNLLAEMGGYDVATAPECVVNRVAVFRSLFALNLGAPDEDEYL
PESGVLELRHEKRVKLHHIDSCFTALSLQNIELHPVDETRTLAVLPSTWSKMESVEERNP
GWLSTHMTSRFPQFFEASVYQPTRACLGANGAFHTTSRALMRIILRSAMR*
>TCRU_1336 hypothetical protein, conserved
MQDTSTQREAHETYDCTGDTSSGSLDATAHSIKRGALRHAAPIVETHDLDPHVISQLAVH
VDPFDLPQSTVRYLGSYTTMLTQVLPLVALM*
>TCRU_1337 retrotransposon hot spot protein, RHS3, putative
MPSERNRVQGGNTKGQASVVPQGDSQRRARSDLEGDTDQPAATCIRVEEAQLRWTMCSYV
SDILLDGETDISDMKLNDFLREHFGSRAAVEEEQNVGMWDFLRSPDAYIKDQQLLEEISN

LKGYQLLTDRRKLEEGNVHNLEDWIEFKKKDTVTPPTRKKITDALTQIRKEVARWKAEER
AKQREEDVMQDTEEKTTKLEGFYESVYGAKWGHVLGFYDNKICEDRMEIHEGKPPQSWTY
KTEDSIFEKDDGVEQFRAPRPRLMVLTSEKGWPYSWRENKPIVDCYVNCEVERVWRIVKD
DLKDSSSPEQRVLVGTPGIGKSMNAGSYLLYQLLHCDAKKIKVVVHCFGEDKAYVFDKTA
KMVTKYVGSKASRSVVSSFPQCGMKGYIIYDVPTNEAQLPISFTPPTGWGTTVLASPKVR
DIYEFLRQRDSRRIIMNCPDEMDVKAMCAWMKRDETPQEQEKYWWMVHRQMLFLGPILRY
IFDAKDFSKRYDELDCVLRSVKSRDDIVCVKHEGMTAWCTENPFYKLMCVKREKGEFGAE
AFKTDLLSHHVGRRAYPFVEKIIPLKEIFGF*
>TCRU_1338 folate transporter, putative
MATDDSAPPRFMSAEETVPARSVHIDARALFSALPCLRRAPIFGTSVEAFGPKCVLSMGL
SEFLCKGVADFLIRCSLFPMFRRRYGVDAMTYQRMANLASMGWSMKPITAMFSDIFSFFG
YTKRWYLAVSCVVGAAFALAFALLPPAKASAAPAAAFVFLTSFTKANVDILTQGHYSRLI
RRIPLAGSSLVSWVWWWVLLGSLVASVIAGPLADASLEWIGVIVAATLQFVTTFFFVMNW
YGENKNRVNRAEDAKFMRAKLLEERRDSSKRLREDDDTAVAEMGNHSMLVLSDEVCAGVP
GEEVDEVPPLPTCCCGVFEVNKEVARRSWRIVLYCLLVAAGSVVMAIVTVLGDIWSLLYA
CIAVSVAVCVFGFFALPLVIAKANLYTFLSMASYIQLPGALDSFYMARPDCLPDGPHFSF
FFYNTVASIIGNIGGIVGILLFRYVFSKKSYRITFVFTTLVMVVASIFDLIIVERWNRPR
VSDHVVYVLGDQVVYQVCYMLHFMPGIILLSRLCPRGTESMVYALLAGFANFGQAVSNTV
GSLLMEFKWPVETDPAVGCDFSNVKWLLIVGHLLCPLVCIPLVFLLIPAARICDNIDVDG
RVVRPQEPPGDCKVEGSRESLSAQEPVLK*
>TCRU_family, putative Se.1
MEHGVTLLEDPMTPRQPIMSGPAPYFFAVEDASVSRVAEDWMATVPHRNAHIFALGCTPH
RSPQQLAWSRIAPRAMRFKDIMLDFAAPEVLVFHLSMQNGFPQLLLPPN*
>TCRU_1340 syntaxin binding protein 1, putative
MPRPVRLRLLLLLVTASSTDELTEAKKQQLIRDGGLTPDAHLFAILEHVTRRAGRVQRYS
TASAQSGRSTSSSASSTATDEDPFLKHACMMMETGRNRTLRVELSLTSVQRDHVTGPNGE
SGGQNSGAAGKNAGPFQGCGGKEKNHPDLGGGGGGGTIALTTPQRIVLLVLCGVACSERR
SVHEISKRYGREVVIGEPRCRVLSLLCAGTWHPTAL*
>TCRU_1341 Mucin-like glycoprotein, putative
MMTCRLLCALLVLALCCCPSARATAGEVKTGEEVNVQVIVSEVLNKLGGEHNSDTDVDAL
RTGKAASGAGQNDNGTPSSNSELLVPIIDTLKSESPKDVQEQALDGVTETAKEGKEKPLN
PEVTDTSDAQQSTSLTAVRKVSENETTAANTTTTTTTTNAPTTTTTTAPQATTTTNAEAP
TTTTTRAPSHIREIDGSLSSSAWVCSPLVLAVSALAYTAVG*
>TCRU_1342 hypothetical protein
MQIAIRVQQVAGEHPIRSIVHDNRSNRKRAVVVRDPKAERSGPFLPRGVPSLSVQCDSPF
LNGRGHEGIHTQAFVPGSSWSCVDPHDLGCPDGLRPRRPHATRLVHPLAIVSMKNHEK*
>TCRU_1343 hypothetical protein, conserved
MPVQRQAPHDKLSAGTPERNGAVHHDGSAASVLVKRQRALHELRTARVRRQRQEPAADAV
AAHRQPRPGHERRRPPQHQQRAIGQRTGVVVHHDGGRVGLHTALEQQSMRGVHLAPRHAQ
HSALRRNHARTRVQRIVATEHSRVQRDTATDKLQHGAVHHHRPPTSHHRHEGNAGHNQHG
PHEPQTRQHQHRCVRRGPRAGKIHKRRRPRGNQQGVRHRQHGPRGERAGQHKRTVQRLHI
AHRQLHRPALVAPRRHRTAQASTAHHVQHGAVQQRHVLHNNPHAVPRHPRHVEHRTHAVQ
HNVAQRHLARAGRHNEHRPHRHALTDKQTRRSAHSGRQRGSTHRHACLAVPAAAAHALAH
TVLHAGRRRPGRVAVRVPVAGVAPSNVDAPSVAKSSSSVLLRRRQDTSTRVVARHPHMRP
SRKRQAQRQHPRQQRSVAHKAQRAPSSGHGASGVEHLPRDAVVHVALRQQHRPAIQAHRR
IKRHRVVLRQRKVAPHKRHGGARHAHSPTHTHRVLAQQEVAVNKRHNGAAAGRNTALHRH
AQPQRRLHRQSVHVQRTT*
>TCRU_1344 Leishmanolysin, putative
MAAREHHDCDDIDGMELQDGDGDGRTLDSHWSQRHANDEWMAPISCVVCRTELTLAAPAD
LGCMRVKWEMVEPMRWWRNSCCALLQRKCSAPNMSEYSQMFSEAGVHAGRCTLDRYSSCW
CVDELSMKGQQNVRWTVAPSLVPHWM*
>TCRU_1345 hypothetical protein, conserved
MAAPYPHPLESVVLRADIDRICALSRQKANLLVRHFVRVSRATSPYAATVRRAPLLPGDR
EMDRPLTPYEIDVTIRDSSLGSAAGPDNMLDEFLHRLGPVACGTQLER*

>TCRU_1346 hypothetical protein, conserved
MRQLVHGNPWLAALLVMFVIRLSSGGVAVTLTHSCSFLDDVVRADGAWATPVVVRDVPLT
AQDASRVCSVVWKKQWALVRKKASTKIPDDSRRHCGFSWAPAMNSHIVAVLGLWLHTRTP
QQGVAASTTETKTTSPLERVPRHARCGELKRSLR*
>TCRU_1347 hypothetical protein, conserved
MAMMMTGRVLLVCALCVLWCGACGGFGEAPTVSVPDLPTADTVAQGITDMTGDAGNGGPQ
DGKEKLQGETKPEVNVNNSENPAGTPSGVNAEEEEDDKEETEEEEEIREDEHNVETEPRK
DQGEEAEDGPGVPNVRPVASAQQHLQPAPSTGSNSPSGSLETTLQATQTGLQQSADTPNN
LPTGNNTINKETGRKTASDKIAEETSPSSLREGVVDIQKDAEGNKEKSENNPHLPATAAT
VQVHQQDGKLDTINETILSGETNNKENMQQNDSSSTSVEAVQSDAGTEGNPTANELSRPS
IEDATPISKTHDFNKDSDSTENVASETAGSVTAPNTDSKPGGTAMPGNSDSSTAVSHTTF
PLLLLLVVVVVAVLAA*
>TCRU_1348 paraflagellar rod component, putative
MPALSERDLSGVEKFSNAMKAALGRQSRQVNLKDTDSQRILSSNPSASDITEMILSERRL
WAEAAVAFWRLASRQQIHLLSHVSTGALRELHRFCEGLLDHIRITFLSLAPTATPLQVLD
CVSSLGTDHILFLLDTFCSPKGCISMEGGEETRGRELHTHVCRVLAEQDHLYWMTRVVRA
FCDESWRVCSEKDEMSRASVSAIHGMLRCLHHCCATSAACAHALIYTEPRPLVAQLLYGL
RLRISSSTLIRPVVRDDPTRLGVALSSTPSLPTPTMRLPATKSALAEYLTSGRTNAFDEI
SREALNVAVAAVKRVAAHETPEVVIGLCHIVDVSSRSIVPSPKDEKKPSISTQIAMYENV
LMALEALFQLLQVKDDEPWSQELVLRQEIVLQQILNHDLARAVERLLRHCPTILDADSTL
FQSIGTLCTPPFTSSRRRRRQSTVSRVENSTQHVPGNGDSLSGSMCSRNASMVSLTSSIH
VSRPALPEGSVARASNLARSLWMSVWPGASRGSGVAGLTHAHAASRPAPSDDEPIGSFFQ
RGTSDMIFDHAPSPTCLSLDFDGEEWWYSNKTYVNLLLSLTQLLNAMFSDATTPLSIRLS
ICKLLTQISMRSSSAFAASEGRLVMRSFLTTLRAHDQGLCTPSVEIHDVPHVEEEKNKKE
ISHEIHLQTHEQSSEKYPCTSIEDDMLYAALAQKIVAGLWHVYHVLHMIPLSGETVDIIE
LPLLSLRWRGEVKEAEEEKVEEGATKSFGGIHKAVLSLFGPVVRVWYTALMSNDDEDLNK
RSQEVFMEVLVRLLKLGGVPLTTMDFLRASTVSIPSTPPTINFNWVASHSENSSFASMEK
DASRAEPSDTIKSMRSVFVILLQRDPGFLSFELLKVLFALLWANDAQLSELGRKCIASSL
DFGEVYPTYAKVIGNADSSLLIQLLLIFSAGLVSSEVSMDAKHERRAWLFQHGCVNAVIA
VLERVLRAPHDVQSFPSLIRQLFCFLSLSESAQGRGPKLEDTRLVHVFTESLHFLEGAEY
LILITQSVLDAATSKYDQYRSRTRRINTHGHFTLPIPNCSVEKPIFIDLVPPLLCYAHEH
AMNVENDLLSALHLVLKMTTNVRSDALLKWALQRKHTALLPYLDLDAAVADRVLPYFGNR
SDIESFWTPILQEAPERTELRFSCTGGIAVTLGRWPEKGFSVSAYFRFEEIYSDIQLFEF
IDGEVLSPSLASIRIAGGDSVNIMHDGKRVPVNEGHALPDLEPQRWAHFIVVMSMAHTVS
VYLNADKVGTCSLPYFRSHSEVVMNIGFVNTTVHDSLFSIGEVVLWDEELTTPQVEAYVA
TTGYKPNVCKILECEIPRELSETHEKLPIEDRIAVFSPYETRDSMLVDVLQKESRGYPIL
AKLTGTYAEAPKNWIDYRTLFLNRGGLVYMLEWIAASTSAEELERYMALTCNCIRGTTSG
GTMDVQTYALLDFHLRCLAHLITPAVCDSLLHLATAKMSVNDEFHPFIINRLVFDHIFRD
IELFADMPLDAALYLIEGVERLFTVASCRFARRNAYYIIPFRFVDSVLNGLVYANVSLPF
VLRGRVISCLKHIMIATDFEPNIVHVIASTAAVLTPVEMEATSRHQPALRVKIPKARFSQ
ISVTLHGAFETTCLILRSLVECCCNGKFAAAFGIIVDLNWYAACVSRFADVACVVYATRL
FFEAMRFNMDLRNEVMHHPAVIVHTLSPHCLNEDLLLLLLSLSVGAVKRIDVLSSKHPML
QQLVGILDSMTPELDAVVLPIFSKLLTLYLTAASSTPACFRPSDISSAMQRRLYCSYRLH
KYFSLARVCSILMLQIYARRLPPQAKKTGHLVSVSTGGRSIPLSLKKDASKEDVTAGNKK
TPVQFGRYRRAFAALRVCLYLWKAIQRRRYRRVRQQNVSGHTPLMVSDVEPEKALFILRT
LQSLPLKPGLFVPCTLSPYQIEVLAFFGTFLKREAVSARFEEKLGLHEGEDLPFTASTTS
LHRLIKENTTGSDFFPPHVKIFDESHLLPVLHDTVNEEETKHDDDGDGDEGDDDSTVEKK
EQGGKWQHGVHQQNKWVEREEKFRVKSEKYFENQLHMPLTASAPRELHVRQTSSCTPNSF
LHGDGDVPKSPPLAATPVSTEVASLPEDDDAPAASGSGSRVDQMTDAATSSEEDRHLSSG
LEQLFLPVEETTLRSEMGGSMSADVSMGSFAPFAKLQSFNAKSLQECAVGVLCNTVLASL
LNLPVTEGWVGGQTYGSCGALLFQLILITGAMCSTEEVTLTLTQFFIDQLLVCVDLSERF
VEPNTSVVSSPKGSGFENSARSFTSLPGSVSDVFIFNICRLNDLLVQFISFDVMRLSSLS
PYFVWLLGWASSSWPQRSQQQLRSQIVKSCIAVLNKPSPNGVTHEQMEIVYMLLQRVLCR
QWIMKDMLECLLRVLFRVYASLPPTSINEAEEARRKELVILTLRLLMRTYEGCKELHKAL

SASALRQRISLYKEFAATLLIEDEAISVVAFETYCKENMTTIHSFISGRPKTKTDMAYKS
MLKERCEYVKRINTFKEAYSKTINIRDKYRPPELVQAYTARFSSCVDPISPLKVSQLHWL
VKQQCSLQQNDPRVRVLNYLDLRGNYTTVISNHVSKDAASTVLGTDNCEQQHNHISTLVQ
PFAICCRPYILHTGTPFVDISCCVTPSALSLLRYLLEPNEVLRFISNGFRIHGIHATPCL
ILLTDSSLKLIGFSRITVEGDIILCQRENDEREEPVLPVHISTTKSSKNKWGSQLTASSF
AASMTNKLQRFLTDGGGKKRRRIETEKLHDGTKIAHSVRQASGDSYRDLFWVYFLQNIRS
VRMAYYMHQDTAILFELMYDDGPMLSLVDTHQSMNTSARDKFLAVLKDVLGTQRCEFFDH
SQRTASIRSMLIRWAAGSVSTYDYITFLNSVAGRTRRDFNQYPIYPWVLADYKSSELDMD
SPKTYRDLSLPMGAQTEERRQKVMQVYQQMVEVQKADPDFEGVQPFHHGTHYSTSGGVLH
FLIRVEPFTTFARIFQGGEFDVATRLFDSIDASFQSCVHGPTDCKELMPEFFFDGMFLVN
ANHCEFGEKSDGTVVNDVRLPPWAKGSTQVFISVMRYALESNFVLQNIHRWIDLIFGVRR
CGKLAVESYNVFQRMTYGEEVLQALKKCDNPHDIDVIIAEVDNFGQTPIQLFQERHPAQR
ELKPCHPMLSTGGTGGAHCTTGGNVLCSGNTYTTAFQEQLPKVLSMVMHALDSPQTWYTL
EDPAPGSFQAMPKSVSDAFQIHESPVLDFVTTRSGMNMCCYKYSLPVDDTDHIICWNHSE
HMLMRFDNTRGHFLSSVRCKSVLRIPASVSAFCVSCRETLILIGSNSGTVYCFSHSRTDG
VLVLSSTLCNHTNPIAGFALSIKYGRIISFTTSGIDCPTVWCVQHCHTVKLHALDLSRLL
PLTTPGDLIVVAAAIDPINANSIIVTRRHLLLFDRNGEPYGVGTLPNADKTAPHLEDEAG
QVGFAVDDSKNFIASMTAVTTYETLEWASGIQLLITGHQDGSLSLWRAVRLPPERMEHGR
IVLVEHHGFLFTGSQTPRLGAITALRQERPDVPVFFVGYHSGAVKVLRFENRTADMTKMT
TS*
>TCRU_1349 GTP-binding elongation factor Tu family, putative
MQGGGGDGDTPPCFCLVLAEPETLELAEWVRDVVAERLTPPVTGVVLRNVGASSGDEYRF
HAAALLSLGQVAADIIRPTGGTRNETAVTASKTVKAVCLVIAVFWDGQSESVKAEFTRLR
ELSDVKKSTDANEDKNKNAEKENDDSQLSQLQLSETQFILQALSAKKGEHTLKENSEALV
SSLRKKLTFSTAVRYVDPQNSESLALSRHMSGTSCNGKGTVIGREDFSESSIAASITEGQ
GECFFLVSGRSMEEFQQRVAQFQESVRSLGVGCMPLVEGPRGIQETGSNDVNSNSPIRSN
HGSGGVGNGAGNKRTKIIAQEFLVRQSESETHIDLRLAMCGNVDSGKSTLTSVLTRGCRD
DGRGSARAFVFKHKHEATTGRTSSISENFLGLSSTGEVVNYTIPALKRLGGGGGISAGQL
AQEVVAQSAKVLTLYDLAGHERYLKTTVLGMTRNMPDYACIVISANNGIQRMTKEHIALC
LALRIPFFVVITRIDSTPENVRQETLKSINKLLKIPTVRKLPYPVRRIEDVVLSAKNLRI
DRIVPIFEVSNVTGDGIQDLIRFLNLLPLRKDWRKARMQPREMVIDSTFFVTGVGTVVGG
IVTQGIFHVNDAVLLGPDSFGAYRTVHIKSIHVKGMEQQEAVAGCNAAFCLKKEKRNAIR
KGNILADPHHPVAAYWQFEADVTILYHSTTILVNYEPVIHSATVRQSARIVYVAQEVLRT
GDRSLVRFHFLYRPEFMKVGQQLIFREGRTKGIGTVTKVIVDRMESLLAKRRPR*
>TCRU_1350 hypothetical protein, conserved
MSSSKTVTRGRFLAPFCKVACKIEKRSARKLNAVDACIAKTIAEHNASGTDAAVSSTKRY
IYEQKQLFHYRVVRFFDECRYLASGEYFRTYSFKDFVWDIRFFTKFLLLFILGTLFGRQS
IFPPIDPDSPLALALETKVNPNY*
>TCRU_1351 transcription elongation factor s-II, putative
MVPYPVYEPFDRVWVKMEGYDWWPARIVSDEELRANGQSRAEGCDITVYFYEGTTTPASL
EHFKSATTSITFFETSSEKAVTADADLLAAIRRAETDETANPLKYEFAAGDRGGGNKTGD
GVAAGAAAVVTATGASVSSTDGMRRGTGNAAANQRPVSKRSRAAELEERHVAAPALNLLR
DEKLIEIAGRINEAVSICDLPSLRIELCDLDTVSVTVRQLETTKIGVAVGSILGRKELEK
LWPLSRAIVSSWARAMPVETIEAIKRHQQYRLGKRQDDANKSSTMELSMMSPLAAGAPSF
ADVNDVSGPLALRSVENGERVRMRFVHRVQKLLDNPTDPPIPGVTSSDLEAVAEELCRDV
VEPEDRKHLQEQLEKPGMTQLRRSLATRAMSGKEFLKLPRSALMTEDERRMEEARITKMI
EDQEKLKLANVSVTSLFKCPNCGKNRCSYYEQQTRSADEPTTKFVTCLECKYVWTTE*
>TCRU_1352 transcription elongation factor s-II, putative
MAFPLNTLVWLKQPGYPWWPGMVLDPAALEMVLPAGYDTCVLCLPSVSSSVAFANSSNAE
ELLRFDPETDAELIEAGKQDADCAAAIEEALNVYEQQAQQAQLEEEGEKNEHEDGGGRKG
THTRKKHMPHKADKHERKRRKRHERNGSTPVDRRYRHEKKKNERRKYMDSDDLESGTTED
LSDNSDDRRRGKRTGKKSGKAGSLDAVDQYEEEAGFLPTHQEDDAAYLERMLRENRHTVS
DSTLATIRHKLETLTEACNHGGVISVSEAAEEELLEVLSPLSLVNVTLDQLRRLKIGVTV
GKFLLRDYPVRVVSLASAILTYWFRQLPPATQQLLSKKSALDMASNDTTIGSERQEVSLG

PLGVQLEACFTDEEVCDTINDPVIVAGNIEKELEAVDDEDVSMEVLAALRDGSNTALRCG
LLDGSITAKDFVANPTNPDLLLKRSDNDGDKTSGVSPVEPESPLEEGSDLTNFTTLYVCP
GCGAREAVANEYSVQAHDNMAVFVRCLKCDETWNVEA*
>TCRU_1353 cysteine peptidase, Clan CA, family C51, putative
MGTLRENNAGFGDEKTDHSNNSIRVSTPRMQPGEAELVNSLDLNGPTNEFAGEQVEMQSN
DSQDTFKKRMSRINAILTSVVIWSFLLLTLLFLASIVFGIVYSGIHYHGPGWHDASSAKS
ACVSPYGAILGAAGDVFAYSNCIRNYDSKSRNNVSSQNKESGLKWDCMEFARRYWMLRGT
PVRATFDSVVGAADIWALNFVRLLDGSKTPLLKYPNGLPRRDGGSAPRAGDLLIYPRQRE
DFPFGHVAVVVGVTKNSVLVAEQNWDNKMWPGPYHNHSREIRMLYSPIHDAYNITEEENI
IIDGWMRYTT*
>TCRU_1354 hypothetical protein
MQELQGHRNCVQHKLISAVDPIFLRCRRRDPFIVYLLQFCGVGPVQPLDVRLAFVLVALG
LRENNFKLFYVRDGGDTAVSNSAEYYGLVEEERGDGGGIGGSSRRVSFSREMETRGAEAT
LLLPTTIRRFVRAMEGTTDMPVACILRVTDGSRLVDGRFSTIMPEERCTLGVFETTGLRP
IEDVEGCAAVAFLFCVFLQLCSCRGGVDGMWCGCGVGGVIHTLF*
>TCRU_1355 hypothetical protein, conserved
MAASSFTDSECDNTNDGKNSDEDGDDLHPGANYLRVLAHESREGLNAVLTIPGLAEPLAF
APSPAVISAHALRLIRLPWSSRSSSRVVVLLIPRWPFRMYFSVAWRVI*
>TCRU_1356 BNR repeat-like domain/Concanavalin A-like lectin/
glucanases superfamily/Trans-sialidase of Trypanosoma hydrophobic C-
terminal, putative
MSRRVFTSAVLLLVVMMCCGNGGAAATEDTSSASGFSPDKSFVWRDKNGGETVSSLRVPV
LVEMDGDVFAVAEAQLKEGESNFTGIASELLEWTDEESKGELDATKLKTQVLEECPSDKK
NCASHSEAQDASQGRTKVRVSRPTTVVKESDIYMFAGTYSFEVTDKAAAGSKAAATKWEL
LVAVGNVSNDESSGKKKICWKDTSVIPWTGFEKEHESLTGLIGGGGSGIQMEDGTLVFPV
EGTKNGKTVSLIYSSATESGKLSKGMSADGCGDPSVVEWKDKLIMMTACDDGRRRVYESG
DKGDSWTEALGTLSRVWGNKKGVRSGFITAKIDNRNVMLVTLPVYAKKEEKENVEKGELH
LWLTDNTHIVDVGPASVEGDDVAASSLLYKSGDNNELIALYEKKKGGGDTPSPGMVSVRL
TAQLNRVKEVLTTWKKVDERVSQLCTTLLAQKDPSAYTACTDKITDGLVGLLSGSFSEDT
WRDEYLGVNATVEGGAAKATKTSDGVTFHGAWAEWPVGAQGENQLYHFANYNFTLVATVS
IDKVPEEGDTPIPLMGVKMNDGKTVLLGLSYNNKEKKWQLLCGGGTKSEGLSSTGEPGTK
SHVVILLRNGNQSSAYVDGQRVGVGAQCELGSTESQEISHFYIGGDGGSAVSAGSREDIS
VTVTNVLLYNRPLSSAEMTALNTESVTIPKLDHAKPEPRGTPSTPAGQQPSELGHLVGSS
KVASGGVEGADPQIRGVNATALSSSLVNVSQENNSDAGTMRGSGLLPSLLLLLGLWGLAA
Q*
>TCRU_1357 hypothetical protein, conserved
MTASASAVTLRGAPQCHGENVEPTARPRSTSECGIMQIFSVTPSCTASEPRRSDSIAERE
SVAAGTRVGDRDAAARRGTVCGHAQWQDTPLGRSMQGCQQLRGEAPQGARLSWESNGSAA
DPDARTARDDEPFVKRPASYSSGEARAASRAIV*
>TCRU_1358 bromodomain factor 2 protein, putative
MRKRGREGTLDKPRCLAFVHQLWDKDKLKMFHHPISAAELPDYHKVINYPVDLSTIRQGI
ESGKYDSDADVQNAVAQMIANALEYNAKGTEWHQQALSFRNIYLDVARQCGLSVDDDAAY
IPSAAFKDDESTLRKAEKKIAENLDVVLKDLEEEKAVPLEELKAKYKRAKKKKGGKNADG
SDSDDSDDSDESEESEESDEIADDSDDDSDDDSDDSDDSDEEKFYYDDESDDE*
>TCRU_1359 MTR4
MSGSDEPTAKRPRREVTPAMTFPYELDSFQKLSIDALEDGDSVLVSAHTSAGKTTVALYA
IAKALREKRRVIYTSPIKALSNQKFREFSEKFDSVGLMTGDTTIKADSDCLVMTTEILRS
MLYRGTEMLREVGCVIFDEVHYMRDKSRGVVWEETITLLPEGCQYVFLSATIPNAREFAD
WVESIHPGTKVHVIHTDYRPVPLQHYLYPCGADGIFLIVDEQGKFRDDNFRRAMSSMGAM
DAEAGGANGADVSKAATSTRGKRKPPRKGTQPIMEIIKLAMDHNMYPIIVFSFSRAECER
NALALSKLNFNNMEEDALVTEVFSNAMECLAEDDRQLPAIEHLLPLLKRGVGIHHSGLLP
ILKEVVEILFQAGLVKVLFSTETFSMGLNMPARTVVFTSVKKFDGEKNRYLTGGEYIQMS
GRAGRRGLDRVGVVIAMVDEAVEPETLKQLTGGGADVLNSSFHLTYNMVLNLLRVEDVDP
EFMMRRSFAQFQRLRNRPVLEKRAEALTEEIESIHVEHEEVFRQFTLCQELLANRRKQMA

DILKQPTFLKRFTHSGRLLRIRRAADDVLFNWGICRAFHAKVPNADPKSPSEFSVDVAVI
CLKANAANPTSLLPCHVNDFTTSTADLYTLTFDFTDIESVARFRVNLPAEPDTESGRAQM
VQTLSKLHRQYDEKVPLLTAEEMGVDDPQFEKLRVQVERLEAQLAENEFVQNPTTELESD
FERFTHRTKLEKELNDIREELNQASRAIFSEELKQMMRVLRRLDYIDKDNVILRKARVAC
EITTTDENEILLTELLFKGVLNSMETEMIVALMSCLVNVHRTPDGFSLPEEFQQPLKELN
EIVSRIATVSIESGVLQEGGVSVERTMPSLMEVTYMWAKGAKFVDIMSKTSAYEGEIVRM
MRRLEEQLRQMAGAARSPAIGSMELHDKFLKGIQLIKRDIVFVSSLYL*
>TCRU_1360 exosome complex exonuclease RRP41A
MSEKKEYISPAGLRLDGRRPQESRRMTLEFGKVLGCDGCCTVMSGLATVCASVYGPREVT
NRLESKYNECIITCEVAIAAFAGEKRRAPQRRSKLSEEMSAAVLEVARSVVLLSQYPNSQ
IHICVEVLRQDGSDKAACINAACLALVDASVAMRDIVYAQTVGLINAVDVVDLTTEEMRS
QCPTICIAVKGHDPSSIVWMESNCRVTSEVISRLVTVAQKSSEGVFESALRGPLEEHAAA
ILKLQGNLGNSD*
>TCRU_1361 Adaptor complexes medium subunit family, putative
MIGVLMFLNSRGDVALSRTFRDGFSVRGLAESFRNRLISTSEVERSPINILDDLCYVHVR
YRDVYVVLVSDGNTNCFACFQYLLQLLGVCQAYLDTISEETLKDNFVALQQIIDETMDFG
YPQTMEAELLKTFIGVKGINIALMKKPEQSERVTARLTGKMPWRKRDLFYRVNEIFIDVS
EELYVLVSQRGQVLESNVVGSVMVKNFLSGMPECQIELNDDFNLNDASYHPCVSLQADRT
ISFVPLDGKFLLMRYRAALASSPPLKVLHTHVREVSKTRTEIDFGLKCDIKEGMRCDDVE
IRIPCPENTADVKLSVARGRVQFDGVQHAVIWKLPSVSQNDEELLLTAEIVLLAPTIATS
EQVWSRPPIKISFTTPSHVLSGFRVKELRVEEPLLRYSASKWVRYLTTTGQYEWRL*
>TCRU_1362 RNA-binding protein, putative
MPDERILLVTGIPSKLCRGEELYKVFGSYGAIQQLRLGDDASTKGCAIVVYELCEAASAA
LEALNDFRVDKDRYLRVSVYDEVRDRKALERRKRRREMKAEYKQNIASEFGVTEEKS*
>TCRU_1363 Concanavalin A-like lectin/glucanases superfamily/Trans-sialidase of Trypanosoma hydrophobic C-terminal, putative
MVSVLLTEQLQRVKDVLATWKKVDERVSKLCPSKSAESASPENACNTVKITDGLVGFLSD
NFSNDTWRDEYLGVNATVKNKDGGETEDTRETGATKTSDGVKFHGAWAEWPVGSQGENQL
YHFANYNFTLVATVSIDGVPAGDTPIPLIGVKLNDSGKTVLLGLSYNGGGKWELSCGGGT
PKELSSALEPGKTRQLAIVLKNGSQGSAYVDGRRVLGDAECDLKNTKDKKISHFYIGGDG
SAKADSDVQDVSVTVTNVLLYNRPLNDSEITALNKKLSIPKAKDAKTVKGTPPEAMKQAT
TEAGTPSILGGQQRTEQDSLRTSENAGSGGLSTSAVSIATTSPAAKESEKQSASGTSPSG
NKNVDVDSSSEGVQTVDAEAGDTVQGDGTQQPSAGTPATADTNAPTAETMAPDGTAVTPE
VGRHSGEDGETVGETDGQKREGEVNAAALSSSLGNISQGNNSDGGTARGSGLLPLLLLLG
LWGFAAL*
>TCRU_1364 hypothetical protein, conserved
MATVMTGRVLLVCALCVLWCGGTVLVLAAGDGDGDGRGVDSEPLLESAESAALLPSSPES
RVELKVVSNPPPKHVEEVHTPAKESSAEEEEAGEEEVTVKSEEGQQEDENSLQVNRRKTP
ELLINENETPQAREGEGSLTAGALEESPTTPGPQAPTLQVPPPPAASGVGEADGGPVGIE
GTGRNVSGGGSRVVAVDTSRVTGEAIKDPKGSEGPISGPPSGGASSSTTSNDGDASGNNG
GAPSTQDTTSLKNNEQSGPTTSSGNAPLNRETPERSTPDAKRHSSETQENETSQVADGDA
NYSAGGQSAVGTKGSSGVSTAASNAPTTPQPQLPAPPVPTTATVTGAPAEKPTTERSPPP
ADSTTEENLTETTTAKRNDTTSPGDSDGSTAVSHTTSPLLLLVVVACAAAAAVVAA*
>TCRU_1365 Leishmanolysin, putative
MNSSNGNCECEDEVVSSAHGIILENEVVPAAVKLHADCLPVQPLEGPLIVPPFVTGSVCS
RFTHPHMASRSTVRHITGAWQSAVGGGALDDCRGGCEGHHDRDDIDGMELQYGDGDGRTL
ESHWSQRHAKDEWMAPIGCAGCCTELTLAAPADLGCMRVKWEMAEPMRW*
>TCRU_1366 hypothetical protein, conserved
MPVDIGLSLLHPLLSVATAAAATSRRSNGKGQAAAALLLPWIGGGAFFMSAYQPPHPTAS
SSLGGGSTAPCAVSAASGLLGPVAVAPPPALSLAASSPNDCPKTDATPSAATSSMSGRSL
SFSASCGAPAVSLGKSTPLPSWHLRRLPSPLATDASGEQPCPHTSVVCHGRSQCFLDRGR
MPVAKLVQ*
>TCRU_1367 BNR repeat-like domain/Concanavalin A-like lectin/glucanases superfamily/Trans-sialidase of Trypanosoma hydrophobic C-

terminal, putative
MSRLVFASTVLLLLLVMMMCCGGAAAVEHAGAAVDPFKGTMPISFANWREFKDGGSKITS
LRVPGLVKVGDDVFSVAEAQCGEKEGTGSCPGIVSKHLDIKDDPMDILTSDISLFCMQLV
DTAASSFGITEVLRPTTVVIGNSVYMLVGKYGRTEPQVEGTNERGLLLVKGTVTVDGEKK
KIKWNETHLVNPQGKGDSHSLTELIGGGGSGAVMGDGTIVFPMQAKDSNGTSVLLSMHFP
KSGNKWELSYETPGKGCRDPSIVEWGENEGDERLFMMAHCAGGYYDVYMSTENGDNWYGH
FQPITRVWGNSHNRSGDGVQSGSTTAIIEEKKVMLITAPVYANDNGKGRLHLWVTDKARV
YDVGPVSREEDDAAASSLLIKDKNKELVSLYENKKSDGSYNLVAVRLTEKLERIKEVVKT
WKDLDSALHSCRSGSSTTVGLPTKGMCNGPVPTDGLVGFLSGSSTGTEWRDEYLGVNAIV
HGPAEKRIGVPNGVTFKGPEAGAEWPVGNMGQTVPYYFTNNEFTLAATVSIHEVPKSGSS
PVPLIGVRMNDTKGTVLFGLSYTHDKTWLAISEDPATSKLVDGWEPNKTYQVVLRMDHHE
WTVIVDREKICHTKYNESLFDFHRISHFYIGGDSKDQSATGGYVTVTNVMLYNEKLWGND
LDELHASKVNIPSLGVEEQPTGQVTGTGLSVASESKSEETTAPLVENGDSEDVGTAPVNA
STTPGGTKIPSESNATTPSNTGILLEHGKFGDLPAISLTGDSTVHGCVSRVLLLLLLGLW
GTAALC*
>TCRU_1368 hypothetical protein, conserved
MEADGTLILDCSVARKTARAHPHRAPRFVRIRGQDALDTITLCRTLQENEKLTNLTAAHV
ERALVPWKATAHSTKRGALRRAAQIVGTHDLDLTRDLAVGEARQSVRISPEYCSIFGEVH
HNADPGVVAGRIDVKGEAAEGQ*
>TCRU_1369 hypothetical protein, conserved
MGMRTRSATRNAVRWMVLFLLLQYLGSHPSFTAAGEEEEGHVVIEIVRYFNYPNSIFFQN
YAGDVSYFSSDDVCHLGGGVLSADQTPAAHVAIADELRKTAVHNYDTSHAYLGGDADHAA
TPTTDPKKSCGRGLNLPTLNCIFKWNQGAFADSSADGLGVAFFRGTLYTLVGVGPMNGYK
AYWPYHYPHHGKTFLMLRLKTRENSEQATWYDWYPAPKTHIDTPYRSFGVVCETQKGYVV
STTTLPPAVVVPWLQKNWYVVLIVVLLPVVVAVALITVCFYRCRGVDDESKWVAHMVLRE
INDNPLYAIDRNDGIHNKDSQMKQDSTLGDGMPTTVPSEHENAEVPTVIASQESTVYDLG
DIL*
>TCRU_1370 hypothetical protein, conserved
MSLAGQFISFCRTHAQPIDEKSCPVILMAIGDVAPSIRPQHARMLRSMSQMYRTPPEMVV
LGLPKIAARSETKQAHPLTTEKMNQFVRSRTDWKQCVVLRPAWMTASCWSDIAVLTPKKF
YTGVGRDHNFVSVRGAKDGEGGSPPHLVVRPDTRAGRVQHYKVMRDTLTQRRTHESYDCP
SETSSGFLECHGAFHKTGCTPQIVESHNLDPHVISQLAVHVDPFGLPQGTVRYLGNCNTV
LTQVSSLVALV*
>TCRU_1371 hypothetical protein, conserved
MADLVRQQGDIQEQLAYMDEKGQSESMMTVSATISGLRWISGNTAKHWKDSTLTGHIADL
RAPQAPNFASQLLLPIMRAARELGARQGLTIAEGRRSKPHYAAADVQNRSAQLQHRMEEA
NNHFSARASNTAVANGLLARRERHLETTPGTKGTHRCLGSAACSLGCGDSPGQRGVSSSR
RGPPDAPAHGYEDSATTPPHHERLMARDAAAPTIPQPSFTGERRRVNTLASLGTVGDRVS
LTP*
>TCRU_1372 hypothetical protein, conserved
MTVGSLYAFGRGVLLPLCGFATGWVAYRAVEERGLIDESYLRRIRLGNLTLQLYLQQHVL
PVAFTENYGYPEEFLKTMIERLGRGDDADNEVERQTFEQILRECDLNGQIAWLEEHVLYD
IPYFYIADIFDGWCALHRSLFTTADEGEGKESNLTRRGDNSDAFASEVLCRGVVEKTLNG
IFPYDVAVRVLCVLAVGHTSNARLLSQLISPTVIVERYEAYLNDLEQRNMMAGDKIPNKE
VTAATLELLRALNTASVHRRWWYFWGRASTGPYPIAAQLNGEMWCRCLGDLPAGTREICP
DGALLFADTLSESFKCGSGNRQLGVSGAGRS*
>TCRU_1373 hypothetical protein
MPALTRPWENASDSSPSTSAVTDTNALVRRNSAVEVGLSSPPNTLTKPPATATAVQSPAD
GSQQVVSSNNTTNTVNRYASGYNSGAGYSGLGMGSMYGGLGMGGMYGGLGMGGMYGGLGM
GGMYGGYGLGMGMGMGMSEDIQRSQMTFMLLGRLLEMCGMFAGVIQMTFGNALQFMGNYI
GMSQQYNQLKSGMYMDESGKWVQLPRRCVTDVDSLKKSHKRKFVNRKRQGRAWVAILRRV
VFLLLAVFMTRRLMGRAAAKELSRLQF*
>TCRU_1374 zinc finger protein family member, putative
MSSSEKEREAAENGVPTSDAAEEREVKQTASNGTGAEGSTATAATAAPLSQQKQQLSGDS

APAEEESQDHRPATLAHIQQNFGVMSRVLEREVFRRGLINSSVEEMYSAFFVISGVLIEI
LKSETDQRLLHMQLLEMLLTILEPTYGIDHARMLVAIMDQSVSDLYLFQALLSSSSLEAL
VQRAEEFMNPPTRSVGAGMSGGIPGNYSGVQNENMRGVQPAYNSVSATARPSYAEFNKDR
RVEPSAEPAMSRGANTYTEGGGAPASSQPEDDAMPAVASGWAAVQRKHTDLDRDVTVHHH
HHHQQQQQQQPQQQQHIQHRVPFNQYLHQQRNQAPNVAHHHQVVVGTGPMGLQQQQQQQQ
QQQQQQESPQTDSFPRAPVPQGQQKRMVERLPPGTQESDEPHVRGPLWRVPPAPSGTQQE
TVTVPQPSYHAQNPSSTPTPHSNQHQAASPNSPTHPRFLIHHRHATTHFVPPGPVGKQAS
PQPATQISPQKPPHSATMTTTTTATTAATASASITNSQRVMIPHHHHHHHLHHHDIPPPQ
LMMPRVSPPSQSVHAVKQEPVSPPETKPVVHNAETAVPQLRPHHFHHIPPTQQKVLRQPQ
SVSVSASLGGGAGGGISTGSGGGGGGGGGGFNGGGSEAPSHGVMIPTHHHMIHHFQQIRP
SVPPPQQQYRQPTTSAPGPMSIDALSNDEINWASSQSAMLSGPQELRYFLRERGVAETVI
NILAKIFFSLDELMAASREAFEERIKKEIVSVNNRQYIWTILHPSEKLPSEKDVPEMNEL
RNFIHNNAIHNNMSQDGEDDTTTLKPGRPNKNILQVPQPRTSQGKQQQQQQQSGKVGECK
GVIWLSSVRSWQIQSAVEELSKYGKVLQHGASSQRPDLIYFKLGDHKSELQMMRKIGSAV
VEDYYRVSPGDVDDGAPPTPTGRPEGADDNGPSKILSQKAGSGMHAYHQHHQQPSANRGG
YGVVDEEDMDGDETRNFRKGTGVKHARGRGRGGHHNYHHHRHPEGGTQMVCRFFSKGTCK
YGEHCQYFHPPKNASRS*
>TCRU_1375 zinc finger protein family member, putative
MTQIITENLRSSVPPAAPKSQRVCRHFARGRCTWGSSCRFSHDVERAAADETQISQTFIG
LQQLQLQKERHVILKAALDGEFEIKPYSVAEEYQRHIVHLRFSSSTIILPRLLTEESLQQ
QLIELEENEQLRIGGLLFLVGEPAVFWSMMQLYRLRRTTTSSKWDTLLENAKRGYVECMF
FRSAVGCLSSDCPFSHTAPPAVKLSTPLQNMATNNALLTPLLGFGLTAPAPATTAASTVA
SVTKTHTNGFRPPADPVRHVGVSPLTVGKMEESVAFRDSVWGACVIPELKPTHEPQESLS
FMWGGGLRSVQDVEW*
>TCRU_1376 hypothetical protein, conserved
MVEKTSPANRPPAAVGLGERLRHIIGNNRKRNLKPTEKSSVAIITDASLHGWGAVFIPDS
GDVKIAGGEWERKPFLIMQAEARAVRLALSVFSAILPPTTDVWVDNTSPQGAANKGSSKS
HAMTWELRRICLFLDSRGIQTSFT*
>TCRU_1377 hypothetical protein, conserved
MDNTLSVVVEKPAGLRRQWIAWPRGKSRDDPYEAIVPLLHISHYLPPVMAEAASCLDVKA
SFFPSLFTAGDSASLSMPRGGRHAGGADTTFNGIQGRPRNSPDYYYLGNCGGDDGGSPLW
AAPPMVRIEVWIDNIRIAGLKSDVTLWEAQVLRNADSCHAYLGGTANRALRNTPFWGYNL
IKLTVRYP*
>TCRU_1378 hypothetical protein, conserved
MDFRKHCQHWEDSTLTGHIADLRAPQAPNFASQLLLPIMRAARELGARQGLTIAEWRRSK
PHYAAADVQNRSAQLQHRMEEANNHFSARASNTAVADGLLAGRERHLETTPGTKGTHRCL
GSAACSLGCRFARTARSFLFQERAARCTRARVRG*
>TCRU_1379 hypothetical protein
MVDPQSTSLEQHLKDERAAYASLALLNIQSSALLDRFYREWFALDSKRQTLMKELEHHSR
AIREHRRQLELTLFPPEVRGWMQSVLPEMEILEEGGSKMNALYEPFSDTPCSRLPATGKT
RAAFEEAITRQVVRWEEGNKGACSEYKEQPAPSRLSRVTPQPGILLGSCFLPYRDPQTAE
LRRRAVGGDVIVMKSPELSVGRVLVSMHMVCRGDLIMVETPLFTSPTSGNEANHEGLLPP
AVRNAMNSVLRQSEVFAVQGWDTRLIRPFYEWLTELAFGEKQEEFLQRWDELGCPVEDAD
PTLLSKLSDLAHFVWMSLPSSLSSIVGSEERVLLFFITLITNAMNYGGGTWADEDVFYSA
ENGVCLGMLETGIGVFGGISLIEHSCHPNAIVVFRHGCTPESIVFAELRATRPIGIGERI
TIAYVPTFIPKEERQKRLRAKFFFSCACVHCTAGYDTTRLMFVSCESLDERVVMCPKGRG
SEWTLWSLKLRPCVYGLKEVGKSYRYDDTYVLDATRDEMLFRQDMNNNELNGDDTLVPAL
RREVKQLTDVLHGEAKSHVSPLHHLFLLRALQVAAITKSRFSRLDAAMIEAVLMLMCELL
KELIGIIFLLPDWFATELLLNERHTGALSDDLGDRLLNDSSNRGNGARQPWQECLTMHPL
LHPHGNLLVSLLEYYAILREESGNIEAAMNAWTACVFLLRYGMCQGGSERCIRAQLRALH
CRHQQQKQH*
>TCRU_1380 hypothetical protein, conserved
MLDYFVAIGVALLGLLVVYFMFHSIKKSEFVAFNASKERKSRPRKTPKAPRRDDSRLDRE
MEALIAQEVAHHTTSMRADIRVVQPVLLENMQRKVAAEKNKAASMKYSSAASEQALIDKE

MGFQKVVNQSKPRKSLSPPPPQRPQQANTDEELSRKLGQFFSNNRKDKKGLKVNLKEEQT
ANTKGNGVHVVVRKDISNARTWSLVA*
>TCRU_1381 tubulin tyrosine ligase protein, putative
MAGKILPPPHNLPTFYPQSLGFLQRDGRDDMDVMTADTTYILHRHQRLVVGKFVPDINNE
DPSNPRYVVQATGRLRPSRMPPLSRSLNGNRLSNASSVEETSSDTAIREELARTNRVATE
KIHYTVVEPLYPFSAPSLFFPIRHSSRAGGKAGDIRSPRQINLAYHGRDRTHYRLEALTV
QKNNLNRMCLYKLGPGAVAFKVVIDAFEAAGMRYTPSNELFNVMWAKRATNYTLAHLGSY
QKVNHFPGTWGIGRKDCLAMNVDKMQRYFGVDAFNIVPTSFLIPKQWKELEEYVKRQPDT
TDDPLILIVKPSASSCGRGIHLFRGMPPMPTGTRQLVCQRYLGNPMLIYGRKFDLRLYCV
VTAFDPLRIFLFDEGLVRFAAQKYPGMDKDLENIHVHLTNYSVNKTAELNRASRGKEYHS
DDPLDIKWCLSDLRDFLIKKNKDGGLAWERIMRGCEDVVIKTFLSIEHDVVQRIRRECSD
KTGRGCFELYGLDLMVDNQCNVRLIEVNIMPSLATGTPLDKAVKSRMLAHLLTLIRVIPH
QRNAPGHTPPNFGEPSSDRHNICYRFGRHPHGDAELVRKPLLSRFNDPRDSDSVLSPSEY
LLLVEAEEELACAGGFRRIFPTAENVLTYLPYFTHGVLRNNYLLASAVRMQAKARN*
>TCRU_1382 Protein kinase domain/Protein tyrosine kinase/Kinase-like/PH domain containing protein, putative
MPNNDSHDAQLLRHYAQFFPNVLFTSEEEMEKYCQDARPDAVKKVLDLPEGEEVPEDAMN
DPRAHLYILKHIVGRNATTAAFVALRGSDKQKVVAKFIMLNDEKQAAYARSEVHCLAACD
HFGIVKHYDDLKSEDKLLLIMEYGSGGDLNKQIKQRLKERLPFKEHEAGLLFYQIVLALD
EVHSRKMMHRDLKSANIFLMPTGIIKLGDFGFSKQYTDSVSLDVGSSFCGTPYYLAPELW
ERKRYSKKADMWSLGVILYELLTLHRPFKGPSQREIMQQVLYGNYDPFPCGVSDGMKAVM
EPLLSKDPALRPTTTQLLHTELMKYVANIFEEIVRNSEVIEKHDKERILRQLAESRAKAP
PPNSIQAGAVSNDVVREGYLLKYSSDMKWKKRYFSIKNGQLRISLTDNPEKDGVSPKSAS
LETVNDVFPVPEAYCRSNPHQLVIWFTNGQKIIAMAKSAEERDQWISDFQRACGM*
>TCRU_1383 DNA replication licensing factor MCM9, putative
MTGEALVGEKLLQKLRPFFLDCCKPPQCLTPASSTESLTTRSLWTQEIDCMRLLAVLPEL
GDLLFCQTMTLIDALRQVCAEMCKSAGRSLNPADLSPRLTHLPTVGAPPPSMPSSRGVLV
SLCGTIVRMNAKRVVPFVHKLKCAKCGDTVEMASSPFDRGSKPKGRCGRKECGGGELKPV
GQVWMDYAECRLQQRSNLSGRLPRTLLVTLEDELTMKCTVGQFVEVIGISFPKWRALYPN
SRPIIEPTVWALNINSVESYREGGSGSMAAVPRRKAVGSMEGSAFTPESFFYSFCKDKLR
RSTALVTSVCPHLFGLFAPRMALLLAVVGGTSTTGKARMHIRSTIHCLLVGDPSTGKSQL
LRFAALIAPRSTSTTGTGSTSAGLTVAAAKENGEWVLEPGALVLSDGGICVIDELRTVSS
ADRASLHEAMEQQTISVAKAGMVTKLRTSCSVISACNPPPTRHGGTEIGVGGPLLSRFDF
IFLLWDKPSMEVDSRVATHILTCSQAGKQPSSVLSQEDISRYLRWVHSHYAQNDGPLLSD
QAAELIRTYYELQNRRGASPLLADCVPVTIRLLESLVRITQAHAKLHLENVCTEEDAALA
IFLMEQSAHSLKCPLESLGPDVYTNSKSLEECFLDPTPEGVQRQRAVLRAIVDVFSNCGN
AHHSSDDTATATWLDKMKFPSEETSLPSQQCGKRGREVSLPSDAVLRSARRLEAEISSPF
SSVQTVVQYTPSSMSQTPIKKSCRRREVDEIMKSLVFRS*
>TCRU_1384 hypothetical protein, conserved
MRQTSVKLLHSKRLEIKQRMLQRNEARRSQLRHAELDRFRAQSTEGGDPEQDAYEFIGRE
FHCDVGDPAVLDLLLSIEEEIRNEQLVSLYEESQNEDWEKYFQHLA*
>TCRU_1385 hypothetical protein
MPLSIPKAIETVRRYVADQRDDATGSVGNTTCLHSSAVEVCEDPQRRILPAFRETWRGGP
NIIASNDGSYFVAADCLWHDRNRILAEEIKDYASLKETPWAGGKLASTISSCARCKIDAT
SAIFSNWPREQVTENLVKQSILDRAKLYRSCRINYIHGALGLQESPYGHAGEVYGDIHQK
LSRARARAICPVKRQAKHTGDSLFGSIPLRQTLCVRRKGGVEPKDHLFGSLHVEEKKYL*
>TCRU_1386 meiotic recombination protein DMC1, putative
MQHAGTRANKSDTAKDAAHCSDTTSFQEDAAHVIMEVDRLTEQGVATADIAKLRQAGIFT
VAGIHMQCRKDLALIKGLSDAKVEKIIEAARKLFDCGFTNGVTYLQQRGKVTRMTTGSTA
LDQLLGGGIESMSITEAFGEFRTGKTQIAHTLCVTCQLPTSMGGGNGKVIYVDTESTFRP
ERIKPIAARFGLDADAVLNNILVARAYTHEHQMHLLSMVAAKMAEDQFGLLVVDSITALF
RVDFSGRGELAERQQKLAKMMSHLIKLAEEFNVAVYITNQVVADPGGASMFVADPKKPVG
GHILAHASTTRLSLRKGRGDQRVCKIYDSPSLPEVECVFSISEQGIVDARE*
>TCRU_1387 TPR repeat/Tetratricopeptide repeat, putative

MSTVEELRQSGNAAFSAGRYQEALAFYEQALALDAENVDLLNNAAAASHSLKKYDDAIAY
ARRSLSVRDNFKAHTRVGAALWALNRLPEAAMEYERAAVLSPGNPSVEESRQTLLRLLQQ
SFGSGGVVLPLCSTGKLGVCLDVAVVTLSLLTMFFLFFMPALAKKTWNFLMATAVVQQAM
IARRQGLLKASKEVLLKWSDHRCTLELLLCSIALIGAVEPQMLLVSALGIYSAVSLASNL
PTLSNISPFLHRLCAPYLQRILANQSMVANYAATLEAIMLFTVMFSGGAIFTLVYIQYVK
NLYLIDNNTRVAFTGIRMNLSRLTRHPYMPLFVDTYMQKFCELIYTIAQRGI*
>TCRU_1388 hypothetical protein, conserved
MNRRHSGGGDTHYKMCKKIAQLTKVIYQLNTQNEDYDERNAEMRRRHEEELRTICSEAER
RVEGLRKAFREEEEKRNGAVEAMKRGYMAELQEAKDVYARKSASMVDELSASKKKFEEVI
AEVRGKALEEAAKKSAANEAETAALCAAKDEETARLRASKEQEISNLVREYNERYKAMLA
EQMDARDALEEKLNLLQSQLDAATAAHAEELQKWEQKLHDSNDVATSMSAELSRWKEDCR
CREAEVSRLQQECDTLHRRLDAESNSLGVSRDVEATLRQDAELLRTHLKEVQEGRDKLER
ALSEKTTGLTKASAEITDLENQKSLLETQQGRLLHDLNDAKKENQQLLSRVEALDRRVLE
LEEAKKAAEQEIGRRSRAERLLEERCDELGQNASELVKKHQEEINALQAKHENALFDIHL
ASQKSNDVFVTQLRREKEELISHHTQELRRQKEQHDQQIEKLTREHEKVLLDLRGQLSSA
DSVGGDLRVTIAQLRETVVKLEEELNTAKNEIQVLVTQKNELAAKCEAMRGDHARRIAEM
EARMNAEREVQIGKRDALEAEIASLRNKLQADAEASERNWESKIRAQAEGYESRLRQIRE
EHNNRLRHLTELNNSANAQLEARHQEQIRNLMEAAKLQKEAERKESNSERKEFAKQLQRG
EEELQRAKDQMDAMRQSAKEHATRFYDEIQSQRQYIEKFTADYELLREELQKSNVELNGL
REEYAQLEKEMRLWKGRECDARQALERLEEASSRSMKEVEALHRDYVSSMEEKFSQKTQA
ALAELRATLTAERDAVQLRADGVIAAARQKCADLQEEVKSLNLKLDASVHDIRKRTEELE
REKSRKEAVHREFTATLEAMRRDFEDEKESQRRDTENQLKEISTNHKMIIDDLMEQHDEE
RTTQNNVIRKLQAALDELRYRYEYRESREEDVAMINRLMREIKQKDQALEKTIRDMKMYK
MELINREENYNKVFGRRPLVEPGPPADKAPRLLQGMRENRDRTERRATQQ*
>TCRU_1389 Archaic translocase outer mitochondrial membrane 40
MLMEWLRGKSEEVSAAPEKTVRRKLLDVDPETPLEPSPAEAASQREEKFRRKLAASAKEG
PTSPAKLVSYESMFRGAQRLLMEGNFGEINEGLTLNVARNAQNVMISSKCVLLSPQMSHW
EVSFQMNGFSDIVAASYNTLSRWSLMYQRVSSTGALLLAQCMAQRQQGMTQGTVVGMLQY
PWVQGGCTAVQYVKNQSFTLSHAQRIIRGLHVGSSLSWDPMTKGTSLSHGFTAVNSLKKG
SLSGDWTPSKGEWKLAITKKDWAHDAEFAMQLDHTQKDQDMTSQLSFGLKKQFIGGSSMS
AVLNGFSVVKAAVGLPFGGERIGLNQMHCRYNVHYNIRSGALKHGLVFTA*
>TCRU_subunit, putative proteasome alpha.1
MSRTGFDKYITVFSPEGSLYQVEYAFKAVTYAGLLTVAIRCKDAVLVFTQHNVPDRLMRP
DTITSLHNINKDIGTCITGRAPDGKSLVQKARQEASEYKYRYGSPIPVSVLAKRVADMAQ
VRTQHAGLRLMGVVMTFLGMEQNDADGSWVPQIYCVDPAGWCGSYHACAIGKKQIEANAF
LEKKQKNAPFHTLSQKDAAMIALAALQSALGASLKATDVEVGRCSVEDHCFRRVPDEDVE
EWLTALAEAD*
>TCRU_1391 hypothetical protein, conserved
MGCVLSNQKLLQNSNRQCSSIVRGKPRVQTDPVSEAVIHDSRGKVINEYDALAFFSDAAA
NSNLVALTDYRGVQVYVTNLVLSKVNSICRWVDSILEVRERNGGYFIDPKSEASSMNRWE
NQTDVEECSATIVQESNSSFQHRFDVSTCGHGASTTFRQTEKSPFSPKQAEYSPASSRNV
EGAPSGEKLHYAEKLCMPEGPMKVEKFSPLDKLQTTPKIDVK*
>TCRU_1392 hypothetical protein, conserved
MTTALLRIARPPVSRLLVAGAHYHTVSDALTEEERRHLEQELPPRELLSELRTKDQPPDG
EGDDDVKVPGDSFGIMRNRVVFYRPIPREVHPGALGELFPEGCHPMRSVNAATLRRLCGR
FGVSLEEAAAAMLEAELEINVAVDLLVRRKGIKAPFGSFGLVGFETYAPETFCMVNFILP
SFEATRDDDVLDAIHELTLSAAELPLDIPKEELLNKFLNHWTIEDGRNCREVMNSFDITA
QSIVMLPYGEYSVQGFYIFNPVKEDVPNIGTGAAACCLDLRTGIHNRFRFHVERVADSVS
EHVLRELVHYGQNIHLLRQAYWFKPEYSVEEYIRFKESLLQPSASIFEMRYAVLTAAPYG
LEGYRNLVEMEKLKVAQHKYEKHYEDFISPGKWLTSDNAQLQTVAGGSGDAPGSILHMQT
NSPSISKTAMETRTAPLRHVLEKSIQAHGDRVFDRFYRMNYH*
>TCRU_1393 Histidine phosphatase superfamily (branch 1), putative
MFLFMVFALVIAVFIAVSPAAQALRMKIGSYISGVQFMMVADDLPKSFTGSNHMQMKAPA
TEGSKRVIRVVFIRHGQSVWNSLFNSFGALWPLRLVKAFVRETIYLFMDPFNSVIIDSPL

SSKGNLEAEELAQFIRTSKGKISFDTNTSLVVCSNLRRAMETAVVGMRPRISSTRERIVV
DSSLQEGSRNIDAQTLSTERGKLVPCKMGKLNFSEQLGTVFDAHLNAGNKTSKINVYGRM
DEFIRHLFDGSQPDSYTPATSSALGNAELKEVIVIGHSSYFRCFFCRFLPSSSQHIAKKK
KLRNCGVVVFDLLRNEFTNEIFIDESSITVLYKGF*
>TCRU_1394 hypothetical protein, conserved
MSGVVDPVDATITRDSWTCPICTVEVSRQNAFCQVCLRSRPMDRCGVPQIFAGCRIHFNG
VIPRTIKHPSHSVEWRMAERHGATCIVDFDVSQVNVLVYRPGYERSDKVRRCVEGGISNV
FVVPITWMLDCLLQSRQIHVGLYRLTSIPAVAQPTGKGPVLPHHQHPYYLMNVEEYAIPT
SFSQNRQKEANGSPNNVLVNANANLPPCFEVPELQYTNVDVFEAVCNAQKGKASVEDDQE
NYEDARETRKRAANIGIFSCEQRHNRVDSFLFSGMKFLLTPSLESNPHLTRALKLCGAQI
VSSSEGSLGKLLRTAVTHVLYDHGEKKCDVMIEAANVKKELPGLTLAKVNWAEDCLILGE
LVPPCGHYVPTEKLLATLSKKFARR*
>TCRU_1395 adenosine monophosphate deaminase, putative
MSRATSFRLDSTNEYGEEDHLQHSAAGENRRLSRHKITISHGNAKFLKYQLQESHMFKVV
IDGDDGGVDMRKVHERIEAALRMRPRYKKVNGRIQMPPLPSRVRISQENGVYKLSDHDVS
PMYSLQSWSTFASDVQHLRLTVGNTTCIKACSHRLNIMQERSRMFFLLNADIEEKHNFRK
AGGVFSKATKVDNSVLLSRSTDAQETLDFILDWYSRYPDAPLRLRDGTNSTLREIFEVYE
VKDPSKLTVEGLGWRPTPGSVENADFALDSAPLSSELRYSFLHLRGSCAPRFLQKRLQRA
EQQVRYVQATEYSIPLYATYPNELADIAMVLNKNNIGPYGRNMWILQVCFMRNPPFGVNY
PVSCNTIQDQLDFIFLPLFKATLYPTVPNYATLAWFLEQVGGFVIRPPANVLEADFDEKS
PVPSEMPYGTKPNELYYLYYMYANLTVLNSLRKRKGLNTFQLRCSGGNRTSMDGLLAGYL
LGDVITRATEIMDYPVLQYLCGLHGVGLTVSPLHDHIQGIVHYQNHPLPNFLHRCLNVAI
ATEAPLLYHHGSSPLFEEYGTAMKICGLSFLDLTELARNSVMISSFPLETKKEWLGENFN
RGVEGNAFELSQVTNVRLAFREESWDLERGMLKDLYINRSLPREAIGSSRWHYISNVKEV
EYHTVLDTRVRFPRTVLNGPHKEVKWAVSAARGIARAMDLRFKYIWEPPNPWEVTQQRSV
EADFQRKTDSFNEDDWTYAANDAVFISFPKNVVHAWPRQLPTVEKFRNDLKELQDICASS
DVKDFSQKRLENLEHRFRLHLALNHANEAGTTEDRVSSNRDIYQAVKVDTHIHMAAGMTG
RQLLSFVVKKLMYSGDDIATKRGDLIHTLGHFFKKNGITKNLTVDQLNVQADHTLFERFD
NFNNKYNPMENPELRSLLLKTDNFMKGRYFAELIQDVFAQYSRDRHTYAENRVSIYGINI
REWDNLALWFSTHGMSSKHNKWVIQVPRVYKVFRAQNLIGSFGQYLQNIFQPLWEASLHP
SEHPLIHHFLNHMSGFDSVDNEATLDLPFLPVSPWAWTSVENPPYNYYLYYLYANIRTLN
EFRASRGFSTFALRPHCGESGSAEHLYGAFLCANSICHGINLRDDFPMQYLFYLSQIGLH
VSPLSNNALFLRFLKNPFPEFFRRGLNVSLSTDDPLMFHQTQEPLIEEYSIAARVWGLSP
NDLCEIARNSVLQSGFDYVFKRDAIGDQWYMSSSLGNDSLRTHLSDIRVAFRFETYHTEM
DILELCSGRPIKRCMLTTNQEEEINEKNFDVEPEKVILSTHDQAIEIMLREVETGREQLR
VSRVKVDALRRQQKSLVDKLTELAIHQQEMQEEAERCNRAEALRYERRPPTPPDAAFMTS
ERATVQRLLRWRPKPLDVLNHTTESSVPLRNPRRLPSLNAPSIGVRRTI*
>TCRU_1396 peptidyl-prolyl cis-trans isomerase (cyclophilin- 40), putative
MGKLCWLQVSIGGKPAKEKILLELFDDITPKTCANFRALCTGNEGKLTDETQIPMTYKGS
TFHRIIAGFMIQGGDFTKHNGTGGVSIYGERFDDENFDVPCDKAGLLAMANAGPNTNGSQ
FFITVNPAQHLTGRHVVFGKVVRGMNTVRALEHTETGANDKPVKPCVIVDCGVTDTLPEP
EPQVGGDTFPDYPEDCTPSLTDAELFDVGEEIRQIGNKLFKASDFENAIQKYEKAARFVK
TINKTTANDVAVNEKLIACYNNTAACAIKLGQWSEARNAASRVLELDNSNAKALFRRGFA
SLSAGDSESAVADFTKAQKLDPDNTEIVTVLQQAKEAEKVRTAKLAAGLKKMFS*
>TCRU_1397 hypothetical protein, conserved
MTLSALICEAIMRMDPWTILLAALVQQLFSMVWFGLIVKTVADYYLAADKGVRRVEHIVY
RYSPVFCCITTFAAGAARAVAVHTIITVCNGKGLCDYQTAGVVVALLTCINQHQFFSCQR
PLPLLLTECGYEFAAALLASVCCLGMQTFVL*
>TCRU_1398 Regulator of chromosome condensation (RCC1) repeat, putative
MPVLATAGSNHHAQLGREGDSSSFEVLSIEGDFVQISCGAHYTLALTRSGSVYGWGRNES
GQLALAAPTVNYPTLINTCFDDRVVKVSCGESHSLFLTSSGQIYGCGEGSCGQRGDGTYH
GECYEPRRVLLDETARDVFCGARTSFAITDAGDLFGWGETTTGMLGPKVWHTPTLAVPTR

LSVGSSRTRIALVVASVNFAILVTTTGDILGAGSNSDGQLGIADCCTYTWRPIRDVGRVS
LVACGRRHTLCYQVERKRCIVLSNKIESFKLSKPTGLAAGDDNVFAWTDGGSSLFVYGRN
GDGQLGVGIIPYVERLTKVPLPIKANVVTVSCGRKHTCLLLSAEEVQRGNYMVFMEDSFA
ADGTYFWETFRESDWKKPSFGWVCTATSFAALITGVLIARFYVCHR*
>TCRU_1399 hypothetical protein
MSRVEEARLYSWNADLEKRAAAVRSARSKLEEKKAACIQLEASLVELANENDASERILFQ
KEQYVASRKAVLEQANAERSSQRSLLDRERKKTTMDAERVENALDNEWRGLEEEEKAAEL
GHHQRMIRFQQQLEELGKQETELIEKLRQLQNAELNVSNILRQLKQKDEQLRSLSEARLQ
HMRAELREREAFLNQ*
>TCRU_1400 lipoic acid containing carrier protein, putative
MRRVFQLSSTLFLASARLYGTRYFTDSHEWVEHDGGVATIGITAHAQESLGDVVYVALPN
VGDQLNAKEVFGEVESVKATSCMYSPINGVVDAVNDRVKDEPALINRSPEGDGWLIKVKC
SELPQGLMTEEEYKKFID*
>TCRU_1401 Kinesin-13 6, putative
MPSIPLLAELGAKAAEFQRLLDSTEAPETVKKKLRPIMEPEELFVSQDSRIFVRCRPIIP
FDQEDMHSESMIRKVEGRRDLVLMVPKVSLAGQCLLDPHTVNLDGVFDGMDDTSRVYRES
CYPLVNLSVEGASTCVLCYGQTGSGKTYTTAGILQLVSRDLASYFTSHDIFITVIEIQAN
KNIDLLTGTEVQVVEDVSGELKIRGADAFECSNEEILWAVFEEAASQRMTKATGRNEASS
RSHMVTCIRIVSKNSKWARPGELFMVDLAGSENTADSATHDKERQMEAKFINTSLMTLKE
CIRTRVLAATSTNHLHIPFRRSPLTLLLRDCFEIAVKRPTKTVMIACVSPLLRDARHTIN
TLRYASLLAVSPPARVVADDPEDPNGFDRKQALEFLVRCSRGRVTEPESILPEGDGRTLV
HIPEAEFIRRILESHSNITEKGAKQIYTSIWQKVVDARTRGRKVMTAARGASFTKKCACT
RK*
>TCRU_1402 protein kinase, putative
MPSGDAMIPRVCRTFSDAFASDEATAKEQGKKYWISRVLGSGATGTVLCAKRRSDGQSFA
VKVVEMEGMSEADKNRAQAEVQCLLNCDFFSILKCHEDFAKKDPADEENVLMVALVLDYA
NAGDLRQEIKNRAKAARAFREHEAGLLFIQVLLAIHHVHSRHMIHRDIKTANILLCSNGL
VKLGDFGFSKMYSATVSGDVGKTFCGTPYYVAPEIWRRKPYSKKADIFSLGVLLYELLTL
KRPFDGENMQEVMQKALAGRYDPLPPTISPEMTEVVTSLLCGDPHARPSSSKLLNMPICK
LFVSGLLEIVQTQPSFAGPLRDTISTHIQSVKQSLKEERRVTVRQMEESQSAAAASTTIL
EGATPMGSVGDLTLYEGIVKKQSGDLAWKRRYLCIRGALEEGERLDVGRMPKFKSLDLVL
AVSKETMRQQCITTPFSELEDVFPVASKYTGSNAQHVFAVAFKTGRRLLFQARGDPERDA
WMQKIQQTLGIDEAD*
>TCRU_1403 Protein tyrosine kinase/Protein kinase domain containing protein, putative
MLNLREVLLNSRQCVSSQQRMERCVLLFPFYAVSPAYSTKTGTPSPKAEEEICERGSRDA
FQLIGSLNDKTVPAGLEDSFVSQVQLVRALNSSAFSTIFLATRGSTQRSDIQKEGSQLPP
LHMNSEVGVSSSSRPCSVSSAAITSPPLRQQQCIVVKFIELGKEDKKLRELANREIQCHQ
SCNFFSIPQFYGVYTRRLCPEAPPHPVKNPITAHVLEMEYANNGDLSQEIRLRVKNDYKL
FSERNALLIFEQVLLAVDYLHDKGIIHRDIKAGNVFLCSNGLVKLGDFGLSKFTPGDVNE
KRNGSFVGTASYITPEMWERKPYGGKADIFSLGVLLYEIFTLRKPFVGKDKNEVRQNILK
QEPKIPSHVSPEIASIVLAMLQKEPDLRPSAMDVLLLPLMRNTLASFLKSVIMDGMNDTL
RRGREIGNGSVSDNFADSTRQSGEKATVYCRLLSCEREKVLLDLSWVYRRILYKAVTLSS
RASQIRLQLSKLNALTRKTSQVLIEGEILKENSGCWKLRHLCLRWATDSTDMSPQQESAH
SGVIELILSLKKDSAESTRTCVSSFIDCFEVLERYAMDGAKYVFALLSRTGKTIFFQASS
EEERQRWISFCLASIQHNNGLA*
>TCRU_1404 engulfment and cell motility domain 2, putative
MLRPSIVSTLSTNAVLLEYRKHHKPHSSGGNTSRSSSVISQRPPDGAIVVLENEENASDE
FSVREAMSPAIMSASDETRIVNTKKNGGLEKSNAQQRIAVDWDWVNHETEQQRVNSNISS
VPCINDQQENISFLDAVRLLKNDRYTWEPYVSAHRFSENKKSNCLISLIERLRDIFCWCR
HSYSRDGIVSSDPDSILHHDTEFTLTLPFVPFDHSYSIHRRLLITIYHNLFRQTKNGSIN
GGTRPGAIDWEVLGFQGSDPATDLRFTGILGLLQLVYLIEYYRDFAMLLWNTCTNGGSGL
LVYEELPFVLVGFNFSAVILDMLKDSGFYTEITRRAQALFYNKTRASRLPIISDSEKALG
REFPLLFICCEYHVGCLFLFWECWHDLKMQRDGKQPTIGDFGVVKAKLCTKLKKNGPQYA

FDACAKAGDPFNTAFHAESAVMPGAMQLSKDDVAIGTNWPQPQEGREGEELWRSEGK*
>TCRU_1405 leucine-rich repeat protein (LRRP), putative
MDAEENPLAADVMMASLDVLTHNEMGHLVYARSNLSSLRLTSIELLGAYKHLQRISLDDN
MLDSLTPLRELRSLVYLSAANNNLTDEVFDDLAPSGTTLERLNLSRNRLASLRGLRLLPF
LIDFYAEENQVTELRNEDFSMLHSLTRLNLMSNQIQRVQLDTFAGFFTVRFLNLSHNALE
KVQFVMHLANNLESLDLEHNNIKSLHGFDILRELVYLRLANNGIESWEELETLSGLINLR
HLTIAGNPILRRPASSSLTKSIPEYSEFQVSADIDFSAGKRSVLFDSLTGAEASNKLVSS
MVVPSARNLVKDVTMLSRRLEEWDVDGFRELERLPLIEQHRFRVISILRQLLTIDSVPVS
SEEIPRALRLFKKKRGTWNFVGGNILPAGSAARAVESQRAKNGSGMP*
>TCRU_1406 hypothetical protein, conserved
MTLTIVAPSPSLVRNPRCWSESWEELRETAVKSRDQIVLTCDSASPLNSDNFQARLVCYV
RTLPRLNDVKSALRRLNTLHELGHSYVRRADDPVSPNRQYDGETQGHRVPRPTIWKVENS
VEGGITVIDALSGAKLMCILEPKQGVMVASMLHAPFRGFINRRLEVIPIENPQDILLVRE
SSLINTDYVWVGFMDGSIRLFPADSRWVREQDRSVLLARGQLADLVYELPRQHKGAVIAI
TRSPCHDDSGTTDLVTANRLSSSIFELTAATSRGCREDREHLSLVCTASEDSCVVVWDVR
KIYRRLEAMRALSQNTRMGGFGVEPSLVSLGGDVVTFNVQPTTDQHGYTVRSTYALVKVR
PLLRLKGGVGGLRTLNWVSSAVTTVGYQKGRDVVAMTRDPKEDTASELIKNRRAIQQTTR
WEKREEHRVMLRLSEENMREVEKELEQLMPPLAPEPNQRRRVNLIIAGDVHGTVHFWNLD
EELERETSEIHSMCSTSRTASASRDSRAGQSPLRSLDLSLNSSRRHPAEPAVTSARGSVE
RRNDGGKLTSRRREFSPRVHTERDTKGKRPNKDAGSEAAGKEQQSSSVPSPRTPGDVKKH
VKARNTAADKGVKTPTRRVSKPPSLNKPGVQLGKQSNITTLRAPHLSPMSSVTADRVKKG
NTLAAPVRMGGRGLSSAKGVRQGNNCFRTSPQAKNSLCQSVRSPRSNLTPLATGRRTPRT
TEKQLLRRTSKCPRQRPSRGLEGVSDVKKTRTGDSGYFSTACMNKSLNMHPCESSANRSP
FRVPRGMPEIETNWTVQWNSPRISNSGSSPSSGRRSPTRHPSPYDATHSARADKEAEFKE
WIGGHRYINDMYSRNAKFHISLIDGVTVTGIAVDLPPIITVTMRRLPDPPEPHYSLLEQP
TKEEIRRRELANSFYSLTEERALFCVFERIQFYVSVERTLMNMQCSPKARVDVLEQRVGP
GRLEEEGVIVGRTPFNFDIVFRRHVLEMHSQPIVRLFIDHPRRQLWVARNHGLLSVFSTE
TKSIITRVKGPSADEAIGPPNAAEWKRTQREMALFGRRPIYLDSHKSFRKHRPGYFTGFR
PISLLQQFELMRTSLVHELDSERELSTPASETVRVHLTIIEGKDALDKHAKNREIGLSVL
LEKLRACRQHALTVRQAQRDHYNALFNGASFRIGSLIGRCATFGVLHAARKIFWSWAHHR
EYFPRQYVLRQLRQRGMKQLSKVTNAVSLNFEMRLRGEYFTKWRVSVQKRRTCRLETWFA
RCLQAQVRTSLRERFANFMGDRNRRQRYWFMWRSLLAPTARPPSLPALCRSVSPLNSTGV
TKIFSPSSVQSMRRSSTSRPRVPERNGVFLDMYAIIRQIYGLRQELISFRGDVGSDSVLD
LEDPWFDAIEAASEEAEAQTAYALKLAVFKFALVPFLESLLSTAEGVLPYIRESPVTEEV
RSMLRGCLLCVDYLYADAEDLTSTAATRRSGRASTGSHLSKNGRASLLLDITTRREQEMR
QLFDDAMEQREFQISIESIAAQRDTIAEFLRVCSGQ*
>TCRU_1407 STAG domain containing protein, putative
MAGKRKTKKAATPGATATAAAAPTDTDAARGSGVPDAAEPVLLQEEGSSLGESLYKALVE
GASADHVVKDLVRIYSDGGKETVCCAILNLVGKASGAAQVELDGNALKDDADVTTLLEEM
YARTQQNASAYPFVRKETKYRVFRTAYRRFFARLVEFCYNANILLDGLLLSTLLRWLIAM
SESKARCFRHTSIVALLALVGALNGLIRDLNHRLPSRGRGKKQQLSALQESIQTMVELRN
HVISQAIHQRPRDVAPEIRLLVFEQLEKWILNYDEEFAENKYFRYFGMALYDKRPEIRAE
ALTMIQKCLVATPESGNRMFLFLQFFAGRLVEMCGDVNMRCAELAIRVMVLILRVYGEEA
EGKEILDNEMIDRVLLTLFDERPSIRHEAGVLLRAFIDSRVPKDASNGVAYQKAAMELLC
AFSATLRSQYGEVMPERYLIDALYNPLQELPPFLENYGSMLDFVLSDDALEVVVGLGFVA
ALLEKLRCKLYLGPLPKEDRRQGIVKKLPPNKQEVIDALVTSLSRDVGVLLDGVLEKHRG
DVAILSGVSAVIAAMEFKAFTSLEQVARINALMTLMRKATAALPRCEEAHLNQITCAWLG
FVVEDHPLKSAAKGQIQELLKQVLKQLSALQKGKKRSESQLEEKELFHVWGRMSILSSIV
SLVDQWDLLKASILRYTNSSTSPELLRLILSTCMRCVLWQLKVEQEAADSLEAATTASQM
IGELVTCIFKVWEMDSQRQTENFTLLPVDGMVYLCDLCALRCYTMSQMEQDVFLGKFSEL
SSVLSKEMHEAKEKLKNAGELFREGGALSEVARSRREVSRLEASQLRVATGVARLLMLNR
LSEDVAPRVFLQWLHAPTKSVSDVFRFLFRSLRDRCNDSFTLEKSILIAAYHHSSDALYQ
MGMKLSSMHWPLPDKYYAACVNMVRFGIEFATTTDPAMLQAVVAYCPKLLKMDALSLAHA
LAANETLAKSTDQNAQLFISAIRRAARLEEAEGATTPVRRAKRVREEEMSEIMEASGEHT

LQPRKSQREATEEGKEEENMFTSPSKVNSRSRSGRRTAGKAPVASRRVLTEDGWRVRPDK
EEVVPSMASSISESMEATRARPTRGLERSHAKAAAATEAAPPTATQETVLSSLAADFDND
EVFIATQEYD*
>TCRU_1408 hypothetical protein, conserved
MPRKETKKRKRASSSTKESVSSTDEQGKRGGALSKFKEFDVSELLQKPQEVPEIQPVVEE
QPCSPCDEVVADPPPLTATDTVEATSEEPAAPIPQLIPPEPLKDLPPDEEFVPLPTIQVV
PMMNRALEAQSGLLKEPAPPASIQRVTQPGALPPAMPTALMELLSG*
>TCRU_1409 Proteasome subunit A N-terminal signature/Proteasome subunit, putative
MFKNQYDTNTTTWSPTGRLFQVEYANEAVNNGSAAVGAKNKDFVVLTALKRSPVAQFSSY
QEKVFKLDDHVGMAIAGLVADGRMLARFVRTECMNYRYMHDSDNPLPLIAEIVGEKYQRH
IQFAGKRPFGVGLLIAGYDSTGPHLYHTVPSGDVFDFKATAMGLRSQAARTYLEKHFESF
PDCGLDELIMHALKALAAATSGGAELNIKNTTIAIVGKGTPFMVLTEEAARKYLDGFKMR
PEDIPPAPEAEEEETLQERSLDVEE*
>TCRU_1410 BNR repeat-like domain containing protein, putative
MRDPRVTEWWQGQIIMIAHCDGSRYCRVYESLDMGTMWKEAVRRLPGIWVNAPSGAYQKE
SLRVEALITATIEGRKVMLYTHKTSHPLEASEPNALYLWVTDNNRTLHIGPVSVDGVVNK
TFDNTLLYSDGALHLLQAKGDHESTDISLARLTDELKTIRFVLSTWARLDTFFSKSSIST
AGLVAALSNAAGDDTWIDD*
>TCRU_1411 Concanavalin A-like lectin/glucanases superfamily, putative
MNKTWETVFDGTKTTPSSTWEPGREYQVALMLQDGKGCMYVDGVLVGGSATIPTPETLGH
EITQFYFGGGEGDINSDVTVTSVFLYNRPLSVGELKMVKKSDDKKGNGGGKKGNGDGSMR
GGRLSGAAVRHCGPLLRNRGEDFLPPAGTYASGHALF*
>TCRU_E3 ubiquitin ligase, putative BR.1
MAETPYESPSDVSEMDPESAVLVPIQQRIEAQLRKHLQEVSQQLYDVKNELVTVQKEREK
YGVELYNAQQHLAKLQETLEKQHEKHVSVQRQYEEKLQEREEISTQADSLRKEIEDYQRQ
HERQQADLAKLTETIVKVQQFNDQLKKEVQVERRAAYKTEEDITNLEREKLKQDNMIDVL
EKRVILVEEEVTTVNTQIESQQKETKRAREILAEALAEMEAINFEKKQLVQQWKSTLIGM
QRRHEAMKKTEEALQQQKDDLQALENEITGCRKDIQGVQTETSKLMEFMSRVDNEVTVLE
KQIDALVERKEKSAQSYAMLKNNLEQTDAETKKLEYEARTHASEVTNIDRKIQKCARDVR
SMENDIVEALSKQSTLKQEGSGTLQEIEKIKGSIRVKELQVTQMENELARIRVDTLQAQS
HNETLKSTLGELEKELQSRGVLIERMQMDIRRRHDEIDRKQKQLDQLNHQYEGLLAAQVS
DKGEHVGPLEATINSLSRAITEKVNENEGLQQEWIKLQTELVNCKNNANEVNEAILELQA
EATVLMQKRERLLANIANEKKEITNLENKANAMHIEMKKVNTQLSKNSEDQKNIANEAFL
LENDLIRRLQEKKREAIELELKVEETRQAKTDLLEQIMNCERDILFWERKMQIAKETEMA
LDPTVGRAEVEKMRKEIGIMEKRMNDLQREQRFLVEEMQRSIDHREIIRTKGQAIQNATK
KNGRGATRLDVDKESARMFKELNEKREEAQLKERLIRESLVEIEKKSKEIETIQREVENL
DEQVTELQAQLLSAQKECDRLEDEKRIKNTTLQRLRDAENGAYNLAVSPEELNEEVTHLE
EKRKMLVEIIDDLTNRYPELEEELSDILSAL*
>TCRU_1413 hypothetical protein, conserved
MNAANALAPWSVQQIFPSLEVSVKYRADLLATASRLPDVASRTLLCPLFHHRLLHHFAQS
SVFGVLSSMCSHALTQQRLHEETTGNGFNGAKNNGYATEDEVKFAATITSSLLEDSMINL
DAALLPIIGNSQGHLLTILKKRLRETEESSLGVKFHEKPTLSMSTQRKITFVPRRKTMEK
VLDQANLPRRHIFSKKLASLTNVAQSADACERRELLGNLGDTPVPFQVRSREEVRQQLRG
QKAFETSMTVVLSDHAVPLPLWKRRRNEVEEITENEGFTAPPTAASSTALNSPQESFVNA
TWVKEGELLLVNGCLIHRDTKRIVEPSAKKEKICALERGEIGSMETPETTTSVKKKTTAE
SERKKRTAKVVCGSFASGRIGTVNTQGVPLMSREEILALVNSLQISDALRAAFLIGLNGD
DNSKIKK*
>TCRU_1414 Ras-induced vulval development antagonist, putative
MATDGFWFPASEPTGEELETTIFLTFLQNIFSTQNVPANREAAQMERKRSRLFQPKRKRD
RHEESAESDQKERGGNLFHIISCIVHAAGIPYEPPLEEQGVAASGFVSMAAETTTITNTP
QPEKDVSFEGDYAAPNKLVQQPRSALASLVEIARLKERLQKTENSERKTRAFIAVSADPL
RSNNEGANIMLERHKSPTASEQSGDDFGVALPLGSFISDDTNDPSKKFEIPVAKHPSQMS

QEEFLRQFKRAPRRGEIGYDAESVAAAEAVGYVMSGTRNKEKQHYVDNIQRKLHEKEARK
LRLQFRKVEDERTDNTMIQGIIAVVNEKTPHEGL*
>TCRU_1415 hypothetical protein, conserved
MQAQLEEKVRQNMETRYALHAALKAESLKWQLSNAQKEKDVDDERGGDDESHENRFGLTN
GEEREKILHAPRGLDSGSGGAHLKKKHMKVRTSFVRFDLVTSHISPHRPQATAVGQPAEN
ATTSACLEIERLQEALYALDQQEEYQEKRAEEQRLQQDAVQLLHQQGLRVVHLRR*
>TCRU_1416 Endonuclease/Exonuclease/phosphatase family/Endonuclease-reverse transcriptase/Reverse transcriptase (RNA-dependent DNA polymerase)/RNase H, putative
MEPSKWLPAERYFYPLLNSIGAYQRYAYRLRAVCDAQRQKLLLSGDIEQNPGPIAVLQMN
VSCLTPSKLATLMAQGADTIAIQETWKSSEQIASMHTGDYVLYAQSRIGKGGGVAVLARK
TLRSKRIPLTIPQHDTSLEVVVVQVALDQNRDLIVASAYMRPPPQVTQSFRRLVNCLPAS
SPLLLCGDFNMHHPQWEPFLETSPSEVAAEFLELCTDAGLTLVNTPGEITYSRGTRERSC
IDLTWSKHLTVSDWSASVSPLSDHYMLTFTLHQAFKDTIPSAPKFFYSWGKCKWDLFIKD
FDAQLPAYDCKKQSTGIRAFTRALITSYRRHCPRGMHKDGPRLWDDTLMEAERIATDSEA
RYLQLPTPDREAEMQRTRSQFFLLLRERLRNTYLRRISKLNPGEPLAWKYISGRKKASLP
SPTSILLGDGQHTYKTARRAANALNRIFLPFHPSHKAVGFSKGINRQSASLNINASFLFG
QQNNSESAATFTSGSSLSSSSEPQDKNEAATTSGLVAHLHSPLDAPFNRTELLAALRNTP
YGKAPGPDEVYSEALRHISSKGLRFLLPCVNRSWSTGTILVEWRRATIVPLLKPGKSPEL
LESYRPISLTSIVSKVAEKMVLKRLLWVWTPHPHQYAYRSMRTTTMQLAHLIHEVEHNRN
HYFQVNLPKKSGIGNQLHYRPHRTLLVLVDFSKAFDSIDHRVLSRLLANIPGVNCRRWLR
NFLCGCYARTRVGHRHSDRRPMLRGVPQGSVLGPYLFSLYVHPLLNLLNSFAGVTADIYA
DDLSIIVKGQSREDAIPTANMVLQKLHAWSQENGLAINPSKCEAAWFTLSTHTESDDDRE
GRWPLVVAGCPIPVMTMGASRTTKLLDMDLDPRLTLNVAATKQCAATSQRISQPRCIAHK
EAGPSPHDLRTFVIGYGASKLRYGSELIWAVATDSAKNEMQKTHATLARTVSGVPSTVDP
ESALLEANMPPLHVLCLRARLSMFENTRACQMDWMRRPPPEPPPRAGFRISPLSRDELYA
SVDAYTKDYGITESSPREERFFRTSIPPWFAASAHRATIGVELPIDHSITDEEELIREKR
RVSEEALALHSHRTWILATDGGVDVPKSAGVGILLSSLNSSKIIEKASINCGARPCSYRT
ESRALLLALEKLMIPRIRHRRKTLLVVTDSQSLLAALNKGPLSQTDWTEDQIWQRLLTLT
RAGWSVHLQFCYGHCGVHVNELADQYAAQTMATGQYTEQGIAPLWHTDLLTCFTTQLTNK
WRSTIRQDTHRYLLCGTRPSDLSGKDLITQEVLHRQELVHLARARCGGSELWGRLYWAVR
DCTNQCRFCNISPERSAYMRSNNDPTAPGTGTVPPSAREEDVSPVRRRTLTRRRKEKCPH
CDSTLTGFSGLVSHCRSFHPEHPPPLPELKCDFCDMVFPTRRSTAQHRSRCAHNPDATRH
RNSSARRRSLLPQDQPASTSTPIGPQETLHHLLLECPGTLAVRQRLGIEQDLRLGKSSQW
QLLHSRKLLSLLDHLLST*
>TCRU_1417 hypothetical protein, conserved
MCVILLLCCVSAALLRCEAAVVRYDVHTVRAKVAALPDDHWARYDETVFERGRGSFVSGY
DDAPYFEPMNISSPFFSRDSVPWHVSSDGFLAPTPSPLCAFFCSDSAPDTLFGQYQFGDA
IYGGGGDWPMIGLYVADLNPSAARLSPSVRVHGVTGADGGPGYERVTVEYRDVPLPACVA
PRDTLTAQVEVWANGTIVMRYRQIPACGRASVGLVLSKGERVVATATPRPNGIAAIRYDP
VVDVCAAFLSHSACVNAADLCGWCAATGACVATSLASSCCPRGSFRTAPPPSPVGGDFYV
VAVEQGAELVRLAALRGHVRVVPAGQSLQLDMGFDFPFYTRDQASHVTNTTHLLSSGLIS
VFSSTQRCGPIWNVCPDGNYTFAAMPFVTAGLWGPGTSITYARLPERTGADVLCERPRCP
PGFVVEVANTSSFASFARSSTYQVYLDASGAVEFRYGLPVATSIASSPVLGFFAFPPPFV
GLFRYRVEDPATVTVPPSLIRTGTRVRFDPTGRCNDCGRHGTCDPASARCVCETGFSGDG
CERCVAGYYGPECLPCRRCRNGGVCDDGVNGTGVCRCREPFSGVDCAVRCEGPFDCSHCN
PRGGYCECGECRCDAASGWGGADCDVADDPCWRHSFDGCEVCNKDTRNGCAFCFDDMCYS
TLLKDTPNGHACSYPAPSGDASRCVPAVQVEVHWFDWGYVAAAGLCLSVVLSVLALLVLA
PFAVRARGVHDIHHAGAAGGAPDRRRGRRRREVVQAVLVQQGAHRGERTHVLGVPLRQVP
LAKLYESQRHGPS*
>TCRU_1418 hypothetical protein
MEQNGLDKNNYYSILGEPSKEQQQLYVQGKLGAVKNTQMRRDNEEANHFRFYELGLSQES
PRFFLLPAISSIVSPEFDNKRNEVIHNWCEFLPRGVPNETTFQKSVSRRLDEILERACEV
FDGQVEKKGKKLRIQPSAVVLHDNANSSGKLHIEKKCISLQKEECVFTSLDRTSNAVTSL

EDLPGVSGLLMDLTSRFQKGQNVTLLTADATIGKHHLALTMLLDFVRLALKRKPNETYIT
FSFVFVRNGRFVYDLLADQETLMASKLSFKCNRLLGPMIQDGVRWHDVQHAHEMDAIIKQ
IEQTLSVLLVRCVSKEDMQSTFIMLTAVAKTVEPHDVVFSSFVGFFVWNEVNLYDFLLRS
QEQEKQVNSAEKEAMRSILLSAVRDRSSFVRTCFVYHTAMPETCAQRFLMVQNRLCGCFS
NEPHKGTLASMTRRQKAKVTGKTTGWRRLLGRGSVKMGEDKSRMVNQLKYFEMILADTYK
AVLLVEGMESKPPSTESPENGGETGKDE*
>TCRU_1419 DIGIT
MPSPPAPPRMIDRRHVFGVCEKVKDGFQYLDDTNVVWVSGKSLVILDTQLGTQQLISCTP
TCQSVTALAISYNHRLLAVAESSKFPCIVIYVCDPSGNPKLRRKKVLQVPDLGSTEYISL
GFSLDGRHLISLGGYPEWNLVYWNVERAKIMATCAVLDENELGSRDKRLLNQCSICPNDF
SLVCVSGHGIVRFLLLRDNQLRRASGGLREPVTNYMAHIWIPSENRLILSTENGDLILME
NNEYKYPLPMSPSDGVSINTLIAYSKGFICGGDLGLIYIFERTDNKEMYRKVRTFKFNND
AENMGPPSELIPVILSLALSPPPAEEYVSFMTSTKQLYSINLPNADFFKSDERVFEPIGQ
PFHSGAVIAVDVSVQRPHAVTAGRDRCIFLWNLITGVVEFRKRFSSDIHSIAMHPSGLHL
LVGLTDGLRMMNLFHNDVREFKNIGIRSCMECRFSTGGQFFAAAHATTIHVYYTYTCELL
GHLRGHSGKVKTIFFVPPDDTRLVSVGLDGAVHEFNLCDFHKVNDNVLKAMTYNCGLADV
GTVWTAGNDQKLRQFDRTKLQPVAEYDLQNASLYSMVISPRLKLLIGGAEDGTVRVFNTY
LGEKLSGMERERENGGEIMIESHNAHSGIVTRLALTFDESLVVSVGEDGAVIFWDVVAPY
RGPHKEVEYSRELFMANSDLEASTKTVSALEAEVNELKQRMQQQQIKRDRVHEEQLLKLE
QEYADEQARRLAQYEALKADKNEQAIQFTDFMAEMETKEKEALKRTKEDYSLKIQSLRDR
ADRLRQLIDELQHEQDAHLAEVREVAAKKRAEDSERQRKVLKELEEENHQLLMERDEDTK
RTENYCALIEDETDVEVTLLKEDVEKRRKEQEEEVATLQANNDLLLSRENIIKAELEAVK
ADVREKLRQQSTLESQIEAAKRDIGALTQEFKDRGETIAEKERRVLDLKKKNQELEKFKF
VLEYKIKELKLQIDPRDEEIRQAKSRLSEMGKEADNYTHSNEHLVLQIRNLRHKKAGQQK
ELDKLAEIMRGFAEYLSRLWTELSCLREETNPRKLKECAKMLFDKYTSTKAVAANKTLAS
RTAADEVREYNRERDHLERNLAGLKNKVNKDAENNRTDKNRITTENVILIREINDLRKEA
RRLAEKAGAWRKTEKCGASLQQEETKRELAMQRAELKRLRALARKLAEEAREKGITLTLP
SLTEGRPGS*
>TCRU_1420 hypothetical protein, conserved
MWRRRPDRSAPGCRGAHVADEPNHAGHGVSGVAKNCGVTRDVAGAPPDKGRSGSVYPQPD
RWKERVVLRLAWMPASCLSEIAALTPNNVTLGPDGAIVLDWPAAPKTARADLRRASRFVR
IRGQDAPDTTKLCGALRHNEKLMNPRTARVERAVAPWNATAHSIERGVLRHAARIVGTYN
SDPHVITQSAGHGGSFGLPQDTVRYFGRRTTTLTQVSSLAAQVRRETPRKDRNPG*
>TCRU_1421 hypothetical protein, conserved
MALVPYTVSEWCDIGARLWDPVRQRGEAQVQITFINGTEMSDETLTVAPSSTGLCLILGN
VTRPWEDSSLTGHIANLRAFQVPNFENQLILPIMGAARELGRVARAHHCRKEALEHEKRP
CRRGRAESKRSASFAAGRGAQQPIGARI*
>TCRU_1422 BNR repeat-like domain containing protein, putative
MIVDCENGRRVYESRDMGTAWTEAIGTLSAVWVNSGSGDSQKESLRVDAPIAATIEGRKV
MLCTQRGYASVENRANPLCLWVTDSNRSFYAGPVAVDNAANWMLTSNLLRSDGSLRLLQG
RGNGECRAISLSRVTEELSAVRSVLSAWTQKETFFSSFSIPTA*
>TCRU_1423 Endonuclease/Exonuclease/phosphatase family/Endonuclease-reverse transcriptase, putative
MSVLTNGTVPPARPATSTWCQGLVLFAREEAKRLLPIRCARRGSRRRTNGSEGHLMEPFT
WLLAERYFHPLLNSIGAYQRYAYRLRAVCDAQRQKLLLSGDIEQNPGPIAVLQMNVSCLT
PSKLATLMAQGADIVAIQETWKSSEQIASMHTGDYVLYAQSRIGKGGGVAVLVRKTPRSK
RIPLTIPQHDTSLEVVVVQVALDQNRDLIVASAYMRPPPQVTQSFRRLVNCLPASSPLLL
CGDFNMHHPQWEPFLESSPSEVASEFLELCTDAGLTLVNTPGEITYARGTGERSCIDLTW
SKHLTVSDWSASVSSLSDHYMLTFTLHQAFKDTIPSAPKFFYSWGKRKWDLFIKDLDAQL
PTYDYKKQSTGIKAFTRALITSYRRHCPAECKRTVPGFGTTLSWRQSGLLPTARPAIYSH
RRPTVRQKCNGQGVNSSSYSESACATRIHAASAG*
>TCRU_1424 hypothetical protein, conserved
MRRPPPEPPPRAGFRISPLSRDELYAFVDAYTKDYGITESLPREERFFRSSIPPWFAASA
HRVTIGVELPIDHSITDEEELIREKRRVSEEALALHSHRSWTLATDGGVDVPKSAGVGIL

LSSLNSSEIIEKASMNCGARHAATGRNPVRCF*
>TCRU_1425 RNase H, putative
MIPRIRRRRKTLLVVTDSQSLLAALNKGPLSQTDWTEDQIWQRLLTLTCAGWSVRLQFCY
GHCGVHANELADQYATQTMESGQYTEQGIAPLWHTDLLTCFTTQLTNKWRSTIRQDTHRH
LLRGTRPSDLSGKDLITQEVLHRQELVHLARARRGESELWGRLYWAVRDCTNQCRFRNIS
PEQSAYMRSNNDPTAPGTDTVPPSAREEDVSPVRRRTLTRRRKEKCPHCDSTLTGFSGLV
SHCRLFHPEHPPPLPELKCDFCDMVFPTRRSTAQHRSRCAHNPDATRHRNSSARRRSLLP
QDQPASTSTPIGPQETLHHLLLGCPGALAVRQRLGNEKDLRLGKFSQWQLLHSRKLLSLL
DHLFGTLAAPYS*
>TCRU_1426 hypothetical protein, conserved
MAEVRFAVRPTAALHHVGFEVETSRKQTQLYVSRRGLVLYVPHPYFIIKNMRRSFWHGVD
KVQFALYPIPLSVVTAFGFGVFLWVLNSPADAWIRVSCVSDILWRLERNFISARIPSRYR
TPALCANVAFGAVTLFTTLQRFVLRKLLSYNRWIYEGQGKLTRKKMLWGFILKTFFMHNL
KRTGAYGSCLPSQPLPDLKITVQRFMKSMVPFYEGKTAE*
>TCRU_1427 Choline/Carnitine o-acyltransferase, putative
MTDWWIKYVYLAQRESLCINSNWFGVAFAKYLPTPLQASSAAALVHNLVKVKKSLDRRTF
SPQFSGLVPLDMNQYRYVFNTTRIPGREMDVLVQHEGIKHIVVIHKGRFYQLEVLHPLTN
HQLTPYQLEMALESILHSEDETDPVEALIPAFTTAPRA*
>TCRU_1428 Choline/Carnitine o-acyltransferase, putative
MEPKSLEEESMMYLCGNGHNRWCDKSFNVIVTEGGHCGVHAEHSWGDALTLANVVEYSHA
SDEFKELYTEDGHVKKLPEDEVALTEGKFKVFVPNRIRFTTDAVMKRAVQGAQERCIREI
KDVDIRVCRFADDGKGFPKKQRCIPDAWVQMAMQLAYFRDQGHFDQSYEAASMRMYRKGR
TETIRTVSEESCAFVRAMEQPSLSAEAKLKLLRVACERHQKTSPDAIAGCGIDRHLFGLY
CASTGFEVASEFLQAAMQSKWKLSTSQLLTRQLPAKFYPPSDEPFETPNGGFGPVQDDGY
GVCYCLYGESLFYFTITSKHSCKSTSSTRLAEHIRRALRDMAALTGWN*
>TCRU_1429 Protein kinase domain/Protein tyrosine kinase, putative
MQPESGTRGATGHILVPGFLGCSGSTDSKYTYVRYLAHGSSWETWKVLDVSDGKPYALKI
TSLKNYREPKLLRVRTEVRCLESIDHFACIRLHEKPETESHFFFVMEYCDAGDLRYQMRD
GGKLLEHQIVHILLKLVLALHYLHTKKKILHRDLKPANVLLKTTGLLKLGDFGFSNAYDT
ISGDVGLTICGTPAYVAPELWRNERYGAAADIWSLGVILYEAMTGRRPFEGTKYEELRHK
VLHEEVLPIKTSHSVDLKRTVYALLRKSSVERPDTTQLLREPLLVAALSNFPEVLRVSDV
EDSLLQRVLHDISPEQLLPRPRLDHSVLFSGMVWKLKPTQDYQERYMELADGQLSLLLRP
GGRVGFRGCIPLLRVEQVACIDPNHFALKIEGADWCFFQTPQAAVWEEQLRAALGI*
>TCRU_1430 Trans-sialidase of Trypanosoma hydrophobic C-terminal,
putative
MAKHRNTDQFGPHRISHFFFGAGNKEDEGNTPVTVADVLLYNRILYRDDLRNLNARTVPI
RHPAAEELDTAVKFTATGEEKQKATQLTTSLADVEDPRIAQPTNSQSVRVLAGSESSAEN
VTDGLSPRTAAKNDTSVPETSHSASDGRMSSEDRLALITDAKLILHDLSGDSTARVCVSR
VLLLLLGLCALVAVL*
>TCRU_1431 hypothetical protein, conserved
MRSAVGACPRSRHRERHRAIAAVRAVLFVVLVLASAAAWMPAVHAVVLRLRGGTVDRAIT
VGHAVDTVLMDGVSITNGVAVVFDVPAMLPGVLRIELRNCVCDGGAQLYVRGYSGEPASD
RSLEVSVSGLSGGYCSLLFVHNLPAHTNVTVRDSTMVTAGPMRYSQLSGLTDAVASPLVL
HATSLLQTQLRVSNTVLRSLHAGGSAVHVGGGVDLLSSAVVLDGVSLEASGGPTASAMRV
ASSSRLSLRSHSVFSVTNVSVVSSGGGLALGERLAVSDSVLRFVGVEGSAASSLVRCDGG
TVGRWVLAGGWTCTTCGRWARRACRSTCRLRGPVVVRGTARVA*
>TCRU_1432 methyltransferase domain containing protein, putative
MKRVVIDLRPKESYAEGHIEGAFNFPWESIRADACGLPPRDVALIAICDGQMDLDIVEAY
LNRFHFASLEVRRLSKNDELVCELPKGTCWSPNPFLSEVITEIEVKNGGPSFALDVGSGT
GRDMVYLASRGWSVVGIENRFRLIEQGVALSKKHNVDCRTLYIHCELKKFFPVKFEGPDL
LHVCRFLHRSSLEMLLKLPRRGGFLVYSHFLDGCQKTEVGHPSSIDGFFLCGELRQILFG
AGYTVIIERETTLPDKRPLVHIFAQRTR*
>TCRU_1433 leucine-rich repeat protein (LRRP), putative,
MSGVGVLGVLPSLCVLDVSKTSLTDEGLDGLSVNNSLRKIILDDCARLTNVSELSSIISL

RDVRLRGCNKMTGISGLGSLPELDSLDLSMTAVTSRSLSGLGVSPSLSKIFLEDCWNLTS
VHTLSSILTLEEIYLRGCIRVTDVGALGTLPVLCLLDVSKTSVTDEGLDGLSASPTLKRI
LLEDCTRITTIAALTSIHTISEVNVCGCSGVKSFLSTP*
>TCRU_1434 hypothetical protein, conserved
MRKADNAQQKTAILQLPVAIAITKIVKRFPPERFSLHVEHLLDEVILKLRTKSDKHRESA
RKILSAMLCETGPGKLAFVITKLRDHLVHGYQLHVLGYTVVTLLYNLYEPQHAMSYRRKK
HVMGAEGAAAAAAIAAKGTPMEHAGAAFHQGDNDNDNDDDKEKVEQQSQTEARQKETTRE
EEEDAEAEEEEEEDLVRQLIPASNKFDWAYGVECLTSCLDNLMSIFLDDYLGEVGEQKEQV
ELMSTMVEVKRNRALQGFTLIASHCEASRVIEVFLEKITWLLTPPSMADTVDVSTLRGSA
RAAALLLQELKTKYSTVSKNAAADFVFVQKVRLLAVRFARALIKNATMEVESSFLTVRDL
LRRHNEVREEKIKTFEAKDGTRRIRGNVHLTIQRAPTATRKDQLDSNFLVDQLPERVDVD
FSAHTVLATQQRQKLRAYRGRYGKEVQQAAKNFYREDPATAVVLDTLDEFLLKYLLSVLK
HVLGIGKERKYTTAAVRLDLLRAQRKKNKNRKGENSCQDEDDDDDDLPEELRERGGKEEN
EEEEEEEKGGDSSSDEEGEEEGTDTLLLLDPQPLDDERGDALDRFATAAQQENEEWDKNA
GEVSEQLQPMTTKKEKAKRHVQEVEKKNKTRRPMADLTTSFTRHHQQLLESLLPVVLTTL
QGEGCDAVVGHALDCMLALVSLRPPLHAVGALHTTLYETVTAFFERGGAIKQRAMRVAAA
VVAHQRFILTEEKAAQLLQLCRTELVDRNEFLPMALSLFHAVLGKHIHVVEVYDLIGVVT
ELMMHTSAKRLLRQRCIAVLARFLTEYRITPEKFRSHMDLFCRNLDYPEVTGRIAILELL
GVLVYRLPTAVLRQEAPLLLIPLAVTLAVSEFYEARQKAGDVLQSLVKNAGVDVIVPTLS
QWLASGQTRPRRAMALQTWAVMIPAVAASYDMSVDEDACEFEACWSWSLQALLDAATFDQ
VATFGTKRKYEELTAAFKRKLELKSWTYVFFGLRCLEGIAMVAPMQVWHNMSVAPHLVPF
LSGRLVLHSHPWIQSVALRLLRMYCHDGVERRCGYLRGIDWVAAGYLVVTSKKNHKKGKR
RKSGMGGNFHATQRASSPVVSHSHEDDKDDGCIPPYVVYFHESLGPPDTVHRALNVLDQM
AAAMRTLLQFIALSDVPNEKYNAHRAMQQPSRTDAVNVALYISRAMFVVSMVLLKVAVFC
AGSEMSYVQDIIAQHFTSLRHQLHSLVKPLIKNGGVANVMVRTTSLVQYFGGFLAALPTD
AETDNTINGRKEEEGDDRNEPDGKVTSREASGATGPVKRAVCWLARNAVGLRFVRDTIVP
TLTVAMHCGERSEKLSSLAAQASTMVREQLERHHVHFGGDFKTTTTTTTTKRKRLEKNKT
RRDEANVWGKETAATTTTGDVDGDAVTVDEVLFTLTAAMTAVRTARKEKTTRRDTIQTVR
KRSHSGGDDAAPLKARRRREK*
>TCRU_1435 Endonuclease/Exonuclease/phosphatase family/Endonuclease-reverse transcriptase, putative
MEPFTWLPAERYFYPLLNSIGAYQRYTYRLRAVCDAQRQKLLLSGDIEQHPGPIAVLQMN
VSCLTPSKLATLMAQGADIIAIQETWKSSEQIASMHTGDYVLYAQSRIGKGGGVAVLVRK
TLRSKRIPLTIPQHDTSLEVVVVQVALDQNRDLIVASAYMRPPPQVTQSFRRLVNCLPAS
SPLLLCGDFNMHHPQWEPFLETSPSEVAAEFLELCTGAGLTLVNTPGEITYARGARERSR
IDLTWSKHLTVSDWSASVSSLSDNYVSTFTLN*
>TCRU_1436 hypothetical protein, conserved
MVPNASMKRPSFANGPRILVASNASGIMSVTCGGREHIAADNTDEVSEHDATGHKSLFLE
NGVAGSGFVAEGDAGGLTQRNASGHEYKPQLREFTAQYSVSGVSPDDCFALATTRN*
>TCRU_1437 Leishmanolysin, putative
MLTWCVWQSAVGGGALDDCRGGCEEPRDCDDIDGMELQDGDGDGRTLESHWSQRHAKDEW
MSPIGCAGCRTELTLTAPADLGCMRVRWEMAEPIRW*
>TCRU_family, putative Se.1
MEHGVTLLENPLTPRQPIMSSPARYFFAVEDASVGRVVEDWMAKVPCRDAHIFALGYTPH
RSPQQLVWVRIAPRAMRSKVIMLDFTAPEVLVFHLSMQNGFPQLLSPLSPPN*
>TCRU_1439 hypothetical protein, conserved
MGVGRQSTVSDQLLLLGLRQLVGAARSHKQQKTQTHWQGHCGIPRQSPHAIQVFPEGFVP
NGDVLFHLPHLRQSLLPVLCHKPRANIDVQRQSGLPLPKLRRSPHRTAQLRKIGLRLHAM
RQSRVIRVEFFQRHRKLLHALRKESMTVGAPVHIFLIHQLLPLRVHTQTIPTGLLIQVIL
QRHQVIKQTPTCALLHQRRHRDTAPINDDPHRVAAAVGAPK*
>TCRU_1440 hypothetical protein, conserved
MMTGRVLLVCALCVLWCGFSLVSADTGDDREHSGQGVVGSVGQPSSPVGGHGDNGVVTDG
SSLSSGSHLPGSAPTGGKPSLPGVGECVGLTKSKDALTLAKEGHEFVRDQHELVPQVSHH
TDAGTGGKSDLSAPEQSPNAKTELENGGGVCGDDAAAAEGEVITTVNEEQLGGSSTSDSH

PPAADRGAKEQEGTVEQAASTAALTPVVGRETTPKGDTEEDSPEDKATTGAGITQDIPAA
GQQQTHSSSTSTTGNGPTSTLGKGRAAEDISNNNERSGKALLREGAEHETVAGSQSQAIP
AATARNTLGTTLPGGSDNSTTITTAVRSDTGTEGTPTSNHPNRQSIEGATSPGMNSDGEA
ASAKNYDTVPQNAGIPTTKAIATAKTNDAASHGDSDGSTAVSHTTSPLLLLLLLVACAAA
AVVAA*
>TCRU_1441 retrotransposon hot spot protein, RHS3, putative
MKGYVIYDVTEKGKPASWLAPCGEWGMIVVASPKVGNYDKWETQARAERIIMNCPDEMDV
KAMCAWMKRDETKEKKAECWKMVEERMEKVGPIPRYIFDANKFIAHSAAIEDALDGIKSR
DGEKHFIPRGVRLWDSENPSQKLVRVVRGRGEVGTDGFLNAPISFCLWRRIPHYFGKRDE
*
>TCRU_1442 Protein of unknown function (DUF1619), putative
MHVLRYIILYNIVLLALFWANAENPVSYPIDWEGSSSTFPDSRNLESSFLGECVCDMTWG
VCDPNCCCDTDCTVEEKQKFLFCLQETVGSSSMDYCYEKQQDEKVTRSSRRYLDRANVGS
SAVCIVRSNNLEDLNSFFVLPKEVPKPREKKSIDWSERKESNGYEVNGDLLLMKRIEVGE
SLYEMRSMGLFRIPTGSRGGYCNPEGRRVRFMNPIDTTSCVLPGERVCTLFSTSTYANLF
FVSPGYSSSKIFTPIQLRIFNHTSGKLLAEVDSNSSIPEVYSSVVNGDSCQNGVVRVQTV
LFYKSNDNGLSLINATTNLYINNINREAIFPMSFQIKFFRSVAAIKLNYFSGTPGYFEGS
RLRAGTLVEKSGKTAILERVSGFSVPSGGRSCYKNKYRTVGFLYDILSSGCTIDFSELEL
RDICSGGGTTKILRSILSINVEHSTDFDGQTKPIEYVARTNDALTNDTTSWIKIDGMNFS
DITPDPYDPVKRKCSNIYVGLHYSFVLSKVGAESNPQDIIVAAFADPIIGSWRIRNTRNF
GENAKSTQHFLFSVSFKWIDSDLQKKRRRRVIAPPILPRLDETVLYPFKAPG*
>TCRU_1443 WD domain, G-beta repeat, putative
MQELYDTVSRYTGRGTPSHFSARVHPRKAASQFVGSSIFHGRLNSFCAALVARFMASNEV
LVVVSRDAVQSISCDGYALQPRSSTPGPTAGEITGVVTSDGFTTVGYSAGVTRYSPSLQL
ERELLFDGYSNSILSIAVTPDGTCIATGGGNATVTVWTRDMNPMHYLSGHTDWVRFVKFT
KGKSQRLQLFSTGDDGLILHWDPIAGVMLSRLDYSHGESVHAFEVSWRTGFMAIACDSPV
ITLYKPRENGMIMPCHPEMSTVENIGQITDAHRLVPTAVRFSEDSQWVISAGEDETLAVS
SVRKLKRVFVCTEFITRRHCMAFMNTFTSLCILASPPETSVLIIAACATDGTVVQWVVNP
ATSRSSYTKKLQLHLGALVAMDIMRDGTGS*
>TCRU_1444 EF-hand domain pair, putative
MQRSLIAGHNNNRHLNYEELENNIGGLPITENKLQELFDSLDTEHNGYLAIEDVKAFYKG
LEHYGLDPTDAEVANEIRKYAKSDDNFMTFDEFCCLMLNFAQR*
>TCRU_1445 hypothetical protein, conserved
MQRGRGQTARRLQRHAVKHHRTRQQVHAAANVHRRPARVQRPQHSVAHTQLRLQQRRRVQ
QKRRRHRVRQSTQLRVAHRSRRHNRRVTDRHVRVCRQVVHKHKRAVAAGQPAHAQLQAPV
TRRLAAVAPHVDLRTAVAHAVPQFNAQRAGKHRRHVEHHSHAVRDGHAVHQHRVHGAANR
DGSVHRAAPQSQHHRMHCGHTRSSCQHQHDEQRHTHSRNGPALHTVPAARTRTHRRPHPP
TQTRTNRAVVVCRTRPVDHNSCPTNPFLQMANSVTQFCQIVIPVLPILILQLIPNTLKR*
>TCRU_1446 retrotransposon hot spot protein, RHS3, putative
MGESSIVKVVKGLSRRGVKGCIVYDAAETDNRLSTCLPPLEWGMLVVTPPEAKMHGHWAS
ERRAARIVMDCPDESDAGAMCIWIWRDQPVQQQAE*
>TCRU_1447 hypothetical protein, conserved
MWNPRNDGNGTRRDGSNAVSSLPLLSSQRLYLLAAALSIPPGRLLRYITEVQRRGPHPLH
HQQREAHCDVIRADVTARSATDTAAVADAFGASQSGHHYHHPRKSKDRLDASTTDGTESV
QEFPLLLHGILSVLVLPFLKLEGSSGAPSASSSQMAAAESVVTQLKDDGGKRLTLHKSGG
SRRVQVTGATSAASKDFISGRPIRVNMQEHFALLKGVIVSQHVSRPSQESLSGAGSNFSS
SRHSSSSSSSRGKMNSTSDDDGDASVSSGSAARGVNEEGDVSVTNKKSKSLKSHVYNLCE
IPPFQREASIVRLLSELAEPFLAERHHVIGNISSSGGGGGGGGGPGNSNDVESQTDLQQC
MRLVPRELHTAAGVARISELYPPFMPLLIEALLKESVHGDAFGLTARRVVVRLIELSAPT
FVSKFFKWGNGASVEASKREEEMYHSMMLHATKRLATTAVSLLEVIEDPVWALNISGSLI
ASQLLHPNAVRALLRHRTVELAKKPLLWSPTVALAYCLVTQHSLRTSVRTEKEQCVLNQQ
TEALPVLNETIDPLDTTGVGILSLISPNSMGKGSILAAASYSMHEFPEHSQEEQQTPPHR
DPFHLSQTQPTPTSAEAASGMPFNLGLSAALMGRAASLTSSLRFSPLQLSTAPEVEHPFP
LRWPLPEKVPPTVTMPNVIVMTCMVLGAGTLGQHTVVALGQWCLRSLTVLPATPCAVGLT

HLMLKYPQFLQKASLRISPGTFSSSTATSSFAATAAAAAAAAAASNNIGGTYGNGQATTP
FCIAVFREGCRLANTVMLRQAKLRTDGRLAMVWQRDKAFASARNEVNSSDFEETPFVSED
NALHELYEVQCYLVMMLQALVMRHGVQLAADAQEELFSLFLTTTRIRIAVGTPKGFEGAI
LPDDADDDGGGYQIPKRTLSQLVVKLVDESFRAVARAVDILLHDYDAGCPRQLCESLMSL
LPPNNSLFSELDRVGAWEKHLDCLETVQAAAEAYREACRSKLSGVQSQEEEGMDMAPSSS
WPSRHNYQRDPLQTHVSLLELQQELELRAKQAAGAVRSRAEVMFGSLISVLPSSGEPLCL
SATPSSGLTAAQQLWWVEHTIAYLVEEWWRSRASTPVRFRLGVDDKDEIEKDIPRLREVQ
KSFIQCCPHPI*
>TCRU_1448 hypothetical protein, conserved
MFDGDPLVQKKKQSVIDVGQRTQLDLVNGEIRRNKETLTNLRLENTKLQVAMRQAVRGQH
QGKDEDYFRREEEQLQNKLYLLKRSLNSVTGKKEELAREIERTMEETNFIMKEGGPVVDE
NSTVGQKIRALENRLDKCLIKHNEVNAIRRTYETLLERLQQEQSGFDTQLAAMEKTLQCK
EKDLADLNSVAADATKGRDAAKAELQRLKSQITKERRVQKKDIAERRVFVMSKREQLQKR
AQTLRDKIEKGEERRLRARLAISNSQKKRLRVNAQKKLQPEEVEQQKRQRELYNRLKEIT
MSNNVTDVIYKLQERRDANAQLLLTVKEADSAEASLKEERKKLQDEWEEIQQQNGGTSRA
LMQQHSQRDHFGMDADDIGKGGRGGSPEKGDTSLAHVVEQRAIARRRVLEEFDDHLSNRQ
NELEEAQRLQDSLGKMLLDVDAGVHHLAEKIACGETLGSITGTASTARPSVDTTVSPRSS
SCVGRGEAIVNLLRSCGLKLQRMLDAMRESELETTAKLVARWHGSLPQSNIRVPLERTAS
PDEKQAHDDMEDPGGAGFFLGSTEKGRSVSDEGEPVIGSHKWLGAAVGRGAIADDFPENE
IHDRNELKMMSITTVERERKKAKKQMQQRSKEEKP*
>TCRU_1449 hypothetical protein, conserved
MPSNPARDVKNNISFVPLVSKGGGTAYSEPHPSNGNEPFLCSLSGACRTRPTSPLPGAVV
GRQTEPSNSSHDLGTEDCEEKKNACSSTAWQCEHSESGLPCHAVDGEGEEINGSRDARGH
KANEGKLSKDEKRTMPQRDNCETSPTSSVSGRRGTSPLLSLAGKPAGGEPVRRIPLQGRS
NTPISLDEKVIPTLSPLFDRLPPAVSMPSPHHWSVFYPDGIDSQPPLLLHYLSLGGWRTV
AGGWDYFSHRRLQDGEVIGHDVRWLHSARPRDRFVFLISPVEPKADDESVGRWHKFRDQI
YGPPPCFAYRKGKLFKRPPFGPSESVLPKLGESASVLGPVSNSYEFSTSATESFSFNAEG
GKHRPCKPHIGRGEKRDHRLGATMNTHSTHNSTAGVCGKATAVGVEVIEVRHEDITATDY
SPRLSSSRFAIESKAVYAYYFSTLEGALTYYDCLDDLRLMEEQTLRGVTNRMPCRPKRDP
YKPSQSLGSATIENGYKGDGLAPATTMNTTPRMGVVGMGKETPLPSRKEMTTELLDLTIT
DRNDVSLGEQTELLPLWNEGQTSLSSLPESPNLSRNTRQVKQRHQYDRSIQHLIPVNTAA
ECAYGPHGEPHHPSYRDVMTPFSTQRSPSRDHAIASEPSIRPRLLPTLTTAHFDTIITVY
SEDPNFNAVLREVLMPEPGTHPYTVSLKEQRRMLSMYETGMPAWTIFLASTGLPYRRVFR
LIFVGLVNIWPLISLFVGLYDLYKHLPRMKTFVATTLAPLLEWIEEHVTFRLSMLTTYII
SVGVTVAASFTSFFSQFYLLELFISPLRILGRLLQTPFSLVFELLWSIMFIIGSFLRLLL
VTLKMIFVGPFLLVTHLSSWEFSSTPVLPAAVEGTSLTMKWWRAWQEFWVTVASPVKNLA
KAWYDSVVHVAVSAARREASIRRWYTPRLRYCVALVAKVREICAVNLAIFWQLCGPGWGL
WLLYSVLFTCTAYLLITGERGGLLDPHAMFGVIPSVVNGEGGGEGFIGASASAAAATESE
GLLKERPSEFPGTATTPMVRSSQAPLFRRHEQEIEDNNGNGFDALFFAWETLIAVWVQLP
W*
>TCRU_1450 ecotin family (I11), putative
MSTAPKTLADYQAPYPEATPNQKRYVIFLEEKDYDSELEEYKLQLIPGRVMKVDPVNRCF
IAGSVKEKTIPGWGYNYYVVEMGPMATTLMAVSPGSPPLRKFVPMAERPFIPYNSKLPVV
VYMPKEGELRYRVWTPTKPKEEIAGER*
>TCRU_1451 metalloprotease, putative
MFACLRSIYEEQTAPPSKPGLIPSPSTERRLRRAVFAAVSSSERRLTQLHEERERKSEGG
VVAAEDRNSIERRLTQFIAHTVQDHLRAVKLESTLVAHSCKQQRNNEDGGDESAARDQAA
LLCSAEELAQLLWMNRTMALGATISIIEGKCPANEALLEAVRQPAERRAPDVRETILCVR
NSSPALRAVLLDGPMIAAGTHVEGGKGKDVIPMEHEDGDAETAPDHHLHPNPQSEAGVKR
LRTDTEETSGLKGWRLGKRQPFALKVDGGLLRSFQDDPALPTVSPSWGNPISNNTIQQKQ
QPLETQREPEAALQGDINLQATSQANCQMKLGLDCGGKLSFAQAPAISSLHTSGVASGPS
PFTVGSEAPRLQPSFTSQPTAPPLFAIEQRPHPLPEGWLTDESVEKAAAEGVKRGINARE
PRGGIRAVISKRRVDETGEAHAKVGGQNNGGFVTAGEQLVADVRAGRTAPSSLSLQRRTP
ALGLRRSSFTPPFQQQRQSTPPPKSDALSATTGVSSATFVGEVLSSIHKNSTKGSHDSTT

RGVRGGHGTAEDRDDSDSECSEFPASLLLPDGSVPPILKPLDPKLVTQVAMEILEHGAGA
ANVGWDDIAGLEHAKRSVEEAIVWPLRRPDLFVGLRDPPRGLLLFGPPGTGKTMIARAIA
NRAQCTFLNISASSLMSKWMGDGEKLVRCLFAVAVVKQPSVIFIDEIDSLLSMRGEGEMD
SVRRIKTEFLVQLDGVATDRGDRVLLIGATNRPDELDEAARRRMEKRLYIPLPDGPARIE
LVKRLLHTMEAQQQQQQEQENHSEKGKVEEHTGYVVHALAEKDIAEVAASTAGYSGADLK
QVCREAAMGPLREVTVRLTDVSLHDLRPIQRKDFVQALKRIRPSVGASEVMRYVDWNRQF
GSFASQTDEATEEEANEPVTTMQ*
>TCRU_1452 hypothetical protein, conserved
MLNGRLGSGKSVDSPGVLTLRERIKLRVDELRASYDRAEREVANELQSLRREFPTCIRDA
NPALRSDDVLCIMERTRQEALAADDADVRAVELIENEQLNRSVFFINDERCVFAALEAYS
IADEELKKACDAKCSRQHIKGLSSERDVAQYKHRLWRARQNRRRLHLKFFTHYREGDEEV
TVSDEETLFAAKAASTLSAEDSYYLYLQQCKTRKLLGAPSPAVRCLTEMMKGRVLQLNTD
VTQAQLAENRVESELQKKYFYLHVPFGDLSHSRIPFEQDADFMRLVAERQELLTPPKELL
QRFPFLSKTINGTSIAQLGLMGDPIFTKLAAEREELLGPLRTASQAVNVKEKQIRERWDA
LVSNAINEENELRAQWPHLMHLPFDVALRELHLESNPEFVALLAKHAALCEDPDRAGGAE
AKRLERAMRDLAKRIAEDVVEARRRALVETENLHEKHPCLPEEPAPGVAIVEVGLVEDPV
FRALSHELDGLRADPTKNAEQIAATERAVRARAMELGSAKLQATEEEQRNYPFLPRRVDD
VLMSDLRLAEDGVFQELVARRDALVAAGPGSNPELLTATERQLRGRASELAAAKKAVDAF
RTDEDEAVRARNPFLESNEVKLVPLRFLNLMDDAKFRDYYKKRLATLGVEEIDLERLRMF
DELLCGRAEIIAERQLKRYDSIWSSMRKRGLSPDLQAAFPFVLLMGSDAMEKALDSLGTG
DDLDVIVRRMGETQALAERGIATELNVLRAQYPMCVRDLNPAVAFDTKFQRLETKRLSLL
QEYTEVELIESLEEEERNRGVELLLDERYVVASQKACEKSKNKGICEMTAEELTSLWRHQ
LLARRSSHRAVSFLEVTNEDWEDAQRPSVRQRRKRSNSCTSASTTSFIDVPDNTPIEIME
ENELLHFPPCSNRTSRIFSSGTSRAEFPVGVDENKNLAVSSNERKRVLMLRRLRNRRASR
YVALDGIEDIDAADILESGAASQSAAESGGSGNGMVDVHASALRWRGPVSQAAKQTKQAQ
MRKETKYVVRRRMKLRHGHKTSFTHLAVSSIPDLPAGELVETGTENLNEGSNVSRNLPKH
ALKQQPEKPRLSNKCVSGSGKEKLSFHSFPRKQRRKSASTSTVMFPAPEEEGVMLLNEGA
PEAPPPHPSDTVPRRKDSAREAYPLVSPVRDQEKSREPNFPHLAMESRPSPGCTLTARVS
MTDVRLHSLSVSDVSIPTPPLRLPKIYPVPSGKSVAEESVAKGASTSHLAKRPSERKRRL
RMKQNNNKSSLVFLHLEEDTYGEIRDTMKDEFPSAGRGFLDSNFRPPFQGSERSARRLRT
ISQHRPSSIEQLDQGDLLSVQDDVTASPTKTRRRRLCSFGFSKAFPSDRREARELERERR
LISEVLLNAQQYLSRSVTAEEIECDSAAMALVFKYDEISQAFAMRRAAIVDAFLKEIFSS
AVQRVIDMSKEPNLPGLVHLPPSTRQQRRQKIVRGAETLGGVSKEEMDSGAFPVTNPRCV
PQPWAHISNEELAQDRRLYEMLNARPKESGENIVHYLREWSERKEAENEEIFGKYPFLSP
LPHGVPLSELRLYEDAEFQREAAEYEMRREDVKLETVLRGIVDRLAKQQAVLRGRENPQH
LSLLERLRAACGDASPSAAIRTDARVELEVVAPYKRSTFREESLRLQFIAQCKRDSVCLL
RRLMVRMKNERTADIPLSIEDIETFRFFFDGVDCGNFGVLDRGDFVDFVMLTICDELSMS
RREVENLAFPTAQKEPLPCVVDFSDFSMFYKSLALREVKRQDSRFCEETGPSPVRGRYLP
PAKEPQKILGAVSKISRGVKMSVNTTALSVILPSLCSQSTCVDTAGGMPLQRRPLTSARR
ELEPQKEMSLPVILPTVRGNMTQGTQAWVKNSCFSLTEGISDTQKSCLAGREWKNEKEKT
K*
>TCRU_1453 retrotransposon hot spot protein, RHS3, putative
MSGRPESVQGGNVEIRVPTVPQGDCRKRTRPESYTDTYHPAATCGGVGKRQQTKWTLDSR
VEDVLLGGKDRITSMGLNEFLCNFLGGRGVVDANEDVSMEMFLTNPMLFSNGETLSCTLA
EFPLCRAMKMLLEAAYTLKGEGVFLLAQWRDFEGKDTVTPIAKGALDAALLLWREEAERE
AEKRARRKAKERTRQKQQIKITLSTNIEDVLLRGRVRAKNMKLNDFLTVELGGRGVVDAN
RNVLLEEFVRDPERYIGDEGLLNEIKTLYNYVRMEIAVRKEMTFCEDLRSLCDKGVDNLL
KWSEAAAEVKASVNGITKHTLDAALVDVRNPKTTSAPIYLEGLYESVYNARWHHVVEVPG
GEGTGLEVREGEPAQPWTYKAVGDTFENNNGVEQSGAAPSRLMVLTSDKGWPYSWKWEEH
KFIHDCYVNSEVDRVWQIVKGDLTEWFSSHGRTEFTPKKRVLIGTPGIGKSMAASSYLLY
QLLHYDIKKLQVVVHCFGATAYVFNKTIQTVTKCEGNEISKIVLYGLWQRGIKGYIIYDV
TWQETPPASYFASFREWGMIVVSSPNLDNYDGWATQVRAERIIMNCPDEMDVKAMCAWMK
RDETKKKQAECWKMVEERMEKVGPLPQHIFRAEDFKARFGAVENALEAINSRYADSRFIL
PGRGLWYSKDPSEKLVRIVRIRGEVGLERFRNAPICYFLGSRSANILAKAMSEKGFLLLV

LGSRKTISSACTDRFCLHALILDGFVSAMAEELKELRPPAHDIQDTVMKANPVAHPTEIC
EIPGVEEINEKQNINYQVLYVPVARDFPLVDCFFFVESPRRTLVGLRMTTAGEHHTTTST
VRQFTQYLSEFFNGWGEFAQGLSWEIIYVQHADSTPMNDWQRCDVVDPPSVGDVDHERIA
EFWETTHQYQFALTECFLRRML*
>TCRU_1454 hypothetical protein, conserved
MLSDPVNRLEELQAELLSRQNALSSRIQSILSETGEKELPELQVLSEDRVQLIKEIQHTK
DEGDALLLQVEGNGEAASMYRVAARVQRFEEFANRANEYVKNVLPEEIERVADADRHHSD
DEVAAYIKELQAKREEELRKIGMLKERTTKRANDLRNGPRVENGELNALCRVTRAKEEEL
DKETKETQEYVDEARLKKADLIAKIKELRQAKSKIQVELLDTKHKNEKKVNEMKARIRHA
ELANKRDIRVCQQLNTTNAALTTNAQTLLGQLNVEHYGIEGAPSEKALITMRKEEERRAA
AALANGRGNNNNGSHPAGNETDGLCPRRDTGNFNGDASAPSSRRQSNQQNPALAVMQRTA
SQRSRDASQKADLDQRRQSGQLSQASRHSATSKDHQAGRTNSRGSSAH*
>TCRU_1455 diphthamide biosynthesis enzyme Dph1/Dph2 domain
containing protein, putative
MFHDSTITPVVTVRRRQKQRPAVRSHYKLDEVAAFILAGASSSPASRTPFQRIALQFPDD
LLEDAIAVTETLKALLAEDPRRADVVGDSARGVEGDLMKTEGVKIDDKKIRLFVVADNTF
GSCCPDEITAQHYGSDCIIHFGDACMSRSTRIPVFYVQDDFRFAAFSTYPQEKERVISVR
VVIRAVKLLRCRLNKLCRVLEENDISAAVSLIVLGSHRSRSLVSASACHWDDKERTAAST
DNKVLVEWCSFDHVQAAPDRSNASFSSFTEGSSWVLNGVRFSRAGSGMLQYFLFVGGSNA
PLLIHLLNVHQYNLFHYPEELRETAFTSDNPHVLTILDEDFGRGSDAGTLGDAESKDGHN
DDDDSVRRMDAALEAHFDRNKGKLIAIQTTLNKRIRQRAFNLELIRCSSAVGIIVASLAI
EGYYETTSLLHQLLRAHGKRAYIIYIGHLNKYKIANFVDTVDCFVSIACPNSREGHFPSK
EDDFPKTVVSPIEVLVALRTEDGDSPLFGHPAFYNTTLDTVLPLLREAMHAVECERAERK
TAVIAVPMGSGSGASLIRASAGVVATQGANGALHRLYERSFVGLDPKKGQTPVQEGILEG
RHGIARGYASEREQQEGNLY*
>TCRU_1456 hypothetical protein, conserved
MDLGQTQRPRSSPGTFNDFEPRRKCAFCCGVRCAAVAGAAVFAITFPCNLVVLGGRMCEG
RGMKGFLDRHLLLPLPVFLIIFTHQTIQSEALWSKNKKTLWKLNWQSAGLNVFLWTFMTL
IGTIISRRYATHWSRQYRLLMWEYQRKRRSCGNKYMPTVLGKITEDLDWYNILWTLSLYH
IVWGMITIMEEREIGAHYAMFYRDCPYSRWCSPRWREWRELEVLKHVNKEHLVGSSRWGS
FLLNDRWRSSGI*
>TCRU_1457 SNARE protein, putative
MDTSESGTSAEKQLRQAKSNVAVLSRLVAEAEKGGSIPYEDVQERMESARVALLALSEGR
LDVNPSNGAVTSQAVWEMSNVHARAAGTQAVTDLQRRVAQQLLAELNLIDTSLQRSRRKA
LQRQTHISEVEKLLGTMRSGGGQDDLSSLKHLENERKSLHYARARVRAMLDESNSVMKAL
RDQGGRLEGTGSKVADVLESLGVANSTILQIMRRNKVDAWLVYGGILLTMLLLYFVW*
>TCRU_1458 hypothetical protein, conserved
MNQSQATLSAVTDGRQREKDAVSDPPHPLEGETETPTENYDPNHQQSYYYYEPSDGNYYY
YSPQDGIANEAVQNSGEIPVTAENLIRSYEFEDWEPQSYNWFADIVLGIFMSLEFRLYFF
FLLYCAGSLLFVLSLNWTFEVLFRMHVSSSGTQFDPSFLLGFFIMFLFLSFTLVTFFCTW
MDMLRGLKQVGREDILFWGMSHYSGKRPPYVIYFVIVIATTLLPFLWGVIASASAGQSIL
FFAQLYSFISIIVSMFLILLCYVWFYWCALKCKCMAFKRRMERDEFILWEKASKHNGLRT
VTKRWYHASTVLEEFGLDGESLRWNAIVFTFGYVPLLSLYMAQALRSYIGNSQVTWGAIF
SITLTCVYVVSWMTLLRRKAQWYVYVSLFLVSILVLVGVVCGGLLASKELVLVIIVLFIT
SHGMLARKRPHKLTRKEQCALFQLPLDPEIQHDAQRRSFDTHLLCCRDAILGCLKCFDVK
TFFGYRHPDIVRAERQYASYNVSILTDQKVLLYWWLIVMFSLAFVIALGNAMVYRFIGNI
AMKSNPIVGENSTLLLCNIRYNKNGSAPLGIYDLSLLAALSYTVAGYGDADFVSWFSQYP
ELIRRYPVRLQPNWDYATSGVTIPFSDYTDLQSDYHFVTLNSNNRGLSLMRNVDEWGTSM
AVQLAGAISPFISIWPEAYRAKFVYGADFLSRWFPSIDALKDVSEYIDGLIADGKKDRIL
LIGDQFNGGYAKILSSRLGLPFVAFNPPGVKYKLPSVVEGVQIFFTHGILSYVDSLEDTL
TTLCLECDADYSSIRCSRIETTIEVISKLCGDPYGRRMK*
>TCRU_1459 serine/threonine-protein kinase, putative
MRLEASRQDARSPSCGSPLYNRTYSHQGLKSPLATPSLQHRVESRGSPTSHHYMPPPPIT
SKAGRVNTVDLDLVTQGSSPVPNLNNAIPCSPSPLAEARYPCLGEPWRPASAVGTRPYGE

LDPIKSCSSPQRAASAVIAPPRTKATYHDGCGIIVEERDSLTREVISRYRCGSLLGTGGF
AQVYEFKDLKTNVQYAGKIIDKRNLTRRGSESKLLMEIDIHRRVKHPNIVQFVKAFQDES
YHYIILEQCSKKSLMDLSKERGVFATEEIQYIMIQIVSAVEYMHRNLIIHRDLKLGNIMI
DAYGNMKIGDFGFASELASASEKKNTTCGTPNYIAPEVLACDKTGLGYGLEADIWSLGVI
LYALAFGTPPFETKDITATYNRIRRVYYIFPRGIAVPESCKELIRWMLQKQPQHRPTPAE
ILRHSFLCIPQPPRTAPRSLVPPQAPRSTSPNARGSPLWASPIAINSPFIDCDRFPVDYQ
KTSLRKEENKKGTEHELQMTLHEFLKENDCISETTSEVRHLRSCGKPVLPKPPLPSIVLK
SSVFCNKYGYGFLSYQCGKQYPVVFLNDKTKLIYDIDSDTVFYYGRARMQAVAPGSGNRS
PLLSEELAAKGFRDELVVFPAASTRLAHRNEGEDATSLFEASAAKKLAITKFFLPFLERG
TRDKRMVAMTCALREWCSSWEKDLFVACGSGDEQVDIVYVKDAVLETLYELTGEHRDTDV
QLLVARMSDYSFQVSVRCNGAPQASFRPKYNVLEASENSLPWCLDVLVYAGFRAVMAMET
AQSSLAFSFWDIRRDAKTVKDGRVYFAARSTTQLKSMMLPTALLRTIAILLRKVRCCNDI
VDCFC*
>TCRU_1460 hypothetical protein, conserved
MVEESFSIMQAEARAARLALSAFSAVLPRTMDVWEDNTSLRGAASKGSLKSHALTWELRR
IYEFLDSRGIQATFVYVRSAENPTDGISRGRVFALQDLAKGWNLRRGAAVSFGCRTPKSA
T*
>TCRU_1461 hypothetical protein, conserved
MVAPLMLDLMDFRRMMCNISVPIRLLFLVQNGREAMLSLCLQELERVHGWSGRLVVSRHP
ENIGYSAAANIGSRLALSLPREEVPFVFVTNSDVKFSPDLLPILLRDVHEMTRHDAARMD
ELAAEAANGPSESSPVLRRGLRVLRSTVDDDRLSTSALLPDRIRYASVREREKAFSKHYG
HFCA*
>TCRU_1462 protein phosphatase 2C, putative
MKGDVNGEEGGITSVPPSQPSSGVTQSLWRMFEYGVTSEQGTRKTMEDQHAMVPEAIPFF
AVYDGHGGTQCAEFLRDNLHTLILSHPDVMTNPEKALRDGIANAEAEFLKKCANGKCESG
STCAVAIIVDDTLVTGNVGDTEIVLCRAGSPLVLSTKHSLQSNGAENERVKACGGRIISN
RVGHPKFNPQVLSLGITRAIGDAGFKLDEYTDGKPSGIIAEAETRTTKLTDDDEFFVIGC
DGLWDVMSYDRVVQFCSRLAAEGVPPQDITDRLCQEALRQGSTDNVTCIYINIKAKRPNS
SDWRPQSAASTSQKNA*
>TCRU_1463 hypothetical protein, conserved
MVCVRCRCWAAAICDCAVAVRGVAAVSLLLLPLCVDGELVCAEGCRQVTGVMAMMMTGRV
LLVCALCVLWCGAGGADASNLGNNAVGGCMASWVLNANKSYTPSGCDKTALTLPLRSVLS
ITAVAASTDPLVTGAAENKSDLAPSSGADFGGAGSPPGRGGEGGGGRDDGAGGPGGVGGA
SGSQARGSKASGGGGGGSNGGSKARVGGSLKSPASDVKSPSPVLGDGGSSPGAPTGSVGS
DGSKGFILGSFSSGGRSQPDVGSPGGDASLSAPVAGGNSPPSITDESDALEKTGAAASTK
GGKPQEAGEQSGANRARNELPELSAPAESTPEAQEDSPSKQEKPESEAAGRAATGEEKRR
SDAEAQGPCTVMSTASTSNGRRRTTNSGGSSGRRKPNGTNRLANFDRRKDKNVTVTKQKG
A*
>TCRU_1464 Trans-sialidase of Trypanosoma hydrophobic C-terminal, putative
MVVDGDNTREQEGVPVTVRNVLLYNRPLTSQEIGSLKPNKAPISPSEDLTAAMVVDTPST
AVGGPVAQKTVSVSTPGGSTVNHESSASSGEDEGTVGETNAQKEEVHPDAGEVNATALNS
SPGNFSQVNNSDGGTMRGSGLLPSLLLLLLGLWGYAAL*
>TCRU_of Trypanosoma cruzi region 5/Dispersed gene family protein.1 Dispersed gene family protein.1
MLPAAASVLSAITGVVAGAAAVAVVVTGGLGSILEMQALGVFARMSCTSAQERASTVALP
YFLSVFAALDPLWMVVGNALLAAVFGCVHCGVTAAFQRWRGVDAASAWAAMRFPSLTYVV
AHAMHLGIFFGSVLALAMPDARVQHRVIGVVGVLYGAAFPAGVCYFIARHVGASFTRYWQ
FSRKPLHERLLYPVGYWHPAAQQRMYGGMLTNMRGSRVYWCVFQLLVLCVVCLIAAVHPP
VGGCHVQYFCMAAVLVAGAGAVAFTNMMRSAFLTVMHTAGFALLALLCLVSAANHLAPSD
GGARAYAAIVLLLTTVLLAVAVYSVVVWYAEDRHWQELREPRRGGLEALLRDDEESGDET
QKPHEMTSSSYASGTTVASSYRPPAPPRPVAGDIRSDALSLFDRASSASCSINYVPLDR*
>TCRU_1466 Leucine Rich repeat, putative
MHFVSTSAAAWQQQQKLQHNIDFNAVLAYCCRPAPYALACVSPWFRRRMEQLNWIGLVEK

IITANYPELLDNTVAKLILGDGNVFREGVAVPRRHLHRMAPGHDHHCAMLLDQLWHKGIL
DSHDTEAVLYLKGTNNNVLVVEDMSSTCHAFISHDAPPPTEDLLYWLSRPQHDKNWWSDT
RNRMPLAVDEMARDTTESSHLLSAQPDNVRVFSRARGLALHITERHLAWLATSLVGELPQ
LQELYLSLDEGRMPPPREMWWWIKCMLAGIPALRVLCLLPRPALLLGQSRKRFDDDDVLA
PYVEEVDCIKTDMRVLGRLQKIHFQTGEQNILSSENVKNYKEGRGPSDTESSFVPPPGAA
VYNENSKLITPTENSFNVSHLLDKTDMEGLEQLSSLEVLYICPSAMESLPFLAHQPSRMK
ELYLLSNPRLETSGIYGLESLPQLEVLWIEGTSIRQLSALNVSTSLRELHVALDSAFSAS
SLQGIEQIPTLEVLHLERVTVDALSFVGKCLSLRELILHACRIYSGNALQGVEKAHLEHV
SLAHSSGIREVKFLSECKTLRVLLLTRCNGISTASIMGLETLPHLEFLELEFTRVDSTSL
LAESPSLRHLRLNNCKRVLRGSIRNLDRCATLQYLSLQDTNVINVEAIYGSCSLVELDLS
RCKHLEQEGVEGVVRISTLKILRLSYTPIISVAFLRTSISLEELYIDNCANITNEGLYGI
EEIPTLRVLSMVNCQASDIGFFGSCPCLEVLHIASMERLRTPGINNIGDCSRLCHLNMAF
CGVDSVSCLANGCPNLRYLNLRGCQRLTTEGLQGLEKLPTLTDLVLDDLDLVCDINSLAE
STSLQRLSAARCTSLTLYGVRDLLLRKPPISLRL*
>TCRU_1467 hypothetical protein, conserved
MEEREEVTATSFRVMVSEAEPYGFQTCAAQQLQSSVRITHITFTPPSSDELEAYMQHKDK
LASATPPSKTDTSICPATFARLRTLCASGELESHTVACFPWSQHDETTTALMATHGNDES
NKNKSINSKNKKGESHLLQSTTTAAAHPLEQVQLAGLFDVVLPRQTDICSNVDLRFDVDG
SVWLEVVGPGSVTFFGEQNSSLIPEWLSHRFFTVSDDDEETGGEEEEEEEEEEEEEEGDI
AAMYGLVH*
>TCRU_1468 HIRA-interacting protein 5, putative
MWDVACWVPRRAFLIRFQETPNEACYKFFVNDVAFLPAGHAGTLRFDLDNCFQSPLAEKI
LHGLPMVEEVTIGPHFVTVRRVDDADADAAARYFAHKMGSRTDTPKEAARRSAALQQRVV
DAMLEGDADGVQPPPTSSSSSNDHGEQCVTNSNGESSLHREDASQLKREKLSEKETEEEV
GTGEHVDEATLRDLLLSTHWSELKLHVSALLTDHLFSGRPHVDPDAPHPHPDTLPQEGDS
EVVLILKELISTTIRPQLQLDGGDIRFVSLEGGVMYVEMLGACRRCKSSKTTLSDLIERT
TRHWVPEVHEVREAEQRKPPHNNFPRRGETKCINQEKKETETRTTVACDAE*
>TCRU_1469 hypothetical protein, conserved
MPASFVSFGAKRSRDEGGENDNLRTPQFSASSSSHAAIIEITHGMSRSAAAEYDKQSVGP
TVPWKDTDALLNSPSFREKTCRFLTLRADGVRPDGPVEMGVIQLLANDGKTLLFTSDAIK
PVNISTADGKRVCIGDLVLFQRFTSDAQEGNGNTFVPAMVEFVALDSSALTDEADWSHAV
SWNAVTMANWMHQLLSVPPGLAQLTCIMDSFWGDVGASVTVADAWLRSLQLPVFIQQLKN
LDGVELRKIQQVKKLVFSMLRLYVAILGNAEAIITLSSTVEGVVYQFLGSPLFVEGLSSV
LPQLAGNVSESTWASAQQAMLEAMKFALRYVCREVVEEEGKVQREAREHAERGFLTVLHR
ISALWLTHEKRAPQPGIQAVMKLVEEMARIGVFRRAAQWMRHKERLAETVSLVDKVHRPP
TLKDIKDLHGLKLSSLREMMMAMNQTYTPYLRQETLRFVECAMLAAYAELYGPVACAVSS
YMESVDKTRAGDGDQGSMMKPLHVTQVSQSSLRLELSEYSLEEVRLHALFGLPLFVTLRV
TSEMSVSSRYLLETHSCFGFILVMLNGKTFVFPVVLCGVVDKTEKESAIISIAYVLRDRD
ILPSLVAASAASSSEHQGIPEAWLFFDSCHVEKESGDDDDDDDALPVDHLGSKTPRFLPM
GTSAVSYFLYILHLQKLRAQMRQEELNSLTAVGRAKGGQGKGDLANKGGVLWWGSDGSGV
AGDMFTQTSSLTGENEMLQRCLSEIAAATLLTASQVECLHTLTTAGPGLRTLVGAVGCGK
TTLMHASSLSRGRMQEALRKHHQPLWHATIKHSAQTLYTRLGELAAAEEMEDLFKASDGS
SARDLPEEVTNAALGHIQEHVHLSEKLRHCHTPMLLRPRAFKQTIPFHSARVLQEENIAQ
HFCSIETLMKQSHQRSLNAPLAAVLNARLKRLKRLGDLLDTIALSLEEIVVPEEGEWKLF
IEFLPWIMSKKERMDTFTEALRKGKTWEAYLSEDLWDASTLVRSTDFDYTPQKTVWKSMG
PAGSAFHAVTLPNASRSLCEYSQAEAAGWRAVFWEKQRDLLSFLQQEIMDEGRHIFLLFL
DLIQAVAATSQTSKATVVTATGGTLLREISFLRKWQPHYLVVDDYDQICDSLYLTLSSVT
SAIVSHQAESSFQHEQRLPVVILRALQRELQGTPLLSTVVLSESFRSTNPDIHKAAQLCR
NASVAFSTALPLGVAGIKEGANNFVVVEGAVGGGVGNDNGDDQTMRKHAAIAGFSSPCSV
EFWTHAVALSAAVACDGVAAAFVRNRLREIEASMTVTVYCGFVKDKAVILEGMMSGSDVD
ATEIMGPVCTIPFDSEAFVERDEECDVAVLCLSGIARRLRRFSSVGRHQAWERLGAERSF
VEQLEWWLWRTVSYVRRGVILVGSREVFSFIEPLHRLERFVLQMREKRQFWLPPEAKGAA
LALRCPDHENFRQLALIAYSDYGGCQVRVMGEKTCRSLCLAPYENCTNPSHACLRICHVS
GRESGALAMEDDEDPSHSNCPFPCMRVLPCGHMCVRRCGELCLPCEFVGLQELSCGQRVV

SGISDVGPVQTLVHHFQRISCGESPRLCEERVTLRCSRCGVKNPMLCHEVVARGGMERAN
LTELECAGCVALYNRVAREHEAAEIASTVPPTAGDLPLKSLPKAAQTRLQELQSITAKKA
QLMLQKEALETMQGRQNSEFYQQQEIYNRMQKQQEEEVRAHRQRVQENIQKWKKILKQKY
AVQMTVNEAMDTEVPLMISEAIAEEEKQRSHLFV*
>TCRU_1470 Ubiquitin-like modifier-activating enzyme ATG7, putative
MTSTATTPAGSRLKFKEYSPHIEVSFWYELERRKLHEWRLQEPVVPLTLFGTVNSAYTAT
PANIVSARRESLGNDTDAIVAGLPQNPLVGVAAATDTNAPFPIPVLLSGELQNFNTLEQL
CKLPRREAIWRVLKEKLLIPLFSGKGSKTIDVNEDRKAAEILVDKVWEGINFALAALFTY
ADLKSHRFHYMMAFPVLDLGSPVDVKHRVNGGYAALATDHGVSYFSSRKAVDRIHAHLLD
QLRRHPERGPNPFIVTCQASGHGENEEVIFYPFTFSTINKMKELSFLVVMADVSTMEEFP
GWPARNVIGALRLARPSITAFALYCIRHNEVERSVVFECTCDPLSYTFEDVMTEGTEGKQ
NAFARAVGWTERKSADSPVSCIDLGAMMDPERLAESSARLNLSLMKWRMLPELSLDGLAS
CRALVLGSGTLGCNVARHLLMWGVTKITLVDRGNVSFSNPVRQTLFEMSDVINPRLEERN
KAVAAAKALKRILPTVEAHGVPLTIHMPGHRIDKQRESEVIAEVERLDTLIQEHDVVFLL
TDSRESRWLPTLMATTHNKPLINAALGFDTYVVMRHGLEPKEGSGGSRLGCYFCSDVVAP
RDSMTARSLDQQCTVTRPGLSAIASATAVELLAQLYNHPLGFACPPYTETEMQEVHLQQP
GDSKATNPTDTNGAVCVLGKIPHQIRGSVLTHYIYTLYGYRYESCTACSDSVVGAYRRER
QSFVLRCVNDPLYIEEVCGVKAFKESFNLEDLDEWDVDSHSD*
>TCRU_1471 tRNA-splicing ligase RtcB, putative
MRRLARERLFIVAAATVKQRCGCADASGQGLLPYDVLRGEGSNVEVRAWIKGVEVERSAH
EQLIQLSRMGEIIRHPIAVMPDVHTGHGATVGTVIPTTRALIPASVGVDIGCGMIAVKTT
LTPEDLPGSLVGLRLAIEIAVPHGRTHNGRSGLDAGSWRGELPKNVVDIWNLHLRKGFEE
ICAMQKSIEASNHISHLGTLGGGNHFIEICIDDDIKNDAQRPHVWVMLHSGSRGVGNRIG
TVFIELAKKDMGNHITQLPSAELAYLREGSPHFDQYVKAVDWAQRYAKWNREIMLQNVLQ
AMRSYLPQPFGVDATAINCHHNYIERVELSPQNSVWLTRKGATSARGGEMAVIPGSMGTK
SYIVRGKGSPLSYSSCSHGAGRLYSRGETKRRFTLEDHEAATADVECRKDTDVLDETPLA
YKKIDDVMRAQGDLVEVVCALKQILCVKG*
>TCRU_1472 hypothetical protein, conserved
MGQLLSFNATHEEVPEDVKEKVEPIEYTPASEENRPQDDRFCANCGEPYFYYFTPSTNCN
WCGGQFCTRCCPNRHLLGGKPGCPECTRRAYLIKRKELLDDHLASLRKKGERPAEVNFAL
EC*
>TCRU_1473 hypothetical protein
MGTIVALHRTDEAEAAVFVGVVPIGRKELEKLPHPTSCPDCGRPFDERTRSRRCRICGEA
LCKQCCRRCRNWKRRPLCDYCMDSCLHDYYRRINGESFAAAHQHEHHLRQRVKKKTASGG
FLRGFFLAALDGEDFLHASKPQGRVERNISLGT*
>TCRU_1474 hypothetical protein, conserved
MLVAGSGGAYGYTLSVASMRDGCAVVLGITTKIILAISSEAGEEVLRVNDGQVVRGVALL
RAPNGAICVAAGGDGKRINVYRANTMKTLLAPDARANDVGAGEMKLLFSFGPHTKRITHI
ATCDGNSILFADKFGEVFRLVIVWGVSDSLTMAQGDAAAPIFLLQHFSVMSALFLSAPIR
QGTPDMKETRRLFTCDKDCHVRVSCYPETFRIEQFLWTESPQSVVTSIAEVTDSSEKKGY
SYFVTGNYEGKVHLWAADNTLTNVLPTEPFTIVGSICAPEQVDMDEEGAAAVVSVVYVTS
SSHGGFDRHAARGFLHGVLIAYANAKDVVFVPLMESVDDHRLLFALEGITRTRLDSPLLA
MVGLATNSALVLGRNGRVHVLQIVGEAGEAVPVSLVESRQVAIETAIGEVERENGGNLFE
KMDIFSQWHYDAVDPRTRKTVGTRNDSEEDEDEKNDDDNKDGGEDVTHNTAVGEKLIGNK
RKRTECRSDA*
>TCRU_1475 procyclic form surface phosphoprotein
MCLFGIVDAVEDFFKSVVACACCLIIGGPALIIAGSILLNQKDLRKAFRDAVNEFDPTPM
NDWTGTINGVPITVRRESLNVKGVDGATSVFAEAVIPASQRSSSRLQVSVNVRTVTPFIR
MAPFNIMRNSTYFCSSSDCKDEKDCQCKRDVRSFGEKCTARGGRYNQAPTWCKAGNICGE
CLETLYLRKLYLVVREVSKGKYAEDTNLRSAMYAFGDLDNDYQPGVPSTVTVRLYSSKDP
YIALQRLTKGTNDLGLNPRTVGIVLIVLGCLFLLLEIGVCTALICYWMRRKKTSSGAPPY
LAPASQGISTPGASGNAAPNFYAQPAQQTGQGYTYGQAVPPGYTYGQPPQGQPIPPGYTY
GQPPQGQPAPPGYTYGQPPQGQPAPPGYTYGQPPQGYAYGP*
>TCRU_1476 hypothetical protein, conserved

MLAFSIISDELRDESSIPTPADLGTSTPPSVASIHERWLCSARASSLTLRFSRTSSSSSV
IEWSIGSSTPMVTRRAEAANQGGMELRKNRSSRGELSVMP*
>TCRU_1477 hypothetical protein, conserved
MSRNIEMDPNAFETNNAPATALTKAVGSKDARKIEAVDGSGERVPGKISGGMNGHNVPGT
PKTHQEM*
>TCRU_1478 hypothetical protein, conserved
MLQGKKASVHIDGRSLGEEEVLLTGQRPPEVLRVRFDACGGHESHVTVTNVFPYSRPLNS
TAMRAPKDSVPAPTREAAPTPPEKIRVVGTVREHVAGNIGEGK*
>TCRU_1479 BNR repeat-like domain containing protein, putative
MGTSWTEAIGTLSAVWVNARSGVSQKESLRVDALITATIEEGRKVMPCTQRGHASGKGRA
TALCLWVTDSNRTFSVGPVAVDNAADWMLASTLLHSDGNLHLLQGRGSGEGRVISLSRLT
EELSAIKSVLSTWVQKDIFFSSLSTPTACLVAVLSDAASDDTWDDEYLCLHAMVEERSEG
QR*
>TCRU_1480 hypothetical protein, conserved
MNVTTHKCAGPYELWWRQFSPDGTIHPPARSGDADRTGIRQLPPPREGKDDTKSGGTTAP
PVNAEPSRQNEDWRQSAAADAPNTVDPSPASNTATNISETKPEFAFVRNTKVDPTPPERR
PTLVTEPKEDPVDDAMSGSINVELPRGVDLFVPRTTLV*
>TCRU_1481 Reverse transcriptase (RNA-dependent DNA polymerase)/
RNase H, putative
MLTFTLHQAFKDTIPSAPPSAPKFFYSWGKCKWDLFIKDFDAQLPAYDYKKQSTGIKAFT
RALITSYRRHCPRGMHKDGPRLWDDTLMEAERIATDSKARYLQLPTPDREAEMQRTRSQF
FLLLRERLRNTYLRRISKLNPGEPLAWKYISGRKRASLPSPTSMLLGDGQHTYKTARRAA
NALNRIFLPFHPSHKAVRFSKGINRQSASLNINASFLFGQQNNSESAASFTSFSSISSTS
EPQNNSESAATFTSGSSTSSSSEPQDKNEAATKSGLVAHLHSPLDAPFNRTELLAALRNT
PYGKAPGPDEVYSEALRHISSKGLRFLLRCINHSWTTGTIPVEWRRATIVPLLKPGKSPE
LLESYRPISLTSIVSKVAEKMVLKRLLWVWTPHPHQYAYRSMRSTTMQLAHLIHEVEHNR
NHYFQVSLPKKSGIGNQLHYRPHRTLLVLVVFSKAFDSIDHRVLSRLLANIPGVNCRRWL
RNFLCGRYAKTRVGHRHSDRRPMLRGVPQGSVLGPYLFSLYVHPLLNLLNSFAGVTADMY
ADDLSIIVKGQSREDAIPTANMVLEKLHAWSQENGLAINPSKCEAAWFALSTHTESDYDR
EGRWPLAVAGCQIPVMTMGASRTTKLLGMDLDPRLTLNVAATKQCAATSQRISQLRCIAH
KEAGPSPHDLRTFVIGYGASKLRYGSELIWAVATDAARNEMQKTYATLARIVSGVPSTVD
PESALLEANMPPLHVLCLRARLSIFENTRACQMDWMRRPPPEPPPRAGFRISPLSRDELY
AFVDAYTKDYGITESSPREERFFRSSIPPWFAASAHRVTIGVELPIDLSITDEEELIREK
RRVSEEALALHSHRSWILATDGGVDVPKSAGVGILLSSLHSSKIIEKVSTNGGARPCSRT
TESRALHLALEKLMIPRIRHRRKTLLVVTDSQSLLAAVTKGPLSHTHWTEDRIWQRLLSL
THAGWSVQLQFCYGHCGVHANEPADQHATQTMATGQHTEHGIAHIWHTDMLTCINTQLTN
KWRSTIRQDTHRYLLGGARPSDPGGKDLITQEPLDQQERIQTARKGCGESELWGRLYWTV
RDCTNQRRFRNISPEHSALMRSNDDPTAPGRTLFPRRQGRPTHRLWRDGPPHSVGRRNAR
TVTPHWWDTRTTSVTVGAFIRNTLHRFPTSTAIATTQSSPHKKGTAQHRNRCAQNPDTTR
HRSSSARRQSLLPQDQPASTSATISPQKTLHHLLPECTDALAVRQRLGIKKDLRTESFSQ
WQLPHSRKLMSLLDHLLGTLMTSCG*
>TCRU_1482 hypothetical protein, conserved
MAISEKKCLFIRVALYLLFIAAYAAGVVVFIISFSNYWAAAGFFLACIFIAITTVLYVVP
DKRLTARLPPGSLSRIIFQSTILIGTLGLFAVALYLLAQGIMLKQRWTGSSYFCSFIAFM
MAAKWCYFIGYRVHGLRNDTRTIGKADPEEIKKFFEEVPDAWSNEKNADIM*
>TCRU_1483 Torsin, putative
MGPCTRSVCGLALLVFFVAALSSRVAYIELDLGEADDCSPRPLCSSHPTLVKRLLCFFFC
YPVNVDGSDYKKHVLDRLRRLTQRNLKGQPHVVEGVIRSIAAKLENPDKPLVLHFAGDNG
VGKTTLAQIISLSLGLRCHDTACETGDSTLVLSGVSYDGYSVQEFRRVVIPKVVQHIIRF
PKNGVIIINDLGALHPDLVRVLLPLLGRATSFPEAPGVFLSRLFVIVTTDFGRQGRTQGK
SLTEMRRIVEDDFKGLYSQLSSSMIETFPFLSAALDTAKEIVWLTIMNYKCRHREAIREL
LVSEDAILWLVDFVRDDLPMENGRCVANAVSALVGPGILRHLLENPSTPVTLRVDINDNG
SVTVLPVRTD*
>TCRU_1484 60S ribosomal protein L12, putative

MPPKFDPNQELIVIVRAVGGEVPATASLAPKVGPLGLNAKKIGEDIAKSTKDWKGLKVTC
ELRVKNRVATVRVTPSVASRLIRALKEPPRDRKKVKNIKHSGNIPFSEILKIAKEAAPNS
MGANMKSVVMEVLGTALSVGCTIDGENPREIQDRVRNGKLKVPN*
>TCRU_1485 hypothetical protein, conserved
MHGRNGELGAVCHVVNAVHTCSNCDASCARKEEGATVAFTVNVTTHKYAGPYELWWRQFP
PDGTIHPPARSSDADRTGISQLPPPREEKDETKSGGTTAPQVNAEPSRQHEDWRRYAAAD
APNTVDRSPASNTATNTSETRPEFATNPSEVMVPNASKKRPSFAKGPRILVALNASEIMS
VTCGGREHVAADNTDEVSERSATGHKTPFFRKWRCGQWICC*
>TCRU_1486 UEV domain/Zinc finger, C3HC4 type (RING finger), putative
MNEIITRFFSSSSHCKPEVLGRILHDVENLCNTFHFYCRDATWGVTQQEKLCIYGGLPIT
IKKSNSDHTSLTPDSREQAPPHRYVLPLQIWLTHLYPIEPPLVFLLSAEQGCRIASNHKY
VDATGRCHTPELAAWHPVSSSLCDVVKKLCQLLSAEGSIPLCFGEEGVSSPSKGHATAVS
QRLRHGLENGDDDKQTDEHERCVVCFSPKETVLVPCGHYCLCDACATNVTHCPLCRGSVK
FRQRVFS*
>TCRU_1487 Mucin-like glycoprotein, putative
MMTCRLLCTLLVLALCCCPCVCGTASGEAQVSTTGHPPPLPQGQTRETMSSGSKAPTPPK
ANTSDEVDLPVTVDAEGGSDVSSTTQDGIILPGKPGSDGGIKSQPGLIDNPDQSVVKSQA
QEAGESKGNKDFTVKTKNTKNTTPITTTTTTTTTTAAPEAPSTTTTEAPTTTTTHAASR
LREIDGSPSSSAWVSTPPVLAAFALAYTTLG*
>TCRU_1488 syntaxin binding protein 1, putative
MPRPVRLRLLLLLVAASSTDELTEAKKQQLIRYGGLTPDAHLFASLEHVTRRAGSVQQYS
TASAQSGRSTSASASNAATDEDTFLKHACMIMETGRKRKLRVELSLASMGRDHAMGPNGA
SGGQNSGAAGKNAGPFQGCGGKEENHPDLGGGGTIALTTPQRIVLLVLCGVACSERRSVH
EISKRYGREVVIGEPRCRVLRCVCAGTWHPTAV*
>TCRU_family, putative Se.1
MWGGLVRSRPTEAGQPREPSNGVCGCVRDYIFHQMLDAVRGLFKVFFCDAHAASALSSSL
RLHDLMEHGVTLLENPMTPRQPIMSSPAPYFLPWSDASVSRVVEDWTAKVPYRDVHIFAL
GCTPHRSLQQLQWARMAPRAMRSKDIMLDFAAPEVLVFHLSMQNGFFQLLLPLSPPN*
>TCRU_1490 hypothetical protein
MQSHEAARPRGGCPLDDDDGKKFVGLIDADETNTKVNLSSMWMDECEEGVDAAASKLTTD
RLLSRILLFSAMRVLKRNSAKGVQARIPCNTFFPSDAHCPDRGRSLKLNCVRGDSPLDID
EYELYDSSATPFLVFAPSVSFSAEKSECTAHVSTTFVSLIVDVDLPETPRFATPHYTPAV
VQWSSIQL*
>TCRU_1491 tubulin-specific chaperone, putative
MSVVRVSITHSSSGIRVPEKRYELSQTIASIKSNIFTHFATPPEQMQLQLIDDRGVKVES
SMADDKQLGYYQCRDDFVIHVVDLRPTQQIANFEDVSQVQKYEITEEAYSKREDNARAFR
QLMMARQREEAEKAGNPLPKELDADSYKETAEKIHVDDRCQCQPGDRLGTVRFVGRVASL
KPGYWIGVEFDEPVGKGDGSVKGTRVFECQPKYGGFLRPDQVTVGDFPPEEF*
>TCRU_1492 uracil-DNA glycosylase, putative
MVQRTLLDFVRKKDPSPNVINVDVESDEQDEAASRKRRMQGVREPALAFTKPRTENKDDI
CCSNNFSALITDSGWRAFLQPLTSTTNFRNIEQFIEGEKAKGKVILPPSDLIFSAFNSTP
LDRLKVVLLGQDPYHNIGQAHGLCFSVLPGVRPPPSLLNMYKELTTDISGFKSPPHGYLQ
CWAEQGVLMLNATLTVEAHQANSHAKCGWQAFTDGVIRLLSEKHQRPIVFLLWGGFARKK
ITLIDQKRHLVIENAHPSPLSAAKWWGSRPFSKCNAALTQLGHSPIDWSIPMSVERRH*
>TCRU_1493 hypothetical protein, conserved
MPPLTLEQVLARSMRLPRKHMKHTATPMASNPAIGISTPPDNSLGRGDKSVAKLFSLIEK
DEESTAAGRPFIHQSNNLIRNSHDLLTYLHTAKRMRAWEDALRSFAEVTKMLLINQTEEG
DGVSLTLREGAEGAEGVTASVSHLALLLDTCASAKQWHLVEQIGEIFRSQSRQTLIDAVA
VLANTKKETIGEGLYGWERALHFLQTRIPPEEQPVEAYNACIAACETALEWEGALAIVRS
MGPNPLQNLDSKVFSGTTGIGVASAGSTVGGVSSSLGTEAREESVLATFPPPAPDVVTYA
TLISVLEQSGKDALASEVLQRLPPVEKEEITASYAALIHVWSEQKYRNHRRRF*
>TCRU_1494 hypothetical protein
MVGSTGLSLDDDIVRFISCPSNSSCCSFAATESHVFFGHTSGEMTVCRLEHHSKGLETVA

SVTTLLPAELNGAKGENLRNKRTMSLAIDCLCVCPYASISGTLISSLHRGYFVTFVTFDA
MSSRSGDGGIKIGRSLELFADETSLQYTNRVTAHCWLDDDTFLVGCLNGTVLAVSASVND
SHRVMLREESPIIQMVWDAETSVLVISSVRRSIVVTYANGSMTFKPIGNKPREEGLFGAC
IWPINHAVYAARHNARVFEYLPSKGEVSRTYRMESDVALGVLKPLRLSSFLAGSSQGDEN
LDGGKEQVFLPILLWSEGPRFISFLLLSATDTHHITRRFDDVAIVSSVILSSIALVLCSD
GRLMYIPLGINPLCLQDDRVDNAREILARPESEQTHDAAITKKRVHRIKVVKRIVRVVRR
RELLGQRVSPELPGRVAPFSDCADSIESSSFTTDDKAHSCDSPVSLKRPLSLHRNNSFTD
EADAEGMAQNDDLGESRTLQEGSGHANNCLFSNLSDAVTTVPIVDGDIPFGESSTTNRMN
FVRKEHEMQEEKSVWKSKRFFLELDRIIRLFNVHALHLGEHEQLYTAVKEFLPLLEERMI
ECGLLTKSFHSVLSQVRVISCGANSPATCDSNREDTSSFIEEEATVNALRNVLKSALAWS
IKDKTGDKENPWVQTLFILYLLLAVPWPAPLFLPRRHATERYVGELESWRSRGIEEEWCL
EEASVIEKTVKRSGADGRYLPLVLALSTSFVSSMTKTQSGRNTRSRLLGVINGHEQCISL
VLQLQVDRRSLMESECPLPMEFHLEQLIVEQSPQIVDAMEASPYPFCFFYVYFPYIFATY
PSKAIVLSMRHYPGTSLAFFEWALSSSVQGAAFQQLRKMGSEVERLSLEDVADGLADLVL
ALFEASGPSICDSPADYLACLRILLQRRFALLWARIKMGQMRLKQMTCGADARETVQLHH
AEEGSSTILRTQRKLQRVERGVLGLLQLMQSVIITIGVGNSVQKEEFKQDVLSLLEEHDF
PEGVVELCWIREYIDYCIKKGCLERLLSLFQIPGATSQKEWAFLFKRAHEENKLEIAMLF
CLSVEKLPDRVQQLLENAFPKRFVSVEDAHDDEQLERVALKLSMLL*
>TCRU_1495 aconitase
MFRSTSASRTGNPQLNPFIKYVATMAADGGEAKYFKLHEIDPRYETLPFSIRVLLESAVR
NCDEFDITSKMVDNIFNWKETCHKNIEIPFKPARVVLQDFTGVPCVVDLAAMREATKRLG
GDLNKINPQIPVELVVDHSVQVDKAGTPTAVKENQDMEMQRNRERFEFLRWGSKAFDNLL
IVPPGSGIVHQVNLEYLARVVFNNKGLLYPDSVVGTDSHTTMVNGVGVVGWGVGGIEAEA
GMLGQSLSMVLPQVLGYRFTGKLAEGCTATDLVLTVAKNLRKFGVVGKFVEFYGPGVDNL
SLPDRATLANMAPEYGATTGFFPIDRETINYLRCTNRSVEQLERIEAYARAVKMFRTGDE
KIEYSHHLELDLSTVEPCVAGPKRPQDHVPLKNMKEDFAACLQAKSGFKGFGIPAKDVNK
TKNYMVDGQEAVMRHGSVVIAAITSCTNTSNPHVLVAAGLVAKKALEKGLKVPPGIKTSL
SPGSHVVTRYLEAAGLQSSLDALGFNTTGYGCMTCIGNSGDIHAEVSKCISENNFVAAAV
LSGNRNFEARIHPLTAANYLASPPLVVAYALSGRVDIDFNEEPIAKGVFLRDIWPRNEEV
QEIVSRYVTPELFKSVYSNITTINEQWNALQVNEGKLYEWQPNSTYIHHPPYFESMTMEP
TPNTVIKDAACLALFGDSITTDHISPAGTIAKDSPAAKFLQDHGVERKDFNTYGSRRGND
LVMVRGTFANTRLGNRLVGEGQTGPFTIYFPTNEKMYIFDAAMKYQQENIPLVIIAGKEY
GSGSSRDWAAKGPFMQGIKVVIAESFERIHRSNLVGMGIVPLQFKPGESAQSLGLTGKER
YSFDFSGGLRPGQETTVQKGDGSSFSTILRIDTEMEVKYVENGGILQYVLREKIRGAL*
>TCRU_1496 hypothetical protein
MDQSSEETHVENAGGNCVIVTTLMSSATDTSNRHHIPIFFQPDVQKAFDPGNRWPPLLPL
APRSNPSAHLENNYAGAFTKTDPCSSSCRVISCENGLSEKRQSENHWRSSSASVSLDPQA
LVPMDANMTSSSWAVKRTAQLGPLVVVKRSKKPNALEHITPLSEVEMMSLQREVDNLRIQ
LKRSNEKLEEARSEAGQQFLERTRELEESHREEERRRQQFQEALSGLRAENEELASRVR
CMQDNAGLAAVREREKLQTEWSNRLSENENQWRQRLAVAQQHWEECVDEQVQEKHEALRQ
VHELAGVVKELEMTLQQVTREKCWVEQELETLLSKQNQGKDRCVCAKGETIDVGTRGALE
GEAMELRRMLKELEGREAALLVQLEMCGKERVVQEEKLEKQLQQVRQELEIERRRGSDVV
KLYSSQIESLHQQLSDALRRNKQLTKELARFQ*
>TCRU_1497 tubulin cofactor C domain-containing protein RP2
MTDQVTEKLIRARLESSGELGKMWAMVVDAALRSISEDSKMLSNLFMPSPTLTAAKKATE
GKQALAIVMDYLEHLGLNYTLNVLKREAGLTENAFEDKGAIMRALNVPDTTGPILMTLIQ
GVCNEDGGDKPKVENTTMGSTTISPSSSKVTVVPKKADNGEDTKYFISNWTGKTFYRCGG
QVSGQQVQLEYLNNCTVYILDPLDSITVDDCENGELIIAACEGSVFLRNCKKMTVYVACK
QLRTRDCEHIALHLFASTDPVIESSHHMTFKPFNLRLPELKSCFESARLDPKVNRFVHVY
DFTEDDQELQRPHFNVLYPNHGLHMTDRCKEKGKPECPPEIEDLLEGRIAPASSSESGKN
KSYNIKTGAHIWKEASPDSIDNADVGRTPVVILPTSTNKRPPQSAPMANPTFSNQINPAL
AGRRGGSDDSYSSFDDDDNGDEGTDDDDIDVDEDSDDF*
>TCRU_1498 hypothetical protein, conserved
MSASLSEYHVISGKRDQLFSKGLLRRTTVAALQNALCAAVGRILIGILKRIGKVGLHSTV

LKQIVKEFFSLDPLKWASIFAALACFPLLKQIILKLGRCCRITEKVAAAISGSVCAVPVL
VMNKETRTDLCLYIFVRALNSFAVRHIIPLLPESLQQFQYYDILVMCLSASQILYSVVFA
PFSLPPSYQQFLSKASMLDNRLVRGHAGLARYQLTPELVELCIERGVKIPQSPREHFRIG
CMLTHPGMTCNHFFISFIGRNMIQVGLPLYIPLKLGAILLYQRKELRRRPFKLIQKTFFS
VLSSALFLALYSASTVRFACVFAQWNIRGGVFFAIFCSFAGIATLLEPKGKRMDLALYCL
MHALRSFVMTQHRLGWIPYPKHILVCLLYIFSVGYLIFQFDQEPDKLNPRLRAFFSKLLP
VEEEEKAEGIVSRMEE*
>TCRU_1499 hypothetical protein, conserved
MLYAFIALFLGVLAMSHSEAFEILPTRVTHVNADHGVWQPSGAVVLKEGVACARAHQDSC
PQCSQEATAVCTSDGARSFLVQQTSYPFCGNRGVVVNNSLFCPSNLKQINSSVINFTVVS
YEWVDSLVETPREDLVMSFHYWPGEEIKDINFKGNSLFFPGENLYIMNAVVVRENSTTDH
VLFYSSDGQVWNAQSKIPLDVVDKTALSQSGKYQIAVSAYTKGEHKIIYSFYKGTRWSTP
KYLNTSLPPTALIVGTGIMIQTGLSNHTTSLELGGSVEGSSKKKLFHIVDLYKNRTEHNE
LPQDFTTECIEESSCLTSSHVALMSLQPGYVTVLFDFVSGESQRKTIAAVTMEVDDSEEK
EEIIQAQKKAEESARLMEELKRKVAQERAERKRKEREEKRRKERERRQRFMEADFVNIEA
AKSRIIEDGEMIVVREVIDEMIDMEKSTFFW*
>TCRU_1500 Leishmanolysin, putative
MAASEHHDCDDIDGMELQDGDGDGRTLESHWSQRHAKDEWMAPIGCAVCRTELTLAALAD
LGCMGVKWDMAEPMRW*
>TCRU_1501 hypothetical protein, conserved
MVDGASATHHDSPVGTCLGRWMWSVVGACPRSRHRVRRRAIVAVRVALLFVLALVAAAAW
MPAVHAVVLRLRGGTVDRAITVGRAVDTVLMDGVYITNGVAVVFDVPAMLPGALRIELRN
CVCDGGAQIYVRGYSGEPASDRSLEVSVSGLSGSYCSLVFVHNLPAHTNVTVRDSTIATA
GPMRFSQLSGLTDAVASPLVLHATSLLRTQLRVSSTVLRSLQAGGSAVYVGGDVDLLSSA
VVLDGLLLEASGGPTASVMRVASSSILSLRSHSVFSVTNVSVVSGGGGIVLGERLAVFDS
VLRFVRVEGSAASSLVRCGGGTIGAGGWLELRDVWAVGEASSVASLSGVTLSGGTVSIAR
CAATGATLVSGPAITSGVVSVQCNRAGGRVLQSSGDYRMAGLPSVSVVPCDGCAAALACF
DALTASFSECVCGCRAGGVGEACLPFDVPPARAGGGGGGSQGCVSGVTLTESVTVGGGRA
TACFDSVVFSGPITVTVDLRSMDAFADALNVTLRHCVLAGGAQLRIGGLSESTARLMPHA
LVNMTNVTSLEGVIVLHGAMPLHSSVLLANSTLRATVGGSQYVTTTRGYEGFRYGPALVL
DGVRLLSTRFVMTRSMLVCGGGSCAAILVERGLGVNLSSVFYMDNCAVLSQSHVMHALAS
DLHVVSGSVFSIQNSSWSAPSVEYYEGACMFGDVAVDGGSVLQVVSSTFRLGFAMLMVNT
LTVTGGSWLVHRDNEFRTAYVVYVDDVDSVAFRDRSVWSIIDNSFKYGFYSALTYMTSNW
SPPSDTHPTIYGMCNEARGSPVTGYQDDLNIGAPVTALDCGACTMDAVCFAARTSSISGC
ECVCAAGGYGDTCLPAAVPDGLGPLPLPDAKDTGVRCVHGGRISSVDYPDPGVRGLCFVN
VTFTVAIALDLLYFDAPQQTLNITLLQCILIGLSIRGSGARVHVSVVSLMMDSGALEFEG
DFGASSLILVVGSTLLMRSRHAIQFQRFSLGANSTLLLLDNLIEGESYAVRLSFVVVDGG
GIVMKGNTLRGAEEEFPLESAVFFESAILKNGGYFDVEDNTMCAVSGIYFYGDIIVSSAG
LLRVADCTFAGITLFFEPALVHLDGLVTLEDGAQWRVEGNNVGAASVMGMPKSLYKMRLL
GSGTTVVLAHNRLVDDSCPFVKMALSNTIVVSPARFVVGCNLQDGREVSYDVVLPEGVVV
FGCGTCNDDAACYMPGTESVDRSSCSCSCKDGWHGASCLPFEVPDTVVPPVAERAVDGDT
SCVVDQTLTDLTLNMWKTHHCYVGVTFSGMGAALTFSLNSMPLHLPINITLTGCTFREGA
ALQFVGGAEAAESSGILIRVSRAVMRSSVVVFALALPLHCDIAVTEVDAMQSSAVQLPES
VNNMLSVMMLRDVVLSASSLLVSNVKACAFGYGSFGLYSTGTLKLVGGSSLYARYCSFDG
YTHLLHMNILIVRNHSVFALLNNRMDSGISLLSQQQDFSVSEHSVLRVVGNSGSVSYAIY
AYNSWTVQQSSWLDWRDNDVGVGAIFKVWDSAFVSIDGSSVVTLTGCKMGSTGLSVSLLS
RADAGYRFVAGCLTVAGREVTTAAELELHGITNVTTVAACGECTKDGDCFAPLTTAVIDC
KCRCAAGGHGDVCVPAPVPAGPPPPLPSPPSPPRTPPLPPVGECISDMVYPEVAQAVGGG
LSWLCYRNVTFSGGGMSLTVLVGAMTGDVANVTFDGCTWRDGAVLVLLGNAYAALGSLNI
VVTGNSFSDALLSPEGVFPPHTNITISGNRFTVTRLIPRSGLVLWGPSCVAMNGLAISND
SAVVLSGNVFQAVTASSSAIHVVESALRVLWRSLFAVLGNTFHMDGGGATLIYLEGSSES
SSLDVLNNSAVVVRDNVVSSQVQHVIFLRWALIVESFSAVVFQGNDMQRSLAVFYSKFLS
YIYYNSWLQLSGNLCRESPSKAFVYIDYTVNIRDSTVSVSGNRFISSAGTPTVLWIPTIP
RELTNGAIVAVCNTVNGEERVQYVIPSVYNAAILSCSGPCTIAASCFPAYTTTASSDGCA

CTCAEGGYGEACLPVAVPEPPSTDGADLCVRDVSVDVEVNVSFGTSVACYVGLTFAADVV
VDVASMSGSVRNVTLANCTFLDGASLYVFGWRSDPPAGERVDVLVSGLESRSGGGVVVAN
LFPPGSRVTVVDSLLIAERRVAYRGAYGLGDASACLVLHNVNLTGSVLTIARTHVAAVFG
DAVGVLVVGGVALSSRGALYVDGLLVQTALGLCVSVEGGVAASGGSVVAFVDSDFLLCKH
AVSVRGAVSVSGSAAALVRNEFSSTEDYAVAFYSTVSLDGGSMLLAKGNVHDGASREMLY
AAGAVTAAGSTLSFVHNRALLPRMLSAGLSLAAGAHLRVACNDAGGRVLSTAEEYAAAGL
LACCTVWRSLLACAT*
>TCRU_1502 pyruvate dehydrogenase (lipoamide) kinase, putative
MFRRCFRFSIPHLQDLIHRLDYFEKEIIELRTFRQMVEAASPEMALAAEAIAKERQRAKG
DIDVVMEYPLTSEEFVELNQFYALQKEKRLPLRNMMHIRNVEDLYVHAKIIHREYLVRLA
QRARALTLAPMGLSQMPSIQELRRSYEWSFHGVNSTKPPIDLESAQEFDSLMRCVFLRHY
NVSSLLSEGMYELGQREMWSERDFSDKSLVETFEELQVFFDEFCTGRVRLRFLVGNYMYL
STHILNVEPKESEKLTTSLFFDHDPDSFTGMICRKCSLENVLKCAIRAANDLYDESDIEL
RVAGDPNLTFVGIPYIMYDTLSAMIDDAIQANMIRQEKYGVACTPVVVTLSQRDENEQIC
VRVSDTAGGMPLEEVKHALKYSSSFKVSEQETKTANTWIHSPIRMPYAYCAARVIGGDIS
VVSIEGYGTDRILYFPRSGIRDIHI*
>TCRU_1503 Trans-sialidase of Trypanosoma hydrophobic C-terminal,
putative
MAAFNPNKAPTPSLVDGSVEGGAIQSSGGGRPEEQRQSLGSSGVNGVSAPTVSSAKTSSG
GEGSATQLVSEKSSDGHKTVDGAFFSDGEPTVETREGGTNGREEEIHAQNGDVKAAALSN
SLGNVSQGNNSDAGTKRGSGLLPSLLLLLGLWGFAAL*
>TCRU_1504 hypothetical protein, conserved
MRTMTICTERRIICVSWHTNPRRFECRPHNSRPCGTVGFCAVTRRNYCPCVEADPFAVEF
TQQLSSVCAAHPALAHSHVSERCLACDLLCVVEAAASRFLLVPNKKHFALRWIFTCVAVD
ARDDAQRVCLLFGGHEGEFGVCRQRREILRRRIEAKLTEACPLHLSWQLRGTPSAQSGWM
DAAQLGRPVFFSALRCPGKIDNPQRHCQLHFTAGDRLC*
>TCRU_1505 BNR repeat-like domain containing protein, putative
MLAGVCMSKGLSDCQVGVAGVAQWGLLLVKGNVSGVDESTKRIYWNDTISILCNAIGDND
DTLTGHIGGGGSGVKMEDGTLVFATEAVKENKVNGTAEDGGNNNVSLLISSSDAANWTLS
KGMSDGGCSDPSVVGWKDKKLIMMTACADGRRRVYEIGDKGRPWAEALGTLSRVWGSNRK
RHEKGVGSGFITATIENRDVMLVALPAYSEKLEKGGAQKGELHLWLTDNTHIVDIGPVSG
EGTMLPPAPCCTTVVEVVKLIIMKS*
>TCRU_1506 ubiquitin carboxyl-terminal hydrolase, putative
MSCFQLDCECDYKEFKRTIYYHAEDFTIPDCSPASHFSVCERVYEKAVKELKARNFDLAA
FYFARCMSIIERANLRPQVSGAKKLMWNCLEALERLDKNELLEHHSKLLQKVRQHEEERK
RIVEQQLLPDEGEQESLEDRHLRRQQELLLNENTVVDDALKRIRNVQIPTPYSQTNKDVQ
PYWHKPSVEQLEIKKNQIAPPRRIPTISPNAYLVWKFNVGSFSVSTPRRGMVNLGNTCYL
NSVTQMLSVTSLGAYFLRDDYTKDIVGADKGEVRLVNSFTYILRELHRTDFATPASPSHF
KDSIGGLYTSFLGYSQQDANEFLRLLLDGIHNSLNDRKRLNNNISEVDTTNGSDVEISGR
TWSQYKQKNNSVIVDECAFQERSSIVCPSCHQISRTFTPSLGLEVPIPSSSGRVSIEDCL
KAYCKEEVLDSESLYTCPSCKQRVNASKQLLIYSLPRILFLTMKRFRSYGDFSNKVLEPV
VFQKELNMSSFMCSPGKNTNYTLVGVVNHRGNIHGGHYTADCLGADGIWFSFSDERVSRA
DSPDFQLAYILCYRRSD*
>TCRU_1507 hypothetical protein, conserved
MRRSCFLFSQIVDVVSALTEKRNYSVKLCKVLLFPSPPLRSLSFAPPPKWTSELSRRLQK
QFPEIKRQHHVLYLRAFIHPSFTTSDAMNSTMNATTSIGEALLASVGECLIVNAFRDLKR
DEVLLLMSFLLSDATLSSVLRYHWEMEDMVLTDASVQLFQGKTLRSTAGLMQWLSSSGKQ
QTPDPYCAGCVKSMIGAVYLDRGLEEAAAFIYKHVLQNIS*
>TCRU_1508 leucine-rich repeat protein (LRRP), putative
MASSITSIGTQESEELCFLEAYEEVAPTKTKEQLIEEQLQDLYNYQFTVPELPEDDPSGK
LAYLDACEKLHKESFMKFPTRSVARRICDGKESLDLSFFGLKKKGSIALSAALRVNLSIK
TLSLVGNHITPAGAMEIARSLSESRTVTLLDFGQNKLGARDPGETVRGGAVVAELLRAGN
VLKTLSLRDNGLSDQHIAEFVEVAIDNTELNSLDLSFNKIGYLGAIDVAQILSRNGDLRE
INLEWNQFQTMGSCHILKEGLLMNNTIKRFNLSSDGLDDNCAQLVGRIIGENAIEEIIIA

HNRIGPIGAEAIAKGLLATSALTTLILDDNPLQTEGCSALIRVAAEASSLKHLSMQQCRC
IVDLLLDAERLTKEARQDIVILISEDCCAKEGM*
>TCRU_1509 hypothetical protein, conserved
MIDDIEATLNRMLAFPSGASSLDAAIKLKNIALALELAEQIPLLTETCMSLCVELAENTP
PQVATVHSVLSLLARFCMREENRGLATRFGVFSLCVLILQDHKSLPEDTIFAVFDLISTL
CMNDGNVRRMLRPSIPYIIEIMNDNTSSLQLAFGGAVALATLTMLDADNATLAVERGSVQ
LLISSFIRAYDRKKEIDCQHICHVSRCKKEEEQQLCEKVMRWSRDALQKLVQSPSHAVDE
KLEEADFGKYGSRVEVDELKWMLKFQRRKVSVIHP*
>TCRU_1510 zinc finger protein family member, putative
MNSAVDSLLMRLKLSDLIPKFKEMKLDRIVNLRKLSEEELREAVPDDEQRNLIIHAISNR
GTHEKRQQSQAANINPPRNADDGGRVGMSFPRGSTRGGMGRGVSRGGRGGGSINNNSNSE
TNTSFPGRQRACNHFFSGECKFGDRCRYSHDKEIYDREMAENGPRRPNDSVSKPKDYSEV
CEIPTHRIKFLLAKKAERLKQIHIQNHTHNEPFKRVDSNVEKFELVVYGPDPQSVLQSKK
MILSYVGVTKQEEQKSRLQYTINELSSNQHAAKLLAACNIKNEGTARELSEETLKSIISF
FRFEKQQDVRHFYLNVNHDRVKLDKVAKIVAQLRGVQAIMFSDQKRVMEMSKVASKIARF
FNGVTPLFLHREIPKEERMRILQEFKDGEENESGIRERLLITNEDYAKLARKTLIPYVNL
VINYVVPRTEEYYLLQSLVAGRSDTVGVSILCVSPYDQSTFHELQQKIVFTAIEEESDFM
ETAVQLVYDTTANPLTDEDADPPRDWKEKLEKKNASA*
>TCRU_1511 arginyl-tRNA synthetase, putative
MARICTGANVELALKDIVRHAMEKAFPTISTPEVLISLGKVTEYQCNNAMGLAKLLSKAT
PPMKMSPINVGEELRKNLEENDLIEMFEPTPQGFINIAIKQEWAATMVSRLLKMGIQPPI
LPPKKVLVDFSSPNIAKEMHVGHLRSTIIGETICRLFEFCGFEVHRVNHVGDWGTAFGML
ILYLKRQHPNFLTEMPDITDLTKFYREAKKCFDEDPKFKEEARLQVVKLQALENESIQAW
KIICDISRKEFSLIYDRLGTRIEERGESFYNPIIPRVLSLLQEAGKLEESSGAKLVISKE
KKLISSLNAKDMAKLIVPHLIQIWRDNTVEFHVNMITVLREVGILTGEEGNETIMLSKKE
TKPLSKFDARTDIDKIVPKLEPYYKNQLSPLFREVFEAAGITDGDTIAVPRFNFPLILQK
SDGGFTYDTTDVTSMYHRFVMEKMDRVVYCTDVGQYEHFRMCAQLAKDMGWMGDATWDHA
GFGLVTGADGKKIKTRSGETAKLKDLLDEAVERSLAILEEREGGERRQGHTKEEMETLSK
IIGIGAIKYFDLKQSRVNDYAFSYDKMLDMSGNTAVFLLYQYARLCSIKRKANVSEDEFM
KAEVVINTPQEKRLALCAFRMESVLLKTVEDLFPHHLTDFAYELVSCFSDFFQNCKVVGD
PMQNSRLCLVELTRITLKKVFDILNIEATERI*
>TCRU_1512 Cupin-like domain containing protein, putative
MTQSWFRPHPLGIYPRGNARKAELLSAPRFGTLSVFQFAAKGWMRKSAIVKESTEEAFVE
LLLHLLSFFSVDDLCRLSATCTGWYCFIHASESFRQNCSLVSSKCLRFRGSWKETAIRIF
LLEATDTASFSVTKRHRTECDTSAANGFQFKHRPVAVSRAFFNDYLFQVWMCTILPCHYH
LLPGVKDDAGASANHGAAWTCEGNLPQQRSSFRSLLKDVPRRSGLSPREFREQFEEPSLP
VILTDVATDWPFFKILQGRFENLAEKKEELFRPGVSPDVSMRCEHTTMTVSDYVRYAKEQ
TDERPIYMFDAEFGTSMAIESLYSVPEHFVCDDFFKVLGDARPKYRWIIAGPRRSGSNFH
VDPNYTNAWNANLAGRKRWILFPPGCTPAGVFPAEDMSEVTTSVSPSEWLLNYYDASVER
WRGFGYECICEPGDIMFIPCGWWHFVINLEDSVAITQNYVSESNLLRVMKFLFAMKSSIS
GIDEDAEDVTSEVVEDRRAHFAEEFAAAMRASFPDLMQRVEVEIKKETEQRRQKKQECVA
LPLLDAGSEGFTFNF*
>TCRU_1513 hypothetical protein, conserved
MGFLTDYYDKLAKEGRLDALLIFGMYDERVYEVVDRHHLANEILNMLPSPRAAVFLSRGM
VYNCISEVVIRDFIKICMMLSRYRSVEEGASLAELIGSCGIPEQYRMNMFNFSETYRHMI
LSRSYFPQRSVLRLLSYIFCDDVLSDAVAQLQYKSDILNHVNKVYVDTPDFVSWRFSVRV
MSLAGGYNEDRSDVEYTEALTRVVTRTIPEFFMYFTVTFFYAMFRHYPRRVRGITPRWLA
RHRALFSAIYGAVLGVYVNSFLEYRIYKHRVLYELRKSQEYRRRRGAARRGIEYPENKYF
NNLEGVTRRIYSMQCTAYSVTVGALLLSMLPLTPVKFPKWMGDVAWRHPFVPRLFPTLLG
MHTASRCMVPFVFAPFVTLTIARFNGWGATLYDRYVECRMRMWYRKVEVAFDTSTDVADV
DITRDSHQKTI*
>TCRU_1514 hypothetical protein, conserved
MLVEGVLLRVKLGKPARDAVDLLQISTEIKSAEAVLKSTRNVLEKARRTASTEGTTENHE
CEQAALCYAAKKRAGMEAFFISEDVFHAFQRVFLDLVEEERVARCRLDDLHADTKKRAAK

ARSAEYFMGTKCVFERFAVEEEAREKKVRLFQEYSGFLSALRFAEEEERQEAISRARARR
RVSLQQSVAQPMPLSSFGQPGVMSPPVGLQQTARPQGPPQFAHQQPPYTAQTQPYGLPHN
FFGRVQQMYQPQPQQMQASGYYAAAPVGRGGYHYGAPSAPPFQAPAQNIFPAQYQAFQQQ
HPPNSGWSGHSQAARGRTHYYGL*
>TCRU_1515 LicD family, putative
MRRPRRWRLYPVRSAVAFIICLCFIYLFVSIESIGALQGQVSFLKVVVRSSAISGANLSL
RVTNETSALASSGIFSAVGSSRLSLLYNADAMIDLAAQREFNQTLCPTEPREKEDPKFVE
FLEFFTKVFKEKYNNIPWWLDEGSLIGVGRAGAIVNADDDFDFFVLLPNATSPCRPGSLE
CTREEFNLLIHQFLLPLWEAGACIRRFNPDISKFDSERRLMYSFQIRKPNELRDPMHCFN
PRAPFAHMHFGMLNADGELETNKWVGPNSHPKDKLPLDLMLPVRRCRLGPSDAPCPFDIV
GYLTLRNRGEYVRRSREGSCLLVKKKWSKARRVNQVKKVQLLHDCGYNSMIRLVKRFADS
DYDTC*
>TCRU_1516 hypothetical protein, conserved
MPQAGQGGEEGQEHTGSLEVTNKLAGNEDSQITGEQNVSSDADPPKQTGSQVPAGGQPED
EEGTKSPQPPPPPPPAEPLPQLPRQPPPGTNTLQSEETHPAITVEKHPKPQDGGHSHETK
MPQTKGSTKHEEGASSLQSPESVSHLSRESTTPLSTSTDSSGVAGSTVENSGANNPTPPA
ASVTQHDFEGNTGPAALPASNTELQSEEIPNAGIIATTNDDVNDSSRAAPTAERPTAGTR
STQTSGDAGDLERANNDNVNDDVARNGKTAEFEAASGDRNTEADNDERYTTVPETATNKT
INTEIPADSAGITAVSHTTSPLLLLLVACAAAAVVAA*
>TCRU_1517 UDP-Gal or UDP-GlcNAc-dependent glycosyltransferase, putative
MVAWVLEKEVKYNPMVYVKVLGCHFHDARNGTGHSQVVPTSMCVHHVQEDDYAALMARFG
NDTSPVARVERASEDVIYPLCD*
>TCRU_1518 hypothetical protein
MLGYNASAPEERSRKQVAIMDLVRQHLRLPENPRSRQATRTNHDKQHKADWRIGHFNTTT
VGWGATETTEDGEGLAGDENEHSDPTAHQTERILTASDINKAIRAETLCTLQATGRAAAL
LAAAEAEPVVFSPKLVKSLDDLYPQEDTSLYPEPAVSALLVTFDSKDVAKMIGSRLARGA
APGLDGWARELLYPLTKDKALLMAITAILTDMANGNVAPEVAHRLRATNLTMLRKPNKKF
RPTGAECVWQRLYHSWRWTRSCQPSQPALRTCSMVSATT*
>TCRU_1519 hypothetical protein
MKPAVAGHTTAPGHGGAGGLLRKGTTGPLVEVDAGSAPGHGAATSPLLHRHHRHPPPTGK
QLPQRQLHGVPGRRHGGGTTGHAWEGMQGDLPGVACPWHRDKQGQDGGPQSREPVDMPAE
YIRPFARVLGAGIANDPKSELITQFVQRKAEETDRLFRAIVELNLQSTRSGGSCRRCHA*
>TCRU_1520 hypothetical protein
MRSPASEGDCGVRLRVSWREGKQRALTDELDAKHQSDPYETLQTSDRKVFVSNTAAGAGR
LLTDPQVHADDKGFSTYLRERLLVRVLPEGQKCVCGADASNEHVHTCTRLQQNPRTTRHE
MINMTFANGLRLCGFQCGMEPRLTEASRRRPDILIVGLDTYAITDVTVTYAGRVTAYVSE
ESMEEADPLRAARDRLTQKRQKYRHWALANGLDFEPFVMLTNGAIHPASRRWLRRILGAR
VLAYAGAQWVAERQ*
>TCRU_1521 BNR repeat-like domain/Concanavalin A-like lectin/glucanases superfamily/Trans-sialidase of Trypanosoma hydrophobic C-terminal, putative
MGESWTEALGTLSRVWGNNKQGVSGVRSGFITATVGNGDNKKVMLVTLPVYAEKDKEKGK
LHLWLTDNTHIVDIGPVSGEDDDAAASSLLYNSGETGDNKKKDELIALYEKKKGNDVKPS
PGMVSVLLTEKLKRVKDVLATWKEVDGRVSHLCTSLIAEKDRASTDDVCSADKITAGLVG
FLSGNFSGNTWRDEYLGVNATVNNKAGEAASAVTSTKNGVKFTGRGAGAEWPVGKQGENQ
LYHFANYNFTLVATVSIDNVPKEGNTPIPLVGVRAGSNGEKTKLMELSYNSEKKWHVLCS
DKTTKEPSSGWETETQYQVAIVLQNGTQGSVYVDGQRVGNEECALGNGESKEISHFYIGG
DGENAANKEGVSVTVTNVLLYNRPWDEAEITALNPNKDPTPPVTPNAQENGTSSALDGSH
LTERRQSMGSSGAGSGGASPSAVPTVSTPSAVKDSLQQVASGKSSDGSQTVGGGSFSDGD
PTVETREGTDGQKEGIHAQNGDVKAAALSSNLGNVSQGNNSDAGTMRESGPLPSLLLLGL
WGFAAL*
>TCRU_1522 hypothetical protein, conserved
MHDFGRTERQTEAGEDLKHLQEPLATSASVMPINASFTAVGGGTSTSPTPCLMRQFLHFL

GDCYNHIDAPLDSALQQSVELARFVESTQCQDFLPRQQQQVRWGLWELLRDEKAREVSLL
EMEKQVRLYGGSTRLGRKREFGSYVNPSREALGNAVGSSGGLGQGGVVNVVFGSAWHELN
EVDEDALLETLRARGRYHARLPRHASHCQRRIGCRKNSSFSQSSSASQLTSSLPPRPNDS
RFMTVMGVMKNNTPQTESSSGSNTFAEVNMTESDDPCVVLGTVETLTPGCSLCIAQGLIQ
GCWVSLHLLQLVNVVSTAHLCAAPLLVPNEALSTSPQEALCVGYHVAQQQLKVAVGFLFS
VSASTSASIQSTSAIQREQAKNSTVDDEKKHHAKVVRVMELLARQGLDVCVHLLAALRLL
EPENAYVVLLHAAVLSFCDRNGVHKAKQLLRRFIASSPAFSERKTMEKGDFDMYDMWLQF
EAALGEKDVMRSDSERIMLLLEQVVTGILFSVFLELLEMKERPLVIMRIPYASGDDTIAK
KTVESSIAMCLRARDVLDKIVQPRLFKLCDAADGAIGTRARQSQSLCWLLRGTLCHILTA
LSLAPGTASMFSHTYAADEALACTREAVKRSDAAREFVGHPLAPAWVEQMLNGPNKGEGR
GEATHGGNNAPFLHAEEIGGNTFYEWRLRVEGDAGAANTTKEANEICSVYAHSADDADNN
DDNDNGGVDVLPAHERVTDVALLPPPAMRLWFVLAQTLFRSTSADLVIARSRDSDGAEKR
DGDTCRESLEASAVPSMCQTGATLASHTGDVASLAPQHRKMKPKKGLAAQRAVMAERENV
RKVMEARRLTEQSPLGFWWRWQEAMTHYCLLSVKTDTTQTSLLRRYRHLLTSVAGEGATL
TTFMRALAITHPILPSESWTFVATHFFPYSLLPSKQTPMREEGSKHAGPSLEIVDVRLRE
AWQRYELQCVQPAIAATTVPTDEENTRHTTTSSAVQPNDDRPFQQRTKQEVSVKAATLPR
PTRGMAQLTNAIAKPTSLQTSSLSQLKTTEEPQRKSFPRQRQQRRL*
>TCRU_family, putative Se.1
MEHGVTLLGDPMTPRQPIMSSTAPYFFVVEDASVSRVAEDWIAKVPYRDAHIFALGCTPH
RHPQQLAWARIAPRAMCSKDIMLDFAAPEVLVFHLSMQNGFLSCCRLRIDGPFWTLRRHV
LWRLFTP*
>TCRU_1524 hypothetical protein, conserved
MGVWCPSTVLDQLLLLGLRQLVGAPRSHKQREKTQTHWPGHRGILCQAPHAIQVFPEGFA
PNGDVLFRLPHLRQSLLPVLCHKPRAKNRPAATVWFASPETPAEPAPHWSTPQDWPSAPR
HAPIPGDTRGVFSAPPKAPSRTAQENNDGTSAGTHLPHQLLPLRVLTQKIPTGPLIQFLL
QRHQVIKQKLRCALVHQRRHRGTAPINDDPHRVAAAVGAPK*
>TCRU_1525 sphingomyelin/ceramide phosphorylethanolamine synthase,
bifunctional
MVLMGPHSALRLLPLKTQAIRFLLLFLLSVLILGVALLVTNARMPDPKVVRPLPDIGFEV
FPKVGWLEHVTDVCIFILNFLSLLVAFKLYLLHRQNEGLDELQPFSCCPLIGKIIFGVWD
RGRQSGIEKRDAHLIVWIRYFTTYFIVMLFRAIVVVMTSYPATDNHCQNPVKITNPVKNV
IMTLVTFGSGFTHCGDLMFSGHTVPITLSLLVQWIYGSMLHWVFRPASVLLVLLSFYSII
ASRSHYTDDILVSFYITVTTFLVLRHSPEGAPWQLQLLIGWWPCCVSNEETEDSDRHPTF
VAVEVFLPHGDYQCAERISEEKTTVGPACGNFGHW*
>TCRU_1526 protein kinase, putative
MQKYRILGKKGEGTFSEVLKAQDIETKRFVAIKCMKRLFKSKEQVNRLREIQAVRRLQPH
PNIVQLIEVMFDRSTGRLALVFEYMEMNLYELIKGRRQYLGEEQIMSFMYQLLKGLDHAH
RTGLFHRDVKPENLLVNEDGTLKVADFGSCKGVYSRQPLTEYVSTRWYRAPECLLTDGYY
TYKMDLWSAGCVFFEIMTHFPLFPGNNELDQIYKIHNIIGTPPPEFLNKLKKYGTRMEFE
FPRKNGTGLAKLLPNASPEALDLLTKLLTYDDEQRGTARDALRHPYFKTLRESEKKARRM
RLENEKSKMMESKTGGNMFPQLSNTCSQVLTHTASKTLLILGTGDATGNQTTVNRASLLP
KLCE*
>TCRU_1527 hypothetical protein, conserved
MTNRVDRSRTPSRTSTGPYRDPAWPRVVGTYCDPPTVARSVEFASSTLEFGGIAPCSTIV
PSSDVTFVMLTRAWGIRRAVISLRPPIRSCAPPASTQWRSVTFSASANASNKRRSTATVI
SPLNTTESRHAVARPPPTVTDSVSVTPLTQSCPPPPPPPALASGTSNGRHASPKPPTRQP
HTQSVNEAVSASKHASAAAQPSHGTTLTEDRPAMR*
>TCRU_1528 ATP-grasp domain containing protein, putative
MEGKVVVLLRTSSVARAPIYKHLREMGVIFVVVHPVRPADAFDGVFHYHLQHDTNEVDAL
EPILRRFLTQHELLPDAIISFDEYGIYPAAVLSQRMGYRPIPLPPEKLRRTCVKSLLRED
CAREGIASPKAGVLTRPQTASVELVVHIQETLKAAGVTFPLVLKPSPGAGSILAKLCYGV
EDVRDHALLMWDAFASHSDMKHLTAVLNPAEGMERVEILFEEYIGGQEVDLDCCVENGNV
VFCSISDNFEVRPPYFLEEGGLCPTALPMYEQRELMRLLDKFVVLHGKDLHGVLHFEAKY
DFERRQAYVIEVNCRLGSAETNSMLRTCYRGLQLGESLVRCALGLSVREQLFLLYPEKLE

EVGSNENEAKDALFPPSCYCASVNIYPFQEGSLVRLEVPVSGKGLVDYGVAAVPGALVAP
PPNSFTFLAWMVAQGTTAEEATMAIANLRENFVQEISAAQ*
>TCRU_1529 hypothetical protein, conserved
MDAAAEAAAAVAPRVVRFITTMKATCQYVFCVLLPGEDRQGIKGFWQTLQQGDNGASSGD
ALLSSRSYRLVLHRLLHRTGYYNVHVGGIMLLVQPVTASTSRSEDAFHDVFENHGGRGCS
AGISAIEIYELAPGSSGEGGSSDMEGGRLILTTKQNVENYSERRYRIAIVARTTYPLTLA
ESRQYSVNMLKDQDHVTAVVMEHVLVKTSGAVLLAELDAGIFKRLGTFWARLQHIIGAIP
GTGRSFSEKQQQRGRQQQQQQQAAELSVLSSPSFVPLSRVSREESDRLERPLGEGQFTQ
LPLEQLTGEGCGGYSPEMQVTTPYVLLRFFTLQLEGLTLEMKFSSSKMPDPDYYGPLTKR
LWEHLRSEAEASFPTTDHSKFSGVDGPYLPLTMSLYLKSLDVSLGSEGVLEVFLGEGQLT
LHDYKERVQSNIMHFVLDPDSKEPCIVVRQRQSRSCDTAWSVARKAHAPFTDAYEKEEIQ
AESNSLISVVVNIRQVHAQLHQDEYLLAVFYLDQVLEMVDTMRTVINSPQKHSENKEYAS
LEEIAGDFNFAISGASNVSEGHLHYGSTEYDPITTSISVRLKVEKGALSVWAPRLSSAGC
TIGDPLWRCIPQGERCGIDKEHLYHLYDVMFSKVSVFVFHQASARLEKTALHGKASDFFL
RERLCKAKLTYVDFPWPAEHASSPNWGGVVTLLQSYSALYNRSHNIRVRPPCASSTESSR
NRHAVGVSLCRHRNVEDHVEAYDVALQFDRISVSHQTAHEGDHWLFVLLNYFSDISGEGL
SSAVAEKEATADTHVTEENDAHSDDGVAIATSADVKITLIDVFVEYRPFGRSSMLVFLAP
RLNVTVSVPSLLKETTTVKVHLDKENLSVFLHDNFQQYLLEYDQEAGLGSLFGSSERGVA
TDLKNIGFVLVLDVAQSAGGKNSGTDDEKDIVTSRSDVSNITLFINPTAEKPVFLEVQKL
RASMFFAYDSLYHFRTVISHFLGCNDLAYLPSPHLLVERSGSRFECIIQSNYVNKGKHAA
VACHRVPDYVPRLHQLLETSVLNIGLASSVVAAGGEVASKAGLPKRSLATNSLEDFDIIE
TAELEASIAPVWETFENDSINLIGRSKYTNFLSIYGAWCSASSEYAQNRRCVPRPLPPNN
LVSMEIHLYDCDVIAHLYGGEDFIHFDVPQSYLHTIQRFGNMEEEVQEPSVLWSGMDSDV
ELHQGRREGRRSGFLGVGRRQDEYVVGRFTGIRLQVDLFVPGKANLLQVYLSVRDGEVVD
CIEKSSVRTLFMASLPPSLRDHDGDLFEMKWTTVVPRSSSMNAGMVVVGKPEEQLIVRLQ
PVTVAVHRRAMNLLSKFIEDPETLSNESIASDDVQSQTLFFSKIVVFPVQLILYVYFEGH
DTSRATRMDMFELCNFLLPSIERAQIKVPLILVTACPLHRVGDRILDLMREQILNFHGLF
MLLCGVQPLQLASNVGTAGVEILFAPLNEYRRNRRFFSALSAALRKFLFTLVSQSLNSAV
AVSRRAHNTTSSVITSVLPVSELSPVSRGSQPVSLLEGLKRGFAELLQGLRLASNVAAYC
LGPHGSPMRLPLAASLVFDGFMRGALEVMCGIRNVVAPELYGQEKKIFKGKRRKIT*
>TCRU_1530 hypothetical protein, conserved
MVAIAEGLFEYKVQEHKLFVVRFPDIVAGYIKAAEKWQSIVAEVNAGKWKARTVLGSRNG
DNLLRVLHRPTAVARVSEAFLLVGSDTVGYDSSDGI*
>TCRU_1531 hypothetical protein, conserved
MKDGMNWLFASNLLYSDGALHLLKERANEKGRAISLARQTEELKTIKSVLSTWAQLDDYF
FKSSTPTACLVGFLSNAASGDTWIDNYRCVNASVTKASKVKNGFKFTGPGSMATWPVNGR
EDNNQYGFANHDFTLVATVTDHKVPKISTTLLGSVLANPSARSSLGCRTARTVSGRQCLM
GRKQHRAARGSWGENTMWRSCCRTATRAPCTWMV*
>TCRU_1532 hypothetical protein, conserved
MTNRVDSSRTPSRTSAGPYRDLSWPGVVGTYCDPPTVARSVEFASSTLEFGGIAPCSTIV
PSSDVTFVMLTRAYGIRRAVLSLRPPIRSSAPPASMQWRSVTFSASANASIKRRSTATVI
GPLNTTESKHAVARPPPTVTDSVSVTPLTQPGAPPPPPPVLAGGTSNGRHASPRPPARQL
HTQSENDAVSASKHASAAAQPSHGTTLTEDRPAMR*
>TCRU_1533 hypothetical protein, conserved
MQRRRGVLHARDGVGGPQLVLVQLQGRMAWRVMPSLRGARRGCAILAGESRRRRHELRGE
PDADENYAEHVEDAPLLRGCDIQRRGCCVYFLSQQYAAASSHQHQAHWVHIP*
>TCRU_1534 hypothetical protein, conserved
MRVPIEAAVPRVQRAASHQRQRPNRVQTKPTVNRCMRLDVADQQRGCSQHNVLQQHDAPR
ANPDVRQMDLRGLHRVNLRDGDVAVQREGK*
>TCRU_1535 hypothetical protein, conserved
MSGSVRNVTLVNCTFAGGASLYVVGWRSDPPAGQRADVLISGLESRSGGGVVVANRYQPG
SRVTVVDSVLIAERRVAYRGAYDLGDASACLVLHNVNLTGSVLTIARSHVAAVFRDAVGV
LVVGGVALSSRGALYVDGLLVQTALGLCVSVEGGVSASGGSWWRLLTATSCFASTRCLCV
GL*

>TCRU_1536 hypothetical protein, conserved
MEGTHPAAYRRVVVSGSKDPVEYFVRTKGDGRTVVPTQTETRTGWLMWPAALRSQLRHWM
RAEQVSLRCLLASRPTRWPLLKGIQEHLFPEGVPAVWSAHCSVRFIGLFLWWEDGLLHFL
DHCGGGGSWGNMPTASVLAHRALVAALCRRHNPPLQDESDVFLSLASNERSYCYVFRAFL
LDNEFANGYEGCVAAGFECRFFFFDGRCLGITGACRCHRRCEGRCFSHPKRGCWEDVFHS
CSGGRHRRGECACSPPYFLYCSAATTGSDRQPH*
>TCRU_1537 BNR repeat-like domain containing protein, putative
MKGNKMFLLAGSTALSDLNGSWKEGSLEIKLVVGEVTNSTDSGPSKRIEWGEIKSPLNGS
TLAAHKGNLTECLASGGSGVVMEDGIIVFSLMAMSEENDGVYSMIIYSKDNGSTWALSED
ISPAKCLNPRITEWEGSLLMIVDCENDQRVYESRDMGKTWTEAIGTLPGVCVNSGSGDYP
DLSLRVEALITATIEGRKVMLYTQRGHAMGENANLLYLWMTDNNRSFFVGPVGMDNAVNW
DVASTLLYLDGKLHLLQRRGIGEHSVISLSRLTEELSTIKSVLKTWAQNDAFFPTCLYPR
RVWWRYCQTHRPAVTSGTTSTFA*
>TCRU_1538 hypothetical protein, conserved
MLWTDEKTLLQLGQGDVDPLLVVLRGGAADTPGGGSGGGRGGPASSVRGRDGTTDIMELP
VICRARACLPRSGQESHRTRAQSRDSMPDVCLHTRNGPCHTDERLACGVLLPFLLDFLPV
QPLDGGVEFHVPGWDASLLQGLCNRDFVVSGLIAWDTAVRWGKYKVCRVNSLHQGKQHNP
NLFSNAVTHGQWGSSECLSCRRGVRCHQDSCGRSGCSQTRRYEMFSALFSGKALCSL*
>TCRU_1539 hypothetical protein, conserved
MRQLIHVTSRSAVLLVLLVVMMFVMCCAGGCFAASLTYSYSSDKAMRADRAKSIPAVVRD
VSFGYEGASQTCTVIGRNGWAPTRINVSTRDLDGTRKYCSSERYCAMNSLLAAASVKVTF
FYAEKREVFVSLYFAATVR*
>TCRU_1540 hypothetical protein, conserved
MAMMMTGRVLLVCALCVLWCGAGGGFADKVVEAAAGVVASNRTDEENLILNWYVLTQEEC
ANESTTGGKLNVTAERICMHKVMKEVCDAFYNKTSGVTHDRDVKFICTYYATIPEEPVEP
PTPQGPPSHSAVSNATSDEGTPKNAPESDVAGRGEGKDDDKEHGNTKQKAVETAAVESIT
RSTDSEVSTAVSHTISPLLLLLVACAAAAAVVAA*
>TCRU_1541 Mucin-like glycoprotein, putative
MKHVNPSLTPKELADENENGTQDIQDPSTDVVSNSNGTSDEPGKTGALDQSAKNPKLEVP
VETTKTTTTTNAPSTTTTTSTTKALTTTTTTTTMAPTTTTAPTTTTTRAPSRLREVDGSL
SSSAWVCAPLLLAASALAYTTLG*
>TCRU_1542 hypothetical protein, conserved
MPGKQSRMAGSGAHGVKGQSIFQSPGGTRAQTVQAAVGIAARATRTVRRTSGLPAAEAEH
RHRPKSTTAQPIPESGDVVPPCLGVSQAWAREGTKLLCTDLQQHGEATAEGMIAVLPFAG
NFSSGRLPPGEAVCASHAAAAVDSACSIPSVQIPVELSRLPHGMLRLAARTVPRCISNHV
ITVARSVSHAEVESFQSAARSARAACFRRGSMPACRGMPSSVPAVWDGRW*
>TCRU_1543 hypothetical protein
MRASVKVWLQEQRRRRNTLPPEEEAHIQRIKDRLRIGSPLCEPYGSHTESSWRKPTPVSS
ATADSLLQHNAVLLSRLEDVEEALRYSEKERQRLRTQLLQASDENRRCAMQLIQLALDTR
RGELLLEEQLFRERLLRHESEKRQKFLWELDRRLSSNRCAHASHNERLRSSPRVSLSKQF
YA*
>TCRU_1544 Leishmanolysin, putative
MHADRLLVEPLTGRVIVPRYSSGTCAQFTIPSSHHTVGVFGADMYLYVSAGPTKGSTLAW
ANFCSQLADGRPVVGVVNYGPSSVTDSVYSVRVVVHEIAHAIGFTLETMKECNMLKKVEG
VRGKAKVLQVSSPKTLEKTREHFNCMSATGMELEDEGGERTASSHWKRRNAKDELMAGIP
ELGTTLR*
>TCRU_1545 hypothetical protein, conserved
MDEGLLAVVAGVVGACSVCVTAAGSPGDASGDADAVVVPMDVSCAQSDGSLSYRVCVAWT
KCSPAAAEAQHENYTFSGFDVRMHGRNGELGAVCHVAHAVHTCSSCAASCATEEEGATVA
FTANVTTHKYACPYEVWWRQFPPMEPSIRLHGAAMLTGRASVGCRHPVKGRMTQNLVGQL
RRK*
>TCRU_1546 hypothetical protein
MPFWCSGDVLTIHSSRNFPRNVLAAAVNGLSLTGIPRAHCLPQRGGERSLRHECIKPSIS
MFQLMAMLGFLLGGVGTTFMFRTKANSGLVSDVLVAVLDAGLRSTVLGTPAAADRRQSSF

*
>TCRU_1547 hypothetical protein, conserved
MRKNPYLAHYFGAALDSSFEEEGINAAANFAYFPNSKHMTQRVFCPSRESEPSNGSSTAV
CTPPSNVTPGIRVAATRARDGFPSSPPEYTNRLPPLPLSASRRSTPVEVYTDRQNSFYTD
LSITRSNSPMGIRTHQTPEHPSIEIFNRGAPGWGSMIRQSHAALAEVRELLGAEKATPAA
SMPVANRVISSVESDGGHDEVGTETKVSPATSLYEGENTRTGTTSSYVPMSCAENHPVDV
SLVPQSTIALPSAEATSQRPAPNASSREEEKNIVTNSISSHDAPNKIGGPDPPALQQSTA
TVPLQVKCFVLTLNPDAVACALKRLSRQPPYVYAMQNNESTVQGESNMPEKVPPVVSTGR
PKSRLHDERKKLKSKGQDRKDGVISVASLYGILERLADVVRPQSESRCVDSNEKRGDLDG
SFQPPHSLNVSGEKGERIDTYCKERSMREPVVAPKVLMGNAPRIEGRDFPLNDHREAFSS
IVPTSQLQSVGVNVQRPLYRQRRTSSARRPSASRAPRTPSYALPTESWLCKGAELNDAPV
DASFIMEGSKTKGALR*
>TCRU_1548 retrotransposon hot spot protein, RHS3, putative
MTLVGMRMATTGEHHTTASTVRQFTECLAAYFNGWEESSRDMSWEIIYVQQTGSTLMNDW
QRCDVVNSNNVSDDESRGIAAFWKEEVRQYQVSISYRDFRRDEALRSVQ*
>TCRU_1549 retrotransposon hot spot protein, RHS3, putative
MYPLGPIPRHVFDEEAFSERCGAVKFALQSINEVTAKEHFSRGGESPWYSEDPSHKPVKV
VREIYAGGVILFNAPISACLEERTLERLPSVAE*
>TCRU_1550 retrotransposon hot spot protein, RHS3, putative
MKFTISTNIEDVLFKGRVRVNEMKLNDFLTRELDGRDIVDTNRDVLLEEFFRDPTKYIRD
KGALNEMQASDAYARMERTVREEMIWKKLYASFAIGVSTIYLGGHWLLRR*
>TCRU_1551 retrotransposon hot spot protein, RHS3, putative
MVWRCWGWLHVVLLRRRWALAVLPTGVAVRLHGAPTTEPCECHAQRHWDSGTKQPRLCFG
ASGTCWPQLGGASGMLHSTGVVMAPRRGSCDGSGAAARHVAGSKVWPQWTMSSTNMRLSD
FLWNYVGGRAAVDERLQCDDGDVCSGAG*
>TCRU_1552 hypothetical protein, conserved
MAGDEALLHIFVGTLSADKTTREQAEATLTHISADPRLITRLIHFSCQELPFHDVPAEMQ
QALQAASIRVRNVLGRSDWNRNPYFTEETKSAVRECIVPLQCGSHVPELVRRQLLAATQN
LICYDYPHRWPSLMPQLRRILDECGAHLCVLSDASSSREVATLRLKGALGILRACCKVYN
NPIKIDADDIEAFIDAFCPLLLSLTEMLVSVWAQELLAQQTPPPPPPQLNGIPDPSFQFS
TKRKEKCMFVLSLWHNELSHCLRIALKCIHSLTEARWPRFLCDQPGMDRLCKGCLGQLLD
ASHKTLLPLYRLRILNSDCGDSNPFSLFHESAVWKLLKWTQGMCLKMMQDLMFPKRCERR
ARAAAKYFCDHYLPGFVQHALDLVRWHATPRHLTSKAYIMSLEILTMAVDGREAYRSVIA
PNAEEIFTSLIFPRLTFSAEDAELWTSNPAEYVRLQTSPAGDLYSAKVVSSGLMLTLAAS
SKPFHDADFVHHVVQYVLERLTTHAKAAAQGDMEAARVVDACLFAIYQFNKVLRSIGFGD
DRVEWLLTNYVAPAAKYPVGFLRARSILVLSVFASKIHWSSPQAFQFVLSEILPLLQDPE
MPVRMQACASIASLICHPYARDVIAPCISDVIQHYFYAMRLMDSEGVVRTLRRTIKHYRD
VLSQWALHLTEMLVQHFLHVLERVLAEEVKDSEFTPFTDNTGGDARLDPLVSDEDAVADI
IMTADEVLETLTTLVRAVSQQEVNSSSKASGEGGLILQMQERIAPLLYVVLSREKGSCLG
FMDASLMLLTTILSLSSAVASPMWRLLPCIHQLIVQGAVDYFYQMLPPLDNFVSVSPEQF
LFFPMSDLCAVPEFAANMASMTPAQIVCMMCDTVMQSSHLLHLRDLSAVPKVYDSILQNL
WLFKQREGGDNTTATAMADSLVEYILQAALRVLNDSSSQEKQRRSLTLLFANNVLSAILA
SAALTVRMLSSAGVLLPFFVHYIRLVQEETMQVMLRSYDRRLFVMAIATLARLQTEQAEL
AASLEEVLCGVLEGEVLEKYSHREGAMVITEVGMRESLSDGDAYDEEEWSGETESDSGDD
DEMEDEEDEEEEALGMENDKCLQSLLHSAADVLKRDAAAADDGDDDEGEEDNLLDETDFV
APIDARNAWAFLLESFRHASAAASTRFCQLVGDANSQRQLQSIMQLSDAVEQLLAARGRP
NAADPHSPLPHPPVREKLKPHFL*
>TCRU_1553 hypothetical protein
MSEVLLLLSMAPGSNSGRFENPSMHSLSATRSVHLGDTTFGEGIPPSVDDFQVKCVGLHQ
PPLYEEERHQRMLIMLECKLEFMTLVMWCFAIRMRIEKELLRGRMQSVQLQEATLQHRMT
RFAHVEDRFGRPVRNPTFRVLLQDEFFEREHIALQETVEFLNVWARMIPARISMMQGPAF
FAEVMASLRSQGHAAFFFEPNVLATAYPDKRLSALRKTVRGLKNDLATIHSEAEVLSFTL
DEEFKVDEAEITLNAMKAGEEGEVLTGRDADYCANVMRLATKQMDKLRGSLFRLKERLHE
TEQSLTSTKRSLLDEKEHSAHLQQILNATEEMAAAVASQCQLGSSRDNSSAQHEDVARPS

GDAPSTYTKGVLQELRRSMLMLKKEVDWQKERAQRGFCDRDAVLEIVYTQVERLAALMPP
SHSLNELKREDINMTNIASIMEEIIAGLMALKNDRSSNCVKDVQTPQTGPEERELAAMKA
TIEQLRSTIELLLSEKGKAGDGRPAVASAAPPVPVLNSQTDVLTMALRRLEEVRGEKIKL
AHELEEMRRHPRLAGWRSRHGTAAAESEEEAEHDDNMWVQSVHRLSEANTLLQEELRLER
EAGQSLRTKLKESERFITELQKQCRRLRLSGRIDLCEVIEQSGSDRDDDDEEEDSDDSDG
DSTARIPFEEKKQREVVKNTEGMGLGEQMSTEYYAQSHSPHSTVLTDTSCSLGPLVGTLG
SSHFMDQENHMACVRDQKRLLKALCGIHGIKLATLLIGKGSQQSLCNMHEILSMFIARSI
SLHTFFKCTGASAQVLRHVAVIYPLPPNEMPFLHWRRMLHPLKYQRGSSSKENNGPSVEV
FDLCEEFQALGRYLRGCMDVCFSHDGRFMYALTSADSAVIARLCSSECLNIPEEHQFRFS
AFLPKGMTPHRSGSTASGNGNGTVSNNVMSTGDSDQSPLPIDMLGWCGRGGICAWALDCM
QFDSAAEKARFERHLKSEYRLVLRLSAKEVQHFVGGSVELMGLTVVGRTVSRLVMSLTAY
EALSPPHRAQILQWYGGPQGILLLNVMNIESLTGAPLRRLLAMVQWHGRWPLSKTPPREA
LERFGFQPHQKQQRRP*
>TCRU_1554 hypothetical protein
MEEAIMSDIILVEPCAFYILGDPVADDTETTCGARKSSAGLLPASHAHAALYEWIRKFLE
SGWGKCRGATAGLCELVEAETALRELIALDESQVYMYRITSCHLTCLQREKQRWERELAM
REAVTKYQLQRLLHLEHHFHPAKGEKSVDTQSTLHQAGFLMKEMQECLAELHAPVLIRAQ
EAQKFAEDRLQALMRVLEHQTGRLRSTQKALNDVCLALNDLAACANVEARCKEGSEIAHA
AIREEDGVFCTPKEVIDAVQERITELEMRRMHTAHEANSTGATNDTFDALTVAVCRLEET
RADFYALGRRLGPVDSRVAPEVLRRLSEVDFMKPPLTPPKCLSPSLSFSPSANVGPKGFV
MESHAYEIHQAVLHEQRELVMMLNDIWGMRPLLLRLCTEGDAEQYAQRPLSLYAGHSLSL
HQWIDVDGTRPTKRATVFYPTPPERRGEVPKEHLESVLVEKAREARDALDIIDLRAEGEK
AGVYFTGNAVFFSADGKFMYCCCGTADTCTDARRTLMLHVVGMLRRRLGVPSANCFCYYS
SSFPLDASLGWVGAGVCAWALDEMRFASHEEKESFMLHLRETYRTVIRLTREEVRNFAGE
CVEIISYSPNYGPTRRRLVVSSHALRSLSEHNRSTLMAWYGEGGCIVPDLSTIERMGGYS
ARSMLLSVVTHGSDTPPPSGHGFFSFIGIRARERESACMEVTTTYKRKPN*
>TCRU_1555 hypothetical protein, conserved
MQQNEFNSVEAELREIEEDFASGQMNAFGTAATKENFVRELRRIADIETQVFYKTCGLLS
ASTSEDHLMRAMMLASADAPCAGGGGGGASSSNNSDEGNTTVATTRSFGRENNSSNSYAT
KSMKKDVANPPKMSGGFSPGSPSESRGPGTGDSASPGFSYAGPPPWSNDAGFTDRPFQDV
ARHFKLLEGEFTAIGEHLRQISKHVLQLNEIAERVRSKGSNLGP*
>TCRU_1556 hypothetical protein, conserved
MHRPDRLVISGSVTPAGHPRRKGTAPGRLSSGGPSQQRRQQQNDLVDSPQALSSATTTTV
SSRTVSSLCPFQFGAMPTVLDPSHGNAVSAGTQSSSPLLPQGRQATAPSGSHVLSVLEGP
LPAEIREIIHSQCSGEPAEVAERHARRLLSTHRRVRVYREELQSAVEEVGDVAQMLQRHQ
TVQDALRVELKDLDAQIERLVRERQLCELQLSQEDEARQRDEVKLRDAKERVNVLRSTID
TITQESLAGYVVLRQLVPNLNVENYVA*
>TCRU_1557 Acetyltransferase (GNAT) family/Acetyltransferase (GNAT) domain/FR47-like protein, putative
MPLIVRFAQGKDNDVFRDAAIIRDSIFMQERGVPASLEHDSHDAYAIHLVLYDEDASDNQ
KQRGGEKKSLPIAAARLRVVRHCGVHAYDTPVVKVERVCVRRPHRGKGYGVFLMKEVEKY
AREKMGISTLVLHAKLPVKKFYKRLGFHETSDEFMESNISHITMSKM*
>TCRU_1558 Protein of unknown function, DUF607, putative
MSPVRTAVVAGGGALRLLRSIHLPCAAFHRCGFSSRGCRRGVLSVPVEDLTPKSIALFHS
YGPPTSGTVPVVTDTTAFTRILNEVLVRDDSGANDVIRDLRRFLRQKREELAPLLAEKHR
IDAVVDNRYVPLLGCLVLAFLVTQFVVLFRWVFIVFDWNLVEPMTYFLGYTVVWAGIVFH
CYYARELTWETMLDTVAHRRRVHLYEKAGIDVKKVERLQREVCGIERILSKYGLE*
>TCRU_1559 hypothetical protein, conserved
MSGAPFIPELSLGTVETGGGWGVGEQFARLPPASVQAESRTPVYTVAPDSGGFNVHDSDD
DSATQEESAEAETIEAEVVPTTHYRRPSMEDDNTFIDHGSTVPPASPSKDLFEKLEMRGD
GGDYSKRGPNSIPSLHVKFLRPVSRMMVGPQVRAMVVAPDGASLWAAFGDDPVTLLDFKG
SELNISRALDVTQVHSMTVVRVPVAKAVTFKPSRGAHRPSYFAASKPQVDEGEEVETYAL
WCGVTRGSIVIVDLTNFSMAGVIKAAHAQTITGMWYLGTGKVWTAGYDKALKVWDPQTRR
RLKSRNIATIISDLCYVRSCKQVWTISDDAYIRVFDAAGNNVQLAHPSPEKPENTLRMRS

EMRFITYYESANLIYVALTRSLAAIDPSTCGMPVVINATISSMTFLENTALVTGYGEWLQ
CTKEAIGLIDLSDPHVPSLLFRGGPLGGSVTPVRMRFLTAVPFAVVAQEAGRSERYLSVF
SYEDSKAFCRSGGVTSIPQQRKITLEAPSTRRPVAASQHAPSPSFAAATSTPTSALSPYP
IVFATGKRGSVMREESNLSSGHPQRAAVTSSRADGNNQRQRHQHSPSAPFSQNFTPIQNS
NSNNAAAAANTAVSSRSQSTTPAMRTSTAIYSEDSRNPSKSGINGSAERSVAVQLTTPPE
LLTSLTKIENKTEDLKALFAQISAPRPLLDDFSKLHALVSRMAMNNQLGLNLDEEEMENI
ERDYHSLEGRVIATAVERLRKTAAASLLSSVPNSTRLRDNSGRRDNVGSVAATSPSVVEK
RANAVTEIMNSSKAEETAGSVGGSEIAPESLLRWVAQITRSGQGERESHRRQLESLQRHN
TRIVERNAALINGIVRVEQAIRTHAQRLLEDADAAAAVISSDASCDSSAHVHAQRHIQLL
NQSLQQIEQVSVSSSPKEITEAMGSFVTLISRLFSTQQVLHGDDIARRVPYDLSSGNAHN
DQVLDTSRASAQNGSTLLTTRSHFSRPTVTSVLSMRPQRMRGIVEDQIASVEEFVKDVGK
FWSRLEETQKVIYVPYESGAKPDLFQDFMQFAVLWHLREAQVMVDVCRKESVMVMAEALL
GDIESREGMVGSNMAGPRRTSVPTALLSSTEKAMHSGGGKNPNSADIKKSNKGGNASILV
VPAAAETIRSDSARLVSIGLELESVASRIRESLKAAEQNLQMGVAKETIGGFFSIPTEKF
FIDATKLRGMLYWERVSMHLLYECLECLEELVFHCDEQPRAFRKDNFKALEQQVVDWRLF
LEDVHGECTQLRSVILSSQQESCTGRMSAAATASSVVYDRSSSLGSREMSRNSDGDTTST
ERTTQWVLLFGLYVQYAERTDATPLLFASHDSGDTVSAADFGLMALLDEVAEIMETVRAK
SAALLRYCQKIQGRIARVVEDVVVNEETGQVFVPACRGGPIAERYCKGFCHDMS*
>TCRU_1560 40S ribosomal protein S12
MAEETPVLEVSPAVVDAVMDATPENLEDALRIVLQKSREVNGLIRGLSEVARALDRRTAH
LCVLAEDCEDEEYKKLITALAKQGEVDLINVEEREKLARWAGLTKMDSTGELKKTFKCSC
VAVRDFGERTRALEFLLSHLR*
>TCRU_1561 60S ribosomal protein L18, putative
MGVDLTGVQKKKRVVRHHTYSTNPYIKLLIKLYKFLAQRTNSGFNKLVHQRLLKSRSNRA
PISLSRIAVCMKRKSVWLEKGKKAPIAVIVGDVLDDVRMTRIPALRVCALRFSKSARERI
TGAGGECLTFDQLAMVAPTGKNTFLLRGRKSGRESVKHFGAAGVPGSHAKPFTSNRGKER
QRSSARRRAFRHK*
>TCRU_1562 Uncharacterized ACR, COG1678, putative
MDKIVRTVYRQLYKKAASVDKDIAMRSVLTCSPQRLYDHRRGVWIAFDLAKQSGWPESRI
FLDSLIRKLNHGRDFFIPQAGRDADDDGCRGGSSEGNRSGGNDSGSSNSSSSAHENEDGN
KSNKNNTHGGGEPSPTLLQVLRKSFEETPFTIGQMNNAFAALKELAYVTEMASKHYGSGV
SPPKEFDTLPKPKLTVVDTVGEMHHLSLTKGLSDVLERLSGEEKGMVSRKSEEDVRVKEE
TKDGTGKGDEDNDDDSDDDNFGLSGKTPGKNPFETPKAVHMLLAHPQLYEFFRYSVMIVV
RSTPNEAAAFILNKPLENDEGMLMQVNSTIRLNHVHPILGKHLGNHTVMIGGPVSRGSFD
SSILLLHRIPDVEDAIPVSQSLWVDGNYDVLQKKLDDGTADAKDIVVICGFSGWGAGQLA
GEISSGTWVVARGSADDPAMDDFIFALARHAGESYCGAPQQPLESASSSSSSSSRRKAKK
MPQGGECRIRSAAAWAWAYSALGPPYADLARNQKPFMQDPREN*
>TCRU_1563 hypothetical protein, conserved
MQQPASLSVSAPPAPVIKRAVPVKLTYEEVRRAAMFSVLDDFASQKQYKCRVQRLKDVGG
NVQVIPNPAVDTVEAAPKGSSTRLLREHSSASWSVYFYQWSLFGLTSISSVATAMGFYLT
IYHNRMFLPLGPMAAAVTWRLWDLLEAAWEQQRYIDNAARLRDSRKGNPMNLVAKRTASQ
EEVEPLEEMM*
>TCRU_1564 hypothetical protein, conserved
MNFEDPFADDLQSLDAVMAQLAAEQGRRSRPPPPESGTSSLSTAIAGKSAAVLEGRTGRR
DAVSIKSLGKQVQPIAAFATATRTPASTAVGKDEPVPWEMYDNPSTTARTHASATTSWGT
SLVAHVGGRDRHDAAVVGRGDGDDDSDKEDPLAFLERAPSLAAPVVEQSQATKPVGADAA
SSPQRHMEDEGARGQAERRDLLSSLDAVGMELDQIYSTLDRLQIQADTELTGLETTIIEK
QTELAIGEDRIASERRQYESYHQRRLKDITDRTTELLQRQVEEVTSMEKEQYEAQLRALT
AEMADVKNRIDQLLQQRELLLQQHRYDEKGIFLALADEEEANEKKTAADGGVGTDAAAGP
TSSTSSVETKMNAALRLLWRHHNERMESLASGVVRFLHQETMDVAREVRERHELAYLQDA
VTRKEELGEFMQEFLRRYREFFQHRSEVRSRKIAALRRGLQEATEKLRLRARQRLEKRTQ
EVAAQMDEAARRFEKLALESAACVRRKAAAVRESDDSVAKSQRADLESRCQMERAVTEKL
QQSEMETLQQQLERMRRLGNGSTSEDDSGVLQKQVAEDICAARAESITGQVRELESSVQQ
LLRQQLLRNQAHGDKGEGQLFLTDEEIFSRMAVQEKEAVVLSLFEEVQKRQNELSATRQR

CGELREGIAFKMQEEVQTVRQQRLAQESRLASVELLRMAWEREQRELLQWGCDMIVTHGD
SASTASSTARTQLPAATTTMDLMSLIVGRNHAIMEERSKLRTRRRQLLHTIEKERENAVA
KQRETEVRWAQFCEQLVGLVAEEHAVSAQRFAAATEIAKMAALRELISHDKELFCRRREE
MQEEVERLKGKLQATLTQQAEYDALQQKIALEQTALAQQQRQLAIQQSRLGRPPASAAAE
EEGETRCPPAKRPPRSDRPLSPGEASLRRPVETLQEVTARDNIPVLREKPQWQQQGREAR
RNTLPPSMARQNTGRSFSHTSVFTEGIGEKASAV*
>TCRU_1565 hypothetical protein, conserved
MPPRLASKVGRAGRRGMTSAGITRRRSGPTPGEIVAATFANVELSFTKASMAEYSKRCER
MSRENELLRHQLEEHESDSIKVVQHLKEKLQCAEKEVTEQRSEIERLLAESADAEEDLRC
QYERILEERDEQIVQYATIVQRLQSDLKGAAQHVHKREIHQLELKRLHDEIEEMRTRHDC
ELAALRFQTVDRKMRLVALEETMRERFQAQVEKESERLLEAKSRALLEDHESLQCERVRL
TQDLEELVQLATVKNMECADVRRKGELHQRACEEALRRIVLGNRRARDSEAKTQRLERRV
KELLREKNEMREELSRRYETQIHELEKTLAETQNSLQCHRNELQRLRRIASTIVGQRSDL
ERFFYVALDDVRRMRSKAPRRKQVASPVQLPSLAADNNGNVAYTGANPRHSQQQQQQAS
PLILEALPSSLATKGGPTFLTESPASAKGNFNTPAASTVNTVPSSIPALSPTGPSSSVTL
PSPPPGAAPGRRCGGSARPTRDAGNSNNNVLLINLGENGEGAYLNDLSWEDKEKIIKALL
FFINQTCYQKLPGSQPVNSEATLMET*
>TCRU_1566 Protein of unknown function (DUF1014), putative
MPSGPKSNKYVNRHAAEARERDQERQANAKAAREKDREDAMWTEDDPKQLRKQEKQRETE
EKMRRQAELKAERKEQLEQEERELGQKVPQKVTRRQLQKDLSKMLSDYDKLKEKERQQQP
GALFKDVGAALPESNPNRMARKDDRNNSSNEIKATGGVSDALSALQSGMGTGGASVPEDR
HIGRRARVVYKKFCEEHLSRLREEKPGLRRPQYNDLLWEMWQKSEMNPFVQRSEQRSRER
LEQERRWMEGEDDDESDGGNEAEKE*
>TCRU_1567 hypothetical protein, conserved
MSAAPIITGRCFWGRRTTPNGPLVYTAVGSHKGSDDRRRTPISFIGNTYYERRFGGLRST
QSTPLGTSLALPSLRNGDILVKMGSATPASLRGRRIVDVTGIQTSFFDASERNARARCCI
DDVVHSMHMHDGHEVPELNRGTVDIGRLTVLCAFPVAGTASLLVLENASQALLKRRSLGY
RSEEEGVQLVRVIAESCRAAALRRICLHPVMRNDCIVGVRL*
>TCRU_1568 ATP-dependent RNA helicase, putative
MPQRNVCQQCKAYDEGAEDPQDGRFYCNSCWAAYNGFSGDTKNGATKGGRRGDKEKKVRA
PRRKELEAMNNLGHDAQGVLRRLEGMYASCSHKRRPETLATSLLEDVSMVMADTDKQQRA
SSFVGQFMQRQRAITVVSHLGEPLSPRGDRVGCVGRSAYQESRKVPLDILLSSVDDIVSQ
IYVNFDESMTQVILNALNRVTTCDTQNQRDAMRVRVIDELLETLGDEHYDLLQCVMHRPR
DIFLRLLEEFCTLEDDDDNTGAGNTSAASPQHALTVRFVKTAKQRSAMREVVFAHNGEAT
DQRWLAHMTQRYRQLMRESDLDQLFKRDLSVIGSSMPSGATVVQKKDHIRIHVPPPTQKV
MPMTERVCVATSLPEWTHAAFQTVTHLNAIQTTLFRTAFYTSQNMLVCAPTGAGKTVCAL
LVMLRCIQEHFVEGTLNREFKIIFVAPMKALAQEMVENFSRRLAPFAMVVRELTGDMQLT
KREVAQTQVIVTTPEKWDVITRKQSNEDLVQQVRLIIIDEIHLLNEDRGPVLEAIVARTL
RQDELHADQQRPTRLVGLSATLPNYKDVANFLHVDLEEGLKVFGPEYRPVPLEQTFLGLH
TGAKDKEHQLDWLAYTEVARNVREGHQVMVFVHSRKQTVGLAKYFVEEATKHDQGNLFKP
SGKLPTEAVKRGGSLQGRELTNLFSSGFGVHHAGLIRHDRTTTEGLFRDGFIKVLVCTST
LAWGVNLPAHTVIIRGTHLYDPRRGGLVPISVLDVMQIFGRAGRPQYDTSGHGIIVSDEK
DVGHYLRLLANALPIESQLQKTLADHLNAEIHAGTISSIVEGSRWLEYTYMWQRLRVNPL
LYGLKIADVRRDPKLKTVRYELVSKVAEELANAGMIRYNPQTGAVDTTELGRIASHYYIS
HESIATFNQKMRRPDETWIDSLDIGSAMNVIASASEFSQLRVRQEELDELQKIHAMLPKK
VQRYAIVGESADETSVEWKVTTLMKAYISRIHVDMHSLASDVNYVMQNAPRISRALFEIG
VQRGRPLTTTVFLTLCKCMEQRCWEFEHPLKQFSLDFTDAVQSHLDKKRPSMMLLQEMTA
REIGSLVHNQRMGGVIAGLVATFPSVSLNIDVQPITHTILRVKVTITSTFTWNSRYHGSS
ELFWLFVEDQDNNFIFHHEAVSLKRKEVEMGVPVVVDLSVPIVPEYDMYSVRFYSDRWLG
SQEDFTFSVAHLHLPDDTQLTTRLLPLAPLRREVIPEAYHAIYKGFPQLNAVQTQVFHAM
FHTDSSIFLGAPTGSGKTVAAEMAMLRVFEQCPPGSKIVYIAPLKALVKERVKDWTARFD
RHLGRRVLELSGDVNPDIAALVRADILCTTPEKWDGLSRSWQVRRYVTAVRLVIFDEIHM
LGSDRGPILEVIVSRMRYIGWHRQAPIRLVGLSTAVANPADLSSWLGVQEKWAIFNFDPS
VRPVPMRVYISGHHGRNYCPRMAAMNKPMYNAICEKSPNKPVIVFVSSRRQTRLTAMALI

GFLLMEQNTAKFVRMDVDEVTALTEKVSDPYVKHCMQFGVGIHHAGLLGEDRTVVENAFL
AGKLQILVATSTLAWGVNFPAHMVVVKGTEYYDGRTKNYVDYPITDVLQMVGRAGRPQFD
TEGVAQVLCHEPKKGFYRKFLYDPFPVESTLHKQLHVHINAEIVAGTITTRQDAVDYLTW
TYLFRRLVKNPSYYGVEDRSPKAVTIFISTLVANVLDELEACGCIASPGDDEEDDLSRKV
TGMSAVGSIDDEKDPDALTCTVLGRLCSYYYLSHKTVRYFDLHIEADSSHVDVLKALCEA
EEFNELPVRHNEDKLNLVLSQSLPFPINPNNAESPHVKAFLLFQAHFERASLPISDYHTD
LKSALDNAVRIVQAMVDITSNNGHLYAALRCMSLLQCMVQGIWWHSSTLLQIPHVTAGML
PMIASRCGNLEHAAQVANSSITALQTLKAVLREDCGLSETQLREAMAAIQGFPLIDVRLC
LSRTPDRTSSSGDAEHSFDGSETAADVAYTLTVHLTRLSVHNKHVVAPHFTKPKDEQYWL
VVGNEKTGELIALKRVNRLVNCVEMTLNFEWDDEWAEFAEGGTVALSLYVVCDSYVGLDQ
QYNFSVPVPVGM*
>TCRU_1569 Histidine phosphatase superfamily (branch 1), putative
MTRVQHAQASKRRSTQQQQEFVGLVDELLLLRHGERQDHVDRAWRATSLLPEADPPLSAA
GRRQALETGLMFLQKRKHRKVRQRALGMLSLLLISPFHRCIETALIVNIVGFDGKLAMFI
DPLLSEWHSTKLFSRPPRLGGSYMFTREMITFRPQWEALGSSLWTFFRSAGNGTEYGIDD
VMTTRWLSVLETQHAQNPSFLVWTSVLMQESLRRNLHSEGGVVQRVLGGASRSDLSGINY
PENKKHMLRRVGEAVELRFDANAGETAGVPACVKAAEEQEAKYLPRRFTHRPRMASAGEA
TSEERAVLLPPARIMMVTHAEVVAMALKHCCPKHHSLDSRFSVPYCSLTSLSRVNDYYCV
LDDGAPDARGPRRASEKSWCVGTVGSTAHLQTTILLR*
>TCRU_1570 hypothetical protein, conserved
MWGSWLDQIQKRLDDVSGLAEQAITLGGGDEEEEEEEDEGRETEKVGNVQGTSGGRSTSP
QPSPEPKLQQGTHGTTFPIHADDTVDRYSLPHEGVALAAAAASMPFQTKNASCFVPATAT
EELTGSAVMTAVGGTEKEPFPVVGLPVRLVESLEAHVAPTMPETVVEVAAAAPKLTTEAT
TPAAVASTGIDAIAQDEAAPHPHSCQQSLLAGDGNEEQLLRFQQVLAEELEVSRLLREEK
CCLQDRIGTLEKELRDLRLFKKNALLADPKKLEALEREGMELSTKLGNERERYKLLYEKK
TQLESRVETLEKKLAQCALKEEEFQQSIAASHTDCEAAKQHAEELRVFVEVQESKLQQLQ
RQLTSAKKSHAELVVEHEKELAQQKAELIFQRESMSGALVSLQREKEAIASSLQRTQMEY
ERRIQELENKLLQANRRADQAELRLTDHERSVLAPLRELRAALDDVERQKRLLMEEVEYW
KGECSAAERSRESEKKHFTQRLADAQSEVQVAHDERLAAEQELQECRGKHSQVHKALERA
LERASDAEAKKEQLSIALEEVVRRMEDAGSSNNNSSTPNSISHAGVGEMTRHSPISIEGG
ANSTRVPLFQVETTESVGDVCRGSRSRAERELVRQAVEIERLRRVEEEGAEAKRKLLALA
AQHDLLMQMYGELQEELQKSQLERPSK*
>TCRU_1571 hypothetical protein, conserved
MSQKLLLHVDVLRGRNYPQTEDDPCACSTLVRLFLYNNNLQETIADVFKTGIQENSNAPF
YSAGVDFDVPAGLESVVLVVEVEETTRRTEPYVLFYGCQPVSMTGKGKCEEVITLTCTNP
VEKPEEAAQEVLLAGEDPTSCPVLTIRYQVIRQKNAEEVRDVHADVELPATPALTGATSI
VPKGSRYVWVNLTSDPRWTEAFRHTLVESWRDRAPLVGNAVAKEGLQASTKGASTSRSSS
QPTRLMDIRSIGDVIIKRWCCCRAHEVLTRLRLCAEAFDRGERTIHFSQERELSYRLQRK
ERVCLLQELAKASLVKMGISELRDTLDHLWIFFSIGIKHDPAGTRALTYEARKKVREASF
DSKDKAILQSSLLNFLVPSLTMDQCNEIAVADMESAERAGNILEETPPTGAKFGSNAMSA
PASEVDFSMALIEYIGALLDTLTETELVAALKAFEPAVVNAQQRIKAKAKEEITSMRSNF
REPMRRELTPYNPGDGIKIDHLDRLGRRKKSYHPR*
>TCRU_1572 ferric reductase transmembrane protein, putative
MMSHLWSDGGDTSVASNSSLEHSIVSCSRNTAGELPSLRLFLTGESACGTLPQRKIDVST
GRIDAAVEISFVIGDADPRTVLVFACGPAGLVRSARDAALSLGVDFHEETFLL*
>TCRU_1573 hypothetical protein, conserved
MVRCRGKCPRECLVQLQVVTPQKLGGNEAGEAVLSSSLDVSALCEAIGHKMSVFCGLAVE
ASAAGLQVEAVRHDTDGVYHVVLRLPQATPLAVTALRASCASTALGPVVGGARRTVRQKK
PRQEETAVTTPVAVRVVHLTAVKAA*
>TCRU_1574 Mucin-like glycoprotein, putative
MMMTCRLLCALLVLALCCCRPSVCAAEPEPEAAASSKATTTPTSINPGANRSVTSQSTPA
TGTPPVPGQSDPSSTGPVASPGDDGGEGSGSTSTEDLGPNGVPNTLQADDNKDTTGPPSD
NGLSEERANSESETSTEETTTTTTKAPTTTTTTTTQAPSTTTTEAPATTTTTRAPSRLRE
MDGSLSSSAWVCAPLLLAASALAYTALG*

>TCRU_1575 nucleoside diphosphate kinase, putative
MFIGATKPHTTLVAEHTFLLCKPDAEQHHRYILQTVSDAGFQVMAKSFIITPALAEEIAS
TFQSVRKRMYAERGVDKLFNGGKTALQNEQNAPQGKRRDIMDTGRASLLPTSFASAFIGN
AGGEEVSNPNNISSRRRSGSVPKRHCSRDETHQGILAEILRLTSRCADGGEGVNAGLPNV
APNNRAVDGRGVSEGDGRQDENRMQQEQKPERRLATGTVLKNTMIEAMSYQDLLNEHVRH
LAFGGTCLAVELSRHNAVEKLLEIVGPENPIDARRAAPHSLRARLGTDLIRNAVSAASNL
AEASHCISAVFGFATRYSAADIRAHTAMPEAGGVENNEQVSGVPAIVRPYCELVSAPYRA
SPRGPVALEAAFRIGKTNPQAHLSYYTGRPLYGRANEKEEDDGTGRRGPSVDSHLWKQKA
EQLEQQLAQERMDLTLHAKLLRARELDLLLREHALENATNNLFAPAQPFKATLEAQSLPH
ISPLSSEEQLMRTHLAPALMQGKQLSPRSKGDVATSRQGLPEPHSPSARYFSTEAYTPEV
RGVLPVIEAEDVSLLLTKPVRLRALYVALKHHCENCQLTQDEVLALYDRSPSIQLLWMDD
HREYFRNYVNLHTEEPVSFDSFVSVLMFLLRQ*
>TCRU_1576 hypothetical protein, conserved
MQTVIQQIHQANLKAVLLSRINLFFIIFNCVAMLIFIVTWSVSIAKEGGGVLARYAACII
SFILLAVATFLSAMVHRKQPQLLLYYAHEAISILALILTAISMGTNDVVVDLCNTKRALS
NTQCGSHTAELLAQIMACISMGFNYASTQQRIVNFIDKGILDGIRGRSGGMTQLP*
>TCRU_1577 hypothetical protein
MRLNNPFLTQRDEMEMGGENGNRRSLQENLERCLHSEGGDELPVDEEDEDDGGDASNDDC
PTPPYVPCRYQFGLSAAAFVIRLD*
>TCRU_1578 hypothetical protein, conserved
MPSSDRDSTQEQVLKSPAAPTAATPKAKNENPGSADAVLVQQVQKVQSGLESGTELRKEE
SVLTTNKQQDGTFDSHAESTPTSRSSEKSVAASNGPDKATEDEISNKKTTGVDAVPEAAK
DDGNKDDNKKTPIKTTAIANGTAPTSDSDGSTAVAHTTSPLLLLLIVACAAAAAVVAA*
>TCRU_1579 hypothetical protein
MGEGGEIFFSFFILLLIFVAFGAVLLLLIYGVRCCRRKIQRRELERRQNAHDERERQRAV
QLLEMLSILREPMTFPASVQGRTASLASSTRSPASVSRTMRRGTRMGNSASMHSEGGWLN
ERKDSNDLVQNVPSEVETAAAETASTQVLRSSTVSFHR*
>TCRU_1580 hypothetical protein, conserved
MAFSGMSATFVSLCVIFFTVILIFVSSSYLRRRHLRLLEGNITARRQQRAEADLRDNDRA
RSRRALMRQAILRLVERGEAYQGIPLVSTANGTEMLADRQLRSYRARNGEHRRPELLLMQ
DGRHATVSSIVSMVQANHTPPPEAPPGPNLLQMLVEISARRQGSEPLQRGETVDTLLEQL
QHLQEEEEEEEEEEAAREREAQPDADEVYGKGLPYLLNPANEGVVYFIDDDAEYDALLCQ
QHLARSKSALEWILTPLLKRKNGPSFENPRRAME*
>TCRU_1581 Trans-sialidase of Trypanosoma hydrophobic C-terminal,
putative
MKDAAKGRSGDSTPSSISTSDSVDAQRKAEEEGDNEMEALNPNEASILPLVNKNGQGNVI
PSFSGEQKEQKGQLLGKSGAGGASALSTVTSSPAGKESVKHLASGTSPDGHQNVDGGSTA
DGYQTVDTGYGDKTQEDGPLKTHTGNLAKAEDANGPTTMGVGKDGTTVNTEVSTSSAEDW
ETVVEKNVHEEIKPQNRDVNGTALNSSLGNLSRGNNSDAGTMRESGLLPSLLLLLGLWGI
AAL*
>TCRU_1582 hypothetical protein
MARLICVQNVCVIVLLPMRACWLFGNLTGTERRDEYLCVNATVHGALTGAERGVTFEFAG
AGTEWLVGKLAHNQLCKFVNSISTVLATANIHGVSKEGSSTVLLGLS*
>TCRU_1583 Trans-sialidase of Trypanosoma hydrophobic C-terminal,
putative
MVKNVFLYNRPLNSTAMTAIKERKPVPKRAPEPQAGGVPQTIASAVSGPEKNSAAPAVPM
TLNSHAVEEASEGGAAKRNASWNEGAQFVVPEVSLNSAGSGLLPSLLLLLLGLWGFAAL*
>TCRU_1584 casein kinase II, alpha chain
MTDQDSSGRIIIVTRPVPASPRPPENNGDVSHVTTDCKSQKNTGENTVEVKRTLTENEIR
HHETGVISHPFWFVNNNMPPSYWDYERMTIEYSSGEPYELIQKIGRGKYSEVFRCRNRIN
GELCVLKILKPVRLKKIHREISILQNLCGGPNVLRLLDVVSISPEGTPVLVTENLEPAES
FRSLMNSGSLSNFDMRYYMYEVLRCLHFAHSHGIFHRDIKPHNIIIDHQRRKLRIADWGL
GEYYIHGQAYNVCVGTRNFKAPELLLGLRLYDYSLDIWSVGCILAEMLFRIFPFFRGQNN
EDQLYRILEVIGTEDLTRYARKYDISLPRFLFGSGGLFKRMKKPWYIFVNDQCESWCDVH

AVDLLDKMLRLDHQERILAWDAMQHPFFDPIRSALREDPQEQYPQ*
>TCRU_1585 hypothetical protein, conserved
MSSSPPATLTAEEVEAVTMSGEALLQLASRLNGAAFVPSSNSQAGGRMWGDHGSGDGGGD
APFVTVEDGAGFMELLRDVIEHCPANLQARGESHYEQREYGAHNEGRSPHAATMLQKRLL
ALHHVVFGLQPELLATVLSLLHAPHMMTDGDAVCHLFQSALLLLQTVVFSCCIPSEHLGQ
GWRGSYVSVEAMELSATCTDLLDRLGAVGIVSDFLYDDFPLTVRLAAAQLLFIMLLRDGS
TVAQAKSSYVRDALSSPERLSILMTVLTSTDAYINSVAAHATVRNLTTDELAMPENGASH
STTLTETGGLSATSATLPDLCLSLRLHIAACVRELANSHYSRLTGPEVLDVLLRLSDTDP
SGDLRALCIESMHVILRHCASSEWATVSFKKELAALCATQLGREGHHDALHATLCLLETL
ILRDALDNSAGDISKADDGEVTHRDLCLCESLLMLFADRALAALIGDAVSPLGERTKKHS
SGRGVNESQPRHRDPTYHSTQDRPQFLQGRKMATRDIFCNVTTVSCLSARCLRLLVQHAP
YYRNGAFYLVQHRPSLSRLLETSVRLAAFHEIRCRENALGSGYDRGISNSIDFEGGGDGV
NVDEASLVLAVELAILVGLCLAQDPRARQLVAAEFCNFHVEAQQMRSALISNLNLVSLSY
FGDAASGDIFEIMDVTGVRLNTLDAVMWDTPDRPSRAAVHHLFTTQESRWNKGELRILSL
SSRINGNKDLADFSDGDDGKQGRENDVNATMNNNGNNGDNREGKTRGGDMYALLQTSDHQ
RLQRLTFIVLSYAIHYTFKAATGDAAAATRFAAAFRGTADDSLRELIATLPSPSFKTTSA
VEEQRRTSRVASTAVATVPAAAIHDSRAQKNLRYYDAYMLDAGKQVPEAKGQKSSAKNSF
LVSARRGQLQRPRDRLTSSAPMKGASSSSSTSTTTDGEEESAKNAPDGPVPVTAETAHLI
LQFHRGLRLFRDLAQFYNKHPTSMLSRTTVYQATEEKGMVRRQGKCEAQRTIAKILQEAE
DASLLSQQPTTTTPTTTTAQPPQSQRGRVTSLGLIGVNTRSWSINELQEGDLFYFCIPHA
QLTTEALQYTRRRALKHSRHLKKVFAITPQSAKSRRWLLHDAIANIMPGVVHALSQFIQW
FDVYGEENVKLPILIYFEDDVGRDDDDNKNKSGSQRYSQDGNGREDSHPAGKKRRSGDGG
GRSAVLPSAPSSMFRPTPSHPQGQGDASMASSLPRAEEVALHAGNLISLLQQVAFYLQRR
DQLPHPEDGLRSREFSTFVASPMPQKGEDTGHPSYASPSITALQHDTGGAGTPIRAVTNS
ASPLSTARNSVFLRDIEREIERLQKLDVYGDENGTEAAGGFLLPEMAVSPYGDTLTPHAF
GKIDGFGRYENDDDIIEEDEEEFDELKGGVER*
>TCRU_1586 proteasome regulatory non-ATPase subunit 6
MATAAVALAPTADAAGSVLEPLLDKAEDLLADGRRAEARHVLEGIVATDVAADDAEGLRV
KERSVYRLADILSLERQVDALVHLLSVIRSFFSLLPKAKTTRMVRKLFDLIRTSGASLDR
QMEVCSDMIAWARQEKRTFLRQRLQHRLAEVQFARNERQESLITLQALLREVRRLDDRAL
LLDIHLLESRIYYSIQNISKARAALVAARTNANSIYCPPLAQAEIDLQSGVLHAEEHDAK
TAFSYLYEAFEGFHQLGDQARQARKALHYMILAKIATDSPDELAALLSSKNVLEYRGQDM
DALRGVAEAYNNQDTHFFNRIMQENKDSPFLADEVLQRRLTEMYTSLLERHLLKLLEPYS
RVQISYLAYLLKLDVETVESQVSQLILDKKLAGIVDQQHQCVVVFDEQDAKVASKNQKQQ
RQQGMVSGGGGERGSSSAAGPTYKNAKSTTEDGGEKPTTTLYQDALEALDKYDRLVSALF
DKASGKFDALVEENLKRRQEMKTDKKKDSKKGEEKEDAEKKKKNAN*
>TCRU_1587 ribosomal RNA methyltransferase, putative
MGRASKDKRDIYYRKAKEEGYRARSAYKLLQLHEEFNILNRDEIQTGVVDLCAAPGSWSQ
LLARHLREEVGGRNEKQSSGAPRIVAVDLQEMMPIEGVQLLQGDITSEATAREIIRLLNI
KEDTASVNDAYVDSEEDKSAIQTDKGGRSTARRPSLAPGGRNSYSDHTNSCEKKADLVVC
DGAPDVTGMHELDEYLQHHLLLAALNITTFVLRCGGTFVTKIFRGPNTPFLVAKSEVFFR
HVIIAKPRSSRNASMEAFMVCQDYQPPAGYRPSFDRPLTTTTRCFTPAAPLPPAFMHLQS
EGRQITQEEKKGVEDQACMMESVIVPFLACGDLSGYDADMCYDRDSDVPVLPPVHPPRQA
PYMTTACKNDEDSVVATNGAEDIMKRRRVE*
>TCRU_1588 ubiquitin-conjugating enzyme E2, putative
MSGLSLAQARLREERKAWRKDRPFGFWARPREKPASSAPLAFSSQVPQDATTLTSTAAPT
ATANASSSTTAGSTGSASRSHTAPTASTGGSNNDAKGLLASSTAAGSGGLDLLEWEAGIP
GKPGTPFEGGEFRLLLHFTEDYPTRPPKCVFTPVLFHPNVYPSGTVCLSILNEEKDWRPS
ITIKQILLAIQELLDNPNIKDPAQEEPYKVYMRDRKEYEDRVRQEVAKHHKKRMV*
>TCRU_1589 Kinetoplastid RNA editing protein 1
MKRLTHRGRCRPAGILTCDAWRNWRRVSRASMAMAHSSHAFMILHNRPRSLRWVSTSSQT
AGETAASCIPSSSAAGDDNSAVHADACGACEAFAPGEARELHFHEDFLFEEVDVHFLLLE
EAHKKHGVLLNVPPQMSPSGTPPEVPEVMFPAVQLERLRGMQLAYEPTHLPPPLHTTGTR
KLIIDDASPMATLVTKTSIASTPTTTKSATSTKGGGGTAILVRHLPPDPTMRFHCTACGK

AFRLEFSAEQHVKLKHPSDSKAAVGTGPGEGEIIEEALATAVTPTSPARTAKETTSKATG
AEKGGPSASENGAAGGAGATESAKTQQPSKDGGLSGAEVSTAKMPYDKATLALPSEELVD
ELIKEIWDDIAARRDDIPKQNDPNAYVPFNAVVEGTADRRKEIEANAKPTARATPEGAAP
GIKKPSMLSSGSMSAARAQKPGMSVPIKELVKKYPNPFGDSPNAVLQELEKEPINPFIPQ
EELEAQLQVARETPPLASTPNGVANGIPLGSKTKTSSATASAAFVTGTDSGRVQDKLKDR
LRASRPSLTRSASLRRFVCPLCAEKKRKKAEAQDSNAIPSFRLLDALFDHVESAHEGEEL
SEEQLHSLYKTQRSSALHAPQPSGNSGTGETSDGAVDPNTHAFKDDDTIGMREETIQDAV
RAASLPEMLQKAAPPAAVEQKALNVHVRAGSNTVLLGRIADVQHGFIGTMAVTQYVLEID
GENATSNKGDEGAKLSPESEKELIVIRCMGDNFPAALLKEQVRLGSTVLVQGTLRMNRHI
DSASKRLHAYPFVQVVPPLGCVKVIG*
>TCRU_1590 hypothetical protein
MILPKGKSDMYGRDAHVPSLRSEYHPSEWIFLLVVMFTATVCGGGGIGFILPVTAMHDAS
MDMTSALAPTPAAAVDVAASYSHAYSADACASYTSCAECVFDASDGILSPSMDCAWCVIT
HRCMRFNVSFLTSNLTPSSSSSSSSFYAAATAKTCPDWRHAKFNANCPDMSCSAAHTTNN
IYICRPITIFWILLACILVLLDLGFYMWLHAIRQLPWKYEPRLSQLLTGMPTPTLQLITP
VRVNQEQAGSEGREVPTEVDMKEQTSNYHHSNNINNHVDDEKGDPHEHNSQHQQTQKQKR
VLSNCPICKMAQPAPLPPGHVCFWCDIARFGFIPLFIGTSTAGLSFMLLFCVSLKPWFAD
GFYAYLLLSAYISYALFGAHVYFRRASRVHVEAARETLYISLAIFLHGRSVLSVMPSLSE
GDIVPSASASTSAQASPLQRPVQPPEGGVGGKALLQSNGLTTEWIRQKKGETAQLLNMED
LEHGIRKVLRQTLSRDECVLWCEKPEVSSVILYDLWLYLDLLAGLALGIWLFVASSVKDS
SYVLVRLVGSTSMCVIGFVLILVFACLLMTTLSSSTRLYVLTDNRLLTIFNGFISPTVTE
TELRSLRRASIYGCKMWTAKPELTFSWEVPVTERKLPPIKSQNFPCVVHVEEFLKYFKMV
APKFMPINDQIRKGIRHERKAWRLHLFFCIVLLHFLPIITFYPLFVPGFLSLLLLIIFGV
VVLATVHRGVRAQHVTHTPLNLVDHWAPCDLRRSRSGSGGRASPLARLVADGNSTAENLG
SRPKTVKFSSGLELPRSRESRTSSPPPPARDRLK*
>TCRU_1591 hypothetical protein, conserved
MWGVLLTLFLVALAPSLQFPFIAAFAVQDAAATNSSATGDHLPGVDLHTLVEENATLILA
GDALRYVYVAPKRYVHMVHLALQDDLSRCFCHFYLQQQFLTGGDLDAETVVPERRDVDAC
KMSYFSLLPGFNDVPEELAVHFTATWSMPSRDYKKNPSGHVTQLTLPQGCFTGLQRVLAK
LAGRSGNHPLRRCTVFLGIHPSSMMKDPSNVLSFNTRLCGVMCGLAIVVLVISGLMVLVT
VLFMSCRRWNAEYGMDEELGQAGAVDGVISSDHTRRGSLSRCRSNEGPERKMPEESQLRK
AGAASGRRASRAPAVGFR*
>TCRU_1592 hypothetical protein, conserved
MVCVDLTCVAIRLTPVWVCLIAYCPNGKRPTPLQALVRVMDYGFSVEAFKRKPIVLLTHM
FVHANESHLAGNLTSLMATLLEFGGPSVQRNVEEHNVWVSVRRMVGSFAVFIGGGIMGGI
GGQLMFNDAQLACRHKRWMSLLSVKGETNGTPLDAVIRRAKSWLERMKYKIDKKRTDSIF
MCGASAGICSLAGFNVSYYKRWGTALTMVAPEVIALVCSLLSSQTRLANTAWLPAGEVVG
HAAHVGGFTAGVCIGLAWRWFSRVYRNARQRKQRTKKV*
>TCRU_1593 hypothetical protein, conserved
MWRAPKQHAATSRRILQLRCIAHKEAGPSPHDLRTFVIGYGASKFRYGSELIWAVATDSA
KNEMQKTYATLARIVSGVPSTVDPESALLEANTPPLRVLCLRARLSIFENTRACQMDWMR
RPPPEPPPRAGFRISPLSRDELYAFVDAYTKDYGITESSPREERFFRSSIPPWFAASAHR
ATIGVGLPIDHSITDEEELIREKRRVSEEALALHSHRSWILATDGGVDVPKSAEVGILLS
SLNSSEIIEKASINRGARPCSYRTESRALLLALERLIAPRIQHRRKTLLVVTDGQSLLAA
LNKGPLSQTDWTEDRIWQRLLTLACAGWSVRL*
>TCRU_1594 Protein prenyltransferase alpha subunit repeat, putative
MHDQRKVSQEFDEKTREQRLAEVQAFTSAYGSLLRQRDNRVYSEAVLQQLSELLQKNPEA
YSMYNYRRIALLHLWGKMREQREEKVEPADAIENTTQVVGDTAAGGLPSSQSDETKQHAP
QGEIEWLKEELKLSSMILVSDYKVYAAFMHRRWIFAQLERLAKDALFANTATNTAGVAVQ
SDSDRVECPEELRFWSSALMKEKKQCDMLLAADERNFHAWNYRRWILREIARMEKLLAQY
FIDLNASTSKNTAGGEVPAMKQEEEEEQQQQKQKPQPRGGSGGDNREPCGSFFIPEELKE
MKFTTTMLRRNFSNYSAWHQRGVIINTALQRLQEKCEDDITRDVALREAWGQLEEDLEFF
ITAVYCDPADQSAWYYVQFLIRAADTLERISPAHHAVRIEFTTRPIQACIELLGEEKRLG
DEAEAYWPRYYLFTVLLSSFGAGNSNTDGKDRTRAVVTAFASEIRRVLCPNDSSGDSREI

EEDCVWCLRELASELVRADPLRAGMYKSLLATVTS*
>TCRU_1595 hypothetical protein, conserved
MGCKNSKAREPPADTVTAAQANIATNKNSSTAPTLSPAQLHVKQTGETKRNNAASPTSRG
NNGEASENESRIEQPKKALGPGGNDVKQAASNSPSKQELDDEGNPLLLPNGDWVRTEGTP
FYYSVEENLYFHPPSCQFYDPTNGMWYDPEKDEWYYDDENGAQE*
>TCRU_1596 U-box domain containing protein, putative
MSIHGAITFFLDQDPGAIGVSALSCLASSLNEPGVAHSLVEQHRPACKEVLSRIVAVAND
AGKNPNARILALESLYGISNVPDVRFRTGDLLDAVQKLNESFEEFLETQSTGNGDAQLEM
LTVLLFRCSDYMRLKAGDLLRQLYNNDDARLVSTLQMIMKKRSLEWNIVHACVRCMYELT
TPVTYFTAPDENLPIETTKVTAFQEKITTILIHFSEGKALQELFTELGARWAIAVGEERL
ERILEGQSPVKDSVCDGVAMRFLGLFRYLAVMLLNLADFCERMDLVRGYQRSFIAQHQSF
LGDTVIPFLSLTLLCWEASCDTTLESQNNPFMNVAISVLRLLRFALYRPSQALQESLALS
LRALTRHVHRLESLLCKEYVGMLVIVLTVEVLCNVNAVNVEPLTADFTALVNLISTDTNP
LRPGAHFTVAQAFTYCLSNETSMYCAPDNESVSLLRQRLRFEDAEMANEAARVVQALEEQ
LGMLQTLMMELAVGQLIGDLSLFASYATGSISSGQAPPVKTGACDTKQMPEKAKKKTKHP
AKYVCMLTRKLMREPVVLRNGHHFELDALQEVVDRVGHVDPLTGEAFDEEIEVDMGLQQE
IARYRIKMAARGDDEAAGQ*
>TCRU_1597 hypothetical protein
MVVTDPLDRHSVDKEDVVTVGEVASLGAQSNVIPPLADRKAEVGDGGALQLIPLAIKREK
NLQSIPLVPNPRCRGQRGGLAVGVVTSPHIFPLEVFLCIVLPIPLRFVIEEDVHRRVVTE
AVVVVRVKNDAEAGEFHRSAHQRAVVARPVSRDPQRKAIGAGALAMQVKPHDNLKGGHVQ
RLRVPQVPHLPGTVCRQADVIVRDDGGAADGPVPQLVFSVVVAQERRGKGVVLNHTLRRA
VVLLRRHPRSIGR*
>TCRU_1598 Leishmanolysin, putative
MNFDPKYMTDTAWSVRVAAHEIAHALGFSQESMREKSLVKKRERSVRGKHRRMVAGKHVQ
EKAEAHFGCDSLEGMVLEDEDGSWEKEIPHWKGRHAWDELMAPPSWCRLLHGSDDGDVCA
YGILPRELEDGGADELGPSQWLRLPGQ*
>TCRU_1599 Leishmanolysin, putative
MFCDAKDTETLRCTSDRRHVGTCTASIVEDKESLVDKDVCPVVSLEFHEVSSGTTYRKCS
DENVDYLRVSLTGGGSWCLDAELLETKDGDSHKSVKGVCTQVSCGEGTVRVKHLGSSVFQ
P*
>TCRU_1600 hypothetical protein, conserved
MELLRLLVSLLVIAQQRLQPATPRLAQLLPVAVLRVPHNDAVHSDRKHHSGQQQHVRRVR
PRSAVARRKMVGRTHHAQHRKQHKARSLHHRQEGRAHHVREGNHARTRKQHSRHAEVQDV
AASHGRVHSSNQAHHTQHRQLEHAPVHVAAPHARQHAAVHPLLRRGVPIPDGVQQALVQR
LPRKLPVPREARARVASNEVAHASRERHSVQHANDTDHAVLHAGVGHRQCKHGAEEYAEV
HRVRHDVRQAREAHRRPRTRRVHAAPPLEGRRHTVVHAAEHSGEQRVAHHHPQRIKGSEH
RQEVRQRNGARTLLRRGARHPRKHAERLHLQDGAQPAGHHHGNSRRTRNNAGDRREGAGS
GGQRVGRDRERPKQHTLAAAKHRHSVAANDYRLVGTNEVTAVHGHSQLQDGGRIRRVAHP
RRVAQHRSPRTAEPARLRRRHRHEAQVRAELRFACPAERRRQRHHQPCRDGAPTAANVVL
RQAPTSRRRRHGECRARHVAPPQSAAHRRRRGPRG*
>TCRU_1601 hypothetical protein, conserved
MIGTAAKIEGGATFEGPGVWAEWPVGKWWRNQRFHIVKKEFTLVVTATIGAAPAGSGSAL
MVAVGGAGSLRMVGLSVTADNKREVAFDGEAGEVQHGKWGLERTYRVEVGLQNGMGSVCV
DGKTLGSSATTVTRGAQLLDVSCFYVGGDGCSGSGCSQVTLTHVCLCNRPLTRQELQMHV
LRGKQTEVDCKERKATPRPGNFAAKLPRDPGVDAAAREEVPVPAKGNLAWETAPLMSMAL
LFYCFKKGLVTAPSMGLCLGHFLALLQGVRGDVACL*
>TCRU_1602 hypothetical protein, conserved
MPSLSRRPLAIGSEAEEGSSTLINEQEQRQQACPPGQTPPLVGRSNSHYGGRMSRNDYHS
ELRNYFYSAGIPAIFDALSTALLEEKPEEPVDFILSWFRRERERVLTNAEEKNMYNSNDG
SRSCDDSSKDESGTSPTLSLSETQLPL*
>TCRU_1603 hypothetical protein, conserved
MTAMEPYSFATVLGDVFGRSVMHAEKRPYVPKGSRVQVSFTTATTTTQLQASQQLGIPRR
VKKEREDDDKGDKKGETRREAEAVDDNLPVPVALTGLISTDPSDGKCLIRRTATDGDDGG

TLWESKVIPSGKRIGCIQIAPDSRAGMLQVLPHAAVFPSPLLYVADTVGNVYAATLPASS
VTAAGVESPAKRPRQEKVNRSPFWGIIVPATTSDSEIAHGACGWCGIVPFFSQQLVCCRE
FFFDIRLVDVPAETVVRQYGTIQPATGTTACPGIPHGVVVAEGPVATLYDMRCPGAALTL
TTDPKPDISLVTSRLTSPTSYVADVCSTCNEYEVAAAVGRSLCVYDVRKWSCVSVSTNIL
KYEIGSISPFASGKAVVVAGIDAEVRIVPLQKASPVAAASAPAHAGGNGIARNEKVQKEE
EGGSAAPNSFRNRIDSAVCCRSTWNGGWVESLDGSNAIGVSADHEVFLSF*
>TCRU_1604 hypothetical protein, conserved
MTRVASQCFLTASHVLESRFTIHKDLPLISTFLDEEVDLFWRFVIICLWAFCIYMRGYDT
ILRGCSPIALETVILLLRVLLLGDDILIKREELMALPNGTGHIHRVHSAPGASVRNGALQ
QRQKERIGPRTAFALELFIWFLLIIFTIDLTNINRASVALFLVNTLYWARLGPTSVVFFV
GIYVLMTYFCCPAYSLRDSFLTLSGQRSWVYRVYSSFGLAFSLTLFLTETQCLQRVRRAG
LWKVLMIPRAFEKNVANTPLRGFSAVMRHLTNYLFFLGFNFYMALSVWFSSMMFEWTSDT
SFTGIVCTPYALSAILGEGWTGDFQRMLREAIACLSMYLVIAIFIVLFVPTDSLSFAANG
VSSAFFVLYMAAHSPRRVHSSFPLVMVWLGMVAVNAYWHVNRVGVNAMHMTWYKKLLCVN
TITSGTLFMLSTALHVYRWEWPIDPSAAALTATVNGVTRLSQSTRTDHKVKEKTNNSTSL
ISNTAEKSAAMMSTPATVSMPGKKASYSIEKVTSFALSLSSSSTSRPVENVASEVYPEAS
INAAELEKCDAMSGGSIRPSSSNQFDKPTTIAFDESVVTENFLCECAHEPRTNSAASEEE
YEEEAQRQQEEEEKAAQYDDDDDYLPTLAASEEVLPTPPVMKKEKKEEKGEVQRDTSFEE
TSSAPLPSQSRRGNENENVLSSIAASDSLSKMEPLSQSLVLSSPMQTSSSVSSEERRGSS
AGNGKKTSERLAATEGGNWKRNKSHDRFAETVVDKIRSSENVSHSLQLVEKVVQPLPLRG
AQQTSTQIPPESTTVSNKTSATTSFSRRAALEPVVNARREHAPNKTSPSGKEPIAVSNQQ
LPSQVSAHRVTNTEFTGNKRQNGKKSSSRGDTTATEFKSQKTLPSQSKRSEDRRRGAETS
AAAKVAASTASASRLGSKPNNDQPSQGNKNVGGGGCVTAPQLRSAQNHLTSNLSSSLTVA
ALKSLTEEKKAWDGQQSSARDETDELSLHLRGKSTSGTKNKFNSRVSSQGDDDNSLDLAQ
LHLASLPESEPERSSSRRRGPTPTFEASRCIPQSTLHSLENFAKAYMPRLDTSGLGETER
QGEAADGKDTQGGSSTAHLSPTTVKHVSLLEAAASAAASRSASGFAESRGAVPQQMMPQM
FAMSSIRDAHGAAIYQQVTPALRPHVAATTQFDSHAMAMPQTASQQQIPVVLVAMGDGRL
TYCPVVASSYSPAPHIMMQPQHMESAAMPQQHQQQGMVYQHVPISHPQQAYPGAWSHLSQ
QLNAPN*
>TCRU_1605 hypothetical protein, conserved
MLDDYVAKKNGKLHDAETTERHLSSVVDQVLNYLRNGVRVTKISSKGSARGRPYFLSEDC
KRIHSCELDHQGFPLNRKRPPVTMWICDIEKVLIGLYTTAFVNHSGEAQLAKTRQEAVFD
NGTHRHDATQNITSSSLGTNNHRAFALLLRGGKSLEMVCEAGSDCEAWLVALKRPLHLRT
PAERLLEERRGT*
>TCRU_1606 hypothetical protein, conserved
MQQPEQELSLWQSAIWTKEQQLEMVQLDGARGREAIMRERHSIEAVRRTVREERCRQRRQ
WIHQIKEINAKFPEQVRPLAEERKKKREQATAKEDAAERALAADTKTIEEYLPRLISLED
IPVSPEETGII*
>TCRU_1607 hypothetical protein, conserved
MKRVNAIESNREEARERQLSVVRERFKHEAGKMIKELERRGRATLDEIERTLEAKKRESG
ALQADRENRIWEYEHTLEKI*
>TCRU_1608 hypothetical protein, conserved
MRVDGNTVRLNCPQPLRDAGFPVAEVLFDVDLALDVPGASTLFRKLCATAAPNFTQERKN
TVAISYGVRSSPKRQLMYGSAAGEGYAACVITEFIRLGAKKGIFALSCHVIGSTEHRADL
LDADNELGVIVGSVREGPRVRMAARIRVSLDGDARAAMDTIVKNYEKCFSPCCLKSSQCL
SWRQCLRICPTPSFFSFAAMMVRRSL*
>TCRU_1609 hypothetical protein, conserved
MTTWQRKNGKLHDAEATERHLSSVVDQVLNYLRNGVRVTKISSKGSARGRPYFLSEDCKR
IHSCELDHQGFPLNRKRPPVTMWICDIEKVLIGLYTTAFVNHSGEAQLAKTRQEAVFDNG
THRHDATQNITSSSLGTNNHRAFALLLRGGKSLEMVCEAGSDCEAWLVALKRPLHLRTPA
ERLLEERRGT*
>TCRU_1610 hypothetical protein, conserved
MQQPEQELSLWQSAIWTKEQQLEMVQLDGARGREAIMRERHSIEAVRRTVREERCRQRRQ
WIHQVKEMNTKFPEQVRPFSEERKKHEQATAKEDAAERALAADVKMIEEYLQRLISLEDI

PVSPEETGII*
>TCRU_1611 hypothetical protein, conserved
MKRVNAIESNREEARERQLSVVRERFKHEAGKMIKELERRGRATLDEIERTLEAKKRESG
ALQADRENRIWEYEHTLEKI*
>TCRU_1612 hypothetical protein, conserved
MRVDGNTVRLNCPQPLRDAGFPVAEVLFDVDLALDVPGASTLFRKLCATAAPNFTQERKN
TVAISYGVRSSPKRQLMYGSAAGEGYAACVITEFIRLGAKKGIFALSCHVIGSTEHRADL
LDADNELGVIVGSVREGPRVRMAARIRVSLDGDARAAMDTIVKNYEKCFSPCCLKSSQCL
SWRQCLRICPTPSFFSFAAMMVRRSL*
>TCRU_1613 membrane-bound acid phosphatase 2
MTAGGVYLRARYVAVWLVFLLLVHVGSGKAAVTLRLVQLVHRHGSRSALVHHNQTQICGD
VPCGQLNIHGKDMLIKVGTFLRERYNSDLNNPFFPSESYDVEVSYTRSTDVPRTLQSAVG
LLYGLFPNRSAAFPVIHTVTRGTDWLLDGGLSPFATVFYLLDDAWKHDVGNKEVDKIIDF
STLQAIAKEAFSEGYCSDPANRWDCAYVLFDIGAALQADGRIGSFKLLKANLERLRSFSR
FYYSSQYAYNSSDEQHVRMGSQGQNLAQQLLANAELHMQGKAKYKLYHYSAHDTTLMPLA
ATLGDIGPESMMPPFAQLYAFELLYDDSTNTHSIRAMRGSPEQTPDTKHRFSWDGFQLRC
MDSNGNVYNASQNTCLYQDFKRFVDSTRPTDPAGLCYLNDRYKALLKCPTREGKPLNKHC
MAYRKLCPAWSCDSDFTLDPVTLQCVCTAAYCMEKGGEKGGKALGASGIAGVAIASFFVG
AILAACIAVIALGWYFRWGASDILVQPA*
>TCRU_1614 glyceraldehyde 3-phosphate dehydrogenase, cytosolic
MTEGKKVTIKIGINGFGRIGRMVFRAAQHRNDIEIVGINDLLDADYMAYMLKYDSTHGRY
GGMVEVREGALVVNGKKIRVTSEHDPANLKWNEVGAVVVVESTGLFLTDETARKHIQAGA
KKVVMTGPPKDDTPMFVMGVNNTMYKGQEIVSNASCTTNCLAPLAKIIHEKFGIVEGLMT
TVHATTATQKTVDGPSQKDWRGGRGAAQNIIPSATGAAKAVGKVIPALNGRLTGMAFRVP
TPNVSVVDLTARLERPATYEQICAAIKAASEGELKGILGYTEDEVVSTDMNGVTLTSVFD
VKAGISLNNHFVKLISWYDNETGYSHKVLDLVAYISAH*
>TCRU_1615 UDP-N-acetylglucosamine pyrophosphorylase, putative
MGDLSECIRRLLGTGQDHVMEILEHGSEKDRSSLARQLTIDLYGVDFHHLNHVLRACLER
EKDHSSNTAIEPPPNNFFFDVMEDQRARGKHVEELETLGYEAIHAGRVAFLILAGGSGTR
LGADVPKGLLTCSGLCEKKSLFQFHCEKIRRREELATFRCGGVPSAKIQLLVLTSIQNDE
QTRQFFQENNCFGLAKEQVQFFTQSSFPCYDEETGRFLMESACSVCVAPSGNGGVYSALA
EVPRGEKETVLQRLQRLGITYVQIGNVDNLLSKVADPLFAGYALKEGAHVVVKSSPKKSP
DESVGVFARLNDEWGVVEYTEIGERAKEVDATTGNLKFNCANISSYICSIRFLQAAAKRM
ETFTRYHIARKRILTANGPTMGIKLEAFIFDLFWLAKECVDPSTGSGDGFRIMQVNRSEE
FAPIKNAEGALSDTQSEASRLLLSLHTRWLSAALISIACGGGVTAQDAKEALAALQSKGL
GVEISPLVSIGGEGLQPYLSRAINEILHGSSNRVVILHPDELPQEPGNM*
>TCRU_1616 Mitochondrial SSU ribosomal protein, putative
MRRRTVISFGCVRVATSAVGVIGMGANHRHHYQQQRPITTRGQFNPIHDFTYAMERGVRA
RDEKKFEKLITNPGPLRIAYSPDYLDWLYRCYKAKGKYMDARAAAEKKFNGNIISGGAVT
TSSGEMIHPLPGAPPPGMFLRPPNSFRRLSGEMKRKHAQETLDEVSKAQGMLDLFERQPQ
FPAIHIDRCTRFHLVELFKEMVLERSLEAVAIWDKALLYRAILSERKASYPASFRYIFKA
VEDTVFAHSSVNCPSLEAYYYFLYLVKKYYIDNAVEAHVVLRCHREPNATDLLFSNPPPK
DEVDVRNVIEALQSEEATFHAASSATSGTAKHDDPAATDEKHTRGENEQKDSANANAKHP
CRFAPPSSYPPIEALWRCEENVPLLEILLFGEFNLIVSENPFVKFPTAHAFLTRPYSTES
SKGPIDGASLANVIAEKRGHLLPSFPMNVASAIDGRAQELRRLQQKHHRDDTVSFQTLLR
STHVDDNPSTFSSYSDWSYFNPRAVRAEERDRLTRKGIDALKEYDSATEDIYRRSFEDAQ
ASNFQRVTEAWNTFPPYLPTLPHFVSIIKKDSHISFLLHVGLPERCSSAEAAAKHKEFER
RIYQLARTLYHTALEFHKETVRRVNRQKVNVAASLLDSFFEQEWVAMLRESESLENSLEQ
GAWPDKKTDMARRLGRYIPFARRSLDENGFPTDARADDYARWMEAPARMKGAA*
>TCRU_1617 hypothetical protein, conserved
MSLADVFTFAPATGHFFSTSERVALATSIPLLMEKSKRRTVLVWGKVYGFKNDYTIVQAF
DDDLVAQPVIYYSIDEGLSFVFLGTTESLMQRTMDEVLNEKKKRMLMYRMRGPFMGDPSY
EYRVVDELAGSTSSYKESLRLVLFIDAHDYHCRVAPRGAYYRELRNTTLPPEIKRNSAFT
GLRRAYNEALSLRNYYHLRAENPYIELIAKEEGTQLHAKSGLERLEEDQHIDSIFFPISD

DLPGGVWRLRYDPVQNAVLGRSAKFVGSVFYHVPGTNKYGTVYMGDGNINHDIAFEL*
>TCRU_1618 hypothetical protein
MGQHNSAENAATGVRAATSVNDDGVILVKAENTKERPVPPYELRAQELYESSVRRIQIVP
PLLRTSAGLSPLSSAAEPTGLNKLYQRMSSIFFSQAVLMEQEALQQQRQQKAIDTSQEDQ
SAAKDESNCPLAQGLREFAFCWRAFETNAAQQANAGSRAIDAALVGVNLTLEGVKGAAVE
QSHIAELYNFVEQESHALLEEVTLAVNLLQNDFAVLLRDTWSLLTDAERTGVQTEFHAKW
KHTTLD*
>TCRU_1619 recombinase rad51, putative
MMSVEHCSCLNPSLKARLQNAGLLWIDEICRLLKKHSDREVEGVTSQQKEGTDASLSSTD
AASRRLLERCPQLTQAEAVEVMGAVLSLYRGNDPADSDYHIPPETRTLEEMLKVEADKES
ERVTTFCRGIDTLLGGGLPVGAVSEVCGAPGVGKTQMLMQLAVNCLLPRELGGLHGSCLF
IDTEGSFVPERFREIAHAAVMQVKEIILRQGPEGVIGDTVVATDEDDLTVSSLIDSGSGS
GSISALVRSRKRGRAEGTGISLAALAGFFTVDYVLQQTQYLRVMDVVSLMALLNVLPTYL
ASHSDVRMVVIDSIAFPFRSFSQLGVYQMGNEAPEGGASATAVTSKHLLWQRSRLLFRCG
QLLQQHAREQNICVVVSNQVTSRTLSSESGECSRVLIPALGDSWAYGLSTRLLLKHRHNG
IMEKETNTEREKCHENVVFIAPAVDGGFDVGSAVNAVQHRVVCLVKSPNQTRGHCCFSIS
QKGVRDIYRVWL*
>TCRU_1620 hypothetical protein, conserved
MAADARTDDGDLRRAYTRSMPAVLRPLWLLEESMRDVQVQWEEIIEGVMVEDVSFSSVRA
AVLHVERLASQLKAIKQELMPRLSHFSQQEEDSEQPFLRYCSCAARPTVNRDNSNCELAQ
GEQTQAARLAARVWVWGVCVGTIMNDLELLSTSLSFAASTCLLDKFAEEVRLTEASTFFL
GKDEGVASPLSDSDLLLTCQLLYHLFTGDDAAAEMTCHRLLSLIEDCEERDDGKECRGCI
LSRLVRIAECVAAEDYGALRVLMKVDFFTGVDTLCSLPPLIIFFTQMMLDTVVHRQLVRG
CFQNVWRPVINVDRPARDESPSVPSEAMKNSPCSSSRLSFTTKRRLMKCCTVFRESLPPR
VPWPIPEDLMDRCFRMGL*
>TCRU_1621 hypothetical protein, conserved
MVVTSERQASAVLGVPPYVAARAFKDVTCIKEFGATNFVSCCILETAHDPSTVGVVRRVT
LDLPGIPQSILRERLASVEEGPYRTCIRMDYVPCDAKEAHRSAFGVEGTKRLLNASTFAT
FTAVSKNPNRCFLEISTKFTVDIDVSPDGVQRAAENPTYHDIDQFWNLYVTRAATALEDY
LLSTAFATTERQIGGGFEKEFVNAENEYCQWAAKNCDKISREEAMQVLEKALNAWQRERC
ELKHQQLVNAQLMADAAGSAAVAASAHRLSLAAMSEMSFSDHLAPKPLRRTTATSIARNT
SAEVITAPKNWVPEVPVVDNAVDLNMLKPVHVPFFSPGGGRVLSVDPAESEALQKVQTES
RDAASPHRPAAKAFQKELLLSFLTEIGTINERVAQTIFNSLDTNHEGFVTERGVSMVLSQ
MDPLGLYEDRNGKMLQMEECRQVVGTGIFRNISISQSFCDTHPAAAGSAAAAAMDAAPHN
IASKGETKDHEAEGRDGFAKELSPLQALIRRQNEQKKVLQDELIKKRTAELMNNYAYRTR
GRLHYDEFCLMMLHLLKDY*
>TCRU_1622 hypothetical protein, conserved
MVSNEGPAAECVIKVLRGVSWAVVGGYGVALCYRFGVFRKVFPKSTVNAIESVVEKVKKR
TEPLGDARRYTIARLHLIHVYFLSSLGIMVAAAGGATFFAFPKVPIILPIALTLVPSTLL
LLLPKGMMLYAGRVACLLTSCFAFGYSFGPVGWIAWDSLGVFLTVMVSTMAGLCVPLFLT
RGMISYVLSSQLLSCTLSLAAVTTLPLVLRSEGVRPAVSFPFASKNVTTPSSNVELLLRA
DVNVLLTLQLLSNWGINLLHTLPTIVRFVQWKGDEGELLDCVDPLKESICICAGAAYVMW
RSFRWACRRLLLAVAKDDTKGTSKEVQDQWGHFAKLSFRTQHVSGVSASVLLVLWYVRAV
SELQRGETVATLEALRRVCAHMSPVGLLVRRM*
>TCRU_1623 fibrillarin
MRGGFGRGGFRGGGRGGGSSARGRGGASRGAGRGGPGRGGRGGRGGGAGAKVVVEPHMLH
PGVFISKGKADSLCTLNMVPGVSVYGEKRIELGATQGGDEKKEYRLWNPYRSKLAAAIYA
GVGSIHMKPGSKVLYLGGASGTTVSHVSDLVGPEGMVYAIEFSNRSGRDLVDMSKRRPNI
VPIIEDARYPMKYRMLVPMVDCVFMDVAQPDQARILALNAQAFLKNGGHYVISIKANCID
STLEAPVVIASELNKLRKDRLKPLEQASLEPFERDHAVVVGVYRSVKKKTE*
>TCRU_1624 hypothetical protein, conserved
MVATHPRATYAARPEGIARSADGLQKRQRGCLPLMGAEDGSPIHSTTSHQSPQASHHTAD
APPDLSNCPTQSSRRAVELIRQRRSTVVRRITDVSALGRLREEGERGGPRNVDAVNVGAE
NSLAGQFTTFLPHARTADQRGEPCGLFDGTVRRGAIDLTAARQEAAVHVANEWNPAGHGD

FEV*
>TCRU_1625 hypothetical protein, conserved
MNQFIRSRTDWRERVVFRLAWITASRWFEIAVFTPNNFKLEPDGTLSWDWSVAPKTARAD
PHCFLRFVRARGRDAFDIITLCGTLQAKEKLTNLTNAHVERALALWGATARSIERGALRH
AAPIVETYNLDPHVISQLAKHFDSFYLSKNTVRYSGK*
>TCRU_1626 hypothetical protein, conserved
MYGSAAGEGYAACVITEFIRLGAKKGIFALSCRVIGSTEHRADLLDADNERGVIVGSVKE
GPRVRMVARIRVSFTGDARAAMDTIVKNYEKYFFPVLCEKQLMPELEAMPSYLPDTVVLQ
LYRDDGEAEFADYMDANSLTFVALGDAGRPALCGSDAAAQLHYEGAQRALVSAAGIVSSI
RRRRLCIPFGMSELSQLLRRAYNTEKGNRSNSLTGSTETALLAHAFSDAAWAEESFHCLS
TTCRVGNVVSTGIRYVTRDLAVDKWRLDQDIMELRDELMIARTAYDHRHCGHESNRPFQT
WRRKG*
>TCRU_1627 hypothetical protein, conserved
MQQLEQELSLRQSAIETREQQLEMVQLDRAREREAVMWERHSIEAVRKTVREERRRQRRQ
WIHQIKEMNAKSLEQVRPLAEERKKYEQATAKEDAAERALAADVKMIEEYLPRLISVEDI
PVNPG*
>TCRU_1628 hypothetical protein, conserved
MLDDYVAKKNGKLHDTEATERRLSSVVDQVLNYLRNGVRVTKTSSKGNACRRLYFLSEDC
KRIHSRELDHQGFPLNRKRHPVTMWIRDIRKLLIGLSTTSFVNYSGEAQLAKTRQEAVFG
NGMHRHDPTQNITPSSLGTNNHRAFALLLRGGKSLEVVCETDSDCEAWLVALKRPLHLRT
PAERLPEGRRGT*
>TCRU_1629 Protein of unknown function (DUF3676)/Trans-sialidase of Trypanosoma hydrophobic C-terminal, putative
MLYNEELLGNELRKLNAGKVTIPSLGVEKQPTGQVTSTDVSVASESKSEESATSYEELTE
SDTDEQEEGIVNGPVPAASSSTLFAGASISESAIAAQSAENSFSEDNAQPSGDETSWQTT
LNEAKESMQRDSDVQPQDLQSEKLTVFSDVEKSSESNDTEEPEEDGGTNDRSGGTTSPVA
ASLSMETAAASVNGEHQVQQSVEPSAENDDVRSTGTVTTGAEESLSLEAGERNSERTMGS
DSSLTPSRSDAEPTSAEDTDNISWTDGAEVSSEDGNGVPQTVDTAPGNTSTAPGDTKIPS
ESNATIPSDHDILLEKEHLGELAAMALIGDSTVHGCVSRVLLLLPLGLWGAAALC*
>TCRU_1630 hypothetical protein, conserved
MAYNHISMDDDGVASFTGGPWHQNGADTEMAPESGMATASIAGVPVIDPTMTPGEFIRRC
QQQYYFEFSWEVPFPRVPAAPLLYLPDAFKELLSDPSPLGVMQAELDAMSEGVLERIPLA
YYLLRPEVVEEARNIAMECEQRVEELQRQAAVDGLPERLTWRPEEVNPYLEQADAELLDT
DIDLNLVLERDKPSLTLLQRPLYTDMGLVPQEQRHLERLARIVPETLRSQSLFYSSQGPH
AMGESLDSSGGIGIPLYYGEEWTQAGFGNGQAAMEAQQKWLDGVRKSFRFARTLDTHHDK
LLELVAQTKLGLDCKNPAAMCLTRRLWQLLFEGTNKGQQRSVWHQLCRFSSNYDDRGEAE
EQLRRAVVDLQLPHSELWLALLREYVDLLKEYTLSLSGGTEKRKEQLQISGKELDPFEQG
AFEPDLQTGATLRRVGKELGADRQPVAIFPVEILPLYPAGFDKAAEEMRAGSPGHIEHLG
ELAKALHHVVLPGALVNEESRIRGPHALVNNTNLLVADKNSARQLSHGLTVSYHTSRENT
FEPLVSENNSTSSYLFQLCTVEGGSPSSLSNASRRHVVYNRLSVRNLFVKSPNAEIPPYE
RYVLMFGLEGERKRQREAHGDVKESPVSTRRVE*
>TCRU_1631 Ankyrin repeats (many copies)/Ankyrin repeats (3 copies), putative
MADFPKLARIKLDDENMEKIHCAARKGQTEEVKRLVEQGIDPAIPNRFGCTALHLACKHG
QIETAKYLSSICDVHGAWHGQRPLHLAVLSNKEVLVEALVEGARERGRNVEIMLNECDDV
SVDDIGEHQKHTDGQTALHWCVALGDNYLPMLRLLIRLGASPTAKNKENVTSMMYAMEFK
NEAAMEEMVKGVKPQQLRLDYQDKEGRTHLHYAILHNRQDYAMRFIEMGHDPQMEDDNHE
TPLFLALRAAMPDLLTYLLQNVDSFSVQQAPFHNGSVMVAERIQWLEFATDEQARTECIR
LFQKRLDEVSFRPEETEKKRAKPTVKKMKLAPSAPLRSRSIGNASALRSRSIGGRISGK*
>TCRU_1632 60S ribosomal protein L4
MTARPSVSVYSASEDKVVGTCPLPAVFTAPIRHDIVQFVHTNMAKNSRQAYAVNRLSGMN
HSAESWGTGRAVARIPRIGGGGTSTSGAGAFGNMCRGGRMFAPTKIFRRWHRKINLHQKR
FAVVSALAASSVPALVMSRGHKIENVAEVPLVVEDAVQGYEKTKEAMAFLKAIGAVDDVN
RVNESREIRAGRGKMRNRRYVARRGPMLVMPDNKGTRAFRNIFGLDLANVNALNLLHLAP

GGHVGRFVIWTKGAFEKLDSIFGTFTKASMVKKGFMLPAPMLTNTDVTRIMQSEEVRRVL
KPKKLQPKKASRLTQPTNGFRNRRLRLRLNPFQKKESSILKGLRNRKNREVRHKSKAVRI
AKAKKSIAGGAKKK*
>TCRU_1633 hypothetical protein, conserved
MCSTEKRVVLGTRRATRATVPEWNRQPSPSWTPWLAELDRAIAAAPHAEGGQRRELRRRE
ALREASESRWHTLRSELEGTDGNRQQVVRPCLSPSFLEHASHEFRWPNAQQTPEGQPPHE
AVGDESNTAPKAPPSATDPDECDGPHPINMADPGRTIRLLLLGPAPGPDGINGEALRHLG
RVARCAVPWLFDIRLRAGAVPRNWQRGAVLLLPNPERTASNRDSPPTCNAEQLPQQAHGT
HPRRTHPGRHRLPVGSAVIWLFAGQFHPRPIAAPQGCHASQCFTTPHGGLLCGPRQCLWH
GGP*
>TCRU_1634 hypothetical protein
MWRASQRSQVWCWTSSEPSAAAPPREARKRTRAAVRGGGHCEGRAASFCPKRRAQRHDEA
VRFSGGVLRVADDGGTKTQSIATRLASGRGCSFASNTASSTLLPLVVHATILLACYFAGS
GVQRDVGQASNTMR*
>TCRU_1635 hypothetical protein, conserved
MQHNRALQRRHVRHVDEGVRHQTRRALAEAANPQLRAARKHAVAQRHVQRVSEHIHRAQV
HRHSDRSAEHHRVEARRRTPAANRHRFRQRHATHAALRSIATGPRRRHVERQARVAHTAG
TAAAHAVGERRSQRIKARQRSRTAVARHHAHGDQAGHAVVAAALQHPAAGTVALHRHDAA
RDRQPGDKRNAGGSATRNRHGTAAQRHPRQRCHRRRLAHRPHVVQLQPPTGTDRPTVAAH
QRRRNRPLDAHEAQHRIKHREALAKHNAAAAAHNRHVRHGEHRVAPQTENRRGRHMHRGS
GRTARRLQQHAVKHHRARQQFHTAANVHRRPARLQRPQHSVAHTQLRLQQRRRMQHKRRR
HRVRQPTQLRVAHRPRRHNRRVTDRHVRVCWQVVHKHKRAVVAGQPAHARLQAPVTRRLA
AVAPHVDLRTTVAHAVPQFDAQHAGEHCRHVEHHSHAVRDGHAVHQHRVHGVANRDGSVH
RTAPQSQHHRMHRGHPRSSCCQRQHNKQRHTHSWNGHA*
>TCRU_1636 hypothetical protein, conserved
MFYCTVCGALFGNPQQLMLHQQSHSVDMQLGGGMQPQYALGAMPNLPIGMYPMAPNLLQS
DSQLLYAGGLGGGLQRGSIDSTALTRRLAPPMIAGNNAGYIIPAKDMYMNEQLLQILMNL
RHPSTEVERLQAELSRFRQQEQEQEKQNQEKQQQQEQKQRQIPQTRLILDSVLNEEKDVD
FDSSTTSCSLLGPATYALRLDYIDGLDIQIPLEDISIGLDEYRLLSGLENHLLEAVRKRY
YASATIEKANSDRILLSLKDPLEFTLRHSHELHVLIITFTCRETTLFWASLRAHKEGMWI
TALRRSPVDVSTCLETTSSCLPETMLCGAIQADHSQDVFVRTAKQMSSTEQPRLILVEDG
KRLILTSKQKPNGELPADKATDESRHEDMPRAVLEDDGDVPKDMKLVKSLLWNLQFDSNG
TLILPRNFKTFSLSRSRLANLSQELKTEASVLLQIRPDGVLPHLEGFAGHGKYNNEVNLN
ISFVTGIPLNAVRSRVLVYLIENIDLETVEVVNKADNPAVLLRPPDEIQYSTVGEGVLFP
TFNVSMLGIFVAEKTHALVIIEYVTSTKSGPIVFGHASFPIHLGSRKGNFLSRIRLGDPR
RPEARDIHSVISLEAERNAMEQYSQDQKEDALNSQALYNSLFEPPGRYFNCCPCGYIMWS
LDSDPDSLYFDFPDEPKPVASEKDLYVIWQGMDPKNIPFSDDWETILKAFMGMASPMGNR
INYILPHNAKRGFYFDIESLHNMGTSHALYAVASDLGEGGPDEIVLTLHRDWSSDVSAPL
FKEPLRMLTGMKYDSYFTAILYLFKLSNLRSIKEHGEKEVKISSVGWSMIRLDLGEGILR
HGRYALPWFSGVPPPEMLEQLSSEPIEKVFPSFLCENKIEFMESKSTVVVSISDAADVGQ
IVGDHPGRPLPQKLFLSKGEKESFPSGVYEGAVGYTQQQMVTAMELNVKDVERAVNACLK
AYIRENLEE*
>TCRU_1637 hypothetical protein, conserved
MGSAVTCVSFGNRRRDTPPKASQDDNEEEDMWQQRFLMEVGKDSTSLTRDFPRIPVYVRT
ATRNNYYEPQREVQRVEKEQQQKTHVEMTGCSEDAAHHCLALRSDRVRTSIELLLELCGE
DTMAMLSAWQTEEERLGVADASSLLLAVLLMSGMLQLKKETEGSEQCENDAPGTSPTAPK
REMSTAETNTTVPNGSTEYGKADAVLLSASPQYIVQSKALRLMQYMVQSAVFFSIQWITG
VFRFPWCRHMHDIVWTAHVYSEKDRNREGMEEGDIELIIVQHKLTLRHYVEPKELRKKPR
FEVDWTCTICIDPRHLLHDTSNPPTMGENAEVRQITAEVLAARVEMPPKTACCVSRVWRK
RRDALKVRLWNRFQVELDLVPALHGRTAN*
>TCRU_1638 cytochrome b5-like Heme/Steroid binding domain containing protein, putative
MDAHNTHVSFADVPCVSGPSSPLQQQPQSPTPQESGADVSPFVNDGTFRIPVTSDIVGSI
PRVKVPRQKGCSMRDWSVCLEKQKQALKQRTLKHQREEESDERCLLAKNCGRRMLPRLSM

REVMTHATHDDLWIVIRGVVYDCNKFQYFHPGGEKILRRCAGRDCTALYEYFHPWVSCEG
MMAPFAVGVIEDEVHV*
>TCRU_1639 FYVE zinc finger, putative
MGEQLPTVGPSRQQPLPSQLSSQIPPLMFYDAESVLCAPVTPQQVISFNPREKGPPSRVM
PDGSEYYGDLLLDQPHGIGIMLFKSSSSFYANGDRYGGGWKNGLFHGDGIFVTSSFTYQG
SWFEGQMHGKGLMTYSRRITDLMLRGISVFSPFDKTHAPLEYRGDFHYRYHRHGHGIMRY
ANGDTYDGEWGSNCRHGRGRLITDDGEIYEGQWSKDERHGNGKITYVNGGEFKGSMMRDK
RHGEGVMMFPNGDEYYGTFYNDKIEGHGTMRYKNGDVYEGMWKDGLRNGEGKYSLRKKGA
TVEGRFVNGLIQGRGVVRHPGVSTFVGEFDRGERRHGTLFWHDSAPGEGACYQGEWLGET
MHNRGLLWYRNGDFYFGRFLKNKRHGPGNIRYADGGEYSGYFVNDMREGQGILQNSNGSI
QAGMWHNDVFIEGYDGEWDGVTFNGIGHLLLRKPPACVPAGGSNSATATSIGTSGGGSGG
DVSPTVATTSAAAFTGMQPTFEHFGLFLHGVRHGAGVLRIAGHVIMGIWKHDVLSCETGS
WEFPSGDLYLGGFKNGLREGPRGQMWFTDGSFFMGNWQLDAPLGNGSFYATDKREPIYAP
EETVADATSPAAAEEENTHSSNYSGVMNLFINLFRRKALPENKEKRRPIGFREHFVLQGE
WDIARLPYAIYQTLMSRLISSSSLIATGDGAGGGSEGGGNSGSNSGGAVAAGTLPSGNNN
GSGVNALFKPPAMIPVGVQEKSGFVFFKSGICLRTEWLHNHPRLMLPHRPDAPLYNKIRG
VIVTDDRNGGGTGGSKICSFCEKPFTFFRKETQCTFCEQHSCTSCLHPIEVRGRPKIEAL
LRLRMGQNSMNSDANNNNNAATGDTFVLPAQMPACVDCARVALLGLEFNVIWIPMHYLSV
SETTTSSNASTDEKETETVTATIASSEASDKAENSLLKFSYSMYSSSLCSNAPYVVYEGY
TCNCIPHIYGSLWWGKEGYYLGAFHEGRRHGRGVQMMANGEKYVGDFSNDEWHGMGIYCA
DDGSAYEGVWEHGKLTSLLYHGELDEQYRRHGRGQSYEADGSRYNGEWQHGQRHGTGILQ
MKDNVVYSGDFAFGRIEGEGKLLMETSVFYGSFHAGKKQGKGSEHFGDCVIEGEWYDDVL
TGFVRIYDVLTETVYETTYQNGNERDDCFVPPVMVDDAYCQNCAQCHATFSLFLRRHHCR
LCGEVVCDSCSQRRASMPLHFKATGTSRVCDRCYRRMEERRMLGIRRYANGEVYAGCWTC
GRWVSRGLFSRADGSVVVMDLAGCPLVNGQDKTSFANTESKLELPLDASPPVEELRKLPP
SSCTEVDAFTLWWSMMLSVAELSLPLELTPCRSFTLLRAPQMKPLRLFLEKDDARDDGND
DGGGTRGNDVRDAAVVRVARYIPSIAPPAPEPPVRLSDDSVLTSSPPPRPMITDSHIMRL
VTDGRMEMFPAVPPPSADGNGVVMLSCYQRGPLLPPTPPRPSPGRNDRIMWDSWQTRPVP
QYRGNPETKTQRGVADWQHVPFACPFLRPVVTDVDRAVRGGNATRWLPTPFVGPQVFTVK
DKSAGGVLVEQKGAPSSSTS*
>TCRU_1640 chaperone protein DNAj, putative
MQHTGDGISSRSPGLRLVNLLFSDYLASPAFGTDVLQTILSTSAGYFTRRRSDPLIHSMN
MIPHLILLRLVECLVLETLLKSGLSIFHDAGVFHVVFQYVQQYRFFHCWITRFLCTLGNI
FLPAGIRQLLDRRIAETVLIPATTSSDPLKAMPVEYRESLRYYRYMYDRSVPYAVFVSGF
VCEMTHCITEQLVWLAEFYRSRHKHTSRWRLLKPYISSLAGNTTQMLLVYSARVVGAWAG
RCVSREPTGRSVFWGESVALLALSPAIHRVTTFITMQLRNKLEMLCPATPEDEREDFHEE
EMREEEEEEPTAFRMQFNNPSPSGTAQVDFYEVLGVKDTASAEEIKRAYRKAALANHPDR
VGHDEAAQKTARERMTSINQAYETLSSESRRRRYDMARQMGTDLGVVKHFENLSTPVVLG
IGLIGILGFSLTASLLLCGQYFAAFHQLTGTGRNPLRYLGIA*
>TCRU_1641 WD domain, G-beta repeat, putative
MGSGGSTSLGGRITTVKEKRKSEPRKLKVTDYTNRPLTARFGRAVFASRMLAELPPPPTA
EDDGQYAWFVEDLDRPGEWESYTPDVSERLDAAWRSGQRECLILSKKKRICTVDLNAMVQ
MAAGSATRSRRVKRVEVEKCGDSTVREKRHFSPDPFLEEALDDAGRGEDMMANENGENGC
HAEEGVVGEADNTDASRLPCLLRSVAAHSTTPYTLAFSSDGRTLLTGTRGELLHWDLETA
IVLTAFDVKSSTVLSAAYSSDGSRVVCGGSSKIAWLFDTNSPDAQMTFAGHTSKIYGVDF
LAGGERLATISMDKTLRCWDVRTSICMQSDECHTAPIFTLATSKRCDWLALSGGDDHVVC
SHDFRVGSNSVTARFCGHRKTIWTCAFRGDENQFASSGMDKALNIWDLRQPSEPILKVFY
HMHPVHFVEYMPHNRGILTCARDWTVRLTDAVSGEPVWRVKAHVGHVFRARYHFGTRMLA
TGGGDGLVNIWRYDNADKW*
>TCRU_1642 Nucleoporin
MDAWRCSVCRKTKELSGKHLEGKTSVRSDCWPCAKKRVFVRESPAVSRSAAQVVTAPFLF
NPNLSTSLEKSTTAASRMPTTGVASNPFIASAAFTNKTPFASAPNTWKGTTASSKPVNAA
AEAFAAAGKQLETEAPARKNAFLPSEKAFPIVMHSAPATDRPRQSPPFAFTSSPTAVMPL
VNDNAKLGNVTSKNGHESSNKPVWRCSVCRKTKELSGKHLEGKKSVRSDCWPCAKKCVFV

LSHPESCVSSEPNLSTFFSTTMKLTTSPNNRTRDVISISHGASPMPSAKRLWQDPVVPSN
DQEANAFIMCHFSQQPSAKEQLDQTRRSVSLESSSPSVLLPSLAMTFEGEVLRGYRQQLV
DGIRTRTVWHDCTLYVLAPENSEGKGPNTAGASQLGAAAVVFLAAEHKLALSPFRRMRRI
FCPDEFTFAAACVYANTFLAGHPTSSQQSSFVVYGPQVDHTVPMTRWGSIIESCTHARQR
VVSEEQRVEGAQSIFRRSGWLTLPLHATAEAIRLLEVQRQQEHAEKNATECLGVDVVFVA
ETMRLEDLPQAAALALPVKPF*
>TCRU_1643 hypothetical protein, conserved
MRRGGGHRVAMSVSATAFPVTASSSFFFYRRCCISTDRCAATAPQDPGPKAFLLQLRRCF
ARGALGVREEAATNFMEESDAVRSHETISLRQILKCCEEVCDAERPLACSPEEWSEAVQL
LLYALARGKRESGDESVTTQDPSAENSTPWCVSLRLFLHTAPSGGESHAEKTPVHPPASC
VSLLRCCVRHGAPVNIVRAVYQTLRLSVPAASGSPVTGWHVPYARFLFMKAATEEMTVSF
SLHKEALRVLLLDAAEDGEMLQPAGLLLSLEVLRRLVTRRKNEEGGKLQENVGMNKVEDA
LWSRLQRGTDVVSVARDVCCCVFYNRHVSHAMKREAFCLFIAVGMRLPLEELRSFLCTVH
RDDVSAASGSEMTPQLFLETLQAVTEILSKGVGGGLASYGAVTRALAEPSNVMHSHSTAV
QRFVLVWLVDHIVKGLASFQRQVITVGSVKESSHCAMESWAECTHAALRCLQRLFLHLPD
RLHGAIKRRKHELYVARQQAFCLLFQAALDGAFYTFVADRASSASERSPFLDVMSVLIQA
YGEHDWRQPSVDVFIECLHAWHRHEVLRRVFCHVCRRDERRWRIGQKCDSTGDSAKDVTN
SSRRWRLSLRLSTCLVVVESSCLHDDPTTAALAVKQTLRSLLAVKFSEQPAQNVDNGEDV
KAHGVNVDSPAENRVLEGRSVAEWTQFIREDIIPRVNEVFCRTGTDTTQQWLGDLDAESY
HSPVVEATPGTTFIHE*
>TCRU_1644 prefoldin, putative
MERYAFAEDYKNARGIPRVEYISDMATFVKNNGGADALISRLILDSSKYEHTEKRLVSSM
ANLDHKIPTIKKTLHALNFLKKRLQEDQTPVRSYYCLTDAVFGEAEVVPQETVHLWMGAK
VMVEYTFDEALQLLEKNLANAETNLKATKEDLAWVQEQMTNLQINISRVYNYDLKNKRTN
GGAAEPSA*
>TCRU_1645 GTP-binding protein, putative
MPAFSGKQKKKQLQERRQRKKQYGEEHEDIQRGDVIHHNGNPHGHDDDRSSNRNEEKDYE
KVEYKYGADRKGIRSAFLKETAEEVAARKKRAYEPLKQRRFMDKNGIPFGSWFNMPGYTD
AALMPFAVEMPTRGWALDEATLENNNQCDDNICSKKNDEVLLENVSLESRELERFEVYLK
AVDNYPLPPALQTLQLSSYERNLDVWRQLWRTVEQSDVVLIVCDVRYPILHLPLSLLHYI
VRQCKKSPLVLLNKADLVPRHVLDKWLEFLPLYFKATGVVSADEAEEASTGIVCDIPLLP
FTSLPAEETAFGADGNGNPARRRKKKKRHAKLYEQLRTGKLQLPETPTRDEYGDDDEDAC
GTSSQDEEENNKSGRVKCDGEYTYADTDNFKGMYKAERELQCDKRGYNELRIVSDMISLL
LQKCRAMCANKTHVTCSTDGEEAFLRIGVVGHPNVGKSSLLNCIRGTKVVSVSATPGHTK
HLQMIPIPSEHVVLIDSPGLAFPVFGLPRSIQAVVGTHQIAQTRDPQSGVAFLAYHLQLE
RLYGLRKVDGADDTVEWSPYELCESYAKKKGYFVKHGKGALDVHRGAIEILQEAYDGRLV
LFFAPPDITWLESGQFRKEICPHLLMELHLPKTVGE*
>TCRU_1646 mitochondrial DNA ligase homolog, LIG k-beta
MVVARIGARTEMWRRCSCLGRAIVQSVDPSLLFGAGRPRTKQQAEKYKELKNTTIQAAFP
HLAACWVGPCWGTVMITPHHISPLCLKMAVWRCSECLQEFEMSIAKFVDQHGVCPLCKKP
QRSLETPNKEPETTNGSSTEGKNGDSNLGSATAMTMTTTTKNELVYAKAPRMIHTNYRSV
LHDNPEWEEINIQPMLAQKWETVAAELLNPGPGEEKELLLASPKIDGIRCLIGYNRRRKE
PKFFSRGGILLECCHGLVPHVMPLFEADPTLVLDGELFAPQCNFEELNGLVRRLEKFTTP
EMRERQAQLLEYFSFDIMHSSQLSSPTAPFSERYRLLKKMVPTCGAKRISAYQHDEEKRK
IIRQKQRPDNAAAEGTIKLYHVPAAQVGAEEMDEVLEEACSQGFEGVMIRRPGFPYEHGK
RSFGLLKYKTMHDAEYRIVDFLPGQGKFRGGLGAFICETKTGIRFNATPKTTYENRLALW
GKRQELLGKYLTVQYQELSSQDVPRFPVAKAVRGASEEEFL*
>TCRU_1647 hypothetical protein, conserved
MAAVAAGGVWQKALAQLQARSWNVGRAPTARCWLDFFRFCATPFSTSTGQNRPSILTSSL
PTPVRLLIAESIIDVVRLARLQQLFSTSPGGMFCDLQDDGRHGSREETAASFWAGGVPED
VNGVFLTTDASPSMPSSEMVEVLSHWCAAHEAPHDVAQLLGWLREAVGFLRFFTPQSISH
LEGGAPQQQQQRRLYLTYSELLRVLHHGLFTFSLRPQANGVEDVSADVSVHEEVALLAIW
LLQKLRNGDAEDGLVDSSNPPSSVHVDRNSNNNNNKKKALNKMENMLQETQMLLASRGAT
QAWLLHLPGVLRDCSENTNGPSSGAVNDTARRIIKMQRRSPVCGIASELLLFTSSSTSSP

NVCVPFVDRFALESEAAVYAMHAQLSSIAFAGKA*
>TCRU_1648 hypothetical protein, conserved
MRGCGVLCAGHKRAAVIATTTCSLGPPTLSPSLTLRPVACAATPVTIPIFPPPMRGSFID
RNPVWASFNEKHTAKSFRHRIVSSADVSLRPPQFYLSNEKSSAGEAAVVQKRAEATDSYG
EQLDEISARWAAKFYGRVTFGPRNYPYPSSRWLARRFKMKKHRIIKRFRFRRYKLAAVAN
LPFAKMIRVGLLPELKSSKTKKGDDVEADLSGRLVSAVRSSGGKKKGNRGRPKSKYQI*
>TCRU_1649 hypothetical protein, conserved
MARYMFWRRRGAMQGVVEGHFWSLTRYRIGRRFLGLDYADNVILFPAYRKKTWGGWAAVT
LGATNASYLTSDTSSANVLEDGYPLHWRASTKLQAFIAEERNDWNKRKEEVIARRAAAIV
EEGEAASSSPSGTSRNNGAANNAGEKGAHEYDEQHFRDAITIKCTSPGFSQRHLRLRPLT
TILYKAMYFYIWGIAISVIVQAYLLFRGWLNPPAREGLKNIENHVLHIPKLIFSLCVSGF
AWLLQKTQPLVDPLLHVLEEKFPRVNWSVASPKVIATKAHQLAGNEEAAQQLAAMEAERV
AAGRAARQQADAGRRTWWKRAFFALFCLFSVLCII*
>TCRU_1650 hypothetical protein, conserved
MTERSLMIRHAVTGRYFAVGMDFPSARVFDVATGSLAAVVEVPPRSRTSLAVLSLTALAL
GEINEPHAAEEQRKAMDVVGMSGACHAAVGLSNGTVLLHNVPKDELLQHLAVSDTQQAIT
AAAFCGRHLFCVAGNRSLCVVDCLRGERVGSQLRVQQDAGAIAVMRCREDDAANGAPDVG
CKFLIFVSGPTNAVYTLCLGSEGAQNTVATMERLVSFASQASRSDFAWMSGTTQHPIVVT
ASAQEGVVRVWDAYPSVSGQTTSSRCRRSLLCGQRIMGISVYDPTTMNSSANGYRNGVGG
SETFVAATNFTGAVLVWNLGSALLTPVADPVPTPPTLRIVSKEISARLLFGAFIQGAAGE
YPSLLVLRGRFAVPHFESVDLREAAQRASMVSSMTPATSSLAEEGAVYELPTGPNSERAG
KIDAVHADMEVLDHAWATHCLQQQRQQRLAARAIYTPSEGFVPPVSFHAASVKDIPQNKL
TLEQRLKKLSLSSAAPSQQQQQQEHSHALGLATVPLYQALHASDASAVMELLTVASRSAE
DIRATVMGLQLPYCLELMQIIAQRVRGATSRSPLFVWIDAIVHYRGVEMYQAQQLFKEQQ
HQEEKKKKSASSSIYSNGVDDPLLPTSTTAASSKANPSPPKEFVAPLLHQYRNMTALYDP
LAAMYGRLSIFKSVRPSEKGVFVNSDAGIIFPTMFTEIRCAGGEYRTMRVRSKRDPHARK
NKKRSAVALLRKARRLAAAEEAGAAAEAKGTSEDNVEEDYDDEIDMDALDQMRLGDDTDG
EEEEEEGESDADKGPGKRARGEQTEMIAEAERGSSYGIESSTRSSEVEFDTASDDDVHSA
AAENDDDEEDDDEESSSSISSGVATADMFDDEEYEDDDDDDGDDNDGMGDDMAELLEVQG
EDKEREGRRHKRVRTEH*
>TCRU_1651 Cupin-like domain containing protein, putative
MTDVVEAPRPATVAEAFANTAKALEQFNIERIRVEGLSCKSSITHCRKHDKNNMEKEEDV
RTLTEQAFMREFVFQSRPCVIVGALEDWPAMQRWRDDRYLFDLDGHLSRVRRETDDAAAP
SSEGTAATGDVAVVPKNNDHDSSDDSTHPREREEEKDGLKRVTVALTPNGRADAVTYVTY
QGAREEGRENDVDGEKDNVEEHFIKTEKLFMAAAEIRATLPELYHLLRENPLYPPVRPVF
VDLRRPAPVVAYAQMQNNCLEVEYQHLHGDIPPELDRFGERVFGEPHEAANVWFGTPASV
SSMHQDWVENLYAVVRGVKEFILLPPWEGVFVPKPELPSAAFSVQRQDGVNGYVFSGTPV
HDGLTIPWMDVEFTPAALEDVSLMPTNMQERLHPLVVYVQPGEVLYLPAMWLHRVAQHAD
DTDRLAREKQLTSSLQSAQDPPLPLIAAVNYWYDMSFTNPAVVMLREFGLLL*
>TCRU_1652 hypothetical protein, conserved
MMEFDLLDDAEEILQPLCLCLNQDSSRVGVGHHKGYVIYRIHRRPAPAGTEEASSSSAAA
SSTGPPFYTTIEAVFPPRRGTQCSTPSTWDVHEGAPSPHAAASSTVGTSRVRRMSGDHRD
EVDHDTLLDALAHSPLLQRMGKPLESREKEENQVTLEGTSVNSNPVGTSATTLASTLLGK
NFLRWTEEKRDAPLPTETGVGAMALLYKTQFVAVVGGGPYPVGPKNVVKIFLTGDMWADR
EICVPDPVEELRLDHRLVIILTTREMRLHSFETELCIFSVPLWADTVPTSRPSTSTSPLF
SASVSLNSTRLQQLNLGASVTTASTSTSAASATRFVAPLAIDYSKKCIAFRNAMVGFSLV
RYDAEASLCNRVNAELLSTVSVAHEHALSSLAMDLNGDIRSSGSNTAYGSMRWHIATCSE
RGTIIRVWRYYERRGESPITVDTTASSAPHSSDVAVKSTIAEEDSSRTLPGYFVRVREVR
NASLPTPIFQMRFLGEAFLFCIAWNTLKIFFVGEQEQPKPYDISTKDPSKTVITQNSYSS
LHHLSVVSAYFNSEWAACECPFPLTDDVFLPKWVNTTSGELRQHLLRGMGSSSRMTVSGN
GVEGETSRVSADPEIPEPVSLLNDIQVSQSRSQSSLVGTVTARSKSQDFGGILRRYVLQM
APMVESAGRVARNYWGFGGESTEAARSGVNEVTNDYSQKRDYTTMTEVAQSVVVWWEQPT
YWQVEYMTKSLSRLSTNTTTTTTNITTTGEVFPNRTKCPRPTVLYCVTCDGSAISFEFDP
VKGKIECSKATAVSSLED*

>TCRU_1653 hypothetical protein, conserved
MTPRTTSDDRSLKRPAPLWDRMALAAGRTACLSLSMRLDPPRCLSPPRSVGLPVGLTAQL
LAGLRGPAERSVFSRSTAAEIYPFPRLAASNRPHEFLSSDTLSAIEALRLGPPAVRGDTT
EEIGAMPPSLADRGRTAGSVFSHDAAESHAT*
>TCRU_1654 hypothetical protein, conserved
MAMLTGRVLLVCALCVLWCGAGGVYTEDDAGVTSPGASASDGKSFQEPPPIVHGGSHNSV
LTASDVNEQTHETDKEVLLTKEVAGSDEVTQTSGEEIPPPSPSPKKSKGGGGPPQEDDLN
LEKVDENVMQPEKQRRVEEGLETLGDGEAGVTKQNEGIGVQPQDHETPVQEEDTINGEGH
QQTHGKDQQTNVEDIPPRKSAGDYSSGEHKGNDGSNEKEEKEGEEGVEGRERHRAQEREE
AAHVSGAPKVNSTDIQQEVQRTHAGETPTGIKQKAGEEKDDETEEEREEQKKQNQENPPG
KEQETITGANATNQLNTMPGDNDGSTAASHTTSPLFLLLLVVACAAAAVVAA*
>TCRU_1655 hypothetical protein, conserved
MHPVPLVAAMGVRCDVDGGASACVLRRAFLLHVFALLCVGKVVGEDMCGSERGWLPASPR
ALGASIHVALLDGVSRHPGCGEEEDGEDLLWAEGGWTGQSPSVSFPVLLAVHTESVNLGE
CGMKGLCRFVRATQSSSLQAVVTVRCGAVPFDEGRTRCHGCCAPRPFIVSAPGFFFLRSH
NILFCFTMNLLWEL*
>TCRU_of Trypanosoma cruzi region 4/Dispersed gene family protein.1
Dispersed gene family protein.1
MEAACFAARTSSISGCECVCAAGGYGDTCLPAAVPDCLGPLPPPDAKDTEVRCVHGGSID
SVDYPDPGVRGLCLVNVTFTAAIVLDLSYFDAPQQTLNITLLKCVLMGLSIKGSGARVHV
NIVSSMLDSGELEFRGVFGVSSQILVAGSTLVTTSGHAILFVKFALSANMTLLLLDNYIE
GNRYAVYLSGGVVVDGGGIIVKGNTLSTTKGDDGVESSVCVTSVDVKNGGYFDVENNTMS
SVNGVILFGDTTVRSAGLLRVADCTFVGGTEIFDSALVYLTGSVTLEGGAQWRVEGNNVS
AASVLSIPYSWQKLQLSGSGTTVVLANNRQVEGSALFARLFKSNTIVALPARFVVGCNLK
GNEELLYDGLFPEEVVLFRCGTCNDDSACYMPGTESVDRGSCSCSCKDGWHGASCLPFEV
PDAVVPPAAERAVDGDTSCVVNETLTNLTLKMWKTHHCYAGVTFSGVGAVLTFFLNSMPL
HLPVNITLTGCTFLEGAALQFFGGAEAAESSGVLIRVSRTVLRSSVVAFLLALPQRCDIA
VTEVDAVQTFAIELPGTVNNMWSVLLLDDFVLSASTLLVSNVKAHATKRDALGLYSIGAL
KLVGGSSLYVRYCSFEGYTHVFYLHSLSVSDHSVFALLNNTMLFGASLLYQHQGFSVSDH
SVLRVVGNSGSVRYAIYNDDLWTVQRSSWLDWRDNKVEVGAMFYDTESAFVSIDGSSAVT
LTGCRMGSTGLSVSLLKRVGAGYRFVAGCLTVAGRVVTTAAELELNGITNVTTVAACGQC
TKEGDCFAPLTTAVIGCMCQCAAGGHGDVCVPAPVPAGSPPITPPPVPPTPLPPPVGECI
SDMVYPEVAQAVGGGLSWLCYRNVTFSGGGMSLTVLIGGMKGDVANVTFDGCTWRDGAVL
LLLGNTYAAVGSLNILVAGNTFSDALLSPEGVFPPHTSITISGNRFTVTRLVPRSGLKLD
CSSCVAMNGLVISNNSAVVLSGNVFQSVTASSSAIYAFGSSLRVSWHSVFAVVGNTFHMA
GGDSTLINLEGSSQSLSLSVLNNSAVVIRGNVVSRPVSYFLLLILALRVESRSAVVFQGN
DMQGSSVVFYPSYSSRIYYNSWLQLSGNLCHASPSEAFAFLSPTVNLRDSTVSLSGNRFM
SSRVTPTVLRISTGSRDNTNGAIVAACNTGSGGEGATYVIPSVYNATVLTCSDPCTLAAS
CFPAYTTTASSDVCACTCAEGGHGDACLPVAVPEPPSTDGADLCVCDVRVDEVVNAGLGT
SVVCYVGVTFAADVVVDMASMSGSVRKVTLANCTFVGGASLYVVGWLSDPPAGERADVLI
SGLESRSGGGVLVANRYPPGSRVTVVDSVLIAEKRVAYRDAYGLGDASACLVVHSVNLTG
SVLTIARTHVAAVFRGAVGVLFVGGVALSSRGALYVDGLSVQTALGLCVSVEGGVSASGG
SVVAFVDSDFLLCKHAVSVRGAVSVSGSAVAFVRSEFVSTEDYAVAFYSTVSLAGGSMLL
AKSNVHDGVSREMLYTAGTVTAAGSTLSFVRNRALLPRMLSLSLSLTAGAHLRVACNDAG
GRVLSTAEEYAAAGFGDAGSIDVVGCDACDRNTHCYAPGTASASMTNGLCVCECGSGGYG
EACVPVGAPALPPAVGTAPSVFVREGVTVRSVFVVPAGASEVTLRRVVLDGVSPVLYVPW
MARDGVRIVVQNVSLLNGAVLYVMGGGALRGARAAGSDESGPVELSMCDVEALNSALVLT
GTFPAGSVLTVTDSLLVAARSTPLVYLPGSRSSPYAPVLVLSGLRLLRSVLVVSGVALVT
VMTGGRTVVVDGAVLEVVGGGVALDAAVLGGDVALYASARVVASGGAVLRVSGSQVYAAH
GLVFDSGVVANASAVVVNENAGVLTDGALLELRGPASFGSGSWLSVRGNNISGKILSLPS
YPRSADLVQSTLTLYGNAGSGPVVMDGTVALVGAGRRFFVGCLTLNGQALRPMDYRSAGI
IGEFRPAACGVCDADVNCFAAATRAMSASCGCRCAEGGYGRDCLPVYLPHVDGCNRTPAM
PPLSHTATLMATRSLTPTWTPSLSATRYSPTQYSQTETLQVTETVALLPTRTPTASVSST
LWWSDVACPTLAVKTTADGGSLTQNDVRGGGSAVAIRLMVALPPPFRWARDPQLGTHLSF

VSVSTAQPRGFGGPWGAMLRNATWVRNATNPSTVLELAVPVHRGYFIGADETIVIRCDAA
AVSGGCKGVLLGSFTISSDTLPAAASALSAITGVVAGAAAVAVVVTGGLGSILEMQALGV
FARMPCASAQERASTVALPYFLSVFAALDPLWMVVGNALLAAVFGCVHCGVTAAFQRWRG
VDAASAWAAMRFPSLTYVVAHAMHLGIFFGSVLALAMPGARVQHRVIGVVGVLYGAAFPA
GVCYLIARHTGASFERYWQFSRKPLHERLLYPVGYWHPAAQQRMYGGMLTNMRDSHVYWC
VFQLSVLCVVCLIAAVQSPVGGCHVQYFCMAAVLLAGAGVVAFTNMMRSAFMTVTRTASF
VLLAALCLVSAANHLAPSDGGAGAYVAIVLLLTAVPLAVTVYSFVVWYAEDRHWQELREP
RRGGLETLLRDEESDEETPKPQEMTSSLYATGTTVASSYRPPAPPSQPMAGDTHSNAQSP
LDRASSASRMVDYAAM*
>TCRU_1657 hypothetical protein, conserved
MESTRPVAYRRVVVSGSNDPVEYFVRAKDDGRTVVPTQTETRTGWLMCPVRLRSQLRHWM
RAEQAALRCLLALRPSRWLIVKGIQEHSFSEGVPVVWSAHCSVRSIGLFLW*
>TCRU_1658 hypothetical protein, conserved
MVSVRLTAQLQRVKKVLATWKKADERVSQLCTSESVGRDTLPGTACSADIKITDGLVGFF
SGNFSDKTWRDEYLGVDATVKKEVAAALR*
>TCRU_1659 hypothetical protein, conserved
MGTTTASPLEKVPRSVGCGVARRFACSPTDRTAQSSFHCGHSVGITRRGGVRRSRSLDED
GLVVFWQLIPSCPSPLRIALAARLAQHRPGMVLMQECPSDEAHWLQ*
>TCRU_1660 hypothetical protein, conserved
MYVVNLCSLTLAISHVAHFASTILRLCPAAVICDLNSHHELRDGRSPSTTAGEDLAATSS
GMDSELSDDPAQATRISGRGVSFPDVTAQRVLRVSHWKSTRLHGPGPPSGIADCGDGGRH
PATGRHAATTETCCLFPA*
>TCRU_1661 hypothetical protein, conserved
MPSVAAAACSVYTASPGGSLLRAEVLCKREDRNHALRRGFSKLLEVRAKQLGALSSPGLR
CLEAWRHHVPVHWDRLCCGTVGTATSLRLGSRHTARPSHCPSRTCYDPHCFLHTLCLRAS
GDWDMDWPFTPCALDSPSATLCMAWHLVPTKY*
>TCRU_1662 chaperone protein DNAj, putative
MGSDVFELRGNRALYDVLGVPRTASDVEIRRAYYKLAVVYHPDKNPDGVEVFKEISFANS
ILSDPARRRLYDSERLRTHIEGQARAYDPMMDPNVELTAEELRQFVERKRLEEEGKKKER
SEFEKQREEEMRRRAEYDAKNPAFKQEYERMRALVKEGVAQRVSTVSVPRHSTTAELMQR
LENKLREEGERRQENSCGAQESKGGVRCTASTSVKRSMMQDFRLRHNGETPAPGTISMNA
NRQATSSRLKFVSDLANNSYSCDLEERLCKFANFDYRSYVENGIVDGGSVLEDAILADAL
GNYDRRR*
>TCRU_1663 50S ribosome-binding GTPase, putative
MRLFYRPLLDKTFRHLSCRPVARVLAQRLLNSPVNAPVMSQREALVARLSDEVFSKGNVH
LKNVNTLSALPEGLHSFPEVCFIGKPNVGKSSIISCLLHNRRLGRSGSTRGTTRILQFFN
VGDAMLLVDTPGYGGWMGRQLSQNLAERASAFAILFRYLALRKQGPLKRVYWVMEARKPI
QPRDEEIFAFLREQQINFSVILNKIDRFKGDKVALQKQVNDVWDFLGSNQVPVLGIRANP
KYPERCLNIRALQHDITYYCTQELVRAEDLTYKGIRELSYAPPSLEELTAVEQRYPVESF
ILPQEDNLSLERFVLMHEEAKAKWLATSPKAALLSAKEKVANHLVESSDKRYKDGGSSNE
KNKNHNTVALPTSCLAVEEGSGPQNVFNSTTAKESTSSSFFEQAETPRRFCVRASLRSTR
QHQPEEDVFWTKRDASTPEAYAKSVSSRVAHESPPSVFPNKKPPLPFSAPLLPQLPTSLD
KETHTVRAINGVPIEKSMITTSVAQLPLHQEGSFEHFAQHSTSGDYDRLLSAELAGQHTF
LEEGEHASLSEGETLSLQRSLRKSARRKQLDRILEKYVSRVRKQRSLYMQAEGYMCPWLA
GAGQPSRVAVVGFAHGYSFMGGGGTLMKGLKRTGFGGKSFSAHTMKNRGRATKKTGYWAT
*
>TCRU_1664 hypothetical protein
MQVTVLQNHLKDASEETQSLPLSTFVKLYNYYDIAVREKILNTLCQYRSSFNVELQQRAM
EYVGLIELGNDELLKVLEPLPSFKEDDFSLVLPGRAQREEANVAVSLNISRDKREKLVAP
RADKGPDKLNSSDLLVINRDVPLNSGGESDVDLEALFTVNSTMQLDRMAKENELLRRRFI
FLLEEPSGVLYSDEFVELHCEQQYRGADARVTVTVRKKMAVGLEKVHVETTGVDVGLLLQ
SRNERGDIASSGVLRVEFVARSWPPFREAPTVRLSYKRPGECTTETKILALPIVTTRSLS
PYDEKDEMAYDTLCKQMNSASVRTRRLSAGCVTHNGAIEEALKGIGFCVIHPVDSAEWKA
MAAHAMQSHGILSYEPAVVKVFSHSPGAFTAQVITKSHVLSVAISDVLGQTLLSKGQTEC

QERFSVFGGNLTPLLS*
>TCRU_1665 Adaptin N terminal region containing protein, putative
MSMRLLRGFPVPTDSLLKEKILLVLRRIVQATDRVLRDAQTQQKQKGTMNRVSAMNAVFS
EVVLLVLQWDLDTRLHNECLDVLSWFVMEKKDSNLRYLGFSLLSQLCSARSSYNDYRTYC
RQYQPQVVVALHDPDVSIRTKALDVTVCICDAETSREGIGALLSYLPIADGLFKENLVIA
ISHLAEV*
>TCRU_1666 Adaptin N terminal region containing protein, putative
MDMRGLAHFIQDVRKATSSPADEKKRVDVELVKIRSKFRSTAEMTTYDRRKYVCKLMFIA
MLGYPVEFGHMEGVKLLALKSPAEKLIGYLSATVFLHENHSLLTLATHMIYKDLLSEQEF
NINLALTAIANAGGKEFAEVMSSRVKSILLSDRWNVHVRKKAVLTYLRIYRKYPDVVDLG
DVIPVVTDLLLSPLLGMSGCVAVFLTGCLNKSNFHLFHFRTQSSH*
>TCRU_1667 hypothetical protein, conserved
MQEELPECFRVVSQQRTCGDEAVGTALEDIHRFVFEKSQRNMETLSPELLSSCDLAVKKV
GGQLGKRNPLGIAHLPMGVAIVEMIARLTRILFWGITACSEMSLPRKLKMIEEDGEELEE
VLLPRFFLKLSVSPIILRDVLMKAPWDVFSRLYGTESALPGEVRECVPVEVRFVLSSLKV
NFGCEFLFHICFPVDLAGSHREAVTTAAEAIDSHMATAFNLGGGLSVAVEADCDNKNIGD
NTNHHLFCDKRCTRSSLLGRSTSRSESWDFKTGRKPLFSSGERSQFTQFIHSRHCLPAYK
LTRRRSGILPGPGAGIECADDLINGAGHYSPATVESHVSSEEHDGETEVLAPETPVKKRV
RAEKGVVPPSPLRPTRE*
>TCRU_1668 hypothetical protein, conserved
MGIGVAPSTRLQYARMLRSMFEMNRTPLDMMILGLQKVAARSETKQARPLTEEEMNQVIR
SRTDWRERVVLRLAWITASRWSEIVALTPNNFTLEPDGTLILDWSVAPKMARADPHRPSR
FVRIREAGRFRHYKAMQDTSRKRKAHEYY*
>TCRU_1669 hypothetical protein, conserved
MPSAGFSADRTYAKVACIPRESKNSYIRCSSHVMACDFELPLFAAPCSEVLCAPTSMVDG
KMAEKADKAKRTARLGLHDKKRLPLPFPSGNFNVAGVWNDNGSPSMERRIRDDGGRCLFR
GIDDSPTIVLDGVSQSLAQANR*
>TCRU_1670 hypothetical protein, conserved
MVMRRVFSVVLVLALLCCCSCVFAAKEEEQEQEVTQVNAECAGDGNVARWQLPGKSLWYN
CTDLSTVVAQMICGMGVGNCAPEASQSRTKGVAKDGVFKINVTSPTPNILESWWKRKVEP
KAATIESGPTTRPEAEPLGPQEHKAPGYTSTTNSDSPPAVAAGASGDPPPPLPQTATPGG
AVANPAAGATATTDPSAAALGASDPAPSGDPQGPANSASAASPAPESPPDPKPTEGGDHS
SHEPPVELREETAAAPPTQLSQTGEDEGAAKGAEEDTAANTTDTASSEGNATAAGMPAPL
SSAPTTNALENSVGTDACFHGTHLHALPPLVLAALTYGTLD*
>TCRU_1671 cyclophilin-like protein, putative
MQGGLRLVRVAHITVLARVRMGAISINHQQSLFSGSSVFYCASASGGHVSDNTLTTRDEK
GNSLIRVKRLPLTLIEEVPLPQRVLTDLELVMMAWCEEHARQYAMIMMAIRVAIFFLLLI
ILYVFYRTQLSSERMIRGVAHMPADLRIGSVVYFDIAENGADIGRVVIGLLNEKCPLYCE
YFHRRCTGSGDTGDSFRGMQLAAIVPRHCLIFGDGREMTHDVTGFNPHYLPTEHLGTGSW
RGALSSIAYGVNKESPNFAIHVSAGDYAPQIFALVIGGFDVVERINAGGSKHGNAPKKGF
TIVECGELCTLAKSHIVPMPWKLYESISKGYDEEKFGERIPRDFLERSDEHSTARVLNST
TGQEKIGKTVPWWRFLW*
>TCRU_1672 RNA re-capping enzyme, cytoplasmic
MLEFEGSSLFRHHIVCSLLTNKPVRVSGIHDEDEPQGIIATEANFLKFIDRVTGGSKFEC
TDNNTTLTFYPGMILGGNFSHEVPSCRCVSYIAEVAVLLLPFAKLDTKLTLVGSTQGEMD
LSVDTLRTVTTRWLQLFGVECSVRIIRRGAHPGGGGAIELQCKALRKLTSVDATTRGRVR
RIRGISFGSRVAADLAHRAATAAKGVLLNLLPDVYVVTDLDNSKTHHGEPSSGYGVLLVA
ETTSKYCVISQETTAAPREPPEAVGKRAAELLLDQVFEGGCVDAHHQMLVLILMALSPDE
VSTVRFGQLTGSAVSAVMLLEAYFGVSCATKGELSMTGENAPSSTLITCVGSNTVNVWKK
SG*
>TCRU_1673 leucine-rich repeat protein (LRRP), putative
MDLSLSHKGLQSFDASTLLLEAPEISLQGKSVPLGERYIAVRTLDLSHNSITRFAGGQAL
SGLAVLDLSNNFLDTLDASSLPGGLIRLRLAHNKLQSLQGLSAFVPKLQELNVGFNRITS
RRISDLPKGLAKLLCRSNLIDSVRPFLGLAHLSSLDLSLNQIFDTNELSRFGELRALRYL

ELRGNPVMSAPEAVPSLLAAVPKLISLDRTPLSQAGENRMFKVNRSRSAKRDQSNSRQRT
SSRLLSNGSGSRRHYGKGETDMELRLLETRVKELARLLKDAEKTELQLRYQKKILKEQVS
ACAGVIDSQAMELERVGRRIKDLKCEEASLREPVAELDQTFEQTHASLVAHRLNQSAGCF
*
>TCRU_1674 tryparedoxin peroxidase
MSCGDAKLNHPAPDFNETALMPNGTFKKVALSSYKGKWLVLFFYPMDFTFVCPTEICQFS
DRVKEFSDIGCEVLACSMDSEYSHLAWTSIERKRGGLGQMNIPILADKTKCIMKSYGVLK
EEDGVAYRGLFIIDPKQNLRQITVNDLPVGRDVDEALRLVKAFQFVEEHGEVCPANWKPG
DKTMKPDPEKSKEFFGAVA*
>TCRU_1675 hypothetical protein, conserved
MALVPYTPSEWCDIGARLWDMVCQCGEIQVQMTFINGNQMSDETLTVAASSTGLCLFHGP
ISKAWQDSTLIGHIADLRAPQIPNFETQALLPAMGAARELGHQQGLITAERERLNIDYAN
AVADVQNRNARLNSQFQEAHHNLSVRESTIAGADNLVLELQQQLEAAHAQLREREFNLGV
LTAQLQEREACHNAQQPVRDAETAVRDESPATRHVANDGVTTLPHQTDILMAGGVPSSAA
RRSSAAYRRPHGNVGRPHFHPSTPLPSRTSPLDAQLPMEEDRSFCAFDVTT*
>TCRU_1676 Protein of unknown function (DUF3720), putative
MGLINAVEAADEGGLGGDAGSSQDKSKESLQDKKLSGNTEGDAALGSGGVVVADPPPPAA
AAPLQKPGGRNTGEEGKQEELDKKGNEAEGKSRGEPGPPKPKPEQSSSSASGGPGTQSDV
ELALEVTSEGNKFSDDALKQENAGKTEVTKLSDGEPEKGKSNMALTETRGSQAEQKEIRL
PTLPTTQVDEHYSSGDTVAKQQLQKTQSGMDPKNNSQTKSAVTITASQHNEPSADHAGST
TPSPAASGDAANNETDKTTEDGIPKNDSAADGEEKREEKRNENMNANPRETPVEASAMKP
TKATTDDSDGSTAASHTTSPLLLLLVACAAAVVAARE*
>TCRU_1677 Trans-sialidase of Trypanosoma hydrophobic C-terminal,
putative
MVPNASKKRPSFANGPRILVASNASGIVSVTCGGREHVAADNTDEVNERSATGHKTLVLE
NTVAGSGFVAEGDAYGLAQCNASGHDNRPLLQKLIAQYSGSGGSPHDCFALRNFAKLTLR
ELTVDGTVRVRVYWVLLLLLGLCGVVAVL*
>TCRU_1678 retrotransposon hot spot protein, RHS3, putative
MAGALENSICRSICGVFSISFTKFIQDEVLLDTIKASPPYQELKKEREEYYVLLGASHKL
KKEHIVTLRHWSNFKRKDTVTPLARAQINKAYSQILREERREAEEKARRERQELLFTIHT
RTDDAVLEGRVRVNKMRLNDFLTMELDGRGALDANRDVLLEEVFKDPKKYIRDAGVLNEM
QASDRYKRMERAVRDEMDMAEDADKLYNKGMYDLLRSLAQPATSTWCQGLVLFTREEAKR
LLFMLQTR*
>TCRU_1679 hypothetical protein
MSDTDGPREPSESVAFVVELEQDMGRTEIEIVEKEERKNIMCRYKDAVSLLTSAQKCIHA
LTCCGCPNNSFWNTNSGGRAALRERWGRRSNMRPYAMDATDMQQRKEGSMAFSLSVPPLV
WPWNPLRAIALQKSATQSENGFSHIARTPAAEARKCQGGLCSAASERLTCGSLSVSLRGD
KQLHEDGKKRRWNSSSNLCSKAGASRSVSLAPNDLVRLQKKTVSHPLVRSQHSVGSQCFP
SRREYHSLNTQTDFSIRLPKALIDDRFRRLNILQDLLRMELEREAIEDWQVLRDEFIRLG
CKPMEVKQLGRNLRCEAGDLEGRAGATVEISNWEREKRRQERRFWALELHNQQVDTVGSS
STVR*
>TCRU_1680 Protein C21orf2 homolog, putative
MKPQSVTLREQTVLERTKAESLSAVRNLNLWGEGFTDVTIVERIPGIEVLALAANQLTTL
RPFASCTSLRELYLRKNDIKSLAEVKYIKDLPNLRTLWLMDNPCAKSSHYRSFVLRCCPN
LKQLDNIEVTETEREEAKKKLSEEDVKHILEHGTVAEADLSNKRQQPNSSAVSKAVEKGS
LASVGGQPSQYGRTKEKEKEPRQGGNWANDAPRESSCTPTSNGVPYTTVKTQQAIITAIV
SLIPELTLGSLGVLQKEIKDEVVRKEKAVRNRSNCPQ*
>TCRU_1681 mitogen-activated protein kinase 2, putative
MSSEIEPHILRKYEIQAQLGQGAYGIVWRASDRRTHQVVALKKIYDAFQNATDAQRTFRE
IMFLQALSHPNIIKLLHVHRATNDKDIYLVFEYMETDLHVVIRANILEDIHKQFIIYQLL
KTLKYLHSAELLHRDMKPSNLLVNSDCSMKVADFGLARSILSLEKEQVARPALTDYIMTR
WYRPPEILLGSTRYTKGVDMWAVGCILAELLLGRPIFPGRTTIKQLELIINVLGEPTPED
IASTNSQFAEAMMKDIRRTHTATFAELLPKASPDALDLVQKLMRFNPNERLTAEQALEHP
YVAAFHKVQDEPSAPAPVTISLPDDTRFTMQEYRERLYQEIANARRRVREAAAALSSKN

ESGGVVTRAHPPPSAAAPVAEERREKVRDAAVTHTSSSTAARPNSSTATRTALANGASRT
TLASRTSDATRPAHHTRPVVSRHGLNGSRTMQK*
>TCRU_1682 zinc finger protein family member, putative
MMRTSPRSIQQPMYRGGQGGFQQQHQNYHQYQHIPGRRNSNLSPVSGGRGGNMGAPLHFD
RHHTIRLVCPRSNTVVCTRLNRVLPTLAKAQSGAAVCEDFMYAQSCPYGESCTKVHVPKE
HTWTYITPDINRSTGLFEPGFIVHCYGPRMTQYYPIQSEFVLNTRGSREYVKMYNEHGDN
FKAKFKLCESMLTRGECSLGEACDDIHAIRNDVWNMESNTTHIAEPELLANYPRLPANIT
VRAFEQNSKDSFVDYPGDQVLLTLGSRQYLTAFEAEGTIPKKKMQHCAHFRTNHLCRRGE
SCRFLHVVTDTAGSPVNSIVGEVSPNSTSRGDSPSSDITVNPRVRHNGPWMSGSQVALMV
AQRFTGATDAASPKVNQGTVGEPMLSNSGTESNNPVFMAREEHQASGEATPSILPSRMIF
PNQATMDMSGKMRMISPTRRNNPYAMYHGEE*
>TCRU_1683 XPC-binding domain containing protein, putative
MSQPGTPLAVELKTVSGRKCTVDPPFTAQRLVDAAVDQLHLERSTIKLFVGRRLVYMWKP
EDSEPNQRLRAQVTVPKGGSVLVVGKPALRQKTHPSRAKGVDTSKGSPAADGAAEKKKPL
LTPVASAADDGMYIRCGSSDRGLYRFLKPLVFMLGRRGLHHLFLVAKGQYGHLPESFQDH
PVMVCIVEYVDRDPGRLKEILNTIQEEEPGLMRWIETHAEFFLNVINTPPQWRDDTSPIW
LIRHAKKVYSAENTKKTDKSELVRAVTPSYKQMELIPEDLADNGILLNEIGLPQDADSSE
VNSNPDSSRTASLESIQSTAVMESSTKDLCSEEDSLLDITIQELQTVYRAWELAKERYGR
TVGNLTDKKRLESELKSRVRDVHSTLMRAMESYILNPPSPASLKKLLLIGHRAWSESEQW
FIEKEHVFNALDTALGRLLFNAGKELLMVRNSTHHQKESNSSNRKSSPISEFQQFPALDL
TIRWITANGPSILAHQLREYSLQQSVSIVMYSQTSCNQVYAYVVRDGDVKIAPIVVDEHA
DRELKELERLYEVMSQTYAPKDAMKMRRVIRSRWAGLLGKLYQRLLSPIEGFLPRPALKP
HVPPSEVGQICIIDTWAPDHIPIPFPALWNPADGASCLLQWAVFKANRPWDLISSAEVPW
SNPVANSPVITRVAAPIVVRPTMSVVEETTCSRTNMVTYVNTSDIGCVSVGDKTRSRPLK
GANKVEYGELWQNVPWEHWIQKALGTFHAGIPTYAFLKGKDSMQNGATALFIHEVVFAEN
ESITSTRSSSPPNGKGKKVLTAREMPYNQSADLFVSHVVGRDSGVGIHRAALLPTFKVAG
RDPAETYASFLKTLVATNDMTAAKAYRLAVLISWHTDCREEPWRSASLMLFNYGGTMNTL
SFLHSEYCKKRYLKSLHVFRGGDANFNKDAEKQQNEAQLLRNQPSICNTADGCYVRKHGI
DAIYEVVLHGLSIARPQGEVAILDNMIQCIENNRSTLEECVKRRREGKDVVQALDTPPAE
LVFAPSLNSDGGASFVSPVRDIVTAFRNGGEENSSSGLPTRDMRRRKGNQPPNSRRPPSD
S*
>TCRU_1684 TPR-repeat-containing chaperone protein DNAJ, putative
MSVRHLGGALRWVVIESLEERLGAMQERLQGWGVTAVMLGTDAASSDLQQCREVGLKRDI
NLAAEYAAAQKEEKQPFVPTEDRSGSQEPLLRRQLRVLEQLSALILEQIAKCRGAGEETR
LGGLEEELREAVELRSSATNCLQACLTEEGRTSSELNTLSSDCTSESSNLASVYRAVSST
SNDHNEVKGNVPSNECRAASAFFAPSRNGELFHRRSASALETDPQFTSVSMEGWMDRKSS
SPRAGTTNKKRPASSSTPRRPQKNTERFYVYGVAMVPERPKTKRPATAKASRRDCMCGGD
HGSITSHCGDLEPKEMKEFNLTDKEVEVVDAAGVGVSVVVRRMLRPSRQQTQSKCRVSPD
SMNKIRMTYRPALTVRRDVRKSRLQEIVPGLRESTEAAAVAVDAARAAVAASSSSAPHTP
CASAVQGSGKQQSSSIGHLHQGSEEWKKQGRKRSGYTTRRSRSFNPDSEFIQVCRERGNR
CMRNKQYEAAVKAYSEAIERDPENDIILCNRAAAYFLLNQYVLTLMDCESVLSRSPSNLK
AHWRAAKALLYTNNVQAARRHYRVAREMCVNLVEERAIAEEMKALRAYEMYYVYMKECRW
IDSVECADQLLRAFGSTGVVGLPWQCRKLEALLNLDSWRTLEDIKRLRKAYPKYAELLFL
HAKCLFYCVHDPNSTGEILELVQAACRQKEIEGCSKDSRYTHLERTVVSFEHHRDRGNTA
YESGDWMEAYTAYTRCLTLDPLNKSLIAVTYCNRAAASMQCGRWNDALSDVHRSIQINGN
NAKVYARRARIYLHFMRDKARVGIDYLQFAINDLRRAVELAPTDENRQQLAEAMKLKERE
ERSKDSRSSDGNEDTDRGRNPNETPRFCSNDANSASGGPRQKTYGHSGDAPNIFASENSK
AHCTKVLGLENTAGLDARSLTRAYREAALRWHPDRWIGSGPGEHEAAEQKFKEIHTAYQT
LKETMVR*
>TCRU_1685 hypothetical protein
MGLAAAKEGNKSGKDVETKRTHSRVCRRDSSGSCEDGVTPFSVVSAKRSNKLRDNFVRPE
KLSTNNAVRSLFFTANPEEWDEVIVISHSPQVAYITTSSNRQASVVGFSNGTAHSERREE
ENIYKGNNNDNNSRNNWSRGRLASRNNSLDETVVQVEQRAVTSPSGFVVFKRPPPHSESA
KVAPSPSIDHCSRKESSKVSSSPIHRKTVIDRSMDEDVNRPLRGSVVSEGYRHSVTGSEK

ASTCPKGLSRRSLLPLRLESIDVTGIRKKSDAIQQWLNEVEREKNRRVFPLNARRPRSPA
RNPVEE*
>TCRU_1686 hypothetical protein, conserved
MPGFSQGFSAFIDNYILLLAARFHAPLDAVSFFEGDPDEPWEKVARCVRDALLHPQTEDE
RREVAHVDFSLFDAQLCRERALYFTWRLREEAANDATKSAAAAGASARIQITQKMSGKPK
NRAAAPSQFDITRDSLRDEVEQIYTAVRKNLPSTFGPRPPKNGAANNSRGNEDVGEQLGD
SSTDEDDDDDDEALDAVEFSLRSEEEMKRDVAIAEARDALLGKIQEMQAEFFAQHRRWID
VPFDIDVVPTHGKAVDGVVPELPPGAKAADHQPLSKSAMREALAPAYTPAVSEKKKTTST
VKEDEAESPLNAGTASPAHLLEALRRSIHVNRPPRPSQMREEDYEADYGDVANAPFSSEE
IRTETEFGIYQKINQVNDSRVPQENSLSRDSLKRNVTFQTTRASRWSILEYDGEADEDSD
DAS*
>TCRU_1687 hypothetical protein
MESLSSNAALEGGRDKREATHEADAHLLLPSRRTWLESSPLPAKHQFFFPSNRSSASTAP
PNGSASPVKAAPVTKQVEGGAGTDAMRKELIRLYDSNAELREQVEKLQACLHHRKMVLAN
EFEWNCCTHLAELREALMAERLRCEEKERIIAALREELSAANQAIEFLSRTQKVPLKESA
GTRFQGRLLDGWDRSSSLVTQDDPQIVLEEEMAALQARLHREKEEQQQGEKSGTGCLLEK
QIGAENFSWSRSHSGLFPTPNSSFGAGNGAGKRTVVTGANNCQRHTTSPTLATLDSSEQT
STLSKRTARTPPASSSRAFVPQTLADINTLTVATPFKTEAEPWYDEEEVKEVVSFSRASR
LRVAMSPRRFLRPSNVKTLSQ*
>TCRU_1688 transcription factor Tfb2, putative
MNPSYFDQVLGYPEREGLMTQCPALALFCYQEFVAAIQGGKRVVDPLSAKDEVAVLLSAA
TVSEVTARVFATVEEGATLHPFWRTLRAALESAWDPVQGGDVHWRLYEVAGNARFYGKAM
PRMPLAQLSFQNTLDASRSILHMTTACALAGSIDPFGEKRGQPLASMLQYYRLVPTSSET
REITSEGLSFCMQPLQQQWWTLVSVALDRILVLTKNSGVTRAELWQLLAVLFALDTSQFV
YPFPEKEKDLAAFQILARLSEVGLVYPLISGGRRCFALSPHFHHAVCWSSTAPLCTAALL
DSGGNSMGRVRREDEDTIITEANFRLYAYTRNPDLLNILNQFAERDAEVDQMIACYRVTR
RTFAAALKRGIGSSHILQFLAVKAHPSMLRHHREGDSKKASGLSVLGAGTRFKNASDIRI
DEIIPQSFCDQLMTWEKECRRLIFSRNLVLLRNVTAVQKELLLNALSSGGERHAVVHQEK
GYLVVQREAYERVLAPLIPTE*
>TCRU_1689 hypothetical protein, conserved
MMRRAPEWFTRGRARERFHQIWPWALSCTCAAMYFINREPFDELAATVVSKVRRPLLKTM
EWIELHDVDPETMALELPPDMIFGPLEFPKQMRFLLAALRADNELADGYLARVLVDHMDL
SLDVPILSESELLAAGGRDLLNFAIDDFLGQGTRKMRKHVFFQPDELLRLINFISVYPTL
TEYFVKKRNGVELVFRALTHSRDEYARVMALRSICLFCFTQREDGDVERRVLQANGVKPI
VEAYKQSTGDPTDTRYITLVLSSIMRHYPKEGGKEFLDADGVQAAVNNLNISRYKGLPQH
IRVLHDAQRLPPEASGKQTVNARIEDADFIPVAIGVLDVFPEFYETAGDILSLLEDIVPS
QTTPLNLLEYRAMPVLSKFFVRWKDDNLFQTDGTRTQVVRLFKMLLDDKTCQKCYDPSVA
SYELQECLRTAKEAIREEEEGVGHGLQHAVS*
>TCRU_1690 ADP-ribosylation factor, putative
MASSEEEKRVAYCKQHNVHHLFELLATKLLLDRPENPFEYLREMLSKVEESEKSKQSYDP
TVLSPPVNGAVEPLKKITLGTFGLDNAGKTTLISALGGQIEVNTTPTVGFTPTKFQTEKY
DICIFDLGGAANFRGIWVHYFHDCHGLMFVIDSAADDAVVRESLNVLRDLSQHRYIKGKP
ILVLANKKDLENARGTKVIPNGFLDKLFGRNTAYRVVASCGIEEDPALEEGVEWLLSVVD
KLHDTLEARVNCDTEEVKMEAKRKAAERLAALKTAS*
>TCRU_1691 cAMP binding protein, putative
MPMTSFSSISHQLNGVQEHRHSPLLSSRPVSSGVRSGYEAIYPSPSKPRRESVFFLAQTP
KPIDFHSAEAQEAMEVLRRLLCPVIMRRVLRIRKRKRWPGENAMRVYTTNSIHHALRSSD
SIIADWPDEVFTLIAEEALYFYLEPNEIIVYTNESYISSGVVVLLYGEVKEQKDHLDSST
SLTRPQICRHVAPAVFCDQAVLCRDVSTSLIAANGYADVAVLPARWVWNVINSYVLQHSG
GCVLDCLKFSIAPICEEIVNKKYFPTSLVLRRSWMWSLFSSRDRIRLARLMTVKVFCIGD
TLFCEGDYYPYMHIIRRGIVKVVVKNQPLLELDLGSSFGEVSILFDEPRCCRAVASTMCE
VYCLHRKHLMKYLRKKPQLYNAVIQTALERRELWLEEGKNRNIMGLAGLLAGVPCLSQST
EKVRISLAMAAKPHLLPPNQTLFAKDTLCDQLFIIGRGSVMLVDGNGDRVVRSTVEFVGE
FCLHPHRWPTNVITCTSVDGWSLSVTEIMSTLSTINAGAQAVEICRQGIELYRAQHGSNS

VIEEITGSGSHLPTVPDAVDAISLAKRETLSYTGDSRAPAQLPSSLLEQSEGKNIDFDWN
AYAMKDKSTLPLEDEEGTDVSKRSERREFEQNVENELGWRVGGMISVPNKKSSSAVDCDF
SADVDEMQQVLVEQIFLMPTERQPILLRQINQSKITLIFDDELEPLEELSSENTTANNLA
LPQYVVPSPVVYTLDGITAYSDGNPLPATEAPPIISPRTGLDFAATTTRRRSHTPEVQLS
PPGQFGTQRLFDELGSLTLRSRPQSGVPSHSRSLRPTSGFSLRRTPPRTASAGCNQRLSR
SVSFCSRTSAMGTLTVSQVSENISTSAQGNASSWTKANSIDLSQKAKITASMFQSALDRY
VKLDDQSYFQEMVRVCLPSQPEEQWFEEDTRKLAKRKEGLILLLMHVRSCKGIEIDKAVK
SPIVKVSTNARVLMRTPVMENLEEPVWPIETASFIHFIHADSEVYFKICDAEDEGKIAYS
ATLPTSDIRENGGVGLRSLPMQAEELGLSESTDDIGARIEVCMMAVTASKYSSLNKRLDD
HDDEDSSEDGTTVYLQILGVHGLKHRIEAIIRVSVEGDGQSREILRTPKVFPKTRSPSWP
GQGSFCAVKSDGVVSFDLYHRDAFLASYETPADILAFGGTGIYTFPLLRAQGGPDEVYGS
LSVSILGLKATSVERQASCRPSRVLLLHLDSLHLTSDLEEFTPDPFVIVRGVNGEVILRT
AFSFSTHNVKWTEDEASCFIMCPTLEGCSVIYRLEVYDNNESNKIGTAEMTVTPNGTRRH
RMELPVGTRGALTVVSHSFPVQEVPRPPPSRRGSLLNESNEEGFLLSIDVRGCDNLQGTG
FDDLQIDPLVTARIGRRRVLVAPLVSGSTVPRWSYPKATFVLPVLPELLSHFTLEVWDTN
IELSDVLGVARISIKDLCRTGTHHFPLQPHKDQEFGRRQNLGTITLKTRFGRMNGEIISD
GSSDLLLTGSSRGGSMLDTRLFQTPGDATEFPVTRVCVHISSCSALQTDASIKFIKVTMS
CLQHVFLEVKKEYDASSSVAWSIGEAHTVIDLRSIHRRPLCLSVACSSFSSVNDYTDVGS
ALVSFSTLLSASEVISVHSFFLTDSPSGEEGVGTIENVDRTRGTSVSLDTPTVTITLLAL
EMARPLSTGSG*
>TCRU_1692 hypothetical protein, conserved
MGPKNNSQSKSVATITANQHNEPSADHAGSRPPSPTANGDAANHEADKTTEDGIPNNDPA
ADGAETAEGKRNENMNANPKETPVEAAAMKTTTAKAGDSDGSTAASHTTSPFLLLLLLVA
CAAAVVAA*
>TCRU_1693 hypothetical protein, conserved
MSVNFCFATGFFILLLCVLLTAFEPWLRCDDVFGLDGDDHISVLRNGDAVFIDAVVNKGF
FSQTNEGGNTSSDDAKPSFSADVDTNQVLAVIVMSAPSLPEGVISSITKARLLHGVRTAL
LAGAPWIIVPGSVEERRSAEKFLWELRSPSEALFPDATLSELPAKVQKLAVQMRDSMSFD
TSLKNWIRHGRLLKRLSHASHVDEEMKVEDMSSPRLLRYGAAEWILPGFLWGEAGQSEEA
ETMTYAEQAVRLMKQRLLAVHADFQNEDGKERPIEKNARAHRGCFNVIVVSSMHNERYAR
AMVYSTIRRDWGASSSLGASQKSVLQIKQQEQQKQIFSECIHVLVTSAGDVEPTWEVPSP
LFVRGPIQSMANFSLQSSFVARFLGVYAHAIHANYRRWFACMQVSLLSYGPLSLIYEICT
IIVAHLTETLSVRDLAYSTFDQPN*
>TCRU_1694 hypothetical protein, conserved
MLYPPQKSVFSAVGQDGTQLHGDSMALLEASSTRYIQQLEVEVAELTQQYDAACQLLYHA
VLVQDASAIVMDECEARGLIVKDESAAHSELLWLFFRVTTMRRQVESAMMPRGSERATAP
PQTESAMNWGKIPECVVNALQGSLREENERLTRLLEAIPQRVCSALTVRDAAGVLPVTSP
SASHDLAQADAARNGSTDVKQHLESIVTRIDEIFKSLRQTGKPDAEPTRTEDLVGIHVAS
LQNVVKSLENLSIRIEEILDRYNNDVNSNSSPRSNQTPKAEELRASQAETSFLTSKVPQD
MPLLRRLAYSSVATLESLFSEYMLAIERMQMMAEKEMHVFSSVQDRWKMLRWNACMEEKA
CEIQRLQREVERLESQSVIRDAKDAAAANTNAATTTNATQATKFPPALAQLYAKFAAPFI
PTNVRIPRATKMKSIVTLRTEAMRAGARPASALSAADGEAALVAPQSTALVVRGADAAFK
GQNSSGTSKPEVEKKDNGVPPPEQPARHASVESAASSASWNATQQEEVHQKKAEENVAPQ
KLDRYASVSPRSAATISSGALPEDVAVMTDDGAARKAASPSVVVGEKRESGTMGIVIRRL
QRDGSMSSCTTSGTDGTISSVQVEGGEAHDDTHGASKPAVVENSFDESSDVPPKTRQSEL
VTSFSGANSKKDRRSDGVDSPAFAVLLEGRGKELASGSQHPINQTVESSSLSSTSITLTL
DTSEKSGNKRQRTAAKRNERTRNKRLSSDWDDSSPRDVVGEKKKGAKSHSPSTRARTVVS
GLKALSFTSHR*
>TCRU_1695 hypothetical protein
MSEKDAVLYAYEARLERAERGSLSALESQCRAEIRASELSKERDVYLRALKELFSRNEEL
GKCVEKKVSSSQSMSEGSVHTPELSPELCEKDAGTELRHSEKYESLVSLFQIRLANSEVW
MNMKSLLVNWMAFVLKKKITAPQMGKIRHFSSQAITSLTSEALEGDDKDISLMDKEEEEE
TGIRREVEIVEEPNAELKATETASEEQKQETEEEENAQKNEGREVSKVVKALEQHLALEP
PEKIYDTTKQSSRSNLHVCTSDLDSLSLAKKNSNPVISQGTADPCLAQEEPISSGVSIID

KKERKAEVEDAEEALSSAVESLHSLQDGSEASAIIATLLSLHRQGVGVWRRRAFLVEQQM
EEWGAKVSTLSGLLQQQMLPYTFVLYEIYTTAMTDKINIFLENFCLMNCASDKMCGAYER
SVMESSFFIMLRYWGKWRLHLEASRLVMLREKATRTVSSMAEQHEQLKAIAQRAMMRIRD
FEGPSGRNEGDVAEI*
>TCRU_1696 tRNA (Guanine-1)-methyltransferase, putative
MRNKDEKGDAADPPSLRKKRQIWTAEEQSAYWQAKKVERKRRSKEAAAQRRALQQKEWEE
LDDKERERRRTEAIAIHERRRALEAKEHAMCVQHLADTSLPVLVFDLSFAWCMSAADCRS
TVSQIKFSYSALRRHAFPMRPVITSINGKETIETSAHSELLRSLCAFEGFRRYPFPIHEE
HWSTIYKKEQVIFMTADTENVLERLDPGTVYVVGAFVDHNARKFLTRDAARRHGVRMARL
PLEESMEVGNRCKVLTVNHVVEVLCRYADRGEWRSAFDVLPTRRVNAGRQKRSRALNGVN
IVSSDNDGAVLHETDERCHDDVMPQA*
>TCRU_1697 kinesin, putative
MADQGVVAAPTDNAVKEAADLTPAQRVENFKDQKKALNKSTNDVPQPVNRCLVYCRLRPG
NKTDFKEGSFKLVSVEGNSIVLKGERRYDFDKSFDDECTQEQIFDHVAVPCVDHAFNGFC
SALMCYGQTGTGKSFTMCNTTPGLEGIIPRSARLIFERIQADPTRQYEVIGQFVQIYRDH
LGDLMVGSGKERVEVRFDENEGVELTGCTSHVLRSPQEFMRFYHVGNERRVVTATAMNPE
SSRGHTALMIRIFSEKLDDPAAGKMRGKITFIDLAGYERFSKTGISSDNPIMKDEAKCIN
ASLLSLGHVVTALSSSGPHIPWRNSKLTRILQDSIGGRSRTSIILTVGPSSDHFYETTNS
LQFGLRAMAVKVSAKQSVVVNYEKLAHNLQMLLDEKQERIALLEIQIAGRDAERAELMER
YNKHRAEIDLRYERDMARLMASNAPPEKIEGLREVYRVEVENLHEQRDEEIQYQEEAHSK
EITKLVREQTEQEAKRRAEMKLAQERIIEDFQKKLDNAREGKHDDIVNALRQLAEKDSLL
ASRANDTARLHEHIEVLTQQIREMGGTPVEEAVFPETFLDVGQVEEIQQRLEAEVERHRE
KEVQLYAEVERLSRICSERVEEINKLHDENEQLRSDLSNKGFDVGETDELTKYLRDRRAK
MIDSSEMETLRVTMRAEMEELKAHNVELKREVERLEEERAQQSLPLTARIFGTARNSLST
ARSVLPAGGAPPLTGRGASQLRQYFLSDDTKSLSLSFNGETSKKAVKELSDQLTFSIQEK
NALLNRIQKLEAELKSHGVESPQPYVPPIMLGSSVLPNIPSLVAPSPDAAPNADVDVLLR
VKDAEVDEFMETIERQQHLLATARSNDEHYQQVISDLRQAIAKAGLPLPESQAIPAPVDC
IAMDDYMNILRAVRDSERKMVVRLAEREGKDPLEIDSVLEEMNRELIFKDELVIENASKM
QFVAKVCIRLKSQLERLGITPCCQLPDSYKELIEREKCEMEEQTEAQRDLEEKLSMLAEE
KQRLNKMLSSMRQEREMDIVVMRSVQERCKEAEEKEIYAAEALSRLTREKSQKERALEET
LRLATMDLMQYQAQLAQIKELENTGGFARILKLLLRR*
>TCRU_1698 hypothetical protein, conserved
METGQYTEQGIAPLWHTDLPTCFTTQLTNKWRSTIRQDTHRYLLCGTRPSDLSGKDLITQ
EVLHRQELVYLARARCGESELWGRLYWAVRDCTNQCRFCNISPEQSAYMRPNNDPTAPGT
DTVPPSAREEDVSPVRRRTVTRRRKEKCPHCDSTLTGFSGLVSHCRLFRQEHPPPLPELK
CDFCNMVFPTRRSTAPHRIRRAHNPDATRHRNSSAMRRSLLPQDQPASTSTPIGPQEALH
HLLLECPGTLAVRQRLGIEQDLRLGKFSQWQLPHSRKLLSLLDHLFGTQMALYS*
>TCRU_1699 thioredoxin, putative
MRLTVPLVVVFLLVYLSYFHVHFFAGVAYASNVANDGERPSRVVELTDETFDSIVMDPEK
DVFVLYYVPWSRHSVAAMRLWDDLSMSQSQKRNYLTFVAARIDGEKYPDVIERMRVSGFP
TMRYYTRIDKQEPFEYSGQRYLSLVDSFVFQNT*
>TCRU_1700 Lys-63-specific deubiquitinase BRCC36, putative
MEKRAPLEKVCVADTVIQACLAHAFTTEQEEVMGLLLGEVTVHHGCGTTAAISSETVRVA
ATVSPAISTGAKSFGGNVNAPTPTPTSPSTVRKRANVWGAWILQRSVRRADRVEIAPEML
AGASDEAERYTKQVGHHTRVIGWYHSHPRITPYPSQVDLRSQGSYQQMESGWVGLIFSVF
YSDATNRNGVSIHCFQTGPGETHEKVEIEIVPVGSMPLRSLPPCEVTHRLLHVFRSEVNA
AVELVRRRCKDAPDAMAAAFALQEVQLYTLEKLIAQPTLRHLRACISYMEREVKRLEKEL
AAQNS*
>TCRU_1701 protein kinase, putative
MWSLDDFDIGRKLGEGRFGKIYLAREKRTKCAVAIKCLSKDMIRYHGLAHQLRREVELQE
YAGRCHRHILRLFAYFWDDVRVFLVLEYADGGNLQTLLDTRTQHRVSEEEARRILRPLLS
ALAFLHERDVIHRDVKPDNILFKAQAVKLADFSWAVRLDRRVPQHSRRYTLCGTMDYLAP
EQISHRSRSQGGVAQRRPMCGLSAY*
>TCRU_1702 hypothetical protein

MESLLCVDEASRLSCEEALKHPFIVGNSCSLHEASAETELPSAVSFQCSPTPEHWDNRFV
RVSLPTYGKAPAVVFAQRRQPQPQQRQQTNNRSGGGGSSRNSINSSNSATCKWVATAACS
RSLSDGSFVTVSSSHSTERITSAPDLTTPNISSAPQREWICSGRAIANGETLKSGSEEVA
TSSQGASLSRPRNTHSTSDRSGLATTQIGNFIGFSPLILCPNDANDNAEENEVDEREADC
VVQLRFDDSLLSLSQLTAASASTVDAAENL*
>TCRU_1703 hypothetical protein, conserved
MPLDLVGKLHRTVYIEGCPSTVREDLLRLLVTKCGTIEGWDLMGERLIVVFSSMNSISTA
ITFNGTAFGDLTSKLFLWVATESPPAGVTQQMAIADRGDGAVGAPHSAEELKSAREARER
RLAAIRSELAPEIEMAESTENKEARMLDLCVRQLKALGTLTSHALVEAEKKLEEISTHLS
ASQNLLESLRREKLGKATALPAEQAEGPDCTADANTAVVAAELPGVLGERDDEVARRVEA
MVAAAEDIGQWRMRFRES*
>TCRU_1704 hypothetical protein, conserved
MWTAVRGTSALRRVNSPILRCRWRRSATFTSSEKAASSRAATLRTGTYSVKGYDFVEGFH
VLGPGAALSRLKATLSMYGLLGVTVISSIAYYLTTRTYELTTDPDPLPKSRFQKFPSDYA
VLQNRWTGKRHVIALRDGDANDNDTREGKQHELEESAILKKRIRVNWLIHSVRLYLHVTD
SIVVVDLDAEFDPYHFALKRNRGSLTTSEVANHVGEPATARLLCRSRLRPIVIRNEYQKG
TLPQYERTVDTVTRELFGERYRQAILSRSGSILHPVFADELLKSGELNGKEMSWTDVVGD
NEAFSAEIQRRVQKKLGEKVILLQFSMRVL*
>TCRU_1705 CDP-diacylglycerol--inositol 3-phosphatidyltransferase, putative
MRPKANFSSVRPTQIYLFYPNIIGYVRLLLSLGSFVVLGNYPGLFLTFYTLSFVLDAADG
MVARLMDQCSHFGAIFDMLTDRASTAGLLLVLNHVLQPMPDWCTTLFAFLVFLDVASHFC
RMYVTLFFRKESHKDTSESIFYLLRLYYSNRKVMFLLCVGQEFSYILLYAYWMYSEVDWV
RRVTFPLLTVLGSLNVLKQVVNVQQLMDGMYHLAVLDAREREERGKKN*
>TCRU_1706 Isy1-like splicing family, putative
MRNPQLLSIETHGEVVIRAKNDEINHLIAKKNSWERRLVALTGEEYKVASSRKLYFGCAK
ELPEAQVEMSTAEEVLTGDALDVSEPESLGSQSSEGIASYFKSSYLEQLAASESDDLLCR
IEKAAEHSLRLEHGKNNGNLFLKRFNEENSLYVVDVDLLPEEEYRRRILDAKRELLKKRL
GTLKK*
>TCRU_1707 histone-lysine n-methyltransferase
MTGLTLRRKRSRGLTPQKQSFDAAVVFRRDDLPARPKKPLQDEEGNGTPHYPFRLPLRNE
PNENGCYHCLTKRCFCVLFDRMLNHVYSNLYCKRITEVAGSRHLCAKSLLPTFVSRMVRL
MEVKSEDTFYDLGCGNGSVLFQVAFLTGARCVGIEISEHNANVAKTAWESLKPQLESISG
RKMPDLKIIAADMTRVLAEKTMFNEEGGKTAILLSNLLFPKSLTHYLSERLRRVPSGTRI
LCFDDLYPHSRSVAAIRDPEAFEIFSMMDYRWQERSVEWCSMDGPFYIHRRN*
>TCRU_1708 Glucosidase II beta subunit-like, putative
MQARQPQWDGSGVALWSLVGAVVVLLVAMLLPWKTLLDDWSNWNEGSAKQTLLQAPPPRR
YTDVMTHSEDTRRQEGESKGRPEPSCYTLEAYSMKFIAPAQRQEATPQHFYRCGTRLMDL
NTEVNRSKREICTAATEKNPTLFSTLRVYLESAFEEKAHLQQRDNVAAQTISLERVNDDY
CDCLDGTDELQTSACSMSGAVLPLAHMRWKQYLLANAHVQLYEEEEAPSMRRTERLLRRL
GGPVLPFRCRSDPDVWLAPSRVGDGVVDCCDGSDETTSLKHHPTHEVHGEDSWRSRVTAM
EPYLGMHSGAASPLYPMASGNRTVAAMLLENGFTSLMSCRRVKEERLNNARTLHEVVKRG
NAIHKDREANGWEKYGKKLVEHITRKKTELDAVSKEFETRRSQIAKKMEKMGKSDPISAG
ISPEEVQAINHMYAELQKRGASLRLVVMILSFRWLGDDFEYYPLADAHFTVPVNRVLETA
RSSSPEVQRVQASRRYNDSYVIESIPVHVDNTSYREFSFYPSFYLVGARNLTREQSAIII
KRAYGLEENESLNVSGTENFDESLPVVFGFWRPDLSEVTIDNFQSVDPSGNGVLRRRLFM
SDPAPVMGGVEQILQHERLSYGGRHRRVMRLGPTDLREQLQQALQGANNDTEIEMPQQKN
TPDLASVRIFSGGISCALDDAMDNSGRLGEEEEEEKENTPRSHGRVVYVCDEEDLVLEWH
RNGKCAHEVVFGTPSACPPWVKEASATRVRQAEEALQNDE*
>TCRU_1709 hypothetical protein, conserved
MNGAGHNGGGGCGTPAGARQGQQQQQQQQPTDMFVACFEAALDEELRGQEISSGFRVCFE
KISSPYGFIRNEGLREALQRLCDLSLEAAEEAARLANKTPGTTTTTAAAGGNTTATQPHL
PYDAIRSAVMPRSAVDPHSTATPMNHAASGYSASWERTQRALNGAHYTMSVFHRVLVPRV
DERSQCNPGGPVTIELSEEAALAWNTLNHAQRTTPPPSPSPVGKKKELDITLTEVTYQEQ

ELALRLLQGLSLVVYDQRRVIAEGLLITFLVEVWQCCLQHIQALFSLRRRITALEKTGDT
VSPTPHSFEEGPVQLVPELETVIVATIDAMEAACHYNPIALTRIVQQGGVRALLDLGLCP
YAPYDIRCAVFDTVSVLMQEVAPFRRAVAAGAAGAAAAGVKKGNEDELIQTMIQQAMAGY
LGRQAAPPPFLMDRASASKFDSAVRDWFSQQGLSHVVSAVLELRDVRGTPIAVGAMPKAE
VARNVQRVNQLREKHLQALLQAVDGKRAAL*
>TCRU_1710 PH domain containing protein, putative
MNLGVDARFPLDVSFDILRRVLDEESLVLAQLAYIVGHLHDPILQSCGHGNVATGPWRSH
GNNKSNAVGTKQQPAILRFAQYWELFANIATLHGAQSTIVGKLEEVVNNRTHLLDQQQEQ
KKEVKSGHGAAKNLHMRSSSSSSSSSDGGTDEGMRGPQSILELFCSAAMRHYIAEHMMYS
LNYSRNIAPRVMELWRLWRCRCGKNTSAHLSARERQELESYMGFLYFLWHAFGGEDGVPD
DPRVIFTRLPETLLVKSQKPKKDRTGHNTAVVPVKMPDAPSIPPNWRGFETLLVLLATPL
ASLRRYMHVARCIIESQSLPIPVRQRLQVDFVDLLVLRLAEEHSIVFDELAKQDISHIMA
LIDEVSPPAPSEAHKKKFPRIRLDEDGSRTLVHYGRLIKRFRRGRHERLVFLFSDWLCYV
EEQTNGRMRLRASISLESLRVVELDDTAEVVNGFDIVTKDMRLTFFAPTLEQKRQWVDAL
RNTTEAYKVKVQQLASPRRQDISQQQQQQQNKSLGHLACQTRVTALPLSHNSRLRRQQR
ADHALQRRLERRSTAELATTNSLVADETPAHVMGIDAGRKQPALSFRPLHGNFDVTYWAQ
QSTETVHKRVRSSDLLRSSWALQSPHDPNACEPKAVAPGMDPLNHGNISTTTDAADCSVH
SEDAPASAGHSPTSPWVPPRAVLSRLLSERFQEPSLHPLYSGGNANSPLPENVSENEGRE
EPDSLYTTLLSCVATTEVHKYSFAAEEDEVVLENDEWVPTCDETPDAIRPEGPAVHISLK
HLVGETDSDHGSNSQSHSNHFEEEEEEEEEEEAMWSPR*
>TCRU_1711 Coiled-coil domain-containing protein (DUF2037), putative
MDVVPSLHGVIETPSDADTVLQKLADGGYHRARVKTLPVFDRIAGGLTWGILSSRHRLHV
EFVENATIREKIRISEKICNALKHMQCPIDLRPHQIHGLDYSSLLPVATWLLKRVEATRA
ENAFFLEKQTAWDFSRRFGTSLQSLLSEKEMTAQSGGDDVSLLPPTQESLCFSRRRPAPL
RQRISEVADRHSVGKPQGGQNFSRLVHVSSALLEYGIRYEMRDSVLMLRHVSRGVVAEDE
EVAREVRELEEEEMREERMLEMIIKKMRENRERMDGKMSLSKVKTLMQTTKISESLQKLS
AEYHARQQERQMQQDSVQAMQRKLQQKQEATVRYRQESETLRLQCEEAEQQLAEAQSRLQ
DAKAKVARRTAKWTEYMHNLQDVYQHVMADPVRGKLLQKLQGLLDQKEEQRLLLEETRRD
GKQRLKAAKANCVALAERSANAEPGVDDIWEKERAKYVAELRRKEAELSAINQKASDLLR
RTDAFPTRLELMQIHMRICELNEEVSSKYEELRAAFARHNTRMEILKCLESEDVIMTQLS
GKMEWCLRQPSWVSKSVSVELIREQERVVTQLRDSLDAVLRRNEDVTSKMTAARREYESL
LVRKQQHRDMLGLLKQTIEEQNARMEPQP*
>TCRU_1712 hypothetical protein, conserved
MEAVGSTVETAEASDEAFNSPESHVWNSGDLKGSGCHATSVSCAYFTRHPLCNGQHPIAS
KNMLSPFSSFWGLHVAVPFLVVLPQENAELSSKEVHQGVNTATVDDAAIDAGAISSPFSV
PPERFLTWHEMCKILVLCGVKRHHCHEAVRQLWEYLLFLCYKGLFESDSKERGNHGGRDT
TQVTDSHTCKPSGAAEDFATLVRYDTLNDVLRSRIHCGGATDKSTVVCFAELVVAASVAL
GNRDLGQLEEVMQAPSLSNRYTLVTAKHTFDRCIARLLINELRGQRITVIPLPRWNVTQA
IVQNRRSLIVFCGGTSGCGKSTLSSLITTNICFNTLLSTDTIRQSLRRTLRREEFPELFL
STYEAHRAKGSGGKTKPRAGKEGNECDPQVVIAAYEKQCEVVLRALDGILEKFICRNQVV
LVEGVHLLTSYMQRKTVELKARGVLCVSFLVCITKEERHLERFRTRAKCMALSPQRNKYV
SQFHHIRLIQKYLLEQAYDHLHLKIINNTNLDKSLMDVHTHLLDAIDCSTSPSWNETEAA
AFGRADATRHAVASDADMHGRLAPKEEAGKSKEGNCGFLHQPFADPNISGKRMLAFLLKW
RSEKKKRRANNTSFYGKFHHTCHVATSLFAPAKRARSAESLTSFRLQSLCVASCSGNTSD
ELLMRAWRNNETQCFYVCDSDKAMAVVDSDGYERPRWKCSPSISSGTAFPIPRHKLVQKH
PQEATHAVSRRGCFLEALTAAEDPPAATVTATAAASRWGKSFRSSSADAAIDAVEGNRAA
VRGNKQKRFLPRHAAHGARENGEEIKEEIPLWVEKEEEMENGSRCEFPSLMGS*
>TCRU_1713 Methyltransferase TYW3, putative
MNEWKAAKPERNTALSLSLSLNLSSICSLQRGFIGVGWAEKDLVIGHMWEEADSSALLCA
VQRGELDEKSPAAQKKKQNGKRPSNRGKELSPQYQQRKSSHVAKQKQCEKEFLAHKQQVM
INLSSNASDHSPKGGVDHKCLPLMHLLNTHDDYVTTSSCSGRIALFHSVIIPEDRGAAGP
PASLKRGGKDALGWLVVKHDVLTEREIDVLVSCLCDDVKDGVVTETATTPLEGATSNKKD
EEKEQQKEGELDGVWVREGVLQASSPPVFGMVALKMEPFVMHVECRTMESAKRLLSAAAT
DAGFRNSGVTPPGKKIMCGIRHASGLGLDVPLIIDGVNYVKGQHAYLRRLLHMANAKMHG

NDVRLHRLEDGVAARLAAEEGAKKGLRVK*
>TCRU_1714 prefoldin subunit 2, putative
MTSTNNSGSNNTPSEEAIVQRYQQLRQECLSLVSRISELENELHEHKLVAEALRPLNGDR
RCHRLVGGALIERTVAEILPELDDNVKGIQEALEQLNKILAEKQAVMEDYARKHGVTVAQ
QHGQNPGCDTNAESAKKPGAAEQSGVLV*
>TCRU_1715 hypothetical protein, conserved
MQSFYGREACNVELPISDVAILLGAMVLANVTATGDINGVTAAKRSCSFVRQSAGRPIHL
PAPGMAWNLLEGWSRLGLRRARSNALQRSSYTQPSPNTATRCDFIASC*
>TCRU_1716 60S ribosomal protein L11, putative
METHAVSEKKATNPMREIVVKKLCLNICVGESGDRLTRASKVLEQLCEQTPVLSRARLTV
RTFGIRRNEKIAVHCTVRGKKAEELLEKGLKVKEFELKSYNFSDTGSFGFGISEHIDLGI
KYDPSTGIYGMDFYVVLGRRGERVAHRRRKTSRVGCPHRVRKEEAMHWFERTYDGIIFQA
KKKKVMTRRRRR*
>TCRU_1717 ADP-ribosylation factor, putative
MGAVMSWFDGLFAKKDATILMVGLDAAGKTTILWKLKLNEVQQTVPTLGFNVQTVEYKNI
KFHLWDVGGQKLLRSLWKHYYEGANAIIFVIDSNDRDRVWEARQELEKLLQEPLLVGATV
LVLCNKQDLPHRLTPAELVEQLGFRDHSTTGLGAAMRGRQWFVQGCCAHTGDGLFEGLDW
LSSHLPDVS*
>TCRU_1718 hypothetical protein, conserved
MPFCNVKPSEVSHLQNVAKKQGANDVATADYTVLLVENMMKRYVEGRSSVCGSTGTDKST
NSLSPSWFDAVKADEARMMRTFPYPSLFLHPLPLEMSEHPDTPSPLLLATQTATPVGDDS
NTEDAPRREYREMVVLDGGASEMVTVLQGECGLAVVSPRCRSTGPRSVYLLSPDATSCTI
VACRVGLHSHLRERLVATTGEMTESGDGALAWRCCKTFTAAMGHLDCAQGVAEVLYEMLW
EAALPAWFHAALRDGDGAVGECGEVGAALDLLERLRRIAQTTTTATTTTESSDSDTPMLV
AEWFLVGGVRSREKTLPVLTAVLSTLFVAEEQDDGGANLPGGKRCILKPSFHLSEVLGGK
ESDGADADLVRIEHRLREDGVCFWSLNTVLRQWAMGRAGELYSCPASLGLLVDVGGGCCW
PATVQPGTRKYPLAALRNALMQPGYRWTCPLEREGTLRVRRGVATALAASYFGLQPVPPT
ESVCDVSLAAYVAAFLRRCEEGGETEEERASVSLPILVRRGVWEKMENIRMLDDLPDNFF
LMASTTPHCEPSDFAALGRQRLGFMSEFCVDDVFEKGTVGILV*
>TCRU_1719 protein phosphatase 2C, putative
MGIPLPKPVMTQLRERHGNEIFRCGSSCVNGFRESMEDAHIIYLQPGWGFFGVFDGHVND
QCSQYLEGAWKEILEKEKMPMTDDRMKELALEIDKQWMDLGREGGSTGTFFAAMKKDDNI
HLQVGNVGDSRVLVCVNGQARAMTEDHKPNNNDERRRIEDCGGRVEGNRVDGSLAVSRAF
GDRDYKTNTTGSQLQQKVIALPDVTHVDLTWGSKDFAVLCCDGVFEGQFSNEEVVEFIKL
QLETSDDLAVVAGRVCEEAVNRGSRDNVSCVIVQFKSGKDYADTDHLEVVPGPFSLPKNG
VFRKVYGIMAQKAGCTIHEVLEKRYDQLVSMDKELLQEEWQWFKGGPPASLKGKDRTQWF
ADLLEQHAAESPSDSRSEQLERIQLLQQQIGVPLPVLLSLMAGQTQE*
>TCRU_1720 eukaryotic translation initiation factor 5A, putative
MSDDEGNFTQQGGEQTGSLTYPMQAGALKKGGYICINGRPCKVIDLSVSKTGKHGHAKVS
IVATDIFTGNKMEDQAPTTHNVDVPFVKTSTYSVLDIQEDRTDPSKPAHLSLMDDEGETR
DNLDMPPNAELAAQIKEQFEAGKDVLVVVVSAMGTDQILSFKNAVEK*
>TCRU_1721 Protein of unknown function (DUF2009), putative
MGYENVLLRLTDTEREDLQLLIAALKVSEYTDDVDDIRHPNSREERMYRCMRELFDTTLG
LCIASGSVSREVREEVARGNTDVRLTISILIGLFEIFRRHKRLNPFSNRSEFGKLTMLLQ
DVQKRSIQERLHISHSLLIPVQTVGMELRRVGAEELLTDRDVDKYLVTHGTEKAAVLQKL
LDRYGGSECKPVVERCLRSIDDVSQFIEGNVRPLRWLRQIILEEFLPLDGNPKYDLSIRA
GVNGAKFSHDHKRHCQYVVESLTLWENVQRNIFDFWQVSEDDMLIDGDGHYTFVNTGQGF
HRMCRAPKSYSRMARCVNEADQEMGGWVGIKVIHLGDRDVPNPLVFIDKYTVIPRIVQPI
MHTIMEIEKIFSPSSPEEHPGLRNFFRAKFNSYKALRMMILSDFFRHAFDGSGDDGGSCI
DGRLTSAWNWCHQLEKKSYYDAFVLTGFSGFD*
>TCRU_1722 hypothetical protein, conserved
MNFGRILLQAFRVRLMTASDPGIPLSDIRALLRTAVHEADAFARATQSLAERRGTQISLR
CQLCHVCGHDTSTRNVRSVARRKPLAAQTAVKKRQRSCPLLMKADDGSPIHSITSRHSAQ
ASHRAADAPSDLSNRPTQSSRRATELICQRRSTVARRMTDIPALDQAI*

>TCRU_1723 Alcohol dehydrogenase GroES-like domain/Zinc-binding dehydrogenase, putative
MATNAKGYAAPSAKAPLAPFSFQRRAVGADDVSIKILFCGVCHSDIHQARDEWGGSLFPM
VPGHEIVGHVTSVGANVKKFKVGDTVGVGCMVDSCERCVECKRGLEQFCVEGTTSTYNSR
ARVSGEPTYGGYSDHIVVKESFVLLIPSNLDLAATAPLLCAGITTYSPLRHWGTKAGTRV
GVVGLGGLGHMAVKIAKAMGAEVTVFTTSEGKREAARMLGADHVVISKDPGQMKAVKNTL
NLIIDTVGNTHDLSPYILALTTDGVHILVGLPENPHPPVSPFLLLKGRKSIGASLIGGIP
ETQEMLDFCGRHGIVSDIELISIDYINTAYERVMKSDVRYRFVIDMATLGQ*
>TCRU_1724 ABC transporter, putative
MHAFRESRLLREAVCLLVLVLLPSCVWCEETGVSPDVFLSWYKKLLLSEPSRDPSGLGFR
SFMSMAAFEESDDDKCMNGGIMRNGDCKCPEDSNFTGLRCEMCQRNEVCAAQLDASSVCQ
HALAIRGSGTQFECSLNSPFLSILGGGRAIEAVVALNCSTEDGTAFAAGEGGVCSLTLFR
IEPHREFVDPFFHCNTSECSMQLVVQNKGNESNDPLKPSRAFKAVVLTGQVVILTICALI
ALLRFVSSTLGYRRKAKIVVGLGVSLTISTVLFISVMLILMRSEASDVYAVYKCKHTECH
CSKDPPEGYDPVCSKSLVGNKILPLIRNSIRYACNLRTRHCQLTMEDITLMFDLECNASE
CVDTKKFPNEPKMVQPETVQAQRDLLFILTLVLTVVVLLITAHALWLRSASRKRAAEFVR
MFGLSEPQPQLEKRCSLDVASDSSGTPAKTSLLEKREPQQQNRLSLRGDSAEEDVSSNGT
ESIGLSFHRQPGGSHTSDARRLLIAMPHEADRIEYFRRLTKSPFELQLMDLAYSLPGSRF
ASKEETRTRPLLNRVNFTVRSGEVLAILGPSGAGKTTLLDLLSARSKQGRTTGTISLNGT
PVNTASAKSIKQYRNIIGYVSQEDTLLPALTVHQTIEYAARLKLPQAFSNTTIKSIVRHM
IEALRLQQCEHTVIGDGSGLRGVSGGEKRRVSIAVELLANPRILFLDEPTSGLDAVSAKR
VMESVVELAKESPMQAYAPHYFAFKPIVIFSIHQPSQEIFELFDKLLLLSRGVSIYCGPA
CIAAETLEKRIERIVGGAKSVPRSADHPNHAEYLMKLEDILDDTTRMEIQAEDVSSRFGM
MQHEGTEFFREPRENNGTNGVSECEALVEGDDILRNASTLHVYYANVYQQLALLSSRAAC
CLISSFHLLLCHAAVTACVGGLMMVLYHDQRLDLPGALNRAGSITFMLLVVSFVSLSCLE
QLVVERKLFVVERENGFYRTLPYLLSKIFIDILPLRIVPAMVLASLIYFPMGLRVDDGVH
FFWFVVIVVLFSICITLIVLCIGIITGSFGAAALLSSVFILWNFVFGGFLVQSETVPAAL
RPFQVMSPFFAAFEALLVNELDGQACTFSPTDETGKPSSTSIPIMCLQYLSNIGLKPKRF
NTDVAQLAGSILLLLFTAWLLLAKFTSIAR*
>TCRU_1725 hypothetical protein, conserved
MQKTLFPAPKSNDYQWRNLYLKQYGICTYAPPMPQNLEVMTANALRLKSREKIDDNNISA
STQTVSLDSEEMEMIEASGSRHSAPSPSRRKGGSVSTQGFRRSFLDVQRRSTVGDFLESL
SDQQLFSANVFSFLSQHEGEANSSQLLRNPLGLDAPSSLRFPSILRNSQVNASNRTAFPR
GSSVDEGGKLVPDPADDLLRLQRETWQLLMDNRIMLWFEREAKKRGRIAAGVQRRLLEVV
TWKLEGQKKTPNKKPGEDKTSNDTAIGTTEEGKGKDGAVKIIPMDSEVEEDKPELGEEEK
LMQTLEQFAPFFAVISAPEVIQRMTTSLLVAKKRGRRHEVPRMVGLPLFSQYNTWRETYE
NRVAWSLSPFSFFLIHDHSYNHAHISALYVLLDLIGSSFHLHTAECLKNENKRQTAIDIR
QRRIRSAKYSLSNVSLCPLNVAQRLTEDILLAYENSTARTTLSMALCVQEFLTELVLDPD
TASSFLSNAALRRQCGTDIVVPGNDAGVFSATSKKNENSFESMEDGTKNEQQEPTKDEAG
VMPAEESQFIDWSSLRKLTDTTFDELFSLVNWFVDTPTANSLISSEARTRNDSLVLANTS
PARGKKGLTSQSLAFNTSLLQESKKSSQQQALSDESTVQSKESGYIVGYVREYDATMTIV
EAITRHELQRQRMAETRSGEQRQRNSVTLAHRKNRRKRLAASRTQTYNDSAAVPDLHAGA
TLLSTPLTFITTYIRLHGATWLMELLERLFSILRRESVLLYVNGLEMEAPRQTSFQGSIS
QLSLSGDRSNDGVSGFGPEVGCWRNSGQKRATHNQRLELLEDLICQDIQYVMGEFCSALF
GKRSVTRLPQGISTLLTNFCTTVHLHLLQQHVSFDITHDKPAIAVRKCLTEMRQKRVTGG
GTVNMWSQNKPDSIERLIRNIEGNRLAKFILFDCWILPTLNNAVEFGYISEEASNHLRWN
LDAFARYLKIVLNSSFSNAENADVYIHTILPRRTSLQSQRGRTTPSVEKKGRAQSEAPLA
PRCDIVKLPPLINGVYDIVSGSLMQLLRDPCDDFVDDSHERFTFGDGSSSVDFPSQKDAN
TSCSLKDTRKCWEDLSPALSYLNESLGITSGDADVEVYEDEVDVTPVRILNIFAARACSN
TSTKVVVTEYEVAPSIAAACISKTFSLVSGEHPIVRNAYLSGALVGQFRSSPYLTCLIGV
LLHPHTASRFLENILHNNRAFYNALLQPLQDENLLNLISPLVTTRNQLPLVDSNELLSLI
AAIPMHSTDTGSCVETKAGDSERKMRDSLTTRETKMKNLQLAAWEATTSNLLQKRARATT
GDILPPAVNFHPQIRRIGPTDSYQSLRATAEILTREMGRAPLCFDTWWRAMVVALTVKAM
NVIEECSETKSMQWENWCNSQLHEMQRESRGLCTYFTKNKGKNIDISKEKALSYTKVKSR

LGSSVLSSVTKVPSARTVEEQKKVTKGKKRVHSSLKKK*
>TCRU_1726 hypothetical protein
MSFRRGSAEEEGEKVMGAEGVPAYASELADAFACKLREMEALHYAAELDTLAGMRCVQMY
AEVLTGLTALGRMRWTTNGGATRDVE*
>TCRU_1727 Aldose 1-epimerase, putative
MTIISSENAKEYSSHVEPFGDGHKITLKSPNLRVSLLTQGATVFSVQYRVPQLAGGDVAD
KDGWVELVLGLDVPEEFAKDKLYIGSTCGRYSGRIENGEFELNGKSFKLLQNDGENTLHG
GPEGFSSRPWKYILLEGEEEIGISFHLISPHLDQGFPGELFVTATYVILKNEPATLKWNL
QAVLAASTPAQSTVINLCNHIYWNLNGVPSRPRRLPEPITNHHMQLHSGYVAEMQELIPT
GSMKSVEGTPHDYKKLRAIREGMEATQAEGRDPPGYDDPVALDKWDSKLHEAGRLFSPKT
GITLQLDTTSPAVVVYTANYLPENASGEAGERFQRHSGICLETQYFPNSPHIPSFPSTVV
SKGEKYDETTVCHFKYTPKS*
>TCRU_1728 hypothetical protein, conserved
MNIVTSYFVVKRLPGSIFLGEASLRHRDARQEEYMECIRRNAAHAEVVSLHILIEGTEAY
RQFRQRVMEDDYFFQSKEMRRKIVPVLWPEAQPTYADLFQHANKLLRGKLTMICNADVYL
PQVGASVRELQKLFDSDPTRRLSVALTRYESEDRRDAPLIEAYRGSHDAFIFRPPTEASF
VESVRHPQNCYKAENIVLYELQRHGYVVVNPCRSFMLVHHHTADLRQWLPAVDEERYARA
PPCTIAEALTMLRQTNRS*
>TCRU_1729 FtsX-like permease family, putative
MRNALYAPFNDTQNSSDTSRPDTRLIRQHPNNSRRLHDSPSEPFFSGSFREKSGLSGRGF
FSLCAILRLSCLYTISDLIHRPRNCCIGVLAVVLLVLFSGAVLLGIWKVPYVLLRLGELS
AGEMDMVIMGGSVDLPFINYTDMGPRIERSPYVKGATPRWIFRGEASKHKNVFTNDNDSV
SPLTEVARVNVLLIDSERERKIGIGRAWGHRNTGFSEAHVFYSLLDYLNVRPNLGDRINI
QFSPKPLLQDDMRSENETLFRIEKPILGNDVNSILLYNFFQTNGITEKSISVWDVLNFDI
SVSVVDGIYSARGKYPSALGNIVIVDYKQLIPAFAEQSCSFGPQSFTPKRGIFLPSTMDM
FGIPTMLSQFNILEKVPTLAVTMTDRSNIYYLPTEDRTKEMVERSNVIMRQGVGLDFNGT
VYFPLSEAMDSFDMLKIILVSSLTCFVIGIVALCALLFYALLNTNAEERRFEFAMIRAQG
LNKTHLVILLLTQSLAFIIPGLATGGAFLVGLNALIESLLSRFTAATPRILFVPLIPLFL
SLGLGLVLPLLTGWGLLSRVLGGSLRDALDVYRQVSNETKVVMVKLEEMGISTWQLILGA
FLVVAGFIVYYLIPFSFIFENMMLLFLLLNLVLVVMVVGICFIVTVAEPYIQLVFLWLMV
WGNEKRLLTIVKKNLYDHQSRNAKAFMMVLISVGTIISSGVMFVMLSNASEDMTRLTNGA
DINIASSAFDVPLDERELNAFLEKRRGLYVEQWAYHSFPLDNYPQMVHSSRIGTIIGTRN
PMCIVAVSEHFFNTVFPEYIMEEAKDSRYSYKLTVDGKYDLVRSMYERKPLPSGTVGQII
GTGLPFGVELPNILNKKTYIIPALVSSAVRDSMGTEVGGSMILEYNYRVSDTIVTTRFGI
EPRGLMRRVSGFPDISPFPNKLIDSEVLLPRSYFKTLVKPWAMDFGDNENFTLAPGTVTE
VRQKKLFVRLRKNISTKQRITFLNEIQAHLDLTFHSASDTHEMLKDLRVIREFIMYFFYF
TSVICISLCTLMLWITFVANVRLNSRTFMVLRAVGCRKGQIARIILYEALSIVLSAFLLG
VVVGAIVGVTLALQLVEVMVLPFRFTLPYELVCVLFGLSMIAAVVGSMLPFRTIANKCIS
SGLRSL*
>TCRU_1730 amidase, putative
MITEKITRQNRQCVPLHGICGFAPGRIPAYSNGSDSIFTNERHYVQHYFMGYKWQCVEFV
RRWLFYRKGLALPQYDFAAQLIHLREVQDVCTGTAVPCQFIPQGSEEPPVADSLIVYPGS
RKNIVGHVGLITHVTSTNVYVADQNRFFHDWGEDTFSAEFPLECVDGRYYIRDPDVECRG
WIVFPGRPNRLDGDPPLVSPHISGPPSLPRCRRIKYVAQQLWSWFTGRETLTFRPL*
>TCRU_1731 hypothetical protein, conserved
MRGTWREQPNILHPRHTGCSFFLPGDATRGQQRSDAPPAASPHDARDRLDPAACSLPDPN
VRSLLSRYRLENERLRDQLKAHAVQEALLEQRLQELSARNTALHLHDFRKKENDAVVEML
KKQLEDRECEVRDLQLQVKEQAGRIAAFEEAITRPLHGTDESPSPSPSPAAAAAAAAAAA
VSVSSSPPLSASSLRTHELLCQVLSSVGFLTRVFNAVKHCQTLPHEGLGCPRSRKECTAR
CLRAAMRGNQETVGDMMGRGHGVSCEALALAVREEVMFCELSAVQIAAQLLAAEHAPAAV
LAVEEAQKETPPLPLEDDHPTATITKIAAAAKEGKKEGEEGEEKRELCAARSISTKEQNV
STVFTAPRATRKPTARPTVNDCEVQ*
>TCRU_1732 hypothetical protein, conserved
MSFEFAFHDVSNDAIKHMTPSEALQKHLENAQLAHRVCVAKALKAEEAPVEKCALTWGEV

LIRYQAWAEYRPPFQDSVAQSKYKKYWTKKRQAEDDKNPFK*
>TCRU_1733 retrotransposon hot spot protein, RHS3, putative
MVLASDEGWPYSWNRKENKSTHDCYVNCEVDRVWQIVKGDLIAWLSPHGGTDCKPRRRVL
IGTPGIGKSMAAGSYLLYQLLHCDAEKLQVVVHCFGGRGACVSDKTIGAVTRCSDEEMCV
SELRSLRGHGRNVYIIYGVAKEGTPPPRHFAPTSGWGMIAVSFPKVANYDEWAKQPQAAR
IIVNCPDEVDVKAMRAWMTRDETKEKQAERWKEVKNKCIFWDRFHVTSLMRRRLMNAVER
*
>TCRU_1734 Cyclic nucleotide-binding domain/PAS fold/PAS domain/
Protein kinase domain/Protein tyrosine kinase/Kinase-like, putative
MWRSSVHPLVETFGATEISLLEFDVIGSTPQRSVENLRGHIRSLVDSVEKEATLVTVIQL
LESERQGLSEPSTTTVDTRGGDNNNLSPASSLMAKLSFNYESHPSGGEAPPTPHNSNWNS
VNTAGRELPSQQERTREFVETEDAAHVAERAEAKRVNEDEMERAAELDGCPQPPPPLIAS
RGEPLVSYGRRAAVSAESLPLNVIRSYSPPVVAKSPEEERIVRAGVMACHLFSNMDSKDQ
SIIVQALKRVTFPAGTDILKQDGASVEKLFLLTQGSCDVIKNGKRVHTLLECSTFGEMEM
MYNLPTCVATIRSVTNCVLYTLDQYTYQNIVLAASLHKREKYEALLSNVQFLREFSDYDR
MQIAEALVTKTYRQGDYIIRFGETGKWMHIIVEGEVAVVGRNIGKKCEILRLKEGEVVGE
LEFLFNDLTVADVIATSRRVTTAQIKRKHFELITGPIQDKLKEYIVNSGRYTHYLGGAKD
RIKSELSLLGKRSKRMATNAERENAFLHQVDVNGEIILPVPKEKVFAVEKGIVLDKENNE
RVNVKDAVSGPDVESSSAHVLFRFPLMPIHKNSLAVIALCEDGSILLWNERMVRLTGYTA
EEAIGQHICSFLLETKDQKTMHQAICDARHYTGDTEGFFDAKGGVYRRAYTFARMDGLTK
VQLKLSVLPSIVSHGKDAAEIVLAVGEETNTESQQVLDKSQLLMDQISSVLSDGECSYED
RLHRIAMVLNGFELTCRAMLASTEHVRVVNIRQMIGQVMMDYGAQCVSSGVAVRQRFEGL
YAENAYLNAKLLPECLRYAMSNCAQHLDDVQVTITAKITENNGMDFLEIEFHDNGPGFPV
DLLDEIHEGRFSEKYAQVPRVRAAMERQGGSMIIESVPGNTNVRFIVPFVPVKERLNDML
EHHAKEFDNSMSLSCVGSVTQASGNTSASSFTLQSCRRLPVWKQNFTTVVVEDTPAHRNM
LCRFLWEQKHAVLPAYKLDDIEQLIDVVDILFIDPHQSIFGETNDTIDPVALLREKARRM
AVVVTGVDMDGESAKAYQTAGFITLKKPCNPIQALQCIREAEEMISQYKMEDERILQTRE
TLSKNSRGAWKRGKLLGKGMFGEVYEATEVLTGGKMAVKEMKLNRRDVQIEQFVNEISAM
CNLRHPNIIHYFYCEENKEENVIRVFMEYGSGGTLQELLKNKGNLEFKEYQSLLRDVVEG
VAYIHSMNYVHGDLKTANVLLSAEGKGKIGDFGTARRVRDGELLYQMQGSPLYMSPECLA
ASERDEDGVLIGYSFPSDVWSLGCIALEMATNNPPFSHLKNVTGPAGVMMYVTNLHDIPD
LSPLFDGPPSIVEFVSACLNPDPSKRATAAQLLHMSIFSEAPDEDMKSALKALKRAQLLH
VLNNFVAFQEPVELERRKSHPPYRYKVRRETEFCDSSTEDKNPAGEEGEELGENYGETGG
MEKEEHAATPTARLYRPSIFEKSKNNHPNMSSTGKGIPPDGCSHKKSGSLVRNSARAEEE
AFFESSLSSFYSPDNEEGGKEASAAPAEEGKGRESKTVITTSKRSSVCPQSPTQVTWREE
FVSSFTEKFGFFPLEHCSVDFMGNSMDNTRVGAAVDVDSQDGPQKTPKTQHELGPREREY
RARTLQDDAGLLKRAFVQPFKRSSQRSSLFKFSATRQPKRGVKNMFLVEDLQVVPERKSQ
DVSSPHASNFGTTPPSFIHYGSVPRQRRFAVAPHLASPSPVMPTRATEENTGLFPSFNAS
TEVYACSPSPCSSGTRKGVFSLSRELTSLQNALSELLERLKDIKPMEVERRWCSQSRQAA
GGMFSQKTSECREDETCKVDSVAALRDLVGRIEGEFMSLKSQISAL*
>TCRU_1735 Cyclic nucleotide-binding domain containing protein,
putative
MFDHRQTLNEELLEEAMECADRSPSREYGDSRPESTSAAAATSTTSEVAAVKPGRRIARV
IEEVMRSIEGRARAVTRKKFWHEEESFETCSSRMNHNAEDGIQMYRNMIPDPVIIKGLPT
HDEKEMIAFLQNTEHFCLLRHDEMREVVHASLRREYLPGEVVLPEGGHAPHMYIVVGGEV
EVFAPNDGGTDSPQRSTRAHPSPPKTQKKWSCRAGGTYCFLDAAQKEDDLQQSRVFHKEE
LCPEVLPLVEECEAMTHCGKVKPGMVFGLDLCVLKDASPLRYVAGITSERTVLALLPLRA
VQQLLGKSPCFSQSVGRHLGEGDIFIPVREFCRYVFMPSAAMNEYLPLWTILDRYTKIEN
VIHTKLKSNTIDTAAWSYALNRLPENVTTTFCFNLVLALPPFVATRMREEAKKADVRHFL
KFGKNKSTTNRTAITYIRTKERRRCTWHLGMEGKTLVLLRDGYTDLLDFLSMLCVHIIES
NKLRGRVQGLVHPPAIDILDEYLNNREADESAGVVRDKALELERIQEVLKNLPLTKEEQK
GLLDLWQTDCLVKIYEVMMHREEFNVRVDPGVSRKFQTNPFVEWALNLRACIMQKMGLNS
HWVPPDDLCIDIVSSNTHCIKNLLSSFHRKHRQAIEEYARRDKRLGPLESWHVEEDAFYA
STTGLLSTERTDLMDDYAKALEESGIMVLVDTAMTGLQVDIIPVHALNFETIDTVLQESL

KRISGCQGRLDSKSWLQMCQPRPLTKKSTDEMSSPFLSSPNHHSRLDRHFIINMDFAFGA
QAEGICRAIFSVFGRRIRSVNVMGKAGGLVGKRGDIQLPKEVLMSKSSLIVEDSQDELRR
CRNQGLTAERLRELAGPNVSVHEGRIVTIAGTMLQNVRMLEFYKCIWGCVGAEMEASFFA
RVIEDFFRQGIANRELETRFAYYTSDLPLASHASKRTTGVSLSVPMTVKEGVPSLYAIAR
GILENILLPR*
>TCRU_1736 retrotransposon hot spot protein, RHS3, putative
MWRCCGWLQVVLLRGRWALTASPTGVAVRLRGAPTTEPCECHAQRHWDSGTKQPRVSFGA
SGTCWPQLGGASGMPHRTGVVMAPRHGSCDGSDAAARHVAGSRVWPQWTMSSTNMRLSDF
LWNCVGGRRPLMKDHNVTMEVFVQEPDDYVQDQRLLEEILNVTAHQALEAITKPLHEGVL
SLGQRRDYERKGYDHSSCKGNTKQSTHAGTDRSEAGGRGKAKTNARNEIYHFH*
>TCRU_1737 retrotransposon hot spot protein, RHS3, putative
MYEEFISALVKELKELPPPSPSKPRSSVLKLNHQGHPTRTVGLGELENGVTRIPMEYRVM
YKPEAQNFPLVDGFFFVDSPRKTLVGLQMTTAGEHHTIPSTVNLFKNNMAAYFKGWKKLS
RDMSWEMIYVQHENSTMITNWQRCGPVNTKNLSDAEKEIVAFWNGNVHEYQFALTTDFVN
KIRAK*
>TCRU_1738 hypothetical protein, conserved
MVPNASKKRSSFANGPRILVASNASGIMSVTYGGREHVAADNTDKVSERSATGHKSLLSE
NGVAGSGFVAEGDAGGLTQRNASGHENPNCENSPRSIPGAV*
>TCRU_1739 Trans-sialidase of Trypanosoma hydrophobic C-terminal, putative
MGEGQDGPAVNLEASARSGGNGETAEETNGQEEKQIHPHDGEANATALSSSLGNVAQGNN
SDAGAMRESGLLPLLLLLGLWGFAAL*
>TCRU_1740 Mak10 subunit, NatC N(alpha)-terminal acetyltransferase, putative
MACDLDETWVECMGEFKEFIGKTGNPWRCYTCEGVEKETMLSAPEVMDAKTDPGCGYGSI
CSLNCLLKDGTIPSAATLTGEALLDVMDLIHLKELNYLQGFSLTSGCLEFSYFFCMDLLK
EQNLTLYTYCRALARCIDLTTRAVMTMRVRSDEEFIPWFKALDPGEDVTEEQIMNELEEA
ACKAESVAIAARLRWRKLFLSILSGFILGSKKSDTEKACATCQEACDLLGSVEFRREAEP
VQDGRFFRDAEVGYWASSFTPTKPLPCAPFAEALQTYKTLLSQLASLKDLYILPSLQCIT
EFVEALGARKPLLLMRSVAVILLFRHDPSESFLHGPSMPHRILQELADEHGAPLYLKIFA
GDEEMLEGVLRYRIQKTMDSSKIPPDQLIFLRQQTVDAVRSWAAEMSRVYLVHLEAMLCN
RGLAHRRLMNALPHLGSLQELSYTTDKSVFLSHIPSASPALEAEAAKRMPLLAIYVNQHV
LHVIQLLVLLTLELNLFTQAELIPALWYWNFTQRAQIENLGLLTPPPATVIPETRINRRT
KVPLYNLALTTRTSGHPDTVRLTILEAERMIADAVFVAACIMEAKGLIDLTSPAKHSFMT
VENTFNHRMKCLQHIRSPPFTPYARCIAAKPDISTKQNADTSTVFLYAQKASEIAVNAAE
ILKQLIASKELDKARKRVIQTHVENMERTARAVGASLASFATVCDNDEALQGYKVSVEYP
FDPAMLCFTFRKKEAR*
>TCRU_1741 hypothetical protein, conserved
MSDDRSLRRTLLPWGRMTQAAGRTAFRLSLSSHLDPLRCHLAPRTAALPVGPHRAAAGSL
ACSCRAECPATEHGHRHFPVPRLAAASQSPTRVPVATGSLSAMEALRLGSLAVHDGVTEE
ISMMPLSLADRGHTVGSVFSPWHCGIPRNVTADADAVVARFLPQGCVNVWHVDCLTAGKR
RNWKGVREKKEGAHTA*
>TCRU_1742 hypothetical protein, conserved
MISMTFANGLRLCGLQCSMEPRLTEASRQRPDILIVGLDTYAITDVTVTYAGRVTAYVSE
ESMEEADALRAARDRLTQKRQKYRYWALANGLDFEPFVMLTNGAINPASRRWLRRILGAR
VLAYAGAQWVAERQ*
>TCRU_1743 hypothetical protein
MATQREEYRALVTGSHYTNTTACLERAHSVEWPTTPATDPRQDPWLQERVPTRRYLHRRE
WPNWLDASRTVMLGYNASAPDERSRKQVAIMDLVRHHLRLPENPRSR*
>TCRU_1744 SLACS retrotransposable element (part), putative
MQREYGTDHTGNGSERAAGTGIGFRATYQKRFEATTHVFNNGGGRHPMRIMRVGLPMEEA
YPSHAETRQEEPAVITLQPEKRAPIHLTQKLLMQQYLSTWISEEHVRTGEQRARHYPKWA
MTATKSHKYGDAVALDFGHWDKQAMGVEEVPFVILLPHHNGTAEYGLVGLVATNTPNHVV
AYIPSARRKDTEWVMIDGMVQKCSGSP*

>TCRU_1745 hypothetical protein, conserved
MSDDWPLKRPASLCDHVPGSAALQLDSPDSRAPCGPHRAAAGSLACTCRAECLATEHGLR
HFPVPRLEAASQSPIRVPAATDFLSAMEALRLGPLAVHDDVSKKICTAPLSLVD*
>TCRU_1746 hypothetical protein, conserved
MASYNGDSRCALGTHPHTLPCLSFGVIAAGHCCERDGTTTAPPLEKVPRSVGCGAARRFA
CSPTDLTAQSFHCGHSVGITRCGGVRKSRSPDVDGVVVAWQLRTSGPSLFRIALLLDWRS
NSPAWFFCRSAGTGVPQRQSSLAAVTSTRPAVGVPVAWPSWCARPCRASACSLWHQLRGD
HRLPRVPTGQPQHVSGEPLQTSTAIAHAALFASNIRRLGPAAVICDVNSHHELRDGRSPS
TAAGDDLAAIPSNIDAELSDDPAQPTRFSGRSVSFSDVTVQRVLRVSHWTSTPLHGVGLP
SAIPHCGDGGRPPATGRHAAATETCRRCSA*
>TCRU_1747 Concanavalin A-like lectin/glucanases superfamily, putative
MKLSYTADVKWVTEIKNKITKSSTWEPNKEYQVALTLHGKKVFFCIDGKLLGEEEVLPTD
ETSLGPAHFCFGACGQKGRNEEREKEDVNSQKMLDQNSPVTVTNVFLYNHPLNFTEMTAI
KDRVFISTRGPESVAGWKNGEKANTTEHAVVVSLSSAGTVGVCGCVRNVRPRF*
>TCRU_1748 Endonuclease/Exonuclease/phosphatase family/Endonuclease-reverse transcriptase, putative
MEPFTWLPAERYFYPLLNSIGAHQRYTYRLRAVCDAQRQKLLLSGDIEQHPGPIAVIQMN
VSCLTPSKLATLMAQGADIIAIQETWKSSEQIASMHTGDYVLYAQSRIGKGGGVAVLVRK
TLRSKRIPLTIPQHDTSLEVVVVQVALDQNRDLIVASAYMRPPPQVTQSFRRLVDCLPAS
SPVPLCGDFNMHHPQWEPFLETSPSEVAAEFLELCTDAGLTLVNTPGEITYARGTRERSC
IDLTWSKHLTVSNWSASVSPLSDHYMLTFTLHQAFKDTIPSAPKFFYSWGKCKWDLFIKD
FDSQLPAYDYKKQSTGIKAFTRALITSYRRHCPRGMHKDGPRLWDDTLMEQSGLPRTARP
AIYSYRRPTVRQKCNGQGVNSFSYSESACATRIYAASAI*
>TCRU_1749 Reverse transcriptase (RNA-dependent DNA polymerase), putative
MLLGDGQHTYKTARRAANALNRIFLPFHPSHKAVRPSKGINRQSASLNINASFLFGQQNN
NESSATFTSGSSLSSSSEPQDKNEAATTSGVVAHLHSPLDAPFNRTELLAALRNTPYGKA
PGPDEVYSEALRHISSKGLRFLLRCFNHSWTTGTIPAEWRRATIVPLLKPGKSPELPGSY
RPISLTSIVSKVAEKMVLKRLLWVWTPHPHQYAYRSMRTTTMQLAHLIHEVEHNRNHYFQ
VSLPKKSGIGNQLRYRPHRTLLVLVDFSKAFDSIDHRVLSRLLANIPGVNCRRWLRNFLC
GRYAKTRVGHRHSDRRPMLRGVPQGSVLGPYLFSLYVHPLLNLLNCFAGVTADMYADDLS
IIVKGQFREDAIPTANMVLQKLHAWSQENGLAINPSKCEAAWFTLPTHTESDYVREGRWP
LVVAGCQIPVMTMGASRTTKLLGMDLDQRLTPNVAATKQ*
>TCRU_1750 RNase H, putative
MIPRIRHRRKTLLVVTDGQSLLAAVTKGPLSQTDWTEDQIWQRLLTLTRAGWSVHLQFCY
GHCGAHANELADQYATQTMESGQYTEHGIAPLWHTDLLTCFTTQLTNKWRSTIRQDTHRY
LLCGTRPSDLSGKDLITQEVLHRQELVNLAGQGAGNLSSGADYTGP*
>TCRU_1751 hypothetical protein, conserved
MEALRLDPLAVHDESTEAICTVLLSQADRGHTVGSTFSPRHCGIPRNVTADAGAVMARSL
PQDCVNAWHVGFLTAVKRRNLKDAQEEKEGAHTA*
>TCRU_1752 hypothetical protein, conserved
MRRGIPRNQGSFDLGSGKIRELFVVSKASCPRILTNRGARWGSALAVFGATDQSKIKAPS
GFSVKLLGIKAAISDQRLAVIHASRKTTRSLQSVRLRIS*
>TCRU_1753 hypothetical protein, conserved
MVCQCGETQVQMTFINGNQLSDETLTVAASPTGLCLFHGPISKAWQDSTLIGHIANLRAP
QIPNFETQALLPAMGAARELGHQQGLITAERERLNFDYANAVADVQNRNAQLNLEFQEAH
INPSVRESTIAGAGNQVLELRQQLEAARAQLREREFNLGVLTGQFQEREARHNAQQPARD
AETAGRDEASATRHAANDGVSTIPHQTNILMAGGVPSNAA*
>TCRU_1754 Protein of unknown function (DUF3682), putative
MASGVLGENGSHMPDGCNKTAITVRLRSVLPITAVEAANEEVSDTSQHANNSGSDTNTGS
GGGEGGGSAAGAGGPSASPAPPPTGGTEGSVGAGDSSVSGGSSGNSTGGHVTGGISSDSS
ESPSDPPAAPGVDSSARSSGGTAGSSGNNSSNTTGDSQTEDQTPAAAEAHNSSPPEGPAG
TTSGTGHTQKEEEEEENEKQQQSGEAQVQQHQQHEHPAESGEESAKDKNALRTNATANTG

DSDGSTAVSHTTSPLLLLLLVVACAAAAAVVAA*
>TCRU_1755 Trans-sialidase of Trypanosoma hydrophobic C-terminal, putative
MPSPRVQAALQQPREEIKAQASTAEEPHEEEPRAAASTAEASIAGKTSEAPVAQATLQQP
QQESNAGQNATVVESANTEHFTVDSNSHAVGETTGDAGTVRGSVLPPSLLLLFGLWGFAA
L*
>TCRU_1756 hypothetical protein, conserved
MAMMRTGRVLLVCALCVLWCGAAAVMCDGGAEMQGVEGSGGSQHVSGDPDGRGGQGETEV
PFPPESDTVIKSLGVGDGRDPKQLNGVVVTPPANPDRKEEREKEKSKEEKVVAEQTENT
VIPKEDEKISQLKSMSEDEGETAPLAAEETPPASTGVTSTAQNGNSPEDLTAIPGKERVL
PEDHSDQPQGGKNPADGLAAKMSREKGEAPEAPPSPLAKNEKHEVDLKATTNNEKDSLEN
GVSVKKTEVLLEDKEVTSNLRKEGLASTTGNQESDPSNSHAESTPPSNSGASNASNDDAD
TDTNEEILNNDSAAGGAATEGAHQGENKEDNTKETTPLRSAAMINETAPTGDSDGSTAVS
HATSPLLLLLLVVACAAAAAVVAA*
>TCRU_1757 cytochrome b5-like Heme/Steroid binding domain containing protein, putative
MQKARRVGAGKIYGVIIASAVVGFFVRVLILSYNKRSDASDWTWGCRFFGNRAKSTIRPV
EKLECRGFTKEELREYDGIQKGDIYVSVKGVVYEVAPQFYGPGQPYHAYAGHEISRCLAK
SDTTAKEANKNWMHDCNEEEIEALEWWAKKFDSKYPVVGWFVADEDFYLDAQNNTLQTM*
>TCRU_1758 ZFP family member, putative
MPPKKRSISKKKKKEVLPDPNTIPNGECDYYIGSWSHYQNIDAISDIAHTVLNQHTSADG
EVGELPRTNILIPACGLGVAGSHYGVEVTNNIVVESVPRTSMKNAGQLKEAGVAGEEALE
PEETVEEKQDVDKSSEVNEPDVGASLSACDVIVHGTVHIDGIVTRIVIKPDLPEPPVVPQ
TKRTSSTKRKKSAKSIEEEQRRLAEAAKIYEEKVNAAVAAAREEEEYRMQYAHPNRWSNV
VFTNITFTGQVIVSHAHVTFRNCLFSARVVDVAQLLVTQYCQVECIRCTFECPEKCSMYG
LPMGKLTFQKCLFTGNPKTLPSEKGATDEARTLRGKAVGLHTDSCCVTVEDCQFKDLETA
ILLRGSHPDATAEKPAMVVRTCKVENIFGSGIVLENVRGVELMKNEFAQCDYYSLDCVKG
KDIRVFQNTFLSEVRVQRHAHVKLMHNRSGTVPLALKEVDNPNWQPVY*
>TCRU_1759 Retinal Maintenance, putative
MSDVDQLIEELFPNGGTEPHSGLNVRSMTHEPPASKADWDASDEERGDATDAKIVSVKPQ
ASVDAKKNFISDPSKSYFDESDGEDFGPAGTVFTFAVVPFPFFPSDLGGACVEKCFVTNM
GSKSLPHPTIARLLLFAKEGYSDRLTQIQLLERKGAFYALDAKFGNGASDRYAENMGCSG
GCPVMVCRKCNYGVIRLQGAAWNDGEGRIDLYLTVRNYYPDWSRLANSFPVGKAKGAEND
RVLGPSVGCAAYCCQCSWLTVKSAQEIIETRLSHTVGYVGKEDSCCFATQLPLMQGERRR
PPLWVCRGHMPHA*
>TCRU_1760 protein kinase, putative
MLADVSDSNRRRLSSPHVENGIPLNGTLLEEARTGDTDVRNISNEKTNPGMWGKANGALK
RRKPAAKLAQTRVTVGMHLKRWIITGRLGAGSFGETFTAVEETKDCGDGDEEDETADNLA
VSHKIRTSSSPVEVCIKVEKENKNVLRIEAAALKKMQSCPQVARYLGSGNTGGINFLVME
RLGPNLAELRRSTPIGTFSHYTTLRLGISCLRAIRGVHQLGLVHRDIKPSNFVIGLGVTS
DPQTCYLIDFGLARRYCRLNGEVRPPRENAGFRGTSRYASLASHRQQELGRVDDIWSLLF
MLIEFATGTLPWRKYKEKEEIGRCKEEVIGPGIVQNLPREFRPFLAHLQTLHYEDEPNYD
FLLSLLERAIERRGYPPDKPLDWEHENGTVYRVDFREEENVLKSPIRQTSTVAANIAVAR
ENPLPRPVPVEYAFRKPLDVSIVPAPEAVNININRPTLAARLMPPSPQSEEEAVEQGLVG
VSDVSLSPGISRRDHLDNGNHTPVCAGTLPWNYSRVTPNPGGATFVSANKQGVGMENKRE
DHGRDEQMLAENMELIDSGGRFSPREGASPMINDNQADKGAHLGSLPLEEMAAAQYPTPE
LMSPQNQRQQKQQQNLHRGTRKKVSFAKDVDLAGNGGVSPYREEKKAEKAKRGINCECSA
M*
>TCRU_1761 rhodanese-like domain containing protein, putative
MSVREAIGEKNFLLRRTPANPKYENVKAVVESGMTAEVARLMKRTGANTERHLGECFLRV
RPKTLAEYILLLKRQEEAKSMGNSDGGFAHPMDEYRSPNARPPKEYLILDVRSEEDYRTC
HIEGSLHYPKRRLVHATNPFLPEMFEFKNKDNKCIVLYDLEEELTVGQSVGKILFEKGVD
NLAVLTGGLREFVQDYASLIVGKCPVPIVPRDNRLLKRAEALAAARSETQRSMFTHKPKS
LSNSLAKPRR*

>TCRU_1762 hypothetical protein, conserved
MADGGAAAAVPVGTRPAARHAPELRAGVDGAADRLWWSVGSDAEQRDVGAQRDESVYRPG
AGCARAPRLLHWCRRDDSHSLRRCGGVWRLQGCATWVFHHRVQHAARRCQRPLGDHRRCC
GCDGCCRGGDRRAGLHPGDAGTRRVCENVMRLGAGACEHRCAAVLPVGVRCP*
>TCRU_of Trypanosoma cruzi region 5/Dispersed gene family protein.1
Dispersed gene family protein.1
MRFPSLTYVVAHAMHLGIFFGSVFALAMPGARVQHYVIGVVGVLYGVAFPAGVCYLIARH
TGASFTKYWQFSRKPLHERLLYPVGYWHPAAQQRMYGGMLTNMKGSYVYWCVFQLSVLCV
VGLIAAVHPPVGVCHVLYFCMAAVLLAGAGVVAFTNMMRSAFLTVMQTASFVLLAALCLT
SAANHLAPSDGGARAYAALLMLLTTVLLAATVYCAVVWYVEDRHWQELREPQRGGLDALL
HDDEESDEELRKHHDRTSSSYASGSAGASSYRPPAPPQWERIIGSNEGNPDTIDRQPQPL
AVDTHSNALSFLDHASTTSGSVNHAIP*
>TCRU_1764 Tryptophanyl-tRNA synthetase 2
MQRTGGARAGNQHRTRGGGTNGIAPFFRRGIVFSHRDFGAALDCVRASFATGTHRAYLYT
GRGPSAQSMHIGHVMPFLLTRYLQDALGLPLVIQITDDEKHFFRDIPVSGERASGLVVEN
IKDIIAFGFDPRKTFIFRNTVHMGDMYPTVVQVQRMLTLSAVKNTFGLKDSDNVGKAAFP
AVHAAPCFSSAFPRVLRRLAGDPPLKCIIPCAIDQDPFFLLARNVAPRMKCCAPALLHTK
FLPALKGAQFKMSSSAEGNGVITLHDTEQEVRKKVRKAFSGGGGTLEDMRAEGVNLHADV
AYQLIRFFCPDDNLLAEVTSKYSRGEMNSAEVKDLAADVVVRHFLSGWQARRAGVTDADV
EHFSGIRSILL*
>TCRU_1765 protein kinase, putative
MGCSQSKTRTKEWGGPQQRRRSPLKNPSERHGEPVVADAEIPPFSPDKELVTWYNAQLEE
GRRIFCAPPAALDKSPLPGTPVGSSHTKKKRSFPENVGNDEALTLYQVSPLNHVIGQGYF
SRVMLADMTVQSTTADALAKNGIEAHLHPRKDLSHMASPGSTTANLLDATHSASRKYLIA
CKEYALAGDSAFFIYNQLRKEVKRLTHLTFNPCLLKVLHMEAVYEDSSTTENDTGGHLKL
FKGMASHFAGEGLAGGSKKHGFRKQGDRSSAGNSHKIPEKVRIYMEYAKYGTLRNMLLSE
IPKKFGKTYMHELTVRAYLREVLIALAFLHENDVVHADLCAKNIYISDHITNVYTTCFPA
YIADIPAGKFATVPAEWVRRVLALMDPACMQPQYYSGEVDKVHNDSVMTHLGWLPSVEAS
GMQNHGDTEVAPAVPTTRVEEEQGHHFHTITSGHAVASAAAVAENSYRQNNADENEAEDD
RCRWVDEGVRMYLQEHSYNDDDNIPLPSPLSSLEGGNVFMSPKLDGVKILRNGSFSRSPL
LPSIPPLLLNQTVSGEYSHNTMKRIMSSMQNSSTSACPQGDESLKQMNPDALAVNSGHHE
KHEGFLVRSPLARRSRKPLIKLGNYGRVRPILVGDSDKDVHKLGRVTHLAPENVQGASFT
SASDIYAFAMTFIELTTPNDGLFADQRPTDMEPPRTRQGKSEYDAAWLANIKRYLFKNDN
IVPLPPQLSEECKAMLRLCLQYDPARRPTAVELLQSKYFLLGHWVNVATKNRKIEAPWLD
TNYEAAAEAAGLPRLPSETGIYDVGTLLRVRAGGKKKPAKHI*
>TCRU_1766 hypothetical protein, conserved
MVMYDPAAPNNYRDVKVMWWKRAREDERKNDISRSLAAAAEAGSSPFDMPDMMRMAPRDE
LAYDILGGSGDTMSLHLSAEELHQRRVRQSAALGITTAMNLEREELEAAHKARVAAGMDR
KGRIGQKLGKLMSVAVTRENATKTNTTSDHVGDGINTSAGEMTPATTMATSTTTSLPGIT
APLVFQRSELAAGGYVVREDVGALEKRKTHYGKPSSTILIRFVRDGPPVEVFDDTSNKNV
GSGTLRNAFLENIQDLCGQCGVVRSLRYMVLDDEKRAALRARLENEREEVSAASADVETR
LAREMIRVLVRFDTVANAFKALEMLQQRHGSNWDVCFFSTQLFDVGALGPAEGESLCCA*
>TCRU_1767 Serine hydroxymethyltransferase, putative
MSKSLVEHDPDLADVIEKEKERQYRSLELIASENLTSRAVLECLGSCLTNKYAEGECGNR
YYGGTEYCDVIESLAKKRALQAFKLDETEWGVNVQPYSGSPANFAVYTGLLQPHSRIMGL
DLPSGGHLTHGFYTAKKKISATSLYFESFPYKVDANGVIDYESLEKISEVFRPAMIVMGA
SAYCRDFEYVRLRALCDSLGCFLFMDMAHTAGLIAGGVLKSPFPYADVVSTTTHKSLRGP
RAGMIFYRKKGRNGEATDFERRINEAVFPGLQGGPHMHQIAAIATQMKEVCDPAWATYAQ
QVVKNAKKLAAALIARGHRLVSEEVDNHIVLWNVRELGLTGNKVEKLLDFVSISVNKNSI
PGDKSALAPGGVRLGTCALTTRGMVESDMERVADLLDLAAKLCVSLQKQVGPKIKDFVDA
MRASELACQLRLEVEQIASSLYIPGLDLGTMKYKGGIPPAA*
>TCRU_1768 TPR-repeat-containing chaperone protein DNAJ, putative
MFGFIKNFKGSLVVTALLVALLAVGNVLCTGVHDDEVTKQLLLAGDAALRQGRSQYQEAL
AKYTEALAHNPSSVRGLYSRAELLSMMRQREECMQDLDRLLQLDTKHHRGLALRSSLNAQ

AGHLQEAIRDVEVLLPLYKEMKKSSKVAEYEEKLRDLQHYTSAWLPLRKKLEAPKHSAGD
ITESEYKTCVALLHDMIRKFAKDNVGMRLQRAECALACGDNQAASEELKYVVQKEPQNLE
AVALAARAYRALGAMEQARAELRMCLSLDPEYAACAQLHKLVRGQMRITKAITEALDAKD
YQRALQQIDEAEGLEENPPYKDQLLLWRCTVTVGLRDTEKGLSVCEEAIEFLGAENPASF
DVHLQKVDIYLMQDDLKSAEEQLQNARRLQPNHERVNEYRQRIENLKRVAGRKDYYKILG
VKKTANDAEIRRAYRHLAKTLHPDKLRSQELSAKERQKADERFRDINEAKEILLDEKKRE
IYDSGEDPTKPSGQGGGQPFYGHPFTFHGNPFGHGGGQQFFFRFG*
>TCRU_1769 PX domain containing protein, putative
MSKWASVPGENDEFGERNEGSGTLFSCSYVVHIPPPVSGPSLPANAIDIVSITLPHVRLA
PDVTQKVVTWYEVLVRSATHQWSVLKRFSEFVELYKAMEKANLLQLARQQLERTIAAPPS
RFFPSQRRQQRLEEFVQSLLQTMKDLLVSLLCSGSPETEVVGDFSRQTASLVTGSAFSIF
IRFLTTTSYNALLVSALAGRDSTAHMTSGATTDCLMMDERTHVPPAKIVHLPKQRFTIML
QLPGVSISTVFVECSEDSREIVVKGAWNNHINGPDSVLSGTIDTLGSLSVTSSGGLAETV
LFTDLCATADTTEVLVDTFPVGDFQMEFEVPPPFAVREWESQYAGGVLFLTWRNDAFNVS
NPTKNT*
>TCRU_1770 tyrosyl-tRNA synthetase, putative
MTAQKPTEMTTEERYALVRSVGEECIQEEELLNLITKKPNIRCYDGFEPSGRMHIAQGVF
KAVNVNKCTQSGCEFVFWVADWFALMNDKMCGELEKIRIVGQYLIEVWKAAGMNMEKVIF
LWSSDEITRNANSYWKTVLDIGRRNTIARIKKCCTIMGKQEGTLTAAQILYPLMQCADIF
FLKADICQLGLDQRKVNMLAREYCDLIGRKLKPVILSHHMLSGLKEGQPKMSKSDPDSAI
FMEDTREDVERKIRHAYCPRLRQKKAEVPEDGAPVSSEEKNPVLDYFQHVIYSRPAASTT
INDTLYKTYEELEEAFVDGTITEELLKDALALELNALLEPVRHHFACDPHAAGLLEKVKS
FRKGGQTPPLTDIGPFEQSETPLAVVWLPTVIKLSVNVAVAISDAVKAFLEQHSDGEVVL
LLPEWTAFSCGEVTGEEKNISAALELNLALLQATYLRKEWVRVVRQSQLILDNPNDYWVA
AINAGRRNMLQYLGDVCGEFKAAGDLVAALMYVADVAMLKATHIISAPHERGYHEVVKKF
FGGRVTAIPAREGTVPPLHRPEALVAGADDVLFLDDTDMDTRRKIKKAYCAPNEAANPVL
SIAAWLLRERGTLTVERAAENGGNVAYTSEAQLRDDAQSGTLHPADLKAAVTKPLLEVCA
VSKAVLSSAEGKKLSQTLRQAEKTLSKRSK*
>TCRU_1771 emp24/gp25L/p24 family/GOLD, putative
MSCFPRSSCMFLQLVAVVIVLTVLDTAQAVRVRSTAQDLTLEVNQEELCMYSVGIDTNEG
AMLHYHVLHGGDDFDVSIRDPKNKTIYVSYAGEHELEDRVYFTKHLHGEYSYCIDNRPYS
GSRKSVTMSIGVTSLKRWKDRIDPLTRMMLRTDSFMLGLHQDQIIFRLRQRNLHETIEES
KTLLLLQGAAETLFILLLSFLHVLLIKHLFARKGMRAVA*
>TCRU_1772 hypothetical protein, conserved
MLRRVFPVSRAAVRGPTLALTAALRHGHHAQTQHDFSGCFLRKLTREEEESLRVLQDNRP
VSAVVPGKLFMRHWIAAEQSTVSVVNRVMSGIISVCLMLWSCGYATLGYNGHNCAHVAGW
IFIAFWLVLHTHCMAMIPLFSLIALMQLVLN*
>TCRU_1773 protein kinase, putative
MEKFTKVRQIGKGNMGACALARNNEDGKCYVIKQIDLRRMSKKERQQSLNEAHLLSSLRH
PNIINYVDSFLLKKSDHLCIVMEFADSGDLSARIKKSYGVNFRESQVLDWIIQLVLSLSY
VHQRRILHRDIKSQNVFLTSQNILKLGDFGIARTLSGTYDQARTFVGTPYYLSPELILER
PYDHRSDVWALGVVIYELLALKHPFNATSMKGLMQRILKVQYDPVPKLYTTELRNIVPRL
LNREPAHRIKLEELLELPILQRRLQEWMLTDVMPKSYIETLLQQNLLPPAIAALRKFNAK
GADVATAEVSALVSTGDAPQGAPSAPPMSAVEAPLLPGRSERISFSVVHRKLQPVEQQNP
QQQHQHQHQQQQQQQQRRPLPPPPPPPPQLQPYSGPMQKPPPDPSYLGAVRMPKRTQPVY
LMYKNINGSYNSAAVPGAGRHGSRVSLFVPRQITSNNAIGRSEPTQAPQLGVDGAKHPSL
SPEPPHGHVDAGGAAYIPASNPFSNRTSSNANAVGGPSYKNQTDTDAKEGGGQQFVEQRA
RKRNQQGSSAVASCLNPFFRQPSIGSSPLSCVPGAPNMDIKAMLQRAALERAQRRAFS*
>TCRU_1774 hypothetical protein, conserved
MEEDWSGGGGISNLISLEEPTASHLPCSVRFRVFSDPVDNVAAREAAWRSALPNSLCVHK
RRRLDEVSTCEREAVVRPLLRVLRDPIAMARRRFETLRWFDEPRAADDICARLELDRRLG
VTPSLAALHQALRRLRSLRRTRLIADLAEDLVMDAPSISVYTLLLIVEGLEGEPQRAASF
LFSLTPLVTNGTVPTSVWSSLCCELDTVARQMPSPLERCIVDLFGQLFYRVREDGPVNGS
IVVYYGQALLKSRAPIRSVVAFVQTELLSGRNTPPSYAVSELRLSAFLSDLIQVLSVRPN

GGADEREEGPPFSARERLLCCSDLVKHAYAGRLSLSQAAFDMLLRFCDEEQDYNLLCILF
LAMCALSTPTLISTARVAEVLCDVEELSHHFAGVLGQPIPMWMLYIIVRHGKSSLFTPAH
RKKNNRKELELHFCDSLARLCARDGDASLCMDVFDAIVEYGNPAGAHVFVCSLTRAMGVT
QRLFGGPATTIDVAALEPFNLETLYYALGTDAVKLEEFRFFIYERPSAFLFPSRKGSCVT
LERALSVLLDSPQVYSTVLDVSAVCALAESLALAEAFVKMMNGYAAKSGALAFVPFDVCA
AIQLTDGGRQLLLSWLANHHWLIMLPLSFTLQRLTGVTSSAMSIPPSLRQQPCTFLFYAL
RRVGHAKAAFVTAASPAAELAKAEGVHPVITVEELKTRLGLR*
>TCRU_1775 WD domain, G-beta repeat, putative
MAVPTEAAKEKPPLHAYPEAKVGTQFFSHHGIYSERYNGVCALTGSIVLMASGRYVMFVD
VVSGKIMSTPGPENGGVGAVAVHPSRRFYVVCEKLPEDPLIRVYTWPSRREVGQFVGGAT
RGFSACCFSADGTMMATVGMYPDYSLAVWNWKTRGMILRNKCYGSDVYTVLFSSFDSGVL
VTGGAGHIKFWSMAKTFTGQKLQGVLGKFGRLEISNVPGFAVLSDGKVLSGSENGLILLW
EGDLVRCCFVREIKKTAVDGAAALAVQTYDYWPCHEGTINVVELLGDGRIVLTAGEDGYM
RYWLASELETAEGEGAPPLYEPKCLFEVLVDPHAHIRALTLCRDMDEWVVLDSTGVLWRV
PFIPPNVILGSSPGRTKEPAQAALRFNGGGITSAAISPIDHTVVTGGEDGTIRLVDYVTP
CEVYKLMMPASTTVVALHFFRTDKTGMKFIVCCENGTVGLLQRGNKAFALLGQWRPHTEG
LTAMAVDREERRLCTISTNGTAFFFDIKRDISLLEPIGFCRLPLKDVRCVAWDDATSCSL
VGCECGKVLAIRAPKMEEMDHTVSFEFNCIYALVGIRQKKKVEKQQVKAATAGRVEGLQE
EEEEEEEEELGPWPVRLICPLPSGSFAVGSGAAELLYQYNLNVRYDNLTELPPLPPTGIE
PPNYVEEPMINLCYRELIPQMASVSTSGLFLVVVCEGAQVILRPMEDNGKIPHKCILTAA
AHDRVDGRITTACTSFDDTMVVSVGLDGLVVAQIREGCRAPSPPKIDAVYPSLFAEEVLA
PQPIPLSISEQKEADDRRLAEEAKQRELDAFLAKLHVVHVKYAELLAENEAAPAHQRLSD
EEMALHPQILKEMEEEKQRRVKESLYENALQLTREEIRTKKMRKRFIDNLIYDRFQVISF
SREFAVSSFRTPNPEETIQRLKGEINELLAHDANEEAIFKADENDKKEGNNDLSKTTGMN
TEGESAEGKDGLLSTASGISQWLLAEEKKWQEKVRMTRRESELLTTTMRQYLNKMDERRE
ERRRRKRGYEILLTYKPDPAVEAAQLEEEMRNEVARRGECILRTDPSYHLTPSAVSKLRQ
LIGLEEMMLNLRSGFTKKLLLLRDEKATLCKSLNVLLHRIREINDVMKEENTQSADVHLL
PIEMPEQRFAIDRDGLDAFVRQRQLEKMREDLAKKAQRGFGADLAGTQTSAKNELVTTVT
STEKGEIQKNTPSQFSIFETPSSGHCRGKNQTVTSIHKASRGYSTAVLAAAAVRERMDYE
LRVKLENVKLTEAEEEEQQMERQRLVAERKRLQCRVDSMMHAFDQRLWKLYEERAELDAD
LCLADARSLLMLHEYQILLVFRQKDRELRLQYEETKRSHEERNKEMVELQKGIGEQAALI
EKLQEKNKAFRHEGEYFIESNFPAEHVPYVTKVFLRQIKRRKHLGDVASDDDDITSDDDD
EDVNEDDLWEEACPSNCSADRWQEVLAMREKRLDYVDAISEGRRKQEQMQRRIEEHKQLS
EELNNALSLSLQAIEDFKGEKRKQLNMLHTLVALRCSQVQCLEEDEKCPDTFCRDDLVVV
SDHALAKLRHRIEELAEEKRDRRTNLKEMKLKLQQLQRERLAKQTLHAQWEEKVYEAMLL
KFGRIVNLELLESISGCREVEELKERLRMEELAWEREIRKREKKILSLRETLQSRLVHNT
HLLHDLGDQEDDRQEVERSLSQATQKVVSRMYDGSNVATAEDRRNLKFLIAAQQEEIDAL
QAEVSLLRSKGGYLYTPSPSFPT*
>TCRU_1776 hypothetical protein, conserved
MSIFGEPLPHEDDTATSLIRREGYPPLGSYDQMPQVYVCPCCSAINPSHERDHLLRTAKE
EKELERRRIMQLDIFDERAKPISTAAPRPRSINIHHDPEDDELNQPVVPPPPMTLDELRK
LRQEANKTRLIVTVHHGKNLQADPHDPTSILVRCGAFEGQTAKVPRGTGTICTWEELFEF
PYPNEEEPLEVLVIDDELPADQDHILGGIVVPPYALRNRARGDEDVLPVCREGEMHRGYG
RMKETPLGSIVVSWYVKYDGQETEPTDGKQNLESPVDCVFVVHRIFQYTDNGTTPFTGGV
LCVLRDTDDNCSASTQYQPSGVANMSTSPYYTKEGFNYLPEPPSQIMQLITEKRLGHVLV
CVPKSDEEGEENEELLVVGAVPLDFGELYRKGSAVLLVESKVKEDVLWGEIALEWCFTPH
ADVQRKLEELQLAEESKLEHEALKDKEMPPTSGSLFVTVVRGLNLTNCEGRPLIRAQVSV
FSGDMEGSTFVAPREVGEDGLTNVMNWNQEVRFIDLEGDPSEITVYVIDEDQRVSSGTFA
VEQDRGYAVVKMHDPYDEEKQMGELLVTYNRHCTTEDAAAKVSGIQNGEANKVNGNGTRR
ISTRERSERSPGSRSHQYSNDDIYERDNENEDNINDLEGYEYDDMHKGDKKHSDEEQSRS
RSRNRGYPEEKEDSRRERSGREGRERGYPEEKEDSRRERSGREGRERGYPEEKEDSRRER
SGREGRERGYPEEKEDSRRERSGREGRERGYPEEKEDSRRERSGREGRERGYPEEKEDSR
RERSGREGRERGYPEEKEDSRRERSGREGRERGYPEEKEDSRRERSGREGRERGYPEEKE
DSRRERSGREGRERGYPEEKEDSRRERDHPEEGLRNAGAGDNIPGDVVARESGKREPVQN

ERSREGDEPNYYGGYPQEYLSERVLKSGHDDNEKQEEMEGQDVAASSKPPRAVRTTLLED
KHRSSRGASPPNGPASASVPSQPHSTPHSDWSQGDTGFEPRGGKTKVGKPPSRPEQFSVP
KVPTVRQPWFPAGSTGNEDHIPLEKRESFINRTDSIRDLERRSRASEKSSGRHVSRQSTT
HD*
>TCRU_1777 hypothetical protein, conserved
MLLGDDSFTREFLVHVSIQDKAPQRLQLKVRNPLSAEDMLRQIGRQLVDPTTSCSEKVSL
RRVLPDSHLFAFSAEENCFVPVDGKPAAVLGNFVHLLVHPLSVLPPLGPAAVAPSRNSRN
SGTRCVSTPLNVDASQTKKGPLQHAQFRVRESLSAGKKQYGAHGQNGCSALQPAEDLLTQ
PRDERDGLDEAQKRRKLSVTLEVMLKVMGHILSEYSSRASDDVIEPTEKHLWNLIEDASC
RSGGVPTPSLDSVAVDAFVETVISSIHRGRLHRTFGSSTHSRPSAKNM*
>TCRU_1778 Vacuolar protein 14 C-terminal Fig4p binding, putative
MSDTLISPQTLKLLVDSKVESRQKGARDVASKTAVIFVKYPLVEAYDIVTRELREAEASL
LSVNSPMHRRGGLGAVRAIVGSLPSRTHPSEVVSLLSSIIFESFTDVDATVRLAALEAAH
ALVRQLRIQLLDVCFMQVFWGLASCINDHDGRVALLAGEVSLSLRELITGNESFKFQTEQ
FVNFITELLEPYSNTTNNEKTFDLLVVNWCVEWIGHVLDLPGDELILLLWRFLKPLLMIS
GRCGGGEVARLLQKCLRDTKDAFYRHMSVQLDSLLLIAAECVEGAEVAVTRKNALEWMLE
LLNIATDELVHLIGRVIMAFLLQLGSKDLETRLAAQNVNHRLMQLVALKQDGVKNVPHDA
VLKSVASQLTGRGNEESRVAALEWIALILHTDAAVVERGFSETFETVLILLCDQSAHVVH
KCIETLCLISGETNFDYFVSRLVDLFHAKADVLLPKAPTIIKQLQLRYQGEDLSQCEKLC
LKISSVLSVHEDKRFLEKVVITLSTLVLTSREFLPLREILNRGVDDERARTTFVGMYQCW
RYNTVAALSLCLLSRAYEHAFQLIQFMGSLEMSAATLLQLERLVRLIESPIFAYIRMSLM
EPSRCLPLVQTLFSMQLILPQGSPQHSLLYRRLKLIPSLVTLERESQARGVSKKESPIDW
SQLLELSREAQRCISDFERRLLLQRLGDTGLP*
>TCRU_1779 mitochondrial pyruvate carrier protein 2, putative
MSAANATQQVIPKAFPKILHRYVGRPRVFECLKPYEKYYVVAYLNSVYNAAPLFKWSLSI
VPLYGIFSGSVPVEKVDFNSSAALSITGLVWFVYALLIQPQNSGSRSLALVNMCLASVNG
YNCYRSWAYKRVKALDS*
>TCRU_1780 hypothetical protein, conserved
MKRCSLCCSSFQRLPFGRRSAAGGINLNKGLLSDRERGDQFTDPTVYRNKKSIVAMDKVS
RKTERLLKEEKQKEGMNALGVDSQMERELSDGSMHPLHREEIAAARVIDEDGLLSSDPGS
KYTTALRRLMEREVDRRDHMMDKFGQPPTAKEFHRLFTRLRHADDESEAIERHQTRLVEE
YGVYPSMRLDAYMLDDDTYFPGWVNALPYSIRDRVKYGSLGLTEEDETLRVTLGRMPLDR
RRQEWERQKKAREYKAAKEEMLTLAELRDARQGKRRFHWLQRKRQKRASMLRRLALRKPD
AFELWPSTVVDYSQRIAFIAQHVENGLDTKGHWPLDPEELARARVRRSQEEAERTFLLSA
EEKKVLKKGNNNGSIMHMLRALDTPEKPFKRLSRKVYANRVNAIVHGDQDEYGRKYRKME
NRAKRRMRPYESLGEIAISKEVRKEPRLYSNGLNHTDDEHWPKHVKSWADGMPSTRYAS*
>TCRU_1781 hypothetical protein
MPPSKVLLADGTEGFLSKGESYCSVTCFRGTVWALRSCGTYFDALDSRDGELLDSVSLPE
GCETIRLAANKLWLLTRTGNIILYELSGVLIGVLNADVGRIDSLHTFENDPSFFATSQKG
AFQTWDLERMAVQRVFPCEGADVLAVFPTRFGTSLLTVQEDRIALWGEEISHPMCVHFLE
SANSAVLVCSQHNAAYADTCWVGGNQWITVFHVEEKFTQMLSLKKERLIPCAKVQQLVSI
SLTRVASFDAEFLVTIWDSCNCVPVRLFKVDSAMGIGRGTSSPVLFLTGVARVATIWVLG
GNILLSWADQEFDDDRMQAAASEMAACTVIRSENAQICKKYACLLEMMDTYRKTVVDIVS
SPNRDRASMAATISALDKCFGEQMTALRSEDDRPRDMVGGNTLRKQVEYWREKYMVEKER
NRLLLSDLRLAVDRLKEVGPDSRVDSQLLDLLALNATSQARESQLRSRVLHLEAQLHKLD
TTDSHNNDFPTHDSQSNSDPYRTRVIELEKELHCALEASEEAVTAQRGIANITDALGEME
RTLHVERGKFATLTKEKSLLERETEKMFEALSYLQEELKKAIWEKDECTELLKRHQLEAE
ENERRASLLLAETKEELHLLRQGMEKLEENRHLASTLSIRDTEIIGLKRQLEFVKSMISI
RHEEGIAASSAYDAMMDIIQTMKHESPITFLLNVLEDIDIRLGHLSSSTRKWMERDDQVA
ARDDEIRVLKNRVQSLESSILRVSSLFQHIPYTIEDVESLLLEVNEYRQRYGRNEEIEEL
VALRLLELAAGCDGEQVAENAA*
>TCRU_1782 glutathione-S-transferase/glutaredoxin, putative
MVLGKGSIGRKLLGATGLIGSLAIGGGYFTYQRRLKENRSCTAEEFNAAQNQEELSWALS
HLRNPKEHPQILLYRYSTCPFCGTAKALLDYAKIPHECVEVEPMFKKEISSFAYKKVPQL

QFNVHGYNGPLVVDSEIIVSTLAKHVGMEKQLKDPEVVKWREWARGPMVRLLTLEFNSSL
YRAWCGYSYINNIDTIPYANKLFLKIVGAPVMYLVSQYITRPRLLKSGHLHEGEDVKGRL
HGEINTFIEKALLGGKKKFHGGSKPDLADLDTYGVLQSVRGHRVYEEIVQSTPIKPWLDS
MDKEVGHVGHHG*
>TCRU_1783 synaptojanin (N-terminal domain), putative
MQAVKTPRKRCAVCVKNGHQILFLSIPKPVDPPAPIELEPLMFFEDTSPSETPCMKKIVA
NGTNKWEGGWVVVPLVVNGNGEMERCRGFGKGEFSSFREAYDFCGPFESLAEYPAMFGCI
LLDTIYLLVATRVELIALLPFGGVIHRVLGTEWIPLFIPGSVPLRLSTTDRSRLKEFQQY
SYERGYFYSDDCDLIYPFPFLPNSAGEAPAFHCDWSEQLRQPFCLHGLSAGCSVLIRGFA
EEKVVQLKNGSVLHMLLLGRQNNLNPGPRYLGRGLNGNNAAGNDHFYEYIMWRPNREDDS
ISFARHTILRGTIPVHWSTQIHRTMVEPSMIFSQNEEEVVRGCDVYFEFLFRQLVSLIKY
DTGTASSDAPAALAPEVRCMSLLRQNPQSGEGVLARYFLDAVRKSDALLRRLFNDAKLDL
VHIDWLNLVKDYGVDVATKIFWESAFEFLAPQKDDAVATVGLLRRDGRITRRNCQRRFLR
VNCADSLDRTNLGCFFTCFQASISMLNSIGIEFSQFVDQRPLPPLEGQEDLSQSGLSSLS
MGGNKKTISPPFVKSWFEARNPSLSPVAVGRALSELYVYNGDIVAQLYTSSAAMHSNLLR
NICGLRSTASNMVIATQRRFENVFEDRSKFRNLELLLGRNKDIHFPSMNQAFLTRPVPLQ
YWSCALVALGVPLSVSTADLAGAVQRALEEIFSLTLPSDQLSRHSSFALGFCISLAEDEV
ASHKEFVTAVSQVTFEQPPPMTEEEGNVIQDNERIAVIEFDHDRFDATSVQRFLREREIL
RVQNCAVTLFPYAYPFQGAGGDSSGLVQKAANSLRSGLKNFVRGLNK*
>TCRU_1784 hypothetical protein, conserved
MLTFTLHQAFKDTIPSAPKFFYSWGKCKWDLFVKDLDAQLPAYDYKKQSTGIKAFTRALI
TSYRRHCPRGMHKDGPRLWDDTLMEAERIATDSKARYLQLPTPDREAEMQRTRSQFFLLL
RERLRNTYLRRVSKLNPGEPLAWKYISGRKKASLPSPTSILLGDGQHTYKTARRAANALN
RIFLPFHPSHKAVRFSKDINRRSASLNINASFLFGQQNNNESAATFTSGSSLSSSSEPQD
KNEAATTSGLVAYLHSLLDAPFYRTELLAALRNTPYGKAPGPDEVCSEALRHISSKGLRF
LLRCINHSWTTGTIPVEWRRATIVPLLKPGKSPELLESYRPISLTSIVRLLGKWY*
>TCRU_1785 hypothetical protein, conserved
MLAFSIISDELRDESSIPTPADLGTSTPPSVASIHERWLCSAKASSLTLRFSLISSSSSV
IEWSIGSSTPMVARRAEAANQGGMELRKNRSSRGELSVMP*
>TCRU_1786 hypothetical protein
MQRRWWTRPVDGGGVRSGWFWRRWEHRLCWVRRLRQGHALLRARDGDGEHEERCVRVRVR
QRRVRRGVRACGSSRTAARCGHRVECVRPRELDGAVGVRCACRRERGDAAPRCAGRRESC
ALRAVDGARRRADRCAERVAA*
>TCRU_1787 Trans-sialidase of Trypanosoma hydrophobic C-terminal, putative
MAPDGTAVTPEVGGRSGEDGETVGGKDGQKREGEVKAAALSSSLGNLSQGNNSDAGTVRE
SGLLLLLLLGLWVFAAL*
>TCRU_1788 retrotransposon hot spot protein, RHS3, putative
MEAFVQEPDAYVQDQELLRRIINLPAYQALEERKILLEAIYKLHHEGVYFLRQWRDYQRN
DTLTPLASGKLNGVLTDIEEKENREAEERLRREEAETQRRAQEIIFTMSTNIEVVLFRGR
ARVKEMKLNDFLTLRFGGKGVMDTNRNVLLEEFFKEPARYIGDEGVLNEIKTSYPYLRME
WVLTEETILERDILKLQQSAGTLAAWSLAAEEVKAGVHDITKQTLDAALEEVRNPAATTA
SIKLEGCYESVYNARWHHVVEVPGGEGTGMKVHEGEPPQPWTYKAVGRTLEKDDGVEQSG
EARLRLMVLTSDKGWPYTWRRESKAIRDCYVNCEVERVWQFVKGDLTEWFINFHLILNPS
PLPRVLIGTPGIGKSMAAGSYLLYQLLHYDVERLQMVAYYVGDESFLFDKTTKTVTKYKE
GTRFTNIVKSLFWRGFKGYIIYDIAKDGCKPSANLPCDGWGMIVLTSPNEGNFSDWEKEI
KPTRIVINCPEKDDVKAICVWMRQYQSKQEQEKYWREVKDRMEDLGPLLRYIFIENEYKD
RNMLCEKAVREMTLPDTNHYLTLGTHKMWDARHVSHKLVKIVRVRGDDGELTLNSLISPH
LGSLMTCKLAQLMMPNDFILLILAIKDDLLSKTLERYSVFTFLSEGFINAIIPKLKELKI
KKDAPPHLCALRVRPHERPFKPLLLPLLKDFEEKINIGCRVLYIPEAKNFPLVDAFFFLE
SNPKIMVGLQITTAGEHHTLTSTVNLFKENMATYFNGWEKFAQGLSWDIIYIQHVDNKPL
TSWQRCDYVNPRNKTDAEKEIVAFWKEKVHQYQVSVSSRDFRRKEALPIVEEEQEQETE*
>TCRU_1789 hypothetical protein, conserved
MPLQSRSKCNRFYGREACNVKPPNIDAAILLGARVRVDVTAACDINGVTAAKRPCSFVRQ

SAGGPLHLPAPGMAWNPLEGWSRLGLRRARSNALLRSSYMQPEPQHRDKVRTPSLGPGEA
AVPPAVIRDPR*
>TCRU_1790 Reverse transcriptase (RNA-dependent DNA polymerase), putative
MDWPFMPCELGVAIRDSSLGSVPGPNSMLNEFLHRLRYVARGTLRTMIHNSFANGSLPGS
WKIEDNIRISNPGKDPCRPGSYRPITLLSVLPKLTEGMTHRRLSALLPHHPRQFGFAPSR
STLDVVTPVADKISRGPCLGCRETRSSVIFCESHVSI*
>TCRU_1791 Reverse transcriptase (RNA-dependent DNA polymerase), putative
MDWPFMPCELGVAIRDSSLGSVPGPNSMLNEFLHRLRYVARGTLRTMIHNSFANGSLPGS
WKIEDNIRISNPGKDPCRPGSYRPITLLSVLPKLIEVMTHRRLSALLPHHPRQFGFAPSR
STLDVVTPVADKISRGFNEFGTVEYERPGGGAHTKNPHHRSLVASIDFSTAVDTIDHGEL
FGMLDRLPRLGPRTKRWLNNSLRGRYVRVCSREQHSRKQITPAGALQGSVLLPQLFLYCV
DDLLHRMGNIYSASALMHADDLALVASGADIHAFAAAMQPSLSLTTTWATEHSPKINVDK
SEAAAFYISSHTRSGEEMVDLLLGNGNLRIQSCPVRLLGTTIDRLPNFDYARFHRCKEDH
ATPLSTAAGRTGWCVPSHHAILFNWISPRCVTVLWRDTCPMPCPHLPP*
>TCRU_1792 Inositol hexakisphosphate, putative
MPPAVDGKGVSGEGKTPQQSEGVARREGVLPPIRPVASAPVGRRRDSSADELLCLKKLLQ
KQESRLNERYQRITLCSESAGGADISRTASDLGDVATKPQGVLGNASLSVRGAHYNPNPI
LSSAPPTAWEQKEGETGAQEARDSREEDEDESELRRLEEDLNRHQTRINHIQNEMARLWR
KPHSHRKTALSPPTPLVMQVDNDREVLELATLVYKFSTFEQQKGGNRATGGKNSSQYDVS
GVVDPWRSFSSSIAFDPERSTTSMQLGRMSMRSSSVLRITDGTIALMESLTNQRDIVLPS
PSEVGVVRSGDVLSANHILVVDLQDALRSHKWPTGIISGAPYFRIVPRLNVAGVAQPNIS
AVRTVVNEVSRAYDGAFVWVNLREEPLIYINDQAHIVRERKEPLNPMIIPNVTGRGIAQI
EEKLKQEVLKEAHENGGNVSVHMEGVNGLMEDQWESADPHCVLTLQNVFDALRPNVIFYR
RPITRNVGPQPQDFDFVFNTCVEYPRAVIVFNCQTGRGKTSSMMLIASIVRFYQMCGHDV
SLDIRLLRSESRGFRFRTIRKLISLIPDGKLHEHRLMILLELIDKVYSITEHIHAAFYTG
TASAEEAMMHLQQYSYFLVFSFYCEQRLWNFNIKIPFSEWLAENNELRLIIASIQSMEDE
LREECIVAPIAEGEAAWAASIVRHRRGNVLSAGRILYTVPMLSGDSQHVNTLRQLAPDVP
IFTCGRLSESGRNLLMAEVRQYFPNEGNILWINLRAEPMVYINDISFILSDYDTISGNSA
ELASTMHVSLKAMEQIEERLRRDVILESQEHKGVILLHHIEGNGKRSTIRVKVRSVRTPK
STMADFATEYGVSYHRIPIPFAGHMLASDVDPFLEYLSKKGGKHDVFIIHDSEGSMRTTV
ALNMLTLYRASRAISLRRLATPDEFREVLRVGQGGVVLPSAQVVSSVAVNPDELPKMPVE
LQLAATICQMLTAGSLLRVVEAAISLGGRGTRWNILHMLNHLKVTMTDAMNKVKIMRNTV
CLVRTYLLVLLSAIYIDSMGDYCLEKPFSDWVEERTEVANIIANLDQRAEQSIKYVEPRT
FMKGSVPHHNGDVLTANCMLKADHFPGCQKKGLRPELCGAPNFRKVDTVNVYGVAIPTLM
GIHNILSLLGASQEPLQAYPGENNDKEVHMGFAAPRLFEPRFSPEELSKPLRGSVVWVNL
REEPILYVGDRPFVLRNLDAPYVNVELTGIAAHKVEQVETQLMVDVLKEATRHNGMFLVS
DEGEPGEIVGIWEPATRETVKTVREVYDELRAQGIRVTLLRLPVTDEQSPSAEDFDALVS
VLLPSIATHMDRRETLSFVFNCQMGRGRTTTGMVICCLLIGLVIPEYYDELNNRYDPLFK
PDDSPLSRGEYSCIVQLKRVLTGGRQAKLQVDLVLEVCAKMQNLRTAIESFALQVKSPDV
TESQRGRAHHHGVHYLRRYFNLITFAAYLQEEYNSMKKMMRSTYSSWLAQRPELTTLCDS
ASLK*
>TCRU_1793 hypothetical protein, conserved
MEFILDAFDSSASTLKAYYEQLQQLGEEGEDCDVKIDALRALHDCFVSHTEKMSFELDVL
QTDEEREADGIEEEVAELEAELLMLTQIAEGCDAGGHRLPFPACDSFQVYGAFATKLCDF
VAKLSVLRDAMQGFLSHPHPLETESLFHITCCGVTSEKAWAEKENELHAAWDVLEAKAGA
VAVTSSDSSIEAVRGLLDEVVALGGRVVDRVADLRQLWERREKVVKQLDGQQRRLVVWCR
QQQVNLDALSDPDHIQEFCASLLEHYKVMSSNYRVLLESAEPFLDCEAVQEKLLEASESW
LHLQVKALERLRHTLFEVHADTILEERVEEHVSFGLQVGPFLEELKNSLVATRNTNSVLH
DRSEQLTEECQLLQEIMPKHDELCKCLLQFANRMRIMREAYACYRAAALSRVTYLASSSD
ILTEAARRKEDFESCVEEIQAWVDHKAKDDSWRDVREKVRGIKALLEREQQFLDQDRKET
ESEEGAKKREQINVL*
>TCRU_1794 hypothetical protein

MSAEAPALPPNTTNGDGSQRAHMEDDVVPAQRVRLEGEEEFSIRLWAARVQYMEMARMNG
NLEGHHTPHGPVLPPGWYININDPVLLTYDPYFRSHAAKADAASAPLFSAKEGRYYAESA
LPLCRVEAHSLKSCPPAGDCLTDLNREALGNVSPCRTGNVVARRRIKAGEVIAMDVSELC
VVEDTLVWRFFPRSSASCRKKKGGRSGKEEKGPIKRNGVRCTPETFSPEIVAAARQIVLH
AHAIRNGVSTLVETGFAQLLASLLLWCGDIDLLPREEEVKTAFKSLETCGNFYIGPVTSS
AALRQSQRVEAFIERWCAVPVESDPHFTALRGAAMLLLQCIPPALLSCAQKDPTEAGGCL
SLQNGNTFVVPHYGFIRRMRLHSPDRLAQLLLGFSREAVPIHVPDLPARSGFFPFLRRVP
HECRPNSFVMFLDSPASHCYGKAGFFDYNTTGRHLDGETRAEAPVSWGFPCSDRVFLPSV
AVLVACRDINCGEHISRGLFQSVFMTQPQRSKIVRQLFGVPCSCRWCVEEPDVARAFRCP
QCPRNCGVICPVGDGSRLKEWVCLQCGYRPDVSEVHRCLDEEQELARVKADKMSGLVRLL
QADTVHYSHAIVFHKIDGWCQKAWQGQDASMCLEYLDVMQKSVHRVLDPCDPQVAQVHEF
TAQVNHAVGSAHTARYEYFLALQIRLRAGMRYAHWTRKTWFMAAEKSLAEFLDSV*
>TCRU_1795 trypanin
MPPKAVRAGMRKAEKVPPPCDPIAELSSLESIHEALKKAQELRNYFQVERDKVNEFWSVS
KKELENCRHAILNADAELEEMERAHQVEMKVYKQKVRHLLYEHKMKVKHCKEESDRLLRE
AEERHQKRMNEIQSQLTQHDRQFEAAMESHEMRIEDQRDSQNYMLTVTKKQKHEKDLARQ
TALYEAKLKTLRDELELRRRAEIHEIEERKNEHINALIKQHEAKFQEMKEYYNHITTNNL
EIIQSLKEEIAQMKKNDEHNETLMYDIDCENQNLVAPLEEAQREVAELQQKRKQYEQDKR
SLGTTRAKLRSLREEIWRLRKEHKDLEERYAEVHREREELKAKFESALRQAMEVVEERNE
VLQQKLIESHALVEERDAQLDGVLQAMNLEPTAIELIASEVDSTLKQKNQMIRDLHFELK
KGEKLVNTTLVELERRCQAVNIPPLSREQFLTARVV*
>TCRU_1796 BNR repeat-like domain/Concanavalin A-like lectin/
glucanases superfamily/Trans-sialidase of Trypanosoma hydrophobic C-
terminal, putative
MLYTQRGYFSGGNKATALYLWVTDNNRTFSVGPVAVEDNMNWMLASTLLYSDGRLHLLQQ
RDNDKGGVISLSRLTEEVSTIRSVLSTWAQKDIFFSNLSIPTAGLVAVLSDAAGDGRWYD
EYLCLNATVTNATKVKDGFQLTEPDSGVIWPVNTQDDNVRHVFLSHYFTLVASVTIEEAP
TADAPLLTAVLRDAGPPYLMRLSYTADNKWETIVEGKMTKPTTESRLWVPKEEYQVALML
QGNKASVYIDGESLGEEEALSTDERPIDVAGFCFGACEMHNFPVTVKNVFLYNRPLNSTE
MTAIKDRIPVPKRAPESQADDVPQTVASAGSAAPGPREVPAAPGRTTMERATTTEHAAAV
SFTSAGSGLLPLLLLLGLWVFAAL*
>TCRU_1797 BNR repeat-like domain/BNR/Asp-box repeat, putative
MKDNKVFLLAGSTDAHKEGLEWKVDNQDLKLVVGDVTPSTRGEPGGRIEWGDPRPLFDSK
YSLNHKNRLNTFFPSGGAGVLMENGTLVFSLMAKSEENDGVYSMIIYSTNNGSTWSLSEG
VSSAGCRYPRITEWEGSLLMIVDCESSQRVYESRDMGKTWTEAIGTHDQESLRM*
>TCRU_1798 retrotransposon hot spot protein, RHS3, putative
MRDNFFPASLEKYALNAFLNVGFVTAIVTKLEELQPPPSSKPRSSVPTLNPQGYPTEAAA
ITELELFDCPQDLNYRLPYLPTFPNSPLLDGFFFSESNPKTLVGLRMTAVGGHHTRASTV
RHFTERLAAYFNGWEELSRDMSWEITYVQHADE*
>TCRU_1799 hypothetical protein, conserved
MRQIQQHRGGEGHVDEAQTTHTGIRIVHGADAATVHAADLRVLRIWERERPKAVRNRSRQ
TRVTVAASSAHTLTAADAARPRCKAHGVHSARTTVQHCHGSPNIELFLVIRYGKAYCLVA
HAVDDRARVRWWWPLARHTCDGRRVRAARELVVKYRPHTPIAERHAVVVGHVHNVGGAEL
VVAVHQPAAASHGQRGCHQHGKAKTESAGHNLQHAAAIHCNVPEHTRAFVHVNARRNPRA
VLYGEHGAAADKQVRRKSVHHVRLRPDSVIVHVEDAGQFHAETALHDNRSARTPATHQRR
PRHDKPRRQKPDTVQNKRGAVPGSRMARGSRHVL*
>TCRU_1800 tryparedoxin 1b, putative
MSGLAKYLPSTINLVSKSGTVSPISLAGKTVFFYFSASWCPPCRGFTPTLVEFYEKFRES
KNFEVVLVTWDDEEEAYNGYFAKMPWLAIPFSSRTELEALRSTFGVETIPTVIAVNADTG
AVVSTKGRERLLTDPEGKNFPWSD*
>TCRU_1801 paraflagellar rod component, putative
MKNSAAAATATAAASCGSNAVGAAADSSQKRLFNLLEAKSKERILRLVMAFRNSTPLEQE
AFAQEIGVSLPREEPQRQGPGPLTRQSLSGPALGVRSRDRSYVALADRLPQLNSTAGIAA
LISPFLTETSVSASFEKHAEDTAASHDESQNQSRASAPNAFSEDQNKVSLTSKGVMTNAD

KETYEEWKEVDSQAHTFFFRSRGYQALRLARGDENAPVTEDYTKSSVFNFTGIPIDEADL
WEWFDTLDVARSGTIGVMSFLETIKQLDRGFGVSQKAEEQWVRDVEALAPDGQLTFEKFA
FLVLRLAAQ*
>TCRU_1802 Etoposide-induced protein 2.4 (EI24), putative
MSTEDAFLPLRRIILGALAIFCAFAFGTLQSLDAVKLFSYLRANVYLRRIMGSCLVVNGV
FFLSIWVASLLFSYMWTFGETFMWSSDAAVIDNESGGGKAAATMNGIGLAGKVWFSVFDV
MWVLPVYALTQALGVRWYSALYTEAYAEKKRRASSLGVQWAAKESGRDDDMCFSGASQGA
EDSFSLRSVAVSLSEIMFKALVTAVYALVAAVVDIIFPFPVGHYLAFIMNSWLYAFYVFD
YRLSSQYLFDRRSRRHIRVTLSATLKFFEIQWAYFLGFGATHSIITAAIRSSWLASSWFS
IASVTSVLFGAHIVISVEATPAPRAPFHVPMFTPFFALCGMVLRMAVGAVR*
>TCRU_1803 Calcineurin-like phosphoesterase/Calcineurin-like
phosphoesterase superfamily domain containing protein, putative
MKVFLCILLLSLGFIWVCGVGEVLPLEAYENRTLYPVIPTYDRLPLLSIGVLSDIQYADE
EEKSRRHFRLSPGKVEHAVKEMNANRSHMDLVMHLGDTVNRDIARNLQVIDSILKQLQFP
FFQLLGNHDFLGLGEEHHDHVYRLLRMPARYYSLQVGEGGVFLLIVLDGTDLSVFATRAG
TARRAETNGMKHRYRHRKNMLDVNGGIGEEQMQWLRMQLEYASKQKMVVLVFCHFPMYPY
DDELNLWNDVEVVRLLSNYSCVAAVISGHTHRWEHEQLVVAHKNGEFTIHFVKFGGIVQS
PFTSWGFIEAYEKELHLHGLNFGRVFDYRLPIRGVPGGAVLRKTTRVLSSGPFIRRRRNV
RVISRCGGDTKWLRNIIHQPVPNKTVGRTENVSSLMWVECVIFLSLVVVIALLRLMCRKN
RGRQSKFVSVRSRGAVH*
>TCRU_1804 hypothetical protein, conserved
MYVVLFFVLLLSVLGVDAEEEDVRRNDEVVARLREELAEEGVEPLPEPPRELGVHAACVR
HGVGCPDEYVEFLESLQRTPHTFPETKPRNDAMSAERSGNMRRQTPLHGAGERRAYSIKG
EDAVIEVQLENMRRLRDVMRERLAKQQSPEQQWKCKEFLWISRGLKMVEAMHSVLLQKYW
NFVMNQLLPCVLLVFVTVWVFTGNRIPREHLRSLLADDPVPVFNEVIIRRKSPQSVAQLV
MERESHNFSSMVDIDDGSSSPSAVPNGFAKEKEDSMRAIINYRRDRWFATRFALLEAYHE
AHIAGVVLRLRLALSAAVLLMLCWTLFSLVLRASEVRRNSDGGIIQHLLVMLFPSWVTTS
FVLYIGWSWIGGMVAYAAWELVSTGEALVRTGERAAALHEKILERWCG*
>TCRU_1805 hypothetical protein, conserved
MTTVDQVVVYADVRDPQLLNQLTAAVSALCKDHTIHSPSTWNSADPLWFLNFWEKERDDV
WTYVNNKVPFLRRFECFVTDSKDQKDGVVAYALYYRQMPVLSLVTAVTGIKEKTEVNEDW
ITTALYCTVARMGCSSPSDAIAVFFTFPKSAGKVLALEGECRALVTEQCRLLRDYILRER
NIPVELVGAGGCDLRSSDLVGKGAATAEEEEEEEESALYASLIDDLLSNPEFLPLREVDP
QLHGALCALNRHHNLRLLRYRLQRGIHVILSRPMSFVLEELLRFCGPQAVPPSTMLEGVV
KSIQNFEHHWLELPRPEVILIRVTGDAATNGEAFAAPRWTACLWDEGQTPALDTLSSWHV
VTVGGAEGGHRQTLGRLHDGVRDAATSVLALAVMGNTYIEGGNS*
>TCRU_1806 Family of unknown function (DUF572), putative
MAKINATYSSGYYLAPDTDYRKLEEEQRRERKRKRQQEGDDNTGSSSASGAIRQTFAMPF
NIICLHCNRRIARGTHGYVNRRPTKEKFMGIRIWELEFRCIFCKGHIYLTTDYETAKLTG
GYHCSRNCRRGEGDFYGTNQINEAIRAKQAAEEAEKGSLDALERENEVMRQIQERERLIE
ELVESRAGTEAEHTRDELLQTIQARKRSKATCQREDDAKENVPFDEGEEEAYRLFEAQMH
DWQEEAERLPKGPSKSHAGDDDKVTAAIISRYTDHGSTRPNVFMGESDSDADTEAEKGVH
GDTTTPMEKSRGMPVAAGARTAAGRRKGSALASLLEDF*
>TCRU_1807 triacylglycerol lipase, putative
MEAVGENKTHVLAESLLAHEISPDAWQQAVRPEANSPAGQEEVSGGDGNQILFSAALASK
KPPGRLVAELQIETLSTTCGRIFLYSWYFMLLFALFLQSMVSLQWEMTNICGATQESLYK
LDKWSSPCVQSRAQRSDAINWSEFAVGGNLHDVPVDILQGSTIGEEEITGSVRNVTIIPN
DFGFRRQRTLRWVGDGQGMLLDLKLNRFVRVILSMASPRDQLPEGVNWEKYPLMIALEES
LLPSDDNESSSSDMQPKKSVFFYNTSTICYRSIPRCSSVVLPQTVAVATNESRITLTIFG
VDEALANVSAASAVGIFFQRAEYTMGTIVCRYVFLFFSILHLIRFIYRKRYSRTLYEQHW
VMFLHLALYLYLDPVFVAGIYETQGASVYHFFEFHVPTYFAALLSCFIYALITASMSWTI
SSEERARYRSLSESEALRETVGPCAVFASSPTLGTAGLAVGMPPVLTSSLQEEEERCPSP
REISRRASFSGLNEIRPIEVVDGAIPLWAKFSMANYLLAVFLLDLAQSYVGGWDWGTDMT
CVSFSCLYIRYTLYSLMLIFIIVCCVLLVQLHHSLGRRPYLETRPQQLACRVFIFVFTTA

LIYFVVQVILIAFLYPQIIGIVAYQPFTQLSPVIVSTFFVNHITFVYTTTVASRRVPIRP
ENPHWRRVVWSSEWYRWLNLHGGILYIFHNERQERYFNWVQNKGKNSTEPCCEANGTAVK
KKRSTREEVGEEANAGAAVLGSEGRTCEQRAAVAENKDGDFSSESSDTGASDNDRDYVDS
QTERPHPLRTPRTRGGVVEFLEKAERHLIDRPAILIDHLEGVLVDPLQSLFLRRRGRMPF
FNLETAIDCLNLSWEAYVATERQGETEGVADVVEFVDEPGAGPSQRSRHCGDSCCPLLGG
GDAGTRNNSDCDNDDGELHLDNGDHCDNHDIRDSVKGDSKRAESFAVFTGETPSPNSRMS
TEQYGYKQLAVVEVREVQVVITVMDTTLPCHHHKHPRLVIAFRGTDNLSNAREDLRFRRR
VWREVDPLRQWGIRQSAKVHTGFLRMWISLKETVLRTVKSYLSANPTEVYSIFCTGHSLG
GALASLCAYSLRRMLRLMNYPLLEVTVYTFGQPALGNRAFQKAYDKAVPRTFRVVNESDA
VSLFTVLGGCHVGIEVDIDRHGNYICKPMFIERWFRPTQGRGFAVANHTLFSYAQSLNAV
AMRNSGGSCKVRCLEPYVCAKGAVPDDTEPHLATPSPPSSSPA*
>TCRU_1808 lipase domain protein, putative
MSEHQHPSHHDHVESCAASSARDSAPVSFEGEPERDLWTTARGHEDHPSETFQQAWGENQ
ERVKSRVIPQAPRRNLYLSSGKTPPVEASKNGTYRSFNFSRHFSSEPVELQIEGLSPLVG
QTMLYMWYAVIIAAAIVQGMEFVSWGLVSICGEEVSDLRQYDKWNSPCVRTKLENATGDL
YTIRWEGGPYGAAFESATVRFPNIVMSLASPDDWGHETDSTRTYNIQAVIPHAANTDEIF
TFPLHVTCTRSSRRCAFVQLPPEAVLNETSSETRSLTLRGVPATLARVANESSVGIVFQR
YAYSLGIFIWRYIFLLISFLHLVRFIVYKKFTSTLYEQSWVVVLQLALFWYLNPLYALNY
SKNRPSLVLNSLEFQFPTWFVAVFVGFMLSAITASMPWTPPPHGQRHVKYNLLTLSGIKG
YLCHSASVYDPPLWTKCLVVVYILIIVALDIVGESLHVKEPLEAWGGGLPSTSYLLAALI
LLGCLVCCVLLIHLHRNMGKKSYLDSRPQQLASRVLILVFVTSMIYYTFHIIMFFLRYKK
IPGMSVTQPMIQLPAIMVSTILVNIMTLIYTTRNRSRLVPIHPRDKRWKMMVWPNTWYRW
LSRHGGSMYIFHTEAEELSFNWNQIGYRIRQHKAKVMRRKGRKGGTSHSRRAVAHGPGQN
SHHPHAEDEAPSEIPTFRSPQATPTVSVTTPYIETLESRDILNMDVTSTWFLPPQTCSPT
APPAIQQSFHSRLPSEERNGSESVDQNRVLRAERCHNIPNSPCPIDWRWEPQMSSSLPPI
APFRETGEPALFDAWNQPLGRQERASGLAESGNTVTSAEGRNVGEAEEEEEEEEEGAPNV
AARTISRAREKLDRIIRGAERTLISGPLQAFEQFETFLLDAAMRPFQDLEYLPFFNLETA
IDCFNLSWEAYGTSHRPEGRMEGRIVRCTPFSISRLCWLCCCCCLSPSSLDGLLRVPVED
SAEAPVGSETHPTENEDGPQIQRSPSGYNEPPPIDVERYGYRLCAVMEALDVQVVISALD
SPEAINNGKAPRIVIAFRGTMSMSNAWQDLRVRRVVWEEMMEGETRPFQHMCFGWKPTVH
VGFLSIWNAHREHVYRRLWEELSANPSTVYRVFCTGHSLGGALATLCAYSVCKMLRQKNY
PLLEVTVYTYGQPPLGNKAFQKAYNKAVPRTFRVVNESDAVGLIRMYGCHVGIEVDIDRH
GNYICKPTYMERLFRPMKGKGFAVENHLMGSYAASLNAIARDTGCPSVACLGETNGDADA
NPVAVPEQQGEPGPTG*
>TCRU_1809 hypothetical protein, conserved
MSNQRRTSPSPSSVATTRDTTNPTGQEILQTARLSLELTRADEVWSAWYKETEDQFVDFQ
SFGDNNEGGLNVTATAGSNIMSGSAQFSSSHRTLSARRVSGQFEEEVMKSWDEDWEDEDV
EDTFDAIMGQIGRYEASRAASVLK*
>TCRU_1810 hypothetical protein, conserved
MWARTILRLCSSKQPGTTAPAAVGSSWMTAAAWASLGSEFAGVSEDKFLRPRPDKFLTPR
ATTDVAVSEELLQSMVEENEERYKGIDVRDPSSMAVYEGDRPRWMTLGSQVRAVSEFISG
HLCHHISLPEWKALFDLQYAEMDLTYWLYVLHVHMVSRRATSIPIEKFNRRREVLEEILL
TMFESWAATSEDIMGRPPLNKIRFYIKDMYYVTAVNFEEALLHDGAGADLMLLGFLMKFC
PLPRPEDVPLYTYYSLVHYVRFHTALLDRIPDDLIAKGNFNFLSPTDPRIFERYDDVALD
RVIRSWTVDGANVGESSDEAGVK*
>TCRU_1811 carnitine O-palmitoyltransferase II, putative
MKRVLAKDLSANVLKLPRLPIPSIQQTAERYRTSILPLKPADTVKAHLVKLDTFLAGSAV
TLQNALVEANKAAEAEGGYPYSYIEPIWDNMYLSNRAPLPVNTNPAILIKKVKNAGETQP
SVAASIVYGIAKWVQNVLKEGVEVRDANMDLSPMLRQFGASLFPGEKADEFLITPFEKLR
HIIVLHDGHLYAVRVFDDQLVPLEQAVIQKCFEYILSITPDTDNTTPVSVLTAGNREVWA
GAYAELVRTPENAETLKKVHEGIVVVCLDSKKWEEDDGLKNGAMLHGNKEESENRWYDKH
QVIVSADGQVAFNFEHSGSDAVQWARWVGDVLQSIESKAVATTTAAAVDTTQVTSLVEPL
RVTFGKTFAGHIRSARTEALDLISNTALGQLQLPFGKTQLKQLGVSPDAFVQMCFHVAFH
KCRNKLAPTYEAATTSCFFHGRTETIRSATQEMLTLAEAVNKETKESTTPKESERAALLA

LIKAASDRHATLAKAASKGEGVDRHLTALNRLAVERNDNAALNFFNDYTYNSCCKWVLST
SNVSYPWVERFIFGPVTPNGYGLGYVVGENEVRIMLSAFTSSPSTNVEDMNAAIGVSATR
LYTVLAGSKL*
>TCRU_1812 hypothetical protein
MKEDLNSFSISSTTSPRHTLLDGDGNGSDVWTEQSSDDTSAAAMMDTLSRWLRSTSAIFM
GTQRLSCGRAAMAAAMSTVGDDHILHGVFTSGTPFLHYSLLQNASVEVDAQEKRHHTSCM
RHYYQQEMHLRGRNGDKLFCCFSGENNEAESIHVPYRMYSKGLRERKGFIPPWYAVHAKA
QLLLSLIDPLWRIITGLFFIPHAGKESGRESQYSQSSTQQTETHVRRLCRRRGGIEKRPL
RMSACASQTFSCMFTESQPTRRKIPDPSEEVCFIPEQAARALWARSHTTREEALDEAPPA
RGHVPTLTSAQLFLKHRSITTAPTEKKLEGVPQSDVAEESPCYPARSSTKSKRAVSGTCQ
REAKTEEHDTTHAATAGSASGARITSVHWLRGSAPPDASDSATSVIVLLLCPSVTQGNEN
GFMARRMGALCEALTNISKLRRQSSYNSFNRRRQRSHQTQLGIDPPDGLKTTTMTDATDP
HFASWHAAIPVVEMISWPSGQSSEHAPLPASSLCRERMNVTVEDIGHILSHLERLRAPSA
YSSRDTNDATMEVHRDVLDSVASMPSMSRSGSYLQSDGRAGPRNLYIVGVGWSNASSPLL
QYVMLYGAQSHIDGIICINQTASSNYALVPETVSNSQRKLRALGRDRQWSARSHKTPFHS
AFLLAPAGATRLLLLAARLHLTADRLLELQKKRQRRCQKIMKGDVLCAENDAAIWDYLRR
TVGNCASGDHESRTANDATFHPALSSTCHLSSRSLLSSAESPLDFLLKVEAQVDEEMNSW
WIDDVQAGNDDDDESTRGRSLSSRGDRAAPFSSSGGWHSLMGLLDWDKLLKNQQNVSAAT
RTGIGLSGRHTLPSSPHVTSNDTLNAEVVETSHPKNDERAEPAKTTAGNDDESVFGEEII
PPSHAPARRATPPFEMARGRPDLLLSAHLLSPLSQNFGDTPRNFVAPKRMRYELKARPVP
SQNSALEVLFYRSASSAHLVSLIHVPTLFVHANDDEMAPLSTLPMRELASNPYVFTVLTR
RGGYGVFLEGISTLYRLPRLTLQEREKAMHHNKSLDGDGDCRTSKTRHWLPQWIPLKRRN
VKLTSKMTVNDSCFVPPITSTNKKKRGMKNDMETVFIIENTTWLEQLIVEFVATTVLV*
>TCRU_1813 hypothetical protein, conserved
MFQLIHVTPRSAVLLVLLVVMCCAGGCFAASLTYSCSSDKAMRAHRAKSIAAVVRDVPFG
AEGASQTCTVIGRSGWAPIRINVSTRDPDGTRKYCSSERYCAMNSLVAIASVKVTFFYAE
KREVFVSLYFASTVR*
>TCRU_1814 hypothetical protein, conserved
MAEAASCLDLKASFIVSLPRETRHLFRCRVEDGTLVELTRLPMGYKAGPEILQIITSAIA
VVTTVVHRLWAAPPLVRIDVWIGNIRIAGSKSDVTLWEA*
>TCRU_1815 hypothetical protein, conserved
MATEWGDIGARLSELVRQRGEAQVQITFINGTEMSDETLTVAPSSTGLCLILGNISKQWE
DSGLTGHIANLRAFQVPNFENQLILPIMGAGQELGRMARAHHC*
>TCRU_1816 metacaspase MCA2
MGFDFGCLLNLCSTVLKRGGAPGSNNYMEIGLNLIKIAAPYVVQYLGPVQRPPRVDVEEA
FQQAEVTEGFKPWEAPTHVSGTFRALFIGINYYGTSAELSGCCNDVKQIIATLQRKRIPI
DEMSILVDERGFPGANGLPTRDNIVRYMAWLVKGAKPGDVLFMHYSGHGTQTRATSDTEE
KFDQCLAPVDFSTKGCILDNDIFRILLSGLPQGVRLTVVFDCCHSGSMLDLPYTFVGSRS
LRSSVAGHMQRIRKGNDCAGDVLMISGCADEQTSADVSNAATCETCTSGAGGAATQCLTY
TILKVSNLSYQDMLIATRDMLRRKGFTQVPQLSASKPINLQQKFSLMTTFEVDPAVAT*
>TCRU_1817 ribosomal RNA processing protein 6
MTGEFGTPGDFPRTKDIVNGAFGLVKEYGKLVSELPWDDYDYHMAFPGFRQQIKRHRESL
VEFMDKCCQLLPEKRRTAFKTGAGTLTEQQRSAVLEAVDSLLENVDGLLDNLKGKRLNAT
DQLSLTFGSELQQSSFAVDGGGCVLRPQLTFEHPVDNSPTPFCPVYYDEKGERHVGQPGI
HPFAELIKNMSMPEEQMIRRVETPYLPLAQCPLRFVDATVDLEDVVALLLKEKEIAVDLE
HHSFYSYQGFTCLMQISTRSEDILIDCLKLRSSMHLLAPVFLNPNILKVLHGAREDIRWL
QKDFGLYLVNFFDTGIALQTLHMPHGLAFAVDHFCQVKLDKKYQTADWRIRPIPAEMVTY
ARQDTHFLLYVYDRLKTLLLNSEGRASIGNLLVHVLNESRRLSLEIYEKPQLDPDASYKI
ALGRSLGGLSSVQMQVAREIFNWRDAIAREVDDSPPAVLHLSAVLAIATKLPTSANDFLK
CCTPVSMVVRTNVTRLVQIVKDSVGDDAIAGNSESDVKVEPSSRETGPMPRIVGVHCPMT
GTLPSIERRPAPVFLKEEAAALRSTEPSAWFNAMRNVAAVLRNKRLPTVALPGQEVVETL
MRAKTVEKQLRDALETREKYDTTRTLSDVKKYGVSRHGEDEEEIAVREEEDTAAENEDGG
EKDGKAEASDEDGSKRAGMKLEEKAVALRQVYGTGGANRKRARMEKNKRYSGS*
>TCRU_1818 hypothetical protein, conserved

MAQELLKEGTDGGGASTTALSTVTTSPAGKESVDLLASGTSPGGTQAVDGVSSSDGNQTV
DTADGGKTQEDEPHKTPADNANAADAKAPNSEGGLQDGFAVGPERTSGEDGETARGTDG*
>TCRU_1819 hypothetical protein, conserved
MMAMTMTGRVLLVCALCVLWCGAGGVCAEGQEAGTLSPPPGPVHGDQGGVLSGIGRMKPS
DTDGQVTVDGGVRDRAESKHLSSHVPLVDAKDVTPAGIGGAGNGAVEEVAPEENESLQNS
CDEDGEMSEGCVKVQGSNGFPGSGGGHVDRGRQQDDRNPPLPLSDQTSSEEINSPLPAEG
KPKNQRPVSRQLPVEEEQTDPVSIRPQKEAATLESEAVRNAANSHSQESAAELTNGTLPA
HARVTEAEDRSKNVFDTHKDAAEKQKKPAHTTGKSESPAASGDAHKATASTAAVDNEAAI
KKAKSPGGASSPISISDNSNNDTEQNNARDTSEEASHSTENATTHSAGIPRAPHTTAKTD
DTTTQEESNSDGGTVKMGEAAPQTAGTAQANDTATHDESDSSTAVSHTTSPLFLLLVACA
AAAAAVAA*
>TCRU_1820 Concanavalin A-like lectin/glucanases superfamily,
putative
MDGGSRGTVTLVDQRAKPSGAKFCVGRSPHSEIWHKECHAVHSEGVSPGEDKANALHLWV
TDNSRARLVGPISMDDDRERTVANRLLHSNNMLYLLHERRDQSTDGIFLSPLKEELDTIM
SVVNTWALMDIFFSGLSIPTDGLVASLSDLATSDTWIDMYHCVNATVTNAEMTPHGFRFT
GPGSKAVWPVNREELNRQYTFVDYNFSLVAMAVIERVPAGSVPLLGVKLEHSNRREFIGL
SCTAEGKWEVSLDGNTTTSSSTWEPGREYQVAIMLRDNRGSVYLDGVVVASSETMPTLET
RFIQLSEFYFGGDEGDRGSSVEVKNVLLYKRILTAAELRTIQWSASSSSGSGPDTSMRGS
IPWLLLCWGCGALRPFTECNRVGPLPRAGTHASLRAPSLDYLAYHVDAVDFILCFFFFCA
RQYIFFY*
>TCRU_1821 hypothetical protein, conserved
MHGRNGELGAVCHVAHAVHTCSNCAASCAAEEEGATVAFTMNVTAHKYAGPYELWRHQFS
PDGTIHPPARSGDADQTGICQLPPREGKDDTKSGGTTAPQVNAEPSRQHEDRRRSAAADG
PNTVDPSPASNTTTNTSETRPEFASNPNEVMVPNASKKRPSFANGPRILVASNASGIVSV
TYGGREHVAADNTDEVSERSATGHKSLVSENAVAGSGFVAGGDAGGLTQRIASGQESKPN
YENSPRSIPGAVRVYTTALPRSTTRN*
>TCRU_1822 hypothetical protein, conserved
MLPIPAIQAEDGLEARESEEGTTLGSVATTGPGSVSGGGHGTVVPGGAGDSGGGPNLKSP
SSVDTPVTHGGGAGSAGGLSSTSQGNSQGVLPEGPKPQHSSSLDEKAAVVAPAVVEPTKN
SAPAGGESVENDAQTRADRQPRLDESTKGKGNLEPTEARALPGNTIQTLQGGEKAVPDVS
KAKEPEPKTQQQTEPSEDKNDRHNTHAPTKSPDVPKGNNAKEGNNEDPAHTPVTPQSTSA
GSQQLAAVSPSTGTPTSNE*
>TCRU_1823 ubiquitin-conjugating enzyme E2, putative
MSADRSSRRKEMDFMKLMNGPRKVHPSNSVSEFWVEFNGPEGTAYEDGTWFIHVQLPADY
PFKSPCIGFSNSIYHPNVDEASGSVCLDVINQTWTPMYELQNVFDVFLPQLLRYPNASDP
LNSSAASKLLHDPIAYVSIVREHVSRHATREVALGSIPLEFRPLQEDAVRANEKTRTVLE
DSSPATSKGLTEAPNSCVDSEEEYEPESIDI*
>TCRU_1824 U3 small nucleolar ribonucleoprotein protein IMP3,
putative
MVRDTAQRASVVKAGLSKSAVKEQEVALRRSKRSKFTPASTHSKGNPIHRALKYHEKKLL
RKHDFMQYPQDNWHEPFCITKYHLEDREDYRRYLRLVGLTRQLLAHLRYLPSDSKIRIQI
TQQLIDKLFTMGLIKEKLGLAEVDKVGVEAFCRRRLSTVIRDLSMAANCKLAAELIHHGH
VRVGTTQVRDPAFLVSKGLEDLVTWMPGSKIKHHVDTFNVQRDDYE*
>TCRU_1825 hypothetical protein, conserved
MSVLGVSSYFLWTQKGKTVLPLVRGSGFLVLFQQLGGFYKYHVLSAAHVTCPVRYRQIFG
DTIGLRAIGERHISTKLLKPGNNGKVEASLDLEFAQSFLPNVDVSSLRCKDEKQINELCL
LPFELDVDPIKEGTELLLFGMEAVEERANPNDDGLHLKDACIKGECRAALVSLDYGTVFL
ASLESPLSLSMCGGPVVRKDNGRCVGVIVAQVLKNAPPSDPQVTPLYRDPWLDLSQNETI
NACGKLDVAFVPVREVYDALRRCEM*
>TCRU_1826 centrin, putative
MESIKAKCTAKNLVNEKPAQKTPLSISISEKHKAQLREAFDLFDSEGTGRIKALDVKVAL
CALGYDVSKEELTQLLKQVGGSIVGGVDFNEFYCVLLAKMMQRESRSEAIRAFRLIDVAE
KGYISVDDLRPIADSLEMDLTDDELAEMVLFAHPPVGFGKNENDIKEPLTVSEEEFLKLM

GRAHVY*
>TCRU_1827 AKAP7 2'5' RNA ligase-like domain containing protein, putative
MLRRTFIFVQKGERGLYFPINHRIVDRRIASGVTVEEADVQSRYRRELRTSFATGETRQT
IPPAWSACERPTHFLSLRLPVRNVLRTRVNEMYNQILFSHQQHAPLLVPLEKLHITLGVM
AISEREETERLASIYDCVSEVFSVIHPLQLRFRGLGTFGFGRVLFIRVVPEADFGILETA
VSKIRRRVGGELKVDMKGNPHDSYVPHVTIAKIRSKQQTQFGSKIPISMWVEYQHHDFGD
VTFSQVDICRMRGSKDGYYHTEGSVHLL*
>TCRU_1828 uncharacterized protein, PH0010 family/AmmeMemoRadiSam system protein A, putative
MVEATPDMAKYCFAVISSKLKNEAIPEAPQSISDDPCPVFVSLKTVDGALRGCIGTFAAE
PLRGQLKNYAIAASCEDSRFRPVELSELPSLSCTVYVLHSFEKAANWKDWVIGIHGIRIR
YKNYSATYLPSVMSEEGWNHTQTLNHLLRKAGYGGDVTESFLDKVEVTRYQESKASVDFL
SIYSGV*
>TCRU_1829 hypothetical protein, conserved
MHGSKSTVEMGFCSVHRRKRTRVDLIPLDGQTGQMRCKFHKECRQRAEIEMVICSLHNRR
RNMNQMKEVRKGIFECLPQFACRGQFNEAILPTTHANVSHPPHFLPPKGSRPQQVFGEAH
PALSASVTWTPTFNRPRNAGVVISSDWAQADRKTWCAKHGKLTSQCEFIKDCYHVCYDAS
TCLSTPIELPSDLLVKGCKELLCSKHNALRLVDFLEKTPDEKGYQCVVGHRCRGTLATRR
ETVPEQAMHPAREAVSSFFV*
>TCRU_1830 DnaJ domain containing protein, putative
MMRFRQVLTLSVRGSIRCVFQSKAFVSVSKELPLTPEGRNPYDVLEITVTNATSFDDVSK
QFRELVVINHPDRPGGSHERMSEVNAAYKIIKEHHNKLIQRLKEYEANTKGNENFQRHKH
ARARSDDDLGRSGGVYRRNAKAAEQSSGLRGTRSLREIESNWRRFQEETEHSVSSMCNRY
ELAVEKGRFFRKTSMLNEITVRERWLRKSFVKNVWEEVHELRGELLHRGARSAQQSQLAE
EMVAFASSTQRKLNEDFQRLTQLSFQSQSQFFLQRVLFALLLLVTFIKVWGWIVTGMFRN
SLTVRFRQGILSH*
>TCRU_1831 hypothetical protein, conserved
MACEGEEATLLAVALAGEETYWLCQPIHGKRLPPEWKNCTCQWLYIVDDVDIRGYIPWYK
GDLGVVLELGVNETSRIERFAVISDSVRARKIVVGEGGDTKTYFFVGNEERDALDAKFDD
LLRRETTVSMRKSSVVGIDGKPPEERRRIISTGASELRNGGRGGNNVVFSWDSFFIVEYG
NRVFYVCSAPHGNKYGFKVERIMRKGITIFLLFQDENAGPCRVSEVQEGERATVSSVLEG
ETGKVMALMTEGREVAVGKSRGDSADSSVFEPPSKAARRENSRMNCPNRRPLRAAGVNLW
VDSDGKEEGAMNLAELFPFSSEGGDSVRTTVFSDGTCVTYNFATASKFAMKKKIAATSTD
CDMVGLLENIQLAKEQFNAM*
>TCRU_1832 ATP-dependent DEAD/H RNA helicase, putative
MSFFGELTTGIKFDVKKHGAAMKRFTPENTKLDHPFAPGNSTPICDIMEVPLPNRTLDIF
GRSRSAGTSEGVGLIEGKAQEDIRNNATTTHTPVKSMSIKKKRNLWKKNELEVEGVDVPQ
PIEHFSELVRPPLSVDGRLVSNLFERNHRIPTPIQMQAIPCLINKRDVLACAPTGSGKTL
SFLIPMFHILKSPNTEAGVRGLIITPTMELAEQIEREAFFLMKGQRWKLVQHGQSTRNKD
IFITTPRRVFTMIEKKLIDLGNTQYLVFDEVDRLWDSATDNLQVVDSILTACTFEEKVVA
LFTATLSSKIEGIARTVMSPDPIRIIVSGRVSANKKISQELVFCGNELGKIVEIRNLLRE
GISPPVLIFVQSIERTKELYDEIRCQGLNIAVMNSKMNHEERDETIMNFRLGKLWVIITT
DLLSRGIDFKNIGTVINFDFPITVESYIHRIGRCGRAGKTGKAITFFTEDDKKRIPPIAR
VMKESGSAVEDWMLEIKSDRRTRRRLERSTPHRMIVSTKKRMLVADQRMERQLRRLEKEV
INAEDEELDGQHK*
>TCRU_1833 Domain of unknown function (DUF1903), putative
MDSNICDEEVTTWRKCIERNLGDHKLEKKCSAELALFDRCIASWRLNGAKEVKIKGESEG
EPPPQCAALSCLIGTCLRNTDYDFSRCKVPMQYFKHCVKSFYGSEYVV*
>TCRU_1834 hypothetical protein, conserved
MLLRNITRCLLPRALCTSYTSARVGTSPTGLASPGLLIEKRMPTLLIDMPKVENLTCGTG
QPAQRGRRKPLVWKRRHPWFDRQWSLDLKRAKKGPLTPQFPEGVPFTAIRSFQSFAGKSW
KINAGRRRKIRLSLKRGRRKLL*
>TCRU_1835 hypothetical protein, conserved

MDRPFPPYVLDVAIRDSLLGSAPGPDDMLNEFLHRHGPVARGTLRTMIHNSFANGSLPGS
WKMGDAISIPKPGKDPCRPESYRPITLLSVLLKLTEGMTHRRLSALLPHHPRQFRICISA
FYVGRGGTCCRQITRGLNEFGIVEHERPGDDAPTRRPRRHRSLVVLIDFPSAFGTVDHGK
LFGMLDRLPRLGLEPNAGCTTTCGAAMLGYALVNSTPGNNSPQLEHRKTASRDPNSPSAV
WMICCAAWAASILPPHSCTLTHSHLLPCVRTSMPVLRQCDRSCLLWPHGLPSTA*
>TCRU_1836 hypothetical protein, conserved
MAESASCLDVKASFSKVSLPRDTRHLFRCRVEDGMLVEPTRLPMGYKAGPEILQIIITSA
IARLTTVVRRLWAAPPLVRVNVWIDNIRIAGSKSNAILWEAQVLRNADSCHTTVGRTANR
APRSKPFLGCSLITHRAVSLSERFVRSVCAVPALNSLTIAEMEVKASRFLYAAAILGTRL
CDHHFFIKTLRRRLSALDRGSCRRHPRRI*
>TCRU_1837 hypothetical protein
MRKEINVRSFGTWVVLLICGVASVTAEDVAESGRSLLPVLYDAERVHALTCCELVERHWP
LLRSWDRCCASSVDATGDNKVHASFEELLHHFWDVPLPGCNSGGISSCNRKEHSLSDEFL
TVFLKERLCADLPATHTELARSVYSVFVVEDPLHEDDLITLMEEACKSVQHKGIILWRRS
LRARNVYEAQEAVCQRHCSAKRSFFDGDVYDL*
>TCRU_1838 hypothetical protein
MEAPGLERWVQNAETFTKEIDSVNNLFRSAERDIDTCEKERKEELCGHFKRVRAKLNVLE
EELTSQIESRSEEQRQSLRNSLAEVQATVEKVNRLIGEAAKPLLNYRAGNIDHRNAVALL
NLTQSVFDNWRAVNIPMYSVVIGWDRDIFNELEGALQPTLATESEGQVELPEAIDVNYLK
N*
>TCRU_1839 hypothetical protein, conserved
MRVASAWALRSAAEVRSACRLSLSSSLKWRVLVPVGFLAVPTHRKAEFPQRLGARHALGV
SHVRHDTNEAAAVLAAHRAVCRPSDRGGVGPAGGGGVSVLSPLRVCCAASAPHQQHVPGM
ASCSGRSSHPYCVGNSAAEAGQCSLFPTSEGGVRARQFPVASYGGAASLWLELSGTNNTF
GQCDDAGGPCVCELREIRRLLGFRITSFAPGHLAVAKDKLHPCARSSVLRARKKGTSTM*
>TCRU_1840 mitochondrial processing peptidase, beta subunit,
putative
MDNLYRCAFDSTSQGLGTPFYGTETGVARVTAEQLKSYRSSALHANRVVVVGSGAVDHTA
LERAAASNFGDLVAASTKSAGFPEARYVGGEYKLWNLRYKTVHIAWGFETCGAACEDSLP
LALACEIPGPFHRSQHELGQHAMHRVLKTFSSLDHSTPTNTHFNEKCIEIANPFLQQYKD
TGLCGMYIVGRPAQSGPGDAGAMIEVFQYTMAEWCRICQKMLHQHELEQAKVNLKSQLLF
NMDGSTNSAEDIGKQVLHYGRRIPLEEMYARIDDVTPTNVQEVLQHYFYSRKPVYSYLGY
CAYIPGYDWTQHWSYKYWY*
>TCRU_1841 hypothetical protein, conserved
MSVEAVKRQILNAISVLNIPLTRLPSEQRVVEACSAAKLLRNRVLREHARVEENIETIRQ
KSLYDNSDSDDENEEFSEFEKTGFTSDLKKLARQDFIFISEMMVMENVVIPLLLAHGMNF
SSTLLPQLLKLLTAMLLPVPFYSMERPRQMDFVCRLMERCGTDEFFTLLIQSVAPVAEKR
SQGVIQKEDVVLLEVVLKMVTLFFTGRKEAMSNVIGAFARNHGIELFLVIINQNYARIES
KRNSSNMEALREEVHANSEQKKCDDSVIVFLDDQNEEEPGIVVLDENVTLERHDKTAEFG
KELDDDDVVAQQVVVEEEVEEGETEGESLDSSDSSTEESESVSNQYNARIIQLLECDEQL
WKWNMHIVTAMAVILRSTSPVELAQLVFVSKFQQRSSHQIMGMFESGKGLRECKKELDRW
RQVARSCNGAISSSGLLVRGKSSCVQGEIGVIGCTSALLGLRSKDPLEKVQDIDLRKRGR
FIKGMYDDSNTAWILPLPTKIQLAQQCLSFICLGFEPLNMMVWNRLAHTITSFESIVRER
NELISGHKSSGEDIEIPTHIDKAVLESAQNALDYLSICASLLRYTREVIRLNQEENEAPS
DDFRRQWQSISSIISLDHICHGFELLRVFSSSPDFRRRFDVVIVVNYLAELFMTLKHLID
GDIVKDTAVVVAAHALASSVLYNEKNITLIFESISRFSAKVLPPSKAGMIVLLVYSVLTL
MERCSFGGRVLFPKCKKKKNEETVMYEAEDRGTAELLESNKRIRIDVLDTLEESNNGSGN
EGDAITKVKGNEIAIHSTPVPISLEDVHLYQGEIVPPDGEGRGNSSENASFTSSSHANVS
NLTSEREVSIGDYFKRLGTVKNMRLILTSLRHWRTNDVDLNNGLVYLIRAFILEGMGNIF
FNAPFLFVMRDILSHGEDFHNALHNVCDQVVYDFFNPSFAKLLDEKRKNRSDMSLPFFGG
AQSFLGFEVALRCVRSLFSLNSTDYSILEEKGLPHLTDFTVIPLKDETQSDKDVVHEEFS
SAQVSPVRKSRRKNKVSLPENRNGIEENEKVFSVDVPI*
>TCRU_1842 transport protein particle (TRAPP) component, putative
MSHNGLVSESVVAALSMELISYVVVRHTFREDSLIDNSGNEIELNIHKGLEGLGLLVGLR

LSERLLYRQPSFGEWTPPGVSQFVARQFWKTAFGKRIDRLMHMNDIYFCLIDKNFRWLQG
FPKMRGERIVSTTVLSPYGYSEETVAEDEGASPRTERLGSVPIHKDVLIYTVGILKGLIR
VMYPTGSIEIHANLNNENETQFVLDFRSPTA*
>TCRU_1843 hypothetical protein, conserved
MSKTQNGVGNDPQLQAVNAFTEAQSLLDIMLKNFYSVERQAADPFLLRVVIAGKKKESAG
RSVGSLLPELLNVAVNTSENPLCEDNEKSAKNFAVLVEYDDYFIELIEGFERHIINFLKL
LEKHCSQKEDIICYDVRLLYLVDDVPSPIASFFVHIDKVPAMSTEGEPQDSTDEEIADTI
AEDVNNLMKLLNMVADEPSTKRKLFLDNLRVTHPLLFPRKSMIKKYLESDIFFTLSEYID
VYGKISGFTRDIELNHPVVDSLKC*
>TCRU_1844 Concanavalin A-like lectin/glucanases superfamily/Trans-sialidase of Trypanosoma hydrophobic C-terminal, putative
MGVRLSREGEKKTFEPSYDKEKKWQVLCDVESPEDQSRTLATDTTHHVVILLRNGTQGSA
YVDGKRVGGDEQCKLETTGSNEISHFHIGGDGDEKGDSEIQDVSVTVTNVLLYNRPLNDS
EITALNAKLSIPKAEGTKTVEGTPPEEIKQATPEAETPSSHVGQQQTEQDPLRKSENAGS
GVLSTSAASTARNSPAAKESEDQSASGTYPEGHSNVDVDSSSEGGQTVDAEAGDTVQGDG
TQQPSVGTSATADTNAPTAETMAHDGTAVTAELGARSGASGETVGGTDGQKGEDIHAQDG
EVKAAALSSSLGNLSQGNNSDAGTVCGSGLLPLLLLLLGLWVIAAL*
>TCRU_1845 BNR repeat-like domain containing protein, putative
MMMTACDDGRRRVYESGDKGDSWTEALGTLSRVWVNKQGEGAKGVRSGFITAKIDGVDDK
RGVMLVTLPVYSEVTEKGDNGKGKSELHLWLTDNTHIVDIGPVSGDDAAASSLLYKSVES
GDNEELIALYEKKKGDEGKPSHSLWSVLLTAQLQRVKDVLATWKEVDDRVSKLCPSKSAA
ASASTDTACSPTVKITDGLVGFLSGNFSNDTWRDEYLGVNATVKKENGAEPAENGVTFKG
RGAWVEWPVGEQGENQL*
>TCRU_1846 proteasome complex subunit Rpn13 ubiquitin receptor, putative
MEARGVTIPPARVGHTARNSLAVKIPAGRMVLQDGIVKPLLGRGLLCLMRDSLMDELVLT
WVPVDGGEEHRFTLPQGKVQVSWVEKCKSGRVLLFDVDEGKQLLFFWMQSRSTDLAEKMM
RRLQYILERHRHYPVNAPNSQAIQMSTFRRILEEVREGAVALDVDLEALLASSKLIEALQ
EDPEFYRSRVVDYLPSSSATVTDTPVDVVSLVQDSQVQWAAVILSTMLRHQATSTYLSSL
FLDAAAPWGLGVVSFIMQIIQAFLE*
>TCRU_1847 hypothetical protein, conserved
MLRSTRLWRFRMKGGDMFVEYKIMSRDHRRSIRVEDAIVDPSVARTVVPLSWLEQLRSPS
LRLHTGYHMEEAVYVPPAYAAVDDKEGRRLSEKSTMTPNAILAGPVVLSITGQSVPVVLN
PYFVPDDTWGIRRNRDEWDLRLGMDAIEQCTLFSELRPGGLLYNKLPSSQNVTRHEPVLA
TLQRYGMKCGLAESPLVPRPWTRMRYMFIDELQRGPKLTEFVGHNPRNGTQWRFSQHSKY
FRIGVWRETIRRNDMNEGLHGHSSWQKSPQQAVPEVRLMAPYP*
>TCRU_1848 hypothetical protein, conserved
METSVEMQRQESGSPKELAGTRAQKAPEAAAEFDYRELGEAAVQEFEEYSSASGAAPGRR
GSIRFVLASIEEKENELRELEAREKAVESQLQVYSEVIGLRKELRLVKEETERVRRQLDS
INVFAEKYGNAVERDLDSAEEEAQRSHVQQVWTSVCRERPNESLGDPSSWTKVDLDNLSR
RVEAARGKLQAASNAAAKVYASQEDMINKNTEERDKAKAKLLESVGVEMAKLVETRSRLK
QLDDDQRYHFRRGTHVKQRHVSSLKEQDIMKGRVYKDESRQFGDVVLLEDECKDLTEWVN
GAKRELEDNKRSYAKEQSELEEQLQVMEDNGKEARELRAFFERENDQLKGLKQDLQHVLI
YVRMRNREAFT*
>TCRU_1849 hypothetical protein
MLSSVKNVICGAHIAPAHDGRSSVDVDAETGDVVLCDGLTGVSRFHFDSVTYHPFHAMAY
ESVLARRVAENFGGGCPYACDTPPCATHGAVIHGSLRIHSVDLFHALVLSAVGHALDVLA
ETSVGDVQLHYSMVCLYSWGVTDALNQQVVAHRHVGVYTHDTDECCRPQLQHNVVKSRED
AALDALLRRACAVPGAHSSIIFSLVAKSANASVSGVLTIALLPQLSQGNRFFQGGMQQLR
ELIAGHPVPHDGPCWLLQHLFPSKEIRSLAMITCITDDSRFYQENYNNCMYGCCPLVNGS
GSEGCFPVCEGSFNILNFRSSLPQFQPSKYRHPVKSIILPGTGLVESCEKKRGRIRRSTC
AIPVTVDDVGATQLPSSSSAETDAAKRLEAELEERTNDLQDHVASVVKGEVAARQVASEL
VSQAETVRSEIVSGERYLVELEGRVRDAKSREDELQQHVKSLEVEVEDLCEAKLIVESMM
RALMQEFESAVVSAESAEDTMLRHVDRAEEVLRLSEADAASREAAAMERIHGMIDSYHLM

APDDVFPLSYESVVSAVADLFDQFVRMKLELLLESLKLWVCRMRAEFRMDAL*
>TCRU_1850 mu-adaptin 4, putative
MFISQIFILSPRGDKLIFKDYRQDAPRNTDEIFFRKYKFWDGAQGHAPEGDCPPFFMEKH
VNFCYVKRRELLFVCTSVVNVSPSLTLEILLRIIKVIKDYLGVLSEEGIRRNFTLVYELL
DEMIDVGVSQELNTENLRPYIFNEVVRVSGSETPTGSSFLGRLRRGEFLDKTRRGDATAN
SILQASSDRKNEIFIDILERLNIVFNSAGQVVMSDVEGSIVLKSFLAGSPSLHVRFNEDL
VVGRGDANKERYASVVLDSVNFHEDADYSGFEGERSLSIRPPEGESTLMNYRLGGRGTPP
FRLVHSMELLTTHRAELMLQIRADIPVSTNGIGFSVIVPMPSICTAASVEFGLGATEQTY
EYKEEEKCVIWYIGKFLGGTEQLCKIRFSTSSPIKAATKRSVGPISMRFEIPQYSFSGLC
IRVLRLEERSSSYNPTRWIRNVTLANSYVFRTC*
>TCRU_1851 hypothetical protein, conserved
MRRVLLGTRVTGLPLWCGCRCSSGKTEGQTTRDDGHPSAAEASAPSAGEARGEYTIPTAD
ILRAVKQRDFNAFQSHAADIVQHTWKEEHSIPAVCLLFFLITWYWASSSRRRVVRSCKAT
EAKVKEEADQTLTLVNTLVRKWRQDVQRADQQLQLILDKNSELTKDIDRMTAALRQCYVN
VPATKGGPAKRVASE*
>TCRU_1852 hypothetical protein, conserved
MHVDSDEEAAAACTAAKWRAPQSATTTNLPNAALGNIVTKLRNLREELVSFDESVPDIVT
FRVDGRLFSVHREVLRKDPQSVLFLMAATHFRRYGDGNPMREVIEIPGKNSALVGMLMNL
LRGYRNPIPEAYREVCLAEARFYGLEHSWESRYPVAATGPFRPFICSDRLLSDALCAAAS
PFYSNGLHFITFGISRCETVGVGVGAEGACLSWMDGMGQCEDLALYWNDGRVTHNFGGLF
TEKTGCSFTSNALIKVELDCEENIVRWSLSGEQSVAVVRLPPKTSFAFAAVLVGPSEVRI
LRGE*
>TCRU_1853 peptidase, putative
MALQRDTVQIITPSALRARHFGFILQGGVKCIVIQDDNAKIPAAAMSIHAGQLNDPEFLP
GLAHFCEHMLFMGTAKYPREDEYNSYISKNGGHCNAWTEDGSTTYYFTVAHDALEGALER
FVEFFVAPSFNSSALSREVEAVHSEDEKNHNVDFWRIDELERSLFDPRHPRYRYGNGNIT
TLRDAPQARNVDIRAELLKFFDAHYVSEAACLAVYSALPPETVLHIVEAPLSKMRVGKPS
ALRFLPKEDPLYAADTRGLWLNVRTIKKIRKVRIVWPVKSSAALWPSMPSDYVSYVLGHE
CDCSVFGVLRREYMATAMVVGPSRVDDDNDLLCVEITLTMDGLRRIVEVIDILYQGIGLA
ARVDSAVYAQMKAEKKINFESSDIGENADHCAALASSANQASLQHCWAAGELLLEDDIPA
SEAYTAQLTPENGVVMLAWGDMPCDDAATPAPVANDEEGEDNEECEEEQGEEEEEEEEEE
KEEKDEAASGPLDALPEFAQLACNSATRFHMTKFSKCRIPDEIIAGWAASRCGPRHPALA
LPPANPFLSTEFTVYAGDGRHATVETFHTPYGVALVRKDAGHHKSFNTSIIWRCLSPCAY
ASPRNLYYMHVLRAILNDAVAEISYFGLLAALQNDIQLSAGGLVLSVTGPQHRIMEFFFA
IFEKLFTPAVLCGSVEMYRNYAEREMRALQSKRTKQPYTLAGDRFMKAARVVAYTFDEVI
EAAVSTSYEEYQAFVKEYLASGVYFDCFVAGNIPSSKDVQSRLIVGAQEILSRLHVSPAC
KEKFPKFCDTYAFPCVSSLPHAPLVDVLIYPPFDETNSNVAVLFDIYVGEETPLLRALCD
STHQLFSSSFFDTLRTRETLGYVVYSQSLRVEGTAHLQFVVQSAVEGVDGAYLLSRIIAF
LAAVEEKLETVCAEAEVKTVVNGLIERRRKLPDSVEKDSLDLLHRHLHPVDFAHKELEIE
ELKQLDSAKVKEFFRTHVANSSNSRRALAVVVHNAASVKNDVLDGADGVRRTVTLPPRRP
GKGSVEEVDNESTESVLQLPDFVVAPMSITVQKHPSPEDFHRHFPIVPGKSF*
>TCRU_1854 E1-like ubiquitin-activating enzyme, putative
MDSLTCTRGEFDEMLEDLSKRGLGWRACRRSDSFGRTLKWLEGPSIIQRDAPCGQAEQLL
VSYFITYSECYSQPQLYFAPEHPMSPQEMCTWMGKVCYGAVERQEYDAPVVSMNFCEEIQ
MPVWGLHPCDATQLMMNTSANGVRSVNLLELFLRSVGHFVGVDERLLPLHVAGQQK*
>TCRU_1855 hypothetical protein
METAAEHCLAALIDSVGSGGPIFRREAIGAIAKLSENGTSAVLRFCVTWLNEHGANSTPV
IQRCGVVCAMATIVGGVGDEELSTREAIALLQCGIREMTVNSDLTSELSQEALRLVVGLC
VMFPAEGCRELLSSLGDNTLPHFYVLLAISHLAEKVPERILPYVKEFLTRLLPPLGNARQ
DNHRMLLSRACGSLAEALLKMRFDARSPLEEYNDLMYSTLNFFLGDYSRSNERRVRTLVV
YALGCVFGVCGDEKRMELAGKVAPLVIYGVRREKGHDVLFPLRGLAGFFSYVTDGVRSSL
RPFVEGTLQAIMQTLEHLGAEDVETKEEIFAVIMAISDSFVSEVLAYLSAFLEAKKELHL
RCAAVYILRMLVLQSRNQKLFLQPFVNDIVATVKLALGSHEDSMVRRAVVECILTICSSE
ADFASAVGYSDMLSYLIRCASLHDEVDEGLIAVRDISRNGLMLVASSKGALDSQLWPFIF

EHMREYPAKPFLLCAFSSICNVITQLASRISHNSSCFYVDFSRQVNVPSPGMLVSVFLSQ
SMLIGFYRVEELLTILEAALSVAPLIDDPYAQSVEEGGGAAMPITELWSTFIPSLQQLLT
REPARELWEMEVERLVDKTMKVKQSEEWAMHLTTECLSLLQAYQQDARMFRSALIIVGIG
CSRTASRDLIASAVETAVNASNHDDPEHVIGLAKCLGYVAKKHVDTTLETLCRLAKGAEK
RGFFHSRDKTKRVFVEGSRSVAAVGFGYACKCMEIAALPSRLDTVVFPSLLTLLREGHTS
QCQISLFEGCRLVGTGIARLDSYFFKCRDEFILSFVRFMQMTSKKKTKEETHPTAFAALE
SIVSCTLLPPLGDNGTAALIEFVKISVTECHMVNAEKDVNVTNLFVQILRHRDCRYAMRM
LEELAPLTVLENDGARCLAVKLVVTVLAACRASRESGADGAAEQPHTLASMGYILGRLVP
RMMDRCPSVRMEAANGIVSGIQTARLFMENPQPMEEEVTEEILKLGKRAHAFVDDGTLSS
EKEAVSVVKSMCTILAQQLQQREDTTALIKALLFNSIFDTQKYAATGACITMHGLIRGCG
GVLDASDVREVFDGLIAALQRDNLPEQVRSGVQTSIRNLTRHHVAPCFVALLRSQLPHSE
AVINSFGAIANDTTLGELLVQHILDFVLNSPTTIAAAPTAKKPSDLQVVSPIVLSAVCAV
GWVAQTDRGASVCRNHRASLYMAIVLHLSVCHRMNDHDAVVMSGTALKHVTCGTVDDVTV
VRFDRYGWKNFFEKQKYLLAIAEAVRYWCREGLGDSNEMDRGKAADADGRFPMEPAEPTQ
LTNELAKFMLPYCSHVSPTHRKAAACVCSELIAYALGDEELVRSLVTALLSMTGCDEEAE
TRETIIGGMANILVHPYASVHGFVPPILSATLACMEQQHTSLAAKSMDIVALFAEVLEDK
SFINGSFVTIATKLRSFFEHDDAQLRAKSFDCLRRFYSLAHRGILERASVNQNVFPYVIP
MLIHLGEHVMTVSTAAKAALHECLTFISVFQHEAGEQIVRMLSKPHMHTDRETDIDDIYD
DVASTWVSHFHGMTREYVLQAVTFTRSPNEALRQSAARILGAFIRCIPRMDLSRCGVEQV
SASLVELVGTGEKSEIVRVAAARAIGCFASI*
>TCRU_1856 hypothetical protein, conserved
MENFHWDEMGIATRDDPDPLWNGYFSRRCGSCGSATSFGDPRGSQCSMRLGSRFSCRSSV
RGEGHTIDETRRKQRVIRYMQEERERAARSKLEREKHEDWMSIRALERGERMQYMTDEER
TAFFQREAMEAERERAAAEEAARAALGRRYSARNPMPRAAKERRGVMEAAIAVSNSHEAS
IAVNRVPVSYEVEHLQKKMAEIQRQEAELQFKLRRAQDDAAAARADAENARQMQRSAEEN
VLREVKRANDESALRKLAESRAESLEKRVEEMRKGVTEMEEEVQRMKREADKNMSMFREN
EKQLASLREQLGDVREKNERLQDEHKQLRVEKNAVATRAKNPPPQSVEKPKPKAKKQQQQ
STRTNASGGKFGPAGSNFSRRPLEAETNSSVDAWGEIQPLREQLERQSSALKRFSDENDE
LKTKLRDETEMRRKLEELSAQSLHPKDDGSLRRQLEQAEKEAERFRAEARAARQELDDAR
VNYGREITSLNAWCARLDAQCKRQMSELQDRAPRDELNTEPTRRKKDMQGGVGTAKAIAD
ASYASDSASSKKIQAELSRMHTRMEESEDQKQNAIAKAESLGQKRGSPPLNEQHDKLCTQ
SHKDALFKINKEVGEGKDAVRGAIAAEGNKVELVQGAKGLLPEKMTPTGAKPLRAQVVED
ASVGNGISSAVTNGLPNDAEMEEACKRTALLQQEMEGEGRAHVGDGSEDAARNARADKGT
TAAVSEKVEKKKKAKCGC*
>TCRU_1857 Archaic Translocase of outer membrane 14 kDa subunit
MSPISERFSEFAEKYRLVQAYRSSLGAAMTAWGWVQERMWPVYSTGLILSALFMLASANE
KQILADHLYGSDVVLQKEREKTVEEARKLFREESVQGVKQVVWMLPSGEFRGEYKERYGN
V*
>TCRU_1858 Archaic Translocase of outer membrane 14 kDa subunit
MSPISERFSEFAEKYRLVQAYRSSLGAAMTAWGWVQERMWPVYSTGLILSALFMLASANE
KQILADHLYGSDVVLQKEREKTVEEARKLFREESVQGVKQVVWMLPSGEFRGEYKERYGN
V*
>TCRU_1859 hypothetical protein, conserved
MLKVLHTKAAAEPILLATTCHNMNLFAIVTRSLVVVYRSTTLTVVTTLPLKMKKNETMVS
ACWSPSGRLLAIGLQCGELYLLDVESGDLIRRFVPRSDIAFAIKDADAKTLSGIIDTSAG
NEINNNEDKDCIDGDKEDDPLRQPPILPISVDGAIVACTWASVAPSTSALHSHNELCLPL
STTVSSPIFEELEREDDIPVMLLLDQRGGITFLPGGMQEVYVVVVNLQWALDPCRMRVDA
FFVAAAPPLSNVKKGESNRCSSNAVMCGENTGSHSAYLVVRERDGETSSRASSQVVRVHL
SDTIARVADREAVAICSIVEYCRMGKVSYQFVRKRWESLIRAVRKDLFLPQNALLLRDAL
MEDIARPSSAELSVYFRGLDLDALVKDTEELSRQFTHLVLQVSNVAYRCYDLALHLSQAH
SGDRQRQCLLMDVIGGLRQRCSSFLRQMRLEVDREKDLVHWVVQRAAPPGSKFFTSHPPL
RAVRHPSLLRTLHRIARGESTAFSLVDENITRGEEDLLYIVKGCLLGGREAVARVVPLLD
DINVESILQRQCEVLVPHVAAESGAECVQLHTVTVTKSRPCQPLMVSYTFEPRGDAEVFF
LAKRGSVSVEIEPLQLAEAGAAFLWSGVVDDGGRCVLLWERAAPSFVGAENMLVVAVVDE

NGAVEVAADAEDEANGEPNDTGIEAGVSRHAVLEIKGISTRHLRASVSRARGFGVFYANE
RFVVVDFYGG*
>TCRU_1860 hypothetical protein, conserved
MKQWEKEEENPQRCRRRIRNESPVGVSDVRRPLPIPLTTALLSSKLRRELQRIWDVTRAP
GKLEHEDHADDSHTEEEVVVDVPPFKVGTMEMYRRFPKQYDIFMRRHDCQAVHEYLDSIL
LSLQQTSATPSDGGNCLAVEGKMCFVRVADFGCGTGRIASMVSRHPAVGAVYCYDIATPM
LRECLINVVCSVAESRQEIRGVCICPSGAACVDGNNDNNNNNALGTTAAAAAEVCVGNDG
VTENSDGVMLLCARPVSFGDVQQGFLDTHTPCHLVVCAWSLSYVMRAQWGDDRWHAAIDA
TVAALIGLLDTSGPAALVIIETLGTDTTKPRRNNTLLQRLESEHGFERRWVRTDYNFCDV
AEAVRLTRFFFGDSMARRMAEEERTALPECTGIWTLWRR*
>TCRU_1861 Flagellar Member 3
MAEALNIPFPDGTLEVYEGLDGRFYLTNSNSTLTPLFVDDTIMKRQEMLRVCECVTDSHE
DTVAVLDKDEVLDAIVESCMDSGTSTITDCLKIVCEHLHGFGVNMCLLKQVIQKIATSDG
YSAAAVAKVEEILFVEMLARSVKQEFYLEVQGKRIAYDDEVLSATLSKHMAEAFTTVDVF
QSRFLEVVAKKYGVVDEDNDIIRGLTTVRLSRKAEIVARICALLGVTIEKTGEKKSNMIW
HVNVNARVLPQLIDPKKVRILAEKYRTIMTQDSHRHAFCIPMRCKVACWYGNYDEALDLV
HATAVAQRNRYGESAIVSIYSRRNVCEVCFATMQKNCIAEGRSLFPGVMRGFEELTCCVT
QGRRHIEYGFWILRIALVYQHDDLVTYRSCVEEAVHHFYQAIGKLPAYLTSEHGSWLHLQ
PYKGLLQCKRLLPSCSVNAKELVERSIELSTIGWASDFFMLYLWDLSLQLEAEGRYEDAI
RVLLTAITVSKKKPTVTLDLPSLLMDGAHIYRSWDPEKYSEHCLTLLREAAEKASELFGP
HSKQYAVLLNNKGAVEIELNRLIHAGETLEKAGIVFAEADVPNDDPDYIAYLENLVYLEQ
RLAARPVVQRGFLRRYPFLATRSEDFPFSDVRPLEDEVFVDLAHKRARMAGGTSDAKNLE
KRMKERIWELLRFIQDGDVLKRYPFLPAVINGVHTASLRLDDDVLFEEFARLLHASKDAN
TRRELESRLREYVVKKAEDTALQRAYMDRMDEEFNEAHEGYVNLMHPIPWLYVLEDERFN
ALTDQMQKCVNTQGNTARIQAIQEKMAQRVAEMEGEMFLWRGELAEWLSPSKLYSVHTKD
LTEDMQLRDLLRVRQFPDSCEGVSVDTINALIRDKLEALWKRATIIRSCLAVDDEELHRE
FPFLVEMPHHQRLSTLMLYEDPTVANLVMRLRTEGNDSHLQTEMMGAIKAIAARRRDSKE
LKKWYYDIDSVMSGSLSLENVPKVRDDYYVHLWQQRNIVQAQQGEKSPLMVKLMHQMQHR
IVQMNSWMRKVSANGFRRRLRLEAKYPFVERRHEGYTLEVLDIEEDEEFMSIVEQRRRLL
TSPDASSSELERLGRHANKRVRWIASQRVKLSEELSEKYPFIPNRLEGVETLTICLEENE
TFAEKAAYYNRLRDAGSNDNEMRCRRLLREMESLALAVAREIGVRNWRESIESEDLHERY
PFLPEEPVRGILLGDVRPVQQPAFRELSNKLDEQRRDPTRNAAAIRTTEEQMTALVVRLA
EERAEATERAHEQYPFLPRRVLGVRLGDIPLQEDELFAAATTERVEDKEPLLKSRAQELV
VCQNLNEAQLADNDDAVLSQHPYFVYTSRKCFPLRHLPLDDDVLFAERLREYENLMQEPS
VDEDVAAIARFRLVLRADSLALHEIEKIERIKQTFVALHPLSLEDLRHLQDHRAFQTYTG
DGAQNPSPVLLEDAKRIIMGSRKDRAARVQELDAVRKSYPTFGRSIDPEMLKDPVIAKLV
SDLEEKLDNAEGNESSVIELEEEINRQIIRHKGQIKGEIIDLEGRHVPFKEQAVDYYVFD
MEDDIVDDKYYQGLMEEYSAFENGQLPEDTSTLRRLAKQIEIRKNQIKNDNKKMAKYHKK
IEQRSATRFSFLNSHVHAIPITELHLEEDTVMLQLEQQKASQRLIDAESPTSIEKKMIER
AEAIACGSDGEGGAGGGGGTCGGISISGAECQGSPSA*
>TCRU_1862 Glutathione S-transferase, N-terminal domain/Glutathione
S-transferase, C-terminal domain containing protein, putative
MSLTLWSGVNAGNTRTHKLLAAATLANVAVTPKTCEYGRENSTAECCRNCSPCGRYPVLQ
TEEGCVFESNAILHHTACIERSGGFLYGLTPLEGSQVDMWLDFSATELDAAPAPFVQHAF
RGGQRLANAMDRVHEVLRALEAWLETRTFLVDECVAVAFAPQWHYRLSGAEGEALTRKYR
NAYRLYNTVVQQPKTVEVLPPSRLCWTR*
>TCRU_1863 retrotransposon hot spot protein, RHS3, putative
MWALDSRVEGDTLLEDYEYVYNARWSHFVEFPGGEGTGMEVKKGEPPQPWTYRAVGDSLE
KDDGVEQSGAARPRLMVLTSDRGWPYSWYRRRNKFTRVCHVNCEVDRVWRIVKGDLTERF
SPHGRTDFKPNWRLLIGTPGIGKTMGAGSYLLYQLLHCDAKKLHLVVYSFGGNTTYLFDK
TIKTVTRYLGRGASKEFLNGLWDLKMKGYIIYDVARQGTPPEEFFASYWCGMIVVSSPKV
SNYDNWMKQKGAARIIMNCPADVDVKAMCAWMKRDETAEKQAEYWKMVKERMDNVGPILR
YIFDANEFIAHSAAIEDALDGINSRDGEKQFTHGGVRLWDSENPSQKLVRVVRGRGEVGA
EVFLNAPISVCLGRRIPHCFWKRDE*

>TCRU_1864 nucleoside 2-deoxyribosyltransferase
MSGVYIAGPAVFDADTGAACQDRVRTALWVHGVVPLIPAGNVAPGAGEMRVKKVEMVRRR
DAVIADLCLFCLDGPGCGTAFEVGHAAALGMTVPTFTLDWRSMREKYGGACDASVMSVED
FGLSFSLVLCDGAEAFDSFDAALHHFLRHSSEWRGCDCGGCVRSWS*
>TCRU_1865 hypothetical protein, conserved
MPRGGRHAGGAHTAPYGIQGRPRNSPDYYCFGNCGVTTVVYRLRAAPPLVRVDVWIGNIR
IAGSKSDATLWEDRESGATHYTFLGVQLDGESGATHYTFLGVQFDHTRQAVSLSAKFVRS
VRAMPALNSLTIAGVEVMASRFYYAAATLCTRLCDYYFFIKAVRRRLSALNRGIVLETSP
ANLPPSEAGLGERLRQMIENHRRRIIKPTEKSSAALITDASLHGW*
>TCRU_1866 Mucin-like glycoprotein, putative
MMTCRLLCALLVLALCCCPSVCVADTGLAESSGTVTSRAIGTPELTLQGSNPSELPAKEN
RPSVELGIGDGAGQNATGTLRADGNGDTSGPQSSTRHIGDQSNNKAVVTADATKKSTTIE
SPENTRKLEANTNVTTLLSPAPGVTPGNSRPEAADDSATDPVPRDIVPEAPHLTDPGAKV
NTEYNTVQKTGTNGTTGQESGTRTSTEQSNKETEYAAPTTTTKTKTKATTTTTTTTTAPE
TPSTTTTEAPTTTTTSAPSRLREIDGSLSSSAWVCAPLVLAASALAYTALG*
>TCRU_1867 Leishmanolysin, putative
MQMSCFYSSSTIPLILLLLLLCVRLCSGLVEHRCTFDKRTRDGAPLPMVRELPRKGQGAF
QAYTASEPEWSPLRIGFFTEDLKNKSRYCTAEGDMRPDYEGNTVKCEKKHILTEEKRALL
INKLLPDSIKLHSDRLLVQPTELFVLPPSLVGACTLFLIPDGHFAEGITGADFILYVAAG
PTHDVNVAWGVPCALRSGGRPAVGALNFGPQHISSRQDLSRAVAHEIAHALGFSVQLFAE
SKMVTKLSKIRGKSNVLVVSSEKTREVTRKHFNCDRAPGMELEDEGGAGTAQSHWERRNA
KDEIMAGVAGIGYYSAMTMAAFEDLGYYRANWGMEEVMGWGRNTGCDFLEEKCVNNGTTK
YPDMFCVDGSFLFQCTSDHLALGVCGLFHFGRDLHPVYRYFKYPFMGGSPNELTDYCPVI
MPSGEGACTNTLNNFTGCRVGPNSRCVESDLLRFNSVTIGAICVEVSCEHKGVASIRYVG
DDTWYPCPEGHRLKPGPPFTRGHIICPRYTDICTTLASAVLKPTLDNNTFQNLLTPVEGP
VQRSLSKPLEMKKTDARKKHFGVDAGSVAVTPSLHWLLLCSLLGALLGI*
>TCRU_1868 cell cycle sequence binding phosphoprotein (RBP33), putative
MSTGDVNGAGDILDGFFSHPSLPFNPSVGEGGDSLTKNEPRQPLPQQNSEIFQPHTPNNT
SSEATTVTSTSSSSQSAVKSIATCSAGENTGSYSHAGSNVVGTHTASPISSTPPNQGYMN
VPSMYTAALAQTTHQQNNMQQHPTHLFSGAVDSPTTATATQPEAGHTSDGSPSAFMQGKE
VMLERLMACLGESKGMAALQLQLAQVTAPSSPAVAALKQVIQHLEEDIEKLLHLRDMPTT
SAFMNGAAVYQKMSTDVPKESLTTSEVQVPNHQYQQQQTDVSVAPLFVTNAQVAATATFS
DRGIWSLSESRNATIKNGNMDLVDMSGSTAGAASMMQQQPYQHLEVITPQSGQMTSYLGD
TGASVTQAYPMLNSALLASCDDAPAMYATSGETGYNFIDANIGSLGFGDAIRGPPRRNMD
EQHPVEGEIVPKFLAFVEFKRHRVRKYECNIDISPGLYVMVDGDRGKDCGLLVQTIKTLP
DGSTSFYCMDGTHINADKIKLEKGRVLGVATAEEIDYLHHTMQQAERVALETCRKVCDDM
GIKINLQDCEYQFDMEKVSFYFNSEHSVDFRPLVRELYRLFRVRIWMENTNPHVKNTMPA
ADLGGIGNSNINEEITAMSEGVASVNYSLSQHQHKHGGGAGLTAIGGNNMTGGRRRGHRR
SLQKNTQGKDGKK*
>TCRU_1869 hypothetical protein, conserved
MAMMAGRVLLVCALCVLWCGAGGVYAKNGDTNALGGCMASEVLGRKTMDTPKGCDKTASK
LPFRSPLPITALQADVSEEEENGLNSIVSSGTGGSSGGGVDGGKVEFLIRGLPVQAVAVK
LEMILQRLLLLVLHLRFLLKLPRPMKVYGPPEVRHPLRAGNLKQQLSSQEKSKRLQAPHC
PA*
>TCRU_1870 G10 protein, putative
MNFIDAGLVRRWKLPGYEQLCCTACCVPGTASAAARTVNKHTNRHSHGRREGNGGEERAE
GTCICRVPAEQRRVKRLEGCTVCGCTGCGSGEKRKRDPVKEQAKDAPAAVVMRVEEEPKS
*
>TCRU_1871 dynein light chain, putative
MEDGEALDLQDALKPEVKERPVRLIKIERHDGTKAFLNSVIEMAQVVLDNMPPDACYKDI
AIKLKTKLDEVEKGTWHVIVGSHFGANVTNDSETLVNFKIDEMHILLFRSGPPERPMTLN
DEGS*
>TCRU_1872 protein tyrosine phosphatase, putative

MNDSNCFPFTKLSVQAQYERVQREFSLLLRQEDPRSISFATSLKNRHKNRYLDILANEAT
LYPQVTDAPWASTPYYINGNLIDLDLPHKFVACQAPVLQGIPDFLAMLYEKKISLVIMVT
KLEEGGFVKADRYWPEERGSGSIAVSGNCGLTISEDPGKAYEVEDELKITRRYLILQRAD
EPPHKFTQVQYTGWPDHGIPQSATSLEALLTNVKNSPTTVPVVVHCSAGIGRTGTLIGAY
AALTHLERGTLTDTTVYDVVSAMRRQRFGMVQRMEQYFVIYLTLMCRLGVDIKALVGLLN
SRTTAAASTAAAGGGGEGKKRSSQR*
>TCRU_1873 hypothetical protein, conserved
MADNSPGRLSSASLVDPAIEASSLLQPSNARQSRHSGRSSSVETKTFVVVVSRRDKTASW
GFLTYNKVFPLLVGEVTSDVAALLRKGDEIVEINKVAPKNYDHAIYLFQRARLHLELRIR
RTVSQEKQRHLARNYSMGTSDVVPHADAAGEAMKNSTSPPPTVNKSATDATPPPSHETPH
VGLEVGASRYPAKMSLEKILRGGRKRRFGWGNADVVDGTLSRSPEHPSVAFNEDIEKVWR
RVFSRKRARGCNIPAKGHCSEVSEVLPRPARQAIYDRRSCPFSAEEGFTSEFHDAVQHVL
TANALTHGREGHSHGLAKGTVTISPGSQHRDVETMVESIRSKEQLRDEEVAKYNAAKGVA
QCHKELEAVHNAMLRVAECHCLMREERRCNEVEKYRQTESAARTFLANYMEAVSSSKPAA
GTKRRSRRGYSRDGIHDAQKYELRSPPSSAAETACDGSATSPPSYQLVSSNVFSTTERVR
HAGGFSSRLLRLPTPPPTPPPPPPPPPPPPPPPPLSSSGG*
>TCRU_1874 hypothetical protein, conserved
MSRVETSMEAVVYEDAPDASARLKRLKVAMLAFAGVPATIPAVTAILVPVELLAAPSSSS
GLRPATLARETAESRLRQVCADRVQIRRWLQHELGITMDVWVVVTVLPRDINELSLLANW
QMDRHRSPLRQADAVSLSLQEVVLLWGDGRCQAPMLEKFLLPHQSLLLDVTCCIGESSRQ
LVNPAVFSPIRAKQIAKGRTCVVAFYSAWLPLSFSTRGLAQLQQQFRDIARHVLLHMKLP
LSCPYGGEVGALQQNLVNVTLTRRNRIAEEGSEQRLAAALLATRSLKDIYSLVGVLFGNP
LANYYGEAMAFCTSVQRRRRAAGFFCYPASVVLAKPAGATGAMRHVDDVSPAEPHRKRGR
KETATEAVDEGLTWCEDTGV*
>TCRU_1875 Plasma-membrane choline transporter, putative
MDQDLKSSKYKAYNLEVLPEAELYSQHTENLHGVAVNGIPLYPNETPGNPRLSRFVVQGC
QDVWAAVIFIAMLIMSVAWGSVNLATYEPIPLNSTGHRTIAIDASNVKVSTFVLLFVIFA
VSIFGACGSLMLIGTFPRQIIFIANVMTAVLTLAGAVVAFISGVIFAGFLLVVACFFQVL
WLYLVRDRIMFSAELLKASSEVLSKYKAVFLSAFFMTATSFVYMIFWSAMCLPAADRTNA
GYDNAGDGFLITFFMLLLFWVSEVVANVIHVTTAGVTATWYFAGEGRMPKNPTLASFKRA
VTTSFGSICFGSLIVAIIRLLRWIMESTRDNQNEFIHCVTSCLLSCLESILEYFNTYAFV
HVAVYGCGYIEAAKRTWQLCKQCFFAAYFNDALIGPTLTILSLGVSALIGIVVGLVYAST
TIGAFSFLGALIVHILFFSVVESAVTTLFVCFAEVPEGLQYSSPELYAALHHADQNGTNN
NAASP*
>TCRU_C Terminus (BRCT) domain containing protein, putative BRC.1
MLRGHVFVAAPDVPQDVRSLVAACGGRVVSSVEASVTLGLVVCGGEARLDCAAQLRSLRI
PILRSSWVRASVAAGRMLPMKHPHTEYDPALFESLRFTTTMLPVHLKRSLVAAIQFFGGT
YLPDLTSATDILLHGEEGRMAGTAGSLKWRCAWEWAIPCVHFTWLQECIASGQLGVLPPP
SSPPAAATTRNGEENESSCSSSHEAAGGNYASCTLFIERQKRPRIDVINVASDAGGNQPP
QWFL*
>TCRU_1877 vacuolar protein sorting-associated protein 45, putative
MSNAAAPNFFTIVSEYVDHMLPADNTMKVLLVDEATLNFMSMACSQTLLLKKGVFLVSRV
DEHRQRKVMKGMRCIVFIRPQMSSVEAVAEELRAAKYESYAIHFSNAASPELLDCLARAD
VESLVTRVSEVFCDFEAHNADAFVSVVSPRALLPAFLSAAAVQRVAEGIAATFLALRRRP
HVRFHQNNAFARRVALELGDILSKNAELYNYKNKDSLLLILDRSSDVLTPLLTPWTYQAM
LHEYIGMQHNRLQFSDATVNEEYVFSQQDDPFFAANMFANWGDLCNNVKTYVDKCKATLN
IDRSTATMDEIKEFMQRLPQTKSLTGSVTKHATVVSHLSSIIKQRGLLDVSLLEQDMVAS
SNAGDHWNRLQSFAAKRHSGGVEATDLFRLCLIYHLRYEKPGQNSRVFTLLEEIDPHKAS
CLRKLDQYSGERSTDELFGATGVMASIVKTFVDVGNIYTQHEPVLKRTLLQLFSGRLPID
QYPYLTPPPTSAANSSQTQQQLSFRPKEVAAFICGGFTYEEAALVNAINAGTAFTGSVAN
NLHQGGVRASIGGTAVLNSEMFLNMLSTHP*
>TCRU_1878 Spc97 / Spc98 family, putative
MLAVSFLAVPAATSVNRPAVGDGNDALSLVELLVELSGSLTSGNFGKYRRGEYGRISAET
DGILRTASVPPSRDPTVIRRKRRDVINSFFDSSTASTSTPVFSTSPAVFRSRLRQLFQN

AKQNIREVTETHAALLELLCSAVAPDIDLKEAAEGAATLSEASEAELMLYIILRTSIAAA
PTWQKPPTIPRCWLSPSITEMVERSPTLQRTMRWSCDPDPVIEAALGVDHTRYNRSAFEP
YMACQLPALLTYHTDYAGVATTAGGGVERKPYPNESLKMDAAHGVFSYWCAGEKEEVRCL
FSRKRPINVDTEGELDSPPHMTHHDSSVVHAPSSPGATVKKLFFSIFKEEYPTDSRFCSL
LFVALHRGIIGQQDPYLLLRLEGGGMGCPPQQSLMITPSKLSFSQFSETVEISSFTCPVS
IGVSPEFLVAATGNHGDEEMRRRSGNQTALNEGGELEAKDTLCARGVVAMLQWSCQSGTL
FLRLHQLCDVSERPEWRAALGQYGKSAIEALRWLLLSLQRLVADMSDRAGGPHGVSFREL
LVAQQRLRYVAEDIAVLSDVFLVHAGANWEASLVLGQISSATLLTSLYRHYAKRTANNQW
RGQHPMVAGASDGKVGRPDVIVELFLAVLDPLQCMLGAWLRAGELKDPYDEFFILPSHGT
TACGFLVDTSAQRLPEFISPETAEKILHAGVSLRVLRAAARHVTLCSTKEKRRLRFQAED
IDAAGLYQEKVEDAEDINLLLREFMESLLGYRSSSTFLEMPEVSVLTRNGSLAYWRAFYA
ACNDVLLSVGDEMEVEQKNNVTFGSIQLARVVPVNLFDGDDTTRSICTEERCGVGGGGKA
KSLVTSLSRLSRTTTAWHEAVSEELAQVAHSTSKEEAAVKTKERAALCSEYERRVLRRRA
QRQLQQWKAVRLSLNMERAAALSGCVDSLLELYARMLSQTPAEEGEEKEDKMEDGRRTPI
GKFVKPLAQIPPSAQAAEKSNATSEGKATLQLDGQLLLGNEEAPFETSIVSVFRVTPPPL
RRGSGSVWMGNMSLSLQSEQKSFTRCEREPQESAHAPEMYVVADINDEEYLMERAGPSVS
QASSREFMVAQYDAAESIVRDCFILSEAYQQACKEAAIKLLQVEGDRSCRVDGDGACMSD
YWWTNPTVDEGTRFAHKGIFSALTRNSHALHKLTEEQAKSLLHCSSYYLTLSSYTASYLT
HKALQVMLLGPYGTLYRLTQQFLDVCLMQSGRVADRMTGIWQRLTKTAIEESKFVFSNFM
AALNGAFVEEWESCVLHGRDTLQVQLALMVPEDTSAGTDVFPAENDEEDGNGAGGSMLFQ
DTAFRKRTLYSAYEHVAEEETQREGDGDGTKGRTGDDAIPLPENPFDFISRLIIVYSTPC
EGFWLLPKNAVAIYGDLFATLLFWSSVTQLVTRVWQVGINSDVAEAFFFCNFSRAVLSAV
AQHMWFLIAGFAREYRHTLRFESGVLYSYHQLESFTTDHAAFLERCRFAAMLTPAFARAR
RQVYGMVRQLEEVEQCLLCAARENAADTSVFRSVSPGGNEKKSGAAAAHITSGAKGRNGK
KRTRASEGGNERDGGGELHGSNRRKEFSPSKTSKSSPSDVSPKVKQKPGRNSGARQLPPT
EEARRRSVRNNVSRRIRSFAGLTEIFVEHLTAVRDNLAEDAESSVVPQDNEPEKHNSARG
TCRTNTCTAALSSLIRTLEMTQAAAHDKF*
>TCRU_1879 developmentally regulated GTP-binding protein, putative
MSNLQKINDIEAEIARTQKNKATMAHLCQLKARLAQLKRDLIASESKKSGGKGEGFDVQR
TGDARIGFIGFPSVGKSTLLTKLTSTHSEVAAYEFTTLTCVPGVVNYRGAKLQMLDLPGI
IEGAKDGKGRGRQVIAVARTCSLILIVLDIAKPLQHKLIIERELDGFGIRLNKQPPNIDI
RKKDRGGISISSTCPLTQLDNETIKAILTEYRMSNADVMFRGDYAADDLIDVIEGNRIYI
PCLYVLNKIDQISIEELDIVARIPHNCPISAHHGWNLDGLLESVWDHLNFIRLYTKPKGQ
MPDYDEPVILKRTPQPTVERFCLRLHRQMMKSFKHAWVWGSSVKHQPQRVGKDHLLEDED
VVQVVKKV*
>TCRU_1880 Protein-tyrosine phosphatase, putative
MSREQRKIATAFLEQLRQLSKRHPGNNRDGFDEEMDELRAVDHRVRSNVDEFYTASLATS
VAKNRFTEVRANEGTRVRLRSLVEENQESYINANYVDGRALFNVPFTYIATQAPLKNTIF
DFWRMVFENDVVFVVMLCGEVEDGKAKSEMYWPKENGVLDIGILQISSIGERRLSDLVFR
SFILHNANGEEHQVYHMQYIGWPDQGIPDTSAPLMEIIQTMGKSELSVQTPIVVHCSGGI
GRTGVFIALHVALAQFQLERKDIDIKRIVHILKLSRTGMVQRKDQYVFLYYSVQREMDRM
IMSAEKGVSLLLELLRRREPTASRQTAPRPVTNTTPQSPVFSSQAAWTTTKVPQQFATPIP
TQAYPHHQSQSRELGPMPRRNFPYASPHSFSAITEGEQRLLEDYLKRQTPSSQQLVALPR
DPVLKLGKETTTKKVPNMSVLATQKESHTGIPNSTTNTEALEEQLQHWQWRNREMRFSTD
RVERREREAATRQAPEAGTKNESAPSKRPSEEKPSKMEALVEPGPTPEEVQNPFSSTRGA
STFSERSAPPVSIIITPQEAGTARNSDPTFHAGSGNSPTNGSYSVGNDSEQHGHSVATPE
KATALTAATAPPATTETKSATAPGDPAEESEASRFDRLGEALADFETLGPFPRRPRTHII
AKEEFDRL*
>TCRU_1881 hypothetical protein, conserved
MAMMAGRVLLVCALCVLWCGAGGADASNLENNAVGGCMASWVLNANKSYTPSGCDKTALT
LPLRSVLSITAVAASTDPLVTGTAENKSDLAPSSGADLGGAGSPPGRGGEGGGGRDDGAG
GPGGVGGASGSQARGSKASGGGGGGSNGGSKARVDGSLKSPASDVKSPSPVLGDGGSSPG
APAGSVGSDGSKGSILDSFSSGGRSQPDVGSPGGDASLSAPVAGGKSPPSITDESDALEK
TGAAASTKGGEPQEAGEQSGANRARNELPELSAPAESTPEAQEDSPSKQEKPESEAAGRA

ATGEEKRRSDAEAQGPSTVMSTASASNGRRRTTNSGGSSGRRKPNGTNRLANFDRRKDKN
ATVIKQKGA*
>TCRU_1882 Concanavalin A-like lectin/glucanases superfamily/Protein of unknown function (DUF3676)/Trans-sialidase of Trypanosoma hydrophobic C-terminal, putative
MMLGRPRENDDAAASSLLIKDKNKELISLYENKKSDGSYNLVAVRLTEKLERVKEVVKTW
KDLDSALKSCRSVSSGTVDVRKKGMCNGPVPTDGLVGFLSGNFSENTWKDEYLGMNATLT
NGEPRVRNGLTFKGSGAGAVWPVGDMGQTVPYYFVNNEFTLVATVSIHEVPQSGSIHFIG
VRMNDTDSTVLFVLSYTHDRKWLAIPEDPANVEDFDDWEPNETYQVVLQMDDEKWTVFVN
RYEIRQKRYDESLFNSHRISHFYIGGDSKNQSATGGHVTVTNVMLYNEKLLSDNLYDLQD
SKVTIPSLGAEKQPTELVANTATLVVPESKSEEITASHEEMNEDDPEKQEEESVDDPVPA
APPSTVDAGSFVSEPAIAAESAENSLSDDNAQFHQGKTAQQATPNEENKSMQRGSDVQTQ
YSQSKKLTEFNDVEKSSESYDTQLPEEEGKADYRSGESTSSVAASLSTETVAAPVDGEHQ
VQQSVELATENDDVRSTGTGTTGAKESLSLEAGDGSSERTMNAGSSLNPSKSDAEPTSAE
DTDNISRTEGTEVSSEDGKEVPQTVDTAPGNTNTAPGETEISSESNATTPSDTGILLENG
HLGELAAMYQTGDSTVHGCVSRVLLLLLLGLWGTAALC*
>TCRU_1883 retrotransposon hot spot protein, RHS3, putative
MEVREGKPKQSWTYGAVGDTLEKNDAVQQSGAAPPVLMVLTSDEGWPYSWHTIQDLPKDV
FVNCEVDRVWQIVKGDVTAWSSTHGGTEFKFERRVLIGTPGIGKSVAAGSYLLYRLLHCD
AEKLQVVVHCFGGGDAYVSDKTTRAVTRCSDEDMCISESRSLREHGRNVYIICDVAKEGT
PPLRHFAPTSGWGMVLV*
>TCRU_1884 retrotransposon hot spot protein, RHS3, putative
MLASKDNILPIFFGWFALKSSTESAFAKAIATKLTELQPPPSSKPRSSVPTLNPQGYPTE
AAAITELELFDCPQDLNYRLPYLTTFPNSPLLDGFFFVESNPMALVGLRMTAVGGHHTTA
STVRQFTERLAAYFNGWEGLSRDMSWEITYVQHADE*
>TCRU_1885 hypothetical protein, conserved
MGGGSSKPELKVGEKGAIASVAAPPPPEKTLSSGKKKSDEFAKYREFRDQLLSFSLGESA
FDYAERLFEKNPSSPEVMALLAETTVLYDKSKNKVFRNHWCDRLDLLQRGVDVSRKCINE
NPDYGPCYRTYVLCATREAEALYFIKSFLGLGLLENYNAIMKRGEKGMELLPSDSDIPNA
LGSLCGRCAYRWYSPTRPYGIWCGVPGWKEMWAKSIEFHKKAVENEPKNLEYACRLAQAY
YQSGDLQNARRWYAKVRDEMPPEDLKDERWQSLAHTELSTAFAKTSWNVPFA*
>TCRU_1886 mitochondrial intermediate peptidase, putative
MLRRVIVGGVCRRAPPLIAGRMVAARYAATIPTSTVASTTISSSPTNRDDASTRAVVSEV
PNLSFLASKTVLAETVEQTIEVCEALLNEIPKVKGAKEKHDLIDSTSNVLCLLLDPCEFV
RQIHPDEEYKKHAAYAFQKGYEYMSKVNSRRELYDVIVELDSPEGRKQLDDEEIKNVVQL
KRDMESNGIHLSDALREKVTEMNIEKEDLAMRFLTEQGAKNPFATLRYLLRCRYELAQLL
GFESYAEQQLRGTMLGNQQSVWHFLCGIAHKYRSAAEKELNLIRQNVGEVRNRQNITDDT
RARVAHSLRRDAEPAKAEEYFSVANCIRGIQCLCSEVFGLRLEEVPFEREEVFNPDAKKF
YVYDENRAFLGIIVLDMYASDMKYCQAGHLTLQLGCKPHQEALARVGLKLPERQYPVVVL
TCNVGALTPAQRLPNGRFDDESTLMHPSEVTTVFHEFGHAMHTIFGQTKVQNLAGTRSSI
DFVETFSQLFEHFLTSHEFLRLWAHRINTREPISFDIVMKRNAAADMFKHLDMLDQVVLS
AVDQTLHGPQPYTVYFPHGQNGHLGKRTLGDSSDYGRGTYNLAKILIDVCTPLSIVTPTE
SGVLGTLSFEHLSGYPAGYYGYLYSLSVARRIWSKKFEKDPLNRAAGKELVQEVMRHGAA
CNPVEVIEKYLGDNLDEIDIWA*
>TCRU_1887 hypothetical protein, conserved
MRRSFCLLAAAAATTAAASAASPTNTKMSTLYKVLTGELHFKNKVPVKECNIVHQFGEDW
ESELSAYAKALSAQQKTVLERQMVRLKITRYTVAELSDYCGDGPAHLDASAREANIEQGV
AFLKEKGIEAFEKYVAEEAVNANWKPADVKKFIDAVKSKAK*
>TCRU_1888 chaperone protein DNAj, putative
MPPKLPLYEVLGVSQDAAEEEISRVYRRMALQYHPDRNPNGEAKFKDIANAYSVLGDSEK
RRVYDATGVIPGGAAETDNEATMAERSAEMKERVQVFYATYAGSPEETEDVISCYKKCKG
NFRRMAREELLFDNKKQGEIQRLMELVRSLVESGRLNPTEAWKVTNTAAVLKQIERSLTR
ERKEAKDALDAMGLSAKEGEGGLHALQALMKRDQEAEWSKMMSNLESKYVKPTSATKGVE
QNGKRNKRKAEATKTVSAPPQKKSRR*

>TCRU_1889 amino acid transporter, putative
MKTNGNDSVCEGLLRESRGLRQGEFTGRQSLTSRISMNVARRLSVIGVAEESRTATVSGA
AINTLCNVIGAGVLSLPLAICEASVVGALVLMFYTAILSGFAVFAVVAACDATQRFSFTE
AVAFVIFPSLTFEEFCTMYSFVPFEGNVEPEKGTMELDNLRRCHKEDEILRRKRRRILMV
LVESVIFLNNYGGLVIYSRVIADSIPPVVKNFLHGSGFFVTRIFWLLTSGVIFFCLSCVR
NMDELKWTSILGFLTIFYIVLTVVARYFTSCHTPPYPNVDPADRGEINWYHVGTGIFRAI
STYGVAFGYHYNVPYFYRELKDRKPSTMMKTVYVAFPIIVFCYATTGVFGYLTFGNLVTA
KTSGGDIVRNYPDDDPVVNVGRLGLFFHFACVYPILSVCARRGFHRILMNALTWCSQTEL
IEPGRDPEQVGRREREYTAVDVTGNMSIIGPDRDVGSPEDTTRLAIVLEAFFLVTTTVAI
AAAVSGISVVIDFFGALFGVFLMLTCPGLIAWFLFSRAQLLRTSPYRFARLLMALSVLLI
FTGIVFSVVGVAFIIKNYTT*
>TCRU_1890 hypothetical protein, conserved
MAMMMAGRVLLVCALCVLWCGVGCGFADKVVEAAAGVVASNRTDEENLILNWYVLTQGEC
ANESTTGGKLNVTAERICMHKVMKEVCDAFYNKTSGVTHDRDAKFICTYYATIPEEPVEP
PTPQGPPPDSAVSNTTIDEGTPKNAPESDVAGRGEGKDDDKEHGNTKQKAVETAAVESIT
RSTDSDVSTEVSHTTSHLLLLLVVVACAAAAAVVAA*
>TCRU_1891 hypothetical protein, conserved
MNCGGVNFPDGTIHSPARSGDADRTGIRQLPPPREGKDDTKSGGTTAPQVNAEPSRQHED
WRQSAAADAPNTVDPSPASNTATNTSETRPEFATNPIDVMFPNASTKRPSFANGPIILVA
SNASGIMSVNYGGREHVAADNTDEVSERNATGHKSPFLRKRRCGQWICCWG*
>TCRU_1892 hypothetical protein, conserved
MDAAAYRRLQHRMESRGRAASSHSTCDVCGYHCSCPPHVRREGHGDDDRLRGAIGDGRRP
TRGAASSVAPNAQAWLLQRVQGVEVDITNGGIDHRRHVSCCCSCCIGYHHLFHARHGCSG
SVCGRVEEREKGERGDSLKESYARRSCSPKSSAGVSHSPMAAAHTHTKSVETDLGDAALA
GNARDAQRGLTFRPSRSSLTVSSFIRLAESVPSHFSSVGENKSNEGGGCQPPQDSTPPHE
YGDVSKGRTEVRQPLRRGTPSLLPVALSAGQTAARQSLQSIPHPASVASPQKIPSPKRQG
TEDISADTSKVTIDASAVTHHAHTTGIAPIVHMSGFTLGSLPNVSVSFSMPQPSSSHVVN
SPPSDCVPVVAHQVSFLESSASPQTSTPVPAATPVVQPTPNTLSEAAGAAMTSAGTAQTS
TPVPAATPVVQPTPNTLSEAAGAAMTSAGTAQTSTPVPAATPVVQPTPNTLSEAAGAAMT
SAGTAQTSTSVPAATPVVQPALVAGTPEPTPADVMNALREKLLLEKADAYVRNLEQQRLE
EKLAYEQEEQRASKTYGQILELLVRLRQDDQEMLEAMYENILEPVEAPFSSPASAVQRLP
TPLQRRTEHTILSQPNLSPFPAEVMTAYKMDAGKKAQSAPQPKPKAPVQAELPPPLPPPP
LVYTAPPSPRVSTATAPSIPELRHELPEAPPFASKGVPWGSAEGTMKAWVDKHFGSSLDP
QHRALLQHVLLSREAEIRRVAAAESQCKKLEAQILGAKNFVPPLAVEETARANLSQWQQE
RDRELNQALSLIYAKEEELMAVRKAQEEQKAKLNELTLLMQTHSVQSEVLGAQEEKEELR
RWKMRCDAIEQQHRFSVQRLEELQAYIEQLRNSAQQAVMNVVSSSAAARPLPSDMRGAQL
SSVNASRPTCCLETASQHSTVPLAKPSSAPRYFSPPRMTDGAFVAAAPQRESPAADASNA
PQPCLDAAPPPTAGLDRAAALKQLREEMDLECARFANETQLWHEYLKHQEERRLQLHGR*
>TCRU_1893 hypothetical protein, conserved
MTHTNTAVTPTGKKADGMSVEEDISRIVEGAAQRQELEQGITAFLRSNARHKVADVLLAL
SAYTFGGLMHVRGQGTALTSLSMWTSGTTFLLNMQVRKVFEIEDSAKRRQVAAGMSLEEK
NVFQLETVASWLWMLSSMQQFKLHKRMKYCGYSSWMGLGCCMYFTLRHTYCMIMLE*
>TCRU_1894 CRAL/TRIO domain containing protein, putative
MVEGAKNPAADGTEGWRLYDESAEKIGSSREQQVLLELKKILGWTPSLTLDASVDAFLLR
YLSAVHLSVSEAVALLKQRRSYEMSLLSLHITAPVLEVLRSGTVSVIGRDVLGRPVLYVR
AHRFIQFGLDAQDMQRFLSVVMEYMLSYCTRAPSSPSHGSRNGETLPSLHQQQIILLVNE
EVSSWHASQAALSNAHAFRSIMKKYYPRFIGLLLIFEASWDVRQGIKAAFGDNPGEEKRV
VQMVTRGNIQKYIDRYVLTQELGGSNTSVESPDNFADAVLSHWFNVRSYLSLEDPKTRPL
WQLPPSFVNINDWNQHLQRREMAATLNPDGNGLPQAQQFLSRCTSSQFDSITCTSLRRST
VLRRGLDEDEGFCSALSEVEDMFPSFNSPRLTPREVSLDMDGNNSSAAVLLRELHKEREL
RIAAEQQLRKVRLGITLDLSMATEVERILTHIHRELNVLVANVVTRARDAAANGSPPTLL
QLLDLTLLAIEMAVHKAQRVPAMKFAVPVTQSPENDSPCCCVM*
>TCRU_1895 Zinc finger, C3HC4 type (RING finger) containing protein,
putative

MFSAIVNYLILLAFVYLALALIGNIVTLLELWRIRRSIHQKRPPIIIPFLVFTTRPVYEV
ALTHMANRAVVLHGLPKVNGLTLPLQLHPVPSAKVLVKLRSTTTARQNDFLLPEVRPEND
TDANEASFNTIPSSSFVHSGGGGSAHVARHSRGCAMETAALPSASRSHQMMGSFGNGGYH
QYRELSLSDLGGGYHKQEGNSGVGRLMARSQLSLQFRVETAARIEAPNLSRIQLGNASIE
AFGRDSSSAFCGALSRSDTMIFEDPKISLVAADDGSEGADLPVKLRCACIFGADDVDIIR
AVTVGTTRTFGRGSFMYHCYYTAEDEENCARERARLLDWHNRQRLGARFQFGDHTPCMPL
SGAFYEDSVTQTNPPSRRRRHLMLVNSMTSVGECLPSRQGSLGSFSTLAGRTRSMCCSTK
SNAFTETFVDVSETACFASGSRTREERGKNEKEGFEMSKYVPASIMNDAAQDLTESNDDC
MFVTAVSPSTSTLPSSTSPPTWTDSSTSDACNEGDSVETGKMMPHTEPQAMNTDEEEEAG
DVADVDRAPLPAENAVTFSDPVNVPVTNVDDAPGTNRNANKAATSKKGHCRRPREFVDIF
LTEPQQGPIIPRAYEGSILITPDALQSDANAIITTTTTSTSAPASLQVDAVDLSLAAFQN
CFKNGPTPASGNEVNAGEGGGGGGEGKDAGGEKAGKKCEKKGEKKGRDSRHHEKKQNDAE
LIPRHPLTFALMLYRRTPQEGRNSGREKRQITSHTRQQTSDCFVCVYSTANAMASLLPKS
ASAMPGGGTPRNSPPGSSLRLNDPNECREIGETYPLPGTEEVQTSLRRLQPGSAKPEKIT
ALLEAMRSKIGAVMSVALQYVRIGNDLYAVTEVSDRTFVQYREERVLMGRCGINDFSSGG
HTNEREDITASERSVWPSKYRMRAASYSLASSPQTGVRHQLVRMCWLCMRTEANVITLPC
AHFALCLGCVETLTHCCICQRSIHATIVLNEDTDQSEFSDTRDQ*
>TCRU_1896 Mu-adaptin 1, putative
MASVFYILDSKGAPLICRSYRGDIQQNPPEVFQRRVLDEEEFRVTPVFEEQGHTYCFIRV
NDVFFLMVSKINICPLQQIAFMHACTKVFEGYFTRVSEETVVDNFVIVYELLDEICDFGL
PQYTEAKVLKEYITQEGLISYLMPEEKLTVKALPAAVTGVGGGTPWRMPGKYKYRRNEVF
LDVVESVSLLASPEGETLSSEVVGQLKMRVRLSGMPTLKLGLNDKAVLGMTRRQGRLIEM
ADVKFHQCVRLDQFESDRIITFIPPDGEFDLMTYRTSKKITPLVHVDCACVSMSSTQVEM
HITARTTFRRNTTADFIDILIPIPGDADKPEAKCSLGKLRYAPESSVLIWSLRNTGGGKQ
FSCLCKFHLPSVRSSDPKALQKAPIQVKFEIPFLTASGFQVRYLKVMERSNYEALPWVRY
VTQSGDYQIRTY*
>TCRU_1897 Protein kinase domain containing protein, putative
MSGTSDEEEDAERSPPIRPITMTPLHSDLWPTVPRSARRTLFGDTESSSSSSSGGGGGGV
TRPIMAVVDTDNSTVDLSEPSPPHVEAEGAEEEAEVETHRRVPWGASDDGKRVPSTAATM
SQWEQRYKTLLPLNWGRQLEVIVMLARDTVDGCLVTTRRYRYCCETDASPAEFLESGDKS
CIPFTAAWKRRIYGLLSLRHANVSPVTDVLIDEERQELLLVHPYVDGMQPLVSALQRHPH
MIHRELTPSMLMRILQEVVDGLLFLHQHGVVHGALSPWTVLLDAEGHASVTGFAVSEVRP
CPRGVVPAGGATHPFYIAPEAKHPSFRWRHVTVAGDAFAVGALTFAMTWERQDAVYVALR
HSMVGVMHGDPKQRMSLHELALLVSELASQNTLNETASRELGGRRLSASTAGDPALLPRG
NEHRSPSLPSAASVVGILQRDPSPVLYRVTGLEKWKRTTNTTAATERRITQRKRSHHSPA
RQVLHRWRVVSTAVLFCVIVVRMSRERRQALAARLFAPARKLQLKKLRFTHSNASPFASV
LSTPRPRKPTTVEKRRSARGGSKDK*
>TCRU_1898 hypothetical protein, conserved
MARVGTTAAAVARCTRRIRAPLDHLIMSPSDPDQVVTAIRTTDAAIVTWYMPLAELQRVL
HRRYEAEQFGELTSPSTTSVSSSTGQQSPNDGTADASDGAGGGTVPLGFLTLVAGRNYPL
KQTWLERCFDPAPHPTLSLRVAVMDRIHWQRTAWMANSICRSICWGRLPQKIFSVNLDWK
NDVCIDATFDAAADRYTAPYELRVPGLGFHLTLQDTGTTLLDPTSRIVDGFLDNESALHR
LSLAREVNMQGLGGSVYRQMLWSTPSLPNNAEVVRFDPGTLFKRCFGVDPPVAKPVAAWL
VQEVEKTLLYRMEGEVEDENDPNSATTYGDRSLTERVQKKAMNRYNGFRDEVRGKVYSDL
DGRELPR*
>TCRU_1899 protein kinase, putative
MTTYRKQIVGKYELGKVLASGYFDCRTRLCTHIVTGAQYVVRIYNKSVLAEAQWMWDRTR
DAINVMRTLPKHENLIETVECFETQTSLYILMQLFAPLQVTKLYTSETASGHRVMIPIQQ
TKHYYAQVVRGLLHMHEHNVVHLGLAPDHVMVNDRDQVKIGYLVSCAYFPKGEMCHDIRG
TTHTVAPEVLRNEGYDPYLADAWSMGVLLYFMLHHGRYPHDGANTTKNIMYNRIRPPDPT
LSPDARDLLARLLHPTPSRRMRVENIMSHPFFAANQSDTDNAEGRGAGRFVVSSPETYCT
LDLATGEDTVNIQIPYGLSRQEEAAYIIQHTYRAYRSRKWQQLSQRKSSVAKDPAAPRHN
KIGRRPSFMSGVANPLLSEPPEVEDVKGFVSHRTDRRQSRGGAIAAPPDMPRFDDASVGS
GSASEIIRDVNDSVAFVLENTVAVPSSAATPALENAVKSKYSPSYSRTHNSTLSSITSPR

RGDAGRTKTASFAPRLEHDARTDEKNVENVVSLMSNIKIDATRPCPLCHRLPMQRLAGRE
PYANTPFVYQKGECIPRVLD*
>TCRU_1900 ChaC-like protein, putative
MDVTNIAKEDTPAELPPTFLFGYGSIMWKQDFAYTRSYPSCISGYRRVFYQGSTDHRGTP
GKPGRVVTLLPSDAPDSWVAGIAYELPSDPTALAAILAQLDHRERGGYRRVEVVLNDLHT
REPLALPQGALCLCYMATEENSEYLGAATEENIAAQILESSGDSGPNSEYLFNLAAELRK
IQAVDEHVFSIEAAARRILEVSGEQEN*
>TCRU_1901 WD domain, G-beta repeat, putative
MMTPRVASEDCGEELRIAILRVRLLLALHGRVSGDDNPGQESAMHAGSVTPSPPRQRTVW
AHSDSLHSVDLEEGSSDRCQLDARRTREQTHEDHSQEVKEEEKGTSITAQTLPLPPLRWQ
LPTLPQRLEAVEKRRERRQKRTDPGQKRGWFASNPVAAAAVAVCGDIDDANDDHQDEEND
RHARTSSSSAFTLTGDRSDRSSSKDTYAASHASTDDVYIHELARGHESPLNASSNFHRRW
GSASLTQGGTNANSLATHLRHHSALLAVAGVHSESTAVTAQLEFDRIGAGEEAPYASYLF
SPEARIVDLSMLPLAAERKHEGGEKKATADESRSGNGSWPLPAPRHTELVASAPEKDGCT
RVCFSPEGDCYIVGTECGKLWFVQVSIKAGHIISTTATSPLPLHGHSGAVTDIAFDDVGT
CFASSGADACVILWNQRTPSKVRRINTDGGVPVAVRFMPKNNNYLLVSLPRHHLLRLYNS
STGFPVTRKGQLIRVEATALVMEPCTYPFLFLGDAHGAITVWRYRVSSVDDIACPSVVKD
VNEARKRSQPTTLPQSLLPQEPHPSTPSSLHSAATAAGQFAVESHLEHEKRGEFMPSTIT
EGQSQFEKVAGCVVEKGAPVASLVASSMNHQQFHCLLRAQRMGQEAHERSTVKKWAAAAL
GGGWKRNEGNMVSNKKDGKISTRSAYAVMLLASFPRDRLVVLSVQPSKSTPHEYKLVPML
VIVGACRIRQMSVGTSFCVDNTRCPTISCTCEEGFVQIIACTPARLPAQKDENVKKSCLT
GQKPVQWSVMATLPVPCGGIPRSLAWSPDMGLIVAVTEEGILYEWRRVRLHLGVPHPGYY
RSGGGGEEHCRDDDDSTGGPRALHEADEWRICLQREKERQMRRYRAQRGTADSDDGSLSG
LDDLWARSGDSVSRSPEQSSR*
>TCRU_1902 hypothetical protein, conserved
MFGTTRVWRNTFLTKSVATPPISVIRTGPRWWADPERMVRQKLMYFTLGVDQLPLRRTAV
IQKDLHRFHMCKPPPRIGDTTGYKRSRAAQLTTWYRRIQYQEYHLQHLFTRHVWGLVRAY
PGNTTKIQGKADDGYVGYDSVPYHRYNRTPLPFPAREIYGRRE*
>TCRU_1903 translation initiation factor IF-2, putative
MWRLSSGVRQLAVSWIGASCGCGAASAMAQTTVRRWQSQSSRGQPGLHWDEGVVDPRYSS
EGNMRRFNPDTVAHYVKATIQNDRREMGLGEVYDWHEFAKDAVYIPTRSGPLWVGSDDPR
CAKFMRRREKMKKSPQQKARPKPGPDPAKQLEDHTLREFFTTATNLRDPLSTANNLHRAG
LIREYDIKHVAAKIQYLPRPPIVSIMGHVDHGKTTLLDYLRKTNVASEEAGGITQNVGAF
QVKISDGHFVTFIDTPGHAAFTAMREVGVAANDLIVLVVSAVDGVQPQTKEVIGLARDAG
IALVVACTKIDRQSKVDPIKQQLRDCGIELEEDGGDTQFVSVCGRDGTGIPELLEAISLQ
AELCEISTPEPSRCEVTVIESRSSDVKNEVAGIVRCGKLKPGQVLVAGMTYGVVRKVMDN
NGNILQEAGPSMPVIMQGFRVHPKPGVVLMQVSSESHAQKYYLFMKDVYKVEGRREDYLQ
LLNQEQHGMIYARKPDNNLVRTYSTKAFVLSCKAATFGMLQALMKTLYELPRLEGVSTEI
KVTEVGGLKDYDIALIGSSGQPGCILLYGESKDTNTLDVPNHVKVIRFNVLYHGIDALKE
TLVESLPKISKTRVTAEAECIQVFRASQAGRNGNAGGMRVTRGSIIGSHLTFRVLRKSSR
SKLTGGDNSPKKATGDGDDAEDARTVVYEGQIKELRRFKEIVPSVEMGLECGVILHDEFQ
FRLGDILQQFEVYEEPRDVAEEYEAAERREKIMRETAALQAQMEEEKAGGNADGAGTV*
>TCRU_1904 WD domain, G-beta repeat, putative
MTTMLLSVPQAHSEVITSLQFSPHHVSVFCSTSGDDTAALWDWRQATAGHEIARLTHHQG
PVNHALFLQEDERILLTASDDRTIAYWDARELSAPVGNMRGFGDGINKMLRIPAPGTNGG
CNWLLASACDDGMVYIYSLGPENVPQRNDQQGRGANASNTAAIGTMVDRFWAATSTVNDL
ALSPNPGLLLTASEDCAVRLWNIVADMRVTTEDRLVASFEEFDNPVNHIAVVHSEAQPEG
HPRPESGFWLYAACSEVVFAVDIDPLTGAFGDAARTLMGHQDYVRGLEFVGRDALLTVSD
DTTAIEWSLTTAEPIRQVKLHEGLVMASATSAAKDVLLTGTDGGELRAWQLPFNTEKCNG
DTTNGY*
>TCRU_1905 CotH protein, putative
MAIPNPRRWRREFGGTRKRRIRILLRFLFFSLCLLVIGVFLLLLLAANEKRTFEKELTER
EIPHYSTNEFFTRVSFVMSSAMATAWRTSSDRLEFPAEMWVERVAVRHSAATTVPLPAVL
QKATVSATLYGKSSLKLQRTKRRSLYIRSDSPIFFVTPNPALSPNPFKRNTTVALKQFIL

LSLWEDIHRIAYRTSVDLLQHLDLIFSYAQLVELRCYPPSQKSHLDHPCVPSVAGVSVRD
GVHVGRTLGVYLAIEPPAPAIWRRMKEAFSAFEELANESLREVIESVSHGSPAALQRREY
ESVMEHTARITYWRQMWRAAKKSSPGKLEHAVEIGSRNFLSFSEPRHDMSHVPSFLKNSS
LLPSYRLLEKDPWHYGLHSETAMGKTVNPKKQFLDLPQYMAWIAINTILQNGDYDDEVFF
YGVLKPIQYGNPKFQLPIYLGVMGWDYDTVFSPCHRKVWFKASVMYCAETTLDKSIYDSE
AFIYRYTDILWDMTQHIDRERFSRSVQVAENELQTILYGAPGTTMATFNQVKRENDAGVR
WIIGAFLEQRQILLRALASYFPVNEVSHCDQTSDRDAKALEIHNREAHLELMKRLSIVVK
VKGSRVSTRWGFLVGGIQSALGVDLPEDEEYQLPDAGIYCARVSAVASFFTSVGLLPPPK
GRGKWEAPGFWFVDMPLVEPSTSRGSAEFGLWSFDKKLPMLYPMTTPDAGATLSIVMPPV
WPQDSKEKRGVFVSRVLFPEQGSENSLFRWSRKNTLIFTIDAPTQKTMIVRGFSAVRFFV
SEKSEEDSFSAQRHEGENTVRGPGLFASNSSTTRLRIGDVDIEQSVPLASVAAPPQALYL
ADGLVDAKGVRFGGSLEKQTVLRCIPENASVAESVVGEGLTLRSAECPIFISGSFEISRG
ATLDVAAGCVVVMRPAAMLRVTGFLRFNGTVASPIVVTSESAENASSWQTIHVDGPTATL
YASFTFFTGSGVKGIRVANTGKHHSHSAAISATNKASVYLRHSFLLELQGAGIAAGNEAE
VQVEDSLVQWAQMGIECVQCTFTSRRSVWTHFPSWDAAYADDDNDAMYLSGGKHRVIDSV
IAHTKDDGIDSGAMGNGGSLVVHNTIVEGCMHEGVALSSDPNGHREVVISHTIVQYCQQG
IESGYASKQHTASITSSLIQKCSVGVRYGDNYHWSHCDGKLTLQNTTLAQNEVNVMNYER
RYQIPQDPEYFHSEGVLSQRHWKLLRDAARDSALPLAVAPPEAVVMLLMDVRRRELPKTG
WEGCKDMFLLPGNSTLIVDEVVDAEAFTEELRVTEDVTVTF*
>TCRU_1906 hypothetical protein, conserved
MTCVSQSIARYSLLRCIVSAAQFAGGDLCFYENYAELVAQDAVGETTTPLRIDYDVLLHF
RVVRQQQLMRIELPRLIVWRWAEANRFASELDGGEILTREDMNRPCTLIEQLHEDDMAHF
LQSVAPMVAASRRARSASRVSTTEPLPLSYFPPNPEADSVASTETPSDVDGDNANLSGGS
QEDNDREGGMDDSSENVSAVWRSQSTVPTTQRNPLLGVGADSVESAGDKSNASRKKKHVD
VAPVTETAASVVSAAVVSEEREEKGEEDRLSQCLEALGAAADTSAVQLTRGISSQSSPLL
ANFTARRRDREAPARGGSNEAVMPNTPPPPSLSSTNLRMPRQLRGKGNTQRSAATRRRPP
HVGIDGEEEAGTMPKRARKEKSPTLFSLPAGATVGDADAATRTTTRVPLVEPPDSGASPD
QPIETPNAVTPSRAGGLPHSTRSDVVSVHRGAATAVKRDATDPNVTAEAAYNFADLMTAP
ENAAHLPLFPEVKKILGELEELIPPKRRGRGAGKTKTPRPPPKKGTAGKQQRKVTAKRRG
GGIEVISKEQTKAAKAESTTAPQKLHPVAGEDDGASTPLPDAAAAALAVAAAANVVVSPP
ANVPPNVKPSYPSQHESFIQAPALPSTQKSQQKSAHDSPEVGHTSDGAAAEAATFCFPSA
CVTPMSDYHLAETASNQHREEHEEINMDTGSGPDSSAYAAPAATAASTPPNRKAISLEQK
MDKQLTSTPLGFHKPLPFAHDSPPLCHCQQRCMRHYGTLSAVVEGDSRKSRWRRVVRQLN
SLSLHLTKARACGEELRGLFLLLLEDAEI*
>TCRU_1907 MORN repeat, putative
MFYTSCSYQGERTPDGKRFHEQGIYTFANGDTYVGAFNDGRMHGHGTLFFTAERGGGQYR
GVWENGRNVSGAFVFADGLVYGSGEDTTTTDNNTAAAAEAENTATVTRLADTTSILNSGV
KGGMDGHPISSNSNRGKGSVRESLAEEWLYCREGDRRLWAEHLREVMPVLPMQAVLGGVR
VPQSSLWAVEPVVAPSVAAEAKIPATFVQGQPRSLADVPAHFWKDPQQRENVMSSMRLPT
PPFKGAQGGAGREETMVDNGDRPAINLSLRIIAPLESEAMRRAIAAAVAASARAATSLEA
RKSPQPESTLSPDSAGCLVTPGDTDVFDKKGANPELALQGTPVHLPGELEGGSALRDDEC
STVERHASQNTPLQDPKLAPCQTNEPSLAPHPLQGLSVPETPRSLTESPEEVTCRGASEA
RVDESAPSPVLQTVMYAYLSQEQPEPDAKQHDEFRQEEEEQDTQEQEHYKQDAAQEHEGE
QQEDLKNEIGSTEEDKMYLSDGNA*
>TCRU_1908 ATG12/APG12, putative
MPRKYRYQRTHTFAERQKEVATIRGRFPQHVPVVCEPASIDTVLSAGPISDRCRLLRDLD
CSKFLVPGDASMQQLMVLLRSRLVLDAEQAIFLFIDDAVLPNSACVGDLYAQRKDADGFL
YMTYSIESAFGGAAVRRN*
>TCRU_1909 hypothetical protein
MERLLKRVGLLGEQGPNTHDKSEICSVGECGSGDSSCSTNADLEFASAVTPKTVTVTSAD
GDFGAAGGSDTVAVGSSFSSGMDKHLLAVARASEFSALKRLELCQQELLQLQRMWDLDRE
TQRWTVASLREEVRHLQKQLEQQRLCGWLQRTREKAIVTRNLSTSPESGVGASFRHAADR
EAGRANGVFEASATSGTGDVSARAEETCLGRSSDGQRTHGVCASSGAEEVEDSLEFYGRL
LHDANGECGELQAELSRVVRRLHDERRFFAQQISSLLREKEEMVREHATTLAHLDETKDL

LMAARAAERAALRRLTERCPGESARVYGGRTADGGEMGTVYCSRCGCSSTACSELLRSSV
DFSVERHHCNSLAVRQSALEEEVCILECEVERDTRNAEGLRRVLERLIDEVDVHARGCRA
ANKDIVGCLREISLLWLAEGDEKDDEDDEHKSDDGMPPSLLLLGGDGPDGDSSSECAPSS
AVRSHLLSAS*
>TCRU_1910 hypothetical protein
MLEKSEEMVQLLEEARQAECNARSELFNREEALLELQRRRDDDLRELEAVTHAHRNLLHA
IRQPREGMTEENAEESFSEAERVQGIERHAVQLESENIALREEMAQRAVAHREAMEGLNN
KISKLMEELEAKGMEYTETLGKLEEFVGLLEAARAAEKSALMALEKREQELFELQAAHDE
EMRELESAMKGLDGGVLPPQGGAADFPAAEMQANTAVADVDKNAVRTMRQQLERLKREKE
ALVMELEVKGRQHEDALNVLNRNIGELMGELDSRIRGCQVSATSAKELLHEIALLRSAEE
ENEEEYNEGD*
>TCRU_1911 hypothetical protein, conserved
MLARGTSAPGETTSLSAEEMHQGIASFLGGIDEPVLCDSIVAYEFLDNNLAAHWSLARVL
SLSDHHALLQRWRAENGSNADILETLKQEKELKEEEVRNCRRSLLQQREELATVRRDTEA
EVARAKDKIEDARRLLEEVEEVRLHEAFSRRLPSAGLCLALEASIAILNQESMEDVVMGW
EDLRAVVRHPDFITRLLEYYPTVENSMDEHQDIINRYQPRLLKLQEQVRLSPRKIVSANS
VSLESVFCDWALAQLGFIIVYPEIKKSQARAKEISEAINNNIRTMKLLETQISQIIEQQH
LYKEGNNVVNCNKADSDTCLSFAEASDIAPITVPRSSITAAIPGKGSGIVIVGKDEAKRL
TSRTAMHRPLIYAALGSALEERARLEAKNTDKTKLIQDLQQEKDNVYSQNSDLAKQLKNL
EEELHKLRKELEQQKTFAKKQEEYHKQQIEKKIKNQQEMSRQLQQSKEEVKQLQQQDKKR
DKENHGYKTQLEKMEEQMEEINNKNKTLKKSIEQKNQEIIELYEHAKDLQLQLEEMHAEI
EQLRVENEELRAEDEHKTRGLQEVSEQAEDLQRQLEELRAENEELRAEHEDKTRGLQEVS
EQAEDLQRQLEELRAENEELRAEHEDKTRGLQEVSEQAEDLQRQLEELRAENEELRAEHE
DKTRGLQEVSEQAEDLQRQLEELRAENEELRAEHEDKTRGLQEVSEQAEDLQRQLEELRA
ENEELRAEDENKTRGLQEVSEQAEDLQRQLEELRAENEELRAEHEDKTRGLQEVSEQAED
LQRQLEELRAENEELRAEHEDKTRGLQEVSEQAEDLQRQLEELRAENEELRAEHEDKTRG
LQEVSEQAEDLQRQLEELRAENEELRAEHEDKTRGLQEVSEQAEDLQRQLEELRAENEEL
RAEHEDKTRGLQEVSEQAEDLQRQLEELRAENEELRAEHEDKTRGLQEVSEQAEDLQRQL
EELRAENEELRAEDEHKTRGLQEVSEQAEDLQRQLEELRAENEELRGEHEDKTRGLQEVS
EQAEDLQRQLEELRAENEELRAEHEDKTRGLQEVSEQAEDLQRQLEELRAENEELRAEHE
DKTRGLQEVSEQAEDLQRQLEELRAENEELRAEHEDKTRGLQEVRRTRSCVLSTKIRPVD
CRR*
>TCRU_1912 Putative S-adenosyl-L-methionine-dependent
methyltransferase, putative
MRLLPPLGRLLCGCGGLTRGAALPLSCRHVATATGSVSTKIGGGGGGCDNNSNGTDGGDS
DGGGLVPSCNSHGHEALVDTGTSFSVAATTNDATAASHHDGVAAVHYASLSTAQGGMPSI
VSSGSHVMSDSRLDELATQEEERRVARHKKLFEDLIVFEGGALADHDSLGTSLIMRDFIH
FALYHKKWGYYPKLHRKYRQLMTSGYFDPVPFASLRNQHDYERYVGKIHESTPACITPTQ
LFQPHYGWVLAEYLVTMMRAKFDPREPLVVYDIGAGTGALAVSVLDYLAEHFSAVYAQCE
YHVVEQNPQLVQVLRNRLITHYHHVKIHNISILNWRQLERRRCVLLGVELLSGMPHDCVL
LSGDGVCNEHWFGFQQHDNLSTVHERYYALKDPVILRYLRYLRWLQEESFHSLKVLCLTG
GRENIDPPPFTSIEPNMNDPLRVIITKLIWTHSPFRTLWLPTVAMLFLETCAAFFPRHHL
FLVDWSGVRQALPGVNGPVCQCKVRVAKDMYLRRPADSLHGNAGMVDICFPTDFEHLAVV
YRNICGAHKDISSLTHPQFWKTFGGDKTALFTTRSGFNPLLEDFEPFHVFATHHPPEM*
>TCRU_1913 palmitoyl acyltransferase 11, putative
MSLLCCDPPEGQWLDVYGDPVRPRRSGFECPLDLLQIVAWSIIILLAILHYTLHVPFLEN
TLMIVVSIISGVLFTVTIALKVSLSLSHIEDPVIFRTDLPRLEQTGLTQEAAPPGTEPCV
FCRRFVQAGSKHCGVCDKCVPGFDHHCRWLNSCVGAENYKSFCAFMGSAWCGMAFILAIG
LYVIVDAILAREKYEDLLELRYKSSNYAVFLLFLFITVALCTMGMCVLGHLIVFHLYLCC
THRTTYQHMVEKREKKRERRRLRGLQEQMQEANGGEGYGLCACLSIRKRRDFRKYKRRRM
ENSNTPQINDNCSAPPFPTDPTWPSAHGPQEPYSSDAHEMAPISPKYD*
>TCRU_1914 farnesyl pyrophosphate synthase
MASMERFLSVYDEVQAFLLDQLQSKYEIDPNRARYLRIMMDTTCLGGKYFRGMTVVNVAE
GFLAVTQHDEATKERILHDACVGGWMIEFLQAHYLVEDDIMDGSVMRRGKPCWYRFPGVT

TQCAINDGIILKSWTQIMAWHYFADRPFFKDLLCLFQKVDYATAIGQMYDVTSMCDSNKL
DPEVAQPMTTDFAEFTPAIYKRIVKYKTTFYTYLLPLVMGLLVSEAAASVEMNLVERVAH
LIGEYFQVQDDVMDCFTPPEQLGKVGTDIEDAKCSWLAVTFLGKANAAQVAEFKANYGEK
DPAKVAVVKRLYSEANLQADFAAYEAEVVREVESLIEQLKVKSPTFAESVAVVWEKTHKR
KK*
>TCRU_1915 hypothetical protein, conserved
MQGGFGGTTGFGQTSGLRPGSSLRPGSQMRPGSQLRGTQAAGVGGSLQQPVAIAAKAPVT
LEGMRAATRSGNAGPGRQVGDRSYYIGLLRPKITELTTEIQQLNEQESLIEKNSSVLTQL
HQRGKNLTEEITKLKGALADINLAVEKSTSQDANSVKDQATKLTHLNVDRRKQVDQMFLN
VKEVETQTKKNLQILEEEMQQLDRRIMAENQDYNLYNTTRNEAYAVSDAVLNLQHRVRTL
TAKQELHMAQLATDTDKRRAAEVLREILRKRQLREELTKQCALSVDEERQLLIKQVKTAR
NDIEVLERQVNETRDALGESKSRCNALDEELKSYSGENLKAFQELQEKDRELQSFMDSLP
ERQREESEKIATVQSNISLLLERISRALELQKQMPAEGASHALHALESEVDARRNQIESD
QVTHQRLERELLDRKMELEKVENLDIKINAELQAHARKMEEQSQEMIRYSDLDGLRRNVE
KRHEDLVRERAFLVKLRDSSKHQLNALSASNEALRKRLNEEAAFRSVSTLEGRLRHLRQS
NFSLEEFVRLKGKESYYLGAKTDCLRLVDEINLLLKDTDRTRGIGGGPVLLLGER*
>TCRU_1916 Ribosomal L28e protein family/Mak16 protein C-terminal region, putative
MNHDDAMWNIMGQTFCSFKVKTHDEKTRFCRNPYNVTGLCQRGVCPLSNAQYATIIEHED
ELFLYIKTAERAHLPRRQWEKVRLDASFPRALAQVDEELQWWDQKIVNKVKARLLRLKQY
IMRRRKMLLEPKVEYVSVNKRQEDKLLRREEKAEAAARLELEVEKELLERLRKGTYDSVM
NCSREAFERVLDQEEDRAEFDALEDEEDEEFPEFVMDEEDEDEEEEPSKTTAGLRPSGRM
PVRRQRLVIEEEVEHEQQQKRSRLDDLDW*
>TCRU_1917 hypothetical protein, conserved
MAASAFSSGFVSSVEDFRALFAPNSDDRLLLCYEAYHDEVTTADLTALEDAVRSAAVRHA
TKVQLHFWDCGSDAFLAAKEQLGKVRGTPLLAVVYRQAIADTLKGPVEVMIEDVDNVCRR
LSSYQRGGKVAVFPISLPRTTADAACISVDVSRLVGVGKRLMTEGKADYAEKFFHRALDT
LDAVRPHCDGDENFDGSTALCLAWVALAQMVQGKNADVQMRRLEKDYVAFCGEIGSDVAR
VRAANRLVTAAPFTWRSETCSQRKLREALARDPQSHEHRCALVVTLFLAGDMERCLTEAI
KLRTLHVPFGTTAISAIGDFIGRDHALMRSIGIDCERPSTGV*
>TCRU_1918 centrin-4
MAALTDEQIREAFNLFDADGSGAIDAEEMALAMKGLGFGDLPRDEVERMIRTMSTDSNGL
IGYGEFERVVKSRMAQKDSPEEILKAFQLFDLDKKGKISFANLKEVAKLLGENPGDDVLQ
EMIAEADEDGDGEVSFDEFKNVMMQMRGK*
>TCRU_1919 centrin-4
MAALTDEQIREAFNLFDADGSGAIDAEEMALAMKGLGFGDLPRDEVERMIRTMSTDSNGL
IGYGEFERVVKSRMAQKDSPEEILKAFQLFDLDKKGKISFANLKEVAKLLGENPGDDVLQ
EMIAEADEDGDGEVSFDEFKNVMMQMRGK*
>TCRU_1920 Major intrinsic protein, putative
MPSTDTATTQPENENNGASNVAIPSSGVTRFAFIQRWYFNEFMSAVIVEFIGTFLLVLTV
PLSGINNPEMAPFSVGFMLMSLVFSFGYMSGGHLNPMVSFAVFLSSANFGKTRMLFYFIA
QMLGGVAAAFYCVLIHGNEFPVPNMGLDFLQMLRAFLAESVFTFVLTSVVLHVAISKQRN
NNFYGFAIGSAVLVGGLTCGSISGGAFNPSVATALIVVRCFVSFSESGCTPMASLWVYWA
SEAVGSIVGTILYLAVQNVEDRF*
>TCRU_1921 peptidyl-prolyl cis-trans isomerase, putative
MPANDCVLMDKIIEGDGKTIPRQGSIVTLDYVGFLPDGRRFDSTIERGKPFVFRVGCGEV
IKGWDEGIVQMSKGERSRLTIPPSLAFGSIGFPGMIPPDTVIVFEVTLLDVA*
>TCRU_1922 cyclophilin-type peptidyl-prolyl cis-trans isomerase, putative
MVYLTRLVRRVHRGPIRNIFSYPLNEVNPVVFFEITVEGDALGQVTIELFHDTVPKTSEN
FRSLCTGERGFIQCPLYYKGIPFHRIIPGFIVQGGDILTKDGRGNVSVFGFPFLDESFKG
KAGKHLPGTVAMAHSGPNQNGSQFFFNMRRNEHLDGKYVVCGQVLEGWDLVERVAALCGS
RCGVPVSRAWITDCGQSSGAKLEEAQRALCGERALHSMPGKEVLDLISPRY*
>TCRU_1923 I/6 autoantigen

MPISPAEAFEERHLQRDDGDKVIPPSLALVAALESGYRFKLSSIEEATDSARYPGFLTKD
EFVSLCEKNPNNCLDARMMAKHVSVLAPNGLFTRVSLQEIAAKTGSSQDALSADEIDALF
DVLDRENTGSIPAERLMEAMYGDEGRVALGKQRKEYAAAKAEEERQRALRDAAAAAAAAA
PKESVPAAAAPKKEEPTPPPPPPPPQQKKKAMCGC*
>TCRU_1924 histone H2A, putative
MATPKQAAKKASKKHSGGRSAKAGLIFPVGRVGSLLRRGQYARRIGASGAVYMAAVLEYL
TAELLELSVKAASQQAKKPKRLTPRTVTLAVRHDDDLGMLLKDVTLSRGGVMPSLNKALA
KKHKSSKKARATPSA*
>TCRU_1925 histone H2A, putative
MATPKQAAKKASKKHSGGRSAKAGLIFPVGRVGSLLRRGQYARRIGASGAVYMAAVLEYL
TAELLELSVKAASQQAKKPKRLTPRTVTLAVRHDDDLGMLLKDVTLSRGGVMPSLNKALA
KKHKSSKKARATPSA*
>TCRU_1926 histone H2A, putative
MATPKQAAKKASKKHSGGRSAKAGLIFPVGRVGSLLRRGQYARRIGASGAVYMAAVLEYL
TAELLELSVKAASQQAKKPKRLTPRTVTLAVRHDDDLGMLLKDVTLSRGGVMPSLNKALA
KKHKSSKKARATPSA*
>TCRU_1927 histone H2A, putative
MATPKQAAKKASKKHSGGRSAKAGLIFPVGRVGSLLRRGQYARRIGASGAVYMAAVLEYL
TAELLELSVKAASQQAKKPKRLTPRTVTLAVRHDDDLGMLLKDVTLSRGGVMPSLNKALA
KKHKSSKKARATPSA*
>TCRU_1928 hypothetical protein, conserved
MAANELLADMRALLEAMEDQEARPIERGEGDASTEENLRTPSPSLEDKIGQLLGALRSNG
VDDWAGKPAQEDLGVRFFSQPREGAGNGRVRENTQTTDGDTINSLMCEYLGAVKQVWRTE
DPLEE*
>TCRU_1929 hypothetical protein, conserved
MKTATVNKSSPLEEVAKEQGVLDTPVVNIKSHSELAAEEGKKKKSTKPVSSPPTKPSPPS
AAVEEVRTVTSYAPTVSAVELKNAPPLTFENAVTLDRFDGSYMELRRPDLKTPWNMKVSF
GGDKLVLTSLPPISAALKTHPFLKSLRPDTNGQINWLVDNVNGTDLTTANKSLRAKALDL
MKKTNKISLLLRPLSK*
>TCRU_1930 Dynamin family, putative
MRCWVAAASLQKGFDHFCFTPRAFWTRGVLSSLAETSCFYPRRRRIRHVPFSPSARVLQN
SCRGFTSTSAATMRFEGIGRNRQKPAVREWDEHGTAKVQQSTETTDTALREIESAISALR
NDILRQYSDVFSFLPSADHRHSMVMLLGNHSAGKSTIINYLLGRAVQRTGVAPTDDGFTI
IQRGDRDSEEDGPTSLSDPRYQLQDLQKFGMHFVHRFKVKTRQLPPSSNVPYGLMIVDTP
GMIDTPVHVKDRTSLEGQLRGYDFLAATRWFASRCDVILLVFDPANPGTTGETLDVLTKS
LAGFEHKFLLVMNKVDMFDKATDFGRSYGALCWNLSKVIEMKDIPRIYTTFIPVKTAATE
ELEATTPLVSSLNLAPLDSATKTIEESAAVSVEDRVAVVAMQEFVRERNEVMEELLKAPL
RRLDNLITETEEGARRILLAGRVCNAILWKYRQKQMITVLASSALLGLGALILTVGGISG
TTMSLAGITALAAAGSLYGCLAYLKRLESELLNDSDAIFDRLFIGREKSLDLQLRWRNIV
KPELLDLASSSATAGRGVITTLPTLSLWGQKSITSILKNDIPRLRLRVADYKNTHHIRGG
KHPASTYSGNR*
>TCRU_1931 glycogen synthase kinase-3 alpha, putative
MRKLSGDLKATTATPLTPPVTTPQPQPDGTDRSKSAGGQATYEPLHVLGQGSFGVVMMAR
VRETGKMVAVKKVLFDRRLHHRELLLLRDHLGPATHAGQAGTRNHDDPGREAGKTDPSST
NNSAGEVGFTPLGDLTSAPTTVSYHPCIVRLLDHFTVAGPNGEQYLYIVMDYLPLDIHRL
HHIYLRQKQQRMPFILVKVIMFQLARALMFLHLRGICHRDVKPSNLLIDPETGVLKLCDF
GSAKVMQPQGVNGPREKNVSYICSRYYRAPELLLGSLYYHFHVDMWAAGCVLAELLCGEV
LFKGASTVDQMAEIFKVLGAPSRQELLALNPQGAEALFHATGAGLEVTPTSAANTATSSD
YLQRYHALRIKALQWQNVLPPNTPPAAVSLVGELLRYTPAERLSAAEVLEHSFFDDIFAE
DASLPNGAPLPVSMFEVTAEEMEALPPWLLERMASAEASARRRHVSAGGSGKVVPPQKRQ
EQEGRK*
>TCRU_1932 pyrroline-5-carboxylate reductase, putative
MKIGIIGCGNMCECILNGLVLSKEYPPEEICIFNRTAANLKRLCEQYGVQAGKDAVDVVR
RSNVVMLGVKPYAVCSVVEKIRDAVTEDKIILSVAAAIKISSIEKALGHARKVVRVMPNV

PTRVGVGLTSVTPNALMTREDTALILKMFSTLGKAVEVAESQIHAVIAVAGSAPAYVFMF
MEAMGDAAVHGGLPRDQAYEFAAQAVMGAAKMLQDSDMSPAQLKDMVCSPGGTTIEAVRS
LEKGGLRSTVIEAMIACADRSKVLEANLN*
>TCRU_1933 TFIIS helical bundle-like domain containing protein, putative
MSSNSDDQLLLGLPTDNLADAPNTADLHGGGEMEGEEDAYLDLGPTSERESNDDEVGDGG
DDEGAEGENQSGGAGGGENDSSVAQLSDKALRRIKSKTEARALGRDLYKRWKKLRRRSNK
EERQRHRHHSKTKRKSRREEKKSKDKKRDRKRRRRVNDDDDDDKDSEHHGGERNDDSTED
RYDRVHKKGRRAAATKKKRKRSRGENPIALGDEDLGAAPFADLDEVLQVEEAAAKNSGGQ
GVKGVHTEPKVKKLTADAQRDVLRKIAAGVVNTMREARLKDEVEMNERRPPLHRVAIRES
VISQCRREALRSFLIEEGILQELSTWLYDFTRNELAAFELRSDALDILLSFPIDGELEVG
VTRNGDEILDAFTGMTREHLIHTDLGSAVNALRQSKQEVHQNRAKAVRLLERLSRAMAGG
VRSGRRGGSDGVRGSQAGEGGLSSTVTWKCKEDPTVAPPFHTVQTGTEAFQSALARPDPL
DPFSYLRLPPRRAPKVHVTNLGGNVGVGG*
>TCRU_1934 hypothetical protein, conserved
MSKQRRESALAVVQRLNASPVRLLPPILHNATDGGLRQSDVLVVGGVPGTGKTTWAYRVA
AQHLSCGGVVTFVFVPGSGNFQMRRFMEYLEMFVVTPDPCALLPLCISSVSVAGDSVMEA
RRKALLGALDRLEVVHCVDINDFFVYCNHGAVGTSSGVGSREVAVPLFIVEGCGGKGSYL
YHLERAIGHAVPLSHWLIGCLQRRRRCALIIIEDWGSEDVAMATRRRSTREESFSPFVLA
VEEESEFLRYLHTETPQKKAAFRHHFAKRQRAGSTMTVTASNAGSGCTAPTQVPLSSDLM
VRDLHSHFAGSLRFFYLYTQMVSVATSGLSPVASLLQWRLTEQNAGGGNNEHTELPTESN
RQERWPPPPPTERTACLRGEVLCVAQL*
>TCRU_1935 Domain of unknown function (DUF4586), putative
MTQLSVYGGLFSDPPLISTDGDPKRTDPYDKMDTIPSRYLGKGMSIGRSGTGQNAEVYFD
KKYLTLASPEQNGNKDLGAFEDADTRMRREAMESKKKNISEKVFLLPSYPKWSDGPGSYS
GCFQDRPYPFIVPGENDKKSRRRKKAAVTEVTDKNKSSLPNIKTNRTKKGTYGVPGTLLD
NPKYNDNWRQEMEKLDALRAKREKNVPQPKPMGSPFKIPGVTHDFLDELHATGVSAVYHY
EPVEEKSPKRKGRVKSPAAVAVFTQERPMSFKRTKSGQEGCIADFPNTWIDPDALERERN
KGKKKNRKPKPEYVPSPPKGASGIWKPNSFPEASVVQSCLRRFY*
>TCRU_1936 RNA polymerase-associated protein CTR9, putative
MAVYSQQARVALEEVHDAFSQGEIDRAKLILDGIDTPNEPSREELHDLFAIRCVRASIAA
MGGERSVASIMRDTVSYALHRPVLLYLEGLAAMASGTSLQTARVKFEEAIRADRYFVLAR
LGLAAVSYHMKRYKRCFSHYRVVLETLGSSCPPIVRVGMGLCAYHLNHLDYAQRCLERAL
EVNEDDELALLVLLVVFLDRRQIPKVIEVAQRLRGILPENATVLLKVAELVYFRAVTQDR
VKASANPIRRLLAEVRRVATVEESAMADYQEGRLCLALGDLSNARLLLESAMQVLPNLLA
ARIHYARFLLLSGRETEAEQLLLRINKDHPNHKEVLQLLAVYASRHGLHEKALEYSRRLT
EIVAPGDIRSWSIASWCARLDKGETKKLMSHLARIRKEVGEPVSMKLMANIAALGGDTEA
LQRIIDCELGADFLGEPNLPVVYVPLVFNLALLLEETDRTRARQLYIFLVKQHGYFRPPY
IRLHVLAKDDGFLKQAVAWLVLLQQVLPEDPTSLASIGEIFFENGRVGAAMTALRSARGR
PLPVALAFGAAFLWCSQQHGKDNRRFLASAKDRFAFVLRRDNGNVLAAHGLACCLGLEAD
YDRCQCLLDRVGEVRPNCSYVRRHHEAHMANVKTLSDSFKQAIDYLERDPQRTPLQTSSL
AFCLACESRYEEAIAALTTVVAENPNLPLLQYNLALLHCAAFVYAVSHKEAITPEKARDL
RHSLATGLSMAFEFIRQEPRSQAMAVAKTFLKTVCAYCLDVNDGSISRLILVGQRDTMEY
DRQSKLWQRVYDEFQTEKRNEEEQRQAEARQRQEQEVQLAREILEDFRRSQLDQPTILDE
EIRTRLEAYRAEFGSAAPPATDDAVQPIDPLGTTSTAILGGMNLEEDGVGWAEAERDEEE
HDVKVEGTDVSA*
>TCRU_1937 NOL1/NOP2/sun family, putative
MSRNQFRKGGRKRGRKEDNWAADTRDLSTLTNDAFISYYKGHVVEEAEWEVFMEALRRAL
PVALRVHPSVSSAEAVLEYLKSRLSSVLPITTIPFIPDGAALQCPISRGDLKRSVELREL
KKVISAMSEGGYLTRQETVSMLPPVLLQVRPGHRVLDMCAAPGSKTSQILEALIPSMEDG
VVVANDINTSRLDVLLHQTNRSAAAHSHLIVTSYDATQFPLLPSESKFDRVLCDVMCSGD
GTLRKSMDMWPRWNVFQGAELHNTQIRVLLRGMMLCKKGGIVVYSTCSLNPVEDEAVVSE
CLLQAKGAFRLMDPTSLVPGLVAAPGLDDWSLLTKDLKARLHTLQEAQVFAETSASNGIS
YRATMFPNKARLQEQNIHYTRRVLPHFQDTGGFFVAVMECLEEYSAPTATTSSKKSETFK

SVSPALQKEVRGALGLPEFFPTDQLLVRNETAREQKIYFANKAVCEILPRLGPRVVHVGS
KVFESYMKYRNDKLRFCSDGVGVLIPRLPSSFVVEIGPEMLLQLSQSKLGESLLPPLPPH
HSFVFKCNMAIVGAVYIAAERACPCQISAKVAEWQVALTKLTLGQPLVECNEEAAADTDR
VGGDKNTD*
>TCRU_1938 hypothetical protein
MSFIDDNDFAVDEEDEEEEVVAESLSRSFEPGEEEEEISAEEEEEEEEEKEGTEEEEEEA
EEDKEEEEETAASELDVSCSDGNNENTLASSVRPNPQKVPEATTVLTSSFGSPQVSKATEA
PVQSSHVISSSSPSLTPFLGAEGAGKSELVVPSPAQAPARRVRLSHRFLDFPGKTASLRD
KRQRCLSLSEETKAAKAVLATFELSLPTTRFDEQLGNILSNLL*
>TCRU_1939 ATP-dependent phosphofructokinase
MENRLRDTSRVVRSHAAPLNEVTQEDLKVERLHGRKYMNPSKKHVMREEFSDKIEHIMHD
PRPQEGVHSELPVSISPLLCELAAPRQRIHFNPPETVVGIVTCGGICPGLNDVIRSLTLT
AVNAYRVKRVIGFRFGYWGLSKKGSHTAMELYRTSVTSIHRYGGTILGSSRGPQDTSEMV
DTLERLGVNILFTVGGDGTQRGALKIAEEAKRRGANLAVFGIPKTIDNDLSFSHRTFGFE
TAVDKAVEAVRAAYAEAISLNYGVGVVKLMGRDSGFIAAEAAVASAQANICLVPENPISE
DIVMALIQRRFETSRSCVIIVAEGFGQDWEGGTGGHDASGNKKLTDIGVVLTKRIQAWLR
KNKERYPNGTVKYIDPSYMIRACPPSANDALFCATLSTLAMHEAMAGATNCIIALRYNSY
ILVPIKVATSVRRVLDLRGQLWRQVREITVGLQDDVRAFKEAEVRRELEAISLVRERLIG
QLSKL*
>TCRU_1940 vacuolar protein sorting-associated protein 4, putative
MSVDFTSKAVDLLKRATTLDEAKEYEEAYRWYMDSIEVFMTAIKYENKNPTKKEMLKNKV
RQVMERAKMIKEYLDGSKEGDSNVGNAGSTAQKTASASKKAKEEEEDKQRMRNALGGAIV
RVKPNVNWSQIAGLEGAKEALKEAVILPVRFPQLFTGNRKPWKGILMYGPPGTGKSFLAK
AVATEADGTFLSVSSADLMSRWLGDSEKLVRNLFEMAREAWKTDGKPAIIFIDEIDSMCS
SRTDGENDALRRIKTEFLVQMQGVGHEDDGVLVLGATNIPWSLDSAVRRRFERRIYIPLP
DVQARYQMFKIHIGDTPHTLTEKDWYELARMTDKYSGSDINIVVRNAMMECIRSVQVATH
FKRVTGPDLKDPTRMTNNRLVPCSPGDPEGFAMTAQEITEPGLLMPLPVTMQDFVKALRT
ARPSVADDDITQHLKFTEEFGQEG*
>TCRU_1941 protein kinase, putative
MVQSQRCERCGEGGATLTNCVDCGRYLCRRCVSCAGQCADCTAKVEQSALGCAICRNMEN
AYYWCAACHSIVCRRCVLQSYKGRPTTVLCKSCQMSSPLPAPVTLCGRCKGVLRDESTWY
YCEVCAEPLCRNCLSSSFECGVLKCLTCCPAPVRLLSGNRQIRMFLDDELKTLVASISRT
KSQTAATSATTGVSFSKYRHLRNREKLGEGGQGAVFKCTTLDNEVVVSKEMTFDDGDRAA
FEARLIQAEKMRMLSHKHLIRYLDVIGLENPLKICVIMPYYAEGDLFKYIKRQRGPIEEH
KLCSIILQIASALHYLHSQRPPLAHCDIKPDNILLLNREEQVLLMDLDLCRSCDVQSSAT
SRVLYECHGNSPTFEYRAPEMVKSAGSTESDIFSLGVVSFVLATLPEFAMLRNGKGVMTV
LNDSDWTPDSLARAVRAAIEAHTRVYSEELIDLIIGMLRHRPASRPQAIEVMMRLTEAME
NRLLRCGI*
>TCRU_1942 hypothetical protein, conserved
MLKDRFAHLRIVDSDSDSDDAQAVRQHNLASLNKPTYKSSTETTEAFPTAAAAAPTSPFS
FPIAANVKKDIASLAAPAGEPGGSSSHTPKATFLNEPPSMVRGSSWKLSSDKEIQSLRLG
GGSVNSFAAASTSGSERRKVQQCERSELPDASSAVPSGLRPLSVSERFEEGTHVEEKQVT
PQGDAEMAATTSEKAEEWGEGLEETHKMETVEEPPTQMTEPMAVVGSKEELRIVATDLSE
PLPAPADDAGRAAVKRFLEERYWEKTHGYDPFKRRMPLFVAIEHCSPKNVPVRSNLPERN
ALEANPEAVRAEEPCQHEIEQNYPDSEGVGKFLDEELAVALRDDRLLKEGGEGMAGPAPN
MASVVSVDDDNAGDLLSSTQPAESGGIRGAFREHQGEAYEEGGQMGDPIITDENVLQEEA
ASEMRTSATNVKKEMEGDENEERTSFTEGSQPHLPGQVDDASKVLLSSKAALNLPGNIQE
NEYAESLKLDENNQRSIRSNVSNFIVESDQKGQSERGSNEEIFLANEELLMNDGINVDSL
REKREVQSANESAVQQNSRTSNKSEEPLQNSELTVESLRKFNARSFEGTHWERYNEGEEY
TDSSLYKTCFSPGTTEAGSFRMGKGLPRRFAGYNDCGGASLRRQELAIPPFHRRLILDME
EKFRREKRSMEHSLKEYTFHPRTTCTPRGHLPQRRRNPTNGGSTESPCQSLAMTPARSSA
RHKVLEEPLPTFKPEISKFARETQRPKIPYHERLYSPRPSPARAGPGGALNGTSPHLSPR
MVELIDGATPPLFRPEISPMAKAIENGAPFYERLYQVKADVEPSREGKSLLQSPPISPRR
PTRPIQLSPRLLERPAPPTETLPYSFHPQISPRAMSMESHGPVYERLYPIKDERSVILQR

QEKKFEDFLASFQPSIAERSNGTNADSRVMGRDVVRRLTEHREQSQRYVDPSLTFHPFIT
QKARDLKNENSAVLP*
>TCRU_1943 60S ribosomal protein L13, putative
MPKGKNAIPHVHQRKHWNPCSSQKGNVKVFLNQPAQKLRRRRLRLLKAKKTFPRPLKALR
PQVNCPTVRHNMKKRLGRGFTVEELKAAGINPRFAPTIGIRVDRRRKNKSEEGMSINIQR
LKTYMSKLVLFPMSYKNVQKGEATEEEVKSATQDRTRFGTAAVGGFVTPAPEAPRKVTEE
ERTKNVYKFLKKNHSAVRFFGIRRARQERREAKENEKK*
>TCRU_1944 60S ribosomal protein L13, putative
MPKGKNAIPHVHQRKHWNPCSSQKGNVKVFLNQPAQKLRRRRLRLLKAKKTFPRPLKALR
PQVNCPTVRHNMKKRLGRGFTVEELKAAGINPRFAPTIGIRVDRRRKNKSEEGMSINIQR
LKTYMSKLVLFPMSYKNVQKGEATEEEVKSATQDRTRFGTAAVGGFVTPAPEAPRKVTEE
ERTKNVYKFLKKNHSAVRFFGIRRARQERREAKENEKK*
>TCRU_1945 heat shock protein 20, putative
MWDPFRDVERLLNRMQSVTGTSFLSTSARGSWVPAMDIVEREDSYKILADLPGMSRNDVS
VEIEGSQLCIGGNRKSMLSEEEHKNVVMAERGSGRFERCVRLPSPLEEGSVKASLRDSVL
LVEVKKVTDAVRKRSGISVKIN*
>TCRU_1946 3', 5'-cyclic nucleotide phosphodiesterase, putative
MGCGATRDVNPLPPPDVDFRSGGQLLKLGGSLKASGGKEPLMRMPGKDKYIKESGAVVMD
DRQKSEGGGGNRASVGMLILFCAPGTDELVISYRENSSGAIYAKRMTDKELLASKDAAGI
RVSWGLFFKSLASDVLNNRAVVRYSTSTSKDVHFNILSRKDRGVKYDYTCELVMVSGLGA
SAEQNAVVEYFVEPMTRMLQVRRRVSACSNRGDALEKLECDHVVKMAALRRYKARLKELL
NTVKPLREEASIISEKTMNLLLEAKSLESKLQMMRGGIGDEQPLDILYEKGGAQYFQHVP
HAEFYYPKEEKVDQGILACIRAAFPLFPGATLDGIMTVLDRPELQPLLNDFSRPLVRDMF
DVFSGLDRWDYNVIELEIITNGNALFYTTYVLLYKLDLVAQFHLDDTVLQQFLLGVQAGY
HPNPYHNSMHAADVTHINYYIMMIAGLKEKCHLSKEKVLAGVIAGAIHDFDHPGLNNNFH
TKTNAYLSTLYNDRSILENHHIACIFELILHPAYNVFAPLSNEQMHAVRETMIEMVLATD
MGNHGNLLRKFKLRMAETTDWRTEKEDVLLALSMSIKMADISNCARPKYIYAEWARNISR
EFYNQGDAEAACGLPISPFMDRKRKMADFPKGQISFMTYIVVPMVEAISEFLPSLCFALQ
HCNENKDAWQSFIEEHT*
>TCRU_1947 hypothetical protein
MANELITLQVVLSAFVCIMVVLVGVFLCCRGRRQDHPATTMSCYTPLLFYQSSSRVAKKC
GDQGPSFMPLSPSDKCRISEWLLEVQRAIDNSGKSLAVPGLSDPRNPTIVDSLAMMATVQ
FCSDTSDKIIMESSASEYATYSPRVSAVEGATEPLTFHLDPLSR*
>TCRU_1948 hypothetical protein, conserved
MDLLLDDQRETPSFSMGLFADRVNSILEAPLPKCLLLKGEKAGERFIGSTVGLSPRGAVS
PRRTTISSSAVTTAVRGNGCASQQGKPIYEALLERGRLMSEKKEAMRLQALEEELSELRP
APSITYAAARNTPRRQQRIEDRLLQRAKEAEMRRELQAKQRKEELLKEMSVIAPFQPNIS
RKGRKATPRARNMAEKTQMDWNRQREQRREAARTAKLMHELSEVRGTPVINPRSECLAAR
RRKKEGLLGMSAVDALIERDRIAQLKRWERHELEKRAEQIPQITLYAATLQRDGDAAERL
YAESYKREERRLRQEEQLISNGAAPFTPRISPHAATTPRFRNVEDELMEKHMQSIALKEE
AQQRECEQESRCHRPIINPVSEAIASKLPESTCERLYRQTRGTPKHRDPASRDLSFSESF
ISGSRVASLPESMAESLQFYEESRQERLRKLREEQERIQHKECTFAPLTNKRDSNFGGPE
DVAARNQQWLARKERKLRELRRMKEAEKVKDCTFKPERESPTVNSTRYGESVYGGDGTPW
GVQEYLERQEEARRIKREREMRLQRRPSSAPRPSTTTPQEFLLGRRDGVSVRSLRRPPQV
SLMSPGKEDDPPIPQEPQHKHESGLSVYERIYNRIYNTVASGGGLGKIGDSDGAETSLCI
ASSFYR*
>TCRU_1949 acyltransferase, putative
MSLSCYLRLPIGLIVVLFLCTQTFGPPVAEQQISESIRSITAQYKKLVERAINETMRSLM
DHLDSNRDGQIQLEEFQSLAAAALQRVRNVRPITQEIGLAFSHITWTELMWRYFLLQLLF
LFSLFVLENLRCLIFTAPRRLLLDDSYLTTTPPEVGPEIALTYDTPWTTYERLKMAFFAA
TGLLFLRIFFLLFFAVLATFFMSLCGWRGRTRRGNPLWFAVWSNAATVLAHIGSVFAGVY
HIKVFGRFADASECKVMIGNHSCIMEVIILFILGNFPSFVTRKENCEKVPFFADVAECLS
AIIVDRKDVNSRQQTADAIRARAKDRNPKSPQLLVFPEGTTSNQRALFMFKQGAMVPGEP
LQMVCVSFPYKHFNPCWTGRPCGGNSFSDLLMRLCSQFVNHLEVRALPVYTPTEEERKDP

ALYAKHCQQMMATVLRCSVSSCKYSDYESISTGKSRS*
>TCRU_1950 SET domain containing protein, putative
MGEVVPTASRDWSLNKEKESGRLFWWNCKYSISIWDSNLNLGQPRHELTDLLVWQAFLHF
QIASVYKLCFVCHQPEGNEDMKVCCFCGLTVHCDCSREATAEQVAWKPANFGFEAYLRAC
FNCEDVHVPVKIRANREASNAHRVASRALSIKEEYPEHILRDILCLAEKAEQPHSSSEDA
SLVASLRRVLTSFYQSEESCLFLKREKRPEKGGGIGVVAAKDIPAFTVVGVYPGYLDVLS
GEHLKLGRPVTKYALKDLNCADYDNVVFEEFQGTFTPFINEPCEFEESNCGWIQEDKHRE
GRLSIMTARKIEEGEELLIGYGPSYPRSYPYRYDVLTFHKTETEFDTPCFALWRWTSTDE
SDARLVSYVGYEKSKDFYFFWGEQEE*
>TCRU_1951 DNA repair protein Rad9, putative
MFLSFSVTGASVRPFMHLLQTVAKQGEGLFFEPHRDSLQLRVVNSTRSTHMLVGIAARYL
EGYRSIHGNSVTNSCEKSGGATSETNLPRHEDDRDEFFLMLPTKALLATVLRQSQGLCSV
HVRYNATSASDMVSCDTLQWECVMSGGIIKKFLLPLAEGRPERAFFSPDRFSFDACAAAW
VWGALLGWFPASRPRVVVQPLESRLELWVVDSDEPNVMQGAHPPLESGKGGGAETKVVAS
QEHFLFMRQHEPRGEGTAVSNETSEVGDARAAPRSRGLSLPGKIVEVKPFKQVCLLADQL
GMMIRVRTGVEGLPVFVEAITVQDAQQAAAAVAAATVKDAVAAESSNAAVQQNVFGRHSL
PSENSFRVTFSMYIAAFDVPQRALDSGEGFTASSRRSTNSATTVTGEERGVVTASSLQEV
NAPLVSASAAPPSSDGVPLSGIAQRTPFKIAENMATGQVIRPTGVTNEKNPVVNSVCSPQ
RVLVPASPTQVPSCALDGPSLVGSTPLRSGGRISSPSNGEEMVVRATPEVGGIVTPHRGD
KRPRGGESVDGRFPQESVVGCTAGTTDTTAMSFAPTASFVAPTNLSPNDRRQAGEQQGRE
GPIPSVSARPSLETRFPLDFNAFMQEIAEEAKEDPEESEEDEDLQRFLESCVSLLVLPVT
PATQPK*
>TCRU_1952 E1-like ubiquitin-activating enzyme, putative
MRQEEESKQKTLDATVRDDNPYSRLMALQRMGVVDNYEAIRQKSVAIIGAGGVGSVVAEM
LTRCGISKILIFDYDKVELANMNRLFYRPEQKGMTKVLAAKQTLEDINPDTEIVPYAFSI
TSTEHWQDFVDALTKNGGVQPNSPVDLLLCCVDNFQARLTVNYACLLHNIPWMESGVAEN
AVSGHIQLLLPGVTPCYECCPPLVVATGMPEAKREGVCAASLPTTMGIVAGFMAQNTLKY
LLHFGTVSEYVGYDAMRDHFPSITIKANPECRNETCVQRQQEYAARRAAMGDAAHPLHQA
NKQREEREAKERAAARAKAKATASAAEWGIVIEARGKEDLAVHAPQPEGVEAVSVEYAYS
SGAGNAAEHAQEEFVMTDGGESLEALMAKMKALQH*
>TCRU_1953 2Fe-2S iron-sulfur cluster binding domain containing protein, putative
MASEEPLNEIPGECPTPGDLGHAGWDVLHTAGAVYPYKPSPLQQEAFRSFLYSWSHVYAC
SHCSYHMRRYLKRNPPVVTDKLALNRYLCEFHNTVNKNIAKPVYNCDPMVVLRRWHPTFP
DMEDQPTIEEQIAEQRRLEQAEAQKQKEKELQQKQVQTSSLGDMKASRERLVGRWRSEKS
ESHEYANNNPNSVGAFATGWSASSRQHENAGDAAAKSAPAPLAESREKRWWIFGKNGSGD
KNAGFSSTTGTPSKVNSSGGGSNSGGMNGSLDDDEASVMAILKRLKACMVYCPDKDEKSN
SV*
>TCRU_1954 protein kinase, putative
MRAGSSAVATVTPAENALLSPGVPFALTKKIATPQQDLVDSGGNSGISSQKRVSAVGSVP
PVRCAVAAECREAEVAPNVCSSLGSFVDLPNSKGNQECHRIHPLRDFLETYKIRRLLGHG
SYSVVFEVVNRKTKERKAAKFLIAKETQLARRKQGAECGNGKDKGSMAIEGGRHISNNSN
TSTSLPESLVKEITVSFMARHPNLVHTSDVFVEDVEDLQRRVQRYNSQLFIVSSTSSSVT
AKRRVKQEPLLVVAKKNAVSHVSSTTCDSFSGRDNGDGDAPEQRQGSFPAKPASSAVVKS
AAETNQSAIEKVRQQLQEGKMQCILVMELLTGRDLFTLVSRKPLNEARTAAYMMDLLLAL
QCLHSQQIVHRDVKVENIVIDPQDRARLIDYGFCEGLHWSSNTNAGCSGETHKVCDGTLT
QFCGSYHYVAPEVIRAAMRSRLSARPTDAKEANNNVSVKLPPISMTGSTGICGNLMTPRT
PAEDKNSRGMHSAASIGILRTLLTPGPMGYGTSVDLWSAGVVMFVLLHSTFPFHDERRSS
LLKLISSGRQTVRPSPLLSSDAKDLLRRLLTHDARHRLTTAQAVEHPWFKRLLQRSGME*
>TCRU_1955 prefoldin subunit, putative
MSILKGKEVEVTQDDQHRICLFARLHRRRRELVAELSRRKEEIVRLGDAADELMITDNAM
YLFGETFMETDNDEAEEWLEKEKGRLQTEQEAAEAELKLVEVALGELKANLYASLGSQVY
LEDE*
>TCRU_1956 hypothetical protein, conserved

MDRPPGPMHDLIRRAYEEMRLYSNYYAAIRIITAGLQLGERDSEENVLERQTSRQPFFGQ
SGTLQGSCSHNNDVFLSDRSGGSFTESTAVGKSRVISVSEGTKIVRVAPQLRGGFNRTQK
FRMLLLRADAYSALKRHDKALQDAQAAVLMSHERSAEAHFVMGREFLRLFQLEASVVAFD
KAESLLCSSPIFSENPCVEEISAEDFWAQRGYHMEDVQRLKLNRCSMEQEEQKAHGVLLG
QRVDDGANGVLASEHALVTSTSSEGLNVANPQLLYWRQLAKEARALHKMSTSHILPIGFL
QTALLFLDHQMSTVRFGVVACLENTTSRNFRLTGCSSPDAAFRNGMQFPDTIPPGCCGIA
LLQPRGRWGGFVCSVCYEVADNAVCCFFCFETSFMGSNRCGVRFASERRSLEFACGLRDP
NSNTSGSSSSDTTTREFVNFKIPNPSLWLPTHVAPLPSGRKLKVSGATRSNSRVIMFSVT
EILSVRLRSVELLTALEFAGPVVLKKMSAVNRRYRELVNNLPPTMFFGSSRCLYPDYCLL
SDRTSSPWIVHDKEPVRWSFVFDGRLMNRELFMVSDSTDPQNNILYFSGESCGSIETVVF
YGDRRTLIAQIKGSWVPFSSTLYFCTSSGRTFASCFLNRNSELTLVWEGASKKNKSEDVE
YVMHRVESVRRTGFGGTTSTVSNLSTVNGFGGTEESNSMLFSSSSGVVLASPLAPSRGNR
GSLVEKYAVWRSQGTNAGSAACGITRQTNNGMELVAEVTINPPSAQTVTKGSVIAEITFC
TGADALLVTLMAYCRFQWDG*
>TCRU_1957 phosphatidylinositol phosphate kinase alpha
MPYNREIIASISFESEPSSRNNSPIRGGLNDNVKARESLDATGEPVSQQNEATRDIIRCM
KWALAQSITSISHPAKQRPLNPKKDFTIETTIEVEVKKNNTLGLGCCGGAAAVKRRSSQV
MSVRESGNSKVNFTYSDTDGADDEEGPQVSFKFTDYSPMCYRHIREFFNIEPRAYCDVLM
RSRWHSTPTPGKSAAQLFFCGKDWVIKTMTTEESEFLRSILHRYYFFVLDNPHTLLPHFV
GHHRLEIAGDKINFIIMQNVFATGNAIHEKYDLKGSTVGRFATEAEKRKQTCTQKDLDLN
RPIHVGPARRQQLIEQISKDCDFLKRSHVMDYSFLVGIHVLPMHDSRRLGTSVMGGKEGI
NMLSSTLAFDGTLASGVLDGQFNQTNNVFHISPNEPVIDGRCFTADEGGMRSTDQPGTRR
EIYYIGIIDILQKYNARKYLETSWFGLVNDSKKISSVPPREYAERFLAFVSSIIV*
>TCRU_1958 hypothetical protein, conserved
MNPPSKLGEFFGIVDDFFKKTFRDDSRVFVAVKSRQYSESFPGKQLFFTAPPPSPLESGK
TDVQTPATAQETAAIESKKENYMTFSPRLTINKDGTGFGKIKLGCGLCIQRVCRFEQGLT
LTSRGVVATNVKLIDLLEGLELKGRVSVNTIAPASEDVSVATIDYQRRDFYTTLGYQRNG
LGSSDLLVDCGTKFFNLLVGAGFERQKLSYLEHHDASAQMDVLYAGLGFTGVNWSIGAKM
VRAHDAWNAARVAFYQRMAPNTMVACGYNFDLLESRAHLSLGFSQGFRLRLPTILQQRSE
EHLDEWTAILPFVGAVKAESDGVCAATIRGIFNGVIHWALIARRNVMLEKSPVQYGVTIS
MESG*
>TCRU_1959 AAA domain containing protein, putative
MADNNMMGKVMAWRTGRLGFKTTVVTLLCILLNILALYLLRRLFPALRGGGDHRHTHGSR
RRSSSRDGNSKKKHKKHHRNGEVKNSLPNLLLLLGIPGSGKTTWINQYIDRCDKSYIVLN
EDSVAGKVVAAIDDSSWEEGVREAFIGEIVRHLNEKQNVVLDDSLRVMDEEFRRSIMEAA
PPCNLLVKEFPIKALFAHARLLKVAEEVTDRRCPTEIEMEELETNFEKARACMKTEGWKQ
LLDSSHLRSKHR*
>TCRU_1960 hypothetical protein, conserved
MPSLNSLTIAEMEVTASRFLYAAAILCTRLCEYYFFIKAVRRRLSALNWGIVLETSPANL
PPSAVGLGERLRHIIENNRKRIIKPTEKASAAIITDASLHGWGAVFIPDSGDVKIAGEKW
EKKPFLIMQAEARAVRLASSAFSAILPSTMDVWVDNTSLQGAANKGSPKSHAMTWDLRKI
CLFLDSRGIQATFAYVRSAENPTDGISRGRVFIL*
>TCRU_1961 ATP binding protein-like protein
MEKLSENALLRIAENIERAVDDEMEHIDNLDDDELMELRRKRLKALREMGERRDAWLRKG
HGQLQEMADPKEFFNAVEQSERVVLHFMRRSTLRCSILERHLRAIASKHFETRFCYVDVE
RLPALAERFNVMMLPTLMLIENKKTFHSIIGFDEFGGTDDFSTDTVVKVLSHYGMVNERG
MFSDDQSAE*
>TCRU_1962 hypothetical protein, conserved
MNVNIQALHPSLQRYLPGHLPKRQRRLYTGKTNSKKFQVAHLADVETEVAQRLPLPGRLA
QSLLFSPTVTRAVVVDETTDQVGIGDSFGTHPRNDNTSIPWASVSDKKDSKAVQNRETAT
SLLAVKATGTEQRFNKETFYFGCPTLGGAGQLEYNDPSDPHGRNHIVSAWNTRHHFLPAI
WFSRATQHPAVADAMIQGSNPLTTNMKSEGPHKNTNGYETPIKELNEKKQLTSVEAQPHV
PTECTPLPNSHTWPDTYNPPNLYDVPPPKLRYQLTDYHSTFHHTKGEPNSFMYISEKERF
RSTKEMPLSTPTNTAIYTYGRRRYPTKDNEKQNK*

>TCRU_1963 histone H2A, putative
MATPKQAAKKASKKHSGGRSAKAGLIFPVGRVGSLLRRGQYARRIGASGAVYMAAVLEYL
TAELLELSVKAASQQAKKPKRLTPRTVTLAVRHDDDLGMLLKDVTLSRGGVMPSLNKALA
KKHKSSKKARATPSA*
>TCRU_1964 histone H2A, putative
MATPKQAAKKASKKHSGGRSAKAGLIFPVGRVGSLLRRGQYARRIGASGAVYMAAVLEYL
TAELLELSVKAASQQAKKPKRLTPRTVTLAVRHDDDLGMLLKDVTLSRGGVMPSLNKALA
KKHKSSKKARATPSA*
>TCRU_1965 histone H2A, putative
MATPKQAAKKASKKHSGGRSAKAGLIFPVGRVGSLLRRGQYARRIGASGAVYMAAVLEYL
TAELLELSVKAASQQAKKPKRLTPRTVTLAVRHDDDLGMLLKDVTLSRGGVMPSLNKALA
KKHKSSKKARATPSA*
>TCRU_1966 hypothetical protein, conserved
MAMMMAGRVLLVCALCVLWCGAGGVYARDTLNNSPGVCMVPGGFGKKTSYLASGCNKNSP
TLSLRPALPIAVIQAEDTEEDAGSDITPGGGGGGSGLNSARQDGSGSSQTDSVPGAGLLP
PAGVSGVGGDPSGSVTPGGDSSPDRSVGTGTSASFSSTLQGESGKKVLQRDEAKDQNHQL
PNGKAPDTPVVETQSRGSDTTRGQDVGGASTLSGKEQRSSEEPEKKDGDSDIPQTVNTPE
DPNKELQEKGKALLELSNGSLPEPKTDHPKTNPEKEKNGGQNTSASTDSPAKQEGNSEDP
VSTSDTAESLSTVSQEQTAATSSNDSFSPFQREISTEKTTVENSQPSDTARTEKRQSVNK
EKIGDSDGSTAVSHTTSSLLLLLLVACAAAVVAV*
>TCRU_1967 Trans-sialidase of Trypanosoma hydrophobic C-terminal, putative
MHGRNGELGAVCHVAHAVHTCSNCAASCAAEEEGATVAFTMNVTTHKYAGPYELWWRQFS
PDGTIHPPARSGDADRTGICQLPPPRGGKDDTKSGGTTAPQVNAEPSRQHEDWRQSAAAD
GPNTVDPSPASNTANNTSETRPEFAANPIEMMVPNASKKRPSFANGPRIVVPSNASGIMS
VTCGGREHVAADNTDEASERSATGHKSLVLENGVAGSGFVAEGDAGGLTQRNASGHDNKP
QLREFTAQYSGSGVSLHDCFALRNDAKLTLRELTADGTVRVCVYWALLFLLGLCGVVADL
WAKECGIATPRRERVPRSLRVLFCYLHFTVGFVVFLE*
>TCRU_1968 Dpy-30 motif containing protein, putative
MKMEQSTVETLGKGIFQSHTSVLVAYADTYFGRRLFSQLYQDSNADKAGKSYKLFACTWE
KDGAQVLVEERVAATLLTGAELDAAETDAADTDFAQALDSSSGAQEASLAPPPVATTVFA
NDPGTDSLRVFWRGDTMAMLHAILQADCIVMEMRQAQDVFDMMHALSTKEHLKRKRVIVV
SSLLTWYATPPIQMDPMDENGGKSNMEGDFEEKEEEEDELEEEEVEEEPRPPDQREVDAI
LGQFDFIEGEDNGNDNINDENGNETEYNVELFTEDQYNRRIPHAKYYNWREAERVVAAWN
SEERNIQTCVVFSGLQYGEGEDALQPFFAQAWNRGKRGLPIYGDGTQLVPTVHIRDLVTF
TKRLIETEEPPALPYVFATDSGTVSWQRMVNAMNHAFGGEKTFHVPSQEYALYSNVELFT
LNMRVDNQTMQNLMPAAEDWVAQSGFVANISKTAFEYVTAHHLQPIRLVVLGPPLSGKTY
LAEALAQRHYHLPTFTMERVLEEFKAYIAALKERLEKFRRHLYETEKKRREEAKKRAFLR
FRKKAGDGEGEDEKEDETEAMEAGMQSENGTRNSSAADEVPGDKKKQRVTVGEKDSSDME
ENLVDFTLTEEELHAVEELVEEWFQQNDRATQLRETIASMERVLAMRIRLQPAANAEASG
ATNPKKRKELAKSKRGNRSGNKKHTTEEEDALNELQENAPFQDKALALMMRWRLSRADCR
NQGYVMDGFPETVTQARLVFGDAPLEIPETMEEAAAGGAFASNSNLSVLRTHEVNAAPPS
KEPCEEARLPSFVFILDAAENYLLDRLKALSEKLNEPPGNEEKRLARFEDAITVYRKQFL
DTDYSLSNFFECARTVAKTLTPGGRGVTVSFLKVDAEPLLSPPPPESQYSVAPPSEVERV
ICERVGKPRNFGPTPQQQYQEKVREIKLLEEERQKELRAVMEQSEREKNEYIRESEQRSQ
VENTRQEIEVMDRNSLELRKLPLREYLQHNIVPLLSKGLVEVCKTRPKDPVDFLAEWLMR
HNPLDDSCFEL*
>TCRU_1969 Mucin-like glycoprotein, putative
MMTCRLLCALLVLALCCCPSARATAGEVKTGEEVNVQVIVSEVLNKLGGEHNSDTDVDAL
RTGKAASGAGQNDNGTPSSNSELLVPIIDTLKSESPKDVQEQALDGVTETAKEGKEKPLN
PEVTDTSDAQQSTSLTAVRKVSENETTAANTTTTTTTTNAPTTTTTTAPQATTTTNTEAP
TTTTTRAPSHIREIDGSLSSSAWVCSPLVLAVSALEYTAVG*
>TCRU_1970 hypothetical protein, conserved
MAMMMTGRVLLVCALCVLWCGVFGIAADDAGGGDGSAGENLIALWLTRLRMECAEEVIRR

KGSSANASAVEECMRRGMDGVRAVLDGRRRWRRQQFAVAAADVAGDVSDPESSSQGQSGS
DDGLKDKVPLEPALEPPPEKLDKAPGNGDALQTPAEIPGLPETRSEKMLNEKNEKEVKDD
RDPVKRIGEGVSIDIDEEVDGAAEISDENPKHEDEEATNTRERNGGEGSLNNEDEISGNH
HVSDDISAGLHVALPSVSHVNGKGTQSVEGTNGTVGEKGLAGQTIVQQAEGSPAPEEPTV
EFIQKEDTSKEAKEKGHQVGRGKEQTTIEEKSTEGAAAGNTAEDSHVTEAQTPEQSETSN
TNGNVEKAEEKKRDESTKNEEKSTKEKEREKNAVKSKNEEKEETKEERVADEKETTTVEK
KMAAVQSLTKINTTTPGDSDSSTAASHTTSPLLLLLLVVVACAAAAAVVAA*
>TCRU_1971 Glycosyl transferase family 11, putative
MSARVARSMYRRMLKIVASIKRVGAEQLALERYAEFLPCTVEVRKGESLHALLRKAFISA
SLSAESLASGFSFIKDATESLPDLELLSLWNAYCLDGEYEMLYGVALLSAALQQAENEEP
THAGELGDIVSRIRGEVRLLVNEMKEAVTATPTPSSVGRHGLDGRPSSLETAVESLWKRG
FDVSERAAEDLTALSLLRKRRASVFLLNVLLTVALHELGVPCTLVGTESHRPWIRVQKSG
SRPLFMSFAHAGVHSAKEVLRLDGHPPTSTQWWRAARWDGSGRKHILSRMLKVQLMLLSS
SLESPLRTRQLKTCRTQILFLLS*
>TCRU_1972 DNA-directed RNA polymerase II subunit 2, putative
MDDELLLQLNETEEFVEDGADYTVDGEHAGSTDIDSEDIWEIISSFFREKGLVHQQIDSY
NDFLLRIPRMARSLLEVVPRQDEQFEPGTQTETEPDQYRLSLEDVVIGNPSHEVSGFSRH
EMHPLFPNECRLRDLTYDANAQVTLGIRVYRSREEQPYRTFLQNMELGRIPIMLKSMRCN
LQNKDEDELPRLNECPHDQGGYFVISGTEKVLIAQERQAANHVYAFSRQKGLLCEIKSIV
EGSLNKPRTLQILMPYKKKGPGHGYENLLCRVAQMDELIPLFVLFRALDMGSDKEILQTV
VPDLKDAAMLEMLRGSMEDASTLQIFTRDEALWFIGKRLGKQDSRENLQRDAQGLLMRDL
LPHMGVDCAADRSKCLFIGYMVHRLLLLALGRREDTDRDFLGHKRIDVAGALLTFQLNHF
LVQVRKEMVKTVHDYSTNRGGVVSFGRILHSRLITDGLRRCLATGNFGDMKSGNIKTGVA
QTLNRLTYSSSLSNLRRIQNPISASSKATKPRNLHCTQWGYICPVETPEGGSIGLLKNLA
LMCLISRGSDHTGVVQAVQARITGFHSIDLPGLADVRVARVFVNGTLIGVHRDPERLLRD
LRARRRGGELSNEVSIVRDIRDREIRVFSDGGRCLRPLFVVERSRIKLQKTGIGELLEPS
KTPGGKREISWNRIIKKGYVELIDCEEEDSLLIAMTMTEVSKNYYYSHCEMDPSMILGIC
ASIIPYPNHNQSPRNTYQSAMGKQAMGIYASNFNMRMDTTAHVLFYPQKPLVRTKAMSYM
RSNDLPAGHNAVVAIACYSGYNQEDSIIMSRSAVERGFFRSAFWRSYKATEERKKDVRET
FENPDRKICRVKRADYTKLDADGLIKPGMPVMGGDILVGKTIPISEAKLENAALNDTRLL
KRDCSIASRTSEKGVVERVMLTENSGNRFTKVKIRTIKIPNIGDKFCSRHGQKGTNGIQF
RQEDMPFNRDGIVPDLIINPHAIPSRMTVAHLIETLAGKVACYKGSEVYATPFCSVVVED
FGKALHQLKFQRYGNECLYNGHTGSPLDHLIFFGPTYYQRLKHLSGDKIHARPRGPLQPL
VRQPTEGRAHEGGLRFGEMERDCMLSYGASQWLRERLFRVSDYYSVHVCNICGTICAADT
EQNVYKCQGCDNDTRISQTLMPYACKLLFQELMSMAILPRLGTGPL*
>TCRU_1973 BNR repeat-like domain containing protein, putative
MPQPRVTEWEGSLLMSVDFENGQREHESRDMGTAWTEAIGKFSCVWVNARSGVSQKENLR
VDALITATIEEREVMLYTQGGHASRKKRSTALCLWVTDNNRTLSVGPVGMENAAD*
>TCRU_1974 hypothetical protein, conserved
MGLSCSHKKKGETVFKGKTTTRSSTWVSKKECQVALMLQGNKASAHIDDHSLGEEEVPLT
GERPPEVLRFCFGACGGHESHVTVTDVFPYSRPLNFTAMRALKDRVPVPTREATPTPPEK
VRVVDSVREHIAGNITEEK*
>TCRU_1975 5-histidylcysteine sulfoxide synthase, putative
MDPEVDIMTDAGFRESVDGIPNGCVKIAEYCKAQFKAHARVEPKRALDFACGAGRVSFEL
SALFSQVVGSDFTARYLIPAYAMLERGKCTYSTLESSSLQRVAKMAEASNYSWDTNRKRV
TFFQSDPTNLHSHMKDFSLIVCWNTLERSSAPAAIPLHMLSRLEEGGLLVIECQYEWVEG
GPSARENRGETSLPHKGFAEEEIYNLLGAESAVERVGEPFEVVVAFPESDSVVKLKKIHL
NTYLKKKSN*
>TCRU_1976 kinesin, putative
MSMGRDMLIARRLESDTVSTSFRSLSRQGEKRFPSSGPATGQSTTGAEAVQVAVRVRPPN
CSEASQELAVTMDPSRSMVFLNDRLDGQRMFSVDMPIWSCVGRALDDSAPVTQSALYKRV
GRPLLAHAIAGFNSTLIAYGQTGSGKTYTMMGEVGSNVMDDNEDVLNKSEEGIIPRLCRD
MFQEIQRRSVKSASGESFSWEVQVSFVEVYCEKISDLLNRNTPVSLREEVVGNETFFALT
GVRRVPVSNTEDILRALKIGNGCRKKASTSMNERSSRSHAIFIVELTEIISIADSEGEVA

SAPRRSLAIRLVDLAGSERIGESGVSGQRFKEGVDINLSLFTLGLVIESLSDPRRRNIKP
PYRESTLTKILKEAFGGNSKTTMICTVAPSQTHRSETLQTLQYASKARCVMNKPHVDENP
SATILCKANEELLKLRCQLLEAGQGGAQHRYLILQLAQAKQRLRQEQESSRRRKAFFEKQ
KEELAAHFRKEEEEREEYSSKLQQLSAEVKRAKLEQKEKERELHHALCKAEECRQENESQ
RELLEKRLNRQSKDMLQRQRDAEGRVQQLESTLGRRVDELLDTVENMKNELKNKGCNITE
RGEELPEQPASRERELNDRLLEARSMIEQLQAKIDATEFDMARRILEAKREAQEKERELR
DEIEKADHDLKLRGTDVQKQWEEEMNTLRKKEMRVLLREAELAELEQELKARTAKQGNQI
ERKEKFAEKTKEMEQPASREHHATLRLQFELQRASNCLYSSETKRRVEEEEEESDDDNNK
FYKLNRRCAQLGKALAEREAVVRIKEEDLNAREREFDRRQQQWEKEAKGLNSLTSAHTIA
DFNSETEAINEAAKTPENELKARFLSIDTGTENLATRLQLSEEALLRRKLWGLECAEGRE
RNALETQEDSELCIIKTQMQLEEKEMRSFKDELRVESLQQRNCQINANWFNCVQELCFAM
EKRFREFLDSSRISEHDLIKLIEDATLPPCQRSGQHEASNHARGMDAFEIRQAELRLHAE
GVVEEESPLTLRTASFEQPQRPWDQEPHWEEDEKKEYLFEREAMDREKMAEHCRTREDFV
ATREQLHRRELALRGYAERVHCMFLEQTAAKRANSIKDWERTEREYIAKMYQLHNELRLC
RDASEGNQEGVQKLLKLKEAEFHEAGNRMRCAVEQLEADEVQWQKRLADEEQQMRMYRLS
LEGDGGQYIRGVEAKQEELERREAALEERSCLIQQEAVEGEQKVRRMEEQFKLQGAEEEQ
SRQERAADIARRRHDVASSLEENERTFLGQEADLRELIAKNEEVKQHIQQCDVELQRYQR
NYIDLRRLLEDREKLLLREHAHRMNGKNSATQLTEDLLYVNELLKKQLEVWIQKYNVLKA
DEKLECERCSWRNTRDATVCRCCGNPNLF*
>TCRU_1977 hypothetical protein
MDCKRSSTSSMPFFAGDGIETASVSFYEESPSMPHDSETAFLQGFPGESSGESMRLADTQ
CSYCRRGSNGWRCAKNEANLLPWHSLEQQCDLAAMQLLEQGRQHHGPNGRRRSQFEPLTG
MTDFIAERSLEEWGDSDNGDDPVLKASPAIPLALSTRPPSVDVQGAAQQQEDTLNELDES
YPEMPENKTKMTIMSPRADSCKEGLGASRRCRLDDLAERMEEGVEQLVCEEGDSFGDASP
PLYIFVAPPSAPADDDASARHREVSPGEHTQINVKGEVQNHDGKCERNPSGSSGTRHFGL
LKPQPLSSPPPWKVELQGTPSSKWRANSLPPQTLSDRFLTHLNEKSFSVDAAWKSATARK
YQQRHLLCTPSTKNIFLDFGSPCRPRSAVIGSWRSLSSHCKRAGLKTPEVPLLLKGSGYG
LFDS*
>TCRU_1978 hypothetical protein, conserved
MKAAGSTAVKFDPNPLKDGVSSIYTPGDGAADVLASSHISRQINFVDDGKLFEGHFTLHT
RGLKSLIQLLVDQQSEQQVIINDLQDQMNTIRQQTSKVRRAVNAAPMFVAPEGGTDTGSV
LKEVEDLRRRVKRLEGFRALWGVHHEEVEALISTYGDPVVTPEEYTAYLLNLQPFRQLRS
DAQNSAVSLVERRLSISAQQRSRESGNRKRISRDDDLRIPTERDARDNSRAARDRRIRED
EPRSPVERDARDNSRAARDRRNREDEPRGPAERDARDNSRAARDRRNREDEPRSPVERDA
RDNSRAARDRRNREDEPRSPVERDARDNSRAARDRRNREDEPRSPVERDARDNSRAARDR
RSREDEPRSPVERDARDNSRAARDRRNREDEPRSPVERDARDNSRAARDRRSREDEPRSP
VERDVRDNSRAARDRRSREDGSRADAERDARNESRAARDRRSHEDAPSGFDDEGESLRQL
ESRVAALEQRLRRGVVAVHSSSRAASANSGHSSVSPIEVVDEQAREDLEELERFVVRRFK
ELEKALSKSRDATPAAATSSVRGAGHPVGRSERLSHGRSVTPTESGGSNAKRSNEAALAN
KDNTNASESKQRKERNAPTDAAVVDQVAREDAFAALDQVANLEKFVNRKLKELSVAIGKE
LGPGPVRPSGESRQAVSLEPQPQGAMVDQIARDDAARSIEAVQELEEDWGKRWQSLEERL
RIIGRASVSKGSLQASAAASSIDRKAREDASMSLMRIQQLEREISGFRRLLQRQETPITS
EEVAPPDARGDSGVVFPVPPKVQRPATFPLTGKGPDGAVGSTDVEYQKRMQDLEQEVEQR
MKEVNRALATLRSTQSGFPVGNSPGATDAFGGKEPQISNQVVFNSRDTDSLELLEPSVSF
SRFPASQTAVLVMKSREAAGVGSPIRIVEPMREDAIVLRSPQLEGFGTPLVKREMPGAET
PSQGMVRNEEEQLRQMRATNGGGTESWQGVYSSGPHLAPHGDGNLPVTDAAGGAAVSTAA
NANLNSVPMTLANADLRSHPSNSRLPVGAVGRASPMDRTGELDQLRSHSTVLHKVSVSPR
GKMGERFLVEQNALGHITPCVVYNCAWCAAQKQGISAQTARSAQ*
>TCRU_1979 hypothetical protein
MPAVDVDLTAIPDMIEAQLTLSYSRLAKLVRLIVDQGNGHDVDIDKLNSRIDALARENAE
LKALVESFMEEKSRESWVQKSLDELRHEISCVSDDCAESSKQMASAMEEVHLRFDDVDQR
LKSESTARANEVNQINTFVNELQEVIRDMRLTLSQCHGFVEVWEGKAEKTEALTRRDAEG
GFQQSTEDRVDYLRSLPVFANVFEEMDVLRQMLRQQAADALSAKSAAGAIRRISTAEVAA
SGVPSTSTVDRTVIDGLQESLGNALKRLKELEERQTPAFVRTQLQQQHQSAAWRGEKEDI

IQLDNRLQNVEDRLRNLSFQASEGASLEKALLTDVDGRLRSLENRIDGLPLARPLQQEQQ
SGGEDRFPVISGQQTSKAQPQSQPSAPAVSTRRLPAPMGKDVPTGETGESMALPYLTTVP
FNGRVEASNAKAVQGTGNGSNSGVRSFVNQSNSEAQRNPKAISYQSLPQSQATVQDDGVM
RRLAQLEENSAVLEIKKADRQELARLEDALRNILQNPAVLPTSALSRGIMPHRPMSQQDA
RGINATESAYCSPPRSSMKSPPPGRPMFVGGSAHQLRDSAGAATPATLSTFHTV*
>TCRU_1980 hypothetical protein
MTALPVYIARCRQCGNSRRTYNDPRLGRSVIECKAACYESHYEWDFVEECKAEEAENTAS
SVPLPSEATPASKGTGASKKHRRMTSRCTVV*
>TCRU_1981 hypothetical protein, conserved
MFALTRHFFRGVAPDELRMQLRLLRGIRRAARRELRQRRCRHRSAQQDCSGFSTASSSSS
ACCSVHVELPPKNDALESAIALALSENRDARTELEFTRHFLTGSFLSLASPVGAWDPYDT
KPQVFALDNYVALPVFTSLPYLRLFCRRFQFTVRDPSGVLWADGVAESAEEEKAGAKGDT
LPLPDVSNSGWWERQVQRHLFATALEPMKSPVNEQEINSVETTGEKEDISWLHADDLFAS
MDATPVEDTSSEQKEDRVLKRKGRTSRVGRRHKTTLKSGGNTGSRRAREAGGKTPHEGGA
APNQDALKEAFWAKVRSTAPFCIKQATPLPVFGPLLRPFFVGYFADVDTLLHNASIVPEN
VDIVLNPVSPIEFVLAREATDRVLHKDQLLHLAYRRVERELRGEFHRFFRYLSPEVAWAR
SACVPFSLPGRPDEVSYELVILLQSDDIHRTFSSLRAAKLHCLLMGHTDLDVLPWDAAAP
YVREASSLFYERGASKDKNCEFQGDLGVFEQRGPIQTINVAQPAESFYHDPTAAYTESHA
VFTEELKLKRRVR*
>TCRU_1982 hypothetical protein, conserved
MASEAGPYPNSPRLGQTEMNDLVRRLYHQQMDRAARREEERRRELSKSCAPPRYIKREEE
GELVRRIYDQQLERFRQSKEERERRIYEETHRCDKKLPESEIQEQVDRIYGQELAKSKAR
REELCKRYLPEMEPKKVSKAKLKESVERLSHVDYAKRDEELFKKHVYPYDPPTVKISRDD
VEAMANRLSTRGGS*
>TCRU_1983 KU70 protein
MSEYSEWLEAAVGMGALDEATDQDTLQQATVYQQRRDAVLCLIDCQLSMFQGGIRSSDPG
EISRIGADNTPGEDSTPAMLNSVATSGCSKGRNTTMRKQSTDVFFWPDVGGEGAERRIPA
GMATGDEKVTDPEVTGNRINNTPFRDAVRCVLKLCQDKVLMSDKDLVALALYNTNKRHNI
YELPGLYIVHPFSLLSVQSVRELEGLAASGDVSSMMYEEFKKNIGHAQRNEVHLSDVLWA
AQHMFLSLHSNLIKYRRVFIFTNDDEPHKGETSEKERCLARVRELYEAGVTIEVFAIGKS
PNTGAGEMGMGRGEANFDALNRATRVADTQLQGGVKQREDTVRFTDCVDAERHFKHEGFW
DQLDRGERFAQGMIEHERLGAVHVTGGVEVFDHLLNAVRRRTHPQRPFQNYALTIGISVG
GSPVPTMAVSVYHPLLPATLPRAEWLDHRTNAIVPRRAQLVVKRPLSSFDSHRLNEQETT
NDAKKAEDSRVIGTPRPNKEGGEETLETVVNPDHLLYSTIVGGRRLFFSAQERQHIFLTA
TAGAPVGFSILCFKHKEDILQHKHVICKSSFLRPNPYDGGEASLRLFVQLTRTLVRGNKV
AVAQYVAQRGAAPRLVALAPSSPAVPVPTCSLPAKGLGLYVVPLPYSEDIRDVPSLPCFN
PATIPSQEDVDLAAGIVSCLSVSYDINAVPNPSLQLRYQALKEMALRQATESGVMMPEKS
GNKPGTMADMSLPDSEGMAQYAPIFHDFNAKLLGPSYDAARLCPQPKRSEFAGEKSLRTG
SGGHREGDDVVDNAAIHLVETAFEQNALPTLTVMQLKAYLSALGESTAGARRKDEVIEVV
THVLLRQRGERA*
>TCRU_1984 hypothetical protein, conserved
MMPSASLPPRPRHTFSLRTVEKLYQRQLESTGAHVRPEQIPTIVENVQRSLLRVEEVLNY
RLQCPASTFHEEKERPLLSKFERVRVGQILANAVLDLAIISMVGGNEMADAVTSTRKAPS
LSSSHVAAPSLHPYGTEELSDECKHQKLESNLHVGAVEETMELPEVNLLLPVPTGGGSDA
GSEASLSVPGRTIVSSKNCRSISKNVSPLAERLPHMPQRVQRNNTITSVDSHGNCSNCKP
ARASLLSQDMVPYQNPLSHVESPLSPVSVHFKPVLSMHSGADSSSTYPLPRQRPRRYYDI
DLQKVSDFTRCLRTVQRLNFTDSDFEKIGLRYAIPGIRELHPKEVQFLLERVDESCKPHG
NPFLCSGEEEFFVKRTISMASTGGGTDADVCSVCLTRRETNPKLVHTPSAVTADPQLPGH
GTLKKTRVSYCQSFTQQQGPGERDTPTNDTGDSSPQSASIKEQCLPLFRGGKETIVQKKD
LQIFLAMVEARLKSVDQEINGRLRFSGTDLASSSGEDDTAYIA*
>TCRU_1985 Protein of unknown function (DUF3676)/Trans-sialidase of Trypanosoma hydrophobic C-terminal, putative
MGQTVPYYFANNKFTLVATVSIHEVPKEDSRPLMGVRMNDTDSTVLFGLSYTHEKKWLAI
AGNRATSKLVDEWEPNKTYQVVLRMDFYSWTVIVDKKEIHHQRYNTSLFDSYRISHFYIG

GDSKHQSATGGHVTVTNVMLYNEELWGNDLDELPDSKVTIPSLGVEEKPTGPVTNTGASV
ASESRSEESTASHELADGDTDKQGENSLHNPVLAASSSTVVGGASVSEPATATESAENSR
QEDNAQFHPEESVQQGTLNEDYKSMQRDSDLQPQELLSAESTGVTDVEGSAESYDKEQPE
EEGGTNDRSDESTSSVGASSDMDTATETVDSGHQVQQSTELSTENDDVRSTGTGTTGAEQ
SLGLEAGDRNSERAMGSDSSLTPSKSDAEPTSAENTDNISRTDGAEFSSEDGKEVPQTVD
TAPGNTNTTPGETMISLESNATAPSDAGILPEHGHYSELAGMALFAESTVRGCVFRVLLL
LLLGLWGAAALC*
>TCRU_1986 MORN repeat, putative
MSMTVPEVILVGQKTRYRHFARGPRDLYAPEEMLPDPPPQHVWSYCVSREHNNTVYFHND
ATGRSLWVLPSLASDGTEAGGGAEEVMRTVGSVESASAAQKHVKFGTTDEKSHEAATVRR
AVMRQSIEKAMSQAETQKPTETVQKKTPTGSVAEILNNENSDSNGKGHASPVRERLAVWR
AKRAQQQQQKEHDGNDPVTSSYGEAEGSRVNHGRPAFSDPNTATTKMTSSLDNKIPSQIS
ECVAASKGSGFKKGGQPAKEVGNLEESGQCQQEQQHNSLEKDVYDERQRLFLEAHEELQR
EKAAALQKERLALEQVARLQEAQRREEEERTRLAAERKRLEQQRLELQKRVLERQRELLA
KVEEEAMQRELAAQLDNFFENKSRLEQQAQEERQRVLSFEDAIAESLLEKAVKNHTTTPK
KERIIDLRLLEESDEDNNNRHGVSGREETCKSRNSKERQDFLQQKRQKEQKQQRRERITY
SPSLVYVGDVTGGECKTPVFLQRQGRGTFFYDIAQKHFFDGEWSGDKRNGSGALSLPHVA
VEGTWRDDVLEGSATFQTRRLKGKGEFKQGVLDGNAVVEIDCNGVFAGSVKKNGAVVASG
AVTLASGDHVEWIRDENSSENNNNNNNRQQTSAHSKNFNNGSGRCRIRFENGDVYVGEVE
NYQPNGAGTYRFAAEGQEYVGNFQNGRLQGRGTYRFANGNVYIGQFYKGLFHGEGRYVEQ
DAYTYEGFWEYGTAHGRGKLIFANGDVWEGEFDHDRRVNGHYTASRLFQL*
>TCRU_1987 conserved protein, unknown function
MGYTRERTHRHFFVARANAFFSRLPIARIQRSLALEAIKQGRMKPWKHTKEQILGAPITC
NFDYNPRPVRLIGTVMDAHTEETSIKGGLKVYARNEETNMMLWIPVGNPKLKYEVTATKG
SFQHYLDERDKWDEAWLTGRARMK*
>TCRU_1988 ADP-ribosylation factor family/50S ribosome-binding GTPase/Signal recognition particle receptor beta subunit/Ferrous iron transport protein B/Gtr1/RagA G protein conserved region/Ras family/Miro-like protein/Elongation factor Tu GTP binding domain containing protein, putative
MGNLVARLRELFSSERKLEMCLVGLENSGKTTLLNVLSVGHPIETFPTVGLNVKMVRKQG
VQLKVWDLGGQERFRSEWGRYTQGCDCIIYCVDSSDFQRTELAKRELHKLLEDKALHGLP
MLVCLNKIDLEPHMSKEECVEFLNLQVLRDNPWLVIPISALRQININEVVDWLVKNSHS*
>TCRU_1989 gamma-tubulin complex subunit, putative
MATHLWANFVTSLESRDVSFQQVQHATDRRAVIVEAGFNDPITIAVLETEWMKRCKTSGP
SSTAKEAVGVPATVAAPPAPLCAQFADYTKLEDVPEEIQDRVVATDVLAAVIGYGGSLLQ
ASRDGFIVSRYVPVSMRSLCKTIVPVADSFVALRKVERAEYMGKSLVAMALGEVVSEICT
SFAHEIANLQRWAQGRAMPLMGVVSEVLRAGHHIVRLRQVLPTDTIMDEEGTLSMVGWRL
LNHIHEQSERYSGSREDDELLLLLLRRASVPYLRLLHRWMHEGLLEDPYGEFFISEARPP
AGKASVSATLNENTTTGMRNVVSRIFPNTAAADDYGEATLEEANAFERRFSMNKNMIPNF
LLKTSKIAKMVFYAGKYCCLLREYNGTLPAFGDLAEHMLVWTDADKLHRKIEESYEIASG
EVLQLFLGPQVDLLGHLISLKCYFLHQRGDWLVDFLDSSEELLVKSPVHVKAHSVRVLLQ
AAIARCCETDPYHSTIGCSFSDGTIEQYVNGSFEPATDEAAPRGSCGRKSAMRIETHRCI
ELLQLEADLKWPLTMVLDSVVLRRLNGIFRLFTWLKICERNLTTAWTDVDVPHQPPQAYG
IKHQLIQFIRQYQFYAAHFVVEPLWGRMMGRIGQADSVFAVNNALQEFFEGVELGLVLAS
SQRFRSLSRILEIATSFSEMGRREVSMRLRAEELGKALQSTEERFLKALSELASPVGPDY
PQLIPLLTWIDFSGFYNRNGVYRVLYSAGISTETVTSAGADRRRH*
>TCRU_1990 alternative oxidase
MLGARRNIMARKTVSAAVRTTVLHVKGPMVALFFSNRCCSVNPAIPTTGGSDAAEVAAKR
EARPALQNDHDQSHGSSLYMPFMGSSSRFTFASPITTISDINALEGERLTHSPPQRFSDH
CCIYLVKLLRWCADKLFRERYLHRATMLKVIAPAPPLAGAMISHLRMILKKENPAYIPGK
GDFATETRGLLAQAESHASHIRILMLMTEITYVERIAAVFLQATHFAIFAFLFLLSPRVA
FRLMGYLGEESVVIWTHMINDIDLGKVTERALPQDAIEYWGLHKLKYTRASAADVKRFDV
REHCDEEATTGKHQQTSDVAEEEEITLRDLILLLRSDEMLWREACHRMADEMNLIRKSSF

MSSFLS*
>TCRU_1991 retrotransposon hot spot protein, RHS3, putative
MSAGSYLLYQLLHCDVEKLQFDVYSFGGSTTYVCDKAIKTVTRHVGKGASKDVLWDCVMK
GHIFYDVTEKGTPPAEYFVSSTGWGMSVVSPPNVDNCDEWEKQVKAGRIIMNCPAEVDVK
AMCVCMKRGLKPDEQVEYWKMVEEHMEKVGPIPRYIFDAKKFVAHSAAIEDALDGIKSRD
GERHFAHRE*
>TCRU_1992 Uncharacterised protein family (UPF0172), putative
MSSSSTPSVVSSVEAHAKALLHCQKYPTQAVSGFLIGKRLTESGASDSVFVADAVPLFHT
IMMTHPHPMLSVAYAQVSSYARTKGLVLLGYYVANERAGDSGISPLTTNVLRMLHEKVST
GHNPLLWTIVGANRAEGVDVRPSYYMGGKNYLPAPELTFGRWNSDTLSCEAITSNTEAIE
VFENAVDAFKQFQLVDFEDHLERVQLSYLEQTVQV*
>TCRU_1993 amino acid transporter, putative
MDTESGNIAPGNDPVVVPELGSENGSGVKWRRTSSGMGALRCFTNIIPYGGLLSTTFNLA
SATLGGGVISLAAGFQMSGVAFSIILLVLVTVCTVYSVGLMMQAVEMTGYNSYAELSKKL
FGRGWDHFTVVLTWLFTFGTCVGYVIAIGRLLEPVLSDPSLPEFWRSEAGNRAVTSAIWF
VGMFSLSLPKEINSLRYASAVGVICIYYFIACIVVHSVQHGFRGGKLRDDVAMFKSGNGA
IEGLSIFMFSYLCHMNCFSIFGEMRKPSARRMTLHTACSMALCCIAYIVAGFFGYADFGD
EVTDTVLVFYDVRRDVLMAIAYAGIVFKLCVGFALCMQPARDCCYYIIGWDVATIPAWKN
CLFCGVMALCALLLGLFIPVLNTVFGLLGSFCGGTLGFCLPALYRMYCGNWSLGTVGIVN
YVCTYLLLMAGVVAVVFGTGASLYGVVR*
>TCRU_1994 Cullin family/Cullin protein neddylation domain
containing protein, putative
MVEKSCKYEEKEDERDRPCEEVVAASRLREAYAALDRESFGTLGVQALSPRIPCASSLLE
TFPSLRELFTVTLNLEVLLRRREGDALRTIREKHLKWYKRIFHTLHGVHDIHALLRYWVK
GVVLYEFCEINRHMEKEDDDEEINNDTGRQNCERGGVMRAADYAMELREALRRFPRLVAF
LFLSKSQEYWAQTLSEVIKEDVGSNLSDGLKLNVLQRLLMKVLQCQRQRRYGVLKCEHFG
TVHPHCHNNGNPLHVVEEVRQKLTVGNPSVDISELIQLFSHPARDAEERLVITVLQSVVH
GCRGFFQREALLSLLEPGINEESRHVFLLKIWPQYWNNELEVMRELPAVEEACVKGILVK
LVFVWETGNGVSVQETSNAPCGLRPYYIFHLMRATLQPLLDALFALDAAAEEGETRSTLE
LLLDIANSMACIEAPPRRTLRLWDQVGAGAALPSSIPREVGYVVMADVCRAFLEVRLPSA
VDHFLSGGEIPEEQRWPRLIAYLIRTEHLFHRLCEMRLLKAKLLDNCSAVPCFYGILKNA
IANHLQRKRNADLFVETLLGIMQTYLLYGSHEGAEVRRVGGGGREKMVLVSLHNEDEVMS
AVRLAFSVSSIDRFLRLYKELLAYRLLFYAVGRARDGSVDAMEARREKLQTEKLVCEYLH
ELLPFERNAIQQMIQMCMDMVTGNNAPEAYDPQALQPEQCGHGADTFSVRPTLRLLSRLA
WPSYPLLTDVPKPLHNVMDQFARFYHAQYSNRRVVWLRTSMETITFTVAYPQAGKVIVGS
LELFNIFHFLSEAGTSGTTWTLLAQRIGKDKTLLQRGLKKLIHDGFFKIQMGSGEEIVAL
NAAFKSHKPRYHFLQPPPRVGALLEENGIINEEVHRSRIASIQAVIIKHMKTVRTCLYDE
LFCWTRQQNPQFQLQNSDFKLALEMLIEKEFVQRDPSQKQRFVYKA*
>TCRU_1995 hypothetical protein, conserved
MQVKEQRAVKQKQAPVNLSVRASAATLFLTSVRRLTEAHGLSFFALQRGETAVRCRALTD
LLTASSMEDVLKQGALEMQRSLATLRQPAEEWSRRWRCPHAEQEQGTQCLTQPALPSALL
RLLVDMGITKASEPFSVVGGLPPAMEFWEGCPRKSACLAAYSPSLHPLTTICINAIEAIT
EITEDLPLESRVAAAKHFLSRQSEKNRIYSEQGDGEDNTAKNNASGTADRSHPAQGPTVR
LFCLSPYASYAALYEAVNKANDSESLFFGGEACEVLLVYICTMTAAGSVGPTTLNDNPEI
WGRLRYQQAGDAESATPGNRRGNHDELFFIAATLGDYLRLGSAFSWVYGWQLCYAPQGPP
PRSLPWLKLFSPTALSAAISTSL*
>TCRU_1996 BNR repeat-like domain containing protein, putative
MSRRVFNSGVLLLLVVMICCGSGAAAAVENSDSVADPTFEWRSITDDEGVTVDSLRVPGL
LKVGTVVFAVAEAQCNKGGEGSFTGIASELLTDPTDKNRWKY*
>TCRU_1997 BNR repeat-like domain/Concanavalin A-like lectin/
glucanases superfamily/Trans-sialidase of Trypanosoma hydrophobic C-
terminal, putative
MLAGKYSRTAAAKDAQERGAGEWGLLLAKGELSDGDSGSNKKVQWNVNQRLVDTLFKREQ
DSLMQLVAGGGSGVKMEDGTLVFPVEVTKKGESENDGEEGKSVSLLVYTSDTSDDSSWKL

SKGMSDGGCGVPSVVEWEKEKLMMMTACDGGRRRVYESGDKGESWTEALGTLSRVWGNKH
EEQTKRVASGFTTATLGDDNKKVMLVTLPVYSKEKGSENKEKSELHLWLMDNTRIADIGP
VSEKDEDEVAASSLLYESAEGEDNNKEEDKLIALYEKKKKGGEKPSHSLLSVRLTEQLKR
VKEVLATWKKVDEAVSKLCPTASAAKSALTDNDCSAGEITAGLVGFLSDNFSGNTWRDEY
LGVNATVNHNGGAEKAKDGVNFRGAWAEWPVGRQGENQLYHFANCNFTLVATVSIEKAPE
GDNPIPLMGVRAGSKGETKLMELSYNSEKKWKLFCGGDTTNELSSHWEAGTAHQVAIVLQ
NGTQGSAYVDGKRVGAAPCELKNAESQEISHFYIGGDGDSAEVREVVSVTVKNVFLYNRP
LTFSGGDADLEEAPHLPPANPQPTEAVPQSVEGDQKAMPPAGKPSEAPGEQATLQQPPEE
RETQENTTVGESATTQQVPANRSQGSVGSAAALNSHAVGGATGDGGTVRGSGLLPSLLPL
LLGLWGFAAL*
>TCRU_1998 BNR repeat-like domain containing protein, putative
MEVVGKAVDPFQGTTPAPYKWQEMTGSEAAAGSLRVPSLAEGAGGVFAVAEAQCSERDGA
CGHAAIASTHMGTGGGGSKAISAMDAGVSLLELVDAARGTTRAREKVQPTTIVSGDSVYM
ALGDYEKKTSGDEAADADGWRLLLTRGTLTEDGGQRKIMWGDIRAVDPVAIGLTQFLKRV
IGGGGSGVVTKNGYPVLPMQAVEKDGRSVVLSMRFKMRIEAWELSSGTTGSNCKEPSIAN
LEGNLILITPCAAGYYEVFRSIDSGIRWDMSGRPITRVWGNSYGRKGYGVRCGLTTVTIE
GREVLLVTTPVYLE*
>TCRU_1999 retrotransposon hot spot protein, RHS3, putative
MSGRPESIQGSNVESQSSTVSQGGQRRARSEFEGDTDHSSATRRRLEEIYRPQWTMSSSL
EEILLGGSILRTDMMLNDFLRSNLGGRGVVDTNENVAMEIFVLRPTIFINDNEILGLITA
SPSYRELEKRKTLLEAINKLNDKGVVSLGQWRGFKSKNTITPFARGKLNEAITQVQTAES
SEVNERMSRKEEERRRRALEMKFTISTTIEEVLFKGEFRYKDMNLNDFLLMRFGGRGIIA
TSENVLLEEFFKDPAKYIRVKGVLNEIKTTDAYLSIEGVVREEMDKGEDVRKLKQAGVYN
LQKWSEATAQVKESVHDITKSFLDAALEEAKKPTMTSAPIKMEGCYESVYNSRWSHVVEV
VGGKGTEMKVKEGKPEKSWTYRAVGRTLERNDGLQQSGEVPPRLMVLASDKGWPYSWNVL
HGTGNDLCVNCEVERVWQIVLDDITKWFSNFDLTLNPSPLPRVLIGTPGIGKSVNAGSYL
LYQLLHYDVEKLQVVVHCFGETMYVFDKTTKTVTKYKGNIASSSVLGGLRQRGMKGYIIY
DVAKEGTPPNPDFAPSRGWGMIVVSSPKVSNYAKWATQVRASRIIMNCPEKDDVKAMCAC
MKRGLEPDKQAGYWEMVEKHMDNVGPIPRHIFDEGSYIDRMDAVNDALSAIKDTDVEEYF
TLGGEEKKWYSEYPCHKLVKIVRARKVEGAEGFFNAPICTDIEFRIADRLAEKKGAKDFL
LLILASRGTLVSQLLENLGLRAFIYGEFVIALLKELKELPPPSPSEPQDSVLKINHQGYP
TRTVGLKGLEGGVTRIPMEYGVLYIPAVQNFPLVDGLFFVDSPRKTLVGLQITTAGEHHT
IPSTVRLFNERMAKYFNGWEELSRDLSWEMIYVQHENSTMITNWQRCGPVNTVNLSDDEK
EIVAFWNGKVHQYQFALTTDFVNRIRAK*
>TCRU_2000 hypothetical protein, conserved
MLASLSRNEFGKVEFDFITCTICPAMKTIFMPTSGSTLEMRILLSNILGLTVESVEFYRK
ETKGFFPVLDESMGVGHYYVLLRIRGGKGGFRKQLEKKGRAFARARRLRGSNQTAAHPTP
TKGAEVDLSKETEGKERELTEKMSSGEEILQSNTVLICKIRDAVRIGVKRTIESLVCTGT
*
>TCRU_2001 hypothetical protein, conserved
MRRVAHSRFGAGGYRCISGVAPMLYFPTNPTSKLTKDEQVKRNTSIAIEMIKRYKGPIPP
QYTRKSTSTIEQLEKEIDALLGGAEKMRKSTTDDQPMDKLTLMERCLRHALWSYHKEEGR
CDFDQIAKWVVYTPEDEVKLAQLKREAESKKKLAAFRKRRAEQGLPSALLPKNNWAQEYA
TVIDREIVTAKRSRYDALAVQTTECDDKQIEAVLQEYRKPAQDRRLDELVDLLERFKPVL
AREAIMQRLTIKHLEGQLGVWRYMDWCPEVRDRAELEVDITGWQWWSPLEERRLLPVRLR
SVNEVREIMAKAQAKKAAEQAEKSPKAIQSGSSDSARDRLLKEVLALQARINQREEIDSS
KNDQKKRAHH*
>TCRU_2002 stress-induced protein sti1, putative
MDATELKNRGNQEFSSGRYKEAAEFFSQAINLDPSNHVLYSNRSACHAALHQYLNALQDA
EKCVSIKPDWVKGYVRKGAALHGLRRYEEAAAAYNKGLSLDPSSAACTEGIAAVEKDKVA
SRMQNPFANVFGPDAIGKIQAHPKLSLFLLQPDYVRMIDEVMKDPSSVQKYLKDQRFMAT
FMVLSGLELPEDEDEEEEKVRRQQQKQKEKEMREEQEKKRAAATELSSEAKEALRKKEEG
NALYKQRKFDEALQKYQEALAKDSTNTVYLLNITAVIFEKGEYAACVEKCEEALEHGREN
RCDYTVLAKLMTREALCLQRLKRFDEAIALFKKALVEHRNADTLAKLTACEKEKEKFEIE

AYLDPEIALQKKEEGNTFFKSDKFPEAVEAYTEAIKRNPDEHTTYSNRAAAYLKLGAYSQ
ALADAEKCISLKPEFVKAHARRGHAFFWTKQYNKALQAYDEGLKHDKENAECKEGRMRTL
MKIQEMATGNSADGDEVAKRAMADPEVAAIMQDSYMQLVLGEMQRDPSRVQEYMRDPTIA
AKINTLISAGIIRFGK*
>TCRU_2003 Repeat of unknown function (DUF1126), putative
MTSPTPGALGHDVPTEYIRTLDPKLLPPRVGHNWLDPAFRKVQDKPQQLELEFRGKRAFT
AKGIYDHTPPKGLGLTAQQLLHALSTRRKGGIEADRRAQYTTLSDKVLRFYAFFTEKAPD
GCVEEYWHRRVVIDFFPEDNTVLIQEPCIPNSGLSGGTFLKRQSVRADPRQREEFPDEEF
LSLNHFNVGRSVRINAVEFFLYDCDVFTREFLTALGVEVGSPVSCPDSSFMSDWKRYQER
MLSGKFGILSQEYHGDEAVRAARFVHDSGKVLRFYSLLDERNKVPVGIVRKLEVLYFVED
DSIAVVERPTSNEAVPSLFLSRGWLPKVGSIAKANELTFAHRVNGMRQPYIGPEGHYTAI
DLGVGITINVLGRSVFLYDCDDYTRSYYLETYGVKLSDAVDCSIDYGISPESRVTAFRLT
STPASVPLTAVASTGGEHAFFSDKRTGPKTKDVLRFLLRLAPPCSVVERQRRFTLTYYTD
TDDAMVFESPVRNSGFVGGCFCSRRRLPKQGGDAEAYYTISDFSVGGFIVVNGHKFEVMN
MDEHTRNYLAGKNDPVMNDERLHLLISAFRLFLGTRFHSFKDAFLGFDRDKDGVISVTEF
VGHLQDLQITERRLDGEALFNHIASCSETGYLTMEDFVRWMSETATDKGQKNYESIDVDE
RAVMRKALLQLCERLEARCLNSLQMFRLASTMPRAYTERRADIYSLANPHRDALVTPVQL
RRCIEEVLGGNPSPREMDALLSFFFPQLPLEEYRSRRDGDLEHALDLKAFQKKYHEMNKL
LMLSEPSKQQQQTQQRQQE*
>TCRU_2004 phosphoribosylpyrophosphate synthetase, putative
MAEEETDIYLSPADSHVAVHEHLMRIESAFYYSPGDAMAAVHPLQGTVSPRRGKDLPPMC
IVSGNGNRPLAEAVAMLLGTPTHQTLVRQHANGEVNVRIDESVLGADVYIIQSTTGNEVI
DVNTAVMELLLLIRKMRLSNARRVTAVIPYFAYSRQDSKKGVRNTLSASAVAEMLTQAGV
DRVTTLDLHSGQIQGFFGNTPLDNLQTNQEFAKYLRNQSWFDPNNMAIVSIGSGGVERAR
KLADRLNIGCIVTILKRCNASGGKTLQPVGDVKGRVCVILLGMCDTGQKIEKACELLQAL
GAAKITACCTHGILTPPCPQRLNDCDALSEIVVSDSIPQEEHLRLIPKLKVLTIAPLLAT
AINMHTRDECISALFDTPPHPVENQLPPPPPQVLSSQSVH*
>TCRU_2005 suppressive immunomodulating factor
MSSAVTLRELDTLERADQPLLERFERCRHEVVRVSASSILHDCIAVTTKGGFVELINIDT
GAFRLFLSHLGDTPLESLLRCFSLVPSLYTARGRHHLLYSLAYSNELLLADMEHGTVEVL
ARFHSRPSVVFCDGDYMVCGEGCGQVTLWRAPCEAAGAKLFWRRPVFSDTVLCISVQRDY
VFCSSADYHFYVLTLESGEVVAALPHEPSPAVAIQSAANSQVSHTLTVLCLPGVLSVYSP
PPAQEDVSAATTIANSTWICTALLRLDVQISCASCCAGLMALGTASGVVLLASMITTNGE
AVERVRFDVGFSVIGIQILKNGELVVVTSAGDVWKWTIEDLLPREADMMEGQEEEEEEVE
NLFVPQPHMRPPDTRWVKTTTTAEAEAGAAGTATSGERVRMSDEEEIVVEVGDEASSTSS
ALWSESCHPVQLEKSTAGLGNPTGDGITEGNAVPLLRSISHSERTIHVNPIHISSSTKST
DTATAAVVVAHGDTATEDHVITVRGVDKSASVAVISVNTDEEKDEVNHSVDDDHDDEYSE
HSFQPSSISIVAGTIGAADAARSVILAPLQIEPNMNSHSVPHVAAATPTTITPTTTNTSP
FNADPSAHRDGTQLHQEDGVVATAQKAKDAPFFPVSTSSACSRALKCLLRGTELGPPAVI
NGLRAGRRMDPRKARHIVENSCTDAAALPEPESQSSRILVDHRQALEEAKFDYAEYTKAH
LLKAESLRYRYPVKLPVHGLHTHLFAAAVPRLQDRPNGNMESAEDGDKRKKRRKQQLQQK
QQQPIIDDLMNATFQNFTRRKDAALEEERLRSGPDIQQHFCDSLLFGPVDPSGTVLFEET
RVQLAVPNMLFLPMPLPPTPSVF*
>TCRU_2006 Exocyst complex component Sec3, putative
MATTGDELRSLVELVFRQRHEEVLTCESVVVIGRGASNKGSKARLLALCRPFAPSTPSSP
SSVSQTTLNVVRVESGRVVKVKLVFVVARLVDVSHDGSFDATFNFGGSGMISVAFESHIQ
REMFVAAVRKVQRDLQPNVTTVSRDVRALLTDALGAEVEADAAARVKSLRHEKRRALTAE
DEQRLQHFLGRHGFDDIRSTEKSLSMRQKEAELSSVEVLLASVGAWEVVRSRIVELVGDV
EELESRIAQYSRQILSKKEQLQRIEHQSNTLQRKQRNLRKLHEHLEGLCGQLSLPPQTTA
LLLRLASVKDEDLVDFFAEGNNAQVLSVAMKHMRSLLQNKKLGTDYPITAVTERRAFFIE
QRRMIAHRSKAYILATIGKYETTYLADRTRYSSKGQLVWRIHSELMAQLANIRDVITALL
YIDLEGYIAVLRRYRVSMQRIYALEMHKFFKHMRTHVKKVGSRGPFLLGTRDYSMDEALN
SRMETATITTAAGGETPRRLWGVGSSVASTPAASWTLTPHTGEGDGGKDGTLCLELPTAR
EEGLRLYDVANVRADAATSAATPGTTPNAIPAAATSYSFTSLASGWTGTFAGKLRPDLAL

AMVLETTFSVVLQEKETLQQCFGIIEKAPTEDDTSGLSRPFDDDPLLACEENPAKLLNES
LLELFGGDKLVQFSPSKAGASSSSYSWPQDGDVGLIGVNKNNNNKHSCSSTIGNNAVQAE
KGCYLLRQLVGIAEYCGEKCDRLYAVPVMAMLGAYRRDGTPTAESSFCQMLLQALEHVVS
STISRSIAEQTTSIATCRKRHVVDPRGMLSCFTKLPVFIHHMETVHNALPPAVCNRSKYA
SITLSFVDQTFEALNYITNFGGTEPRREKKLSFIESIEKRASSWLEGLDNNPVKRGIIQQ
YRHHAFFCTFYTSLPSTAYAVELLRERYESSRRLRDHYEEVYLTRILLVKDFPEFGNFAL
VAEDLAQSHSREELRHHNALSVEAMGRILQCLPREVRSGISSSSRRMKKHFLRDVADRKE
EKLFHCALLQHVWQHFSTLLLQKIEFLETLLRWPLYAGMEMTITRAEVLSLLQAA*
>TCRU_2007 hypothetical protein
MDAAGGGRALRVELDGANRLAEFRLQKVIELKAEVLRLRRLLLSLRGGSEALERVGLKKK
KRNSGESLATCMAESTKSGVTRLTQEKRRVLSSPSPCIYTATAAAVSTEHVMSPGPRVFS
VASCMSRGASRTPIAAQRRQESSAARCGSHRLSSPLLLTSSSSFTKAKPKATTRPFIKLH
TGVELRGPKTFRRVPPPLSLSSCLEGAAHTTSMPTATTTALDVFSAGATTPPASPSSRFC
SSLAIRQEHEGVLEALAMLRGIVLRADRSMLSTLTTLSNRRHIDHALPTALAEFKQCLLR
VHSSLGDKLHDGEAWCIDEAERDALEVMEVPGMAAALRDVVITYDYTACIHQNILHKEDG
MVGVRLGKAFHEFHANIYVLLALAQCVPAALR*
>TCRU_2008 Thiopurine S-methyltransferase (TPMT), putative
MQRCVLRRISFSLPSGDELEGQILLNRFRKRYGDVKRRSWVPHAITASRLQGWIVVGGIL
LGAAVFIGPWLVDEYREMLGYKFVPVPPSAMPHTSPVWWENEWRKGNPLWRAGESTSTFY
RGPFDFVRAVTGRDMYDGNTFKRGRDRKRRPRALVPLCGDSPIVKELAVRGFEVDAVDAS
ETAIRACVERTERALEVDVYKRIHLHWRDIFAPELWQGPLRDVKFDVIYERQAMTSLNRE
QREDYAYLMRQALAEDGVMYVEGIFRTGRVHGNTVRGPPFALSQLELKRLFPETEGFMVR
CEETNDAITKLSRENRILQRIPKELYVTPFHCAVFRAKSVDAAAAAAAAESAATLP*
>TCRU_2009 guide RNA binding protein, putative
MRCILRTCLHHNHHLRQRVVATTNTTNNAAAAALLFGVRCQSNSLIGTIHGGELSLPSDP
QGGSQLSARNIAMEALQMKKLHQERGGNPMLAQQARRVLFATSIAGQSLDARSVALLLNT
AVYFGMESDAKLVRECIDYCLKNDKLITVDVLPIVVTACATLKSRDAREVIEMQAQKAAR
NAKYLDSKGVANIISAFSKTGINHEKLFGFLSKRVQTLARVGEFEAAHLVIIANAFSRLR
YRDKFLFGAIARRAMSLRERVTVNELVPLIVAFSKIGLKDPKLSKRFATKAMEYVDQMNA
EQVASMFMAFAYFGIRYDQLFGVLTNRAVELIDEFNAQFISTTLNAFQRIGINNPELFDN
LAERALAVVQDHDARDISKTVTALAHFGLKDEELFKRLASHAASIADQFDALGLVNTAHA
FARTNFLQQDMAVALSERSVYVCRQIDAGEARRLLWSLAKFQVRDPKILTPVFNRCLALH
QDFFSDPTGSEEIEEIFDIYGPNFCPPLYQLYVSRGTAPQM*
>TCRU_2010 hypothetical protein, conserved
MLNTKKNATNNTPTAAAAAAEEEAGMTVRQRLRLKRELEEKRKFGLAPLAVDEVSGNEIS
PNVPQLISQAPWYYGTTGPTLDHQRLKCVDTMDRLQDQHDTVVVLGKAKTYTAGACGNCG
SSTHKTSECYKPRKKVGAMYSGKVTSVDMHVERTEKSYAQKRDRYAMGASGVDLLQQASQ
EGEEETQANGNLHANGEMIVKKHPRLQDVFATQTAVTGEGAEIKELPKHLHNLDDDSAMF
DPKTGSMRGNPNAADPTRTFQGDLQRYRTGDYYTYLDMQLRFLNGESKSIVDFRLDEQLQ
KQRRQERQQQKDEGNSNNNNNNNSNNNRSVDTEEEMTRDRLLRSLYGESSCASAENSLRM
REAIAGFAAASAASLSSSPQVASSSSLALSSMSTPINLPQVRIGSATRNGHACVYGSYFD
REGFRWGYKCCKRLGKDAAACQSTPAEPSVAAKAEGEVLGQAS*
>TCRU_2011 Putative intraflagellar transport protein D4
MNVYFSKRVAVPTSTVITSIAWNEEQGWLACGGRGGLLKVLKIDNSIGAKGALLVNQTLE
GHSADVSAVAWNHQYRKLTSSDESGQIIVWTLHKGMWMEEMINNRNKSTVCDLSWSADGT
KVCIIYEDGAVIVGGVDGNRLWGKDVKRELAKVTWSPDDRYILFGTKNGEVLVNESDGGH
AVCQLRISCLEGVESLSGLSWHPAWVDSPDPPATLAVCYANGRMQLMRDINDNRPYLIDS
GILVSCIAWNPQGTTIVVCGVISTAAMMGDRPASQPSSPSSSLVVAQFFNTEGVHLRTLR
VSGQHCGGVTWEGDGLRVCIAIDAALYFANVRPAYKYTYFSKTVVFAFYRSDRAEQGVMF
WNTRSNERFIRYIRGLFYIDSCKDVCMIVNTSADGSHRVVQLLNAIGSPLEVRMMDLEPL
VCGMNSSHIVVSDEENIYVWQFRDPDVVLDLLDPISVQASRRETRDRIVHVDDIVRPDSP
PSLVTHTAVTNDLICSLCLSEEFLFVARESGLLQLYRFSPLHLVGKMVLPSRVQSMAANS
NSTRLSVVDVSGIMTVFHIDPMKLSLVPQKIAPEPNFEHKDVWNVRWSKDDPELFAVLEK
SRMYIFRGTEAEEPVQSRACICKFKSLKVRALQLDDIMHDPERPRKEHVIEFETRELREA

RQALQNLSMKEAYMYVENHPHPKLWALLTEHALAQLDFDHAEMAVVRSKDYPAIQFVKRI
KTLDDPQKQRAEIHTYYGRFDEAEKIYKEIDRKDLVLDLRYRLGDWFRVVQLVQEGGGDE
SHMQRAWENIGDFYADRQKWTKASQYYTQCRQLHKLAHILFLQEDYDLLAQLIPSAEHDK
GLLLKIGEMLFAVGLGDEASRAFLAAGEARLAVAGCVQLHQWDSAVAIAEAHKLKDILPQ
LARHTKSLIESERLPEAIELYRKAGQHEEAARLLARLGQRAAATDPLRAKKFYVLSALEI
EKYRKKKVALSDDGTAAVDMLLSEERAATSERVLDAAWRGAEAFHFFLLCQQHILHRNLP
HALNVAMRLMEYDDLISPVDSYSLIALTAYLAKNFSICSKAFTRLEAAEQKDAQQQNQQE
GGTPAVAGQLQFIDMTMDLDVSKQTGVAHRNNACSHGAAGGGSTGLSGTTTSLQLTNRSK
GTALATQFKYPTVSLREPRRRFADLAAKIFTKHKPEDNSADRVKCPKCDAFVKEWASSCA
RCQKQLGVCIFTGRCITTEDFWQCAVCHHCIIDVEGDRFRNCPLCHTPMKHGARG*
>TCRU_2012 MIX protein
MLRRTSRRFVSAKQFFGTEAQPLEPYTVKSLFLFSFIASIAFLSVLSVKETKKAGPIELP
DELEAEKRRRNDPRRPPWPLLHQRIVLLREGKAPHDDLALLWEQTRHYYPADWLVPLEVT
QVLKYTSGAYLQNYVADPDKLRKDVLMQLLNIKYGRVKDPNGGRINRDVEEIISMAIEDL
ENMDLSPSADAALVPTHT*
>TCRU_2013 hypothetical protein
MPDAEMPETRGGLVLFPRCLSRAFRCLRDVPIMHFVAVTKVNRRGKRQRRFLVITSMHLY
SCVENGSIQRSVPTRSVAEVHAWNGELPQVGLRIPSEYDMLFFMDSAADVTGVVAALQSA
TRDGASGRGDGGALRLVEATEALSVAAYCLERPKGFERGMRGTFGWLIGSAGDVPAAGMD
HDALRPFVSANAKGLFVKSSTAAAAAASSSPPSSLTAAGGTVPAAAEETGLPGASNGTRC
LSKDYSDDEGADAVSFSVPPPHSPTIGSAEAGAEQRTKKKGTGEEKTAASVISRHDNNMG
GAAAALAGGPALRDDRRQLLEEREQWCLLLAAERRDTYRLRQAISDLRGQVLALTSDNIK
LRTELLNQRVTWHPKQSS*
>TCRU_2014 poly(ADP-ribose) polymerase, putative
MSPKKLSGAGKAPAAKKASAAKKASAVRKAPAGNKASAAKKASAAKKAPAAKKASAARKA
LFPAVNNTVKIIPAKGAAPAAATAPPAATNVGGNSLTPATDITGAKVQQMLRKGRGVVDS
FSGKVDTCHVYEKGSDVYQCTLNQTHVAANNNKYFIIQLLEDDAESIYHVFTRWGRVGYG
GQNKTVTFSSLGQALYLFEKTFCQKTANHWRSRAQFSAVKGRYELMDIDYGAAEDEAEKS
GESSAARGPAEKPVISALPGEVQGLLKLIGCKETMKRLMAEYAINTEKLPLGKISQGQIT
RANSVLKSLEAEMKKANPRLEELSDQFYTLIPHAFGRSRPPLISSESILRQKMEMLEALG
KLEVAVSILNAEAPEEIHPLDKLYRRLNCEITPLSRDEPDFRRIVEYANNTRGPTHKFHV
EVKQVFTVRRKDEDDLYGSFKRLGNRQMLWHGSRITNFIGILSQGLRIAPPEAPCTGYMF
GKGIYLADVCTKSANYCYPSRDTKTGLMLLCEAALGKQMELTDSKYMENPMPGTNATKGV
GSYFPDPAGAEVVDGVLWPKGRVRESRISSSLLYPEHIIYNVGQCRMRWLVHVGFKYH*
>TCRU_2015 hypothetical protein, conserved
MTSAICPVKGTAVELPWKLLVAAAQPYQTLSGPALSVNFHRQPTVMPFQRLPFHHCGVSG
RYYHSLLHSLAEFATTPRPVRDSHVRMLHAPMEDFWHAVGFFNCTYLTTYAPFTHEHPAM
GAVELAPSSHRSHKDLRFSLYSGEHQPISTMILTGLPSGAVDGKEKDVIAPLPHIEKDEE
SLRVANIGDVKRLADGSILQRAIFTRDSQQSRDVVPPWWVDVCVAEFCQTLALANLLAPH
VAGEGRNCTWNIAAQQALTQGELLTDAPFDIVSAVPRVFAAYRTPPWRHRIGLPLFSADR
QPLCVALRVELRQHGCTLTAGTYFLVEVEGHT*
>TCRU_2016 Zinc finger, C3HC4 type (RING finger), putative
MPKHRKGSRGVEAKDIGNFQYERQSDSPGVARRPTAAAATRRDVNPAVLKRAYMLHNFQF
VLRPDVYEMKAMPGLPQGKRRLNEMLLSSDAAPPWDAVTAVVVRGTMEQFQCPICLEPPT
AARVADCGHVFCLPCMSQYLSRLKQENKQRTCPVCHNFVTLGMLRPCIFRPIEPPCLGQR
VGFTLLERRGGCCVLLRKDDPRWHDAAPMEDELPLPLFHEPSATFSRYILATEESECARR
RLDCTAIMDALQQLNAQPRPLTQFDDGVLRFAEEALEMVLQETNAQSPQSSVRNSPPIAP
KRPNVDGASTYYLYGESEGQPYYMHPISFKMLRDDADTRGVPLPNTVYAPVEEIVTFTQD
EASRKHYKALAHVPLHGTIKFCLLDLSDVVLPSTLKTFAPSLARMREARTWRERGEESSG
EDTSWQEYLRRYRAISPHCTEDSGVSPEPVPSEYSGVSDLLISSVVTAWSEPGGLSGDSS
LETRQRATKRHDTPIPSCWTAENSRRLFATPPMRPAMPTWGGHVFNPHKPSSAGLHPTQR
PGC*
>TCRU_2017 Concanavalin A-like lectin/glucanases superfamily/Trans-
sialidase of Trypanosoma hydrophobic C-terminal, putative

MDGDGYPILLGLSYNKEKKWQLLCGGETNSKDLSVFLGPETKEDHVVILLRNGNHGSAYV
DGQRVGDAQCELENTDSKGISHFYIGGDGDKTRNQGDVSVTVTNVLLYNRPLTFSGTRAE
VEEVTDSSSKDILSLSGDPQPTGTVSQLVEGDKRMMPPATIPPEAPVEQTTSPKPQQERE
TRKNTMVGESANTQHVPANTSQGSVEKAAASNSHVVGGATGDAGTMRGSGLLPLLLLLGL
WGFVAL*
>TCRU_2018 hypothetical protein, conserved
MMTMTGRVLLVCALCVLWCGAGGGGCSETPPGSPGTPPHEEPQNDNGAVGVTGGGVQTGK
QAVPQAPALPASGLPEVPARPSQGTQPGGGEASGTPSTTKDSMVQNTEVKKKEEDEEEHG
GNEEEEEKQEEEEEEKKQEEEEDGTEEEETKKEEAGTGTTEVTSAGGQEQPNLSSGAAGA
SNKTNPNSTQTTGDDDPAADGAGTPEGKQNENKDANPKETPVEATAMKTTTATTGDSESS
TAVSHTTSPLLLLLVACAAAAAVVAA*
>TCRU_2019 mercaptopyruvate sulfurtransferase, putative
MDIVKKVKIDLPVSLAPMTRSNACGHQWKVFLTVDDIARSFMEYQVFDVRYNLADGNYGK
NAYAKGHIPSAIFVDVDEELSGPLTNTTARHPLPDAAKFVEWCKSRGIGSGKSVLCYDDI
SGAMGACRMWWMLHALGVEAYVLNGGFQAYCAAGLPLETGLPESRTQPLETWPYGVEFRR
ALTIEEVPPNAIMVDTRSKNRFLSTVRPYGADTLPGHIDNAISFPWDSILLLTDQLKCLK
SEEECRSNVLEALQSVWDAGHSNASNCVFYCGSGVTATLNIALLCHLGLGDPFLYCGSWS
EYAGQFSFSIARRIVGEHGMLFKMMSPCLGDNPKAPASDAATVIVDGKIVPQPDAEISSA
IAHMHLGERAQVFFKSRRVVVIEVLPK*
>TCRU_2020 hypothetical protein, conserved
MSNTLCLLEFVCNLRWVLSYAVQRLDPVLEAHYAHVIERARQAESDVFRSYSSVKSLVEV
SRDALTLAQEGIVYLLVAEGVLGHCPSTMHSFLLRATASSDSRALSDCTCQSDVSSLSQL
QSARNVYQAHFLFIRWLVAEGLLTDEELLDSLGDEADASACGQKSRFELHVSMAKSLSSG
SPFALQAHVILTRSLILHHCCIILSVDDILAHVRFLDPMCSGTLESIEDALVFWMHHVLK
VMHEKKMINDQLYGFAAKELGDSLYHSVQSGVVLGLIVHFYKQNILPLSHICTASGEGEE
TTRCRERRLQNWGSLLRVSEELGLWPCLYADEVELYGAVVLQLHVLRFVGELFAVLATQA
EDLEEHGEDAYCMPLQQKKKNGTTAQSQGIMDVGRERKRNEATLKNCRVEKGDDIISMGK
GDEANVGSSQNSSSGYPSVHSRRQYDVMMASLQMRGVDIGVSGPPKSYSSSMKKRESAGA
AKAKSGVRVLASTASMRATAAASSSSSSTSSGTAGYLSRADSRRFIPEKVTLSKNLSNME
LVQSHLNSAEGVMRSDVVQASKPIAINPPRLPFVTNANAVTTSPISSNSPVTTALSAIAP
DLSQESFHATLYMESVLRAFNLCDQVSFLGAKDSGKLFSPNSTVNGIHSNMKERASCTTT
ESSFPINSAGNLGSPPQSRKMETARKLTSLSRSHSNLRGQGDMESEGVSPMTPELATDQS
DAHSQTALTSFNQQHLSSLRSQCQRRRSEAHRKLEERLKDLLRDLSAVDHSSVQSTAAFQ
TLLRTLEKQQAIIKRLSSRLAAMNGGNPLWHDDVWSGNEGAPGVSEGPGSTHLLCCEFNC
GSDDSGQHAETEISSIPDAFLDDEKALLDH*
>TCRU_2021 PIG-P, putative
MSLKSVAPVETASSGVSKGHNVAINGFIAWLLIITVFVTYFFWAFLPRHVLDRTLMSYYP
DKYWAVALPAILVISTVYYLSTSFLLVLHRTNPLTDGFCVADADAKEDYHGLESLSDAKE
GVPPITEIPVSVASRLLFQPWT*
>TCRU_2022 Endonuclease/Exonuclease/phosphatase family, putative
MKSHLVAVLGLWLHVETPQQSVAASTTETKTTPPLERVPRHARCGELKRSACRLSDKTAR
SFRCGHSVDITRCAELQKNPETEEDGFISVWQLNVAGLSPVKKIASAARLEQHQPDIVLL
QKTNTQSATAAKLYGYNSFLQSRTGRGGGVATLVRHSLPCECLTLPGTDLLETIAVRATP
QDRPSICVVNLHSPRTATVQSSSFITAIRCSGPQLLSRVN*
>TCRU_2023 60s Acidic ribosomal protein, putative
MDAFKTLLGASVATEYEFDEFDGKNLRKAALEGNLGGGVADAAAAADTGAAAAPAAAAEP
EEEDDDDDFGMGALF*
>TCRU_2024 60S acidic ribosomal protein P0, putative
MPSVSEAKREYEERFNGCLTKYGRVLFCLMDNVRSQQVHDVRRDLRGLGELVMGKKTLQK
KIVERRAEDKKASAYDKLLYNTCIEKKLLCGNTALIFTNEEIPVITAVLDKHRVQAPARV
GAIAPCDVIVPAGNTGMEPKATSFFQALNIATKIAKGTVEIVSDKKVLSAGDRVDNSTAT
LLQKLDISPFYYQVEVQSVWDRGMLFLREDLSITDDVVEKYLLEGISNVAALSLGAGIPT
AATLPHMIMDAFKTLLGASVATEYEFDEFDGKNLRKAALEGNLGGGVADAAAAADTGAAA
APAAAAEPEEEDDDDDFGMGALF*

>TCRU_2025 hypothetical protein, conserved
MPRVLIVLLTYDDPECGGAADALAEHLQRDCAVMMDRCQLTVKPIAILHNASHRDALYRT
LQDLFQVKPQDIYVITFLKENNFEEYRKVRELCNGVKPCCIKHQLLTHVANYSDVGLIIR
NLVRLVLEEMRREA*
>TCRU_2026 hypothetical protein
MSDHYENASEANSLSPLLQTPLLSTRQYFVSPRRQNQQQVPTDGIWSLFPWFLGPSGDSN
VSARAQRNRSLPASDGGENSHRVLPSLELANGMDNGNEVKHRQQQIHPSSLSFSLAGFGS
QFDDMQPLVTLLRSSTTSTLGNSEEYRSTPIRRLRRCVSPVHFFHFPTKNFLPQTRRQAA
IRIVRWWRSREALSEKRRVHALKLIQRVGRGYLCLKQISRRVEVTRDSSSFARSIRLCSS
ISMWLNKLGEKSNTRFPADKLGVVLCESRGQFNPGQSYCSRCCRNTSGSMSRNYVSFRGL
GKGVRGARLVNFDPEKKTASAQAEESKNAVSSDANNLSGSFINENADFIYSPALSRNEGA
DIFYLPRISQRTHAKKEDEETQPQNEAFFCTFNNRVSLEPFSVKVARSLRSRDYNELGPW
SLQSARRSTVERVCPNASML*
>TCRU_2027 centrin, putative
MSTTRAAGGRLTAPVRTAVPAALPRKRRFELTDEQRQEIREAFELFDSDKNGLIDVHEMK
VSMRALGFDVKKDEVLRMMQDCAARDQHNQPLMDLAGFTDLMTERFAQRDPRQEMIKAFQ
LFDENNTGKISLRSLRRVARELGENMTDEELQAMIDEFDTDQDGEINLDEFLAIMLEDED
Y*
>TCRU_2028 hypothetical protein, conserved
MMRQCRRLFMMEGERKFTFPGTPASHSTGGVPFYGNIYAGRVDTQNYIPPMQEGSGLIKP
KSSLWDDPQIRAKMDRCPYFALCDQVLWHNMDPAYLDPGLLTAEEHQLLLLFSRAYYEKW
KAIHMRPPNTLPRVRIKYGDTHLLDEMLELLDVPAKVRQSLVKEFQLDDASLIAMNPDCW
ETAYLDPFNRDHFSGRVLEFAGMDEPLREKFFLLLQQMRRFVVVRKDTLEEQRIVLPFSS
AITLFDLYKHLGLTPYNGQLKMYDYVDLQQTQHVLTLGDEETYFDLLVTTFFKANIRNAT
LSDVADDTDYTARYTITVEPYSVPEKRIGRWF*
>TCRU_2029 Aminotransferase class IV, putative
MQSELSGILSRIPATVIGTILADESCETVWFFNPKSREVISMDALRSLPNPPSNSGADAT
ERHLVYGMMRVRNQGVMFERDHIQRLYENCVLAATSKPLTDEATLPFPVEGVTQSIRDYI
LSEHKESSDINLKFVTWLPPFSNSLTTAEAWQRFMSDFSYVVYFIKSFFPPKEWYTEGIR
ISLLYNARRHTPNAKIIQAPLRSRAKSLQDSSGAFEVFFVWDKEAHFLVPEGSRSNYLLV
TEDGRLCCSLQKDILRGITLHTVKRVASTTGLADIEHRPIYVGDLCLAKAIAMLGTSPGV
LPVREIQFYYDEESKANFISSLDDYEQCMKEKGECPSLPRETILSNGGRLELDSPHNTML
AQLREAYEAEAFR*
>TCRU_2030 dolichyl-P-Man:GDP-Man1GlcNAc2-PP-dolichyl alpha-1,3-mannosyltransferase, putative
MNEFLSYLSLNLGCSFLFVGILLVLRLRIHEAALRRFRPEAAETRNPTDVASQKEAQRPL
KVVILHPDLGIGGAERLIIDAALALQKHQRVTPVSVCIVTNHHDPKRAFPETVDGTVKVV
VSGAWLPDHFLGRAKVFCATCRMLWAALMICYCYPDTDCIFVDQVAAVLPLLHFVATPIP
RLFYCHFPDQCCDANRDAQGRFLKPIWRVHKLYRRLFDYLESWALSFADSIVSNSKFSRG
VTVRVFPHLDSVIDAESDIFYPPVSWATKDLLTDTTPDLENDELAPLHNSLENRTVVLSI
NRYERKKNLPLAIDAFALVVAAVSNATSRSPLLVMAGGYDVRLKENVEHLKELKELVAHH
NLTDDQVLFLQNISDVTKGYLLSQCRCLVYTPAMEHFGIVPTEGMVCAKPIVAVNQGGPC
ESVGEGGTLCDPTPEAFAEALTAYVKDEALAKRVGAAGRERALKLFGMDVFGEKLATRLV
TLWAKKNGELNSAQDEEGVKKQKSD*
>TCRU_2031 hypothetical protein, conserved
MGSLVTPGETYRILAPSSSLREHLTDSYWCSAMEEVCDGKLDGVAVRYTGQNLVLMQFRP
KRLSPAGYRYPLSMSIPVEYLVPVASRPVDESTSVGGSFYSSHEPGNSSLSANGFARLCI
VCGRYNVPDMVRRRHGFKCRTCVGKKSSAKLRLEGARGKDELEI*
>TCRU_2032 Sec7 domain containing protein, putative
MKANGHVSSLSSSDGAECWRRDCLVLSNQTQNLLVAIRSNGRFGANARFANAGRITEHPL
LRQLKALQREIMTPTLVRYISEEAILRPFCDLYSSEEMGDTIVGVALASLSNLIDLRCSF
ITRNGLEMILKIVHGKKGEASDISTYEVVLARTLQLYVSCAGHPCARGLPEDLFIRILRR
ACKVALHPMVSPLLRRTAEQAMKSIVTSLYTFVVKNRPHSSPLEVKNETAVNNEAGEEEE
SFLTGANMLRYICTLIMGSDNERGSNSDIQRSVNKSSPKAAAVQLEGLWLVQAMLLVVND

SLCEPGWEELLHSVQQDLCRALLVTGVSTENIIVLSLILRTVHIVVKIASIHVVPQTFSF
IRVLHLDPIMRMTEDLRSGPSSPRRNISSPSMATSPSMPTGSQAHRSITQMLDLYERREL
LLESLVEFCGDADFSTFCYAQYDLSRRFPPLFDRLCAVLVENCFYLVDEDSSSALRREER
TGASFDFGAEGFLTQMDALALEAMFGLLRQTSLAVSHPLPPPAEFTSLILSRLEQKKLLM
EFASLFRRNAIKEGIPFLLGKATRVPAGSSYRNIFGSGVADTPAFVLVEPAGGREVGACL
YYLSDVLDKRALGEYLGELGRELPAPASGEGEEGAAALAAWESEREKSHLQAGTVRFYEE
QLKGFLQEFDYRNKSLLSCIRETAYRMCMPGEAQKIDRVMEAFAKIWAKSNFDAGKEINP
FCSEQGPFILAFSLIMLNTDQHSGKMMKPMRKEDFRRVHRDSDGGNKLPDEFLDRLYDDV
KAHPIIMAEMIDAGFSNDVTWALEMRELALAKDVISKDALMRCSMQVTGSNPKDQAVAQA
LHAFVFSSLWNNCLTAFSNTIYMCGISLEQYLMAEGDVELESTKLDSSLTPAEEAYNVAL
DGLCFLAKTATMFGVVSAVNHVVLVLLSQLPLDMQDANGSLVRLGNQRFSLHCLEKLFFV
LHDCMPMVIDAWGELGNFFSKSFLLGVFAKEPNVTSSTSSSTDCTEVWEELRHNPRIVVA
GAKRATSEGGWFAALWGSSISSERAKAQEGEERRAMERVKMLFPTMEEFLRMIDKLEDKA
HLQFFRALCETGRMKSKGAADSYAASYMMLLITEVVIRRSTDNVVLECYMKLCQQVTSSI
FATINQPKSLRSQQSNNNSCNDGSGSGCNSNSNSNKNNTTSAAIEYESSLTEPSQPIRPE
AHEYWLSLAIRVVKALLRAFKHFICHYLQQEVGPQVLDLLTNAPSKVFTSVVAPLLSSTL
LELLSEPQYLPHVPTHSILVRLLNTLCMMATACSHPLVQWRTRKALTIMVKEEHYDILRD
GEDVINALVTYALESYSEDEEAVGCVANASEVDQGENLESIADDVTTICRRLVAAHDLGS
TDNENTEWHRVWIASLRGLCALVVLSRQYRDRSEALLCLQHCLLDSDIKRLPVASISLLY
EDVVFPLTEQICAPSSAADPAPSRSTKTPADTAVNPTSTQTFVTSLLSSFSPRPTAKVAT
KNSADPSSAARERNQIVAIDLKCRVVGLLPKVLLHYAKSLRETPEVLQELWQRILGTLYA
LYTAPTESSCNTVTADAQDARELRRCNALTEEENVLREAIQETIKNMIYVLSATLSEPEC
EEVMQQTPYFWTSTKNLLRTFDFAGPLLAHIDALDG*
>TCRU_2033 IgA Peptidase M64, putative
MRFIALLRGLLLACLILGVVKTCRAADGGPKYREVQRVSVDWRTRSITFLSAGYTAEEED
QFQSDVNKCVDALYGRVRGVVSEPWSRYASSLNIYAVFQPSVGSDNTVGKNETLRNERLD
LECSISHFYPFYIYCNLSAVFALSTHAPTKDLIVVLVNDPKSVVGEGGKSLAIFTNAPMF
MPFFVIRELTKAIASVSSEYVLGVEEEHDVYVPNCAPSIEAARARWDYWIDQGLADKTPS
QGCFFNNYYRPSESDCMMRNSSIEGLCAVCKEQVNLALLFADPNFSRPTTRCTRTAQKVQ
LDKSKDIQLNIGEITGMTNMNVSWMLGDGAIFDAAHSSQLVVPKELWKNWSLPTELCAVI
TDNSPYIRPVGDTKLFRRILSYELFSDKSAPFEMKMCGMSDSCTPCEGSICDDSSGFNLR
DVEWKKKGVAKAGKRNYVISIVVCSSVALLVLLIVTVLYLWCYLKRPHELFSTLFMDILI
RKFIFILSLIIVGFSIFSFASVLLFLEADIILYKQLLIPYVVITVWAYLITLLNIALVIF
RLYAAVIVCGALLLVNGLVHTILGALWVIVAIFEGDFLFGYLASRWMKTAIKNPSEIELI
QSRLQCSGFFVSCLEVTSSSCPKESESNMYAKPCEEFFMLDLYAVHGTITAPVFVTGILL
VVVGLLDIVFLIRSFYLSKIGIQRRKYHSDPKAAVLPLTFAEARKLRRMFYRVSGKTNRT
LAGRSVTRFLEKVFRTKLEPEEKKKIEDAGPVTFDKLMSLFFPHFITSRMDPRQLTPEET
QEVQGVFELQNCQYEKFCRFATASGALSPETLFKLFLFYTSDHFTVDEGEFISLIKAEAE
TNCPDASMCRGLTAFELEGLRHVWTALNPRILGDLNDEQIALFYQWTHSEPLSGDSHFQE
WKNSLDVRQRGSIGWGEFCYPFAQRALLWKARRFLSNMEKEVPPEMITKNLVEQRFGCEV
ANAIFLPYEDEIPIERVLEYVLLSSKSK*
>TCRU_2034 GTP-binding protein, putative
MDQHDEPKAVTDSVESALEKRAAATFDLHRRLAAVVLADSEGCGCLTEEDDDGDVEYKWR
LTDVTPARFKHLVTQMQFRVGEGNGQCLYELGIANDGSPRGLPRCEYQETVETLQKMAEA
LGLDFNILQEFLLRSEPIPLWCGEILITQRQAELQDGKVAFCGTAGSGKSTLIGVLLTGY
LDDGMGSARQLLFNHKHEVSTGKTTSIVARAIPIDSSEGKNKNSTLCRLKGIDSSSRSLA
MIDLGGDVTKHMLFGLMSRRPDFTGICVSVEQPVVEVTLYARVFRAMRIPFFVVVTKLDV
AFDFEVDAFLFELAVEFSLIGCDSVLLADVDGVDSFQRSWNKSNQVPVLRVSSADGSGIE
LFRHFMSSLPIGCMPVTPDGEFEVFLDGSFFVRGVGPVVHGHVVKGSVELGCQCRIGPDS
EGNFYPVIVQGIHVGGSHVTRVRPTDEATFALSELPRSVDMTQKGKILVPTSVSVCWEFE
AHLKVLSQGMTPRLQPILYTGNMRQAVKIISPLPNDADVFYKGCLVRFQFLYHPEVLREG
ASVILQWNPEGIAVGEVRTVFSHAPVPVVG*
>TCRU_2035 hypothetical protein
MPGQTPEKSHVIETLAMQLKAAHQQRKVLFDTLHKLEMEREKTRAFCEAELVPLREANMA

LVSSTKTLKSKLDGGNQRCFSRRTPKEDKMVGTSDHASDHQALLSNVSQPERTVQAPSRF
MSLCTGEKREENGTLPGMSCDFSGTVSEPLRDTLMDGPAKQLSLANHPPLGHYVTSHPLH
DGPSELLQETEESTTSASVVNALLVQLSLMELLLRNYREAFVSRDSLQIHSTNILSDFYL
MARDVLGGEMASAVVQEALQGMLLSNEHEVIRKLFFTLFHRYRAAVSHFPSRSI*
>TCRU_2036 Coatomer subunit gamma
MPSDRDRYRYEEEDEEETLPFEGLEKTSALQQCRVFNDVRLDISSCLRSMTQCLYLLCTG
TTLTETEATDLFFMSTKLLQSTHPKLRRLHYVLMKELSPLVEQRFIASNSLMIDIKSNND
ASKCNGIRTLFKVMNSSLYASMDRTIVEALTSQSSNVVCAALVTGLHIAQVNPEMARKWG
TQLTEVIRSCGKAQYAAIALLHKMRKNDRLSVTRLIDQAKSGVIRSPMALCLVIKMCTEL
MREDFEGSLDIYKFVTSMMHNNNDLVVFESVKSICSLRNITAKEVSPAVMVVQLYLNTQS
AVLRFSAIRVLNEVATLHPAAVSPINSEIENLVTDPNRIIATLAITTLLKTGTEYTIERL
ITQLSTAGYLRELGDEFKMVIIDAMRVLSAKFPAKYNVFLGFLSKLLAEEGSSQLKENVV
DVTMEIAKSNPDSKESVLKHLAEFIDDCNYSQIVRRVLMYLGEEVPLTENPKTFVRYVYN
HATLEGPEIRAVAVSTLAKLAAYVPSLRRSIVVLLKRTCNDADDEVRDRAVLYTRIFLNN
DENIIRSMVCDVANTVAMSRQARIAARSAVLNTVAEDIGRQKAIAKEAESSTEETSAVSH
AVLQGREKMRKIKQLLELGEPIVSSEPVPLTDPDSEYVVTVMKHTYISNIVLQFKVKNTM
EKVTFKNIAIELDTDEVGVEPQYFIPIESVAPASTSYGYAVLRYEEEQYPSGTLECRFKF
AMQEEGGDVEEEEEYPLESFDINVSDFIAPVNLGGTFETQWEKMQLEETTGTYSLSSMRN
LTVAARELVEFFGMYVEGGKPGKITTASHTLQMSGTMVNRARSLVLITARVFIAKDKSVA
LQLMLRGANAELREYLSAALLA*
>TCRU_2037 hypothetical protein
MSNCAEREFGRNVTNAPVGKTAFVGLPLTANAPQRSGAVERIRQPQKRNDREEGHEDAAH
VVTAVGAKRRCTEEGILCQPPCGAVGQVEALAVKQRSVMNPACSMSGSVSSIEAYFDEGV
EASRESSPACAELLRAVEEAETAVMVDLRCVCGGAPGSLHSPLLLESSSSPPVIPLLY*
>TCRU_2038 molybdenum cofactor biosynthesis protein, putative
MFRLATLLLAKKKPLKPPSRRGRAAAVATTARRTMKSPARRRLPSRTRTPAKRAGGKGET
ASVIPNIDAPNRQPHHHHDQKSSGDEKVGTNSNMGSGVANTSSSGSDNVAVQTTAMAKTT
EATVDTSEKKPPYCLATAVSTIVVPPAANFLLENMKQTLAPSLQRPNDGGVSSSSSEETR
SPVSQMEEFYTSKKGPLFATAVIAGTNAVKQASQLIPFCHPVRIQKCSFTFRRRVVAGLS
RTSALPHRVVLRKRVSPPPPPSRARGECSVLYCFCTVASDDSKAGVEMEALTGASVAGLT
LYDMLRTLPGSQEDGLSIGESFVLAKRGGKSDFTKILMSELENVPEPPNAPAPTPPATRD
TGGEETAGMLTEGGATPPSAAAAAQSESEGNDGKSKSDAPAPSSSGSGHEQPQRDSGGTP
GDADDNAATPDELEEKGAWWKSTAREKKLQQLHPRRRYESQEKTPMEDESPPTKKTKSTP
ATTAAATTAAIAVRRNKNTEYALAKKKMENKTRLKMKAKVAKIRPKRLAIPLAKRRVESS
IPEDDASAAADDADDHEDVVVAMKKKKKKKENREVAVASNNKRAPLKKTAVHDSADADDD
SADVENDEEGEEEFAVDTFENEGDLAPPARREKKRPRRNVRETAKMNDNVADEDKDDEAD
EDEDDDEESFHEEEAVDETREYDDDEEEEELPARRQKAAKKRNKTPPSPPPRRARGSEVT
RPVRHDSWDNETEVESEFDQDDSEAHEFFEEEGEEEKPPPTHRKKKTGKTSRTQRQTARR
*
>TCRU_2039 SelR domain containing protein, putative
MTHCASHAETDGSKDIDDAAWRRKLTPEQFHILREKGTDPRFGPYNDHFEPGEYLCAACG
TLLYKSEMKFRCGCGWPAFWDCVPGAVREEPDSDGVRTEIVCNACNSHLGHLFRGEGLCN
PPPNERHCVNSTSIRFQPSP*
>TCRU_2040 protein phosphatase 2A, putative
MAELNEMIETLYAGKLISEQQVAQLCDRCKDLFLEEGNIEVVYAPVTLCGDIHGQLFDML
ELFRHGGRAPDTSYVFMGDFVDRGYHSVETLLLLLLLKVRYPDRMTLLRGNHESRQITQV
YGFYDECYRKYGSANVWRFCTDLFDYMPLLALVEEDVLCVHGGLSPDINTLDEIRVLDRK
QEVPHEGPMTDLLWSDPDDINGWALSPRGAGFIFGADVTKAFNHRNGITLIARAHQLVFE
GYKSMFDDNCCTVWSAPNYCYRCGNVASIMKIGEKSRTDRSFITFGAAPAEERGHLTKKP
PPEYFL*
>TCRU_2041 hypothetical protein, conserved
MAMMMTGRVLLVCALCVLWCGVSVVAADVAGGGDGGADEYFVLRWHAQLRRECAEAFGRR
TEGRKDAFAVDDCVRRGMDGVRAFGDGRRVWRRQRSAVVAGVDTTDDDSTRGSSSEVQTE
TDSGSLDGRPPKPSVKPIPGEVPDGQESGKLSEASEPTINGTGGTLDGEGEEGDVSQDEG

QDDKDVKDEVVDAAVGNGERETPHKEHGEEKEGVKEEKAGSTTKEREGDENNAAGRGPAQ
GAAASRPPSPVDVSNPQNPVLDQDGAGGERGGAGPVVLPQAAGLREAEGAPAKLAPGGDA
AGKETERQNGTAEQVATKEVQTAVEDSSAEAAAAGKESKGEKEGENVSEAEKEGSHGNVD
EEAAGKNGGNATKKLQKEATDEAKTKKRNEKKEAENANNTKEEPDEEEVKKATQEEEAAD
KENTTSARILTKINTATPDNSDGSTAVSHTTSPLLLLLLVACAAAAAVVAA*
>TCRU_2042 BNR repeat-like domain containing protein, putative
MLLRGTLTLRGIVPLLLVRPIKYTWRAHTVLGAAEGKEGLGVVHHSTTVTKGHGVFLLAG
GSDVSYEGGCWREGGLELQLVVGDVTNPTGGKQSGRIEWGGPKPLSKLISSASHEGERTE
FIASGGSGVVMENGTLLFPLMARNEAEYVCSMTIYSTDNGSNWALSEGMSPAECLDPASA
SGRDHFP*
>TCRU_2043 hypothetical protein, conserved
MASVTIEEAPSGNTPPLTAVLANTASNRTMGLSCGHNKKRETAFEGTTTTRRSTWEPKKE
YQAVLMLQGNKASVHIDGQLLAE*
>TCRU_2044 BNR repeat-like domain containing protein, putative
MEGGTLVFPVMAVNEAEDVCSMIIYSKDSGSAWALSEDISSAECLNPRVTGWEGSLLMIV
DCENGQRVYESRDMGTAWTEALGAISAVWVNARSGVSQKESLRVDALIAATIEERKIMPY
SQRARLGGEKGHCAPPLGHGQQPHVFRWTGCRGQCCGLDARQDPAALGWQFASFTTEGQR
*
>TCRU_2045 CRAL/TRIO domain containing protein, putative
MEDVLTAELGPVSVAHRAALGELRRRLGICHTDFDCWLYAFLENKKFDVDETIAKLQRRA
AMEVQELGAYEITALGSSGGVDGKACCTSDGMARGVIQIIGCDRRGRVCLYVNTVRDTCE
KSLRAEKTRTFDAILSYATRLRRDHKSCRVVMLVNQKDASLLKNIDLPFQVSIALRISKY
YPGLVERIYVVNMSRLLAVVAKSVFKGLPRDVSERISVVSETEMKQGALLQWFDAAVIPV
ALGGTMDRDTPEHWRAFADTVMRHFRHLQDAVVLRGMSVKEWELEELQRDGAVGGGVEES
SVAHVPRLFDAAEQHRNVSGALLAPRASLSSGEAAWRAWSPGDLQLRECLALEQAYAIFR
NEIVESEFLQRSSVVQPAWEVLRRQQWLQCHVRSRHHRLAVSLAEERRWMVHVTGAAGAL
VAVVSVGVLYFCCLLHTWVWCLFGAWWVPAG*
>TCRU_2046 hypothetical protein
MPGTDTSTRSAGPLASPEGDPSIAVYPDTLVGLASAPNGPLDCPFSTATDPTVRCGPPTP
ADENDVDGRTGATIHPAADCLSKQQQQGDAKQNSSSSSLTNALDVWREGRSVGSGSPSFS
GSSPCLDECPSQSPYLSEDADDLDRLLNLLSYFLPSMQSSDGGPIAVRADPSATTGRFGQ
PKTNSLAKACSYPSVSTEEARWTDGAEDTNTSFSVLLRYQFPTEDPHEDVKEDRAHAPGI
CGTRPVHPQPFLVSDSRSRKGGGGCNGNVFSGTCSVSFGAKNSVIERQ*
>TCRU_2047 hypothetical protein
MGHNNGKHKDPEPTSPPVTTIPPATTSPPEPMPTFSPKGTRTGTPAHTASPTLMETEART
ATLSMTVPSPDPIPDGDSRAIPRKIIFIVCSVFIVLFIVIIVVVFMHCLRRKKRQAGNRT
WEDSVGSSHGGSNPLSVEEEVGESKEIQQPHFYPYMPAPPVRLSEGRRLHLSEGNREAFR
SIRMHQINHMNEDEATRFHLLRHAETAFVEFGPFVDSDSD*
>TCRU_2048 hypothetical protein, conserved
MLLAKDNVHDGGSREMLYAAGAVTAAGSTLSFVRNRALLPRMVSVSLSLAAGAHLRVACN
DAGGRVLSTAEEYAAAGFGDAGSIDVVGCDACDRDTHCYAPGTASASMRNGVCVCACGSG
GYGEVCVPVGAPALPPAVGTASSVFFREGVTVQSVFVVPAGASEMTLRHVVLDGVSPVLY
VPWMARDGVRIVVQNVSLLNGAVLYVMGGGALRGAGAAGSDESGPVELSVCDVEALNGAL
VLTGTYPAGSVLTVTDTLLVAARPTPLVYLPGSQSSPYAPVLVLSGLRLVRSVLVVSGVA
LVTVMTGGRMVVVDGAVLELVGGGVALDAAVLGGEYAFYASARVVASGGAVLRVSGSQVY
AAHGLVFGSGVEANASAVVVNDNAGALTDGALLELRGSASFLSGSWLSVRGNSISGRLLS
VPSYPHPCGPRAEHADAPRECRQRARCDGRHRCAWGCRPEVRRGVPDAQRAGAAADGLQV
RRHHRGIPSRGVWRVRRRRKLLYCCDEGDVGVVRLPLC*
>TCRU_2049 predicted C2 domain protein
MNGKSQFKAHDTESSSFSLLHGSARRSRHSERSFHSSPRRNGDFSSSPSPHRRQRPKNGH
SGHDPFHYNDTLPPNAVGRSVPPEMLSEDDEYHTPYAAPLDADDSNANSKQKLELRVRIE
GVKDLVPDSRAMQTPPSTFVTVQTAYGRGKTPVIYNNSNPPFNDEFMFQVNNPETEEITV
TVVAVTSTGGKKIGQCALSVGNLMQRMERRQWVALVRHPGTERAYECGEILISLYSENSG
TDVYSSPLKEREFREKLRALFRKDAPQELHRLEWYVGKCVEDYDGAYAAIFGRYHKPHCE

SAAFQLTINSVADLTMKNGSPTTRETCYVKVTSSRFKKVTKMVPYHRRAVFNEKFDMVID
NPKADSISLAVYSANHKYGECLVSLNGLQPRVPKERTHNIVYAAGTGDACYCGTIKIVLC
TVNYGTESPVSDAEDEKRRIRLRNYLWKYLRDDLHRLDPIVASISHMNSFMGKLVRAYGP
EPRPYMMMLRIQNFHRNEKEEKPKTYAVVVRMGPEIYRTAGVRFSQDFIFSNETTFPVYT
AEKGELFFMVVDEADANDAEVGRVVISLRGIVRESLFQLQLPVYQEALTADAKVVGTLSI
EGIVHGYGLVKEVISPNKKKYFCQRVEALLSHYDPSQLHRVEYLLGEYVGQEERLIKELT
DTLGPETGSAPMRVCIVNINDFAPMCSCFVKVYLDDVLVLRTKDHHAAKSLAFDIEMKNE
TTVSIANPLHSTLRFKVCEHRLFRASRLLGVAEMSLRNMVRDELNLCSLSLFDEGTREEI
GVLAVEVQSPGFVKGTVTMYGKPRNGGKGSAVEEVTRDVTSLMRKYLPQEVPHAQPLISK
ATSLRNAHRELLKRFAPDPIDFTFYVHIDNVNMFRVEDKDAVEKGNISISASFLNEEMRS
YLKVKWEDRIDTCKYFPEMRMDISFLRGSVSTRRVPPLEIVFYEKSAPAAQASSVVSKRN
TIYKSLLLPERELGRILLSLRALLTRRVYRLGEAFTVPILRSASRSVTPGGLGGTVPEVI
MGEITLHITTPAFEHIPHRLRFAPKKMEHYNRGYVRHFEKRIAAFYRLYDPSSLRELHFT
LYERHVASARWPRSLYDWLLMLIKQHGPEPINEFGPPPKLSFDDDSEEEDEEDEEEEEEE
EEEKRSQENGRHATGTITRREEMVNSLPSSRLQSRRHRLIRPNTN*
>TCRU_2050 hypothetical protein, conserved
MRVRLRVTEGGNARCAPCTTEATITWVAPPYDEGKAIFRDHAQVCTISATSGSLLPVVPW
GGDVRVWSRTVSPASTEQALLRASVPDPESIGTGRGTPPKYVDTAAPQKHPSPEEREVAS
GATTATECSCPATPRVRRRRRRR*
>TCRU_2051 Leishmanolysin, putative
MPIRINVSTRDLDDTGKHWISERYYAMNSLNGNCECEGDVVSSAHGIILENEVVPAAVNP
HADRLLVQPLEGPLIVLHLPRAAFAVGLRCVLGIVSSVLPMLTWCCAWQSAVGGGALDDC
RDGCEEPRDRDDIDGMELQDGDGDGRTLDSHWSQRHAKDEWMAPIGSAVYRTELTLVAPA
DLGCMRAEWEMAEPMRW*
>TCRU_2052 RNA-binding protein 38, putative
MPVHPEFIPLAKQKEMFAISSWRDSPQTAAAAQPMVPSPAILPEAQCDKDGASQVKGGHR
SRRGRRGKNRLVQRQQDVDDFFLNCVLPKGVSFATLNSELQCNATMPLRAYDIGTPYALF
TRRNCGALSGGMEPGNTENASGSGATATAATGTKAPMPPGGDHSSSTNNNTNNHNPNTTN
TVTIAAADARIGTSSSEAEAAVVAIASGDGLATTTKKSNHPVVTFSPFRTPMVGASGAAV
LTPADDSDDSPGDAPPPDLVLPPSVPSEILSKGHLISLLYAESGEAEKTSEWLQKKWPQA
TVMVVARNRSILNSSLVLKGLPSLAKTERIIEEVEKFIPQKPSYIRLHRGERGVFKNVVF
VKYPNREVAEESKLRLERFFIGSRQLKVEFKKREKGTAERESEASLQQLVRDLRVSTEHE
GFIYQRSDLTKDELKLLKQLCHSYGLSFDLSEQKVTVKRILPGNDRQSPALRPSQTTPLN
WTQATPGALRPMDFKGIRHWGEMRSKYSSLGIARPKGPHDVPPFASGRGRPL*
>TCRU_2053 protein kinase, putative
MYDYSTLVTILSCTAVFKQGCHPRYNSGPVALKVTAAPPLPLASRRRGEEEGLRVDHEGD
RERATALLCDGTVHPTIHEARVYALVCPHPSIPVFVECIEDAFDTSRFLAKEHHRGVRRL
PVLVTQFVKAASLDRFFYSFPVLRWSIAVQAAHQVANVLQHLHSKGVVYGDLKLPNVLLG
SEGHVWLVDFASAVILHSGVAVSEKRSAVEVEELARGITFHLRAPELFSTTQTPGTEAVL
KHPFMVDFWAFGAFLLELLTGRPCLGTFAECSGKYTREELHARAMEGCSLARQRYGGAKG
PHNADRVWDALGDLLAGLIQHSPEQRLGFRGGWGDVLQHRFFDLVGPDLLEPFPEEYAEE
NDELMLGL*
>TCRU_2054 hypothetical protein, conserved
MRRSSLFWKSKTLAQMFRKLKLAGAHPDVVWAVDAQKAQHNGEEMIRLETMMHRRSSAHS
NSHGPQRIAESFTFWLTKQQEAHHGRRATALRSVTCTINPAAPNNTVKQQLSQLFRDAGI
YEDFDIDGTGVEMSLESVLRHADERQNEHLHQSGVLYIRGDKAVEMLRRFGVLLIVEENG
ITYLVTLPEMHPQPQHQEPLDRSSSTYTPAKSLVDPNGEIDPDVQEYMRERGLRAFDLRM
AGSHSSVERFVSAFERLERMYDVHRGRSMRPCVCIVFSLNSPDNYVAEDGCIVLSVQRMP
AWEEFLLTLPQEVWQNCVQMHRDWRIGNAPKLRARQRQLMKVADMFHFFRLQLDSGIGSR
VDWQEAFILRLMKEEMMIRNTVRKYNLKSELLKKRGEIHVAFTLRSAVDPTKEIGYRIGG
DGRLFFNFSTMTTGQMLRVLKDNLHRLETFQKQHDNAVATLEHLSRSIPVDFSVDTEWKL
REEGNLVNCLQRFVHTIKANQAQLTAFLTMLLKNSTMVGVPKKRMVWIISARFDTMPSGV
VFVPWDVDFESIKKNLLPRG*
>TCRU_2055 hypothetical protein, conserved

MSDGNRRIVPGTQLCYRALYATIVGDQSNDCLPGTTVKMSSDGPILVDHVMRVARRYVGG
NATSKMHKVVAFAGMDSASKAMKAELLSVVEELRRQNQQYQRELETRTQQLELRKQEVQT
LQNKLRVVHSEAKKRGYNFRDLADRGNQRGVEHKKSFDTDRHHGGRDSTPPRSSRIGSGM
GGTGEHTRPPLPPLRVDDALSSTAAPPPSPSLQTFQLKHSHSPPSYSQESRLVAPNGTAN
AAPSLTSPASLDGGSVAENGGGSDMSVGASVSKNALPQLGLEEDVRGNCRDVESGSLDTK
EDTPGNHPPLTRKNGAGAPILPKQLLHENPGAMAFCHRCRLFLAPHPVSVTNHLQSEGHQ
KSAGASPASGTTYTQFGKLVQKMPPFIEPITVVSDEQKRKVTATGNNFFEGGLDIVNSTC
SICQEIITTQSYKVHESLPAHRRRAAEMKADPAV*
>TCRU_2056 WASH complex subunit 7, N-terminal/WASH complex subunit
7/WASH complex subunit 7, C-terminal, putative
MLTSIDFGVDIGKDEWEAFQDKLHFFVSGHEKMLMEIEQTFDCRWDPVLWDPHTDTVSLQ
LGAQAARQGQLTTSIADTLGRSQLHNRLFYRITLCFAGLVLEMDALTEEAFDVLIPPLAM
FGDDSCLEVDITDEEAQRGAGRMLGLLQDLWKWVQRVRRVVQQTVRQLAALYSPLRERDK
YRPFTHVRFLRVWRSLTDIFSALLSVEEVLQTHDTLRHGLSTYRKLLAHVSRNRDKFDCD
DMTMELFCRLLNKIDQELLDDGVLQRLVMQSYEVPEEQVSVTTNNRLCAEFTEVLDHLIC
AVEEKVGTPREGNARRQYIGILGMYYFHLHLFQQAANEVTRRDLCKRVFHLHLQIPVIYI
HGFHLFRPLMWLSRRVPDELNRHLWDPSKEGMSAIKKACEKSGMEFLPQLNQLTTSVSAW
IAEMETTLPVDPSHYKAFLHDTALLVQRGILLAEHIQRLVINYIVLHNCADVAIPLQHVD
CVSHGVELLMMIRAAFHAKTGVIATTYNLLISSITYVMERHLYEMYLLFGDVIQTSSEDV
TDQYSAVRQAIALLHKPQTVENLVCLDLVLSTAFNRCQVKDGSVAMAQKKYEDILIAFSQ
LHRLVFYQRSLRVATDCDFLYWHRETFYPLFFERMYQRPLRSNYLPYLLMAMHDCRTSIL
SSRHVEDAHTLLDAYISYIQKCIQNHLIKTLCIDIENDLRLYTHSVVLGQPFRKVDAAGR
KCDVGCFTRLPPLRFFHEWLHIATEVEHYLDAQFYNLNALMANDWKTYEEMRNLALVRYG
LRICDGYLPGSIVDQGLDVLVITENIQVFVANYTYNMNEQLFVQRPSTTESKHLHTLHIR
HIANSIRTHGTGIMNTTVNYVYKCLLKKLAILSQFLYDDHVKSRLIKDAKFFIAHKEELN
GEYPLSRAENFIREMHKLGVADDGQTFLDKFRHLVSEIGNALGYMRMMRSGGLRAIADAA
VFIPFIDNVVRLERVIDPDAIGSDEEEEEVEMMKEEGMDEEAYKAPPSTVEAVRVVDSVI
HNMRMKLSDGSEYFRMLLEAVTKRLKDADKYEHLRNFHMIIPPICVLHVELMIREKEQLV
KKNKDGIFTDDGFALGCSFLLKLFGLSDLFESLHWFESVGKHYVERLLTMQEGIAERTKE
AQKRKKPQRRDASHEEEEINNMHLTATMVDYAIREYTALEEAFVSSKVFFHSAIPDGEDG
ETEEEEEN*
>TCRU_2057 glycerol uptake protein, putative
MNGSRKRPRNQDHNAKAPAFLQKNSVEYLFSALTLCLAVLYFIVIDIGGISYWNSELLEN
GLAPAWPPFCWLGSKGMDVRDDQWYQFTMVLPIIVPAFGLFLHVSRRLRRIGGGNELWSL
YMLHIIAGLLIGFGVSGPGFVFTVILLFGNFYGLNMLHNRAPFKVFMAIMWGTHVAVLFL
NSYFGGYKFAWIGLQFLDKLKTPILPWTVHYNMSVLRMIAFNTDLHQAIVESSERRAKVI
KKHDATCIECAQVREAHAQKGGGSIPFGLEVGGLRCYKFRTEYPLDVREYNLLSYLGYIF
YPPLYIAGPISSFNAYVSYLKEPSRAINEKGLQRYGLRTLFSFLVVATTAHYIYLTSIIM
TPSLFSTISFPRKAILFYLQLGFLWLKFNCVWKFFRFLALLDGFDVPEDMRRCFSNTVSI
QEFWRDWHASFNLWIVRYMYIPMGGNRMKHFNIFPIFFFIAIWHDIELRLIHWAGIICIF
FLVELLFTHVLFCQGAPLTLMLARRPLLWRHLRTLGACAMMLQLAIVNLVGFSIGLGGAK
QNLREMWMESPLSFRLLMLFYFYLAASIAIQARDQEKFEEQQRRIKYGLVSARDGTSVLV
SAVAVDGSG*
>TCRU_2058 hypothetical protein, conserved
MSSAQVEELERRASAAEVAIEELKKSMQARPQSGAENNAALELRLRELLKLMEEDRLECE
VIRVQRDELKEENERLRAQVMKHEYRIKHLLQTINEIESGKQQN*
>TCRU_2059 60S acidic ribosomal protein, putative
MPKSKRAKIVPLTKTRAKTREDKDKLINRIRKALDDYTDVYSFTLSNVRTNILQQIREER
KGDSCLFLGNNKLIMIALGRDEESSQRPNLHKLCKFLVGSCGLLFTNLPKKEVKSYFASV
GAQVYARTGQTATSSLVLRAGPLPQFTHSMFDHLSKLGLPLKLDKGVIVLLQDTTVCEAN
DTLSAEAAQLLKLFGIQSAEFRIDLTAHWANGAASRIVGRNEENVTGKA*
>TCRU_2060 hypothetical protein, conserved
MVACIIPFVALIFACVAAAGGVFYCAVFLLLLSLCVDGELVCAEVYTQVTGVMAMMMTGR
VLLVCALCVLWCSAYCRCTEEESVSLSRSGIGDSRTGSGVSGENGVKTDKTKTMKTASEG

DSHPSERDVSDGGSFDQAPPDKSMCKEKNDGSTVKPLTTDGEVLLDSQETIATHLKGTDT
ELADPNINQNHKNPKAIDGIDTQLPGAPAAPKAQPSPSTVPTANKTVSSKEELTAEETPE
HSRATRNPNEDSETKNTTDQALKPDAGGIDTLASEDASSPSIDDAATSEAAANTARVGSP
TASLNNTDGKNEGLGDGESPTPSPSAAEPETITTA*
>TCRU_2061 zinc finger protein family member, putative
MQLLAPLIEAKEGYTYDADGTLCMVVVDPATRKLLIPCNFIYATRAQHRATIPSLCQLFL
HGRCRQGTQCHQVHASLDAVVALRSRVGKLPCCCVFHGDEDIADVLNERSWLSKVVLYVP
EVSFEGGYVPLSRVGYTVPISKILNEVSYEIAHSLESYTEAIREGRVPHEESPVVVLEAG
DIPICRLHIRERCRFAEECSFLHLCKDVAGGDPELPLGRRSRLKATPSEPDCVVSESHDS
NMPHKAFLSPAAGISAGTNACPISVRGFPLVQRGGASVYRSSTNKLCVNGENSLWVRHMH
GSGSIDQELDSSTVEVSTVPTPVPKLRSLGCSDIRPLKRRSVVRKKLSSRVIVSCESLLH
CEGDPEAAEEGTPELRNGGFTEGCRKAFSVRGTMSNSMAASSRDEGTSTSSMSLSTSHRC
CAGGKWRHDPYNLSRSQMGC*
>TCRU_2062 C2H2 type zinc-finger (2 copies), putative
MPDITALRASCKAILLRHQRGLSLELPCIVCRQRVRGQESIMQHYVDAHGIHTTNCDNIV
DLNGFLVHLRELLLFRDDERMHCPVCREDCGDEASLLEHIEKEGHSRWDIDTIPSLASFC
MADVNKSEEAGEEEEDSANGTNVVESDDDGDDEDWGVEPAVCLLCDTASEDCLGHMIDAH
GFDFRAAVRSHEGVQDVYDVIRIVNVIRKCVARGLCPFTYGDELAEAEACRCDVAASSLE
EHLRNHPLHNLPRVVPSSDRELIPVLCADAFISSVVVGGDLLARGEEDPEYPMVPTNAEM
AKKQTARRQSEGNA*
>TCRU_2063 hypothetical protein, conserved
MDDTLIVKLLRLVRNGRSPLKGCETFTFGDLHMLDRGPCIADEETLQAHQAKKGLASQVA
LVVYVKGAIAEIWRFQDFRVPGVVSPNSKMPSAIAASQGLAITEQQGNSLKILIRVIFAY
LRIHELFVDITYVDAGDIVLRLVVDMDELPRLETDSCVKKVWAVDGLVSVSVTYNRLWRA
SLKQEGDLILVSNTATNVKGECPAEQRVGSVLAHCSDSVGSGRSFPTPHFAPAPLPSSVG
SSFLLQKWPKSPTLGMSEFSLPLASTSMTDNNYSYYKTPSQGAVAVAVAACGTRTPAVRG
FTFASVPTFAAHVNDSASEGESDGTSNQEQLHELLEICEVSLQEATTGVQEVLDILEKPL
LP*
>TCRU_2064 cyclosome subunit 1, putative
MQSAFTIRVGEKQAGGVRDVSVLHNGEVHFSVSHRSVKWAFLTRFPAPSRRKRSLDPAFV
LPESTCANSELELNLDSTGGERTGAAAHCGSEAVQEEYLCVFHRDEPKNRHSHYTAHQSS
TICSAYNTVHPAAVTHFVLKNIAPTTVVPGLRGVFVHGSQQSSRGSSSRALQVEGFLFHQ
AADLWWMASQLTFRPVYVSAGGSELITRLPSNPIVTMMSVSSQVDSGSATPGYALVDAII
LTQFQTYKGRLMTVAVRGSEICLATTVLVEHLDSLLWVWWPQRLHVPQLHVKATDVSVTI
SESPDAPVQALFLFDAPHTLLHVLRTPNLLTGKGALELLFTISTNCPPVVLPSLRPCMMQ
PIMVCHYPSMNEVTLYHVPTILEHPSSSNSVATISLATTLPKDFIVNGVLYQTGSAVAFH
ATPRSSGNESERGEKHAHELKQKTCVVSFPSLLEEQHPLVHFILEALELVLEANCVASLQ
CELLRRAWESKHLGNVWDFGIGCIELIIEVVRTSMIGRSRRSNILENNNNNTGAGGCNEN
DGRYFLKNALGHEEREEVRLCDVLLDPIALNADLLQRHSLCSPCAPEKNGREAFQYGPNV
IQLWTQHQCGLSVFVLHLLCESLKLQERHWVLLEPLAHMNLELSKLMRWTQYVWYYSTCL
CVPETSGFANRSSVVSQPSPAHEFQRTLPEHILLERFVFYAGSSSDAVLSGAPPILYTIF
QRALAGKARMSGGWPAIKGVLSTSPLSMANRLFFMLVDCFDTPQAQHNHASRWWFLLCRG
LLKYGIDPKFVSLELCVGVAQPIERALAIAKDQVDDTWDNDFNAIIGRLDRLQQHAPTLL
RYADSSGDVVRAAQERSIGRQYCATLNDDDGVIMRPDFPKHWGDPRMDIVQTMLNTAAPI
ALPSQLDGADAIYASLKTLSRRATALPVGRGIFTLCTQNFRLRDSVPIPRLNLEGRTSDG
ITITNSSDEFSSENLIWPLFHNGCAAGLRFLPLPQGHGHVKEEQSITRHWVLYQTRNITC
LASRSGLLLAAGLLGHLKLLQRTDIYSLLVSPQSEFSGRETVTIAVMLGLSCSLRGTCNS
IVFNCLSMHVQSLTPATEDIEVSLDAQTAALVSIGILHQRCPDAFLAEMLLVQISRLPSD
EHFRDREGYALGAGFSLGLIFLGIGSSHGVPNVENRLLKFMEGARRETVPSTCEGLEVFN
ERNPDSGHFLTRALLLRNTKDSFRSHCTRVYEGDCFNTAVSSPAAVVALGFIYMQTDDAS
MAKKVSPPNRLVGLQGIYPELCLLRCMMSSLILWSSIEPTCEWVRQSIPSCLMRLVKSPR
QSGLAPAQVSYLMMNLGHCLAGVILALGMRYAGSMDADAKALVLGELKGFMRGRIGTTGA
AIITIQRSTGAFQPCLSACTVALALIMAGTGDAQCLSVMQKLYKRTNVKYGDHLALSMSI
GLLFLGGGQLTLSNSLSSVAALVMAFYPVWPDTTSDNKMHLQALRQLYCLAVVPRVIETV

DVLTNRSVSVPIRVIVHRGRLGQTEPTSVVKELWTPLPKGKEDQAVRMVTPCLLPDKSTV
TQIEIRSAQHYNLTIYNTGSNVIDDRGLVVRVLEKNVVHTDSETFLRSPSEELVVHWIKR
LFHGEPQQRPRPIEATVILDNVNLLFAAQESFLSEIGSTENDFSPDFVMNARRTLAKRYG
GLLQHCGAMSSCHPLAQIIMMRKSLYSAAVSLLQSLRDGAECSCDLAPVLESLRFYNTAT
EAETEGGNGGAGLVTSVVMKWLSQALHFYGLVSSMGSLSQVLADHASFLQRREQRFLALH
SINQQLLLHPRVLEDLVECCVEYVE*
>TCRU_2065 intraflagellar transport protein 20
MEDDKLVMFDAHGAIRMYDPEKFDQLMKTIEVEKRFTGRMDDFRNVVNQTMSIVERLGEA
IEQEKLRAIGSRNIVESEAEERFRTVQEAKVRLREKQTELDRYIAEYDSLCKVEQEQKLI
IQRLSQSARD*
>TCRU_2066 GPI alpha-mannosyltransferase I
MPCHSDRSWIDGVNIRRLLQLGGIVRLILIIYAHFHDRWFRVKYTDIDYMIILDGAREMW
MGGSPFDRTTFRYTPLLAVLMLPSVLVANPFGKLLFALCDLGAAYYSYIVLLKFATERSA
KWMVSLFILFNPIMVVVSTRGNSDILVTFMSLVVLAKFAQSKYFQAASVLGFAIHFKLYP
VIYSLPLVLGVWEETKAETFFQKLKRAVPTVTLCGLLVVIFFTIPTMMCYKWFGQQYLDE
AFLYHLYREDNRHNFSPYWLLMYLNMARSHLGLESEYAPGLFAFVPQAFVLSFVSYKLRR
NVAHACCIQTMLFVAFNKVCTVQYFVWFIPFMAFLFFHPRGAEGDGKRPGLLKAVAAIVL
WAATIPLWMTTAVPLEFYGRSDFARLWVVSCIFFLATVSLSSWFARIAYRLQEAVALQHK
AKGEKTTKVA*
>TCRU_2067 calpain-like cysteine peptidase, putative
MTTYFAGLSLREIYEKKCVELHCRKNSAICQLLSDVPDEYQSLVFIDLSKNFLGPKGILP
LLEIVRCCRNIRVFDLRAQQLDNVAIDALCLALRQHPSILRINLSDNPLTMNAGVSLLQL
VRENPVIEFMTLERTGIGNSIVQAIKCQLERNRRVKEMVAERELGLLASPGSRDVNSSTN
EEKSGRERITPAKIFRAAAAAAAANGGLNELPPDLAFLAPMYEDSFERKRLKAHDIEAAL
LSFSHSVHEFLYDDNPVSVIDQLCEQQYAWFYDHQFAADDVAVQHRATAMYRIAGWRRIG
DIYPSARLFPDKDEESRLVLPSQSPPSFSWIFVCVDAVFNDLESLRSTVLGSNSLGHGVY
GLRVHIDGAWRYIIVDDLLPVNNKDELIFTHPVNDKYFWPCILEKALAKLNGGYPALSLS
APDYPRSRLSSRTERINRFSITTRGHSVYSFDGTSFFTSSNYQGNNERQTNCAKTLSDLS
GGVGIVRFLHHEQFQLDEWWLTMSDVHSAGAMMVAVSRSANGTLPGIEPSHAYRILQLQQ
LSGARLIELSSFWTKMQWKGDWSDESVQWQRYREVDAALRNQRSVAKNSSFWMSYTDFIA
AFAQVHLCHLFQGFHQRTIEDEWGRNSAGGSCHEQNWHLNPHYRLKISQESPFFINLAVP
DTRFTPTDIDSLALHIIRHEQYPVRYASDREEIVVATDYVFTDSISFEGIMEAGEDYWVI
PSSHTGGVMGKFLFRIFSQSGFIIWKEDPSYHWNTLTYSDTVECSGEYRTGDDNAQVALC
FGSHATVGNMENKTGSLIVKAQAPDSDETALGMFLVKTTLVEGRPSRSVGVLRDESVVAS
SPFITDEIVYLEAKVHPGERYTLVVCVHPVGSQAKLKFTIWSSLPLPQLTPLPVWKKKSV
IVSWPQGSGSYYDVTRNPQVEIYPARILDTFVIRMQVKDCSYTDPSIVFFVLANDGRQGE
GIKGQIPADRVMVRSQYVRHHVVQCELLMTESMDSLLVVPCLQPAGSFGECTISVATESA
EFMIRALSTG*
>TCRU_2068 ferric reductase transmembrane protein, putative
MEQPHLLPAVPALEQDHFLNEAQGGSGATFCMEWLCRSVWTRRFVSISSIFLFVFVVARA
RTTFWTDVFVYHPILMAIAFVAIIPELLGSIFIIQGYARDPRMRCGLMQAHRRYALILKI
ISAFGIIAVEWSKFRRSKAHFVTWHARIGGVCELSQVLETLLGLTIYYRLLDHRITTSQR
AKMRLAHRYLGAIVVVTGLISMSLGMLSHFALRVFEVTFLWLFFAILPVVLAVWAYYGVS
*
>TCRU_2069 hypothetical protein, conserved
MGKLGAGLDVNERRRPVGSLFMEAMDRDSIRPRVRRTLHHSLAERSLPNTPFSPRPDEPG
RTRAFSAGGRRHFPDRSCGNPIVPGPPEVHSARRAVPKQMASHLDSAAQAIYEEKPILYA
RQRCKQPPFDEKSPAVVPRPHALNTSPLKRSHLSLGSLAPKDEPPKERAVPPPMIKKPIR
FGRRHTPAPRSKSAEHRRVGIKMVHPLPHWESVTEKHAAFTSYGIDAYAKKDIHHRNQSN
WEIGFIPPAPFRRSVSTENRKNNAATPLNMNPWRGRRYSCSPRQRDYDIISWTPRRALRM
DEETGIEEGERNMER*
>TCRU_2070 Domain of unknown function (DUF814), putative
MVFYFTLASDPSVFCYMGRDKYENEELIKYGWPEDIWFHVDNHSSAHVYVRMPKGKGMDD
LTPEMIEECCQLTKANSIEGCKLNHVRVVYTPWSNLLKTKGMDAGQVGFHVESQRRYHTV

EKKNNALLNKLEKTKITKENVDFKALREERDAEERRAERKALLEKQQREKEEMEARKRAA
DLKSYVSLMDETKMHTNQEGVPDEDDFM*
>TCRU_2071 hypothetical protein, conserved
MTSPRLVLRLACVRRSLLAPTFPHCQSRASTAFSSDAALCRQTAKVLYSLERFHRQNSSQ
NERRKVERQAWRELQKLPDAVADSASIHDVTDIVAAWCYFSRFWALGMHGPEETADEKQK
GGLLERNYIDVPLVERRAPKARSPTVDEAPPPRANPLDEVIEF*
>TCRU_2072 kinetoplastid kinetochore protein 16
MEREIDRLLRMIDELAAGVEIWKQKALSSMDFIDKEQKALGSMVQQLSIRTAARKEEMMK
RQRQRLEELRSACMERFKEVELRF*
>TCRU_2073 ribokinase, putative
MNRLHVLESHEGSNGPTVVVVGSCFLDYIAYVDRIPRIGETLTSKSFSKGFGGKGANQAV
AAGRLGARVAMVGIVGSDGDGDEYIANFKRNGVDTTNVYRERNGATGLAMIFVDTKTSNN
EIVICPNATHALTTEFLRRQSDNYNRFLSQSCRFLICQNEIPLETTLDVLREAHKRGIYT
VFNSAPAPSSAEVGVIKPFLPYVSLFCPNEVEAAMITGMEVSDGASAAKAAKALQALGVR
NVVITLGAQGYVICEMGSEPIHAPGLRVKAVDTTGAGDSFVGSMVYYMSKGMSLSEACRR
ANVCAAFSVQRKGTQSSYPTPDELPADARA*
>TCRU_2074 kinetoplastid kinetochore protein 7
MTEVCGEETLVPSYPVSPRPRRQPMASLSTNQRSYSRMSSKGIFGDESPLLLSPLPKYPN
RRRSVTFADETEATVREERPYYTACETGNTPGKHPSESPFRRPIIKTTSRTNNATDAVVA
VNMNRRNDTTSHAPVAPATSPTIKRGRSSSRQRRAARRHQAELYHGFYDDSFVEDYVLKA
KKEIEGEEEEKIVEENLKQQEKELAEAEMRAAQATVKISALQQAKEYLLATANARCFSPA
CSSSERRDSGFASSRSCHHSLKKEPSLIQQADGEVEGVGDIEQRRRHLPQKPVKHSMPLV
TQMDDMDEKGSGKRIRQASLKKGGHSLPIRHDGQIHTEDDDDENEDNNMSKRKKAKLERI
IARVIEQQAKRRHGKRSVVVIDWDCDGCGELLASDADVEEEETDNDDEEAAMTRAAARSC
KEPMSKKPSNAASASATAASAASTASRQKKQSNTRHVSVRAPSRKRGANSASVAPDLGAD
SLLLEEDQPVLLRRPATRRRAPTRSISYMSPEAEDDSVPDHAVDVSRKPPRRTPAPKATG
RGRKAPAPTTGTALSPHRFPSLVPSSLAVAHVSQSFTSARHNSHANVEPLDDPPLPPVNS
RRRCRTTSAASRPPTTVTSSDDPMAVFFSASFPSPSKFEEMMLAAGGLQETRKVGRGHPR
QPALLLPDSILRRR*
>TCRU_2075 hypothetical protein, conserved
MSALQPLALLVPKQIQSLVVDGGAEIVVERRPDEKRCLFLRFFDEAEREEAVRGKSTCAH
IHEQVLEDLKWARSTLWNDRRLQQYFYSRDWMTWPSLPELSSMVELVRSAKETDEVPDFS
GSNALKKALGIIYMEVTYPGLHALKEENLAGTLLAFGTTRRDNRIVGDTMHALLGRGDEQ
RIGSSLWVLDLKTPSFTPDEAILLSLTARVREKCLRVMQPELGVADCGGSWSAVLAAHRR
GIEELGPRLFCGISEGLLQFAMAPVYLAKAMCEAAERGLRVRLRVGIYLTVLQRTEVFVM
TRGSCQSAATDDKLEVKAAFVERTGRSYDACAAAGLFLYLLEGETLEVAIRLRFLGPLAG
KFDIMETMPLTATTQITPVLVRHNPFAIVHGPRVVGTFPENCEVRKALSELLLSRDSSVL
PATPEPSQQSMAMKTNYTSATLTGEQLRRGVDDAIFRTLSQGPMTRAALSAHDNMARFRG
LANFEAILKDSLKRNAVHQGKKYYLRE*
>TCRU_2076 ribosome production factor 1, putative
MGKGARSKRGPRGPAKKQHAPRHRAAEVDPALVELYRSTRQPVKKSQLFEQLREQKKDAK
RVRREARERERKQLKENAPAKAIPKTKERLRRPDVTIVEEKDDAEIVRDEADDEFAAYYN
EARKPSILITTGEHPCFRTKQFVKEALWLFPNSTYRPRKDYTLKEITQFCINREFTDLVV
ITDRLKEPYNLIISHLPEGPTATFRLSNFMAHAQLKDPAPRTEHYPELIFKNFDTRLGRR
IHRMLECLFPATRDYAGRAVATFHNQRDYIFMRTHRYIFDSLEAVRIQEMGPRFTMRLLS
LQLGTFDTQFGEYEWFRKKEHDSDKLEWYI*
>TCRU_2077 Vasohibin, putative
MKSLATIRAIVEELAPKNPYTGLKDLPEIPRPEESSIPRELPSLQQIPYVQNVLNMLSYN
FLPQTFFCLEKHRSLDSIMLTSKEILAEALPIRCLEATFVGLYLTHGMKGVDRIPLSFKS
RARGKSFHHIVLVIRCDSLYGALGLSRRSTLMDKPMIYKNLFELIMEYKEQYEAIGHQLV
DFKLGLCASHESHSRKAPCWRYIAVSFAKWNSPLHLTSPHPSDELPTSENSKVSSEDGCA
EEPSVLANVSNLLDRYSELLTIISAQYEKCTTPFLHGSTRESHPICLRDLHEMEETTARQ
ENMRRLEAICNGRLPCLVFDVGTKKRAASRSTSKKRIETSRKTATVTRTSATKCTAAATK
KYSKKDIPADGQKETQHKQSQMEQEQELQQLPLVMEHTSSLFSSLALSGRLNNSTSVTQR

SERSGLSPSKRHSLPFLENKGNEFGSLVSQNGDSWRIPFLPFDVNGVNFNDLL*
>TCRU_2078 glycosomal transporter (GAT3), putative
MGVPVLNSLRDGGLVVSGTLAFLICLVAQRAKLHRYDAAILDIRTPDDTRVTRSFFGDFL
YVAKLALPTVWCKEFAGMTVFIILFLVKSILHVAQSNANGQILKAIASDTPENRVQNFFR
VLLLRAAVSMMAAVCSGSIEHLRPFLIACYRERLSRYLQRRFYSHLLYYQATVLDGRLET
ADTVIATYCGEFAEHLAELPYYFLLPMFGYMTSMVVLTRNVGAKAAGLACAAVAISVLIL
QKISPAFGRIHASLLAREDDYRRMLTNSLNNVEYIAMHNGGSYTRQKLERQLGKLKRALD
HFALAKGHFDFLEISFSALLQAISSLVIFRGAHQMKTKSMNDIYVEIQCMQDLIENVKSF
VVNFREVSHLSTFTVKLAEFDSTLTSIAEGCFIRSSGASPKSDCNSDFPVSYTHVNYIVR
PKWKKDFTLFAFSNLKLMTPAGQLLFNDLDLEIKSDQDWVILGENGCGKTSLLRMISGLW
RPTEGSYSMERGIKLLFAPQHSYMVPQCTLVEQVLFPIVSSKLGAKELDSFKEAIRLSGS
HSVIDVLGGFDSPYVGADPRTADDTYDWDSLSGGQKQRITMARVFYNILTMDRSEQTPVV
LLDESTSMMDETEQAVLLNLRKLQVRMISVTHREEVITHHTHALRILPGGNWTATPVTTR
VNID*
>TCRU_2079 hypothetical protein, conserved
MDVHSRCWLHVEHSASFSFDAENPIKVESIHVRLTLKTGNEEGIMPRGGGEAKTSEMKCV
LWAKCVPTITVADKLFCDQPPFIVASFLLGPEGVATATSLSWEIGENVSVSICLTGLTET
PEFLSTPSEEPLLCPTNMNNTMEPIKEANKSSHLELESIYNVTLTFAGVKANWGFQQSIW
QPLLCFRWFELQGHIREATVPSLPLEKRGKRLNDTRAKKRRMITGDIKEASAVSSCGNYV
LWRDGTLGLRQELVPYHRVYTPANAEKRVRVRRFPHRFADIVGEIPFGSSVEAIGRQVDG
YTNETYVLVILLGAPDAATVAETYNLQCIEPNKWIWGWSKISSRNGLLLLVEVTDTARSS
LPDYGHVERLGEPTYYTSVREERSVRIRSGPSLSTDVVKHLEPNEVKVAVAIHHFTSIND
DSSVRLLQQFVEWEEGGFSLLRNNDRVYLVPVHLQQQPRHFPVCPRPAPNSPEIIPLQRK
RCRTVSPANNRKRPDTGTTPPPDLWSPADIPETLAAGLKKGSVRLEELPKINLNGSPTHS
SASSNS*
>TCRU_2080 protein associated with differentiation 3, putative
MALPVSCLERRETQREEDDCGGTERPSAGDEVANEPAAAGGPPKKVETDVDYIAPQYQTT
FLQNLKTLKLWAFLCSFFCIAGSGFVIIYNASFLYAALADEEVDNAIKTLLTVLNGVGSA
AGRLLMSYFEVWSQKRKAEDRVSIIVSVYFADVFVILSLVLFLVVPRAALPLPYVLCAIG
NGFSAASLVLVSRTVFAKDPAKHYNFIFLALVCSTIFLNRLLYGEWYTREARRRGVDVCL
DCACVQLPLLVMLGFNVTAFISNAYVHWEYVKFNRQVLDERRRLFEEQQEGGGLWA*
>TCRU_2081 Bardet-Biedl syndrome 4 protein, putative
MEGGVTSSASHARGDLVSGDDDAGSHVQNAEALNRDVTRKASSSPAPTNSTATAAAAAGT
FSVAKSLLDIRERRNWLIHQFYVRQEYADCLNVIETQLQESGGVCEYALYVKGLLKRRTG
ELTESFNLFQAAMLINPQNPANRRQVAQALFFLGRHRAAIDMFHDVERLCEANGMGNDWV
IQYGIGICYMYLKDYQKAEAAFVNSITIQNHDCTVMQLGKVLVLQQDYTRAISLYEKYLL
KFPDNADLLTALGLLCLKVENPPQAFHYFGKCLTLNSSNPTAIMAAASIMQDNGDFSVAL
NKYRVAVSKLPHSARLWSNIGMCFFAQQNMHAAVACLRRAAALGPFEWRIAYNMGLVFLH
LKQYASAFHHISASTYLHGNYAPAFMHLGVCLALMNDVDNACAAYTRALSIEPDPLILLN
YCITLLNCGQEEKAREQLAKFMQVWHEKSETQQQAFGPTFLRMVRLLTERLCGRSEDANS
SSRLPTE*
>TCRU_2082 hypothetical protein, conserved
MRVHPGLEMDAVSLIREEIVRLREETERSSCLSDESLFRDARSSLLGLLNKEYGGSYFKF
VVGFMLVPFFVLLTVILLYLSAKMLTEWIALRRISGRMSCHPLIREEMHARELSQASRRS
ARRRTAFVVTLKCVIASSVLFLFVGSRVMMEVLTRGWIKGLLVPLSTDGSFAATEDDFDP
WLELYSGLAMLLSVDWFRDIQGLLTFSLLGSSLMVVGLRLLRSASEDVVSMRTDCISSLK
LLQKIDDKQKERGQEIMRRQMATLISMLAEQAASIPEVPRNGNVVPPLSVVENGENSSGE
EEANGSSAENNREE*
>TCRU_2083 Mitochondrial ribosomal protein S18, putative
MNRMGGSVYANAMAQFAICRQPWNEYINLLTKQDSTPYHVEPQEKPAYRGRKRGREGWLF
GQQVQLHYHRFPDEQLLTNLTRWRTGETVGDIALQQFRNAQPFDIEDKDPQGMQRPSPEV
YMKLNYKNPATISRFLTRTGHMYPADILPLNPEAVAKLRVAKAQAVRIGLYPRFGNPFWF
RSQKFRPKAYQENYDPTTYSTKHTMEHFAYNWVQTDRIRRYFKELEELQKNASNGARGGS
ATTAEQKQQNQFYAPENQPISMHRNNISYMAEVERSMKNPTVPGLMSTKGMKKKFHNLYS

STSTKRMGFSNPTLGIKKV*
>TCRU_2084 Glucokinase, putative
MNIKEFSLHELCEELKTPAWNVPLTFVGDVGGTSARMGFVREGKNDSVHACVTRYSMKRK
DITELIEFFNEIIELMPASVIKRVKAGVINVPGPVTGGAVGGPFNNLKGIARLSDYPKAL
FPPGRSAILNDLEAGGFGVLAVSDAHVFSEYFGVMWEGTQWRTCEQEPAGSVIGRGRCLV
LAPGTGLGSSLIYYNPMNQQHIVVPLELGSQTIPMRKDIDYIQTLHAELKLLPNYENMVS
GAGLEFHYRQVVRGSRPPCSAGEIAKLASEGDANACKAMKKYHEYLMRVGSEASMALLPL
TIVLVGDNIVNNAFFYRNPQNLKEMHREALNHEMERFGFQSRVTYLRQKKLLNLNLMGCY
RCGLDLSVGKKQKAQL*
>TCRU_2085 hypothetical protein, conserved
MRAHAGRAQPGVIIVCEGLPCGCHNGATVFLTGASRAGRHRSCQACRQTRHAGSTNAGTR
DGPGSSATASSRTPRAADPRRTTGDSASSTAGMPSSLGTGTATVCGTAPARTTVRLRQNT
RHFLRTALALLTDHKEPSVSVVPSLPDCGDRPASAPAGTSSRREHRNSLSTSERRIDVLL
PNLRQSALVRPQYEGTCRTCSAWRYNSLRGPPMRMPQWLDRLPNRRLARRSPPKLPSMPA
GKTRRRHKRGHSRWTRLFRHRLITDATGC*
>TCRU_2086 Mucin-like glycoprotein, putative
MMTCRLLCALLVLALCCCPSVCAAGSEDKAEKAGQGATLQDSWGSSGSDNPGAKVTEDDE
HSSDNKPIPSEGPSNTASAPPTPTSAGTEEGPSGLPTSQDGGGASGQPGGSEENNLSPGS
TGDSDKSAVKNPEHNKEVALAGKDVKEQAPTTTTTRAPSRLREIDGSLSSSAWVCAPLLL
AVSALSYTTVG*
>TCRU_2087 hypothetical protein, conserved
MHEKLKDEEEEEDKDEGEEEVDDGKEEDGGEEKKKQEEKDDTGTTKRISSGGQEEPISPS
RVEEASNKTKPKSTQKTGDKDPAADGSGTQEEKQKENKDANPKETPAEATAMKTTTATTG
DSDSTTAVSHTTSPLLLLLFVACAAAAVVAA*
>TCRU_family, putative Se.1
MEHGVTLLEDPMTPRQPIMSSPAPYFFAVEDASVSRVVEDWMAKVLHRDVHIFALGCTPH
RRLQQLPRARIAPMAMRSKDIMLDFAAPEVLVFHLSMQNGFFQLLSPLSPPN*
>TCRU_2089 syntaxin binding protein 1, putative
MEEPLAALKKLHEYHPGLVQGVEPKAKLAELGSSVRAPPEFRERQARLSLHIDICARLVA
QYREKRLAEVCEVEKDIVVGHQPFRKNLNGVRHLTRDAAMPRPVRLCLFLLLVAASSTDE
LTEAKKQQLIRDGGLTPDAHLFANLEHLTRRAGSVQRYSTASAQSGRSTSASAGNAATDE
DTFLKHACMMMETADRNRTLRIGLSLASMERHHAMGANGASGGQNSGAAGKTAGIFQGCG
GNVENHPDLGGGGGTIALTTPQRIVLLVLCGVACSERRSVHEISKRYGREVVIGEPRCRV
LRCVCAGNWHPTAL*
>TCRU_2090 kinetoplastid-specific dual specificity phosphatase,
putative
MGNVACTDAVPFTLEDIQLSRQAAPARLMELLPEAVIEKFTRYQHNLKAFMFRQGAATGD
AVKRMDSSQVYLQPLDLAQAKIKLTESAVYVLLHVQCKPPAAIDSNYHATVGTRGGDRPH
RESDGTQCSNEETLLWARVLTDMFTPRGRATAFATNIDKPSFSALPSTGLENAGVGAGGN
SYGFTASSGMGGLDFRFSVHILTGNKADQLVASVALLHALRIEQSLMESDEVVRRLFFNY
KPSQGMSDAKLASLSCMPIGEIVGDRSFSHRSPRIMEECPKNAIFRILHNIRCKTSFSTI
LPTPFSARGNLSRDSTPRGMLNTTTTNNSSSIYQQATMQSRAGVASPSSAQGPPVLEIPR
LRFAAEAARQRAVDMPLNAPRQSTAQTPRLTQLEGASSVSNATSTRTTTATVGSTLLPVI
ANLHLNETLNRRCATELSLENDEIRMTEERVKKLRAAAPEATEVLPWLFVGGEEAAGDRI
QLLAKGITNVVNTVAFSMGNVHSDIFRYLGLYLSDSPDEPIFSLFPVVIRFVEEARLKGG
KTFIHCHQGVSRSCSFVIAYVMWHQGICYDRAFEFVRSKRQVCSPNTGFYVNLLLWENQL
STPVFNKIYAYVPYMEYLFPFSFRLALTFRGSQRSQDDDSQIVNPFVHVVHGTDESYTVD
PRLSYGILLGDRGSRSEDRVMPIKSYFFAGSDCDAIVWQEARVDWDAFIKYSFYQGQKKR
STNSKGEVITFTPMPPVEQPTQCLWELRDLGRVINEGVFSQTCRASGGTRYPNVVQGQVQ
CWNKLLARDDLVFRLSTYLAEEQRVNLTSQSRKRMREEERRRLSKLVSSASTPRLSSYKG
GSGRNNAGTSSVVVNAANSTNTTNNAPLTAHTSFSIAAPSSRVAAANTAAMDLLPLVQPL
PTPPLVTPATTTTKSAAASAKTSLPRKTKSSIPSTARSNSLGGEVEIYAYPFTGPPLTDI
LDVADMEPDGCYAVVVPATAAAATTSWRHRVFLWLGRTCSVGEAEAWQTYRRSLKIDKDV
RLWNKTLLQSPLEEVAHDGEEPDDLILALE*

>TCRU_2091 hypothetical protein, conserved
MNADDGGAIESATSHQSAQAAHRAADAPPDLSNCPTQSSGRAKELICQRRSNVVRRITDI
PALGQLYEESERGRSRDVGAVNVGAWNAPSGAIHNLLPHARTADERGELCGLFDGNIRCG
AIDPTAVRQDAAVHVGNGSNPAGHDDSGVTKTCGVIRGEAGAPLDKGRDESSNPRPDRLE
GTCCRTTRVDNSESLVRNCGLDTQQS*
>TCRU_2092 hypothetical protein, conserved
MLVHGPVSKAWQGSTLIGHIADLRAPQIPNFEKQALLPAMGAARELGHQQGLITAERERL
NIDYANGVADVQNRNARLNSQFQEAHYNLLVRESTIAGAGNQVLELQQQLEAGRAQLRER
EFNLGVLNAQLPEREARLNAQQPVRDAETAGRDEPPATQHVANDGVTTLPHQTDILMAGG
VPSSAARRSSAAYRRPHGNAGRPHFHPSTPLPSRTSPLDAQLPMEEGRSFCAFDVTR*
>TCRU_2093 Concanavalin A-like lectin/glucanases superfamily, putative
MLKGAGAGAGAQWPVDMNGPTVPHHFANREFTLAAAVSIHEVPESGNVPLVGVGMSDPNSTV
LIGLSYTHERNWSFMTPSDANPDEPALFDWKQNTTYQVTFEMNTDGCYVFVDGRGLYMKK
ADEESFATLLEKHRISYLFIGWGSARGTESSRMTVTNVILYNHTVYGEGLEQLNASTVPI
PQSGVAKQQRKHEGDPSCGDSTVHLSVSPVLLLPALVLQLWGMTALC*
>TCRU_2094 BNR repeat-like domain containing protein, putative
MSSCGDGQHQLCVSDGHGTSWYPNRVSINRVWGNSHDRKGYGVQSGSTTAVIVGEKVMLA
ALPAYSKDNEKGRLHLWVTSSVRVHGVGPISREGDDAAASSLLVKGNNDELLSLYENKEK
SEDGSDGHFAVRVAAWLERVKDVAKKWTDLESALQ*
>TCRU_2095 hypothetical protein, conserved
MDGQMNPQQSSGTRHSPSLQQSAAKAAGNFSEGLDMFDIQLQNDCANVPGSFMGGLGGNA
ATVGLSPSPPVTLSAAMFSSIATATHGLASTMSNPQTARRMVQEVAQVILRFISTPDEPQ
PENLTDEYGRQLILQFADELQRSAAALTTGMTSGGGDGRLTIDKEVVLSLKNGREGLPPP
GADVARPHHPYTGYFSTMNSASPRLTTQPLWRGTPPNFSNGLHSSNSASGNTNAATTATI
INNNCNARGSHGATESFSPAGVGTTVHPQANLTAPSLSKRVGGGDVFRPRPQHPKSGSRT
LMAHRRVVTSTKKVLRSASSAFSECSAGETGEVSSSMSRTTVGTSALAK*
>TCRU_2096 RNA-binding protein, putative
MAMRRAVLLCSPLSPPFSSSPSSSSSSSAAAFFNGRSATVEEQKQTSDGGRDVDLALRLQ
DQLAKISEKVAHDNDVIRQQATAFAMLQRQQVVLERRVVAVEEETVQMQQKIAETTRVAS
DVVLQHRNVEALVRQMEKLVLSHASSPKNTKSSSGIASQAEEAKSNALGVSTNPKLMTTP
SYTSPSFPSSASSMGNSAVAGMGAQISALSTRLEALQARIEQLTLEKLVLNNVQLHAQKL
QKDQQQQQSLVEEAVSRVAAAAEEECGGGSGTGSGTVPTGHSTKSLATLLRNTGAFPFKD
STGATRVSSQKVLVRGVPVNFGASEVRDLFSCVGSVVSCVVRRMPVAPVATKARPHVSRY
RSQQKQQEEKEACEGETTDGPTSGGSAANFTTSTAEENERVFEVTYQKVEEAVRAILQLD
EYQLHGKHILSVEPVVSADIVAAMQHLERELNRR*
>TCRU_2097 kinesin, putative
MGKKTSKAAENIKVLVRCRPLNEKEELQGYKSCVDVDLTEHTVTVKSLVGEPDRWTFDAV
INNTFSQRDIFTQFIRPLVDSVLEGYNATVFAYGQSGSGKTHTMTGKIGDAELQGITPRC
FAHVFERIAEIKEASPNKQFSMYVSFVELYNGKVQDLLARQQVPLALKENKDKTFYVQGS
HIPQVKSPDDIFRHMEEGAERRRVASTDLNADSSRSHSVFSLIIECTEISEDGDSLSVTS
KLNLVDLAGSERQSKTGAFGDTLKEGCNINLSLSALGTVIDTIVKGRGHVPFRSSPLTML
LKDSLGGSSKTVMFANIGPSEHNFSETVSTLRFADRAKQIKNKPVVNMDTKDQKIAELTE
LLHELREKLKRYETEGTGSLEREVEELREKVGEMEVSLDSAVRAREADAVDFENVKAKLN
MERQATTSRVIELEELIGKLQNDMQIMESSVTDERLQREEVLRLCSQYLCGEEDGGKPIA
TANELEAVLQENSHGKHSVELARLQNKIQKLEVEARQMRKEHKTVTRELEEKLQEALASL
KSAESKLRKANRESNEAGDVRKGTSQPPGGNGASSEEVAGLRVALENAQQELQEKGIAAK
LETKLQEMQDSHEKITTAYLTQLKQAKEALGAPGADDSLIVQQLRAILAEDDKTYDFALN
HMSNMHNVMHGLKSAMREAQTVPLAAISGNADASRETIKKMGNLDAHEAVMYAGMLSNLH
DAVDKAQTHRNRLLDAIAALPDTPVNFKEELSKLVSENDELRKMYKDAIKVTETAQARVV
EEQRRTVSEESVQAQIDSVRKEYEARERELLAAMKLDENSGDGKAVLALLNKNASGGGAV
SEAAQKEIESLRSDLEEERVKCEKLVQSKEAQQVELKALRQEVAESESRYVDLEGKMNEM
VSDYERQLANAHGSTEKIADLTARLKERTDQLDQMRGLLEKQKALIVKSNEKCEYLQQKL
REVNARAERTEEIYRQKLQERDENFQSVLNQRLEEYARDHHDDVTKQEEKSKKLRKKIKK

IENEVDRLKEEYDCKVCECEDLRNTIEEQKVELMRLLRRMGQTEEEAEVYEKKEKIQTAL
ERAKEEQRRKKDLFALGEVANVRRGEY*
>TCRU_2098 guanosine monophosphate reductase, putative
MSVSGVGSIPVALTYDDVLLVPQKSPVRSRKDVSTTTRLSRNIKLHIPIVASNMDTVCEH
QTAIAMAREGGIGILHRFCSIREQCEMLSKVKRAQSFLIENPRMIMAHQTQEEALQGLQW
KGRKGGVSCLMVVENFSTRKLLGIVSKNDLHFADANEPVSKLMTPLERLVVSTNTAITLE
EAREMMREHRTSNIPILGKDNALIYLVTLSDVLKLTGKKHASLDARGRLLVGAAVGVKKE
DITRAAKLVEAGADVLVVDIAHGHSSICIDMIKKLKTDPRTNKVDIVAGNIATGEAAAEL
ILAGADGLKIGVGPGSICTTRLVAGSGVPQLSAVMECTRVARKHGVPCIADGGIKMAGDI
CKAIAAGADSVMVGNILAGTEEAPGRVLVKDGKKVKVIRGMAGFGANLSKAEREQSLDED
VFAEMVPEGVEGSVPCKGPLAPIVRQLVGGLRSGMSYCGATCINEMQQNARFVRMTGSGL
RESGSHSISKL*
>TCRU_2099 cell division control protein, putative
MSRIRFKGGAWTNSEDEVLRASLTVYGLQNWERVASMLVRKTAAQCRERWENYLDPRLHI
REAWTAEEEEQLVQLQSLFTNQWNLIARELRHRTGMNRPAWLCEEHYHTLLDALEYERQQ
KEGGNNKRNESLTLEDFLAERRRHRAYHQGHETRASKADAVNSDVFEKEMVEFAVSRLAN
QDGKKGLRKERKKQLQHTSFLAKLQSNREAIESGTLSAKAKKRMERAMLEDRAGPSDTRL
QDEILEDDDVGSSGGSDVGNDESKRGQKGVFRPIDLGADKQEAGIQAKHRELVKNLSADM
TRLQQEATVSEGINVELLRLASGTSGSANRQVEDKKTPPITSKSSAAPGASPATADLDHL
FASLPEAILPEQESVVGARASVDELFGTLPEPLLGGRVSRDKFNVAVATPEDYEAEEEEE
AEVSYTVSPSEPSQAPLTALNFSTEEGRSWLREAKRRVLVESESVFLNRKRVRHHMSSPT
EKTQLMMTTKNGEEEKGQLEGERKEKEEKNDDNDESTWCIVRALVEQQLPATARDLLEAK
AVPDGFLAKKAKREEDSSARYNNTQEYVKEKGGNEEEKDTTIELAACVAAVQDEDGADFW
KDVGATEATVQLEELVHHQREQVAANTANDTEAYQHLERMVRICFAGSRKELRGEDIINR
VCVYWGWRLEEAQRRLAFYSAIREVERQEMQRRINDVTSRLTEIEQKERTLQERYRSMRL
AE*
>TCRU_2100 Putative methyltransferase, putative
MLRWVVKEVESPYGNVATVHWAVDDGDDDNVEAATDAFDASLQQQLENAEDQLGAVLWNS
NAVALRYLHEHVLRDEASAYRVVELGAGVGCLGIALAMAGARVVITDLKELLPLMQKNIE
MNAARIRLRSNGQGSCTALALRWGPPPRLKKRMKKQQEDEISLGSKPKETWPYAGPSPSF
LALQQELDRVDMVVLCDALYGNARDWPQLLYTLSEILAANPSCEVINFCEQRVKNVEGDF
LRLLQQENSKSVASDAEDGKENEEESLESALTRMRGAYEWWSATEVVREGTSDLGMTVCA
TRIRWVPRRKRQELVSSVSASSSSLTAVVGASECDEGLRGKRRRTKL*
>TCRU_2101 hypothetical protein, conserved
MTEMCFSPCSIFPGEVEDYYCTSCHTTCSGLSLLVGPHTGHNRVPLIDAATYLPAALQRD
ANALVMRIQEEYIRPKTTHCNTLHATLAHMQQERQKKRRELEELQRELGTLDEKIDNLTE
ECAMALCHWSHQRDGFLSQVRRLLRGADALARTLGIRENQPIGSERRVDVDIAAPTKFPP
PFALRAVRKELYEVRRLLTQPLEWLEQEDEEDKEERSEGGKELRLSRSVRNGADAVVSHR
SKKEDYGQLSGTSRTPRHVNDHTLVSSDAEAVLQQLLAAHMKRQGEPHTQKSPSARAYVA
EVEGTSSCNSQKDVQVTAERRRWKNDYKRRERLLHEGLNVYMREGFFPGRTTTKIAGGVE
TDVNDMSSSDSVKEAVAHLLRNSQNDIFINTTPHQQRRPPSCALASEED*
>TCRU_2102 hypothetical protein, conserved
MLRDTPVCRFGTLSTALANIALFTANSIGVCDPFSVLVVGGTVFRMLLLPATIYGDCCLA
RVACALPELQSAHQDYKTIIDHPRAILWEKKVAAQKLKNDRERIFRNHHTDNVRLVLLHL
VAMAGSWYSLCIPAQQLSEFVGTSAVTSPMIVYAFGVTMDPTIGVAATLTLLNTRDHLQR
RMGFNDGLDGWIRQIKRHMTLTWSAFVAVSLLGQCFAGPVFLSYLFPFQLAPVWLGISMM
SSCKIILVNHTAPMRALFGIQDYPLTHGMHGALTTAEAHEYRLEFTGVDAEERRDMWQTQ
KKALDYECNVRLHRMLKQIGLFDSVEEAEHEAENLRRKLDVVRDRQKKRKQEQERNDAEL
SSSMAFCEADIVGPSAESVAERHFDEIQAQNNARRQRGRGL*
>TCRU_2103 hypothetical protein
MGNAFFRWEACEADAAVDTLTFLAEDPNAPRFRLKHAAVGTALDSHSTGFCFYSRYVFLE
VFTALQVLLAFFTLYLFNYMGTIRHYYAPRTVLKEEEGFFFPPKEKEVLVPHKREALAAV
YLAVVVIGLCFILLVAAYILLTNSGWWASLETKRWEHDKQAAVSSQPSHGELQHRVRSSG
YHLRRLGIRWISWTRPYYAFPRRYLPDFDYRHELHAKFGAPSTRGDPKKMYFVFLSPFVF

MLTFMDIMTVDEMRVTEMRVSLLQGLLLACGPLFLFYVASILFMSMMMSQRSCFGDTMGV
LYA*
>TCRU_2104 Protein kinase domain containing protein, putative
MSEIAQVGNLSGLNATTRSLSRLTESKEGEWSELDQAELERFFIETQQSSATPESLCFMS
ILGGVQITTPHNETPIFGATETNKTSCSSTQPNSTVACSLPPVWYGLPRSTPNTSFRLEP
LPEVAMDSLQEKKGEKRRWVDPLSSSTLRDSLRGMQPVSLQNETKGSQTEAWKSNPVQPL
ESRLQSRQRCNLWTCGRNKLPVSPLEALARCGCHLSAHEKEEVLGFQHVYYCGAHAKCAA
LASMPPSGLKPAKNEPSPLSFDDHDHNYRIIPGDHVGYRFECMEVLGRGTYGIVVRALDY
AARPIPKQCALKIAKNLPRYAETLRREADILDFLWHNKPEVRRWIPQVLTRLTFRSHEIL
AIPLFGLDIKDLLRVNLFRPMPMALTRAMTAQMVVMLELLSDANVTHADLKPENIALFDR
SLSDVAFPPDIQRQISLEEQERDGTKLDDDGDDREHDSPLFLDSINSLSSLHTRGVPGMA
ASEEIENDGEDDVVTSHIAILDFGAAHVGKSCIYPVQSTFYRAPEVALQLPYGPAIDMWS
LGCVVYELATGVPLFVAESDKELLQRHITVLGMPPPEVLQRLHEMRTSSGDVEEKKGEEE
EGKMRDLFASLQVERTPNAERPSAQFLNLLQLLHIQEREEAIISNKQTQQMRQQKKHTDT
HLAKEAYPLVMATSPSSYDGDDDDDAGETVSTLLLLDFLLGCLMWDPEERLTPDEAKCHP
YLVPCLKEDFMQCDEKAPLVRTRSTGMYYLHTHALTRGEVRILEAAEREYAQRRAKGKVV
TNGMHPLMRLNVRHAIQPLRRFTSQRFGDDTSETPLRVSCRSIRPVRIAVGDSLQPYTTK
FFDPIEQVEVSIMYFDEI*
>TCRU_2105 hypothetical protein, conserved
MERDFIFPASFAGISDQQGVISVLQRHDAVRVRAAHRIPSFSVAVEVVYQFLRSLPPPLR
WSIREVRVVAAPSASNFTVELTSECQRHAELDVAKLISCHGEEAERMSDLTAVSQEVRLT
CAVESSMQRTRGIQDTAHSTEGMGILFEGHWKGKEINKGEDHWNGIPAVLTTKLQAYLRL
LAKKQKEHQPSSLFFHLRCEVRNLFYCMPVRQRCAAGWSYTSYRLRAERQALLLTAYRMA
VECVFAPLYLSLNEGAGSSASFYLHLSNSGDGRNKAANDNADVFYVHNYPVQRSKEMVEQ
LKKRRRMEPESKNDDGDRSCTLRTAHRILFSFFPHLRLCGESLECSNGIASSRLREVVVD
PGKFVVLFLIPAWDTHDATRRVGRRPSALGTVVVCRHTESTACYVVDESHRIYLQVLNSF
TTLCPIIVFLEGNLLESNDATLRRMFQSAERKYLCGLGTDTPRAIQTPTREDSLSVVGKL
METKDASGEQKIVGTALRVSFLAQRLSSVNGTKRARCFGGAPISSFPRRTPFFPINLGRN
LCGTSTLSQVRSAMHLTIDPSLFAPRAYSGPSVWPGSGEMENKERTPCPFRAKHLIAQWD
RKFLLLQMNDVGEPSDTSPFDEIGSPSSTSINGMPTMGVPPVLCKQAQSHHLPPPALYIV
DPHAIHERLRLEFFLTTAESYVRRDIGPISVPVHIPEEIRRDVTTYEDFLARWGWRFAHE
NLQCTGLRSAMASKSRYTQLSWCCVAVTQWPHLEIEGHVLQLETIDALRKTVEELVTVLP
SWQMPAATEKESSKECDWIVESPGARVVPSAVLEFLVTRSCRGAVMFGDSLSTATMAQLI
GALQAVEQYTLCSHGRPSFAVIHRRKM*
>TCRU_2106 hypothetical protein, conserved
MAMMAGRVLLVCALCVLWCGAVFVSAAGDGDGDGRGVVGEPLPESPESRVESKVVSNALK
EHVEEAPTPAKESSEEEEAGEDEATLQGEERQQQQEGENALQVNGRKAPEILVKQNETPQ
APKVEGIPTAGALEESAKTPGLKAPTLKVPQPPAASADGEADGGPVGVEGPVSGPPSGGA
S*
>TCRU_2107 BNR repeat-like domain/Concanavalin A-like lectin/
glucanases superfamily, putative
MAATHGGHDGKVHDSGEMGKKWMEFCGAHLNVLSGALRSATSGGRLDIVTVFIEGRSVML
FALPRQNNIRRATSVLLLYMTRFYPIGPLRADGVSKNADTLHYKGGELFALFEWYAGQGR
VVLAHLTEQLEEIKTALRNWDAVDKNVTTPCTSTSKEGTAFDDACSTPMPTDGVVGYLSN
EGERDCWVKEYVAASATVIGTAAKVEGGATFEGPGVWTERPVGKWWRNQRFHIVKKEFTL
VVTATIGAAPAGSGSALMVAVGGTGSVRMVELSVTVENKREVAFDGEAGEVQHGKWGLER
TYRVAVGLQNGMGTVCVDGKTLGSSATTVTRGPQLLDVSCFYVGGDGCSGSGCSQVTLTH
VCLCNRPLTRQELQMPLLRGKQTEADCKERKVTPRPGNFAAKLPQDLGVDAAARDEVPVP
AKGNLAWEMAPLMSMALLFYCFKNGLVTAPSMGVCLGHCLALLQGVCGDVAFL*
>TCRU_2108 hypothetical protein, conserved
MAMMTGRVLLVCALCVLLCGAGGRCEENGADVVSGGGPGTGNDDKNSEALCSPDLSSLER
QEEGAGGAAGGFADAQGGKDISTSSTGTNILKVEKVDLVVDPPNPIENQNKSKDSTKAGT
GTINQTASPSKPQSPPPAGPPPPPLPEAEGVKLTKTPEKQDSSVGHQSTQHTVPQVKELK
KQQQNTQNQIINEKHESERLQVQEEKEKQRSEEQLEQRERPHEEEREREDLQEEIRDQQK

KQEQQEEEHVDSIKQEKEQDQQHQHQQHETFAKNSDGPIKNETAVGTNATASTGDSDGST
AVSHTTSPLLLFLPVVACAAAAAVVAA*
>TCRU_2109 hypothetical protein
MDVEKWRRTLQGFVSSSALQVRPAPLQKVLQSARLEVNHPPLPMEDWMKLYQNMESRFST
VQAGLSLQAQRLRSEWSGLQEPAPGTAVDDHADNAATVLAASIERLRGVLLNACSSLPGN
AMESNADGSA*
>TCRU_2110 eukaryotic translation initiation factor 1A, putative
MPKNMGKGGKSFKAGNAKGIMQNQKRDIVYADPQEGEEYAQVKKALGNLRLELQLADGSK
AIGSIRGAMVRKVWIGQGDVVLVSKRSFNKNDIVDVIHRYNPAEVRALVKDEVIPRDFRS
SDERDAKNAHSDYVFVAENDDDGGADDDDQNVLDRNKVVLDDPLATFDEL*
>TCRU_like histone chaperone, putative AS.1
MATRVELRGISVVGSNPAPFSDSIVLKVLLEVFEKPPAHAIDVKFTWKLIWDFPVDQELD
EFEVGPLTTLGVHELTLQSDPPDFSKIPDPTGPTALIISFTYEGREFLHIGYNAVVTCEG
EMPDVFESAEQLRRHLTQCFPKQLSIVWDERPADAVGNPSDDDSGSFDVDKAEECAGEGD
MDGEHASDSGDGAPPLKRVKT*
>TCRU_2112 Sedlin, N-terminal conserved region, putative
MLMVIGPDDASLFECAKSVDGESTHLSRQLMLYASLDLLDDVLWKTGDFLLPAIDRPLEG
KYYVSAYVGFAPIKLLLMQEQEPNKNTRQFMNEVYGLCVRYLLNPFSFPNTPVRSTLGEK
VRRLYERYS*
>TCRU_2113 6-phosphogluconolactonase
MSFKPKIIVCGSPAELSGVACKKIVEIIYASERTNWPLSIALSGGSTPKMLYSLLHEEHL
HLLKEERALRFFFGDERLVPADAAESNYNMARQALLHDIPEDLVVPVDVGCVGKVSKVAC
NDAVKSADAYEKKIALLLGTQKVEGMEAEIPVFDIVLLGLGSDGHTASIFHGSQAESEMH
RAVSVGFPSPTMSPKVWRVTLTPITIIHARHVILLATGKEKKGVLNGIIADTPTEVPVSR
FLRNCKGEVTFILDKEIAGNLTC*
>TCRU_2114 BNR repeat-like domain containing protein, putative
MGRPQTTPETDLFASHEGDWTEFIASGGSGVVMEDDTLVFPLMAKNEAEDVCSMIICSTD
NGSAWAFSEGISPAECLNPASPSGRDHFTWLLTARMARGCTGRVT*
>TCRU_2115 Nuclear transport factor 2 (NTF2) domain containing
protein, putative
MLGRDTVAGVACSFLVTYYREFVKEKEVVGLVELYGESSLITYAGYNEETTVVAQGRHEI
AQHLGKMDDALGRRKVEVRFADFTPLPGGCIHIVCQGILYLRGQRRAFFQVFVLAPTQYR
TNTYHIAIDYFRVMVVEVEQIPEDSIIMTPAQVAQHLLEEHERRKRMEESREQLRRQQAA
AEALRLQQLEEAARRKEFHQTRGEQEPRHGRHERQNNGTRNDGYGNNTSNRRDRVEWNDD
RRGDRTDMRTLIRNEGSVSRNNRPERAERNNGRYDERNERNGERRWERHDGMRGERNDNV
RAREERGVLEEGPAPQSTRPLRSREQPSTAASKSENGGSKPSIARPVPRSKAALEKRLEG
RAEIEENNIINRNEAVASRRVPEETAMKEETRKKTAAPRVAAAATAAAAATGSTRDPARG
RPQFNRTGSTDYARLVRVPKYVTLTDIKAAVTSVLGAEPIDAYWYGRSSADCVLQLRSSN
AVEQLVRDSVTVLESRLSAAKFYP*
>TCRU_2116 cytochrome c
MPPKARPPLPPGDAAKGEKIFKGRAAQCHTGTKGGANGVGPNLYGIYGRRSGTVEGFAYS
KANQDSGVVWTEEVLDVYLENPKKFMPGTKMAFAGLKKPQERADLIAYLATLRD*
>TCRU_2117 hypothetical protein, conserved
MSYQLYLHRSFDIQLSPVAAAIIDWAPTRRDGQVSSDLSSGLSSSGESTNTMGNRKGSKP
ARDGENEDKMGKLMSRSHAVESPYATGSSETVSLEATQKAHGPTGASMPDGGPREVRSSH
KRMYPWLGQRRLFLLNSYAVVPLVTRGSLSLNAGLFAGFGVTGGASSPASSIGTSCVSPS
TGRASIYASDVLQLECIWGRMQWAGVFNVVCFSWPSASLPLCEFIGMTPRTDWSMQVDRL
LFNWSGFCRVFVRRGRHCRRDSLISLNSPADSALSSHGDVNAAQSCAAKNNPPPTPPVTL
TSRGDTAISGQDEHHAHPRAVKSPHRTERPRAAADNNNNQTNNKNKHHHLTDDDETPPD
SDGGQISKRRKSRDTTAWSHLARKSSQTTSHGGEGYRWWRLEELRCGAGLAVKNDLASGV
NLYCGCSAHFGRALTVSTHVDVLRRACCSITSTTSSLDLAARLRMNLITWHRTELDFGVG
WRPFKQVSGLTCRLSHSMGRTSVGITIKDVGTQYKLLRCSRCAKREEHEERQEQEGRSWP
WRRRDSHDDSMSQENVGGTTSVPTLSTTWSLSDWYDPYVGWVWRLGGSIFSSASLSSSPS
AASPAEGVAAHAPGLRKRLSQAMRAGLDYADALSRQTEFDLTLGLAEERNYSRKPRCFVV

LSAH*
>TCRU_2118 diacylglycerol kinase, putative
MQTKSLDILHACEQGDKKHPLGKCPPENFSLSCYSALEGGNCTENGSAEVPRGNGSGCMQ
FRETSNGPNGFETLPVMVNSPSRGYDSVEIANLKTDYRPMTSPRDGDPEYEYVLCIVNPG
SGERGVAEHVLDSVRAVIGADRVVTLDAALFADPTPLLEAIRRHAVRSISQERKKSALTR
VTAGGNSDADARGDGVYSRGLERRGTVIVCGGDGTVSFVMEQLDAVRDMMQRTERGSFEA
GARARGGALKPRFVLPAVAVLAIGTGNDYSNCVGFGNGYSRHKLSCLCCCMENAIEPLIR
NVVTAPAIPFDRWRVQLVPLTAIWEQQERQRQKKQTPHLTLGTKHEELVLSVNPPRGDME
EKKIQEGKKEEVTVTEDTVDLYALDWDALEADETCRKYNFINYFSIGFDAYVLQKFDFFR
RKHPKFCSTRMNNKLVYGVYGLKAGTRCSSLRPCIPQICVPQLAQHSPIHTEASQRSSGQ
TTETKSMMTLSLPKGSKTLLVTNVGSYAAGTRPWKEGKGKLYRDDHLAQPMIITPVCVND
HKMEVQAVGGIFQMGLLQLGIGKGASKLAQTREMFAFVLCNPTDISRRENPRYGEEMLAG
KEGVEEHTPLCMQLDGEAIGRINRPTVVHITELHRPRVYVRCRNPRVVRYPIKDEEDAF*
>TCRU_2119 hypothetical protein, conserved
MGCRQSVAKRPVAPQEAAAAAQYGESGKFGEHCNASSATAMELTMRVSAKKKVHDAGNNT
PHAGEQQVTRGKVRWVSTKKDFAASHRELLGRFWETHDMERLSTLLVRNVPVSLQETTTS
TSFAWTSTQREDGSSMTIFSPNAITVSSLAPLDGRRTRSARRKRALPHHSRWVSLLHTFS
EPHLGGGETGEHVSTSCAPAHAREGAGACKLSGMRGHMHPLSGSGSPNDADVGSCSAHVD
HGAATGCGYPGAKKLFAFRLSEMPSTGKGTSFLRKRKMNPCMGVHAQRWDRVEFFSPNIH
SIAIAVAQTQPNSANSFFTNVTSPGSPLSRLKESHATFNASFDTTKVLAPVLLTESLSST
VSQISLGSPRNAEHSTRFPESSLYCHCLALPASHIQELQAGGELIPTSFLSPFASTSFGG
NALEKTTVDMDGSPLHDATICSGNANMTLDNNKKSASTANIEANVPRASVYGTQGKMELQ
KLNLGVRELISAIDEKKKASPLREDTGGHTTNMCSSSELQQSHAPKTILSGIIVSSFDLS
IDPFRWREEQYKCNTIKYNGARSCVREQFREGIVTRVMYGGVFVVECTSHLALQKLEKAL
KLKKWSDRKKKISALSKITTFPRDHNSNHVRDGAGRVTSSSCYNSGMECSNGGSFLEYKS
CNDANQLELYRVAYCVGGMQIKWESYPKTEEQSPRSDMIGTHTGCPTLTPWINLKTVMKL
AHSWARHLLSTPELAEPLAVYVQSFVGIIPALRLVSDWNCQLMQLFTESEDQEDDDDELS
TDCGAGIERGGLPGMENNFGEASPFEDSSDLSTKTGGGLSVPTVLFTCRPMTQYFKSDGH
VSKSTLRSSANISLITSKRTSETARRPSSIESLQKHPRPERSRRATARAIHESVPKVDQC
KSSARQREGGFLTPRAKGDNPHVPRTAKPNEKKEGTNERNVPVTDPMMNEQTKLEFLTPY
LPPPLSFEGEDGEAFWERSMLEAMALEKELKDVMDARRKADDEFYRLGGIIHSLQCAFLP
NSQLGEIEPLCAYLQTAIDDPMELPHRCCYFAGPDWFSCLAAVLTNPRNSIAAVDISYRL
TFSSLPQLGMLAGLLFHECAMPFLQRLSLQCRRIFFLKCGEKNELSRNEKVGKLWSRIIS
RHVSKKDHERRPEFSRPTSIGFDEPRRGPPFFLTPKQAKRVLVILTESLLDNARQPHFTL
QLREFPYKSFERDSKVFHKVFHVFSRNFMRHATSNDNNNNNSHPHHHDTDGAGRKTKKKK
NRTEEENALLRDRESTTSSWTDPPEVVFSNDPHPIQCAWDEAERQPHCMLMRHREFLPTR
WSAHHKNHPYAGRHEEKDQNHSSCYEKMWVYGLGSEYLEFAVFCELGAQLLEKAPEIFDA
VQAALSHRGAGKGTKKGSNGGKLLAQTEFLLTC*
>TCRU_2120 U6 snRNA-associated sm-like protein Lsm2
MEGALVQTIPSVLAAGGHVQHSLMRVELTNGIVLTGRILQLDAVTMNMKLDAITDIAVRR
RCGGGGGKKIEWETDPAALRCTSSVVIRGCHIRYIDFIDEEASGGRGLQELITAARTVRP
TLV*
>TCRU_2121 hypothetical protein, conserved
MLRRLVSSHGCSNGGGTSGGRCVRPVKEEPPVFSSLVLQRRFSFKYATKLQHDEMRQPFY
IHEKRHGIFSNEKNIKKSRRGLPFITPLYTRHMNLWETDTDASNNRFFRGYVFGQRELHQ
LLGRPHGFEANNTDGSNDISAYEMTTDQRYKGIPRPAITNLHYEPEWNYTLYRAGTHGSQ
LSNPRSPLTAEVLGDELMKIRDIKSFDHCKAWFDRLQYLIKLHYDAVGDIGEFKSRHTQH
VHEFFVAFHDALSSFDFGDSYLFEQFHAARPSELTDLFGIFLEMESNYVHEDYCPRCSLP
YSTTRYCGEGDANTPFRKHRGRWAPHQRWGREWYAVVARRAEALWYRATEDPYFGTPQHT
QRQTEALLRVYVQTKQRGKAIDFMNALRGSKEFLLGSICITPEMQESYDRLLDTTPHPHL
LTNGFTLESNAAKYTGEVQKVPFSPLQFRIDMEMNKYRRQQKEEGAVRVPPAMWRIDTSA
IVPYKVDPKTKRVINWREVKEGIEKSFLSTGLPKEAYTGSEWREMLHLKSIIAGRAAKAA
ELERRLHTDKVKLLEVASKSKIASETNTSTSSSIIFPDKDGYHVFDTSVASLRPFGVSQS
GAVFQSVTHTYPSPHAVPYNDPVHGKQFILDTTNESCHLFGGFEHGDRLLIRARKSDKND

NNPNSVLAVKGNEFEVIVLGVSKEGSDSEWQLCAMHVDPALQREHGIVFLGTDCVEIHER
WASVRYAAAAPHVKGRVTLLEERRTAREESLGQIVGVRDGVLFVQWRLLRGGGSEMDRSV
AEPIGTVEHVRNTYQITEQGVEELMHPPSWRTPFRNDFAEERLEELRQAPFKRENWVSLI
QGRYTPKRKRFGYTQHTTMDDFETKEYKDRLLSKQFFHNPQAFEVIPDRRDRAVTFGGKW
EYQRTHGLPTVDRNELENGWSEVEAVTDAEMHVIEQALRDISGRRPGNFIKSPTKKSTLQ
LNESWWEPLEFGWEQHNKEQKALVDPTEQRLIDSTSLPFGGKIPPFGTTIGIGERIREIA
EDYAKGFGLGPHGHSPSHDTSQYNTLNAEEDRVRELGYKDALVRLFDEKMAGKDVHQWAV
EQCADGEADVRQLLLSLHEWRERGRPPSLMLLQVLSKYLEQEIAAFNEGVPASVPKLSLQ
TVDGTLSPSGNSGERSGTIWADVEPTAYALQYASQANHSSLDEPFILQLLKSAQLGGRNA
QFTDPFYNAYLENSVVSEFQLGLAALAGKGVSPSLLAQKTSQLHRGSVRLSGNVIPFVKS
RELAHLLERMGLSSENIAVVTRGLANCPEQESVGDDFAVPVSVILTWGGPGSGSSTSVAD
RKGNATQSRNEQQRKGSAALSSAIRQLGQKRSSSKNWQNEDKMMVHVIEELALRDDGLVM
DIQYIVRENRRNPVLRHEFFAALLPVFAGKHEKVAQLYDEYCEGKYVPNITLAIEAFIAF
LCNVTKHADVYPGSSYFDVDTTNGPHAGQYTSLKLLDPLDGPFIFDNIKAEHIETVERFK
QHGIQVGPVRAPATGFIAANSKSLSYFTRRPEEVVYVSTDADQGLRRSLERSAHYKTIAA
SPAMQFLLHTQNGAGLVATFNRFFYRTMPMLSFYQRILKHYSDNVQPLRQKAQNSVRGLA
RVLENERSAAMEEFRRNSERYWRNVLEGRSVEQAMDGSGGSGGGGTTPPVSSSPSQSSQE
AMATDVARAIGSGRTGDQKGGAAKQQQRQQQQRLQKQQRTAGDDGGVRTTFASRQGGSRS
MTDLLSKLNKPKGSNTSGTAKGPTKRGNPKSHTDGGRGAKP*
>TCRU_2122 cullin, putative
MPTSLGDLISIRGSSSGSSSSNGSRSGSKHDSGGCRSDAKRVDCSCIRERDVFSRLQDRP
SVDDLWPGVESYLVHAAHTIRTGSAEMMDRLRDVRHRMNWYAVIYSVCSGHPQKSSEVYH
RLSLFLLNDLEVNVLRQFLRRNAPCGSPPSSSLCLQFVQQYKLFMAFRRVVLSCFSYLDQ
YFTAKFRLDPVTALCVKLFYVVVYEPMKEALIAEVLELADAARTAYLNDKAVSSACSDVQ
QALLSIVEILSTVKAFSAPYDANTQSALTHQKKDNSNPCNYKRGVGEGRNMLAAFAAKVG
RMQSPSTAPNRDPPRTMGESPRSPLNKSHNSGHSRVEQQNGSEGHSISSSPLAKDWDAMA
YRSLDGIRMLRERLERRSFGDLLAAHVMRSSSRNKSSHHQHNNGAGIGITSGGCEGSANC
SVDSAGVVIVEITQPLHFLIFAEFGLRYVQAAETYYRNRREMQFSTPEGWMDYIPWVQGC
LAIERSLMRDVNAPLLLSALRERIHHVLLVEVHRSIILAKEVGFRAQLDAWDSNTEQGAG
DMWKDGSSSSSSIPPMGAAMSGTCSDVDAATSPAAFFSAQSTNPWEQLSREELGDRIKEF
VATFVSTQDEACWTLLASEFASKVVVDTAALFFTYMSQLEALAPQQLQQGEKERPGAQQS
ASSPPVQPRKTVIPMRRRRSLRADGLPSDRNFLTQQDLAMALIKGLVDTSIHYNKLICEK
FNEHPPMQVAMGDAMREILNPERWGQLVVGGAMHGGVAAVNRGELPSVLLNHDAAKKTVA
VLNRLATMRGSREIAVPQFLAMYCDQLCRREMDDGVTDPTDHILYLITLVDDKDVFLEHY
KLLLARRLLFLPSAQRNMDREHVMMHKMHHALGRTLTYGLEAMLRDHEVTASMNEEFSRS
DVALALPTRLRVQVLTSVHWPTYRVVPLTPCDSLALVMKNFTDYYIGVHPSRMLHWIHTL
GTATLHAVFPKGDKEVIANNLQAHILILISDAYNMDRGGAASSGANDSHTAMASRRSLSG
REIATAMGMEFCDIYMYLAPLVKHKLYNLLVRVDPSAVAGTGQTTQSSPPLSPPNARNAA
AAAVDDDDDAAATPSGTAPGQLLPEDHFTLNVEYTHKLRKFGLPVARPSRGEGFKGSVVS
STHVERTTAALSDEIEASRRIQMDAAIVRIMKSRRSLRYYELFDLTVQQLSKQFVPRPRS
VKMQVEDLVCRGFLRRSEKDTSVFEYMA*
>TCRU_2123 PPIase, putative
MDLKATSRLGCTLTRGSPASGTRYASHSRPIERTSCWMELACFGGFPKVPSLSGSMVQQR
QDLLASTKGAGEPEIILRIEFELFDDESPKACANFRHLCAGQSTSRKGQTYCYQGLTPCY
RGTYFHKIIPAFCVQGGDLTMRVNKGANHFSSFGRGWFSDENKRRRLNEVGLLLMANNGP
NSNGSQFFITTSDSEEKALNGRHVCFGRVVRGLDEFLREVAPYGEINGNPSRFVVVVDCG
VGPLPETLGITTSNSWAEEQKRHKAEGDNSHEEVGFEDGAATVAGAANAAASVEEEEEKE
KELEETTNATEANKQPPAMNGTS*
>TCRU_2124 Domain of unknown function DUF221, putative
MDLSLKPRSTWDDQGLWFTLALNSILLVVLSGLSIFCLTAPLLWLRRLYRTNHTITVSVG
PSGFPAATTPLTPSSSSPSASPTPPRQRVGRVLPYRCSLFSLPSLRDWLSIEEEVANLES
VVQPLSTIASPKDDEDDTKRHISGEMEMSKVNKRENTAVEDTFLVSATKAKVPFDPQVAL
YLAFLKLFATVFVLGGVFNFWICVLSGTDNYLEKYMVRRDIHGCQSLGDNETACNDRAPF
CKYVETDCVPVRLRGLYDLSAQNISPHSWRLWLVGILDLFFSVIYVVAVLFFLYRFDAYI

KSVMHLQMENALGHRVAIVRGLSADMMSEKRFHQGYLVEEAYFRPRGGKGGRVGNGDSGN
DNNGNAEEDELDVYYQYHCTGLSCLFSICGLGYYKTTRRDAIFTRDCSVRQLLFPREPPE
GMYESMERTETAMKELQEAIADQKAYSKKQRERMPVARERGEDVEETLMMCAPFPSCCSK
VPKVEYRKRVFIAEAKRLNQFIDATPARDAKGSAFIIFSDALEAYEFVNLFESRRGGVSG
AQATIAGPPDGIIHFNLTTDRYTGWLRTAVFFFVYILLLFFWAVPVGFVSSIDNLVYIPY
IGKFVLWIYMKVPETLRGAVTSFLPVVMLELFNLILPFMIRCFVIAMGVVNRDSLRGGEL
FLQYLFMVLTGVIFQAALQGGLSQLADVLKDPSGEAIIGFFLAIVSPNGGYWYAKVITAA
CISTWLNLVDPLPLIRAFLRRKEVSVQRVYDKLFKPCSFDWPQFYSFDLTILAMGLLFRM
TVPLLAILVGIYFFVRYMTQRCRLYDRYCPRVHPLHDCTVVSASAQVMRAASWLYCLGGI
GGVLVMSLRKHVGGVVLCSISLAVGILLLARVHSVSRRWVNSLANARRFLKHRAVPQTRY
GAMAKRKATQNRTQRTDKPSAPPQSTNVLSEGCSMEQLATELVSQTFETDGDVDNRAVTD
RSDSSTDDNNGVVGGDINAREGATSFGDQVPIPATELLPHNPNIISRYQPKHQRLLHIDA
EQEIQRMEKTVFHVESYWNSSFELIDDVGMPL*
>TCRU_2125 coatomer delta subunit, putative
MPVIAAGVVNRQGNIILSRNFNEVSRIRIEGLLSAFPRLLESSVNKQVTYIDAGTVRYVY
QPLEELFLVLITTKNSNIVEDLETLHLMGRLIPEFVDTVTEATLEAKAFRVFFAFDEVIV
GGKRENTTVEQIRTYLEMDSHEELMVREEKRLQMERAKKDASRKAHEMREKRRKGMNPYS
GISSEDPGYGGFGNTSAPVIVSGGGVDSAVLTHQFEGVSSAFTTSAAAPAPKRAGGLTLG
RAKKADITSRVLKEAGLPSPAPANAAGAASSQQPSSLTTGAGGGVESKKGVHEGTVEGLH
ILVEEKISAHLNRDGEGGAVDVRGELSVLVSDPQLATVRLILSPMSSKFSFRAHTKVNKE
MFAEDHVLSMRENKPLPVQQMVTILRWCLINSDNARPPITLSCWPSAGSITVEYELDRAN
DAIDALRDVRIAFPLHGTRAQEVTPSVGTWDIVNGCVVWSIPLLDAQNHPNGNVEIELAD
GGDGAASGSEVFFPVDVSFTSCRTVAKVQVVEVVRTETGTPVRFSQESRLVTESYTVPA*
>TCRU_2126 hypothetical protein, conserved
MDKLASRSDAAQPSSSLDEQLLRLLQQQVAIEECLSGQRALMQRITEGFSVLREWASRHA
HHDLSLHDLLVLEAYGGGSLCHNEEALQEVEKEGDEGDIHRGGYRHCGPNDMAIPAPHGD
RNENGNAHHPEYSKCERWQQQLQEAKRAYRSFIRARVLDEGCAAPDNPAQFHALTADSFY
LLLPQLTEMLKELVGITPGAARVQQPSSSLFSLSTSPSFASLADWCASLARVLRFLELGL
KHFPEVLDNSPLRPCLVQSLLQLQEDGNDRSHTREEAVLQLRQRAEKLCAHLHRLPVDGV
HNYE*
>TCRU_2127 hypothetical protein, conserved
MQTVDKSADAVHNADDVDMRESVNALKGKEPAAALAVASEPHIDKADVSLFPSPAHGKNE
NGGSRGGHPAYTDEGRPSHASSDVRKTNTNEGFKTNRCDAAGGPYQNEGISNPSEYLRWR
QRRKNLTSADYWRAAEKERVIGLGTVGLFQDQQPDSPPPGEASACVNGTIIVGPPGYVRK
VKDYNDWREKHSELFSTLRNQDERERQCMRLRRLHEKARQREEEEYEYASAVRRLRRRGW
SVLGNAPRQVDHTRFNQIKAEREAFRAECEGNVVRKIQEASKQRENILQQVAMYKEEKAR
QENIVRDAARQEREEQNAKREEYLTLLKERAANTRAIAEEEREFIRECAATESKYRQNRI
DILRERVFAKTYTNGLLTNLSSTLRGAGRQYPPGDWRNTFKSPLRRLPNASDDAFRKQWV
TELQEERRAVARAEVELMKRLQTSAQREAAYRDNCERAEALRKEKDALRERKKQQCEEVQ
LFHKVLRQQEVEGQKRREEVLRALRESKREAASIIRQAIEEEEMMFQTLEDEELRQLQER
VAQMKKNKTFPPRMALSPPAERVL*
>TCRU_2128 hypothetical protein, conserved
MSMNTSPERTGLKIGTALRDSRLRRSPERQSVPVSQELSFIYDLKREFDRIADSGAFIIR
AVDSLSKTDAHALDIDVDVVLNDIEREEEYIFSPIPHYLQSEWKRMVSENESAINDVVTK
LDTVGRLNEDGKLRADYSESARIVIDPSVPLLMPFSKERYHASPQRKTAPSCYHSLFFGE
GEPVVFVPPENNKRKDIEKLNKEREYWKKKFQRAPRSDVVEQKKTIKVPNDFVPSCAQVD
HSLVQQELQKRNEIEKNKAKKETPVKRKTVSPAH*
>TCRU_2129 dynein light chain LC8, putative
MSADRKAVIKNADMPEDMQADAIEVALQAMEKFNIEKDVAAYIKKEFDKKYQPTWHCIVG
RNFGSYVTHETHSFLYFYFGQVAILLFKSG*
>TCRU_2130 SCAMP family, putative
MYKFGGETKDLRNIYNFGDISQRETEPPKDLSLAENRAYLVDVEVHSDNNEEEMGHRESQ
QPNSRVSPTAQGVPQSSAFFPEFSHSSGLDVPQKPSMENTSDQKNSKEKQKENSKVKIAK
EVLGINKKNTSGMSPEEKERVLIEERWKRAMAEENRLNALEEQVTHREQATNSSGLLPNF

PPKFLCIKPLVHHDISSVPEVRRQFVRFNFINWIATCVLLLVNMIIVIAVVFASHKEDAK
KFNTSQNTVLAILYLMGAPLSFIVWYWQIYSACSTGRHTKHLLALSGLVIALAFDIFMIV
GRTNYAACGVSLAIDISKTKSKFAVLPVIIVLFFWVVEAVILCYCIAKQWMYYRLDVNAQ
EEVRRQMRNVIGI*
>TCRU_2131 hypothetical protein, conserved
MAMQRARGVRLNGGDNRSEGTELSISRVIRAVQGSKSHKLSSINALLQTVVQSAKNVVKG
TDSQRRFNAQFSEFCRASLVGISDLEDAVYTLEKADGVFGNDSSDEQGVKSIGAVEFVKE
VAKFISSQTSALGVQYLEHVGISAVAAATKTQQLTTTETKRGSKRGRFDDTRQRHVDYRS
SPRKRMGASPSLAPAWETDALLLSSPANKSTLARGGATDSYHGAVKLSGIPELSMWLQPW
NLSLPNAHGVALDRVMTERAKSLATAEDSLKPPDEVSVFDICTAVATAEVLRLWEARECL
LSTGLDRATASNPVPTGKVFPPIAEEGKPMFFPKSDLRARAWVPTQVMVDLVHYGISSLA
MPFKDVE*
>TCRU_2132 hypothetical protein, conserved
MEIAAYKLFCLVESYATHPGHAVGKFVNLSGEKESFGIAFDAVQLFGHLICTSEDGMELL
CTVLRKFVEIHEVTVGTACRVNFITYVVLHAYHTLAKEAVMSQRIGGGLSNLGWGTCSPF
PSHETNSEDCDARCCWRIMNNFLQELRAKGHSLDPTDVLEEVASSIVSAGEGMCDTDGKK
AKFLSPRVLHEYLQRTFAYDPLSWGYRAGKLAVDCFLHIRSRLIEFVVEMETDKTQSGLS
SLHTFVEKSLYRVSNEGIDWLPIGGNEGCVVPGIAFIRTVRMKRSSDALLGTCVCAPTNA
FGTLVENEGFRTHLLLMFSQFPDAATEESDFGFRQLALMVKNVEKGLLVIAVQSHVGEET
LRRMESWGNHTRTVLFLVALGKKIMTQLALAFRVLPRPLGLDQQSFLQGSLFVKPLFGSH
SLRHCSGVELRSFLLALNPLNMEGVQHPIVSVVFGGRCAADQVLVERLFSRQLHHLVNML
CLPAPLNVVMPAGGVVEAYAIQHFDKKLREKSMESDISLRCFEIRLIEALRSAIIAYMES
VLQKSGGLTVEATLEHLALSQERFAAMDRDAENRESNCNGFGAFAMRSALPNPLYSRFVP
TPPLTEVSGATSAEKYRAKSTLLLADLREWEAQVELVSTYLTIGMCLDHLCFTSIMGRRS
AVRDSKEDTQMDELFFFPDIV*
>TCRU_2133 hypothetical protein, conserved
MRQLIHVTSRSAVLLVLLVVMMFVMCCAGGCFAASLTYSYSSDKAMRADRAKSIPAVVRD
VSFGYEGASQTCTVIGRNGWAPTRINVSTRDLDGTRKYCSSERYCAMNSLVAIASVKVTF
FYDEKREVFVSLYFAATVR*
>TCRU_2134 hypothetical protein, conserved
MPSPLRIALLLDWRNTIRRLGPAAVICYLNSHHELRDGRSPSTAAGDELAVIPTGMEIEL
SDDLAQATWNSGRSVSVPDVTAQRALCASHWTSTRLHGLGPPSAIPHCRDGGRHPATGRH
AATTET*
>TCRU_2135 hypothetical protein, conserved
METTVSASSSERLRLPFSRCEARDERVDPDALRLFLEHARQEAAERHQRRVSQVESQYLR
HLQRCSTLPGASMSRLQGEKGDAAPVDDGEVIEIELEESDENNDDAEDKKNDEESKSWAS
CGFTSNFSTATTTDFTRALGKTSATQHSSPTPSTPQSQSNSQGISPEVYTPLISTPEEVS
STNSASAAKVSSLMRHDGANDTDAEVWRANAAPNSAAMFPNYGRGPEDYFGGMSVQKLCA
ENAAAQQTRSLHTVDECGDQHDATRCLMHAFGRHLDVEFQQVEQDAPCSSPAGVPLETEL
EEFLAWQAAKPLRRRERLQGALNCGSHLAKVYAAVYPELLSLALFDAPGKKLAEPLTPES
PASRYIQWIASDVDCYEDLLLHLTSASKEVEQTLLEGVCEAALNAYITECVVDSILHNCK
*
>TCRU_2136 kinesin, putative
MTYRDPQRGEISPAHGTPLRNVRPSRQVSNIADPLRISPSTVARHENDDEYMSPTSLSNN
HSPIYRNNVPANHSGFGGDEGSESNFNVVIRVRPPVQRELNGYRPYVDVVRIPNDSRSIT
LCETLDTEDGRGGVYSRQTFTFDYVYDKQTLQSEVYERSAQPAVFSVMQGYNATLMAYGQ
TGTGKTYTMEGFTSEDQRGIIPRAIEEVFTSIEVSRHENMRFLVRASYMQIYNEVISDLL
KPTTARSLVVRHTPQRGVYVEGLSEWVVRSPHDVYGLIERGTSLRATSATKMSELSSRSH
AMFTIIVEVMEGDETSPTSYRVGKLNIVDLAGSEKLRQVGATGQRLEETKKINKSLHELG
NVISALANKSGGRNRHVPFRNSVLTSALRDSLGGNCKTTLIACISPALESYTESLSTLVF
ANRAKNIKNTAFINEDLDQSTLLRKYERELKTLRQQLQERDATSQQAGGGVDPCILLELR
QGMRRAEEDREAALEALHESSRAHQEELVVRKRFEERIQELEMMLQNGGFKGVPRTSDEY
ALRLDELDRERQVMEEEKAQVDRYKQLLLKQRDIMLNLTTRLNERDEMILQLQEEIDAYD
THIQTLEEALEALQRQQQQQQQKPEPNGSSKSHFSTPFSRARAEEYLQRTRVQNGDEGGE

LRYRSDANPAVLLTAEEKMLELLLAFPHVPSMAPQQPTIQVNEDMKRQLIELVRERAEKM
VQRQIDDYFWQQCREMELLKSQLLESEQKSNVLEQLLKGVKAQHPPFQDVFARTRRHIDE
QQEMVRKTYEGRIQALQQQLAQEREQRLRLMNEVDVLSMESRKALERCTNAAQRVEMNNV
CMALQQLEERIRTETKNGIEAAVLARVPAARDSSHGNNFNSHGGMIHVHGTNNNNGRDVH
GHNTAGVSSATSSTSEVSPAPATVTNELEGQLKALQSSHAKKEEEWRRALQEKDATVSQL
EERLHAVHVDMSEEIATLTKSLNTHKKDRRALQTIMEQRVKVKIDTICELVAAGPQLPPQ
EQIRLCNEVRVLQNLVNASIKAMES*
>TCRU_2137 acylphosphatase, putative
MSAPKSAIVVCHVFVRGRVQGVFYRKYTQRAALERGVRGWVRNLEDGRVEMMAEGPKENV
DSLVEWCRVGSPKSRVDGVEATIVSSGDAYTFKTFEVRR*
>TCRU_2138 Trans-sialidase of Trypanosoma hydrophobic C-terminal,
putative
MVPNASKKRPSFANGPRIVVASNASGIVSVTYGGREHVAADNTDEVSERSATGHKSLVSE
SGVAGSGFVAEGDAGGLTQRNASGHESKPQLREFTAQYSGSGVSLHDCFAPLNDAKLTLR
ELTVGSTVRVCVYWVLLLLLGLCGVVAVL*
>TCRU_2139 hypothetical protein
MRDSDRRCWVRQPPQTVANPRFDVTAQHSLAAQLSHQILCHPSLHGVAATDGCPSGQHRC
SVQADGWFHRGKSTPPQFIRAGVFMCRHIHREGNGGPFLFHCTAGSTVATCVHRIGHMAH
STQLTISAMHAHIKATESVILMLSLSNGSRTILPRPYTHAIAQAAQLGAHDTSTGTTTAS
ASPDASPGLPAAVTHTEQAAAAQERKDQHGAAPSSPVVESLKSVS*
>TCRU_2140 BNR repeat-like domain containing protein, putative
MLLRGASTLRGIGPLLLVRSIKDTWRAHTVVGAAEGKESLGVVLRRTTITKGHRVFLLAG
GSDVSYEGGCWGDGGLELKLFVGDVTNPTGGKQSGRIEWGDPKPLSKLISSASHEGEWTE
FIASVGAGVAMEDGTLVFPLMAKNEAEDVYSMIVYLTDNGSIWTLSEDISPAKCLNPCVT
EWEGSLMIVDCENGRRVYESRDMGTAWTEAIGAL*
>TCRU_2141 hypothetical protein, conserved
MLQGNKASVDVDGELLGEEEVPLTGQRPPEVLRFCFGECGGHESHVTVKNVFLYSRLLNF
TAMRALKDRVPVPTREAAPTPPEKI*
>TCRU_2142 BNR repeat-like domain containing protein, putative
MVAGNYSFEVTGTAGNTAAAAKWGFFGAVGNVTNDGSSGKKIYWKDAFVVSLSDFETKHE
SLTRLIGGGGSGVKMKDGTIVFPVEGTKKDGNAVSLIIYIATEDGKLSKEVPDDGCSDPS
VVEWKGKLMMMTACDDGRRRVYEIGDKGGSWTEALGTLSRVWGNKQKGM*
>TCRU_2143 BNR repeat-like domain/Concanavalin A-like lectin/
glucanases superfamily/Trans-sialidase of Trypanosoma hydrophobic C-
terminal, putative
MLITLPVYSNRRGNGKGELHLWLTDNTHIADIGPVSGNDDVTASSLLYKSAGSGNNNNER
LVALYEKRGDGKPSLGMVSVRLTAQLKRVKEVLATWKEVDDRVSKLCPSTAARNPSTGND
CSAGKTTDGLVGFLSKNFSGNTWSDEYLGVNATVKGTNGGATGYADGVTFRGAWAEWPVG
SQGENQLYYFANHNFTLVATLSVHGEPKGDTPIPLMGATMNDKNTVLLGLSYNKEKKWQV
LCGGKTTKELRSNSEPGTTHQVAIVLRNGEQGSAYVDGQRVGDASCELKNADSKGISHFY
IGGDGGSAGRKEDVPVTATNVLLYNRPLDDNEIRVLNANKISIPKLTDLKTVAAGATGVG
TARHFGANGDGSAVCGGGLLPLLLLLGLWGIAAS*
>TCRU_2144 translation initiation factor IF-2, putative
MRYSRIHLLKFLDTFYPLPSVMDACSFVKLVRKRTSRLAEFLEKEVLASNANQRSRSFDA
KVHVDIAKSLDALLSLKEKDVERVIWEEHKLTDERLLTCLVPYQMAANIIMRGLPQNVRK
MCVEEIRDEEDYYLKGITKKGHNKFIQWIERDVFVSTGLNYLRRLSPLRHARIPVIAVMG
HSQHGKTTLLDVLQKTSFSEEESRGTSQSIRAFTISASESGFSSVTFVDTPGQRVFTETR
FHAQIIADALLLVVSLVDGVQIQTLEVIKVALNIDKPILVVLNKLDLYSDPGKADEAMFN
VLMDLREAGLNVVMVHKPKDIERLRACESSTKLDAQSSKEVQNTLQLFAPMKKVDAQYKG
SKTHPVVNLKRSCAGVCISAKKRENLDLLWSLIELMSSTLSPFCHSRMGNYTSHSCSHQA
VILESSKHLFDEESFRLRKNVQRLQKRQDATNERQKLRLEKNSVSARIHSALNNDRKKLQ
SSNPTSTNCLVLTVIVREGCITKGLLFVADQTRGRVDYMIDAAGNFVDKAYPGTAVIIID
VHSNTGCPGVGTHLLSMPFVDERDRVFEYRRWLQWFIECFPNRLNLLRPRGMDVSFAHLG
DYGQIGNTHCLEYQLLYGPPQNAFSTGINSQSTEELSINGSKPLEKSIAEYLSEKNLEDR

ERHNGSPLEDGSHQKILMEDDLEVTLQTTWTQLQPRKRPETQEMHDEMIKSCIQIGVLFK
VDSWHSARMLHREINRLGTRKVAFHVVGMRFGELTADDIMFFGRAMKIAVCYRTPLGLST
DLDQYLEINDTWVLQTDDITEVILFLKWCAVALHKEHAPDDYGVTERSHRSYLVLMKDKS
GKSPGKKMDQQKNSRHRRLLVTPNF*
>TCRU_2145 IQ calmodulin-binding motif containing protein, putative
MDTAEDGDYLQFATKLYAASKPFLAIKAYERAVSEVEAAAAATSDPKPLPGNKLGSGNAL
FKDFILHCNAFGVKAATSDTKMAGDFLRLGFKELQRRNYLQGDLDANDPSQFFIPIQEDR
GIAFLVSLILSNWACLLLRSGNTREAAAFLQLALTNSMAEELTRVVLLNICAVDVHNCLF
SQVRENAMEALFEDGETEEEGQEKMDKDNAAREDVNGLLCAFAYHNVAIAEEYLQPQEAE
SHYNKGKEMLASSPYQRWSSVIECARRRFLDIVQRRRYEATRRREAAEAEMEAAKQVEAT
VRRKVRATWSATARQPSAKPGNRGKKYKKRISGLTNDTKDESIELPPISEYLRQKMQTQG
LSNVVVPILRVVDVRKFVFGGIPAHEDSFFPFRESVNLNNVAMVLLCEETPLQVVDEAEL
KDTGPPRKTIWTPFVPRRDDTLIIVPPKPKPGAVLSRDAANIAKSFMPPPLSEKSIENAQ
RSVTAFKKILNHRLLMLLRAENAFEEKWKATGIVKRALVAFNLAQDMLKLKETIKWKRET
RAILENASARRIVRFFRLIIERKSNSAPIMREARVKYFEEKAAIALQKYARRWLAVKELR
SLQRQRRAEILRVMKIQSTYRMHATRSRYIAYKEEQNEALRFEKETVAREFAAIQIQCAY
RRHAYRLRRWYEGGEKRQFILHHFRSSREYYATLIQKTFRGFLVRRVYGPAVYAKRCYGR
NCYRAALVNTCATIIQSVFRGYRVRRATRIPLQQHLRQCVVQRAREKVESLRRIQNEAAR
VIQCAYRSYSARRVANELRTLRERERLLRRNRVYPSFRLEEQVY*
>TCRU_2146 High-temperature-induced dauer-formation protein,
putative
MGTSSSTLEVGKAIRLLLVDTPVDDGIFNLLLNTALSKSELERALPFSTLRVMRHYYTRN
FALLLFKCIEVVSQIRNASLESASDVQSASQGLQNALCLMRRIIPIAMEGGDTPCAAEEP
EKRGAPASNPPVEGANLPAEEAKDEKKRTLFTATFVRSFFEENLACNDAQPEQMLPFLPG
QNTTLSKFVGRLLVDCCFIRGLTLAASSLPAKESESHPCVDESLLWYPGVGGKQVEGTST
QPMNGTMHALRQELLYTLTALLSYPLFSPPGTRDTLFSEPLLNVDENPLLPTLVASTLNA
ILAYKPFGSIPYTSHWVGPEEMVVLMSARFLSALICYPGALLDGSTQTRHTKSPEESDKN
EPKENVEAKDREDGTTTEQLNSPPLRIVHSARDLISAITPEEAKYVIERLKNTIGLKVYS
NRTVLPDSQRCFITQDEFMMLLWRLIELSPECQLAFAQQPAALNYIVPFVDYALNAKRDP
KYFPHLQLVLFLLLRLSETRAFCLQCNALFHERIPFEFESFVGTYNDLLIIMLCSFILLR
HELIRPLHVTCSAIISNIAPFMIKISPVTSEKLGLVFTSVSTRYLAYKALFASNEVILDD
AEIKADQVVLLNIVEAVAGVLQYHEGAAATLLAAFIKHKSLVGEVSDIFLAEGSNAFRVR
LTSPFLIGTVRDAITAVEGAVAAAEEAGIMDIVSVICKTSLVGALPTPHRIIVRRLYSNS
SMEQWAMATTWSSLYMHTPPGTFGGSKSIKMLRFF*
>TCRU_2147 Zinc finger, C3HC4 type (RING finger)/EF-hand domain
pair, putative
MPPLILATECNEELRCALCLDSWKDPVELVPCGHIFCAVCVRGMNTCPICRQSVANSKRP
NRTLVNLALAVPVKCASCSWTGTREQGNSHTCRTDTKMASVPSALAPGPKYAFVEPTSQE
PWHQFGLSREEYDQIMALFVFFDNDESGGLDRAEVGRLARWLNFARTDHDIDRMFFLMDT
DGSGTLSLNEFLTWLSHNRPDPNALYGLSQSEYNTIMMQFRLYDTDQNGLLSIDEFARLA
VRLGDVNNIDSGRRLFRCIDTDGNGVVDLHEFLTYRVRTRRK*
>TCRU_2148 mismatch repair protein MLH1
MRRIKRLSDNVVNRIAAGEVVQRPSAALKELLENALDAGSTFIQVLVRDGGLGLLQVTDD
GHGIHRDDLPLLCERYATSKLRSFEDLSRITSFGFRGEALSSISYVSRVTVTTMRRVDKD
EASSGTLAWRCQYLDGAMQGEPTPCAGNPGTSIRVEKMFYNSAVRRRALNRPSEEYSRIV
ALISRYALAFPHVGFSCRREEGSGGKAEVFFPKDSSSLANIRLFHGPAIASHLHELKCVE
AGAGGGSPETVLAKGGEAGEDCFLITGYTSGMALLNRNPYLCIFVNNRLVESAVIKRAID
TVYSGILTGGNRPFTVLFITIPPDRVDVNIHPTKHEVCLLDEEIIVAQLSESVRLAVMES
AARRQLDTRHILSKAAALAGDRGAHVSQPSPLPSSIVVAGIAPAPCTVVRVEPQRGALDA
YCRRMPISKKEEAGEVSSENAVEKQRPETCSHPDPLTAVKSEETIIQEEQHAAGTSIVCT
ASNEDVTASLITSIMKFGSTDAADRDEGEAKKRLKEEEREEGEEEEEELGYVMQQFKRQR
KEIQRAAGIVTTAEAAEEEKEGAEAVYASAASSACEDAVEPVPDERGDLLLTSVSTIVSQ
IRQTTSPTVQSLFEKLVYVGVINGHMFLAQSGTTLYAVDTLRLVRLVVYQRIFMRWSIAS
LPAPPQMLLQEPVRVTDLLWFALQHDVPPQTDVSLAESTQRTVRRMDRCLRQWRCMLLEY

FSIEITHDGYLLALPFGLNSSWPPSPRVVPLFIWRLAAEVPYREDEAACFTAIARHIADT
LYGLRLHDAWVEPRVSPSPGVEPPEGGSVPSLSDAIRFALLPCVTSLKLFVPPADALIDG
TIQTVVSVEELYKVFERC*
>TCRU_2149 kinesin, putative
MYADDTGGETPLEDFSDFPVERSGVFSPLNKMHLPISRRRMVLPVVHIRTRPILPYLGEG
KNQRHVWCLDHQKIVVRSNKLVPSRCRTASPLKSNGTPRSAPTSPFVSSPPRQHSLSGWL
SESLPPKMLFARKTPRKHHSESSRSRGTTASATKIGNKTFASATPEVTPLNNSGVIRREE
HFNFDFVHGEDATQEELFEESVLDFVDMALLAQSVAIVCYGPTGSGKTYSMMGSTPGQSL
KKIGKVVPAVDGLLLLENRPIFDSKNRGKVVPLAERLSNSQRSNLTSTGTALSDTAALVS
TTASTAIPVRETLTPAVKIEEEQQQPHRQQQQQRNFISPRRGADANDMEGSSLSSGLSQF
DLGFSTYMDAFGGTRNEMSLTGISSGLTESMTVPDEGNETMGLLPRIVLILLERRGERVV
LGQNEPSVKATPGNRSGNGKEKSQETQKQRHNVSLTLKELTLYGVELYMDEFYDLLDPAK
RAIPNISDIGGLDAFCQRLNIPNSLAVSSSCGGKRASGGGTGSTSFRGGGVRVSSVEDFR
RCYKLATRNRVMKGHQRNDRSSRSHAVFILQLQFDLADSQNAGSNAVTQSIYSYVAMVDL
AGSERVKETKVEGVALREAKYINKSLSAFSAVLLALYQRSSHIPYRDSKLTRLLRPCLEL
GRVLVLVHVAPCSAEETISSLKFAEQARYTNVQSHSLLNAATEVVALFDDMTDPHAEELM
ETYHRTEVEYAQLCAEVRLAHLSRETPDGSLTFAESPGENSFTLQELAQNKKRDYVIGTL
VKRHMHRYKALEEKRLQESVAEIEREWAFYVENVKRSRKQDMENLKLSIEKLRSANARLA
EENSKPVLRDDHVMAIQQRLKELSTEINNYAREKLLLMEGMRAIRQRLALQTDLERCLDQ
QLREFQQVAAGGGDNGARLPVDSIVDEELGEIYHQQLLLSREMSLLRRESTCFVRGDRIW
EGIWARVMRQELLLAVSIDMEMMEGILLRPQSLRWVLEANGMTPDAARDVVFPTSSVARN
VKILLDTLETLKKQSASNPYGSGESDARDPPLPHVPLLMSNGDEVRPVPTLIGRYGDEAK
LQEAAVRKLVEEGIFCEATFFSMGVEELVRRRLPATGLMQQTNYKVHWGCLRLVHPPKDT
SSYSLELFQRTYSGSDKVRDRRVISIPLSEPQLRIKLHVIEAEGIVHTSPWDDGSVATEA
ALPLIALELQGVHPVTNTGAQQYQEREKDVKSRGHTHYIAPCATDTGGAHNGNGLLANGA
ESSSARDFLLSKRLDCGGELLLPSGCMAACEEGSGFFLFHFPERIVARKTRTEAVECIVA
ALSGLTLPFFPSASRCPSSSSMNADTSIGDISVVTYQHVPYLLLSSNGGSLRGGDAKGLP
VLRRVGVQSLGYFSATQICFYFQKEDSRRMTADGDALSRFPRSPLGRVAMPLSTACAMLG
RLGALTPFSGSGVGSSSNGHAHIGSNNAGAAQSVDETESMMWRMQQLYAFRRKTRARVRQ
QIFDAEDIKAELLYSYSDSEFMRGSYIKDVLKEARELIDLQQPQTSAALALRGMIRATPG
ICRELCGAVVPWTLWHWAHRWKALQLTHSHQQALADAYDMEDDEDSMDGSEGAGRLSMMA
SSGTPRDDGGQAFIVFEELPCFLACME*
>TCRU_2150 PH domain containing protein, putative
MDSDEDLQLPPPKTPINLDDSDDDLPPPAALAVKGPPPVKPAGPALTSPKNKPVALNKNG
VSSPLMGSPLNNTAAQRGAQYLDPQQQQLPFTSETRARVNPAKVYKEGFLFKQQPSWPYS
NQKRYCVIKNCRLYYFDSQESMKEGKPQGVIDLTSAKLVDPGNEAKLPSSFGIHVASDRQ
AEGGGSVKKERTFTFSVMHAHELGEWKAAIQSVVGDPAEDEKVHWFEKMVQGVY*
>TCRU_2151 Protein of unknown function (DUF3682), putative
MAMMMTGRVLLVCALCVLWCVAGGVYARDVDTNALGGCMASGVLGENGSHMPDGCNKTAI
TVPLRSALPITAVEASTGTDNAGGKKNDPNSSGTSNAGVNGSDPAAVTGRGSGGVPGGDS
DASSGAGAPAAAGGGGSGGSGTSAGGHATDSVPSDAAPPPANAAGHSGGPPATAPGVNSS
AGSSDGTAGSSVTNPSNTTGESQTGDQSSAAAANNSSLPEGTEGTTSGTGQKEEEEEENE
KQQQSDETQVQQQQQHEHPAESGEESAKDKNALRTNATANTGDSDGSTAVSHATSPLLLL
LLVVACAAAAAVAA*
>TCRU_2152 hypothetical protein, conserved
MLVPHGRVSGLLFTVNPRQESKALRELQLYLHPLIADLEEAHKRVEEHVDDRGRCDNATT
EKQSSSKMSTDAEGKITPSTSSLLAAELAEYITTRGGQRRHEMRRIPMKEEKSDVSDDED
EKKSTDNNNNNNNSDILLNRSSDDGSHDTDAGRQSSARKRQRHETEGSKRSVINSLRWLA
PLETGCKGHLMVSIPFASQEQGENEERETGATTAETQIVKSEAVEASTPRTASVSGAASR
GQGEPPHSILYNPLVHIVVERIFKDIEENPRPLLRNCFRLMPCELTCCPTLPEMREALGQ
LVGAHFPPTKGPQRLHRVSLAFSVKNNTGVENKKSYFHAALETVFPVNRFVLIPAARIKG
PDGETEAVFCAFVAHATCAMGVQRLFSHRGGYNLHALSAKQLELSSSV*
>TCRU_2153 Mucin-like glycoprotein, putative
MMMTACRLLCALLVLALCCCPSVCAAQDVPAQASFQTMTTTAEKPGRKKKRSEVLFKENV

ITTTTRVVDSKPEDDTRGTATTLPSPSAEGERGKGKTGTSGVVPAAEQEDTARDGQTLTG
PDSYSIVATSTLQREGTDGTSGTQSRTSTAAEQATEEAKDPAATATTKTTTKAPSTTTTT
TAPEAPSNTAMNTEAPTTTTTRAPSRHREIDGSLSSSVWVCAPLVLAASALAYTTLG*
>TCRU_2154 protein kinase, putative
MEESHPSELQLPAREGDEFKGGGDDRGGEGDAVGCTQEAGRAELLDYKVDWDEVVGEGSF
GVVYRGRHVSNGTEVGVLTLKGSLELREELCKEAEKGVMLLLNQWPGVIQLLGTFDIAEK
DTVTVMSILQLRGVEDMQAFFMRDVVQKEEKKVKGELEDDPASIAPEQSSSFAGCCGVEE
GACSRLFNENEAKWKREEEETDKGEAKTHRAEEKKYAAAGVAGEKTQSLPPPASLAQRQG
GALSVGKYMVQLGSVLGRGKFGTVYRGWHREEGYEVAVKILKGPVSKRAKMLIEPRELGR
LQKLDHPNILRMFGAFRHEEKDRNEMTLALVLELCQGGDLKEFLARNDPLSERQAKHIMR
QLVDFLCFLKSNRMMHRDLKPANILLTTTNIDRAVIKVADWGMAKQIPTFGSSICVPDAN
VMFESAVGTVAYMSPERLSRELYDFQAEVWALGVIMYELLFARHPYLHSASSVRTPEELL
NAITRAEELEMSCNVFLSENKRKEGNVFNQHHSEVSPFSRECYELMRHILDPDSKTRYTI
EQVRAHPWISQPEKMRATEEVKAVTNTVEPAVAHGENLNTVKVIKAEDVVAATTKILKAE
ESVTTEMAATGPSAGVTGIPLRVPSSEDEVAGPLHFQGLGSNWSPLQRQQIIYQAFREYF
YILRYDVLEAESDPGRGLVLLSYAWELLRAVARAALLEEHRLSGGTVCLDASSSICYSAR
SFSRFALPDDIKLMENYIHETAQRLKRNLPCLTTHISMGAETLTFSTGSMCGMEASGESL
TPLPANPQKLTYPSAHTLLFERTVSLIRAAATEELMLSDSHEQDNVMEEEEVRPEGERYR
QKRMYEKAIAILRLILQQVVVRCQSSLHYPFPPVVTDTEESNGMQLLTIPLVPLEDEADQ
RTVQALLHTVEKHYRRLVNVK*
>TCRU_2155 retrotransposon hot spot protein, RHS3, putative
MWRCCGRLHVALLRRRWALTASPTGVAVRLHDAPTAPPCECHAQRHWDCGTRQPRLSFGA
SGTCWPQFGGASGMPHRTDVVMAPRRGSCDGSVAAARHVVESKVWPQWTMSGTNMRLSDF
LWNYVGGRGAVDEGLQCDDGGVCSGAG*
>TCRU_2156 hypothetical protein, conserved
MGALPSRETQRRGVYSHRHGRNRRNEVVLIPLTAEFFPDEHSPLFERLYQARHHRTAKYY
YRAVRPANTDWDSDYYCQTIAHAYGLDLSDPDGPDKDGLFEKSFTDSIEGLYHRLIRETE
YCMCESDEELRQRYMVQLQQQQHQRKQQQQQQERRRSTRQFPSSTHEEYFGTGLNAYVS
GYMDAVMYMQGKEQHTHRNKDGTHVTSPAAFYLRPVPCAIISRYDAQRPHAEEGVEFLGT
TVKVLGMFGLFPTTIPMTVNGETVEQHMLKATMFVDKDVGDQILGNVVMLAVHMYVRQCL
EARIRAPYIPMVPFILRVPISNIPILQFVREMRVRLQSLLDAGTPRSGARPVRGGSSGSG
GGKGGSSAVGGSGAASTTDCADIGDTYVGKFDADAFLFEEDIPSATSPRQLFAEPSEMKW
LRNHTSQNTNPEGTLPILMMSGVNVQTTPLKHLSMTNMSHVRLPAWVTEASNEGSLPKTT
TSQQQKQSKLAFLLACRRSRFRIWVEDGRLCVWASAAYARRGCLLLAQQLSVTVDSKAFP
LPRFWNYRGLLRGGMTVPEPMLHNDVFVVAHDMPSVGLYQGDILRCSTPREIAERQKQQQ
QQQQLLLSQIKHSGGMSSTAATSPSSEAASTRRGFSNYGVLSAKEPPSPSEQSSQKANYT
SNHTSNIFLQDPKEAPVGAFWVHVSFDRENSGPSNSEKRRRGSAPEAQQCDFISYRQTNV
EEVLLSWIKKICIQARDFLEPDEHWVRDPITRALVHVDRYVIRRVEHDGMRYFIGITPRF
VGQQRRLEKVLVELKAMQGEDPSYACGDGNNDAEENSEADEGSAPEQASMVHGGDASVAL
LVEEVEEEEETTIHASEAVPSTTQEGVAPCEECNDMNVRLK*
>TCRU_2157 J-binding protein
MLESAPPQLLPKKGRLEISELAPQQRTIRTAEEIETAYNEAVRKHPFYDNADHTIDFHDA
TVFRDARGVVGGVFLPGALPAFAATMAADVLRPAAVRTSLRSNMFGGFAPLSGIAGYFDY
RGSPVELKCRKTSFTYENVHSWPNVFPMIDYVSAIYKAVFPEQWAAQDAAVPDIVRIHGS
PFSTLTVNQQFRTASHTDAGDFDMGYGLLAVLEGKFEGLSLALDDFGVCFRMQPRDILIF
NTHFFHSNTEPELNHPRDDWSRLTCVCYYRAALGEPACVAEYERRLARAKEIGASPPPAV
DAILQKDNGNNFNKPAPTFPYLLTPFGGAASVCSLHCCTAKLLRLHELLLENPTLEVILF
GESLRTDDGLPRREKEQLISVHLPVVVKMSPSGGFSELGGALKAAEEKQYFFEEKYLADE
LGPDLMSMWTQSRAHWLRLVKEDWERLCRRDPERTKFTWNNSSAMNAAFFDLCEVAKQMM
IGLLNKETPSSAENHSFWILFAAHLNYACTTENGMPRDAVGMHKLNVKLKDFHFGGTRYL
KDMPPEEQERRLERKKRIEEARRRGSSAHETHTDNWLLNDTFDYQQEDRKVEFEENGWMT
PEAYVKHLGLKPCGDVTAAASPTEPIHVLVVLPRPAAATTAKDVKRDVPLATSEESIRLL
MNPAAQRVLRGKARNVALPSPLSFGGVKITVLFDGDDIDCIHPDFVILQHLLATIEEDEA
AKARVKYWARVARYCVFVVETDVRDRRHFLLREEVRVAYEDVAEDCFRSLHAAAYSTKYN

RLRTTPSLIALCNRKNIGLRFKFRGSPLNTIALVVVGERLD*
>TCRU_2158 hypothetical protein, conserved
MLQTKDSSEHEEGETISSDTQRSASKEIPEATTPLSASKSGSGIAGSTVGKGGVNNPQPP
AASVTQHDSEGDTGPAALPASTSELQAEEIPNAGIIATTNDDDSDSSPAASTAERPTAGT
RSTQASGDASDLEQSNNDDVNDDVAHKDKTVEFETASGNRDTQADNDERYTTVPENATNK
TSNTEITADSDSSTAVSHTTSPLLLLLLFVACAAAVVAA*
>TCRU_2159 Endonuclease/Exonuclease/phosphatase family/Endonuclease-reverse transcriptase, putative
MEPFTWLPAERYFYPLLNSIGAYQRYTYRLRAVCDVQRQKLLLSGDIEQHPGTIAVLQMN
VSCLTPSKLATLMAQGADIIAIQETWKSSEQIASMHSGDYVLYAQSRIGKGGGVAVLVRK
TLRSKRIPLTIPQHDTSLEVVVVQVALDQNRDLIVASAYMRPPPQVTQSFRRLVNCLPAS
SPLLLCGDFNMHHPQWEPFLETSPSEVAAEFLELCTDAGLTLVNTPGEITYARGTRERSC
IDLTWSKHLTVSDWSASVSPLSDHYMLTFTLHQAFKDTIPSAPKFFYSWGKCKWDLFIKD
FDAQLPAYDYKKQSTGIKAFTRALITSYRRHCPRGMHKDGPRLWDDTLMEAERIATDSKA
RHLQSPTPDREAEMQRTRSQLFLLLRERLRNTYLRRISKLNPGEPLAWKYISGRKKASLP
SPTSILLGGGQHTYKTARRAANALNRIFLPFHPSHKAVRISKGINRQSASLNINASFLFG
QQNNNESAATFTSGSSLSSSSEPQDKNEAATTSGLVAHLHSPLDAPFNRTELLAAPRNTP
YGKAPDRMKSTVRHCDMFRQRASDSFFVALTTVGRPVRSRLSGDALPSFHS*
>TCRU_2160 hypothetical protein, conserved
MAMMMTGRVLLVCALCVLWCGLSVVANGGTDDLVSGRDEKNAEHLSSSDLSLPVRQGEPT
AAGGGRGSTNSQEGKEASMSNTGIRLPQEQEVKLDAAPHKPTGNEYENKGPTEAGADTRN
QPAPPPPPISLSEEEDDELTKATQDQDSPVEQESTQNNEILQEEPGNELNTQNPLPQEQH
TSEQPQVKQEHGKEKQRSGQLEQREHREEPKDQQQKQEKQDEESIKHEEKQQQQKDEIQD
QQHQQHEHPTENEEESAKDENVVRTNVTAAKGDSDDSTAVSHTTSPLLPLLVACAAVAAV
VAA*
>TCRU_2161 Endonuclease/Exonuclease/phosphatase family/Endonuclease-reverse transcriptase/Reverse transcriptase (RNA-dependent DNA polymerase)/RNase H, putative
MKQYENIRREDYPGSAGHLNVVPGSSTLAREEAKRLLPICCTRRGAGRRTNGSEGHQMEP
FTWLAAERYFHPLLNSIGAYQRYTYRLRAVCDAQRQKLLLSGDIEQNPGPIAVLQMNVSC
LTPSKLATLMAQGADIIAIQETWKSSEQIASMHTGDYVLHAQSRIGKGGGVAVLVRKTLR
SKRIPLTIPQHDTSLEVVVVQVALDQNRDLIVASAYMRPPPQVTQSFGRLVNCLPASSPL
LLCGDFNMHHSQWEPFLETSPSEVAAEFLELCTDAGLTLVNTPGEITYARGTRERSRIDL
TWSKHLTVSDWSAPVSPLSDHYMLTFTLHQAFKDTIPSAPPSAPKFFYSWGKCKWDLFIK
DFDAQLPAYDYKKQSTGIKAFTRALITSYRRHCPRGMHKDGPRLWDDTLMEAERMATDSK
ARYLQSPTPDREAEMQRTRSQFFLLLRERLRNTYLRRISKLNPGEPLAWKYISGRKKASL
PSPTSMLLGDGQHTYKTARRAANALNRIFLPFHPSHKAVRFSKGINRQSASLNINASFLF
GQQNNNESAATSSLVSSTSLISCISEPQNNNESAATFTSGSSLSSSSEPQDKNEAATTSG
LVAHLHSPLDAPFNRTELLAALRHTPYGKAPGPDEVYSEALRHISSKGLRFLLRCINHSW
TTGTIPVEWRRATIVPLLKPGKSPEVLESYRPISLTSIVSKVAEKMVLKRLLWVWTPHPH
QYAYRSMRTTTMQLAHLIHEVEHNRNHYFQVSLPKKSGIGNQLHYRPHRTLLVLVDFSKA
FDSIDHRVLSRLLANIPGVNCRRWLRNFLCGRYAKTRVGHRHSDRRPMLRGVPQGSVLGP
YLFSLYVHPLLNLLNSFAGVTADMYADDLSIIVKGQSREDAIPTANMVLQKLHAWSQENG
LAINPSKCEAAWFTLSTHTESDYDREGKWPLVVAACQIPVMTMGASRTTKLLGMDLDPRL
TLNVAATKQCAATSQRISQLRCIAHKEAGPSPHDLRTFVIGYGASKLRYGSELIWAVATD
SAKNEMQKTYATLARIVSGVPSTVDTESALLEANMPPLHVLCLRERLSIFENTRACQMDW
MRRPPPEPPPRAGFRISPLSRDELYAFVDAYTKDYGITESSPREERFFRSSIPPWFAASA
HRVTIGVELPIDHSITDEEELIREKRRVSEEALALHSHRSWMLATDGGVDVPKSAGVGIL
LSSLNSSEIIEKASINCGARPCSYRTESRALLLALEKLMIPRIRHRRKTLLVVTDSQSLL
AALNKGPLSQTDWTEDQIWQRLLTLTCAGWSVHLQFCYGHCGVHANELADQYATQTMESG
QYTEQGIAPLWHTDLLTCFTTQLTNKWRSTIRQDTHRYLLCGTRPSDLSGKDLITQEVLH
RQELVHLARARCGESELWGRLYWAVRDCTNQCRFCNISPEQSAYMRSNNDPTAPGTDTVP
PSAREEDVSPVSRRTLTRRRKEKCPHCDSTLTGFSGLVSHCRSFHPEHPPPLLEFTDFCD
MVFPTRRSTAQHRSHCAHNPDATRHRNSSARRRSLLPQDQPASTSTPIGRQETLHHLLLE

CPDTLAVRQRLGIEQDLRTESSLNGSCFIAGNFCRCSTTSSALRWHCIARRAGKSRKKNI
GPISREGDDAAASSLLVKRKDELILLYEKRNGDGTYSLVAMRLTEQLGRINSAVKTWKEM
DSALRVPTEGLVGFLSSTLDGTAWKDEYLGVNAAVGGGVTSTESGVTLKGSGAKWPVGSM
GQTVAYYFTNNGFTLSATVTIHEVPKAGSIPIPLIGARLNDTASTVLFQLSYTSERNWNF
TLHKKDFLVKPSGDVGNWETNTAIRVTV*
>TCRU_2162 hypothetical protein, conserved
MQHDRALQRRHIRHVDEGVGHQTRRALAEATNPQLRAVRKHAVAQRHVQRVSERVHQAQV
HRHDDRPAEYHRVKARRRTPAASRHRLRQRHATHAALRIATTATGPRRRHVERQARLAHA
AGTAAAHAVGERSRQRIEARQRSRTAVARHHAHGGQAGHAVVAAAPQHPAAGTVALHRHD
AARDRQPGDKRRASGSAARNRHGAAAQRHTRQRRHRRYLAHRPHVAQLQPPAGTDRPTVA
SHQRRRDRPLDAHKAQHRIKHREALAKHNAAAAAHNRHVRHGEYRVAPQTEARRGRHTHR
GRGRTARRLQRHAVKHHSAQQQVHTATNVHRRPARLQRPQQSVAHTQLRLQQRGRVQHKR
RRHRIRQPAQLGVAHRSRRHNRRVTDRHVRVCRQVVHKHKRAVAPGQPAHAHLQAPVTRR
LAAVAPHVDLRTAVAHTIPQFDAQRAGKHRRHVKHHSHAVRDVHAVHQHRVHGAANRDGS
VHRAAPQSQHHRMHRGHPRCHSHKRQHNEQRHTHSRNGPAPHTVPAARTRTRRTPHPPTQ
TGTNRALVVCCTRPVDRSSCPAKPVLQMANQETQFR*
>TCRU_2163 protein kinase, putative
MPPKRPVLENLRIDPPEKTVSITDTLTLVVKGDGGLEMRVKESFMASGPAFPAQAAKQKD
DKNDVSMEKIRFEDLRTCQELGKGSQGRVRLARHLPTGKRYAVKYIRLEEDTDGMRQALE
SELRQVKALMHKNLVTSHEAFFRDGRVYIVLEYMDAGSIADVLRRHPNNFNEVMLAYVAR
ELLQGLEHLHASKVIHRDIKPVNALANSKGEVKIADFGVAKRFSGGDVETLSAQGSLIYM
SPERIQGQPYSFNSDIWSVGLTIAECALGAYPFASMKHSLYDLMQAIATRTARVDWTADG
REHSSELIDFVDQCLRPVSSRPTATELLHHPFIQKAANVDPAEAGRWFVTQS*
>TCRU_2164 hypothetical protein, conserved
MPPKENKKNSKSDKDLQDDEQQLNEDIVKMGIRLSALKAEYAGQLEEIFGLRREKKELEF
VCAKFEDDLRLATLNRVDVLTDFVRQYKTEEDEKIRTCTDLDETLNRLEEEKIRLTEKAK
KAETEHEDAINSMKTEYDSFSEVD*
>TCRU_2165 hypothetical protein
MHTNLEENASTVSLPVHFDTHGVASYEAEGHGAGDLDCVGEMQMDWKMGNSLHFCGKDAE
LSPVNFPFLSHHHKSSRVTDWEKLFQIESELQYLGEEVGFFLQKLGRSLVGNDPLDSVDD
TVELGEALQMVESRLNSIFAGCDDSGMSQRFLSKDALHKATDCSRALMAYRGVAEAAESM
KKLLTGVFNYRVLLPFGADAMKRAREVSLLLDDMEKVLDSLEARVETLRELSETRLDVLK
RGLDDLSK*
>TCRU_2166 cullin, putative
MLEEDKEAIRRMKADFEAIAELTESEFKLNTGFARRMNHYSAVYTAATRTTNMRSFSDAF
GYYVDELLYTDFQEMLTRYLLKYRDLQDSSQMRLFGKILKLWNHYKVLMKWNMRAFAYLS
RFYIVNCSKPSLQQVALNIFLEQILKKNVHVISRVTQDLLCLERNGESVNRDQIRGAIEL
MSSVSVEKKDEIYTEQFLRPYMALTKSHYEGLVTEWSKSFAPSELLRQIEQAHNEEKARC
MCYFLPDDRKIIMTHVEEVLLESPATVEKLLKSDDGFVAALRGRDEALLEKYYNLLSCRT
NCLAYLSNLMRDEIIAEGKERLLQHDSQKKEMDFRSCVGDMLQLQDDFMQLLARCFNSNA
IMMKSMREGLEKVFGGSVHASRGPQRSVPFSELLAYYVDAVLQGNEGSTVEENLEKAVAA
LAYVTDRDTFLAHSRELLAQRILFPRKKMDEATERSLIQRISQRCGVSSTSYLEGMLHDV
DIAEGFGVTEKLEAVGKAPNFAFSVLVLKKGIWPPRIQGECFVPPRVIQEKLSAFQKIYL
EGTTGRVLTWSYSNSSGDVCAVFKKGVKILSMTGIQCWVVLAFNELNELTPNDMMSLFGM
SLEDAKPALLPLLKCSILRGESDATTIQPQAKFFINEDFSSKWKKVRVPMPTCRRDGLLH
GEVIAKKIEEDRRPAIDACLVRIMKSRRVLSHSSLVEECHQKLSQLFSADQKLIKQRIEE
LIRKEYIERDHKNPSVYQYTA*
>TCRU_2167 TFIIH basal transcription factor complex helicase subunit, putative
MRLFIEDVLVLFPYEYIYPEQLEYMTELKKGLDQGGHIVLEMPSGTGKTTTLLSLLIAYI
HYHADEKRKVIYCTRTVEEMTKTMGELRKLLELWNTDELGSNRPLRGLCLTAKKNLCIES
TVASQRYPEDVDAGCRSLTAPWQTSNRCAYFDNLERIPFELPPGAYSVEDLKQFGEAHQL
CPYYLVRKALRVVDIVVHSFLYIVDPIVAEVTKGYFNENAIVVMDEGHNVDDVCIEAMSL
IITKEDAAQAKENVKQLNAAADHLKATNRQQLQDEYDRLVNGLAMVEMARSVDSMMMPVQ

APSIPSEIAEEAIPGSLRQANHFLSFIQRLVDFTHRVVCRISRTYVADPLTFLSKVRDEC
AVDIRHFRYLTERLKVLMNTLQITNSHKFRNVSLIAQMYMTLSTHYTDDRYEKPGFVVVC
EAHDPTRPAVPDPVIRLVCVDASLALREAFSKYRSVVLTSGTLSPLDIYPKILGFTPVIA
KSFQMTLSRKCIAPVVVTRSTESVNITMEEITSSFVVRTNPLAQDIVSSAYEDLLRGLGK
TVPDGIVCFFTGYQYMGEVLLGWHRSGFLKELAQYKLIFIETQSVEETSIALANYRRACD
IGRGAIFMAIARGKIAEGIDFDRHYGRAVVMFGVPFLPPNDEPLRQRICWMETCLGIPGN
EYRNFDAMRQASQCIGRVLRNKTDYGMMLLVDRRFALNDKRKKLPIWIQQCLKENTNLSV
DAAIAVARGFFKEMAQPWEHKKDLGTTLFSKETLALKGFLKPSKRSFPCRVTEKCSNSAE
ELVHGEGDAIRAVKEPEHRKRLRG*
>TCRU_2168 NLE (NUC135) domain/WD domain, G-beta repeat, putative
MPVRKRQRRNTVFTEAGAVDEIEEKDKNVMVRLVDEKGVLTGAQLLLPVTATPKQLDELL
CSMLDESARGTPYAFFIDGDQVHDSVRDSLFRRQQEAFVDKMMKEGRRFRQQDVSKLEFE
LPEETVVDIQFKPQAKFMVRPVTRCTGALEGHSEAVLVVTFSPDGEALASGGGDKEIRIW
DVYTLTPTEELKAHTSWVQVLSWAPDGKHLVSGSKDGTIIIWSHDGMYGNFKGKRHKAHS
SYLSHISWEPLHRNIDCNRFVSASKDTTLKVWHNVTGVQFSLSGHQACVTCVKWGGEGFI
YSSSQDRTVIVWDASNGTPRHILRGHAHWVNFVALSTDLVIRTGAFDHEEKKFDSREEMQ
KYAERRYQEVVTRFGYHERLISCSDDNTMFLWAPQEQTTPLGRLTGHQGAVFHIQFSPDG
TLIASCSADKSVKLWNAADGKFITTFRGHVAPVYHVSWSLDSRLLVSGGRDSTLKLWSVS
KRELVEDLAGHCDEIFSTDWSPDGQRVATGSKDKKVLIWVH*
>TCRU_2169 hypothetical protein, conserved
MTNMETTASAFSTASPIHLDCPAACPFGHGANNGDNSRTDSHDDSMHRGSSFPPVISHNL
CDELGKCFMQPPIALLSRRRYQDPAVMRLHAALAFREMRSSANESPEPQVSSSWTTNSAA
SPKNSLSIGYDDDAMSSFLSTPANGSPIKFFRRAKSFVFPSVRSSASLASGDEVNHVSSV
VETTTKLKNCPFMNCYWNSQQQRQQQGIEDGQEGLIETEKQEETIEGVQSPCFVFDAMAD
PSQGAERGMSDSDVKFRGVPTDSSADIVAAWEMDDRERKSHDVDDDAALSHHEDDEVVGV
TAEGNFIYRRDIDAEGSRFGRGNVQNHQDDCPFLDECRDADTFPAFIPTAPFCTPERNIF
ATVLSDRAAVPNSHEERVGVWKNAAAASTTASLTNGAAFPRGGSIGRSRQKKTPEWTPND
AEWCSLLLRYRVCENADETEYKELISAVNKWHEVRLRYPMEALERRLREGESYDRPPFVL
RW*
>TCRU_2170 BNR repeat-like domain containing protein, putative
MGRPQTTPETDLFASHEGDWTEFIASGGSAVVLEDGTLVFPLMAKDEAEDVCSMIICSTY
NGSAWALSEETSSAECLNPASPSGRGHFS*
>TCRU_2171 Concanavalin A-like lectin/glucanases superfamily, putative
MRWWRNAVKDSDGLQVTRLESRAIWPVNTRGDEVRHVSLSHDFTLVASVTIEEAPNGNAL
PLTAMLANTESNHTVGLSCSHKKKWERMCEGKTTTRTSTWVPKEEYQVALMLQGNKTSVH
IDCRSLGAEEVLLAGGRPPEVLRFCFGACGGHESHVKLTDVFLYNRPLNSTAMRALKDRV
PAPTREAATTLPEKIRVVDSVREHVAEDITEEK*
>TCRU_2172 hypothetical protein
MSHANSTVVVIILILGLLLLAGTSDGQRMSLPRETYPRPAKDGVNPAAMFLRWAQLVNSF
VEALLNAAGPATVADKLKRNAPTDMEGSELRAISISPNYLKALQNGDVKRAVDHDDGLKY
LLPPKRTLPPHSCWSGYLAVPNTTNFTCGIDGSKTADEYSNMTDYQRAGCRHDEKVEAVC
LCPIGTVWVKRRFDREWVCYPRLLLVSARLEDKHICQDSLGGSLGLRSSYNPSDFCIHTQ
RKATLLFNINVSYRWTENDELADILAAKNNHLVFGKLQYDETYITLLRGISGLPDVLSRS
PKLFRFGVTPNASHLDAFGVVEGHPLQRDGAFEHVVYPFREMTLLYHGQVVQKLSDSEVM
NTFFSGDRNFTLQQVTRDLSRLSDSHITSGMMYMEIGFYGTGIPFGARHSHLWITFKGTL
PVKGNYNYDQSPSPAIIAAMTFGGAVLVITMVTILCYLCRRQKDIDDPLLANALEKQSQM
MRKKKD*
>TCRU_2173 hypothetical protein, conserved
MTNRVDRSRTPSRTSAGPYRNPSRPGILGTYCDPPTVARSVEFTSSTLEFGGIAPRSTIV
PSTDVTFVMLTRTCGIRRAVLSLRPPIRSCAPPASTQWRSATFSASANASIERSSTATVI
GPLNTTESKHAVARPPPIVTDSVSVTPLTQPSAVSPPPPPDLATGTSNGRHASPTPPARQ
LHTQSVNDAVSASKHASAAAQPSHGTTLTDDRPAKR*
>TCRU_2174 CRAL/TRIO domain containing protein, putative

MHSTGSDLCKADSLVTCYSGMAEEYYDDVSGTDLGSELLREADEWKKVMRFFPRSMSLFF
MEELQRWRIAVDSEEIAARYKILDEHIAVRREMDASLPEINVKATNWYKRVPVQLRFLYR
FGFVALGVLNGVYFFAALVFLAVLSANVIATLFFGFFVRWNYIFPLGATLAMAVIQGFAL
CSRAVEMLVAMARGKVIRPFETIGTKLGSVAQMCLFAVLVAMQFIVFCVYATFYSPLRGL
QVSFAVGWLSAVFIVAICHLVFFFDWLNARQLRRGRHIIPLSLFVLLNLHEAAERDLDV
YAITTSSYFICGIPVLLSLFLGIGFLISRIVGFAAAVCAAAVLASFTATYYLDSLSNALS
GKLLRTAVWIASLVWVFDCYVFGFLNDGRKWYVPVVIASIISIWFLFISFICLKKDEFHV
LLRLTFVSLVILFCATWISSFPIVGWRIGVFCVCLMIHNALGMLVSRAELTNIGGVFVVS
AAALLIIMSCTLLGWYGTSPHCTLARSLPREAAATPLTPLAEYHRYPVCSLRTASGLVIT
DLALLTQLVNSYGTDAFFFDFENWFNSTDFSYGGVVQEFFSGEGKWLLHRFDSTVYNQTV
LVLHNGDALSSMLAMTAWLGSISLSPLHIFLPASWSNNIVYIISFVTQMIDFPWRRYEQQ
LVNYMQGIRAVSTKGLILTGSGVAGGIASIAGAHAQIQTVVFGSPGLLHLLRLTGLSLAE
YHKYVLTVGASNGVLDNVGGQDITMSQKLYCNGNAVTCLSMEYISRELIQSCDTSGRRHI
*
>TCRU_2175 hypothetical protein, conserved
MAMMMAGRVLLVCALCVLWCGAGGGGCDDTPQALLSGSGGGTVVGTGAGPSGGADNGLFA
SEPNGESEGDRLERTDLGQINSSPRGGTVGLDNKLAVMPVGSPVTRNQVLSNEALSLQSP
PSPKPSAPELQDLPEGPPLSGGRQGGEADNAAGGVGPSGSSSGSGVKSMVSASFSAGTLS
PPIPLTGMFCRAARVRQPP*
>TCRU_2176 hypothetical protein, conserved
MPVGTARERRRRRCSAGRRPCDAHTPRRCARSALVPFCHGQASPRRRPCVVALIEADGGG
VPAALTGGGGPVPRSPLAAQRAAGGPTTPRAACVLPVSADASSVPRFPGTSQAAVSKGGA
THRSEREAGCGYKIAQQRIQYTAGARGQDKRRVAKGQFKRTQREGPNHIPATRGHASTKC
ADGSGDRGKWDSDDATDERVSLLPDFVGAYQRYTCRLRAVSDAQRQKLLRGGDVERSPSP
IAALQMTVPSLTPAKLATLMAQGADMIAIRETWKSSEQIASMHTGECVLHAQSRIRKECV
AAVLVRKNLRSKRMPLITPQRDSSLDVVVVQFALGQNRDLIVASTYMRPPPQPTQPLGGC
*
>TCRU_2177 hypothetical protein, conserved
MTMGASRTTKLLGMDLDPQLTLNVAAASQRISQPRRIAHKEAGPSPHDPRTFIIGYGASK
LRYGSELIWAVATDSAKNEMQETYAGLARIVSGVPSTVDQ*
>TCRU_2178 hypothetical protein, conserved
MPVVTARERRRRRCSAGRRPCDAHTPRRCARSALVPFCHGQASPRRRPCVVALIEADGGG
VPAALTGGGGPVPRSPLAAQRAAGGPTTPRAACVLPVSADASSVPRFPGTSQAAVSKGGA
THRSEREAGCGYKIAQQRIQYTAGARGQDKRRVAKGQFKRTQREGPNHIPATRGHASTKC
ADGSGDRGKWDSDDATDERVSLLPDFVGAYQRYTCRLRAVSDAQRQKLLRGGDVERSPSP
IAALQMTVPSLTPAKLATLMAQGADMIAIRETWKSSEQIASMHTGECVLHAQSRIRKECV
AAVLVRKNLRSKRMPLITPQRDSSLDVVVVQFALGQNRDLIVASTYMRPPPQPTQPLGGC
*
>TCRU_2179 hypothetical protein, conserved
MTMGASRTAKLLGMDLDPRLTLNVAAASQRISQPRRIAHKEAGPSPHDPRTFIIGYGASK
LRYGSELIWAVATDSAKNEMQETYAGLARIVSGVPSTLDQ*
>TCRU_2180 hypothetical protein, conserved
MRVVSPVSQELRRLPSAREVPLIVLSEQRAAGTRWRSNAFNTPPGPGPRQAKSRERTIQA
HTARRAKPHPGHQGARKHEVCRRQWGSQQWDSDDATDERVSLLPDFVGAYQRYTCRLRAV
SDAQRQKLLRGGDVERSPSPIAALQMTVPSLTPAKLATLMAQGADMIAIRETWKSAEQIA
SMHTGEYVPHAQSRIRKECVAAVLVRKNLRSKRMPLITPQRDSSLDVVVQFALGQNRDLI
VASTYMRPPPQPTQPLGGC*
>TCRU_2181 hypothetical protein, conserved
MCSQYGPLRNSSQRGGPIAVSVANSCLRVAATQKFSKPPSAVHPRNIGRQATEDSIASEL
KGLAGVYQHQRSPMGSAVELASNTALLGKVRLEVIGSVMLHFVYRVCQLHRRGTHTTARM
LVRTRRPQPKQSPQCHFLSDLTHDGRRADGSI*
>TCRU_2182 hypothetical protein, conserved
MAKGFLRVNWRACGGWLILQWRRPLPGTTERLAVSGLANGTTPLVAGLGSKACQRVRVPY
GCDSMFRVLSGCHGLRRMLSRRLISMHSTMSVTKLPVHGPAGTHQCQETLPDPVLVEGR*

>TCRU_2183 hypothetical protein
MGGCVPLRQRKHGKCGEGSERFEVCGGWRRGALAGEPAGAESTVSFCKPRVHAGGVGDDS
RGSERRESFAGCEPGLFWWQKTPGALVRQEAPVAANIRINAGDADGIVGDG*
>TCRU_2184 hypothetical protein
MPTISHVTVNNVLLYNRQLNAEEIRTLFLSQDLIGTEAHMDSSSDSSAHGTPSTPADRSA
HSAPSTPVDSSAHGTPSTPVDSSAHSTPSTTVDSSAHSTPSTPVDSSAHSTPSTTVDSSA
HSTPSTTVDSSAHSTPSTPVDSSAHSTPSTTADSSAHSTPSTPADSSAHSTPSTPVDSSA
HSTPSTPADSSAHSTPSTPVDSSAHSTPSTTVDSSAHSTPSTLAGNGATRMFLILPDGAA
ISAFSGGGLLLCACALLLHVFFTAVFF*
>TCRU_2185 hypothetical protein, conserved
MAKGFLRVNWRVCGGWLILRWQRPLPGTTERVAVSVLANGTTPLVAGLGSKACQRVRVPY
GCDSMFRVLSGCHGLRRMLSRRLVNMHSTMSVTKLPVHGPAGTHQCQETLPDPVLVEGR*
>TCRU_2186 hypothetical protein, conserved
MCSQYGPLRNSSQRGRPIAVSVANSCLRVAATQKFSKSPSAVHPRNIGRQATEDSIASEL
KGLAGVYQHQRSPMGSAVELASNTALLGKVRLEVIGSVMLHFMYEVCQLHRRGTRTTARM
LVRTRRPQPKQSPQCHFLSDLTHDGRKADGSI*
>TCRU_2187 hypothetical protein, conserved
MVCVRCRCWAAAICDCAVAVRGVAAVSLLLLPLCVDGELVCAEGCRQVTGVMAMMMTGRV
LLVCALCVLWCGAGGADASDLGNNAVGGCMASWVLNANKSYTPSGCDKTALTLPLRSVLS
ITAVAASTDPLVTGTAENKSDLAPSSGADFGGAGSPPGRGGEGGGGRDDGAGGPGGVGGA
SGSQARGSKASVGGGGGSNGGSKARVDGSLKSPASDVKSPSPVLGDGGSSPGAPTGSVGS
DGSKSFILGSFSSGGRSQPDVGSPGGDASLSAPVAGGNSPPSITDESDALEKTGAAASKK
GWKPQEAGGQSGANRARNELPELSAPAESTPEAQKDSTSKQEKPEGEAAGRAATGEEKRR
SDAEAQGPSTVMSTASTSNGRRTTNSSGSSGRRKPNGTNRLANFDRRKDKNATVTKQKGA
*
>TCRU_2188 BNR repeat-like domain containing protein, putative
MSRHVFNSAVLLLLVVMMCCGSGGAAAVENSALVAAPTFEWRSITDDEGVTVDSLRVPGL
LKVGTVVFAVAEAQCNKGGEGSFTGIASELLTDPTDKNWWKY*
>TCRU_2189 Tir chaperone protein (CesT) family/PDZ domain containing protein, putative
MNANDVLTVISPDDGKRFRINIKGDIGRLTIGRLKQCLATASSYSVPVADQVIKFNGTPL
TQEDEVCAAYGIMNGSTLTVEHRANVDPTTDHTASFVSPHRQALEYQTIRQQEEILGDEV
LGYDNTLQVKIDQLQDQVEAAEKKKMKIAREKEKAERELARLRLQEEVAEKQKRRLTQLR
EREAQRAARRAADLAARREQLKAEEEATRAVALQKLDNERRRASLEQERAVYEAEKERLA
KEREEYEQRAKEREVNIRKKEIEIEQQILAAERDRRELELSRLVSHQNRLLYHERVGRPP
PPELTHEGMRRGEWSGNDPFSLPVATPEDKNVAWGKTQEINNNNPPNQQWQLNQKQSYRQ
SDARHPSGPIKSEENATANAQFEDSYQNRPRPPRAAENVVKMMLPGRDNFALAHGGNDVY
NARENAIDNLVCMGRDLGLENTLEFDNNNTCVISVDGQYNLIVTFDATTERLYIYSTLMT
NIPHDPVLRLRVYEFLMEGALLGREMCGGGVGASIKNDFILLSSSIYLPTSLPTTLSTLA
PQFLFSLNKWREKLGELLSTVDMQKMTSPSKNGHSTIEDTNELDSRHTPEEKASRRIASS
TRSSRTHSTESGAGRPVIGIEATDSVMINGVPSRHNGGVLVVNVGGPAALAGIKPHDIIK
KINYKRVTSLRQFQREVSRLIVGKMVPVVVERSDVLHTIFIRVGSA*
>TCRU_2190 hypothetical protein, conserved
MRTGSFLRKKASRVGCFLFGGVTVAASSHIHRASWHTSTRCIVSGGVKNEKAGKPAVSSV
KRMPPPPPPAQKQQQKLRASSSHIYPEPPLASQRPLLDVEDIIRALVEVFVAYQKHLQNC
NATKTATISNMNKGGGTDVNVPFYPPVPLEYVEEQFESLLLRLLPAGLTRGDGDASKVVR
FSDNSADADKNRRQIASRIQSSGVFHLTSTRRRNSQREGASRDDGETFFLRIRPGVRELA
VEIAAFLFSLEPHNGTVRKAVPLTVIRRMSLSPANVAFVRHELANDVRRLLLVYTHSVFF
LTKNGTMVQLLEPSVGGGESVLHAVSLNTTAATREQAQELVASTTQVNNIPEGTNSTETT
VTTGPGQLSVHFASGENDTVVHMGKRFIAPTAIERYRLSQRLIPFLEFVPLTRTVINTNN
GGNDVSSAVADTEDGFVDFVAVRNRAVEKYPSLAPLLAFDYNDLPQLCDGPLFSALRALG
VEFCLGRPLPPQCQRKRRQQQRSTTAMSSLPVEMGLLFLRRIRTSNEAAASTPISPTGEK
MPLRLLLLDLQAELDATVMPQWRRKWWPWPSHIHPVEYGITEGEATNGAERHEEELWGHA
FLRVHAAVYEELAATPAQWMPYDSTPVISLKETSYPVGVSANSAFVQHLLRQLTPRQHEK

LHDILLRGVQGRVPPLIQHEIEESVACLFFPYGQSSRSHGNIIEQNSNNSSGDSNREAQQ
ELIVFNKYVEGFYDKNLSLQLPFVRPRTLSEEPPLVYRRLLPAKLVLEILCCLHREQLRH
AEEIGLEVIDDDEQVADGDDKNCFLSFVPLRQTLQPASRTYVRDYIGDLAMIPLLVSCFP
RYFCVPAIDTVSSWSSIGLTREGRHLAQLLEASSKASDGRPLPYRSQRTGCFFTDDTRQQ
MEAFLLWR*
>TCRU_2191 WAHD domain of WASH complex, putative
MPAAMYNDSATTVCGYVVPVPLTNGDCVTMLSVIHALEELDQISSCVFGNIEAAVRNSRE
TLEGLQERIETCTARVKQLQGRREAMVVKSRSRFPNGCYRPMHLAQAPRERRGRFLRHRQ
DVMPVLLPVDESDSDSEYVLENEGNAALNNGESNGVSGQGKRGNLECKNFYQRPIRRLEG
TVEEQEPVRPQHPLQRVELGDVMPLAASWRYHTVAERGLGRLPRQLMSVGSLLLFNTREN
LYKEYHELDVLKQQREERVVAAKRRLGGAADVHRDYMTRLSSDEYAFIPLMEEVANLMDD
LPEDLPLEGIAEVAWHAYALEAADNIAPSWQRRLGDESEVATENAGPLRGGKKAHGQTLA
ITLGPAETPTTSLINPSSALEAPPPPPPPPPAGKAPPPPPPPPGFKSMKAPPPPPPPPP
PPAMMMKTAVQPARTMTAIPPPPPPLSAEAAPLPISPIAPGPPAARSLPKLGNPPPAPPL
PPPPPPRKNAVTRAPSKADPRPAPKVAPRSNLDTLSSLLANRRKGILGMHSDEDEDDDVP
QSAPSKGMRGVKESASQASTAPLVSVPKSTPPLPPSATGKLIPRRVDDDDDDDW*
>TCRU_2192 60S ribosomal protein L10, putative
MARRPSRCYRFCKNKPFPKSRFCRGVPDPKIRNFDIGKRRATVDEFPVCIHVVSRELEQI
SSEALEAARIQANKYMVKRANKEVFHMRIRAHPFHVLRINKMLSCAGADRLQTGMRHSYG
KPNGTCARVRIGQILLSMRTREAYIPQALESLRRAKMKFPGRQIIVMSKYWGFTDILRSK
YEELRDAGKLQQRGTHVKLIVPKGKITQYNLLA*
>TCRU_2193 nascent polypeptide associated complex subunit, putative
MSTPQVQNPPVEDDEVPTLEAAEVPQVAKQTKRYAKAMAKMGLKPEPNVVKVHIRKHGSL
SFLVNQPELYRFPGTNTFLVFGEAQLGDTAMEAQEAAARAVSGVVPETESRVEEVPTTAE
TPETPVPAEKTDDADAEDGGELDEREIKVVMSQGNTDRAGAIRALKNNKGDIVNAILELT
M*
>TCRU_2194 chaperone protein DNAj, putative
MLQRTCSGSLYAVLEVARDATPQEIKKAYHRLALRLHPDKTGGTTTEQFTLIQEAQSILS
DPRQRRVYDTFGRMGIESLRQFGDGMVVMTTAGIRCAFFIIAFWMLLWLLTLVLAIVRLD
YNKGWPWAAVFAPVWVALVPLLLIGGLLVFHGVTRREIASTLLGLMCFLVTFAVAMFVVG
LSGALTWTIALAPSAAIYVFQSCFILRYLLPFQFRNGFAEFIPPGSSVCLSRMYWGFLLE
TVFEKLCCFSFISLALLRAAQTGGEDTSKLISFWIVFTPLILYFGCMTLVSAARRFFAGT
VEGQSCLLSRLCRALVAAMGYASLLFMACMLAAKCEAEQNHKAVSLHPSAAVALLPLEVT
LALAVLLPSCIFYNAELLFGDVSTMDVAEDDGSLAPGGDDAFDRGGFMHNFMWNNYAKNG
GRGETAAGSSEAYRGEWAPDPPNLKTTYQGI*
>TCRU_2195 hypothetical protein, conserved
MGSDLSVVVQACAPVPHVIHETWKRRGSSFGAEKNHALPMSYTELRLVCRESLNPEMLTE
FPKEEVTPSPVRPIVTDTTPHFLSFLDLEDAALFSFLHDALRCKRCRGRIGFVVDPSVCI
GQRLYEAEQRHIFTQSFSIFLSKLVAVMKFVAPSCHDDWTQGTEGESSLDGDLVDTRPNT
PNKPHSWIPHEFFLLEIQQGLLDDVSGPWLLRDIVDHMGHVLHYHSAVSFLCGSSNQSVI
SSGDMQKKLMSGETSTSSCVLTRVRAYDDINETSLSDSDTAEFVFCRSLQHVLLSQNNMS
RVGLRKCLLELLNSIEQDQLSSARPLQMIDVRENDESDATQRFIAELSSYTGVQIIIGTS
GKRWRHRAHYFISKSLSHRGIGGVGMTHAFPEDAPYSRLNMEESILSGLRGDQSLLPEKS
EPHSLGNTGDSVQMMPHFECEGKSLFASYSEEPRNEEVNKLNAAFTFTAAAAAAAPGKKG
GKMEPKPSDSTPRESLLLDEDDVNGGEVDEKRMHSLRLDGAGELEAEAGHHKEIQELKTS
IMSDSPATPPVSFSRIQKDETGIVSQDVISSMHEVEGTPADCIASALPVESLDNLESSVS
VPHIVGRDQTSVADRVASVPPSQLEKKTRPKPRNAPPAAKGGKSVRASKRLQPNRHDTAL
KESKFP*
>TCRU_2196 hypothetical protein, conserved
MGVSARSQKPYVPIDTCSPGGTWIEEKRYNKVRHESHKRQACKVLCGNWQEEKSLEDDLL
AQQPLLPGSVPADDFVQTAVLQASLDVIREGKNGRYAGGYQSTPYLTIDARNPHLRLLQT
TQRHDYCEANGDVRRLRRPDMGARSRALLEVALKAAKEEKEACDREQQTRLSRLAEDGGG
IYLSTYHKSHCREEELPIINELRNDYLSDEPVTLYTGNPVTGCTMTVHGKTPVEPVEGRS
RFGRHTYFTERKYML*

>TCRU_C Terminus (BRCT) domain containing protein, putative Pescadillo N-terminus/BRC.1
MHRKQVWAKRVKKERLKKKFLTRMQATRLLQVDSVHFRRLCILKGIYPRALARSKQKQSG
NDKQYYLAREIKWLVRDHVAERMMAYRAWEKKVRRAEAMGLADELKVLRSKKAKPQYSLV
ATIKERYPFFIDAVRDIDDAMSMIALYAFLSPEVMSETTIETHHALTSGLHERARGIIAR
WNRYVARAQVLSKGFISIKGYYFEAIVRGERVRWLCPHEYAHKFPPGIQQYITLSFLEFY
VELMQFVLYKLEHELQRDVAEQQEAEDEENNTHAEDFRLPDKEMTQSISDVKKDTLKKGE
LMQEELRKARRVFDGLTFYISREVPHKHATLIIEACGGRVATAYAADNITHFVVDRPALP
PGFERVDRIEYVQPQYLFDCLNARMLLPPNGYRIGEELPPHVSPFTVAITNSPEDIAAVE
EAKKNHPKIVSYVPKRVHEIRALVNPQYTPLDPEGKASDLLLHDESDEDHALVPELGADD
DVSLSGDELLEASHRPKWEEEEVTENVQRSKLSALKVAKQRKMNLMNAPTDEVVARRRLA
QLKSRESRQQQEPSGQRLKRKLAEMKRQEAATRKMQLQVARKKAARYYKMVNSVVQGSQR
REKMLEAKAKQVADGRIAKKEDGRELVNVRVEAKRARAAASGKPLREKKAANPYKKLPKW
VR*
>TCRU_2198 Peptide deformylase 2
MGLFPRRNDACRSALRAALRCSARLNSSRTAYDNNNAAGEAAYMAGNTVHVEHPIVQLPA
RSLWCKQYPLDPAQVRSGGFAEMIERVRAARQYYQYPSLCAPQIGWNVQMFTLFDGSVFI
NPVNLDLLEVEAAGSRSGMPIAEAEAQWVASCRREGKTCFAWEPCASCCFLMHYIERPAT
VRIRAIGADGHPFEVTLEKMRARMALHELDHLSGVLFTRRIPDTNHVVPLEGFSTMSEWS
DDYPSLEARSTYLYTTYTPPYTFTTDNVEDAQLLDRKFEDGIYPGCEHDRRMRIESAALE
ELQRAQWRMEKNCSEEGDSTQCMRDTDDEQQHEDEETEEDVTAQKGTL*
>TCRU_2199 hypothetical protein, conserved
MERASQRSSARSSKVNAVKGGRSSPSAASRSRADSLKGNRWGNGNPQASSPANNTSNGCA
KNSPRGDIDGGSQRNLNGLAQGRGKNGARRSSQRPKEMSREELLSLLFRKPQLKEMRPEM
HRLDLSQRDDLRFLYEMQHRVDPELVPVRNRMYRANWNKMHQSHIFDTEPASKPQASTTP
VKKNDDGLGSLARFVLCSEERNASPVRGRRHGNYSAGRTTLRLFDGNSKESELRAASQTP
RTGVRRVGLKPADKLGANLQSRDLLCEVGASRHGVRTMEAVKKFESTQPKLTLPPQKLSR
RFAHPARSFSASDNDIFGVRKRRERMMQRASSCTSARTTEPYGSEDLEGSTRHRQSSLGS
TSVIDEKRSNHSTIQSKNVPFGEEEPRNGGTPTRHQKSRRGGSLASRSSSVAPSPSVRSW
SHPRSTRGSNSEMLRRHNDVIGKQRNNTSATTVSSKISVISEGGEYSASLFPTPRVRTGR
ARVPGHSITKSNIVFGDLNLLNSPRHVQRRIR*
>TCRU_2200 atypical dual specificity phosphatase, putative
MNEVLQLLINVERHPIYVHCLDGRHIVGLVIMGLRKLQFWEVNCSHLEYQRFTRSVQDEV
AFITDYSGPIAVPMRIPSWLWKGTWFDALGKPRRIHVGIRLKFPSVSRNMESTEGSGSVP
SWGREGVNCTSTNPSTRNLTPERLTSPTRTAEGPTSPLHMAIVTPNTGNGAESGVAYVDL
PSITPAKLIGPAIGARHSAFAIQTEKPLAADLRLLSLRTSAETVSGSSAVTAPPPLSSML
TLSSRGWDIGEAFSVAGMVPQGSSSAVAGLPSTATAIGSELSAVSRSTTQRGTPLAGGMK
LWEFDDDTNGSHFSMQHGVHGMVNTSLQRWNEWNHSSASSTRYSSRSRRHLQYFVAEAAG
YGPSSESVASTKKTRRRNSL*
>TCRU_2201 hypothetical protein, conserved
MYVVNLCSLTLAISHVAHFASTILRLCPAAVICDLNSHHELRDGRSPSTTAGEDLAATPS
GMDSELSDDPAQATWISGRSVSFPEVTAQLVLRVFHWTSTRLHGPGPPSGIADCGDGGRH
PATGRNAATMETCCLFPA*
>TCRU_2202 leucyl-tRNA synthetase, putative
MTPRAARNMAYQDYVINGKTGVDPSPLFEVEGAKLVGLPLSAPLCPYATIYTLPMQTITV
PQEYTLVKLKVQNATEHPAFLAFKHIIGDRPVILPGATLRVETVCGQTNCWISPNFSYKA
YTILNKTGEEEIFIMTPRAARNMAYQDYVINGKTGVDPSPLFEVEGAKLVGLPLSAPLCP
YATIYTLPMQTITEAKGTGVVMSVPSDSPDDYINFTQLVRKPDYRSKLGIKNEWVDFDMV
PIIEVPGEMGRESAKFMCEKLKINGPQATDLLEEAKKLVYQAGFYQGVMIAGPFTGEKTS
VAKVKTLQLLTKEDSAIRYYEPSRLVMSRSGDECVVALCDQWYIEYGKKEWKEAVLKHLE
NMNMFFPGVRNGFEETLNWLADWPCSRTLGLGTKLPCDESGTMLIDSLSDSTIYMAYYTI
AHFIHTSTEGKLRLDGRHDNVLGVTPEMFTDETFDYVFLGKGTPESVHAVNGLPMDAAEK
MRREFTFWYPVDLRCSAKDLIQNHLTMFLYTHAAIWPDDTSNWPRAIFANGHIQVDNEKM
SKSKGNFMSLAETIDLYGSDATRLACADAGDSLDDANFVRETAAGFILKLTTAIDGAQEL

LGKKHTLRSGEYNIFDKIFSNSMNFIILQTERFYVGMQFRGVLNLGFFELSNEFSQYKLN
CDELQIHADLAERYVEVMTLLLAPLAPHFAEYIWTSVLGNKKSVVCAAFPEPTGPVEYAI
LVANRVMTDVVKEIRSQVAKMQKKRGPVDEVFIYTAASYSEWQIKALRLLRELYEANGRS
FPVDFSKKVMSHKQEWMTKEILPDVMAFLSFTKMNVERCGEEALAEAPAINDAELLKGVL
ANICKLSGVPTVHILGHEDERYPEHQAARRKCRPGEPAIVLPVKKK*
>TCRU_2203 hypothetical protein, conserved
MKRSSPWSVGESAARGVCDEAVAEEPGPSRVPAFVLPACLACWHAWQLRWRPAHEVPAKK
TVAPLRHPHVRPVKPSERQAEHVGHVPSRCGQTGALRRRLGRRMSIRRSLVGKELRSFSR
EEVPAEAEAGGSPQ*
>TCRU_2204 Leishmanolysin, putative
MSARKHHDCDDIDGMELHDGDGDGRTLESHLSQRHAMDEWMAPIGFAGCRTELTLAAPAD
LGCMRVKWEMAEPMRW*
>TCRU_2205 BNR repeat-like domain/Concanavalin A-like lectin/
glucanases superfamily/Trans-sialidase of Trypanosoma hydrophobic C-
terminal, putative
MSRRVFTSTVLLLFVMVCCGTGGAAASDGKPSEPNYKWKDVKSEDGVTVNSLGVPGLLKV
GSDVFAVAEAQCKKDGKYFTGIASQLLSMETDNKPEEVLKNAKDKTQVLEEGVSPSKKVD
VSRPTTVVNGSDIYMLVGKYSHEDAANCQAGTENIKSGILLVKGELSGEGDKKIDWKTTD
GVPCTLGDQHKSWTGLIGGGGSGLKMKDDTLVFPMEGTGGRNTVSLFIYSPDNKSWKLSK
EISADDCSDPSVVEWEKDKLMMMTACDGGRRVYESGEKGDSWTDALGTLSRVWGSKQKGN
EKGVGSGFTTAKIGDNRDVMLVTLPVYSKKTEGVSANGKGELHLWLTDNTHIVDIGPVSG
KDEDVAASALLYKSGDNNKEELIALYEKKKDDAEKPSLGMVSVRLTEQLKRVKEVLATWE
EVDKRVSKLCPSEGALEDTATDTACTTDKITDGLVGFLSGNFSNDTWRDEYLGVNATVTG
GTTKVAEPSDGVTFKGRGAWAEWPVGRQGENQLYHFANYNFTLVATVSIHGEPEGATPIP
LMGAKMDDGKNTVLLGLSYDKEKGWMLLCDDGKNKEHSSYLETKTSKHQVAIVLRNGNQG
SAYVDGKSVGDESCVLKIADSKEISHFFIGGDGGRAEGQDDVSVTVRNVLLYNRPLSSAE
IGSLKPNKVPISPLVKEPSSPSPVASDSIIPPITPVNKNAQIAGTSSTPAVTHRTQQRQP
MGSSGADSGGASTSAVSAVSTPSAEKDSVKEVASGKSSDGAQTVDGGSTADGEPTMEKRE
GTDVQKEEVQPLNREVNATELNSSLENLSQGNNTDAGTMCGSGLLPSLLLLLLGLWGFAA
L*
>TCRU_2206 hypothetical protein
MSEGSSHERSEGGSSVSEGSSHERSEGGSSMSGGSSHERSEGGSSMSEGSSHERSEGGSS
MSEGSSHERSEGGSSMSEGSSHERSEGGSSMSGGSSHERSEGGSSMSEGSSHERSEGGSS
MSEGSSHERSEGGSSMSEGSSHERSEDGSSMSGGSSHERSEGGSSMSEGSSHERSEGGSS
MSEGSSHERSEGGSSMSEGSSHERSEGGSSMSEGSSHERSEGGSSMSGGSSHERSEGGSS
MSGGSSHERSEGGSSMSEGSSHERSEGGSSMSEGSSHERSEGGSSMSEGSSHERSEGGSS
MSEGSSHERSEGGSSMSEGSSHERSEGGSSMSEGSSHERSEGGSCRHKGVSIVILLLGIC
TFAVVY*
>TCRU_2207 hypothetical protein
MLRHCEATRGTSTGSGNESGVGSSMSEGSSHERSEGGSSMSEGSSHERSEGGSSMSEGSS
HERSEGGSSMSGGSSHERSEGGSSMSGGSSHERSEGGSSMSEGSSHERSEGGSSMSGGSS
HERSEGGSSMSEGSSHERSEGGSSMSGGSSHERSEGGSSMSGGSSHERSGGGSSMSEGSS
HERSEGGSSMSGGSSHERSEGGSSMSEGSSHERSEGGSSMSGGSSHERSEGGSSMSGGSS
HERSGGGSSMSEGSSHERSEGGSSMSGGSSHERSEGGSSMSEGSSHERSEGGSSMSEGSS
HERSEGGSSMSGGSSHERSEGGSSMSGGSSHERSEGGSSMSEGSSHERSEGGSSMSEGSS
HERSEGGSSMSEGSSHERSEGGSSMSEGSSHERSEGGSSMSEGSSHERSEGGSSMSEGSS
HERSEGGSSMSEGSSHERSEGGSSMSEGSSHERSEGGSSMSEGSSHERSEGGSCRHKGVS
MSFYFWEFVLLR*
>TCRU_2208 ubiquitin carboxyl-terminal hydrolase, putative
MPDLTSIMNGREEEGNEMHVVSLRTTPASASLFETPFILCPLVNLGNTCYFNAGVQLFAN
CIPFVYGLRNSPFRHPFIGSRALKYCRTGGKASQALFQAFAELLYDMEFARLGQGEALSP
LKALDCLAAVHPAFEGRGQQDCPEMVNVLIANLSEEGRQNVELEALLRSFEEDALTRAAS
DAVLVDVVGRDNIPNSHSTVGMDFRSMPHEHATGVLIKEETLKLCSHSAMDKEEKIEIPP
SFLFAEKPNMLSFNFPGSWWTYNTFQLMQTVNHDNERLDRKERARKDRPQQNTFCPPKLH

YNSVTDCFTGYMLSEVQCHTCNSTSRIVEEFSSLTIDVSSYSQRMQYARRHPEARCSAEQ
RPFMQQKSRGPLQWWNPFVWIGALTRFFFSFFKGFSDYIDYPITLKECLDIHFEPVVLKG
SNSYHCASCDNVSEATKREFLLSLPEYLLLQMKRFEYGTCFNTKKTDPVIFPVSWDIGEA
GDADALRLSDYVHESVMRYMAPSVPCRSIISTDICTNAIENMTNPMHTQQVDSPISESME
GAYTEMQSPIYTYTLENVVNHHGSIMGGHYTVYARKKTEEENVWLSVNDEEITRVGVNEV
ADSEEYLLLYKKQPLQPRSEAVLTLRRKAQCLLSLPPSDGKVLQKEEFTDRHDKRDAKGK
RVVYISRPWLQRMAFMEEPGPILNRLCYCIGEDQGHKANSRQEVQGSHNRRGRMSHGLPV
EWFYVPLRCDEYDAFYKMYGGNTALTQREYEELYEAQHQVS*
>TCRU_2209 ATP-dependent protease ATPase subunit HslU2
MLRFSWCRLCGGSSSTVAATSPLAPDVLAVAKAQSAQLDGLSPRKIVSILDTYIIGQSEG
KRAVAISLRNRWRRRQVKDKELRRDILPKNILLVGPTGVGKTEISRRMAKITDAPFVKVE
ATKYTEVGFKGKDVDSIIEDLYMNAKTKAKRRLEAERAEEALAMTLDVVYGSWATRRRAS
GGDARSKYDEDGEDGGRAPFTDVTLEEFKAKYKTEFKDDLVTVDIVPTPPKHNRQGWSKA
EMDIAGLFGSASEVRLKTRVTKRVEEAVPLVLQDILERLLDETQISTLARTLAEDDGVVF
IDEIDKVVTDAANNDADVSSTGVQQDLLPLIEGSDVTLKDGTQISTDNILFICSGAFHLA
KTSDMIAELQGRLPVRVELQALKEEDMRRILREPKFNLLLQQKELMKTEKVDIEFTEDAV
DELARVVTTVNANGQNIGARRLHTVIERVMDKYSFNCQDYEDKKVVIDANVVKEATSTLH
TNIDLAKYLL*
>TCRU_2210 catalytic subunit of the vacuolar transporter chaperone 4
MPFSKAWRSAVYPDFREQGAYINYKATKDILHRMKEDIANPSTPDELYNSLLLQKCRVYK
WCEKKIKELLTVAEALMAASDYLTEEDTKTNLSLVLNTLGSGNMKCLPPNEARLLADSIT
HELLRFVECCNLNTDTIEHIIGRMYRYAVLGPTGKHWNNIMTEYDYHKLSIHEIFYLLSK
VYDRVTETENMRIVKRSGIPAGTVGSQVFDRRSVKYWVHLQDLPFVIARIIPHLPHSTFK
ETYQTCKERNIPFTLGSPVSSVYWDNNEFLLYHRRLERLEGATLIRMRWYGDPLESDWNK
LGPNDSVFMEIKVHHEAWSGERSNKRRFALKEKEVNSYVHGELDLNPALEKLRAKKASKK
ELHNFMDLSTEIVTKIDAYDLKPVIRTQCSRAAFQRGIDQSIRVSIDTDLRVCAEDFGLG
HHWRYSGVDAPVSYFPYAVVEIKLQCAENERIAPWIEELMSCRYMESVPKFSKYAHGIAS
LYGHTPFIKMVPYWMHQLDIDIRASTKPEQNQWDPTVGLASGCWERTTDRAIFGVGHAQT
QTVGASEAQFLPRTDYTRVYQKALHGIRGEEVATPATADNFQDAEAEGGRASGTAGSRKQ
QLQPRLPAHTLQYDVDRRHKAYTTFHLYPFAEYGVESLCFTPASGKNAAAEVLLGLIPWQ
TGKRIRVPQKYDPKTLLTSERYMLKWTEHATRLGLVGLGVIQFGNSMTLPGDVTQLSSFW
RANFHIVLGIALVLVALMTLMYALMTFKARSRRVYARKKIRFDDSWGPTVLTFFLAFGIC
VIAMMHILGRYGPMLTGDDNF*
>TCRU_2211 Domain of unknown function (DUF4586), putative
MFSPSVYLSIGDEYDKPSATPSLGVKSGGRSFVVPMKRSPHITDAVFQKPFISLAVGDEY
VAVPPHGTFRQPLDEKKAGMTVLVPFKPPSRPHKSNGSGTYYGTFNEGNPPRHEAELPLI
DTKKAGTPAAATTPEWPKRRIYTNPGKKGTYGYVGLTIGKAGEVQYIADPIAPYTTSTRS
SSPRSQKPFKAAVKSGGCFDESPNGFTTVYSISRPLPPKKPPMPRPPLDVKPWKPAGALL
REIIKPPEYQEDPYDLKEKRIREERQKEQSYKPWCPAGNDPYRHIFHYPVRFDPPPLNE*
>TCRU_2212 NRDE protein, putative
MCILAFVTQCSPRFPLVLINNRDELIGRVTHPVGVDKSTGLLWAADGVAGGSWLGLNVHN
GRFAVLTNCSRCPTAPLAIDGISMPSDWRGAMPLAEVVRRMKVHATPAEDGGTDVFLTFI
PDLSRGCIVGDFLKEGRVPGDCLPSSSLLTEEAMNVPPSLLSSPYFDGYNLLTAESLFDP
HQVQLLYSTNRYAAQHRSPAEHGVVHCLQNSYLDNWMEPKCTLLRKRFEEALKTFFPTGA
TPYDAEYVADSLASYCLCLEPNFDLQQEIQGGNLSPEVVQGYQQVLDAPLPYKGYNGEAE
LIQLYGAGLQPPVHFPTDSYKEHINFQLRNIFVSNGMYCTRSQTTVVVETMPDGEGTVVH
FSQRNCNAGKAHGPWKHFTVQSRSII*
>TCRU_2213 FHA domain containing protein, putative
MRGRKGGRKGVKGKCAEKVSDPLQQGAAERISSWEVEQPHEETQKDSIEESWERLAPSAN
GPLVARLVPLSADGAELSAIDLYRDVGRMMLGRSQQLFAECRIGVASVSAQHCELAVNPG
SLKVTIRDFSTNGTFLNGNRLKRGMEVELRSGDRVSLSNPAKESKPDAPSVEFVFQRVSS
EIKVDVLVEELTCPVCRGLFIRPCSTIPCLHVCCAACISQWIDAGHKSCVQCRANIWEVR
PAHKIQSCVEELLKSNPQLARSDAELHEFAKNDTIPQGGKNLRKRPHDEASEVNSNTSSF
PQDSDGSDSFHYSEMRSNDSHVVDGNPRQSCRHCRGASSVDGFRCPTDAPHRHCRACGTS

FPYRPLCSRPQNCQMCGATYCNLYYEGEGGCRAFLNGHLVRPKDGTPIRTMPRTVFGGNT
VERNILSDYLSTKGISLGMVWSECMEKFRAREWVPNIVCANGQLTEDSPLCVKCMNAVVD
SLLFFYRLKVPRHELPESVTNRPNCWYGDKCRTQFHKVRHANRYNHACYQEKGKE*
>TCRU_2214 kinesin, putative
MENIRVVVRVRPFLPNEGSSRCLTVRGNQVVIGDERQFAFDEVFDMDTMSHTVNDAVAMP
LVSAFVNGYTVSTIAYGQTGAGKTFTMSSLSSDVVRRMSAALKANVTAEGGEKPEFRVSA
VELYNENICDLIASTISSSSSIAMASVASLSLREDPRCGVYIQGLSEVAVESEEELLAWV
ESSSANRRTASTMLNATSSRSHALLTITLTHNGVVSRFGLVDLAGSERFKKTYGRQRRRG
SLEEFGNESNCMSTNDKAKGSEVASRIREGISINTGLLALGNVIVALCERKAYVPYRTSK
LTRLLQPMLSGNSKTTIIACVSPDVSSFEETLNTLKYADRAKAIRTTPFQVASGFSSLEE
AEKTILSLRRQLGMAQQQLREEQLPIMYSFPVVDETQLGELQNLLTRERNLTRRLQEDLF
NAEYTAMMEVEKRKQIEKRLLTLEAQQRCRLSQSIETDNTQLLDANLGEDGAGHTSYLAQ
NPAEHDEPQVPKAEEAVDAERLSMPHEIISVSRNNIDESKLENLSREIQSKERLIHQLTN
QNEDAVRELEYAKRRQQEMQDVKRHLEEELARAEAKLEATQMQRQGKEEERQKLQTHYQL
RLRKAEEEAAEYRRKVQEATTIISGREANAEMMKQLQSQVVEMSDELNRQRHALREGQQR
LHKVSASHSQEVNQLQKKLRDSEAQVARLQVQLQRKEREIARVKKGGAGVANRRQQTPTK
TTTQQAHASNTPSVCLSEDLQKEIDVELISLINLEKELDELTADRDELRDSVREAQERAG
GMTKWKDAMNGFMMRLQQLERNLHSEVLNEEIRLEYNREKASIEEKLRQLELLEPVLAQA
SKQLEEMDNNIDSLQEARKYHLRRVRQMQNGTLTPVNPTVRASELLRRGVANHTEMV*
>TCRU_2215 hypothetical protein, conserved
MTVGVSNGDDDVVSFLQHAGERYKEHKELLRRKHLEDERKKCPFKPTVSGYAEQVSRQSN
IRRSEAITKRLHELHAKRLQTVEAQRVKSRKRQEEEMTAEMRAPQVTERARSLVSRDPVD
VSRKWLEKREEKFARLREEALRRDLATLREVPRISQYAQEKSIVERHLGQRIEDYFMAKE
EARRERMYWLAEKSVGMNSSFGSSSFFSHVGDLPMQKELPQPPFTPRITEYAKQLRRSGN
VVDRLLADGRKGRRTPQDTSCTFAPRVAPASLEMSRGYYSDPNTLVYERLYRNDASKLRE
MRRKRMEIDLQNDRTGIPRISETSRLIVERKRSEAMEQARQKSPTQRLHSSSRDVHADAR
EKQRLMEARERKEKENCTFKPRLNAASKKMWRHQLQLLQGDSDARTAAAARELLWKRSQK
RMDEEIRRRRAMVEAREMAECTFHPKVGRAPERRVDRELSVSDRNEMWQLQRAQRLRQAR
MELERTQLSECSFHPTVDPVFPLPAHDASAVSGYEAHILRQEESRRRKQEAQDWWRPKVV
PPGGHGASRHTLSVASEPRSSHQRKQRNLRSEGQSRALFSPRSRDDSSADMDGIQFVVYQ
APSCASSVSPRSVASASPPSYKHHHHHRNYHGDFLHSSDNGIAAAVQSAKPSPVDFMINH
HRAILDSSRRR*
>TCRU_2216 hypothetical protein
MRRTDGIDMDADGLMVNAVRSAAYRQKYDETNNTRWGGLLVDGDHDCEEEDNGDLPPRRR
SISLNQLLYSEPLQRDFMARTTTEKLSPSVNNHHDDDNNNGGLTAIQRLIDAQYDSCAIR
VNNHRSGNSERKKNNKKNNRNDDDDIYSEVLEGKCDWDSALPPRPVCYSKETILKETHMR
LGDREDAEASRLPDKRETCSVGVQCEMRAAKKAATLRHLEQLRQQSLSGDPESIARLLAR
HIRELEMERFGHSSAFTSALLQQL*
>TCRU_2217 hypothetical protein, conserved
MLCPRRCATVAAGDVYCRGMRGSGRPQASKSSTHALHSLYRRLRKEGEDRLTLERALSTR
PLEKNIQLGLRLLHYHTRLVNIDREAREVAFWRIVRQLQLAAARRYFAWRIFSLRLRLRS
TAAISDALVYFLFLTVCFMLYQMYRVCRIGVTRAEERYMTLAIPIKQTFEALEDAQERRK
SLRREMEKDVLQQR*
>TCRU_2218 flagellar protein essential for flagellar pocket
biogenesis
MLVINVAADIFNSKVNFELNFPHRPTLLEVSRATEASFSNEIALRRPDNVPPHNFHVAKI
KIYDEERNKWVDLTSEGQLMDYCQLYAFQPENQWHKESQKEIPAAVKPPTSAQRHPGTHS
ASAVGRGNSNGGVPQAGTIAAYTGNRTPVSQRSESQSFRRASLHTSTSGALVPKINAEAS
QEEKHRVLFAELDSKGSRTLGLGDFKQGFQTFNLDLSAETVEDLFEKADTNRDGTVSFSE
FERFCRLYPIMTDCLFFRSKAFWDEEQLKREIQAERQAVRQAEQVVEQARKSLEDAEQDV
VAAMDAVTVAETDLKDRTDRLRDLTRDMDNAKREKERAIREKKECEKELMSTKEREKEIR
KESQDIAREAEKLDRRAAALSSEAVGADDKVRQLEKALEEARRAAERAHLSAEQAARDAD
SMKQRVRDSTKDVDDVLRQIARAEDAVRGAERNVVDADNSARELEGMGRDLSREAEEEASQ
RRYQYEKAVEDAKNSVRVKEHQLDEAKRHVDDRDALARQKETELMEQRKQRDLISQHERA

LIEQELRLREQRDSLEERETKLMSEASSFLGNLRSQLQGGRS*
>TCRU_2219 Unconventional myosin
MNEPAIERVAVQQQVYYYLPQKGWLRGVVATVLDGEKKAHVVNATTGNKETVPLSQIHGY
MDQAFDPSDPDLFHLSDLHVATTLYCIKERFVQLKQQYSLMGEMLISVNPFRLMPFNSDD
HREKYLNSAQNHLLPPHIWQVAHRAYSQIVIRGFDNQSVVISGESGSGKTENTKMLIYYL
GELSHMHSSNAMQKSIADKVANCLKWSNPLLESFGNARTVRNDNSSRFGKYIKLYFDPSS
GVMVGGQTVTYLLEKSRLLLQSVGERNYHIFYEMLAGLSPEEKKELGGLKSAQEYKCLSG
GNTFVRRGVDGKTLDDSLEFQNVLHALTALDVDADVQRSIFKVLASILHLLELRFKSDQN
DMACFEDEAPFLTACSLLQLDPALLRECFLVKSHTSLVTIYANKTEAEGFCGAFCKAIYV
GLFDRLVDFVNQSIDAHGEVSRCKYIGLLDIFGFEKFTRNSFEQLCINYANEALQNHYNK
YTFLNDEAECEAEGIEVPKIVFPDNSECVQMLDQKKIGVFSMLDEECHFKAGTVERFTHN
LWDQWANKSKYFVLPKSTVPNQFGVNHYAAFVNYNTDEWLEKNTDSVKEEAYQCILNSGD
PFIRTLTPKDSLLDKRKQTVALRFQEQLTALREELESTETQFIRCIKPNMEASPDTLDNC
LVGNQLESAGVLQTIALKRQGYPMRRTIEQFCRYFFLIMPRSAIKLYKQNKLKAAAKDFL
AHYQKLYGWQKPHYAVGHTKVFVRAEVWSSIERLLLRRKAWLLRRCVPFLLRWVYTNREQ
KRLAEERRKEELRKAKEERERLIAEIKARGFSLEQMEWVEELATLFSNVDVSTLIDIVAI
LPKRDESLATLTEMQQQRVDGSLPSTFMRVMSESEVRPSVVQALVEGGVATMAGLSQCDQ
AQMKQFGMTDAEVAAVTRTMLKEQSARVKLERLQSSIGTAQLEKVVGEVKRRVMTQSDFA
TKVEKLVRMGFPRSHAELTLTHYNGDVQKAAERLLHGGVEKHLHEEPPHGSKGRWTSLDP
EVQRLVKLGVPKGAAKRVLRQTSGNVDEAVRLVFPPVS*
>TCRU_2220 retrotransposon hot spot protein, RHS3, putative
MKFTFSTIIEDVLFKGGVRVKEKKLNDFLTMELGGRGVVGTNRSVLLKEFFKDPTKYIRD
AGVLKEIQITDAYARMERTVRDEMDVEEVVRRLHENGVDNLLGWSLATAKVKEIVHGITK
QFLNAASEEVRNPTTTSASTYLEGWYESVYNARWSHAVEIPDGGGTVMKVKEGKPEQSWT
YKAADGAIEENDGAQQSGAPRLRLMVLTSDKGWPYSWRENKSTRDCYVNCEVDRVWQTVK
DDVNKLFSSHGKNKLSPEPRVLIGTSGIGKSMNAGSYLLYQLLHYNAE*
>TCRU_2221 RNA polymerase subunit, putative
MSIFYSYVVRDVLEVEPVYFQGRVTVSSTSSFAAAAAKVAEEDVEELLDKQHYQVTFEDI
LMHRLTERYVGKIVPGCGLCVAIHDILQYTQGIVRGPSASAWLTAQFRLCIFAPTPGARL
RATISAQTREGIYLTLEFFQFVFFVPGDLLVAPSSYHEEKKCWVLHVEEEDDDDVDGGEE
KGVLESALNPYENGDEVIVKVEHVIVRELVDFHSNVGTKSQNNNINININSGVSVDGKEG
SPTSQSPMEVRGSFVGTGLGPVLWFEK*
>TCRU_2222 eukaryotic translation initiation factor 2 gamma, putative
MDENDIADFTMDENTFVTNEQNERGLATQDINALNLDKINAETFEVMSRQATVNIGTIGH
VAHGKSTVVKALSGVKTQKYHREAVMNITIHLGYANAKVFKCDKCELPAAFHAFPSSQPD
KTDCPTCGSPLTLKRHFSFVDCPGHDVLMATMLNGAAIMDAALLLIAANEPFPQPQTLEH
LKAVEIMRLANLVILQNKIDLVGEVHAQDQYHKIRNYIDSTIGSNIPIIPISAQLKRNID
YLLEYLCHIPLPTRQLNCPAHMTVVRSFDINKPGEVDIENLRGGVAGGTVTRGIIRVNQV
LEIRPGQVHAQTGGTFSCTPLRTRALTLKAEDNSLQYAVPGGLIAVGTTLDPTLTRQDKM
VGHMIADEGSLPEVYAEIEVQYFLFEEMVGRSKQRDRNAKRVQKLNLQETLQINVGTLTA
GATVVNITKNPDIAKLTLVTPVCCTLDEHIAISRLVEKNFRLIGWGIIRRGVPVKLN*
>TCRU_2223 ethanolamine phosphotransferase (EPT)
MGNSTNVVYHIRVLLLTVAFLTGAFIFADSMLSKKVMLSNMSTEVCRGMQTVDQVVILLI
DALRPDFALPALSEYYSTGNDCNASTTRGVGAFQRRNTTLTYVEGKLKDTTHPSHGFFFL
ADTPTVTSQRMKAMMTGTVPAFFELKANLNTEAVESDSLLHQLRRRSILLGDDTWLNMFP
DDDDDTTPWKRTHAGPPYNISDFETNDNNVTKNIFPTLLSETLEKAPSDYAKLIIGHYLG
VDHVGHRHHADHPEMDRVLARIDAMVHNLTHFIRHQRKTRMRTLLLLFGDHGMTGAGDHG
GDSFQEVETFLYAELFDGNNASSSSLNVSAEAVAKSLRRRSELTEDRWTDEFGEGTIKEK
SCRNMAGVPPQKLGVAQQTDLAPTISILLGVPIPFSSIGRVIPEIITLADPDADLNTVEA
CNWRQLMSFFNQAGVPVMDAWKNKSLTWRKRISLMSHFGRKSRNEMRRSGMFIGSSLFMA
SALSWFRNKDVLGIIRRERLIDTWALIIFLFRACAVFANSFILREDVEVLSLLQLLLLTA
LLTTTHMRVAMFLFALQVTLRVMVPLLYRSRLQISHTVAMPSLLEQWVEANAAGKEWEFT
GLFLGAFVFLCASSCTLDRLWVVGQSLLMFVCHNHFPLHHIAPLISFMLTLFSRNGSGLR

YMVFVLWASSLCGEKYSIGAATAFYGAVLPSVVRATQHLPGFSQAVLLHLFSWVAFFTQG
NQCLFSTIDFNATFVGNSSEHIFFGGVLVTVRTLNAFALAPMAVMHVYRAQRTHGWHVCY
LLVYLAVVQSAFSSFNGYIQKAHLMLFSIFCPKLIFDLLIFLMTFIGYAVASCI*
>TCRU_2224 aspartate aminotransferase, mitochondrial
MRSFFSAKIVSAKAPLEIRRAASSFFASVPLGAPDSILGLSAEFQQDSHTPKVNLAVGVY
RDDANRPFVLESVKRSDTGSDMEYAPINGMRSFLKAAQKLCFGEDSRAIRDGRVASCHTL
GGTGALRIGGEMLRNFVNDCSNIYSSDVGYANHAGIFKAAGITLPPYTYYSPATKGIDLP
GMLKSLEAMPERSVVLLHACAHNPTGVDPTQNEWLQVVDVIKRRNLLPFVDMAYQGFATG
DLDRDAFLPRYLVENVPNVIVAQSFSKNFGLYGLRCGALHMVVENPEEVGRVLSQYALLI
RTMYSNPPITGARIVNSILNSQELTALWKEELRAMSGRMQDVRRRLVKELGECGSVLDWS
HIERQIGMMSYTGLTKEQVEMLKKKHHIYMTLNGRAAISGLNSTNVSYVAKAFHDVSK*
>TCRU_2225 hypothetical protein, conserved
MGSPQRNNGSHTAVYGLSEKDLVDPVCSSAFPGMRYKGLDSGRPFQVEARPDLSSSEQLE
LSSLTSEEPCALPATVLIGGASRCVGTVSPGEYETMEGEDEEPESSSSLDNSGEYAPLGF
TVVTGGPLRALYKPKTVSYSADESEDSNTELSFSDAPLVMTVQENRGQASVQRQKERNIE
VLECKPEAEAKEDVEEQGQLNAIVIRNLVRRLRDPNVQVAASEFENLEWDDPNFEEELQT
LKQIASYTEVKETMMKTAKDGILESSSLMDQKIKDAVMMLPEITVVGVKDKFVEQVGTYG
TLNKIGQEFVSPNSPKKSLLGAPREKIIIVGNNGKVAVLSKEAYTGSRFAGKKKMTESMI
FTAGGAEAAISFKESRVGSCNVEKKKRKDHLLTSQKFTIIPKEAAFGEEEKPTRRMRRED
LGRKSVVIYEIPELINGEIVETEYMDTEDARNGDKDEEKQTMRHSRNKETTRKGRGWSVI
NEIVSDAAQNGYAANGDENLLSKKGRVPATRRRRRNNQNRSITFAELLEVEGEEVPLEAL
GTQTISLPSSTNPHDRETTETIVENEQATHTPLGYAQVFSQDSCKQRNSADVLQPLIEKN
VTGVVERAKDGKSQQKKKKRYRPVSDRQKSRELEYSLVVDEEGTEIAESEDALGLNDGRA
STVSRLREKHSLDHQGAAKDANCQLLPPLAPATSWRHRIRSKKSLNASLSVTELPEPTVS
EIPDTTMDPSKVAKMSNQGESALGMTHNARKFRRARSNNLNRSATSFVFVPDEEIIELKE
KVEESLDQTTSRGNTLNEGILLPVLSRPRRRKHSSAAASMTSLDIAESSTMEFDLDDTIQ
STWNRKRSK*
>TCRU_2226 conserved protein
MGRNKKNKSAAAYGTEEMPSEEKSPRDVPSDESSTHSDNSSVVVQPVAKKKVYHGNKLSR
AAGGKGKKEEQAHQLPVPAAKVSNMEDEKTTREVEVNRKDDCNAGESADGLAGQLEEDLA
IACFEPVEVLYCPICTFPAELCEYSGMYEQCRPWLLEHAKELAEAEERGRKRRALTERER
LERLVQGRGTKKAIERIVLVEVTQRKGRKMTTSVKGLDLFGFNLKDLSREWKKLFSCGAG
VTSSEELKQSSIDIQGNVAAQLIEMLPERYNIPKGAIYQMEDKKKVKCYSEK*
>TCRU_2227 hypothetical protein, conserved
MPLKYFRRLLLSALVSEGSKAPLFQRVAKKTARMERDAPEKVAVWTRAAYVEARKDAETL
WVKLREVLYGDTNIKRVEGDKDSQNDRRE*
>TCRU_2228 hypothetical protein, conserved
MISIKEYIHLIGDSVNDADTARLAEEAVNAPSLFFYGSLFELHSVQKLRNNPSFAWIPQL
MEILCYGMVDELHLLPDSAKKHLTPCIYQKMNKLSVLSLCIRDGTGGMLFIRDVQGAIGA
STPLEAEELLIEMMSDGLLHGRIDQRGGVVILQDFAAREVRIEEVGRLREKLETWCRNCD
AQIEMLEEMMKE*
>TCRU_2229 hypothetical protein
MTFDGKAVMESDLPLTVEELVANYQSIRHLNEELCAFIDNIRVECDSIAESSPCAAQALR
DLFLNFSQGKPAVASFLNANDPHSSGAANGSTSSACTGGGVVLEGVVVKNQKCNEISHSA
AGGAKKSRFLRFVEEATRDYKEECGVLRLKMMAMESEQKLLLQARKNLREQEKKYATCMS
ALNDCRNDLALSRSHCTFLLGVLKNGESTVTASTMKNIIEQVGECMLVENGGCGGSTVTV
GSTEAEVDSVHSLAVRLVEAEIGLQESQAAVKSLDTKVERLSYGNDQLKARLETLAEERD
ALKSHNTQLEGQLNRLLLLFHDDAEARRGMQGTEEASSVVTCRAEEGPERDELKERLQRL
SCDNRALQNTLLQLQRQINDSELRAEELYSEGGGEVLVLKEEVARLREALREKQFELQRN
HHDWEKAATRWELLDKVHRTVIYHMSMRLMHDVARRFLPRSQEDTSPKAPAAVSEDTKEN
SSDLALSDDTAVAVAVSSSRNSGNDNRVKELLSLIESKDSEHRLALRQWQHVNQELRSAL
ASLQKDLKQKEGHIKVLEREKRSSDIHTTTCLEGVASGGHGDCGGDDLLNTQPKDLEQMS
GLRWKEIQKENETLLQRVAAMQKENSSLNVLLGDFRARCTSLEQELAKRVGAVSRQLVGG
AALTDTIPIRNLQRILHDVLQENLILVARVKELESR*

>TCRU_2230 Fcf2 pre-rRNA processing, putative
MASSSVKAIMAKAVTTSPTFFSDSNSVKGRKEQKDAIRKERKERAATLSQWYGMKKVNLG
AEERRELELLQFRNFINPELKHQAPKKSNTEQSEFVEFGFFAGTGRNKRRRLKSFADEWI
AENPELEQVVARRLKRNVRLNRKTKQLMAKKAAREAARVKAKKSSKRFKKKDMIL*
>TCRU_2231 retrotransposon hot spot protein, RHS3, putative
MRLNDFLQNYFGGRGAEEFNENVIMEDFLSSPNDFIQDEVLLSKIKALPSYQELKKEREE
FYVLSGASNKLGDERLLTLRHWREFKRKDTVTPLAMAQISTAYSHVLRVERRKAEEKARR
ERRELGIYVSTMIKYAVFKGRVRVDKMRLNDFLAMELDGRGALDANRDVLLRSFLRIPRS
IFAMRKYWAECRHPIVI*
>TCRU_2232 DNA repair helicase and transcription factor protein,
putative
MIVGANGSLFVNNTHPAYPHLVDFLTSCCEPVSRTLRMSEYVISPSSLSAASAEGTYSTA
MMRNFIRYFRLDEQHRLPVDLAKLSALERQLENGEDVAMGTTHESEESKGKKQQKQEEDE
DRLAPKRAKKENGERVSLVKQEATEETAAPSRRFFSLVKAVTIQPEPLISHALCFPDALR
PLPPDLEQMLLEEERSSRVRIVLQPQLRSFQRQQPHHHSQGQIHHEDEGRLVYFLVSQDR
QQMEFLVSRLQEFLVPVSLHGVMRWVLSDVDRGVEEKSVVESGRAKTLRRLFDTPSVSFA
SAMDDVGSCAAAAANSTGRKYSRVVYKSQVKDGRLRNVRERLFKELGVRADLFYDYVQDG
ALDVRDLVLAEHVRLRPYQVASLERFRCGNKAHQGVIVLPCGAGKTLTGIGAATILKKRT
IVMCINVISVLQWQREFFRWTDLSEDQVTVCISDKKQMPGDVFITTYSMLVAKRPTAPEQ
EQGEDARLTSRILASVEEQAWGLLLLDEVHAALAHHFQEVLNKVKYKCVVGLSATLLRED
DKIGDLRHLVGPKLYEANWLELTRAGFLARVECAEVQCPLPLPFFREYLDSQAEEDPFAR
RGTSSLARAVVCFNPYKLWCTQALLEFHRNRSPPDKVIIFCDDLEGVQYYARHLNVPFMD
GKTTEVERENLLQYFQHSNDINAIILSRVGDVALDIPCASVIIQVSGLGASRRQEAQRLG
RILRPKPPSLDNTCSYFYTLVSQDTHEVQQSYGRQSWLRDQGFAYRVLQCDSLLKELRRT
GGKPCCVGHPQWWYETLERKLPSAVAAKGSIWLPFSQEASRRMHRFFVNGREVCELDSTV
LNDTPRAAELAVSGLGEKWIVRFSASNAPETFGTVQMAEDNPTLVRRICYGPLDAAHDCL
KGEEECIQYILGQMRYRIKRRSNPR*
>TCRU_2233 hypothetical protein, conserved
MGTRARDQNCEISTSVSRVEGTIGISSEFVETDSRNGGLPGKEMDLQRRTRKLRDEEAVS
AFTTPKEVKLATAGEWKQRNKQSNSKRPQGRQRPGPGSRSMGDEGAGAGGSTDNFSGDAT
AGDRKVNRPARKGSEAGTQRSSIFHSLVFPGARVDCKQKDVIAADGGGGVEGGENGGNQP
NSVSPGTTQPDCLFDDAIEGESSVQEENGLMGEEFLQIIQSPQMRGLGGELQAEAARRAR
REKVQAAENRQQRSLRRLQLVEEMFAKALERRMKAMQRIAADPPADRLIGNHAMQHIPVS
MLPGELRRTALAFMDGVTRDVNSTAGTVPFAADKNEVSRKKFFLTDVDEAAAEEQKKEPI
DAIDAGVGLQETEEEEKTNFPNLNGMEEEELLRKWRELGYTAVYLPATGRLVYPGESSVR
NRSSKKPYVPDRWMSRQPLPPVHLVQRKLPPEFILLHGGMKSVGRRRPLPVVCRSGKK*
>TCRU_2234 Ankyrin repeats (3 copies), putative
MRAMIPPDLRALARVLNDVDSMQVSELFTPVGPNLPVHELEMLLEEINGAPLSAKEFRQF
QMAIMEERHHLRGVDLTTVAESQVGSALSLTSRLDATCTEAKVPLAHVFPQMKEAHNFFL
RITSFKCSVKQAPVFYRLSGALCSLLLCALEELQRYMDEMASSTSKSGGLKLRAEGQNYL
EGGGEEDTMPWLNEGAHLPAELECILADIDGGVGFHLAHLRWYLEVSQQNTYFDSGAYLG
DVSVRKIWNAYVGKEKPACFADTFSPALQFFPEWSRVAIMKVVNYRECGVVSLYTLKRLL
SVWGPFLLLDRNMRDVMQVGLFELEHSLEYHKKAFARRADAQEGDCLATLTDIPGEVVAL
VLRCQSEKQARTDGENGLATRTIRFTQETGAWVAKGLSGAEYETVYDAFCAFPELFRRPC
GKCYQLGESSEGDVQWGEFSGAVTNTASGLNRACFRNNSRYVKTLLERGSALVVNSASSD
PAISTQFAWTPLLCAVNNPNSDPGDVVRMLLEAGADAELCDDAQCTALYYAICNGYVDAA
RVLLEHLPQLSTSRSSVPLLVALGAHHFHACESDIRRLCDYIPSVDMLRVLLPYQTSLPL
LRLCVEIIRGKLRGEERLRQPTDDTSIWCPDGELELRTPEEEKFLLDIIAHNSRLCQQFR
EDVSSAARLLYNHCYFLSCREAFNQNPVDDQASSQCVESAPAQESS*
>TCRU_2235 exonuclease, putative
MGCSGAGTGVDRVEGGSSRVSNGSGSSGSNGTKSCNRVTHRRNAEGQWEDIKLYFSTVHR
LVQNREELTFVLDTVLGTGWCVDVETSPKDSESSCSAGVTLPLSQAAISGIESPRAPSFS
DRTTEHKPKQQGEADEEHQQMVVIDKLKVHSTLNHKAVPFESRLHSVLASPPLSAAPYYF
SGRSASTPAADPHTNSPVSFSTPPLHQCGGVAMEMASSAMPSASTTVSNGTTPPAGPSLL

SSNTAFKLESREDMIRRRTAAAAAAAANYAHTTSGNYYGNRSRGTWSGSVSPLLSFMNPA
LSHSSCPYDYLLVLDFEATCEESTPPSYLHEIIEFPVVMVDVRLQRAVAEFHHFVKPKVN
PKLSEFCRQLTGIRQEDIDNALPLEDVIRRFERWHAQTVPPGSRTMLATDGPTDLKEFMY
IHSVSRQGIRFPSMFYQWIDVKRFFAHFFQCQQGKIKAMLDALNCPFEGRLHSGIDDARN
VATIVIRMLKLGCSFCEIPLSRLPYGATATTASLVAPEITTTATETVKLDKNDTDASSS*
>TCRU_2236 exonuclease, putative
MQSARTRDLAAEVALIRKELRPLPESALDGTDDGAEDDGGGALLHGTGTRFEILMRGAKR
RTAAQSAGTSDAVDRHNGSSSQSKKRLVPQIFSHLLVCDFEATCDSASVAYPHEIIEFPV
VCVDTAELRVVAEFHSYVRPTKNPRLTSFCTALTGITQAQVDAAPRLPEVLRHFDLWLRE
VVYPLCRRWRKEHLEGLNKCLKASQKRRFVYDETHNPGWIDCERMICMVTDGPWDMRKFM
YECSVVRDGHLFPPLFYRWVNVRHSFAEHFRMRPRKLNDMLRKLGLSFHGHPHSGIDDSR
NIARIVIELLKRGYRVHHVSTINYENAAAATAVAQLLGESDEDGTKPPHQRAEGRKKGRK
NPTTTTAARRRGEKGHQAQ*
>TCRU_2237 hypothetical protein, conserved
MSHQAKELFRVNVSRGSAADNPSTASVTAHSSFKGGSTSPWSSPLAAGSETQGNLAEKTL
PRTPSGATLLHESQRQEKDQQNHHHHQQPNVRTAPNSSVLSLRFESPSFVMFRPHTPPRI
RPTSELPPTRDATSVLRGMPTVLKASDDTLHDVPARPPQAPAQPRGLDALHTIDTSTHET
RAASQAAMPVSSTVAVMGEEKTVRGTICRVCSLCGISVILVGTFVEHQQQWNEHVDSWAH
QRNVQLRALARRDDFISMESTDVASSPPLSQGMIRTLGDISASPVLLQKEEASAMAISPL
RAIFFPHRADGIQADEDIFSPHEKRRGNVSPTAAPSHVFKELFDEENLAATPDAKSNEED
HVDICRKEERPDALWKVGSRRRRKAMLRDKRGGESSRLAERTSEVVSVNERGLSRVKKIP
HTDYAASSEHSGSASPRRERQLECTLAKFLIQKEGRQLLACFRRWFNFVLVRMMSDSSLL
IRASSKRGPAAAADLVEEAFSAVSAKPKCGEESLLHSSAVTLEKREDAALPAAPSHIEND
QQREDQMLNKTATGDGEREGPFMASRVKDFAHFIGSSSSSSVEMEMARTDVSWYVHRTVR
PLHRGSGGGSGEGLRTPPWESDTSIEEMLSTLTPALQERVRAVLSGEPERSRAFPKTSEA
LNIIRYPSLGPNLSPGGPRVLDTDGNHSISSSFPMAHVTRSGSGDVMQGIIDPQKGSSSS
FSTHVGPIGRRPTGGDAKPGNQEVPILMDVEALNFPSPPAELSGMPPLRQEEPLGRLSGE
TAPVEVRDSPSHLRRQKSLEGYQKASSEEKPSPSPPSKVCDFIGQDGDEKTDSAPHASYF
SQPSVPRQRRGEQEGSPSWRQAWREELDVGKDGANEEFKGIPGEYYCYYNDAYHRVLDPT
CEEYYDARGRRLPIFFVRRSTRTRAFSTRRLIATTRPKSPLGPGRLNPYCSVCVARYFLI
VVDEYMRPITVTPRRQRGASGGNGEAHTCACPGRSAKSSPSRGMRDAFTRKKALQSPRRT
SSWERQDSSFPSPLPHSFPLAMKSDSTGATTSGHASASPKLVSAAQLGDSKAPETSQDPV
PEENKRLRRLERDLRRRVKSLLWRELPQCRGSQQWVDYLTSLKEVLQMLEALRSAASVQ*
>TCRU_2238 hypothetical protein, conserved
MQGEGSRAALASSACRAKTPLLLHLGRVITPQSLRRARQYVDLLANPPSPPHPFGVFLEE
AARLRSRAAAAATHNTARCAENGEGVSSGSAVVGAEDADVSSLLELLRREHGYSTGSAMH
RIWVQQPREYLAGMAQRIADLVLTRVMPLVASERKGGEYSHSQSEANNTSRKTQFVKQER
GNYDIHVRFLPSSASSLQVHAPIAKVFCAEITVRDAEALRRLEEDADLLRTQYTSVAAEG
QTVQSALAFWRAIKMDAAAEEIEGPSADDALSAVVLGRARNPADEKCARRLSAGGADALW
MQPPNKRPRQAAASATASAGINFSSRRKLAAPQTTGLEPPRVPLQSTAGLVAVFYLAFSG
TLYTGVLCGAAEEGDPYPAPVPHSTYAASDGSVTGKRVETTATPLDAEPVFFLPCTASNS
FVRGLLGL*
>TCRU_2239 cofilin/actin depolymerizing factor, putative
MAMSGVVVSDECIKALTDLRQKRCRYVMLHIIDQKNIAVKAVGERDATFQQFVDSIDKSS
PCYAAYDFEYETNDGKRDKLILVSWNPDSGLPRTKMLYSSSRDALNAMTEGFQPIQANDV
TELEFEDIVRKVKSHRQC*
>TCRU_2240 hypothetical protein, conserved
MTNATARERNKAAVQRLFARGRSSPAPWTCPPVRCADGTLLPDMRLSKQWQLAQEVGCGH
GDAAAAAAVSAPHICAAGPGSSSCSCYIMNRKIARHEWETNTKEKRRLQEGSPRSHEREK
EQRAAMDREQAILRYELSIPRRRVVDHFLHLTASQLDLCHTMPTAHTMDRLVEQRRGRWV
LQNPYRQLLLRGEPVMTRIMRGEFYAPQRNETKATNFPQANKRHVRIASNINHAMHFNRG
KRGSVDRVQQSAEKSALT*
>TCRU_2241 hypothetical protein, conserved
MPTVVSLVATWAAEHSLKINADRSGAALFCIVSHRQSDEETADHRLGDENPRVKSHPVRL

LGNAIDRHLNFGVHVTVAARQTVPRRHQLRLGAEAGASRHTTRSFLVGCVHSALHHGGEA
IAPCPAPVHLHSLEVRHRDSCTASLGPRASKKDTSVHLAANLAPLRRIIGFRAPTQHERL
TLFRCIEDVRGAI*
>TCRU_2242 hypothetical protein, conserved
MNKSVGIASRADVFLQEYFDACNDEEKSEEEFDDESDQDSPNYWRSSRLSPLRGKARLSL
HQRNSVQQALMSQESLSRRGRDAAAAASLLSRPFIATAEMREGMTGSATVSLNNTMEGTA
AEGFLFDAFCAFRQSPNGSFELSPTPREGGPPVWSLRKGIVVAETWAAISFSLRSLMEAL
LAGANEVARAQWLLQLLGGSLSQEGVSVMEDAFTDYSQTCTGEEGLLLAARLVLCRFASV
GRGLVQRIMSWTTQVQEKWNSINPNEFMKPDLHKCIERLLLPLRRCIHLLERSCSLAGER
PSTIDSNLTGTNVNTVTRATTLMDHLIVRMSALQDSNTMDIYRFYMVLLIYCGWPYVLLM
TSAIFGFVTDIDADAWRSNIPRIFRLSFSHIRVGDHGRNDRKVVMLPTDILSFVLLCVGY
EPVEADGRCSEAGGKPKRDVNRARRRAAFSSMLSSRSFILRSFAAFTRKKVLLGWSRRRS
ADWRPTNAECSTGDEWLGFIQKGEKTTSHSVSTAAALSLTLCGDGEDEFLSLWPNFSTWT
LCVEEKKTHHDFILDDGTAGGNGKPLQLWLHTSLPAARWVTASLLVPIAQVVKRLNERRL
AELLSNEIARHRVGYHSENNLENTDDVCYDDDDNTPAIRFDMNSDAWNVGSSTVSMRRGG
SLSLIGGGRGAVATKLLPNDVTFQDAVRLIIDIALCRDSERIVYTFLYRLFNEPHWWYRR
DAVGYKYTGTASTFISTTFADAIRGKALAQFVRLSVAPTSESTLSQGTGDDSALEVLRTF
ASFELVFSFPWDIELILLPLNLSVSWGGADAIQEKYASYFWKNRRCGEEAKVRRGNTEKA
AGKTGAQEERQQQPLDKQAVKARESWSYCFGYLCSLYYAQISLREQRKRLQQRDIFSHNI
MSALGSSEEGKKHTVRLTRGLGSAYFELSFAVDSLLSFTQNIVGIVARELEGELTKLGKV
SSCMALCQRIDALLLRLCTVCFPVAPGLEDTCPMTDSLPIRESITAVLAIALNPTSLPLR
HITSRTQNAIEALVAVVRALPATSVLKEKLSSLLVLLTFNRFYGK*
>TCRU_2243 hypothetical protein, conserved
MEGCPGGKGGRTHRVAGACRCGEDSIPGVDPDIGPRVAALRTGAMRLLPALRQACTRPAD
HKEPSVSVVPSLPHCGDRPASALAGTSSRRERRNSLSTSERRIDVLLPNLRQSALVRPQY
EGTCRTCSAWRYNGWRGPPMRMPQWRHRLPNGCLARGSPPKLPSMPAGNTRRKHKRGYSR
WTRLFRHRLITGATGC*
>TCRU_2244 hypothetical protein
MLENDSHNSSEHVFCMEKETERVAREESPMERYASLLLCEADRHQRLRQALQKRLRHVQF
VPNSVSAGLLGLLVSPSKNGFSVNTADDADASSAILKQKATHNDADIVPQLTSELISHVT
TDWSLVEASQEEVIKHICELPTSTDDEFQLIQLIQRDKIRRMEDMYLLLEMCFKRMIMSS
CSRAGEESAEGPVAFPVGAHNDVVKGELLSNEKRVIGNTNFFLPAVPELLDLCDTMDLCL
SQCQETRQKIQHQKNVVCGAFGVLAMKRRRVHLTTVIRMLHFIEQRHKQTHDINIFLKAG
RFAEVVLSLHQLRIQYEMRKPVSGSEEETLEMMCEATTLGQTVLKKHLYLCTQGVKALGL
ALQNRLFQVLDSLQDTCLVWSKDLVCSALRLAYGAHALCCWKGKPTRALNQHSSNNTGNL
TAGRLMEAYVMDKLHEVVNENIKNSEIGGGGIAVLAESVTLSEVRRCTSQLLTKVSSFLR
AVLLLQRCFEALFASESRDNCMKDDCLTFEQAIESFLGDDFAFCKALSSGDEAAVRNYIY
DVISLKKVYEDCCGIGRHAVKEALRLVLSYQTHLPLENLTLLELLLSYRLFFHFVDICRP
VFPTDDDFAGNITAEMEARLAMLVKRNYLTAQADTLGQLLRNDTGIFVGATEDDLPPLYP
LLPCELELEGNAAHKYLLVPPVRFCYCSKEKVLISHITCSEEEEMVMLNPFLNTQAFLSP
EAIPAADTFRIRLFSSRDALEVFTFSSLMGVKMMVENTMYAQMFRRVALALVYVNLGITS
LYVWYILSYFVSSGDQWFFRSTAVDGELKQFARLLRDEYLPRSFDWNKVDSSPSLVWRSV
VNSRQHLFAAVERATGLCSVLSIGAAFKAILSALKPLLPVDELGQLDSVKELMGRLCTCI
SQNGLRLLSNQLLSSNAFAILMEEFHWDGSAESFPIAVSKVKGSCSFSYPVRQVMEAFLR
CQHELRELYRDAPSVSLRRLLDHMILNVFTGFVEGVSRIRKFNSFGEQQLLHDAEYICHE
MFCAQPTLNRRPIIFYVQRYALAMTLSSGLAELVTTMHHLYTTRQLIALTARLDLSKRRD
VEQLIQRTHVRDEIPYCLIEEIA*
>TCRU_2245 hypothetical protein, conserved
MMRLTRRCLFITKISKISRPPASGYEIAELPNEKVFMEFLKYYNTKCVLAVLHSGSLRST
AGEGGAGTKIVEDPLTRSFISSINAMNMGNPNETKLALVPGPHAPRMVEEYGVLTYPTVL
LFMNGKCVERVVGARTREVSIKALFTLRNAGRNIFSRE*
>TCRU_2246 Protein-tyrosine phosphatase/Tyrosine phosphatase family/
C2 domain of PTEN tumour-suppressor protein, putative
MGPCTGTCLKVPNEGTCGKEGNTSAPNGSTCVLPELCLNDLNKLLGEEFNETDVHHDEGN

EVIQEYTKMKNTASETCLSTTMMCIRGAREMEEELGAAKKQLEDLMLDQVEFFSHSLENV
GNGINTAAGQIDLNIIDEILSEDSFDSDDAVFFRMAEEDPVPKPTEIGSSLAVLVSQNSA
LAMMRSKVSQLKRRYKMDGFDLDLTYITPRLIAMGFPAWGTEKYYRNPIDQVELFLESKH
GGHYRIYNLCSERPEYDSPKRFQGKFKRFPFDDHNAPCPISLVIDFIRDATSFLEEDAKN
VVVVHCKAGKGRTGVMVSCLLRSLDPIGIPDAKEALRVFGNARTYNGLGVTIPSQCRYVY
YYDRIVRDFGGQLPPSRPIKLYQIIVDSSIKSCGQVDVYFTVEENGVMKIDSRHLFHGGL
KRDPKGFFFLFEEEPTLQGDLRFTFHQRHRLFNEELFYFWINTSLCSNYERFSLKDRELD
GRLSKGKDGDTFCKDLAVNVIFVD*
>TCRU_2247 hypothetical protein, conserved
MSKVVPLPGITFAGASYARNERVFDSLPKNDKRRIRRGEVIRFRGAPRSFERPRWNISVL
DEAHPFPQLPIMHSLHSFEGVEVLHREFPEIVRGAKHLETKCRPGLWTTRVKKGSPRDVD
SARERWNSYLSRVIASECSERKKELRSGWDSSKKSWTFTGRIGDPKESQRSYVELNKTKK
ILNMDAYNSPLSRVKKMNESIRAAKRNEKMRKEQTSLTHVCASGAPAVFKMSNIDEWWHL
DPTLTAAETTAASAVNVNKGSEQ*
>TCRU_2248 EF-hand domain pair, putative
MAVLYASKAKCTRFKAIVERTRRLLFTGASGANGIRALSRSLGIAVDAGGKLVDKTTFVE
CLKSNDVPLDEEDVEAIMSVLDRTGDGMLDPVDFIAALRRELTPVKRTWIIRLWYTFRQN
TNGTIFIEDLVNAFNPAGHPSVLSGERSEKEVREEFQGTFNTTTNPDGVLTRQEFEQYYS
CVAGSCLDDASFVALLRGVWPALAGKSGQHVTVNDERENICGATFKASQTAVQKGAVNKV
RQIAADFDGIIRTSHRPAVMASPLAARQVSLLLRVKDAEGAFFLTREDFLATLWQQRLYI
AKPEEALEVLDTRGDSSVDYLLYLTMLLPQLSPARMMMLERLWELFPKDTCGTIDVLELH
NSFNAKDGEEKNAFLSAWDVRLAIQRRVTLEEIVDWYIPMSATVQLDKDFEAVLKRQWNL
A*
>TCRU_2249 Trans-sialidase of Trypanosoma hydrophobic C-terminal, putative
MLQDGKGFMYVNGKIVGSPETIPTLETQGHEITQFYFGGGEGDINSNVTVKNVFLYNRPL
SVGELKMVKKSDAKKGNGDGSMRGGVSRVLLLQLLGFCAFVAFY*
>TCRU_2250 hypothetical protein, conserved
MEATTKSGDKITLDTTHDTGFGFHPGEIVHFTKSLRNGKLALIRGVADGLLWFSVFRTVE
EAEAAEALRAPVDTASCRAKEEFIRQFGWVLDLKTNPAARGGGV*
>TCRU_2251 PrimPol-like protein 1
MFWCKSTFVTHFAADDCQLFPTQQKLFDFIDAQNEEEGQQAHSAPKWLPFSIELPSEEEV
VRELASRSLRASAAAIPTHSGSGGARGRLFRKRPLSMHGEDTRKTDDEAKYLCEGKRQRR
VVLREDYAVFYGVTPLSSQTRMFLAATVNGLARVVREIEPLQQHLYEVIREGAPCHMYLD
VEREKDYSAWLPVVSVVDDDSEENRVIYECRRAEEYSAALSTALWKPPRCCAWNCRLRAD
NSETTTVLLTELRRFLCSTYPALVKSRDSENHGAFEDEDDTVLPTDDNSTAAPLGPGVEV
MVMRSLDATEKEGGRGGKFSQHYVVKFGGQWFNSNADVGRIVGQFVEYLYERAVADPCVH
VALFYHDSPKDFAVLPPSQPSRHLPFLPLRCVIDTAVYSRNRMLRCLGSCKLHKRSILTV
ERNVSANFPTGTIPTTADRVALFFDSLVTLQVGHREVILVPAPTISTFGAASSNEGCKYG
SGETQLTKDVDRARYDALAEHVAREWSIVGGVPCRVSSVRQRGGRYLFFLLDGSRYCSNV
GRQHLSNNVYITVDLQQRVWVQKCFDPDCALYRCAPRAIPDDVWSTNRHDWTSETPTAAL
ASHASLRTIFQPPR*
>TCRU_2252 hypothetical protein, conserved
MVYRRSRLSLLSRAVPPSKLQALFLRCGLRDIVEAHGDNFAREVAMRSKYQLFSPLFAPY
FTMESHHLTMLGDTIIDDFLSQAMLRYAIHAGLVLTTNATRQLNAVLHNHFTLRLFAKEL
HFDELAVPLNLDSLLADDGGKTTGDRLAFLEPQYVGLREHDVAGTSFQVTPLRAGQSPLG
WMFSHFIGALQQTLGSDVVRRVLSHTYGDVEMNLPSGAAALLLRTLQHFPAGNVTEAVLA
AQGLPLRFVTKTRVLSDEATREGEEDGDEVAGKKSVEEAVLCSGFGVQDATRDAMTIDGI
AANPGVLASLAAGPGGIDAINTWRQRAQRELQEGRGLLRDAIRDEHDDDDGWLHPEERER
YRRGAAFYNAVDFTAVANYFADLYGIPQPVNAQRVTLLKFRNSVRDPLFYDTRSDMRNGV
PFSTDGEPLAQYLDRFNRPHRRLFEVSLVVGSGEGRTAGRAIATRYTVARESACRAYLGA
VLHDLHTMKERNSTAV*
>TCRU_2253 hypothetical protein, conserved
MEENPAEEDVNEINTTLLELQDAFATALRNAGVLGKLRAQLRAAAISVIRGDPYLRTAAV

GKTILPAALSPEGRVALILIEEFARVHGLQQTLGVFEEEGNLSLVGELERNTARRLRGSL
ESSALEQLIAAAMAREAPQLQGKEPLMTSGSVLPASTPFVSDEREKEREQHAGARQQQQP
KKEVRAELETPCHPEIAAGLKEYEDSINYSIVSVTDLPGVDESNYDKIENF*
>TCRU_2254 WD domain, G-beta repeat, putative
MDRPFETHIRAEEVEEEEEAEDPREAFLDLDDPNNEILDESKMAEIEDDDNDDDGGGRGE
EETQSCGARDVDEVGDAEDLNGIPDREPDRDDALCVFRGRDGHPLHAVTAHPHDTQIFAA
SGESDEVYILQLASDRRTVETRAVLQGHSDTVSLLAFSPDGTWLASSGMDSAVALWSTST
WELRHCLRDLSGEILTLLWHPSSLVVLAGGEDAQAAMWNVSKGTLAMYFAGHGGPVTCMA
WSPDHKKLLTGSGDGGVIVFNPKFGEQEAYITKGLSPDRASVTALCFLGDDDRCVVGCED
GTMHVISLRAGRAVASMQEKHSQAIESLCISHSSAAERISSLPLLISASCDCSIAVWNTA
DLTLRVVFNVGESVIPAVWAHGHFVVAGCSDGEVKVWDGRSLDQQPLLRFMGHRRMILSL
TVREVSGVVATASDDGTVRFFSLGLQ*
>TCRU_2255 hypothetical protein, conserved
MAQRVPYDVVYCTSQDDAFPIDGLGHGLLPNAAAAANVTNASMLRNCKGWQSARNAKMPQ
ALVLRFPGNVELTQMRILSHECKIASKVEVLFFALKGEEMEMHPPSFRAIHFTKLGAVDF
NSNEQSNYRSKERKTIHMHAVAYFVKLLFHTPHRNAQNVFNQVGVYSIECHGQMLSRVWC
SPDKTPVIGNNKNKNGRSMSGGDLTESVRSSEQLHGLVLDPTRRRQEQQKREQVEEEKTP
LRPFLRQELRSPYSAQQQTRQQLPQSPPLTPVREIIPHGNDIPVFRSVRIVEFEEFFIRR
SEELLALKDQAVAVEDFDTAKLCRDRLNLMNRRSKRIYQIEQDKVQAIIEEDFDAAKEAK
RVMDAMIERVYKDAQLPRPQPDFDQYECIGETFHENLNYNPPRSDMADVSEERTRDKSPR
DTGGRDDGADHSISSNGMDADDHRYKNERDSNNNNNNENKDNIRMDIDEYRDSDAHEGDT
MPMVSKFAQRMVDMVRDTTYEIDKVDPPPQEKSDDNPPTPKIEESTLQPWERRVAQAIMA
ASSETNGPAVLNPYVKNTREALDLLGVLGTFTTCCLFSKRFKLREAVLDVLMERMGELYQ
ASPSAMEEALLRFLDMNGHGLQDTIPNVVAAGCAFVRMVLADEHKCLSSVLAPVVNLLPR
LLCCAADSNQRVRDEALATLTLCIKTHSLTNTTILTAVLAHPLDKERRRLPSCGPRVQVA
RLTVFEQLVTAGHMGLRNHTESVWGKLLRPCINHQSREVRDAAVSTITSLVQERKILLTP
KHVNMIDNAAIRDQIKAVVEKSNAARTSGEAGDAVGAAAGAGTKCRPNTQGGKAGNGRGK
PRTKRVNSDVAV*
>TCRU_2256 ZIP Zinc transporter, putative
MVSCSEEGIGVRVVRGDLLQLLLENCAWATARMNDVALFKIGVALSLTSVTVLGMCTPFL
CVQNAKGNTPPRPRTQLMTSSRPLSLANCFSAGTLLAISLVHFMPCALATAGNGVSPTAL
CAALMIGVLFPALIERNITESVAQSGYGHSHGLFLAHESYQRLTLMPLIMLPMCFHAAVE
GVLLGLEASVAALMGSAVPLFVHRFFDGVVVGVCIAKEVRAEMEAVDEVTPLNAAEKNTT
AGDFAATFRRRACRFPIILWLCIMPTALLFCVVLTGDFHVHGVSEALHRGGLTRPSDGAE
SHLRGSWHKESKWLALTQAVGSGFFLYAGLTTLTLEELKGTRASLALFAGVLFTSLLGAM
ETH*
>TCRU_2257 hypothetical protein, conserved
MSSDPFSITLKTAVNTRAPLSDRVSWRRQVKEVVSAKSALHRGIHTTYDAAYNFVPARTD
PSDGNRFLSLLRSRVGAAGSGKSARLEIVTAPVAEEHLQCERKQPAVPRHSLPSQPEEEL
QVSSNLRFFEPVLTRHMPSEWETEQQASSRNVLPVASHGFYSTARKIEHEDDDRRSGLVG
NTQRTVDIMRKRASNPLLCPAANKYHSFRPVRPPCVTDESGRPASYCLDIVDCTQLAPTT
EEQLGFDMGPISRLPNTATLSQALDVQQRVITHMGTSHPLFVGTAKVMETAVPNYTGHAP
CHPRNIAAIRGNDTDPLRLWSKSFMTLAEHGGGVDAGNNCKNPLVRKRGGIRAPQSKELP
PKSEKGIRSTVEGSMLQATLKDTLPAEHAINQREDKALISLA*
>TCRU_2258 hypothetical protein, conserved
MRSALVWQRGSSKCVGDVLDAIIDKAGICSRGDKDERTAKRDGEGVTRWKHRNRPPEGII
AAVMRGSNPFFVSLRPLTNATSKKKETAEGPRVNEAVTRGREAVPKREKRRGPKRTAAGC
GNKQRINVPVTATAVQSCVLSAAGASVASTTPPSPCRARRLTARKLRWLSNGVTKATALF
KLFKTLSLYGQSRLVKNRRRHCCRSAEKGNGVDGGEARLHAAFWSALASVLQQRRLHKKQ
WHALWEAQSDGQCDVQLLRAFPLFGLLVVVERMRWRQDNEDPCTVIAEVVGSALGVVMHE
SRAFIGVALLREEKKLRVLSNHPLIGEGMRRKSTDNALMGDEELPTGRILRVSKMFPGGT
GTAAELRCPRETTRCVVARVAPEELYDNFTAFSPLVGRYL*
>TCRU_2259 histone H4, putative
MAKGKKSGEAKGTQKRQKKILRENVRGITRGSIRRLARRGGVKRISGIIYDEVRGVIKSF

VEGVVRDATAYTEYSRKKTVTAVDVVNALRKRGKILYGYA*
>TCRU_2260 Zinc finger, C3HC4 type (RING finger), putative
MPKKTGGKKKGQSSPDGSEPRKRKNNKKATMEPRDVDEMQKLQELLGDEEQPLGVSNKSL
EGLLSLRQPQELAVRLAQSLSSLRARLAELELERLNRGSEAPGLSNIVARRAQEKAEKLE
LEIQKTERVTRRLKIISSLVGHIIRLREKTLTETHTAMEAEVQSLQEKIRVNEELIRERF
VSRVNMLHRYWLWRTLQELGDQTVGWTFEEELARGPRYRTLGVQNNIVSETLEQQLSWLL
VFAEKEKIFREHVRRLELLVEELTDINDALEEALTCRVCGLLFEDPVLFWPCGHVFCLVC
FDTLSIAPSLFRCPTCGSMGSEGYVHNLLIAESVAKWMFKDAGYGDIHGALSLIRLHLSK
FRKEVISSRVAQLHQQLTEARQKETKVEELSQMDITYRDF*
>TCRU_2261 hypothetical protein, conserved
MTSKTPSRRLTDILRSNSKETLLRNAAELARVSTSLPAEKLQTRFLRNMHLISPKSLRLL
CDDAVLSRWFAFAVYKPPFCPMRRAEANKNYHHVSVESFVEAALRSRTVQPVLQRELQPE
EVKVRVLNDVDMYASGPVVVTLADRHTFSTSLQSVTMRYDVLVAGHMPLKEPCSVEADHL
FAPLRRDSNTEDICSYPDTTARATRLPAEAAPSSCVCTYQVRRNAYYSVHPVSLLEFTIT
APPTPLPPRLEAFVCDHLGTFVLGDSDTPATTRRTKRTAKTPSTASESTDRAQAAAKASR
SVVAMHGDCDFPRVFTHLREVSIQTDVADVAGGFAAKKPIEFHCRKCFEPILQRERIYSL
KGRRIGLLDGSWTPEAEFL*
>TCRU_2262 prolyl oligopeptidase, putative
MGVGGRPYDADEYDATIVWVPSDYAFHGDKFTSPLRPIPTKNPVAVVRIPVYLCRKKALR
SCGGNPMLLSVYGSYGECCDVVFENERLSLLDRGFVWGFAAVCGGSELGAAWRKASCGLQ
RATSVNDFVSVSFYVQETGWCAAGQIVTHGSSAGVFVATAAMCVAPSLPLAVVALVPCVD
CLTTLMDDSLPLTVSNWEEFGNPRDDADAYRLLRAMLPMDNIPSVGVALPHMLLLTAWHD
TRVGFWESLAFTARLRAREMEEGAATR*
>TCRU_2263 hypothetical protein
MFTVCSNSYSEVHVIPCDAGVEKCVSEVPVFPTTANDKGEGLQNNTCAVHCFADRNFCMD
YDIDHHSSLFGEGVDAWVVTATKSSKGLEKFRVAYVLDEDEKACRKDAVGGGNRTLSDPS
CWQSLFAYDTCIEVEDVECMRDYLLLSVGRAASSTVLLLPVRVLWDRWRDCHGRNMPSLS
LQDDTVDLRQVASRPFHSSGEVRFSFAQLLQEQRLKKDNNSNEEKENEGCAHRFY*
>TCRU_2264 Prolyl oligopeptidase, N-terminal beta-propeller domain containing protein, putative
MRANGASQGKKGRGGKRTSKNTGDHGRRSAAGILSLPKYPRKEHRIVHFGDVRTDPFYWM
RDRRNPEARRLLQHENAYVESVFAKLGGDQLFRTLFSEMRAHWKEDNASLPF*
>TCRU_2265 GTP-binding protein, putative
MQSLGKSFQRSKKASQRSQREKYRGLLEGERNAAILQEEERQQQQRQKGAPLKSIWETNN
LEEFLLIADAREKGYEAERDIRVVVDGTPHVVTNDKVLPMSKESVDWLKLSELLTIPRRP
KWEYGMSAEAVQAMETSAFFDWRRLLAKMEEEHKVVMTPYEKNLEVWRQLWRVVERADVV
LMILDARNPLVFRCADFEAYVRSMCNAAGKSKEIIFLLNKSDLLTESQRSEWATYFAERG
EPFIFFSAAPAAKTSPETTGEKDGSRTATQEGKQNEEADDGDEEEDEFLDADGHQVDESC
VPELLRHRREKRRHNKKSLRAPVEVANPYELVEQRRAEKARRSQEKQQASRVAPPPTENE
KARDARVSQLQALHPWAVLDPVQLLDRLALWRTVYGIRDANTPLMVGLVGYPNVGKSSTI
NAILGCKKVVVSATPGKTKHFQTLVIPNERRVALCDCPGLVFPSFASTREQMVCDGILPI
DTATDALSAVNVLCRRIPREVLQKQFGVSLRAEDDVDESHSLVEHFLNALARRRGYLGAH
DRPNKSRAARDVLKLYVDGALLYVEPPPNYAPSKDAGVVGLPAAHVDGKDSDGWEDVECD
SHDDGSDGDEWDDLDSAAEARAISEEGDVSMDAHADKNDEEDEDVDKTASLMFYARPSGI
RGVLTAQEAFNAEANANRIRTLTSAALRRKKRTNHQLDPVMDTFVNNDGEVELIIDDDDG
IIELVTDNSRQNAEGKQQQQTQQKRRSKRQIRRELKRLGAGPVNPTSRKMQVQGY*
>TCRU_2266 zinc finger protein family member, putative
MADSEENPFVVDHTDGNTEPFLTAAELPSPAAAGGMEGGRVKDEPQRQQQQKDVEEAKGD
VSSLIPLCVQHASGPQINSAVFGYDIAQMQKRPWDDPAVNLKDYFNYGFNETSWRLYCAM
QAGGEASLLTGTNAFLSQPAGPAMKNNAMEEVVEGDGASYYVPPQGSSHAYYAAPRDNFL
KTKLCQRFALGRCVKGDQCSYAHGHGELRSAPPVQHQHHHQQQQQQQQQQPPSCILPPFH
VEQGYMPSMGMPPTGVLEPTVAGNYHVMPKRQRSPDGAEGQVYDASY*
>TCRU_2267 dihydrolipoamide branched chain transacylase, putative
MRRIALWPHRICGARFFHFSRPRWGTIIPYKLADIGEGIQKVDVVTVFVKPGEKIEEFDK

ICEVQSDKALVDITSRYAGVIRAVHITVGESTLVGHPLVDIEVDDDVKDDASGAEPQPQE
AAAVAEPTTTSSSSSPGKQKIKAASTTPTTTAVKLLATPATRGFARECGVDLEKLSGTGE
NGRILKKDVLAHAQSHGNDEGDVVVSLLTGIRHVMVSTMTEAGKIPSFTACDEIELTSLL
KFREELRRNLTPRSPGDATPKLSLLPLFIKAASLALLQHPQINSHVSQKCETFIIRKAHN
IGFAVHSPKGLIVPVIRNVEQKGTMDIVQEVNELVELGRKNRIPPEHMRDGTFTISNVGT
IGATYATPMILPPQVAISAFGRLQVLPRFDVDGNVVRANIVHLSSTADHRVIEGAAMVQF
NNALKGLLENPQQLIA*
>TCRU_2268 hypothetical protein, conserved
MVSLRYVREQQEAAVRRARFEEARRIGSAIGVQRARAYAAQKSAQAVKVREDMRKQWECE
VQQQAALIDRVLYDGQSRLGEGMRGAAELTAAQQRNASDEINAWVAEHALERERHRIARA
TERVTRALQDEPHRSIVGRKKAVREVEDKRSKTRIAQPSPRLPSFASHGSPLAGALSHTA
IMQARTPTPVASVVMVTADNDDGHRRAARGEAAAVQFAEERKRELAGEREKLQQCQREAA
ERAAAYLSERDKEAKEAREAARRRAANMAALEQRIVAEPQDDNSVSGKVLETRRVAMLKA
QEEVEFGQLFLSGPLPLNDELSLKEDNDDADEMRPFPLSSVSCLLPGESEVVVQLHPRPF
HVYPIPEEAPADADDAEEKATSEAGSNAPNAASVAPQPTPMSPQLVVSTKLNSSPSRTLR
SSEQSLPLEVPPSSSLSLPPPLSLFPSAATAKKPDVAQLNLSTHDEPPNLALHHEQFLRN
LKALQDKLAKVSSDSADVNETVDVSTCHGNVTLSSMSSPLSMARRPGKETREDEYEAGAT
SIDTSANSGSSSNGGSSSTSSSGPSTSSVYSRSYPTMTAEQLKAALLKLKLRMRSA*
>TCRU_2269 hypothetical protein
MDSAREALLAETKRLLAALNDENRELKAENTRLLARCKTAAERLTSVSVEKEGLEKDLKH
INFNLMHQLRVKVGAVKEHERRAAQLQATVTGLEKQLQESRLAQESAVKESKMQREQIAV
LESRLQQLTERLFRTEAEAGKYADDLNAAISGHREAVAKIQHDGAERLKQVEEALRVSRG
NEAGLERVVEELRYELGRAKEVLRQSHCSSSEGQRRTLSARSEALYAHEQVLTACNASCQ
QLRELQQKLRGLESLATNASHHVEQTLERGEARLREQMEHFIGTDEALVAFLCQRIMDQE
EALRTAREELARAHHRLRESEGELHKVRAEKQSYEAELKSLTALQQEMQTIRAERQAHGR
IEEELRAEVGRWRHEAERSASQVTEFRRLLQEQREQSEAADRIHKREEDDVDEQLRGFSA
AVSQALRKFDSTTRHRRRLSSSAGGVTLSHAPSTQLTEYAPDGNMSSPSVVRVEPSSGAP
SHT*
>TCRU_2270 Nudix hydrolase 2, putative
MYRKNVCVAIFNEDLRFLGCRRIQDNLFQFVQGGIEKDADLLHAAYREVYEETSLCKEDL
HFVGEVPPPSGDPREFRYELRRTSNLRNFGFQGQEQRVLLFFTDSGNIEKVNVIPPKETC
AQQEFSEVHWMELREIIDRCPREKIHIFLAVAKVGVPMARAFLQSRSALKKDSI*
>TCRU_2271 Putative methyltransferase, putative
MLSSRRHNILLAPKLMFITRSSTAEQQKEELHDAVRRHDSLVADNLKRRNGSAHATYDEE
VAVHFPAFQLAILPPTNGVHREGLRGSLATHVTEEFRRFLVQPWTSAAKRHVLIVGSRSV
GYLQRQLAMDTGHSVFVVDHALSALLDAATALVPRYGPRVHFVRSDAPFLLQRLMPPTTA
DVCVVPMPVPFWSESGSYRRLVTREFLCLVHRVLRVREGPADPRGIVTFTDTTPLAEFMM
EQLEESRLIVPWTRKDPAEIYGRWLPIQSPPDREFQQQRLGDVVAWAASKSGPTSTQAEE
AVQSFDYKRRYYRALSRQEA*
>TCRU_2272 hypothetical protein, conserved
MVFCPSSMALGRALLLSPFCHQPVAIPAAPSLRRLLLEVKPRSESSREEVVAALLEKLPA
HCDRVKKDMLHARYASRYYRSRYGRGMETRGKQLRGLLEMPDDEVRRWAQSRCVPVRDPL
APR*
>TCRU_2273 kinetoplastid-specific phospho-protein phosphatase, putative
MDPPSVPSSPQAPPEITPPGMSHSFRTEAFITLEMAQAVTINREKLSEWHHKIRSRLFSL
VSGFFVRCRVEDSTDGRALYKVGRIKTLKPDWSVELDLITTEEILSGRSHTNYDCISNAK
ITPTEFNSFISKVPPELLPNIFNTVFKMEERLRMLESVILVEPAFHGKRRDDKRTQEGSQ
VGGGVANLPEKYVFSQNVLLCEDDFVNLPQTVTIVDLLQNAVGQKGVAEPDTPRAGVAPG
SGTKPRAFDEAVASHSTMLQLQDMRNYMNSPTQPPVDAARLIETIRRAANPTHNGFSYEN
LPEFCKLVISVCSQCREVVAKEPLFVRLKSPITVFGDIHGNFADMAYFLEKVVGFDDIML
KYTTTHLLFLGDYVDRGAFSLECVMYLLALKVINPSKVTLLRGNHESPEVNGDMDVYGYS
SFKYQCLDKFGRERGIDVWVAVNEVFQFLPVIADIDKKIFCVHGGLPQYTGGEDKRLEIL
SDPSFPRVPVVQCTDPTNVAQRMMVNDLLWSDPAPPNHQLDKYGFGPNPRGPDIKTFGSR

AVDMFCERYNYQYIFRAHQEKADGLRLSDNARVVTIFSTSDYAGHQNGAGCILVANGKMR
MAIKKPQRQESKEVKGPVSPRTLGKSVPGFVPRLKRL*
>TCRU_2274 Transmembrane amino acid transporter protein, putative
MRKPSARRMTLHTACSMALCCIAYIVAGFFGYADFGDEVTDTVLVFYDVRRDVLMAIAYA
GIVFKLCVGFALCMQPARDCCYYIIGWDVATIPAWKNCLFCGVMALCALLLGLFIPVLNT
VFGLLGSFCGGTLGFCLPALYRMYCGNWSLGTVGIVNYVCTYLLLMAGVVAVVFGTGASL
YGVVR*
>TCRU_2275 Trans-sialidase of Trypanosoma hydrophobic C-terminal, putative
MAHDGTAVTPEVGGHYGENGAMVGGTDGQKGEDIHAQDGEVKAAALSSSLGNVSQGNNSD
AGTVCGSGLLPLLLLLGLWGLAAL*
>TCRU_2276 hypothetical protein
MADNATLVMALLFAFFVLLFFLLTANVYKRHLEDKKQRRRQERLRNCQRAEPFAPPVSLP
VVVDAVVVFPSHEEAQHRIEEDGAPITRVPSELPGDGLTTSHEYFASAEPVEEGNALPRA
RSGDVLQGVPVDRSLRLPDVNAASRRLNTLSGATYRLRPASAVYGSASYLPRSSSSGSGM
TAGDVPGVLQQSDAASCPETASPVHVCEAPPRAAAAVNAEGRTVPTTTARRPPVEEGQRT
*
>TCRU_2277 Cell cycle checkpoint protein RAD1-like, putative
MSVYCRVAQPHILLKALAALTIAKDSWVTVAFTEGVVVLHVEGKDQSITSTARLPKALFS
EYSAKDVRFAVHLVTLRDALLLGGPALLAPVVLARVVLAYPMGDARLLVELTDGDCTLQS
TLATRPVQERLLDLRFGETSVANQMVLLGDVARDAIEDLVAAQCPHAVVVLDPRSGVTLR
GEGGPYGAVTVQLQRSSEAILSALDEGTHAHTRVLRSHLALACGVRPGGKLWRASGAVAD
VAPGMQGTSGGAAQPTFGGFERLLFQVNEARQLSVVHMPRDHDVPVTVTVTVSPAHGLYD
E*
>TCRU_2278 hypothetical protein, conserved
MLQGNKASVHIGGHLLGEEEVQLAGERPPDVLRFCFGACGGHESHVTVTDVFLYNRPMNF
TAMRAPKDRVLVPTREAAPTPPEKNRVVDSVREHIAEDITEEK*
>TCRU_2279 hypothetical protein, conserved
MVMGGSSHPHFVSNAEGDGVSRGDARELQLTWTPTHSQLPAAKHSAAELISGGNCESAQC
GIEEHSNAAPDVIVFLPEIEEVTSLVRPTVRDACEQKAEVAAQHRLKYASFGSPSTSSSF
MHNGECFSFYEECLPIDTAVNDRICACEEQSILTTSIKAEDPGIISSQGAIARVGRNCGE
GLAKRCSPISHPLTRQETSGMMSGGTSSTNGTAIHQGTLVFAPRPDTEGSLFVAPSASCG
IAGPREDMFLFQAPDSRRESTLSPENLIAVATPRAIDPFCLDSSNVQNNLSLPLVVADDS
SDVKLNFTRSFSRTDNDPAVGRQVNTPNLGCELYRQSGALRSFGGADEASEDM*
>TCRU_2280 Putative methyltransferase/Methyltransferase small domain containing protein, putative
MHYEDFGVITAENGASSLSFELACATAMDHYIRCTAPHDKQETDKYMSPCSTGLRVYEGA
RVLAAFVARFGRVLLPYTFEYDTFRYPSSWVVELGCGCGLVGFTAATLFPELSVAFTDAS
VDCLNLIGASAERNGLTLIQINGKGFSGVFENNEHRVLVAYPLEWCEDDTVRLISTIRRF
SNSPNHVKTGDIRMVLGSDLLYYRVDIKALLTTCKCLLQSSEETASKDQCNTSLAPRLAI
LAHFMRIPDGDAKLQMVAYELGFGIVRVPLEAFVDADVIKNRGWNGISVVLLFLRSLPHT
DLHCLNIGSLCEQHEEEDLAEVKRIFSVCHISFPSEALVCYSKKALGNNADAFVDCEKGS
DLDNLPFFF*
>TCRU_2281 hypothetical protein, conserved
MQDTLAQRKAHEYYDRPSRTSSGSLECAVALHKTGCAEARCSKRGHIQFGLTRDLAVGEA
RRSVRPFPEYYSIFVEVHRTADPGVVAGRIDVKGELRKGRNPG*
>TCRU_2282 hypothetical protein, conserved
MRPTPLRGHSGLYTQRAQRNRKNILAAQTAVKKRQRGRPLLMKTDGGSPIHSTASQHFAQ
TSHRAADTLPDILNCPTQSSRRATELIRQRRSTVVRRITEIHASGRLYEESERGGLRNVS
AANVGRRMPLAGQFSTFCRTHEQPINEESRAAFLEAILDVAPSTRPRHTRMLRSMSEMNR
TLPDMMILGLQKIAARSETKQARPSTEEEMNQLIRIRADWRERAVF*
>TCRU_2283 hypothetical protein, conserved
MACVDGLVSEREYALAGSEQLLEANRAQLQERKCQLGLVNAKPQEREARVSAREAQQAAW
ETEMAGREEAPATWRGPADEPVTRQNTAEDGVTKKPHYTRRLMVGGAPSRTAQRNLTRER

RQVIPQTSRGNFW*
>TCRU_2284 hypothetical protein, conserved
MGVASAWAVRSAAEERSACRPSPSSSLKWRAVVPVGFPAAPAHQEELTFHSAWERVMPRT
LAMCVTTLARQPQCLPHTGQSAGRRIVAGWGPAGGGGVSVRGSLRACGAASEMHQQHVPG
MAGCRGRSSHPWLSRTPVRRRAGAASTSRVRAVSMRVGPSRLP*
>TCRU_2285 Protein kinase domain/Protein tyrosine kinase, putative
MGKEKRGEKHVRKQLEESSVAANHGGTVIPGENVDGRDALSWTQETQRIVVDTLVSLQSS
SNVDNTSSVMPATVRVPRRSEDKVASAAKTRHIDPLSASDHLFREKERHTNLFAFPSVLS
EGHTEKELLCHYPYHREGSLHQSYGGPKKRDSSVFMPPISNDPQTSIGSLSGITAPRIRD
RETGELHDTEEHCVVKRRNSKRENQKLTCMTHGPKPFCMKQSDLTLAFSDCAKKTADVAE
TNKKTAAQALNLRTTECCNDEASFECLGISLLPSQHENSSRASVPPRSPHLESINTLGYE
MSPIPREQEETGRSKLRKKHFYCCFKELPLSSVLIPYFLVLMCLSLFCLLTPYYVTTVII
RESVEEFHEHARLEINSQLEGALNVPTLLSSIFVALYVVDERKPRPNDDVIPMGFEMSQR
FCAALQNMDRYKLLTFIYMASLPRDEIAHCARGFHGHYFATAVRRKSDGALNALVAVDPE
NVTLVEPPLVLKKFSKPFILKDLIYNFTPTKKLIDSWKEDVIAGNEPRMQMWNVENFLSP
RYVYTYPFFEKDGTVSFFKVALDLRRVLGKIFAEESDPISLVRVALLENDGNDSNMTVIA
TNFNVKWQGESGEGEYEFVFSKGGNGRVRTIGKAGEYMVPSPKVGNIKDPLMREAFLHVN
MNEVMSIVRSGRNFFTTFPYDGSVGTFSVCLTQSELNATMVLAVVVPPFYGERFEILHVV
SLIGAFVSTIGATLLVYFVFSTLVSRPLHSIAAELRVTAKVDVTLARTSINTPRIRGIRF
ALLEIDKLQSVAASLKNQMRHLRSFLPEGVLPLRYATDEERKAKYMDGGIQSEARNADMR
DGVEGAQASSPNFMCGKGMDIARSLLQDPRENINVFEEIMCSVVSVHILDIKTILEHTGE
TANIVVEAARKYGGGVDVFLPELFNLNFPRRGDSHLDKLDATFCAIEIHRNLPDAVRRCC
AILVDSGVFHFGICGGPEKKEPVLWGRATSTELIHVQRRYGVSLAILQNTVPFIRSRVHV
LPFASVLVDSADMSSKVTFHIVIEDIVNEGSWQCVEETYLNGFRYLTAQACTEARMCFES
LVASPYVTPQLMRLLRCQIAQVQQCDVKKRSRFPSEWRVGGSDRVDTLTSKFSPASVDNS
ESIVSGLHPPSLTFCGSRRRLTPSALSTSASSTHVSSAGQAIAHEFWDKKNVRWKRASEN
FPESGRFPVFLGISDTGTLAALKFIPVSSLPDCGYNDMESLDLALTRAQTLYHENLVQLL
GYGQTKEHTCLIMEYVPGGTLRDTVRRYGRALPLMAIKRFLVGILRGLEYLHDRGLVHGN
LRPEDVMAAVEGLYKLRGVSMACTAEAAAAMTEERLRAESCLNRWAYSSPEVFTTGKKGA
AADIYAVGVILLELITNQTPWRWMEGTVTRPLQNISPAEDENIRQQNTKKTLVELTSILS
DYDRMKVAQMERKISLVEIPREGDALIQEVLKSCLVTDPEGRKTASELLQLLSSMQ*
>TCRU_2286 Tim10/DDP family zinc finger, putative
MQGQMMVMHAMEHYSMLDLANDVLEKCWNICFDVNLTRKELVEGDLPDSKLRKMEACQRK
CIARHFEVMKLMNGARELREKEALQGLPPGSLSAE*
>TCRU_2287 iron superoxide dismutase, putative
MLRRAVNISIARGRMALMSYATLPDLLKPSGAPAELPKLGFNWKDGCAPVFSPRQMELHY
TKHHKAYVDKLNALAGTTYDGKSIEEIILAVANDAEKKGLFNQAAQHFNHTFYFRCITPN
GKAMPKSFESAVTAQFGSVEQFKDAFVQAGVNNFGSGWTWLCVDPSNKNQLVIDNTSNAG
CPLTKGLRPVLAVDVWEHAYYKDFENRRPDYLKEIWSVIDWEFVAKMHVQAIK*
>TCRU_2288 50S ribosomal protein L2, putative
MWRHWAASIFAGGAVYRGCLRFLHDIRGGTVGSPSLVCAKITRKGTTADVHFFAPALSVA
RRGVKLSNPSSKQGSQHYGVEPKLEAGKDVQLKHTEMYDGYTDDFGVFKEGPPTTLRLEY
VRSPEHGYSQQLLQKDIWSPFQVNANMMIYTPFYYDWKGFASRDYWGHRSGTNPGSKVLL
GHYRRAEERSWGYRVMVNHKPQKRIPKWLACIVHLPRKKTFVGFFLYANGAYVAELLTYK
QLPRICYNKPFQGCMPTIGQTVALSEVTYGKELHSVEMYPGHGGVICRASGTAAVVLRGS
EPNLVPLLLPSKEVRLFDNQCHAVFGRRAGVMYRYQRNFGKRNVEENMPHRPKVHSKTKR
VSSHPAGGGNGGSANLLVPLDWRIHPRNCVKTKYWLSGYILRGRQYNKSQTVADIKSKTY
SWANRDPVYR*
>TCRU_2289 ADG2, putative
MPKKDRDVWDPPEDRKMVEEAPSAFMQHFQDRAVRVGKDGSNAQLVRVKKTKPIQWSDGN
NRNKEGRGSIPGPLGDETSMHKWKPMMLKLPLRNRSHVRGIVAPDEPKAIMPPRRLQAIN
HTICVESGNGLGRHTRPFFDAHVVNRMDAHDAPNPAMAISPDCRIENMRQTVAAATVMSP
RLELSDVTSISVDKSQSPEVLSVECVDVSQPVKPIPFSRPLSDTMGLVDAGEGLRKSLAR
LPPRKISGSSGADEATMMPRPKNTAVSPPLPLERNEKKKKKEDRNDVLSREDEVDDFSVQ

FEDDNSEFRACDSTDIPIYQPVGFLMRPRPGAVKGENKTSIIAAKKRNPSTGTRRPESEN
GACGTRQDYHLCTHRKKGSSTGIQEFATSAPVKTSGINSHPSRLQPLCLPLPPRTSTVSV
TSSGALTETKESEQEEEKKKNPAYKSYNLKDYKLMMEQVTHLKLGGLGPADTDEQRQAKE
KMHRQCQYGKLVEKKAVERIREKQQRRIIQKLKLGEGGDADVVLTGTPNKMPQQPPPPER
IQAKERRSRALDYARNLPRPELPASRKPRDENSISTLESCTPQWRSQQDEAAWRREQRLK
ELEARHRIDRERVAAVKRQLGY*
>TCRU_2290 hypothetical protein
MSAFSNSPVYKQILRPLRSNSMLGLCVMTGCMSVFVYWWYARSIRATLRQTQESCQITAD
NAFAQSRQLLKSLGDDWAQDMKSRDDSVRRLELQNVEQTRSIARLDAAMKMCIAPGPEIS
Q*
>TCRU_2291 calcium-translocating P-type ATPase
MALLSLPATPSTMDASAVTKSLRVDAKRGLSADEVEERRRQFGSNELPTKPSTPFWKLVL
AQFEDTLVRILLFAAMTSFVMALFEKNAGDFVEPFIILLILVLNATVGVWQENRAESAIE
ALKSFVPKTAVVLREGKLVTVGAEDLVPGDIVEVSVGNRVPADMRVLELHSTTLRADQAI
LNGESVEAIKEADAAIGQQERFPSSMVYSGTSIVYGKAQCVVVRTGAFTEIGSIERDVRE
QEEVKTPLQIKLDEFGMLLSKVIGYICLAVFVINMVRWYSVHTPTPDEPWYERFIAPAIH
CLKVAIALAVAAIPEGLPAVVTTCLALGTRRMARHNALVRDLPSVETLGRCTVICSDKTG
TLTTNMMSVMEIFMLGVDGNPREYELKDSRFNVMPNVVTCGGKPVTSALETDGALSMLTN
IAVLCNDASLHYNTKNGQVEKIGEATEAALLVMSEKLAHATDPAAVCAFRKLAEQKWKKN
TTLEFTRQRKSMSVHATSTAGAKLNSLFVKGAPEEVLRRSTHVMQVDGVVIPLNDALRSR
IIAKIDAMSGSEHALRCIGFAFKSTQPVRELKLSDPSTFEQIESDLTFVGACGMLDPPRA
EVREAIDNCRTAGIRVVVITGDRKETAEAICRKLGLLLKTETSGLSYTGAEFDAMNPAEK
RKAVMSAVLFSRTDPSHKMQLVKLLQEQKLICAMTGDGVNDAPALKKADIGIAMGSGTQV
AKAASKMVLADDNFATVVKAVGEGRAIFNNTKQFIRYLISSNIGEVACILLTGLCGLPEA
LSPVQLLWVNLVTDGLPATALGFNAPDEDIMQQPPRHVDEPIVNGWMFLRYMVIGVYVGL
ATIGGFLWWFLSHGFNWKDLTTYAACTDMQDAKCAILADPETARAIALSILVLVEMLNAL
NALSENASLITARPSSNIWLLLAIFSSLALHLMIMYVPFLAALFNIAPLGVDPLIVKEAQ
PFSVLVPSNFDDWKAVVVFSVPVIFIDELLKYITRHMQASRNKKNN*
>TCRU_2292 Ribosomal protein L9, N-terminal domain containing
protein, putative
MRRTGIRRVAPWVPPPRHDVKVTMPPPPGGEVGGSFGVSIGYSDRIACTPYWKRMALSNY
ALRMKENATRYPMSEHRPGEYDIRYLPKPYPCTIRNRPLLEVGEPRQIPSIQIPVIFLVN
LYNEEKNSWFGRKYETVYVSRQFMREELMPQRYAIYATPEAYALLGLPVVDHGIHKEIPK
TPREYEKLLERQKYDEERWKYSIEYLFRKYEAGPPELLDRAEDTWDGTEVLAVSSTAGAA
RHGETVQRKGAIKHRKARKIKLF*
>TCRU_family, putative AB.1
MRAELACIWTAAVTHDTPTLKQVAKRFGCEDYALFASCFLQHPYEAFSAETNLTTKATME
MMREQVRNRMHEVNNIVSGLPKEYALVLRNIMAVKAINRELQRPVNRPLRMLRYSAVVAR
RTSSRWQLGFMLLRAWWSEIISSLLMLYAEWRYPQLMQMLDDSLQVQISG*
>TCRU_2294 AmmeMemoRadiSam system protein B, putative
MVYSRRASHANSWYEGSAKVLKATVDDLFERAKDFQLGGDETLIGIISPHAGISYSGPTA
AHVYKALRNYIYGPKGSNVTHIFLLGPDHHKGFDGVEMSGAQQYETPFGALSIDTSVVSS
VFQSLKAAGVQTTRTSQSTDEEEHSIEMQLPFISHILYHPPAGLTGAKNRIQLVPMLVGW
TDRKTEERIGAVLSSYSQDSHNIFVLSSDFCHWGSRFQYKYHYQKSEYPAIADAIIAMDH
EGMEWLEKRDMNGWYDYLNKTHNTICGRRPISIGMEAVKNDRSTTVRFLHYSQSNRCASL
SDSSVSYASALITHR*
>TCRU_2295 hypothetical protein, conserved
MKSRQRRAPLSEGVAFSYELFGSVDIKNEEFLNFTGEKGPGHNSEITEFLRNKSFAHGRE
PVARQPSYLQFQQAVDVGNGTAEELGGWNNSFVSLEDAAQHVWFISQRSSGAMPFARNFT
TADRFLKYADEHSLFPTEPQEAEMEAPNEPLTAAKWLDIQTTNKALITELLTQFPVSEET
INNCHYLDGMDALSAHSALGYFFFNLMCTPIVPEEETGRSRQTKSVIQRSLEAAMEDKPR
SSVPAAVPVAVIVFPRWIITVHEKPFHELGDLLRFLHINCSDHGSSATPRRRRQVMTAPF
IFSSFFQIVVGHQLDTVSLTTALDRMGDYVFKVEEKAKEREEVIKRITNVRRSFAECAAE
LTRREHIVSILLQPHMMNSFLTQERVVCQQLESARAHLLRLSKDVGDSRDTVVVTNWYHN

ATRTWKIICRGNKATRQMLLLLEIMNIIYPVVAFQMLYTMNVPVPFDSGGDPPVTNLYAF
FVVMGIILLYLLLCTRAGYALFKRKRWSTRLLAE*
>TCRU_2296 heat shock 70 kDa protein, putative
MLLQALLVLSAVVVAVAAPDGTGKVEAPCVGIDLGTTYSVVGVWQKGDVHIIPNDMGNRI
TPSVVAFTETERLIGDGAKNQLPQNPHNTIYAIKRLIGRKYSDTTVQTDKKLLSYEVVAD
KDGKPKVQVEVGGKKKQFTPEEVSAMVLQKMKEIAETYLGEKVKNAVVTVPAYFNDAQRQ
STKDAGTIAGLNVVRIINEPTAAAIAYGLNKAGEKNILVFDLGGGTFDVSLLTIDEGFFE
VVATNGDTHLGGEDFDNNMMRYFVDMLKKKKNVDVSKDQKALARLRKACEAAKRQLSSHP
EARVEVDSLTEGFDFSEKITRAKFEELNMELFKGTLVPVQRVLEDAKLKKSDIHEIVLVG
GSTRVPKVQQLIRDFFGGKEPNRGINPDEAVAYGAAVQAAVLTGESEVGGRVVLVDVIPL
SLGIETVGGVMTKLIERNTQIPTKKSQVFSTYQDNQPGVLIQVYEGERQMTKDNRLLGKF
ELSGIPPAPRGVPQIEVTFDVDENSILQVSAVDKSSGKKEEITITNDKGRLSEEEIERMV
REAAEFEDEDRKVRERVDARNSLESVAYSLRNQVNDKEKLGGKLSADDKSAVEAAVKEAM
QFLDDNPNADKEEYDEARDKLQSVTNPIIQKVYQSGGGADGDERPEPMDDL*
>TCRU_2297 hypothetical protein, conserved
MESGQYTEQGIAPLWHTDLLTCFTTQLTNKWRSTLRQDTHRYLLCGTRPSDLSGKDLITQ
EVLHRQELVHLARSRCGESELWGRLYWAVRDCTNQCRFCNISPEQSAYMRSNNDPTAPGT
DTVPPSAREEDVSPVRRRTLTRRRKEKCPHCDSTLTGFSNLVSHCRSFHPEHPPPLPELK
CDFCDMVFPTRRSTAQHRSRCAHNPDATRHRNSSARRRSLLPQDQPASTSTPIGPQETLH
HLLLECPGTLAVRQRLGIEQDLRLGKFSQWQLLHSRKLLSLLDHLFGTQVALNSYTHL*
>TCRU_2298 hypothetical protein, conserved
MTMGASRTTKLLGMDLDPRLTLNVAASKQCAATSQRISQPRCIAHKEAGPSPHDLRTFVI
GYGASKLRYGSELIWAVATDSAKNEMQKTYATLARIVSGVPSTVEPESALLEANMPPLHV
LCLRARLSIFENTRACQMDWMRRPPPEPPPRAGFRISSLSRDELYAFVDAYTKDYGITES
SPREERFFRSSIPPWFAASAHRVTIGVELPIDLSITDEEELIREKRRVSEEALALHSHRS
WILATDGGVDVPKSAGVGILLSSLNSSEIIEKVSANGGTRPCSRTTESRALLLALETLMI
PRIRHRRKNPACGYGQSVSSSGSKQGPAQSDRLDGGSDLAASLNTDVCWLVRAPAVLLRT
LRGTC*
>TCRU_2299 hypothetical protein
MESLHAVVDGRSRWRCQLSAVSPAAAEDVKGNANTGKVVGEENLNTVEVDKKRNNEVSLP
RQSNELLVNSGQPATESQKKLPHSETDLVPPPKETSDQGQHSGEKRKEEEEAEENDGESE
HEEEDAEDTEEEEEEREEVDGGGEKEEKEEEGGEKDETDGVEEEGKEDTGEEDEDGDAEE
GEKANEREEDKKEEESEKNTKKEEDEEKDDDNTSEGMPAGGQEKRNST*
>TCRU_2300 Mucin-like glycoprotein, putative
MMTCRLLCALLVLALCCCSCVCTASVVAEHVVTSEPLKPPQGTSLGIKSPDLIILADQQD
VGADALSPPVKAVKEGKPRVSSNAENGEGATEQAGSEDVDTKSRGSTENSSKSVVKSSEQ
NPEVLRPNEEANLPTANTKTMTTTTTKAPATNTTTTTAPEAPSDALNSAEASTTTNTRAP
SRLREIDGSLSSSAWVCAPLLLAVSALACTAVG*
>TCRU_2301 poly(A) polymerase, putative
MSTVDKAFLAKKVISSDGPSPKEEEHNVSLVDWCVAGIEPEEEANRRRSVLEHINNIVRA
WIRSTMITEFRMPAEAATQVEGRIFATGSYRYNVHASGSDIDIVLIAPNRITREHFFNTL
APRLQHEPRITELHCIRDARVPLIAMVADGIDIDLSFGSIRQDRVPEVITDDLLQGLDDQ
SVLSCNAVRVAHNIMDLVPHKASFRQALRFIKAWGKARGIYSNTFGFPSGIGWAILVAFV
CQCHPNQNAAGIVVRFFRIYHSWFSPNPQESGRENRAIYLTETMRVKTNLGRGWDPRESK
SDAMALFPVLTPAVPYGNACFNVTLTNLRQLCTEFARGHEICSSSLVTSAEEAKSRFGPH
GIWSRLLEPVKFFGEHKYYLHVLVSCTDAEAYQGYVDAVESKIRFLWAGTAESRGYALEN
VRHVRIRLNPRRYEAPEEVETRARLLKKTSRSSGGGNGSMEVSSHQTSGAVDDSSAPKIF
SCHYFIGMSVDPKAPSGKIDLAPSIRAFHGIVRQLRQYKEGITRLPVITVEDISKIPAFV
KTAVGFKETVVVPDADHADDAASPAKAHDENGARSTVAGEKRTREEGEVKHDADAAIEST
VTEASSPSVANISGVIHGRTKSGAANNKAVDEDEPDLEEALGLGF*
>TCRU_2302 Myosin-like protein
MSADDNDSLWLQKSIHISDPIPETGEKAHLQNDGKQEEPKSASKKTADPEIELTRTPFSI
NSLRPMHCPSQRLSCRLSMANSAFASFQELDELRARVSQMTRERLKWSNVQRDLEIARFE
LTQAREELKTVRDYVKTMREELDGANSRADRETRERQTLETQLNDVMEKRRAETQFLKQK

LDDVEAEHTRRLAEMAERTELECNSRSETLRGDVAKLSEEIEELEARSIQLDRERMQFYK
ENEELRGQLKGIQEKYDDSRRAVEEYSRRCIELEKMHAEFVKETEKSHAEVLNSHMTLSD
ERVRQITESKDEMVKDLRDELQAQKEKSRALIDDLSSLRHRCQIAEEELKQQGLDHQKEI
KRLLDEHRLALCQQQLQTESLLREAKGGRLSMEEERSSLKRQLTRTSEELSTVTTILAQR
EKHISHLEKELATAKDTALRMEQENISLSKEAEAAQEALGHAEERVQRLMEQQDTSDADY
AKDLRELKDQLATTQEELVRVRGELSSTAQEKMRHETECRGVISGLRTKLEEARYDADAM
QQLTREKKHLDEIAEEYRGKYLSMCKKVENLDIELTAATNRCTLLEKRLDEELRRSLRNV
STSPTPQRQNGVEKRLRSASSVNVPLSLTTHPRKKSRSVEPRVFTISGFDASELLTEIKQ
LPHATVAECRSNTPVPSNLTHLITNGQLTIKLLTALVRGCWILPDVYVRDSVRDQRWLDE
SAYGFRHERPPLFQKRIGFTEGFISSRHYSTAKLITDEGGATVETELRVADIVLCTHAEH
GTLEECTHAMTWDKVVELIYPVKIGDTQ*
>TCRU_2303 HECT-domain (ubiquitin-transferase), putative
MCSKEILSACAPTFEDIALENRKLLELLGILPNNRAVLHSLMQMMSCMGELSNLAYNNET
SLSPFEQGEVRAVVGKMAVCLATFGEAFDINVGQAAMDTLADQFHRASNSTLGRSRAERK
KSFLSTLVAEDTAKEEGKSHAHARPNILLSPTSNRDFFGFLDELVTYSRSDFEKAELKWV
APLPDGTIQELVENGSAINVQYNDLPKYLECVQRYRNARTNEANRNKPEAETAPFVTSQR
VKSASVPILPDNSQHDKERPISSPGVMDGGLMSPGTLMFPSASDTSVAKADIGDFSSAEL
CSSDADFCAKVEGLKRGQVTGLQIAQLNLTFSVPRNGRLVELIPNGIHVKVTSANVAEFL
RLLGDKSVNQCGRIRRMESIKKIEVTGAGSQDLSREKDKFSPTHFSKDIFAPYEDRTSFL
VDFDASDEILPIFIKEREQQQRRAESQYVRVVCQGDLKAWRAIIDEVQNNPAALKKYGVT
FCVPAAVVVDATEEEQITKVHELMRGGISTPVEDSQLWLFSRLIEQFSHSFL*
>TCRU_2304 hypothetical protein, conserved
MRSCSTYASEDVFGATARGGCSGVPAGLCPPSRVPTPPRSMDLLDELFSSPPVSVPSGSH
LVSGVPVRDDAAAATCLPADDFAAAPVSRHSIGDSVGAGPNVCDVLSSAHSERLEEVVLV
LERIMNDDVPRWYEASVILQSLLCRSDSGSMPNRVASDGMGGDPQGSSVWNDGFVNTHRN
LVSIFPFLEFVQTFKHVSVFPFLQVPRLAFKLRQCVRDWVESHGGVRAVLSDSSLVLDLQ
TLLKNVNEVFVAVVATSPQSASEALLLSSLTDVCRYCIQLVCTCLPHGNHLFFATTMQSV
MHHNSSVMPHLEGELKAQEDILQLLVVARSTAYHRCSEDIQASVSPLVAELFSLLDTFSS
SSMVPLDKKKVPERRCKLSGLRLNSSDTSEVIDAFVKRYWNVAIGSPIPP*
>TCRU_2305 retrotransposon hot spot protein, RHS3, putative
MDAGSYFHYQLLHYDAGQLPMVAHFMVIGAYLLDMTARAVPEYMGESSTVKVVKGPSWRG
VRGHIVYDVAEPDNRLSTCLSLLEWGMIVVAPPERKICGHWASERRAARIVMSCPDESDA
EAMCVWT*
>TCRU_2306 hypothetical protein, conserved
MLRVTSLFRARAIIKRRTPQLWGAPGAPIIRMRGHHVVWKFQSYDLFVEHTHKRRNSDAR
LLHYLGKHCPHPQKSLWSPDTPVAQDRHLFMLTTVDVDAFKYWFGVKRCRLSMRPWALLA
KAGLLPPSLRQNSKIMPKPIFDKEQLMRYYLANRKEEATIEREDYLNYKNSLVKSEEERA
AERPVAPYL*
>TCRU_2307 Archaic translocase outer mitochondrial membrane 46 kDa subunit
MESPINPAVWKFLFHGTVAALSAASLFSLYHERHNILCSLDTPFTALRKAKTDEERAAAA
EVILNKLKEDAAQKKDSSRMSAMRHGGTLLRLATLSKDGSNVVGVSMAIKAIVRVFGADA
EGCRKLYMLGGYRTLLTTMSEAHRQGQQALMEEIAQALQTLTQVNDAEVVLETDVPPGSE
GAYALACIPATVKMLRLLDPQSPILFLDALTGIFANVCALRVGALAVGRGVEGRSGMSYF
LRLLEHSNQGVVEHCVLNIRYLARAGIGHEELAEEDNLQRLAEHLSVNGDPRVTNSILTI
VLLMCDSHHGAKFFTCLADKTDIISGLFEIWCRASEKMLRDRAEMLVQLLARVPQCTAAV
QRLLERYRVQIAERRAKDEEARRKQLQQMRQSQMMQQMMMENMGMGMGGGEGWW*
>TCRU_2308 oxidoreductase-like protein
MSRIVDSAEEELPSNGQEEGKPTSLAEREARWGFTLEELLVATKVVRTLFHNPSLFVGDN
YLHESRLYTMITRDRKTKRENREVYKMIMNAEKSRRKRFKRMQDIEAIRRTEMKREREEA
LNALMQRPTTPMLAIEGPSETKPTCERPELGEGDMLAETQREEDALDSFSAYDRQIIRLI
GAFENIIRLEETYDVNPTAPDWLKISQLVAQVYRYLPHAFGSQMPSALFRGAAPAMTPPA
QREKYTAVLKLRIAHLCLEWMKLAEVTKNNEEDDENNTSDGRNNTRIRNILSTQEDVFVA
DSTLLRKVSSLRKCGDVNSLSSDVLCAVQHIVPGTDILRSLEAVLAEGVADDRFVSHDGN

GASTFSAEASLNLFIARRLYASSRVREWGESPFKVNTCPPTSEDEEPDSYSVFDDARMYA
AGQTVPSDEVLQLNRWIACHTCRVRYNKLHPYYYSLCHLCGEYNFNKRLLTRDLRGKVVL
LTGCRIKIGFAMALSLLRCGARLVGTTRFVHEALARFQREADYATWGDRLHLFALDLRDL
WLVTQFCAFVRQHFPKIFAIINNAAQTIARTREYTAHVRHVEAFPPEELHRRLYEDTKAG
EWHRFFLSHTSVTIGEPLRIEHHPHEQPFLDDVKAVANGKDMVHEENVTGEKQQSIVATA
RTADTHSLIFDRYDTQTEESDRRETNSWMMRLAEVPGSEAAEIMAINALSPLIINGKLKP
CLLNREGDAAPSEERFIINVSAMEGQFYRYKQVTHPHTNMAKAALNMMTRTSGEDYAQDG
IFMNSVDTGWITDESPRLKKERRAEQLLLCPLDEIDAAARCLDLIYTNSREYGKFFKDFK
EIPW*
>TCRU_2309 glutamyl-tRNA synthetase, putative
MSASNANVESDKLILTNAEEGKVVTRFPPEASGFLHIGHAKAALINSMLATKFKGKLVLR
FDDTNPSKERGHFEQAILDDIATLGISWDIGPTFSSDYMDLMYEKAEELIGKGLAYCDKT
PREEMQKCRFDGVPTRYRDIEVEETKRLWNEMKKGSAEGQETCLRAKISVDNENKAMRDP
VIYRVNETPHARQGTKYKAYPTYDFACPIIDSIEGVTHALRTNEYHDRNDQYYWFCDALN
IRKPIVEDFSRLNMEYTVMSKRKLTQFVENGVVDGWDDPRFPTVRALVRRGLKMDALRKF
VQAQGMSKTVNFMEWTKLWFLNTQILDPSVPRYTVVSNSLKVRCTVEGQIHLQECEKLLH
KKVPDMGTKKYFKSDVIFLDTEDVALLKEGEEITLMDWGNAYIKNIRSSGELSLITDADI
VLHLEGDVKKTKYKLTWVPENPNAEVMELVEYDHLLTKKKPDLEEKIDDIIAPVTKFTQE
VYAEEAVQTLKKGDTIQIERRGYYIVDSVTPKKVLIYIPDGREKVNHLGAKAQYLKSLSK
DGISSAANDLAAKRAAKAAKKALQKQNSQK*
>TCRU_2310 pre-mRNA splicing factor ATP-dependent RNA helicase, putative
MPSKKPIRFEDLGLSTPLPRVLSTTREQKKKEKHEIKKEKNARLKVAEEQLSLVKATVEK
HKAFVKKEIALQKEKKAERRKAIFALQECIRQKREAEEAIQKQNSFIAHENYVSNLKIGR
AVDEKIQEEVQEAIDRETKELAKYAPKNVHILVCRNPEIEKTRKDLPVLREEQAIVEAIN
NCSRTCVLICGETGSGKTTQIPQFLWECGYGDPKGSVFGREGAILITEPRRVAAVSMAKR
VAEELNTSFGGMVCYHVRYDNNLSDNFKVKFATEGIVLKEIQSDFLLKKYSVVVVDEAHE
RSLAGDILIGLLSRILPLRNDLHLEELRKHNGIVSKTNIKPLKLVIMSATMRVEDFRENN
RLFSIPPPLLNVDARRFPVTNHFAKRTELRNYTDEAFRKICQIHRKLPPGGILVFLCTQQ
EIESLCKRLREHYAKTRIEYYEQSYSKHSLVVQRRNDNDERDFDSEKDKSASFDEKDEFG
LAVEDYALEETVEKKDLNFSRKRCRDTADEDEASFEEDEINGELNSLHILPLYALLNFSK
QQEVFREPPRGKRLCVIATNVAETSITIPNIRYVVDTGRVKTKTIDESTGASCFRIGWTS
QASAEQRAGRAGRVGPGHCYRLYSTAAYSNLMPKHGLPEILRTSLESVVLLMKYLGIDNV
GSFPFPSPPNEDELKRALTHLTLIGALDSQIERRITTLGKRLIAYPIPPRFSRIILEALD
RKLPDCILNMVVAIAAVYSTTTNVFTSDGNGLKWKARDLKKPDDERRPFISSLLNPGSDL
ITSLNAFGVYLNDSSSGNCYRYCLVQKSLSEARQLKQQLHVLVRQDAVVEQVGDTIDAND
VGAMDSGPHITNIFLPTLSKEQELLLRKLFIYGLKDQVARRATIQECRDHGVEYKDKKAT
KAPYILLGRSMISYIHPSSSIARTFPPPEYVTFAFLQRSVRSETKDPVTMMLGLTVVTKE
WLSECGVILE*
>TCRU_2311 hypothetical protein
MSTTNGNNAGWVNSSESLSLAPLKKDSPLAATLDKENLKTVSDTCGCVNTTTSPLAVVNS
GDNTVEELQRRLKCAELNNSILRGNIETLKAVVNQLAAQLDLANNRLALHEEKENVHALC
HCGREGKDTESIDDEASLTTADYERRIAVLYDTMLEKDATITALQYEMESREAEASALLR
MSLEKKDKEIIRLMGELDQLRRLHNMERYARVRK*
>TCRU_2312 hypothetical protein, conserved
MAPNQFVELVDTVEKYATRGSNECGCRIDTREESIPSNTLVGYPFANAEGVGVHLSYRKG
DITLSADVSSNPKATWRDVLPTFSWTRSLRGQQHEVLLRLAMTPLFAYSFSHPQFTCSSE
VNFMDGFRSKFTTSTQLSMRNQLGACVEYDPSRSGVIDYTVAFLRAGCSAVLGGDFVVQY
NATRGLGVHTRIPVKPFMGAVALVERGRVIIGTEARSPCGAQMMMNLDLVNSRVMLAVVR
NISTVWKFTFNYSAPLPGTSSAARRFGLVFTSQDAP*
>TCRU_2313 VHS domain containing protein, putative
MVVLSFLENVKDAASRLIPTPYMDIVVEATKPELPTPQYESVAFLCDSANSSGDAAEDVV
RAVRRRITDSDAKVQLLTVLVLGTLIKNCDNALHVEVASQKGLLRDLQNVATRTPCVTEK
ERMAKEECASCVCGCAIWFTGHPDGRLYILTTISDDVRHAIGPSAFEGIQHDTTQLNSSV

MRQQQHNPRFNRQSRQTGRQQHQQQQRQQGLVVDAVGINFPTEEDLAAMLDCCMTLAEYL
NNAKVQPDGSIKPDDVISGFRDKILADHKRVTVLLSSDLKLNNRDTLRDVFDSQSALIQR
LQSDVKLHPDQTPAPSQVTDVAPTERYLPQVTTPNPPAAAAASSGGQGVGMTPAMNPPMS
QVEPVAVPTALEAGTREAAGEAEGSQPCPAQEASKVMENLFVSAPVTESLAPAAETSSRE
YNDNVDLLLTKADMPPSEPIPSVPEKKEEVLMAGKEENQSKQEETQKQPDPHEKSPEEKS
KQPAGEDDEFDAFLEGRLSK*
>TCRU_2314 kinesin-like protein, putative
MEEQNRKPTKAKVFVRVRPFTEAEAQVCPEDSPVPREVVVWDKHRILTVLDAANGFSPRK
NGQFDIDNVLWSFRDEERPKMVLHTQKDVYSAVVAPMLPQIVDGYNAAFCIAGASGSGRI
YSLYGDDVDGVNRGVLPRFAENIFHVFKREKREDSSLTCEMEAIDVLPNETYMDLLAQRR
KSPANAAEEPKLTNDATEGMKLQGATRISVGKAADALSVLRQLARVVPKRSSCHTVQFRF
VETFDFADPEQQDQVISKSRRINVLFVLLRNMPAAFQRCIDVAVEHDSGENPLAKVPTRE
TAFTKLFPELLQQGYNLSFLCCVSPFYEHTRETVQTLTLATKFMKLKCKPRLIQDEALME
LRNLSDEVKNLRTEVVKQTESAQIVQHELNARELELMRQEAAHHEAEVKLRRTERELALA
KHALAINQFRTRGHRKQFAKERVSKEKLLGATKAEKDETDKSLEAAKKQNEQLESGKNEW
EKKLRAQEEKNAAFQARLLADEEKEKRIGKMRKFLAASPEERSRILEEEAREQARNDVNA
QKAEKELKFLQGAQEKADGRCRVLEKDYAAVYAAEKGTREKAELQNNIAQLKKEIAEAEA
AIARMKAELDKRPPGCNCVLM*
>TCRU_2315 Pre-mRNA-splicing factor CWC22, putative
MTRYVPPHRKREVSDVDDGKSTGNDGGKTTSEALQRESWQALSRSITGIINRVSGENVQL
SATELFRENIIRGRGLLCRSLMRAQLADPDLSDVFASLVSRVNKEFSIIGLLLVKRLVVQ
WWRLHRRGDWTALRCVSRFLAWLYIFNVVSVDVIYQIILAHLTAEKRCDDDVDQAAALFR
DTFRAMSQRDVAEFHAHVLTPIRDLLAMDDDALRLSPRAQTLLETCLGEVQKWERVKHTV
SIIPPQLLLVEACEQKCHEVDLDDAAKMNAEEQLDRFVFDADYDAHEDAYETARCAVLGD
DWETELLEQVAAAEEEEEEEEEGEGTIAREEEARGQERSAQEKQSSMDAAKQVVDERERQ
IRKDVYMAMRSSVRADEVVHKILKSMPSQTERTVCFMVIEGCCEESIYKRIYGMVAERLC
KSNTKFQLFFAEAFRTRYEHAEDLVEKQIEYTCKIYSHLLRTDSLSWHRCLSVLDIIESD
MSQRLMIQWLLQGMVEALGMRAVQERLEKDKELRSSTVKLFPVDANEEGLERAVNLFEAM
GLGELGTGVRAQLEKVRRSRRMTLNTNSAPNYNDRFSGGVANDQKGHKRSRKNMMEYI*
>TCRU_2316 vacuolar sorting-associated protein-like, putative
MEVAFTVSPDERQHVEYLADLFAVIVAIEKVEKAMLRDLITQEQYDTTIRRLLEKYKSSV
SYLEQGRNPYYTTIDSFWENYGSKCPAARARIQLGLANDHQQKDGHTNTTLVLECGQYFI
TLMDSLKLQQTAVDQLYTLLTDLVQGLRRLGASDQEFHQRLVAWKEKLDTMKASDELNER
DTREFAFVLECGYQAFYAYLSDTSRTSSGSMKY*
>TCRU_2317 SWI/SNF-related helicase, putative
MGHRRKRQREEEESVVVTATHAPNEPAAVVPAPAQQQQQESLLTLSKSCRRRTAPVTSSF
LFAPTMFLPQWQRVALSTLRQLHESQAEEHRAATRGRTLQKDLTRYHEDLHRREERLRAQ
QREARARLGNKLFTAIEKQWSRRGDVFQQLLCVEFDDVKQRHTERRQEDLLREAENLTRS
LLDGIFSGTKNHGSHSDGDDSRGIDADEAVADALRNAEMTGGASGPVLESSLSLLDTQNG
RRPLRDYQRSALRWMTNLYTKKLNGILADEMGLGKTIQTIALLAYFAEYKNDWGPHLIVV
PTTVVLNWKAEFQRWCPGMKVLVYIGTPKERHRLRKGWMGEDALHVCITSYNLLVKDRCV
FRRRPWGFLVLDEAHQVKNFMSKKWQSLFDLQAEYRLLLTGTPLQNSIMELWSLFHFLLP
FASAFSSNEEFREWFSNPMEDMVTGRTFFNEEIVRRLQSLLRPFMLRRLKKDVEAQLPSK
TEKVVMCRLSRRQRLLYDDYMQLTETRERIRGGAGGVLGVLLALRKVCNHPDMFEERRTL
SPVALDCLSEITLLVPRDILLQDNDFRGCYRFQKWRLCIEDVSMSVKYDKEMVFDDTWLD
CLGLRLLWNDQAFECPLSRYSPENFRWPENVPAEQVFGADIKDSMSDTMWSMLCQQVEER
QAWVRQRHQTTATLQAQRCRGIETSMHVWYPRSLEIVSHGRPGFLPSWLHPNVSQRVCAI
LPIAEKTAVYVPRVAAVHPPRLHCRLPVVHYRAFCSHTRESLAPLLALGVSSSNGHARRG
VFDASSFLSELWPLHVRRCFSFPDKRLLIHDCGKLQFLQHALKQLRREGHRMLIFTQFVH
MLNILERFLAIIGIPYLRIDGSTQAERRQAFVDRFNEDDRITCMILSTRSGGIGLNLTGA
DTVIFYDSDWNPTMDLQAQDRCHRIGQTKPVTIYRLISEHTVEENILQRARERKKLNNVV
IRGGQFHAMASVDETYEDSAAALAALSDPVRLRSFFHDLDEDATIVADEQQRGEAHASKT
DMGEPVDIREEMMRLEDQEDREAQQNVEEELRELEEQRRNDEAELGDEEEEDEEYEDDDV
EEEGGGRSRRRKRKRELTGAELEEGLMDVSADDNALDAKNADMSRGVLPMVIQRRRDILR

RQRTPLDRLLSVRFGTCHAADARKRYDALCENYATRVGEGELPAFKTKFRL*
>TCRU_2318 hypothetical protein, conserved
MTAHTVLRALVRRAAAYERPFNSQDSLLIVRALQALRYRSDDVRGVCEKCAFAIASSRIT
VPVERAIEALSGLLSLQAGALPVVHEKLAERLTEVTPQTASPRYVQKAAVVIMKLGRRYC
KPAIADHVFDVLTQLLPSHPNDVALRLAVISVFMYTSSALKYEVMCCKGTARNEDLKEEK
DKRVLTLLEAATHIVDAHLLGALRGNSTLQAFHIFPVLSWRSLIHDTSKRNGQACIDGMN
SPADHDYDRMLTKWERVENAFSEELQFSTVVPGGYAQFMFDFFPTLGRNNTSGVAFNLAL
LDVVCHENFRKALNSYDVAKIAMSVSHALLNRRSSAGPRTGRTLTGVERAKDEEGGGNEN
DPEKLEERTLLRVVGSLVQQLCFFDAVAGWSRPDGGRVDQVAVGAVWRVTQKFVCNFQPG
AGEVQQTVAQLVRLTKGIASKLRDVWSWVLYSMSLCARTTVYNDGLRDGMEILVQESSLA
RRPEELPLRSCVQALYAVSILMVPSTEEKELMLRATQASMRVQERVQVRRAQWTPEHPLV
VSMLQRLLNFRFREYSGDDIPMLLQSLSNLLEVTTAQRAQPLPAIQQMMERVFYYMQGNR
NLDWYRMHGVSTTYTLLSMAEFFPVDCKQLRDAVKQFFFYLESEEMIELSVLRRWLELLT
LQKRRGVNTTWLCEEILKLLAVQVHCACTGRKEPFHFLMNVLESAHEIGASIAFVNSSEE
VSNTRRVTIHLDLEPFLHDGMLKAVDAAQVLRIMSYEVQSAGLVRVHGDHIVQLRRIAIG
DEPAAVMRVETLLHLLRANATLQASKALFDTRTLEQIMVSLCHQLTENRKALIQRGDAGK
IVYALLSSQILLRLGCSLRSYKGLAEAVGEVLDTLEATGEERPETLFQLRLLCGQ*
>TCRU_2319 hypothetical protein, conserved
MPSLRPNSCFLHAPVNAVTVGTRWICLAVGNRVLVGSVDGESNSDFKLVAELPRGAVITA
LEQHVVDDANCKAGVILVIGSGDSVFVLNHLLLGWRAGDHHLTQLVLEDMARVLRIRSLG
PGAGVLVITAISSAYILCEESLSWVRASEEAPLLVRYAATGLGVALAADAVLERDGDGMT
VFTGTYAGSIAVWRLPVAAAQKQNNEEETEATRGALIPFSWINAHRQGCAVFAVKAITLR
DRMEADQMRFCVATCSDDRTATLYISDDINCLANMMEELKWTCCWRGAGASFSRRRVFDV
SMYFTAENNGTLFFATGSEDGSVHAFVFNFQELLNAASTKNLKEITKKNAFLATTVGRRL
LHRGHQHDGRGVYKVALLRNPTFNTIAVVSCGFDGAVYYTPLSVHASRRGEIVSQKEKPK
RHVRGVVCDDVGSLLACTEDELIIFPLDPKGKERRLSIPITDAKKDIPSCIKVAAGSIWR
GRQQYFLALIGTTGGNLYGMWYNLTGTQPKSQEEVEEKELVNIFVLLHASSSRASKVLLV
QGMHWQERVLIAGIHADGNLILLEAHTLDERASEVRVTRRCAGVHTTALSICIFASPMED
DEKEHRTPSPKKQKRRRQQQQQQQEHQRTQPRYLGIFVGDDEGGLSIDLVRINDYAYTAT
TLLFREPLFRESIASVIRETRHINSPCERITVLSTQGEWRVVLVNVEGQCVDIVPVSSPL
RLPWRISSVLACSETVAVTMFGTDISVYRRRLSRCSWHLVEEYHGVRAPRLLSATIPADI
SGAGGLSVFVCHCSDGMQAEWFACDSLSETRILSGGAIIGREYNTALFLPSPAFAVICGS
ENSMLTVLLTPHSPRMGMDAPLTITGPHDSNILAMAMCVTNGDGSMMRFVTVGGLATLVL
WEWSTYHNWRVVAHVSQQHESTSAFPAANDRKKEGRGVPRFLSVCTTFEEIVVGGSDGIL
RVFSSNDSLRLLCEARFPTSTPKPMMAVDTLNARNRLVVVGDTSGTVGVCNVANGSFTTH
FTWAKSAVNAIAVGRRIGFQSQKQEEQQQRYGQKREEGEKGKGVEDTFAVWRVLAAMDSG
DVVLLHVGVLAIGVICVVRVGLTSACGVSWITGSPNDDTDDDAAVAVNGECLTYLQTCGV
QSAAGGTEMLRVAWHTRVNVRGVSGLAVVSPTRRSACAVVVGEGVEAIPLYADRDNT*
>TCRU_2320 UDP-Gal or UDP-GlcNAc-dependent glycosyltransferase,
putative
MVGLVLEKEVKYKPMLYVKVLGCHFHDARNGTGHSQVVPTSMCVHHVREDDYAALMARFG
NDTSPVARVERYSEEVIYPSCD*
>TCRU_2321 RNA editing endoribonuclease
MRACVITRPAAVARARGRIKCNYADIFSWGSSPPEQDGGVSKGEMNRMKSRFYNSGQRRC
TLCDEYLEASFSTHCCHVGHIARVGILERATAILQKETAEVMTSSAREPASNVAQMVRVL
LETWWKRLNEVEQETLLDYKRIASLSSPSARRRLWRVRFLLEFLRDREVIRDCLTLAKGT
PSNEGSFVRTKRFERSEMIGDNIVKVVVPDRLVRLFPPTEGGVTYKLACIQQLLDSNEGL
LQIYDYIDLNSIIGTRLPNNKTKSDVVESLFGELQTFLWASEVACGLNQYPAIPNAEHRY
VRALVEHLLNELTHMVIMWRIESTLENAKEYLEEQIQLKLQSSQQCALKGGAGGCTVVKE
VECDRGRYAVLPLLLSFPYPSASAESRQHRQRPSTPPPVRRVGENGTPQMLFTTSIPKPE
LMVSHYRPRLRVVRSSQEYRLEAVKALTMQRNLFTNILPASTSTLLGSRVSAKGEKQQLG
HLSFPGLAEGKYPTWAREVRIAEVRRSVEEQMARRGVYVETIDWGSMLAALARTLTGFHA
IRAPRLQSEPTKAPTKPLSPPTVAAAAEVFPPVEGKLKIVEEMCIPPLKPTVE*
>TCRU_2322 40S ribosomal protein S18, putative

MSLTLQSEQFQHIVRLLNTNVEGKRKVPFALRMVKGVGIRFAYMVCKKAGIDVERRAGTL
TAEELERISEVIADPAKFKIPDWFLNRQRDPKTGKTEHLTSAMVDTRLREDLERLRKNRV
HRGVRHAYGLRVRGQHTCTSGRHGKTVGVSRTK*
>TCRU_2323 hypothetical protein
MMRPTLQPIAGASGMTAAPPPAAGAVGLGTRTRQTNFNQMVLAALSASRLPQRPTTPPAS
TEIDGTGKALTECQDTSCLPTSLLVKQPEELTTTSFTSNDKGTTTIADSDSSTAVSHTTS
HLLLLIVTYAAAAAVVSRPV*
>TCRU_2324 hypothetical protein, conserved
MRQLIHVTSRSAVLLLAMVFVMCCAGGCFAASLTYSCSSDKAMRADRAKSIPAVVRDVPF
GSEGASQTCSVIGRSGWAPTRIDVSTRDLDGTRKYCSSERYYAMNSLVAIASVKVTFFYA
EKREVFVSLYFAATVR*
>TCRU_2325 hypothetical protein, conserved
MGTTKASSLEKVPRSVGCVAARRFACSPTDRTAQSFHCGHSVGITRCGGVRKSRSPDEDG
LVVFWWLRTSGPSSFRIALLLDWRSTIRRLGPAAVICDLNPHHELRDGRSPSKTAGEDLA
VIPSNMDAELSDDPAQATWTSGRSVSFPDVTAQRVLRVSHWTSTCLHGLGPPSAIPHRGD
GGRHPATGRHAATTEACLRCPA*
>TCRU_2326 hypothetical protein
MRIVRLEQDDYLILFRGLMLASAHFSDLREATEPVKAETDSGWNDDDFDEDEMQFSNPCG
ANLAASMSVGRVQMEEADDDEWGDW*
>TCRU_2327 heat shock protein Hsp20, putative
MSAESLPSDESEAHQQPQPQKKSTDIKKNPANSYYYWHGHEKERAKVGDVAPMPTPQLVK
RSEACEAPAIAATTSLVTKYSWCDAENAVTVYVDTKVEGQESLADGTLQVEFKKRTLSLS
YVANVVSSKATPLQRKKQLLLHLSKSINVDKSEYKVKEPSGQVVLRLVKEKPSTWWELVK
KNAGSGDDSEEDSEAAEEGGVSQNDAPA*
>TCRU_2328 hypothetical protein, conserved
MAAPSLTGSECDNTNDGKKSDEYYGDDLHRGADYLRVFVHESQEGLNAVPTIPGLAELLA
FALSPAVISAPCVEADPFTVEFTQQLSSGCAAGPALALSHVSQRCLACDLMPVFSKPLSP
VFFWCPIRNTLRCGGYLFAWPSTHVTMLNGCVFCLAGTRETSVCADSTAKVFVDGWRRK*
>TCRU_2329 hypothetical protein, conserved
MRTGGRANASSVEECVRQVMESLHAVVDGRSRWRRQLSAVAVAAAAEGGGKENPNTVEDE
KTSETLATSGKASTGKTTAVASEQTSVNPAPAIVPPARETLVQGKHNETNAKEEKEEEGG
EKDDSEEEEENHKEDDEQEDAEEGEKAEEREEDKKGEESEKNTKEEEAKYGVEEDEEDKE
KDDDNTSEGMPAVSKEKRNSTLGPEEAPNKTNTEGTQTPGDIDGSTAVSHTTSPLFLLLV
ACAAAAAVVAA*
>TCRU_2330 Ring finger domain containing protein, putative
MYFCYVCQQFRNQHALEDVATNCEVCQSPIIEIVRDQRHYNELQAIVGRQQEQQQENQPG
NFLAEILHPLWPRLAENLGEHIVIQFTIDEGPETSPRPCVSVDDLIGSFVTLPNGLQESD
RLEYPEAFVSSSCPICLDSIVGSGVPVVILPCRHCFHKNCIREWLREHPECPLCRAFVVP
SQDTVASQ*
>TCRU_domain containing protein, putative RF.1
MYRYRRFVCGLAGLVHRTAGRRLVTGGGGKPNDKASGSEGSNDGDAYGSSSDIPPGIDAQ
KLRAMQEMIRSQPLEKQVEMMKKALEFQKALGRIPGVGKLAQKNIEVMEHLVKTVENQHR
HMQQNNPQDAPKPPEFSDTKSGLFTGKNNTFSGLFQAVNAAAANAVAAGGSGGGRESRQG
SSGPTIDELKKVNLGPEIEELFAELASMRAKKNSYREKLHAKEEECEKLQKELLQMRETD
ASLRTKLRKVEQDVVLLNSENMELKEHEKEWRQVRKKNELLTDTVQKLQKSDAAQDRHRS
DMLQTQLKEREENLRSLQRKLERLRRRDPLLKFSRLCSDVVRMCDTERDSAKELSDGAFA
SLQSCYEREQDAAWKTAAMQEGAGANVYVAVVRRLFLSRLPHVNYDALVNVEGDVAVLKS
FVEESGYELQHVTAERYVIAAPAAATSSPTFIGPYGVGVALFLSGKMDESTYRLPFKLTA
VCPNVSTTLFSNAHRATVHYDTARSSGPGGQAANVTETQIIAKLLIDGELAYVAEAQDSR
SALSNREAAMERLSKVRRQQYNEQLAKQHRAERVERQLVQMIAEHLKMNSDTSAEVMTTD
GIVDMVRTAVEKGYVSRADLYLVYGLQYLSRAATSASVST*
>TCRU_2332 hypothetical protein, conserved
MRRCFVKRHGRLKFPLGNGFLTVGHELHPLVPSNELYYDPPKLRHRLDKDGYLYFKNIVP
RIVLNEAMDDLGNQLLQCGWTTVEDRAEQVSRDGVALGVPFPSTGQLPPPQIKYTESLRS

AVSGTGVMALVRQVFGGAVNVTDVQSLHLAAPKETFGLRMSSVYLNKGTKLALVALVPLH
DTPFQMGTPVVVKGSNSTESYAALRRTYGQHEVESGDIRGDGCYTHDPQELLPLGKQGGI
DETTGRSITIDVNPFVSTAFELGDVLLLTVYTMYAFLTNTTNCWRIMVEAVWTMEGDDVG
PDPRYVGADAPGLSTWYADRDDPSKYPRTMNEAKQAWGLLPGVPSEA*
>TCRU_2333 dual specificity protein phosphatase, putative
MRVAGRGAVPASELTAGTRMVIRDIEVENFKSYAGKHCIGPFHKTFTAVIGPNGSGKSNV
IDAMLFVFGRNAKKIRLERLSELIHSSAAHPNQAYASVTVHFVRLAETPENEGDPEYRQE
VPESLLSIKREVYKSGSSQYFIDGVKTTQREVVEKLIAEGVDLEHNRFLILQGEVEQIAL
MKPKSEREGEEGLLEYLDDLIGTNNFAERISQASKAAEAAQQTRLEALDRERKLRAEREA
LDDAKDSAIAFVTKDNHLQKTLIVMCQLRMQTLEEKLAEPRRLLTEIDERIASMKKTVET
HSKEKAKLDEDLLKRKKELAEATKERDTVRRKKETADAEVERIKSGADEQTKARKKAQEQ
LKKAKEELQKAELQQQDADREVIIHQQNFEESTQRVEKLQKEYDSTTEELAHVFVPLRQE
LEKTKAAFAPYEKAVVEATEQLDTARSRLASLDNGRLMTQEQLRKIDAATERNRNRVAEI
EELLQDANKNRYKEELQRLQEMLAGAAERKHSVNNAIQEIKNAYREGEADDRAMEFLLAQ
RSLKGYYGTLRQLGRIADEYDIAAGVASNAWGFHVVEDRETAVAALELLRKNNIGRATMM
VLKEIEREVGDRMNAPFTSPSPKATRLFDHIVPTNARFRVAFYQAVRDTLLVRDLGEARE
VAFGSTQRARVVTVRGELAEPGGSITGGGNTPRGAKLKAARSPQDKEAARVALQRLQGEL
VEAVEEERAAQEQLHKMRQTQRHLSSEQISKLRVELGTLRATLESDAKKRTALLQEMEEN
SSGSQSRREAILEEVDEAEKSLQAANKSRLKHQSDLERIEQEMDAVGGAGYKALCAELKL
QQERREAEENAIRECRRLSHRLHATQQRKQSDIAELEEELKRINEEAAQGASGALAAAIA
TSEEWTRRYNGANAKFAKAQSDVEDVKTAVPVAHKALIEAQKLLDDEERFRQVETAKMAD
ALQELAKFEQKIEGCEEKMRENAELYGIETLQLDVNSGTDGDESIDEEEEKEVENEEYEE
EERTKRSKKNKNRNNNRRAEEDEEELRRRPGKQQSNCIDLRNMSFRLSPEELARCDYEHS
VRLAKSLSEETKRLHSEIDFRAVALWHERDAEHRKGKSHYLSCKEASDAAERGLLELKEE
RRGRFMECFVRVQERLREVYQLLTHGGDADLELVDANDPFEGIHFVVRPPKKSWKQISNL
SGGEKTLSSLALIFALHDIKPTPIYVMDEIDAALDFRNVSIVANYLLRQASGAQFIIISL
RNNMFEMAHQLVGVCKVKDVTRTLVLMPRGFQRMVEAELLRLQRKRQATDMVTFSNGDGE
ICRDEGGDEGNDEAEGGNDARNHTAEAMNNGKRVKKKRRAS*
>TCRU_domain containing protein, putative P.1
MSAKEGAAAKWDECVSPATEACRHFSRPPPYPTPSSAWINRLEEGNVHETAAGRHAVEGM
REAPVLGYRAKRRDPVYPENSQNCTANALVELECILMYVETVTKSLAQRSRVPIPSESVR
VISTSVVTSRRLVNAEQHDGAPQYSLAMGLHDSANSLLGCLAKLEYMSLFQKNELDDIER
KVNTTAGDVNIALDAMVLRYTTHELSDVKEFFLGNQPTIEECRRMVHGVSQTAHQILLLR
RAVVGRDLALAKKLFSEHQTEYQKKGGVTRLHSPFFTWMGKRWFPAMEAWRRIVAQAHEQ
AETSDVLSAIAKGHQETFSQVDLISDILLPGETLSDLLRKELDEPFSHLAESLKKTRHHV
EALRVAVAKPQVGKLTECVCAVEELVAAGRVRSVADRELLKEGKRLIIRLQQVENVEAAL
RLAAQVGETVSLFRTINHANEIILQLGIAGDAAATKEIVARCSALLEEMKSSKGIPPAEL
IARRVDAASITVAHVPSQIWSAKTTMAYQAACQSLAANTAQDLARINLERVLKGGEDCQL
NTTGSTFDDASNISLNSHSMRVAAAKGPFPKFKGARVADLRTALKRANELGLNGQLIQDG
EAVLRSVEALKLKVHFDAQLRVVLIADPENATFDEIYKKVSELCSRQEGGGASNSAGRKL
RLRYQDSDGDCISLLTQEDWDVFVGEEAPNGVCGAKLELYCDYMQLPHAHVTDTLIETQP
AVEEALSSPEKVAVESHVQTCIKKTNPRSARATPQRRNVANNGTRFFRSRSAAVGLRHSE
FLFRYRTPQRTNFHESRVQKPHKLLDAVAEREDAVNVASVGESSQTTGQERGGAPAKDAL
PGTKPGLSGRGRVNSLRLNSSLSSSTNKPKLLIPIYQERKSRTLTKAAVTASSAAGNCGG
GGDATASTYATTSTNQITPVSTPTPTATSSVVTMPTNTNDHKNNEATAAVNTTPSASPPI
ELETVKKWNDDDAPLELRTVVSAASTSKRAVISVKGGENSVKQLAKKRLGGGFTVTPCSR
GIGNSSKAVGTDGVPNSNTPNTASITVLEEKREWSAEMFMRSDICTVVSEQTNQPPSVTN
CVGRQRPTNVTSLINTTRVDPAIVPPSNASSVKKKAEVLQQLRHLREENQKALKAALNRR
RTGK*
>TCRU_2335 hypothetical protein, conserved
MAVTHWVHGGVVPPPLPMAKRVLWLVLMMSLYVDAASASETLKGVAPFRCMRPFESLPRF
SPANMDSGENCRLGPFRVINAKAMTQCVRDDDDTARAASPTCSVTLRVSFQTEGDKRRHR
VGGAALAGSDEAFGSEAYRFSLRLRAFNDSIVEYKGFDQNGYSMCCHFIEGSECSWEVKE
EEGNSTQQEAATPQKDDDGRASHAPRRREAMVVSCPIRNPVVAGIVFHGAITKPLHRLVI

TDWEARLELWRGRPHEKVFLGRVLVPFRLTEDDIASAQSNAAATTTTTAMTATDSSASGK
MAGGVLLVTEDVVKERNGYEDL*
>TCRU_2336 hypothetical protein, conserved
MGTRRAVPFPSALLRQGARKLTEDSSRRRVIAEGSGNNRGRFFCKNRRIDNTLLVVEEKT
TGLRRRWIAWPRDRNRHDAYEADAPLLHISHYLPPVMAEAASCLDLKASFLQVPLPRETR
HLFRCRVEDGTLVELTRLPMGYKAGPETLQIIITSAIAGMTTVVHRLWAAPPLVRVDVWI
GNIRIAGSIGDVTRWEAQVLRNAVGRRATIGEDRESGAAQYTFLGVQFDHTHRAVSLSEK
FVRSLRAMPALNSLTIAEMEVTASRFSYVAAILGTCLCVYCFLIKAVQRRLSALDRGIVQ
ETSPTKIPPAAVGLGERLRHIIEDTTVSESSSPRKRRRPPSSGTHRSMDGEPSSFQIQAM
LKLPEESGRGSLFASCRPRRTVRLALLAFSAILPRTIDVWMSNTSPQGAANKGSSKPHAT
TRELKRIYGFLDPRGIKAFFAYVRSAENPADGISRGRVFTLEELAKGWNLRRGAAGSCDW
RSSNSATP*
>TCRU_2337 hypothetical protein, conserved
MPFAVFPSHQCEQRRHLGQHCGVPPQISNSILGKVERVNVHRQQREHVWVKIACLHDWSC
VPQRTLFCGVRRGIPRSQRSLHVSSRKICKLSLRRSVLRNVIVSNASCPRILTNRKARRE
SALAVLDATDQSKIMLQREIVGS*
>TCRU_2338 hypothetical protein, conserved
MTLVPYTPSEWCDIGARLWDMVSQCGETQVQMTFINGNQMSDETLTVAASPTGLCLLHGP
ISKAWQDSTLIGHIANPRAPQIPNFETQALLPATGAARELGHQQGLITAERERLNIDYAN
AVADVQNRNAQLNSEFEEAHYNLSVRESTIAGAGNQVLELQQQLEADRAQLREREFNLGV
LTGQFQEREARHNAQQPARDAETAGRDESPANRHVANDGVTTLPHPTDILMAGGVPSSAA
RRSSAAYRPPHGNAARPHFHPRRCPPALLPWTPDYPWKRTAVSAHSTSPRGPRVYGMRAP
PESLSTSGAHTNAGNRRTSRLNLPLTIWSSGSARWKACRIPPIES*
>TCRU_2339 Scd6-like Sm domain containing protein, putative
MSAERAIGSTITLITNSLIRYEGTLGQIDGPNNTVSLTNVRVFGTEGRGQEAGLAQIPPA
DQLFDQIVFRGSDIKELTVFEEPHNAMMDPAVVTALPARNNSGKTVSVNQRNGPSSGNTS
AQYHQTHHQHQHQHQHQHQQQQRYGGGGYRRGGGSGYRGSRRVDGHTGQDFRPATGAAKE
EFKDDFDFSKSREEFEKKKSEFEKAKEDAKVHSKAYDKSSFFDKISCDQQDRALRMDREG
VKRADAETFGSEMVGNMRGPRRGRGGRGRYNGRYN*
>TCRU_2340 hypothetical protein, conserved
MPLLNAGKNARTELSGASNLANDGAPPDYSGPLSQNFSDNPTAVVGKDTRPSNSSKSTSP
LFTATPTTVSPILEAPTKEEGGKKDCRSLRTRARHAERRKKDGLITATSLAGAGRRAAVW
VEQWDRGSRATRLLILEAFISLHSSSNTNRMETDLGDSSMLFFTRITAWLRLTCKLGHPL
RLLLAAISLFVRGVRYLTCLVEVGGAQTLIDTLATCRLVPDDRREIALLLLYMANAGRVY
REMLCDDDDIDLLLHAMRCESDLEILDLHAALFLVLGEGNSPRVTSRVQSGVIGMILHDE
TTKGAMLQAARVLRIFQSSRDRMYADAIARDLLEDTGTVSVVGIESNFSTESARQLLEAL
FFLLSHEDLSLRVEGLELLTMVAKNIQLTGHILTRCFDVLDEDRLAIEPKDNLSAVMVFR
RNQISFGSAAVNIMLLDTKCEARQRLTFDIIARRSAHFSLLKYLRLLESGYTTSILDCCR
ALQLICRGALMQERHGVTGGAEVGVPLGKAANYIREVVGDTLYSVLLYEDLTEDEAFSIA
RSVTSSEL*
>TCRU_2341 DnaJ domain containing protein, putative
MNVSGFRAIARCGIQRGFNDCVNRGLPPCARRSLSASLSLSITSVPHASLFAQSRTQFAQ
VPGKRDQEAFLSQYNPLEVLDLDESCTVEEIDEAFKRMSAKYGPNGPRPDAQLLDRVFRA
HAILKDPGSPFYLRAHSSETDRHRLQFQLLPKSKRRIIGFQVGILVLLLAGLAILVFVLI
LRPMKRSIRAATR*
>TCRU_2342 hypothetical protein, conserved
MMRAGTGGKFAAVVRRSLCWCPPGRGRGPALNVQRRTVITGQIPTSPTGGDAPASSTPGW
ELRRGHHLHSSVGLHTSFPTACGRTFNPQLYYEVLQACCDQQWLQAIQRELRNDKEWMDT
LRREGGVAKAMNALTESTHHDDEFMMKLRDTLKTDKKMSLTLCAIQESYERIRNKRDMHE
TQVAADGGDDPYAAMRRKQQGEFGHKYPSF*
>TCRU_2343 hypothetical protein, conserved
MISPLLLWARAHQDQKQQNEPRGEDCIITPFVQCFYALGGCSRINADNTLQSTKEGQDDD
SKWIIYARHRRRLYLHHARELSQFSDAHDYYRLMMWCHDQRLFIHPAVCMRRHPSLYRDH
IFAVSQDVDRLTPLLAIPEALAIGFKDISSAGDFDNDLRNEKREKEFNELNSGEGTDADV

CRFFFDSLNMIVSDLVAAKSSPLTDPRWVFAELLGKVRTLKNAPYFEDDVVLDSETTGLA
DVLLQMIRNYINGGPLVGKLDRAELRWAVSVCLSHSTPLVIASTRSIGMIPIVHLFPHGG
KETNSFLVARTSRPNSTERIAAFFSKHHGYDFMRDREGKWIFLVPSRPLQSGEEIRLQAM
TPVCGKDSEAEKMWRLSCAAAPQEYMTSSAVAHMQRCLTRELIERGGDILAERCGGAPS*
>TCRU_2344 hypothetical protein, conserved
MAGLYSVDIGAASSSACGKFASVEMNLSEKLISWAQSLGKPLRCTSCLLKIQTTEKTVMY
RLRTKYHVGDVSERRFALIFPVSGCSGAVTSIMARINGRLVVGQLVSVEDSASGNLRRFK
MATDMPDENFTDIRQKHPVSIYSITPNASDVLGGKAAEVGAEVIVEIRWISKEMVWCEPH
ALQVLYPFVCAPRLPDEIACRAVFSSPVKMVSSPNCRNGGGTLDWKLNRRCCKVWLTPRN
AEKVLRREDDVFILTFYFQGALDAKYQPGTLAPVLLVIFLGIIVWMMLTKDLSV*
>TCRU_2345 hypothetical protein, conserved
MDESPVGTPFYAAFGEFKDGYNREEAEAGIHEAFRLRQRARQKNYVKELMLGSGLGKAAK
RTVQSGLYQPQTALYLPTNVVSAAAAVADAGDVSEECEESGDADEDPIGNFLMPPGLALL
SNSPSSVMNKFTRTEVEVQTLVEVERQLEENIKVAEESVARECASFSFENLLESLSLALA
VVEEGDAQIQVKLPVQEEDAVPLHGAEAVMAYFREHMAPITLTLLATLYATEWMLQYTMT
FINSMFIENEKEAQLREELLARCVRWLGKINRFVAFAFALEMASESYEAFAEMQSLAEQH
TEELLNGIEGDINSALLLSFPKRRQGLLGRDGRIKKRLQQLLQLLHDVETSWLPTVKLQR
DWKDEFLTRIYSRIHTVIAPGYLSAQLGFIDESLYEFVADGTVLRKQNAMNATRGSNGQR
TSFRQSLPRILVATQVERRIVDEKLTFQQIGMLVRGSASRVIGLQLCMGTRFILRQCDTE
YDAQWGFSMSHSQDSPNAVRDAVESVSCFMESLKMQLKEQMDYQMGILRHFLI*
>TCRU_2346 hypothetical protein, conserved
MSFTTDDAPAAVRNPTRSSGGNGDCQCEEQVQADPMPPVVAHYPLGSWERHLLEERLRAI
KDCERERLLREELEKECTFQPRLATSRSGEDEPQKDFVSTDQKQRHVPVFERLAHRAKER
EARLASLAEEKRRNEEETLRHAFRPRVNTFSEQRVSFASNEARIPVEERLLHYGRSVEQS
RRLLQEQRQQREIEEMRQKAVIRRAQSNASRDKRQSDIAGRSKRLLIEREAQRAMAQNAL
LNAHSFRPKVCTTSDAIDRTLKAKKSVRDRGLALYEEGMRRQQQRQAETVQRQAKESSGL
HKPATNPLTDDWIHHGQHRALFRQDFIKRQEIYQRVKDEHQRNLLSALEQSERAEVPRVN
QEMLEQQVERLYLGAQEIRKESRKRMEEHINSRECPFRPQLAPGTAYVIARTQREEDVVK
RLASVPRSRRVSQYSIFDTAYADQKENEKNSPRKSKVIHPDEAAEFFYRQRKALEDREVQ
LREQKQRLAMEELCACTFRPRTCTDEYFHRRQREATDQAQKPIEARVSGVSAYLQRQAEA
KRRLKEQESRYSNLGRGLPCNGAGSTVITPFQLSSGRGRHPHGLNPRWNELKLFNGEECD
IRCHVDPYSNNEISMALRGKLVEGSARSRGQ*
>TCRU_2347 hypothetical protein, conserved
MNQSRDRKAVPADRDVRARRKHTHRAEQRVTESVADDDNVQRPNSGVRVAQQQQHGAVPP
RAAIKPDIRHVARHRTNQHRQAHAAPAERHVAVAQPRQPAAHCMRHLGVHHVADALTNWR
RWRRGQHWRRRRRWRASRHRHRDAHIAVPSSSALAPAVNYRRRQRSKAAAILRALPAHRN
RRHVVDAVQFQLCRRRHHTSRDRQTPSNEQVAGGSTR*
>TCRU_2348 hypothetical protein, conserved
MCTQNRCVYPPKLQYRTYSELPPTSVSVPVEYKPNKPCLGAFALTLLTNSEDALNTTFFS
ITTLHMLFADSGNRTSENCTASTSVTAMSQCCGRANANKATEDRITVWLTRIRTPADSDA
SLPPTNCSTAP*
>TCRU_2349 procyclic form surface phosphoprotein
MCLFGIVDAVEDFFKSVVACACCLIIGGPALIIAGSILLNQKDLRKAFRDAVNEFDPTPM
NDWTGTINKVPITVRRESLNVKGVDGATSVFAEAVVPVSENFLSEFPVSVNVSAVTPFNR
MAPFNIMRNSTYFCFSSDCIDGKDCKCKRDVRSFGEKCTARGGRYNQTPTWCKAGNICGE
CLETLYLRKLYLVVREVSNGKYAEDTNLRSAMYAFGDLDNDYQPGVPSTVTVRLYSSKDP
YIALQRLTRGTNDLGLNSRTVGIVLIVLGCLFLLLEIGVCTALICYWMRPKKTSSGAPPY
LAPASQGISTSGASGNAAPNFYAQPAQQTGQGYKYGQAAPPGYTYGQPPQGQPIPPGYTY
GQPPQGYAYGQPVSPQGQHGYAYGQVPPPL*
>TCRU_2350 Leishmanolysin, putative
MAAREHHDCDDIDGMELQDGDGDGRTLDSHWSQRHANDEWMAPISCVVCRTELTLAASAD
LGCMRVKWEMVEPMRWWRNSCCALLQRKCSAPNMSEYSQMFSEAGVHAGRCTLDRYSSGW
CVDEVSMKGQQNVRWTVAPSLVPHWM*
>TCRU_2351 hypothetical protein

MASLVVYTRGPRSAGQRPPVATEHGCSSQSSTLPPPRSSESTVPNRYDWEWNGGSDIERV
VNGHGRRGSSEYVHGNVRAVSPVSYGGSNTSERNVSGHLEGLDASPERIYMEKDRVIRQM
ADSVGRSRRNSYDDDSFNEDDYNSDEEEEEEEDDDVPRIAIIGGRHNEQFVREILYGTPR
AGESPKDPHPHQQQQERYHTEPFDREFSTTGTGYGESGLRSDSKEERRVVFLQRSSQMRQ
EGHQQQQQLHYYTQSKRFPSTEQLPDKPERIVKLSPIRQRPQAEVTRTRPALLSAVKSRS
FGVVRSPKRRESFSEPVHGRHMSYDKGGTRGHDASLSPRRHSRHEDSSLRRRSVDSNVKS
PSVSKDKNRRNERERQQRLRKLKQIRYSEEVDDGSRVRRLPSPSTGNDFGVKETKMKHRR
TRTMSGSTSSGSGRENSRKSRKKRRAAGVEDDGNETQKAAVQFKPVFTAVESVPPKAKKE
KVFCDCCRGKKEKSQKEKQEADDSAKREVVRRPNEKTGNVEDEKPSALEKAVHATRKTKS
QPQVPPRRRKRRPSMPPSDAAGSAVKTRERAAASAS*
>TCRU_2352 MORN repeat-containing protein
MSLPARATQSQPADVTFESHQSGIQRVMMCPIEFGTCTGIQTEQLGKNAGIGLKQQNVCL
RHLYTGEVVRADSNHNCGNSSNVFIPHGVGKLVCFFYAKGGTVGFAGKPGSPVGLPHTVV
ISEYGEVEELRDAILLTFSMYTGSFSYGRRQGKGRLTVYPHYFIDCLWDNDAPLLDRTPC
VLCFFSKDSMSPTDEKSQMKPTRVGGNSGKDMAANVKTQYIGMISLISSGLTPHRGTFAG
LAWAEQARFVPDGIGEILYPQGIRYCGFWQCGQRHGFGVEYDPNKSTVYMGGFVYDQREG
SGTLHYCTTGVIFSGSWKKGSPTETSNAILPTSPFILQGGLWKSYENWSFEHGALMRSSV
PLSGIWEPLFLAFDEKLASAPWDHQGTSPTTKTTTATTMEGLDNDEKDHADEKNILLLSN
MMQSSEFKALLVPFQRCYYFLYKPCVGRGRRNCNGSSSLGEDAGHSLTQWAFPSFSSPAA
ARSAPVSRSSLLTAYDDENRPLSNSHDILRSRWASLRCFQQEKSYMKSLERLSPLVLQLR
ELGSDYRQGFGEAPGGGCWSCPSWCEEMGVVGGNGDNSKSNSSNISNGIGSGAIRCFHTE
SVVESLAKRMSPVKMFERAMRDLASFVSSVRLRLLSYVAAHPLACDVSVSKRVLAVCWDS
VYALVAPVIHELAAAAEAEAVIAASLALQRCTSLGPHDFIPMAVAKNFDYFEEDRKVTKW
LARLFSSPTHHSRRVVEQTINGQLLCFSPHVGSGQETPAVYFSPACMFAAFDSVYKISLR
LFPQHPLFQERWMSYSVLEAFNYAEGIVPRDALSPPAVLRILHFLAAEVNPRYPFPGGST
ELRDDAITSKGWEKALHVTNGNGTNRKDGRDPSAIMESDTVDLEPWLNSFTALYSAPKEA
TEVVSLLDFFRDGVDRLKSVYPSVRVNSLRPSAMDATVLEGGVLGIVYPLDELAVRTRFV
LLAASEWLRNRQQQGGKECEWNINDGKEVEMTTDGENGDTSRKANGLIRRVLSLPISVMQ
PALSQQADELSYEAIHMHEDGLMHSVEQRRQQEMSSIANLSSEVLLWIVECIGRVLESPN
FPSAVTSSMAPRRTSMQPRETQQLRQPDPLPFNLGNKERCDSPADFTVATPKESSWGGAV
SDQDEGNVVGSQYLRYQWKHFISNNMTFPNRSASLPLGDTNESHANTSVSAAATTSELLT
EAENLQILLLQSFLAEVGLWITLDMHSCYDNEEDVSSTSLAPYAISPREEKTVRRCVGKE
AILSLQVGLSFLGSRAWEVLADAWLAACLRLRANASAFPHARRKCNPIDALKCVH*
>TCRU_2353 Replication Factor C Subunit 1-related protein
MSLPPRLSSCSLVHTVSLSAFFADGFRLASSSSPCLSQHHTGDAGQCCTTAEERNVPSPC
GGPAEYRAGEKRIRLSLSCTSTQDITPSSEGKMESADLLSGDGAEAGKLLPFHKDDASLL
LEPAAASLIRLEPSGSWLGSLPRSVPEKCGGVSGEEQRWTPSLPKEGNETANSAELASLL
HTALLECYRVEDELLKRGREVAVSKKETPHCAPKLVTEEKMERAEMRHRPSPSTSSSSGT
FERMVGYIHAQKRRRLEQSERLEIQVRDIARVKELPVLVEDAVRYAQQLFEWSCKCGGND
KGAFLAEAGAVHSDESKLDDTCSPSGTTPVFAGSTASDNREANHMLYAKFPPPLFHTSSF
AAAVGRLLRWVRTWQRAMPGGVGVTESIPTKAGDKTQRNGSTKGAASRKMSTKGVLNLSS
ASCQQRKRHVRQLETRGNSGFLNDDGGEREEEDDGRVAMASNRKKEEGHKHKRTALQSYR
DDWICMADQMLGNSNRHITPSPFRSGSPFESLEEKEYGLATDRVIYDEYGNRLRSVRLAK
KMAEERALYEEAQKAREQRRGYFARAEEETRSTRRALFDEVSDSGSDEMSDDSNEDVTNI
AVLHGPCGVGKTAIVYLVAEILGYRVVEMNTSVRRCPKDIDRLFSEVTRSRRLSGLAAGK
AMVSIEEELKKLKSEHAAALAAVSATASEISKTTSSKKTKKRNVISAKAVAAFFRPRVRA
KSSEVILVDGSPTVSATVPPHDKQVLNHAEDCKYGKRDGEANVTVEPMCGGHEGTTRTLL
LFEDADVILGDETMKSFYTAVRDLAQRSKVPIIVTISSSASSSSSSPATTTMQEPEAVQI
VPLDAAQVSHFFGKRTPFTAIEPMSRAALFAQLLVVAAVERGLVMVQGGAAPHREENDEV
ESSSKHNKRRTAEPMLPSTPLELVMIKDVTKFRQIADAIRSEVYNDDLETPSRPLNDPSD
VRLWLNRLQYLLLVEQWPASATSMGSRHDECIFGGTCILPSKTDACSNAMQFAGVKSHWD
TQLGRVLRAPEYERPAVSSIWEWQEFQYAEEIAADINANYKCSDGVIGETRGSNNSNGSV
VLTTPQVEVPIAVELAEVLQGVFPNDVFCQQKRKGGVEFVDGVRRRCTTTTRERIEAFGA
WWRRTKKTDAVKFHVAARSATAYEDLIGFSCLLQQASPVGKGISG*

>TCRU_2354 WD40 repeat-containing protein
MLEESVRHISGLSPTPRSGSRSDTSVVGPRKKTDQKAQLLGKMTLGDGLICVDFLSGPNI
LAIGSTKAVHLVEVTPMNLPSQAAGADVSSTPHLASVSFAMRSCGMFGNVAKVEALAWYP
NRNEAVLAIVQLSRSVTVLFDALSKKGDAYMAQQWIHSSRCRTLVSSTVAGSEPLSAAVV
SAASMPSTPFGTAMQEVQPSPFVERDPDRPCSMVEVVINTGFLRVERITWDPHNPYTLAL
TSNATHFELWTIPLRDKKVYPPRLILRPPAHDERSVTRGVVFSPFDPNLIVVVVERANAG
KVIVYDRRHVEVLWSMETTGPCLSAAFHPIFSDLLAVSFRKAKTKTNSRVQFFRVQSDVI
AAPSTTETNHSGGEKLASAPAPLESTDVLPPIDSIDSLNRLRWRPCGNAFQVTGGEACVE
TSDGSIDATSSKLWFATTALNGQEISVWDAANGFSPIFSIRTVCHGREFVRQGAHHMNPT
LQELPLQEPTDCVWVNALTLVSIFKDGGVVLTSLLDEKTTSRDTLFRCITDPNTQLSAGS
DVLSLSAILPTAAVVADYFGRSFAVRSSSSLLREYYTKIINAEKRELLEELASGKKNAVG
GGGVLSSPSPSSTAVSLGTVKHHKDSSRATPTGGMMFDTRYSPLPSHWSTQTPLQQASFE
RRFPSSLASAHAIAGSPNSGRRLEFLFPLLFSLVPSPSPVLTVATTTTRAMGVDRVVAND
DAETKSADRFLRRSPYLVAFDSRHRSSRGGLETQSESGLEGKACSFPTRHGLDGAAATAK
RGLFGARSASANEQSLSEHRGIVSPMISLMNALAMTSSFSPTQSEEGNARRTNLFTPVGR
TSGGAVSLLQETQAGCANLTSEYGAEGGSVEKIPAQPPPLGRRSLVKPVIDCFILSNETC
GWAYVEQTSEEDTFVRYALNWEMGFDTSRKMRAHRHAATEEIAKTETENVDQCFARMMGH
NSKVCRSRERELQAATKAISEGRGNVPGVDGATANSGKGTGNSEYPLGSVDPRGRLWAAA
AYAWRSHNVTLITSLVCSYLEYTSLVGDVQFCAVLYLLFCLWWKQRGMSVRSYACPLFPT
DEKPAGNAQEHEKTREATNGSPWQWKMRALQWVEQYVTRLYERKLYVPLNELLIIVPEVM
GNFSAGLPRAEDVAQKLFTCVYCGTCPKSELVPRAPKKGERLFLHTATPATSVDVSEGTN
ERLGSDDDSSDSFESSLSSSHFCMCVQDEPADSGACNDSVLVAKEKVNGGGSAHPLANSG
VPPRNAVCSRCRNAPLMTCVICEEVVEGMYLWLLSCGHGGHVHHIKEWLEVSNECPRCGI
PVLRPEEKLR*
>TCRU_2355 retrotransposon hot spot protein, RHS3, putative
MKLNDFLTMELEGRGILRANRNVLLRDFFSDPTRYIRDAGVLNEIQTTDAYLRMEGTVRE
EMGLEEAVRRLHEKGVDSILGWSEATARAKKSVSNSTKQSLDAALHVARNPTMTNAPIYL
EGYYESVYNARWHHVVEVPGGEGTGMKVKRGKPKQSWTYEEAGRTIEKNDAVRQSGAAPP
RLMVLTSDKGWPYSWEQGVESLPDCYVNCEVDRVWQIVKGDLNKWFSSHGKKKRSPERRV
LIGTPGIGKSMNAGSYLLYQLLHYDVDKLQMVAYSFGDEASLFDKTSKTVSIYEGGTSIL
KIVKGFSRRGVKGYIIYDVALKGHQPSNTLPCKGWGMIVVTSPNTKNYESWAKLVEAEQI
IINCPDESDVKAMCVWKEHNGQVEEESKEEAKEETEEETEEEEEETEEEEEEEEEEEEEE
EEEEEEEEADYWKKVKRRMDKVGPLLRYIFDDRKYIGRIDSCKSKVKSMVLPDTHYYSIL
GSNEVCDDNHISHKLVKVVRVRGGKKSELPLNSLISSYLGNLVTCKLAKLMAPNDFILLV
LGIKDDLLSKALEKHSVFTFLSEDFVSAIIPKLKELKKRKNARPQLCALRVCPHGRSLKP
CILPCLGKNKKKFNIKYRVLYKPEAQNFPLVDAFFFLNSNPKTLVGLQITTAGDHHTIPS
TVNQFKEKMAKYFNDWEELSRDMSWEIVYVQHNDNKPLDGWQRCGPVENNKLRPAEKEIV
AFWDGKVHQYQVSVSSRDFKEKEEALPIVEEQQKQKKKQIRR*
>TCRU_2356 hypothetical protein, conserved
MAAPSLTGSECDNTNGGKESDEDGDGLHRGADYLRVFLHESREGLAAVPTIPGLAELLAF
ALSPAVISAPCVEADPFTVEFTQQLSSVCAADPALALSHVSQRCRACDLLCVVQAAASSF
LFVPNKKHFALRWVFICVAVDARDDAQRVCLLFGGHEGDFGVR*
>TCRU_2357 hypothetical protein, conserved
MEAGLPAEHRAVARISLIGGVTAEPSNLLDSDILLRLMGHVIAVAEQPSDSLDDIMARRV
GVSLPLYPTEPALARCCCVDDDAVTEGIAVVYSR*
>TCRU_2358 hypothetical protein, conserved
MQPRCKPHKIVEAFRFPVAEAPAHLLWQPGELACEPRAERHTAQTVRDDGLLRRTLHCVC
VGTPLPHPHNSPRLIAAVQHRTLTVKTTRHAVSQDCVFVCPQLYCRLRRHNVKTEGLQCR
HAARGIGTEAFMRGRTTAGNGVEPQCLTGPVVDEVSTFPQNTIKACGAPRSAQIVMQSSG
PSLRQRTQRVSPDAHHTFGNRSASRARVA*
>TCRU_2359 hypothetical protein, conserved
MLPRRKHVALVLRDADCDASTSACVTPLATVTTWLEMRKCTIPRGSRDIPTCIWTDGMEH
AESTAAAAWDAHTASPGGSLPRAELLRKWEDRNHALRRGFSMLPEARAKQPGALSSPSL*
>TCRU_2360 Adenylate and Guanylate cyclase catalytic domain

containing protein, putative
MKARRVQFVSGVVTDAMLEVESVTFIDPLLLGSRLNRFRRNVIRLMPTLEQQFFVLANYL
GDTSRGSAHAVIRSGEASAMADLLRRSLLTFRWSLEPAKLLAGGDALVDHLPVKGDVFLV
GLAAGDAGAIARHVASHVGVRVFVVFSEFALLYAEFVAAFHGCEGADRVVFATSLPHWND
ENSTSVTTKKFVVAVPEKKRTPLSLMGFAAMGLMEVVISRMDSVSAELLADFFYKNVAVT
VDDMLYGPFADGRACASTESGAGCGVNYGAKRISVWPLARALDPAVPLLFHAVNPSMKYV
ESAAGGLTARQLIGVIVGGVAAALLFVAVVLYFCRDSRNNANAPKNRIHPVTLVFTDIES
STALWAECPEVMPDAVATHHRLIRSLVAKYRCYEVKTIGDSFMIACRSVFAAVQLVGELQ
QVFLQHDWGTSVIDDTYRMFEEGRAEEEGEYVPPTARLDAAVYRQYWNGLRVRVGVHTGL
CDIRRDEVTKGYDYYGETSNTAARTESIVNGGRILLTRASYFALSTAERKHVEVTALGGV
ALRGVPKPVEMYQLDAVPGRVVAGLRLDRDDGAVDDYSDVGSGDGSGSTIRGGPSGYVAS
VLGVLFGTFAAPQRLKALLPLCQRWSVPVPSGAGAGRDKEACRAAMERLAAKVCGVIEKR
AYGTSFEEGLRTLSLSDNAFFWNECGLRLRNSAFERNGLPVDFRRRSLLHQASLSAAGEA
WPAEEDPVATIHVRRPV*
>TCRU_2361 BNR repeat-like domain containing protein, putative
MEDGTLVFSLMAVNEAEYVCSMIICSTDNGSTWALCEDISPAKCLDSRVTEWEGSLIMIV
DCENGQRVYESRDMGTTWTEAIGTLSAVWVNARSGVSRDLSLHVDVLITATIEGRKVMLC
TQRGHASGKKRATAHCLWVTDNNRLFSVGPVAMENAASWMLTSNLLCPDGSLHLLQGRDN
GECRVMSLSRPTEELEEIESVLSNWTQKDIFFSSLSIPTAWLVAVLSDAASDVTWNDEYL
FLNATVTNAVKDNDGLRVTGLESRALRPVNTQGDNNLRHISLSHNSTLVASVTIEEAPSE
NTPLLTAVLADTEPNHTVGLSCSHNKKWETTFEGKTTTRSSTWEPKERIPSGTHAARQQG
LCAH*
>TCRU_2362 Glutathione S-transferase, N-terminal domain containing
protein, putative
MSLTLWSGVNAGNTRTHKLLAAATLANVAVTPKTCEYGRENETAECCRNCSPCGRYPVLQ
TEEGCVFETNAILRHIARLDRSGGFLYGRTPLEGSQVDMWLDFSATELDAAPHRLCSTPS
VAGSGSPTRWTACTRCCVRWRRGWRRAHSSWTSAWWLRLRRNGTIV*
>TCRU_2363 hypothetical protein, conserved
MVCVRCRCWAAAICDCAVAVRGVAAVSLLLLPLCVDGELVCAEGCRQVTGVMAMMMAGRV
LLVCALCVLWCGAGGADASNLENNAVGACMVSWVLNTNKSYAPSGCDKTALTLPLRSVLS
ITAVAASTDPLVTGAAENKSDLAPSSGADFGGAGSPPGRGGGGGGGRDDGAGGPAGVGGA
SGSQARGGKASVGGGGGSNGGSKARVGGSLKSPASDVKSPSPVLGDGGSSPGAPTGSVGS
DGSKGFILGSFSSGGRSQPDVGSPGGDASLSTPVAGGKSPPSITDESDALEKTGAAASTK
GGKPQEAGEQSEANRARNELPELSAPAESTPEAQEDSPSRQEKPESEAAGRAATGDEKRR
SNAEAQGPSAVMSTASASNGRRRTTNSGGSSGRRKPNGTNRLANLDRRKDKNVTVTKQKG
A*
>TCRU_2364 hypothetical protein, conserved
MAQQRIQYTAGARGQDKRRVAKGQFKRIQREGPNHIPATRGHASTKCADGSGDRSKWDSD
DATDERVSLLPDFVGDYQRYTCRLRAVSDAQRQKLLRGGDVERSPSPIAALRMTVPSLTP
AKLATLMAQGADMIAIRETWKSSEQIASMHTGECVLHAQSRIRKECVAAVLVRKNLRSKR
MHLITPQRDSSLEVMVQFALGQNRDLIVASTYMRPPPQPTQPLGGC*
>TCRU_2365 hypothetical protein, conserved
MCSQYGPLRNSSQRGRPIAVSVANSCLRVATTQKFSKPPSAVHPRNIGRQATEDSMNSEL
KGLAGVYQHQRSPMGSAVELASSTALLGKVRLEVIGSIMLHFVYQVCQLHRRGTRTTARM
LVRTRRPQPKRSPQCHFLSSLTHDGRKADGSI*
>TCRU_2366 hypothetical protein, conserved
MAKGFLRVNWRACGGWLILRWQRPLPGTTERVAVSVLANGTTPLVAGLGSKACQRVRVPY
GCDSMFRVLSGCHGLRRMLSRRLISMHSTMSVTKLPVHGPAGTHQCQETLPDPVLVEGR*
>TCRU_2367 hypothetical protein, conserved
MAKGFLRVNWRACGGWLILQWRRPLPGTTERVAVSGLANGTTPLVAGLGSKACQRARVPY
GRDSMLRVLSGCHGLRRMLSRRLGSMYSTTSVTKLPVHGPAGTHQCQETLPDPVLVEGR*
>TCRU_2368 hypothetical protein, conserved
MSGTSLADGSLRSSWETGDTTRIRRTREERMPHGELSTLPLLSVLLGVSEGLVHRRLSAL
LARHRRQPRLTPRRTVSDVVTPVRDKTARGPNERIAVECGRRRGGVPRCTLTVAALSQSR

PASRLRSAPLTMGECWLRPAASRACTVEPNTGCAATWEAAALG*
>TCRU_2369 hypothetical protein, conserved
MDSAVNWELASTLLHSDGDLHLLQRRSNGEGRAVSLSRLTEEASAIKSVLSTWSQKGSFF
SSVSIPTAGLVAVLSGAASDGTWIDEYPCLHATVTNAVKDCDGFRFTGLESRAIWLVNTR
GDNVRHVFLSHHFTLVASVTIEEAPSGNTPPLTAVLANTASNHTMGLSCGHNKKRETAFE
GTTTTRRSTWEPKKEYQAVLMLQGNKASVHIGGQLLAE*
>TCRU_2370 eukaryotic translation initiation factor eIF2A, putative
MQPPALASLPVILRKDDSLVVASFSEQQRTLTEVEGTLMTGDYRECEVRFNPVKQTAFAH
ATTTHVRYSELVPTQDEYLWGLKQIFEVEERNVVALSFSTLGTVLVTYAMMDSRRPEGNM
VLYDVNTGNVLRRCVQARWPGMVWTSDELYCVRPVQGFLHVMDGNLRKGEVPVLFKMDLQ
LPQDKEIELSMCPGSISLLALFKPFHKQVQGTLFIYRLPNLLEGLIYQVPFGRAESATVL
WSGSGNHIALLVKSESDKSGRSYYGTVAFFLVNVMGRSIKEIKFPSGESVHDCQWSPTTD
QVMVIHGTMPRNKTTLYDKTGMALMTFGEAPRNTILWAPNGRYCALGGTGNLAGDFVFFN
LHPSDSSNNMKNVHKDKNTRVPMAPKPVAASSSEAAVMIGEFNEKSSVQMWAPDSHNFFC
ATIFTRLRIDNKLVFFKKNGEHLLTQKYPMLYGAHWVQTQDTRLYPVRSASPRKETDKSS
KPQPYRPPHASAAAAALLRRPDSSAPMQVKPTVPPGATVVVQKKKRR*
>TCRU_2371 hypothetical protein, conserved
MVCVRCRCWAAAICDCAVAVRGVAAVSLLLLPLCVDGVLVCAEGCTQVTGVMAMMMTGRV
LLVCALCVLWCGAWLCDAAETSSSAPHRDNAEETMTPEEVTEAIEKLAEGVLDMIRVSCL
MSFITSVNATLPAHNDTEVEKCVNKTAVGISGVTPRGRPAAATTDATFASPLPSSSSATS
A*
>TCRU_2372 hypothetical protein, conserved
MYAAHKKDINRHKSFHPLTYRNLSKVEQRQQEEEAKKKSAKERKEELRHDQEQRRYDDLI
LAASADSVSGQLAKFRQVENIFAAEGKKANASSSAPPVKDELASIATPLVRSGALAFKKE
NTEHDGDEKTEGRKRGREDAAGLPVGGDGSAVDGPREGKKAVTGFISTSNAAQLRKELDK
LQKERHDPLRRIEQFQNRTVAAEAKRRELGTGAASVSNCVDTEKDAIRSRIHELIQMKRQ
K*
>TCRU_2373 phenylalanyl-tRNA synthetase (beta subunit), putative
MPTLAVARDYLFHLMGKNYTEEQFEDVCFKFGVELDDVTSEREMFSREQGMVGGANLEEL
SDEVIYKIDTPANRYDLQCAEGMAVALNVFLGLMRAPAFKVLNRCSPLYRMTVDKSVRNV
RDYVVCAVLKNICLNERSYRSFIDFQEKLHSGLARRRTLASVGTHDLDKIGDNNFIYTLK
SRESIRFVPLNQRGRMLDCAGDGLAEFYKDDRQISKYVPLISGLSHYPVIMDREKQSILS
LPPIINSDFSCISQETKNIFIECTAPDHYKANVLVNQIVCAFSTYCEEPFTVEAVRVEYE
EATPDGTKVEVTPNLDPAVIPVYIPKVERLIGIKLDSPQHCGQLLERMLHRVAKMEGDNI
FVEVPATRPDVLGPTDLMEDVAIAYGYDNIKYVECTTRGKVTQQPLSKLSHLLRIEMANA
GYTELLTFSLCSRDEAFAHLNREDDDVAVHIANPRTMEFQICRPSLMPGILKTLNANKSH
PLPLRFFECSDVVLIDNEKNFPPLIRPHSEYPNCGACNQRHVAALHCCSENSGFEDIHGL
VEFIMKKLSISRKGEQNAEAEEDTYTLERGTDGAFFPGRCMDIFLHRKDQKVCLGGFGVV
HPNTLRAYDIPFPCSYMEINIQFAQ*
>TCRU_2374 hypothetical protein, conserved
MIITEAVFACLREWHDATLARAFFADDEGVFVLFSAMNVLPGNVETNEAGESATALELHT
RCCQGSRLFLESERLQVARLLRFRPLFQEKKSVSVSDGNDVDECSTASLTSPDGRNIIAR
LLLELHASSCWWRQGEFVQNMLRTPRFSLADTAIIRHRCAAVVVTDETSSASQLKSLDAM
PDGDSHAKKLLLPRGSPPSFLQGVLSSYLVKWLRAKRTQSLSHLTSNTPMSLLRVIFFLK
RPRSSEESDEHSNDESDELIPCVSLLMALFGYTLTQTPPRNTCDASEAHKAWRQLCVDMQ
CHFCGGRPRLTLEKTVAEEPLDQPQLQEQKNEEDVMEGEHNKRMESGETLMIAEPTSRQE
PGEPVGLENRAQLELETSQTSINPDENKNVLAHVAAVCNSDKKEASPHSRFCYTIEVSEQ
DEISGHHNTCPWKALFLSAVLADTTSSFPSKEVLFNLCNADEEGGSLQMCFKLPEVAKLI
KCWRRSLYAESVWKEMYIRHPLPAALSSVIPSPTDSGTEAFLEGLASRSAEGETFQLKPL
TPLFDDRSPWDSYFRVASFLKASAIVDYGGVEVRGKDTTEIRKPPKVEEERHWKAVYDMG
RRCMLAAVEEGGSESAVELSEKFKEQYRHCAQQDATGGHTSLPLEGVMHKLLHLLNSTLS
VDLLHGRKVSLGGLFEGPKIAAQEQKNVHSFLDALDDVVENATHEPPATEPTDPAKRPLL
IQGNNNDTTLKKGGGGGKGGTSGARQKQPKQPQKQKQHQYHHHQHQQHQQQHQRQQHQQQ
LRQQGMPMLMAQHVLLPSSSPTEGVFPFPARGSGSGVLPTPVPVFQQQQQPPPHLPFQPA

LHGIPFTNCNQKTVLGGDSIGVLDDAYMQRPSVMGQCQTTVAKPPQARMGGRGGNHRGAR
GGGRGGGNTFKG*
>TCRU_2375 hypothetical protein, conserved
MAMMMTGRVLLVCALCVLWCGAGGADASNLENNAVGGCMVSWVLNANKLYTPSGCDKTAL
TLPLRSVLSITAVAASTDPLVTGAAENKSDLAPSSGADFGGAGSPPGRGGEGGGGRDDGA
GGPGGVGGASGSQARGSKASVGGGGGSNGGSKARVGGSLKSPASDVKSPSPVLGDGGSSP
GAPAGSVGSDGSKGFILGSFSSGGRSQPDVGSPGGDASLSAPVAGGNSPPSITDESDALE
KTGAAASTKGGEPQEAGEQSEANRARNELPELSAPAESTPEAQEDSPSKQEKPESEAAGR
AATGDEKRRSDAEAQGPSTVMSTASASNGRRRTTNSGGSSGRRKPNGTNRLANFDRRKDK
NATVTKQKGA*
>TCRU_2376 TFIIF-stimulated CTD phosphatase, putative
MPSHISERFASVSTSLVPGNRISIQKQGHASEGSVRRVPLRLANLTTGGNISGRQGFTLN
PSMPAENSLKSYIFPRGSLSKRLRCIPASNNLSSSLSLSHAPSNARLVRARAAAGAADGF
FIPPVRAVDEGKLVVVLDLDETLVYSRSGRIILRPGINRLFDVLRGRCETVVWTAGEKSY
AMEVLQLIDSSHCVQHCIYRHEKWWTERPGYVKDIAALGRPLHQTIIIDNTPDCLRAHTR
NALLVRDFRGPCGGSSRFDNTLFVLADVIDDVLNGSENSIDAFHDHPQLQLRSVECESGS
TIDVLTLKQDNFLDPPKGNNVIPYNTQRASRRRTMVGM*
>TCRU_2377 kinesin motor domain containing protein, putative
MAELAGTAGRIQVGVRMCPPRQGEKVIVHADSDDQRAVLIDAEGGRASTMFKFDRVFTGG
QDEVYETIGRPMLKEAFEGFNVCLFAYGQTGSGKTHSLFGDLNSKEGYGVAPRFAQDMIE
EAQLRVESDSAATIKFFVTMIEVYMEKVRDLLAPRARGQEPESLEIHEDSQHRVYVKGAG
VHSVLSLERMLELLKKGNANRQTGETKMNETSSRSHAIVQITISQKYGSLDMRDVESVVL
LVDLAGSERQSKTESTGVAFEEAKKINQSLLMLGRAMNSFSDRKGGDAFISLRASKLTRL
LSESFGGNSKTWMLATVSTAANNLTETISTLEYAQNAMAITNKAKVNDTKKNIELKRLRE
LVASLEGRLDVLALEKQRKQEEIGRLTQERDKLRQEVAFADSVHDARDKLELALNNIRLS
NIALRRRVEAASEGFIHSLDNKSCFLFFKGRCSITLESVLRGQRRSFYIGLLTESGVLTE
ATLHIQLFPCEHHANERNDPMQFIGKSLRFCLHVVGASGIPKAFVAHTFCKFTLLHDREE
RYFTTSTAENTQNPRWGYVKVFEIPELTAEVIRCFCEHTVFAFEVFAFNA*
>TCRU_2378 kinesin motor domain containing protein, putative
MGEASKAASRIQVGVRMCPPRQGEKVIVHADSDDQRAVLIDAEGGRASTMFKFDRVFTGG
QDEVYETIGRPMLKEAFEGFNVCLFAYGQTGSGKTHSLFGDLNSKEGYGVAPRFAQDMIE
EAQLRVESDSAATIKFFVTMIEVYMEKVRDLLAPRARGQEPESLEIHEDSQHRVYVKGAG
VHSVLSLERMLELLKKGNANRQTGETKMNETSSRSHAIVQITISQKYGSLDMRDVESVVL
LVDLAGSERQSKTESTGVAFEEAKKINQSLLMLGRAMNSFSDRKGGDAFISLRASKLTRL
LSESFGGNSKTWMLATVSTTANNLTETISTLEYAQNAMAITNKAKVNKTAKDLEYDEIQK
LAWVLQIRLDDEAKGISALDAQIQGLRGEIDCMKSKLTVASDSKVETELKEALEDREELL
LSLRKGLIEQRKISGFSAALVCFSGTCKTSLANIPFGSYEVTTLVVPCYMLAGPPPFLIC
HIHVYLTEESSVAVLLRLRELQHLPDYATGGVQVTVWFEGHYSSAVQTPIVRPNGGNPQF
SLRQMYLFGPHTEDLERFFQLDVLNFRVEGIMSDLTS*
>TCRU_2379 ubiquinone biosynthesis protein COQ7 homolog, putative
MIRRTFPRILGLPSRTFANSARKIKIDEVLRVDQAGETAAVRICGYQLLWMSPLDSAVPV
VKEILKDELVHEKTMNDLAKKHSVRLTALDPVFHVGGFAMATLTALLGKEAMMCCHAAVE
ITIVNHYNDQLRELEELENGGVNLNDDDAKALKEIKDIVAKFRDEEEHHQRLGETNGAAQ
AFCYPLLYNGIRLACRMGVNFAKKV*
>TCRU_2380 nucleosome assembly protein (NAP), putative
MSTYPQHVVDAVANCNEKVKSLQTETESQANQTRIEYRKKLELLFEQRQEALDKIEGFWS
GVLSATETPLRPLFNGTIDPKIVRAITNFKVTTSVKDGFLCRNVSIVLRSNMFAEQGTIY
REVNTQLKTISLGPIKWKSGTERARQDSVFRFFTVECNDEGFIDETLDAFDTVFQNPFLA
LESTEY*
>TCRU_2381 hypothetical protein
MQSFRANKRDVENILHDFSTFLRQFQGSNGLRCFLDQGKLSLLSAIGEFKQSSDEIYDRR
MKVLSQTRVSLMKLSDHFDAVGNAIETLFAQLDGLLSELHNFKEICENHLEMATETFSEQ
AEFISINSLKVKLRALSQELTSSIRTNCCGIQSSSIEQLDKAYLDVKCEVSRTQTAFDVW
RRDAEVCESKLLKSRSGSSHDQVRELEFKLRRLLERLHESGDKYHEALQGSMNKTSFLLE

QVSMATWAACNVFFLHLSVFFKDTMNVFELVASALSSVKNNRSVSRRINLEKQAAAAAAT
VATASSLCTTTALPRLVEEKTAPSTSPTHELEYLFSAQLGEGSVSHINLTEDEINRGTRE
FTDIDDIFK*
>TCRU_2382 hypothetical protein, conserved
MLKVWLREPHQSDDEAIKLMVDEEADVSDVLLKAPELLRVQQLKPSEIRAEFEDTVLSNR
LLVSSVFEGKKEGTFVIRPRPGVVVPPNGVPKVSSVPAGRGGDGKNAPPLAQNNVHPPPS
SRRNQFDKSPRVNSEEFKRTGSLGGDRRKPPVARSTSRGPTPPRIVGEGTPRDGKANFDV
HQKERNITSRKSTPNRGTSVSSFDRRPAVRSRYRDPKNVASRFRQEAEKVRVPSKRNQVD
AVCDSFKPQWGKPLCATCNHLKQAHWEAQKIKSSGVEPNIPVENDGSLSKNAQLRCPTPN
ENVSAELKIDGGNNNMNMANSASLPLREEY*
>TCRU_2383 mitogen-activated protein kinase kinase, putative
MKKLGLVLDAAAVEKSQRDLQSYQIKNNNSLQLRAFLLNESGMFTKDGRKLPGSVTKEVI
DDTSKRIIGRGASGAVYFAKLKDGGPVALKHIPITSKPHRDEVDRELSFFSSQSNSPFVM
KNLGAFWDSEEAAIVIPMEWMAYTLKDLSCFWEGIEETMLRDIFFQVVSGLVYLHDTKRV
IHRDLKPSNLLIRDDGYVKIGDFGVSKLVHTLDVSSTYVGTMYFMAPERLEQGAYGFNSD
VWSLGLTVLSTITGKNPWAPPEEMNIFQLLGRIAGDTTPTLPEKPAYSEEARDFVKKCLV
RDPQERPSTAELLKHPFFKGCTEASAVANVKQGVDYMTRLINSDAKKNGLLKRSPEDVTK
EVNAKLDRLGDL*
>TCRU_2384 enoyl-CoA hydratase, mitochondrial precursor, putative
MLRKSLFLLNSMDPIVKYAQKGAVVTLTLNRPKQLNALNAELTNALAEKLLKCDADPSVS
VLIITGEGRSFVAGADIKAMANQTFVEFYKHNMLRGLDTIAAVRKPIIAAVNGFALGGGC
ELAMSCDIVVASEKAIFGQPEIKIGTIPGAGGTQRLTRLIGKSKAMEWILTGEQYTAEEA
ERAGLVSRVVRHEELLPTVSAMAEKIALNSPLAVSLAKDCINKALETTLAQGMAYEQRTF
QATFATDDQKEGMAAFVEKRKPNFKNA*
>TCRU_2385 hypothetical protein
MALAMNDAKTYVLYFTEGGQQMLLPVCPGTSAGMPPSAASQGTPFVWLPFQDATELQHQR
PAFVATKSGAGAPLMAVPTNYILPWAQAKQPIGASYLTPGPNGSAVPVVPISPNTNLQNA
HENYQHSSAEKGVKSLFPDAKNPRGARTGDEEKSANRFEEILSVRNFTSPVGSVLVRGRK
MSMNVPPLAKRRPPLCRNPPGASSSAVNSNTPINP*
>TCRU_2386 hypothetical protein
MPSTSRMSVFDSSVTTSKMASLFRFSLISFSLMRQFRSEAPANMEKVWKSSSSRLPKVRG
SLRPLSVTMSSLLINWATATIDPSYVTGMQRIVLVRYPVALSTSRLKRSSEYASEILTVC
PVWAT*
>TCRU_2387 hypothetical protein, conserved
MVCVRCRCWTAAICDCAVAVCSVAAVSVAFSLCVDGELVCAEGYTQVTGVMAMMMAGRVL
LVCALCVLWCGFSGIAADGAGGVPDGSAGECLFSRWRAQLRRECEEEVSRRTGGRANASA
VEECMRQRMDGVLAVVDGRRRWRRQRPAVAAAAAAENADELGKENTSSERNVLSGGNGSG
SEDAHVRGGNLHSESRDGVVRPEGATSEELNVNSNSHTEALPTKEIRQVIPESISPVSSP
DSKEVEEDTASKDDKTQEILVSGEQQALQSSSSLGGNSPASDQGAELTAAENPQNDKKGE
AEKEKNLSDKNVPTGIDPSSTNSNDNAPTKLQAETLYRHLPMRIFLPHITPTRVLRRRFW
TPLLFLLPQ*
>TCRU_2388 Trans-sialidase of Trypanosoma hydrophobic C-terminal, putative
MNCGGVNFPDGTIHSPARSSDADRTGICQLPPPREGKDDTKSGGTTAPQVNAEPSRQDED
WRQSAAADAPNTVDPSPASNTATNTSETRPEFASNPNEVMVPNASTKRPSFANGPRISVA
LNASGIMSVTCGGREHVAADNTDEASEHDATGHKSLVLENGVAVSGFVAEGDADGLSQRN
ASGNEYKPQLREFTAQYSGSGVSPDDCFALRNDAKLTLRELTVDGTVRVCVYCALLLLLG
LCGVVTVL*
>TCRU_2389 hypothetical protein, conserved
MLLCKQPGDTGVFATATQHKIKIYRLSSSSQLLRETKAGAATTTTTTTTSVAVGEGMQTE
SNTAGNEWGSAFTRTAPSSPIASVPPSMTRKRCGTPIDASCVIPLSQFREGEVLSVAGGA
ARGFGRLYISHLQPNPPEGKTSGDESLANVPLTLSLSCARDDTLRFLYSGKTLGAWIGAE
NTVSALCTTSVNGAPYDAGYHNHRAEIVASGWDPLDASALAIGRRSGVVQLLNIENSNGM
LSSTSLPLNLVAAESFSVSNGCQASFGTSPTYPSAVTRNTERMPLSVLSVNVVQERLLKG

TLTSLDWAPRSHTTVVAAERSDGIGHCVEVMDLRSPDNGIRYLGMPFETTSPASTMSLAG
TGSSISEVCFAEFVACDCSGIYVATVGSQSRRDVAQLWDLRMTSRPVCRQSHSRVGYTSL
NWSTHEIPTVIATTRNGGLRVHVFSDFRSETEEDEGALTETEGNRTSGRSVYAPFYSSLS
KRTAKPFDERFHPQPRVPAASVAWLDGCRSHVRCSEGAMAEENGVGKQGEQRAQHGPSQR
VAGGSRTDIPCLLLLDAETGELYPQVFFPVGCKATMIVDVPLCSAGPNVFILDSRCLFEG
QPERKSVEPDGTSFNNNSMGSAGTHINYDPYNEMEKGGVAFCNSAESTPTNCAGMATLAA
VNATAKGVYTGNTHVDRHLHRLDRLRAGFVPQSDKVLSTLLKEQSDREAYVLFRYGWYAQ
RYLYPGRATLPVPDVIPGLLELLERERRPINSGIPEAFCGVRKLILLAAGWVTDPREERL
MLTPAGGGGDDDDDGHVERTVQLPSRLFSPSEPTLAGFPPQHQPLREKSTTVSSNLCSRG
SADEGMEATDLFLPTSQGELSTPSASSLKPADHVEMASFQEAVERRVAILVFMLRLNEAA
ALLCHYSHLHPLYSTFAMLLLAATANRNGVPPTLTLDVSECSFWIELCFFFMNAFWTSAT
PSTVPEERKKDYRAKKGVANNNDGGDVDEDHIGEFYRSPPSARRECFASFVRCFPQIPLS
DRIALATLLLLDPSPTADGESQCNILSEVLREMVQQQFYTNTTGGCSLFLAAAVEGVRGS
CVPLQRYVDETGDVQTPVAYVALYGSGKGASWRQWNEAYRSQLNAEGHAILRSLHDLSCK
KLARLREQQSGFSAKRHAVPPPEAPLGRLVGLSSPAVGLMGVPFSGLAGGAAAETKTKPK
RKNKRNVELRCKCGKVVATSPMESADTVTIMGRRQQSLCGSINCLTPMCVVCGEPMFALA
SGYDVDSSFTWCTVCLHGGHWSHLRDWFAKHRKCPAEDCLCTCYCPSLSP*
>TCRU_2390 Trans-sialidase of Trypanosoma hydrophobic C-terminal, putative
MNCGGVNFPDGTIHPPARSSDADRTGICQLPPPREGRDDTKSGGTTAPQVNAEPSRQHED
WRRSAAADGPNTVDPSPASNTASNTSETRYDFATNPNEMMVPNASKKRPSFANGPRTLVA
SNASGIMSVIYGGREHVAADNTDKASEHEATGHNSLVLENGVAVSGFVAEGDADGLSQRN
ASGHENKPQLREFTAQYSVNDVSPDDCFALHNDAKLTLRELTVGSTVRVCVYCALLLLLG
LCGVVAAL*
>TCRU_2391 retrotransposon hot spot protein, RHS3, putative
MKQPRVSFGASWPQLGGASGMLHCTGVVMAPHSGISGDGSDAAARNVAGSKQRLQWTMSS
SVEEILLEGDTLSTDMKLNDFLRSNLGGRGVVKKK*
>TCRU_2392 hypothetical protein, conserved
MRAVEAADGDDAMNDTSSAENSEESASGAEGKGAAGAAAPSLDAGVSEADEYKKESNAFK
PNGNAINTGDPTKGAANEF*
>TCRU_2393 superoxide dismutase, putative
MRRTLRHGYHAYVPKGALLKTDMLASSPQLVTAVFRETEAAAERADRANDDAGFFSRPRL
NYPVASGIPAFISRRQFDVQYNIFHHDAVATLNRHTLGTSLEGHSLETVIRRASFDATQA
AAHTAAAEHFNYCFFYKSLRPWGTAVPKQLREAFQLQYGRDGSVDVVEEVKRLFTVVVLS
HQERCGWVYLVWTGKQFDVVEFPHGACPIGSDLIPLLAINVHEGAYSLDYGPSGLEQYAQ
NYFRACNWFLAERYYLQATGRGVELRCMNVSG*
>TCRU_2394 hypothetical protein, conserved
MLQGTGRRLFSTFALNPETTVAPHGPPRGLINRYVSMGLPPWAAWCNKINRYSLYRMSGV
APRTLLPKAPHEMDVIWLNERVRERVRTSRGVQNVYRQLKYPYVKTGIHYSDVLCHWVQV
PMVEAAMFEVERDGGFDNFVLKRSGPELRSTYGERVRRHILVRQKEIRKNFVLDQQAKHL
ADAICGEVLSLRDESAAEAVLAKYGIEKKRFLEELARIVVARKEKANAVAVTAEEPLP*
>TCRU_2395 hypothetical protein, conserved
MMKSLRRDAAGRGRQRKDRSAFWVAPPQRYVINRHSPKAVPHSLSVVVVVDPHMVEPTAF
LQPIAPLANDKRNCGAAGNGDAIFAEPDSASDQVVPSFFRLDVYVWDDTTLREILEEVLA
SSAAARQVLLPSDAPATATAEVVGEASRPPASTGAEKAEKRGGRDGGGDDGGNGNLKDDD
DDGASVELDDGDDSVGGDAADEEPASKRERTEGEKKQRDETRMGAPSAAKRAQPRDRRPP
PTPAANVRLFHVFVDSDKKPQVRDIALLRVDRPVFHAGDYTTMQEMRTFKTGGRGWKNGD
LLVLSPTTRHYRATPM*
>TCRU_2396 ABC transporter, putative
MRTQSDGDAGASREEALSLGKVVNFSWENLTYRVPVEDDDGNVTYKTLLYNLSGTALGGR
VLAIMGPSGAGKTTLMGTITGKLYNAKAKLDGCCFLNNTIYSQRYKKLVSYVSQDDIVMG
KETPREAIKFACRVRLGLNMEESEKLVDEVISRLHITECQHTVLGIPGILKGVSGGERKR
ANVGTELVTNPCVMLLDEPTTGLDSVNALRVGKMLQELARRDMRTVIATVHSPSSDLFDV
FDDLLLLAKGRVIYHGPTEDSVAYFASLGYQVPPRTNPSEYFMNILQLPDEELSQLWVAW

EDYVMSPAANNNHCLMVVQGPITRQDEFLESQLKVKKSSCAVQFFELWKRSLRMFCRDPA
AFFGRSFQTLFFAVFLGLFFFNLKLNQQGVQDRAGALYITLINNFFGACMHGISAYPPER
AVFLQEQANDSYNAVVYFFAKYLAEIPFQMLFPTVLDLITYFMMHLYRSPGAFFVNWFIL
VLLATFGYTFGLMFATFFESSTTAFAIIPVIFLPLLVVAGLFANTQRLVPYWVWLNYLSF
PRHAYLGVFVNEFQRLRVICDPVTPHCTYPNGEAVIEQFGFENWQPWRSMVALIVYQFGL
AMIGCTSLYVQGMRRRGKLAFKKNLGSRVASPRAIASARSNDELSDSDATPMDEIPTPSV
AYTDTVPLDKDDPSRPGGGGARGMLTTPIVTPKADTRGQS*
>TCRU_2397 arginine kinase
MASAEVVSKLEAAFAKLQNASDCHSLLKKYLTKEVFDQLKGKQTKMGATLMDVIQSGVEN
LDSGIGVVYAPDAESYTVFAALFDPIIEDYHKGFKPSDKQPPKDFGDLNTFIDVDPDKKYV
ISTRVRCGRSLEGYPFNPCLKKQQYEEMESRVKGQLESMSGELRGKYYPLTGMTKETQKQ
LIDDHFLFKEGDRFLQAAHACEFWPTGRGIYHNDAKTFLVWVNEEDHLRIISMQKGGNLK
EVFGRLVTAVGVIEEKVKFSRDDRLGFLTFCPTNLGTTIRASVHIKLPKLGADRKKLEEV
AAKYNLQVRGTAGEHSDSPDGVYDISNKRRLGLSEYEAVKEMQDGILELIKAEESAR*
>TCRU_2398 hypothetical protein, conserved
MISRTAVRTAQEKQGRVHRLDSATVQRLFPPSQIAQWPQGYLPACGVARFLDPKGLPLSD
VIDCYSEMKADQRHELQRDFAADYALWCRGVLADFVRRLGDREGRRFRRYTARTRAMHDA
EVRELFGLSSYAPPHAKAGRRYEFTPYAATQTYAMLPVSRCDGPSAKAHFTTFPQVRNVM
HTEFDSMGPSRALSLLTGDDLVPTAEMLQALALYLRRRLEEITAGEETFRRSAHKSAGSN
GSCRSSSTPEEGDNSELRSFSRETPVACFFGNGRLAWLLNNSELLPFRVLPLQPPSYAAR
RERQQRHLLARSGILADLTEKGVISRSFSTIFPCESISVQDALRKYRPCIALVEPHTDRD
WLCDLRGFYSVREVLLLGPVDSPAMGSFAFPLLTFGVTPGPMSYWTYSNTLQRVTAASRI
QMPMDPPHVLQGYTRHAVDDISAYLLSPNDCPAFGCQYRSLSFVRTVYPVVIGQPARFPT
ESSALQEE*
>TCRU_2399 Protein tyrosine kinase/Protein kinase domain containing protein, putative
MKTRVPRLLLEESLFDDRSHLISIHNAAPEENSLRAMQPTHMNAAMMGEKSERTEKLAEF
FALSRSQQAAELANALLRQFAFRTYKTSTPDIVPAFNNAFPRTAHSLTRKSLVLALLDEM
GLTEERVASAEDAETEHFSSTCNPSRRMTSVENSTVSEWEREDTVLERLLRHVLRQFHAQ
HPCWCQSVNPDSSTTTSSSTGRNRNSRTVYLREWRKDRKNCDFVSNGLDSMAHVASPVEE
KELDASALRRFFRDHPTEDEQVFVVANELLQQHIRTPHRKPMETPLSGDVVSEREFSSSS
ECVTQPNMSRVDSLNGSICENRKEDGVKLPRERLVLIELLKSVHEASISSRVLPAIFEKT
VAWSSSNAMRERTLDTTVSNPTSTYSSGTSLEALSRQIGSNTTSGGEIQDAQEFCGREKR
RRLPSLLSLYLQSHLQEKSPSMENVNKRMLRMRRDSPKLTSASVSFRESPSACVFSPVMS
SDAHCNIGSVTHYKFSSPSNIVSVRSMEGMTRIGRGSSGELFAMRRVDTGELVAVKRIYI
TDLSREFVENELSVLRRSIGLSDDAGGGVNGNKSDGAESVKPPTSRETCDTDKDDDGNSE
ANDCRAVIRCYDAFFDGGSAFIVMELMDSGSLMDALNARFGRPFSEEECRAIAFQVLHGL
RYLHETIRQLHRDLKPHNVLLDKQKGAVKLTDFGLCSEQLQSMGFNQCGTYVGTLAYMSP
NRVDPASVYGKESDFWSLGITLIQLALGKLPFFPNVFVIAGFATHPPRLTTAFMSGEGKD
GTRGEKDEDSISSLTPFSEAFEDFIAVLLPTSRERNHVIPLVPAEEENNDSTQSCAYAFS
EHCGDASSYSPADVEYGADEGDETTENFGGGVGHCSIAPFTRWILSRSSTPDSHTDAMQE
LPPPALPEPRGMPQVKGSGNDNANAIRQDDAATEEGHLTGELTSTALLQHPWLRGMTWEK
SRAIIAAVSLEP*
>TCRU_2400 hypothetical protein, conserved
MSADLLHFSETSPRHHFESATALRNAVLSKGDEYSEYERSILEEINELRRDPVAYSFVVE
SEATVGYPFVRDDPEPAECTEMTLEQLRVYIEDFRRERREVRDFLQHLQKEWADAESAMK
ERWSAEDVERSKKSKRGSATVRKQRVLVHKEEDYAGERQRAAQELTDHYTQQAREANSKL
TQIERSCRWAVDGANLIIKCAHELREANAVPELEYSRSLTLAARDVGKECHGAPAIGMQS
PQASLFSPLLPLSSTTGDEDRIRFTQPPSTASTRQGSSVLRSSIVPSSPAEENRHAALPP
KNSIRHDAGSVVDALFTRREENSERAPTPLLKVGTNSLQKEPPHGFHSHDFSSECDYLAK
LAMKACGKYGYYSAAIRGVRICGAFSPRKMLIQMLLGTRVPQFTVPAPTAPSDSCEPFTS
SQTKQTKATPLLWANGRLLGIGWVRAINGTVSVTVLVATSFEELSLIHERRDFTLPQLHR
ILNTSCSAYTRTEQIRPAVMHIQSLLGVQVVEPVAHPILVDKEQRVARLLVRADPNCVEV
TATVSCVSEAIPSIPLLDRELLLVQRSRDDIEKVEILLDTQAAWHRWSGHPLLVHMFERD

KTARGVKSFKNIGFVRVIPLQRGDGRFMDSRQFTSPSSKREKDTAVVGVPPLLVHKRYVG
PMEGQAESCGWPLVTVNFQELCATLIEPLRGKWIACGEMQHVAIQIPKVAYLDKSIKNVR
VLLEKEQAFFEWEATSGSQERMNEVITSKMATLKVAENELQEKEGPMQQELAQLQKNMVK
KRGKEMAKLKRQEEELRQRMDDMKKNVEELRQSLSNAQEDLVQLGREYALRVKRQRNLRK
EYERLRDRADRTKPLIVEVVLVTDETTRSTVAENTRLQPTNNACTLYEGDVRLTLGFKGC
ALLLVNEQEVVRWEVRPG*
>TCRU_2401 hypothetical protein, conserved
MFYGSIPLRIAFPVHRDTTKVPRMLKEVGTWLYRQETVDAFRVWCKANGVRGHNNVKVVS
SMHYARCLRAARHIRPGQAIITCPHTACFNFLVVAKEMYNLNGAAATHNFPLEVNWMNYD
ERCKFLRGASMAELVTAGWMCRIASLEESSFTPYIRWLLEDTRGRDGVANGMSKERGEDS
GLVDHYFSEMATDACEDPEVFLENLFRSFAALHLRAVPIESAAICLFIPGTNFFKAKSDE
MFVPTLIPLVDAVPQLEDDAHNTVVQYFPHDRSDKESLARRCRELFLPEEEMRAMEARLS
QGDGDSGFFALRALCPIEEGDHLYLRGVPKLGDAAKESMTVKVMEANRLMSND*
>TCRU_2402 WD domain, G-beta repeat, putative
MEQKEGTGRSLVKRHAFGIRPDVYGCLNWVEEGTLLYPVGKTVVVHNLNSNTQRFFDAGV
HSSGLTALAVSANKKFIAIAEGGIAPQIQIIDSVPRKRRKTLSVQDLGSESFVAMAFSVD
GRHLATQGGAPQWNLFYWNWERSKPLALTSVIADLKVEGMNESTSRFSLLHDTRTKEPHG
ASSGPPPVSCVTICPRDPLLIAVSGLGMMRFYRYVDGVLQTQPPINYDLEGVSNFLTHTW
VTGDRVVASTQTGELMIIEGGVYKRLLPVPPPTRAQLTNPTVLSLVPSKGGFIAGSDQGT
VAIYENASGSSGEYSIVYTVPVPTEEELHTKKCALEGDDTRPSATTAAGNRAALDSNASG
SSLADAPDPSSLKPWATKGSKRIFHVNTGVAQKVTAAPAEKTRPQSGIQGMARMDVLREN
SVVHLSLDQLEETMALVTHSGQILAFNFSQQWAKRTADDPPALLPICQSFHVGGIIGVDC
SVRRPFLVTSGVDKSVRIWNTSTHRLEMCQYFPSQPGPVSIHPNGLYVVICFTEKVLVMS
VLWNCLHEHRVLNLRNVTDVKFSVGGKYFALAHGNLIHLYNALTCDVHGQLRGHPQKISC
FQWAATSPYPTDNGMVSCSLDGIIINWNISELRKDGEYADKRYQYRVVVADDKNMWAVGD
PASSAAETQCKSMLREMDRFSSVEASSPTTTTDYEFRDSTITTLLVSPKQRLLFGGTDDG
SIKFMNFPLQLGLQEVPIVAHMGKVARMVLSYDESIIYSVSADGTLFVIEVREDGRPAQR
DAGYCGDEVLVLASDVEDRQIAIESLTHTAGKLKAEIEGEEKRRSHEQNTRMRERAEEFK
SEVSALEAEYAALWSAKAEQERSFVAVRLEKEAEAAPLLEELERAGQAEVQQLEDECTEL
QHQLDWSKSKYMQEVSDLEAQIERERREEEERFNDVVAKRKEGMQKIRRQVQRAKETNAE
SRRRLELDTDAELESIRERNRQDLEAIRERYLHMKGESAIMRKNALRMEKEAEVHRNELS
ILESAKSALMAQLGELNQRMSQLHQDIEERDAVIGEKEKRIYDLKKQNQELEKHKFVLDY
RIRQLKSQMEPRQREIAREHQLINEKNVELENLHQNNITLRQSIEGLKADLAQQQLQIKQ
TMNHMKDFETYKSRVKTDVGELAPAMQDPALLREVVERLYQRYVLARDGRLAAQVDQEIK
GEFRSQLEYLSTSVDALRRKCKADQEQHRCKVSEMMVENLSLIREIHDLRAELVDLRNLS
VAAADEKKRRARMEEFKKQASMSSRQNVSCSPTATSPTRPEVPQELKENREEIQRLRAFV
DAAQQALGATASTRRATGGTCMFPPITQ*
>TCRU_2403 TFIIF-stimulated CTD phosphatase, putative
MLYRECCRPLPRYASTGAYGAWMASCRMYTSSLSSGKNRGVSDGKLFSRTQSSIHMYANK
ENAWKDYTDDRFYGAKRLKDYWRRMLSDRGVRYYLFGTIIILAVAGSKLWRLQEQQKSTF
GLIGPKKKAYRSRLTIVLDLDETILSYGDKAFRMKAGMVVRPYLAELLDYLSSIDAEVVL
WSACSERYMRQVLNVIDPSGVRVSQYIVRHRDWFTQDNYYEKNILWLKRPLEDTLIVENR
PLSVRSCNANAILVDDFIRGEYMDNGQDYPPKDHAMRTLKEIIQDLEESGRSVQEYLADA
KHRNKDIKEIPCHLAFRQLPDELARGVFYFIGGKYRPGADMRGRG*
>TCRU_2404 Phosducin, putative
MLGSGKSSGMVNEATNLKANQNLTSAIAEMGTKEYMSNRDMDDVVPTHRRRMEEKPRDNG
VPVYDPVAEKEREKQERGAVPRDIVERDDDDDDAELLELRRRRVAQMQRQQTKEVEWRQK
QHGEYREISQDEFFGIVVREKGGSDDVCVHFYHKDFETCRVVDYRLSELARTVLSVKFVK
IDAEKSPFLVERLRIKTLPCCVLFHNDVAVDRIYGFDGCMTEDGTLDPVLLRDRIVRATN
PGPEE*
>TCRU_2405 kinetoplastid-specific dual specificity phosphatase, putative
MSLSHSGLPSAEIERVFAVLRFVSQQQEASLSSLSLPAAGGSEGDTLNAGTVAALAKGKA
GKRVSDPLIIPEDVQNKLEAFRMAVTNIPVQMEKVQSARAALRTALRDAYPNKLEDVDQL

CDCNVFVLKATAKGTPHFMLRDVYALQAMHEVVPGLYVGSYHPASERELLHRHKVTHVLC
CIDVMPRFPSEFTYMKISAQDMPGYNIAKFFPQTFEFIEDALIKQHSAVLVHCGAGISRA
PTIAAAYLIKKLRMTAVAAIELIQSRRHVASPNLGFRQQLRAYQEELGIFPEGAEGARPR
AGMSLQINNKG*
>TCRU_2406 hypothetical protein, conserved
MHGAGVESCLASAYERRADAVLRLAEELECGSPSAGQCSSPHFFRALVTAYLVQNDAVNA
TWALQRWATGPAGAGEQEEEGGVRAMLERVARHCGRCAYGEAFREALGAVGGGTGRDVEH
LERWLLDYLAARHVHQRRTFYGESGGMEKLAVGLGVTVADLEARLQRVREDELRHIGREV
SGGPCEKTRDTLCCMLQVGKAV*
>TCRU_2407 hypothetical protein, conserved
MLKAPYMRAAAVGRKIPLLPLEYYSGGRHGAAFMLAELRRLDDRKDSSISNTAPSLALWL
YRSSLLRRLWLEGIVCDAVVADGFTAREFTALLHRILQTPRFLRGENKATQDEAVKLVWG
VQKEFAWRVLGAIVEENQWFWTLREAWDCATTLVKDSVFPDDVMSLSLLLPLARRMATRE
GSGCTLHEGLRDLHAQKLHGGVLWFLARRATSSPRPEKQLLEAATKAACAVPTFLIIKSS
DGKLNGDDEKQMASLLQMTHGSGETSQESVFSPFWHVLLYEMVYYAHCILCDATAKERHQ
VGTLLSLRRVLCSFCRHSLVRPVLEFHCAITAPWRKGLEKDPSALNARYGPLVYREALLL
DAYALMTLGHVGETAIDVDDAAVKEVMMIQSRCLAHLEALAQNEPDAGIDRDAMGLHRGA
RAGVERRLCEGIRLSFKLLLRRGAYEVIHQILTRCPSLGGSWEGGKALVMLGKHMEAVSI
FTNFLESTRKQFASGIPHHIRCMMLEAVEGAARSYLKETSDVTGRLLSLYEVTRNWGFPV
PAAVTFAAILRACCTGRNGTEGFRNWGRKKRLDLMNALLRHHVTVAAYIGDGLLLETIEI
FVKLLAECREGALSSNLDLLPLLQRAHPGLPVYRVAILHFLRVEYKDAVNALLKHIAPSS
ADTTVLLTPLQELPLAALEALSRHVAQAESEEQRVVSAVLSALEYRRFAEEQRLPWQCHL
CRHWNKKNASACKLCGSLELAVVRCHACGGFSPTSASGCLVCGAEKRRNTSDQGVSHVRE
DCTIFPLRRWRCGRCNHTNEPQHLFYCSKCGSVQPEMVEALTHTSFDCRVCSHHNPLGLL
RPWCPACGTLSTVAAAGPPKTLWRCVECRTLTPWILTHCHGCGGSKPAPTLRFDTPWFTR
VCMSCEAINPAWSVVCHGCGVKLATDGGKKEHEVEEREQLVVHSARGEVSAGEPTSTSCP
HCGNLYPDHRDMFCRTCFVLRPNSTCLLWVCLHNNCLGVNVRYGERGERGLLVCSHCGVS
HNIAAAGGYHSNPLRYRELTSVEKRRGCGKDPPTGDESLGMQSSQLPSALLLPVPSAPRV
CAFCGAYLQLQNIARICHVCFYCGYSLPHSSDASAAEHDIALRLILSAMLQTVHRVKQGL
LSSTTVLSPLRSMLMALLRAQEVQLPWRGCDLLNGMRCGLQSRVPVLENDTVHSSVCDVA
EIIRTVCEIASSTTVDEKATAKEGTKSSWTKKRPWVHLALDLVDLMNRTTEYDELGFDVL
AQLCMEVRPQQRSHIYRETHWTYLRYMKLPREFIVNDVVCPTCLLPKARGTGEAGNTCRC
TVSLTDSGATDASVMIKRA*
>TCRU_2408 hypothetical protein
MHSQASAPTPSFSTASETVVSSSTLPTSETRMEVLQRTLHLLRQEVLVPYFEFVERLRVT
NNRAVRVQDVVETLYTASFREKGSSA*
>TCRU_2409 UV radiation resistance protein and autophagy-related subunit 14, putative
MSSSVFPDEFRCFRHITHIYARNLPLHPMGATVQAADDGGIAGSALDTPTLCDGARLGCR
TSQGQRCVSSYFVLSLPPGKVGKMAAEGGNRRIYTSEVRRETQQPVWDHIPKETIEQFKG
ITRFVFSLYYCTTHPEEAIASPETDCLFYEMLIQTSHVQYVAASMTKADSLPNLPYHVEN
RPNSFVVLLRCMDGVFFPKHQGVQMDEGGEGMDFIKTPYLHEDSDPTRLCVDAEVMRARG
KSINAPVEKITLGDLKTFATATLAWTYLSELAVGRRGERQAYIDDMMDSRRGEVAIMAQR
AMLEARLAKAREDLGCRSSELLELRNRYKEEQESMMLQMAQLEKLRSSTALEEVQQQEAN
KMLNEQELPLSTLRAQLGTVRRLRFEELRTMFPIHVDSAGLADTICGHYLPVKGKEVGVT
SDVMHDWGLALGAAAHLVATAATIYGCSLPHPLLLCGGRCCVMAKPGLPVAAVVSPFTGP
SDVKLPLYCARTADRPLMSAAMQLLSEDVAALARAMGHHDIAANARDNDGRLAVILKQLL
LLL*
>TCRU_methyltransferase, putative EMG1/NE.1
MSRHDLARPARRSVKRPRNTSNGSGARSDVYHPLRYTDSTKQAMVPPPTERAVVVILENA
GLLLGREGVVDAYDRAASTEIANASLKDVRPDIVHQCLLALFDSELGWQRRLRVYISLFA
RSGRTIEVSPALRPPRTFRRFKGLVAALLRDGAVRSVDGTVLMRILPGSVAPVVPCGAPV
IGLTNAVAAPVRTATQLARKALADPVDDNLQGGVKNVAGFFCISCTDEAGLDSIDYVTQQ
ACLSAYPTTAHVMCARICEGFARAAKTIDPAASKSE*

>TCRU_2411 hypothetical protein, conserved
MLAQGVAPRVAQAPAVRVPTLEPSTSSSSLFTGTRMFRSNPNERQMQASTLSDHYRPAQV
GTDQQYCPHTAQLRELESPINTVKIEELLRSVRATSTAAVAAVGELTDDSPPPRQITSSQ
QRTPCEGCQRRSASPDRVHTARSSPMKRTPRGESPARPAWNQNRPTPRVKPTVSAPRTEP
RRVTRDASVENRSRPWMEGMKLKNSSRCKLTAERDKNHQKFTPVIQSQPSHQVPTCARKA
SSVGVGMSSRTVSGGTGGLKTGNDEVAFQPRVEFLEESLRDVQQRAVRAEARCEELEAAL
QALALEHRESISRLEGRNVQLNARVQHVLQWIEHVDATRVAREVFAQEVEMERTNESGDV
EVPSQSLSINNDVGDSPGIEKAVKATPVRVPPTPPMTLQIQHR*
>TCRU_2412 Meiotic cell cortex C-terminal pleckstrin homology, putative
MDSIHEAHLERILENRRRRGGNANYNNRRGSSTATQTQQWDAIKNVETPTLIKYELSPTP
RGVPGHSTADEAGQCENVARATRDEADSGLGVCIEKRENKHYKRERLIANSSGSEHDTGS
FKYSSSPRFGDRDNSNRGRCRFRGNENLQLRWRPLGNFSSFFVEKNVEFGRRNGFASARS
PSRTVPLLTYGSSDSPTLRWRGVSSRRGRTVSPHALSTWHVGAQLFQRQDGLRAGGLGND
RRSNCRGGNGENRSYGRHDSSPKIYMDSPRGVKQRSNQRRHSTEAAKIPFGPPDERNWQA
AGSVIYTGNAAKEEGSPFVSSRQRRRPPGSPLPQGAPISMADRNSMTDPNNDVVGLSSTR
TTIDIRTSVSILAAGDWFYKWNGNGTSVSPRWVWVDSQSHLLLWAHKETHESTFAGAIKL
EKILQVVFRELERRNAEGVQLTYYVLLVETSRRILQLATERRMKADTWYEALNNVMSYLR
SRKFGASFTSPIS*
>TCRU_2413 transcription initiation protein, putative
MAEELNVSELADLLGDDELLDVTSPLVAALPQSATSAELDGKDVGSKKKHKRKGKRLRHE
KKHRKLSEIEENNESEDDNNETPRKRSKYVIDAAESGDSESEGDDFIVGSDEEDEDGDYV
GGIPTYEPRKTHIFREGEENMTSEELARAIEERHRASKKEGGKTETLLLAGLGKGNMSSL
RYTSHLLPQDTDPRVFAVKCRPRMARLLVARIVNKCYAYRIGRNYEKRKVDLGIISVFCL
DHVKEYIYIEAHRKVFVENALNGLDGLFRYKITLVNPSELMQMMERRPSPEKLRVGSFVR
LRQRQYRLDLAQVVEVHPISNQVTVKVVPREDFVGKPFNKIEIRLPPRFFVPTLAAYVQE
RGDFYRWGDLTFDKEGYLLKTVSSRMVISGTKMKNPTVEEIALFFNNDRERVREAVARFA
SVGHRGADLRIGNTVRVVSGQLRETVGTIENIFLDTNTVSLSCPVPGRKEPIKVRVELAA
CVKHFALGTHVIVSSGERAGESGTVVNSSGDVVYIFSDRATASRELTVCASDCHQSNLIG
SFGHTSGSWRLFDLVMLADTSSVGCIVRINRNDICVLTDRSETRYLSPMQIKAAVASPRQ
TTDRLNNIVTRGAEVIIQTSPNTPYHLVGQTGRVEQLFGSTLFVRVRSVKENAGLVVLDA
RSVLLIGGRTSTKHIQQPKQLPAVQLNPHNATKGDVSVPHPRLTSEDWATNSEWYEIDAS
*
>TCRU_2414 MORN repeat, putative
MQKENDRMRKKKDETREEEEISIFHCDDGSVYEGRVTRREIPVELPLGMPTPAPASTLRG
QLPPEVMPQIVPIQHGKGIFKDAGGTIYDGNWIEGAMCGEGVLQFPSGATYTGGMRANEF
CGTGTYNWPDGSRYEGQWENNRMHGFGVYVDAQGGRWAGKFYRGCGVGLLGEIHL*
>TCRU_2415 GTP binding protein, putative
MPPKKKDEKSQPPRTILLGRPGSNLKVGIVGLPNVGKSTFFNVLSKKGVPAENRPFCTID
PNTADINIPDDRFEKLVKINKPASIVPAQIHIRDIAGLVRGASNGEGLGNAFLSHINECD
GIIHMIRVFEEVEVTHVEGDLDPIRDLEIIFSELIMKDLQVVNGLIDKITPIVNRGIDKS
KKFDLEVLMKLKEHLENGEQIRCCQWNGKEIDFLNTLQLLTAKPAIFLANMSEKDFIRQR
GKWLVKLKEWIDQHTGEPLIPVSAEMEANFLNMSPEEAEEYCTANKTKSQVHKIVTTAYH
AINLIHYFTAGSDEVKCWTIQRGTKAPQAAGKIHSDMEKGFICAEVIHWEDYDKLENEAA
CREAGKQHQEGRNYEVQDGDIIFFKFNAAKGGKK*
>TCRU_2416 mitogen-activated protein kinase
MEGYETLGILGEGTYGVVVKARHRATGRIVAIKKYKQAEDDNHVRKTSLREVRVLKQLRH
PNVISLLDVFRRDGKLFLVFEYVENTILQLIEEKRHGLPPDEVRRYTYQLLNGVDYCHAH
NIIHRDVKPENILVSKDGVLKLCDFGFARQLSSKGKYTDYVATRWYRAPELLVGDVFYGK
AVDIWAIGCIFSELSDGQPLFPGDSDLDQLALIMRSCGPVPDRMVDIFERNALYRRVAFP
RTPMEETLQKRFKSCKASWLELLTSCLRTDPAERPSCAALMNMPYFTEDNFRVEYEAELR
ALFTQCQTPAVDASNSSGLVAVQPKAPLFPGEPEETGEVFLPLLAMPPRVLESEDGWSSG
NKRTSRGGASLGNGCSGASPFQQLGTLWSHYLGTAINGGVNNSGELPSISTRQMQTHHTM
ATPIFNGNKNASKPENKANKEMLISSIQTLKMAGGSTQVPLNPPSTTSQQGSKVSGVLQG

TSTHSRKNWKNQGMASKKADPMGGWKLGSLKKSIKLGYPMFPALPSGCSSPVSTAFTTTS
SVALNALSVLRDANSNTVPKSMPKTHTNTVPTTTTTNTNTNTNTTTPTTTVNSSNGDGVV
NNNHPPKSKRKQSKRTPLPGGTPASPPRVSGNTTSRHNVNGQKCSLHDVPSKR*
>TCRU_2417 hypothetical protein, conserved
MTQAKNEDTDEFAMAIAPGRRMRVYSPAGSLVALARALQECAERIHLGPHGLLAASVSVD
GELRLLRPRANDVMRNTAGLEAWQVLWRVFLADNSTPRTRAGSHVNRGAYQRLSDGQGRL
LLTCASLLRQLLSPNTHYPVAGSPSSLRNRLRLVRVLREITTPGEMRYVSPVGDDEDDDG
AEQQRGDDVIGRMVRASLRDLQPYVYGVAGSKVTRRVVEGCIHFIMCTHVEDDVLTKLRQ
LLVRFLWSSSFPNGEGDLHSIVARARDVLIDPTSYIVPCQGESVLHSRVLPFGTFLLPTV
SPEISPHQGTVTPFFTLERHGDGQAERVMFLRIDDQGGLLQFEHSNRKEKIDTDSDMDAG
DCSNDGSRVVERQWTTTEQEKAQWEAARMLYEWAWMHGVTAVLTPSHVPPVMKTYGLRYN
VTGNRDKERECRRPIFMVDEVDEVGFDALLRRLQYWRHEKLTERCGAQEDPVWCPTAAVL
RRDVLELPGRAEDVLFPFSRLAHGPLLEVCSSANVKEFMVNECSNVRVVLLELDQGTLLE
ESARGLRRLQGLGSMLLVAPTAYQLRMYTDLLLKCLASVRAAMDASPSSGDASPECGLGF
VRAGGALPIALSRQLRLSAEALKCQMRRLEGTSHVDDAHATANLPVVAAVLQMLSEVLLE
LPRRLAELITAHHQPLRHAWLQLESDAISRAKSHSANSLTSLREPPLHVCIREDVSFYRR
ARMPPPCEVGHGDGERSYFCCSDTESSDSESDEDEDACRQERLRGPPSLGFVEPIATTAG
ALRAAITLLRLTLMDDSGALETAGL*
>TCRU_2418 dynein light intermediate chain D1bLIC, putative
MKGRSDAVERKGRSGFLDADMEPSGANGVLQGDAAHLDGAALPTEDGPGMPQVTVDPTKD
LWQNIILNTRENQVTQTEVNVLIVGTSGGGKTSLLRRIFSGVNTSSSSVPKSFLRVKPTT
ALDYSFVRRNDRHIAQVAHFWELAQGMELSQLCEVVITPENVHTMVLAIVVDCSEPSTMW
DTVVYWMKRVDRRVIEIFQKMRAKGSATPDKLLSRAKRLVGMEHPDLNRLRLSGVPTMII
CNKLDAFAGEMTMLKTLVRSMRFVAHIYGAYLVFTSDAEAIKLRAVMNHLVFVSTFDLKH
IELDPERGAVLVIPSADTFADIGEPAVSDMGGLQSTGDAELDRWKAPLDAMFPAKQSEGR
MQNDSFLKRLYDTGENGFGEPTVDAVRKQKEDELEQYRKSAFKREKSTKDDRGKSKEKKE
KE*
>TCRU_2419 hypothetical protein, conserved
MMPCRADCCSHENEVVEAQFAGWDSCTATQEVPAAEEECLDIDYAAEIEESSRYVFSQRP
YCRNTALAVCGGDGEQGEFCGVREEEQEQQVRELQSHVFVTRGERVQLQDDTLSPSDRRA
SLVFLMGEARRRHLALLSRHGASEGSVMEAIRQWRAFARRFLDELDRWGHEHARRMLSNM
FLFELPGKSCNLLDSGARVRELIRNKERVLQELRERNQLFLTAEANLPQMDTSPCVSMAS
EGEELEMFPTAADSLSLMAMAQLNDPSFAKFQEGREVELCEFFSFSLDTSLKSGEGASSS
DCSSDTDEYADACRSPPSTTGDLWLSAPCQ*
>TCRU_2420 Rab-like 5, small G protein
MSEERVKILVLGPSRSGKSTIANFLAGLRETPTKGYYETQPLRILEVEIGLDNLKVGGRH
VVGPKKAIVQLWDLGGSSKHQAGWQAVAANADGIIYVFNPEVRGAEKELVLWHKNFALDQ
DKLDERNNFKVRVPDKHSLVFSHHSSMPELAVGDNAIPSLPKQLRGVRAIETSLDYQSGN
FKEAFDAMVEQIVVSRIVAEEDEILQKEREAGDAPRMVR*
>TCRU_2421 hypothetical protein, conserved
MDGKMTALLQQLQHVLATWDAVNGEGEPGTIKLSRNRGGANHKNLFLASVDAAAADNNGN
NTKKDHHADDESCGVVGKSSETYPPWLTEAECNQSGKEVVERRDGDAALAAALNGTGFPD
MSVDPSLSRQRITKVELARAALALFTSNSGIYRPILQKTLGFIFGLIDELLEEKMDLPCR
EETPQNFLPLWMATENEEVATAHERMRKMEVRMAKLVDEAATQRDHLKAEIAATRAREAK
LEGLLKHQILLSHAKGMADEVFLGPGDKETDALYVRRLIARAENEKAIDELQGELRLLQN
ERNELSNRIQQLMELNGKYARQCVELAARLSVLTDHNIAIAVDCKRHQNEVLVEKQRSER
YQVEIQVIRNVLMTLFMNRNHSVQSWWESLREKRMKDATQVLLNLIEASKRFPSTPQTSE
GSKTSRHTTDERRQGSELEEEDDSDAKNKETSSPYQAADENSLCHMWIPSAGNNPETPEH
LRSLSSVEYIRINPPVAERAVYVILSEWACDAELRPLDIFTRDYVLRAMKSSDASPIFTL
QKSDQASAAEEANSHLPITYALDALSRSCYCGSLTYAFGLVTRRRVNANLFRMLELDAAI
LISICRYLDIKRSLPGKPRGLIPVVQFASLLVAMYPSYPLVRLQQLLDAARNDAGNQMDS
MELLNYNVLLPERLFLRSAFITVENAGALWDTHFKHLLYKFVCDDVEGSWQAVEDALCLY
ASRVNVASESQLLYQAMSELPKEGRKYWLPSIRVALSCWDDLRADTASVLLDGMPRVPVK
AAASYLRGQLLIRRGTCPNSLRENEELLSGLQERWNKFAGSYEKLNFQYYAQLVEILDGK

RHAVWGPASMATDDVMRHIGPSDDLSVGALLARLQRTKETNK*
>TCRU_2422 Golgi transport complex subunit 5, putative
MFADSALMSPDFDEEAYLRYALHDPNCQAEQARLASCVKAVREEVHKILSENAEDMLQQV
TAACRAQRDVAAVRQATFSLMGSTNRLRQTIQEPYRVISANITKLGNMNTAINILRSILK
FIGLTTRLKSQPSQDLARASRTLREVEELLQTSNIKGIEVVDNRMDTVERAAVTIRTKAQ
EMLRHGDAQDASGVAISLQCLFTLGLLPRVLGSLMTEQKREVIRSLMRDLDPQTIVDEVN
HGGSSATNDMNLRMREVLFSRIKSALANVSQHSQVVVSVWRVLVKKVDPVTHTSYLSVVE
SPTSVLGDYWHLVTEKLCERLQAVQKRPNFLAVLAKDVIQYQNLLTGFLGSMNELLKILD
RLMEMDAAALKRTSGSSPSSKALHTSTNTTTNATASEELKRLWLSQVTQEVDERFAAHIM
DRHRERLHHILSKLSTILPTASGRSTVSMKVDLQRPEVPAAARVLDTRGYTMLATQDVVT
YRQNPHTLGIVLDCILQCLASFMQPVTETTGKWPLPPLPSVSGDATASQMLHICVSNACT
LLCNDLTALLASLPEGELFAADFLNDWWASNGSRREEQGEKSGSATNDQKLVVEKYHQLR
GFTKRFNSISESVVQPFFRSVSVLFLDSILMCVDGTLEQEVAGIGQLHSQMRHFMSCFYY
LFEPQTPTLDEHSRRLTDNLLARLIVAVTLTYPFTTKMQKHLMNCLQQIPRVVLSFGPAF
GHGTVGRSTMSLKGQIHQLTVWYSLPEEVSEKPIGEWHTILLTLPPILARLLLLQRVLHP
GIKTKKPYTLMEVTSGGFMEMVEAIVLEQLYTSDTPPRSIRDGTDGVPRVTRLGEVLVAV
EKCFQESVAVSDEDASRGPTVRWMESLWAQLNS*
>TCRU_2423 NADH dehydrogenase subunit NI8M, putative
MSWRARFTPSVASLTVWLNPKDPNCFGVRNWWRNNLPELQLLNPSCTFTIQELSFGEPHM
YINYTPTDQRMIRLAGATEEECEDIMEACITYGMNHAVIERPRTDDGGDLVNQPAIISFG
YTESFMSKLEVSPPAEIGQRTPEGVDDPGQKPRMYPRNVGCKLMP*
>TCRU_2424 Amino acid kinase family/Aldehyde dehydrogenase family,
putative
MATVPVPVERKRLDHITSRKDLKFAKRIVLKAGTSTIVTESGFPSVRRISNIAEEIFALR
SEGKEVVFVSSGACGIGRNVLRRQDVMVCSAFDRLHRNCRPFSSIGNNTFAAAGQANLMN
LYQTVFQQLDITLAELLLTHHDFLTDDRRENLQDALDNLLSLGIVPVVNENDTVTANRNN
TSNDPFTDNDGLSSLVARTINADLLIILTDVDGLYDLPPSDPKARLITTFAPDNAVVFGA
KSPTGRGGMNAKIAASLAAVRGGVPAVCICSGHRMGTIGELLQGKPNVGTLFVRDPTDLI
ASEAAAAEGRRTMEKSAREAREGSRKLNTLSYLERKKILLEVANALDTNREKILDANAVD
LRVAVESNLAPSLLKRLELTHGKIDTVVSGIKAIAEDKDPLARVLSARMLDQNLVLCKET
ASIGVLLVIFESRPDSMPQIAALALSSGNGLLLKGGREAEHSNAAIHSVIVEAVTKASEG
KVPSGVISLVTSRADVYELLKLEGFIDLVIPRGSNEMVRNIQRSTSIPVLGHADGICHVY
VHPDADMNMAAKVIIDAKLNYPAACNAAETVLFHRATVENGDAKILVEKMKEAGIKIYGG
PKAIAASLARAGAVSMHTEYGDEQITVEVVEDMDAAVDHINKYGSHHTECILTSNVDTAS
DFVRRVDSACVFHNCSTRFADGYRFGLGAEVGISTGRIHARGPVGVEGLLTQKWVLRPID
PELYATVAEFQSGERTFTHEDITNAMILQEEN*
>TCRU_2425 Serine hydrolase (FSH1)/RWD domain/50S ribosome-binding
GTPase/GTPase of unknown function C-terminal, putative
MVRVLCLHGKRQTGEIFEHQLNKLCRFLSGVAELEFVDAPCVLTGEMVTGDAVVTRSWCE
PKDGGTRDSYAAGDDFVRRCMNSGGSPYDLLLGFSQGALLAARYVMLQKMNGEREYGPPV
KAVIIAASPDPRRVFPEFVASYLYHMPQHDINRTGFLGAVPSLHIVGEKDAIVDPAESCS
FAEACRPQSELLFHAHAHSIPQLRSVLVSIKSFIERIAVYTGERGETEKTEATLERENDD
DDDDDSGSLDAACEEEIGLVTSMYGEECVQRNKTPVVFLPLLLDGVSDEASDMSLSALRI
CMTLPRRYPAELPKLDVVGGPATHHVFFERWKLQLITKTLAYLRDDLGVGNAMLLPAMMF
AGEQAASDVAFLRSVMSAKASASAAPDGSAAQHLQQQQQEINAWWDADGDDDDDRSACIV
DAEQNAQQTLEGNPPEQAFNELSAAVFDNHHPNSRGGVLELTIGLIGKPSAGKSTFFNAV
TDPDSESKAAKVAAFPFTTIEPNVGAGFGAVFCPCGILSTSSSPCDAMYGHVPVGGALCR
RHPVIIKDVAGLVQGAYKGKGKGNQFLNDLCDASVLVHVVDGAGATNADGSACPPGTGST
CDDITWVRAEVHSWIYDNLRAKWGGIRRKPEKLRTMFTGYRSPPSFVDGVLRYMGITSEA
ELMSQLPVWRQPELHRLVALYVRLRFPIVVALNKSDIQSVAGELHADLQRRYPHEKFVPM
SAKVECHLLRLRQSGLVEYVSGDSSFKEKGSSGDGRGSLMSPMVSMALAEVRQFFGSGRT
PTTTGVQEVLAAALFLCPIVLVFPTVSMKLPLPSLRECLVFKSGSSAGDVFESLLRANLL
DGKLVRFEAVCAERLREGHEPTSLKKDAALPGRHVLVRVLTNKRQLVL*
>TCRU_2426 hypothetical protein

MRRAATKDPAEPASLPPDDSLQFTPIGGVESTFIEADEELIRTAGVATLEADRSQREVER
WRLEASAQMHCLTKVRREKALLLREVLQLEKSLEEAREAAAVPTSKETCSLIHGGRSILG
DVCDPHGDGIIVVPGTFTLETKGGVTAEEVELRYLISRAISSLDSLQAEIAALRQQHADL
WHRKREITEDSKRLNATYQRQKKQREELLEKKLFVEAAEADPAFREALQKRMELKDLVRH
AADRRAALKQLRMERASMIGHLLEGAMSRERDACNLHVRIAKKKGFIGHPMHDMLMALQK
ENKEIRAKYLQMVRGSKEEDAVLSDYLDVLEERLLLDWMADASHDKAV*
>TCRU_2427 hypothetical protein, conserved
MEPTAGSAAFSPSFRPGFTSGTHRRALTVDLMGLLDSKETDGMGWEVATKGRAPVYVAFD
PERLWQIPDGGGAEEVGNNRAFHGVMVVFSDGPLCGDEGLPEVGGDACFGGCVLKFLMLR
TDFMRHLSSFSAPLCSSTCEHREEEEEKEEKRERDGFRAVLLFTDVKMEGTDDDRALSVV
YVQLAKGWRHAVDRVAVQRRAAQVIDAALSLFPSSLWVIFWVDFTGILDAKDSLLPHEIR
DAWSDLAAASASTPSAPRVTVLVEPAAALKLRQDGAKVTGEAAPILAADANLSTVKETLC
MSSGRRPFDDSQRGSSASERNCMDPHEPLVSGDVDGNGEKKGGNSSDDGEDELIWKKYKR
TKTNAQDANDGSTSHLGHPAHEESDDEEDELLWRKFMCVLKEEAEVSMNNATQGLSCLFL
ECPGDDLFSSPGDDASIRELFASWSSLGSGHLPRTIVLIAGDDNLLERTLLSWVRVPWSQ
NALILPVFGVSELVTGQHSQEGVELPPSRSSLPSAAAAASLSMNRDAASASHRFFRILNF
AVRYFGIDVHHSACLSVTQPLMQAAWLLDFRVVGDYARVFSWVMQLRAGNSAVQGLLDVE
RERERLFAELCATNELARKLRESPRRPEILLRVMGSMRVGVVRPVVYDAVALASKSLTWT
RVRRSLDTALSLSPEQRSRVYRYMASFFTKNVFCVSVDIVRSCDSDYFLGYHFSAVGYLR
YELSSWVRRHVFVELQVDFQPLLEGDPLGAETEPHWSRVFRRLSCTCTPRRHEVLLCTET
GEKKCRHLAQLLYWSLRPRPSWNTSFFKKTFADQNHAS*
>TCRU_2428 hypothetical protein, conserved
MFELWDKFGKRNIAEKEEKNGDDCYLFSALRRLHAKLLAAQPEFTEEDVIIIAYPSPGGS
PTHSPSTSPRDRDADCEQDDSSWNTGGEKSSKSSTPTRDTGNGNVALTPPGQGSTSSKIT
AMGTGKELTSRDFLALVRNFDEKALLRNRKAASDALKLLSDAYALTWRDKRYFTSATLMT
SVGSSRLMAASARELGGRGNSSVLSTVRRPLFTPSSSFRAPTHKPPQNLVLLMGKCVESL
LVYANESLSLRTMISSNVTLLRLLCFVCGESRKLESSSCGSGWRDIDEELNELVSHWQLF
SLSLPLLVALQECYLLEFLLDAALPPTVCSSKYDGIALVLHEKKRRLMTEMMSSRTGFGS
ITRGLPLISLAAIVSMSIFAEPKLRARVLDRMTGEGLLQTIRDELESTVNNFKRLMTGES
DGQSPRTASCSPSKRSGGFFSFFQSKARRVRREEKMLTGMQDSPLRRLGSCLEILQNLTA
PNLAQEAAPATTMKGVVRATAWASQIEDVREICTSLLNLSSIMAGSDTPFSFLRPLQEQV
MECVRQIASYSAVLYGVVVETVDSALSEYARERTNGQRDITAPLLPLLVELLCLKQHAEA
NGECVGRWFTCLTECLVPQAFDVPVLTCNRAGNRYSWSKGVRRKSSNSRCDAPVLARNLR
REEIISLSPYFRFASRLILETKWRDGAEPFLARIALLAYNVLLTQARRPNLIFGQASACY
DAVAPLLETPNGVLPGGSSSGTRELCGTPTRRSGKTPDSSSSPGRKRCRSVSGGATNTGD
EVFVPSPSQSPLFLEAYSSFSETEKGRDSDGVSRGEGSPSCALTAPSWRQRLSLSAFLGL
FGEAFPE*
>TCRU_2429 Miro-like protein/Ras family, putative
MSFEGDAALHSFTEGGAELAFHDSQVDALITKNENCAVFPTLTPNESESLCISQSVPLVD
GGDLPLSEKIAVGRTAEQEGHNSEERRGPLISDEEEKAANPSCMPNKILFGLASVGKILD
PSSVNATIRREGPTAGGMRHCSTCPESSLCAQSAEDTAIMESDENTTAALKPQDVQEMLQ
WLGVKRRGDLLSRYLVMGSVAATQHGHGVNDACSLGSKEKHVDPQMRNLLLQEVQRTGSR
GRFPNKQIFQRKLVLLGHQEVGKTSLLKCFESEPYFLKKLPDVKTTTGVEVCSQTICVGE
DCVQLILSDFAGHEVYHSHTRFLTDRSIFLLVWKISSVGQDFQSSGISVKEEERLYKWIA
EVYAKFPRAKMALVATHLDELRVQGQRSVEKILLRVEKKVTSFMQRIAATDPTTGKIVTN
EIVGNFAVSCKTREFIATGGLRRFSVRKLSALLHFFAEVARRDCMEDTDFPAAAIPGRHL
KLLEMLMNEKKRFPEKFFMPLRDFIYSAVQFGVASDEELLQIARLMYSWNIIYLFNPYCF
SDNSPIMLRPLWLSRLAAALFSYAQVIRTPLHLRSLIGRLEYTVSVAEAADMHLMSKGFL
RWPLVRVLFRAPLFDILGHEPDDLDYTVAVRFLISLSLLYPVLIPCDELALLEEETPIDI
EAGQPIVREPKITRYFVPSLSPYFVPDSLRRLAPVLFKRGPKLRFLFNLLPDEAWWRFQF
RLHSYQQVVVLQEPCGALLADNKGDDDDGGDAFDEYRLLEADEEHNRWNDAMWLSGDCCR
VFLYREGLQVIRVFSAETKPHAAESVLEDIERTILSLLGEYRGLQRTVQIECPEPNCNGW
LMSSELLTGTRLKCQICKQMISTEDVVAFAAVECVEPRRSHCFSDTLLKEAGELLCFSLN
PMGCMQFCEYLGLDHTILCGAISKDSCNTDGDEEVTQTEQNPDYMYALDKVVRAAMLRQL

RERVEEEARRRRMLEPAPTALQVSF*
>TCRU_2430 hypothetical protein
MQHDVPSEAAKGDLYTKRTFGPGSNDFWAGTGGTSSSMSGCERAAWVPLSAALVVMLWAT
SPTRRAVDTVGGNELARRATVLGAQLAPGKNGTRPYFFNATLFLSLRIALGLRKILAAYG
RGPVPSTASKKGLSQDGRVVAFGAAGGKPSGQRRIVSAAWQKVKKAREERKTIVVPPDRI
*
>TCRU_2431 protein phosphatase 4, putative
MDSIDQFLETVSKGAYPRESEVMKLCQTAHNILDAEANTHVIRAPCTIVGDIHAQFFDLI
EMFRVGGEVPDVNYVFMGDYVDRGHHGVETFLLLVALKVKYPTRVVLLRGNHESRQITQV
YGFYDECLRKYGSVNVWRYCTEVFDLLPLVCVVEEKILCVHAGLSPMISTMSQMDAIERR
CEVPHEGAMCDLLWSDPEDIEAWGLSPRGAGYLYGEEVVRTFLETNRLELLCRSHQLVME
GYKVMFNDTLVTVWSAPNYCYRCMNVASILELDEHLNKNFKLFEAAPDDAREPSKPSLPE
YFL*
>TCRU_2432 BNR repeat-like domain containing protein, putative
MRDGALVFPMQAKGKDGTSVLLSMRLSKSENKWELSYTATGKGCRDPTLVKWKYEDEDDE
RLFMMAHCAGGYYDVYMSTPNGVSWYTSGESINRVWGNSHNRTGYGVQSGSTTATIEGKE
VMLVTAPVYAKEENGKGRLHLWVTDKARVYDVGQVSRENDDAAASSLLMKSGRDNKELIS
LYENKRSDGSYNLVAVRLTEKLERIKEVVKTWKDLDSALKTCRSGSSGTVDAANEGYVQW
SCSH*
>TCRU_2433 Concanavalin A-like lectin/glucanases superfamily/Protein
of unknown function (DUF3676)/Trans-sialidase of Trypanosoma
hydrophobic C-terminal, putative
MGQTVPYYFANNEFTLVATVSIHEVPQSGSIPLMGVRMNDTKGTVLFGLSYTHEKEWLAI
TEDAVDLEDVDDAEDVGDIVKWEPNRTYQVALRMDCDEWSVFVDRKEIKYTEYNQRLFDT
HRISHFYIGGDSKNQSATGGHVTVTNVMLYNERLLSDKLYELNASKVTIPSLGVEEQPTG
QVASTEVSVASESRSEESTTTEKLTGDDTDKQEEGIVHGMVPAASSSTVVGGSSVPEPAI
ATEIAGNSRPEDNTQLSEDETSQQATPHEGSESMQRDSNVQPQYPQSEESTGVADVETSP
ESNDAPEEDGGTNDRSGESTSQVASSLSMDTATRPADGEHQVQQIAELPAENDVRSTGTG
TTGAEQSLSLEAGDRSSERAMNSYSSLTPSKRDAETTSAENADDVFRTDGAEVSSEEGKE
APQKVDTAPGNTNTTPGETGIPSESNATIPSDHDILPENVHLGELAAMYLVGDSTVHRCV
SRVLLLLLLGLWGTAALR*
>TCRU_2434 hypothetical protein, conserved
MERSPDSHRRRMPWTAAKECVPGVVHGSKGKMVLDGARRVDVECVDRASQVYPLEALRTA
AATCEYDIFRGKNIFNWSLRRNQMCSGNEFTARSGRRGRTTKTPLAPSFISVVARPQRSP
RQYDLRIFRGGMRCSVCRHCSPSPSVGADVPVDASSCGFRTHPLIDAADTPAHCWCFWHD
RCTAPAAGAVGMGTRTQQTRFNQMVLEALSSSRRLQRRAQKSTGQERNQMTAREAILFYI
TPSQTAGRIHNNISH*
>TCRU_2435 Mucin-like glycoprotein, putative
MMTCRLLYALLVLALCYCPSVCVTTEEDNPSTGSDPQEGEGRTNGRQKGTSGNPDLTTKE
SEVESEGGTQDIQGRTTDVISNSEGASGASGEPGKTSDVDQSATNPKLGVPLEKTTTTTT
TTTTTKAPNNMNTEAPTTTTTTAPSRLREIDGSLSSSAWVCAPLLLAASALAYTAVG*
>TCRU_2436 BNR repeat-like domain/Concanavalin A-like lectin/
glucanases superfamily/Trans-sialidase of Trypanosoma hydrophobic C-
terminal, putative
MIAFVEGRNKNKRFSEVIDLSSSDIVAGYIKAPETWQSLVAEVTKDDWKAHTVLESVDNW
NHVGVANLPTGITRGNKVFLLVGSYEERRENDGDIWKTEAWNIKVIEGEATQSTEVEPSQ
PINWSEPKPLFQTDSPNNKGELKEFLGGGGSGIVMGNGTLVFPLTAKDENDKVVSLITYS
TDDGQKWEIPEGVSSAACHSPRITEWEEGTLLMVTYCEDGRKVFESLDMGKTWTKAIGTL
LGVWLKSGPELLEVSLRVDALITATIEGRKVMLYTQKVRHSLEVDEPNALHLWITDNSRT
FHLGPFSVDSAENMTFANTLLYSDDELFLSQQRGFRAGTAVSLARLTEELNTIKSVLSTW
AQLDASFSESSIPTAGLVGFLSNTSSGGDTWIDGYRCMNASVTKATKVKNGFKFTGPGSR
ATWPVNSRWDIKQYGFVDYNFTLVATVNIHQVPKGSTPLLGASLRGSKRTKLIGLSYGAD
GKWETLYDGTKTVQGGTWEPGKEYQVALMLQDGKKGLVYVDGVIVGSSAMLPTPDERWTE
VSYFYFGGDEGDSGSNATVKDVFLYSRPLSVGELKMIKKVEDKKGNGDGDGEDKKGSGDG

DGEDKKGSGDGKKGSGDGSMREGMSRVLLLLGLCGFVELYGA*
>TCRU_2437 chaperone protein DNAj, putative
MRRISPVIFGVRVVSFATTSVAFLQARAGGGLTDEEQRELEEEEFRVMDAAREEAELLPI
AHRLAAKADALDAELRRLSYVLFEKPQLVMDTRSATLTGRPTSILTVMRRYNAGSRHTSC
PAGWVKPLGSSSWIVQAYKGVHSAGMRLCGRAKQEESKRPDQLVTLTGDLDYIRRMKHLH
ALNFYSRMNLTPKRHIGFSLYALLWNVVVAVQNAVGCVIFGAMRGVRDNEACVGIIKGTA
IGILRALQFLAYGLLVSPCIHIPCGLCNSLYGVWNAVSGTLFFEVSAGRWQYSTALDAVW
LRHDIQLEQRAIRGVGRLEFRRKNMRAEERWQQRLASMGFSFDKINQKMRRTGDTRRPSS
AAWKEGEHAERGGAEEDPYEVLQVKRSATQTQIKAQYKNLAKVFHPDVVQSRHGGPLSEE
ERRDAQRRFESISQAYQILSNPEKRKSFDLGGAQALRLHESKMGRFMSRTPEEMIQSVFG
GEPFREKILGSLLRSHWHLRNEAQVSVSLHEFEELQCLRCRELTAELAQILDVHANSPLA
TGYQPRGGGHGRSEVLHSLLSELGGSAPKPKSRKSSQRSAKNNATTESMFDLTPGSNEYN
CFSRDFEERCERFTRRLSEACFGRELMYEAGQSYVISSQRFLGIRPFYAPKLHVYKKIFS
GVDRVYAAFREKIDDRAKNNKEWLAQKVMIEYFSMEFDAVVADVSVILRYAAQNVLQDVT
LNEEQRRRRCYALWYLGEQMVRKGAPWVAMSRDDAELMAYLQQAANSASTTSKPGPF*
>TCRU_2438 macrophage infectivity potentiator, precursor, putative
MAFCLLGVLFLSCITSVQTASGDAASHEERMNNYRKRVGRLFMEQKAAQPDAVKLPSGLV
FQRIARGSGKRAPAIDDKCEVHYTGRLRDGTVFDSSRERGKPTTFRPNEVIKGWTEALQL
MREGDRWRLFIPYDLAYGVTGGGGMIPPYSPLEFDVELISIKDGGKGRTAEEVDEILRKA
EEDREDM*
>TCRU_2439 dynein light chain, putative
MAGTVAVVKESAMSRELQQDCIDCAAYALHELGLHEQTAIAQFIKRELDSKYGSRFHCIV
GRSFGSYVGHDGQYFVYFLIGDCAFLIWRTVDTFEERVFYVPVDDLAIGRLQ*
>TCRU_2440 transcription factor IIa, putative
MTKKAICKGHTLAPCGTSDVYEVAMRNFRRFLLERVAQQEDEVFAARARFQLQRAGDREH
FDRTQSRRIKSEGTRKTGLDNFVRNGMAATGSMERQYAAEQEFLADQQKLYVEHEEELQR
AYPLAFLDDESAGVIAFNLMPILRGKFESLAREQTRRRKELENPTDKTKMLSDLTVPSLP
QSHGCVLVNNVENPQIIRVKKENEPDVDAGLEAGGEACVNILQTTTDQSTSSRETEDNRD
APLPREESVKDVNDGCVSVDAGRGVVNPPWVSHIASGGEVEFVRVALMCRKKSLLARQEE
KRRCLKSLLTPLTPCNLQQLAKEKLFLLKLEEEDGARKEAEIWRLWRATRDKINSNGSLS
NADTDGEGVAAATGNGENAEDRRGEGTLPCSVEETGDDKTTNGKDWNEYYSPSRAIEENR
RIRINCLFSLGSQAASSGMRNSLFDFNTLRWETITLDDGATTIDGGNNSSNRLESMAESY
LWPQHIEAEIKNVLFGEAVRSRRRWLSFLEPQGTATQEESEEAEDDGSAGDGVPTRVNGT
KGKRKMPIVLAFPRCGRKVWLKLDGLEEFVARDVRFYQGYLHP*
>TCRU_2441 CAP-Gly domain containing protein, putative
MAQARGCELMDFVSVLPVPPQTERRRGVLRFVGSTDFAPGLWMGIELFGTIGRNDGSVMG
KSYFSCPAGQGVFVRPELVVPYMSEGEGPSGTVRKGDEFLAVVEPLRAELTRMQEELQQR
EAALREAREELRNVMALHDAKSVELEREREEKSRMIMLTMTACRNNVTATTQTDEDEAVE
VKDLEIHRLQQQCADLKDLCELQEDEIHRQREHADDLAQIVENLRREHEEERVRMVEKIR
SLEEEITQRQKATAEDSAKADVEDMHLHAFQQQQEKEALRILKERYEQLYAERESERQEQ
EAKWKRATAEYEEALTQQREYKNRLLNEIRQLQHEIKDMRHSREREREVLQVPLSTLERDE
YESQIYGLQWELMGVHEEMFSKRYDHFLAPVSSWNLPADVMRALSEEIMSLERVLPFTTP
EKCQQSRMVLFQMHPRDAEGGTIAAAPL*
>TCRU_2442 ADP-ribosylation factor family/Signal recognition particle receptor beta subunit/50S ribosome-binding GTPase/Gtr1/RagA G protein conserved region/Miro-like protein/Ras family, putative
MGFLAWILEWIRGLFWQQEMEVTLVGLQGAGKTTFLAAITDGTESVRLHDTIPTIGLNTR
KVTRGNVCIKVWDIGGQPRFRGMWERYCRGVQSIVFVVDASDISSLEEARRSLHDLLGRP
SLYGIPLLVLANKNDLEGACSAETLISELYLLKLAADRETACYSVSAKNYTNIDVTLKWL
MRHSKASSSS*
>TCRU_2443 hypothetical protein, conserved
MLTALHITLPHRRDHRDSQQHLPNRCLVVNEDTCEASLISTEASLKYASGAAHELYESRS
RFRFGHVAISPHHGNIFVTQVAPLIYDLCRVFLRPLCSNEPRMESRPLTPTTETTTAAGS
DSNGPGISQKGWDTVTARRCRRICKKTWSMSRTVIIYGSHSATERRALFLHLAKATSLAL

IQETSAMGTETLEKNMCQVSFSLVDLYHAWGVGDALDPNAVMHDSIQLQRLKREEPRRSL
NEPKGMDFVEPTRQTLEDRDSVSTCLLRALQRIDPPRALRTNAEKEDHAVKSTLVLTFII
SRNSRCRVGVPRPRNVHSAKSEKLKGWQRDGFPKEARFHLILLPDSTAAAHGKQGAKELG
ALAGALQACTMWQNEIVSAEEEEEGEEEEDDARTQCANCVTATDGHMDASFSTTTSTPPQ
FSWVPLRESPLLLYLFESTIYYSHQLLQSLRNLRLSKERRRLFSDRSPSGSSNISTHSQN
AARVVSFSTNTSMEKGSCWMSTDTNSTNVIQCIAAGIGQCLLIVCVNSGWDQYYQARSAF
LFAQRATNQFAQPYMSLNSKTADVSSIFSTEHGKGNPHKEATPTLTEIQPPQQLRQKQRL
HESFLSLEKTGTEVERQLWSSPRRREKTEPRRPQAEDSPVFELAPSEVAVTESEDIVGRD
GNDVPSHNLSPPTLSQGIGMNPIQQQVKSKTGLVTKITGPTLSLPERNTSPYPVQQLFRC
NQDDPKGAINKVFDESGEENASNSWEQPETARKNQIYPKYSGLCESPSSTKGVNLGPVPE
ASSTAINDVLQKQGLLLQENERLRALLVTLSSRQADAGREQRESTTLKDVCDITLLESRK
AARSSDLDELRTALSITTKKARTLQKTLSRSIDENRKMRRLIHSQMKKCCVEFSRIAQGT
PAEQGSNGSTVAEERIYRWEMLLENTVKQILRSVRGDSFVGSDDDNEEREEEKEATRTGN
GDRFGSQRLQVKNKENSSLANCGCAHMQDGMQPCSSCNGSHQFINGGADLSLSEKAQGRN
CRAFKRDAEVEDAVNSPQYRKIDVQLSQLLHIAYSLFLGESSSANDDDDDHSVSGGNMHK
TAEGAEVEGCNRTHCSLSRSVLDILSPHQQGTRAKDGRAKEEEEEEEWRLVCKRSPETEVC
WRCHSSRRTRCNAAYTKFLEILRFLRSHMTINGSFASDSYRQGMKEAEEKEEINGRDSAS
PSRHARVSSLHSRFREKLADGSPAGASSRDGGTLLLKARQLSDSLQKRTNDRVNEQAAVQ
YMQSELDKEREWRRQLQDELLRLRLCLSKCESKFDFIDETVKPKLSKDIAQLEQKISERK
RQEETVRKRRAMFMSEIQKRLQKVVYENDLTLLPSSSSSFSLSPRRL*
>TCRU_2444 Vps53-like, N-terminal, putative
MADGSKVRFSDAVEKALAEVCPPRDPFDEPDFDPVDYLNSRFPDESSLAALPAFLEEMTS
RLTKTENDLLKAVEAQATNAATADRDLHNAKAAVVALYDRVSDIKAKTAKSEDTVRELCH
HIRELDTAKTNLTVSINTLRSMQLWMLQLQVLSTSFERRKFIQCRDALMEAQKYSVMFDS
MKHLSKVREINDKQAQLCRRMEHYIRHTVFGDISLDSLDETLMAEACAVVDLLGKESIRK
IRDQFIEKMLEVYSLRFKRGSEDAKLERTERRYVYIRNLLEQNEGLFLNVFPRHWCVPQE
LCVTFCLRTKAELDYLLREAAGNIDVVVLTYVLQKTIDIERDLTRMMAWKEEFPGRSEVP
DYRYNGMILSSFKAHMDLFVRNEDKLMGDALSHSLVGSSSDVESIATGSTNAMAGGAVPG
WSIEGDIHVGATLPVAEDLFIFIKESLKRTIRISQQDVLLEMAAVWRKHLIRFAETITTL
LPSPAASREDMRRACVIVNTADLCQSTSQDLGDEVCARGEVPAKELGFDQVTEAFSTLYS
GAIQCILQGTELKLAPFLVEYSNAAFVNRRPEEQDIHDESIPIRSMSAVLHDMVLVCSAV
LPSPILRFLLDKVANSVIPMFTDTLYRMRRLPPDFALGLMRVDSAALEKSFLQLPNYNDP
DRFAPSHLMGYMKLVRREFDRLNRTLKVLQVDASVDTFVDVYYEAMLPDDRSIQNFVRLV
ELKGRKREDVRSWIANLSKKGVVEATKRDMQRESSLGIATGTTSQSAAGSNGISSGIKIS
NIFSPTTANTYEHPSTGGLSNAFSNLVGRAGSNSNASTTTMPTAGATSAATAEESFGSRF
AKAARSTVTTMNFLSNFKKEGGNTGSGAYGPLR*
>TCRU_2445 ABC transporter, putative
MGKPKYLKKKQEATAVTGQQQKSSSGSQANPELNVPQSSGAMVSFANPVFRDGVSDILVE
KIDVSYQGVSILENATLNLVTGHRYGLVGPNGCGKSTLLKVLGYHEIPFPKHVDRYFVSH
EVEASDITALEAVVSVDKEKENLEKELEELALADQEDMAVNMRMDEIYKRLDELDADTAE
ARAGKILFGLGFTPEMQQRLTKSFSGGWRMRISLAQALFINPTVLLLDEPTNHLDIEAVV
WLENYLSKFKKILFMVSHSQDFMNNVCTKVAHMARGKLTYYDGNYDQYCITRAEKESNQM
RRFQWEQNQIKSMKEYIARFGHGSAKLARQAQSKEKTLARMVRGGLTESIAKDRQVNFGF
PCAGPLPPPMLQFREVSFAYPGRDPLFRDLELGIDMESRICLVGPNGAGKTTLTKLMCRE
LEPTSGYVAKNAHCVIARFHQHFVDQIDMSLTPLEWMGQEYSSVSDPTILRSALGRFGVS
GKLQMTPMSTLSDGQKSRVVFAWMAFKTPHLMILDEPTNHLDIESIDALADAVNCFEGAV
VVVSHDLRLIAQIADEIWIVDKGSCRKFDGDIADYKEHVQREVNRMTEDYAKRK*
>TCRU_2446 signal recognition particle protein, putative
MSDVAKWEKQLERLLVDGSDSSKILTTIEKILSVNPKHVFALRCKVVCFLHQDKHAAALT
VLDQIAVVDPALPQVSQDYAFQKTYCLYRALEHRQAQHVLMNDAPHTAEHVPSRHLLAQL
HYHLEEYEAAAGIYEALLTEGKFRDEHEKTELQTNYAAACSAIDAQRTQSIVRSADEKNA
DLLYNAATAQLEVQEYAAALQTLQQAEFLCAKAYPTSRLRSFQDACSRSDTELRSLLDVK
GSPERLFFNDVAIIWVQTAYVHYAMHNEEDATALLNLVLRYRPSSAVTLAVASINWTAIQ
RHRDFFDTHRKLKSAQNPAVNNRLTSRQRLLVRYNTAMLLLHTGNFNAFKRQVELIANEY

PDSDLTHSLKLALAVRESKKKRQPDEKTLTEYLKNYQQSVRAQKEHARNPSVGKLLPLIA
AQIFLENGDLEHAIESLSSAPEEIRKKPCTLMTLATWKVQLGDVKGAKQLLTEFAGAAAK
GGTVVKPILFWAVQFLTARGLYADAADVIRDMQSALPVLQNDKEARALLALCLSHYDMQA
ARTCMETIPEAGFTGTQTEKKLLASSIAEATLQQPSRKHIEALGYHRISADGHDGNNNGA
KVKRSRRRPMRRPPKNADARLDPERWIPMSHRSYIKDLPERRKRELKRLRAIEQEQKRHE
AEKRKAAAH*
>TCRU_2447 transporter, putative
MDRFISSAVTVGKVILVSVVGIWISQHFHNKEKSLKALSYISVKIFLPCLLFSQLAKDLS
WDMIHKYYWACILPLIPMILGFCTALAFRSFIPAELHGLLQLSCTFQSIVSYGLGIVLNL
DISWWSKEDRSEAQSYVFLFNLLHSLFLWSFGTMIVEKGAMALEEMKATAAVATAVSAED
DGDDAITSMRNTSGALVEMEECFGAESHQSAGQLEAYQPAPEINSTRDVVERENKSTADL
TWPEYIRVQIPYLLSEQIIASFLGLLVALVPPFYLLAKNPVGEVLMGGISFLAPGAVPLQ
LLVLGVNVTADDEDDSKKLPIRFLAVVILLRLFFIPAICFCIIHFLVVNALMPYDKPFIL
VMLILTSAPTAINTSSICSIYSYKVKEYTKVLLFMYMACIFTTTVWLTVYVWYLDS*
>TCRU_2448 hypothetical protein, conserved
MAMTMTGRVLLVCALCVLWCCAGSVYAGDRDNNAVGGCMASEVFGTDLSHAPSGCNKPGI
TVLFRSTLPITAVVASDEEVTSVTMQNDLGSRENSNAAGPAGPAGPTGPADLADPADPAG
PADPAGPAGTAAAGVPGAPGGGGGGGTSLGGGGTDEISSVSSGGENEPPPPLLKAENEAI
RVPTVGEDEAKKAPQSHDNSVEQHSGENQEHLHEEIRDQQKKQEQRQEQHENNINHEEGE
EKQQQQEQHQQHDHSAKNSDGPTKNETTVGTNATASTGDSDSSTAVFHTTSPLLLLLVVA
CAAAAAVVAA*
>TCRU_2449 hypothetical protein, conserved
MGSVLQPEGQRDAVRDGEADPRVFAAHGAREPICVERCAHNWGRVAARHCGAVCVVRGRS
MAAIVRDVEDTELLDWEEVAYVAARRECITDCLCWRGACWSERGVPVRA*
>TCRU_2450 Leishmanolysin, putative
MASRSMVRNGAVRGWALSVVVDSTTAAMAAREHHDSDDIDGMELQDDDGDGRTMESHWSR
LRARDEWMAPIEGAGDCRGSAPHRT*
>TCRU_family, putative Se.1
MRGGLVRSRPTEVGQPREPSNGVCGCVRDYIFHQMLDAVHGLSNVFFCDAHAASALSCSL
RLHELMEHGVTLLEDPMTPRQPIMSSPAPYFFAVEDASVSRVAEDWIAKVPYRDAHIFAL
GCTPHRSPQQLPRVRIAPRAMRSKDSMLDFAAPEVLVFHLSMQNEFPQLLSPPN*
>TCRU_2452 hypothetical protein, conserved
MVCVRCRCWAAAICDCAVAVRGVAAVSLLLLPLCVDVLLVCAEGCTQVTGVMAMMMTGRV
LLVCALCVLWCGAGGGYAWDFGSESLNEHYYGANGAFCKILPKMYSCELRINASKLQEAA
SQKNNTQSGTGTGQPNAGGSSGRQDQTLEESGSGQPEGTAGEGTQASQPPGEGGTETPTP
LSSPPTATATATAAQTSATRTPDESDGSTAASHSTSPLLLLLVACAAAAAVVAA*
>TCRU_2453 hypothetical protein
MQPASAPCSRRHPNRCHPLIPRVAPRPVRLQRPPVPIAILLLHAINVVAVVVLPRCHRGS
RRLHHHQQRPATHAGSIPHHAAACHVRAGKPDSMGHQVRGDAGGEASVKQSGRAMFMAPA
TGRPSSSVAHGSAHTPPGAAATHSTVSELAAPAEQCSAGTVNRLQTLPVASDGTTNEPSS
GCTSRRSACSLTAAGTTSSATAFPCADDTTPFLQPNAPSNAFAVQHQEKPQRLLLPHGSA
VGALMRTGTHRSLQTTLHTCNASCAPRGTSPTTPGDAHTPPARTASSRKQGYVRMTAGHP
PAIHIAGIITSSSSCRPRGDVDQPTHGACGRKNVMA*
>TCRU_2454 Kinesin motor domain containing protein, putative
MYEAAEQQLALVSRKTHRLGEVLRQQIDALKELRTARMAAFAERDELRQRLRVVEERANA
VDCKHNEAECCLRRTAEEFAATQLLQKQYENTLAELKGRQRVVVRVAGAAPRSGGPAWEE
ASRDEYPKKPPQRTQEKQQQQREGEEEERNYRARIAVTDECTVVDLDRRRTFVVDLAYAM
TSLRRRMPSESALAAAAGISSGAAASEAIPAEDKTTQNFMIDEDLFATLRLEHVIADVLS
GVNMVFLSFGPAGAGKTETLFGTKAAIIQHEMLQKRGRKQQQQQQQQSSRHSRRSGSNGR
GMSVEVLSKSPQVCGATAGLLSLFVHGLFNSLVSHAVTHVSISCSFVELAMNQTVDLLLR
QPGRPVSGSGGNNTTLVKMFSAAAGSASTASISCGGPGEEVTAVRAETAEEVMRLFFEGL
DRLGRWRRSFSSFLLMGDDNGSNTNGPQPKDEHVVMAHRGGVSHAIFALRVENFNDKGHY
RRSMVSFVDLCGPPPSGAGSVMRPLTPEEQWVDESLQAVCDAIAATSSVKPNEGKNDAER
AGAAFDQLPSSHTNCMARFLRPIFGGNAKATLVCCVDVEMHSKEQVLNAISYAYFFKRIQ

HQAVPYDIPPELQRLNLQMSAFLSGEGGDDDGEDEDNDNDVITSSVGLGTARNSLAPVDA
NWHCEGYSRA*
>TCRU_2455 BNR repeat-like domain/Concanavalin A-like lectin/
glucanases superfamily/Trans-sialidase of Trypanosoma hydrophobic C-
terminal, putative
MLYTQRGYFSGENKANPLYLWVTDNNRSFSVGPVGMDNAGKKEVASSLLYSDGNLHLLQR
RGSSEHSVISLSRLTEELKEIESVLSTWSQNDAFFSNLSIPTAGLVAVLSNASASDDTWN
DEYLCLNATVTNATKVKDGFQLTEPDSGVMWPVNTPDGNVRHVSLSHNFTIVASVTIEEA
PNNNTPLLTALLGDAGPEYFMRLSYTADNKWETIVKGETKPTTESRPWVPKKEHQLALIL
QGNKASVYIDGESLWKEEALLTDGTPLEPFGFCFGACDFADDDEEDSPKKIGKKPRVTVT
NVFLYNRPLNSTEMRAIKDRIPVSTRAPEPQVKIAPKPAVPAVPAVPGPREVPTAPGRTT
VGRTANTQHAPAGRLTSAGNEGPAREKGDGGANGDAGSAYGRELLPLLLLLGLWGLATA*
>TCRU_2456 retrotransposon hot spot protein, RHS3, putative
MRRAVKGEVIFDEDIRRLCDKGVNNLPGWSLAAAEVKAAVHNSTKHFLDAAAEEARNPTT
TSAPEKLEGCYESVHNARWSHVVELPDGVERKKTGTGMGMHEGKPEQSWTYRKADDAIEK
NDAVQQCGAAPPMLMVLFSEKGWPYSWNMIQDLPEDVFVNCEVD*
>TCRU_2457 TPR repeat, putative
MSSNQKEESRAPSRQAVEEWLERIDDIAAAVEEILREDPMEATIRRQEREARRRQMQIDE
RKEKVKMRYDPRHYVRFESDEVIDKLLKEAEVRGETTRREVKDESLYTRAERVSLEEAIR
LKDDAARAVKASEWERACELYTTAIKLDVVDADLQRTLRNNRALVQLKLKRYLDAVDDAS
YVLREEPMNLKALLRRATALRHIHRPVDALKDVEEALKKDPANQEAGDLAQWLRRVKREQ
SVGAVFQHRQPEEAKCLENSIRMLTSAVKELHNCGAIPTGESLAKKESEENAEKKPLIQA
VESVRRCLGVVQMFHQGAAVFFLLLDGLNPLIELVCKAIIGGWSELVVSIAAGSPEQMST
CGMTFVVSARLLALVLLGSETGVEGIELSTVTSLVRKLADVLSIALQQKSLGHRDGATLT
LTVAILQVLEGLATRCPILVHAHCVSVLVEAWKVMKKRQPPPQLLFFYCGILEALIKEPS
VCASVAKEMEEILPHAIEVAIEAGPEQLKEVGLSLAVRATCVSSACAKSLGSSELTRTFS
KVLPLSQGESRDSPVSPRLEEGLYAVVYNVLLQAESRSQYVAQWKAVTDPGSSGDFSFSL
HTWRTLREQVVAEGKSTRRITVHAKMMAVLAKFSPHDEALRGAMLEGEETLWAMLWHALS
GLEPPDVGPSAAADNQNFANRTEDEEKEGEVEAVIAAAAWELVEHATTCIASHYSQNHLL
REKGLSTADRIAVLMHIIRRAGPQHVVALGNAGLIASFVPSASCSHFAAMNGVDVLLEGL
RSVRDLIFKLEHDGKRGSTQWIHGGAAQKNLAIALSRCCVVESQRERLRELKGFETLHAV
LEPHSA*
>TCRU_2458 hypothetical protein, conserved
MPHVSYQHNPRSIADDPLYVAAGSAPYTRSDTPVSFRVFPNGTSSFGSDLLTSNESIARR
VAETREWLGRMREEVREAKRKCKAEEKSASLLCSVLRASSALMTERAKGRRSSRGRARQF
RFALYSFLDGTPFLTPQALCRYNTALAEMLSDDHLELIRRHVFNYDEEAFANLLYADEVA
ESLERRAPYGGGELVPEFVQRFNETSRRALERLFAAPGGLADATINAAHRVPIVADDVEN
VAVHGE*
>TCRU_2459 Type IIB DNA topoisomerase, putative
MLGDDEIIGAAEAVVHAYLVVLLHRTDAAVTEASSQTHHRHSAPCGMGGQKMPQFCETKC
TFTEKKMNALRRTARVLYVLSCVVDMVHQGLTCTERDVYYQNTSLFRNGQRDVHTSIENL
CRWMDSTHPLRNADDPESIGRRRYTREGLRIAASGKSILIGYISFDIPKRSPVLSSLSQT
TQMTLGNVSQTQFCFEKTSNYLTGTVEMNGMRHSSGILVNMAVGMRGCHFRGAEGVGSLP
SAVILVEKESTLRTLVVAEGVLGLGAPLNRCSFLCSKGYPCRASRTLLHKLHCELPQLPI
LVLVDGDPHGMRIALTFLGLFGGVASTRRRKQKKRPFASAEFISTLLPARWIGVRPSALP
HQATGRAPLTPLDRRVLSQMMARVVEALSLLEDEVVDATCSGEEINIIRGTLNNILREAE
WMKEKSLKCALQAWPDGPLHLLMDSLEPGRPV*
>TCRU_2460 Flagellar-associated PapD-like, putative
MEFLAYEDGTLRSEFYRTSDVVEEPIFNPRRVYHQESKVSRLVLNGVQEFTGQEGALGFI
HSPKSVLFDNFTVGESYTMVMVLTNCANRSKTFRVLPIPIKYRDVLRVEYAPPPRIPTGL
SWKVRVIFTPEENMDFETAVGFRTEERVFFVPVKAYRKRSCVSVDPECLDFGVVIFGEKS
TRVLTLRNDGALAAKVFISGSFRKWMEVFDKDPQNDQESLVLSIKPVANQLLVNPFSTCR
LEVTFSPHREINIDDEIELRYDSDGKDHIQRVPVKGFGGSLPVYVSSASIIDFGWCFYEN
RYCEKILITNTTNVSVNVVPEIPPTLASSMIFSPETACIQANSTFDLYFLFTPFRGMESN

ICSAIKMIVSGQTSPIAVTLKAQITERGFSLEPKKLEYGTSSMNEEIILPLKIRNNSDLP
QTVGFLKLPVNVHVTPFQTIILLPKEVFEVKVGVRPPTLGQYTQMIKMTNEYGDSQSVQI
TGYGNKNVVQFLKPCFSFPACPFGGETACTNVLENRGSVPCGFRLISPTDVLRVYPSSGV
LYPGKTIPIVLIFRPPEPQPIIVTVEENVSRPGGKGALKKRKERENLNATTGVLDQEVVS
LDTLYKDWESNVIDEEWSRHCTFLVKCLSTMGTEKFLNLLQVNCTVVKPTLIARYLSRSL
QKIVVQKVSSKRNSKKSPPLVNLTEETSVVEMSGGGTHLDIDFGEVPLKQLSEKTCLLQY
NGERSISLQVRPVNTLSPFRIVKLPPAKLSQKEECTMGIRFHPTEYGNFRDTINIASVGA
NDIALSLSGICRPADLLITLSSDITGDRFNSIDHFSFDTVLVGQELRQSLYIHNLGSLTV
EVTAKLLSTEGKELKWPESHSFIIESDRFTITGNCKVSIMSIFCPRVAGTLSAFLQLSAG
AFHNTILLKGRASENLVFYTFPEQSTQMTETSFVCTGLQPEVFPPEGSSPAYPFQVLCVA
GETKTITVGCVKLGASFECIVCGWSESYHENGWKLEPMKVNVPSGGNVLFTLNYNPKREI
GDVSFCTFFFTTKSSQPLAETKCHVRCTGTMKV*
>TCRU_2461 RNA-binding protein, putative
MVPYQTFNSAAGQPVNVYQQIPPTSASRDPRTNIHIGSKLFVGQVPAMTTEEQLRPVFEP
YGELLEVKIMRDPLGRSKGSAWVRYETNEMAMNAINALHEKHTVPPQTNPLRVQFATPNS
VRHQQLQARYAVELKVPMNQYNFGGQMGGGTQQSGMVMLRSGASPNVVPPISANVGGRYM
SAPFGGGPMESSSIMSGPYTSSANCFDGVNSSGNRRDLYSPLEQPQGTAPQLYQQQQQQ
RGGFSSPVVTPPSVYNQAPSTRQPTARNNNTKSGGGISMGSFESGHGMVNPNSNGAPTMA
NEMSTLDAGIALWEARNNNGLQGQEPREAAARVRSGERKEE*
>TCRU_2462 ADP-ribosylation factor family/Elongation factor Tu GTP binding domain/Miro-like protein/Signal recognition particle receptor beta subunit/Gtr1/RagA G protein conserved region/Ras family, putative
MSSKACASSIKIVLCGPSNAGKTSITKRFITDTFDEYQDTTVGSAFFSKQLYSKAHNTNV
RVEVWDTAGQEKYRAMAPMYFRHAAGALLVFDETVLKSFEELEAVWLPELLPHMNDSTEF
IVVCGNKCDAVLTEEQRESLQKAQEACRARGITFLHTSAKKGHNVHESFGGLVDRILTRR
EKEAAAALAAGANGGDGRAKSTLATRIGSEDNGGIKRSNCC*
>TCRU_2463 Zn-finger in Ran binding protein and others/FYVE zinc finger, putative
MSFNLDDLFDTGSRQQQQQQHGGPTYMPPPPPSDPPPPPVFMQQDQFGVNATALPPPPPP
PPPAAAAAAQTVQPFEAVGHAPIMTAPYQTYYAPSPTPQAYGGNGYALSSPPIPSSESPS
ALLPQAAYYTAPTPKPVAYGSSYQTSPGAPIVSPSTVPPTLQSTAMPFVPSPSHPEPAIN
APTASNSTSVFAQEEEDRKQLEKIIQLRKELEKERERERKKREELETWGCPSCTYRNSLD
VNRCEMCDSNRPGYHPPPDVVSPSTIKSDAHSSSSSPLKKVAISSEVAGPTAWVCSICLA
PNEAHNSRCKVCRSYQKNGKPITGGNGNPTGATSDVYMPPTAWLCGICGKKNAATAARCG
DCNSYQSNGVPVVDPEPKAQIVAETVPTTWKCSVCTLENSVSAAVCEACQSGQRPRHLAP
PKTNEKKKSGNEPRKNDVPKTWSCSTCTYQNAIAKEKCEMCSNERPSQYKPPLPSGAKKE
AEDSGEDIQWQEDEVARECNRCHLPFNFTRRRHHCRACGFVFCGACSSFQLALKKNGPLE
RVCVSCYEGQK*
>TCRU_2464 40S ribosomal protein S27, putative
MGFFDSDLSYPTVRSERMKHKRRRLVQGPNSYFMDVKCPGCNAITIVYSHATSEVQCNGC
ATKLCRPTGGKAMLTTGCGFRKKADH*
>TCRU_2465 Dpy-30 motif containing protein, putative
MSVKDGGEESDEMLERLAAHLREPPTFPSVPIYALPDQQYLEATVLPLLLRGLEEVAKAR
PADPLAFLAAYLLGNNPQRTSHSLMGDEGRRVPLQGIAQMAADIISRK*
>TCRU_2466 ATP-dependent zinc metallopeptidase, putative
MSDNHQLEYKRGLEDARRHRSRTEDNWVRASVGPLLWFGVPFAVAWLYLRRQAPASAKIN
PFEGMMEQMMPIKKRQFRVDVKGTKFEDVIGIPEAKQEVQQYVEFLTSPNKFTRLGARLP
KGCLLTGEPGTGKTLLAKAVAGEADVPFFSCSGSDFIELMGGSGPKRVRELFEEARSSAP
AIVFIDEIDAIGSRAGKIGGSVSSEENRTINQLLAELDGLNTGTDAIIVIAATNFQDNID
KALLREGRFDRKVNIEMPDKAARVDIFKHYLNRVGTGDPRGRKVDEEGEPLPTNENVDNL
ELARELADLTPGVSPATIATIVNEAALQSGIREKRLVDKESILEAVDNTLVGRKHRNRQS
VTSLRRTAIHEAGHALTAWMLPSVKQVLKVSVVPRGHALGYTQRAGAEYHEYQTNATLLA
DMVVMLGGRAAEETILGNPSAGAMDDLQRATDLALKQMMAFGMDANAGLLSYHPESTQAG

RIFVNYSNTAQRIAEEEAKKLVSLAYELAVGIVKANKAKLDAMVTELVDRKELLTGDLER
LWGERPVSPTVEQLVGKLMTLQGN*
>TCRU_2467 60S ribosomal protein L10a, putative
MSKIPPAQLSEAIQAVLTNRKERKFKESIDLQVNLKNYDPQKDKRFSGSVRLPHVCRPRM
TVCLLCDLVHEDIAKKNNVPTMNQEELKKLNKNKKLVKKMCNQYDAFLCSESIIKTVPRL
VGPHMHRVGKFPTVCAMSESLPEKVLEIQSTVKFQLKKVLCLGTCVGHVEMTEDQVRQNT
VMAINFLVSLLKKNWQNLKSAYIKSTMGKPQRIY*
>TCRU_2468 serine/threonine kinase, putative
MVSSAKLGEYFLGKKIGSGNFSTVRQCTDAKGRKWAVKIIDKGRLRQENMEDQMLREVAV
MRSLRHENIVALRDVMESNNHYYLILEYVPGGELFDKIVQLKRLDESTARQYFHQLIAGI
YYCHSKGFAHRDLKPENLLLDANGTLKISDFGLSNLQQDFLLQTVCGTPNYVAPEVLMER
GYNGLSADIWSCGVVLYVMVAGRLPFEDRNMNALLAKIERGEYQMVRHVSEAVKDLIARM
LTVDPKKRITLEGIIAHPWFALGWDPQRLHEAAETNWA*
>TCRU_2469 hypothetical protein, conserved
MKTSLGLQPSEEDDIKCFLADIQYRLSLHCCRVLAQLTAHTGKSFLTLARRGRSPTRLEM
GGERAEDIGKRDEFSMHEEAYRSVLSAITASCTLGHRSCHALWGPRGCGEHRILRLVAKE
CRQRDGTLVLYLDGDTLNGDEDGLSSIANQILTFLKSPQSSRLRAADWSLRTGAFDFGLL
FGFPKLLQRDVAASGERKSDNDDANKTTRRRTTANRQNKSGKENNKKATRKRFRSPSPID
SGSEDESIDGDESLLLVTSTMACATGGVSSALPALQRSLLLMKSYGTNLVVCIRRVERFG
IWCDQLLYVLSGLMHESDGRGGGMSLVMTSSTPDIRQLEKRLSSRLTCETRCIPLLPWTA
DRVTRACLVEVKESLQSLLESRAAVKKRESKKKKTLSSETSLRSRVETLLFPHTGWRFDV
VSAAQEGWKQQGSETLGPFTASSNAEAQLLVETMLEMLTAAMAELDSAFKLQDESVNDSC
FAGRITQASGQLRSLGATAGRVMASVTSAFGDVCSGSLAFLHEKSRRNIILWLTGRLKKS
GGSKSLRGNFTTAPEAVRALWLSTSSKKPFRNGTGATAVTAASASSLSFRGPQPLFDDLL
SDCRLVDLGYCSRETFLVLVYVYLRHEAGVARTVVDLLEDVSSSMGTQAAAALDRAAFRV
AIADLHRWRIISIAGGDDVGIVSLRGSSARLREFLQSVLRRADYCASVLGLEAREMTRLR
SLV*
>TCRU_2470 dynein light chain Tctex-type, putative
MSVETPSDTTSAQDVAVDPPIPTQSKEKAETRPEISAEEEQPAEPKKVSVTLTPVTHPGV
GLHDDPDSADPEEAEKASDHDNAMEISGIPPNDVKNIILQVLSPYFDDEGGEDDAQRYDH
IKAQGWIQLICDGIMEKLLGMRRPYKYVVHCMIMRKCGAGIHVCSSCYYNQADGWVNHAH
DLSAHIYAVVTVYWSVI*
>TCRU_2471 Elongation factor Tu, mitochondrial
MFRAAIRGALLAPKKEVFHRNKPHLIIGTIGHVDHGKTTLTSAITTVLSKHGNTKALDYF
AIDKSPEEKNRKITINATHVEYESEKRHYGHIDCPGHMDFVKNMITGAAQMDGGILVVAA
NDGCMPQTREHLLICSQIGLPALVCFINKCDMMQGQEEMIELVEMEVRELLEKYKFPAEE
TPFVRGSAVKALEGDAENEGKILELVKKCDEWIPDPPRAVEKPFLMAIEHVFEVGKDKKA
VVVSGRVDQGQLKVGADAELSGFSAKKLTVKVASIEMYHKILEDCMPGDSVGAKIVGSGE
TVNLSKENVERGMVLSAPGATTLFNKVRAQVYVLTKEEGGRHTAFSPHYRPQLFFHCADV
TADINFPESEKLAGELNKKYGRDAAEQKKKETELKEFEKTLVCMPGDNREVLLTLAYPMP
MEKGLKFTIREGKITVGWGSVTECLALDPKVSIEGIKHTKAVTGKGKKK*
>TCRU_2472 Iron-containing alcohol dehydrogenase, putative
MFRFSRPSAAASTFFMPKVSYLGVGALDSATSKMTKLGFKKPLLVCDTGIEKAGILETVM
DVLKKKCQMDPVVFKDVHPNPTAHNVETGLELLNKNACDSIIAIGGGSPQDCAKGIALVK
ANGGTIYDYEGLDKAEKDQYPLVCINTTSGTASEITRFAVITDEKRHVKMVITTDTVTPL
VAIDDAQLMMGQPPSLTAATGMDALTHAVEAYVSVGASETTDACALYAMRLIKQHLSNAV
KDGKNLVAREGMCYAQHLAGMAFNSAGLGYVHAMAHQLGGFYDLPHGVCNAILLPHVESY
NSFVCARRLGDVAVQLGVSTTGMDDKNAAAAAIDAIRALSKEVQIPSGFEQLGMKEKDIP
ALAESALKDVCAATNPRQGSKEDLMKIYRESM*
>TCRU_2473 hypothetical protein, conserved
MNTPTKLGDLVIATPSSLAAGKSCAAEVGTTATSRSRALVVQHKKETAKATVKEVDVLHR
GLKRLETRMHELESRVQYVNPWVVQYMWELLCELRDRIGMNYSHQREKAWITGLRRRREN
SRGDQPLTESGMKTPLKQGSTEARTGTAGSRGETLLTTPTHRFEYSNEGVEGEQQEDDDS
ILDTSVSDESDDGRLPFRENGVVTTEACAQGGADFVQSSPSIDDSKRLKVGRTPKTDGRR

LARHMATLGARWEVYRITQEESTSRLGIALEESTQRSLLISSLSCSLATVLHQREVVLAS
SASSNSGNVHGMHRNATQRQGGIATQRRRERFDDLDVPSPPRTEIPVGLSPIQLAQSPSD
FFSPERDNGGRGGNQERSRRAVRSATDVVDDDSDFFSPNNRRQKSRREHEMTALACSVLE
APEFMGRVVNEVSRQLAAQIQQTDMSGERHLSSSQSLPSALVLTAREAVNAIAGAHDEAL
YHQLRECLAHEQRQRTTKEESLWQRVTEYEEMWERRTAAERQTKEEVLQEHQRLLALGRE
DAESCSEIRRELLKLCGEVVRAVEGMPDTFAASRPSTELDDRQTNGTDAIRDEKVFLRLE
TLSAAVSSLYSDLESLRNKSKGELAPQSEWCKQTESRISTLQESMMDVQEAVSIMARKSR
AAEERLDASKAEKEELTKWTERIQGLEQQVEALPSIFTTAMSPPLPPPIETTATTGIEMN
LMSVEDAMICVQRNILSRRAKLETSTDSYFRALSALCATLQQLSHLYNVGTGTFGVSLRL
IFAMPPLSVPSSLATACQAYLREHSAAELKEAMEALNVTQQVAKVLYDSYYWMQSHILVV
DDKAIEASLQNMEDNIVLAEGGNFTERAEPLSKSNVVSSCRKWQLRSATTDGKCRTEALS
RSKEASFLQEPKIATTNLTVTQKEPSEKAMPLPSLQNNTCPESKSGEEDENHQQERPTGE
LTSLTALESLAMASPLRTMDAHCGVMHHYHHHFNCSREEGHNFAPQFLREKEKERTFSPK
PHASQCSETQWDARETRWGNAAQHIGVLYHDGSDFAEQTTPLPVSSLNRSHIDVEKGIGR
MGERMAQNGCTSLDLPPTPSVVNECLTLPTAVSSFVDPQQATHTSGRGSLPLKDLQPFYT
AVTRPRPNSHSNFDENNSDGSAHERATRFVSRLVGDEKDKGTRHSLHRHQNLHLNLQKKF
*
>TCRU_2474 Zinc-finger double-stranded RNA-binding, putative
MFHTPVCGRSAGAFYCPSCNVYCSDSRTAALHRSSLKHKKKSGELEMERQLYKEDASVTV
EDVMALVERKRVELGVVPWSQLRFTEEETHAD*
>TCRU_2475 paraflagellar rod component, putative
MGEGRCESFPHLETDLPSRNEACQNAVGVLEQILSISDLHMTARATLHETQCSSVPTLSS
MTETIAHNSQLVCERTVALLKKLVPEPREVSEGEVAGGRQLSSPKIADLPSFVQKSRLLH
ERVQRVAVTLGGLKHSRLLGEESAGRRIITERASLAIKQLLEVLLAVEATVDETPLSSRA
GQGTARSRYFVLDSQRSSEPLEAPSTESSHAASEMLFSGEINKGNPPKSISFENGSQGKK
INRNSPRYLTTTRLRPTAEKREEREGSKTCTSCCEGKHLPFLQDERKESSSNSFYSCFSA
SVSSGNADTMGQSSSLRPYDKKGASPTSPKFREINCGNADSLETIASTHCIPLEELCSIN
TQLLAFKNKLLPPNTTVRVPIRNEDLSTAVPTRVNVHRRIFQGKLWRELLCNEDPTFTSR
WKELFIEEVVLLFGIPHRWVSDVQLANDTQTTLTGGDQAVKFRVRLPLSLEANEVNTRIQ
NHEFISMLLLYEELQKKKEEEGIKEISQSDTNSFLSVASRDNPESMSPPEFANTAVPNSD
AGSSDSRSGSGFDDCHNCLSNKIKNDKLLLDASSAIIQKETLSRDGLIVQEKYTRKLVMK
KSTQNQHMPGHEQQGGKKPVDVSLPTYRNHTGQQEPQIEASDALEEVSPFRFPNTHPACV
LPPLPPQSFAFPRHEHVSASTVDEEKTELSASSPSPSPVRTDCEVNDADYKEKSIFGNGS
LLMNPHLPESSEREFVLGPRKAWEPTVQWFYTSHEKVISGERWDLVLEKERDELRRTFIA
EVAALFELPEENVTAVEFVLGSLHATFNLMHDRYLKAAEIDALLTVFDFPQVRILYHRCV
EGVNAFPPVPSNKMSGSGYERQNVNANDLRMQLPPDNSIAGKNTSSFRNECADEKLVRLA
SEESLFFCGRQLFPVRLSMNSLQENAATPDNEPITFAHLALLHDISTDALVRANPHLDAF
GVNDPLPSSAHIVIPRLTLDDDACGFKEGYEEHNSNGTTPILVNQTTPGLGTANADEKSC
SKSSMNLKDAAREMDSNGQALRQRSTHFHSDGNLDPISRNCTVNVSVGSLEASDSPVVLP
AACASYQSSGTLIGMREESPTAVVVSGSDGEKRTVGFGLESTPFKQLTQESADGNGHALA
AGSEPKGVHKRWHDFASLHQVVKQHLLAPPQRSRPFMRHVSDLRVKPSFSTIRDAAVRRD
QAVLLMLRSTEQFILLRFFARWQYVARIRKGHCEYLALQRVPLEGSPTKSPIQETESLNS
RFREKTLGGTLTPQHVVPSKTRSLRGQNAELIAKTIEAIARTEKRGVSSRNAHQNIPVQK
KSFFPAQQNMQEVSSGTHAISVSAPMRTFGGDHSLQSGYEFAHTGSRESSRSPSSCSSSP
HLAFSGMSSDHSSYSASAFVHRPNVVPDLGISERPPLGLCVSSLLTVTEVHGEAEAAGIQ
RGDQIWEVGRHRVRTLKDFRKILASTDGTHICVGIRKKSSGALTRIRLRRGWRGPLESEG
SPSTNSSPLEPRIISMLGKAGSAFLASAMNRAHSAFVPKSVQQSYLPLNNTEHGS*
>TCRU_2476 DNA-directed RNA polymerase II/III subunit, putative
MGDESNAIGQEALLYDKMFRVMRTVGEMMCDRKYQVADKIIPSSVEEFKDWYTDPREGVI
YRDRMTVPCERIGVSQRSRAMVFFSEGLSMETMKRYHTQASEANCDRVIIVVQGRINAIV
KRHVEDINRSSLGLKIQLFDEDDLVVNITHHELVPKHTQLEDEEVKEMLQAHALELSALP
RILSTDPVAAYLGLERGRVVRIERKSMSAGFYVTYRQVV*
>TCRU_2477 DNA-directed RNA polymerase I subunit RPB5z, putative
MGSIEELKIVRLYRAYNTAIQMCMDRGYTVRHPSAVAQAVRDPCVYSYEHGLDYDWFLQR

FVTQRAAASGAERAEEDEDDKVSVPTNQNKLLEENKKRILMRGAMRLVCSAELDGATDDE
EVSLSKRGEKKGSLMLVFFSASPSLSMSELQEFREKALLKEARCMVVVSAGKIPPMVRRG
ARELGGFLRAGTGEEIMSIQLLEEDELAYNIARHDTVPQHEVLSPEEARLFLQQRQISLS
QLPRILKEDPMVAYLGLARGSIVKIHRDTKESGPYEMYRQVI*
>TCRU_2478 30S ribosomal protein S8, putative
MLRRTIVPLAKVYGAAPPNGTGQKQPVRHRLLHGKRERQGSLFAIANDVKYDEKRLRQQL
NAMLEEERLPPRTRLEHNKANGGEALPQRRLVDLPGVERRRDLPADPITRLFFQHKGDHA
LYYGTYDNPSLQDEDRIQIEKREPRYWTYNVFTPVYDFCHRIREATEQRKRFVIVPSTVE
TRGCARVMLGHGLVAGFRDFYNDRAFAVELKYFQGDSTINVIEPCAYDGKTEFEWSPKMM
RRLMNTHGIHNRLVVYICRTADNRVIDHIQAVKENVGGRGLIMVH*
>TCRU_2479 ethanolamine phosphotransferase (EPT)
MDLFLSVSPTDDRPLVPPQLSYLTEHVLTPFYDLVVLYYPASWTPNKVTLFGIFMTLLSS
LLLLTGMPPTTLFEPPYATIRPASFLGEDSKWQNSFGPTPLHPTTLRPLWSSIFPSGNWL
LFTCGILNSIYCIADNTDGRLARRLKKSSCIGEYLDHGLDCVTSLLSTCVALSVFGISFS
NISLAVVTVAMATVFSHTLNYEKHIFIWGNRFVSVDEAMIFFCVVHWIPLLFPGLASAKV
SPALLYAVLPEKWAHALLPLRYVEAIMVIYWASQVYVILDIALKNWKMIFRITTLALLLN
TVLMLGVIPYQTAQLVKVDPAGYVWGPFSYVAIWFITVSCTSSTVVHILIYSHCARLPRV
DPTALAGLLFVWLAFINCPAAGMMISVVWHIAQILCYVDGLQNRHLEGSQKVKVV*
>TCRU_2480 hypothetical protein, conserved
MDLSSIVLALQGNLAFLPRAVSVLFNHANPLSEREAAKLSEFTREQQETGAPVTSVPDNI
KQLMERPLHRLSAVTVIQCILGAARVPSLPVKYQRRLQRHLLPLLCKSELLSLEEWSHVL
RQTATLPMGHSAIPLRCAEEVLKAAVPALFLSKNKSAEEESVNEVQKLFLKTFSVVVSHY
AKRYTRCFQRSATQKMPTSKSNNKRNFLYSTPFRRLQRILDVNWGHTREAWLEIVETLSP
AEVLILSLATPWNYPIKNGTLMQVLKGSPTLLFHLFSDRPSPGSLWVECPNDAIQTCIYN
MGLTNCSRDNALDYLVVLLALSGKDKFEFFKSRGMDGFVQLLTPFVNNVKTLNVAVAQEW
QELYALLFLHMKAEEELFPLFVQAASDVVVNRCRAVTEKASIITLTASVMHAMSDAARSK
AAAGKLTRMIERELASPAVDTAAAAWRLLREARLEDEVADEIRSALKSVKNNEQLRRVMI
IHISPQRAPADVITSLIEPLFEECEEAEAYRPDAFLALRIAAQRRFNAVAKLCGILCQAD
VVAQLGTVADELNGLMETAVALAQISLFAPMALLLTHRSGLLRQQLSSYVATISTDASAI
IALWDSLTRTVFGLEAKEKPKILSSNAVGALLTVAPALLQHTAKTPVSTTLCEVLLCCGH
DHIQGTDPYYFHTKSFLLGRRRTSSGVVQSQVFERLFAGEHAHLFKLHSEALVSKICENF
NGPTHLGTAASRGLVLLLSFITSPLSLQQAFENLLDATKKSVDYLISLDARDRAIAASSV
TALQEYETKALRERQLLKTYSDKPPKGMSEDDYEDMKRRDAIALEKGREVLRIEIEKRRH
EIEEMIRKNKVPLATIRTLGATGRFPIEGVAVLYPYLQGILNRNDVPEVLERLIVDAISG
LLSKTIFADMGENMACTVAQLHDKPSLTVGDVSRLSTMSLHLRQAITHMIPAPLFVVLLP
FSRVAFKAGRGGNAVQTTIPVATQHQIMGLLLQNLGQPKLPQPTETLQLLTNILQNFPSL
FKSVQQGINLLMALIPASNLTALELGLFNPADAVKEVVASAYHRFSHFLTCRRALVLAAV
SLHDSSLEVVKSMQRVTQDPVYSFSLDPMDWNDLLYFLRNYGQHQASHAVRIGETMRELF
LLPSTTVAQQKLWLEDLKKIAGQGAIVSIQVLSSSLMNDAFQGVLLFLCEMAESPSTEPL
MRLILSCGRVVLNDCAIDVLKSLAPTLQTRLGKPPKDLTDAHKELYFATSTVWLTIISCR
LKETVLLESIVEQQSSTLNKSSSAMVHRAVCTSMVEITKNEEVRASPKLDEFVEKCLKQV
IHASSYIKKKAHAYGLVGVLQGLGLTSLRRYQIMEIMKKAVSENRTERTGVMLLLEVLSE
EMGAKFEPYALAMCGGLLEGVADKDQRVSECADDASRVMVRSLTAVGLQQLIPRLVDSLS
AEQAKRRVPPLNFIGYVAFCSPKQLAATLPEITKHINACLFDVNHNVSTAAMNALRRVAG
VVSNAEIREHVEVILLALRSPSTETENALDTLLYTRFVNAVDPASLALIIPILSRGLSSQ
MPHLRPKAAQIVASMVNLVNDPKSLKPYSEELVRLLEEAAMDPMSEARTTSAKAVAALSA
ALGGKMVDDIVAWCFTILHKSHVSSIEKAGAAQVFVEVVESCGDSILYDSLPTIAAGMLD
ERPLVREGFLHIMVYSPSTLSPSTFQRFLPLSFPWVLEGLSHFSDRVRDVALVAGSGIIN
SYGTRNLSLVLEPLLSGVVSEVTTLRQSSMLLASKLLIHLVQQIRKRMRISLAKENLAGD
QDKTEELERILEQEAEAAEDAENCGVLQMEAARDVEKRGISILGSLEEILGTEGFTQLLS
AMYCGRHERNLNVRTDTNNAWQTCVASLRGAVKKIFNGLVNVLVLFASSENLDCVEMAVK
TIEFTSRLNEMIEQLIEALCDRYHEKSRRSKLGALTCLANIVVYIDSRRLIGVGGQIVGC
VLPGMQDTDPRVQECARDVFAKVSKSVGPRLIEDAIEAQIETSVRGVVEVVKVKPVAALD
IIFKYLAQRSVYTQDNLELLDAILDVDEAYEQMRRYSDDMSRTLLAFLVQGLEGASETYQ

KFIEGLSREFEHLPVEHWEKGLRSPATQRGALLAAEAYGLGVSFDSIETLTAVFRAAIEA
LGSDDDELRSIAVSMIPRLIASLEQRVVESLEEEEQQDLTTSKRAAGRYLLQYLDAFQNT
LGVTARAFITETEPEFNVLGEGEPARIFDVFMNFYNRGLDYGTSVQKVQAVECIQDLLSF
APRHVSAGAANTVAGRCSKVLFTRNEGTVVLALVRLCLQLMDYPASGKEKMVEGTMALAM
FNASLCDKGEARVLALRVVIRLLQKSEKYAELILGTVVTKKNSVDTALLRGVMCRFVSVV
IRYSHFTKALSHITKLMDFVLPTWEWAETPATAAFAGVAVAALCKSASTTDGQLAELQKK
ALVMISSKGSAALGGFAFAYSLVTCCMERADPTFIRAAADGVRTAASVGASDKLTITWLL
RAAAALASTGLIQASDLKPEAFVPMLRRVDVNDELMMSTAQYYYEVIVERFPEVAGALAG
IPREQSTQWGIVGHFDADLDDEEAADTVY*
>TCRU_2481 periodic tryptophan protein 2, putative
MQTNFQLASVHGMLYTGGNVAFSPDGRQLYSPVNNYISSIQVQQEGHRSLPCSNSTIQCF
DLSPDGDLLIAIGRRGVGFFYAISAGVVLDTIAFPPDCEVRCVKFSPCGKYVAIALENTL
QVYTSPAQRVVAYHACHRVENLHAALTLPIMNVEWSPNSEHLLLCGQDARMKIYPRQGRL
QQKGMAIQQNALVGHRAGVHGAWFMDDACSCVVSVSADNVIITWKRTNVTRREVLQAVAT
AQMKARIMQQEDESGDDDDGAGRAPKSFLEKRRLEQLKLEGVRVSVADDTYLPDILRCAY
EIDKKYLLTHKGNVSVTCFHRIRGLVAIGYNSGIFAIHSLTTADEAELTLVHLLSISAQS
LTAAAFSPTGDFVAFGSAQLKQLLLWDWKSEAYVLKDQAHYYDISRVAITADSGSIISCG
DDGKIKVWKASSGQCYVTFTEHTAPVTGVVTCAATNAFFTCSRDGTARGFDLVRYRHFRV
YKTPEQTQLSCIAVDPSGEVLAVGSGQTDRIFLFAVQTGKLIDQLQGHEAPIACLAFHPS
GTALVSGSLDHNLVFWDLFNRGDGGDRMKGDAEVVDIGSEVLSVTFSNSGRRLAVLTMRQ
EISVYETVVPTEPIIIKTFQTSFDAAGGWNKTVGPRSANYNARFNVIAFSPEGEKIVAGG
DSKWIVMYHATQGYMLKKWPVTTNQDVQGAEEQYQWRQMTEAGHVDDIDVDDADIHLRRG
KIMEMPGSRHRHFATGKRQTALTARTMDLAFSCTGTEFVAATTDGLLMFSTRVARPKFQP
LQLSANITTQQVRDQLDRGEPVMALMGAMKLGDKMLGVECLRRMPRDAIPVAVSSVPSVL
FPLLVQWVSTEVEESRGIEQALLWAQSLMLHSNECTGGFAQERSAVIPALKVLQRGLQQH
RALTDLAKENYFSLRYIIDMTKMQKDILQPAATVE*
>TCRU_2482 hypothetical protein, conserved
MRGQSTEVPLRPKNLQSSLGISSGINTLPGITSIMRNEFLPLMQCLEQGNDVLCKTYIVE
LALTLSRFDALLKEEERRVFVCAFDALVRRLLKDLHHLIELESFLHQDLLMLMELAERCR
RVHHLYDCHTRSSSAGDAGGRSRGVRSTSSRSSVAGNALDVKGQRGTLDPPEACVANCTS
AELQQLFQSDVGRQLHPMNSMTASGTWSAGGNENTTNQPSSSTIPAQTSVQPFRLLGMVL
PRNFISKRASCWTEDFWDFLEGRIPKGEIAALARLLLSEGDNKNENFTPFSNAHRDFPIH
ASTKPTRTGSNNRDASASFVAHVDATLPKRMMQNAEDNDSLSREMDGSKCDEAGTAFLGG
RAMNQTAFFLNVLVEGLLFGGSSSVSSLNSSFGSMEDAEQNSAVTDAVNESRCSGAVPQG
DMIGRFITLPSPAALSLMPMLFPSLRNYAEDFTRLAEGFVEAVEGNSGGGNSSVAASSDI
TRRAQSGFQRIKGVHATATSGGPFGDNSMDPLHPATTLQMMTDASGFVRPNDVVLYVLLR
SLQRVDGQPATTAASTHLKTGGTEESTPKDFSFEYAAAASKTQMQDNAATPTLFSRIGVE
LESMIKQVQEYITSSQTMQEQIREEVTLLIFRVISTTFDFLLPAVQFVDLRVSILYRKWL
CDMYRLLWEEDLLDRFSAIVAASLASSSNDNVCGVSSSSSQPQSTTPRRGGGESKRHLGK
GDSTGGNTTPAVGGITSKNTSNHPSYGKGSSIVASSETLVKKREEKLRWIAPGNAYSRAA
RGLTGSGSEFMSLSSTLAVHPSKFRSINKTPTDVGLYGFGVFDDPTVLMAELLLEDKVEE
IPKSNGFVMTRLMATLLRDGISDKLCHLHLAPERSLRDGGVAAAALHDSQSSTVRKTTTP
SARSVKQPTRKKNTISPHHSPMGVRSAHSTPENKSSVPQQRPSPRYPAVTNVNDVKALYY
SVLCDTEVALSPLDPIRAAVVQNTVDFLVSGMRLPKEAYELLDAYLDDVGIEPIQPPVTR
RVALDGTSEATRGGAAGGDNPLSVSDSPFSVTTIRRARQMPSDTHSGTGGKTHTSHASHT
PQVKRGGPVDKETSAAALRLPLIPKVIPSWNTQEETEQFLVILALLRREYIVLRAELGLL
QPAPPKQSKEVFSR*
>TCRU_2483 hypothetical protein, conserved
MPARRSSGSACTKKNAGECAPQSQTAPANLPPVNSVLQELLMDLGLTETAKMLEEESCRR
LQFLTSATVEHNNFCGNGVQTLDVANTGEKSARGNSKEAKTTLEPKGKDGNGSPLNVPCK
TEGGTETQQPSSQPDTARTAFLWNFMHRKTGGDDETKNAGALQELCRIGIALPGWDAARA
TTTDGGDNNNNTTELVALTPHLRKLFNVFLDADSCETEHGEKKSGAQSADILPPEWVSLL
EVFFSYVEVLWAQICLVEAVSWADFTSQGWFRHRKNILERLSLLHEKLQRLLLDAHQLEI
NRHLFESFTPSWGENQKADGLFNSEEIKKDPSQLYSSANEVMITTKDIHSGIIALFCRGA

STFRKNIEEVSTWIILHCGQYNKRMESIDVSERKLKEPISYTESGETSSSEVKTEMEGSS
ACKPWHMLMGVVRRAVLMGSGEVSDASFDAGEPSFISSPALRWAQVFLRIQILAWVVGSV
AEFADVWLQNSQGFMEMTNCKAEGCHETRRRLSIVSASLGFWYQNCAAQISEIIDGTKGL
RKDSPSYTDNGQGKGLSQRLPRSRRAKMADEKENIGFTLVNTLKRAVHEFLAGTTTLVAP
LRLSLLGHEWQSETGANLPNHKEFCGTPYLHDNMNNEVDIHMALLHVLATGQPYQLGRAP
KPRRTPLYFLLKKVLSAPKILLDLQNERVPADIQLRSMENPAANMFLMSSQRNGVASKFF
PNGNQGRATQTEKAMNEPVTVRTYEGEISVSGEATSGFLDSQQVLSGGTGIGAVLPGISE
ESVEPFMFPTLSLPIHFQALVSRMAHQANEFTQSEGSVSVGSDEEAVSSTEYNLDRPSDV
ILNEVQIVSVTPCGSLLALLTTKSRLVVFALRAANGLNQTNFNDQLQDSFCEQLVLDVVL
MKGLKDPYWYEQLESFLSFSPSGQFLLCSVQNAPPFVPEGDEASRSHHATTGKVFLYSLH
RAECGQIDASDVSRHGAVGEEDRLYAAFRIHKTLITVARWLDPRFWRGNSSSSVLPLEEG
SPLWKHEAQRRLAALQCLSSGRDNLILRWSPADGSIIQSIATFPVFDILFSPLMQAFYTI
NQYGQLSMYDAWNERDIESAKDNVVVVSQRGLPATLAAMQSSTDLRERSFTQKSAAPVKD
GEMQDAFFVGTRIIGFDRRSLARGGTERESAVSRMSRLVQEKGLPAGTETKEGNRLGRRI
VQQLLRQDGDHLVPHTLAVIEPSSGSDKDFDGDDDDDYNHDGQATRGHRNHQGNTNGGVG
GGFNSMPFLRARHFRGEGKKLPTLSNETKGNNDVSISGEAVLYRTGSRLVFEEISQLLCQ
SACIWPGTHEPRYAPQQQPYQLLSMAASGHPLWSEGADESSSTCNEFIVHEKQRKSASSL
PTFSYYSDVPKRRSNSDQNHQQQSNNHSSGYDNNIGKLSQVLGTAGRSSSGMNNSRRSER
NFALAPVAENGRYLCITASVGPARALVPYDRPLEYRAGMYACLVFDVLYGGVVRVIPVCP
VLPRTALTENGVSHFHSYTKRAPIYILPCAVTVVRHPHRHQSDGVSSSASASRRTNCNTM
EGMSGLSYPLCLQEEEEEMRNEDKSGHEIADRNSDNNGDSMVLVAVGALHSTTYVFDALT
GSRVKVIGLRKQAGLTNALNSGQTRLPIKRARCSYVSLADLGVFSNCQSQRDDGNDDDDS
GSRNDDESSTASSELPSPFGDEQYGYQRQLLLNHLVEQHGMDTLLRAATELLRVVPLPFP
PFFEKLQEDCIPRLSEGHRSNGCSRQQAHGHCGAPDSASSILSFPSPLPHLKLMMSCPSF
LGQLKMALGGVSPANGFQSAASRSDAEGKANTATLFYDVPFAHVAQRELCQALLSNETPM
SVGENGLPSTGGVAPAVAVNTSMNSASKPQKRFLCGVVNSVALFYDHVKGGVFVFSSDEY
GGLFMAGGLIPSRV*
>TCRU_2484 hypothetical protein, conserved
MHGRNGELGAVCHVANAVHTCSNCAASCATKEEGATVAFTMNVTAHKYAGHYELWWRQFS
PDGTIHPPARSGDADQTGICQLPPPREGKDDTKSGGTTAPQVNAEPSRQHEDRRRSAAAD
GPNTVDPSPASNTATNTSETKPEFAYV*
>TCRU_2485 BNR repeat-like domain containing protein, putative
MEDGTLVFPLMAKNEAEDVYSMIVHSTDKGSIWTLCEDISPAECLNPRVTEWEGSLLMIV
DCENDQREHESRDMGTACTEAIGKLSAVWVNARSGVSQKENLRVDALITATIEEREVMLY
TQRGHASRKERSTALCLWVTDNNRTLSVGPVGMENAAD*
>TCRU_2486 hypothetical protein, conserved
MGLSCSHKKKGETVFKGKTTTRSSTWVSKKECQVALMLQGNKASAHIDDHSLGEEEVPLT
GERPPEVLRFCFGACGVHESHVTVTDVFLHNRPLNFTAMRALKDRVPVPTREATPTPSEK
VRVVDSVREHIAGDITEEK*
>TCRU_2487 hypothetical protein, conserved
MRPHIVDGRAHSAAGWAGGHQSAARWSAGSCGAHFGKRFWRRQRRAGLLSEGGTASPTQR
VICDTEGTRRHVEEWAGVAPCLCAEPPLGARCPGCARNRVLATPTGACHTVYAPFLFSWA
SLQYGHLTAVKKPTCQIGLSSCGRDRAIMASSSAASLRGIPQCDGENMMFTERPRCIKES
SIIQISSVTQSRTASGTTRSASILDRDSVATRIRVGDWDVSKQKGMRCLMPSSVARHSAL
YERASLPAAARWTSTGRSFVVVEPYNSAADPDASSARDEETSVHNSKSKSPREE*
>TCRU_2488 hypothetical protein, conserved
MATIRRKAPRLFTGAVFTEEISRGLMTFSDRHNLTSDVWVPKKLVGKMKAGATLLPNAIL
CDVSLIPGCGAVDLKTIGFERVLVNAAHTTDVEFFEGYRNDILNGLAGALPLTVTGAPHR
DVRLASLAVDGSQRGFQSPYWLRRWGRKRQWVNAEETPFPGRLNPTDCTSYEPHTFTNQL
FSRAVVRLMRRRASRYGYVSRTWITLKEGEAHGTQLDAERIKDGPPIFVSQFSGSGQVQA
VEFFCADQFEDCTVFPTQREIDLAASGVFIGAEKVRGYPLLASLAVESSSSDRHRRMAEE
TAKLSDPVVFGATRALEPQFLGSEFKESLRKFSLLRGFSKPHFFFHTAETAKYLRLKPQE
QGILYNPPATSIGPRPEQSRHCVFNVQQFEEPAVVEEIIFRTPTFFFTQVPIAGPMVVEC
ARAQCRNREYSQMWLPARALDYLAGAWAISPGAVVVPRDETLLAFSPKMYNASEVSIPSE

ALEWWPNYKPHNMHNNLYWGQVRMLLSLRAWERQYKSPLWITQKLMSRLGITTKRQTRWK
LFLTGKPFEKPPTVLIDDDEYTNMEEIKNPDVLMASMSNADSDINVLAKKG*
>TCRU_2489 hypothetical protein, conserved
MNQFELFVASSESARSDAWRYLRRLRELDSRIDENMQRLREIAETVVKPTVTQITEERKR
KNGLPHGNEKRGRSPPGTVTSGAFFRGHGEDEDDVSSETSVVLLEPSEAKLRVEYRACRH
RVLRYALEREAVAEELKACGSEMKKCLAVRMAKLRQTLRGVEYSSPGTSS*
>TCRU_2490 Papain family cysteine protease/Protein of unknown
function (DUF3586), putative
MTYTGGVLTNCVSKKLDHAVLLVGYNDSATVPYWIIKNSWTTQWGEDGYIRIAKGSNQCL
VKEEASSAVVGGPGATPEPTTTTTTSAPGPSPSCFVQMSCTDAACIVGCENVTLPTGQCL
LTTSGASAIVTCGAETLTEEVFLTSTHCSGPSVRSSVPLNKCNRLLRGSVEFFRGSSSSG
RLADVDRQRRYQPYHSRHRRL*
>TCRU_2491 hypothetical protein, conserved
MNTAKRATTRKAQVPSEKTVSETETRCHSALLVIGTYHSVMAGLVYRRNKFGMLFSVKHH
EGCINSVAVGERYMATSGTDERVFLFTNKNQKSLTPSERLRLRAAGESLSVRLADLGHIS
PPSEVRCLLFIANSQQLLCGCTDGQLLMYRTRDWSVGLSLPLHEKGVVGVATHPGSDGAL
AVTIGADRHVAVIDLARGRLLSKWRYNASLEGTEVKKRQEEEEEEKDDDKKDGTPRVKRV
KLERRDGPHTVKFSPSGRYFTVMSSHAFAVYETATVGVVAIYRTPSPQPREELHVFAFVD
DDAMIFGNESGELLACRGPWKLEETQPGECTLHPVAIHVPSESEQHADEKKKSLRHPTRH
TTRLKALYCVERTVFSLDATGTVIAWLVEEVADATMTLKFNCSANCQGRVTTMDVLLL*
>TCRU_2492 metal-ion transporter, putative
MQSEVELLLRSGGAAKADYDTTGLRPQGLCPTPVERAAIEKVLSATEERRKRESKFLYGA
LIFCFVFMIVEFACGVFAHSLALLTDASHLLIDVGAYALSIMSLKAASRALCGKYNYGWH
RAEVIGTLVSVFSIWALVVWIVIEGMTRTWNVVSCSRMPAAVAAITQQRGGSGGEVVADA
VLASSRRCEEINSPIMMLIGVLGMLVNIVCAAILYCGGSHGHSHFGGHGHSHSHGEVTNC
ESDLEISINGHTKDHSGHHHTNDDDDCGHGNSDEKEEHDYDHNHVEKKKSKKGFALHAAL
LHALGDCVQSAGVIFAGVFIYFANKHAFGVPSYQYSIYNIADPLCSLFFAVVTLNMTRPL
LGDLFGILMESTPPGIDYLALNQALLSIEGVVSVHDLHIWSLSSDYVALSVHLVADDAAK
ALRKAQYVCEVLFGIRHTTIQVDAVANGADLCQGSCASPNCGKEYQTIT*
>TCRU_2493 hypothetical protein, conserved
MSSTAAEMRSDTGIVKQEINECVETGREAVLGAKASLEKPVRSAVEKVKQGVRECTEVGR
ETLNGAVTKTNQSLNATKEKAQKLADEGKQRIGDMIESAASYVSGAGEANGEDGGSNSYA
AKIISVADQYVESGRKILNDAKTSAEAYHSAAVEAVQSKITHGRQQVETYATTAKDSVEP
LVDSARGLVNKSAKRLSKTSEVIREQKTACMDKLDTVRTKVRIAVDESKPHLQTICDNGK
SAVFKRDARLALTAVAQLFAGVILLMNAFFEEALKLTFVKNTTSYVQNCRVTQRTRELLT
AANIPQKVDHLPIVGHRVVVAVTIFLSEVKRSMLEMKKKNRCVSPKPAGAHDAKELREDA
IPHGRKTQRKEKLEA*
>TCRU_2494 hypothetical protein, conserved
MPPSSRRKTASAAKREKSGRIKKANAKYQNQKYVPEQMMQRGRGGAHGRSGGQQAERGNG
EQRRQRSARTDNIDIHLAAKVSAELRVDLSKFKCQLNATEIFGKELSQIFKFVPPPFVRQ
HLPEDVKSLRSYISKFERGAPSIVHAFIVWYEWASYELNKGKGHSYSSKKNGVEGQDASV
AKEDENDEREEQDGEEARKEKDHGKVSQARAALSKTVREREHIRALLQMCLRHNSSRRKE
GQHAFLNHLKAKLSEDVDCHSLKSLRETSHHAVPYALSRLLLGMVTEDMAMILLHAHTLF
LLLRDTTVTPSVILSLIGEELALDVRLKASKARLEARAAKKDTLTQEQQQQADEELDDID
PDKINDPTRGERNQQMIATVFATAVIVANPRKLRLEDAQRLVHYLCFAYIEQKALRVLTA
NLFFNIIEKFPSLWEDKTVLEWLSYAFFLYPKLEYYRPEGVQLLLRLMMMEEKPPGMKRG
VPTVVMKYAAMDPLEPSTVEQIANALFRKEQVTMVYPMVHPVWTDWLDIVAQRCAAGESM
KEHLSTMVHNAIAPYRRGNADTPRRALFQQLVSRLGQLAVQNDDAEQRAEILQMASKTVG
YGRRTIAKATDIADLRNMPLELLDAKVRDLLRHYRVTRDSDMSASANRTWVLRELRSCLY
VPLREGVKCTYIDDAAEALLEFGFYPPPPHRDTRSMNRAIFLFAEVFSFTYTAALSRPKC
TLSPLDIIRAYLEAEKREATRYTTGVSQSVFRKARNRIVEALENSSKRSVLFYAERDMHI
LLVLLFLVLSTDDYTNDEAKALATSTVPDICQFFLTGTLETIDLFVDVLMALTMRSTAPI
HVIPLMTCIRRIATGYLLKFARHVRERLTLDLILAPLREAFHTDDRERSRQAKARGETEK
EDSDDDEDDDDDNDNDSNKDNDEGSEELREDENEEEDGGDTTGTSDTSESEESVATESTA

ATAPDDEAEEETTSDADELSDKEDFMEGDQEEGEEEEEEAPTQQYIDALKGMIGNVDLQF
IYPVDTSNKDKSNVVRAIQIAARVGASMRCPLMLHIFQVLLAVCRENVKSPDDVIFNSAV
SSTQILMMTKNRYFGRFLVPEELFQLLSDIQSYCRKLDHVLVRKESQSAKHAVTIRRRMA
LLRDLALRVFHFVAFLAYKNHAGEDVRVTLLEFYKSIFCDRGWNDKKRLPGIKRDMHHYR
HGFGWVVLPSAFEKFGEVEGIAGPQRVRVFKGCCQMIEAMLPRLSGMGASLKTATGTAIT
SFLQSTTVRAVYEMKYTLLYDYFHCLKMVAKYNSRVHLDTAWLTSVVEEAVNDDSLQMSA
ASIRLLAGMERLLGLTPRAKETKAPVPVKVLYQQFEKQGRKEKAVFYKRAKRVRAKVVKA
LVAYRNGDLTDAERALKRRRREAMKIDDRLRRQVLREEKSRVLTKEEKEEKRKRIMIAKQ
ERIAKNRERKRRLHEHREKAFQRWREQKLASAAAME*
>TCRU_2495 hypothetical protein
MADFAPDGASASRYFQSDSNFGECGVCLCQWVCPLEIFPCRHIFCEACVESVDRCPECSA
TIEQRRQPNVAILELQKHSKGRCSVCGWVGTFENFNHDHVTCNAKPSFSSSSLNVSSTSP
PLGSRVGRGYMLDRPETNDAGQSSPDFSSQQEQQQKQQEVRSRVSPPKLNANALRPVSSI
SLSAEGIRRWKETVSCVASDYGLSEDEFKFMRDIWPEFATLNSTRTNLELQWCDACRMLR
YMNLPSHPNDVIDLFNMAMQPIEGGCVSFESVCLWSSVYRRDPSRMYNMNREQYSCLLKY
AQIIDIEKTGLFNEDQCRGIGEQMFLRELTDLEWRSIRPLVVARAEEFRGNIPCAYPTAV
SQGQQMKLSLHEIILLYNRFFDPTKKSTGMAGGNNNGLSEALQQRIQFMLKH*
>TCRU_2496 hypothetical protein
MSLYSAEVVPCEVQEVPLSDVVCPDVPETNKERADVPSAGDDVARSTESAVPLAVDASDV
VEGGVSAMDEEERTEENEEQLPQNGEEEQNCGFVAADHEADVESDGAASKRRRLESPARD
STDLKSGGAAMDAAAAVPIEEDGLTKVISANASQTLDGLAESPTQHA*
>TCRU_2497 hypothetical protein
MPTKGTGAEGMTHRLLESAVFLSVVAAAKIEELVGDTTVTLKLVLETLPQLSNQKDAMVA
VSDETMSTETLGLGKEEVLPLQLSHADAYQLLAELEDVLEALSQKQF*
>TCRU_2498 translocon-associated protein (TRAP), alpha subunit,
putative
MRSLLSATIFPLLFVLLALCLIAAPAVRAEDMGEGGAFTTGGDADDDDEEGLGDAARKYP
WASARVIFPDKPMNLLHTLPAGSEKNALIAYRNNQLSHQHTVVLIAGYLTPLNLYGQVIQ
NFSVVRHARIVQPTDTTSFQYTFTPDAMLEPNDYTLILGLYYQDNVTNHTYFVTAFNNTV
TVDESLETDPKMILLYFNLLLLFGGVAYFVALKLGLVALLREKRGGDSSRRRVEVGTNGE
GYDPDYVSKEHHRYKEALLQRHSSSSPTKKRN*
>TCRU_2499 Tim10/DDP family zinc finger, putative
MQLEVKQESFRIEMLMSRLQSECFNLCCKDLTTNELSMQEVRCVDRCSQRYLRTHDIIAN
AVDRGQSSGGKIKL*
>TCRU_2500 hypothetical protein, conserved
MRAFCPAMRVTGRGALRCTIRWSLTVKEDEAFVEQMRVLLRRYRALPLSRITALFSDEML
DIMSNISGGLRRYFQERPSRFRLEILPSGVTVVMLAGDLRSVGPGLPIVGQALAHLAVSP
QQQMTVSQLFCALPALEQRKCGNEKYLESFLLKYPSLLTVQNGMVRMAPTSFMQLRGGVP
APLPTSRARVVKLQSPVPEPPTSSNTAVEDAKMNKLMALLYGVVPFSYYVPLSFVTGHSR
SYMAFSPGMPVDDVLEELQRVTATTLDCRVIGSGENDVFLRMMDAERRPYINDETRLSSR
YEVLPLGAPLLEAFLTYAAKGPDHIQRLRSGIAMSDLKDILPSELLKRLHLYDSTQEDAA
CIFVFDRLRHLFDVNMTAYMVRPWEVLAAHAQPSSLTWQTTPIPVVLRHGLMALEKKPLS
PEAFLNALPSTSRKQLKCAYASVADFAAHHSLYLFLKDGLIWTPYLAAVSQGARVPASSR
NASSGRHLTDAAKAKMMMELLPLHHPVDWSRFCTQPSVRDLPFEAGNMRQEFFERHKECF
RVYEPLGTTALIVGRRDGIPPPAKLLRPPCNSLGDLVRQVALLSIDGAQEGNILNQLSKE
ARLLVKRYGTLEDIARQLPMWFDVRNEMRDPGNALISYIATDQSRPPCEKQKPADE*
>TCRU_2501 FKBP-type peptidyl-prolyl cis-trans isomerase, putative
MYDSCSSVDGEGGFSVPVDKKNIPARKSMRSLFAQDALQSKQGVTSLRYEDQSKKNKTVS
LSVMEGNSAPGYPPNAPSPLQTAVQAVTIAYSGDTSMGACVVAVCVPHANAPIATPLIAI
VDREKRPRCRVPMDGGLQFLQNESSPQYASLYDPSLGSHWTLMFKGRRECTEFFSAVHTT
VHYTEIDIGAAPRFVEWNNETNAEEGALQVKLASGDVASISYMAWLLRRMPGTRFFSIGK
MVEEVPPEAPCEVLVGDGSLMAGIEEALVGMQRGASRLVFVPPKKTSIRMGMGNPEVSPS
DTVVVHLTCQDVAVRSDMRGGHKATVASPLEKRFSNSGTQAKTTSPAPVTQTEPSSKPVP
DAPAVVGGDLSTLLQAILLQMLQQQQNSSGSSISSSNNTNNNNNSTGCDFGNGVSGQWSA

LERSIDRVHIQLSSLYEKIDRLAIAETLEKNNAAIERIVKRVVGKAPAGEVDVEEMAKDR
DGLLATIERLKKRLEEETNNYHRALEAMGRHKDEVHGLKNDLRIEQETHESRMRQLEEHN
RLKFVEMEVRHRQAIERVAEEKLREGREIGLKEGLQLGKQEALEAAGGHSDREWKDRFFA
SEQRALQLEAEMQERESHHISERRGLQEQIDALHEMTEKLDQRAQHARITRQTGVEETSR
QCKLLRRAMNAAYTHMEIQLSGMDETVNVTDVLQMLMVALRSETDTFIDEIKKQAEFFCG
LDSQSEVPMEGDKSRVQTDENASDHLEATEAEAMDKKRTTALFPGKEQKTSMTTELAEDE
CVKIDELPPVPPQDLSSFMCDNPPNVFLESVEPVAKDDDISHDFVRKDENKNGDLCNVAA
VTSRLPKSESNDDESDW*
>TCRU_2502 retrotransposon hot spot protein, RHS3, putative
MDHWVLYEPKVQNFPLVDGLFFVDSNPKTLVGLRMATAGGHHTRASTARQFTEYLAAYFN
GWEELSRDMSWEIIYVQHADSTPMNDWQRCDVVNSNNVSDDESREIAAFWKEEVRQYQVS
VSYRGFRGDEALRTEEEPSK*
>TCRU_2503 retrotransposon hot spot protein, RHS3, putative
MPGGIVRVCVQCEVASCGGSFWRRGDGNGSEGGEAPQSWDYKKVDDTLEKDDGVWQSGAP
RPRLLVLTSEKGWPYSWRWKENKSTRDCHVNCEVDRVWQIVKGDLTEWFSSHGGNDFEPK
QRLLIGMPGIGKSMAAGSYLLYQLLHYDAEQLPMVAYVVGSQSFLFDKTTKTVSTYRGDP
RIDDVVNIFSLRGVKGYCIYDATLACRQPPAGLPCRGWGMIVVTPPDKNDYERWAKKWTL
LQS*
>TCRU_2504 retrotransposon hot spot protein, RHS3, putative
MGLNDFLQNYFDGRVVEEFSENVYLKDLLSSPNDFIQDEVLLSPIKALPSYQELKKEREE
FYVLSEASHKLNREYIVTLRHWREFKRKDTVTPLARAQISTACSYVLRVERRRAEEGERR
ERQELGIDVSTGIKYAVFKGRVRVDKMRLNDFLAMELDGRGAVDANWDVLLRSFLRTPRS
TFAVREYWAECRHPIVTIRWRGL*
>TCRU_2505 Concanavalin A-like lectin/glucanases superfamily/Trans-sialidase of Trypanosoma hydrophobic C-terminal, putative
MLYIQRWYASGKNRATALYLWVTDSNRSFYVGRVAVEEAVNWEFASALLYSDGNLHLLQR
RGNDKGRAISLSRLTGELKEIESVLSTWAQKDIFFSSLSIPTAGLVAVLSNASANNETWD
DEYLCLNATVTNAKKLKDGFQLTEPDSGVMWPVNIPDDKVRHVFLSHNFTLVASVTIEEA
PTADAPLLGATLGDTNSPLTMGVLYTADKKWVTIFKYEKKPKSGTWEPKKEYQVALMLQG
NKASVYVDGKSLGEEELPLQSERPLEYLRFCFGACEMHSSSVTVKNVFLYNRPLNSTEMT
AIKDRKPKEEKGSGDGEDKKGSGDGSMREGMPRVLLLLLGMWGIAAI*
>TCRU_2506 hypothetical protein
MATSRNYTDVEEQLRAWQRKQEKKIVVSCMDWQNIESNLPLLIPIIGITLVTCFVFMLIP
FIGRMKSGKQNPGLACRYSANLGSSRALLFASRFNAPLFSAFLTIWLLLCLANVVFYLMQ
VAAKDPTWAERVLTTPDGFLEPMVLLYIVQYWGISSLLIHVIFTSHHYMVGCMASFLPFL
VSFYQATRLQGDEVDDWPVIAIKAALLFSFFATVAFTVPWMRNEYRIAMREIDAHTSRAL
GLRRKTGKMPERKK*
>TCRU_2507 Protein transport protein yif1, putative
MSAFSPYNSASPQPQPNSPAPWGGNPQEIMLQMGLQYGQSMLQGGEQKFMRHMPVISNIY
RYFRVDNQYVKRKLGILLFPFTRSFRKDEQGPGCEQEVTGGSAGFGGDSAPFSPISATGR
TYPTTSTALPTNNVYALDLYLPLMGAVTYIILSGFVHGLHHHRVTNEDLLGFASALVFWF
LGEVFVLKMVSYILRIVPDINVLELMALTGYKYLTVSIIVFLRELLQFESDAYYIGTMAT
YILFANGVFVVKNVMRMYEREGRTPPNARLLAYSAAFLQAPLVIWLAVKPFR*
>TCRU_2508 chaperone protein DNAj, putative
MLAFSVPEIPRLEDGAEEISFRVDPKLFFGLATRVRVLPRGLALQQQARTSAQVWVEEQL
QHSRRHGDPHAMSGSDLDESDDGSTGGNGEGRRQQTFRRKKFVKLTDEDLGVDWYEVLQL
SQNDGSTEEQIRTAYRRRCLETHPDKQKDHSDEAFKKVQRAFDILGDSETRLAYDSSRPF
DDSIPNETLPANADFYATFGPVFERNKKWSSERDLPSIGDDNTSLKAVYRFYDRWAHFQS
WRDFSHLVELDEIDDSMCREEKRYYARGNERQLNHLRKEEQQRLRTLVERARKNDPRLRR
KREEDEARRQREKEERELKRRQLREEEERRRAEEAERERLQREEAQRKQLEIKNEIRQAQ
RDLLAFLEQHGLLDETTTNKLLPHTVRRPNVVWIFSKVSTPAQAAAILSDVMSCSTERRP
VDTTRGSEGVGNRIGDDDEEEGLTVEAVLRFNALVEENERRIGVTRYGEPIKKHPPSSTD
VVEVAKKITPKPTANWDEEDLVRLQKATAKYPPGAVDRWRKIAEMLRGKFTEEEAMRKLN
EITAALNHSNNSGQNTRTPVQKQPPLSNTSKFPPSAGGAAAAVPPVLSTEDWTVKQQKML

EQGLRELKEYKERDKFQKIAAMVDGKNAKECFERYKYLCTMNKRK*
>TCRU_2509 hypothetical protein, conserved
MLCSCRRAAVPGCGDEWGPNTERLSRRRGATHCGRTRPEGPHGVLERPSLCTQPQLKAKE
HGLPCSDGDVQTKNEKSINRVTEQAHRVELHAWLPTAQAMVGRVLVRLSV*
>TCRU_2510 hypothetical protein, conserved
MGTTTASPLEKVPRSVGCGAARWFACSPTDRTAQLFHCGHSVGITRCGGVRKSRSPDEDG
LVIVWQLRTSGPSPFRIALAARPAQRRPDTAFLQECRHQGAPATRRVGCGDFHQTCSGRA
GGVAFLVRHTFPCQRLLLVAPTAWRPPPSACAHRPAPAFTW*
>TCRU_2511 hypothetical protein
MKLTALFQRLLFLVVISGSALLHGVSARASSESELLCSPLEIEPYQKSFCVINVRDSQQE
PTMAFELEEFLVTPQSSVPQADLKLSPLQRGVDVTTLTFDVSASEGTSITVNVMYISGNQ
SFPIRNSGVVVSVLNWPAVRMGPISCNVPSSGLVLRGTTTCGAALYDASNRPAAVHQSDV
TFLEEKDLGFFKFISGMGKLVFNFTAASFPSMTAAYFTLLLTLKGSKDVYSTNFPLLYPD
LFPAASFSTLSCYNETTPIVCTLRALDEAGPVRINMDYFRIAFDMPDNNVERGKWVDNTK
AFDVYSRAWAQEDTVFLSWKLKVNNFINTERLHVYVVSSTPGNGGKRVEEVRGSPFLFVS
GVMPHAAYVSLRGCRVSSITSGNRTQCFIDLHNGVTGDVRYFNLSSTLGAVFENITYLPN
GPIYKTPVVSFVYVAPVLNTRSEDFITLIVGGQSAIRSPFHMNVFPRRSSEKNEDSETVR
GSNALVIGVGLAFFGVIIGTGSFIALRSTLRLNRARQLRAMQKVQMVEMEEEAGCSATGK
VGIEPVPQPHHPQESESD*
>TCRU_2512 hypothetical protein, conserved
MASDDLADENNVLRTRVSVLEQELKQRTADCTKLRDELRAFEELKKKNESQRELILALRG
ELDFLRAAHLSAVEALEKKKKEDRDREHRESIQSINARLGADASPKNGSPKASAPKLGAA
GGLVPTSSGPQVLLKSSNISSLGFAPVRRPVDFLGGQQNSLQQCGTAGSGNGDEDEAARL
MEMVHDEALLDVKYAEVADQEEKELLERFEALRRR*
>TCRU_2513 zinc finger protein family member, putative
MSSALCFVCSRCELPICSRKDLLMAPVAGGTTETAFEYFLEDVLALDISVPCYSGDEVAE
VSVTVSEEILASVLPPGAQQLALEAVVASQRSEQQRQQQVEQNDVGTSLSSPRGGSSPGS
STAALPHSSSSSSGDNAEVPVEIAPEEANSHNATGTSSRGALTSENSSYQQAIQALARYK
RVQETRVDLVCVREDVLGCGLSRCTRDRVAQSSSASSPPASVEEAQSSSSPTQWFSHPGN
FIVEESFIKVRRRTKTARYPWFDAYDCRGRLECPDCHLGLGYLFVRRSNEHPGSEAVAVA
APSAGHAETGDDGTAEEVPEPQQKRERKEEGNDNEKKQKQQNDGDENVLQYPSTFLGLEL
KKIRERQWGLRDFQKRYQRSKELKTFREMFPEAEELENLYSRLTALRMQSELYNNLLRKH
KERNDVQSALLQSQKERIHTYEEKLRTMQQIIEAQREQLEMQGRQIKHQEELVKNHKSQV
HTQQQQIHVEQLLLTEQSRTIESQREQLNLIQAHLRARLMKEKLDERCEQLTRLLYDIGG
ERRSQILTTDTATTTLQRLSRHPLPPPHLMGVVGYDTREVRETETEERGMEYRSTDGLCQ
RPTSDAVAPSRDDLVTSGQEGVGAAVSETRGCPSRASPAFFTPKGLDGRSAFGRSPDVSQ
RTAAIMRRITEERARRGVGKDSLPSGDISLGSAMANDERQNSDGACALNSRNVSLLLSKK
KE*
>TCRU_2514 CW-type Zinc Finger, putative
MRGAQGAGAAALHSAGDGGEKNVASTARYIPMPESLWALRHRFPNVTRSVLLQCVRCHRW
VAQPVTKHPPDELATYPTLLGKAASKTKGDRHQKPRKEKEEWRSPRLRQHYQKVVEAVKP
QKPRIQEEPKERISLYIPNPANFTCQWSYCALEDVWADLRDNWLGDLYATLDGAMGNEEG
GNSSSKSTRLRRSPQSQNERINEELTRELHGKRSERLAKKPYNGMDVKQENSESNGSAVE
PPSRGRIAQQVDKLIHHDEAVVASFCWVSCDSCGKLRHVDQPFPGGAPFVCALALNIGSC
SVPEQEGLGPIFSSETDHFSAGDKSVAITGDAALRVAIEAQAFAATSTAAVASTGNEATK
LTARSEHRGFRRSAGGLPSACKSRASVELSLPLLRELTSALRKRALGTFVKNIMMIPEEV
RAKREDVARASLVVSEDTRHMDSVSPKRECGESDVKDVQNSNSQREERDNPNQEQEEEEK
RSGATGEEKDTNGASDGTVTCVAETHRGEEAPRPYRRLGCPPRRALCKTREGTCPEGALT
RVDLLPLPLRETTRSAEVAELPTTVKLEAVRMPCEVSNSIRTIHESPNGNSAKREASVFP
LEAPTVSSAEVPIRESLTKNISGRKRTRRQSNLKDVEENNSDDNKIHTTQAAKPQGVKRR
GRKPRRVENDETNNTVNEEEVEIIHWVCCDLCDKWRIVPEKISEDTEHWYCNMRADGTTC
SDTDDEIRMKRLEKSGRRRGRKKKI*
>TCRU_2515 guanylate kinase, putative
MNPPLDAIAVCGPSGVGKGALLGRLLRDFPDRFGYSVSHTTRAPREGEENGREYHFSDRA

TVEKMRDEDGFIELCEVHGNLYGTSVRAVAEVRSTGKVCILEVDIKGAQKIREKTGLLNA
LYIFVTPPSMEDLRERIRKRGAENEEVLQHRLRTAEEELRFVEENPTFFSHIILNKDLDA
AYEELLRVFNEAFLRCNMSKLERNSE*
>TCRU_2516 YEATS family, putative
MRQGDRGAAAVEQEHVLYTGAMLIPFCVGSVALPLSLCRASSSSPLLAAPLSVPRRKSKT
RRRGEKKNQQGTSHEGDGDNPHDYNAGISRCTHIRYSYLRDGSSAAQDLAYRRALYTSGD
VRMTSVSTSEEKAVTTASQLYAVVDRVVFKLPNSFPEPRREIRHPPFFVVDDTWAEHLVE
MEVHFHPWLGIAPFTVSHMVLLQKRADFTPQLLSSTFGQNKPPPTVILESSDTGVLPSSS
LAVATTRCHSKQREGPPSEAHPLVVGEHDTDNDEFYAEERGICVKFEDLEEFSIVSERRD
AIRIFHPTLNVARFLATVRTMAQPELEPPPSFCFFPKQKSAPSHVQLNNENVPTVDCCRT
AEKRNRSNDTDGENHSGALDGGLLPPWCFPFEAIMHAHEPKRRDGAVDAMRAVLAALRKE
QSELREGIEQKISLAMNNAEELRGVIQKMRETCAILKKPVR*
>TCRU_2517 chloride channel protein, putative
MSLRALSKDCEEVEGRCENLPQSCFTPFIYKIGLVSPGRDLWNMLLKKRRRFFLPSERQK
MERYESVDTYEPATIVYKDHLESRNQEPRWFIWVFVFGLGIASSVIAIVVVSLLHVLNGI
RYELLGFGLRGFSFNDSNRYAMTNRTDLDIAALFEGVGVGLYGIEPRSLKKGYLMWISFS
LVLSVISSLICLLVPESIGSGLPEVMAYLNGVHYPMLGSFRVLLAKVSFIIFSVASGACT
GHFGTIILTGAMLGAQALQRRRYFRCDSVHIIECFRNPRDRRVITVIAAAAGVASAFGVS
IGGLMVVVELISTILPVRFAIYVFAASLVSSLTLQIYFSFFSYFSLRDRHSYPHGELISE
MVQFFVSCVSFEDLVRMHIFYFIPTTLIGLFCGVLASVFVRLSSFALGLRRWLEQRLETK
AVRYVLPVLFTGIYVTIHFWVAAAVGSGGWSPPAPSPSEPPRIGSDSIRLPMLQQTMSNE
SNPAGLHGAMGGPCVAVPSTLLTTLNVTSISYYGANGFFCSAPTAVEDQVHEERWGMKPT
LSVLHAYASLAFANADSAVQTLLSWHTEEMLGAPVLVIFLSIYFLASALFLGVSLGGDTL
LPGLVIGAAVGRLFGIGVNYAAVSVVGAEAASSWADPGCFALIGAGSFLGGTTGLTFSIC
TILMESTSDFQHLLPLMMGITIAKKTAELFTENINTILLQMRCVPMLDFENEVHKYPMFD
ARHVMTSHVVTLETVCTIEQILYVLRSTRHNAFPIESVRDRTYKGIIVRQQLEILLWHVY
FTPYPSICTYELGKKVEARLFYDKLLGTLPPLETRLDVRIDLSPYIDHSGFCVLDKTTLP
RTYTMFRTLGLRHLTVVNPENRIVGIITRKDLVTDRIIEGIFAAEESRRAIIEGKSLALP
DGALITAGGSDSNSISSNKRMDKTDGNASYSSNVLESSARRQQIAGKEYEFYRTGDWLVL
GRYDGVWGDVAEGIEGLMHPSASTVAAIRAQAATRRASFVQEPDAGGPNEVNPRHVAKRE
SISLLDCLPDVIQTRKTP*
>TCRU_2518 hypothetical protein, conserved
MFPRRTPYENSPARRPNIITVATPGVTSSRSQISRYTGVDGVDVLENACSWQPSNNCITN
EVTAVEELAAFRRGRGNYVLTAEEDPPFLYHASCRVAPRLLHNVMASPEMDEIAYNKFSL
NPLNWFMMLKEVIVSALFSISSKRCMYSQARMTNLGGERDQAACQREIRRAVRIIKERQS
VTQGDNDVTAHSSPGRPLHLKLVLFGCSRGATTTFYTAMKLPLELAQYVSLVVVEAPFDT
LHDVIASSCWFPRFALWFFCNFCDYGGNEPYSFVPGRVQLRCPIAFVMSVKDTRVPNKLT
RRLIETVKNECPQIPAVEVLELKHSRHPLMAVGNREDQDAYVAFMERLYDTYCS*
>TCRU_2519 hypothetical protein, conserved
MLPATAKSHMGAYVSESPAYLSIHGNNRNSDTGASQEEGEVLENSSGNEEAQCPTAPRLI
DDYPEPLTGKQRELLHRHTATMVHRFEMLRKGARIIERGYNSLQSLLSRLEEEKTRLRAS
LDELAKPAETQILGTSHTEPTANPTEVKATPGSRRPGGLLRSAAFANMHSILNAAKREEQ
QQRSNVTLEREKIGKTTRLKIIEDDLAEQKQYLAPLAEKYQKSCALLEATKGKVLELLRL
HDEMQRMEVSYASRFFLRASGFHESEGVKGTASYDVFFAPAKYTDEVQDMIREQVEEILN
EYLAYRSRVDPVLESLAEEQRNREAKRKDQLLQLIGIDTDNIEVAASKDLGFTTNMVTKT
TNDAAPPALRRKDGAAAVAGQISDVEDDNDDNDIGGESQEKKQRDQIQSALAALDDFEEL
Y*
>TCRU_2520 hypothetical protein
MQLHSPDPYFYDNEIDRMLEEMRAQRQQQKRMNKQRKECADNGRKKRGFMPADIFSSLLE
RQKSMKKTQQRARSLPKTSFLAANEGEKSPPLNDFIAHTTPQSQEERDATTDAFNDDSSV
ELLRRELRIARKENALLRRDMYTTLTTIGRYLAQFAGKNSVRRKLDVDTGEAENTAGLSD
NDSPTLRLPSATSVWELCERGALRLGPEEAALVRSLNEVIASSLERYVGLEKQFAQQGPI
GLQPEREDNCVMRQQHTSGEEGVADI*
>TCRU_2521 transketolase, putative

MNNSKKDELVANCIRCLAADVVQEANSGHPGTPMGMAPIAHVLWSEVMKYDSKDPSWMDR
DRFVLSNGHACALQYAMLHLAGYNVSMEDLKKFRRLGSRTPGHPERGFTTGIEVTTGPLG
QGIGEGVGLAIAEAQLAATYNRPGHNIIDHWTYVFCGDGCLMEGIGQESLSLAGHLGLEK
FVLVYDSNHISIDGSTDLAFTEQPKQKYESMGFHVIMVDNGDTGFDAIREAFEECKKVKG
KPKLIVLNTTIGFGSGLAGTEKVHGAPLGDSDIQQLKRRFGRDPSKKFYVEEEVYDVFKQ
HVAVCEQKHEAWKAAMKKYAAEFPKEAAALEAQLKGELPSGWKSQLPLNDASKIATRKAS
ENTLGTLFSIISGLIGGSSDLTPSNLTRPNSAALTDFQKATPQGRYIRFGVREHAMCAIM
NGIHAHGGFIPFGGTFLNFFGYALGAVRLSSLSHHQVIFVATHDSIGLGEDGPTHQPVEL
LAFLRAMPNLLVFRPSDQTETSAAWALALENAKGPSIICLSRQNTVPQSASSMDGVAKGA
YVIHSADKPQLVLVSSGSEVSLAVDAAKALAAELRVAVVSMPCQELFDQQSVDYQKTVFP
DGVPVLSIEPYVNYGWEKYSHYHVGMPGFGASAPGGELYKQFNITTEHVAAMGRKLAAKY
PNGNAPEKRVHL*
>TCRU_gamma, conserved domain containing protein, putative
Elongation factor.1
MASSGDDSRCAPGTHPHTIPCVSLRELDELKIDYSHTDTRTVAAPYFFQHCDAAGCTTLW
YNKYNKYNKMQCMTANLIRVWLQRMEHVGQYALGAALMFVEQRRHDIVALCVLGGRACRR
F*
>TCRU_2523 hypothetical protein, conserved
MMGDCENTSAFQKDTVNESAVRECKREAKKALCDFFYGDHSGEHDASHLHSICSPDVGSG
GAVDQQAQEQILTLRETGKPAPPTTPQGLSEESPDTPPTPASAGASSQAPPIQASTEPTV
LAKQAEPNTAGSVPPKQSQGGAEAMRPPQEPPKVSSIHDTATQGDPPTEDRAEQTLPPAK
ANDNSSDHSEEGTAEESTVDTPASGAVQTEGNESETPGSGLKKTKTPHTTVEGSNTVKPG
DSESSSTAPPAGESDAATTTTPNNHDARRLNNNGNNTFTEEVDQKEAAIKPKSAPDGTDT
AAANSEASTTAINISTNTTNKTTTGDSDSSTAVSHTTSPLLLLLVVACAAAAAVVAA*
>TCRU_2524 hypothetical protein, conserved
MMVPNASKKRTSFANGPRILVASNASGIISVTCGGREHVAADNTDEVNERNATGHKPFVL
ENGVAGSGFVAESDADGLTQRSASGRENPNYENSPRSIPETV*
>TCRU_2525 retrotransposon hot spot protein, RHS3, putative
MRLNDFLTRELDGRGVVDNKRDVLPEEFFKDPTKYIRNKGALNEIQASGRYLSMKRAVKG
EVIFDEDIRKLCDKGVNSLPGWSLAAAEVKAAVHNSNKHFLDAAAEEVRNPTTTSAPEKL
EGLYESVHNARRSHAVELPDGVERKKTGTGMGMHEGKPEQSWSYREADAAIEENDAVQQF
GVAPPVLMVLASEKGWPYSWHTIQDRPKDVFVNREVEQLWQIVKGDLTAWSSPHGGTDFK
FERHLLIGTPGIGKSMAAGSYLLYRLLHCDAEKIQAVAHSFGGSTTYVSDKTTRAVKRCS
DEDMCVSELRSLRGHGRNVYIIYGVAKEGTPPPRHFAPTSG*
>TCRU_2526 PAB1-binding protein , putative
METTYSSNADRLEYLYLNLVGQVVQVRLVDGSSLEGIFVSRTDVDADTEAGILLCCTRCL
PSSKHKPLDPSCLELLEDTIIPYRDVVMMEVQNAKIRTEAPGRTDTVRSDYSMNKIDWAD
EGANELLDSEQHQTGAWDQFEANEKNFGVKTTYKEELYTTRLDHSKITEEQRAHADRVAR
EIEVSATRGIAHRMEREEFLKDDGGMDEGTLYSDVQRPQEKKKTYVPPPVAAARKAAGEM
QTAAPAAAPVTTPAAATAAAAAMAVDDNAMRHRRMEEEKIPMPHDNHPRSPGFNPAAVPY
TPMKTGAASAPVLDFMTLLADTIMSNEQCYESLPNWPGTCMYYDQDDSSYSQQNYENAVV
PPSMGAAPQMYVTPQQGLPSHHRAYQGGGGRMNPSQQVPMHHMHAQSFHQQHQHQQHQHQ
QQQPQQQPFHVYGNYMPQHQHNAPMQEYPPNKGAPPGPMRNNMRQPKSSSASRVEPQQPV
TANTSVAPPPVPTVDSAPTPTPEVKHAPKLQRGRGGTAPQRGGDGDGTGDAAGGNPGAAN
PPAGPKKRSGK*
>TCRU_2527 hypothetical protein, conserved
MQRAGCGIVRPGRGCHTTPLYCSLATISTGVFDHLPFQHRRQHAFNTLPLHDANHFGGRT
AYLREIGPVNIKKSGRRFKKDLRTVQFNVDIWCAQQTLRKRWKQRDWEVIEVPFRLAPAE
QQRVIPEMYTDVPPMTDPERHDFSNIRNKVYDREELQGVLFGASGPLPYPPLQRIDRQAM
TLDKFL*
>TCRU_2528 hypothetical protein, conserved
MPHTTPTAARREEEAAGEKRDVSSRSGSESGSKNNTNDNDIAVEARRPSRRALHRHAVKK
FNERRKNEERRRLADERDGKLLDRNNATETVAGGSEGTPVSHTDGGGELQATQLLRVNPM
EKSILINKWEFEFFFRTIPSELLQTPAESKGPDTAVVPPSSSSLYNEQEQSLVVLNDDRH

RNSGGSISLKTLMQSSITKSIHVRRVEGVPPLPMKTPMQPGTDLDQPTRNYLLPDTTYNV
HQHAPGFERDQNAAKRSLLACAVQEWRGGEENVLLLSTGEALRSVFESTYCDEKPLALEV
RRMGPTLLIDSHNERDFRTRVRDMREKALLGKALYRIHNDVFSWKETDDDNYEGAGGGVL
SLPTHRVGSLALSKEIRTLSRYSHIFSWEIGLMDVLVGIDTPIVIDRRDNTEFVLKLENT
SVVMTPQEIQRDALRCWFDATLANVPHVGIYVHNDGIIQRYEVRKVQELLGIVEGSMATA
AMNFTMNVLQWLVKQCKKDGTTYAVIRNYESGYLEIYEYSRDERLEKFLPDDGACGKGNF
VGVNDVINNSKFNRGREKSGVEEITETQRAEEEKSAEENERLNWGLATMCFRMGMHLKDS
EQKAPDAVSLLLRSFAVYFMQRAKMDEACSHVCEIAKCLPALIGRLIKNRKAAIEEAGGI
IQLPEIYREAFLACGRFEMRLQEAAYDETLSVAIRRAFLRCILPCSAALCVCVARTMEEF
AAERRVYMQLRAENNRGSHVHIHSLIAKDLLQVVVEGLLRLEQMNRILKSGAALTKGMPL
GTNVESMALMDTKTEILDVTPLETTVWELYTDVVLLVMSDRTPFTADVLVELSRRIKARE
QRRRCGGGSKDCDHEDNHNSSGSGSTETIVAGESTLAWLSALTPDVVSLSFTALRFLTKA
GLQSKRLLSKTAQVYYHVGHHYFLTDRYTKALESLHRAQSLFKAMQQAPADTVFGACCTS
SATVTLRNVQQSLGELYIRMTRLKTRTVPTEMKLSLQQPLLMGEVQELSQEEENFFRHAV
DSFTSCEAKEQLASALRKYASRQIGQVAFSGQQADVAKGRHIFSMLRKVGELTSNDVEID
WEVLRLFTCSAHHAALQHRAELILSQWTAGGGTPEAGAVRMPLKLMSTVHPVECALQMAL
VSLSLLENKKKVKMHQVMGLLRASVAHLMVAIKDAHAASKTPGALLVRAMSTWKARCTHE
WVCRISIMMSLRAMRVMVRLLTGDKASEAKQLLRRLIEMEEKHSEILNKSEICTGKCQFW
RLSETLMESLQDVAKW*
>TCRU_2529 Zinc finger, C3HC4 type (RING finger), putative
MGNRQTVQPLIGGPRTFYLVSGGPRIRYVGHPSSAGPLIFHIETDLMDDNEAVDRAETIQ
LPVAVPSRQKFRLVQLDSKNRESGFLLSFSIVMSEPGQTLRVLAGVDIEYRAGEGVQLMQ
SEKSQNPFCLLEFVSPEEFREEIEVSEVLHPEKLNKAIFRYSENGARRLTYAPIAIELVY
EAPTDRAPSKSPDCISPSSAKSVFASRQECENSMSHNSHASFAAKLLPQTERMVQYTFLD
IPEGALGRTKVSLLPKSEPQEGAARIVYEAKILRQLLQHGTQVYELDDVFDLGGDGSENN
LDGNDEEEEEIDLCVICLLNPKDTTLLPCRHMCLCYECASILRFQQNNRCPVCRSNIDRV
MTL*
>TCRU_2530 hypothetical protein, conserved
MISTRYKVIEKPGGLGEVVQVAVKDIGVNTGENTPALFVSPLQTLTTTMGVNSWSQKETT
TCAACARRQERMFQLEMQLQQLETQLRVNNILNALMNSMEAQGTAAGESAGWDYTSRPHG
TRRRLQRQSSPLNQEKEDEEPNETLLEMMREELKRQDLLHALMDLMERPTYAERQEIKKF
VPKNVKALLFTPSKNVFSIPTPEKSM*
>TCRU_2531 Ras-related protein RabX1
MSSSSAPGGDCDYIFKIIVIGDSGVGKSSLTVRLSEDVFYKDYASTIAIDFRMHQMNYMD
KRVRLQIWDTAGQERFQSVATAFYRGANGVMLCFDLTHRPSFLHLDQWIERVRQQALPGI
PCLLIGCKSDEARSARQVSKEEATAWAQQHGISYIETSAKEKENVQYAFQEITKEIFLDM
KDRNGKPMASGGKSAGRSESVRLDSQGGRRKTSERGRCC*
>TCRU_2532 small GTP-binding protein Rab7, putative
MSEDLAYKIIVIGNVGVGKSNVSSRYCDGTFYEDIVPTIGMDFKYCHSTTLEKKPRTVRL
QIWDTSGQDRFVTLTTAFYRNCNGVMLCFDITNRVSFEDVDEWYDRLRTNCPNMPPVILV
GCKLDLVQREDVNDIGISLGSQRQVEKSEADAWARSHNCLCYLETSAKENRNISEAFQQL
ATYVAQSTTPPLEGGRSGINGRHGRPINREIEGKKGCCTAF*
>TCRU_2533 hypothetical protein, conserved
MILGVGDGTFAILFVTVIGIIVTLIGSYVQPRLALPIGLVCFLLPFLLYACILSAPHGRL
LPPLPDTPYNRERRLPPDAVVVDQLLPVRIGVMILLFLAFFAAVGYHVLLVIWEPPYKAP
RVRCLREQLKELHPTWYR*
>TCRU_2534 flagellar protofilament ribbon protein, putative
MNSAAQNAPAISPTATMSTGPLDTTSKKRLFMMQRAERLRDPKVRHMGIDKEALDDQVRE
KEALRRLEKERNEFFDRQALLMDRHAQALQKEVNEIRAGREKELQDYRETFQKKHMRREW
DLNDPTWKVKDLPARVGDDDPRNGVSSLQKFEGEDLDFKNRRREQQLQQRDWAQQQVEEK
LAKKWMEQEANHAFDERNEEVNRRIYEVERNIAEQRRLSHKNQAEFNKALAEQKRREAIR
DKEEDTRKGLEEIRYHLEGDFLNETETMVGELGKKVKSDRYKGMTAEQKEQLYKDQALQR
DAMRRRRLLEVEEEKHWGQQENLQLRMANALERQRERERRAEREAVAAEQKKQKEAAEIR
RKKLDELYTNAVDEDYYKYWDLCM*

>TCRU_2535 Zn-finger in Ran binding protein and others, putative
MQRCVASLAGTAFFGHLRGPSQTLALGCSSRAYMISATPCCLGRRIKGKRSRHREPAEGD
WLCQCGELNYKSKRECFKCGAPAPPLPPGVRRPSLPGEDPHDWACPCGQMNFRGSVVCHK
CLQPKPAPPPLPGKEVTLWTCPKCKGVNRSIRNYCFKCSAPSPLLTFKPVV*
>TCRU_2536 hypothetical protein, conserved
MMSSRLNNLESVEGFSSLLYGGDKAALQLLTEIGWSGMAAAMGLSLMQKVLQESTSAYAI
FFVSQTLRQLVESKFGTADLVPLESFLSWLIVQRHNSLNASSVDVLIRLLCAVVKQGFCD
APELQSFPHRVTAALKLEESNCSDGHIALSCAILVAFIEEAERAEGTVRSLINHKRMIKC
LRNACLLPIFRAASQCLKRLQQTDGKAICGLVALVKRILLFDFTCSCDEAADDVMTCEFP
LEWAADLVDQGLLVKLFDLYATPVTDPSFLCDVLEAITPLVSVNASLYSSRQQQAEWMNK
ILSATLSIMESRSHLEEATVLREFCRLLNRIKPNFTIDEMRRAPCYQRWVRAVAEFTKLC
FQNWHHARQSFLSLTSIWAKLVGSQSYCKDGCTLFELLAPDVCLSYMMSHQEQAVRFATS
GEASLFGEYILDEDAETSSLEFEFVSQILRFCGEEAEKYIVKEISSLLEALKIPEVRSSP
ELFCVCERLAFIITLASSWLGSYRFSRNGRALDSTVLLACLNVVSQSSQMNFSRSLPSAT
SRHFHRSLLAFLRFAWHITLLNRMDGSKKLQESLQLSLAAKDFGTLATFILGLVVDEVLD
CVSSCSEQTVFEAIHLLSEMAQSPSTAVVLRKLPQFDGTRLVLDASNVKTSRNAAVFYRV
EYHLSRIRAQVHFLGCNSESSRADFLRPLFEELEMCLRRGMAVADGQLDFFTRVICLWRG
VFRSCVGQNEYNMLLRRFFPCLFLLTQQLQNHLGTVCGVQLLRLINEITENRYRRINFGA
NGVEGYHLFREVSRSLEMAVHLVRRALGSGELCLGEWGLKCLRILIHTGRNILSGGYCNL
GVLRLYEDKALKTCLTVLWQAMMLVDRHRFCQYEKLAKAYLMLAGELLRDLHLWFLCEVP
VGELLHVIHMLEISLGYCISGSASLASQSADALGIFTGVLCCAECDSVEHRDRVCGSLLH
TDPSLFSRLLRLTLDVVLSRKCPSSKAEVLLRSLIALDGESFRRLAGEFAEIALSAGKDA
EVRAAFELLGSSACESVRKNNRSLFTKEFQHFSTLVTGCLG*
>TCRU_2537 hypothetical protein, conserved
MPPPKKSVALRHRAHMIASSVEDQEAALMEWRARAQQGKETLQRIRSKMDAHKKSAETTK
RGGRWLAEWASLQREAVSRDLELSKAYASCAHLLPQSLRNKLTEQEEQKNELLGYLSERV
TRIRKELERLHKQKNQPSQEALRTLSESISALRKELLEKKVTDVKAEKMICGEANGETDK
QSPMETLQRLIQSTMKTFDGICESIDGKINAALVETYRNALQTEGENTTSQAAACIDRRV
GTVLQSQNDLRTVSLILKTYDSEVESGTTAAAISDELYDRVQRALPHLSKMSARRVVDEA
LRQKRGRVYMRSVILQYKKRSTELLESFKKAVMAEEEIMKMRNAISEETRMREERQQKTQ
EELERLRIAREAKDASRRAEEEAKKMEEDEKHQKLLCSREAEFQERLRRLREYQEEQREL
QEKERVVQQALAEENALKKAIQKDHNAKRVEERKKEYEEKCRLRKKHQKEMEELRKAHQR
TLETFFKGIERRLGVTYDAERVLQPTTSSQQSVPFVSFSEASQFNLHGYTVDDIMKDPRF
RLQLALMEAGLHQTPYGREVISRGYHVPAAQRPSEENPLSMEY*
>TCRU_family, putative Se.1
MLDAVHGLLKVFFCDAHAASALSRSLRVHELMEHGVTLLEDPMTPRQPIRSSPVRYFFAV
EDASVSRVVEDWMAKAPHRDAHIFALGCTPHRSPQQLVRARIAPRAMRSKDIMLDFAAPE
MLVFHPSMQNGFPQLLSPLSPPN*
>TCRU_2539 hypothetical protein, conserved
MGRCPITYPAMDVRDIETVIWKGEYLSAMSGDKPSSWCLDTLPNNTTDARDPKEAHLGAV
MHAALRCLGMRVRLKVTEEGNIRCAPCTDGATIAWATRPYDEGNVVCRDHAQVCTISANS
GRLLPVVPWDGEERACGAGRLHRHLLNKDCQGHPSPLRRALRPAAAHHRRMLSRLFPRSI
R*
>TCRU_2540 Leishmanolysin, putative
MRADSTYWIRVAVRDEPFLSSWCAATIHRHWEGWMPIRINVSTRDLDDTGKYWISERYYA
MNSLSGNCECECDVVSSAHGIILEGEVVPAAFKLHADRLLVQPLEGPLIVPPFATGSVCS
WFTVRAGHCSAGVANAKTVLCVAAASGGVWAPRSATLEDGRPFAGAMHFACQFYFTDASP
PASPRTFRPMLLGSNARTWQAAAW*
>TCRU_2541 Concanavalin A-like lectin/glucanases superfamily/Trans-
sialidase of Trypanosoma hydrophobic C-terminal, putative
MVSVLLTAQLERVKEVLTTWNEVDKRVSQLCANSLAQKDLSTDTACSADKITAGLVGFLS
GNFSDNTWRDEYLGVDATVKNGAAEAILHGGGVKFHGAWAEWPVGKQGENQLYHFANYNF
TLVATVSIDKMPEEGDTPIPLMGVKMNDGKTVLLGLSYEKERKWMLLCGDATSNSGELSS
TGEPGTKRHVVILLRNGNQGSAYVDGQRVGNEQCAFGNAESQEISHFYIGGDGDKTRKQE

DVSVTVTNVLLYNRPLSDNEIAAFNPNKVTIQQLKDKSSEPSPVSSDSVDTNASPATAAA
QQTGTLSTPDGTHPTEQRQSMGSSNAGSGGASTTAVSTITTPAAGEEPVVQVAPVSSSDG
HKNVGGGSFSDGDPTVETREGGTNGHEEGIHPQVREVNATALNSSLGNVSQGNNSDAGIM
RGSGLLPSLLLLLLGLWRFAAL*
>TCRU_2542 BNR repeat-like domain containing protein, putative
MMMTACDDGRRRVYESADKGESWTEALGTLSRVWGNKHEGNAKGVRSGFITAKIDNRDVM
LVTLPVYAKTESEEKEKEKGVRHLWLTDNTHIVDIGPASVEGGDEVAASALLYKV*
>TCRU_2543 tRNA-methyl transferase, putative
MSCTAVRVAIALSGGVDSAVAALFLRRCVPRWGDVEALWRLERPLALCEVVEALEQQRPL
SEVVALAPATGNSHDPAAAVKYFPLFMKNWDDAFESDGVGRSWCEAAQQDYNDATDVARA
LGLLHSREILPLHNYSACYVEKCFQPMLDAYARGETLNVDVLCNEEVKFLALLDALLTAG
RAAYLATGHYARTVHFPLLSCENAAPTSLRVIARPFSARNDLNDQTVFLSRLSKGQTLRA
IFPLGHVFERKSDVRAVAEHFGMKRISKKKTSTGLCFVGEHYRRAGRGGSCAGGFRSFLE
EYMAPNCTALQKLTAAGQQTTFINAENGNVVEASGLALHGDGDGVLLPAYSLTIGQLIRG
KSEDGKTVRYYVQGKKLFSFRENYSKGDNEDDDGGYVRHLHTVWLVDRWDHPLLYGKVAK
IYDMKLSVHPQWLGNRERSVRLHCRCCARHQESLRPALICFDWSDVSEKNGCIRVATATV
LFEEPVRALTPGQALVAYISLSKEGAEDAAGWFVVASGWIA*
>TCRU_2544 hypothetical protein, conserved
MKGNTLRGAEEYVTLESAVLFELAVLKNGGYFDVENNTMIAVSGIYFYGDTTVSSAGLLR
VADCTFFGITLFFEPALVHLSGSLTVEGGAQWRVEGNSVGAASVLSMPNSLCKMKLSGSG
TTVVLAHNRQVDSSKAFAKLLPRGTIVTSPAWFVFGCNLQGDEEVSYDGVFPEDVVVFRC
VTCNDDAACYMPGTESVDRSSCSCSCKDGWHGASCLPFEVPDTVVPPLPERAVDGDTSCV
HLV*
>TCRU_2545 retrotransposon hot spot protein, RHS3, putative
MWRCWGWLHVVLLRGRWALTASPTGVAVRLHGAPTTEPCECHAQRHWDSGTKQPRLSFGA
SGTCWPQLGGASGMLHRTGVVMAPRRGSCDGSDAAARHVAGSKVWPQWTMSSTNMRLSDF
LWNYVGGRAAVDERLQCDDGGVFSGAGWLRKRPAA*
>TCRU_2546 hypothetical protein, conserved
MMVPNASKKRPSFANGPRILVASNASGIMSVTYGGREHVAADNTDEASERSATGHKNLVL
ENGVAGSGFVAEGDAYGLSQHNASGHDNRPQLQKLTAQYSGSGVSPHDCFALRNFAKLTL
RELIVDGTVRVRVLGVAASSGIVWSCGGLVSRRVWNCDSSRRTCAALHACFLLLFIFYVV
FFLE*
>TCRU_2547 BNR repeat-like domain containing protein, putative
MKKPYNTSARWLWNNFVRNCWAGSWGVWGNSYGRKGYGVRCGLTTVNIEGREVLLVTTPV
YLEEKKGRGRPHLWVTEGARVHDAGPISDAADDAAASSLLYSSGGDPISLYENRSEGSYG
LVAVHVTTQLERIKTVVKRWQELDEALRTCRPTATIDPVKRGMCIRSILTDGLVGYLSGL
SAGSEWMDEYLCVNATVHGTVRGFSNGVTFEGPGAGAGWPVARSGQNQPYHFLHKRFTLV
VMAVIHDEPKKRTPIPLIRVVMDDGDRTVLFGVFSPMMGGG*
>TCRU_2548 Gryzun, putative trafficking through Golgi, putative
MEFLSQLYNDDAYRDYLDANPSFSLAVLGRKNLRIAEGLSYFFDSRVRFRINYFSDVKAF
LNKGVNVSLGAEISGILKSNWCSKHLRLRCSCVLVCLDWDQLGETAIDEVWLSGVCSQLK
EYTRSFRSKVIIALVTENTMRTSANSTAQIEKLQYDLKMFFGSDFKNFLPLYESGMGKES
AEKLFQMAVGLATKYHKDEMSRLKNSKLEPSYPLVRRGFKLGWHCLVLNDTKNAQKHFEN
AYRTLRKIAPFWPAMELRICGTILLFRVLHIVALESAISSDDMYCRLCEDHIMWIGRAIP
CGSPEFKPMAGFLRLILVGECYEWLLRNCRYVADETRRDYLFAAMGAFEEAIKLNQPSGS
SKRHTVAPVFVGTECHLESHNCIFYSNNSMRTLSVRVKDLLSEIESHNSLSLDAFYISVG
VHASFHAWEQVFVCLERFGRLRIGTYKEAEAVHKIWMCAMQSTGGNLKENIQEDVLFSYV
SLCFGPAVGMEQKNYMKQFTELMKQNKLNHFSLSYPRRGHFAPFTILCRFQDEHHVCATK
TEIQFSLFTASIEMIIVDSLYVVVSWLSSDGVEDLSMYLVSDKGLKLSSNTPSSASLTLA
LDKPGTYHCKSVRGRVNCAGIQINVEWRLEKSLGGNPFLRGTEMLGSISGLLYHKPWISI
VRPVCGVDIEIPRNIAGIEGEEVNFDIVVNSKQDLTGNGTLVIHDVPSLYEFVWDACQEK
KMKMGEGLRKGYRTVILDNIAFISCDHPLYVTLNYRCLRAGKYMVPILFYYKSGNYSDVE
ILKRIQLNVFYPFSTTYTFLKVLPWMVTPIKGDVIVGSETTLRVPEFSRVSIQHERFTLV
HPRYSTRDDVPTIQSNRDSLVYIAHTEREDHSDNFASSKGEEIILLASMECRAPQGLMVK

NLDVVCTQDASLSSVVVSLLPCHVEHLEVLTLSIKFRLLNAGKFSLGFIRMILAPGNGAQ
HIISDFPLPEVKVEDTPFVLSLRGPSIAIIGSPILISFDITNKTENFQNCELLMEKNNGC
FLMNGRTQWSFCLAPNTFKSTEIILQPLCIGTIKLPSVYLRHSTGLASGTCAVCVSDSQR
ICVLPS*
>TCRU_2549 Dos2-interacting transcription regulator of RNA-Pol-II, putative
MSGEKVDNIIERAISMGLNCDEETFEKLRQVPLLQLCRMMSRYLTDEKHIIEALFVLSLA
TTRRKTLLDEEAVVVLKFFLEKLSSLQTIETSVECITRVIEYLSSQRPCSKIVFTELANG
FLPNVRLQALPTRVRRSGFKILKYMVSEATVSWLTTPVLCLLLEAMDEEGEPELVLHMFE
LYYFLSFFVDKNNIVPLKEQYFDSISSYFPVVFSRPPGCSVTREELKRSLTRCMTCPLYL
DPCVSFTLSRLSSPSSFVKQESIAVLLELFSPESAHDMNDLSPHILPVVSHVRNEVIKGV
SLGFSEGDSYIRDCMNLLSFIGKRSHGVLSPVIASWIEPAISGALTSLNSGSATCSAYAT
MFYHLARSDASCGTSLLSHFLPLLLMNLNEIDGGKENAFIILSAVFTGILDLCTTDEVHH
GLSSTLDDVKRSLELSSPELFRLVETLLLALKMAESSSKIIMCELLSSLLSLDVYLHPWM
PVESVQLSYENLLLISLQGNEEIHIKVARCISRIGHLEGNGLNNAIVHLLEKETKFNATG
IITLFNALLMSSVPVALLAMELLLNPLRSLVESRLSESDVFSLCRRALSENADFSEEDIL
HLLQIVVSKNLPASLEFLCELLVRISLCSIEKTVLQMVDGKRWSILVIALLSSCDSDVLH
VSETLEHWVTEMLNAVQEEEFRRVTIQGMSAICAHAPNALEGFLTRSRGLDPELQLAVHA
AAARGLIKAGLAMDGEVKGIILRLLDAICSGVDIDEVLTATFFSPSLGSNKCVTLLVPFI
QAVRTSNIPMPDKVLNVILQLLGKEAIGLEFDWSSILEVCENLARQKPSEGHVIGILQLL
DCVLLRIDSKVFFMKRILTNDGALFQMLLSAVRSSDLQARCTALRLLSEVAMFAVQLTAT
NNEDELNYKRIIAREKEGVLHLTQILLADHKRVVRRAAAQCRHQWYKLR*
>TCRU_2550 thymidylate kinase, putative
MKIVVFLDVRSEALCTAVASEAATVGDSVELVNCHNSVVQVLRRKNKQQETVNTFICLIT
EKGSLKDAGVVYALFRRRIAVLSLEEGSIASTSIPLLETISSLHVDLSGGLLQAQLLAVK
AFFSFNASVSQVIVFEGGDGVGKATQARLLVDRLVDEGHRVSSIEFPSERNRYGELLREV
LSGKKGGIQDLDPKLFSLLFSMNRFAFLPELQYWMCRGTKIVLDRYYTANCGHQASKFPE
EERAGFIGHLQLMEVSWLRLPPANLVLYLDLPPHAAFSAMKVDPNRGSLDIHETAQRAYK
ENVRKTYLWCCENMSNWFHTDCCDCAGSRLSREETHNKVYEVIERQLIPIA*
>TCRU_2551 hypothetical protein
MGASSSSEELSGTAAFFGAGLIAGDALAFCAGFRRGASSSSEELSGTAAFFGAGLIAGDA
LAFCAGFRTGASSSSEELSGTAALSSVLHCFLEGFSPSDDG*
>TCRU_2552 DNA replication licensing factor MCM2, putative
MSPHRKRPREEKRTRHTHESDDDRDGEDEEELGEDLFGENYERDYLNPEDESELAEDDVA
DDDDWIDDTSDVSVISESGRLAVDALLDRRKEMEQRLREEQRELEKGVFSDVDDEESVIS
AESDYYNAADPDDDGGEEDEAGRNRVGLNNGEDGDTDYTGGNKDDGVYVRGDMEAMDFDW
RRPQCDLIEWLAQELPRRVVKNRIYNFYLNYVENGVSVYEQKVNLMTRENEQSFELSYSH
LGRAYDSVLALWLVDVPDLMIELLEDAANYFVFRLFPHYQKVHRRILVRICDLPLCDPIR
DFRQIHMNVLVRVEGVVIRRSPVYPQMQAVRYDCVRCSYIIGPIYQRGDKEQRVSMCPSC
HSKGPFRVNMTLTEYRNHQTVLLQESPGKVPPGRLPRSLEVILTNDLIDRANPGEEVDVT
GIYRNNFDPLLNSRQGFPVFTTVLHANNVVRRSAEVGSFRLPDDERARIMELAKHPNLKR
KMLRSIAPSIHGRDDIKLGLLLAMLGGVPKDVGGDQSHRIRGDINVLLVGDPGCAKSQFL
KFVEKTANRAVFTTGRGSTAVGLTASVHKDSVTGDFVLEGGALVIADRGICLIDEFDKMS
DQDRTSIHEAMEQQTISVARGGIVTTLSARCSIAAAANPIGGRYDPSLSFDANVNLTTPI
LSRFDLLFVVRDEVNVELDEKLATFICHSHIRNHPRSQEENKRTERELHQQLSRLRYALE
NASTEEERHVIEGQLQELRHHLQEKPREEDEDPSSNKPLPQPLLRKYILYAKAHCHPRVS
NIDANTIARLYTELRQESKHGGVAITVRHMESVIRLAEAHARLYLRDFVRDEDVNAAISL
FLRCFIQTQKYSLRSAMENRFRKYLDSEMEPIPLIQHHIRVAVHSIRAFERQISGGVEPT
KVRIDVAQLEHCTANISSEALNTFFESEEFKKDYKLICDPATGARLQIEHSLV*
>TCRU_2553 hypothetical protein, conserved
MPDANSVQSFWRENLIPRWHELLKNECEAEYSKETNLSLKDLAVNCCLHHAMHELCKDLY
PKLFKKTEFSNVEGVCKEYAAEPDEVKCPKRQTQPPPATDNSVKVSVPEALGKEGDLGET
PEEAPLADLPAKPTKGPPSSPMGGQPTNADDVPAPKSEERPVSTRPTNDSREGDTDTITD
TDQEETSTSEAESAAPTPPVASDNDDSNETDKGTGEEVPNNAPESDDAGTEEKQDENKEA

NPKETPVESTATKTDTTPGDSDSSTAVSHTTSPLLLLLLVACAAAAAVVAA*
>TCRU_2554 retrotransposon hot spot protein, RHS3, putative
MKGMYDSVYNARWHHVVEVPGGERTVMKVENGEPPQSWTYKKVGETLERNDGVRQSGAAR
LRLLVLTSDKGWPYSWAGTEPVCDCCVNCEVDRVWQIVKRDLTEWFSPHGRTVFSPKKRL
FIGTPGIGKSMAAGSYLLYQLLRYDAKKLPLVAYIIKKSVYLFDKNKKTVSDLGSEDVFV
ELLKDHTERGVKGYVIYDVAEQGREPHVGLPFTVWNMIVVTSPNINNFKGWTKQNGPTLI
VMNCPDESDVRALCVWMKRNEPRGKDEYWKQVKERMDEVGPILRVILDAQKFKNRKKDID
KVINDVDGSNAKHYVGVMSDKLWEAVNPSHKLVQIVRVSEDSEDETYLNSPASRVIASKM
YIHLSTKMHSREIIKLLMHPDVFLLSNVLEILGTATFMCRGAVNIIKKKLKELKPPGRRA
LQLAALERNLEGHPTEAVGLPTKKGVSGKKLARYKVMYKPAVGNFPLVDALFFMESPRKT
LVGLQMTTAHDHHTKTSTVKEFTEYLSWFFKNWGNFHRDLSWEIIYVQHPDSKPMNGWQR
CDLVNSRSRDEKEIKAFWDKEVRQYQFILTPADIKKLRAI*
>TCRU_2555 hypothetical protein, conserved
MGMGGSMYGMGGPMGMGGSMYGMGVPMGMGGSMYGMGGPMGMGGSMYGMGIPMGMGGSMY
GMGGPMGMGGSMYGMGGPMGMGGSMYGMGGPMGMGGSKYGSVVDAASRRDGFLARNELIR
ANIAPVKHSNNNEEKNNEKAPSPVPKKYEKAISPVPKKDEKAPTPALKKDEKVISPALKK
DEKAPTHAPKKDEKVISPALKKDEKAPTHAPKKDEKVMSPALKKDEKAPTHAPKKDEKAP
TPAPNKDEKVTVADSGSSNTTNLASKNDKPETAVANNAASEEKAAPKSLVSTSLHSTENS
ADKKEGSAEKPATKEDKKKEESKKETLKPPTGTNIRGVVVIVKTAASASSAAVTMKNATT
VTTSGKDEFSFDEVITHTDPSKTMESVMLEDLRCHWLAGHNSSLLIGTTKGEMKDAFNLG
TKFITNTLSVLEKEGCKFDVSVTMVAVKGGSEVKDLFKSSGAAYENIQLASSPVFGPALR
GMTSKKLTKSSECVKLLEDGLGKHDKEKELLVAFLVLKQMKKSSTGDDTVYLSTLCLVVA
GEEVSHLTGIKEKKASEPWTLLRYAVGGASNTVCLLAVSAEGKKSSNVNAVLEVGKTMRE
VRNGIPRSGNLMHFVEFTKRELTKQRAAAVKDESVKRVVVRLEKMLKDAEEFLANPDSEP
KTY*
>TCRU_2556 Protein of unknown function (DUF3720), putative
MGLIKAVAAVDGGDGAGNDSLENKDKSPQGGVLAGTKDNEAPGAGGVQGAAGDENGQLPA
AGREGNENKDVDPGIKAATDSQDKTNDQLSSSSGSTPSRSGEKPSSTGTSEMTQLPKDTV
TKLDADLQDKRESSEGIKAEETEEEERQKIKAHTGSSESKNNGGETTPPPLTSSDEESPI
SDQENSQNSKKVKNEATLSESKTASEAPAPPSKDATQGQHSHDTDTEDPTKNAATGSPVE
PTTSSTSASGSGDHVHNKADEDDAQSSEEQHDGIETGNTNVVPTLSETASQTPETATDAR
TNDTTTVGDSDGSTAASRTTSPLLLLPVVACAAAAAVVAA*
>TCRU_2557 Trans-sialidase of Trypanosoma hydrophobic C-terminal, putative
MVPNASKKRPSFANGPRIVVASNASGITSVTCGGREHVAADNTDEVSERSATGHKSLVSG
NGVAGSGFVAEGDAGGLTQRNASGHEYKPQLREFTAQYSGSGGSLHDDCFALHNDAKLTL
RELTVGSTVRVCVYWVLLLLLGLCGVVTAV*
>TCRU_2558 Concanavalin A-like lectin/glucanases superfamily, putative
MGQTVPYYFANNDFTLSATVTIHEVPEAGSSPIPLIGARLNDTASTVLFQLSYTSERNWN
FTLPKKDFLVKPSGDVGNWETNTAIRVTVQKDNDDEWFVYANGRRIYETDEGEMAQDSNA
DQFGPHRISHFFFGAGDKKDEGSSPVTVADVLLYNHILHRDDLRNLNARTVPIRHPAAEE
LDTAVEVTATEEEKQKVRQLTTSLADVEEAPIAQPTDSQSVRVLAGSESSAENVTDGLSP
RTAATNDTSVPETNHSASEGSMSPEDRLALITDAKLILHDLRGDSTARVRVSCVLLLLLG
LCALVAFF*
>TCRU_2559 hypothetical protein, conserved
MQHDRALQRRHIRHVDKGVGHQARRALAEATNPQLRAVRKHAVAQRHVQRVSERVHRAQV
HRHSDRPAEHHRVEECRRTPAANRHRLRQRHATHAALRTTAAGPRMRHVKRQARLAHAAG
TAAAHAVRERRRQRIEARQRCRTAVARHHAHGGQAGQAVVAAAPQHPAAGTVALHRHGAA
RDRQSGDKRNAGGSAARNRHGTAAQRHTRQRRDRQRLAHRPHVAQLQPPAATDRPTVAAH
QRRRDRPLDVHEAQHRVGHREALAKHNAAAAAHNRHVRHREHRVAPQTED*
>TCRU_of Trypanosoma cruzi region 4/Dispersed gene family protein.1 Dispersed gene family protein.1
MPGTESVDRGSCSCSCRDGWHGASCLPLEVPDTVVPPLPERAVDGDTSCVVNQTLTSLTL

NMWKTHHCYVGVTFSGVGAALKFFFNSMPLHLPINITLTGCTFRGGATLQFFGGAEAVES
SGVLVRVSQTVMRSSVVVFALALPQHCDIAVTEVDAVQSSAVQLFDIRINILSVLLLRNV
VLVASSLLVSNVNAHSLRYGGFGLHSVGTLTLVEGSSLYARYCSFAGYKHLFYVNILIVR
NHSVFALLNNTMSSGAGLLLQQHGFSVSDHSVLRVVGNRGSVSYAIYANNLWTVERSSWL
DWRDNDVGLGAIFYDSGSPFVDMDSSSGVTLTGCTMGSTGLSVSLLKRVDAGYRFVAGCL
TVAGRLLTTAAELELSGITNVTTVAGCGECTKEGDCFAPLTTEVIDCKCQCAAGGHGDAC
VPAPVPAGPPPPPTPPVPPTPPPPPVGECISDVVYPEVVQAVGDGLSWLCYRNVTFSGGG
MSLTVLIGAMTGDVANVTFDGCTWRDGAVLVLLGNADASVGALNIVVTGSKFRDALLSPV
GVFPPRTNITVSGNRFAVTRLIPRPGLGLGRPSCVAMNGLAISNDSAVVLSGNVFQTVAA
SSSAIYVVESSLRVWWHSVFAVMGNTFHMDGGDSNLICFEGSRHSSSLSVLNNSAVVIRG
NLVSRPVRYFVYFFFALRVESFSAVVFQGNEMQGSLAVFYSSDSSNIYYNSWLQLSGNLC
RVLPSNEFAFFSSKVNLRGSTVSVSGNQFLSSTVLPTVLRIPTVSSDLTNGAILAACNTV
NGEEETNYVIPSVYNATILTCSDPCILATSCFPAYTTKASSDGCACACAEGGHGDACLPV
AVPEPPSTDGADLCVRDVSVDVEVNVSFGTSVVCYVGVTFAADVVVDVESMSGGVRNVTL
VNCTFLGRASLYVVGWRSDPPAGERADVLVSGLESRSGGGVVVANRFPPGSRVTVVDSVL
IAEARVAYRDAYGLGDASACLVVHNVSLTGSVLTIARTQVAAVFRDAVGVLFVGGVALSS
RGALYVDGLLVQTALGLCVSVEGGVAASGGSVVAFVDSDFLLCKHAVSVRGAVSVSGSAV
AVVRSEFLSTGDYAVAFYSTVSLAGGSMLLAKGNVHDGASREMLHAAGTVTAVGSTLSFV
RNRALLPRMLSVSLSLVTGAHVRVACNDAGGRVLSTAEEYAAAGFGDAGSIDVAGCDACD
RDTHCYAPGTASAGMRNGVCVCACGSGGYGEACVPVGAPALPPAVGTASSAFFREGVTVR
SVFVVPAGASEVTLRHVVLDGVSPVLYVPWMARDGVRIVVQNVSLLNGAVLFVMGGGALR
GAVAAGSDEGGPVELSVCDVEALNGALVLTGTFPAGSVLTVTDSLLVATRSTPLLYLPGS
QSSPYAPVLVLSGLRLVRSVLVVSGVALVTVVTGGRTVVVDGAVLELVGGGVALDAAVFG
GDVALYASARVVASGGAVLRVSGSQVYAAHGLVFDSGAEANASAVVVNDNAGVLTDGALL
VLRGSASFASGSWLSVRGNSISGRLLSVPSYPRCADLVQSTLTLHGNAGSGTVVMDGIVA
LVGAGRRFVVGCLTLNGRALQPMHYRSAGIIGEFRPVACGVCDADVHCFAAATRAMSGSC
VCRCAEGGYGRDCLPVYLPHVDGCNRTPAMPLLSHTATLTETRSPTPTWTPSVSTAHCSP
TQHGPTETLQVTETLALSPTRTPTASVSSTLWWSEVACPTLAVTTTAAGGSLTQNDIRGG
GSAVPTRLMVALPPPFRWARDPRLGTHLSFVPVSMAQPSGFGGPWGAMLRNATWVRNATN
PSTVLELAVPVHRGYFIVADETIVIRCDAVAVSGGCKGVLLGSFTIRSDTLPAAASALSA
TTGVVAGAAAVAVVVTGGLGSILEMQALGMFARMSCASAQERASTVALPYFLSVFAALDP
LWMVVGNALLAAVFGCVHCGVTAAFQRWRGVDAASAWAAMRFPSLTYVVAHAMHLGIFFG
SVLALAMPGARVQHRVVGVVGVLYGVAFPAGVCYLIARHVGASFTKYWQFLRKPLHERLL
YPVGYWHPAAQQRMYGGMLTSMRDSHVYWCVFQLSVLCVVCLIAAVHPPVGGCHVQYFCM
AAVLLAGAGVVAFTNMMRSAFLTVMHTASLVLLAALCVVSAANHLVPSDGGARAYAAIVL
LLTTVVLAATVYSMVVWYAEDRHWQELREPRRGGLEALLRDDEESDEDTQKPHDLNSSSY
ASGTTVASYRPPAVPLHPMAGDTRSDTLSLLDRASSASRSINYAPLER*
>TCRU_2561 retrotransposon hot spot protein, RHS3, putative
MVLISDKEWPCSGVVGRCIRDCHVNCEVERVWQIVKGDATEWFSSHDGTDFEPKRRVLIA
TLGIGKSMNAGSYFHYQLLHYDAEQLPMLAHFMVSGTYLLEETSRTVSEYMGESSIVKVV
KGLSRRGVKGCIVYDAAETDNRLSTCLPPLEWGMLVVTPPEAKIHGHWASERRAARIVMD
CPDESDAKAMCAWMKRDQPVQQQAE*
>TCRU_2562 hypothetical protein
MADMAEGIATLPVLLAAREETRVHEAVRRRFKNPGDTEMCMEAVERHGCVAEALEHAGEH
CRRGVEALHALHTSPARDCLEAAMGLILTRQV*
>TCRU_2563 tryparedoxin peroxidase
MSCGDAKLNHPAPDFNETALMPNGTFKKVALSSYKGKWLVLFFYPMDFTFVCPTEICQFS
DRVKEFSDIGCEVLACSMDSEYSHLAWTSIERKRGGLGQMNIPILADKTKCIMKSYGVLK
EEDGVAYRGLFIIDPKQNLRQITVNDLPVGRDVDEALRLVKAFQFVEKHGEVCPANWKPG
DKTMKPDPEKSKEYFGAVA*
>TCRU_2564 SKIP/SNW domain containing protein, putative
MSSFSEDGGAFAECFFAQYPLGMGRKPLPPVVMEKIAGRRDVRATDAIAGPTHGISSHPL
FAAVPNTEALIVGSSDLSAGGVSTLSAKGSVVQDSDAEALAETTEKTKEALEEVLRGRQA
QKEDFSGRNIRRVPKTKPGLEIVLPGDVGVTGVHDPAPIVRSPTRAKKDAGNSGEGSSSS

SSANVAAVPFSIGNWKNKRKLIIPLEQRLAQQADTQAGSGGGISEQVMNLAVAMQQARKE
VEAEQAAREKARQEEEERRQAEREAEATQRARELLEKAASSMMQTSTERRKESREERLDR
IRLERELREREKQQEVRQRRLAKAAARLGITVEALEADKDLLKTVDEAAAGGHGVSVTDY
ATPSSSSYLGAKQEGSSLEQKPPSLYADDEEDGTRPARGIRKGVFGSAVISNEGIRREME
ALARMEEQEGGIKDGIRLRNDDESTDDDDDGGGDGLGLDQLMKKRRRL*
>TCRU_2565 Acyl CoA binding protein, putative
MPLFDSSLSQDAAKARELCTWTTRFPRNISGKHFFLDHELGDLEATARFEDRQKLLFYAL
RRQAEHGPCTGAAPSIWWTRERTNYNAWKQLGDMSRFEAMVNF*
>TCRU_2566 Concanavalin A-like lectin/glucanases superfamily/Trans-sialidase of Trypanosoma hydrophobic C-terminal, putative
MVSVLLTEQLKRVKDVLTTWKEVDGRVSNLCTSLIAEKDPPTGDVCGADKITAGLVGFLS
GNLSGNTWRDEYLGVNATVNNKQGAASTLASTDNGVKFTGRGAGAEWPVGKQGQNQLYHF
ANYNFTLVATVSIHNVPEEGDTPIPLVGVRAGSNGENKLMELSYDSGKKWHVLCGDKTTT
KLSSTWEAGTPQHVVILLKNGTQGSVYVDGQRVGSEECALGNGESKEISHFYIGGDGENA
DNKEEVSVTVTNVLLYNRPLDEAEITAAFNPNKANIAVQLDGSVEGDAIETSPGGGRPEE
QRQSMESSGVNGVSAPTVPSAKTSSGGEGSATQLVSEESSDGTQNVGGGSSPGSDAPVET
GDRSTVQGDGSSQTPVGTPATADAYDPNAEAMGHDGTAVNPGASASSGADGETAEGTDGQ
KEEGIHAQNGDVNAAALSSSLGNVSQGNNTDAGTVRESGLLPSLLLLLGLWGFAA*
>TCRU_2567 BNR repeat-like domain containing protein, putative
MSRHLFYSAVLLLVVMVCCGSGAAGVAGKSSGPQFKWRGINESDDVTVDSLGAPSLLKVG
NDVFAVAEAQCKKEAGGVSFTGVASQLLTKKTAETPEEVLGKPKDETQVLEEGASKGQEK
KVDVSRPTTAVKGNDIYMLVGQYGSKDCLSSDEGGCELLLVKGSVSGGGDTNNKKIDWKD
TESSLQRIFGTQLDSWTKLIGSGGSGVEMKDETLVFPVEGTKKAEEGTEEDVKTVSLIIY
SSTDNTNLKLSKGMSDGGCSDPSVVEWREGKLIMMTACAGGPRRVYESGDKGESWTEALG
TLSRVWGNNKKENVKLVRSGFITATVGNDGDKRNVMLVTLPVHG*
>TCRU_2568 hypothetical protein, conserved
MEVLGRRGRGIMSLAASFSAPTAFASLEDTGAVTLWDTRLKRKDYMCVDDGAIKGNGEAT
ELLLASDRHMAVVLAENIVMAYDLRQQTLLHSFKHVSEVVAFLSGTHPHGASTTLIADEN
GAIIPFDLSRFAPTTCVSERYFGVEKTLSKPSFGSLSNYCCGLGLLQVKQRQGVCALGMD
GRGAFYMDPIDPPVEFCLMDDLATARGQFVNPPLPTTCHFLGEYIATGRANGIYSIATVD
ADDNSVVEVFAAPGHASNGLSVVLWTHDGHLMTCSICGEVSMWDVMPLLLQDPPVDETEE
GELPSLEKACNVRETTSHHSVVNCGTILGSGVFLAGDTMGFVTSCPILQA*
>TCRU_2569 hypothetical protein, conserved
MNSNPVAVLGLWLHIRTPQQGVAASTTETKTTSPLERVPRHARCGELKRSACRLSGKSVR
PFRSGHSVDITRCGDVHKNPGTEEDGCITVWRLSIAGLSSVKRLLLLCDGSDIKLTPFFC
RK*
>TCRU_2570 Domain of unknown function (DUF4201), putative
MYEYDDQQIKEEALQSKAAAVIQQRWKEYCARREAEAAMLTTSDRGKLVAIAAAAAAAAT
AGDTAAAGGEEAGEDPAVYLDEYVRLVEKRRDVIGRSLACQRQLARYFAAQRQRKGEERP
QPVSPEAEQQYWVAVKQLQEERRVVQDRKEASGTLLSEVKARHQSTIEEALAQEEQFRQY
IRQLAEESVFVRTHQRLTAEEIDAFLKKETEQRREVRNARIRHILLCHELKKLQKTAEQR
DQQQDGMNLIDFEQLKIENTNLNEKIEERNEDLLKLRRKVTTTIHVLTHVKEKLEFMKIE
NAQLRRQVSMTEDELNELRDKLAQTKRRRDHFITSNIKMRESMPMVGSEKLLLDYENRKA
ACNTMRQNVLDNAAKHKELLAATGRNQVVLNRLQKSLASGIPANVGEKPFNMLAS*
>TCRU_2571 KH domain containing protein, putative
MPAAPTTLLSSPQLDLIADPHARSLLEEERGLRERCDKNRAALRKILREKSSCISEHAPK
VNSFVAEITSTIDALVLKKDNLVVELDKLKSLQFELSNLRRDLRVLEHDISTLAQAARDA
EVLLESEMCELKRMREEREARVGRMRFATVEQAEKELHRLERELAKAGDENCNTAVNLSR
RTQAARARQERQEIAALDARVLAQDLRVEKCRAEIEARTAERQEKEEAKKTLKEHLQALN
EDPAKREVQFNAVRSELAKVQQELAEALRRRKEKRPASNEMDSLQKEHDALVAEMQADDK
RLADLRKQLPCRTFAYPPELKMALVGKGGATLEQLQVDFGVAVCIDGVENGCGFVFGGEA
DVNAAIVAIGEIIAAEEACSHKVVLRYDAALRTHLIGKRGTNLRRIELESGAKLDVERDA
VTLRGTQKAVEAARELVETFLKGQHRAEMPLLADEIPHVIGKNGTVINQMEKETGVRSLR
VDREAQKIIAIGEQTRVEEAMRRYKELLADVRQSQPVAECDKTTQGDVAGSDGRTVQDVE

AATGVFGHVKDSMISPSGTSQSAEAAHVLPHKAQRTEARVPMERSLMQLLTTALVEVNRG
PGGDSEEKRELEVLANEENREEESVRVKGAGEAVEKKEMRHDKGAGSRALTPLQVIHQRS
QCKHVKPVHSEGIIFLCGEPKHIQHARMMLEEFALQYRPRAIHVSVPEILFTCITQPSGK
KPSLVSEWSKGYSPLLSIELKPKEEEVIVGSFIAEEARAVAETVREFTKAKEESLVRRVR
PFPQNRIGTLLGKGGKKLRELEKKTGTHIVLRRDAGEVQVFHADGDDAALAEAVDLIMTV
AKS*
>TCRU_2572 Cilia BBSome complex subunit 10, putative
MASGDAPVEILPTSGFVFNEVEEPAELLCPPKLLPIKSFTLERLEKLEKRVTDEAKAKRA
QRQQQQSAPMWSTNVAPTKEAVGDATSE*
>TCRU_2573 Thioredoxin-like, putative
MTDAIDVTTYADDRALGQVAPSLEKLVPIRGAKVAIEGGKVYVLYFFNTFYRGADPVNEE
LTVLSEKYGDRVVFVAISNDADREKTEKYLNKTIVDENTKKPLRLAPPHILFDEKKATGK
TYAEASNLTVMSCPMAFIVGGDGKIAWRQQFLQTFTISQSNFEAQLNHVLAGEALESNGP
RPVVQVEEEAVEVEEEMSLF*
>TCRU_2574 hypothetical protein, conserved
MRHGYVSGISGRRVNPDDGAVEYSVQWSGEDGVCDTWEPRCNLPASCAGPVSEVDAREET
EALHRIASWAKRDGTFEDASDATPPAPDLTPPLHGIVVHSSSNNNNNSAGDEGKSLVSLV
ALGERVLADATSHGLNPTKKRRLQGQNEHDPSCGPALLLEKCWREQHAVSFMHTCAISTC
RTPAAQEFELGKGSDKCRDASSIEIYGIAPAALVRSGQVYDNAKNCDPLAGHAEAETLSG
QCNFKSMPAHTTQSSVRGKQEQLVVRYFIMENNGENDQTLLTMPLCVFRRYYPQLLLDFL
LRHALVMESS*
>TCRU_2575 60S ribosomal protein-like
MRRIAFLFPTLGRVSAARGCAKMTSNSNPKLDELASAYSQLTLKELSELQRLIFKKLGHS
DEFYEKALLRGLGGGGGGGVTMPAHAVAAAAAAAAAPGAAAQEEAPKAEKKKVEKATYDV
KLAKFTPDIKVKLIKELRSVTSLSIADAKSAIEKCPGLVQTNMRKEDAEKLKGLFEKLGA
TVELI*
>TCRU_2576 histone acetyltransferase, putative
MSVFEARQKVYATLHGTFHAAIVQEVAVDAETGQYLYYVHYVEQDSRMDCWLPASELKER
RQGRQASKQQTNKSGGGGITTRGQSRLVAGKEDEVSRGPNTPTSSAKRSNLQNCVKVSTT
RARKDSSFFSRPKNIHSICMGPYEVATWYFSPYHLARPQIQQGLKQNAELFSGKMELQLR
QENKKSAFQTAVVPTSFVLHICPFCLHPFTAHEDVVRHLQIVCPRHPPGNEIYRDPVRQL
VVLEMDAVVEPVFCEHLALLSKLFLEHKALDHDMTPFLFYVLCAVQPHGLEVLGYFSKEK
QSPEMYNLSCILVLPQFQSRGIGRFLIELSYELSRREGRIGSPEKPLSDLGEKLYLGYWG
DVIVSALARAIEENHCATLEYLVQATYLSQADVLRTLQYLRLLNGTQIVVSEESIERCYS
RRLHRERSGKNYVFYPHLLSWSPHMYYELKEQDVAPPIYVTQSEELEQNAASKGQRVF*
>TCRU_2577 nucleoside hydrolase, putative
MVRRKVIIDTDCGGDDAIGIMSAMTHPEVEILAILAVWGNVDVEQGMENLGKLLDLYGRD
IPFYRGAAGPLVVNRETVQWGGFGRDGFGDAGFPPSPRVAAQSKKHASLALVEILRDAKP
DEDVVYQLVCLGPLTNIALAIRLDPSVFEVLGSETESAVTVMGGTSEGKGNSNLMGEFNF
HCDPEAAFVVFHSTGIKHPIQMVNWEVTVNCAMPWRFYDEWVGRRETSEGLRPVNQNKTQ
WFIEKMFQRLETFTRPDEDGTRADTGDAEATQDVTCVIPDAVAMLVALDPTSLEDSFLTY
VTVELQGRETRGATCIDWYGTEQSMAKKGRWRNCNVITKVNCDTFLEVMQRIVDFPL*
>TCRU_2578 Cornifin (SPRR) family, putative
MSIPPPPVAAVPVPPTAAVPAPPTAAVPAPPTAAVPAPPTAAVPVPPTAAVPVPPTAAVP
APPTAAVPAPPTAAVPAPPTAAVPAPPTAAVPVPPTAAVPVPPTAAVPVPPTAAVPAPPT
AAVPAPPTAAVPAPPTAAVPAPPTAAVPAPPTAAVPVPPTAAVPAPPTAAVPVPPTAAVP
VPPTAAVPAPPTAAVPAPPTAAVPAPPTVAVPAPPTAAVPAPPTAAVPAPPTAVVPAPPT
ATPLSASSVPPVVSVPSGAVPQLSVAAPFTGTSIALSLPAGIGVPSQQAIHEESRRSLEE
AAIQQFKSAAGDHYLDLDDVVRDTICKLFFLSNYDVGTMMNSVAKSLHCLGRYDVGLLRN
DDSTAFTLIQSPPFNEAVVTDETQSVEQCRRAIQEAKIRIERADTRSKARFFDAARAGTK
PKEQPGRVPSTCVRKEAKVKPATFRKTSYEQIQEMLRAYQASRVGRGRGASASPGVPQNA
SPERDGYLLWRYGGNYPKENYYTHYRLHGLKK*
>TCRU_2579 Calponin homology (CH) domain containing protein,
putative

MPLPKCWQTISLPELAPSRRAAGGTAAAPGAAVFVPPSVSSLNFDEEAVVDTDRKIERHR
YAKLLLHHVARYQQFTSTDLFAETFLRDIALQDGVTPQARAAAATWVMKLMCSSSSKKWL
MEPLVDALFPAIYRDYDEQKLDHAPPSIQALVNDRTLLRDNPYFSHSMYVQDIVTINATI
ETLSRKIQRTFPAIMQWKRLVVILAEYVRNTIKRRAFSAWRELARRRRTDDLLRRNVIIR
HEKNIRSSRLQAAFFRWKTAVETSRTEYLTERLHQAAFQLESAKNQFQMQCFRSDKLQLS
HEQGQVEIQNLIEERDELLRSVEELKETLVLREKEHQDRMKNCVREVVTLVQQQRCILRR
AIDARLCVEEITAGWLDEAISEFKSKAADEEALPPGYKFLLKWCNQIYFRQRTKKPLRVS
NFAADFANGEAYLLLLQYVFSGDQLATIKPPPPPMWLSKVNKTERLNRVCQFAASTPLAI
ALEPVDLSEKREDKIAAAVAEIFLYHIREKRVMYTKRALQIVSDVALRSEPGANNFAAAI
NTPTRSESASGKDSASDSVGVFDDGFGGAASFHDEETLRRQVMEWGCELEQWEAELQRNV
VQENKLQHCGVTIAHDAAHMMRERSKGQPLYVVTSSTGLIFTSINAKNMEDLRTKFKMPN
PQGWKTLVRVALKNVLWKHLRLIASHFYHFAGVDARKMSEVQFWRFVEETQVAAEPLAPL
VAMQIFDAVVSPQIASIWRVAGKDEKREKMLEAVQEQIEIRFLKPNQFTEILVRLAVARY
KGGIIEATEHFLSELRLPVMEHILPITLLFYEPESQRVMSYFQHDLARVFFFYMNHQLNE
SLKGRSHKMQGCGRFMARLSYTTYIKMFADCDFILPEQDGESGSVGYQRKTRFMSIDEVL
KMLEDVQRRIPSISPGELCFSLFLETFGVACTYWWPDPAVPLSRKLAAFLSRLVEKLRAV
FASDVLILGNPPCVSLEGGAHVELF*
>TCRU_2580 DnaJ domain containing protein, putative
MEAVAESYFTSGDMKAAATFYCNLMVLHPRYVKMARICRVLADPSAVPQPHRYADIVEAL
LEPTVTHEALFGVDAGPADINKAYQRWTLLVHPDKNPYPRAGDAFKRLFTLKTMALETAN
AMKERKDCENLLHGGRRRGRKNSNKKSDAPAARSRHDANASLSVQSSVIDMTLSELKKKQ
VILKSLKRKDIDDNELPELRAALRRVRERRFCSRPTTPSVLTAPCMTTPSLSPEQSVLST
ASCPPHGFHPATEKQKSLTPPPLPKIGTLLKTVALDEQIQSSSTVNSSQCVLSDTVLEEQ
LDELFTEEVRPGQEQTTTDVEPELPENEDVKKLNEKNGIENTCSIIRKPEIANSSPSPPA
SVTDDGVPLKDAVKGSIIELIRGIQEIKVNRTKLRLSCDLTFATYEREKQQQKREADMKG
KFAE*
>TCRU_2581 hypothetical protein, conserved
MFCRLRAGSRLGGEALRSEVLVSQRRLLNINRSVQTRWEAENQAASQKRTDYDFDKEQWS
KQMRFASMDCIMNPDITPIRYTTITGLNKAFRRFMTMRKLVERRPDFNPEDLKKLFVQLK
TISHSLNPEDLKLLQRITTHGEASRIGKEIKLGMNEEFSKKSWKSLKMQAVQNTYQIELD
YFQLVNCYMGQMAQEDWLQITYKCEYRERPDEASEWQSMCEYPVFEVRLGDGVQSSNTHA
FIVVGVMRKDGTRYGKDSQDAAALRKQFDRSRKWF*
>TCRU_2582 Leucine Rich repeat, putative
MHGKEHRPRGCSFDGPLYDISGYLLSHPLAFLAASPITRQVAERIVCETLLRSTVMHQTG
KNQLEGELWLPHSGAWCGQLSNLKYTLWWIRRQADANDGKLAALFFAYRSSIEDLTDVKD
LRGLRFLFIPEAILLNKTLSPLASCPTLKHLWLRSCRTLTRVEALSHLHSLKSLDLSRTG
VTDDGLLALTACHLLEEVDLSGCDFICALPFMKSMGCLRVLKLRNSGITDRAISAIGAAT
ALVHLDIAGCFLVTSLNPLGGLKRLEWMNASWCGIRDGGVEGLSCCGNLEYLSMARCWDI
HNVNVLGVLSKLQVLDLCGTNVDDEGIAGLSRCASLCSLSLSDCFCIYSVRALAAMPSLK
EMDVSYTAVTEESLSLLPSWVRVVRKPVCVVSSNDSITFFRSPRTPLLSITPFVG*
>TCRU_2583 Miro-like protein, putative
MPSPLTVYVLGSPASGKTSLLRQLSCMCENGTLRTRPTHDAPTKGQEVYSLVIQAPQPKA
LSCTVEIRELGGEMAPVWEKFIQSFMNSIKGKGGPNCALMFFVDALSPHLLPLASVMLSR
LKGSDGVCRNWPTVVLLSKALAMNAMTEEELWFFLPADTCKNIQVFSVDSWNGLGLGDVL
AWLSGVASSC*
>TCRU_2584 SNF2 DNA repair protein, putative
MPVPSHVSVATLQSVLQTVCSTGEPAIIAPSSFLGELDTVVDEVKRHGMKLASIPHGGIT
ILPPVPISGTELFDFCAEYCKAQTHEERLAVRHKINNHNFLMQDPLLRMPCRQLYNPADY
VLRIVHLCSELVSASEEEYHGAYGVAPLLHINPVQDVCGKLRSMFQSGSLYVKPWILEEE
EERREMDESNRGVLFNSDSFGNGRGGSSISSSERSVDENDVADEDLTGEEVVDNATDTVV
EYLSEKDFDVVTESGIFYDDSGERVHAIYLRGGIKKELCQRAAIAIEEAATTKNLRKAVN
GGKTNPETGIVGYYDYLNNPTQRKCRETEFTRRNWSSVVDSCEPFLVALNKLYSECAPTH
YKLQRIAIPHHYQLFNTVFSTMTVNRNFRTAVHTDRGDFRSGLAALCVIDGVFEGCHLAI
KKLGKAFRLETGDVLFFDTSLEHGNTEVHNFDYCWKRVSVVCYLRNGLMSQICEMERRRW

LQKQMLKQRLLDRSRQSVINLNATDPNLPPIYLPGRLLEVLSPVQQAALGFVVDRLSKGN
GCVIALTMGLGKTLLSLALCYSHMYDQNPRDVLILAPKIVLTHWTGEKQKWEKYGLVFSH
FVVSDGTDSVSFEIALKRYKQQLNGELPRTSHVFVINPEYIRTVLKKLTGFRPSLIIVDE
GHRVSSKGSKLKDWLEGLRCTARVILSGTPVQNNAEELYRLIGWINNDVHSVLPPRVFTD
LAGTINRYINGDDSALAAAVSAQRYIQEWMCSYVFSVMKTDLPPLNDYIIICGFSSIQRK
MLEDHFGMEGIDGLTSIKASEHRPYHLSTHPLCFLGFISGVYKSLNGNHKLTPEAEEELE
SQEYASQLYSLTEDDIGLIDECLSLVNSGFLTEFVGLSGKMTVLISILHSIREKKEKAII
FSQYVGSQDFISRTLTSFDIVSSTIRGRDCHERRRRTIEKFREDEKITCLLLSTQIGAYG
LDFTAANHVILWDSWWNPQVESQAIARAYRRNQTRAVIVYRLASEFEDTIVLKTQIRKLA
LFRCIMNEEASRAVPPEELLDCVDTEEDEGRRFLWRSLKKSYLEGGAPAVSKVFRHGDTV
RSESWS*
>TCRU_2585 hypothetical protein
MAWWNQLTKQMLLRLSKHPTVHRYARQGMNYVRDHPSGRKVSSAMSNSWVEIRCRVESST
SAFRTLGFDGNNKNGGYAKQVFTLWNEYKGRVFSFIVVNFMGILFFFQFGSALWQMFKQM
VSTLFTEPEKKLKKNEGSSRAINVYVAGAEGIEEKKKKVILQQQQQQREQEEEKHPVEME
NVDQFSFVFSDGTDAHAKQSMLRLERDIFGDVKKPDFSTSFKFRMGEDEEFRPSAKK*
>TCRU_2586 phosphatidylinositol 3-kinase, putative
MSSPSELFEGLKVDFREELEAEDIYGSKNSEGFERLFERSEKCKRNVTQLSTILQSLIFQ
ENEIETEGELEAIIEYIVGTGLYGQSSSLEAKEFRKKIRASFEYEWQEFQKENSFISSSR
AMRTLPYRFSLPSLEKAQLEMLTELSSRGMSNGSLGTVLLRIWYIDLVGCTCSTLRGFPF
DMKASDAILITMERLFLITNFDKSKSQGIMYVFKIQGRKEYVYGDEILINFCYIRDSVTQ
QREVNVVVEPHEKTLLTDPIFVPSYSYFSKTSDENHHVFYKSESPDDISTGISVWDIEGK
LVVKIGENLCNLVFTSERAKEEGVREGDLLYLALLVEVYHGAKLVCRPQMTKWTVCNDLV
ASNELTTSSSVNWGKETKLTFDMLVSNAPREIKLCISLLATCGERFCSVNSLSAEEIVQK
AEERIGVKKMEECHLSPVFFLGYASVQVFDYMACMQTGKLDMRLWSTKKKANPMCPIPKN
SNKNDMGFSLIFPSFNKPVVIPNVAPPDSKRRDWEQQLLDSDLCLVGNLRESKIEQLRQL
KRILASDPLTELTTIDKVLLWKHRGELILRPRALAKFLLAVNWLRPYAVYEAHTILPQWA
PLQPMDALELLDIRYADIVVREHAVKYLNGMTDYELKGCVLQLVQVLKHEPYHDSALFRF
LLRRSLQSPHMIGHYVFWYLVSEMENTSVSERYGLLLEELIRRMPERKVYLRQLYLADQL
FECAMRVKDAPKKDRIDCLREHLQGLRLPRRFTLPINPAMECSGLDIGKCKVMDSKKFPL
WLVFRNYQEEGDPFYVIFKAGDDLRQDLFTLQLLELMDSLWKSCGLYLHIIPYGCITTGE
GVGMIEVVLNSDTVANITRKYGGAQAAFREEPLMNWLRQFNRERDEVERCLWNFICSVAG
YTVATYVLGIGDRHNDNIMLRQDGTLFHIDFGHFLGNFKTKFGIKRETAPFIFTPMYVYM
LGGQSSPIYLYFVDLACHAFNVVRRFGGTLMTHFMLMLSTGIPELQKIEDIGWLRTVLLI
DRSSEEAREHYRLQIALATSNFRTLFNDYIHIMTH*
>TCRU_2587 ribosomal RNA processing protein 4
MNLTTCIVGDSICGSLGDSKLLEPEGVVFLRGYSTYEGNRPNEVAAAQEGGGEIIAAING
LIEVTDRVVSVKGISTRYHPEIGDIVIGRVKEVSGNRWRIDISAYQDAVMLLSNATEPGG
ILRRRGRNDELTMRHIFDQDEIVVAEVQRISSDGVVSLHTRCGEKYGKLTGFGMLVLVSP
ALVRRAKHHFLKLVILPVGIILGTNGMVWVNPNVAQRGGTDNSLYFNEQTDVDVRRSVAR
VANCIKVMNFARLPIFAKTIEASVKLSIDVGIGPFDVLHNSNYDLITNAVMEAITKRKRQ
RS*
>TCRU_2588 hypothetical protein, conserved
MPLAVFPSHQCEQRRHLGRHSGVPPQISNYALGKVERVNVHRQLQYHVWVQIACLHDWGC
VPQRTLLCGVRRGIPRNQRSLHVSSRKIRKLSLRRSVLHNAIVSNASCPRILTNRKARRE
SALAVIDATDQSKIMLQREIVGG*
>TCRU_2589 hypothetical protein, conserved
MGTSGGLLSLCPPPAGARKLTEDSSRRRAIAEGSGNNRGRFFCKNRRIDNTLLVVEGKTT
GLRRRWIAWPRDKNRDDAYEANVPLLHISHYLPPVMAEAASCLDLKASFLQVPLPRETRH
LFRCRVEDGTLVELTRLPMAHKAGPQILQIIITSAIAVVTTVVRPLWAAPPLVRVEVWID
NIRIAGSIGDVTRWEAQVLRNADGRRATMGEDRESGAAQYTFLGCSLITLTVRYP*
>TCRU_2590 hypothetical protein, conserved
MEERVPPNAWIALQAFVEDGSVEAGAFLRDLLRRAAFTANITRRSLPPLFFREAVMKFYE
GTLSASSLLLVTILNRLFCMQFIPDAMARPVNDERPSDLADLLQFYVDSLGAANLPAPVM

EQTYLCVSSLLLLQSNVSVVEALLAVFGGAVPEVARETRSLMKLLSGIMSVLSDHRVSIG
PVRRSTQRMRIQNNMHLLLISIIEEDVSAQASIITQGVSFLMEVSVHEPQEIAPLFWAQL
PSSTFWRLALERLHQGTANEDILRAVCAVLRAITVLDASAVSLLLSALEVVLDSRTHAPL
LHVCRIITSSLESSVEEVVVRFGTDHVLYQALASGAQTLKRVLEQPEAVVASSSEVGLAL
CEGLSVLSQVLMVLPVPEMDSTDDPDDYAMFVEEIQRRNTQKKEAFGRLEGFLKGCLTSL
LVWLGRLTAHDVRSIAAYITQNDSDEMAVWHDELSVALFTTYERLCTLLSPSQDKEIRAL
AAVGCLTVAAWDAELSYIALVPYGDAAIQHQQLQHAPLMPVVVSRHQRKWGPEQRECVVS
SLMALLRVAVMISGDVAAAAAIGESLRELNAPFLPCIAECLWQGLYLPCGEDKTPRCVLA
SYLSNILEQGDISLPTEAFENSQMDDYSLAELRSCVLRSLQDVLRVMDLLQRMEPEIVQS
HRVAERITKWVQKTVATGLLEIELYTRALQQWYIVNPFHFCITWRLIRPLEPTSAAGLLA
DALERFVSCKNESGDMNWPIESDCFSPYVLDTIAQSPYAPRLYRVLYSLISSNTYMGDGS
AHFRLRAVVRGGNTLLQSQYCSPTLTFELFYAASKEYVKLRHELSAASSCLGTDNDDDDD
DGDLLRGISMLGKVVRMDGVRIPSNAPAWLHEVRNAVDEFEIQGVFKAI*
>TCRU_2591 transmembrane protein, putative
MWRQSFLRLCKGATATATGSNGGGRCVTEELEGRTNNIFSIRRAPNSLYEDKMHSNSLTD
HPLTHRTSRDELAYRKRKLTERGNYEQAAKAEEEAERRDTEGRAGAETREYYSAICFVVL
GYLTAHGVVFKRFYPDDLRPYYSPDRGYSDDVAARRQDLEAVDELIGMNVMESLYSKRKE
VFKSQMA*
>TCRU_2592 hypothetical protein, conserved
MLEPREDYTSMLALTPQHVAEEMKVISLLRQELRNTHVNYDENPVAEPPFEPAHRTVDAK
SLRTLKCTSYPFIVCEEAPTSYQAPIPYQLESVIERLREEFRSLEENLKNAEALRREFTG
RQRSTSPSHGSHSRDGSRRHPKEQGNQTRKEDALQLFLEVVGRDARFANLSKEVSMVRWY
TRWVSSHSFICLEEALEQVEYVLSEGGLTTEADLVEAMQQFSFVEAVSTLENDRRDVVEA
ERHRMHSHSRTFREHVSPLAITAATSSFLTPEEKYWYQECYNQTEHVMTMLHHVLHVQFT
SKGRDPSLVSLGKFCPINLEVGSNLISMKETTSVGEESQILSDAAMRALRQERDVITNLL
QKIQDRQENGVMLGPTRDQYRNSLLAAMVVYKCLVDDMVMLRSQKRLAAEFLAEYDDWIL
HLCGTNDQAVIRRLGITPTGGSPPMPLMGRNTPRGNSKSHSREREISAPLFCYSPIGQLP
RHVEEVCESLMSLLKLSRRSSGTDNSTGVRETVYRRWMCLSLRYTCMQIVRGRWPGDLLL
DCMDDIANNLIRLVSEYNSNPGAVQSHLTELNAVLRCFLGLVADGAARRQSQREFPGINN
GTASSLALASATAPTGATSVARHSFDVSVAFSLDRDISSMERAANISDEDEDVWGAIFDA
LWFQADPTGCAHGGFLSCKDFGMDVEKPFVFLRLGSITLPDRLPKTAEGRRRFPKVYMSL
HYVTHTADMSPARSLAEVAEGDATLMASTSVVTRYGYMRRILYIFSILEQRQLLQDYACR
VEEVELVPIENAVFFSRKRDKKTITTRLFYSALPPPRNGDDASTGEKYATVVGLIPPAAV
HLMRYNAIPTGTEYPVSRSKRSTLTTLNLLLGNMLKKIALCRSPLLDKMYNMGDTLWELG
VGAAEEKDNDDGSQGKPLVLPILRAFDEYISSPGFEKSVKEADDEMRQQSAQHVNFYHCP
Y*
>TCRU_2593 protein kinase A regulatory subunit
MAEEENTLSLFLEACRKEGVKQPNTILMDFFRSNPSPSQIEEIDLSHNYVGNRGILAVLD
LIEKLPSFRFLNCSNQKLYNTDLSEDSVRGNATVDRIVEVFKVHPTANALDISNNPISNY
AGRKLLVLAQVNRRICRVELSETRVDFDLRKRITQQCEKNTIALWEAQASEGADEERGFG
EGPVWVPKQAPADLTTIGGGRNRRRTVRSEGIDPEKAKLYQAPYFEKSEDEMNLITKLLT
HNVLFSFLNTKDIKVVAGAMQRATFKHDDCIMEAGQTTCNKLYIIQSGHADIIKEGQKVY
LKTEGTAVGELELMYDTPVVATVKVCTDELIAWVLDRDTYRNLVMGTAIRRRETYIQFLA
NVPFLGGLDSYEKLQLADALSSEEFSPGEYIIHYGEEGEWLYIIMEGTVEVIGRDADGEP
TKVCEFTQGDHIGELEFLNNHRTVADVVATTHVITAKLNRRHFEMCLGPVIDVLKRCTND
PKYEYYQNVLKTGAAQPSYVDDV*
>TCRU_2594 hypothetical protein, conserved
MMTTTDRVLLVCALCVLWCCVAAVAVSAAGGAGGDGGAGEYLIAGRHAQLRRECAEEAGR
RTGGRANPSAVEECVRQGMDGVRTVVDGRRRWRRQQFAVAAADGGSGNDGRNSVSSQKAV
SGTGTVLPDDPKLKSPVAPGPVLADTTTGGGNLLIPAGSLETAKDKNGEPSRETDKGEKA
TVENRSNEGTNGDEQVDNAAIGKPNEDPENKKGETLQVEERIVKTEEHEVKNGLEENNTE
TDEENRNKAAEDPEGDDEVTILSSGGQYNEEGERQTEKDSGSIKKEKEVNGGGATDGISA
VAPVALQPASPVDVTDRRNLEKKNDGDTGGNRDAGRTQTQESAGPSQTEKLAAELITEEE
AAETETGAPGKKTQPEDAGKEQTTVGAKSNQETPVAAKEANNKKEVPREEEDAETAATNE

NFDKKRTSGKDNAHNGAETTRQENENEVEKAEGTHAEAEGNEEQTGKKNFADKDRKLNDT
ATPGDSDSSVAAFHNTSPLLLLLLACAAAAALVAA*
>TCRU_2595 chaperone protein DNAj, putative
MVKETEYYEILGLEAEATEHDIKRAYRRLALKYHPDKNPGDQEAAEMFKRIGHAYEILSD
EEKRRIYDQHGKAGLEGGSMDEGGLDAAEIFSMFFGGGRRPRGERKPRDLVHEMRVSLED
MYNGKTKKISVTRDRICGACEGGGIKPGAERRTCVACRGQGVQTFVQELFIGMHQRMQQT
CQSCGGEGTTVREVDICGRCRGSGIVKDQKILEVHIEKGMKHQDVVRFDGEGNEVVGVRL
KGDVLIILAQKPHDVFRRVGNHLIMNYTINLQEALCGFELPVQHLDKRLRLITIPCGQVI
DPGAAWVVRGEGMPLPNTGGLDRGNLVIHFEVEYPTRLSAQQLKSIAKALGVTESFPRVT
GQKLTLSDVSQRQSRRRSGSQRANAAARRRQMQMAGGMDEEGFTAFHGGHSGAQTVQCAH
Q*
>TCRU_2596 Leucine-rich repeat, putative
MSDGVVLDGTTLCAVACGLTSLDMIPLLCGRTADGQTEDAPPPPPRSITEAHLSFNAIDS
RSLCRLSHLAATLQTLVLDNNELQEVSSLPPLPGLQRLWLNNNRLVGLDDVLTVVSMQCP
ALTYLSLLRNPCCPSELDGHLPIEYTRYRLYVKYVLPKLAHLDTDSFTQAEAKEAKKKGK
FARPARVKTNNGAAEKRPITVRVNNNRDTEQSGNNSVEREDLFASFDARRHEGAGAVTYL
SEHRTVYVGRRSEGNRFICDDML*
>TCRU_2597 Ribosomal protein L14p/L23e, putative
MGKEKANVKGCRFRVSLALPVGAVVNCADNTGAKNLYIISVKGYHGRLNRLPAAALGDIV
MASVKKGKPELRRKVLNAVIIRQRKSWRRKDGTVIYFEDNAGVIVNPKGEMKGSGIAGPV
AKEAADLWPKISSHAPAIV*
>TCRU_2598 hypothetical protein, conserved
MSQLWVKRAKAEVSRHSRLLETLNRVFPMPFEERRSRVGMVTYGTYRWFRYFPIVLVPVV
VMGAILETRDIPQEHVFTSLHLWLADHGMFRHDTVKALNPAFEDERRAIEWKANDRKWRF
TGIDMPSEKELREFAAINIQRQGLSSSR*
>TCRU_2599 hypothetical protein, conserved
MKSVEYKLARLEEQNASLRQKLRHQENEISRLSVRESQLKEARRMPLLQKRLNTALTERE
ELECQLAETRGREARLSDEVKLLRMFIKISENPEIAETARQYVSHERIQVELKVLGEQLR
SIATERDEYKKRVLLSCEEAECYREKWNASLEEKRRLEEYIKKLVAEQPLVPTVIPSPSC
RMVERCASCGADTATRKTNEDFDLDEMQYLRRSLEEEKARSEALQETIDALYEQQRGQRR
SCSLERRITSPPASGSKPDIAGKDGTSAGIYTLRQKIDFVESECRLVKNRWHALREQNKI
LLSRISELELTEAAGRGQIQQLSGECEQLRRELEFVLKARKLIACHHDEPVDNEAPKEKN
GLTP*
>TCRU_2600 hypothetical protein, conserved
MCARLARCDPRLLDFVTWAVLKGCRLFQRVHITPLGLVATSTIPAYDFVAVVPTSATLSV
LTLAGDESFPIKVSPQNHGDELEFWSDLTLGSFALIGYFTKALRTGSPRGVRSYLDVLPF
DPGMPIGNVADAAQKSPGYAEMIKPLKNFCKAQDVEFDLTFRHAYCLFRRHAIPFWTNTE
AGSGGHPDFQHSPFATLGHGDILGMVPVLDLALHSPEPNASIGYPDADMLQWLAQEKKAG
IQVEKGYFAMQAQRDIHKGEVITVNKNAYFNFDDATFRAWFGYPNVSQLHQSAETVKIPT
ATCDDIMETDNLELTDM*
>TCRU_2601 hypothetical protein, conserved
MKHRFHENCSVATGVIPSLLPTCYTTSSLTPKWVTAIGVVSFDLELGQILTHLFPPLPLS
IRQETDLAFLCFPDTNHTEYGHSLHHFIFPLNKKDCDNTRRTVFRMPRSTALSSASREMP
QCSVLHCKLCDEEYFCSSIYLQQRDINNERGTQQKALVFISLYGFPKIFETILEMVYGLV
LDSCNVAEKLETLYNDICSWPAPLPTKLYTGLHLLGKPIQSFRTPYYTRDQIGFRWCEDG
LGREEKCFLARIRLLEALEKPCPDPPTIQRCLASCVLGPSAIEGMGEYIEKQLRLPLDVR
AWRPSDVLAVIFAVRMRKYEEVDVTPFPEGILDEDSENIMNDDVRHLARQFPRFCQVTPQ
VAAVQNQELGYCRRLAKRLLFEHKDFIVNVDESGAPAMFSEFDVQGILLPHLNRLWKLWE
ILVTGEPLCIVGSNLSRVSAAGFCLYSLLAPLSLSVVLRPYLTINNSEVDGLLPRAGPFS
PVILGATNPFFLRHWSSSPHVLCLGATESYELQTHKTLPHSSLKKSNCQFDVKTNMFTSK
HFLVRSEKHLWDECTANCSSVGVLNLPIAARSVAGATLNMDHNESVRKTFLKTTEQFLLP
LERAVEVEFEKSIPLFFYGDYVPSRVQIFSVLERSSDSDLPLHIFKGRRGMLKVYWRFMN
TSTYSCWLRQKVFSLKRALLLRSNSFENVLLLEPDTCRRYEVFMSLQYMLDAELRRPLLD
VLLLKKLLYLTKSFAILRTCLTSDNSNDEGASCGF*

>TCRU_2602 proteasome beta 2 subunit, putative
MPGFSFANVQRNLNLQRQGLQPPKTLKTGTTIVGVVYKEGVVLGADTRATEGSIVADKRC
KKIHYMAPNIMCCGAGTAADTEAVTNMVSANLALHRLDTGKQSRVHEALTMLKRHLYRYQ
GHVSAALVLGGVDVEGPFLATVAPHGSTDRLPFVSMGSGSIAAMSVLETGYKENMTLEEA
KELVASAIRKGIFNDPYSGTQVDLCVITKTKTELLIGYDKPNERKYPKQEIRFPPGTTPV
LREEIRQLVTITDVE*
>TCRU_2603 T-complex protein 1, eta subunit, putative
MIQPQLILLREGTDTSQGKPQLISNINACMNVVDTIKTTLGPCGMDKLIHNGRDVSISND
GATIMNLLEIVHPAAKSLVDIAMSQDHEVGDGTTSVVVLAGELLKEAKACVEDGMAPQII
IKAFRAALSVAMKTLQELCVPFDPNSEQGRKNLVRCAETALNSKLINTECLFFAKMATDA
VLTLDSDLNLDMIGVKKVSGGSMRDSILVDGVAFKKTFSYAGFEQQRKKFESPKVLLLNV
ELELKAEKDNAEVRVKDPKQYQSIVDAEWKIIYDKMEKCVETGAKIVLSRLPIGDLATQY
FADHDIFCAGRVSTDDMARVALATGGVVQSTLSNIPKEALGSCALFEERQVGTERYNFFT
GCKNAKTATIILRGGAQQFIDEADRSLHDAICIVKRAYKTGSVVGGGGAVEMELSKVLRE
YSRTIRGKEQMVIGGFARALEVIPRQLSENAGHDSTDTLNKLRQKHYMAGGAGKWYGVDI
INGGICDTFERFVWEPTLVKRNAIQSATEAACLILSIDETVTNPESDAGKKQAAAGRGGA
AMPISKAGMGGMFKGAPGVTKMKGRAGR*
>TCRU_2604 nucleoside diphosphate kinase, putative
MTTTSRYAFKVDYYDPQANLTRQYLLLYYTEDATIEMHDLKTKRVFLKRCAYPSLGVQEL
FIGATVNIFSRSLKLVDYGDEATRQHFSAAMGEFILIITENGFRSVGTIVDKIISQGLRI
KNIRLVDLPDNMCLQFGISSRCIVILVKGSEAAEKVVLLNEAFQNVTVVVSNPTDVGLLS
DVAFGPGKTTAMMKNCSICVIKPHAITSGYQGAIIQRLIDEGFYITALGMYTLSLTDAED
FLEVYNGVVPEYKRLVEQMSSGPCWAIEVYAENAVTALRAVCGPHDPEVCHVLFPHTIRS
KYGIDRTRNSVHCTDLEEDAPLESEFFFSLLQNL*
>TCRU_2605 predicted WD40 repeat protein
MVKVKTISRSHLEWTKDRNGEVPRVNRNFDTKYNPMAKQTEFTRAIRAAKLDRMFAKPFV
GALSGHHDTIQSVAVDYTSLSTVVSGAFDGGMIVWDVFTKRPKAIVDAHRHSVDGLVLST
DGVACFSASRDKVVKMWDLDFPSDDSKVEPLAEYLGEFPFSSIDHHFHKSIFVTSSDAVH
LWDVNRTQPLQRFSWGDDTVSCCQFNKVETELVACCMSDRGVFIYDTRTKASHSKIIMEM
CCTSVAWNPMDPNTFVTGSDDRNCYLFDMRVPGRPKSVFQGHINGITSVDFCPTGKKFVA
GSLDSTLRMWDIHQTTKSNSTEMYHTKRMAKVFSVKWSPDSSYIYSGSEDAILRVWKADA
SKPVRPLRGPERSSFNYMRSLKDRYSGFVEVRRIANQRNTPKAISRAQLRGKKAEKREFV
KEASRRKHDDIKPLAQRKVYQSLN*
>TCRU_2606 calmodulin-like protein, putative
MSRAALMKESFELLQRDGKIPKASVPTALRAAGLNPSEERIKEIMNTVTDLDMAGYEALV
AKHDDKTDTPEAVKEAFRVFDKDLDGTVSVAEFRHIMTTMGEKYTEEEFRDLIQGFEENG
VIHYEKFVNKMLAPFTDHGNLEDVI*
>TCRU_2607 hypothetical protein, conserved
MASRATVFQTDVLEWLLSNKASLSRLRKERYALEKGVMDYLYEFRDMFRDVKEGESKEGE
KKLEGVQESVGSRSLSQFYESEELAKRMKWKRVTWDEDADYNSFSRRLLDNDNLLKNVPA
FSFLSIHVTNDPSSIQFVRFPVCKKSIELGTLTNWVLQERDPISVEKYLPSRKNKLRSAF
TVDWVAGEVKVIDTASVQEVLTFLHTVAPRLQAVQVRMEEQQTALVENVQDAKIRVGAEV
KFNEHDTTFWDDPKRKDNLDYVTPDDVEKFLQGLLKSAFLYRWFLKGQGLRVLPPGKPYF
IDMEKKEIQIPANFAEFNWRAAHKRFEKIERIFSTMRAFWWLWFSLAMVIIGDVELF*
>TCRU_2608 hypothetical protein, conserved
MGLEKQIASLETFFRHVNYPLQPLHYETKVVLDTFFEKGGFLNLCDLTFASYGTKGRAGG
ARSLSWMDIGNLKPNGKDASALFRLLAPGGHLCNLLEAVSLPSKDFATCLKRSWKENLAG
ESEIKFPFYTFDIPISALYSLSYELLAESADTNWYTLSRSRCPPLFLIPFWNPPLATRFE
AEHGQVLTSFPCSPLSYFILRMFFYLVRKVEESGVEMVCLPRLDTMGKRVVSWFNILIDY
FRDVPCVELPLYHRLLTAYIQYFVSFSFIGRLSSKRVLEDVQWSTSDVMAALLLSAPNLL
CMKGFRRPELEIYRNSCRDVFSAALLFKMIPFLTECILALRKTDSEESFANMSKYSILPQ
KEMRLDSAVSSAWDILFFDLSFYRNTLIALRQSLISFEKFRDCRREHFNNSLELWYLLIK
PSENKDVRETYVVHHYEIYSFFLIDVFSMVLRSSFLDSIDVAGASMLLRCFEVFSMASIR
NVFHELSSNDTFSRSSILDNIIQYLTLNWADEDGTVHIVKVFGKEILELAADFCLAVESR

IEKGDLDPAVTFQLKGLLDLLLNVFEGLQPVVEKCRQMHASCRKPNADVRTNSTPTEIKK
KLDTEGEKELFFKGIRRSCGFSGPLNLRFATENGLLPGTSYPAAGTSLGNEFPCLLAVTR
LLDAFFEKILEAYYSVWIPKCSRGHGMWLLSSSKFACINHPEESAVWECVFCEEVYGACC
RSNPYGGDGKKMVRAENSQSDFKFCSECYAFIPTNTVFYTLSMSGTVLCPECASRPFKRR
SFRFFAAYRTWTIICISCLLVTFFYII*
>TCRU_2609 Sperm-tail PG-rich repeat, putative
MIKEELPLFRAKLPGLYTGGGTGPGVGPGSYNVTSNLSKPIHGEAPFGITSERFYSHHGT
AVPGVGTYDIITNDYRRGDITVVPFFSSLDRFFCPRADEVPGPGAYPHDGNKWGRCGRSH
AMGAACSKGTENSFSIGPGSYDPNYGSGRRANSRAAHFGGYSGRDAPRFNDIPGPGHYDI
AAPDNSLYNNKPSSMFSTKTLRSTFGGKSVNPGPGSYNLPSYFQSLDEYRVANPESFSAF
GSSAPLHAPGPTDSPGPGAYTGDIAPRRPKAPPKTKKACTFGSGTGRNRMPPGTNIGPGA
YEPTKPNKLQKYQLASVPFRSQSPRFPSSKSTEVEGIFEPRRPVVRVPLRIHPPRPRDSA
INVAAKMSSRKAPLDPVYNVNYDWPKPTCTSGSYFGTAPRFEKKDSKTSIPGPGQYDISE
KPSLGGGGSLNGTWGTDGRFTSGNEVDNPGPGHYRYESTFYKKSFNSTIGSSEKM*
>TCRU_2610 Chromosome passenger complex (CPC) protein INCENP N
terminal, putative
MFRRGLVHRRYPFNKRGPRERKSWKHHVLTDPPKPIQWRDPKVWTKDLTTMKSFDAPQWD
LWQSRARSEDIDEALQPFMDMPQSLKDRRYDIPWWANPFGAWYLQNILSVELLKLPSRTN
AEKVAIYRNQKHSLSSKKKGEAAQDDEILANIIKERWRTLEFGDRDAGYPCTFSDYIQFL
NEWFKSLDEEGMQRLREHFDRRIRPLLAVMSPVDILWLEALTQNSPHNKEQLQRKIAFQT
SLGTPEFFDMSKRLRYEINEDYKVRDELGPELFALWSKAPERWPPERLSKMYGLDFTLVR
KILVWHHFKACYDACVEPDWTLPKRLFALEWIRDVRARKHGLFYGKMRFAEQKITFYSDR
FLFRDLVNRREASYANVWEMDDPYRFLQTEQDYEDYWGDNYDVYRRMFPEMIGKSGEPVQ
QYGQMPVWTGPHRQHANKSQHNWMFAEIGVNVGHEALKKLELDPTNEKRRRFVIRQPDGT
LRSAKMSEMRAWYWKEEWADFRFWAPNMEWGIENTPSQEQYQEHVPDTPDADFRKQRRIQ
SRPVKWFYESHYTRTGNFAGFQPLRFMQRRTEREVRWPDVINAAVQIQKRKPDAYIFKAI
PEI*
>TCRU_2611 hypothetical protein
MSTESFEVSRNEAVEKSGNVYSPPPFSYYVTRKEEDSAEEPISSTSKPGNAGQNMETLAG
AGELPLGAAAQPFSARNVGRFLEQQRYLREINRPHTISLWDQACVGHLFPGMFEGTIDAI
ELFHILKMLVMIAINSVTVAVNVFYGTTSVSSLEWAKPMSRVVFLIEFLVVYFFFFFLIV
VILHAIAYGNVGANLAGDIAALSERITSFSSLRVVAIVSPDFAINNIFPIVQNAVSVSEY
LRATLFFVLWLCACVLAMAAVVLKVTQIEFASTLSISSWTFGEFIQLVGLTMNLARVNDS
YETETQVLLDAVHRHFCSPGMPKDSFNRVECLYTRLCDYLSLPRPKKYEFFDAIFDYHFK
LAQEQKNIPLPMKLRKMLNYLAFILRTDNAVLRRLLQMSQSPLPFFQMGRDAFFQCFNEH
DELGMLVAVWRSDPASLPVKLFGMENKGVFNITYKCEDGRHAWDADNLSHILSHSESEKK
FHKDVVDACL*
>TCRU_2612 PRP8 protein homologue
MGISQDGLNMRCYTTTREGISWALENYAKALEQEPTEVAPKHFAPCRATLSEDLQGFLLV
PTGGDWNYAFKGATWRESTEFDVSADIPVFFFAAQHRPDHFLNFARLSEEEETIDMADMD
DTMA*
>TCRU_2613 Leishmanolysin, putative
MPIRINVSTRDLDDTMKYCISERYHAMNSLNGNCESEGDVVSSAHGIILENEFVPAAFKL
HADRLLVQPLEGSLIVPPFATGSVCIRFTVRAGIVPLVLPMLAWCCAWQSAVGGGALDDC
RDGCEEPRDIDDIGGMELQDGDGDGRTLESHWSQRHAKDEWMAPMGSSVYCTELTLAAPA
DLGCMRVKWEMAEPMRW*
>TCRU_2614 Mucin-like glycoprotein, putative
MTTCRLLCALLVLALCCCPSVCVADTQSGPEKPPNTTTTTTSQAPDPTKSGLGDDVNTTS
LSLSLPGSPSKEPVVERGKTVGGEEGLTGQEAPTVTEDTKEVKNGTTGPISGNNPPAKQT
NEESEASAQTTTTTTTTTKAPTTTTTTVPEAPSTTTTEAPAVSTTRAPSRLREIDGSLSS
SAWVCASLVLAASALAYTALG*
>TCRU_2615 hypothetical protein, conserved
MSLAGQFTTFSRTHEQPMNEESCAAFLMAILDVAPSTRLQYARILRSMLEMNRTPLDMVI
LGLQKVAARSETKQTRQARPLTEEETNQVIRSRTDWKERVVVRLAWVTASRLFEIAVLTP

NNFKLKPNGVMVLDWSVAPKTAKADPHRAPLSDRRRGQDAFDIIKLCRTLRRSVRLTNIT
NAQVERTLAPWDATAHSIKRGALRHAAQIVETYNLDPHVISQLAKHVNPFDLPQNTVRYL
GKYTTMLTQVSSRVVLM*
>TCRU_2616 retrotransposon hot spot protein, RHS3, putative
MDEVGPILRFIFNEKKCKDRFNECEGAVNSITGSMLQYYTGIGTGKSCESNHVSHKLVKV
VRVRKDNIESHLNILISPHFERETLAKMKREMRRMDFTYLVLSIWDYVIPQILEDEALDA
FLNEDFLRAIRSKIEELKLTGRPGSHSCALKVHSNKSFTRKEVLPENPSKRIAIEYWVLY
RPTAKNFPLVDGFFFVDSNPKIMVGLQITTAGEHHTIPSTVELFTERMAAYFKGWKTLSR
DMLWEIVYVQHKDNKPLDGWQRCGPADPNKLSRAENKIAEFWKKKVYQYRVGVSADDIKQ
RVSSVENDQQGKSAN*
>TCRU_2617 Leishmanolysin, putative
MISEVPNVRGRPKVSVVSTPNAKAMARQYHSCPTLEGIELEDEGGPESVLSHWKKRNMRD
ELMTSDVGVGLYSALTLAAFEDMGFYLANYSAAEMLWWGNNSGCGLLEKQCLTDGISEYP
DLFCNWFWSAGYDFCTYDRLSLGRCGIKWYDEALPEEYQYFADPRLGGDDLFMSRCPIVE
AYSDTGCTNGVPLMPGSIVGPNSRCVKGQDLQFDDEYVGDVCVDTRCGDGTLSVRFLRDD
AWHECRAGETVTPPSGPWRGSIVCPQYADVCTAFPNISGYPIPVVEPPLADDPTSAEVAE
*
>TCRU_2618 clathrin assembly sigma-adaptin protein 3, putative
MIKAVIIINTAGKIRLLNVYEKDIPLGVQQELVRRIHRLISRRSADLCNFVDNFRDWPTP
DTRVVYRHYATLCFVFIVDSSESQLAILDLIQVLVEALDRTFENVCELDLIFHSDRVQLL
LMEMVMGGMVLETSREEILRANTEMQRIGSSKAGTGAAAVTGYH*
>TCRU_2619 hypothetical protein, conserved
MKLVKFVNCDAPNVPADMLQLSVEEQRICVVSNQRSDGEHETKSWHLISAAPSMDGVYWL
LNDDNTPIGPINEILLATRDNANSARTKVPEGVKECHIDGVDLRSKMGKQLQRTREEYGQ
TFKTAARNAALLMDADRLRVGREEKVRKERKRVVEAIENGEEPIRKTRGKKEDGNKSNGG
SKKH*
>TCRU_2620 Arrestin (or S-antigen), N-terminal domain containing protein, putative
MGVFNRDRVEVELTLHGGGAAYIPGSTLSGVVKVFVKKKILCRGIRLNIRGEEYAKVHCG
GVVRQDTHIHYDRMYALARDVSNNNGALDSPSLLFPDSDDEVEADMRKADVLLPGTYTHM
FELILPVFLPPSFDVRDSRGVSEVRTTYTAKVSVDITSGFDVKCIRKFYVLSAIPEMQRR
QWMEERMKAALVSYAELPQYHVMERFLCPRQGRFVLTEVSISPTVVLMEDCVGLANSYGD
NGEIRADVRVFNDTQTVLKTVRVQIKNVVEMKLQDVKCVRRVNVGEPFIFSEIIHPGEER
SFVASIRPPLFFPYSSLEPHENDSVDFNRYTFLPLIPPRKQKQYILYHQPLMNMVTSFVD
SRCVVSVDFPGVNAKGTIKANNVLILATTVDGNNRARPIPCECDKLSPISGVILNNTYSS
QLHQYDHDRDSDEMRSESPWNYRHVKDRTGIFPAYTAGNWNPGKEALT*
>TCRU_2621 DNA repair helicase, putative
MYDAGSFRVPFPFQPYPLQLHAMGAIRDGLAGGDVVVLESPTGTGKTQVLLNSVLSHVFE
PVLNTEGEDTASLQKRSLPSLENAAPPPPSAEVVGRGHKRQRRQTTSVNQQMKDVTNEVD
EDAFLLEQNTYEEACERQRQHKIMGGASSCFSSSSSSIGSVKEEEENVAEWKPFLLRLQK
PKVYFSSRTHTQLQQLTEELRRTVFAKYLVRPRLRQHVAIGSEQMEEPLEPALDEAPHML
RYVHVAGRQQLCINSLLKNAAGNNNERLNELCLEAMKYEYSKEGREARKEHEIRNKCSSL
PDIEENVPLGEVPKGCGYCRRERLKILRDYVDISPRGISEMREIGQRVGACPFLATRELL
RGADVVLIPYSYLVSPEMRYALLSGTATNECETVANMEEVTTDQGLDGNAAASHVHNDMT
LRRGSILPPNFTGDVIAVDEAHNLVDFCRSATSSTVSEPDLSLVRRLLDAYYTRYEKRLL
TRNKQRIREMVLFVDKLLRYLHMKSVTQTNSVAAEVWNFSSFVFDAHVDNVNVYRFLAFL
NESRLLTKLHGFVAFLSMQKKEHAEQKQHVPIPLGPIKQNGGISHSAVSETFRDGSNNLS
LLAFEYNSKSVTSALYRFDAFLRWYSRSDEHTRVVLRRVPVEKESESNEAVLLQLLQLEP
GTHTFFPLLRQVQAALLAGGTMKPMALTCDLLLRPPNPHVAPSATTEEKKSMMNLENKPG
QSLTEKKVFFIEEGHIVPSSSIAVFTLGSGPSGHRVELQHATRHQWNRQFEEIAAALLNF
CRVIPDGMIVFFASYDVEELFHGFLQRSGFYDKINEVKRIFRESGRLASSKVATNSGVEG
KSLSHAVDVMLAEYTRWIGTEGSGGALLFAVMGGKLSEGINFNDELGRAVIVVGLPYANP
SDVELQLYLSHIANTRLLRDAGFAASKTLAVDTVTIDRSVPFNSSAEWSLFTDLCIRTVN
QSMGRCIRHADDYAVAILFDARYGERPDIRRRIASWMQPSIHVNRNFGECFRGVRDFFLA

RRLVPVAENVLCDSHRH*
>TCRU_2622 arginine N-methyltransferase, putative
MPSRKNLPPYELGQGQQRKKKVFNTKNVTNGACSPLTRTVANESLLATEGEKNATSKSLY
GDPAARISSNLYSISDRIRLRAYQSVLKGIKGKSLLHIGCGMGLISLMAARNLASAVVAI
DHSAIVDAAQVVAKQNGLQNISFFRGSLREMMGNFPVKKFDVILCEWMGTFLVNDPLLED
ALFARENLLAENGVMCPDGSSIHIVGVSDYAFYLDTVEYWSNVYGFKMEPMKGLVRQEVE
TCAIPASNIVTSTSLIHTVNITEFRDADNKDADESGFTVPFSVRATRNTTVNYLTFFVDA
TFTNPHDPGANFVIGIRPGGNNAWTETSVGLPQPLPLKCGETLSGELRVRVLNRERGVTL
VEVTAQTDGKVLAEKTTGSYTYQRF*
>TCRU_2623 RNA editing exonuclease 2
MLQRCRILWTADFRTSPKISEEFNNVLNRSGWGFDHVAIDMGALAGAAGRLTRDLIGQDR
DAETVKVLHRQVTNVILRRIQPKKSLAIFIDGSEPLWKVRHMRLYPGKKFEGKFYRSAAS
PMVYLLEDKFRQVAVDLRTPPKEFVISGAGVPGPVEGKMSAWMLDLASRALPDGAVTLSP
PLLSSTPAEVTFNDTICLIGGTDLFLSALGATPFHNITTLTLQQGEMKSLSMSESLEWLA
MDHLLKGEAATQGDQGTGRQELQKRLAAVRTDIVLLYLMANGISATDLSGLGVNFKELME
AYMAEESAGASHEHIFEETPNGTSLLLSSAVLERVLSRATRKTGGSPKPCQQSADYLEVL
LQTHAMICGGGIRNFCWTPDDNDCVPEKPPKIPIERFLAHLKHLTTLTREGGKGGGGEVQ
GDGAEKKTTAIEKEQCRVTAGVEASVDWTFALTGLETLLLTAPRAELIEQIVPVFTRGHS
LPAGIADDIVSTKNVMEAAQKTRNILQVIKGNNSEEGGGAVKDHPALDHHPSHYFVRTPG
CRGPPPGWSYQSINLGVRAAGFGVRYRTGVGASAATSRVLDGVGVASKNTLFVYAADKNG
VWSEEPCGEIHSPVLPTPTEELSTLRVLTWNVQFNRHSGERTPLGRDGIDWCTPTRYIAL
AKTMETLDADVISMQEVEPAWCKYLCEQQWVRERYALSCNEDSHAIRPWGVMLLVKRHLR
VEGMHHANVAAFAGHTSVMPEVTITVSKNVPVTIGSLHLLAPYNKNNVSNRTTQLENLTK
RMRARPSAGGVSTGAIVMGDFNDYANNYFTFPPELGFKDAWSLLNPENNKNEGYTIDGDR
NPYAGQIIEREFFGRADRVLFASRHLQPIHAELVGTTSVRALGITKQVNCDKAVPEYLYP
SDHFGLLVEFQVM*
>TCRU_2624 hypothetical protein, conserved
MAAGDGGNSPQQSSHAHSISHASIGERNDDEFRTENEEFIEEEGYSSVGAVFSKFLERVR
RTHPSRIAWVLFLIFLVTSGNAMQVVGLNFWLRSFPKDGAPGNFTTLAVSSLLFGLIFFF
MLVVYIIVARPSLAYLRCACGWRILFLIGLADAVNSYMAIYAASHTPEVLQALFTSLVPV
YAAGFTKWILEDTRKYWNVWILTSFFLIVSGVVVASAYHFAHGFGGDSKGKAWWSLIFFF
SVPPTVLMNIWQNLYMIQFTYDPSPEESHFFPPQPRRPRRGSMMFSDSVKKNGEEEFNAD
GNDGDAAFSPCETTHAPESSNGERHRFGEIEIPSTQSMQGTDTVVKLVMLACGTTIQFVL
IMLTLPMDAIPWWGGSKSVHDAWTNFYEGIRFVFHSSKNSLYCMVYTLGFLFTYVGSAYL
NQYSVTLCSMIGQLSSPFTALILIIVPKWNLEKGYTPWYLSLLAIVFLCVGSFIYTLWEE
KTDEEKRCGERRLKEKLMLEMQHSALSTRLRSNSTFIFTTEE*
>TCRU_2625 hypothetical protein, conserved
MVIGAKVREKSAAAARYWRHLRFKTLRRQLVTPHVGEICLPSASFEVDGSPLPPVRMKVG
TSHEELLRWFQLEYFGFFQSKSSNGGPENDRTNADVCFHVGPPKSLGYPYTLVSEVNNFT
EAVRRSEEYAAWEETDTAVGPHSTRWITQQLLDGFVSRRVVAHVGLACTNMSQTLSTACR
LQLELAPSDVSPYYFANDLLTSWGIFGVPDPKSTEFRTDDVTRLLQFAHASTVLPMYRAL
WINGAALCNEKGDAVLILGPRRSGKTTLALHCLAAPSPRLRVVGLENFHLADSSTLANIK
PSLDGFTALLMGIPASVKVGVGALLGTLRPNPTLAEAAHTFQLSPSTIQQLIRNNELTIW
NMGARHHIHIAEAFGRQRWCPTIIARLKGILLLNWDVRELSMPHARVNPQVMKWESKEKS
LGLLKTLAETKRGSLFKGHYLLRSLYDETTAMEMLENLFNENEALPPPLYELQGSVSFDT
AAQLVCNHILKQNDS*
>TCRU_2626 leucine-rich repeat protein (LRRP), putative
MGREQSADFEKAFADLLLAGLRRQGEVHGDERVRSIMRRVLVERGGDGREETGTVASAVF
SLAGAGLGDKGAIAVAEALLKLGSPQMVKSHGVRRWTVLLNDNGLTDRFSAHVSLLVSRC
APPVVLLDARGNAMTPAAIQRIEEARELSPAPCALRLPPTAGTAGVAAAASLRESASSAS
TAGGWRRGLDAEALRKRRSALFASGAASSTAVGSELGGRGRPRSRQSEGEAPRDVAAVEV
AARAPSSASGPAPEERSRRMEASGRRHSAGAGKSEKREEEAPPRGGDCGEPLDETANLSP
LLAVVLDLSNAVPTGRLQALYGTDDIWGVVASSGGAVSHAFPPKQTPFLRAGIKSTVSFH
SLTVLLRANKNASKKRKKKGRKR*

>TCRU_2627 ATP-dependent DEAD/H RNA helicase, putative
MSSRYRELIAIFGEFLEENDFHVRISMDRVGEVKTLFSAKTIPVESETEAAIEVVTIEAA
RRRDALRRTCNAVLRTRHSDQHVDVGTIVADEEERLAWKFLIGALAAKNSEILPVVEAEL
LLAAVEYHYATARPELCDMFVHLSASEMFFIDGDSLFMAALSPQSVDWDLIQPLHVIYNA
QKLLYDMHCRGARFHVVFFDSLLWIWESAPAKLFMRENLRKTLMSLSENNAKVGLSISNF
SSYYSEAFETYVKQWEPEFILMSDGEQLGRLNPLQAFFVRPSAANNTHDSRRKANYTDAD
EANPYRLRRQYRSIVEDEAVGDKAALYYRCIHLWAATRRLKVAYSSRIIYKENAMVVFTV
RVDGASFERAVTIESDVQALAQSMEHEVQLPAISSASLGLLDEEDLSCRERVVYAALHAY
LRASARSEQEHQLCQALAITTYITGYLNKEARAQQVRPNPILAGFLNEISPFLLAVWRHA
DCTNGNGQEFDLIDGHLFSAVSQQLRTASVSELFDEYGVEDIESTWGVLDDQGSADIVNA
PLSYLPVIDSVDVEKLQPYPLITHELVERLAKGFGISTHRADAPYPTDFGAANELAGWDI
TTPFDRLNDVIDAEGDAIAKSMMTEKEAKNVQEYYKKFVRNALKQAQSMGISGFAAHELA
MVCSDNDSDDDAGGNSANNKTAGAKKVNKEHAGQRNKKERSKEDEIRERSNVIAATATVA
EWHKQMNHLLHAVDMSHGRTTNRDRDESINTIMAAIKRLSQEKFGKNFDPGYTLGGSTNT
AVPLKLEMWRLLVAASQLREVEFAFAMEDPALKDSKGASKKKDSKSEYKMLYGFHVINQF
VEREAVKGNQWGQLDPLRKAKPDMTIVEARSYLRWVYLSFVEMHIQLKLKCRVVKLQLEN
WRAERERARLAQESPKIALGIPLFLYCHHHVLAVIRDEGPRISSEDIDTVRSALKHFDLP
DSYYNKLDQCIARWQNMTLGTLLPSLLPQDKQLFETPEMLQLIHMGHLLERPFVREHDYR
VAFNPDNWQRELLDIVDGRGSAVVCAPTSAGKTFISYYCMYKALRRTNKKVVVYLAPARA
LINQAVADVCARYGSKKYKNPGRYIYGALGGADYHQFHDSCQVLLTVPETFETMLLSPKY
TDWVELIDYVILDEIHSMESNGNGDVWERILALLPCPFVALSATLGETQQLCSWLNRVQG
RLKEQTEEMSGKMRDFEVHLLPSEGKSIQRWNDIKKYIYLPPPGAALTHKKIKAQYDNCY
IRDLHPLSILTADQLQRGFPPDISLVPSEVVSLFEKMHSKFNEVVWPNYSSLQLAKTLRA
QLMLIEPSKYFEAETYITQERARQYEAEVKNAFAYWAYLGHEGCELPENLVEEDLDDFSA
SMNMAVESILRTFAQKLNEDEALLERHAADGMEKKKRMLLRQQHLQLLQQQEQENEPNQE
ESMEQKSEEQGGAQEEEQEKETVGSVSFPGSRQFIREHILNVLRELIARDMGPTIVFSFE
SEDCGDLVKYVVEQLEEAESRYRKTNEFALYKARIERAAAAQEARRKQRESTLKQKRLTT
GDDGDVEVADRDMSDGEGEDELFVVPDVLPEFTFIGEKCTVEPEVVDSLMEDCEKEGEDL
LLRALQRGIGMHHAGVKGKLRAHVERLFRGRHCGVIFSTETLALGIHSPCRSVVLAGDHI
LLNPTQFRQMMGRAGRRGLDYLGHLVFLGITMRRIKRLMTSSMTVIKGNVQMDPISNLRL
LQLYDFNTLRHLKNEAGWKTHVLKLAERLFVNPLFFQGRNSVAGGNMEGFTVEWLQMLLG
YFQREGLHFSDHASSLGSILQDAMYVFREAHVGNEGFTFIRMLTSGVFDKAHYSPLYDKK
LNSGVLDEPLAELLAYLFSTHQTCGVPLEMHRSALLDPAVSTLWEGKTGPTQHRAVLSPI
DVCSPTIHAFDNTDFFALLSAFYNYLASHLAPQTGAALRLPCMKSTNKKCRIFGGGSTEF
LLKQKLQESSVPYKARSPFVAISGCGDLFTSVDDVTFTLRDGLYCDRTLLPILDLADGWR
HDGAQILINACLLDFLRAKAQIDTTRKNYRFTLLEELNGLSQSLSYAVLNRAEKILSNLA
GLVRPTKLPRAKVLTAIMPDESEEGIFMAGAPRLLEVAERLNSLQPQIQKRLAEELLTAK
WAKRISEMNAQRKD*
>TCRU_2628 GPI inositol deacylase 2
MVFALSEFGEHKGHKHTHGGAVAVPFMGLEESHDGAAHYVRLEEGHDGAVVLTKRVVRAF
VWLAAVAAVIFAGSMLLFQIASRELIKKNRALSISSVVGNYWLNYERVYPVRDEETERNG
EEAAWPYALYRLFCTCERNLASSGKKAPAITSSKLLVFFIHGNLGAYDEASQLGCVVSRL
ISVTSEVYTFDFLQQANIHRGKLLQQQAAFVAHTLGLRRWGSESKNITSAMDFQGEEMAK
STDHLEKQDDGTGVWIVGHSMGGVVARLAVEMMRNSGTSIFLEGVITLNTPHRYPPIFMD
AAMFRIYTNLWQPSDRDLALKNSWHRAPHFYSLTSGSLDLQVEQWLTNLAGLDENRFPHT
MFRTEDPTVCGKSLSHTDIVRDYCVLEFVANIIAKKITGLRHTPIHARNPLASFSGGVTA
PLWWANKSVTSHTLPVAALSFYTALVLSAIYPTVFPPMAKQRFISNHRISCFCWGHQVAL
PRGIPTMGGAVIGSLFIGICSRIFFVQIRCFFLPCSSICGLPWVADDIACISEARCSHLP
SSFVAWLGPALLGSWVGLLVYYLICGLLRLSLLIWTVLGSCHLYKTHLLRHIRPGVNMWY
CFFMLVYPVTAATSTVFLELYQRTIVWLAFILLMMPLRVFVENAAANTPQPTASADASAP
VAVYALLHALNVQPTFAWKNKKLSGTADSNNILDECIRVSEVFLHVLLLTHFLWPIFRSF
VLQKSGHLPLLKLSSSRGSFLCMATSVVFILIYMSLFAMNGLPVEVFRVVWTLLWGFPAF
LFVSTVGLDGVLVERFAPTSLRVI*
>TCRU_2629 hypothetical protein, conserved

MLTSDLQRLQRLFAYFKKNTSSVLGGCCALIELIDEDDLTNWYVLLKYEDENKKEFFVSL
RIYFFEELQRMPLVFVIAPRIIATFIHHGAICSLELMSQQWSVDAEGIALLIRSLHATLN
PFSHDSRVAVDELNSGRDGGNQQNRLLAAEGIGATAAPAVSLSAYTREEHELGIRHIRQS
HPHLFRACTRKDSQPQQQHGSPAAKSVMNSTPSPNSISLSTSYAYHGGAEKAATMKAIIH
SQEELQELFLAGGLVDAELDVGYHCSLDGSLRIGVDAPSVKCKLPTAGNPILNYTMKISP
YGWEKNDEKLTTTTIPKSGIDIASDTTQAVGECSLTAHHSDVPHKWKGEDRKKFVSRQSF
LTPAISADDAEQENKPHYHSKHRGKEKGSTCMGIERSKERRKVVLFLEGEAVMDTGDSNT
LSNAQEGRRPEAPCTIPAGCTLRLEHLTVYGAIVVLGTLEMHDCKFIGHFSSEGEGRVEI
SQCRVCVDSAETRREGILVLDASTLEIRDGTTVRRREYTFTEGGESVPVCAAAPFLLQAF
ILISNSGTLRIHGGCHIMPYGTEKTIFAEQDASVDIADCSIMAGYSSAVSILGCHAFFSN
TRFYGADCVELQVSDLNRPTGLNVELGGLVTARHCVAEYLYFGFSVIAHSVSHFFGCHAE
HVVNGYTIDASSVTIENSSARSNHVGVFVLNKAKCNIMNEDDHVLSFDERCRLLLKSREN
VERQRKFSFVETHLGEEDAETSSSFSSVSSCPIVLQRSPAAAVGDEEKMDHPNVGISALA
DMNRKLFMSHMFSGETATAITASASIFPLSFRGGLFSLEVRDASMVAKGLLLVDAQETSI
YVYDQGVLTLTDVIVWKTLASPQQPCGVKIVNSRANVQRCLVADYSFGFAAIQGTVVRFE
ECMAVGGTNGFTVDDAHCTLHHCGANTEHVGVFALNRSTLRMDNLNAPKCKEGWLSCVLK
GHLHGLESRSSDTICIGVTVQRSLDSGFTCYNGGCLRLEKCTVDISTSGSTETNIGDISC
RHFSPSGRDCSNPAFSSGAAAAALDGSEGGLNRAKIGNTTKANSPHDEMPGTSLAYSSQK
NCGVKAWSGSRCLAIGCEVRHVTFGYAAIGPDTELEASHCTAKHIVNGFTVDSAKCRLSR
CSTNSNHVGVFVLSHGTCTVRRGSYTARMYSIENRNGIVHIEGHVKLSGFSRIGLYVYDG
ARLDTLTDSMLDIKVRKDHAGQQLPPELNLTGPSASPSGLFPSCFAIDKGNAVIHNAFFG
GGAQCAVSCGDGANVYLHQCTAEFCNIAFNALSGSQMYLSNCCARDVRHYALIVHRGGVS
RVNRQSPIPSAGGILQGRIRIEGKCFLEHVILHPTTDVDSEQFETIQPSLPLLSSSSSSS
APLLPYGSEFFVEKTPLNPSLPKSNFLIYVTLNGTLVMDCCLVLLEEHHRVLEEDGGGGE
SIANSVIVAEGCRATVQLRNVLFQRLTAAGTGSLGSFEGSMSSHGSVLLPSNTHGSVISR
KKTTWYGVVLLQGAKGDIHGFSEAALISGSASTIRGAAADNPLEASQARTHFSPRSSVEG
GDDAISLLENHNVPTEYEFLYRSLKKGRSVLSVGEIVPLRVYLTRASSLTTENVNVRKLV
ASGGSTIDAVCCFFAGADAVILSGGSTFRAVESRFVSAVFGPAMRVESATVHLRKCRGYS
THKEVIVARSATLTFVESLLFPLNLVNNETSGTSFSAGERWLGISSRNDEECEIGKGDEV
LILPHRRRRDGGGGNEGAIVPKSAKQGDTSMPKTAVPTCLALENTPIERRVAVPLGVPLL
SHARAEETRSDTLPQDVKCLQPHDDGIVDHAPLVLEGVQNELFRGNCPTEEGPRRRHIST
REAGVPLLQCKKGSCVTEEALELAESETGSYLGRRQHFDVRLEHSYLYCCGATRIEGGVT
ADAFSRIEASRSTWFSGLISAARKVLARVYGT*
>TCRU_2630 Aminoacyl-tRNA editing domain containing protein, putative
MEKVKRYLCSCGADYIIPKICEFPESCATAELAAERLSCSPSMIAKSLSFTLMGKKGGVD
CMPIVILVSGDAKVNNNKYRNKFARRPVMIRREEVEELVGFAPGGVCPFGVNENVRVYLD
ISLRRFETVHVAAGTANSTAPMSIKELERFSGFVEWVDVCDGWTE*
>TCRU_2631 hypothetical protein, conserved
MRAVASARINGFFFSNSLAGVSISVRCLSLSELAKAFTELSESNGAPVATGPAPAPHEAR
VRAAFDMKSAPLTLGGNDLGPLDKASGLRLGSSRDDSNNGAVTFSTAYSERLSNMSGAQL
AFALEKQCRQQHVGQMLQCLVEVWRRLRETSLMSVIITDEEARGEVGGNNVAFTSFRSDR
LLEGVLRALAKTNLDEVQQSSRLVRELGHDGISVEWVTEFGYLLMQWLAARAASLSPFCS
ATFLHLIAQQKVFHSDAVLETLRDNIEVHIARDAVRQAGIAFSSKISSSRVTSAESVREF
DVCAFAVLLDAIARWQAKFVRLLGGDGLGPQIRLHDGRKVTETITRRQHPILNASFYNVV
VDGLVRGVRDGSLHLTRQGSPSTFFFLTLALAKIRWLRADCVEVLLPQLHEALRVFSGQY
LGVVFLLGRREVKTCNVETTDLLLQTLLDAMQKRGKRYISADERRRHSSDAVALPTSLST
TQSADASSWISTSVGHEVDLSTAMAIADMDTDDEDLQLLSASPHLESVGDKGVRHFSETE
AINSGVSSHSSPVVFATSFMDIRSMPVLLDSLNHILTTTLEHCSAQGQETQRNAVELKAR
TLYEALLNDTHGGVKSLYALQESPVLVEKLLSSVLRIPYETHHPLIIELAYVFTRHVGAR
HVVPQDIRKSTVAAPHWQWRTMSLVELLVQHGVILPETYVMTDSVIQFAPRVLSAVEAEK
KRLLERHHERQQQCGKQKGREKKKKTVRTSEVFARFSRVVLQLKQQM*
>TCRU_2632 hypothetical protein, conserved
MRRVVAKSVLSIRRTPRWTAAHFATSRPLLNDITVPHDVTGENQTRNEGRAKPWRHQRGG

GPKSSTHGHNGPVKDRFPPILTDLSDPRRRLAILLDASSVEPRVFYNTVLPAAKKVGVPV
LVRIFAVQLSSEWESHVAGSDGAKMSGRDLSSSSGAEAGEKTKICNEEQEEKTTGNTSLC
PPVEFFRVERFIPVSMQMEADANHIFDFRRQNKIEAVCFVCTEVDRGVFEGFLPNIAGNG
FNQYVLDELGMAKEVLEDGRSADGSP*
>TCRU_2633 Protein kinase domain containing protein, putative
MGEECPSLPSDVSDRWMRRCGVREIVDHLIMDLVTAHPEDVMGFIHEWSAPRRTGGPMHT
GTSYPDGAQREEAVSSERRQSHFSEELASFEEDKGCVEDENKDAGARLVRATEPTVQELS
SIYPDARGSPFTPLLSEEELEAYFRHSCPVSLGKSSLGICLGRGNYGFVIPAIVKSEAKE
GADQKDENEYQSDTNALTGVGNGRLVAIKVSRIQAIWSLDEAESLRRVTVCRQSLEDEVQ
MLQKELDALRDAEANDALQSQQEEQQAAHPGCNVSMSLLESRLHAQSFLLQGARLVTSLV
GDIMYDVRRDAFWFPLEFYPSSLRECIQARSKLCTGSPPSGSSFPDISLNDLGETVMALL
FSVQEIQHVARSLCRALQFLNVSCSLCHLDVKVDNILLSTPWVPGEPELGKANSPPAGEL
SGAQSNTAVPTLPQVVLGDLGLAQPLDAPIMQLGDFSTMAPEVYWASDCGNANYAAYRTS
VFCASSDMWSVGCVLLQMINGLEYSSWGPSDMFAALEENYVSPALRHPEVWPMELNDFIA
RCFERDPRRRMRAGEALRHPFLLFH*
>TCRU_2634 ATP-grasp domain containing protein, putative
MLRLALQRSGVAIAACYAVPSASMCGTAWSAMMVTAARAARFESRRYKASSAPATTPQKS
AGSSRGHGSGTSHAGSGQVTGSPSTAGHLSGNSSHNGATASMTVSHASGTNNNNSNNSNV
CNSGGSDGGGSSGGGNGNMNNSGGVSWLRRKKQTKKVLKRKAGLKKKQVKKSVSVANTAS
AHIGGGASRNAASGGANTTSSTTPTVTASRAVNSRRIKRKLEPKKKKMATPKLRVCVLNS
SYEGSDSVTADIDNYHCSPAHWVKDKKRYTFTEVSLRKSESYLRVRELVTSNNFDVFFNL
CDGGKDEKRAGVEVVEALEEHNAAFTGTDSHSFEPSKIDMKLLVGAAGVNTPNFVLLEST
EGLAKKCRHLRFPVIVKHLSGYASVGIHKDNRCDTLDELKVKVRAFLAEFNHALVEEFIR
GREGTVLACADRGSLFGVKVFKPLMFKFLQNDDDFAYFEKKWTMECNENAYSFLPHNDPA
YPQIIDTARNAFRHIMNGVGYGRVDFRIDPKGDVYFLEINPNCGMWYNQKDGGDFADVMV
QGDKSWDHDRFIRNAIIRAVREQAARRPWYFISHDSKGNFSTRASKTVRAGKSLFSDAIH
PIPVVAQALYKLGEEDPTVGCVILRGDRPHQAVAIRHSCEPNMQFMQGRTLTLVSKRRIN
VGEELTVDYATLCDENMPPFACSCGTSNCRSVIFPSAPTPRTLEGKNIRQMLREKKKAWY
KEKEDREAERILKKRASKGKGNSGSSASSPSSSPSVSSTSAAGVSSNAATGVNGSSNSGS
VAHGINSKK*
>TCRU_2635 CHAP domain containing protein, putative
MIHTARKKQFGLSALALFVLLLFLLVCVTLGLHWWRDTSGPALHPPRSRFDPLAHCATPH
GTLLGYALDIPAFSNCHRRWNSKSQAFVTLEHPHDVRQEPFLLGTLRSRSTGVRWVAAEF
IARTYYLHRGMSFIYGAPDAPEYWSALEFHGPMVREREYETLELLNSAEATTAKERKRLA
PRVFDVVVWSPMEERDLPEGHIAVVVQVEDDVEAAGGEDRLRELRKLRLQPRLLYIAEQN
FDNTHWGGKNYSRVLRFQWRNGREAVLQDPLGLPIIGRVRPGRPKQNTDVEDDL*
>TCRU_2636 CHAP domain containing protein, putative
MAAGFVENKSLGRVACLAFVLICGVLLLWGWRGNKRNPLSAEGNPLFDPLKHCRTRYGTI
LGYALSVPAFSNCHSSYSAERLVITRFGYPELVADHNDGSKGNYCSGSPWSSMEYTARIL
FHYKGIKYFEMPTPQEVWSTPYFFNPLEAAERDKERRYEPVRVANYEEATTAKERKRLAP
RVFDIVVWPAQMEHELPEGHIAVVVQVEDDVEAAGGEDRLRELRKLRLQPRLLYIAEQNF
DNTHWGGKNYSRVLRFQWRNGREAVLQDPLGLKVLGLVRIGKRTVIFEDEDGGDL*
>TCRU_2637 CHAP domain containing protein, putative
MIHTARKKQFGLSALALFVLLLFLLVCVTLGLHWWRDTSGPALHPPRSRFDPLAHCATPH
GTLLGYALDIPAFSNCHRRWNSKSQAFVTLEHPHDVRQEPFLLGTLRSRSTGVRWVAAEF
IARTYYLHRGMSFIYGAPDAPEYWLALEFHGPMVREREYETLELLNSAEATTAKERKRLA
PRVFDVVVWSPMEERDLPEGHIAVVVQVEDDVEAAGGEDRLRELRKLRLQPRLLYIAEQN
FDNTHWGGKNYSRVLRFQWRNGREAVLQDPLGLPIIGRVRPGRPKQNTDVEDDL*
>TCRU_2638 CHAP domain containing protein, putative
MAAGFVENKSLGRVACLAFVLICGVLLLWGWRGNKRNPLSAEGNPLFDPLKHCRTRYGTI
LGYALSVPAFSNCHSSYSAERLVITRFGYPELVADHNDGSKGNYCSGSPWSSMEYTARIL
FHYKGIKYFEMPTPQEVWSTPYFFNPLEAAERDKERRYEPVRVANYEEATTAKERKRLAP
RVFDIVVWPAQMEHELPEGHIAVVVQVEDDVEAAGGEDRLRELRKLRLQPRLLYIAEQNF
DNTHWGGKNYSRVLRFQWRNGREAVLQDPLGLKVLGLVRIGKRTVIFEDEDGGDL*

>TCRU_2639 CHAP domain containing protein, putative
MSFIYGAPDAPEYWSALEFHGPMVREREYETLELLNSAEATTAKERKRLAPRVFDVVVWS
PMEERDLPEGHIAVVVQVEDDVEAAGGEDRLRELRKLRLQPRLLYIAEQNFDNTHWGGKN
YSRVLRFQWRNGREAVLQDPLGLPIIGRVRPGRPKQNTDVEDDL*
>TCRU_2640 CHAP domain containing protein, putative
MAAGFVENKSLGRVACLAFVLICGVLLLWGWRGNKRNPLSAEGNPLFDPLKHCRTRYGTI
LGYALSVPAFSNCHSSYSAERLVITRFGYPELVADHNDGSKGNYCSGSPWSSMEYTARIL
FHYKGIKYFEMPTPQEVWSTPYFFNPLEAAERDKERRYEPVRVANYEEATTAKERKRLAP
RVFDIVVWPAQMEHELPEGHIAVVVQVEDDVEAAGGEDRLRELRKLRLQPRLLYIAEQNF
DNTHWGGKNYSRVLRFQWRNGREAVLQDPLGLKVLGLVRIGKRTVIFEDEDGGDL*
>TCRU_2641 hypothetical protein, conserved
MLCVDAVHRDVLVAKRLAACSETPALGRPTLPLASGSLPITVSDPLPHSNGTIVVTICLE
RAFAGNRQANPHPASVIVRMEWEVFFSVAYPKEPCVWRLVDGLPAQSSTDVDYRVVRLQS
FLMEEVLSRLPKAKKTYLAAYVGEAGGGGGATYHRSSRGDTATLRSQTELSTGTTLAEMY
PGLGSGGRNNKNNKKRQNSSSCSKQDNSHNNGNNSNSNKGAVGVVVSPFLSDFAFLCSWW
AALELDVGCIISERQNFILRNISSGSMVLLEEDAGSRSYIRQGRGVHFLDEVGGNRVQNP
GRGFAAVFMPRGDVVAWGLRQLPQIAKKKKEEAKSNTVTGTANPSIFGTASHEMVPQRCG
GSWSGYCTGYMETSGRPGYHNQDDQYHLQSRRAQQALRITSVRSDMAGAVLPSLLVSSNI
LRTGVYELNDICLYANDPSVALCQNALILREGKALKDVSNAFLMLRHLIKGVSPHAPSRY
ALQLLAPALHELVKSMQEQRKPFWAGVALCCVLLPAILNDRPMPFEITAHLIDPVECYRC
VSIVAMIFSTVRATNKFREAELTRSAILSAYRCKKPPMSLIRKSVDCEESKSNSGKIPPV
YQIPITECAVCGMPLLRDTRRGNGTATGNPFNTGLGSGDDAVPASARKDGCIIVQCVRCG
HGGHVAHIIAWWNDKNVRCCPKGCDCLCIY*
>TCRU_2642 cytochrome c oxidase assembly protein, putative
MSSDPFRDAPATGGDGKGVVEGIRQRREKLELYQSHSHKMKSLMMNSAGIRSGLSTKNER
ALNPIWTNFPTKCVKPIRSVEEMREKLQYFTKSDDVMVVRYHQNRCTACNAVDKSFEFLC
HQSADRLPNLKFYEINKDEVPELTKNLVRYPQVKGFSGGHWTDLEFKPPADFREELYMAV
EREVKRRKDEARPVTALEAEEMYFSAAGPSMLEVLEESIVKFYAKSQVRLHNYWKQVSVR
RSWFFKKFIEPKVEERLREEWQMKSLFGEKIMYGPEPKIEEF*
>TCRU_2643 50S ribosome-binding GTPase, putative
MSKALPPIKPLSPRGRDAYNGSGRPRTALQTPLTPPEGNARVPTATTTTGTAQSSTIRFL
SREVGFSLGPVQDRFLPPYNDLEDPHLAQYWARREVLIRELAERRHELQRQRRLERRRRE
MLRRRFERRRRRELEDLSAKKGYASQRREIEEEEQERQRSARSRQPHPPQTNRSFSGRNR
SWGRGYDVSAPNNRPATASCGAIMDYSKKTPPSQRQTSARKANQESSLHKAVHHRPSREM
PTETKKTAKADTSAHKKRVNSAKAVAAAKIRAAAAKAAADTLEREPSESQSSLNTPIKCK
YPQEADTRTEAPAQPKSFKKADVFSNFTASTSSSSLSSSSSSRRFRRNGRRQRNNKYNSN
KKSAASARSAKKRAASPLSSKEFQTVVNARRTTPRYDEYDDDYESETPASAKKRPAVLSS
KEFQAVVDARHATPRNDEYDDDYESETPASAKKRPAVLSSQEVQAVVNARHATPRNDEYD
DDYESKTPASAKKRPAVLSSKEFQAVVDARHATPRNDEYDDDYESETPASAKKRPAVLSS
QEVQAVVDARRTTPRNDEYDDDYESETPASAKKRPAVLSSQEVQAVVDARRTTPRNDEYD
DDYESETPASADEQPAVLSSQEFQAVVNARRTTPRNDEYDDDYESETSASANEQPAVLSS
QEVQAVVDARRTTPRNDEYDDDYESETSASANEQPAVLSSQEVQAVVDARRTTPRNDEYD
DDYESETSASANEQPAVLSSQEFQAVVNARRTTPRNDEYDDDYESETPASAKKRPAVLSS
KEIRTQVKYKASSSSSISRSSTSSRSGDSDSDESGRGDPYHELGDASGKLKGNQKKKVTA
SANGLSPGVSLNKSAKFSTSSPKSNKKGLSENHMVKDSSHVHDEY*
>TCRU_2644 hypothetical protein, conserved
MAEAASCVELEAVFLQVSLPRETRHLLRCRVEDGTLVELTRLPMGHKAGPEVLQIIITSA
IAGVTTVVGPLWAAPPLVRIDVWINNIRIAGSKSDATLWEAQVLFNADGRHATIGEDRES
APRSTPSCGAV*
>TCRU_2645 Reverse transcriptase (RNA-dependent DNA polymerase), putative
MHCNAPKHRTATDFVGHANAFDTVYHEKIILEIRQLSIPNHIVRWSAEFLSGTGAVVRID
KLASFPNTSVRGAPQGTVLGPTICSIAMNSLCSCLSNAPSIRHGLIVDDPTLIAQHSNRD
TINSAPQKGLSAVVEWTERYFMKANAGKTGCTLLGNSNTAPLRLA*

>TCRU_2646 hypothetical protein, conserved
MDSTERHVTSQTQKAARATVPERNRQPSPRRRHSAAQTPTQRSATRSIQKPLAHAPPQAQ
GHGRQQPADSTPCVRSSSPERASHEIRFSNAQQTPGGQPPHEAAGGKSNRTPKAIPAATD
PDECKGSRPPITMAELDRTLRLLPLGLAPGPDEINGEALRHLGRVAKRAVPRLFNRRLRA
GTVPRNRRRMVVIPLLCNPGKTASDRDPPPTCNDGQLP*
>TCRU_2647 Endonuclease-reverse transcriptase, putative
MTAEGLDGQLCAGGPQINGADVKAHTEAWNRSIPSDPRGDTIARRCIENVLQVVNSGECT
RYAKRQGTSAPNIALSKDCAVTHWAAQPSTDSDHCMILLDGLIGDDDTPAVVPRRARKSL
AWKKAN*
>TCRU_2648 Trans-sialidase of Trypanosoma hydrophobic C-terminal, putative
MVPNASTERPSFANGPRILVASNASGIMSVTYGGREHVAADNTDEVSEHNATGHKSLVLE
NGVAGSGFVAEGDADGLDQRNASGHENKPQLREFTAQYSGSGVSPDDSFALLNDAKLALR
ELTVDGTVRVCVYWVLLLLLGLCGVVAVL*
>TCRU_2649 Vacuolar iron transporter 1, putative
MSEAKRIEVKAITDYKSTARARTAFLKGDLEMSRREHAKPLPHEEFHSSTASDYIKSVVF
GGLDGIMTTFAIVAAAVGSNNSYVTILIFGFSNVIADGFSMGFGEYVSGEAERENALAER
RREEWEVENAFDLEVDEMVQIYEAKGLSHEDATTIVNIISKDPKLFVDFMMTEELGLLLD
LSDVHGPKKQGAVMFISFVMFGFLPLLAYVPGKGRGVDVVFVFSCLLATVSLLVLGSLKG
YLTGIGMIRSATLMVFNGVISGLVSYSVGMAMESLLRARIGDGP*
>TCRU_2650 hypothetical protein, conserved
MTDADDTLADYWAWCAQNGVVSKKLFLRRGLHGFMPQFSLHVAESVRAGAVVATVPYLVT
LNTQTIRGDMKPAAVPPIHAMCTFLTRRRRMDLVTARSLWLASCLACYRRLLAQGHALSI
APLLSRLIMPELPSPFTSTHIRCYPAIVASLPEGVDVAALEESVFSSLWKRVEGQLRVTH
AALHFYHQRRVTNIPRHLVPSFDELSMAYRMVLHRSLLLPIDCVPSSPGDLADLFTEQPD
LDLLPTLVPVVDVIRSSAVARTLLEAKGGGVRGSQDDSTAANCALYTCTQSDFVSSGTRR
RVVFETEPLSSRRVVVCATRDLKEGEELLLDYGSE*
>TCRU_2651 hypothetical protein, conserved
MSGALGRGSYRSVVAGTRNVPHRLTYYPCAYELMQLHKAHKEVIRHFYVRDKIFDNKFPT
TALANGLFKFVPNRRESYHMREVMESIRRRSILMHRIQQQRAINAKVVEELEKGYGKESA
AAMLCFTTPDSDAYFNPQRYQSVANAWPNYWQHPSTSHVVPKPRWRRVPELGGITRVQDP
LTEQANDY*
>TCRU_(FEN-1), putative flap endonuclease.1
MGILGLSKLLYDRSPAAIRERELKNYFGRRIAIDASMTIYQFIIAMKGFQDGQGMELTNE
AGEVTSHLNGLFARTLRMVDEGLRPIYVFDGKPPTLKASELQERRQRAEEAQQLFDTAKE
EGNDELMEKMSKRTVRVSREQLEEAKKLLQLMGIPVVQAPSEAEAQCAELVKKNKAWAVA
TEDMDALTFGAPVMLRHLTYSEAKKRPIAEFHLDEILGITGLTMTQFIDLCILLGCDYVP
KISGIGPQKAWEGIKKHGDIETLLQSLDAGRHSVPEGFHYEEARQFFLKPEVTPAEEIEI
QFREPDEEGLVKFLVEEKLFNKDRVLKGIQRLRNALTRKTQGRLDQFFTITRPVTKLNTC
DAKAGLKRGHSAIALSGTLQQKGSSGHKKVVKK*
>TCRU_2653 hypothetical protein, conserved
MDVSVVLAADVLSSSDTRHALILVGQRSVAGGSGNQEQVVCIVSDAFPCTDMDVIMEQVE
CLEQVLPCGIAFLGVFLPGDGVKDLAALRHSLSSHLQVSFFFVAKYDNEGRVQCRLLQSG
RMLSVTTPDTKPVLVTLACYFFSPLGQFPFIVRSKDENITSNVVLDVTSTVLMEHNQIWD
SVDALYAVQLGSTEGQEKDMLCVHVTFFPFLSCGQGVYRAICTLLPKVERRPQCVMVRVG
SRRYPSLVYQWIFTPTERGSAALSVKQWDELKELIEDGVGEEVQPSQIVTDTFLGAPSAT
ASTTIKGSNNMRNKTQASEVSNTFQREKAKGDFLRYMPPLLVLFCSLFLYFCCTK*
>TCRU_2654 hypothetical protein, conserved
MPRRDSVAGEGTLPGIPPSAPGGETTNGGPDREGRRAESRDNDDGNGDVAAGNSTARVGE
EIVEPSIEDLVEEAEINLDAPCANLPLLRAQELKIIGRCVTFLGETTVEGERVLLHYEGV
VAMITKDTVMLLQVRRYTKEDFLKYQEMIEAEKRDEHKKDDHQELHGEMDRNGNGRAHAG
TEEMMNFHAASEATSNDDVHHDMHIVDCNILAAQETRENAEMPEPDPPLTRQNRLLGTRT
MGPVPFMTFSRRRIHNVVFGHDPQNSLYSIFQNPSRRYFDMQCLRMFVRRYLVHTSQGNN
PRKINLRPFIEWRCNCKGIADDLLVNVAKQELAYLIKTEREMAKAAERLANNRRNFFHTY

QPTHRLFRATGILFLTRIPAQTFSLAILEMLVSLLLLGFSAYSFATAPPILIYGYVKDYT
FSVTFAGIVSLLASGMTAFHSLRMRIPLTFSIHTALRLVAATVAIALDTMALVMIGGAVS
YSHILGYLFRLAQESAELCYYYALHNCTGFGEICGPDNMSPICMQHKCPSNRTTTCTQPL
TATLIRIFIPFIAISMVLVALFMIDHLLHYRLLQASRLLMARM*
>TCRU_2655 ras-like small GTPase, putative
MDHVDVAGTRDMGSAPSLTALQVIIPYGSNSNNNNNNNNNNFYSGGVCFTRCPSITANVT
RSLNVSCGGDGSSFSNVWSLAKPTLDPSQIRFCPEGCWVPDAQVMTCMAHECNVSFSLFN
RKHHCRLCGRLFCAACCSHVITIRSTRGGLSACCALSGAEGQETPTSLPAILGATDDNSA
GGTSGNPNPSSLYPSQAQLVQQQQHSTSHLEPTATTTTHRICSACYYEIQLVVPRRDQNG
EVRRRCRGELKMFQWSLLVKVLSYLSMHDLLEASLVSSDLYFISRDNVIWYQYNMAQRRQ
KEVQLRIPPRRQNSSLSLRRRFLQRQKEPPRAFGIEFGDVSSSDASKRVISLHARYNFTQ
FLDFARRQEMARCRGLSSFSVAARTLFSSPLKIAIIGSMGVGKTLLVRHFVVSHENGGSS
EGRISPKLGFVRYAKMVHLTGSLAADVMLHIYDISGELRFEELRRFLCSNCHAVGLCYDP
RRKFTLVEAADVMMGVEPALGPQPVVVCGILCPAEPARQDLHEAAGTQELEVTEVDASGI
TVRGRGSLQCAWDNSECLFQQLVQSLLDRLAMATATSSVVSAASTARLAGDEKEATVNCN
IAQELLRITLDPSPLDVLMDSN*
>TCRU_2656 hypothetical protein, conserved
MATGQYTEQGIAPLWHTDLLTFFTTQLTNKWRSTIRQDTHRYLLCGTRPSDLSGKDLITQ
EVLHRQELVHLARAMCGESEVWGRLYWAVRDCTNQCRFCNISPEQSAYMRSNNDPTAPGT
DTVPPSAREEDVSPVSRRTLTRRRKEKCPHCDSTLTGFSGLVSHCRSFHPEHPPPLPELK
CDFCDMVFPTRRSTAQHRSRCAHNPDATRHRNSSARRRSLLPQDQPASTSTPIGPQETLH
HLLLECPGTLAVRQRLGIEQDLRLGKFSQWQLLHSRKLLSLLDHLFGTQMALYS*
>TCRU_2657 hypothetical protein, conserved
MQHDRALHRRHIRHVDEDVRHQARRALAEAANPQLRAARKHAVAQRHVQRVGKRIHRAQF
HRQSDRPAEHHRVEARRRTHAANRHQLRQRHATHAALRAIATTAAGPRKRHVERQARLAH
AAVAAAAHAVGERRRQSIKARQRCRTAVARHHAHGRQAGQTVVAAAPQHTATGTVALHRH
DAARDRRPGDKRSAGGSAARNRHGAAAQRHPRQRRHRRCHRRRLAHRPHVVQLQPPAGTD
RPTVAAHQRRRDRPLDAHEAQHRVGHREALSKHNAAAAAHNRHVRHGEHRVAPQTETRRG
RHMHRGSGRTAQRLQRHAFKHHRTRQQVHTAANVHRHPARLQRPQHRAAHTQLRLQQRGR
VQHKRRRHRVRQPAQLGVAHRPRRHNRRVTDRHVRVCRQVVHKHKRAVAPGQPAHAHLQA
PVTRRLAAVAPHVDLRTAVAHAVPQFDAQRAGKHRRHVEHHSHAVRDGHAVHQHRVHGAA
NRDGSVHRSAPQSQHHRMHRGHPRRRRNQRQHNKQRHTHSRNGPALHTVPAARTRTHRRP
HPPSQTRTNRAVVVHRSLPVNQKSRLANPFLQMTNPEKQFR*
>TCRU_of Trypanosoma cruzi region 4, putative Dispersed gene family protein.1
MYALASELRVDGGSVFSIQNSSWSAPSTEYYKGACGFKDVVVDGGSVLQIVSSTFRLGFA
MLITNTLTVTGGSWLVHRDNEFRTAYVLYVVNENGVAFHDRSVWSILHNYFTYGSYSSTI
LLMTSKWSSPSDARPIIYGVCNEARGSPVTDYREELNIGVPVTILDCGACTMDAVCFAAR
TSSISGCECVCAAGGYGDTCLPAAVPDGLGPLPLPDADDTEVRCVHGGSISSVDYPDPGV
RGLCFVNVTFTAVIVLDLWSFDAPQQILNITLLQCVLVGLSIRGSGARVYVSVVSSMMDS
GALEFKGYFGASSQILVVGSAIVSTLSYAFAFPEFSLGANSTLLLLDNYIEGNRYAVYIS
ETIVVDGGGIIVKGNTLSTTKDEGVESSVFVYAVDMRNGGYFSIENNTMSAANGVHVFGC
TTVRSAGLLRVVDSTFFGSTRVSNSALVHLSGSVTLEGGAQWRVEGNNVGVVSVLAIPHF
WHKIRLLGIGTIVSLAHNRQVGSRMPLFNIFLASIVVASPARFVVGCNLQGGREVLYDDV
FPVEVEVFGCGTCNEDAACYMPGTESVDRSSCSCSCKDGWHGASCLPFGVPDFSLPPAAE
RAVDGDTSCVVNQTLTSLMLNMWKTHHCYVGVTFNGVGAALTFFLNSMPLHLPINITLTG
CKFREGAALQFVGGAEVAESAGVLIRVSQTVMRSSVVVFALALPQHCDIAVTEVDAVQTF
EFDLPGTVGKTLSVLLLHDVVLSASSLLVSNVKAHALRYAEFGLYSFGTLTLVGGSSLYA
RYCSLDGYEHLFYVYRLSVSDHSVFALLNNTMSSGTSLLYQHHRFSVSEHSVLRVVGNSG
IVACAIYAEDLWTVQRSSWLDWRDNSVGVGAMFHESEFASFAIDGSSVVTLTGCGMGSTG
LSRPLLSQAEAGYGFIAGCLTVAGRVLTTTGELELNGITKVTTVTACGDCTKDGDCFAPL
TTAVIDCKCQCAAGGHGEVCVPAPVPAGPPPPPPPPSPLLPPPPPIGECISDMVYPEVAQ
AVGGGLSWLCYRNVTFSGGGMRLTVLIGGMTGNVANVTFDGCTWRDGAVLLLLGNAYAAV
GSLNIVVTGNTFIDALLSPEGGFPPRTNITISGNRFTVTRMIPRLGLDLRRPSCVAMNGL

AISNYSAVVLSGNAFQTMTTSSSAIQVVRYALRVTWHSVFAVLGNAFHMAGGEGTPIHLE
GYAESLSLFVLNGSAVVVRGNLVSSLVQYVIIFVCFFCVESRSAVVFRDNDMQGSSAVFF
SSHHSHIYYNSWLQLSGNLCRVSPVGAFAVLNPTVNLRDSTVSVSGNQFMSSKGTHTALW
IYSRPSDLTNVAIVAACNTVNGEEEANYVIPSVYNATILTCSDPCALATSCFPAYTTTAS
SDGCTCACAEGGHGDACLPVAVPEPPSTDGADLCVRDVRVGVKVNAGLGTSVVCYVGVTF
AADVVVDVESMSGSVRNVTLVNCTFVRGASLYVVGWRSDPPAGERADVLISGLELRSGGG
VLVANRFPPGSRVTVVDSVLIAEKRVAYHDAYDLGDASACLVLHNVNLTGSVLTIARTHV
AAVFRDAVGVLVVGGVALSSRGALYVDGLSVQAALGLCVSVEGGVAASGGSVVAFVDSDF
LLCKHAVSVRGAVSVSGSVVAFVRSDFASTEEYAVAFYSTVSLADGSMLLAKGNVHDGAS
REMLYAAGAVAAAGSTLSLVRNRALLSRMLSLSLSLSAGAHVRVACNHAGGRVLSTAEEY
AAAGFGDAGRIDVVGCDDCDRDTYCYAPGTASASMRNGVCACVCGSGGHGEACVPVGAPA
LPPSVGTASSLFVREGVTVQSVFVVPAGVSEVTLRHVVLDGVSPVLYVPWMARDGVRIVV
QNVSLLNGAVLYVMGGGALRDADGAGSDESGPVELSVCDVEALNGALVLTGTFPAGSVLT
VTDSLLVAARPTPLVCLPGSQSSPYAPVLVLSGLRLVRSVLVVSGVALVTVMTGGRTVVV
DGVVLELVGGGVALDSAVLGGEYALYASARVVASGSAVLRASGSQVYAAHGLVFGSGVEA
NASAIVVNDNTGALTDGALLELRGSASFASGSWLSVRGNSISGRLLSVPSYPHSAELVQS
TLTLYGNAGRGPVVMDGTVALGGAGRNFVVGCLTLNGQALQPMDYRSTGIIGEFRSVACG
VCDAEVRCFAATTRAMSRSCRCRCAEGGYGRDCLPVYLPHVDGCNRTHGIPLLSHTATLT
ETRSPTTTWTPGMSTAQ*
>TCRU_2659 hypothetical protein, conserved
MALVSYTAPEWSDIDGRLSELVRQHGAIQVRLTFVDENGPSYATLTVAASMRGLRLVSGK
VASRWEDSSLTGHVANLRAPQVPNVEAQVLLPIMWGGPGVGAPTRARQR*
>TCRU_2660 hypothetical protein, conserved
MLRSMSQMDRTPLDMAILGLQKIAARKETKQARSSTKEGMNQVIRSRTGWKERVVFRLAW
ITASCWSEIVALIPNNLTMEPDGALVLDWPVAPRTARADPYLALRFVRIRGQDAFHTIKL
CRTLQENEKLTNLTNAQVTRALAPWNATAHSIKQGALRHAAPIVETYNLDPHVISQLAPH
VDQFDLPQNTV*
>TCRU_2661 Concanavalin A-like lectin/glucanases superfamily/Trans-sialidase of Trypanosoma hydrophobic C-terminal, putative
MLQGKKASVYIDGKLQRQEEVPLTGERQLELVRFCFGACGEDAGQKTKVTVKNVFLYNRP
LNSTEINALKDKAHTLNGPGGALAGWAALEGAAAREHAAVVGLTSAGSGLLPLLLLLLGL
WGFAAA*
>TCRU_2662 BNR repeat-like domain/Concanavalin A-like lectin/glucanases superfamily/Protein of unknown function (DUF3676)/Trans-sialidase of Trypanosoma hydrophobic C-terminal, putative
MRDGTIVFPMQAKGKDGTSFLLSMSFDPSDKKWELSYETPGKGCRDPTLVRWEKYEYGAE
LFMMAHCAGGYYDVYMSTPHGVNWNGHFQPITRVWGNSHNRKGHGVQSGSTTATIEGKEV
MLITAPVYPKEENIVGKGRLHLWVTDKARVYDVGPVSREGDDAAASSLLIKDKNKELISL
YENKKSDGAYNLVAVRLTEKLERIKEVVKKWKDLDRALKTCRSGSSAAVDPRKKGMCNGR
VPTDGLVGFLSGNSTQTEWRDEYLGVNATVTNGEKRVPNGLTFQGPGAGAEWPVGDMGQT
VPYYFANNKFTLVATVSIHKVPETGSIPLMGVKMNDTDSTVLFGLSYTNDKKWLAIPEDP
AGNVEDFVKWEPNRTYQVVLRMDHHYWTVFVDREEIQKTKYNESLFDSHRISHFYIGGDS
KHQSATGGHVTVTNVMLYNEELWRDDLYKLKTSKVNIPSLGVEENPSGQVTSTDVSVASE
SWSEESVSYEELTEDDTDEQEEEIVDDLLLAASPSTVDAESSVHESATAAQSAENSHQED
SAQLSGGETAQQTTLNEAKESMQRDSYVQPQDLQSEKSTVFNDVETYPESIDTEEPEEEG
EADGRSGGTTSPVAASLSMETVAASVNGEHQVQQKLELSAENNDVRSTGTGNTGAEESLI
LEARDGNSERTMGSGSSPTPSKSDAEPSSAENTDDVFRTEGTEFPVENGKEVPQTVDTAP
GNTNTTPGGEGIPSTKGAARHSDNDTFTGEIADLLAMGEFAESTVHGCVSRVLLLLLLGL
WGAAALC*
>TCRU_2663 hypothetical protein, conserved
MAMMMTGRVLLVCALCVLWCGAATVVSSAPDATRAQSVWPEDLIQKWHELLKNECKVENP
YENNEDLKELAVNCCVRDAMNELCSAFYGRTVMENKDANVEGICKVYAGKPDDVVNCPKP
KTNLSPAAVTPVKVSDPEAPREKEEELRKTPEEVPKAPPEEVPTPLAGTPLTNSPANPTK
VSPSPPLKGQPTNAEEVPVPNSEEGSVNTGPTNDSEEGDTDTVTDTEQDEPSNSQAEPAT

PTHTVASDNGDKETDKGTGEDTPNNTPEPDVAGTEENQEENKNNKPKERTLQVAGIKTTT
ATSGDSDGVKATPPLLLLVVACAAAAVVVAA*
>TCRU_2664 DNA replication licensing factor MCM5, putative
MEADAGLVHVTNFRGGLLAGDDEGERQPDEAVDLKLIFKDFIERFRISNDHLYMAMLRSN
LAGGLFFMEVEMMHIQQFSSTVFNALHSTPTRTLPLCEHALWELAHERKLFPPFARQSTI
QLQLYWGVPPTPLRQLAQASVARLVCVSGIVVKASPCHARCVRAAIQCTSCLSKTYINGG
RSIDLPPHCLENLGRANGALGFGAAAGGVSGTQRKCRPNPYTLLPMECEYEDQQIIKLQE
LPEDVPTGELPRHVTVVVDRYLVDRVSPGSRVQVAGIVSVQEKRGSLEGGRKKGKGMRGV
AGLRAQYLRCVGLMYITAKEGGISVQSVNLNFSSRVRSQSVMSWQAEDEASFKRFADQGH
VYEKLAQSIDPAIFGLEDLKKSIVCLLFGGTRKKQGSNYLRGDMNVLFIGDPSTAKSQLL
KFTEKVAPIGIYTSGKGSSAAGLTASVISNGNGDFVLEAGSMVLADGGVVCIDEFDKMRE
QDQVAIHEAMEQQTISIAKANLTTMLNSRTSVLAAANPTLGSYDPLLSNEDQMDFQSSIL
SRFDLIFKVLDPRNPEIDSRLAQHVVNLHKGGSGRYANTKSSPVVERSFFTKYVSYARAT
RHPRISEEAMSVLLDFYVNIRREAHQQILEALSSSTSSGSKPQTPIIQITARQLESLVRI
TESMARMRLDVLANRADAEEAIRLFKVATVDAIKSGVTDQTMTAAQSELVLRIEEALRRR
VALGATVEQNRLLAEMSRMGFDAKLVDRALYAMVKREELEWRKQRTLIHRLR*
>TCRU_2665 hypothetical protein, conserved
MSVFGLPPQNPPGAAPSKAVMMPGPFASVFPALADTSQQKLPHPQPQQGKHAPLYGLQPV
FFSPQPNSPVLSSHEQNTFNAPPSVFSRTAGMQQSFGSQNTLTVTPSIQKTVLPSNSSAS
GFSAPVFGVSSAFGTCPIPDNVSARSTAPASTSVFQQFSQKGDVLQKPQRSTTFGMPKSG
MNTLNPAVALPQPSQPFTMASPVFSKEESGNVTFNPMASGAKRGRGGSTPFGGRGAGRVD
VAPPQFGEHSPRRARKEPPPTPPQQKQKFSSAFVAPVPAPATKGSWQPRQPVQEQRQMTT
GGIATEQLLSTRVFSAFMRKILEESDVIREEAADFLEDAFFGVVGSHGSGEFPTALQQML
AVWHEVKETQPQSSIKGEHWVVLFALGCHVNFLTNTILAEVNEQHGTADADNNNNDNTNN
EGSNDDNGGAGDQKDIVLGNKWAALTNSCTYANIMCDIFLQLFSNQAENHVPYSVLPFVL
RRVRNTFELANDEGVLAVQSNMKTTLLKLYRYVRVPTLQQEQHSVVEQQRTAAYACVLGE
LMLHSYAPSGRDEFVQSFHQHCSNMTEIRCALYHHYAHNLLQESLTDTVIQEAMELLARA
FVILPESAKENKRLLFLKLTACGLALGRVPSPEEQEAYNVAELEDLIVAVRSSNWMLFEI
AMRNNSEFYVQCGIHNVLQVVGKRISLLMVVKYYVNSFSSRLHVQDMIDYYEMPFTLYEG
CHVWLLPLLVEKRINGVMESGVLVLSSANPFDAYSKEALTALATRDQGE*
>TCRU_2666 par1
MTVYHEQFALVPPQYPRRAVQEAENHRALAALYELVENAIATAENYVAYTEGRLVPLSSR
GSELLAASKELRERYRHEAPCGWTEQKVMQHCEQEVTVEEMAELLLRPPLDVSGIRSILR
TLQETRTQLPRGRFLLMRDNLSALKPHQPPDLVRDLSDVCGALYEIQYVDLMAELRAELS
ELDGERDKVQEKLKDAIHLYERALAKGDVVEVERAHRQLIAARYEFVNACAKLMHQILGE
DMASQESGFAAEMEALRRDATDSISRFVEALHERRQAFRNDLHNCDKKRLEEDGNHQKCL
EAYNAEERETAAQIGQIVEQKKKLVEELRQKARELRDITLKQKEMVEAQVRAKRAEEERV
TAYNEFVNMEEQQKHRLLRCLAYFDGMEELTADLRSYVDEMVTRIPQQNLRQVIDQLNDI
EAEAFMNAYSGFVSCCGELTVKKMHRLDTLERQARLLEHNRDSAMESLDPNMSNYRLELD
DIIEQMKGVSGVINALNATQDAGEQLFQSVEKGVLAKYERSGTPFVHPLQEYGIKSVEER
NRFVDRSMHYVENEERKVFEKRNVLNRMRQAVEEDEAATESAIRNLPAAPEY*
>TCRU_2667 hypothetical protein
MEARSRDIAEALRTAEATNAAEQARLRALFPMCADLPVSMEADNESLNLLRRRSSLLSDA
FGNSRAIDYIDRSLRRRVSLLALAPEETTIEFCQESEVVAFDVRMNRNVGKHRRLRHTTA
ANQRDGDVRGAVRNEGKAVLEEQGAKSRLEGDELGSELALKAGENLAAVLVDGEEEVARR
TIQQEEVLRRVEHCLSAVDACEAASREGLMMDEKSALYAQFLSSLMEQEELARNVVDNDH
NSAMMKLLHEKAAEEASVCRNEELAAALCGMMGEEADDRARLQQSEENKRNALLDEWLQL
EESALRKLVELNEVQLLDELMLEESDSRRKAALGGTDRASYRARENLCDEESLDRNRLHD
VESGEMNDLHKMALTERHSTISKAKIEELCDAEAQGRIAITANEEEGDVELGIMKLVVDE
MLLRQEIEEEGEHLLASTTNAFDELCKLMDTLNEMLLAVGSTEGEERAKLMDEENKAREQ
ITFEVEEGKATARDRTINTLNELLLTVKKAEREERAKLMDEENITRGEALLEAEKEEIEL
RRRRKMEEELRFSMDEEKKCRDVIVANEQEDINLLRRELEVAIAVEESVAKMIGSEEEAR
RSIGDTEEYEWGQLREEVGKASLEAIRAQEERETVEKEAQQMKRRQDAQALIKSVVFAYR
IRRRLANRFQNRKAQENKNAIAEILRMEFNTRSAICDEERLECQKIDEAKRSLLSLIGTR

AARTIAELEKEEDDARHELMDDFAFHLSQLQRASRKALAKRQDLIPSLKQLQEFEENEDR
MHATTVFTATGRRVGLSLSDDTGDEGDEEEEFDTPLTTGKYKGYTLDSIGTFLLQYERDL
QRFKASLEKHRVIVAADHDMLKKARDNATQDMVHGGTGALWIPTRPLPLSDLVRGPSAQY
RRGRIVDARGQRPVSRTTDAGGRAATPSDVHFFDGLVK*
>TCRU_2668 hypothetical protein, conserved
MKSRDFHSGRSGDVYREIPRWCLRKIPLLSGEEVESIVHMYDDNTGESEAMSEATHSTEA
TAARPTAVSNTTRQDSSLQLRLAGLPMFLQTEALVLRRHEQQYRQYMKELIDLSECITMV
CDGTLRHPSPNSASHVAEDSPAARRKKDDALQRMFRRAEEVEVCARNAAVERVRSAQRML
LAAAVAKHKKRARREKAHEEEEDALRVRRRRL*
>TCRU_2669 leucine-rich repeat protein (LRRP), putative
MYSRVLGLTSDGNEKEDGADGVVLLRNEVSEFTPAEIQALSRLELSDDPTPNAGHMPVDG
CSAPDVGCNGSRRKRRVNTRAASSFLRGEHQNCCRSFKSLLLPEERVGGKDSMADAVLST
SQLHAEDRALRDTSVVECFLNLTHLYVQHNHITSLEGLSLLVQLTVLVVHHNEVESLRPL
ADLGALRFVDARFNSISKLDPLEDLPRESLRYLSLSSNPCCRGESAVAAERDAYRQSILQ
CCPLLEMLDDLPCQISSSSDGDDSVVEGKEEEEVPDEGRTSSLPTSHDDAPEHCSHFRTR
ISHLMRIHRTPSMNASLPSPAVRNSSGSNDATTTARLCQQFEHRSGNIRKQAFDHAFKLP
CPDHSRWAGESVKRNGRDDDRDDDDESDVLPLSLMQERETKSRLYADIHFALGTSNAQVQ
QLMGNVWEDVGKVLRTRQALVRHRRERMELQRKQPSGAYTESLALLQKENRTTNLDKYRR
*
>TCRU_2670 hypothetical protein, conserved
MVAIYKPVSDCVWHQMPMDVRERIRAFRERAVSLERYHTRNAWSADFINAPPPGMVGRKP
GLVAFQERPKRPELLSLGNGLNDGGVAIVQRSSSMSSSKFGKSRHSLHFRGELWESVVKT
NEFGLKASFITDVVNATGLDGSRIEDVAFVITNVLIVTFTVSLPENPERMTEVHAALQRC
NFPRTTATYMRHRLALF*
>TCRU_2671 nucleolar protein
MAAAKTELAMELNDVVCFERLCADREWRNTLGKVIGFPTSTHVRVELFERAEQTNHSVSI
VDEKLQEMATDTGKLAVWEEKLELDHQLQQAVEEEDKASEKLFKLRDTTQQRQSKASARV
RETMEALDEARKAMTRVPPRQWRDVRAPGRPTESLVSICRAVMLALQEDRVKSWDGMQAV
MRQQNFTERLMHLDCTVTPLPLARRKKIVAELGVFRRELHGGNSGGEIGRREPSKLNRQP
SAAHATQIEMAMRDWLLAQVACSETREAEEHVVGDFFLEYQEQTNLVREVNSMRENISAL
KEKILEKKLAICGDTPLAALLIKDAEASKDTMFIKCMSNGRHVTEMLLRSSVLVVFGAKK
EEQLEDDENALFLSLTPEEVMQLRDAARAANEVHDLEEIEALYALSVREEEEMQELKTRM
EELRHKENFTEDDEEEMRQLDAALTDVVRRHETTQWRIRHLRALGRDKRFLKVRDVQDDM
VYSELNLTFSGDKWGVLLANTEYLMMVETALCNDLSHALGLPPGNMSNFRFAIKSLLVKL
TVKHSRDVTAEQLQALANEAEFLGLRGLYERITFRKSYPLNTPSQRLEYEEILRMQRIKE
NFAGAGLPEKLEDFADDSEGETDDENYRRPVVEIATVRYEYGLEARYGYPTLAHVGVPAG
FEALFEKGRTQLRAAMYPEEEEEAAEAVENAAVEDAEEKLDSVEKEPTAEEPLGNTEAEA
TAVEAEDVHAVHTDHVDAADHFEHAELSEHAEHSEHAEHSEHAEHSEHADAVGSAEGFLE
PKETLEAPKDGEQRDPDSQDKETHEL*
>TCRU_2672 microtubule-associated protein
MRRFEGDDWDLVVDNNRRKLEDAFSRETAAALGVSPRQVVLLDCRVGSLLMVFKVLGCAM
SDAEITERTEAHPYDEVYALYRPRIRSFECVDAAVPRGFRADADEVWTRSRVEFEGEDWD
YVLAMREADLLRAFSNATAEAFGVDEDHVRDVSARYVAGNLHFDFELRRPAALTKADVNA
RLKECAYAAVWALYEPRPLQEEKKAVTTHELGFEGEDWDYVLERRRAEVKDVLAVETARA
LGLEREDVLEVEVDAVPRSLIAFVTVRHPSLLSDRQVEETLARCEYRKLWALYETRPLES
SVLMRRFEGDDWDLVVDNNRRKLEDAFSRETAAALGVSPRQVVLLDCRVGSLLMVFKVLG
CAMSDAEITERTEAHPYDEVYALYRPRIRSFECVDAAVPRGFRADADEVWTRSRVEFEGE
DWDYVLAMREADLLRAFSNATAEAFGVDEDHVRDVSARYVAGNLHFDFELRRPAALTKAD
VNARLKECAYAAVWALYEPRPLQEEKKAVTTHELGFEGEDWDYVLERRRAEVKDVLAVET
ARALGLEREDVLEVEVDAVPRSLIAFVTVRHPSLLSDRQVEETLARCEYRKLWALYETRP
LESSGAVRPRLIVAEEVEAELDSITFLRNALRRARRERDEYLALLDPLLGLRGERQRILD
SLDAA*
>TCRU_2673 microtubule-associated protein
MSLEHVAEVKGGPGALRVGEMVVVMTRRVEEQTAHWTLGSVESAPYLYDVEIRLWEKQMD

VDWQEQFNVGESQEEGELIMHIKKTKEELEGSIQHVTELTKKLRQRRRSILREIDDVENH
ASQSRAAVKAAYIDVKGISYHHWHELKSYRAPSRMVLTILRAVMLLLSEDDAKTWPQMQR
VLRDPHFKSRILDYDPDKELSRERGDYILYECVRKKSFRYDRAVQGSQAMGPIYYWVLAQ
LDRSEATGKQYQLEKRKQESQKELRSVINEIHKQQQRMAEYQALMDSADDQLHAYRALQR
ESASLRGGQLTETRLREVAQQRYETSFAGAAGKEYLRAAFYTWKPTEYRIMVLRKSVLVN
FGTVPQDAWDKNNYTLDEPQIRMLDDALQQRQSVEETFYEKESEQQQQQHKKKEEEEIEE
GLFHPVDENYVPKSKMSVDWLERTFEGEDWKYILDRKRLAIMDTFCDETAEALQLPRSDI
SITQMTCGESNNLLMVSSEVWHDGSRSRGDLMDAMNAYLYPKLSALYSNELDGFAKLEKR
FDGKDWQYILTDHKNDIEYAFKNDTATALGKTMEDTEIQNITATAEALIIKYLLYIKENQ
AKKAKKAISNYKYPEICQLYSRLKPNTSTEDKIYSTKRIELEGEDWDYVLERRRAEVKDV
LAVETARALGLEREDVLEVEVDAVPRSLIAFVTVRHPSLLSDRQVEETLARCEYRKLWAL
YETRPLESSVLMRRFEGDDWDLVVDNNRRKLEDAFSRETAAALGVSPRQVVLLDCRVGSL
LMVFKVLGCAMSDAEITERTEAHPYDEVYALYRPRIRSFECVDAAVPRGFRADADEVWTR
SRVEFEGEDWDYVLAMREADLLRAFSNATAEAFGVDEDHVRDVSARYVAGNLHFDFELRR
PAALTKADVNARLKECAYAAVWALYEPRPLQEEKKAVTTHELGFEGEDWDYVLERRRAEV
KDVLAVETARALGLEREDVLEVEVDAVPRSLIAFVTVRHPSLLSDRQVEETLARCEYRKL
WALYETRPLESSVLMRRFEGDDWDLVVDNNRRKLEDAFSRETAAALGVSPRQVVLLDCRV
GSLLMVFKVLGCAMSDAEITERTEAHPYDEVYALYRPRIRSFECVDAAVPRGFRADADEV
WTRSRVEFEGEDWDYVLAMREADLLRAFSNATAEAFGVDEDHVRDVSARYVAGNLHFDFE
LRRPAALTKADVNARLKECAYAAVWALYEPRPLQEEKKAVTTHELGFEGEDWDYVLERRR
AEVKDVLAVETARALGLEREDVLEVEVDAVPRSLIAFVTVRHPSLLSDRQVEETLARCEY
RKLWALYETRPLESSVLMRRFEGDDWDLVVDNNRRKLEDAFSRETAAALGVSPRQVVLLD
CRVGSLLMVFKVLGCAMSDAEITERTEAHPYDEVYALYRPRIRSFECVDAAVPRGFRADA
DEVWTRSRVEFEGEDWDYVLAMREADLLRAFSNATAEAFGVDEDHVRDVSARYVAGNLHF
DFELRRPAALTETDVNARLKECAYAAVWALYEPRPLQEEKKAVTTHELGFEGEDWDYVLE
RRRAEVKDVLAVETARALGLEREDVLEVEVDAVPRSLIAFVTVRTCTSTSS*
>TCRU_2674 Domain of unknown function (DUF4457), putative
MSRLPIWGDSQQVWPGSTGRRVLHMHTDSATGRLSSGDSFTHCHALPLPMEIVNLGNVEG
ISDWSKSPGQYAMKCIQRYTGSPTFHTSDTIPFSSDSPRRGNSSAAPAITTILAPPEEPR
GQFDVCGGTRGDSGGLASYLAEEMPRGRFIVMNLLSTWGAPEEVGINGMEFFNENGDRII
PPAECLKKPSSSLQSPPGSTGGKIPQEKSKIVDGYHTILYTTPQSSLSLMVAYPIEGSSS
DSANASAGTDEVPRSDLANLVNGIHNTHDDTKLFTMPFNPDKHHLICFAFPKSVVISMVR
VHNYGGRGRVHTSKGVRLLEMTVDDELVFRGEIAPNNGDVVPFHRVGLENCENILFTEEP
RVLRRLLACPATETSVQGRAWGKERRTTIGVLPREIIGHSTTISTVLFGTVNEDKSKRMG
GERSVLIPAAVPVHAKGKHIIVQNSLGNFENATATPVTGIGYQTSGSLSMFSRYPQNVTS
LCFLLLGTWGDTEKIGLSGLRFRNAQGEMVSQRIAHWYVRFPSQRHESDVGEKWEDQLVH
LFDENAETAFTLPCEQGIEVIFIFDTPLPTLGFVEVANYSLGEHTFSGVKEARLFLSSAT
PNWSPDALVAAYSALWEINTATSCTTLNLHGIFEATPEEGVSLRKAPAFLSIPRFQVYDL
SLAAELRAFEGGSGALRNSLTHSLSASMTIRAEMALRRARMALKPRPEWFLEYQSYLTPL
LPVAYVLKVSLGICARSAMDLRKYAKKWMVNPLRACTFADENGETIQVFSRDGAKDTEGH
GGDVAGGVSRSENTHAFVTECLFTPRPNALQEDEEAAAVAAQAGIRVSLFQASLSLVYVF
DSPFCLSLLTLNRPLLQEEECAAWVRQIRVFMDDALVFDSGDVGVPRRAPPATVGPSSPL
RPAGEGFLPFAVGGGGAGNAVHRMRLKPLVFFTLDTVQLEEARREMMVAG*
>TCRU_2675 TLC domain containing protein, putative
MGRYGAYDLYVAPFVRDFTVLLPMVAVWFVLQWAMPSLYTRIFGAYFTRLSPKKREDVVV
RSVSVLNSFLMTGAAACFLSNLYAHNFTLPGEYYGDIPGYCFFRISIVSYFVWDIVVCFM
YGWGELWKIHAIASLLGAYLLSFPFSDHYASYFTGMFELSNAPLHLSVIMRTLDVKALNP
FVMAMEGAFALLFVVIRVLGGTIVAAAWLKLSCIRLVANYYTGGRLLHGEVPVVIAMTLI
VVIQLLQYIWCVEVVRRVIEVLAPSATTRWKAKQVRTDAIQLDK*
>TCRU_2676 deoxyhypusine hydroxylase, putative
MTQDFAAYEEEYKRLLDPNEPLDSRIRELYRLKQSIFRTPAGVHVLAKAIDTTDSVLLQH
ELVYNIGQCGLEEACPILERVIHATDVYDTVTRHEAVESLGAIASSSSKPILERYMDPAH
EPHKAIRDSCELALERIRIRELRGDAALKLPCNCPYVSVDPAPAFCDANTDGPVPFTVEE
LERLLCDTTGATSLWRRYQAMFSLRNMGTEAAVMALTRALRGDTVSALLRHEVAFVLGQM

EHPSSQAALIEALKDETEAPIVRHEAAEALGAIADPKALETLANYAEHKEQIVRDSCVVA
IEMHKYWSRFRQHGEAAA*
>TCRU_2677 hypothetical protein, conserved
MENHGVAEVDLLAEQLYADPDPTVRRRAQATLGVLAKEETDSHLYYAILRQSNSQYTLLL
IAQSIVSWFRSTRKWMSSEEQRELIVVHCGGCLTRMFDTGAPKHVVSSLLTAYARLTKLA
FENEPFLEAAVDFPLEMLIREQKGSSAAVLSLMLLNVMVQEFSRHDTSKSQTYLSFVAHR
HCSGNFREGPLLKIFLAALKQLEGITTDSVHISEVVKLLESCFLFDFRAIMMDETEGLPF
SLFPTSWKSTLLNKQNLGTVWGQHARLPYPHCATLLSGIVNICSVHRTLFDSSEERGEYL
EYTLTMLTEVTMIQDGRLKIPHYVELFAEACTRVLTSFGYRELRLLKAFEPWVNALSILS
VEVVSIPFGQEGSFSTASSILSFWATLTTSKRRSYHDASQRDIEDVVMQLLQNFVKARIC
NLEVNAVDPNFLINDDSHDLNNSVIAQSASYATICLLDPLTCISDLVTHLNQHFVAPILS
SPLSTGWLFYLVGSVVGPVVAAVEEGGVAKCSHLLSFVVWCSTNRLRAGDRDPAMFGPFV
ECGSLQFLTHLQTVFSGARQAPLATIVTNVFGSHSQMFQFILDSAGHNLLRGAEDARAVD
IIRTSAAVITAACRDATPELLQDLTFDLPPFSELPLAQSERTYKLRTDLMKALWFIRRSE
PTTYEQMTSYLSGIEANMQQTLNNELTSTSFVAGWLRDLRGACQAMNDEGNLFSDFLEWV
CGHSHVFVDVLNKSGESPVIVNALMRFLCELVATGRYGRFYLRPSGNSAVGLVLFVNLCA
FIEKAEEAVFSDYRIASIAESSSDCEKTLKPWMLSMNIMTKCIRGSFVPFGAMMYYNDRK
YENNAFHLVRKLTMLEPNVFRERAKFRAAALDLLRSMTEEQLYFPLTHLESNELLKLVEI
VVSICEDVDTPPDVLLHGLSFLGFIAGLVRDVKEILFTPTMQPSRGDAAAPPPLSLSHSF
VPILSSPSPASRAGQASPMSRFTREANGKLARLLAPHQELWQHLISVAMGIIAYRDHAVS
ASSGVVYPIFEAHPPFWYNFVEQFVASYPKRKQLGIREALSILTNASESQEKFFSEVFTF
RQALRRLNT*
>TCRU_2678 Sedlin, N-terminal conserved region containing protein,
putative
MPETEYPTWAIAAVAVFGRHTTPLFIRTFISPREVLEHPDAATCANLYAGEEDVVRLHFL
LFSSLDRCDDILRERRKQQQLEKGSSIASRQGPGSLRTVVGVSAGADVRFLGRLLRNHKF
TSYGFQSASGIRTILAIVGDIPLDAVLPLCRSTYEAASAALCNPFRTPRMHAHLLQSWSK
SGSQEPTILPAEYAENLRAAPTPERVAEEPSLAYSHGFREQIRTIIEPFTVTARSALI*
>TCRU_2679 Domain of unknown function (DUF4460), putative
MYKKALHSFFLKVHPDFFHHNRSQQLVNENSVARLNELLSWAKAFKSGTLRPPPATSITL
AFYTKPDDGGVGEDAPGGNQQRAAPSSSFSQGGFKAEKSSSPFLIESTFELADNFTPDDA
HRGSVERSVNKFLRDLLRRAACIDGITESISSAEDATAERAEARPLRRRPGPHRRKVEKP
KSLLDEATESMTLQWSITPAPTLDELMEADQILFARELSPLQSASALHTLRQHLGELRYP
AWESMPLIISNEFNIGEVAGTLTIPWDFTPLQFLAFLARNDKAILHCREAAMRFANTLEQ
LISEISHELDLDDILISCSHKDAILVLQLLQRNKELLRGYGLTDLTLEIGTRHAMRANGM
VIISCELTADELRPWLKKLQPKLALHKRLYRISKQLLEATMWHLKEFRTMAEPGGIDAFN
NDYTYAQRLEWAKELFRIGSSLAPWDWKEITFVLAPDVDVDWAKGLIALPYNFNGDAFVR
YVEEVQHEAKRRKREELLEDDAMRRQGAEDKRQQEHDEELMVQDDENKESGEKLSQDYLR
DMYRRTSPHMEEYLASSSHRVDTLSVERPLSHAVTFNSDAEAEDQLKWEGFYADPYVDQV
PTGDLDDVAHTFMLTNRWHREEAAKKMLEQLRDSYGKKSRRFDYQKMGDVLEINNAKVQP
KGFPTIPRGLRPTQ*
>TCRU_2680 hypothetical protein, conserved
MPHRMGIGLVERHRGGVSRELPGPAAAAAAAASGVGGGVIDEGDNYDEGPRPSSFVPTPE
PRVPATASTAASASFLHAGISTSPRSTRAERNTLGGSLSMSPAMDGASLRRQVRRRSSVA
HVRRLDLSTTVMDGVEWSWLCRDVLPTMSSLIYLGLAHMGLTDKRLVELIRECHLDSVSR
STALPLSSSSSLLSRRSGGRVASKSIMFGDASFSPTSREFSAGRKKPARALPTATGARGR
RELRGLSVLDLRGNHLTHRSAAPLGKLLLCVADTLDELHLSGNPLQDYGLQILGIYLAKL
KLTSLRNEQHFFSPSLVQQYAAILKRKKREAEHEVRLRHGVLQGPTHTKTDEWLRGENGG
RNAKEEEDDACGEAKIHLGISFLDLRDCQGSARGISEVLAGASRAHRLETILLAGNGALA
AALIPPKSTCTDGEMSPLATGNRGEGPSETNPSPTANRFSSFQELQYPCALSAVNLCRVP
LSIMCTPIGCRELFLNIFFCCPKLILLDVSRSLDFSKLPNSLLQQVLMDGREPSSDPRTY
WRLQRELEGVAEFTLDAVMIGRQSCVGSILCELVAHAAFNAQRRQTVAPSAFCHLKELYL
NGTGVTDVAVRGLAIAVSSSGSTGVLSSLTVLDMADNLLTVNGCLQVMHAFILDWPVTPS
VLSTLALQGNAGIYHDDNDTILLLQQVAEAAVQKRCEERQRRDPVDGKGFPSPLSIYLGA

VHAAIFSISSGGVTASTGVPRTHTTRSSWRTEGDNIAAAAAEEKETVKMARKREQGEVLA
VHESDFDREFPTFQLGEVGPAPSPQKAVRPLMEGPVGASFISPLGAQMAAPCDVSSIPPG
ETPRQFLFSMPSTPLMPQRTADRLRPPDQQNRTPLRQEIKEGGQRRRSFPILSPLVVDDS
SFIAGGSTTEFIAGETHRGSVFNIQPPTGVSSPSRGAGVVPRLTLAKGDDRRAMDVATAT
HGRYCNGGRGITPPSSTTNDHPVLQDRQTAVQRETSFTLASSPNSKAFKFGCLFASMDVS
KGRDSNVEKGNALTRSRVSGEEINKYNDGGGGGETDAGEKGARVMPETAKVLTLHGDKIL
GLKEAEDGESPVTVSYGGGAVGRERQTASKVEVTSSAVDGKWRMNTAARLGRLQKQFLSE
PLDESDRPVMMMTSTAHFRSDKTDTDDSTAVATAGKDATVMKPEPIQDLLLQKNGIGEEN
MRSDPVIPALGNLDDMETASLRPIKGRREKSFVMSYYHPMGHILCRGWRLLYRNDSVGED
ARGCVERDVWTLLQPLDTEQGSAVVSSVSFVVPSAEDMDNDAEYGITRLKIVSNERRSVL
AERLQHNANMENGIIAFPRLIAAIRRAEEDTVDVVAAMLDAAHGRLPPEYVGCTAEELVH
GRHGNEDELMAELGAAIAAGGSDEESQASFNEEECAADQLPSFSSTVDAFPLAEEPVEGI
AEDSQLLQSQKQTTELSEKSEEAEEEAIMPSAALSHESPLFNVADRPQATSVSAEEPPAD
TPETASPAGITVQLETGPATSAHAASATTEGVGEAMNQPARSVVGRMKTFLFSFPHREGH
CLCRGWRLLHRPDSVGEEARRLLEADVLSFLRPATEEEEKNADNDTRKISGDVYDDASAM
KAVESVSFTWEGSSSSTMQLQVVTSERRAVISQRIQQNVVGDEGMLPRFSELLRSNANYD
TIHAVETYLQRQASVDLRERIANRYRSIGTLVHYYHGDEAAMLVEIGAASMVDVEATVAP
AAADSAAVTSIPSREILEGESERGAKEVMATGDGEEAHRLLECGDGVQGLNESSLHLQET
LTGIHSNENVVEKEDHHTAENTPLSRRSPLSFLTLSTPIQREGDGRSVELTPHGGKSGSS
QHIHNSAANKSEKTVTKNEDVSSPSAHMQEAFAERVRRLQYLAYSAMAKGAILLDKRQRH
KLRICKGKWGRQPVTIAVEWDVFLVVYFLRGTRFGMKSHKTLVLVHPVASGVRCCVDVDN
NVGGRVHAIRIRLERAHSPDELGVPLQEIEERLHSVSMLNSRSRSMASLHASLIPMQSVT
NMSIGSKSSSVDTVDSVLSAPSTERLLQRSVSFQVTLSDTKKAREAVAAICGVVQHAVKM
IQQTISEQDRHAVARPLPATRTN*
>TCRU_2681 Reverse transcriptase (RNA-dependent DNA polymerase), putative
MDRPFPPYVLDVTIRDSLLGSAPGPEDTLNEFLHRLGPVARGTLRTMIHNSFANGSLPGS
WKMGDAIPIPKPGKDPCRPESHRPITLLSVLPKLTEGMIHRRLSALLPQHPRQLWICNSA
FYVGRGGTCYRQITRGLNEFSIVEYERPGDDVPTRRPRRHRLLVVSIDFPTAFDTIDHGK
LFGMLDRLPRLRPRTKRWLNNSLRGRHVRVCTREQHSRKQLASTGAPHGSVPGP*
>TCRU_2682 retrotransposon hot spot protein, RHS3, putative
MSGRPEEGLYGNLESQSSNVSQGGRRRTRSEFEGDTDYSSTTRRRLEGIYRPQWTMSSTV
EDILLEGSTNRTEMKLNDFLRSNLGEEWVVERNGNVTMGMFVLRPTMFINDSEILGLITA
SPSYQELERELKREQEERKILLEAIYKLRREGVYFLWQWREYEGRDTLTPLASGKLNGLL
AEIQKEERREADERAKREEEKLIREAEARVRQEAEERVRRREAEMKFTMSTNIEDVLFQG
GVRVKDIKLNDFLLLEMEGKGIVDTNRSVLLEEFFNEPTEYIHDARVLNGMKTTVRYLKM
GRAVMKEVDMEKVARMLHEKGVVSLEQWRNYEGKDTVSPLAKGTLDAALSRVQTSTSVVK
STVLEGLYESVYNASWSHVVEVSGSEGTVMKVEEGKPKQSWTYKEVGETREKDDSVQQSG
EAPPVLMVLTSDKGWPYSWEEDEFIRDCHVNCEVERAWRIVEHYLNEWFIPHGGTNLTPG
RLLLIGTPGIGKSMNAGSYLLYQLLHYDVEKLPVVLYVIGNDAFLFEKTTKTVTKYMGDF
SIDDVLRSLSIPGVNAYIIYDVAKGGRKPYQIATTRGWGMIVLSSPNEDNFDAWEKYKSP
KRIIMNCPEKDDVKAMCVWMKRNDSSVQAEYWKNINGRIDEVGPLLRYIFDDSKYNGRVQ
SCKDTLNSLSRIEAEYYLHFGTKEMLGGDKVSHKLVRIVRVRGVHNIESTFNGLMSPYLG
NLTLCKLAELMPPNDFILLILAIKDDLISKVLEKHSVFAFLSEAFVNAIIPKLNELKMKK
NKDAPPHLCALESRPHERPLKPLPLPLLENFEKKIEIESRVLYKPVAQNFPLLDAFFFME
SNPKTMVGLQITTASEHHTIPSTVNLFKENMAKYFNGWGEFSQGLSWEIIYIQHADSTPM
TGWQRCGPAKTDNLSDDEKEIVAFWEKEVHQYQVSVSSRDFRRKEALPIVEEEQEQETE*
>TCRU_2683 Amastin surface glycoprotein, putative
MSKLGAILYGVVGFLAFLFVLVGTPIDQFRAKEKGATGNTPCMTLWGIKEDCHSTKYEFT
VGEDFRECPSVLRLFRMAEAFSIISILLLLAATALGVAAHFCLKSLKIFATLLLVVSIVT
VGLVWIPMAYFYNHDVDNCLGTPLKTGSKYGGGFVIIVIGWCLIFVAVVLLRML*
>TCRU_2684 tuzin, putative
MHGVTPLFVLKIRDGSSPLRIYNEVVALACDRRLCHVIIEVPIESLTIAMTALPRLDFHL
VPNFSVSEAFRYTHHLIDPLELTHFVEVVGTNSNDLDELLAAVRHAHMSATTYTNQKLVK

AMRQLQAAWAKDPSLREAVIKLARFPFEEGQSEGYDYSSLRNEALRDIVMYNAVADVWMF
QQKVFHTAACCWQ*
>TCRU_2685 BNR repeat-like domain containing protein, putative
MSRRVFTSAVLLLLVVMMCSSGGASNTATSNSGNVQLPHTVDLFLPNQTLVVSKDGRSQG
TKRDAFVSPSLVSAGGVIAAFAEGRMETEYQGGPSIKPSSDVVAEYIDAAWNWSTLVEKV
NKGTWKAHTVVCAAEEEESLGVVLRPTTITKGNKVFHPAGGSDVSYEGVCWREGGLELKL
FVGDVTNSTPGDPGERIKWGDPRPLFDSKYSLNHKSKLNTLFPSGGSGVLMEDGTLVFSL
MARKENPNEAFYTIVYSKNGGKK*
>TCRU_2686 BNR repeat-like domain containing protein, putative
MSPPNCYNPRVTEWEGSLLMIVDCENDQRVYDSRDMGTTWTGTIGTLPGVWVNSQSEDYP
DGGLHVDALITSTIGGRKVMLYTQRGNYWKGQTERTNALCLWVTDNIRSFYVGPVGMGNA
GEKELASNLLYSDGYLHLLQRRGNGEDSAISLSRLTEELSAIKSVLSNSAQKDVFFSNLT
IPTAGLVAVLSNAASDDTWNDEYLSLNATVTTNAKKLRDGFQLTEPDSRVSLPVNTRANN
VRHVSLSHNFTVVASVTIEEAPSGNTPLLTAALGDAGPEYFMRLSYTADNK*
>TCRU_2687 hypothetical protein, conserved
MLQGNKASVYIDGELLGEEEAPSTDERPIDVADFCFGACDFDDDESSTKKKPSKKPRVTV
TNVFLYNRPLNSTAMTAIKERKPVPKRAPEPQAGGVPQTIASAVSAVSGPGEVPAAPAMP
MTLDLHAVKEVSEGGAAKRNTSWTEGAKFVVPEVSLNSAGSGLLPLLLLGLWVFSAQ*
>TCRU_2688 quiescin sulfhydryl oxidase
MWLLYHSITVNVDAGGDTNPLEAIQEYVRYFFSCEECRQHFLEFNFTRDEDPVLQLWRAH
NSVNARLAPVKEGADPFVPKRQFPDAEICGNCRNSLGAFDESEVAVFLRKWYEWDPSTIS
TSTNESGNSSYFFRPGRPMPLYKDPKQRHRDGVRYDSVPLHVFLTVFVTVAVLNAGIFRT
RRRSVRSLLHQHCRQKI*
>TCRU_2689 hypothetical protein, conserved
MLQGKKASVYIDGKLQRQEEVPLTGERQLELVRFCFGACGEDAGQKTKVTVKNVFLYNHP
LNSTEINALKDKAHTLNGPGGALAGWAALEGAAAREHAAVVGLTSAGSGLLPLLLLLVLW
VFAAA*
>TCRU_2690 hypothetical protein, conserved
MLQGKKASVYIDGKLQRQEEVPLTGERQLELVRLTKPRCGEDAGQKANVKVKNVFLYNRP
LNSTEMTALKDKAHTLNGPGGALAGWAALEGAAAREHAAVVGLTSAGSGLLPLLLLLVLW
VFAAA*
>TCRU_2691 hypothetical protein, conserved
MCVVNLCGLPTVIAHAAHFASDIRRLGPAAPVAVDLNSHHELRDSRLPSITAGEDLAATP
SGMEIELSDVPAQATRISGRSVSFPNLNAQRVLRVFHWTSTRLHGLGPPSAIPHCRDGRR
HPATGRHAATTETCRLCPA*
>TCRU_2692 RNase H, putative
MIPRIRHRRKTLLVVTDSQSLLAALNKGPLSQTDWTEDQIWQRLLTLTRAGWSVHLQFCY
GHCGVHANEPADQYATQTMESGQYTEQGIAPLWHTDLLTCFTTQLTNKWRSTIRQDTHRY
LLCGTRPSDLSGKDPIAQEVLHRQELVHLARARCGESELWGRLYWAVRDCTNQCRFCNIS
PEQSAYMRSNNDPTAPGTDTVPPSAREEDVSPVRRRTLTRRRKEKCPHCDSTLTGFSGLV
SHCRSFHPEHPPPLPELKCDFCDMVFPTRRSTAQHRSRCAHNPDATRHRNSSARRRSLLP
RDQPASTSTPIGPQETLHHLLLECPGTLAVRQRLGIEQDLRLGKFSQWQLLHSRKLLLLL
DHLFGTQMALYS*
>TCRU_2693 peroxisome biogenesis factor 1, putative
MQQSSFEIFIDSSRTDAFVTVSAKFLNDCLSPFYGSKTPPVVPLRLSDGKRNLYVGCLTR
KSHNLEPCKLLMSVILGRDLELENGAIVYCVPVYHPIKASRVLVSPASVDESEVAEQNAL
RIETQLLRQVQVIFPGMTISVVVFSDVHARVVVQRIEDSQGRELSSGCAVMSEGTEFVIA
TRSRQVAEKGTKPMWAILRCHPYGTYEATITSLTEEKASLFLNPATAERNHWCDGLVVGF
WDLACMSQILESKEVTPSFLRTNAWRAPIKYIKEMPEGVCCCTVFGQTSNVVVFPNPEEN
LPTVTDKDVAEDHCIFSSCKEAGFDRVAAVHGELSQELLRHLSGWFSRETELNTSLGEVN
GGNGNVLLCGGKGSGKTALVAAVLSRLRDVHVCVVQCGSGGGSRQLVSLQQALAECILCA
PSVLILDDFDAIAPVQREGAVAAVTGKTKSTLEEMLHCFSDAFTFAGRGSVVVLANCGNR
EDVYERLRSANCFAKITKVEALDRKARLALLQQIFPKESADTLEQLAALTENYTPFDIKK
VSVRIKSCSTDGDFPSLEKAKEVIVRFTPLSHTGIHFLRGEKQSLDSIGGLSNAKKILYD

TLVLPMKHPELFARLPLKTRSGLLLYGPSGCGKTFLVETLVNAAELNCIVVNGPEVFGKY
IGQSEQKIRDVFERAEAAAPCVVFFDEFDSVAPQRGVDNSGVTDRVVNQLLCYLDGVDER
KDVYVVAASSRPDLIDAALLRPGRLDKAVECPIPSYEERLEILLFCLDKISANLSRQEVE
DIARGTVNWTPADLSGLVSSAHIFVTRRVIERLSLDVAEMDFERSFAVANVGRDTTLEKI
EDTLKPLFGTVKKGQDLNLFTQMEMEDMKQALAVTRPSLTSKDILEYERMRYMFTGGDTG
NKRSKVGRKLAMR*
>TCRU_2694 SET domain containing protein, putative
MEFLFSEGPPRRFFCANRKTYREVWPYLLSRRVPAARSFLRWAARNGVAVHCALKLQFHS
GRGCQLVATRRIPAGRTLVSLPLTLSLSAAPPSEGKSFVHRWDPLETLTGVVVRELHNPR
GFHRRYLEFLHDLYNVDTIDMSTGRTLLHRELETMYMGNSMQAQGVSNGPCLPKEVFVSA
SQRVEWVRLHSLLRRMEQSLPHFASKSVPWGLSMVLSRAFSDDYGGLTLYPIIDFCLHSF
EPNSAVCFTSANSAQGKGVGLRWHDTDQFCAHLLTRRAISAGESVTLMYSPRRVVSTEDA
EYWKLRWGYVPN*
>TCRU_2695 ruvB-like DNA helicase, putative
MPGIKIEEVISTTKRQRVAAHSHVKGLGLDADGSAKPSADGFVGQVKAREAAGIVVELTR
SKKMAGRALLFAGPPGTGKTALALGIAKELGTKVPFCPMVGSEVYSAEVKKTEVLMENFR
RAIGLRIKEQKEVYEGEVTELRAEETDNPLGGYGKSIAHVIVTLKSVKGSKQLKLDAAIY
ESLEKEKVSVGDVIYIEASTGAVKRVGRSDAYIGDHDLEADEYVPLPKGDVHKKKEIIQD
VTLHDLDSANAKPNQGQDALSIVNSLMKQKKTEITEKLRHEINKVVNKYIDQGVAELVPG
VLFIDEVHMLDIECFTFLNKALESTLAPVVIFATNRGSCRIRGTDIRSPHGIPTDLLDRL
LIVRTTNYSIEEIVSIVDIRSQVEGVKVSDASLELLGQIGERTSLRYVVQLLTPALIIAE
TNGRTVIEAEDVMLVDGLFKDAKASAQLLHEHADEYVYQ*
>TCRU_2696 Tetratricopeptide repeat, putative
MKRILTFHSYMLHAATLDRLVGGMMNRFSVTSAAFAVRVCSTQPPFSTRCTVEELQKSAA
EFFERGEFMNAIKQWESVIESMDKADNNNNNNNRQYVLMSCLNNLACAYGEIGDYARKLQ
LLERSRDMVERVYGMDHPQYGMVLYNIASAYEEMGRYQEMEDLLLKSLEIHERKFHSKHA
KVGRVLLLLAEAHGHLGKHGAQLQVAERALEIVRRHCGPEHIQTTIALLTFSRAHGANGN
HAKRLQIAQQAYDIQERKLGALNPQIALTLLELADAHGANRDYFHQKDLLEKAIELQRRA
FGSQHTQLVSTYTALGDAYGNLDDRKNQFASYREALDIARQRYRGNHIVIGAASNKCAWG
YLRIGKVEEAKTLAEEARRIIEGMVSEKHPAAVELRATLQELKRVTGGTGKNV*
>TCRU_2697 hypothetical protein, conserved
MPVTPNDDIVEISRRCDFLCMALASAEDGVKTNPTQRYMYLCKASGERPNHTFLHFSKGT
CGTRLDLHRAYLSQRAILPILLTLPFCPWLEHINLREERLTTTLVELLCESLRNLPCIRT
IDVSMNPFGSFGVQALLRLVLRKRSIVDCYVGDTQSVGSLLRRLQMACERNRRDAVDMEE
EEEEEEEDEEEEKAFSTLPAECPGS*
>TCRU_2698 amidinotransferase, putative
MATLMMLQSRFFGSKKRVHDNKFITVTEVSENQAQQDNCLVIDLQRKIEERLLNDAKARV
IYLNLNEEPRTMRSAYENKVGALFVNDSISVHHFTDARGRITRRLIFLYPMSKLRRGELP
KVQLLKKLDKTVKSEEPSGLELVDLRDFESHDLCLEGMGAMNFSYNGEFVYMALSDRSSE
KLLDVVCSPENLNIPKEKRFVFTAVLPRFSGENKRCVGEDVVHHTNLIGWCGKGICAWGL
NFLRFSSEEKKEAFFEHLEATYKKIINLSAEEIRAFAGNACEIALSSEEEERHVLCISNE
ALNSLHHRNYQILEEWYGRENIFVFYAETLERRSGTSISSLISCPVTHGEVLPAPGEVTA
LEVAHVDEKVIANLLNR*
>TCRU_2699 ZFP family member, putative
MFGGGEEYFREPPSIDRDTLRRFALFCARLRAAEKPGKKTVAESAELARGVGDNEMAFEL
VVGALFRHIKSWTGNKQLACWYILDKLCKEDRDKYGYTASKYILEIGRDYIPFEDPVLGP
KYESLVEHWEGVFQSHVVDALWLAKKDRLWAIEHPNEVLQQKEEEERQWKLEEMAMEDED
GLNDYGQPCMDYLQGRCFWGDACRLYHPPGEEGTMPLECRLGDWKCSSCGAINRHFQRRC
SSCVREKPQYKKGRKPTAEEALLSSPDPNVYAVLRRQFGYDPNVVEEAVAHWKTRLETTS
IEEYKEERRAAYRMRILGRAPTSKLEESIRLQKNYPDIDIGLPEDVVDPCAAPGRMENES
LLPAGTSPVSAVGLLAQTIVERGARDPKLPQLLFELAHQVKQVARDSGIKLSLTQCETLM
TACKIVFSAWGADRGATPFVPLFFKELRHTEDGLGLSTEQQEHLTSITKEFCTH*
>TCRU_2700 hypothetical protein, conserved
MRLSRRVQLNPEVIDSSLQRLNCVSQNIIEVDISPTVLALLTSVAPDVVRQRVAELPSSA

AGDIGKEEAIDFGGAGMNTALALSLHTVLLSRNRLTTTLGLVQFKNVVRLSLLGNRVRRI
EDCEALSLLPSLQYLTLEFNPVTSLPHYRAHILRICSWPDTLNSKNCRLRKLDTRSVLPA
EVERAAQCLQREKVTLGELVHRMRFLALLSEAEKRIALHNEIRRRGFMLQDSFLTLTVES
IVTRCSAQLLALVDVSTASHLARRLMGGKCRICAVTPESSFDDDNSNEKELSQLATSFGH
SLSSSKTLSLQHPYQAELTAFCKDWSRDVFRRATTSLDHRICGLLLNIAHALGEELTVMD
VDYLNKLWFTQIAKVDSCKFYQFQNPKEKPVANGSAPTVAARSEKPCEGINGSMKESTWE
EFVRVPLSPSETHPHAPRVEAMIGNEDVLSLRSISPITQQTVKCSDVVANCERQKSSCDA
PEHSFQKLPEIERVPNDGNNGDELKKSPKVEKCNSDWASIEEFPLMAPVPFPAPKDELAL
EQELFHKLKCHVRRRILRVWRSKVRRKQQETLCANFIQRRFEGFLALSAPTPHAISSTLQ
MLPRKEQKRVLFQLWREQTRMSYAPRLLRLAQLFSRWRWQTYQQEIINYFAAKRRTRTLT
EVWHCWAEALHVHIADRRNRSGEKIQNIAVLPVATSGFQSPVRKGCSRMMMSSDRRLKET
HDVFLEDCSSFKVTTDATPGLTCSTKPSVNGAFNVPQMPHLEGTSATASRISLPMAIPKM
VHSPSVQPLFSCEKATQTLPPSNDGVAEECKDELCLEAKKAVEVMQADRLRLVDRLIALE
QRVCEESLRVQHREEEIEDLKQKLKAMTVREECVGSTIGDYQREIEHLKVIVRALREERR
ELLRPIFQ*
>TCRU_2701 DNA topoisomerase IB, large subunit
MSKKEKNLVKEDAVVKEEDDDSDRSENREELNWWELENVQFSTKGQKRWDTLCHNGVMFP
PEYKPHGIPIYYEGREFKLTPEEEEVATMFAVMREHDYYRNEIFRRNFFSSWREILDRRQ
HPIRRLELCDFEPIYQWHLREREKKLSRTKEEKKQLRLEAEKEAEPYKWCVWDGKKEQVA
NFRVEPPGLFRGRGQHPMMGKLKKRVEPEDITINIDEFSPIPKPPEGHRWKEVLHEHNVT
WLAMWRDSILGSFKYVMLAPSSAIKGQSDMLKFEKARELKNHVDEIRRCYIEDFESRELM
KAQRAVAMYFIDKLALRVGNEKGSDEADTVGCCSLRVEHIELKPDHVVKFDFLGKDSIRY
ENEAVVLPRVYELLRRFTKGKKTGDDIFDMITPTVLNDHLKSFMAGLSAKVFRTYNASIT
LDRWFKEKPVPPSASVADKLAYFNKANTEVAILCNHQKSISKNHHNLMHQLTQKSSYTRT
IIDALKRAQETTKKKSLEQAVKEYFAEMDEMQYKWLEAYGTEEQKKEYDEIVKNRGMPRQ
SSSTSKSNGSRSNGKKKKRNSGDGERGKKKRAKSAKKSKKQKKGVKKSKKTTSHKSRSRA
KNAEVSESEDDMSVADIVKKKSKIDTSNKDGRRGRVKRDRGGNPKLKAEASDDEDVPLSA
LFGA*
>TCRU_2702 mago nashi-like protein, putative
MTTEVPCEEGAMTMPEETMESHLYHLRYFAGHTGRYGNEFLEFDIRDDGTLKYGNNSRYR
NENIIKKQARVSPAVLEEIKRVIITSGVLESDDERWPQPDRNGRQELEIHLGNTHISLVT
NKITSISEVEQTDNSKGLETFYYFVRDIKSLVLALVSLHFKIKAC*
>TCRU_2703 serine/arginine-rich protein specific kinase SRPK, putative
MPKFNKKTRGWCAKKNGKSAPTKKKTKYIDPCGTNEVDASINTAIEEVESHEEPLGQPHF
SKIKRKKNDKLQLQEIGRLTLKPGMTVAQAHEKANKVYQQKPLQNIPDPSFEEEEEGYEE
GEEDHGGSDEDYSDTANERSVEYRKGGYHPVVVGEVYHDRYRVVRKLGWGYFSTVWLVWD
YVTKRYQALKVQKSAKHYTEAAYDEIKLLGEIMSSDPDKTCCCARMNDYFEHTGPNGVHV
CMVFDVYGEDLLSLIDRYEYRGVPLPIVKCISRQVLVGLEHLHSLDIIHTDLKPENVLLS
SPKHAIISLMKRYHPPPLHQRLRLVERDPKTMTKSQRRRYYKKLKAIEQNGKKNENISEK
GNQCATAKTHKNSIEQNREQDQAEAISESETDSDWEIERLHHVVLADFGNSCWTYRQFTD
EVQTRQYRCPEVILGEPYSTPIDLWSAACLIFELITGEFLFDPRKGENYSRDEDHLALMT
ELLGDLPESMRLGEGKYRSQFYNSRGALRNIKDLNFWSLDDVLYRKHKFTRKKAEEIADF
LLPMLEFDPQKRATATEMLANFEHFFEIKEDDYSPFCFVSSDADREMVEASTSNEEDSET
SGDSNDADDESENNSLGREKTRCGNKEMDSAQFWDEHPLLAREFLEKRGLSLLDIHDLLA
GKELEDPAAHKSALEVIHLLSEEAADARGEGHADRDGDATSRVEELNNSIGDAGSD*
>TCRU_2704 homoserine kinase, putative
MSDPPKKVVLRVPATTANLGPAYDVLGMALSIFMEVTVEHADEFSVHLEGDGSEHISADR
ENLMVKACELAFTEFAKKAMPPLKFTMKSNIPFGCGCGSSSAAAVAGFVAGMKLCGLTMK
TEEEEALLQVIARFEGHPDNAAPAIYGGIQLGYKDNNGRFLTYRVPTPASLLVVLFVPKN
KMKLNTHATRNLIPSSVSLEDTVFNISRSSILVLALCTGDLRLLKSCDDKLHQKQRADAL
FPHFRPCVDAAMAAGAHYAFLSGAGPSVCALVGGRHGDPLTQPRGERIAESVAEAMIQAA
NAVGVAGRAILTQPSDQGVHCVGVTTIRPDMEYISI*
>TCRU_2705 hypothetical protein, conserved

MAMMMTGRVPLVCALCVLLCGAGGRCDEEERAVSGSGDEPPPGSAEHGTPRKETQELKVG
APGVIETASPPSLKPTGEDKDDDDEDDAGGDEDDDETDEAKEKRREGQSNKGGTVLPDTG
SGERNLTGSGQETGQSNVPAGGISPSGSQLSNANPLRTEVEEKNDTDKNTPAAENALKTA
NGENTLPGGIAEGNPPSPPEEGFDSREQDGEDTTSEDEKNAPPPETAATPQSHRDEGSEG
TGEDTKATTVTANTTDTTNTQNSDSSTAASHTTSPLLLLLVVACAAAAVVAA*
>TCRU_2706 retrotransposon hot spot protein, RHS3, putative
MERGGAWPRPRLMVLASDKAWPYSWGACEFNRDCYVNCEVRRAWQIVKGVNKWFSGHGGT
DFKPDPFVLIGTPGIGKSMAAGSYLLYQLLHYDAERLPAVACFICN*
>TCRU_2707 retrotransposon hot spot protein, RHS3, putative
MNCPDESDVRGMRVWEQRSKPPQQQAEYWREVRGRMDKVGPILRHIFDEQSYDDRIEKCR
ETVEETISPETQYYTGLGNSTMWGGSSVFHWLAKVVRIREEACKGGFSLNLPISDHLCNR
TLCMLAKLMQQDDFNSLIETQA*
>TCRU_2708 hypothetical protein, conserved
MVPNASTKRPSFANGPRILVALNVSGIVSVTYGGREHVAADNTDEVSEHDATGHKSLVLE
NGVAGSGFVAEGDADGLSQRNACGRDNKPQ*
>TCRU_2709 Reverse transcriptase (RNA-dependent DNA polymerase), putative
MDRPLTPYELDVAIRDSSLGSAPGHDNMLNEFLHRLGPVACGTPRTMIHNSFADGSLPGS
WNTVDTIPIPKPEKDPCRPVSYRPITLLSVLLKLTEGMIHRRLSALLPHRPRQFGFTPPR
STSDVVTLVIGKIARGLNESSTVEYERPGGGAPQATLVTIARS*
>TCRU_2710 retrotransposon hot spot protein, RHS3, putative
MAAGSYLLYQLLHCDVTKIQVVVHCFGGGDAYVSDKTTRAVKRCGDEDMCVSELRRLRGH
GRNVYITYDVAKEGTPPPRHFAPTSGCGVIAVSFPKVTNYDDWAKQLQAARIIVNCPDEM
DVKAMCAWITRDETKEKQAECWKEVKKHMYLLGPIPRHVFDEEAFKERCGAVRFALQSIN
EVTVKEHFSRGGESPWYSEDPSHKPVKFVREIYAGGVTPFNAPISACLEERTLERLPSVA
K*
>TCRU_2711 retrotransposon hot spot protein, RHS3, putative
MKEKDTITPLARGTPNGVLTQVLTEARREAEERLRRTQEMKFTISSNIEEVLFKGRVRVN
EMKLNDFITRELDGRGVVDTNRDVLLEEFFKDPTKYIRDEGALNEMQASGHYLGMKRAVK
GEVIFDEDTRRLCDKGVSNLPGWSLLLRR*
>TCRU_2712 BNR repeat-like domain containing protein, putative
MSRRVFTSAVLLLLVVMMCYDTGAAATNGAASCPVSSPGKHFAWRDVKEGEKVDSLHAPA
LVEVNGGVFAVAKAQCTKDEGCVFTGIASELLEFNGETLKEELHESKLKTQVLDECSASE
ERQCPSQAVKPDSSQRVMNLRVGRPTTLVKGSDIYMLVGNYSRTAANNPEESV*
>TCRU_2713 hypothetical protein, conserved
MTLVPYTPSEWCDIGARLWDMVSQCGETQVQMTFINGNQMSDETLTVAPSSTGLCLFHGP
VSKAWQDSTLIGHIANLRAPQIPNFETQALLPAMGAARELGHQQGLITAERERLNIDYAN
AVADVQNRNAQLNSQFQKAHHNLSVRESTIAGAGNQVLELRQQLEADRAQLREREFNLGV
LTGQLQEREARLNALQTARDAETAGRDESPAT*
>TCRU_2714 hypothetical protein, conserved
MSEDEEDIIVLQVCANQRCLGIEDLEFDEESGEMYCVNCRELYARAEDEGFRLLLTDEDM
PLINMIFNCFDGGKRYWTYEDFDRFRGYTGQSSETAINSHEALRDFFKEEYDIEISKGAT
GEYVVYKQNLEEMYGGYIYNNINALIADCDSLEDAGMIRTATLE*
>TCRU_2715 hypothetical protein
MLEVSTVSSPTSTATLRRKEVQLFHRAQERWKRRIAAVSVHELRLEAEKDHLRQLRRHGS
SRAHQTKSPGLFQEKGKCEPAVTVTEENETSREGHAEARDEPQPHLASAPPRLPPPPPPP
RGK*
>TCRU_2716 PX domain containing protein, putative
MVSKYPTIRLNVVDASPSGEDIVLKLCTMDPERPTRILSVIEQRYSQFLLLHATMCAFLA
EIELPPLAVSGFFGGFDANYLEDSRVSLEVFLNSVASHRLLCNDPDFLSLIGLEQDMCRE
QREEENPKREEYKKWAVICFMTVPGRAIFLG*
>TCRU_2717 hypothetical protein, conserved
MKERMPSVAEVESLIALTRRSLAIPPRNVYEAENAYLANVERLKEARMKLGQFTGEYVER
LLREMDKLEQDLNDMVIRKSNPVDALLDVDFDAEGMDNDLDEVAAAEKEMEKKRDGRKKQ

GGRHTSNKKTQAKADTTNKKEGIGGTSDLTPEGAVAQEEEKDTATVAETADVEVEVLAKY
NECSSSEIPQEVLGPGRCLMVPNTPLVRLDFTSPVDYDGILCQCRVTLHRAVVTALTYGV
SSTTASNCVAAENSLEIVKADGDDEPPCNVSGAIRHEERQDAEDEAELEEMDEASVIAKL
SKQLVAEYDAAQGGKEDEESGTNLLPLDVPPKLCDSLRSFTFLPWVILMCHGGYFAGGVF
LDKKPVVHKSFQRYVVRKKQGGKQSSNEKEGGSYGSVGSQIRREQEVKWKTDVRDILLSW
RNYIDAAAIVLYVAPGPHNRAVLTDFSGLPAITTGNGERAVSPVSLKDPRVRKAPLTTHR
PTFQEVQRIYMTVSTCTVEYVRCIV*
>TCRU_2718 hypothetical protein, conserved
MADESFFYGLLSAFFRSAAYSCLTGCLHSAPFEALRSFIQGDVTIIMAAPSLTGFECDNT
NDCKESDEDYGDDLHRGADYLRVLVHESREGLNAVPTIPGLAEPLAFALSTAIISAPCVE
ADPFTVEFTQQLSSVCAADPALALSHVSQRCLACDLMSVLSRPPPPVFFLCPIRNTLRCG
GYLFA*
>TCRU_2719 hypothetical protein
MHRHAWRGQIFWDFDVRQRHGGSPGGAAAGRGESCIGAWSLLHGLAAHGGAAARRRTGLA
AGTSVGAAARAECAAASGAQRPTAGAARGGAARRVVFCCEGGGGLCARIAGEGADGAAAA
RGSC*
>TCRU_2720 hypothetical protein
MQNGFSQREVPSSVIDSGRDSPFPSQLPFSATRVPLLDNTVNRDDWDNDDNDDNDAGGGS
SNSSFVDLDQIAVQMNVRVEDLLRALQNGLGGGGGYTAPENRLRSQFSFAGSTSLNRGDD
NNNGTRNNCSGIDFFSYVDHDNESNHADNRSGENESGMFGVLRQHLSKEALSKTQLLSFA
VEDSNLSSQEREQQIREGTGTETVTAPIVFLSTSSTRGSGLHLSPIPSTEPLMRANAPSP
LPSPSPQPPRRIRFGSRVVTSVCVYSVNNEVYFGLQFRQHWSSWAALLCGFLLETVFEVH
LDWFAKVGSATTHDTIPIAHWVYTYRSLFSVVYGLLWLLCGAWRDGVRSLVAVTAEPLGV
CMKSLAVTLVSSFFFSLAAASMVLTNEMSGSSLLFTTTVMHCLWILFYRVSRAQMVFLVE
VCGSIMLIVGNVLCNVDGMRLMGLRDTIYGNLLMSGGSLLFATAFLVMAYGLQRGLCSTT
GLTVAVGIELFFTTFIMGVCYGYSMSSGVGDSLIAGFQHANAMHSCLLAAEDLFFNVMYL
YALYHLDVLSVSACFSLKVAVVPIVEHFLVWRVAEKVPPVVRARWGPLLFVGVGVVAVAA
AFVMFFASVRRRFVARRLFHMRGLRRVPDPYQKKKRGRHERRQRLVAPSSSW*
>TCRU_2721 hypothetical protein
MSITLRGELSQSRNADIRAMETRNCTALVTTVGIIMKGMRMMARSEMAGKTISGVSSCPR
MWYRRSTTAIMTGGVASLMNDVSTRQTCTPLRYCSSMSRFASMFAMKRGSHVINFAMRIP
EKNSFVAFTRASVVVARRLRSRIMRRITSALSGSVVLRAATATSALHPTSRYKRKMENTA
TIGELQRYMQNCRNSSARSTSVLTILIIILPVLISGIANDLGATTRHFSNITAVSALRKRR
PPLMLM*
>TCRU_2722 60S ribosomal protein L19, putative
MVSLKLQARLAADILRCGRHRVWLDPNEASEISNANSRKSVRKLIKDGLIIRKPVKVHSR
SRWRHMKEAKSMGRHEGAGRREGTREARMPSKELWMRRLRILRRLLRKYREEKKIDRHIY
RELYVKAKGNVFRNKRNLMEHIHKVKNEKKKERQLAEQLAAKRLKDEQHRHKARKQELRK
REKDRERARREDAAAAAAAKQKAAAKKAAAPSGKKSAKAATAPAKAAAAPAKAAAAPAKA
AAAPAKAAAAPAKAAAAPAKAATAPAKAAAAPAKAATAPAKAAAAPAKAAAAPAKAATAP
AKAAAAPAKAATAPAKAAAAPAKAATAPAKAAAAPAKAATAPAKAAAAPAKAAAAPAKAA
TAPVGKKAGGKK*
>TCRU_2723 hypothetical protein, conserved
MSHVTADLEYFKCDMCGVYLHKDIFCDHRRECKGLDSKELKKSQCHQIGMALDKEARHRI
ASRMVDGATLVPVELAERHQQARVRRNVANSYQAEIDKRLQEQLAPERMRALSAFLSE*
>TCRU_2724 hypothetical protein, conserved
MRRSLPWSVGDQQHVASVVRRWRKSRVHLECPRLCFLRALPTGMLGSSVATSARGACQED
DETIAASACEASLTIITPGRARRTMCRHAADKRAHSAADWAGGHQSAARWSTRCCGAHVG
KRFRRRQRRAGLLGKERDGTTDTGSSL*
>TCRU_2725 hypothetical protein, conserved
MTASASAVTLRGIPQCHGEHLKPTVRTRSTSESGTTQISSATSSCAASEPRRSDSIAERE
SVAAGIRVGDWDAAARRGTENCLWPSSVAGHSARQEHARLPAAAR*
>TCRU_2726 Protein of unknown function (DUF3522), putative
MRPRYNRNEASWVTFITCCLSHLALLPAIRYLQKRGMHYEVCVSVFSTLVSFLYHSSESL

RTPLFLSTIRWHRLDNIGAISSITIVCLHVCSFESEKLVNYLKYFFLFVTIVLQEADPWN
ILYTAIPILCALAMVLVAMLLSPIRRRRIAGKNLYVGMASSLLGVICFLRGLDEDRDTYR
AWHGLFHVLVGMGIYNIFLALGDWGKGSYSRKMSMTSPSFDV*
>TCRU_2727 Protein of unknown function (DUF3522), putative
MRDEPAWALFATTVVSHVPMIPTAVLFYKRRYVYEACVAAFGLASSLLYHTCQSFDVEMF
LDELGWHRLDNIAVLAGVGMWFVFLCTFKDPVVERSVKYFTLLASIIIEEKDPWNVAYTV
VPITLFACIPVAVFLYTRQVPAIEYRQFIIAAIFMCLAVPFFIAGLNDTEDPCRIYHGMW
HLFGGISSYFMWLMVKTPGATGPYPKFGSITVRGDTLL*
>TCRU_2728 retrotransposon hot spot protein, RHS3, putative
MVLTSDDEWPYTMNAPHRCGNDLCVNCEVDRVWQTVKGDLTKWFSSHGRTEFKPKKRVLI
GTSGIGKSMGAGSHLLYQLLQYDAKRLQMVAYSVAEQTFLFDKTTRTMSEDVVEPDTLNI
VDGLLDRGFKGCLIYDVAMKGCQPSTSLSCKGWGMILVTSPHAS*
>TCRU_2729 retrotransposon hot spot protein, RHS3, putative
MNCPDESDVRAMCVWKKHNRPAQRQADYGHEVKGRMDNVGPLLRYIFDEQAYDDCIEKCH
EAVDGMAFLETGYGFVLGDSMTWDDNNVFH*
>TCRU_2730 BNR repeat-like domain containing protein, putative
MDVARKAGYDSFRAPSLVEVDGVVVAVAEAHYKNPKESELYAGIAAKWLKGNEKKWMNGK
VVGMNHFDTKVDRLLGATSIVYYGEMNALFGGCGM*
>TCRU_2731 BNR repeat-like domain containing protein, putative
MIGSFRIIRLPRTASSLWSLNGGQRSPHDDVVCGRQPQGLGGGRKGESVDGGIRPSFTRV
GQLTDASGARRAGWLCQRDDRWTERRPFQPAGVFLEGGEEAGRVDLRLTDMQRIYDVGPV
SAENERFAASTLLYATDEVRSLLVEKEWIKLYYSHGVAAADDECNIAFVDLTEKLEDVKR
VLVAWKEKDA*
>TCRU_2732 Trans-sialidase of Trypanosoma hydrophobic C-terminal,
putative
MEEEAPSSGVLAPALSSTPSVVSRQEVLESKIPVSGGRPEGGREHLPSNAAALMMGQAGK
ASEGSSHNGYTDGWPQRSISRDFFFESVHKEPSTANTLAGDKQHDDPEGETHACTAVGTN
SGPDSFSSTDMTPADVATAAPEPGADPATAHHNGNVLDGAGAAPAHSSTTPGKTKVPSEF
NAAPALGSDPFDLESVTALVSVGLRCGNTVHGCVSRVLLLLLVGLWGAAALC*
>TCRU_2733 hypothetical protein, conserved
MTTKWSPPSDTHPIIYGVCNDARGSPVTNYGEDLNIGMPVTVLDCGACTVDAVCFAARTS
SISGCECVCAAGGHGDTCLPAAVPDGLGPLPLPDADNTEVRCVHGGIIGFVDVPDPGVRG
LCFVNVTFTAAVFLDLSYFDAPQQTLNITLLQCVFMSLLIKGSDARVHVNVTSSMLDSGA
LVFEGHFGASSQILVAGSKLLTMSSYAILFLNFFLG*
>TCRU_2734 hypothetical protein, conserved
MQRRCGVLHARDGVGGPQLVLVQLQGRMAWRVVSSPRGARRGCAALAGESRRRQHELRGE
PDADKPHAEHVEVAPLLLGCDVQRRGCCADVFL*
>TCRU_2735 hypothetical protein, conserved
MFQLIHVTPGSAVLLVLLVVMFCAGGCLAASLTYSCPSDKAMRAHRAKSIPAVVRDVPFG
SEGASQTCTVIGRNGWAPIRMNVSTRDLDDARKYCSSERYCAMNSLVAIASVKATFFYAE
KREVFVSLYFAATVR*
>TCRU_2736 hypothetical protein
MLSCRGRGHSHSVCGGAPRGLSGAQRLVGIAAEEWLKGYGCHWSLQRCVVRFLLVDTLDI
FGSDLRVRHLLGDRGAVAESTCEAFCNDWSARVSASGVCLHIVDGQEDAAAAWAGRR*
>TCRU_2737 retrotransposon hot spot protein, RHS3, putative
MERTVRDEMDMEGDVRKLYKNGVDTLLKWSLAAAEVKAAVHNSTKHFLDAAAEEARKTKK
SSAPIYLEGHYESVYNARWHHVMEVPGGEGTGMEVREGNHHSHGRTRQLAELSKRMTVCG
DPVQHFLGRWCLPRRRDGRTRGIRYRIFLMTFL*
>TCRU_2738 retrotransposon hot spot protein, RHS3, putative
MAPRRGSCDGSDAATRRGVEGTRQPQWTMSSSVKDVLLKGKESITNMRLNDFLRNYFDGR
GVEEFSENVIMKDFLISPNEFIQDELFLRTIEASPPYQEQKKGREEFYMLLEASNKLSHE
RIFTLRQWRDFERKDKVTPLARAQINTAYS*
>TCRU_2739 vacuolar assembly protein vps41, putative
MPHVGSRIANVARSKANDVWLMTAVVHVATSDLGKHYETLYRRRGAAIRVDPRMAVCSFC

HQPTLADVVIFGCSHTYHANCVIGYLAGEGVLLVQPADVDVGRFFNHPEEYLRPGSRKVS
PRCLVCCEVRGAVK*
>TCRU_2740 U3 small nuclear ribonucleoprotein (snRNP), putative
MRRSVIRLRKEFLERKQNERVHEAIHARKEQFREAVSNATPLPGHLRKDALALKKFTELD
DDQTRTLQTSVDDEYANAGVEDPRVLVTTSREPSQKLLEFAKEIRLVIPSAVRMNRGNLS
VRQLMDAARRGQYSDVVVLQESQGVPDSLTVSHLPLGPTVVFTIHNLVTRHDIQDVGTMS
EQHPHLIFENFTTRLGRRVRDVLKFLFPVPKPDATRVLTFDNQNDFVSFRHHTFRAVKGR
EVQLTEVGPRMELAPYRITLGTLEMDDAETEWVLQPYMNTAKKRRLL*
>TCRU_2741 L-threonine 3-dehydrogenase, putative
MLRFSLPLFAAAVPRVLITGGLGQIGTDLTLTLREKFGADSVLTSDLVTPPEKHPLAGGK
GYQQLNCMDRDAYEKLVKDFKPTWLFHLPAIMSVRGEAEPQLAMNLNINSTRHALDLARD
YKMRCFIPSTIAAFGDKCGKVNTKDDTILNPSTVYGVTKVFTELIGTYYREKMGTDFRSV
RFPGIISAATLPGGGATDYAIHMYHCALLGKKYVCPVRRDEPLPMLYMPDALGGVVKLME
ASDSCLKRAVYNISGFSFTPEQLRASIEARMKRPLEVEYVEGPAQSIAHSWPDSLDDSNA
RQDWGYETKYDVDKMTDDMLRQIPVLHGLPSL*
>TCRU_2742 zinc finger protein family member, putative
MPSKGSAANTTTGSQLEVFDSNFKCLYTVPSHLVVHTPPARFNISRFVICRNYRCGEADS
CPKGEGCRFVHADVDYSTLEGQPIHVNYIWRDEKLCIYERLPPGDVLEVLSHSGSEKPDL
IPSERILVTRRALSYREDNSRRLSHCVHYYCNRMCNRGENCNFIHAVYVDPNVEGDFKRP
PAQSMSQFNSSTRSSQTDASDSPLEWKSQMNSSLRSYGRLPSNKHFGSQVFPKTQSADSL
PLNVVPQELGPFCMAASQSANEAVTSVPLNGSNMSFNRNNNSSVEYNKIFEDAHILRIER
GTRGSGMGSFTPMSQTPMMSSLGLSCDSSGALPIPGEGAKNIPRVAYKTFEGSIGQGFLG
SPCGFTVSPATPMQGAGNFSLSFTTPSPCQMPLNSPQETVLAACGGVITRNKAADRHSGH
MDKLLRVYRHNPYKPIN*
>TCRU_2743 Long-chain-fatty-acid--CoA ligase 5 (EC 6.2.1.3) (Long-chain acyl-CoA synthetase 5) (LACS 5), putative
MNTVTASNLLENSVFSLMDQENIRRMVPHDKIQWIWQYGAQNEPLPGTETENMSAVYRRV
GTNDAEHAKLCHEWYYGDNMPQRFKKICEEGGSRNAFGYRPVLRVSKEVLKDQNGLERAY
DITYLTETRYIDFDTLWGYITAFGRGLAELGIRPNANVAIYEETRWEWLATIYGIWSQSM
VACTVYATLGEAALAYAFRETECSAIVCNAASVRNLLRMMREDLIPSAVIIYLGELPPDV
EAGSHRIFSWHHIVDNGRISEEPLRITTDNDQLAFIMYTSGTTGNPKGAMHTHGSLVSGV
LACADRVNDLIGPFEVGEAYCAYLPLAHIFEFGVVNIFLARGSLVGFGSPRTLLDTFTRP
HGDYFEYKPVFTIGVPRIFDTIKKTVEARLAPRGSLERRIFEHAFQSRLRALKDGMETPY
WNEVVFGPFRYLLGGKMRTMLSAGGPISAPTQTFLNVVFGLMVQGWGLTETVCVGTKQLC
GDIETVVAGQQERICEMCLLDVDEYKHTDTPEPRGEILLRGPFLFKGYYMQEKLTREALD
EDGWFHTGDVGSITTHGRLRIIGRVKALAKNVLGEYIALEALESMYAHNALCMPNGVCVL
VHPARSYICALVLTDETKAMMFAKEHGIEGSYPDILRDPAFQRKATESMQATARKADRQK
FECVRHVRVVGDEWTPENEILTAAGKLKRREIDKRYADVIQLLFVEEC*
>TCRU_2744 Endonuclease/Exonuclease/phosphatase family/Endonuclease-reverse transcriptase, putative
MAIQETWKSSEQIASMHTGDYVLYAQSRIGKGGVVVVLVRTTLRSKRIPLTIPQHDTSLE
VVVVQVALDQNRDLIVASAYMRPPPHVTQSFRRLVNCLPASSPLLLCGDFNMHHPQWEPF
LETSPSEVAAEFLELCTDAGLTLVNKHPW*
>TCRU_2745 BNR repeat-like domain containing protein, putative
MSRHLFYSSVALLIVVMMCCNAGGAQQTIKAAPDPRSLPKTYFVWRDKKGEETVISLRVP
SLVEVKGDVFAVAEAPCKDGGEKVFTGIASELLTLSDEESKELGTNAVKTQVLEECPFDE
KSACHIAGQDDSKTQTRVLLSRPTTVVKGSDIYMLAGNYSWKRAAGVQGADGGYWGLVLV
KGNVSNEKSQKRIFWNDTYAIPWNYNKKNESLTRLIGGGGSGVRMEDGTLVFPVEGTRKK
TTNQVGTEKDEKTVSLIMYSLKDTNSWKLSKGMSDGGCSDPSVVEWKDKKLIMMTACDDG
RRRVYESGDKGESWTEALGTLSRVWGSKQDKEVKVPLEAGSPQRRLAVMKITEM*
>TCRU_2746 BNR repeat-like domain/Concanavalin A-like lectin/glucanases superfamily/Trans-sialidase of Trypanosoma hydrophobic C-terminal, putative
MHDDTLVFPVEATKKGDAQKDVKTVSLIIHNSKDTNEGWKLSKEVPYDGCGDPSVVEWKD

KKLIMMTACDGGRRRVYESADKGESWTEALGTLSRVWGNKHGEEAKAVRSGFITATIGDV
DDNRNVMLVTLPVYSKEDSKKVTGNGKGKLHLWLTDNTHIVDIGPVSDDDAAASSLLYKS
AASGTNKKEEELIALYEKKKEDEKPSPGMVSVLLTEQLERVKEVLATWKKVDDLVSKLCP
SGSTEKSASPDNACSPTVKITDGLVGFLSGNFSDNTWKDEYLGVNATVKKKEDAGETGAT
NTSDGVTFRGAWAEWPVGKQGDNQLYHFANYNFTLVATVSIDKVPQGDTPVPLLGVRLND
EKKTKLMELSYDSGKKWQVLSGDKTFKELKSALEPEKTHQVVLVVRNGTQGSAYVDGKQV
GGDEACALENMDSRAVSHFYIGGGGDSAVSTGIEEEMSVTVTNVLLYNRPLSSEEIGALN
PNKASIPPVVHEKAQGTQSQSSSAGQPPSGPKLLNGNEGVGGGSTSTSAPSTVTNSGKEQ
SEIQPPSAISSGGSKHVDVASSSDGDPRVGAEAGGAVQGDTPPQTPVGTPATADTNAPTA
TNVAQVDTATTTDVGVSSGADEETAGGTDGQKREDIHARDGEVKAAALSSSLGNVSQGNN
SDACTVRGSGLPSLLLLLGLWVFAAL*
>TCRU_2747 hypothetical protein, conserved
MAMMTGRVLLVCALCVLWCGLSGVAADGEEDAEKKKDESSGGPGPDADDGSAGLQAMQFS
VKGGPEREDHSLGAQTGNSTNEQSVVVISPPESSIGPEEQAEEEEEEGGETVQKEKLEST
TEEEETPKLTLPQEGEGPPAPLTTRKNEKPAETSDATPGKGQSSSGDVSTRPELHKTPSE
EQPSSTAPQAPASAAAAQIKNESTTSADVVSGQQIKEESQHDMQSEGASGKEENVPTTVT
KKDGPTESNAESTPTPPSASKDAITNDDEKSNEEVIPKHDQTPDGETMNEASQVENNEGN
TNKTQTVEAAAITNNTATPGDSDSSTAVSHTTSPLLLLLLVVACAAAAAVVAA*
>TCRU_2748 hypothetical protein, conserved
MLTSEDPAAKHAGQAGQAIRPPPSPESDGTRPSGSSRGDSSVSPLSTAGISSSSVLDAPL
VQSTQPSEKGLQSETMSLGTALPNEQPKEITESKAQQGFSTSEEVAKSPDDSGDAVEKKK
EKGDVGPKATTSLSSTTSNPPVTQTTPRPPSAEPSPTETELQAGEETSTENVTITMRNAT
ANTGDSDGSTAVSHATSPLLLLLLVVACAAAAAVVAA*
>TCRU_2749 hypothetical protein, conserved
MRRRLDSIHFVSAFMHADDRTLVFLDAYILACGAAMKAAVSLVATWTAEHSLRINADRSG
AALFCIAPHRQSDEDTADHRLGGGKPRVKFHPVRLLGNAIDRLLIFGLHVTVAAGQTVPR
CRQLRLDAEAGAFQHTMRSLPVGCVHSALHHGGKAIAPCPAPTHLHSLEVRHRDSCTASL
GPRASSKDTSVHLAVNPVPLRRIVGFRAPTQHERLTRLRYIEDVRGAICSETTPSSIHWK
AATTISLPGDAVLKELRSVFRHMGISRTTSAPLMLSSELLPGRCEPQRGGRLPATARQGR
PGRCQTIGR*
>TCRU_2750 hypothetical protein, conserved
MGVVSPFPETSQAAVREGGVAHRSECATGSGCKMAQKLIHCAAGARGHATQTVAGGQFKH
TRREGSIHITATRGHASTKCGDNNGDRSTCNADSVTEERFACCHGRAQTQSQEYCSTADP
RRQGRGAAMSRGITGLGSTGHQAVSHRPLAAWRNHRRGHDCGPPVCGEIQLEVNCLLLRL
CARRMLPLQWTRHAPFRQSTSLWELSRLQHGMLRLAASKVPRCISNLVITAARDVAHAKV
KASRSASRRTTATCFRRGSMPVCRGRPSSVPAVWDGRWWSDSM*
>TCRU_2751 hypothetical protein, conserved
MAWVDVVTSVLPATSIWELAPKPPSNSNAPESSIEDVTFTCTRAPLPLTDRPMRTHCSSV
MLSVCSGASKLHRSSTIAAVKVTLTKHRPRTPGAGTSTELMLPPCTQRTSVSSASGSGSG
PRPSGTAAGRHVSPWPPAAHTHPQPLTLLVLAAKHTASTVQAPQSNTVTGEPILSSSP*
>TCRU_2752 hypothetical protein, conserved
MTKRVDRSRTPSRTSAGPYRNLSWPRVVGKYCDPPTVARSVEFASSTLECSGIAPRSTIV
PSSDVTFVMLTRAWGIRRAVLSLRPPIRSCAPPASTQWRSVTFSASANTFIERRSTATVI
GPLSTTESRHAVARQSPTVTDSVSVTPLTQPGAPPPALAGGTSNGRHASPTPPARQLHAQ
SENDAVSASKHASAAAQPSHGTTLTEGRPAMR*
>TCRU_2753 hypothetical protein, conserved
MHRGRGRTARRLQQHAVKHHRARQQVHTAANVHRRPARLRRPQHSAAHTQLRLQQRHRVQ
HKRRRHRVRQPAQLRVAHRPRRHNRRVTDRHVRACRQVVHKHERAVAPRQPAHAHLQAPV
TRRLAAVAPHVDLRTAVAHAVPQFDAQRAGKHRRNVEHHSHAVRDAHAVHQHRVHGAAHR
DGSLHRAAPQSQRHRMHRGHPHSSSSNQRQHNEQRRTHSRNGPVPHAVPAARTRTHRRPH
PPTQPGINRAVVVCRTRLVDRASCSANPFLQMPNTVTRFRQVVIPLLLLLIILLLLLRVI
TKTLKR*
>TCRU_2754 BNR repeat-like domain/Concanavalin A-like lectin/
glucanases superfamily/Trans-sialidase of Trypanosoma hydrophobic C-

terminal, putative
MSRRVFTSTVLLLFVMVCCGTGGAAASDGKPSEPNYKWKDVKSEDGVTVNSLGVPGLLKV
GSDVFAVAEAQCKKDGKYFTGIASQLLSMDKGKEKEEVLKDARVTQVLEEVTSSEGEKEV
DVSRPTTVVNGSDIYMLVGKNSRTLQAGDQAIGWGLMLVKGHVSGENQRDKRIYWNDTYA
IPWTYKGNQHSILKRLIGSGGSGLKMNDGTLVFPVEGKKEVDTQEDGKTVSLVLYLQGTE
SWTLSKEVPADGCSVPSVVEWEKDKLMMMTACDDGRRRVYESGDKGTTWTEALGTLSRVW
GNKHEGNEKGVRSGFSTATLDGVENDNKKNVMLVTLPVYSENVEKGVLHLWLTDNTHIAY
IGLVSEEDEEEITASSLLYKSGKGNNEQNDELIALYEKKKGDGDTPSPGMVSVLLTGQLK
RVKEVLTTWNNVDQSVSQLCPPSAEKDRPTENDCSAVKITDGLVGFLSGNFSGNTWMDEY
LGVNATVKNNDDGAATGYADGVTFQGAWAEWPVGRQGENQLYHFANYNFTLVATVTIDGV
PKGGSIPMIGAKLNDSENTVLLGLSYDKEGKWILLCGGKTNKEHSSDWEPQTQYQVAIVL
QNGNQSSAYVDGQPVGGDEQCKLETADSKEISYFYIGGDGSSAEGQEGVPVTVTNVLLYN
RPLTSEEIGALNPNKAPISPPEDLTEAVVVDTPSTAVGGPVAQKTVSVSTPGGSTVKHES
SASSAEDEGTVGGTDAQKEEVHPQAGEVNAAALSSSPGNFSQLNNSDAITIHGSGLLPSL
LLLLGLWGFAAL*
>TCRU_2755 hypothetical protein, conserved
MGLIHLENLEQCQDEWRRKTEARVNQETLMDPPRLDSPRSLRACALCGIDPATSLESLSL
RQHFEQVVSGAPWVDRSVSTSVRAQDALNACEESGILTGSTQQKMQAYSNFLRYEENRRD
ALMLAQELRRQLIAEEIFERRRRVHVDRGSGLVSSLCELGSESRLEHDVLQVSLRPNGGT
LAAEAWPVPPSGGTAMVPMEPKKQAKGKEWQPLERRWLGSPPDENTTVSAAAKGTLTETA
LEDDHKSMRTAAATVAGTAVEAKEVQILEENAPPAPPISSREQDEVFYYYCANAMVTGRI
SKCRETSFF*
>TCRU_2756 hypothetical protein, conserved
MKDSFGSPSFALAGEVMVAFAEGHKDYNGPQHTSDLYTSEIVAGYINATEPWSSIVADIS
SDDWRAYTVFTKESSKDRFGVALLPTAVSTGSDVFLLVGSYYLIRDATGRRWIDDGWDIH
LLVDKATQSTDAKQGELIEWGAPTSLLASIT*
>TCRU_2757 BNR repeat-like domain containing protein, putative
MRFVGGGGSGIVTEEGWLVFPMMATRKNDRGIESMLFYSKDKGVNWDVSRGIPAADCVDP
RITEWENRELLMLAYCVDGQKVFRSRDMGDAWTKASDLLPRVWITTQAIPRGEGWRVGAL
ITVELYGRKVLLYTQKVGYPPGERNDKALYLWVADSNHTVYFGPLSVETGAKRAFANTLL
CSGDALYLLQGMGNGKMDGVFLFRIIEELRKIKSIVRNWMEVDAFFSGLSTPTNGLVGVL
SDSSDADKWNDMYRCVDATVLGAIRIEDGFKFTGHGAGGIWPVNSRGDNKQYTFVDFTFT
LVATVVIEKMPPGGSSVPLLGAMLEDGMGTKFLGLACAENRMWEKCLMGKRGRATFGCRK
KNTRWHSC*
>TCRU_2758 hypothetical protein, conserved
MELIRKRRSTVVRRITDIPALGQLYEESEQGGSRDVRAVNVGTENVPSRAIHNLLPHART
ADERGELRGLFDGNIRCGAIDPTAVRQDAAVHFTNGSNPARHDDFGVTKDCGAIGDEASA
PLDRGRDESIYPQQDRLEGTCCLTPLTEEGMNQVIRSRTD*
>TCRU_2759 hypothetical protein, conserved
MQAEARAVRLALSAFSAILPSTMDVWVDNTSLQGAANKGSPKSHALMWGMRRIYEFLDSR
GIQASSAYVRSAENPADGISRGRVFTLQDLAKGWNLRRAAAESCGWRPPKSANS*
>TCRU_2760 TFIIH basal transcription factor subunit
MSELGEILNTAQISKHYKVIQFLLSHANVEYTLTQLDFLIPRGASLQRFPEAWREYMEAG
RCGNQCIEVFHRIVDVPDVGVTDDRAVRNVRSKKELVLVCRRPELHRLEELERLLLSPAK
VPDREGCVALHTNQVILQEKLLKESQQRGLLYYLPDHYMKAYECRLVKGFRSPFKRGESL
NVFLCDDEADAVLGADGTAGQLELPVGVVAQSMLYTRRGVPVRGKNSIPTCLHVGERVLV
IFDRVLSPSHDSSMSFEAPALRVSLGKPRKNLDGALPVDVRVKASRRTGGTQRSFMDVRA
QAKGNVAVSVFVDEMETIVPVEVIDEVTSMPGVMIGREALPALPVPDGICIHDPALWGGR
IAGQGGEGEEFRGEGRKLAGTVLAWWLNPSPAVLKVRDLTIPDSETMSAASRAVWIPHQK
DEAALRVVRKTLQEQHAAEVARRIRRKRNRNRELRNCHMVHFGFDASVPFGGEMQDR*
>TCRU_2761 hypothetical protein
MQCRGGNSTGSGGLKGSFLPLGAYRRVPREATILWKTTMDTAKEQVAVVWETVSSWEGSE
VTSSDSCASGINDPSLEDVGLWEEHLQIRMELIRRLNPFRQGPPVGGAAHMTASAERNGM
TGTKLGSIRTQRACGSVTHGECVGEDILFPNHENLCTAPKTPQTYQKVRRNPCECTCRVR

RLAPAEWSELLRLGSEDVVEL*
>TCRU_2762 ribosomal RNA-processing protein 8, putative
MFGGSKGRKGGKHVRSAKKENSAASTERHPKKVHRISTDPTKKSHNGSTLKGPVVSHSGG
NASRDFANDRQHIPFKNKLEERAYKKANRIPLYTPAPGGKKGDSDATAKRSSGASSAAQR
RAIMKAKGDDAVLEHFRTKLSSSTFRLLNEQIYNSPVAFASQLLRDPSTYADYHNGYRQQ
LEQWPLKPSQVVVDALLGDRRGRFLANKAKSMPGYIPTSWVIADMGCGDAQIAQAMRPKG
YTVHSFDFCAVNDYVTVANATNVPLDNNTVDICIFSLSLMSTDYVECLYEAFRVLKPKRL
LKIIEVRSRVPHPKRFSELVESIGFYLDYYDTVEKYFVAYDFIKREDQAKANRGVKHDPR
EVLVASLYKKR*
>TCRU_2763 glycerol-3-phosphate dehydrogenase (FAD-dependent), putative
MGRRSRRFAARVAAVGVAFVSSWALYHRWQLRRSAVVLLPVDTTMNMPQSHAMRLQLLGS
STKKEPFDVLVIGGGATGLYTAVDAAQRGLRVALVDADDFGAGHTGTSPPLIPGAFSYIQ
RALRQRDLVWLRRGVEAWRALTVWSNVAPGLLENGVSTLVLSCHLLELMELTTAAVIATL
LSAFSGDWRPFRFVRGNVLRKELQEAGKGMRGGILVEDAFLDGNAASIALARTAEALGAV
VLNYASVTSITNVETTAELKGQANFAVIVNDVSTPSGVGELAPRAVTVYTRSIANCAGSW
VDEVKQLVPGNAMDAVPSAVKRHQVRSYLVLPRTAVQTATPNNADLLFGADAFSVAPTSH
SFSSVMMLPWFDGCVLIGPSLSSLFRLPSKSYAVAPTTVLTLAGTKNNNGCNIHDVYISQ
RKHLFRALQVSGVKVDEERVLSCLSSIVPIIASPPHVPLAKDIFMEGCHISSGKEQPNVV
HVYGGTLGFARDIAEKALDRLLHDSGAFSTAETAALRPCQTSRLALVGAQSRDATSDVVS
PVKRIQRIVREEYAARLLDIVARRKHTAYSSPAEALAALPAIAEIMRCELGWTAERTKVE
LEVTKAFIRSISFA*
>TCRU_2764 Transmembrane protein 231, putative
MLREFCFRNAALFHEHYKVRYIPRGLGSWIFYILCLLAILVAPVFIGLAMRNFWLRSNSF
MQMPHVYFTNRCVLRYTTVKGQEKLWTCSNEFNEKHINVQEQLAVLPFFSIYEDDRDGDG
RVDFVTMILSLPINDVGDIEKDVTPSDMGDAVFQVDFLPEFFYEFDHHIVTLKMRTAALF
SFKRDHWIPGTEGGPLSAITEGDLLFHSTEPLIASPNVRYTRTYIDSPFDNVTDVDELLN
VPLFASQYTLRNQSLQFKPKLETVGGLALLRDENYNRVLGEDLDALGAFTWRIKLHIQRA
EVNYVPSIQEALKWAWVQYFCIAYVLQRLLWWLRGILVKTGVLGSTAFFHRGWKSS*
>TCRU_2765 zeta tubulin
MAVVVVQVGQCGNQLGDELWRQLKLTTNSGALPSPFFTRDRRARCVLVDSEPKVVTAVYE
RQKDVLRAENVIHGQSGRGNHWALGYYGVNDPLSHRAAENAVVARPFQVVKDQRRCDSHV
VRNALRAIHAETRRTDDAMEFEAIIVLHSLVGGTGSGMATRLLEKIRYYFIEPVDSSLTV
DECEEAEMMRHDGLDGMMMEKRRASYLVSITVAPQAVGELSTQGLNAALTLHALQENANA
VLLLRNDDCIRVQEDSCAGGRPRGRGNTSFSTPSFSSIPSCATFKEINEVFLTMLLPILL
YGRSTNAVGELVLKCSPNPREINNILTILPTPQRHYLRFKESVTVSRLYVLRGAKQSMPG
VYPSVPIELIHTARSLRNPNLVTSREEVQVPKELLKGLKETAATRRSVSVALLQQMEGVL
VLNEARELNAAVLFRLLRSAAMKVKAGAFLSTFEDMGMTAECIQAAIREVATRLSAAEEI
*
>TCRU_2766 kinetoplast ribosomal PPR-repeat containing protein 3
MRRVNLFARGGFFIGTQCRRAVISPVDRISGANRHSAPRTSYQFAVRDEERGLYDHGVTP
DARILMAEAEAQYRASAHWLHAPPGGVAPPELRLDSDGGAVSCIAAPFAEEYVPSHKERY
AHRGHVGELSMQRAAAPKFLLNEHASLNLRKERQFCELMSSPMPSLQKAMELERLLHDFY
LQRLALSSRNVEAIIALWAQCSVEERLAGRAQVPRLTSSSSAGETHVNSVAAVDAVPFLP
AMKRLYFHARQTHVAPTPLVLECLMTTLAAVTSPDSSVFHFSNRLLLDADKYVVLPTRTT
YAAFFTICSLHNAMSFAMARFKDAVVELHISVDATMATALLKGLCQNGLVEEALALLARI
EHVPLDVQFLNASMEVLLLSAQPQACFSAFDAMQSCPVQPNSETYALLLLACERSGLWCR
TTAILADMQKRRVRGDTKTLNLLLKGLLMERLHSYAKQLYGTMVKKNVEVWPALESQMRL
TGKNRRKEKKPKQPSPMASSTQTSEPC*
>TCRU_2767 hypothetical protein
MAILPHGFIEAIHASPLRCVLYVMGAGSSVIAQLTSVAGCSRTLLDARVPYAMAAALQML
DDRPPKMVSATVARQMAQHAYHRAVEYSNGDDDGTLVGIGSTSAVQTDRIRHGKDCAFVA
AWSERQVVEFAMELPGHLARAEQEEQVTLLLFKALAECAKVPFEISFVVEPKRLSYIVPD
SPLRSLLQGEIKFLVFNTYGELRADFVPYVAETSFAVKTENSWKALLFPGSFRPLHWGHT

ELARAAVRAMATMKGKLNSTSTDISSPSWNVPDASNVRVTYEIAVDNADKGIIDSDVELK
KRVQQFLQRGARVAVTRARLFTEKALLFPGHGFIVGIDTMKRILDPKYYDNSREAMLRAM
QFIREQGGYFVVAGRASGGKDAVWEDLTSIEVPAEVESMLISIRKEDFCVDVSSTMLRER
SKSTC*
>TCRU_2768 SSU ribosomal protein, mitochondrial
MFGRTAFLFRKYRQKSKAPDLKTSFSKTRNFKKAEKIAIETVDGEVVKGRGKLRRRSHSA
LKKAIGEMEHPSIWLWYPWRMNPEPPTPGMPQRRALKNLHGAVFTDLTPVQKKRQEQMLY
GINIPETRQMKFEQEHPLLASALRQLDGQPKGFPFWYKKYPTRRHAYGNRFSIPDEMLEG
YGEEMKKALSKEMMSIQEKQFAQEAMYMERYAEHDFDTTSPAVLAVKRALKCRVLRNHLL
TNPHNNIIKAVLANTEKKLSHALRKLRKVDFKKYWEIIRDHDVQDVLQPSNLVTYRQGAY
WKYDWNAGLAISTNLADVLDPRGLNGCVETGRSRSEVARDLGLSYTRPLQENEKKQLSHQ
ALYYERLAKFKMEQPEAARALERERFVRKFSGMFAKMDIKSGAPDFPSTYRKLLGTKVVR
WASKRHGPA*
>TCRU_of Trypanosoma cruzi region 4/Dispersed gene family protein.1
Dispersed gene family protein.1
MDGTVALGGAGRRFVVGCLTLNGQALQPMDYRSAGIIGEFRPVACGVCDADVHCFAAATR
TMSGSCGCRCAEGGYGRDCLPVYLPHVDGCNHTPGMPLLSRTATLTETRSPTPTWKPTPS
ATHYSPTQCGPTETLQVTETVALPPTRTPTASVSSTLWWSDVACPTLAVTTTAAGGSLTQ
NDIRGGGSAVPTRLMVALPPPFRWARDPQLGTHLSFVPVSTTQPSGFGGPWGAMLSNATW
VRNATNPSTVVELAVPVHRGYYIAVDETIVIRCDAAAVFGGCKGVLLGSFTIRSDMLPAA
ASALSAITGVVAGAAAVAVVVTGGLGSVLEMQALGVFARMSCASAQERASTAALPYFLSV
FAALDPLWMVVGNALLAAVFGCVHCGVTAVFQRWRGVDAASAWAAMRFPSLTYVVAHAMH
LGIFFGSVLALAMPGARVQHRVIGVVGVLYGVAFPAGVCYFIARHTGASFTRYWQFLRKP
LHERLLYPVGYWHPAAQQRMYGGMLTSMRGSRVYWCVFQLSVLCVVGLIAAVHPPVGGCH
VLYFCMAAVLLAGAGVVGFTNMMRSAFLTVIHTASFALLAALCVVSAANHLAPSDGGARA
YAAIVLLLTAVLLAVAVYSVVVWYAEDRHWQELREPRCGGLEALLRDGEESDEETQKPHD
VTSSSYASGTTAASSYRPPAAPLQPMAGDTRPDALSPLDRVSSASCSINYALLDRRRCFA
*
>TCRU_2770 hypothetical protein, conserved
MRELCNDVYARSLKETDDSQVEGICKEYSGKPNDEVKCPKPQTELLPGTGATVEVSVPEG
LGNEEELGKQSEEAPGAPPEDSDTQPTGITASPPVEGSPDKAKDVRVPNSEEGSVSMEPT
KDSAEGDIVPIADTDHNEASNGKAESTTTTPPDASDNANNKAENGTRENIPNNTTESDVT
GTEGKTD*
>TCRU_2771 hypothetical protein, conserved
MKKVLAAWKERDACIAREYGCGNEKNDKKRSGCGGDFGKGLVVFLSSTSTVNTWSGECPC
VNAIVHLAVTCTPDGGLTFQGSGAWTEWPVGEQGQKQLYHFADYKFTLVATVSVPEVMEG
IDSSFPLMGVRMNDNKSTLPFICCGRRKISGR*
>TCRU_2772 BNR repeat-like domain containing protein, putative
MDATRVVCWDLLRVTFLFGVSGVVVAVAEAHFKNNKDGKLRAGIAAKSMEVNGGTWANGT
GMMFDYYDIKVDRLLSPTTIMADGETCALVGGYGESGAPWTKVAVDGYWSSRYARSTVLS
EDDDKYKREFEWNQ*
>TCRU_2773 phosphoglycerate kinase, putative
MSVAVLGSKRAVYDIWPLKNKKFFVLVDPQNILGETTDSNATLNTIEYLLKKQGAVVVGA
SFGPLCGIPLNLPRQQREDAIVAFRREGGRGFSNFFSSISSRMKMEVLKGVPGLVLPSTC
SVDGKTGKTLVFSQLENATKFDALHRVFPKAEFPPVSTFPFVEELAKLLPGVTVKFASDC
LRPPLHQLKPGEILVLENLQFYRNETSSDQAERMAMAEVLAADIDVFVNESFATASKSHA
SNTAIPKILYHGAAGLSMDRELAFFSKFLTQPARPIAVVVAGRNIPEKLLMIRSLIGRVD
KILIAGLVKIPFLSAKGLSSGKSLNLPEVVKRKLLNSRGGDFEEETITCTQLAEEIMSLC
EAKGVELVLPVDHVVSKRLTKTNDASAAVVESMAIPSDVYAMDCASNTIALFAKYIRECE
CVYWAGTFGWTSLGYHEGTRAFATILAQERKLSIIGGRSTARAVQQFGLASHFSHISGGG
VSCLELLQGNLLPGVEALSDVAPELDSKSTFSVDELLRNLPLFSRCTSHQLKSVARKFAR
RIHACGDYLTYRGDRHVSMWVVADGGLVARFGDNTITIPPRYIGRGQTVAMYDFITQSFA
TETVQAALSGTITYQLTSSSLNDLLHEHPDLAAQFLQNVSEPLCLMANEEYQEQTSLCPA
LRRAAACSRVPSSYGVPKEWCLVEDIVQDVVSAVVLQKLTMQYIDYPALPPNHQVKHIWD

KIKCHGALFPAVGASVLRAFVYHKVLPLGLMHASIVTAVAAAPLRLVAMGVTWRGLTYKA
ILDEAMVSVAVSWAPAVAYAGFLVTQRRLELLKRSRCSRIVQLLIMAIIRVVLGFVVFSL
LFRRCDEIGSTSLLGIFRKDAFKAYELKQVISVFLRYVVHLLLGVVRKTSAYTKSIQHGR
QLLDRK*
>TCRU_2774 hypothetical protein, conserved
MSCCSKIPQTADKELDENGVYLENITKTFKLFEKSIAETLNTYQMLMNSFEKVAQCFVEI
AGGSSKNMVGLADGFKEGMRKLRDGPTLSGLQMDITRYVKEGLPPLMAERNKVYKLYKRA
KELKKAEDTYRYKIGEMEKDFARKNRPLGESKSYVKLGKNRERATIKYQGKKQEFVTAME
MFKDLVERFLGTSLPGYAACTSSFSRHLGQVMEAYVSGEHSISSANGSPLNQSKHRPSQR
NSDSTVDQLAHGNQGKNPLSLEEQYYQVE*
>TCRU_2775 hypothetical protein, conserved
MMVPNASKKRLSFANGPKIVVASNASGIVSVTYGGREHVAADNTDEVNEHNATGHKSLVL
ENGVAGSGFVAEGDAGGLSQRNASGHENPNCENSPRSIPETV*
>TCRU_2776 Concanavalin A-like lectin/glucanases superfamily,
putative
MELSYDSGKKWQVLCGDKTFKEHSNTWETEKTHHVVILLRNGTQGSAYVDGKQVGGDVPC
KLETTDSKEVFHFYIGGGGGSAVSTGIEEEMSVTVTNVFLYNRPLSTAEIGALNPNKDSI
PPVVSDNAQGTQSHSSSAGQSPLRPELLNGNEGVGGGSTSTSAPSTVTTSLGKEQPAIQL
PLGISSGGNKDVDVASPSDGDPRVGAEAGGAVQGDTPPQTPVDTSDTADANAPTATNVAQ
VGPADTTEVGASSGANGETAGGTDGQEESTCTGQGSKCHGTQQQSRKCVAGE*
>TCRU_2777 hypothetical protein, conserved
MLDKIKTFVSRQAGQKARNALVSSSGAPPSLKEAFDRAMQMTNTLFEEREWQCPCGHKFR
ASGEWVATLPIYCEVPSCPNPKYYVDGPGRALLEANPSGVKLQEVKDAPSLPASSSSRGV
GARFHNRPK*
>TCRU_2778 hypothetical protein, conserved
MRSCLVGCVHSALHHGGEAVAPCPALTHLHSLGGRHGDSCTASLGPRASAKDASAHLAAS
PAPLRRIIGFRAPTQHEHLTRLRYIEDVRGAICLEAMPSSIHWKAATTISLPGDAVLNGL
RRVRRHMEISPHRNHAPHAEHRIVVWGNGSCSGLDVFQPRHARDALDGVRRSAVEAACAS
IGLCVLDPPRCHSTPRTAAFPVGPHRAAAGSSIVPIAHTSSCCHRYPLGDGGAMPWPAGG
ARRIHRGYLYRAAFTGGSRSRSFLHIFPMTLRDPTQRDSGR*
>TCRU_2779 Endonuclease/Exonuclease/phosphatase family/Endonuclease-
reverse transcriptase, putative
MDCDCLGLPQTARRPVCDGIIATFVRTALALLTRYCDIIRQQQDNNSICDECWPLAQPAT
STRCQGLVLFAREEAKRLLPIRCPRRGRRRRTNGSEGHQMEPFTWLPAERYFYPLLNSIG
AHQRYTYRLRAVCDARRQKLLLSGDIEQNPGPIAVLQMNVSCLTPSKLATLMAQGADIIA
IQETWKSSEQIASMHTGDYVLYAQSRIGKGGGVAVLVEKTLRSKRIPLTIPQHDTSLEVV
VVQVALDQNRDLIVASAYMRPPPQVTQSFRRLVNCLPASSPLLLCGDFNMHHPQWEPFLE
TSPSEVAAEFLELCTDAGLTLANTPGEITYARGTRERSCIDLTWSKHLTVSDWSASVSPL
SDHYMLTFTLHQAFKDTIPSAPPRHLSFSAVGGSASGIYSSRTSTHNFRHTTIKSSPPAL
RLSRERL*
>TCRU_2780 Reverse transcriptase (RNA-dependent DNA polymerase),
putative
MHKDGPRLWDDTLMEAERIATDRKARYLQLPAPDREAEMQRTRSQFFLLLRERLRNTYLR
RISKLNPGEPLAWKYISGRKKASLPSPTSMLLGDGQHTYKTARRAANALNRIFLPFHPSH
KAVRFSKGINRQSASLNVGSSCISEPQNNNESAATFTSGSSLSSSSEPQDKNEAANTSGL
VAHLHSPLDAPFNRTELLAALRNTPYGKAPGPDEVYSEALRHISSKGLRFLLRCINHSWT
TGTIPAEWRRATIVPLLKPGKSPELLESYRPISLTSIVSKVAEKMVLKRLLWVWTPHTHQ
YAYRIMRTTTMQLAHLIHEVEHNRNHYFQVSLPKKSGIGNQLHYRPHRTLLVLVDFSKAF
DSVDHRVLSRLLANIPGVDCRRWLRNFLCGRYAKTRVGHRHSDRRPMLRGVPQGSVLGPY
LFSLYVHPLLNLLNSFAGVTADMYADDLSIIVKGQSREDAIPTANMVLQKLHAWSQENGL
ANNPSECEAAWLTLSTHTESDYDREGRWPLVVAGCQIPVMTMGASRTTKLLGMDLDPRLT
LNVAATKQCAATSQRISQLRCIAHKEAGPSPHDLRTFVIGYCASKLRYGSELIWAVATDS
AKNEMQKTYATLARIVSGVPSTVDPESALLEANTPPLHVLCLRARLSIFENTRACQMDWM
RRPPPEPPPRAGFRISPLSRDEPYAFVDAHTKDYGITESSPREERFFRSSIPPWFAASAH

RVTIGAELPIDHSITDEEELIR*
>TCRU_2781 RNase H, putative
MIPRIRHRRKTLLVVTDSRSLLAALNKGPLSQTDWTEDQIWQRLLTLTCAGWSVHLQFCY
GHCGVHANELADQYATQTMESGQYTEQGIAPLWHTDLLTCFTTQLTNKWRRTIRQDTHRH
LLCGTRPSDLSGKDLITQEVLHRQELVHLERARCGESELWGRLYWAVRDCTNQCRFCNIS
PEQSAYMRSNNDPTAPGTDTVPPSAREEDVSPVGRRTLTRRRKEKCPHCDSTLTGFSGLV
SHCRSFHPEHPPPLPELKCDFCDMVFPTRRSTAPHRSRCAHNPDATRHRNSSARRRSLLP
QDQPASTSTPIGPQETLHHLLPECPGTLAVRRRLGIEQDLRHGKFSQWQLFHSRKLLSLL
DHLFGTQMALYSQRCR*
>TCRU_2782 hypothetical protein, conserved
MPCGGRHAGGADTAPNGIQGRPRNSPDYYYFGDCGVTTVAQRLWGAPPLVRADVRIDNIR
IAGSKSGVTLWEDHESGATHYTFLGVQFDHTRQAVSLSDKFVLSVRAMPALNSPAIAGVE
VVASRF*
>TCRU_2783 hypothetical protein, conserved
MLSHSSQLCSLWIESARVIRLDHCRCPGRLVSTGLGVSSEIRKTNLNGKSYTLDPVEMEP
VFGVNRASDFSEKGGLCSTNETNEKNVLLSGGDIVSRNQKEVDKVERVALVAEKEHEEEE
PLARNLHNTIGPTPYRVSWYDYGSVSPSKRFTYWAYRRLKELPTDWQEYYRRMLYQEISA
ARYVNQTWDCFMMVVDGYRKAKWILKKYGISIDESLIPKPYNNFWEETTHEERVWAHRRS
HQMKELQALYRDEDELVFGAHTMDRREVSHGSIKNVQTGMQGGTPVHALDLPAPCESEIK
TSPQIEDMSSALMSSIEDDKLWNPVLRSRIHTRRSREEVELYSFEDDEEDENSPS*
>TCRU_2784 calmodulin, putative
MADLLTMQQITELKEAFAVFDKDSDGSITVEDLGEVFEAIGQKVSRNKLQLMMAEADLDA
NGVIDFPEFLTLVATKLNDPEEKELELRRAFSIYDLGKTGFINVTDLKFVMGRLGCPLST
EQAFEMVNEADIDGDGRLSFNEFRRVMNEGWP*
>TCRU_2785 conserved protein
MMIQSLLHYSEQNNVDDDGDFPPLLRSVIRPASHCPLFDLKIEEEHTWPCANLLNGNARY
RVQYQNGAHLVMSDNRLLVVCNSEHFYCPPWNTPIRDACVQRQGANGNSILAVGLADGLY
LALLQRNPQLQVTDDVFLTMKQSVEKIVFLRDGEMALCYGNAQVEIYRINTENLQKVSLV
SINRNHTLNLFQAVASLWDTRRYRDSAYDSGNGRMFVLSDIDLTVWAYKSTDAFAAVCSV
RIQENVVAVLPSSQLHRYAMLVFNDGGRQPVIVEETFAKRSDETRTVIRLGAVRPLPEDV
LLDTVELACQDAEGNTMLYDSRKCTLVMLTVASPIYEDIFDVVEVVSPLRLSTRAVGVAC
VSELQDLSASFIVYGKGGILCRIGVRSLGYMFYGLLQKQGLTDVIRASLHRLGPKRGIEA
LVGAAFAGASNEVLSPLLQEFMQPSFCENEMRVAPGVNGIISLVNREITLAECLWNAPFS
WHLIPDLERIALQLWAWHEKLEALLRPYGWLDCPKQLNLSWNGFVATSHDHFTIRTALNT
QAMLLETLLKGLRDAGVLCWLYSLLLRGKPGIDTMRQNRLKPIVWGDNPSSTIASLCMET
LSAADGFVMSQLEARKNVLPIRARHAISIHLCISGNQPDAALAYACDNVRSLRHEQVFEY
VAEKLEGTFPERMPHLRLLLCWLRYNRGAIVELLEMLERYRISESSEQLKLRLGVVLQAV
TEYPALQHAVVRWMVNYPLEDDRVMGFAELLEEHSVVIDEPQTLTALFFVSWANRNRRPA
LAARGFCDIARGRRRLALPSRILCIKLALEFAPTVSEQLVYFVLLLQEELAEAIEAAWRA
DAAQSDSWREGKVEADVDELRHSYLDERRLFQLAGEYKEQGGAKVQLDLLKVHPETPEKV
TVEVLHDLLEFLIRKGMSATEAARNVVREYYDGYAAGLPLLPFVALLAQHGVSTEEIATL
LQSSGVPTYAVVEFFFHFLDERSEGLTFKKGSLVTTLVAMLAQLSGESRDICAAYLLERI
QNLLEGEQKAMAATITTNKILQESDIMQLQRAESLLKRPRTVSPP*
>TCRU_2786 hypothetical protein, conserved
MRRTTAFLTSQLLSLATTKIASTASEGTIVGAPNIGGGGGLFFADRAVKMTRLTTPGFSL
ICKPSILSCESPDAAAAVRLITEEPGKGPSARIQLVGMCTVSFFRSHRIVFHCTGELGRV
QRHEIGLKNGDVLAVLRGCDLMRDWKSVERTLRSVAGEDPEYMAEEILLNLASDARATKN
GNALNNAGSSGIDDDDNINTNASVDDKEVSTSDSSMPNGICIMTVVRNRPSEELPQPPSY
LNWSMACGGEKQNGSQKKAGWGR*
>TCRU_2787 hypothetical protein, conserved
MASVYHRKITISAGEECTISGSGAIELQLLSGVVDVAGVLLKAKETHVFFLEADKRCVIL
YTLEGGSVQATAGFPFETLKTATGMASVVQLCRRSLSAERRTKVLVIGKSHSGKTLTAHT
ICNLLRDDSAGELTTAPAAAVFLMDLNSESNCLHAPGCVSAVQVEHVLWPGTTSAPTLLP
FSFFIGEATPPSAENILSFLHFVEQLQECTESLLNGMNHERVHLVIDAPAPASDIKESVY

FRKLIELLRPTHVVTVSPQDGSETWSTFLQEDVQRVLPDCEFSHVSPVVRRCTTSLREQH
LREYFAGTPQFPLGCAKVVLPLTQLQFVQYEAHGVDEEVAVSCRKVAMTPTFVGCICALS
HAEMIEEVPLAPVAGLLLVVAVDEEHEELVAIVPACDDLPRRIVIVPNETRAAETPTLSV
TTAMASIEESVAV*
>TCRU_2788 Protein of unknown function (DUF498/DUF598), putative
MRRSLLHQSGSTVNWKIRVRRGVQRFVRRYVPDPRDDPLTEVPMNAETYKKTYLQQEGGV
RVGIRPGQSIEDYMAERDKKWLEEQKLTTQRLVEEQQLPTTSDGQLFTGAGTRASYMNVD
AANKLVKTPTPSDYEADEELILMRRQLQVENEAEYRRFVAEKKRSEMTMEETQKRRHPHP
SDPNYDPFKITPGYRPQDSVALAQWLKRQRESGKTASGLSLQTKEGQERYYNEEKMATQF
HANPFAARVEEPRGLSKMRVTAYSPDSIFVNDKEVIGSCIVTDKAYYHWNVSSFEEVDER
TLALLLHLYPVPDVIFLGTGRNLHFIDEELRIAFQKRGSVIHCLTTPQACGHFGVQLSVS
RRAALAIINPIPTNGYGVECFGDFVENDMFSLSDTQLGVPPIKQFSSTMFKPNKVAEKYR
HMQGTGFGPKYHQLSDGRLVRPGTSGTKLRPLLEPGEEVQWERLPSYYHWYPKEHLHDYI
ENTTMREIQGKPTGDPTERRLYQSMRGGKPDKENAPTPDLAPWDSASIPITKFPHERNDD
EIVVEDPKTGRIIGMDRGTYERWRIMMQERREGKPESDPVEYDQERFVTNKDGVVFDLSK
MKYRPIFEGRWNPRRPQSTGRTNPIMT*
>TCRU_2789 tRNA intron endonuclease, catalytic C-terminal domain
containing protein, putative
MEKLEDGDPRQRRHIGGDAAPATRKTDKCEEERGGEANDDEKIILRIIRTHGPLFVCENP
SLFLQTLPQARKKHYRSDHLSIEELEYLGHSFDIVFKNEQDSHRRACLRERHATFCRVYR
YLTDEMGLRLRHGSQFGAAFIGYRDVSGHGEYLVYLGPLSRLEEVAAVRVARSVAKEAWV
AEEQQDGSGFSFTRLSPSVSGRVSRETPRQLVMGTNGPKTVS*
>TCRU_2790 hypothetical protein, conserved
MLGGRKLRSAGACETPSSCAVRPHRQLGCPGTQVAAPRIQTHAQQAVRPAAWAIRPQKGA
GRLYGLSSGVVVRAVVGTPWLEGARPVAAHSVPDGNAVLSVRGANVVRGDHAVADASRSI
EDGASRQRENRRCLPMRRRRRGFRGGYAPHVLNISEACEPFVLSGCAEPNDPPQRCWIGF
QVDRSIPR*
>TCRU_2791 Glyceraldehyde 3-phosphate dehydrogenase, C-terminal
domain containing protein, putative
MATARATMASLKEWRGGRDATWKIVPSATGAAKAVEKGIPAPNGRLAGMAFRVPTPNVSV
VDLTARLEKPAAYEQLRAATDAASEGKPKGLLGRTEAEVFSTDMNGVALMPVFAVKAGIS
LNDRFAKLASWYDDGTGYSHKVLDLVAHTSAH*
>TCRU_2792 retrotransposon hot spot protein, RHS3, putative
MLAAGRPLMKDYNVTMEVFAQEPDDYVHDQLLLEEILNLTAHQALEAITRPLHEGVLSLG
QRRDYERKHTITPPARGKPNGVLTQVLTEARREAEERLRRTQEMKFTISTNIEEVLFKGR
ARVNVMRLNDFLTMEPDGRGIVDTNRDVMLEEFFKDPTKYIRDKGALNEIQASGHCLSMK
RAVKGEVVFDEDIRRLCDKGVDNPPGRSLAAAEVKATVHNSTKHFLDAAAEEARNPTTTS
APEKLEGCYESVHNARRSHAVELPDGVERKKTGTGMGVHEGRPEQSWSYREADDAIEEND
AVQQCGAAPPMLMVLFSEKGWPYSWHTIQDLPKDVFVNCEVDRAWRTVKGDVTAWFSPHG
GTDFKPKQRLLIGTPRIGKSVAAGSYLLYQLLHYDAEQIQVVVHCFGGGDAYVSDKTTRA
VTRCSDEEMCISELRRLRGHGRNVYIIYGVAKEGTPPPRHFAPTSGWGMIAVSFPKVTNY
DEWAKQLQAARIIVNCPDEVDVKAMCAWMTRDETKEKQAECWKEVKKHMYLL*
>TCRU_2793 Concanavalin A-like lectin/glucanases superfamily,
putative
MCTGPVPTKGLVAFLSGSPTETEWRDEYLGVGATIKNTAAAVNGVTLKGAGAGAQWPADM
NGPTVPHNFANREFTLAAAVSIHEVPESGNVPLVGVGMSDPNSTVLIGLSYTHERNWSFM
TPSDAHPDEPALFDWKPNTTYQLMFEMNTDGCYVFVDGRGLYMKKADEESFATLLEKHRI
SYLFIGCGSARGTESSRMTVTNVILYNHTVYGEGLEQLNASTVPISQSGVAKQQRKHEGD
PSCGDSTVHLSVSPVLPLPALVLQLWGMTALC*
>TCRU_2794 BNR repeat-like domain containing protein, putative
MSSCGDGQHQLCVSDGHGTSWYPNRVSINRVWGNSHDRKGYGVQSGSTTAVIAGEKVMLA
ALPAYSKDNEKGRLHLWVTSIVRVHDVGPISREGDDAAASSLLVKGNNDELLSLYENKER
SEDGSDGHVAVRVAAGLQRVKDVAKKWTDLESAPQ*
>TCRU_2795 hypothetical protein

MSDAAVPPKKASPKKAAPKKAVAKKTPAKKTAKKPAVKKPAAKKRAAPKKKPAAAKKAVT
KSAKKHAAKKAPKKAVKKAPKK*
>TCRU_2796 hypothetical protein, conserved
MTRHDAARMDELAAEVANEPSEYSPVLRRGLRVLRSTVDDGRLSTSALLPDRVRYASVKE
REKAFSKHYGHFCAYYKSSCFTSVILTRLAIGTVEYFDENFYPDCVEDVDYSLRLRLLGF
QERNVLYGKLLHRGSSNIRFANEMETPDALWYRRVRSLSANKPYAMMKWGPQRACSGGYK
KPYDGMVPAEVWVKDETRIQRIRVHGHDEEQGIPKVECDRTLLHPVRTKGR*
>TCRU_2797 RNase H, putative
MQPHDGIQCTAPGHGKAECPSYPAQAKTLHVVTDSHSLLAAVTKGPRSHTDWTEDRIWQR
LLSLTRAGWSVQLQFCYEHCGVHVNEPADQHATQTTATGQYTEHGIAPIWRTDLLTCINT
QLTNKWRSTMHQDTHHYLLGGARPSDPGGKHLITQEALDQQGTDKHCMSRVRGIRALGSP
VLDCERLHEPTPFLQHLTGTVCIYAL*
>TCRU_2798 retrotransposon hot spot protein, RHS3, putative
MAAGSYLLYQLLHCDVEELPMVAYVIADRKFLFDKTTKTVTRCSAASSIMDILDGLSDRG
VKGYIIYDVAMKGRQPSAGLPCEGWGMIVVTSPNTNNYESWAEQTEAEEIIMNCPDESDV
RAMCIWKEHNGQVEEEADYWKKVKDRMDKVGPILRFIFGN*
>TCRU_2799 hypothetical protein, conserved
MTRRAKRTRPATPQDEHAIPLQQQVNVPVLNLEWIQSRLHPATLESLMQVWGLVGRLTFP
PSSSGKACEGSRRIPTADAHLLRKAGIIEDASSTITGGWIIPFSVVEEKTNGLRRRWIAW
PRDKNRDDPYKANAPLLHISHYLPPAMAEAASCLDLKASFFRVSLPRETRHLFRCRVEDG
TLVELTRHPVGYKAGPEILQIIITSAIAGVTTVVHRLWAAPPSVRIDVWIGNIRIAGSKS
DATLWEAQVLRNADSCHATIGRTANRAPRSTPSWGCSLVTHTGRYP*
>TCRU_2800 hypothetical protein, conserved
MMTVSATISGLRWISGNTAKHWKDSTLTGHIADLRAPQAPNFAFQLLLPIMRAARELGAR
QGLTIAEGRRSKPHYAAADVQNRSAQLQHRMEEANDHFSARASNTAVADGLLAGRERHFE
TTPGTKGTHRCLGSAACSLGCGDSPGQRGVPSSRRGPPDAPAHGYENSATTPPHHERLMA
RDAAAPTIPQPSFTGERRRDNTLASLGTVGERVSLTP*
>TCRU_2801 Histone methylation protein DOT1, putative
MTVQRYYLLGGALFFFFVYCLVRPGSLRRLAFASGIGFALGYGLPALNNVRKHSFKAINE
PIHLLTPPADFRSMYHRIFEHISERYDELGQMEIGEEHITVMQNVFAQIGTLDFDGIRKM
WTSVIIPQLRSSISGGALVNDSQNLPTKIEESVVFCDIGSGVGNVCLQILAETHCKKVVG
VEVIPSRFRNAKTALANAKLYYPEYFDGKEAIFFNDDFVNCVSCLKEQQVNVIFAHSWMF
DDELMEKLTELVTSVPTVLCVVTSRRLTEKVLQISPIKLVSQVYLTADWNEQAPFYVYTK
N*
>TCRU_2802 serine/threonine-protein kinase a, putative
MTDTKRMANANSGLPQPPAEGDAAKSDPLEGILGTHPDCKYIKKKLLGQGSFGSAWRVEE
RETGTIYAAKVMDTNNMSSKDRGFVTNEVKCLSRCNNANIIRHHVSYERGGMLLIIMEYA
DGGDLYKQIKARQQSTRYFKEHEVLFIFLQLCLALDHIHMNKMMHRDLKTANVLLTTTGL
VKLGDFGFSRQYEDSLSNPVGSTFCGTPYYLSPELWRRAPYSKKSEMWALGVVLYEVIVL
KRPFGGRNMDELIDNILHARRQPLPNIYSEDLRNVCDQLLSLDPKYRPSLRQLFQQPFIR
KGLETLRRSVLNHNRIPQNVQKDIACNVDEVLNSDIQDYESSRVVPRRGIVTRHTADRGW
QDCELLLDEEAVVMRDVATGSEERVELNALTSVCPIDAAIAHEKFVFAMKNQTGKAFWFK
EVNETSYEEWLVALQNALPF*
>TCRU_2803 squalene synthase, putative
MEYMEELYAMVRMKWRLRRIEKGIACNDEDLRFCYDILQAVSRSFAVVIMELDEEMRDAV
CIFYLVLRALDTVEDDMSIPVDFKLRELPKFHEHLHDTTWCMSGVGVGRERELLERYTHV
TRAYSRLGKAYQDIISGICERMANGMCDFLTRKVETKADYDLYCHYVAGLVGHGLTLLYV
SSGLEDVRLADDLTNANHMGLFLQKTNIIRDFYEDIREVPPRVFWPREIWEKYTDDLHAF
KDELHEAKAVECLNAMVADALVHVPHVVEYLASLRDPSVFTFSAIPQVMAMATLSLVFNN
KDVFHTKVKTTRGATARIFHYSTELQATLQMLKTYTLRLAARMNAQDACYDRIEHLVNDA
IRAMESHQKPNGESVARSMLMRYPALGGHLLYTLVDNVVGYLGK*
>TCRU_2804 hypothetical protein, conserved
MNSLGDRFRCVVALLSGDAGNDDNTAAYHVAIVMPAMGAAALEEEVAANTVAFGAANKMK
IVDWGSDTAALLAAAHGPALAHVSLLRADAVAFADARQQLLAHHDPTLLLLELACEKAMP

FAAEYTAGDEWSWIRPPSSYFAELTESERDAAVCIPAVMRCSCPLVRATASLLSPSLWMN
SAVTLNGMLREVGYKVGCLSKYGS*
>TCRU_2805 inositol polyphosphate 1-phosphatase, putative
MVSVDLLHVLTVCVRGALAAQRYIALELLKVQELETQLPALPIMDQDPQTKKVLLALLHQ
AVTVKVKEQLSNKEDAAAMDLVTTADVVTQAVVEHVLRDAFSDMPFTIVGEEEVASVSVK
SLAERCVEKYYRELTLVPYQTELEAHACRRGSQVTAATWKELRERVGVFIDPIDGTNCFV
EGLWEVPLTLVGLTLDGVPVAGVVNRVFGFSVAQMISENSSRSSTSLSYVWNFGLKEPFI
VHEGRRVVPAATYKIVSANSVLRVIGSSTTKSQCFADVLEKLFPIEMHGARGAGNKLMFL
ISSMLAGSPSVQSCDVFISPEDTIKKWDTCAPHAFIVALGGELHTLRGDVVRYSLHGKDL
ARLSSGVLGVSRRSKLEVARRIKWSSSSL*
>TCRU_2806 hypothetical protein, conserved
MSTNPKYGREALRARFADAAKSVTELYRTAVVSYDAGYRDALLFVHRYILLDTPAAMTTD
VVLPHSGQPPAKTASPLASARSPDAERLLRFIQNTLKRHEAVSAASRGMSRRRKRSPGWI
ERPACGVCADGESSIMPDGGRGAPCRRTSFSARVFEESGAEVHDMLSNVRIAHPAESDSG
ISDDEFAAER*
>TCRU_2807 nucleoporin (NUP54/57), putative
MFGVSSSGFGTAANTAGGFGTAANPTGGFGTAANTAGGFGTAANTAGGFGTAANTAGGFG
TAANTAGGFGTAANPTGGFGTAANTAGGFGTAANLTGGFGTAANPTGGFGTAANLTGGFG
TAANPTGGFGTAANTAGGFGTAANIAGGFGVTGGLGFTAAGGFGAYAGAKNNNMVGMEQN
IQEFGSQSTAGRYLLELDNAYNALHPNCRFRAFLYNMCAPGQSTMAVERERFIAAQAGGG
CKEEELLRAQERNPDPLRMYPSPIHFMQELKARAEKQQEVITAMTKHVDVLISKSERFRE
MDEANVAQYRELMLEQVMLQRRWYSLLKKVETLRQLGLPLGEESRIARIVKTLNLQLSAP
GVYRTALSELQPFLDAESATVASLLRSRPGDEAREPVVAKEDGMLQRRTDPQLLKNWTRF
AERIQEGVEALIELLEKDSADMRAIRHRVGLT*
>TCRU_2808 Region in Clathrin and VPS, putative
MATVWRSFKFFELEVLKGPLSRMEMTSCCYGHKAIFVGDGEGFVYALNRGGTTTTLEFSA
YKGAVTHMKHLRSRNVLVTIGDDDALNTGIMRVWNLDAVSESAPPYREHRLFNAKHPPPS
ETIVLRTNYNTELMNSLKFRGRDGDAVNAIPATAFRTVVVDFDVSEDLQNAAVALVSDEI
VVLRGDLEHDPTVKICRIRSGVARGAVAFVGFPGAKFTPRAAQGKQHLFGLGIGNPNANA
NSSSINSNTAVAHALYTVFADCVTVWQVTGAAEYKEHRCDPPFGAAPECCCLTDDGQLVV
TSATNDQIAVFGSEAVLSPSARTSASLPVFDPTLFGAVHFAEVEGHKRRIIAHRSYVVVL
TQSSTRSEKFTLQCYDILNGLRCLSRTQESSCTNVAWVIADAADILVVCQETKHEEFVAQ
RVMRLVETELQARLEQLFQKECYDVAKRIVRRLHAADASQQMSIQKKYGDYLVSKGKYAE
AIDQYIEAIGFLEPSYVIRIFVNGQHTAELTRYLEELHHTRHGNLANRSHTTLLLNCYIK
LRDEARLSEFIHRDDIRFDAHNAIEVCRQAGYYDAAIYLAEKYAQPHDYVIIQLENLHNP
KKALAFIRNLCVDDAEAILLEVGKDLAGMEPRACTELLAELCIQWKGPARRLADPARTHR
SSPQDYSFRLQQQQQSRPSHRSEAKDFMHVFVDSPVCLLYFLRAVVESGVLDGGGESQRV
VYNTLLELYMTRELKQCIRHQVTPEGAEVYTVEPYERRLEQALTFIEAYAGSYDDYHALA
LAEQQEFEEGVLLLLQRLHLSSDIMQYYAKRLEEGATPAIRRAAREKLIETCRSRLNRDG
TDNDNNSNNNNNNTIGGPGADHSRANSGSSRGRTDESTKELWLSLLSLLVRSPDTEWQDL
VQVLQYIEEQDLLSPLSVVEILSTNPRLQLCTVREYALRVMRKDEQRTEAFHGLIDTRLK
KLRELHGEIDALQTKATVFQAHKCFHCHAALDLPVVHFMCQHSFHQRCLNDITECNVCAP
AHRRLAAEQREAEERYGDPKIFFERLGAAHDADGFSVVAEQFGKCIFAAPQLRKEATLFF
GDPMVPGAVSAAGACGGRSGPAISSEDEDDYDDMRRAAMEDGEEVYDEDGELLRPEAIEL
W*
>TCRU_2809 hypothetical protein, conserved
MGKGREAVTLETTPDLNFVKSGHLNMLIYTNKEGEQVKVPVNSLEFLEDRRVVRSRSMDQ
VNFNNDCVFKVTLEFMEPMACLEETAVRELTDWVLCSCRGHASFYSPVEKRLVLQQCFVC
LQSNIPELLDPFILVLYLEKDQWLVERVLR*
>TCRU_2810 ribosomal protein L7/L12, putative
MRRVQTHTKAEILFRWFTIGGVTGTAMPRGMQAFPLRDSAKTIETIAEAYVNMDLATMRK
FHESVLKNTTRTCGTSRLSYEQALLQGIGAGNSWSSTGTVGVGDSALSSASSPTREEALA
SDNAALAKKVAEKTAFDFTVRKYPSANKVKLIKELRAVSGLPLHEAKTAVEKCPGVLVKA
MARADAEKLKALFEGHGAEVELL*

>TCRU_2811 hypothetical protein, conserved
MAGLLGLFIATVELLLFFIFDLILARVLNTLKLDHPELEECAPEDLPDLDPAAIVDTLRH
NLEELYDHQHRRFTGDTKEYTSFNETNVEFAPVPPPPPCNREKILHGIILVCGPAAPLFT
CLQFLFPTAFGWCRFLLPVWMQYAALIPGLAAVFVFAARTTHVAQYSDVSKQQWILLMWF
RLDFMCIMSVVALCGAGAWLVAGCMFAVALYLAHRVMRIEKRLAHLTHETQLVRGEVDIE
HVYLPGSEQAERALASSDVQGYNALK*
>TCRU_regulator, putative Ma.1
MDFVPLASFEFLNSLLQGVEAQGCLMTVRLEAFTCRSTRKKKQLAASMAHYANANTPPLA
PASHAGLSPPPLQLDRRLPVVFAPVDPEDIDDRLVYLVAALNSIYGEDGYDFSVLTEEDF
VVCNEAQVRAEVDVTLHSMSDSCRPAVEQFWTLVSEQVMDASQGCEYFRFMCPTCDPMVS
RSLFSQHYFLYNRRSRLLVSLLIFAEGNAYRGDDGVIVGNGFYVERDDADTDEGNNSSRG
AWQSSPEPLSSDDGVGGKNLHYYYGH*
>TCRU_2813 KH domain/Domain of unknown function (DUF1771)/Smr domain containing protein, putative
MERVVQITRVQVGHVVGRGGATIKELQCTTGASVEIIDGPSVRITGDSEEKVLAALERVQ
AIVACQKNPEYEGPVGAKLRREADALGARRSKLFDEATARRNEGDAQAAHKLVEEAKEVG
RQMEEKNKEAAAAIAHYNNEGKGKGDDYFDMHGLRKEEAMTMLQFRVQRLRAKPSGTVTE
FELITGAGHHSAPGKKQLLKAATVAYLQGEGIPFTEVSAGSLMATIIGTGEAAVAAPTAT
AMAADAAAKKDEKKAAASASASASKLQQKTEKRKEKGKCGGLCTVM*
>TCRU_2814 hypothetical protein
MTNVVLLDDALVEMEGDACHLLYREEDAGVWKVAFVTPPTLVSLSVQAPPTAMAAGDAKA
DDADEDALEFFSD*
>TCRU_2815 hypothetical protein
MWRARIACSGFVLYFARRSVVVPSNTDAEKLFQRIVHPNYDEDTNRRLSGSMLVSCVAAI
EFRRQRMEVKGKLGPMDAFLGNDMLEFLVALSKMCDQRGSLAVVEGICSNVHDDLQESNR
VAFAVKTLLNALDILCNFSNGAHQRSFAVDAVKLLQLELSLLEVLVRVEGGAHGNVQEAC
YHMQRMQHVLENRGIYTLSAVEQRRWMKRMRWLTTVTYSHLKDCSHEDEEAFKRTLRSLY
GEEGTFGYINKLRYAVGVLLPFLLKNAATMMKGRKWQRKRNAACKVGDKLPPQK*
>TCRU_2816 hypothetical protein
MPSRRRRQPAYAAAEQRKKTTEMQQQPEAASCEAGAPDEAKKTEAVAPEVSPMAPIPPPT
AARQGGQAAEVPPTATPTSVAPTDAKRAVLTKMVTKRKPKMAFLFDNEETKGAEHKEEPS
GTKEAVPKRKPKLPFLFNEEGTHDGAAAQKQPQPEVPQANPVVETAPKTNTRIDEVKKET
IEKISAEADWSGAAPTRTVAATPPRQKMAISKNVKLYRRMRREPHEATNNTAAVEKAEER
SNNSAPTNSTYVQVEMKKTQQLHTDDGKASPPFTEPTEKTLHESSGKERHEHTAEKFSEA
PKEDVKRKHTATMSKLMRHGRHFQNCKTAVDSPSNTSENAQTQTKEEVEVPEVIEPGATS
EIAQRTPVCNDQPNLPQSLDGIRAVENSKTAEMPRHTTKKHEKKKTKKSLVKRKFMKEMA
EKQAASKQAEEEAARKQAEEEAARKQAEEEAARKQAEEEAARKQAEEEAARKQAEEEAAR
KQAEEEAARKQAEEEAARKRAEAAARKKAREEAERKRAEDEAARKRARREARERAKEIVK
QRRAKEEAARKQAEEEAARKRAEEEAARKQAEEEAARKQAEEEAARKQAEEEAARKQAEE
EAARKQAEEEAARKQAEEEAARKRADVKKLSEKGPKTRPH*
>TCRU_2817 kinetoplast-associated protein, putative
MRQSNPVTDKTVVAAAVAQEVMDVPLVSVHACGASSASDASAAVPSESKLLAEALEFPNS
SVTIDRFDDNELVARRRSTDDAWEIGLRFDWTIKTLAIGSMPSFSLTDPRRLHPFMQRYQ
SRPVWFLEEVNGAKANNIREVMDILKKSLTARFVFRK*
>TCRU_2818 kinetoplastid kinetochore protein 8
MRSTTPPLYYKRPEGVPLPYCTAAGPAIEVPPSLRVPPATTANNWGYPTAPNAARVGSVE
YTKYGTIPSFTNTVPSMPAVANPFNPTNPSPRTSLAADNPADLIQQSAARWSTTANQSAQ
ALSALEAEEASILLEEQAMKAKLAELQLTRQRQSEGQLDLSHGWAALLDREERYFTEPLV
DLSEEIMAREHQCALLRDQLQQAEAHLEALRREYQEYDTVVEEKNKLEAEMLRVREGFLE
IEGRRKACRMRAELLFDVESKRAVKASHHVRELDVQLKEMTSVGPLSELQSAPPSRSASR
GVTFAPKVMHLHVGSHAKGEQEEEADDEDAGLTQGFGGTSVCLQNDGDDDDVGGIGGRS
VAYSLSVVPKRQRVEVPTT*
>TCRU_2819 Endoplasmic Reticulum-Golgi Intermediate Compartment (ERGIC)/Endoplasmic reticulum vesicle transporter, putative

MRWLGQLDVFPKFDTKFEQDARQRTAVGGIFSLISLLIIAVLVIGEVRYFFSTIEQHEMY
VDPDLGGTMEITVNITFPRVPCDLITADAIDAFGTFAEGVERDTVKSRVAASTLEKISEA
RPLVDEKKKITKALDPSGAEKENCPSCYGAEPEPGACCHTCEDVRRAYSLRRWVFNEDDI
SVEQCAEERLRKAATLSSQEGCNLFVNYKVARVTGNIHFVPGRMFNLMGQHLHDFRGKTV
RQLNLSHIVHTLGFGERFPGQVNPMDGLVNSRGAVDATEEVNGRFSYFVKVVPTQYQSAS
ILGVGSVVESNQYSVTHHFTPSPSAELSAAAAGSSPVMVPGVFITYDLSPIKVFVIEKHP
YSSVLHLVLQLCAVGGGVFTVAGLVDSVIFHGVRRVQRKMQQGKQS*
>TCRU_2820 emp24/gp25L/p24 family/GOLD, putative
MRRLVTQCPHAPRCTAHASVVIVWLLALFLYGPYGAVGFGTKIDAEDRECFVESLAAGGS
LAFTFRVTDGGSFDIDAVMYATTVAPLNSVEETTRLHYSEALIKMRENTTTVIVNEWRRA
NEGSQSYTAPSAAETKHGLPAEITVCFDNSFSRLSPKWLLFQFLKRDVPEVDPESTTRAE
AEIETKLHKHGTVLFELATAAERMRQMGESNRVRYESLASIVMVGLVGNIVILALMAVYQ
YMTLTRFLIRRRAK*
>TCRU_2821 retrotransposon hot spot protein, RHS3, putative
MAAGSYLLYQLLHYDVEQLQLVVHCFGGRDAYVSDKTSRTVTRCGDEDMCISELRRLRGH
GGNVYIIYDVAKEGTPPPRHFAPTSGWGMIVVPSPNVANYDEWAKQLQAARIIVNCPDEV
DVKAVCAWMTRGGTKEKQAECWKEVKNTCIFWDGFHVTSSMRRRLMDAVER*
>TCRU_2822 retrotransposon hot spot protein, RHS3, putative
MKRAVKGEVIFDEDIRRLCDKGVNNLPEWSLAAADVKAAVHNSTKHFLDAAAEEARKLTT
TSAPEKLEECYESVHNARCSHAVELPDGDERKKTGTGMGVHEGKPEQ*
>TCRU_2823 retrotransposon hot spot protein, RHS3, putative
MITYKTSGFLKKFLTSLRTKRWGPLLGLFTKVCTLSGNVGTMKEKHTITPLARGTPNGVL
TQVPTEARREAEERLRRTQEMKFTISSNIDDVLFKGRARVNVMRLNDFLTREPDGRGIVD
TNRDVML*
>TCRU_2824 retrotransposon hot spot protein, RHS3, putative
MSERHGEGNDDNETSPASTVLRGRRARARAESDGDSGQPAVPRRRVEETRRPQWGLFSWV
EDVLLGGKARGNYMKLNDFLRGHIGPSAVVDERYNVTMEVFLVEPRNYVQDQKLLEEIFN
LSEYQVLQEMRGLRRDTYNVSQMGVRSLRQWRDFERKDMVSPLARRELDAALRGVLKEEK
IQMSEGFYDSLYNARWHHVVEFTDGEGMGMEVREGKPPQSWTYKAVGRTLERDDSVEQSG
APRLRLMVLTSDKAWPYSWPLGPSIRDCYVNCEVDRVWQTVKGDLTEWFSTRGENNFTPG
RRVLIGMPGIGKSMGAGSYLLYQLLHYDAEKLPVVAYSVAEQTFLFDKITKTALHYVGES
TFLKAVEEFSGRGMRGYFIHNVAEPIHEPSVRLLPRGWCMIVVAPPEESARRGCKKQLRG
RRIIMSCPQESDVKAMCVWKKRDQSAEAQVEYWQEVRGYMDGMGSILRDLFSERDTNARL
SAAREPWNGLPHQMLMNALA*
>TCRU_2825 T-complex protein 1, beta subunit, putative
MLFANQAQQVLREGASEEKGERARLMNIMGAISVADIVKTTLGPKGMDKILQGMDRTQSV
RVTNDGATILKSLFMDNPAGKILIDMSKTQDDEVGDGTTSVTVLAGELLRNAEKLLDQSI
HPQTIIEGYRLATQVAREALVASAEDHGHDEKMFYDDLLRIAKTTLSSKIITVEKDHFAK
LCVDAVLRLRGSGNLEMINIMKKLGGTLGDSYLEPGFLLDKKIGIGQPRYLENAKILVAN
TPMDTDKIKIFGAKVRVDSVAQLAEVEASEKEKMKNKCMKIIKHDINCFINRQLIYNYPE
EIFAQHGIMAIEHADFEGIERLAKALGADVLSQFDETEKVKYGFAQKIDEIMIGESTVIR
FSGLPQGEACTIVVRGTSQHILDEAERSIHDALCVISQTINETRTVLGAGCSEFVMARAV
EERAKKTPGKRQLAMLAFANALCTLPAIIADNAGLDSNDLVTRLQASHYQGNNTFGIDVV
KGDVADVKALGITESFKVKNSVLGYAAEAAEMILRVDDVLRAVPRRRTQ*
>TCRU_2826 WD domain, G-beta repeat, putative
MSNKYSAPEKASGAQKKEIAVTNNVNTDITKVKICSGHTRPICHINYSEIIDGTFWFVTS
CHDAKPMLRNGETGDWVGTFEGHKGAVYCSVFNQDATRLVTGSGDYSSMIWNAITGEKLH
TWSHPKYIKSCDWIENKIATGCFDGLVRIFDANDYNAEPLSFDSPEKVNQVKSTYFIDPT
TMVTACENVIMKWDLRDTSRPYLRKEIPGLNFLEYTHHHCIVAAHEKSVSFIDTTTLEIK
SSFTTNEDVECASLSPNGQNVAAGSKLKAKEFTLDGAELESHRGHHGPIFHIRWAPDGKS
FASGAEDGMARIWPSHDIIQVYDGDN*
>TCRU_2827 hypothetical protein, conserved
MMMMTGRVLLVCALCVLWCGAGGGCEEVPGVFPSGVSGGIERERGDSEDSLRSEPPGAKK
LAPETPPPKVQQQEVISPPPKVPPVGSEDGAGGDKEGKAGEEGAKDVGKENRGDATSKEV

KKPENHPKSVKSPQSRENEGLQSPSLNGTHGVGTITSTSDSLTVNTNQQDLSSPLQPPLP
NSSPHAIPSSTSAAGPDNEKSRENGNGTKNSQMPVVGEIQERNGTCVKAVASTSGEENSR
LSAKRVEEMSDATKKDNSESSTTATAALRSDAGTEGTPTTNHPNQPSTEGATPPETTSDG
EAASANKYDTVSQSAGSTTAPTTNAKTGDKTKPVDSDGSTAASHTTSPLLLLVVVVACAA
AAAVVAA*
>TCRU_2828 hypothetical protein, conserved
MQHNCALHRRHIRHVDESVGHQARRALAEAANPQLRTARKHAVAQRHVQCVIERVHRAQV
HRHDDRSAEHHRVEARRHPPAANRHGLRQRHATHAALRTATTATGPRRRHVKRQACLAQA
AGTAAAHAVRERRRQRIKARQRSRTAVARHHAHGGQAGHAVVAAALQHPAAGTVALHGHG
AARDRQPENKRRASSSAARNRHGTAAQRHTRQRCHRRRLAHRPHVVQVQPPAGTDRPTAA
AHQRGRDRPLDAHKAQHRVRHRETLSKHKAAAAAHNRDVRHGEHRVAPQTETRRGRYIHR
GRGRTARRLQRHAVKHHRARQQVHVAANVHRRPARLQRPQHSVAHTQLRLQQRRRVQHKR
*
>TCRU_2829 chaperone protein DNAj, putative
MHYRTLGVKRRATQEEIRKAFRSAAVRVHPDKPDGSVELFQSLQTAYEVLSNEKKRALYD
AELAKKMSKLRGFKRPPPLDNIRLPVHYKLADDEYYAFETAPDCLKCSFKHGDIISWNNK
LGCFIGLAGDNFLYWRQDDQTHASKLCQMGSFGMGGIKVVVRANFMLARGRFTSSSLPSR
SADTANSGSHRQVAGKQHDDAGKSAPVTHGEAMKKGRPGRLSDAERLRRDIMRRERERYY
WESMAALAEKEEKQRVRLIEKIEADMEDELQSFRNWLEAGGVAVGGEEPAWVRHARRHAF
GK*
>TCRU_2830 protein kinase, putative
MSSPAWSVYEGDWGSFCDDADNVPPTLALKGCGLRQKCLLQRGAQGAVYLGEDADGNVFV
VKRLFTQRSEFGVRGVSEGSLREATLLTYITERSRTLGPEPLFGVIRLERIVEAPFQELC
LILERCAFNLSRMVFYSRKRPYGAAVLFPETPTRCPILAKMNVIQYLMRGILKILQFLHE
ECHIIHRDVKLGNLLVGEDGRVRLADFGSARFMHEAVEKMSDARSGEYTPISLRTTVIYQ
APECLLGERSYTAAVDMWAAGVVFAELLLQQHLFHSCSELAMLSDIWKLLGTPSDDPSFS
SGEGAVTYAMRTESTIARKFPEGIVSAEGLDLLKRMLETNPKKRITDSEALRHPFLCGPA
VESGMEEARRLWNKKVEACMQETVAQPAAMGMRSFYVDNVEFDEESGENDDGDGGIATLP
TCLDCFPHILSDVNGGYDI*
>TCRU_2831 Intraflagellar transport complex B protein 46 C terminal, putative
MPPRRAIDDDEEITEEEEEEELETTSGSEEESSVEDSAKGLGAGTTGPKEALLVNKPNDE
KYVVEGARHVQTPPDNVTGSKVLYKSRPESPRGMREGNKPETSLGSQSLRQAQSPQESLQ
IPSDMDIRKLRNNPNDEMVRVEDALNIPTPRGKGQEEAGGLLLRNAPHDEAIPLSTRGSS
VGTPRKQTGEKMGAMSPTDEGSSADEEAEEVETQSRGYINQRPEAIDTEAHLKTNVIPNL
EYKPQDYAKINSTASREMQELFKCILDYQPVIPDLPAKLRPFIPDYVPSIGDLDPFCKIS
RPDGRPDGLGLFVLDEPSVSQSNPAVVLLELRATNIHSSGGLAEVVDSFEDAANRPEVID
RWIADVKKVHYKKPLPTTNYQMPMPEIESLLQVWPPVFEDFLNSDIQFPPPTIDMDLDQY
VRTLCCILDIPTYNSLVDSLHVMFTLYQEFRANQHFQHE*
>TCRU_2832 cytoskeleton-associated protein, putative
MGHEEQVDMPMVPGAAAQSVEDFSNLPLNALLAHKSWKARKEGFEQIRNSPKSLKAHLLE
KPRKLMCESNAAAQEALFEALSALVDVCDDEELNILVGEPLRVVIEKGITGRPRAVHASF
KFVSDLVGAAKQAEVFDALLPAFAHKAPKNRMAATQLCAQIVGEYGVAGLPTKAILRAMQ
PLFNDANAQVRKEASSLCCQCYRFIGAGIKGFLTDLRDVQLQELETLFEDVILGEAPRRS
IRGLLPAASTKPTALTVSLVSHNGNTNDISDDAYESHDEVPVLSRLPRNFYRVALDKTAK
WQDRVAMVQDNLLPLIAAAKIRVKDDYHELAGMVRELLLDPQAPLMLLGFKCIQELARAL
RAAFAPHARSYLNPLFDKMKDKKTSVVEHITTTLEILMRYKCITLEQCQDEIELTLQSRV
PNQRLALIQWLLRLTVKLDHSCFNRLSRAQVILGRLMNDEKVEIREASCMLISKLITFLG
ETNFQSLLASLDEKQRIKLAKAINSESNLQCTPTISPAKKTLRVERREGSSVAVSRSLSI
GETHSPSHVSASLRGHRHSSLMARHSSPELRRQHPIDDSVSLESTLPSKEGASNRMLGLM
NGDTTVTQLLRSKDWGNRYNGVMKIREMVERWSKKECTEYLDTVLVYLRVDPGWRESIFQ
VFQGMLSVIQELLLRATEVSAGASYAIINGCVCRLTEPKNKAYVRNLMTQIAKFQGISFV
TRHLMGEVTSVKTPRLMQECNEYMTQLLQTFPASHVDAKGIIDYVRIHCFGQQFPAVRSS
GVALLVALRLHTGPLVDNYMNNTIPALRTAYEEGVSHSNGNKISHRGTGCDVHRAFSPVS

PSRMQKTENRLSEKGTGQHGAVVNTAPSRVENRTGFLSRGYDQTQHITENEESPMRADVS
HQLAPLIKQITIAKDWRMRLDAVKRVEELMYASNKNIAPNLVTELLRSLRSRFEEANKNF
VIDVLRTISLVVESAGSEACRPGMKSILQGVLGMLGDQKMSLRDEATNVAYLALDCLGLD
LVLQCMQKPLTSESHTAHQTALEIIERGFQKEPEAVVSKQLVISLVPAVVRLCMSRILEV
RVAAEGVIGKFLSLVGDEAVLKAMQSLRPAEQQSVMAPIERQVQIFLRTANEEEARRTST
LASMISAQVPASHTPRSPRSPRAASKNLSISHKSVDFSQMSQGAPASCTASQRTSMSKEA
LQPFAPSLVQPSASVAQSTHPQVRREELLSMQEIMVGLRSASASTAASTCVEFLERVKND
EDCGTPEIIQVMVERLYENIRFFDLGLALGLIRCLKAIFERPRYTQWCHSSLLFRILGMI
FDCLLSETFSLQEEVIKALNNMTLKLLEGCPGNDVFSALMSRMTRYSAIYTENGKKVDLK
YIQVTVKCLMRLDFDAVSPDNVVLCCHEYLLQHPPSAFRNLDDLSIRTVKTILQDFSRRC
GTPLLDVSNRLVGTQDLVTHFIRACLENQERAARAISENEQKKAVDANMQLQSHPQQQQQ
KQRDASPSHPARTRRGQNVYVDPNVPVTPSEAPLGPRGVALVAMEEKSMASIFSRIRNHL
TSKQGIEELYAFLKQHPKSPEFEQQFRRCSEAFRSYIKRKLDRQLQDDPTKPPGFSLPDV
LRPMP*
>TCRU_2833 hypothetical protein
MDPTVPPFCDGVSFTLCDDDVYAFGGAVPEIIWRLNLATHTWHEIPCTGSVPAARKNHAA
ASFAGNLLLCGGEPLHASFLQPPKLMPYYELSLDTMAWGLIDTIGAIPLNRSHHACSVVG
ESMILLGGKPLLSKDEGVLTNERLNEFIQAGFYDVYIFDILPRFWRAVVALGPAPLLWGH
SAAVYDERYILVFGGMDVSANETVVISRYGSPHHRHDPPVAEVSDKLYILDTEQMKLRVV
SPAVQYPTPPRAFHAAHIDGVRMYVLGGVTVTKHGNLVPFDEMRVYDIIEGKWNLMEFPL
RFPAAVRRLSFAYESQLVVVPNMTSMWCLDVHKQAASKGWKEVPTSHVKVAAIPATSEAD
EYNELLNPRRRTAYDDIPCGQTDGVELSQSMPPSTSPRAPSMQFDMIMDEIHQLRELLTQ
RGPRQPSPHSPQQKQRNKEERPPTATVTEGLQTAGNEVESTKMRERRRRGREKIKKFTHQ
KELILQLTRRLEHLLERKKEEKQEETTGLFAVGQQLSQRTSDEALEADPLICSLAKHCES
DASVPSPQSPLHDTLISFQREGVGKKGQISFNDQDGTLEKWTRLIEILKKSPMKPSSKAF
RASVVMI*
>TCRU_2834 hypothetical protein
MSLSVTQSVRDEMVAALLLALDYTQGVLETGIDTKNVNVFFRNVLSRVACRWLPCILSIF
ILLLVTLQKRVLNARRRQLPDVIISSERSKNPPSNTVGAESSTGRNIGAEESTGMRRSHE
AQRVIDAYFGPANNTVVDDFDADGHVQKLLKHFNPSNTTTTSES*
>TCRU_2835 hypothetical protein, conserved
MEEFYNNPFRHDPAVDDVCYASVQFPSMTASNTETESCRERDFFSISSALRFQTQLSEAL
RANSTLRSTYEAHIETQRNEIDRLSVENNELQMKLTRLEAQLRSLLLEKDCSTMAYKREV
EKNALLEEKVGCLEEELNSLERRTRELRAAYNRTPQQKDGCETTFSRTRPNLLQQQRESH
YTEQGTPSSLVSASDLHSPKSLLESERNNRSGEKQKWRAAEEAAASWRTETATSMKTGLG
GRTPKPCSKATPYPTQVMDEENDVVVKELEKRLLRECQTRDEIEKRLQKMESTRIRSGSE
RAKKIALEREFIAAQRAVGETRMRLRELSALVR*
>TCRU_2836 hypothetical protein, conserved
MKGLEEWLASSSSLDDSNNADAVSAPPVRGGRGGEGASSTDARTRNEEMRERMRNLLGDR
VVKAVLEETSGVGKAAAPQSVSTADNYCVEEDGGRRGDRHHEKNSRGGSDRCIRPSSREY
RRVEVMVVDRGTQTTSTVGCQTDPVENYAPYMGYPLTHCCTCSCGKRRPGGGLLPREEYS
GEPPLRFKEQLDMIKNSIDIMIARYNLPPPPLPRVE*
>TCRU_2837 small GTP-binding protein Rab28, putative
MDSGSSAPEDTEPEYKIIVVGNGTVGKTSLIRRYCEDGFGQGYKQTIGVDFYSKKVEIPG
SQAVSLQIWDIGGQQIGGTMIANYVHGADAVCFVYDVTNPVSFKDVEDWRQSLLALQGNG
EGRKPLMVLVGNKTDLPNRQVTADMHAKAASAYDMESILISARSGDRVNALFVMIAAKLA
GVDISQQELDMTNRVVLSIDSRLQERTQGLRATEGKEKRKSSNDNCWLM*
>TCRU_2838 hypothetical protein
MAHKSDSYPSCPVTDISVLLDGSRARGQYAAAMVAMNRYYTMLYNSLLSLSFGLGLKFDP
FLDDELAEDDEGPLLRCLGMGSICSHDGNAVLRVRYEEECRQLEAQRSFLGAALEKKRLE
CEKLRAQGRAFRMEVEHMLPLTLAVNRKVRLSVPSDGETLLLSMRPAENPTAPLPAKSTA
AVSKNTPLLTMAAAIAAVEDQIRLQEQEVLRNGKRFSIKGTRHVVLQALREDLQDAQEAL
QRDVMSLEAAKIDALEAEEEKSRLILELEMVTWAKNCGEILFKEISERSAVLTQRVAKVQ
RMHQALQRVLSAGVQVTGSTSPRTDVKHNVTKKEEEEEEGEEEESSLENLQDSVRQLCTV

QESLFALNNCVVDLLDDVEEIETVLNELKTHSNALSSSTGTHLKVQKWRSRFLQEAYNDA
VLELLSWQARERLTVTAAAKLLKQFRRLCIVNNIFSTHFEALARAVMDSGTLWREVHQML
PAAQMALSIVRNRNC*
>TCRU_2839 AAA domain (Cdc48 subfamily)/AAA domain (dynein-related subfamily), putative
MGGGLSRQEELFVLGQWPELLESAMGTDNEASKENADGSKHASSTFRMSFYEWQALISLL
DSVVPSEVPSRLCTSISGQQLLNPIVVVMVHACCWVDGSVSEQKFAERLTRLQEDLQSQQ
DIPRWGRRSKEEDTERELVNKALRVFTDILAQRGKVPTPRRGDRVGIAAVESLISWDHSK
RPVVVLAHAFIVGALACIHPEPCYAESAIHSFRNLQQAMDEAVSSIWRGKRGVKGAACKI
GEWQAVRRLETILVRRQREADVADFLAVEDRWWLPSNTMVPHASSKASLEERWEAVSSHY
IGQQHVWAALKTHFLSSDVFNAEKPTVIVLFGPSGFGKSELARRLACVIHGVEVGELETS
GKLVYIHLPSFCTRDTIYSLVDPPAAHVGEGVLLSALRRNDDAVVVLDEFEKGTADAIQN
LWLSAFQKKGTLRSLKEASRSISTEKVTFVLTCNIAADTILHRTNEYLRGTAEEQAAMRK
EWTEVCRDVCRKTMHDPFVNRVDYFFPFVPYTEEEKRRFIHLQLRRVLLDQRAKDRRIYV
TPRMVKALARKLQTFHASTIECVVRPLLVEAVQKGWDTAVLTVVELVSGTEFVALPAVDK
SDGVTTWNSLPGAKRALEVCELPYGATGALPPVEKRRTDMVPGGNSGGGGGGEGSKSNIG
SLRATADRATQPIFRESNVVPDTRVGVDRKYALRLETAVEKELQVELNRVKELLVMKEKE
IAYLKEKVFQLEKLLAFFLATTLMCLLFLSFLVGMKIVFVMGLVIFMGLWLLVEMPLQLL
MGAIRVLYGVLGPVGSVIAVLVASLWGSQALRHAVVC*
>TCRU_2840 hypothetical protein, conserved
MGPKKGLKKTEEKALAVEDPVVVGCTRSLAKWTERVTANGRKIQQVQYVLSVLSSQMEVC
NKELADVYDCANLLTALRIVEYRQKLNPVPPAQPAKKLRGKRDGSGRLALKPLPDENSTE
ALVAAMDEKKRQKVRSILVSLFRRNNIDSPVPNTPTNTVASLGAGAPTASAETAVRQEVN
TSLCLPADAASISTSADLRRPKDVPPELWDEISKLRVKRVDLEGRSAMLMSGIDKQLARF
DLLVQNHGIATYAAEDLERRIQELRVGKSTARAL*
>TCRU_2841 CRAL/TRIO, N-terminal domain/CRAL/TRIO domain/Divergent CRAL/TRIO domain containing protein, putative
MTDTMSTATQRELECVAYLKQKISPTFLDPEDAGFLTDNTYLRFARARNAHKEKALAMLS
ACLDWRKEFKPYKITHGDVANAMKQFTITPAGRCRKGRPILVMTVGVPNACEVDERVKQL
VYLLEEVGQRCHEGITWIIDFSELGKHPRDARSSETRKTTMKILQDYYPELLGALLLYRT
PWYVRLLYNAVRPFIDKRTRKKVFSLGHDENLLLQCVSRDQIPESLGGTFRTDASKPLWE
*
>TCRU_2842 PH domain containing protein, putative
MMSGGMQQQRQRHACRASWLFKQAEWRFAWDRRFFVQDGLRLAYRVGENEAEKKAGYVIS
LQRTVEPPGSHTFRVWLKEGECWTLRAETEEEHQRWTSSIAAALMQDAAEELHDVREGFV
LKKGAWTGQWRQRYLELDEYRLAYRMNKEGEVRNRFVIVSADASGEEGREEELLVSTSCG
EQFWLKFASKEQFDDWTGVIMRGLRRTTSWHWKTLVSPQSSEYASVRVQYHASGAGNGPC
VYCYGGTYTLSSRRYFEASKLFLPDIHRVSVGKQLSFVQLTGEHKCISLDILPYEEGPHA
KLRPPPLFGAALCVLRSPSESLSSPVLPNFYASTPPLECLLLVGGRSETSVFRDVTVDVW
WCVLQGRSSRWQRCTYDANVLPGRTFHTLTAVSSRSALLIGGLDSMNRVCADCFLIFWPE
DETEAFITPPVVKFFCYLPEPRAFHVCLALRDVLLLLLGGRGVREAAPCSSLFVFLSSES
EWRELTVEPPLPVMNHVCAEVVFSSSGEQWVFVLGETCTSYPTLRLFQLSVQSKLAVSRE
VAITVGDIPRRCCGATMHHAGGYLYLIGGCYDGNQRNDQGQAVLFRDPIRMLIGDSEGGG
PVTTLRQ*
>TCRU_2843 Putative papain-like cysteine peptidase (DUF1796), putative
MRRWVSLGGWCGPGLMLSKLGIRPVEEQLPFDMARCSFDGLLEFTRNGFDNGFFPGPLQR
RPFTPDPASVWLLFRGQHACITHFDINADEVVQEFKRRFDEWEKMITCPTRPVTFLRTCI
AENARNEVELVPQWHALLREKSAGKLDFCTVMVMHDQGPTTERVASFAEEDAAGSPCVVW
NLAFDKQLPVEASLFDKCHDGYAQIIREMNRNEAWYVSTSPLRLVSPKPYKALCLVEGVP
ALRGSCTGFGTTHSALLGRCLYCGSTNGHEVVRDAFDSKKPWDNAEDTTLLAKWITSNGD
KVAAVEATALELKRGANEVLLRLQQLIQS*
>TCRU_2844 pseudouridine synthase A-like protein, putative
MPVAPNGLSVGDFVGGPSKKMTRKKNREERPFLFANYPSRKVAIRLAYHGHLYDGLAKQE

ETSNTVEAYVVAALKHVRLIPQDGPIDLARCGRTDKGVSALGNVFSLTVRASSWPTDSPQ
KPALDYCAMLNSALPVTIRIVGWAYVDDTFDARFSCVGRTYRYYFCHRGLNVEAMGNAAA
RLKGVHNFRNFCKTDVVNVSNYERDVRSVGIFASEAFPELVSYLEIHANSFLYHQIRCTM
EVLFLVGRGLEDPEVVTQLLERGDRKPVYPLADGTPLVLWDCHFKNLQWNISKRAFMAVE
HELQDIATALFLRASLANSMRSQLLTWYNGHTDSEGKTIMAQLPEVQAADRWSVTGCDWT
DKRTHANMRVRRYDLFALSRQREHGASGHLSSRPYTKLLERETERTFDEEVEALSQKKRA
RFESNMEKK*
>TCRU_2845 hypothetical protein
MAGSLATAFAPSETMQEIDEETAVYCRSHDVMWLGFVRDLLSTLRKAAHEVQAKSEGQLE
SRHLCVGDFGQFSRLSTALELLQDCCLKWEECRAEAEKGFLEYYFAMHSLNSGTEPKTGA
VPVGKFMSNLFAGVPGTVGGDRLAEELRVERKRLCLADALGLPLELPRGGDEQQLPQGML
ALERALKSFRVVTDETLPGYLYCTIQPLLCEMTSCWLLNGIFADTPLRLIGTAEGMRLAK
ERLELHASSLRFLLELAENEFCRCHTATALESKKELEEMLLRVTVMQTAATRDGVVGQLA
KEWRMAAEEARAVLASTEKTLLGVLPGQLMGEFDKLFQLSPHRVMDHIVHSFQLPLTSTA
PNHDVDRVVRCDDVPTAVAAVGALFDGMSLEEVEYLLQPSDPDNMSTTQREEGTFLAKIQ
AHFYRLFKLLLRACCLQGGEVLVMLWPFFELQASPFLCSVPHARLAAMLQRHNYALFDAL
LGTPWRVKAAVLLGGGWVRAARGHFAQWLSERRGEGRELLPIVVFKQLWREDDKSDVENG
TQNPFCISLLRRIITGCGGRLMNAKVAVLVPAGGATVLSRRYSVFPMMRHASCEVGGTDA
DETRGGKNNVSCFMMTEAKSRPLLVALLQATMMPVFSYSVCGATPLLFVSDGLDSKKQMH
VLDAGLLAGNGVG*
>TCRU_2846 epsilon-adaptin, putative
MSKLYRAQTQQGHSRGFFEYIRSVGESKSKQEEDAIVTRDLAELKKTLASNNIEKRLLKE
YVVRIFYAEMLGVSAEFAHIHCVNLSSSPDLLSKRTGYLGTWLTVGPEHDLMYLIVSNLQ
RDMKSSSFLDIAAALTAASKLVRLELMNAINTEVVGLLRHPNALVRKKAVSTMHAFYRKS
EGLIGDTKIFRQMLCDADPSVMGAALPLFADVICTDPTSQRELISIFLSILKQIGEHRLS
REYEYHGIPAPWLQIKLLQMLPILIGDEPSLARKCEEALREVITRADNGLVIGYAVMCEA
IRVITLIPTIPTLVELAAEAISKFLSARKANLRYAGIQALSQIVRLDPKYAHEHQHVVMA
CLEEADDTIRRKTMMLLLAMCNEDNVEVIVTRLVKSLSQTTDKYFREEFTRRICDVVERF
SPGAVWYIETMNKLLLCAAEHVPQMTIQGILKLIVEGEGKDGEKDAAFRTFCVEAYFDLL
EGSQKNLPEAFCRVAAWVIGEYGFLAKRISRTMLLDRLCDMLERAECADTRGWIIMAMIK
IVAHAGAMPDNVEDLITRFKDSRSVWIQQRCYEFTELVKMPVLMKKVLPLDGCCEEVDLD
ESMGFLDGIVQEALLAGARPYEKRKILLGVKEEETLRTDAYELVRTDVVNENELDPENFK
TEEPHKLVIRPAVKRWGLKNLEEDITPTEGNPTGGPEASEATGSMDCFSEGAVLHHVDPH
APSSLSTPSAAVNLPMPREVSKSSKNKKFLNDIFSVGNKKKLTVRGHNGATESLVHAGKG
SEENDTGSLLSIHMEYVRELATTGVVVRIVGKVGVENVVISMRAPPNCTLRVVSCSATSA
TVDGTAVTLNKLQANQPVEVEMQVLPVGFPKEGRVLVEITYRSAVPGDPQCASVSRALSA
RDILRPPAQMTTAEFGQTWVGLREECKLQMSSATPLTPQSLQNLLMERASLRVVEAIRNE
LITAAKLIGTGKPVLCHVDVVGPNAANLTVRADDKGFAAYVAHALSAP*
>TCRU_2847 ATPase family associated with various cellular activities (AAA), putative
MDAYTSLLNLNFMSAWRTGNSLIDMLIAMTIPFVSNWVARFFSVIWPAYVKKFIAWILPR
DAEVIIQHGLTDRYQRAVDLTEGSVLQEAVERYISHELKPFYSSGEVVYNMVSHSDGPDR
KTMAQVLEEDFRLFCRPLKESVRLGNGIAFFIRESEDETPSKSKGAVANGGGEKGGRLRT
KARMREVFLREKSGGRDEGDKVLAFVQAALKWHVKSLEKTSTRKRYLYQPTRNKDALGGN
DGALTRVLRYPLYDMKSFSSLFFPEKEKLIALIDQFESKTGRFAVPGFPHKLVLLLHGPP
GTGKTSLVKAIAQHTGRHIFSIPLSQVRTNQELISCMHDQLFEVCKNRQFWKVSLRPEKL
IYLLEDADATSDFMLKNKTKSTAKKSQGKQATCPPNKDAITTKGLLEAFGGILDAPKRII
VMTTNHIERLDSAIVRPGFVTMRLYMGKFTKEYAAQMVRHYYGPEAATEERLKCLKEVLQ
RASDTEVYFSPSELEQLCAEYEDIEELIEVLKKGSREEVF*
>TCRU_2848 hypothetical protein, conserved
MLHEAYAPEDTRSVAWSRQKKVAFLSLISGMRDGTRPPSVLLLYGPTGSGKLTSVRAMLR
EQRDAVGRPLLVEVLHSCESTPQQYYSFLMDTFTEHQLAGNDPCESHLSASSVSADDSSD
VKGRAQRPCEAQSRQQPRVVARVMKLYGESLISPLHARTLRFLDHYDAWRRAEFLGKCTG
DGPNALGGSILRRHFVLFVLTTHDTHSDKVALGKLLPSRVLTHPCIYSFHCTPITTRNLT

IRVKDVLRMEQRRQTCGVRCHISDEHVDFLCEHSNGDIRQALLQVQWRCLLGERGLNEAA
FSSTSAGTPKKKGPRPKLKKPKNVMDLVDDHENVSAETCTLQSSLAPEKGFEEEVEKAVL
FRDEYLDVAHATARVLTQKYTVSSVAKELNIQPEKLFSYMTNNMPIYFRPDQMQEYAVCA
AAASTADAIRSAEVFGRRRQTGAELHTARLASDDDSDGMGVIGLDLMALVLFGKTYEVCH
KDVCIPAALVAQRPPPYQPMAYPRLRDVEPTRKRFRNDGGGYIEDEFGRKRGRDEEHKVD
FTEQEWRQEFLRRIEPRAMGGGSWQGADADILRETLPAIVNRCASLETVLLEYAPYARCI
VLDWSPPTASRGVRDGNATALSSNVESFKKRPVETSFKPPGPKKTLFSFAERRAPSLQKR
PCSLLRYKILCCGLVERPPVRAEHFFIVKRHDDNGDDGDDLLDDAASDPNAPLPLLPDGE
DIEEYDD*
>TCRU_2849 RNA pseudouridylate synthase, putative
MAWCDASFSVIYGNEELVVVNKPHDVPMDGEACPVTVEKWAWAYLAKGGCADNSAGGGVE
KEEDTTRTAGRGRGEKKMVKFVHQLDYATSGVLCVAFSREMAARLAHCFEMRTTRKAYLA
VLLGMMPTTSAMQKKLDTDTGVDFLRGSPYASHVRRISMDDVPGVCFIRKCVIEASGHEE
GENADCCATLLEVNLPVGYDTTDHEGFRMTVNGRDARGSVTYLSVLQRGHMTHPTSGCPV
PVTKVALFPRTGRRHQLRVHCHAIGFPILGDVTYARIPTPRVASKVTGAEEVATVGAAES
INAPNEIDAIEAWQRMMLHAWRLSFPVSVEPLRSGSERYMQKKKRRRETLGLAVAGGDDT
TVWTHFETEDPFQLVRPE*
>TCRU_2850 dendritic cell-derived IFNG-induced protein
MDGVAFVDGKPVPCVMTLSQVQALFPQGLVGLCECFGGRSHVFPDAEGTFHLKGGASYTL
KLSGAAKWPSQGEGKGEKQGPCAWAPSLSTSTSTAAKPIVDSTALYNANAEDEQCLFRSA
NASPCYAEKSVRDIFHSASPPDALSDARAGKVSYNVGTASSLAEDDAPRTAVDCSGELSF
CLPLLGTNDTPGKHIQDRVHEYVFLPTIAIRIVDTLEFQRLRSLKQLGTTVFLYPGATHT
RFEHSIGVAHLASQMVRQIALCQPELNITRADTICVTVAGLCHDIGHGPFSHLFEHLVNR
IREKKRIKDTWHHEQMSIRLLRRILSRINLWEYGLTDEDARFIELCILGLAPKSPWPTNV
GRPPYKRFLVDIVANKRNGVDVDRLDYFLRDSLGCYGRAALDVHIPRLFSACKVLCYEGE
YQICFEEKMALSLSDILNVRAKLHKHAYQHRIVKVTDYMVSDALYEADPFFKVRGTNGKL
ISMSECVEDEEGFCQLGDWVCNAIAASSDPRLAKAQSIIQRINERSLYRVVGTAMFARFQ
VRVTEESIREEILSYCMEIEADLVLRDAIDKTLIVSFVVITFGSFDDNGLPDDPINKVTF
YNPKNIELGAFKLPRTRVSPLFLPSEYGERMLFVMVREEALADVVTAAFEAWRERYERHL
GVAVPTSNRRKDESPGKGSARPRRFSENRDVLSGNDFVSFEEMQRQVQTAACEQEAHQPQ
PQSQQQQQQHFQRGVKSARSPSCKLGNLQGERTDGNDENNERVKRICTSVGRGNVEEKHT
DTCTHEGGAVE*
>TCRU_2851 kinesin, putative
MEQRINVAIRIRPPLQKERFDPLCAHKADDGRTVIIKDEESMGAPSTFQFDDVFDGGDDQ
CQLYTQSVQELVDDALQGANVTVLTYGQTGSGKTYTILGSLSSGDLTEESGIFSRVFHDI
FAYREAVKRERHLLVFISAVEIYVDDVMDLLSKRKRVKLRETPEGALTPGVNIIELRTVE
DVIQTFNMANSYRSVTSTKINDCSSRSHALFFIDIFQMPLSASSEPPAREQLIDASGIPI
SGDIKGLVRSRIALVDLAGSERVKRTGVEGQAMVEAQAINKSLAALGTTINAMYMESNHI
PFRESKLTRLLKPLFVDRNSRLLLIGQISPPSVSAQESRGTLRFCDRVKGLKAGSPAFFL
SQEDEESYIRSLRQQEELTAELRIAGAECYYRPMRPLFMAYVKNISVDEARRASIDLLQR
DASSIVARKEEECMRAMEAEIRAKQMERVHAFADKMNQMIDEYEALSHAVKKEKKDAKRR
KEEQAEEQEKKLIGAKKAKKTRLKMEAKVEKLNIALKEMERHAEEMEAAFTSPLVEDTNE
STEELTTGETPKDDSVQLLVENFHSHATELNRLQDLYAQWLGATQRQRSQVRRAKLFSSS
IIMDGTLVYDIIDFIIDRSVDIADGVSHAEKKFSWNDIDGFSSLLRSGERLYPPLVTTPS
SKAEVCEPSAYHNVTFLSSDESDEENSHHREENRMLRGDSRSFSSSEGPNTPIKGQYDAL
GDKKAQGTFKQPEWNGLQQTANLEGEFILQGRDGSMADANGDDTDDNCYDEEKKGEGEER
AGKQPRQNKEENTNDEEAQSLESTRKRDVRDNEYLMRVYDSPTLVHDLIRFLRGGTVMLK
HGRSGRPHFRYFWVTISQNSRKLVWTEPESKITAERSSIDLDDVSFIQLGCFSKVFRRHP
VASTDPSFYRSFTLGLKGGGRTVDIVAGSLPDFEAWIVGLSNLVRVDPVWGGKLDITKEA
HFEQLNCFEASLCEMNYIYPSQYILLKKRVKRIAMRTLGVLEQCGNDATKAYKILGGIHP
PAVNEKGAVYLTKGELRFIGLNDMDILRITKVWILFQQMNLVYDENFVPATTFGVTERH*
>TCRU_2852 hypothetical protein, conserved
MTFRQPRLSISNSLLPTAVDWAVMRAMREWHNGVEAADASAISSEGHVTEELSPFWIPKQ
VVGFLAEALLRERQSSQMPFHTPVMDGTRMTTRSGTKQVESVYFAKGTRSVSVFGFSFVN

AASTPICSAVDSCTCVSYELPISESGDLIRFLMGKMSFAAPSRLSSLKASCGDNHSLGKG
CFPLKFGSPYWIEGEGLERRWRGVTISPNATPLYVGRSSRWLINAEQTNDASRFDSLSCT
PFRRHQCLTENGVHDVSLDFSARLAEAFLEMKRDPEKTHSTPTKFCEPNTTRRTCHGHHC
FYERQQQMTDSPSNLWVDAGVIERSGWSLQDAAVGVELPPHQLIGVERNAVSGVNSATVK
AKPIVVVNAEFVCERDSLLDVIMYRPECIGLQRNQFLGSRATTQLALLAHTMNYTSPTWI
QATVAERAGLCVKKGRAATGVEVKIKESYQNETSTVVFINADELDLTQERIAELRNGSV*
>TCRU_2853 WD domain, G-beta repeat, putative
MQRRTTSPSRGLGGRLQGTASGVTRPGVSRPSAVVRELHKNDGTNPRGARGRQIASEMES
LMEDVYGGAVTQRPPPSSGAGGEYARFKTTFNASGVPRSWQSGPVDATGRCFNASDRNLL
CMDVYMRNKLCVVGSADHGLKVFDIKTGKEKKNLYTKNYGHTEWVTCCKFLQNTGQIISG
GMDSKLCLWEATGPVRCSDLLGHTGSISQLEVNDSSKIVISSSYDRTMRLWDCGGDGRCL
STLAGHKAPVMMFSWCGAQVLSGDRQGTAKVWDLETGDCLATMATKRGQIGSLWHLLHTS
VGLLSVIGDQGGVVTVLDFPRLGSKPIFQDVLHPGGVVSGIRSIDETPYLVTAGADKIIL
TLDPRANFSTVHRFTDHRDFIYSLETFGPLVLSGGGNGWLLVHDTVTGKCCYGLGANAAA
VREIFASPEHLVAAGDDGNAIVYDYL*
>TCRU_2854 ESAG8-associated protein, putative
MEVTHQQPKSCFSPFSPFLQEITNTNVLDHENKENIPPALFGKSHNEGGSGCKSGWKNGG
ITQLLNNSLNSCCCTPVTNRQRAHHTGLTPGNKCAAGPRGVSALVSTNTNIADISLITES
DAGDLKESNFFITPVKRWDGAGAGATTNAAHAAATPCFRQGKLCELFNESVSLPLPVSTP
NVSVESTLLPRSVLLESDDDDSVKEVQSIKKPCVSEVPRSVVPRQLDLRSSQEDPSQQRP
VEGQKAFPASASMHGNAHPAPQHHAVQHSGPPPAYPRHEKRQNVTRVVGKTASLIAVGGR
RVPIDKLHLLLRPATSEGKSGAKVNDSTTGGKGSVSSSAFDPITPAPLAPSLRPTPTAAK
AVKVVSPPAAAPAPLIRRSLPVHQHCDYVILEFARGVVMRLQVDSPHPLPVIGRRYLAAI
KSSRSPYDNVAYEDVGVCVYLQRHHGDANTTSGPVQRSALYDGTIIREVGEHIAADAARL
QELKKACNNAIVECRKQFRFLNLPFELVDVCYTFDRSICIVYYNIQPQEGTSSQPNVSRL
LRMLQFNLRTKVFLKGITSGE*
>TCRU_family, putative Rio2, N-terminal/RI.1
MVKLDVSLLRFLEEEDFRVLTALEMTMRNHDVAPTALIERIAQLPHGGCRKRLRCLLKHK
MIYHENTMYDGYAMKYGAYDYLALRTLSKRGTVAAVGHRIGCGKESDIILVKDEAGHECV
LKLQRLGRCSFRSVTRNRDYKGGGRTRHGESWFYLSRLAAEKEFSFMKTLYDEGFPVPKP
IDQNRHALLMELVDGTLLNNITALGDPEKVYRRSLDLMIKLGEHGLVHGDFNEFNLMITE
DMRVIVIDFPQMVSTNHPNAAELFSRDVENLANFFHRRFKVTTLFYPTLEKNVERKAELD
RVVFASGCFSSRQQQDLERLLQTQLTAQDEVDSEEEDGQEEDEEEATTLSGNASVDVVSA
GGANACGVHAADGSEGEGQAQRHPPGEVGDDDNGETSSVARSLDEVVDEAAIERRGQRQQ
NVNFNPDGTVNEEYVRSQVRRNIRRQDNHQFNLGLHRNVQKGRQKQKIRRQIKQGMSNGF
FD*
>TCRU_2856 Kinesin motor domain containing protein, putative
MSVKVAVRCRPLILDERKDNSVVIVRMNNNEVTVLDCLEGVGGKFCFDEALWSAQVVVEG
NANPHASQEMVYKSVASELMNHVVTGYNGCIFAYGQTGSGKTYSMMGTPTDFGVIPRAAK
SLFTEVRGLASSGVESCVEVSFFEIYNEKVRCLLCPSVDGFENSTLRVREHPNFGPFIEG
LTKFVVSSEEELLRLLNDGNKVRTSGTTAMNAFSSRSHAVFVTTLTQKCTNGRLVKTKTS
KLNLVDLAGSERASKSMAAGKRLTEGVNINKSLMCLGNVISSLAEEQDSGRPRHIPYRDS
VLTWILKENLGGNSKTVMLATISPSSLQYDETMSTLRYAERAKRIVNRAVVNESNNNEVI
AALQKEIEQLKSQLRGASGSDRDSLSEQLAASEKFQKELQMTMDEKLMESRRLMEERKKD
MQRLEEQLEAQRGEIANLRQDNAEKERKIHELLQKIEVVKKRLGGGDDTEVEELLEQVAV
LELEQNAVSTKHVNTAADASRVADDTVLRLENLGNVRPQLIEGGKITVRALARAFEAVGR
ELPAAGSAAALCEIKGNDYFAQNVNAVEKELRGENPLSSLTTADFDAGIDEDLLLEELLE
FDEETVQEHGDQMQEEGTLDSAGVNVNGDFDSDLVVDEDEEIELDESELEFDLDLELDEN
IDGNASVAEKEGGEEVGEAFSGGICQSENDLNDNDINAVAARQELSRAENVVNDDDYEKA
SHVHEETTPNVTQVPRETSINSNIMIEVAAVDENGGNNATNQSVVKDTSHTTTKNAATAT
DTLMDADKFANASTQSTGNGLLSPFSTVTTLVIPSHALPNNRYLNEVFKVIKRNDAAILG
NRKDRIWLVDFFKRRFANLDMAGYMSFQYPASNLFRVEKDPLNSKRLTLHFFDAAHPYEL
EFTSTGRRQRFYEISMTLRRNSVMWCPSLCLPNEKNVILHVQGTTIRRPNAKTVNVSGES
KFTVSRMPYEVLDMWYGCFSMKDRPLPRSAAVLGSFIPRGSHEMYVIGIMDVPASFIGND

VFSKYFLEYLGSSLYYVLANTAVFSKRRQNNNALIVIVRNSFILRTSHIEAMETTPVFTD
DIPKDAFTSVGCTLRVNEATIGIILVNAKPIDCSLQRRAAALRSMMSSYPFGDTLVDIGA
RFDYFIVSGAFNFGADFRHDDMLLAQIKARNLMSGLVEAAPSSAILNATEPIRIFFAFRP
GVCNLDVKQYTTSRALAAPNAFISADVFCQRAYLCVFGEQVPRVQLVLDKLVLASPNLRI
PSVEAPELRISADWIENSPLLTALFKGGDAYKLSGPAVPVVAPTTSNTVFLRLQTITFSM
LGIPGSAKRREKTVIASGSLPLKNMLYLGEVVEFSTPLYYRTCYVGNLTGTLFLSAYERD
TADLAGLESERDVIVVSCYENQILEGKNWVSTIFSQHHAYGFSTEDATVECSREGHTLPD
STKWVWLNLWRHEVFPHDAEGWTYSEGFDQPFFSIKPSKTTIVRRRRWTRAMQANDPVTY
HNFLMLKREGSSFS*
>TCRU_2857 hypothetical protein, conserved
MGKRRRTISDVTCFIESILSCRRIEEPDASFSLSRVSDGTNSGSSRSSSCSRSRSRSESM
NGTSTNDRSAVSRVLTIPSGSMNPSDSQKSTGGAFATEINFFCRAEEDLADMSLYLARVV
SSARTPDAFVALVEHFSKYGAETVMLSLKRSGTQPKLLGHLCSVPDGGLTKGYQQLFLRF
LAFLVRLCDTEVLYEEELMKFLLRCCQSHAEASVNSAASSKRPSHWSQGARQAAVVPARS
LKLSEIKRSVDALCGEEEDNSHASLALRVLLEIVFRQNRNTSHPSDPNFFIVFAELGGFK
IVSALLETEECADALHLLEAVTLCEGLRGVYASELRITTAFLVRLITENEKQICKVAPRC
DEKNAALRVLTNVTGLVPSVLSGSPQLSRALAIVAAKTLTDAEAVSDTLAFTLCFTTNIV
KWEASQKCDEFTSVLVESPSFLTRVACLAFSAYHAEDMEKNVLAGYYALLLAVLSLCESP
KAQLRVPVMTAVAHATRGTAALKKVEDKPMTLIVAILQEFLLFQGSAGSLTRDSLVSMTG
IIDSVAECNGIEVSSDE*
>TCRU_2858 FHA domain containing protein, putative
MEQYFLVDTASGKRELLRPGKNVIGRSATVSAVDGVSFVSLESPSFTISRVQAVVEVASN
GDAWISDCNSTNGTFLAISEGNGIRLDPHCFYQLKPGNRVILGDVEMRLETSACSTNEMG
DQKGVMPSSALSSLNTSHPTNLSGAHSEILCASNDTRVATTANATNTIEKPSDAVESEAN
GAVLQKSHECLSGKCSNDRVESTPYHEGSVFLGRKRIRSSSDITPPSGASKTVSSLLRVH
WVCFSGMDGAEKADAQKLAKKLGWQTTDRIVDADILVVGSPVTRTPKFLIAVGRGIPVVT
GNFLKDGDTAQLEKYVPALSHGSHTYSAASLRKVIFRECKTPILQGVSFQLGALPRKLRA
VAREIILGCGGDIVRAQSTDALSLTEESLDSVYDSVLRGESL*
>TCRU_2859 hypothetical protein, conserved
MRQTGWVKDQFLNRGVNHFVVFSSLGRVMESSGDFEDEEKQQLAYTILQQTSTLLRSGES
VKRLSMTFDDFVYIATTIAGEGENLGVVLKRNAADVLATT*
>TCRU_2860 hypothetical protein, conserved
MNKQLLQTLPLAKGKDIHSRVAKLALYKECIALAKNVGDPLVRQHTLVEVREKWVQNRGC
GGHSLQLQIASALDRISYGRMCVSKQRLRLIPNASEKYDWGVSNPLEYHERRLTKRSEKE
SPDAFPHGAGKRDFVPMTNWGYGNMDPDAVMRHKELTDRQHFIGPHWRGKPKPMVLEDLS
FEEQLHVHFQSKPKMKKTPKKHY*
>TCRU_2861 hypothetical protein, conserved
MSSALLLESAKATSILLASEVRLPREDVGAAFESIGAAEKGYEVMYTVTVVDNRDWRRIF
LCRNATEATKVRDSFDEAQFDVYALRKYNDARKFEPRRSVPLLVPSSAVMQREYRVVTRE
EAASRVEGKSGAQESMKTANTSLNCSGEPMTRDAAPQEKPTIVNNVSMGNKLPPPPPPPP
PPPPNESCRSPIRSPQTLLKTPLSGKKHTRTADTAKKQSEANLKKPCVRNKKKGKFEETR
SLMKLAKASAKNKIGSDSSRISGPKTANTTTTFACSSILQNLLDKDEENGDHCSNSSNSG
ISFERRDEELMDEELPTLDVAVSSKENDDIILCDDAPPMVFCTPEPVAGTSKKEQQQIQH
NQRRHESLGSVTQPKLTGFFQRDVIEYQKQYIREVMTDTVIENGEYFCKDKVCFRHKETK
EVISEEEFYRRSADIMRSVRSDSQNVNSNDRDTTGQGSPSTPAGSVKRVSAEAARGSKPP
KIEKQLRSQPKTLFSYFKPTSS*
>TCRU_2862 Calcineurin-like phosphoesterase, putative
MDLIALEEKVRRLETLEVNEMRLLLNTVMDLFLYESNVPNVPLPVTICGDIHGQFPDLLH
LLALAGEISTTSMHYIFLGDLVDRGFNSVEVVTFLFLMKVKYPEKITLIRGNHETRQVST
AYGFYDECNRKFKTAEVWRHCTDVFDCLPIGALVEGRALCIHGGLSPDVNTIDNIHLLQR
KQEIPTEGALADLVWSDPGNIEGWGISQRGAGHLFGANVTREFLYRNGLNLLARAHQLVQ
MGFKYHFDEEFVCTVWSAPNYCYRCNNFASVLRVYEDESREFVVFEKVDRQYVEDFPEKL
QLSYFL*
>TCRU_2863 Protein of unknown function (DUF1193), putative

MTLIGLLSWMILLLFGEYNIIHPHAPWPQNALQNGSSTSEPLASPHSLPPSSIPRKHDVN
KARASPFLFTPPEFEEALQTYENNLGIVEGLMGNLDQPTTDFTDIIESTGPGVPLPYDSS
RVANAKEWEACDQRNAEFTPERDVLCQSYLAELNNMRYIKAMSSRLLYGRTIKFRVRYAH
SEIEAIVKVSQRKFYFEATSEYLAFSIDRALNLSRVPTSAFVPLPLNYMKVATAYSPFFS
QWINRFIFTYNYTTRNFFPCGFSGVSPDNEILCAYVTIQLWMHDVHSALSSFLSLPYEYD
SAFAAKYYKIGDKLWPPKSSRLRAIGDLLDRFIFDFLIGNTDRGMNDHNNFVYGGCSFKT
TCQRPPKAKRILGLAKYAFLDHGSSFYSHREPKGNAFFGEEYLIPICRFRRSTYESLRVY
RKRNSSGPHHPLTTQIRQSLSPYIFRVTSISVFEKVQGRLEKILRIVERCLEQYPPSEVF
SLPEYWELRIPEEEEEDEQEMSDSVNSE*
>TCRU_2864 Zinc knuckle, putative
MYGNKGPPKKYSNPRGVTDQSGASRVCQKCASTEHWTFECQKAADATRKTTARLSRSQML
RWGIKRRRQEFVPEPTEREAYTAHAKEVERMLLTEARRDVLQRQRLEKSRDVKDDSDDDG
SSRGDDTWNDVKSKESKQSAILKDEAYIKEEKKDHMEM*
>TCRU_2865 dynein heavy chain, putative
MGRLNSAVAENCVAVTEKVLKRRLGITANRAHQSERTVEFVFTASNDRLGEISSALFREF
VQAACGSEQGRTKLGSVDVSVDAQKGLQSVLFTDVVCVHNFRFDELPDDSPAITAVAEAT
YFRYLAKHSDAQAYAVTEFPKCLTAKGGPRLDVIIAGYVLFSLLSDDGDEVKLRMYIKNI
DEVLGTMCTSSFASTVLPHSWSNLDRLEPHQLVDLLEETQLAISDFWTDSAQDTRARSQV
IFLMTTIGSELREYFSKKTLAAGGIFGDSKSATEMALSCCDDWVNMCRNLTTIDWGAVWG
GRFEDLQLRVVCDRLRVVASLRDLVGEMVELLNASGELHFLRKETLWEAMESIDIFQTTA
AVEKQWDAALSAFYRRLEPVEHRCAAALRDFFGERGNLAPQTILNEVVKFRQLIRRPVVA
KELVSERDALLAKLNERLQGIRLEFEHRSESTEDDLFLEDEDRRCQTGRFMPGVVNNMIW
LRQLRGRVEEMIKMCKSLLLDLQNAREFVLAADTLLEEIGDYELELYKHWAMDVEDNSHA
LILDANAPLMDIDANGRVEVNYPERLVQLIREVRIFRGLGLRITGEIQRMVDQGVCFYRN
GVSLKQIASTYNSMTKDIIPCTRAMLLEPALFFENIITASGDRKLTWRNVEDAERFIGKL
RTASQSLTDSNRRLHRLHKEIEAIVVELFSVDLLRSRERWMGKVHTIREKMEISGFKNME
NWKLFWDVQLYKAMEYQYQLGLESLHEVVAEMKADIVYDQETGLAALRPSLEVIRGQYYQ
RIKDFMTFPLGFRGCGENEFFKEMPARNERGIFAVMQHAAQLFKKVQQELKRFHPLLIIG
QCGRNGNPSLEEIVGKTLTEVQHWEQGIRLLKQKGKEINGEELFIKCGCITLCTASIKGT
VEDHLYKLSEVLRVTLRRSAENHLRRIDAYLVEVSGSLDSTLTKLDEIGAANVHHAYLVE
QRPAMEVEFYHFYNKNMLLQSMANRAGLDFAKTRDEWDRVMRRLDSYESEMEEQMDKLKG
VIEESVKSWQKKLERFTNQWHELKPKSADSPNAVQFVKDQQEKFKALEAEGEECVQQCKY
FQLEEPDIEPLHDLGRDIEEYALMWSMVGKFNEELGVLRAEPWLALRAKLYRFDDLIKVW
QEKLKEIPNNPITVQIRTLLDSWSRCAPLFKFVRGDGFTPGHWVELFRLLGTPNATQDTL
QFGDIIDRYENILRHEMELKKLHSRAQGEAQIREALDDVRSWGTNAKFTLCAHPERPGVM
LITEWKDTLSALSDNQALLLSMKESPYFSIFASDANKWEERLACLDEYLRSMNQIQRKWV
YLEPIFRRGALPHEQERFARIDKEYLQVMHNIAKDSRIAPLATHKEYKEVLRNVLEQLDR
CQRALNQYLEAKRDSFPRFYFISDDDLLEVLAQSRNPLVIQSHLKKLFMGIHGVRFDTQK
EHILQIHSLEGETVQLEEPVRITDEVEEWLSKLDVAMKDTLRVHLVRCLEKLDIGAYATQ
ILCTAGMIDFTKKTEEAIRESKAGGLLKLKANLQSQLRDLTIYTGGSSDVVVVLKLKSLI
MDLIHNIEVVDTLIRNGVEKETDWLWRKQLRYYLDHNNNCVLRMVDAEFNYSYEYQGNAP
KLVHTPLTDRCYLTLTQGMQLGYGGNPYGPAGTGKTESVKALGNAMGRQVLVFNCDEGID
FKSMGRIFTGLVKCGAWGCFDEFNRLKVDQLSAVSQMIQVIQEALKNGESHCHLLGRDID
VNPNAGIFVTMNPAGKRYGGRSKLPDNLKQLFRSVAMSAPDNELIMETILFSEGFENATE
LAKRTVEVFKLAAGLLSYQQHYDWGLRAMKSVMRLGGSLIHQYLMDRVGGKVKLTPQDVL
QKECEILIKSLRVNTLSKLTFEDAILFNNLIADVFPGVPIKEIDYTELHPAIVESIKELK
LQFVESQVQKILQLYEALKQRMGVVLVGPGGSGKSTLLRVLRRALQRLGVSVPLYVMNPK
AMPRTQLLGHMDNDTREWFDGVLTEAAKKVVKEENDVHSWIVCDGDIDPEWIESLNSVLD
DNKLLTMPNGVRIQFGDNVSFLFETHSLEFASPATVSRMGIIYLSEEDVNPKMMVATWLL
EQPEDSRNQMRSWIDEFFYKAIDNLLATGNLIVETTRTGLVASGLSQLQNCSNKAQFTLA
LVYGLGSYLPEENRREYAKDIHYMTGERVLDMKDPLGTRYDLQHGGYRSFEFQPAFELSV
EDLYRYPMIATIECQRNMEVMRAWTKPLRPGVYRPFILVGPEGCGKTMLLTNLFAGISGT
RVATVNCSAQTEAIHVIQKLKQTCQMFNTNQGRVLRPKEAERLVLLLKDMNLPKPDKYGT
VQLHSLLQQLILYSGFYDTDLEWITVERVQIVGSMNPPGSMGRYPVAPRFLAIVSVLCMS

YPSRESMQIIYSEFFNIMIQSHKLKFELPGKGAADIARIMTIVYETIAIRCTVDVSSHYI
FTPRDVTKWALNLLNYNSVDIPDAIGYEGRRIFIDRLISPDERTKFAKLIYDNLIFLVGH
RAGITEKETTYYVSWMDVASSRVKKLNATSMDELKKSAEHLLLNYSREFAELDVQLIPEV
CAWIARVDRVLSQERGNLLLVGRSGVCDRAIIRLVAYNLHMEVMTLAITREYGMKQFNAE
LKSVLCKAGIEGQHVVLLLEDHHFGNNPLFLEYVNSLLSSGEIPGLFTQEEQEALLAPLR
EEAMGEGMSPYAYFVDRIARMLHVCVVMDPTNPNYEVHCRSNPALFTRCNVYWMGTWYSD
SLKLIPRLMMSNVFKALDMRSDKKEFSLTTEIVHIHRGFAEKFSPQHFKTLCLTYQRIFN
EKSTAIAEILDRLQSGVSKLDEAQENVDQIATDVADKKTLMEAKQREADEALQQIQANME
EAGNQKKNIQRIHKDLEKEQRAIEERKRVIEERLSGIQPMLDAALSSVRSIRAEHLSELK
SMKQPPAVVQDVMEGVIILIASNFVTDINWTAIRKILAGDIKSDILNFDLDNVNETARWK
LERFIQSHENSFRREVIGRASKAAAPMAEWLKAVVEYGKVLQTVAPMREELKGYEANLKS
GNEKKKKYEVKLSKLEEHVEELKKNFGEKTMEAERLKDRLEQAEQLYQSAHDLLVKLASE
HDRWASQIRAIRADQHLLPKRCLLAAAFVMYLGNEAEDVRRRTLDGWKERIKQLDDFNFF
TFLRPQSTQLQYKAEGLPDDELSMDNAVMMQEQVTTPLIVDSSDQAVNWLRNNLKGRGVT
VDVCSVSDERLVSALELALRFGKSFVVTDVDRIESFMYPLFRKELRMEGSKRVIQIGDRR
TVDYSDGFQLYLLTPSTDLRIPPDVLSYLTPINFSVTLSGLEGQFLGTTIQHEQPELEEE
KLRVLQKEEKLKLQLSTLEEALLNDLASSKGSLLENKTLIDSLNEIKTQAAEISEALEKS
KQVQEEIDMKRNVYRPFATTASETFFIVKSLKGLSHMYQFSLNFFMGIFRDTLKRHTEDH
TDAETKIKALKTTFVRLVVSSVSISLLKEHRPVFGIYLARSLHPKECTSAEWDFFLDRAI
ASEAEKKEVHMPTWVLPDSRDLFRSFVVLFRELLPKMNLQEADVWLQWMRTTTPEAEYPR
FLMQLTNFQRLLIVKTLRGDRLIAAVNRVACDLLSVNSWGDNRTLASFVKRTESNTPLLL
ITTAGADPSQELQSLAYHQVGRDRFHQIAMGGGQTDEAIRLLRLCAETGDWLFLKNLHLV
IPWVPVLQKEFNLLTPDSKFRLFLTSESHEEFPSILLSQSLKITFEPPPGVQQNLLHTYK
EWEGTAFDSKGTRQKEVLFIAAAFHAIVQERRSYVPQGWSMMYEVTSADLNSAADIVLQQ
TKAETDWKAIRGILENAIYGARIESEFDMRILQKYTQHFFDPSVLSDAKRQANLFATLRV
PGTGKLADYIKVISELPESDVPSLFSLPPNADRVVQLLKVRQLTDNLQHLNGSKAESSLS
REEWTSRLTPVLQAWAELTQPHSNLLTLSATESQEVTPIIGFLTAEMISSLRLVSLVDES
IGDLRRVTEGTALLKEDRRAEAAAMISGEVPSHWDGYFHGSPRILPWLQSLLRRATTIVE
WRKMAVNRELLKSDLSISSFFRPQTFLHALRQETAHSLHEPLVSLKLAISVSSPPEGAPL
PVCLEGLLLQGAFIDQSDMMQPIETSDEPALFPMPKVYVAWMSKPPAAKTSVAVPLYTNT
TKEKFIMDFLLPCTSEVAVKAFILAGVSIFLEP*
>TCRU_2866 Replication factor-A C terminal domain containing
protein, putative
MDALSRGFCASAIKGVLPHGPGESWALLWPSPRVQVVDIQRYPKIVTEPFVVSRFYVAVS
DSASLIWLVVPPGSEMEELIDTFQIEVGSCITLNEYSVVAAKNALNVVVPLSITYSGNEL
SLLGNPTIEETLFCLSTEALTKRRGAVKPNFSRAIESYIKISLIDLVDTKGHELEDYAMD
LRIIWKGKPRHLTASGSMGRFVFDCIGVDRNGDAVSISCMGQSGLYDIFSLGDCVCVTNA
CLTSRSEAEDTMRLQLNERSFVVLRGEEAVSNIPTHPCRMFGARSVTNLINYSNVGDIVH
VEGVVTSVSSAALVNTRRGRVERSGISLQDPASSSLIDVTLWEEFSRSVRPNVGERWCFC
GFVVGEFMRRLTLSSRYGSGAFQMKMTPEKSLLPVNRTEKIPFMSLENKDSLQVVLDLEE
ASETLPVLAKIKRVRVPLTYVACRGCGKQIRSKALSCAACGGSTLEERFFVRLELSDGIC
AVSAVGFAQIGETLFGMDLPTLMKQREKSPGFEGNIARDVVGLPLLFWLLRSPDDSLLHV
VKCQHIDMARCAATLLGALAQMMGPNFDASRTE*
>TCRU_2867 hypothetical protein
MPYNFLRPNRLYTSEEKVAEEENKECDDDNNDVKIQRELNAWRLHMREERRRRLLAAASR
AERTRTAEEPSASHVSPRESSHEAGRGGESHHQELTALQQYRRELGEVTSGVQKGRNSLW
NLRVPIRRQRRVKTNRVTYHEARSVLRDYIHRPPPPPANVQKTPIGTLRAYKDALALSAR
ACSEAPTSPPLWHQRPLRNISVHRRRNDFSKFTSVSTTRSTTLRRKHTESSFANRHVDRK
LKWCQCDVQRKRNGDPSKRSFRPVLEATQAYQSAIKQEEEKYNQHRQCHEKRFPFEKEPS
PQSSVSSQCLSSRRSATQPMVTHAIDSPQARQPSHISYIRHGVEPEEPFHNPVVNPPAAE
EAVSPQTSALSETGTASHYEERSRRESSPLLRKLLSKLTLSSPSLYRPDLYQGNTIPFTS
PVVRTVEPHPWPPVGAKGQG*
>TCRU_2868 PQQ-like domain/Protein of unknown function (DUF1620),
putative

MKCSTPLFLLVLFLFAVVHPSLAIHEDEQGLRDWILRFVGHVDHASYQPGLQPNHVYLAS
KPGAVAALSLSDGGLQWRKLLSEPQVCVAASKHEVLVSSRSGTVHLLNAANGEIERTFKL
SLPIGSTVESCAIVEGKVRVAAMDTITAYIYEFGLVTEDDMMKPRNNFPIGTDVHALRIS
GKHIWALRTSSANRYSLTGDVEMTDVEGGSYGSSVTASGDAVVFSGTRIAVVRDDGKTEN
IACENCDVGVLYSTTGKFEGTVKTTADKLGFTVFFPGSNVHVAYDSAAIIAPTVLLAVRD
RLHGAQVILRTYNGHILAVSEKYGKMWERLEGLAHLSATVIADNPAHEDHFSFNKVALAI
STYGVFYAIPISEMGHNIRVLSDISDVILQKTAAFSMESVKIERMFLSGTNVVTVVVASP
EKRVSLAVDYSTGSVMEVKVHENSLFVSPTFEVTRSLELHGAVSYNNMHFFTANVSSGLI
EGYAVSESSEVVPLWTVRMPYPLVAYATGEDILRTKTVNHLRVFPNKTSQADEVRRKYPT
RNVFVVAHYEPSEDELTTLVVTAIDSVTGSVLATARHRNVEGPVHMLVVEHAVLYYYMDM
EKVRHCIGVWEMFEEEPGRVLRKDAGATIPQVIASFFSRFKRTFSSRATRPPVVTVAVLG
VYGGNLATMGVTTSFNGISRKSVVLAFDSGRIATVELNRLLAGGQLPLDDKGNQATHVII
PSTSLASHKLRVAKPSLIATTPTNLESSCHVLVSGLDIFYVRASSGKAFDLLNSDFNKNL
LITLTCGFGVLTCAARYFVSRKALNLLWK*
>TCRU_2869 Alpha/beta hydrolase family, putative
MNLVRRVFSSGSTVRALPISESPVLLNYTLSTFRWHLDSRPSSKAILVHDLFGSAASWQQ
LLNDDLSRLPLSQFTPTTPLELYAVELRGHNHSRGLPCPKDGDNYTLASAADVVLHQKQV
LRTEAKLVGLGFGALVACQAALHSPKSGFDSLTLIVNEPSQLLCCDPSNYSLPSIIRGMP
RGMRSLGEVEEYLKKTVNNAVERALIFAVVELQEDVPGFRFSDDLLHFHGPFKGAADIAE
DITFERRVTVVVCGNEAVSQEARERFTKHFPRATFVQFQGGSNPGIAGLYKQGTNFICTF
LEALEMLGTVDGGEKEEK*
>TCRU_2870 PX domain containing protein, putative
MEFLVADAERMYTVNGRLQHLSYWSYKILARSFLESYQNPPLLNSIRAAQNFSKGSGYVD
FCTWHRYSEFEWLAAQVELEFPGMLFPPIPSKETDGTVDKFTELLTSVKENNSTTRENPL
VKKRMRQLQLTFNAIAQLHELHESPLLKAFVTLDELSWCAFREEREKKMKTSILTIFKAK
GLSIIAKLRSFRESSNHDYTLESPLDSVATRQSDLGELLNTCFAQVEYINKYCISEWRHW
SDVNKLRGSIQGSPMPWTACYGGHLVQHSKHPGLEGVVREINNDAAVVEWNGQGGRLSKV
PLDELNYPSSGISDPAIFALQELASQVESYFMYLSRSSGVAVLRDVEDLLWFLSTYANRC
VRVIRRFREMRSEVRYLTRVVEKKASVEQFEQLERLKQILSKGTSRFMEQYNNFYRCFMR
NSLMEVAQKFGQVMTVIMSDDQWNERLSVAKTMLTLSLPLLPEEREEIFAEDASKRDSSP
MKQVNGLEG*
>TCRU_2871 hypothetical protein, conserved
MVHIVGAVKQQLRVSLDERSVAALERAFNTLQGLRTGFVFGVLPSSTAFNELHILCCEAA
IGLEQWGMAESCLKIFAGKRAPTRALDARALYCEGLIGLHNIPISFRGRAKVTSHLRCAT
KIVTGVKIALEEWPKEAHTLAVGVEHLWLAIRDLFIPGSFDSVLDIVAFVLALHEKLGLG
GPFSQLQWSTRYAMCLRGVGRHQEAVSQLSTASELVSRVGSERLQLQFLRVQIAFWASLP
ATRTKQDYTKPVLQALYVAQAFFCGMIDEFSATADLIAAHEKLLLDPEKKEIKLKSRKTM
EAEITFSDPALVMDVLSEVVLGLALCGADEVCKISLPSLLQGDSVRPRLFGEFAQAILRA
RACGALGLAKALDASYLTRTMADALFNCIKAVEGTMDNARMIEDPSERAYTLQIGCVILW
NLSLPLLQSETRGKLRHTLKRIVVLLTNYGSCLTGLLVHVAYESSLIEFEEDNPISAIEQ
IDRALSLNHCVVNAEGKMVFPMDFALLSLRRRCVVRKTIDSSVFSAREDQVIHLIEQART
SNSASNRLVLLQSAVKKLPPLGQVLDPGTLAKERGPFLRERSKIGRAKKHSVVVEPPEGQ
PPRVITNPAAAHIYYLLLKEAKEEKNPSMHAIVEKVASSLTCIDVLNEADSKELIVMKAE
AHLSMAEVLLKNEKSSGGTSSDSTADALSHISAAAKISIKLCAAGNREEWIAVNACLSLI
GWYSELFRKGLFLPATVALGELYVALNQTHVDPNREYKLFHDICFAYIMSVLQSYISSKG
EVDGELSCGNLAETLRKTFLYPICEANNPMLRRAEEVCREVMGKWRLPSQRKMFCTLFVC
IQRLLDRSPEDSHHPQEQLLFLLGKLNGPVSMEEKRILVNKESLDLLRMDPSVELCGRLA
THSMQLPENERVTLELCRIADQLYQDGKLGWGTKTLIGNHTGKEKGHSPNSASSAAVLAR
GAFPKPTEDDWYWYSDILLHQATVLGRLGTGTERSKRHELEKRVIVAITNSAIAASHGPT
CTRVSQITDAYYAFCCTADYFSGALRQLLLPSLRILLSPSMLSILPLSSRGGGSSTEGHA
EREALLPLITNMAGIFLTTLSENGAHEEGIKTLNNLLAVLPTKCHKVFRTYEAQFRSNLG
LSTAQLVNKVRGGDPEIEAAVWVAIAKNAAANADSTKSWLKALEVLERKPLQRADTLLQM
AEWMATRNAVSRHELITLLLSALDSVERFSDPQMSRLPEEGMRQEGSSRCLRQTMAFSLS
GRTICLPDAGSMRSGNNLTGGVGEPASLREIMTAIRIMYLLFVVAPEKPLEGHTFTVCKR

SCASTILHYVFCMWNIVSTLIRSKDAGKENALKENLLEIPEEFHAWQGFCADPQHIDFIR
VSCQDDAPQNTDKLWGMLFDVCEFLMEEHWEQFTFMIFSWIEFAANWRYGSLDPRTMAVV
RAVRFKCLLAGARSGWGNLSSMYQVEAPTSEYWSAIQEILLSAENSSHFSPWPDGALRFT
DFRGSTCYCVLSEAEDLFLLGRVQEALRWSGKVLNCALHIGNIDAVVRCRLLEARARILR
GEGAAVAKQLSEIEEEFVGLTFRLWTELNLVKVDALISCGHVEKAISLARNLPQALGLRL
TKIAPDGLAGDISNDLLKDCTRRILCECGRQLLAFLRLSLRRSSFPGESEDAARKYLAEV
GDQLQKMDDWQSLSTALCIKQNLNPPMDEVLLTTDVEKGKELLELLYTEYCEALRVTELI
RSEMNVVAPFSLYTKSLATHPSILRAYDEVLVIRARNCLERELLRDFIILQYDQLSMEQL
GIPTGGPKILDTSVLQYMRDPAAYEQMKKNMTAGSGKESPEGGFYEEYNPTRDLECALNH
FFIPQAQVEGASSAEAEAMLSRTVKLDGECTPLQLEAVICVAFAEFQRRLKALLRAEDAT
LAVTLTGVAQIREDKGIGFRENDMLTRKWVRLIPNIGQLDTRRTSRKRALSVSRKSRKSL
LTEPPKKEEGPVNLWVNEDSSLPSMIQLGAKHARRLLEYKELAKCYICLAHNLVLSDDYC
AAGTAIEFAQAAEMCGFLLDIQSALLQSSPESNLIRFLERMKLKASFLLNTSAFDSMRNL
LMNTSSMLKLFDLGCEWPAEPNKPQPLYSDSCALSVVREESTKYFLVALRCPEGGVHSRR
VQLDINSLIQCGDEFETFKQTKKVEIINKNASVKASLEETSAALLKSLLDRVQYLLAPLW
EDFNGILSWLAPNCHLFLCLDPILQSLPLERLPPCTQFLSVQRELSVFNIRNKISTRGGK
SSSGGSLFIVDPFGEHETSLQTLFGPESRPKGATSEVISSVRDKYGCFCNPSQQYIRQAL
TANSRGILLVDLCGSFTDIVSPETLMELNLEHFLAVLVAEGTNDSSLRREQKQRTSKTTL
SLYYERQFIFSLLLLARGVQCVVTNALLPLSPEECGLICRRWLPAILAGGKGLFEAVRGG
TGEGADSFASVFLFYGVPPTVKQK*
>TCRU_2872 hypothetical protein, conserved
MYYLRSLFFTPEEVFSREHVRNEIAREKLSRAKLQRKSEEETHMAEMERLDNELREKREL
FAAAAKPLLEEFDDVSLAQHYYQEISNIVTSHLHIVSQLADREFSEFGYVSKKLISVSLN
LAALKCCMMGGVPFERELNTLLKDADSSDLSIVATPLCHVRSIPPAANVRATAFDLARAI
EEVGKDGPTREPVRGWLDLFKFRTSFSPTMLETRQVEARRAAKQFLSHIERGEHDKALVL
AEDAYVKVGHEVSAANAVLLESAFGAFRDTVLPVIASDVFLRYARASLDASRFACVEKML
KE*
>TCRU_2873 GRIP domain containing protein, putative
MEEISGGCAEDAGGEVGRLQEELRKAEERMRLWKEKTQVAVNELRDRIVKLTSERNELQE
AFTELKNHQEQQRQQDVDGGSKYTADGALSSSSYSSLVYVPAVTIVRSIVQWTITTVDVF
FPILLKKSEFALGLAACTSQELDKCKRRTAQTLRLQAKNIEALQSEISGLQQQLNEALAE
VRERNHAIDSRDQALSVLQNRLEDLEAANLRLESSRVLLSNKPNAEQINMFEERLEEELE
NVRREFVSRENVIFDQHRDEIERLLAKHEEEVAELRAELEEKILAAETAVDATAAAKVDA
NRAERNGEGAYIDLMRDFEALQDELKVTQGENEKIMAELREVRGKLAHFNGNGNSNDNSS
VASQKEPFTLPEAAVRIAELESGMMRLSEELCETKKRLIAAKHQANARDVSRSHVLEGQQ
LSYLKCIVVKLLGAAECGDVGRNLLPVLSTLLNFNSEDLQELYAAHPDWVKRRF*
>TCRU_2874 chaperone protein DNAj, putative
MRRTARFLVYEKFEGAVSGDRGYMTYQQACSIFGFQMNDRLEAGEIKKRFNKLVLRYHPD
RGGSSEQFQLLREAHKILLTHRHDKGDGASAKGSFINFRRVNYDNVTNTIHRQTAENPEY
RSFSLQDMAFFFFFVTVVMGFYLYRAWRTQMQIIRSRWSYTEDRLTTEATHHDEKLWHPW
RSDQGTRDMMDNIGILQGSVKRELVEEKRQLAPSVYMPWQPGGPFASGQRVESERDTQLS
GEKH*
>TCRU_2875 cysteine peptidase, Clan CA, family C19, putative
MMLYESNDELADGMEEKLPYVGLVNQGSTCYLNSVIQALFHLPSFRQTLYFMAHPERDPI
ILALRNIFSQLHCATPLVSTKELTDAFGWGANDAWVQHDVHEMMQKLFDRLETICKGTPQ
ENFIRDLFYGELTYITRAVDGVDYISYRNEGFYDVELLVKNMDTIYESFELWAKPERIEG
VSLEITKGGPCTSHTVERSQRFVRLPPVLLIHPNRAEFSMETFTVRTLPGKWTFPTSLNL
EPYVLPMELEKNGGTGLSPFYELRSIVVHQGIAENGHYFAYVRFGDQWVCFNDMYVFRVS
EEAVMTSASCSSDTENLNKGSNERASLLVYVNTSVSKDILTEDFVPTHIKAVAEEILAER
ERIQRERCETVTFRYITERESLVEALDDPYDPPVNVKSTSVPTFFSIEEVHAAIAEDIGQ
SPDRIRVWMYDGGIAADVFEAHLHNDINRREILFVEILPDKKEKFSQMSVEKAFFALVRI
IHEDSLGPCKIIHTRKDLERLMGESEEENKCLICLQGPSLWEVSVDRVLVGGNVILCPVS
MKKEHALELLRQRKLKEVEVLLLDRRGTGMWTPTGTIRLDAKSSYADAQEKVYEHLVAAH
HSPPISPEYIGFHCAGSHARNPSIAVFPPSFLGSMKEGMFSLLWHGEDTMELFVTLLPAP

LSSIDTLRVVLLNVGGGIRPAVHVEGRSYKLGELFRLAMQQTSSCLPTDLFNYIMYRLQK
RIPVLRLILIPNEKNQRVFENEDDEVLECIGLYVMDVLAEIRKGFFRVDVVFYDRRSGEG
RFGFPTNIALTESYEETGMEIAQRVAEKIKAAVSVEEIGRWIVAVKGKSGVIRTVGPKEV
LKSIMKDLNEELECFMIDRPRSLLLDGVDDVRQGIEHSIIIKDPSLQDSRSKSAQHGEM*
>TCRU_2876 conserved protein, unknown function
MEGVEKVAAENPLLDRLREKHAELFRQRTSELQKTLETEHSFASRKMHNLPPSLDLISDE
IIIALNEIGTRCEEYAVPQVGQLLRDVRTMMNRQLREAYASLEAGYKSAFHKLEEEAEGS
RRRPEASSTAQKSVGRAMWKLQAESSPPNEESSQSTSSFKERLGKRNTVPKTLRLRDTFL
ATLGFFAETTAVLLRCEVVRIFLYDEYENLKCCARFPFNSTLGDPTSGNDMELMLARELH
STVCKKCIAVNGKEARLVAMRERDRDALEEELQQLGWHSMTSCLIFPIFSSAGCGRPFGM
IHAVNKQGVSFDRTGTFDENDEVFMSVTARLLGCLLTRYPTKLFVLSVGEKLRNCIPSSL
KGNEESHLPPILQDEVKGAAEVGNKANQMFTRVLLYRAPINVIYQTRSLRKEVRKLESLN
VIDRALSTVEFDIAALEELWRVGREENIIMYQQCQRLNEQLNALQLLLRNVLDAIGASRV
LCSMSDLVRYIQALEVYARQENISMITELISGVLMSPRTGVSVPPESGETSSDRLSPSEM
IRIERRVAQTPLKLKFGAQAPSNVRTYSYDPEKKREKVRFLNQLAEERETDFVAKFAVKP
VARHEAETKATIAKKVNQPVPFLVQFGPTDYASKRPFQIGNS*
>TCRU_2877 hypothetical protein, conserved
MRRAALAGSLVRLNLVVKKQQQYRFSAAGSKGSPGLVDHCKLLQKIHMDQQPYHVKRKRI
QGVLRKMETNYIHWLRECGTKESLLVLETLAGVSSYLKKGRSATVNVQQRDKEEGEEWTG
ASPYFLPGVNDWLCVLLERGARHASRPDECMTLLWAAAELELADTPTLQHLCRQLLKEEY
TGSAAPGELVRVIQLVSLLVKRCRMPIPQIDYLLCRLSAASLDSRESINVLSSLLRMRER
NSVEVVRAVSRRAVEHVATYTARDVIYALEAIALLNCCHESYAGAVLNRCIALHAELKPA
ELGSVCKYVAMLNVSRRNNETAFFCSKELRRLLPALLRRAEQLLGRFSLRDARCVLGCLQ
QHKVHHSVVFSRLTPLVSEN*
>TCRU_2878 hypothetical protein, conserved
MLRSVKGIECLFTGNVTALCATPRRKHSRATTTHLDEEPSSHMEDVGKKGGGEKTKGGSE
TSEESFGKRLFTSWQQGVVASIGAVMGIGAIGFFLYAPVKEDTVHHTAVVASEALGDLRL
REQAIQLSKGVVENVLTDPKSLDLVVRLVVQVLRQDDTRIAVSSFLRSLFEDHYTQEVTK
KFVLMTILDPWIQEQLRGIAKDLAKDLLNDPEVKRALVNLLTDSATTSLRDEELRYGAAR
AMRSVAKRTINPWS*
>TCRU_2879 NADH(P)-binding, putative
MPAAVNFEVDRGALVFERPRYLGRGVEVFNADDVPITPYGSFIRPNEYPKKFNINEASSI
ACQESESVQMPLDLFASHEDAGSAMQSQPLDQSFVPSTTGSLVCDERRLKHQLHRILLIG
SPGLVSTHVAAGLLKHGYRVRLLERDYRGKIFLYGLESLIETRPSRLSLFYEDDLSLAVA
DCDGVVYVNGPDTSEISNENDIERLFVSSIQDVFLSIKRNGKSVRRVVLISPASSIFPVE
TTLVAKTRGLKRGQAAALKEAERLSYKAGVPLTILLPAMMVGPSLLGEGDGNVQALVLLA
EQQRRFTSPIYYNIVDVRDVAEACALVLMAPRAEYQKYILSGGEMSLAQIACALQQCIPR
VSPPKRHLPVWLTRVLLHLGILNFLGFEFCERVACERVGWSYVLSSLKAQRDLAIQLRSA
QTAVVTAVAHAMNVPVAVQQPNVGDTHTSGKVRNQLRQSRLWRGLQLVGVSVLFATIGFF
SGKRLFSRSKVI*
>TCRU_2880 Peptidase family C78, putative
MSDEVQFFLSLKDELPLLILRKSGQTIAVTRDEPSVGSILPFSPRVCDLVRVSDEDVLRK
TYEDGYFTAVARNWRIKAKVNVSLDPIEERTHWLLFLGRNKGTAEKSKTTVAKSFKGTEK
DLREVVNWLKSLSVEGYCEVYLASEPSISDLRVTGEVGRLCLCRGDERVNEQVFFGDSMD
EIAICLEELLSVFFFDCVREAKAVFWPPSLAHPLNIHYGAREMRVAEHNAALLPMRPLLH
LGQRYKEWPMTINLPTVLAASVPRTGQSWEKNLICNIHAHLSTNSVINGGEKFVVSGAYD
YYHYRVDGFKDDGWGCAYRSLQTILSWFQHEGYMNEPIPDICAIQNILYAKDPDKMNRKE
FIGSKEWIGSFEVMIVIQHFLPGMDCMIRRMESGSDLETDPSVQQTLVNHFRQKRACPVM
IGGSSYAHTILGVDANLATMEARYLVADPHYSSGETSLKTVVKKGYVGWKEAGKFFESNS
WYNLCIPQLATYDPR*
>TCRU_2881 Rgp1, putative
MLLSASLHRRWYAPGDVVRCEVCLHGFSNSSESVSNGVAMRFGRMDNYFADIDIDAPTVV
RVKSLVAQILGACVVDSRRIQYDLGGDRAMDERCRIVIEDTLSKDHNVYGLFCSDEFSLV
SNLPLRERESRALVVKFTLPQYLPSSFRGQCIRFYYALFLHGVYCDPRNSNAPVKLRIPI

QVFSAQAIVTPLLLPVSFPLERFDFQEKVSFLQSAPSSALTGPLQSHLVPSEVEDFHAHV
LSKKALNTQANQLSKQRTPLQFPLSLDGELALTVVVASTTVMIGESLSGIFVVQGNPKFQ
PVKILGSLEFLECCSSKHVKKGIFRHQLPGWRKELVVAQTRVMEELDWVVLDRATVPFSV
PFTNPNTYASMHTDVVTLCWQLRLRFLWCRLSELRQCRATCREELKIEKEEPELVVPLIV
VPPSQSEQHPCRGATLQVLC*
>TCRU_2882 50S ribosomal protein L7Ae, putative
MTDKNEEQQNTEEYDYDRQLYRCPISWPITSDKPKMTKKLYSLIKKTVTRNKKGILKGIK
DVTKAIRKGQKGVLILGADASPYDVVSHFPVMAEEAKIPYVWVPSRQDLGTATQCKRATS
VVLLKPEPEFQSSYDKIVLAIEDLNSDE*
>TCRU_2883 gamma-adaptin 1, putative
MSTARLRDLIVAVRRCRTSAEERALIKRECAIIRESFRESRASLRTRNMLKLLYITMLGY
PTEFGQVEVVSLLAQAEYAGKRVGYLTLQMILDENDEVLTLSENHIKQDLAHDKPLIQSM
ALNAVANIASEVMARDMLDEISRLALSSNTYLAKKACLAAIRIVRKVPAYAEVFLELFTS
LFIDHSPAEMLSAVTLVNECLRLPQGEPFLSKYRVMANAAVRVLKQLVLSSRVTDQDVLG
VTDPFLQVKILEFMRIIGKGSAVTSEALNDVLAQVLTNTDATRNVGCSIQYECVKTIYAI
EGDEGLRTLGINTISRFLSSNDNNQRFVALQSLLDYASRDADAVREHQDIILDCLKDVDI
SIRRRALDLTVALVTENNLRLLVPDLVSYLTISTEEMREDVTFHLCRIIENKSPSTEWRV
EYSLRVLRLAKRFAPVEFATRLITLLSNESTEIQTSAVVSMWDEASYPFDALHQSRKAFL
VAAVWGIGEYVDLLLDAKGIQPEDVAKCVAEITTNTVFNIIKCYGLTSLMKIASRYPSAK
PVVLPVLSNYTTSLDCELQQRACEYMTLLESFVEEAAFCFSRMPPICHVEEDVEVQPVPQ
VNLPPEFLQKKAAVALEDLFEVNSKPIVMTHPVENKNRDKENNVVAIDDLFGLKTDVSSN
TAPLQTPSGAATSAEMEPAKPPPEVHVFDCDDFTVSVSGVVQGAINANIIVFSRLPTAME
NLSIQAAVPKTSMLNIGFLTSTVIPPYGRIVQQLIVDNSNSNKNPRLLTLRVKLLYTVDG
APRSQMFQVTQEL*
>TCRU_2884 ubiquitin-protein ligase, putative
MFFAFDSGPGKTPYENLLDLCTVLFISEEGSELPVQRVFHAINDSLQRAEEDGDVLVLAA
RALALILDRFSRSLLISGNSDASRAVKKCAETVFSIVKKRHVNFRYTKMSEKELSEELLT
CFSLARNSQEVAAVAPSTAEQLKFCFNALDESKWTSCRVLKHCIGLMRRGELKTAGDHEK
ILQLLQHHLASLCYVDIDHEWDDLLRTVVEGILTYRVWCATQLPLRKRRRASGGGSNAMK
TTGIIFDGVPLNNTTEALLAIGERILFSGTAECRMELTLACLASLVETSSLSMEATEKAI
VWVSRLLKQNSTRVAGFSNPFLRSRQDAAKREHLRTSPFQALPNLQWIVPPLTWSSLVLL
SKLCGANFGFCGEYMWAWRGEGGEYYPYLRGTRQKLTAMFFAADKNKKVLQKRHVIDLCT
MTDTSALTLVVSRVYFQPIPSVVVFDGALSIPPVYTHISEELSALLQDALRPLSFGHSKT
AYLARAIRLHVICGGAPTNSEDVITAFRSLSAEQKRPVVEAISSALMQHDKRWAPVLLEA
GVADALTAFPASAISKKAAVTTSSEQKGPPVADLIRSAGSSPRMVAMKIPPVTELPSFLS
HASPAELLAFIEALEDSTLSISSLKRVCGEIDLDPQLVCRMRDAAHNYVLRILHQTSVAH
QEVKSLARRVSGFSHFIGICRPTQGGMNHVLCPEGHPLCVHFSVNWRCNVCANSNSFGSL
ACRECDYDLCSDCSNTKLSRMDVNVSALVGDIIRYWREFRGDELSDEGSSQRGLKHSVLF
TSKGVLSAGLSSSTLQGEDVHYAEMGNCCECNISVPLLVANSMGHDVLDSPLQIFLRTFG
PLHQWGEIESAIVEVLESCGGEIFESGVSGIPVSVVRVLQAISPYISLSFKRDTAHFLSV
GCYRFGLYHLQDVNASVRGTVMGEIQSNGLATKFTVKRETGAMTQALFNAFVQYPSIRNK
VEFNFEGEEGTGEGPTQELYTELSQRYREMATLWHERNDGALEAFPTLRQVHKKEFFVLG
ASCGRAFVDGYTMNVSLIPLAWPFIRSPSVSMKNKWTLLIELEPELANTYKRMVRSTDAE
LKEMDLEDENGAVLTTATVKAYVERRVEESLSHAILNLYWFSLGISSVIDLRAFWFLSDD
EMSVILCGASRESTEEQLFGEEALRSAIVEAHGYSGGSREVAMMISVVGGEFTREEQQFF
LEFLTGSPRLPLNGLAGLGRKITVVRKELEGSGEQTLPSCNTCFLYFKLPPYTSRAVMKA
RLLTAITEGRKNFSLS*
>TCRU_2885 FAT domain/Rapamycin binding domain/Phosphatidylinositol
3- and 4-kinase, putative
MTDAAEPSIDRFFKESASALGRQFSIIFRNLQSAPESRLRRVNECLSDLLDLLVAHTKEI
IELGLLDHIFQLVNETFDGSVKRGIQALRVVFDAHLGDQHEERSRCVALLMRQAARGKCE
EDCAFMLNELGEVLQENVRSTSDMLGRHLNRAIVMMRQGATVHERAVGCQILATMVETSS
SVIKRNPALLDSLNATCSPLFLLRETVESFSMWHRHALVGLLKNIVEKHCIEISPFMATQ
MSAVMTEFMENADENVIIVGLLTAIETVAASPTLQSAPWHLKKIHSFLMERDWNTYPKEV

CIMVPRATGKALLLFRDDEGLYYGIMRCFLSYLSDWEEDVAVVALSTLYDILGEVNIAPH
VELAVDVIAHFIVKFPFYQLPFDALERLRAQCGEQSVARIYTLFDIIGIDMEYTNLQKLS
ALLRFMGDRKQSIWERFRLLQIDISPHVPVDVLVRALPFLATLGRTEQIGELIDLMFHEN
LKVRQAAVDAVVILCEKLILQSRSNDNIHQLTQRLDNTVTSAVGKLLDVAVADRDAELRY
NVLCRFSPLFDAYLSLQDHLDALLMARNDVSRRARDRTLVLLCRLHPCNPAIVHSQLLRQ
QEYMLREIEAKDGSMSAAIYQASLLQMTAEHDRLLLQPRTVELAVLARLQREPFASKSFS
VALLNLLRLILDRTGPHNHCDANQLLQPVIHIVNGSTSSRRRKAALETLCSILTTIRVTK
QSSYVAVYLALARIIRSEAEEDEGVNYAAMKALSVIGAVNPVKLRFILKTLEDDEIEEAE
EVVTSALACYKPRMRVHPHMTERYPSIVLYLLVKTLQQSADPEQQAGTIATINSMVREIP
GNQKAILLTHFLPQLQSWLADPERDHLYEGILLLMTELATLLRQFKDVIPSNTGSELLHS
VRQFCLLPQASQKPLNAGVVELLDEVARGLSAQEMRDHRWAVEFIHQRLSQDKNDLDLVL
RAVKSLESFLAVLHEKDLQVVLPHVLQCIEPEDATPSALRPKAKDINAACFDFLNQIMVK
QPLLVKDCCAEIVHTVMWYIEKSKSQDEMEMGFNTLAFLVEVVNRQAKRFILPIRRLAEQ
KGFSEAMFTDLLNSAARGFLKVRVPVTKQDKLDPDLPLSVVSHLPGLSRKDFEEEMMNTL
NLGGDDFEVMDVSHPSGQTIINLRFFGGDDSAANFRSFTRKAQETQSVLRRNLGILKLDQ
TQLNECKVDEALLQKIATIPVANKKKREQSWIAWLHKTSVEMLANSQIQPLRCTAELAKY
NSDLSRDLFSFAAAAFIAQLDKNQRALVMDTFTRALEMSPNDIKQILFGFAEFFESERGK
NELVLVKRIETMNCVVEREDTEKKFGINYDQNARGIAVTKLEPNGPGAKAGVPVGALLVA
INGKKVSAVSEIVSNIRGEKKISLTLKYEVEEQRKPESEPLMDLEVLARVAFDSQMHAKA
IFLNEVLFDQLFNGLGEQVSGRQDAAVRSALQVAERLIEFYDHLGLNMVAKGLVKKMSAK
FSGKIIAPEQFGFDEVGALEQLNWWGEALARYESRMMNTDGSINTSSLLGVLRCLDALGE
AIEMEVLINRNWHQLDWESRFEVAPLKAKVALSLGRWQQFDELTEQSGVMERLGMVERCA
TLFRQGQYEELLEFTRERRESLFDLFSESFMESYNRAYDMLVELQHLRHFEELVSFVNSG
EERQKMLKELWQTRLAHMSSRPAQVKTLITINSLVLTPAMDLTSQVMCIRALSKSQWTSF
ADHMLNLLLGPTGDYQALCHQDPDVIHVYMKHCYTTKNRRETLRLLSEILARLQVSPGDD
RAEAWGSCWLLLGEWTMHLFPDKGDEAIEQLKKATELCPRNHLAFHSLGILHYDMSRDPN
TPVEVQTAHHVGSITALFKSVELSSNQGNGVMEDVLRILSIWFSHSALLEINEAIKEGID
RVPDYVWLKVIPQLVARIGITAKVARSILTELLVRVGSKYPHALIYPLTVTEKSPEAIRQ
LMAERVLKGIRVSNGPMVGEASLISNEMVRIAILWAEKWHSHIQQAAGKQNDVNAILRIF
GPLFWELENPKTPNECAFEKTYGPTLRHAKAALQNNTLDQAWSLLKQVYVQLNKSTGERR
LLMGDLSPTLGGIKESIVAVPGSFVLGQPLITIQKFHQAVIVMPSKQKPRRFGLDASNGQ
KYRFLLKGHEDLRQDERVMQFIEFVNTIFFADSSSSAIGLSIPQYAVIPLTDNVGIIGWV
ENTETIYRMLETRRKENSISIYEEVRMIVRNGALRNIEEYHHQSKEQRKNLLNQVFNNTP
DNELRRIIWDKNDTCEQWLEYRGTYGHTLAIMSMVGYVLGLGDRHLNNLMLQEKGSVVHI
DFGDCFEVAMHRSLYAEAVPFRLTRLLVSALGISGVDGVYRLTCELVMKNLRRHCENLLS
ILEAFIYDPLINWRLANVGDGERSSSRAEALVKTGDNTLLNPGETNTKDPQMQLSYSVAR
VREFVACATLDQLNEEETRNMQGDLALARVRAKLTGEDFDSMNGSSFLQNSRREFCDESG
DASSCGSQSYGDSQKDSFAIGNPALTTYLSLRAVNGSANCLDVPHQVDRLIQEATSLDNL
AEAYITGWAPFW*
>TCRU_2886 TFIIF-stimulated CTD phosphatase, putative
MSTGRARNTRSAGPPGSGYRNFRRGRGFLNLERRRKRKKQRLTNSSRVCSSASAAELCLI
ATDDFHDRGEQDSSVLSDFDAINDVIQEFLLESDDEEEEGGFNHVSIFHAVKPFFGYSRT
TKESHRRQARKVVSMLPSRLATSGASVTPGLMLAEAMLPGIGGKGFRRRQNKALLPSDKG
LPLSPLCIVVDLDETLVWARRGSVYLRPYLKEFLDACHMEGCEVVVWSAGSPLHVNNAVR
AVSDVLQRKDWFHHIISRHHKWYRDEEDDAKDLSILGRPLNRVLMLENSPRSIRQQPRHA
VLVEDFNEPTKRDNSLRIILELVRRLVAVLRVASDSSDVDVPRLLAEDVALVDLSFSLLD
NSKNERNIFCKGLLYSPQDTFACRSYGGENPSVPRGNGA*
>TCRU_2887 cytochrome c oxidase assembly protein PET191, putative
MEKERKSICWRPKNALIECIVGTKCFEEKGKIEDCISANECFLERRNWTLCKVNATNPRY
RIRGNPYDVATEDQKKIEARDERIRRRELEEEGIFTK*
>TCRU_2888 Histidine kinase-, DNA gyrase B-, and HSP90-like ATPase, putative
MTLPRRPYEALRGHFERCLATPLRPMNLLTLAGNDKGYIHTAVEQLGARTARSYRAIYSE
VQKRSLDREINARLSPLESICRREFECCVELSEVAEVYLRKMERGEICDADTSLPNEIID

DAQVDSIMKGKGYGGSGNSGGDSSVKRLIDVLESLRPHENLFDIVVNEEWICSPNFNNWL
SRFCTRRVSWRVIHEHLLHLVGPGRYSPIIRTDPNVGSILRHTGTVVVEQYVRSIQSIHG
GSMFIHMEEQPDLFTRVPRGRLATSSKVDCPIFPTTSMPPAPSSRVIYSVEGHLEYVFRE
LMKNACLAMRSRTNSIELQVCFASNDTHVVVDVTDAAGGIAPDAVEKLWLFGWTTNSSFD
NKMGGFGIGLPASKCYMDLWGGRIDLYTTEGVGTTVRVIFPKAPTEVFVPKGTSSSSD*
>TCRU_2889 hypothetical protein, conserved
MRRNVEAAGVHLADCTLLDYRRELELEEKAAATKRSQQYSTAQWKENAKCPGKTAKQLVS
PYIRVDGELISTNIALSSLLRAEEERKERLALEEQQKKETEALFQLHDGMRTTIIDQWSL
GMEKLVLLEKRDCIRAQCQERCRRASDWWDALAEQHLADEILLWADEEQVMARERDEGRA
AYRQRSVHTRK*
>TCRU_2890 hypothetical protein, conserved
MGHNRESHAIPNYREVLLMQQRTASFLMVFSVIAIAFFVLIVSIQTQEAICQTRANTAAF
NVYAKAEIKNGSFADALIDALKRNGVHAEPLEIKSQVRTRIDVFDTLEKTLFNSGFRLLR
KPDEFGVTWRLRYLEHSVCQTERRISMEALPNVDYEKVSYDVMAINTHGDRAFLYEAEVK
TKDRDRITTFPQLQSLFPGFNARTASPQMVLTDSVVTLTAFAAELYYGSTSLDIELQMEQ
RSREKGGKLYWRVTLLTKNILAESNLKGVHRIVSEVVQNRSVLCDPASCGSAFGDPFLQ*
>TCRU_2891 dynein heavy chain, putative
MSAESRNGQPRDEEDPRLAWLAARVKSCLYGVDDDVIADLYGDTDSRKVILEIFDNPKER
FLVISSQEATTTEGNAVGGRLSMATPRKPPPKLIATTRAIKNSNITGSTNFPPDRTRCIF
FLRVQNVQLTRELVHNWRTETECPIHRAFEISCMVVPSLSSFHTILKYAYGPIVEDVVSS
AGRRRCVATKKVANRQLYTEILNWVHRVDMQMTGFRNQVCAERRLPMPQTLVSMGYSDSG
LKSALANEDVIKQIDITVSQWQAEISYAMSLDPQKEGPLGEIEYWREKYSTISALYEQIN
SPQAKFILKVAKEAECNSFHLISTTIQHFFRLYAEAKDNVKFLGTLDRHFRTMHGVTPGS
GSLQPIIDTLSSMMTGIRMVWIISRYYCTEERMVGLLEKVGKLIAQKVSQHIDFRSILGL
PFSEAKEKVVEGQQCLLKWKATYVNVQEEINSSEREQPWNFNQKRLFETTDYMSDRCTDL
LEVIETVEYYNTVLGAQLKTVLTDTSGIERILKDVERVKRPFETLTFDPFERKATHNWQV
VYSNFVNTVANLDHEVSEFINKIFDDDLRSAESAFELLVSFKGIVSRPQRAGEAFDTASL
LSEKADRILAQYFSEVERVRSIFVENRDNPPLTKNQPPVAGAIHWSTSLFQRLKKPIVRF
QQEGMLVTTMGRQVRAKYVEVARQMKEYATSRFIQWSEGVRQTTTASLKMNILRKGNGDT
YVTNFDVALFNVIREAKYLDRLGFEVPQEAINVTLQDESYHANVDALKAMLLNFNCELQG
ALDGPERVILAKNIRELRLALEPGIHDINWTSLGVTDFVANCERAITKFRNLSREVRKRA
DHIQTQVVNKIGSTKLIPEFEQLLQAGGELPELQVFVDTIERRRTTLVDACLRAYHTAKP
LLTKMESQLVGTHTGKCLILESYYLHWEQRIWKALTRMVLNSLLAFAKMLGYRGSSTVKR
PPLFKVLIFLTTEPTFSPPHQEITSAFHKVQSSIIESTQRFQRWMRGTCIEFTQGELVPR
PAEGEQETLFTYYKDINSLPQVYKLQGIINRTIQTHLSALATNIKMLQRYRFIYLADKKL
SVEQQAKSQFHWIDYDAKFQLYFNMIADFDAEEHIHDFGFMRCDESAFYAELVEHVHQWI
AMEGAQMNDTVRVRMQKRLESIVRINKDLQRPCENIEDLKFVLEVMHDTRASSLDVEQDI
SEIKYIYESIMHFGVKVDPQELKEALSFQGLWDCTLARLHELEKDLEPKKLRFREYTRAE
VANFLQKGKSVLKEFREKGPSRADIDLQEGNNLKKEWREHLTQLQAKRDQLTKAEKLFDL
PLTSHVHLQQLNEELTKVEAVYDLYEQWVAILKRWNRSSWKDLLLEDLQTTTDEKVKRAR
LLGRTHGDVHPFKAVQQVILNFHSSLPLLAKLKSPALKQRHWVELMRVTGKEFSIEQINL
DDFIAMELYCFADDIDNIIVTAAREQAIEKDILAVKEFWSKAEFSLVPYEPRKGDFRCSV
HSDTSEIQEAVDDNILKMQSIANVKWAQPFIDDVKLWEKKLAMINDVIAVWVTVQMKWQY
LESIFKGSDDIANQLPKETAKFNDLDKKFVRIMNETAAGPNVDICCNATGRLEEFRYLEE
KLEECQKELSNYLETKRYMFPRFYFISDDELLSILAASGAKSVQDHMLKMFDNSAHLIFK
DGEDETICGVESQEGEVVIFDRPVATMGAPVEQWLQEVLEESRSTLRDVLKAGVYYYPRM
GRLDWLKKYHGMVTLTGAKIWWTFEVEDAFNSLKKGKKSSMKDLSGKLTRQLIDLVGEMD
KDIEKQYAKKVNTLIIVDVHGRDIVDRFVRDSVTDAREFDWESQLRFYWERDGDTCVIRQ
CTGRFNFGYEYMGLNGRLVITPLTDRCFMTLTQALSFYLGGAPGGPAGTGKTESVKDLAK
ALAIQCVVFNCGEGLDYKAMGTIFSGLSQTGSWGCFDEFNRIELPVLSVVSEQLRSIQSA
LKAGEEEFLFGDKVIRLVPTVGVFITMNPGYAGRVELPDNLKALFRPVVMVVPDMELIAE
NMLFSEGFTTARELAKKMVTLYQLAKGQLSKQHHYDWGLRALKAVLVMAGQLKRGAPELT
EESVLMRALRDMNTPKFVAEDEPLFKGLIGDLFPGLDPTRAPQENLSKAATKVFKEKGFF
VDARQVDKVVQLYETMQTRHTSMVVGPTGGGKSTVIDCLCKAQTDLGLPTKLYIINPKAQ

PTTALYGVMDPLSRSWTDGIFSNIFRAINRPNESGARERRYVVFDGDVDAKWVEDMNSVM
DDNKLLTLPNGERIRLRPTSSLLFEVGDLQYASPATVSRVGMVFLDPVNLGWKPFMYAWK
LKRPRDELDTLNELIEKYVEPLVNFMFDGIDGEVVTDPPKLVIPTNELNMVRQLTTMLHT
VSPQQVALEPKALQCVFLFCCVWSFGSLIATAEGRVRFDAFLKKVSGWNLQDVGENFLTR
FVGSGSLPERKTLYDYFFDLEENRWKPWHILVQPFERPPGAKFGSLLVSTVDTERNMWLL
NKIVLNRTPVMLVGESGTAKTVTIQAYLKQLKQRSLEADTAADEDVHLEEMLLELNFSSR
TTSLDAQRAMEDNIEKRTNTVLGPPAKKRLLVFVDDINMPRVDLYGTQQPIAFLKLLVEH
HSWYDRKDLLFKNVRDTQFVAAMAPPGGGRNALDPRFVSLFTIFNILFPSDESINTIYGQ
ILRDAYKTMATELGDLPSQLTSMTLSLYQQLFAALPATPTKFHYIFNLRDLSRVYEGLCR
ATPEMFPDTKSIVRLWLNEISHVFMDRMADSSDKEFVALLARKEITRHFPDAAEFALAEP
LLFGDFGDFEPDSDVERLNIYEDFGAEFTRARSLVEMMLEEINTPTKKMDLVMFDMALDH
LLRITRVLSLPRGNCLLIGVGGSGKQSLTKLAAAMYRMGVFEIVLSRNYNEDAFREDLKR
LYARVGVQKEKVVFLFTDSHVKEEGFLEVINNMLTSGMVPALFTEEEKEPLYSSVAAEVE
ADGLAPSKDNKWTAFVARCRDNLHVVLSMSPSGNILRTRCRNFPSLINNTTIDWFQKWPS
QALEAVGNRMLQEEEVSEDLKRAIASHAVYVHLTADELSMRFLGEMKRHNYVTPKNFLGF
LANYSRLLITRREEIDDLVNKFAIGLEKLDRAQEDVKVLQEELAEKEVILEEKQEVNERM
TKEIKEQKERNEKRKAEALLMEESLNVQSAEIEKESAEAQEVLDLAMPALEEAMEAVKHI
NPKSITELKSFAKPPASVVAVVRMVCVVKGVPATWDSGKIMMGQNDFIRSLIDIDSLAPT
LNQGKINEINRILKEFPVNSNDLKKVSLAASGLMIWVEAMKAYWNTAKEVLPKQARVREL
QEAKANAERQLSACQDEIGQLTVSLQRLQERLDAGMHEAQLLQQEKAVMERRLNAARRLL
DGFGSERVRWAEQKRTLADVRNCLVGDCLAGASFLSYLGAFTFQYRQEALESLWLKDIHE
RGIPLTNDFQIQHLLTDEVSVSQWASDGLPLDSLSVQNGILTTMSTESIGKGRKSGRVRF
PLCIDPQMQAVNWIKRQHKSNPRFECASFSDSDFLKRLEFAIQYGNPFLFEGVDEFIDPI
IDSVLDPQFREDSGQRVIRIGDKDVVWDPNFKLYLCTKLPNPEYPAEVFGKTIVINYGVT
EDGLESQLLNYVVASERSDLQRQSEELVQTMAESRAQLKELEDTLIRELTLATGNILDND
DLIATLENTKSSASEVQQKLEQAKETARVTEISRQEYRPAARRGAVLYFVISELSMINSM
YEYSLSAFLHDVFGYSITKSDASFEIKDRLNNIIRALTYNLYCYVCMGIFEKDKLMLSFQ
MAIRLLTQENRMDRSELEFFLRGCVLASKDYPSNPVEWLTERQWNDICKLSTTCQVFANL
RNDVSENLEEWHQWYALDRPEDPSSVIPCGYTERISEFQLLCLLRCFRGDRVYTAATNFI
SNCDLLGERYVIPPILRYKDVLDKSSSTAPIVFIVSPGANPTEEIIKLAKKEVGIQKFRS
ISLGQGQGEEAMKLVEVGVVRGHWVLLQNCHLLTKWMKDLEKVIEKMDQTPPQEEFRLWL
TTEPSKDFPMGILQRSLKVVNEPPNGLKMNMKNTLLKVTEEQLDACLHTAFRPLVFTLAF
FHAVVQERRKYGKIGWNVVYDFNETDFSVSMRLLDTYLTKAYKNGDSIPWDTLRYLVGEA
MYGGRVTDSMDRRIVKTYLEEYFGDFLFDTFQPFYFYVNDEVSYCLPADSTDSNKRVTLQ
QMMALVESFPKDNTPEVFGLHPNAETGYLRNAAEGMWASLIELMPRSGKAATLGGESRED
VLLKLTDEILSQLPEPLDRKRIMRKEKERANEMGHSNLQPTQVVLLQEIERWNRLVGVMI
ISLKELQKALSGAIGMSNDLDDLASSLHNGQLPWSWRKMAPATRKNLGRWLAHFHLRCKQ
YNEWAMNGEPRCMWMSGLMVPESYISALVQVVCRRYKWPLDRSSVVTKVTTYMTPQEIQE
RPKDGAYVSGLFIEGARWDPNKRCLAPQRKKELISEMPVMHIIPTESSKVKSVATFRTPV
YVTSDRRNAAGVGLIFEADLSTDAHSSLWTLESVALILDSDD*
>TCRU_2892 TPR-repeat-containing chaperone protein DNAJ, putative
MGRTILKRHGEATLEYTYLPKPRKSANTEFFSGGLATMPTDWTLCLRQASDEEGACRRLL
IANEAIERQRLVRGLECCLPEVNASFTRNEERGIRLLEAKDYGGAYVSFTRALQQKPHSK
IACYKRAVCSWHLLFYDECTKDSRRGIEMDLDLVSLHCRSLLLRERYQEARQGYEYALGV
LAPLMNDPRNIKWRAECEAIPTLKKFRNSFKEGKWEEVLRLRDAAKPLIDDTPLVLLEAR
ALLHVSPNMARLRLLSYVPTIPRPVSANTEMSLEEKLAWRLVEEHYLQAAVLLAQSNAYC
GSSFLEIAAALVQTCLVISPSFGPALVLGHYLVSLEEILSRVRSLFAKERYAEAIPLIHD
GLLLDKSNQRICSTLYCMRAEAYASLGKNSNVINDCTAAIGMDSGCVKAFVLRAEAYRRM
NQRAEAAADRLKAVNLDPSFRHILHGDEEQFLRHTEHPRSSHGNPGSQKRRSNWYDAFAT
KSEIPPKSGATTKDASSDAERASKLGAKTTLYDVLELPHGSSISEVHAQFKKLTLLYHPD
RLVKEGEKKQQAGLERFKLINHAHSVLTDTNEKCMYDFSLGLNVSPRV*
>TCRU_2893 small nuclear ribonucleoprotein SmD3, putative
MSTFSIPLKVLFDAIGTTISLEVSNGELYTGTLSEVQDNMNVLLISAKKTKKSGKTVDLN
SVFVRGSSIVFFQLPDALRTSPALVKAGQVLTNALDNRGDGKGFGARRVVKRGRE*

>TCRU_2894 Major intrinsic protein, putative
MTFSPAMSEIVAEFVGTFLFVLTIPLASLGVGSLAPLPIGFMLAAMTFSFGYISGAHFNP
AITFATFINGKIPFRKMFFYMISQILAAFLASLYGSAIVGLRIPVPSTDVALVSVWQAIL
CEMVYTFALASVVLHVNYSRQRSNEFYGFAIGMTVLSASFAVGGFTGGAFNPAVATGTQL
VGCIGGNCKPLIFFWIYWVAPLGGSFLASIAYQILDTHDRVAPIELSGQVF*
>TCRU_2895 hypothetical protein, conserved
MEIAALLAEYLRAPVSSRGELVASNPCLFAPDVVEALDDAHDMMAQVQKQARDACRTIDD
VMGNLDLPSLPRLTPTERSELVVTEEVMIAQFVDELSLLLSQRETTPCSLKGTLSASDEA
CNFARPWHQQRSVVLEILSFLPVECTFNVAENVCRLWRTWLCVPADSRAFWAGCVQREFP
ESMQTLIQLQEVDLYESDWRTIAMMCSTDEEKDDLMAEEEVTAS*
>TCRU_2896 hypothetical protein, conserved
MTALPEQPSHGGCDGSTLPMPALTARPFDWGALVADVRAEARESRRAVEKRTRRLATLNM
RLRKKVEENAGTVLAIQELDEKIENAVKQEKCLLRLITAAKKRKAQLQADVEEAEKRLEK
MTPCHQQSTPTAAAASVVVKNSLSRARKSEMEDCVVVHENCEMGNLQHRLHVTRLLYRRM
QEENNQILAILADLNFEENLTTAKLNERSSSNLTAHHFEFSNALQDLIQSFKDVTHAAYK
PH*
>TCRU_2897 TPR repeat, putative
MSVVEELRSDDEVDPCPVSEVPAGCERVVDVVACERLKKEGNALFVSGKVAEALEVYRDA
LKAAPIKPIQKEPKSKKGGDSRADGRPSAPETQASSAPAEGGGNSGSTACNENDTKNDVL
DEESNDGGVDYTLTSQIYCNAGLCLTKLEFYEDAVTDLTEAIRHNGLYAKAFIRRAECYY
LLGKWSNAYGDYEEYEKLGGVLDADGRAHKAAAKAKVDEEMKKMLGDLKDMGNRFLNYFG
LSTDNFKFDKDPVTGGYSMRFEK*
>TCRU_2898 50S ribosomal protein L14, putative
MFRRWGLRLAATTTDAAAKLPAGNPVNKSWFRYNLIIRRKASYRSRWGTGAEGYGTGVPF
SDQVKLHCVDNTNCKHVRLISKATAERFAHCRVFPAVAHRVSVQRFKSGRGEVSRHRVKP
GNIYWVCLLSRRQTNTRMSGLQTNFDRNTCILLNDQRVPLGTRVMYCAGRHVNHKYHLKA
VVLANFFI*
>TCRU_2899 hypothetical protein, conserved
MRGQRSAKFAEYTATYAILKRLGLHDTVLIEKRDASVGYGVFVRDACDAGTPLLVVPSRR
ACSVTTLERLGADVRMVETGALLKLHNLNDGVVSRVLGCSANQWATLAWRLAIEQQRSLS
PWWGWLNVLPSFTSFRTMEENCERLCRLHQAPLLPYWMDARRRIEEEVVAAHEIFAEENV
VPSFCYFRGAVDIILSRAQHLPLCWTTAEGHPVELGILPFIDLINGHDGTDRKRNARVEV
AFAVEELPVWYRSWFVQESERGGIDGEKELRRLMEEHFFAVVVLERGLIASEEVILEYEL
QPWDTGSLGSEEELLLGRLLRYFF*
>TCRU_2900 WD domain, G-beta repeat, putative
MNCMHSVLGAKIIPGKDASLNAGAAPIDDGRNWPYQSAEYGCQDLLAYSSYCHGSGDVFL
VDMNSMQLVQTLHGHNAPVTCVQWKPPPASYLQHGLFLITGDRGGAVAIWDVAEGVVLNS
IQVPNSQPIHAFSYLAKQHLLVVTRDGSNFVYDSHLVGKEPFGEFEFPVRTEPTDSFVPL
RVCTSKLSSTQASCFVLGDRLRVIGSLETAPGKSGVALQMKDLVYDSEDGHETVLDATFS
EAHEDVLYFATRNSVGAYDWKTGLLLNEQVLWHTKSDVEFRRLFSSSPSSWTESPQELPF
LYSFGTDQRLCAWYNSQKEKVTTVSADVRGARIVSKTVSNVVQSRTTANLFAVIFADGSV
ARWRYLVERRRWSLEGFFSFALLKPTRLCVVGDNIVCCALENGHMVLLDVMHNVTLRRIN
IVHSGGTRIILLSPHRWGESVWMVTNKSTQLRNFHQVTLFDCRSGSPLRVLRKPTIPDST
RMKEITLSATGTFLLLTFLDGTFEVWGVEEGDLIYTFEGMGVAGVSWAPQSFLSCLTGVQ
GSPQLLAIIFTDGGMSLWTVYKDRVVKNRDVTSLFSGGSIPRVNCVPTEEALILTHGMKL
PLVVSLGSFGFSLEAFKSVSPNNPAIHVAVSKGEGMCANNTRTEKNNSVVLVAIAFTDGA
FGVWNASSKERLSYSRATLINLTAKQLTWIADKLFVLTTNGDIAVLDKSLTSANSNVSCK
FHRRPLQTSAFFLPAHRTYIQTLLETQTMCESPGAGNLQSASSPSYLVQSIDSQRRPCRG
PFGQVITEGITTLAQELDLYKETMVPRHIREPMQRAVAQKRTEEVAFWVARFFGQEEKQR
FWLQFCVNKYFCHFGSKAKDEIQPSDGVEEERKWASPFFYSHCNRFSEAVANPDVIRSNR
MCINEHRIAALEATKSQNTEVSACRLIVARELLKLQEAQRAIDVLMDANLESDHFSHLAN
LAVMIAASITARESSSFSLFSNTTKRAAALHLARRDLDAAVEKFILSGDYYEAGLALQSC
GKWGEAAALAKVTSMTPNQKKEILYRWCSYYAKRGEIMEVARMLFSMSSPSEALVLLSES
VQLIDVAGLLAIVLLEDSFFSSWESLQKVIPSPLRDEDPSGALSLGDVVLNVLADYCSVL

NSVGNVVTERMVLEIIASLKRGNRQGSTLSA*
>TCRU_2901 Phosphoglucomutase/phosphomannomutase, alpha/beta/alpha domain I/Phosphoglucomutase/phosphomannomutase, alpha/beta/alpha domain II/Phosphoglucomutase/phosphomannomutase, alpha/beta/alpha domain III, putative
MSVEKLVQEWISWDRDPETRGVIEKLWKDGDTRELQILLGNRMEFGTAGLRSKMGAGNSQ
MNCLTIIQTAQGLSAYLRATFTQEKLSSGGVVIGYDGRYGSKRFAELSANVFTNAGINTR
LFGQVVPTPFVPFAIRLFGCVAGVVVTASHNPKEYNGYKVYWSNGAQIISPHDKNISKFI
LENLTPLGSSWEAAAGVSDPFQEVWDQYFATLKAEYKPLAANYPVKVTYTALHGVGCPFT
MKSLETVGVPRHCISVVRAQAEPDPEFPTVKFPNPEEGAGTLTLSMETAAEAGSNYILAN
DPDADRLAVAERRQDGNWRIFSGNELGALLGWWTVFRIRRLGEPLENCYFIFSIVSSMIL
RSIAKKEGMHYAETLTGFKWMGSLAESLQRESSARVVLAFEEAIGYMFGTRVFDKDGVTA
AAVVADMIAFLEKEEKKRLSEKLLEIFKQYGFHFTCNSYVIARDPARVSEMFGAITKMQN
GSYPKEVAGARVLHIRDLASGLDTQMPDGRATLPISASSPIITFYFDNGVTMTIRGSGTE
PKLKWYAELITEDPLREKELAVFVDKAVEELVRPSYYNFEYRSNL*
>TCRU_2902 hypothetical protein, conserved
MTSRGFNAAAARNRKQYLQLLRETAEVAVSEGTTSFGVNGVLKTSSRIRLHEAALKLPEN
WVRNSTTRLQTSLEGVVPREKLLTNDLFLCALIHPSYVSSRSVRASVAMPIELTLTGSST
LRLLKEVAAVHGMAGLLATEEIAGIPSGLHVEDLLLYEKSMFEPCGQIPPEEVRMAAATA
LCGAVVLSEGICSATALLDRLREGKKE*
>TCRU_2903 actin, putative
MAVVEVGWTIRAGYIGDARCTRQSSSICFRDGPMETSFPSILMAAEATGVDIIQSVWTVC
SLERGMNQNVEQLEYIMGNKLQCSKDDPLMLVLPEALHERTDLVRELFRVVLEAGFTSAL
YCFRPSVAWTLASGRTSAVVLDVGHNQATTTAVLDGYALRNTVASSSLAGRSVTGRLIEM
LDREQLDSLESIKRYRVPSIRNWALDDMVNDIKRMACFVRVGHSNFSLKEEPLVLRAPDG
NSVTVSASARTEPYEMLFSPPTGFGVSLAELLVSCKKRLDPEWLQQAVPHVFCGGTSQAP
GFCDRIIKEAQKRDSCYFRYEKEGSFHVFGAVDGAWTGASLAAASSSFASLWVTRADLEE
EGDSVLYRKLLY*
>TCRU_2904 hypothetical protein, conserved
MAFPLEIAVGERLLSLSTVSRSTDIGHNGKQPLSSFYFVGERLPDGGIQWFLSPLDGYKQ
KRLRVEEAVVEKDAGNRDVFLRLGDSTFRQYTVKGSPSRIQAIVTNRIRQDELNDSSANS
RQSSYFLSSSSATPSMVSQPGTQEACLILPGKDANLYDGLEKKSLRVTLRLPSDEGDGCK
EALTKQEVEGEKNDTLHLPPVRVPQKPATRINSNVRPTGSIHLRRLLGGQ*
>TCRU_2905 serine-palmitoyl-CoA transferase, putative
MSMSDFISLVLTQIVEHPFHILIEIVFTAALTVALWRKQARRRKNHSKAAAFEMPSLEEQ
ELRIAQFQSQMFRADCKTTSNAVVPNALGTAEVDSREGCYVTIRNVSDGSLLRCIDFATE
DFHSFSTHPTVLEVARKTVMAYGVGSCGPRGFYGTIKPHMDAERDLAKFLGVEDAIICSL
SFATVSTIISCFAGRGEYIILDDGVNLAVAEGCTLSRATVHKYRHNDMTHLESLLKKVAE
TEVAAKKLSRRFVVTEGLFMNSGDICNLPRILELCHTFKFRLILEDSYGFGCLGPTGRGT
HEHFGIPATSIDLYVGSLSTSLGAVGGFCAGTRKLVDHQRLAASAYVFSASLPPYITAAV
SMALTILHEDHTYPEKLQSNAKLFREALRQARFNSDKIRLLECEGDVSPILVLRPTEAYL
KEHSQSVEIELEQVVQLARKMKVMLLRHLYSADEQCENYPALRILVKGKANREEIQNAAK
VVVDAVKTVFP*
>TCRU_2906 hypothetical protein
MRSRRWGPTSTKDCSFLEPLNPNAQSGDMTTKVLLGRETLATSKHDAFLSSQVSAEASLR
EESTTPIALLFFERMQELLSLLRRYMKRWRRFAREHNILASLEGIQRGAVIKQFLRMFYK
WFLFASRSAAVRNMEHSLIKEASLRLASRYLKLWRMAVELRQKRSRQILVLRGIRYGAER
SLARRQYLRWRAAARRKVELHRRLAVLSESRTIHLAHFFFVTWMRWRQRNFYTTQLGLIK
YAAFRSLARCTLQRWGLLISRRIVARDMERCSLERFVGGFLLRWRRYAHTCKSLRCIGHK
AYWRVARRAFGKWKMWLVRIVVTLQLEKRNTVLLVQVYFFRWLMFYRVHRLDYNAFLSSL
LL*
>TCRU_2907 Protein of unknown function (DUF501), putative
MQPLEASNKRGAFGTAASQESYRVADLELASCKLHGGAAVLCSTAGKVGRKARSAAAGAT
LFWLTCPYLNIIVARLERHGAIGALQQLVDENERVAAQHVASHARYEERARSLLSDQRWA

FFDSHFVSIPESSGGRKYGNGAVGHAKDLKCLHALVAQSLCGAPNPIGDAVLQYIYLFSK
KVEALRRPTKSSYLEEGGDEAEEENGPASASFFDDISVFVDFVEGWAKKNEEERAKFPVA
VDANDYCSAACSVLTFLEGRPPRLRKKHRIN*
>TCRU_2908 hypothetical protein, conserved
MDADDGGVPAAAKLTGEQEARLVARLHDVSMSHKQETLRLLEARIYPTMPSKRIPKEAIE
GSVMRQVDQEMMKRQAAREEREKRIQEEMKPLKVPSETVERSTCRLYTESMQRKKANLEE
IKKRHLYAGPPMVQKKFSEIREYVTRLAVPKKREFTIDEINKIYGLAAEPVNAMGVSGGA
DAAGGEAHNES*
>TCRU_2909 inositol-pentakisphosphate 2-kinase, putative
MRLLGMGCSSAVFSLLDNEERGGEGKTKEGETIALIIEQIFESGRNPLHGLIDRVALRLS
RAMTFACSTCDQALADFIGFRHEISLTDELRKCCAATVSLSLQEHCRGAGHAHIIPNFAT
NPAVLIGNGRGSRWLVVEIKPKGVWRPPRVVGILVDGVEYYIHPVKLYQCRFSLMQLLKY
ERRGTGVAPPSDSSTAYCPNMLLIGDQPSIKKGIRHLIRYPANNFRLFFTENSSGEISDA
DLDDLSTALHESGILKVLENLQLFGCGSHSDWEVLDIELLYRWSSAREISDVQWIVRESD
ASSSLKVECACQRGTIERWTDEGRIKDISTRFIAPPIGLKECINRFYASTTAKDVSVLVS
LLNGNTMEAAAASPAVGGPTGSRPRLEITQWKSLYGGVFVDFGFPNASICRVGAVDVDAK
VHKSLSHYFILDKNVLAAWERWKSTRILSLR*
>TCRU_2910 hypothetical protein, conserved
MRQTLWLWQWKDLLSHWKMYFHSGQKLTRRAALQLLQKTVDAARSSANGPGNSPLAKKRN
SEMIWKTIARHNVHWADCLALMNVQWAVLSPEQRHFIQEGFTCSGSGRHLPCLMADVWHP
ATDLSSVYARLLVSETIVQFAIVVYAAVRERPHLGDVLPVTPQVLLRCYFLGRSQPEVAL
QCCRVIALSSTKDISRIEREALAFEYIMLHAKKGQWSVALYLLNSSREIRPSAKRLFRLY
TFQASQWLRAIASVVPMEPHIVAEAITHAPHWSYALRTFERWESAEAICALLARPDCVAA
MGWEASLRLCAEARVNSYSLIRFIFTTVPSYCPQLMQLHATAIRPAGEVMYSGTLTRRAY
KFVQSGCWAEAIGLLCGCCYYDVALPLALYAPPGFALPHSLLKYEEAVHRIKLSTIVSAD
ASFEEQLAVALHGDGTRLKNLHTNNRLRSLILAQRLLSPNAAGKAMDGAEEEKCEVQCFT
FQSEKISRLVCRSAGIKAQRYFMQVIPRALRRHHFGVEEIPASELYTSERYMTFRSSNEI
VSEREACNMLCAMVVEHLRASGKLTGQVFLREIAHCLASGKVRLAAADCHALPLAVLRAA
TYFHRLDAVVTTRIALSTPSQHLMTHPEALERGMNALVLTGRWQRAISLLQRTKRPYPES
FAVVAYAAPRSLAIKALNMLWKDHAKSQWVLLLQDLIQGDIRLAQEELIHCSSRINSHVD
EGKILWRRRVLGACCALLQSSDSMQYVVRAANISSFCSLDVDEHGLQRLLPLLSWNQALT
AITDLKERGEVVEEHWALLVCTKPSISLDVARKIISWFPYSFLLNSVFLHQYAVLHGDLV
TSIKAMARYHALVITEYRRCLTYLRPFLVFLKNALHQFDDEAWKKASVWSLVRRLFNQVV
EDPKFVHWGRQGRKSIPFSLREEGPLAALFIVGFLYHRLSRALQMPVPAAIMSRLLRSAA
LSTNDSQCALYFFKSLKQPNDVERSLLVFALRDCDDAMTVLLNTGRFIGPRPDQVLLWSD
PGLGSEKWLEAIKLLSQSPPSQERLERLCASWTKEESLRALKLLQRTHGNSAEVRSYVEL
VESLQQKKVKSE*
>TCRU_2911 50S ribosomal protein L13, putative
MHRASRIAAAPFKSVLRRHKHHPEGLPLYNEMSKQWLCREGERWWILDAKGEQLPRVAAV
AAQYITGQHRPDFTPGMISGDHVIIVNIKDVVMVGDEWIRVPITWQTAYPGGKYRIRLTD
MYERDPCMLMWWFLKDEVNRHFVRKLKTRVAPLEKAWLYEDSVHPHGEKNPRPLCWSDPV
VRGWKYTDPQFIRRWKPNEFIT*
>TCRU_2912 V-type ATPase, A subunit, putative
MARDHNPYEAEHRMGSVKAVSGPVVIAENMSGSAMYELVQVGSFRLVGEIIRLEGDTATI
QVYEETGGLTVGDPVYCTGKPLSLELGPGIMSEIFDGIQRPLETIYRMVENVFIPKGVQV
KALNDQKMWDFKPRVRVGDLVTGGDILGTVVENSLMYDHSIMVPPNFQGRVTFVQPSGNY
TLVDEIVEVEHNGTKRSLKLMHRWPVRTPRPVAVKESGNHPLLTGQRVLDALFPSVQGGT
CAIPGAFGCGKTVISQALSKYSNSDAVIYVGCGERGNEMAEVLMDFPTLTTVVDGREESI
MKRTCLVANTSNMPVAAREASIYTGITLAEYYRDMGKHIAMMADSTSRWAEALREISGRL
AEMPADGGYPAYLSARLASFYERAGRVTCIGGPRREGSVTIVGAVSPPGGDFSDPVTSAT
LGIVQVFWGLEKRLAQRKHFPSVNWLISYSKYLKVLEPFFNTFDPDYMRLRSVVAEILQR
EEELQEIVQLVGKDSLSESDKIILETAKVIREEFLQQNAFTPYDKFCPLYKTCWMLRNIV
TFYEEGQRVVAESAGEHKITWNYIREKLPHIYNGLTEMKFRDPAEGKEANIDYFKKQNEE
IINAFASLLQ*

>TCRU_2913 Nucleoporin
MLFRDRQIIDRGNEILAYISHLDAAISSAEASKERLSELKSKQGAIAAMMQRVEDAVQPI
YAKVRPNFTRLDEQREATYTAVINLFDEVEAFRLRLSQSVAQHNRSLRQLRECDDLERMA
GLVDCQLSALERCSKRAEELESEMDLLLGKNSAM*
>TCRU_2914 NADH-cytochrome b5 reductase, putative
MKFLAFSAAAFLGAAFQSYTATPDRVAQCRQENVFGTKFKPFVLGEVINLAEDVAVFRFL
LPKPEMEFDLMPCSTLQGCFKEGINIVDQPMRFYTPVTRNGTKGYFDLLVKKQVNGRFTE
HLFSMNVGETLLFRTVQYKLKYRKNAWEEVGMIGGGTGICPLLQFLNASLDTPGDTTKLS
LLFANRSENKILLKGMLDKLSQEHSHRLKVYYTVDSIENEDGSYNGYVGYITEKMLQETM
PKPAPKNLLLVCGPDPMMEKLVGAPPSVLKAMSSGQAYQPAGAVLNNLADVEGTLGRMGY
TKDIVYRF*
>TCRU_2915 Endonuclease/Exonuclease/phosphatase family/Endonuclease-reverse transcriptase, putative
MEPFTWLPAERFFYPLLNSIGAYQRYTYRLRAVCDAQRQKLLLSGDIEQNPGPIAVLQMN
VSCLTPSKLATLMAQGADIIAIQETWESSERIASMHTGDYVLYAQSRIGKGGGVAVLVRK
TLRSKRIPLTIPQHDTSPEVVVVQVALDQNRDLIVADAYVRPPLQVTQSFRRLVNCLPAS
SPLLLCGDFNMHHPQWEPFLETSPSEVAAEFLELCTDAGLTLVNTPGEITYARGTRERSC
VDLTWSKHLTVSDWSASVSPLSDHYMLTFTLHQAFKDTIPSAPKFFCSWGKCKWDSFVKD
LDAQLPAYDYKKQSTGIKAFTRALITSYRRHCPRGMHKDGPRLWDDTLMEAERIATDSKA
RYLQLPTPDREAEMQRTRSQFFLLLRERWRNTYLRRISKLNPGEPLAWKYISGRKKASLP
SPTSILLGDGQHTYKTARRAVNALNRIFLPFHPSHKAVGFSKGINRQSASLNINASSILG
SRTTTSLLLHLLQVLHSHLVLSQRIKTKLPPHLV*
>TCRU_2916 hypothetical protein, conserved
MQNAYATLARIVSGVPSTVDPESALLDANMPPLHVLCLRARLSIFENTRACQMDWMRRPP
PEPPPRAVFRTSPLSRDELYAFVDAYTKDYGITESSPREERFFRSSIPPWFAAFAHRVTI
GVELPMDLPITDEEELVREKRRVSEEALALHSHRSWILATDGGVDVPKSAGVGILLSSLD
SSEIIAMSVTKPRCTPMQLQDGIPCASSSPREADDPSYPTQA*
>TCRU_2917 C-14 sterol reductase, putative
MPRSSRGASRSRSRTPRRATSLTRVRAPEPKQATPLNPRTVEYEWGGPVGALALTVLLPA
FVLIINVQCGEEQCAVTGIYNLPTEILETIRASLSQLPFAIGLELAWLLLHALLYMVPIG
GRVKGTKLRNGKTLVYNMNAVYVFVFTHAVLGGLHYNGIFRLAGLAEMFAPLMIASIIIS
TGMSIVLYLASFRAPTVLLSLGGNTGNPVYDFWMGRELNPRTGSLDWKFMCELRPGLIGW
SVLNWAFVLKAVEAGTCTPSIIIIALLESFYVFDGLLLESGTLTMMDIVHDGFGFMLCFG
DLTWVPFTYTLKTKFLAYHPVKVSNAYVAFSCMLAVFGYVIFRGSNRQKNKFRQNPHDKA
VMNLKVMETSRGKSLIISGYWGICRHPNYVGDWLMTFAWSALTGIEAILPYYQPVYFAVL
LIHRQLRDERQMAEKYGDADWKKLCSIVRYRLIPYVY*
>TCRU_2918 hypothetical protein, conserved
MPWIRLEAENGLRVMAPSFSASPRMEGIDKLFEYPVRARSFHCFRGCAMPLEKDDNVDSR
LCLMEEILVLVQRQQMGGKLGEVTIAVGGPAASRDELTVVLDSAFDVVRRTNYSGARLIF
DALCTSSTFAPAANDEVVEYDGLPTLQESHWKERRVSDWRDSDKMSSNKGGRSEVRSSLF
VRRRALLFFVTQDVVPVNLLYCRVSGVKSHGCVSSVAKAAQHYRKGLQSLESSAARACKN
LVDVIDRTLVAAYNVNKSAKKHQSLWGGNFDKITFVAVFSINGLLFASPLFFQLTDHRNR
LFIAAVAVDQLSRWPTERSLSKQTPLIHVCDLLLKCGKDGLVLNLVFLGVGNAVSCVFAF
RTAGEEAFWDAAVLLRFIKEHLRTPRAALRLGAGTDDGDDDEEEEEAAMKKEAREFAPHF
LLCTASIYPTTWFHRLLYEMNKLEEEAPKTFLQGTNDVSNGKLPPRLHGTCDCLPFFGQH
GKKTVKSTPFVWSTDTVDLPLDIAWCVASSHIVKEEANACSEGREMGHYALFSILSGIAG
NEGELGRYGVGWFNHCWRHTSIVGTRGDKENGVFDYYTTEADPTKFLGGLASIGGGPCAP
GFYEVREGGGCFFRQASRFEARAGDDGPFFTSKLVTFPYSLLFEGNGAEDDARTDEENGG
HLMTALYTMNTHKVKDAECNALLQEMLLMEGDLTEWEEELIETLRRRQHRSPSVNAAFAL
CHEAARCVLEDVWRCSIL*
>TCRU_2919 actin A
MEATLWDEEPAVVLDNGSGNIKCGFAGEEIPRCVFPSVTGVSMNARSSGSSSSQRVYVGD
EALQEKGLRYFYPMEHGIVFDWDQMERVWRHAYEQLRVPPERQAVLLTEAPLNPISNREK
MAETLFESFGVPALHVQIQAVLTLYSSGRTDGLVLDSGDGVTHLVPVFEGQTMPQSVRRL

ELAGRDLTEWMMELLSDELDRPFTTSADREIARRVKESLCYIPLFFEEELQAAEEDGINE
DVKGKEPFTLPDGEVIHVGRARFCCPEILFNPALAEKPYDGIQHAVINCVNSCPIDLRRQ
LLGSIVLSGGNTMFKGMQQRLQSELAALANKRAAEDVRVVAASERKFSVWIGAAILASLT
SFASEWITRTEYAEQGAAVLHKRCDSLSFVSK*
>TCRU_2920 hypothetical protein, conserved
MHSPSSALSCDSAESIYNLLSAVQAQANADEEEIKTRRLHIANCEREMERTNEEIRFLSE
QHEEALDEVRAKRLRGEEQKLRHAGAVEMLQCTREEVAGVVDALFACELPLHPVAAAAGA
SFETPQESLPPAMIRSATTSLKEKIRAATEQRQSQVTEPQRLFTAVAQEASLGNDAGNVF
AVRFRQDAASEASSDPTNNTAIVSECAVVEVVSSPSERPRRKVITFNDAQLEGHIEKNHS
SPSSSSSSTAALSEAPPRKGLEQQEEMRPAAVVSVYGVLNMKRLPRRTFWQKEPRLQK*
>TCRU_2921 hypothetical protein, conserved
MALRLTLLALGSRAKGYENRVMVYAHRRHRSWYQPPKLPHARSPLANKPPEEYGNTWDPR
SGVDWYYRMRRRGAYRHWPWARWSDDPVRHHRETPFNRTFSHNSHGSNDGVPLWNYYAEV
GQEYNTPSHFPLHYMAPFIHQYTGKMWSRNDIERHLKLIEERTGLISIQDVHDNLPRLQE
WGEVEMGTVPHGLLQHVEMLANDIVLQNRKKGFRRKLHENGVLRTSTMERYYALPHLRSG
PPMPTTLQQPSGAFPWGKYTHMLGGTKIHPLHRPDGFLKENMYPP*
>TCRU_2922 ribonucleoside-diphosphate reductase large chain
MLATDLTVRKRDGSLQSFDANKLFARIERVSHGLDPNYVDVKILTEIVVGGVHDEISTVE
LDKLIAETAAYSVTKHPDYGLMGGRLCVTALHKTTSASVLETFSILHKHKIHDTGRAAPL
LSDEVWEVVQRHHEQLQRMVNYDRDMRFEFFGYKTLERSYLLRVDRGRGEREIAERPQQM
FLRVALGIHGEDMERVKETYDHMSLGYFTHATPTLFNAGTPFPQMSSCFLVAMREDSIEG
IYDTLKQCAVISKSAGGIGVHVHNIRCSGSYIAGTNGQSNGLVPMLRVWNNTARYVDQGG
GKRKGAFAIYLEPWHGDIFNFLLLKKNTGKEDHRARDLFYALWIPDLFMERVKSGGKWTL
MDPHTAPGLSDCYGDDFKKLYEGYERENRGVRTVQAQSLWFAILESQMETGVPFMLYKDA
CNRKSNQKNVGVIKCSNLCTEIIEYTSRDEVAVCNLASIALPRFVDKERRIYDHQRLYEV
TKIVTKNLNRVIDRNHYPVPESRRSNELNRPIGIGVQGMADTFILMRLPFTCQEARTLNE
DIFETIYYGAVEASVELAEVEGPYARFAGSPASQGQLQFDLWEQKPRSGRWDWDALKKRM
MKSGMRNSLLLAPMPTASTSQILGNNECFEPFTSNIYVRRVLSGEFPVVNKYLVMDLIKR
GLWNEEMRNQIIAYNGSVQNIRGIPNDLKELYRVVWEIRQKDLIEMAVDRGKFIDQSQSL
NLFLENPTSSQLTSMHFFAWERGLKTGMYYLRSKPAADAIKFTVDPNTLKGLVKEDDRKQ
VSSATPSNEDCLNCGS*
>TCRU_2923 hypothetical protein, conserved
MQRSIDRPLGRGGNEARDKRPRKHISFARDGVKVCFVKPPPRSMNHCYWFTETELVAKKG
LFFEVEPDIDVSVDVMCRMQLTSVASQPWKYPRLFSKGGGAGEKRPKGRSSERVFLEIKA
TPSLGSIGVSRRASRWLTVASVEEGQLEKVRVILPAGSYALRCVGPRPVQVFAQVWDVET
RLD*
>TCRU_2924 hypothetical protein
MTFCIYGTSHHERLAVALFRGSSLTVYRQTSVHGKSDELFTMEPVMVLRHCHEGGGVEQV
VFVQNGEGLATSGRADGRHICLWALPPLPN*
>TCRU_2925 hypothetical protein
MNAIKLLTGYLLVSFEELGVVGSLFLAAAGAVVVCAWTAWWLRPRHSVASSEEVKFDKDV
GEAALTGKSETAEAPARRKEAKARSDKDATGRMTAPGEPKGEDSIQPTSVLRGYPEVDSI
MVSPCQRYLFIHCRNKRRARLYPQNRNRAFMERGKELQEKHFVSVDEAVISATGGDAKVE
LYAAAFSYDGERIMISERNSDTFFLFSIGTKCQLTLQSSCKLPHRRLVSTLPPWGVAGAA
YDLIVMTYDNGCEVEVFSLQSKEFLGKAKFKVGNALAWAQRDLTIAAAGSFLSRRNFSGR
RSSRWEMPWPGRSETSPLPRLGHFSRNRGWRRCRFALMETGSNY*
>TCRU_2926 calpain, putative
MNACRYNPSEEEFLYLPQSVFLNNFSSMQRCHINCGDRLTAIGEWDKTSCGGNPKFTTFR
NNPIYLVENKSSRPVRILAELRHQAPVFYDADSVGHYHQTGLALLQHDGSVSVLSGIITN
STHNFIQKGIMLDTREVCSRMEIPPTSTCILIPYTMKRGCLGKFSVSIYPGDSSVNFMPL
TPLSVTHGFCDVDVILTPGSREGKRIEFVVNGACDAHLLLRQNKITDPASIKKGDVLAED
DVMMMLYDEYMTRLASTGDATSAREHSLALQLPSAGRYSVLLACPNKPVTGNCPCSLYIY
TPKQVATRILPRPTNGTPQILPFLSLPQSSKGAARGNVKGKVIGAGDVATGTGNRPVETQ
GMKLPNPPRNGRPTYHR*

>TCRU_2927 hypothetical protein, conserved
MGEEAFSYHAAEVRAVHLALSAFSAILPSTMDVWVDNTSLQGAENKGSSKSHAMTWELQR
IYEFLDSRGIQATFAYVRSADNPADGISRGRVFTLQELAKRSGGVLWLEDPKVCHFVINN
I*
>TCRU_2928 hypothetical protein, conserved
MELIRQRRSTVVRRITDIPALGQLHEESERGRSRDVGAVNVGAENVPSGAIHDFLPHARK
ADKRGELCGLFDGNRSRAIDPTAVRQDAAVHVGNGSNPAGHDDSGVTKNCGAIGDEADAP
LDRGRDESSNPQPDRLEGTCCLTTRVDNGELLVRDCGLDSQQFHNGAGRDLNFVLVRGAE
DGEDGSPLRLAVCQDTRAGRVRHYKAMQNTSRKRKAHESYDRPGRTSSGSLECHGAFHKT
RCVEARCSNRGDTQFESTRDLAVGEARRPVRPSPEYCSIFGEVHHSADPGIGAGRIDVKG
ETAEGQEPWLRAEERFFHDFYHNHAATITRRVARTRLATPQDEHVIPPQQVNVPVLNLER
IKSRLNPATLERVMRVWGLVGWSPFPPSSSGKAREGSRSIPTADAR*
>TCRU_2929 hypothetical protein, conserved
MAEAVSCLDVKSSFIVSLPRETRHLFRCRVEDGTLVELTRLPMGYKAGPEILQIITSAIA
VVTTVVHRLWAAPPSVRVDVWIDDIRIAGLKSDVTLWEAQVLRNADSCHASMGEGRESGA
TQYTFLWVQFDHTHRAVSLSEKFVRSVCAMTSLNSSTIAEMEVVASRFLYAAAIFGTRLC
DYYFFIKAGRRRLSALHRGIVLETSPANLPPAAVGLDERLRHIIGNNRKRIIKPAEKASA
AITDASLHGWGAVFISDFGEVKIAGGKWKKSFLIMQAEARAVRLAFSAFSAILPSTMDVW
VDNTSRRITAA*
>TCRU_2930 hypothetical protein, conserved
MASSDDDSRCVLGTHPHSPPCLSVGVSAAAAWSMAEAVGAAERDEAPRNKRSNLLDETTM
ADESCFVAFASYIARSVPCRRLTGCLHSAPFEALCSFIQCDVTIIMAAPSLTGSECDNTN
DGKESDEDYGDDLHRGADYLRVLAHEYQEGLNAVSTIPGLAQPLAFALSPAVISAPCVEA
DPFAVEFTQQLSSVCAANPALALSHVSQRCLADDLMSVLSRPPPPVLYWCPIRNTLRCGG
YLFAWPSMHVTMLNGCVFCLAGTRETSVCADASAKVFVDGSRRK*
>TCRU_2931 mitochondrial carrier protein
MYPGSTYAMLYKVLQRVIKFAGQSYMRDFLESRFGHIFLGDAQEQSKQQKQQKQHTHRRN
NHGAMALEATAGCLVGVFEVILLPIDRMKVLNQTNKGAIKNRSFFRILRQEGVMRMYAGI
GTTAVRNAPGSFLLFGGVAWTKEYVFHLEDYSDATVVQNLVSSTVGGFLGVWFTNPIDVV
KTRIQNKRFEDGRVTGWSVMTETFRREGVTAFYKGMLPKLLTSAPRLVFSYTMTQFFIRC
FRGEKPKPLRESK*
>TCRU_2932 Transmembrane adaptor Erv26, putative
MHYEYEYYNFRGSGKPPYMRYGPFDVLTFLMSCITGIAILFSVTCAIGYVADVAEEFPTR
AKKILHTMTIGVLFVHGLILVLDQLSLWCSLVSIITNVLYLRILSGFPRVSLTHPLTILT
IVSLVVELLSWYLFFLFSPIMPEHLGSYRFFSFTAFLWLIPLGLLVSLQIEPVGLPGIGF
SASPQRHTDIAAEGRKKTVFNLLRGWLTPVMD*
>TCRU_2933 small GTP-binding protein Rab11
MEDTNLTFKVVIIGDSGVGKSNLMTRYTANEFSQDTPATIGVEFMTKSIKIEGRDAKVQI
WDTAGQERFRAISRSIYHGAKGAMLVYDITNQTSFDSIPTWLQELRVFVPATCSIFLIGN
KCDLEHLRVVKKEVADRFARENGLSFLETSALEKTNVEKAFEWLAKSVYDLVVAPVDAAG
GRRPPLNAAANAKRPVNLGASNDGQAAGKKENTSRCC*
>TCRU_2934 ABC transporter, putative
MDFVKNFPPLLRDIEGLGEEYGVQSYTLSATTLEEVFLKIAHGDGVGAIPAVETQPTVLK
GDYSEMDEKGNKPVELEGDFSTTQKDTPRLVAHPSIRSRSNSGIETMSQLQTIWEAEVIK
DEFRLMFSQLKASLMKRLLYALRDRRTQFIQIVFPVVMLTFVLLLQLVEFFSYPSLLLSS
NMYGERVLIDLAACNGTINVSIPFAKSTSFGIHDIADTGDLYKYLIEQQKTPSLNRYTSI
SCNDVVYRERKILLYNGSALHSSALSLVGYYGSVLYAQLRGASTAYQEDIFLTRNFPMVQ
TTREHATSEAIKTVLMGILAIIPFSFIPSTFVSFVVRERECKACHLQKVSGLRFSIYWLS
NYIFDFFCYLITAILAILIMLAFDRKEYISSEKIGATFVLLLMYGLSCIAMSYAVSFLFK
QHASAQNVMLLVNFIAGFFMVLLVFILEMFESTKTAAKGLQWTFRIFPSYCIGEGILNLS
LLKGKEDVRGKRISPWELDVVGIPAIYMACEVLVFLLITIFLDHPTRRMRTKRALHRGNG
EAEEIADEDEDVAMERRRIQETENNPSEDIVRLENMRKVYSNGKVAVRNLSLGVKSGEVF
GFLGTNGAGKTTTIAILCQEMAPTSGRAFVCGKDTVRDSRESLRCIGYCPQFDALLELLT
VEEHLNLYAGVRGVKSGERETVVRDLMGLCELTAYCSTLARQLSGGNKRKLSVAISLIGG

PRVVFLDEPSAGMDPIARRGLWTAIQGISCSCSVVLTTHHLEEVEALAHRVAIMVNGSLR
CLGNKTHLKRKYGSGFEMVIRMADDEMRERTEEFIAMYFPAAKLNEVRGNRCTYALPATT
ILSEAFGLLEAHRNEVGIADYTLSQTSIEQVFLRISEQAQQEPDSVNVTAA*
>TCRU_2935 hypothetical protein, conserved
MRLSNYCLAPTLSKATRLQLGNIKTEYGYVSTAAGSQEKWGFIFKVRRYPLLSCGYTLCL
MSIAIGVVGTYQQYRDMVIMTEHISYEDVKDRCLTPIPGWARIRNVQIASPLGPMTYRDP
TPLDWLPFELKLGHVKQASY*
>TCRU_2936 proteasome complex subunit Rpn13 ubiquitin receptor,
putative
MQRKGRGGECVIQFRAGKMNFKDGKVEADRRRGTLSFFKMPNNDVKMTWISGTDEEQHDL
PRGEVKFSQVEKCTTGRVFLLDFGNRRPPIFYWLQEKTTENDSTYFSTIQELIGGDLRPS
DRKRAEFEVVLQGILRNMRNGLEQDVDLADIIGSKKLLDALHEDPAFFMYKLHQFLPEGT
DPTADIVEQVRNPQVSAAAASLGLALRDPLGFLETCSAFGINGSNPCVFGFLSGVMKLFK
KGDDNSQ*
>TCRU_2937 DNA polymerase epsilon subunit b, putative
MQDYRAEIQFMARAYQYRLHPSALKSFSDHLSTIGDSEKRRDALRDLFALLHKLCAGDRF
VDDKKMRLAITQQSVRDRGISEREGVSTVFPVPLEEIPYVYMDEKTGEIKVQRAERDIDR
FTVLRQRYLFTRRRCLRSGLFRRDLNKQSLNQDLPPLLSTIALEGIDPSENVAVLGMIRR
GFNEICLEDLHGRVRLTFLGGVPPLIGFVGDGFFVLVRGQWSSGVLVVVSIELPPAERRE
DTLRDIGSDYDLFGYRPTDMAAAHLREKNSLQAVIIVMSHVHLDQTSTVNKLVCFFKEMQ
NRTESELMNTTLVMVGDFSSSTIHYDDISHLPDPFEGSNRYKLLMDTLAMVITTHAPSVA
QHTQVVLVPGSNDVTGLLGVLPQPPIVSVFTRTLQARLKKVFFAPNPCRLRFFTHEIVVT
RRDFFRSLQGSGRAFNRSVVETPNSISSFESVAKTILDEAHLAPEVKEGILWKADGALSL
LALPHLLVLCDSTEQWECTYKGARVVNPGSFSLASTFLWYTPADGECSLSSIDY*
>TCRU_2938 DTW domain containing protein, putative
MKRVREVYTEGAEERRRKIIDFIRSLQFSVPLYRLRQQEGRRPCANCGKRRQYYCYDCLT
VVHPESHPPPLFLPLNVYVILHPGEIRGKSTSLAASTISPDLHIVEYPKVPSSLELESTL
VLYPSSQSTELSDIKDLDSVKNVVFIDSTWQQSKAIARDERVCRFKKVRIKSQTSLFWRF
QSNDPTYLATVEAIYFFLREFISNKRKHCAEESKKPLYRGDVDDLLFYYINQYVAVQQRY
SHDTTMQYTTRHFDGYILPSSSWDELVAPPPKDLKEGNVI*
>TCRU_2939 trypanin-related protein, putative
MPPKERAVATRSKAKGAANDARKNKSIAAVPSAPPPPIPDGRELWLATDTLQKEKAMRNY
FQLERDRIISFWEVTKKQLEDLKTELRQRENDKAEAQERQEVEKKVFKQKIRHMMYEHQL
QLAEMISEAERTLAIREEEYRQKERNSGGEIRDAKLMLRAQENEHRDMTSALTLSHDQEI
AEQQLAFERKNKEMHLIYEKKMKDLRDEMDQRRREEIGLIEKRKGDHIAELRETHERTFR
EMKEYYSEITSNNLETIRTLKDEVYARKRTEAHNERAMMDVAQRNKKLTEPLAKLQRQKR
ELEQELINYSSDRDKLREMKMNVREREQELRTLSWENEVLSQRFVKLEEDRDIILNKYNN
MLQDIQRKATFRRVLVQNKLELVQSQLEGRDARLTELLKRVNIDPEGISEIEHRVRDLSL
EKDAIIGDLQHLITQLAAKQQVIVSTYEAYLRGNGISGVSG*
>TCRU_2940 rRNA processing protein, putative
MPVYDVAARKKRPAVVKLNGTPKEVNEKNEARRKGKMAPNDKKKNNAEGEKTAAVEFSEP
AQWVERMALTSTKSLPADLNADDDPRREEVFIQQALLSVSRGISLLEEAAVPWRRPADYY
AEMFKDDVQMGRIAAAIEDSKARVEAQAHRRAMKDQKKYGKEVQAELLRQRAKYKREMGE
RITEWRRKRKGNEELKEILDDGDAKGIQSAHTAHRVRTPKQKNLRPGGVGGSKRRPGKNA
RRRR*
>TCRU_2941 hypothetical protein, conserved
MSSQWLPDFVLQRGQEAESYSSTRGRLSNPFDLVTEDACIISLERKSRHLTEVFAVRDRI
HKKFRGFVDNVASEFVLKSSLTQLGINAEDIKINLDRGALRAEIRMNLVALSPLGVLMLD
YIGEGAYIGKLFALEEVRRVRNVSYINRLLKTLDQAGHFLLNYGDSSEPDWELKIVDGRV
VAFLPILEGTFAYSGEVHGLLPTIGAALKHRTRYKELLRLHQEFRLNYTRVASPEGVLLV
RGLAMHLRTLFGRVVDELLPKGLKSMSSRVIEPDTGLKQKLQKRTFVFYGESSVELTHVP
IEFFTLESYREHVPFSLRKTLSQRCGCKADVLSVFRTAPRGNECCCTYICKGGQFSELTS
ADWVVADPKPLPYVGYEDPGRQQEIAQQAVYQECEYSILSAIAAGDITSDGVLMTRYFPS
PSLKSLILSCSVGKKVRAIFFTKASRHHGQFFSQEDSGLLCDLNTFGILVFYVDEAHGEI

YQFIRRQDRDCGVFVPVERRQEYLLATFFGVYGSNLMQGDFEAELRFLFNGILRLRQYCN
HPLLNPGKTLALVTGGGPGAMEVGNRVAKSLGILSCGLFVDFGSLSHKPGATINEQRNNP
YIEAFMTYRSNKLVERQSDFNLDFPIFLTGGIGTDFEYALEEVRRKVSSVPPNPVILFGT
LEHFKNKITGRYQENLRSGTIKGSEWICSIPWLVTTGAEALEIYKRFFNGQLPVGPGHPL
NDLGFVVASEYLANHPM*
>TCRU_2942 hypothetical protein, conserved
MMAGVLGPEVIKELKAIHCVAKALGAVKTSPHASFSPDDGTITVSQQSGEDTAAVLGDIG
KKLAAAAERLADAIEAQMGEFFNHMREKEASSVVGLLRTRSVVPDFFKVMGKNEVRAGDT
NAASRSCEIPEINGSMQKNINSLTSHQSDLRGATTLLQTCLFLMMESVLTRVNAVRAAVY
IGDVSENPRVLCRVAHIHADDKLPLEVSYANSSTLITAVQGGMAVNFNNTKMHPNKDDRE
ANGHSRPFEAHLRCHLRVQSGIVVPIGNYGCVLVAEKPAGMPQGFTSFDEHHVWALAILC
EGLIPRYPRDVFVKMSWSPAVRTLRHCSRLPSILKPLARASTLPRTGLTVTSAPSLDTLA
SVPRKLTILRASGNSPSIIGDPKGIPRKHMTTEGIFREAAEYVRNIEDLWQKAVHEANQL
QQVVEEHVRNLEEKTHQITLLEEELRRLKRHAFGL*
>TCRU_2943 60S ribosomal protein L5, putative
MPFVKVVKNKAYFKRFQVKYRRRREGKTDYQARRQMVLQDKTKFGTPKYRLVVRITNRDI
IAQVVQAKVVGDEVVMAAYSHELPQFGIQHGLTNYAAAYATGLLVARRMLTKLGLANKFE
GTKEADGSYSAVRTKADDQGDDEKRFPFKAILDVGLARTTTGARVFGVMKGAVDGGLAVP
HRPNRFPGYDKEKDSLNAKAHRDRILGLHVAEYLKQVREEASSNPEESTCQFSKYIKDKV
VPNELEGMYKKAHAAIRADPMKRRARKERKEGAAVKKYNTPKRTGAQKKAAAKEKIAFLV
ASIRERASK*
>TCRU_2944 protein associated with differentiation 3, putative
MTGEHLIEVLATGPQKAIYEPRRFALLLLGSYGCICSSLSYAFNLIAPEMQSLYNLTGRD
ISTISTVGLVVGYFLVPYGFIFDHFGPKPIFILSMVLFPLGALMFGLSFRGTIEGSVVRL
SVFNGMLILGCTLFDLAYMMTIMSHFPISRGPVVAILKSYIGLGSAIVGSIQLAFFDGRP
DHYFYFLMVLFLVTGVAGFFFVLLPSYHLTGYEEKHLGVEEKQRRLARKSVYLRQKPPTI
RLAIGIAFVVLLVIYLPLQSALVAYLEWGRTQRIIFASILIAVIVALPLMALPVSCLERR
KTQREEDDCSGMDRPNASDEAANEPAAAGGLPKSVETDVDYIAPQYQTTFLQNLKTLELW
AFFWSIFSIMGTVFVIIYNASFIYAALADKEVDNAVKTLLTVLNGVGSAAGRLMMSYFEV
WSQKRRAEDRVSIVVSVYFADVFVILSLVLFLVMPRAALPLPYVLAAMGNGFGAASIVLV
TRTIFAKDPAKHYNFIFFSVVFSTILLNRLLYGEWYTREAEKQGGNVCLGRSCVMMPLLF
FIGLNFTALLSTVYFDWGYRRLSRVVLEERRRLKARAAEGLLAVNTSPVVGTGPQEGEED
AGNRTTTAPNDMQAVRP*
>TCRU_2945 hypothetical protein, conserved
MLQMDRTPLEIVILRLQKIAAQSETKQARPSTKGAVDKCIRSRTDWKERVVVRLAWITAS
RLFEIAALTPNNFTLEADGALILDWSLAPKTARADPHRASRFVGIRGQDAFDIIKLCRTL
RHNEKLTNLTTALVERALANWNATAHSIERGALRHAAPIVEKYNLDPHVISQFASTSTRS
TFPRILFDICGDTPKC*
>TCRU_2946 rhodanese-like domain containing protein, putative
MGQDNSFENIEQKKALSLFEMEALVKSKMDGENSDVYIIDVRDGFEVEEFGSIPFSVHIP
STEVRRAFRLREAEFMGNYKSRMPRKFDKIVFYDQRSGRAATAVEVVEAMGYQRATYFSD
GFSEWKSKSGRSADEDL*
>TCRU_2947 rhodanese-like domain containing protein, putative
MFGLTRWLRVKVTVEQVAAVVQAKLSGAQSVQNTLLIDVRSTGEVAATGVIPTAVNIPLK
LLEFALGEEVEAEEFEKTFGVQKPQPGMTQVIFYCTHGVRSAIATEIAGNLGFTDAKNFA
GSFTEWQRHHGEPCDNGDVPLK*
>TCRU_2948 Rhodanese-like domain containing protein, putative
MQNSLDYAAIKDIVANKLRGELKTTHLIDVRGRDEVQSTGLIPTATNIPLDELESALKAD
HESFQKTYAVPKPLQDDTLIMYCKKGLRGLEGEKRARQCGYTNTAVYPGSWDDWSKNTKE
QKEE*
>TCRU_2949 BNR repeat-like domain/Concanavalin A-like lectin/
glucanases superfamily/Trans-sialidase of Trypanosoma hydrophobic C-
terminal, putative
MSRRLFTFALLLLLVVMMCCGSGGAAAVGSVSEDEHLSKWVDIFVPNKTQVLAGEDDSKS

GVGVSFASPSLVSAGGVMAVLAEGIISYNPSSATESFVYHSDIVAGYVNAAESWPSLVAE
INDGTWRAHTVLSTVNEERHVGIARFPTTIAKGNKLFLLVGSYIMMQDTKDDFWKLGGTD
IQLVQGEATKSTEGKQSKSIKWDEPKSLLGQIAPQAQGDSKVLWAGGGGSGVLMEDGTFV
FPLQKRKENGEFLSMIIYSTDDGSSWTLSRGMLPAGCRGSCITEWERGQILMITDCDDGQ
RVFESRNMGTTWTEAVRTLLGVWVKSESEFFWDKSLRAGALITATIEGRKVMLYTQKRYP
TQEQKDKALYLWVTDNNRTFYVGPFSVDSAENKTFANALLYSDAELYLSQQRGIKTGTAV
SLSRLAEEMNTIRSVLSGWAQLDASFSESFTPTAGLVGFLSNAASGNTWIDEYRCVNATV
RNAVKVHNGFKFTGPGSRAIWPVNSREDNNQYGFVDHNFAIVATVTIHKAPKKSTPLLGA
SLGEGHPTKMIGLSYGAGSKWETVFDGKTTAQDSTWEPGKEYQVALMLQGGNNGSVYVDG
KLVGSSETIPTLETTVLEISHFYIGGDEGDSDSDLTVKNFFLYNRPLSADELKMVTKRED
SVRGDVSRVLLLLLLLGLWGIAALY*
>TCRU_2950 hypothetical protein, conserved
MGTVTGELQGDAFLRANLEYLASLEPPAICPVLPRLPGGSGLLPDDFQLPPTATAGEAAS
IILQVNAVEDVSLPMAQRLRAMAPPATAAVQDNETELPTAVGDEAQQISVDEVLRSMESD
EPASQRRGRRQRLLRLTLTDGFTRVIAVEDCRKGCDALREGVAWGTKLRLYAPLQIRHGV
LILKSQQTVMVGGFVLELQEFWERHAKAMLEEMSGRPKRRIQQLAEVPSRVAPVQIHNGT
RTNVEHNSASLPPPPQQRLQEAGAEENAAAPAAPSHSNTMASLHSANAVNQPPPLSFSLS
ASQPQSLSAWQSHHPPTAPFRTVAVIREVTSELTIREVSARSPEASKRFSLLVLLSTPPV
DVNSDVPTGEAELVVDLGHNWLQQALGVDPDAFRALFLSRQPDDVAKLNTLVESVGWELE
NFGVAVFVLRQRETDGVVEVVKVERL*
>TCRU_2951 profilin
MSWQAYIDDSLIGSGHMHSAAIVGLSDGSYWGYGGTYIPQPDEVAHILKCLGNFSLVQSS
GVTIYGVKFFGLQSGEEGEMKYIFFKKGAAGGCIYTSKQTAIIAVYGNPGTSSSLQQDLE
KKEGAEIAVNPADCNSTVKRIAEYLISLDY*
>TCRU_2952 hypothetical protein, conserved
MRASYSPFKSKSQWTVENLAERIFISSPRLFSFSHRVLVSLRHPYTSVVVDLVDSDAYRK
KNRLEELLGALLQSRDEERKWVRVATMFDTKLTAEEIDSFRRKISLHREIRLCIEALWAD
LGGISNGAILEDSYKAFHFRLYSYILRIDNVSVVTSTSAAIAEDFTYDKRGSVGVEFGSF
AVSILELADNWTKTREVEDYIHFLRSIHDNCIVKNRSYSLKEDTIPSYKAPVFFSSGLVC
RVQEGDNVVYSKKKI*
>TCRU_2953 Ras-related protein Rab4
MAEKYQQLIKLILVGDSGTGKSSLLHRFVEDTFSEQQAQTIGVEFGSKIVQLAGRKVKLQ
IWDTAGQERYKSVTRSYYRGAVGCLIVYDITQRSTYESVPQWLNDVRQLAGKDVVVMLIG
NKSDLSDGRTVQHNEASLYSLENGLLHFETSAASGEFVSEAFLKVAKTGLSMTAEEVNIG
GDRAMEVAAKQESRTGYSCAC*
>TCRU_2954 Protein of unknown function (DUF2870), putative
MVLIECKRLPKDEYSKDYDVFLVSIPGVTNIGEATDVLQQMRNTRVRIKWMMAAAKQLAK
DFCSEEQQHHLLGPVADAERYMSLERAERRLTCTQKELDDLVDAFKGGAMILFPAECSGT
DAVKRLAQLLDDDNTTDTERSKAHRLLSIIDDGATTEDILQGVATMWWSGKQLDRAADFT
KYVGKNEKTKITVKLTAKDAPAPPREPALDAKAQSELMAYYFRKQEEMKKMIEDEDLSYG
NSAWANPQGLKSQLHGFDSVKFKPG*
>TCRU_2955 hypothetical protein, conserved
MVTRDAWLQGLSAVGFFLTGGVEELQALADVIAGVRAGVSATRSVAVGKTCFTLAVPRPW
FPRLHARHHPTLCTVLPRRLDLIASHIEPSPCGRNVVSKRSRGRRARHLHSFVEVPWERN
ADLSASGVSLLLLPYSVSPWVLSGAPAFYWMIGAAEMWRVFAAPSFSTFFARRVRDGFRL
PHCMGCCKAIHYATRLCCGCRAVKIFDVVEVPLEGGVAILRRG*
>TCRU_2956 hypothetical protein, conserved
MTKLAAHRSLRNLPLHERQGCPRKGVGLINGGVKTGAQCGERDAGIGCNCELHPECIQQG
PAQIHDSIGGILQAFQRADPLDQIVKGKFNLLVVLLPALVCTPEVDNDSGGALIPYTRGP
RGDVECAETAVLFHG*
>TCRU_2957 hypothetical protein, conserved
MTLVPYTPSEWCDIGARLWDMVSQCGETQVQMTFINGNQMSDGTLTVAASPTGLCLLHGP
VSKAWQDSTLIGHIANLRAPQIPNFETQVLLPAMGAAARELGHQQGLITAERERLNIDYAN
AAADVQNRNAQLNSQVEEAHINPSVRESTIAGAGNQVLELRQQLEADRAQLREREFNLGV

LTGQFQEREARLNALQTARDAETARQDESPAT*
>TCRU_2958 hypothetical protein, conserved
MDGEEADVTKETKEGGPNTSHTFSVDAAPFEPSVNVSSNLANGWQDASSALTEQLSALGA
NESWPYPAQYIDMFALNASMRAMVEAAAMMNVGAKRYSGAPIVKTTSPPKKGSNASEASR
KREKGSLSIHAKPWSPSPETGLNQKEGDTYTHLKKGWVKVPYRGEMVIAPVAFKEIANDF
VHSLTVRPIEKPRIHLELYNYHCARCATHSFFGRLSCRDADVILKGIAGDIPPLCVAHLI
EQVTGAVVCALFTKGDCLDYEIWLETPDKAAHFAKTISDAMWTCPMFHGYAVHTKTEEEK
AFLHEYISSLKDDFPEETPYPMRCVEAIASSTGKVKG*
>TCRU_2959 hypothetical protein, conserved
MFITKTSEPNVSGFPVVDSLQEFSKVLTDYGTAFSDFSCFTRELIDPIVLPKGKTVLDVF
RVCFDDNASLLEEYHRDRKDSNQKWEPWRPAQTGSPQFSGQRVFTCTTTIKALVSKSCPF
TEYQRYAFMNVGGHEPTLVVQLSGQAEGLMYADVFRAEALLVFTQSDSGVAMRVFGYIQF
LRDVWVKGKILRTSLDNEMPICYRKLGSMLIERLQQEGGISSAEGPEKSSPLLPSSPSAN
AAEVLKGSSHVSGLVWRTVVYIEFVLEILSALLCLNSLFKTYFFTASKDIAGAQIPVLAT
GEISVNLTDAEQTLNTEILSLLVVDLLRPVLLASLLCLLFVIQLFLRKTVFRMKR*
>TCRU_2960 EF-hand domain pair, putative
MVSNRSLSAPPGGGSLANDSNHFGYRWGELSVGGFSPSPPPSNPWPNRPQTFSRYESSAD
WRKSPSYGGNRHGAYFRRNRGGAYGRVSHEFNRGYSSRSASALEEQDLPFPPQGRNGRFN
AHRSSSTPVWSQGPSYPTYQTPSPRYNGRRSGSSSSVSNSAKGKAEIPLLLASAFSEKQT
TRVRASLESSSTFSQAGIRLKLLEYLGYWMSDVLTGAAVEKLIMETYREADLKDTHTSVG
ERKKYSFSEFSNVMNTGKIAGDRHVQEGEKVNGSNRATSHLLSPSSSFSSLSLSSVSFHR
SESKNTSEGAAVRREQETQGKWERGTGTHFRRDSGTDASSNTPEGRVFSLVRSCGVESMA
TKQVFLTDEPKILPQQTAVLQTSSLIQATYSHVPRFYFPKGAQKTTEETLVGPMKKKHEN
PHLRVLEGMVIPTAVHTNSEERAAVMSDDVFKRHEVKKVPIAPLKAVEDKCVHMIIQKEF
GRLPVPPKIIHGTRPQSSAAKGFIGLNNGMQTRQGLNYRIQLTQAMKRICTQCFGLPKYF
AFLIVRILQANVDFGNSGVVKSGRSSSTMALSSPATGLPSASQTEVMVRQLLDFFETYLR
GRCIVRRVFEVLLLSSRVATSGSETSTRIGNDWRLVYDGEKQQQRSYLLPDDFRAYIEVL
LEHHPGLLFLKQTPDFQSKYLETVTHRIFYDLDRFNRGRITYAEMERSSFLDSIRQVDAT
NDINSVLLYFSYEHFYVLYCRFWELDKDRDMLLSRREFMNYAPEGVMNPIIADRVFTGAG
RRKACTQKDRINYEDFVWFCLSEEDKSTPTAIRYWFRILDLDGDGLLSAYELRSFYDEMK
KTVTSYAPDGAVPFEDILCQIFDMFGLETSCSLRLEEFLAKPEAAAVTFNMVTNVVRFLQ
FEQKDPFVTHQNRLEGGLEQSAWDRFARAEYGRLSNAMRKE*
>TCRU_2961 Alpha/beta hydrolase family, putative
MRTVRLTLSTATASAPPLVKEAGKVAAATTTDKPRREFTSLRIQPWQRNCLGASREQMLP
PSLFYHGTHLGPRPLIILDHTTKNTAEAALAAKKYEAVLSQLSWEYGAVYIPLHVDFPYT
TSGLIEQTCQQVCAVMDALDVHWTHFLTYSYGCLVAAYMAASEGFPHRIGTFISLDTPLV
TRELLQNMEQREEIAKAERDVNVPEANLSFAKHNLISSLEEPLPCPAGADKSLYYDYLFN
PAEIFQSDGLVRCEERYVPVKHLADVRHPMQLVVPSANALADVTVHKEFFGLRRPVVLKG
CQRHEEIFRDESAKEIAKVLETWLQRFEPDAYIAKRYEQAAAEMSQLMMSSAPATTQADG
AEGGDQRKKKKEKKKKA*
>TCRU_2962 hypothetical protein
MEVPIFSPHGYTGYAKKVFGEDCYVYGPTTEHCEILLSNVDEDVSLHIDGGTPTTVYAND
VALLDSWMMTPVLISSSGFVTIRRVQATGGKGAASSRGSGVLPPVSTRGGSGNYGELSFH
TTASSPVVAPSLPLGVLAPMTPVITCRVSTKCWPRVFPHGDNSTDTQLVQYFKEAVVVSV
RTMRLLGVEEGSMVQVDFHSEASVLAASKVVFSVTSDFVGEEFSFDSCLRHFIAPFFLQR
CRAISVGEEFEFKNHDYGQLHVTVVAIEDGLLKCGLVVDSTCIRLVAQPEAPPKLQGHGS
GRVSESSTPRMRVPSARKSSGSVRGSSLPRNSHSRMSEASPRPKLPPGARSSTSGKPPPI
VGGPPRVHEPSPRRASRDVEPHGGIASFSAYSPQTFDASPTLIINQMHSPTSFTALKVAR
ADRAPDDDATAAITIGTTVTAASAPADVSKVAVVNPAARTSPSSRVQDSDGVKIPPVPSP
QHRDTLPTTEHAPDAINPSNFATESPTEHSVSFLEKPQETESALLLQSGVDFLEVDPQED
LEARRFETHSSRKSETNSVDNMASAASSPTGGSWSQREVHSAISTTDRRIVACSGNLSVA
TQHDAGSVEFRSVEVQTDRESPVKEIVCDISLGNTQGALQEPTEAAPRQDEDEEEGGAKN
VLGLSSIHLPHTVSNLTVSSTRPALEKYGMGTRCEKRNEGIHSLEAEEEGVSGKDSAAEP
IADDFNEALNGKSQESAWSVPNQEEEGKGPKNDFSSNDAFKILTHHSGSTKATEMRLEQP

LTVTKDVAFRAGRIVEKWLDVKAKWQGLSISHLLKRESSAVIQEFQHFLALYKELKQRHA
DYLAYLDSPYGHTVRGDLVLRISLLAASNMEGDGGGEIKPPWRPAELRGESAETPSEKAN
DDFKNNNTCDANSKSCAAALATELLKLSVREVSAKLGCGVLHIFRVGGTCPALYCTLMKP
YADGPGEITLMGESFPVEVLQTTILEDLCEAREESRRDWVQLSTLWKEAISGVLQHGVFT
KHMQKVRQLFFELAFLLHCPGEIRLSVRYQRHMDPFPPNSAECVAQTEKTEGDFNLTGTS
NRSPSTTSSTERKAALSNSCKEFLPHLQELQDTYYLYFELLEPFLLHSMQHLDTVSTLTD
AMKCEELVDFLKVSHEAQMLEQRAEETGTPLPLAVVEEERAAFLKLVKARKAGEIMPLPQ
EWWEADNLSSKNKFP*
>TCRU_2963 hypothetical protein, conserved
MSDTADLVEEMPAGRDGELQQPPMEERQPVRRIVPEIFFETQNYYQPQPQPQPQTISRRT
NGGRMRLDGKPPEAPQIQTPVQPAAAPRLLQLSRRGRGPVLERMKIRAASEASRENGSGG
KAAVQQKRFSRGGTATRRSPHGDGRYGAYGKLLRDAARELAMRSPSGVRSADGGSPASSA
RKKERGRSKTASCGRVSPEATPTIHSFSVSARGSTSRERRQQNVSATKDSPGDIGEAVAT
ASRNGGRQSSSSVSALRRVVHQPGPIRPENGCVLPLPCQMARRKVGHGRLIVFDDLLWKR
EKEHRWPLPNGETKVIAPSRVPQLTTEAPALPAVLEVALADHYLAVLMTCPQSEVERVVA
KLSPNGFSEVVQYLRQTYSAYFCPNTRNYTSVQPQPTQSAQRAEWLIKHRRHVQGRTRSI
ASGNAKKSNANGTYNPISKEVRQFLKLFTREVCMTFMDEGFAACTMRRERARAEQPQEVQ
ARGVPLPADVLDSLYSQVVTQLLFQILGWTEKEASRVLQTVVRQIKRCFPPSPNLSPQM*
>TCRU_2964 phospholipid-translocating ATPase, putative
MNEAVEMVRVYNNGNRDGSDDESSLCLPIPPMKPFSYVRLVRGWLNMDVAEANEPRTIAI
GCDPAVWSGRSYPKNAVNNRRYSLLSFLPLALFYHFRSVFNVFYLLLSLSQLIPALKVGF
IVTYFSPLVFVLLLSLIKDAVDDIQRYRRDQIANKEKVEKLFPDGSTGVVLASSIQVGDL
LVLRSGQRIPADCVLLRTSEKSGTCFVRTDQLDGETDWKLRYALKLTQTLNNETLCRFRA
NIRCEPLHKDIYKFVGMLDVAGLDAEAICLENTLWKSCVVASGTLIAAVLYTGVDTRSAM
NSSKPSTKTGLIESELNFIGALCFLFLVVVSLFLVFLQQFEGDWIVMFLRFLILLSAIIP
ISMRVNVDVGRMWYSYEMFRDSKIDGTVVRNTNIPEELGRLQYLFTDKTGTLTKNKMEFR
VIQVGVDTILNYNEVDRFRRIMEAFFVERHETGGLNAMDVGKGGGLLSQRTFPRDVTSVG
EALLALVLCHNVSPVMEDGRLEYQAASPDEVAFVKFCSSIGMTLTHRDVNCVRFTTPAGR
TVHYDIIKAFPFTSERKCMGIIVRERRGVGEGGYKYLMKGADTKMASVLRLTDWLYESCQ
ELSQVGLRTLVFAQRTLTEELFETFLAKYNEANADLGDTRNSSLDAAMRIIERDMKLVCV
TGVEDELQDDVTTSLETLGMGGIKVWILTGDKVETATTIGHSTRLIPRNGVVEVMASSTE
EDAMRFLEKLAARYALLSRGACVSRPWTLILDGTSLSFCLSESVSRIFVEVSQTAYSVIV
ARCSPTQKAAVIHTMRKYCDKRVRMAAIGDGGNDVSMILAADVGIGVEGLEGKQASMAAD
FSITKFAHCVRLIMWHGRRSYCRTCRMSQFIMHRGMVYSVVQAFFSLLFAGTTMSIFNGY
LLMGYTTIFTMAPVFALVLDEEIREKDVSEFPQHYKELLKARSMNTRSFLQWVWISIFQG
GTMMFLSLELFSEELFQLVAVAYTSLLLTELIIVASTAHLRILWEQRRRHLYLFIAAECF
SIASYFLAVVVLPETIDRNFFFSSGCWWRVTVITLAIIIPIYTIWLVSKYLLFNQKLAYS
LL*
>TCRU_2965 BNR repeat-like domain/Concanavalin A-like lectin/
glucanases superfamily/Trans-sialidase of Trypanosoma hydrophobic C-
terminal, putative
MMMTACDDGRRRVYESGDKGGSWTEALGTLSRVWGNKHKGNEKGVGSGFSTATIDNRDVM
LITLPVYSNRRGNGKGELHLWLTDNTHIADIGPVSGNDDVTASSLLYKSAGSGDNNNEKL
VALYEKKGDGKPSLGMVSVRLTAQLKRVKEVLATWKKVDDRASKLCPSTAAKNPSTGNAC
SAVKTTDGLVGFLSKNFSGNTWRDEYLGVNATVKKGTNGVATGYPDGVTFQGAWAEWPVG
RQGQNQLYHFANHNFTLVATVSIHGEPKGDTPIPLMGATMNDNKKTVLLGLSHDKEGKWQ
LLCGGKKTKELRSNWEPETTHQVAIVLRNGEQGSAYVDGQRVGDASCELKNADSKGISHF
YIGGDGGSAGRKEGVPVTATNVLLYNRPLDDNEIRVLNANKISIPKLTDLRTVAAGATGV
GTARHFGANGDGSAVCGGGLLPLFLLLGLWGIAAS*
>TCRU_2966 Asparaginase, putative
MYLWDHNSLTLEDSADPSLEVGPDTEGEEVLLINARPYTLTGVSAEKGGLSQGFIDIKKN
KDLQKAGVPPFRVMELDVLRHSADQNHDDWVSLAALIRENYHKYRGFVINNGKDNMVATA
TALSFMLENLGRPVIFTGSCIPPERLYTDLYRNLIVALLFASCSQINEVCILFDERLFRA
NRTIQISRSAISPFDSPHFPPLATMHGKMSIHRTFLRPYPRGQFRVMDNMRDVVLCLKLG

PSMRRRVLLRSVELTEARAIVIIAYGGGNAPTRGGYMKEIVRKAVERDIAVCVCTQNLYG
SVDLGTYAAGDQLLEVGAVSSRDMTIEATIMKLKYLIGVGLSTKEIKRFFGDVSLRGELT
PPASQL*
>TCRU_2967 hypothetical protein
MSVNEASQPNLKNKETKAVSDILLACSVCGTSNVLPASSANVLKGTNMTMFCKTCGGATI
QRVVVSYFLGDHSKDSVDALAAPGKSSADSYSQASETDMDESIWRRLQDALVNSGVSMAE
FFRCDTSTRARLISAVGFNSAEIPRLLEFADANATRSMLSNVMERMQNPFETTAAATLRI
GAVAGEQAINPGPMKEVKNRTSAEFFRNSAQDERVFVNTPHHSAQIDGSMLSLKIKEDGT
LIARCSVYPHLPAEFWCSLCGVLVSSRCHVSGIHKDHPFITLRQAAEAHVRDLSTWSERC
RNQLNVANTIISNLQHGRDVLNDSAKHEEEALDRHFEEIVQGLTQWCQGLKSSMRLQIDA
QLENINVAIKRTNKLIEYYAERLKRCDPLLQSIPPMHQEDKKSEDWSLRVMDLVSQLKTA
SQEPIPMPRITVPEVKSFATVTTYMELLKAVSVPAGIRIPDVLDTGYLNFPTPSVLGRET
FTLDVPKDAKTRGILIYSGRTLTRSQDITPTHSLVCASQIFYTGITAWEVHVDRLGAGPG
RILAGVLMAGTDGEGVVWDGQRIVGPNEGECRALSERYRIKTGTTLRFVLELEAPSYFLV
CYYEDEVVARVPLPPALNGWIPAFSVFGPQDQLTVVPISAAVAENLLTDAKPEEKKKREE
EMKVRLGEQEQRLNALHQQLLAVNSRIDQDQYEKLNHASHARQEIRGDGSNDKGDADPSI
LSSPFFWRVSPQVPGDGYQSANNKHRLDSMHGAGAVAATGELTSPSPLLAPNVDSGQRNE
KNKRNASYSPELQNLLNFVDSIK*
>TCRU_2968 UDP-Gal or UDP-GlcNAc-dependent glycosyltransferase,
putative
MAKPKLRRKLKRVALLLILFIAATTVLFFRLATTDDDDAPEPNNPRHSRAAAADDAALAY
IPRSAVEKWGRQEYLVVLGIPSPDDEARRTRRNLQRTTCWRFPGVATRANGFTGAMLVLY
VLGRHPSHGYDYSAALLEEAAQWHDVVALPMKEGRVTTKKKIGGSGSWGDEAEIGVTRKT
YMWFDLALRLFPTAGYIAKGDDDIFLRVPLFVAHLRLLPRRGIYMGGHVGLAIFVKRGLP
GSTFMVGWCYTLSRDVAEALVSYKPLRRLAYLPYSKEREEEFALLRFHCEDIMVGWVLDR
VIKYQPMIYVKVLPCHFHDARNGTGHSQVVPTSMCIHHVREDDYAALMARFGNDTSPAAR
VSWVSEDVIYPVCD*
>TCRU_2969 hypothetical protein, conserved
MNADDGSSIRSATSHHYAQASHRAADAPPYISNCPTQSSGRATELARQRRSTVVRRITDI
TALGQLHEESEGVSSRDVGAANVGARNVPSGACQLAWTTASLWSEVAALTPNNFTMGPDG
TVISDWSVARKTARAHLHRASRFVSIRGQEAFVIIKLCRTLQHNEELTNLATAQVERALA
PWNATARSIKRGASRHAAPIVETYKFYPHVISLLVRRVDFFDLPQNAFRYFGSYTTMPTQ
VSSLAALM*
>TCRU_2970 Alpha/beta hydrolase family, putative
MNIRVSGNCRGRTVVFLSGWPDTSEVFRDNIMSALAPRYRLVGITLPGFDRMSPQVQRVK
RSRKKEEAAEKGSLSSKMDFLRIPRMGYSFEDLVDFFEIALLAVMEHRPLERPLLVVHDW
GCIIAHEFLLSRPYFFSRVVMLDVGGDISDPLLTKGGECDDGVGGGIWAHWWRCVHIFIR
HAIVIAAFLIIPTSVGKGILRFILQIARWPSYKYTRHLSALGNDTDGEEEVLMVPICAQK
STVDVSRKPTRFLDVFNALLRFTVFGGDSEQKQDVSTLAASFYPSQFYYAKSRRLYTLPP
HPTASGIHYTHYRYRSARTQKTNGSASKNTSNSITTQNGVHPPTAPSHDDEASLNFVEVD
PLNGWIYLRYWIFWISRLLPSSRRFFIPISVPVLFLYGTEKPIMLHSHEWIKYIEAKGET
DGSEVVSVAGGHWFFAEDRNKGQVTKRIAEFLDADKFEAQISQSWKGHLYGGERDSYTSY
VSE*
>TCRU_2971 hypothetical protein, conserved
MEVPRLLRFSNLPPLSSAAAGPRDRRVGSDAVSAPLSPPPLPHAASVIGTEANRSTVATQ
TEESAHAETIHRAYSPQQRSIGTWTAPVKSTRATSPIPTVLVDAANNTEMSGAQWEGQSH
ELLQKTTLLQEQLCRNNFLETRQISMELAFTSAQERIKQGEERHKAAISYLTAARDEHRL
ELERLELTCRQSLFDHEAELRSMLHTLLGSWRTATLSTEQTRNGHERFLRAARGYSQFED
NTFHQLILDEVRHREEVKDSEWQSLLSLRQLRELAENCQRESSRTTAAINLAEEEKRAAI
MAKKAVEEKLRDVLEREKELRLQYLHVLRLPTTTIVSCSHQSNAATSVEEDIPVHARFVR
AHKEAVDAVRAQRRHTIGQIHIF*
>TCRU_2972 Haemolysin-III related, putative
MHELLTVQQVDPWLQFNPHIVSGYRPRMSVRAALRSFFEWHNESFNVWSHALAACFVLYL
ALFPPTMEISTTSTVSGCGVGTSGIAATDVSLTPHDRRWLGSSSEHLLGASSTTTFRCTC

LVFFFIFICSVVYHLFMPCTTSESLYRRLLSCDVFGVVICITGTSWSLVYRGNACSQNWS
CHVGMGALLLSALFVLYGAVFNASCGSFGRFKAIGFHSVLRFLILLWVELPKVQSQGYHQ
AVHCHAMSFVFLALGAVINALRFPEKHLRRATRLLPTSSSSPEATVTSRCWRWLGRFVVS
GEEIDYTWNSHGFWHYCIILSTTMMLLGCYYDLEEFELAKC*
>TCRU_2973 hypothetical protein, conserved
MTMAVEELPPLRSTPAIDRLRDAKRRFALSESQFASSRLGDRWSASPRMTAQESSLKQPC
TSSSSSAANAATNGSGGQREGTRHATLPFSGLSSIHRHVVGDSDTNSLLTWTLFDSNCRN
TPRGAAPIMTGVDLTPTVASSFGVPGSEEAGSTAPQVVASSGDGADSGRVFGSDGPAAMP
TASCKKQKHYLSDVPTHDIYRQRYNSQRVEMSRREDDYRRRRDANRAEVARLISHTTRLG
CMMNLQRQIYNEIMKRPLRTLATTEPVMNTAAPLPSKRSSVSPLFLSVRREASGPTKVES
GVQVALKLLKSRFASGCYLSTNMYRKPLFSEPAAAPTLETPLEAAGELPSAAGATSTEKV
AGSGGENEKSHNDDNTKRECRGVNEEVVHASSSPHEHEEAVTFHSSLLINPATAPTMIYE
SCIHHASGRKEEENPTSANQRRGSSETLLTEEEQRGLRLLSTVRGLRRQTEELTTAVLHA
AGEWGCWDSPGASVFRGLFLSPRTATCIVEEEKAAELRLMHMAALEEAARGEVHPVTAAL
RQQCPFRRSLPPPPSVLVQMEMFNLPGMRQQIRRCQEQRQHRQLQRQEEANAKDGNSREA
IWRPFVQHVLLPEAAEHFSKTEEEAEERGKQRLQQSPVCVEQDADPVDAPVDVGPPSQLA
TNLTVIKMGEQHDHQGSFASLKPRHVHPFISSSSSSSSSYEFSFSSSYLSSQSSTEDGRQ
TASAYSYASSALLSTNMKFSVAALSEGEESASEAQRGIIKEKKENEEEREMSKKENIPYN
SQREVLPLDRRGLAMTKSSDNPTSTSLGPRVAGELSGTAVVPIFRAEAACEQSHRGNKKR
KSLAKRLLSFFGACSCGGSRGATVSGRKRRSAAS*
>TCRU_2974 RNA-binding protein, putative
MDLFFGNIVGDGCGNNYIHNTNTENFSSISLGKTNEFFDALGVIGDERECAEEGFIGGEI
KAEATHSPPLNSCATSFLHSITSNVADPRLPSGVRGGSAYDEGAWMTDVVSSTLPMTTLD
ARTSVESSFAAKNSEAEASVTANLPTVITPKSTPTNVAINSQQTSATGDTMRLTVRNNVY
VSELPAHWNTDKLRSVCSAFGTIISAKVVHDGATNKSREYGFVMFETEEQAALCVESLNN
CSMAGRVLACRLAHEKAMPAFAHAESISWSPCTTTNATPSTGFITPQEGPIESGRHFNPE
FFEASYTPPQNGQPNSTIDQNAYSGLRKSRNVFIQGLPLHWNTDKLRGLCGTCGRVLRAK
VVRDATTSLPCGHGFVLFETEEQAAACVATLNGFVAEGRTLTCRLTREKRSSLQFSMSNA
DGLTTTPGMPPLSPSGRTSVNTESLVTPSTNVLAPIGIVEAVATPQNAAFMSVPNYGKWP
PVGMVPNCAGGALPQTLQHAGLTSSPSFLGHATLATPQSYATVATAGSIPGFAAQPTEYG
IVPGMEGQFASLSNVSNAYIITLPYSPAGMGQVTGSNSSLATVALAATALPLPMGTAGNS
TGGASVVPSELVGGYIGRP*
>TCRU_2975 hypothetical protein, conserved
MSGTAGDAEITPSCAEVEEEGWLLLLAAFIGCFVDNREGREEDSLGACGAVGGDTEPSGD
MTGTAGRYVLDGVRRALRYLVHDSDALRAKRELEHVVDAVSEGFWRQLRSTRRPCRRGDD
AKNDLGGDDTTRDEEKMLQLLYRLAGVLPHSTTTLNVVLPAELLYVVYYLAYTQCGPQAW
QVETTDSRWVQLFFGPSLCACSTMMKSSMTNDFAASTALFSLHAHGMEPGGRMPQEENMR
LHMTRFFSRCTAHGDTKNVQLFVDAYHAWLMRDYHGVLEHTEKLFQLLPFGEENQSRLLW
VMLQSSHFIYVFSSIAITNTSHPAAREELKGRLQRVRVESNSDSYERYVSSVWGECTSLH
TCPSLLCAAYNLVYCEISHSGGNHHGEEEGDGCLHDEQQPQPSTSLVCWQEMFLLTLRVW
LRAKRLTEEGCMTESRDLLLHTLERLYSHSCRTRNNNSRSVITVFSPPLTAGANAIDPGG
AASGGGGNDATKEDASISAGKGETHVVILLMSSMWLRNLLLVNCAALSDTRTPLHLMDHL
RQPLTAAELLYLAACPTIAAGGVHSPIFALFPIDGAGEMKPATGVNESLERMPLPVRVPL
LSHRLWKLRQQLLQELPRSPDENCECEDHRVSLQREMIHLCESLAMSARGLFGSLDLSFT
DCAPHRIVFDAIMMACDVQEDILRSAALSLANASTFSPPTSPCSSSSGSGSDKERTGGIM
SETSNIVDRETNTDTMLTSYIMCTSAGDSPSFISSVTRVHDKSRSSIDDNNENNNINDDN
GDDDSDSWIMKNRAALLISPSELSQASSFVACTAVAAQELYWFSYRKSHEWMEILQHFFP
QLPLVVQVCQLRVAAAAFDRGIARLARNLLAAFHENALVASHVALALFASLHVVPAERCI
HEALQKHPHSAEMRRLYRVICGHNNISRQELQGNGDEQEAKGWVRKHLIRPDFTHRYRGV
LPVKYVACEQRGYKAVYVPLVMSLVCIGVLVLCFVLHLRDVDLTTERTILKDDAVFRWSF
ALPSAFLTYLGFGVLLYGFAAAALVPFLTSRRVGSNNRVQHVAMFLHALMEWNCLEHDTP
NQLVFCLRGLPLVNLLTGVQLVVSNALWLKTHTGKPCISNEARIIPDECNRVFTGWGAVQ
LLVIVVLLAIAVLFYGRAWRVLRYRDVLVGPPATFITIFFIDVAALLVFFPVIFLCCVLL
EPLLFFITVVFSVAFGSPLLTEHLPTVPTDMVLASTVLPRIPTATTRAVGRWGGLTALFL

EQVTLQIRERLRASMPPLHWFPSRHQDCSILCFHEVLQPLSNDGKDASNSTGGFILQFLR
LAASRGIAVCAFLQDIINWRIHRIPQDSEAAVQLALLRESFTAVVVPAPANVTSTLNDLW
GVNTKVELDTCATRFATPPRWKVSSTTPYG*
>TCRU_2976 hypothetical protein, conserved
MPLDVVWTRLLARGAFFLFLTLLLATSGGAAHSLAVYPIVEDPLVNYSVVASQRCEDAGM
RLAAPDSLEKAKHMESLLSRCSSSTGSVLFGAQVVECDATISLFLFKDVFSEPSSPVAGD
AELCDSLAGAAGRKNVPGMFGGTLGLPFYHVDFKDCKHPNCIRPVLLDYDPAQRYGFEKP
QSNEMNLLVWKLKKTEDGKTLFSWEGVCSKGTAGCPFKGVQKPFIPNQVICESDGPMTDL
AAICFESYVDTTEAKKPVDGNLWIIVAAVGSGVLILALLVIIFSTNSWRQRRDMKRREES
EDISAPYKSKRENGSSLKLQHQGSLAGEGMQRQGSFAGGEMPRQGSFAGGGMQRQGSFAG
GGMQRQGSFAGGGMPRQGSFAGGGMQRQGSFAGGGMQRQGSFAGGEMPRQGSFAGGGMQR
QGSFAGGGMPRPGGFPGGGMQHSKSFSGVGMPRPGGFPVVGMPRPGGFPVVGMPRPGGFA
GGGMPRQGSFAGGGISRQGSFAGGGMPRQGSFAGGGISRQGSFAGGGMPRPGGFPGGGMQ
HQGSLAGGGMQHQGSLAGGGMQRQGSLAGGGMQHQGSLAGGGLSRQGSFSDAEEIRGETK
DDVKLKRRVSFTGTYVLL*
>TCRU_2977 hypothetical protein, conserved
MMESPLSRGGYVSPNRVGTGCNSSLAPGGRSVSPGTNSLTLPAVNQKVAVEGWCRGGRLR
HHKVNPSLFQEGETSLLFVDDGKGGKRESVTRPLEEPLVDAKWQCGMKNPVERKKRESTT
SCRGMLALGNNGGLSRQQLLTLTEASKGGTTVPLTDSSSQCTETSRDGGQQRTRRVAKNG
LEYDPKTYGELLPATLRMHKAGCMQAGIVSSAVVNELLAERDAQQANRGALAMNGEGFYT
VSLQELQGFLGEMNQFQERGKHMRRRWRRHLLCVEAPSSAAIRVVNEDDDTSSESLQEPD
SDDKLKLTLSKRQQQTSEATRAAISTWREEINALQLRGGNPRGAVLLRNEIRKSQRAVEK
ASQGVTLSKILMIQNETNVKTPKEEEQREPLRGQSCLSAEEISWRTENTMSQRTVSPTPP
GLGAAGVVERTNDGGSYHGDKRKGSDSDAVHRRLDTQLVSLLNRGKGTTVYGLDITRRNR
SCGAQQHVPPANSLPSLQPASGKSSPMDFSVTWTGDAKKNPELKGRHSHYSIQRPSRPLP
VEYGVGEQSSLRLKALITQWRLEDCQMEVRNQEDIRRTRELLQELIPKRAALLDESLKCS
RENARVILQKHRQHIFKRVAEKDSEERKQACFGYVDLLEENYSGVVAEGSLAAIRKALQK
TRDLIKVSPTRCNARSFQSILKENFLIAHLVEVPVRELLWRLAELFGMTRSQYKSAMSNA
YQLLNTPRDYQARFREIERTIVCISTPAERRIRLTLHRCRFFLANNTVGTSHVVSAIGGE
RSRELSEIYVQIKSENQVFISTAVRARISNDDDTFSWDVSQRDGKYHSLCGANFMDQAFR
FNFGENAELVVALLHNGLVIASGKFCLSDCTFNPRGIALLWLRLSGEGGAEAEIKLTLAM
LAEKNQQKKTLRN*
>TCRU_2978 hypothetical protein, conserved
MRGGRRTAAIAASTFFLAPRRRRFPQLTTLVQQPRYCSSESSAKKIFHYRPHQLSGQSER
NVELNLGASTKKLSSVHITLDSLAPPSTLTADTAVEYLAALRGLHRSLGIMASASRVRWQ
AREFLIRGVPKPNDNETQALWTMTEELACAITVSCAHFEISILECPYGNESVEFLQHLWQ
ERQSSSSPLSSICSCEEAVLLGRLTMAVLDLLVNRVLYLDADGYELLSQLLCRTLLPAVM
CVLQDDVLFTPPKGEEGEDKDLTNTQAAALTVLLSSLTKAAVAQWARRSSDSEDGENVAL
FGTPTSLPPSVESGVRIPRAIKVPIVPLSLAKLARIVQHYATGLTPLRVRETALPLLRFF
TLTQCVPLFVEPHDECGPHGTGEGSSGGCEQGTNMASLVASVTRVLQASMDNRTFFVSHQ
VSNITSTYARILSFSPIQSKKEVGNDGHAVVASTSWCLFEILMMGLKSNILYHVEPLSPT
VVMPQLCECVTAFSKKYLFSDLAALLNALAKIFHVLCEQHNNLWIQQQQQQLQQKQDQPV
KVNGTRSRFERRRTTASVVRSSMLPKDEGGQSSLTSPALPLTGVFFVSNDDISEIEGELE
VLLDACVAVMERHMLSFWNTRQALVPRSGIHSSTSGASASDKSLKVLKEERGIAFSGVGV
SLAKALGGRSRRLKKSVSSRELVMIAEGVHALSWRPFVAFQERLLALLSSSVGQSETTPS
QYACLVRFVLRAPPVLPSVIFHSVGDRLKRVIHAEAVTTAVSKRNGNEDGKEESTNMASS
AEEVPALVIAAAHARSLQDTPLSLYDACAVVFAFAQKMKKSALPLLHDFFVRTGKRVFSD
CETSPEPCQDAATCAALMMFVASATWVLCQSAPILPGDHGLTFIAASRDYAAEILAASTP
EEKLNAAKSFFSLSDLAYACMMLEPSCDSQGSAQLTQVRCFLQETYKAHTSQCDSMEVTQ
FITDYLAIPPLDISYGDAVKMKQKEEENEKKSKEFLQLSPWMISSLRLRDRLEGNTRLST
VMFFPLLGGGRDDISRVLNVTRMYCRTFATLVTTLRLLAAADVELLKDDAVLEVVFRKVS
QLTRRTARPLDLFQFFELYGDYPQMASACLSELLPEARCFEAKNAFVPIVQVNAAVRAVR
RMRKRELRQRWFATMTPLIVSAVTFSREPLDVVLELTEHIGNDDPELLQSVLKSSASMQM
IHETFRFTVSGDVLLRLLQQLQNASTSHPTVRGLHAASRELLQKTNAHIPLKQLAQAMLL

PSVEGTTLGRRLKKLFVFRVTVLLGITRPRASRPTGQSAVKSEEDPPTTTLRCSHWDESL
DCLELEHWMLLLRFSHVAEPSSYRLLMTIKHDMDVAARRMGKLALSSTPAKGEKAAAPEA
AAGSSHEGAVLLLEGKEEDPGYEKGVEEWSDKRHRSLAFFYAFSDHTILCLRRSLGAFQF
ITLVCELLHQASIFSPDLAAFSRESNSHERGCMYDGSASPLSDERVWPFLRLAIGLVEEM
SADLGGFLRREREQGRRLNHGEPRTAQNERNEELEEISVDIGAILKFLNVLDTYDPRRRE
RRLLLLSSASPSRLSLSHDTPNVFDDVMQIPHRYLYRLLLQLPQHWGKVTPAASFVDTPR
VRLQNISSVFDYCSAPTEFHSPFGTPDASVKAHFCLLSIFGADRPLISDVWMPIAALQSQ
QRLIDRSVKAQLFGALMRCLVRDGESLAATRPRLAELGPRDLARVAQTFLGGGGGGAVVA
AGNDDHYREEYDKWRSGRRAEAVIALELLTDMLPSASAEDLHDIVMMLLPPPSAEPKWEG
DKRQTPHALLYEEVAEVRRFLVHVPVVMGNDTERFPLPVLYGILLRLHEPHRLIAPAAAQ
MMLTNLHAVDETTKGQELPEHEGEEEDVGEQVLEDAVSGPRKKQHYKATVALLRRVVSSS
SSFSL*
>TCRU_2979 hypothetical protein
MSTAPGGAVDDEIQSRLSKLSVYKRAEYHCVEGIAALRESLRDVVRLEGEESGTNDHGAS
KAVQNSSGSTSGGGGRAELAKARQAARRAHQQLDKLAKEAQRAARREDGGCDADMKELMR
HVESAKRWYRECFRVVPPIEGGYERQGDTGGAPGLDDDLHARQCTQHGATSMTTPLLAAN
FNDVNSNIHNGRDGVTSSRNASVTAPQPTVSDEEFLLFLDQVRRNDELADVALDRISHGL
SRLHENALNVTSELRTQEGLLVAVEEKVETTHANLSVLNKRLRKFLAEKNKCDCFMYAFC
CALLLGVVAAIAVLLKK*
>TCRU_2980 lipin, putative
MISSISGFLSLNHLDMTSGANDVIVVRHSNGRLFSTPFNVRFGKVKVLRPSDKVVQVEVN
GKPTSAVMKMGSDGEAFWLKPTPWSEYVSGRPESPLATATAYMNGGFSSHGNSGSRSPNR
NVENLQPTEPVSESMNSPTINAKAENSVAKEPLLEEDHEGHLKVNAVARQSGVDITEVQL
AEETVVAAEMAMQRIAQMTIEYDDYMRETVLLEKDEGILSTDMDKLECRPGALENALLSV
DDDTNAPPAILANTFRRSDRVYMETAGKDGDESGSVSVEIHPEKKECGHETPTARVAKES
ENTDKEVLTTPKKRRAMGSQKSLAASDEGLDTLDDYLFEDPINPEEYGGEFGGYNTGGTP
ASVPVVQEAGSEFPNLPLKTSTPDEKQCEEPSLSREKLEEVVYFTRTLIPTEADLRKLNL
VEGHNQVRYITHSSLRGEVAVDANVYLWDSTDRLVVSDVDGTITKSDLWGHLMPLIGRDW
THPGICSLYSKIDRNGYKFVYLTARSVSQVSMTRNFLWNIEQDGFRLPKGPVLTAPQRFF
TALTQEVSKKSHVFKIACLKSVLDTFPAQSKPFYAGFGNRFNDVVSYTATQIPQHKIFII
DPNSVLHVYNVRQTYKNLAHLVDVTFPPIVKRIHHHHHHLHHTPRETSDNLQSPDTLADA
EDGSENFTAALVGTRRIGSPGPAFLDVRDSQLLYDDDGSRLTSSPFDMRPLPEDNVGRVH
ATASTTPLENHADLNIDQEFHSFLYWRVQPADLVAEKKETKENENVSKGPKQHGNRKGSR
WLTFSLGTSSNHNNSDGLEGKSEDELNTRHVVGASESTGAQHSAPKAVAAAEDGVPKTGG
TTQDSSRLPEGNSTQERSENDNV*
>TCRU_2981 exosome-associated protein 3
MDFDDDVLPLMREMDAKLIRMQSELLPLLASLNEDVLVTKYSVDEQARVSLATAFILVLL
TYAHDRMQHVGGKKDMDARVQLKLERVSGYIKKLREITQLDNQLQGQKAVTAAATGGGKG
ARKHRREEADGGDVERRELTDANAADDPYGDAILFTEIGRGAGRPLSSLVQSLLRHVTDS
SAS*
>TCRU_2982 BNR repeat-like domain/Protein of unknown function (DUF3676)/Trans-sialidase of Trypanosoma hydrophobic C-terminal, putative
MMAHCAGGYYDVYRSTEDGVSWYTPGEPITRVWGNSHNRKGHGVQSGSTTAIIEGKEVML
ITAPVYSKENDKENKQKGRLHLWVTDKARVYDVGPVSREEDDAAASSLLIKDKNKELISL
YENKRSDGAYNLVAVRLTEKLERIKEVVKTWNDLDGALQSCSSVSSATVDPRKKGMCNSP
VPTERLVGFLSGNSTRTEWRDEYLGVNAKVKNGEKRREVPNGLTFKGSGAGAVWPVGDTG
QTVPYYFANNEFTLVATVSIHEVPQDGSPVPLIGVRMNDTSSTVLFGLSYTHEKKWLAIP
ENSGDPEDVYVWEPNETYQVVLRMDDDDEWTVFVNRYEIRQTRYDESLFNSHRISHFYIG
GDSKNQSATGGHVTVTNVMLYNEKLLSDNLYDLHASKVTIPSLSVEEQPTELVANTGTLV
ASESKSVESATSHEKLNENDPDEQGENSADDPLPAASSSTVVAGSSVSEPAIAAEIAENS
LPENNAQLSEGEESQQSTLHEVKESMQQDSDVQTKDLQSEESTEVTDVEKPSESNEEEMP
EEEGEADDGSGGLTSPVTASLNMETVTGPAYGEHQVQKSIELPAENDEVRSTGTGTTDAK
QSLSLEAGGSNSERAMNSDSSSTPSKSNAEPTSAENTDDVSRTEGAEFSSEDGGEMPRTV

DTAPANTSTTPGDEAIPSTKGAARHSDNDIFNTSEFADLLSTALNHDSTVHGCVSRVLLL
LLLGLWGSAALC*
>TCRU_2983 minichromosome maintenance (MCM) complex subunit, putative
MSISGIPQENVKWLWQMYCASLAPLSFEEEVVVEMWRLFAAFLAPLSLQPSATASNQASS
AIATFTFTVDLRSDGLTAEMRTALVNQPQIVVPLLEFFRAVLDYQQNSEEHRRGALGSKR
LVCSVIGDDACATSFDALGASLLGQLVVIRGTVVRMSPSRVVCVKMAFRCGLCGAIVEVS
TEDGVLIYPGSCRGRCRGYKWSPLVEQGKCEEVQVLKIQEQSGVYDGSTGGGNIHKMMTV
ELRGIWIDAATVGESVILCGVLFARRSEGRADATQQIAVRAYSIEGSRHDSERSVLLSMN
SLEESAQFYEMARHPQWFARLCASLAPSIFGLQHVKEAIVLAVVGGTAHTMRTRSNIHLL
MVGDPGLGKSQLLRSACTIAARSAFVCAHTSSSCGLTLTLTRDPTSGETAFEAGAVVHGD
GGITCIDEIDKGVTEHRALLEVMEQETVSMAKAGMVFSVPVHTAILAAGNPAGGQFDESK
AVTANLNLSPALLSRFDIVVCLRDPHTECTRSLSEHVMRLHRRHDEVGARLAGCKSGGIT
LPVELVQRFVAFCRHHCHPSLREDASEVLKSHYLKSRAETKDDELAVTPRFLQALIRVAE
ARAKVELRHEVTAEDAEYAVELLRQCSGSLRGNPHTLSVRPAAAKKMSQREMIINALKAE
VCRTGVNALPYPVVLSACEEAGCRNANAMLHQLNEVGVILQIGDKYQLRGV*
>TCRU_2984 Trans-sialidase of Trypanosoma hydrophobic C-terminal, putative
MGTPATADANAPTAKGVGHDGPAVDQEMNASIREDEEAAGGINGQEDYHAQHGEVKAAAL
SSSLGNVSQGNNTDAGTVRGSGLLPLLLLLLGLWVFAAL*
>TCRU_2985 hypothetical protein, conserved
MTQAAGRTARRLSLSMRLGLLRCHLSPRTAVLPVGPHRAAAGSLACACRAECRATEHGHR
QFSAPRLAAASRSPTRVPVATDSLSAMEALRLGSLAVRDDVTEEICTVLLSLVDRGHAVG
TTFPHDTAESHAT*
>TCRU_2986 expression site-associated gene 3 (ESAG3)-like protein
MNQTFKGAFFLLQFFLVLALLIAASNWEKDSSLILSETLAKDDVQFVKAESESERPRVVF
LVIQTRKSPGWCRMMLSSLLSEVSVVSIGFGKRYNHAKRPLWILDYIDNEGLRDEDVVVA
FDGGDTFITGSFQVKQAVDRFLEATAPTAAVFNVTAVHRGEATAPLLFSSEGLCFAPLMK
ILISSGPRKHARKCVWYYSKVWEAMNASADQGMVPFTSAKGLFLNAGGMVGRVWAIREAL
KAYKKVLGLTDQWWCDQSIWSLLYVWSLTRTTNVSLDFRIPYGILNLDYHHNFFQCPYKG
LVAHPAVIHLPGGQRDWAPSMPELMNHTTWFSKLKSSLQMTRDGLTQLSNLFVKIFTCDG
ETRFRRFGALCSPRKVIDPDWLLSPLRK*
>TCRU_2987 retrotransposon hot spot protein, RHS3, putative
MVLASEKGWPYSWHTIQDLPKDVFVNCEVERAWQIVEGDLTAWFSPHGGTDFKPEQRALI
GTPGMGKSVAAGSYLLYQPLHCDVKKLQMVVYFIADRTFLFDKTSRAVTRYSDEDMCVSE
LRSLRGHGRNVYIIYDVAKEGTPPPRHFAPSTGWGMIAVSSPNVGNYGEWAKQLKAARII
VNCPDEMDVRAMCAWITRDGTAEKKAEYWRMVKERMDNVGPIPRHIFDANEFIAHSAAIE
DALDGIKSRDGERHFSHGGVRLWYSENASQKLVRVVRARGEVGAEGFLNAPISVCLGRRI
PHYFGRRDE*
>TCRU_2988 Mucin-like glycoprotein, putative
MMTCRLLCALLVLALCCCCPSVCVGRSTEETIVVREVDDVKEPTSSSTLSSESQVEDNSH
EERGSGTGSSEEGKTKVPVTPGSSGAVTQSNEAKMPVQTGEKKGTTEDENGTTAPKSQSK
GSTVEQGEADVGGVKSLTAPKRQSGDAIDTVHEEDGSTTGPQSRTTKSADQTDRETKDAA
ATTTTTTTTTEAPTTTTTAPTAMTTRAPSRLREIDGSLSSSAWVCAPLVLAASALAYTAV
G*
>TCRU_of Trypanosoma cruzi region 4/Dispersed gene family protein.1 Right handed beta helix region/Dispersed gene family protein.1
MWSVVGACPRSRHRVRHSAIAAMRVALLFLLALAAAAAWMPAVHAVVLRLRGGTVDRAIT
VGRAVDTVLMDGVYITNGVAVVFDVAAMLPGALRIELRNCVCDGGAQIYVRGYSGEPASD
RSLEVSVSGLSGSYCSLAFVHNLPAHTNVTVRDSTIVTAGPMRYSQLSGLTDAVASPLVL
HATSLLQTQLRVSNTVLRSLQAGGSAVHVGGGVDLLSSAVVLDGVLLEASGGPTAFAMRV
ASSSRLSLRSHSVFSVTNVSVVSSGGGIVLGERLTVFDSVLRFLRVEGSVASSLVRCGGG
TVGSGGWLDLHDVWAVGEASSVASLSGVTLSGGAVSIARCVATGATLVSGLTITSGVVSV
QCNRAGGRVLQSSGDYRMAGLPSVSVVPCDGCAAALACFDALTASFSDCVCGCRAGGVGE

ACLPFDVPPARFGDGGDGAQGCVSGVTLTESVTVGGVRATACFDSVVFSGPITVAVDLRS
MDAFADALNVTLRHCVLAGGAQLRIGGLSESTAYLMPHALVNMTNVTSLEGTIVLHGAMP
LHSSVLLANSTLRATVGGSQYVPMTPGHAGSRYGPALVLDGVRLLSTRVVMTRSSLVCGG
GLCAAILVERSFVVNLSSVFYMDNCVVMSRAHVMYALASDLRVSGGSVFSIQNSSWIASS
IEIYEGACVFRDVVLDGGSVLQVVSSNFRLGFAMLIAETLTVTGDSWLVHRDNEFRTAYV
VYVIKENGVAFRDRSVWSILYNKLTYGLYSSTHAHMTSNWSPPSDTLPIIYGVCNEAMGS
PVTDYGDDLNIWVPVTVLDCGACTVDAVCFAARTSSISGCECECAADGYGDTCVPAAVPD
GLGPLPLPDANDTEVRCVHGGSISSVDYPDPGLRGLCFVNVTFTAAIVLDLWSFDAPQQT
LNITLLQCVLMGLSIGGSGARVHVNVISSMMDSGALEFEGDFGTSSQILVAGSTLVTKSD
HAISFFGFNFGATSTLLLIDNRIEGNGYAVYFNDALAVDGSGIIVKGNTLRVTGGVDSSE
TAIYFDVVDVKNGGYIDVENNTMNAVNGVYLFGATTVGSSGLLRVADCTFVGKTEFSDFT
LLYLDGSVTLEGGAQWRVEGNNLSTDSLLTILYSWHKIQLSDKGTTVVLAHNRQAESSNV
FAKILPWSTIVTSPARFVVGCNLQDDEEVSYDGVFPGEVEVFGCGTCNDDAACYMPGTES
VDRGSCSCSCKDGWHCASCLPFEVPDTVVPPVAERAVDGDTSCVVNQTLTNLTLNMWETH
HCYVGVTFSGVGAALTFSFNSMPLHLPINITLTGCTFLDGAVLQFVGDVEVAESVGVLIR
VSRTVLRSSVVAFALALPQHCDIAVTEVDAVQSSAVQLPDTVNNKLSVVMLYEVLLTASL
LLVSNVNARASKRDAFGLYSIGTLTLVSGSSLHVRYCSFGGYTHLFYLDILSVSDRSVFA
LLNNTVFFGTSLLYQYRGFSVSDHSVLRVVGNSGSVSCAIYASNSWTVQESSWLDWRDND
VGVGAMLHDSESAFVSIDGSSVVTLTGCKMGSTGLSRPLLSQSDAGYQFVAGCLTVAGRE
VTTAAELELHCITNVTTVAVCAECTKDGDCFAPLTTAVIDCRCQCAAGGHGDVCVPAPVP
AGPPPLPPSPSPPPPPPTPVGECISDMVYPEVAHAVGGGLSWLCYRNVTFSGDGMSLTVL
VGAMTGDVASVTFDGCTWRDGAVLLLLGNAHAAVGSLNIVVTGNTFRDALLSPEGVFPPR
TNITISGNRFTVTRLIPRSGLGLRKPSCVAMNGLAISNDSAVVLSGNVFHSVTASSSAIY
VVESSLRVSWRSLFAVMGNTFHMAGGDGTLIHLEGSSESSSLSVLNNSAVVIRGNLVSRP
VKYFILFFWASRVESQSAVVFQGNDMQGSLVVFYSAFSSHIYYNSWLQLSGNLCRESPSE
AFAVFNPTVNLRDSTLTVSGNQFMSNNGMAKALWIFSGSTDPTNGAIVAACNSVNGEGEA
KYGIPSLYNATILTCIDPCTLAASCFPAYTTTVSSDGCACTCAEGGHGDACLPVAVPEPP
STDGADLCVRDVRVGLEVNVSFGTSVACYVGVTFAVDVVVDVESMSGSVRNVTLANCTLV
GGASLYVLGWRSDPPAGERAEVLISGLESRSGGGVVVANRFPPGSRVTVVDSVLIAENRV
AYRGAYDLGDLSACLVVHNVNLTGSVLTIARTQVKAVFRDAVGVLVVGGVALSSRGALYV
DGLLVQTALGLCVSVEGGVAASGGSVVAFVDSVFLLCKHAVSVRGAVSVSGSIVAFLRSD
FSSTEDYAVAFYSTVSLDGGSMLLAKGNVHDGVSREMLHAAGAVTAAGSTLSFVRNRALL
PRMLSVSLSLAAGAHLRVACNDAGGRVLSTAEEYAAAGFGDAGSIDVAGCDACDRDTHCY
APGTASASMRDGVCVCACGSGGYGEACVPVGAPALPPAVGTASRVFVREGVTVQSVFVVP
AGASEVTLRHVVLDGVSPVLYVPWMARDGVRIVVQNVSLRNGAVLYVMGGGALRGAGTAG
SDESGPVELSVCDVEALNGALVLTGRFAAGSVLTVADSLLVAARPTPLVYLPGSQSSPYA
PVLVLSGLRLVRSVLVVSGVALVTVMTGGRTVVVDGAVLELVGGGVALDAAVLGGEYALY
ASARVVASGGAVLRVSGSQVYAAHGLVFDSGVVASASAVVVNDNAGALTDGALLELRGSA
SFLSGSWLSVRGNSISGRLLSVPSYPRSADLVQSTLTLYGNAGSGPVVMDGTVALVGAGR
RFVVGCLTLNGQALQPMEYRSAGIIGEFRPVACSVCDADVRCFAAATMSMSASCGCRCAE
GGYGRDCLPVYLPHVDGCDRTPAMPLLSRTATLTETRSPTPTWTSKWTPSRTLWWSHVAC
PTLAVTTTAAGGSLTQNDIRGGGSAVPTRLMVALPPPFRWARDPQLGTHLSFVPVSTAQP
RGFGGPWGAMLRNATWVRNATNPSTVLELAVPLHRGYFIAADETIVIRCDAVAVFGGCKG
VLLGSFTIRSDMLPAAASALSAITGVVAGAAAVAVVVTGGLGSILEMQVLGVFARMSCTS
AQERASTAALPYFLSVFAALDPLWMVVGNALLAAVFGCVHCGVTVVFQRWRGVDAASAWA
AMRFPSLTYVVAHTLHLGIFFGSVLALAMPGARVQHRVIGVVGVLYGAAFPAGVCYFIAR
HTGASFTRYWQFSRKPLHERLLYPVGYWHPAAQQRMYGGMLTNMNGSHLYWCVFQLSVLC
VVGLIAAVHPPVGGCHVQYFCMAAVLLAGAGVVAFTNMMRSSFLTVMHTASFALLALLCL
VSASNHLAPSDGGARAYMAMVLLLTTVLLAVAVYSVVVWYAEDRHWQELREPRRGGLEAL
LRDDEESGDETQKPHEMTSSSYASGTTVASSYRPPAPPRPVAGDIRSDALSLFDRASSAS
CSVNYAPLDR*
>TCRU_2990 Leishmanolysin/Trans-sialidase of Trypanosoma hydrophobic C-terminal, putative
MDSCPIIDPAMDVRDLEAVFEKEEYLSARPGDKPSSWCLDTLPTNTTDARYPEETYLSAA

THAELKCLGMQVHVKVPEKRNVRWLPCTEEETIMWDTPPYNKENVICPEYAEVCTISATG
GSVIPVVSWDDEERVWGRTVRPEFLRQSSPEAPVPVAESIGSGRGTESENVDSAALQSKP
LPQGDAEAAVAQHDHQAGNNNSTPTGSEREAAPAASGENNNSPTAAVAGAGDFETTHSED
FFTTEVESPMGEEEQSAPEEGANPTTLQSRETTGDGDAQPVQESVAPLAGSEGLLPKQSE
APVPENNHSESGSGASQEDRLALLNDAKLMLHDWNVDSTARVCVSRVSMLLLLGLCGAVA
VL*
>TCRU_2991 hypothetical protein, conserved
MAMMMTGRVLLVCALCVLWCGAGGGYAEDVGVVSGDPGIRGERGGGDKSSLADGSHGGSG
GSNGDSSHESESEPSDSASNEKSLNPQVDEDEDPNSSEQPLRQGKGGSKGKQDHSQSVPQ
NGSPHKVNGAETLDSAAQVQLSATPNAELNTNVADGEIANGGRGNTGHNTGLSPNSSDSS
APPVPPAMTVPTGDTNRTASKDLQNINETTSMTPPSHSKTAAEALQTPSGNGAPKQHRKE
TDTPDLKDAPASHTAESTAKSIYTMGSDGAQKNEEKFDSGDQRSNGKEPQDGLEDRNTDD
APTASETETETATTQKNATPKFGDSDGSTAVSHTTSPLLLLLVVVCAAAAVVAA*
>TCRU_2992 Mucin-like glycoprotein, putative
MMMTCRLLCALLVLALCCCPSVCVTAQDPDSPSSSPEGTGVAGAEGGVGTGTGAEPEVVR
KNEDGPRNKETTTPNTEAISVEVQTLNTEAGVAGQSGSVNTKGQDLEEIPGPLQKPDAAS
AREAVKKANTEIKPSNTTTTTTTTTQAPSTTITEAPNTTTTTTTTAPEATSSTAMNAEAP
TTTTTRAPSLLREIDGSFRSSAWVCAPLLLAVSALAYTALG*
>TCRU_2993 HPP family, putative
MLRNDSEEMQEGQQGGDGNVVLMPFSRDDDDTGMVMEAIGEEFVNAPVSTSSPTLGPREE
SASSRFFLVYLRRLRGAGEKGPPFFAPSVDLIPTFLFTTVIMLTLGVADVYGFEPHGLQA
YLPAFAASCALTACFTTAPGAQPQALLGSHLLAALIGLSFAHATHSLEQPLGHLLASVFA
VAFLAPVCHIFGWFQPSSAATAVSAAFYENGQKDGGYIFVIFPVLIGVSIIFVLSWLLNN
LIPWRPAYPVWL*
>TCRU_2994 glucose-6-phosphate 1-dehydrogenase
MSGSENRCRFQKEVDKFFDTLRERTLRDHPQELCEYLMENAKKVAAELRGEVCERIPDAV
SPELRSRALTIVVLGASGDLAKKKTFPALFQLYCNGMLPRDVNILGYARSTMEDVEKWKK
DTLAGFFTRLDERGCHVGNFLRRISYMTGSYDRDEDFARLNERILRMEEAFQGPEKGGNR
LFYLALPPSVFVGVCRGLSKGAMQKPELGWVRLIVEKPFGRDTETSEQLSNQLKPLFNER
QVFRIDHYLGKEMVQNIIVTRFANRVFSALWNSNSIACVQITFKEKIGTAGRGGYFDSIG
IIRDVIQNHLTQILSLLTMEKPRSLSAEDIRDEKVQVLRQVVPANPAECVLGQYTASADG
STPGYLDDPSVPKGSHCPTFAVLRLHVNNDRWHGVPFIIRAGKALEERLLDIRIQFKDEI
RPFGESTQRNELVIRAQPSEAMYLKLTAKTPGLLNDTHQTELDLTHERRYDVTLPDAYES
LIHEALLGNSTNFVRVDELDAAWRIYTPLLHAIDRGEVKVLPYAAGSCGPEEAQEFFRSS
GYKTTNGNAYKCSNA*
>TCRU_2995 Protein kinase domain/Protein tyrosine kinase, putative
MGNTLPACSNGMRRFLSYLGSFCCSSKHPTRVVVGGVEYNVERLLGEGGSAYVYKGRDTR
SGNFIALKRFTLKDHQYKTQCMEEVALHRSLCPHSSIITFYDSDIVKRPGPILPELWIVM
ELSEAPTLANYINIRMAVKQSFTIREVYEISHVVVGVIAHMHSQSPPVSHWDIKAENFLF
EDSQNLKLCDFGSASRLYYEPQNALHVSIAEAELGERMTLLYRPPESLDLWSRQRVDTKC
DIWSLGVLIYLLIFFEMPFEANVMEIMDGIPRRFREGNWEEVPREEFKPVVEIVRTMMLV
KDPVKRADIFDVSQRLSALSHIPPIPRPRPGYQRPQDSRF*
>TCRU_2996 hypothetical protein, conserved
MGYKAGPEILQIVITSAIAGVTTVVRPLWAAPPLVRVDVWIDNIRIAGSKSDATLWEAQV
LRNADSRRACMGEDRESGAAQYTFLGVRFDHTHRAVSLSDKFVRSVRAMPAPNSSTVAGM
EVVASRFSYASAILGTRLCV*
>TCRU_2997 hypothetical protein, conserved
MIENNRRRIIKPTEKASAAVITDASLHGWGAVFIPDSGDVEIAGEKWERKPLLIMQSEAR
AVRLALSAFSAILQSTMDVWVDSTSLQGAVNKGSSKSHAMMWELQRICFLILAEHRKPLP
TCGLRKTPQMAYHAVVFLAL*
>TCRU_2998 hypothetical protein, conserved
MGLFTGGRGGGGGDAVGRGAVSSLPNVADAQTVVLKQTRGEGREEGALSPSLIDRSKSQQ
LVIRKGDARVGFFDTDAVDCILRRYEKEMTVADQLMKKKQRKQQEQGLFHLYDYEEDRRY
SQWLKDQEHVRATLKIHWLDMIIDKPLLCLTYFARVGTTAGLFHGAGRSVYLYRTMDKVY

IKLHGVSFAGIALHEVSLSVIKGALVAAAGTVGVVVGEAATSIARSAATGDVSVPERTWA
NVWVCGTSSGFFAGGAFAALHASILTPWGMAVAVTGFTALSSLAGLALARIAYHPFASAR
QGRTYDPYWHPWYTRRVNDGGGTYMRGKYI*
>TCRU_2999 Gim5A protein
MSACAHTYLSDTWNRDKVMAIVQFLPMALEGPVRNAGCDSLAESLGNLSKMADSYRAVTR
LSLLLNALSSKTLKDLAKPKGDALVWRLEQVSHAFHIGFCLNEHTAVLAGRGVLNSGLTR
FGGVAVVCWLYTLLLGIARQAYLLAKHSPRGSCKALLPEDAEKKVVPYTHEECKRAVVNL
VKMSCFAVFAMTCLPEGRPKLLQDVCGPLVPLHELIRAIAPNKLHLSDTVRGLLAATASL
CDFY*
>TCRU_3000 hypothetical protein, conserved
MPRGGRHAGGAGTAPSGIQGQPRNSPDYYYLGNCGGDDGGSPPLDCTSIGACRRLDRQYT
HCRAEKRCDIVGSQVLRNAAGCHATMGEDRESGAAQYTFLGVQFDHVHQTVSRSEKFFRS
VCAMPALNSLTVAEMEVMASRFLYAAAILGRRLCAYYFLITAVRRR*
>TCRU_3001 DNA polymerase theta, putative
MQPVTGNGGETTAEQKEDDGANVSGCQQEQQQRTVPTETIWVHDSPATSSRSRADAAVAA
TSNVSKHRGDYCRFKDVLMVCEPLLQGLLGPVGIDVEMNGTTAMLRDPNGFATALDSLEK
ASCDSLRHFHDSIQLFFIIRGDSFYTLDSSVCSPLVFLAKLLSVPAVTKIVHQAQPIYRL
LFMFLGTDRVEVRNLVDISVWSAVVERVCGPRPYTKGFADSQSLWQSLPETSRESIEVQL
EKAKQERWCEGTLKAEEGEATMVLSSSSPSSSGEMPLPQLLGRFKSLATLYAYYEAYLQS
VESSGATGNVFMSLFTVCSLETRVSFLCEVMSYHGMFVEKNMLDSMVADLEQQIKALIEK
GNVALRAMATASNESIEMETVDAWNLIKTLQSQFGGPWTFAKGNVEEQMQQIGAYLDEKQ
SCADSNVARIWLALHERREFKRSLMEGFTKGASMHARLIAVPEEMASTEGGGHSNPHHVG
AQYRLFFSVHPKWSVHHATTSRLFCSRPCLQTVAKNPPISRTAPSSSPPVTIFTNPEWTL
RHLYGPPPGCTLVSLDFNQIELRVMAHLSGDPLLVQQLRGGSDVLAEMALRVMQLPRLAD
VTPYLRQGTKVVVYGLLYGMGPESLDLRLRQLHKETTPSAEQPPLSARQFLRSFNQCYPA
VQGFLKSTRLRAFHEKRCATLNGLRDLSGEPDGNRRKQMAVALAIQGGAAVILHHAMVEV
HEKRHELVPFLPAAAVALVLSVHDELVYAVPDGAVSFVAPKIKQILEQQAAALSLSVPLP
VSMKAGKSFGSLQSLPV*
>TCRU_3002 BNR repeat-like domain containing protein, putative
MRVGDSYGRHSLERSPREYPVADTAAAGHIFRNPHLVNFDGLLLAIVGAQFNGSVRSGSA
SMQLMARISVDGGRTWRSYAGPEDLDAFAALPHRMSFPSSFGPLGSLLAFVEGYDLRNGA
IIRFTDGWGGSGLESVIHFLETGPGRSGDLSMSSVSMSIRPPYPYKSDDLIGFLNDASTP
ITKMTDNTLVFPVQFLTRGGDTASTIMYWNSDQQHWTLAKSATHAGCTNPSILEWEAGKI
IMITSCKYGRRRVYESTDKGEHVEGGSGDALARVEQLIGRFWAPHSEWIYHCNYWQKEGD
PPHPAGIFQTREQGRDSPVANRHEPHLPRWPATHWVWCNLQLPVVRE*
>TCRU_3003 Paraflagellar rod protein 2-5
MSAEEATGLEAARKQKIHNLKLKTACLENEELIQELHVSDWSETQRQKLRGAHLKAEELV
ASVDVGTKWNLTEAYDLAKLMRVCGLEMSQRELYRPEDKAQFMDIIGVKKVLQDLKQNRN
KTRVVSFTQMIDNAIAKMEKVEEELRRSQLDATQLAQVPTRTLKQIEDIMNATQIQNALA
STDDQIKTQLAQLEKTNEIQNVAMHDGEMQVAEEQMWTKVQLQERLIDLIQDKFRLITKC
EEENQPFKKIYEVQKQANQETSQMKDAKRRLKQRCETDLKHIHDAIQKADLEDAEAMKRH
AANREKSDSFVRENEERQEEAWNKIQDLERQLQKLGTERFEEVKRRIEEVDREEKRRVEY
SQFLEVASQHKKLLELTVYNCDLAIRCTGLVEELVSEGCAAVKARHDKTSQDLAALRLEV
HKEHLEYFRMLYLTLGSLIYKKEKRMEEIDRNIRTTHIQLEFCVETFDPNAKRHADMKKE
LYKLRQGVEEELAMLKEKQAKALEDFKESEEALDAAGIEFNHPVDENNEEVLTRRSKMVE
YRSHLSKQEEVKIAAEREEIKRARLLRTGGGGSGEQPRIGNNTAPARLE*
>TCRU_3004 mismatch repair protein MSH5, putative
MSGICDTEEEIATYEVVSLLVFYHEVGLASYDSATCQVSCSESAAALNRGEEMFIGTLMD
VPGDIVWVAQFLLSKKPKVVLIPSGGSQILIDVANLCGVNVVLTAPREFDEGRIWDLLGV
LWANVTRLEWHSRICPHHHVMLMALSALLPYLQRSELPIADVAEVPPLGLLYIEQETLSG
LQLLRTEPHPMDYQGIGRAKEGLSLLSVVDRTCSVLGRALLRQWFLLPVRDESELRRRYD
VVSFFTMQENYDLMMQLRRALRHLRITNSIFTKIRAAKHTTNDYESLLRTVRGFLRIASL
LTPRAHFSPMFLRIVASCQTNQLEEISRLIDEGVSFSRDPGAALSKTYVHIRPGFDAKLD
ELCAHFIHLDEVLANVAQQEARCLPPLWGLCSVVCVFAPCWGYVITLPRFSLPVGREDIP

GWELLFQTEEGPFFKTPLTRRMDEEIGDLRSAILVREGEIQRELDKRLIVLSPALIPLHL
CAELDCLMGFALAALEGQWSRPEIVKEPGVLVIERGFHPIFARTAAHQVVPYSINIRTSN
QRVCLLTGANGSGKSILITAVAHAVFLAHIGCYVPCTHATIGLFDTLMSLHAVSYSSRDR
TLASTTKELHSSFGNELVSMSRMLQRCSTRNKEGGAGSGRSLLVLDEFGKGTLSLDGAAL
LAVSIRSFISFGDRRPIVLLATHYVEVLQPQLIPREEIMVLEMQTTLVPTARTGGEGGED
VCEGFIGGSQLVHSYRVAPVQALSSMDTIQQKGMTSHALRFALQFSVPPLLLQRAWDLIV
TESL*
>TCRU_3005 MORN repeat, putative
MPGARHNFGRGFPNGDQYDGETIEGNINDGKCGFFETTGGAKYVGAQLLGKPHGKGKYIV
PGAMGYIYEGDFVNGERSGNGVCTFSNGRKYEGDWLGDKMHGNGTLYGAEGVDDFVEYVG
PFVEGERSGADGRCRYVNDNIYKGLWLHDKRNGRGELVLGRASSTPGYTPRMGEPHIVAY
KGKFENDAPSGMGEFLYSDGSSYVGECVGFSRHGEGLHRKLNGDTYKGGFQLDRRWGKGV
LQNKYGEYNGEWRDDLLEGHLIFRGDAAGAVATDLIFYEGPCVSGEMVGNEVVAKYRDGS
SYKGAMVAGKPNGIGILEKKRIKIPGVGEFMLRYEGDFMGGVPKGSGTGTLTLLTADPRT
APSPPISAEVRCFIPQLDVSGQYKGMWLNGLPNGDGDWQWVNEQSYRGAVKSGIPQGSGV
FITPKVHYEGSFICGLPEGYGKITWRGTEEKKEDTYEGSWKEGYLDGEGKLILHDGSTYT
GGWKSGIKEGNGVDSVVKKYHYSGELKEGKRHGKGTISMLKEGFKYEGEFVMDELTGNGT
MTLEDGTVIIGGFANGKPHGNVKITLPTQDGFQGEFKHGVVSGSGTLFYNGGKYEGEVAD
SGNPLPMRHGRGVYTFIEGDVLECTWKRNVLHGDGIYTTSTGDRSTRSYVDGVLKAVSPQ
STDAPPSQKGFPIELSEEEQQQQQLHKKQVVSIRRSLPNRSPSNSFQRSLARQMSSSGT
TRSTVAMPKVKSSSISIMTTPKPVSPRSAAVASPSFSFKSVLRISESPVKPLSAMTIVRR
RTVTGVAGNGIEAGGSSHLTPFSRFHDGIKGLPSCTESSEEWKGAQLNTSYPVAGGDKGL
VQSTVVRREAREGEIHSLTEALRRINEQIWQESFMAGLPVTAGNEGLKLTKASRLDQLES
LQLERRNIVDKLSVILR*
>TCRU_3006 thioredoxin, putative
MKVAVGFLQHCLVRRRTGTSVTTVFSTAPAASLSSWTCATRRHIATFRGHSREFLDEVNR
EKVALVYFYAPWREPRNRVYVKLEELLNKGRPAVRGGSEATSSTSLASSSLSSPSAAAAS
PSKDPAAEGTLQPGALADPKSRVDPEVLLKSVREFCVRARANGRTVRIIPVNTDENPKLG
ALHDIRSVPTFVTYRDGRIVGRVEGFSEDQIEQLVKELLQEDSGDTPVTDQAKRNWKSKE
CEETKKGEKK*
>TCRU_3007 serine peptidase, clan SB, family S8-like protein
MLSGTPPFFLMTLFSLTLMHFLLSGVGTCAASAGDAAAAAAAAASKRPLAMRRSPLQKWL
HADWLKIHETPLHWFVQKHHHQYPHTGENSTSHVASACDSCRTRRPFLTARTAGLSGEDA
SLLDSDDGRYVVVKLDKLRWKERRNVRSSLMRHRLEDTHIIDELHMLVYVDSERAAQIRA
ALEAEDRGVSPLTSLTVLDVYSTPTGARLKLSRAIRFSMHRCGARHDKSAVDNKPMEEMD
LRWRLPLDVRTALGRENVVERSLQSVSSKTRNVSAKCQWNIISSIAGKGLVRWVLTLSSG
GKKEEEKEMDVVCHCSAFINTVVSHPAVRWMEVAIEHVAPLNFYATALVQNGNQDEREPF
RPFWDAGIDGSGELVGLADSGVDFDSCFFSDPKESVAIYPEVNHRHRKVISFVPCDLFPG
ERLIGDKKRGHGTHVAGTLAGYVLDDDENSKYNGVAKGAKIFFTSLGVPSQPLLTLPIDI
TEVFRPAYKEGAHIFSNSWGFFAPDEYTAVEKSMDALANQMDDALIVFAAGNSPNSGLLT
PCRTKNMICIGAHKNTFDPNTQNTIAKFSSPGPTYDHRVKPELVAPGDTITSAFSDGNIS
TNQCAVVSMRGTSMATAALAGSVALMRQHLRTLENISSPSSALLKALLIHCTVNLNNSRL
SGFGRLDMSLFFTPTGIIGWFRDRDSIGHHASKVHHFTLGPNLLDVNEIRVSLVWTDIGS
AMEGSAISLVNNLDVLLVDSKGAVYFAGENKTLDTTNVMEQICLPISWELAAGFRVFVYG
GSVHEGNNQPYALVVSGPGLSYVDNASIMSGVVCPNNCSGNGNCFGGICECHTGYRFIDC
SFCDEEAMCNGHGTCAAKDLQCKCDNEHFADEHCSSCKPGWYGPSCSSNCTCSERGKCNM
ETGECICQKDKTWGGSGCFSGPNCEYCCEDFAGEKCNVRSYWCREDGWPFEVNDTAGGYI
EVNGYGTYSPMLVCRWVVKAPRGRIIRLQYLRFEVESPTDIVVLYDDASDEAEPIRVDTG
TSAQGLLFLSSSNALAIVFSSRWSHVRKGFLMHYSFEGPNNSNCTVPCVKNALCDVWGGG
FCVCNEGRAGWDCGVEVGSDNKVIELQEEDKRGATYFTPLVNEELQLNLKHQSPNWVRTD
VYLALESSIFTKQQGEMALRAAAIRSTPSGAISIKKEKKIGWTSNCRYARISLSYRLSAP
KGELNISFTLLNNASLLSAANGNASYRAAAYLAKSEPQDIYETLDTDDRLLLISCSSDLS
SEVPLSVFGDFLNRTNDASEGRTGVSAFVAPLLLFVFFASGFFWVPLFFTWRPRQGLALA
TIPTEEVMVLTSSSVCIGAEDDARIESGDGLFSSISVFSETHEVGETSTGAA*

>TCRU_3008 ABC transporter/AAA domain containing protein, putative
MGDCGEWRSNPFNEMDGDGGNQEERNQPNILVPEVSDAASEEDGNEVVLRLRGIEKSYPI
EGTEERVVALRNLNLCEDCGRDGFGPIRRGEFVMIRGPSGGGKTTLLSIIGTIDLPTSGT
IELMGTVINGSTPDSLLADFRLRKIGFVFQTFNLISTMTALENVELPMTLLGALSAKKRR
IRAFRLLALVGLRNRLYHLPSELSGGEQQRVTIARSLANNPQLLLLDEPTGDLDTLNTIQ
VMDLLVHLNRITKTTCIMVTHNPDVECYADRILYVADSRFVKEVRNSCPQRLVYEAYVKN
LEKREAESTAVVCSFQDP*
>TCRU_3009 Zn-finger domain protein, putative
MSCSTSQGEEASKEAGDNTRMLPSTGEERNTEHSGVSQDEEEREEQQQPQRHEPAASIFH
EDAHETPDADHDGEMECWICRSGGGTTQNPLITSVCKCRGSVGWVHRECIDAWVFSRRRA
SCPSCGATYNILAISDVSLPQTFFEQLCFFFWDLILPLSAKALALLLGIALNGFVVPCII
GVTFYHKCFFNNGDAATRTSEYSSNSSETALLQGPEEGASTFLDVWLWINVMAYGWICVT
LWRCTKTGWCQWRAFFEDTDAVFATATEEVQEEEEDDDEGEDEDEEDDDDEEEGPRLQPN
TVVGMLHWILDWSLERTGSSQKMLRPRILELGTLVPLSFLVNFTLGKLLLCLILALGLVL
RRLLYPRKRINDPTRRFAEAQERKTSATEIDIALWFITYLSELVLFSILMTTFGGIVVHF
ALSPYFLTFPASLSAFAGELTLTRLLAYWLVGSFCSMILLHVETAIIVPLFAPGVELYFV
RSVDLNLEDDTAYWKFVLAQVFDTDPLGVSLDFIRFCCIEMTLLYVFLATPIYVMFAAHD
VMIKDGANSTLTSPLAPGFPVIMVTPAVDGISAWLESYLVPEKSRLLESYYQFFFSLIEL
LGSCFARFSLGVILWFTRNPYWLKCVANTLIVFGTGTAIVCVRLFPIKHMQLKVLRHVVM
FIGRHVVHLEDYLFDKERLSLLDSWLRGDGQSDIPLPEARLPSVFRRREWALPPGTAHPK
YLKLRVAIFSFLFFVTSSALFWIIPMAVMTPLLTVLPYSVPLLCLSANIIFFVFNPKMYF
TAAAEFMLLCIIFVIGVLLQPLFFCASLRNISRLQLIQETMEYYHKLRRTVGVYNGEAME
AA*
>TCRU_3010 hypothetical protein, conserved
MRSSFNAAFGRLLRPALFHALWPGGCRAFCITKPTRPLLCLSGGTPEVLEVELLLPDDVD
EGRNNKSRTNVDGDDVDDFPELEEYEASKEAAAETITTIDTFARAKVANFAKRMPHSACE
TSSSASHLLAEVREVQTAGDEEKLFHARVRVPLPPEYGERWGEGLASTEKEAELVAAMHA
ERVIDAMGFPMFQLTSKQKRHAEAARNAGRWAPMPGDDPRPPSTPSPPRLQLLREGATKE
VERRLQIDKMEFSNVTAGAFTSMKLTLASPFLLDYGSVNRIKQFFAAYNCNIQRYSRVTP
LHKCVSKEKFGGSGSGEAFSHTGADNNNNNNNNKMFVAQIRLPIDGRFGERIACGKSPTK
KEAIALACMHAELIIDALGLALYPSDKEKQETHADECRKVNRWCSGPCGYEYHFTKPSPP
PLQLVKESSVKTAAEVDSAASLESVLLSHAKAIEKFENVTELQRIDPAAHHIFVSYMAQY
REDAKEVGSLFFVESLGVKGHEVYRATVTVPVPFVAGTTASTGSASVHDGDDVAVSSFVA
IGIGTSEMEAETTASMHALSVLKSLDRPLLGSSTVGREKFQQRNLTRDTVIPPPYRYVVA
HLGRIVVPGLSPARNIGGLPLAARQRGFSSEYHEARIQGARAGLPKSDWSLDADAEGYII
QNPSVNVQETRNYVHTLPSVRQADRFALVRLRDYLERHGKRLEAAVTSSMLETDEGLKRW
VKKVVLPVPEAFGHVVAHGEAYTEEEALVMCAVHAELLLDELGIALYDLQALQERHCDAA
GMLGRWAPYETHSTRRAVPTPPPVRKEHADSCLWARLSKETRKEGTAPCGEGIKQTVSST
HPPDGNYKAADGENHTDATATRKAEVVSPSPGVTDDALFDLAGLVFVHPSEFFRHAPRLL
ESYCGRQGVDISRCMRQYNVCNPLYGFVHRAIMEIPVPPQFGRRYALGCALTKKDAFFLC
CMHAVYVLGELGIPIYIGPKQAEYAAIARSKGRHAPNPGDPLRPPNTNSPPGLKSLPDMH
MEKPPLPRPPNRRECHKSRVWQGYVTACRGYIKKMKEQVVWDALFTKGKAPRSGVDLEDN
GLDVVEALPLFTNVKSRLPQKCAAARLPAPPPLSFRFEVYGRPPHRRYLVEQPVLGTPFV
ARGMGEEGQEAVTRAAMHYEYILGIISGTVQGNSETGNFLRNRHGDLFDPTLRDFTPRGK
LAIMALHSTLRAPFLPLRLTLKERNNSAAGVQLVYVSLVEIEDESGLRMAGKGEDRIHAE
ESRNRAIAELYKQLQRKATFQAVAQLLQTYPALRAEGAAHMLSETKTMRDLRSVLAGRKD
PIPLPLEEFPEGAGVATLLRGGEEHLDSPELRLLFNIVERELVSMPTPAPLNLPVAAQHL
LRTMGLVKTQGGDEKNKKTIEANAIRVAVALFSLCLPPLLPMREGASPLVIGALPLQLAK
LLFAGSLLDCLESCIRVVALVLSSFIGENDVCRSGDVVELAALELSQEAKRLATVIAARL
RELSKQLKKLTSPSHILTTLVDSSCVVSEGTWGPLQEGWGKKDEGRLRLAVSFATFPRAF
IQKKVAADGVLRLITVESRDRQRTAQIAVPPNLAKSCNASSDPASSSLSPCHSSFPWFCA
FDGASAGEGEELPMLGTFVPPCGLLLASAPCDESRMLVIPDDVPNMAIVDGFIPVVMKST
ESLATITELRLTLKDHWAVLKPLPVDTQDIVDKLLVSEHSLANVLQAELLD*
>TCRU_3011 Integral membrane protein S linking to the trans Golgi

network, putative
MPIPLGAPLSDPKFVFLQILYTLSMFFIMLGVVRVVLGVLSLTFIDSSALSVPLFTSLGY
CFHVPLRSLFAVRVEDVSDGSAMRFFLMHLLTACFFSGPLALTIQRRKFALDFIFTLYAM
YYVFCCLASWRLTGGGFAWWVSVLTGFCVLYGLTFLLCGRRELQDIFFPLPTAPAVAVGG
SGGICNVSNLAGGGDGDVMMATLGAAAEANGVDVDGCSFSNGCSSPSAVTEAVLMGPGSA
RKRRQ*
>TCRU_3012 hypothetical protein
MQLPHGIVRVSTPSHHGSQRRRARSAYPFTRSFLADTITSNDATISPPLAATAYPWRNAV
PGKCILKTPRARDSSSIVVVAAAAAKGHRRRVHFDEGSILTKGINGGVLDRDDAIGVSLP
ISGINAPKKSLNASVAAEVPYTVAWGEDSEWFKELFGLKAPPSRGSKRNVGPIIERDLTL
HSNTKEDPARCTRFVGHRAIISLSHAMPNTPLGGASWGNTSNFTDSARSAVHVSSSRSRP
RINAVRRVPEDHAHARVAVGRGGESNRFVTPPRLRLAPKVSSTKNCFWDRPKSLSVEWKS
EWQVIPPQRRLCNFTAPQLCFVNFGRDGGKNGRRRRGEWLVPTNIHA*
>TCRU_3013 hypothetical protein, conserved
MDLPPSENPALSESGPLAFRARTALLEQLRDLHRQEQQQQKRRGQLRLSNNGPQTGTESN
NDDDSDDESDNSAPMAVRFVRGCPPLTDPSWAMEELLRIDPNVALWTGTGLSTAVWAPPG
SGHCPFGTTQADFIRHR*
>TCRU_3014 XPC-binding domain/UBA/TS-N domain containing protein, putative
MKLKVNCSMGGEGKITKELVIHNPAATLSNLRAAIAINFRLPVHGFRMIHDTADFACEKV
LLRDSGICDGDLIVVDAKREREDETGGVNKEQKTGTEVGPNQQQRYEPKQAAAAPTALVA
NTVTPASAELTVPERNANAAEEPDGLGDGGTAAGDDDEEEEEEEEDGPAELLSHLLETVP
NLVEMRQQFLANPAEIMQQIQERDPRLLELITSNYQEFLDLVNNEDLVETLQREQEEAHA
AIFGEEEELDEEVEEAVSSLLAQFTVRGGLPEEADDESTEAQNYLERPLTEEEEAKVEQL
MQLGFTRDQCKVAFFKAKRSLERAANLLFEDPPQL*
>TCRU_3015 hypothetical protein, conserved
MSSVVQTIAASVGGRFLLLGCADGLRAYDVHSLGIVKKIIPSAGETPATRGCRAVGGPVR
VVAVLGETPLIAFVLEGGVTSELVSEGDLEGAAVSGRSTSSVASTSRVLCLYDASKGQRM
AELHFETEVVGIQMNTRRLVVALVECIHVFDLLTLEHLAELRTASPPNPNGLLVLSELTT
NVHGEAVSYLAFPQSDQGYGDVWVAQITEKPMDREGEWTDVVNASGGKRCGFVERVSIVK
AHHRAVVCMAMTRDGTRIATASERGTTVKVFEPSSARLLCHFRRGVKAARMLSLSFDTSC
ASSGLRLAALSNRGTLHVFRCSSTGGSELVVHESLLPYTGSEMRSFAQVTVGKASTASCA
SPSLGLNCKMGNELQCCFFSQNGQFLYVVLPAPCVMEEGTARIQDAAGSGTSLLSERRAD
VGARWMLQKYAIHMHGCELVNSHVLN*
>TCRU_3016 Rab-GTPase-TBC domain containing protein, putative
MKNRVRTGALASFWPEETTPSDCSSGGCGDGGERAALDEIPGRGVGAHAPDPVDTSIKWT
EMTPEVFSPVDWASEERLGFPPSSNLEVRAAEMDAEPLLRLAAQRCEKHWQSYSRGLPLL
ATANAKVEEFMRRAMWHHGIPQHLRGVLWLTISGVASKMDENQGFCRALLKRHGYIRGEY
AEAIEKDLHRTFPEHRYFADGGLGIRKARNVLHALCWRNPLLNYCQSFNYLVAFILLVVD
DEESTFWLMCHLLENLLPNDLYSESLIGTKVDQLVLQALLQERLPRLAQHFSEVHFEVET
LVSAWVMALFINVVPVQTLLRVWDCLLAGWSHSSEHSCVPLEVVLAVLKLHQNELLRCND
AGDVLMCLDHATKRLFDAEKLVRLIREMRLSPSNVRQMRRSARPTVAEEQYRREKRRQEL
LEKNHLGYEKKNGGIIQMWRSEEKEKDDVVVTVEDPQPISEAGISGQHREFVPAPPVESR
QKEYRERFAGTVFVEVNAWHQIQKGKSRLGNAGGAVDEDSERPALSECAVGTRRWRRREE
SVGLEMESICTANRDQEEAEHQ*
>TCRU_3017 hypothetical protein, conserved
MARGRRESSAVAASSSSSKSPPGKKGGRKLPVVKSTSIAAALRQASCDSNESAVILRGKA
GVGVKGQGACTADVQSHPQGSRLRLQQSAPQISFWKGICRPTLLVQALAKIGTLVEAETA
LLFANSRVRYPFSALTVERVLTAIQAIVDAAELLRTVGTDGNRAVFSNDAMCQRWRVIGK
LIELAEAAVPMKVSLLAGSTTACESVTAGLPPVSLWALNGRLKCLQRLLQLRECHSRILS
TLRLCKTRNEVSAALFGPSMIEAATIEPMPGVAYWHQGEQFVIKGLMAEVNDITPCSESW
GPSSITPLRVAGGSYVLHKHSRFETLACLLRVAEFIGLPPFLRSIRVAGGRSRVFQPQPS
KMKSVDDGSLHIYKESLPLLFPSSDDLDPRHFEAPASNHAQLVQEELVKLAGQHCCFGAL
VGEADQVVALLHESAACLQRRKMASCDERTVAIKVEREEDDGGGRPLRRGVMDASGDDPL

RAYAESLLQAAKEENSKKNRVRWLCGNRWSPTMSYYALVIATALPHKTNDVMEAEEDREA
GRRVDFVASYYVAAVLELTRF*
>TCRU_3018 hypothetical protein, conserved
MLRITSACRGGYMMYHRKSMGTMKYSKWKGAHGGVSHFYGRTPMVEEVKRNEPITLIDRR
IMHYVHRSRLRHFQLFRSYQQKSNATECKLREGEMLRRRWHRKLQKSFIAFMQFKTMKVL
EDQAKLVNTYGQAAVNAALGDSWDAVSDKEKDRKYVTIRRQVKALPVVSVVPKHVATMKQ
IHNDRFNYRWRVN*
>TCRU_3019 hypothetical protein, conserved
MNQVIRSRTDWKERVVSRLAWITASRLFEIAALTANNFTLERDGALILDWSVAPKTARAD
SHRASRFVRIREQDAFDTIKLCRTLQENERLTNLTTAKVERALAPWDATAHSIKRGALRH
AAQIVETYNLNPHVISQLARHVDPFDLPQNTV*
>TCRU_3020 retrotransposon hot spot protein, RHS3, putative
MKLNDFLEMELEGRGALDANQDVLLEEFFKDPKKYICDAGVLGEMQASDRYKRMERAVRD
EMDMEEDVRKLYKNGMYYLLNWLAASAEVKASVHAVTKRFLDAAAEEARKTKKSSAPRYL
EGLYESVHNAKWSHVVEVSGGEGTGMEVREGEPPQPWTYRAVGGTLEKDDGVEQSGEARL
RLMVLTSDRGWPYSWNRKENKSTHDCHVNCEVERVWQIVRGDLTAWFSSHGRTDFKFERR
LLIGTPGIGKSMGAGSYLLYQLLQYDVEKLQVVAYIFGGSTTYVFNKTIKTVTRYLGSKT
SRNVLYGLWHLKMKGYVIYDVTEKGMPASCFAPCGEWGMIVLSSPNVGNYEKWETQVRAE
RIIVNCPDEMDMKAMCAWMKRGLDPDKQAEYWKEVKERMEKLGPILRYIFDANKFIAHSA
AIEDALDGIKSRDGEKHFTNGGVKLWYSEDPSQKLVRVVRGRGEVCAEIFLNAPISVCLG
RRIPHYFGGRDE*
>TCRU_3021 kinetoplastid membrane protein KMP-11
MATTLEEFSAKLDRLDAEFAKKMEEQNKKFFADKPDESTLSPEMKEHYEKFEKMIQEHTD
KFNKKMHEHSEHFKAKFAELLEQQKNAQFPG*
>TCRU_3022 kinetoplastid membrane protein KMP-11
MATTLEEFSAKLDRLDAEFAKKMEEQNKKFFADKPDESTLSPEMKEHYEKFEKMIQEHTD
KFNKKMHEHSEHFKAKFAELLEQQKNAQFPG*
>TCRU_3023 hypothetical protein, conserved
MPYRLLVTKIPLHVGEEEFYDYVRAHVSGEAVVNALLIMLPGGGGASTLISSSGAALVDY
SAKNAADAARRVDFYVDGVAVMMKAVGSVPTPGRHTELLAEEGRVAYQRLRRGLSHADRC
TARTDAAAGQKRQRKDENVVVVDDVDTNMKGMRETDNQKAEEVELRLVGIPHDVYGSLFD
EGVAGSGSHTTRQSTVDLFTDVALTIRRATLGELLSLRRITDDEAVIRVFADTADTLLGR
AETGLQLLIQSTATTEDAKEFLKEPSDQRILFVRRPRPQVRITGDNFALATKHDVVGAMA
RIMELTNAKVAGFIDPFGNLLLPSLN*
>TCRU_3024 CRAL/TRIO domain containing protein, putative
MYSPDSTLELRLPRDLFLFPKQNAGFQIPFFLSTEEEAAKVRPPAVVKTLEERLQEFSEG
QREILSSFKKAVRDAPWYSEAEDDDWLLLRYLIARNFDVKKSFCMLEKSVHWRRKKDADN
WVCEACLKDPNRHMMQFVGWDLQNRPVCFMAMRWGPDRKEPLKHCVATFNHLVKLMPLGV
EQWVCVTDFETYSHIRDSSPKMGLSVIHAIQDHFPERLGLMILVDAPKAFSVLWKLLSAV
IEEKTRKKVLFTYKKSKPTIESEFSKIFPPALAEYLCESYERSRTAALPATPVWYPAQSN
E*
>TCRU_3025 membrane transporter protein, putative
MEETYVRLPNGQLSTDCGHYSVPTQRQLLGDLMRIGLPLSASQVSQFSFIIVMFFFAGHL
GVQELGSVSIAIGILNATGFAFGSGLCGALETLLSHSYGLDRRSTMYGVYAQRMFIILML
FSVPLAILLSCLTPILIAIGEPTYVAEEVGSFCLICVLGLPAIMLLELLRRYYASQHRSN
PVFLTLAMAAILNPLVQYICVFFGGYKGIALGWVILLLLMDITLVIYLRVSGLYRHTWGG
WNMAAFQNWIPMLKLAIPSLGMAFSEWSAMEVNSVCAGLMSSVQLGAYAISSQLSNLCWS
VVSGFFMASTVLVGNSVGEGKPQLGRRYAMLSFIVVFALSFVNISLLYLYQEQIAYIFTE
DPEVVSIFMSITPFFLVYHLLDSIQSNFLGILRGCGLQVIGVFIVFVGLTLVGTPLGLFL
LFRCGYDVKALWIGPIVGCTLLAIPAYLFVFFRRVEWEKLKPHIEEQFEGSINFAISRET
SSMAF*
>TCRU_3026 MATE efflux family protein, putative
MSVNIHSVANDSFATAGCAGGMEEEKPLSVDITTLEIVKKFLVTGLPLSVATLIQFFIIT
VILAIVGRTLGVNELGGAALALGLINATAFAFAAGSCGALETVLSHTYGMHKSSGNSGTM

YMYGTYAQRMTIILLVVSIPLGVVLIYADMFLEYIGQTKEVVYYTGRFCHIAAFGIPSTQ
LFQLLTRYYACQHETKPLSFVMVAASFFNPISQIVLIHFFGFEGSPVAWLFLYTFIDLSL
VGYAYCTGLYKKTWGGWNINAFKNLKGLLKLAIPSMAMMLSEWVLLEVISICAGFAEPHE
LAAFYITMQVFGLCWSIPSGTMIIVCVLIGNAIGEGKPLLGKRIANVALVMVVITALLDI
TICWLLEDRIPYLFTSEESVVVVYRRLLLFVLPYHLFDTFQSTVMGILRGCGLQKLGALI
IGCALCLVGVPVAFYLFFHVKYGVESLWIGPFLGVTAVGFPSYIYILYWHIDWSKLKPHH
DEGIVVLEEEEEGKGGSEEAREVTVEAREGVASCS*
>TCRU_3027 Fusaric acid resistance protein-like, putative
MPGHHAEKGNGDGRRNNDKNTTISEGLISLDGEMSSFDDAIDKQNYHKGNGSFGLPRYVP
RYPFDQSRRESVLNVPWHLRKPGGLEQCTFFDSFRSVKFWRQSEFALRITLFAVLLPASL
IAAELPNSPFVSKTHCLSAAVLASKVTVGEGLAYVVTWTRAGCIWLPLATIGAAANLGNY
IIGWCFFYTLLLFLMAIFTENMVRRICLLLFNSCMFGILMDPQRGTVYPSRVMSDWCIGT
GLCLVATFLPYPVFSKNKAHRALAEIARNTGTALTGMSSCFWSESNVTRNMAMTKVRMMT
AAIDLLMQRFRTAHEDSFYEFIFEGFEPWEVREAKVKLFERLRANLRGLSRVLDILESKP
WVVDDSERSRAFGALIQQHVLRFSNSLDKLTEELASTSSFGQIRQLHGHFVELNDVTVKL
QHEFDQARRRLFYENKPGTLEEFVPLMTFFIFSIVNFRDTMLQFDSEVQRRKHLLRNSIK
RVVDKTMLEPIQYTFRFCRRLVTQWRRREIQSLIEAAKVSAAMILTVGFSFLIGIDKKSL
SGPNIIAFVSGANPVEALQASIVRLTACILGTVMGFFAGSYSSTAVQKVASLCVLMFLGT
FFRTDKDYGIMAVYAMFVLIPLDTVGHTTTEDTLARMNQITFGILIYVSISVIVLPLSPG
LILRKKRINILLRVSEALTSLCDMFSEPLPIDSMHCSTLPIYRESIVLDEHYESSNLPDA
DFLKNASTRLMVRVDRPMLKISELLDEIEGRLEATYPFMGFARAERGIVDADYPAKSCEK
TTYHLYRIVSLLRTAWCSWEVLRTQKHYAPDTRHVLHCLQPIAWDVCRSFQRFVGMLCYS
LRNPQVSLEMELIQVVLELIQATEELHLRKNQMMLFIICNSVEKHVRYKKEPDFFTADVT
TVQSAPHKEKGLMALGGLGRKRVDDGGSILPLLRKGTKANLEEDDSPEPPESVALSDNFV
MPITGADAEGLHSFTLSMQMFADETKKLLMCLEEMLDHLRKKM*
>TCRU_3028 DNA repair protein RAD2, putative
MGVHGLWRLLDTFGEVTQPSDWRGKRVAIDASIWMSQFRAKCTSGENMEQCILEGFFLRI
LKLLFYGIEPVFVFDGTATDLKSTERRRRAERRAALERSALTRRAQQIFKAQLASGLLNV
QALDQKGPVKTAGRPPSPKKETDAGMLQRSSLTNISDTATGCCSTNPRSLLRKRRREIVL
EPDAISRTLTNSFLTEAEEWIEERKRGELCVEANLLFYSSTSLFMGPRGVLEDKINGPIK
AAVLPEKDTNPPAYMSLSSLSSEEASLVISDDDKTSINDYETVSTGITSSRGSVVIVGGH
TETFPDRLREMEEITIHSLHDDSCDTWLEKDMATCDDILSFCNEGDSSGGSGHSNSDHDD
SVQLWNPGTQLICQSSAETTRKHREVSDDGIERPKAASIGSVEETTSEGSSSPVTKNLMG
AVDVTRSLETPSPSHPPQTLLPKPITKGAKAHQVIPFELLEILELLDCCGIPFVLSPHEA
DAQCAFLSQQRLVDAVFSEDSDVIVHGANVVLRGFFSKNRYVVVYRQSDLAMCGVDKDVL
VALALLLGCDYAEGVDGVSLLEALRIITAFWHQESVQGPSHVLDMLKRWRDAVAQRRFVW
EEEGVLLQSYLNWRRWSTLRVAADFPQVPVVDAFFNATVDADMRPFAVASPEWNRLRTFA
GMHGLLGDVTCQQRLELAQKAFLARELQRNNERKQQQLRLTDFAMPSHAKEKVAYKKQPP
KFAAALSALRAARRSGVRVDCCHY*
>TCRU_3029 methyltransferase domain containing protein, putative
MPAEELHCSEETRKRPRDPPFVVDYRPYTARQLAHALQKRTTAQKEHWDVYYRHNTVNGY
RDRHYIISEFHELRETLERLKEESSLPATDIIWMEVGCGVGNAILPILEEYGEVNGWRLV
GFDISFVAIALLQEKRHSLPENCQEKLSFCVLDPVEEDISVAGSTSASPLAIAESSVNFV
SMIFVLCSIPVEKHLVVLRRVAFCMAEGGTIFFRDYCVNDHAEKRFGTHRRVEENTFTRS
NGTLSHFFSLAEVRQLFKDSGFEEVELLVVEREMVNRKKGTSMHRKFLQGRFRKTGGAKT
TKITKLDKEFLAHTRVKECV*
>TCRU_3030 cyclophilin type peptidyl-prolyl cis-trans isomerase, putative
MSVVISTTRGSLLIHLRYTDCPKACFNFLALCASSYYDGCLFHRCVTNAFVLTGDPTGTG
KGGESVFAPEQRYFDDEGLGTVHHDSRGVVSMAHRGNKPNTNASQFFIIFQPCPSLDTKH
TAFGCVDLAWNDGESERTLKKLEELRVDDKYNVLDKDAKIVGTTVLYNPFAEGHIKLDV*
>TCRU_3031 Iron-sulfur cluster assembly protein
MRRFTSTPLAANAAVASLRTLYSALVEDHYNNPRNMGKLDKTDPNVGTGLRGAPECGDMT
QMQVKVNPDTGVIEDVKFKAFGCGSAIAASSYASQAIRGKTWSEALKLTNKKIAEELSLP

PVKLHCSMLAQETIQAAVENYLSKNPSLKSKVQKKQSTEASTP*
>TCRU_3032 hypothetical protein, conserved
MSTSAYRVASLLRQENNKLCSDKEKNTLARLTRDVEAFLDHAADVPPVVMETPYDDGETD
LMEVHLSVVAGVLEPCEPASDVTAVDGVFMPTAKNHVAMDRKRVREAQAMLNLLGALSTD
CKMPTASVAAATRCRLSLGSGEKADFESDDESIAVFDADELENDTSSSDGESNVHGRPPP
RILELN*
>TCRU_3033 hypothetical protein, conserved
MPPNARLRIRLIGPSGHKSEYHCATLLGNWQEERMSFGLKDPAKEPLEGQTTYLASYRGT
NNDEELLAAKPPGCFCAEAPVQLLFYHGDIVAPPQSQFTVHELSYTRPTRDVGANPPKTI
LDKEGISQRREKTEALRRAMGFSGHRTAVGAPSSLGRNTEAAPLAYSTRCATERDNEYTR
TPRRGHYQPAKLPPIGRLQAKERFLTTKNVTFDATGEYLKDNIDEYPAASSECWGQFMAY
KDDPMNRTRLREEYER*
>TCRU_3034 hypothetical protein, conserved
MEGTPPLAYRRLVVSVSKDPVECFVRAKGDGRTVVPAQTETRTGWLMCTVTLRSQLRHWM
RAEQVALRCLLASRPTRWSLLKGIQEHSSSEGVSAGWSAHCSARSIGPFLRWEDGLLHFL
GSCGGGGSWGNMPTASVLAHRVLVAALRRRHNLPLQDESNVFLFLCQQWTFFLLCCLGVS
AEQQVCKWLRGMCDCRV*
>TCRU_3035 hypothetical protein, conserved
MEGIGSSFPLMGVRTNDHKSTPPFDLLWAKKNKWEIIFNGSRLALPADAKWELNTTHRVG
LEMDYFKELLVYVDGLTVYDGGGEDYSFFEETKTVLGFHSVSHF*
>TCRU_3036 hypothetical protein, conserved
MKTKAVAASEARVPAPKGAPESSHASESSDEPANGSVAMDEARQYATSSSPQRQDSPAQP
LGNKKGPTVSRQTFSSDVIGLATPAAKGEMKEEAASSGGMAPALSPTPSAGSRQEVLEAR
KPFGGERPEGGREPLPPNLAASTMGQAGKADEGSLRNGTEDDWTQHRISLYVLRSVHNEP
ANADTLTSEEQDADHEEDTHVRTAVGTNSGPDLLNGTYATPADATTATPAFNRDQTIVRH
KDNV*
>TCRU_3037 hypothetical protein, conserved
MAMMMTGRVLLVCALCVLWCGLCGVAADDVYVPPGDSGTSGKNGVGVGVQPPVLSESPGA
NEDPTDKDTEESEGGSLESDDLDGQSLLTETVKDGVVADVSKDGLPRQEEGTIVTPGQPE
PVPRVELPSEAKATRPLAPNPQEQLPNTPTEDEPLSTGLGGNRPSGNSRKRPLYLLQRTA
VLPAIKLPEAPRTKVCTMIPCMGPRLRCKTTVRETRN*
>TCRU_3038 retrotransposon hot spot protein, RHS3, putative
MTLFGLRMTTAGGHHITASTVRQFTECLAAYFNGWEELSREMSWEIIYVQHADSTPMNGW
QRCDVVNSDSVSDDESREIAGFREEEKHQYQVSISSGALEETQLSEVRNSHGNGKRNWGG
KGAVLISGKN*
>TCRU_3039 retrotransposon hot spot protein, RHS3, putative
MDVKAMCAWMERDGTTDGQAESWKMVEERMEKVGPIPLHIFDASKFIAHSAAIEDALDGI
NSRDGERHFKHRGVRLWYSGDPPQKVVSVVRARGEVGAEGFLNAAISVCLGRRIAHYFGR
RDE*
>TCRU_3040 MSP-B, putative
MCHTMLLLVLLLCCVSGSVAVAEHHCISEEIEKKVGPRTTAVVLELPTRGGGMMRALTAS
APEWAPIRFQVFTEDLNDPSKHCTAEKQIRPDFIGRNLECNKRDILTKEKRSIILNSLLP
RAFRMHTDRLLVRPLTGRVIVPRYSSGICAQFTIPSSHQTEGVSGADMYLYVSAGPTRYS
TIAWATACSQLIDGRPVVGVVNYGPSFVTNSENSVRVSVHEVAHALGFSVWLLQERNMLK
EVLNVRGKAKVLQVSSPKTVEKTREHFNCVNATGMELEDEGGEGTALSHWERRNAKDELM
AGISGIGYYTSLTMAALEDTGFYKANWGMEEPMSWGNNSGCALLTEKCLMNGVTQYPEMF
CTAETTLISCTSDRLALGYCTIYLYTAELLQQYQYFSNLKLGGSNSSLMDLCPYVQPYSN
TRCSNGEASIMHGSRVGPRSKCLKGDGLVDFMGRIGDVCAEVSCEKGEVSVRYLGDDAWR
QCPEGSSITPTGLFTGGKILCPKYDDVCIIFDPLRGGGDVSSLLSVFPSISVILLVFIFI
SMC*
>TCRU_3041 BNR repeat-like domain/Concanavalin A-like lectin/
glucanases superfamily/Trans-sialidase of Trypanosoma hydrophobic C-
terminal, putative
MLVTLPVYSKEEGNGKGVLHLWLTDNTHIVDIGSVSGKDEDVTASSLLYKSAGSGNNTNE

RLFSLYEKKKGDGESSLGMVSVLLTAQLQRVKDVLATWKEVDGRVSKLCPSTTAKNPSTG
NGCSAVKTTAGLVGFLSGNFSGNTWRDEYLGVNATVKKGTNGVATGYADGVTFRGAWAEW
PVGSQGENQLYYFANHNFTLVATVSIHNLPKGDTPIPLMGATMNDNKKTVLLGLSHDKEG
KWQVLCGGKTTKELRSNWEPETTHQVAIVLRNGTQGSAYVDGQRVGDASCELNNTDSKGI
SHFYIGGNGGSAGSKEDVPVTATNVLLYNCPLDDNEIRVLNANKISIPKLTDLKTVAAGA
TGVGTARHFGANGDGSAVCGGGLLPLLLLLGLWGVAAS*
>TCRU_3042 phosphatidylinositol-4-phosphate 5-kinase related
MFTRRAVRFCTRRLPSGLLLDGTFNPKHPDFLLEGCATTPDGRVFRGHFDAERGFPLGGS
ELEDDGDLYRGSFNAQWQRHGDGEAWLADGTYYKGRFCEDELVEGVVRIPNGTTETVFEG
LLRDEAFVRGTLTQHDFTYEGEFLDNKPHGRGRLRFATGAEQEGTFFAGKLHGIGCKMKL
DGGFVYVGDFVDGCIQRGQLFTPTYTYDGEFNEHGRAHGEGSQTYLANEPRLIFAGIWEN
GALVRGTCTDEYGAPVDWKDNYELQAKVFSEDGGDVAVAINSYGAAKLKEADTLHREMNQ
SYVKDAERVRQETGRYPSKMSLGYEGSIQQERESLERASRKNVEDMRNIRDSMEASHADV
NCMCEKALNDATIPINENTARMQYARQEGAQQLAAERVDEQFERFMKTFSRETDGNASRR
IIDGNSPWKAYTPKG*
>TCRU_3043 Metal-binding domain-containing protein
MDDKTLQCTLEVHSGDGECTRRTFTVEHLPRTLREVVELLKAQDLRLQRGDFEFSVICGE
SGSPQQLLADRDVNNLFSSFASERLLFKVTLQERVGGLVSSMLADDSLVHLRIYHRGNDL
VTEKRFIPPRRGVREALVIAVRDAIQQPPPASVGMQLYAIVGEMCDQTALKDEDAINTFL
RSCMVTRSVCRLTYQFDNEGVINEADASIQTKNLGVNNAQMPPSDPIQANSATPQMPKCN
DGDDVLCETPSRAEDESDAPCETPSRAEDESNAQASSVAPVNDVNSALVSKSSSHNGSPA
SEPLTQMDAVSVPPETAVSAARNASPLRELAKNGRQLLDGAGHLKQNDGDPPAVTLTAKY
EEAPRPIMLVAEEKVPAVAVSGRCHAVDAKIKKEKLRIEIECAFGDSTVVFPFSWRRDSA
SVLQSLREACLIALEVPRDENVNLYDRKGAVLHTEEDTCKRLESILLSGCTRIGLVLWSG
HPCTSGLPLQCCVSWGTQQIVMVCTVEREMALMDLRRAILDEYGMDYRTIDGLHLFLSNR
EMEAESELTSSRAYEQLKAASLHEELVNIRVSIRDGPSLCLSMERNLPLKTAAFVEEALL
VLGDRPHSNDVLKIFEECSGKTGALEKDAKDFIALVIVMLSEPRILTRERLTSFLLKTAA
PCNEEGVLEVLNRCVAIAQLRVVRRPFDVLKRFFRRLYRVIQIPEGAKGIPLTMVTRQLA
QAGLSDAKGLLRRDRTILAELQENDYTELFLRLYFTDANMITRAVVAARLSGIGKIRSRR
VMTSRGQEMLREDFMHEIRHAFDGIRSTDVLKFLRSQEPHPEPKFRCLLSVMGSLLATHP
VQHPDHWFVERFRGKVADALDVKLRTFEDSVVSTPQLYRLCWDVLKPDMHHLLLLPESPV
ASAFACWAFFAVQLICVNRHLEFPPVPLVDLQKFSLVGLPISPVEIARDDALNVVSPAAA
ASGGNDGGAAAALPFRTNKPRIKYKYSYLCHDLCRDVRVERPLK*
>TCRU_3044 wd40 repeat domain-containing protein
MLASPQCTLGGELLAANVTPISAVYCDPNGGLWAIHASGMCVFYFSASAVADIISSTSSS
PSASFSGVAAIGAKSKAPSAPIFECGEEDQILAVVFPTTRNASRLFACIATTEGRLVLVD
TEEPVNVLRQCDIGTLFTAVTPVPMDAACEAAEDGAGVLVSSFDGTFSEVVFVHWEGDDK
AVVSATGLFRIRGNLHNVILDEASKRILTITQHGEVDVWNWRKGFDETLSFGMLAYSADE
YGEPSCSILLSGGRLWIGTMMGYIVVFHLRPGVSDGSPRHVWQAHSNAVAIRSLLLMSLG
RHIWSYAADRQALVWDAEAWTLQGSFQFPGDGFSKLHGGLRCMDTIAWGIDETAGTVTWF
TVKEPFLRADCGLPFGRDECVMPRGEVERLHLLETLVSHLCSQLLHAASSDEGTAVRERS
SSLNDEHTPKNGVKEEEEEQNMATPPHIDLALQQLDEDHPSARLLPPAVASLVVGWRMVR
RTLADAGMRSRSFLEDVQVLLQDNRRHKELQAQVRRCLAELRQELQHGDACDTLDDVVDI
FRALVRRVEEDSPAASPAHSAIDRNRQTSSRTHSGSTDDAIKTVPSLVEASDDVRARRLE
EDLMYERQRRERSEMQLTEAYDEKRELQRQLTQSMRLQEDYEERLLSLERSLKTAKKAAA
VTATEAARFSEMEANLNEAHHTIHVLQDKLEGLMRERQESLQTAQENKSLHAEVQNFQAK
EQVARRALASFIETQDFMLDKLSDVLQHTNTDVELYSEVSSLYETFCSRVEMQHGFLVEL
KRGYARQRDKDT*
>TCRU_3045 leucine-rich repeat-containing protein
MPVERTLGKEVNVDLHTSAGQPYDVQQLQSEFHAISPAEAYGELCQRFQCHAIAAVAAMF
PSGVGQWNGTTELNLSRLYVGPKGVRPVVEMCKRLPALRSFNCANNYLTNDSVYFITRMA
MFHPALERIELSYNEFISWTGGTFLTELVVRNTNIKEVGIRSTAIPTRVAEAVFEQTRRN
CVLAYQAVGRMPKPTNHPAAIHMRTMKRFFMDIQENGTVPVSALVDGFRERLRILGQERD
LSKYTESFFETLCRQVPQDRITWEAFIVTLRTDGSLYDAEFVKKVQRVFLEFNIEPSAGT

EGFVEVRDLAAMFTRLYGEPPTSKELASMRSLLGLNDTMTLHWDEFLPLMYIRGPKDKCM
AMGWNLSPLYIPTMLHF*
>TCRU_3046 hypothetical protein, conserved
MDYRDRVIALYQCYAPEKMAGVDAQLSKYEGREEEFIQAIINKYGPEPDSAEGIAENEYN
AQEGEEDEWYAYYRQRLVNFYTIYASEKIDSVDNQLKKYAGREEPLFEALVAKYGPEPEY
ENADENEKEAADKTNLSYENVGENENKTGSKEKLSYRDRVVALYNLYVPSKLGDVDAQLE
KYRGREEEFIAALEQKYGPEPSVAESASADYRSRVVSIYERYAPGKLGNVDAQLEKYRGH
EEEFIAALEQKYGPEPSIAESASADYRSRVVSIYERYAPGKLGDVDAQLEKYRGHEEEFI
AALEQKYGPEPSVAESASADYRSRVVSIYERYAPGKLGDVDAQLEKYRGHEEEFIAALEQ
KYGPEPSIAESASADYRSRVVSIYERYAPGKLGDVDAQLEKYRGHEEEFIAALEQKYGPE
PSVAESASADYRSRVVSIYERYAPGKLGDVDAQLEKYRGREEEFIAALEQKYVYGSGSAL
PSLYVSGTLSPVVSEKAGSSHQMVLMGMALTTIMEEESARRLELLEAFTAGLMSLSMDAE
SERRLKVEHQEQLAAAVFEEFFIRLQIIAEEAMGRPVLEHPHFPFVSDKSADAVARFFAA
LGTTHVNGKAFIMASACRLLFFEAVGEELLEEPQGELVSLEEFRQLLTRACHCEKDVVAR
LQSFVSMAKRQERPTVLAAPQELCAGFWAYRCVKPCVAEDWQRVWVVLENDTRLVIRRPN
IKKPELTIPVGQVIKCQLSSMLGTQAPRSCAKNGLYLQLSSGVNPVLLLLCPQRVANAQA
LVKMFFRGFPQKTSGMTRDVVAEAKDTLMNKGEVKISAPAAGEICRTEDACVATIVWCCR
GGCSKFVRSVWTLVGDSIVHQAVGEKENFTWSLATVRDVYLVNELGMRPPVPTAKHGFVV
LLEKGSLFCCTETAAARDAIVGYMKRVVFKHFLQSNQKRASALTSLMTEESKEGKGGAPL
ASAKHRVPGVAHSSSRR*
>TCRU_3047 hypothetical protein
MNVLVNGSLYRSRTGTLEIKRRFGFGAVLVSYEGNRPCIAPVDALGRFLVRDGCSYDVYS
LQTGESSRMTRLALQRDARLSQRETLLSEVPSQLPERKRARSITAKTPRRKGNYVRFVQI
FLKSFQSVSFADAAKEWRLFSEELKRTAPVEELLERVRAKGAYQPSLA*
>TCRU_3048 hypothetical protein, conserved
MMMGYDYQALKERLLSDVHLLSVDSIIHMLSQAYGTSSDGSDEANYLIQHLLSRPLKPVV
CRELLELLATVGNSEVAQSLRMKAVECIRGEYGQGSFGKHDPIRDLATVSIAFALERQQT
LAVSAGTRMLLEELIARCHGMSDGVWDRYSGRVTGVLAVALDVLASFVQTGKKSPSLTGI
YHQLEQFPESWERGSSPSAVITECAACVCSACHLLRVFSLLKTPFSTAASPSSVETTTLG
DWNGTHGSLTEALLRRCTDVFSREGTLLPRAVVFDMWRASTGVEGTSSAVLQPLMELLLN
YTEKHPADFPLREVAKFFGTDCHRFPAEAFELFVRCLRNESDNVVSSHLDMLVSSMEGVA
ARGDKALLQRLQLMLFLRPIGHEKALIHAVLFKTPQLVRLFNCCNSASDLTASMQATLLE
LMVSSCGRCTTMEELNTAASLLPHIERGSLKDALAWALAANAQRMHPTVTPDVQKALLIV
YLQKGGVAVEEDLLQALRRRA*
>TCRU_3049 conserved protein, unknown function
MWETRLISPPRRSLSAPCATIRHVCSFSADEDVERELRAEVVEAASSLFNPRLRITRAAF
LFPSFTASASSSGETTKGTDAWQALLNSRVCGRKEVVGEPILFDRLRTANSQINLLNSMS
GYTLQRCAELEEENISLSRTLKQLLDENRQLSERLQSCLRELYDERKLVAVLQSELEASQ
HEKSAREVSK*
>TCRU_3050 hypothetical protein, conserved
MHTTEVESVPRRNPFKNQWRTNLPSFHDCLASLGSRENFRSFGEVLAAVSPPPREIVGSP
CALNSPKMNAKLDFSFFTLELSDTTLPGTPPHPVERYQQSCASSTERTPRISDEDGQGEG
ISLTPRQLPPLCPNASPIAHSPSAVGGSCSAPRTEPQEVWLTHSVERAHPALLKEKGNLE
TLLVMENSPASIDHCARCGGMTPLISGGDFFSGNDNGVGQWGCINQRVNSIIPPLCEWSD
GKWLRDPINCYGDFAYHHPLIYRVSAGAARDDVIREMRFL*
>TCRU_3051 ras-like small GTPase, putative
MPDIPIVVLGGGGVGKSCLTIQYIQGHFVDKYDATIEDVYRKPIDIDNQPAVLTIVDTAG
QDAFGAMRDQYLRKGEGFVLVYSITDAESFQQLKRIYAQLCRVKGDQSVFCVVVGNKIDE
VNYRVVSTEEGNQFAAQVRCPFLEVTAKNRGMAEKVFETLVRTIRNGGTACEQRVANGVG
KPPCTDARGSGATTHPNMKAAPQVVDPPALPSPAYPKRKKRRCVLL*
>TCRU_3052 DNA repair protein, putative
MARQTRDVTSARAFTCLPPDAPWTLSQHFSPIVTLAIHRIGKVEVSIGKKKGNDFDSHRN
GDCMNKVRDYVVVEDVEMVGEEEDVVCVEPATSQRLLLPRSDEVDPCMEFANRVIQGWCR
DTQRGRTAGNASNPVFLLIVFGERALRRYVCRLTHTLEDFQTFELNGFVAAYQARHGADI

RRRAEAPATEKTTHVNLRTWFLANNDEVMEEDEEVAGNHNNDDEDVGSEVGNAVKKGVGI
CIEKTPTRTLSQGPSFLFSQSSRLSHNPAISNDGSGGAESVSLHQLILSQAPLTPPSEEE
EKQQNEEKQRERRHPGTLLCLDSVGAGIALLSVASSDCNLPSPSLRVAVVNGSLLCASEL
ISMVKGVHDAFALPTRILVVEQELRFLRMLEMTQDALEPQSLRQLRVQVLVEQLHESPVT
DRVLEEEREAFEALAHAKATLTGSLLADRDTLRTVQENLDSGLILQKNLGRRQQGLPGLC
GNELREVQTSAVSGSAPTTTTALEAPLVVFDERELRSLLPYALYRRGIELVPLTLATADY
VLSPLYALERKSAPDFIQSLFSGRIFTQLAALSRRYEFPMCLVEFDRSAPFRLSFSSPSP
AATSDVSGPVAHFEGGGLFARIARLYASFPRVQVLWSRDPTQSAGIIHEMKRTCAREAVD
PSTPSLTRGNIDPHDSAMEKEASHLAARVLSCFPGITPGNAARVMAVCGSLAGLATIDES
ALVEAMGEENAKQLYTFLHDDLVAKVF*
>TCRU_3053 retrotransposon hot spot protein, RHS3, putative
MPERHGEGNGENGTSPASTVPQGLTRARARWVSRSGQPAATCRRLEETRRPKPRLDSEVK
DVLLEGELEVELIRLNDFLREHCGPRAVVDEVHNVWLGVFLMDPDAYVQDQQLLGQILDL
EVYQLLQRSQKLVMDTMKLHERGVHRLEQWWGFERKDMCTPLARLKLDLALKVAPKEGGE
EMLEGFYESVFNASWHHLVEFPEGEWMGMMVRRGKPKQSWTYRAVGGILEKDDSVEQFSP
PRPRLMVLVSDKAWPYSWMLENSTRDCYVNCEVERVWQIVKGDLTEWFSTHHGESDFTPE
ERLLIGTPGIGKSMNAGSYLLYQLLHYDAEKLQVVVYRIAERSYLFDKITETVTHYAEES
TFLKVMGELSGRGMRGYIIHNVAEPILEPSVRLLPRGWCMIVVTPPEESDHRGRRKQLGG
RRIIMKCPEENDVKAMCMWMRCGSPGRQAVYWQEVRDYMDKVGPILRDLFSERDTNARPS
ATSEPWDGLPYQMHMTTLA*
>TCRU_3054 hypothetical protein, conserved
MVHTAVARALTPRGATTVSRHDAACPDACPCRLHYANNNPTREASLSHGFSRQYGVLNPG
AKTSQSSMKSQEESMMEPAKKYRLAPNGMRRPTLRSHTECTLYSIELMLRPSLKNASVSD
EPSNTIEENKLAVMMRHRQLQ*
>TCRU_3055 DNA topoisomerase type IB small subunit
MTEVTPLKAPLVTGAPTPPASYPPTKVPVKMPYVLPKPPFAPVVPMRPPAPRLKRDGDDG
HLAVPGLADTSKRSRVKDPERRGRAMSESSNSESSSNSDSDDDSDSDSHSSSSGSSGDDE
SFSSSTEGSGSDETRSGDELTLFEIAKARGLVNERVDNKKEEEEEEVAPVGPPPRPPIVR
SFPNNVDLALQRYEERLNREENMIRIKDDNKAVSLGTSKVNYIDPRIVCSWAKEYNVPIG
KIFSATLQKKFPWAMGAENFSF*
>TCRU_3056 Protein of unknown function (DUF3720), putative
MGLIKAVEAVESGEGSSGDPDQPQDKSKVSSPDKKLDNNTKGIGASDSKGGSVAVQPEAA
PLGSGGSDTGGQERGSNVLGPNEEKEAGRMDQKDGKGTGPHNEGAEQSSSLPGSPGPQSG
EDHNLENTSESDGASDIQLKKEDAVEAEVPKISEKEEPGGGKENENGKALTGIPDSQTKM
KETKTVQPPPLLTKTAEEARITIGIPAEDSTPAETNGHSKPENTVPVQQLQLPQSDMKSN
SNSQTEGIASITANSQNETSDGHAETKPPPTENGNFANNEADKSTEEGIPNNDPAADGAG
TREEQNENKDANPKETPVTATATKTTTARAGDSDGSTAVSHTTSPLLLLLVVACAAAAAV
VAA*
>TCRU_3057 hypothetical protein, conserved
MARSGRSFFVSAAVGAFAPDLSARWPQRVAVTLCSRRYAATLLLVCCFGDNVPAAVRLPS
GGKCVSAVVESRVGCLAAEGIAHPTQHRSCRASSITPQHLSRRQQHAARQRMPLPPRVRL
EENASAVVFRGSHCVAQWRAQNLFAESAHDTGHWPRSVCVVEHTAVESLFPPRGRTPDFP
SFSHPGGWLRDDATTRHLFSQWRGNICGAMHWEYERRMQ*
>TCRU_3058 hypothetical protein, conserved
MVRGRSNTPCKRPESGRGAGTRIALDKALFSVVSAAGCDAIHPPDDAAALLCRLVPSTPQ
KEGGNGAAKNFALDVEVLILDAVEIVLAAYNEFTASSAKEGEETFKSLLMRASLTNLRSL
ATLLSHRPPFSPPVLAALFQLTILVVKHAPLQALDEEGCRMLLCLLELLGCLPRGVETAG
TPSIQRVARITARNVMRRVGPRLCPSAFSASVEASEKVAKSAVLREQLERDIIPWVERHL
LPLHAVTGAAAQADLKEDRRLALSLLEFMLLSDGDACGPMSSQRISIYYLADTVASLLEE
EDEGGIDGPDREVALAFLHRVGRTLSNPAAASLVSTSREDGAHEGCVQSPPLRVRDVREF
LRAKDAAYVAEFTQLHERIEEEQENQAILQWQLGELTKEHERVQREIQEGILHSFNADEG
NTSVCTLMKGDSTHNTTSAASLSLGERARNILSFLRPVEDTTIALNEQKRRRSLF*
>TCRU_3059 RNA-binding protein, UBP2
MSQQMQYYAPQQQLQQAPAQMNPEPDLLRNLMVNYIPTTVDEVQLRQLFERYGPIESVKI

VADRETRQSRGYGFVKFQSGSSAQQAIAGLNGFNILNKRLKVALAASGHQRNRNGVSTGF
GAYGGYGGYGGYGAYPSAANPYAQQQMMTMQHPYMMTQSVPSVPRQ*
>TCRU_gamma, putative elongation factor.1
MQQPKTVEVLRSQGATFGPVKAERKGKDAAAPARAEKKPKAAAAAADGAEEEDEAPREKK
KPNPLDELPPSPFVLDAFKREYSNTDTRTVAAPYFFQHYDAAGYTTFWCRYKYNEDNKMQ
FMTANLIRGWFQRMEHVRKYAFGVALIIGEERRHDIVALWVFRGRGMPAIVEDVEDTELF
DWEEVADVAAQRERITDYLCWEGPTIPRPVLEGRVFK*
>TCRU_3061 hypothetical protein, conserved
MRRQMYTREELIALREALALDTFADPVPLDCPMRTDSDLTLHQTTVRKRNKEDWKVMSFS
KRISAFNGTIGENNVTTRPDIHRANFGEASITLCRSNRTEEYTDCDDCGCKVIMPPRLQI
PFVTIDPMRRHYNTSSRDYKKKAEVNIDMERNMVARLRLRQQRQPTKKNPTEYREKQSRY
KEVPDSCMVFATSSPPKTEDASRGAFVHITQMIGLGSSTVPQQEVGSAVSISNVSLFNSM
LGSRYLGFTRSMGLWHYLSKTFDGKYLHVLEETADTGPSNGATNNNPIGRHHNGNESSIG
PDDSGTTPAGEEKSGSEQVEETHGNYEVLKQRRPHRRRTNRTHAEKRVNRQLKEFFSEMK
TSGEDCG*
>TCRU_3062 N-terminal region of Chorein, a TM vesicle-mediated
sorter, putative
MFIANYISSWLSTYIRWFTKHWNRNSISVSILKGCIELKDIELSTDITAFFRASIPLERA
SVGRLRLTVPWTALYSCQCELFLEDVLAVFKQHFQEEVADDEGNPLEGKKRIVELLDAIR
YKSQGNTETKRQGFLERLKWVIFRNMKLVVKNFTVRYELIGTSPLSATIRLQYLSCIAAN
AMNEPFFVTDDECDLYRIILFQGLSFTVASLDCIETSNRSWRTDSLHSFTSFLIKGMEKE
VEKFGYVLKPSSGRAFGSLQFQHPKLQLGAFILVHSCGIDLDFKKIQGLLMMTSYVVSFW
PCDKDEKKLRPIEKPSTVIPGSCAAWWRYSIDRIISNTRGCVKRTAFHWATYQDWKCHSE
EYYKHYLLTLGAPWLFGLDVNKTILEEALSSFSIGYLIQIRQEARFTLEEFRLTGRVFPR
HYGDTISDRGFGYKVAVVEVLKDSVVNSPMQLEVSISLGVPKLPLFLRGHNMHFNIDLTD
MSLRVSRSADLLQILFSAYSIQAFSEVSGFNRVNTVFIIDGTKTTKGVVVTYLRNGKNKD
LSFVLTSFKIRYNQCIAGKLIAEFYMLESENALGWDNFVLYSLHDVAYPLKTQVNIGNTA
LKLGKLEVCFEDLSFEVQWRCSRLFVSEISNSPFIDLKNVFLPYTTDSSIFIEESYICGD
LYSFYVLKNMIQDMMVIYEPIMYWLRKRDTIYSTETMNIFELKSPFFMVTLPGEGTFLKI
RQFAIHLSLGPTWILRASCIGMACDGVLSDTNMHLMVLGGTNKKEIFLDGPLKNSDSDLI
VYATFEEGHLIVDNRLVGLVEFLNDLLFAMLFPAEINPPVFLDESVMIPSLEVLISCSNL
SFDVLGESSGTWNLPFRLSCNGQESLLVEFKRFPDTHMNTTVTFGRCFLCSVDEFTLILP
SYSNKGVLQLVMDWRPPMELVTEEKYSTLTASLFVVERYFTFHLPALHEFLHQFNRNGSA
LYRLRSLGSRLHFGLREPNKEVYSAPLWPHRTIIKLDAKNIIMFYHPWYKKELSDLSSNT
EFFVRTSFSSCKANWSFDGFKGHILMELFLITGLPRVMKSSCSCASNVLSKNNVLLVELR
QDVTYDLEKQKEAFSIVMRGKDMMKIPQMFKTPIGAKPSSCAADMMPFMVSFEEAYEFIE
IVNAAFDFSSPQQVTVEISPRHERVLKINVAPFLVYSPSLFDVAFVSWEGIDFMIQKDGN
FEGTMVGISLLYDASLRAIENQEMTVLWDHLHTVKKATAILEVLGNQDTEEDFITLKKKS
DMDSVMLHIGGVFLHITEKKLPLLLHQFMEVHNFFCSNTLPLPLLPRENVIATSELNYAL
MNIQFDEVHLGFRWNSRMNGIYGELHCALFAFTWRSNGALTLKDGVVQLKWFDAAPLGES
MGSVDLLLQEASETVPLIEHISLSTEESRLGKILVKISPIHMNLSFDTYLSLVELVVYHL
RLELRRPARRTSGDFVSVETLGKGTLDMKKSLSSASYLFSCGNGDTLTSFSGAGFLLQSG
DVTPTWQFSLELAKVRLTVDTERRVKNGDRKNFKPLLWLNLTNIIFDLFPNGTSFALDEV
RLSFLRETSVVSLSGLQFSTSRASDSMPSVPEMVYMVQSMITRFFTPDLLTCFDRLIKTP
AEELAELYWETTHPSGSDILCRFHTEDSTRDNGGNGDNRSGSKKGNYRKMEVLTVGTPVW
ELTNDLVLSRNGGTVLQFSNVNTNNIHVEMNGHSILIETPRKAGAEIVMVVDGGISLTFC
NGRFFLPCLLSEETRMRRLERLGEEALLLPFIALGECSYIRCFKVRYATTERHNLQHFYH
EEQKQQKEDGVIESVRPQRWKMHAGIHSVVLHVSSERQKGIVKATVAVEAKISLHGKKIE
RGEISLENFSVVTRTMDSTTNNTTNTTTTAVSRSSSPKSNSNMSASAMDNVLIAPVSIHL
CISESRQFAFSLGRVVVDAMVGDLLLLSIMARELFLFARHVISFQLAPTEQEYRLQIEHV
LGWNSVGMRGDQHEEGGDARQRGEAPNEEPTNKMPVLSLALFLPMMEVKLSNLKYPLVQL
SLADIVWKVNMFCTSRTTLLQSQQLNIRVYGKGRWDTLIPPKAVISLEAVHSWSRRTRTE
QVELQCDGFVLYASHLLLSKMMHIHREWSEFYSTIFELSHHAQEASPPIGLSNILNPVST
ATHRFINVFDDVFYLFVGKDLENGEIVPLPSSTAVDLTLPFNRAEDVRVYLYPRHCIELN

ENGRHVRNNEGLRRDMQHCHVVASQLQYGRAFGLVLDNLLVVISCVETEGIQKGMPPLSS
VNARCQQYGDRGAKPLNLTNQANLLPPFRKLPMASFGGIDEAGLVIVRIHSRRSLYNGIG
MMIEVRQYILADSEETPMSEDFWIVPPNSEYPLVALCNRLELRFSLDGDVYGTSFSTPSL
ETGVVLAFQRIGNAVSNRENSGFLPVSVFIALRRFPESGSTVIHLLPRMTVLNHIGVPIK
LSLWQEEGTEEEKEEEEEEERLLNHRDDSVSSTSNHGERGSSITANSGQPSSRWPHWRRR
SSRRRLISLGEHGALPHQGALSICRCSYSSGLLLSLSFTQTGGETVQTERTTKVCSSLKR
RVGREPCLIQLRDSSERSFYVQVTVLHRTIILSVGYWVINLTEYPILLKDSYTSRHLTAG
QSMNTGIPPSRGLPFLIGSRPAEFTVALLKIGLDEEWSEGIPTQVGSHGVAESPRRMVGG
VTRSCNYVIQFPRVQEGRPAVLLLTPRWVFINNANRRLKVHFTFPVAKKMPSLKKLSSRE
QRSPRASEADTDSLPFLTLRPGEHQFSCIGTATGNMASFQDTLEDAVPSVTSLFSDTVRH
KNLAEFYEAHRTASISVDGPGHAVFNLWASLKRQPAVPSVFYGARIANHRSSSLLPQSSS
DCPEFDEDVAFVTGRISVTVQHIDNVLAVVIKSIHSTNMVIQNRARSVTLAVRQKGSQRR
TVIPPCQNRFFLWEDYRTDPVISLHVIGFKGAWFEVDFSRGSYQVQRRHTSELTENVLSP
FSFRACISTRNAQRVTILFTDENFPPHTSFDDAWHRSVGRTISFPSLELLWLMEGQSTGD
KPHLSLVLRDLQVQTLVAGNTHAISIILKRLQVVDVRRQAIVVLHRANPATSNATEVSTG
GRILSWLWRGEEVPQQDFQAVYAAMMSPSGVTRVTELSFVFTPFVLNLSDQLVVSLWQEY
RGLFSFFKNNGSKTTLVSEALSSTPVSHHEFSQRLMARMQEPENCTAEISVGNTSLTSSS
AIPIVNYFNQSETHEGPALLFIDQAHFSRLTVFLTFSRQKPDLLWELLGMYTLMIPKRLE
QKEFSWNALVVENGTTTWGLLLAQAQRWLMDGALRQWPKVTRLGDIVDTFKRGSNRRVTL
AGTPAPRLADVNNFSDGDDDKKEKDKRENEALSSSRRML*
>TCRU_3063 PUB domain containing protein, putative
MDVAEADWILERSPPDALNRAISTVFRRLLANIVLEPENPKYHVVREENKFIRDTLNPLP
NSVVEALFAGIGFKRAQNANSETIYVFTGNTENIKEADYFLSHLEDRIEKGQRKEAEMHF
VPPETKVNREREERRQQVLQEIQRDAEKRQGETTGSEPPTLINAFYPGDYTLVENLIEKS
RRTLLSTGRVRNFLFEGRHFTLRRMAHGRVYVCKEGCGTDFLEAHWHLFTGKNLMYSYVA
HLSPDASAMMHLGVEHGYQYNSLPGTKNFRRTVHFSAKRINETNGLLEYIEHPDRPSETC
IYCGKPFSELFL*
>TCRU_3064 Glutathione S-transferase, N-terminal domain containing protein, putative
MSLTLWSGVNPENARTHKLLAAAALANVAVTLKACEYGRENETAEYCRNCSPCGRYPVLQ
TEEGCVFESNAILRHIARLDRSGGFLYGRTPLEGSQVDMWLDFSATELDAASAPFVHHAF
RGERGSLLCHFFFYFSRIDCMARVAVTRAYGLYGVAAAPQQKVFFSGGVLCVRPCANNRS
VGKALLYFWLTIELCPCFAKK*
>TCRU_3065 Mucin-like glycoprotein, putative
MVTCRLLCALLVLVLCCCPSVCAAGSEDENEQSAVPKIDEKEVTGKGTDPRPTTLQPQTR
EEVEGSEKQETNTPTHTSQETAKATGTAESESSSNNTQDQGHEDGQSTEEGTENTNTNTS
NEQHKESNAPTTTTTTTKTATAPEATSTTTTEAPTTTTTRAPSRLREIDGSLSSSVWVCA
PLLLVASTLAYTTLG*
>TCRU_3066 Trans-sialidase of Trypanosoma hydrophobic C-terminal, putative
MTAHNTESVNIPKLDHAKPEPRGTPSTPAGQQPSELGHLVGSSKDASGGVEGADSQNRGV
NATALSSNLVNVSQENNNDASSMRGSGLLPSLLLLLGLWGLAAQ*
>TCRU_3067 hypothetical protein, conserved
MRAVEAADDDDAMNDTSSAENSEESASGAEGKGAAGAAAPSLDAGVSEADEYKKESNAFK
ANGNAINTGILRKVRQMSFNFAPPVNFLLNPAALPLHRLGESPFRG*
>TCRU_3068 hypothetical protein, conserved
MPMSQPRFTTNPERIARLEENYNAGVEREKIPVAEREKLDLECLNVSSAKLRFLYQNSME
SYGPDVKKNKVKEYNLKHGSDLSRKWFGIF*
>TCRU_3069 3-oxo-5-alpha-steroid 4-dehydrogenase-like, putative
MLKRHGGNLSSTWAWLLVAVVFLLITTVCMVLLQQYVLPLGSTTVIESASYFHLVMAAMT
YFTLRFVEVAPFGRHATSTTAHVTMPAPLSWALQECPTLINVVYYLLMEYPCHALKCGSS
QWIRLLFDDPFGQAAHAISEGITSLHVGLLLFVIHYLHRTFLYPLIIGEGAPVPIKITLS
AAVYCLLNGRLQLLANIRDGPSAPRFPLLFTSHMALFWIGIVLFFAGMGVNVLSDNRLVR
LKKQPPRHSRKIPHGGLFEYVSCANFFGEIVEWCGYSLVVWSTTATTKPESGLAAFSFSV

YVMANLLPRGYAHHEWYKKQFGGAYDTLRRKAVIPYVY*
>TCRU_3070 hypothetical protein, conserved
MYVEQLRRGIEEQERRVSALRQEKQYRDSLLSEVRGLINHVEPHLFHELNSDTQELLHTL
ENNVSVRGPPTNTLRGSGPHDLDSSTIPLFSKEMEERIMQIEEQPPHRERRPRHQRHGER
KKKKGKRGATRRMETSPLALTELEIKKREALMAALDSLQLESFETLPAEKGVLLWLSDAG
TLHQQRNVEEVAQGEWVGEEMQTYYSDDFEELTDVER*
>TCRU_3071 hypothetical protein
MSAGQSVVGCGDVSTADTHGPGTSSKFLPSPLYGTRAEVSATDGGASPLIKPPLYATNTS
GGGPMTAKTGMDESTGFAHDAEEGETPARNPARASSIAASPQSLCADEEDFSDRNAAAME
ESYFRRSAAMSRVQSGAVNAPVQFEGSFLGQTVMLGQSTQGRANTMSPSVIVSRSLPQLA
GNANCGDGGAQEICEECADGPSITVSLANSTNDGVGPWKRVQLVSFSAPGIAGASPMLTI
PVELASDAESPNKQRANPPRHRSIKDPAAPIGMHNSQQTAASSISFSDRTPQREGSPSRP
PSSACSTMDMSRQLNISGMSGSMSFRPPVGGVSLADLPKGSPLVFDSSMNSVTPTRGTPV
LSTTVQQPKPCPVNRTTSAAVPQFVTTFCGRSSGLATGNGLPLSRGVMGGSGGNPLLSSA
MSHIFTDPHELPAAPKSQKPPTAPMSDSDVFRPWLPLGLEQDVAAAEVTGWTPLGNGSEM
PEQSCNGPILAGNIVNDRNNDGDDDNGYGEKSMSPARPEAVGSGFSVNEDDGNPFFGATS
TTLMEATLLEMNLQDGSISVSVRPPPSNQAAKGAEDDRQGKNSQEARENRVVKPTTCKDK
VGQSTRGRRQPSLRNQSESQMNKETHGVVKPDTTVAGTTPAVLSTEEPTPTRHE*
>TCRU_3072 protein kinase, putative
MVGGKDAAKRAGNAKRSDDKKNGKGESGNKIGPAAHATVATHSDNKREHKTAKAVHAKPH
PQKKGPEKPGKSKNKSKDAGKSAENLNESLPLLPPSPRSNGVVEEAAVAPANVTNTPIVE
DLERNDDNAGVSFSGMQISLGVQSVPPVRREKRSGSGSTLCKDGMAGLFRADQRAGESLM
DNSDGGARWPKEAPTSNWTAGSTEDRDSWVNAVGGVTAKNKSRKETAGSEDDDDDEDAEE
EDDEDDEEDDEEEDEDGEEEDEDGDNDDDDEEEEEEDEEDEEDGCNDTVNKGEGWKQEQI
TVKISREGDRNVKVLYVTRRTKFRAFAKDIMRRFAYNRLCDFDMYCIDAAGDHVDIDVSQ
DFKNLITQFLQTLRLEKQKNAAVDARPSSKSSQSQHLPPKASRRFSARGVLRRSASLSRP
GSQTVETSPVADDGERSSTSVLRLYVRDSYRVRKEEQRRMHPSIATGQSQLVQLGLSSQQ
QTHRSLQITGSVGEDSSNEFFTPGHADFLPIGSSNISVNFSSTARFFSQTINFREDEDVQ
WSKMGLLGKGSFGSVYEGITSEGKIMAVKVLEISLDEDAENVASIQREINLMRSLKHKNI
VAYYGCQTKELSSGARQLEIFLEHCHGGSLSHLRRKFERAKERFSISLVRTYAKQILEGL
AYLHSMNVVHRDLKGDNVLISALGEAKLADFGCSKRIGTATMQQDGCGEKGAGYQTMVGT
PLFMAPEVVKCEGGYSKPADVWSVGCLVLEMLGRQPWIFRSNANAFQIMYQISKSTSMPT
GVPNNCPADLYSFFTRCFEHDPNKRATAEELLKHEWITCPDSKLQEVLEEAGGE*
>TCRU_3073 hypothetical protein, conserved
MMSHVRERQHRRSFRQALLRAAVTFWVLFSLCGRTLAATRTLEISYDMLTPPPPPPPTLS
ELNERLRLPHSSSSSSPFLRDGDGTSLCSVDHGCPLSSMAWASGTFTMSDVLTPASFLIA
SNLTLKLSLCCGYKYNGTLLRDEALYDVFLQVLLQASPFQVLTREEKRRMFPREHVIIMD
TVRQRRYDANYVLTTEDFRQQNDGDGDVILLVSGAVGEGILRIPLYRAAEDLATAAPANI
NLKIANVAIKCKCPHTLLVMPLLQVRPDTLLPVSVLGLGSHCGVHPLLPPTTPHHGENIV
QLREKAWAASVAVAPSQLPATRVQWVVTDTRTGSIVLDELLHVPTRTDSSGALLRHHANC
SYFEVLPNHKYEVNLTVSRGTAVQRATGFFILRLPIDRPQDVERGPPQLHFGFERRLRLS
ERTRTYLELQGALSPQHQPAEYFGYWENTPLNDQPLPQILKRIRHNPYAEETALYPMFVG
RNEIFFYVRDVDEVCDPVLLEVQEVWALQGILLQPSISPLTACHDDVPMEALPLKHVVAT
IRSSLQLPVRFQGTSWEPLQEQNFLEGRFDCTERGAGPCDIRTRVANGGTSGVLQMRKRG
VADLAWVVRVEVNETALVPPHSLDPSVARIVANTVDEGGVVVVEVRSRPAVVVRRMSTMP
NNVNVTLAMRLRRMKVHPPRSLEIAVDEVPIGHWLVAAPLLLLSVDDAFVAELDTSGVPN
VPQRLREGTLRFVAEDPGEAIDLGVPYTSRCPPRVVEHTVTVVALAAPPIVPALVPFYTE
DDCVHLRAPRPRWKDEDARWHVPHCEEAPQSPPYIAGLEKGKRAEHNVSSLEEEEVQEVQ
VCGIQVYRWCDVVWTARNPVAAVTQTFSLRRCQSPPQLLFHKDGLQEPRDISLSVYALPL
IEWLDGYDDVPDDMDVGGSAKNTTAQVPRYTARRVIPVAGYSATVGYSFNYSGLQQDFEV
TFKYAHDAAHQLGVTQQHFERVGDDGLSDEMAAAAAAAPLLLRRSLLSLDGDKMRDDRND
RNTNDNDNNDSMADDKSILRVGDTEGAVNLEQQVAERARERDYALDLASDTRFFFTHLSG
LRKVWFNGLDNSSHYPLLYKVWPSPTATGPSRDAAEFARRGMCLLQRGNISVFSVNPRGV
VPVVENGVLPFCYPSFLFSRHPWLSTKYKFDFSWSCEDDGVIVVERRTPTASILRHNGPA

NDTRCSLTVRNDLTETVQKDTFLLRRVYPTPLPPTFAILSGNAAMGECFANGTTRSKQTT
RLETNGSPDTTHRRTRTRWIFGKKLILHVDAHVGGRTSRWTALSVVPAVPSPVGTGRLLD
SIHFLPLPRHPLALERGFGDATVLVSGIRVPGLYTFGYYHPDPMYPGVCPNSSIVETLHV
VHAQVIEKELTVCGDSAVLKAVPIPREIGCEFFGQWEVVSLQTSENETWTSGANFTGIRF
DHGRRPRTVVRGLPFGILTVRWAVYAVSPTDPDTQRNDEERQEKQRGLLVDYETVRLLVL
TENLPSNRLVTLSQSVYLLATTSTLHWVLTETEASSSPLTLRRPRLRVETVSNESSTGSR
FGAGHQVVIVTNLPIGLTRLQYELHLSPRAFGPMLTKRCPYVPRQEFEIERVSAFLSANT
TLSHECDAYSGRGQISICLHAEGAQSRGILFTSSSSLPSFGARLVEPASFEAIVKSGAAR
LEARGCSLPWWVASQTRWSNVHADATGRCVSVTVTAARHLHPQEVSHHMFLAQEQLGSSR
LCPHGGDFMTSEIIDGATTWGDGGSTRNSLFRGGEIAQFLPFLHFATLTREERSMRPMLS
LLGVPDIVTTELDISNEGKAVVVLLRLDTAQPKQMGGYVHAAESSEDGRQRHSLLANCAR
GTVRASDVLNFWTEVNRPDGAEGRTAREWLEYVAAVREKHVGCEPLFVSLAHLLPRPDDT
EATLRRRQLYRVPPKEQRLVTLDVIEQQLEGLQREYRWTREGIDVTGLPPELQAALARRR
ITKRQLDEIERERRAKRAEMKQAEIPALLAKLTSEERFERILRLYEGVVEEGVAAIRLTL
PPHEPFTVPHNLFLRAAVHKDVLCGTNVSAGADPASLVLFGRVEVIDVPPAVGASPAAIK
QCEVEGGVPVIQVSVSGTTFAAMDVFPTDRVLLEEVHNPNSTGLTETSPEVGLLPCLQQM
HFGMRVAASSMTKKHLYLQLSPCPFFQMLSHADYDEQQDSELLQKKLFLPRLLRITLRGV
LSEDSHTKGKTSVSFVVKVLPTLARLHLRTLPAPEEGTARSSWSGSIARRPLLMCEGDVR
RRGAEFGIELIGDRWHDRVGGRGIHDDDITSLPENAWYENGAGRIGISGTISSSRSAVDG
ARYRSPHNVALINSFSIVEHHRLDSAHHLLLRHGGLYRSDFVNYLFSIMARPEITQRHQF
VYRYNDTFVSVRIPAAQSDLNDLFHGDEYDGTFDDDHLPPIRSFSTIEEILFAVPGIATE
CRGCLLADTTYFMAGDLTGLWFFPPATSPPLDNRVLVEPLRVPFYAASRVPREWRFSTLC
PTEAWDNLQLFLRHWQPPRLQRGSAPYLQFELVVYNLRDILGGEVPLSRRPNAILTGLPV
SLTKLLKEGRGRGNSLAEVVFQVNTTAAERAHATMAADFSRYGRRHLHTVHDYLRDHRGS
GGALAVNASVFLKVCVPSTAAVPEAQCSIVSVTHTLLIMEEPRLMSYYGRPRQGSWRSIF
DPASLLHHAGTSLTGYIIFPRLPALRRTLSLGNTHDVAPARIGLTVALFVAVVFLYAMAT
RLWAVICTLLWATLLFLVPEIIVGIYFVMHFLFWAFVM*
>TCRU_3074 chaperone protein DNAj, putative
MRRAAILRPAIGVFCGNNPALWGATLPLSASRRLLTGAAPYGSVPADIEESRHAQKPHEE
EVVRGVTRQTVTGAGGGRAASDACGDGGEEPAAAAAAGYNPWEVLGVKPGVPSQTLRLRY
HELMREVHPELDPKGVGNIPRLNQINKAYEIITKSPTLDRRYRNLVSDTQYFYYKFLPEW
MARNVDEMPRYWSWVKWRTPGAFQVFLWCCGCYMIGRFYAAFPLLTTVFLVSVAFDILFH
TMTAPATCSMLFLYAIMTSQSHDMAWLTSPKSFLRRELGY*
>TCRU_3075 Colon cancer-associated protein Mic1-like, putative
MTDVSTVPPFMVFRKPVFRFCTTAAAAGGGAGGNDASRVTRIYIDEVANDVVVFSRASRQ
VCFYQLRLREGPGNYEATTAEIASNSSLLQPDKNVDAVADCRKDPCEKVRENASSAANDV
EWMRHHPALPADAIALTVSSREGDGELLPVRYAKRCPIPLHCGYHDTMTCSSDSYHSGDE
RAYLVALSIDTRSVLFVLCHAYMGALPLYALATFQCAPKSLRFRDDFYPIQAYYWAEELH
GTQSPSCNFPTRRASSMVDLPGGKERQQHERRNVPDDDASSLTPAKFLSAATSRRRILLC
VTTISLDLVGVDEELLPCATEEKVGSRELCVLLRRFPTHSDYWHYCPAARTLLSVNRLKS
RILKSFEVSDEGLRPLPALRLQDEDSDGGGDEDWAISGDENSEMSLLFSSPACMDRPSVR
YPVGLLVLYRQLFLCSLSRHDGGAMLYRYISAENGSEESFVRYALLRPDFSLNLRRDPVA
LQVIDNLVVLHVLRQGISCAFDIACGEREHDVKSSLSGGTSESVATSMPASAPAAARIGG
WHGTDAASSQCGGSGSKSLWASLILEREEPVSWITRITSGLRGGDGSGSSNNVGRRSGSH
ARGGGYETFLPVLQPSLSAALMMYPPLSANTSTCIHMDDLYSHVFVYGTMPLLLDRCHGT
VHLVGANPFAIAATMPQTSQRVQFYLHRQHCAGGAVQSLLHTMLSKQESLDLVAHAFEIV
TTHHATQQLLQISRQNGDKKKTGTPSWAHDPLFLSVTAAIIPQTNGHACFMGSTAALRRC
LPVEDEEGPLPCGPPWRRWGGVTLQDFLSTCMTSFDGFPVPGGGGDGDGRVVLSTDVSIG
QLVMQREVFAPLADVVLVSPPFAGGCSGSGVRVAARSEVETATTKTQAASAREDCEEETI
DARSPSESRNSKGQLYFARYLFYALFEYASCVMRTGLELVDELQRLLIRLLHAAELHCGP
LRQLLLPGMITDQVPTAMVLLPMGGEYRRLGIDMLLRLRAHLLVVQVLLAERRPLDAMNY
VYANIQNSQDTGDELLNPRVMNDILQCALDAVVDREGNCEGCGINRRRSLEFIIVFEMIT
GALMQQPSIGRMLPPDYHTMRAKYMQLLAEGLGG*
>TCRU_3076 Fructosamine kinase/Phosphotransferase enzyme family,

putative
MPEPLEETFSRVLSAKVARAKRVFGGDINETYRLEMEKDDVVYFLKLQRGTTADFYAAES
LGLSLLSDVARTPRVVQTGEVNGDAYLIMTWIQSFPSAMRGGVRRNMAELGRMVAIVHRQ
TSPNGQFGSSVAVPLAGCKTSAGWSKDWATYYIESLLAPQVALARRKGYWSDRREAMFVA
FSKRFCAFYASKKNVQPSLLHGDLWGGNVMYGESGEPYLIDPSSYYGHREVDIAMTRLFG
GFGSEFYTAYEGEMPLEAGHASRVPWYQLLPLLCHLNLFGEAYGSSVDSAMAHVL*
>TCRU_3077 hypothetical protein, conserved
MSDGSANTLPPPPALRHRRLVAESTSEMNSGNWVSESASNHPMSTAQSPNSPTVVVATTV
NSTTTTTTVAAAAAAASAEARALLEFGEGYHRILEELGNPGYHSIKALFYGEEGPPGNTVP
SITVSFELFEAVQNRFATTKATLERTKEDLHTRHQQYALLEGQHEQLQGSYNLLEQRAND
LERKNTQLLQDIAKQGVMSELLRQQVTRMKSEAEMIEEKMAKRDEDLQVLHNRIAEGLVS
LSQKETVIANLRRQLGYGGRLKRDGVSGNLVGESDEYSPEALKRIADDVEGRSTEARLRL
CVVELEAKVRSVIDERKYVMLRHTMYRNHVQIVTESYEALCMSREDAMGSHVCEAPPSFA
VMAESTEDRMMLNNNNNNKFMRSSASRLSPHTPSLSERHARNLSPPFQESDFERRRSQGI
ASKRNSVTSRGDSYERTDMSPMTAESDTQPQRSPSPQNRFPVASMRTSSLENNAGDKGRA
SSPSYTKSVSPASKAKEGTRPRESRTKRKLHPATTGSMDHSGASLPLVIQHGESQGQKSP
NDYSNRGDNTATPPNKIKKRTKNAGARDQGTQTEWHAGKRPVNNPQLPPHPSSAGRHNTT
GSHSLNPRSPSLSLLAAAQSADLSSTGNSASDKAWERAGFAPRESASTGPFGSMLPVFEQ
QFREKGEDSKNVSPIRAISEERARAGRVTQDRSDISERTKSATRRIVRSLAANYGELHAA
LADLHEFLFPLKGNEQQGGTKPIPFYLDANNADAATQAEYLLTVVAADTRVLLHILNRSQ
RDLSVSGGLHPSPKKMPRGGLQRVLSGQPSETSSSPNEKPLVIARTGRSPLNGAFDVRPV
DVDGAGKTPSRRREITETTSGTNSVSASGKIHAADKLDTSLVADSPGGLSPAKRLSESRR
TVTTGNVSSLLSGELAGAGENEARRSGKGGRISASERTTSGNLSRVSQSLNSGGVSRVGN
RGSVSQRLQEDGGASLISSASRLSNSTYKKKSNKLLPTNASRNDKRGDAERHPPQLSTGE
ENAGAEQRGHFRQSSSLVKDSEALVGKQKDLQTTSQTAKTTPFLSLPELPIRGGDDEGSK
GQAINQEGVTAAADPGVGAGAGTIGSKSAPVLPSLSTRQRNMTYRGADRGQQGSKRLVWN
AAVVRFDFSKLFDPKMEGQAPSHYPPGRQQPSRSFIPARSPKETFFRRALNNKGVTGRRQ
FSAHHGATPGIAFDTDKGTGFANAFSPAPSNYLLPGFCFSPYMHPTTSIPSKNTAEGQFN
DLVLGGTAMFTPSSTERVRSLSHSPSPPRSRWPQDPVMETRKSNPNDFAVTDHWLGDLPS
YSNNAAHQPPAAGFASRVGICTQEPPYWNKFYSNEQVLVHILHRDPEFWRLVRDSQGRAR
QPPKTFSYWMRRRLFRGRQLRFLLGLQHPRVHPRCMPLRLFNYQSLVRPSDLYIGSSRNS
TLAPLVLGRQWYAKRRPRFTSLVKRVHPFPMKNIEPQKEFVPLLIRGLTLRPLPAKKREH
*
>TCRU_3078 hypothetical protein
MAVTQHDGAAASTRPATSLRVLELSDEFVKLLGVERDAAESLQIVFAARCRQLLPVRRGM
HDDRNKGTNEKFSTRVASRVVLLTGSGTLFVLKNRLANGCRMVERLEFGAHLHPHDRQAR
RRGQMTRRQRKTHSDEDEDDEAFHFFGYGSTHRGWPLVRIEVNVDEKPIKGMQSICITGW
AHRSRKWVPQTAGPFFPGAGQTVRNPKTGSSATAAAFVGGGATSLSNPSSSARRHEREGK
GNNSCNTMKVRFVLALQFYARLGLLGSVLALKIKQIATHFGRLGIMTFTLNHFSQSLHHA
RQNMALFAAESLSSLPSPPPRGDGSLAFLVSLRHLFRLRDVVQWVKKRKRHRRISLKDVV
CNGIPMPPTMTVYESEDGEEKDASLRSSSSSVNSSLSSSELAAMYTTDEEAEREAGLAEL
EDRCFSTDNTLLDDDIFNARIIGLLDDITRVEGILMAQRPQGQ*
>TCRU_3079 hypothetical protein, conserved
MDDIYRDGESTADLSQLQCRPSLRLASFPSTPPPPTPLKYRDTNTLGNSKQWRSLHCRRG
LVATARTAPTSPTRSRPRAAVVRRPLTPTFSAIPPLHRPVMTEADEDHVNSNSSSRFSLV
CDVPVAGGTNERRVSAGDGEGGDGGNEEQEESLAQSSRWVSFSFASALTSRRQSESRPSS
PSPQPRAAIIPSNFPNLHLSEDEYNMAEGVDATTRCSPTTTTPLLSLGRPPVVFNDTPEE
ARENREEDGDGGEGRPIKKGRESLLLTSAPFSHTAEVTHFLPTATAGAMTFPIGVEGLPT
LQSRGKRERSALSSPLTTSFVLCGTDSSFQGAAESATISLGRGEGDVVGKAMWPNTDSHL
PRPRRLVETTNLEESVERSVLSVASTATMRQPIPTAEGGGSLVLVDGPTPTPWRGRLAPV
VTATAESKPCGSMVLDVRCSDAAVAPVALFTDVLAGCSDDGSAQKGERSENDDNERAVSH
DSRCCNATGMTTCEEKDVTRMEKTVGDGCGVVDDERLAMPNSIVRPSEELKALIESAREE
VRRASKRILAALHAHRCASCDKSDCVQSNGKGSTSGVADDDCTWVGDSPQLAFGRSLTGP
SGDGAGSPWISKDTVVMRRNLAARAGCILRRLRGYDPTDVGILSLAVVIRVVRLVLFNDA

TADALPVCRSEGSADLFWGLTSSSSVCSGTPERKRTRQEVCAAEESAMELCMSLWRCFRE
HFGERYAAKRFGICGDVDAENSGEDNSCSGSQSPSHAFATCFPRVRLQLGLPGQSLPPSP
PLLDTRIDYNVFAASLAELN*
>TCRU_3080 hypothetical protein, conserved
MINYVLKSWAQLDSSFSESFTPTAGLVGFISNASSGGDTWSDDYLCVNAIVTKNAMKGKD
GFEFTGLGSGAVWLVSSREHNGPHTFVSCDFTIVATVMIQNFSKLSTPLPGAGLDAPVST
NFIGLSHNADSRWETVFYGIKRASGSTWEPGKKYQVALMLQDGHRGFVYVNADLVGSPET
IPTLETQGAEVSYF*
>TCRU_3081 hypothetical protein, conserved
MDSCLIIDPALDVRDADAVIWRGECLFAMPGDRPSSWCLDTLPTNTTDARDPEETYLSAA
MHAALGCLVMQVRLRVVEKGGVRWVPCTDGATIAWATRPHGEGSVACRDHAQVCTISAAA
GTLLPVVPWDGEARAWSRTVPLAITGQGLRGGIRPRCGEH*
>TCRU_3082 BNR repeat-like domain/Concanavalin A-like lectin/
glucanases superfamily/Trans-sialidase of Trypanosoma hydrophobic C-
terminal, putative
MSADGCGVPSVVEWEKDKLMMMTACDDGRRRVYESGDKGDSWTEALGTLSRVWGNKKGVK
VELVGSGFTTATIDKKKVMLVTLPVYSKGEKEEKGELHLWLTDNTHIVDIGPVSGKDDDE
VTASSLLYKSGGNGGKKDELIALYEKKKGVEETSSGMASVLLTAQLEKVKEVLATWKKAD
ERVSQLCTSESVGRDTLPGTACSADIKITDGLVGFLSGNFSDKTWRDEYLGVNATLDNEG
VEQVETGVTFTGRSAGAEWPVGKQGDNQLYHFANYNFTLVATVSIDGEPKEGNIPLLGVR
LDGEGEKKLMELSYDREKKWILLCGGETHKKHSSTWDPETKKKDHVVILLRNGNQSSAYV
DGKRVGEDVPCALGNTDSKVISHFYIGGDGKSAVSTGNGEDVSVTVTNVLLYNRPLTSEE
ITALNTNQLSIPKLEDLKTEARDAPPPAGTEPHTLGAVSSTIRGGHQDTEEKLLKISNDA
GGGGASNSAKTTMTTHSVGSQSAEQLASGGSHDVNKNVNVDSSSNGNPAVGTVGGDTKQG
NGSPQIPVGISDTADANTPTTEGEGQYGPTVNPEAGVSSGENGETVKGTNGQEEEIHPQD
GELNAAALSSSLGNVSQGNNSDAGTVRERRLLPSLLLLGLWGFAAL*
>TCRU_3083 hypothetical protein, conserved
MTFPTYEDDFPDDALRDPQLFYLTTRRNAGLVLSDEGLPSRLVPKRRAHEEACCSRLVDA
CPVRSLFTTVERLIRLIAEPSTHCTVLVLLSNADRTDRMMVFHCWKAL*
>TCRU_3084 hypothetical protein, conserved
MESTYPVAYRRVVVSGSNDPVEYFVRAKDDGRTVVPTQTETRTGWLMCPVRLRARLRHWM
RAGQVALRCLLASRPSRLLIVKGIQEHSFPEGVSAVWSARCSARSIEPFL*
>TCRU_3085 Leishmanolysin, putative
MLTWCCAWQSAVGGGALDDCRGGCEEHHDIDDIDGMELQDGDGDRRTLESHWSQRHAKDE
WMAPIGCADFCTELTLAASADLGCMRVKREMVEPMRW*
>TCRU_3086 SKP1-like protein
MPEEFDSQEVDFTKYCVLQSNDQPPVEFKVLREAAMMSGLLKDMLDDQGDMEPIIPIPNV
SGRTLRLVVEYMEHHYQNRAEPIEKPLKSKIDTIISPWDRDFLYTHLVKDHDEKQHEVLI
DVIMAANFLNVKDLLDLTCACVASMIRGKTAEQIRALFNIESDFTPEEEEKIREENRWCE
EV*
>TCRU_3087 Organic solute transporter Ostalpha, putative
MASEQTPHTDGENSSAGKIRGLDMTWALNPNLTYAAGVCVAIACIISFADLRDHLARFDY
PQLQVLVLRIIAMIPIYGLFSLLSLVLLDMRFFLETIRDTYESFVLYMFFILLLKYCGGE
GQLLRSLKAKRYKGVHLFPFCWLPTYPLDTAFYLRCKRWVLQCALIKPLCSFLAMLLNPL
GVYVVGKFTLNNAYTYLSIIMNFSLTVSLYYLVLFEVELEKELHYAKPFLKFLCIKTIIF
FSFWQSVMVNMLLKVQLLYTGETEHERENVSAAIEDLLMCFETLPVALLHRAAFGRSKLD
EEMAAVPMYMKDENNNNIRSNIDTALSINDVIEDTIATIFYRRGKLVDQENTEDDYANEN
ERGNGSKKRGGGAAAVPEADDGFVEAVVAYGRDPTLEELVHHAIASDYGVRADGLLHYDD
DSDREERNRDMNFADTDVIMRRTRYEEASEAVKVDTDLLQSHQLQLQTNTPQIYCVVCGR
FDREMVRRKNGYKCKECIGVKSKVLLRTYQQEVLEQTASASHGVVPDA*
>TCRU_3088 40S ribosomal protein S15A, putative
MLPTASQGTKPAKEKMTMMSVLANALRCIASAERRGKRQVLIRPSSKVVVKFLQVMQKHG
YIGEFEIIDDHRAGKIVVNLNGRLNKCGAICPRFDCTTPDFEKWVKNILPSRQFGFVVLT
TSLGIMDHEEARARNTGGKVLGFFY*

>TCRU_3089 hypothetical protein
MAGRRRRRDDFDGGTPLRERAAHLRSRTGHDAEFAVGRLLHPFRAEFNDHFETSLEALRD
IAVVVDQLRLLLRPSAPENFVVYDPYYCAGTVVRYWNTLGVQRVIHANRDFYKDIAEGDD
PDDYDMLVTNPPFSGDHIERLFNYLVARKKPFAFLVPDYTATKDWYRTAVRRHFTPVPSS
GKGDINASRHTRPKLPAALLQPPPFLKMEQTASAEIALDDTKNKKQKEDNKCDNEETIPI
GTEPFYLVPRGRYDFSHPKGVGKDHSHFRTMWFVWTGRHTTEVLRGTKVEFARRHREDST
QRQVSPDVVHGLDALAEGHYVQSGLRLNPQRRAKQRNGRR*
>TCRU_3090 60Kd inner membrane protein, putative
MSFHAVVRRAALHHVAPLCGTTGAKTSAAAASPLTFFGGFGGRRFGTETILVSVRCISWN
PANWVGNSPVGRESHRQIEDELPDVFAQQPQEVDDFIRPEKSVFERLEDFWDWIVSFMQP
VEKQLEVMRSLRHSGFLGFDFGTWGNVFFFYGIVMRLCTLVPSLYSHRNALRMSYIGPQI
SEITNNQNRVKNDRTLSTAEKRVIKDGYNRMKYALMKKHGCAQWKSFFTMLTAPITMSAF
FSIRRLAVYESDLEMAPFLWVKDLTMPDPTYGLPLICAGMFLVNFELNQQMQRGGRSSSG
IYIRWAVRAASLVGVYVFASQPSAMFAYWIGLSTAGLLQPLLLRWQPFREFFQFPDPPQA
AKSQIIAEVKGLSMVERLFLSKEEKLRREKDRQALWEERSKKKFERVEDYDDVVFDVDED
LSKSTRKH*
>TCRU_3091 eukaryotic release factor 3, putative
MSGWAQPGGFNPNANAYNPQGQQGYYPPQQQQPQGGGYGGQQGRGGYGGYSGAYYPPQQF
QQPQFGGYSGAYYPPQQQHQQPPVGGYSGGYYPTQQQHQQPKGEYGGHSGGHASQPPVDN
IGNTAQQKQQDQQKVSAVESGKLSLGGAASEPVVVPKKGKAGTLSLGKKKTEDDKKKNED
TKAAEESKKGTSAAVTMSDEKRDGTPSNSKMSTEDFREYVKREIAKQREQNKKEFVRDPR
PHFNIVFCGHVDAGKSTISGHLLMEKGLVDQREMEKLRREAEVHHREGWEYAYVMDVSED
ERTKGITRETGAAYIETEKRRVTILDAPGHKAFVPSMIGGATQADICVLVISSRKGEFET
GFEKGGQTREHAMLVRTCGVKQMICVINKMDECGWDKGRYDEIVGKLKPFLRQNGYDEEK
AKNLCFIPIAGLTGDNLIHHSSDDVCPWYKGPTMMGFIDNLVLPETKSENDVLCIPIVGS
YKDDGRVFIYGKVESGSIALGERLQILPTKLEAFVEGISIESTEFEKCYPGDNVHLRMRG
VDESDIHAGYVATSIPTTLSAVEYFQARVVILDVKNIICAGSRVMLHAHAAQEEVSFHKL
LAKVDKKTGDVLEKEPTHAKAGDVVIARMELERSLVLEAHKVFDKMGRFMLREDGKTIAI
GLVMKLYESTKESLAKSGKA*
>TCRU_3092 protein transport protein SEC31, putative
MRLKSTPLSCAFAWAPSHLGTPTFLAAASLAGAMDEEFSNEAFLEIRLVDVTQTDETEMP
VVARTFIPERVFRVDWCSFGSACGMIAVACCNGAVYVYDASVMMKNYQLNPHSNTEEPLL
CAITEHRGAVRGCQFNPSQPNFLAIGAEDGKWDVWTLEDPRDPQQVPVMSDAAHTAAIVH
LQWHPKYPHIIATASSNCVVNVWNLKSQTLAVSLNVSKGGKSGASANAIAWHPVVATQIA
VAMDEKDSAIQIWDLKKSMVPLREMRGHESAVTGLAWNPGDASMMASCSADGKTIWWDPS
TGEKSGALQQENSYVIDVQWSPVLPAVLATSSFEPLLCVSTAEDVSTAASAGGPVPKWLK
RPCGASISLGGFVASLVPNSTKKVGLTCVLGKSAMSPATSQDLAFCDTLRKIPRDSEERV
KWLREQNYSLLAAAAACKDDRTPILAYLNEETSTHDEGEDPFETMEASQKSFEDVAAAHI
AAGRIREAVEVCLDSCRFDDAFVIAYLQGGTLLQRVQQEYAHYTLTQSRQKRHVVYAAAI
ASGDFGSLTESENVPWKEALSVIISFVGDGFSDACDRLGEALKKKGNREGAMTCFVCSGN
VDAVMQLWREDNTSLGRIMRDALLLEEVTGRRVTSAFFSDCLYEYGMKLVGEGVLTEGVP
ILQRSASIGNHSAAVMVDRLKFHVPVGQVVFPFAMMPLSDSPSPSCATFITQQQQQLRAQ
PQPQPQQQQQPQSQQRFPMPGQYQQPPKPLQQPPMPGHHHQQQQQQQLPPSLPQQQQQPM
QSPNFYLQQQQQQQSQGITATRSVGATMPQGPTEAAPQPRLMPHPISSYSSMKQGAPVSS
GSPLAMASAGYSNGMPSAPPRQQPQFAPQGGYAAPQQNYVDPSRPLPPPPLPSGLRGASV
PGGQALARPTQLAGIQQMQQPMSSQGTGVAPPPPPPPHPGAPVVPPQQAGGFVGSSHPLP
RVSANASTYSSGVNDDTSVGNINSQGAISSLYGNPSASTVPLAPSTNAAASVTAPPPIRP
MMPVPGNPPFGVTTREDSMGGSLTNSYGIGNATYQASLTPLENKVLHQNNMSWPPKAAVT
PSTYGLQPNANKSSMPSPYGSSAVPSAMASRVPGSVPFTASSTVDDADLSHFDITSLNPS
YHALAQKLCADVQQVSNAQRRLVISKAALELFKLLQRGALSPDVVFLVSNYVNLMGTPLA
KNAWRQLSDAHFDVVQPFLNLKFLSNNDGVPN*
>TCRU_3093 MORN repeat, putative
MNLVKPALHARGTGLRDSSLNFPAFSGSSLSTNSTVFLTSDVNAVIEEPISLLVSADDSG
ALGEQHSLKKRAAVLYAGEVRPYTADEVRATAGTEFASGKAVHVFLGHYRQSASASGLQE

RSIKEGPGSMYFADGGFFLGNWVNNYRCGMGAFYSSCGYTYEGEFEDDLPQGHGVEHFPS
GEVYVGEFLEGRPHGRGVTYYPRNGSIYVGEYSRGEKHGRGVIFYENGDVFEGSWVGGRR
SGEGISTFLETRDRFNQSKRCNFRRCVRAQWHGDKCKWETVSSDPTHEAPSLSRYIDEGL
VKPLEKLEIPFGCSVGNLYMAHELVREIPPVLLRRAERCFDHLDAMCTGEVPFSTLVAAL
ESSSSREAIVQSILKAIPLSYAASRDASPTVHLDFATFLRGFFPHISEADIQRHVIFELP
LEKLFRLRGVLAGMVSSKKDGFLYLTHGFQEDALFHIASRECDGMRQFAVGGPEQTRRES
VTSAGAAQLTMEELNAAKGFLGGVRVPHGLFLWKKIVSGIDRLSFRELLEGLYPCVTLQT
LRRCELTFFSTHVFQGYLQAFSKLDEFHLGSLSIQAMDAAQREFLAARAKGHERIPRPAN
ALEKFVLSGRRRSLVQWEIADIALSVPFAKYMDRSKSGSVTLADILRHAYPNVPCAATRA
RLSLAALPPRRDPHVTEGQMNGTAAAGRQNSSGSSISLLQCCRCCICTMCAAHGHPLGAC
*
>TCRU_3094 60S ribosomal protein L28, putative
MTHSTDLQWLLVRQNSKFLQKRNGIRLSSDPFNNNANWTKRHAGFLNTKAAVVKTKGDRI
LVTTKDGKAGNKPKSMYKKAVMDAGVEASVVSKAVAAVRPDLASIASRRARKMASTLEHM
KKVRAARKERSSKITFQRKAVRPKRH*
>TCRU_3095 sterol 14-alpha-demethylase
MFIEAIVLGLTALILYSVYSVKSFNTTRPTDPPVYPVTVPFLGHIVQFGKNPLEFMQRCK
RDLKSGVFTISIGGQRVTIVGDPHEHSRFFSPRNEILSPREVYTIMTPVFGEGVAYAAPY
PRMREQLNFLAEELTIAKFQNFVPAIQHEVRKFMAENWKEDEGVINLLEDCGAMIINTAC
QCLFGEDLRKRLNARHFAQLLSKMESSLIPAAVFMPWLLRLPLPQSARCREARAELQKIL
GEIIVAREKEEASKDNNTSDLLGGLLKAVYRDGTRMSLHEVCGMIVAAMFAGQHTSTITT
SWSMLHLMHPKNKKWLDKLHKEIDEFPAQLNYDNVMDEMPFAERCVRESIRRDPPLLMVM
RMVKAEVKVGSYVVPKGDIIACSPLLSHHDEEAFPNPRLWDPERDEKVDGAFIGFGAGVH
KCIGQKFALLQVKTILATAFREYDFQLLRDEVPDPDYHTMVVGPTLNQCLVKYTRKKKLP
S*
>TCRU_3096 palmitoyl acyltransferase 7, putative
MPQTNSTEWRVFTSLGASLRQLSVEEIANHSSHSFSASLAHSRSILRVTGNNVARSTLSG
GGGGSGRQSSLWPSDNRVELKIAEVGEKKEQQREYSRCHQNDNFHVKADDHGVVSGADGE
LGISMQHKHQKGAHSRKGGYSMEVSRELVPSAAASVVSGVRPESITDAVEAFSSGEQKQI
NEDGIERQFLQDKAEAKKKRRKRRERIHSVMSIKASDVDKGESEVAGMSSIARHSVECVS
RSEDPPESVFFDGDGTGQIPNCVHFSDVYTEDTAVSPFRVVAHRRVELCGHGSKGGDEPE
GLEVAMNGAGESLAAKGSMSREHDIFFPQIEPKGCLAEEFCVLMPAEKQQNCPREISSPG
CDRDLIPSMETGEYIGSPQQFISSSPEIIMRAARGSSCGSFSPTEETVNSFQVSQTESDL
SSFMVRYSKPPFASCCVDRSNPDGWQCNRPRRHAFQWPLHFLQIIALTLTALSTILFGAS
ILPTYVLLYRSGGFSNCLPEMVVLVTLTGSAILSTCILWAVISFRENGDISNEGEPCTYC
RRLTHSDARHCKACNKCISGFDHHCKWLNMCIGEKNYRIFIAFLVSSALSMLLAFISGVV
LLARWWSYLSPYSLFFRIGPIVLCVLMLLAVPPLLHLLGFHIMLHRLGLTTFEYLIQRRR
VMRDPHAAASATDAGKELHAHEVREP*
>TCRU_subunit alpha, putative protein transport protein SEC.1
MKNYMMMAQPILALLPEVSRPQRIPPIKERIMWTAAALFIFLICCQVPVFGARPGQTSDP
FYWMRVVLASNKGTLMELGISPIVTASLIMELLSGVKIISYDVNDKKERAVFEGIQKIAA
LFITAVEAMAYVSSGMYGDVREIGTIMSGFIVLQLMLATLICILLDELLQKGWGLGAGTS
LFIATNICDTIIWKSFSPSTINTGRGAEFEGAIIAFFHLLVSRSDKMRALKEAFYRPQLP
NLTNIFATLVVFAVVVFFQGFRVPLMTKSRHAAADRQPYMIKLFYTSNMPIILQASVVSN
INFFSQILSRRFGQHNFLINLLGRWEERAYSQSGQMFPVGGLSYYLTPPATLYELLNDPI
HGIFYVVFILFSCAIFSKLWVTISHTGPRDVAKRLVAEGRWLAQARESEEDMARLLEKYI
PVAASFGGLCVGALTIFADFLGAIGSGTGILLAVTMINQYFEILQQEGKDLGYGFLKQKL
A*
>TCRU_3098 hypothetical protein, conserved
MSKQLFIISAGASAALLQAATAIVDKATAGKLSTTMTSMAEKNAVVVGPETPVGVHTSVT
AAPASPDPAYAGVKTVLVRAVLPRASPEKVQLRDALDVFFAAGINMDAEVKAATESFKKS
AEVAVANAKAIGVNRVTLVLKQATKYNNVNELFRKVSTETIEAAGMTTEIQSTSVATNHL
IMFPESLGVVLLNDVTSTEKIELAYAGVLGGASRTYHTVSGNKISAGHSFKSVALAVAQE
LRALGMGSEATKVEAAAAKNPRAVLSSL*

>TCRU_3099 hypothetical protein, conserved
MHASGRDGSRNHPSQSFAGVPDGCAEGNDYRAHVMHGDRSPLEVMGQLPREPLRVDATNY
SALSRTGWRPFGLPITSSIEYDAAASRGTSHGLSVRRVSESHAALVNNLTKRNSSSLLTS
NMQNLTDRGMLWSDLFAFGDDAQVEHQKHVTNVNIEEICLWTAPVSAGRCVLPGAEVGIV
PTRPKKFTRAATLSDFQREPATVSTTQGADNNAATTNFDDSNRGSVSCMRDVLNDGASVM
SSFSSENSLLTQRVATPFSANISQRLDIARRRKRGVFALFGTIERGYFESFSRMFRRNQL
VLEVFLENVLKSNQDLYPKEIDFLDFLRQGQRTPDEQFFMDPLISWPLHLLYEVSCFYVS
AKLHGYHDTFETIRQQGGLFASGKGIFAALAVAMSPTEEELVHHASRMYRATFFVGLVYS
KMEKRLQTHLENSPQRSFALLLVNISTISLQLLDEVNKVSFGSGNCDGCCGRNKCCVIP
MSKLEMTRIISSHSAIVCGHPLDLERLSTVLVCYAESTGVRVHMEYLLTTVPENSTFYNQ
WQHLELLQMWKDNGVELEDSELQLTLYSPVDGQPWKNSSKCTLADCIASTTTCSSQDLTY
SLQHMRGGDVLLDFSGATPCMSQLIAWTRRNITVLSEPADCVSTNVFPSPRRSPEDGIIR
ETLARCARINTVLQNMKWEGKPYRACVLDLTTSFVDLGLLISSEEFLKTYSGVSYLKGST
TVRKLSGVSTTRYHQHELSADVRSNSGKDMAMNSHNDDLFCAAGGHSASAATGTIRHVIQ
PNEKLPFVVDIYQDSPVVRYYEMQSKGFLTRGAVEFTQRLSLCTGIAFPPHTLLMCPTVL
ALMELWDTYGFLRHKISDDE*
>TCRU_3100 inositol-1,4,5-trisphosphate (IP3) 5-phosphatase,
putative
MRSLLDGITSALRIPQQQQQQQNEQSSQGPVGGTGVTVAPSYHQGGSTGASAAGSLPGSS
LYGGSNISCSFAAEKLPDLPFTMLLLTQNIGCICSRCDGGNGEEVAGGGPETATTKGISD
AVRTRVADFLLELRLLIHEASSREFVLHLRKEKLNAEGGASSAADAGGPSSEVPPLLDVI
VVHFQEVGGKGLNTAFNAFFAEALKTLLPEAAWSSGLLMETNSDMQRFTALGSIVFLSSR
MCPISSILSFRHRTFVAVADDPATYGSSPTFLFHGGKFSGAGSGRKGFLLTSLRFGTVVV
NYLNVHLYHDSRNTDAAMASPSLYSVQRQEALLEAMAECMVFISPEDPLFIFGDFNARLD
THNLLKHLKETQQIDVKILVKEVQAPDRFWELFTEPQNAGMIKKFDLELQRLMDVVAQQS
GVELAEFAIRFPPTFPRIPEREFAKSNQKQQIEMSGVNVEASEYGVKDDDPKLGNERRKK
AAGKEKGTGEAKPSLVLPRITAIPTRGYTRQRIPAWCDRVVWNPAGLELMTGQRSLSPSV
SSALGKSDGATDSSPRYAYRSFVLAHTDHDCVALLF*
>TCRU_3101 pyruvate phosphate dikinase
MESKKFVYYFGGQKADGNRDMKELLGGKGANLAEMVNLGIPVPPGFTITTEVCGIYQRTK
GLPQEVVEQVKANVRRVEADMGTNFGDVENPLLFSVRSGAAASMPGMMDTVLNLGMNKAT
AEAWIRRAPHLERFVYDSYRRFITMYADIVMQVGREDFEEAIGHMKEKRGTKFDTDLTAK
DLKELTEEYLVLFQRKTGFPFPEDPMTQLFAAINAVFRSWGNPRAVVYRRLNNITGLLGT
AVNVQAMVFGNINDRSATGVAFSRSPSTGENFFFGEYLVNAQGEDVVAGIRTPQQIGQSL
SLRWAKSHGVSEEERRHRYPSMEETMPENYRLLCEIRAKLENHYRDMQDIEFTVQDGRLW
MLQCRNGKRTIHAAVRVAIDMVREGLITKEEAVLRIDPLQVDHLMHPNIEPGAAKSNKPI
GKGLAASPGAAVGQIVFDADSAKEWSARGKKVIMVRLETSPEDLAGMDAARGILTARGGM
TSHAAVVARGMGKCCVSGCGDLVIKGKQFTLSGRVFREGDYITLDGSKGLIYAGQLKLQS
PNLKGDFETILQWCREVKRLGVRANADTPNDATKARSFGAEGVGLCRTEHMFFEGSRIDA
IREMILADTLEGRKAAIRKLLPVQRGDFLGIFRAMKGLPVTIRLLDPPLHEFVPHEDAAQ
AELAKKMNVSAEKIRNRVKSLHEMNPMLGHRGCRLGITYPEVYNMQVQAIMEAAVAVSKE
GCRVVPEIMIPLVGKKEELTFTKQQAVKTAEETLAAAGQRVDYIVGTMIEVPRAAITADQ
IAEEADFFSFGTNDLTQMGCGFSRDDAGSFLRHYCSLGIYSKDPFESIDQEGVGELVRIA
VQKGRRVKPMLKMGICGEHGGDPTTIGFCHKVGLNYVSCSPFRVPVATVAAAHAALKEKR
QMEQKYKAIAAKL*
>TCRU_3102 Adaptin N terminal region/non-SMC mitotic condensation
complex subunit 1, putative
MLSVNQLSGSALFSRAFDSTIVREDLLSQNTPRQLSALQQVVAFMTIGRDMSGHFSDIAP
LCSSTNLAIKRLVYLYLMHNSHAQPQKAVLQAGVFVKDTVNDSPLIRGAALRTMTSLLVP
VMVDFITAPLQRCLEDSDPYVRRIAAFGTLKLFYIAPNVCEELGLLEKLKNQLHDENACV
VASAVAAILELRQRHAPISLEEAIVENVSRVLEAASDAPGWYQHYLIEGVAVAFKNNSLM
LDMERAEKIIDGVMPFLSSFNVATVMSAVKAMTSFLLQASALFTLSAHGNDGSNKVEKAS
QLRDRYGPKLVGACVSLLYECSLEVRYAAFRNIRLLLKTGLVFFFKRHLGPFFVKYDDPI
YIKLEKSELLLELADIEVGEIILSEFAAYATDADEELVRKAVRLIGFLAAKLEPLAEQCV

ERLLGLIDTGMSHVMQEAAVVVQTILRRYPNRFLRVVRKLCEILDELRSPESKAAVVWVL
GDHAEHVENAGDILEMCAESFSTQPEIVQFALLTAAMKIYLSSECKDMGRSTNFLQRVLS
MATQSPRPDVRDRAFMYWRLVTSDTEAAKKLVFTFSKGLSFTMADTLEKRRLQSFLTDVG
SLTAVLHRPLHLIYSNETGLNENDEEEEDAGDHFGEKFIRRVVCDDEMAGTMANNLVSKG
PNIRDKESMHVALPADEGNGLEVRMCWSQLGNKLLLNCHFSLVSGEGYTRHAVVRALQIN
RNIHALGLAQECPVVELEVGEKEPETLQLMTGTNNEKSPMRDLLVAVNVDPIGTRYFLAP
TVPPAMLLLPAASIDYGFFAHQFQQLHAPSWTMPTTLTPTRTDQTRYTANALRLHGLNLV
YTSKLHGSGILRLFISAETIAAQKLFMEAAIQDDLVVYLGVRCKDPQVPPVFGAYTLSVL
SVAEN*
>TCRU_3103 HIT zinc finger, putative
MGFGNAQDIFAQSLQEERLAKLCDDNAFVNVTEVFKRQRRDGALVNAVDIEVNFLEPSAD
FSSAPPRVHLSRCLMEQHRAYTRALEEDRRDHKKEDGASAAEPSSSSSSSEDGDTDMTTN
RVKRPRDFESRSRGRKKQAREELHDGGVHVNKISVNVVQTVSDVGRERRNLGGWLQSRRS
IPLELTVPGPSYMAMAAPPPLHLGGYHLCSVCLLPASYKCVRCRGALFCSIKCHVTHDAT
RCMKFIV*
>TCRU_3104 40S ribosomal protein S5, putative
MSAKAPKLFNKWSYENLQTTEIALSDHISKTATYYVPHSAGRWQKKRFRKARIPIVERLTN
GLMFKGRGNGKKPQAVRLVKHTLEIIHLLTDQNPLQVVIDAVSKGAPREDSTRVGSGGVV
RRQAVDVSPMRRVNEAIYLMCKGAREAAFRNLKTLPECLADEIVNASKGSSNSYAIKKKD
EVERVAKANR*
>TCRU_3105 40S ribosomal protein S5, putative
MSAKAPKLFNKWSYENLQTTEIALSDHISKTATYYVPHSAGRWQKKRFRKARIPIVERLTN
GLMFKGRGNGKKLQAVRLVKHTLEIIHLLTDQNPLQVVIDAVSKGAPREDSTRVGSGGVV
RRQAVDVSPMRRVNEAIYLMCKGAREAAFRNLKTLPECLADEIVNASKGSSNSYAIKKKD
EVERVAKANR*
>TCRU_3106 hypothetical protein, conserved
MRGGFRRGRFDRLPQPGAVNSYSEAIYPPRVYHYYTQQSGVAASSSAGVIVPSIDDGVGE
TRGREAIPQGVGPVMDKKSPPLLRDFVGCFIDGNQHVANSRAFAILDSGKEPLNVLHYPT
LLGVNRKDEDAGAPIADKNAAHVSTAGDHEIRRWEDSLGLPAELRIYGLAKSTGDMSTAA
VDRLRIRPSKRKRIVKPIRRGTKVVVLPPACTEDAQEIKKVRRIVGEEGETEGGNRAQPR
RENDDRNEEGSGGFDREDDEASLSYNIDDDDDGGDLDGPEGSGGEEDFF*
>TCRU_3107 MRB1-associated protein
MMQGNDSAKSFLEKLKYGGSNTAFIDNSAPPDADGIIVRRLERQYASEPLSVLGIKISDD
GEVLSTESGSPAAKANILPHYMIIYVNNQFVGCRADFEDIAAQHLTLIIKLQTTSAMSEL
MAKILDDEEKVETENGKPSTDINELLRTTPRYGFLSAEHPMFLRWNHRIQTQREARQLLR
QATREAEEQLAKEAFEKIRRELEAEEAKQRESQQVAQPAVNPVPVPAPKPTEEFLLKHVQ
DESSFGRVEEFTEKMDEVQEEASAEELLKLIGAQPNEEKAPVNIVPENDGEVRFFIIPTE
EYKLSNGMIATISVKKRSGPFPKPPPGKPPKGLRGLAVNKPNGAVTSMKSFESDVVCSFC
YLRGHNVDECAEKKEQERRLAFVPKERQICRDYLRGRCLRSDCMMRHVSKHSQGRISPPR
KRDRERRHRSRSRHHRGGSRRRHHDGRRRGHRSRGVSTERHRRRR*
>TCRU_3108 PUB domain containing protein, putative
MASLSEEIQRLSELFASGALSKEEFKAAKEVTIKSYTPLAATAPLLGEVGRSREEAEVHT
PFPNVNISEFGEDAAGQPQREKAYTVLEKILRNLLQFPGEEKYRRLRLSNPVLHSQLFSV
PGAMGFLQSIGFARCASDDGDGDEWLRLPGMPCRPFLEEGLRAIHFLRTRSREVVARNQD
FLRLRRTLSLEVRRERCEKAKAVGELRSYIVSEFCSDDAGDGLYSSQINLDKLETILTNV
LRCPSEAKYRRLRLGNPAVYAAVLQQRGGMEVLAECAGGEMVEEAGEAFLLLREGTVTEG
KLRCALQAVHAARLAVREVQEKTEQRVREEARAAVRSQARLEREMELRRHCPQHNSEGAE
LCEGIQRVGGRRVPVAEALRFLMGKGRAEEEEEEED*
>TCRU_3109 AAA domain (Cdc48 subfamily)/ATPase family associated
with various cellular activities (AAA), putative
MVITRSQSRKEARSLICTSNGNKDDAPGVVGDDNQNSTQERLGRGCRIPREPPASYDSMY
VRKALLPSAMRRAENEELQINSRYPQRDAAKRAVLRMSSTLHSLGERTRRNFWPLEDNNG
DGDDESSSNDGTEDGTASFQSLGRKCNGNRFTNLPRKRVREPSINSFITDDSLREGSNGR
GKDADEELLVEDPSSLSSAEGCISEGEGRRVFRKAKESTVKHIPINHYLDEVMKASERKT

KKGDKQKCKTHRRRMEDMFLGLNEAHGGPVGAGKGGEGRSITETPAPLGDITPLHIDTSV
SFEKVGGLPGHIVMLREMVLFPLLYPHLLQTMNLTPPRGVLFVGPPGTGKTLMARALANE
AASCAQRKIAFFMRKGADILSKWVGESERQLSLLFEEARRRQPSIIFFDEIDGLVPVRHA
KAEQSQAALVSTLLALIDGLDDRGQVIVIGATNRPDTIDPALRRPGRLDRELYFPLPDAA
ARRHILSIVTQPLLPLDHPDRNAIMEELTTRCEGWSGAEIQALCTEAGLNRLRTSLPQLY
VTSRRLQIPNNALTIQKEDFFVAAQRFKTSVRRGTESIVKGLEEHLEHLLYSTRMKLVSH
VASMWPSAAKALANERRDCSNAGDAVQELIGFPIPHTVKPMLLFLTSSVERNVYGVDSYE
FALEKAALAFIKALPSIRIFTLNLPNLVVDRGSAPMMVTATGSAVPEGGAGNFEELGFPL
NNSFTSGHIYSCIMAIQRSSGPSLVLLQGLDLWLAENGYPITNEEGNEGDGTDEERAQHK
KQMESFCYYLALLRDSDVLIVAPTLHCEVLSRFFGGTRLNTQIHKTVGNVPCAPSVDDLQ
RFLQYVFHVVELALLHCTHTVWEELHEDISPPPPPSMEEVRAARHGTLELWRRVEYRRRQ
LRYVLSKWLGQYLVSRKFSILCSADLDFTPSHDLYDEWRQHTQGSRIGLCDVMEKLENEE
YTSLSQYHDDIDMLVRNVRTFFRTRSSTDQRYRSRALELKETTVLNLYKINRNVVQFCEQ
YKDLAEPDVSLSSISPNDDVKPRIEEPTESRAVQSTVKGKRKPYRYYRDRRRRRRKVRKT
PKVRENDNLHKIGDENEEETTREEGRSANRDELKTEPAAAEALPRGEEEVDDHAVIVERN
GINSSDFSSSIAQCDKAAEIRVSMEMLRGLSFLELHGVFQAAMSLLEEEVSRRRNQQQTV
SVSPFTGTSEVGSTSFFLWMLGEAVRAAVKERRTSD*
>TCRU_3110 leucine-rich repeat protein (LRRP), putative
MFVSTSYESKLIGDEGLYAAIEAARLARSLRGATIRPHVRGNPLINPNSILLANTPKRWE
ARLLSEKAEQEILEAISGGDKAVSQYLQQVQIAIPEEKDLEKLRAVVPRIHHIQPHPVMR
FDEAKRLIQLMSLHGANITCLNFGNGALMPNDHTEASPAEVMAKMEELKQRFPLPTPKPK
KNTDSEEEEEEEEEDIDLNNNEELQKWCQNQQLEYTDYNQAIRLRAAYELDAFRNICEML
MHDKKVRVLLLGKNRLGAPNEEERVSLIPLRLLAKVIDANETIKVLDLSSNELGAHGFGI
IGKALTKNIAIVSLDLSDNQLGMPMPDLDEDPEYQEEDPVFGETYNGLEAISEVLKKNKF
LRHLRLRYNQIHSGAEGEEPPVADPTEMDPENDATTPDVDGWQDLPLWRLVGPLREYHRL
RALDLKGNALGPEGARMVATALAENHSVEMLDLTDNGIGFRGLHYIAKLVLTSPHSVLHT
LILRRNNLAGKKGSKAQRKMALAAMEAMSVALNGNTKLRRLSIAGNYLGPTLCAAMLSTI
STASALEEIDLESNDACGDVTAPHDTTVMHLIALALYGTALRGNRPSLAVLRLGSNNIQA
AGLRVLFPSSATMPTSLREVDLSRNSIRDGLEAVNHLLISSPVLHRLALAYNSITTAPAL
HRGIDANESLCELDISHNKLGSLKEQYCSDPQAQVRGVEELLQVLIKHPSLEYVNLSYNE
FEGVHGPLLAELCSGNKSNNSLRRIDLSGNPEINQQDIDQMVQALATKPGIEAFYISSSY
PAQLEKVATLRPVGQDAVLDPAAQRHQQLSLLKLLQETVYQSPSLIDINCDLQKGVLSEE
DTGTDEGEVIGEMRRRLLLNAILMPYGK*
>TCRU_3111 hypothetical protein, conserved
MRGGNSAAAKGSSNSGEKKKRTQTHLCETGVASAGKKRSKISCFPLPPHDSTTDNTEPPS
VRVAAARQHVMSIDKDSSVTSPVMGTTISSATGSRSGVLVTDPWVILQPQQPPFLRGWAA
QRSNTRDSNNAKTAERCVLPRTTTKSTATIPTRMSLCQVAHEILKSNTVENDACFVDGRV
RKLQRLELHFATPSERKQDFTRVVDPYTETMRRLEEEHHRRFRRALYVLQQMHPDPWWRL
QSSVYYDVGVPDLLACDENLLFSRVAGEVRDAGVTWLSLKNSTL*
>TCRU_3112 START domain containing protein, putative
MSSKKNNEGEITSNGSDSSSVVYQSPFPITGPEVQHGGRTYRLVTKKDFFTFREFADSLD
GFEMQYNRQNEFISWNKKVAGEPMRIIKVFGVLPNVIPATLYDMLQDATFRALWDTHRID
AFRVVKLDENNDIGYYAAKSPVPGVSNRDFVNQRAWINSGNNEYAIFNTSVPHRDVPIDY
LKVNKVSSDSVVRGISKITGYLIRPWVDNKGALGSCITYVTQCDPGGWIPAPLINYISTK
VAPSTLKTVAQAAAQFLEWLPKQENYVKDWPETADPFDTPVKNETIAFAKKKWSANGHLE
KKNASDERLVQ*
>TCRU_3113 MIF4G domain containing protein, putative
MTSSTADHLYERAKLRRLNLDPAPQRSVFAARSSGLKFDVKKATALAKRFKNYSAEASEK
TILMQLDTVSFGKFLSEAAHAVVQSLCHGTKMKPADAHSFTVVCSEVHQRYEDFWALVRK
ELKENIESVVDTGSLKLAVERIEEYASTTTTTTATTFISGEGNVSTGTTVVSSGLSAPPS
AAAKGGLTPSLSKSPVDEFNRLRLVARVFCEWWLVGLFDDAKPLLNLLKLLQKLAERNAA
TPNSPLAVATVFSITALMIREVGIEILGKGNAVLSSLFTEKKGDENGPERLRDLLGHCGV
FTIDENSEAGRKTPFLLGPAGARLAFNATTKKVGPVENAVAHQAEEVSWARLCEIRNASA
ENYCNDEEKRHFFGLALASTKACLRAYKQRKEEIEKWWHSVNDQAEFRGKTARFNNDEAR

FRFFQEHVERLYVVCENLLGMLGFSPPLPVDLSIVEQREEVEAVRISVTRKFHPIVGAET
LNRFGDDEQRIFYEYVPEIETLLDEVFLSSLTAEKAKWELFSRHSVIGGAIASGTGATTS
SGLSAIGSGGVGAPGGGDGGDSEEDDMDVEAAANTMEEGKGQHNTETVKLMERRRLRLAK
LGVPAALFNDYAEVLKLLEELGECTTAVAIDEWCERFVGEAIVPLRNSSENVVGARLFFT
NCRMLLTLELRHFLRFRYPEKLPFMARCAAIFDQYFHDVGIYLGARLEAQWRREYSTSTS
KRETSTSCSTRSDEFLQRRVGATGRYLCELMKFGTIPPSRVLNLLNIAVEDLKQPCSVAT
ICAIIEHGGLFLVRNHATRRATEKLIQKLKTNMMNEEMKAECSHMVTEAFSFLQPATPSL
VAFPSPAQKEESPLERYVRSLLHERLCPDGTKFVLDGLLRMPWDDVEKREMLIRALRSVH
QMSLDVVPLVVDILKELAHADHVHVVQRILYEVAEDMRRDLEVGAVTPELVSGSSRKSMM
ISEPYTTHRPLQWRLADCFFLANFYRVRLVSFRFVCHMLLMLLLYFPFITTHCNDYTRLR
CFVTLFRECIPSLPWIVNSRTGDFNRPRSRTSRSAERIATLHQLLRKLVAQLYLYRFSLA
EPLPLQLQVCLEELLHLLRDNIGRDERYRETWNKLQEKLKAREKGSQKEAEDTSKNKNSQ
LGIKSSQKLVFPETLEEAHAYAKSVAEETVLPANAWYEKVYEAIKGSQNDEPLFRIFAVE
NQKELSLCTSEVVHAEGADAENSRHASLDLLPEDMEQGDEVSTDRSSDAYEDENSMVRGM
TRGSCSSVGSISGSESLSDSDNVDTSVALSATVSDTTSTGEEEEGEEDEDEEDEEESEEE
DEDEEDEEESEEEEEEEEEEEEEEEEDGRFAAAEALRIRLEEADLDAQLRALMSENPRETT
GHAASAAVARGTSVERLMEQDLVMGIRMHRQHQRQCAKNTFLLPTAETAKEDTVEEVRFT
LLRRPPPNTSAAGKTGTAGVAASTSSANTAVASANVVKKSDFAVLSIPRGAKFAQNAMMS
QERHRQQQEELREITRRINHMHEVERSVHAAKPKESK*
>TCRU_3114 hypothetical protein, conserved
MPSTSTRRERSNLGKGSTGSESASKRPRREVTSDNSNAILSGTVLHNKADALLLPWLFEC
HRVLRAATPPFAVSAGFIKELLSPRYLKSSLNGVQQLTGCLLSDIVRLSSGHASGEERAN
MLPFDTNYADDVLCCLTSPFGQVARGEAPLKTCEQLIERASASHIFGYLIPHCRQPVQER
LTCVFVGVQQAAAGAFTGNEAPATSDNTITIVTAAEMGRILIDILNATRSITSDELAPLL
KEITAASPTLLRRAEANRKTGNTVDSNPLANVARKKTRAPGAVIAARVFLEKLDVIHPAI
ASYVVEMVEQGVGEVAAAEVTGDDVEKKRHGLRGVGRAMECLVALMELHVDLVGQLIPGI
LPYLEHEITDIRLLILRGFFMAFGAHEAAISTYRSAFVTLLSRFNDPKHTLRIEMVQLAA
SAISASTHVSASLAEERVCELLPFVEMRLVDPHALVRRAAVIAYSDVVSAAPSLVTSDRM
EKSLGLRVADKNPKVRQAAVERLSSIYQTLLYPWMPNVVMQCLNAEGGVSLLESSFESML
PPPSRVMPSDASFTTFSSSTSTGRRSAQASMALFDFEKESATRERTYVDGFAKMCSHLNF
KSFNKLLTFAAKKAQLRLTILRLFQMRAEVRNTDLKSLEGQEMINNIHRLLNFLQTMTHS
ERGEWDALFRSKDDKVSKAFLSCCADRHFHYAKERELLVKTMKGRVEGHVFRFVQESLSR
QMMLPIEVEHVNELLARLREALNGICNDARTKVSAEMRCEVEGLLRALLVFGHTAPSFLS
HCAVPLVDFLELVCQSSNTTIPTSWVILLLDCLTEWAGYAKTTGVDNKEHDGGGASVNMV
SSRKKGLFTTLGRLCVCGHEPLLRDATPEMVGAVCKHAARCFTALVGVNGAQESKALSQL
VNSIFARIRSTTTPATSSVVGWLKALTAFAKNRTVAPLLQDESLTRSITELLLAAVRDKT
DAEDLAKVKAKRADVLPMSLAGAVVDAATKCLTAIARCQSADGIVTAVAPTINILLAAYK
AASERESNTVGACRRRISINQQLAKLVIRPSSEIAKELAVAVVLSAEEEMVVRRSVQKKM
TFHIMHNQCDMRYVALLILTVVSEETKSGYQHLRLLLQQVGDHLRSKQRSSGATLSSPEA
LTCFLEYTIPFLVFFMAHHTFYSSEKENHFVAYQRVWHLLFEELYRHGTQCASFVVELLA
RIKQADEAVDRESHAARLLCDLGSRVMQACLGQRQISAEALKRYPGRVLLPTFFIPSKSA
TEALNTIFLDAKVHIFPHAPFRAPTAAATGGGGGMAGDKTPSLARVAEADVGDDNLVLTA
AKAKFDLPPNVRQRIDRALDAFIGSMTQEEIVKVLWSEVKQRLREAMVGGDDNGDSNDDE
DDVDEANVMEYAKEQLRQIYERAPPQ*
>TCRU_3115 hypothetical protein
MEPELITLSPLFIRLLRVGTTTAYALRPCFATRLDVYSASTGSEGGWTPCALLVSQHGEM
YLFSAEDGRVINAALLSPGPIANTEKARIDIVTQHTLDAETDTQSVYIRCRQQLQLMNNG
GEGKGKGLMPTQAVAVSDKAPGALFPPFSYLMRFHCRNAQVMLQFFRIITNFADPKKREF
NLRKANISLRGELDHSLFIDPLITVPRHQEREHQKQDTNKWSNALSKVSPLKLLSRLPGG
RNSVYMRDVAKFEKKRLLDAGERIELPLQDDPSLLLKPNSVCEVNSANAIGREASAEVSP
GKNEHAGAMANALLVKEGDPYSTVLPAVAKFYGWIMDKIPQFTLSSSEDILCMYGLPGFE
SQFAVFFVNPRIQNIVENSVGQLLASFPGLPDLPQYRIIAREAVREEMRWNFGVTYRELC
DAARKLIDDISIDQLVIDEERAAIQKSHDVAVEALLAESQARWQKMLEEEGHVFFDANHE
PKSVTTGQLLAPLCTEETEWRNEVAEAAEALEVLELGIQRIRNTIVADSDEDNDDSSGKQ

DSLGNNVGGVAFMPSRGGKGGNSSHFLSLQEEAAFLLQEIQRERETLLSMQLQCSSGDRG
PKDIVEDDVEIKSMRSEYNARLLQQQQQIDTLKQELQKSAAMFGINARWMEEQNRRPGLA
QELLNSTYVV*
>TCRU_3116 hypothetical protein
MRRGLLSTHPVPSPATDRKNHETTGDQGDRGTPEHTLARRAAMRSTAGMLQDNWPREGKP
RVTLRDLNEKVALSLSIDSGLLASPTNVTKMATEFAEAIERVQLRNSLLSVEQECNLRDE
LSSCLQRKFEEVYHLALEKERQHQRNTRDSYNSELLIAYRREQQAKHQEQLEDLQQNLEA
EFREEQKEQRRRAYQRRVKVLLTQQGEKKTEYQNALEQYYTYREGGQPASGTFSPRDGFF
QVRFTCEEEIALRTSAESMSRDQVMCLRIALRRLKKRVKMTKEHTQRCLSQRQKSVFAAR
NRAQERYLSIIADQQNELRMLQHWHNEMQKLEREAKEIMRSYTEQRLRRQEHMQREKEGQ
VSSVTGSNGEEGTGKGGSALNAKCSNGFQRGLFDLLL*
>TCRU_3117 hypothetical protein, conserved
MSQTEGAKQSGKSFRGTDALPLTLLRRSCPEFISAIASVKEHQVKKASNKNRGSNLEFGQ
ELDAAVPPQCIRSGTTRTNTDEPFNVRDDSASAAPQASEGLAERMQEKNASTMAPKKRRR
DGMISLSTTAAPSSSPTAASASHRIDDNNIGAAAFVASGSRNRRALPESPRSFLREALVN
CSRREEDAIPDELVANFDGMAFFQLLQQCSGGVCVSLERAEEAVDGQNGSDYSTSPPSPM
TKSSSSLNGSGNLPIGRLSKRELQAQLSAALAWASSAVHGVEDENDDGGDDNDDDDGDDA
GVMVRLPRAILETASQIEHPSLVFELLHCKDSKGRSKGAGACCGKNTYTDDTEEDDDDER
EEEDEEDRSFLGDLDDLKETTEKVNSLSLHTINELAAWWGIPVEGGNGEVYRLTITEHFR
LLAFTICAVVSNTLEPQPLFSSYAGGNWRQDGNNATIHSSGHKKAAADAANGASDAGVKE
MTPRREDDQDGPMRGLLSLRHDICTLSAEHLRYFANIFGFPDRCCSPEDIFAAISAVAVV
TFFSPLPTPLLEKVCHELSISTVEENTESVDAHQLPELLAEKISHYFYSPRYSVPSVSKL
SFMPQMQIHEHAPGRYTCTVDNAKLMKVIMLQRLISNRFSCNKIRWHALLTVINDKLSFY
VWHRNTGPITAHMIVRTQDPKKKRKNNGISGGDNNNKSKNSDFSPNPESSLRESSALFLE
REVEAAPGELMGFENFVSLTVALHSSTMSQSGYRLYNRVEDRLVFQYAMDLICPNGAPPV
EEKRKKSSSNHQNGKSTCANRGLENSMEQGRVNQQATTDSAATATANAENEKRRKEWEKV
VIKLEKSESQQREAVDQAWNSGYRQLLSEHGKSYQKALQKKKDRERKMLLAKVGPSPELL
RELETLTQTVSATRAQVTKLAKEKSKEEAATKKLQEQIEEGKLEVEQLRQQLKSSNELLS
SLEAEAAACITRFKERGEAKRRKQRDLAAALRIPEMSSTRHETLAINDLQSFWAPSSTNG
ANSQLLLLSTLSATSSSPARSPQSSAIAAPGVNPNLAFSFVGGGGSGLCGLSIPLPRASE
ENSCGVFSPPLQSTAEEVMGVGISTWASMTDPLSFGEGGKGGDTANTFGTPSAAVLFSPQ
PPPLQQPEAATHLAGVDTAGGSAAGHSSSFTVTAPFTSGGAAPRFTLDANACPFTPTTMV
TGRTSCNSAPHTSFGVKTGAAPVSLHSPLSTSPTTVISGNSPVFAGGMFPERPRYTSVSA
PVSGGVASNEAFSTDPLQSSAPLFAPPGLSSASGKFLQPTSKALSLNFTTAPWN*
>TCRU_3118 hypothetical protein, conserved
MPPRTVTANSHNHNGGSAKNNSKPNAKGSHDHPRSKRDEHAAEETAPPSGPIIKTQITNT
NKASSGKSFLDALRVGASKRSNNNTSAAVEKPTVATAVAKTETVAAVVEPKTVTPEPEVT
TVESLAVEVNAASVEETAQQLASEQPVVEQNSATPTPPVQQKEQQQQPVTLPESDAHFSW
ANDDDYSFVNQFTQPHREEQVQAGAPAYLVQYPQDVIDAQEKGTRVIFKAPGENQTMASA
VAALQKESRELQDSRRVFEDYCKQREAELNEKRAQLSKFERQLQEQSENLQQERAKILEQ
QQKLQQQQQQQQQQHHQQQQHHHHIQQSPVRSAPAAPPAPTQHGPQPAPGYTYSPAMQE
NMQWCPPPRPDNWALGHGPVVPPYEHNRYYQNMNYQPPRGKSFVPRSGDIPPHMGRGNPM
RSNPHMQSNFNNRPMGAGEMRGPMPDGGVNPMFNQRSQAGYMQTNNFHNQW*
>TCRU_3119 hypothetical protein, conserved
MSALESVLQRVRVRVAQRRLRLDDFFADFDSLRSGRITAAQFRRALAINSIQLSDAEFET
LTSAFAALPANKTTGSRTPRGGASNDVNYVAFLHALRVEDPPAELLTTLRRKGKMLSDEE
ERTLETTFRSLHYAVRVRGIHLRKLFEDFDPFHSGKVSASQFRRCLPFEGLRDDILKLII
KKYGDEDGGVLYAAWCRDIEASTSLKEERLSASISPHISPHGVSAPLSLDELLSVLRSQF
SMYRLRCDDFLKAYDHFKTGFVTAPQFESALGQLRFVNFHLTAEHIDTLTRAYADARLGG
TVSGTNEEVFPRVNYVQFLADTNPRREDATPGLTNYYEMTRTPGQFLSEVDADEKERAEF
VLNKVRRLVQSNRIHLIPTLHDFDRVRKGIYEHRTCTQSRFVRSLATNKIFLSPEEISLL
VKWYLIRNADGSPSDEVNYYQFVMDVDNSHSLQLTSARLMRSTGMGELQNQKGGSYSRPA
DTLSEVLAKVAMQAEERHLRVNEFFIDFDPLRSGMVQTEKFSVALGIAGLQLHPQEIELL
QREYKSTKARDHVDFDRFITDIGEFAPTAVPSVTTKFKFTASQSTKGALSAISKDALYNP

TEKLTDAEREFLPRLLARLSHDVATHHAMLTPFFSDFDRLHRGKITKSNFLRSLARHRFV
LSPADTSLLSRYYASPEDAELIEYTRFVRDVGQGEMEQGIREKKTGVSSSGMNDTMMLRT
NTASVCLGDGSPHGATVDEEFVERVLTKICCFLQERKARLSEFFPDGDELRHRHVTNTRF
RHCVSILGLHLTEVELQALEAAFAHSELKGHVDYPAFFSVVSEKLQAGVGIMAVTQRRRG
LYTSTDTASVPAAVPANEKSVALKEGTASGFEKSTKILEDLQAELYQSAMDKVRRTLASR
RSVSLSAFRQYDRARKGFVKEGQFFATLMSLGVQLTPAQSDAIRKVHSIGGGEMGYTKFS
LATDDERFA*
>TCRU_3120 hypothetical protein, conserved
MSLEDRGVVAIWEGEWLWLRRQLSTVRQTVWMFGALLGPVFDLAAYLATAKVCVARADRF
VTEQTLLASEQLRRRHWILPAIGITSASLFVLFKSSPWGTYRAFRNGAVTAALLTCFLLP
REIIDAVDAELPFSTRDTRGVEGNARDIGEK*
>TCRU_3121 FHA domain containing protein, putative
MEIVLLATHNLQLLSDSGGICAVPKKSSDNSEKWNELQPTYVRHGSVNNYKRCRLRACFF
LKNDGNILSLESEAKSANGGNTPMILLLPLGRDPVGNSAVITFKMAAQGPLLSRSHCVLQ
LSSSRLLRISHDPHEWCAMSVRDCFSLNGTFVNGRRLASGACSPPVTFNPCNFRSWKEPL
MTLELGAGAKLSAGESLLEKQLFLRFDVFVRFVGERKDVGLIRRCLEGSCLANLGTKDIA
TCFLRDGNSACRLFSEPAAVNISKDVLANEECVNTLQAEEVLTLLCENCSAAARRIQLLN
SAKAPGSTTAVQQHIPLACSTINNSCQCLYSTILTCRRECGTNNDDAETLTWRPASPNCT
SDLPLHASEHPTEVGAAKGETRGNESMAIFLTNRRFGDGLQGSLRDVLLTPHKAGSKSVF
GRGKEEPGNCASSGIASNSTKAVAHSVPGPCRDGCKQNYFLREPPLDLSPVLVKKKLLAK
SPLLEPPAKEDCMTNHCRLVSSFEEMQRNEGTNVMFPTRKPPEVIEATNAGLTKDKDKLA
EASKAVQQQLSTIHTEMEQVLSWRSGIDSLPSSQDDRAIVLPRHGRVKEEGEGELVPYGG
FITVKKSPPLCDLAAGTGQAEWQAQTAKKRGVKRKRVVDMKETMKKFVAELIAGTDGASP
HE*
>TCRU_3122 hypothetical protein, conserved
MAWNAWRPLGTPSRQSQISMDLFDKWRSEQDMSIADRGSDADPAKEEEHNSFMMRQNLNA
YICYKQLDEYTSCLAKHHIIEHTDRGHEINTKNNINERKCRGTHKAYVACMGSQKNQETL
LHSAVLHNNCREFHAELMCCYDKNRELETKTSEPLCIPFYRRLLRCGLNHLWNDYWRALT
RFGEAEEFHLYELSRDDNKKQEFLRVITSTVEQQQEYLRNRREQEKGYFLPRPDKDVESS
DEKMKAAAAVLAQERQ*
>TCRU_3123 40S ribosomal protein S21, putative
MTTIGTYNEEGVNVDLYIPRKCHATNNLITSYDHSAVQIAIANVDANGVLNGTTTTFCIA
GYLRRQAESDHAINHLAISKGIIRIKTGKKPRAKKLKNVKGLGVRGLPRGALQQRGARVL
PTQRGVAQRGGAQKGNVRKLQPQQQKQRSQLNQRSQQQKQRPQLNQRSQQQHGARPTRKE
EGGRMQRGGRDAPQARKQQGRNEPQARKQQDREAPQARKQQGRDAPQARSTKSGPNRK*
>TCRU_3124 hypothetical protein, conserved
MSYPNYSLTDSIRAALAAHGVRNARVVIDLLPGNGQVGVSGNENGMNGDWHVVQTDDMEI
IINEREGVLLMEGTPAQQPLEPPKTLPPPRAHSPFGTVSAPPDLLVGGGGARQPKKHHSR
EKVEGGNGYDFMENVVEPTTKSDDALPHGSFVGDAPSSGSFERKVPIVPLEDLHKVSLSS
DLPRGEGAGEGLPHDPTTPLPSSSLLKSPSALFPTPQAPDDRRELRSNSSLLPATQPGSN
RKTSVVLVEKLKAPAPPVKTTPSGLMPVFSILNTEHSEQLERLPSNASSRKVSSVHLVES
PVVEEPKHQPHVVEGSEAARKPGILVTGASSKTGQTDSKPGVVGGNGELKFPHFGEQSSK
MSVSSEIDVSVNGKMLKTPTPHVDFWKLSGKQRLQEMLQEQREMEQQDRLSRAKHEEIIQ
EAQKVLQRLEQNKMASKDEGELPSLPQISLRTGPASSRPVAALCASPHCSSLSPSNANGV
NGIKVLRFTLPASGKAKEEVVSQLSTSPGGSQHLSSKDKYSMLLQEQERILRQNEEGEDE
YKKLVERAVAFHEQRKEQRRLHEEQGSEESEEDDPVKL*
>TCRU_3125 histone H2A variant
MSLSGDDPLQQNAMMGPGSATADQTSIVSGGKHGGKGAAARGKGKGKGKGKRGGKTGGKA
GKRDKMSRAARADLNFPVGRIHSRLKDGLNRKQRCGASAAIYCAALLEYLTSEVIELAGA
AAKAQKTERIKPRHLLLAIRGDEELNQIVNATIARGGVVPFVHKSLEKKIIKKSKRAG*
>TCRU_3126 Zinc finger, C3HC4 type (RING finger), putative
MAVRALLQVAEKCLVCRVCYTLLRSPVVFSCGHVFCKACAERFIEERPRCPLCNHAVMSR
RAITSLPTVASLMGLVQDVAKSLRLQQTAHVAAVRAGNIAPPRLTEKIVGIQTADEKCFP
TAAVRDSLASTPTVDDDADEKTVRVPTIHRNVSMPETQFLRTSPPPVPVLTDDFVAWEDA

ETQRISLRSPIASSFSRSLQNHCYQCVAVRRCIGELSVGAYERVGCCFLCGLDIKNRTQV
RQYLQQILNSDPTCDHMRLRRLNEVTLSDFLGSMWDLHLPTQIVKRAASGMCRKHGRSGT
FLSSSTLLVHQACLEWCVLHERVRDAVALKVAVMGTQMQQLDGSLRASTCFFCSNRGKCL
ILCERCQRKSYHYPCALLTGAGVCNILSETMSLICATCLQEQEEQGEGEAEEEEELLVLF
*
>TCRU_3127 ssm4 protein, putative
MPVESTASRGSLPAIVIQQAITQQVATSTSTDVSYQTSSLSPSRPPSALRVEAETRQGYV
YQGKLLSLDDDYNISLAEATSWRDRLCDVERALLAVRGFPAPAASPATRRRYVGSVFIRS
SNLLMVRFLEYDEPGANGRRDNSHGAAGLRSAFKSMVSKVKRQINMERKRNRMERQKRLH
LAQTPVPTKAANDGSGMRSRKDPKAGSNRRRAGRFSS*
>TCRU_3128 hypothetical protein, conserved
MTALTVPFVCSPTEDTVPSYVLYSPQGSRPLAGLHVEGGTVSRYYDKETRSQLIRTSPSA
HISFLRNTKSRVGRISSSSSSSLSTNGGGSLASSQRVVILQIFCEESACTAFSLTVELTL
QDKLGRHRILFSNTFQKLQRCAQHVKVPLRCVAPGVWSNIVLDVHQIHATLYANSGGRLS
TAELINSRRLLSVTLGCSSVCRIRRILGMPQLPPSYRELATAEWPLSLRLPPEVHAYFWV
IRTCEEEFLQQRIADGPYHHLEAFIHVNSTLDPSISAPVQKKTLQKPVPAVPFQSLSVSS
VVESGVLRAVPGASDRGGGVDPHVALMSVAAGENGLSASCTKGCQADGSEPKWQSGTIEL
SALAGSPPRSVPRCDASVQTSLPAAVVTAKKGGATEQLQVVGTRFFDTFASVTESPVKMV
NLIDMTHVVFDDEEEQREEEAGDGEWWPRSKNNGSEVNKKETVSVPVAFVAPTPSRCLDS
PGRPDKKEVTNMRGAQPRETVLNNIDKNVPEVMHAPSLNNYANTLNLMRQRVKCIQALIS
ESREHDVSLNEGEISPDDDRCATSGGNNNNEHNQKHGGCEDNDHQGERPWPQLSAEGKHV
ILCDGSTQKQQQGLFSSRCFIVPQEHRPEKIRGVPFQNTLTPCSTMRTTDEGVAHVCTYI
PNGGEKNMVGTDEEDEEDEGFTVNVCTSGDHERPAVVVPGESNALSKQAPVSSLCPKPHE
VDRLCFDSSFLENGFIEPSSQRPVHRTTQLVEDDSMVLSGPLSPDYSGSITSPAPAAAPS
VTQAQRTRATHHRGRLGEQARGEIVKLEAFQALASTETNTPPPSPRHGNGGEEVESSIST
AEYRRPSMSRDGIAAGGFSAAAANAGRFISVGSNCEVDGEKDKEEDDARQPFCLPSLPPK
SPSKKQQQYQHPRPLASREDSSNLLSSPLVPLTRRCQHAHIGDTKKECTVKSANNYMSNI
RSMPLSSTQLTKPPKGADDAREKIENAGDSLVFDPLLQCCLDLRSNTYVVTIRSPEGTQH
K*
>TCRU_3129 hypothetical protein, conserved
MSAAVPLAARIAKNRQLKLLAAQSRHLAARFSDIFGDASVRTTRLNDRHFESTVFYSFSG
LCTLRLASEIAESLSEAVERVRGAALALFYSKADLRHMLQAVDSDVFAELNATLDSMDAK
AVIHLARSKKEMLDATASQFMVTVTAQLPLKDEEITEVGESGGKEYNIMTDIAICRSTGQ
CPRDAIKRGFALSLAELDEYAVEEQRSRGEEAWCYLQSVQRDELQRDWESVALSVFLSRS
LGDGNLDTIEWLPPREETDTVRVACFPRKESRSLTLTIRDASGNETLLTLPLHSPPLAFQ
RVLVGDDDGSARIVHTSALAWPEKWIASSALMQLCTILNAGVRAPETISCGTAGVALTSQ
EISFLSVRSFAEAHGTLPVTAVPYSIERCLFMVNNRVALCFAKKCGREYFGGSSMPLICL
GVVSNEAKKLGQGIHLQLEVKPSEGGAECQKYRNFVTTLLRRAKKEALRGRHETKLQKVT
AITAGLLGHLWKLFFVIAGLAEKEGMNPLRDISVWSPTEIPFSGNQQKKQQELSSLLCQL
FAVDAQVVKKPSWGGSFPNILHLSSSSLGVQALPVCHTLETPEMTLKRLLDDASPSMAEV
VEIASKTDPRRTVEVAAQQREIEQSSYYCPDILLMIQEMRLELHMPQRSFPTLEFFQKAV
HDVPQRVQEIAMGTYGDVPVYTRVVAHWGPNLRVEVESDMMGLTDNYGVYRFDSPHHNHC
GPLGLLLSVVYTMYERVFPHRRTVPLIGLNSPNRRRSIDFLLYSWFMASPQIRCFEISTR
AARLAAATTAMAKKSSDVAEMTLSHSVGNYIIRAMLFYDFCGQRVLFAETHAPTLWEAVR
RVNELAVSLNIPKQDFFPTRATTRRDSRSFYTPPDGCDSEVAFVLRALMSATRSQLRVFF
VRTNDSTALWIEGMIELQVPIRQIRCAVSVTRTRGVVPALVLACREALRCASDEEEAVRL
LRSAHDAGPLIPRDNFPRFSSRFYTPVNLLGEVLQGVAGKYHCTYTIDSKTREVVCLLHV
PAMAGYGNAARDSVPFPLGIGRGRSKKVAWAAAASQALQANFPDVLPQVERHKKLCELLH
RPDYLSLLDCPEGFKIEVVPDREKESGGGYKCTVISRQPSNVSDVNTTAEVSDVEHPKIL
FEYSADRSVDAYLAAADALLDRLQQQRQERTAAQMGDSVFHLWDFTSNYGKSVWHACCGA
LGVAFGGKVSLRFEGGREDASVWESEVPMRVQLVVRTWSPADERVIETPLHMLNERRIVG
YGSLTGLYEKGKPSAEQLIFASTHLLAEAIISYTDEQTRKCLQRLLNLFQQLKEGEQSCS
RLNAKRRVESYMELTLGCQCRVVVRQVACGMVTAELGAWLPGHFRESRVPVVLSACTENT
TGKALSGLEESVRQVVESLWAIVRRSCAC*

>TCRU_3130 hypothetical protein, conserved
MAQAVYATLLLICLLAAHSAAGIFIVDSRPSGDYCGGYMSLVNGRITVHPTTSEFDISLD
VFGEKYLCKEEKYSYNETTGQMFLDGVNDPDDCLGTILRDNGLKLSVTYLQGEDAILLDF
DVVTVKLSRCS*
>TCRU_3131 WD domain, G-beta repeat, putative
MSLEINRQFLMGDGSKVTEPVIPYISIDTTKILEAHKKPYLTKAEHVKFTGEISCLCTVG
STIWVGEVSGKVSVVEVDTGKFLNIVLDHRGAAVIVMRTARGYVWAGFADGYLRVFNSLT
IELLFEKKFHKDCITDIALSQNCDTAYSSSLDSYVCYWNTSFFAIESPRVLGDCRGAVRS
LVLFHDLLFSGGDDSKIIEWNTVSKERKHVLAAHSAPILVLTICNNFLWSGSEDGVVCIW
SLSDLDGGSPECIHMITNPHEGPITILKTVGLKVWSVSMGQVYIWNSSSFELEVHCRLSK
EHHAFLNVMETVSQAFCSKTWIAYKNTGDITIVQSDDLLPLPLQNLCFWGDVKDTKDKDQ
FIDQLLESQKSLTSELLVLRAKLATLTPQQCQKEATDRFELAKSDGTEKATLLNAVPKSF
FFDSNEIMDNMTNLTSLRSNKDDHGLSNIPFSIEGTNYSNFASHMPRYAMVDTHALLERL
IYFREQVGEIIGSSEVLYENNCLYLTDEKGRPPIPIGFITLSELTENERITLMNICGGSR
PPFLIPALTPTNQAEICQFIRGITSRLRGYRSIPSLASSEKRILINDSSNSVHALGKPTK
CLSFFESEPDGVGFPPLSFYLNKIICEVMVGARRISQNYNSRLSNKRVDSMGKAFQGLKQ
RRDAGQKQSSLSYGIKETESNEGLAFFSPDVDVLSLKEHFLPLNDGPQSKGSDDDRRNAT
LMQRITFLKRQLVGCVTSHSSKQVKTCARNLPPLTYTDEFSDMESEMEELSAISFGLIRR
LCRLVEKLETEMQEQKVLKSIFGAVDDSCRKNHTDDTRQQFPGLVSLFNRMQESNLVAHE
FVIQHLLEVGGKIALQYGCLMETMLQRQNPSAYSSISWGYESGRSNVQSSSLLDSLNEYS
ILVEKVHAVEAVFFADRT*
>TCRU_3132 hypothetical protein
MGSWRSDACGAKRPAALKCEWRAFRSAEVPSLGRIGASSQLHSEASIISERRSPLVAGRK
GSLKRAFSRATQTDEDDFVLSEVFSVVMATELGSSTADCLDWAIRMNRMTLSRIQRAERL
SDFPLSSGCGGHDANCALQSSANFPLETILRIAHFQNKKHQLLSVCFLGQKANSLF*
>TCRU_3133 vesicle transport v-SNARE protein, putative
MSDVAMYDEELKGLEEQISSLLRALDSATNLDQRLEKYNKAQDIMKRLHKTHHQFKVEVR
LLEGNEQEVYEKRGQIHLLRINQLKMDLQSKKVEETPFSAARENSVLSSNSPSNDGKDEA
RAVARRVNTIQTSTLGSLAMTERLLNDTETVGIDAAAKLRSQTEQIQRVNENLNELDSDV
NRAKKELTAFIRRMMTDRIIILFSVLILIGIITIVVLKVTKK*
>TCRU_3134 hypothetical protein, conserved
MAMMMTGRVLLVCALCVLWCGAVFGHAEGLAAVGGSSGGGNSAGSEGGSRGGPDVNSPLE
PLSVVEGSKGNMISSLETSPSVGGIPPNSLSEEGDGSQSPGETLTSEDELSSDVSGPSAQ
TKTPGQSDSPIVKAPEKSGPETKTHISDKQGEAGSTEANEDATENEKGQSEVVKRDTFSA
PQSPPTPPVPAFPTEETPLAPADLRNSVEEDRNTPPSQTKTDLEAPEPSVEDAEAEKHGE
GATTSDSMNDAAKGRSGDSTPSSISTIDSVDAQRKAEEEDDSEMETLNPNEASILPLVNK
NGQGNVIPSFSGEQQKQKGQPLGKNGAGGASAVSTVTSSPAGKESVKNLASGTSPDGHQN
VDGGSYCWWLPNGDHRIWR*
>TCRU_3135 hypothetical protein, conserved
MNQVIRSRTDWKERVVLRLAWITASRLFEIAVLTPNNFTLEPDGALILDWSLAPKTAKPD
PHRALRFVRTRGQDVFDIIKLCRTLQENEKLTNITTAQVERALVPWDATAHSTKKGVLRH
AAQIVETYNLNSHVISQLAKHVDPFDLSQNTV*
>TCRU_3136 retrotransposon hot spot protein, RHS3, putative
MSSTVEDILLEGSTSSTEMKLNEFIRNYFGGRGAVDTNENVSISVFVLNPEMFINDNEIL
GLITASPSYQELKIELDEREILLEAINRLHHEGVFSLEQWRDYEEKDTVTLIARRKPNGV
LTQVLTEARREAAERLRRTQEMKFTISTNVEDVLFKGRVRVNEMRLNYFLTRELDGRALW
TPIGMPCWRSFSRTPISISVIKEH*
>TCRU_3137 retrotransposon hot spot protein, RHS3, putative
MEVREGKPEQSWSYRKADDAIEKNDAVEQSGAGRPRLMVLTSDRGWPYLWEWEEDGSTHD
CYVNCEVERVWRIVLEDLSEWFSPHGEADFKPVRRLLIGTPWIGNSMAAGSYLLYQLLHY
DVEKLQVVVYCFGGGDACVCDKTIKTVRRYVGKACKRFLRDLWDCGMKVFIIYCVTEKGT
PPAK*
>TCRU_3138 hypothetical protein
MSRNSRTKTGTNRTMTSSTTSRTRARNSTRNGSTGISSIMNSSTKTGKSSSTITGNSNTR

SRSISSTNRSRNSTRAITGNINTGNSSTMTGTNRTMTMTGRTRSSRNMAGNSRNMSGNIS
TRNRNSGTGTGGTSSTMTGNSGTGTGGTKIGKSRNKTRGST*
>TCRU_3139 hypothetical protein
MSSGVQSICNRDRSILAYRRRVDVATSIANKKTAELSSLIGRSCVQQKFVNCPLGTFSAP
TLAAPTSLEPPRSLPSYSWSKAGMSVLRRATVERRLRISPSALLVDCVGQFQISDEASVF
RCYVCAGRWEVMEWMGLPSSTFISSGQPPCRFLTAVCTASDSFWVRLGGYI*
>TCRU_3140 DNA repair protein, putative
MRVCSHRFLRITCISRVVKQATLPTTDPAPVQGFYSSLQYDDFGALDVPDLATTLLPFQK
EGVYWMVQRERDHIGGIMADHLGMGKTVQMIGLCLSSHHFNKAIDETHIQTIQSKTANYR
LLTVIRQLQRINVIANCSRINRPAMDLRALMNKVEENAAPSDESMDSVRQEVDKWLKFAS
KFHPSYEKRATAFLYDERKRSFELIESRDLRTLVVVPAALMLQWKSEIEAKVKPSRGLKV
FLYHGTNKSITNTELELYDFVITTYDTLTSSAQFALTPIFDDKNMSFNRREAGPLFHVRW
KRIILDEAHMIRHANTQRWRAVKELQGFHRWVVTATPLHNTIEDLQNLLHFVGLPRLPLL
PGFNPEEVLNDPVLQRGIARSIQPAFLRRGPVMIRNGKEEILVKLPPKTENVVMKRFSIH
ESKQYNSILARSRTALATSERKEGAFHIFAMMTRLRQACCHPWISEGRALSVSVCGICRS
EAVSTVTTKCGHYFCYECLLLRFREAVDGEEMAVRLPCPTCGTTITNSSVFKSYTLTSSE
RIAKFKKRDLEMSTKLRMILDSIDTMKKEYPDDKMIIFSHFTSFMDVISVALDKLEISHL
RLDGTMTLTNRNTVIRRFQASDDVRVILASKTATGVGLNLTAANHVLVVDPWWNPAIEEQ
AVHRCYRIGQKKHVHVTRIIIEDTIEQYCHEICQRKKEFGDAILRAATKGDSGASVAASR
LRELLSRLNYIPEKDKETVEGTVPGVER*
>TCRU_3141 hypothetical protein, conserved
MVDGGEQTLLGGTETGNSFDNQKSLSNSGSLKEFLSSRIRDGFASSAYWGATGEVPPREP
NDVAGRKPHAFEMEINKKLVPFPEDKTSLPRTLDYTLVGLHRLKGDGSEIKPQLNAAQMR
FLETSSNSLKDDTNGGKKPNLMETTTTQNRTIGKGKFDPDVLDALSKGLEDLEDSSKQRD
HLKNMPLEGPAPNHYRPTSWDYCDMSGIDPSSYWVTKRDPNAPGGDGFLPVYKSRYNLVE
KEGPVRREKTTTMLEQGRNVDKGPLKDTMKDMNTKSLPEGYQPWSANEWMSTTHDYHAPY
NVAEAAATNRRSATIPLPRTYHTLSPAHEQSVLSLSQRHMMRHSGNWATEYSDSYLNRFD
QAEVNKEHSKRSIFDIRYGTYTMHHSAHHPRDDTATGEMYTPSEVVPGQYTTMSQQPLHA
RNALSQRANNS*
>TCRU_3142 hypothetical protein, conserved
MLRLTLFLQRKTIQSYPVPPKTRHFNRRWSSSRTNSYNRQYWRIVMNENYSRPSFWVSDF
RHRYLMRTGSDFQGQVPASPQPGLYQGFSDVHKLLANHPKPQRESRHLPVMPMTPRVAFE
HANEKKIDFAKKMRSDRRRVEELKTLEFWGWYMKLQRVRGRWCREQGVSSRGVYGPAVDA
AELWG*
>TCRU_3143 Sas10/Utp3/C1D family/Sas10 C-terminal domain containing
protein, putative
MPKKNSLKKRTRREVEEEEIVEDLDLDVDEYNVEEEDVIHNRRGKAKRDEQAELAAVLPS
DAHKFEGYDDDDDERGAHLEEETLALEANKSMRRRVIESDFDVGGITSGMKRKTNGESDA
ELNVAPVVEAVERDYAALSSSERMSIVQKESPELIKMLEEMKQYLAEVKELAKPLQELFF
QRRFSDADRNLIQFLETKVQLMLSYCMHVTFYLLLKTEGKKVSGHPVIDNLVEIRVYLEK
LFPLEEKLQYSLNRLLSGKTTAAARLDALRPLQHNERGVLATNTEAKKRRKQLEAMKEAE
EIEKEELTTMNRIQTKKSATLKETSSFDRKTMVSALGYREDEDQYFAKLVAGGEEEDEGT
QGLSLIERLRRRQHSFADGVAGNPNYSSDEEAPVVGDDDDDEANSGEDDLLGEGEDGVEG
DYEMLLEEEQVRLMRAKTEESRPKPKLVEPEVDRRKTSKKIETHRGLTKSRPKDRKTPRT
AQRRKYEKGMRIHKAQTRTCQPEPEGGFIGLPAIKSKVTQSIRF*
>TCRU_3144 SNARE associated Golgi protein, putative
MERADAILAREREEINLLREPLRVLWLALVMCVKYVTRYVTTHLTPSVLRVVVPVLLLMT
ATSLFWIDSPAGHAFNALDTDRDGLVSPEDLETYYTRVLGHQPGIGQQARSAFPQNSSHL
NKAQFVGWWEEGQGDMLRRDAFFTQGTWREVEYLLADALWWLVLGILSSVGLGTGMHSGL
LFLFPHIYLACSSADSCGNTNFWTYPVNRFYGPRDRTFTCITPHVTTTVPPTLARLLKVV
PACIIWGAGTAIGEIPPYALSYAAARQGKRHAELEELSKYDVLNRMKMWTLDKIQRYGFW
AILFLAAWPNMAFDLCGMACGQFLMPFWSFFGATFIGKALIKVNMQAVFFVLLFSGDNIE
RVIRLAGDALAKVLILPAWMSSHGTKGIVEMSIAPIVKARENIAARARGTEEIDLEERGG
SFLMMFMHWIVVAAVAWFAKSIIEAFAQNEQEERDKWTLERVQKMISHRRTRADIKSEEL

RQLIELARAGEVGTSRSPIESIFMALFTIMAFLGFVMGFYPVFVLGLSLLGHTLMFGLCT
NESINRWTFCGVRAALTAAALFSLSTQFTVQG*
>TCRU_3145 Domain of unknown function (DUF4539), putative
MDSQLLLQREASTRGLTLTSLCQVGTLPTSQKVACCGGLLETIVAESAQDCTVLLRDATG
TVHCAIHGAVSSRYPDVLTAGALLLLRDVTVLVTSSLMRPVLIVCLEHLAALMLPEGPPE
SSQSHAVGFTSTASPFTEVPPQGEEYVSNMCQDTLQEVFHETPHEICLGPVTSVAATTLP
KDSLIPSGFDNDEDCLQLADDF*
>TCRU_3146 btb/poz domain containing protein, putative
MEGEAPLKRLRKGRDDGRLAREREPGGTHGAGSGEQSSSAPSSRGDMRVCINVGGSCITT
LASTLCSEPSLLSEWVSNDFAGLPRDANGNPFVDRDPENFRHIVNYLRGYELPLATEKIV
FLAEDAEFYRIEKLRALIDPPARWRFVSGPGVSKDSTLFSTENILGICGNEPLPTRGKSI
LVLRVDKCELVSIGLIGTEKPFENEPLQGQPHSVAYCNTGELVQCFETEKTYVSGTGFKS
HDIITVHVSFSPGLTAKITFLCGTVKTYETEWPAPTPPLRFAVSLHGTSAVTLERCVTGD
MSDEEGREEIGGVSEIV*
>TCRU_3147 protein phosphatase 1, putative
MSLDVDAIIDKLLGVRVSKPGKQVNLAENDVKNLALRSREILLAQPALLELEAPIKICGD
IHGQYYDLLRLFDNGGFPPSANYLFLGDYVDRGKQGLETICLVLAFKVKFPENFFLLRGN
HECASINRIYGFFDECKRRYNIRLWKAFTDTFNCLPVACIIDDKIFCCHGGLSPELQTMD
QIKRIARPCDVTDAGLICDLLWSDPEEGLSGWGENDRGVSYTFGQDIVEKFLRKHEFDLI
VRAHQVVEDGYQFFAKRQLITIFSAPNYCNEFDNSGAVMSVDADLLCSFQILKPSVKKPK
FYQ*
>TCRU_3148 Ankyrin repeats (3 copies), putative
MSGATAVEEDNYHREATTIAEAFFDCARYNDGDDAKLLLAYLSATPSLIGAKDEQGRTAV
HLAAANGHIGILEILMEFGPSPDVPNHEGNTALHFAALNNQLEAARRLLEWGWHASARNV
FGKKPIQLIYGKKYEAMETLLLGYDDEIENYTRGTVSLVENGDDPPPAPAPTATATLAGS
SCLHNTPVHQLCEDLSPEENSHHAEAKSTLQAKHEDPTRLLGSADVDGIE*
>TCRU_3149 hypothetical protein, conserved
MEPRVWRPKFFNPFSERRTLRRHLPIYRGFFRVMPLHRWSLKDLQAILGYAALHPSLFGF
PEQSTCIVNTAYFLPSHRSRSRGIWVFGSERGCKSIIVPASCNTEPFDALDAKLMEPLFR
WNPGVEYLYLSRFCQPMALYLVPYLQRCTHLRTLTLEGWDDATAIHRVVMACKNVDVLDA
FSLDDPPRKWKNELSVQSLNTLIEMHPKLICVKATRVYLADWHEATQFSRCKHNIALVTF
SCDYVLLHYGLFLLLLCIPCYFVFKGMWRLLEGMLIPAYQFFWSVIAAVVVFAALLVLDV
GVWHTYGRGWVQMQKYVILARRRWDAAMRLQQKSLTVS*
>TCRU_3150 protein transport protein SEC13, putative
MQLDAVFDTGHSAPVTDTAVDAAGKRLASCGEDGVIRIFAINNESPDEWDLLAVLEGHTG
TVVSVSWAPPAHYASTLLSCGEDFQVILWSDFGNAKGWAKVYFTTLASVPWCVAWAPHEY
GKMFAVGCASGSVMIFVGKDQQWSHTEFTAHPNGCSSLSWAPSLPPGMLLMAPLESELTA
SGAHPPAGMPLAPPRIVTCGGDRSVTVWTLCGEEWRPQELPLEVEASWREVAWAPGVGMP
FTYIAAGSDEGFVVVWSQDGSARGEWNRMVLPQQEDGVTKLSWSLVGTFLLVSCANGTAS
MWQECASSQGWERACELLPSGN*
>TCRU_3151 ER lumen retaining receptor protein, putative
MNYLRTFGDMLHLLAIIILLGKMLRQRSAAGLSLKTQFLFALVFTTRYLDLFTSFLSLYN
TLMKIFFLTTSWHICYLMRNKSPWKATYDHENDTFRIRYLIVPCIVLALLFHGKPRGGWL
MDFLWAFSQYLEAVAILPQIFLLEYTERYEALTSHYLAAMGAYRFFYLLHWIHRYLVLDR
VNVVSVSAGLLQTVLYVDFFYHYLTQVVRRAKQRYDLAR*
>TCRU_3152 protein kinase, putative
MNNYQIYDEVGKGRFSIVYKGRRKKTIEYYAISSIDKSRRQRVLTNVRFLRSAEHRCIIK
FHNWYETNNHLWVITELCSGGDMRQVLNLDSRISRQAVQMFGTDIAEGLMYIHSRGVVYG
DLKPSNILMDSTMTMRFYDFGLSCDFNTIRGDGTVGTPLYMAPELFTKDGIPSIASDLWS
LGCLLYEFGTGKPPFNGSDLASLIRNIMSEPFCPHEALSDELNSLLESLLVKDPIRRATW
GDIVTHDFWQGRLQLPTAPFPPQPAFERRQRMAVAWASGDWPMSGEELRRHVEWAVESAK
RNYTVSQSASEGGPSRCPAVTEEIDLQDHSAVTDATTEGGIAASRVDGQKDGRRLRAGPT
GAALDATNSQFLRKKESFRGETANDSHTAANCDQNATINAVSNAEVSIVMNGLENLHLDE
LMAHTSDAHIRPLVMNSRIERFVEQKYDSEKMGFEPPAKAEIKTFTEQQQSDFIKMIYRT

LSSSSLSHEEKLNVLCYFEDVCTDAAIANFIVNSSIMTLCLKMVSQRNAPSNYRATAASI
MSILVRHATFIHTDLAKANIFSSIIEVYGEEDSPRVKRKLTACLGELLIYIAVQRERDRN
VWGINANATLQLYLTVLKDADDVLRHYAVKAIENLASVSDHGVALDVFAKKEVVTALLTV
YALPPASIRSEHMRSSAACAALKLAVLKEDLMPVVMESPHLKVKYYSEVIAEVTAPKTAQ
SLLTFLNMALVKSLVAIRNPIMERWGKDTINIFSSSSLAQEKGKKIIESISGVAEAVVSG
LCGVCERASVAIKGKMLLLIVLLGCMDGKLLLRLSSNKLIGCIDGVLRDKDSYVQRCAAC
LASYLGVFLEAQLVAVAKGAPSGTTSTILDAVCNVLSARNLGMMVELSDSVFVSLGTCLE
KVMSSPVYVTYEEEFNRVVGLLTQNSERVLKHRRAVVNELFPLYIHMLGGSNIERRFSAL
RTLSAFIAPLVDGSGLVREEKIAEAEKLDHLMQTVAASLPELLKESEPIPILALRLLATC
GERRSKAFANIATVELVGDLVQYMLRSKQSDLSVPLQLLLLLALQTERAAEIMDYLAAQE
FFTAVLLKTLTMAVAKELDHLLEPCCELSVFFLKHAVNNTSSRMAQQCLLLLPQHGMETL
WLPLCDSPVRSAAGNAAACVYHFVQLSPAAQQDLMTTESLTLVREIISNTRDNPTAVLYV
LRALRYIWERVRKRHVQKLSEWLLAALKAIVREEDPDRELYSEAVAFMEFIGG*
>TCRU_3153 dynein heavy chain, putative
MLEHHLRPDRKFSLTNAQGEATLSAFSLTRSEETLSRKGVMSPVRDKQQATERRDPTSPR
SLIGISATYNKSAILFPDAHKAAFEPKVQVPFTYQRGRIPRKIEIERRRRQYEFHDVQEL
VELAGLTLKQLAKKDSQELPLEVFDDTSYDCRNPSEWMEIAGQNENENGRYLPAEGIYEF
INEDFRMRPCRVIAWDAAKNELKVLWGPTPVPGETPDVVPRFFVRLLAEDPIVYVKRLVS
AQQQREKAMAWIRYRLCCDAMPLEGLSGLDSGLGARLRHLGTGMPHLSKAVFPDVEERAE
RLIAEFTLAWKRSHNRILLQDRMERDEVLLRTVANATQMSLEELVRGPNMAIQRTDKGKG
DSVIPIGNFDFEERERSFTFSTYYTQPEVVTALTGVRSECIKVLEGSLFNLPKARQMQLA
KFQQLQEDHMSSMAKYLKGEWTENICDVIRKSFTSAGKGWLNVHESKQEIYEMSKLKKFF
TTVKFMMEDTLFALVYTSLQDFTVFFEEVSEFTVNVIDMNHVENKWPGSDADDCVEKQPL
FMIDLAERDGSFTYSISFEDFEQTIIQLFNDAIYCTDAIPQVEKFVMSQYFWRREGEGPF
LDSVKQEEERVVLLRNRVRDALRNSMEPLRDYLQVYDELLPLIRLDKKEFIMFYEQQGHT
TDEMKEEIRQHLKAKKVVAQKLPAYITVGNYVVDCHSFGQIMANKEQELAKLVMELICKM
AKARTTYIREEFTKIVRTVEKQPQNPEKLYELKNFILSLPERISELSVEIEDMRQYYNIL
DGFQYELSDDESRQKWEAIAWPRQLSLRVQEVNKQLEKVEEELHARLQKYSEDFSKKVDA
LQRVVATFSKYTDASEAEKVAAEVKVNSIEIRKCIEEARSINSDQRLFGDKLTDYRNVFD
LEKEFKPYSDLWLTTYQWQDCYRRWHADPFDSLDPDEIESVVANAFKTMTQLSKTFKDKN
ATLKIVEEIRSRVEEFKPWVPVVTSLRQHGMKERHWKGLSEKLNMNLVPGETIILLDDIE
PLLNHKDVIVAHCEVAAKEAQIEKCLKEMRAKWETRMFLIESYKNTGTFIVKDASEIVEL
LDEHLNLTQQLQFSPFKAFYEESITDWERSLNLISDIVEQWLECQRSWRYLEPIFSSEDI
ALQLPRLSRLFERVDKTWRRIMGTVNHQPNVLEFCIGTSKLLESLRESNRILEEVQHGLN
DYLAEKRQTFPRFYFLSDEELLEILSQSKEVKRIDAHISKLFEFISRLAWNEKTQISGFF
SAEGEHLPSVRPVVPEGNVEMWLQSVEKMMKSAVHDQLRQAFYDHTKTPRSRWVLRWPAQ
CVIAVSQIFWTNGCEEGLNVQGSVKKFFRLLESQLDELVDIIQSPLKPRERINMGALITV
EVHAKDTVELMKRNEVSSIHSFDWIKQLRFYFDPDDELCHVKQVDAHFVYGGEYLGNTGR
LVVTPLTDRIYLTLTGALALCLGGAPAGPAGTGKTETTKDLAKALAKQCVVFNCQEGMTC
NSMAKFFKGLAWAGAWACFDEFNRIDVEVLSVVAQQVTDLQQACLTKQYRIIFEESEVVV
DPTHAVFITMNPGYAGRTELPDNLKVLFRPVACMVPDYALIGEIRLFSYGYKKARSLAQK
MVMTFKLSSEQLSSQDHYDFGMRAVNTVISAAGLNKRENPNEEEDLLLLRALRDSNVPKF
LIDDIVLFEGIISDLFPGTKVPSTDYGMVVDVLRQVVTADKLQPVPTFISKCLQLYDITT
LRHGLMLVGPAGSGKTMAYTSLQKALSECAVMQSKRNDLCSREYMKVYTHICNPKAVTMD
QLYGVYDENGEWRDGVLCVLFRRAAKYGDEGNQLGKHWVMFDGPVDALWIESMNTVLDEN
KKLCLVSGEIIQMSRDMTMMFEVEDLLVASPATVSRCGMIYMDPTACVPTSAIAATWKAS
LPAFMVSKNEYLAELANTYLDKLVEFIRANLKEYVPSTNIGLVHSFFRVMDGYIQSFAVP
KPQPGQPVMSPERAEILEKCITPLFFMSVIWSLGATCDEGSREKFSDMLRTVAQGNNHAD
SLPAEGLVYDYCFVYSAVPEDEEQPRWVHWDDLCDTCEIGRMTKFEDVMVPTIDNTRQKY
VLQHLLTQKVNVVAVGPTGTGKTVSVSDLVLGGLPDRFLGLTFTFSPQTKAGVLQNSLMS
KFDKRRSHVFGAPIGKHFVVFIDDANLPQKERYGAQPPLELLRQLLGHGGFYNFTGGIRW
NAIIDTSFVMAMGPPGGSRTQVSNRLMRYLNYVSFPEMSEVSKRTILNTILKGGLSQRGV
KSEVIDLSSKLVEGTLSIFRRCCKTFLPTPSHVHYSFNMRDVMRVFPMIFENDGNSLPNE
EVLVKQWMHEMQRVFYDRLICAEDRQQFISFLNDELTQLGYEGGYTSLVPEGRLIFGDFM

SERERSYRQISDMNALSIFFDEQLSAYNDANENVMDLVLFLDAIEHVCRISRVLCLPNGH
CLLLGIGGSGRKSLTRLACFLIPDMDVFTIEFTKNFGVKEWHEALAKLLLDCGKDDKKRT
FLFSDTQLVHPTLMEDVAGLLTSGDVPNLFEDQDIELINEKFKGVCMSENLPTTKVSVYA
RFIKEVRSNLHVVLAFSPIGEAFRTRIRMFPSLITCCTIDWFAEWPGEALISVASAQLAE
TDMKMEDMEMEHLSECFKSMHLSASETTERFFLETNRRSYVTPTSYLSLLNNYIFLVENK
RRFVLEQCSRLENGLEKLYDTENRVVELETQLKAQQPILERKKTEIQEIMERLRVDRKDA
AEKETSARHEEVIATAKAEECSKMRRECADRLAEAEPALQEAVKVLSKIKAAEISELNKY
QSPPKGVQYVMEAVAVLLTFGNCPREFYTGPPGGKKTPDWWLCAKSYMKNANQLLDTLVQ
PPEKGGFDREAMDMPLIEKVKGYYDNEEFLPEKVRTVSVPCMAMCQWVRAMYNWFFVNRE
IQPLRQRLSEAESELRRVNAALAETRKKLDAVIEAVVALEREFTEAVDTQTQLENDVEET
SQRLHRAARLIDGLGGEKVRWMELVEQYKAQEKCIIGDMLIAAASIAYFGPLTGPYRRSL
LDTWSGILRGFDIKTSEQMDLVATTGDPVQIQEWQLCGLPKDPLSTENAIILTNARTWPL
LIDPQGQANAWIRNLHKNDNLQVCKASDEKFMKVVEGAIRIGLPCLLENVGDSLDPALEP
VLLRNVFLIGSTPHIRVGDSAIPYDKRFKFYMTTKLPNPSYTPENIVTVSLLNFFITRSG
LEDQLLGKTVEKERNDLEQEKQKLTRDCAEKSLELKKMQENILRMLEEAEGDILDQEELI
NTLERSKLKSTEIKDDLQRARETEKTIDETRNKYRPHAYRGALLFFCVSELSMVDPMYQF
SLQWFINLVLFAVDKTEQAEDIDTRVKNLTEYFTYSFYTNVCRSLFERHKLTFSFFLCTS
ILQQEGSLNGDEYRYLLTGPTGRGGNAANPAPEWLTENSWNEIQFISANLPGFSGFDKHV
TENCEHYKKLFDSFSAHTFPLAEEWKNRETNMQRLVITRCFRKDKLAPAIQEFVKHYMGE
RFIIVPQFDLMDAYKDSTCLTPLIFIISPGSDPMNDLLRFAEQMRMSKKMDKVSLGQGQG
RKAEELLTNGRERGQWVLLQNCHLATSWMPTLEAVVESFTPETVRKEFRLWLTSMPSESF
PVSVLQISVKMTNEPPMGLRANMTRSYYGLNDEDLDHASKPKEFKKMVFALCLFHAVIQE
RRKFGSLGFNIAYEFNDSDRNVCLLQLRKFISLYDEVPFDVITFLTCEINYGGRVTDDWD
RRCMLGLVKTFINPSVLEDGYAFSPSGLYRTVEPFDRLYYLNLLGSWPLNPEPEVFGLHD
NADIACAQSESANILSTILSLFSREESGSSHTSREEMLMRTAQGIQEKLPRLFNVQEFHE
KYPTRYEESMNTVLVQEAVRYNRLLRFVQKSLAEFSKAVRGEVVMSAELEAIGSSFFINA
VPASWAALAYPSLKPLSSWVDDLVQRVQFIQSWYENGVPKALWMGGFFFPQAFLTGTLQN
FARRTQVAIDSVSFRFGLLDEKDPARVESPEEGTIVYGLYLDGARWDAKERTLAESRPKE
LYVEMPLIHLDPKVDYVNDPKDYVCPVYKTLARAGTLSTTGHSTNFILAICIPTNVEPGH
WVKRGVACVVSLNF*
>TCRU_3154 hypothetical protein, conserved
MAQKRLKGKSHEKSGGALRKSVGDSKKKTVYGKRRKTIEAKARANYASAVESRVASRVPS
DQRGRLTVVKPTAGQTSQREKKKHMKKPLTRGRRRKDAKKA*
>TCRU_3155 hypothetical protein
MASALFSENKQLRRRVRELEGEVSFLQDQNYHIQRVNDTYRGKVLQYIESLRSKGRRGSS
PAFATAKDLEELVRRLRAHDITHSSSRPQVEEMTATVFPKGSITREGDDGLMNSTSTLSR
ATQEGEGGVHVAGRSNAPRKEAFSPKGKKSQGNESATVKNAQRATVLNLTAENRKLVSLV
DTLQSRLAAAGRLNSSLEKKYRALSSLMGCSGSSGSGRQQDVTRDKMPGLPELEKQLREL
GEENEKLRSENDHNKELCQELSHYRILLSRKNVEFEVLRQRESILLEEINDLKGKLAKTT
ETLQTMGTALRLGKVREKRAISAMETLQKKNEEQELQRRQQVQETRMTASEIQPAFLSAF
IARVAAALERHHFVLSEYTLACHMLHVILHGRLTGQRAQESMWAIDFGWKSIVTETLFLA
AEHLANLIKIFEETTMIHIASVTSSSLAKSGMDAPVAKASEDGDRRGCLDNPATMNNNKR
LQPPIQLFTDDGNEVALKSEDEDALLSSNPVRRESRSIEGSVERSHAQMVDAATITVSPV
QISLAVGCSLPVSPQFPSKGTETISPLLISRAVGEDFGIEGCALVFPVACAASFQHDGRR
EQ*
>TCRU_3156 hypothetical protein
MDSTSRKEAVETLLRTCSRGGDREDVARLLLLQQITGERVKDVAGELDGASIVDEVLFHG
EGHGTTLPRITPPPLFTIGAKELWEAFPSWCRDTELQRRRCGGDAVHGHSFVCCAWNLGR
VVGELSFPSIRGWAAAYRSPWCHRYQLPLLTRDGSGGDNNNNKNGESGMRCTTSLEEEAN
AIEAAQREGRLMEALDYGEPGAVIMELQQRLNQRLEKYGAQYFYTPCGAAGKEMGVRFAS
PWATSAFVRNLSSAEEVISGFVTLEVIALECVDIAEEERPVQAASSLFDGDEMWVKGGRE
EDEWPSFSEMTNTFSSVGRRRWSAGHSFQLRVNPTELLIRHDTAHLIANPKYHGDGSVGN
AANDSSRMWLFHKDQGRPETLPFPRRTCRNHDLVTTLERGEEEWPTFLENLDLDALVSLL
CARVEKSDNAVVPLGVSPIVRVTLPLVAPSPKRMWERALQKRSTVVAAAAAGVAAMVRNR

SGRG*
>TCRU_3157 Modifier of rudimentary (Mod(r)) protein, putative
MLRMQPYVDELKSRFGKVTVIHNSSAETLLQVEHVIPDRGYAAVLCVTLGVHFPRTPPIV
TYFDGRKISLASPDGSAPDAWDPSKSNLVDAVGNAFANLANLWGSVVPPSMELLTSQLSS
LSDSMLQDIVSNPNCLESYAYQLPFFKAIRDASCQTIDDIERVANENLKLQPVVENLRAE
VEGLQRSLEQNVQSMQKMLRATPLLNSIGTPESLAKTLATDVRTLDAQCEEIAKKILQLD
CATDKFRFDNLLEEYREKAKERHFIDLKRRAYCASLT*
>TCRU_3158 methionyl-tRNA synthetase, putative
MSLKLFSEKANTQALKALLCCHHVKRPVELTLSGAYTSPVLHHPALKQPIFSANEMVRLI
LHDKWRDDSAGGSGGKKTGCYDGHWASVSLEEEAWLEWEATTFTCAVHPLYTQRRVTPEA
AGAFEYLDNKIHENDCRGPCLAPVGEKGTSTKSPSFLVDCIIWCAVLPALCEGGLLGEKE
REKVPHLLKWFESFQSAREAHIAGAVESLAVQEFADFLRAPRVYKLSPKLSKVFFITTPI
YYVNAAPHIGHVYSTLIVDVIGRYHTMKGERVFAMSGTDEHGQKVAEAARQKGMNPYDFT
CQVSREFIDCFEQMGYHMDYFIRTTNESHKKVVHELWSKLEAKGDIYLGRYEGWYSVSDE
SFLTAQNVTDGVDKDGKPCKVSLESGHVVTWLAEDNYMFRLSAFRERLLEWYNNNPGCIV
PEFRRREVIRTVEKGLNDLSVSRTREAVQDWAIPVPGNPNHCIYVWLDALSNYYTASRLR
LGKSGEELELVEDHRVLERFPADIHVIGKDILKFHAIYWPAFLISAGLPLPKTIVAHGWW
TKDRKKISKSLGNVFDPVEKAREFGFDALKYFLLRETGFSDDGDYSDRNMIARLNGELAD
TLGNLVMRCTSAKINVHREWPTPDAYNAKDEALIQLIKELPGVVDHFFLIPDVQKALAAV
FDVLRAINSYVTENAPWRLVKTDPDRLRTVLYITMEGTRLATLMLSPVLSQKAPVIFDML
GVPEANRVGVENFGFGVVPSGTPIGDAVEGKVLFPKISLDKAQSA*
>TCRU_3159 hypothetical protein, conserved
MDLSLKHGRILLLLDILRIYGRLRSLILRHKCSCPLWGAARELGHQQGLITAERERLNFD
YANAVADVQNRNAQLNSQFQEAHQNLSVRESNIAGAGNLVLELQKQLEAAHAKLREREFH
LGVLTAQLQEREARHNAQQPARYAETAGRGKAPAT*
>TCRU_3160 hypothetical protein, conserved
MPGSDAIVSRIRNVFSRDLPKIHEAIGGILPALQYADPLDQNVKGKFNLLVVLLPALVCA
PEAENGSGGALIPCMRGLPGDVKCAETAVLFHS*
>TCRU_3161 hypothetical protein, conserved
MASGVLGENGSHMPDGCNKTAITVPLRSVLPITAVEASTGESVSDSTDIYPDSLSVPPSP
PPVTGLPEALKAPKAPQDPPESDVGQTGVGDRLSLVPNDDSSGAGGGFEENGNSNKIGVS
ASEFSTVVGASRSSIPPEVGGSENKSGAPPNLKLESPETNELRERILVETNKDTQKVSKL
EENPESSRKKEKANIRGTSEEAGKNEAGRSGVEAHGPSSVLSADGQLQRQGTTVTMTQPP
PPSPEQQTSAVSPSPGVDTPAARSREAGDLEEGKGIPPARTASHNSTDEKRKAPPLRSEK
ESAAPKPPSADGVLEQNSEDTTTKEAIKMDAHTDGPAATSEQSTSASESGVVKRNESEDG
DNAQRPEPKGQHNDPDAVNTKAAPTASEAASYTAKTVPARKNDTVTYGDSDSSTAVSHTT
SPLLFLLLLVVACAAAAAVVAA*
>TCRU_3162 hypothetical protein, conserved
MAMMMTGRVLLVCALCVLWCGAGGIVADDAVPAELEKENKSESLKDDPSVDKTDTHEPQE
GMIATQQNGLALKSTTNPLELEEDMDEDEEKEDGKGKGKAEKDTRSTEQTETQKSLTKDG
ETSKLTSAPGTPSTPIPPEREGQETPSETPPGPPTNGKPAEETGPPALTGSQNAAETTST
TSPSHSKTAPEAAETPSGNGAPNQHREDAETPDSMKDGPTSLPAETAVSSVSKSGIGGTQ
KKEDKVDDSDQRPNTKESQDGIEDGNTNVAPTFSETAQKTPETITVAKTNDTTTTGDSDS
STAVSHTTSPLLLLLVACAAAVVAA*
>TCRU_3163 retrotransposon hot spot protein, RHS3, putative
MDLECGVLYLLAVGNFPLVDALFFMESPRKALVGLRTTTANAHHTQTSNVRLFKERVASY
FNGWEEFARDLSWEIIYVQHADSTPISDWQRCHDSANLTEAENREIAAFWEEKVHQYQVT
VTAEMLLQNRPPEVSNN*
>TCRU_3164 retrotransposon hot spot protein, RHS3, putative
MQVFVQEPDAYVQDQRLLRIIFNLTEYQVYKLHHEGVFSPEQWRDYEGKDTVTLIARGKL
NAALFQAQTSTKVVKSTVLKGVYQSVYNARWSHVVEIPDCEEEQTEMGMKVEKGKPKQSW
TYKEVGDTL*
>TCRU_3165 BNR repeat-like domain/Concanavalin A-like lectin/
glucanases superfamily/Trans-sialidase of Trypanosoma hydrophobic C-

terminal, putative
MEDGTIVFPLMAKSGKGVVYSMIIHSTDNGSTWSLSEGISHPKCFAPRITEWEGSLLMIV
DCEDGQRVYESRDMGKTWTEAIGTLPGVWVNSQLFFRDLSLRVDALITATIEGRKVMLYT
QRGHASGDNANTLYLWVTDNNRLFSVGPVGMGNAGEKEFAGTLLYSDGKLHLLQRMASGE
GSAISLSHLAEELNKIKFVLKTWAQKDVFFSGLSIPTAGLVAVFSNASASDDTWNDEYLC
LNATVRNATKVKDGFQLTEPDSGVIWPVNIPDDKVRNVSLSHNFTLVATVTIEEAPSGNT
PLLTAVLANTEPTHTMGILYTANKTWETIFKGETKPTTESGTWELKKEYQVALMLQGNKS
SVYVDGKSLGEEEALLTDETPLELLYFCFGACDMKNSPMTVKDVFLYNRPLNPTEMSAIK
ERIPVPTRTPEPQVKIAPDPIAPAVSAVSGAPAVPAVPAVPAVPAVPAAPAGNEGTAREK
GDGGANGDAGSVYGSGLLPLLLLLGLWGFATA*
>TCRU_3166 hypothetical protein
MKVQIRDSKFKGAYEPVNDHPRRRIATPTLQEHLNDVKYFHRMAPTTQSRGIKYRLAAIE
NRLEHGVRQRYQRKVQQIPSTSQQFQSSSKRQEHQTVPTDSPNMASNGSETPDAERFAFL
PRDTSTDECKCRVLRPLDRFETKLRPQGDGSMWLAIRSSRRGKTVQKVGW*
>TCRU_3167 hypothetical protein, conserved
MQLVLGHCGVSRNEACDTKSEKASDLSQLTDTWSTGVVALAERIIRARQLKPEAHRSAVR
GDWELTRTDPTLTRGVEAALARLRTGVSHKYGWLLRPLQPAIPRTCCWCGSDAAQQTRKE
LRTETPPPPAGPTPPRSDGRQTARCAASTAAASLVS*
>TCRU_of Trypanosoma cruzi region 4/Dispersed gene family protein.1
Dispersed gene family protein.1
MEYRSAGIIGKFRPVACGVCDADVSCFAAATRTMSRSCGCRCAEDGYGLDCLPVYLPHVD
GCNRTPAMPLLSHTVTLTETRSLTPTWTSTWTPSLSATHDSPTWYGPTETLQGTETVALS
PTRTPTASVSSTLWWSDVACPTLAVTTTAAGGSLTQNDIRGGGSVVPTRLMVALPPPFRW
ASGPQLGTHLSFVPVSTAQPSGFGGPWGAMLRNATWVRNATNPSTVLELAVPVHRGYFIA
ADETIVIRCDAAAVSGGCKGVLLGSFTIRSDTLPAAASALSAITGVAAGAAAASVLVTGG
LGSILEMQALGVFARMPCASAQESESTAALPYFLSVFAALDPLWMVVGNALLAAVFGCVH
CGVTAAFQRWRGVDAASAWAAMRFPSLTYVVAHAMHLGIFFGSVLALAMPGARVQHRVIG
VTGVLYGVAFPAGVCYLIARHTGASFTKYWQFSRKPLHERLLYPVGYWHPAAQRRMYGGM
LTNMKGSHVYWCVFRLSVLCVVGLIAAVHPPVGVCHVLYFCIAAVLLAGAGVVAFTNMMR
SAFLTVMHTASFVLLAALCLVSAANHLAPSDGGARAYAAIVLLLTTVLLAVTVYSVVVWY
AEDRHWQELREPRRGGLEALLRDDEESDEDAQKPHEMTSSSYATGTTGASSYRPPAPPLQ
PMAGDTHSDALSPLDRASSASCMIDYAAM*
>TCRU_3169 hypothetical protein, conserved
MLLAKDNVHDGVSREMLYAAGAVTAVGSTLSFVRNRALLPRMLSASLSLSAGAHLRVACN
DAGGRVLSTAEEYAAAGFGDAGSIDVVGCDVCDRDTHCYAPGTASASMRNGVCVCACGSG
GYGEACVPVGAPALPPAVGTASSVFVREGVAVQSVFVVPAGASEVTLRHVVLDGVSPVLY
VPWMARDGVRIVVQNVSLLSGAVLYVMGGGALRGAVAAGSDEGGPVELSVCDVEALNGAL
VLTGTYPAGSVLTVTDSLLVAARLTPLVYLPGSQSSPYAPVLVLSGLRLVRSVLVVSGVA
LVTVMTGGRTVAVDGAVLELVGGGVALDAAVLGGDFALYASARVVASGGAVLRVSGSQVY
AAHGLVFGSGVEANASAVVVNDNAGALADGALLVLRGSVSFVSGSWLSVRGNSISGMLLS
AAAVSAQCGPCAEHADAPRERR*
>TCRU_3170 hypothetical protein, conserved
MWSAVGACPRSRHRVQRSAIATVRAALLVLLSLAAVAAWVPAAHAVVLRLRGCTVDRAIT
VGRAVDTVLMDGVSITNGVAVVFDVPAMLPGALRIELRNCVCDGGAQIYVRGYGGEPASD
RSLEVSVSGLSGSYCSLVFVHNLPAHTNVTVRDSTIVTAGPMHYSQLNGLTDAVASPLVL
HATSLLQTQLRVSNTVLRSLQTGGSAVYVGGGVDLLSSAVALDGVLLEASGGPTASAMHV
ASSSRLSLQSHSVFSMTNVSVVSSGGGIVLGERLAVIDSVLRFVGVEGSVASSLVRCDGG
TIGGGGWLDLHDVWAVGEASSVASLSGVTLSGGAVSIARCAATGATLVSGLTITSGVVSV
QCNRAGGRVLRSSGDYRMAGLPSVSVVPCDGCVAALACFDALTASFSDCACSCRAGGVGD
ACLPFDVPPARAGGGGGDTAQDCMSGVTLTESVTVGGVRATACFDSVVFSGPITVAVDLR
LMDAFADALNVTLRHCVLAGGARLRIGGLSESTARLVPHALVSMTNVTSLEGTIVLHGAM
PQHSTVLLANSTLRATVGGSQYVPTTRGHAGLRCGPALVLDGVRLLSTRFVMTRSTMACG
GGSCAAILVERGLGANLSSVFYMDSCVVRSQTHVMYAIASDLRVGGGSVFSIQNSSWSAP
STEYGEGACVFKDVVVDGGSVLQIVSGIFRLGFAMLIANTLTVADGSWLVHRDNEFRTAY

VVYVANENGVAFRDRSVWSILGNNFTYGSYSSTIARMTSKWSPPSDSHPIIYGVCNEARG
SPVTDYGEELNIGTPVTVLDCGACTVDAVCFAARTSSISGCECVCAAGGYGDTCLPAAVP
DGLGPLPLPEADDTEVRCVHGGSIGSVDYPDPGVRGLCFVNVTFTAAILLDLWSFDAPQQ
TLNITLLHCVLVGLLIKGSGARVHVSVVSSMMDSGVLVFQGDFGVSSQILVVGSTIVTMS
GHAILFVKFTLSANMTLLLLDNYIEGSCYAVYFSVALVVDGGGIILKGNTLSATKDDDGV
ESSVYVYAVDVRNGGFIDVENNTMSAVNGVILFGDNTVSSAGLLRVADCTFVGGTETFDP
ALLHLSGSVTLEGGAQWRVEGNSVGAASVLTILYPQYKIQLSGSGTTVALANNRQVGGNT
VFAMLLPWSTILTSPARFVVGCNLQDDEEVSYDGVFPEDVVIFRCGTCNDDAACYMPGTE
LVDRSLCSCSCKDGWHGASCLPFEVPETVVPPVAERAVDGDTSCVVNQTLTDLTINMWKT
HHCYVGVTFSGMGAVLTFFLNSMPLHLPINITLTGCTFLDGAALQFVGGAGATESSGVLI
RVSQTVLRSSAVAFLRALPLHCDIAVTEVDAVQSFEFELSGTVNNMWSVFLLGNVVLRAS
TLLVSNVKAYATKRDALGFSSIGTLKLVGGSSLYLQYCSFEGYKYLFYVHSLSVSENSVF
ALLNNTMLFGVSLLYQHQGFSVSDHSVLRVVRNSGSARYAICNDELWNVQRSSWLDWRDN
DVEVGAMFYDTESAFVSIDDSSAVTLTGCKMGSTGLSVSLLKRADAGYRFVAGCLTVAGR
EVTTAAELALHGITNVTTVAVCGECTRDGDCFAPLTTAVSDCKCQCAAGGHGDVCVPAPV
PAGPPPPPSPPLRPTPPPPPVGECISDMVYPEVAQSVGDGLSWLCYRNVTFSGGGMSLTV
LVGAMTGDVVKITFDGCTWRDGAVLLLLGNAHAAVGPLNIVVTGSTFSDALLSPEGGFPP
RTNITISGNHFAVTRLVRRPGLDIWSPSCVMMNGLVIANDSAVVLSGNVFQTVTTSSIAI
YVVRSALRVSWHSVFAVVGNTFHMDGGGSTLINIDGFSQSLSLSVLNNSAVVVRGNLVTR
PVRYFLLLILASRVESRSAVVFQGNEMQRSSVVFFSSHSSYIYYNSWLQVSGNLCRESPS
EAFASLYPKVNLRDSTVSVSGNRFMSSTDRPTVLRITTGSRDITNGAIVAACNSVNGEEG
VEYSVPSVYNATILTCSDPCILAASCFPAYTTTASSDGCACNCAEGGHGDACLPVAVPEP
PSTDGADLCVRDVRVDVEVSAGLGTSVACYVGVTFAADVVVDVESMSGSVRNVTLANCTF
VGGASLYVVGWRSDPPAGQCVDSAGLSRVPAAACWWRTDTRRARASLWWTRC*
>TCRU_3171 damage-specific DNA binding protein, putative
MCILSHLHTPRLVVGLLNGYIADISLEAVLMGSAARETLIRLQPVQLFNLESHNAVLCLG
EVPLIIILCDTGFQLTGIDFNDVAACAIIEGSHISSKYIFLSQSENSLAFGNIVDLKKLN
SCFLGLKATVTRVKYIAWWNVFVMSVRRNEKDQILLLMGHELANSWVPQNELFSIELFSI
ELLENERCVFFETVVLGGSNESPNGNEADKDKAILLGTTFAFPDEQLPRSSRFIWYSVEQ
GKLISERPQLRQIGSKDVEGALQCCCIVPNYVGRIALGINGCIALYSWNAADSVFVAEET
ICIGTIVTRVLPIFQGDASYMVAFDARHSCFFIQVDTFQGSLEIVARDSEPRGVMDGAVF
QLGNIYNICFGDDYYNFFCLSRGASTLPSASNATSATVSSGKLETSAQYHLGDMVTAMQL
GSFAPCSVTNIAVPIPTTLIPGICGPQVVFGTSHGAFGTITPVSNETYLLLKALEVAVSS
VVPALGGFEHVTYREVLRAGQERGYSRNASFENANVVSSGVFEKRRKRYLSRCVCSGDLI
ESFLTFSQTIQQRILREAESRIKSCYFSMNSTLGEFFEHNDLDNSFAERSDACLDECKTI
GDVNALFSESGLPTLPLTREAVNEFLQNMQRIH*
>TCRU_3172 BNR repeat-like domain/Concanavalin A-like lectin/
glucanases superfamily/Trans-sialidase of Trypanosoma hydrophobic C-
terminal, putative
MSADGCSDPSVVEWKDKKLMMMTACGDGRRRVYEIGDKGDSWTEALGTLSRVWGKKRKEN
VKLVRSGFITATIENGDYNKKVMLVTLPVYPKDTDKGQNNEKGRLHLWLTDNTHIVDIGP
VSGEGDDEIAASSLLYKSAESEDKKEELIALYEKKKGGEKSLGMVSVRLTEQLKRVKEVL
ATWKDVDSRVSQLCINSLAQKDPSADSVCTDKITDGLVGFLSGSFSYNTWRDEYLGVNAT
VKDGAAGAILHGGGVKFRGAWAEWPVGEQGENQLYHFANYNFTLVATVSIDKVPEEGDNT
IPLMGVNMKGAGNSVFLGLSYNNNGKKWILLCGDGTNSGEHSSNWESGTKRHVVILLRNG
NQSSAYVDGQRVGVGASCALGSTESQETSHFYIGGDGGSAGSKGSRGVVLVTVTNVLLYN
RPLTSEEIGVLNPNKALISPLKEEPSTHSPVPSASVVPPTPLVAATAQQTGISSTPDGTH
RTEQGQPIGNSGAESGGASASAASTVSIPSAEKDSVMQMASGKSSDGAQTVDGGSTADGE
PTVEKREGTDAQKEEGIHPQVREVNATALNSNLGSFPQGNNSDAGSMRGSGLLPSLLLLL
LGLWRLAAL*
>TCRU_3173 hypothetical protein, conserved
MELICQRRSTVVRRITDIPALEHPYEESERGDSRNVCAVNVGAEDVPSGAIHDFLPHART
HDERGELRGLFDGNRCSAIDPTAVRQDAAVHVANESNPAGHGDSGVTKTSGAIGDETDAP
LDK*

>TCRU_3174 hypothetical protein, conserved
MVRYAKRPWYTPVGFMSYAPHSYGALLVFVGTTAVFPLRYKISEYFERQRDGPHVELRRK
AVLYYNEIERMHRRQALQNLSSMQEDESSHRLSATLQGEALRMGHVDQEYNYWYAHQRDA
LRAEKLLAEVRELQERVSQAQ*
>TCRU_gamma, conserved domain containing protein, putative
Elongation factor.1
MHFLTTNLIRGWLRRMEYVRQYALGVALIIGEERRHEIVALCVLRGQGMPAVARDVEGTE
LFDWEEVADVAAQQEGPTIPRPVLGGTCVEVGVACL*
>TCRU_3176 hypothetical protein, conserved
MALVPYTPSEWCDIGARLWEMVCQCGEIQVQLTFINGNQMSDETLTVAASSTGLCLFHGP
ISKAWQDSTLIGHIANLRAPQIPDFETHVLLPAMGVARELGHQKGLITAERERLNIDYAN
AVADVQNRNAQLNSQVEEAHKTYR*
>TCRU_3177 cytochrome oxidase subunit VII
MPRPFGVWAPATTLAEYRARVPNLSAFSLKWIYSAKREVYYHPEALAHFKDHQQYKDAVD
TVRAMSVSDKFFGEGQKLPRRPVIKLMGALLVEGLLVYAVIRYYYGVYVPANNPTWRKVV
NKEWEEAINTSPWDHMSHVWQYSDQYAAVMGDTASPGPRKFYIPA*
>TCRU_3178 ATP-binding cassette protein, putative
MPIQGVCESEGLRQPSPVQSEEPLLGRGMSSVQFLGKQKESTSKLALTFKDITYTLPKCS
GRSEILCGISGYVRSGEMLAILGPSGAGKSTLLDILAKRTVSGEVGGEVLLNGRAIKDAA
FRRITAYVQQVDVMQCFLTVRETISYAAQLRTPPSFKRREVRARVEEVMRQLGIDGIQNK
KIGSDLVRGISGGEKKRCAIAIELVASPSLIFLDEPTTGLDAFTALHLMKIFKELTSLGT
AVVFSIHQPRSSCFALFDRLLLLNGYGEEVYFGPAGEAMSFFAQIGVVPSAPENPADFLL
DSISVPPEEELLACGDEARLCHVACGQSAPNIAAAFRDRLLEGIEREIDAIDDTFMQIGS
ETSMAKDVSPYFRSVWTQIRVVSMRGVINKIRDPMAVIVTFAASIFFAVLTGSVYFRLGL
DQPSIRNRMGVLFFIVMNTSLHSVSVLNLLMEDRPLLLREHRNGMYRPVAFFIGKIVQDL
PIKMVSNFVFDTIAYFMVGLQPRVDKFLLFCLICFIIMLNGYTFCLLVSTVSKNIQVANI
LAPLVVVVYLLPSGGVLMSVEAIPLFWKWIKYISFVRYGLTTLVINEFDGLKFNCSPEDV
ICVRDGKLYAEMQGFYTKDFWRFIGAAAGSVGVYLLLAYIGLVLVRTRGSG*
>TCRU_3179 hypothetical protein, conserved
MTSTANDEAATLWVQHAVECLHKNDNGCDDGGEVRYADLLDFMQSAPPPPNSLHAMWEQC
VWRLQDEPEALYLLRCLANSSRGGVYSDASFLLDARASLPFPCVSQEDAGGAEAGNGLQR
LVARIVALRLVPSTTDIIQLGWSVSLAGPETTPAASSSLRPMYCTRTHVVIQLASGRHLS
AWAYGGVGLRLRVGTVFCALLYPSRTENQEMAYVIHNVSEITAADDSVGSLAWSRSIRRD
NFASYPSMPGQFSFEQLVESFSPEMAGQKLCKSLLLLVVVHTMYRAVRCRARRPLHALLL
GAPRSGKSALLRAFLHLLGPHATLVGAHVVHGQQRSAAISQAITATYPHVRQQLLLGGAV
STFDALVVDEVSGRGCANFVEGFITGVCPIFSGGGGVSATMIRSHVIPTHVQVTAAANDD
LLAAAALIPKFSVVARTTAQLSLHNAASIGEGVIAASVASSQSLSRTPSRSASLCESSPG
RLDSGVFCAGPLCEELLRAAVEEAPPASVLERMIPAEAFTSFYLSRLREHWHGTKALGTT
TVTNSTPPLALVLSVLWELNLARLVLEHGCCHGERATGWSELLAEEVWQCYTHHLWALDT
AAVATARERIKKQAIPCGADFSAVGSGKKRKISKKAACVALLRMMAHEQRESGADSVSEA
STRAFFQQLGGEAMTGQSFSDVIQQLLDAAMIIQRLSAYAVVAEA*
>TCRU_3180 hypothetical protein, conserved
MEEFGASPPDADLDIASRDYARRVQGLRNDAYRQAVAEIAGELYGAVYMRAYRDALEMHR
GDWGGTASSSPSGCA*
>TCRU_3181 hypothetical protein, conserved
MRRWQYCLVPIACNSSARAITTTRGLLGLEQLQDVPASTSRRPRGMHRGPGKRQTTEKEA
ERYRFIKRWDLQMREGWDELEAFKGLPKPKQQFGNEAAEIVWPYAVLLERVVKVHPFTKS
IYVYYGQRQSTPLGKLAAETAKRFAREFLIPITFHNSQVYVETEMLLEYNETPWVVVHAL
DGGQRILPVHPAAEDTPIDAGVTQLLREVVKTCEALAAAVANPKEVTRMLNERPLQNQYV
RVNYQWFGDTPDERCSHLVRWDFDPAEVEPIMRQRTRHVLNWLNHDGNLPNHNSVRVNVF
REKTRLRSPQTTSGPKTFFNSVGSRANARSSRFGGQSAVGK*
>TCRU_3182 PHF5-like protein, putative
MLRHSKQEQQMCRKMPGASFGYVCDQHDGRCVICDLQFDEVLPSMREVHVCDDCGFGKEG
EERCIMCNSGKTTEVAYYCHYCVALEKDRDGCPRVLNQSRHQRMATLRTR*

>TCRU_3183 hypothetical protein
MNINEKISSVLGYVRDSVNPYTRLVHSAMNTDKRSVPFYQMEEVREASFQSDHACEMIMK
ALAPSLDESSSIVLSKALSIFRVLFVEGANGIRIRMARVRGSLSRIANLQDVGRGTLEAS
NRDLARAFLEAMDGNSDALVSFPTVQMSGAREEEYTSPQSYKSEFQTMHEKERRMYRRQC
EEEKRRSAVIVRGRIYGTFDGSLSPEKLAEQVVACPKKRFAQTELDSFVSAAIATERVTE
VCAALDQHLQGARQTLQNRYKVLLVVEALVSSGVKEAQDYYRENNVGICRHLTMNSVENP
VKAEAAQSTARRITQALQAPLLSSAPAPTVPPRHVIAPQQPTVSQLDDLFSGVSVQKKPA
ATSFSHVPLLQDISADAVIEDIFGPAPRLQSAPLPPTATPTGNGTFTPTPSLPQLFEQNH
KQGEKQQQEQSTLSFISQNFPPSGGNFISAAQQSTPDFIGSFQGTSTAAGELASQANSFG
TSSEQRVGDARSDADKLRLMQQIQAQMEMTRRMLEQQQSALLALQAEFQKK*
>TCRU_3184 NUC194 domain containing protein, putative
MTVEEISFALRADVAALRFLLVGLQGSASDACHVFDNMERRLRSSKIALFSAHETVPLLE
CVLSNEESGLLRVLNDYCEKMRFSGTDARDARVLKRAMSFLNLFFESFQESVTIPQVEEI
HSTVWHTFMLLHRLSKHVEVQAECLGVVSALLRSCGQRLSPARLDVAKVVEVCVLILERK
ITSGLRLRSNAMRLLGCMCGVYTTLMSPHAMRILHAMVSVGQHLLASDEVEQVIGEGLLL
AFSDYSGGFSLVFNGPDRDTLMLMYHLIKKAILLAGTCQNYRFCKAALALLGKRADFLKE
CLMNDMQDCFTALRHLWSHRNAEVRRATHAATAGFFRALALSWSGDSQRHDLAPRLRYML
HALEATMRDKQGRHRDLSFAIMAFGYMARPLVSCFGVDYLAITFREMVKRCEVLFTLTSG
PVEDVVRLLPYFLSALASMLLELPPMGSELPQLLNFTLHTTLFLYPRGMCSENIRSTFGS
ALLKLLTAIRYCHGVEFGVVLVARWLLALACVPFSRWDPKKGLPTAVEDVTMQKSYILLW
ESLQSHSHSVLDWGSPWNLSISDAMLDSLRMALRRELVSAELEMLRQFDVSLLSTEEEEE
ARDGGDTDFHWPVVDELGEMQLCSMEDCNVFLRFTSFFIGSLDATFSEAIGLEEVAAVLV
ESLVSFATRHTHIAGGYTCLHELLQAGWRAGLSLSHAWIFLRSFSRTCLHCLTIYTDGLQ
IQSALCMISMNHFGLLETAQLLYPIEVVIRHADDTAAVADALEVLDCCLEKDRALVMPPF
CALLSQLTVFNPVFGQLHSLLVRHGAQLGDGVQRGVNDEVLECCHGTTPALPCLRRHMDG
AQLPLPMGSTTLFLPLGSLLPRATMLSFQERDSQLRLAACELLRAAVGWCIEHNVDDLYG
ELFDTVIVLAGASAAGLGIQVMFGALLRQCARCFALRGGRGAKVMENAIFRGTAVPHNAS
IRRVSIEALAEYVQWSLKYELRLQLLQEDGAQESQLKSVLLWVCLTLRGPSAWQRLGAAG
VLDRLLHLLRCERAVLPFVVPFTRALLAALASMRMNEWDGGDEREGDGGGRAAVSLAGNL
CRCVRRLEVYVVTNYELLVDMWQDRNVAEENEAVLRDILLHAYREGRHSTSGAALLRLFA
AICRCMPGYTEGDVGPRRWLLTHLSVLRDFDLLETIPGRRLECGLQMHYMLMSNALFPST
LKRARTEDASLDEESTYFRRVLEELDRYVAESAAGCSDSVCISARVLFIARHLVRVLSGE
VGTVASKLLLLNRSLPQFAVRLLLAPTSLGITPGPYYEVQRLCGRELVAALSVSLRSDEG
RFRELVDSLREHVPATLWLTPQPTLLEFFLSDPHTAVHTLRCLAVLVDLGLWAEVLLLPS
LSHVIQRRLLEGLLEAVRDECVVNSQWGTIFCHMLLLLLLSPLPPPSLDDAKARKDGENV
TNVPRGFGLREVLSAVQQDEERFVDRKTSPVFLAAILRGDWRDAGKNGATLRCLLTELFQ
VCGELLQRSGESSLQSAPFVALRGLLHDFHQGPKPAAETWALAMKRGVDALMHIGHGGSP
AVLAFFLEVHRCCLRVGGSHFLACYEYDILADISAAVDGERWTMRPHTLLTSRVAVHLFV
QGVDVLCDAVAAASAGNERPTQSYATLVEQLMRRAINDELPLDWREVDDGSINGIHYAFV
VRALIRLVRCTAALDSALLLSLVPLLHQTDMPQYTALVTAVSSVLQKLPSEALVELCHKS
RELQQQCGLPWAVWWGLTHRLLLPSLRRLLPAVRDKFFLQHVVGLVEEASAPPSGAATLQ
RKTRALIMLEAVFDMTAASVLRGPINAAFTGKKDGSSGKELIIALIKACCAARVFVPPAT
FSPEAFHDALNYRQSAHRCLVAVLVATQEQEKVFTRLLLNSPGGGVWNQIVDTSFKHFFY
VETNFPHMTECTAKECSDWEVRKCVATFQRLAESNTGILRQGTEGEMQCVYAVENAVDHT
FLTANLPWLSHALVMHDEGDDKPHNRKREEKENGAPRVKLDGVWISTCFGALLRVTDFLL
SRFAVNPADGWVKEIIGLFSSTAIHVNVRLVLARLIQMRASSFTPAANALRDSLVSLVCE
EEMGEGGVHYVARDLLELLFHWGETGAMNFTTEAARKLMVFLFRGASHPSEDVMQHNLDL
VRRLVSLLKPLSPVCDRDLLHVLRGQLLEFSFGIRMCGLLIDNGVLLSSVAPASLVDLYH
SLVSCFKRNKNIAKVEVEELLVVADVVGKELRGLRDFAETDALLLRDTVRSALLCLWSRS
PGVALRVLCAIVQHDSGVVEDIPLSTLLANFMSRDATEQLAALHVIKCTLSRTAEAYATL
HDIFSGHLFTLSAPVRVALYHLLVELFPLLAAAEKERFMNAFLSPDAMQIMCGNEDTNLA
FFKLAVRVYEVAPSSFLLARVAEGLKEPLPSVRFMLLEFLDAHVLPRDSYLSRLAALKEA
ITPQTVGHDVWLKNAAVLTLSLCHTAGATCPVTGNLIASPPSFAFLGSCFLNDPLKRHGV
PHDLCPRASRGGLPWAHSESEDLFLQKHREEGAEKPEASITRALPACSEAFVEFQIPSE

SFVAPLQLLCMHDTETARCFLGHIEASVAV*
>TCRU_3185 Concanavalin A-like lectin/glucanases superfamily/Trans-sialidase of Trypanosoma hydrophobic C-terminal, putative
MNDNNKTVLLGLSYDKEGKRAVLCSGQTTKEHSSPWESQTKHQVAIVLQNEKQGSAYVDG
KLVGGDEACALKSKDPRVVSHFYIGGDGDKVEGQEGLSVTVSNVLLYNRPLSSEEIAWLA
KNTITNPKPEDPKTSVVDAQSSATSGSPVQGAVSLRLRRRQLPLEEKPLKENIGAGAGGV
YSATSVATTPSSDASQTVVTGGGDMMLGNGSPQTPEVSVSSAEDGVTAEGTNAQGEEKGI
HPQVRELNATALNSSLGNLSQGNNSDAGTVRGSGLLPSLLLLLGLWGYAAQ*
>TCRU_3186 hypothetical protein, conserved
MATEWGDIGARLSELVRQRGEAQVQITFINGTEMSDETLTVAPSSTGLCLILGNISKQWE
DSGLTGHIANLRAFQVPNFENQLILPIMGAARELGRVARAHHC*
>TCRU_3187 hypothetical protein, conserved
MGEDRESGATQYTFLWVQFDHSLQAVFLSDEFVLSVCAMPALNSSTIAEMEVVASRFLYA
ATTLCTRLCDYYFLITAVLRRLSAHNRSIVQETSPANLPSAAIGSGERLRHINEDNRGRT
INSTEKASAAIITDALFCG*
>TCRU_3188 hypothetical protein, conserved
MELRCFLVHCGNPSFGTSYVDIPPPPSCGSERHHGAVKYLGRHSPELYWRLGNNPRYSRR
MMSLWVENTSPEEIPLTARVHPHVAQRLVLKCIGKNTITVSRRTAGENRILLRCNESVTL
RVGDKVGVAHLVWMQVVLALVSVNGAVKAPVLVQQEEGNDAQYFSACRTLCPRLQDPPLP
PVVRVNVAWMKLPSAIRAALAISVWERQLAADSGAVATAALTSLRPTTFAASPEAGDGME
EAAAISNSSQVIQEGGTITTSSETRSKRRLHASPSGSQGVESQLISLDVPPPQKKRRTSV
AETAVMDNNLRVTLPENNTYYETFDERLDDAVRFLESRTEITNSLIAAGPPVPELLGESP
PCEGRSVEVKKWARRSKSATVSRTELRHGKKQKEKELQLSSGHVEGEAAGGKNPFAGESQ
VVYYRR*
>TCRU_3189 phosphoglycerate kinase
MALKEKKGIDQVDVKGKRVLIRVDFNVPVKDGKITNDYRIRSALPTIKKVLECGGSCVLM
SHLGRPKGVTMAAAAACGDDSVPGYEAGATLKPVAERLGELLNVPVVFAPDCLKAASTVE
KLSPGGVVLLENVRFYVEEGSKKEEERDAMARVLASYGDVYISDAFGTAHRESATMTGIP
KVLGHGAAGYLMEKEISYFAKVLGSPARPLVAIVGGAKVSDKIQLLDNMLQRIDHLIIGG
AMAYTFLKAQGHKIGSSMCEEDKLDLARSLLKKAEDRKVTVLLPVDHVCHTEFKAVEAPL
VTEGVDIPDGHMALDIGPRTIEKYVEVVAKCKSAIWNGPMGVFEMAPYSKGTFAIAKVMG
DSTQNHGLMSIIGGGDSASAAELSGEAPRMSHVSTGGGASLELLEGKTLPGVAVLDDKE*
>TCRU_3190 Phosphoglycerate kinase, putative
MTGIPKVLGHGAAGYLMEKEISYFAKVLGSPARPLVAIVGGAKVSDKIQLLDNMLQRIDH
LIIGGAMAYTFLKAQGHKIGSSMCEEDKLDLARSLLKKAEDRKVTVLLPVDHVCHTEFKA
VEAPLVTEGVDIPDGHMALDIGPRTIEKYVEVVARCKSAIWNGPMGVFEMAPYSKGTFAI
AKVMGDSTQNHGLMSIIGGGDSASAAELSGEAPRMSHVSTGGGASLELLEGKTLPGVAVL
DDKE*
>TCRU_3191 Protein of unknown function (DUF563), putative
MILGTLLVISPGLKELKEDVWPEVSDGAFPDNLIQRGPGGGYLLQSRRGREGPPLQLEHL
RSFCSKYAPGLENAWWKRENIVNFPIRSNKSKDIRLPPYPIFPGPVCFLGGSSYFDGDDL
DRRRNLGGARVKIFPSWRRLTDNVLMDMQNNSYIRSLTFVPAIAIVFLNFFSLGQMYHVL
NDMVKEVMVIMTEVGNETTPWKYSELGKSPTQFFALPRAELLWTRENLATALPQTTHFLG
AFSHPTAPTRYFFNDIPVLSRDAVYCTCQAMFTCEVPPDETFEQKTVAFRLIGDLMVRYM
NETPHESAQEVSDDLKKKQVVPLSILRRFSHIPQIAKRFWETELKVMAKYKNREPGDKTQ
EPFVYRPRLLLINRNFREIHDYQAVAALAERVGFNVQVVYFEKMPLEDQVHVSRHADVMM
GMHGMGLTHVLWMDGRRRPRCRALLELMPFGCPRTLIHFYKLFADTVGIHYEYIEAADMY
LKHMGGLHTSKAQQRDLEAVKAVMRACKFDYMYRGFTNQVAMYNMSVLEQRLVAALQRLK
QCGVA*
>TCRU_3192 lysophospholipase, putative
MIGTPIENLTVSQIKEELRTFYGIRDFSDVVELKDLQAKLTTTRNSQLITHGLQYGPLLQ
VGNRKNPNGVVTLLHGLGDSAHGWEPVAHELAGSLPHLLFLLPTAPVRPVTINGGMSMNA
WYDIKEISAATDVSRQDGETVLISADYVKSLAYTTTQRYCIPKNRVVYAGFSQGAAVSLA
AGITSRIAPAGVAVLSGYLAGGNVVLSRLCNKEIPILMCHGTEDGIVPFEAAQQTKKALE

AAGVASITLKSYRMEHSSHPDEIRDVVSFLKKVLPAIDSKA*
>TCRU_3193 beta-ketoacyl-ACP reductase 2
MLSCASFSCVFRRCAGGTGAAAAIAGSLLTSGQCWVSVRHYHQHTTCLLLPRKLSQAKQQ
PLFPGMCRHVKRFEGEHTYNYGHHTFTIVREGSTFFILPLGFLIAYAATLFVPMSFQLEA
MYSVLLIAFSRYFARGRKNRDWKDLKERHVIVTGGTSGIGRETASQLAAMGADVTVLSRN
GPNVDVAMAYIKKFAKSDEQQIRFTALDLADFIAVRDYCKRAQQQRMAVDILVNNAGLMQ
QKQVMSRFGDDEQLAVNLLGPFLLTEGLLPLVAENGGRIVYVSCSAHVAVKRNIVSTYLS
GRGLWSPRIRDKFDGLEQYGFTKLGCIYHAQDLAVRSYTNASKPFASMTPATVASILTPS
NGRAHKSNSNDGNSNTPRYTTCVAVPGGVVTNLYRYVPLATTFRWCRYFYLLFMRTATEG
SQVVVNCCVREELVNGGYYQNCRYSPTGLSATACSVEERKSVLAWVRLKMQPYMRWES*
>TCRU_3194 hypothetical protein, conserved
MVKISHEDTQRIKTAFLSYAQGQDKVTEAMIDQLICGAFPGLSWEQLQEKKKGRAAANGY
DRSAFFSLVASDEQYVRFIAQHFPCAPEEEKPPEIDALELKTQKGF*
>TCRU_3195 RNA-binding protein, putative
MDDNLAAEIARIEAELEADRQFRELQQRQQQQQQQPFGAMDATSAFERASAAENNPHPA
QRQRMFAHGGDPMAAQQQQPGVMAPTSGQPHQFSKDSDGRSIFVGNLPRGENGGPTTTPE
ELAQFFADCGQILNCTVLRDRGTGELKGTAYVEFASYTAMGKAIDTKNNASFKGSTIIVC
*
>TCRU_3196 Leishmanolysin, putative
MLWWGNNSGCGLLEKKCLTDGITEYPDLFCNRFDVYDFCTYDRLSLGRCKMKRHEEALPE
EYRYFSDPRVGGDDLFMSRCPYVRAYSNTGCTNGNPSVMPGCVVGPNSRCVKGQELQFDD
EYIGDVCVDTRCGDGTLSVRFLDDDAWQECQAGETVTPPSGPWRGSIVCPQYADVCTAFP
NTSSHPIPVVDPPLADDPTSAEDAEGDEGEVPRKRPRRL*
>TCRU_3197 hypothetical protein, conserved
MADESCFVAFASYIAKNAPCRRLTGCLHSAPFEALFCFIQGDATIIMAAPSLNGSECDNT
NDGKESDEDYGDDLHRGADYLRVLAHESREGLDAVPTIPGLAEPLAFALSHDVISAHALR
LIHLPWSSRSSSRVFVPLIPRWPFRMYLSVAWRMI*
>TCRU_3198 hypothetical protein, conserved
MEHAECVAAAACPELNCSANGRTAIMPSVVASPCCQRSVRNSLVSSRAQACDAPRHGGAT
SLPSGIGCAAVLLGPRLCSASAVTSSRREHRHSLPTSKRVMDVLLSTPRQTGTHPADYKQ
PFMSVVPSLPHTEEAGLPRPPPEPPPGVSTAPPRRTTSSGSTSPCPSCSSVFSSVHNMKA
HARRTNPRTPIVAEGLTCGFCDSSTVFPTGASRAGHHQKCQAYQRAKNGQRTSASSNDSR
SPSATPLLQPPPAAEPRQSTGGPAAHAVGMPPSSTTEHPTIWEHTLSHDSTIQKGKLATS
LRTARRPLTNSGCKV*
>TCRU_3199 hypothetical protein, conserved
MGTTTASSLENVPRSVGCGAARRFACSLTDRTAQSLQCGHFVGITRCGGVRKSRSPDEDG
VVVVWQLRTSGPSPLRIALAARLAQHHPGMVLLQECRHQGAPATRLTGCGDFHQACGGRA
GGVAFLVRHTLPCERFYSWHQLPGDHRLPRVPTCLPQHVRGEPLQPSYSNCSRCSLCEHH
SSSCSYCG*
>TCRU_3200 hypothetical protein, conserved
MMRRVFCVVLALTLLCCCLCVFAAEEEVEEEVLPVDAVCAGDGENVGRWQLRGRPLWYNC
TAEASGLGKMICGMGAGNCAPEDVKKRVEGNAKDGVFEIKFHTPTPGRVKSWWESNVEPK
PATAASGPAKPAEERPPVDREEEAPRDTTTISSGSSPAGVGGSAGALSLPPTTPRDAGAN
AAAGAAGKTDPSPPATLQAQNPAPSGELQGAAHSASAASKSPPDPEPTESGEHSSHDPPA
GRQEETVDAPPTRLSQTGEDGGAAEGAEEDTANTTDAATSEGNATAAGMPAPLSSAPTTK
ALENSAGNDACFHYPRLHTRLLLVLAALTYGTLG*
>TCRU_3201 hypothetical protein, conserved
MAGNFGAASWMDEGALARVAVVVGACSVCVTAVGSAGDVSGDADAVVVPVDVSCAPSDGS
LSYRVCVAWKKCSAASAEAQHENYTFSGFGVRMRSRDGELGAVCHVVSAVHTCSNCAASC
ARKEEGATVAFTMNVTTHKYAGPYELWWRQFPPDGAIHPPARSSDADRTGISQLPPPREG
KMTQNLVGQLRRKLMLSRHVRTRIGDGLRRLTDPTPSIGVPHPTRRPTRPR*
>TCRU_3202 multidrug resistance protein A
MQTEIGEKGINLSGGQKARVSLARAVYADRELYLLDDPLSALDAHVGERIVADVLLGQLA
CKTRILATHQLHVLSRADYVVVVGDGAVRFAGRHEEFMGSPVYKEVVAQDARQQQHQEKE

VREGEGDDVLDGAPVLGNGAPPGAGSPGRAKGAMMTSEEKAVGSVLWSTYVTYFRACGGV
SVVVIVLIIFLLTEMISLSSNVWLSMWSTQRFGLSTQTYLNVYIALVLFGTVTVPLRFSV
AYSAMRQGSRNLHRLILRSVSIGTMQYFDTTPLGRIVNRFSRDVDCIDNQLQMTFLFLLR
VLYSIFSSLAVAIDSQPYVILALLPTVFFYYKLMVLYNVTNREIRRVGSIVKAPMISLLG
QALVGSSTIKAYGCVASIMKESLRRIDRVCASSFLENATNRWLGVRVDFLGNVIVITIAL
LGVIGTMMSFSTHDIGLVSLSLTMALASTSQLNWLVRMIGTMGADMNSVERILHYTHNID
HEEVPEMDRLVDELREKNTEGGDVTATVLVEGVSGGSEGRHPETTAGWLEFREVEMRYRA
GLPLVLDRVSFLIEPRQKVGVVGRTGSGKSTLLLTLMRMVDICGGDIIVSGRPICAYGLR
ELRQQFSMIPQDPVLFDGTVRSNLDPFLDSTPAEVWRALELVGMRERVELESGGIDGRVQ
EGGSNYSVGQRQLLCLARALLRRGSGFILMDEATANIDHALDQQIQRTVMTAFNSYTVIT
IAHRLHTVAAYDKIIVMDHGVVAESGSPRVLVSDPSSRFSELVAALGKQEAAKFMASVGV
AAAS*
>TCRU_3203 hypothetical protein, conserved
MNVFLLSFQCWVQATQEEAQSPRSAMPSTPQIDEVLPRGQQRQAEEAADRDKQQQQCHHR
ESEACPACRPSRCFGPCRREKLLAVCQGILAGSTSPLSTDSYEEQPCLHPSVVCRGRSQC
SPQRGRMPLAILVQ*
>TCRU_3204 glycosyl hydrolase-like protein
MGPSSPATATLREVGREPQRVRRPTNVMTDVPLNILTDAPQEGLLWPHQRHDLDALYSTQ
TCKLVADGPNEANEGDVLMRVRLFRPNCRRRVHGGGEGKVRKGPCDDSAHPIVGLMPHQS
AASFFPEEVLMDETDVQVGPSTLRSIVEEEKEEGEEKGMRWCASPTASPSLRCRLRARGD
AEGKSRLVWDSGEFFVGISLRMTATQDELSSGLDANFSFNPEYKMRFFGLPLSHASGLRL
PDDGEYRLFNQFHLNQTLLSGTRMHGSVPLLYGIGRAPIRYGKKSLYGQRRRAIGILWLN
GSPLHVRTATGGVGGETTLSFRSTAGATRVYLLPGPTAEDVLLQYYTLTGFPMMPPLFAL
GYHHAHRGYGKMDELLNVSAAFLQAQIPVDTLGIGLHYMRGKRIFTWNLTRFSNPVKLQD
DLWRHGGRFMVLSTVPYVQVDPRFSLYIEGKRHEYFVRTNADSDEIFIGFSESGANVWID
FLDSRARHWYGGLMKYKRFLGSTNHTFFSLEENEPFLQGGVRGTLPMDAGHRGAVPHEVI
HNLYGMLHAMAAYGGQLRRTQFYRRPFLITQSYFAGTQRHAAVRLGYNAASWEHLRSSVE
LCLAHSIAGISFVGADVGGFYFQNVEEELLVRWYQLAVFYPLFCTDANENAPLREVWRLV
PHVRARIRDAVQFRYALLPYLYTIFWRAHLDGELIIRPLFFVYPQDPLAYVEPTMLGQRF
FLGPHLFVAPVLTSVGTRDVLHTVQIPQEDLYNFWSGEFVAAGTTLRVSKNISYSHLPEE
EITPLFQRVGTVLPTFTNTSGMRSTHDAANYTLTVTLPRLEPFFSGGGEYGGSGISDQIL
AQGDLFVDDGSRHINYHGRNANYHAFCALALECVLFPGKGDLVLRWRAIANSSCCDVLHV
LRSSTTDVTDGESFLLTRLRFMFATPEEKKHVTLVESLVDDQRMTLSLGGSKPSLLEVSG
VALPLLAGDCHGNGADSRGSSRPVGEVHLKLNFSHDSWP*
>TCRU_3205 Leucine Rich repeat, putative
MCDVQQKRVEKAPSARRLHEMITAKLLHRIRSNDPTLCTLRMCPELITSPGMLDALLRNS
TIRSVVFVNVTAKTVSEVLFAKFLACLYHLPLYTLLAGGNLRLPAHYMRHLLELVEYSCG
VGKGCKWSSEENKATVVKGAASAADEVVNQATNSSTDDLKPMAKLEFVDLSGSHITDSHV
MWLGRMIFHPRSSLRHVDLSECRLGTVGVRSLLLEELTLNSRLTAEVADNTQLEVLDIRW
NGITEDFALRVFLDSLEMNSSCTTCIRAHGNYISSELYQRIVDREASMLSIIEGKRQRRR
QTRRTEAEELRRQLQQQKEGQQQQQKQQVVSSSSGALGFLLSVVSDSPSSLPCLSLLASL
LHTQE*
>TCRU_3206 hypothetical protein, conserved
MRRTIAALTATPERFSILGTTHPKPKRTGFGRNNKMRSKPSDNVAWYDKGPVEWLPRPVR
LTYDHLDQLRDWMMRETLDGKTEEFNRIRDMHREWSQHPLMPVLGDVEPKFPLNLFKQNH
RAKRRFLVRWHKANTPANWLWMPRGPTVVTPLHHTNPSQYPESWRQMVRKKK*
>TCRU_3207 Cornichon protein, putative
MDETHRFREAVAMQIDWVHVLITERHRPHTTWKIRQAAFFAFMHFCFLMVFLGHFVHNIV
TWVLAFLLQVAALLLSIFHISSVTDYADKMNNAMELEQQLNPLIIAELSIRCFALLHCLL
MGGWLMGIAGLLELAYDYWIFRRGDFLVDATTAWKKLGALEMDSRVRLAYQGVMVIASIF
SFIHFLVSM*
>TCRU_3208 hypothetical protein, conserved
MSSEGTPQLAALKQPSDTTIIDRTMHLFGTTQQSSHECEIESLPLEFLSTNRVVSVLEKA
IERLELLGLLDTTAPDEGATRPFFPAIGSTTNVGDAGRNKSSSFTPRKAAVMVARKEEMS

TRTVSELNSALLAVASTQRTGRPTVADLVEEQQRLELRYGELLRRAKKARPSPNEPELDP
SCFAAVQDTERAAVMEELRQTSRRLREHNKALFIQLKDNPNDVDNWKKIGNERSELIQLL
QALIRELTSGYNAAKAAVAQGGLPRPNSRRKRSGVSESIGTESREATMSLVKNRFGSTIQ
MKRSVRGPTGPRIPLTSFFERFAKLISDEQNAQRWADELLLKEKEMNQNVKQLQYDLKAE
KVLKEKDIAERRQRIAELKVKVRLEKKHVKEQSEVVRAEVEALNEARQRAARDEERVVLE
QIRGLEQEEQMESRAHADFKEHLIERTEAMDELASQWEKKNQTEIKRVEARKIDVEQMRQ
QCAERLQKALKDKEFELQLKSQRDEERNQEEAERTAAEAQANLEYDAVSQIQSAIRAMFT
RQALIILKKKNRKKRKAEKK*
>TCRU_3209 hypothetical protein, conserved
MGRRTLSMQRRKRCKPEGKKEKEEVVEFMAATSPTTTRHPQKGRKTRMTEEETAALFLAD
LAVATTTNGDAIAARETEGGLSEAVKKGENASTTKIKSKKSSPMAPKTTRSRKRVPADTR
KTSTVKPCVLGSMDLSAMPLEEGEEDPSVSAEVLRQTAAFLARLTPARSQQFWAMRQMAR
GELSGRSAARMRRTELTSDDNVREWDEEDVEFTSSEGVYDREVSSVAGKEEMSSYSEGEG
EEDFAWPKIKENKTHAAAKCKNRVTNKKRPRGGALFSSNPDIWDD*
>TCRU_3210 UDP-N-acetylglucosamine-dolichyl-phosphate N-
acetylglucosaminephosphotransferase, putative
MGASNSVFVALCNIVKSFCTHSFELSAIIFAGVVAYVICMRFMDPVCMKLLERRICGIDI
NKTTLEKRRKIAEKPLKALEEEERRLVVPESLGILAGAVYLCVLVLELAIVFGPSMHKLD
GAITAITVMLLLGFVDDVLDVRWRHKLLLSAIGTFPVMLTYDGSVSIAVPRPLLPYFSTS
FVYLGVFYLLYLGLLCIFCTNSINILAGVNGVEVAQSIVIAFTCVVYNIFQLRLETEFQD
GLGGSGDMYVHKMETDGGGSMHELMAIALLAPFIGVSIALWHYNQYPARIFVGDSYTYFA
GTVLAVAGVTGQYGKTLMLFFLPQLINFALSLPQLFHFVPCPRHRVPRWNAKQDVLQNSG
NYTLLNAILWVYGDMHERALTNAVVKMQVCCCIFGLLVRYIFASYMYDHIQ*
>TCRU_3211 GINS complex protein, putative
MSTASSATATNGVGIHFDNDTAAVAAVGGGVPGGRGGGSSSFTAYHFASFAAMEVTVIIV
PRFTMPRVSTAFGGRYGPFAPNYPVELPLWLALHIRQTDTCTINPPPFMTIPYLRNVVEK
EKENEATFEALPFYFFEVVKKLCENSAAAEDVPHVAEVIRLVGEIKAIRRQKLQRSMAVF
EAEGSPVYIPGIKLTNIVNNELEYLRRSFSKVLQQAAEMKRRREQLIGGAVEGMTPLRPS
RGAPSTPAFSSAAVDARMSTHSGIDGSFITSTAEDTTAPTATLHGAEQQSVSSQGPTTTA
SSAMEPTETLTTATESIASPPVKKRRTLRQT*
>TCRU_specific WD40 associated putative domain containing protein,
putative PQQ-like domain/WD domain, G-beta repeat/Utp.1
MEGFSPFRVVGAVCGEVPVSSTVLYGAPVLMVATGRTFQLFRGKELSMLRGGPHFHSRVR
AVAQTGKYRFVAEGPHVHGFTHHKPIWTCTHHDITQSKVNHLLAADDILFCIGQDKRVAV
RAVKSGKLLGEFLIERSEEVRAAILPTGYTNKLLAATAEGSLHLYNFKTGVCLWYAQRES
DAQILSLAAGRYKDIIAYGTSCGRVVVLNLLTNEEIMMFEHKYQGAVSALAFRVDKSILV
SGTTAGEVVLWDLENRCLEGVLTRSKQVRSEVEVFETPHTDAIHSIVFLPTDGTTMVTAG
ADNALMQFRFDTVDGLGMLVRERRGHMGSCTNALFYNTDLLLTAGTDRALRVTHVFSDRA
SWELSQGKLGRRGREKQMGREALKATPVIAMASCTARNYQWSSVVSLHEASAKMCCWRMD
TRSLECKLSGITTSMHTARTVSLSDCGNFAVVGYSSGNVAIISIQNRSVKQLFDVALQPN
DLAHTSSVECVEIACGNSVVVTAGLDSRIKLWDLLTGCLRCALQVDCPMSKSCLHQPSSL
FIVAQHFKIRVYHCNPDVGFDEDFLATPVREFDGHNGPITALALAPDSYRYVVSASGDAV
LLVWDLAAAACVGQYRLASPATSLSFHPDALFMVSTHAGERGAFLWSNNLRYGFVPEVVT
DPKGRRIEQLPLLHFPTAHGGDELQEDEQEQHHQDEEEEEVSGGMNFNGLQPPGKLTTTM
SGRKEEEEEAELFDASKDSALIRRQRKQEQWAQLDGIVSGGLRLAGVPRSLWFNLTLLDQ
IKEKNMPLLPPKKRDVPFFLPTTQELRPTFLVVASSGKAGEAAASQTHFVGASLLERTPV
QRMLMQGEHEKLMLHLLALKTPQAIDLEIKRAVEYTDGVSYSKEEIDCIKSCVCGLFSFL
AVWLRRRENVDLVQGILADVIRSHGTLLTKCGGEMVAVLEEVAQLQNAVRHSMDHLVGYP
SCLVGTFSGSLF*
>TCRU_3213 hypothetical protein, conserved
MQDTSAQRRAHESCDRPSRTSFGSLECHGTFHKTGCVAARCSNRGDIQFLPTRDLAVGEA
RRFLRPSPERIPIFWELHHNADPGVVAGRIDVKGETAEGQELWLRAEEPFSHDFYHPATI
TRRATRTRLATPQVELTIPLQQVNVPVLNTKVDPEPVEPGHLGETNASLGTRGAVPFPSI
LLR*

>TCRU_3214 hypothetical protein, conserved
MGRRCNQDRLRPRFRNHRQVLGELEPYSTYCESPGTSSDNFRDSIAPSHRVETARELGVR
HWLITAERERLSNDCANAVADVQNRNAQLYSHVEEAHNDLSAREFHIAVASGLLAGRRDA
VSRRSAHLSERAAR*
>TCRU_3215 Leishmanolysin, putative
MNFDPKYMTDTAWSVRVAAHEIAHALGFSQESMREKSLVKKRERSLRGTHRRMVAGKHVQ
GKTKAHFGCDSLEGMVLEDGDGSWEKEIPHWKGRHARDELMPPAVGAGYYTALTMAVFAD
MGYYRVNWRMAEPMSWGHCSGCDFLVNKCNTTDNLAAKYPHMFCDAIDTETLRCTSGRCH
VGTCTASIVEDKESLVDKDVCPVVSLEFHGISSGKTYRTCSDENVDYLRGSLTGGGSWCL
DAELLETTDGDGHKSVKGMCAQV*
>TCRU_3216 Sphingosine N-acyltransferase, putative
MGTLRFADGTIGLPANMYAMPLDRAREYGFVSHYNHTGDPAMDRHANNLTFTGEQLWWLV
TNPVSLQMHLNGWGGVGLDAAALPQLLPCLLWAVVLIAVRLFFQRRFAWLGVQLQVVVPG
TSQKKVCAGTGANAIRLNVGQRKKLRKFQTQLWLAVSYTASTVFGYMVQRGEPWFGLPLS
EANRISILSPHPYNPGRWILLYYQYGLGFYLSECFSHLANHDIKRSDFLEYVIHHIVTIA
LIVFSHCSYEHRFGVYVLFIHDASDVMLAVSKTLSYVVKAAEAREQRAARNGGNGAAPAG
PPACRLYRLVFSSTTVLLSFVVFVALFVFFRLICLPFLALASVGLAVKIRTFTVCYWVLV
VLLQVVLQGLHLYWFALIVKLAIRALFGGPLDDIRSEDDEENDGH*
>TCRU_3217 hypothetical protein
MLDTAPASRRCGRRSACLHSTPCCGHVEPAGVPDCAGHPLCGRRDEANTTQPAADDVLAA
PRSGDEGERLHRRDARAVRPWAALVARLWIFRRAAGGGGGVFFSEGASEGETAGGVGCRS
QRLMHATRVPCHLSHIPQRRAGLGRAKVS*
>TCRU_3218 hypothetical protein
MDQLNVSASILWSNARHDLFPADVVLGDVAVVASAPATIQFLGNSEMSFEGGMALVESCC
TGRNSQMFLLVFTESDMSLARIFVAVQSCDIVAHVEPLGGLRSLALCVDMDGQLRYLVVG
PLPTSAMMEACFATGSSAKSESEIADPSTRYPLGHESSVRVDLPRVVDGSSSCVTAEGGR
NCRGGSSADDVVKISVGLLRDKVLSVLSAQRPHLFEFPWNVSKGMMTWLKRREVLEALSP
PSLGRAVYLIAGHCTFFASSEAACNATACHASDRRPIGALRGLVAVIRNALVLLPLETNC
GTSPGTLVVRWNQLLGYQASDVLKCTRVFHVAMSDGWKKHDRGAFLPCGIWLSIIFPSWS
DCNAMEYALRTAYISATGIECRVLERGKNVTVGDAEVWGGVWPPSLKGTKSAKCNVSSTM
MMMEPGSAVTTRRNLARKAELMPPSPKITTDLVVDCDGRGEACISRAGFVWVLIHQTSSV
LLLVDHVACRSMTATAASGGSRNPSEIADIFIDAFTELRSTPTPGGIVGNGVDISLVHAA
ICAECSLRRIPPASMDPCLCCGSVTKPVRLSPLSSLAVESHETIENFFPMIMSPMCAVII
VRFIMEHTSEAWDAVRERVLPAREMAVNFSEHIIPNLLIALDEMREFPFILSFVFKNFFM
RLPPLPHIHDSCEEGSAKSQAAALHLVVQLYFTGLCLHARETYGLGLQTGNLWEAFHSLL
KWCGNVIEAVLLGEENDIRSVKLPEETMRRALTVRDPPADHPLRRLESLWIEHAPSMITA
SLALTAGGGAIGDEAAIHPLADAADAAERGQAKNLQKGPTKPSARQPTEYLSLGPFCMPP
ASGKEIFFADRKRSLRRGERKQELMNEVSVQVCHLSSHADLPPAAAQEGVQHQNQHRCYY
PRYGIETVEASVQTVRTSSQEEALLRSHLQLQTKVRYLERVCADQRPTSSATSSMPLAAD
VSVQFPYLKAYRSHLEHVTALEKRIDELKRLSDERQEELFAVQKENYALKKALCEATSPS
GNQPAPFHVAYLMLCREASQFMLEREEHERDTIIFSECKERTALLFSLLRSFS*
>TCRU_3219 GTPase activating protein, putative
MSKVDFVADQPRPLEDFIAVTGLKLGHAKECFERFGGYAPALASFAQSYPSLPTDYFEEV
GRVEKNAEVINAELKLIYRRAREIKSQSGKEDEGVWAVYYTRPSTPESKPQNFNASSADK
APKPFGSFFSFTSTTSAAPPPGPMNFGFGAQPAPSSSEAAPVSTTFGGFAQKKTTEEKKK
TTNNERTVKYCEATHFTGPLFDMPLFWREEVEGRCPLLNLDKGFLEENRDVLRKRLEKWV
KKAEFFVKPVQYLEVDDEDEETIRVIIKDADRTFFHPEHRNKFVNFLTAMHNEFNAYGQA
MSYLAGLCLLVLSEEETAAVLRYVANVHIPGHWAAEAVGFSTTAWVVEGFMKRKFPDVAK
HLEELKLWPDTYLQKVLTGLCIHVLDFKDLFVFLDLFMDGGVKFLIKYCLAIVEHFRSDL
LRIKSATAANEVYEIMRIDAKVADSHDVKAILRRAPLIDLGAEGDLIDILRMEAYNTHVA
PRLQRAPKTEAFEPCAVCNERKPIWWNDDLGAVCTECRDAAPELSYTKY*
>TCRU_3220 rib72 protein- like protein
MNEKTVYNALPKLPGFSFPELHHPPNHSRRQYCSIHDGTRAVLDARPIRRRDGDNGEGGE
CGGLSRAKIFARFPAWNSMDDKVLRFFAYYMERVDESMVERMRVRKVKIYFYLCDGTLVV

VETPAVANSGLPGGTIVSRCKIDGVDFFSFYIGSTIQVRGVEYTLVDCDAATRDFCAAME
VPQPEPLGYPEDAFEASVKVVKAPMDELHATMRRNMEVAAANATGAHATLLSPEERSKAR
NFFEHDREVLRFFAVWEQRLFRLQYYIADQTVSVMLEHARNDGRDPISVFIRRTLIPKDP
KAPLRDTETLNHPLGAPVVYISEEDLRTGTRINLFSREFYIFDCDNYTRAYYEAKGIAQP
SFPKPPTEADILAPKPKSPPPTATQLPIIKSVQGASTMVFEDHSSHKDRLKLTRYTNDVF
RFAARRVNPSPEDEGRMFVFCYYLADDTVSLFELVVANSGHVGGKCFARAVVPEINEPGK
LCVGARVKLAGVTYELIEMDERTKRYIAMGMPLMDEDYFRTQELVHRVRNVILQRFSSVT
DVFRRFKSTVDGLDKEDVKGLFLECGTRLDAAELDRVMARLDQDNDGIVNLSELTENLLL
LQFVSDFVPKNETFRSIEKGPLRSFRNLEASKTAAREADEALRKLISKSEARRILLIRAF
RITSNSTYDGHLSVEDFRRTLKDRMNFSLTDSEVESLVYKFYYLPGLEDWTARRLPVKEI
QRLIMC*
>TCRU_3221 ABC transporter, putative
MSCCRAEVNEPVTPSSASSLESDDQIAPKQKGNEPQIEDYSIIAPGFSIKGSVAQFDAVE
QNKSSVSGRFSIPVSWNNLSYSANGTKILCGLTGTALPSRCLAVMGSSGAGKTTFLNAIS
DRLTTSRTLKLTGKRQLGDLEYKRHYRRMVGFVAQDDILSPRATPEDSLRFSLRVRRGTS
ISETNKFVEETLEELRLVHCRETIVGIPGLVSGLSGGERKRTSIGVELICDPKILLLDEP
TSGLDSVTSVKIVHLLNNIARTGRTVIYTIHQPTAETLTYFDDLMLLTGGRCAYHGTMAK
SVEYFESIGFPCPERYTPSDFFMKLLQDPEISKVLVKKWKSYLKHGVRTPHTTAVELNPN
PSESPTAKNIESYLGRFGSTSCIQFQELFRRFSMDLSRNHVYIFSHFIQAAFFAVIVGLI
FLNVKDDLAGMQDREGVFFMVTMNRAMGQTFIMVNSFMQDKALYVREQMVGSYSPFIFFL
SKTLVEFPMRVFFAFLECCILYWMVGLYRQAGAFFYYFAVIALLTEVASGLGFAIGATFK
SLVVASGTAPVILLPLAMVGGLLANTDRLHPYWYWLEKPSFIRQAYILLARNEFKHIDHI
RCDGRGKPPGFCKDKPQNGEDILRQLGFQQKQYENWVLWLTLALLYIAFRGWAVISLYSA
ARTKF*
>TCRU_3222 40S ribosomal protein S8, putative
MPQNEYIELAQKRYGRRLDHAERIRKRDARKAHTQAKHLRTVRGIKAKIASKRRYAEKAE
IRKKIREHEEKETKERVKEKGPKNALPSFLMDRSEADRAKVLSNSLKQKRKEKAGKWAVP
IEKVKPVSEDEMMKAVTSGKRGKKSWKRMVNKVTFVGETFTRRMPKHERFIRPMALRFKK
AHVTHPELKATFHLPIIGVKKNPQGKMYTGLGVITKGTVIEVNVSDLGLVTPSGKVVWGK
YAQVTNNPENDGCINAVLLV*
>TCRU_3223 hypothetical protein, conserved
MSSRSERHNQSSGNGTLHSGYDMSQREIPVTKSASEGMSRVEQRLRIVKSSRVRWTTDTQ
GETLDPSPQPSVSSIPTAVEPPCATYPAFDLARLSKHEQHIVDALLAEVVDAVAFPSVQD
ELAQ*
>TCRU_3224 Domain of unknown function (DUF4496), putative
MLSAQGAITNELVMIECTSSQEQTAKEIFLLSELERVWTALSTVLRGHLIEGHNVRIANF
GSFWFETRPVVSDGVERYYARRVHFGMDSNFALRYNLDSNKVPQEPPRLVYVKISMADVV
SIAEVPARTASMALREFFSYVGEGLFRHKVFKITFLDVASLLIKREKSVLEIDPSFRRDI
FAIDSRKWPPRVREAAESVVMDIGRPATASSVLSRPSTAQLSRQIAASRPIEPRPAFVTS
APRDRLFSEIEKEPPRKLPPRSSFPVEDSSRSTDNDAAACVKESIFDALSSDEYPPEEPE
IEVIEELPRELPPLRAYEEEAARPVVNTGRRSFHDHSSVRELLYGKAIAQVEPLMRGRRR
YINRDSDAVAALMKSS*
>TCRU_3225 protein transport protein SEC23
MDLNVESMQQQQQQQGVEHLPRWSWNLYPANRIDAARMVVPLGCVYAPIGSSCKEVYYEP
LRCVCGGILNPYCVIDFRSRTWGCPFCETKNNFPQQYVHISDQNLPLELAGWNDTVEYVS
IVKRDPPVFVLVLDTCIDTESELEGLKEFALDALSKLPAEVRICLITYGTTVQIHELSGV
TDYPRSLVLRGSQEVTVETLKKVMPEPKLFVGVLSNVKSVVTVILEELQQDVWPVPKSHR
PLRCTGAALSAAASILEIVSPNTGSCILGFISGICTEGPGIVVETTRERFIRGHADIRDS
TAAASFWESSCTFYDSLMHRIVKQGHSLSCFTASLDQTGIAEMKLCIQASGGVVLNDESW
RKEPFRQSLHRFFERRDDGTLKMGLNVTMDVITSTTWKVMGVIGQCVGTGKKSSSVAEYE
VGMGGTCQWTACMMDCTTNFAIYFDTTAAHPSEAAKRSVRYAQFITKYEIGNEVRTRVCT
VTHQAQCTTSMPELSASFDQETAAVLLAREALHKADTTPLFDVLRWLDRTVVRLVSRFGD
YVKDHPSTLKLPPQFVFFPAFMYHLRRSAYLQVFNCSPDETATLRLMLLKSSVQDSIIQI
QPTLYSYRMDAPPQPVLLDSAAIQPDNILLLDTFFEVLVHLGSTIAAWRRAGYAELEEYA

YFKEFLQVPVADAEILVAGRYPTPRFIYVCQDDPDARILYNRINPSRSYGGENDQKYGTN
EGELVYTDDASLGVFMEHLKKLAVSQ*
>TCRU_3226 hypothetical protein, conserved
MMNREKVGRRLDLVNSSAGSIISLAAWCALQASSADVIFAAIDERMRNASTSKETRCALL
NVIHELLLTCAANGVPEAGKRSVLMAVSKTLPGMVQAVRGLAGADAEAFEQNLSKVVSWW
SMLNIFPRAWIAQLGVKKEAEQPRDTTGGASMPVQLSYIANLMSRYNEMKEECLRVSHAS
PERAASLREEAIQQLTKIREVVHCKLDGGVSLVAWLDAERNALEGNLPSMTSSVVGQKEE
DDVLGSFF*
>TCRU_3227 hypothetical protein, conserved
MGQGLTCGATLGVGLSLSVLPARLSVTDHLGICAAVAGCGLCVDFVSDCCAKEGRVIIHR
DVLLQAQDVLRHVSTFYTMCDSNPIARFFRQKHEWLVLESHGRRYYTVQKNPATGDVLMD
VRNSLRAANDVGLSVAGRPIHTGEIRLHRVDLEFDLPNDLQVAYMIAWLRKEDPRWSFST
ENSRHFTTRARYALNDF*
>TCRU_3228 WW domain containing protein, putative
MREAIFYVPPRRSYATSLGTERQLKTRSPPRGSSSPAPRPNSQPLPRHIAAGTSADVGAP
PLPRSASYSLGGEIARQSGAETASEMRELLHLLEQEEKKTTVLLESQEMRIRALRLMEET
DAPLSLRQQAMTISGVHFSCNGQQKPRTESQEMGGKEAVQTVCDGSYASHERPPLVLREI
QPSPHRDQHQELSLYTQKPSSGSVMPTDADVKCEAVTPALTSLGTHNGSNEELNGARRMG
SQHKTEQQRQQQLPVDGLSDSEALAREEWRLELSYGKEEAATTEPQPLVKRNPSPAVAAA
ATKAAEACSATPAEGGGVLCSNWEEPRQREAGRTYDSQTATQEARGKPPSEALEEKKGGA
APQREQQEVQEGNAAKTKGALRVCGTWKEYRDANSGRLYYVNTETKQRTWKIKETPFANE
*
>TCRU_3229 hypothetical protein
MVSRWVDENSLSKLLEALDNLLLDDLKYPQTGVPSKLHVTQAVLTPRYSDIFLGIFPKIS
RRIASSIYDMATCANPSLLEWFYLLNANGLLDGMKVMIPSGINTHENILMLDCVELGRCQ
IMDGTTETNAVGMCVYLNILRASRLFEDSSAAPSLNEG*
>TCRU_3230 Got1/Sft2-like family, putative
MTIPVEVVDQEVVNDNDEQCFQGLSWSSRIQGYVLFTALGFFSSFMGWVSLGIGYYWKYS
VLSTLGSLMSLASTFILMGPRAQLNYMFDEYRRTSSLLYISSLIITWIVAIAYKSALLCS
LCGLLQYGCLIWYSLSYVPYGQEAVSGFIARLIFSR*
>TCRU_3231 hypothetical protein, conserved
MPSQTGLRAGRGQSQTVTVHILNDRAFPQWMRRSLPWYVGGPAARGVCDEAVAEAPVHLE
YPRLCFLRVLPTGMLGDFGGDPRARRLLRRWRHCGIRMGGPSNYRNARLSTSGMCRHTVD
GQAHSAAGWTGGRQSAARWQARSCGAHVWKGFRRMQRRASLLSVRERHHRHRGFIVIRRA
REGLSKSGQESHRACAQCRHSGTDVRVNTRNRVLATPTGACHAVCAPSFSSWTSFQCCRL
TAVRQSTCQALTQSCGRNRAMTTSVSAVTLRGIPRCHGKNVEPTA*
>TCRU_3232 pumilio/PUF RNA binding protein 7, putative
MLQYGAAGGVREELVNALLDVFSEIHEMLSFAQGSRVMQKLVAYASDEAVLTVVNALLRE
AEEERKKSGTIDEEEKEEEQHEGAETDDDNDGNGDGEKKADQPALKAKPSRREQREINRK
KHYKVTSRAIVSYALHNHACYVIQAILRECRSRQLEHQRKQLMDELKPFVFELAVSPWAG
RIVLETMFQSGSAKLAEVMKNVVFLKAEAWLSDVPEKNTRGGSGLDPTMRQALRRHREEP
DEGRKEKFPDDSSKKPPRKKLYRTIKK*
>TCRU_3233 zinc finger protein 2
MSYPNRYGRGMAAPYGTVQLPVGWQMAYSAEGDPYYIDHNTRTTHWQIPAEVLQRMNQGQ
VYRGRVRRGIDRTKLKTKMCMNIQRGGVCNWGDNCAFAHNSEELTAVPHGGNQRGGDGNP
YRMRGPANNNSNNNLTNNHSMNNGNSAPNAAELKQPKKQEQQPQQQGIPAPQQAMPAQTQ
*
>TCRU_3234 mitochondrial carrier protein, putative
MQTRKVQQNENFSANAGVDTEKENLLSSERPCEQHLVIKRDSYTTLATFFAGGVAGACSR
TLTAPLDRIKIIVQEGHLVTGPYQRISIFKSARLKDVFNLIREDGGWRAFWRGNGVNCLK
AGPEFAIVFSLRRYLLSLYEDGVEEEERRELEFISTGCSPLDSELYRSAVSVLPSPFDRY
GTLTGVPRLFVNFSIGACAGFGAQFSLYPLEVVKTRIAVSKMTEFRGGIREVVMATYRNG
GIAEFYRGLTPNMVGVFLYRGLEVGMYSTAQQQIMMYRMRNYGMSRHNAALSSMETAVVG
MFASMIAQTVSYPLNVVRTRLQTQGINGREIKYTGMMDCFIKMIRGKGLASLFSGITANY

LKALPASSCMFVVFELVQKLLVGDD*
>TCRU_3235 hypothetical protein, conserved
MARETAQNDGQADLPVAEAERKRGQVQCGAQPIPEDEGAAPPRHEDIATSGWVRLQVFSR
GLSEAQRSNGVRLDFVPPFFGYIRLEAGCLRVRRCVCFVCCFGGGLGLFHFVCPYLFGAV
TAAARDMALSCS*
>TCRU_gamma, conserved domain containing protein, putative
Elongation factor.1
MQCVTANLVGVWLQCVEHVRKYALGVALMFVEERRHDIVALCVLCGRGMPAIVRDVEGTE
LFDWEEVANVAAQRERITECLCWRGAC*
>TCRU_3237 hypothetical protein, conserved
MHKDGPRLWDDTLMEAERMATDSKARYLQLPTPDREAEMQRTRSQFFLLLRERLRNTYLR
RISKLNPGEPLAWKYVSGRKKASIPSPTSILLGDGQHTYKTARRAANALNRIFLPFHPSH
KAIRFSKGINRQSASLNINASFLFGQQNNSESAATSGLVARLHSPLDAPFNRTELLAALR
NTPYGKAPGPDEVDSEAL*
>TCRU_3238 hypothetical protein, conserved
MKVIPPSLLALQQHIASLPFDVLIRRIHSSSQGNPARSLEAHFIGARSHLARGKGQGFAE
LPQVSGEQLASTSSQNAETVVVSSLQLRELLSERLLQDVQQPRTRLQLNVDDLSLILANA
VRHKFSFRLTNTTLRRFTEALNGVWGKDAASQQIEALSRVMEACCDGLEGNDLMTWDDLA
NLVAMVEQRWSLFTKSNSLCCLLLQFFSSVIAVEVSTEAPMPGILRADPVLLKERCLQLV
HDATDILADTLEAGIPLTWSPAELQGMCRVVKQLRHYEHVRSSLKTSSLGRVRFWKRTAA
TAPAEPADAVPVYKQLSRLVTTQVLSNSRLVTDVEAMTLAQVAGVFSSVSFACGTEAILR
FTVPVQNVLKRVVVADATSMRHVICIGEASLHVEGASLLFVEAVRSISRFAFVLTSAATW
DLLCRVCALLEGAPLSLRAVVVMCLCSLQGSLEKSRRLPSSASPEFASFRLAVAMVCTLL
AQHKVQTGQRMEEIIHSVVEAAPMMRDGGKIGASTLVRCFFFLTSTRSHANSERQARAIH
LAMAEASRLLNEKNEDHPIQLTNAEQSMLRNSLKRYREMLAHSEAKRNTFLQGAETQHDA
PASSPRDADAPSAAAASRQERFSPSASKTAKTTGRI*
>TCRU_3239 hypothetical protein, conserved
MAGVADSAALLIPQLLAENARLKMKIGIPDNERVLQSVLQGQVQRFFDDARRTLENVMSN
AVVLGGKAEEEGKEESEGETQRVSALVETLRVLGAQNEEMVQKLSEMQTQCVAGLNGKLE
VLTCERDTLRQRVLQLEEELAAASAEATETGEQPPSLLAELRYLRASDALLRQQLEELRQ
RSKASTEEANKNVREGLHDTLKLPRLLEHLGEMRTRVYQVETERELFRLQMEESQKRGGV
WHALREAQLEEKVALGQSTIQNLLTRVAALEAREAEAQTTAETHKETIGRLENTIQSLQT
KHTSSDEALASKRRRVEKEILDNPDSVRRELITFWQEGSDELRQLREEVEELREKERILR
TTQEKLTRMERTRSTVTNKLVTLAEEIGRVREENQSLRAQCVGLEVERDYLRSSLASAIS
HHMQEEELQQCSAAIRDAAVKAAAASLSVVADPQAIQQAMRQLQELKNEVAQLTKQREKI
HRYITLHEERITALITREALLENEVTRTCNSVADNGSVLQRVKRAEMQAFDLLTGLAEET
SVHTEGGMRRKETFLGMEQRITEYEQQLETYQEKLSKVIKERDDAKKQFEELQAEMRSTL
TARETAMTETLNSNAALMKSTDSLRLENQQLRASYNVSVLFSEKLSTALAGLLELLRAEM
SFAASLNNMLTELRAEIVQLTRRSANAWNTREGEVQEVVEAVKRMCGYILQAVEQQQRGH
TLDETVHIRHLMETLKKQEQRLKETQEMFTHACQELGEGLAQRITAALQSADVMWKKHTA
TLLEERQHLQAQLGTEKDLLARIEQSMAAPANTNTTNSATTTTVDTGVKAAEESHASHHQ
VLDAINQFLAMSSQEVAEKVGEEEQVEEEGGEYGNPSRGFSGAVVSVTDDTGDIRAAEEE
EGEEGEEDVTQSSPGQR*
>TCRU_3240 hypothetical protein, conserved
MCSLLAAGAATIRLVSRRQRTIHGDAVPIFTSTRRYVNALYRAPPVQTVPTHNSSGEEVD
TELFIRQFKELFYALKQTQLVEQIHEMRTCPYYECLFTADLIFQHYDIVLPMPKYGREHR
SSEISETLRYLRACGASGDFNHILQDVKGGAASGGDFVSTSHGILMAYGTARTNKLSMMA
LTGSGAADEAAALQQRALNVVPIEVSPDAPPLADLLAFAGKRTLIVQDTVHGRHAAEAAA
SSIPKIPWQILRVEPGCSFLSHLCGVNAVYDVLVDQDYPHSMERIGEAGLNPFPVEWTEP
RKLGITMRSVCLIARFARGTMSAGGYADSDGHRAASFTYHSRGIAKNTRLFQNGHRRHGD
NGAPLEAQLRSGELPEPVYQRPPRYAPPMHRPGGLMTSDAEGRE*
>TCRU_3241 oxidoreductase, putative
MGAVHSQAQEKQGMEEKYNNNGTAPEGHTIRCEGWTIEKPIRSWSASAMVYVDNVMVPPP
GPGQVRVKIYAASVNPVDVKRPRFGRNNGFFSWCNENFGKVGKNSNSNIGAGVSEIVSTF

TRHEPPEFPFPYVMGTEGAGVVESVGWDNDDMEQRERYGLQVGDRVAFLADITMANGGTF
CQYALAMADAVGKIPSPTTDDVIGFLEAAVMPCAVGTAYVALFDKLRVSSGRSIFISGAS
GGIGSAAVQLAKYAGMYVLASCSTVNVAYVKSLGADYVFDYTASDVVGECLAHTRGFGVD
YVLEVADAALAVHHADALRFGGSICVLPEHMPISSEVFFRRQLSIGYVCLTGLHADPLTR
GNLRHVVETCMKLYQLGAFKLQLETVPVERADTALDIISAGHTRGKIVLTIFHPDDMPHG
DDMMGTRRRKHAKVQSGQAAPAVSIQTAAPPPAQ*
>TCRU_subunit A, putative Chromatin assembly factor.1
MDRLASLLSGMAPGQIEQAVQLLQRYTAAAEANRQTVKGEEAPFLSSPNSDAKTDDAGGV
GREVGHPSPVKAERDGLNAPAGGLDAVSSVIALALEQTIRGGSAVAAASGDGSNDDDDDD
ISLVALAQKRQREAGKKSPTAAPKKKNKAQNVTAEDKESGNITALEDHERKSQKTDDSGT
PSRKGTSSAKAAGSPSSQQQQQTTAIAARGLASFGFVSTIKRTTVTVGDKKKLFTPFVQD
RRLVPSALRFWCHDKDAPASQKEPSVPSCVPREEMPMGGQQVIAVDEESSLMHLPVTSCG
DNEKAQDAQRQRQFEGAARRYVSFTAMIDDTTNFIDKGTQQEPPLQCPCAYHVSIAAPRA
GFRDEVIFCGFYAIGYDPCQSRPPYFGTYNSQDSLNMEELLQLARFDTGKEMPQMSNIDY
EYDSGDDWDVVENDEDLGASSSDTEDEEDSDYSSTSDTDNDFINDNDDEDDDSDAELQRK
MVEERQRRLNRLRHKDKLVPVFSGPFVGIPRLEHPLRECDRMERLACTLDGASFTAMMEQ
EMRGLGSAAALISLSDANRSGYGGGGTVPTENATAESLDERVLQQRLVAAALRNRRDMLP
DEIDAMHAIIAANGKIFPKAIVEALQRQQLCAGVARAEILRTIRRHYELKHKMMVRRPEP
WSPTDERLFKKRLRQMAGGSNVNRKGSTNNLTQSASNKDDNNNNNSNNFDQERGRDEEEE
ADDYEDDEDVPLLEIAIKRPRAACEAIEGKNE*
>TCRU_3243 hypothetical protein, conserved
MPPKGKKTVSRGKKKKLEKILDPDERYRNALDKMHYLSKESRAEITNSCLRRILTSIKAF
TLAQPPLEIESLMSREEQVNKEIATGVPMKVLPDIVRSLGLIPTDKQLRQIGWMVLNHEE
LQKQQRPQPKWEGVFAKFPQDNAKEVEDEYDNDVQGKLTLEDMMENPFSETVEWAAVEGK
ELIADREKLEAVLLDIMHTGLLVFDPANVQGGESASKFERVVTVLEYVNPEVVDNIFDVL
WSTSYRQKTGDGVRFIYSSDLTRVLETVEESEKSGEPPFESDELEDLLLFVGDTGRGTIS
EDAFALIAL*
>TCRU_family, putative AB.1
MTSAAHVRAKATRRSFWQLAGVSTLLVGSTGGGLFLYYRYARLPEQWRVARLSRMQPPVR
RTRPFVAGTSNRGNEDINPCDGYGFSNELLLTGEPLWGISAVYFYGLVLLRSVMLLTRMV
PVIWYAFLTYGLHLCGEQVLFEKLRDALTAMGPSYIKFGQWMATRPDFFPPTLCAALEKL
YDQTSAHSWSHTEKVLRRTLHEATPSDGIIEDNGMTSLPDVVEATKTSRKQVAMKQRHNA
LYYLQEIETVPVNSGSIAQIHRGVLREEVDGIPAGTEMAIKVTHPRIREHIAADVTGMRW
FVNILTFLWPSTVYFDLKRSVQEFSFFVQSQLDLRQECDNLQQFIFNFRDFPGVIFPRPL
PSLCSQDVLIETFEEGEPLQGIQSGENYYDLAERGCHMFLKMLFEDNFVHSDLHPGNLLL
RTNPGAPVSSVSASLLLPSSDAYYPDGKMKLLHELVVLDTGLVSTLSKKERNNFIALFAA
VACGDGKLASDLMIDRMPKQRRPQSGVKREKFRDDMQAIFDIVPPGKSEGFKLSKVRIGP
VLAKIMNTVRENKTPIDGNFASLVLTVIVGEGLGRRLAPDFNIFSEAAPYLVALLEDSEL
YFLASKLRETYGTPALLRDSVHFVRPERTATYAEMGVKKLVKTMGHMWRQLTGEATDSDV
ATVEK*
>TCRU_3245 retrotransposon hot spot protein, RHS3, putative
MWRRFCRLHVALLRRRWALTASPTGVAVRLHGAPTTEPCECHAQRHWDCGTKQPRLSFGA
SGTCWPQLGGASGMLHRTGVVMAPRSGISGDGSDAAARHVVGSKVWPQWTMSSTNMRLSD
FLWNYVGGRAAVDERLQCDDGGVCSGAG*
>TCRU_3246 retrotransposon hot spot protein, RHS3, putative
MIAVSFPKVANYDEWAKQLQAARIIMNCPYEMDVKAMCDWMKRDETAEKQVECWKEVKKH
MYLLGPIPRHVFDEESYIDRLGAVNGALLAMNPSDVGEYFTLGGEKKWCSEDLRHELVKI
VRARTDEGAEVFLNASICARIGSRTADRLAKPLPRRIFCCCYCDRMVLLLPKLWKN*
>TCRU_3247 hypothetical protein
METTRRQVIAVALLGSSEYDGDEEEGEEAWNMRGSSVYRNGSDSTLANQMSLYRISLATS
PPASARRGTRSRRGALIQPASTAQEENRFACSEDESAESSEGVERLPQNRLFNKVRSRSW
AFVTLSGSVHGMFCVQEGSLNGSVKPLNDLADQTDSGASLFLLFEQHGWARESGVNGVPC
RCSSGSPLSKGRNLSLLT*
>TCRU_3248 cyclophilin-type peptidyl-prolyl cis-trans isomerase,

putative
MNLFIGGSVHAAGAYTSPYPQNARNSVVYLDTAVQEGYTWFGSASMKPIGRVEVELFDDT
VPITARNFRELCRGYQNKTPEGKPLHYKGSVFHRIIPGFMIQGGDITKGNGTGGCSIYGV
RFKDESFDSKAGAHKGPGILSMANAGRNTNGSQFFICTVSCPWLDGKHVVFGQVLRGFDH
VKAIEEVGTPHGKPSKTVLVSDCGVLKEAS*
>TCRU_3249 ribosome biogenesis protein, putative
MSSIGGFRRRAPKTLKTRHALKKYEPKVVENPKKILFLRGSRTSSVVNDAMTDLAAITKP
YNKKLKKRNGFHPFEGREHLEFLGFKNDCSLFCFTSDNKKRPHNLVLGRQFDFHILDMVE
LGIVAADRLDLERVKGMDVASLGGKPFFVFEGSEFAADPAFIRLKSLIIDFFRGGTETEV
NLDGCDRVLAFSLRSENGRDACVAPSTECYGSGPQQERGNTVLCMRHYALQKPSTAAGMP
KTLSSNIQLVDIGPNFDFSIRRAAFATPAEFKVATRVPKEVLATLRSTAENVSSDAMGNL
RGQLHVGKQDVDQLNLRRFKAHRKRGRHGDGEDGDGGEKDEEAPQKRRRRRHGGGDDADD
DARPETDISETDI*
>TCRU_3250 hypothetical protein, conserved
METSGVQRVPAVLLLREELRLMDEVIRGGGRRRVYRHHLFFRALLALRMALEKMLPHFAQ
RRATPPQWAITRLLALAVRCGEAAAVELSVSRIDTLSLAALSLAISSRIGCLLGALSSRE
QDVFSGLAPADEGSSSSNNNKKKKDKKREGVNFLVTIKRGRQKRSRSPEGVMSLQRPTIG
CVIEAVLH*
>TCRU_3251 zinc finger protein family member, putative
MQKQQLTLDAAYKLTLWYSLFCEAQSRQERTTECRSHDTRVRHIAFSLLKASLPRKSLKS
CCLSGACGRDSLTHKRVHFGRPEICPASGGRDDVCQCLAHATAFVGCPVRLMVRDYSSSA
SLTGDRYGGLRCMLRHMTLQSVTFSGTSDPHDAQRVVEELSQTLMSHTGTLGRLSLGGIP
AHVVISPLLRAMTMVDSLTELRLEDCGIQEVDDIVSYLNMAKHLRRISLRGNSGISEGMA
KLGGAVATCRSLLSVDMGLCRLRDDHLYHFLNNLHGFLGRASFTFDVSNNFLSNLSFEHL
RDAPAAFRATVSELDLSGHNLEKCGVVASAMLLRLTALRGILLDQCELGSRDLQRLCRVL
SQKERRWNLLSFRGNALTTHDIKRLALVSLAAGSSLCVAGNSIGTGVHKLNLTLVLPFLC
ELDLSICDIRDEGLLQLANALEKVGPFSLRVLRLDGNDIGVEGKKGCSGLQFLGRALRAS
YAPRLEVLSLAHNRLLLRPLLTLVEQVSSTLRELHISYSAIANDYGEVMSLVKEIIQRQK
GGLGFEQLDLWAMRAADADAFCSGELETWLEGQRAVRVITGTK*
>TCRU_3252 hypothetical protein
MNITLESLAQALTVLQQATAADLSGETETAVEMYVAAKNELDAVAPYLPRGHADVVRGHA
EEVKRRIDGINCNRWAKEGKSEFPTFPLEYVPKPVPVEEFRVPLNPTFRVLWLMRLLERS
IRQGAFVAPDLYVGKEVWYQNGGGAALTHTGPKIRYLTALCGAMEKLRAVANLNNTETVT
KSLRKYLRTAEELTTALEMEIGIRTDGGASPRSRIERGMRGLFHKGQQVLRTWMNQEPNM
ELCLIWAVNTLGQAQLFERWIIYYSQVHMSPAVTEIMGFLHRAVGLLYSGPCSFLLRDMA
ILIERHQEKCRKSITRLLPVDVKLEGGTFS*
>TCRU_3253 40S ribosomal protein S33, putative
MTEVAATKKEKKAPQEEVVVQQTDQAQVGLIIKVLGRTGSRGNVTQVRVRLMAEEGSPDA
NRTIVRNVKGPCKEGDMLSLMETEREARRLR*
>TCRU_3254 40S ribosomal protein S33, putative
MTEVAATKKEKKAPQEEVVVQQTDQAQVGLIIKVLGRTGSRGNVTQVRVRLMAEEGSPDA
NRTIVRNVKGPCKEGDMLSLMETEREARRLR*
>TCRU_3255 CDP-DAG synthase
MNKGLSKRVLSPLKRTASGSLSSIESLPPKTEAPEKLRTYKDFINRTIFTIAMAYVFLVL
ISLGVEVVIPLVIAIECTMFYEITRINQRARKDRQLVSMFVLKLWFLGSTIFLVTSHSIR
EPLVATFPWLLRYYANFGMFAFGLPMIGIVGFVLSLRKGMYRYQFIQFTWIVMTLLFTVI
QGGIQIKNMMRGMIWFLLPVSCVVNNDIWAYAFGKMFGRTRLLALSPKKTLEGFLGAFLF
TMAWSFWFAGFLGYFPELYCPKVDFHSSMHCEKDPLFVQRAIVLPSFIQKLTGNYLTTIQ
CSKAQQHALVLAAFASLLAPFGGFFASGLKRAFKLKDFGDLIPGHGGITDRMDCQGMMGV
FTYVYLMSYVYSDAKCPSLHDLSSCALRLSDEGKIRLMRLLNESLSKGF*
>TCRU_3256 thimet oligopeptidase, putative
MALRIFADVETASKCAVLFPKNVSACQVVAEEAKLRAMEKLNAIYSFEGNRTFDNTAVAA
DIAAAEFDVSLSLLSVIMSVSTVKEIREEANKQIVELKSFEIDNFQSNKRLFEALKEVSQ
SSQYKEKYLEGNKDKEYSYWLDEELRNFRRKGMELPDNKFQEVVALEKELSALCTTFSRN

ISEDQSEILASAEELKGVPVSIIDGLKKADDGRFILKMDYPTFFGVMKNCEVAATRQSMA
RAASNKAYPVNERVLKEIIAKRQALAELLGFSSYSHLNLSDKMAKSPDVAKAFVDELIPR
LQQKWKHELELIKKNLHESCILTEDGRLREFDVAFMMNQIKKIHLNVSETEIQEYFPLDV
TVKGLFDIYQAFFDITFTRIDNGFELWHESAFTLQVKDNATGKTLGHVIMDLFPREGKYS
HACCHSVVPPVLLHGSEDEFSPALGVVIANFPAPLKDCPSLFLHDDVTTFFHEFGHAIHG
LMGRSRMATFAGTNVKWDFVELPSQMLEEWVWEPEILQKITSHYKTHECLPSNLVEAKVK
SRNAFIGRDSLRQLEFATYSLEVFSAPFAGGKDPNKLDTAELMRSIRSRISPDIKCEDDT
HFECGFGHLTGYGAGYYGYMWSKVFALDVYEYIKSRNGLLDPVLGNHYVSNIIGIGGGQD
PTAMLKKFLGREPNSDAFLKTIGV*
>TCRU_3257 hypothetical protein, conserved
MAMMTGRVLLVCALCVLWCGGAVLVSAAGDVVGGDGRGVGESLPEGAQHAVLLPSSPDSS
VALKEGTNPLQKRLEKVPALAKQSSEEDDEAGDQDVEDVTVPIEGQQQQQEEQEMYGVET
SGVLIIKKEAPQAREGERNLTAAPMEGSPTTPGPQAPTPKLQPPPAPAILRPEAGEERPS
PPQASGVGEGDGGPVGIEGTGRNVSGGGSLVGASDASRVTGEATKGPKGSEGPVSGPPSG
GASSSPISNDGDASRKNGGAPSTQDTTSLKNNEQSRPTTSSGNAPLNRETPERSTPDAKQ
HSSETQENETSRLPDGDANYSATGQSAVGTKGSSGVSTTASNAPTTPLPQLPAPPVPTVS
TVTGAPAEKPTAEQSPPPADSTTVEGLAATTTAQTNDTATPGDRDGSTAVSHTTSPLLLL
LLVVACAAAAAVVAA*
>TCRU_3258 hypothetical protein, conserved
MDTTHLHATCAAQPEGLLAAQSVIKKRRGAAGCLRGRKKTAPSNPPPPTNPHTRRRTVPP
TLHPIFRVARINRREGQWSLSAKKFTVARRISDIPALDKLHEGSERGDSRDVCAVNVEAE
NVPSGAIHNFMPHARTADERGELCGLFDVNIRCGAIDPTAAHQDAVVHVGNGSNPADHGD
SGVTKDCGAIGDEAGAPLDKGRDESTYPQPDRLEGTCCLTTCVDNGESLVRDCGLDSQKF
QTEDGRDLNFGLVRRAEDGEGGSPPRPAVLQDTRAGRLRHNEKLTNLTTAQIERALAPWN
ATAHPTKRGALRHAAQIVETDDLDPHVISQLAKHVNPFDLPQNTVCYLGKHTTMLTLVSS
LVALM*
>TCRU_3259 hypothetical protein, conserved
MVALPFHISQYLSALWDEAAALLDLVAPFYRRLFWRICTGEFPLSSEICGSRRVDSPPHG
PRVLSGSSPHRCEGLGGRPRDGKTAVCGAARGKETSVWISSIRTSGRRKSVVRRDQCAAK
KWRQQENRRSLPRGVNLLAYRSAARRRVSP*
>TCRU_3260 BNR repeat-like domain/Concanavalin A-like lectin/
glucanases superfamily/Trans-sialidase of Trypanosoma hydrophobic C-
terminal, putative
MIIYSTDNGSIWALSEDMSPAECLNPRITEWEGSLLMIVDCENGQRVYESRDMGTAWTEA
IGKLPGVWTNSQPEVSQDESLHVDALITATIEGRKVMLYIQRGYASEENEANALYLWVTD
NNRSFYFGPVGMDTSVEEELAGVLLYSDGNLHLLQQRDNDKGSALSLSRLTEELSTVKFF
LSTWTQKDIFFSSFSIPTAGLVAVLSDAASDDTWYDEYLCLNATVRNAKKVKDGFQLTEP
SSRVSWSVNIPDDNVRHISLSHDFTLVASVIIEVTPSGNTPLLTATMADTNSNHTMGVSY
TADNKWGKMFEDKITTESRPWEPKKEYQVVLMLQGNKASVYVDGKLLGEEETMLTGETPL
ELVRFCFGACEMHNFPVTVKKVFLYNRPLNPTEMRAIKERRHIPRIEGVRAKAPAGSGLL
PLLLLGLWGFAAA*
>TCRU_3261 transporter, putative
MFGVTVLAVTKVVVAICIGAFASQRIPNASVTVRDFTFVIANILLPCLTFYNVASSVNAE
LLIRCSVLFLFSLFVIGSGLLCGTAASKLMFRVSCSGIPHGLRDSLRYTLIYQDAEGRHV
KRAARGRRHSPSLKPVVCVLVSGALETQQVEPEEVLPLLEPPGVEYEEQPFYQYASLIAC
SLQNTVTLPLSLLQALAESLAWIDLAAGTSFIFIYSLSTTASLWGFGPALVDRAQKETNK
RRKIRELLELHKKLQGCCDVATQTEVNEEFSLIASPIGLNAPPFLSGKQPNAKFLSTIRE
AASKKILHEEEGEKNASKMMAAATAHSTGEQTDIPPGAWRWCLAAAPATVETFHYNWKER
GLIRVMYARDFEDETEAERQKRNNFFPFLRALKNIGVRLFKTVAFTSLIAGLVVGLIPPL
RWLLFEGPLSMLTDSIALVAQGSIPSSLLLLGANLVGSTSLTAPAPEVRLREAEQNTEFP
LDDTSADPKDTEDYNERHEVIEFDEHASFTLMSLQQQLQQAPVVVSTATSSENSTADSPD
TFFSGNGWMSAVRRVFALHGVWKSFVFGIIFIRLLVIPAAGFSLVLLLRRTAPSLFGEKR
EQNVLILVLLGELAAPTAINGTILFNSRQYMPGVWSRMLFFQYLICTLTIVLWCSLSLWI
VT*

>TCRU_3262 N-terminal region of Chorein, a TM vesicle-mediated sorter/Integral peroxisomal membrane peroxin, putative
MLEKYLSAILVPYFSKYAENIDSKQLNVDLWSGKASLNDLILRPSILDALLQGESGSDVP
TSSGVASTPSCSSSTAVHTTAASTAVSSCLPLTLQRGVCKKVSIVVPFTQLRSKPVVVEV
DELFICVCGSTGSRDAVLSKAAKLDAQAAHKARELEQFEAERRRMRDVAAAAAAAKEGGN
NTAAASPSGPAGTRSGHGSGFMADPGDGVAPAPSLLTPLTTGRDGYFSHLGELVVNNIVV
KVRSVHVRYEDESTKSVMGVILGGVEFTTIDSITGEATFIDPSGLSRMHKRLEFKGMQFY
CDDPLRYEREPGDYFISKMTDVSEWHAAMRNRVEAGDIEMSTVLGPVKGHVDVNLVFKKF
IKNLLHDPYLTARMKLGPVTAKFNRAQYVSLMRTVSLLSNWTGGVELFPRRPTAPILQDP
AVWWRYAIRAVRTITGAPRRERLLQRISEVCIVDYHVLYRDVVRKAEMSADKQRAYRFIT
RFMTVPDMIAGRKYVYTQIANAIQLKRKDNEAKKAEAQSNFAKKNTGWFAWLRRGPKTPQ
DIEEEEAAFEELERSYGINPNDPEGAETTMVDVSALPKSYCWLDAQFDLPMFSLRLDMGK
RETVTLMLHSVRTHLRKFNAANSVQLRFITENLTLSNPVQDNALLERLPSLVEGIPLSSS
AASAWKIDPTSRRSSSMSSSLLPLSPKQQGGVPLLECSAAFNPIDAQKEPQLKNTQLDFA
LDLRLLPLRIVAEPSIIDHVICFFQFPKGLDLASIVRSTKSLAATVGQAASSELRRAMAH
SKSFSISVDAAAPYIIVPKTLRGSVEEPALAVSLGHVQFETEPLTETEKQSRLASTTTLS
EMNEDLMYYSSSATFSNFYVEIAPIGLSLERPGKGFMLVPEVAFSAKVLQIIDDSIENRE
RFIVRMEVPSLCMACSVSQAYILSNMVESWLLYLQATDSPYADEQLPPSLEATLRGGERA
SQSAVGGLEGPHGFTHTNNALMRNRKGEESESDDIPASPSPLGRSREDLPYVRLHLKVKE
LGILIYEDEPETLQPMKTPRFTMAFSTAEVTLHVRTLQKCVSMILEQPYCRDAKEPDQLI
LSATVIRCGVKTAPDTPIRVDVELEPSLRFCFGTPCIQLLETLMDIVNLVGGAPRVLKEG
DGEEKAFSSQLLRHFTGSTAPRGTTTIRPTSQHSQVNLVEELCSTYDTNVVKLSLHVAGT
TVVDIMDRRRKKRVPRKDDSNAESGEEKKTAEERVENEKNDKEEGEKEEEDYPFAHASFT
EVTLSLTKNGATLEVSGGVGQVTFALAEAHDVAPAHRTIVQYKASPSRTPLRPALQQDGP
FLDTHLVRHESHEHINFSYRTSLPVVPIFETDCKGKRHLSNAAELRFSSFVEIEVSSSTV
LLDVYSILTVTQYFSSGLFARVTQLTSRPRYDGRVTVSSPPVQPPTLLTSLRVYARDLFF
ILPIDTRTTGDGYFQVTVDHVVVQSSLLSAEGKQTMNISLREIRLLHTSLKEFAAVATGS
EGENEEPHTLMPTTTLDMSVKTPLDPLSDEPLHVDLRGEDVTLQITETDIVELCRLASGN
LRRMPPPPSVHAVVSAPKSPQLVESSLAPSISPSSSTMHMPERPADISFVASESGLNLLP
IPRAVPPYMDKENTGREVRVLWRASKVCLNLLDESTNKMRFRVEGNGFIFQVFVPSNDVT
VGWESLELNDVYEVTQRASMMLCGESRVELRNILTSTEILQKNFGMRSSVTQTSDRSVWD
LWSGRVSGTEGYSVDIDTELLEVPTVVVDFTLRRFAVSDQWLAVHDFVCNEAVMAALQPM
MGAAEASPSSSLPLSPDGNDAEAKRRFHCLIKTRTVNVPFLTTSREEFIEADITSLFVDF
LALPGVSNVTVRMQDLEVRHRASAERILYRQSDTAPNAGGSQEELLRPVTDAAPIPGVAF
SGSVEGEASAAEKASGGDILAFSGSFDAGAERQKVTVSLGQLTALFSMPLIMQLVEYCTR
PDQPIAKISNLGVMRERREWLARAAENLVQSGAFSLSLLWRQPRIILAGDAQDLGNNLRS
IEAQLGIMRSTVSLDKRNGNYTVAMKVTDMAIPDMLEKSSLRLSYALKDRRENVDVVLDK
TAALLHPEELEKLLWVLHRNLMAPTTTISAQRDRAGMKTEASLEAPVSPKKKDEKEEENE
KVQEGVTHVEEATLRTIHFRAEGVVLAIHDVVGINAHTASLSGIDVEVAPSGDVRMTVGS
LTILEHFTNRQLAQSLGDQPITVCMSSAEKTVNVTLADMRFMLIPKALGSIFNTLLSVQF
PPPLEEILVIETGTELSPMDTHGKSPVITATEAPTTESQTMTEPSSPVLSRCILVSLNHC
SAVAVLGSRDVCVAELKDFIVDWRAYTDASMRLTASIEQVIVRDAFSTSTLYPEILQPLL
EGATDTKGQTPSGVIEFSFISLPHRNLNSNKKISSSGHIGEGDADRSVPSYTYQLHCQIP
SFAIVLVPEVTSAIVRLLSEVKEHISDVNREKAYDYVADKTVQTVQERQELTQVELTLHR
SSIILVDSANSSKTMEMCPGNFFVRNEICCHRSDESTPPAESYAEVFRLHINRMSLNVLG
APAFSHNSVIEINVLRGIGENSASSLMELTSSAELSSSDTTSVSLVIPTLSAHLTRGQLE
CLLDVMGAWRNSGLSGIPVEGVPVVASSTLQAAGGGSGGSGHPEGAASGATPLATSLLGT
MAMKKKSNSGNSALLHSGNTANVTPLSPDTLTPSNRGRSGSGRGGATGGSITLKANMQRF
EANIDGVFQLDVDQLDFKYSTNPGVNTTATTIALDTIALRHHVCGVSDLRADIHLFVMRK
AQLTSTVTVQVSLAARMSETNTNITMDSLQMSISPLVLLDAREMLYGPFCTKVLRLPLSP
IPVCRLTTDVHVLGSDLVLDSHHIIITTGKTRCSYKLDLNQHKLYLSGTPTAQIVLDDNC
QLILTNGCIVVPGMYALSSFVSFGLNATLFTTDTCIIEKQVHHGRNLAPFVKPSKRRSKA
VPDSYLLSGNKNAVPSSLPSVKPLPQSGVLSPPIATITPVHAGSPSNSTSNFMGDDVRTI
ASFQCKEFTMRTLSEEVEEISVTVTASTSFLYLQQTENGVLTQRSANISLKDMHTSEEDE

NTLFPTTLELSLAGADTICIVANLSGLELCLRMALLRSLLEFGNDFMTAFIGEAMLQRHA
PQVALENEDLQPLVDLPVAGDCLSCGSPGASLGESKDATGVLCYHCCTGRRTLPATEINF
DVPLVDLVLLGGQGGMLQLYTSSGIAVTVAPDKEIQLQMKLQVYNLNPHANIWEPLVERF
EAGISGNVTSSTYKIKVDRFDYVFSPDNMKLLSQLAAEFSPTSKLHRQLRKKLRDNVHKV
LTGEKPLKDGMGGDTLRSMMNIQQVSLVQSPHYNEDDKICAFVVVTNYLNESLEVDGKTV
EPRGGRLEFAATSSSGVVRRCGVVGDGMTIHWAKSPLYVRGHDMLAEVRLEFSRDEEAFR
LHCAVRLHCYPVHRSNMIICFENKISTFVEVVRGLPAVSPHERFYFHSNFSLDEKLYLRP
VRLADGEEYTAAVTKQTNVTPTLRMLLNASFITLVCRGSKNTHNDFIFRMKEQQDGVRAG
IPTFLISIEPQLYIQNCLPYDVFFSIGGAGSKKQQRYCAVVESAQKADVVLGDVGIDELA
LHLELQQNHSSTGGMTTTYRTQRGISLSRGFTRVTLTAASKETQEMRIHVQCMGNIILLG
TPYSIVNFTPLDLQVWESNSLGELVSKKRTNFGILPKKMQRTYAASPSGKDTTEFFVNIS
VGDMWASAVPFHVQGRGLITMIERNVAVSRVGTRSVFHFVYLTQVDMYGTRFVTIVPRWI
LVNCTSRSLYVAQALKQSASAPGATTTASSSSSSATAAVAVGESSGGMSSRSSGDYKNWS
ARLRKSGTLLSPIPTLSGEGSLYACSKEIMQLAPANSATPFFSTLNSTPDVGYGAFLLQD
THAPIRGDPVSLEEISSTLVVVHAAGDTPEMDGSDRIVEVSVATQGPYTFVTMQDPVVPP
YILINRTEKTLDLYDTQDRFLAQVKPGCVKPFAMDVRNGNSRTRVSFARDFAATCFVGHK
SILAAVNSPVATAEAKTTMEIDKKGEEETLSFVEFNFQRRMTPQEASNPLGIEYNLSLGP
FGQQIVEIVYSTGSTERTLHKYQPSPPSPLDVVLNINVLSISMVMTDRDVLFGAIVDTRV
HFNREGTKEVFDFSIKNFQVDNQTEIKPFFEVAVVAIRNSPDVTCLSGHVERIVVPAKAI
LYLNEVRLDVVPVALRISDNVLVALAAFGAQLSTELPSRQTSPTEEELFDKAQSTVSAMD
TRVIMERMVVNPLVVRLWFEREVDGHDFIREHIQIRTAALVSMLVRSCEDVRIATPGIAV
RKRSSSAGLMLKWLIQVYLDGIRNEIHGILFQYASSLPLLGVPIKLVSGIGSGAMKFFQE
PIAGLSRSPKAFAMGFASGSSALVTEVMGGGLGAVSNLAKTGADIMGAASGGRERKRTSL
LHGITSGVTGVLTKPMEGAAESGATGLVRGVGMGLIGVVTRPMAGLLSDVSKVTGAAAQL
CDESYIPDVRRLRKLREFHAMGAVAEYESVLSVYEYERGEHDGLRWKGNTFIPTDGPQWY
PYTKEQAERARGVSSSKWSLEMANTNFEGWRFSTKYYGVYTREQVPTARVRRRRWVAVLR
SPLTSRVVRLVQRGATTVSGSHAAGGDESRRGGDTTTNTAPTRSSTGAANVLPAIQGPRS
ASGAAVSHEATTPTAAPVTEGQQQQQQQETLPEVSVLPRETSLPNEQVVELYEYESKLPL
IGWGSRFLPAGCSHWQYRDGRAAPRKSDFQLRQGWVWASAWALASAGRSGGASSSAPPDG
WEYVQRENTSSLPTLRRRCWRRTARRVQ*
>TCRU_3263 hypothetical protein, conserved
MGAPNDAVALRKYGRVPFFPGANCAPSTVARRANSFPPTVTAARRVGEVAHGITTSAALR
GTHAARPHPYIDDDVPPVPAQKSLLPEPKVLFVCVYRPPFAASLGTPYCVVQSNHVCGIV
LHWAGARHAISLLSEPPGGWAVPFLGISTAP*
>TCRU_3264 Leucine Rich repeat, putative
MPPRPVKPTGKKAGKSVRIRHVAPRELYIRACAELGLAPNSGLIDLLSMYQESASVEFID
VSRNYLGDKGVIPLLAVVDRCNNLREIILSENGLRNNAIAALCNSAVRHPSLRRIDVSDN
YISEGAAAHLQRLLEDNPRVCELGIDNTKIDVEWRLKLRDLLRANATAAAAAAAAADVSQ
QDVKIGPN*
>TCRU_3265 hypothetical protein, conserved
MAMMTGRVLLVCALCVLWCAAGGRCDEEIAKDPVGPSGAPGNLGKDGMEKADRSAVGHGE
LGLVPEPPKDVSQGGPSERTLGGTGEDAVDKNKDVLSSGRGYGFKSPSPEKQPPGTDDPA
KSIEEEVELQQQRNDGKELENQPQLQVPVAQQEHGVLPLSLPHPQSQQSHQNTQQQPQP
QILRKEGGMPHEKGSEKGTQELDKLKEESQDTELQNKVKEEKMKQELKNEEKGGEHQPQL
RVPVEQQEQSALPQPPQSMQQPSPPQEQPRQQKQQPPHGKPADNEQESKTDKNAVGTNKT
ANGTATPADSDGSTAVSHTTSPLLLLLVVACAAAAA*
>TCRU_3266 Mucin-like glycoprotein, putative
MMMTCRLLCALLVLALCCCPSVCATEPPEKPKVDLNTSVPSPSGTGVKEAPQANSKSLPQ
GINSGLDQDNVGGEIRVLTDRDSQTDDAHNSVNREGTDGDTGIKSPRDFSEEKTHEETEG
RAKTTTIKQSPEDDRKAKPEANTNATSPMPSAPVIKPYDASAHTTMTTATREPTTTTTTT
TTRAPSNTTTTTTTTTTRTPSRLREIDGSLSSSAWVCATLLLAVSALAYTTLG*
>TCRU_3267 hypothetical protein, conserved
MELEVASDGDVYARSLQEARWDILQGLNVAKDWGRLEERHPFFRDVILDAKRQAKLLASV
LTATEFFLQRLLWCCENDTAPSFVDANRVKQWRASLAVFISLYESSPVPTRARWLAESRE

RADGTCAVSVDGDEKYNSYDHNKVRGMDDDDVDDDDGSFVENRLKDMVLQCLAIGRMWCR
QLDEEDEMAVKVRHALSVMDAFAAPKTLDW*
>TCRU_domain/DAK2 domain containing protein, putative Da.1
MVTTKFIDTPETAVDTAVRALCMTRPHLQFIENTHVVVNRKIDPTKVLIVCGGGAGHEPG
HVGFVDCGWLSAAVSGDVFASPPTIHVTAAIDYLHAKQGPNGPGVLVVVKNYMGDILNFQ
FAVHEAQTRGINVEMVMVADDACFGLDDINCRRGIAGTILLYKILGAAALKGENMAALKQ
LAGRISSGMRSIGASLSSCSPPGSKPLSTVPDGLVELGLGIHGEKGLYRIPFEGAKTLVS
HLLGILLCGGKKRGEHKEGETEKEWKGAKVALLVNNLGSTTDIEMGILTHHALRQLQQAG
MDVVGVSVGRYMTALEMHGFSFTLLRFSIQDDIAFLFDQQQTPLLPFTVPQFSISPAVGP
RSALQLAQEEKCGLQCNGLLGRVLENVFETLKNSKDYLNELDAAVGDGDIGSGTTRASIK
ALEILPHLPLETNVSKALVLLAKAVADVFGGSSGPLYGAFLLGAASAVAETMCRDGASSV
DAIRAALRAGSANIQRVARSKKGDRTMVDVLEALNENPQVNDANSIQELARVCVPEAKKA
AAATALLPAKHGRSRYLQGKELGQPDPGAELVVLWIEAAANTFLSGGKL*
>TCRU_3269 poly A polymerase regulatory subunit, putative
MYAGLFKKRPREADAASHGNAPEDSGDASSRAAGISSPTLSPRTVYHYPSMLPFPTVIDS
EFPAESYRSRSKDVKAAIHWGQRKLLLSEIQLLSIYARPNVSYHIVYAGSAPGTHLAFLD
ELFQSRHTWELVDPGRFDRPVLESRPNFSLRNEFFTNVTAYGINARRLLEVLPGLGTVYR
CVALDGAWSTKKEIHQKLQSIVGTIDVARGTEDIPSMYEPRLDLPFGFELLCYVGMERSK
PLLFVSDIRSGSVNLPNFEDHVAENMRAQECWCQILQGEYSMLKFRLPYTHKKIGFGDSS
RKVNSSLIGPDGTVNYLRGDILLPIWTRPTSTEGRLVVPRGAHRVPYKVAQVENCFFFFN
ARVREQVHFNHILLPDENLDHHYDAAAEVNCLLKYLQFLHPELREASAAVLRREVKHIST
SITAHLRVNFQDVVRRREYLLLKQARSGQSEEEGDESDNEAVDADIAPNCGAQLGSSWET
FAKEIIQAASKERARVVWRRNVEETGSDVRSGFWVTTIMRQ*
>TCRU_3270 hypothetical protein, conserved
MLATRVGVGRLWCGDRCLPPRHLAAVVRCMELLGDDDAVDALIREEESRVRQFKLFHCTD
EERRRQYGAVSRPMLPEPSPSPPKVKLTGIPLEMLPVEAEEMLVHEYEQHRSAKHDVDID
LRRRGLPPPYYDGPLLTLRTRGPYTRFEVLACRPPMRPRVEGKKASQKDCSQHYPRPLRE
KPKEDTVAMPGDSLRRWKYSMGLLDE*
>TCRU_3271 transport protein particle (TRAPP) subunit, putative
MLMPYRDSVRSSKSDKASSRNTNEEAKVSLSAFSFLFSEMCTRAHNTPSKASNVEEIEQR
LTSLGVLVGARLIMLSSLKDPLELQRRPITIEGALKLFQDKLWSRWFGKPANDLQRETGS
DRFFIFDTEPIVLRYVYPSPEYLDGEGRWSINYASFMGGIVEGALKAIGFAAEVLTYHQP
EPSKPHQSIFAISFAKHVWDRERRLRG*
>TCRU_3272 leucine-rich repeat protein (LRRP), putative
MAGVLTENLVLQKTKVDSIQRVRKLNVCAAQLSDIGVLRRACNLEVLSLSLNDVSELGVL
ENCRRLCELYLRKNRVEDLNQVLHLSDSPYLTVLTLAENPICQDPNYRRFVIAAVGSLRR
LDDIDILPQEREEACRVFPNLRAIAPPPSMYCDPARGKVRPGSGPTSGTVSLAKLTAQRS
HHNSVSAYADDHAAHGNGDRRGSLSRSPTRTHATKRRSTHLEFSHHSQFEPMNHSIHTKR
ASSGNGGMGAIHLQPPQIGPTETGVVQAVKVLLSELSAESLEEVRRFIDALK*
>TCRU_3273 Ribosomal protein L31e, putative
MTRAGFKGKVLGKERKQALLDARKKAVASRKARDDRRWKRVLAKMDSEKRKKYHGVGNTA
EHSRVRGITRAALRKRTGRKPDNITMEATIHLSKLLKRKTFHKRAPIAIKRIKAFVARLM
KTKDNRIDASLNTYVWHKGVKGVPGRVRVHVERKSETLEGGKRKHFYTVISNVPVASFKN
LTTKVTEQ*
>TCRU_3274 hypothetical protein, conserved
MAGESATAPANVEEWAEDDGGFFSAFAEEAAGGFSRRKRNRQAHEREVEQQQAVQRAEAA
EDEAIVMARAEEARRQIAAKVFSRASPREVRQALKKAKKESIPKKPHGEGDKARQCNFSS
LQKRQKKRRK*
>TCRU_3275 Peroxin 19
MKNPHDDDELDALLDDCINTMDEQERRHEEEVRARDAAREAELEKGTADSNQSGELMGLL
QALMNGENGDGNADPETLMNTLNEEITRVSSLIDSLPDASEEERASMTEVREIFDRLTKM
SSTNEDDAAGGNAMNSDETLAEAFKKYMADIEKTSGSSAGNAAASETPRNTSSTEAPTAT
ANAAEFEDVIAGLSGMVLDCLLDPGMLRIMKVMQRTYPRWLAANESSTSVNDLARYKKQY
DVVNSICDLVGDGPIDRQDETKTSQLVSLTHEFASLGPLPPDLEKLDAEEEQCE*

>TCRU_3276 replication factor C, subunit 3, putative
MTNEGRNSTGHAAKSSTLPWVEKYRPVTLDEVVAHEDILSTTRRLMDSGNLPHLLFYGPP
GTGKTTTIKACAYYLFGKDRIRANVLEMNASDDRGIDVVRQQVREFASTSSFYFASAPAA
PTIAAFKLVILDEADQMSGDAQAALRRIIEKYTKNVRFCILCNHINKIIPALQSRCTRFR
FAPVKKSAMLSRLKFVAQEEGVRFTDEGLVAAFRLSNGDLRRCLNTLQASAMSAGEITEE
SVYRVTGNPTPADVRGIVEDMIAHDFAASWEKVHQTVSEKGVSSTDLVREVHHIVMAMDL
PQESKCFLLMKLADVEYYAAGGAKETTCISGILGAFQLVKEALTQKRHVFALANF*
>TCRU_3277 hypothetical protein, conserved
MSSSDDEWRNPPDLLARRTAGRRGGEDAMLAARRQAVQRQHGSAALAPPPTQPPAQEPLM
VPEQLARKTMPPPKRETGVTEEMLESLRQELEAKKKVLTAAKEKENSLREKWRALRAQKL
AEIAQLDVKLHHHKDAMEDVKASAEREVREAEDAQQQQLEEERIKVASAVREQYEPQIVA
LRSQLEGLQQQEAKLQAELGVKDTVKDLLHKCVGSALATILQRVNDLFAAESSTMESWED
EIQRLVRHEVRSSFAVTTGSEAQREREDCQKLFQELLEFWRKAEEEEREHVLKMDEQLIL
DMQSMVHDDLDRLQREELSMEEMYVESREAWASQHQEILKRELDAAMSRRTAEHEEQRAL
RHQLHLERLKAVEENHHDMLEKQRWFHEKHMALLREQHVKEERLAEQRSRVNAAMQADAS
RATEEVNRTVHAVEALLKRMKEYRAAIEDGRAAIDSDRRRALEAREETLTMLQELVTKQN
VSVGEEYNSLMATLSRLEGLRRTMEQHLEEERLWLGKQEANFANGKTEWEREYRRWKQMV
EDEKSNIQERFGRVLAELREATVQLAEEERDAKAEQASMVSWFRTASESAHAEVSQLRQR
EEDLRGRHEVMVKLLNDFQRTTEQVDEQWRQLRQERQGLMKESEALRNDEMQLRQSRQYL
HLLQEQLDAKRIETAEGHGRINALRRELQLSKEAVENLPRQIPEARHQMLPAAPPNFCHA
MPAARQNANRLPLRVLHELRGMLGKNSRQIPVRAVVSESPEPAVDLTFASTAAETSPVHR
RMAPQHEQPRSYQTGKPEHTYVDPVDSSRDTSTHLYDTSNNNFTSLINFSDTESTRHSLH
TSS*
>TCRU_3278 cystathione gamma lyase, putative
MSSQKHLVSDFTEGSGSWQDQTYGFDTVLVHGGVKPDPVTGAVLTPVYQSTTFVQESIGK
YQSKGYSYTRSANPTVSVLERKLCAIENGDYATVYSTGMSATTTAISSFMSAGDHAIITD
CSYGGTNRACRVFFPRFGMEFTFVDMRDLKNVEAAIKPNTKLVFSETPANPTLTLTDLTE
LSKLCKAKGLIHVCDNTFATAFIMRPLDLGADVTLISTTKFVDGHNMTVGGALVTKRKDL
DEKVRLTQNILGNAMSPFVAYLQLQTVKTMSLRVAKQSENAQKVAEFLETHPAVEKVMYP
GLKSFPQKALADRQHLNNNHGGMLWFEVKGGTAAGRKLMDTVQRPWSLCENLGAAESIIT
CPSVMTHANMTKEDRLKVGITDGFVRVSCGIEEAKDLITALKTALDAL*
>TCRU_3279 EF-hand domain pair, putative
MISLNYHQRNTQAVKVILSELYATWSTSDEGAFDIQKALEEILNAAHLTNKSGHVDGGQA
QHRYASRLDDRDDNCQTELRALEVSSSHHTDPNVVSQPLSPKRSTSPPPSSPGQMRRRLS
DEYQRTPSPVQTPRFFGVDELDYPHRAGSPGCIAPSSPSASSHAKPVVDPMAPVPHVSNN
SGATVSPPAAHSLGDLNLDTIINAAMVSGDVFASIETVTNGSVSQSQRFVHSSGSFALTP
PENVRRATYNDIPTFYGKAEMRGALHAISETEEEALRSFFAIKGSALNRKLLSGSTLPID
STKTVRKGQFGKLCQDVFKIPTWMKDALFRRIAAAAGVSESAALTYEQISSYYYNVFGPL
TVNRRLFELIRGDSNNEYLTLQEFKDMVRYLVDSHPGLEFLKQPEFQEYYCRTVAIRIMY
SLERQQNGQVRWFAFDRSDLPEVMKELDEKDVNLVLQYFSYEHFYVLYCKFWELDTDRDQ
LVGFDDLCKYGQNSICPSVIKRVVEGSGRPLSSGKLEKLDFEDFVYFCLSEEDKNNSPAV
YYWFKVLDVDGDGILSGYELYEFYRENHQRFLEFSESPEGDLSYADMMCQMIDMMGFSLS
KEHQFGLTLSDLRACPTPSNFFNMVFNAQKFILFEHRDPFAEHQQKMRPEKTDWDRFARA
EYDRMASEAQ*
>TCRU_3280 RNA-binding protein, putative
MGKTLGLSSLELKALSVPSSLRFLLGVEYDVLLCVTEVGKRRSCTIRFPVLGINFQKLLE
LCAERFGLCCTQNEDHSLLSGTAIIPPLRYIDYLPGALHAEELLGCLGVKPVTNPKRYGA
SVHDFLASFIDYSGYNTDASLADVHGTGFYLTLDHGLSTATHSHLVKISFHKGCSLEEAL
REFEEFSGWTTFDIRYEDVDDKLAGGVVVMPSVQDACDLFEKYEDEDSDDEGGLLPFMLR
PVSPLTSVPSVEDSALKRLRDHQWGEIEKFWERFPKWKNCVVVSNLYESASLEEALALFK
DLTVTASSLVEDNSPARRRRVFVTFATSDEAKTALFVDGKSTKGKTLRVQVSPPYLNESR
RGRVLESRPPSLSEPPGQQPATEKRPVSLGSPAHAREKRDAAASNNPEASTPSGRNRSSK
KAGDDKNWNAPVPKSRTPIQDAASAPVPGAAAAREGGDQKKTATPPKQSQRSPIIVASTM
NANAREFVPSSTYQASTTSPEYYHSSIPMAYLDENYGQPPPCYPAVALGPVGMARPMGVV

PRSLRSGAAPPPYSSPPPYSMVSGQSMHMVQPPPPPPPPSS*
>TCRU_3281 Histidine phosphatase superfamily (branch 1), putative
MSEKKVHITASGNVIRSDRPSHLEPPEQIIVVMRHGERRDGAVGSPPEVDPPLTENGIVQ
IAEVAKKLQALLGVKRARKLLLIVSPFLRTLQTAQELQRCAIGELHHQIIDNTLCEVYGP
VRIKSSTAPLLSDVIVKNGLGELPLWGETIELASKRYVNCFFRNCHRYSDKNLLLVTHGD
AISAIMAALYPLRTVYETEYLSFIVFQRDSKSVSRGDLESCQLITSSGVQWLVDGPETSL
AETNTLEADGFSGSGLLVRNSKPPPNAGMCGYGCVDSNVGAIDGEWMAINDTHGTNRHEL
ASVSLIFRCMVIVSQWATIYLWSNKGNAAIYMIMVIAFELVSVLLHFNNLNRLRLGASLS
RVELFHRLTRNQFACVFCGLSPPFQRCTFSLCISLLKGLGIFVLCMAAAFIASLFQNSGF
ISMRHSYAEIFKSVAKVSVFALFFLIDTCRRLSDLEH*
>TCRU_3282 hypothetical protein, conserved
MNADGGTDETTARLVSRLAQRSTLSSDAEPFIPSFLRAPATTTETQHKNGEENAVELKRG
KVLETGLKQLYCNAETYTQLPSVESLATTAAVASTALCTFGSMTEEQLQELEEYLWNPSQ
NNNESFQDDERVFANDDDGMDPEEEEWLFNQMLEAAGVTQIEETTTDEIGGS*
>TCRU_3283 protein kinase, putative
MDRSRSDGEKPRVFSRTSTQGSSVFESSNSQFSMGLGIVEVPPDSKKHYDVSGFQLKKNN
CSIRPPTGNEETQILSFSPTSSSAVSTRPCGPISSSKTTDGQASVHRALFLRQAFFSPIR
SPDGDRGIHSDTRSNELWDKSKQEIADGDVMMSSSPLANFSGSSHLPKARPVDGAAIAVT
SEFVPSNPGRDELEIKRLLPNVKSSSETPFFNSAFPRFSTGENYMGSSYMRDEVDAAAPN
ITLRRYILSRPKPAVRGRGLYRRLYFMLISLVLLPFIFIALLFGLHYWASTNVAEELHHY
AGASLSDDSFCDRNFLELQHRQIGPISKEGFICVMGILLALIIIVIGGAFILWLFSWRCV
ILTYLKTLEFIRRAAPMLTQIEPLGPEQLAGAPKPVPSFFFWHCNRGENNKKEVLSQGIV
EKVVLLGNTLNELKKYIPSTVQSDVISAVLAGHSLDSSTAVSTMERKGRRISGPSPPQED
VLAAAGDLNPLQIKVQAHLPLYVKKKAATFTGGLRHSHGSGNNFGFTQTAGQRGSSNKLS
PPHRRFICPAGSLSSLILSTSSSVTLTAENDRALQSVGNRGECAVSEAAETRITMLNDYG
FSLQQTTFLVCRLFLPKLELNSDASAIRDSVNQVREMSQRFLRIVLRVAKEENGFSFNIC
LDSVIVVFYTPSGTNCVNLFQPRNCALRLVSELSKLESEWAAASSTPLNWGIAMHMSLLM
IGVSDIGSHRFAYLYGEELQLAYRVAELCMILHCPLIMLQSCYDLFRVCMMAVPVDVICR
ETIRGKDRIYLYELKIPEEGEDLDTKREYYKPLTVAFGLMCGRQFADAAQKLAGIVEMDH
NVSRLYRLCEYFLQMQEKGTINTILSPTENYVRKEPRWEPLESKAVKFLDKYCVREKQQC
IHRRREVSGNNEGNEENGTRDYHAARFFEDGHEVNGLRGEKFSETHVRTPSVRCDDITEA
DDFLYSTNTPSELNPSFAVPMSPVIATHDTEVKRTSTIPLQSICNPGEPQQVRVRYISRS
TAASVTFPEVDSGNNDVPHHWTDVRNLQESSKNDGGNEGSKYVFDVYEPLGRGTFSTVYR
GLHPNGNIVAVKKYPFRDRGENNSWAQSVRFEIEVLSSLRHKNVVRYISSFFQDEYLYII
AEFVSGGSLSAIVRTFRRLPYNTIRRYTGDILRGLQYLHKRQVVHRDISPNNVLVNIDGV
CKLSNFGGAVVFAMQRNEFCSNSSNATTAGIFAPRSVVHASDDSGSATGTVILKNCFGTP
ACMSPEACSGVVDPKNDIWALGITLCFCFSGRFPWPEEELNDVNAFVDKVRRGVLSPQVP
FDVMDSQAADFISLCLRRNTSSRPSATELLFHLFVVG*
>TCRU_3284 riboflavin kinase, putative
MQNHSNKAAFKPFLLSGKVIHGYGRGGTQLGFPTANLELDDNVRAQLNPYKNLVLFGWGS
VIAIPGKENCGEGPYPFSMSIGYNPHFHGLDLTAEVYFIHEFAEDFYGAVVNILVLGDLR
EMGAFVSLEALVDAIKDDVRKTEEALQKPELKRLKQHSFLAPSLAPSEELPQFELAVV*
>TCRU_3285 hypothetical protein, conserved
MYRSSGELRKYAQCVFALYCLDDGLSISRFRLAWYVLWGKWPEESVVQMLFDKKDKSTGG
NEDPTREEGGIVGSGGLAEKDKHHDGGTPLAKSFPFSALRRRINEKEFVRFAVTYHDTMG
ADAVGFGVTEEDGEYATVDMQSAFSGVVRARHWLQFHALAGSKGYVTLDDLIATEKVDWL
YPPMSGGSASGLMVEQAGDDSSTMPQDDGDRRLAVLSHVFAIIDDDHDGKLNFEDVTRHL
AKTA*
>TCRU_3286 class I transcription factor A, subunit 2
MPEFGTQVYWHNIPACPPPWKDEEERARTWRISQHIVGAISDQRHLLEPPRAEILAKRLL
GRFAPKRVHRYWGIVCEPYLSPLHGLNMVMSELQVRPIAIEEAKEAVNVVCEDYNAMTQQ
QQKSTQSGIPSQGGVKTEISTQQSNTAAMDLREAPLLQVVKILCASRGLEVSSFSRAELR
SKGRLGVADLLPRRRVIMAFDRSKPKFVFATAVSVKKKKDAEKSTVCYIQLVAPTSNPVP
WRQLLKREIPYLPPNECVVVAVQRHHDDEEYDDDDGDEKKSGDDGPYLGDANVRSEVLLG

GNESDKDDLGEEGDAQPRQQHEGENPQKRHKCGYEDKIDFSDTSKVSESSGSTTASCRSD
EAKRKKFVGREPIFTAEEKLQNDPLFETPVAVLRKLPRKTRGVHDWIQRLINPVARKRLY
AINY*
>TCRU_3287 inositol polyphosphate kinase, putative
MAACESASHAPGDEPIVVGGHKGVIQRGPKAPNDSATITKKSTGWEVLMYLSMALRIDET
MCELAEMAPTLVAILSPEEENSGVWITVEKEENRPLLEAFHEELRKASVHTHGYSEVMDT
ARWSVCLIDVTDGMLLPCVADIKIGYIRHSPHTPPEKMARIKKKRLIQPLAIRLCGALHY
FYRTISNEQCIERETCEKDIGYLLHTEEDHRRCLRAFFSSAVSMKQEGSGNKCGESEVLS
ARLKACCRHVEKLLSFFTSSVGCALLERTAFVSTSLLLLYDAVSRVSDNVGNNDAADVRV
YLIDYARVSDRRLNFAEENIGFLRGLENFLFLIS*
>TCRU_3288 hypothetical protein
MQRKLPRSESPRNDAPSTMSIATTTHGGLTRSTGLSSLSLEEEDSRQRGPLQARSQHVMT
FVGELPWVPHDDTDNEQPPREEDVVTGNIPRQIFIEDENVFLSRFVRPARLVAPAPAEGQ
TSGTNSSGGDGRSRRRWLPLRMDYMG*
>TCRU_3289 dynein light chain type 1, putative
MAASSTSNVTGRRRSGLRSSTGRLSSHSSMAMIPEMEVDPRVANLSRVAVYIDVPDDTLK
KIIDIAVEAYKRFVLAPTRNTWRKEEGARKELERELERTALRDIAEMIKKNVESTFGGTW
HVIYGRSFATYVTHQTRSFCHFQLDGANVVVWRHGG*
>TCRU_3290 WD domain, G-beta repeat, putative
MWAFDSPVERGIVLRAKDSDKCKISPLLAENIHDLEALKEEFAPYPSGMTMDTLFNVLLR
HIGKKRGDSDGFGLSQSNWLASRGETPASPQMSPRGIVKGTVPKRPQKTSMLPAGRPSNS
FSSMPQKDALGALRDGLYMSPWKVGASHQHISGSWTDINAFENLFNRLDSDSDGLVSWDD
IMQLAVEEATQSITIITEEMRSYSLSRISSQEKLVRLVHTLPDHKSVFAIASRADPLLLV
SKEDFSVVKQFRLKDFDNNHPQLIEYLPVPDVLLAYSVNDKTLRGWWNAVSVHRMGTLSP
LYLGDTIRRIRTQPWKFPYSFFTCSSNGKIVHWQLPKQRSLLEISRVHAYEVLHKPDTGG
ITDFTVTDEWIFSTGFDCRLFSTNIETGRSILVGLPHDTIKFLEYNRNCDCLPTVSYSNR
VLLWDVRSSSATPGATFRDEGGRPHTADVIGLCNAPGLPQIITCDSVGMMKVWDLRALRC
SQTLYADGTAMEDMNVDYMYSDQAINRSDRSFLFQALRKGDPKCIRSIGFFPNTQEVVCC
SPEAIYCLRYNHRRDGHTADVEVVHAAFYDPYHSTIFLQGSARTSVWDAVDGFRRRLFDR
SSHIKVPYWQQEVIAVCLDYPRNRLFFAIAGGTVEVRSSKNFVLLETFAMHEVNAQEMLF
SMKHKMLVSISNNGFLTLLHEEDSGHSISNIGLSSLPLRALSLSDEAGLIACCDENTLFF
VDYKQTKLTFFSVKLNVPVRTAAMLGTYPVLATSSDNGELTLWSTPPAEESYTPLVSFFV
GQSSPSFWRRKTVKNFSFSRSSPDRREQAEVSSFSAPLRSATEPDEEYPHGESSDFSEKL
GEKEKQKMGFTNLLEAIAADEKASGASTAISFDDVLHRLFVADQKGSLFIYSCCSFLQGF
GFRRCSFENRSRYALWGMNDDFRTLHFPILLQTLSPHNGYITFMKWFPGLSAVVTCGSDH
LVKILDYDGKETGQLRLDRLPPRSLLDETDGTEIKAYAEKTPQCFTIPFSLPPPFPALKR
CKECARIEVELKDSRNSSLKELAKESLPLPAANMYQCGSLAPLGETELTLPQPSAFVGVA
SEEVNCNRGKETPSPPSVSLRLVALEEKKQWRPRREVPCLFRERKHASATTRTSFSGETV
SGRGSARQMCRTTPLRGMSSTIEWWRPRSVPTPPSFKTKQHRGAPTCSNDNNDNNNNGNS
GSSGIEGARDDFKNFSGELCFSGNSVSPPSPVSLKLPEDLSAEGPKPVLDSSQNILRESG
GRFLKRNQKKKWTFIRTNRSEDETRAEMTEQSGSFLDSVLRTYEKELRRCVRGRNVMKRL
CGNSPTARMTISSPTGHGASR*
>TCRU_3291 Major Facilitator Superfamily, putative
MFRTYNGIRFFRVFLTLWITYAAYTMVRRPLTVARADIQRETGFTSAETSLVDTMFVFSY
ALGQFFYGRLKGRCGNKEMLLRGILLSSAALAILGLSSRLPAFCVAWAINGIAQAAGWAT
CISIINVWVFPKERGRVMGWWSTNMAAGGVIGNAFAAFLIGRGLSWRTAVEAEVGLLLAV
GGVVLLALVEHPNAVGFPSVQQVEGGVEFAKLSFDCNSAPLSKDGEMYMNSPRSTNSSLE
ENMNFRHKDYDDHEEPFSPTFLNIIQLPGLCGICASYFLYQLVRYGFMFWLPYFAVHELN
YTTEFAGYVTCAFDVGGVVGIVASGYFSDWMFHGVGRTRVILLLTVGMVLGSCCLAVFSR
HFVENALFFMVAVTFVGFFAFAIDSLVSGSFLLDHLEHIKMVKQAGAISGVVGGVGSAGS
TFQGVFTAVLISRSWPTLFYGFGVAGALAGVSLIRPLRSELLRARKRSVVF*
>TCRU_3292 ATP-dependent DEAD/H RNA helicase, putative
MSFPQTEKNGITSESPAREFNTNEHGDLLNSAGEPWRGGRFQERGRGRGRGRGRGRYEGD
GEWRRSDNDRERGNERFSWRRGSGDAGGGGDYEGEEGYGGGWPRGGRSGYNNNYNRRGGG

GRGSRQYGPGARKEQWSNWRQGDPKRDAGDDYSEPHRGGGDNYDVEGGRGGRGRGRGGRF
ADDDRELEERAQRRSQGATDDTDQQEPAQQSEGQQQQQPPAQYGRFDVDSYERPRGRGRD
RPYRGRGGYFREVEEERQQQPQPQPQPQEQQQRPRVSEDPISTNPSRTQRAPHLRKNCHD
EREIEQLFEHHHQQKGISLDNYANIPVDIIPNNVEPVESFEDLYVEPALALNVAKCGYKQ
PTPVQRYGIPVCLNGDDLMACAQTGSGKTAAFLVPVVHYILKHGVSPARERVSYPIAVIM
APTRELALQIYDEVRKLTFRTDIFYDVVYGGTPYPTRFENDILVACPGRLKDIFDRGIVS
FSRVKFLVLDEADRMLEMGFEEQIEYLVASRYTDMPQTTERQTLMFSATFPQRILNLAKR
YLRQHYYLLTVGRVGSTTKNITQKIVRVQESEKMDKLFEVIYQQKQTDLVLIFVETKRAA
EELHYALKSEGIPSATIHGDRRQFDRERALRDFKDGITPILVATDVASRGLDVPNVAHVI
QYDLPKEMDDYTHRIGRTGRAGNKGTATSFYNKNNRNLTVDLYNYLKEHEQDIPLWFEEE
KENVEGERFLGMSRGRGGGGIGGRRGGGGDNYRAAPTLSNSTWGESAPSRGRARGVGRPQ
NNSNVDDGGF*
>TCRU_3293 aspartyl-tRNA synthetase, putative
MRRQRWVLQRHCSGSFTATAIGRATIRQGTSLSSLTSLGATAAKPATRIRGRVETTRVRG
KIAFIHLRQPPCHSIQVVASAADIVRRVKELTPESIIDATGTLVPAERPVTSASCKNYEL
HAERVDVVSRAATPLPFPIKDCNTRLDTRLNHRVVDMRTPLTASVLRLVSAVCQCFRKQL
LARDFVEIHTPKMLGAASEGGSAVFTIDYFGQRGYLAQSPQLYKQMVLMGDAMRVFEIGP
VFRAEKSLTHRHLTEFVGLDAEFVIEHSYTEVLDVLESTVCAMIDHLQEDHAALVRQARE
SLADMDAAEGSPSALGRNQEKEEASIVCELSEETLGAFGCLTTDATEAHLTTDCYHGRVG
VGSIDTQRRNPRQPKVLRLTFDDAVRLLLDHHVVDQPPTDFSLPQERRIGELVRERYGVD
VYIIDQFPLTARPFYTLPHPHKTESTCSFDMYLRGEEICSGAQRIHDITLLLQNMERLQV
DAASLKDYVDAFRYGAWPHGGFGLGLERIVLFLLGAKDIRQISLFPRDPKRLAP*
>TCRU_3294 Aminoacyl-tRNA editing domain containing protein, putative
MPTSLETRLASLAARFQEIERSIMKLRHMSHGTPNAPSAAAATVTPTVAAQYRDPSELQE
LQPCARDTPEVAALRKWCLKHGLFSARFRWVPSDYYQQSLQWRRDILEAPSIYHLCKSIV
LENTHCTHTDCSHRENSRYYIAVFPYTERFDDRLIMRFVMDMNKGMGKKKFNFRLADPQK
ALQLTGFGHGAVAPLGTTEEIPVIISDRIAQLSPPLFWMGGGHVGCKMCVDVAEFLQVIH
PFVGSFTSPLPVEELERLVD*
>TCRU_3295 hypothetical protein, conserved
MTLSRRQLLCPVLLDIGSSRSVTLPQFHQEVRSSLLREVDRILARQGGHSRLPQQSSAAL
ATSPELWIRPCGFSAEQPLLQHDSWKMPWQAHIHAMRQRGQMPSAAASAPQIGRETETVP
LFGTLSLPLSVRVSNAAAKGEECVTMMMEFFRVDAFIPVHVQMYHVMRRILERRDLWGVA
AVGVVVPTHHTEYFMDAARRMHERVSRSTLSGCDDVPTVLVYNPKGLQFQSP*
>TCRU_3296 katanin, putative
MENDIALQQAIYSSLRSQKKYNQQCQSRSAPVNRQTETIQPRNGLRNALLREGIMPAVPR
SAQTGILAPVAVSHYRFSPPRTECVGSTDENSNSAGSPIHLQRVSDRVPGWTQMCSGGRI
QRQNTLGSQLVFIGTEAHLEQTPKICCSDLLLLRAVAPLPNDQSMSCFTAQVQSLVSPGH
YAMFGMGVTPNAGEELHGFPLILIHFDASGAAFISVERWHMTENDTFERLVVLEPMKALR
LIRDRVTLSLVITSQCMSLSINGELLYPMIVAEGVRLGEALPILLSTGGKIIIRDVTVTE
GDKPPVNRLYGRPSSLTVSPKKSDATKKGQASLEDRHSPGTRASRSENRVPTTKRPTATR
NTTTKEGKSRPGGDSLPSGINADFAERIESEIIERSPNVQWEDIAGIPDAKRLLKEAVIL
PLLVPELFTGVVQPWKGVLLFGPPGTGKTMLARAVATSAKTTFFNISASTLISRYFGESE
KMVRTLFQLARHYAPSTIFFDEVDALMSSRGGNEHEASRRVKSEMLQQIDGLSTESDRRV
MVLATTNRPWDLDEAMRRRLEKRIYIPLPDAEGRMELLKKQTSSMSLDPSVDLSIIATSK
TVGFSGADLNLLVRDAAMMPMRKLIADRTPAEIAAMKEGGKMVLPAVTMRDFEEAAKKIQ
PSVSQQSLKQFERWSEELGSV*
>TCRU_3297 Programmed cell death protein 2, C-terminal putative domain containing protein, putative
MTGVLLGVFDGYMSLGRNALTSETKIGGTPTYFPALSDAEKATIRGWTTCGVCGHPMFLL
LQAFSPLPSAPDLHHRMIYIFCCNSAACVTQPSCSWCAFTLQVDAVDENAAEDEEPEDMS
RCDPLRSSELPPHTFPPCFVEIVAEPKKEIVVPTELEAEMIRASEEKARNAGITEADIKE
LERTIDLKDKPSDHEFEKFRRRMAREPRQVLRYYVRDPLGKKKNGAPAPTASPLFMHPRN
VGNRARIPPCDSCGAALMHELQLMPTCVYYLRVGEYIATGKSAADEGVDWGSVTVFVCSK

DCSKDCSGVVLRRAFVLVEKAPELEDEARETDGRQNLRSFINSS*
>TCRU_3298 hypothetical protein, conserved
MSRDAKPFFRRPTLADLVAGRQGSNVSQESNSAGTCQGVSVSSVGSTVPPVSVNTGGRPI
YAVEEAGAHDDSFARMEQSGQWKLEKINREKLHDMFVKENGIRRDNVIRLLKKIGINSSL
NDAALQVLFKENTHTESSILPEQWVALVEKLLRVREDATEGTAQSNEVLASKPRNDEQPK
SVKAMQRKDTVSSQRNREVLPERKDENKNNVSINNYVLNGTREHPGEVFEKKWITGSEAT
DIALLKLFIHCGALDRKDRGIELLPEDFRRVTYLRRNILPPKPIVWDATSKIADWCLQNS
HSTSYETDDLCMKMSNFLDLLNDEFLDLTGGKVWNDKAANSSPPTVCMVLASLAPVPLQR
LFGKEVVDHLCSPSTVQNVLKALGGSRGLFSTSNEPMSQNGKEFGIPPPLPPPASNNPQR
PQARLLPFSSSSACIIGMGGGGGEGCFTGMDGAGIKKKSPLVSYSVGSRSSSTLRGANGK
NGQPRYMEPRPLDEISASIVARSKARRLIDELRKNAIPINRYECFARIDYVQRAIFDDAS
LGEEEEEEEEEVGGNVSRDAGARAHRRHRYTRYRARPPDVTGPLLPGMYKMGGLGQPLQR
RARQETFERLYRLRFGQVRGVGNGSGYRSTSLKSAALLASRSASERGTLAPGQINPVKVE
DSKKVGCAEERPMQQADDAPTPLYQRRQIRGKMTRRGSRPPLMLANLFAKQIVFSPPSSQ
PPTPNCGTVSTRKGQKRNG*
>TCRU_3299 hypothetical protein, conserved
MSLLTRVAPSRLVPGAKGVFITRPLKKFTPVEVAPPPDAIAPPYLVNLLRMPPHKISLRH
FLALRHQFSSGMYFNVATLGGWYLIVPGSHAPLADPAGLRAWRERHHHRYEQKAAVRRRK
TDEVVTATHGSTAKKSRTTLMDSCDGGKRNSSTAVQGDNDTNMEGENEENEEEEVHAGPS
ATASSSVSFGETVSRASLLSESRIVIPEGHLFEINDGVLWETPPLNDLGNPAYWKHHTAR
MQLYEDTSISAEQVEFIASPLNTEGIQNVSGYTEQERHVQQQRLFQVLTHKANVRLNVDD
NTRLLTLMPIIDLKEGDELLLHYGREWWSQRLLSTLFMSVPDNEMRDIRWIEALFKKPTD
VSKPFPQLCSAVARRKMPKRGKRGEDGKASDVNDGSNVDAATQRDNLVLYNAVTRRKATD
AEALIFAVRRSCVNRNFFTALVGTGGAGVFDVSNCDDEVPLRRLRLTLLQSLHLEHVSAD
MTGVPAERSEVQFAEDEEDGSLIL*
>TCRU_3300 intraflagellar transport protein 172
MQVKHYKTLLASQPGFARVQAICWSPNNKRLAVADNNRFVNLYDEFGERRDKFPTKAADN
SAGKSFVIRGMVFSPDSSRIAVAQSDGMVAIYRIGLEWGEKKAICSKFPQSNGVTCVCWP
NTTQGVELVVFGTLEGKVKVGILKSNKSQSLYAHDHAVVSVANYADGQKVICGHLDGSVY
QYTFDSEEANEVAGSKKIFHHSCAPYVLLWGEHICAAGQNGHVTFYDRNGQKVQNIDYKV
EEEGEFTVGCFNPSGQTIALACREKFRLFDYNLRSRKWEEGIVINLPNSYSFSAMAWKYD
GSRIVTGTLTGAVDMFDACLKRYRLRGAFEFTYVSHNQVIVKRLATGTRIVLRSNLGYEV
QRVNVYHDRYLVAYTSTTLLVGDLISCKLSEVPWRLSGREKFVFENPQVCMVFAAGELCL
IEYGKKELLGTCRTEERNAHRISVRVHEPNDNGGGGEQQARKFIAYLIDRQTIQIDDLGS
GITVARVTHQSRIDWLELNYRANKLLFRDKQHQLFLYDVEEQTRTTLLNYCTYVQWVPGS
DVVVAQNRVELCVWYSIDAPDRVAVVPIKGEVEGIERGNGKTEVIVDEGINTVAYGLDEA
LIEFGTTMKDHDYNRACDLLDQIALTPETEAMWANLAIVALREMKLQIAQRCYAALGDVA
KVNALNHINELAAAAAKASDGLTDGYDHYTVRAELFMLNKEYKRAEQVFLENGKIEEVMA
MWEEMNRFDESINVAEARGWPELANKRAQYYNWLMETGQFEKAGEQKERDGKLIDAINLY
LRGGTPSRAANVVSANDLRPEPQLLEAIAAALFKAQVFEKAGDFFEKLKMDDRAIEAYKK
GHVYSRAVEFAKRAHPDQVAALEEAWGDYLVGQKHVDQAINHYIEANKYGKAVKAAIDSR
QWSKAAHILEGQAVGADNDKTVKGFYKNIAHHYEELHQYGDAEKFYIKAGSVNEAVEMYS
RAGMSDHMYRVAQRHLSQQQLVALFVDQAKQLETKGDYAGAERIYLKVNEPDQAIVMYKK
SRDYTNMIRLVQAYRPDYLLKTHLSLAAQFEKEKNFKMAETHYVAGKDWGRAVNMYRDRE
LWEDAVRVAKVHGGANAAKQVVLSNAMVVEAEDGVRLLMKFSLVDAGIDAALEAQKFDLA
LQWSQLAQPAKLPYVYLKYAMYYEDQGDFRMAEDAFLKSGKPREAIDMYLHQQEFENAMR
VAEGYDQTAISSILQANGRELFQKGNHREAEALFLRANTPEPLLKMYMDSRLYTEAQRLA
KEHCPEMLGEIAKRIALQSNDPKEAGAVLEEHGEYQLAVDTYIGATPERVPNPNVLASLW
VRAVKVAQKHDRNMLKGVLRIATEKLKDAKRYVEAGKCLEDCEDYKGAINMYVQGKKFDL
AEYLAKRISPELEDYVKRAIVQDSIENGGMKDAKVVEEIDPEFALKAYIANNDWENAIRI
AKQLTPEKMKYVAGLQMKYHLNKDELANALDVVEELPLDTGDFRFYDTWLDMAEKLVSMM
PLKAESNLKLDGFHRSLINVSESMATSGQKPEDVERANALAHIIHAYYMAPRMLDLSLGD
YAMKLLLGLPRWIPYIAPEKAFFDAGMAAKNAGSDAIAFLYLNHFLDITEKIAEGATDSS
SIDDSKFDCTDFPKKYLLPKSSSVDVAAEEEVNKWVLTISIESSFDPHLPTTMDPQNHVE

MFEGALRSPAGEKFPECAVTGYPIIGGGLTRCRNCQRPANPEDWNRYVVLGKQCPWCGVA
DSPNFSM*
>TCRU_3301 trypanothione synthetase, putative
MDNTDKQRGVHASETEHVSFGGLIGVTDCGVPVFSNGEKKFFGERISLTLDCRRIEAATP
TTVIGTYDTLGYKWLCLEFARRYLLMTKGVWLASVPTAEDIWEIEEIATSVPTGTPVPVE
REKHGDTTNMPSVGDLLVWSRTEDSPYGHLAVVTHVSDNGVCVAEQNYEFKRWQQGKNYS
RRFDCEKREDGVTVFVGETHLLGWVIIKAPPYDFSLGDLPDKYRHIIGPGHIVRRHLEKD
VLLPWLNPSQRCDFFLKRSLTVGGYMGEDAVAEVHSVPDGFYMMDYDMWCRFRFATKNLH
AVAVEATRLILNSRDSETLLVQYFGLPKELHLQLRRSFETIPSMCGRFDFGYDGKEIIML
AYNCDSSAAMLECGDTQEKFARHYGVELGTSTGSFLYSRIANYFNCLIQNEFLCPHHKIV
HFMIDDDDEERYTALYVMNAAESAGFRAKLCVKLSGFRFGSTTGEVDRDASPTERRSVVD
LENEEVLLVWKTWAWDTVLQQYVEQRTQEGHGVSTKPTLSDILLNEHIRVLEPLWKAVTG
SKAILPFMYAAAPHHPNMVPASFYRTKEIISAPYLTKPVSGRSGKSMTMYDTEEDPEGVA
VAPHTAAAPVLGRPISAVLFDDAPAGTHRVPFDNIQENLTGRLFDSVVVYQSRVFLSRYE
KKYSPIFCGWNVGGEFGGVIVREETFNNTKPSSLVIPSRVVRQHIPLHAMQESTTSGEV*
>TCRU_3302 hypothetical protein, conserved
MEEKKRQRAEEKKQYDPGEDDEDTEDSADDPDHKEGEGQKEGDAGSSKKGKEVNGGGGAD
GTPPDAPVVLQPPSPVEVTDLQSIEEKNDDGTGGERDAGRTQTQEAAGPSKAENLTAELS
TEENAAETETKTPGKKTQREDAGKEQTTIGAKSNPETPAAETEAHNREGVTKEEEDSGKA
TTNENFDGRQTAVKDDDHNEAEKTRQENENEVEKAEETQEEAKEKEETTEKKTVAAKGLN
LNSTAAADDSDSSTAVPHTTSPLLLLLLVVAAATAAAVVAA*
>TCRU_3303 hypothetical protein, conserved
MAMMMTGRVLLVCALCVLWCGAGGGAADGDGVVSGASSGGVVEAGGGPSVAVESIAVDAG
GKDATSNEHGTRSSEPVVQVDNSSSSNIGQDASHVLDPPVKI*
>TCRU_3304 ribosomal protein L15, putative
MGAFMYLNELWKKKSSDVMRFIQRVRAWEFRHQHTVVRLRRPTRPEKARMLGYKTKQGFS
VFRVRVRRGGRKRPVHKGITYGKPKTSGVLGVKLNKNSRAIAEQRLGKKYGNLRVLNSYW
VNADSTFVWYEVVAVDPMCRTIRRDPRINWIVNAVHKHREQRGLTSAGRKHRGLRHKGHK
ASKLRPSVRAAWRRNNRMVFLRKR*
>TCRU_3305 leucine-rich repeat protein (LRRP), putative
MSKGYTVVSVMKQTQETVINESLIRDCIYLPPACTTEEERLRFVGVREENQRHQRVKTTM
ETMELHKVTTLLASYRRIGKIENLVGLCNLTKLALDNNNISVVCNLGHLKKLQWLDLSFN
QITEICGLEDLVELETLSLFSNNISVIQGLETLKKLTSLSLGNNRIEALEDAARYLHRLG
SLRILTLKGNRVEKQPHYKLRLLAFVPTLQFLDGCVIDPNEVVSAREEQRENLMPVDEED
ERAAEVAKVEQELEKARKDYERFNCPDETNFYDELFHLELDGRGVVELLRVDIIASLSKE
LLEKYQMEFTEKARELTEAMKAIRTRRDEDESAFNTASARYRQKNADACKAIIKQFEKEV
KAHIPRTMWCREVDTGAIPSEVLSQLQIRLREVRHQLLEKEADQYDALESLNAGTIGKWK
GDGIDVILQTAFENFLKMEADFQVALRQLFDSVFEMRQKQEHATDSYHLIKQDESIMAII
DNKEEYQKVLGDWFELRRKRLEEMEQMYISNEEKLLRERSNRILDEEQNRHRARLNEIHE
FVEQMKGLLECAW*
>TCRU_3306 hypothetical protein, conserved
MENTRRKEPPLWLNPAVHVWITKNDIPVVNLRDISEKLRLPRSAFRADEAPRTPDEESCP
AESASSNGEKTPEPTKTESLNALLGIAQRRSEKQTTPPTQLLAVPSNPPASGGSGASKEP
EAKATSFPKTQPAAAMDVEFVQGVNLPGEKNTVTPVQPQPDGVQSVALFHDPVWGEVGSS
YDIDCLAPREEEKIDVLSSLKFGILSENVNKNELSTTGTATTTTATPPAFTASNPMSEMM
YQPPPHPPAMTSSPQQIAYPQGISLGASGWTTAPPPGHGVAVHPLQFVGLPFPFMSQAAV
MAVPQPYRVPYQFYQMHPQAPPLAPVPPQAGAYQTSPNPDLYQIAAQKLANQQSSSPRPG
TNVGAAPFIPGGKSV*
>TCRU_3307 Protein of unknown function (DUF773), putative
MEQQVIAVHYQRMEEWLSDRKAVLGKKHKKEFFRLMELAPKLEQLVRYEIPAQHKQNKRL
SSLLDDAYGSIEDATKARKNLEERKKRFFQEYGLVESQGAEEEDLEAVIEAKIASSTTSL
NEALREFGRSPHLESFREAYVRAAKMYSMGMYDDNVFSLHLPWLDRVYQERFYKSKVDIH
HHEQHQKQRAGERDQVAVKASTGPCNIDWGDEDTVDLVDVGETVEIKWDEEALEVAVEGA
VRNCSENNKRMDDGCEEKPTDTVKGIFTIDVSNAAHRKNILTELQAFACFASERSSMDDE

SLAEPAAAVEVLVKKLTASTEAVFVRMKTTPTLRFSFMEEVRRLQRGIAAASMRKSNSQA
RVHQIQEEIERLGPQLDALLIAAGNCRDECLAELLKMFPGRTVTIVGDINKYL*
>TCRU_3308 ATP synthase, epsilon chain, putative
MFRAFARRLVSRTLPLLQVPNHDLPEGFEFLENKVVDKDIHATHENLETLRFTLTRQDEF
LMRENPVKCVTVTGVNGEYGIYPGHAYKIVQLVPAPLTVEFTDGTTKKYFVSGGFAHINN
EGSCDVNAVECVAMEDLDLSAAEKALAAQQASLGSAKDEKAKAVIEIRISVLESVISALK
HHH*
>TCRU_3309 glutamine synthetase, putative
MSGLAEKRIACRLTYIWLSGRDSHHDIRSKDRTAYLTTEEVAKHPKELLADGIFPVWNFD
GSSTKQALGADTEILLNPVNAYHCCLPRLSTTVPWILVLAECYLPNGEPTNDNSRAVARR
IFERAPEEHPWFGMEQEFFLIKDGHPYGWPPRGFPAPQGPYYCSTGCLCVHGRRYVDLHY
EVCLQMGINISGTNAEVAPGQWEFQVGPCEGIEMGDQLTVARWVLLRILEDDGLDADYRA
KPIRGEWNGSGLHTNFSTASTRNENGLRVIYEYVERLKKTISKDIVLYGAENNERLTGKH
ETSKPNEVTAGVGTRGTSIRIPNAVAAERKGYMEDRRPAGDADPYLVSARLFASCVCIES
PELDLISSFHERNWMKFGDLS*
>TCRU_3310 hypothetical protein
MELCARNPTLILCNGGRDPANRLAELMDCSSALLWVSMPEALQLSEAKRRQSRSRSPGFH
ERLWAGLCIGARRIVVIPACHGLASAQGDRCLFSGGIDGPSHPSHVPHLSLSAPPPWQGL
AGLHVERGSLG*
>TCRU_3311 BNR repeat-like domain/Protein of unknown function (DUF3676), putative
MRHKINTESASLRVPGLVKVGDDVFAVAEAQCGERNGAGSCAGILSKHLNISDDSMDILT
SDISLFCMQHGDTAASSFWKTEVLRPTTLVLEDSVYMLVGNQSRTTPPVEGPNERGLLLV
KGTVTVDGGNNKKLVWNETHVVEPQGKGKSHSLTELIGGGGSGAILRDGTLVFPMQAKNK
DGTSVLLSILLPNLGRKWELSLETPGNGCRDPTLVKWKENEDEERLFMMAHCAGGYYDVY
RFSESGVSWYTPGEPITRVWGNSHNRKGHGVQSGSTTAIIEGKEVMLITAPVYPKEENKG
GKGRLHFWVTDKARVYYVGLISRENDDAAASSLLIKDNNNELISLYENKKDGAYNLVAVR
LTEKLERIKEVVKTWKDLDGALKTCSSGSSATVDARKKRMCNGRVPTDGLVGFLSGNFSE
NTWSDEYLGVNATLTNGEPRVRNGLTFKGSGAGAVWPVGDMGQTVPYYFANNEFTLVATV
SIHEVPESGSIPLIGVRMNDTSSTVIFGLSYTHEKKWLAISEDPANVEGFDEWEPNKTYQ
VVLQMDYDWTVIVDGEEIDRNRYNKNLFDSHRISHFYSGGDSKDQSATGGHVTVTNVMLY
NDDLWRDDLHKLHASKVNIPSLGVEEHPSGQVTSTDVSVASESKSAESATSHKELNENDR
VKQEENSVDDLVLAVPFSTFVAGSYVSGLATAAESAENSRPENNVQLSEGETSWQATLHE
DNKSMQRDSEVEPREIPSTKSTEVADVEGSAEGYDTPQPEEEGGTNDSSGGLTSSVGASS
DMDTVAAPADGEHQVQQNIEHSAENDDVRSTGTVTTGAEESLSLEAGGQKFRENNGLRQQ
PHSFRD*
>TCRU_3312 hypothetical protein, conserved
MTEAAGRTAFRLSLSMPLDPLHCHLTPRTAELPVGPHRTAAGSLACTCRAECPATEHGLG
HFPVPRLAAASQSPTRVPVATDSLSAMESLRLGPLETHDDVSEEICTVPHSLAD*
>TCRU_gamma, conserved domain containing protein, putative
Elongation factor.1
MRCVTARLIRGWLQRMEHVRQYLLGVALMIGEERRHDVALWVLCGRGVPAIMRDVEDTEL
FDWEEVADVAAQRKGPTDCLCWRGAC*
>TCRU_3314 Vps23 core domain containing protein, putative
MGEDELRRCMSMDSLREHYSQAAATEINKYISDFMVYLGNFGNISCSFSMKANQRLFVLQ
CPLFVTPPRNDNTGAQEDNKEFPPLFVEVYFPPRFPSVPPCCSVNSTNYKYKNVEKWSIR
QPSRIVTSNGSVNLPELSLLSGATPPYSLLEILLALTEQFEIEFPLIPANQVTQSKANTQ
TQGHRRPSTNTSSERRSALERRAAELLMLHLLSKAEGYLDTREQSLNQFERLYRCGGAMR
EARGLLEEKKTELLAYSPAVDKVEDLVGRLTQLPDNLEVHSTCIEPMDDLQTHALELLSD
IHALDDALELLERSLKAGQLSCEEYVRRVSDMGRKQFEARFLFARVAEAVNRSSAKTAQR
LPSIPPRVYSRPVLGDEPIKVPTKLNSLDLLLKEFPEVGSEMVKAVLNVVDGNLSDARVQ
LRAMLS*
>TCRU_3315 WD domain, G-beta repeat, putative
MKEDSSPDSSTDAIAEARRLPLPLPPFEENPNALVLHHEASKLSNEIHFSAPVAPLGMDE

TENTSMSFSGKLSINMAEANSEKAMHDGAKLWGDDKPLKLPEISTATGKSEVDSDAELPS
RQRPKSWKEDEVTTQTALASQLHRATFIPLVNTHHPLDTHSRVFQSGRNVRSRPSVLDMS
LLTIDDPHEEPLGSETEFYSGATWLALTILGMSPLHPNTSIVHPFVRIWLVNGVTGSNLV
QIGGGVAPFVLTHPIDLRLRGTRAPWWGAKFNLRVQSNIVHDVSSNAVLLLEVLEMGTEN
ICGFSLRRDGLYPICWGFLRLHDAHGRLNTRNRLHVQMFPFPQRASYLTKLMQLLPSFFF
PSSFSFSADAIRTVAADGSPASPNSYHGVPDKTENFSFKTSTPPDIFFVYRDPRNRKVPY
SAGMLISLEVNKDAHHPGETAIFPYEEYLLNQMSVPNEFLPKSIVHSLSPFSHPMRRVRD
VSLSLVNTAALLKSYLREKEERVLPPTDVLQRVQLRGRVSCVAFSGGTVWLAVGVTASME
HVVQLRHVLMPEIPVIREFRGHVGHIHHLFFRNDDKLLLSCSSDKTVRVWRCDFLSSPMD
ASRDLQSSCICTLPHTFPVYDGIFYQSHIITCGYDPHLFLWRYDKEHPSSLEEMRLNSTL
LRSTCDSRGNFLGSSIFKTDLAFSPGGESFLGELLCKTPCEEEAIIYSVCASQLHRVWSV
DAFGNLLVWRVLYEEAKDGKRIWKMSLRRRFKCHGATRVESHGGRVLVKCDKLPVAYLFD
DSSQQLLHKISFHQKPYVPAVTLLPDGEAVVAGTNNGRLLCWECGSGILCTPGDGYGKAQ
VNFSIRSIAWSGAQQLCVVLGTEVGIGHVIHGTETTMTLGAVIGTPRTMESVILCKDSSA
SELFRARFAGELPAQQRNRAFSRSTSVAVNLATDRNTRFYGVSRASDKNVSGDKETRIRQ
IISMWRGFVGQHSRKKQEAVGEAQSDPTAVYIIEDR*
>TCRU_3316 T-complex protein 1, epsilon subunit, putative
MSLAFDEYGRPFLLVKEQAQKERLTGVEAQKSNILAAISVSNVLKTSLGPRGMDKILVTQ
DNDVVVTNDGATIVDLMDIDNEIGQLMVELSKSQDSEIGDGTTGVVCFAGALLEQANGLL
DKGIHSSRISEGFEKGCEVACKRIEEISETVPVSREEYNYLLQTARSTLNSKVVNRERDR
LAKICVDAVLSVADMERRDVNLDLIKVEGKVGGCLEDTCLVNGIVLDKDFSHPQMPKQLK
NPKIAILTCPFEPPKPKTKHTVQIANAEQMSEIHAQEQEYFRKQVQLCKDAGAELVICQW
GFDDEANYLLFRNDLPAVRWVGGVELEMVAIATGGRIIPRFEDISGSKLGTCGTVREVGF
GTTKDRMIFIEDCPNSRAVTILIRGGNKMMVEEAKRSLHDALCMVRNLIRDNRVVYGGGS
AEIAASLAVLNYADSISTVDQYAIRRFADALESIPINLALNSGLDPIKALSLARIVQVEG
KNPYAGVDCMDCGTIDMKQQQVFETLQGKCSQLRLATQVVKMILKVDDVILTHVEEEQ*
>TCRU_3317 protein kinase, putative
MLDEKLYALKRIDVKEVTEATNAVERQGRKRGLVKELNMIRLQRSREPPRHLMQVFNAVA
TLDNERQHLYVLMELMSFSVEDAQKMLSRFPVHEMMKMTQSTFRKHLAGSALVQKQTEKL
LKQQSASCMHLTGRSSYNVPEDWELSIDRQTPAPEIILSILASDVLKGLRELHEEYSIVH
CDLKPANVLLDFNKRCFKIADFGCGCQMDPNTQLVRRTGVDLGSKLYKAPERLQNELAFA
EIEDEMLLVEFTPAADVWSLGIMLLELSNGVNPCSSFKSDYWNYVNNLKLSRMVKPLAWS
SAFYDFIVRCLVRDPSQRWTVNMLLQHPFILRYSGVPREKLNSFMERLKNESETFQRRQQ
RELLEKQILLSTGKKAHDRYQKQSWTRWKSFTGFLSVAPQFHDTEKFPLLS*
>TCRU_3318 septum formation protein MAF homologue, putative
MSASHSNGPVMVIGTSSRRRADILVEHFTGVYKEFITISPEIDEKEWRSTDPFDLTKLIA
RAKMQAVLEKLKDMHPAPKHGVVVTFDQVVVKDGEIREKPTSVEEAKKFIASYSNSSVRT
VAAYVVYIIDSGKIKVGEKETVTYFSAYNDNVINRVLARGVCMNTAGAFVVEDEDISCHV
VRNLGTLEAVQGVDPAALELLIKQD*
>TCRU_3319 hypothetical protein
MESLLRRLQTVADVNDDDIDSLGVLEEEEELHNMPTDRGELQRRLRHALSRIRRASERWE
ALQSESCKIEFELAALRQRFITSRKTWLLVHQFDRKTIEALLEERFNAESKGQSVTLDGN
GNRRNVKHGTDGLSHDITYLSSLQEQTATLKELLCKYAEERDKYKLEAQKLRALQGTQGT
LSEGGPIATLGKYSENSPFKEDCTLQDYVEQLEVELRRQSELMSEMELQRRVHWVAWEDT
RGEREAQLQPKRKMERGLVASRQGEDAGNGGALSNFLDSTEFLKINTMESSCESTAPAAM
KKRDGDEKVNLSLDPTSNAVEELLKRLEESRAESNAREEALNALYEDVHFLRERNNQLEQ
QLADVEAQLEQLRLDMMMTQDECRLEKGRNRELMEELECVQQQLQRQGRELVSANEEKRR
LRLQASFPSSCGEVHRAFLNMNSLPNVTAYGSAESVSGCGSGSVRNGKQIVDGVVPSMHQ
DVGRTPWGGVKSGGWLDQNRTFRGVMFLIGRRRRLIIGFFLSLFIAMALIMQARLGAPVP
DVVLSQLQSDLRACQTQLRLRDMTN*
>TCRU_3320 vacuolar sorting protein, putative
MVLVLVVGDLHIPQRAAAIPEEFTQMFSPGRINIVLITGNVGCREMYDYFRTVAPEVYCA
KGEFDQWSHTQLKEIHVITVEDLKIGLVHGHQVVPCGDRDSLAILQRKLDVDVLVSGATH
HCKTFEFDGHLFINPGSITGAFTPAHLDVTPTFVLLDIKEKTVTSFSYVYTRREGAAGGE

NFTIKRKVWTKE*
>TCRU_3321 dynein intermediate chain IC70, putative
MTDVSYSYSRPRREFGRMPEFHANEGEILSDIPPNRTVKNYYTKLDPCETEIQNVPSLSE
SSTNTERIKLRHRAQSHVEGGWPHVVDPTEFEEKMKYCKKVEREESYLSSCRRLVQQCTD
KYIKQNNAIDIYGSYFAEGAPPEDESLGPASAKIVSVFKDPNTEKRTAAAISWQNDGRKF
AVAYCRLRFQSSTPTMNTNSYVWDVMNPNTPVETLVTPSPLCSIEHYLKDPHIIAGGSWN
GVVQYWDTRQPTRPAARSLIEESHKDPVWSVKWLQSKSGELLSVSTDGEVYVWDCRLPDK
PVELHKLPEDSLTLLPKSNEGGSKGILGGLCLDYDPQVGGPSKYMIGTEQGTILSCNRKG
KTQAEKIGPNTFNAHHGPVYSVQRHPYFSKYFLSVGDWTARMWFEDFKFTPLFSTFYHTS
YITSGAWHPVRPGVFFTTRMDGYLDIWDLMLRQSTPALSVQVSDYALHTAKPTVEGRHIA
AGGIDGNVTLMELSPSLQTIMADEKAAIGQMLENQSIRDKNLDRAVKEKRAVAKQRQRRS
THVQAVTKEMEDLDEQLEKIEQEYSASINEERDRDLRLQEELEVQRRKLLEEVGDGIDVD
DEK*
>TCRU_3322 Mucin-like glycoprotein, putative
MMMTCRLLCALLVLALCCCPSVCVADTDTESAGVPEDEDGGRRADQLATAVVSPHEETVP
SGLPVKEPGELQGTTTREGKRSTDKLPKSDEADDTVQTKGKNGTTGTETDKNTSTDQTNA
PTKTTSTTTTLASNITTTTTTTTTAPEAPSTTTTTTTSTTTTTQAPTTTTRAPSRLREID
GSLSNSAWVCAPLLLAASALAYTTLG*
>TCRU_3323 Mitochondrial small ribosomal subunit Rsm22, putative
MCIRFCRLVQVFRLRGSPIVISAAQLNQRNLLSSLSSSSTVSEGGHHMREGAMHQPGESG
QQAGSSSTIAGKETWSQFSAKMRHGRRRIRVIDVAAKMSYEYQMLRKMCKRRPSMRQWAV
RDDFCDMHPGVVVMSPSMQAAFMKVFRLKDKGLIRQCMRDIIPVIEYRNREEPMRLQVNA
AEMIPENEPDTLNQKKRELFERREKGENFAELRLHDKRSQAKLRFKIRQRLLKFQRQLAV
ANAVASRTVLYSADDAIGYFLFRGAAMYAGMHRVFFELSKQLPHFVPKTMLDFGAGTGTA
TLVAKEVYDPGALAYPLYRSLRQTMQGNESSRIHQLAELRYDLKRLQRNNAEKKKVRFMA
VAALLERGEVDPAELPEDLRREIAEVAAAAAAAKKERLSREAHARHRDVVDGTEWESGDP
LNVEHAILEDPNDVMDGEEHDELHDGGSIHKKTWWEQFVDLESETAQQRAAKRLRPLQEV
TAVEPSPGMMEIGTMVLHDDVPNVIWKRYLLPEDEVIQHDLVVAAYSLSEIATVENRRRV
VQQLWKMTKGVLVLVEFANLNNFNLLMEARDCLLEEKDVGLWDWQPTIVGPCPHEQRCPL
RHCKAGVKRKRMRVCSTEAHYRATFVEVWARHMPLKVGVEPISYLIFARNELVPERALRR
QEQMKKAEEAKRQERDAKQRELHEASLSVKDVVFERLSDEALHRPETGVPTPLQHALSTD
NVEPLTPLAEALRDGAVSTGEVGHMPTDVPRLVKTSNTRYNRLIFPLQMPPATHKFNRAF
VDAGYQRQRAITPAEMLVVRAEVDRMRERVMRMAPKYMRVVRDPRCCGKVQADFCTPDGD
LVTGRVYRRFYGDRNRVSAHSTMRWQHIGGWKLLKRLKRGSLFPHDVPLYAVTKHPQVEF
PNTLLDARHSIVEQTAMQHNDPLSLVETPDDQLDREELRLKRRAQRDAELLQKVEGKLEE
LFGAKVKHDTDLGNGRIDSRREITEQEWADAVRRAKIRTVQHTKNAVPFAAKQRAAQRAL
QMRRRNIKREMASNRRR*
>TCRU_3324 hypothetical protein, conserved
MSDGSEPQWLVARLGNLLTAWTKNGGSSELAVQLLDVYWRFMRAVHHDVVLKETQGAALA
TAACIPVWEMLHFYGGSFGPLCGSRKAFAVTLLTALYPKTRDRRCLYQHLGCITGLGFPR
VPLLSRLPMWTEIMKCPMDGGHESDAYSEVVAYVKNTVSAEACILGGSSLHSLKVKIKVL
MEDVLPQCQFVRGEAEAAFVTANCPSAIVISMSDEPSLSIRSHEVLQQTIWKYLILLHVH
LPEERRTLSNYLTTSAWSQQSLKLSDDSVVTYAAIMAPIVHEAAVCASLMPSVASDASDN
APLIHPRVLEVLLPEEMASKDPRPFREDLTKAFVADRIKKRKISVAQSTVVGVRELLAES
IPQEIAFFGESSSCRDCDKLTHFCRNVLLSPVTGNTVAGGGLSLRVMRCMLFSRKRRRDD
GFYEGPVFLPVVSSLFEMLDEKAENASLSTLERIATAPGFQ*
>TCRU_3325 hypothetical protein
MAGAASLLIFVLCVPLSVRAAGSVRATVPALELFRVVFSGSDWQYVYETVAQHHDTSVGR
AAASTLINPVSAAVAELLTTSLSTPPGVTGLSVRGCEIDATGGLSVVYAVTRFGGGGELT
NTQINNLVRTADAFYLRSFYWRNGGRVTESIFVRDATLSAQRETCDLGCQYAVGVAVPIL
CCAVICIPVFISLVSARRASLRLDPPPPPPPPCAQVCCSRHAATLTSSYRPFGAAASSES
GGVANVSLIAPEYVPVTEGLEGCPTGYRPPVERTETDDVYYTRVAQQAFEAEKRRREDEN
CWRRANGLPLLPEPSNSTRKPRVLVVEDRQGTVQDTVMGLDPEERAAVGCTTPEEQRRQQ
PRRFEPHVPLRDGVAGVSGVLQAAESFHASVSTMDPYRPNRRPVVPSSTVVPMHALGVRH

EQPRPSSVASVLESTGCGAEHNTPANSLHTPLPMTSGPEDPQTGILPLTAAERRKMPLRD
TGLIGGEIVARAPSASEVRGPSNGVRSIVRPFLTEGKPQW*
>TCRU_3326 transportin2- like protein
MNSEEIYRPTPNDVVNVLRLLHDNGVSVQNSKKAYAELQQYQANPSFCILLSYVFGAEAC
PIQELQLGGSWIQYRRLAGITLKNNLEKSQYSLGLVAVKEAARCTLSVLGNPSDVRIARV
AAQIIVKITALTSFGWWQECGMGNLSHLLLNELFEAGDLRTLSALYTLQYLMEDLPKEVG
AASKDIIERVVTLVLDQNTKTNLQKAAFRMCFNIYEQAEQLDWNVEGLSPLQTGLTAASG
TFAGVCTALLEQGCGGDIPLLIQVLRSCAIMLEYLDYFTPMPDAEMTKLIDWWIRRTIDL
VSSRVDVGEYNLQLKTAAMDLLTAALKLYDRMGGNGPISFLMEPIPLMLPDLVQALVTCV
PMSIEEMNGILANDDYRLRDATAVNYHLNEASKDISEDTFMDDDCGAATLRASALRCIDA
LCVFSSKETFPHLIEQIKILWGADRQLKEASIVLFGTIANGCYTEIELTVPDIIRQLIDT
VMSLSEDIFVVSVAIWSLSRILEWAFICDEKILSDIVAAFVSRMQSTSKRVQQAAVSALN
TAFVTGHNMGATARLVETFPYLSESIIACLPIYCDGNLSLLCDLALHLIAVAEDLNMTSR
IGAAFKSNRIERIKYFETSYVALYINGVPNTHVDKDIFSIDRFVAGFLTRFPDSETSLKI
LGVWHAALKDAAERDIREDEDLLCNMMLTCCNFLGVTSTAALTEWSRQTQGSLASLGFQF
LVTLPKQTVKSAAVTLLHRLLKFIEKSAYPNGIGEKLLVALTSSIRAIDDSYDKLDLVRL
ITLMVEMYFSEFPQIASVAFAAAVDALRSDAYNDSRWLFSQMAYDICHTLDAVPGLLVQS
RPDEISRIMAQAENTCDKSSATISLMNALLSAEDIASAIFPDVMRLIYSWQQAACNFPGT
REKISSMLLFYSSKHGEQLREHLSALHPNLREMILSTYSIS*
>TCRU_3327 metallo-peptidase, Clan MA(E) Family M41
MRRYYGKFESVLRQQTLALRFKLSPGERLTTVIIPHSISIQRRSYHDYSRQTAYPPPPSP
SSMQQPPVPPYVYQGGPPVPPIFNNQRVNPTGFLGAAVPELGTKERPIFVLSAPQQASWA
TRLWLLLLLGIGFSCILNLLDELSTRIQENNSSVRASAISRGAITGMFGSADVKPVDLND
LDVTFDSIRGCDEAKKELEDIVEFLKDPERFYYLGGRLPKGALLVGPPGCGKTMLAKAIA
KEAGVNFFYATGSEFDEMYVGVGSRRVRELFAAAKANSPALIFIDEIDALGGKRSRTDHA
YSRMTLNQLLAEMDGFSSKESIIVLAATNTPDALDKALTRPGRFDTTISVDPPDMKGREE
VLEVYLNKVKADASVKAQEIARGTTGFTGAELSNLVNIAAIRAAVLHKTAITVEDVEYAK
DRVMMGAESKKIIPEEERRVTAYHEGGHALAAILLEKEGADPVHKATIVPRGNGIMGLVQ
QLPEKDKYSQRKKQCLARLKVCLAGRVGEEILLGVDDVTTGASSDFHQATQMARNMVRRF
GFSEDLGFVDYESSDTPEGAYMSEETKRKIEKEVASLLKDSYKEVKEILLVHRKELDSVA
QHLLQHETLSGEEMKRILKGEVLPARPTKENLQASSTQKPPKGSDGMKGRRRPVPIS*
>TCRU_3328 hypothetical protein, conserved
MRRVHSQLSISSLAGGPLSSASCRCPPIAACCGTQARHCMGPSREKQRSRPNADPGTGNQ
MSLIEPPDLTQHIQFGMNNLEHVMVPEVPEERRNRTDVEKEHEMRSRFGEYGPGERLTKS
SQQMRRDMLQEQQDVDGLPWEVRWRKGLIPTAEEMIQEVRDRFDLYIQDPIEREQEWYLH
WKDRSFKIQKDRMIWPQGYTDYLDHYDEHGRRRVLPSDQRWSDTSWKHLADTKYKDRMWL
IEGEERKAVHESLREDRTLMEEEEQRQLEMGNVYNGIVSGVVDLDPEQTYQALSGTADPL
EVARTKMKIQAYGAKQALQDGRKPGALEVDPISGFPKADMEPLPTGITVEQGASIAAEAN
IRNEMLEQTADMARQAGHDAVPVLMKTHHNTSKQHGDRPVLRSLVEKKIEETKRLQKTHG
ACKLETMENVLHNIESKIVPTLDAQDKGALPEEDLLAASKANSTSQYGVLESETKKEAPS
GFIHVTKRPQDAEPNIPVELYNTPKEYETETIPEWYRETLVETEPLIQAYDLTHDPLETR
EAVKVEYYQPPKLTEETRITNDEAIGGISAFDEMKMHKLQSLPELVEGYKPLPLFRELRK
GDQKEGETGLTKGATEKKKSEKLRKERQERLKKAKVQYDPDILLPSLPWETDSITDPYRG
VAAEDAVDFNKPDMEKTWRAYRDTFRQSMTEFGSLTQVTTEEKCEQMLMDTMDRFRRGDV
GEHPTIPEEHEAIFRMIFEAHTKHFLADFYKFKGNRVTEAKTTKAEADEILRRASAKCKL
LGPSFMNFIQEMTSLELDSVRKNPAQRYVIMIRDRKYESLGKPFMNWITNELGEFILTEF
KSVEQLETNLEFLRKHVNDAKFKCPMRVEGVTNSARDAAVEAFFQWCRGALCFYAGKQLH
QMYIEFADTRFRTRAEDLFEDSIECFSNYAKAANGVVQIPIPDSDPPYEYDEKEFLDGEH
AVYAEISGQLDKAEGLWHSGTSKRWYPVTDETEYMWYNERRGRLTLAMDISDRCLENVNK
KTHPSIHPFPERARPFLEGAPLTQETAEHLWMRHMGDLYNEHSEFMFRQGRFQTGRSYRE
KCLNFYHLAIRTARERLPSSWKAPLLTEAKYLMRVYEPGCDVERHLREVEVVVDKLFAAK
EPRHWLEPFSDLPYRIALVHANLAAFGECITNRVNARLKNRPSNELLLRGMEKRAHGGAY
LPELEEYFSVANEVAEEVFRKKVLRWRSQEHEGSDPYLFWTHLYFAFRVQPKNADEVRKM
WDMYEPAYLYIYTQGALASRNRGQKLINDALRRMCQILLSGDESEKKILLRELTNVREKL

AHLIDRRELDRTIYALQAHLADPTHSTFTYGSSLLYRDAVRHERIANNPNLRSDIAAKEY
AELQKRRESLRAHETTLSPQQAAKLQERQSSASSMNEEALGSQPDLSTREIFEAEGR*
>TCRU_3329 hypothetical protein, conserved
MTGVGGGVPFGDFLREVGHGIDTHTSRMREDILLLLSPYIGEQLRLMRESLRAEAERREQ
VLMDEKREIEASLIVAEENIQKQKKLVQALALTLGDAHKRLWWRRRFRDWLDWILKKRQQ
RQLCRTMIQTGNVMRKYHFYVQWRLFAASRRQRKHALAEESRWSRREAELLAEAEALKGI
VEEERRRSDGLEEKMKAAFVRGVCALNKEAVQVLRGAQTVEEEVEESKGVPPRLYDARTV
RLPSARTSHTPSTTARFEGPPIQKTTTATTAPAATATITPIGPVCSQEGYSYQYTGVPQQ
ASSQATCPVHHFCPPGPPCHVCYAPDTCAYNVRSSPHQPFVVSVDPSAVRSYGDTPASQR
PPHVARRSGRTERPRVSR*
>TCRU_3330 hypothetical protein, conserved
MDRRLNHTVMTHVLDALNVQRKSALLSLPFEAFATTSFTGNKTSSSRLLSGQLDTEKEMM
PFVRDAVLRAVERSPQDCGGKKTAEWPEVGTLMEGRRLVGIDLACTRLSGSFNRSDLSGA
NFTGAFADHSTFNLTRMQGCKLAGAQFHSCTFLASDAAGVDARRGRFSQCVFCRANMTDW
DVRGATFYCCVFKLSDLSRWVYDSQTTVVEPVDWGCCRRLGWMTPSEDCGDCLVVGKGGG
FPSLGPREETSPRN*
>TCRU_3331 hypothetical protein, conserved
MLKIENFIRILSAMYMTQDNETRRVATMEVLKAEDQMNSDEMLQIGMGLLRVSDHGAAVQ
AYGAVLLRHAVASGCLAAEKVPCGDIMMWYLNEPSLGRLLCGDLVDLITECMIYEWPERY
THLMEELCPTQAQLSKQPRKLRLLRALVVRFMDPHFGNVPVSRMKKLKSTLSSCSRVILA
EVIQALFDLYTAAGGEGSHQLAESALELIVDCLTITVHVASSLAISQWWELGLGNALSVL
VRWLPVAPEALAATAALLRCDGVSTATATTMNQQLLALVTAVLDCVEAYVMEVNYGILEE
IMELLLDLPDSIVQAVVAPVSQSCLVVLRVPSIYLATSACHLLKRLGDAAFAHVSPLELM
MRFASLIPKNKFHPEFGTDDEGKRLSEQQYGSIEPFERGFAEFRSLAGHLLTSLARIYPV
VSNQFILQILTTLCDGRGTLADPRTQSGFVTQQSLTFREWEATQFLLTHLSESFKYSSDY
VSQTIAALIAKQTEDMVLYPVYLNMLSSFWSCKDDAALGVWDGTLNIIFSCLCKRQKDQY
DVDELSARKRAVTLLVTASSQHAARFVDLCEPFIKRIEQLLMMPSTTPHERNFLYEAMAA
LTSVLPADEGLHRLQSFFTPVVKMLIQRVQSLDQMKFNSIIVAQGSDLQNERNMLRDSVA
IVAGVMRRCKTSPYLVESFTEWTPYIVRLMEFVHGLRADQLPPEYACILDMDGGVREQYL
PGRSRKSVPRQSNKEKARTALMDLRMSLYQVVGALSTFLPSEALRGMLQMLTSSAELLPT
HTMRSLIVHCVFPIGTSHTDLIASILPICGVFFARVARRISARPEDEIIDTKQLFYFSKD
IFTFMRQNILEKNLLSGNVPLTNLVIEVALTILESGSNVYDVPRFINATIDVSRSGCSGN
SAMEAQVEEFAVLAFGRMLEFSVRADDAVLSFNDRERLVFFLSDTYVDRYPTYTAALYSR
FSRDQIDALHTELVMTNRFDTKRRRFKEFVLQEAAKNTRIS*
>TCRU_3332 dihydrolipoamide acetyltransferase E2 subunit, putative
MFRRRVVQQLSSAVASLRLLTITPIPMPALSPTMEKGKISEWVTKVGDAVASGDTWCKVE
TDKAVVSYDNVSEEGFVARILVQNGEEASVGDTVCLIVDEADGINSDEVKNWHAEGAAPS
RAEEPSAAAASPSTGPAAPVTTSPSTSGARVKASPLARKTAQELNVSLEGIIGTGGGVGR
IVKKDVEAAAASGSARPSAAAEAAETKVQSIPKQMPAPDVATVAAASKPTPAVNDNYTDI
PVSNMRATIARRLTQSKNVDIPHYYLFEECCADNMLALIKQLNAKGDGKYKITVNDYTIK
AVARANMLVPEANSSWQGDVIRQYNTVDVSVAVATPTGLITPIVKNAQARGLADISTEMK
ELAKKARDGKLQPHEFIGGTVSISNLGASGIPGFTAIINPPQSLILAVGSAKPRPKISFN
EETGKYQVGTEVEMAIKFTASFDHRVVDGAVGAEWCKHFKDAIENPLSLLL*
>TCRU_3333 hypothetical protein
MVSKGKQLSVLIAGANGSIGRELVSLLVSHPDVGRVVALSQTPIPINRWRSSFPRIHPGD
ALRCLSVVSVDWDKIAADAAHVPASYVRAGVWSNESEANPGLLDAWRRIRGSAAATGLRF
SRPRNNGVKGNGGPRKAARGSNTGGPCGGRGGEKNGTNRGVNGDEALGRGGRNASRFPTT
SGSRSKAGYVKMSMGDPTASAEARNKFLNELLETNFYKSVFSGHHVVINCLSSRNRFMRS
EVNAVDHQYAIAFAKIVRIFNCMVHAEPSEEEEMLMDKHATKQNSVLWGEIYAACYGHAD
CRWFYTGEELEEKRDGEEEDVLPTCPEQRLSRGGVGTLRQFTQVSVMGGSLWNSLPYFRA
HGEFDKELLALFNRNEDDIQDNFMQRRGTRGDEDDVDDDRDYALGEEASVMARRRAEMLL
RRDVVKLWDNSHLTIWRPGLLQRDNLQLVEKILGVFTSRVDVKHLAEAILEDIIASLEDE
RGFNEVGTAKVIGGNNVVVRAKMKEAMEWSWHGKRIVTTSADS*
>TCRU_3334 methyltransferase domain containing protein, putative

MANAGDAQEVSPVRLVEGDVLEREHVLKVYDAIASHFSSTRYKAWPKVQAFIESLPKYSL
VADVGCGNGKYFTCAQCYRKATVTHIDDVGVTVEADDLEDACRYVVGIDLSEGLLRLAQE
QQKKLASASQVISRTDLLRADGRRTALRGGVFDAVISIAVIHHLATQAGRVEAIRELLRL
VRPDGLVLISVWAKEQPRKRSRNDEADVLIPWEMHEKYDKEKRVYQRYYHLFVQGELESL
AVEAGASVRESYYDKENWCVILTPT*
>TCRU_3335 hypothetical protein, conserved
MRKNNRRTEAQAALIVSPEWLDRTADAIASRNGVLLTDCVTIAEEGEATELPSAAVLKNI
EAAVEDLRCTLMERVESSHSRHRGTPGCCADDVKERQLYVDVLVRVAVTGALARYQRMEP
QSGHSNGAGEDNSGGEKPPQRYFDTLARGMLHVFEAFQEVHSMQRSHYTPRRRGGSTVGT
VGWDTLLLLHFVHRIPRVARDASEHVIDEATGQIVRSWRKLLLSLQTADPQESPEHSRRR
GALAVCNGLLSILFHRYNTHQCRILVNSVAQSEKIAESTGDWGKSILRPAQHMIAEVVNF
CYYKGRIMLYDHRFEDAYDALRKAYYLLPPPGEGTEVQQRNKQRVRFYLTVAGVVHGKVV
PEEILRTDDLIAFVFMPIFLAVQRGDPHAFANAVDAFGTLLRKRGVFFLLQRAKLYCFLV
LLARTRTALGMCSGVDNSRVPLPVLTAAYAHVVDEGRKAATAQTGAAGSNQKRPKRHREE
NDENKTSVMTDDEMTWWTAKLIASGLVRGYISYEHKTIVLSRQTPFPTLGIAPP*
>TCRU_3336 hypothetical protein, conserved
MLQGNKASVYVDGESLGEEEVPLTGERPPDVLRFCFGACGGHESHVTVKNVFLYNRPLNF
TAMRALKDRVPVPTREAAPTPLEEIRVVDSVREHIAEDITEEK*
>TCRU_3337 BNR repeat-like domain containing protein, putative
MRGIGPLSLVRSIKIHGGHTLCLVQRRGKGVVLRPTTITKGNRVFPLTGGSDVSYEGVCW
REGGLEPKLVVGDATNPTGASEWDGSMGRPQTTPELIPSASHEGEWTEFIASGGAGVVME
DGTPVFPLMTKNETEDVCSMIVYSTDYGSVWTLCEDISPAKCHNPASPDGRDHFS*
>TCRU_3338 Mucin-like glycoprotein, putative
MMMKCCLLCALLVLALCCCPSVCDAKDTPVQASSQTTTSIKTEPTDTNKHTEPEDLSTKS
VITAKELVVNSETEDEANGPATTLRSPPEGEQGKGKTGSSGSVPAAEQDDTARDGQSLAG
PDSHSSVATSTLEREGTDGTSESQSHTSTAAEKATEEAKDSAQMTTTTTTTTTEALTTTT
THAPSRLREVDGSLSSSAWVCAPLVLTASALAYTAVG*
>TCRU_3339 BNR repeat-like domain containing protein, putative
MLVGIYNLDGASTGEDITDEWGLLVARVNVSNDGDRGNRIYWNVTYGLPGTVFKKEHGSL
TGLFGSGVSGVHMEDGTLVLPVEATKKGKRENNAEEEGKTVSLIIRSSDGAGWTLSEGLS
ADGCSDPSVVEWKENSS*
>TCRU_3340 syntaxin binding protein 1, putative
MPRPVRLRLLLLVAASSTDELTEAKKQQLIRDGGLTPDGHLFAKLEHVTRKAGSVQRYST
ASAQSRRSTSASASNAATDEDPFLKHACMMMETADRKRSLRVELSLTSMERDHAMRPNGA
SGGHNSGPAGKNAGPFQGCGGKWENLPDLGGGGCGGMIALTTPQRIVLLVLCGVACSERR
SVHEISKRYGREVVIGEPRCRVLRCVCAGTWHPTAL*
>TCRU_3341 BNR repeat-like domain containing protein, putative
MCCSGGAATAQVGSNADASTPGSALTGAIAGEGSASGGVEGLQRVDLFVPQKTQVLPKTG
TTDSGRRDSFVSPSLVSAGGVIAAFAEGHINAKKTHTESTKPSSDVVAEYIDSAWEWPTL
VGEVKKEEWSAHTVLGKAEGEGSLNVVRHPTTTMKGNKVFLLVGSTALSDVNGSWKEGSL
ELKLVVGEVTNPTDSEQSKRIEWGEIQSPLNGSTLAAHKGNLTDYIASGGSGVLMEDGTI
VFSLMAMNEKNEVDV*
>TCRU_3342 Kinesin-13 3, putative
MSSSRICVAVRKRPTADVDKDIVVAQSPHLVVNEPKVKYDLTPYTERHQFTYDQVLDENS
NNALVYQRCCSKLIGTLFNHGNATCFAYGQTGSGKTHTMLGNDHEPGLYAIAAKDIFSRA
APLNAEVYISFYEIYGRKIFDLLNNKERLVAREDAEKVINICGLSEHKVTDIQGLFDIIS
RGSTYRAAGQTSANSESSRSHAVLQMEVRETHNRRAKTIGRISFIDLAGNERGADTFDCD
RKTRMEGAEINKSLLALKECIRALGMGKSHVPFRGSILTEVLRDSFVGNSRTTMIATISP
TSAHCVNTLNTLRYTQRVKDLGGESKPVPAERAERRPAKKSKPFEAPPPIKSRPEWVDNF
LSPEGDHAVEDDGNSGNEMAKPPPKQRASRSIGGKGRRSKSEVIVRDPKIATIVQNHIEA
LEDGSDEEEEEEEDNAVQLGALAKNEEKQVRKVHAHVLEEIAKAEEKLVAFHRRHIDTKM
TGIKDEIRAIQAFEDSDSVDEYVNRVRTLLLRQKQDVETILGLLNGITGMLREEEELSST
LNTSLKGGK*
>TCRU_3343 Fibronectin type III domain containing protein, putative

MPQEENIVCHAVVLRMGELFGEFGLEFDSSHLANDVAAVTKWNLHIDLEDFDGSPELCFD
EEMSIEKPRKVVKLRAGRFYLARVRVFLADTLTWSAWSDTVRTATLHAVLGQVKEIGEDY
VRVQWNRPPRDTSHIIEERDIQATQAIADILDLELRIMREDEMVQEFCDRFAVGVRTYTV
RGLRPGAAYIVMIRYKTLINTMKNWTEVGRLYTKSRNIISLVSRGEDAIKVSWHPGPSPV
DAVGYIVPETTVHLYEVYLEGEDTTTNTVQLPEQQREYRVEGLSPGTLYAFYVRSLTIEG
IWGCRSEPCKVRTAEIPELSVVASGETFLSFTWMRGHEEAEESRVEFCLQSFTSRHKQQE
IIQVGPRAEDKVIHVRGLTAGTEYSMSIRTFLQGEWGRWTEPQRFRTQFKVSLSFLERGE
DFFTITLEDQNNTMSDSHFNVMVMRVGKGDERFVVLDQEFKREPNEPGFRVGSLQSNTVY
EVKCRRWQHNCVTGYEGWGDFTAPIQIQTLQHLALHVLDIGEDFAQIVWRRGVSDTAPLN
CLTTQTFPDLKYEVVMGCVDTGEDYIVHKQVLQTNYTITNLQPATTYIVSVRACNELDQW
GLWSKVRLRTLASVVTSVHEIGEDFVRLMWQREDIDDENVKKDDNCNDNNCRPIESLVNA
DAFVSRYAVFVCSSEDGESIPSTLSGYHMGGNSEVARYEIVTSEHTSLRMNDLLPDRDYV
AVVRASTATGKWGLWSHPLRFRTNPQFRIPVNNLTIGENYVNLVWSRDAHPVVDKDVFLG
DLSVTGQQLRIHGVDTPYSKDHTLPADLRELKIYGLSPATAYTIQIRVCGKGGDWGRWSP
PVHILTRDTIVTRAVEVAEDYIIIAWERRKVANPKNYPTGRGIVTSYHLRVYNNSGIHSE
VFLGDGDSPYLISNMTPDTYYCVEMKANYNDEEWGLWSTPMWCLTMKTLEIQTKLISEEF
SCIVVKRPYQQKRLPEDDGNRASDDRIIAFGQFRPNLMLCVTSPILNPTPHISAGTTRPR
HQNSSLPPDAADHRLIYQTEILCASDDTDHTIPNLRSNTVYSVSVRSKLVNGEWGIWSQP
SLLFATVPATQVEFTDIGEKFLKVDWKRSAQRIPPQIKNPSAVKCGLGTISASRVKVREI
GGSLQRTYELNNSLKTLRIDALSPASTYAVSVQTYNDNYEWGVWSEEEKVRTIPVIDIAV
QHISEDAVWVAWKRKSDVSNFVNYDTALNVDVIPCTYEISIVGGGYFNYTKEMDTNTLFF
RGLQPDTLYNFQVRAQSFENQEWGIWSTQSFRTKPRLRVTFGNVGEHFAIVEWRRHLPSS
IGERDVNTVVESEDVVQQFRLKVERAGSKETYVYDLSPYISSFRLKSLQPSSEYRVWLCA
KGYEGVWGFWNDEAIFRTLPKLELEVTAIGEDYVTVSWKRGKWVGETVADTDAVVREADG
AVSGYKVRVLDINGMEVVSQFIGFRETSCTLSPLDLSSVYSVEVSAKDTYDEWGLWSDAK
HFMTLEAVDLRIISVGEVFVEVEWGRCRELLRRRARHGRRRRRNSILSSDENRSGGAVG
MNETEGIYNGGNNSADGSSKEENSSGMELPDTDARVSASNRTNDQEGPYQNEAHNIAERN
DGDEDGEEYEFDYEEEEEGEEEEEGEEEEYYYYYEKTLLHGDDGVLNWHLRVQGQRVFGG
QPGAEDFAELCIAADELQCVIDSLLPYATYSVSVRGQDKGGAWGHWSEPRKLVTCPLLAL
TADSVTETFIHVTWNRPPATLTLPEGFICFPPANDIHNYQVHIEPLATETPFDEQDEPLV
DGARVYETSQPSLRLWNLSPGVRYRVVVREQMINAQGEFVPNAWGCFSAMQVVETVNPMK
VVPIEIGEDYCLVGWHRVQRPADIASDISLVTGVAKVTAYELRAVRLDEMAKKEYQGPLA
LDAVVPLEANETSYHLVNIVSNTIYALSIRAQMERYWGPWSAVTKFVSQSRLEVKVRAVY
EDTFVVSWSRPLPEWALSRADSRNLNGETGPRREKKPDADFVVEEVTDENEATGDESINN
NNDNNSKGGNLSVESSNDFFATVLIGDYSVDRYELYVDGITCDMEQCLTLPKEQMSARIG
GLKHNQIYSVCIRSQSAKKHWSMLSRRESLLTLTPMCVDLSHYTETMTLIRWFRAPQDIM
VYDALLEGRRAEEERRCDERERQDLFELKRRAQGMEESVERKYLSDHLAVRREHNAARRM
DALHHLRIENVVLGDAEVTGYHFKFMGKVGGCMPSIPGVQRMLLQQRGTHSSRRRRQESA
KKSAGRMRRQQNLEETPTHTLGDAAAGNGAAPVVADDEFPSEDASLIDVQLGSHVTSVVV
KDLEPCASFSVQLRARNAAGEWCPWSQRESITTLKSIELHQNRFGEHYINLYWHRLPAAF
MQQMEDDEAQLHSLNEEFGHMSAKDLEDKKRELPELEYNVILERLKGWCELKRQVAHVRS
QLSRGLGDVIVHEKTPVKGFQLRIIHESGKFMDYYIDNKNNIHHDNDTNDQANNDKTTMD
TTDTVVTSTTTPMNFTFTVTGLVSNTMYIALLCADYGVAWGPWTTPLKFMTQNLIQLSIT
YISEVFVEIEWYRAPNKRIPPGEENDVLTSDTVSSEGRVCQVRITYEDEENDGKIVEEYR
TMRSCNMFRIDKLRTDTKYTFAVREWDAEGDWGLWCAPKKCVTLPGMETTVKELGEDWAE
ITWRRKERRVDYDDDIDVLQYDLEKLSYYLRVQELPENDDEDEAHAVPLEEMRENTTELI
ISDEMEGIFTSNHMTHGVLPLQEHPAEFEGEKDGLQCLIRRFGKDVVSFRLENLRPDRFY
NVQVMCETAENRLGAWSSDQYFITMSKIKISTQNIDEQYVDLVWQRLPPRQHPNLNMSRV
FTGHYMTIAYVLEVKGADDFYLHEQLQGHNNTSYHLKGLSLNTIYSARVYSINDVDMKSL
WSETLRFVTLDRVKVQPFEISEQSIILVWGREEQRPSCAETGGKYDPTISVGSRESGNYL
LRVYQGCEFSRCLLLEKNFPGDVHKFSLASLTPNTPYVVSVRATNLLGEWGLWSEERCVY
TMKLICAEIAAVGEDYIRFLWKREEPDELQIGDSLNSPKQPNKSGHDVFTEELAEAEAIA
INVDTTDNDVIPERRTSEGVDGATGSVCVQPSTEANVEMSGQKRHSMRGVNRKKYPLMDR
PERKGVVYNSANTIIDVYVVSVRRADEAEDYTIEVPGDATSFTVPSLRPDTRYSLAICAR

YGSGQWGVSSAYVTSGTLNLLKVELSGLGEDYLTAAWQRLPITYDTSTVSHGRKEDAIFY
ELAVHDFTDVSFGEEDVEDLAPRLRRTVFVPAKVWSCTVKELFSHHRYRVCVRRWYKPLE
AFINDAPCESPSTDDEQILDAIRRNHGLPGTWGECLYDVTLRDMMCFLTDSAEDFFVVRW
ERDPRAPALPVLNPFVQMPVQGYHLRIDEMQWDGVTVVTSADSTHIDQSLDANETNYKAQ
NLRPDTLYRIDVRCCVDNVWGRWSRNLFVRTLPKFFIDIANIGEDYAAFSWQRPRRSSCL
SDGTEVFCGNGDNIERYHLEVVGIEHRFHVSKKFKAARNNYRIKQLEANTVYSVSVRSFD
TRFDGWSLWSERVLFATLKPMHLTVGTVAEQYVMVEWDREEQTSEEYSEIVGEGGGIVQL
GNPEVIAYQLCVFFTQHTPSLAVVDKQFPKDVTRYCLGSLSADTPYVAVLRACNTESRWG
LWSKEGCFQTQRILELCVITVGENCVRVKWERSEVDFHEDGRGAGTRAEPIIYQLLLKAE
TETIERLIQPIDCGVTEDDFKLPTYLLEGLTPNTNYLMALQPGYEENRWGLWTPTINFKT
LQPISITPTTIGSNRLVISLCRGVETPSTEADIVATGNEEPERTAPSGSIVYYQLVLLKT
DEQQIAQEKERESEPNFLSTHATLFSTVATQRTLSISAAAVGAASVAAADATAAAAVEPN
SIVAEDAGEVDAEEAMYASPTIPAASVAVDAESLAATTDEKPVLPKGRVASVVIEEEPPV
PEGYDRGSLSRSAFFCPPAKKDNVVLTRDFEVNKEDPTNTICELEELESSTLYKIQVRAL
DENGMWGTWKELNVETAPFPPHSVAMHRINAQFCMLQWEPPDNRYLYRYVVEQAFAPSDT
RGKTKGGTEWRVADTVEDTFCRVRFTVPPNKMRCRVKAARVGEGHEFSEFSEPVKLSSGT
PPEPVTQLAITAIAYNFMTVEWAQSRSEISGTSRQARTLTYRIYLGIRDHTPIFVTTVSR
TTSYTLEDLEPSTEYTIQIVVENADGMSYNNPTIRAVTKSEQDSTVTRHEPGEPPLGSIV
ESISSLRRTLLPEIPSARPTLVPQPPSRSKRKRISGRSTPGAATATSSTAVTGTAAVSTN
ESRGNYRSMSRTGSAPRIMEMNSKRGTRDRLPSLKRKR*
>TCRU_3344 Primase zinc finger/Mcm10 replication factor, putative
MTRNEAEDNADDDEDLFAVFNTPEPAVDLLAAPAALRQSDSFFGGGRSVSVTEALSKSAS
SPSPSIMHFVCASTQDGCEMGSVTRQPPAERVHAAAVREPNSGINIRHSTKSCEQLPLVL
VEHPYSTFIEMRRSLATAGTPPLLTVIGVVIRRTDPKQKNGKGAYGIISLWNMRGPFPTP
QDELSILIVGSAFDAHYTKLAIGLVLALSGVQRMEGRKVTLSGNTKAENTTLTNSNRPGA
NSSNVCSTNLELLKVVKGDQIRVLGYAVDLHTCEGTQQRSGERCNRIVNGALSKFCSNHV
ANLRRVARGKASSPSTTNPQKPLRSNVSSTTFRAGVTRVDPALTNPKGVIASKKALQSMV
RQQPGFLTVKGSCGLPTTTPAGSDVVSAAGAYAGVVPGGALSVTGMCRDVGRVFRSPQAL
GVTARGRAVLAAAVDQEDAKERQRLLQLALKPSELGAKHPRNEGTSTPKSPRTTVAEDVE
AVREQYAPLTGGSSSSSFAAMRCNGFEEVCRHRQDYQHHVITKRANGTTSSIVSAVEQYL
RGDGVLAKTRASIVQEETSQQKNTASVSKSLLGTVAGSIESVNDGLRATDERRRFEQRME
RRLMQEKALEALVAVTEQKVNGIYCRQCERWYLRRNERCKMLQHVLETRATIRRFIECEH
CGYKTSLLGDVRPSKMIPRCPRCCADAMWKASNAAPEMAAPRKDPPPL*
>TCRU_3345 peroxisomal membrane protein 4, putative
MPVSTSDAPGLRRVALTSEAARLIDDMIRSGKYKELLDAIKGFRNGFVYGARIRAPHALV
LNLVWTNAPYRVMARKIYQVTKRHSIALGCTSLTFSLLRAFLRKLHGGPQNWHSALAGFF
VGVIFWGEQNPVTVQMSMYMLSRILSALLFILAGKFGWKFSPGAFRIYSGVLWMIVMPLF
LYYGDAMQPSMRSSMRYIYQDNEKYSSWYDLLCVNSDTSF*
>TCRU_3346 hypothetical protein, conserved
MRYSFEQVAKKETGGLCRAVAAANATRSELMKAYMQLAEAEIRALPKQRRLLERLLVQSE
EERRAAANERRQLALRRRDELKRKLRQFKEEGDGDGASKNSSHDEYARQWAAVYGGLALR
REQLAHVLSCPSTAVHVTTFADAVDSLAPFISAVHRAITFLEDKKDTETYTVPTQLLYTQ
LEQVTKMQLQVTSLLNRERHTQSLLRENTAEFFKGQERILAWCVEQREALHDLKGLKDLR
EFCASFMSNVAVMDTNFLVLLERSEQLASNRAVREALVAVNRTWMELAIAVYHTIRQVIR
REHANSGVEAACKWWVDTFEQRLRRILVEARDIADHMEMQSEPLMQTIHRRSIEVLRDLA
LPHTISQHISEFTEREDSVAPHEEALRDALVMRLSLLTQTFVGDVKYDGQREYADRLREV
SDYLDAQATGAVYTQLLERVKKLRSMVEEQLRLLPAGKIVTAADA*
>TCRU_3347 btb/poz domain containing protein, putative
MESIETVEVVSENGARFRYHCPVKLLRNMSLDASHYSPQFTVGGYTWRLHLQRRATAASG
EEFLSIHLQSCTNAAVVAQFKLLLQCYEDPPSSKGHIFRCTFKKSGSAWGMNEFIPMWRL
IQQENGFVYEDEGSGALYFDIDVLLKIVDANGQSYGNGGFETNVLPGRSGSPPAHLNFHQ
DPNRALSRMRSPMEYRGVGPTRSTSARGHLIVAPENGFIVGGGQAVVRAPLLYPFEHLEA
LTDMTFDIQGVRMKAHRCVIAARMRPLLPENVLPLQPGCVVTIAVTTDVFSAFLRYVYTE
ECPQQGVLPPEALLDLYLLSSACEFYELSGVCLNFVRPLLTNENILHITLTRYNAGDEVL

NALYLRVLLENYDFLIQDPKFEEIPGHLFRRLSLILRDKEPVPQVTIPTSKNTLGRQLAW
LAESGEYSDMELVVGPKHLIVKAHRYILASRCVLFSQAINPRSSVAIPPFTAAEFDFSQR
SWQKLLIGIYRHHLESYRDFSAEDVAIVFKMHSVFGMDGHLKREADDAFNHQNALRLLIY
AVKHQVPELHERGISYVASNFNALIQNDPQAWELVSELPQHAVVALFRMVVESQR*
>TCRU_3348 hypothetical protein
MPAAVEFPSLVAVSVVLVLSPCATAAADGGVPLGVPSAAEFSPGPLAAAWPPGDGDVGSA
SPPAGVLRVAPDAAAVPGSGEPSPVTPWFPAAGNCDCSCCADCGSVVTPEVAGSPGSADL
GKFIGPVGAGGSGDGGAPPCWFNSSALPSSSFERPEPLPSEPWASLPAEPSSLGSPLPSP
PRPAID*
>TCRU_3349 UDP-Gal or UDP-GlcNAc-dependent glycosyltransferase, putative
MVGWCYTLSRDVAEALVSCEPLRRLAYLPYSKEREEEFFSIGTQREDVMVGWVLDRVIKC
QPMIYVKVLPCHFNDARNGTGHSQVVPTSMCIHHVREDDYAALMARFGNDTSPAARVSWV
SEDVIYPVCD*
>TCRU_3350 UDP-Gal or UDP-GlcNAc-dependent glycosyltransferase, putative
MLVLYVLGRHPSHGYEYSAAPLEEAAQWHDVVALPMKEGRVSPGKKVGVRGAIGTEAEIG
MSQKVYMWFDLALRLFPTAGYISKGDDDMFLRVPLFVAHLRLLPRRRIYMGAHCGGNILM
NNRSLSAHFMVGWCYTLSRDVAEALVSYKPLRRLAYLPYSKEREEEFFSIGTQREDVMVG
WVLDRVIKYQPMIYVKVLSCHFHDARNGTGHSQVVPTSMCIHHVREDDYAALMARFGNDT
SPAARVSRVSEDVIYPVCD*
>TCRU_3351 hypothetical protein, conserved
MGKMWSQLRDLNPPAEAARCKSLRRSRRAPPAGQGIAPPSPRGSLWRQERLWAIGTLLPG
AELGSVCDHAHWQDTPLGRSMRGCRQLRGEAPQGARLSGESSGSAADPDTHAPSEAYAAS
TANRLTPSKASLACRGWKTPKPAAAHSAPDNNAVLSMRGVDAVRGDLHVAADASQSVEDG
ISRQRNSRRRLPMY*
>TCRU_3352 Acyl CoA binding protein, putative
MPLFDSSLSQDAAKARELCTWTTRFPRNISGKHFFLDHELGDLEATARFEDRQKLLFYAL
RRQAEHGPCTGAAPSIWWTRERTNYNAWKQLGDMSRFEAMVNF*
>TCRU_3353 hypothetical protein, conserved
MRSYCRHCMFHVCVFLVCTAVGAHAGGRLASSESVGCCSFSTVTGDTAVSLSVPSLFRRV
WLHLRHGALLRFAAEGVSTPYAFFSFSVSTPRMLERCFPWIGVLHDAASRYFGPGVYLDS
RWRSPEVADRVWRAMTPKGFAAMWCCRCVTGVLQTNCFRTRNGPAEYLFLVSGKEQASCE
VHYSGEVNAADTEGEREHEPSCMMSWLRPLTLFVMRTGKTSMVVFAVTSCASRTNLSWLP
FPCAGGPQYVALCYTEMTLMLLSLSFFIFSPPLSLGSLTAATP*
>TCRU_3354 hypothetical protein, conserved
MLFTLPRQNNIRGATSVLLLYMTDMTRFHPIGPLCADGVSKNADTLQYKGGELFALFEWN
AGQGRVVLAHLTEQLEEIRTVLRTWDAVDKNVTTPCTSTRKEGTAFDGACSSPMPTDGLV
GYFSNEGEKDCWVK*
>TCRU_3355 Trans-sialidase of Trypanosoma hydrophobic C-terminal, putative
MNAIKDRKPVPTRGSESQAGGVPQTIASAVSGPEKNSAAPAVPMTLDPHAVEEVSEGGAA
KRNTSRTEDVQFVVPEVSLNSAGSGLLPLLLLLLGLWGFAAL*
>TCRU_3356 BNR repeat-like domain/Concanavalin A-like lectin/glucanases superfamily, putative
MSRRVFASAVLLLLFVVVMFCGSGGAAATEKANAGKNDLINIQNLQRVDLFVPRTTLVLP
KKGTGPVKTRDSFFGPSLVSAGGVIAAFAEGHMNAEYQGGQSNKPSSSDVVAEYIDSAWN
WSTLVGKVSESTWKAHTVLGTTDGTDNRVGVVLRPTTTMKDNKVFLLAGITDAHKEGPEW
KVDSLDLKLVVGDVTNSTSSKQSGRIEWGDPRPLFDSKYSLNRKSKLNTFFPSGGSGVLM
EDGTLVFSLMAESKENDGVYSMIIYSTNNGSTWSLSEGVSSAGCHNPRITEWEGSLLMVV
DCVNGQRVYESRDMGTTWTEATGTLPGVWVNARSVFWDVSPHLDALITATIEGRKVMLYT
QREYTSGKNKVSALYLWVTDNNRTFCVGPVAVEDNMNWMLASSLLYSDGYLHLLQRRVSG
EGSAISLSRLTEELKEIESVLSTWSQKDVFFFGLSIPMAGLVAVLSDAASGDTWNDEYLC
LNATVTNATKVKDGFQLTEPNSRVMWPVNTRDDNLRHVFLSHDFTLVATVIIEETPTADA

PLLGATLGDTNSPHTMGVLYTADKEWVTIFNGKKTTKSGTWESGKEYQVALMLQGNKASV
YVDDKSLGEERLQTLLYWVRFWWTIIPGIPWESCIPRIKSGRRYSNTRRQTKVALGSLRK
NTKWHSCCKAIRPLCLLMASRWGRKKRCQQMRGHLMSQAFALARAV*
>TCRU_3357 hypothetical protein, conserved
MEMMMTGRVLLVCAFCVLWYGLSGVAADVGSVEEHGTGKDDKELKGISSSGLSLSKGKEK
PAAAALGGRGTPDLQGGKDNSMSEEGENILQEQELNLYDNPPKPNEGENENKGLTDAGHD
TRNQAAPPTPLPGTSGESPEPPILPAVKGPPASAGVSSQEPPTQASTKLTLPVQPAELNT
AGSVTPVQSQGGPETM*
>TCRU_3358 Reverse transcriptase (RNA-dependent DNA polymerase), putative
MIHRRLSALLPHHPRQFGFTHSRSTSDVVTLVNGKIARGLNESSTVEYERSGDGAPTQHP
RHHRSLVVLIDFSTAFDTVDNGKLFGMLDRLPRLGHRTKRWLHNYLRGHYVRVCTREQHS
RKQLASAGAPQGGVLLPQLSLRHVCDVLHRLGNSYSASALMHTDALTLVASGAGIHACAA
AMPPALSRIPTWAAEHSLKINVDKSEAALFHISSHTHGLTRTWSISILLAETYVFSHAQC
ACWTPRSIDFLILVRTLPPLQSRPCHAATNYGRSHRLVRSTTIRNPFRLDTVMVCHCILA
MQLSLALPPPASIIWKYCTATVVKHPLALVHQRKIHLSN*
>TCRU_3359 protein kinase, putative
MMERSEYGSALWWWEKARAVEQGALAEESPLCRSSQAARLEKECVMRMSRGDYAPFARRP
RLANGENDGVPHSDVVSTVTGKETVSQKTASATSSFRFGPLVTAPESDAVSLEVAAGCLP
HRFRDRLGNVWKRALNPIEDPSTDITPVYMAISATGTLAVLKVCQLTDAAARLTRAEVDA
ALEELMGLRVNDSLVQYLSYCYVPPHRVVLVMQYVPGGTLREMKSRYGRKLPLTAVRRNV
ASMLRGLAHLHGRGVIHGRLCPENVMVGVEGRWRLKGMLLGAAPLILHQETYYVSPEVAA
GGAKTAANDVYALGLLLLAMLTDSHPWQWSANAALDRSRNELDSLLADSTAFREALRNGL
LTPVPTPADTDETLRTVLEACLRTAPGDRPTAADILFLCNP*
>TCRU_3360 protein kinase, putative
MRFAGSDASSRSSAPLHGDDDGHASRPGRQVNQSREEGSGVSEGASHKRQPSRALETMHV
ASARRHNYGIRDSRGSDVVFLRTCESQARQGPSVKADTKKESANASVSELQKTEDVATRE
AVSASSRGVASSSAVVYKNEDCLGADKVFRLSDNAGKDGRSSRISRLSGKASLTVCDRWG
AMDSAVAEGEVPALPKMTNLNAKSQEMSEVRMTAASFPREVLSRTDSAHLLIRHSPPLRS
TLTLSLRCWVLCPLVVILLVVAAVTMALSFATSKSSSESVFLSLHDVVLDSIEESVGDLT
LTKMARMAVSTGSMYFSKNTFPNLRADILMPLEGGMMSRLCAVLRDMDPAKMVSSLGAVS
LTRQQAAVCFNSRSRPGNFVGTVSRNGVIKGFYYVDSVTLEYKTPLEPLEKVDPPMSIDD
FTKSQRFEKVVEKWKTAVEKGEPMLSEQYWVTPRHPPNYVVFVYPFFEVYEDGSTGVGYI
YSTMFTDDIASIQWYSPTESGIRVMLVDPDVRAEQLLVVANSWGQPLANVTNAWLATVRG
DPVKFMHVEDVEDPIMREALKHVDLCAAISSGSDQNASFLYGGFDGRITAKRIDVQGGVK
FLLVVVTSRSYYLGPVILYRNVAIVAGIVVLLLVTVACVAFVECCLVIPLRATRAELKLA
LGGAPVSARSRRRAVLREVRELEDVCTTLRCRLDKVRLYMPDRFNARVAAAGYGSPFRSE
CGIDKASLDGSNSEELKRVTCSVAYVYYTPRTVRNPTDAVVELIMRAVVKIATDCGGRFE
VQRPDYCVISFGAQLRGKEFAAEALEAVEFARRLREELTACAELDGLYRVIVESGVFLSG
VISGGGRSHFVLLCRNLHYRLGGFLPHTGVVAAVTEETALLVRGRCRLLPFETVYLEDAA
TTWWEKARAVEQGALAEESPLCRSSQAARLEKECVMRMSRGDYAPFARRPRLANGENDGV
PHSDVVSTVTSEETVSQKTASATSSFRFGPLVTAPESDAVSLEVAAGCLPHRFRDRLGNV
WKRALNPIEDPSTDITPVYMAISATGTLAVLKVCQLTDAAARLTRAEVDAALEELMGLRV
NDSLVQYLSYCYVPPHRVVLVMQYVPGGTLREMKSRYGRKLPLTAVRRNVASMLRGLAHL
HGRGVIHGRLCPENVMVGVEGRWRLKGMLLGAAPLILHQETYYVSPEVAAGGAKTAANDV
YALGLLLLAMLTDSHPWQWSANAALDRSRNELDSLLADSTAFREALRNGLLTPVPTPADT
DETLRTVLEACLRTAPGDRPTALRLQEVRCDRSL*
>TCRU_3361 hypothetical protein, conserved
MTDLQEGSIYARVAILQWAADILHGEYASFGNIPAKDIAVLLHAIFNTPCEGGSMDTGGT
ALVSLHNIQFCEHPTPTTLFLNAKRVLSLVQTLSSSVVDGNGDMNVAEGCGGCSRPLSLP
AVGNMTVSAWMEGKAFVEELKMWRWIRAEAFRRGLEKEELERRAQKFLHGAANPSKVPSS
HESTTTGPALELESGPSSGSVGKHVTSPRKRSREKDTGAPNAPEAALDSGNGASNQVKGP
AATDIKPESGRDGGVRNCMRTDIVVPRGRRGDESTYISSQYDKVPREDKGEAVSLPHMTC
DGVAVAPKLLNVLEEVKKELKERKVAAEASAAACGSITGSITTASRGSEPHVIEEACGSG

HPVVGCSSCPAIFSEALQQNYSAIERLETARRLAVAACLKKDAAGLLEALAFI*
>TCRU_3362 hypothetical protein, conserved
MLRRPCVSAVDMFAGASSAIVAVSPRRGISTEQKDITRSGQPHPPAVPHNEPILPMDFNP
HIEWFEYPKEIFDPKYPYTREESIRMTEESWESTMDEMASRYGMRLNMTTKPTYWMAWSI
MVFYSWYTLWGCYRAMGTEPGWPHYRQIVLNQPNVPTLEEDDIYLDQWIRPEMREKHAKF
DLFWNWKPLVNRDGAKTTWQIPMQYHDESEWRHMTSPN*
>TCRU_3363 Protein of unknown function, putative
MREYGRGLNADDAPPPRSGGTLLAPLRLTQQQQQEQRQPSEVVPLLQHLTSSGEGLQQVR
MKMGAKPLQPIGTMATGAGSQRAGQAMTEHVPLNSAPTHLSESKAINSFNSSSHSPLQSF
EPKADGNDTMNSTGQLKQAPFFPIRLEEPRGAALEPSIPRVRQTVVFVEGSSCSQPPPET
ANSREPTPQLSHEKEQKINANSSTSGWNANYSPPFSEANNTSLPLNDVCSVSNPTVGGSR
TLPVTRPEPQINENPPKIAASIKKLGDINLSAFTQWCSENVLDPNGLTERRLGRRRSRRW
ISSTWDLVSATTVRSRKTKGSSVSMGGATKRTTSNGSNGGIFTPEDSTLKIPPRETREGL
NRRLKKDAQSLYSLELPTFDEETGTLGDACVFPRSLAVDVAVEHVISQERGELRTKATGV
GILRFLKEFDESCEIIYLYFWYAIAHIRRIKSEKMLIGRYINLERIVRGAFPRYKAEMPT
IKQIGCILAKYAPTDESISENNSVAAAREEVEVEEAVPPFFPAISNVHLLGGPGSIVSEE
SGRTAFVSQYPKKKRPRTGSGGADIIQAFDEAVDKSLLQDPAIISLCNEAINAFTVVQME
VYELAEFAQRCFHRLAVLFGQAFERLAEEKDEVLTAFTFIVPHVVYYVFVYCFPNDVVAG
IFNAALRMNLYRIFYFWCSGLVATYVRVNGWPKPLTDGKKSLLKFIAQQAGSPTLSQSTA
SVISPISPKRGGSAFIAQGESNSQGVVAAIGNSKSLSSPTAMGEERLEALADDTFEEVDV
EVANGHYRIALEFKKYAKHVNYLLQELQKRTEDMHREAAHSSLIPTPTDIENTSMGGMAQ
PDRFFNKLPSVQMSASPQLRTVSWNGGSAKKKMPETRQLPRKSTLLIPSNNHDAAGNYDE
AVLPAISQANGAVKKTKALPVNNDCILSGFPLEHTAIHVKQYMMRPTGVAPAKVPLPPLN
VPSTEGTNSNEAASPHADKHHTLPSLRTVKVPLPVASPFFQHYSSKRLASNAAGINEKSF
PRKSTRHDKPHPFFSECVVPTSMGSPSSSISHATATRNSVGSLSSSTLAPSISQKRGVGA
KNPTNSLMLPHVRRKAIKLMLIPPSDAQLRPLKFKSLTREVTRRQLEIERQMDRLSGREQ
ILRRKYDAETQQGSRRVHQLLSECHSDKQKIEAYAGFLSDKRKKNEDTMRRKQKFPLERI
KR*
>TCRU_3364 hypothetical protein, conserved
METPRDVPSTQQHPFNFTGQQPEETAHRSFHFTCTLEDREKRKRAAAVRWTSPFQDTAVK
TSGRKHCTEVPSVNEPPPPKTTSLHVVAASNDTTKAIASGDGPLSLEILLHESQQQLSAL
LWQKRRCRTLQAALDASLERQKVLENALVESSGESQETREQYRAYVATTAEELRQTRKSL
RASETALRVIEDEVGGLRRENERLRQLHQQAEEDHTVRRRQQERMEDQLSCLLAEKERIQ
GELSTSRARCRELETLQNAKETTIDESAEGNDETKNMDKKKIIHGNDDNGGSDAARRDEI
NTIITLLEDELDRRRAEMDAMKESTEAFREEVRRVFGMACKRLTVVSQRGAQLTSVLACD
SKQLQRGMEEDVENGGDMDSLFATLEIIVRRLEQQWCCLEEKVMSRESESQKQEELQNQL
DAMKKAHLAELQTIKSTRQEATERLHQMEIRYTQLLRAMERQSQMVLAATNTDMRISRGN
RTSRTKEKPRNKQEDGDKNSDDSSSNNNSHEEYMEGRGEERGKRMACEKETFQTYEWHFG
RKEFMTCETILDPSYDRMVYTLLRRTRQATRNAIRQLREARSKHDTLLKLQEEKQRQSMA
ISEERVRYQATVKRLQTSLKAAHDTESSLKTKIAAVMRTAELERNSLVRRAEAAERDVQA
KSKQERLLQRQVREMLEEYEAAKQELAARDDKIAELSAELAEGQSTTREKQFDLNTLHHR
CEDLVRMTDVLETRVRREIQNQDVFYELLRKLCGAVAVLTLRLSTVAEERDALWRAYEAR
ETDCRAALQVLQEFMKKEEEEGQELELGLQKIGRVLPSHHGRSFYVIVAVVIACHRMQHL
LSSRYSRRHVMFAVYNEDDNSTNLRSSGRDLSGFALSPWHMLKSRNVLSLVASVPRVSAA
LRRQSGGTRMPFVHLPPLKTLLSVIASRDRNGTAEHEVNDTLEQILALAELNNNVEANRF
WLEAHISRGVAYAASAAGGGGGGGAWTRRGLPCVNSQPIHCRGMLAEKLHYVLHTVRDAA
TEFRQKMRQKDASIQIAEEESHQLRSMVQEREGIAAGFAQQLREYEEHVATAFVGRDVYQ
ELQDRLRAAHDALQEEREARRKIEEVNAALRQREIELTRIVQLLRDEVRSLSIELAEQTS
HDDSHCQRIDAYLSTLRLSPSRRKTMNTSRESNNTQLYVPRSVKLTSLPFTAISSMNTHP
NTNNEDGANLDEAVRQVLSGLASRLEQAEGNPLQP*
>TCRU_3365 WD domain, G-beta repeat, putative
MAELSPVALLPGGCQQDCALPVISHANVIVYCSAAAIYMHTPNTITLPASAGAAVGTERV
KNENETFHNYPLTRIFASGVVGVIYSFDFNDEYMACVTSTTNTVVWRIADGENLNDKRVP
SCVGEFFRREGGPSVVRIAGKYHILYGTKTGWVVSVNMNDGTTMHQLRLCVTYKSVNSIS

LSTPASVATKAAGGHWEGTETQVGYVTVVDVSISRPDTIVVGTSEGYLFVLSIHPANGLR
QTASLRPFSAPAVFEDKPAADSTNNGSDALNEMGWLPVTAMAFDPNNPNFVAVGSRDGGL
VLVDIVSTTIVQTYAVNGEPVISIAWLAGQAGEFVTTDGESSKLHVWSVSTRTAVAVWHP
VAGCAIFGIAAFAPEHLFIALRSGSVAVFNTRTGQIEMQTEAGHTDVMHTCRYAHHDKDQ
LATTSTDGTIRLWNTKQLVLQNTLDVGRVIVRSMDWSLSGKYLAAGLSNGEVVCYYVHTQ
REHWRVSVTTVSTVYFVSWSCTESGGHIAASHRDGLAVISARDGKILRHYKTKSPVLGIE
FDRKHPKHLAAACQDGQVLVFHISSSREEPALILTGHMAAASSVAYNPTVPNFLLSCSHD
TTLRLWDLYSGASHTASVSCRVLRGHTNSVNAIAWCSIAPYIALSASSDCTVRLWDVRGG
SNLATVRAHNAEVIALSTHPERPPVFASVSHDNSIIFWHLGLLRQVYLEAALGRIEHCIV
SDGASLLASAAASSFVDPAHLVTGDVVQELNKELKEANLAPHKRMERLVRMFEFPCGAVD
LARAAGFSVDPQDVSNAKCTVVPTSRLVEVHGKWGKTNVEKSHGKTVTNAGEEYKKSRII
EAAEFMLQIGKVAEYCQLMAEVGEWDRAIAAAPVVSRDFWRSLCLKAAETMEAAGNTRAL
RYFIMAEESARAALLVSRQSNKNWDLAAVIAQTCPQRVVNETSTEPPHNTTVDTNGVSAV
VKGLLNARATALENAMNPRIVAATKLAEGANDDAVMDLIHSGDVVIAHLLVHTVPLQRQT
TIDAGYRLSMLLSCRQRQWDTALICATRHSNPHDGLATVLAFYQEAEEKTRSVDTNGVPA
PVNERMKTFHEQLLLECQRLQLPLDAESIQRKHATDGLGSINQLAAMALSPKTPVGTVTS
EELLHTMSNFIDSVLQAALQDIDSPNAVFYLNQAFSATCYVSLPIQSASKGANSTTPTGF
STTITGNGTHSAAVRRFLAQVFLLAALMCVKVYRFPNFLNPTFTKARELAEGDNHLMVLL
SKAQQSLGSYSPHSVEVGCPTLGASIPSYDIEDGIQLKSILTKEPLAGAVHVLEDGVSLM
GKSEAFQWMLCCAFSPLASGARFLPL*
>TCRU_3366 hypothetical protein, conserved
MHGRNGELGAVCHVANAVHTCSNCAASCATEEGVTVAFTMNVTTHKYAGPYELWWRLFSP
DGAIHPPARSSDADRTGICLLPPPREGKDDTNSGETTAPQVNAEPSRQNEDWRRSAAADG
PNTVDPSPSSNTATNTSETRPEFANNPIEMMVPNASTKRPSFANGPRILVAPNASGVMSV
TYGGREHVAADNTDKVSEHDATGHKTLVL*
>TCRU_3367 hypothetical protein
MEEKLRNASREELLNHVLTQRLLIRHLHRRIVELERLVPLTSNNLDSRGGNEANLSPPVP
QRDPPPPAKLSEGLREIDRILRAHATGAVRLPQEVVAEFLYIQKELSAVESGQNTAESTE
TSALPVTFSSDAFLVSRTPVVSTSAVSDASIHSTPYGQRSGVSRRFSPPSSLIQRTQKHL
SMRERNELP*
>TCRU_3368 hypothetical protein, conserved
MQFVVVDGSWPVVPCAIPRSQLLARSVKDTDRGEDGGGQSVLPGHALFTLCQEVLDTSAS
KTKAPPELATLDMTFLRDPQSGPLGALRLYTFKSVSHQFFAVEDLVSSGCLIVFTSHWAS
LTSAALLTGVRTTHKEKDDSANSSRRRIENNTGSGTREVSASRASSLMGRGRSVLQKFSR
AETYFTSVASATQRDFEMVNELLLSGTIRMNAHLESFLCSVCGNLPRRTMTTSCCGAVVC
LVCAPTLSSSSVLREEVVCAVCGEMPLTNPEAHVSRDAEVMRLVRELRVLYHPQVSAVAC
RAMPGETQPPPTPFNVHHAGLPVPMCDMNEPTGSVLGSVFMGPS*
>TCRU_3369 BNR repeat-like domain/Concanavalin A-like lectin/
glucanases superfamily/Trans-sialidase of Trypanosoma hydrophobic C-
terminal, putative
MVAFAESLFGYNIDEHHLLGIRPYEIVAGYIKAAESWPSIVAEVNAGKWRAHTVFGSRNG
SDRVCFLCRPTAVAKDNKVFLLVGSDTVGYYSDDDVWVQDGWDIKLVEGVATQSTDGRQS
TLVSWAQPKSLLKHIPNHTRDQLRDVVTAGGSGVLMQNGTLVFPLVVNGEKYPFSSITYS
TDNGNNWVFPESISPVGCLDPRITEWEKGHILMIVDCVDGQTVYESRDMGKTWTEAIGTL
SGVWVRSQPGFRWDEGLRVGALITATIEGVKVMLYTQRGYASGKNRANALYVWVTDNNRT
FHVGPLFVENNVNETLANALLYSDGALHLSRESIVGTRWGISLARLTEELNAIRSVLSNW
ARLDAFFSKSSTPTAGLVAVLSDEANDGTWVDDYRCVNASVTKAAKVKNGFKFTGPGSRA
TWPVNSREDNNQYGFVNHDFTIVATVVIHQVPKESTPLLGAGLGDGSGKKIIGLSYSMIK
TWETVFDGTTKAQKNTWELGREYEVALILQDGHKGSVYVDGVIVGSSEAITTLETQGHEI
THFYFGGDEGDSDSNVTVTNVFLYNRPLNSTEIRALKGRAPVSTRGPETQVEGMATTEHV
AAVPGPVELPAAPGRTTMERATTTEHAAAVSFTSAGSGMQPLLFLLGLWGFAAA*
>TCRU_3370 BNR repeat-like domain/Concanavalin A-like lectin/
glucanases superfamily/Trans-sialidase of Trypanosoma hydrophobic C-
terminal, putative

MQNGTLVFPLVVNGKNHPFSSITYSTENGSTWVFPESISPVGCLDPRITEWEHGQILMIA
ECFNGQTVFESRDMGTTWTEAIGTLSGVWVKSRSGFRWDVSLRVGALITATIEGRKVMLY
TQRGYTSRKNKANALYLWVTDNNRTFHVGPLFLEDNVNETLANALLYSDGALHLSRESIV
GTRWGISLARLTKELNTIRSVLSTWAQLDASFSKSSTPTAGLVAVLSNTASDGTWVDDYR
CVNASVTKAAKVHNGFKFTGPGSMATWPVNSRGDNSHYGFVNHDFTIVATVVIHQVPKES
TPLLGASLGDGPGKKIIGLSYGMDKTWETVFDGTTTAQINTWEPGREYEVALVLQGGNKG
SVYVDGVIVGSSEAIPTLETRGAEVTHFYFGGDEGDSDSNVTVTNVFLYNHPLNPTEMRA
IKGRAPVSTRGPETQVEDGTERRHIPRIEGVRANAPAGSGLLQLLLLLGLWGFAAA*
>TCRU_3371 hypothetical protein, conserved
MTGMDTAWIPFAVLYILYQIPPTMMEVYSYYSRGGLVSIEDIKYDDTFTFHFQWLHDKDI
YLKRYCDRPISEAGKAFFSWLDWSAEAYDRSVYVMRRNHARRDGEVSMWKRYIYGPFVFN
WPKKQWLRGDDSCGMRMPAWGVGGEARARQEYFAAKAKGFDKNWHYQIMRKIRREQARKA
SADAAKA*
>TCRU_3372 HECT-domain (ubiquitin-transferase), putative
MENFFTRIVVDAESSVVQRKMMKEIAAARDNINSDDEGRQTVGLLDLCNLLNMATAATIA
SIRPSVFVPPVLACLKKEHNVDLMLLAARALTYMVDAISSAVYVLGSEGGMEAVLRHLLE
VKDIELSEQCLTCVEKITQSSHGALMGLQKGGVKSLLAFVDFFSSSSQRKAWSSVAAMCR
RVDVTTFDRVEDSLNDIRSRTNHDDGKIGDKAIACLYRIINGVRTNPELVALAYGDVSPS
LLCVLTRSDVSENTFTMTLSLIGSAISYSTEVTRKVIESGLMECMLALIAHSSKHQLPLL
QQQPQQQLQQTWRSFWESPILGTQNPPGSSFSQDVSLLPNSTTTQTTAAFRERRLTMEQT
KTLCIALAGLLPRITEGYLAYGNILTELLAERAQGMAHRRGFFSLEASYEEDEEEEEEEE
EEGETSNSDEIRLRIFDEGIPLEKNTKFSRCGLTHMCDSCGKLCRPGDWFRCNKCADFDF
CGECLLMNWEEHTGNSAQHTFCDMLEVFPGFKRSPTPAKARKTLDDALNTERKELYKSHP
QLLDTILGGLTKMVALFNESETHIVRNHSLAFIDRAVCLASPQQLAGSGLVEASVCELIL
SAIADPSLVLNVQVMLLCRTLLEKLPNVYKNAFVREGVTSALIKAQQQNQTSTRRSTEHS
EEKRPLIERLVTISDWRELVVEEAKSMLSMFSSLSEETHVMRLAEFVSLMEEGQFQEAFD
ILRTALLNDITTFELASSNVLRSLRETLTRVNDIKVVVTLVKTLAKEAPNPPCALTRFVR
HLQTILSQLDQFRPPSFGEVSSIHVHIPVHLVPHASEQQKTKPSFLAKGGGVLRRPPAAA
PSKESGAPPRSTRIPATRRSSGTNIASTLAPSTSNSTKGPLEGRDIMVSVEPLTSMDALM
SFVASNVLSGEMEHSDSRRRIEDEDQVEEVLPELGREGRDETSVSGKKLTQEKAQQTVYL
RYESHVLPSSMTILQVIQQFHSNSLSGTSLSGRKKKKSGQGSGTANATTTTNTNASNTTT
AGGSNGSSGGGRSRQINSMVEELRRGTGEAITLHYSTVPFGPEYTMELSRSTVSCIAPTD
PIRVRLPSKDIRPAAVVEVMCALHQDYPFSSRFLTAAQKDVLTLLGTIYKTLRCWSELLL
YLGYCGEDEMDGGVWSPSTSLGEFIHHRLNNKAMRHSSNLLLAGQHLTTWAVNLAMDCNF
LFTAMTRKFLFEISFCGTARSLVQMQENMEKYGMRDLLNIDHQFIRSYRLHRVKKRVWRH
HALMCAMEIMGKKQVNDSVLLEFEYYNENGSGSGPTMEFFSLVSDELREMKLQLWRRTDE
TPDEKYYHPQKGVYPRPLPPDSPEIDRVEPYFSLLGRFLARALLDKRIVSIPLSPVMLKM
LRGDECGIYDLIDVSDSLGSFIVALSIATHHGATTIQLPGSSTSCSVEELSLDFTLPGDD
AIELVKDGANKSVTFKNLFDYCDAVTGFMLRTGVERVIKAFRSGFHEYIPLCALRLLSVS
ELHEALHGHADLVTVEDLEANCQADHGYTMTCSHVRQLFEIIASFNEEEQRQFFLFLTGS
VHLPVGGLASLRPKFTIVRKTSSEPKVREQDQLPSAMTCQNYLKLPAYDSKEQMERKLRQ
AIDEGCGAFLLT*
>TCRU_3373 exportin 1
MESILDTSRPVDVQEFDRVVQYLSTGTPQEIMKAQGVLTMFKDRPDVFAQAGTLLSKSQN
LTTRFFALQIFDETILHQWNKFTDVEKGDFRVFVINLIVGECKSFNQIRSRKALLTKINS
TLVSIAKREWPLRWPSFIYDICSSAGPNEPLIENNLNLLRLLGEEIFEFAEKTLTSRWIK
RKKEALKNDFCLILQLCLSVLGTSDLALLKTDLECMEKYLAWMEPTLVFNEEVLMYIASL
VVGDATVAPVAVRCLSVVCSLDTDEGAAGDSQAQIAVRVFRTAFNNILNALPTSHLSVEG
RIVQMYEMGMDDDCGFISGVNILLISFLKRYYKLILYDDILLLSTHQMLVGMSHIDNKEL
FKSCVEYWWWLGEKLLRFPSPTPLHKKLSGVLSNVRFVLIKKMAKPEEVIIVEEDGELRR
EHMSDVEGLQLYNLMREALVFLTNIDPYDTQHIMTELMQKQLDRSEWSWHNCSTLSWAVG
AVSLALSEEQESSLFVVIIRGLLDLCKEMLGKENRAVIASNIMFVVGQYPRFLRNHPSFL
STVVRKILEFMKELFPGVQEMAVDTLLKIASQVPDQFVSLDNRKKSIAVDIAERWTELTS
LLQPQQTQTCFAAAGLMVQSESLERRALLLNLFLQDVNINFKAITERAAAQGASFCRDSS

SMMELIHVLRTFSSIASTCGTAFIDEMGIIIWDLHGFYRTFFAAQNALIAEGGPAAMVQQ
QDARYLRLAKKEILCIFERFVDNTEERDFVAANCMPSILTVVLEDYRDSVPEAKEPGALA
LVTACVNKLTTRLSGDCAAILDHTFDTTVAMICRNTEDFPEFRVNLFKLLQALNRHCFDA
FLSYTSRKEDVIAGMLWVIKHTDFATMEVGLLALDSFLENVAKSELAKEFFTSFMQRIFV
EVLVAAMDSLHAAGFSLHCSILIKLFSVSDMFPPQTPVVGRVALHEFLVENLSVIPTLTP
SLITDFVSFAYESYCDEAEFRRRFADFLIEMQVWGAEEENRLQAEEERRLRGEINPGFAS
PSTGPPEEFRPSSF*
>TCRU_3374 mitochondrial carrier protein
MSSTYWNELLASSKNKNVDAVVAHSNAEKEKTDLHASPLSHTVSSQFASATSTLIYYPFD
MLRVRFMSQDGTTYRQHNGQTYHSIRRALVTIYREEGPRALFRGCHVAVLGSVAAWGIYM
YTYRSLCCMADVSSFLGRTGVSYLASLTSTLVSSPIWFIKTRMQLEDATQSRNYATFRKG
LRHVLQTTGFCSLWRGVSLQLTLIIPNALNYPTYDFLKEIILRTRLEHSTKRGELTVTET
LVCSTVTKVLLLLLSHPIMFLKVRLQDQRWNMGDVKYTDIRHSLLLILKREGTRGMLRGL
NSSLLHSLPRAVTHYMLYEKTLGVINGYI*
>TCRU_3375 kinetoplast polyadenylation/uridylation factor 2
MRRWITEPTRRFALTIELRRCSTATKDNESLRPSRSSGKKGTGFFGIDQSKLDLALKLHE
IDFDTVVKATREALAKERARKDTVFGDGMNIKDLIPEGEKESPSAATSLSPASSAGVAGP
SAPATDENVRDDSSKRMERQMFRGIVGARDTHTTNSFAVVREQRKAEGARYGLMDALRDC
LLAGNWVKAMHLFESAVETSCKAVQNSFSANIVIETEETERLETLRRINATTPPRSDSTG
SSMHPGVLRWSGGHFYLLLKLLLSKHRVAEAERVWDVMKRIGFVEIHMDARTLNQCMKLA
RNTTLPDIVSPLKESNRHTEDRIKTFRTTFLLELEELAKKKELSLDGRNKELTRAARLAE
LVKNGRERVEDKGEVDPLNEGGLKVGDFSGLLRRCNSEESTARVLQMMDKLQMPRGGEVF
GALIAALRQPHYILTPHDMTGTVVGTKEEYDAHRKQRFERACQWFEQCPEDERSADVYNE
MLQIARGDEKADEKFQNVLLEFRGAPLALANAPTEGENKDTNNTDGEQGKKKKQVKLLPP
HWRVSPNGRTYEILIFRCKYLQEWRLLWALYDEAMERRVKGTQRLYQTLLEVAKYHPPQG
CDSNAVIMELYEEMKGRGIEVTGIKGTVSVLNAWCATRRRRRW*
>TCRU_3376 hypothetical protein, conserved
MAALESLNFNAETAIHYIHCVLSNVPMTLALSEVAPMVVPNPESPIVKELEAIGCRIVPH
QLNMFKEPLERKFGFVNFFIREALRAESQGKVHELVLLWISRELADQLASVNVSVTLGQP
HDCYSAVPFLRALHKECSFLDASKVSDSKKLETFLLSKIHFDDAGSNLHKNNGVDGVDAE
EEKKGDGARRAVGSFRISSLGWKELLWLSRGHSDLPPILITNGNISTATCDQEALRIFFE
GALFPLIRVLP*
>TCRU_3377 Ankyrin repeats (3 copies), putative
MSFKEFVHAKGIVWKQRAALRSIDHDGAMKRVVNVIKDGNESLLFDWMNTCDDINVRDSW
GNAALHWAAALGKLNAVTHLLLAQADVNVVNMNGATPLHCAAICGHSNIIRQLLRSGADV
MATNHEGQTMIDLLRYMGWDNASIQQEHSRNVSPLSPSGLENFGEELSFSDGASLGSLKA
YAPPDSPYVYGFNVEVKNHHHDLRTKEIGELSCVENDASNDVAGEELVCMQWHAEMEQLF
SRESAERNELISKYAKWMLQVTDTMKSDHSAPTERERKEVGVVLDAKYSPKGSDQFLLHL
AGDAESEELRVKADDVKDSPVAKAYVEPLETSTITPNISSEIIGGDVLLAREHSEDAEQP
ELPSLLRRIPCGNSQISKSAPVSNAPKASIEHLPPLRISPSTNSSPALFVNEKKPADSLS
LPSCNLVTGILSQNVAHRDTPHNSILTISTHSDKNKIKSCSNSSPANHIHCGEKALPPLK
LNKVNYNAAASVKEASKFRQGGVFCGEKRNANREYLSEEEKRIYEEFLRRSALRYLGRIV
ELRKA*
>TCRU_3378 hypothetical protein
MRTTHHLKSLINDLEVYERELKEKVQQKEEELKQLLQRIGPVTVIAGLPPSSFSGPWQLH
ITREADGKTDSEGVLSPSPCITEERRHLQYTVGGGDSVGKHRSEKSNAKQRKDTLLSIDI
DAVSSISFASTSSAESVETLEMAQQLLTACDRAKDRQARRVRFVSRSPQVLSNAVEALAA
VTAVEGETGGKARGTPLPLSQAYQALTGEGGDTDNKDVDALMLIRNITATSNMGLEATNH
ADLASRVHEDNEEPCYIRGRRKPQEQQQQQQQRQKEEVQLHPLHTVTSDASPTNAEDILA
VVELPKVNSTPGSCVTRSLRRHRSESGQTQQLCSGPPAKRSQISVSPTVSAAWPHHVSDT
QMEGRGAGIILACGPTLFFE*
>TCRU_3379 proteasome regulatory non-ATP-ase subunit
MAEEYEIGLTVRKVAEEFLKLKMDYERKNDDGCKRLLESIKKKLILLPTFLNPTAHSRTR
SEELTLTREVLEHGVLVSARLGDLDSFELYFNQLNVYYTDIDSSELPESPLYLLILGLNL

VRLLVRNRIAQFHSELEKIPNQIHGTNTYIRFAVLLERYLMEGSYKKLLNSRHQAPSNEY
IPIVGMLESTVRQEVAECIPRSYNTLSIDKAQKILMVDSAEEVRQIGRCHNWQLSADGRS
FVFTREEDLARKEVPFQEMLKNHLHFAVELQRIV*
>TCRU_3380 retrotransposon hot spot protein, RHS3, putative
MDATAIVTNCPEENDVKAMCIWMKRNRPLQEQAEYWKEVRGRMNKVGPILRFIFGKQACD
DRIKACQQAVDGMNASKFEGYLDIGNCPTAATCFKSL*
>TCRU_3381 hypothetical protein, conserved
MARMCASRVTWGQRGRRLPEHSRAFGSGHDQDSVGTKACVWKPSSPRPLREGHALHSERV
RLGGNATALYLWATDNSRTFHVGALFVGNAMGETLANALLYSDSALHLLQKGVNEKGSAI
PLARLTEELKTINSVLSTWAQIDASFAESSTPTAGLIGFLSNAASEHKLIDD*
>TCRU_3382 hypothetical protein, conserved
MKAHERTKTELQQAPKIAALLQRPSCDKQAGNTGGLATQVRQKHPDSKKGTTPKMEIKYP
CAVKLEAQQEEEQIHTKTLLPQRRRMLLRHSALCAQNDTYASVHKYNTVADSMMARRSGR
REGATHPATTPRWDHHRSNFAVQHAAFGAAAVTDRPTTAQHATEVDLTL*
>TCRU_3383 hypothetical protein, conserved
MLANTESNHTVGLSCSHKKKWETMCEGKTTTRTSTWVPKEEYQVALMLQGNKTSVHIDCR
SLGAEEVLLAGGRPPEVLRVRFGACGGHESHVKLTDVFLYNRPLNSTAMRALKDRVSAPT
REAATTLPEKIRVVDSVREHIAGDITEEK*
>TCRU_3384 hypothetical protein, conserved
MGGPLQPQGQRDVVRDDEADPRVFAAHGKRAPLRVVRCAHDWGRVAARHFGAVCVVRGRG
MATIVRDVVDTELLD*
>TCRU_3385 mitochondrial carrier protein
MSTSSEKKHTPWVNAVAGGLGGAAAKSLLSPFQRVVVMQQLGEHKEYSTLQLARHIREQD
GWKGFWKGNLTSMIIRVPYSGIQFVLYSQLKFFFQDICERYVRPRKGSAGDAHHRGETLE
LFLMKCGAGGISATIAGVAVYPGEVVRLRLMSGERQFNGIFNTIRCIYAETHSLRNFYRG
LGASLMQRVPDILVSFAVYETMKYRLIEKDFLSSHPSYKNIVSTVAGGGAAALASILVAF
PLDVAKRRIGMSGKGKSGVVYAGVRDCLRQVYAQEGIRGLYAGARVEAIRCVPQVVLMWF
FIEGFQSFLTDHCVH*
>TCRU_3386 phosphatidylinositol kinase related protein, putative
MSLPVNNQTFSTSSPSLIGFFDTPAASALAAPVMVADTAMLKARLTAIVNDVQMPNPKEA
QKSLSDLRLFLDAVAEQSGTHIQNIFGQHVTNNILFVVMDSVVRPALTQYCSAAGRRRSM
ATRAAGRGANATSRRQHELTALAAASSASLVAEEELDALRNNKAKKLCTDVVRPWRALVE
TLHHNLPSSLLLAQASSSLGTSSNADNEEARMAAVGAAAIGTRGGQGGHAKEESAASNAF
DGQWMTSRAAANRTEMSSVADLLSTRHLWARTANHTSLPLLWKNAVPLFSITRHALEVLQ
SEVLVAVCGQEYAKLLLHLFLFNGYLSNLQPGIIMELIMRLLELIERTVYEANSTDTTVA
TSTNRTSSPLLAEMSPHLDVGNQTRHGVEGHVEDDVYHPHVEQGTTFAALLVQLFRAPQL
MSAFTPFEDEATLLLSTSTANNGTMPFIMQRLVRLTTQRHMRPTSALRLETHLLIALKLL
VTVARDDCPLTSAVPLELVRPLCHFFYTTTRRDGWRMEALTFMTTQLCVCFSAVLGRNVR
ADIERHRPHGVRCSQEAAPNETNNEKGLGGNMSEGTTSACDSVLVSPYSLTLLHETVLPF
MLQLFKVVRSEFGHVSRRELFHFPCPPLAPLFEFGAVVFYLACSTHEAYMKLPSLSSFVT
PAASPAPAASASVTRIPTVSDDALSKRVFAQEKQREQKGIKRSREEEDVMGEKTGEHSRR
KKRETPPQPGETLLQLFRSLFFLDSVSDDATAGTTGKTRGTGGKDDVGNLTEKDTETERD
TDCLLRVSAAAGGSLSAAAVQDLVKDGSLFMLHLLAHPGTTLGCSESQCTLLVEHGVLPL
FPYYGVRLETLLLAVLLAVTPRASLMCQQDVFRWLVRRYMPTVTTQRDDESQPKEHVYRL
LSVLLGRGVVGRWPLWINGEERARLQAAAHFSAISTRIRAVLYGVSKSLPKTVANVPQHT
AALPPAMMASLIATATAGHRSKTGLSTPLFMAMLQFLSLYQHYGAISAEIIAAQRMARKK
HLPEDDLDANHVTTALKDHSLPHLPSAAVCLSTGVAFVISGLSHAVCFLPHDESVVWRHD
TDTAAAASTCSSSFMQALCWAEEYLLAACFTVTENELGWQHLPIAVGGAMSDPAKNKETS
RGHGGGGGKTAASLQRMKQLLARSRYVMDAMFIAPEKHVPITVCDAVEGHWLLLHEFFAS
STEEPLAGIDAVRTEKKGVGNVTNEDEETDVSELFLGRLRRGDVPLGFVEILSGEVMRAV
GELAERAKVIGAACVGLAGGSSLQQPPSPPSQQPQHQQKQREVDEDAVAMAHRTLCIHAV
QLMHLLLRLFRLGIWQLPQGHRSCPKGCEGSNSDNTNNNNNDTDDSNKIGKNSSEDDLCI
LSDIPHMQLFDHPLSILRRLSPEGRLYYAVMWICRHLATRLLRGSVVRDRVLMRSLLILF
HDVGTVLLSQSFALWEPRQVVLSFVGVVSRRALDEGLVKLASWQPTNVTSADAVAAVHAT

MRLSCSLLSAILTLVRRTHVLAASADPLVIPARVTAVILHILEKDGLIPATMEAPKGHRS
EHLMNHGSETEMSSTQNVSNLNPFLPFSLAVVDAVLGLLQVLHEAYGDTQARLAARQLLE
TLFARYRYSLAESIFLTFSHYAPPHHAVVSSTVADGINYDEESEEEETMSTNTCNREDVE
EVAHRVGQNLQTPGGTMRRGKEKKRGRNAHDDFLFEEGQLLEVFVDQLTDVSRNDMPVLT
PQLQCAVLSSIYGVLFRCESSVAEKLGKYLFSFLSIKTAYVVRRHTALQVRWLFNRFTHR
TYAVLQELLYRAREGIASISPMHSATSLRALGEAAHAAPMLEADIICTLLECWATQGFAH
KGLLLECLEFIGKNTRLRERGFATATAMTGTAESETWMSAQELCHYHCERLLFDWLWRLG
HPMEAFPVVCFGYAGLQQMWQALMPTLLPLALLCSARQNGAEGTSVFLHPSDLLWRLAAC
HKRSGDATSNNSRTRELLVGCVRAYFPDVMARLLVFAALSKRHGRSGGVGDVQEIAEAAR
IALEWMGSTFSTEYVPFVRHYADAILCRLVEIVGTMTPFVRVRPLQEAVTMLCDIIANAP
GLPSTTGPGVGNTALDDFHHDDELSSLATMTTVDRFFASDGGDHAYVLLQQLYAGLTHDV
HRGTRHTMLALLFEQIVRHWWGRELLQRVPHILHTVLRLCGNILLTCVDVRARACNVLLY
VWTQAVSNTAHLLSSSLPTRIVSADEAFISTVMLTLAEDSRVVASTWNAICRADATLEQQ
DWKLQHARYTAESFVQNEDVDEHEVIVITPGANEEAEEHHRHVEKKGEESACLHLEKNDG
QTLSVMEAIRQVTRILPLLKSTVHVSGAPASSFYTDAAAAVDRARPAFVKLIRVHIREGE
AQSRLQGRVGLVKKDEDVARRIQTVYEMCCRAVQHLLTLVCMGDAFDLSSQTMMATPLTR
MRTTTTPTTTTSTAFKLLQETTREVQLSVFRLLRALCWCFIEAGAEGHVDALLYPVGAMQ
RMLQGWPFDKVLHCVYRQHVEQLYRLAFDVDADVARVASTTLRGWLCGITTDNNGGEAIG
EGMDGASENVIMSTRTCAVASITHRRRGVGDEGDLIRRTLEEFSGDETKQEGDVQRASSL
PPEEKEEKEGDVKGSHASSVILLKDILPFRRQLFDAQPQKHATVLVETSLKVASLLDEVV
WDALRVHEDESQFLRLFLVALIRHYNLHHRFSTVATVLHHVLLRPFSSRSGGLILFIESY
LPIVLLHTMLVRESETQRSQRAVWSRALETHLFRRACGFLHMTRLFLRALNVCHSVLMTV
VRSHGVWSENLESNPLSRMSPSELEAAAASLTFPLFVRSEYGELPELSDVRDSYWLSDID
PLVLASAAVTCHEPHLAALFTELSGESLLGRRKGIPSAAMTIVGQPKCSVLFPSKRRPSS
REDASREGHRRMLRGDETEAEMTQRLATYRAAVYAVLVDVETTLRFRDGSDNGGMVISDS
RSSSIDGTSVGGGGRNGSAAVGNNSSVMQTAGDGGDLNFYMSPWEEAFFLQHPARLMERE
KSRGNWHAVLLLIDRIEGAMRPMRVSSGIHRSYFACGFSSSRLLVEKANALMQLGAADTA
VQMLAAALKRHAPPLLFAAAGDKRRGKHEVKEETGAEEEEEDYFLQLRSALAATLWRLGR
WDLDFLEETEGTHRHHKQRRDLLLPSLVSVEEGIFYAMRAAHHGEHHNVRRWTTQALQRL
ACQFDETNWFQSMLQAEALHDIEECGRLFLRPHSSLSSSTSSTVEAKRAGGMRRPPPWVR
ASLVHVHAPYRELELLDSVHHRLCVLYRQPQWWLSHLENSSERALRSNRPVLVHQWLSAY
SEFTGGDVESQCGVKKEENEPFQQDGTIFEKLITSHELPAIAFLALVQARAEFVGGRPHR
AIAILQNTERSSSEEPTSVMNGLLFPMPANAPQMPAIVQQLVVWAREARLVPLSQLVRDP
FLLLSVKNDRTGVCCLQLARLCHTLVRDITARLHSEEYRKLQESIGASHRIRVELEQQQQ
MSDGLSDAEKRILQRRIRTLAVETTREEDEWQREVEMYALYRRSALNAYARFLERSGPAE
SDSLLAVFAFVSLWLQQDERLDERRLKAAGSAASVTSGIIQKAIKNVPLHKFLPLFSQLV
AQLGTSLDDTNLESLVLHIAMENPLHAVWPLLALANGENFGPAVATMAHGGGSSGLHVVD
EKKVTLARQILRTLKSPTNRHDDEDEDGAEDDRSTMKEEENKNKGRRCGWREKIVTEAER
LSAAYIQLAFYRVYNKHRPDHRIPADFMIIEKGVRDLLIPPPTFTLPLFSLASRSALPAL
LQQLPKIVRYENTFTTPGGINLPKALRCMLSDGRIVRQLLKSEDDMRQDSLIEQVFCLSN
QLLAQDKRTCRLHVFTYNVVPLAPTVGIIEWVDGTIPLGQYLNGDPNGGGTNANRLRFQT
GAHERYFPGEPTTLECRARLQEANKSQKHGVLLDLYAEFSPALHYFFLEHFFTPQEWLER
REAYTRSVAASSIIGYIVGLGDRHGNNLLLHTQLAELVHIDLGFAFDQGKQLPVPELVPF
RLTRNIVDGFGVQGTEGSFRRHCEGTLGVLRGQRELLMTILEACAHDPLARWAVTVLPPQ
RQQGRPQEIALQVGEEEEGEAVRRTTPRQRDLGVSRGKPTYADAERVLSHVAEKIEGYES
GELLSVPTHVHKLLQTAQNTELLAQMFVGWSPWL*
>TCRU_3387 Zinc-finger of C2H2 type, putative
MSSVKALNPSLQARRDPYYKRNHMGRVTCTLCDVCCTDDNNFLKHIAGKTHCTQLERMER
HARREERLAVEEELNREACRRASQEKAARELLLQQQDQQAGSRGGATGTDIASFAPFGRP
QFNYCTENDPELFQTKVWIEFFFPQAAAGVRPLHRWRSAREQDMERPPDDSVVYLLVACE
GYVTIAVKFPSKLPRTSASSSVAMTAKTMASYGSEVAVTASDEEEGRYHCSWDPLKKVYS
LFFILG*
>TCRU_3388 Cyclin, N-terminal domain containing protein, putative
MQEEHGKAVGQNGIMRKEDVMKNTHETILSLCRKILGDPTVTRRAQLPCLSFLWGLPWTE

PLIFIYEGDSWPSVIYPCHLFEVSGGFLPVAANNGATAIQHASLTNFETGASGLSIEDED
YAIKVSRFELVGTVFSFESYRIFSRNTSQLNPSMKPEYNRPVSSAVIRVITNESGTPSDE
KAATHRIMQLKRRELWQIFFYSFIPLIGVTREHLEKRWSSCLTNGLSQSDMESGGISSSA
HSSQALNEERSLVSPNHHPGISIFHASTRQSSTLKANSLKFNDGIKNSAASFLTTEGAER
NSDFSLRWDIFRHLFDQEERYRTDVERCWGYWTNGWDKVNRFDVVDSIDCIITSLGSSRE
AAHVAATYLDAFVCMSQTVVRDATYFSGVLLACAMLSCKQVDRFFPSIKAFLRYLRPQHS
ITATDFHRYEMHVLKTLNFCLQPVTSLEIVEVLLYLCGGPAAQQDAERRYRLRELKRWND
GACGGGGGGNNNVYDNNANNNEKNTSDGYRKWTDDSFTNTYHLRWNDLCKLAHFIADLMI
RNTRAQEFRHTIMGLAIVAIAAEKTSWVLPAPLVELLPESCRVSIHAGEKAELDCESYEL
LVRYAATMRCRCEQDGTETDAVSPLWRALALVHECCEEFGTGEQIANSVLQRRYNINHAA
VMSGGF*
>TCRU_3389 hypothetical protein, conserved
MWGGVKARAHHGDIFRGWSASQGLENLRRKLYREPRPWYLAWSLHHHKHLLSDHHCARLL
AWCTTVMDLEKRKTAKRQAYMYSQHVFQTMVHSHKVGSETYQEFFRLCAAGRDLTTAFRW
QQYLVETGHPFSLSHYTWLLHIAALSPNDESAEDMAEAVWEMYLARYFHLVKHEVTDGNA
TLQFQTSDEETRELEGLFRAFKQLRSKIYHCAKPELLEFIDALPDGSSNSVSNDTWPPVI
RHHTFRHLEAGASWTPPTLSRPHLRDSILHESFTNDLEQAAFAGNVQRVVELVDEYLRRI
SAEKSKESAKKHQAGERDIWHCFSDPSAVAFRKPLVEAGGVTAELYHYLIVAISATKPSL
ALRSLRCMEESNLQVLDLTRAVMIVRCEGSSADQQALLREQIKEIELRRKIDEDYDLTRE
VELFWKFCYTDFFHYRNALSRMELYLILMEGLGPVRVQELLIEAKLHESCSMEDVVVVDE
NFRAAAAVYFRSCCGEVDVNKALDDITEKMPKLDISLVGSIPHFSDYALPEGEFVATDVS
ALRNKLVGFKRIFLLDSSFVETSEHFITVGQASDVENEGSSLLLIPYCCLQQMSLTIEQE
NDIVTMDEALQEANKAEPFIASQRLRSLFALLRDVNKNRRTRVLHFSECLMAHSVMESQI
KAAVLDPAHSDNDHLFLLLAMIRTIAPEEADVVLCTDDAALVKIFHDDKSASFFVGKISV
MSSQPPPQVLQNGEDLIDDNPELCMSMDFEPKLQTSTYLGEKNRVLEGTAMLPSKTQLPE
VDEDVLGTAWLDMLDGEEQREKPMDLASRTSYSQFEDSKTGAAREMKHVGEKENEKEEDE
GEDDLAARENERLMNTYDSEHAVVPVGALMAKASSLGSVFEQFDVVGPDAEVERAMASPA
PYETPGQSSLRPRRRTLLEMEARRNRGASNRQRFRLARRLSNISGGRVPFNFRYRVLEVN
IADPKNKSFVDAYKKGMAKKRESFKRKFKKSYLVGGISP*
>TCRU_3390 Pestivirus Npro endopeptidase C53, putative
MEVSCSIADKPQTFDYFTERYYHQYVVRNCKGVEDNNCRLLVHSNGLCVLCLDGSHAAIQ
SHRKGFCTDEELDASHPRIESVTFGSGRGNAKLAPQNVCLVGKRKKNATKCQEDTNICVI
TMTDGTLYKIPACVAGFVLELNPAVQERPELLVTAPHVEGFIAVISPNYNKLDLGKYQKV
WEATGGDIVDDNDADDCTKKYEPQ*
>TCRU_3391 hypothetical protein, conserved
MKSNNNSQTEGVAPITTNSQNEPSDGHAETEPPLPTENGNFANNEADKSTEEGIPNNDPA
SDGTGTREEKQNENKEANPKETVPTTNAATGDTATAGDSDGSTAVSHTTSSLFLLLVACA
AAAVVVAAWE*
>TCRU_3392 hypothetical protein
MDWLPVPLHRTDNNKLGLCTFIRGKYLVSTVLPRDVVAENSAAVTRGGGNGGADGAGRRS
GRCVHVAAEVCGATLLKREIELVEIRWSSNATSLAGTFLFAIGRCTSVVVLQPTGDTHSG
KLMLQVVHYIEAKGVVASPPFLPFPPIAVGAARSGHTMLVVSHGTSVAFYQAPEFSTKLF
VAVDEVVTCVGSYERRLTMGLASGAVVDVRSGMPIAKLQSPPLLIDRIEGTSNDEETWLV
ADTSGECTRIYRRRQGDAEPRTECVLSIARVRRAADQPAALIMGCSRMSESLRITSDSAP
QFVQHPLQHLMIARGPLLAFFTDGVWLLAPQ*
>TCRU_3393 glycyl-tRNA synthetase, putative
MRTALPSRLENLVRAEFEDTCRRRFFFGLAFDPYGGSAGLYDLGPPLCAMKANLLSYWRQ
HFVLEENMCEVDTTSLTPEEVFKASGHVVRFNDAMVRDTVTGECIRADKLLEEYSETRLK
DATLPIETKQEMQTLRNRAAGMTPAEMKAVIEHYQVKSPKGNALSEPFPFNLMFSTSIGP
EGDRVGYMRPELAQGIILNFKRLLDSGNAQRMPFAGACIGTAFRNEIAPRANLIRVREFT
LAEIEHFVNPNDKSHEKFESVRGTEFWAWSRELQDANADPVLMTVGDAVAKKIIDNETLG
YFIARTVLFLEAVGLRFLRFRQHQRDEMAHYAQDCWDAELLTSYGWVECVGIADRSAYDL
TQHSAASKKDLFAREEFAEPRIERQLQRKLQKGAIGKAFGKNAGEVMEYLNTAPEATCVA
LGEKLASGSVEITTANGFTATIKPEMVQFEFKEVKVTGRNYIPSVIEPSFGVGRILYALL

EQSYWVRRDESAKNEKRAVFSMRPVIAPQKVAVLPLLVKPELLRVVEEIRGDMVLRGIST
RTDDSGASIGKKYARVDELGIPFCVTCDFETDGCVTLRERDSARQVRIPREAVADVVAEL
SRPLRPREWSSVETEFPAQSTAA*
>TCRU_3394 Snf7, putative
MLSRLFGRGDKQAKAREKQQSGGPGSVSQTLEKLESAYELLEKREGVLEKKMEMELERAK
QYHAKKNTQAALQCMKRKKAFEEQLTNISAQKQNLETLKFTLQNQNMNNELLEVQQRVKN
ELRERNKKMDADRVEENMEELQEEMEKANAVAEALRQPLDSQLLDEDELMGELMAELQTA
DVKVAESAPALPAMPSVPTGKLPKLSAKEEEDEDEEALRALEAELQS*
>TCRU_3395 Leucine Rich Repeat, putative
MLPIVKEYMTECAQRRVEPYYGFIEHAGAGEIVTDLDFAPLHSVRVFALTLLRCGPRSIA
VRGPKLQVLALRYDSNKGMHHPPRQSKRKVSYTTLLSQRLPEDVIVAAQNRPVLRSLIDG
VRVACMTYTGHLVRVELCGLPLREIREVVTRLFDVLQHCGLLRVLNLNKSKLTDQLFTRL
TTTVNTFPSLKEGHFSECGLTDGSARGIHSLILLNRGREQNAMWRASLRDGGGGHVATFA
RGLEVLDLSGNCLSDATIRRLGLAIAHDASLRVIDMSRNTVTTAGLKEFLQRGTLQESAV
ESLDLSKNLIDTKDKFFVGDNFACQHTYGEQLLLTRLDSRERHRSASTAPQLEVSGAAVP
QLRSSAADGAPLEIHHPRAVSPEDVGAGGLRSRESSPAAVLDTREENAEVCSQLLRCPGM
ERPQANRSTWTASPEMFDRQSNASRRNGDRKASERSRFSYQETGKQQENQPRLNQCPPPH
QTAVPFFFPPLLFAPTGSGDGSQQQQQQQQWCGVPLYVPVPVSSLGAMAMPPPARSTRDVA
VGTSPQPDTMQQSTSRSLSTTPLADGDVTDDGTAFEKMMMEPLQTNDWELADWRRNEQRF
LESLIVRLESHENATTEMLERNYQATCDRLGAIQEEFSRQIHELLQEQRVGAERFRGELR
EELLSERHQQQQQQDPEAAMAEQLAQLIDTGMKRIHDQMERRSASASGTAAKAAGVDLQQ
PTQEYLRVVKERLSSLGW*
>TCRU_3396 eukaryotic translation initiation factor 3 subunit 2, putative
MNIQGLALHGHMKPVTMIKFNREGDLLFSTAKEPNISVWYTKTGERLGVFNGHSAIAACD
VNNYSTLLVTGGMDFKAKLWCVESGDELANIMLKSPARAVGFAHDDSKVMVSTSRKMGEK
SAIQLYNLPFAVPKEDYIVHPVKTAFNPCAEFVSEGDDITFAVWGPTNDTIYYSMSDGSV
AILDVETMRTIRTQQPHEETVNRIGFDCNYYTLITASKDKTARLLDSRDMSVIQTYRSDV
PVNDASISPCGDHVILGGGMEAQDVTTQGGQTSFEVKFYHKVHEKQLGQVRCHFGTINAV
SFFPDGRGFASGAFDGLVKLHRFDENYEAAPGAKPVWSPDSS*
>TCRU_3397 hypothetical protein, conserved
MCIALGIVAEDDEAWRIASEAVAAEKEPVFSGNNGPFVDVWFGEQKLFGLVQRVAPNDFI
QVAQECGEKSDDAAATLRMRVTHNVSFVLHLSSVPHAMLQARGAPEDKFVNFMQLVVDYA
SLLRRGMKDEFLGVDPESDAEYIRFTPQ*
>TCRU_3398 S-adenosylhomocysteine hydrolase, putative
MTDYKVRDISLAEWGRKAIEIAENEMPGLMELRREYSQSKPLKGAKIAGCLHMTVQTAVL
IETLIQLGAEVRWSSCNIFSTQDNAAAAIAKRGIPVFAWKGETEEEYQWCIEQTLKGFSG
DGFPNMILDDGGDLTNHVLDHCPHLVDKIYGISEETTTGVKNLYKRLQRGKLPIPAINVN
DSVTKSKFDNLYGCRESLVDGIKRATDVMIAGKTACVCGYGDVGKGCAAALRAFGARVVV
TEVDPINALQAAMEGYQVLLVEDIVEQAHIFVTTTGNDDIITAEHFPRMQDDAIVCNIGH
FDTEIQVSWLKANAKERVEVKPQVDRYTMHNGRHIILLAEGRLVNLGCASGHPSFVMSNS
FSNQVLAQIELWTQRDSGKYPRGEKAQVYFLPKKLDEKVAALHLGKLGAKLTKLTAKQAD
YINCPVDGPFKPDHYRY*
>TCRU_3399 hypothetical protein, conserved
MLAALLPSVVLFVLALVAFSGAVTIVHGGFIRLAIGALIVALLISIIFSSCAVLLLEASS
SFPTDVVPVIFLRGALGAIAQPTIAGIVLLAMLVVLAMSTSRVCGLVEEIQILLDAHKAV
AEAEVISSTKR*
>TCRU_3400 hypothetical protein, conserved
MDALYLMSRAQFHQAATHISLYREDASPGYRTLGEECLRLVGLNPSRYVYWNVPNMSAYF
GKTVPVDVHGGYVLVDEGAAGRLATSYGVLRYAYLSAAVRAREGGRWRYDFMTMNITLAV
GVAGGFAALSVGRSRWAWMRRHPVGGIAVSLLVFLTGTVASRQAIRVLGVGIVTAHNSHK
KALTKLNCADCFDDVNLYTAQQVEDLRKQEIPRQPGMPPPPEEFVKRFERGTQLQIKMLQ
ADMDEVRAEKRRIGSHFCDVHGRLREDEGYAASVVLPISPVDTQRASERLRAERTEKKAE
*

>TCRU_3401 oxidoreductase, putative
MSAKKRAFDVLVLGAGAAGCAAARAIALRYPNATVGLVEQGTRRPLPLVMRVPLLTPLVA
SVRSTRGFLRKYHGVPEECIGGRQLKYVRGCALGGSSCCNDMRYMRGTTRDYAAWGDPMW
SFEQLLPFFCALERNSRGGSLFHGNSGPLDVTDVPRANVGFELNILWFESCEALGIPETM
DFNAGAADGFSALQSLIAKGVRVDVFDALLERDRHLFPNLTVMPETRVQRILFDGKRVSG
VENRVGRDVEVLFSGRVVLCLGPLESPALLQRSGVGAGGRVAEIPDVGRNLIQPTSATIV
FGVTHNANLRSKSVSWRNAPYLLRQWREYNEERSGAFASLAEGAAFVRSTPTAAWPDLSL
TFFATPNVRWCGWRPFDGFAVRVAHHYPESRGEVDAVDEHNIRITSRMFSSRKDVQCMDE
GMRWVGSLFNDARSAFHIDEKGRPTSPFASLGVHVRHPRNALHTQQGAAAFLAEHAQGTG
DLFGTCALGKVVDSTLRVRGVDGLHVADASVVPTPTCGASCVIGAAIGSRVASILQV*
>TCRU_3402 replication factor C, subunit 4, putative
MSQQLVPWVEKYRPKSMEDIVGNADAVSRLRVISKEGNLPNLLLCGPPGTGKTTSVLCLA
RTLLSDQDGGDTTALKEAVLELNASDDRGLDVVREKIKLFAQTKKTLPQKVNESSQQKIN
LHKIVILDEADSMTPAAQQALRRTMELHSSTTRFAFACNNSHKIIEPIQSRCAVVRFKKL
THADILKRLTYIIQQENVTYTDDGLEALLYLAEGDLRSALNALQATHSGYGMVNAENVFK
VCDQPHPLLVESIMDGCLQKKDLPSAHKELQRLLGRGYAASDVISTFFRTAQNPKLFRNE
QQQLAVLRIIGETTMRIAEGVGTPLQLASMLARITMVNTD*
>TCRU_3403 hypothetical protein, conserved
MTSYAELLAACTEFQENFASLNGKYNAAVQEVQNAKEANERLSKQLEAMRSSEDKLQHTI
RELKKELHEAHAYKDRYLAANVSVEHLKGRLENARRAVADKMLEHEQQVEQLQGQIRDLL
QRVSVCADAQTTQKLKKQVLELEERCAEQSGHLLEEKERGSQQLLASHTALREQQARNLE
LEQRCRTLEAEITQMRSVVRRSADLQNEAAVAREKSASDARRAVMQAESVAAELRETRAR
LNAVVAQHAEEMEAAKSAADDEKSQLLERVSQLSAAVDDAVASRNAEQERYAVLQRSLHE
RVEAVRTETRLEVTVLQKSLSDARAEGQQLAWRVEQTKMELEESRRAVQQREAKLESCAA
ELRQLQTRLDAVTQREAWLSAERDHQLAQLAAAREQAQHLAASQRAHEESNLAGERLRIQ
LQFLEEELQGARRLAEASDSHVRDIEEAAERRLRALRSELKSVKKNARAERAKADAVRRR
LVQALLEREANVLPQICLQTQRPHETVATAPEQQRPAVGLDVLTLLRGQTEQAAALHHRL
VELSR*
>TCRU_3404 14-3-3 protein 1
MAEGIKWHNAAIQDELVPKKSPLEFKLPDSAKELVFMAKLTEEAERYDEMVLCMRKLVKL
NSELDTEERNLLSMAYKNVIGSRRNAWRIITSIESRESAREKSENLQLIASLRKEFEAEL
AAICDDLLALLDTYLIPASQGGEVKVFYLKMKGDYHRYYAEIAPEAGQRQAALDAYAKAT
EVANSSLASTHPIRLGLALNFSVFYYEIMKEHEKGFQLARQAYDEAVTELETLDDEAYRE
SNLIVRLLRDNLNLWTDEQPSS*
>TCRU_3405 DNA topoisomerase III, putative
MPTWLNVAEKPSVAKELSLVLSSGTCRMGNSLSRFNPVFEFDFEGKQMLCTSVAGHLMND
EFPPETRSWQNYPLVNLFTASITKHVKPEMEPVKKNLEALGRRSSNLVLWMDCDREGENI
CFEVMQVVQGVNRSISIFRAHFSALTRRDLLSAVRSLKAPNKALSDAVEARQEIDLRIGA
VFSRFQTIKFRDAFSGMPRVLSFGPCQIPTLGFVVRRHREREGFVQEDFFTLVMRHGQTV
FHGCRGSVFDQLAATLVLETMLESAGPNAEALVVDVVRRPTRRHPPVPLATVALQKLAAT
HLHISSEQCMTWAESLYQEGFISYPRTETDSFSFTDDELREIAGVQRENPEVADYVGAML
DDSSHKFRRPLKGGHDDKAHPPIYPTKLMRAKHDNRSALYNLIVRHFLACLSPDAVAATT
AVTAVFGEEKFTTSGTAILEKGWLEVYPYERWHSTCLPAYQQGERFVPKDISLEKHRTTP
PPHLTETALIALMDEHGIGTDATIAQHIKTVLDRGYVKREGSFLVPTALGIALASAYEVI
GLLSLLQPQLRAQMELAMSDIVSGKASKAQVVDAAVKLYTEVFQKLSLGSKEMYEELCCH
LSPAAPQTSGTHAYGTGRIEKRRLVHCGVCKNPMDLIEQTEGERDTWCVRCHTCLKDYRV
PNGRLNQLSSCGQSCILCGFGVLEIRNTEKQTSYTVCPYCFTSPPPGTDMESFTGFRCFQ
CLADCPLAKGIESVAIARCSACKQNDIRLCTGSRGAFLSCRGFPGCTFSVTLPWAKGVRP
TPTMRCASCQAVMLQFNFGGMPSVPGLEEGDCVCVFCDGRLQDYISVKGGFTRDNDHTAD
REHANQQQQQPQQEHPAPYSLPSLRGREANNRSKPAKGSATGTLCECGVPAKQLVSRKEV
SKGKRFLTCAARKCSFFQWLD*
>TCRU_3406 hypothetical protein, conserved
MSDILGKNQQTTPVGVPVTYYETCRRIEYLASLGMPQVTPYTEPTKPLVATLPEPRELLL
GEGTPLSSPSPVLLPVEPRRTVTPSTTSTLRGTCRKKTELEKFDMDAAYAAFIQRLEQRL

DEEEAKNSKELGSASPLQ*
>TCRU_3407 CobW/HypB/UreG, nucleotide-binding domain containing protein, putative
MSAVEDGEDCPELISAKVPVTILTGFLGSGKTTLLHYVLSAEHHLRIAVIVNEFEFGKTI
EKGLTLKSSQKPDDEWLELNNGCMCCTAQSQTVKALESLMTRKGTFDLVLVETSGLADPG
PVAAMFWQDEAVCGSLFLSGILTLVDAKNICRYLHDPVVSGEAARQILMADRVILNKCDI
ATEEEQGRALAEVRCINPVVQVIHSSFSRLENLQEILFLNTTRRLVELGHLHQDAPSGIT
AVSLEFFEQPNFLALRDTGDVDLICRDLLYKDHDPPFEVVRSKAALWVKKAGQYSLLQLQ
TIGELFDVRPMEGQSVPMGCSRVLILGKQLDKDVLRNIFLRYMRVDADP*
>TCRU_3408 tubulin binding cofactor c, putative
MEERFLKTRAEREEARQQKTAVPSSVTWDKLEFEQQARDFERDVMQFIADGGVRAAQEGV
NRFKLLVQEATSNNVLTSHEMARSNATLSRLQALIDAKRDTTNRAKAFKFSSQPKAKGAV
IMEMQAAASATQRSNEEQSHASNVYGYARDRTLFVGPSKAVFLRACENCEILILPVPGTV
FISDCKNCRVYVACHQLRLKNATNIDVYVWCASTPIIECCSEMRFGPYSCWTGLLHSTVE
GHNYATHEEWVGRVGEQRIDEHTANSYKTVDDFQWLRKTPSPHWGVLPQEEWAMSTERFI
QGEPPAK*
>TCRU_3409 ADP-ribosylation factor GTPase activating protein, putative
MNPLARRVERNRDEVRKLSQKCGNRFCIDCGIRGPLYVVTNFRVFVCSTCAALHRSLQHK
VKGISMTEFTDEEVACLNVGGNDRAARVWLASYENNKPPHGSDIAVRDFIVNAFENMAYV
NREELERFQNDLKAVGTGASCCHLPHTMNLPLPEHGPQRPLTSLPPEQPTKEKPLPPEPS
SSSTGLQDLSSAPRPQVELFEQSTPQTSSSAPVNFSAPTTDDDLFAEFVAAAAPPASSVA
ADVPTAQRPLDGWLNSVTSGHPHVNIPVSIPPGVTMGPGPYSTSQPLSAGKMEYPPNMLS
QPGFQGPFYVQQEGYGGMTRGNFFQLQPQPQPQPQQFQYAFSMGPPAGPNGVPYQAPTAQ
GQTPSGGSATLMNMCGVDFFQEPPANPWGNFNSSESTKTEPVKTKSEPQNDIFASLDPFI
GKW*
>TCRU_3410 hypothetical protein, conserved
MANSAMPWAYQAVVSYSRMNAAANRGGVLRAAKCGGLSLEQQSPAPNLLLSQSANDVRRA
VWLLHRPRGVLGLVVSPRPGTHYGSMPHEAAPLGSPGVKRVMRHLRSLSFRVAGDGPTCP
PMVVHKNHLIGWCLGGKAAPLYAHTIDELARLKPGRIKTLSPENAFVCVNYYEWDDWREV
SMEPGWSWHHADEHVANGLVSGQLSPDELHNLAAEVA*
>TCRU_3411 hypothetical protein, conserved
MKDVKDEAEGVEGRKPPTFSEDEDEEGSEENDEDEDTEAQEKRNEREGDQEVAVTSDPNS
GETNLSRSGQEKDTAIVSTQGISPSNSQESIVNPTQPEVEGKKDTDKSRPAVEKALRPGK
GENTLPGGVAEGNLPSPPEEGVDSRKQDGEYTASEGEKSVPSPETAATSQSHRDNGSEGT
GEDTKATTVTANTTDTQSTQNSDGSTAVSHTTSPLLLLLVFACAAAAVVAA*
>TCRU_3412 E3 ubiquitin-protein ligase KCMF1, putative
MDGESNVASTPIPWSLMLEYERADAVLQLFRQFSFFHADKLLLIEREEVICSPGMDSERM
EYLVIHGSVGNARKEAFLKLLRLARLTLVQDMASQVVVCPQIGDLLPNTMLHPLWETSQV
RSFKKWNRIYWAMRCGLLKKIKRRREMIQAHVEQVLGLKAENLVQNSSVEAAGISHVNEE
AFYNSFWNLLELEQEEQNAPLELYYTACSEERVNLSTVIATVPTLILLWASWDITSMEWL
RENLFNVTKILKANTLQTFSSTYSSSMKDDPWVETVMRFVQLPRRIVMYRRTRLKRYLKR
AQIVLISVDREKAAAVDALNGLFSEVSGWKSPEVPLIPLWCGPDGLQSDLATELNIAQLP
FFVATQLPDKQTRKLGDGRFPRICYITSHTEGEIAPDACMKNNEGEEVSERGFRLMNWHA
IEKKEREIVMSAISRFLAASDAPLRFIARVDRTYQMNPASLAQVKKRLKPEVTSFVTMSG
TISTLDLMKLKEKISVLSRVRNFFFDVKIMKPSIPLLIQLNPKTPTRYVLGSARNITCSE
CIRDVLIDKEHHFRCIQCDQSEGVVCWMCFAAGKHPQHHVLLRVPTFSETTLNLLWGPSN
VSPLALFCGKLVSNINETHVGVYCNGCSQLICGVRWKCAMCYQYDLCDCCDEKRNRREAL
KCEGDASTPSGGISGPIHTFTPPSTHPKEHLFLCIRHGCGADGDACLNPVIEKNSLPLFI
SQ*
>TCRU_3413 developmentally regulated phosphoprotein
MRRFSGFLRVAAACLCRNKVDFSDLQRELQEVKKRVGVIEERQTLQSLLAFYSSRPMQKL
SNLNEVIYYCLKTGYNAEIFCHMELPVLLARLIMAIDTLPCGLNAMPSVLLVRNTYLDSF
KKLIKCDFPDNDERILHFRRVVADIEEAHSKRDVLGTMAMGLLELKKLLSRHGQYVFSQN

KKPSGSAFELTLDEALMDVTKPMDNFSLRMVNYNFISRMLLTLEENDDDMVGMVDLKIDL
ERVVRNAVDDAKEVCTQHYGDCPDVKFIISKDANTMRFPHMSSTITYIVVELMKNAFRAT
VESHMERNSAGMVDCSNLPPVEVLVNIKKNAKHACICVSDEGLGMTRAQCELAMTYAYTT
VKRPIIQHGADEDSEEERNGVSPLAGYGFGLPMSRVYAQAFGGDLVMSTMEGYGTRVYYY
IKP*
>TCRU_3414 Enkuring domain-containig protein
MSGENIYNVLQEGAPVTTGPYTGNYEKHFKERPKPAYSTFFEKGKEYDGSHVRYFKQRHA
VFGPVIGDTVDPKNFLRSGRGVKHSVPPVQQRKIFTKPLLDHGISKSRNQGRERVAAKPN
ILSAGEEADELRDANGLEGGYGRNGEKSGVLTTKESGAQSGEEILEDQGNIDWKHYNGPT
GYVKESGNGQHGNEEKDFAAHGANIDQSLHSANAGYLGGKDLVDGKKDFATSNIIQVSNM
VPKRRKDQPENPTSRKTFGQAPGYLHRVKREIDEEKMRLKSIEEMQIQQKIQNMKKFVHR
LDKKEQLQLLEKLRIKLNEKSAELIKMPFLKDTYTQILRRAELEKGIKQIEVAIAKLDKD
AVFVYNDDPRCIHWTKNAALEEATLFASEREN*
>TCRU_3415 hypothetical protein, conserved
MGARPRAQDYGPRHAQLLSLEREQHRVVTDRLYQKVQKLSLKWAQEREAHASTTLLLFKT
QMEVCILQEESARHAIAAAEAEDRIGFTQLEGSREPVGSHRKGADSCVLSQFFSAAPRKK
PHILYAYDSDLLRALQRSEAYIAHLERYCHEHLPCFSPSAFFASHYLHGSPCSETWSSAL
AGTALDGDTASYVPLSPYIRTIM*
>TCRU_3416 rhomboid-like protein
MASRLLQRRGVSLVGPAKSTFFALYNAQKQGMCVSACRCPGRAFSSTRTIQEQKNTGDYL
AGFREVKTYEGKGEEGEGGKEEEDPFSAGPGQGYLPFPPPFHTFNVVMLLFLANVITYFL
MNFSGSDSLRDFIVDHFTLSHENAGKIYPLLTNAFYQENLLQLLIDCWLLWQFGNTMLGF
LGNTRMTFFAVLCTLGGSFIHLARQKFELYYGMDPLEVRGRCYGPNPFILGLIGVEGLIF
RHLNFIQQPPVPFLVLTAFVMVIDVWRIFTTRPEEHGASTGGALVAYFFWALPTRMLGLD
KLTAAL*
>TCRU_3417 Protein of unknown function (DUF3808), putative
MSTEVSSGSNDGPSFHHHIQDAVHMIWNNQCSEAEEILFPYKEIHPRYALEYANKYLVQT
LMNSTNESREILLELFKVADSLASAAKYSEPMFDFDVENDGDGTGMSMCLPSKAERKISK
KSFNGRRKAAEKAGQSFVDNWKLECDVIYADALLMRSVCQLMMNSYFKGGVNLRRTWGLY
HKLIQVVEADRTNQIPNELKMCVKYGTGAFYAFLALVPANLMRLLNVIGFISDKELGEQY
LTEVFESNGVRSPFAALVLCTLYLFLPTGLGRVEETLAKAKRILDAMNQKYEHNTYFYGY
SNFYHRKRGETAEALMAIQLAETNAERAGLVPILIRYLHADTLFMDLRFEEARDRYAALL
DHLAKTKESFAYTGQVVLSLAACFVMLGDDAQAIQWLKKVGSMYNSRSKNDANSPKFASR
VLANPRLLPLCGVYMLYINRDLAHMKVEQAERLLEELTRVTKGKDLSGPEPENMFTLFVG
VIHKGCDRIDEAFGYMSKIFANEKRVPSDSMVLPYAYYEAGELEYRRGNYENAKALFTKG
QSLKGDGNETLMNRYNIALKQLKRTMEERKGA*
>TCRU_3418 hypothetical protein
MMTAPAGREQIIAYDSDSDDSVVEDVAPHRTVAKRAEMILAQQQRFRSVGEREAGKRELN
SLGGAYGDFCRGAPLSMWVAETACTPRKLSKSDKNEACATAALQTPEKNRNARDDEILVP
SPKTGGRVCGYRSPLPKKTTSPSIVSDISLASACKGGAWGGQESIGRNSPTASEVVPLTP
DGSATSSLLSQAERSAVELLACDIDSVTRECWRASQEQLLPSAIYAEEDRLAAAAAHTTS
PGGTAKRKRPRGVATPEKVNDGTARCLQFETDSSMRSLRPSVSEMALKRLNKQTQQRLAS
RCLMLPY*
>TCRU_3419 Protein of unknown function (DUF1637), putative
MLNFKTVLQRKGGQFCSASITKALAQLTLLDLKSYVSEEAAQRCMADCNAAISEHAFSLH
DALHSSGPNCWKGKLDGRVGCSTLYETDDIALCWFVVPPGGVLPLHDHCTMVVWQRILFG
RIHITSFDWAQGFEPVDTQLCNRRGGEAIVVFSGTSEARSNASSPASLVTSFGPHEGGVL
HEIENESTEPALFVNVMTPPYNKPPNNIECMYYSLSRLDGGRATPLESQSDAACRERLTV
GQRVLLTPRPTVGKMPMNVFIPIQSPW*
>TCRU_3420 procyclic form surface phosphoprotein
MCLFGIVDAVGDFLKSVVACACCLLIGSPALIITGSILFSQRDLRKAFSDAVKEFNPKPM
NDWTGTINDVPITLRRESLNVKSVDGATSVFAEAVVSVSEAPPTELPVFVNVSAVFPFLR
KAPFISVKKLFYRCFSTECKYGKDEKCKRECRNFEAKCIGMGGVFVFTPTPCNVGEVCGE
CRLIFYLRQLYLVVREIGNEKYVEDTKLRSARYAFGDLDNEYQPGIPSTVTVRLYSSKDP

YIALQRLTKGTNDLGMNPRTVGIVLIVFGCLFLLLEIGVCTALICYWMRPNKKSLGTPLY
LAPASQGTLTSGGPGDTAPNPYAHLVEQTGQGYTYGQPLPQGYDYDQEAPPAEQQDREYG
VSLYTQDTHGSFSPPNADNK*
>TCRU_3421 Cathepsin propeptide inhibitor domain (I29)/Papain family cysteine protease/Peptidase C1-like family, putative
MWNLICCFFFVATATTAVATAVSAITVTSNEDYLAQYTFEKYIADFGKRYADPEEHRKRA
AIFKENLAKVRAFNGVLGRSYRLGINKFSDMTKEEFNAKFNGRVAVPQSTRSPQRASYKH
TKATFPEALNWQEAKDPVLTPVKDQGSCGSCWAHAATESVESMYAISSGKLLTLSTQQIT
SCVNNTRKCGGSGGCGGGTAQLAWEYIMNTGGITLDAEYPYVSGGTSVTGSCVLNRSMPR
VVNVYGYASLPHNDYEAVIEALVQKGPLAVSVAASDWMFYTGGVFDGCGKDGENITISHA
VQLVGYGTDNKTNQDYWVVRNSWGEDWGEKGFIRLLRKKQDELCVFNNAWNTVGGGCADD
PNITIVACGMCGILYDVAYPVVTKPPGRGRFLWAAGTAFGGLCVVMIVLVILSRYRGTTP
KVCEAFREESEALN*
>TCRU_3422 Monooxygenase, putative
MFHPSLRALSPVIPRNLFNVVVCGGGIVGAASLASLQQLRSRFLSEISREDSTAGHSVYA
SRLSQLLLVDSVPRPQYDAQNVMHTLRTVSLTPVSSKLLDNLGCWRRLQTKHPYYRIALR
HEQTNSPFFPKQNRPVSYFMSYLLGGNEATAEPLLEFTDLNRPLGFVSYNAEINTELINV
IEDQQKLNDANVAKDRIFFGSTVTSVSLPPQDVVDGALGGAVLRSTSGDDSLNFSLLLGC
DGRGSSLRELLATGAVQHDYAQTAYVCTVRLEHMDDGNSCCFQNFFLNGDVIAMLPLSED
SANIIFSTTAARARELMAASQSDLVSELNGRLHAFAPKDIPRILGVHEGTVNGKQVRGQG
TFPLRLNFVLRPYSPRCLLLGDAAHGIHPFAGQGLNLGLYDVCALIEVLEKAIRSGQDIG
GVCAVGQPFASEMMLHTSSMITAMEGIYTALNAAPSLSCAGMKVLQGLPLVSSFGKQCIT
NFSSGGLFARRHSDCFLLL*
>TCRU_3423 Transcription factor e(y)2, putative
MKHTECKVEGGPVSSGARIYEEMSNVQKQLLRDYLSCRLGTASNWRKAVSQRVEEVVRRR
AQSGESLDAHDVVGEVLPFSRSIIPSEVREGLFRQISDALRLRDERD*
>TCRU_3424 hypothetical protein, conserved
MSGTKGEIANKIREGAQRRGEEIQRIRQLRRERREELKERASNASSAGNSSFIPYILISL
VFLSFPLSEPKVDWLLVCLFLALNGTWTIFFRSTSWINWAIMCVSNIVLVRWSPFIPDLP
RIISQLSPTHVAVYVSGNIFLCIILYFGYVERRAPWNASSRKSGKKSTRSDKGDGEQVES
LEEFARRRDWMNRLDLAASGMILGNVALLIVLGVVPLDLFMQSLRQFFSFVR*
>TCRU_3425 hypothetical protein, conserved
MAPKKRLPRRHHAADGHPNLENAAAERIDAVTAEPLHPLKFVEIMSPDGTLRLFYNTATL
IRIATDRGMFMQPPHFREPMLPSLQRHVEEIEGRTFRFERSNVLIHDLGDGNDGGIHVLH
RHVYFDQIMDEFYLLNPAELYVCPSCYLHWIGTRLIPHLTVTRRRIEYVDGDPNPVIDPL
DVFAHMQGVQNAAADETDNSDTADVVEEVDRVPLTIPSMEAVPEWDSPLVHIVFRRAISW
RRHVRRHHNDSGTAAVDYRLKEFLSQYISQYNRLLEMKNRKQRRRGEGNLAGVPEMTVSR
YWHINARYNRLRYNRIVETVERAEPHLEECVNQTAFPNENVMSTFHSNDNDSSAGDFICD
SESSSGTSGSGDESLQSSCEEADAEGTKQNRKRRRAISQSTSSSLSSVEEDEEEEEVSEG
QRRLFKSGILAPLPEPYRNYTYEERRVLDTLSRRVAKPTMLYIPPPQDEIDDDRGDEDVD
WGQIGETIESASMYLREKQQRQNSATATSPADHRSGREGSTRGQEEPTTHECAATVKQMT
GALPVAFPVHRLMLDEDSISPTTTVQDVFVLEGQKRLLLDDSD*
>TCRU_3426 hypothetical protein, conserved
MSEWNAVVPLIVSGASDAHEVTHAELCAMVRGMEETVENTKRRLKQVDKIIERQRVVVND
VRISTSRFEDMFTKTNSDLQYIQQQLEYVVVSLEKERGIVADHTEQLRLFAMESNRRDGR
SADVLDDGTSLLFWLATWLYRPLVHFAKGCYTLFSPLINTLQSLSLFNTDVLMRRSEIGV
RLRHVETNEDLLKRLQKGSLDPTSLPKKK*
>TCRU_3427 cis-prenyltransferase-like protein
MHASVCFEDDARVLGTLFADTSDPVFVLNCCRFCSVDALTEGFEVIVLFFVAILFSVCLP
CACCSLWRKPALRAVSRYLRMCEMLLEAEGQDMCLKPQACPCDEGANSGKDATQHLSILP
VGCSLNGILNHGVRHLAVIMDGNRRFGELTSLSTVSVDAVTKLCDSILEEKNPPGSSVER
CSALVSLLRHTTLDGHRCGGEKLIEFIDNCLEFNIQMLTVYAFSTENWERSALEVNVLMA
LFYFFFERVRSSAREKGIFIRFISPAFERIPISLRKLMLLVEEESRGHRPRRIVVNVCVS
YSGREEVLEACNRLLRRVDKSIPISNAEFMSHMLRSITQEGHEAEDATVLADGGGAEPQV

LLRTSGEQRLSNFMLYECAYTEFFFTKKTWPEIGREDFIRLLCDFSQRGRRHGR*
>TCRU_3428 WD domain, G-beta repeat, putative
MDEPPTARFVSEEESKQRSEVGEAISIPSSTLVEFNPRSDTIRGELLHVVVGWLIEQGLT
SSAQILRDEAAALLRQEHNERKALRAISRAIHEGNWDTAHAQLKKLESITKPDGSANSEV
PLRIVSLLQLMPFLLAQQQFLEYVDEDDGQRAHVFFMRRIKPLEGTISKDHFQQLIYLLT
CRNVSEAGGMFPIWRGWTPTIGRSQLQSLITKFIGYCDSSPYCRQYEGNFDAGPHIAFKG
LEQILANSLSYELLKSQHPSLLRGLATHSVHSILQPLGEQLVPSNLIASIDVNEVRCGNA
AARGKNVRLTACEPFLQVHAVVVGTHMGSILWVPLEKKRVESLSGASCDACLKLYQYNTP
VRGMTSHERQILLSWSACQAVVLNLGSIMGSASDLLGTSECWMDCVANTFTHTAEVYSSC
FFPSGFILATGLSDGTVAVWDMLTGAKMFQRSFSTSAIESLTSNRSGSCYFAASKEGLVR
VVDVTTGVLLFTFASPVASEISSIALSPSASLLLVSYRSGMLRLWDVLTGDILPQRFEGV
ESHTRKGASVTFGCVDSHIISGHNDGYLYIWDIGTARSRESSILLSSTAPNGTAEPFQRE
MAGLHSSSIPGRYYWPMGEAVPPPVRTKLHRSSITDVKFQHHYVVTCSDDGMVCICSGLL
NHSR*
>TCRU_3429 adenosine transporter 2
MAVIGFQSWPEFFVYLTFLFFGVSIMLVSNAIKAAPSFITGYYKYAQGDKNAKPADPVFW
NNVLTYFNVAVFGTQTVCEAFMLTPLGRSIPLRPRLIVGLLIPFGEMLALIFIPLARTSE
IGAKITVMIIAVVSGLSKTLCDSTTGALAGPFPTKFYGSVIWGLGVSGIISSAMSIIIKA
SMSKDFESVQTQSRIYFGLAMGIQLISCTLLVVMPRNPFARKYTAEFRYMRAHSNERAPT
SGELVPMGSVDTKEDSAPQVEYDIPSSDAQCKKNVMDAEGDADNMEDIDQVANITSTQQM
LNAQIATVFKCIWPMLLSCFVVFGATLLIFPGVFFAAGTSKDWYPTIIVAMFNLGDFLSR
FLLFFKRLRPSPRFVLAGSFLRTLIVPFLVLCLRGIIPGDVPPYIMCLLWGLTNGYFGGM
AMIHCTRTPSLTMAGQRSIAGIMASLSLLLGLFLGSSLAMAVMKGLPR*
>TCRU_family, putative Mn.1
MVEKVKATTRVALESANRWTDNVYLLEHHISQRMGLTPRELRMRLQLPADVDYIEFDELV
PDREANPKTTAATLERMSSSPQRAAENAAGGVVEAPSRSPAAEEADTPAAINTSSTHKEE
EEAAPRNGRKDTRNTQQRIKRGRDDGTVAAKQSQSVVADSPRNDASEDAPRRTVARRGRK
KKEL*
>TCRU_3431 BNR repeat-like domain containing protein, putative
MEKGELHLWLTDTAHIVGIGPVPGDNAAAASFLLYKSGKDGGNKGEELIAVYEKKNGDGK
PSSLGMVSVLLTEKLERVKKVLATWKEVDERVSKLCPSKSDADCASTGTACGALKITDGL
VGFLSDNFSDGTWRENYLGMNATVSNKEAAEQVKSGVKFKRHGAVAEWPVGTHGENQL*
>TCRU_3432 hypothetical protein, conserved
MGLARLLFRLFFIVLFVYASAVGAVLVPFPPSFFVTTGTTIPFPLATPVAQSGAIYVSNK
TKQLRIDNFFLGSQYSFIADGERRRGYVLESHAPGSFGASYEEKGSFCRTFKMTGDVTSF
GVPDEFLKHAEGNLVRGVEVTRYTGYDRDSTGPLQQVDYYVRNMTFKLPGEKKGTVEEFV
FTIPWRVQTQRQQKALKELTGAPVTVPNWRYFGGPFFDEVITPDEPFVTHLQRLMEDTIV
TVDFYNFVPIAPDPSVFKVPSDCEEVDAGTASSNVDISLAQRLLVDLSFYTTAGRQTLRE
KSKDT*
>TCRU_3433 hypothetical protein, conserved
MYQLDAVPGRTFAALRLDREMPDLDDGSDLASGDDSVSTERSGPSGYIASVLAVAFGTFS
APQRLEALLPLCQRWSVPVPSGDGEGREQDVCRVAMERLAAKMCGVLKKKSF*
>TCRU_3434 hypothetical protein, conserved
MSTYRDRLVAFYEKYAPRKVGQVDAQLEKYAGREEDFFSALVQKYGPEPGNAAGTPAASR
GASPALSESATPTTMDRMSTYRDRLVAFYEKYAPRKVGQVDAQLEKYAGREEDFFSALVQ
KYGPEPGNAAGTPTVSRGASPALSESATPAVAGAAEKNSLADHHQPTSSEAIKNTVAQKV
DANARNISDEDRRERLRALAAQLGVAVESDDPLQVLSALVDARRGRNFGTTSPVRDADAR
VCRVLNESMYAPSTSRLTASVKAMMMMDSRSPTDLSVMLDAVNEEEAESRTTLELLAADD
FDIILAAQRRALRSVGRDGALSSLRASAEAALLQRVWRGWHDTAADYVKQAMHGRHHHRT
RYQKMLFSKSVAMYYDRIARSKRQQERRRRQMDAANAQANAMRRRRLRETIAEHGNLVNR
NIRAVLLRQSPGAHNNRDTPETPEQSPDAVFTHGKAEPQRTHSQRLAQSPYWHGDSANRH
GSGDRRNNRSLSFYSYGNRSARLLAIEKDFDRLPSDARARVVRRLQKWDILPKSPTQHFS
RGFPHLQSMRERGEQLARRLDTLDAFAAVADEEDTPPQWWKSETELRQYLARVLIDAFDA
EEREMLGLPYEVL*

>TCRU_3435 Phage tail fibre repeat, putative
MAAGIRAVETDVLQRVRLAADRILRVRLAYEAVGNALGDGSVRMATDTSDGEDDSVVAKI
RELHVCLTDSLESLVAFLGDDTGGATRRLHDGVSFEAMVKELMKECRSASAAFVDITSLV
RDGVEEKEEVPGGEATTITTTNKKNNPKPLVCCYEDLVVCVRRSLTRARGEREKNKRLCE
ELRRVLATTTGTVSVAAQSSLLGACLSHADVATDNISSFSDSLASQKAKQLFSKEELVGS
GDSDSCQSPLGSRHSFEIVHQVRSHIASKVAEVQELRTAVTTSVEKLGGTVTSPSVASHV
IANALLDVVRETSASIADVQALIDPDAATVRSGPPRLRDLVSCLQVKVDEWETSMREARE
LVNSVMNVNDPNYSLAASPGRSLTRKSKPNPVVAGRGDESLSLNRTFPVSSESGSIGQLP
DGRSVADASPRKNRAFTPFSSEAEGGGMRGKFVQDAVLEIRAKLIGIRQLTNACATAVCL
MGGEEGVERQALDILPVQLLQRAQEVNEVIQLTQDAVSLLDGTEGEVGGTAGGVAASSCP
SIRTRVRRLVEGMKALKDENESLRLQEIASLKQKAFLLQDFEAMRDKFASQEKRLGEEYQ
KLLGECNELRGTIDRRMQEIQERDDTLAKQERRNTELEEERERLHAECDQLRRQITTLRV
TRDDLQVLEAGPKEAAIILRRASAFLYGRLQKAINQISAVSSSSSSTSTLWTAAEEKVTE
NHLQEDKPEGIVKALDVNDASVSAAQLMAESGRELRRCGQAIQQIELHLRTETQLRHWVR
WVCVRTKEEVVSEEAAVRTLLREACRNGAVLLSEKMRVLKERDEMRCLLQQRLEELLSKH
DEERAAWKRRLQDLNETQQLPFCSSSAASNVATVREGACEVQREGPRAHSRLEQWIIENT
TVPLPNASLQRAAAAAAAAAAVVVEKTTEMCGAPLECVPAALEFMPAVGGDGAAGEWRPE
EPLSDGVKCVQKLAEDLSRCRVLLQEQLLMMGEDNDDDVSRSEERRDDGADQRASLSRLM
ERHKSSLKRLVSERAALHVAWQTAQSEVLRLHRELHEGEARYTRDTEEAGRRIGDLRAIV
QQKLVADAKTEREMEEIQSVMDQHLAVLYSYAKEEEAVTRHLHELCRLMRSKTNVQKQHA
RLHF*
>TCRU_3436 hypothetical protein
MKRKVRRPVRRPRHVGLSLPKLANPAQETETDDEDGNAPLGTSFSPSLQAQQSVRLASTS
FKFSRAVSLSRSNTSATENIEGELWYVTSNMGPGKRATLAMQWVTCDDHKLTVSSGWSER
GRIIDMMQFDGIRVIFSFSHVHEHFASSRQAPITRIVRRDDNGNSIYVNVCKKDKEYSRN
DYQPPRRAMREFRLCEGKLRPRYYYFGIEFSEHRDNTGAPRRRQRRLIIFATDIKSDQQA
WLDYFASLGDLKCRSISRTSNTHGCFSVQDESHCQSLQEPQSVGRLNLEERDWGSSRSYE
PLEFAATGRPGSESSPFLVDFDELSESHASAFPRSRHNEQLRNYLIAQLTDEETRLRKTL
FVEEENDRRRLCAVRQAFFLAFRENNSFSAWTSTRLAKTGTTDDATLAACGLFFPQCEEV
SPETSQCEEFITDTIKMLQRTITEKNNHINDIKKELEELRRQLTVDKNKIQTLEEKHRPN
VCNKDVNGKKGNKEATTPPNKPGGQEADDRVRHLIGTVQELKERLRATELARTVLEKQNE
RRRRELEEQLAQLGTERDALAERVRELDTRLGSATVSLHDAEHECELVAGLLGVPTVEAG
RESDASVAGRLRCVSGALCVSWGSRTSADAVSWRSNWRSWAPRGMRWRSVCVS*
>TCRU_3437 60S ribosomal protein L8, putative
MGKTVLTCRKGNGSVYQLHGHKRLGPAKLRILDYAERHGFMRGVVKTIEHEPGRGAPLAR
VEFRHPYKYRRVKELMVAPEGMFTGQSVLCGVKAPLAIGNVLPLGQITEGCIVCNVEAKV
GDRGTIARASGDYCIIISHNHETGRTRLKLPSGQKKTVPSNCRAMIGIIAGGGRIEKPVL
KAGNSFYRFRGKRNCWPKVRGVARNPVEHPHGGGNHQHIGHPSTVSRHAPPGQKVGLIAA
RRTGRIRGSRAVKGAWHPEE*
>TCRU_receptor peroxisome targeting signal.1
MDCSTGAAIGQQFAKDAFHMHGGVGVGPTGNSEHDVLINEMMMVQTPTGPAGEWTHQFAA
YQGQQQQQQQQHPQELAMRHQQNDAFMLRQQQEMEEAFCTFCTTHPHSHAHSHAHSHQPQ
GLVGPAMMGPQIMPPMMFGPGTGGFMMGAPPMMPYASMKFAGDAAMAAANNTTMTQGATA
TSTTSVQQELQQQSSDNGWVEKLRDAEWAQDYSDAQVFTLEGQSEQTMEEHAKNSEFYQF
MDKIRSKELLIDEETGQLVQGPGPDPDAPEDAEYLKEWAAAEGLNMPPGFFEHMVQRPQG
NNEQAEGRLFDGSNDALMDDGALDNAADVEEWAKEYAEAQEQLQRVQNETNYPFEPNNPY
MYHDKPMEEGIAMLQLANMAEAALAFEAVCQKEPENVEAWRRLGTTQAENEKDCLAIIAL
NHARMLDPKDVAVHAALAVSHTNEHNVGAALQSLRSWLLSQPQYEHLGLVDLQEVAADEG
LDEVPEENYFFAAPSEYRDCCTLLYAAVEMNPNDPQLHASLGVLHNLSHRFDEAAKNFRR
AVELRPDDAHMWNKLGATLANGNRPQEALEAYNRALDINPGYVRVMYNMAVSYSNMAQYP
LAAKHITRAIALQAGGTNPQGEGSRIATRGLWDLLRMTLNLMDRSDLVEASWQQDLTPFL
KEFGLEEMAV*
>TCRU_3439 threonyl-tRNA synthetase, putative
MSKAATTSAGEKNKKGAAPSASDAATVDLKSLQEPEFWKARDALFDELLAAQEAKYNSMQ

APITITLPDGKQLPATSWLTTPIDIAKRLSNSQAERVIVARVNDELWDLTRVFEGDATLE
LLDWDDPEARHVFWHSSSHVLGYALERVFHTRLSVGPALEEGGFFYEGDTGRPLSETDYK
SIEIAMQELVKMKMPYQRLTVSKADALRLFGYTEFKSKILASKVPENGFCTVYRCGHLID
PCRGPHLPDTGRVKAFAVTKNSSSYFEGKAENAVLQRVYGISFPKQTMLTEWKALQAEAA
RRDHRNIGRQQQLFGFHEVSPGSAFWLPHGARIYNALVEFQRKQYRRRGFEEVVSPNMFS
SKLWMVSGHWDKYADNMFRIVVEKEDHGMKPMNCPGHCIMYAMQQHSYKELPIRYADFGV
LHRNELSGALTGLTRVRRFQQDDAHIFCRMDQVQEEIRGALVFLDDVYRTLGFKFFLKHA
TRPENKLGTEEMWDQAEAYLRAALNSFCGIPEHLPDPFKEGATFTFDGRRDTVKKLRALL
KKQAANRENKDTEKKEEKDDEWKGPTHAEAWEENTGDGAFYGPKIDIVVEDALRRRHQCA
TVQLDFNLPQRFGLKYTLPTSTVEGGAGAEAKEHHTDPNAPNVGTDATTTEGKPEAGDEC
LSSYEKAARELCIDRRLDANQARPVMIHRAIFGSLERCIAILCEHYGGEWPLWLSPRQVI
VIPVSAENTAYATKVRDAFYADGFHADVDSGNATLDKKIRNAEVARYNFIFVVGQVEEEA
QTVNVRTRDNRRHGTKSLAEVQRWLRELVDTYDLKY*
>TCRU_3440 RNA-binding protein, putative
MPSGDKKIDTRNGDGHDDNRSDYCDDDYVPPAQRGVLFFSTIPRDMRPQEVRMHFNEFGK
ILRQKFTPFPKKERRPGGPLLPLQFMNGYLEFAHAEDARRAAAAMNGTPVACKRRRKCHG
QLWTVKFLDDFTWDTLLEQKEETRRTRRQREAEARSAERAINEAYRAAVLGAMRRKHTRG
EGGASPKEPNECRSPKKTERREQNVTGRMLRDVSVSSDSVGAAAVTPLEVVKKKRRKE*
>TCRU_3441 C-1-tetrahydrofolate synthase, cytoplasmic, putative
MVVGVPTAALLQKEDNFLVTLCSRSNDLEAVKEVSRQADVLVTALGIPRVVTADFVKPGA
IVLDVAINEDCASGGEMPSGSRRVCGDVDLASVREKAAAVTPVPGGIGPLTVAYLMQNTL
KAHLLAKNHINLFSPLASTQGNAEWNNEGHTEADFKRMGVDTHDCAGVGKVG*
>TCRU_3442 SLACS retrotransposable element (part), putative
MEVNLPLAPVSALELPIKNDSKLRRMFSATVVAGAHCEACGWALPMEEAYPSHAETRQEE
PAIITLQPGKKAPIHLTQTLLMQQYRSTWIFEEHVRTGEQLARHHPSWAMTATQSRKFYD
AVALEFGHWDKQAMGVEEVPFVILLPHHNGTAEYGLVGLVATNTQNHVVAYITSALHRDT
EWVMIDGMVQKVQRRPINTHKVILCLYRRLMPETGLEEDGEDDEQELHTHKRERNGRTLV
SAPKGSRHFQSQKSEEAQDDSSMEDGFGKNGYGQPPGSATHFSPLASSLPSTQTITQDSF
LTPSAPRKRGRCGTEKEVESEGGGNKGRGFVCEEDAGGILQPSVPPTSSQPNQQTPQGQF
LNEKEEEITMRRAQPQETPYNNKNEPPPHQQSSLSQKEEKREEEGVGDEEGELLTPQHTT
GQEDSRHPFSHKTTELPDPVWCLAGGCHHNFSGPRRGEHPRSHIHAVHRKQVRMDITNEA
LISQGLVWCDACGEVCLASQRARAAHRPRCGHHTCRKENLATQREEYRASVTVSHYNKTA
ACLERAHSVEWPTTPATDPRHDPWL*
>TCRU_3443 hypothetical protein, conserved
MLSENVTVKEKLPDQTAEDGSASRPSENFTVSPLTSAEGAANETSHNSNEGDSQSDASGE
GNSTVAGTNPLRPTRDESANESTEDIAALLQNFVGPDGSDVTTVWVRTPLLLLFIALVCA
VVR*
>TCRU_3444 hypothetical protein, conserved
MTEEALAQQVMQDFMATRHAGSSVELLRSVFFARLQQTIAERYPLAYNRLLLAWHWRGKW
RCFADEIVGLRCFRYMLMDCAETRDLEVHIAPSELRRCLHGGNHCAVQLTDGSVGALLRE
HLLQKGALHCWCDETVRAAQADGGAGCADRALWRETRLRGT*
>TCRU_3445 p22 protein precursor, putative
MYRLFSRCKTNKLKRVFSHALDASIKNEGVAQQRKRQVLPRRHRKLHKLTIREENEEGLR
DFLPPKPVVPTGWKLEHAKGSNRFDLRKSVEIRDCGKEHLHAVAMMEVKEYEGTYRMDNG
EREEEEYLFFTLFVQKNRFPGALEFGLTSIDMELVMDSLAIHNNREELEDAYGALSVSTS
RDGISNRIIEKSNLSCRSCRDNRYRGPMLSELDDDFSDEILDYLDERGVNNGFAEYMMAQ
AHFFEQEEYINWIRLLRQFAQ*
>TCRU_3446 Ring finger domain containing protein, putative
MDSAVEDLIRAMDINKAKHDSASLWRKIRNMREESQTVDEFLFKRVLLCSARCVLAKLEE
SGGAEEQWIGYLDFFMEAVRSFGTRYADPLLGTCEEVFHLVLGYPEKPRDLFHEYLFCLS
AQRHQCMGMNPNLAGTAPKCPMLENKSTEVALVPEVPLNEVRQYVNDLPQRLTFPLQNGV
VRMRLGNPLPIPDVGYVRGGYRCDTCCISNIQVAYQAMLYDDMDKAGVRSAVHFRNLANR
VGFDMCVACAVYFYRDAVLRLSQFLGDHSRTFRVCPDADVQLHSFSTEGNVVKFTVSILP
WGARPIVWIADKEEYNPPAAWRSAVKIESCNQYDPSRRNGGSDDDQCAICLQLLANGTPV

LETPCKHCFHVDCVQEMRSMMDDECPFCRRENVFTSCVNLTGQLNMYKVQVDLPNEAKEI
VLAVGSLLTSDGEYNNPTNIAACRSILVRHSCIMDFEAEWERNSPVS*
>TCRU_3447 SPRY domain containing protein, putative
MENPHVGFDSTPATSTFTPNKDETAFRHAVSMNFGKVALSEMEEEEGGCEYEHLVGREER
EVLCVPRGAASTLQGFGWKANYEPKKEESLFIHLNDAKNKTYETSTVADTTFRQFDHSPD
GGRTERQERVREAVGAVKASDPGTGMAHEEEEEEERGIQKLGKDPGTAWGATKRKDDPEEC
AGRGVFAFQHSMFLNKPQTVAFFQNYFVPTTQSPSSPTPMCENKTPIDYTQRGKSHSLFS
GENEPVPTETEVVLSLMNGEESVSIPPTSRSVGRYLFSTQRECNDKDDLMPTAEGTHPVF
LRRELMELEHNAAAFLVEEEHVARCSLEEEEVNGRDLLLNAVQLDLDTLCFGEVDMEGDQ
RYTTPPVNPKPYSPVLDNMDDHNELFLRNTRIGSNIDDDVVTPLEKLLRESSPNARNLST
FNSPANGVYANIYEPPSGKVEVATSKLSSGASRIGLLAKNAPKPLSFEAAQSHLSPWCLL
ETQRHQLEMERRLTQRDRDNLERALDARHQCIQIPSDAHAQLRFEPSDSFTRERQLILGS
FGITGQKNSQADTSTPQKEGKERGFHHHLHHHQQREQQAVLQPQTHEEGMPGVGVSPISP
HTFVSSPSPPKLLAQKQSPTEATIFAEACEAKYKESLTAMKELIELYQAFIKKKPQSKIL
SGESQIQVYNSNVNEPAAFSIVTYEKRLARIKKLCSSFNTKTSVTHYYRRILHTPPLPLS
LLPENVADGISQNYMSALVKGTIIPHRSFVEGVENTRRTDKSGAAKYPSHRFAGFCESGV
GYVVQKVKPTPFVADENNERRGTLYCQNADSYLPPHRRIKTPPASLGWEKQISTKEVSVL
WPVLRWDAQRSENILISRNGTTCMVDASRLLQLIALEKEQTGKAVPCAKIPMFALGTVGI
AEGEFVFEVRIDAHPSSTHTPPSVFAVGITTKYYRGVHQKSPAYLFRSDGTIVACSDDET
GVPYGCPYHCGSHITVYLSLIRKELHFSLNGQPLGVAFRFRGVEDPEPLFPLVVLGGEGH
SASFVPPSAPQLPQHVIPPAHWSMHVVQSH*
>TCRU_3448 B9 domain-containing protein 2
MAELHVIGDLMSGENFGRRSYFCIFEIVTGEHWSVVEGRTSGCTHIMQSGGEGSVLWCFP
LDVHFTTNSIEGWPKISLQVWSIDQYGRKDLEGYGVAFVPPPSIEEQEVVVGTWKPCYWS
PSFFTRIYEALRLVVLGGNPVLRDKALIHTNDERFKLQTKSGGTVCLQLNVISRGMQRLG
IRYS*
>TCRU_3449 repressor activator protein 1
MSSSMDGTRSTWRGVQFFVDVDVLLTFRTVTLSDLEGQPGQPQRRIAVVDGKAVSVVSYP
AASFFLKTLRATGNSVVLVTPWKTEKTASLLRLFGWDAFDEIRTILREPGHENFRLSCLN
CTLEEYPFSVVVTTSRAAWHTAVWSQVVEVRHETNYPCQSLALLRALSCCLRIAECRVRF
PHTAVSSCISFSRARLFSQYRFYLSPEVTDPSSVVMMINEHGGYLVSKYEDATHYVIEEG
ENSFSCTLQKPRLSREDGERSSSLSSSDSEGSDSSSAALIDVERESSVSLNSDSGEEDNG
GASGEFKNFGVNEEQEKKTIHVAMLPPVTTNESMTATEFSTDSVNSSLILPVVNGCHETE
EHDWTTWTTPSTSAKTTTAITVTVLWLQDCIDGLLVYPPQVAHSSNDNSQWNISALLLAA
LIPPSDVEPISLSCLNNIAEDYGFRPLQLSQMGGEVNLATVLQKQWGSVLHVEMRGGELF
LRQPSIPPNEAGQEMERHLAFIAEQFCDAWTNAIRNNRLRNMTDSGTQTAAVLMATSGAN
TDVVLLNSEGATVDPSPQGRLLLMHALGPAEWAKEVDASHMTYNAIRVTEESVSLEPSSS
LALVNEPPTGYESPPEVKEVQAISDEKAPARFAQRGVEAEKGQPVVDLAKPPPGGEREFV
KCFIPSDGLGDERETLDKDVLLRQPPFRNYPMKLTVDQQGIHCLFVTTFDAKRFYQEGYA
EVRNRFLPILPEYAEEISSTVGTRKRRRSRSPVTKRSASNTSRGNRKDAFSSNRDSQSFR
GAPGMEKNVDTGGSTELSKSDLELLGEIGATFDFACQHVDVVERYALNELSDPVFRKQQH
QLLRVVVKLKRIIKRGN*
>TCRU_3450 ATP-binding protein, putative
MKTIALISGGKDSILSVLLAMRYGHFPVVVANICPTGDEGPEEVHELDSYFFQTVGHEAV
ESIVHCMGLPLRRAYIRAGQSKVQDLHYSKERDDEDEVETLYRLLHSVKEEFPEVEGVTT
GAILSHYQRYRVEDVCGRLGLHSLAFLWQRPAEEVLDIAAVLHVHAILVKTASIGLDPRI
HVGLSLEDVRPTLEKAQKLYGTHGAGEGGEFETILLDCPLFRDQCLGVSLLERVIVDDND
YSPSGYARLKVKLRDKTMEERALSKELLLRLPTLTFPSDRMCHLPRVDQLINVYNEELEW
EKSFRPSNIDAKFWGDNCCDVYESDVLHTEDEVEYCVTGVLQRIVEDMSGRNREVFFMLV
FSPSIRYFVFFFEAFAHFFPNPQPPGCAFAEVSDKKAFCLEVLSAPREFIQRATLQVRSR
SCCGPVSMGPHSFSNLVNTNAERCVIVSGCIGLVPVTQRLAVTEDMPEILNLSLSRLSRA
LGLEEDAVRTFFVQFAFMYANSVIGLTHFGAGVPFATHATFFLRDMRFAPLVPFLWRWCA
GDFARLLPWDNPRACIEKDGVLFRILHVTRLPSNSEVEIVLERRGLRLEED*
>TCRU_3451 RNA-binding protein, putative

MNYDYMFVYVNGEKMGLLEDGRLALYQRFPVGSVLYDPSELPPREVPISADAAEYGVTAC
KLIPYGHYEAYIPEPATGDLHPMTGENVCVYVTNLPPNTTAEMLGRYFEAFDMVAEADVF
TAPSGECTGRGWVVFQDPSKLLMVPPVLEFFPRLFIYTSLSDKIPTRTTLQLSSVPETLV
TGTDIASFSEPTLNHNRGGNHRRMNGKASGNFLKISTAANAGVINTSAYYFVALIRRDEV
EKSVEEGVFWTNPANRRAFYATLERGPVIIIFLLQEYPVIFGYARLLPDGSADKNGFSSC
TVEWMKHHVFLKEQEMRNIPSVPISKMGDGVPLKPEIGESICHLAEQHPTHSTIPERLGQ
SLPNNGRHVGRGGGVVISGNGNVGGFNSVGGGGLFHSIGDRGTLRSGIGITSLPQPLGTR
PATRGGHIMNNRIPQRRRPYNDNSNTYVGYGGSAVPPRKL*
>TCRU_3452 hypothetical protein, conserved
MAAREGSVCLCGGRRSHDAETVFAGGSECPNSPPIDDFTHVLRSAHVNFGHVSPGASSIT
ASGTVREQPSPIVERITQGLLASSLGCARCAQIDGIRESLKCVEASYLGEIAQLRRMVGQ
IDGERMDAVNRMEQTLLTFERGRQQSLSKDCEIQSLIGQLQQMEDRSQRLEYELDSLRQL
YRREIAFYHQREMFLFLENERCARAALSSTEDGWRSALETRHLSVVEVDGKIHRSYPHQN
FLFLDFSKENEGGSSMRGGLSPGLVRSLPPSITRDTRKNRNGGGGSKTSDIKGGLLTELW
QSKRTLNELNEKLQRELRAREDQISTLRQQLSVREESLVQSSEERAEEDNLKREMQEEID
DMLRTEFFLTQTCERHRLETEAMEGRAHLLQWLLQRHVLFSQERNLMFLERTPEKQGLTE
KVWTPGTLPFFISSNDDVKPDVYVNRSKYSLSCQQQQQQQQQQPDELYAVVSQAVSETLQ
SVLTHQPFLIKRTAEAICATEDELHKNSNNFVQKMHYDEDDNVINAAGINASHEKLLGDV
RRVLQEFCAANPPQEIVDLSPVIDALKKVARLTSHNAKVMDDKLNDMLILQSAVARELAL
LTTPSAGRRVDESPSSNVSATASLSIVDAGVTGANNKASPISRRAHPKPLNLDKSPAGRG
EGNAPVILQAKPSHEWDVAESDDESIPPIPVFSSLRKTPYDADEL*
>TCRU_3453 Nucleoporin
MIAGRKRGRDEVDDALLEEVTAAIQSVAAETEDIVGSVGKVNGAVLASLLERFRQMEVAL
NASGGSTVQLWKLIVQSQRNRVADALNHIAVADDARRKGHLIAALQEQLLSSASLAAEGV
SSGLCRAHETAVALFSSQLAGERETNNKLILHVQELLRHSLEAIQVSSRRAMKAELLSFK
RDILVARLQSSLSFYQNASLRQHEEVMALIKIVRILASKGKDADLAETKLRQAKTHVKLL
ENHLELAGIVEAILPPKFVPPRQGTEPPNEDLKRTAPEYFALRLLEHKDMTLSELVDSWQ
KQEARIMAAEEKYRELELSLESLRRESLTVHQGFLEEKRRREVVEQRLVDLVRERIRPSS
NDALVQQLTLLRADHTKALDDLAQMATLVNVTRERLFAEQQRNALLEKKLQQMQDDSAND
EVANTLASLRHYYEEELDRFRNGLVDADTRSAVAAASSEIQQKASKNASNALMVTEQTLM
DMSAALNRVEKQLLIDQEACGFSSSERIAESAVKVLAESEVHLNRTIPTFKRMIEESEIR
QEKDMAFFERQVKQLLESLQSLLPTVSHSEVTTGAEIMMVQQQQEVQSMMREALVHALQF
VSDNLVTNEISKTRLLDTSISDARQISLLFEKVQKLTVDRDAALERLARCERIIDQHSLT
AVERVLMHEVPVEESLDAMEAAEQITTLRKEVSSLGAARTAALAELKELRKELKENNHER
EFAVARFELLEHHNSRLIKSIEECLAREVVLIEQVHQLQRNLQEGKKMELPVLPTGDSNE
TTEMERFMPLMQQLEQLTGGINEMKEQLSQVQRVIFSSPAAEGDVTEKQLLCNGVRQVAD
ALREALQHAELFRHALPLEVTKDAALSIVGSSAETMSNNDGHEKAETISELGRSVADTPE
MDERDEELVRLRSKVETHEQIEQALRKHERDLQEQNEGLQAKIFRLLQINRQLLEELKTT
RASSTTSANKAPQG*
>TCRU_3454 hypothetical protein, conserved
MLVGVREYRREEIQRICGTSAEALVSYYTLLRNKNEAIMADVTDARARKNMNFDYYMTTA
NSLFEEAVRPIIRTVLPPISSPQVVVVVDGTETTRFHTLLDPEEGILALFWRLLMDAAEF
RSILEAVYIAVVELDDNNRLRDITCAVTAEKEPIIFEDAEDLFTSVEDASYMEIRTEADI
ARLMQHALRVALPERHQIFSFILVPQKASGLPNCTIQFVALSVSEMARGMRSTRHCITTA
ASLIESRSPSMTFSGTKLSFLLKPALLGQQPGLWISCFAPTSVAALKQRETFKESFCIAH
TLCRVYSSRVGASVSEACQRIGFKEGDYSNENCDEQVELQPRKDVPKPSLLGPWEKTRRR
LSKTTSLHPSLSVSLLSSSFSLHSTPNAGQQKKGEERGNSPTYGINDEHLSSLAAPQQKP
TGLLVTPSERRTKSLDSNKTGFKKRRDVTSNGRRSLSRPSHDRCSSLEKHPYNCSARYEL
ETYKRVMEPAMNRLREDIQHYMTLLEDARRQIRRIKRVNNEEVEVEKLRDSLLTAKRDCA
SLEAEFTRREEMLQSRVSELQTRCEELSQQLRGQTSCAAPEGEKLPHKSISTQTHNPVPY
FPPLSSSPPPPSAPLPAEDGLVAFRNFPKDSPRKDVQHASQWLEVVNYHQRELEDYAERE
RSYRNRILLLEQKLSERESEMAQVRAELIRRDCEISLETVSNQRREHEKKMDQCQFEDVE
YLRAKLFEANNVSSRVEQHLESCINELKEERRQREASEAELRRLQGLCAQHMETGAVKEM
IQMVKESYERHVNHLQREVEELRGVAHQAVSNEMISRANSSTTSTPRQRPQGHQHGGMVS

PAIETLQAKSMFPSKPSMPDETNATNDVPSTHSKTIPARSGVRRFEDFMKEEPSYTIRLP
KALDRANLS*
>TCRU_3455 Pre-mRNA-splicing factor of RES complex, putative
MKRPRYDSDDDDDYAEGSLLPYALENKGDFLMAMGVGGRESSDESLRSVEEVSVAPEKCK
EKERQDAEQKEELMESFFSPSMVVDRLQRQALRVDHDVPEHWKGLLSEGLLQVYFTTLSY
AEKKRMVDDKLLSQYLPHDAKRFPPLGRPTDRKWDNAFGIKPGWRWDGVVRGVGSL*
>TCRU_3456 phosphatase and tensin homologue, putative
MSSSIYSREDEKRLSELLERVNWGGELSEEERLELARLKRLYQEFIEEGERRLRLRGGHE
KAAATNSDGPGDVLLTLQESVACAGKAAYFWGSLVATLVPGYFGRKVGLTSGFLHWNFIT
DRLILGALPVVTRVGSSGNHLVQIREQLESRKQKLGLVIACLEDAEVQGFGLQMISFADE
SSWHEYVSPAVRYIRLPMPDTTANISFGSVLYAVKQMHHCIKEQNCVVYVHCKAGKGRSW
MVTMCYLTSYGGMTFDDAEQLIRFTRSQINPSPSQRAFAAQFASRIPAQISGS*
>TCRU_3457 hypothetical protein, conserved
MDLSLGETSDAIASLAEGCSLEHIAELATQCESHLASVEAIMDATENALRTSQGPRRLVL
WFFIDRLAKQHANFLDALRPRLRGLAATAAPAVGTAEWDDFKTLLKSSIAVVFGAPLVSL
ILLEIDPDTQKETASTTSTTERSQGILAMHSNAMRTSGAFLTKTIGTQRPAAAKHTLQVK
TIDSQMAMNMASSYAPARPQEIAVPEIHPDADAPHGHVKALPSDYLKNYENTRRKRLREV
MERNRKEMDRRQEEELLHRVRGTAIQNGKMPDYSDVVMPLEFPRDEHGVKRGFFPLGVRF
IREAIRSCGGAVELDVLVNRLSTLASKETVAEFGDVREFILIHRPTFRITNERNRWIVRL
AGDESSDPTWESLQCPLCSKVMRGRNLARHMGCRQCVTTQIALGLHGEVHGPISELSFVA
KSIIDRGNEYDDWDLEWFADCLERSANCRRFKLSSQSKFAPILKAMRVVRNRWLARKGVA
NVSEAAVDPSDAAVANLFRTIGRNVNRLPIPWLEMGDMVDICCRFSDLVLPPFNPPPRPA
DPRISLHNEYPGFLLCESEVDDEDDPSDDEDAFSDDEAPTFVFAPPVELAETVFTAGFER
DTKRLQHRMRTAPPLVLQRVLKGDRSQTQREMYAVAKDASGLNTPS*
>TCRU_3458 mitochondrial carrier protein
MNTDDVKEVCAGSIGGALATVVEYPLDTIKVRLQDDPSRYRGSLSCITEIARLEGFINGF
FKGLPAPVIGAAVENATLFCTYRVAINGIQDVFYEKRFESDTEPYSVVFSAAAVGGLVVS
HALTPAELIKCKMQVQNTLPGHKRALKNSLHCAMSIYRSSGVRGLYKGHVSMLARETIGC
GVYFLTFQWVIRNMLHEGQSFAEASPFVHFLGGGCAGVVFWTSIYPIDALKTKIQTGKGG
YGELSFVRGMMQLYMREGMRGCMRGYTVTALRAFPGNAVLIAVYEKVNLLWEMSHRSRCT
SVCSASCVGST*
>TCRU_3459 wd repeat-containing protein 19
MGVQKKFELSTKELGGGVILFSWNSSGTQLAVYSANGKLQILSPDGKKLNTSSFPGVVRI
AWDYSSDTLALIQKNNSNVTLQSTGKQSSDSIDSGLHELCFLAWSRRGSHLAVGSTKGTF
VVINSETLKKLPVVGTHTKAIVDGKWSVDNTLVMVGEDNVLSISEEDGVLRRQVSLKLMP
LSVTLVDLSQRGEKKSPISTAALINFGKTLEHFNLETFKAQPVPFESKLGTVTVCDFISG
GLVCVGFSSGAVALVNASVKGVKMCGVVRSFHDTVNALACCQNFSMCAAVGENALKFFRI
ADGSIVENAMERVELGGTYGTLRGVEWSKDGSNVTLSTEQGQLISYKLNVSAVSTTKGTF
IYFLSSSRVVTAKSLQDDRIIYNANVDIEPAIIAAARGILAVCAGHNVLFYTTEGNEFSL
LRRADFPSAISALKVSSRIAAVVCDGKIELNEIRSGENSATFPENGTANIISLDMTEVLL
FYATTEKICVVNLIDFQCVVEHTPSIGIKRAVPNTSCTRVALIGKNDALYIFNPVTLTLI
QTEGFEAEHKNILWDQGDYGILVSYDSKTMVTFVYSPHSRYGPTSESVVIKDTNTENLVT
PIPVGYTPVALFKGKVTFQTSSGSLDFAHLKSHNQAMTRVPNPEAFYTNFSLNHLRWASQ
NISTSQEAEDLAVKALHVLDVELAIRLYRQLSQPSLVLCLENIKHINEKNLLIGHISMIM
GFFKDAQSFFLRSSQPLCALQMRHDLMQWESALALARQMAPERVFILSKEYAQQLEYRGE
YAKALEMYRAGERAMPKGHASTQLTAAQEEVKSHNAVCSKGAARCLLRTGSIREGVKLAL
DSNDEALIMDCAEILESTRQFEDAAHLYEKASEFEHAATLYILEAKNLKAASRVIPKISS
RNIIGMFAKAKESEGAFKEAELAYTQAEDWDNVVRIKVEKLNDLYGAYVVVRQTRSAAAA
AIVAKMCKKRGEFGSAVEFFVLAKSFQEAFELAKEKNCMSTLEGALLNQAQLLDGIAPAS
NQAEFAMIAQYYEQNGKPGQAGLFYHIAGNFPLALRNYLDAGESEDIEKAIQVVGKSRSD
ALTNRLIDYLMGETDGEPKEPSYIFKLYMALGSYEKAAKTSVLISTKEQEMGNYLTAHKT
LVDAYRILRDKKMHVPNDLRRSLMLLHSYVIAKPLMKIMKDDETASRMLLRVVRNIHKFP
KHISVIYTSTALQCLKSGFRKSAFDNACIIIQNGKYGTELDEKTRKKIEGIVRRRGKEEM
DDPPEKTSPCPFCDAQVPETELDCGACKNTLPFCIVTGKHMVKENYSECPICQFPALYSS

FVTLLRSSQTCPMCESIIDVNKVQKQSDPDLKAYLA*
>TCRU_3460 ubiquitin carboxyl-terminal hydrolase, putative
MAKKWLPIESNPDVLNTYLRNLGVPNPKVEFCDVYGLDPDLLVFLPRPVRAMILLYPITP
EIDAADAKTVEMQAAEIKAFMAEKDVFFSKQTVENACGTMALLHAVMNNLDSVGDVRKDS
PIDFLRREGLHGNPEERALLIESDPNLDAAHMGASAEGVTKNQPVNANIDLHFTCFVHAK
DQCVELDGRKQSPLLHGACMDNEQFVSVTAEAIQAKISRNKESFRFNIIALVDKSS*
>TCRU_3461 cleavage and polyadenylation specificity factor, putative
MLRASASSIKLTNLYGAPTGDTYHPSTPFANLIEIDGVRILLDCGWNDEFDVNFLDALMP
YLGDVHAVLFSTPELVSCGALPFVVEHIPTGTCVAAAGSTAKMGLHGVLHPFLYLFPNVK
TWRLENGLDFEMTVDKVYSAFRSVTEPYGGKVTIRHRDAEVECYPIFSGRMLGGHGWLIK
YKIDELFYCPDFSLKPSYALKRFLPPTTSTLLFIDGSPFHLSGNTGRKYEEQLNALIREI
LGTLRNGKDVLIPVSVVGRGLEILTIVTHLLTEKGGDNYTVVFASIQAAELVAKASTMTE
ALLDEIILSERQLFANVVTCKTAEEVLSVAGPKICIADGETLDYGVSAELLGHFLQADAD
ERENLVVLTGAPKPHTNAFTMAAAKKGDAIDLRYTIRSPLGKEELEEYYLQIELEMEEQR
KALEGGAYEVASLEDENSDNDNDAGKEDEKQLRVTQQCTPGLVLPSYMTFVSKHLQFPIL
ETAASLANAMFKKVDYAYGLPISEEMQFLMRRKAPARIYSDEGPEGIQMHNDAQAEANIP
SKTFVNTAVVRKNSRVFMTDLSGFADAAIMRSLLKSRFSFAKKIVLIRGTVDDHRALYQF
CRSEKVMKCGENVFFPRTQRTHLELATHVYSYMVQLDPTLANALPSALRRVKESRSSGFW
DVGWVDGALESSFFSLTPEDDERQSVKRLRAEGNGGEIKDGVFTLVPLFGERAQQCARER
ELRGMQRGLFYVGDVDLHRLRDVARNEMGLRGEFHKNAPMLVFDAGLCVRKSANGNFSLS
SMISPSVFALRKTVYGQFSQTL*
>TCRU_3462 hypothetical protein, conserved
MSDTDVLLLNLEVEAAAARNADLSRRLEMITEELGCSSVHMTGKPELVSQGTQTGFDILR
EVALNLDDLLFEIAEIMFRLYPSTPIDLSIRRGDGSRNLEELSHRCLGEMRSLRVAWETK
LEGYNTFIEELQRAIGSYYRSMEEMESQMQEKELEFVTLQKHNVELSRQCADMEAQLRAA
REKNESFRENIASQMRRTRDLEEERDQLLKRETVLANTVASLRAECVLGPWKNTVTKRDT
LAENSVHSFNVLPVRGSAKPVVGACAGIGVADPAINAMWACQGSAVNRAFPEWCVSAEKV
VDFSCKMRNSR*
>TCRU_3463 hypothetical protein, conserved
MEIHEGRLDGWIDLVNAGDATSLIAVFREVLSSTPLDPSALGTISTFLEAHLFPSALEVD
LPQLFSPMPTEYMEAALSEARAHALDPREILSELLAIGEALAGYALVVYLRLIRASFRQV
FFDTLGRKKGLCASVEEQFSKTHEIMLAWYPGMDRLFSRIISTICQRDAAPKMVRTVNNF
GHGDVDSDDVDEKEVDDLGNMPLLVEPEALQVLVLLSLDLLLMVGEPHKIMPLLLMVRTS
IVAAQPYNESGEFLHRTGKDLREWANFFCSVGDKRTELKEEPYSTQNSPIVATIKESERM
REKIARAVLEISPPCELFSHLQKCWSNEFTKDLMDSVTYEDNEHGKDASDARAFATLHAL
KKEKDSLHILSDDDYEREECNDEAFSASLSLQKIGAMLILCDFMTLEGFSDQQLLLSKSP
ESFFFLTGTMVLECLKSSSLNVVMTGLAFLSTLVPQVPPYSVCANGELNTKSGEIHEFGQ
KQSAGCTWGFHTKRFELIFEVVKSLMNISATSPCECHREVSRGIFVLLLRRFASKLRMLV
YSLFLALTPFASICSLMLHSLREEWNGSQFSPNNSEFDFLQNTLPTLLLKAQKNWLSKMR
FGELTFVEPMIQSINFVRCVIIEDRKRRPEDALFRFDPNCAKVQLREEDSGGGKGDRWNI
YLCQLLKDVIPPLRIMVLESPETSATAMGLSGGVTLSPLDRFALSMSMDGLGEQVSLAA*
>TCRU_3464 DNA-directed RNA polymerase I subunit, putative
MIRSHLIVVDEFASAAESLRVTPVQERRSSAYLTKYEMVRVLGERARQTVNGSSVFLSSR
SRVNERSALELAERCSDPYSSSVDPIFIAKMDLLQGRISMIVQRTWPNGKIENIPVSELL
IDKTMLNMQA*
>TCRU_3465 elongation factor, putative
MRRFLRGVTRAQCRVTHLCSLRHINFREDLRNIAVIAHVDHGKTTLVDSMLRQSGTLVEA
RNRVMDSKDQEKERGITILAKNTAIFLPKRTASQSERRINIVDTPGHLDFSGEVERALQM
VEGIVLLVDAKEGVRPGTRYVLRKALSLNLKPIVCLNKIDRDDTSIKKTEDAMQDLFLET
AENEEQLEIHFMFGSGRDGYMNDTPKKGGTLDPLFEKIFSTVPAPHCKGDDALQMLVAQV
ELDESTGHKIAIGRIFSGKVSVGDIVTVALEEKEVDALVKNIQLFVGVERSNVQSASYGD
ICILTLQEPIGQKVPVKIGCTVCQQGVVDRFPYRKPDEPTYSLVMQASEAPWRKKEADER
LGTILALQKRLEREAMVNTALQLSGLGTPHITLMGRGPLHLSVIVEDMRREGYEFEIQAP
RILTRVIDGVSCEPYERIHLEFFENLVSDMVSFLSNTMGEIGEIRQLSNGRVLMDCVMPV

RFMSQVPLRFHTLTKGDGVLNHSFETYRPMTSVQVTRETGALISVENGEATEWSLSGQAQ
QGRFFVLPGDAVHYGQIVGENSKTLYQNLGINICKRNEQLGGLRSNANDKAIRRRASFTA
FRATFEDCVAWVTEEELVTVTPKSIRMRQPKFNGKTTMRNAAQKR*
>TCRU_3466 electron transfer flavoprotein, putative
MFVRTIPSHFTTTSPLWMRVMVGVKRVVDYTVKVRVKDNKVQCDGLKMSVNPFDEIAIEE
AIQMREKKLADEVIAVTIGNKKAEEVLRTAMALGCDKAIHIVTPDGEAEGLEPLVVAKIF
QKLHEEIKPDMWILGKQAVDDDLGNTPQLLAGLLEIPQGTFASKVEVSDGKVKVTREIDA
GHQVVELQLPCVIAADLRLNTPRFPKLPNIMKARKKPIDSRDVSSLGVDTKPRLVREAVM
EPPLRKAGIKVKSVEDLYDKLHNEAKVI*
>TCRU_3467 hypothetical protein, conserved
MRWSLALRKVTRVPTRSVVLSDALRYHPRYSVPRKMVLSNSFNTVDEKNRFLSLRLILEK
LSGNVGRRQYRMMCDITRHFERLKEEGIDWHELKWLNRTELMDLFDNILSLSRTERAVLL
PAIEAKICGVLRRSDTRHSTSICVNQGVAEHGWRCGLYGHTNDVYFEGKAKANPTVLQIY
HRSSVRSVERSGVRVEVRTAPDFSVVGRFSHPLSPRVWSTPENPVFQVSTIGYEFRVHPE
DPRVVPQIEAAAREWELHAGVTQQVVWEMLEMYAVERNPQPLELKSGEMGAPDCPSLYSS
AFTVRHDSIAGSSDEQGAVTEQRMISTDGHEVPWFKEPLTQKFEGGIPVILPFAPSIVVK
SSFRQVTRTFPVDVERQLLQPVVDVTCFVHPQACFWWSEEDEERCLNHIVEYAKRIPYAL
PFNLYLRVDTTKNFRDAPSRVEEMEKRLAEKSHYFNLRRFRESTAANSEK*
>TCRU_3468 hypothetical protein, conserved
MFRKIYTPLTREVTTTMCAASLSLFSCTRWETSGRHTPPNAGDEDEEDFSMIFNDDFHFL
DECFGESGLDVFDYIPEHEAALLGHDKR*
>TCRU_3469 Mannosyl oligosaccharide glucosidase, putative
MEAHVDLFSWVALLSSIISDVELHLGAAETVPKRLWTVWLDTVHWDAANQRYADRAGCPG
DSFSPYIGYVNLYPFLLGIIDNKGRALTIVELAKTELMTRYGLMSVSYDSVRAARDAGLR
HENRWMGHVWLSANVLMLHALRTKYIDILGDPAGELFKRLRLCMLEISGGSPMMQEAYNP
VTGAAESTVSLVGYRAMLLGLLEDSR*
>TCRU_3470 hypothetical protein
MGEVDKSPKRIGVLSADNGSSSDRPNKFYHWHSGSQTLPFIKNETDIVQWLLEVDAARGK
DTPRDMEQEELVAFFKGRSQQGGTLMASVSTTANASIPPRGEEDEPHLTRSVARLSPHIA
FSAAYHGKPMREALSSAPQTGRSSLPCGTSASRFSGGGVMMKTATAAGITEGLGGYSPGF
VPKQQKQHHQQQSPLVLAASLGRGFVHHLVTGNSSSCSVPDLSLPPSLGTGAPVTFCGTP
VKRTVNGETTSTTAVLLETTCGGSDQTTPLRQRILPVADRIRQPPSAPHHDRRRRDMGLT
LCCNGVTNTKSRSLQQLRLNNPPKLCPPYAPRLLALVTECPTTNTTTGDATGPAQHLNSL
LPGRRGVGVLSKETTATQGR*
>TCRU_3471 glycosomal membrane protein
MSDFDKFVKLLGQTDGRDKIYKFVGGVLKALAALDAVECRRSAYKSVSSSITDGRSLMRM
AKWMGDIPKMRSVFAKCAEGGRMEMTALIMFLRVLGNFLYILGDNVAFLMRYKLVPGQPK
RVQYHSKVSQFWGFFFAAVLDLLALRTALQKRASDAATSCKEAKSALINLTKDASDVLVT
MAAVGYMKGVWHPGPVTAGALTAVSGGVATYLNWKKIK*
>TCRU_3472 hypothetical protein
MSVPFKYRQHIRDIHESFHDGKPISEEELMSVMDIIASAEHQALIDQYRAKCPLNSNSFL
SLMYELQKASKISYDDLVWNYIRGALKRGVFNGEDRTDLKTLRSTLDPLMRAVRLDVPNL
YKAHAPPDGEGKITVDQLCDVFRAMYPANQIVYLEMYKWSRTKKVTKGRGSITELAKNFE
VMTPPPFAVSAPPAVPTSLSVSKPPQPREDTPSEMLKGTTNAAVVDDSTETASTQQRSLR
TQEKEFTKEPQEELEKLSQLPEEGPNLPDRSVPTEKWKITSEADGEAKKPAVEIQLPDVG
ETKLRDSTNPKVEESAEASSIKETEANIQLTPSLFSEKKLENGTVSPESLPAATFPSPPL
DPHDQQEPLEGEKDAVKNFIASERPSVSVTSLGSFPYCDLPPNVQVERRPGLNFNPSYIR
DMEPPVRKQKSFSDGDNKPSLPPRLRGHEEQPAWEHEEVLLDAMLSRVVPGKPVDSQVME
YLKELARQEEELESALRAEMENLTSNMLACEIVEQRMIILRAQRQAQCFDRMESRRCKLL
EEMEKYLSGSRKAHGQNKVLLAGIKESGMVSCRKDGHSSNHDGNTTNAREKPTDFVSMFT
PPPPLLSAERQRRTLKRDLRLGEKLQRMQSRSPRPASMRRVSLPQDPHFQAQQLPSCMEP
EFLQGSPTSHSFGNTSQRGRWAKKTTPRQPVPVVHSRPIQQQKQQHLSPRHFGGEDFTIL
VNSGSRSSQSPLPRQTPNRGVHPHLTTRMAAEVLTSSQLQRKIEERLEARFSKAFQAY*
>TCRU_3473 histone acetyltransferase, putative

MEKKSDRKAEEYPRQSSILGTPCSVRGPDATVRRGQLLEVRYLWTPASLHVDTHAPLFTP
PLYGFVRLKDLNHRLSGWFSLEDIGLKEAKGGGGGGNAAAGTVSPPPCGSTRIHGPILTS
VYREVTLGMIRTRREEKLLDFFEERLLRDCRTPTTIRYFVYLCKFVFAPWYYAPYGIFHR
EYDPLAPISMADQERQGLRLSEETGGINPFIRDAYLCPFSLRIYSTLEQMQHETRCYRAG
RLRPPGREIYRDESRGFSMFEVNGSQKVTYCRHLFLLGKSFLENKLAGHDVHNYFFYVVC
LHHRHFPQYTDDPSAMYFVGYFTWEKQVADNNLACIVTLPCFAGGAAQQKDSGGSNGRPQ
LGHFGQFMIAASYELAYRRKHVGSPERPLSDLGASAYDRYWRRVLVAWMHTLVTESTTVV
AVSDGDSTDDQSVIVEATTPAKGRRSKRSRAGSSDRNSETDDDVDVRAAQTETLKVTSVR
GIAQQVRLEEPDVLRTLLNMGFLHLNAEDRSLRLVIPVPFVRREYERAREQAKDQTRAEF
EASLLTSSGAAAVMASSRQQQKQPGVAR*
>TCRU_3474 Protein of unknown function (DUF2826), putative
MLRLGDPHIVVVLTNSRGTAHTDALSGRSEASFEALQGAECVDAATTCMECHDGTAGCGW
WNTCFSEATERELLLLLASLLPFVASHADYCLSAVHGWCVSTSDVVCRHSTAPAKHTSRR
MLWLWIWCCECSRHYFARLLFTAFRQMDASPPESFTAAPCAVLPAQDSPVVWRLADSQTP
LRWDMQGCGLPLTVLGTAVWMRQPHERMYCGTIKCVKYAESQRLQ*
>TCRU_3475 Mucin-like glycoprotein, putative
MMTTCRLLCALLVLALCCCPSVGASEDEPEGSSKTTTKNTTQPPVLKDKAGPKPNVGDVP
PPPPSDPSHPTAGQPGMQSNGSAEQPRNNAGYGGSSNVTVPQLNGEPIELQKDDKDSMTE
DA*
>TCRU_3476 hypothetical protein, conserved
MARDAAAPTISQPIFTGERRRVNTHTSLANLERPHFPHSENSPSRSPDSEEVSGSNEVDL
DFLEFDVTTWVPRIWNGGILGILFEIRSACDYWHRGDAHTQIAFDISGFVDARRGYIGNP
TSSLWTWAGLYRGPSGCRL*
>TCRU_3477 hypothetical protein, conserved
MTEAASCLDVKAYFFSCLFTAGDSASLLMPRGGRHAGGAHTAPSGVQGQPRNLQIIIASA
IAGVTTVVHRLWAAPPSVRIDVWIDNIRIAGSIGDVTLWEAQVLRNADSCHASLGEDRES
GATHYTFLEVQFDHTHRAVSLREMFVRSVCAVPALNSSTIAEMEVVASRFLCAAAILARV
YVTTNFLSRQCDDDYPHITGELCWRNPGEPSAGSDCLGRAIATHHPEYNRKRIIKPTEKA
SAAIITDALFCG*
>TCRU_3478 hypothetical protein, conserved
MNQVIRSRTDWKERVVLRLAWITASRWSEIAALTPNNLKLKTDGTLILYWPVAPKTARTD
PHRASRFVRIRGQDAFDIIKLCRTLQHIEKLTNLTTARVERALAWNATAHSIRRGALRHA
AQIVEAYNLDPHVVSQLAKHVDPFDLPQNTFRYLGSYTTMLTQVLSLVALM*
>TCRU_3479 D-isomer specific 2-hydroxyacid dehydrogenase, NAD
binding domain/Acetohydroxy acid isomeroreductase, catalytic domain/
NADP oxidoreductase coenzyme F420-dependent/NAD binding domain of 6-
phosphogluconate dehydrogenase/TrkA-N domain containing protein,
putative
MYSMLYFYRQTWRSLASRAEHKWDPFLMVELRGRKVGIIGYGDIGQASAKLLSAFGMEVT
GVKRSASTKEVDEYGVRLVHGDAERERVLRESDFVINILPGTEETKRFFNKELFSMMKPS
AVYISIGRGITQNEDDLACALRDGVIRGASVDVFEREPLPAKSPLWDISDDKLLLTAHSA
DRTANLVPDSVRRFIGLVNEYAKTKRLDTYLVDPVRGY*
>TCRU_3480 hypothetical protein
MPSECRGGGRAATKVSCYEATAIERTFRGEGPVQCPAVDAQPRCLDILEADGAKFPAGRL
ACLWKLANPAAAEIASWHCCFDAVWRLGCVRSDFCAVRCFSVWGKPRHRNHI*
>TCRU_3481 BNR repeat-like domain/Concanavalin A-like lectin/
glucanases superfamily, putative
MGGEPREWIKWGDPRPLPKLISSASHEGEWTGFFASGGSGVLMEDGAIVFSLMAVNEKND
GAYPMIIYSKDNGSTWLLSEDISPANCADPHITKWEGKLLMIVDCEDEQRVYDSRDMGKT
WTGTIGTLPGVWVNSQSEDYSEGGLHVDALITSTIEGRKVMLYIQRGYASVENKATALYV
WVTDSNRSFSVGPVGMENTVKGELASALLHSDGKLHLLQRRASGENSVMSLFRLSEELKE
IESVLSTSSQKDVFFSKLSIPTAGLVAVLSNASANNETWDDEYLCLNATVKNATKVEDGF
QLTESDSRVMWLVNIPGDNVRHVFLSHNFTLVASVIIEETPTADAPLLGAILGDAGPEYL
MRLSYTADSKWETMVKGDTKTTTESSTWVAGKEYQVALMLQGNKASVHIDGKSLGKEEAL

LTGETPLEYLSFCFGACGMKNSPVTVKNVFLYNRPLNVGELKMVRKSDGKKGNGDGSMRG
GVSRLLLLLGLGFCAFLALY*
>TCRU_3482 hypothetical protein, conserved
MSLLETVHAEGNYHDKYEYLSDVVGHLFRSVQGLVMYAEGSLNSLESLRHGLCGVWGRHD
NEGDAGGQKTVNPPLPNSTLATGPFPSSLPSSTVSAAANSPMRQPQEQQRLAIFEEERAR
LEMLAAQLKYAQRHCCEVVEGLGKGKEELQRQCTAHQQQLEHLQQVSLRNNTQELSRALK
ANEKALVQLREDLQQSFATERAQARERERELEQRCVHQSSEVRQVTEMLALAREQLQMEV
ERRNQAEQRVQEAEEAAAEAQRHCGVAVSELHRDVEAEKDAMEAKLQHLQRELIARRAEA
VCVRQESIALDANVTKMEFLLEHGYERAAAEQHYTLLLTREAARHAQIQIATEQRALRER
EATAQQCYEDLCSLLECMREDEFKAEAQQREAEADTEARRLIVSVETHTGIIPKKKKNDE
CGAVEEVDDGNDDGAGGKTQVLLHRYGTPTTLTGVVTCLQNAYEVVRRHQRYMQSTVDVA
KERVRQLQQEVATLRPRLEQLMAEYEKQQVNFKRIWNEQTMWERQETLKGLLAKQEALLA
TVNVEREALQVRWSTLSEEYRALERRNSQLHERCAVKEAENARLLGFLRAKQIASPSSFS
PSSSTPQQPTSTSSRSPGVVRESPAVATTNANETAVGNSPDTRRTETSTLPRHPVSRGSS
SVAGLSGQARTSCSSFLDEEAAVSNSAVGVVLPLGQHPHDRGVWRTACRFGRSVRTTAAA
TATMAEEEPCKPN*
>TCRU_3483 hypothetical protein, conserved
MSSKTGTSAIAGQPKAAALVATVFQTSLDNLLLAATRHRSMITLRDAVQSARIKGFSAED
IFHTFVLACDTLHGKRPYRPLLMQTLSDLGKMASLRMLPMSCNVIHVICHLVQKLLGRCP
SGNVNLLQSSIRSTTVRGRSGFDSIASSLLLDGASTLDAEFREGSTSQYPGSDSAIDTHP
PDEELHMRILQTLMAYLSACEFGNAALADIMSILFLVYTQATPESMVEATCAATIEERTQ
ALLRCLRSSSDAKQLEDDSASFTAQIQAVAYAKDLCALCSGATPKWLKLSLPRLGSVSGG
PSDYIFAGIWTPPRRLRLLLLRTLITHFTESERIHSIHETNVFFTQYLNEDVVSLVVGSG
RKPLHVQRHPEEKNVEQNMGTMNLVDLVLPDEQPNNEEFYLTQQLGVIAISKALPVMRHA
MPILLRHHVMLVKLCSEGEDGELGGNARRMAVVLTLWQKVIADHEILQQLLLLTGSNSVL
LPNRPPTVSSANIPSPKKQGLRRPTRASEDDAAALLLDSPPDQGGAMADSTNEKHPFADL
VTVTAELLSDIIVASGGMLNLDNVRANDNRSHPGASASAAAVGGGGGGGIAAAGVMGGGS
HWSAISMAFSRPLHLSSTPKATPNQEDGESSSPERALTSIMAQRHPATGQAADILSFLTI
TSQSFARVVEASKLGSSPTKTCSGPTTVSAREELTALFISLHSPLLRCFALAAQHLQGDD
VIHMVLKGVTHLVQTSCNLALPAQRESYLQIFVSRLYMKRDKQLLEGELVGTRSKNRVAE
DSALPAKVTGAALGVGHGRNLPDKVTPQMKHATVLRRSLQSLQEKLYVLNCVISVANCMG
ASLEEGWRPVASCLILTEPLLKDYHRLMEQQQKQPQYGAVDEITLQDALRNADRVRDALQ
LLFINNALLPDSLALEDLLKSIVMETCNCAPSIYLLSIRLVSECCSLALMTVAAGRTDVS
FAQLRRLWGTVKPLYLHVFDPVNMVKLVRVCLHSERMMEMVAGIFEQLVEDIAVVTAQFC
QRLNKSFSDMRAKEQKQRGEEKDATLTFHFAAGPYATAALVARLAYLCSSPTRIQQGTTS
TKCLVHDNGDVAKDDVETERRQLLASPTELLAVIYGYLQQPRQRHEKMQQEDVEEDVAKN
DREAVETVTRGASLMVLKEVLKLVQGFGEELRGAAWEHLLHLLRHTAAPSGAKRHPHPTT
APLTSVKQSIGIAFRALETIQHSCITSLDDEGLRQLIRCGGAFMTQQFPGLEHRLNINLS
AVQLLWSIADYVVSREKGDNKNDDILWCTLLMQLYDGCLDVRPEVRQSALKTLFSLLQTY
GGRLSAECWKCVFIAVLTPLMEATVQAANTCVVNSGTLSMPDKLRFRRCSSVKQQLRRGS
SDSGLTSFSDSAQILYGGYANEEEQHIAMLLAHLGEQPALFESMRVTIMDAVCRVFVSHY
QAICTVLFVNCSDAQQRTDREMLLRETISLFIGLCETSQLVARTTSGESSALSAVRALHV
LMTAPGMTLLTENELESAWRAIEQLLRRDGDGANTMRKQCTTAVVSTIVTSLGDVVAMRI
AEREQQKQQKQQQQRHQTDALVPATNVKAPTSSTFFEQLCHAFSSTPSGLRGGNGGSTGG
RARHDYFPDYIHIVQESLCSFAVADSYFFPSRVQTAVIGVWKTLWPMLNEDERGAIIKIA
LQQFPTRHSIMSFITQGTNSRGRKNREGVEEKITLTVTMAEPSPCQKEEKEERQISLSDS
LPPGAHPSFLLALMDFFRFVATESEGEAVAENQQQKYVMFNAAPALIRAAGVLLLLEYAS
PALLEVPGSGLLFRIPADFFDRAERCLTFFLVDTLWGGKSTPLCKAPSSTPPTVMYGFSA
SLATSRREGLLAFAFLFSSLVSIASNQLGAFTKNGLHDVSTRDETPEHLRCASQRLESLA
VLIGELIPNVIRRSGDLASTTELLSVLVPSSSLESPLLAAVAKRSLDVLRRWSVSSSPSS
PLLSPSSEESAGARGKVTAVGAEGSSSSRQKDDDTEGPQMTAAADAVALSVEGSDLSLDG
EELSNRLKSVARASMGARNLAVLQRFLSNTANEETRQMMKTTLQTIVLLYPPNLENQKPP
SGWHSSGVMPAAIEGNGASDEVKNNRKKENDTGEAREVLQGILVELARLVEHTGDDAELR
SMLSRAMLGICASLGFVPSA*

>TCRU_3484 kinetoplastid-specific phospho-protein phosphatase, putative
MSSNVKQFNALRKEELVQRVLELEEENLSLRQEMEQLRRRSTVIRHYDTNSLPSLQRYSD
EPQPTVSPTGIGRPSSKGKKGNTYTVSFSVMLIENGTAISVPLSSVIDKSYMRPDLRDAD
TPVVASSLMRDDYDTESFARHSMDSLCVDRSLSASADEPLGRSSELHGVSLACATTSVAG
GWVCNGSGGRPSVLLEFIPTDDANEPVPQDPASIQSALVSSQPREQQERCQQGQDGRHWS
VRHDIRQLSLGRVLNKRSISLDPDALVSHHTLRTFHKQLLDGCGVLSPLSSYGNAIYHYI
VKTFAVRNGCRHSSSDIEGFGRTLCSLCGEVKAVLQREPRHGSVVSPCYVFGDLHGNFLD
LFYFLDNLISFQDLRYTPHRFLFLGDYVDRGEFSVEVLAYLFAMKVLAPEKVLLLRGNHE
DTLVSGDISGYGNNSFRAQCQNLFGHALGEELWHRASETFAYLPLTANIDGRIFCTHGGI
PRYRGGVDDRLHILSRSDFPAFESFFQVPEEDTAYMRMCRQMAMDTCWADPAEDESTLDK
WGFGANPRGSGVIVFGSVAVDHFLLNNNFEYIFRAHQEKSDGLKLSKSARVFTIFSTSAY
VGHENGAGVVLVADGKIRLIIKSADDTARTPVVAEEEEDKDEEEEEKEAEIEAEEREEKK
NEKMRKSSRLVR*
>TCRU_3485 hypothetical protein, conserved
MRVSEDKRLRGQSLVPGQRNVHLEPGLTVSSLFDTHSGHKEKVVRVFDEKGCLTTREVGV
KRFQFARLSIFYMIRSLTWPVLCTYLLLLYIAILFAFAMVYFIWGNVCGAWEDAKWVTAL
YFTIVSLAANGGYFGEPTDTMLQPDHTCFTGRTVLVMLLSYTNIIFVGIVAALVVGKAEY
GGRLSQRIVFSDFCSLTSVPGRRDRCWRLTFRMANSDKQKPIAHGRLRLFIVTAESLKDR
RISRGEMRRQSLAELPEEQRSSKGGGKSWRQRLPHEMSIVSMEQEEGGIQLLRASASSLM
EDLSPADTSLAVTQGQLSPKSPRGRQRRGKRRRHEEQKPPGTKCELATGRGKEGDEDHAA
QTKKTCIGSAPTCGSTIAVAISEQGRGISDPSPFAEAGIEAAQHQRHQERLPLKHPASSH
PTSSFAAAAAAGVFAAGGNHEDGFERVSIHVEELRWTSSNEQYLEARQGQLSLWFPVSIT
HFIDRRSPLFRYVDQEKMDPAMLLSQSLSSASPTLKTTGEKGNTIGSLRSFQIVATFDAT
EMESGATIVAKRTYTLNDIITHYRFSNKMVQMRPGSNEVLIDYHYFNEMIPVSGMEPTYT
STSESDTTL*
>TCRU_3486 hypothetical protein, conserved
MEKYRKYGDAATGINPFVLMRTPTTFSVVVAAVLFPLRLAGALLFLTALVVLDVVAYFFF
LIPGMSCVSWCLLAPCQRELLRCLLFALGNVGITRASPRATAVANAAGGDATPVAGDVVV
VNMQSVWDMCVLEVAGRLPLCVVAFYIGGAAGANSGGDANKKKTDHDSRHDTLLVMEPSP
FQRWRVWWHIYHTGSLEFLRAAAGGDCGDYKSNTEPVLHLAALQQRCERIGVPIVLFAEG
SCTNGKGMLRTSPLWVRTAPRRVFVCAVDYDTTAVHTVLRPHRLLSFIFLMSASLHGSRD
PAWYSPPFPTATVRMVALLESSLSSDSILATKTTAGDGATVMWEEAKVRQALCAASNSRR
VLAVGLHEKCGYAEACVGVKAGSVSN*
>TCRU_3487 hypothetical protein, conserved
MSQPHNGYEQLPWWKRIALDDAYRRRIRKLATQRYREELHLLQEFPREIAPAAWDDGRPI
PQSSQHICVEDGTRAWATKYPESIVKVSGFQKLQTAAVATVISGPRAEEHEAYILEEVRR
LYNEEKASERLYGLSYFWNRQLPPNGFIRVWPSPLSAMWPSLLISEPFTPMYKTLSAPGS
SAELPPGMNPTCMTDPLFVHVFRRNGEYLGAWRQSLFLIRYKVICWALHEEERRRTCGAV
GRPVLASDKAFPDVATTQMLKNVPEFQLPLLRLQRETVTFSAKTCRLNLQSLSEPLTWEG
IVPAPTTYRAEQAYLNWHSLHRSSAVVRQAVRDVFNLLPQNEDGLITKTTYVEFFLDLLN
LFFPMHISSANISIAEEEWVYRGTTELMRFETFYEKFFSFPFIFFRDLSKVTRDQYVEVW
CLIRVCLLENEVSVLSCLQDTAVLGPPNSVTSGGLSFNTLSPAANPSAPRPVVDYSRTRR
PKRILEATLCSLQKLKDLGRKKAPPLIGKILGVSYDDILRLGGTRFHNTDNYPLHSAKQL
AAVMVKENLQKLMPSHAIAVVHAKGKQLLAETSRARRQQELQEQLQKKDNPGRSMDSEFS
SVLTIASLSKNENEGEETEGSSDLVIMNRSNSFRGRSLAEKRRKKMMGWSHVTTGRHATL
EKIEEARMKKKLIETSVFYRHRRLRRNAEHAKILSHISRSHTAWEVCRHDIYEIPPEKPD
EEDKLLAFVQDLPDDFFDDENSLRVRYGIHAALLKERAERSKNCYPIEPVLSTSSMTMAS
SRDTPKGNEQDSPATCMYTALTADDTTNGSSRGGAVSSVHVFRTRRRTPNGEGASRMSNS
PGLTSLVGDGSSPSSSCRMNSSSTPPSSASSGTMRGDPPEERNGSRENARDMASLEEWSK
RKEELEPKRAFYIRLNFKQNMDVNYKKLLQKKPHRLFPLSMPTARKGAPSTVVKSRLQEG
LRPSGAQQTF*
>TCRU_3488 hypothetical protein, conserved
MTAGGGVLTPSSAGAQIIHLASRLVVGVRLHGTIAAFHPRTRGQLKCRPFLRYFAETLHR

LRCDVTLFVPVNSRTGPQQVAELSTHEFPCEYRVVHDPTSGAVDDCSRGQRRGGGGASRG
NFQDLLNRVATEQQTSPSRILFIESEINYRFSPVQTLVVEAFQAHRIRQRRRSMGEAAEA
EERRQAGEHHAALLESEYSWTQGGRVSRLRHAQPSAPFSAELHGDPQEEAKKDDVRSAAL
AINREDYTLVALAGMLVELAAADVAVADYLRVEPLVEKLTVPFHGEVNYLPMENCDDMEL
WDWAAVEVREARRKEEAETAVPEVEEREEHRDLFK*
>TCRU_3489 leucine-rich repeat protein (LRRP), putative
MNLHAGGLSCGASSIKSENTVATTTATKLKGSAELTSLVKVSSWGKGSSNLCDCSSCQKK
TSENFMTEPKFDLSLHKSPLLPQMMTHPSAIGPHTVSPPQSAKTQDSVELPSSGKDAATY
SRTAANTSLLPPCTVERQQFHQYYSHPHQQHHEQPPASRNLLASGGVPLQRYRAKLEECA
DLHCRLQDTCVELSVTREHLCSVKVDRNNLINEIERLRDVLFVTTTELQSMQQRVEELER
ENKHMSVELRGLQSLSMCPSRRMEIITTLRATPYDIDAGKSENIYGTNTDSFAPGVMGAR
RRLTNSAAALCLESNPSLLRRSLTCADTVKPTDILASYVCECFRTEVVPHMELLRSLYDG
SRLMAVDPPVTHAQLSALKRVLVEAAAGTTRIGGAGAASIRRLEKRPLPRVGGRRLNPLT
SAKRWNCQVELLSFRCENWHASTSVLLYMIRSLESVRHVEIFSLTDVAFLRQLAEALLMA
PHVEVLSLPELSLADEGLAVLFSFMVRQEQLVTKAPASIVETKSSIRTKYEPLGPNEPQK
GIGVLSHNRDHKEDEDDDGAGGIGGKDEEGEEVSCKLGDYSAQFSSEGRYVKPQFSIPCL
DLSRCRIDHPTGLFRTFRGPLVKVLVLTGCSALRDAHVRDVLEACPQLHTIDLSGNEGLT
TACVRYISQHERLKVLRLENCPAIKRLELANVEVLFSSLAYVTRLYAPELCRLPVPLTQS
HVLFDLYAPKLREIILKGIVVDRGTLAAFMHNGSNGFAGEREPSSSLRAHSSSARLVEKN
FLEEQQQQEQQPPREGEGEDEEMEEKQEFQEEQQEQQQEEEEENQHEPGDVPQLLSVGFI
GCLIASASELNSFMKQQTQLLRLSLHGCHGLVDSSLAWLPHTLMDLDLGGVAQLTGSSVD
VIATRLPQLIKLSLKNAGTSIQNADLHRLRGLGNLEVLNLLGLPQLFPEVVAAVVEALPQ
LRVLYHETVVVARTHPTTVLQVNRIDEEDTTHHDIQHCLKELLQLRNETALALWMEAQLP
KPAQLLPCRNGARSGTEMGAKQPPLTINTTFYEATQLELSQQGRFTMALENEEEEEPSTA
ENGENTVEQEKPDVLTISSDHEEAMPHDFFDGEGDNDEDDENEKSKQSLYDTHRRTESGM
DEKSRLAIEQTPLGPWGAADIVAEHRQNSGG*
>TCRU_3490 hypothetical protein
MDAEVEETGKPNSAVVSPVDNEERQHVVDSEANGDPAAMKAKSSATDPKKVPPCDEYPTE
APKYFANNHHNNRSGSRRKRKGDGGGGGKKEAAEEYPIEESGASSQRLKGADVHPTTSPR
KEWKGDCRPVRKRESSQKNPRISPQKVYSAPIASVSRHSEKLTQHYGKASPTARQKRGQQ
QQEQQQQQQRPEYLLSMGKNGEYVSQTPPAEEGGRRGRVRNQAFLEHYTSVYMKTPRAPR
RQQKLRPINLGGEGGKTFLNEAAQAYDRKADDLHRDDEYPVEDTQDGQVPLLGHTHKSDN
NMESDSVESKAKWIPFSRGVNHLSSMSMVNASSVKKEFMEDNVPSRDETQRRISSQRLLQ
TRCQNDEFQRCLHELIGLFNSRSEKDMVTSLGFSYACILAGKRRQKPSEIPEVWFMSRRA
LLLPQHRGAAERAQETTQLPLIYSNSNNNNRRNESSASFAENVPGTKKSGCSNERRGNGL
QSVRPAQQESQDAFAELELEECEQGRNTDGFKNVESTEKLDDVHCEGEKHKELLAR*
>TCRU_3491 retrotransposon hot spot protein, RHS3, putative
MLTRDPHGDFDFGRLGTEIIGMEGHGFLLNTQRLMRGVNKFYCEGLNSLRQWRDLGRKDT
VSPLGRANLNEALSQVLNEERQSRRHKKANKLEGSCESVQNARWSRGVEVPGGEGMGMEV
KEGEPPQPWTYKKVVLSLEKDDDVLQSGAERPRLMVLTSDKGWPYSWKWKENKSTHDCYV
NCEVERVWQIVKDDLTEWFSSHGRTDFTPKRRVLIGMPGIGNSMAAGSYLLYQLLQYDAE
KLQVVVHCFGETTYLFDKTIKTVTRYVGSKTSRNVLYDLRHLKMKGYVIYDVTEKGMPAS
WFSPFNEWGMIVLSSPNVGNYEKWETQVRAERIIVNCPDEMDMKAMCAWMKRGLDPDKQA
EYWKEVKERMEKLGPILRYIFDANKFIAHSAAIEDALDGINSRDGEKNFTHGGVKLWYSE
NPSQKLVRVVRGREVGAEVFLNAPISVCLGRRIPHYFGKRDE*
>TCRU_3492 retrotransposon hot spot protein, RHS3, putative
MKQSDFILLVLRFWDYVPPCLIEKYAVSAFLNKNLLRAIRLRIKELRLPGRGESHRCALK
VHSGKSFARKEVLPPPERLSNPVAMDHWVLYEPKVHKFPLVDGFFFVDTNPKTLVGLRMS
TASKHRTTTSAVRRFTECLAAYFEGWEELSRDMSWEIIYVRHEIYRPMEGRQKFEVVNSD
NLGDDENREIAAFCREKVRQYLAALSSADARRGEALRR*
>TCRU_3493 BNR repeat-like domain containing protein, putative
MVAGNYSFEVTGTAGNTAEAAKWGLLGAAGRVTNDGSSGKKKIYWKDAFVISLTDFETKH
ESLTRLIGGGGSGVKMKDGTIVFPVEGTKKDGNAVSLIIYIATEYGNLSKGMTDGGCSDP
SVVEWREGKLMMMTACDDGRRRVYESGDKGGSWTEALGTLSRVWGNKHKGNEKALEAVSA

QRRLITGT*
>TCRU_3494 Adenylate and Guanylate cyclase catalytic domain containing protein, putative
MAMRWVAAPYLFFALILLLALFSPQGAASQAAVTDLHVRLWLMRKMNQVRLKGTPLAFYS
GMNASLWARNCTVAEGVRVEVVEKEEVQEGNIAVLEEEIRKDTNVLALVGKQSGEFLQLV
LPLLKKYDLVSFAPFTGSSLMRGWNPNLYFLRADPAAELLALLRHAWTHLRVHRLGFMYL
QGVSFGDREYEQAQRVMLGMGYNLSGVFTVKSSMSRGAKKEVFDAAWKAFAKTQPQAVIV
FGPPIHDTVKFINRMLTDNRTAGAYLLAPLMLQDFFLRAWHGAVAGGVEFEPGQVITTGT
SPLAKDIQYAAVQRFQKVMQDYLAHSGQKDYVDKDHFLKDDGDGAMMVAGWIAGEVLSQA
LGNREWVKNRTSFLTSLYNQRRYMVDDIVIGDYGGECKSEAASQGATCLCNQGGRTVHID
KVMGAHRLKPFKGVMRFPLSNCNVSYVKIPPVFIGLYLLMDDNVFAFTACLQFDIVFAAA
SSLHERWWEQGMLLLDFLQSNTYDSAGDLRSELQSRRAHAVLGVVTDAMLDVEGMTFIDP
LLLEPRLNRFRRNVIRLSPTLEQQFFVIAKYLGKTSGGSAHAVIRSGEAAAMADVLRLSL
ETFGVSLASVTLLAGGDVLADHLPAERDVFVVGISADDAGAIARHVASNDGVRVFVVFSE
FALLHAEFVAAFRDCAGVDRVVFATSLPHWNDESSTSETAKKFVIAVPEKKPDASGNDGL
CCHEADASGFLADGPYQR*
>TCRU_3495 small glutamine-rich tetratricopeptide repeat protein, putative
MMSDTAEAHSTTSQPLTEEHRKLVFSFMRMLRGVNSPDPERVNTIVKLLGDEYGVNPAGV
GGCHDTGVDVFEAFCQALQSQESKENIQQNEKFKAFLDLIQKKGYFAGAEPGTEEYASRL
EKAKQKFEMRNNPYQGMSAEEIKNKGNELMGMAKYKEAIAYYTKSIEMEPENHVFFANRA
AAHTHLKDYDSAVIDCERAIAINPNYSKAYSRLGTSLFYQEKYARAVDAFAKASELDPTN
DRYKEDLKQAEEKLKLMGNASSGSSAAGGFPFGGGMPDFSQIAQMMNNPQFIETTTRMLE
NPQFSQLVANMASRFGGGSANPEELLQSLGSMGLSGTNDDGTMRTPFGNINRERLEQLQE
DEVKNNPKFRAIMEDVRQNGFQAFQKYMGDPDVMDLMVRFQNTIMGGNNANSSP*
>TCRU_3496 Concanavalin A-like lectin/glucanases superfamily/Trans-sialidase of Trypanosoma hydrophobic C-terminal, putative
MNGDENPLMLGLSYDKEKKKWRFLCGGKTTTEHSSDWDPETTKKDHVVILRRNGSQGSAY
VDGQRVGGNAPCGLENTDSKEISHFYIGGDGDKTRKQEDVPVTVTNVLLYNCPLSSEEIT
ALTPNKAPTPSLEEKPSDPSTVSSGSVVPPTPLVTPNAQQTETPSTPAGTQLTEQGQSMG
NSKGAGSGGASTTAVSTVSTPSAEEESVVQVTSGTSLDGTQTVDSGSTADGEPTMQTRGG
TNGQGEEGIQPRIRDVNAAALNSSLGNVSQGNNSDAGTVRERRLLPSLLLLGLWGFAAL*
>TCRU_3497 hypothetical protein, conserved
MPSDRHIPGNASYLSGELLLVEVPKMRYGSGLPFLQHRAKTAWEWLRHGLCSLRRRCDRR
AVSRSRPRSSAGRWFRWDFWRLRLTAKAKFPQRLGARSAMGVSHVRHDTSAAAAVLAAHR
AVCRASDRGGVGSSRRRWLFRAKFFACLMYVIRAAPTACAWDGGLQWSQQPSVFVRNTGA
GAGRCSLLPASDSGSRARGLPVACCSGAVSSWPELSDTNNTSSQCDDAGAPCVCELCEVG
RLLGLCITSLFPGHAAVAKDKPHPCARSSVLLARKKGTPTM*
>TCRU_of Trypanosoma cruzi region 4/Dispersed gene family protein.1 Dispersed gene family protein.1
MDAFGDALNVTLRHCVLAGGAQLRIGGLSESTARLMPHAFVNMTNVTSVEGTIVLHGAMP
LHSSVLLANSTLRATVGGSQYVPTTRGHERFRYGPALVLDGVRLLSTRLVMTRSTLVCGG
GSCAAILVERGLGVNLSSVFYMDNCVISSRTHVIYALASDLRVVSGSVFSIQNSSWILPS
TEYGKAACVFKDVVVAGGSVLQIVSSTFRLGFAMLIANTLTVTDGSWLVHRNNEFRTAYV
VCVAAHYSMVFRDRSVWSILDNNLTYGSYSTIANMTNNWLPPSDTRPTIYGMCNEARGSP
VTNYQDDLNIGAPVTVLDCGACTVDAVCFAARTSSISGCECVCAAGGYGDTCLPAAVPNG
LGPLPPPDARDTEVRCVHGGSISSVDYPDPGVRGLCFVNVTLTTPIVLDLSYFDAPQQTL
NITLLQCVLMGLLIRGSGVRVHVSVTSSMLDFGALDFRGDFGASSQILVVGSTLVTTSGH
AISFVKFILSANMTLLLLDNYIEANRYAVYFFNGVVVGGGIIVKGNTLSTKKDDDSVESS
VCVNSVDVKNGGYFDVENNTMSSVNGVIFWGDTTVSSAGLLRVADCTFAGGTEFLDPALV
YLSGSVTLEGGAQWRVEGNSVGAASVLIIPHPQYKIKLSGSGTTVALANNRQVDNSYPFA
DLSPPDTIVELPARFVVGCNLQGDEEVLYDGVFPEDVVVFRCGTCNDDAACYMPGTESVD
RGSCSCSCKDGWHGASCLPFEVPDTVVPPAAERAVDGDTSCVVNQTLTDLALNMWKTHHC
YVGVIFSGVGAVLTFFLNSMPLHLPINITLTGCTFREGAALQFVGGAAAVESSGVLIRVS

RTVMRSSTVAFIRALPQHCDIAVTEVDAVQSSEFELPDTRMNTLSVLLLGDVVLSASSLL
VSNVRAHATKRDALGFYSTGTLTLVGGGSLYARYCSFDGYTHMFYLYGLSVSDHSVFALL
NNTVFSGASLLYQRNRFSVSDHSVVRVVGNSGSVRYAIFNDDMWTVQRSSWLDWRDNDVE
VGAMFYDSSSAFVSIDGSSVVTLTGCKMGSTGLSVSLLSRADAGYRFFAGCLTVAGREVT
TAAELALNGITSVTAVAVCGECTKDGDCFAPLTTAVSGCKCRCAAGGHGDVCVPAPVPAG
PPPPSPPLPSPSPSPPPPPSPVGECISDMVYPEVPQAVGGGLSWLCYRNVTFSGGGMSLT
VLIGGMTGDVANVTFDGCTWRDGAVLLLLGNVHAAVGSLNIVVTGNRFIDALLSPEGVFP
PHTNITISGNRFTVTRAIPRPGLELDCPSCVVMNGLAISNDSAVVLSGNVFQSVTTSSSA
VYVVGSALRVLWKSLFAVLGNTFHMGGGDSTLINIEGSGQFSSLRVLNNSAVVIRGNVVT
RPVKYFLLVILALRVESRSAVVFQGNDMQRSSAVFFSSYSSYIYYNSWLQLSGNLCRESP
SDAFLFLNPTVNLRDSAVSVSGNRFMSSTGMPTVLRIPKRSRGLTNGAIVAACNTVNGGE
GVKYSISSVCNATILTCSDPCALATSCFPAYTTTASSDGCACTCAEGGHGEACLPVAVPE
PPSTDGADLCVRDVRVDVEVNVSFGTSVACYVGVTFAADVVVDVASMSGSVRNVTLANCT
FVSGANLYVFGWRSDPPAGERVDVLISGLESRSGGGVLVANRFPPGSRVTVVDSVLIAEK
RVAYRGAYDLGDASACLVVHNVNLTGSVLTIARTHVAAVFRDAVGVLLVGGVALSSRGAL
YVDGLSVQTALGLCVSVEGGVAAIGGSVVAFVDSDFLLCKHAVSVRGAVSVSGSAVVLVR
NEFLSAEDYAVAFYSTVSLADGSMLLAKSNVHDGVSREMLHAAGAVTAAGSTLSFVRNRA
LLPRMVSLSLSLAAGAHLRVACNDAGDRVLSTVEEYAAAGFGDAGSIDVAGCDDCDRDTH
CYAPGTASASMRNGVCACGSGGYGEACVPVGAPALPPAAGTASSVFFREGVTLRSVFVVP
AGASEVTLRHVVLDGVSPVLYVPWMARDGVRIVVQNVSLRNGAVLYVMGGGALRGAVAAG
SDEGGPVELSVCDVEALNGALVLTGTFPAGSVLTVTDSLLVAARPTPLVYLPGSQSSPYA
PVLVLSGLRLVRSVLVVSGVALVTVVTGGRTVVVDGAVLELVGGGVALDAAVFGGEYALC
ASARVVASGGAVLRVSGSQVYAAHGLVFESGVEANASAVVVNDNTGALTDGALLELRGPA
SFVSGSWLSVRGNSISGRLLSVPSYPRSAELVQSTLTLHGNAGSGPVVMDGTVALGGAGR
KFVVGCLTLNGQALRPMDYRSAGIIGEFRPVACGVCDADVHCFAAATRTMSGSCGCRCAE
GGYGRDCLPVYLPRVDGCNRTSGMPLLGHTATLTETRSLTSTWTPSHTTEHYSPTQYSST
ETLQVTETVALPPTRTPTASVSSTLWWSDVACPTLAVTTTVAGGSLTQNDIRGGGSAVPT
RLMVALPPPFRWARDPQLGTHLSFVPVSTAQPRGFGGPWGAMLSNATWVRNATNPSTVLE
LAVPVHRGYFIAADETIVIRCDATAVFGGCKGVLLGSFTIRSDTLPAAASALSAITGVVA
GAAAVAVVVTGGLGSVLEMQALGVFARMSCASAQERASTAALPYFLSVFAALDPLWMVVG
NALLAAVFGCVHCGVTAAFQRWRGVDAASAWAAMRFPSLTYVVAHAMHLGIFFGSLRALA
MPGARVQHRVIGVVGVLYGVAFPAGVCYFIARHVGASFTRYWQFSRKPLHERLLYPVGYW
HPAAQQRMYGSMLTNMRGSRVYWCVFQLSVLCVVCLIAAVQSPVGGCHVQYFCMAAVLLA
GAGVVAFTNMMRSAFLTVMHTASFVLLAALCVISAANHLAPSDGGARAYAAIVLLLTTVV
LAVTVYSVVVWYAEDRHWQELREPRRGGLEALLRDGEESDEETRKPHDRTSSSYASGTTV
ASSYRPPAPPQPMAGDTRSDALSLLDCASSARGKIDHALL*
>TCRU_3499 hypothetical protein, conserved
MHRGRGRTARLLQQHAVKHHRARQQVHAAAKVHRRPARLQRPQHSVAHTQLRLQQRRRVQ
HKRRRHRVRQPAQLGVAHRPRRHNRRVTDRHVRVCRQVMHKHKRAVAPGQPAHAQLQAPV
TRRLAAVAPHVDLRTAVAHAVPQFDAQRAGEHRRHVEHHSHAVRDVHAVHQHRVHGTANR
DGSVHRAAPQSQHHRMHRGHPRSCCCQHQRHKHRRTHSQNGPAPHTLPAARTRTHSRPHP
PTQTRISRVLVACRSRPVDHSSCLSNPFLQMANPVTRFRQIDIPVPPILILRVIPNTLKR
*
>TCRU_3500 Transmembrane amino acid transporter protein/Tryptophan/
tyrosine permease family, putative
MKNHSQTAPYGDDEGAGNIFFPVLVTDRNSFSKAHRNETSLMSCCSRERIMEMARSTKNI
DGVLHDFEEGPPTAEMLRQRKRKSLPQRILERVFPPGSMLSSAFTLGSSTLGGGILGLPS
AFNMTGYILSIILLVVVVILTVFSLWLLARSADVSGKRTYEDVMKVLMGRFPACMVAVLM
CGFCIGGGVGYIISIGNLLTPVFANPGVPYFLTTKPGNRLITSMIWLVFILPLCLPKQID
SLRHTSLLGCICILFFVICIIIDACQYASKHGFRSELEMFGTGNHAIEGLGMVMFACLVQ
INAFEVYHEMSHPSPRRMVRDSIIAMSGCGLLYILVGFFGYMRFGKAVTDSILLMYQPGE
SVLFAIAYVGIVFKICVAFALHQLPMRDGIYHLIGWDVYLIPWWQNAVFCTFLSLVLLLA
GLFVPNINIVFGLVGAFCGGFIGYIFPSLMFMYSGDFTLKKKGFLLYFLTYFLLLAGCVA
AVFGTGASIYGVAV*

>TCRU_3501 hypothetical protein, conserved
MSVKDPQSRDNEGLRPPSLTGTRGVGTTTSTSDSLTVNTNKQDLPSPLQPPLPNSSPHAI
PSSTSAAGPDNEKRHESGNGTKNSQQPVVGGIQAKKDTGVKAVASTGGGENSRLTVTTVE
EMSDATKKGDSDSSSAALRSDAGTEGTPTTPDSSQPSTGGAEQPSATPDREEASDSTENG
ISQSAGSATAPTTNAKTGDTAKPVDSDSSTAVSHTTSSLLLLVVVACAAAAAVVAA*
>TCRU_3502 retrotransposon hot spot protein, RHS3, putative
MKLEKGEPPQSWTYKKVGETLERKDGVQQSGTARLRLMVLTSDKEWPYTWAGTESTRDCY
VNCEVERVWQTVKGDLTKWFSPHAGDYFKPKKRVLIGTPGIGKSMAAGSYLIYQLLHYDA
RKLPLVAYIIKKSVYLFDKTKKTVSELGSEEIFVNLVKDFTLRGVKGYVIYDVAEQGREP
HVGLPFTLWNMIVVTSPNINNFKGWKKQNGAMGIVMNCPDESDVRALCVWMKRNEPRGQN
GYWKQVKERMDEVGPILRVIFDGDSFTERKNDIEEVINAVDSSNATHYIGVMSDKLWEAV
NPSHKLVQIVRVRKDSRTERYLNSPASRVIASKMYVHLSTKMQSRKIIKLLMHPDIFFLS
NVLEMSGTAIFMCRGAVNIIKKKLKELKPPGRRALQLAALQRNPQGHPTEAVGLPTKQGV
SGKKIAGYKVMYKPAYPTFPLVDSFFFMESPRKTLVGLQMTTAHDHHTKTSTVKEFTEYL
SWFFNGWEELSRDMSWEIIYIQHPDSKPMNGWQRCDVVNPSSREEKEIKAFWDKEVRQYQ
FIFSSEDIKKLHAIQESTTEKKQEAAVGNH*
>TCRU_3503 Concanavalin A-like lectin/glucanases superfamily,
putative
MKAKDGFKFTGPGSGAVWPVSSREHNGPHTFVSCDFTIVATVMIQILSKLGTPLPGAGLD
APVSTNFIGLSHDADGRWETVFYGIKTASGSTWEPGKKYQVVLMLPDGHRGCVYVNGVIV
GSPETIRTPETRGLKSHSSTLGATRETSTAAM*
>TCRU_3504 retrotransposon hot spot protein, RHS3, putative
MDVQGSWRHSRKDDGVEQSGEAPPVLMVLTSDKGWPYSWKWKENKSTRDCYVNCEVERVW
QIVKGDLTNWFSPDAGTYFTPEQRLLIGTPGIGNSMNASSYLLYQLLHYDAEQLPMVAYV
IGSQSFLFDKIIRTVSVYMDDHRIDDVVNVFSLRGVKGYIIYGATLACRQPPAGLPCKGW
GMIVVTPPDKNEYERWTKKWTLLQS*
>TCRU_3505 hypothetical protein, conserved
MIDITTVPTRSLTCPFPLVALDAAAFHETPTSRLLLLLVGRDRACVASLQDHKICLRRVV
LQYHRTLTAGACRSMNSKDGHIFALADTSSTLTVHRVPNLSSGVVPVDQESGTVFLGNAW
DKASRIDQVVSIQGSSQVDVICTRQGETNVAWAISFEREEARMIRVDGDLERLFPQQEIT
PGCFVGGTPHGVSLISPATGETLIQLKLPSGNRPAEAVQSGYAGNSYYYAVVTACGTALM
YDQRRSEKPFWSKPLWDSRSVNENGDAGTLMAYCCEGVWAVRAAERLHCLRCTEGSVVCR
SFPLPGGLHSSQFTTAGLHFTNDVLHFVGTNNF*
>TCRU_3506 monothiol glutaredoxin, putative
MFASRLLLLRLTRASHGPEKVAPALAASIKDIILKDRVVIFLTGTPEEPRCRFTAQMVDM
MQQMGVQYSFFNVLDDDDVCEGLKTYSDWPTYPQLYIDGELVGGYDVCKNMLLNGQLTRL
FQEKKLL*
>TCRU_3507 formin, putative
MERETLVMKEVIIDPNRERNVGIVLQFLRLPIQTIEASVRTFDELTLGEEHISGLVKIIP
HAEDLRPIEAWMRRNPKASVARLHQLSIPVRFFMMTMKIGHYAERLQCWNLKNEFKGRID
DLEVKICRTLEGVSAVMESTQLPRLLQVVLAVSNFLNTGSRFQEAKGFRVTQLPQIIEFP
TTNNNRVLLDYLVEIIDQQDPALHNWTEELLSAAENASNFDVPGVAGELKSVRARLQKCA
SLVRAIPDDKPWTTKLGKFIYTAFPALDHVEQLMSELNAKIELMPAYFCENSSSFSMNDT
MRCLANFAKKYNRKRDQLAAKQMRLSRLAAKDAENSENVTEDPNSRVNVNESHKVQHSLQ
EPKIKVEKERSKPSSPAPYALMAKGDRKGTVGNGRGVGRRTESLNNGSCSNSSNGETFSE
PRLLLFQ*
>TCRU_3508 hypothetical protein, conserved
MFSTRRLLTGAGGAEMTVPYTRGSYHIVPKKYTVGKRIAVRSYLDRNRTELSDRTYMPQK
AWFEPYTPRRFERDHEELSYNFYNLETKLIWTAFDTPEKIGMLIHDETIKGAKHLYNKEF
LDAALHWTREGRYWRCIGITKPFYNKKTLRVHCWHDRGVQIGTLVLSQAMRHALMDLERA
VRRKELGLPPNYIWDRWGPIGFIDGSKTDYLPRFSHNPYIDPDGVEVTELDVAPFNTHEQ
IKERYSEFIEPDLTPFSGVFRSPSHGALTLDDVPEQAAVELFRQLKSKAGTPVFSAGGAE
IAPADMRVLFYLSADPQRMEKARGVDSWQEVCEMLAPVQEVCDEKIEALRLLQNTRHDVN
RVRTFYEEKCGFADFMYTPDKVITAAVLCYLQELHRICTETEWGKPLALALTDMERVQVM

GREAFLVYRHIEDAILDKRRRIWATRFAGEANEESTLDYLLENFGRRTEQTRNVGTTGAE
FDREQEPIGRQVQRRVLDSDKANKLAEARQKRGKMWSQKKSVFDSLHQKQLQNVTYGVR*
>TCRU_3509 hypothetical protein, conserved
MDADGVPVRAHLSMPSAAEPSLAASTIDRGKFASPPATPFQFAQPIPTESAVTFYFVVFL
CNRSPIFHSNRIPLRDDTPPYTPGDIVYFGPSEEVAQQKLQELAFPLGFGCKSSLTRNGP
LWNSRLESVQKRGKVRYHRLMIASTFVVSVAVENGELRTMQTKYAAVALVVTCPADRPYR
QFLEENFDAVVRNMLHVVLSFAMKATQLFFFRLSPTTVVGDLVTELERDPQAIQTYGTLG
AILVNAFAHSHAIPRLHSRISCTDVFNLSRSSSSQPHNTTETVGDLLSGALRVLCSFSET
FTSRFIAGLLSLSNWERWGSGQSNVDPQQQGHRIGDDGDRVDIYGDYWNRMEMVERSSSG
SGDNINSIGLVSPVLERCKNIGNNNNNNNNNNTSSYGYTESNAATAGAQVLHRLDFNSVS
RTTSAVSTTAHSTAPNTDVVCSLSSERVGRVVIFCTDPELARCLLILAAFFFRDHRATVG
MSSRLCDKAPFAGKVLSCIYPSFPVQWVMEEYDETRMDRLLSFPYSVDNLLLVIAPRVGV
CNRIKLEKKFSLTPILVEKFKKGFHPVCPFQQRTLSVVKMLPDKMISSALREARRLQEKS
HGTVNCAVFFEFFMLWLVRQSQLCHSQQRFAEVEATSVSVASTPLKVEGSTHVSSRIGSH
VTKLFSFFSRKNGDVVDRRCFPHRVFSGTASPSIDVSATARPGRVFSDNGSDGMFGCVHE
NSQLMSLLTKCWTSSRDGP*
>TCRU_3510 hypothetical protein, conserved
MATHISVSNEHVSQPAALAFQCCRCNQRVICCPGCGEANFILVDGVSSPTCFTTNAARGR
GSTGVIINGRGGVTWTRRPPAKSNGASTPALPHAVPTAFELFCRDAEVQREVGMDDNRIV
EVVGGELEDESLYDRRRLHAECWLAADAETRKRYELAASALAAAITSKVHRVAGGDAPRN
VKKGKRRSAALESPVVATLLPCCGDVTDTSSSVTTANVSNDDGKMLGAYRSPMTAFRFFC
KREQQNYKNKGALMKAWRSMSLADKKPYDDEAAAIRVATRGKRSTRVCKN*
>TCRU_3511 Sec8 exocyst complex component specific domain/Invariant
surface glycoprotein, putative
MAWRYSGVAGNGTFGPSPTSPFSTASMRNNGQDNGNTGFAVGSGALFSGASANILGAGAF
LRSNALSSFGTQKQEIDEELRSKGEDSTVESEGRWPRREGNGSLAIADAPPVFGRSQAPT
RSIAGALMSNLARKGDILSVADASEAGVVLADKGRTVQGSSKTELLAKKLFYHPPNVRED
EYDDDEYEEDGDARHVDREFVRGANFLFSQVDPELLLENFVPFERGVEIIANEGGTEATE
TEIDERLAPYYDLQEDIDVMLGRYIEAHHDEFNEAVAGFSKMVLAISGSDEMLARLKQQL
TMAAASFDNLAEQLQVLRMRAAKALLMAESLKRLEASLSCESEVKQLTANHHYLAAVKLL
RQHKARLEMPEVSNIRATQMLRDYVNGTLENMHVTVVENLMSCVFRQGKLYEADAQLVGH
MLVDGASEKTVGAIDALVALHEKSHYSDIKADKTTLGFSDVINSIDDDPYDNDGDDVTRN
YLRFIPLCVKTLQELGKLNCCYETFFSKATRSIEQTIAHFFRLYGAWRQRAVKSTSIAGS
LDAATVRVMKLQAEGLASPTVSYDEAIALIQQPELIAMLNALFVQIEKIVRNADYLIKVI
LVSYFPFLSSCLMKPGGFYPDTTYHDWDVSQTPLQNPDEMREILTAALSTTLSNYGAQQK
DDGNNAEMVALKRLLVKLDVDSTFERLKALLGRSVCLRDVLDLIDAATEAVIRENPGDSE
EEVMVCRNSLSTLTTDMVLEAFRRDVREMHSQWDLQVLSSQLSYYIELIFRVLCDAKELE
EDFGDEVALKPFLKHRNLVGRVHRTAELMSSGAAAAADNLFSLEKLLSTQDTSGLLAMME
QTGYVSFSIVAAVKYAQRELRCADEGNRKQLRDRSMAASGNFVYSWRSGSALLLQQAMTL
KNWFVFTPMNILTCHESVRQFYRRCAVRAPRFVEASREEIKNSLFHFLQKTYIPSLYVFH
LQQLRQLFADMSEAESFTDEASAGEGNVRKSPVRSPSSFLLPIEPLVHHQKVQQEGIKWP
IVTLRHGGENGYPILTCVHYVGQVLQKLAHICRTLPDKLAEAIQQNVTETLLSELTIVLK
KKFYLISHQTLYHCLLHTEFEEAFAGRPSARWQALSEETRLWDAHNAVDADLFRYYAFNN
ERRTSGHQYNHVSHDFLGCDVMENSKEANESGVDASIGAIPPAEIALKGSGVVVALAMLC
YSMEWLCDVVLQTVWQNGWHYGISTDCLVPGTVFFKRHDRWSTSLAATKPSDSTRWTRLG
GIMRQMYTISQVCLFYLSLEARFAAFQYIPQLRDESYNIVATTRSPDTFVQAYNRRIINF
FALLKLHLSEKKLKYVGITVAKPLSELIMVEIAHLREKAISHAGLARLRANLLSIETALQ
LILPTDDDVRGAVKHYFLRPATFLRHLPNKDVANDLITSGDYKHFSMHEMEELIRLVFRR
EASDEFVTAQIQLLHVKLKSVAPAVATPEMTPSASLTDENLLPAEHNPQQFERGEGSEEE
ETEEEGEEEESDTVLQPGGRGPSRHVDPTTKMATSPSADSQNPVFPTGPKLAVTRH*
>TCRU_3512 40S ribosomal protein S11, putative
MTAPEQYARVHSVDLTLQHEKAYQRQTAVNENSHNPNKKHVNMSGHIRYSKKIGLGFKTP
KSAINGKYIDRKCPFTSDVSIRGRILRGVVHSTKMKRSIVIRRDYLHFIRKYQRYQKRHR
SLTVHCSPCFDPKPGDEVIVGQCRPLSKTIRYNVLEVVSKSAPGKLGRKFSKN*

>TCRU_3513 ATP-dependent DEAD/H RNA helicase, putative
MSSGLADFDGDDVKVPIVSAQPHMGVGMGTHSAVALGGFQDFCLKSELANAIRENGFEHP
SEVQHQALPQAMLGADILAQAKSGMGKTAVFVFALLEQIEKVPEGQKPHCQGIVVVHARE
LAYQIEQEFKRFNKYLPYCTTGVFFGGIPEDENVKQLKKEVPAIVVGTPGRLSALIQRKA
LDVSRVKWFVVDEFDRCLEDVKMRRDVQTVFLKTPKEKQVMMFSATMTDELRNVAKKFMN
NPTEIYVDQRAKLTLHGLAQYYMNVTEAQKLRKLCDILDAVEFNQAIIFTSSVERCEALS
RQLQALKFPAMAIHSRMEQPERLRVYESCKSNHTRIIVATDLFGRGVDIDRINLVVQFDM
ASDADSCLHRVGRAGRFGTKGLTIAFLTDEEKEIKRENRKYTDHGVMKELQERFEMQVRE
LTDISTQLNQSQYMNQ*
>TCRU_3514 LETM1-like protein, putative
MQGSWATLKKNCRNFFPGLALFAKQTQEAVDIWLRIYNRQLNYGPKDFVEQSETFSPDYH
KRFHSQDKNMWVDMELSKEVSQEEIAKLMRYKLDMWRMVHCTGSVLVIGGYALPLALFWL
ANDTWVPSAFNCTAEELKAWRHAQDLYRYRSIPSYLTDTKWHFDFHAYPWNETQERAWDD
LFEKNDVWRDPKLVRHAADMYDGFIKFELIRRKSLRHLCRSMNIPTYPMLTRMCNGTRVR
DYWNLAWCEDYMVITQKLHEKMTDEDLYDYAWRRFLAPYDKNLTREQLMERVEGYFTFLG
PEFLEKGKAPNLVILTNYVMGYYNDPAFLEEDISELDKNDYDHLASWGKDAFLRRLEFEN
GPLRDQVEAHTQKLLAEREAVAKKAASSADPVKTG*
>TCRU_3515 hypothetical protein, conserved
MATDEMAELPTVTLFRYPACFAIPTADPHALAVECMLRFVGAHFVKRDAPLPVALEVPTR
DGSAVGCHENGGVVRKTGVASCLQQIEDSVHVDKDVLPVEKRHLSLCVKILVVNSLLPAF
VYLTHCDLDLFDHSIRKGVVPKVATLWQRLLGNYRRGVLERSGCCGRYSSVAEALEEAEK
GLRALQTLYVGNSNPPGTFFLGTERPCSADALVYAAASSFLHADFANHHASLAVVKMQKR
LQGECTTLVKYIERVRQMYFEEYSAFYTLRAAPSCAAGAHSGNVAPEPDDLFKKGRRMVV
AWTIGFSLLYFTLSNANLILEALSELQVDEEEEEEEGKEEEGKGGEKK*
>TCRU_3516 hypothetical protein, conserved
MLEGDAAPSASGTVAGIQADGSGPEFNDATPSNLRKQFTIPKLDEKKLDMKAQEPHGTTT
PFVVLDGGMSSGTTTTTVTAVTETSSMPLNSNAPAFVFGTGSRYTGGNVIAVDSPSASAA
YLLSMQCQNPQYQLAHAAPTDQEIFLTQSVLQMCPREVLLWVLYSLAEESPVLAPFIRMR
CEKALHLILQQQQQQQQQQQQQQQQQAAAAGHFATHAAGAGATAGSAPWVAHLPASSYES
PRANSFTGNASPHVGSNRKGRGGKVREEGICSLHNNVRSMNHLQLNKQTGLYECVHGFHC
LEDGGSAVATPCNFNNVSTTSNANQNVNDSHASNPNSGRPHLVKMSSGQFRQTKDVKNAT
SEKTGGVKKIFPNTTRSSSNSSHGNNNNNNGKSQGAESGQDGSNGTLWHELSELRKKDEN
SGFTYEMDTLQNILQTVRELAAKEGE*
>TCRU_3517 choline dehydrogenase, putative
MYRTLTLRRANYNVAVVGGGLAGCLVAGRLAMENLSTIVLEEGDDIRKKPRWYRSIPCSL
LIHRFARRNYESCENFTTPQKTHRAEDPLAPVMIPTPRVLGGGGIMGGRSWNLGDQNDWD
GAGWSFREELLPRVQRLENLEDPPPHRGKRGKFFISRSQALSPFFKVFCEAMSKDTALTS
AFNGKEYHVRPGCGRAESFVDQRSGIAHTTLQSYLIDAIALKRPLEVVCNARVIGINAVD
NTKVTASGVTYVRPNGETEHVGADAVILCAGGIGTPRLLACSQGTLSIDPAVGTNFWDKP
QVKMQFRTRIPLSHNCFMEPIVQMCIGINLRYGNPLHALCSAYDDMICYWSSSGEQTPDV
KFIFQPFTMNNDGSTPKGVEHGVQIIAQLVRPKSRGCIRADGTIDPKYLTHPDDEKALCK
AVARIKELTKMLPFSNIFTDMIAERFESASIYGGASFPVLDQATFRVKNTDNVYVCDESV
FCSPLIGDSLPYMLALGEKFSDGFLRKPDMRSKSEAAMRGMNKARIIY*
>TCRU_3518 NIMA-related protein kinase
MENYIQIRVLGKGSFGSAWLVQRRSDKAQFVAKEVRLGGMKPAERESAKHEIEVLRTLNH
PNITRYVDHYEKNGSLYIVMEYANGGDLYTKIRSRKGVRFTEKEILHYFSQICLALLHLH
ERHILHRDLKTQNVFLTNDGVVKLGDFGISTVLRNTFELKRTVCGTPYYFSPELCLNKPY
NNKSDVWALGCILYELTTLTHAFDGNNMKALVQKILKGVYPPIHSSYPPNLSKLISAMIQ
IDPKLRPNVSQIILLPYVRDSLSSLQRVVEVAHYNQKPCDPSEERKEPARPSQLEAMRQA
EDRRKEEQLKAMKFAKERQLLRQQIEQRNRENDERLRQIRAQQRQAEIEQNQRRKELEAR
VREQRREAVKRAKAQALVNKQREEEWERNLREHAEEYKRRQREEERCVRERAEQEALHER
LFKNNNNNNGNGYVEKPAPSVAETAAELYREMRRQAALNKQRMLEDVRAMPGRADSNVVC
VAEVQMPKSPPPARQHRVNSLGKMQPEEAVEVRRQVYWQMRREAEENKRRALGLDEAPDV
ALPKRPAASPQAQQVTPQHEPKQGQEKPQEPQKQRQPEEAAKQNQSVVYAKRVVRNDAPQ

NLPPSPAAVEGIGHRAPDGEPETANDDDVGRKRDEDYSALQTAIDAALTQDEKRADAKDF
NDEAFFDTDPSKFMLDGQTLILPNVQATDPLMHRIESLRLFLENKLGESALIASYRQMNN
IAVDDDEAMQRVADMLPEEHQRFIPLIAQLIVCEDAFNRHLLQ*
>TCRU_3519 Microtubule-binding protein MIP-T3, putative
MSEAYVEKTQTELGAIISSPKLTEKLLLKPPFRFLHDIVTSFLSATGFPKGLYPDALLDS
SNVTEKTIKIEFLTQLIAAVEAGTGKKVSAQPTKIVAGQEAEKTNELLQLLASCAKLTAE
QKHAAVQKAKGGGGEQPAADKKAAEQESKEKEKEKEVEREQKAEERRKRKEEEKRREEEK
RREEERRREEEKKREEEKKREEEKKREEEKRREEEAAAAAEEGKKKRRKEDESLTVDESP
KVEQQRQEAANASGKRQKSGELHGSHNEEKEGKKKHGETLEERTQKTGDEERRKRHEERR
RREKEAAEREEIPTADESLTTVTRAPRVEQRIASTAPTPNAAASAAGKAPPRTKPTHEVV
DGSQLDAVPVSGVIREKKRTPGGKAQEAEDEGEWMRVAEQQNAKAVTSGSGDGGGVNEAE
AKGYLGQQALKAKREQEEETARRAQEANARALQGGSTGGGIIIHSSKGGKSGAAVAESEL
SKLREQLQLLTKASNPLGKLLEAIFDDIDTMARELEMWRSEARSQALAAADARRETMESL
QEVNAQLQNLEDAINDQILKTHNIRRSVINNGNAIAGMVRMIVSPEIGKR*
>TCRU_3520 hypothetical protein, conserved
MPGREAVPRTGNANERRRPAGAPYDTALTSSRQKAIVQKPNQSKQQQEPLAVAPACEMCL
ALLQSSQKVRNALEAVLTNARMVFLDDTLHHQMDDERWVRVLRAIGQDKSMYYARNVTMK
DPPH*
>TCRU_3521 peroxidase, putative
MWRSLRKSYALFSCCTSFCASASTSSAHAGETKGNRFRSPASVGSSTGGSQQREANPYKS
WEHLNHKWLLMMCVGCLAGGLAAGQVVDVDDNTLRPPFLKESVLADIAKTFEFRPDLAAT
SIRVAFILAARRAGFATDNIDESCAVVSGLEDIAGVLRHLSTVHGLSTEDAASLLAVAAV
KFLKGPCDVIESNWRWGRNDTDTTLPRKKQLAEADSGLIALPTILEGLGGLTDEESVALM
ACHGVGEFHEHVSGIDGVSHIGSQYKLSNDYYKFLLANERRFFELEVPRTEENKVIQRLP
KDFVCVYAHVGKKQKKRQCVFNRREVEALLRNKKWRSLTERYATDEAAWAAAFQSAFTKM
IECHFKRLRPYNKSSEQMHTE*
>TCRU_3522 WD domain, G-beta repeat, putative
MDESIIRVPFYASTSAWRTKAQLQGLLRNHPSVIADSGLLAAPGTIFSTARLSLHNSESA
LALGMNPLGGGGSVGLMSVEGPEPTLQGATHIRWLDLPQDVCVSSIDWCDDHLLLGSTRG
RILLVKAGPYSVTESGEALDPSRCLASGDTQPMVGDIIVAPSSHAVSTLVRSVRCNAEVN
PSSVVGVVESRAMVWDLEGDFHPVRAWTPGVSTEVKNNGQHEVLLFANWAPHCDSIVLTG
GYEGSLILTDTRADNTRGHGLTLPMRRGYMARCADFNTLLPFVVAAASSDGTISVFDCRF
PEHAVRTISSLQGDVASLRWLRLHSDLLATGGIDGTVAMWNLRFPPTYCVGRAQYKYPVV
DLAATLSSVEQRLFGVTSGGELTLTGIALPALSALASWPSSRAKPQQVGEEKPHLRERES
RGVGLLYARRLREAYEIITDCAMERFTRKDTAVAMALVALTDVMLVPKFDYKEMIEEKVG
RLFALEETSHTQALEVFDELLTRSSERLCMTLPLRKVRDLMKPNPEDVKKLEALRLNLLL
QRVLYTGNPEAVIAGVQFFAANAGNLELVDTDTACGIARMLLRENYVEGEKFIRAFLSLL
IQQDGTVLARTLTRRLLIVVQEPLMTEGLPPRQAKRREERFLRNLVAAQEAVHVQLTILG
MGIEKHEQVIATVNRYEKACLEKGESGIFGWLGMKPILLFLHSLTADSNYATFFWACVQL
IEVFAKCPGSRQVESLLFITVDRIHSAAKHIQSQLNQAAETPRFSLPALREVGNTIRSAL
DFLLVLLRVQLECENVAIESGMLEIPPVMGRIFDILSTSSSDLLEAWATLLEKLEDCKMR
DLVRKCCLGVVRNFSSQIEELMKVSSKKEDDEPLNEILDTCYHFIDTFLKGDDE*
>TCRU_3523 hypothetical protein
MSSFDRIHEKTAPSLELLRAQLQHMRSFDPHTAISVPCHFGQRGIPVSESGEVSVFLSPL
SSVRDAFLMSQEKWDANDCMLQQLDSRLRLYQENGAKKRGDLVIAMDKAHTEMEQLQNYA
AQFWSDMEENLRLLRQLLADSKNMAEDTPGRPEARITKIIRGLGDEPDCCADNRDEDECL
MSRLEEMRRTQDVIRQQLEESSASGTPSSIAAAVERIQAELQKERDGADAQLSALKESFV
IVVQKTLDELEVLRRDRLETEALCLRILS*
>TCRU_3524 hypothetical protein, conserved
MSLSSSLASSLGSIFSRGGFSGDTELEKRVALPRVAPYVNCLPIEILHRLFMEVIENDNY
NFAWIGVLSSVCWRWNCACQHAVIWEFVSKRIFVAYPLVQRLHSAPLDQSDVRLLNKMRR
LGAPEQFITQHDTMEPLIAPTNRTLRKAMEEVRHYLQSRSYYEYVQRRRVFVLQMFLSWA
LLSFSLFLGTTVCSAEGVALGALCSPSMAFSLLWMTYLSIVTIVIANVVMETHFEPKPLF
YRLRKNKPLIIRSATGIIVGIACIAFPTLLVQINFAREERFPWLLCGITPMVSLCMWQLY

VLLSYVAPEVKQHIHEQRTGFRPMAIMTFLLSNVPYVYPAFFAGTIFCIFQYVEHGGRMY
VLLAIIPVLTSLVVLTLLFMLDFFLTRRAKDFITCGCLFLVLLFPLSLMWKEFRGLSLLP
VATASFLFYVTHLKLMAMRALQELIDGALGYRNMRRIR*
>TCRU_3525 hypothetical protein, conserved
MEVVIEANAGDSAFLSASEKREMIDTLAEMIASTIEGASVTDVHEALASHNITPGGWREL
VQRKVEARRQRKGSDVVSATDSAPVTQDTPLPPTQTPTTRTWECPNCGTQQEVVDARRSL
RHSGTSLAVRIFSDTSPVGCSVCEGVISHVR*
>TCRU_3526 Trans-sialidase of Trypanosoma hydrophobic C-terminal, putative
MGEEEQSAPEEGANSTTLQSRETTGDGDAQPVQESMASLAGSEGLLPKQSEAPVPENNHS
ESGSGASQEDRLALLNDAKLMLHDWNVDSTARVCVSRVSMLLLLGLCGAVAAL*
>TCRU_3527 Mucin-like glycoprotein, putative
MMTCRLLCALLVLALCYCPSVCAAGSSQDPAKDIPSPLPTQSQGTGVSGATNPSTTSSSA
VAGAASPGKIEGSGTQGFSGAGQALSEHPSVVAAPAGSRGPTIPVSITKEVPGGSGSSGS
PGPVADTVQENGDSDTTDSKSGNNPSTDQTNTNADDSAQKITTTTTTQAPSAPTTEAPTT
TTTRAPSRLRGRRQPQQLCVGVCPAAACRIRAGVHRSGLKRCVRAVRASTQSRGFVCVYC
GPM*
>TCRU_3528 ras-like small GTPase, putative
MGGHSKKHGKGGGGGGGVGRNRPPEKRKKLTKDLGVPDLKKVGQNLTRTARNRNRSVFSI
PFLRGEAKLPSIVARTVAGSGEPPQTEEERMASERRSMRMLALQCAGKTYQYEAPKQWLD
EAEGQDCMYDEMDRRCADKSLRRFYKEFQKVVESSDVILQVVDARDPLGCRLTQLERNIR
SQFGDKGKKMVVVLNKVDLLPSKEVVDRWIHFFESHEGVECIPFTTTAKGTVGHSYVANM
FRRLRALALNEGTGAHKSIVVGVIGYPNVGKSSIINALKQKHVVGVGNMPGFTTGNTEVE
LRSDIRVMDCPGVVSPGEDSGDVVLRNAVKVSNLADPFTPVQRLIQRCTQVDTDDPHSQQ
LFAAGVHPLALFYNIGTFDTNDTIGFIRLVGQRRGRLRQGGEIDEEGTARMILQDWNDGR
IAYYTLPPTSDLFDRETLTTMAEEETAEGVMLTAAERLCGPQVVDSLASGMQWDGLPTFH
ISWERRDGTSRRCTRYDKMNTQNGVES*
>TCRU_3529 hypothetical protein, conserved
MHPDPLHCHFTPRTAGLPVGPHRTAAGSLACTCRAECPATEHGLGQFPVPRLAAASQSPT
RVPVATDSLSAMESLRLGPLAAHDDVTEEICAVPLSLVDRGHPAESTFSHDTAESHAT*
>TCRU_3530 hypothetical protein, conserved
MRRSAFCLQSGALAILINAVSTRRVELGPEDVYKICALLKTKEDALLLQKNRAFLSGLHA
EYQKMDPDAVTPFQRTFIDGVFAALPSALLTFSDPSYSSPSCIDDERGARKSDVTAVSGK
QNQPWSTMTTTKETCANGFATDINNTSNDENNSTSSGDNTAISRGNAVVRSDEEPTVEER
IAAIWEVVQEYQSTNFLGTDGSQKIQKHCKALELQLRQMKPFEVASLVRALATVNYQDYT
FTNLIARRGCEVASKLSLSELCRTYFNLSKLQSHDSMVAFVNQIEAQMDKLRHEQIQFVA
MALERQPQIASAPARMVPKLLARAVSHFSEVDSVLYHRALLIVAARYNLSRHPAVLKTIQ
DSSKHLENIAERDLLAILHSLVDLGIPAGTPGLAELLGKAEAIVTIIDIRHVDVLMDILS
VLPIDTSGIMTKLMDRLIVDGGKLSIPQITFIMDLLSSYPPAKDNACVAALAFSASLRAE
SFDREALEQVVLSLARLNQFSDDFYTLVSVLQKNKGGFRSFDNFASLMKWCSREVALDVR
GQEMITKGILGLAPSMNDEQLAEARKLLTQLGVNDKNVHQMIFRRAKQLQRESGSRWTRR
GYSSATDDFT*
>TCRU_3531 BNR repeat-like domain/Concanavalin A-like lectin/glucanases superfamily/Trans-sialidase of Trypanosoma hydrophobic C-terminal, putative
MSRREFTSAVLLLFVVMMCCGISGATPANEEEVKNDLINIQNLERVDLFVPLKTPVLPKE
GNGSVTTRDLFVSPSLVSAGGVIAAFAEGHMNAEYQGGQLTKPSSDVVAGYIDSVWDWPA
LVEKVNEGTWSARTVLAKAEVNDRVGVVFHPTTIMKDNKIFLLVGSSDVFYENEEWGTVD
LELTLVVGDVTPYTRGEPGGRIKWGDPTPLFDSKYSLKHKSKLNTFFPSGGSGVLMEDGT
LVFSLVAMKENPNESFYTIVYSKNGGENWLLSEGMSPPKCYNPRVTEWEGTLLMIVDCED
GQRVYESRDMGTAWTEGIGKLLGVWAKPRSVFWDVSLHVDALITANIEGRKVMLYTQRGH
ASGKKRATAFYLWVTDNNRSFSVGPVAMGNAVRWELASTLLYSDGKLHLLQRRESDKGRV
ISLSRLTEELKEIGSVLSTWAQKDVFFSSLSIPTAGLVAVLSDAAGDGRWIDEYLCLNAT
VRNAKKVKDGFQLTEPDSRVMWPVNTRDDNVRHISLSHNFTLVASVTIEETPSGNTPLLT

AVLLDAGPPYIMRLSYTADNKWVTIFKYEKITKSDTWESGKEYQVALMLQGNKASVYIDG
KLLGEEEALSTDERPIDVADFCFGACDFGDDDEEEASTKKIGKKPRVTVKNVFLYNRPLN
STEMTAIKDRIPVLARGSEWQADDVPQTIASAGSAAPGPLELPAAPGRTTVGRTANTQHA
PAGRFTSAGNERTARETGDGGANGDAGSAYGRELLPLLLLLGLWGFETA*
>TCRU_3532 hypothetical protein, conserved
MGLSCSHNKKWETAFEGKTTTRSSNREPRKEYQVGLMLQCNKTSVHIDGRSLGEEEVLLA
GERPPEVLMVLLWRVRWARISFDGDGRLSVQPPTELHCDACTQGQRPRSDKGSGSDTARE
DSSG*
>TCRU_3533 BNR repeat-like domain containing protein, putative
MIVDCESGQRVHGSRDMGTAWTEAIGALSAVWVNARSGVSQKEACVWMPSSPRPLRKGRR
VMLCTQRGHASGKERATALHLWVTDSNRTFSFGPVAVDNAADWMLASTLLHSDGNLHLLR
RRGNGECRVISLSRPTEELSAINSVLSTLTQKDIFFSRLSTPTACLVAVFFDAASDDTWN
DEYRCLHATVKECSEGQRWVTIDGTCVQGNMACEHSG*
>TCRU_3534 hypothetical protein, conserved
MAMVMTGRVLLVCALCVLWCGAATVVSSAPDATRAQSVWPEYLFLNWHELFRNECKVEKP
YENNEDLKELAVNCCVRDAMNELCSAFYGNTVMENKDANVEGICKEYAGKPDDVADCPKP
KNNLSPAAVTSVKVSVPEAPREKEEELRKTPEEAPTPLAGAPLTSSPANPTNVSPSPPLK
GQPANEEEVPVPNSEEGSVNTGPTNDSEEGDTDTVTDTEQDEPSNSQAEPTTPTHTVASD
NGDNETDKGTGEDTTNNAPEPDVAGTEENQEENKNNKPKERTLQVAGIKTTTATSGDSDG
VKATSPLLLLLVVACAAAAAVVAA*
>TCRU_3535 hypothetical protein, conserved
MAMMTGRVLLVCALCVLWCGAGGVDASNLESNAQGGCMVSGGLGAKTSYSVRGCNKTVLT
RPLRSAFFINAIQAEAKDVKDISEEGDNLKLGSIPQPPAPPPAPETPPPPDASDSDGIGR
SELGADGQTGGGGGGSSGISNGDEDSTLKVPVPDDKSSSPIPNAGDGLKNTGDSSVTQKN
NPSGTDEPSKKPLNVPAQPTVQTPEALDPDQNENTPNTQGEISVEARGKGASGGGAGESD
VNSKGSSSVSTAKSNPTGKTTPSVPSAGETPTITATPNDSENPTGKNDSTAASGTELDSE
ATKKSSKDDAAEQHSQERDKADLVKSANTGHPAYTAASSISTYGKGDAQGKANENGDDPE
RHDSKGTHDALEADNTNVAPTASEAAPEAVNSTEKNDTAPTGDSDGSTAVSHTTSPLLLL
LLVACAAAAAVVAA*
>TCRU_3536 cAMP-specific phosphodiesterase, putative
MSQEKTGLQRPVLAQLSPIAIRGHSDEGVKKLRRRNDDGCFGEALFKGNAPARSLLFGDD
ACHRRVENLYLLDNNPTNWSGIKTNLKREIPLLQDPTETFARMNKVMHQTLKYVKEVESI
NFSSRGVIDFNGEVMEDHQRIFLSVCTSVFANFSFSCQMDVEKLLSFLMALFENYSKENA
YHNALHAADSVQMLFLILREKPATLIFTENEVILAFLATLSLGFVHPGVSNAFLARIDHP
LVLVYGDMTTQQSAGLTALLYLLNREENRFIDLFANSGTQPFSQFLREILVETVLGTAPR
LRSSLLRDLEKVAVADAVELNDIPILLAAVVILADNALALRPHAQFVNLAGLMVTEWLRE
AYEEEQRGMDPLVPNLRKQIYENGLGMAVDYCKVWLRPLITNIRALVPQDLYDNMEGNTE
APSMSEIQDFLVHLDSSNERWNDGSVPVIDIIRRITMHATSLDRKASKRAILNAASNRAV
SGFLSRSSSMCLSPNGARMSAEASEKKINQQPSRCEHYFSFLRLYDTYEKEGRPAAEFAA
QLVFLALQLNPEYIGRYTHKGIDVCSKEECEEIAELIMKKEEAPTTAEVIASPLRDGEQT
DGFILRLMEMYAERDRERERNDTLNSAANPTSNLRRTLKCYNPVYGGMYSGKKS*
>TCRU_3537 serine threonine-protein phosphatase PP1, putative
MSLAHNLLEKMLTLTKSATQQQILIREEDIRSVLDSVRDVFMSQPMLLEIRPPVRICGDT
HGQYYDLMRIFEKCGFPPYSNYLFLGDYVDRGKHSVETITLLYCYKIVYPENFFLLRGNH
ECASINKMYGFFDDVKRRYNIKLFKAFTDVFNTMPVCCVVSEKIICMHGGLSPDMTSLAT
VNEIERPCDVPDKGILCDLLWADPEDEVRGFLESDRGVSYLFGEDIVSDFLDMVDMDLVV
RAHQVMEHGYGFFANRQLVTIFSAPNYCGEFDNDAAVMNVDEKLQCSFLIIPAR*
>TCRU_3538 hypothetical protein
MSVLQRENLETSIAVEHDSTLLSSTDSEDSCGEGCGDAGIIPLNVMYAMTGAIEKQERDM
SREQRAQKITQEREMLSRRQLLRERVELLRHRLASTREPFEEGQEEERRANAVGISMELS
ISGSEIHAAHAIRQTAYLPWSTRLWHSLYNKVRYFFVAASFITGGNVYASEALNEYYQQE
TYD*
>TCRU_3539 3-beta hydroxysteroid dehydrogenase/isomerase family, putative

MARYDLGEWVQSRVLRSVASPFHGVMREESTRHECNLERGRAAASPDMDAAGCATMQESA
RHYIFFPRQNTMDVSRELDTPAAFSTVTLIGGTGFFARFVADQCLRRGISVTVLARDLEK
AKNTLLPRLRCNVTKGTEIVEEHNMDLMEGDCFLRYTSSMKRGNFAVPLQLEILKGDVTS
PQAVQHAVRQASIVVYMASAKCSSWWRPWAKSNVPGCVKVDVEGLRHTIDACSLVDAHLV
AFVPLFNRSSWASALQWYRTLFLYRVGYLKATRRQEHLLIDDDGSPSALCLQHGGPMRFT
LFRVTDIVHPSFNERVAIAKNNNIADPVHLIPLRRGDVDANLLANILLRAVALCRSSIGS
RIDVTGCMRNGVGLAHVDSLLHGLRNE*
>TCRU_3540 hypothetical protein, conserved
MWRWRLCDGLEVVGLPVTFGLSRRCCHSNCPQPPSWVLQLRSDAKESKTASSYDVKIYDE
LAWRKMRFIFDKELWDPARMSSILRRRLCLGNEEAEYTIIGEAFAFPDREDAPVVNEPQH
ACRLLWDHNSPSEKLLVQLSEHFPPLLWHAVRPSLDSLKRIFSEFLEVDAQHMKKHLPYY
ESVQNSIETATKETLSGTLSPVSIKDRFVEDMRRRDPKTLELDYPHEHNIFLGTSPHFRL
LEDKMMKSNPFVFGWPLLLSDGNEHLEGTPLRMAAFRTIYSKSLLMFHTRLDLQVDHRLA
RLEEGDEEEILLEVPVFCTVNYPQNTRLCGGRPLVQRFNDVMGTSYPFDTPVDVLAALAK
ETTTKGVRELLEELKFLTVASTRAPEVERVIRISEDQLSSNRILGQLAYTIIYLALLSYE
RFVEDVFEVYKGHKSDLVRVACAKGAGIVERPDLIEQLVAAEPEGSRCRKMLQASMHGPP
TTARA*
>TCRU_family, putative CBF/Mak.1
MNAKITAALKRCGQCATYQELCGRFDATVRPLLESCLSAALVRPVRDHVSPDECSPRVAE
LREALRAANAGDDVWFFLFRATATFVERSAKKRGRNGDTAISAIVVNVFVLFYGRVIPDL
SKLRLQWGFLKKERSKFEANDSYVRSNAAERRKALMTSVLSVFSERAHRHYFTALWLPCL
HHAAAAALHLHILHRIGDVILPHLTNPLVVADHLSGCFSSGGLVAVLALHGIFLLMLDHG
LEYPQYYQQLYTLVTPDAFASRHRYDLFRLLDLSTSSLRVPSYIAAAFVKKVARVALLSP
SPVLYFALPFIRKVLQRHPNCLALIHRSTKEAVEAADGAAETGEVEQPNKSGKIAKEEAQ
RLTASLFDGNDPFLEEASLEACHALHSTLWELTALERHFIPTVPLMVSAFGSTAEDQAPL
RYEKTYARLFTAEVTRPISKSQLPTVAYREPQEEDGTSAVLLV*
>TCRU_3542 protein phosphatase 2C, putative
MFACSRRLFSAASVQPLLWFGRRGAFAVPHPSKAKNGGEDAFLVHTSGIGVADGVGGYAR
IGVDPAVFTRNIMRFTRQALEKDQNRGTISALEALNYGFAETQKRGKPGGCPVSLVTLVD
GRFASVLNLGDCGTICLRSSKLFFATEAQQHRFNCPYQLPEDPPSAGDRTTLEVSEGDVF
LCASDGLLDNVEMSDILRRLENVEREGCQRVAETLVEEACKNGADEKFDSPFAKNARAMG
YRYTGGKQDDVTVVVAQLTRLDIEPNVCPGDIAEFLKLPTE*
>TCRU_3543 Iron/manganese superoxide dismutases, C-terminal domain containing protein, putative
MLGITSALLCVTAGRAALVASELQKLVPVLLPLLDPPIAKGVNVSAAQLIVASGHTSRRN
DVHLPDCPVMAHALPLTALAEHSAFLRHPCRACLKRRVHFLSSEHFCMHLEEKCSQSGGG
MPVEWESIAAGQFGLFTHFTDEFVMYATSRREPGWTWLVYDKERPEGHRLVVVNFPANRV
PLVLGLWPLAVINMTEGRLLSAIGARTKTSAAESSPAPWSRAARNPLVAEASTATVAAMA
AAVTDTVDDLHGSLSSIRAQVAQEAVLKMNWGFVLQQWKQAESYYKSAERERKVQAQRTK
MEREAALAEMSKLRGSGATILTSDTVEITVAGSDAAPEKASRDLKASTQTTKEPRKEEEE
TANVGTATQDPHCTQLEDGTWQYAYKNGDLTLLRPDGTRVFKKSDLTTTVFADGSTLYEY
PNKTSILDRVDGVRVTTFADGTTKEGRYK*
>TCRU_3544 hypothetical protein, conserved
MRRTFSYWASRAWRVHSELIQGWIAAASSEPRKLQQARSYLYDLKLEDAIEEVEDATDLV
YFMHRVSYPAGSHLETKVGQFLSRLSEQLTRENLLEIVKRLNEQPNSGPLWSRVLFEQPW
FSGRVEELLEGLTLEEHVTFCAVVWRSLLPMLQSHSSQHATRGAAVAVSQQLALLTAQRI
ERSNDCQAVCLCAQLGVPLQGESLAVLRELILSLTRGGKLSPEQMITVAEAILAHHSTDA
EWLDALQVAICGKPPGSVSASQVIAMLRAVTSARSGHLSKMFEDLLLRVLPEMSPEDMLD
CCRSLTAMPNAASCLRKELQRLLCSKTSLGKGTSDRYDNALLLQLRGMSLIAPSEATKLL
LRFVDQLKGTEKISSPSMTQILLRCMRDLQLFPPELVSHCKNSFLQNTPSNFTQEDIAYL
LYAAAHAGEAVDGIFFNELLNRFAATRKFDRRKHKAYHGKLLSIPTVTMVLESFNVAKGG
RLSVESKVYAVLRDAIEQQQPPEGIKMEDAMYILKLLVHLGVDDRGLTTLLLTRLSKAIG
SMTPIHVRTLCEVLVALKSRDVLMFRALLSHLSHISPDHESITSTALTARKLKFVPYFEQ
SVLVEHVTSIEGWSLSDIVLVACTCSEKQRKAFLALPGAEVLSQARPEELSTLDLFLLLS

ISNEDREKTAAMAATLRSRPPIAAGDLEVEDVWRAFVNVADDKEALAAVCRSGAATLQTA
DENTLMRFLEAASNVPELPNVFFRVVGRTVLRLANNMTVENALRWLELYVGHQIRDDSVG
KALLSKVRTRSHNAASLVDKTLRKASTMYGKTYNTQGKLRKNKEKVEWRYFHQD*
>TCRU_3545 Predicted membrane protein, putative
MNVGTPTAGGPSLSFQLLLYGSAGWSGIWFVVTLGLLIYKGSMLHFPPAALPMEIVSALL
LLVIDFAALSLGTRGNLAEEVGTSCLAIGLLLVAAVGAIYYMWLQTYVMMLDLAFSAILL
GLNVLAVLAGVYAVQGVIRAKHSPRQRFAPQPHGLPSFMRDKVKRHKED*
>TCRU_3546 Uncharacterized conserved protein (DUF2039), putative
MPSKRLVRHGARGSQQVHMNKVKFDPYRFKVNDQRLPPVVLAFMTSLCCRRCCDILQWKV
DYGKYIPLERQRKCNCCQERTVTIAYHHICQRCATERARCAKCQKPPTLGNSTTGTENRG
DDTNAEGNANADIREEEEEEEDEAAEAMSAGMPKGELSGKYVFVDQEDSDEEFWPLRGLD
IRQLKQRKKELARLQEKDRVCRLRERERRTVLRGALRGPGAEEGADDSDEEI*
>TCRU_3547 hypothetical protein, conserved
MFVGRWVYNKGQTVGCLSHLALRQKHDDNEADAVANTLYGCVLFLKDDVVNCFAGGGGEI
VSVATEGDLMNTVLQWARGGRGLCGEVPEVTAPGASLPFYSTSSGPWFVIERCVGDPCSF
GVIAGFVCRYVHNGDDVDGVGGCNLDQESCGNGFLWEWGELINCTTISQLSLYLDSLPLK
SVK*
>TCRU_3548 flagellar attachment zone protein
MVLLNTLKRDTPQLQNGQTVAYEYLIDDEHWQWTLGTVVSLKDYTAVVQQWSINQGDDAA
LRNIVSNEIEKEMKRMRVFQERLSTSRDKLAAIRSENEDSVSAARSVFEVAKEQVKAVDE
VHMREVTSQARPSPVAVEVLKAVLAVAKNDPAINDCFSWYDIQMEYRKPGAVMDFIYVDV
PGRYYPTPDTILSSLHEPRFSLAAAARDSEAIRSLHQWVVSALAYQEAHSRLTSDMRIQA
QNDAIGAAISGMKACRMKVMKLKEELSRRNSSAARGQVTSFIKTSVHSTIPLSAVISVVP
IDPSVTDCALTDDEVDVLYEDASWLRFQLKDQLSRTMNEYIGAMAELHCLSMYTTELEEK
RLYLQEHYTRNVIRSHDRTILNFHDRDTSGKNKELQDIIDDLKKHDERWLFADGLKVTTK
HAKKYSGKDWGKLLKNKPEEVLARFKFEQALACHVPDDCIQNVEFHLEPDALVTSFNVRH
STELTTGEIDRRLEQFPPREMNRLYHDPNGAKVSLDRAIVEVCRAIDIPETKFQGLYFDE
FVEELGGKGHLVDKDAYESEIGDLLMLLDKIHNENRSLQCTLEKSAEEFRRQTASTLREQ
EALRQRNNELHAEIGRMRDLVEKLKDLADKQASELELFKLQKNQAIQMRTQRNLSTFKGD
NTAEPLYCVTLDELHEQMKQCELLENEAAQLQKQLEDLNQTHDNLLVHLNTVTQEKKGME
TENEQLKEELQIAQKAASDAAQERSELLHELARKRAECEELAKNLSQISDLLDALKTSEK
EALEGIEARDEEIHDLQKQLEDALNNQNVGARALELKERQNKELMEINQRKQKELNAHRQ
KRRTAHEARSLEPTLQLYTVKDAGFEDALDPEELLREPLLSITMDEYTNQIQRSNQLQQE
NDTLRQQLQQLSDDMETINSQLREAAADNQNLSDQLRAKYEEFVKANNTIQSLYRQNETQ
ENELQQKNIENAKQAEELEKLTIENEKLADELEKLATDNEKLADELEQKAAENERLADEL
EQKAAENERLAEELEQKAAENEKLADELEQKAAENERLAEELEQKAAENERLAEELEQKA
AENERLAEELEQKAAENERLAEELEQKAAENERLAEELEQKAAENERLAEELEQKAAENE
RLLDDKKSLEEELERNVLERGRIESECRSRELVVGGLESKSRELEEALVALSAEKYNAVE
TIEKELTDILVQLKVVEGVNGALRRLLSDKEKELVFLRAHCELWTDPTEVKEKVVTRHVK
VFDGDEWGKLLSERPEALMAAFVIDAGNACHVPGDQVSAVSFFTERQ*
>TCRU_3549 BNR repeat-like domain/Concanavalin A-like lectin/
glucanases superfamily, putative
MVAFAEGLFEYNVHGHEFFGIKPYEIVAGYIKAADSWPSIVAEVNAGSWRAYTVFGSGNG
NDHVCFLYRPTAVARDNKVFLLVGSYTVGYDSDDYGGRWVKDGWDIKLVEGVATQSTDGV
QSKLVSWGEPKSLLKHIPNHTQGQLIYVVTAGGSGIVMQNDTLVFPLMVNGKNYPFSSIT
YSTDNGNNWVFPESISPVGCLDPRITEWETGSLLMIVDCENGQSVYESRDMGKTWTEAIG
TLSGVWVISQPGVRLYEIFRVGALTTATIEGRKVMLYIQRGYTSGNKRATALCLWVTDNN
RTFHVGPVAMDNAGKGELASTLLHSDGRLHLLQQRDNEKGSAISLARMTEELKEIESVLS
TWAQLDASFSSLSIPTVGLVGLLSNAASGDTWIDDYRSVNATVMNAVKVHDGFKFTGPGS
RATWPVNSREDNNQYGFVNYDFTVVATVTIHQVPKKSTFLLGAGLENSNSKKFIGLSYSM
NKTWETVFDGKTKAQISTWEPGKEHQVALMLQDGHKGSVYVDGVIVGSLETIPTLGALGH
KITYFYIGGDEGDSGSSATVTSFFLYNRPLSVGEPKLVKKSEGSMLGGVLLLLLLLGCAA
LWPSTGRNAAGPLPPAGAKCTSPRTFLNN*
>TCRU_3550 Paraquat-inducible protein A, putative

MYNLTCGTDGFYCSLPCTNGIELLNLRLYGLNEWDRLVVNYIGPFVTQRINELFSSMIIP
FCNTTDNRFQIPIKESPDIISVPPTEVIVPSVLVGFALVFGSTFLSIRKHRKSPVYLLDG
SIIPLWRVLLEDTLLILMIVVNALAFIWSNCTTAARIVAGDEVTLLSFSLMETSTNLYKA
GLRPFAVLVFVFSGLYPYLKLFTIMTCTLVLQKPESMILKVIDYFGKLSFLDSYAMVVMS
SGLQMEGIANVKIIAGFYVFLAATVLCVFTGNYATTFWRCNTSLRMRSNSIPRPMKEVPD
EALLLAPLTVNERHIVEMPNTPRSNFRSLFWRVLNALFVAACILPPWITPCLSYRVRGAA
TIIQPNDRNMTLYELSSASTSLLLTCVFTICIAPLLYAAFYPRLALLASWGAADAFLLAC
VAGIFQLERFVEFVIGTNMRSLYTAEARLLWPMIPLLIASVWQWVLAAEHVFGLSEFIRN
YRAKPLEALAS*
>TCRU_3551 Protein of unknown function (DUF933), putative
MAHGWTTKVGATAKEASGEIHSDMEKYFRRARVMPYSEFLSKPNLEAAEMQMEFVDAKYI
MKDGDVMIVEHNTPI*
>TCRU_3552 hypothetical protein, conserved
MRSAEGACPRSRHRVRRRAIATVRVALLVALALVAATAWVPAAHAVVLRLRGGTVDRAIT
VGRAVDTVLMDGVSITNGVAVVFDVPAMLPGALRIELRNCVCDGGAQIYVRGYSGEPASD
RSLEVSVSGLSGSYCSLVFVHNLPAHTNVTVRDSTIVTAGPMRYSQLSGLTDAVASPLVL
HATSLLQTQLRVSNTVLRSSHAGGSAVYVGGGVDLLSSAVVLDGVSLEASGGPTASAMRV
APSSILSLRSHSVFSVTNVSIARCAAAGATLGERIAVLDSVQRNRAGGRVLRSSGDYRSA
GLSSVSVVPCDGCAAALACFDALTACAAAVPAAWARRACRSTCRLRGPVAVVVRGAA*
>TCRU_3553 amino acid transporter, putative
MSTNLRTYASDDPLNDGVELVEDLQGQAGLPTGDRMDVAKIRIEKLNQERQERVARRRDP
QSCLKRGLHRIIPYGGIISSGCNLASSSLGAGILALPYAFNTSGLAMALVYLVVVGLLTI
YSFTLLGIAGKRTGLRNYEQVTRALLGKGADYFLAFNMWLFSFGAEVSYMIYMGDLIGAF
IDSSQSAPDYVKSSSGRRLITSMVWLVAMLPLCLPKEINSLRHISVLALAFVFFFVICVI
IHSVRNTITHGMRDDLVYFRTGNSAIQGLSVFTFAFICQLNAYEVYEEMYKPSVARFTKS
AALGVFASFTMLFCSGFFGYLDFGPALTGSVLKRYNPIEDKMMGVAYAGMVLKLCVGYGL
HMIPVREAVYHVLHIDVKTIGWWKNALVCGLMATLSLIGGLFIPSITVVFGLVGGFSGGF
IGFIFPALLYMYSGNWTLQSVGYFHYIGTHFLLFVGVIGVVFGTAFAAYGEAMH*
>TCRU_3554 hypothetical protein, conserved
MRRSLPWSVGDQQHVASVVRRWRKSRVHLECPRLCFLRALPTGMLGSSVATSARGACQED
DETIAASACEASLTIITPGRARRTMCRHAADKRAHSAADWAGGHQSAARWSTRCCGAHVG
KRFRRRQRRAGLLGKERDGTTDTGSSL*
>TCRU_3555 Mucin-like glycoprotein, putative
MMTCRLLCALLVLALCCCPSVCVTTSEGKAGESVSRVDEKGELDGATDQQSATSSPQTRE
EEEEKEEQKSSTPTHTSQETAKATGTPESESNSNNTKDQDHGQSTKEETENTNTNTSNEQ
HKESNAPTTTTTTTTKAPTTTTAPDTPSNTTTEAPSTTTTRTPSRLREIDGSLSSSAWVC
APLLLAVSALACTTLG*
>TCRU_3556 Protein of unknown function DUF84, putative
MIIWAVGTTNKAKLDSVDIAVRMCFPSYQHDVRPVAVLSIVRAQPISAEESQQGAQHRAH
EALRLVPEAHYGVGLEGGLEKISGRWFECGWMYVIERSTGRCGIGSSARFEMSQKLMSKI
LDEGKELAEVMDEMTGETDVRSGQGAMGVLTAGYLNRAESYSHGLIFALAPFLSDHKYWD
*
>TCRU_3557 diphthine synthase, putative
MFTLVGLGLGDASDITLKGLKAVHEADVVYLEAYTSFLINSSAEELANVYERPVLVADRE
MVESGEVLQEASDKKVVLLVVGDVFGATTHFDLVVRCHEQGIPCRSIHNASIINAVGCCG
LQLYRFGQVISLCFWTETWHPDSWYDRLMSNREMGLHTLVLLDIRVKEISDENLARGRNV
YEPPRYMRINEAIEQILEVEQKKKGGAVAEDGSTMSIALARVGSNTQQVVAGAMRELRGL
DFGDPLHSLIIAGDIHTCETEHVNLFRLK*
>TCRU_3558 hypothetical protein, conserved
MRGATRPLRYLCPTGVLMNFFPLGMYSGPINAHVFLPNNNLDGRATNHMKKIDGLTKDPQ
LVFSRNDLKLQCEYCRFQWVHDTLCVRCPAQPNHNKREMWLQPTWMWGKQQPYQYYKYMP
AVRNPRTGMPLAREDASGMNNERRGQGLTTKTLNIEKERRGISRAVSNIGKYNTRWQTRF
PFPA*
>TCRU_3559 hypothetical protein, conserved

MSADPDAPADPNRRSGGHIVRNMIYRHDSARNLEIVTGWQDQGTREYNQKIVPPAKMELH
SKHPCHDLNTQLLRCSLDCPAEMKLAGRIATCNTERKALMVCLTKQKHWKPEPSSAWYRF
W*
>TCRU_3560 cytochrome oxidase subunit VIII
MLRRTGPAVSFTTSHRALMLRTSRPLLGGDMNSKERFKAAWDEMPLHLLGASRKQVFEWH
WKCMYQLGIRDTTRMTKLRVLTNWVGLFSFMYLTYISVFYSSFYHIYYEDWPEEFKRENA
RAYAQSKGSDVWAADGKFIRPYFHISPPMLTMTAEDI*
>TCRU_3561 hypothetical protein, conserved
MFAVNCEAALLWHPVVHHSLLLLRTLRQRHTLVLEPSCTTASRPDEFWRLSQAGNYVSIN
ATINSDSVFLEGGEEEGRGLALLHISEGGLHRVQNPQHILYFISSSTLLEQKQQLQTYLD
TAFVTDHVLRAVDDVLLALSTDEMEVTRQTHSNLMDLVARLETFLESENDDVMQETEKWS
TWPLFTTLKFLVEEAGLLLGPFPRVAKAYRQLQDSKPVVAHTRLVNRTVNLALSGEGLFV
QPPAWPYRGFLREVQRKLADYTADPAERMMEGVLGEKGPLKARVSGARFGLQPVSARMPW
NMQREKMGRS*
>TCRU_/ S25 / CI-B8 domain containing protein, putative
Mitochondrial ribosomal protein L.1
MSRNGELCLKKVIISYCPNRGSPNTRQFLATHLPRFHAKYPSVTIDIRPRLWAETSITGL
YRDGSERSYKTKYMSSMGIWLRFHRLVNTANDYDLPFSASHLHFQRRSVQGTWNPWLWHY
ETDRRRTETPQWRRKLSEEEWDYYLGQYSAQMKQEEEAIQQRVAEHTEIPLQNTREVQER
WKQYVLPRLQTDMEFNLSHYKRQHARGQRHEPVTMGEYRLFSVPDHREIGQDAVDMMRRR
EAKHQEEWWQHRKSQLKPPK*
>TCRU_3563 protein kinase, putative
MQKSLSSAVSLVDIDVVLTPQTVLSEVKTGVVPLIPITDAIRCLGQNHGSVRDCGSAASI
ATSPVCSLNNTLQSLSAGTLRGTLGTSLNVPDRMIIPLERLRLFGGHVLGKGAFGEVVRG
ELNMLCPTVGYGSAMSMTSCVTLSPQSFTNSTPGQPISFGEICSEEDHLPTGRSSAGESV
VGLLRSNGQYNHYCSRSMEENNLTLPLYCGSFSTDKSARKLLLLTPRVHSRTISRELCGG
PSTMHDDVLTDTSNSFQQHTLRHAANRGDTVVSFGPRNEMASYASEEGVIVKSVAVKRVD
KFRLWRRQKFVKSFQSELNITATLRHPSIVKVYGVAETDAELFLIMDVVEGGTLEDYVKR
KGLEDMNIMAPRFLTDVVLGLEYLKTVGVAHRDIKPCNILLTEDHHVVLADFGSACYLHD
NAANTFAGSAAYMSPEMVGTGKASATSDLWAMGCILFELFVGRPPFDSENCMLVMRKIKE
YQDGHLEYPPYFPEAAKDLVQRLLRRDPMDRLGSDATGGFDVLKTHPFFGGIDWKQVLRM
SNLIFRCADFSADLQQSILMEDENVVHCSMVLKESRVFFKRTLRRRLLVLTDLPRLFYVD
MSTRTVKGTIPIAPDLYVEYDTVEAFRAVTKKRTYVFCDPNKHAYIWAMRVNDVVKRLST
AGNCVDPATRQKKDRGKIFHRRYESR*
>TCRU_3564 hypothetical protein, conserved
MGAAPSAEILREAPFRPARNDSARVISLIPMTREFFPSPDSLIFGQLYDSRCDPYVRYYM
RRDEDTDIRLDVIPRIEFTRDEYYEKCMELSKSCQPCETNKDLRQKYIAEPSQQHLLRPI
PCAVAFLYNDRRDAPEGSVPLGGAYVTVIGGIGCELKESDASSEAMRLDLMIFLRRSAGD
HNMVRAMLLASWLFYTQCIDICKLPCVPMLPVTFRALKTNIPFLCFLREMRSRVGILHEN
TVEHCMWIDGAFICGFITAEHAVELCEAFAEQYRTQVEFKSSSKLGIKPLPVTSRTTNSP
TSSEVASSVGTEETSSACRGLTLSRLHKQFFNLAGFIEKDEFIIPASTHSSNEVYVVCVD
ALVRVEQCEDAATSDAGGKDGSDASLLKVRRGDIIRFVPEAIESQHTPGLAAGGTAKGAT
EEAGVTTEVVEDADIFHSKRGRWQVDPVIHLENVLLSFVHEECKSAREAQPDDPHWLRDK
ETGEPVSDGGFRIWCFDRKGVRYFYGTVPRFANAFRNRSEPARRGASHAEAGAGDQAAGE
GMAPQRKGTSRKKLSGKGGLQAHLARDKGMRLGYYKDAGKAQFEVARRMPLMIMNDMNQG
AVCPFYPSMAPFAPVQAQYPESPPLMMMSPALTYGSLPAASSGEPSESCDGGIQSNVGVF
NAMDASSPVPCVPMVQSMGVSSAPPVQYVPYAQPMPSAMQPMQNAYYCVYPTMGHTGMQS
VQQFSYVPAQVPQRPDGNPLFIVRQ*
>TCRU_3565 hypothetical protein, conserved
MPPFLFLFLLEAPIFYGDDCFCSPLRWHEGDWAPACWVHSEGSGWCAPCLTVGVGRHPPQ
PAVRRHANLSWQREVKDFALELDALRPFSLRLCACGAHGSFLQCDVDGVAAACAFVHVLC
GGLLFLSHGTALVCGRLGGAERCEELSVGGCRLRPAHWGLPHTRGFSRQPIAAPWPWIER
CEGLLHAEGGQRDAVRDGEAGPHVAAAHGARATVRVERCAHGWGEVAARHCGAVCVVCGR
GMAAIVRDVEDTELLDWEEVAYVAAQRERMTAACAGGPDDSEACAGGTRVEVSVACL*

>TCRU_3566 retrotransposon hot spot protein, RHS3, putative
MTTSAPRYLEGCYESVHNARWHHLVEVPSGEGTGMRVEVGKPEQSWTYKAVGNTLEKDDA
VQQSGAAPPRLMVLTSDKGWPYTMNGTHRCGNDLCVNCEVDRVWQIVKGDRDKWLSNSDL
TLNPSPLLRLLIGTPGIGNSMAASSYLLYQLLQYDVEKLPVAAYSVGGSTTYVSDETIKT
VSE*
>TCRU_3567 Trans-sialidase of Trypanosoma hydrophobic C-terminal, putative
MSVREGTSLQEEVPPPLGTEDIPKADVERPIYEEEATSPEWATERQTQETNAPLVENGDG
EDVGTAPGNASILPGETKISSESNATPLSDHGILPEHGQLGELAAMYLIGDSTVHGCVSR
VLLLMLLGLWGTAALC*
>TCRU_3568 Concanavalin A-like lectin/glucanases superfamily/Trans-sialidase of Trypanosoma hydrophobic C-terminal, putative
MVSVILTTQLERVKEVLKTWNEVDKRVSKLCPSENAKEDPSPATACGAAIPTDGLVGFLS
GKFSDGTWRDEYLGVNATVKKNDGVEKKENGVTFKGRGAWAEWPVGAQGQNQLYHFANYN
FTLVATVSIHGLPKDGDNSIPLMGAKVNDGDKNTVLLGLSYNREKRWELLHSGEKTSEHS
RSWEQGATYQVATVLQNGIQGSVYVDGQRVCESLQSNLENTKSKMISHFYIGEDGGNAGN
TGGDEGASVTVTNVLLYNRPLSFEEIAGLAKNKINIPTPVAARNSSPAVSRPKRSSESLA
GGASGVGTARHFGANGDGSTVCGSGLLLLLLFLLGLWGFTAL*
>TCRU_3569 Concanavalin A-like lectin/glucanases superfamily/Trans-sialidase of Trypanosoma hydrophobic C-terminal, putative
MDDNEKSVLVGLSYNNKEKKWILLCDGGENKELSSHLGTENSEHVVILLRNGNQSSAYVD
GKLVGSAEFECENTNDEEISHFYIGGDGDNAEDQEGVSVTVTNVLLYNRPWTEEEIGALN
PNKAPITSPVTENAPETVLQPPAKPQPSEHEPLTTNIGAGGVPSAASTATTPSSDVDPTV
VTGGGDTMRGNESPQTPEVSASSGEDRETAGITDAQGEEEEIHPQAGELNATALNSSIGN
LSRGNNSDAGTMRGRGLLPLLLPLGLWGFAAL*
>TCRU_3570 hypothetical protein, conserved
MTYSVRKLRLLVDDPGGTLRDNLAIAQSFFAAEDDRELEEFRSLMDLEVHYANFWGVARG
PHAALAVFDNERSALRLTWPSRVMALTANTFERRGYVHFLSGGVASIPGIGNWLTRFTQS
RVRESLVVKDGKVKFRDLSFEWGLAVH*
>TCRU_3571 hypothetical protein, conserved
MRHTRKHFKKRGRDDLPMVDMEREVRDVASSNDVRTLVQLFPEEIEFLLMRLPPVTKVED
LDTLSERLSGLGGKEELGFSPLFCPLLIDAACQRGIFPLALSAGGDRYIFAPKLHYHRCL
TQLEPPVEGFPMANDAKEGKFKVGLLQVSKKYLSGRNEMTRARSFDVFINRKEDIAPALS
LIVSQHGENWMCRALRACFVHMFLNQERYRTKIVVVTVRRHCYGADSREKAKTADERVRD
FAEGDHVEEGDLVAAEIGFIAGDIYTSATGAYSLSGSGTLQLAVTGEAMRSVGCKVWDLG
MAMAYKSQVLGCVTFPREKWLRLVREHVADTSLAEKIEGRLREKFSEGVAVRTLLSLADK
*
>TCRU_3572 ribosomal protein L36, putative
MPARKKEVPKAEAPAPRTGIIAGFNKGHKTTRRARAPSSNDRYALPHKKLRAVKAIITDL
VGLSPMERRVQELLRVGKDKRALKFCKKRLGNFKAAKKRRAKMEEALRHQTKKK*
>TCRU_3573 Domain of unknown function (DUF543), putative
MTQRVSTVASEKVLLPRESLTEAEKWRYAIDNTLKLCGLGFLSGAAFSLIVFRNVGPRIA
VTALGGGFGLGKSYVDMRYVFGHEVVAEAVWTAEVVKRTNHGTAAFNEGETAGPQ*
>TCRU_3574 ATPase subunit 9, putative
MMRRLALQPVARRAVATSPSALVVRRASTVAISVQGLHYVGTGLAAIALAGVGLGIGTIF
GNLLVACARQPNLTKMLFNYAILGFALTEAIGLFALMLAFLMLFS*
>TCRU_3575 BNR repeat-like domain/Concanavalin A-like lectin/glucanases superfamily/Trans-sialidase of Trypanosoma hydrophobic C-terminal, putative
MSRHVSTFAVLLLVVMMCCGSGGAATAQVGSNADASTPGSALTGAIAGEGSASGGVEGLQ
RVDLFVPQTTQVLAKGGTSQETERDSFVSPSLVSAGGVIAAFAEGRVYTVNAGRQNEKTS
SDIVVGYIDSSWGWSTLVGKVNESTWKANTVLGTTDGVDNRVDFFYHPTTTTKGNKVFLL
AGSFYEHKQTVGNQTYSGFKTQLELVVGDVRESTSSKQSERIEWGSLSSPLNGSTIAAHK
GNLTRFLAAGGSGVLMEDGTLVFPVMAKSGNGGYCSMIIYSTDNGSTWVLSKGISPANCT

DPRVTEWDGSLLMIVNCKYDQRVYESRDMGTTWTRAIGTLSGVWVNSRLYFWDLSLRVEA
LITATIEGRKVMLYTQRGSFSGEKRERALYLWVTDNNRSFYFGPLGMSSAVEEEFASSLM
YSDGKLHLLQRRDNGEDSALSLSRLTKEVSTINSVLKTWAQKDVFFSSLSIPTAGLVAAL
SDAASGDKWYDEYLCLNATVTPNAKKVKDGFQLTGHSSGVLWSVNIPDDNVRHISLSHNF
TLVASVTIEEAPSGNTPLLTATLANNNSNHTMGVSYTADNKWGKMFEGKITTESRPLEPK
KEYQVALMLQGNKVSVYVDGNSLGEEEVPLKGESTLGLVHFCFGACDQKEDAGHKTDVTV
KNVFLYNRPLNSTEMTAIKDRIPVPTRGPETQVEDGTERRHIPRIEGVRANAPAGSGLLP
LLLLLGLWGFAAA*
>TCRU_3576 hypothetical protein, conserved
MSEMPEEFTDSADEWEAPVQNRPEVDDKPQRTQPLLDKLDGEERELAGEVVHVEFHLPDG
RIVRRDHLMGQTVACLKAHLEDIDGVPYEGTTLFLGDRPLLDPLSLNDLPFKGGECNEVT
VRIAE*
>TCRU_3577 U5 snRNP-associated 102 kDa protein, putative
MDAAPTLLPQKDKYEQLLAKYDWSSPFPPRGYQFGVGRGAKAFSTSAELSSAVGAAAAPF
LLSTPETSGLLDALDRLEEKRQKRRRDDAKGARQAATAHESKKPKTLLSMEDLATVGSGV
AGHSLVKRKARTEDMDVVDNHVFADETSVVTAGDLLRSQTFTTKQTLENILGMGSPTEQT
TWITHSRAFREMGLSKKAHQTLIEGCRLTGSKGPLIWKERLEHTQDPAAKRQLLEEAVKA
CSGCEELWLLLLEHEPPHGQLHWLQQAVMVCPSSEKLWLRVLLHISAPRDQKKIIRKALE
VTPKLSSLWAMLARLESYETGKAIFNAAAAEHLSLQIIVEAAKFEEFHFSSRIKEGKRTE
EGSLQVLGNERIQQFVHQAVGSFLIVEEEKSRREWLEAARAAAFERYVLTSAYMYLYFVS
DRKTKSLFVPATWIEDIVALVPDIWRTHETVCAMWAAMLILHQIHGKPLCGDTHIKEKEV
EVMTVALRLASSVAIDAALSALLKQMTFTDASNLGNQSVEEEEEELGRPGTTELALSPPN
GTTTSATAASNSLPLPLTCVFLCTLLHMPWLTTAEMTIRIAKVLYDRGFYEGASHVLRLS
ISQHPTNCFLFPAAAKTLLAIGREEEAVEMLLRGTELVSAESDIVWVKLAVHQRSKHKDI
LPLIDKALCLFPKSEKLWLLRLEAEGARIKRLLDDAAMRGVSTLPFIIELRQIYSKALSE
ANCRFSPTVWCYVGERLESVLLSSASAARALLLEGVVVCGRKQPNKKAEIQAAFGLARCQ
VERIHSGRETALEVVKETLQLLPKNNGSFTVPVGELVSLSIDLEAPAARGRAAAQAVQHW
HVRDPLVFGSIAKVYHAAGKYDKALEQAMKAVKMSEGSCGDAVALWLKLASLTAYRRLVK
ALMCIADEGELSNTEEPIPMSLMLSWLWNQIALDSDDSGNATDAGSNKQHTPKINGGPLW
VLVAKIKDPSNVTLLGYRDSVESMLQKVMDLIEL*
>TCRU_3578 hypothetical protein, conserved
MSSVFNTNAFLRLTTDTVRHDMRTMLHWDPVGNRVHCAMRDAFFLVIDTPRFFTYTHSSY
GAMRRYIRQARINKGKVNPEDFRDVDTNVLRESLIRYAQWKGPLQKIDFRWFYWMYALMI
GYVLWQGLALQKILDEKVERSGVSLEERRRMFDDEYEEDVRPR*
>TCRU_3579 hypothetical protein, conserved
MTLAVPQCLLRLLEDYTLQALDAAHDVSDEFEEELTDLTSRAIRIQAVLQLIRECEKSRH
ATSSLASSKAIMRNTPEAFTQFALQALGQMSSELTGIPATGARKPQFNAPSFGLLPIGRP
VSLSEAALELFAHNTKVVFRRVEKRGRDGDAVEGEHRQESLKKSVFSRLAAEQRKRRLVL
YTARTALFPRAAPLSSLSVEDIEENLPPFVPPAVREFAALNMEAECEMLTAFYRVDAKVQ
LLRELPGLGSLVRQLRESLRAAGVLLQCASDSMNEAGEQPWFEFPSVSEGVIRIVVRQSL
VLDVTWESARGGQWRLLALHWLLRVQTLPEILQVKTFSMHTCENEVAKGLFLYGGSLRVQ
PAHHEATLRYLSSCLETGLESGCMGALRVVNAVAMEVIEAQCKVLREKFFVGPLESNFML
DVRPGTHVAMTVQLPSTMGSVHGGALHVKCNLCMGTIVVERRRGTDTQTMQQTDFYTDSL
ARLKHSPVVVVDMESLLWQFAFA*
>TCRU_3580 cyclic nucleotide-binding domain containing protein,
putative
MRADTTQAFLPSGEKLYSFPERVVKEVRGIALAVIRCLFCPILRRHLAGMHRRRVLQLMR
GSGELEAKMLHALEQLSAPAQVAHAVEMGAELARVSWVMKQVAASADYRVFTQREFVQYL
GEPGNAVVVLLVGSVTRRVRMARVRGKSVLEKTIFRSFTAPGSAVQGDETSQVSRASSAL
SLRRSSVSNVYSEKPSQCTVVCAPAVFGEYNTVGGYPWTEYIVAESPLVVTASLSKNAYC
EILGAFPRHIQKQVLLMALQSREKLIPLFAPMTVGRMRLCPLLTDLSEENVLHLMDYLVP
HVKAAGMQIGELNAPQHIFFIRRGTVYIRNEELFLADLSSATQPKCRSLLVEGHTFGERQ
CIFREALGDSFYAVTNVDLYALPFSVLIQFMKQQPDARKIIYASAKAASLVLENDYERMR
FVPSSVEEMGTVFLGSALMSKLFHRRVSISTIGSRRITPLAALAGNTGKMDGKVDQSACG

VSPHFIEQIRRIPLLDLCSPDDAFYAECASHWNMFLYDAGEYIVRRGGECNRLLLFPQGG
AAVVLNEKNASQCLVDSSTPFFRQNFSLVPRECIIGYTCVRRHPWALSVIALENQLEVWE
IKRVKFLELLRKHQLERKMQEIVLQLLQPLMCLPSRSTVLDMQPLLAPAPNSLWSEHRIP
NLHPVSLCEHLRFPVWKEGDLPLGSYNLQNRRNTI*
>TCRU_3581 adenosine monophosphate deaminase, putative
MVKELQASTETENGRSAPGNTDEAGTDIITQQLARARLCSHNKTNNNSNSNNSNTPTKQN
PSAGTSAANNNNNNNGEGGTGKFAFGNFYNVSIDGDDGDRDFLKCAEMIANIILSRQSYK
SFDKGEMEVFFGSDATDFIVQIDVQQAGEESVLSFDGGIFRFKGMRTKIVPWETYVRDVK
AVFSAIESGPCLSTARTRLTTIAEKSRLYSLLNGGVNLSCSKKTFGGGIYSICARVDNAL
HLPTSVTAQNLLDFIITTALEQPRTPLFYDEKGKAVHLMEYLESHGVTDPRELTVHGLGM
QPPHHRNKFQYYNVLDTNLNPSGKFGAHLLQAVFSTNGPKEGDLFGALIRPELEKREFKD
RQVVATEILLSFYGQNAEELQRLAAWVRRQGFNSFEMNMWTLCIPRSAPLRGPNEQPVTC
VTFGDQLRHIFYPMFMATLYPTDPAWADVAALLRKTGAISISPERICHAENLLTDALDPD
EIKYEDVVNDHCFFYYIWANLVSLNALRTRLGLNTFNFSPSVLEVAPAYDQLVSSFLLAD
VVYHVTSLEKSWIMQYLFMYCRIGVVMSPLRDNTLSMSYFDHPFVRYFAQGLLVSISTSD
PLYFHHSTSPLLEEYATLMKLHSLLPMDVRELARNSVINSNFPLEMKRKWLGDDFQLIMN
RGNDIWRSGVCDARLDFCTGCLIHEETVLNLVLSQVCKKGEQPLSLSFLSTRNPTRIPTG
ATQQLRDARRANYTDWRIMYPRIDIFRNDTQVPATEAVELLCDVISLRKKYINFHVREVD
VNVEDVFTDSKFDESKWEYNSYYGVFLLFRIGKSPPWPSFIPPVREFIRDVATVREAVMS
HRGLQHLAEQRLHLLERKFLLHLSLNVSKEAGAKEEKEWNNRDFFTAHKVDTNVQTAAGM
NARTLLEFFVEKALHHGHDVVFEEDNQPVTLRQMLERYQINATRITVDELNHLINSNSHI
RTIFLEVDNLMKGRYFAELTKRTLELYQEDAFSFSENRLVIRGESKDEWWKLAQWFDRYG
MASSQNRWMINLPRQYRRLRQEGAVRNFGEYIDNIFQPLWEISLHPAEDTKLHYFLTHVS
GMDCVGDESKIDLPLTATYPHDWDSDLNPPYNLYLYYYWANITTLNEFRASRGLGTFSFR
PQCGELGGVEHLIGGFLLADGINHGVTLSQNPVLEYMYYITQLGVAMSPLSNTAAASAYL
SNPFPLFFHRGLNVSLATNEPLYFHFTREPLIEEYSIAAKLWQFEFNDLSEIARNSVTQS
GFPPAWKENALGKLYYLNSALGNDVRKSRVSDIRVAYRYEAYQSELNFLSGQLSGGRQMP
RAMRLLEEEIAIYEEVMRTKVVMPAAGDEDDGREEIRSPAARAVRIKAELDALERDMQRM
KVLAMQMGSENRGIVEAFHRIRARLKTEGLTVMGNLNDCFAPLDTNGAASETINRVSENF
VKEAA*
>TCRU_3582 GPI transamidase subunit Tta1
MSGEEDWRLRVDMPWAVACVGALLVALGVHYKALCEDRTDLSLPELRASMEAKCRLLGDV
FERPVMEPFLHPVPSQFGVAVWVEREQWASPVHEALDRIEEAIGRGSYKADAAGSGARRL
RLIQRIVRVATSRPHSGTQDDSLSLVSVATAQQLGLGLAQHVCTVDLRGEVIMHGISFFR
RASSSAGDSRVQCFSFDERQAYCTIPEDVSSTWLSREVPAAVATLMSRWLQLDSFRDAST
VRRWALEREAQSCKYALRALQQLELSVNAHPEMPAPLDVAETIDRLQSLIDAGELIQFAR
AADDLQFHPLLLPQLYIPWDQALVIHFSILLPVMMTGILGVRITVAARRRQRAVAKLQEA
TEAKKND*
>TCRU_3583 hypothetical protein, conserved
MTKSSEPPRGRMRVEPSIPLDFSSLPAVQEMIEENNRQTRPKRRLKQFEFINEVFTRLGD
RAINDAAAVPVLSSFLQECVEKHGTLPRSSALAKVLYLWSENGLVGEGQSLLGEVLQADP
ALRKNAFDEKVATVSLRLLSRSASVDVDLMNKILAMLPEGAHKRRLFFPLLQHAARTKDT
ALAFNTLRMGQNKKIEFWDADYHQLLCTIQHAATEGGNTTKLLDELLEAMVDHHPVVGKR
NAEALKHMMGGEMAEINDKTGECSRCGTKLFSFDFTSIDRATLLSDIETKLIAPRVGGEN
HYEPERIVTPAEKDRRWREFEAFKETLTNCDYDVVIDGANVGYYGLSSWYREAKELCLRA
RGVDPTTVPEYQLCEVPLPVDVPPKFSLIDEMLAQTRCVGRKPLVMLHKRHVESPSKESS
EWLLKWKADSALIACPGFMNDDYCWLYAAICKPDCLFVSNDLMRDHHFMLLSRRSFLRWR
QRHRVTYKALFQRATGTATLLLAMPRPFSVWVQRGLLSRTHWHVPVLLSADIIDQATNRA
SGKQVEVDKDGDDSCDAWLCTAAKNLLNRSE*
>TCRU_3584 hypothetical protein, conserved
MLLFKRPHLRACESHADIAVDLAPLSQLRNYAEFEELLREELQKIYGNAPAEFHGVITYS
TRDAPQSFRGCFTERQLETLHQYDAAVEKINHLSSEYRVALEEHERLVEGNKDRKPTQKR
IREEEKSRKRLRAMKREVVAAEYNKECLSLKLKNLFSIDVIRVPLH*
>TCRU_3585 hypothetical protein

MAANSLKCPFSSKMSVSDGVVALDSDEILVSPGAVLVLPGAVSTVCGTPLPSSEETSAPS
VQEMLSVSSADVGSASLLEGVRLLSESIALSEFPSPASRLRLCSAPVVPVPVDHTPSFSA
EGSVLFCTWCSLSTVSVAASISDDAPTEDVDSPLLSLVPPSSTGISSSLLSEDLSNSVTP
VNSSDGSSCGCGSESCCIELLSSFSVA*
>TCRU_3586 hypothetical protein, conserved
MRKYTIPRGSRDRPEESRRTECSRPSLLRRQHATSVLSYREAACPELKCSANGRNAIMPS
AVFSPCCQTPVRNSLVLSRAQACDAPRHGGTTSLPSGIGCAAVLLGPRLCSASAAGNQLV
RHTVRVALAAILTVVRTPCACVPSGDWNMNWPFTPCVPDSAIRDCLPGMAPGPVGVLNVF
HRLLAPAARTERLSIPPSLTAACEVPGKRGTPLPSANAGRTNAAQGELPAHPAALCPARG
ERRTGPPSPSPFSRRPDRHLDCIRHR*
>TCRU_3587 hypothetical protein, conserved
MDRTPLDMAALRLQKIAVRSETKQTHPMTEEEMNQVICSRTDWKQRVVLRLAWMTASRLF
EIAVLTPNNFTVEADGTLILDRTVAPKTARADPHRALRFVRIRGQDAFDTIKLCRTLQEN
EKLTNITTAHVERALAPWNATAHSIKRGALRHAAQIVEAYNLDPHVISRLAKDVDPFGLP
KNTVRYLGRYTTVLTQVSSLVALM*
>TCRU_3588 hypothetical protein, conserved
MKASLEFYSKSGAHAITGTVGTLTPGSPLAFLVLWMRALQLHREPNKQFMDLGGIVLRAF
RMRIVIKSDPSIPIQKLRARLRTAFHEADTFARVAQSFMDRRGTKRTVRRSLLCQLCLIY
GHDASTCNVRSKRVFPELQTKV*
>TCRU_3589 hypothetical protein
MKEEVEDGSTGTKTAVRLDVKVSYLSFMIRDDEESSVLFTVESSEFWMIQHSMKLTAQYT
MSNASIQSTYKSKNCHVLKPFCFDMTVRDVELLINAISACSAQVNATRLELELSDILLYQ
FYQLAAHRQDNSWKTMKKKGSKGGDASPAIAVPFISPFSRNRRIEINSVVICEICVIPTY
DRRVRPPEDVLFRGSPIGTFIPSLRRATIRLPTVRFRDVSRKTLMEVCNMVQEVLFFEFL
KQIPSFISSVFLLPTLSPLGNILTRVGSFIFGSGGASSELPGAALI*
>TCRU_3590 hypothetical protein
MIRLEVGGVKYAQQSAPFPEELSGVFSDCDMDSDTSVVACEKYTHAVVKNWNVHLKGVSE
ADAVKELLGLWMELRHMMNSIQRRNVVNCTLLRGGHYNLDRTGPWFFDSPGEMVLVEDCT
FLSCTSGMHMVVCSGTHVCFRRCHFSSFFQHAITVEDECASFVCDNCIGEDFGGESNATG
AQAEVEKKTVQKFLFFFHRGAVCLTLRDECKLTLNLSTVDLRFFTCLKKTVSHLRLQRGR
LEFMEKGALHGLVSRMDLDLDWTLRHRDDYATLSSSLRVGSVSIPVLLFGTMRCLRGVWN
AEVPFSEASVREYPSPVKTRGFSVARWKALVRVSIIPVELCPPSGGAPLISFILDSTSVV
SRRETTGEAFLEARSGIQQLSLPGNFLRNRMFHFYGICSLCWWLLARRIFLVRHVTCLSP
HVSFLSPAGKSSAFCPS*
>TCRU_3591 N-terminal region of Chorein, a TM vesicle-mediated sorter, putative
MVKTEPTLEAGAELMKVIVITFSGVAVCANAAKEQPQKIVEIDHLSMRMTSVYSSAGILC
RKNVAIVFDGVSSLFFDEDALRSLTRCAVSHGIMKEVPEYCRPFYKLRSLSSRWSHAKSC
VFAFIRDRRGRYNFNSSHLHFYAEARTSYLALLDYCHRTRDIAEKREQLADIEGDIRYRD
VVLFLRRKVQERYSTTVLAGSTGAGVTPPPTQPSDARQFVVVSSLHVDAKITCVRLPAET
TVWLRDVAFVKRHGESNFSVGNVTLVAGGKSQLLRAPDSGEPFFSLQQTESNGMTFVRTT
LEALRFITTSNWMLQLVQPIMVAMPHVLRAIPETNCNAPETKLSNRCEKTTSFVFPLIDF
HVDDLRLHLKQLSLTHIERTSGDSRVGLLMKELSVRFGEETDVLSPMKVEMTEEEDILVS
AVTLNVSSALWEYLCHVRTESGTMPYYGLVTCQALYQECSQQRVAH*
>TCRU_of Trypanosoma cruzi region 4/Dispersed gene family protein.1 Dispersed gene family protein.1
MSSTVALNVLRISTVSSDLKNGVIVAACNTVNGEEEVQYSIPSVYNATILTCSDPCALAA
SCFPAYTTTASSDGCACNCAEGGHGDACLPVAVTEPPSTDGVDLCVRDVSVDVEVNVGFG
TSVVCYVGVTFAADVVVDVESMSGRVRNVTLANCAFVDGASLYVIGWRSDPPAGQRVDVL
ISGLESCSGGGVLLANRYPPGSRVTVVDSVLIAEKRVAYRGAYFLGDASACLVVHSVNLT
GSVLTIARTHVAAVCRDAAGVLFVGGVALSSRCALYVDGLSVQTALGLCVSVEGGVAASG
GSVVAFVDSDFLLCKHAVSVRGAVSVSGSAVVLVRSDFSSTGDYAVAFYSTVSLDGGSML
LAKGNVHDGVSRGMLHAAGTVTAAGSTLSFVRNRAVLPRMLSVSLSLAAGAHVRVACNDA
GGRVLSTAEEYAAAGFGDAGSIDVAGCDACDRDTHCYAPGTASASMTDGVCVCVCGSGGY

GEACVPVGAPALPPAVGTASSMFVREGVTVQSVFVVPAGASEATLRHVVLDGVSPVLYVP
WMARDGVRIVVQNVSLRNGAVLYVMGGGALRGAGTAGSDEGGPVELSVCDVEALNGALVL
TGTYPAGSVLTVTDSLLVATRPTPLVYLLGSQSSPYAPLLVLSGLRLVRSVLVVSGVALV
TVVTGGRTVVVDGAVLELVGGGVALDAAVFGGEYALYASARVVASGGAVLRVSGSQVYAA
HGLVFDSGVEANASAVVVNDNAGALTDGALLVLRGSVSFVSGSWLSVRGNSISGRLLSLP
SYPRSADLVQSTLTLHGNAGSGPVVMDGTVALGGAGGKFVVGCLTLNGQALRPMDYRSAG
IIGEFRPVACGVCDADVRCFGAATRTMSRSCGCRCAEGGYGRDCLPVYLPHVDGCNRTLF
RPPLLSHTATLTETRSPTPTPTPSQSTTHYSPTHYGPTETLQVTETVALPPTRTPTASVS
STLWWSDVACPTPAVTTTAAGGGLTQNDIRGDGSAVPTLLMVALPPPFRWARDPQLGTHL
SFVPVSTVQPRGFGGPWGAMLRNATWVRNATNPSTVLELAVPVHRDYFIVADETIVIRCD
PVAVSGGCKGVLLGSFTIRSDTLPAAASALSAITGVVAGAAAVAVLVTGGLGSILEMQAL
GVFARMPCASAQERASTVALPYFLSVFAALDPLWMVVGNALLAAVFGCVHCGVTAAFQRW
RGVDAASAWAAMRFPSLTYVVAHAMHLGIFFGSVLALAMPGARVQHRVIGVVGVLYGVAF
PAGVCYFIARHVGSSFTRYWQFSRKPLHERLLYPVGYWYPAAQQRMYGGMLTNMRGSRVY
WCVFRLSVLCVVCLIAAVHPPAGGCHVQYFCMAAVLLAGAGVVAFTNMMRSAFLTVMHTA
SFVLLAVLCVVSAANHLAPSDGGAKAYAAIVLLLATAVLAVTVYSVVVWYAEDRYLKEPR
EPRRGGLEALLRDDEESDEETQKLHDMTSSSYASGTTTASSYRPPAPPLQSVTGDIRSDA
LSLFDGASIAS*
>TCRU_3593 hypothetical protein, conserved
MWKTHHCYVGVIFSGVGAALTFFLDSMPLHLPINITLIGCTFREGAALQFVGGAESAGSS
GVLIRVSQAVMRSSVVAFVLALPQHCDIAITDVDAVQTSEVQFADTISNKFGVLLLLNVV
LNASSLLVSNIKAHASRYWGFGLYSIGTLSLVGGSSLYTRYCSFDGYTHILYLITLGVCD
HSVFALLNNTMASGTSLLYQHSRFSVSDHSVLRVVGNSGSVSYAIYSLNSWTVEQSSWLD
WRDNDVGVGAIFYATESTFLIIDGRSVVTLTGCDMGSTGWSVSLLSRAAAGYRFVAGCLT
VAGRELTTAAELELHGITNVTTVAGCGECTKEGDCFAPLTTAVVDCKCRCAAGGHGDVCL
PAPVPAGPPPPPLPPVPPTPLPPPVGECISDMVYPEVAQAVGCRGCATAT*
>TCRU_3594 RNase H, putative
MIPRIQHRRKTLLVVTDSQSLLAALNKGPLSQTDWTEDQIWQRLLTLTCAGWSVHLQFCY
GHCGVHANELADQYATQTMESGQYTEQGIAPLWHTDLLTCFTTQLTNKWRSTIRQDTHRY
SLCGTRPSDLSGKDLITQEVLHRQELVHLARARCGESELWGRLYWAVRDCTNQCRFCNIS
PEQSAYMRSNNDPTAPGTDTVPPSAREEDVSPVRRRTLTRRRKEKCPHCDSTLTGFSGLV
SHCRSFHPEHPPPLPELKCDFCDMVFPTRRSTAPHRSRCAHNPDATRHRNSSARRRSLLP
QDQPASTSTPIGPQETLHHLLLECPGTLAVRQRLGIEQDLRLGKFSQWQLLHSRKLLSLL
DHLFGTQMALYS*
>TCRU_3595 hypothetical protein, conserved
MLAFSIISDELRDESSIPTPADLGTSTPPSVASIHERWLCSARASSLTLRFSSISSSSSV
IEWSIGSSTPMVTRWAEAANQGGMELRKNRSSRGELSVMP*
>TCRU_3596 Endonuclease/Exonuclease/phosphatase family/Endonuclease-reverse transcriptase, putative
MEPFTWLPAERYFYPLLNSVGAYQRYTYRLRAVCDAQRQKLLLSGDIEQNPGPIAALQMN
VSCLTPSKLATLMAQGADIIAIQETWKSSEQIASMHTGDYVLYAQSRIGKGGGVAVLVRK
TLRSKRIPLAIPQHDTSLEVVVVQAALDQNRDLIVASAYMRPPPQVTQSFRRLVNCLPAS
SPLLLCGDFNMHHPQWEPFLETSPSEVAAEFLELCTDAGLTLVNTPGEITYARGTRERSC
IDLTWSKHLTVSDWSASVSPLSDHYMLTFTLHQAFKDTTPSAPPSAPKFFYSWGKCKWDL
FVRTSTHNFRHTTIKSSPPALRLSRERL*
>TCRU_3597 hypothetical protein, conserved
MHGRRHGLQSDCATHVLNTPETREAFVRCGCAEPNDPPQRCWIGGQVDRGILICCAWAER
CCAAVAVPRFQAVEVSGGRTRCGRLAAVVQRTVDASNQKGSRGLPGRPSRSAEPQLTAAW
HGLPCSGGDVQTKIEVSINRIAEQTHRVELDALVPTAQTMVGSVLVGLSV*
>TCRU_3598 Concanavalin A-like lectin/glucanases superfamily/Trans-sialidase of Trypanosoma hydrophobic C-terminal, putative
MHHYDGVSVYVDGEEIYYTKDYEDGEDYNFTQRLRTLLGSHGISHFYIGGDNKSNSEDIN
VTVSNVLLYNRALNEAKLNALMKKDAFAAAAAKVPAQEVAAQTTNVSEPSRHPANVPVIT
PEAQQDATSSPRIQHSPAQTSESESGPADFKQTFPDIIVPSTSAGVGMAEEDSPQNGNTD

DWAPYGTSLDVLEGVHDEPSNANTLASEEQYADRDEDTHVSESVGTNSDPNSFSSTNVSG
GADAAPAPPSTAPGETKIPSELNATIPSDHDILLEFRELAAMALIGDSTVHVCVSRVLLL
LLGLWGTAALC*
>TCRU_3599 BNR repeat-like domain containing protein, putative
MLEDNSYVLPIQALTRDGKNVSLVILAKGTHYGWEFSKDTSDEGCIQPAVLEWNDEIIMM
TSCDDGSRRVYWSSALGKWWMEEYDTLSRVWGNSLNRTGHGVQGGFVSAMIDGQKYILVS
RPVYTEKNGKETGRLHLWLTDMQRIYDVGPISAENENVAASTLLYATVEAQPLKVEGPKE
EKKLYCSYEVAAGGDNYNITFVDLTEKLEEMKKVVAAWKERDAYIAREYGCRNEWENYWR
SDCHEALTKGLFGFLSNTSTDSTWADEYLGVNATVNKGKPAADGGLTFEGSGAGAEWPVG
KMGQNVPYYFANYKFTLVATVSIHKEPKGSSSIPLMGVRMNDAQGTLLFGLSYTHKKSGR
*
>TCRU_3600 hypothetical protein, conserved
MDAPETGGNSQSAGSPAPAQNNLDGQDTSITNAEAQSSIKPHTAGDQNFNDDETGGENER
KAAVTGEPPSKQQPTPLQPVQPEQPTTDSHTRPKEERDFEEEQSPPPAPSPTNSREGIPA
TRELEISTEEKRQTSPSETQKGLVGQEKTSMDDAEEHNTEGTTPSDSVKDAVASGS*
>TCRU_3601 Trans-sialidase of Trypanosoma hydrophobic C-terminal,
putative
MLGNGSPRTTEESASSGANGETAGVTDVQGEEDEIHPQAGELNATALNSSLGHLSQGNNS
DGGIMRESRRLPSLLLLLGLWGFAAL*
>TCRU_3602 hypothetical protein, conserved
MDSDHHLPSHTAGTEDGIPRQAGMLPRRKHVAISLREADCDASTTACATPLATVITWLEM
HRGTVQAAAVTFPQESVRTEWSRPSVLQRQPAPS*
>TCRU_3603 Nodulin-like, putative
MVQLVDDLFRMRMLMAGVYLGLGISSTYGFSIFTDHLRHKYGYSQSDITTISTVGNCVGY
CSFLAGMLFDYAGPMVVLPLGGLLGCLGFVLFGLTFDGYVVSNPSVIHFSIFNAILYLGC
PSMDVSSVMPLMLQFPLERGYVVLIMKTFNGLGTAVLMAYFNGWFKAANSDQPEDNNYSG
YAYFVGGQILLCSLIGTYFTRLPMYFPCSWRKKRLSSEEAAEREKTLDLYMSQQAPMRRL
RIGVMLVVAMLIFSTTQSIATAYVDTSHVGYLAISIVAVLLMASFSVIAMPFQFLGRYTP
VCPTHMEGIGKATTGLEHERTGETICEGAVADGNDLRANVVAVPAPQYSGSFWSHLLTVD
LWAVWLACFGMWGTGTVMQMNAAQIYRSKNNGKFDTRTLTLYVAIMSVGSAAGRMAMGYL
DMKLSALQRAGKTRTLTTIALPIGPLLLVAAYLFFAVLPGSVLLLPFLLGAMGNGVGWGV
GVIALRMMYSEDIGKHYNFCFTSGAVATIALNRFMFGEMYDAEARRRGEFPSCNRPRCVR
NQMFILLVVNVVATLAAALVHWRFSRFTRARLDERETPDSLQDGKDMIGMGSGDQATEED
HLQS*
>TCRU_3604 AAA ATPase, putative
MMTSAAPLFAITGRGNDVKHARELIASIGGVLTTKEQVADYLVVVKGAGRRRVEEYCQHH
HHHHHQKQLQHNGGRGSGNLTGHTTSSNGIRDLHSRGGRLLLLDMLEQWSTRGKVPERDV
SVEELEGMNLLSSAGAERDQRFYDIPEGFLKRSRVTLDPHGNAVDEADNSVLVSSRRAAP
PLTATPPPLGKTATSPALLRPPMVPAAVPLAGAVVFKTPLQHDVQRRGVPRDDESVPQKA
AGNVASYGPKNNGVVNPMRAAFQRRPKPEWDTYFADAPGSCGAGDAQWVGDATTNKKKTN
DSHSSSKVHVAPQNRAATAAAAPSPSECTPSNQAYPSSTARGEAAVAPKITRQKKNSVEL
TFDLSKRGTRCPEMALRGAKSNSEMNAHESTTKKRSDEKATVEEGARKQQQETKEKKQEV
RTKKIRRDGPKSRFYHSALNSKPGKSRSVELPTSRQSKVNHDDAPCRRTAGSGVLASDGS
FGPPPPLPPRPHESAGMRSTTGAATTTTQAGCKSTPAASRLARPLSTSMPSHPPAVATAA
VTTKAANNNSTRVTHGNSLLQRVRQSSYCKGIPEETCLAVLQQVVDRACPVSFSGISGLE
VCKRILYEAIILPAKCPQLFTGLRRPCSGLLLFGPPGNGKTLLARAVSRECDTTFFSISA
AAITSKWVGESEKMVRALFAVARALAPSTIFVDEIDALLQARGGAHEGEGSRRIKTEFLV
QMDGAGNDNSEARVLVMGATNRPFDLDEAIIRRFPKRVFVPLPDAPARTQILQSLLDTEE
TPNSFTPAIWQRIVAMTDGYSGHDLRQLCEEAAMIPVRDLLAEKMRNGEELTAQAYHHDL
LRPLTLQDVETCIKARHPSCCPKQLKALSEWSETYGSN*
>TCRU_3605 chaperone protein DNAj, putative
MSWHGGSHTWLFFFLLRGVLLLLLLLPLEAGEVVVVAAAKSAPKVEEEDFYEVLGLGKER
DDASERDIKSAWRKLSKKHHPDLAGESQREVYQRIQRAYEVLGDRKKRKVYDILGIDGVK
KIEQPQEQQQQQHMHSFFSFFGGGHQQQQVDRGKNEDLVLLVPLEDVYSGAAHTVKLSKT

KICRTCRGTGARSKDHLVRCPHCNGEGRVLRRVQLAPGFIQQMEQPCAHCNGQGVFISEK
CLTCKGKKTVRSTSSISIDIEQGIPDGHVLTYELEADQQPNQVPGDVLFTVVTASHPRFT
RSDNDLTVTVVLTLKEALLGFSKSLTHLDGHLVELEQSGVTQHGERRKIAGEGMPKHHVP
SERGDLHIIFEVEVPSLLTKAQKEALERAFG*
>TCRU_3606 hypothetical protein, conserved
MGRSPDPHCRRMPWTAEKECVPGVVHSSKEKMVLDGARRVDVDCVDRASQVYPLEALRAT
VASYEYNTFRGKNIFNWLLRRNQMYSGNGFTARSGRRGRTTRTPPALPFISVAAAVTQPA
PIRLADFPG*
>TCRU_3607 AAA domain (dynein-related subfamily), putative
MAATDENPLPVTEAEYELPEMEEDHSDNADSDCEAPEDTSRRRRKPAVDTTLNEESDDEN
EEDSKKDADKRRGKRRVKVVAKIHNDDDDDEREKEEEEDVNGFEQQQDTLAEGEKMSRGR
KLWQEQVAAVQGLSHHLCEQLRLILAPTLADKLQGDYKTGKRLNMKRIIPYIASQFKKDR
IWLRRTKPNKRNYQILVALDDSFSMQCNNAGVMSCRAVALLAEALRQLEVGELGIACFGK
ETHIVHELHEPFTGESGPRAFSEVTFAQKSTSLKILLETSLDYLDAARERMHGQTRSTTQ
QLQQMMFIISDGQITEDRLELRRLLLRAEENRQIVVFVLLDVKAAAAPTAAVGGGGVASV
GESCVAQKETGSLSTAPLAAKDLSQLSPAERLRRLKAEREARLHRVQSSSVLDMQLVEFK
GGKVVRRSYMEEFPFPYYLIVRELETLPGAIADAMRQWFELLNVQQ*
>TCRU_3608 hypothetical protein, conserved
MESTCPVTYRRVMVSGPNDPVKYFVRARDDVRTAVPAQTETGTGFLMCPVRVRSQLRHWM
RAEQVALRCLLASRPTRWPLLKGIQEHSFSEGVSVVWPEHCSARSIGLFLR*
>TCRU_3609 hypothetical protein, conserved
MADSIKALEDELNRQLEILAEERKGLRALQRKMNRAAGGPNEGDEDSRGRQLLTANKKLR
QDLERLKREGAVPESSPQLIGVRNKIAAAWKEVDEVEQEVDTLLLLLRDRKKVLERERKC
NRSVDVLRGRQREEQQSLRAKMKELEAKLKRLEKRDVMLRNRFTQLQNQVLLNVTEKDAT
ALRKKHKQQMRTIGKLQKRREEQASSWIKVLENDGQRLTIREKMAKSQMEKEIAELQALV
LERNEEIAQLRHRLAKSFF*
>TCRU_3610 endonuclease/exonuclease/phosphatase, putative
MAKSSPTSVCHISLTNGEDITEASRHCITKPSGLSLFHHRDESTTCASPRAENGCKIATT
RQLTLIERREKKHYSTSFPSLSAETLLDESRDAVAGDAAIVSSTLRGWSVRHVGKNMSSL
VLDGKVKIVSYNILAQRLVSTELYPHCPMFALAEDYRCSLLKRELADAAPDIIALQEISV
DVFEKPGLLGDWLSSNYCFAGDHVVVTDVKGRPRYERDELPKKKCDVHVFKETDVSSQRR
GGEHASWVTSPPPETDGPLQKRSEMEGVCVLYLKDRFDSCEVVPIRFNEIAAADKHLTQR
ERRSLQVGSHNVALISVLRDRVMPNMIYVIGTVHLIWQRTECQLWQMHHVLREMEQLKKK
YEKISTGGKDEPALVSLVLAGDFNSDAGSPPIRYALSGTPPPGSDVMNYWKLPEEENSSG
CEEAGSPPLSPSDTTSEAAGVRHIGSKRRRYRITDTVGHSISFTDSYATYREQHPWHVST
VNPSSNGEGGVLDHILVEEKHVVCTSVMRLSGRTELPTKDYPSDHYPVGVIILPRSSLK*
>TCRU_3611 hypothetical protein, conserved
MSGSTPGIEFRHTVDRLSRGVCAFLFLLRFPPIPSPDCSEEAHVPGIDGVLREGSSHDGV
GVRRHVAWIPQCHGENMEPSARTRSASESGTVQIFSVTPSRTASEPRRSASIAERESVAA
GTRVGDRDAAARRGTGNCLWPRSVAGLSARQEHARLPAAAR*
>TCRU_3612 RNA polymerase Rpc34 subunit, putative
MSSHVANAKQRILSSLSLAGQEAVLFTTLQRDLALDQRTAADALRGLMEGGKVVLRRGNG
DGSVYVALSNSMPEGPSLVLDAIRSSGNSGIDQATLCSKVRLPKSEVIKSLQLLVAQKRI
KERRCFSNRAKRIYLLFEFEPSDEITGGTFYGGEDSREMDVWFVDGMRRLIVALVAQRHS
VSLEQITQHLHESHGSGAAGEATLGVGDAVVVVTALPSGSVVTSANDNKGGNKRISQRDA
QLLVQTLVLDGVLDCVTPSVGAPPQYQLATGKNVLRHFTATPAAVGGWPSNGVTTPLEDG
REGHGDVFAGGLWNPAPVAQPSAWAMPAVGLPCLGCPQLSVCFASSNSVVNPCNCAYLNE
WLS*
>TCRU_3613 serine/threonine-protein kinase, putative
MWQHHNLSSPTLGPREAKPTVQTSRRGCTRSDAWCSSLAGGGHDDPSESVPNSRVSPRAS
SFAPPQRTAPSPTESTSQGLFELQHFNHHKQQQQRRQKQQLVIRQEDIRDDDFQRLEVL
GDGSYSVVVSARHLPTQQLVALKEVSRRRLRELKLEKQLQWEINVHRTLRHPNVVRMLSY
YITPQTVVLVLEMCPGGTLLQKLKTMPGGRFDERRASRYVRQAARALTYLHGHGIAHRDL
KLENIFLDAHGVARLGDFGWSKALVEPTVEQDLSSHHDNFGTAPCSAVKDVSPLCSGGRG

RLTVCGTLDYLSPEMIIGMPHTFKTDVWSLGVMLAEMLTGAPPFYCVSQQDTLNAIREAA
PHLGDDVQMSSLARELVLAMLQKNPDARPTMAEVLQHPWTRQNGEKKKKK*
>TCRU_3614 hypothetical protein, conserved
MPPKRSKGVTFKLVHPSYDDAEYEGAKNVAIMVEEQELRKHLPTTATRRRRQRAEESDEE
ESATVSGWAREWATTTSFASSTAQADAGRLPEEDAYMNRHNFLRAQDSEDDEEDDDKLFA
DQEEVEFTDDFLREVVFGDGDEEDEEEVRSAGGGAKSVTEHDGFVYVQHDVTKRGVDRQF
TQMMREFNVDARLNDAYTDDPRAHGALGVDQYMRALEEFVVERAGVDYGTAEPRRNKGLI
HQLQNMALSAGTDMSPKGELYTTTVLPDKQARFLEEFQREAEEIRQAARERMQRQQLDGP
AGNFEDAVQATEQDDDDHEVYVLREVEDKENRLDCETAVSAYSTYFNQPNVIRAPSPGRR
RRSHGDFSAAAATEKTTENGKPEDEDVVSKRGKQLLALARHKGETREEKKMRQQLAKQVQ
RERRDKKRELKQAYKTLETDEAKRVKESQTAKKTVHFF*
>TCRU_3615 RNA-binding protein
MHTSEQEKPGDIVEHPSDEMYSHANLFIRHLPRVVDENELRIFFSPYGQILSAAVMRNIH
TGENLGTAFVRYATNEQARTALHECNGRFLHGRAISVHWAKKRHDNTPIGDARKKIFKLF
VRNIPLDVSADELARLFACFGPVGSVSLHKDTAVPRNKWMERRIGFVAFLAEGAAEKAAE
AVHNTRPFPGSGSIPLMVKLAEDVPARNRRPGAAVTDSIRRGGAPNDMVRTNATDRTMTR
APVDEGRVNCCRSDFSTWSFLRWSRCNSPQPTQSANTSSTNYPCNLMPQPFYGCSAVPTP
SPMRDYMFFHEANRQRVCAPGMPPQDHADAFLQQAPCLPQPNFFTHAEKDAATFHSLGAP
QKTLGSSCSSLCPAESEASTPIAIQYFNEWSARPATHDGSAASRQGAPITASTSFCSAYG
TNIAEESSVSNSTPTATEGRKRCTRYTHNPYRLHQQRLSECPTPPVPV*
>TCRU_3616 Cell growth-regulating nucleolar protein, putative
MVSFTCNYCHDVVKKPKVNGHAAQCGVASFTCVDCMEVFDLQTIKQHTSCITETEKYQGR
WRQNRAPAAAARRCVDGSDDGDGDGNDNNKRQPLRPPRPPMNLSSDSDSDDDTWVTGGKK
KAVMEKNKGRNGSPNGCLQKRAKTTSPSNKNMEAKSGLKRQKHEELLELPRAVTAADCIV
PSFVLGTDEEVAEVTRWIIEEAGETPLTVRALAKKLVERYNTRIAKHVRHAIETAIQNGK
LLVEGGTVNLP*
>TCRU_3617 Major Facilitator Superfamily, putative
MGDLPSNERIGSRVVFSAQRSPASVLEDPLAQQRESADFALERTRENSEGSGHNASNILL
IDPKKISDFMRFRVMFIGMLLFISSSTQGLHALFALYMEKVYMYDVRTMVDIFAAGTAFG
LFVFPFGAMYDFFGPKIVIGIATAITSLSHLLFGLTFAGHIDASKTRFCIYFAMMNWGCY
AFDVAALPAILTYAPRDRAQPTGVMKTFSGMGASLISCMFRGFFKNRYDHLMYFLMAIVL
GFGLIGTFCLSNAPYEVTRWEDSRISLKERLRRHLIRNRYMSQLLPKRRFYILSCLLLVM
NVYLSIQAVCAAYFQKEMTEGQYRGIAIGAIAIVLGILLLLVPLHALDGDTPQDHTVLER
AREKERKLAELRRLRLAREREEKDAEKEEERAQKAVAHQNAGDVAIEVKELPRILVDQSS
PHADGHSVLYDNSLQHKDGEKDDEDGEETQHRRYLPLDPEVSLFEADDLIPIEARDDDAS
RNSRQRLGNTSIATRVSVSNVSLAARRKDNEGAEMDFITRELSTYDRPHVETITVCGEVF
VTPVYETSFLLSLTYVDLWLLFYTVFAVWGVGITLTANWNIRIMVGSVFKGLDYQTYVLF
ATLAGISTAFGRVAIGGYEVLLLYIGKRRGVMLPATIALPVPSVMLSLALIFYLSFPGNY
SLLVVYIIAAVAYGFSTSMTIYVIGIIFKRDIGMHYGFCFLGAALGIVLLYRVLLFHVYD
HHKSGLTPSLQVNKEGICMGKECLQKTLIVYLILVFLSIGTSIWLHYRYWKLVHGKLKHK
RVITHFVKKRLRRINRRREKCHATEEGAQQQQQEEEEKR*
>TCRU_3618 hypothetical protein
MKPAVAHANPDRADCRDFALRIPVLVPQMISEDAETVHGRNAKREINAGYQHPHAEQIIY
QLYHDFLSPRSTTSKEGIQLEEPDEETAHATGRKNCLPSCWKGQP*
>TCRU_3619 hypothetical protein, conserved
MPGISVSSSAATTAVRSEASTEGSRTISDLSPSSSEDDARPKNTPDGDDASHITEDVVLP
SAGTPMPHQTAAQTDATATPADSDGSITVSHTTSPLLLLLVACAAAGTAAAVVAA*
>TCRU_3620 Tubulin cofactor C domain-containing protein 1
MSGRYAGNTYYGEGDDRYVDAYPASPLFSTNQVVFCVNPVLFMNCGILLDAPSGLTTSAI
MKIRQESSNSAAISLAMWRDIAVNVIGIRESVAYSVFEVVYALTGSFVQTPVAPQVNGNL
VSAKSIAEYRQEGNRALLKKMGGSVDEARWPAASVSRQVSLPALTIFLLAQLILEHPPRA
MLGEISQELIMSSVRQHLHDYITAVAITRPGRLTVGDAQELKILLREFVNGVEQPFGTSI
GFLWPRSEKTIDITILSQFIRPRIMLPSELAATTTSVSFRNNVVVRGLRGTVYIPPCPVM
PNNNTKLMISSSYTIEKCSQSSLYITSDLPHTRLSQLVNCTVAIGPVGGVLCIDRCENCN

ISALCAAIVVSQCKNVTIFVCTNTPPVLSSYDGVNTLENVRFAPYNSHYSTLEEHLASSG
VNPKLNLWNVGLPTSQYVLPPDDFTPICFPVAPHTSAVITTRTNPCPLPRLYAEALERRL
QRFHDTSAQLQEAYQRLQAEGRKDLAEKLRGKVHTMFMEWLRRTGQEKGLISLLHQGTES
HSQN*
>TCRU_3621 chaperone protein ClpB1, putative
MPVYVTEPSVYECISILRGLKERYETHHGVQITDNAVVVAVQLADRYITGRFMPDKAIDL
IDEACANVRVQLSSRPEEIDQLERKKRQLEIEAKALERDKKEKSSQERLRIVKGDIQRVE
ELLQPLLARYNEERQRVDELHEMQTRLDEKKTKLERAERMRDMELAADLKYNAIPVIQDR
IRSLKEKIEQQKASMVQEKVTEVEVAAVVARWTGIPVKKLSQTDRERLLQLSSHLHRRVK
GQDEAVERVSEAILRSRAGLSRPHAPTGSFLFLGPTGVGKTELAKAVAAELFDDEKHMVR
IDMSEYMEQHAVARLIGAPPGYVGHEEGGQLTEPVRRRPHTVVLLDEVEKAHPSVFNVLL
QVLDDGRLTDSHGRTVDFSNAIVVMTSNLGSEHLVHVGNSPKGLEATREKVWQVVRSYFR
PEFLNRLDDIVLFRRLGFGELHEIIDLIVAEVNGRLRSQDILLEVTDEAKNFVLENAFDA
EMGARPLRRWVEKYITTEVSRMILAQQLPPNSTVRVLVNGSQGKLAFSVKRSSL*
>TCRU_3622 Major Facilitator Superfamily, putative
MSEDARGSSHAQSTFRGDWEALHNPNVRRVLIFCFANGACFSLWLPQVFQVLISRTFGDT
TVGWLSATSCTAQVVGAVIAGLSDELPRQTIIRLGAFSGITAAALSIYALSVLQVHIFFF
ASVLWGMFSGMVSTGVEVLFADSVETGRRDFVYNLKWANQTLCDCIGSATSLLMLVKLGN
EWKLSSMKVLVYTGISLHPIAQLFLFTLKDKYVLDESTGGHVPQCAVVVEDTDAPDLTTK
PKLLPGASESFMDSKESLESPRQPLVSENGMKNEDLSSVSVISRSVASAGAVVLQHEAVI
AQLTMRSAQKYHEYMEAQERRNPAARLVAAVWHHANKLCEWSTVPYLLCLCDFFLVFGSG
MTMRYIPLFFVNDYHITPVLLLSASIVSSITTATLSFLVRYMGERFVSRVAAVFSARFIG
ATLLLLMGVTHLHLSILVPIFVLRNALMSCTIGVTRSVIMDCVPKSSRAKWSAFESFASF
TWAGSAVIGGYIAGAKGYQFTFFITSMLHYVGICVLVPAAIVSQNMERAKRARWRNEKAE
EKERKKSLRRLRSTGTASLLAAAEGCEEKDQLLGRDRTSLQ*
>TCRU_3623 GRAM domain containing protein, putative
MDKWTGRVQAVSNSVAAVDVSVGDVVKVLLAVAEQYAADMSPVEAMGRAISQGIANASAT
LVSCLTDLATMSEAMKDAMNANPKEAHSLMMNLFNVFVKQLTVYYKENYEAFKKAEVTLR
QQRALFWSQKSEMETNNDNTSDFTLLPAEEPSPFNISLVAFNAPKEAKKKYADGKKKKTR
DSSKDTSVVLPSEQHSEKQQGKPAKGEGGGTNVCVDAPSTNVAEKNETAQQGNGGDIPSK
VKSFSTEPVVTVKEPIYLHVASTVEDALGRLVLSSRPNVFTAPEVIENRAPIATYYIKLP
APGSMGPLLLSNYIALGDDMQADLMCDILQSDCVEVVPFESPAGEIIVENPSDLHELPAC
GWRVGSSEDHRQAFYTLYILVTRGEKMTPSLLIQESLLIDSEPQEVMHEITAVPDAPQIA
KVTVRGFGPAPARSASPTKRYTSDRLLAIVNITKSTYERNGFVEIKKEELRALVRQMHPH
ELQLLREEELLTRPNSNLTTAAAAFQGTVELASLEQPGEQQGWGLTDVAKGAVRGAAAVV
LAPVSVGKVVGNAVLDASGVTGAIKQQKEVLFRKSFPNLSSEEIIETFSCALVDDGNPIP
KQGFVFITPRWLCFQAGLLSANFSLEWDEIRDIQKQRTVKVLENAIAIQTHLGGTYFLTS
FVQRDQAYADMMKQWLRR*
>TCRU_3624 hypothetical protein, conserved
MSDHTVYPKPLVVTEERLKEAREKQLRQQALKEALEEQIAQTRRRRIDHHAPNKKNNVMK
EDGVRRQYTFTYEDGHGRFIQRDNSPEPREQPTRTITGTSTVDMTSDNATALSIISGHRK
PLEPVPSKNSQSNSLPPNFSMSTSEANPLVPKRTEHSGQPLPVDSGSSSRKQNGGMNNIS
PRVRDLFPQGASPKSGLRSAELVDEGGCQKNCIPRNFNLEDVKRLLQGPQASPRPAQPPA
GGGRSPFPLRNIPQKHHTGHLPPLDPLEGANGRQRSEEPSTGVDLAFSPPHDFLGAHPPN
VKSPRLLKLKQLSTPRRLSNEGRGRPTVNALAPRNGQARVTENTVERLQKELESRDVQMA
KMREKEKNWEEQVKQLKIELKSAKKKERDLNRLVKEGPRRAETAPDQPFLSPPTLSAPGL
PAKKGFGAPSPPGQGKKQKFARSRVFNQQTFRPISAPSDAASSSYVPQEENVASLMQESF
KCASVLTRKTAFGLPEENANRPVPIEYEHLLQFVKEQIITQQQADALWRLFSNEEPPLTL
LRRQSTVAQPSDTTVITNTDNYLVLENGIENSIYNASDAKRKEKIFNEEIDRKELVCSNG
HGHEELELEGDTFETFAELHLEESFSQEEAGDE*
>TCRU_3625 hypothetical protein, conserved
MIVNNDERSIDFSQNPSGELDDEVLTSLRDALQGNDNYCKLVFDNNRLNDDAIGILAAIL
KGSTPIEHLSLSSCQFRDVDFTHIANALCVKKVLRHLDLSKNPEITSAAAADLSRVIRTL
PSLRTLLLIGTGLQPKNCSNILAALKQSPSLKVVELPYTVGFKVLDSFRKLLEERNGDEL

EDSQKKKKKKAAKSEAAPTDSGDGAFCCSKCDTWKATSMPKLVLPPIHASQTAGQKPLSN
SCGPSNSLRMMEFRCWADPAVKNAAVHLHVLDKRCHLWEVYQKEKKEKILARRGVDEKNR
AVKVRSRYGGYDACPNL*
>TCRU_3626 SH3 domain protein, conserved
MRYFRVLHNFDAQDGVELTVKKGETVFTAESEVREGWIKVEVLHDPRRRGFVPFNYLRET
TESLSREQEEGSRGIGRELGGMSTAPMTRQPSAVPRETSVTFTSGALDYGDRLQNMYGVG
ASAPSPVSQRSRSAEQGMRRVNENSMTTATKGAVMESLLPSQKVPSSPPSGADPVPERSL
LNNPSAIVEAFMKNELHFKQLIRQRQDALAQMRTTVEEAMSELAACKEKNFTLARKLREL
DQSVEKERGRWKERIMEEKAYISRSMSSE*
>TCRU_3627 hypothetical protein
MTELLEGKCACMEEFAASAIPTVLLLWRGRRDDAAVAHGSHLDVVHQSILLYLLRTLDGM
CGSDVKCVEQTESALKLRLLLWISFKALYLEPPGGGHEFPGEWRSEVLRDSKAAERILAS
DIFLGNGGAVHCQKVEVLSWLVVEVLLQYRCYWEHVDMWGSVEGCRTHPFILWAVGNLSE
VGTEGAHPWTLSCVQQRAVEMIVLLGADAVLLERNRELFTDAYLRTLVAAGREIFTPELF
GGLTSSIRDGLIRGDRLYHDDLCVLLETLRRHVSVDTDEGRVRDLRTVIDVLLNTACPFS
CEKEIMRGVEAVCAWFELLGLQEDYRHVLTEGAIDGEVLRSGLSLHEVQQMGIRNEMDAA
SLLRVLAARPNRRVMPGDSRM*
>TCRU_3628 hypothetical protein
MENVSDNHMNGGKGDDSLEDLYACDSLFDESGSEDDLFLAANVEEQLLMPFSCFTEKDLL
SVFNVSAPYGDPVRLHPEEEPPKETRLEGPGVLRGVRIPSPKKKRETCTHRKNKSSTEKN
TRPAGKRKEKPSNTSSNQTRRLRTKGFIKALTPDDLKDDEDYKR*
>TCRU_3629 hypothetical protein, conserved
MMSQSLNALRSGTACFPTTIAQQRRWITRLYTSYYPGVLYPNQLVQHKQRLPPGVSLSTA
LKARTQTRQEQSVAGREVPEEKETRGVSWTDASSTDVMHTGNARRQEKVSVATGRSYVPL
GEVAKLELQGDYCMEGGLFQEALERYGVVAKAYSIAYPENHSQRIGILLKLASAFRHVGR
LDSSKVNIENALKMLDASSRPSLELICEALLELGLTREAKSMKKEGAEAYEEAVEVLNLF
HNWGESHRMLRLLPRLGRRFNLNFEEKFVYFSPFDYDRTFAIADQCMERAEKVYRETGDS
NGIIRVLQCRKEMIDKKFFNLRDFAGRIHTMRGHWMRRAQHLTNAPTPDELLRYTPTIHQ
VYRDFKYELTAPIGREKEVMPGVNRVVLDMGNPYRRRGRLSNKMLRDADHNFANYVRREE
YNA*
>TCRU_3630 hypothetical protein, conserved
MSNTVPPHYVRLRRQNLTIFLHCDVNHDTVQAIKERYEKLTGRLFYTVRLYLGRQSLDDF
STLYNCGIEHEGAELIVVHSKALKTDGTGEYIWEDLEEATQLPQPSREEAKETKEGETSL
GSREGGETAGEGGSASLLTRPEEKSHMSFEPNVGFVGV*
>TCRU_3631 hypothetical protein, conserved
MHRHILCEALLHAQYGRRDAPCIADFPTDVSRKEVEEVYLPFLTSEEAQLRQNLILGDVE
IIGYDSGSLLAVAGDAIAAGLVLSDMLSEVPASTVFPTSVGPHSSAR*
>TCRU_3632 replication factor C, subunit 2, putative
MACISVAKKPRIESKPNATPWVEKYRPMTLSEVKSQEEAVCALRASLQSSASMPHFLFHG
PPGTGKTTAILAVARELFGPDYVHSRVREMNASDDRGIQVIREKVKVFAQTAVGNVGHKV
QSDGQVYPVPQFKLIILDEADALLPDAQAALRRMMEDFSDVTRFCILCNYVSRIIDPIAS
RCAKYRFKPLVRETLYDRIREIAKMEEITVSDASLEALDRVSGGDLRSAIMYLQYAQKAH
GSDLTKENFLEVSGSVPMELLEKYLKALVTKDFDGMYSLTKEIVGQGFPASQLLSQLHQY
IVGSSCPLNSLVRSRIALKLCDVERRLSDGGDEFLQLLDLGTTICSL*
>TCRU_3633 RNA-binding protein, putative
MSSKPSDENGVQFNTEMDPEAELQEMQRQVSDLQEDLRLKTLQETAAQDEGHRKSTGFVA
TKNTSIFVGGLDPRTTEGELRVFFSSCGTIKRLTMLRDKFTGQLKGNAYIEFEAPEQAAA
GILKDGQSLHGKPLNVAMKRDNIPAFQRGRGSSGFSRGGYRGGGPGAMQQQMAMAATMMA
ASMMGGAGGGFNPFGGMARGRGRGRGRGGVH*
>TCRU_3634 leucyl/phenylalanyl-tRNA protein transferase, putative
MGLHWRAGENYLDVLSLSPFTIHGCQPADAEGSFLSEQKFPLHARFQESSGEYMATLWAL
DTGRAYLVGVGPSTEESSTRDTNLESCLGVGRNGVDAPVKFFIVKTCINRGPLAFLAANT
ILDVRLLYRDDFLDCLLSQRSSWMLIEHFGWENTTLLQHLFYHSLFAIPDAIREAPVYTL
PNGSKGRFCLDLKQENIAWRKSKKVRRIMLCDLFAVAVNRDIRDSLCLAREYHLEKKGNT

WLKESYIDLLVDLAACPEYGVKIMSVELLEKSSGNVLAGCLGFSLGCVHHDFTMFTMQRS
PEGFGTFATKLLGEALQQCGYNLWYWGFRLKYMEQFEGKYGGKIICKADFFARWAQNRDV
QPNCTLEEFFRSGRGMLPYFVSAE*
>TCRU_3635 Hsp70 protein, putative
MIRDSESHAESDRLKRELVEVRNNAETQANTAERQLTEWKYVSDAEKENVRTLLAELRKS
MENPNVTKDELSAATDKLQKAVMECGRTEYQQAAAANSSSSSGNTDSSQGEQQQQGDQKK
Q*
>TCRU_3636 Hsp70 protein, putative
MAADNQMMGQFDLVGIPPAPRGVPQIEVTFDIDANGICHVTAKDKATGKTQNITITASGG
LSKEQIERMIRDSESHAESDRLKRELVEVRNNAETQANTAERQLTEWKYVSDAEKENVRT
LLAELRKSMENPNVTKDELSAATDKLQKAVMECGRTEYQQAAAANSSSSSGNTDSSQGEQ
QQQGDQQKQ*
>TCRU_3637 valosin-containing protein homolog, putative
MGPKKARYKPPIRPNADGTGFVRESAGKMEETPTRERGREGHPGLGAVRLSLDLSTPPCM
GPSDVSGAALSLQIRSNNSLPSNEARLSPSSFRALGIVSGAVLHIVAGNSKAYAEAFISS
SCHAQTIELANEVAAALADKQANVSVMKQPLPSAPLDLVVLEIAFDPPEETAAMQLEGSV
KLPFLQVRSAFRRQYRNRLVYRGMRAIVRVATQPFCVEVVQCVQFGADVKDQRKKKQIQA
MLGFLLDCTVIRDGGCEIHIENESSENSQDLLKGTCNLQHVLVLGESGTGKSRFLQQEGR
DAATLKCRHVETILVQELPQGEMSDVSTATVLREAFARSKVAAPSTVLIDDLHLICGTKG
SSTAGSLWAATVVAATLKEELDDIWGRRLDVCVIASATSTESLDRSLLTPERFGKRVTLL
TPGSAEERYVCLKTCLKERDALDSVSEETLLTVSQEAHGFTQCDLARLVEVAMVENFKMT
GNVAVSDEALLAAAKLVRPSALKQFEVSVPNVTWNDIGGSEDAKKTLQDCVAWCLGKQQW
IFRKFKLSPPQGVLLYGPPGCSKTMLAKALANESKMNFISVKGPEVFSKWVGDSEKAVRD
IFTRARAVAPCVIFIDELDGMCAHRGSGGVSDRVISQFLTELDGLPAALNEKSDALVFVA
ATNRPDNIDTAVLRPGRIDRKVYVGLPDVSERRAIVSIHFARIPVATDLDADYVANRTEG
YSGAEVVAVVKEAAFLRVTMDAKAAHITREDVDAALQKVRPRISPSDVAWYKRWSMGRSL
ALR*
>TCRU_3638 E3 ubiquitin-protein ligase, putative
MSYVEEEYYESEEYAVDYEGNGGEWALVSPAEALRRPVGARAEAWLFTTLSISEVLGDLQ
RDVEKVNEILGLTPEAALLVLRHYGWKMNDATLEKYFNEMDKVNSELRITEAAFHSGGAG
AELIRGDQPIECPICGDDVPAGESVALGNCRHFLCVNCLRTNLLCAVKHGHDLLDKRCPI
RGCHSLVGLNLFKELLPAREYGQVQRRFLNDYFISNRHMCCCPNEATCEGVICVKAIRES
SLEVQCHVCKLKFCFNCLCAPHAPATCDMMQRWERMVQENEPSLALIQHMTKGCPNCAVR
VEKNMGCNHMTCVRCHHEYCWVCLGPWSEHNAGYYNCNKRSRSDKKIGKDFFLDCFERWD
NHKRSIALEAKSLEECSKKVRKLTQHHKDTSTLDRTLSVLFNTQRVLHDCRVVLMNAYIA
LFFSEKTGTSLHYRIHQLELRTEETSRVIGVSPELIHADEIECQSHQALHWCNVLRGDGF
D*
>TCRU_3639 kinetoplastid kinetochore protein 15
MAENDGTICRFEVQGRISSMAYGDATNACLVSTIMNGTHIFNMDSSCGAPTRYVAQLESF
PHQIIGGDPIISSCFLSSDSSLAVGSSVGGIAVVSRSSRSVVRCYNFQDEAPVLSLKEVP
HEPSLLLSASPVGVQAIDVERSVVRLSLAVPKPRVVGAVAITRNSFAVANYDGKVMLYDV
RCGIDPVTVLSVPDQITSIAVSPDTRALVAGTVGGRVFVMRCLVSKLREEAFGTGKTRSP
VRCVAMHHGRIAAGDISAKITLLDSGDCPAPTMYWTAHSLLPHVVATSSWGVSSVLLRKD
SLWGAFTSPESAASHVVMLPP*
>TCRU_3640 heat shock 70 kDa protein, putative
MFARRLCGAGSLAAASLARWQSSKVTGDVIGIDLGTTYSCVAVMEGDKPRVLENTEGFRT
TPSVVAFKGQEKLVGLAAKRQAITNPQSTFFAVKRLIGRRFEDSNIQHDIKNVPYKIVRS
SNGDAWVQDANGKQYSPSQVGAFVLEKMKETAENFLGRKVSNAVVTCPAYFNDAQRQATK
DAGTIAGLNVIRVVNEPTAAALAYGLDKTKDSMIAVYDLGGGTFDISVLEIAGGVFEVKA
TNGDTHLGGEDFDLCLSDYILTEFKKSTGIDLSNERMALQRIREAAEKAKCELSTTMETE
VNLPFITANQDGAQHVQMTVSRSKFESLAEKLVQRSLGPCKQCIKDAAVDLKEISEVVLV
GGMTRMPKVIEAVKQFFGRDPFRGVNPDEAVALGAATLGGVLRGDVKGLVLLDVTPLSLG
IETLGGVFTRMIPKNTTIPTKKSQTFSTAADNQTQVGIKVFQGEREMAADNQMMGQFDLV
GIPPAPRGVPQIEVTFDIDANGICHVTAKDKATGKTQNITITASGGLSKEQIERMIRDSE

SHAESDRLKRELVEVRNNAETQANTAERQLTEWKYVSDAEKENVRTLLAELRKSMENPNV
TKDELSAATDKLQKAVMECGRTEYQQAAAANSSSSGNTDSSQGEQQQQGDQQKQ*
>TCRU_3641 chaperone protein DNAj, putative
MRRSLRILALDPFAVLGLSRSSTKAEIKTRFRELARLHHPDAPAGNSEKMEQVNKAYNML
IKEGAYERMRMKSSGVKARADVRRPSPFSQESERQPQQREEKETPYEALSEEEVAKLSAL
DPTTERVTPTGTYMYQSRDDGSWTELDRPLVRAHQPRYASYAAQADMSEELRRRSIEKEK
EENSKSILQRVADRMSDSADLPSRNPAALRFYFILAFVVLYFTYQRTFAWGKHRSIRADF
YSSVEQKREELLEVYKENRDALETSVAAAAIVFLVASQRKQETDPVVPPTPEKHFREIRP
PREHFYVISGG*
>TCRU_3642 hypothetical protein, conserved
MQKPPPADPSWSDEEELDRNGSGRDSSSDESEWGTVTRDFRINGILEDEEGGGLLGGDDE
KQLYLPEPVVSLTTTPKPELPRLSTENLPTGCHVWVVDEDDVHLLLREHWDDSYRAVCNG
RLCGTVVRHSVNVTLVSFYDEHLEMNYGLSLPRICLSNVPVESYNRSPGGSNRLIQSCFG
LGNLDPRAVRFREDAAAPKVNRQFYESLSVMEVQVPQSLPQLAAVQKSLSEGAYEEALKL
LDKLPLNAVNRVDILVLRSRANIFLGRYEEALKDALEAIEEEPRWVKGNLAAARAFSGLC
KFEKAAQQIHRAMLLLPHSHELHKIGELNSFMLSLQMGLPRNDLDLFLDSQYDKRLVSSR
RFKKDEIVYKGDQVIFAMESIFSTPSGRCCVCLKTEGKMMRVPVDLDGNSSEELACYCSV
ECQQRSSLFFVMELGRHRVAVERARDLISCTLPQRVNQVSLDLTYMATRLFLMICVTHDR
LLSIRRSQRRLNDVTNSNASGEESDQSAASDKTEFLPLQAALKHLGVYPLPTDQLSPSAR
DKMYVLYNTLTAFFSDKDKQTYSSALFYALCEYVRTFAVAVKVREAESMLYYLPKVMGAI
RHVEASEANCTVVINENGTDLALVASRDILPNEMLSVPDWNAHNTS*
>TCRU_3643 hypothetical protein, conserved
MAEAVSCLDVKSFFIVSLPRKVRHLFRCRVEDGTLVELTRLPMGYKASPETLQIIISAIA
GLTTVVRPLWAAPPLVRVDVWIDNIRIAGLKSNAILWEAQVLRDAAGRHATMGEGRESDA
AKYTFLGVRFDHTHRAVSLSERCARSVCAMPALNSSTIAEMQVVASCFLYAAAISCTRLC
QYYFFIKAVRRRLSALNRGIVLETSPANLPPSAVGLGERLRHMIENIIVSETSSPRKRHW
LPSSRTHRSMDGEPFLFSRLRRR*
>TCRU_of Trypanosoma cruzi region 4/Dispersed gene family protein.1
Dispersed gene family protein.1
MLLAKGNVHDGVSREMLYAAGAVTAAGSTLSFVRNRALLPRMLSLSLSLAAGAHLRVACN
DAGGRVLSTAEEYAAAGFGDAGSIDVVGCDDCDRDTHCYAPGTASASMRNGVCVCACGSG
GYGEACVPVGAPALPPAVGTASSVFVREGVTVRSVFVVPAGASEVTLRHVVLDGVSPVLY
VPWMARDGVRIVVQNVSLLNGAVLYVMGGGALRGAVAAGSDEGGPVELSVCDVEALNGAL
VLTGTFPAGSVLTVTDSLLVAARPTPLVYLPGSQSSPYAPVLVLSGLRLVRSVLVVSGVA
LVTVMTGGRTVVVDGAVLELVGGGVALDAAVFGGEYALYASARVVASGGAVLRVSGSQVY
AAHGLVFDSGVEANASAVVVNDNAGALTDGALLVLRGSASFVSGSWLSVRGNSISGRLLS
VPSYPRSAELVQSTLTLHGNAGSGPVVMDGTVALGGAGRKFVVGCLTLNGQALRPMDYRS
AGIIGEFRPVACGVCDADVRCFAAATRAMSGSCGCRCAEGGYGRDCLPVYLPHVDGCNRT
PAMPPLSHTATLTETRSPTPTSTPSLSATHYSPTQYGPTEILQVTETVALPPTRTPTASV
SSTLWWSDVACPTLAVTTTAAGGSLTQNDIRGGGSAVPTRLMVALPPPFRWARDPQLGTH
LSFVPVSTAQPRGFGGPWGAMLSNATWVRNATNPSTVLELAVPVHRGYFIAADETIVIRC
DAAAVSGGCKGVLLGSFTIRSDTLPAAASALSAITGVVAGAAAVAVVVTGGLGSILEMQA
LGVFARMSCASAQERASTVALPYFLSVFAALDPLWMVVGNALLAAVFGCVHYGVTAAFQR
WRGVDAASAWAAMRFPSLTYVVAHAMHLGIFFGSVLALAMPGARVQHRVIGVVGVLYGVA
FPAGVCYLIARHAGASFTRYWQFSRKPLHERLLYPVGYWHPAAQQRMYGGMLTNMRGSHV
YWCVFQLSVLCVVCLIAAVHPPVGGCHVQYFCMAAVLLAGAGVVAFTNMMRSAFLTVMHT
ASFVLLAALCVVSAANHLAPSDGGARAYAAIVLLLTTVVLAVTVYGVVVWYAEDRHWQKL
REPRRGGLEALLRDDEGSDEDTQKPHDMTSLSCASGTTVASSYRPPAMPLQPMAGDTYAL
LEGPRLCCAPQPAVVNGTYSVVGRCFYRCVSILSGLFQEFFPFRDDYVEGDCTVALVAPV
VVLYRTVISWSCVRGTQRSFYAMEEGSSESPSGSNELQLCLTVDLHAGGVTALRSKAAPE
*
>TCRU_3645 hypothetical protein, conserved
MNGLAISKDSAVVLSGNVFQSVAASSSAIYVVISSLRVSWHSLFAVMGNTFHMAGGDSTL
IHLEGSRRSSSLKVLNNSAVVIRGNIVSRPVKYFLLLILALRVESHSAFVFQGNDMQGSS

VAFFPGFSSYIYYNSWLQVSGNLCRESPSEAFAFFYSTVNLRDSTVSVSGNQFISRTGTP
TVLPW*
>TCRU_3646 hypothetical protein, conserved
MARGRRHVLRPANRRTQRRVRQQHTRVQRHRAMQHDRALQRRHIHHVDESVGHQARRALA
EATNPQLRAANKHAVAQRHVQRVSEHVHRAQVHRHSDRPAEHQRVEARRRTPAANRHGLR
QRHATHAVLRTAATTAGPRRRHVERQARLAHTVVAAAAHAVGERRRQRIEARQRSRTAVA
RHHAHGGQAGHAVVAAALQHPAAGTVALHRHGAARDRQPGDKRRAGGSAARNRHGAAAQR
HTRQRRHRRRLAHRPHVVQVQPPAGTDRPTVAAHQRRRNRPLDAHEAQHRIKHREALSKH
NAAAAAHNRHVRHGEHRVAPQTETRRGRHMHRGRGRTARRLQQHAVKHHRARQQVHAAAN
VHRRPARLQRPQHSVAHTQLRPQQRRRVQHKRRRHRVRQPAQLRVAHRPRRHNRRVTDRH
VRVCRQVVHKHKRAVAPGQPAHAHLQAPVTRRLAAVAPHVDLRSTVAHAVPQFDAQRAGK
HRRHVEHHSHAVRHVHAVHQHRVHGAANRDGSVHRAAPQSHHHRMHCGHPYRQRNQCQHN
EQRHTHSQNDHAPHTVPAARTRAHNRPHPQTQTDINRAVVACRSRPVDHNFRLINAFLQM
ANQETLFR*
>TCRU_3647 RNase H, putative
MPPSMHGKAATSILPPRDAVVNGLERVCNIIGIPHSHNRAPLIQHRIFPWDTVSCNKVEF
NQPRFAQDASDDVKRTALDFIYSSLGDFDFELWTDVSSSLAELASGSAALLYGSTTTNDL
PVEVHRAAAGRLACSYRAECLATENGIRNLIPFCLETSQSPTRILVATDSPSRTEALRVV
PLAMRDYVTEESWIMLLSLIQRGHYVDVMFSLSHCGIPRNEAADEQAMIARSLPQEDSPI
WHVDFFTAVG*
>TCRU_3648 mRNA export factor MEX67
MSAPYKKKNTQNVPCIYFKRGACTNQRCPYLHVRGKELGENADAAPIGNGSTDAATNLLS
TMLKLVFEKQQHRVYDASSGMLDLSELRKFPDLKDVSNSINFNTQSFCRALCSSIKSLIV
PPPSAMQLKGNDITSFFHFASQMENAGLHLSLRALSLEANDIKTMEALQGIKKFSNLQEL
VLVGNAVARRDDYRAEVKKAMPFLLGLDGEAIMAPPLLLPWPRFTNSDYTDAQQHVLQFV
QCSLLNPLQAGDAAGVPQGVDAVSDFYAQKAILSISLSSPEAAVSTPARTVKSSPVGGTQ
RNVVREIVSFRLKQTESNHNLLHGVKSSVVACGRTKVCSQLEHWLYPKNFLVEHFVHSSA
SASFLDNTYLSGPSPAIMKVPVTVVTLHGVMAWTHLSPQNQSSNDRVVIYRNFSRVISVQ
QNDAGRWLITNDMVSLYPFPGDAASPSTKGDANASTQISLSECRILFSPREDRSRAEVLG
RKKNVPLEVVLALSQHVSNDAELITVLNDIGGVPLSMYEHCATLAGENILESIQVCRIGN
RFGLAPEEGLELLRRVGGNWCAVEEAMAAAVGVTPQAS*
>TCRU_3649 Concanavalin A-like lectin/glucanases superfamily/Trans-
sialidase of Trypanosoma hydrophobic C-terminal, putative
MGAKLNDSDNTVLLGLSYNDNENKWILLCGGGENKEHSSHLETKKSEHVVILLRNGNHSS
AYVDGQPVGGDEKCKLNNTDSKGISHFFIGGDEGNAEGQEGVSVTVTNVLLYNRPWKSEE
IAGLAKNKVTISKSEGPKTSMDTQSSATSGSPVQGTVSLSNSARQLPSEQEPLKENIGGA
GGVSGAASVTTPSADANTPASNGEGKVEPAVKKEMSVNSAEDEETRGGTDGQKEEVQPQV
REVNATALNSSLGKLSQGNNSDAITIRGSGLLPSLLLLGLWGFAAL*
>TCRU_3650 hypothetical protein, conserved
MAMLMAGRVLLVCALCVLWCGAAVEAEGDGSGGSAEPLPESQEPEKSPEGTQDFGHEAGG
LKEELTPTSADEDDEDDDDGEGAEDEEEKQIEGPSGEKGNAAPDPVSGEKKLIGTEQQTR
QSIVSAEDIPLSGNRESNAILTQTKTEGTKDSDKNPPAVENPLTTVNREQTLHAEGNLPP
PPEVDDSREHDGKDTTSEDKKNVQPPETAATPKSHRDKGSEGTGDDTKATTVTANTTDTT
NTQKSDSSTAVSHTTSPLLLLLVVACAAAAVVAA*
>TCRU_3651 retrotransposon hot spot protein, RHS3, putative
MKQSNFVFFVLSFWNYVPPYIIEKCDVSAFLNEDFLRAIRINIKELRPPGRREPRSCALK
EHSDKSFTRKEVLPPPERLSNPVAMDHWVLYEPKGKSVPLVDGFFFVESNPSIMVGLRIA
TAGGHHTTASTVRPFTECLAAYFNGWEELFRDMSWEIIYVQHADE*
>TCRU_3652 hypothetical protein, conserved
MSRHLFYSAVLVLFVLVCCGSGAAHAVGSVSWCVRLLQCAEIFVLQETPVKPKDGSESKV
KDSFASPSLVSAGGVMAVLAEGTLARCPTKQVYPADFVAGYANPAWGLSSIVAEAIKDTQ
RAHTVLSTVNETGRVGVARQPTATAKDKRAFPLVGIHE*
>TCRU_3653 hypothetical protein, conserved
MSISQSDDSGNLNGGNSKLNRLKNCLLANDSNGAIHRSEVRSALCSGKDMRTNSPFITTA

KSLRDDKGGKDFSLDATSEKCDLESALKNYQEDSEKLGKQLLTLKKELEAEVSSKRVMEE
KLSMLESIVATQRKEILLLQRKVQSGVSELSEKRLESALREIAFLKSKLGGVGETENSSQ
GDVFLQLQSNLNKLVAEKDELLNCVRKQSMLLDVLERQKLHLEAAILIDISEKELERYFD
VARP*
>TCRU_3654 hypothetical protein, conserved
MACITGDDTGLVKVWNISRSTGATLAFSYGEQSRKRGIAGMCWLDSSMTKVVFSMNDGIV
SVLDLEERSLLLSKKLGFVAGLPNSLIFLKENFVMASKDGKVLIADASFEEKKIFECNGP
VEAFHIHRKFGMFSMGGKDNALCVYDVSAQEVSVPVFRAKNMRDHVLDVPYPVYVTGTCI
INPFVFCTTTAYHQVCFYDRRSSERPVQEYEINREIDRRPTTLMQWNCNKFLIGEASGDI
HLYDTRRGFASRAKLRGGVGSVRSMAKHPAGHQLLGVAGLDRKARIYHVPTGRLLLTMYA
KQKVSSILFDRQLPLADNTGAFSGIANNKQPSKSAVLGDEIWDSMDPVVDDITEVFPLGQ
NDNGNN*
>TCRU_3655 hypothetical protein, conserved
MASCRTGRLPMPDDPLFNPIHMGGLRSSARIAGVGLLAVIWIMYQKLYHSHHYITYKGPD
NPFARIRHRRYPGGGFLFGWGNNGLNRDCGLKEWECWAGYTGKEYTY*
>TCRU_(PEX11), putative Peroxisomal biogenesis factor .1
MQNDTLRVLGSNPFENITAKTRLRQLLQTHQGRDKLFKVVQYFLRIKLWMDSVSYNQNYL
PGAEFTAVERNLMTIVNTRRLFRVGRFVGEFVRMRLTLIKCSELVYIPVHGGQWIALFIQ
CQMICDMFARALMCVKSLCEDVAFLTQKGFFNSQVADQLISVSIRCALPVFLIDLFLNTL
RLLQGIRDASQKPAKKELLLSMETFSLLSRYDHVDKLRRRMSKSNPLGKGKVDDDEEEDE
EEEVQMLEVGEENEIIVNSYQELLWKDFELHWIFVTELKLLLDLFVALSNLNQWKGVGGL
VSIGGLFSGLLSVYRVWTYGR*
>TCRU_3657 hypothetical protein, conserved
MAMSAGKAQRLPKVAPLSSMAYGKRNEGDWKSTLKLFVEEVSRLKEETDALKSTRNDIEA
SWQKAVEKMKILTDENCLSQHALESLLDEERKKSLAVVKEKEDLLVKLKCADEKCAAKEK
EVRRLADMVETEKIRHSVSVALHEKTMEKKESQYRAELHLQEKRLEEMQHELHQQLKRQE
SELLAKDILLEKERKRWRNWHEGYENENSSDMPQIKGASCACRLSPEATMNFADISSSTP
FMAHVPFSVPPDDDDARNVSKKSRLEAARQQLRRINGKSKEDVTLISLSKLNHLQEHFRH
*
>TCRU_3658 N-acetyl-D-glucosaminylphosphatidylinositol de-N-
acetylase
MSVFFAVTFLALILAVLLQWQRRSAALRPRILGDVLLVFAHPDDEAMFFSPMLEHLKRYD
VKVHFLCLSNGNYEGLGALREKELGLSAQFMGVHRNNVKIVNHPALQDGMNKMWDAGLIR
QEVLLYLQKARNVRTVVTFDQWGVSHHPNHIATHNGVSLVKENMPPGIVFLSLRTRSLLG
KYSGVLAAVQYITNFSLLGHQHRFVFLVPPISFLTSFLAMRLHRSQLVWFRYLFLAFSSY
TYVNELEELKAS*
>TCRU_3659 endonuclease III, putative
MKKHAFKPPPNWERLYVRVKELREGLDAPVDTLGCSKLFDKAALHETRRYHILLALMLSA
QTKDHVTAAAMHALIRIGCTPEVIAKMPEKTLDGFISKVGFHNKKAKHIKEATDAILKRH
QGRVPHSYEDLIALPGIGPKMAHLFLQEADGVVLGIGVDTHVHRISQRFLWVPSTVKTPE
DTRKALESWLPRKYWGEINGLLVGLGQTICTPRLPRCSECPASDLCPNAFKEAKRSGKRG
RVPDIEDVDTR*
>TCRU_3660 RNA helicase, putative
MGPNRQRKPRTTKTHRGRGGNRTPTSSTAAPSMLSRAGREMIIDVLHHAATGGAVPVPEK
HFDANAGIRITLQRNSPLTPYETLQRLYEGLGFPPDLTTQYVTDLQGASKIPAFTSITEL
LAEDGPYQSFLVNSCFELFSHMLFYSRGEVDVIMASEEEDAILENELETIQSLFDESFIG
VRQIDDKEFEGCEMYFSFSSGSGKRVVVCVRVPDQYPADPPLILVQPCGNQVGTLPILGV
FHADTKELPAALRRSILDAAVETINGLAGEGCLVALMSSLHGVIASLEVMPISTCNSGIK
EEERPGKEEKINLDAQRKAFVNALTATPGVNPTSTEDHHASYPMPVKIGLKTVGCVQDEG
DIVRREYLKTDKSLDAKLMAEWESLRTSGTMKKAREQLPAFQVREELREIISKHQVVVIG
GETGSGKTTQIPQYLYEFMCESGMGGSANIVCTQPRRLAATSVALRVAEERDEAVGGVVG
YTIRLESCVSRRTQITYCTTGIVLRRLQVEKFLGSVSHIVVDEIHERGVDTDFLLILLRD
LIQRRSDLKVVLMSATMDSELFARYFGGAPVISIQGRTYPVQHFHLEEIIPMVGYILEDG
SPYANREAKREERRRNNRKQAINIDPEEVDDAREMELSGQSLTGKLNAAPKTLETISRMN

LDVINYELIESVVFYIDTVMKVDGAVLIFLPGMAEIMRCMEQLKSNPRLSNSCLMYNLHS
SLGSSEQHGVFQRPPKGKRKVVIGTNIMETSITIDDAVFVIDSGKAKENRYDARKSLSQL
VTVNVSKANCRQRQGRAGRVREGFCFRLFTSAQFEALDDHQLCEMHRVPLEGLILQIYSL
NLGDEVEYLQKALSPPEERAVRGSVRALTTLGALTLDKRLTSLGQHLANLPLDVRIGKMI
IHGAILHCVDPVLTIAACLAVRSPFLSMMDYQAEVEGVRKAFAGEYMSDHLVSWFAYASW
VFMQYNEGSSAAKKLCARYYLSLPALKQIQSTKRQYERYLYEAGFIEEAPIHSSHERFLY
DPVITLEDRLYESGGRQFNANAGSVKCILSCVVAGLYPNIARVQTEAGKKGGAFLKLTTF
DGSEVMIHPSSVVGREKRFLSPLLVYVDKIKTSATFLRDVSVVTPLHVILFSGGGLEYLP
KYGELVVDDMVAFKCRGEDATLLKHLKDQLDSALSQKINNPSESWESTSSVVVRAILRLL
KDDLESVRHLTVVDHRQPRTSLTAPLAPPEEAVARERQDAKRRTQQRCFVCGEADHMARS
CPRQAASLKGGPSVRCFVCGAWHHPTECTLASRVS*
>TCRU_3661 hypothetical protein, conserved
MAMMMTGRVLLVCALCVLWCGAGGALLRMSRLLSVLLRMLVDVSRNHHNLRRHRQTSKAQ
KYHQQMFVCQCSRLPIHPWKEPMEMMSKKSNRQERVMKEFEHKNNTVNTIDKMTNMSGKE
RNADDPKSGKEGEAPGKDPATERGLQSTPEDGSPSHLPNDARRGNAQEPRNASPPGNDDR
RQDLLNVSQDAPVKFAPDTILSSSSTGGHGADNSHGNQREAKKAQQTSAGVKQHDAEFGE
KSAAPTKGEGESQLPVTADETKSNTTMPGISVSSSAATTAVRSEASTEGSRTISDLSPSS
SEDDARPKNTPNGDDASHITEDVVSLSAGTPMPQQTAAQTDATATPGDSDGSITVSHTTF
LLLLLLVACAAAGTAAVVAA*
>TCRU_3662 BNR repeat-like domain containing protein, putative
MVALCGSGRKVFESRDMGKTWTKAIGTLLGVWAKSKSGAPLEEGLRVGALITATIEGREV
MLYTQKVRHSMEASEPNALYLWVTDNNRTFHVGPLFLEDNVNETIANAMLYSDGALHLLQ
ERANEKGSAISLARLTEEPNTIQSVLSTWAQLDAFFSESSTPTAGLVGFLSNAASDHK*
>TCRU_3663 BNR repeat-like domain containing protein, putative
MSRRVFTSPVLLLLVAMMCCGSGAATAVGSKSGEVQLPQWVDIFLPNKTQVAEKNGCASG
VKSAFILPSLVRAGGVMIAVAEGRTKYNDTHKSGRLDSSDIVAGYINAAGTWPSIVAEIA
KTEWRAHTALGGLNGKDHMSLFYLPTALARNNKVFLIVGSYERTYNYDYETWVQDEWDIQ
LVVGEATQATDIQHSKPISWGEPAPLLSHIPQRIKDDLDQFAGSGGSGIVMGNGTPVFSL
MAANRTNHPFSMITYSTDNGKNWVFPEGMSPADFFYPRITE*
>TCRU_3664 retrotransposon hot spot protein, RHS3, putative
MLAAGRLLMKDCNVTMEVFAQKPDDYVQDQRPLEEILNLTAHQALEAITKPLHGGVLSLS
GNVGTMKEKDTITPLARGKPNGVLTQVLTEARREAEERLRGTQEMKFTISTNIEEVLFKG
RVCVNEKKLNDFLTREPDGGGIVDTNRDVMLEEFFKDPTRYIHDAEVLNEIRASGAYARM
EGTVREEMGLEEDVRRLHQEDVYFLEQWRDYEGKDTVTPLARRNLNSVLTQLLREESREA
EERAMREEQVESTLTTTIRGVLFKGRVRVMDIKLNDFLTMRFGGKGIAATNRDVLLEEFF
KDPTKYIRDKGALNEMQASGHYLGMKRAVKGEVIFDEDIRRLCDKGVNNLPGWSLAAAKV
KAAVHNSTKHFLDAAAEEARNPTTTSAPEKLEGCYESVHNARWSHAVELPDCVERTKTGT
GMGCMRGGQNSHGRTGKLTTLSKRMMLCSNSVRHLPC*
>TCRU_3665 hypothetical protein, conserved
MTMGASRTAKLLGMDLDPQLTLNLAAASQRISQPRRIAHKEAGPSPHDPRTFIIGYGASK
LRYGSELIWAVATDSAKNETQETYAGLARIVSGVPSTVDQ*
>TCRU_3666 hypothetical protein, conserved
MCSQYGPLRNSSQRGRPIAVSVANSCLRVATTQKFSKPPSAVHPRNIGRQATEDSMSNEL
KGLAGVYQHQRSPMGSAVELASSTALLGKVRLEVIGSVMLHFVYQMCQLHRRGTHTTARM
LVRTRRPQPKQSPQCHFLSNLTHDGGKADGSI*
>TCRU_3667 fatty acid desaturase, putative
MAKSSNAFFEQLFEPQGRGDYYWSKNPQPHEMRRKLMLKKYGRQIKELYGPDPWIWRLMT
PFIFLQLYLGIRASEMSWPIYLFLAYFVGGTISHSTFLAIHEATHNLAFKTREYNDYYAI
FLNLIVPIPYAMMFKSYHAEHHRYLGWELVDADVPTLLEAKLLSSYMGKFLFLTFQVFFY
ALRPCMIRKIELEKRHFINYIVQIVFDIIVYKFFGPGPLMYYLFSTFLGTSWHPTAGHFI
SEHYVFRGEGRQETFSYYGPLNWLTWMAGYHVEHHDFPNIPWTRISRLHKIAPEFYDDLF
VTESWPGALYDFLVDTNVNQCSRVLREKGAFQRANLLPNVTEDASVG*
>TCRU_3668 cytochrome oxidase subunit V
MKRFASISLVAVSTCRHFFGKGWDNASLDTIYSCMLRKPEVNDRVRSQYASTMDPRDADV

LRRLGEVAKENKTFIRVFLPPHLGDPHRLLKCYSLMAYPILDDKGGQLKVEMDGHHLDAF
ADPDDDYAKVVIPHIELVEYLAKSLLETMKWEATPRGAASLLESLYRGADIPDHVFQTPA
VIERIDEFKDGNKVTQ*
>TCRU_3669 protein kinase, putative
MLPHPILATEDDRDYIERSILSMQEHVENFFALLELVYLESKSEQTPRAHALFHSIQSTL
KVLGTVPSSSYRKNDSKLTVDEELPKGLERMRRLFFDVVTVGPESLVWKKPVTRLAQEAA
QHAKNIQIKFPSSSTLVRKGVSRNSFCFLLLILIFVSALILALVTMVVIPFIGPSPSVAA
AVGILSAVFLVGSGSCIIFCFVMAVRALQELFDRLLGERMHANLIYQPSSGLSDEASPCN
TTTNQLRVKRGRDTRRGYYAIPYGDCVGYIEGKHVDAHVVMIGFDSNYLITRWNMAAEVM
TGFLESGCIGKPLSDLLITPSGDVPHDLSTLQASKGDVLKVKLRAFATAPVTLCTIAAPI
LNLSGKTIGSILICANAKDNLGVFRSYIRDYLTSEVSVSLSELIEKNAVDTRGKAAISSL
QSFLSCALDKQVQEMAREMLTEWEWTTAEQLLAQALRSFAVRHEKQIDSMFPATLCLHPN
VPKAISTLLDTIGVSCMVKLEVLSQTVNVFALAVTITPHCEESELCLNEEELQDAIMPLI
RSICGNMCKDGKDINLRFPCQVTAFLEDGEDAFKAIEKRGEQSIIDQTRAIVNCTVNVLA
LITDLVDQHNLSMILLKTMFVSLTTVRQRDDLERRLKAAPNDVDVIICDRGWLNSARDLI
ISSDHSAIVVPMADQGVFFAPEEFQYVIRSPISGNEVRKVLMDIGTAVSLRKNAATAREE
RERILTLRQDSPWTKGKLLGRGSFGAVYEAMSDLTGGKMAVKMFYFSADCDESINKLLNE
IRIMCSLNHPNIVHYFHCERKENNINLFMELCDASLGEIIIGRKPVPPTLTVVQILRQVL
TALTYLHTRGVAHRDVKPQNILLKGDTIKITDFGTAREGKASKDVQGTLRYMAPEVYRGE
AHSSPCDIWSVGCLACELFACPPKFMDNSLILAEMTSAVPYLENVTSSVTLNDFLRKCFQ
DDPEKRPTAADLLLHSIFSPEFSYEVESLLTVFKKKLMRNYSFSITSQK*
>TCRU_3670 hypothetical protein, conserved
MPSPHSLNSVQVFLGNLPVESVREDLEVEDVPQNDEGLRLMAADANWSGVLLLAERLGAE
ADADTIQGLACKLRYTLVQVTAYFSMQRYAAAKKLVDAVGDLNSDRFTDPVTKESIVPFS
LRFISALLPSYCGSAVETQCRLYVLLEECQKHAGVAVSPLRKARVKRIQRALVVSYYQTE
KYADAIRLSEEMIAAERYDDGDYEGADFRVLKQMLQLQQLATLCLHCGNIFLAEDVFRAI
DGLSTADESDDVQNFHRFLTTINRALWLTFNDRAGEASQIFREVARGTSEFCRIPNAAEK
SSSVVPTVSSQRQTSTAPLSAAKTQSTLCHKAFHQLWVSAATSHLSCIPYEATLNKNPTT
LMASLIDTMEGYLRQNPVELLHSDAFLGNSVRLYTLEGGAHRKKLDLLADMLEVFRCERG
CIPSLENMV*
>TCRU_subunit 2, putative Biogenesis of lysosome-related organelles complex.1
MRQPSDTEEPLEPKGSFNSDCISEPGSAPSVSVVNKGAEALDQLRRAHAAMQQYMTAEME
AYAEDWKLLGKCNELMESRCQEVRASAKDAVKELETTAGLMEHSLSQLASIDRIEAQLAT
LTEAVNRIDEYSKALADRFKCT*
>TCRU_3672 hypothetical protein, conserved
MVFSSAGDAEQPLFVRPFNCTSSHWAAPVVDPCGRGHVKSSALCIEESVRKRFEPWELGL
IKHNALQEYNLLKNASERRDLEREESIAREEIVNDATSLLAAVIEWRVEAGEEVMSDRAL
SRLLERLRKRATTQQRQYEINEDALREEGRMGWRREVEEKARSKQREEAMARWRRECEDS
LRAAAAFLMGMERRERRDEVAEEEAAWAALCTEEVNARAEAERIAYENFMNSPEQVALRK
EQERIEAAAQAAAARQLKKFLREQEHLVKSCRHARGAGSVFETLPSRNLCVSCGVKFDET
VGYYVRMRGSTLVPPETWSPPKDTSLAQRNSRSSIAKLPPITQQTKQQGRKSLTGAALDG
STRVSRVSAVKKA*
>TCRU_3673 hypothetical protein, conserved
MEGTSQLCGLFGDDREITTRSGSCASGDAADYGDGGGDGGNGSDVGAAAVVVDSTASSTR
KQASLNSFEQRHMDDDDTMTKRHCYYDCDPEKTTDRPALSYNIWPEVKTVTVMVPTMFTT
AGGRQICVREKRHIARGSPYWRLLFECPLHEGAALSSHTDEEEGLNKKKKDEKVEGGEES
GAAGPHRGLLPAWRRRPREANTYSTPSLATSLRGPRCRSSPLPPSDEHGINEDCGDTSFT
FSYSSFTRADGSMIYLSRAPT*
>TCRU_3674 hypothetical protein, conserved
MDYNLEYSEEQREYLERVGMREYLETFVAEVVRQKPNDIYAFLHDWASAHCQKQTKMTPT
EASIKIQCAQRQNLAIKEMRSRQRKVNELLEQEETERVGKVEMEG*
>TCRU_3675 Cytokine-induced anti-apoptosis inhibitor 1, Fe-S biogenesis, putative

MSSKPTQAFSLKTRQAVDEDALLTEEDRVVKEATKGEDCTTRRRACKNCTCGRAELERKM
LAEGKKVEMPQMPAGGCGNCAKGDAFRCATCPFLGQPAFDNTSDGKVKLNLTDDI*
>TCRU_3676 BNR repeat-like domain/Concanavalin A-like lectin/
glucanases superfamily/Trans-sialidase of Trypanosoma hydrophobic C-
terminal, putative
MKDGTLVFPVEDTKKDDAEEDGKTFSLVIYSSDTPSCTLSKGMSDDGCGDPSVVEWEDKL
MMMTACDGGRRVYESGDKGDSWTEALGTLSRVWGKKHKGKERGVRSGFTTAKIDGVEDNR
DVMLVTLPVYSKEEGNGEKGVLHLWLTDNTHIVDIGPVSEEDDAAASSLLYKSAGSKDKS
DELIALYEKKKGESSLGMVSVLLSEQLKRVKDVLTTWKEVDERVSKLCPSSAAKNTSTAN
ACSTTDKITEGLVGFLSGNFSENTWRDEYLGVNATVTGGTTKVAEPSDGVTFKGRGAWAE
WPVGKQGENQLYHFANYNFTLVATVSIHGEPRGDTPIPLLGVKMNYSENTVLLGLSYDSG
KKRAVLRSGGKTTEHSSTWEPETTHQVAIVLQNEKQGSVYLDGKLVGTEECALGEAESKV
ISHFYIGGDGSSAEGQEGVPVTVTNVLLYNRPLTEEEVGALNPNKAPTSPVVPGIAQGTL
SQSSPDGHPPSGPKLMNENEGVGSGSASTSAPSTVTTSTGKEQSAIQLPSGIFSGRKKDV
DGGSSSDDDPTVVTVGRDTMRGDGSPQTPEVSLSSAEDAETVEGKNVQEEEGIQPQAGEV
NATALNSSLGNLSQGNNSDACTVRGSGLLPSLLLLLLGLWGFAAQ*
>TCRU_3677 retrotransposon hot spot protein, RHS3, putative
MVLTSEKGWPYSWNRKENNSTRDCHVNCEVDRVWQIVSGDLTEWFSSHGRTDFKPKRRLL
IGTPGIGNSMAAGSYLLCQLLQYDVEKLPVAAYSVGGSTTYVSDETIKTVSE*
>TCRU_3678 retrotransposon hot spot protein, RHS3, putative
MIVVSYLEVRNYGEWVRQVRAERIIMNCPAEMDVKAMCAWTKRDETAEKQAECWKEVEKH
MYLLGPIPRHVFDEEAFNGRCGAVSLHWN*
>TCRU_3679 vacuolar type h+ ATPase subunit, putative
MRFSAPVTLGAAIGVCMLSAMGVFAATRREACLARMYPQSAGFYGALGAAVALVFANLGS
AYGAAKAGVGVAHLGLSAPEKIMRGIVPVVMAGILGIYGLIVAVIINNNIKTEPHSYSIF
SGNLHLGAGLAAGLAALAAGLSIGVVGDTAVRAYGKQDQVFVAMVLMLIFSEALGLYGLI
IALLMNNQANRYGALCEVE*
>TCRU_3680 kinesin K39, putative
MMGADPNAIGGADAGVTPRLCLELFQMRERIEAEGHSKWSVEVGYIEVYNERVSDLLAKR
KKGSKEEVFVDVREHPTRGVFIEGQELREVSSLDEVLGLIEAGNRVRHTAATKMNDRSSR
SHAIFMLLLREERSMQAATGQTITTAGKNSRMNLVDLAGSERVAQSEVVGQQFSEARHIN
LSLTTLGRVIDMLADMTKNKDSQWSMPPYRESKLTFLLKDSLGGNSKTFMIATVSPSAMN
YEETLSTLRYASRARDIVNVTKVNEDPRARRIRELEEQMEQMRKDIQGKDPAYVAELEEK
LRLLEAEAQKRAADLQALEREREKNEIHEKMLRATEAERQELLSRAGALERQVEESRRQA
EYHERANQRLKEEHAQRERELLEQMRRREDEMERIRRRKEAEVMSGQEQLRKTMEDLERE
RRDREEALRVLGVWKEELNAALSDSRQSQEQRAELERQNAQLADRMRQLESEREAQQRLL
HELELLRDEHESLQRAVVLLRTEVEEMDQRHDRTTYHLLAVMELHAECYEAVVARLGVEH
ERQLNQNAQWDADERAALERRNEEGLAERDSALSALQEALAECQRRLDESETAEEKLRVA
YEALEKEKGGIHENMRLLQQAKTEVEQSLEAMTSERDELRENLLSLKKDYDFVVTSCARL
RVDMSLADLEHQDELARLRIDNDAFAAELRRVLWKLNASEVRLKYLRSLCKSAGLGGSAE
SGEDYQVTRSVLAMRRT*
>TCRU_3681 retrotransposon hot spot protein, RHS3, putative
MTTTGEHHTTTSTVRQFTERLAAYFNGWEELSQELSWEMIYVQQADSTPMNDWQGCDVVN
SNNVSRDESREIAAFWEEEVRQYQVPISSRDFRSGEALRSEEEPPK*
>TCRU_3682 Glycine cleavage system P-protein, putative
MSQGTPPVILRNVIENPAWYTPYTPFQAEISQGRLESLLNIQSMIIDLTAMNLANASLLY
QATACAEAMYLAFHHGRKERMTFFNYRGTSSHLA*
>TCRU_3683 Protein kinase domain containing protein, putative
MTHEVVSRWYRAPELLLGQRHYGPEIDVWASGCVFAELLRGHGGAFFAGDGDIWQLSRIF
DVLGTPTPTSWPSAVLLPDWGKAHFEPKRPLPLREFFPDASECALDLLRRLLCLDPQKRL
TAAGALRHAYFSEYPT*
>TCRU_3684 zinc finger protein, putative
MDDGQHTGSLAFLAAPQDSLRYSEGSDVIHVLIYVSAARCQVILVAVIDGETVLMLAKRL
VDVRFRLGRGSLKSIRTAAREILLQKLRFSSESIVVENEANASDVASHCKHLVLSTLSAS

ELQHAHDGDPMEGFFCSELRNENKDGMIC*
>TCRU_3685 hypothetical protein, conserved
MEQAEFAAEAAHEAHTLPHPETAYFEPNVPEKREERNQALRRCSAVLLEARAKGLGASPS
PRWRYIHGVVAPYPHPLESVVLRTDLGRVCALPRQQANLLVRHFVRASRAITPYTAAACC
TPLVPGGWEMDRPFPPYVLDVAIRGSLLGSAPGPDDMLNEFLHRLGPVARGTLRTMIHNP
FANGSLPEDSMSGSDDGDSSSPSQVDISWLPLSSMVKKSDGEDSLGQ*
>TCRU_3686 Concanavalin A-like lectin/glucanases superfamily,
putative
MARVCSSRVTWGQRGRRPSGHSRACGSTHDQELPGTKACVWETSSPRPLRERNSCCTLGK
GTPSGGTEAKALYLWVTDNSRTFHLEALSTDHAVGETLANALLYSDGALHLSRESIVGTR
RGISLARLTEELKTIKSVLRTWAQLDASFSESSIPTVGLVGFLSNTSSGGDTWIDEYRCV
NASVTKAAKVKNGFNFTGPGSGATWPVNGREDNDQYSFVNHRFTLVATVTIHQVAKGSTP
LLGAGLGDGAGKKIIGLLYSMNRKWEKVFNGTKTAPESTWEPGREYQVALMLQDGNKGCM
YVDSVIVGSSATIPTPEMQGHEITQFYFQGDEGEINSSDVTVTKVFLYNRPLSVGEIKMV
KKSGVKKGNSDGSMRGGISRMLLCGIVALY*
>TCRU_3687 hypothetical protein
MGPAPGADGMRGEALRHLGRVAKRAVPGCPKDDCTREPSRGIGDVESSCPSSIRGKRIQG
VIEPQSTLQTSGCWPVNFTSDQLLRLRAACAAMLHNTAWRLSLWNAPRPSTRWTMKASCW
ECGDCRSVMERNAGQQRSSIAKALWFASTNSHLPPRSPHEARRKEQRCAPSCPSSS*
>TCRU_3688 hypothetical protein
MERARAARPHEEPEQRLLHLKLILSVAWRTKRDTLRAFHLALVQAKAMYGIAVWHWGASP
ASRSTPNSAQYQRSKMISGIPKGSRMEDAVLETEPRPLPDVVLGRRFRCALLCETRGSAL
RPSAPIVRRPAPGL*
>TCRU_3689 hypothetical protein, conserved
MSRALWSHDAEQRLHGNGCGLGCAVCGGGAVGVSSLVPVLAKVAGGGSGGIPGRSGTPGD
LALRVASAATVLLAARRAFCRGFASLRGGVQRDKKRFSCEVFCVSVVGRPGRTNGACVVW
RVAVVVAAIRICAEAGQRSLFLTSEGGVRVRGLPVACYSGAAGFWLVPSGENDAFGQCDH
AGGPSVCELREFGCPPDCRITSFFPGHAAAAKDKPHPYALSSEPRSEQEAHDSTKYRVRC
REGARSQGVEEEGLACED*
>TCRU_3690 ubiquitin-conjugating enzyme variant Kua homologue,
putative
MSSVKEGKTGVFEDEFSIRKANAKKLAAGYTKQKRFMECCYLSFATYLWFTQFIIVARYF
VHSGEVNLVHLAWSPLMVFFAMTLADFIGGMAHWGLDTWGTPETVVFGKFIRSFREHHVD
QVAMTKHDFIETNADTTLPLIPVLLLQRYYLFSANTHGKYEYNIDSENVGRHVFLLTLTI
FIAITNEVHKWSHMAKPPALARFLMRCHLILTPRVHRKHHRGNFDVSYCITTGWLNGFLD
AVNFWRLAESVVTALTGALPRANDQNLLGK*
>TCRU_3691 Myosin head (motor domain), putative
MAEQSVSFLPGMHCMCNHPQHAWVPGIIDSFDGKHVSVSTMTPAKEQVSKLLPDDVFVCE
KNVVDEDVHDLLNLSVLHEGTLLNCLKSRYFRDVVYTNIGAIILALNPFNFNIPWYMENK
MSDYLAEGETIQHNIPHSWVVAHSTYYEMRRDGGNQSVLVSGESGAGKTEAAKIVMKYLA
AVSALSCTEEEKKTGEFVGLRMFQSNPILEAFGNAKTVRNDNSSRFGKLMRIKFNRKGLL
TGADVTKYLLEKSRIVTSAPNERVYHAFYLLLQGRDRERYGLAELSRYRNVIAGKAPTIE
NVDDAEEYDSVNKAMEICGISQANVDSVWRCVGAVMSLLNAEFIATSTDTCAIDSKTNHY
IANGCRLLSISEEELKKELTTTTLIIREGPVPTTLTKAKADDARDSLCKALYDNLFDFLV
NAINKTIDTEDYTSWVALLDIFGFEDFKVNSFEQLCINLTNETLQRHYNYFIFSRDMDEC
RQEGIDVTAVVFPDNSECVELIGGKGGILSILDEDCLLAKATDNSFLDKVTNVFAGKCRF
FERPRLAKAPCFRIVHYAGTVTYEVDGFIEKNRDTLKDSFKILLNTSGDALVQTLFPMPN
PESRVRYTVGGFFRNQMKELMVVINATNPHWIRCIKPHPAKKPLHFDGVQTLAQLRSCGV
LGTVQIRKAGYPIRIPHVEFARKNKVLVLSEALDFSDAVGVAHAVLKSVGFDSRKAQIGR
SRVFLKSDAYQQLEVVKKQRLQVFLRVAVGGALVSLARMRTAALLRRRSTEIVEVFLLWR
NSQQIYRKKEYSFRREEIISNIFQLLKLQKNEMEARNAFVEERENFYNDITAKRSRLLEE
LYEQWLAEKPHHDAMALEAFIAAEECSRKEMISDALNELRQTLVLLEEDYDASRRREEKR
LATEFALEAAAQREAQRREAENARLEAERRKEEERCLAIAYWDERKKSMEQQVARREAEL
KRLREGVFDAMTSLNISLALKKQEPVLQARQRAEYPTAQFRSCGVDLLRDPSLGLCGLQD

GVTASPFSSAYNNVKKAPLRSCSRRENITNEDKFKQMDSFSEKVRLGKERVCPGRTGSLD
IVRRLRHLQAMGEKSFVTKPGDLEDSLDPYSPNWKPPIDGVVRFPDGFSCNLREAALNGI
SNQKSERCDGTSGRGRYISQSTMDFGF*
>TCRU_3692 hypothetical protein, conserved
MLKEHSLALRELFDKSCPYIEDRDNFVALFALRAHPFYRAFVIVGANADAAVRVTRSRMK
AALRAMEECNPAIADLEGALLMHEAELLSASNTIKDLRSSIKEAQEKVEKSEKSVWNAFF
LKGEEGKEREMRSTLSAKPNLQFLEHLEAALEILEGTVGRWRLRLALCKEELALYKQEMF
IFGRAGVLSCSDPGKERERDDERPLLGENGNRSDDVTKKYMFPPREEGKRNSPVGVVYVV
DRVIKKAARKVTEAPLHQGVHLWSLMSGGRGVTGFRGPSSADAATPQFVYSAEEEKRLTE
ANLVLQDQQKTASAEDAKAVEASVRELSQLTSLMNEQVVQQKEQFSILLKNTETAHNNMH
KGVIEVQKTLSKFWNSTRQLILCLWVSTLVLLVANWIIR*
>TCRU_3693 PHD-zinc-finger like domain containing protein, putative
MVQNRKRKKSKAGDVPMRIALNYEAVLPLVLVDNMDEVRAKYKPYKIIEEGLSEDSADDE
HTVSKGRLATSQPSRKRSRMPSTVDAVAKGFALPSARVGWGAQALRESTPYGLLRRRDLF
DRCCYIMDLEDYVWCHEKKVPIPFFVDAITKLERKYVMHLEAAARGSEAPTNVVVFNGAN
NNNNLYLSGENRVELCGVCSLPELSEESECFLHCRRCGCQAHVKCWILEALPPNAKNWLC
PCCVVARREKGVGRCHFCGRKGGVMLPFVSGVGWKSESSVGKSLFCHIVCSLALEELAID
AKKRVVHPVKRSRKSNHLARCVFCNSESAGACERCHHPRCFDSMHPSCAQQAGTLECYRA
PHAISATGPWDGCRFYCRAHFGVVVESPSGFAGIGEFAEAEALRHVNFADFVDEKRPVRR
GRPPNARVTPAALDAVKKYWVEKRLQRLHDGVKIVENVQGGRCDIVSVSLRPEIMKITPT
EVRLARFVCLIPELQHYINASVEGVLPIPDEEYDDVEEYRKAHSRTRYAGNTRKLYEKMQ
SVASHINVFCELAELLHQRAVVWRELLETDLQVLEARCHWGAEAN*
>TCRU_3694 hypothetical protein, conserved
MASRAWVDDADRDVEEQLPDFVKQSHVLPAWATVSSSSKLMMAAQKRFRDEDLDELLART
EPLLTSKHDRKYAALPPVLSVLPLPRDSARTVQWHRNGQLAVVGGNHHLYLFNAAGRFVE
QLSKTDVEKRIECTALEASGEDVLIVGHQSYTPDLLVLSTEKLIPLKFLFTRDSAVYRNG
RRDNGKHDFYITNVALPATNSSARLVGISSGATVTVGSLSSGSVTHRIEMSDPVVDILFS
GGPHELTVATRNKLSVFDLRKSAQFLRETCDDGTVDITTFATSDSALAVGSKSGIVSIYN
KGSSGPVKILKNLTTSISHLAFGERSNGDATLAYSTKGQKSGFRLAALPECRVVPSFPPI
SQRHQFIQSLTIAPTIPVLSVGEKQKVTNYAL*
>TCRU_C-terminal conserved region, putative PS.1
MQQSLKNQEHIWAVLQKMRMEMNECFAEHERMLRQAHGQLKERLDKIESAFVARNGGVGQ
LVGNSSSSLGHLDHATPVYTRFVSDKKGSPAGMRVTMLSTPPNERMQLMPPDSRGARSDV
LPQPVDPTDWVALAAPSRQEATPAVSPHNRWTNDDSIQAEKTPSVDATCFEALNNLMNDD
DFQHSTLESVIPSYTQDDDSKQLQSQPNLGTYTKKNDIIHMGAPGSSQTQHFTKSLKVTY
MDGRPQAVPIPDGPPDEPDKLGIDVNAQCKVLVEFKRKRVLQFESKEYVPPGSYVMVGGD
RGEDLGLVTYTWCETTKSLKGELGEFGNGSNKTVVLCVGLTGSTLTRSIGLGAGTVLRLA
DDSDVKQLHTVQVELERRAIDVCSQRVLERGLPMTIVDAEYQFDKKKLTFFYEAQQRMDF
RELVRDLYKSFRARIWMELVEN*
>TCRU_protein, putative AB.1
MLKRSGTICSLQSVVRGAAKIAAAIVDASVGNVNVVDNSHNGEGGTPARAAGRSHTSSPP
EVLPRSVVTQLHESKDDLRKNAVYMSVPSTRITRAAGFASLFVQLGWDHLVDRNGEGILS
SQSHKRVVNTLRRMRGVVLKLGQMLSIQDPATVPQYVLQLFEQVRDSAYAMSREQLDQTL
AKEYENINWRKDFFLEFDDEPIGAASIGQVHRATIKGEGDTQIEHVAVKVQYPGVATSID
SDIANLKMLISLHLLPPGMFVDRILQDLRQEFLLECRYKIEAAKQVRYAALIKQEPELQE
VFVVPKVYESLSTENVLVTQFVTGIPVDRLANISGVQDLKNYVAERMLLLTLTELFRWRF
MQTDPNYANFLFDAEKNKISLIDFGAAREYSKEFVKDYMDVVAAAARQDRDRIIEKSIKL
GFLTGNEMKPMLDAHAASVELLGRPFRHHDKPFDFSAENLPSKIQEHVPTIIKLRLRPPP
TPVYSLHRRISGIILLATKLKATVNSGEMFWNIYNSCRTNYE*
>TCRU_3697 hypothetical protein, conserved
MDAILVIPNASSTMVIDAEAAAVVELNTLLSRSGLHFSTASTQLHIMPETVYFLSREDVA
VLSRFARVLVKNASVQCDFSALWGVLWGHAKEVENVLNQHAQQPLDKEGRPQETALRQLV
PHLMLLAHVFHTLRHIEEPFARQEVKDAVNIVQKEVEMVVRLALKVTRVFDSALRNPQRT
NENSLRAVELCLAALEMFIASIASRKTIDVSPVLAFFNSDLVWRFSGVGVIATESYCEAI

RRLIVAIFLRQDDFVGVEEVAVRLLRHRLTNRPPFDWEIFRRLYVLRDAELSSVASLTPQ
YGILRYMSIVQLCVESLLLSDESWTKSLRRQTVKSLHQMNKKEMLSFFQVSLLGAVEGMP
EMNFSDDAELQRRSVVTHLTVQNTSKDCILQPSFLRILLAHGYIVPQINHGVLKRTSIIS
LLRAIAEQLFQLPLIQSGEKNSLTDLTLIPPVLTKRVLRLIVDAAASDVEMACDVMLEVH
QITWVIYEANISQCASLLSAQRMPVPLRRLSVSAMELLAIFFEPNAILCSAGHSMTLESL
ARVFAVLAFYSSAKKDAGNMEKKATLRLINNLGMKLSSLARMMTAEEIKSFFHTVILPCT
SKEKLIQKNRQQYALQEAYLRAFSSSAVALAMDEATILRHWVDTALRCIRNTLSGALSLA
GLDFFTAIFLSRRAIAPLFVPTYVALMIPIKNKTRYGEPSLFLVRHFAKGVRATCQALED
CDEQILAGMMQNPNSSLKTFLLEIYGEGDAAPSLDNVRPISCVLLIVSALFDKVCLILGH
TAKAQTTIATASRQERIARFQAYFSALINLLRCRSRPVLHRVCASVEAVILEHLHGVPRA
QLQWMKYTTATVDLIEGTGKKELVEWLLMLEEKARGSIPHSQL*
>TCRU_3698 retrotransposon hot spot protein, RHS3, putative
MTVVGLRMTTAGGHHTTASTVRQFTECLAAYFNGWEELSRDMSWGMIYVQQAASTPMNDW
RRCDVVNSDNVSDESLEIRCSGRKRCASTQCQYHPETLEETKPSEVCKDNNNRKID*
>TCRU_3699 hypothetical protein, conserved
MDKARPFEYVYRLPATTIEELRYAECVYDRQVLLNETWHSYALRKGRINEVDNEHVKWIT
RTWDEEEKARVDQRSFLDKSNAQGEKQIFAREENLMLKRKKSAAIVAHIMAHQEHLLSDR
IVCRHNLHVDFDLLQENKFIGEQNIIFIEEEFLRLFQEKGLDGFCDALFLISECHRIFCL
MKLNIRLRREEEERRRIREEQERAIEEERRREELRQKELEEAERARQEALEQAKMLERKK
REEERRAKARSERERLKSHHGVHEIGLSEDKSQTLAFVDEVLPVTENLSSVLEKLDLNN*
>TCRU_3700 BNR repeat-like domain/Concanavalin A-like lectin/
glucanases superfamily/Trans-sialidase of Trypanosoma hydrophobic C-
terminal, putative
MLVTLPVYSKRVSANEKGELHLWLTDNTHIVDIGSVSDEDAAASALLYKSAEGEDNKELI
ALYEKKKGGEDEKSRSLWSVRLTAQLKRVKEVLATWKKVDERVSKMCPNLSAEKDNPTDT
ACDAGKITDGLVGFLSGNFSESTWRDEYLGVNATVKGTDGGAEKKAEVATVDSEKGVRFQ
GAWAEWPVGKQGENQLYHFANYNFTLVATVSIHKAPEGVNPIPLMGVKMNGEPKKSFGLS
YEKGKWKLLCGDGPKSGEHSRDWEPGTTYQVAIVLQNGTHGSAYVDGQHVEDAPCAVETA
DSKGISHFYIGGDGGDAGDEESKDVTVTNVLLYNRPLTVTEVAALNTKKPSLSRLDGENE
LAGVAHSPEKGVPAPRGTVSRPILGDLQPTAPEPLRTSKDAVGAGASTTAVSTVKTFPAG
EGSAKQLASVTSSEGTQTVDGASLSDGGQTVDTGDGGGTREDEPHKTPEPGAGSGENGEP
TEGTNVQEEGAQPQDGDVNATALSSNLGTLSQGNNSDAVTVRGSGLLPPLLLLLGLWGFA
AL*
>TCRU_3701 hypothetical protein, conserved
MSLAGQFTTFCRTHEQPINEESWAVFLVAILDVAPPTRPQHARMLRSMLEMNRTPLGMAI
LGLQKLAARSETRQARPLTKEGMDQFIRSRIDWNQRVVFRLACMTAIRWSGIAALTPNNF
TLEADGTIILDWSVAPKTARADPHRAPRFVGIRGRDALDTITLCRTLQENEKLTNITNAK
VERALAPWDATAHSIKRGALRHAAQIVETCNLDPHVISQLAKHVDPFVLPQNTVRYFWKY
TTVLTQVLPLVVLM*
>TCRU_3702 ubiquitin carboxyl-terminal hydrolase, putative
MATVKVKWGKELLELTVSLQSTVGAFKEKLQQLTQVPAERQKIMGLKANASKDDATLSQV
GVTDGKTLMLLGAAAEPPQTKEPPPSPAAASNTTAVQAPAAATRMPTGLRNIANTCYMNA
AVQMLHLVPELIELLQSRKNDTLLHSMGELYKTMEANKDPVTPLLFWNALIMQNSTFGEV
DEHGHPMQHDAQEVLNTILQQLNNGIQETHKNLFSGTMKRTSVCKEIPDDVSEASDLPFL
MLSCNINAEVQMLETGLYASFNETIPVRSEKLAREVLYSRTSRISILPEYLFVHLVRFSW
RADTQKKAKILKPVSFPLVLDTFTLCTDALKESMQEERGRVLARRDKELERRRQVRQKTQ
FDVTTNAEEVSTGAMAGDAHNIGNKSGYYELCGVISHKGRSAESGHYVFWGKPEDQWFVY
DDEHVASVTEEDVKRLSGVGEAHIAYVLMYRSRDPRTKATTIPL*
>TCRU_3703 Protein of unknown function (DUF3676)/Trans-sialidase of
Trypanosoma hydrophobic C-terminal, putative
MDGRSKEPEQVRCGLLATWGRLCRTTLRTSATGGHVTVTVTNVMLYNDDLYNNELYKLNASK
VTIPSLGVEKQPTGQVAGTDVSFASESRSEESATSNEELNEDDIEKQEEGSVDDLVPAAP
SSTDAEGSSVPEPAIATEIAGNSRPDDNAQLSEGETSQQATPHEGSESMQRDSDVQTQDL
QSEESTEFNDVEGSSESYDTPQPEEDGEANDRSGKSTSSVASSLIMETVTGPADGEHQVQ

QSTELSAENNEVRSSGTRTTGTEQSLGLEAGDGSSERTMGSGSSLTPSKSDAETKSAENT
DDVSRIEREEVSSENGEEVPQTFDTAPGNTNTTPGETAIPSESNATIPSDTEILLENGHY
SELAGMALFAESTVHGCVSRVLLLLLLGLWGTAAPC*
>TCRU_3704 hypothetical protein, conserved
MPVGLRVVCLRPVMRSSIAVAASPLCCTPLCTARRLQASDQFHRYLHNPHIVQPDGVDTL
PRTKGDQQQWLNTEQWVGYSDTYLKRHMQYPPTNEQPNAQLNSSHALLYKCLRTCYECYG
PLAEELQALHEDPLHPRFHEKIAHVKKEKEKIAAELDRVYAALHPTVKTVYDAYLVRRFF
HLNDWVKHVEQKRYNLLCRLSPDHIEQVKRARGVSAHFIDRLQRLEAAFTHNPTLGLLEG
EEGKQYTHDELVMLRQKAAYFRNMRQLGASQQEADVHTH*
>TCRU_3705 mitochondrial exoribonuclease DSS-1
MSAPRRVVKLVQFSGSYLNGTWTRKFILGSLLQRYSPTPEAGGTTPSPSLPKVTRESVAL
DKELLRLQRMLGEVWSLPVRPLDALSEARIFRLLARYAAGEGVLSLEALSELNRVLACIH
GAPQSVEDPQQMECLLQVLKYVEPGDNLRRVAFSGELHYPPSALSHMQPYLTEELQQDGG
DPFDSIRQYVDGPAYAIDSATTSEVDDAIGVHTDPSTGEEYFVVYVSDATVYCPFDSPLE
QLTARLLTTTTYLPEGVFFMLPKPIVEAATLREDRPCRTFNIRFQIDEVTGELKNYSISV
GWLRKLRRITYDEVQVLYDAKEEGTEEVARPSWMEAEDVRALHRIHLAARKRLEERRARS
LEHFIDANLPEPHIKVEAARVLSVEDQVVATKDARLAVAEMMIAANEVCSRVAQANHLPI
PYRGTRELSMDHEAARSYSEPQGVTEVASLDPQYVYFAEAIQHAIRQLSAVTRAVYFHTP
IYHAGLNTYNYTHSTSPLRRYADMLVHHQLKMWLWRTSAQNRGRAVPTSSPKRTVVAMIE
QPIAEHTMATLCSMISNKQEQSRILQDNSQRYWVLRHIEQNILKKNRQYRFICLVGDTRG
VESAPEYTRFVDSSHWDQIREDKEFGDYATSSNLQSVKRTVPFIGRWQQRRCEYRYVSDI
YIPELQFAHVVLHSLPNVVIGAVVECEVLTVHPTQGQLTLKVVNVWSGGDERQFESMWKK
ALLPSLDS*
>TCRU_3706 hypothetical protein
MKVESCREANVAPLLPLSVSFPFARQELHEKGSCIFGNWGRCLKAQPNDSPVPRMEIQMG
NRWQGESSESCVHRALSWSHREESEKPCGIKAHRCRSRSASPMAVDAIGGSIPVLGTTDN
GGDIPSASGENSVENGKKEVPLGRCEALLHAREDRAPVLESAYCSNAPNDRCLEEKCEEN
FSFASSRRSTAHHEANPHSTASMPTSEHVHGLAQLMDGLQHAILQLSGEMRELRLAFQQM
RGSFMSGRQKTLSRPPLVGPMAVTTPDQPQEEPAPSSSSSSLTFPRRTEDLSFYSASPPL
HPEGSTAFLDAGIVNAVSDGLKELEALPTFLPFEHRVVI*
>TCRU_3707 hypothetical protein
MQFDEFNFGSPGYQISVSESSSGSLQRSLPAPVLPLAPTVGGRVVLTSKNCNHDAFIWED
KEMDLLYLQCAMQHMLVGSVLRETERHLIVRFDIPYRCLPPVISSRLVKRCFGEVRGEEG
ASDKRRNKVVDDDSDKVEKEEGGDRLVGKEGQDGEGDKSASVADVALPRFVPETDGVGGG
GVGCPTSRPTRFVVGIVPVLALTHETVLGSNSRSNAGGTLSANEASEKSQLIATTAVSGA
YFINENQNDVLHVSLSVTRSGCVSLLQNLLEQHDKLECVSLAVANRDFKSAANHIMAVID
ARQNLLKQQKEMCKGIAESRQSSPTWLQPNPQAPGTDARRQTEEFIPEDRRSRFVMIFDQ
ENESLSCRGFQPHSLTTPNEEECRCLSKENVSAFNDKGSSSIVQSKTDVVDLDSASINKM
HKLHILWNSGKAIKELAPLYAFLSFLHFQDGHYEQCLEDAMTALAHDTICIEAYTRVISG
LMALGLEKEAILTAAVAVKRCLVLSPQFARLYQLVHVRFFYHRSLGNHSIVDVSLRLRHS
SIADLGSAKDGSNISKTNRQPRRPTTVRVPGLSNILTSPLPFYCVPPPSLRKILSGSSPP
MDYFLRNLRVRAARGFERDETVFSEEVSLQVLLWSLPDCEYENSSKPFSDDNADLANPWM
LCRSRFCAYCGHPLVSKSVLLAGIREKTSRAMADRVRWNFSEKKPLPCLAGCGDHYCDEI
CRNRAALEYHWIECALLPVDESESEDETPPRDGTMTGEVTRTHDFFSPAAPCGGDDTHAS
LLEILPYAKVPLRRTDVRSLVNICRRLNNRQSCLGIADLSIGSLRESLWQEFSTSSVNEA
MMERERLYLSSIQACCSVIPEIRAVFGSYYASVGTAILEKAPNAITAPLLVMGRLLASVL
HLIMPTAATKNDIFSRAKNHFIGLEHRCRERSDAVKKIVARYASRGFTSGGASPSDRHDI
HGLCLAEEVLQQIRVPFFADTNLLQPSTVWELLSEPPAVKSTANNDDASSGAAATTQSIR
RGFDRAVELLRAINDIVQSRLRDEVVAFSLSKASDPLQWLSPESVSAGGDSRLSKSQSDD
ATLASRGSDQSFAGSLPFLTDVGSLAVWLVEEHSKVGFHMAKRPSVWTFGRLLGFLGKRR
FMEQFWDFCVCSYCVVPKCVLTVGVGDSVPEHLQEGWKKICREYTVLVAVMGPLTSLVTD
LSAFGGDVCSRFFKDAAPSGQRNPLHLRKGDSPAASMLSINSLTHESEEPHSRATPQSMG
GGENKASPQADAIFSAAMPLGNLELCSKTSFDRSGERRVTMKAQRPITPGEELCYEGS*
>TCRU_3708 hypothetical protein, conserved

MADNVVPRQRKKFIKGTNIIVQKSATDGVEDAHLRMMYAIAADDKNEVLWWRNTLRPERN
LTHPSEAFLQGIKALRPSARFNTFDIEKARLRRLVKQKKDKHRFLERRKREVAPTAAMKA
SINPHKVPRTGGQAPEEEVRFLSRGRKSSKQSAPNETEKKGYFHKWKKRKMQKIGKGL*
>TCRU_3709 Right handed beta helix region containing protein, putative
MVHPEVAQTVGGGLSWLCYRNVTFSGGGMSLTVLIGAMTGDVANVTFDGCTWRDGVLLLL
LGNAYAAVGSLNIVVTGNTFSDALLSPEGVFPPHTNITINGNRFTVTRLIFRSGLAIDRP
SCVAMNELAISNDSAVVLSGNVFQSVTASSSAIYVVRSALRVSWHSVFAVMGNTFHMAGG
DGTLI*
>TCRU_3710 hypothetical protein, conserved
MRVASSSILSLRSHSVFSVTNVSVVSSGGGLVLGERLAVSESVLRFVGVEGSVASSLVRC
DGGTIGAGGWLDLHDVWAVGEASSVASLSGVTLGGGTVSIARCAATGATLVSGLAITSGV
VSVQCNRAGGRVLESSGDYRMAGLPSVSVVPCDGCAAALTCFDALTASFSDCVCGCRAGG
VGEACLPFDVPPARSGGGSQGCVSGVTLTESVTVCGWVRPAGR*
>TCRU_3711 hypothetical protein, conserved
MHGRNGELGAVCHVANAVHTCSNCAASCGTEEEETTVAFTMNVTTHKCAGPYELWWRLFP
PDGTIHPPARSGDADRTGICQLPPSREGKDDTKSGGTTAPQFDAEPSRQHEDWRQSAAAD
APNTVDRSPASNTATNTSETRPEFAFVRNTKVAPTPSGRRPTPVTEPQGILLMMR*
>TCRU_3712 BNR repeat-like domain containing protein, putative
MLVTLPVYSSKGGNGKGELHLWLTDNTHIVDIGPVSGDEEDVAASSLLYKSAGSGDGDNE
GEKLIALYEKNGGNEDTSLGMASVLLTAQLQRVKEVLATWMEVDERVSKLCPTESVGRDT
STGTACSADIKITDGLVGFLSGNFSDDTWSDEYLGVNATVHNKEGATKTSDGVTFQGAWA
EWPVGSQGQNQLYHFANYNFTLVATVSIDGVPKGG*
>TCRU_3713 BNR repeat-like domain/Concanavalin A-like lectin/glucanases superfamily/Trans-sialidase of Trypanosoma hydrophobic C-terminal, putative
MEDGTIVFPLMAKSGKGVVYSMIIHSTDNGSNWSLSEGISHPKCFAPRITEWEGSLLMIV
DCEDGQRVYESRDMGTAWTEAIGTLPGVWVKSQSFFRDLSLRVDALITVTIGGRKVMLYT
QRGNFSGKNKATALYLWVTENNRTFFVGPVGMGNAGEKEFASTLLYSDGKLHLLQRMASG
EGSAISLSHLAEELNKIKFVLKTWAQKDVFFPGLSIPTAGLVAVLSDAASDDTWIDEYLC
LNATVRNATKVKDGLKLTEPDSGVIWPVNIPDDKVRNVSLSHNFTLVATVTIEEAPSGNT
PLLTAVLANTEPTHTMGILYTANKTWETISKGETKPTTESGTWELKKEYQVALMLQGNKS
SVYVDGNSLGEEEAPLTDETPLELLYFCFGACDMKNSPMTVKDVFLYNRPLNPTEMSAIK
ERIPVPTRAPEPQVKIAPDPIAPAVSAVPAVSAVPAVPAAPVAPAVPAVPAGNEGTAREK
GDGGANGDAGSAYGSGLLPLLLLLGLWGFATA*
>TCRU_3714 hypothetical protein, conserved
MRGADVAREDPHVAAEAPKFLEDSISRQQNTRRCLPMRRRRRGFHVGYAPHVLNISEACE
PFVLSGCAEPNDPPQRCWIGCQVDRSILL*
>TCRU_3715 hypothetical protein, conserved
MWKRRRRHTPGSRSGWTPAEDDEIGRLSRSHSTEGADRIDVYGFLREVKQQERDFRLASL
EDSGHLSSEAGAQPSARSSLQWSPEYLDDVSVRDRPKRATRFLYDGKANRSAGTHTGSSG
RVRPRGRNVPPFVRRPKSKPAGQLLVVDDDGSEYDSGELSSLFANDDEDLTLFLATFPEE
LQHVRLRTEARGVLGELKETSSLLVNYATNVRHRLPEGYVATAPHESLSSATAMAEAAEE
LKTKVRIDELRQRFELEGYEEHPELWRTLQDLRQKVLEQEAFLKESLMDVVNLSRPLPGE
DRDAFAIRLTREVNEYAKSHRLDQRDLGKNTDIKDDYTAAVKGFLESFALTRGRRPGHDV
GCQCNPFLKESLMDVVNLSRPLPGEDRDAFAIRLTREVNEYAKSHRLDQRDLGKNTDIKD
DYTAAVKGFLESFALTRGRRPGHDVGCQCNPDDLGYVDEEIRRTEEEMEDLYFHARGLFS
AIKLTIVAVGNVMGFHASLELESTCKRCFYIFESPRTLWPCGHTFCQQCLPFMFTEQGEL
ICEECGSLCEVGYTPNLALELVASYQVMQHTDYDDDDDDDGDGEIGCVVQRRSIEEVLTA
MLKDLMSTQTTIADAPRKTAGKAAPHPLELVM*
>TCRU_3716 retrotransposon hot spot protein, RHS3, putative
MIVVSSPKATNYDEWEKQTKASRIIMNCPDEVDVKAMCTWITRGLEPDKQAEYWRMVEKH
MEIVGPIPRHILDADEYGRRTKDVMRALRWINIGDQGKDFTLGGEKKWYSEDPSHKLVRV
VRARAEVCAEVFLNAPISFCLERRIPHYFGKRDE*

>TCRU_3717 retrotransposon hot spot protein, RHS3, putative
MTTTGEHHTTTSTVRQFTERLAAYFSGWEELSQELSWEMIYVQQADSTPMNDWQGCDVDN
SNNVSRDESREIAAFWEEEVR*
>TCRU_3718 universal minicircle sequence binding protein 1
MADNAMTRGSRACYNCGQPGHLSRECPTRPPGVMGDRACYNCGRMGHLSRECPTRPPGVM
GDRACYNCGRMGHLSRECPNRPAGGFRGVARGACYHCQQEGHLARDCPNAPPGGERACYN
CGQTGHTSRACPVK*
>TCRU_3719 proteasome beta 5 subunit, putative
MIADFESVLRTDFSLDDCPRIGPFTWHNVPGLDGSDEGDEWDAPTLSSYGSIETRPRRND
DFRVIPSTGEMLTEDPLSTSNRLQTDMRMWKLMKTCPVPRSVPKLDMKKGTTTLGFHFDG
GIILAVDSRASSGQYISSQTVMKVLEINEYLLGTMAGGAADCQYWERVLGMECRLWELRN
NCRISVAAASKILANITYSYRNYGLSMGTMVAGWDQFGPSLYYVDDKGTRVKHELFSVGS
GSIYAYGVLDQGYRKNLTVEEACELARRSIFHATYRDGASGGIVTVYHVHQGGWTQISRD
DQTKLYDRYVL*
>TCRU_3720 tRNA pseudouridine synthase A, putative
MRRLLFPQRVLLVTLLWQQQQRRWESGGEKRSGVRGASPPPTSPKRQKKMSPAASELSRS
EDVAKREERLDVERHFPVAMSHLKATPVSSHVAEEGRKRRWREVTRVVNADGYAPPNEDA
QAPPSFFNEGSLDIRGGTKKRGARERMSSRRESISPDVLQLSAGNTRQHGLALAEESNLS
PADPTIEPEGEEGRSTYGDTPVQLTDMLKERLMELKAEKVREDRNDTYLPRRLRLLSDQE
VQKRLEEKKAQSEIAAKPVSTAPRGRITTLADVFKEHAGNDEAGPGQLMTSAAATNNSVV
MDAIFRRTADFIPGETPYDPLADFAKKSLGGGIGKSGTPDGSVFLLRCLPRAGFCSRREA
LSIIASGQVRVDNVVERNPFRLVRAENSIHVESHSGRLRFAPPRLWMYHKPAYVIVSRND
VAGRTLITKHARILGMDHLVPVGSLPMRAHGLLLLTNDGELSRLLENPKTMIQQTYLLRV
RPAIDPLLAHKLNTEGILVNGKRYNNVEFFVNPAMKSRHSLKVKVRGETMPVAHLMQHLG
RTVERGGRISFGPFSMSGLPVGSIREVTVPPYYMQHSGAVWKEFIERDWPFFRRQRVSRL
RRLSRYRELTPRELEELDNFTYEELKDALSFESQELKATSEEELRRMSVRPTLNEEPLPN
EFASADVDGNGVFPAEEEIVEDITAVI*
>TCRU_3721 hypothetical protein, conserved
MVRVGGLERPASSKRRQSASRLSSSSLQGGAVAGRVLRKADGAQRRNRSEGQSRKLSLKR
RSLKAGGSTNAQRKRNGRSNRAYSVRSHSAERGAAAGAQGKTLGTSGRSRPFSRAVMDAY
VSRIQRWFRRLVLGGVVRCRGLLVMRIREILELQKRLLELRVRMAKRVMSEAFNTYALSI
HSDSEMRGIRKYKPVVTLVNAFMRAKKDCVLRSEKLHIIYQRAVRVIERAWAKCVLFRSI
QQASVENKMQWLLIQQEEIERRDYIRQRLLFLVECHQQCYNHPLLMKAVIAVRNTECWAG
IITESPLHLTDRICSFVNLSNEVTLNTSSARDFRHSIMMQTLRSQASSRFASPNFGRTSD
SQRWSCLNEEKHKGLIFQTTNEQPSVKHRFNVRADTAFVHGLLQEMGKLDFPSSMGSRPR
VLASPRDLTDAAVSTTCTARVQSTEHASELTNSRFVWVGNVILRECLRSSPLFTEFCIWN
NSKERGWIKGGKATSSSYVFEDAVNACFLHPAPRRKSRERTLSPMDFSVSVASMGKAWAD
FYVFMDLTGDALKSFSCNNTTSDSSPLASLMVSRRSKGRALLHFLLGTGAASVTRTSGKL
HTPTPSPADQTQANWAPTKIFGSEFDIKYEIERLLVREYHRRLKLQDLYDAEVAALGWII
QRGTATTRNAGEARTNVKESRQQFKKARVPLHLPPTLC*
>TCRU_3722 Peptidase M76 family, putative
MVALWGRGKENRNDVEEGEEGVKASLLHQQCEAAVSEVLATVNTVQYLIKSIEEITGKPF
HRERIKCVTLNRGIHSGGAVGNNGSGPLDGNVMAGYMWRRAREDCEKGDILLVEELMVGK
AGNSGTNIPDKRTIETVERNLRHELIHAFDDTRGVIEASDCIHQACSEIRAARLSGDCFV
GEELKRGRLDPLRSGIRCVKRRATLAVEKNPVCRGFSERAVERVFQRCYSDYEPFAAPIY
AMGSYGEEKFK*
>TCRU_3723 hypothetical protein
MATHSYYGGYSLIRSSAYTANSNGCGNSCSSKNASSTRDGTRTAGLARMNFASCNSDWMP
SLATSSAAEVRSTVMPVDAVGDVASSASAPTRTPLIQALLAMQNFYRGVPLCVTNTTARN
ASTTTTVTPPAAPSAKTEKFVAATTAEVSKRAEAKKMLPTIPMPIGGDTWTALSDTVLAA
KTGVVASRHALKRPVAPMAVGVTDADLDREMRSTRKGPEPLFRDLLAFSSPSETPTQSAF
ACNTNNSKRRDPVPADELFSFQQHEQEQKQHCPVPPAPISVSPLLSPNRENSLAPEEKLQ
MFQKQSEAPGGFYDEGHYALRSSPKELQMGLASFSSTPEERQQEKSTIPVLSGGINGASR
QGIQRNQLLREGDEDKPLFDEEDEDALEHIRQVINIVLSVNASENASADTDASCKPKKGL

EFGMEWLNTRYHQSEQQNSFFQGF*
>TCRU_3724 phosphatidylinositolN-acetylglucosaminyltransferase, putative
MSSERRRTHTATSSDEFFAGVSVHWASSTGSAARGWRKVLYERQPFEDNYVDPEQFMRDL
RQNVNLKTYEYGKVVMDTFVVVQQLSFVALFLFAFAKMYSEEGNAYALAWINATLFALSF
VFYVEMQRQQAMEQGETTTPLLQHVVKLGRQVLPLVAVIILLSPALQTLTATYSNDTIVA
LSTFAMCIHLLLTDYSYLNCYTERYQQYTAVSAATFGTILVASRIKNFFCSGPLITFGIL
CFALSPIPRHHLKRTSATAHVGLTFALCGLAAGCLMQAAIFAVLYCLLVVAVSLFIPWCF
VTLHGSMKKQINGPWDEAKPTNSAAAAEWANAGLLR*
>TCRU_3725 neurobeachin/beige protein, putative
MFKFFSVIRKLSGGSANVNAGLRGQEEEEEEELLAEFTKFLTLPSCEGIRVELEKLECQC
EVSNHVHHPHSPDSGLERSVLHLLRTESFQPALPSRLVALLLRRLESCFTGSDETEITGF
LQDSLPTLHALLMTDASRFGTTMLALGIDNHLLLVLQFVRAKLINRTLSGTKAPMNITAT
QTKEQEENSFETETNFSLNTTSQPSLARRCVLVRILCETLRLLLAIHVSPTATAVSVTGI
SHILLEKGVTRYRTDSNFLTSSAAPRLISPSTTGVTFGGWGSGDSLATIDGMLTECGHLF
LYFSKGALDLHVPADEEEEEEEEEGELVAEAICSLVLLISNLYQEPSELIIHITLSGIGV
QLARFLVAEEPGKSLLLLRELSFGLLLVSKFMRESPLVTVQAMSSVYFFDNLVGTLQILG
QQYECARMDESYASAREDALKELLSVANLEAFRVRKLSEKVIHLSRPRFDRLAEDNTVDE
FLHCYIGLLETQRFVKSKMEVRRDPAILRVFAEIELTLIKAFLDSPTSLSPASCMYETLM
KYTIASLPLHEMGKPEKRHVTLIRPLINAGLYRILFYEPTFKSFRHDPRLYYAAVYLLHY
MLIYPLLSLEDHVEDEVGIILDAFLSNEAEEHSHELPEKSAVMCAILLSAVCGPNATHVL
NSLMQRNGIAMFAAIITRSSISDSRKQEKEGGGSSGGVIVGEWVTYLLTQLLRQPEVQRS
VLVDQADVAFSLLSVRGIRKNVEQMVVSLLSHRCVEAEYSQRMTPLVNCTWEFVSQCMKI
QQGEPLIATLIEADDEDSLLLSILYCIRASIRALAADLPCWGSVRQLQSVLCGSVTDAPD
PFLCLLHRVVLPWHGVRASHGLSSILHTITMLVQGNPALRVRLFQTMDAEQLVTCFQSAW
FSSRGGSWLQFVHSILALVYEEEGELREGDRPPKSKKKSGAATLLADVREDTVIQNPELL
LPLLRIFLKLTEFSTKHRDALEYLLARLLRTLKASLVSLWMLANAGLFEVLTALIPVVTG
TALLEMVLALIMNITSHHIGVRETKQFLMGIAHTQSEEERRRLVPIVIEVLSVSSYMFLN
QQTERQNYIAFREFSGQTGVKAILSEFPQDGYTVCMWLRMERREDGRMPQCIFSLQNMEK
RTILELVATAHGLSVIFLNEQRRTLDLDLSCSIPSQTWTQLTVVHRQAAFPFSGNEFLCF
LNGMEVAAVSGVQYPPISLGFFYIGTRGEDVEQRSSRRSFIGQMTAVYFLMRALTPKEVM
EFFVGDTGVTSMKQYATHIAVYIDPRFCERGQVHNLATLMRGKASELPLITYEGTIACNT
NSIIDSICVLGALHTVVVPLLVLLVNPQLPFQCRIPPAKRKSVPETTRKAVDDLLKLVES
LLLSNTVRADVLEVGLFPMIAHVLQQFMEYHCSNLAQRMCNLCSALLPNGAIFDAAYTVL
FLSGDLLHACSEETQLALIKTQTAMCGANKELCRRAHALDLSNFVVSEIIHVYNTSSPHH
ASMRREMFVLLEVMMVEPITMNDADALLRLINFAVKQIESMVLLEILERCRVLIATRKPL
LAAYMGKKDFAAVLLLLLEDSRGMVQNEALLLFCLLVGRSRRTQELLNPTLLSAREAAHV
ACDISLSWLREKLHAVQVDVGLYITLRAALTGRFDAPINDCTELKRDDKILFAPAFTPLL
QMMKRCQEKSLKIRAMSDIAALVQQDPSAWKSVVSVPGWYASVVDLYLSEDDLRDMQASD
ISSFLASTTMIFVKVLFHALLHEAYGASELELLVAYLVQQRAHVLLNAVLTGVVKEYSAL
FSVRHNLLGGAHFGLGSQQAATNFTTLLFLVEDVLFYSVTTYQAYGAARPLESLRKRGYT
EEEELVLGPMKRHARDARRKEQGEGGEEVEEEEEREESADALGGQFFIGDKAMRFRTDPD
GVWLHATLAVCTMHILTTHAAVLNAGGTNSGGSLDFYMRGRRVRKGGFLRLFVRLFRVTC
NFILRDAEQVEGVVLMVGRWIRIVEKEQSSFLLLRRQSTEQREHSPLSGSMIMILGLHDL
LARRLRFSVEGSSTRFEDLNYEILDCIKCLCILYRRTLAQMQIFHAQSPAYESAPRTRKD
TLDWLCSRKRERSIVEFVEVASRQDYDGFIRHCTLVMERDRLTEKGIVRLIEEEHTKVMA
HLHQIITGLSVARRLMLDALEQYLQATSKELAETETLKERFAPQTARKLATVVFNTVWRR
FLERCKGTIWDFDPAGQHSTKYVRLLEVEQQQLVRRKLVFDSNGTDHANITTMGSASLVL
QQAEQQQPCPRGGEHMLLSTENSGNDEEDEEEEGKDEEEQMEEEEGMELSGVAALPVATE
ANQTVHFSMACEVPYMMHCWSATMVIRESDLCIFFDEENKAYNQRIAEEAESLLIKPGSI
IYPSGHVKILAPGRRFRMRRSAVELWFLDGMSVLINFATFPDMRAAVNNIRTAAGRHKLL
HSPLYIFQVNPKKDPMLWRRMEQWRDREISNFEYLLWLNFFGGRTLNDITQYPVFPWVIA
DYTSETLDLESPSTFRDFTLPVGICGGPQSRARVEMRYEATKQLGDIPAHYFTHYSSSAV
VLYYLIRMEPFTTLQIILQGGHFDHADRMFHSMASCWHGVTTNSQDVRELIPELFYMPEI

CMNTNRVRFGCKQGGEAMDSLELPPWSHGDPYEFIYRMREALESDYVSSMLHHWIDLIFG
YKQRGKEAIAALNVFNWHSYEELDKNRDTDVDRQLLIDSLDNIGQTPIQLFTQNHVERRP
YDLLDPISCSMSVKTIDVQRTCSCVARVALFASDRVLLVGGNGTALLFRLIVAPLTKRAR
RACSAGSVGSAAATGAGENSVAASAGSGGLPSLSNMTPSASGRCGGISRGRPSVDMLEEV
ERRTAPLPTGVVPNTSKKAGGPCETENVAVLSFDNEVFVAFGGLFDNTFVIRPLDSSSVF
AEERLSAHRGRVVRITASSNSRYIVSGAEDTTFVVWSCQLQQGRTRLRVELLFTVYGHED
NPTAVDLSPVLDLVATASRDGVLMLHSLTSSRLERSLRHPENFSIDRVLIQASCYLPNIL
YASALDHVIHQIGVNGVALRSVTAPGRITSWCVTPNQYILVTTKSFTNLNASDESGGTVF
FLHSFYLSFLNSVPCPLRSFGATLTCCAAHPSNPQVIVCGSSEGSLSLLRVEL*
>TCRU_3726 Sec34-like family, putative
MLSDAEFSHRLDVALAQMPKIFQHAIPFSDRMLEFGGSLLDTTVRFGSSINRASSLSPAV
ELRDPQKEQEAQAMEEMLRDIAKAESESRVEGLQRWLERAAEGRRMGEQCLSVFNDLSSC
LGSMCDVTAAVQEQSNLLSSNASALMVRKTKLEMVQEELERMIKHFTRIEVLTREAENPV
LSANSTRYPSMLQEMEEEMQFLSRNPNFLSSKVYATRLALSQQRAFQCLKDAIVESVRSA
QNLTVGSDVYRDAFYRRQRGTSNDAANTEDRALKTTTILPLPGVDGSSDAQRAVADALRR
INAIFCEKLSEKASLRRMLEAIGGDGESKDEVNDNEIFDAYRESRVLVVGPLLQNWLEAW
CSVDVTAKPMPKLVNHLNSLMKIALAGEKEVFDEIWLREDFSARVFQQLVAGLSNEVYHV
FRSQLLQVDELEELARTIEEIQWENTRQDNVAELSELLTKLIQDTQERLIFRTSVFLRQS
IMRGGPTKAMLREFQSDGVKSDDSYIPALSNCVALLRILYPSLEFSVFSVFAEEAIHCAL
TQMQELARLMSQQRGENTAIKGLMCQLCHLIHLREELSRIDENITVVEKRIDLSKIAQRR
VEIVQSSRESKKDVETEIKVCCERLVQMLFSGVSAPLSGVARKKPDEIRDAVSALEQNWT
TQKELLRLFIPNATTREVLLRPVRARIDELLHETDSIQQQQQQQQQQEQPEQRTEGGTK
TVVSAPASVPTAGNSFAPGQVEDGGTAELLQQETERQTTMPEKNNSFPARRFGSGLV*
>TCRU_3727 hypothetical protein, conserved
MLRSSYIWFHGQAARSNSVLAALLRIRQQHQRPSHPINRRDASLQVISPSRDLAVSSSLE
RQQLVDEAMKILRKEVRGGDALSDVDAASAAFCVEVGKLLSVAAVFGAATTTPRVAEALQ
WVRTNKKQLVSTRQVMSICVPLLNLKDGKTAGRRFVIEELSGPLLQALESPASPTVSSES
DLKQHRSAIVSVFSLVGRLLESDGGDADGGKKAMGEESVFPVPPVEDRPMFYQRTLLFFT
KAVDALLTLAEQSLPPLEISESVYILQACQRLERDSEGKSIEEVRAGLNRVRPMIDAALG
PSTAGTTKRPGDVCQLLSVVSKLQDTHHMTDLSRAALGLLPLALASASAKDICAALQVLV
RLSGSSPEVTEPLLLRRVLEAVRPKLFLIAETHPASMRHVECSLLMSTLSRLEEELADDL
VNLLCGCFTASMDLAQPAQLIPFLQGLFRCSEAASRSSADTHNVPFWEVARLPSVRPCIE
KAADRVIAWAAADAITDSESAQLLLLFSQLRYTRVLAVYVALEKKLVLTVDHLSRGLSND
DSNGNVAESISSSGRIGNGDDDVAGLVVGGDDGSSELKHKSLAAANGIALTSVLMNALYA
FQNEAVTIVREEEECLQRAGKLYETLRQGAVESLTRTRNPKAFVMLLHVLMPEGKRGRGG
GGGRSATLESDVTAASVVNSASRPLDAVAPQVCLLAPVSNAYEVGAIAKAVGEFMAHGLI
GEEGAQRAMEAILERSEAVELSSYDAQSLLEGMRRVRVVSIPPSLLHRLATQLQNTPQKT
TMMLRRLLPVLKTITMKPILQEALIKLTEVAMANACTIMFTNASSPPPLRDTALLAHVLA
QLYDLSLEPLVLNLVVKEEMGMTEEAEEETKEDENGEGEADFLDDGTTDEPDEFIHHQHH
HHLLTVTKKAFLALGDVACSSLQQNVDSADGLTCTLIPPQEIVMLVQSFEKVGVRHHALL
YEVVPLVRDMSGSMEPLELSLLLNAFARLGAWNGRVLNTLASSVAAQIKTCSLKQCQSIL
QALQSSGFLRPGAFLSTTEYKTTLLRDWRPACTPTTQASIDPLVVLATSVVERMTTLVER
PESVSAMLESQSLEDVAAVVRVLGFFVQPPQPAFDTYFEMAMKKLVLDSKDIMDDSSPLR
QRKWLMVALFLKGHLGKLRRYHRQCISAQAMADSITAHETTLLSLTPGLRLMAEEKVADL
YYTCCVFAMVYNVKFSPEAASTILKVISEKLQEEDVARNPRRVSSALRHLTKLSSKHIGA
PGIVHKIALDSAWHLVAQLQGNAAEERNCSSPECSSSAAAVMLEVLCMSHLLALPYLVEE
KQKLQQVLFFFSNHLRKNDISRLSAAECSILVLSTAVGTAMQICCQVNVAWLDEVQHGIL
LKEKHLSVHDAVNILLAEALCCDESIVQAETVRCASRVLVGQMGKTDWTGLRLLPELYRL
ATAGDACFMAWCKRLQQVAGDDTTAPVAGGVLEVFERVLPALLRALRSAEKGTTPTLCWQ
CRVDVAEQYQRCAETCGDERGLADALAELVKKETCASS*
>TCRU_3728 aldehyde dehydrogenase, putative
MRRTSCTFCKAVFDLKNAAKLTTGVVLDRTVAFINGAFVTPASAATITVEDPCTQKAIGE
VPNMGKEETLQAIDAAKSAFEGWKNMMPRERAVIMHRWAQLVLCHSQSLGIILSRESGKV
LAEGVDECLYAERYLEWYAGEAERVYGDIIGGPREGVVTTVVRRPVGVVGVITPWNFPSS

MVTRAVGGALAAGCTVVLKPSELTPFSALVLAQLAAEAGVPDGVFNVLTGNAEPIGDALL
DSFDVRKISFTGSTRTGKHLYARSASTMKKLGLELGGNAPFIVFDDADLNRAVIGLIASK
FRNAGQACISSNRIFIHEAIHDAFVARLVEKTRETLRVGNSLDETSKMGSLINGAAVDRM
EALVKDALAKGAKLELGGKRIPGPGYFFEPTILTRVRHEAMDCSKREIFGPIIPIIKFSS
ESEVVQLANATPAGLAAYIYTQDYRRQSRVSEQLQFGMVGVNDVGLSSPCAPFGGVKESG
LGRDGSKYGIDAFVDIKYVLESRI*
>TCRU_3729 hypothetical protein
MTGKWQEMEVDDVLTEMEAFIDASGIRDVALIAASDDAFTRDHLRELEDELDRQQPRPTK
ELILNPVVKRHLYFPLTHSSGGHITQLPLPQDQIFRLGARVVNCRAVGSVPFGAVGTIVR
LLATSHDAEVVYDEPFTGGSHFDGRLRQPRGGLSKLSSLLVLNRPGDDDGGGAATESERT
TVSILRALMQKSQQQQQQQQQQQQQQKRGSVPMAESVSVIPKPRSATEDASVSKTVTGMA
PASAAVTISGGAGGIRLSDLVGKLKASPEHSDAASITPPTVPEEQKDARPSPVSLKPQVA
ITTNTSSVAAGSNDKKGNNGNLGVNNTPPQVETRTLIPVQSGNENNAVVENRLQMHLRGG
GSPEEKNSGKRLKMTFTVPREIASGEFTIRRGEAVPFFRRLLKLRLSEELGKTTTHR*
>TCRU_gamma, conserved domain containing protein, putative
Elongation factor.1
MVCSKQKDDKKMQCMTANLIRVWLQRMEHVGQYALDPALMIGEERRHDIVALCVLRGRGM
PAIVRDVEDTELLD*
>TCRU_3731 hypothetical protein, conserved
MAMMMTGRVLLVCALCVLWCGAGGRCAEEKADEVLVAEPGPSLSGGGDSPTKQSKNSSPV
PGPLVLPPKQQKDDKSEHLHAKESLESEEEVPLKTQEGENKTQVPQDGVQNGNQSIPQEQ
SPHQALPPHQPPSVGSDAVSPGGSSVGSSSSGVISSPHGGGDKGTTDIILSNAPSSSPKG
VLPAEEAETEHHSLPNENAAERGNVGRAAAQGNEDPFPLEERRTQDGKSEAHQTEESSKD
LNKQPQGNGKIQNGSPNADTPQLQTPHGRKTPETKGNNSHNTDISTNLPDAQEPKYVKES
NNENPASTPVKTQGTSTGIQEEAPASHPNGIPSLQQENFTGMKTTENVQPPEVAATNKLQ
TGDKSKDVNSDGSTGVSRTTSPLLLLLVASAATAAAVVAA*
>TCRU_gamma, conserved domain containing protein, putative
Elongation factor.1
MCEGGRCSHTFASSTHVAPLGSLSQHPDRGSRGVRVCYKHKKDNKMLCVTANLIRGWLQR
MEHVRKYALGVALMIVEEMRHEIVALCVLRGRGMPMIVRDVLDTELLD*
>TCRU_3733 BNR repeat-like domain containing protein, putative
MMAHCAGGYYDVYRSILDGVNWNPDGEPINRVWGNSRDPAGDGVQSGSTTAIIEGKKVML
ITAPVYAKEENGKGRLHLWVTDKARVYDVGPVSREDDDAAASSLLIKDKNKELISLYENK
KDGVYSLVAVRLTEKLERIKEVVKTWKDLDSALKTCRSGSSGTVDALRKGMCNGPVPTER
LVGFLSGNSTGTEWRDEYLGVNATVTNGERRVPNGWTFKGPGAGAVWPVGDMGQTVPYYF
ANTKACACGDGVYPRGAEGGQQPHSFDWREDE*
>TCRU_3734 hypothetical protein, conserved
MKSHLVAVLGLGLHIRTPQQGVAASTTETKTTPPLEKVPRHARCGEVKRSARRLSVNTAR
PFRSGHSVDVTCGGDVQKNPGTEEDGCINIWRLDIAGLSSVKRLNLRRDWSCIKLTSFFS
RK*
>TCRU_3735 BNR repeat-like domain/Protein of unknown function (DUF3676)/Trans-sialidase of Trypanosoma hydrophobic C-terminal, putative
MLVGKYSHTEKQVEGKNERGLLLVKGTVTVDGGNNKKLVWNETHVVEPQGKGASLSLTEL
IGGGGSGAVMRDGTLVFPMQAKKKDGTSVLLSMSFIPSKNKWELSLETPGNGCRDPTLVK
WKENEDEERLFMMAHCAGGYYDVYRSILHVVNWYPYGEPITRVWGNSHNRKGYGVQSGST
TAIIEEKEVMLITAPVYPKEDNGKGRLHLWVTNNARVHDVGPVSRENDDAAASSLLMKDN
NKELISLYENKKSDGSYNLVAVRLTEKLERIKEVVKTWKDLDSALQCCSSSSSGTVDVRK
KGMCNGPVPTNRLVGFLSGNSTQTEWRDEYLGVNAKVTNVEKRVPNGVTFKGSGAGAEWP
VGDMGQTVPYYFTNNEFALVATVSIHEVPKEDSSPVPLIGVRMNDTSSTVLFGLSYTHEK
KWLAIPGKSAASQFVDGWEPNRTYQVLLQMDYDYWTIVVDKEEIDHKSYDKNLFNSHRIS
HFYIGGDSKHQSATDGHVTVTNVMLYNERLLDHKLDELHASKVNIPSLSVEGQPKGQVAS
KDVSVASESKSEESATSHEKLNESDTNEQGENSAEDPVPAASSSTVVGGSSISEPATATE
IAGASLPEDNAQLSEGKTVQQATPHEPKDSMQQDSDVQPQDPQPAELTGVTELETSSEGN

DTEEPEEDGGTNDRSGGSTSSVGASLSMATATASVYGEHQVQQSIELSAENNDVRSTGTG
TTGAEQSLSLEVGDRNSERTMNSDSSLTSSKSDAEPTSAENTDDVFRTEGAEVSSEDGKE
VPQTVDTASGNTSTAPEEEGIPSTKGAARHSDNDIFNTSEVADLLSTALNRDSTVHLCVF
RVLLLLLLGLGGIAALC*
>TCRU_3736 trans-sialidase
MPRLLLYVLVLFFLFNFCPNVPAHALAPGSSRIELFKRKNSTVPFEENGKVTERVVNSFR
LPALVNVDGVMVAIADARYDTSNDNSLIDTVVKYSVDDGETWETQIAIKNSRASSVSRVV
DPTVIVKGNKLYVLVGSYNISRSYWTWQPDGSDWDILLAVGEVTKSTVDGKTTANITWGS
PVSLKEFFPAEMEGIHTKQFLGGAGVATLASNGNLVYPVQVANMKKQIFSKIFYSEDDGK
TWNFSEGRSDFGCSEPVVLEWKNKFIVNTRVDRARRLVYNSGDMERPWVEAVGTLSRVWG
PSPKSDQPGSQSSFTAVTIEGMRVMLFTHPLNFKGRWLRDRLNLWLTDNQRIYNVGQVSI
GDENAAYSSVLYKDDKLYCLHEINSNEVYSLVFARLVGELRIIKSVLQSWKNWDSHLSRI
CTPADPATSSSERVCGPAVTTVGLAGFLSDNATQNVWEDAYRCVNASTANAEKVPNGFKF
AGVGGGALWPVSQQGQNQRYHFANYEFTLVASVTIHEVPRDATPLLGASLDSSGGEKLLG
LSYDEKHQWQPIYGSTPATPTGSWEVNKKYHVVLTMANKMGSVYIDGEPLAGSGQTVVPD
EGTPDISHFYIGSYNSSNMPTESHVTTKNVFLYNRQLNAEEIRTLFLSQDLDGVEEDKDS
SDDGDENDGDDSDGDDNDDASSLSAGGRNLVYVCALVLHLLVLVYF*
>TCRU_3737 hypothetical protein
MKAKCLLFVLEIIVMACVAQAETCRVNTSDTGESISLEYLSNYGNVSVMLGDVKIDLGVS
FCWPKKFSAFGEECDFGYMIIYEKGCEGTFTHSDNVSLENDTLFFTIRSPNTLMADVYVE
CDESINGLRALSANETEITKYVFRFASTTVCPGYQPPGRDNFPSKGFLITIVTGIVGVVV
VAAALFQWKSRSPSVEDYSQLI*
>TCRU_3738 PPR repeat family, putative
MLLATAKCLPKALSPYAKALRHVALRGAVAFGPSAKEMELDMYRKGTLPPDYRPPVQGKW
DDTIERWAYAWQFPAEEEQEDIVGAVERNSSGMQALLEFGNRLLQSPPSAQPHSGKYSKV
YPPITGGPNNGLTGLISHSSNYEHLEQAVSELIISSDTESFLHDTMTAAELSARADVPVA
YIDDNSELSNASKELACILEEFGLEYTEDGLITVISALSQQQYSSMSRAIFDFACTVGLG
PSAELYKALMRHASRRGDVNESMALIEEMKAKGITPRIGNWHELMRAFHQANDYPAVSQI
VDNMKMYANIEPNEVTFALQLKALAKDSSQINSLAESIQLFDQMENVYGFIASRPHYDAM
MYHLSQSPKPEMRLRCEELAHKMELMGIVWNANTYLNLIRSAQVVGDISAVEKYLSKMRD
DGIVVGITHLSWAIQAHAQSIIRLDYAAMREKSEDPLPIWLEHLETCFGIYELVVRRGWT
MQLPFVNALLRLCCQTTMLAMEHSPDQTTTIGRFEEQSNKIWNQTFEEWHLKKDVYSYEC
YIALLAHQQRIDEAEKLFQEMVLKEDLTPSKRTYDCLIFMHLSSGEEGGTARALRYLEAM
ERAGIQIRPSLLKKIVRVNNAAGYKRDMKRRARRIMQAREEYFARKEQNIGFNEDAPSSS
VSSKADVDSEGNSVLTPLPISASSTLAWWEQWKRETASKHELFAEENTDGTPKGETFDEK
NEALRQLGINSAFPTKDHLPRLDRQKILPQIRSEEGEMAGSLWAMDGGEFSYPKDGGGPQ
GWGIRLWRERQVVKQEYQKVIDGSQPVPQLSAIGNAVRTTSTQLEIEKSGARTPGELSDH
HQFPHHCFDSGALKPPLETAVPVPLSGELVWQGESRDNLAPYKSDEEIALENDNTFFSDL
SEEAERKMDASVMAIQKREENALEIKGKGVTRRSKYDYLEKWREMYRHGTLEVPEGPTLR
FGRSPDDHKDTMAALVREWYLRNRKTPATLEDLQRWESDSQREKEASISRKALKKRKRIR
SHHKGE*
>TCRU_3739 F-actin capping protein, beta subunit/WW domain
containing protein, putative
MRESTNKAEDVAAAGASLLAQYPPRKGRMILDNIVTLCKYRDAEAVLNIIYRQVSPVDTP
WPVLQCPSAATQWLSATPHYFIAFEYNRMGDSYRCPLCNKYVSGDKFVSFPLGSSGLLRQ
MESAANRVFVEYAELYYGKESTASVYTWECDPANTESKMGLAVMIKHRAKSPNGNRPGHF
SLREKRGVWQSAHVGIVDISSGAYYFQSSFYMDAFVPLGSLPNKPDAVGRFNGSVASRLF
SFESREAIATPDLDGLIASIGEQVQLTENSFYGRLFEIYFSKAQFVAEGMRVIPQSTLFL
PPQSRWKEQPAASRVTEESSQQADVHVIGQTSDTNAMTHSGAVRLDYQFAEGSEEIASPL
PRGKESTVVDDWKEAYDDDGNRYYYNEVTGETAWEKPDVGKLVLDILRNLHLMHSPDKYY
RRLKKYFRQYETELNIDTFYSLTNEDQMLELDDGSTMTALEYIVPAYGDRRKIEVYIAQN
ARKDA*
>TCRU_3740 amidohydrolase, putative
MNNINVLISAVQPEVVQWRRHIHENPYLAYEEQPTADYVASVLTAMPAPLEIRRLTPNSV

IADLRGGAGEGPIYALRADMDALPLQEESGEPFSSKRPGVMHACGHDAHTAMLLGAVKVL
CQVKDKIRGTVRFIFQHAEEVIPSGAKQLVQLGVLEGVKMIFGLHVSAATPAGKISTRSG
TLYGACNDFDIVIKGAGGHASQPELCTDPIVIAAEVVMGLQTIVSRRIGALTAPVLSVTT
FHGGTGSYNVIPDTAHLRGTLRCLDRDVQALVPGLMEEIVAGIAKAHGAQHEISWLEPNI
VTYNDHAAFLIAKDVISEFVGADAFLEKEQPLFGVEDFSEYVAKTPGCFCLLGIRDEASC
SVYTEHSSKFKVYEKALEHGVQMHVGFIVKLLMRST*
>TCRU_3741 BNR repeat-like domain/Concanavalin A-like lectin/
glucanases superfamily/Trans-sialidase of Trypanosoma hydrophobic C-
terminal, putative
MMFCNAGGAEQAEEQASERNYKWKDIKEGEGVTVESLGVPGLLNVGSDVFAVAEAQCKKS
LVSFTGIASRLLTTQKVNTPVEVLNGAEDKTQVLEEDGSEDPKKKVDVSRPTTVVEGNEI
YMIAGKYSPTAATDQDGGAEDGGLLLVKGSVSEESGSGKRIKWSDANAVPRASVGELDSW
TGLIGGGGSGVKTDDGRLVFPVEGKKKEVEAPNDKKAVSLLIIYSLDTNSGVLSKGMSDG
GCSDPSVVEWKDKKLMMMTACDDGRRRVYESGDKGESWTEVLGTLSRVWVNKKSEGGVRS
GFITATIGDDDKKNVMLVTLPVYPEDASKKETDNRKGKLHLWLTDNTHIVDIGPVSDDDD
AVASSLLYKSGTGDNKQKEELIALYEKKKGEGKPSHSLWSVLLTEQLQRVKDVLATWKEV
DETVSKLCPSESDAKDVSHDNACSPTVKITDGLVGFLSGNFSENTWRDEYLGVNATVKGN
VGDTKAKKTSDGVTFTGAWAEWPVGEQGQNQLYHFANYNFTLVATVSIHGVPQEGSPIPL
MGVKMNDDGKTVLFGLSYENGGKWQVLCGNGTNSQEHSSAWEKEKTHQVAIVLQNGTQGS
AYVDGKSVGGDVPCALENKDSKKISHFYIGGDGSSAGVSEVQDVSVTVTNVLLYNRPLDD
DEITALNPNKVPIPPLVVGSAQGTLLQSLSAGQAPSGPESLNENQGAGGGRASPSEPSIV
TTSGEEPVRQSASRASPGGSKNVDVASPSDGDPRVGSEAGGAMQGDTAPQTPVDTPDKAD
TNAPTATNVAQVDPTDTTKVGTSSGANGETVGGTDVQEELHARGGEVNAAALSSSLGNLS
QGNNSDAGTVRGSGLQPLLLLLGLWVFAAL*
>TCRU_3742 dynein light chain, putative
MASATSVKEAIARFEEAEYRRRTSEMSEEEKANAPRVVAAEEPRVLLIGMLPPIVKMDKD
IATLVNCEHLGLSTNGIEKIGPGLRELKKLKILSLGRNVIRKIEQLDIPQLEQLWLRVCD
GASCQSLWPLPREAGGRCGSSCGTRPSGPAGGTGATGRCSTASLGVLAA*
>TCRU_3743 hypothetical protein, conserved
MAEAASCLDLKASFIVSLPRETRHLFRCRVEDGTLVELTRFPMGYKASPEILQIITSAIA
VVATVVRPLWAAPPSVRVDVWIDNIRIAGSKSGATFWESQVLRNADCRHASLGEDHESGA
THYTFLGVQFDQTHRAVSLSDKFVRSVRAMPALNSLTIAEMEVMASRFLYAAAILCTRLC
D*
>TCRU_3744 hypothetical protein, conserved
MAALRLQKIAVRSETKQARPLTKEEMNQFIRSRTDWRERVVLRIAWITASRWSEIAVFTP
NNFKLEADGSLVFGLVRGAEDGESGSPPRLAVREDARTGRLRHYKAMQDTSRKRNAHESY
*
>TCRU_3745 hypothetical protein, conserved
MVHTAVARALTPRGATTVSRHDAACPDACPCRLHYANNNPTREASLSHGFSRQYGVLNPG
AKTSQSSMKSQEESMMEPAKKYRLAPNGMRRPTLRSHTECTLYSIELMLRPSLKNASVSD
EP*
>TCRU_3746 hypothetical protein, conserved
MQAEVRVAHLALLAFIAILPSTMDVWVDNTSLQGAANKGSSKSHSMTWEPQRIYEFLDSR
GVQASFGYVRSAENPADGISRGRVFTLQDLAKGWNLRRGRRGLVVGGPQSLPLRK*
>TCRU_3747 hypothetical protein, conserved
MSPATGCKNGAGGRYDDAAVVPEAYDNDVMSWSFCVSSSPSSSSRSSASSPPRRGSRSSC
QWRSSTYHTTTLYTVAKSGTVVSSSTIAAYALAPPSLGARWLAAPTTHSAARSTKLAVCI
TVRKAERIMFVKATTPAPASSTAAMQKYWTWQPPTGGCTAAIRPTTHSTDSWNTHQYTWL
PLMFVSMPPYIRCCAAGCQYPTGYSSRSCSGFPENCQYLVKLAPTWRAIK*
>TCRU_3748 hypothetical protein, conserved
MPRCIACATTYVRLGKRIAAHAVAASTPRHRWKAAVTPQCTHPNTAASSALPTTIHSGSR
AANTDRKYGSATVLARSCAKAHGILANTPSACISRTEPSPPVTTTATAAAPATTPVIAER
ALAAAGSVADLIVKDPSSTPLQPPDTAAASQRMTIVSSAAMK*
>TCRU_3749 hypothetical protein, conserved

MRGVHLAPRHAQHSAPRRNNARTRVQRIFATEHSRVQRDTATDKLQHSAVHHHRPPTSHH
GHEGHAGHNQHGPHKPQARQHQHRCVRRGLQAGKIHKRRRPRGNQQGVRHRQHRPRGERA
GQHKRTVQRLHIAHRQLHRPALVAPRRPRTAQRSSAHHVQHGTVQQRHVLHNNPHAVPRH
PRHVEHRTHAVQHNVAQRHLARAGRHNEHRLHRHALADKHSRRSAHSGRQRGSAHRHARL
VVPTAAARAHAHTVRHARRRRPGRVALRVPVAVVAPSNVDAPGVAKTSSSVLLRRRQDTA
TSVVARHPHMRPSQKRQAQRQHPRQQRSVAQKAQRAPSSGHGASGVEHLPRDAVVHVALR
QQHRPAIQTHRRIERHRVVLRRRKFAPHKRHSGARHAHSPTHRHRVLAQQEVAVNKRHHG
AAAGRDTALHRHAQPQRRLHRQPVQVQRTT*
>TCRU_3750 hypothetical protein, conserved
MPKPEAEEQSSTEQDTEDSPDTEEVTTGKKNVQNTVEMGIPSSSSSAASKPPVQQTTPIL
AQSPAAPSPERSALAPSEKESTAPSFSAGEGSRRAANVAQNSKEEKNEKLPSENETQSKA
VEQPSGNDVAEQDSPARTTASPIPASGGADAQRNEDADNSNAQALKSEGTHKNSETGYTN
PASTAGDAATQTEKALADAKTNDTATHGDSDGNTAASHTTSPLLLLLVVACAAAAAVVAA
*
>TCRU_3751 hypothetical protein, conserved
MGGFGLRAFIFFGEFVGALLEELKELRPPASCGGQYAVLKAIPQGHRTELYGLGSLKNSS
ERENLKYRLPCTPETKNFPLADPFFLCGLTTEDPGWAADGYGGWAPHDRQNCEAVQ*
>TCRU_3752 retrotransposon hot spot protein, RHS3, putative
MAAGSYLLYQLLQYDVERLQVVFHCFGGGDAYVSDKTTRAVKRCSDEDMCVSELRSLRGH
GRNVYIIYGVAKEGTPPPRHFAPTSGWGMIAVPFLKVANYDEWAKQLQAARVIVNFLDEM
DVRAMCA*
>TCRU_3753 mitochondrial glycoprotein-like protein
MQRSMRGLWLLRPSVPTSSVSRLPPLLLLLQKQQHARRFMSDGGLNGVDASSGEMAVARR
ALPPSDFPERSFLKCIEKEMQDEALRLDKEEGPPPPPSGWEMYHAPGTSLFYGRRWWVPP
TPVTPTGQRVAPRVLERHTIRVQLTTRDPSLDPECDVRGEHFPFSLFVQRVADGAGTSAA
TVHGSSSSPPVNHAERAHEVLGEEEERGLYEQSIEVRADFVEGELVVDHVVFHGTFKPSA
AFKSRTNTHSNATETQETEGNKNESSRVSTESEVHYNNMFGGYPGPNLDEAEEEVLDGIQ
AWLAERCIDDQFGEFVGQYSVWVEQMEYERWLRQLRDFVTA*
>TCRU_3754 hypothetical protein
MSDARLRGGPFCSAVLDHIAATETATSELEEYLKGLRARHGVIEQELKELAEELLKLGFR
PSIAALQERSQQLQAAFHKVDELKETMDQINELILIVFAAVKAIDSAPDVMGKASSFLAS
FGLALKSERANAGLWSRVPSYAMLKSRTPKEFLKGVRTVFAPLCPNDGSRGDCNRL*
>TCRU_3755 hypothetical protein, conserved
MHESQNKEGAPQPPPPPPTIPSGGPAPSSTAGEGGPLPDQENLQKLNKAKKENTPSRSKT
DSEAPEPPPGDATQGQHSHDTDTKDSTKNAATGIPAETSSTSSTSASGSGDHVQNKADED
DAQSSEGQHESLETGNTNVVPPLSETASKTAKTTTDAQTNGTMTKAGDCDDSTAVSHTTS
PLLLFLLVVVCAAAAAVVAA*
>TCRU_3756 hypothetical protein
MSTLTTGEYTPCPSPPQFSTPVLPSTSVFSSLNTWVLVDPSHIESPRTFFDESFSAIVPP
AVSTANQTIRSSSAGSNLTCGVHHQEDFSSGWSILSTKQSQLQSGNDLCDLVASEHSFSN
NSLINRKNMDGFQSGESNDEIDQFGLYNSMPFASREYPCGKNLRRRTTLPFRKCSGFYCP
TSISCLAFHFTYLSEMGNGGATYTLFRNVVHPMGAWHWREKIPSESSDGPSPEMSVGTMP
VHNHAKGWGGNSKNEPSFSHHSDDATGENSSNRSNDSSGEETSVVSFFLCMPFLRFVLKT
IAMMASSNENVTRRSGHRRRRRRRRGGSGRTGEGSDSRPCLGERIFGEIEALVTCALHGI
LVFML*
>TCRU_3757 hypothetical protein, conserved
MAGSQVEGGMSKGEQLIGNLLVDALDDRFRQERLNVEFAFVFDTTVDTVLDDLVEALAGM
GLRLAIPRGQCALLYDNSCNNASNGSATHAGESLHKGQEVDGVGGAELHVEGEEEDDSRR
MLSGADVIFNAPEVEAFYNLNYRCGLHRVATFRIVKRVRAPLPLRGVSRGTSGKENSDGS
GDQAATEGLSVYLLHRKLQFDDLSNELLAPTNIEGNNEAHGDYKKDLLRVREMVVAAERS
ILTARQQHYQQQPPREPHWSVQLSKPPSRHEWLCAVEAEATAYVRLTAKLARDTTSVVTR
QETARRTLENEVAEFHQRIDEVKMNVVRREALQERVDRLKIEIEAQREKEQELLRSLERA
KISEVKRTPPVRPVASSFFGSYAEQHPQRTPGVPPASEAGALRTHRDVPLSSRLLSPPRP
TPPLTPPPPSSLPHEFVSNYSAIQGSNTGIGAATGSQFFSPSLREDVRQALAPYMRSSIN

AQPYQSDLQRLRQLAVELSHHLRSPNPDPVERRRLMGEIRSLRQEVERQREEESRVTPNP
*
>TCRU_3758 UTP--glucose-1-phosphate uridylyltransferase, putative
MKMVPDGGEPTCEELDALRERLSSPELDQRHLFNGWPASAEEYTEEQRRLMLELFRFRDH
YSGGVEQYVRNAQRLFKGLKNTRHEYAALELPPYVYEAPSLFDRSEELMNLEREGLGYVK
KSVFVLVAGGLGERLGYSGIKIGLPVETATNRCYLEHYLRWIKHIAGPNAPFVIMTSDNT
HERTEKLLRGLGLNMTNVHLLKQETVFCFNDITAHLAIENGKLLRKPHGHGDVHSLLYRS
VDRSSGKRLVELWQSQGYSYIVFLQDTNATATLTIPVSLAISAKHRLAMNFTCIPRQPKE
AIGLLCKVRMFGSNIERTINVEYDIFESLAASLTELGGDRAAPGSIYSYFPGSINTLILN
MDDYIPLLTEFCGVVPEFINPKYTDDSKTTFKPCRIESLMQDIALLFGPEEHRVGALRFS
RFTYQPVKNGLQDGIKKFAQGLAAYCAATGEEGFYEAIRLRLQAAGLNLPTRPKDAYDVD
FGAGLKVRLFPIIVADAMAMGVSVEDITQRLLPHPEHVKVSARSVLLVEGCVRIESLDLD
GALRLVGPTDENAAPLVINAMTVKNAGWVVRPLSADESADEIYRIRGYVIEEKEMQAVHH
AKL*
>TCRU_3759 XRN 5'-3' exonuclease N-terminus, putative
MGVPKFASWLTKKYPEMVLDVIPADVHGLYIDLNGLIHPCCHSERDPSVAARPEEEKLQC
ICFEVEVLLATVRPHDILYIATDGVAPRAKMNQQRARRYMSRAKIVSSGTEVVEEVVREF
TPNEMAEVDDDLDDIRQLLMQDALYGGVNFQDDTSETLNGVANIADSLTPVTPCHDKSNN
HEAHAEAWAAFDGLTDGTLEVTEFDSNCISPGTAFMVKVTNAILTMVQDKLANGDPLWAG
LRVVFSGANTPGEGEHKIVDFLRTQSSYPGFNGKGAHVIAGLDADLIFLSLSLHIPHILL
LRDRDRNPYAVRMHDRHREKIQVQPHKLHTESEKNTPDLARELEERTEGNEIHVDADNEN
SKNSSSEGSGEGKPPGVLEANTNYEYYDIDTLGSCIVSELHGLCQLKNFTPNNKKAFSPE
LLVSSNGYHFYRHAGCADGSLRSVSKGDDKSHHDKESWRKFLPCASQANSKVIDDFIVMG
MLMGNDFLPRLPGAFCGESVMDNLLELYVSNVLPYGFLTSGYHEIYLPQLQRLLRSYAKI
ETVKFRRFALSNKKMTPKEAAEPLYSPLDEQWRQTYLTSTGLTGNLEEACKKYVEGLRFV
WRYYSGNASTCSWSWHYPFHHAPFALDIADYLQRCDLTNLPPPPLETTPPDMFLQLLCIL
PPTSHALLPAVLGDVMLNPPEELRETFPSMWRVDYTAAYGKDYLAEVLLPFANVSGLREV
VESSRPYFTKEEQERGRQINYHLVLGSRTSGFEINGNICELNANDAAIWDLQREGLRYMQ
LWDSPPERGRPKTYSCTTLVPWVTCDARGRVHKRERLSRSERRKHRDDVLSDASVTLINK
GPSFAAYLFGFTASFLLMAMTLFPWLSLSILQVLAVNLTAVAFSFFIGIAVHDPIVGSGV
QRNNIRFTFVDWLCAGCLSLNFSCNDKCFCCRLPFDSKRCLAVFSGTQSVTPPLYDPNHA
AYMETACLEIPGRKPTDAS*
>TCRU_3760 WW domain containing protein, putative
MAVIPKAGQVFHGEDGTFSIVLPDTCDPNNEPTQEELLDYANWLGIDAEKEPHLLWIARQ
GLRTPLPAEWKACRTGEGDVYYFNFLTGENSWEHPMDDVFKRKVEEERAKLVDGEGGSSK
TRKKSKSSSTAGGKGDRGVEKRRLEKGAAGYVASPERSGKGGKLSAVMSPTSSTSTSPMT
KGLPATALRFGRLGALGTPEATQRLTPDVSGLLKPSNSGDRSFTRFGFGGANASTSSLGL
AAGGISGGIGSGKLSLTKPPDGVKEMEAQIRRRIDEEMDTRLRAMRTRHEKRIMEERTAM
EKELQKVKEDGECAVAAAQQRHAEGVKQRAQAELESLRRALEKEHKETKGRVESLRQTLQ
RERVSMESTLQERLEEVRKKLQLSHSEGLIRQREASAARLNKEKARLLEDLARRLDAEAN
ELEKKTQTTVENFKKTLEAERAKLKEELNEKGMEHGDRSASSAVAEAEAQRAYEAAVQKA
TSDAASAMEALRREYRLKEEKLRAAKEEENCQEKESSTAMTSFGDANKNDGLKRREAEAE
AERLERALKQKIAQYEAETQRLVATRQAAMMKNIGPLRDNAESGDQGCSNKTSSKNAKVG
HFATAAVQEQRRFEVASRRLETKHAQSMDRIRREHEMILREKNLFNPRQSSGYVEKIQEE
HKSWLKAHPPIALTMPQLEPVPAVPGLMDVSPVPMPGQEEQQKSVDIAVTGAKKKRAVED
SKKLAEMEAALSREMKEAVAAYHEKRLREIEAQLTQYRVAQAEEYQRRSYQMAADIAEAV
SPQSSQLRGKAEDPVPRLNEVSLEEAKVREEEIRRHLQGRIDALEELKWHARDELRQRQE
EAQRKTLTLTPATTALQRPPPVPTDTFFPGCQEQKISPRPCQQQQQPHGLLTPVLNRTLS
TIAPANSLWSSTRPSPTKPYHWLSADNAASSLFWQENHAPSAGAGNVEKVLPSSLSQRDV
VLEDRLHRASLLLKKKKEELRAQQARMERARDAWRRDMEECRRKRDRKHALMLRELKEVL
EEKAVQLNHEVMELKRATQWLRKRTVRYKQTLNIQTSNLASAEETSTADHTAANGSGRII
SLLKDILANTELLGSYVASTKKTDAKTLRHGRCRSPASPPNSRTYPHPSRHGDALSSWKN
AGPSGVTRWLMEQ*
>TCRU_3761 membrane transporter protein, putative

MPSLNNSFVGGAAQQVDIPQSISTREIFKRIIVITIPLSFAQLAQFSINVSVIAVVGSLG
VNELGGASIAYGLINATAFGFASGFCGALETVLSHTYGRDPMSKLYGIYTQRMFLLLMIV
GLFLSPIILFADRVLLLLGQNPDVIVFTGQYCRISVWGIFPTMVLELLRRYFACQHLSTP
LSVNLVVGAVLFPFLLIACVHTMGFAGAPTGWCLLMTLMSGSLLLYLIVTRKYKDTWGGW
DDAAYRNWGPLLSLAIPSMGMMFSEWVSLEINNIFSGFGTREELAAFGITFQLSGICWAS
ASGTFIAASVLVGNAIGEGKPLLARRMAFCCLFMTVTIALLSLIVVSLAKNLIPRIFTDD
KKVISIVHSLLKYFFVYHVFDAFQSGVMGVLRGCGKQKLGAYVILLVYSIVGVPLGIILF
LKTGFGIGALWFGPAFGVMVAGFPVYMYILLYHIKWGELKAHTDSQELSGVIVEENVDEV
LVCSNDSSEKSENGEEGNSKNDDKKTILNGKTSQNNTPTLTRSPSPQPIGRQKRYQVETN
VAREGTE*
>TCRU_3762 hypothetical protein, conserved
MWSGLKLERLIYTFPETTTPTGWSQPDPHLSSLLGEADTNEGDSSAVSGEDVNVEHRHFN
NGERRRKLAVRPFTVPASAMAEEGGEEGSHVHLGFSFPGEGKFDEEPEAVLWRPQGNCQW
LNEESDCSQRSAMSCPARPTKTTIQLGDPGAPIKKNRERKYICNDADENSNSRVGPAMYH
QLEFFQESNSVSFPCESSALSHITPLDETGESVDLANHMSHLTVAVSPIQKRLLFTGVLD
DAVDAPSGRDVGDDKGVGSGLQSLRREKRFLEEMDTNRDAANSNSMACTMPREPHRKFRR
IDAGENDRKSLDNHFHSRR*
>TCRU_3763 Concanavalin A-like lectin/glucanases superfamily/Protein of unknown function (DUF3676)/Trans-sialidase of Trypanosoma hydrophobic C-terminal, putative
MCNGRVPTDRLVGFLSGNSTGTEWRDEYLGVNATVTNGEPRVRNGLTFKGPGAWAEWPVG
DMGQTVPYYFANTEFTLVATVSIHEVPKEGSIHLIGVRMNDTKSTVLFGLSYTHDRKWLA
ISEGAGDVEDFHEWEPNRTYQVVLRMDDKEWSVFVDGEEIHNKKYNTSLFDSHRISHFYI
GGDSKDRSATDGHMTVTNVMLYNEELFGNDKLYKLKTRKVTIPSLGVEEQPTEQVASKDV
SFASESWSEESAIYEELNEDDTDEQEEEIVDDLVHAASSSTVVAGSSVSEPAIAAESAEN
FRQEDNAQLSEGKTAQQETPNEDYKSMQRDSDVQPQDPQSEELTGVTDLERSSESNDEEM
PEEGGKADYRSGGSTSSVGASSDMDTVAAPADGEHQVQQSVELATENNDVRSTGTGTTGA
GQSLILEAGERNSERTMSSDRSLTPSKSNAETTSAEDTDNISRTGGAEFPVENGEEVPQT
VETAPGNTNTTPGETEIPSESNTTTPSDTDILLEHGKFGDLLARGEFAESTVHGCVSWVL
LLLLGLWGTAALC*
>TCRU_3764 DNA repair protein RAD51
MNTRSKRGKRKGVEEVEVHEIANTSPDPVAAPEQQQQQQEDQQNVDGANNGGFRVIQVLE
SYGIASADIKKLMESGFYTVESVAYAPKKNILAVKGISETKADKIMAECAKLVPMGFTSA
VVYHEARKEIIMVTTGSREVDKLLGGGIETGGITELFGEFRTGKTQLCHTLCVTCQLPIS
QGGAEGMALYIDTEGTFRPERLVAVAERYKLDPQDVLSNVACARAFNTDHQQQLLLQASA
MMAENRFAIIIVDSATALYRTDYSGRNELAARQMHLGKFLRSLHNLAEEYGVAVVVTNQV
VANVDGSAQMFQADAKKPIGGHIMAHASTTRLSLRKGRGEQRIMKVYDSPCLAEAEAIFG
IYEDGVGDARD*
>TCRU_3765 plectin, putative
MDERLRKLRLERGQALLESCRMRDFDRSPEETARLKQRGRVLYEERLRAEEKGSPCVEMR
YHSDAVIDLCSIPVKEGQAATEKTHGLSYLPPQSVELLSESRRDGDNISTSHDCTTAIID
YSILDSVREQHIRDVRQIEILTKESHLLQETIEKQQEALTKLAAELAQRDAQIQCNLKND
ARKLLPPDEKETGVRYDGGSHHMQEMVPAMRPRAQDNRRNPHLETSEDLHDAESLLARKQ
VEIQELQLKLQEKILYCNKLGEENKNLNKNNQQLQEDVVALLKKVCMIQEELINTTEEGN
LSQKTVIYLEHKIKQMQSEYKPHTPVPESTHMEEELERLARQCADAEATMHRMEAEMAAE
NAKLKEELCRMQQSHEEEIEAALLEASDRIAEHDDLLRHKLEEQEAELEARFLEQLNSFK
EPGGDSDALHSLLSLKEENAKLKEELCRMQQSHEEERQAAGAAMHRMEAEMRSQQQTQLA
GPPRRAAASSTNPFASMGALSKEPGSFPDQGDDGDDVFGVGTEQHPTATGDAVSVDRQAE
PEGLVTELRGRLAELEAALSAPHWPGIALWERWSVLHGSAPMRRRPCTGWRPRWRQRMPS
*
>TCRU_3766 plectin, putative
MQQSHEEERQAAGAAMHRMEAEMRSQQQTQLAGPPRRAAASSTNPFASMGALSKEPGSFP
DQGDDGDDVFGVGTEQHPTATGDAVSVDRQAEPEGLVTELRGRLAELEAALSSATLARDS
AVGEVERLARQCADAEATMHRMEAEMAAENAKLKEELCRMQQSHEEEIEAALLEASDRIA

EHDDLLRHKLEEQEAELEARFLEQLNSFKEPGGDSDALHSLLSLKEENAKLKEELCRMQQ
SHEEERQAAGAAMHRMEAEMRSQQQTQLAGPPRRAAASSTNPFASMGALSKEPGSFPDQG
DDGDDVFGVGTEQHPTATGDAVSVDRQAGPEGLVTELRGRLAELEAALSSATLARDSAVG
EVERLARQCADAEATMHRMEAEMAAENAKLKEELCRMQQSHEEERQAAGAAMHRMEAEMR
SQQQTQLAGPPRRAAASSTNPFASMGALSKEPGSFPDQGDDGDDVFGVGTEQHPTATGDA
VSVDRQAGPEGLVTELRGRLAELEAALSSATLARDSAVGEVERLARQCADAEATMHRMEA
EMAAENAKLKEELCRMQQSHEEERQAAGAAMHRMEAEMRSQQQTQLAGPPRRAAASSTNP
FASMGALSKEPGSFPDQGDDGDDVFGVGTEQHPTATGDAVSVDRQAEPEGLVTELRGRLA
ELEAALSSATLARDSAVGEVERLARQCADAEATMHRMEAEMAAENAKLKEELCRMQQSHE
EERQAAGAAMHRMEAEMRSQQQTQLAGPPRRAAASSTNPFASMGALSKEPGSFPDQGDDG
DDVFGVGTEQHPTATGDAVSVDRQAEPEGLVTELRGRLAELEAALSSATLARDSAVGEVE
RLARQCADAEATMHRMEAEMAAENAKLKEELCRMQQSHEEEIEAALLEASDRIAEHDDLL
RHKLEEQEEYYEKILADKTRLESLSVDNSVLGDALFWKEEHDKLYERFEQLQTELRSYLR
DTTVHQDVEAVVRQGAPAEEGKCVDGPLQAALNEVDLLKGQISVLQRTREQEEEKVAMQE
RDLQELRERLRAEEKSRCEFSQMYEDAAALLRDGATSVEMLLQEKRARIEELESQTEKMS
QQVLSTEEQLRFVRVELEQRQQEVDEGTERYFRLEAVHREMGAELREVKRLLEERQRTLS
QRLERVQNDMEKQMHDASEEIRRVCCEHEQQENGLRREIDRLRAELQAAQTLAATMPFTA
QTNNHQLVHLRQKLDEALSEKMQVVAELEAMQQLLRKKESLSRGRQQGAEDGTEAGDSGQ
SLDARINSLLQRSESLEVRVQQLTTEKETLLQEKDALELEVQCARRMVEVRELACQRQTD
EVKQSRTQISAMKDDLSFRIQSCNALRQELNEAQEQAAELARAYEDLQLVTFAERRNLLL
REYMEAILLAKAMTVPRVMQDVLHGIMQAHRQLLRRCSENQRNLLARCDAIEQTATEAMV
EGERQSNELLSIIEDAKVEQQQLQEIIERLEMELAAANCVARESTEEAAAVKKISTEATK
DAREEASKAKSDLQALQLEYNSAVNTISLLETEKANAARILARANSRLEELAAQQQEEVA
RLQESLIEKTCLLRDAQEVAKNKLEVVEQQAEEYKAQRDDATKRAQAAQASLDRVLPRLE
RLEAEQAAREAELMDTTQQLTALSQRSTSTEVVSRRQVEALNASIAELRSAHAALSEECT
KAHEHITELKNQLTASEAEIGRLRTRKAQQEQEAEAAKARLTELESLYANDTSDAATWRR
GAERRSAALESRNSALQEELQASQKQQCVLQEHVDALEAKLRQMSDENHSKDQQQRRFIQ
QLKERCEILEGEIGKLDEAHANLSAERDTLHRDWLNAKQDVGSLENHLEATNQHNEQLHK
ELRRQKESHDAELQMVRRTLADAQEEISRCRCTLVEAEARENELNHVVYALKKEKARCVE
ELQQMRGLAESEQELLVRERSQRQEQVAELQEEVVQLQKDLHAVRQECKGLECKCTQHAD
EAELLRRQLTDRTDRCRTLQQDAAAQHEQLTATVKSLRAEVATLQSKLEEALDTLAQKQR
EYEQCEERSLRKMEVSRLSEEILRKEVADLQRDVQQLQQKLAFTATAAHDAEKTGAQQQA
TISKLTEELERQNTQSTAYLKEREQLQSALRENTSQLERVARKATADLETALAKTNELHR
SNVEKLETALRREQQTAQEVRTARDRALESLEERQGECEFLRAEVARLQQMLRSAQAQVA
STQAEVRSTVTQVVVEAGLPQEGELSLSSVVNQLLLRLNRLSHAAVLLDVEVSQFKCFEK
ACEAHAAALRCVIDATRSSQQVPASSTAPTSPLLPMCESSSGSSTHSLRQRSFSSRSRVL
QDIISSVSTFAHRLMDHCHTIRGMLDNMEAAVVQCETKFVGDPAVTHLKLVVVATLSELQ
LRTSRQTAFLSQIFDGAVMSTALQEAIRFLRDDETYVLRPFNELLMPSSSSPLSTPRWGG
GVQESTFFTKTRVPAADTASMSQHLWRGSAKKENELQSRIVVDPRQQMESARMPGEEPRG
PSIPATSGAEASVASVTTAVDASTGPESSSANWCVNHEMQDAA*
>TCRU_3767 hypothetical protein, conserved
MKSSDIFHAYRYTPVFLKARQHDSGVNQYGLKPVNAYDFINPTNLVNFGRGTSFDNLGVR
RAGRGEIDSSPSLGGSPVFTQAKLVGLSGEEQLTMCQSETMALRVCMARGGQDTCERESR
ALDACLSRVGHLRRAMSEACGEFNDWFIQNVSDNHTKPFQHRPHDWRHFYAQEKLVRERQ
QNGHAYGRRPKQFSFGARYVKTEGYGKRPRLPYNK*
>TCRU_3768 hypothetical protein, conserved
MGNPKDASSLAHILYEQCKGDATSLNRLLRQRVSPRGNRFERDTPDTFLYIERSKNRNIV
AYAARLLDAVTQKSVSSGAGRSCIMDHSNPVHAYFIVLESAKFKENQENVVMSEIKELNF
IERRLAYGCSAKPICRTMSDSMKEGIGPWLKHFDTHALTYVALPGHPLLLLLLPPLGGAT
ENSAEAEGTGQTNSGGGNEQEATDTLAVLLGFVGGVLSVLQRVYVCSVEPKHFYQLPTVK
YIEFFGVSLESGLDTYEKRKN*
>TCRU_3769 metallo-peptidase, Clan MG, Family M24
MPPKGGGTNGKHQHQQQQQQQQRQGGKKGSKKSDDDEFDALLAKAVKETQAALPKAENGH
HQKKQNSGAGKQQQKKTSATEEVRDDGTGVKVVSTADHVDNPYPQVAPGMKRQTWPEPTV

PVCMQFTSGNFPPGEICEHPGEVNAYRHSSEEKRAMERATEQEVQELRHAAEVHRQVRRY
AQSFIKPGISLISMTDRIERKVEELIMKDGLTRGQAFPTGCSLNHVAAHYTPNTGDKTVL
TYDDVMKVDFGTQINGRIVDCAWTVAFNDEYAPLLEAVKSATYEGIKQAGIDVRLCDIGE
AIQEVMESYEVEIKGKVYPVKSIRNLCGHNIGPYVIHSGKSVPIVRGGEAIKMEEGELFA
IETFGSTGRGVVQEDMECSHYMMVPGGDKTQLRSEKAQHLLKHINKTYGTLAFARKWLDR
DGYDRHLLNLNQLVEAGAVNRYPPLCDVKGCYTAQFEHTILLKPTAKEILSKGDDY*
>TCRU_3770 hypoxanthine-guanine phosphoribosyltransferase
MFIRLFAREEEGEKEGKICHMPREYEFAEKILFTEEEIRTRIKEVAKRIADDYKGKGLRP
YVNPLVLISVLKGSFMFTADLCRALCDFNVPVRMEFICVSSYGEGLTSSGQVRMLLDTRH
SIEGHHVLIVEDIVDTALTLNYLYHMYFTRRPASLKTVVLLDKREGRRVPFSADYVVANI
PNAFVIGYGLDYDDTYRELRDIVVLRPEVYAEREAARQKKQRAIGGADTDRDAKREFHSK
Y*
>TCRU_3771 hypoxanthine-guanine phosphoribosyltransferase, putative
MISSQQAMVHVVSRNADGVIVVDGKAYPMAEELVATESVIQRSIKAVAKQIADFYRPLSH
RDTHGGGGVAPISDENPLIIISVLKGSYIFTADMVRYLGDYGLPHVVDFLRVASYRGTSS
TNKMKLLAETQFKALRGKHVLILEDIVDSGKTLRYILDKVQREHQPATLKVCVLADKPGG
RRVTMQPDFVCLTVPNKYVIGYGFEVNDRFRCFRHIFTLRPGEARRYPAHL*
>TCRU_3772 serine/threonine-protein kinase, putative
MSVGGGSLIRSETFSSRSFGSLREAGNVNVSLERCASACSDSKNNHADDSGRVISQWDRF
PRSHQIGTGSFGNVFLCHDMLPGSPWYRQPVAVKAVPLDALTDSGVMLAMNEVAILRHLR
HPNILHYIDSFLDEEKQMCLVSEYAEGGDLTFLLRRSWELSGGSNNKGSCHSHQWEEHER
EDMREGSQSANLASGSKNILTTRRAVSVLDDTQHGQARPYNWIESYRITDIVRQCLEALS
YLHQCYIIHRDVKAANVYMTKNGSVKIGDFGASKLVNLTDPLANTFIGTPFYLCPELCLG
EPYSFGADVWALGVLTYEMYCLKLPFVADNVLAQIHVVTEGQYDREALYRAHTFSPSDLQ
SLETKYGAAFTQQERSLHMLVVALVERMLVVDALERPSASQLLREFFFADPHSSSLGTVS
RPISRESLPRPDSSNTRESASQGLISRCLTSTTESNKETREATIEAAALIASSVSQSYGT
LLEKLPKERRNDIIQAASRSASLAGCLRRGSSALQATIDAVELGDTVCLASHEVISETVD
KIPWLRHAEAFSLLTLHEGHEDDVVQVDWIDEEHFSLLAPRKRRCLSLSCARPDGDKEVD
LSETRGLTPTAGRKMAGRGEDAEETRAVATPAIHTFHVDEKVRSPPGSVGRCQGLSTEAL
EKILRKKIMASYVHRQWRLKAMREAYAVHEEERSRLRAELNELYAKSFFEERRGMMTCGE
DMEMSSTMNMPGNLESTQSPHSIAAAAALFVTDATLGPVIGGDFTKTAGIERTLRRLTAS
PGYSTTGYRDGNTSLLTNEVVRIARDAAAVAERCKKWAVQPPARLDFSKRQVDEVDRRSA
SSVTDSERGTNMEVYDTPVTLCILPDESYITRQHKSPPPPPVSGSAVAVGSLVLPMTVTM
KPISRRTRLDGIIWRVKTTLKAHGLDHVLFFPLDIDNANEVDAEELGIRYFDCVGDIIEI
SEASDWYYVRRDWYQRKGLPWMQLYMVIQ*
>TCRU_3773 hypothetical protein, conserved
MFRRLFQTEASDASQLSVAVSLGASRPKASSRGAAAAESKVKEITVRLLRRLRSFFRTHK
NILLGTCGAVVVGYMCQWIRRVCGERRNIRSAQDNVKRPASGWGLRWFWHSPFGHMKGFS
GDTVGDSGQSPCCPSGRSIFRQTGEDDDEINFVDATESAFRMFQRRVERTHEAPLLLVLS
ELESLGRRWQSQLSDTNTALEEEHEEAGNMNAASAGIRTEETVDETLREAILRKAVEADE
LLTQYVVQLDLAPVGTSSALKKERKASIVAASALAERIAKWMSLPSRHQQP*
>TCRU_3774 hypothetical protein
MGATCAKDTADGNIAHASSISTRSRGRRRRGRRSRMFAQSESNIQRCGTSSVVAFSGPDA
PSHAREELLPEQEDVLSIPSVPFGITLTAPALENVGTNCSSAYHTKTKPIVTGATAATTG
MGESAVNDTKRGPEECFRQWAPVFREVTAEPESTAVCMNGMSDGPSPPSSRVNDKFAAAS
SGSTPKTTTTENYTECSVEGLSAVVSMKRKYSVGFSHTSALFTCISCHEEEHTDAPSPHF
YSSALDAPGDADTKSRGKKRSLLPTLISKNVAVQPDGDNNSKTETKQQKEEDEEDDEITA
KKETLIKDEMSGRALIEKEELDVREAFLCSYARERAFPQRLDQEVTPTGAVGAASASQRT
SHLGDSRSAKTSSLFTFSSIGEEFLMHAESDLLRPPVVGENFIEENSMWSSVVSSHEKGV
DETCQGSNSHQKSLQFPRWEGSGLLSPTPPPPQNNSTMVVNIMSHDDNDVNPNTHFGEQV
RVERLCGERPYYEPEDNERKTQLMGLRADHKEASRVRVLRSPSLVCLRDGYFNAAEMDGN
DWKPSCKLSLSAREFHEGLIESPSLIHSDLPGSCGAHYQRYGASLHTKSLTSLDTPVLER
TGTAQHKVLPCRRGGLIPPPPSPPPSSLSRRAEDEERESRGSSRHMWQRSYTSPRPERSD
GSVSSFGYLYSPRAEPVLSTCTVVSGAVAGMATTAIPCGVVERKSAKHFCRWCDKPYECR

EVCAVAGESHAVLRLRRKHEKEVKKRAQGLLRCGRIREAISELKAAGII*
>TCRU_3775 Costars, putative
MVELIIDTNADIDLLLRELQRLNGSLKKIDAGRGAVLLWGEIYSDSTLQDLFQERLASVM
SAAKKQRAIHFPRERLLIGNYEEVEVELLAPHLRILPESAIRSKSKQEERKAAFKEAKLR
ALEAHKEKMLRLAMQGIDLPSGKSGSPQTTAGGAAERGDENDVNPPPAPPIVGDGGDVIA
EAEGKKSDDNLLPPPPPPPPADDDDDDESESLEKEGDDDEEDPPPPPPPPEDDDDDGEES
DEDDEDDEDPPPPPPPE*
>TCRU_3776 hypothetical protein, conserved
MSFRIVAFPYSPPAGAPYADACLPAQPGERLRLVRRVDPWAIVESGGRFGLFPIVFTRDD
YQGCPRMRTAARENLVETISLSPEDKIVLEEKDSDNVLNHAAGVAPPHENKQDKAEVFVV
PGCGINSDCKNELDNSNKISSSGVEDHHPKYVDCNAAAEEEGWRTKKDAPFLKMVPRRCS
GQRVSGRRRDGDCCQYETEGRGNPHGECLADARWEKNEEKEGEEETVNCSRHVRVSVIEG
KEQLVSPPQQARPVVQDCSSDALQELRRQYMELDAEMGWLSHVTENVRAERQRVRDAHAS
DAQQRAAQELANTEEMERLIRNIAAKDAELKAARKYAAHLRSILLANNITGDFLKSLPPT
PPPLSFSLPSFLQQIQEQCNDDKENKDDAYNNSHEEEDDDDDDVSEPVEELARNPNAAAE
VNAIKECIKQEQETLQDYHEQHNQLIAHMKRLRRIQAAIEEESCALRESEEALEAVAPTG
SYEVVRDGGFISGGPLQPVGSGKASVLTQGGAGEVDVAMREGFAWPAKLPGLTAEEQDAL
SNYQRLFNKYEAKHQYWQKQHEEATETKLSSVNEKLRAAISKGDMALNELLQRTENLKEF
VTKYGPVAESIREQVAVSQRILERQREKCGDACAAYEAAAASKNRDQKR*
>TCRU_3777 hypothetical protein, conserved
MTAVHCVGGSRGYGDDDQPLHLSVVEAIRIQRELKRHGGEPIATEQGGRMFAFQYASPLQ
MLRRSTTVRNSVSSCLQAEKEKGQRRRAGKRSTFSWLSASYCRAEPCIGRRKGRGLTQVL
LAREARPPPFPSVAARLENDIALGLAASMWQSGNVVGHCNDRLAHLLVLSSTVVQWVGHE
VQNGGEPRVPLELREAFMAAIEHFVRLECVVARAMLLTSVEKELQRQQGEYAQRQDLPEG
SPSGTDVTKMSPAAFSVAVESAARVIRGLVEPATLAAAAVAPTWQYSLVRTAADVPNRYD
ILDAMAYVLQPCRHLTVRRTFCAVTRQLALEVLWGAGNEKFGTRGGAAALRILQHINTRP
FFLTVEERMAGQRFLALLERKLRSLMALLPPNALDEASQPTRELIDAVRLFCTPLV*
>TCRU_3778 ABC-2 type transporter, putative
MSTSRVAGSSYFSASSASSLSLTLGFENLTIQRDKRVVINSASGIIYGGRLTAIVNCSGT
QSDSFLLGALTGREECASGTIMMNGIPLDAPAYLHRTVLIDEGFTALEELTVRESIEYAA
SMRIATSAFTVQEILEELMLESVADAVVHRCSPYVRRRVSLGRELLLNPLVLCFDQLLEG
LRTHEAQEFMRLLRRIAAPMAVSLDLITSRSPFLLPPRPSSSPASVVSSQVLPNNAGTAV
PMTDGATLAPHSLATNSQRERMRIVLVSMSQPRWAILKFVDDVILLERETCVFCGTVTGL
FSAIRVDPTAGLAENAINSLYRLASSSEKSLVDIFAPSGNAERVHQSVVQFFHNCAVGRE
MLEGPPYSSPGAHVRLYYMFKYDLLKVRHNLLLKGVVFLAVFLLGVVLAAVYNSQEGQSG
MQNRIGIIFFLVSSTFLYSILSLDSHRREYNRFLRHRTHGYYGVFTYLTYAVLYCIMERV
FFLSVLTFAGFCVSNVVEKWNYYQFIMELVLIIGVLSFCSHFVVFFFCTVIWTRRFAMFA
LFAVYTLNLLLAGIILNLTTLPKTFQFISNVSLIRLAYESSILSQFLGRDFGCGESNETM
CYTGPQYAAFLGFKKSRMWVNVWILAGISVLLIMGSLCAMLWHRPPRDFC*
>TCRU_3779 protein phosphatase 2C, putative
MGIWQSIPVPPEVVQAAANNTSNWGVPPEDIPEDEKDVEELVLQTWAFCKARPSPAQNYR
SDVEERQRRRFVSWRKRASFIELDCGEDSFFVSNTYKVIGVADGVGGWRDEGVDPALFSN
SLMENAKLFAETHRKELNPEIILQSAFDKVLNDRKVKAGSATACVAALRKEDTGKHVIDV
ANVGDSGLLVVRNRKVLHRVHEKVHGFNAPFQLAVVPKHLRGRAFSDKVSDATREKVEVQ
KGDVVIAATDGFFDNLFNAAIASDAGWVGKVEGSVFERVPLVGFFLSAIIADEKVAYIDP
QRVAQRLVQNAYKISVDEEAHTPWASMLRTFGAADAKGGKKDDITVVLSRVTTRDELNTS
NIW*
>TCRU_3780 transcription activator, putative
MGFDNPFATFLGKRRVPRTGLKRHRIEGDVSLESTQEYRTTAEDVVADAASATASPNAAE
LSCAEKTSSVDTAFSSPVKVVLLSDERDLEEEEEEEELRLLLGGHHKVSVRPKEVAAVSS
SEGADVAAPTATTPVALRDLPWTTRSNAVLAEIAERFAVQFRWELRHNVELVRVFEPFFS
DKAVRRIEAETRGPQFLPAPVATPENTGAASGYMLRSYQLEGVQYLLNNFHRGSSCILAD
DMGLGKTAQISAFLHLLKTQHTIDGPHLIVSPLSTLTGWTRELARWAPTLRVVKYHGERK
GRASIRTTRGNRHCVFVTTPAVLNQDRGFFRKRAWVVAVIDEAHVLKGRDTIISYVARKL

TACFRLAVTGTPVHNNLGEVWSLMKFLYPSYCASYDSSGDDDDPIKAAEDCAKLLQYVML
RRTKGSIELGIPPRVDEPTCMIEPTRLQRQLLFRMTEQALNGDKANATRLQAHFTHQRIV
CCHPLALRILGVEVRASAQAGWEERLASAGIELDEASIIAPSAKMIELDRMLREFKANGH
RCLIFSNFTSILDLLQALCVLRGYGFERIDGSTSRVERELSMIRFNAPGSACFVFLLTTT
AGGVGVTLTGADTVILFDAHYNPQIDRQAADRAHRIGQTRVVRVHRLCLKNTIEERIHNV
ALRKASLGDFIVDGGSCKGDDSSAPALSAHQILLLFGNENRIINYPRCHLSHMDVVDFDH
DNSVEETMVEALLCNEVEGLPRSHATTKKIRGVRSAVVGVTHHCFVCGEIMRPLEPLYHC
AVCPKAYHADCIGERRPKAGMIGPRRWTCPRHSCVSCGKVQGFDGAVFMCTECPSSFCFD
CLDSRYFELDDTRTQFVHIRREYPGMEAEGMNSRRSIYYIACLRCCGLLSSSSSSASSSS
SETESDAVNDDAVVPEDQNDMDSMEEELVEG*
>TCRU_3781 RNA pseudouridylate synthase, putative
MHQVRGLVQRSSAATLRLLEKNVTPHDVRTCFIDTVFVDGFGAASAVQLLHPVALQQLLA
ELPKSEALVCVQEARKVVLASKNDTAPIGTWLKKLNHLLCIKDTTQRTVTELRCRQSRFL
AKTEGRLMAHDFSLVEAFTNRLLTREIPQQEEKELFLPDEVAMYRGVVEELVRLNPQAAL
ALAMNTKLPSLGSGASAALAALVNLDVPAATLSFLESFSAKDEMSAASETCATQVAVCLR
NKLWELRELSADDPKRVALRESLATACRFNVDPGVVFPTDAGAIQRWGSQFLHEEMWPME
RGRVISDIVRRKRLMPRRVSLDCLSNWCLTDLERKWVSAMLADGQGTNMIGGSDTAVYCS
ALSCVLKDPKRCLVDSRLLRQYALLLKRGELEPSHSSFPHFIEECGECHGNDERPEELSF
LMPPQSSPSQSSSLKSQTLMDALVALAPQCSYWRTLLAEKQEGVEAFVAPQGHIHVTVKL
PRVHECVEAAMQAARSSSTSSFSFASCLPVIFEDNDILVLNKPPHLASSRHALSCTQMND
KRATDVVSLILASTKNGETMRGVFRQGQVHRLDAETSGCLVMAKTDCAADSLRHQMGTSA
AYSQCNKVYLALCAVMEPDLRRIPLRGVLRDPMDPKISTKYRVIRFFRKARVALLECRIQ
QGKKHQIRRHLAAAGLPILQDVEHGGAACTTPLIDRVALHAAALTLVHPRTAEVVTFVAP
LVMDFAETIRQLQ*
>TCRU_3782 TFIIF-stimulated CTD phosphatase, putative
MSQDAASFFRGVSAKISAANQSYISRRARRRVEEEEEDEPQQPIASPSLSTLLSTTQNSS
APCVATSVVGRVSKTAMTTNVAGNTFAIGRSSSKIVSSVVKDEHGDGSGGDFSELSMQSE
QISSTLMTQVRQIPRSSRRESLSLTGNIFLHTGREPATGSNGASSGGDGSVSDKNENGAN
NNSNNINNNGNNNNTGSSSIVSGGGNSGDTEVRPKVHLSALLIRTRRIQQMPKNTSPIIA
KNHASLLPLQMRQYHGKKTLILDLDETLVHSSLTLQPKQHDLILSMKTEPEVTTIYVAYR
PFLHEFIQAVAGLFEVVIFTASVSMYCNPVMDAVDPEGILGSLRLYREHCSILNGAYVKD
LSLLGRELSQVAIVDNSPVTYLFQQRNAIPIPSWFDDPKDNELKRLIPVLEALAQAAEVY
DVLDRYNAVLQLQQEQMRPENKFARR*
>TCRU_3783 hypothetical protein, conserved
MTSVTAALLRQQDDECSTAVLHLLCDNDAFKRLAASEGEAILRRDKEDEEKLAVGIGIAK
AKGILSGNPDVGSVTRISVAGMTAQQVTEEIMKYLPSKKGNAIVMQGLSGTGKGTTVNKL
QKLLPRCVTWSNGNVFRSYTYLSLEALKGEPIRTELLTPELLAAVEKQVTFEQVENGGFD
IVLKGSIRVADIQNTLLKRPEIEHAVPTVAQQTQGEVIRFATMAVGKLKDAGYNVILEGR
AQTLNHIPTPFRFELVIDNITLLGERRAAQRVMAKAFSDIKNHLDGATNKMVEEAIFRAL
SEL*
>TCRU_3784 hypothetical protein, conserved
MSIFGKKTWRVQDIIRTDGAQEIVSILKITHPFRRQRIVVVPAPRFAQESYYNDWVYQPY
AKEHRMYVSNDIFNPTYVYLARILIRRGVFPGYAYFHPMGFPDCIDLNLTRREFIAREQP
LKTPTPLILLTPNMFRYKRHPWIPRRVINIVGEQYVTHPREEHQSMLFVLPPEYIPDAVN
TLQSLGFQVTDHTTAVAGEAKTLKKLHHWSDIAQLVVLGYLWFMVALFFLNESQRMRRMF
HEYKREMVEKAGKDPDEMGL*
>TCRU_3785 HpcH/HpaI aldolase/citrate lyase family, putative
MSAGGAEFKAELRAGKPKFGVFLNSASHLLAGQFSHSGYDWLLIDMQHSPVDALTLSQLI
AAIRTGHAKVMVRVGSTEDRPGIQNALDSGADGVLIPYVKTAAELSAAVSCCFYPTVGTR
SVYAPQQCMNAKGLLGYAGEANKNVVVAFQVETADCITNIEEIMAVKGIDIAFLGQNDLC
MSMGLYEKYVFPDMYTSTELNDATEKLITAARKNNVILGLFLFGTDRVGEFLQKGFTFIS
IGSELHHALTQAATHVKVLEQIANVNGKPWKHQPSALV*
>TCRU_3786 retrotransposon hot spot protein, RHS3, putative
MPGNQASAVPQGDVQRRARPESEDVTDQPAATHIRVEEARQPQWTMRSSVKDILLEGSTL

STNMRLNDFLRNYVGGRVAVGEDSNVTMQVFVQEPDAYVQDQQLLRRIFNLPEYQELEEK
KILLEAIYKLRHGGVDFLEQWRDYEGKDTVTPLARRKLNAVLTQLLREERREAGERARRE
KQVELTLITTIRDVLFKGRVRFMDIKLNDFLTLEMEGRGVLHANRNVLLRDFFSDPTRYI
PDAGVLGEIQATDAYARMEGDVREEMDMGEDVRKLKQAGVYNLQKWSEATAQVKESVHEI
TKSFLDAALQEANKPTMTSAPMKLEGCYESVYNSRWHHVVEVSGGEGMGMEVREGEPEQS
LTYREFDESLERDDAVQQSGAPRLRLMVLTSDKGWPYSWAGNKLIHDCYVNFEVDRVWQI
VNSDLTELLSSHPGAYFEPKRRVLIGTPGIGKSMGAGSYLLYQLLHCDAEKLPVVVYVIG
RQAFLFDKTTHTMIRYMELSAMEHFLAFVSSRGVKGYIIYDVAKGGRNPSVFFVPSEWGM
LVVTSPNVDNFEEWRKHKGAAPIIINCPEEMDVKAMCFWMKQIQPTGEQAEEQAREQAKY
WETVEERMDEVGPIPRCIFDVLEYGIHLNAIDATVKDINASNATDYMGVGGDKIWIDEYV
SQTIVKFVRVRAVPGIEVGYNAPVSHSARVKITHHLTNMTPPVDFFNLLLRGYGYFLWVV
FEFAGTAAFMNPHAVDIIQRKLTELQPEGRSRSRFSVLSNKPRGHPTRSKTLKKLSDNPA
GMDLDYGVLYLPAVGNFPLVDALFFMESPRKTLVGLQTTTANAHHTQTSTVRLFKERMAS
YFNGWEEFARDLETFRWWMPFSSWSHRGRHWLGCRRLRQMHTTPKPAL*
>TCRU_3787 protein kinase, putative
MEHIRKTNKKLGVGARGVVYEGYDEERGRFVAVKEIPFFDDGGVGTGGGDDAAEEDPTLV
EERRAIVREITLMERFEHPNLVVYYGARRSAVGVQIIMELINGGSLDAIIRRQGSLRESV
VRSYTHDILEGLAYLHDTARVCHRDVKPANVLITVDGRCKLTDFGVSKLLDDNVSMRTTV
GTPWYMAPEVVNGNADEDVEGVEDTKRYDGEGSCKKRVHKNSYTTAADIWSLGVTVFEMI
SGRKPFGGEMKNPAAVLFAIASSDGTPPRLPENCEASVSLRAFLDLCFVRDARLRPKARE
LLSHQWFMETSGQTCHGLLGNNMRKNSSSSNNNNNNNCSKVPGVVMPPPGSNQTSLPAPL
NMLDTADIMDAESLLSPKKCSPKSQRSEVKRRIPGEIGPAFASRALDPSAVRDKNRDGGF
FSSDGHYVDLPISLPLVLSPLRKREK*
>TCRU_3788 hypothetical protein, conserved
MAMMTGRVLLVCALCVLSSVAADGAAGVVSGGEDNSLKELFIPVARLKARQEQTLKESAQ
TATEAAEKAAAKATAASEAAETARVAAAEAALKTAAAAEAAAEAKTLAETAAEAAEKAAA
KATAENAAEKAKAAAEAAETARVAASEAAVQTATAAEASAKAEEAATKAKTLAETAETAT
EAAEKTAAKATAASEAAETARVAAAEAEAKTATQKAATAAAKAETEAEKAATAEKEAATK
AKAAEEATAAKDEAEAAAAEAKTAAKEAAKKAEAATEEAKTAKTAAAEAATKAAEAAAKA
KTLAEAAETAATEAATKAKAAAESAETAKAEAKAAEEEAQAKAKEAAAKAAAESLRGTTV
GEEEAKTATHDQDNSVEHHSGKKQELLKEEEPELHEKEQHEKQQSQQREHSAGNGEESAK
DKTANGTNATAIKDDSDGSTALSLTTSPLLLLLVVVACAAAVVAA*
>TCRU_3789 retrotransposon hot spot protein, RHS3, putative
MAAGSYLLYQLLHCDAKKLQVVFHCFGGGDAYVSDKTTRAVTRCSDEDMCVSELRSLRRH
GRNVYIIYGVAKEGTPPPGHFAPTSGWGMIAVSFPKVANYDEWAKQLQAARVIVNCPDEV
DVKAMCAWMTRDETKGKQAECWKGVKKHIYLL*
>TCRU_3790 Protein of unknown function (DUF3720), putative
MMTGRVLLVCALCVLWCGAVFGHAMEDYCGEGGGNGLRHTSNGGDDGVSLKADCGLLFTR
MVLIKAVEAAQAGEEGLSDTLPPGNSKDKALEGNTNGSEAVGSGDVGVAGQAAAPAAPTL
GGSGTEEQERGSNVSGTSDGNVAGAKKDHEGANAGDSKHQRAEQPSSSSSGSSDTEGVKQ
PVVNENSKSRETSDSPPQEEEEEEEEDLRTKPDSREEEAEEELKNDKMGHEAPKETPGGL
TKHTEIQTATTPLNGQSSPEALEDVKQSEEVQDERDSNHNSQTKSVASIAANQQKEPSAD
HGESGPPSPTANGDAANNDSDKSTEGGISNSDPASDGAGTAEGKQNENKDANPKETPVTA
TAMKTTTATTGDSDGSTAVSHTTSPLLLLLVSACAAAAAVVAA*
>TCRU_3791 glycerol-3-phosphate dehydrogenase (FAD-dependent),
mitochondrial
MVSAARKCLGAATAAVAGFIGFSYTNPSWTRRRFDSRKTLPPLCTENPSREECLTRFKAF
DSPETPMDVLIVGGGSVGVGSALDAVTRGLSVGLVEMNDYASGTSSRSTKLIHGGIRYLE
KAVFHLDVQQLKLVAEALRERTIMIHQAPHLCHEIPTMIPCYDPIDFIMFWCGAKLYDLI
AMWERGTLKYSGFLTPYDTMKKFPQIRYANKKGELLYGSVQYYDGQMDDSRLCLSAAMTA
ASHGAATANYAKVEKMEIVQNTAGEKVVKSVVNDRVKQHKFTVYSRSVLNAGGPFSEEVQ
KLMEDKNTLSIVPSSGTHIVIEPKYCPKEGATVFPSTDGRVVFGVSWLGGCIVGTTDKKC
EVTEDVRPSEADVRFLLDSMEDYVGKVPREAVLSAWSGIRPLATLNGGSENSTQNMVREH
MIAVDKDRLILSVTGGKWTTYRKIAQDAVDSLWDTLLKDRIPFKPCVTEEIQLIGAHNMA

SVPAAAPSDIPEDVHRHWRSQYGDRYNVLLEMVRRNPAALKKLHPDEPIVEAEVIYAAQR
EHCERVQDFIARRTRLSFLNVDHAKAIIPEVTRVMAETKRWGRSKKNEELANAFASLDSF
RAN*
>TCRU_kDa subunit, putative NADH-ubiquinone oxidoreductase complex
I, .1
MATAEEIGPGGVNIKGKPIFLAVEHTRCAPSTERELSDQELLAWKSFQLWRSTLSVERRD
NVYKSLTNSDYTNFRLAHHIMGTPFPVVDTTPGIFDVGKSVRWYEHAISVAVALAYGYWI
RSKASTRNARMMPMSRSAITLLTFFSMDLCFFYRSMYRLTGYLPNDYECRKYGVMEDKER
LERKKVLWEKYAKYKKEWCRRFDYHVYGIRPGENWSFFSACLFPAWAPMFNTCTNYPPRK
NPYFLTATPLRNIFAESPFTYEIPKDETVPLVKERPEFKYLYRGPEREGIVNGKN*
>TCRU_3793 hypothetical protein, conserved
MKSPQEDRPRSLKALCEAVITNSLGEGGKRPYHKKRKEEENEEEEVKRSLEALFSSGNPV
LGGLPAVKGLRDAVRLFVKKHAYLIKKTGGNDVAGASNEPVNLRDALLSNQEAMEKIRGE
WKEILENMYAVEGDDSTTPPGETLETDTNTPSNVLFLDAGHTGAWEELQLEEKTPCSDAR
GFLETCVREWVEEGLPIEKGKPGAFVRALREVIDTELDSIVFDDHWEAWVSSLLMCVELR
TKSSSSTSWEVLTLVELIYSRCTPQRKLLLLDFIAKEIQQPFEGVPILKDFLLQLFHRVL
LCTAENIVGFMDDEIAQLVLKGLRVIADHLASFDTMDPQARWLKHILVRPSLVRDIASLP
SREPELINKLTAAMPAPHAVGLMLIMLPLWMKEPGDEWTSTRIFDKLVDALLNKHVLDDL
VEDAMACVDRCLRGLHTEKRHEHFGKVHSFLLSQEYSEVQESVGRLLRLLLCFSIPSAYD
PGKSYRLLPTHLRSLEEHLLRRLEKWTRKNAAHKIVVGDFWWEILRWHAADLPKVVARAV
QAMCRDERHVGSPWDEGHLVAISYSVSSWRGLEEVLEDEQHAGSLNYVALLLNICAVASR
ASSRSQQERQQQQKQQQPPVSLNPTACWLLMRWCTNACARLGLFLATTEVLRSLTDDKDV
TEGGLLAIPVEEFDSITHMPHAIPAQELNQKCMLATILSVRLWIATLSPLSQTPSVAELD
VTGKLAQQLQTYWVNYDSNVSMPDKPFGEDSLLLLLCVLSMNRLTRVRKSGNHSQEYILQ
DSLKRLLAERRGHDPVWHLLQFLLTGVGSEHLAAILVHRFGTAQEVPLQIDVDTYETSVL
HDVGEKELGAMKDIIGLKRGDKERETNLLPVLQLLLQLHGGDVSRAVAAFKKYPTSRWEA
YGRCMDLAAGVVERLIKSFPYLEERMRCEGVDVYYLSLLCTRRWLRDSVKLPDLARVSIE
KFMRLGQAAWEGVVADSLGKHIEALWEQFSEADGLLRLCAESVILPVSPFLALCLATDFS
LKENV*
>TCRU_3794 SBF-like CPA transporter family (DUF4137), putative
MSSGGGRGEGQSSQIWRPSPEAQSIRDLSSSCTGEDVGFIAGSNERVVRLVVGSLSVVDW
VRGQWLLGLFILSIGMAYYHPVSAGIAASAWTRILVFISFALSGASFPDIDRSATLRFMG
PTITMFVMSFILSPILGAAIYVIFRSSSGEDNYLLVGLMCTFCLPPSVILSVAATRSSQG
NEALSQYLSAVGIVCGVIASPLLMLACMLFSYSSKFPVTADLMLFPATAIGPFFFGVIAQ
KIFAWASHRRITGEDRESLRIAKAEMACSELCNGSLFYWRQRAVQLNSLVLLLVNYFMFS
SFFVQSIAPSLLTWRGVGLISFVELTLYTVMCIVGWLLASLFACSPEERIALFFALITKS
EVLVVPIILQIFHENRQAAVILLPSLIYHLIQAFTTGMLSFPLRRWRCQYNCRPGTTLLP
LRLSRVNRSPSTKP*
>TCRU_3795 phosphoadenosine phosphosulfate reductase-like protein
MRSLKLVGTDVGDIDVAQACMNHALTRVELENCDRVTDLSALATVPTLEEVHIRDCRRVR
CFGPLGQTQTTLRKLVLSGTPVTKAQLRELTRLGQMELVVDNCGDDPKLERPAQSLVKSS
IDMIREVAGRFKPEEIGVAFNGGKDSVVMMDLLECALGPEMLSRFCVFTLGASGREEFGE
VVAFREAYLENHGLTGVKTDVSLSMKDGLAQLKESKGIALVFMGTRSSDSVHQKKSVEPT
TAGWPEMLRACPVFHWGYEDIWGYILAYSLPFCILYKMGYTSLGLRGATAPNVLLRRGDG
TFRPAWELHDDLEERNGREVNSS*
>TCRU_family, tetramerisation domain containing protein, putative
Sk.1
MIRSGAAFNPMRRTPPPPPHIDTFQLIPDDYIKMTSADGHNFLVHCNCARASPLIRKCFA
DRVDLDLPAVAISWGETEKSPPTIHFPTLTAARLEVIIQYMYYKHRYEGETEGRPSFDIP
VEAALEVMKVAETLQC*
>TCRU_3797 hypothetical protein, conserved
MSKKARAAPPSEPSVVSPIAFEKSYMICLRTADDRLFLVDRNCAMVSGVIRSAMRKESHN
GDAVTSSVNTPPEAYEMTTIGTENHETITLEEISGDLLELGLEMMYFKYRYDGDPERRPL
EPQHSPENRHKLAALSVLLDM*

>TCRU_3798 HIT zinc finger, putative
MHGSEVELLDAELDDTSGEEMRSLSSAADADLEWIPEENEADESQLIHEDYYAEGEENEE
DEGPHFMASALSKAFARDLVNQKLQQQEGENTGLGSPCTAEQEAVEGKVKKAKICCICSD
AAVYTCPGCGARTCSNVCVQMHKKEFQCKGERDIAKKVSLSEFTDGQLQRDFHFLEDVSR
IISNCRRGFSKFWRYTFRALPPPLHALREAAKKRGVVCQITSEGMTKRNENTSRFDRRTE
TITWRCQFNFHDPDFTVVTDWGSERHRLGDIVAFCWSTNPPLPCFHINRRYNRASRWIGT
AEHNEEVVGEEGRQEPMTEEGEGETRVENVATSSRVFLDNKAHCQEAWSPCQVSITPLSP
AEARNEKAVAAFLEMEPVIILSRAERLGMRQKYFRMSPSCTLNEALRTLFFINEFPVFDV
IHVSALEAYPLVTEADKEFIRESFRAAPRPARPECRPRPTKADLSPEEAARFAKVPCRMF
LAGSCKLAEGACPYWHCAYNEVPACRNFVKFSICERGSSCIFRHDTAAVTAARKRMREER
KHPCERRSKRRYE*
>TCRU_3799 hypothetical protein, conserved
MQEAEKTFRILQEALEKAVRFFSIGVTVDGVPLTQQQAVDVLDVYDTLLDAEEPESYDAR
RYVLCRSLECGFMRFLAEILTQPFQHLHEFDAQVTQPFLPLRMKAVKALQRLLLACSDMG
EDSCVSCFRVAVEEEIIPLLLRALSESPYEPLRLGAAETLFIFILRISHGRTGFVSTGGV
NAMCRSLMGDGSHTVRSMCASILRELVNTHVAEFNNPATVKVILKSLEDTSADVRTLGVE
ILEQTLRLCTSSVAYVLQDAQSVLLPLRRILDDDPSVEVVVSAARLLETCCEVADMMHLH
TFFKFVVSMGLVKVLLLRIRDGGEAGAARARGLRLLIQHTPASYELPRQIFHDTEVLAVL
LKGIVDAGRGRTTPGEEFAAFQIKSLELAICVAIILTQSPEYREVLMEELKEYPQWAAAI
KNAVISLLNAASLDYFVNIELRDVTGHHLNALHGVPWEADNTPQRVYIYRLFQEQAQRVI
MMRENALFDEEAPSSSRADARDILEEEKQGKKVRLTFILLVFATNITFPVESQAAVKEVV
DEPPSPQPRPGSRAVPRFPPSTLAGSKHREHVLSPNGLVSPTKWRNKIQSKKLPDEKEAM
AIAYDKFNSSMSFVMKFAQQFGKTKQKADDVVETEDGYFLRMSRLKNPWHAIVKHQKLKT
WQVKDLRVGDLFYFSIPFDEIGIKALDVVLYKARRHMMYLKKELLITPHNSKGRRWFLDD
MARNIMPKALEYLEDLKELIQSRGADGVRFPIFLFREKELHFGERALHPGNIVEVLDQIG
FYFSQSMDKIVGVDKSRLQQLAEQLDNIEGNADMPDTSIHFCSVGPHDTTAEGMKLDTEE
ANEEDYDGGYGHGTISSDTESDF*
>TCRU_3800 Amastin surface glycoprotein, putative
MSKLGAILYGVVGFLAFLFVLVGTPIDQFRAKEKGATGNTPCMTLWGIKEDCHSTKYEFT
VGEDFRECPSVLRLFRMAEAFSIISILLLLAATALGVAAHFCLKSLKIFATLLLVVSIVT
VGLVWIPMAYFYNHDVDNCLGTPLKTYFKYGGGFVIIVIGWCLIFVAVVLLRML*
>TCRU_3801 hypothetical protein
MTALPRLDFHLVPNFSVSEAFRYTHHLIDPLELTHFVEVVGTNSNDLDELLAAVRHAHMS
ATTYTNQKLVKAMRRLQAAWAKDPSLREAVIKLARFPFEEGQSEGYDYSSLRNEALRDIV
MYNAVADVWMFQQKVFHTAACCWQ*
>TCRU_3802 hypothetical protein, conserved
MLQGNRVSVDVDGESLGEEEVPLTGERPPDVLRFCFGACGRQESHVMAENVFLYSRPLNF
TEMRALKDRVPVPTREAAPTPPEKIRVVDSVREHVAGDITEEEWRGTTPLEKNLHYKESL
RRVWSGVSPDDRLASLNGVKLTLRDLGGNNTVRVCVSSVLLLLLGSYLGRGSLLSRRVWI
CDSSRRMCATLPALFCTTVFYFYFIVFLE*
>TCRU_3803 Concanavalin A-like lectin/glucanases superfamily/Trans-sialidase of Trypanosoma hydrophobic C-terminal, putative
MCLTSSAAESTSTGTACTSVVTDGLVGFLSGNFSDGTWRDEYLGVNATVTSGVGAAGTTD
GVTFRGAWAEWRVGKQGRNQLYHFANYNFTLVATVSIDKVPEEGPISLMGVKTNGQGNPV
LLGLSYDSGGKWKLLCGGGTSKEHSSTWEKETQYQVAIVLENGSQGSAYVDGKQLDGNEA
CALGNRGSNEISHFYIGGDGSGAGSQDDVSVTVSNALLHNRPLTFSGGNADLEKDVAAPA
EAASHTVAGGQMTTAGESATAQQLLPSPSQGSVGRTASSSHAVGGATGDGGTVRGSGLLP
SLLLLLGLWGFTAL*
>TCRU_3804 BNR repeat-like domain containing protein, putative
MSAGGCSDPCVVEWDDDKLIMMAACDDGRRRVYEINAKEDSWTEALGTLSRVWGSPKGAN
VRGVGSGFTTATVGNGDDNNRKLMLVTLPVYSKG*
>TCRU_3805 BNR repeat-like domain containing protein, putative
MSRRAFTSAVLLLLVVMMCCGSGGAAADEAASGEGSSPETYFDWRDVKKEETVGLLRVPS
LVEVNGDVFAVAEAQCTKGDSVCDFNAR*

>TCRU_3806 hypothetical protein, conserved
MESTRPVAYRRVVVSGSKNPVEYFVRAKGDGRTVVPAQTETRTGWLMWPAALRSQLRLWM
RAGQVALWCLLASRPTHWPLLKGIQERLSSECVSVVWSAHCSVRSIEPFLCWEDGLLHFL
GSCGGGGSWGKHAHCFGVGAPRTCSCVTQEA*
>TCRU_3807 hypothetical protein, conserved
MGEESFTYVLSPVTSTQNFAHRVADENLPSLEESMEFTEKMTGTLLIDYDGFIEDLLLGF
CRVPNGAHDSRVGVGRGEYAAPAGAILPTTIKAVGLDPSSSASSLFQDGAWPFVWAQFER
DVTRETLIIDGAPCNDALIALQRIQKLLADAYDELPSRITAAKTHGHGKADECGGLASRF
LNTAWGFLGNVQVAYKKMVRNKKLAEAARIAILIAQQTVMAFPVELLHAQFSRSSQGNFK
DKDAAESKIVYIGERIASKENDDEKSDPPHKEESRRMIVRVERRNHGSRLPYARVEKFMH
VFTLDESANASIKLELCIVIEVSLFTSDQVELNWEWRRPPHTFSGDAAAAPPQPPEEETQ
RLNKGEFIHAFADF*
>TCRU_3808 Amastin surface glycoprotein, putative
MGRANHAFSASRNESTENSKSVPSQPEQEAVSPPPPPPAGDLDISPEIVTLAFVLNSVFL
AVSLGTAWFLREQYAAGPNGENVKEAFKLWKTTRWVGSVRTSMRSDAIACTPVRQRLLVL
QILAVSALFISLITLVAAVVRRYFLQGNSGIRLALIYCVAICFIILTVESAIGINVYTFS
FVACGEGSSYHSHAFELSVGFALTIAAWAITAFAGIVVYNKVPFPRDERIVAYGIQAFDM
LAFIAFLFSTVACAIPLWFYKDSARRILTEVFLWKERYSILWATNASSNSTSVKKLGCDS
LSHYFLAAESFGIVSVLLNGTTFVIGFLLVKGLVGVTKYALMLGYISTMVAFLQWILLIC
IYYGDWCSGRVAYHRKKYVMAAGFALSVTAGVLMAVGTVALTLTESIRRRHFPSAGPCKK
IKEIFVEMCQ*
>TCRU_3809 flavoprotein monooxygenase, putative
MWKRCWRLLGSTNRYTDGSGRRLDPSGLPKELELPPISNRAYPVRGENWQPVLAGEPSPH
KGNVIISGAGVVGLTVAALFALRGWHTSVIEQNVSALKPSPVVAQPPVPSTGTNVEMTDT
AQYRFGPFLGLQHALATRRAVDALEAAGLNIRELRQCGVPVSGVMDHPGAYNSWLTRGLT
EHHPFAIKMLSMDLLTLRQLLELHVHALPTGTCRIFHEHVIEAVYPLKQQLVVKPLRESQ
THAWRMQQKSACKVNPSEDNAGPGTREREDDAAEVIVRRGYRMETSLTLHRLKWEKKFST
EAVDYDLLVCAEGADSQLRDLIDVEGFAADGDFGMRWFLLRSKKLSHAHIHRWLHKRRKS
PKQVDVCSPFQVPIAIAFPRQDRESNGDDAKEKESSFALLVYGPSSDIERLSDEEALRSF
CPDLAENDTTCQIISFTRTVVPYPTIYCENLFNSVGLPSAVVVGDSAHTCNPFWLQGLAM
GLEDGVNLLNQVDAYSKHFYDAIKQYSQERGCSGDALREITDRCLYYERMKHINPLVRFQ
NSFQLAMNMILPRSFNEYYSGASVNHLYSKSIEEMLNGRGYTSYEYAEKQQAKHRAFFHL
GRVYT*
>TCRU_3810 hypothetical protein, conserved
MRRCICCDAVITELLSRASLRVQRRRRGASENEAKKQVFRDVLTMSLLRIAAATRGGGAP
VQAVRDAPPRREWQANDWHSSLSDKAKSSSGTSAEGTPRFLRALLLKSRKENAVVHDAKR
VPHAPTAAKLLTDAEDGLCASETLSSTATEGADAVGAVATWYAAFQLFSEAVEQHTVVPS
IEHINVLLGITVREKRWQQMQQLEMFLEKILVSECGMCLSETPCKMDSILDVSGSQQTAF
PLQPNSETYEWLMAAAVSRGDWVEALRCFDDVRDEYLPVSDSLLKLALEAYRIGGVHSLI
DPGGNGHTNKKTTLAPLRTDYRGPFLHCAASARPASTSHGVRTKPAAARYLWEAALMLFS
SFLHRVQESDTVALFMSMMADAGKHQVLLDTYRDRSRTVNFGEEILPLVSNASREVGDWA
LNLCLLQHILASPADTERAAVGRQLLVDALLVLRRTQHYQKIIGLHVQVPSRMWTSRARV
MVAQAAMVQRDLPLLLWLFEEEETMRVETNRDDDGGRRTSHIMDTGLCYPFGIAAVSPVY
PSFPVEMYDIALRTIQLELIHRRFHSTPLRESAVSRLNIPQDLAALSIAIYEKFLRDSKH
QISTNEGVPFDASLSVYAPFVKLLQTSANEGAGVGRCTNGLPEGIWEKALSHVALIRRPD
TIVVSLVTHLLQRENQWEAAFNLLSHVIRSEEVSREADGPSRRRHSRLSSPNGVKVDEAN
HSDRYTCTAITAVAIRYAVWSAVRQPSAAVHLTQRALATGWIDGSMAMQLLEGVEGILGK
EEQLQDHWQDHDGNLSYASNDAVHALLKLVAMHDKREGGLGSFGSLFLKTLQLLQQLQTR
VEPPTSLLSDLTGNLEEVVSGLAKEGNDELRAKAELVVPVVLHAVWHQISMSHMSRWTLW
QECLRCLRWFCSPLSQHSSFVLQANESTLRVLRAFIRRCDRPEEGFVESVAVSWLLVEAS
PSALEKLLSLLDLLPDLVDRAVWLSHELLLDALVWCGRHRVKGLLRRLARILLPLFLKIL
QRGVNSTARKATEPRHCLRSETLTALTVAVQCLTSGPHEEVLHDWPLVHQLLSLLLQANE
FITAADTAQTTTPGRITALAESLAAVLDKLRRTFHFLTMKISLDAHRRRSQGSEGISFSR
DDEAKHLTSVLESGAIEYMDLCEAFAQRFFTEAGVDWMALGAEMAACLEKVMNCVWDVNY

TLYVWYTTFSPFQSGSSVLKPLQKRYRWLMCLCVLFLPASCITPLRVGRWLVVMSGDGES
AVTLTQSEIKGTETETNMIAALLHDITTLEAWQRCQFTTISHGGEGSCHQSRQLIVPLVL
EAIHSITIGVSEHVPSSFSQPTTNLAAAAVREALPPLKNGYSARTPFDDHIALVSPWQLR
TMQKAGCTLIYMLLCEGQAELPQMAKKAMSWSCCSHALLEVIVSAAQSTSAETFKALWTP
VALVDVFGLLKAQNCFWALFGRPQTAGGSQASRVLSLLTASPAVRAATRMDEDICTAALL
ALDPVDVMGDTEHVVAVRHTVCRLLAAFPKILEKRLLLLLLAGQLDDLQLTVMDILSQHI
VKVTGGRSLGCSPTLMRSIAMWLLLRPPCVAPCVMPCNAAPLQMFSDIMISGELRSADHD
RRMTGTMMRKEQAQNVSSSSREKSEEMMKAAHIRRWLGWEKAVQLLNDALQAETPHVDLN
KAFFGAMEIYGTYVSRRWEDDFFKALLEQNKRNPSSCNHTSSLRRLMGELIDHVAYAPSL
QQSLFRHEPVEPSELRQVGEKLWKCLGTACKEQTVTHADVWKSVFILLQRVLQNNLPFSC
QDTGELVFSLFPVNCLMVERLVPHLLRHGVVRNPSLPEKQESVVDIPLVASSMLLMLEMA
FPGRAVNMVLYRLYEKVLGEEAGCDSGGVTPPLSSSPAPVTSRQGAWRSLIECAVYFLTL
PGLLDGSITENIFTSGVAAGNNNKGNNAARVRDAFSTTGAVVGLVFRFICDDAVYVLSTD
DKLSHMREDNMQECLLRLLELSGRCVASLATELLLLSGTKRMEEDSAAMKRIVALFLQSA
LLMGSWRDIYWLSSRLLVSVSATLQLQRGEEAKSLFVSSWCWIVGCLPAMVAGSSRTSSS
LHSQVTALLKMIPAAFLQQNYLATAAAIVEKKGFGDGCVEARQRQLFYEFLMGLHERILL
KAESTLRDEGKRASSFTLRLSVIRPVVQVGVLLGLHNVGATEAGKLQSSLLFFLADDFQW
RRLLEAVKAECKEATTSKDLLKLDAANGGSAVSSWQVALKLLCLADKELFQTLSLKNGER
KAGAEEIQKIKDALHVFHVSCFAQAVPWEVAIDLFHRDDGPPHTALVFPASYTEQLIRHC
PSWSMALELFKYSLSRVPHPTKVQQLLAYLILYRMRTDPTWGKGGQTLLPHSYNSGGKDV
GLLSWEVACWCYNQLTQDRAAPTLPQRNMIEILRHCVFSPSAALSMYEAYWKQEYRLTEG
VNVFLSLLRAARLARNEELVLRAMWDYIRCCDEHDGVISKTSCGSVRHHMFTAGGNATLC
RSFTHVCESNIVSRQAAMELVTALKQRGRIGEMEELLMVTSYSRGVADATEERAR*
>TCRU_3811 tRNA synthetases class II (A)/Threonyl and Alanyl tRNA synthetase second additional domain containing protein, putative
MSTAPTFVRVGELACQRNPFLRGLRARVVLCEPVSMNKPAGGSGKGGHNSTNTNPSYNVV
LTDSVLFPEGGGQPCDHGTIVYGDEEVPVRNVQRRGDTCVISTPCPLEVGEEVEVRVDWP
RRFDHMQHHSAQHLLSAVVEKASQCRLPTVSWSLTHPYSFIVLPTGNKIDSDVVAHLEKV
CNDAIARAIPVRCDMYPSKEAYEQALREKVEKEDSGENFRQSRSIPSDVTGPIRIITVED
GIDQCTCCGTHVSNLTQLQMIKLLHQETKGDTLKLFFVTGDRLCRYYGDMYFREKELMKE
LGGVRPEEFVAAAVKRGKEYADMEKRLKNLTLELMKAESEKLIAEAKASLAEGAGGGIVV
YRRDDVGGDFFNALRDAIRQACPECLAVLAWGSPVATATAGGAMGRAKTGQFMVIGPTDR
VESLAPSVCIALEGKGGMSKYGYRGKGNLAGWDELVQKLRLS*
>TCRU_3812 hypothetical protein, conserved
MDANTTEKVEKKRQEFLSKDAIEEMRNFSDQTSSRVRHGLLPRDYHQTVDKAHEEKMHAY
ISPRRTWRQNVYVLRNFEYWRLRAEYYPYIERSDKYSDNFFIRGISCHPRLSDYPGCRAV
IQDYFICRDKNKVMQLFNMCAPLKEQFCACINEVFIKNHERGDKKFNANRKEYFENQRER
RFSKMLLQAEESADQRKKLQD*
>TCRU_3813 hypothetical protein, conserved
MMRAVMRAACSADSVNWGEASLLAADVLSVLLASQAAASLKTGLRFLSDAVLMVPRLLFF
LRGKADSAMLDRVAYAFLQPLWRVASGGHPLLQCPATSTDDAATQLRLSLPSLFELRVPD
DEAPGAYVTVDGVLSRCTERTRELGTHLLFYLCGEAPCTGAAPPAHTTLASTLFQR*
>TCRU_3814 hypothetical protein, conserved
MGLSCSHKKKWETAFKGKTTTRSSNRKPRKEYQVGLMLQCNKTSVHIDGRSLGEEEVLLT
GQRPPEVLMVLLWCVRWARISFDADGRLSVQPPTEFHCDACTQGQRPRSDKGSGSDTARE
DSSG*
>TCRU_3815 hypothetical protein, conserved
MPASCLSEIAALTPNNVTLGPDGAIVLDWPAAPKTARADLRRASRFVRIRGQDAPDTTKL
CGALRHSEKLMNPGTAQVERAEAPWNATAHSIERGVLRHAARIVGTHNSDPHVISQLAGH
GGSFGLPQDTVRYFGRRTTTLTQVSSLAAQVRRETPRKDRNPG*
>TCRU_3816 Concanavalin A-like lectin/glucanases superfamily/Protein of unknown function (DUF3676)/Trans-sialidase of Trypanosoma hydrophobic C-terminal, putative
MKDNNKELISLYENKKSDGSYNLVAVRLTEKLERIKEVVKTWKDLDSAMQSCRSVSSGNV

DAHKKGMCNGPVPTDGLVGFLSGNSTVTEWRDEYLGVNATVRNGKRSVSNGLTFKGSGAG
TVWPVGEMGQTVPYYVANNKFTLVATVSIHEVPQSGSIPLMGVRMNDTKGTVLFGLSYTH
DKEWLAITEDAVDPEDVGDAEDVGDIVKWEPNRTYQVALQMDSVEWSVFVEGKEINYTEY
NQRLFDTHRISHFYIGGDSKNQSATGGHVTVTNVMLYNEILSNYTLHKLNASKVTIPSLG
AEKHPTEQVTNTDALVASESKNEETTAASHEKLTGDDTEKQEEGIVHNLVLAASSSTVVA
GSSVPEYATAAESAESFHQEDNAQLSEGETSRQATLNEAKKSMARSSDVQKEDLQSTELT
EVADVEGSAESYDAPQPEEEGGANDGSGGLTPPVAASLSMAAATAPVYGEHQVQQSVELA
TENDDVRSTGTGTTGAEESLSLEAGERNSERTMNSDSSITPSKSDAEPTSAEDTDNISWT
ERAEFSVENGKEVPQTVVPAPGNTNTTPGETMISLESNATAPSDAGILPENGHYSELAGM
ALFAESTVHGCVFRVLLLLLLGLWGAAALC*
>TCRU_3817 signal peptidase subunit, putative
MHSVSQRVLEILTMSVSVGFVGLLIVVASSFSPLGPKNVSPIISVFNVSMSPLMRMPMPV
IYAQGQKPPPLRDYVFFKFTLRADFSPVWDWNTKAVYVACVARYRTENYVLNEVILLDTV
LKSKAAAAQWSLENAQKYTLEDAHPGALAGVQVQLSIRYQLLRYCGHSPMFEVSPVGKEY
PVLELPKTYGSVKMKDWAL*
>TCRU_3818 hypothetical protein, conserved
MLANVVLQENRALKKRLKVLQELMEKERVAYESLRIASSGLFPGDVALPVGHTTAVAVVG
NDGEEKGGGGVGGGAKVTVAAYQQLKAQYEELRCTFNTLQKERLRQGDA*
>TCRU_3819 40S ribosomal protein S17, putative
MGKIRTKTVKRASKQLVEKYFSKLNKDFYQNKRVVMDVTIARSKKLKNKIAGYTTHIMKR
LARGPVRGISLKLQEEERERRMDYVPEVSHVDQAIQDGVHVDKQTFSMLKRMETGVPRHV
ALTTVVAPVTKKGGRRAPARK*
>TCRU_3820 hypothetical protein, conserved
MVQLAGLSRAYAVLRQLELLRQQDVLDPLPPDTETETEAGSNLSDTGRGNGKNAGAPTTL
TDCCAAGKGPRLESSPIITTAAKSSGDGSRENTTHAPQCTEAYVKDLPSLQERMSHQSVD
SPMVGNGERERRLVEEYSYDTTESVTSGRRLSPHPSMDGRAQALQRVLQEIVARRRQAGE
KAVTPDHDDVHKLTQQDPYHRAGVAFRPSGRCSKKISSRLLSPSRGIDGEEDVFFDESPL
SFGICTRGTPGREAGQRIYNSHEKSCADEEETRNDVPHAKEEGEEYSPRAVPLVYSPPRR
RKDLLTYSAVSEYTKSLLRQRRQRHEKIKAETQQQQRCRTVDAQRRLDTTPLNATLSPIA
CIENPDKDVSYNLSRNLEFDSLQEETVIPPLPLFIQELLVMGSAEMQEKLRGMEGCCNGS
AMWCRDWFALTALVNSPDAIRHVILPLLLLELLEQNESNEQDASDANSRMSHILCALIGL
GGLAAGVLSLLLDMLTKSLGCPRLVALAIRAVGGDEGLHALCRLARCEQMDQGVHTAAVY
GIGTLAYPALGHTDVYCVGAPGLNGTIVFYQPPVLVGYVEVGNDGERRRETSLQCPPPYR
PTHVILSAELVRNKLLQFTVSRNFRRATGIYETLPLVLRDFARDAPEACRLQGLSDDVQE
TLRRCALELEDVSHFSARVLPFSYDPHYAGRKEEVFAVEEVLMNVLYNRKTTLGVVEQTL
VSIASLPDFATTHAAFPVLDYVIKCVTRFEREGDAAVEGEKAVLVAGLVALGRLLRNEGT
PPAVIDTACVVLLHNLDSSSCRLRHAACIGLGEVGAVVNNPGVVARALTDALSDAAMNHE
TVAWALTRLGLRGVRVLLDQISPGDGKSSSRTSGDWQEKKNGSRHHAAAALPISTRMICA
RALGKVDFLTVSLGIHEDARRLQEELVHRLAAAITSAQMEEELALECAYALAEAVSINLN
GKTPTPCRDEEAASYYRSETLNEAFQVLKGLVESIPLPLSVNKALFYSICLYGGAHGELY
ASQTAIQAPLVALRAAATFGLRACGGRVIRTVVMALNDDDAAVRVEAFDTLDAVGVKDVL
RVLRTRPHKHTQQVIAALRDSLLRDMGRGAKRQAARDLYAALLRA*
>TCRU_3821 DNA repair and recombination helicase protein PIF3
MTRSRVRVSPLELMRRGASAFAAASLQAATNTRIARRSCAVLPALFTADVSDGKKPAKQS
SQRNEPALLPHRSHVVSPEGEEAFINPFTGRLTSSSSRVAKELLRVGFLQSAVNPFRLTW
DAKKWHAAGAAAVKPFQSSMVGWRKNLAALLAREDAADAVVEEVQRLYSELTSAYLPPKL
DTMHDPQLSAMEMTPDQENAIRCALRGYNMFVGGGAGTGKTVLLKAMHRRLTEMGLRVAM
TATTGVASVQLGGCTFHLAFGVPVGAGENSKKRWDANAFRAVDVVIIDEVSLLDAELFDA
FELEARMARLQPLPFGGLQVIACGDFLQLAPSDSTFSGPCYQSDAFRHLIAVRLVTQMRQ
AKSDPLHKLLAQLRVGKFDRKSFALLDRHVPGDTDNVTYIFPRRREAQLLNEEKLAELRS
EEMTFTPQRGPLQLVGKFTPPGLIEFARKKQPVSRDEIVQVLTEEVKRVAAVDVVDHNVV
VMPTRSDKNGMLIRLRNPDDIVAARANGVGGNAKDYNSCSNTGGNTSRSGNMRDVHEISA
DRWKTILEATAKRLKATLRRIYEEDPQGFVPISVSMMLADVASQPNAEHLMPLRLKLGCR
VMINRNLSRTVSNGSVGVVEAFAVPNPDLFPRRHETPVRTMYGRLLEKNMFPKLPIVRLL

DGEVVQVPPLSLMVGGTPATFFYGHELYLLPMQLGYGFTVHKVQGLTLQGTVVLDCKHFF
ECPHLVYVACSRVRTLDQLIVKNIKGDMVIVKRSALEFSEKLRDASVITNLLPPDGCTRA
SWVDRLRPRLMGLSK*
>TCRU_3822 R3H domain containing protein, putative
MSAAGSREEMLEALQKAKQAMARRVPVVTLQETPSEGGELQLQDAETQPSFSRPRPHVPS
EAKTNRSHFFDTWCRKIEMFVADPTKTVLELPNDLAANDRRELHSLAEKYNLSHQSRGSG
ATRQLVLKKDALHYRMPDARPDNIETIKQNAGSKQSKFHLRRFLQDPDAAVGSIGAFADE
ATANMVRRLDRATDEYRRAVELGYSQEELLAIEAGESVEEILRRGGDGRLLLPSAVEQAS
GDVGSVPSVATHGGNDALHAIPPDNRKGKMYEEVCLRCGTRAHVDYDVQKWECNGYCAQC
ATQTIWKLVELDGVKEELVHPSFKRDRKRNFQEMEMEAAPAMREPEEIDKDNDEDDTITV
EDVVDMASMNDFCAADVNWIRSFATHHQPRGSGNILSAHIVFCIEFEDLFSMRIFRDFLS
LEDKKHAMKERKRTREESNAADQKDKGHCGWFLRLFEVKETGVPLSTLLEEISLCIPERY
DCLAVAFPNMSVYGAVASCVCLIQSNKVEESSLEILQRKYGKRSIQFARDLCAVLLNGGE
TASVS*
>TCRU_3823 eukaryotic translation initiation factor 4E, putative
MNPAAQEFVPRKKCAMDSNAIHMMGRNPPQQQQQKQPPQISPPPATTTKLRATAPAFVPK
GSLFPMATPPPTSFLQPPVSIPPPALAPPTVPQYADPNTAVGSGLPGPKSFSPSAPPYTP
ANPKVVAKFSSPPKTSGSPNPDPKKTTRTPSPHPEQQQEEDQPNEEKQPREEQEPKPDQS
PLAQKAQTLGVANRIDSARQEDSFGSTSAADETSSNALQQTGALGEAVENDFSGSVLPSV
FEVSVVAKPPPAEAIRFNTVWALHADDHPTPFGAPLAYDPVLVHLVGDVECFWRLWRYLP
PPSALLPAFTYHWFRRDIKPNWEHARNKNGGTITIVIFDRDKPGQNSKQTMDDAFMTMLM
ACCGETLAESTTNLNGIMLKVRQNKPTTVQIWTASSDQRKLKALAGSLRNLLEKIIGAKP
LQKLEYFSHQQTQVGGAGSLAGRMKGKPTRITPDFTL*
>TCRU_3824 hypothetical protein, conserved
MEAGDILHDIMGGNGPPLPQLQAPHEQRHESLPRRSKLVFLYSSDEEGDVDNSEKTRGVG
VAKVTEVLTLNLLPNDLREMRLEAELAAQHHPHAALQSPGDTEQQPLLFTEDTAGENVSF
AVQGRVKVVDALPLRECISSFGNKEVPLRRHLHAEETKRLAYARKRFRDDGDNEDRSSVE
DAGQCGAASGNVVVAEPRPVFDGDGLVRAIAVGGYRLTVDEHELQVSGVGEDVVTGEVDI
GHDTLEDDYFDQEDDDEEEEDEVLEEAVVVAVVEQLVRCCEVGELDESLREAGERFLVKA
RSCLQRLQTGELDLAAFGDEVQGDILRLSRAFQRFRRPKDAPVVIDGVVMDM*
>TCRU_3825 membrane-bound acid phosphatase, putative
MVQAAAALLGGIYASENATIVPTIISTAPERDALLNVEAFPSSTITRMINAKAFNATLES
VVDEQFPDASVVEAMGAEVGLDAALCSAPATRMVCCQQLQKLAVMYAAMGATDSAPTVMA
NKAKLDAVAAAYFHVSQGYNASVPQDVARGSLGLPLARELLRNMRAKMLPVGDANRNTKI
MMQYAHRVPIQTALGHDPSDATPLGETFLVDLLRDDATNAYFVRLRYAAATNGAPAAAFF
PFRCLSAADVPTDATTADGVICPFDDFTRFVESSSGTSAAGAACYLDEETRKKFGCSVEG
AAPSPECARYRAMCPAQACPGGQVYDVRDDSCKPRVVLSDVISNGAGVAVGIAAVVLGFL
LSLFLMHLCPMLCLTKGASEMHGDCPGNVEVSTVPRQ*
>TCRU_3826 BNR repeat-like domain containing protein, putative
MTATIEERRVTLYTQRGHTSGEKRATALCLWVTDNNRTFSVGPVAVDNAANWMLAGNLLY
SDGNLHLLQGRGSGECRVISLSRLMEELSAVRSVLSTWTQKDIFFSNLSTPTAWLVAVLS
GAASDGRWNDEYLGLNTTVTNAVKDKDGLQVTGLESRAIWPVNTRGDEVRHISLSHNFTL
VASMTIEETPSGNTPLLTAILANTESNHTMGLSCSHNNRWETTFEGKTTTRSSTWEPKKE
CQVALMLQSNKASVDVDGQSMEEEEVPLTGERPPDVLRFCFGACGGH*
>TCRU_3827 hypothetical protein, conserved
MAMMMTGRVLLVCALCVLWCVAGGGFGEEDTQDEFCNNTYLGVLTLLANRTDKEPAEKYC
KNAGDKTECVDTLKKNIEVALENEKKMKDNAPDRERNVAHGAGSSVGNPTTTSSTTVGAD
NAGVSSPLSLKVEGGVGAQIHSQAGGASRQPHSDDGRSEAERSHRLEAEGSNQHDVAVQG
KTIHNKDEETPQTGVKSTEAREDTDGAPAPSQDPPNTTESHEEKSRRVAAPDTTPALNTS
SGSNQEQTSQSSPPSGSETTGSSDSEGPEKNSKNAQISDAQLKDEEQHHESTAGNEEGTT
VESAAVQSNTTTPADSDGSTAASHATSPLLPLLLVACAAAAAVVAA*
>TCRU_3828 BNR repeat-like domain/Concanavalin A-like lectin/
glucanases superfamily/Trans-sialidase of Trypanosoma hydrophobic C-
terminal, putative

MSADGCSDPSVVEWKDKLIMMTACDDGRRRVYESGDKGDSWTEALGTLSRVWGNKHDENV
KLVRGGFITAKIEDRDVILVTTPVYANTESEEKEKEKGELHLWLTENTHIVDIGPVSGDD
EDAAASSLLYKSAEGEDRKEELIALYENKKGDEEKSLGMVSVRLTDQLQRVKEVLATWNK
VDKRVSQLCLNSLSQKGLSTQSVCTGKITAGLVGFLSGNFSDNTWRDEYLGVNATVAEKD
GAQKADKGVTFKGRGAGAEWPVDAQGENQLYHFANYNFTLVATVSIDKVPEGDNTIPLMG
VKMKGEQEKFFGLSYEKENKWQLLCGDATTNSGELSSIGEPETTHHVVILLRNGNQSSAY
VDGQRVGGDAPCTLGSTDLREISHFYIGGDGVSAEDKESREGVSVTVTNVLLYNRPLASE
EIAELAKNKITITKPEEPKKLTTRPRSPAVSATGVEGTVSLSNAAGQQPSEHEPLTANIG
AGVLSSAASIATTPSPDADPTVVTVGGETVQGDGSLQTPGVSVSSGADGETAGGTDAQQE
GIHAQAGEVTATALNSSLGNVSQGNNTDAGTVRGSGLLPSLLLLLGLWGFAAA*
>TCRU_3829 hypothetical protein, conserved
MAASSFTDSECDNTNDGKNNDEDDDDLHRGADYLRVLVHESREGLNALPTIPGLAEPLAF
APSPAVISAPCVEADPFTVEFTQQLSKGCAAIPALAHSHVFQRCLACDLLCVLSRPPPPA
FSWCPIRSTLRCGGYLIAWPSTHVTMLNGCVFCLAGTRETSVCADNSAKVFVGGSRQK*
>TCRU_3830 hypothetical protein, conserved
MVLLWYNIYNKECEEKNKENGSLNDSAMKSCMRTSMKEVCGVFYNDNSGETPDPESDRIC
KEYAGDPDGAFQPSASQAKPLPTAEKPAVAPTPETSKDEAEEMGLTPGPPAGAAGDLPAK
PTEGPPPPSAAIDAASNEMGNGTGGKVPAGAPEFDVVGTEKGKESEANDGSTKKTPVETV
VMRGVTAPEDSDGITAVSHTTSPLLLLLVACAAAAAVVAA*
>TCRU_3831 P-type H+-ATPase, putative
MLPPSKGLTTAEAEELLAKYGRNELPEKKTPSWLIFVRNLWGPMPFALWVAIIIEFALEN
WPDGAILLVIQLANATIGWYETIKAGDAVAALKNSLKPVATVHRDGAWQQLDAALLVPGD
LVKLASGSAVPADCSINEGVIDVDEAALTGESLPVTMGTDHMPKMGSNVVRGEVDGTVQY
TGQNTFFGKTAVLLQSVESDLGNIHVILSRVMVVLTSFSFTLCLICFIYLLAEFYETFRR
SLQFSVVVLVVSIPIALEIVVTTTLAVGSKKLSRHKIIVTKLTAIEMMSGVNMLCSDKTG
TLTLNKMEIQDQCFTFEKGHDLRSVLVLAALAAKWREPPRDALDTMVLGAADLDECDNYT
QTEFVPFDPTTKRTAATLVDKRTNEKFSVTKGAPHVIIQLVYNQDEINDQVVEIIDSLAA
RGVRCLSVAKTDSQGRWHLCGILTFLDPPRPDTKETIRRSKQYGVDVKMITGDHVLIAKE
MCRMLNLDPNILTADKLPKVDVNDLPDDLGEKYGEMMLGVGGFAQVFPEHKFMIVEALRQ
YGFTCAMTGDGVNDAPALKRADVGIAVQGATDAARAAADMVLTGPGLSVVVEAMLVSRQV
FQCMLSFLTYRISATLQLVCFFFIACFSLTPRNYGSADADFQFFHLPVLMFMLITLLNDG
CLMTIGYDRVVPSKLPQRWNLPVVF*
>TCRU_3832 haloacid dehalogenase-like hydrolase, putative
MMLGVGGFAQVFPEHKFMIVEALRQYGFTCAMTGDGVNDAPALKRADVGIAVQGATDAAR
AAADMVLTGPGLSVVVEAMLVSRQVFQRMLSFLTYRISATLQLVCFFFIACFSLTPRNYG
SVDADFQFFHLPVLMFMLITLLNDGCLMTIGYDRVVPSKLPQRWNLPVVFARSIILSAVA
CSSSLMLLWIALEGWGDETYPNSWFKALGLAQLKQGKVVTLLYLNISISGFLTIFCSRTG
GRWFFTMAPGLVLLIGATISLFVSSMVASFWHTSSPGGLLTEGLAWGDTNSERLLPLWVW
IYCIVWWLIQDAVKVGAHMLMEWMDLFGCVSKAYGGKVVEQYMENKITEPAN*
>TCRU_3833 hypothetical protein, conserved
MQDTSRKRKAHESYERPRGASPGSLGCHGAFHITRCVEARCSNRGDIQFGSTRDLAVGEA
RQSVRPSPEHCLIFVEVHHNADPGVVAGRIDVKGEAAEGQEPWLTAEERFFHDFYHNHAA
TITRRATRTRLATPHDERTIPLQQVNVPVLNLEWIKSRLNPATLERLMQVWGLVGRSPFP
PSSSGKAREGSRRIPTADAHLLRKAGIVEDGSSTITGGWIIPFSVVVEKTAGSRRRLIAW
PRDKNRDDPYEANVPLLHISHYLPPVMAEAAFCLDLKASLFQVSLPRETRHFFRCHVEDG
TLVELTRLPMGCKAGPEILQIITSAIAVVTTVVHSLWAAPPSVRVDVWIGNIRIAGSKSG
ATLWEAQVLRNADSCHATMGRTANRALRSTPFWGCSLITFTVRCP*
>TCRU_3834 hypothetical protein, conserved
MDGVHMTNGVAVVFDVAAMLPGALRIELRNCVCDGGAQIYVRGYGGEPASDRSLEVSVSG
LSGSYCSLVFVHNLPAHTNVMVRDSTIVTPGPMHYSQLNGLTDAVASPLVLHATSLLQTQ
LRVSNTVLRSLQAGGSAVYVGGGVDLLSSAVALDGVLLEASGGPTASAMHVASSSRLSLQ
SHSVFSMTNVSVVSSGGGIVLGERLAVIDSVLRFVGVEGSVAPSLVRCDGGTIGGGGWLD
LHDVWAVGEASSVASLSGVTLSGGAVSIARCAATGATLVSGLAITSGVVSVQCNRAGGRV
LQSSGDYRMAGLPSVSVLPCDGCAAALACFDALTASFSDCACSCRAGGVGDACLPFDVPS

ARAGGGGGDTAQDCMSGVTLTESVTVGGGRATACFDSVVFSGPITVAVDLRSMDVFADAL
NVTLRRCVLAGGAQLRIGGLSESTARLMPHALVSMTKVTSLEGTIVLHGAMPLHSTVLLA
NSTLRATVGGSQYVPTTRGHEKFRYGSALVLDGVRLLSTRFVMTRSTMACGGGSCAAILV
ERGLGVNLSSVFYMDSCVVRSQTHVMYAIASDLRVGGGSVFSIQNSSWSALSTEYGKGAC
VFKDVVVDGGSVLQIVSGIFRLGFAMLIANTLTVADGSWLVHRDNEFRTAYVVYVANENG
VAFRDRSVWSILGNNFTYGSYSSTIVSMTSNWSPPSDSHPIIYGVCNEARGSPVTDYGEE
LNIGTPVTVLDCGACTVDAVCFAARTSSISGCECVCAAGGYGDTCVPATVPDGLGPLPLP
DADDTEVRCVHGGSIGSVDYPDPGVRGLCFVNVTFTAAIVLNLWSFDAPEQTLNITLLQC
VLMGLLIKGKWCARASERSLLDDGFRSVGVSG*
>TCRU_3835 hypothetical protein, conserved
MVYPEVAQSVGDGLSWLCYRNVTFSGGGMSLTVLVGAMTGDVVKITFDGCTWRDGAVLLL
LGNAHATVGSLHIVVTGSTFSDALLSPEGGFPPRTNITISGNHFAVTRLIPRSGLDIWIP
SCVAMNGLVIANDSAVVLSGNVFQTVTASSIAIRVVRSALRVSWHSVFAVVGNTFHMDGG
GSTLINIDGFSQSLSLSVLNNSAVVVRGNLVTRPVRYFLLLILASRVESRSAVVFQGNEM
QRSSVVFFFKPLFIHLLQLLASGERQPLP*
>TCRU_of Trypanosoma cruzi region 4/Dispersed gene family protein.1
Dispersed gene family protein.1
MSSTDRPTVLRITTGSRDITNGAIVAACNSVNGEEGVEYSVPSVYNATILTCSDPCTLAA
SCFPAYTTTASSDGCACNCAEGGHGDACLPVAVPEPPSTDGADLCVRDVRVDVEVSAGLG
TSVACYVGVTFAADVVVDVESMSGSVRNVTLANCTFLGGAILYVVGWRSDPPAAERVDVL
ISGLESRSGGGVLVSNRFPPGSRVTVVDSVLIAEARVAYRGEYDLGDASACLVVHNVNLT
GSVLTIARTHVAAVFRDAVGVLVVGSVALSSRGALYVDGLSVQTALGLCVSVEGGVAASG
GSVVAFVDSDFLLCKHAVSVRGAVSVSGSAVALVRSDFSSTEDYAVAFYSTVSLAGGSML
LAKDNVHDGVSREMLYAAGAVTAVGSTLSFLRNRALLPRMLSASLSLSAGAHLRVACNDA
GGRVLSTAEEYAAAGFGDAGSIDVVGCDACDRDTHCYAPGTASASMMNGVCVCDCGSGGY
GEACVPVGAPALPPAVGTAPSVFVREGVAVQSVVVVPAGASEVTLRHVVLDGVSPVLYVP
WMARDGVRIVVQNVSLLSGAVLYVMGGGALRGAVAAGSDEGGPVELSVCDVEALNGALVL
TGTYPAGSVLTVTDSLLVAARPTPLVYLPGSESSPYAPVLVLSGLRLVRSVLVVFGVALV
TVMTGGRTVAVDGAVLELVGGGVALDAAVLGGDFALYASARVVASGGAVLRVSGSQVYAA
HGLVFGSGVEVNASAVVVNDNAGSLTDGALLELRGSASFVSGSWLSVRGNSISGRLLSVP
SYPRSAELVQSTLTLHGNTGSGPVVMDGTVALGGAGRNFVVGCLTLNGQALRPMEYRSAG
IIGKFRPVACGVCDADVSCFAAATRAMSRSCGCRCAEGGYGRDCLPVYLPHVDGCNRTPA
LPLLSHTVTLTETRSPTPTPTPSLSTTHYSPTQYGPTETLQGTETVALSPTRTPTASVSG
TLWWSDVACPTLAVTTTAAGGSLTQNDIRGGGIAVPTLLMLALPPPFRWARDPQIGTHLS
FVPVSTAQPSGFGGPWGAMLRNATWVRNATNPSTVLELAVPVHRGYFIAADETIVIRCDA
AAVSGGCKGVLLGSFTIRSDTLPAAAGTLSAITGVVAGAAAASVLVTGGLGSILEMQALG
VFARMPCASARESESTVALPYFLSVFAALDPLWMVVGNALLAAVFGCVHCGVTAAFQRWR
GVDAASAWAAMRFPSLTYVVAHAMHLGIFFGSVLSLAMPGARVQHRVVGVTGVLYGVAFP
AGVCYLIARHTGASFTKYWQFSRKPLHERLLYPVGYWHPAAQQRMYGGMLTNMKGSHAYW
CVFQLSVLCVVGLIAAVHPPVGGCHVLYFCIAAVLVAGAGVVAFTNMMRSAFLTVMHTAS
FVLLAVLCVVSAANHLAPSDGGTRAYAAIVLLLTTVLLAVTVYSVVVWYAEDRHWQELRE
PRRGGLEALLRDDEESDEDAQKPHEMTSSSYATGTTGASSYRPPAPPLQPMAGDTHSDAL
SPLDRASSASRMIDYAAM*
>TCRU_3837 hypothetical protein, conserved
MLQGNKASVHIDGESLGAEEVLLAGGRPPEVLRVCFDACGGHESHVTVDDVFLYNRPLNS
TAMRALKDRVPAPTREAAPTPPEKVRVFDSVREHAAEDITEEEWMGTTPLETNPHYKQSP
RSVWSGVSPDDRLALSQRSEIDTA*
>TCRU_3838 hypothetical protein, conserved
MKVPENQPEADISLLSSSGGRAAGIPGQESSEFPSKVSRSSEASGTGGDGQYKNTNGTAK
PAVAAPKPVSVTQPKPSGIAGAGAGEGERLEDPLPEKPVKETAPAAPSPKASSRTASSPS
KKKDTESTKPADDPQKKDKITTTAATQNATTEGHGEAASSSSAEDDSNAVANNADEGNVE
NSNDGSASRAAVPDEKQQREREDGSEKETESVPTPKAKNEDPGSADVVSVQQVQNAQSGL
ESGSESGKEDGVVTTNKQQDGTSDSHAESTPTSRSSAKRVAVSNGPDKATEEEISN*
>TCRU_3839 Mannose-6-phosphate receptor/Autophagy-related protein

27, putative
MERGKRKVHLVLRFFIYTFLLAGLLSCTLVARAEVASCVVGTYNLSKVPEANLLARERNS
DGTSQGPYLWNIVLCVAQSGFVKQYSKTRDRLGKAMTLQSIIVNDGYVQAVYSQWYRDWG
YQMNLDIKCGQEMELAWPSADYEVRTYYYYDTNTEMLNATVLTKAVCPVQPSGGSARGCG
GGCAFVVIFFVSLAAYVIVTVLWYYLRQGKRGKDLFPHPEFWADFPYLLKDGMVYVYTKI
RLCIGRGSSTTQYEQM*
>TCRU_3840 cyclin dependent kinases regulatory subunit, putative
MSARDFFSITDPVQKEQARHMISKLQSKILYSEKYYDDEYEYRHVVLPKDFARLVPRHRL
MDESEWRQLGVQQSVGWRHYMFHKPEPHVLLFKRPKTQ*
>TCRU_3841 hypothetical protein, conserved
MPLMNAGDGSPTTSHQSSQASHRAADAPPDLSNCPTKSSRRAMELIRKRRSTVVRRITDI
PALGQLYEESERGRSRDVGAVNVGAENVPSGAIHNLLPHARTADERGELRGLFDGNRRVA
TSTRLQYARMLRSMLEMDRTPLDMMIVGLQKIAARSETKQARPLTKEEMNQVIRSRTDWK
ERVVLRLAWITASRWSEVAALTAKNFTLEVDGTLILDRSVAPKTAKADPHRASRFVKIRG
QDAFDIIKLCRTLQEKDKLTNLTTAQVERALAPWDATAHSIKRGALRHAAQIVEKYNLNP
HVISQLAKHVDPFDLPQNTVRYLGRYTTLLTQVSSLIALM*
>TCRU_3842 BNR repeat-like domain/Concanavalin A-like lectin/
glucanases superfamily/Trans-sialidase of Trypanosoma hydrophobic C-
terminal, putative
MMMTACAGGPRRVYEIGDKGESWTEALGTLSRVWGNKKGGQATAVRSGFITATVGNDGDN
KKVMLVTLPVYAEEGNEKGKLHLWLTDNTHIVDIGQVSGEYDAAASSLLYNSGENTDDEK
LIALYEKKKKDGGKPSNSLWSVRLTEQLKRVKDVLATWKEVDGRVSNLCTSLSAQKDRAS
TDDVCGADKITAGLVGFLSDNFSGDTWRDEYLGVNATVNNKAGAAAALASTKNGVKFTGR
GAGAEWPVGRQGENQLYHFANYNFTLVATVSIDKMPEGNTPVPLMGVKMNGNENNVLLGL
SYDSERKWHVLCGDGTNEELSSTWETQTQYQVAIVLQNGTQGSVYVDGQRVGNGECALRN
GESNEISHFYIGGDGGSAEIKGDVSVTVTNVLLYNRPWDDTEIGAFNPNKANVPVQVTPN
AQETGTSSALDGKHLTERGQSMGSSGAGSGGASPSAVPTVSTPSAGKDSVMQVASGKSSD
GTQTVDGGFFSDGEPTVGTREGGKNGQEEEIHAQNGDVKTAALSSSLGNVSRGNNTDAGT
MHESGLLPSLLLLLGLWGFAAL*
>TCRU_3843 MSP-B, putative
MCHTMLLLVLLLCCVSGSVAVAEHHCISEEIEKKVGPRTTAVVLELPTRGGGMMRALTAS
APEWAPIRFQVFTEDLKDPSRYCTAEGQIRPDFTGGTVECNKRDILTKGKKSIILKSLLP
RALKMHTDRLLVEPLTGYLIVPEFLSGACAQFTIPSSHHTEGVTGADMYLYVSAGSTKGS
AVAWATSCSHLADGRPVAGAVNYDPSFVTDSEYSVRVSAHEIAHALGFIVRIMKERNMLK
EVEGVRGKAKVLQVSSPKTVEKTREHFNCMSATGMELEDEGGNGTASSHWKRRNAKDELM
AGVSGIGYYTALTMAAFEDTDFYRANWGKEEPMSWGNNSGCALLTEKCVINGVTKYPEMF
CTAESRLFSCTSDRLGLGHCTIELYDAPLPPQFQYFSNPKLGGLPNTLMDFCPYIELHEI
TKCSNGNKALMRGSRVGPTSKCLKGDGLADSEGFIGDVCAEVSCDKGEVSVRYLGHGKWH
KCPEGSSITPTGLFMQGRILCPKYDDVCIVIDACRGGGGVSSLLSAFPSIPLILLVFIFM
SMC*
>TCRU_3844 hypothetical protein, conserved
MTCLRLISAKIAKQLEGSTVAGHFSNRWAFQAPNFDAQVLLPIVWGRPGSWGASKGSSLR
KGRYYARTTPMPLRTYGIGLLRFTFRRKRHPTTARHVKLTLRCLAVFLRKAETLFQREAP
TSRRGPPDGPTHARRWRKYTEKSTASFDGLGRVSIRVFPTQSCGGETAGQHSEFSEQFGK
IHFSFFFAVASPPSPVKENFGLEGQVLHFLALDVTIWAPRVWNEGISEVVVGPRSAYSYK
CLHQADAQVQPAFDGLVSCRRALEIERDPADCFMDLFRAPLQTFQLQMMTTESSVPISEL
RAGPRTQQQRSLRCRLCRICGRDASTCNVGDATRGSYSQRRWPSKNGKGAARRL*
>TCRU_3845 hypothetical protein, conserved
MELICQRRSTEVRRITDIPALGQLHEESKRGRSRDVCAVNVGAENVPSGAIQNLMPHART
ADERGELCGFFDGNIRCGAIDPTAVRQGAAVYVGNESDLAGHGDSVVKKIAARSETKQAR
PLTKEEMNQVIRSRTDWKERVVFRLARITASRWSEIAALTAKNFTLEADGALILDRSVAP
KTARADPHRASRFVRI*
>TCRU_3846 hypothetical protein, conserved
MHRGRGRTARRLQQHAVKHHRARQQVHAAASVHRRPTRVQRPQHSVAHTQLRLQQRHRVQ

HKRRRHHVRQPAQLRVAHRSRRNNRRVTDRHVRVCRQVVHKHERAVAAGQPAHAHLQAPV
TRRLAAVAPHVDLRTAVAHAVPQFDAQCAGKHRRHVEHHSHAVRDAHAVHQHRVHGVANR
DGSVHRAASQSHHHRMHRGHPRRRRNQRQHKKKRHTHSRNDLAPHTMPTARKRTHRRPHP
PTQTRISRAVVLCRTRPVDRNSCLANPFLQMANPDTRSARSLS*
>TCRU_3847 hypothetical protein, conserved
MQRRRGVLHARDGVGGSRLVLVQLQGRMAWRVVSSLRGARHGCAALAGESRGRRHELRGE
PDADEPHAEHVEDVPLLRGCDIQRRGCCAGVFL*
>TCRU_3848 hypothetical protein, conserved
MQRRRGVLHARDGVGGSRLVLVQLQGRMAWRVVSSLRGARHGCAALAGESRGRRHELRGE
PDADEPHAEHVEDVPLLRGCDIQRRGCCAGVFL*
>TCRU_of Trypanosoma cruzi region 4/Dispersed gene family protein.1
Dispersed gene family protein.1
MVYPEVAQSVGGGLSWLCYRNVTFSGGGMSLTVLIGAMTGDVANVTFDGCTWRDGAVLLL
LGNAYAAVGSLNIVVTDNNFIDALLIPEGGFPPHTNITISGNRFTVTRLIPRSGLDIRRP
SCVAMNRLAISNDSAVVLSGNVFQSVTASSSAIHVFRSALRVLWHSVFAVVGNAFRMDGG
DSTLMHLEGSRHSSSLKVLNNSAVVIRGNVVTRLVKYFMHILSALIVESFSAVVFQGNEM
QGSSVVFYSSFASNIYYHSWLQLSGNLCRASPLDAFAFLLPKVNLRDSTVSVSGNRFMSN
IVAPTVLLISSVSSDLTNGAIVAACNTVNGGEEANYAIPSVYNATILTCSDQCALAASCF
PAYTTTVSSDVCACTCAEGGHGDACLPVAVPEPPSTDGADLCVRDVSVDVEVSAGLGTSV
ACYVGVTFAADVVVDVGLMSGSVRNVTLANCAFVDGASLYVVGWRSDPPAGHRADVLISG
LESRSGGGVLVANRFPPGSRVTVVDSVLIAERRVAYRGAYGLGDASACLVLHSVNLAGSV
LTIVRSHVAAVFRDAVGLLVVGGVALSSRGALYVDGLLVQTALGLCVSVEGGVAASGGSV
VAFVDSGFLLCKHAVSVRGAVSVSGSAVVLVRSDFVLTEDYAVAFYSTVSLADGSMLLAK
DNVHDGVSREMLYAAGAVTAVGSTLSFVRNRVLLPRMLSLSLSLSAGAHVRVACNGAGGR
VLSTAEEYAAAGFGDAGSIDVVGCDDCDRDTHCYAPGTASASMKDDVCVCVCGSGGYGEA
CVPVGAPVLPPAVGTAPSVFVREGVAVQSVFVVPAGASEVTLRHVVLDGVSPVLYLPWMA
RDGVRIVVQNVSLLNGAVLYVMGGRSLRGSMAAGSDESGPVELSVCDVEALNGALVLTGT
YPAGSVLTVTDSLLVAARPTPLLYLLGSQSSPYAPVLVLSGLRLVRSVLVVSGVALVTVM
TGGRTVVVDGAVLELVGGGVALDAAVFGGEYALYASARVVALGGAVLRVSGSQVYAAHGL
VFGGGVVANASAVVVNDNAGALTDGALLELRGSAWFAFGSWLSVRGNSISGRLLSVPSYP
RRAELVQSTLTLHGNAGSGLVVMDGTVALGGAGRRFVVGCLTLNGQALQPMDYRSAGIIG
EFRPVACGVCDADVRCFAAATRAMSGSCGCRCAEGGYGRDCLPVYLPHVDGCNRTLAPPP
LSHTATLTETRSPTPTWTPSLSATHYSPTWYGPTETLQVTETTALPPTRTPTASVSSTLW
WSDVACPTLAVTTTAAGGSLTQKDIRGGGSAVPTRLMVTLPPPFRWARDPQLGTHLSFVP
VSTAQPRGFGGPWGAMLRNATWVRNATNPSTVLELAVPVHRGYFIGADETVVIRCDAVAV
SSGCKGVLLGSFTIRSDTLPAAASALSAITGVAGGAAAVAVVVTGGLGSVLEMQALGVFA
RMSCASAQERASTVALPYFLSVFAALDPLWMVVGNALLAAVFGCVHYGVTVAFQRWRGVD
AATAWAAMRFPSLTYVVAHAMHLGIFFGSVLALAMPGARVQHRVVGVVGVLYGVAFPAGV
CYLIARHVGASFTRYWQFSRKPLHERLLYPVGYWHPAAQQRMYGGMLTNMRGGRVYWCVF
QLSVLCVVGLIAAMHPPVGGCHVLYFCMAAVLVAGAGVVAFTNMMRSSFLTVMHTASFVL
LAALCVVSAANHLAPSDGGARAYVAIVLLLATVLLAVTVYSVVVWYAEDHHWQELREPRR
GGLEALLRDDEGSDEETQKLHDMTSSSYASGTTVASSYRPPALP*
>TCRU_3850 hypothetical protein, conserved
MEVIRQRRSTVVRRNTDIPALGLLYEGSKRGGSRDVGAVKVGAESVSSGAIHDLLPHART
ADERGELCGLFDGNRCGAFDQTAIRQDSAVHVGNGSNPAGHDDYGVTKSCGAIGDEASAP
LDRGRDESSNPQPDRLEGTCCFSNCVDNGESLVRDCGIDTQQFHTGAGRDLNFGLVRGAE
DGESGPHRAFNFVRIRGQDVFDTIKLCRTLQEKEKLTNSTTAQAKRALAPWDATAHSIKR
GALRHAAQIVETYNLDPHVISQLAKHVNLFDPPQNTVRYLWRYTTLLTQVPWLVALM*
>TCRU_3851 hypothetical protein, conserved
MGTVCVDGKTLGSSATTVTRGTQLLDFSCFYVGGGGRSGSYCSQVTLTHVCLCNRPLTRQ
ELQMPLLRGKQTEAGCKERKATPRPGNFAAKLSQDPGVDAAARELLPVPAKLEFGLGNGT
ADVNGSTVLLFSK*
>TCRU_3852 C-terminal, D2-small domain, of ClpB protein, putative
MREKVWQVVRSYFRREFVNCLDDIVIFRKLEFGEHHDTVDLIFAEMSRRLKSQDILLGMT

EEAKLFFFLVNDFDVEIGARPLRCCAEKYIATEVSRVILVQLLLPNIVVRVLVSSSQGKL
AFSVKRSSVA*
>TCRU_3853 hypothetical protein
MDAAKLLEPSRARCELRTVAATFFEEYRENVETDAVLGRRFMPVYVTEPGVDGSNSIFRA
LGERYETHRGAQMTDNVVGVAGRLADRHAASRFMPDKAIDPIDEACANVRVQLSSRQEEM
DQLERTKRQLGIEAKAVGRDKKKKLLQERLRIVKGDMQRVEKPLLAQCNGVRQ*
>TCRU_3854 zinc finger protein family member, putative
MDLDYASGLHAVISWSNELNNEVACMKTREDAAAENFGESILSNGAGTGVCQSNISEDEN
WRPIPMRGDALPTSLGSSLGLTEAVHTITNKLASRNYSGASASQNSLNAFTVLLENGVQH
SQPYIEFDDIWGSDATQSNAVVKDNESCQEMPQALFIPSSASSGLVGSVGEERRDGITEK
CGEEKVIVVVDPRRSKLRVPLSAISPTKALGIRAQNPSLCLLFQSGRCRQGVNCHQVHVD
PEIVQRLRRIVDSLPCCCTFHGDCNTHCWDAKANSKRGVFISNVWVPLSHVAYTSGLARF
VTNKVQRPLSFGVLCRLHGKTGGCRYGADCWYLHVCREILETKFAFMTEANTKDSKTEGT
FTLLPNAAPMFPSVMTEVQLAASSRQQQQEQTSSFLPAVTQFPMMITSPFSTKDYKSNGG
INNVFPHRDTAVASAVPCMWVHSASPPQGSALAFSTVAAGAGAGGAGMHHEGLQHKTSDE
KVLPRRVSSMSQNIPNESAISVSIGPPSSPAAFAFSLGNGNTNPSPVSSLPYQLVEQTQV
SGMNVMPMNPSPSAAYSAMWFSHQQQQKQQQQPQQQEAYMPEQTRNITVPSAFVASSAGY
NLRSRLEEFFQPQNNPGPSMTLLPVISYLL*
>TCRU_3855 hypothetical protein, conserved
MYQRSLLFLLFSKPITQGYVAQLPQPAPTRRCTSWRRAFENNDEVRKKYTPDAERYEEWY
KATYGLESLSDGCAASGATSNRRLDDTQNMESVPSLDDFCAVEEEVEFCSRQFNHNTLVR
*
>TCRU_3856 NADH-cytochrome b5 reductase, putative
MKALFAAAATGFASSFCYNRELATCSAAKSPFSTTEFRPFTLMNFYDESHNTRVFRFALP
EADMPLNLEVSSCITAKYTGKNGEAVIRPYTPINKSDQRGYFEILVKRYDNSKMTTHLFS
LKKGDTLEFKGPWVKIPIKANQYRHIGMIAGGTGITPMYQVARNVLRVPKNTTAISLIYA
NTRKEDVLLGNELNELMETCPLFSPYYVLSQAPSDWMGGVGYVNKEMIKSVMPPPSSAAD
SIILVCGPPPFMEAISGDKDFKTSPPSQGELKGMLKELGYMQKMVFKF*
>TCRU_3857 leucine-rich repeat protein (LRRP), putative
MIEDAEHAYRRAVEIIEVATIPPDPDAPSLHMWRLRPSEEAAFLIQRYLNPPPQWGIKLS
LSATFLGPERCVSLGRKLILNNTVTTLDLSMCDMQEEAAVEFFHCLKRNRCLKHLNLDGN
NVGDKGATAAAECIHNLETLHMSCNDIHDKGAIAIACFLRNSSKIKTLNLRANCITFYGA
YKLISALEPILERLSPALLERISIEEPVSSSDYDDVLNLSARPSTQGTFDVNGDIATEES
AEKASHGKQNRIPPEDVYNETLHTLWLRHNRDIPEELFKIVETILAKRFPQPPKGISKKK
IALKSKRE*
>TCRU_3858 hypothetical protein, conserved
MKKEQVLQQEESDSALARRLHEEINGPGNVQPGAGSIPNQASGVKLIECPACTFINTITE
FVPGRSYTCEQCFTPLSVPASNETIDAPDSNQKMVMCNACQYMNRIPLGNFDAIVCGACC
HKLKGKTPVSETQHPQLPPASTRPLQVRCGECSSINALQVGMEVSTVRFECGSCQTVNEV
SL*
>TCRU_3859 protein kinase, putative
MGRVGEYEIFETVGTGAFSKVKRVRHIPTDACFVAKIIPKSNQRVENDIRVEISILRRLK
HRNIVQLVEILESPNNYYIILEPVFGGDVCSLVLSQDSSLEEEVVSGLFFQLLAGVRACH
QNGVAHRDLKPENLLLTEGNVLKISDFGLSRLHKQSNFHAQIDEYAHTLTGTLAYVAPEV
FCGTYDAFKADIWSMGCILYVLLTSCFPFGYTTDARELEERIKMGRICKLPDSVSEEAKE
LTLWLMSLHPEDRPSLDQVALHSFLKRYVPMKHILALIGSKQPPPLAANPAEFSSEVVEE
EKQVNERGRPLFGKEAPREYSLKPCSGGSSKPMGQSGKD*
>TCRU_3860 hypothetical protein, conserved
MLRKRYRPLDPAEERSVDVEHKLQVGLLYDEQSYCFRWPDAPLTSMLRRSNASLTRAVHG
NMVSFTQSIIAQPPIFTIDSIATEHGKRDFFASVSVDRPIISFSSGNVSPSARYTETQRY
KRHPGAPIETSTEDSIFSFNAQQLKDLRDAGAFKAFREALSRSAPDSVNLDNEAELESIA
RREMENELTRLEWQAFHESEKTAPALQRIFLLTTAVPFMEDTKKHGKEASTQVSYGWQRI
VYDLWIKWRRETPMVIGDTLTDTNSSPWGSPLLTGAFISSLAKEYFLTGNGEPNGAIKSG
RLREKRGGKDKLMFPLTSPRRRGAYGYLRWRIDLYEPRIRSAWGGSRGENGDRGSAKMLS

AAEVNKLINEKLPAALADVGCDERMDPVDALALTDIRSEVETWRHWLTYV*
>TCRU_3861 hypothetical protein, conserved
MSKSSQQSTRKWIGQRALARAEDATNRKANAAVPPPAPASHEHNPSSPPPTLVQLPLSLI
FERQEAVDALSNLYHNAETVRKVETGDPIFDLRDASKVHEEIMECLKAGGCA*
>TCRU_3862 hypothetical protein, conserved
MDANDGAPPSEPQGTPVLRGDKEQPVQQTANELQAPRVEAIEEANENIQAVQSGAVVEEP
LQQEEREASEGGEELKPNLVGAEVSSERKPREELEVIPAPVATIPTSIEEEEIMQPQQPS
HGTQVELGEDHPSPTPSVPAPSASPAAVTSAAELSSNGYIVYAQNDVIVDATVYPTGFRI
LVKAPGSPGGKIATRFVYESVASNLGTHPEVIKVYCKGKRIRFGETIDYEIGELQAKGRL
WVDVHFNEDKMPPHLQKLRKDDNVVRCLQLRSRPDDISPADLVAARKRGLTFDEAINEFR
QRNKSKDIVSVVLLHEPTGTRMPFLGGYRLKKDPSRVFLHASTQLRAPGDTSLEDLQYGP
LPRDGVSRRTQTYGVSRSCQTLRECSTQTARPDYEADDNYDEIVIARRYFSSVQLLALQT
EKAIIMQKMYRKWKARQVYRELFAARQAFLERAAAQAAAEEAEKKLREEFERRRRATPRT
ADDFSTLRRELEAWRAAETTRILTDRTLSEVAKRAALTELTKRELHLLQELETLRRGVVQ
NRRARRFEDILSLMTAPKQCGIVSVTTRAAERARELRELYVALTETPSSVEARLDVLLHI
KWTVKEFDSPLTKQIVELVDRESDLLQRGRTACSLKGLRTRLEHLFKRFIATPEYNPAVD
EVVKGPTFGADENGEAASMVAARRRLKPSNAV*
>TCRU_3863 Prp18 domain containing protein, putative
MSFAGLQKRLKNKAVATTTTNSTPPSSDAKTDTIESSSSVYDVAAREARANHEERKRQRS
PHDKENTAYNPTINERNGPADMLSDHPSVEKASSKTLTETEFLERRHLAEEVVLQCAEAM
SRNMELAATSIDKIDIKKESEVYDMSGLPLPLCQFLEQRLQRSSDATAMLISTCEEEEHN
LVSALRSPSNLPMLFPPQVEDFVTTLRTLWLALCWHWVMALRGKEGNEGKEVSENGWSYN
VYLLSRHALPITVRNTRGRDVALALQAWQEAYRVRQNMLDFLAYILHDMGVVLRFSGDDA
SKGDENLRIVPPKIADSIFCMVQHLRRRDFTAVLQAYVELTMGTANWKLGLFSGGEVHMR
RSMERIERRRIEHLLHNERAVRLLHVLRELIEFVQKNEVMLLHTGIFAREKSMKEHSP*
>TCRU_3864 hypothetical protein
MKHLRVTLRGEGAHELNQLLKVLKARVARPRVGLEQRQHEANLSIPNPLPGVELKIVGTT
KPENDYFDGARTKEFYVLRIDLKHLLYRRVSVTGALRKGQPRLGEARNEAPRIGCPSRGG
TKQQSCLLQLHGEFATRNACEQVSHKGDNKTFLRLVTGNAGFVSAAILLAADAKAEAEAA
VAGVTVLHAVRDFHA*
>TCRU_3865 hypothetical protein, conserved
MRLYFADQQRGRTQHDVPQHHNAPHVNPIVQKLDLRGLHRINLRDGNVTVLREGECKHNN
RGSHHRLAHADQDASRLRRLSSPNKLQHGTLTEYASSERDVDGEMQRHTVE*
>TCRU_3866 hypothetical protein, conserved
MDNCVVRSRTHVMYALASDLRVGSGSVFSMQNSSWSAPSIEYYEGACVFEDVAVAGGSVL
QIVSSAFRLGFAMLMANTLTVTDGSWLVHRDNEFRTAYVVYVANENGVAFCDRSVWSILD
NNFTYGLYSALTYMTSDWSPASESRPIIYGVCNEARGSPVTNYQEELNIGVPVTSLDCGA
CTIDAVCFAARTSSISGCECVCAAGGYGDTCLPAAVPEGLGPLPLPDADDTDVRCVHGGS
ISSVDDPDPGVRGLCFVSVTFTTAIVLDLSHFDVPQQTLNITLLQCVLMGLSIKGSGARV
HVNVTSSMLDSGALEFEGDFGASSLILVVGSTLVSTSGHAIQFQRFSLGANSMLLLLDNY
IEGSNYAVRLFFVVVDGDGIIMKGNTLRGAEEDFPLESAVFFESAILKNDGYFDMENNTM
SAVSGIYFYGDIIVSSAGLLRVADCTFAGITLFFKPALVHFDGLVTLQGGAQWRVEGNSV
TAASVIGMPNSLYKMRLSGSGTTVVVAHNRQVDDSCPFAKMAPSNMIVVSPARFVVGCNL
QGGEEVSYDGLFPEDVMVFRCGTCNGDAACYMPGTESVDRGSCSCSCKDGWHGASCLPFE
VPDAAVPPVAERAVDGDTSCVVNQTLTNVTLNMWKTHHCYVGVTFSGVGAALTFSFDSMP
LHLPINITLTGCIFREGAVLQFVGGAEAAESAGVLIRVSQR*
>TCRU_3867 BNR repeat-like domain/Concanavalin A-like lectin/
glucanases superfamily/Trans-sialidase of Trypanosoma hydrophobic C-
terminal, putative
MSRRVFTSAVLLLLVVMICCGIGAAGVAEQSDEAKFKWRAVTDGETVDSLGVPALVEVNG
KVFAVAEAQCKKKDNETSVFTGVASQLLSTEKGKEPEEVLKEAKKNTQILEKGASEGQKK
KVDVSRPTTAVKESDIYMLVGQYCSKDSQNSGAGGCELLLVKGSVSGEGTNNKIDWKDTE
SSPQRLFGAQLDSWTRLIGSGGSGVKMKDETLVFPVEGTKKPVGTEEDGKTVSLIIYSKD
ATNWTLSKEVPDGGCSDPSVVEWKGDKLMMMTACGDGRRRVYESGDKGDSWTEALGTLSR

VWGNEHDKNVKPVRSGFITAALGDGDGKRNVMLVTLPVYADNDKEKGVLRLWLTDNTHIV
DIGSVSDDEDVAASSLLYKSGETNEEELIALYEKKKGDGDTPSLGMVSVLLTAQLQRVKK
VLATWKNVDDRVSKLCPSESAEEDTQTDNACGAFKITEGLVGFLSGNFSNDTWKDEYLGV
NATVKNNDVGGKKGEATSTEKGVKFTGAWAEWPVGSRGENQLYHFANHNFTLVATVSIDG
EPTKEGSIPLMGAKMDDDDKTVLVGLSYDKENKRAVLCSGGKTREHSSTWESQTQYQVAI
VLQNGTQGSAYVDGEHVGNVQCELGNTDSKGISHFYIGWDGGNTVGDEGVSVTVTNILLY
NRPLTSEEIDALNPNTAPISPPEDLTAAMVDTPSTAVSGTIAQKTVSVSTPGGSTVNHES
SASSGEVAETVGGTDAQGEEGIHSQDGEVNATALNSSLGNLSQGNNSDGGTMRGSGLLSS
LLLLLGLWGFAAL*
>TCRU_3868 Leishmanolysin, putative
MNGALSSAHGNAVAEEVVPAAVKPHADRLLVQPLEGSLVVPSLATGSVCSRFTVPAEHCS
AGAANSDTVQYVAAAPGGVWALPCATLEDGRPVAGAMNIARPPYFTDASPPASPRT*
>TCRU_3869 hypothetical protein, conserved
MAMMMTGRVLLVCALCVLWCGAGGGYAWDFGEKQELNEHYYGANGVFCKAFPTMYSCELK
INASKLKEAALQKNTAQSGTGTGQPNAGGSSGRQAHGSEESGLGQSEGTAGEGTPELNKP
GEGGTETPNKHEQPPTATATAAQASATRTPDESDGSTAASHTTSPLPLLLLVACAAAAVV
AA*
>TCRU_3870 BNR repeat-like domain containing protein, putative
MDFARKAGYDSFRAPSLVEVDGVVVAVAEAHYKNPKDGELYVGIAAKWLEGNEKTWMNGK
VVGMNHFDTKVHPLLSATSIVYYGEMNALFGGCGM*
>TCRU_3871 BNR repeat-like domain containing protein, putative
MARYASGKVSAGCGNREMKAFEWDYGSKSNPTDLLRYCKPHPGRFKQFLGGGSAGVRLED
GGRYVLPIQLLNGGGRSVSLTILAKGFTHDWEFSDYTAHEDCIQPAVPEWRAKVSA*
>TCRU_3872 Trans-sialidase of Trypanosoma hydrophobic C-terminal,
putative
MTPADVATAAPEPGTEPATAHHNGNVLDGAGAAPAHSSTTPGKTKVPSEFNAAPALGSDP
FDLESVTALVSVGLRCGNTVHGCVSRVLLLLLLGLWGAAALC*
>TCRU_of Trypanosoma cruzi region 4/Dispersed gene family protein.1
Dispersed gene family protein.1
MVDGARAKHHDSPVGTCLGWWMRSAEGACPRSRHRVRRRAIAAVSAALLVALALAAAAAW
MPAVHAVVLRLRGGTVDRAITVGRAVDTGLMDGVYVTNGVAVVFDVPAMLPGALRIELRN
CVCDGGAQIYVRGYSGEPASDRSLEVSVSGLSGSYCSLVFVHSLPAHTNVTVRDSTIVTP
GPMRYSQLSGLTDAVASPLVLHATSLLQTQLRVSNTVLRSLQAGGSAVYVGGGVDLLSSA
VVLDGVLLEASGGPTASAMHVASSSRLSLRSHSVFSVTNVSVVSSGGGIVLGERLAVIDS
VLRFVDVEGSVASSLVCCDGGMVGAGGWLELHDVWAVGEASSVASLSGVTLSGGTVSIAR
CAAVGATLVSGPTITSGTVTVQCNRAGGRVLLSSGDYRMAGLPSVSVVPCDGCAAALACF
DALTASFSDCVCGCHAGGLGEACLPFDVPPARTGGSGGGAPGCVSGVTLTESVTVGGGRA
TACFDSVVFSGPITVAVDLRLMDAFADALNVTLRHCMLAGGALLRIGGLSESTARLMPYA
LVNMTNVTSMEGTIVLHGAMPPNSSVLLANSTLRATVGGSQYVPTTPGHERSRYGPALVL
DGVRLLSTRFVMTRSLLVCGGESCAAILVDRGFYANLSSVFYMDNCAVRSRAHVMYALAS
DLRVVSGSVFSIQNSSWSAPSNEYYKGACMFKEVAVDGGSVLQVVSSTFRLGFAMLIAET
LTVADGSWLVHRDNEFHTAYVVYVANENGVVFRDRSVWSILYNNFNYGLYSSTIAHMTSN
WSPPSDARPTIYGVCNEARGSPVTDYREGLNIGAPVTVLDCGACTVDAVCFAARTSSISG
CECVCAAGGYGDTCLPAVVPDGLGPLPLSGAKDTEVRCVHGGSISSVYDPDPGVRGLCFV
NVTFTAAIVLDLSYFDAPQQTLNITLLQCVFMGLLIKGSGARVHVNVTSSMLDSGALEFE
GDFGASSQILVAGSSLVTTSSYAVAFLDFVFGSNTTLLLLDNLIEANRYAVYFSIGVVDG
GGVIIKGNTLIATEEEDGVESSVCIYAVMKNGGHFDVENNTMSAFNGVYVFEDTVVSSAG
LLRVANCDFVGSAEFSDSALVYFGGYLTVEGGAQWRVEGNKVSTASVLIIAHAQHKIELS
GSGTTVVLVHNRQVDDSCPFAEMSLSNTLVDSPAKFVVGCNLQGDEEVSYDDVLPEGVVV
FKCGTCNDDAACYMPGTESVDRGSCSCSCKVGWHGASCLPFEVPDAVVPPLPERAVDGDT
SCVVNQTLTKITLNMWKTHHCYAGVTFSGVGAVLTFFLNSMPLHLPINITLTGCTFREGA
ALQFVGGVGVTESAGVLIRVSQTVMQSSVAYFSLALPLHCDIAVTEVDAVQASEVKLADT
ISNKFGVVLLQNVVLTASSLLVSNVKAHASKRDAFVFYSTGKLSLVGGSSLYTRYCSFDG
YTHLFYVNILSVRDHSVFALLNNTMASGTSLVHQYSRFSVSDHSVLRMVGNSGSVSYAIY

SLKLWTVERSSWLDWRDNDVGVGAIFYATESTFLIIDNSSVVTLTGCKMGSTGWSVSLLS
QADADYRFVAGCLTVAGREVTTAEELELHGITNVTTVAVCGECTKDGDCFAPLTTTVSDC
ECQCAAGGHGDVCVPAPVPAGPPPPPRTPPPPPVGECISDMVYPEVAQAVGGGLLWLCYR
NVTFSGGGMSLTVLIGAMTGEVANVTFDGCTWRDGAVLLLLGNVHAAVGALNIVVTGNTF
YDALLSPEGVFPPHTNITISGNRFTVTRLIPRSGLGRRGPSCVAMNGLAISNDSAVVLSG
NVFQTVRASSIAIHVVRSALRVSWHSVFAVVGNKFHMTGGNGTLIYLEGSMHSSSLSVLN
NSAVVVRGNAVTRPVKCFVLFLSVLRVESQSAVVFQGNDMQGSLAVLFSRSCCRIYYNSW
LQLSGNLCRESPLDAFAFLLPKVNLRDSTVSVSGNQFMSSTVTLNVLLIPTGSSDLTNGG
ILFACNTVNGEEGVRYIIPSVYNATILTCDDPCALAASCFPAYTTTASSDGCACRCAEGG
HGDACLPVAVPEPPSTDGADFCVRDVRVDVEVNAVFGTSVVCYVGVTFAADVVVDVASMS
GSVRNVTLVNCTFADGASLYVVGWRSDPTAGQRADVLISGLESRSGGGVVVANRFPPGSR
VTVVDSVLIAERRVAYRGDYDLGDASACLVVHSVNLTGSVLTIARTHVAAVFGDAVGVLV
VGGVALSSRGALYVDGLSVQTALGLCVSVEGGVAASGGSVVAFVDSEYLLCKHAVSVRGA
VSVSGSAVVLVRNDFSLTEDHAVAFYSTVGLSGGSMLLAKGNVHDGVSREMLYAAGAVTA
AGSTLSFVRNRALLPRMLSASLSLTAGAHVRVACNYAGGIVLSTAEEYAAAGFGDAGSID
VAGCDDCDRDTHCYAPGTASASMKDGVCECVCGSGGYGEACVPVGAPALPPAVGNTSSVF
VREGVTLRSVFVVPAGASEVTLRHVVLDGVSPVLYVPWMARGGVRIVVQNVSLLNGAVLY
VMGGGALGGAAGSDESGPVELSVCDVEALNGALVLTGTYPAGSVLTVTDSLLVSARSTSL
VYLPGSQSSPYAPVLVLSGLRLVRSVLVVSGVALVTVVTGGRTVVVDGAGLELVGGGVAL
DAAVFGGEYALYASARVVASWGAVLRVSGSQVYAAHGLVFESGMMANTSAVVVNGDGALL
VLRGSASFVFGSWLSVRGNSINGRLLSVPSYPRSADLVQSTLTLHGNAGSGPVVMDGTVA
LVGAGRRFVVGCLTLNGQALQPMDYRSAGIIGEFRSVACGVCDADVRCFAAATRAMSGSC
VCRCAEGGYGRDCLPVYLPHVDGCNRTPGMPPLSHTATLTETRSLTPMWTPSVSMAHYSP
TQHGPTETLQVTETTALPPTRTPTASVSGTLWWSEVACPTLAVTTTAAGGSLTQSDIRGG
GSAVPTRLMVALPPPFRWARDPRLGTHLSFVPVLTAQPSGFGGPWGAMLRNATWVRNATN
PSTVLELAVPVHRGYFIAADETIVIRCDAVAVSGGCKGVLLGSFTIRSATLPAAASALSV
ITGVVAGAAAVAVVVTGGLGSVLEMQALGVLARMPCASAQERASTVALPYFLSVFAALDP
LWMVVGNALLAAVFGCVHCGVTAVFQRWRGVDTASAWAAMRFPSLTYVVAHAMHLGIFFG
SVLALAMPGARVQHRVIGVVGVLYGVAFPAGVCYLIARHVGASFTKYWQFSRKPLHERLL
YPVGYWHPAAQQRMYGGMLTNMRGSHVYWCVFQLSVLCVVCLIAAVHPPVGGCHVQYFCM
AAVLLAGAGVIAFTNMMRSAFLTVMHAASLVLLAALCVVSAANHLVPSDGGARAYAAIVL
LLTAVLLAITVYSVVVWYAEDRHWQELREPRRGGLEALLRDGEESEEVVQKPHEATTLSY
ASGATGASSYRPPAPPLQPMAGDTRSDAQSLFDRAPVRAAALIMPFWTGEDALLDEEISA
VSPFGV*
>TCRU_3874 retrotransposon hot spot protein, RHS3, putative
MIVVPSPKVGNYDKWMKQEGAARIIVNCLDEMDVKAMCAWMKRGMDPDKKAKCWRIVKER
MEKVGPIPRHVFDANEFIAHSAAIEDALDAINSLDGEKHFTHGGVRLWYSENPSQKLVRV
ARARGEVGAEVFLNAPMSVFLGCRIPHYFVKSDE*
>TCRU_3875 retrotransposon hot spot protein, RHS3, putative
MVLTSDKAWLYSWKWKENKSTHDCLVNCEVDRVWQTVKGDLTKWSSTYRGTEFAPGRRVL
IGIPGIGNSMAAGSYLLYQLLHYDAEKIQLVFHCFDGRGEYVSDKTTQTVREYAGGKASK
EFLYDLWNCGIKVYIIYDVAK*
>TCRU_3876 hypothetical protein, conserved
MSRRVFASAVLLLLVVMMCCGTGGGAQAGVGSNADASTPGSALTGAIAGEGSASGGVEGL
QRVDLFVPQTTQVLAKKGTDSSRRDSFVSPSLVSAGGVIAAFAEGRMNAKYQGGQLTKPS
FDVVAEYIDSAWDCSTLVGEVGKGRMECTHCAW*
>TCRU_3877 Myosin-like protein
MEQQDTSDADYAKDLRELKDQLATTQEELVRVRGELSSTAQEKMRHETECRGVISGLRTK
LEEARYDADAMQQLTREKKHLDEIAEEYRGKYLSMCKKVENLDIELTAATNRCTLLEKRL
DEELRRSLRNVSTSPTPQRQNGVEKRLRSASSVNVPLSLTTHPRKKSRSVEPRVFTISGF
DASELLTEIKQLPHATVAECRSNTPVPSNLTHLITNGQLTIKLLTALVRGCWILPDVYVR
DSVRDQRWLDESAYGFRHERPPLFQKRIGFTEGFISSRHYSTAKLITDEGGATVETELRV
ADIVLCTHAEHGTLEECTHAMTWDKVVELIYPVKIGDTQ*
>TCRU_3878 hypothetical protein, conserved

MAMMMTGRVLLVCALCTLWCGAGGGGCTEDTAGSESGLSNRGSDEVSEGASGQTASGEAG
NPPVQQSRLQHVGGSPSGGSRSQADLQPQSPAIQKPSPSGSEPTDSLSHSQSSGEERQDV
PDEGPPGEPGISPNQEKNKNVSIVNQQSIDPPSHSGNGDVVSSNSEERTEDTPSSTEIIV
AAPSEEGQERENATPSLEQPRETSTAAPAITTQTSSTTISDVSESNTVKMSEASPQSTGT
TQTNGTTTKIANSDSSATVSYTTSPLLTLLLVVACAAAAVVAA*
>TCRU_3879 hypothetical protein
MGSAAIRSVSPRPSDSRNAGSNCSQHVVGSHNHTTGNASNAETGHTVPQSPNTHEVTLLP
ILFFTEPKNAQSNNMSLGVSSDKKDQHVSSPVVPGDEVSVGLYADGNIVSAERQSTYNSH
WVRQASRNEISDSFEGRNSDGHISNQQSFQKDGRCEGLLHKRRSNEFHRTGPRVCLGSSL
SAPEMHHNSKIGSLSSILQRGSSTRHVRSAGASGVFLSSEDGWNEDDNNPAMIKTPSLKC
LQEAESVVVALEDAEEKERQRIIKLIEEMKEHKLQLISTISSAFLPPCE*
>TCRU_3880 hypothetical protein, conserved
MLSGRTLRSAGSRKAASSCAVRPHRERGCPGSQVAAQRIRMHAQRETTCRPAACVIRSQR
GAGRFKGRSSDTVQGVLGVPWLEDAHPAAAHIVAEEYPGLSMRGADVFREISM*
>TCRU_3881 retrotransposon hot spot protein, RHS3, putative
MGAKDLLLLILGSRGALASRALEQLGLRVFMYGELVCALVEELKELRPPERNEARDSVLK
VNHQGCPTRTVGIRELEGGVTRIPMEYGVLYLPKVENFPLVDGFFFMESPRRTLVGLQMT
TASAHHKITSTVNLFNERLAAYFKGWKKLSRDVSWEMIYVQHENSTPMKKWQGCDVVNPN
NETDAEKKIVAFWDGKVHQYQFVLTRDFLSKITEMRTQ*
>TCRU_3882 hypothetical protein, conserved
MLAHGPLSKAWKDSTLVGHIANLRAPQIPNFEKQVLLPAMGVARELGHQQGLITAERERL
NFDCANAVADVRNRNAQLNSQVEEAQINLSVRESTIAGAGNQVLELQQQLEAAHAQLRER
EFNLGVLNAQLQEREARHNAQQPARDAETAVRDEASATQHASNDGVSTIPHQTDILMAGG
EPSSAARRSSAAYRRPHGNVGRPHFHPSTPSPSRTFPLDAQLPMEEDRSFCALDVTTWDP
RAYGMRAPPESLSTSGAHTNAGNRRTSRLNLPLTIWSSGPARWKVCRIPPLES*
>TCRU_3883 hypothetical protein, conserved
MVAPLMLDLMDFRRMMCKISVPIRLLVLVQNGREAMLSLCLQELERVYGWSGRLVVSRHP
ENIGYSAATNIGSRLALSLPREKVPFVFVTNSDVKVPPDLLPNLLRDVHEVTRHDAARMD
ELAAEAANGPSESSPVLRRGLRVLRSTVNDGRLSTSALLPDRIRYASVKERGKMFSKRYW
HFCAYYKSSCFASVMLTRLAISTVGYFDENFYPDCVENVHYSLRLRLLGFQERNVLYGKF
VHRGSSNIRFSEQLELPDALWYRRVKSLMTNQPYAVMKWNGLKACCDGCKGPYDGMVPLD
VWVKDEARIQRIRVYGHDEEQGVPKVDYDRTLLYPVRTKGR*
>TCRU_3884 BNR repeat-like domain containing protein, putative
MSRRVFASAVLLLFVVLVCCGISGDTPANEEEVKNELINIQNLERVDLFVPQKTLVLPKG
GTGSVTTRDSFFAPSLFNAGGVIAAFAEGHMNAEYQGGQLTKPSSDVVAEYIDSAWEWST
LVGEVSESTWKAHTVLGTTDGTNRVGVVLRPTTTMKGNKVFLLVGSSAVFYESGGNWS*
>TCRU_3885 hypothetical protein, conserved
MQPLPSCSISDTRYSLPGSHVLLGVVFVPSNTVSHVLFMPYDKPNNLPAETVLQTRTQQR
SALLRNMVDGHRRHNSESVVCKAVLVIILPAVHRPCCPGPRSREFEPVFDLRCLLRSCIH
AALIVDPRVVTGRIGQSCHQTRRGYGRLGEEGVQSRPSTEGGIDRIQLISQAGK*
>TCRU_3886 hypothetical protein, conserved
MLDDYVAKKNGKLHDAEATEHHLNSVVDQVLNYLRNGVRITKIPSKGNACRRLYFLLEDC
KRIHSCDLDHQGFPLNRKRPPVTMWIRDIEKVLIGLSTTSFVNYGGEAQLAKTRQEAVSD
NGTHRHDATQNIIPSSLGANNHRAFALLLRGGESLEVVCETDSDCEAWLVALKRLLHLRT
PAERLLEERRGT*
>TCRU_3887 leucine-rich repeat-containing protein
MGCKESSLRSPDVKKEDDELEAKKAGGKPVGTEAPINLDGDSSNNASLSKLLPLNGRTRS
PLLERPHGSFELFTHEPLLDGREAPNDMQLVSFLANEDNGFRMDDEYFLLSDSGTSTPLL
FRTEPLRRAQTGEGTWEWWYDQVENLRITKDNSSRARLQDIPNRLTRQYDAKKDQVELDL
GWCYMERSAPYVLGRLFVSPYLREIRWVTSLRLDGNYFTDDGFGNMLAVMGVANESKTVF
PLLKNLYLNNMNLDHRSVYGILFYVFPIDLSSAARGTQVPRIAGQVAGLPAEAYMPFHGA
PHAPLFPSLEVLSLCDNPGIGNNGLAEILRCFVAPHYERRNLPVLDLSRCGVDEVGVCYI
NEYLEQLRIADKRGDYIVVPRRVVLFGNRHSSSSKSSVDGNTAGSGVTLLM*
>TCRU_3888 conserved protein, unknown function

MYSKGRDALRRLDMVLESLRQGALKETRATREEVKRSLIREDVHRQNEERRQQQQQQQLG
IEQEMSSSPSVGRVGDPSMASGGFSTSSLVGCGGNDKSSGMGGAKGLPAWYDGDYTTAPR
RVEKTKNGRVSTADCDERESSNGGDAKLHDEESAMVVDVTQRVSQAERKLYPILLADVAE
CANADEKNRGGNALDVGGSEVIEWDDRGAPVRLRSTWMPVSPQTLVPVSRTVDESKESAR
SDVMHSRRTSAAKTMTTTTTMQTISSDKKLCTQLHEYTWKEDPLAPMPMAGRPAEEGPSP
PPVGLSGTSSRGAVNRMRQALPFSPNRGNSTRRRNRPPLSTEGTAVAESSASAIARQIRQ
AVVKQPTASVEVNKKKAGSESFSSFPVIHGSSGRCSVLYEPDEAECVITHVLNPVTAFFC
TSCGVCVAREIVTGEPNSCTACGEPFEALPPLPSPRLNGCLLDTVVTETKSVVSLREEGV
PAGISASVKKCTATTSTMTTTTSTTTRDISTLPMTAKVTAGTQAGTSWSMPPLAATAVQG
LYFLRLWDQAIAL*
>TCRU_3889 Isoprenylcysteine alpha-carbonyl methylesterase, putative
MLQYLVRQVLWTISVSAQLLAAMGLGFKWISMAIRLLVFALLILWYFIPHICAYIMSPNI
IRRVSYRSSKRTRQRNLQIFLQQVKSIPPVRRKENGREVSEDPAEQRSGPDMDAVELLAT
KLNPADGRLMMGLPRRLSSPTRTTEVLDDEKVMKIGSFSDTVAFIQSYYATASAGEHAAS
NGVPAGEHKNPDWASDAAQEPNSTGDSEDEEDEEDRNNLHNRAALDIYLPIPLDSLFRMM
EQEKKISPRSRLQHKKFPVVISFNGGAWIVGFYLWNFLLAPLLAARGYVVFCPDYRNFPQ
TDMEGMILDVSDAIGWVINNAGRYGGDPEDITVIGQSAGAHLTMMSILSQAQLSARAASG
GTAPSDMAYNVPRYNPRVSIRQYIGLSGIYNIRGLVKHFDRRGLYRNVLYQIAGGRSNLP
RYSLNSYFDERRCGDTGEVLPENIFDFLPQRMYFIHGDADCSAPFSESANLAFFMRNAQR
ARLAEQYTNGGQTMGICLAPPVDIKYILVPGATHTDAIIEECLAARESHVVDFLCYYSTD
NEERDKCLNNSNGFRDVNNKRDEDDGQLDPSRHPDGVLPSPAPHNERPMLIRLSSYVCPF
*
>TCRU_3890 hypothetical protein, conserved
MMDVDDVITRHRARLDEHRKFLHTSPAGSTNPPKSPDGINSSGILKTVTRFHAVGDNTFS
PLPAWPGSEACFDVSPQPDINSLKPGRDKKGRVKSEPSKKLLREPKTLMGSADVTPEKLK
AQAASFSGRALEAAPRRASSGPVLMLSSDNKNNNNNRNRKLHDWLAEEERFFDSQLKAVG
RYYVTKPEAMYAKSQLWLESRERSLQRLQEESQRDVLGECSFQPNVAGSRSLTRRSSSTH
AISSFCEDPSVAAHLERMEEARQLRVEAERRVKGKKAGTWTNSITVPKEFEFAKKVAVIP
SLRKPLVNVVPLHDDDEVKEEGAKPRTDGNCAGVACGDLLVNTKAVATQRKRHGGEEEEE
EGRKREEAKVRDASVRSAKAVSQLKVVPLLEATIVQLKEELARKDEQLQAQKGELALLSR
ELEVAKATVRRLSLQDVVKVEEGEEERGTTPGPTSPMDCQESTESNA*
>TCRU_3891 RNA pseudouridylate synthase, putative
MRALEDGEHSPGDRYPLVMRATHVYDAPNNNSSNDDNNNNNVGFHETAPVRVLAVAPYKF
TFSAPVKGRWLGLPLLTVFAKEFAYIPPVAAGNETPRASRDPRVTIPAYVEELRKGLLCL
RGREEECRAAGDAYADELRHLRPLPRPNENADATRGSPVEGMATCSDAETMTLLLSAVSP
RLLLRQKDVILHHVLRREAPMPDGDPVHILRYDHVPCGSPPRRLLFVRKPHGLPVHPSGR
YRKNSVTSILEDVFGGTDAHRYRAIPLKKEDKRRNVPQPADSVECVAIRHKRYDFELVRV
WVGDGYVTRDAWEELRSRMTRQTPHGLKVFVVHRLDAATSGVLLFGLDSRSARMTAELMA
QKGVPDDKDAFMPVAGCTKQYIARVHGRFCADKLAETQHHCTYGDRKLFASSSLNESLGK
LSARPACWLRVDRLIGCLSYHKSLYWCPDAALTESWLNEKEKWEKDAKEQQQSASMVKGH
AAGRKRGAPFGDAAALEAKHERMRQLTRGGRAKATAASDGEVNNDSRHPEESTSKIQMQR
LKESMKQAITLIRLLAYDAACDESVVECLLLTGRTHQVRVHLASLGHPIRGDKKYIRLAA
DSPSIPLRTDLRDEEEEDEEEEEEELKGYLSSHGIFLHAWRYTLRYAAGEAPEVLEAPPP
AWALTGEEGGAITDPMASSCV*
>TCRU_3892 60S acidic ribosomal protein, putative
MSSKQQLACTYAALILADSGKTDMDSLLKVTKAAGVDVSKGMASAFASILKNVDINDVLS
KVSFGGVAPAAGGATAAPAAAAAAAPAAAAAAKKEEEEEDDDMGFGLFD*
>TCRU_3893 Asparaginase, putative
METLPPASANGEAVVSRNPILFVSYNESGGRRYTDDLFPRKDRRVLVLYVGGTIGMAWTA
NGVLEPCKGYLTQVVRGMGELQQRPEIAPFDIVEYDELLDSSDMDGRDYMRIATDVARNY
DAYDGFLILHGTDTMHYTASALSFLLMNLAKPVLLTGSMVPLVEPYNDARRNIVVSLMLA
SNPSIREVCVFFNDCLLRGNCCCKLHHTFGAFCSPNYPVLGMVESTGFHLREQLLLPQPS
GPLKVQSNMRGRVVVFALHAECDKKVMKQLLLEDEVAGGTPTTTPLAVQEESPSSSTEGK
DNSEADPDNKKKRKNTQDADSVAGDGRRINPAVANTCPGPKKLMDAVLLEIGGVGALNSE

SSLVLRELASVAKARDIVFCATVQEPFGSLSKSEICRLRGVSPDLIYLHMTTAAAETKLM
YLFGKGLSPAAVRKLLPRNLRGEVTPGAACEANL*
>TCRU_3894 hypothetical protein
MEAGSGQKDRNNSEASYFLQNELARLREAKAGGNCSRVADDAGITMNQSRMPGRENETPR
AQLPNEFDRAHRLRQVQQELPTSQPSFRQAESRIDSVRLPSEALRRAVRAAFGPGRFVLD
DGTAVMLEYAHDFIVLRGPQSVRHSFMALLLRTLLKHVESLPQQEQGNSGKERQNLSSTH
VISSSMAEVGESREDDPTGPPLYVLRRAIGMEKMEREKAQLAQHDAEHECALIKTAWDVE
RARYREEKQRLEERVRQLETDLKRNRDSLQIALHRLQEREPQKREVSGDRHADDVEALSC
QHGEDIRSFPGEGGKSKIHGIPSVSLETGDTSSVLPCSEGNDITTTNTAGNTSHADFSSL
ACGKCGKTMNLLQREMDLLRNEKDILSRQLQVSVGVVDELRNRLQNIEAAAVQLFSSVLS
SSEVVLTVLKGAAAAPRLFLHRCGGVDEPQVQALVQSLSADSTAAPPVEQRLAELTALLE
IEHRALKASLDFAVHERDAALQANREEAFLTSPEKAAVSAPLTRRTNLDGRIANTSSATT
LEKENTELRIELRRSFEDIRQLGQAIERSHAMYQELQEKLRTADRCMREKEKTARSMGHQ
QEIALMEAIKRERSLEIQLQRLRQQNKMSAVHHSNTPIVHSEKQEKTFAHLVTTPSKKSC
AASVLTLDESRNKYPFHHRHNTDTRENDSVSRNDGLQSGPRTTPTPLKGTGAPTPFSPSI
VLRRENFEEALNAIHPS*
>TCRU_3895 hypothetical protein, conserved
MDNEVTWSSLFLPLEEEVVFASFITILCPLQRQRQQQQQEMFSRRETSSEGDEQTRFAS
GSILYHAADEDAMGSPLFALYGERADAIVCVLANRSVVFFDLTDGHEGPKEIRRFSMGKP
HGVNLEKATCASMIPLEVVYMCRVPAMAKPPTVAHHTSSNLGTTALASKASVSDRTMYLR
GVVGSSDGRVDVFSEYGFVFGFVAHDAPVVAVAVIYCPEDLDEDRTHPSSGGTNVYRLPL
SGREAERWFLNVTTGMGFVTCSSEGAVYVWKQQGLIMKSEMLEKSVFPSRTFKWHVVQPS
NGAWLLGGLGGAQVSLLVHTTPGMEHELRVRSTVTFEDVEPRVKLPGSQFTRTTAIASDE
KLALVARGRKVYTFVFATARCELLFTARHTVTGLFLSKYGLVAAACDSGGLLYVVIPKLM
LVLGHYNVRGGGPIRSVSLHVVSRLLTIVAGNGSVEVVLMPETWNDGPLSRLHYLTDLMN
STPAAKALYSLVSLQKRQDDSSSDLVNATQQSAAHESLLLARMAIPEETKRYLRPTTGLA
*
>TCRU_3896 hypothetical protein, conserved
MLRRAPCLLATNIPRSAWDPAHFNTNWSDSYSTEIAARRHWPAKKWSIGLEPRTPRDWLQ
FSYRNLAYAYNGALRACQSFPEMLVCYKEMKQRGVKVDVDTMNVLLTRAARYERVQVDDV
FLLFDELTALGARPDIAAVETLHTVLDHSAAMPYEWREARRRQLVELYNCLAMEEIERLA
PHRVDRLLKEQIKRYRDNLRALKASLSPSVYRRYLHTMHSASMLLEEVHNFLWELVESDH
PAMEIPALQLRIPFVGSVMRRPETDTNEKLLKYTDFEDTDVCSVFLAAAERAVDADLHDT
RAVSERRIFLSLLTMISYSGVLYTSDLMAQLMEMVKYSTHASSRDSDAQRLLRYAVRGSS
AAQDDLYRSLWLKVEMVADSRVLGRYIGAREPWSPIRVCFDERGLFKSYPPLVHASAKKT
SAIEADATSGSGSTTMEEEQRQQRERQGMSTEITSHTSEGGEEMVEGVRGAVVAEASEAS
GVKDVALQEASHGEQRTVEALDLRWDDIHRLIERTRALTSPPTERHPQQEKMEIFTGIAV
YLRTIATGRRYCSGEEEAGGEYPAESRERALFAVGYELDVWARLFELVQEVRHDMEKFIT
DNAVHHVEPEFECWEALLITLRCILDFCVVRTQEKGKEERAAVEELFEKTMKLRNELVEE
SRTRFGGRMRILWLQEA*
>TCRU_3897 hypothetical protein, conserved
MGMASRWMPRPEEPNAEAPPLRFGGPLLMQLVRYLPLLMRLLTHLPGFTAGFILTCLFFA
VVGRDTLRNEYAPVAGFHVVALMLLRELVAPWREGKKKAAFVRQRVASLRSFADDVQLQW
RDVLD*
>TCRU_3898 lysosomal/endosomal membrane protein p67
MRRERIVKDVIFGVTLLLLATAILATGGVRAEFWQNDDVTTVKVRYNMKTKEFSVTEGEL
FGAVAATLEVHDTFLQNGWDVVNAEADKEFLHSGPIETRDEMLRIAYQAMGYGEGYTTQK
RMEAQLNNSFLGKGGLNEILASSPEAVKWIQEHVEYMDTADAVDAYGRQLKNLLALLDGM
VAGYNARVGAGGVLLNRTWLFYLNFLAEIGDVVASVSSQNTIAGLRETNPRHFLDLHCSA
LVKVTEYDIYFSHATWSSFNSMLRQYKTYRVEHRTVTMSSYPGMIHSIDDWYMTSERLAV
METTNGIYNDSLYAYLNPKTVSEFLRVMIANFLATDSPSWVKYFSLNNSGTYNNQWMVLN
MAAVANPKTPMPANTFWVAEQLPGSTYPLGVTAADMTEHLNKYGYWASYNIPYFENVYEI
SGYLKKEKEFGDYFSYTNCSRAKMFKRNESDVVSLESMKRLMRYNNYKEDNLSLIQNCTG
AGMNDTCNPPFSAMLTIASRGDLNPAGDAKNYGPLYEYLGRRDHGATDAKIATWSGMVMD

LHHYTSHVICGPTNEQQPTFEWKDDLFTPMPPTYGLPKVYNFTFMKYVTNVSPKSPSIDN
ARNKWVGIAVSTAAAALVFVSIAIVLVRSRRRAKYLHEDMLLAH*
>TCRU_3899 hypothetical protein, conserved
MIITSAIAVVTTVVHFLWAAPPSVRVDVWIDNIRIAGSKSDVTLWEAQALRNAGSCRASM
GEERESGATQYTFLGAQFGQTHQTVSLSDEFVRSVCAMPALNSSTIAETEVVASRFCMWR
PFGARVYATTTFPSRQCDDDYRHFTKGLWRRHPRRTFRRQRLVWARDCDTPSRTQP*
>TCRU_3900 Protein of unknown function (DUF3720), putative
MAMMMTGRVLLVCALCVLWCGAVFGHAMEDYCSEGGGNGLRHTSNGGDDGVSLKSDCRLL
SARMALIKAVEAEEAGSGLSVTLPPGKPKETSLDNNQGGGAPGLGGDLGVEGQPEAAALQ
PTPGGSGTEEQVTELEVVDPHDKNVAGDKKEQKDVNAGDSQHQRADQSSSSSSSSGSSGT
ESVKQPALKENSKSNEIPDKPPQEEEEEEEKDLRSKSDGREEEAEEKETEEDKKKASNQA
PTETPGGPTNKEEIQTATTETNGQPSPEALGDVKQSKEVQDEKDSNNNSQTKSVDSIAAN
QPNEPSADHGESGPLSPTANGAAANNDSERSTQTTGDYGPAADVAGTAEGKQNENKDVNP
KETPVTATAMKTTTVTTGDSDSSTAASHTTSPLLLLLFVACAAAAAVVAA*
>TCRU_3901 40S ribosomal protein S3A, putative
MTLGKNKRISKGGKRGKKKTQETMSRKEWYDVVAPKNFEVRQFGKTICNKTQGTKIAADY
LRGRVYESNLADLNKTQGDDDAYRKVKFVVQEVQGRNLLTQFHSMEMTSDRVYFLLRKWC
TTIEAAVETKTADGYTLRLFVIAFTKKQSNQLSKNCYAKTRLVKWVRHRITNLIRQRLSK
VNINEAVTLLTRNILRDRLAKRCNPIVPLRDLRIRKVKVVRTPRFDAQALLNAHGEIPAS
AEGEARVVEEAQEAPAAEATA*
>TCRU_3902 BNR repeat-like domain/Concanavalin A-like lectin/
glucanases superfamily/Trans-sialidase of Trypanosoma hydrophobic C-
terminal, putative
MLVTLPVYSEKAGNKKGVLHLWLTDNTHIVDIGPVSGEDDAAASSLLYKSVENGDQGNKE
ELIALYEKKKGESSPGMVSVLLTEQLKRVKEVLATWKKVDERVSELCPSENAEHDSSPDN
ACSTTGKITDGLVGFLSGNFSNDTWKDEYLGVNATVTNGAEKTDNGVTFKGRGAWAEWPV
GKQGENQLYHFANHKFTLVATVSFHGEPKEGVTPIPLMGAKMNDNENPVLLGLSYNNKEK
KWILLCDGRENKKYSSDWETKTSEHVVVLLRNGNQSSAYVDGQPVGGDEKCNLENMDSKG
ISHFYIRGDGGSAESREGVPVTVSNVLLYNRPLSSAEIAGLAKKKITILKAEDPNTLVDA
QSSATSGSPVQGAVSLSNSARQLPSEQETLKENVGAGAGGLSSVASVTTTPSSDVDPTVA
TGSGDTMQGIGSPQTPEVSVSSGEDEGTVGETDAQKEEVHSQVREVNATALNSSLGNLSQ
GNNSDAGTVCGSRLLPSLLLLLGLWGYAAL*
>TCRU_3903 twin BRCT domain containing protein, putative
MLVTTTAIYGEELDRIKKDLHQQGVTYLAELTTSTNVLIAGRSEGGKCRVARERGIPCVT
PAWVRLGRCFISRIGEFEMGRSLLGLEVCSTSLSMDEVGFVRRACETHRAKYNAFLTRRC
AVLVVPNGKFVEDQSEKLHFARKNRIDFLPYDKFMKRYGFATELQQNLAPVSRQQPLGNG
IQGLVVYCSPESLMTETISSLFEKVGVQRVPVLTPLTTHVLVLEPTDEVFPQRPNLEFVS
VAWLEECAKQQKQVAVESYRVSVFQLPVITFTGVPREERMAILSCLEERGLPFLMQEDFV
LGRMADDSSGQRNTTHLVTGRSALLQSSKVAALSCRVHKLRRKECFLVDVEWLRRSLEVG
SWVDLTKFSLEVPPISAFGWATKRTRKTLAAATAPDINNQMAEDYDNKRGLSSPCSFARE
KGDEMLPSRSLELLIEELESKTSGRGSASTTPIIAVSQSVWPKKSSFQERTEDHSCKGAH
LERSTASLSLEANLTSVPVESQVIVYKRSELELPKKLMRQQQHFVLPCTEKTGGDADGF
LLTEGAVYKTEESPRRPSGRFCCIMISKALMHMEENLERLQALGCTLAKTVEECTHYVTG
KPSRTEFFLCSVAAGKWVLAPSFLEETLREGRIVPEEAHEWCPEIAIAALLRNSVVDLVR
ACSLQRKRTVRSFSSWRVALCCATESRTESFSRVLRSGGCRVIRPYSSPQILNTLKSDFE
ELRDLCFVLSDDNVWEASQLDILAVHLPVLRMEYVAHCLCVEVPEPDLYLVQGDSGRLRK
RLKVV*
>TCRU_3904 diphthamide synthesis protein, putative
MESAESELGLPSNYRFEIDKCVRRIKEKNATRVALQFPEGLLIFAAPIADILEAKTGAEM
VILGDVTYGACCVDDYSAVALGCDFLIHYGHSCLISIKDCLISSMMYVFVEIEIDIQHFV
DTVRRLVSPETRLACIATIQFVSSMRAGMQMLRDHFQHPVIIPQNRPLSSGEILGCTSPV
VDPATVDMVLYVGDGRFHLESFLIAHPTLNALQYDPHNKTLTKESYNTTEMRTLRKEAVK
KGMSAKSFALIMGTLGRQGSPRIVDRIIQMAERQGKTVNLLLMSEIFPEKLSRLQDVDCY
IQVACPRLSIDWGYAFDKPLLSPYEAEVALKAVEWGNEAYPMDHYSKDGGRWAVYTDKSL

E*
>TCRU_3905 ubiquitin-conjugating enzyme E2, putative
MPSTPTPQCVRRLQKELSALCREAESFFFTRPSAKSILVWYFVIKGPADTPYEGGRYFGK
LNFPPDYPMKPPEIIILTPNGRFETNKSICLTMSNYHPENWSPLWGVRTILTGLLSFMVG
DELTTGCMTSSDELRRKYARESRRFNAEKMPVYKELFPEEYQKDLEELKREDNEKNGRTS
GSAGCGANTKGGGVMESQEKEQWRGLFPALLGLFAVLMGAYFWPW*
>TCRU_3906 hypothetical protein, conserved
MRCGGRWIVERSDGFVIEVEKPGVSVVFGRDIGLPSAQRMDGKYVSRQQFSVEWKASSLH
VRQLGKNPTFYSMEFHTLPGDGTAEKELVCCREEETREGKRMGDGRKGLNVILVPPVSNE
KFLASLRGGAKGIEPATLHFPGELNLPRFTVRYVVADEKSSEEKTSREMLTVPLGSSKSD
EVEDDERANQPWMTGSWRGILDSVLHEQQQQAER*
>TCRU_3907 dihydroxyacetone phosphate acyltransferase, putative
MARSDTTGVTRRWKFRRSVIVSGDSTCWLSAACAISLATSPIFAPNAPGDPKSIVVMNRF
DVSRGIFYGDKCQAIGSCTSSSFAAFESLCRDLTKGVEIMKHVAFAVDAPQMNETECFVA
DVQSDLYIYISAWSDADSCGHAPLSSLASLRKSGGYLVILCDGATQRSAWEAAQTHLPGW
TVLGFCVPSFAPPSFDLHLTRSFVRRCHAMKGILECVSLGVAMGTVKHLPVGFRDPLRVV
PLDVALITAMTAVLLHKDNGLGFISADAAMMKLGCAADNVLVWGMVAEYLMDYYGRIGPA
ALGDLPFPQLLDTQPTLQLSANVSDWVNYGVSLMMPFRCYYAHQVTQRRKRILGELPDST
TTEYIRGALGSIDAAVKCITAVAAARVSPSHAVDYSDAAKPPSASVSFNETDYQLLLQIV
TRDASLRELLPLILLRNVQWDLYIKVIAQAVLEGVALQIFKRPLSLPPPVPLYHNDIVFH
GTRRVPSTSLIPRRWFSDVHWTARVGVKPDGERFAASPGLTKESMSRILAHPSVLRIMGE
VAKNENSSEGAVRERAMAILRTVGDNLNHFQLRLFGFFVRRVLFRLYDEVSLNSGAFKRL
YNLVNTPRVGVVLLPSHRSYVDFIIMTYLLVVMGFSPPHVCAGDDFLRMGPIARLMRGSG
AFFMRRSFRDDQLYYTLFREYVRQLVLRRRTLEFFIEGTRSRTGKTLCPKLGILRFITDA
FLESKGELDDLYFIPISLSYDELLETRLYADEQFGVSKPSENMGNLFRARSLLKTRHGKI
HVYIGEAFSLRHFQDQPLQCPAPFEPRGEKDLDASPRKNRITPPRVLLNLAWHATHSIEE
NTVVTPTALVAMVLNVFAPTAGTVPLAEVYRHMTWLHTNIVRRKGALSDDCANSDVETMT
RKGITHLQEFVEVLSDDAEKIRVRQDVGSFMGITICSNQLVHVFRDEAVVAVAARMYGEV
QAAGNVLRVERQHLKEGCRQLRQLLSSEFQDYLPFCPYTFDSWFEHTLLRLLSCEESNTE
SEVFLSLGGTTAEAEKRKQDRGDNLSIERPNNVFIPMTRLYRFLIATLSPFMESLFFVSL
ALSAVVEPGSERVVYKCLLLKACQACILELYDAKRVAHPQSGGSGLLKSALEGIAMLHGV
EIDPVAKEAAYLIPATADRTVLLERLQQLMRHVCDMRSSSPKLLRGEEQKKAREDILKEY
VKQLKRMKRPSKM*
>TCRU_3908 hypothetical protein
MIRLTQLKNNGGIKFRRRTRRTLVILVAALGALAWIIAVSLLRHLGGSATTETAVKTSST
PVTRSRDPTASTNANTGRSSPMFVKGNIAKGENSISLPHLYPMHRHLPANRSAVVEAIQE
KQMRRQEHCWVTAPHSPLFPSPSSVGATLLRPLHVETADAMLQQEYFFARQKPTGSVSTG
TFYSGYDTPPTAANGTAFPWWVSKKSHHHLPCTAEVQKRLHEYQNEAPCSARRFLLSGIK
EGGHGLGSSLIVISFDFLGALRLGRTLLIVAPKSDKKWQFVARGCLRAGRRSLDCFYLPP
SRCKLPGGSVRVVRRGRDAASISSRVIRKYSVDIPGLDRNTIPSDEAFFGPGHQQWACFS
RYQKWVKNPANMAVYGTFEKGIDARLTFMLAQVFTYLTRAPQPWFQAMIEYHLSPLGLIA
PHMVRVDPTPRNRCIVYVQDRGEVAKMREYYNVFGCHTVGLSIYRDYVAAISTSSKSAPS
QNSCRVFVSGGTPRQSFLWLKKEFEDDSYEVLSTWNLSTLKAGSESTRWGASSPASSWVD
LYAGVASTNWICAVQSNWCRMINFLRLTHGRVDCGFIDIGVLMLTSVEAREKYCVISDFP
TKPFSNVIGR*
>TCRU_3909 hypothetical protein, conserved
MWNALSCLEAPPLVGPKKVDTPAVVVPVSACEEPPAVSEAHFADAAQTEAVVIEKKSDFC
EEPPTHADFATLGDASTEWDAFSRRRLGIAFHRGAAVLPGFPVLQRELRANTPHDSPSRQ
VMDVDLWNSYWSAFSDRFASQIDYLQEYLKAHDGESERRRVLVAASLDAEPYMESFKCSL
TAYARSFGALEHVSRERCVVMLQFTSPRSAEIFAAWAFSLTLEDIFTEADASQQAGAHCQ
LLVRLISHDAGKITSTLRWGSNIVLSTPFVESLFKGVFDAVSVTYHDGSFLILFASDYAA
KTALHSLQRSLLEVFGTTLSFSEQQPELGGKSFLMDKLGSESNLPR*
>TCRU_3910 Shwachman-Bodian-Diamond syndrome (SBDS) protein/SBDS
protein C-terminal domain containing protein, putative

MSARIQVPLSQRRHTNVAVVRYTKNGVRLEIACYKNKVISYRGGIETRMDEVLQVERVFT
NVSRGLYSSEKEIEAVFGKGTSEKEALQYILDHGELQVAQQERAAEIDQMFIDIAVIISQ
KCVNEVTQRPFPSQVIEQALRSIGAAVKLDQPVKKQALAFIHQLIDAKTIPIARTRMKLR
CIVPDEPSLEKLVEWCEINGTSILQKVVEVGGSDAALRIHSLLILLQPHLFRDIERFVKT
EMPPGGSVHVIENAAMDVGEGDVMDAELIARANAHMTGAGGVGNADKSHCTGSSSNPHAE
SNRGRRKGKGGSRKPQGVQRVLGNDDGTDQQTVTTAELSSTPAVVGENDDELKVALSKLV
LDETHGSRDEDYDGKGRRGKKKAKRRQGEPHQKQQQQPATPQKAVEVDEQKESDEEVLVN
RKQRKQAAARVKVKDDKRNEYWNDADDDDYDCGYEDEDAGEGRD*
>TCRU_3911 Spef1
MTVPLTEEDLHELYLWVDDIPLSRPKRNITRDFSDGCCVAEVVKFFFPRLVDLHNYTPSM
SMKLKRDNWETLNNRVFRKLHFEVPPEEINDITAGVPGAIERFLRALRTKIAQIKARREK
MSALEMGHGGSDDENNDGGDDRGGGGGGWTTSGRPWAAKRQPKYPEHRPGSGVGADPAAY
RSPSNVRNVSEMQPSNQSSRDNKRNDNGDDATTSFRLLLDEKDRTIFELRETVSLLSEKV
TKLEELVRVKDEKLNAYREKLGRH*
>TCRU_3912 tRNA-dihydrouridine synthase 4, putative
MERLNDDDEREDLYCVDHGRLKYKHTDLLAMLRDAQSAIIRFSEENHDIIADVVNFASTS
ERKNTLRKLEEAYLGIVKVQAPMVRCSRPAFRKLCRLWGTDVSYTHMIMADSFTCSDAAR
HADFSMYAGEERLVTQIASCSGPVAAEAATLLAPYCDAIDLNCGCPQRWIIKEGLGSALL
QKPEVVADMVRCIHNGLSGGVSLPCVVKMRVDNDVGRSVDFARHCEAAGCGWITVHGRTP
SCSAHAPVRLDAIRTIRECVSVPVVANGGVNNPQTALKTSLTTGAGAVMSATGLLANPAC
FYVPREGELLLFAPQRHGIDVALEASMGPCRGADEGASTAGSENSAWLKGLPGCPVEVIS
DFIRLSCVTDLASKATSMHLLKMGGNYLSPTERVFLAQMHSSFSVVSAFQQLGLYTQDGR
FRVVESVT*
>TCRU_3913 hypothetical protein, conserved
MTLLGAVLAFYEVSGVPLALICTSPSVRQACEKNAAAEVGLWKLSLESDGSRRVIWQPSV
PVRHCHKTDVMCSVAWWCNRLMPIGGKAPPTRLDLGAAGRLLDTHLPIVFVFRSLVELTL
RRAPIKDVNVLGKLPMLKRLDLVQAQVVDGGIIGLGESRSLVEVDLWGCTAVTNANTLGK
VPTLRRLVLAETCVSDDGIAGLVDGLSINEVDLEACAFVKSVGFFGRLPVLRRLLLVSTS
VDNNGIMELGSSMSLVEIDLWGCSAVDDVNSLGGIPTLQKLDLFGTSVTNEGIKGLRRCP
SLEEIDLTRCEAVSDVETLTKIPRLRRLVLLHTQVQDVLELVSMGVTVII*
>TCRU_3914 Uncharacterised ACR, YggU family COG1872, putative
MKVSSFISQLRPGWFHLTVHAKPGARSSSLACHPAVTDAAIEVRVGAPPVEGKANAELVE
FMQMLLEQELARVRAAQQHTPLESNGAVMNCVYGGHSKKDKRKNKPNTKMECPVNYPEKV
RVSLVSGASARHKTLEVAFPGTQEELISVLQSAYVS*
>TCRU_3915 hypothetical protein, conserved
MLRRTQRWLLLNVSGESPSFRSKPVRNLQMDVQMSPYKEQFKDYWSTPVPWADREYGEKP
KIGAPIPTRRAATAIVVGKNKHVAVDKVERGEDNDYKVLMMFREGKNRFVRDQFIFPSFP
ISLEDSSEDWGKILRRRGVKTAWNDLHHRLCAMRALFAQMNILLVPKEGGGLVEVEGPPG
PKKWHLLVHSQPTAMKNLVDVLELPMETCLSQLFPFRCITTPTTETFRFDNLVYLVPFEK
IPDVRFTISTVGEKLVWVSPMEAMARFNAGIMNMPTPNIIAISELNNECPTFADVIKRKP
HDDYRSILPELYRHSQTKVATVLLPGDLMHPNTTGEDKAERYIRRFVYEKDYPFGVRAVF
EERPATPEEMNEPLIQEAPKLLEEANEMDMVYADTPYPKREKQPGEIGRTRFPVPSYSFQ
EGAENEEGVIPVRNRNIESVGDLYSHTN*
>TCRU_3916 hypothetical protein, conserved
MEIIDRPLSELIKEQKIGESLRKKRRGHGGQQNTRNGGRGTSRNGRGNRAMGRGSRGTAG
NDQGRRYRRPLRFRSNGGRFRRLGIGFDDRRGFNSRSWRRNALDLGDDRRDRSVKSTATQ
RRRKAAFERNDRVRELRRREALSRARSRFSRD*
>TCRU_3917 hypothetical protein
MSNDPSDAAIICATSDTTYMEEIILQNEALLLEVEQLRSVVKGKGLDQLQQLRGENERLQ
QLLKLSESQLAERTHQLEVLESAYNRIDGVHSAIHELTEQQKALSEMKERCISLEVHNTA
LKTELVKTSESEEELRKALQEAERSRHVLEEEVNKLRASVTETKSRLENVENLKCSAEEA
LEGAKASIAKLQAHSEDLEEKNEAFKRRLRDAEQEACEESRIRNGRHTLIEKELEDLRAK
EAIGAKRLMEAERVQARLQEELRLAREALAAKEQDTSRTLLQQRDQVERKLKLEFGNLQR
ALEERERLLRQQVEQNIRLKEELTMLSAEKDILMRRKQGEPVDVLQLQNRLKELSDETSK

KEALQQRFQEMLKSSTATNSDIKHLFAQMLDYQERRDEEMRTMIAIERIKTEDMKNMYEL
NLEKLRAEQKSLVYEIQTLKSEGDASVTRRIAPAEAEISHEGTQYGAVAVGNSLMPQANA
NPPMFVGPTERVHPVARVGGSDVGGTSGSIAHDLWQRSLTRADRVQTTAINYEPARLRFP
HEERLSLLACPRCTFLNQPGITKCEVCGASL*
>TCRU_3918 Enriched in surface-labeled proteome protein 11
MTVWYFLQSALVALFFFKDVSALERCRPIERLKGLNQEILAMPRGGFYAGYEYFCAEDAP
RQFHCHCGVDTICKNKTDAWGRNVGVCACCPWWMIVLFAILALVAVVAISTTLYVWCCRG
KWWFDGYPPPVKAIMCRRGAATVVPATGPLPPNLFRGYRTSEFVSEPTEQPPSRVPLAAR
GGEAPRYLYHENRRHSSPPQNPPHSLQQRLSERNPLSHR*
>TCRU_3919 hypothetical protein, conserved
MSASGSTSKDASPYVCLSAHCDLLHFIGPVPHPYGGPKPQTIAQRASQYVRFSCESCEKA
PTVTVKCEHPLFPPIPITRTSDGVPSFPLTGIELARWAYRENFESVASQSQLNPHEKKVD
VPHPPLQGDSARVAHESALPEVASTAPLEEPEPTMESGRSSCIVNFHALRRIFLQTLFIL
NLPHRSPAVSFRLSLHDPRPFGVTSFFLLPRCAEKLAALAHPAKVTLTVARTTGGDTLQC
SCDCLHKKDEEEDRITSLQLLWELKEGDEREQYAKDPVELDSFLTRTMIPVLFALPEGLA
QLIESEGVQTSRAIGWSQRRSFHLLALIAHCVWCEKVAWCIMNRFSVSMGLCRLQKARLK
LRNSASLESTDKWESTLFFYPTAFNDDRHQTTRGRLEAIDVTATSCAALIQVTVRGCDEE
MEWEWNGEIPHRSSAGLCDFTELIKKRY*
>TCRU_3920 ATP-dependent DEAH-box RNA helicase, putative
MRRLLCKSGLLAPAREMRHERDFRGRNRQRRQRGFGEGRSSSNPRNNDDIGILQIKFHTI
PALQVDMRAETQIESFLRSQLREDGSSMLLSTNASSLPIVRVINVPRSTLKRAVLEFHVR
GIPVRALGQSTRAKRARALCFMHAARLIDHFSVEGTDNSAAPLPLEEGTELNAQKKYLKR
VMSIRKTQAFGPPPQPEEYDCWEDYVDESRRHIERQEQERRNEAFQLLRIPHTGNPLVDR
AAEMTEQERCTDRLALQKLNNELLGVSKGVNIVRISHRVFVATLTLDSVTNLVATGVAES
SQEAKQRAAMHALNILTLVKEQRQASAVDGKASAFAGRGSCASPDAVSLSLTPRYSKMLD
FFSLFFGVTPAPVFTREGNSYICHMDLDGVKCTGQGINRFEAERHAIENALGEMELYDER
LQGINSIIAQYPNIQPRCVPTAKLPEALRNEIHAFVQRCRTELHFPVDGNTVGNEGHGGT
PEKILTDDVLDAETRAAIKALEDVRRDEAYARRLRDRLCNLRADPKYLEKFHMRRSSLAI
ASVEQRILDAMDQHRVVVVCGTTGCGKTTQVPQYILDREIMEERGDRCCIVVTQPRRLSA
FSIADRIASERLDVVGKDVGYAVRLDARPGRHITLCTTGVLLQMLSGMPSLDTVSHLVID
EVHERDINCDVALALVKELLMEDKNKRLKVLLMSATMQSEMFASYFGDVPVISVEGAVYP
VKVCYLDHVAHLFQHSSPPGYYSPMFDALSSATTSQGRNTYRGRGGYRGARWQLTTPPKT
DYALIARLIERSVEVDLHGEVEGKSVLVFLPGWKELVAAKQALEALNGEQRYHIILLHSS
VDAKKQRECFSPAPEGFVKVVLATNIAESGITIDDAAVVIDTGLIKQTSWVSRATGTQQE
HHASSYSTQLALQYASRANCTQRKGRAGRTQGGICYRLFTREVWDVLPSFQEAEIHRVPL
TQVLLTLLSLGHTKPKETLQRFIEPPSVKNVEASMRQLRSLGAVTADEKLTPLGLYLSRL
PCDPRIGKMIMMGAVLRCMDSALTMAATADVSPFVTSREVSFEVRQKRHAFSMGSQSDHI
SVLNAYNAFCARQGDGVFASEQYIHRNNMRIISRYKQQYRDILRRSGFIRDGESFSIEAP
EDEFIIPNDDAVAPLHVDSGLLSLDAADVTLVKACLCAALFPNVAVLDPSPLLQGGKRAK
TLVMRTAAHKNISPSKDSACRRLGPPRAHGVLTPKEFFGRDAESGAEAPVIPSMFYIYQS
IFCVQESREEFLTQVSSVSLWALLLFGVGEADMRFDPLLQLCIVGGWIGIHIDASSFATL
HALRTTLHTCVWRKYQKPDDEKNNEALEVLRRLCKEVLQSPPNDKEQYLNRLVDTGRILS
PSKLEPMTTDTNDENFAWE*
>TCRU_3921 hypothetical protein, conserved
MLRRWPVCAVATEVPTIVATTTTAKLWGSVRAASSSATGNGTGENAPSVAEAADGAAYRE
SLIQAILSRDKEIFELKRQHELSMLRVEQNQRRILKDQEDRGMYYEQNCNVHTFDTISVG
LYTQRNTLYHTMSVERLRNVKLLLTLLVTLLTCFYLYYRYMINPNWVYVEKPLKIIGSRV
GALNDIRAQLGSQEEETMAMRSKLRE*
>TCRU_3922 hypothetical protein, conserved
MQGLPHDVDGHFGIRVAARSSKLRARQEANGRAHVAGSGAQVPLRTRTILAPRLQLRTYH
DNTAMRDALEELKKACGHFPLDDAEEMEKMNPTNSSAGLTPSQLNQMPGHLIVYCFTYCD
LESLCELGRVSLRFAALSNANLLWETHARLRNVTTSAPESAREDLRRAVVEQHERWQAEV
QRHEREYEALERRLRERAAAELAAPVDVNATLANNRPGRFFGGNSNGRDAGNAADQEAFS
AQLEQLETVKLDLLRGIREMKSELAQQQRQIEELQMWVAKTEEATCEAGNTTAACEAEGI

TFLALQAFERRICRIVLSAVNDLPTVLRRGVDDFATMELLCLFGAGDVGGRVRQRWAAFK
RFFPPMSEDYGTVRYLLMTGEPPSSDRARECIASLGGVIRRVQRMTDNEIVQIIM*
>TCRU_3923 Endonuclease/Exonuclease/phosphatase family, putative
MTVASSDDDKQLDRSHRSDGSCCSISSSISSSSTSTSSTHGDDGIPHANGVVRVHQGRSL
FSGIFMCYLSLFCVIRYLARLLFSGFATVRAARDSETYAMQKKEDDFLCGNQKNSERGGG
ITAKGIQALRSQFRLDSDTDTETTSGRSFSSAAVSGEARRRLRANLLIRQFFKKEKTESM
LRALRVHACTWNVDRQPPPNGNNGFYEWLLGRKLMGKVTTYWDLSKEARENSNETTPPFP
IEEFPDIVIVTLQEVEMGGVVLVREYTETGVMWTEAIVDALNKISEHRVWYKKVKSVQLV
GLVLVVVARVEHINYVTNVRVSLTRTGAMRGVWGNKGSVGIRATIYGKRFLFIAAHFVAH
KHNERTRTLNYHASLKDLMFEMPAGVDSELDVLETFSTAARDSNMEDGIIEKRSRLTPFF
RNLRRTPSALLEKEVLDKYDYVFFLGDLNSRLHGLKGTDIRRLVRNKEYDRLICHDEIRQ
GMISGDTFDGFQEALIAFPPTYKLDRKTDLYDTSRRKREPAWCDRILFRVCLPCEKSDDD
DSNDHNVDGVNRNDGNNKQEGENRALYGSFPYLNSSTKLMYSMPEFSVPVDNVNGPLNRT
DDDVVSLQSSTSSMSSEGAVSLLGKSDSVLSDMHYRSCYLETLSKMRAQNLQISSFTHRD
VSLDETESLPTFLAARRSTCTDKRFKFFPMIPNKMLVFDYHHVQALRHSDHRPVCAQFRV
SVLSFEKDLVDSVLQKVNEAFYLEENYPLHD*
>TCRU_3924 hypothetical protein, conserved
MPQEEGDFDGVNPDVQARLVDLIIFSDQARRRIAELESTVETLEQTLSELRRNDTHSAAT
LRRPLSAPLIDQPFKENGLQDICSWNEQLVETVNALRGAMLKMETEAKVQIVVLKDEVER
LRRESDSLRRENDALKERVADDVLRQRQTNRVEMAVEKAKIASRVRAAGLRRLEFMEAVL
MEAHDDLYFAQRFDAAKLPVRDVTFVIHAVPNVPTQHLSAEEFTMTIYQNFLLEVAKKYG
GYHVCGLREIEVFAFYSATAALHFSSECHVGVKNLPWPARTTDIPFFAPVIDDGELLYSG
PRMHTCMYTCNPGSEVDPINGRSVYYGQEVKDALTTALQGSPIGEIVANKACVIMLFKEA
SIMNDVHDDFFLEMSRVRAKLGAGWSIIASERTVGNLFCSILPKSLERRRGIPPYYLHPS
PRFPRGNIDVDTLLPQLIASMKGVTRPCQGGTQQKPQAQLQIQSQSQQLLLHHDGWVDGI
YRSRKRAAEKRDSNSEDVVSLYLLRRDRKNIISLYNKLEAVSAFHEQTLMEAEDWYLARF
QSIDPSETVYLCTVDVGSNSSWRKITQVSMGLEEHHQLRQQLFSSALLHGKNNFGVYVNG
NNTDVFTFAFRRTEHALRFTAQIYTVVKQKCEAFTKSETCLMRAGITVGKLRPSNDSHTA
STNTASGTATDCKATRMVLCRGKALVCSGHLCDLACRGEILAVAEVIQAFQSNKSNLLSM
EYNVLKKGGRFLGSYSTLVDVYSILPKAYAYRRQLLKGGDDGSCRDECESPRSSVKAALQ
LGKRIMSREEVEQLLLQHQQLLEKSEAAMMAAEDNAWEITTQQALSLPWPVVSRSGVRST
TAERGFLYCDAVDIVAISRALPEELYKNIMGQYNYVVKEALLAYDGFIAKTDSTAAYIVI
FTEPHCALEAALQIQRGLLAVDWPQRIKTLEATLHVKSCKANIVLFDGIRARMAVHVSKD
YQSTRTFCGDDKEFVEVFGPAIETVAELGFQACGGEIIVTSFALSSIGSTLHGSMLLLQV
AMRVARIQSSNKMLLASCVPQRLWERLHLFVPAAPVAEVAKQGTGSKLRQLKVAERKKSW
WREGNNSLLSLPFSSQAPWGERVSASHSTFAFSVQASLGGAVGQIRRELSSSIGLSTHRE
VFLNIVCEILMNMSHLFRVVEDGVSRANVLSPLPPLETGGAAAISRDASPNKFRQNFSPE
NRSSGTFNMKLMHSGDSTSSPSVSISGEPHKNAVQMLDNLLRGALHRMARALGGTGKPHL
ASFSNRMSTFKSVSRRSSPKPSGQLRPL*
>TCRU_3925 hypothetical protein, conserved
MVWEWVFRWLKPRSGVEDKKTSEEILAYETLRKAQTELKRLESDIETNTETLRQCEEALD
SLFHKELQLREGLEGILMEMHDHTSVDLENTIGNNSDGIASLQQILMHIQRKRGKLHDQG
YRLHIKLQCLKAEKKGVVKSTNMLLSRLLGNGLAVQNGMGGEDGKEGEQGDDDDGSWRAV
VHSPPVSVSFIEEYREKEGS*
>TCRU_3926 hypothetical protein
MKPLCGVLFTVLFVLCFAPCSAENVPNTGTSQMAENISRAETIPNGETVGMTQTVSNTQT
IPNTEALVMTEAVSNTETVVTTKNAPTAANPPHGAVPDRLSLALATRERLPTREDKTRAD
AVAKWFHDRIKINKQPSGIGEPTLHISSADSSSGRSTCFFTPLLLMTVLASTLMS*
>TCRU_3927 G5-interacting protein
MSLNPNAMPWNMPESGIDPVMDTNTRMGQQDPRFHGYPSYYPNHHAHQMPPVMIPHQMPR
HMMGHGFSNPNHMPPMPQNEPVQSYNHPRVRSQQYNAGIMNPMHSVSTPSYKKLRPVVVV
FGYRRVGKTTVAKRIAEARNYKYISLKPKEEGAVFSPTDYVAPLVELLSRKNSFEGIVID
DIVVRNKYEPYYVHSLLQKHNLKFDVIVVLDNDLDNTKERGVDYDDSLQRQLHPESYEFC
ASYLQGEPTLVVECREKTLDEVVTDALRQLGEVNFTKDSNISLKGVELMPGCPLERNAAL

VTAILDAQTQQLKLSEGSLFPFSEPNYLLEYAIFARYAYAFRSYMITPWIQGDKASLIGY
QGAVYVHLPSYNIVFCFIDAPSTLTQLAKGNVDNNEVGFIFEATFAGDKLYISDLLMMGE
MKASEMLVTERVELLKKKLKDMNSSTVELLPWYPVQSIEACESENPQAAGVLFVNPDGVM
FGGYDSRNFLYPLQRKKSVELRVWNGNFVGDSWTFDAFCEEMGNENIVEGIPVHVPDTEV
TQHCINDGNILECIRQDCHAGPNKGRRGAKRFVFQGRCQWKVRPTTMFKQEVFVSDPKWP
FERMAQACASVKHIPPTRPEGITTADG*
>TCRU_3928 LicD family, putative
MRRFSCAIFISHRYVSVFRPHIHLSPCVICDAAAGDSQETFGTSPSCDYHPPNFFPPPDV
RNDCVVEQTSSGWSTTLERRREGLKKWLQKEHEWVGGHRRVNSAYLESCWEAFCNPARTL
TKDEIAGTIDFLAQFCEVKLDDVPTETSASSNFMRQETEQPQCQNSVPSRVCHGKEEMSK
NVFLTHVQRSISALAEIERQLLFQRTLLDFFGVTNSLNVPLFLCCGTALGAHREGYFIPH
DNDIDVGVFYEDLQRLGGTMEEDAQSAVVSLLSHVALDGHFVLLDICGTVEKGLELRFLH
HETRVALDLNVYYPPLADDAALISQFGPFVWTASHYEDAGSRRHGMYRYRHASFREALIR
LPFCDPAVASRADGFLVPPVSYLVEYFGEDWRTPRAYTYTEGLQNGEYKNIIEE*
>TCRU_3929 cleavage and polyadenylation specificity factor-like
protein, putative
MLHAAVKPSLPASGVTHSLVGQFSAGLPAGVRELILFKKNKLELWRISCGEEWVSSLSYV
ASTQLNAPPVSVAICRPPGFAVDVIAMCLDDFHVSFVQYDPVRMRLSTLALVQLDDREVV
NDVMPLEPIMRADPTGRFVAVLARRRHMFLIPLLGLTRSENGDCVTGIHLEAENNTKLKE
AMTPVDDWGDEDDYKELAEETDPSIPPEKETTVHIEPEKHDSRVADAFDGLLHIGHLSMF
SVSLAVNGVIRYVRDMQFIESSGEPIVAFLCERHPTWAGRVKLVEWRTKAVESKMLSSQI
VWVQISAASTSNRKLLLIGEVDDVPYNVTHMTPVGPFSQIPSGVICYGINTVMHVTTKRG
YGVYLNNGGMEECANSKSSAMSYGKVGWCDPKMEASTALFKVNLSLANCTASFMSIVNEM
LHLLVVSEEDGVVLTLSITAQSSSVQGIRIAILGTGCYCSGIARIGDQIVFLGSACGDSC
IAKVDMFHSDVAKRFQIIESMEAIGFISDVDVVDCTSMDETDPNQLNGVNNSSLCDTPYA
ELLGRTILEPMPSVSIAECRAVMDLAVCAGRGGTGSLFVLRQSVRNKVFRRENVNAISAF
FLETSQVHKRSRAEHEGEEGATQHPPQPSPPHLLLTGLSFTLLFAVRGESLLHVRRSEFL
TAYRTVFATEIPWLNALLQVTEKEGRLISFDGKTLLQKFNFMSEREAGKKRFVKAASIAA
DLMLFFPLLEDGTLFRLALKAAKTAPTKEVFAEGVTAFALWPEQRRVVIFFTDGTMTIKE
MESCEVCATFPQFANLPQYSTPATDESNKEARDDEALPQVSHVEIVTMRDDNTVEATVLV
VILTTGELAVYKVISSDAFGPLRLKKSMHHFLDHKAEREVIESIEMKRKRLQRERGVVEN
DTQLMRQYSRRIVPFDAIGGNTGAYVCGQHPLFLFWDRRTRELEAYRHQTLGPVRGFVPF
RIINSGYIYCCEGFVDFASMDTYCRPTGQGWLTRRIHLGVTPHFVVYHPPARSCFVVTSK
KEPFRPQRAPFDVQLNIVYDEESGGVQSITTEAPVSNMPPIAPNAGIRVPMADRFEIRLM
STTDWACTDTLLLEENERVLGAQMMEIQCERDAEGLHTAPVCVVSTAFPLGEDITCRGRI
LLLATMCTKKKRKIVLFHSEPLNGPATAVVGIRHHIAVAVGGTIKLFRFDWSNRKLVVGA
LLYAGTYVTRMSSFRNYLIYGDLSRSCAIARFNEENHTLSVLGKDRNAVSVVHCDMMYHD
RAFGLLCSDDERNLLVMGYTPRVQETEAGSPNKVLESVLSLDGEYRLSGGCLVKSLRFRS
LAGNSSVTLYVTNYGEIGFIVPIGEQANRTASWLMRRLQIDLPHSAGLTPRMFLGLSQGS
PRTAMRAKEMLVSASLLNEFFFLDIHSRKTIASAAYTQLERVTNVASLVYEECNLF*
>TCRU_3930 PPPDE putative peptidase domain containing protein,
putative
MTGNFEKSHEPFASSSTDADDGLTASPKFYSYRRIIALPATPPDAASASRVNGTASTQLP
SFCPPQRECSSLLAADRVTFVLSQVTPSPRAVEFTVILHVYDLSRGLVNRHSEELLGFNV
PGLYHSAVVCYGMEFIFGGGIAVMGAGHTRFGKKYKKILLGTTKKTLSEFMTWIREREKD
TYHLNAYHPTKNNCHTFSKDAVAFLLGPNGSIPSFLTTVIEHLVKTRLGQGVLEVLTHYL
IYPKCITNRLLREHMLERKKSAMSIAQSAVACELMSQPPPCVVLFHVDDAAKCRQVFEGL
MPYVEQLKQRGKVDQSAKMILTSTFIISTGAELIDPTIASDYIELVVRILMYTHTTLWGP
VLNSLRIALLHKVVLCACVFHPTLLALLANGVRDFLNMTPEGKLSFLRVLCNLSSGLHGA
LALNSSRFALMWVSVVGLALADYRNDAIVYTGASLAVNLAHAFVLTTIPRVSKDHGVLFS
FHRIRQLMTILLFYLRHWPPKRIPEAAMNMMLLALFFLMSSYPENVEFAATHGCRLNYAC
MLQRAQTNESKALLCLMHALEQCGHMSCVNVIS*
>TCRU_3931 hypothetical protein, conserved
MAVDAAYANLFGVEGKAAVLGCDELGLVPRDLLHIPKKSLERAGDDKNAVLVRYTLIERA

RQNNIRKVLAIKNHLIADGDVQKKWKERCKLFPDAPGVPKIPKAVMNKLMTELDTETDDD
DNIDELGLTKPMPRYGDPPASLVADGANREASNASSSTSSRARFSPDRLRRWNGSKASNP
LSLSSKEKPLKKKGHTRKLSKFWIKHAADTNSGKISSGRLSGDKRDTFWRPKPPLDPPFL
PPLPYPRRDAVTLKMELEELNEYRQFLLRYAHDSSAMAQEYHDFTKSLQEDNNTREKFRN
RAATYTVEGSKFVNEDELTPLGPADGGNVTKSTFTKGGISNHKRQLDAYVRLARLEIRRN
ISNYKKWNGFMEEMEGVESTGTIAADVRARLNVGKLPIFEEYVQMVRERTKRRENMLLND
KRKQERLNAFCEAKDRHTEENIEKIKRTQTNLFMESQVVQSEIKRRNREYENRRRRMREH
[truncated: 528,899 more chars]
